# Integrated multi-omics analyses reveal homology-directed repair pathway as a unique dependency in near-haploid leukemia

**DOI:** 10.1038/s41408-023-00863-1

**Published:** 2023-06-08

**Authors:** Yunpeng Liu-Lupo, James Dongjoo Ham, Swarna K. A. Jeewajee, Lan Nguyen, Toni Delorey, Azucena Ramos, David M. Weinstock, Aviv Regev, Michael T. Hemann

**Affiliations:** 1 Department of Biology, Massachusetts Institute of Technology, Cambridge, USA; 2 MIT Koch Institute for Integrative Cancer Research, Cambridge, USA; 3 Broad Institute of MIT and Harvard, Cambridge, USA; 4 Dana Farber Cancer Institute, Boston, USA; 5 Present Address: Genentech, 1 DNA Way, South San Francisco, USA

**Keywords:** Cancer genomics, Haematological cancer, Cancer genetics, Genomics

## Abstract

Whole chromosome losses resulting in near-haploid karyotypes are found in a rare subgroup of treatment-refractory acute lymphoblastic leukemia. To systematically dissect the unique physiology and uncover susceptibilities that can be exploited in near-haploid leukemia, we leveraged single-cell RNA-Seq and computational inference of cell cycle stages to pinpoint key differences between near-haploid and diploid leukemia cells. Combining cell cycle stage-specific differential expression with gene essentiality scores from a genome-wide CRISPR-Cas9-mediated knockout screen, we identified the homologous recombination pathway component RAD51B as an essential gene in near-haploid leukemia. DNA damage analyses revealed significantly increased sensitivity of RAD51-mediated repair to RAD51B loss in the G2/M stage of near-haploid cells, suggesting a unique role of RAD51B in the homologous recombination pathway. Elevated G2/M and G1/S checkpoint signaling was part of a RAD51B signature expression program in response to chemotherapy in a xenograft model of human near-haploid B-ALL, and RAD51B and its associated programs were overexpressed in a large panel of near-haploid B-ALL patients. These data highlight a unique genetic dependency on DNA repair machinery in near-haploid leukemia and demarcate RAD51B as a promising candidate for targeted therapy in this treatment-resistant disease.

## Introduction

Acute lymphoblastic leukemia (ALL) is a hematopoietic malignancy where abnormal white blood cells accumulate in the bone marrow, and is the most common cancer type in children [1]. ALLs arising from malignant B cell precursors (B-ALLs) are often characterized by gain and loss of entire chromosomes [2–4]. The severity of chromosomal aberration spans a wide spectrum and may result in extreme cases of near-tetraploidy (81–103 chromosomes) [3] and near-haploidy (24–29 chromosomes) [2, 4]. Near-haploid karyotypes have also been found in chronic myelogenous leukemia (CML) [5], although at much lower frequencies. Interestingly, ploidy has emerged as a strong predictor of clinical outcome in several ALL studies, with hypoploidy being associated with shorter event-free survival (EFS) after treatment [2, 6, 7]. Near-haploid B-ALL patients have a 6- to 8-year EFS of less than 40% [2, 8]. Hypodiploid leukemia cells have also been found to undergo whole genome duplication to give rise to diploidized, or ‘masked hypodiploid’ populations in patients. While hyperdiploid leukemia has a favorable outcome in general, masked hypodiploid leukemia does not show improved patient outlook and may be a source of misdiagnosis as hyperdiploid leukemia [9]. Furthermore, a rapid initial response to chemotherapy followed by relapse with the original hypodiploid leukemia is often seen in patients with masked hypodiploid leukemia [10, 11]. This suggests that therapeutic resistance originates from hypodiploid leukemia cells in these patients through yet unknown mechanisms. Given that massive chromosome loss is poorly tolerated in normal human somatic cells, these observations lead to fundamental questions regarding how leukemia cells thrive under loss of nearly an entire haploid genome and why such karyotypes are associated with poor patient survival.

A recent genomic study of 124 childhood hypodiploid ALL cases revealed recurrent mutations in receptor tyrosine kinase and Ras signaling pathways as well as in the *IKZF3* gene in near-haploid ALL, and implicated PI3K inhibition as a potential treatment strategy [4]. However, it is unclear whether these alterations drive pathological differences between haploid and diploid ALL or are mutations later in tumor evolution that imparted a survival advantage. Indeed, frequent PI3K pathway aberrations are also found in other cases of leukemia and pathway inhibition has also shown therapeutic potential [12, 13]. Another study by Olbrich et al. [14] showed that a p53-dependent response posed a survival disadvantage to the near-haploid Chronic Myelogenous Leukemia (CML)-blast crisis cell line KBM7 (haploid for all autosomes except chromosome 8 and 30 Mb of chromosome 15, also bearing the BCR-ABL translocation t(9;22)(q34;q11)), manifested as deficiencies in chromosome segregation and failed mitosis. These investigators showed that p53 deletion in KBM7 and haploid mouse embryonic stem cells leads to increased viability and reduced diploidization of near-haploid or haploid cells, a process that is thought to occur through aborted mitosis and restart of the cell cycle [15]. However, the molecular link between p53 and haploid-specific disadvantages remains unaddressed.

In this study, we leveraged single-cell RNA-Seq and computational modeling of cell cycle trajectories to infer cell cycle states in single near-haploid and diploidized leukemia cells. We performed a comparative genome-wide knockout screen of near-haploid and diploidized KBM7 cells that revealed genes showing high essentiality in near-haploid cells but not in diploidized cells, and collated these genes with those from previously published cell line knockout screens to filter for genes specifically important to near-haploid cells. The two analyses converged upon the homologous recombination (HR) pathway as uniquely essential for near-haploid KBM7 cells. We then show that the HR pathway component RAD51B and its associated expression signature are a hallmark of chemotherapeutic response in hypodiploid leukemia using single-cell profiling of leukemia cells in a patient-derived xenograft (PDX) model of near-haploid B-ALL, and further confirmed such a signature in childhood hypodiploid B-ALL patient samples.

## Methods

### Cell culture

KBM7 cells were a gift from the T. R. Brummelkamp laboratory. Cells were regularly tested for mycoplasma with negative results. Near-haploid and diploid KBM7 cells were cultured in Iscove’s modified Dulbecco’s medium (IMDM) with 10% fetal bovine serum (FBS), 1% penicillin-streptomycin and 50 μM beta-mercaptoethanol. Cells were passaged by diluting cultures 1:5 every two days. K562 cells were purchased from American Type Culture Collection (ATCC) and cultured in RPMI 1640 media with 10% fetal bovine serum (FBS), 1% penicillin-streptomycin, and 50 μM beta-mercaptoethanol. Cells were passaged by diluting cultures 1:6 every day.

### Flow cytometry-based cell cycle analysis

For single-parameter DNA content analysis, Hoechst 33342 was added to healthy KBM7 cell culture suspension (<10^6^ cells/mL) for a final concentration of 10 μg/mL and cells were incubated in a 37 °C water bath for 20 min before flow cytometry analysis on a BD LSRII-HTS instrument. Acquired DNA content signals were analyzed in the FlowJo software (v9) for the proportion of cells in G1, S, and G2/M phases.

For double-parameter cell cycle analysis, cells were pulse labeled with 10 μM 5-bromo-2’-deoxyuridine (BrdU) for 2 h, fixed using the BD Pharmingen BrdU Flow Kit (Cat. No. 559619), treated with DNase to expose BrdU epitope, stained with an FITC-conjugated anti-BrdU antibody as well as 7-AAD for total DNA content, followed by flow cytometry analysis.

### Bulk RNA-Seq of KBM7 cells

50,000 live haploid or diploid KBM7 cells (3 single-cell clones each, with flow cytometry-verified DNA content) were sorted by FACS into 20 μL Buffer TCL (Qiagen) containing 1 μL of a 1:100 dilution of ERCC spike-in control RNA (ThermoFisher Cat. No. 4456740) for lysis and RNA extraction. RNA-seq libraries were prepared with the SMART-seq2 protocol [16], and amplified cDNA libraries were sequenced with on an Illumina Hi-Seq 2000 instrument using 75 bp paired-end reads. Note that one of the 3 clones used in this experiment was subsequently used for the remaining assays (single-cell expression profiling, CRISPR/Cas9-mediated knockout and immunofluorescence assays, etc.) in this study.

### Bulk RNA-seq data pre-processing and analysis

RNA-seq reads were mapped to the human (hg19) reference transcriptome using STAR (v2.5.2) [17] and transcripts were quantified using RSEM (v1.2.31) [18] with default parameters.

mRNA expression values from all samples were normalized to the average read counts of ERCC spike-in transcripts in the corresponding sample, showing top 5 expression across all samples. Differential expression analysis on normalized data was performed using the Seurat package (version 3) in R [19].

### CRISPR-Cas9-mediated knockout screen

An optimized lentiviral genome-wide sgRNA library [20] containing 76,441 guides targeting 19,114 human genes as well as non-targeting controls was used for transducing Cas9-expressing KBM7 cells (see above) at 1000X coverage. Cells were transduced at ~40% efficiency as determined by puromycin selection (2 μg/mL for 2 days). More than 35 million post-selection live cells were harvested as input samples. Both haploid and diploid cells were cultured for ~20 cell doublings before being harvested for genomic DNA extraction. During the screening process, library coverage is kept at >500X. At the end of the screen, 100 million live diploid KBM7 cells and 300 million live haploid KBM7 cells were harvested for genomic DNA extraction, library preparation, and high-throughput sequencing. After sample barcode deconvolution, the STARS software [20] (v1.3) was used for generating a background distribution of sgRNA ranking and scoring sgRNA depletion/enrichment in each sample compared to input samples with parameters --threshold = 30, --num-ite = 1000 and --use-first-pert = N. GSEA (v3.0) [21] was performed on genes with guides depleting specifically in haploid cells to determine the REACTOME [22] pathways they participate in.

### shRNA-mediated knockdown of RAD51B

The following guides were used to generate vectors that allow doxycycline-inducible expression of shRNA against RAD51B as well as the E2-Crimson fluorophore in the GFP-expressing miR-30 shRNA backbone [23]:

Hairpin 1: TAAGAATCTGTCTTCTCTCTGA

Hairpin 4: TTCAGTGTTAAAATATCTGGGA

An shRNA vector against the Renilla luciferase (Ren713, guide sequence TAGATAAGCATTATAATTCCTA [24]) was also generated as control for knockdown. HEK-293T cells were transfected with the above vectors (20 μg each in a 10-cm dish) together with the pCL-eco (12 μg) and pMD2.G (2.5 μg) vectors for retroviral packaging and VSV-g pseudotyping. KBM7 cells were resuspended in viral supernatants diluted 1:3 in culture medium, and polybrene was added to the mixture for a final concentration of 10 μg/mL. Transduction was performed by spinning the above mixtures at 2000 × *g* at 37 °C for 2 h. Cells were placed back in the incubator and are diluted 1:5 using fresh culture medium the next day. 48 h after transduction, GFP+ cells were sorted using FACS, and doxycycline was added for a final concentration of 1 μg/mL. Forty-eight hours after doxycycline induction, cells were sorted for either protein extraction or plating growth competition assays.

### Immunofluorescence staining of g-H2AX and RAD51 foci

Coverslips were washed with ethanol and coated with 0.01% poly-L-lysine for an hour at room temperature, and subsequently washed in ddH2O and dried. Cells were cultured till confluent and diluted 1:3 the night before transferring to coverslips. 2 mL cell culture suspension was added to each well containing a coated coverslip in a 12-well plate and cultured overnight. The next day, coverslips containing attached cells were washed, fixed with 4% formaldehyde, and blocked for non-specific binding with goat serum dilution buffer containing 16.7% goat serum, 0.3% Triton X-100, 20 mM sodium phosphate, and 0.45 M NaCl. Primary antibodies against RAD51 (Abcam 63801) and g-H2AX (Abcam 26350) were then used to stain cells at 1:1000 dilution at 4 °C overnight, and secondary antibodies (Alexa Fluor 488-conjugated anti-rabbit, Thermo A11034, and Alexa Fluor 647-conjugated anti-mouse, Thermo A21235) were used at 1:2000 dilution. DAPI was also used for nucleus staining at 0.15 mM. Cells were stained for secondary antibodies at room temperature for 1 h and coverslips were washed in ddH_2_O, mounted onto a glass slide using VectaShield anti-fade mounting medium (Vector Labs H-1200-10), and cured at 4 °C overnight. Images were acquired using a Nikon fluorescent microscope at ×40 or ×60 magnification, and DAPI signals and foci were analyzed using the CellProfiler software v3.1.5 [25].

### CRISPR-Cas9-mediated knockout of RAD51B

Cas9-expressing clones of KBM7 cells were generated by infecting cells with lentivirus produced from the pLenti-Cas9-blast vector, selecting cells with 20 μg/mL blasticidin for 5 days and single-cell cloning followed by Western blot verification of Cas9 expression. Cas9 activity was determined by transducing cells with a vector expressing both GFP and an sgRNA against GFP and monitoring the percentage of cells losing GFP fluorescence over time.

The following sgRNAs as well as a sgRNA against the *LacZ* gene as control were cloned into a tdTomato-expressing sgRNA expression vector previously described [26]:

Guide 1: GGCTTGTGGATCCCTCACAG

Guide 2: ACATTACCCACCAACATGGG

sgLacZ: TGCGAATACGCCCACGCGAT [27].

HEK-293T cells were transfected with the above vectors (20 μg each in a 10-cm dish) together with the psPAX2 (12 μg) and pMD2.G (2.5 μg) vectors for lentiviral packaging and VSV-g pseudotyping. KBM7 cells were transduced using the same method as described above for shRNA vector delivery and tdTomato+ cells were sorted 48 h after transduction and incubated for a duration of time equivalent to that required for 70% cells to lose GFP expression as determined by the Cas9 assay described above. Single-cell clones were then isolated using FACS and clones lacking RAD51B expression as assayed by Western blot were subsequently used. For PCR amplification and Sanger sequencing of the target *RAD51B* genomic locus, the following primers were used:

PCR primers:

GGAAAAGGTATAGAATGCCAAGTT (Forward)

GGGGTGGTAAATGTTAACCTATGTG (Reverse)

Sequencing primer:

GGGGTGGTAAATGTTAACCTATGTG

Mutations were identified from Sanger sequencing data using the TIDE analysis method previously described [28].

### Single-cell RNA-seq (scRNA-seq) of near-haploid CML and B-ALL cells

Healthy KBM7 cells were resuspended at 2 × 10^6^ live cells/mL before input into the 10X Chromium 3’ Single Cell Platform and processed using the Chromium Single Cell 3’ Library, Gel Bead and Chip Kits (v2, 10X Genomics, Pleasanton, CA), following the manufacturer’s protocol. Briefly, 7000–20,000 cells were added to each channel of a chip to be partitioned into Gel Beads in Emulsion (GEMs) in the Chromium instrument, followed by cell lysis and barcoded reverse transcription of RNA in the droplets. Breaking of the emulsion was followed by amplification, fragmentation, and addition of adaptor and sample index. Barcoded single-cell transcriptome libraries were sequenced with 38 bp paired-end reads on an Illumina Hi-seq 2000 instrument.

Near-haploid and diploidized B-ALL cells, bone marrow and spleen cells were harvested from NSG mice bearing PDX leukemia, and each sample was stained with hashtagging antibodies conjugated with sample-specific oligonucleotide barcodes (BioLegend custom order). Hashtagged samples were then pooled in equal fractions and resuspended at 2 × 10^6^ live cells/mL before input into the 10X Chromium 3’ pipeline (v2 chemistry) for RNA-seq library preparation and sequencing similar to that of KBM7 cells. Barcoded single-cell transcriptome libraries were sequenced with 38 bp paired-end reads on an Illumina on an Illumina Hi-seq 2000 instrument.

### KBM7 scRNA-seq data pre-processing and analyses

The cellranger software (v3, 10X Genomics) was used to map reads to the hg19 reference human transcriptome, quantify UMIs for each gene in each cell, and cells were filtered to retain only those with less than 5% mitochondrial mRNA content. The MAGIC software package [29] in R was used to impute genes with zero reads with parameter *t* = 3. Genes were filtered to only include those with non-zero standard deviation across cells to facilitate downstream correlation and signature analyses. Differentially expressed genes (fold change >1.25 with Benjamini–Hochberg corrected *p*-values < 0.01) between haploid and diploid KBM7 cells in each cell cycle stage were determined using the FindMarkers function in the Seurat package (v3) in R. REACTOME [22] pathway enrichment analysis using Fisher’s exact test and Benjamini–Hochberg multiple test correction was performed in the PANTHERDB interactive web tool (http://pantherdb.org/).

### PDX B-ALL scRNA-seq data pre-processing and analyses

The cellranger software (v3, 10X Genomics) was used to map reads to the hg19 reference human transcriptome, quantify UMIs for each gene in each cell. The Seurat package in R (version 3) is used for processing single-cell expression data. Briefly, cells with at least 300 genes that show non-zero UMI counts are included from the raw UMI count matrix. UMI counts were log-normalized, genes with variable expression were identified and expression data from different batches of experiment was integrated using the CCA method [19] to remove batch effects. All code for the downstream analyses performed to generate relevant figures is provided in the github repository: https://github.com/yunpengl9071/NHLeukemia_manuscript.

### Computational inference of cell cycle stages in single KBM7 cells

Cell cycle stage signature genes (G1S, S, G2, G2M, and MG1) were obtained from Whitfield et al. [30]. Signatures were filtered to retain only genes with non-zero UMI counts in at least 5% of cells in KBM7 scRNA-seq, and have a Pearson correlation coefficient r ≥ 0.5 with the average expression of the signature genes. The average expression of the retained signature genes was defined as its score. The signature with the highest score was used to annotate each cell’s phase.

To infer a cell cycle pseudo-time trajectory, an elliptical manifold was fitted using the least squares method to cells in the 2-dimensional plot of G2M-G1S signature scores. A rolling circle with an empirically determined radius of 0.5 was used to average expression in cells along the elliptical trajectory. 95% confidence intervals for the trajectories were estimated using the theoretical value of 1.96 × standard error of the mean (SEM) of expression around the mean.

### Patient-derived xenograft pre-clinical model of near-haploid B-ALL

All work involving human patient B-ALL cells conformed with protocol (13-351) approved by the Dana Farber Cancer Institute IRB. Near-haploid B-ALL cells were obtained from the bone marrow of a 2-year-old male patient, and chromosome spreads showed a karyotype of 28XY with trisomy in chromosomes 14 and 21. Cells were transplanted into 6–8-week-old male NSG mice (Jackson Laboratory) and re-harvested upon development of overt leukemia and cryopreserved as a patient-derived xenograft (PDX) line. Diploidized cells were also observed in serial passaging of PDX cells in mice and subsequently isolated through FACS and cryopreserved. In our pre-clinical model, 2 × 10^6^ live PDX B-ALL cells were transplanted intravenously to 6–8-week-old NSG mice (Jackson Laboratory).

Mice bearing PDX B-ALL were either treated with vehicle (solvent) or combination chemotherapy according to the following 7-day regimen starting when peripheral human CD19^+^ cell percentage reached 5% as determined by flow cytometry:

Days 1–5:

Dexamethasone: 5 mg/kg body weight (dissolved in 44% PEG-400, 54% saline, and 2% DMSO) daily for 5 days

L-asparaginase: 1000 U/kg body weight (dissolved in saline) daily for 5 days

Day 6:

Vincristine: 0.15 mg/kg body weight (dissolved in saline)

Day 7:

Doxorubicin: 2 mg/kg body weight (dissolved in saline)

Forty-eight hours after the final administration of doxorubicin, mice were sacrificed and bone marrow and spleen cells were harvested for either scRNA-seq (above) or cryopreservation.

All animal experiments were performed in compliance with ethical regulations by the Committee on Animal Care (CAC) at Massachusetts Institute of Technology.

### Definition of a RAD51B-associated signature

KBM7 scRNA-seq data were used to generate a RAD51B-associated signature: the Pearson’s correlation coefficient of RAD51B with each gene was calculated, and genes with r ≥ 0.5 were included in the signature. Signature scores were computed by averaging the scRNA expression of MAGIC-imputed PDX B-ALL samples after CCA-based data integration (time step parameter *t* = 7).

### Analysis of B-ALL patient microarray expression data

Affymetrix microarray expression data of B-ALL patients showing hypodiploidy or masked hypodiploidy were downloaded from the Gene Expression Omnibus (GEO accession number GSE27237). Data were normalized using the limma (v3.48.3) package in R [31] followed by principal component analysis (PCA) and inspection of RAD51B expression.

### Statistical analysis

All statistical analyses were performed in the GraphPad Prism software.

## Results

### Near-haploid cells display decreased proliferation rates due to prolonged G2/M phase

**Fig. 1:**
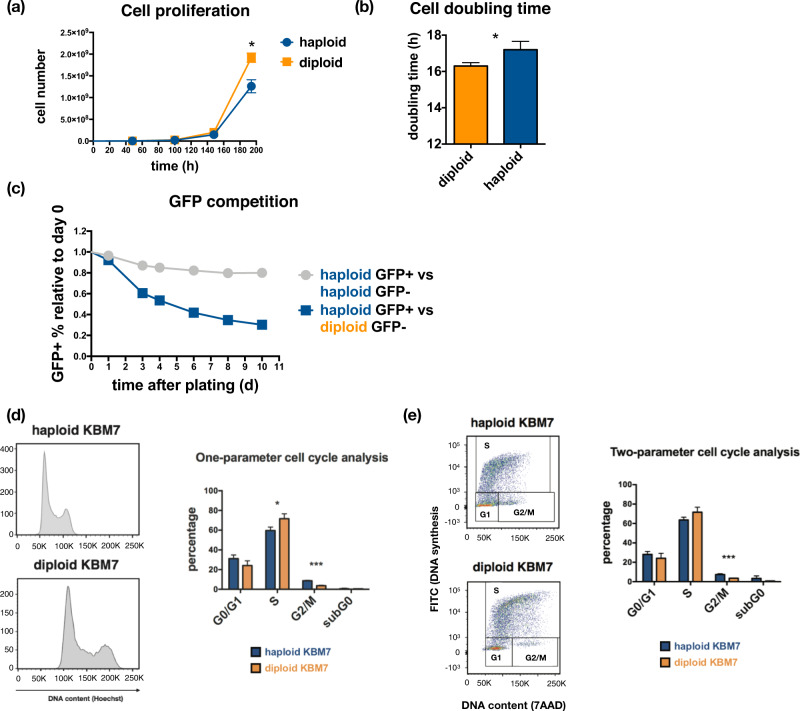
Near-haploid and diploidized KBM7 cells show different proliferation rates due to differences in cell cycle profiles. **a** Proliferation curves of near-haploid (haploid) and diploidized (diploid) KBM7 cells. *: Student’s t-test *p*-value = 0.0234 (*n* = 3). **b** Cell doubling time (hours) quantified from data in (**a**). *: Student’s t-test *p*-value = 0.034 (*n* = 3). **c** In vitro growth competition assay between haploid and diploid KBM7 cells. Shown are relative percentages (average from 3 technical replicates) of GFP-labeled haploid cells in a mixture of GFP+ and GFP− haploid (gray curve) or diploid (blue curve) cells. **d** Cell cycle content analysis of KBM7 cells using Hoechst live-staining and mathematical modeling of cell cycle stage distribution in FlowJo. *: Student’s t-test *p*-value = 0.026 (*n* = 3), ***: Student’s t-test *p*-value = 0.0002 (*n* = 3). **e** Two-parameter cycle content analysis of KBM7 cells using BrdU pulse-labeling and total DNA content staining with 7-AAD. 3 replicates are used for each sample in (**d**) and (**e**) *: Student’s t-test *p*-value = 0.0015 (*n* = 3). Data shown in (**d**) and (**e**) are mean values of replicates.

Near-haploid (referred to as ‘haploid’ hereafter in the text) KBM7 cells spontaneously diploidized when grown in cell culture—allowing us to definitively compare the physiological states of haploid cells and their diploidized counterparts (referred to as ‘diploid’ KBM7 cells). We first quantified growth rates in the two cell types and found that haploid cells proliferate more slowly than diploid cells (Fig. 1a), with a mean cell doubling time difference of 0.5 h (~3% slower in haploid cells, Fig. 1b). Consistently, in vitro growth competition assays revealed a substantial growth disadvantage of haploid cells, manifested as rapid depletion of haploid cells when co-cultured with diploid cells (Fig. 1c). Using live staining of DNA content with the cell membrane-permeable dye Hoechst 33342 in conjunction with estimation of cell cycle stage proportions (Watson Pragmatic model, implemented in the FlowJo v9 software [32]), we observed that haploid KBM7 cells show a significant increase in the proportion of G2/M cells (Student’s t-test *p*-value = 0.0002) and concomitant decrease in the proportion of S phase cells (Student’s t-test *p*-value = 0.026) compared to diploid cells (Fig. 1d). In an independent cell cycle assay where we pulse-labeled cells with BrdU, fixed cells and stained for total DNA content using 7-AAD, we confirmed that there is a consistent enrichment of haploid cells in the G2/M stage compared to diploid cells (Fig. 1e, Student’s t-test *p*-value = 0.0015). Additionally, since we did not observe a significant difference in viability between haploid and diploid cells (Supplementary Figure 1a), the decreased relative fitness of haploid vs. diploid KBM7 cells is best explained by stalled cell cycle progression. Consistent with this hypothesis, an estimate of cell cycle stage intervals based on the relative proportion of cells in each stage (Fig. 1e) revealed that haploid KBM7 cells spend ~0.7 h longer than diploid cells in the G2/M stage. Conversely, diploid KBM7 cells spend ~0.2 h longer in the rest of the cell cycle stages, suggesting that the difference in doubling times is largely explained by the difference in G2/M length.

### Cell cycle stage-specific expression differences in near-haploid cells

**Fig. 2:**
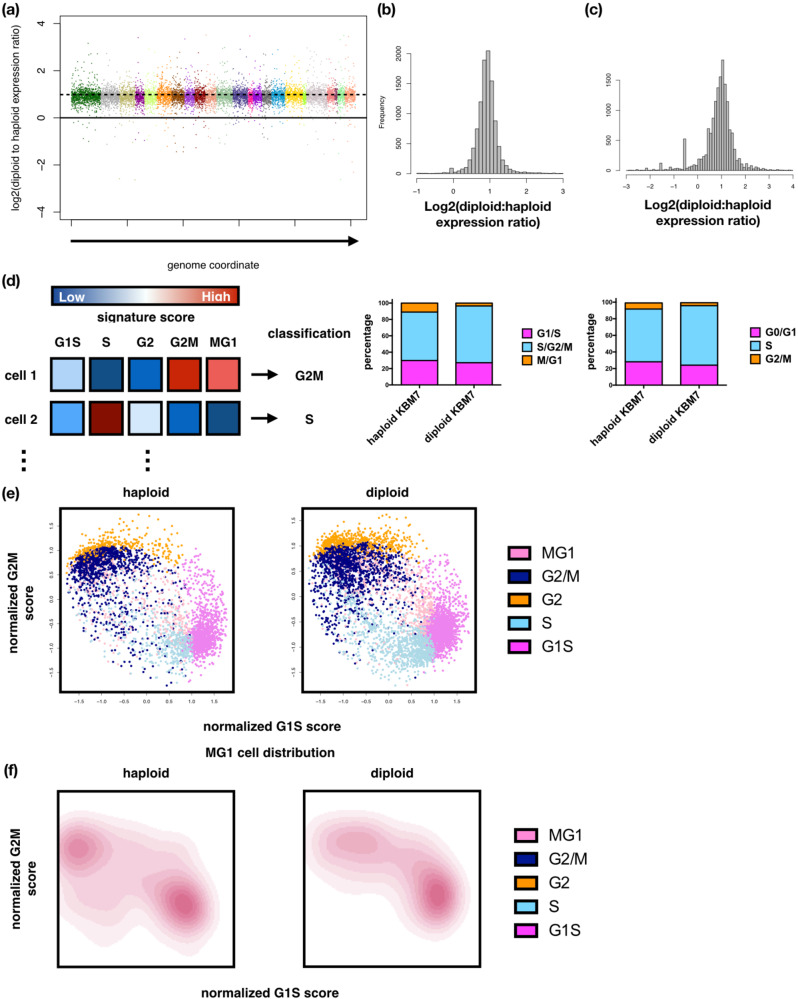
Single-cell RNA-seq of KBM7 cells and computational inference of cell cycle stages reveal aberrant cell cycle expression programs in near-haploid cells. **a** Bulk gene expression ratios between diploid and haploid KBM7 cells. Shown are log2-ratios computed from the mean expression calculated from 3 independent DNA content-verified clones in each group. Each color group represents an individual chromosome. **b** Histogram of expression ratios shown in (**a**). **c** Histogram of log2-expression ratios computed from single-cell gene expression profiles in diploid and haploid KBM7 cells. **d** Left panel: schematic of method for assigning cell cycle stages using single-cell expression profiles. Middle panel: computationally assigned cell cycle stages. Right panel: flow cytometry-based cell cycle stage assignment of KBM7 cells. **e** G1S and G2M metagene scores of single haploid (left) and diploid (right) KBM7 cells. Cells are colored according to cell cycle stages. **f** Density estimation of the distribution of M/G1 haploid (left) and diploid (right) KBM7 cells.

Bulk RNA-seq showed no significant differences in mean expression of individual genes between haploid and diploid KBM7 cells, after correcting for total mRNA abundance using spike-in controls [33]. However, we observed a ~2-fold increase in global transcript abundance in diploid cells (Fig. 2a, b, Supplementary Figure 1b, Supplementary Table 1).

Given the cell cycle differences between haploid and diploid cells (Fig. 1), we hypothesized that cell cycle stage-specific differences in expression were masked by averaging in bulk RNA-seq, and would be detected by scRNA-seq. We thus profiled 3745 haploid and 5542 diploid KBM7 cells by scRNA-seq, observing the expected 2:1 global expression ratio between diploid and haploid cells (Fig. 2c). We imputed drop-outs using MAGIC, a matrix-assisted gene expression imputation method [29] (“Methods”), and then scored the imputed data for cell cycle stage signatures (M/G1, G1/S, S, G2, G2/M) [30] (“Methods”) (Fig. 2d, left panel). The distribution of cell cycle stages by computational scoring of scRNA-seq (Fig. 2d, middle panel) was largely consistent with that from flow cytometry (Fig. 2d, right panel, constructed with data from Fig. 1e). Based on the cells’ G2M and G1S scores, cells lie approximately in an elliptical trajectory following the order of events within the cell cycle (Fig. 2e), with different density of diploid and haploid cells in the different phases (Fig. 2e, f, Supplementary Figure 2b). For example (Fig. 2f), Haploid M/G1 cells occupy an unexpected subspace (left panel) that is usually occupied by S/G2 cells in the diploid population (Fig. 2e, f right panels). These data suggest that haploid KBM7 cells display altered cell cycle expression programs in which cells show expression patterns that are mismatched with specific cell cycle stages.

**Fig. 3:**
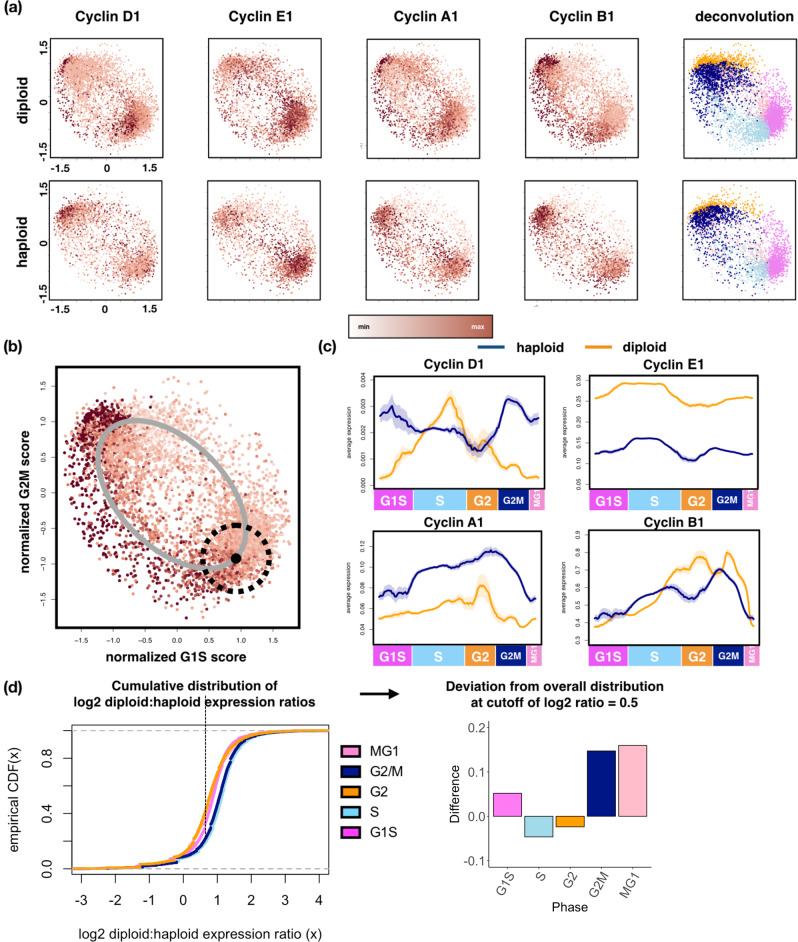
Near-haploid KBM7 cells show overexpression of genes in DNA damage repair and mitochondria function in post-replication phases. **a** Relative expression of key cyclins in haploid (upper panels) and diploid (lower panels) KBM7 cells throughout the cell cycle. Cell cycle stage assignments are shown on the rightmost panels. Horizontal axes are G1S metagene scores and vertical axes are G2M metagene scores. **b** Example of elliptical trajectory (gray curve) fitted to diploid KBM7 cells, with the neighborhood of cells used for smoothing pseudo-time expression estimation shown as dotted rolling circle. **c** Inferred cell cycle pseudo-time trajectory of cyclin genes shown in (**a**), with estimated 95% confidence interval shown as shaded regions. **d** Left panel: empirical cumulative distribution functions (CDFs) of diploid to haploid single-cell gene expression ratios in log2 space, grouped by cell cycle stages. Dotted vertical line shows cutoff of 0.5 (~1.4 in linear space) for identifying cell cycle stages with ratios substantially lower than 2:1 (1 in logarithm space). Right panel: number of genes in each cell cycle stage with diploid:haploid expression ratios less than the cutoff set on the left panel. Colored arrows point to the cell cycle stages with the largest number of genes below the cutoff.

Strikingly, while four key cyclins (D1, E1, A1, and B1) exhibited the expected increase and decrease in expression in diploid cells, they had significantly altered patterns throughout the cell cycle in haploid cells (Fig. 3a). For example, high cyclin D1 expression, which is required for a successful G1/S transition, was observed in G1/S diploid KBM7 cells (Fig. 3a, first column, upper panel), but did not peak until late S/G2/M in haploid cells (Fig. 3a, first column, lower panel). To better analyze expression dynamics throughout the cell cycle, we developed a cell cycle pseudo-time inference method (Fig. 3b), by fitting an elliptical manifold to the G2M-G1S score space and using a rolling circular neighborhood along the manifold to calculate the average expression of a given gene in the neighborhood (“Methods”). Comparing inferred cyclin expression dynamics between haploid and diploid cells (Fig. 3c), cyclin D1 dynamics were the most altered, and cyclin B1, responsible for mitotic entry, lagged in expression in haploid cells, but retained the same overall pattern. Moreover, G2/M and M/G1 phases had the highest number of genes whose expression in haploid vs. diploid cells deviates significantly from the expected 1:2 ratio (Fig. 3d, Supplementary Table 2, reporting Wilcoxon rank-sum test *p*-values along with Benjamini–Hochberg corrected *q*-values).

### Changes in cell cycle expression dynamics of homologous recombination repair pathway expression in diploid cells

Genes overexpressed in G2/M and M/G1 phases of haploid *vs*. diploid cells (after normalizing against total mRNA abundance) were significantly enriched for pathways in energy metabolism, regulation of cell cycle checkpoints, mitotic spindle organization and stabilization of p53, among multiple other pathways (Supplementary Table 6), indicating that genes participating in these processes may be of particular importance to haploid cells.

**Fig. 4:**
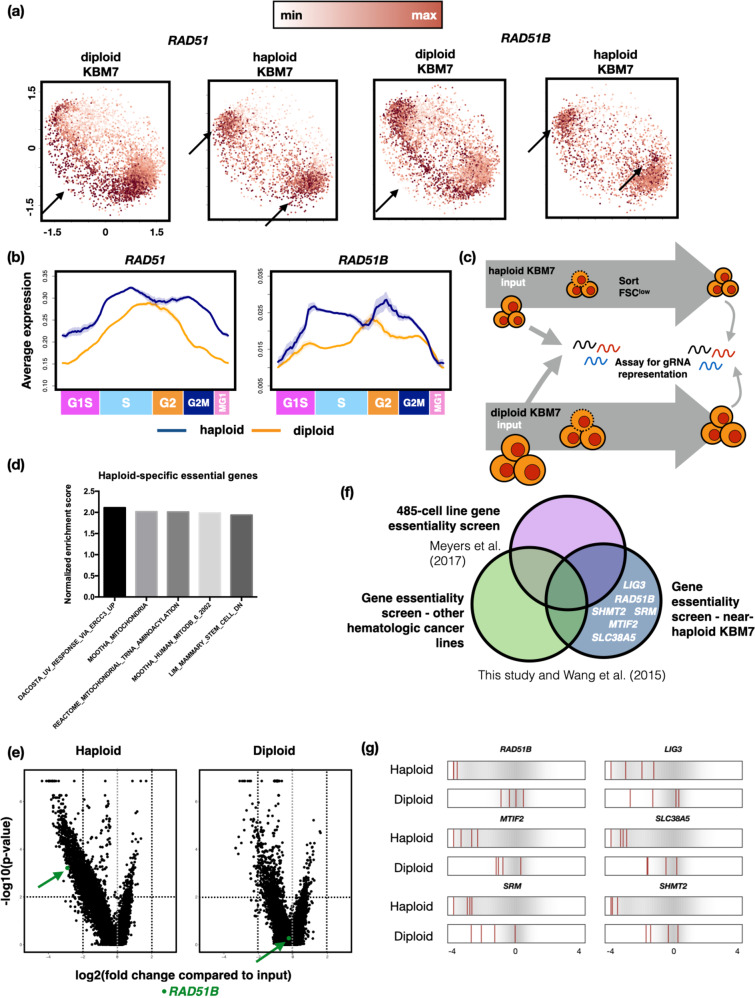
Near-haploid KBM7 cells show elevated expression of RAD51 and RAD51B in post-replication phases and unique genetic dependency on RAD51B compared to diploidized cells. **a** Expression patterns of *RAD51* and *RAD51B* throughout the cell cycle in diploid (left panels) and haploid (right panels) KBM7 cells. Arrows highlight cells with peak expression in each cell type for each gene. **b** Inferred cell cycle pseudo-time trajectory for *RAD51* and *RAD51B*, with estimated 95% confidence interval shown as shaded regions. **c** Schematic for genome-wide CRISPR-Cas9 gene essentiality screen in haploid and diploid KBM7 cells. **d** Gene set enrichment analysis (GSEA) of genes essential to haploid but not diploid KBM7 cells. Shown are normalized enrichment scores. All categories show significant enrichment (Kolmogorov–Smirnov test *p*-values < 0.01 with false discovery rate <0.25). **e** Volcano plots of relative enrichment/depletion scores of genes (horizontal axis) and the associated significance levels of the scores (vertical axis). *RAD51B* is highlighted in green. **f** Venn-diagram highlighting RAD51B and other genes as uniquely important to near-haploid KBM7 cells by comparing essential genes across a panel of cell lines [44], hematologic cell lines, and haploid KBM7 cells (this study and Wang et al. 2015 study [38]). Genes shown were pre-filtered for those showing higher cell cycle stage-specific expression in near-haploid KBM7 cells as identified by our single-cell analyses. **g** 1-D density distribution plots (darker gray denotes higher relative density of values) of the log2-fold change of individual sgRNAs. Log2-fold change values of sgRNAs targeting the 6 essential genes specific to near-haploid cells as shown in (**f**) are highlighted with red bars.

In particular, genes involved in the homologous recombination (HR) repair pathway, specifically *RAD51* and its paralog *RAD51B*, were overexpressed in haploid cells across both the G2/M and M/G1 phases (Supplementary Table 2). RAD51 is a 37 kDa protein that forms a multimeric filament that coats single-stranded DNA (ssDNA) generated through resection of double-strand DNA breaks (DSB). It then mediates homology search and repair of the DSB. The RAD51 paralog RAD51B forms the BCDX complex with other paralogs RAD51C and RAD51D as well as XRCC2 (BCDX2) or XRCC3 (CX3). The BCDX complex facilitates RAD51 recruitment to ssDNA. RAD51 and RAD51B-mediated HR repair is mostly active in the late S to early G2 phases of the cell cycle when sister chromatids are available as recombination substrates [34]. Consistently, RAD51 and RAD51B expression peaks in late S to early G2 phases in diploid KBM7 cells in our pseudo-time trajectory analysis (Fig. 4a, b). Conversely, in haploid cells both genes show bi-modal expression with peaks in both early S and late G2/M. This suggests that the DNA damage repair machinery may have altered functionality in haploid KBM7 cells—and that they may exhibit enhanced dependence on specific DNA repair mechanisms.

### Genome-wide CRISPR-Cas9-mediated knockout screens reveal that RAD51B is uniquely essential for near-haploid leukemia cells

To systematically query which genes and pathways are preferentially required in haploid cells, we performed genome-wide CRISPR-Cas9-mediated knockout screens in haploid and diploid KBM7 cells (Fig. 4c, Supplementary Table 3). An analysis of fold changes of guide RNAs at 20 cell doublings after introduction of the guide RNA library into Cas9-expressing cells (input samples) revealed target genes whose cognate guide RNAs are significantly depleted (and thus represent potentially cell essential genes) only in the haploid KBM7 cells (“Methods”).

RAD51B was one of the top-ranked genes among those with altered cell cycle expression in haploid cells that were also haploid cell-essential in the screens: it was among the top depleters in haploid cells but not in diploid cells (Fig. 4e, g). Conversely, guides targeting RAD51 were significantly depleted in both haploid and diploid cells, consistent with observed essentiality of RAD51 in vertebrate cells [35, 36]. To ensure that the dependency on RAD51B was specific to near-haploid cells, we compared gene essentiality data from the DepMap cancer dependency project that scores cellular fitness upon losing each of the expressed genes across a panel of 485 cell lines [37], and from a CRISPR-Cas9-mediated gene essentiality screen across 4 hematologic cell lines [38]. We also filtered the above gene sets using the set of genes with higher near-haploid cell expression in specific cell cycle stages (Supplementary Table 2). Interestingly, among the genes with higher expression in specific cell cycle stages of near-haploid cells, RAD51B and 5 other genes were among essential genes in haploid cells in both our screen and a published one [38], but not in our diploid screen or the DepMap screens (Fig. 4f, g). Only three of these six genes, namely RAD51B, SLC38A5, and MTIF2 were up-regulated in haploid KBM7 cells in the G2-G2/M stages.

**Fig. 5:**
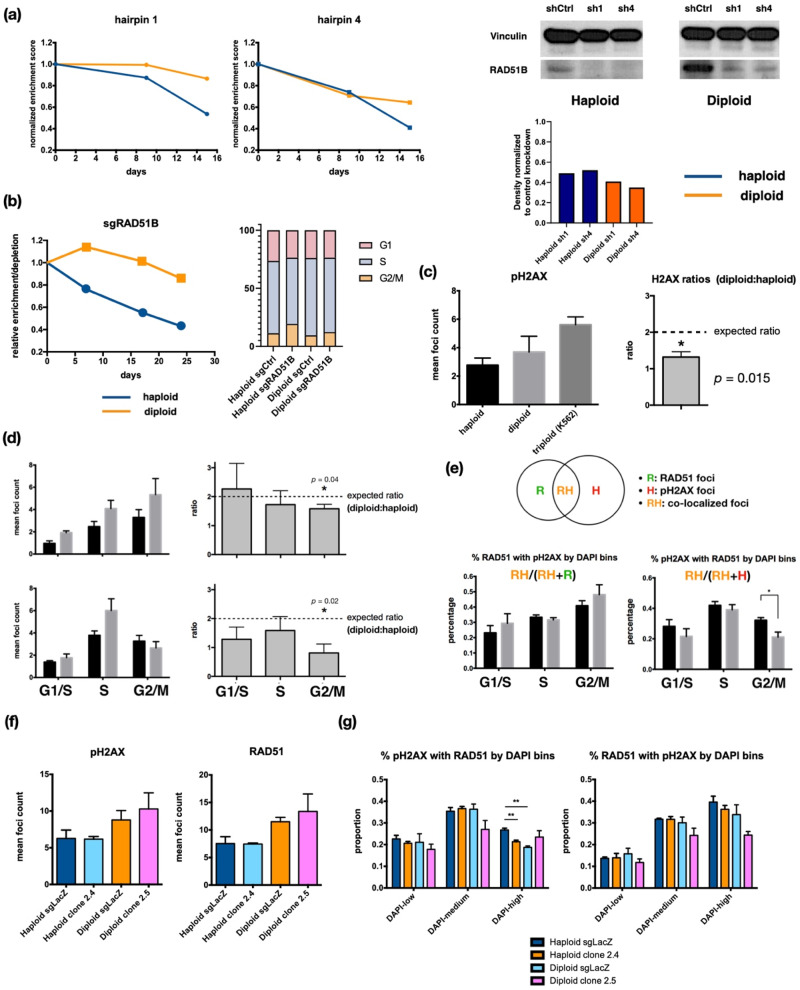
Near-haploid KBM7 cells display survival disadvantages upon RAD51B loss due to impairment of RAD51-mediated repair of double-strand DNA breaks. **a** Growth competition assay between haploid (blue) or diploid (orange) KBM7 cells with inducible shRNA-mediated knockdown of RAD51B expression and those expressing hairpins against the firefly luciferase gene (shCtrl). Plotted are relative enrichment/depletion of shRAD51B hairpin-expressing cells (shRAD51B:shCtrl), normalized to data from day 0 of the assay (72 h after doxycycline induction). Shown are 2 different hairpins against RAD51B. Western blots verifying knockdown are shown next to the competition assay curves. **b** Left panel: growth competition between KBM7 haploid and diploid cells with sgRNA-mediated depletion of RAD51B. Shown are relative proportions of cells with sgRNA expression in a 1:1 starting mixture with cells expressing a control sgRNA against *LacZ*. Right panel: estimated proportion of cells in each cell cycle stage using DNA content by Hoechst 33342 staining for RAD51B-/- clones 2.4 (haploid) and 2.5 (diploid) as well as knockout control (sgLacZ) cells. **c** Left panel: γ-H2AX foci counts in haploid and diploid KBM7 cells as well as a near-triploid CML cell line, K562, across 3 replicates each. Bars show mean foci count +/- SEM. Right panel: Ratio of γ-H2AX foci counts in diploid to haploid KBM7 cells. *: One-sample Student’s t-test *p*-value = 0.015 (*n* = 3). **d** Number (left panels) and diploid:haploid ratios (right panels) of γ-H2AX (upper panels) and RAD51 foci (lower panels) in haploid and diploid KBM7 cells, grouped by DNA content (DAPI signal) bins corresponding to different cell cycle stages. One-sample Student’s t-test *p*-value = 0.04 for γ-H2AX foci ratios (*n* = 3) and 0.021 for RAD51 foci ratios (*n* = 3). **e** Co-localization of γ-H2AX and RAD51 foci in haploid and diploid KBM7 cells quantified using two different metrics of co-localization (schematic shown above plots), grouped by cell cycle stages. *: Student’s t-test *p*-value = 0.042 (*n* = 3). **f** γ-H2AX and RAD51 foci counts in Cas9-expressing KBM7 cells with gRNA-mediated knockout of *RAD51B* compared to control cells (expressing gRNA against the *LacZ* gene). **g** Co-localization of γ-H2AX and RAD51 foci in haploid and diploid KBM7 cells with or without *RAD51B* knockout, quantified using two different metrics of co-localization (schematic shown above plots) and grouped by cell cycle stages. *: Student’s t-test *p*-value 0.004 (*n* = 3 for sgLacZ cells and *n* = 4 for RAD51B knockout cells).

We confirmed that RAD51B is a haploid-specific essential gene by short hairpin RNA (shRNA)-mediated knockdown in haploid and diploid cells using a doxycycline-inducible expression vector [23]. We co-cultured cells with RAD51B knockdown (KD) and cells expressing a control hairpin vector, and followed the relative enrichment and depletion of KD cells over time. As expected, RAD51B depletion conferred a survival disadvantage in haploid KBM7 cells (Fig. 5a). Importantly, such a disadvantage is unlikely an artifact from differential knockdown efficiencies as we observed better depletion of RAD51B in diploid KBM7 cells compared to haploid cells (Fig. 5a, lower right panel). We also depleted RAD51B using CRISPR-Cas9 and observed a similar decrease in cellular fitness in haploid cells compared to diploid cells (Fig. 5b). To rule out possible biases introduced by outgrowth of RAD51B-proficient cells within bulk knockout cultures, we have also performed an independent assay of RAD51B loss’ effects on KBM7 cell fitness by sorting Cas9 + KBM7 cells expressing sgRNAs against RAD51B or CD19 (control) into single cell wells and quantifying the number of clones growing out among haploid wells compared to diploid wells (Supplementary Figure 2a, bottom left panel). It can be seen that there is a significant reduction in the number of haploid KBM7 clones growing out upon RAD51B loss compared to control, whereas no significant difference in clone counts were observed between RAD51B knockout and control groups in diploid KBM7 cells. Interestingly, among the haploid clones harboring sgRNAs against *RAD51B* that grew out, we observed a higher fraction of clones showing predominantly in-frame deletions in the *RAD51B* loci compared to diploid clones (Supplementary Figure 2a, bottom right panel). Taken together, the above results suggest that RAD51B is specifically important for haploid KBM7 cells.

### Elevated dependency on RAD51B is likely driven by impaired double-strand damage repair efficiency in G2/M near-haploid cells

Since RAD51 and its paralogs are core components of the HR pathway, we hypothesized that haploid KBM7 cells may acquire increased RAD51B dependency via an elevated DNA damage repair burden. To test this, we first stained haploid and diploid KBM7 cells, as well as K562 cells—a near-triploid CML cell line, for γ-H2AX, which accumulates at the sites of DNA damage (Fig. 5c). We hypothesized that with a constant intrinsic rate of DNA damage, the amount of baseline double-strand beaks (DSBs) should be proportional to genome size. This is indeed the case in diploid KBM7 cells and K562 cells, where mean foci count is approximately proportional to DNA content (2:3). In haploid and diploid KBM7 cells, however, the ratio of γ-H2AX counts is close to 1:1 instead of the expected value of 1:2 (Fig. 5c, lower panel, student’s *t*-test *p*-value = 0.015). This suggests that there is a relative increase in the frequency of unrepaired DNA breaks in haploid KBM7 cells.

Because both RAD51 and RAD51B expression peak in early S and late G2/M stages in haploid KBM7 cells, we wondered if differential DSB burden across cell cycle stages could contribute to their altered expression dynamics. Indeed, as the distribution of DAPI signals in cell nuclei resembles that of DNA content as assayed by cell cycle profiling (Supplementary Figure 2c, Fig. 1d), we could estimate the number of γ-H2AX foci in each of the cell cycle stages (G1/S, S, G2/M) by binning cells according to their nuclear DAPI signal strengths (DAPI-low, DAPI-medium, and DAPI-high, respectively). We thus counted foci in cells stained for both γ-H2AX (Fig. 5d, upper panels) and RAD51 (Fig. 5d, lower panels), where a subset of RAD51 proteins is recruited to damage foci to initiate repair. Interestingly, the deviation in diploid-to-haploid damage foci count ratios is only significant in the G2/M phase (one-sample Student’s t-test *p*-value = 0.04). Additionally, although the dynamics of absolute counts for RAD51 foci seem uncoupled from those of γ-H2AX foci, the ratios of diploid-to-haploid RAD51 foci also significantly fell below 2:1 only in the G2/M phase (Fig. 5d, lower panels). Additionally, the percentage of γ-H2AX foci occupied by RAD51, which represent sites of active repair, is significantly higher in haploid than in diploid KBM7 cells only in the G2/M stage (Fig. 5e, lower right panel). These data suggest that an increased amount of RAD51 may be recruited to DSB damage foci in the G2/M phase in haploid cells compared to diploid cells. Of note, the percentage of RAD51 foci occupied by γ-H2AX foci is not significantly different between haploid and diploid cells across all cell cycle stages (Fig. 5e, lower left panel), indicating that RAD51 is likely performing similar functions in the two cell types and that higher occupancy of γ-H2AX foci by RAD51 may be a result of increased RAD51 expression instead of enhanced recruitment to DNA damage sites.

To test if loss of RAD51B further exacerbates the DNA damage burdens in haploid KBM7 cells, we knocked out RAD51B in KBM7 cells using CRISPR-Cas9 and generated single-cell clones depleted of RAD51B protein expression as confirmed by Western blotting and harboring potentially functionally-disruptive mutations as seen by Sanger sequencing of the target *RAD51B* locus (Supplementary Figure 2a, e). Here, RAD51B depletion led to a slight but statistically non-significant increase in overall or cell cycle stage-specific damage foci counts in haploid and diploid KBM7 cells (Fig. 5f, Supplementary Figure 2d). However, we observed a higher increase in the percentage of cells in G2/M phase in haploid cells (19.3% vs 11.2% in control cells) compared to diploid cells (12.3% vs 9.5% in control cells, Fig. 5b right panel) in the single-cell clones used in our immunofluorescent foci assays. Additionally, loss of RAD51B resulted in significantly decreased co-localization of RAD51 foci with γ-H2AX foci in the G2/M phase of haploid but not diploid cells (Fig. 5g, left panel). Similar results were observed in an independent set of RAD51B- clones (Supplementary Figure 2d). Thus, RAD51 is less efficiently recruited to damage foci in the absence of RAD51B in G2/M phase haploid cells. Furthermore, there was no significant difference in the percentage of RAD51 foci that were occupied by γ-H2AX foci in haploid cells but a decreasing trend in diploid cells (Fig. 5g, right panel). This result, coupled with the slight increase in occupancy of γ-H2AX foci by RAD51 in diploid KBM7 cells upon RAD51B loss, suggests that diploid cells may co-opt mechanisms to compensate for RAD51B deficiency that are absent in haploid cells. Taken together, the above data suggest that haploid KBM7 cells are intrinsically deficient in processing RAD51-mediated DSB repair, which is particularly sensitive to RAD51B availability in G2/M haploid KBM7 cells but not in diploid KBM7 cells.

### A RAD51B expression signature is associated with hypodiploidy in B-ALL samples as well as early responses to chemotherapy in a PDX model of near-haploid B-ALL

**Fig. 6:**
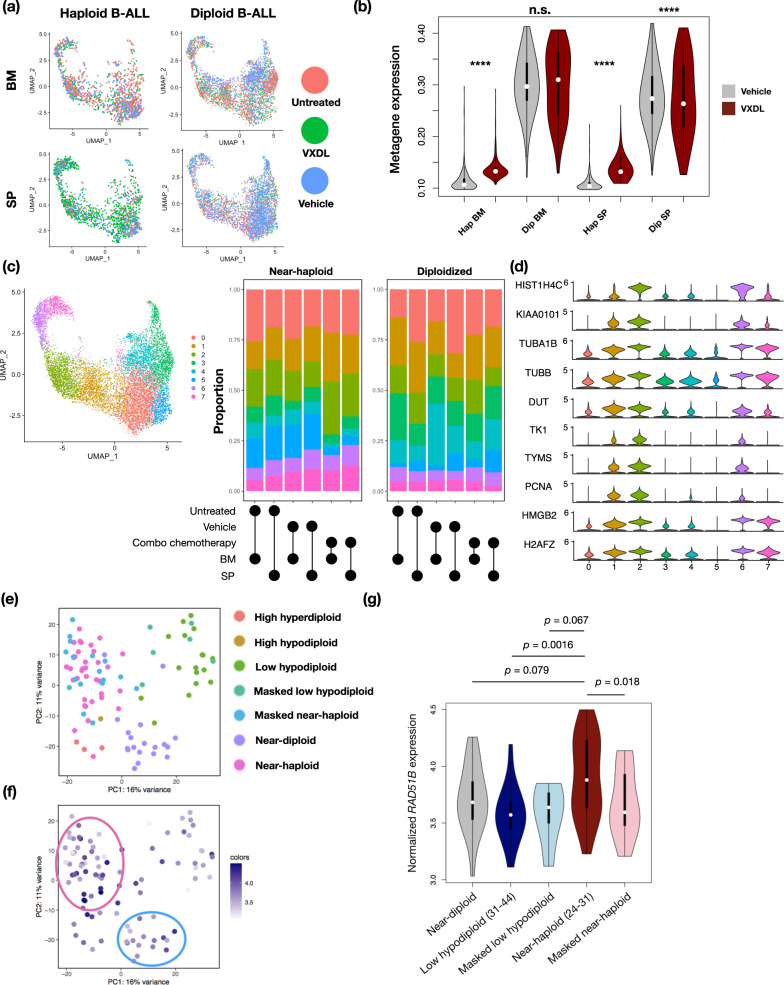
A pre-clinical model of near-haploid B-ALL shows in vivo up-regulation of RAD51B and G2/M checkpoint signatures in response to combination chemotherapy. **a** Uniform Manifold Approximation and Projection (UMAP) plot of single-cell transcriptomic profiles across PDX leukemia cells in different conditions. BM: bone marrow, SP: spleen. Cells are color-coded according to their treatment groups. **b** Violin plots showing single-cell expression of a RAD51B signature program in response to combination chemotherapy (red) vs cells treated with vehicle (gray) in a PDX model of near-haploid B-ALL. VXDL: vincristine, dexamethasone, doxorubicin and L-asparaginase. ****: Wilcoxon rank-sum test *p*-value < 2.2 × 10^−16^, ***: Wilcoxon rank-sum test *p*-value = 0.0017. n.s.: Wilcoxon rank-sum test *p*-value = 0.424. **c** Left panel: combined UMAP plot of all 11,922 single leukemia cell transcriptomes in the experiment, with cells color-coded according to their labels assigned by unsupervised clustering. Middle (near-haploid B-ALL) and right (diploidized B-ALL) panels: participation of each sample in each of the clusters, shown in colored bars representing percentages. **d** Violin plots of expression of top 10 (ranked by log2 fold change values) Cluster 2 marker genes in each cluster. **e**, **f** Analysis of microarray expression profiles in B-ALL patients showing gross chromosome gains or losses. **e** PCA plot of individual samples colored by karyotype groups. **f** Same plot as (**e**) with individual data points colored by the relative expression of the RAD51B signature. **g** Violin plots showing normalized *RAD51B* expression in B-ALL patient samples in each karyotype group. Wilcoxon rank-sum test *p*-values are labeled between groups. Number of samples in each group: Near-diploid: *n* = 19, low hypodiploid: *n* = 17, masked low hypodiploid: *n* = 6, near-haploid: *n* = 35, masked near-haploid: *n* = 17, high hyperdiploid: *n* = 4, high hypodiploid: *n* = 1).

Given the potential importance of RAD51B in processing damaged DNA in haploid leukemia, we asked if RAD51B and the HR pathway were involved in the in vivo response to DNA-damaging chemotherapy in near-haploid B-ALL. We utilized a patient-derived xenograft (PDX) model of near-haploid B-ALL [39] and its diploidized counterpart, and treated the mice with a combination chemotherapy treatment regimen consisting of vincristine, dexamethasone, doxorubicin, and L-asparaginase (VXDL), modified from Samuels et al. [40] (“Methods”). This treatment regimen elicited a potent therapeutic response in NOD-SCID-IL2Rγ (NSG) mice bearing near-haploid or diploidized B-ALL cells (Supplementary Figure 3a, upper panels). Consistent with results in KBM7 cells (Fig. 1d, e), we observe that haploid B-ALL shows a higher percentage of cells in G2/M phase compared to diploid cells in the bone marrow (Supplementary Figure 3a, lower panels). Interestingly, upon combination chemotherapy treatment (VXDL group in Supplementary Figure 3a), diploid cells show a significant increase in G2/M percentage whereas haploid cells show a decrease in G2/M content. This implies that haploid G2/M cells may be more sensitive to chemotherapeutic stress than diploid G2/M cells. We isolated cells from the bone marrow and spleen 48 h after treating mice with either vehicle or the VXDL regimen and profiled 11,922 near-haploid and diploidized B-ALL by scRNA-seq (“Methods”). While expression profiles of cells from each group spanned a full spectrum of states, the distributed differently across the expression state spectrum (Fig. 6a).

Because the HR pathway is a key component in the G2/M checkpoint, we examined if a RAD51B expression signature of genes co-expressed with RAD51B across single KBM7 cells (“Methods”, Supplementary Table 4) is associated with the elevated G2/M checkpoint response we observed in near-haploid B-ALL cells. Indeed, near-haploid B-ALL cells, but not diploidized B-ALL cells, had significantly higher scores of the RAD51B signature upon treatment (Fig. 6b). The same trends were observed in spleen cells. Interestingly, despite elevated RAD51 expression in response to therapy in near-haploid but not diploidized B-ALL cells, both RAD51B expression and its associated expression signature score are relatively low in near-haploid vs. diploidized cells across all conditions (Supplementary Figure 3b). Consistent with our KBM7 studies, this suggests that both RAD51B and genes that act in concert with RAD51B, while induced, may be expressed at insufficient levels to support RAD51-mediated homologous repair in response to genotoxic stress.

To further understand the expression phenotypes of PDX B-ALL cells in response to combination chemotherapy, we partitioned the cells to 8 clusters using unsupervised clustering (Fig. 6c, Supplementary Table 5). Haploid and diploidized B-ALL tumor samples populated each cluster with distinct proportions (Fig. 6c, middle and right panels), suggesting that tumor ploidy directs different cell states in both untreated and treated tumors.

Since the bone marrow is a known chemoprotective niche, and given that a larger amount of bone marrow cells compared to spleen cells were consequentially isolated in our study, we asked if bone marrow leukemia cells displayed different expression responses to chemotherapy. Notably, among the clusters with top expansion or shrinkage after treatment, near-haploid bone marrow B-ALL cells showed a more prominent enrichment in Cluster 2 (26.1% in treated vs 14.9% in vehicle) than did diploidized cells (16.9% vs 11.0%).

Interestingly, Cluster 2 cells are characterized by high expression of genes involved in both mitotic spindle organization (*TUBB*) and DNA replication initiation (*PCNA*) (Fig. 6d). Specifically, near-haploid B-ALL cells bearing such a mixed G2/M and G1/S checkpoint signature were enriched upon combination therapy, while those expressing high levels of HNRNPH1 were relatively depleting, suggesting decreased relative fitness compared to other cells upon chemotherapy. Intriguingly, Cluster 2 genes significantly overlap with the RAD51B co-expression signature genes in KBM7 cells (21 genes, hypergeometric test *p-*value = 1.59 × 10^−6^, Supplementary Table 4). Taken together, these results show that there is a haploid-specific RAD51B-associated expression program that is induced after combination chemotherapy. Near-haploid B-ALL cells have relatively up-regulated G2/M checkpoint signaling genes as part of a co-expression program with RAD51B in addition to G1/S checkpoint signaling programs, whereas these cells are both lower in frequency among diploidized B-ALL cells, which remained relatively stable before and after treatment.

Finally, to interrogate the relevance of these findings in a larger clinical cohort, we inspected gene expression profiles from 124 pediatric hypodiploid B-ALL patients [4] for expression of RAD51B and its associated signature (Fig. 6e–g). We observed a striking enrichment of the RAD51B signature (Fig. 6f) and higher expression of the RAD51B gene (Fig. 6g) in near-haploid B-ALL compared to their masked counterparts and low-hypodiploid B-ALL. Given that these expression data were collected using microarrays and are thus normalized for total mRNA content per sample, they further highlight the increased dosage of RAD51B in near-haploid B-ALL. They also suggest that RAD51B and its associated genes may be induced in response to an emergent dependency in the haploid state.

## Discussion

In this study, we integrated functional genomics with single-cell transcriptomics to systematically dissect genetic dependencies in near-haploid leukemia. Through filtering of cell cycle stage-specific expression profiles with gene essentiality scores in comparative genome-wide screen, we have unveiled a set of genes involved in mitochondrial functions and DNA damage repair pathways that are important to near-haploid but not diploidized leukemia cells. Interestingly, despite the key player in the HR pathway, RAD51, being essential to all cell lines in our analyses, its paralog RAD51B emerged as uniquely essential to near-haploid KBM7 cells. RAD51B has previously been shown to promote HR through recruitment of RAD51 and assembly of the RAD51 nucleoprotein filament [41], though the exact molecular mechanisms of such a process is poorly understood. Our DNA damage foci analyses have pinpointed the G2/M phase as a key stage for RAD51B function, as loss of RAD51B led to significantly impaired co-localization of RAD51 to DSB foci (Fig. 5g). However, the RAD51-DNA damage co-localization in diploidized cells did not seem to be sensitive to RAD51B loss, which together with the fact that RAD51B does not score as a top essential gene in other cell lines, indicates that RAD51B may not be the primary mediator of RAD51 recruitment to sites of DNA damage in the diploid context. Surprisingly, we did not observe significant changes in total DNA damage or damage in each cell cycle stage upon RAD51B loss (Fig. 5f, Supplementary Figure 2d). Thus, the physiological consequences of losing RAD51B may manifest as slower DNA damage repair and subsequently prolonged G2/M checkpoints. This is further supported by our observation that an increased proportion of near-haploid KBM7 cells, but not diploidized cells, are in the post-replication and pre-mitotic phases of the cell cycle (Supplementary Figure 2a).

The haploid-specific importance of the G2-G2/M phases revealed in this study is consistent with the idea that HR-directed DSB repair relies heavily on the availability of sister chromatids for accurate repair of DNA double-strand breaks. Indeed, gene conversion using homologous chromosomes as templates are highly inefficient for most chromosomes in pre-replication near-haploid cells. In this context, it will be of particular interest to investigate why near-haploid cells seem to rely more on RAD51B-mediated HR repair. In addition, our gene essentiality screens have pointed to genes involved in cellular respiration and mitochondria function, including the serine hydroxymethyltransferase SHMT2 and the mitochondrial translation initiation factor MTIF2, that are also overexpressed in G2/M to M/G1 stages of the cell cycle in near-haploid cells. Given the extensive crosstalk between the nucleus and the mitochondria in response to DNA damage as well as the established roles of the mitochondria in the resolution of nuclear DNA damage repair [42, 43], RAD51B and the HR pathway may have previously unexplored interactions with mitochondrial processes. It will thus be important to profile mitochondria states in near-haploid and diploidized KBM7 cells and understand their potential association with the HR-directed DNA damage repair pathway and pre-mitotic checkpoint regulation.

We have also established a pre-clinical xenograft model for near-haploid B-ALL and investigated leukemia cells’ response to a combination chemotherapy regimen similar to regimens commonly used in ALL patients (Supplementary Figure 3a, upper panels). Using multiplexed single-cell transcriptome profiling, we captured expression programs that are activated in early in vivo responses to chemotherapy (Fig. 6). Here, we observed an enrichment of a subset of cells bearing an aberrant co-expression of high levels of both G2/M genes and G1/S phase genes in response to chemotherapy specifically in near-haploid B-ALL. This is consistent with the hypothesis that near-haploid cells diploidized by aborting mitosis and re-starting another round of DNA synthesis, which could potentially be a coping mechanism for elevated DNA damage stress accumulated throughout previous stages of the cell cycle. Together, these results imply that near-haploid and diploid/near-diploid cells show differential requirements for RAD51B expression and sensitivity to G2/M checkpoint regulations, and that RAD51B may be a promising candidate for targeted therapy in this challenging subtype of leukemia.

Despite our observations of consistent phenotypes in near-haploid myeloid (KBM7) and lymphoid (B-ALL xenograft) cells, an important caveat is that due to limited availability of culturable hypodiploid mammalian cell lines and xenograft human samples that can be stably propagated in mice, further case studies would be required, should additional samples become available, to extend our findings to a broader setting. In addition, it has proven extremely challenging to genetically manipulate human xenograft B-ALL samples both in vitro and in vivo, which limits our ability to perform RAD51B loss-of-function studies in near-haploid B-ALL. Nevertheless, we anticipate that future multi-omic profiling approaches would yield deeper insights into the role of RAD51B in near-haploid leukemia cells and help validate its potential as a therapeutic target.

## Supplementary information

Supplementary Figures

Supplementary Table 1

Supplementary Table 2

Supplementary Table 3

Supplementary Table 4

Supplementary Table 5

Supplementary Table 6

## Data Availability

Processed high-throughput sequencing data can be accessed at Gene Expression Omnibus (GEO) using accession number GSE201080.

## Code availability

Code for analysis and modeling of gene expression data (bulk and single-cell) is available at https://github.com/yunpengl9071/NHLeukemia.

## References

[1] Ries LAG, Smith MA, Gurney JG, Linet M, Tamra T, Young JL, et al. Cancer incidence and survival among children and adolescents: United States SEER Program 1975–1995. NIH Pub No 99-4649. 1999; 179 pp.

[2] Nachman JB, Heerema NA, Sather H, Camitta B, Forestier E, Harrison CJ (2007). Outcome of treatment in children with hypodiploid acute lymphoblastic leukemia. Blood.

[3] Lemez P, Attarbaschi A, Béné MC, Bertrand Y, Castoldi G, Forestier E (2010). Childhood near-tetraploid acute lymphoblastic leukemia: an EGIL study on 36 cases. Eur J Haematol.

[4] Holmfeldt L, Wei L, Diaz-Flores E, Walsh M, Zhang J, Ding L (2013). The genomic landscape of hypodiploid acute lymphoblastic leukemia. Nat Genet.

[5] Kotecki M, Reddy PS, Cochran BH (1999). Isolation and characterization of a near-haploid human cell line. Exp Cell Res.

[6] Pui CH, Carroll AJ, Raimondi SC, Land VJ, Crist WM, Shuster JJ (1990). Clinical presentation, karyotypic characterization, and treatment outcome of childhood acute lymphoblastic leukemia with a near-haploid or hypodiploid less than 45 line. Blood.

[7] Trueworthy R, Shuster J, Look T, Crist W, Borowitz M, Carroll A (1992). Ploidy of lymphoblasts is the strongest predictor of treatment outcome in b-progenitor cell acute lymphoblastic leukemia of childhood: a pediatric oncology group study. J Clin Oncol.

[8] Heerema NA, Nachman JB, Sather HN, Sensel MG, Lee MK, Hutchinson R (1999). Hypodiploidy with less than 45 chromosomes confers adverse risk in childhood acute lymphoblastic leukemia: a report from the Children’s Cancer Group. Blood.

[9] Charrin C, Thomas X, Ffrench M, Le QH, Andrieux J, Mozziconacci MJ (2004). A report from the LALA-94 and LALA-SA groups on hypodiploidy with 30 to 39 chromosomes and near-triploidy: 2 Possible expressions of a sole entity conferring poor prognosis in adult acute lymphoblastic leukemia (ALL). Blood.

[10] Stark B, Jeison M, Gobuzov R, Krug H, Glaser-Gabay L, Luria D (2001). Near haploid childhood acute lymphoblastic leukemia masked by hyperdiploid line: Detection by fluorescence in situ hybridization. Cancer Genet Cytogenet.

[11] Raimondi SC, Zhou Y, Mathew S, Shurtleff SA, Sandlund JT, Rivera GK (2003). Reassessment of the prognostic significance of hypodiploidy in pediatric patients with acute lymphoblastic leukemia. Cancer.

[12] Canté-Barrett K, Spijkers-Hagelstein JAP, Buijs-Gladdines JGCAM, Uitdehaag JCM, Smits WK, van der Zwet J (2016). MEK and PI3K-AKT inhibitors synergistically block activated IL7 receptor signaling in T-cell acute lymphoblastic leukemia. Leukemia.

[13] Polak R, Buitenhuis M (2012). The PI3K/PKB signaling module as key regulator of hematopoiesis: Implications for therapeutic strategies in leukemia. Blood.

[14] Olbrich T, Mayor-Ruiz C, Vega-Sendino M, Gomez C, Ortega S, Ruiz S (2017). A p53-dependent response limits the viability of mammalian haploid cells. Proc Natl Acad Sci USA.

[15] Fox DT, Duronio RJ (2013). Endoreplication and polyploidy: insights into development and disease. Development.

[16] Picelli S, Björklund ÅK, Faridani OR, Sagasser S, Winberg G, Sandberg R (2013). Smart-seq2 for sensitive full-length transcriptome profiling in single cells. Nat Methods.

[17] Dobin A, Davis CA, Schlesinger F, Drenkow J, Zaleski C, Jha S (2013). STAR: ultrafast universal RNA-seq aligner. Bioinformatics.

[18] Li B, Dewey CN (2011). RSEM: Accurate transcript quantification from RNA-Seq data with or without a reference genome. BMC Bioinforma.

[19] Butler A, Hoffman P, Smibert P, Papalexi E, Satija R (2018). Integrating single-cell transcriptomic data across different conditions, technologies, and species. Nat Biotechnol.

[20] Doench JG, Fusi N, Sullender M, Hegde M, Vaimberg EW, Donovan KF (2016). Optimized sgRNA design to maximize activity and minimize off-target effects of CRISPR-Cas9. Nat Biotechnol.

[21] Subramanian A, Tamayo P, Mootha VK, Mukherjee S, Ebert BL, Gillette MA (2005). Gene set enrichment analysis: a knowledge-based approach for interpreting genome-wide expression profiles. Proc Natl Acad Sci USA.

[22] Jassal B, Matthews L, Viteri G, Gong C, Lorente P, Fabregat A (2020). The reactome pathway knowledgebase. Nucleic Acids Res.

[23] Fellmann C, Hoffmann T, Sridhar V, Hopfgartner B, Muhar M, Roth M (2013). An optimized microRNA backbone for effective single-copy RNAi. Cell Rep.

[24] Zuber J, McJunkin K, Fellmann C, Dow LE, Taylor MJ, Hannon GJ (2011). Toolkit for evaluating genes required for proliferation and survival using tetracycline-regulated RNAi. Nat Biotechnol.

[25] McQuin C, Goodman A, Chernyshev V, Kamentsky L, Cimini BA, Karhohs KW (2018). CellProfiler 3.0: next-generation image processing for biology. PLoS Biol.

[26] Maeder ML, Linder SJ, Cascio VM, Fu Y, Ho QH, Joung JK (2013). CRISPR RNA-guided activation of endogenous human genes. Nat Methods.

[27] Platt RJ, Chen S, Zhou Y, Yim MJ, Swiech L, Kempton HR (2014). CRISPR-Cas9 knockin mice for genome editing and cancer modeling. Cell.

[28] Brinkman EK, Chen T, Amendola M, Steensel B (2014). Easy quantitative assessment of genome editing by sequence trace decomposition. Nucleic Acids Res.

[29] van Dijk D, Sharma R, Nainys J, Yim K, Kathail P, Carr AJ (2018). Recovering gene interactions from single-cell data using data diffusion. Cell.

[30] Whitfield ML, Sherlock G, Saldanha AJ, Murray JI, Ball CA, Alexander KE (2002). Identification of genes periodically expressed in the human cell cycle and their expression in tumors. Mol Biol Cell.

[31] Ritchie ME, Phipson B, Wu D, Hu Y, Law CW, Shi W (2015). limma powers differential expression analyses for RNA-sequencing and microarray studies. Nucleic Acids Res.

[32] Watson JV, Chambers SH, Smith PJ (1987). A pragmatic approach to the analysis of DNA histograms with a definable G1 peak. Cytometry.

[33] Jiang L, Schlesinger F, Davis CA, Zhang Y, Li R, Salit M (2011). Synthetic spike-in standards for RNA-seq experiments. Genome Res.

[34] Zhao X, Wei C, Li J, Xing P, Li J, Zheng S (2017). Cell cycle-dependent control of homologous recombination. Acta Biochim. Biophys. Sin.

[35] Lim DS, Hasty P (1996). A mutation in mouse rad51 results in an early embryonic lethal that is suppressed by a mutation in p53. Mol Cell Biol.

[36] Vainio O, Imhof BA (1995). The immunology and developmental biology of the chicken. Immunol Today.

[37] Tsherniak A, Vazquez F, Montgomery PG, Golub TR, Boehm JS, Hahn WC (2017). Defining a cancer dependency map. Cell.

[38] Wang T, Birsoy K, Hughes NW, Krupczak KM, Post Y, Wei JJ (2015). Identification and characterization of essential genes in the human genome. Science.

[39] Townsend EC, Murakami MA, Christodoulou A, Christie AL, Köster J, DeSouza TA (2016). The public repository of xenografts enables discovery and randomized phase II-like trials in mice. Cancer Cell.

[40] Samuels AL, Beesley AH, Yadav BD, Papa RA, Sutton R, Anderson D (2014). A pre-clinical model of resistance to induction therapy in pediatric acute lymphoblastic leukemia. Blood Cancer J.

[41] Sigurdsson S, Van Komen S, Bussen W, Schild D, Albala JS, Sung P (2001). Mediator function of the human Rad51B-Rad51C complex in Rad51/RPA-catalyzed DNA strand exchange. Genes Dev.

[42] Fang EF, Scheibye-Knudsen M, Chua KF, Mattson MP, Croteau DL, Bohr VA (2016). Nuclear DNA damage signalling to mitochondria in ageing. Nat Rev Mol Cell Biol.

[43] Leshets M, Silas YBH, Lehming N, Pines O (2018). Fumarase: from the TCA cycle to DNA damage response and tumor suppression. Front Mol Biosci.

[44] Meyers RM, Bryan JG, McFarland JM, Weir BA, Sizemore AE, Xu H (2017). Computational correction of copy number effect improves specificity of CRISPR–Cas9 essentiality screens in cancer cells. Nat Genet.

