# Supplementary material for: Integrated multi-omics analyses reveal homology-directed repair pathway as a unique dependency in near-haploid leukemia
Source: Blood Cancer J. 2023 Jun 8;13(1):92. doi: 10.1038/s41408-023-00863-1 (PMC10247733; doi:10.1038/s41408-023-00863-1)
Supplement: Supplementary file 1 — Supplementary Figures [file 41408_2023_863_MOESM1_ESM.docx]

**
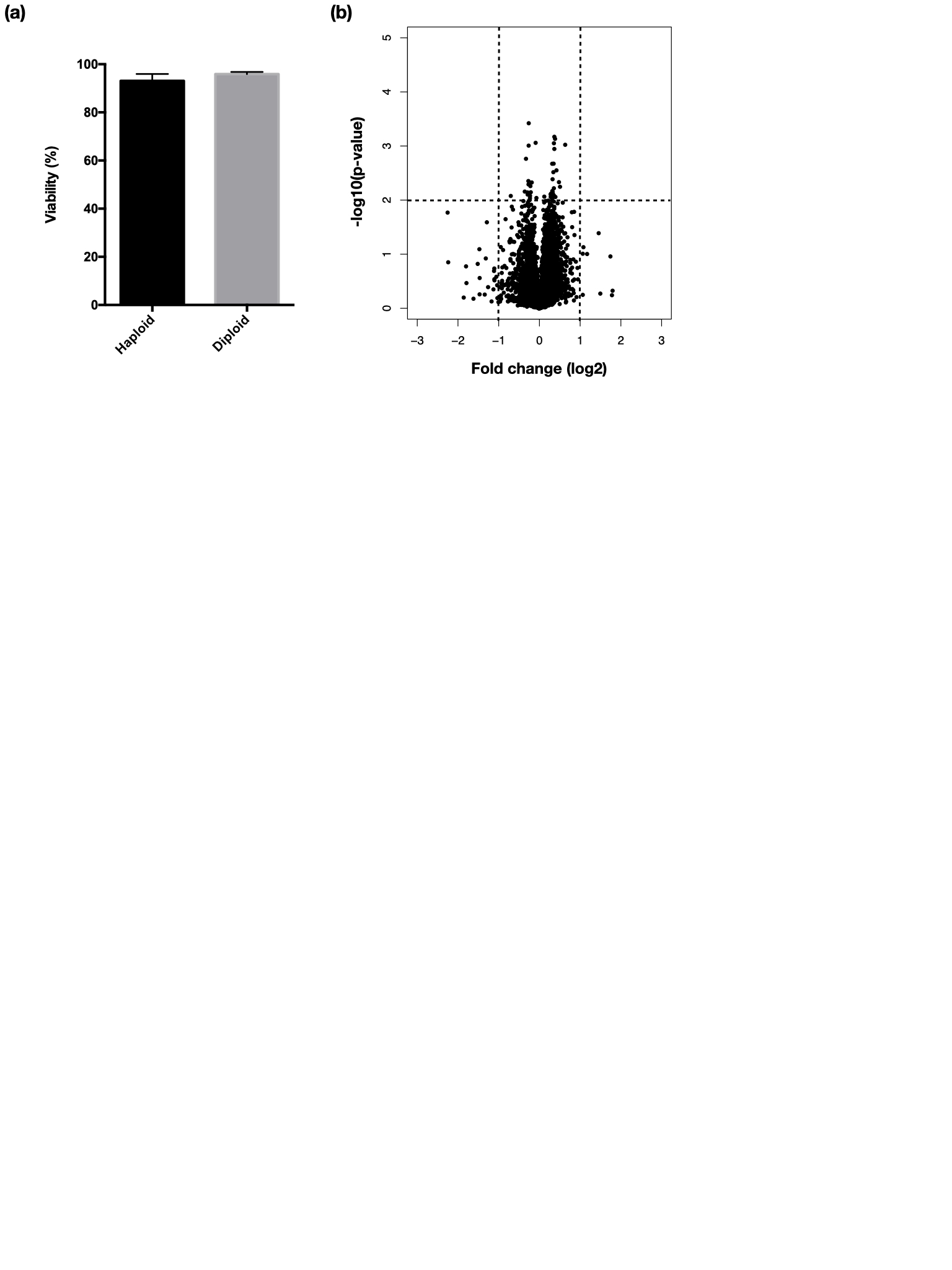
**

**Supplementary Figure 1.** (a) Viability of haploid and diploid KBM7 cells quantified by percent PI-negative cells. Bars represent mean+/-SEM. Student’s t-test *p*-value = 0.18 (n = 3). (b) Volcano plots of differential gene expression between haploid and diploid KBM7 cells after correction for total mRNA abundance. Dotted lines delineate thresholds for differential expression.

**
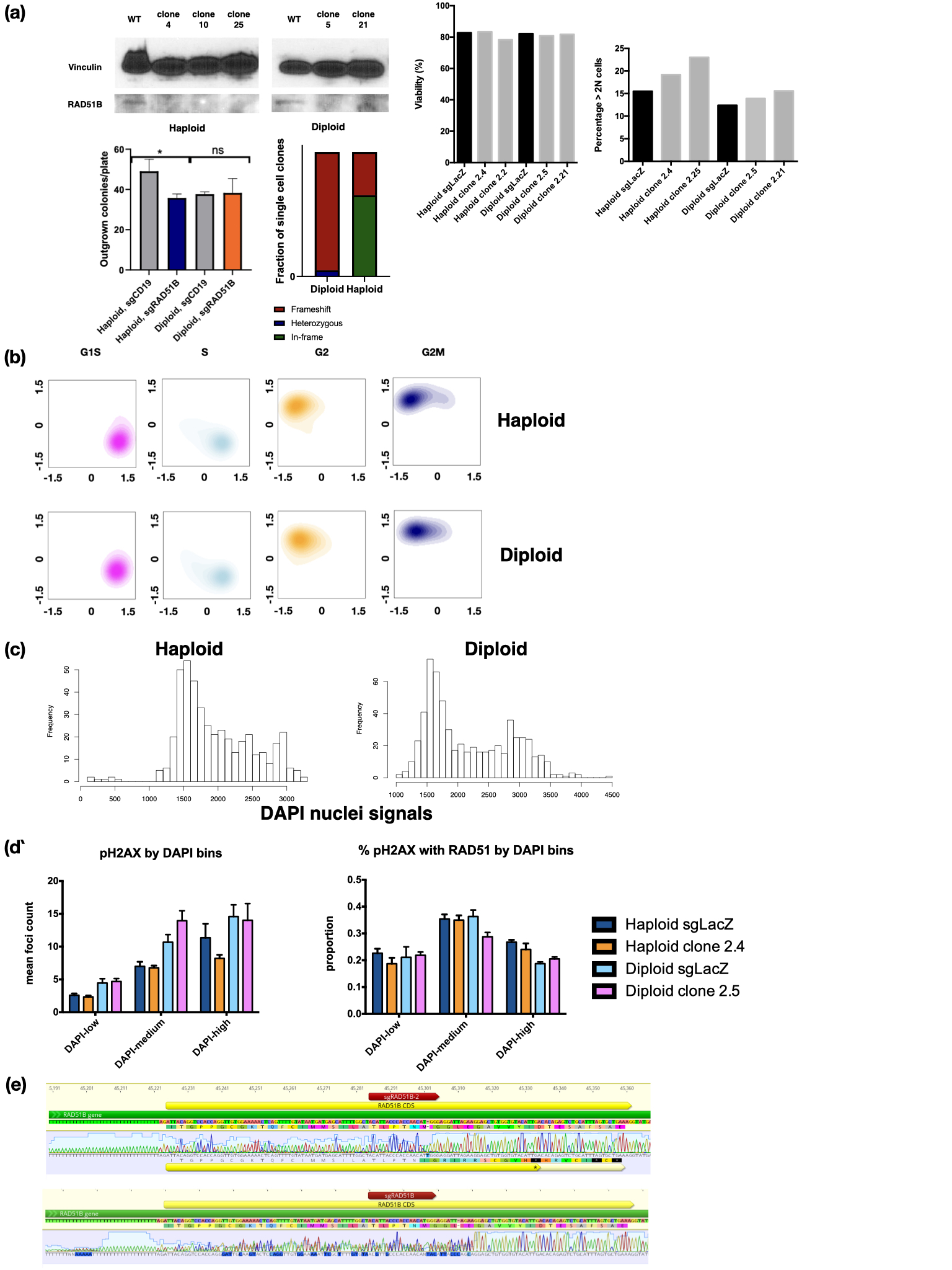
Supplementary Figure 2.** (a) Top left panels: Western blots showing generation of RAD51B knockout clones in haploid and diploid KBM7 cells. Top-middle panel shows viability of RAD51B knockout cells. Top-right panel to the right shows the relative amount of cells that have doubled their genomes in each of the 6 knockout clones generated. Bottom left panel shows the number of clones that grew out in control versus RAD51B knockout KBM7 cells (*: Mann-Whitney U test p = 0.0238, n=3). Bottom right panel: percentages of clones carrying each predominant type of mutations as determined by Sanger sequencing of the *RAD51B* locus. (b) Density plots of the distribution of haploid and diploid cells in each cell cycle phase in a 2-dimensional space. Vertical axes show G2M metagene scores and horizontal axes are G1S metagene scores. (c) Representative histograms of DAPI signals acquired from KBM7 cells that are also stained with antibodies against γ-H2AX and RAD51. Note the resemblance of the histograms to a 1-parameter cell cycle stain (Figure 1d). (d) Left panel: γ-H2AX foci count in WT vs RAD51B knockout KBM7 cells grouped by cell cycle phase. Right panel: percent γ-H2AX foci with RAD51 foci co-occupancy in another pair of clones of haploid and diploid RAD51B knockout cells. (e) Chromatograms from Sanger sequencing results for the *RAD51B* locus targeted by sgRNAs clones 2.4 (top, haploid) and 2.5 (bottom, diploid). Shown are tracks corresponding (from top to bottom) to the region targeted by the sgRNA used to generate the clone, the RAD51B coding region, the RAD51B gene locus, reference DNA sequence, translated AA sequence followed by the chromatogram of the sequenced amplicon and base calls. For the diploid clone, chromatogram shows a bi-allelic mutant with widespread mutations and indels near the target region (blue bases). For the haploid clone, chromatogram shows a frameshift insertion that introduced a premature stop codon (annotated in the bottom-most track).

**
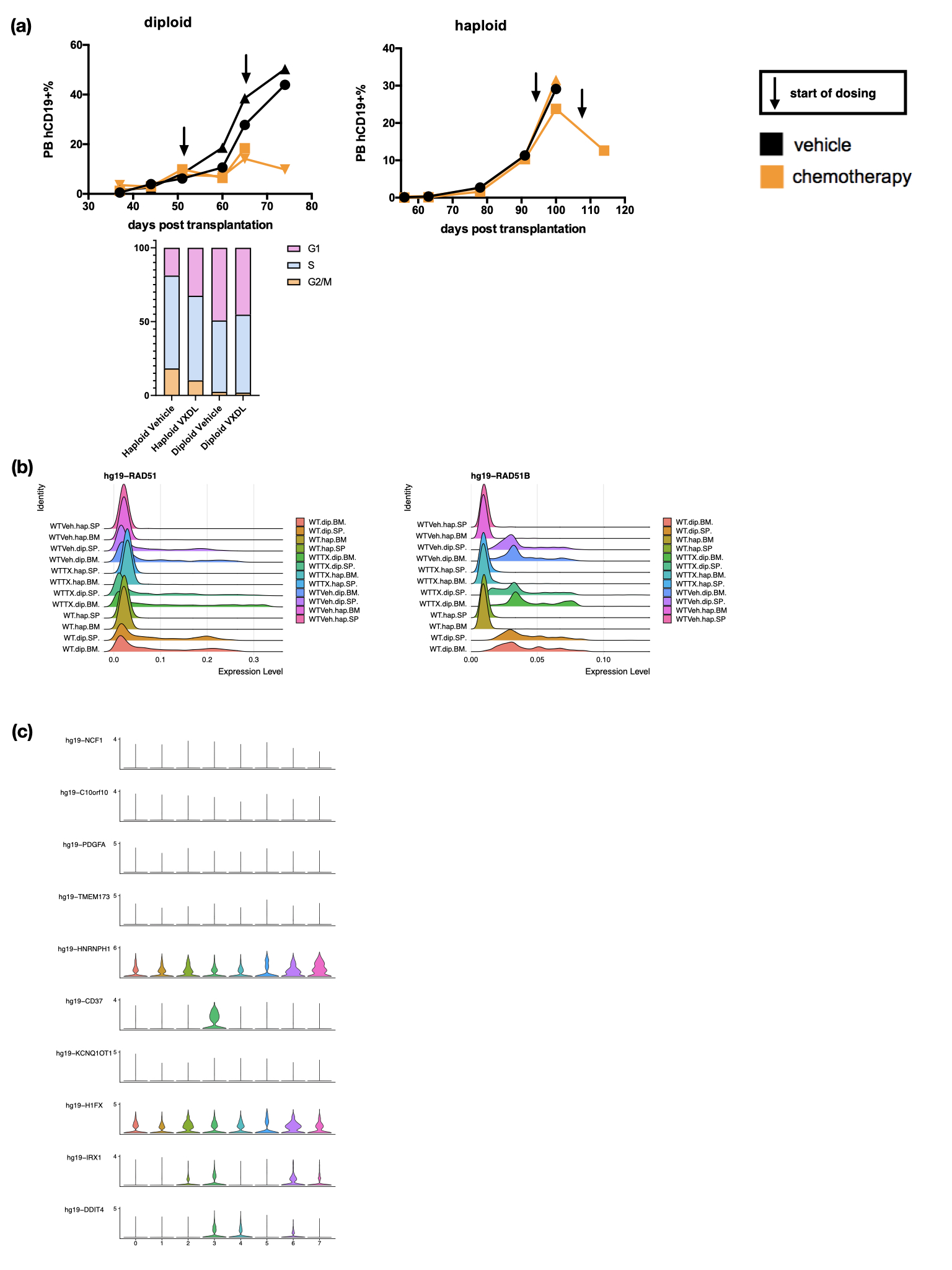
Supplementary Figure 3.** (a) Chemotherapeutic treatment response of near-haploid and diploidized B-ALL cells *in vivo*. Upper panels: peripheral blood burdens of human CD19+ leukemia cells in NSG mice, lower panels: estimated percentage of cells in each cell cycle phase in each of the bone marrow samples from the upper panels. (b) Ridge plots of RAD51 and RAD51B expression across different organs and treatment groups of mice bearing near-haploid or diploidized B-ALL cells. WT: untreated. WTVeh: Vehicle-treated. WTTX: VXDL-treated. Hap: near-haploid B-ALL. Dip: diploidized B-ALL. BM: bone marrow. SP: spleen. (c) Violin plots of the expression top 10 (ranked by log2 fold change values) Cluster 5 marker genes across clusters.
