# Supplementary material for: Integrated multi-omics analyses reveal homology-directed repair pathway as a unique dependency in near-haploid leukemia
Source: Blood Cancer J. 2023 Jun 8;13(1):92. doi: 10.1038/s41408-023-00863-1 (PMC10247733; doi:10.1038/s41408-023-00863-1)
Supplement: Supplementary file 2 — Supplementary Table 1 [file 41408_2023_863_MOESM2_ESM.pdf]

gene	haploid_1	haploid_2	haploid_3	diploid_1	diploid_2	diploid_3
A1BG	0.25	0	0	0	0	0
A1BG-AS1	0.43	0	0	0	0.02	0
A1CF	0.02	0	0.02	0.01	0.01	0.02
A2M	0.17	0	0	0	0	0
A2M-AS1	0	0	0	0.02	0	0
A2ML1	0.15	0.04	0.1	0.13	0.07	0.15
A2MP1	0	0	0	0	0	0
A4GALT	0.14	0.08	0.26	0.07	0.5	0.25
A4GNT	0	0	0	0	0	0
AA06	0	0	0	0	0	0
AAAS	49.7	68.42	68.67	68.29	75.18	67.87
AACS	10.34	8.52	6.74	6.53	9.6	10.44
AACSP1	0	0.03	0	0	0	0.03
AADAC	0	0	0	0	0	0
AADACL2	0	0	0	0	0	0
AADACL3	0	0	0	0	0	0
AADACL4	0	0	0	0	0	0
AADAT	0.51	1.22	0.16	1.41	0.5	1.13
AAED1	1.11	1.66	1.3	1.37	2.16	0.75
AAGAB	38.05	29.34	23.59	22.43	28.02	26.21
AAK1	2.59	0.93	1.08	0.72	1.18	1.07
AAMDC	48.89	57.27	44.54	49.95	55.87	50.56
AAMP	102.77	100.83	93	89.11	88.93	95.97
AANAT	0	0.24	0	0	0	0
AAR2	30.54	33.76	35.61	35.72	33.48	37.6
AARD	0.22	0.15	0.29	0.05	0.09	0
AARS	49.1	37.59	44.1	42.51	44.37	38.83
AARS2	8.43	8.94	8.66	8.82	8.99	8.22
AARSD1	36.57	37.95	32.29	32.32	37.71	32.48
AASDH	1.94	2.97	3.06	2.12	3.08	1.77
AASDHPPT	29.04	29.49	26.23	22.02	27.92	28.35
AASS	3.44	4.47	4.04	3.59	4.11	5.24
AATF	68.59	73.61	70.07	79.58	66.93	75.98
AATK	0	0.01	0.09	0.13	0.27	0.55
AATK-AS1	0	0	0	0	0	0
ABAT	0.6	0.48	0.28	0.29	0.39	0.52
ABCA1	0.01	0	0.02	0	0	0
ABCA10	0.06	0.03	0.17	0.02	0.02	0.01
ABCA11P	1.7	1.95	3.13	2.64	2.58	3.01
ABCA12	0	0	0	0	0	0
ABCA13	0.04	0.03	0.01	0.02	0.01	0.01
ABCA17P	0.07	0.05	0.03	0.07	0.08	0.11

ABCA2	2.32	9.68	7.81	4.09	4.45	8.2
ABCA3	1.01	1.14	0.93	1.83	1.2	1.53
ABCA4	0	0	0	0	0	0
ABCA5	0.13	0.06	0.1	0.18	0.07	0.01
ABCA6	0	0	0	0	0	0
ABCA7	1.31	0.55	1.12	0.51	0.68	0.73
ABCA8	0	0	0	0	0	0
ABCA9	0.49	0.2	0.34	0.22	0.14	0.28
ABCB1	0.08	0	0.2	0.05	0	0
ABCB10	1.07	0.68	0.41	0.34	0.62	0.42
ABCB11	0	0	0	0	0	0
ABCB4	0	0	0	0	0	0
ABCB5	0.39	0.28	0.3	0.16	0.37	0.4
ABCB6	0.39	0.02	0.18	0.02	0.23	0.17
ABCB7	30.06	34.22	27.37	28.94	26.19	33.96
ABCB8	3.3	4.41	4.32	4.77	4.8	4.89
ABCB9	4.2	3.5	2.44	3.59	3.07	3.91
ABCC1	6.07	3.52	5.61	5.41	4.88	5.38
ABCC10	4.76	2.87	3.25	2	1.93	3.51
ABCC11	0	0	0	0.02	0	0
ABCC12	0	0	0	0	0	0
ABCC13	0.03	0.1	0.04	0	0.02	0
ABCC2	0	0.09	0.03	0	0.07	0.01
ABCC3	0	0	0.11	0	0	0
ABCC4	16.27	4.44	4.33	6.8	10.69	7.18
ABCC5	7.16	4.73	2.66	3.9	4.77	5.08
ABCC5-AS1	0	0	0	0	0	0
ABCC6	0	0	0.03	0.06	0	0
ABCC6P1	0	0	0	0	0	0
ABCC6P2	0	0	0	0	0	0
ABCC8	0	0	0	0	0	0
ABCC9	2.01	2.47	3.58	1.84	2.04	2.61
ABCD1	8.75	5.72	6.11	6.68	7.69	3.25
ABCD2	0	0.03	0.06	0.06	0	0
ABCD3	10.99	11.68	8.53	7.23	8.49	8.8
ABCD4	5.46	6.12	4.38	5.77	5.07	6.42
ABCE1	35.22	36.68	30.83	29.92	31.5	36.58
ABCF1	27.27	23.81	21.43	24.7	27.78	20.44
ABCF2	39.02	35.37	38.42	42.08	35.86	35.49
ABCF3	16.06	14.19	18.29	18.13	16.65	17.92
ABCG1	0	0	0	0	0	0
ABCG2	0.64	0.35	0.88	0.49	0.54	0.21
ABCG4	0	0.25	0.02	0	0.02	0

ABCG5	0	0	0.07	0	0.05	0
ABCG8	0	0	0.05	0.02	0	0
ABHD1	0	0.29	0.05	0.25	0.15	0.05
ABHD10	11.49	12.49	9.81	8.39	12.99	10.7
ABHD11	12.09	13.92	13.22	11.07	10.93	12.77
ABHD12	13.37	19.47	23.39	22.15	17.41	11.79
ABHD12B	0.21	0.24	0.14	0.14	0.05	0
ABHD13	2.13	1.89	1.72	1.39	2.55	1.97
ABHD14A	28.02	46.62	48.56	50.06	43.54	56.06
ABHD14A-AC	1.79	0.95	1.9	3.09	0	0
ABHD14B	79.53	69.16	71.43	93.05	85.96	80.53
ABHD15	1.06	1.52	1.41	1.19	1.2	2.12
ABHD16A	57.31	35.94	28.59	46.13	49.66	27.36
ABHD16B	0.38	1.38	0.73	1.3	0.67	0.51
ABHD2	25.75	13.04	13.1	20.36	21.23	12.69
ABHD3	6.83	8.28	7.04	4.68	5.47	7.38
ABHD4	15.45	3.98	5.4	6.97	5.07	2.9
ABHD5	6.26	2.74	2.44	3.21	3.26	3.22
ABHD6	2.45	2.25	1.26	2.51	3.33	1.68
ABHD8	1.2	0.58	1.03	1.34	1.03	0.79
ABI1	18.8	18.11	14.28	14.8	16.32	15.27
ABI2	9.91	4.37	4.4	5.59	6.5	4.49
ABI3	0.46	0.08	0.26	0.63	0.17	0.79
ABI3BP	0.21	0	0.03	0.01	0.05	0.02
ABL1	7.06	5.12	4.46	4.64	5.67	5.67
ABL2	1.6	1.83	2.24	1.55	1.76	1.82
ABLIM1	3.04	4.94	4.44	0.62	5.22	7.42
ABLIM2	0	0	0.05	0.06	0	0
ABLIM3	0	0.03	0	0	0	0
ABO	0.28	0	0	0	0	0.05
ABP1	0.12	0	0.03	0	0.2	0.3
ABR	7.44	9.05	9.07	6.82	7.31	7.87
ABRA	0.13	0.21	0.19	0.13	0.03	0.3
ABRACL	65.33	108.77	103.1	58.03	73.99	105.27
ABT1	34.29	39.64	32.1	32.01	36.47	28.93
ABTB1	2.4	1.12	1.95	1.68	1.8	1.63
ABTB2	3.62	9.49	2.86	2.63	1.59	6.5
ACAA1	35.51	38.47	33.25	40.48	32.68	31.03
ACAA2	53.03	56.42	55.26	52.41	50.61	56.85
ACACA	13.21	14.76	13.91	11.76	13.51	15.72
ACACB	0.45	0.3	0.41	0.26	0.37	0.14
ACAD10	5.78	5.95	3.71	5.54	3.33	5.45
ACAD11	0.9	1.75	1.35	1.39	1.31	1.32

ACAD8	14.12	9.06	5.14	7.85	6.82	5.38
ACAD9	24.97	33.4	25.79	28.13	30.27	27.38
ACADL	0	0	0	0	0	0
ACADM	15.61	13.79	11.71	11.27	13.53	11.92
ACADS	11.12	20.2	19.33	21.64	19.42	20.27
ACADSB	2.5	3.47	2.87	1.96	2.19	3.17
ACADVL	33.87	30.51	26.27	31.18	27.62	23.29
ACAN	0.02	0.01	0	0	0	0
ACAP1	0.23	3.31	2.14	2.53	2.1	2.75
ACAP2	3.86	3.58	3.61	3.76	3.18	3.87
ACAP3	0.48	0.46	0.39	0.45	0.18	0.68
ACAT1	76.12	92.36	78.94	77.2	83.56	79.4
ACAT2	80.64	76.87	77.59	75.51	101.36	81.16
ACBD3	3.81	2.76	2.33	2.36	3.01	3.57
ACBD4	2.12	3.86	3.07	2.63	3.73	2.45
ACBD5	4	3.61	3.02	1.77	2.43	3.87
ACBD6	48.25	40.82	48.86	51.62	46.33	38.75
ACBD7	1.75	1.82	1.95	1.23	1.78	1.52
ACCS	2.76	6.72	7.92	7.18	3.96	7.38
ACCSL	0	0	0	0	0	0
ACD	18.08	26.96	29.07	30.63	22.69	24.57
ACE	2.34	0.3	0.78	0.88	1.48	0.51
ACE2	0	0	0	0	0	0
ACER1	0	0	0	0.19	0	0
ACER2	0.49	0.3	0.66	0.56	0.6	0.49
ACER3	4.38	2.17	2.35	2.87	4.04	2.64
ACHE	0.3	2.06	0.27	1.11	0.54	0.81
ACIN1	12.93	12.67	12.21	11.55	12.57	17.2
ACLY	70.47	71.09	72.89	61.77	67.15	72.3
ACMSD	0	0	0	0	0	0
ACN9	13.73	13.28	10.74	10.64	13.48	13.61
ACO1	20.18	17.77	14.59	16.19	18.14	19.49
ACO2	65.87	65.87	64.56	67.54	67.81	59.38
ACOT1	0	0.04	0	0	0	0
ACOT11	2.08	4.54	5.68	4.14	3.43	5.16
ACOT12	0	0.03	0.03	0	0.25	0.08
ACOT13	15.19	14.96	16.15	14.33	13.71	15.18
ACOT2	0	0	0	0	0	0
ACOT4	0	0	0	0	0	0
ACOT6	0	0	0	0	0	0
ACOT7	73.21	89.35	100.3	100.97	94.32	85.87
ACOT8	36.8	29.06	29.64	28.28	34.5	25.18
ACOT9	40	28.56	27.7	25.5	34.56	24.81

ACOX1	12.93	8.41	9.68	8.79	8.44	9.84
ACOX2	0	0	0	0	0	0
ACOX3	4.83	6.31	6.08	6.26	4.9	5.86
ACOXL	0.03	0	0	0	0	0
ACP1	112.66	86.17	73.71	83.67	90.76	93.7
ACP2	16.11	12.79	13.04	11.5	14.84	14.28
ACP5	0.1	0	0	0	0	0
ACP6	8.04	10.87	9.43	6.84	7.71	9.92
ACPL2	1.83	3.12	2.65	1.83	3.35	2.46
ACPP	0.87	4.02	3.69	0.45	0.55	4.42
ACPT	0.11	0.22	0	0	0.05	0.17
ACR	0	0	0	0	0	0
ACRBP	2.79	0.03	0	0.97	2	0.08
ACRC	0.27	0	0.06	0.05	0.04	0
ACRV1	0.23	0	0	0.06	0.3	0.08
ACSBG1	0	0.08	0.02	0	0.07	0.3
ACSBG2	0.05	0	0	0	0	0.03
ACSF2	8.1	6.6	8.36	5.38	7.16	4.82
ACSF3	17.9	15.77	17.46	11.43	13.93	15.87
ACSL1	10.78	13.05	9.02	12.93	9.05	13.17
ACSL3	15.1	11.82	9.58	7.84	15.38	11.97
ACSL4	20.1	12.52	11.62	11.92	14.73	12.31
ACSL5	12.18	23.58	23.23	21.13	15.35	24.77
ACSL6	0.17	0.24	0.18	0.12	0.21	0.22
ACSM1	0.54	0.09	0	0.17	0.1	0.07
ACSM2A	0	0.06	0	0	0	0
ACSM2B	0	0	0	0	0	0
ACSM3	65.85	23.3	24.87	69.7	73.67	18.86
ACSM4	0	0	0	0	0.08	0.09
ACSM5	0	0	0	0	0	0
ACSS1	1.14	3.14	4.71	2.47	2.83	2.84
ACSS2	14	14.61	14.27	14.35	12.54	16.2
ACSS3	0	0	0	0	0	0
ACTA1	0	0	0	0.03	0	0
ACTA2	11	7.71	6.85	7.93	4.03	4.75
ACTB	3339.09	3387.32	3526.63	3339.38	3654.86	3060.55
ACTBL2	0	0	0	0	0	0
ACTC1	0.02	0	0	0.01	0	0
ACTG1	1155.76	1253.67	1464.29	1345.54	1363.07	1341.56
ACTG1P4	0.15	0.06	0	0.06	0.25	0.3
ACTG2	0	0	0	0.04	0.12	0.25
ACTL10	0.4	0.46	0.37	0.05	0.1	0
ACTL6A	91.32	94.4	71.43	85.38	94.5	75.47

ACTL6B	0	0	0	0	0	0
ACTL7A	0	0	0	0	0	0
ACTL7B	0	0	0	0	0	0
ACTL8	0.36	0.66	1.06	0.33	0.56	1.53
ACTL9	0	0	0	0	0	0
ACTN1	36.65	41.62	41.34	34.58	35.54	39.74
ACTN1-AS1	0	0	0	0	0	0
ACTN2	0.03	0.03	0	0	0.03	0
ACTN3	0.05	0.02	0	0.03	0.02	0
ACTN4	84.65	42.53	39.36	52.69	66.94	44.24
ACTR10	21.13	18.07	13.05	14.98	19.69	14.32
ACTR1A	52.74	39.99	45.07	43.06	48.82	42.32
ACTR1B	7	12.08	11.54	12.66	9.96	9.46
ACTR2	30.88	38.25	32.92	23.62	30.15	47.73
ACTR3	105.49	100.35	103.57	91.29	106.27	94.55
ACTR3B	1.48	2.22	2.9	1.83	2.47	2.16
ACTR3BP2	0	0	0	0	0	0
ACTR3BP5	0	0	0	0	0	0
ACTR3C	0	0	0	0	0	0
ACTR5	10.48	12.53	10.26	12.12	9.74	6.46
ACTR6	10.58	9.4	9.65	7.75	9.12	8.72
ACTR8	7.64	6.49	8.92	6.05	7.41	7.39
ACTRT1	0	0	0	0	0	0
ACTRT2	0	0	0	0	0	0
ACTRT3	0.81	1.25	0.98	1.62	0.52	0.93
ACVR1	2.65	1.95	0.84	0.89	0.96	1.39
ACVR1B	1.57	1.23	1.07	0.9	0.81	1.55
ACVR1C	0	0.03	0	0	0	0
ACVR2A	0.59	0.88	0.6	0.57	0.95	0.55
ACVR2B	0.37	0.46	0.89	0.63	0.86	1.16
ACVR2B-AS1	0.41	0.02	0.15	0.2	0.17	0.19
ACVRL1	0.16	0.04	0	0	0.02	0
ACY1	27.1	26.72	26.85	36.64	38.35	31.01
ACY3	0	0	0	0	0	0
ACYP1	4.96	5.61	1.84	4.32	8.13	4.56
ACYP2	4.16	2.01	1.25	1.46	1.06	1.82
ADA	11.4	70.42	77.41	54.39	38.64	53.01
ADAD1	0	0	0	0	0	0
ADAD2	0	0	0	0	0	0
ADAL	0.02	0.03	0.02	0.03	0	0.02
ADAM10	23.42	22.55	17.99	21.79	25.08	19.33
ADAM11	0.61	0.88	0.98	1.97	1.83	2.08
ADAM12	0	0.03	0	0	0.01	0

ADAM15	51.87	75.04	85.21	74.35	59.04	90.77
ADAM17	6.74	8.67	7.48	4.98	7.13	6.41
ADAM18	0	0	0	0	0	0
ADAM19	3.48	0.02	0	0.02	0.29	0
ADAM1A	0.15	1.36	0.8	0.55	1.19	0.62
ADAM2	0	0	0	0	0	0
ADAM20	0	0	0	0	0.07	0
ADAM20P1	0.46	0	0	0	0	0.02
ADAM21	0	0	0	0	0	0
ADAM21P1	0	0	0	0	0	0
ADAM22	0.33	0.4	0.51	0.51	0.41	0.53
ADAM23	0	0.08	0	0	0	0
ADAM28	0.52	0.75	1.61	0.39	0.21	0.71
ADAM29	0	0	0	0	0	0
ADAM30	0	0	0	0	0	0
ADAM32	0.03	0	0	0	0	0
ADAM33	0.02	0.05	0.03	0.01	0.03	0.02
ADAM3A	0	0	0	0	0	0
ADAM5	0	0	0	0	0	0
ADAM6	0	0	0	0	0	0
ADAM7	0	0	0	0	0	0
ADAM8	0.19	0.15	0.08	0	0.05	0
ADAM9	4.08	4.27	3.26	2.64	3.93	2.06
ADAMDEC1	0	0	0	0	0	0
ADAMTS1	0.25	1.33	0.24	0.64	0.48	1.08
ADAMTS10	0.02	0.48	0.52	0.15	0.21	0.38
ADAMTS12	0	0	0	0	0	0
ADAMTS13	0.15	0.18	0	0	0.31	0.07
ADAMTS14	0.9	0.02	0.19	0.15	0.47	0.01
ADAMTS15	0	0.07	0.06	0.1	0.33	0.06
ADAMTS16	0	0	0	0	0	0
ADAMTS17	0.49	0.81	0.34	0.38	0.26	0.46
ADAMTS18	0	0	0	0	0	0
ADAMTS19	0	0	0	0.01	0	0
ADAMTS2	1.49	3.29	2.24	2.17	1.92	2.71
ADAMTS20	0	0	0	0	0	0
ADAMTS3	0.78	0.43	0.58	0.29	0.5	0.26
ADAMTS4	0.81	0.64	0.59	0.71	0.64	0.82
ADAMTS5	0	0	0	0	0	0
ADAMTS6	0	0	0	0.06	0.02	0.01
ADAMTS7	0.13	0.21	0.5	0.23	0.53	0.48
ADAMTS8	0	0	0.04	0.03	0.19	0
ADAMTS9	0	0	0	0	0	0.38

ADAMTS9-AS	0	0	0	0	0	0
ADAMTSL1	0	0	0	0	0	0
ADAMTSL2	0.06	0.09	0.07	0.05	0.06	0.14
ADAMTSL3	0	0	0	0	0	0
ADAMTSL4	0.31	0.24	0.09	0.28	0.5	0.13
ADAMTSL5	1.09	0.3	0.32	0.46	0.41	0.44
ADAP1	1.46	2.49	1.97	1.89	1.73	1.98
ADAP2	0.88	3.81	5.33	2.89	1.66	2.08
ADAR	24.01	24.88	25.54	24.15	21.4	27.68
ADARB1	0.81	0.51	0.42	0.19	0.18	0.23
ADARB2	0	0.04	0.01	0.01	0	0.03
ADARB2-AS1	0	0	0	0	0	0
ADAT1	3.89	4.86	5.43	2.79	4.79	5.23
ADAT2	3.43	2.55	1.52	2.44	2.36	1.98
ADAT3	1.56	3.07	3.95	4.08	2.95	2.37
ADC	0.37	0.13	0.22	0.7	0.25	0.07
ADCK1	11.59	5.94	5.87	7.18	7.83	6.19
ADCK2	1.12	1.42	1.96	1.7	1.76	1.67
ADCK3	22.11	31.08	30.99	29.06	23.3	36.54
ADCK4	4.16	4.42	6.37	6.74	4.14	5.47
ADCK5	8.71	9.48	7.9	8.77	9.58	9.08
ADCY1	0.02	0.07	0.07	0.03	0.09	0.1
ADCY10	0	0	0	0	0	0
ADCY10P1	0.02	0.15	0.04	0.01	0.01	0.02
ADCY2	0.06	0.11	0.06	0.01	0.07	0.01
ADCY3	2.63	1.35	3.58	3.02	1.25	2.45
ADCY4	0.15	0.02	0	0	0	0
ADCY5	0	0.06	0	0.05	0	0
ADCY6	1.59	0.6	1.41	1.14	1.12	0.61
ADCY7	5.56	4.19	4.7	4.34	6.41	6.2
ADCY8	0	0	0	0	0	0
ADCY9	0.52	0.96	1.16	0.57	0.92	0.73
ADCYAP1	0	0	0	0	0	0
ADCYAP1R1	0	0	0	0	0	0
ADD1	10.41	6.02	5.9	6.26	6.11	7.35
ADD2	5.81	9.15	10.7	8.75	7.57	10.16
ADD3	26.02	10.32	8.24	12.5	8.97	9.93
ADGB	0	0	0	0	0	0
ADH1A	0	0	0	0	0	0
ADH1B	0	0	0	0	0	0
ADH1C	0	0	0	0	0	0
ADH4	0.07	0.15	0.2	0	0.07	0.19
ADH5	51.77	47.48	48.12	47.99	46.62	42.58

ADH6	0	0.04	0	0	0	0
ADH7	0	0	0	0	0	0
ADHFE1	0.04	0	0.04	0.08	0.04	0.08
ADI1	126.82	132.31	130.66	116.19	127.69	134.2
ADIG	0	0	0	0	0	0
ADIPOQ	0.16	0.11	0.15	0.13	0.14	0.16
ADIPOQ-AS1	0.13	0.02	0.11	0	0.02	0
ADIPOR1	56.62	47.05	47.05	51.55	54.73	42.41
ADIPOR2	3.73	2.3	2.21	2.64	2.65	3.64
ADIRF	0	0	0	0	0	0
ADK	27.2	44.69	41.95	35.35	29.69	42
ADM	10.57	0.82	0	3.68	0.49	0.21
ADM2	0.11	0.12	0.17	0.18	0.13	0.19
ADM5	2.06	1.84	1.95	1.44	1.88	1.34
ADNP	7.24	8.23	7.22	6.39	6.22	9.74
ADNP2	2.29	2.26	2.71	1.95	1.8	2.72
ADO	1.54	0.89	1.27	0.66	0.97	0.85
ADORA1	0	0	0	0	0	0
ADORA2A	5.39	1.03	0.99	2.16	1.72	1.62
ADORA2A-AS	0.09	0.03	0	0	0	0
ADORA2B	15.17	8.93	7.6	9.68	13.97	7.99
ADORA3	0.95	0.57	0.48	0.27	0.56	0.26
ADPGK	51.91	59.94	47.14	45.43	44.87	63.83
ADPGK-AS1	2.45	4.66	2.99	3.11	2.83	3.28
ADPRH	0.02	0	0	0.01	0	0.6
ADPRHL1	1.39	1.23	0.4	0.46	1.41	0.26
ADPRHL2	29.63	48.81	38.92	40.41	29.32	33.57
ADPRM	4.73	3.85	4.6	6.59	5.5	5.13
ADRA1A	1.47	1.22	1.67	0.76	1.07	0.77
ADRA1B	0	0	0	0	0	0
ADRA1D	0.41	0	0	0	0.03	0
ADRA2A	0.26	0.06	0.09	0.09	0.16	0.32
ADRA2B	0	0	0	0	0	0
ADRA2C	0	0.09	0.07	0.08	0.04	0.19
ADRB1	0.28	0.1	0.02	0.05	0.07	0.21
ADRB2	0.33	1.09	0.23	0.02	0.17	1.02
ADRB3	0	0	0	0	0	0
ADRBK1	11.52	16.13	13.31	13.37	11.7	17.85
ADRBK2	0.66	1.14	0.71	0.56	0.65	0.82
ADRM1	146.34	147.17	126.91	132.6	133.2	133.27
ADSL	185.12	163.47	152.14	173.63	146.09	157.57
ADSS	26.32	27.13	21.87	18.36	20.07	23.81
ADSSL1	0.21	0	0	0	0.16	0.04

ADTRP	0	0	0	0	0	0
AEBP1	0	0.01	0.02	0.04	0	0.11
AEBP2	2.66	3.47	2.66	1.91	3.25	2.96
AEN	46.12	56.18	59.75	59.51	49.39	61.63
AES	8.58	7.18	8.02	8.34	10.52	9.59
AFAP1	1.29	0.26	0.18	0.41	0.6	0.36
AFAP1-AS1	0.16	0.09	0	0.01	0.01	0.01
AFAP1L1	0.12	0.03	0.03	0.05	0.05	0.04
AFAP1L2	0	0.03	0.02	0	0	0.02
AFF1	5.31	3.45	2.71	2.34	2.91	2.88
AFF2	0	0	0	0	0	0
AFF3	0.15	0.14	0.18	0.13	0.1	0.22
AFF4	10.89	5.66	4.85	5.79	6.84	5.67
AFG3L1P	4.64	4.91	3.65	3.03	4.7	3.7
AFG3L2	16.3	19.44	20.2	20.03	17.99	16.55
AFM	0	0	0	0	0	0
AFMID	31.74	26.67	21.81	32.68	33.06	26.15
AFP	0	0	0	0	0	0
AFTPH	3.31	2.4	2.26	1.5	2.38	2.5
AGA	8.94	10.11	8.09	9.12	8.25	8.7
AGAP1	1.41	0.74	0.72	0.82	0.96	0.75
AGAP11	0.08	0.05	0	0.08	0	0.06
AGAP2	0.88	2.68	4.21	3.22	2.83	3.85
AGAP3	4.55	2.47	3.11	3.7	3.82	3.07
AGAP4	0.66	0.12	0.06	0.03	0.22	0
AGAP5	1.11	0.8	0.48	0.54	0.24	0.63
AGAP6	0.29	1.01	0.59	0.13	0.91	0.52
AGAP7	0.17	1.14	0.33	0.67	0.52	0.6
AGAP8	0	0.33	0	0.03	0	0.54
AGAP9	0.49	0.75	0.2	0.1	0.15	0.08
AGBL1	0	0	0	0	0	0
AGBL2	0	0	0	0	0	0
AGBL3	0.14	0	0.15	0.03	0.19	0.15
AGBL4	0	0	0	0	0	0
AGBL5	8.24	9.72	11.29	12.9	11.92	10.29
AGER	0.36	0.77	0.42	0.21	0.55	0.63
AGFG1	2.47	1.47	1.29	1.04	1.81	1.29
AGFG2	0.66	0.63	0.45	0.67	0.13	0.3
AGGF1	9.68	7.83	7.04	8	8.22	7.43
AGK	14.57	14.65	12.6	13.68	14.99	14.24
AGL	5.18	2.38	1.75	2.26	3.23	2.75
AGMAT	1.25	1.31	1.73	1.14	1.4	0.76
AGMO	0.04	0.15	0.16	0.13	0.1	0.09

AGO1	5.34	6.33	5.3	4.59	4.33	3.47
AGO2	10.58	9.51	9.62	8.23	8.27	8.8
AGO3	3.03	4.15	2.93	2.17	2.78	3.45
AGO4	0.4	0.73	0.57	0.41	0.41	0.6
AGPAT1	42.4	25.9	29.84	34.43	33.61	20.56
AGPAT2	62.8	125.9	142.97	118.86	80.64	127.56
AGPAT3	4.01	2.98	5.24	3.84	4.28	3.65
AGPAT4	0.17	0.32	0.28	0.53	0.54	0.36
AGPAT4-IT1	0	0.04	0.11	0.5	0.08	0
AGPAT5	36.04	19.26	14.14	14.57	24.41	17.81
AGPAT6	30.32	19.42	23.11	23	23.72	22.41
AGPAT9	2.82	10.96	10.56	11.39	6.06	14.66
AGPHD1	6.37	4.44	3.48	5.58	4.24	3.44
AGPS	3.39	3.44	3.49	2.84	3.11	3.22
AGR2	0	0	0	0	0	0
AGR3	0	0	0	0	0	0
AGRN	1.54	0.81	1.14	1.7	1.25	1.91
AGRP	0	0	0	0	0	0
AGSK1	2.22	2.44	1.74	2.22	0.86	1.77
AGT	0	0	0	0	0	0
AGTPBP1	3.03	3.37	2.13	1.94	2	2.37
AGTR1	0	0	0	0	0	0
AGTR2	0	0	0	0	0	0
AGTRAP	2.38	0	0	0.58	3.12	0
AGXT	0	0	0	0	0	0
AGXT2	0	0	0	0	0	0
AGXT2L1	0	0	0	0	0	0
AGXT2L2	3.74	5.43	6.05	6.21	7.07	4.53
AHCTF1	2.78	2.41	2.43	2.04	2.29	2.35
AHCTF1P1	0	0	0	0	0	0
AHCY	213.41	271.54	312.9	326.1	265.54	304.09
AHCYL1	9.57	9.5	7.35	6.82	7.93	6.98
AHCYL2	1.13	0.85	0.9	0.66	0.61	0.9
AHDC1	1.26	1.26	1.6	1.12	0.97	1.15
AHI1	2.35	2.34	2	1.37	1.55	1.76
AHNAK	14.53	9.8	11.67	10.54	12.66	12.63
AHNAK2	0.05	0.04	0.04	0.09	0.01	0.03
AHR	4.93	2.8	2.09	2.26	2.91	1.07
AHRR	3.76	7.58	6.95	5.45	5.36	5.32
AHSA1	186.8	193.81	194.98	175.02	196.01	186.96
AHSA2	0.19	0.32	0.24	0.23	0.23	0.37
AHSG	0.09	0	0	0	0	0
AHSP	0.66	0	0	0.12	0	0

AICDA	0.32	0.64	0.74	0.42	0.59	0.32
AIDA	5.82	4.06	5.66	5.17	4.19	4.15
AIF1	38.67	144.14	181.39	166.8	144.94	224.8
AIF1L	0.76	4.58	4.01	5.88	2.99	6.16
AIFM1	81.48	104.39	82.98	76.69	81.64	79.63
AIFM2	13.19	9.42	6.71	6.3	9.94	7.03
AIFM3	0.29	0.13	0.03	0	0.12	0.1
AIG1	5.6	0.43	0.1	0.41	2.26	0.66
AIM1	0.12	0.17	0.16	0.04	0.05	0.07
AIM1L	0	0	0	0	0	0
AIM2	0	0	0	0	0	0
AIMP1	17.54	19.48	16.3	14.2	16.93	14.92
AIMP2	77.03	88.89	80.68	78.42	80.94	73.33
AIP	65.28	74.4	92.64	101.95	109.98	68.27
AIPL1	0.53	0.69	1.09	0.33	0.45	0
AIRE	0	0.06	0.12	0	0.12	0.19
AIRN	0	0	0	0	0	0
AJAP1	0	0	0	0	0	0
AJUBA	0.86	1.67	1.99	1.08	1.01	1.14
AK1	0.2	0.14	0.36	0.3	0.27	0.51
AK2	89.91	101.49	94.48	98.05	97.5	101.7
AK3	14.99	11.24	12.55	11.12	12.04	12.29
AK4	19.16	11.87	10.67	10.57	13.91	10.89
AK5	0	0.05	0.08	0	0.05	0.04
AK7	0.26	0.02	0.04	0.02	0.15	0.02
AK8	0.07	0.06	0.03	0.07	0.07	0.07
AKAP1	3.54	4.8	3.85	4.03	3.79	3.6
AKAP10	6.05	2.88	2.84	2.54	3.02	3.57
AKAP11	4.8	3.98	3.52	3	3.5	3.72
AKAP12	2.01	0.07	0.24	0.68	0.67	0.13
AKAP13	5.85	5.12	6.56	6.42	5.87	6.82
AKAP14	0	0.19	0	0.06	0	0
AKAP17A	1.34	0.98	0.83	1.03	1.78	1.1
AKAP2	16.09	7.07	8.15	10.38	21.88	7.66
AKAP3	0	0.02	0.18	0	0.11	0.64
AKAP4	0.23	0	0	0.05	0.02	0
AKAP5	0.52	0.48	0.68	0.62	0.63	0.77
AKAP6	0	0.03	0.01	0.02	0	0.01
AKAP7	0.35	0.69	0.74	0.36	0.33	0.69
AKAP8	10.51	13.57	12.96	12.62	9.08	12.21
AKAP8L	7.05	6.21	8.07	6.72	5.7	5.22
AKAP9	3.81	2.86	3	2.64	3.44	2.75
AKD1	0.43	0.63	0.16	0.3	0.63	0.29

AKIP1	29.33	25.13	18.17	18.99	25.84	19.82
AKIRIN1	22.23	24.54	21.41	18.6	22.91	14.91
AKIRIN2	15.59	12.88	13.11	13.23	13.96	12.44
AKNA	1.07	6.13	8.51	5.7	3.87	8.46
AKNAD1	0	0	0	0	0	0
AKR1A1	68.52	91.65	79.3	88.07	74.2	68.17
AKR1B1	119.04	102.82	86.68	111.72	106.65	109.68
AKR1B10	0.27	0.49	0.31	0.24	0.22	0.23
AKR1B15	0	0	0	0.03	0	0
AKR1C1	2.37	0.04	0.05	0.58	0.83	0
AKR1C2	0.21	0.13	0	0.14	0.13	0
AKR1C3	20.41	8.16	0.12	54.15	32.87	3.88
AKR1C4	0	0	0	0	0.06	0
AKR1C6P	0	0	0.05	0.41	0	0
AKR1CL1	0	0	0	0	0	0
AKR1D1	0.14	0.06	0.03	0.02	0.02	0.1
AKR1E2	0	0	0	0	0	0
AKR7A2	36.85	52.57	52.17	52.28	47.06	45.11
AKR7A2P1	0	0.36	0	0.47	0	0
AKR7A3	0.06	0.2	0.39	0	0.12	0.06
AKR7L	0.1	0.2	0.17	0.22	0	0.43
AKT1	4.45	5.02	3.38	4.06	4.35	4.59
AKT1S1	4.64	2.36	3.68	4.39	3.65	3.82
AKT2	6.88	9.7	8.39	8.26	7.13	10.03
AKT3	0.25	0.04	0.09	0.05	0.12	0.11
AKTIP	1.07	2	2.18	0.55	0.61	2.83
ALAD	2.54	3.85	2.41	1.24	2.95	2.15
ALAS1	77.9	30.86	26.16	23.2	36.58	31.24
ALAS2	345.33	0.43	2.01	111.96	77.16	0.37
ALB	0	0	0	0	0	0
ALCAM	2.88	8.02	5.39	4.09	2.87	8.8
ALDH16A1	6.89	9.56	13.81	16.17	12.39	9.28
ALDH18A1	37.38	43.31	45.85	45.39	42.7	61.85
ALDH1A1	3.29	0	0	0	0	0
ALDH1A2	0.09	0	0	0	0	0
ALDH1A3	0.12	0.29	0.29	0.16	0.2	0.08
ALDH1B1	31.07	28.49	36.37	26.12	31.19	33.24
ALDH1L1	0	0	0	0	0	0
ALDH1L1-AS:	0	0	0	0	0	0
ALDH1L1-AS:	0	0	0	0	0	0
ALDH1L2	0.24	0.13	0.11	0.07	0.1	0.13
ALDH2	0.08	0	0	0	0.07	0
ALDH3A1	0.29	0.36	0	0.17	0.18	0.21

ALDH3A2	8.87	12.12	12.24	10.75	12.06	13.32
ALDH3B1	3.87	3.6	1.96	1.59	1.28	2.92
ALDH3B2	0.03	0.07	0	0	0.03	0.01
ALDH4A1	8.5	7.64	8.62	8.39	9.96	9
ALDH5A1	6.51	11.78	8.47	9.56	7.7	3.76
ALDH6A1	9.31	16.62	15.3	11.48	15.68	17.85
ALDH7A1	0	0.04	0.03	0.04	0	0
ALDH8A1	0	0.28	0.48	0.05	0.2	0.4
ALDH9A1	35.92	34.19	32.23	34.25	37.24	36.01
ALDOA	1647.44	1577.77	1633.27	1727.5	1562.45	1528.97
ALDOB	0	0	0	0	0	0
ALDOC	15.84	3.59	5.7	11.16	14.03	4.79
ALG1	22.16	24.32	23.95	20.96	20.69	24.17
ALG10	3.7	4.74	3.63	3.13	3.38	4.7
ALG10B	1.31	1.32	1.69	1.28	1.33	1.37
ALG11	8.29	8.8	4.67	5.84	8.04	6.9
ALG12	11.48	15.64	11.39	11.49	10.95	11.77
ALG13	7.02	8.53	5.61	5	5.54	4.96
ALG14	5.51	6.86	6.1	5.11	5.01	5.35
ALG1L	0.81	0.54	0.89	1.09	0.43	0.88
ALG1L2	0.39	0.72	1.15	0.26	0.64	0.92
ALG1L9P	1.85	1.54	2.02	2.22	1.73	3
ALG2	17.66	18.67	17.62	13.39	14.82	20.78
ALG3	165.74	196.92	197.73	182.94	179.38	195.71
ALG5	57.43	60.62	72.69	55.24	69.37	65.49
ALG6	5.39	11.76	10.67	7.6	8.99	9.73
ALG8	56.53	74.01	55.14	65.56	68.41	67.27
ALG9	4.94	5.82	6.49	7	5.32	6.87
ALK	0	0	0.03	0	0	0
ALKBH1	6.34	8.68	5.83	6.12	6.28	7.45
ALKBH2	17.68	16.58	18.8	16.7	21.55	20.38
ALKBH3	19.19	26.51	19.39	21.36	18.76	19.89
ALKBH4	14.05	14.57	14.2	15.3	15.67	13.98
ALKBH5	9.02	9.63	14.74	11.88	9.92	6.88
ALKBH6	15.27	10.68	8.75	12.27	17.87	8.8
ALKBH7	49.13	47.08	39.88	50.37	45.23	41.53
ALKBH8	1.57	2.17	2.53	2.3	1.31	1.64
ALLC	0	0	0	0	0	0
ALMS1	2.29	2.35	1.81	2.33	1.93	2.36
ALMS1P	0	0	0	0	0	0
ALOX12	0	0	0.06	0.19	0.06	0
ALOX12B	0	0.42	0.51	0.38	0.66	0.68
ALOX12P2	0	0.04	0	0.11	0.2	0.13

ALOX15	0.04	0	0	0.04	0	0.03
ALOX15B	0	0	0	0	0	0
ALOX15P1	0.08	0	0	0	0	0
ALOX5	3.11	0	0.11	0.95	0.26	0
ALOX5AP	6.26	0.61	1.1	1.3	1.38	2.08
ALOXE3	0	0.16	0.14	0.25	0.23	0.3
ALPI	0	0	0	0	0	0
ALPK1	0.69	1.32	1.61	1.29	0.87	0.9
ALPK2	0	0	0.05	0	0	0
ALPK3	0	0.03	0	0	0.03	0
ALPL	0.25	0	0	0.35	0.18	0.21
ALPP	0.03	0	0.02	0	0.05	0
ALPPL2	0	0	0	0	0	0
ALS2	7.58	14.97	9.57	7.42	11.55	10.45
ALS2CL	0.12	0.78	0.82	0.37	0.39	0.12
ALS2CR11	0	0	0	0	0	0
ALS2CR12	0.15	0.21	0.07	0.03	0	0.15
ALS2CR8	0.44	0.68	0.33	0.49	0.26	0.42
ALX1	0.7	0	0	0.08	0.33	0
ALX3	0.41	0.48	0.23	0.07	0.05	0.31
ALX4	0	0.02	0.04	0	0	0
ALYREF	5.92	8.74	6.57	8.08	8.57	8.09
AMACR	1.62	2.63	3.37	1.85	1.71	3.7
AMBN	0	0	0	0	0	0
AMBP	0	0	0	0	0	0
AMBRA1	7.2	5.95	6	7.75	7.34	8.06
AMD1	54.33	58.08	59.42	50.34	55.61	58.8
AMDHD1	0.21	0	0	0.05	0	0
AMDHD2	13.29	12.8	9.92	11.75	15.18	7.53
AMELX	0	0	0	0	0	0
AMELY	0	0	0	0	0	0
AMER1	4.87	5.84	7.45	4.86	5.66	6.32
AMER2	0	0	0	0	0	0
AMER3	0.01	0.09	0.11	0.09	0.03	0.05
AMFR	8.55	3.9	2.71	4.11	6.44	3.51
AMH	0	0.17	0	0.15	0.03	0
AMHR2	22.12	0.26	1.08	6.66	15.9	0.63
AMICA1	0	0	0.28	0	0.25	0.04
AMIGO1	0	0.42	0.03	0.04	0.31	0.06
AMIGO2	6.8	0.79	0.79	0.9	1.29	0.27
AMIGO3	1.45	1.85	1.93	0.4	1.69	1.84
AMMECR1	11.41	6.09	6.15	7.51	9.49	6.61
AMMECR1L	5.58	4.69	3.63	3.47	6.28	5.17

AMN	0	0	0	0	0	0
AMN1	1.69	2.17	1.83	1.32	1.91	2.32
AMOT	0.06	0.16	0.05	0.39	0.07	0.07
AMOTL1	6.44	5.63	5.97	7.3	6.51	6.92
AMOTL2	0.29	1.01	2.36	1.47	1.15	2.1
AMPD1	0	0	0	0	0	0
AMPD2	13	14.56	14.31	16.17	13.34	17.61
AMPD3	2.51	1.7	1.42	0.85	2.56	1.73
AMPH	0	0.17	0.02	0	0	0.02
AMT	0.11	0.18	0.13	0.08	0.41	0
AMTN	0	0	0	0	0	0
AMY1A	0	0	0	0	0	0
AMY1B	0	0	0	0	0	0
AMY1C	0	0	0	0	0.04	0
AMY2A	0	0	0	0	0	0
AMY2B	0.07	0.19	0.06	0	0.06	0.07
AMZ1	0	0.03	0.22	0.11	0.11	0.18
AMZ2	16.9	8.49	11.48	12.72	11.9	11.71
AMZ2P1	1.98	2.85	2.46	2.27	2.11	2.31
ANAPC1	9.83	11.03	13.23	9.01	8.91	10.7
ANAPC10	9.39	8.61	7.79	7.04	8.43	9.02
ANAPC11	226.86	185.51	167.03	206.3	209.2	160.74
ANAPC13	50.41	48.48	33.8	38.85	48.79	39.43
ANAPC15	65.69	90.04	86.13	91.8	94.04	77.82
ANAPC16	23.44	24.28	21.11	18.84	22.26	20.15
ANAPC1P1	0	0	0.06	0.08	0	0.25
ANAPC2	2.38	4.5	3.55	3.04	2.15	3.64
ANAPC4	6.19	10.22	7.54	8.23	6.2	6.84
ANAPC5	93.54	125.69	119.31	111.01	104.69	113.28
ANAPC7	33.91	34.48	28.11	25.2	34.36	27.39
ANG	0.11	0.29	0.2	0	0.61	0.43
ANGEL1	11.5	6.17	8.46	7.47	7.17	7.68
ANGEL2	3.83	3.87	3.64	3.08	3.71	3.51
ANGPT1	6.3	1.23	3.97	1.54	6.71	1.11
ANGPT2	0.69	1.18	0.75	0.52	1.11	0.75
ANGPT4	0	0	0	0	0	0
ANGPTL1	0.02	0.03	0.02	0.01	0	0.02
ANGPTL2	0	0.05	0	0	0	0
ANGPTL3	0.03	0	0	0	0	0
ANGPTL4	0.46	0.03	0.04	0.27	0.08	0
ANGPTL5	0	0	0	0	0	0
ANGPTL6	1.85	1.29	1.8	0.65	1.35	0.87
ANGPTL7	0.03	0.05	0.06	0.14	0.12	0.07

ANHX	0	0	0	0	0	0
ANK1	6.89	0.02	0.03	1.94	3.28	0.14
ANK2	0	0	0.01	0	0.03	0
ANK3	0.03	0	0	0	0	0
ANKAR	0.21	0.41	0.22	0.17	0.17	0.22
ANKDD1A	0.58	0.83	0.48	0.43	0.44	0.49
ANKDD1B	0	0	0	0	0	0
ANKEF1	0	0.02	0.05	0.04	0	0.12
ANKFN1	0	0	0	0	0	0
ANKFY1	4.05	4.66	3.99	3.56	4.61	4.88
ANKH	0.74	0.99	0.66	0.74	0.63	0.7
ANKHD1	6.85	3.99	3.98	3.74	4.58	2.87
ANKHD1-EIF4	1.55	4.89	4.83	3.38	3.02	4.06
ANKIB1	2.12	2.18	1.77	1.5	1.68	1.7
ANKK1	0	0	0.01	0	0.03	0
ANKLE1	21.78	23.89	27.58	23.8	21.36	27.27
ANKLE2	3.92	2.92	3.67	3.18	3.73	3.1
ANKMY1	2.82	2.23	2.23	1.95	2.56	2.4
ANKMY2	6.22	7.65	6.69	5.36	6.17	6.24
ANKRA2	2.21	2.3	1.04	1.08	2.15	2.41
ANKRD1	0	0	0	0	0.14	0.04
ANKRD10	14.22	13.09	11.38	8.34	8.9	11.65
ANKRD11	5.04	4.22	4.43	4.5	5.17	5.04
ANKRD12	2.79	1.79	2.08	1.66	1.89	1.85
ANKRD13A	3.49	3.4	3.34	3.13	3.36	3.53
ANKRD13B	0.87	1.39	1.64	1.58	1.17	1.46
ANKRD13C	4.7	3.26	3.94	2.62	3.54	3.37
ANKRD13D	1.9	3.96	2.05	1.87	1.92	3.55
ANKRD16	0.99	1.98	1.55	0.89	1.32	1.7
ANKRD17	14.67	12.03	11.99	12.54	13.13	12.3
ANKRD18A	0.08	0.23	0.54	0.04	0.03	0.59
ANKRD18B	1.41	2.18	1.44	1.78	1.9	1.43
ANKRD18DP	0.18	0.69	0.58	0.42	0.38	0.48
ANKRD19P	0.71	0.63	0.66	0.51	0.69	1.4
ANKRD2	0.17	0	0.05	0	0	0
ANKRD20A1	0.08	0.41	0.16	0	0.21	0.21
ANKRD20A11	0.33	0.29	0.16	0.15	0.19	0.07
ANKRD20A12	0.34	0.37	0.51	0.17	0.16	0.26
ANKRD20A19	0.07	0.18	0.07	0.09	0.03	0.06
ANKRD20A2	0	0.14	0	0	0	0.15
ANKRD20A3	0.19	0.3	0	0.26	0.64	0.17
ANKRD20A4	0.26	0.21	0.42	0.49	0.28	0
ANKRD20A5I	0.18	0.27	0.2	0.23	0.3	0.35

ANKRD20A8I	0.08	0.37	0.14	0.14	0.08	0.14
ANKRD20A9I	0.99	1.2	1.21	0.76	0.83	1.05
ANKRD22	0.28	1.37	0.1	0.08	0.1	0.48
ANKRD23	0.45	0.51	0.13	0.47	0.08	0.09
ANKRD24	0	0	0	0.06	0	0
ANKRD26	1.43	1.17	1.19	0.63	1.07	0.6
ANKRD26P1	0	0	0	0	0	0
ANKRD26P3	0	0	0	0	0	0
ANKRD27	13.81	13.85	15.91	10.95	14.52	13.13
ANKRD28	3.04	1.4	1.3	1.2	1.21	1.48
ANKRD29	0	0	0	0.07	0	0
ANKRD30A	0	0	0	0	0	0
ANKRD30B	0	0	0	0	0	0
ANKRD30BL	2.62	3.12	9.32	5.57	3.21	3.88
ANKRD30BP2	0.19	0.22	0.23	0.19	0.19	0.3
ANKRD30BP3	0	0	0	0	0	0
ANKRD31	0	0	0	0	0	0
ANKRD32	2.55	3.29	3.41	2.48	2.04	2.46
ANKRD33	0	0	0	0	0	0
ANKRD33B	1.47	1.17	1.58	1.86	0.99	2.14
ANKRD34A	0.22	0.25	0.2	0.08	0.04	0.04
ANKRD34B	0	0	0	0	0.05	0
ANKRD34C	0	0	0	0	0	0
ANKRD35	0.65	1.96	1.88	1.14	1.35	1.86
ANKRD36	2.02	3.79	3.12	2.4	2.23	2.61
ANKRD36B	2.42	2.41	3.18	1.92	2.08	1.79
ANKRD36BP1	59.02	69.75	64.65	44.03	54.74	64.47
ANKRD36BP2	0.09	0.03	0.08	0	0.13	0.04
ANKRD37	3.2	0.94	0.24	1.98	1.92	0.6
ANKRD39	27.7	23.84	18.37	19.91	23.27	17.83
ANKRD40	6.62	6.99	6.55	6.36	7.37	6.35
ANKRD42	1.05	1.46	0.76	0.49	0.67	1.04
ANKRD44	0.95	3.44	2.2	0.98	3.23	4.59
ANKRD45	0.19	0.26	0.26	0.02	0.18	0.34
ANKRD46	7.97	10.51	12.17	7.9	10.38	11.45
ANKRD49	5.13	7.62	5.05	6.5	5.32	5.9
ANKRD50	0.75	0.84	1.28	1.06	1.08	0.91
ANKRD52	16	12.69	12.94	14.94	14.57	14.32
ANKRD53	0	0	0	0	0	0
ANKRD54	6.67	7.94	9.2	8.63	7.46	5.76
ANKRD55	0.85	0.9	0.67	0.22	0.41	1.07
ANKRD6	0.47	0.54	0.79	0.34	0.43	0.63
ANKRD61	0.21	0.33	0.05	0.11	0.55	0.27

ANKRD62P1-	0.13	0.04	0	0	0	0.05
ANKRD63	0	0	0	0	0	0
ANKRD65	0.55	1.87	1.26	1.41	1.14	1.63
ANKRD66	0	0	0	0	0	0
ANKRD7	0	0	0	0	0	0
ANKRD9	0.46	0.43	0	0.19	0.39	0
ANKS1A	1.28	1.8	2.2	1.48	1.14	2.72
ANKS1B	0	0	0	0	0	0
ANKS3	2.92	1.73	1.62	0.83	1.96	2.36
ANKS4B	0.5	0.58	0.54	0.3	0.47	0.89
ANKS6	1.02	1.9	1.63	1.76	1.52	1.84
ANKUB1	0	0	0	0	0	0
ANKZF1	6.3	10.53	9.36	6.76	6.47	9
ANLN	8.65	9.67	8.16	7.32	8.19	6.85
ANO1	0	0	0	0	0	0
ANO10	9.21	5.28	9.67	8.67	9.15	7.42
ANO2	0	0	0	0	0	0
ANO3	0	0	0	0	0	0
ANO4	0	0	0	0	0	0
ANO5	0.94	0.54	0.6	0.57	0.68	0.62
ANO6	6.89	5.54	4.99	5.19	5.54	7.64
ANO7	0.7	0.49	0.85	0.65	0.55	0.55
ANO8	0.02	0.07	0.02	0	0.02	0.02
ANO9	0	0	0.02	0	0.29	0
ANP32A	42.04	48.12	49.35	49.22	52.64	56.28
ANP32A-IT1	1.23	1.17	1.48	0.74	0.63	0.87
ANP32AP1	0	0	0	0	0	0
ANP32B	252.92	225.7	217.52	229.64	245.78	231.27
ANP32C	0	0	0	0	0	0
ANP32D	0	0	0	0	0	0
ANP32E	45.85	52.81	42.93	37.82	51.64	58.72
ANPEP	14.91	38.08	33.18	22.73	20.54	23.36
ANTXR1	4.79	0.51	0.25	1.32	1.21	1.64
ANTXR2	10.41	3.99	5.26	8.32	7.31	5.59
ANXA1	46.23	2.25	0.34	1.22	3.4	1.3
ANXA10	0	0	0	0	0	0
ANXA11	24.43	30.7	30.15	28.03	27.11	23.38
ANXA13	0	0	0	0	0	0
ANXA2	333.16	87.35	81.5	107.44	124.01	68.75
ANXA2P1	0	0	0	0.24	0.05	0
ANXA2P2	0.41	0.07	0.21	0.01	0.2	0
ANXA2P3	0.06	0	0	0	0	0
ANXA2R	3.88	1.6	0.38	1.81	2.53	0.91

ANXA3	1.94	0.62	0.21	0.73	0.17	0.19
ANXA4	25.99	17.99	17.6	15.39	12.15	18.83
ANXA5	94.89	84.76	94.38	80.9	86.96	82.24
ANXA6	18.55	34.55	29.62	26.85	23.81	24.93
ANXA7	71.01	62.62	50.46	44.05	50.59	49.55
ANXA8	0	0	0	0	0	0
ANXA8L1	0	0	0	0	0	0
ANXA8L2	0	0	0	0	0	0
ANXA9	0.08	0.26	0.07	0.11	0.19	0.29
AOAH	0	0.02	0.14	0	0	0.27
AOAH-IT1	0	0	0	0	0	0
AOC2	0.19	0.11	0.13	0	0.21	0.36
AOC3	0.11	0.21	0	0	0.14	0.04
AOC4	0	0	0	0.02	0	0
AOX1	0	0	0	0	0.03	0
AOX2P	0.02	0.02	0.04	0.01	0.02	0.02
AP1AR	7.86	6.75	4.33	4.52	6.16	3.48
AP1B1	55.36	37.8	33.37	44.37	43.04	42.26
AP1B1P1	0.28	0.07	0	0	0	0
AP1G1	12.59	11.29	11.47	10.97	11.63	13.15
AP1G2	8.96	13.85	11.59	11.03	10.44	14.2
AP1M1	56.65	45.98	44.18	43.28	38.69	40.99
AP1M2	0	0	0	0	0	0
AP1S1	10.19	11.43	12.79	13.06	12.67	11.11
AP1S2	60.23	14.93	15.29	24.16	29.33	15.61
AP1S3	2.57	4.51	3.48	2.49	2.93	3.85
AP2A1	15.96	17.55	19.36	16.26	15.59	16.86
AP2A2	2.31	2.41	2.4	2.28	3.04	2.65
AP2B1	63.17	46.23	47.36	51.72	49.02	48.09
AP2M1	226.79	172.11	164.1	186.17	186.98	190.48
AP2S1	210.76	233.28	224.8	214.19	215.74	240.97
AP3B1	13.86	16.7	15.94	16.2	16.74	18.32
AP3B2	0.25	0	0.13	0.01	0	0
AP3D1	7.94	6.47	5.83	6.39	7.5	5.99
AP3M1	16.74	15.36	15.59	14.94	16.63	16.75
AP3M2	9.92	11.23	10.33	9.1	9.09	6.89
AP3S1	42.68	54.5	46.52	44.84	51.41	51.26
AP3S2	9.54	8.43	8.11	7.66	8.27	8.68
AP4B1	4.45	8.86	6.95	7.14	5.5	8.85
AP4B1-AS1	1.83	1.6	1.23	0.66	1.56	0.86
AP4E1	1.96	1.3	1.61	0.93	1.01	0.99
AP4M1	6.95	6.38	4.76	8.44	6	5.78
AP4S1	8.09	3.53	3.44	4.04	5.07	2.63

AP5B1	6.6	7.83	6.26	6.39	6.1	6.93
AP5M1	11.89	13.19	11.98	8.73	8.39	10.4
AP5S1	9.29	12.54	11.72	12.51	9.58	11.16
AP5Z1	9.34	6.62	6.87	6.54	6.81	7.85
APAF1	2.17	3.12	2.58	1.93	2.6	2.79
APBA1	0	0	0.03	0	0	0
APBA2	12.01	7.17	6.7	11.15	7.66	9.24
APBA3	9.52	9.73	8.47	11.42	9.02	7.11
APBB1	14.34	9.55	12.15	12.83	13.43	12.4
APBB1IP	7.88	10.82	14.61	11.45	10.61	12.86
APBB2	1.44	2.76	2.98	2.12	1.85	2.37
APBB3	0.03	0	0.09	0	0	0
APC	3.91	2.48	2.31	2.64	2.38	1.88
APC2	0.01	0.02	0.01	0	0.04	0.07
APCDD1	0	0	0	0	0	0
APCDD1L	0	0	0	0	0	0
APCDD1L-AS	0	0	0	0	0	0
APCS	0	0	0	0	0	0
APEH	60.28	85.42	83.88	76.33	67.78	79.41
APEX1	175.89	294.02	256.01	252.02	227.37	239.62
APEX2	78.44	49.98	38.48	70.98	65.7	43.55
APH1A	32.05	38.67	42.54	45.01	37.53	48.41
APH1B	5.36	4.43	5.16	3.69	6.53	5.04
API5	36.75	35.63	31.91	26.16	31.51	31.91
APIP	29.75	33.28	31.96	24.82	32.05	22.42
APITD1	20.1	19.05	16.3	16.77	18.38	16.16
APITD1-COR	3.66	3.62	2.67	2.55	4.21	3.64
APLF	1.34	0.84	0.45	0.84	0.55	0.29
APLN	0.14	0.2	0.1	0.68	0.36	0.32
APLNR	0	0	0	0	0	0
APLP1	0.03	0.09	0.58	0.27	0.37	0.12
APLP2	50.98	54.66	34.47	39.86	37.72	49.03
APMAP	60.64	58.6	46.68	44.65	51.54	51.22
APOA1	0.35	0.34	0.24	0.3	0.08	0.7
APOA1BP	93.08	100.53	97.78	107.46	95.23	91.09
APOA2	0.18	0	0	0	0	0
APOA4	0	0	0	0	0	0
APOA5	0	0	0	0	0	0
APOB	0	0	0	0	0	0
APOBEC1	0	0	0	0	0	0
APOBEC2	0	0	0.17	0.03	0.09	0
APOBEC3A	1.7	1.26	1.95	0.56	0.99	1.34
APOBEC3A_	0	0	0	0	0	0

APOBEC3B	55.33	49.71	35.52	31.76	47.06	34.47
APOBEC3C	104.89	58.73	55.94	78.12	111.03	58.84
APOBEC3D	7.7	2.83	4.55	7.04	8.57	4.11
APOBEC3F	8.6	3.87	4.14	6.8	6.57	3.92
APOBEC3G	9.96	2.47	3.96	3.48	6.72	0.87
APOBEC3H	0.38	0	0	0	0	0
APOBEC4	0	0	0.05	0	0	0
APOBR	1.2	0.78	1.19	0.75	1.17	1.03
APOC1	2125.41	1.87	7.43	373.39	759.64	7.62
APOC1P1	14.61	0.56	0.51	2.73	4.34	0.15
APOC2	0.22	0.69	0	0.3	0	0.22
APOC3	0	0	0	0	0	0
APOC4	0.06	0.1	0.12	0.09	0.25	0
APOC4-APOC	0	0	0	0	0	0
APOD	1.01	0	0	0.09	0.13	0
APOE	288.5	1.73	3.18	64.96	152.48	1.43
APOF	0.05	0.1	0.12	0.07	0.03	0.09
APOH	0	0	0	0	0	0
APOL1	3.91	2.62	3.37	3.17	1.56	1.37
APOL2	14.21	10.01	11.05	11.51	11.2	8.86
APOL3	1.15	0.29	0.57	1.29	0.16	0
APOL4	7.91	10.56	11.71	8.53	7.09	7.9
APOL5	0	0	0	0	0	0
APOL6	1.34	1.6	1.86	1.23	1.16	1.54
APOLD1	0.25	0.59	0.37	0.3	0.46	0.34
APOM	3.62	3.14	1.98	3.03	3.46	2.71
APOO	39.09	38.88	35.29	39.22	44.26	32.63
APOOL	16.46	14.27	12.2	12.31	13.37	11.76
APOPT1	24.09	19.38	18.16	16.73	22.48	18.65
APP	35.03	43.14	39.22	35.48	33.26	43.26
APPBP2	1.78	2.86	1.93	2.15	2.89	2.21
APPL1	3.34	4.92	6.03	4.52	2.95	4.98
APPL2	1.13	0.6	1.01	0.74	1.27	0.52
APRT	426.12	391.95	368.78	418.87	466.52	403.6
APTX	23.38	27.67	22.54	20.74	21.54	25.4
AQP1	0.84	0	0	0	0	0.24
AQP10	0.37	0	0	0.51	0.51	0
AQP11	0.17	0.65	1.32	1.06	1.13	1.32
AQP12A	0	0	0	0	0	0
AQP12B	0	0	0	0	0	0
AQP2	1.49	0.03	0.05	0.2	1.37	0.18
AQP3	121.98	7.21	9.24	44.69	75.28	11.3
AQP4	0	0	0	0	0	0

AQP4-AS1	0.42	0.25	0.44	0.06	0.46	0.53
AQP5	0	0	0	0	0	0
AQP6	0.94	0.91	1.13	0.62	0.62	1.5
AQP7	0	0	0	0	0	0
AQP7P1	0	0	0	0.11	0	0
AQP7P3	0	0	0	0	0	0
AQP8	0	0.27	0	0	0	0
AQP9	0.1	0	0	0	0	0
AQPEP	0	0	0	0	0	0
AQR	10.72	13.52	11.85	10.35	11.91	10.22
AR	7.52	4.52	5.23	3.71	4	4.43
ARAF	41.89	50.77	59.17	56.08	48.72	54.55
ARAP1	7.1	5.65	6.43	8.02	9.11	7.16
ARAP2	0	0.07	0.2	0.12	0.21	0.18
ARAP3	0.58	0.03	0.04	0.02	0.08	0.21
ARC	0.1	0.35	0.59	0.46	0.09	0.48
ARCN1	29.15	28.03	28.03	25.08	25.36	27.87
AREG	0.67	0	0.22	0.51	0.41	0
ARF1	417.8	347.14	305.56	317.03	362.61	309.48
ARF3	26.02	32.43	38.32	32.47	31.63	38.55
ARF4	113.22	68.99	61.35	79.11	83.74	65.2
ARF5	127.35	119.64	83.21	111.48	114.95	92.43
ARF6	14.44	9.37	10.78	8.38	10.78	9.33
ARFGAP1	14.15	13.15	12.97	14.32	12.92	12.64
ARFGAP2	34.65	36.8	36.05	41.11	36.26	38.98
ARFGAP3	10.93	8.2	6.51	7.97	5.87	6.62
ARFGEF1	4.07	4.13	4.69	3.31	3.37	5
ARFGEF2	2.69	3.71	2.31	2.04	2.58	3
ARFIP1	6.74	6.16	6.23	4.41	7.11	5.21
ARFIP2	16.28	11.69	10.97	12.49	12.27	11.01
ARFRP1	18.15	16.88	14.1	14.69	16.74	15.26
ARG1	0.2	0.12	0	0.04	0.1	0.05
ARG2	10.02	18.26	21.56	17.71	16.05	21.14
ARGFX	1.24	1.56	1.49	0.96	0.94	0.8
ARGFXP2	0.13	0.15	0	0	0.12	0
ARGLU1	23.34	28.93	18.45	22.42	27.38	19.19
ARHGAP1	42.6	25.04	26.6	29.15	31.52	24.41
ARHGAP10	1.74	3.36	2.49	2.03	0.94	2.32
ARHGAP11A	12.34	16.6	15.84	12.68	11.38	14.61
ARHGAP11B	3.15	5.71	1.99	1.61	1.52	4.16
ARHGAP12	1.33	1.7	1.25	0.66	1.4	1.87
ARHGAP15	34.38	45.99	40.09	37.44	48.04	41.82
ARHGAP17	13.95	16.83	14.97	12.35	13.32	18.08

ARHGAP18	28.65	2.93	3.4	9.79	12.47	2.52
ARHGAP19	4.37	7.12	4.47	3.94	5.22	3.61
ARHGAP19-S	6.73	2.3	5.93	4.77	4.59	5.49
ARHGAP20	0	0	0	0	0	0
ARHGAP21	2.89	2.36	1.6	1.64	1.41	1.57
ARHGAP22	0.53	1.89	1.63	1.33	1.15	0.95
ARHGAP23	0.34	0.83	0.68	0.44	0.91	0.76
ARHGAP24	0	0.01	0.09	0	0	0
ARHGAP25	9.07	6.96	9.18	9.29	7.47	6.43
ARHGAP26	0.34	0.53	0.3	0.44	0.3	0.65
ARHGAP26-A	0	0	0	0	0	0
ARHGAP27	1.27	5.01	4.7	2.91	2.48	5.23
ARHGAP28	0	0	0	0	0	0
ARHGAP29	0	0	0	0	0	0
ARHGAP30	1.72	7.05	7.8	7.93	7.13	9.19
ARHGAP31	1.38	1.28	0.83	0.87	0.6	0.87
ARHGAP32	1.24	1.32	1.1	0.87	1.03	1.27
ARHGAP33	1.34	3.21	1.59	1.92	2	1.53
ARHGAP35	4.03	3.63	4.08	4.22	3.99	4.77
ARHGAP36	0	0	0	0	0	0
ARHGAP39	0	0	0.01	0	0	0.06
ARHGAP4	43.99	62.41	54.12	52.92	52.16	51.56
ARHGAP40	0	0	0	0	0	0
ARHGAP42	0	0.18	0.1	0	0.1	0.02
ARHGAP44	0.25	0.51	0.23	0.16	0.22	0.38
ARHGAP5	2.26	2.97	3.17	2.03	2.85	2.72
ARHGAP5-AS	1.18	1.54	0.76	0.81	0.5	0.6
ARHGAP6	1.23	0	0	0.29	0.42	0
ARHGAP8	0	0.04	0.08	0	0	0
ARHGAP9	4.85	29.67	31.7	23.2	17.98	19.91
ARHGDIA	34.57	42.25	43.89	42.08	39.86	40.97
ARHGDIB	239.27	549.91	506.7	463.33	451.02	478.37
ARHGDIG	1.64	0.06	2.03	3.63	2.41	1.24
ARHGEF1	12.24	11.66	15.69	11.27	11.85	11.97
ARHGEF10	1.34	2.24	1.89	1.31	2.26	3.12
ARHGEF10L	0	0	0	0	0	0
ARHGEF11	1.56	1.5	0.75	0.59	0.85	1.26
ARHGEF12	1.68	0.05	0.08	0.35	0.81	0.14
ARHGEF15	0	0	0	0	0	0
ARHGEF16	0.63	0.16	0.23	0.16	0.38	0.74
ARHGEF17	0.29	0.55	0.67	0.28	0.14	1.04
ARHGEF18	2.13	5.22	3.78	2.87	3.97	3.88
ARHGEF19	0.96	1.95	2.31	2.02	1.61	2.92

ARHGEF2	15.27	23.15	22.99	20.27	22.96	18.23
ARHGEF25	0	0	0	0.05	0	0
ARHGEF26	1.37	2.18	2.46	1.8	2.43	2.6
ARHGEF26-A	3.25	4.57	3.74	2.63	2.74	3.85
ARHGEF28	0	0	0	0	0	0.03
ARHGEF3	0.46	1.72	2.44	1.34	1.58	2.64
ARHGEF3-AS	0	0	0	0	0	0
ARHGEF33	0.03	0.11	0.03	0	0	0.03
ARHGEF35	0.1	0.03	0	0	0.03	0.07
ARHGEF37	0	0	0	0	0	0
ARHGEF38	0	0.01	0.01	0	0	0
ARHGEF38-I	0	0	0	0	0	0
ARHGEF39	7.22	10.5	8.45	7.02	8.16	7.81
ARHGEF4	0	0.03	0	0	0	0.02
ARHGEF40	0.59	0.69	0.44	0.79	0.82	0.19
ARHGEF5	0.27	0.15	0.41	0.09	0.13	0
ARHGEF6	34.17	41.21	40.04	40.21	35.78	53.63
ARHGEF7	10.17	7.52	5.73	6.67	7.68	6.28
ARHGEF9	3.79	3.26	3.28	4.22	3.39	4.14
ARID1A	7.18	8.7	7.77	6.07	8.81	6.89
ARID1B	2.91	2.38	2.72	2.54	2.6	2.69
ARID2	5.84	6.45	5.68	4.6	6.15	6.21
ARID3A	1.35	1.39	2.19	1.16	1.72	1.71
ARID3B	10.63	11.48	11.56	10.1	8.97	9.77
ARID3C	0	0	0	0	0	0
ARID4A	2.78	2.59	2.55	2.18	2.22	2.53
ARID4B	5.17	5.59	3.26	4.32	4.22	4.4
ARID5A	5.97	12.27	10.54	10.09	8.69	9.32
ARID5B	4.46	5.44	8.24	13.17	8.61	8.13
ARIH1	9.36	7.8	6.28	6.44	7.58	7.31
ARIH2	26.66	22.11	16.72	21.89	24.64	19.18
ARIH2OS	2.34	2.01	3.46	1.37	1.35	1.68
ARL1	14.51	13.37	13.32	10.19	14.43	11.89
ARL10	0	0	0.14	0	0	0.06
ARL11	1.41	5.43	4.83	3.08	2.92	4.98
ARL13A	0	0	0	0	0	0.14
ARL13B	2.38	2.03	1.1	1.57	1.61	1.27
ARL14	0	0	0	0	0	0
ARL14EP	11.42	12.31	10.09	11.88	12.68	11.54
ARL14EPL	0	0	0	0	0	0
ARL15	28.55	4.37	7.07	8.13	17.09	8.8
ARL16	25.22	31.73	32.82	25	30.6	24.79
ARL17A	1.65	3.72	2.81	2.3	1.98	3.19

ARL17B	2.51	1.85	3.16	3.18	3.52	3.26
ARL2	88.36	80.59	94.18	104.45	118.12	82.82
ARL2-SNX15	0	0	0	0	0	0
ARL2BP	33.64	26.11	23.85	17.96	24.43	23.41
ARL3	5.84	3.2	3.44	3.32	5.18	2.97
ARL4A	22.69	7.59	5.24	8.92	10.55	4
ARL4C	0	0.06	0	0	0	0
ARL4D	1.04	4.64	4.92	4.15	2.49	2.95
ARL5A	5.83	5.47	4.71	3.51	5.44	7.16
ARL5B	2.51	1.78	1.93	2.39	2.29	2.37
ARL5C	0	0	0	0	0	0
ARL6	1.06	0.7	0.49	0.31	0.58	1.09
ARL6IP1	167.48	108.52	104.22	117.53	119.25	97.56
ARL6IP4	156.59	172.35	172.58	177.81	195.56	176.82
ARL6IP5	33.67	38.42	34.97	27.29	31.09	28.87
ARL6IP6	9.28	8.52	7.78	6.41	8.46	9.31
ARL8A	0.7	0.27	0.87	0.51	0.62	0.71
ARL8B	50.49	31.85	27.06	29.6	37.68	27.41
ARL9	0	0	0	0	0	0
ARMC1	29.71	31.07	27.15	28.75	29.08	29.58
ARMC10	5.82	6.32	6.94	5.95	6.88	2.81
ARMC12	0	0	0	0.27	0.49	0
ARMC2	0.09	0.45	0.53	0.18	0.38	0.28
ARMC3	0	0	0	0	0	0
ARMC4	0.08	0.51	0.37	0.84	0.13	0.16
ARMC5	2.04	3.31	2.66	2.1	2.39	3.31
ARMC6	49.97	57.75	60.05	55.86	59.96	50.93
ARMC7	6.76	6.28	6.57	6.63	8.54	6.77
ARMC8	15.73	12.16	11.11	11	15.47	12.65
ARMC9	2.1	5.19	3.54	2.37	2.53	2.66
ARMCX1	0	0	0	0	0	0
ARMCX2	0	0	0	0	0	0
ARMCX3	15.21	11.56	9.24	10.6	10.96	10.66
ARMCX4	0.01	0.05	0	0	0	0.03
ARMCX5	10.91	14.67	10.38	13.26	12.19	12.57
ARMCX5-GPI	2.29	2.5	1.45	3.13	1.55	2.43
ARMCX6	57.1	38.06	28.57	43.8	45.58	32.2
ARMS2	0	0	0	0	0	0
ARNT	10.54	9.03	8.68	8.66	9.99	8.38
ARNT2	0	0	0	0	0	0
ARNTL	2.57	3.33	3.84	3.55	4.44	3.43
ARNTL2	1.12	1.3	0.88	0.9	1.14	1.28
ARPC1A	103.05	94.09	102.04	114.37	121.33	94.97

ARPC1B	189.39	184.65	167.7	142.12	153.35	154.59
ARPC2	199.42	313.13	284.28	247.5	242.17	283.26
ARPC3	384.5	402.68	343.05	342.31	406.83	399.3
ARPC4	105.34	110.99	98.32	95.05	108.68	97.79
ARPC4-TTLL3	1.08	1.32	0.81	0.53	0.73	1.48
ARPC5	63.27	53.53	47.08	30.73	43.49	48.29
ARPC5L	45.02	43.17	44.99	38.79	41.82	36.8
ARPP19	22.04	17.37	14.76	14.3	17.42	18.01
ARPP21	0	0	0	0	0	0
ARR3	0.06	0	0	0	0	0
ARRB1	13.48	8.93	11.95	12.51	12.09	13.58
ARRB2	2.94	4.08	8.06	7.42	5.44	8.78
ARRDC1	17.42	33.76	26.93	23.72	24.88	25.79
ARRDC2	3.86	3.8	2.15	3.11	3.7	2.81
ARRDC3	3.65	3.76	3.95	3.84	3.07	3.5
ARRDC3-AS1	1.93	1.96	1.24	0.9	1.43	1.33
ARRDC4	0.71	0.08	0.24	0.2	0.32	0.63
ARRDC5	0	0	0.04	0	0	0
ARSA	4.14	5.02	4.39	5.42	3.59	4
ARSB	5.38	10.65	8.58	5.49	7.21	7.25
ARSD	3.57	2.24	2.15	2.75	2	2.52
ARSE	0	0	0	0	0	0
ARSF	0.03	0.03	0	0	0	0
ARSG	0.47	0.25	0	0.18	0.08	0.35
ARSH	0	0	0	0	0	0
ARSI	0	0	0	0	0	0
ARSJ	0	0	0	0	0	0
ARSK	2.67	2.64	2.25	1.6	2.12	2.35
ART1	0	0	0	0	0	0
ART3	0	0	0	0	0	0
ART4	0.82	0	0	0.68	0.1	0.17
ART5	0.3	0.33	0.11	0.79	0.06	0.25
ARTN	0	0	0	0	0	0
ARV1	32.31	27.1	23.62	27.85	28.53	22.37
ARVCF	1.06	0.51	0.76	0.57	0.51	0.45
ARX	1.6	0.9	1.02	1.03	1.22	1.21
AS3MT	0	0	0.97	0.91	0.93	0
ASAH1	177.46	49.61	45.34	56.42	82.46	41.34
ASAH2	0.24	0	0.08	0.14	0.04	0.09
ASAH2B	2.4	0.78	1.17	0.64	1.03	0.78
ASAP1	3.82	1.99	1.78	1.8	2.86	1.52
ASAP1-IT1	0.26	0	0	0	0	0
ASAP2	0.31	0.54	0.63	0.44	0.52	1.12

ASAP3	0.92	1.6	0.64	2.22	1.75	1.63
ASB1	2.18	2.04	1.53	1.49	1.88	1.79
ASB10	0	0	0	0	0	0
ASB11	0.2	0.86	1.49	0.63	0.5	0.61
ASB12	0.18	0.74	0.27	0	1.05	0.65
ASB13	18.67	28.26	27.29	26.54	22.52	30.12
ASB14	0	0	0.09	0.03	0	0.07
ASB15	0	0	0	0	0	0
ASB16	0.22	0.54	0.19	0.29	0.36	0.43
ASB16-AS1	6.5	6.16	7.57	5.64	4.49	4.07
ASB17	0	0	0	0	0	0
ASB18	0	0	0	0.04	0	0
ASB2	0	0.21	0.1	0.04	0	0.19
ASB3	5.09	5.1	3.2	5.75	3.89	4.69
ASB4	0	0.07	0.09	0.03	0.11	0.04
ASB5	0	0	0	0	0	0
ASB6	7.89	9.26	9.29	7.87	9.17	7.76
ASB7	4.23	4.08	2.17	3.8	2.29	3.53
ASB8	18.41	20.23	13.74	18.68	17.06	16.23
ASB9	0.44	5.43	11.16	10.66	11.11	5.9
ASB9P1	0.14	0.18	0.26	0	0	0
ASCC1	11.92	10.97	11.55	10.99	10.92	12.4
ASCC2	29.56	27.32	22.17	26.1	22.62	23.97
ASCC3	5.65	6.39	5.63	5.13	6.16	5.37
ASCL1	0	0	0	0	0	0
ASCL2	0	0	0.04	0	0.04	0.04
ASCL3	0	0	0	0	0	0
ASCL4	0	0	0	0	0	0
ASCL5	0	0	0	0	0	0
ASF1A	19.84	15.35	13.6	12.88	16.05	14.4
ASF1B	41.8	81.21	65.85	65.7	60.5	67.26
ASGR1	0.05	0	0.32	0.41	0.05	0
ASGR2	0	0	0	0	0	0
ASH1L	2.48	2.18	2.56	2.12	2.14	1.95
ASH1L-AS1	1.47	0.84	0.62	0.84	1.61	1.48
ASH2L	79.01	68.25	53.65	89.39	75.12	74.51
ASIC1	0.69	2.1	1.74	2.25	1.05	2.3
ASIC2	0	0	0.19	0.23	0.02	0
ASIC3	0.03	0.08	0.21	0	0.03	0
ASIC4	1.9	0.02	0.09	0.32	0.63	0.16
ASIC5	0	0	0	0	0	0
ASIP	0.14	0.55	0.51	0.1	0.4	0.28
ASL	15.06	22.14	18.37	19.47	19.8	17.53

ASMT	0	0	0	0	0.04	0
ASMTL	63.96	55.93	66.04	68.86	51.31	42.84
ASMTL-AS1	0.33	0.55	0.72	0.15	0.28	0.78
ASNA1	90.56	72.48	63.84	77.74	78.22	65.37
ASNS	44.7	14.99	16.28	21.98	22.59	18.1
ASNSD1	22.14	22.76	20.32	18.59	23.1	20.96
ASPA	0	0	0	0	0	0
ASPDH	0	0	0	0	0	0
ASPG	0	0	0.07	0	0	0
ASPH	10.44	9.21	7.8	5.31	8.88	9.9
ASPHD1	7.46	3.72	6.92	5.63	2.92	3.9
ASPHD2	0.55	0.88	0.34	0.62	0.68	0.64
ASPM	7.04	7.71	6.68	6.29	6.5	6.08
ASPN	0	0	0.15	0.07	0	0.13
ASPRV1	0.17	0.05	0	0.14	0.29	0
ASPSCR1	4.1	6.55	4.82	8.61	7.67	5.29
ASRGL1	0	0	0	0	0	0
ASS1	0.62	0.29	0.61	0.4	0.63	0.55
ASTE1	4.48	5.5	8.65	6.39	5.65	4.36
ASTL	0	0.05	0	0	0	0
ASTN1	0.04	0	0	0	0	0
ASTN2	16.03	16.88	18.64	12.73	14.27	16.24
ASUN	22.35	27.41	33.49	26.17	28.08	30.52
ASXL1	2.72	3.77	3.31	3.45	3.16	3.57
ASXL2	4.61	4.21	3.59	3.08	3.02	3.36
ASXL3	0	0	0	0	0	0
ASZ1	0.19	0.11	0.21	0.22	0.14	0
ATAD1	16.77	15	15.64	12.75	16.27	14.32
ATAD2	17.41	21.66	17.27	14.64	19.33	19.05
ATAD2B	2.7	1.98	2.27	1.9	1.98	2.44
ATAD3A	10.41	16.05	14.58	14.18	11.42	12.09
ATAD3B	10.62	13.28	12.43	12.09	11.99	8.28
ATAD3C	0.57	0.42	0.59	0.33	0.73	0.42
ATAD5	4.96	6.42	5.56	4.83	4.57	5.31
ATAT1	4.33	4.13	3.7	3.73	2.77	5.33
ATCAY	1.34	1.33	1.8	1.35	1.49	1.59
ATE1	5.2	5.9	5.73	5.39	5.6	5.23
ATF1	6.11	4.95	6	3.04	4.47	4.76
ATF2	13.34	11.9	8.14	9.23	11.52	11.01
ATF3	1.89	1.09	1.13	0.37	0.87	2.08
ATF4	225.85	207.86	156.97	148.66	181.13	179.69
ATF5	3.15	3.83	2.82	2.79	4.13	2.91
ATF6	10.25	9.27	8.94	7.97	8.99	8.68

ATF6B	47.41	54.23	45.8	42.52	41.59	47.96
ATF7	13.46	13.62	14.26	11.91	10.73	10.5
ATF7IP	25.78	28.23	31.54	28.66	27.2	26.31
ATF7IP2	7.53	2.78	2.78	3.73	3.29	2.12
ATG10	3.47	2.55	2.07	2.78	3.92	4.13
ATG12	11	10.66	7.12	6.47	9.07	6.8
ATG13	19.14	12.79	11.41	10.79	11.5	15.19
ATG14	2.21	2.49	1.76	1.62	1.96	2.31
ATG16L1	6.24	3.8	4.61	2.81	3.56	2.17
ATG16L2	1.44	1.81	0.91	0.99	0.78	0.73
ATG2A	3.85	2.65	3.15	2.17	2.98	3.36
ATG2B	1.61	2.01	0.95	1.19	1.47	1.58
ATG3	81.92	80.12	80.63	70.27	75.94	70.83
ATG4A	13.74	7.15	4.71	5.59	7.67	3.44
ATG4B	23.49	17.74	15.73	18.15	21.22	16.78
ATG4C	3.94	3.59	4.71	3.17	2.99	3.19
ATG4D	2.22	1.46	1.25	1.24	1.74	1.12
ATG5	11.66	11.14	8.81	8.59	11.57	11.37
ATG7	5.98	7.94	7.77	7.2	5.13	5.74
ATG9A	32.82	17.34	17.25	22.21	22.5	18.17
ATG9B	0	0.2	0	0	0.04	0.02
ATHL1	1.56	1.05	0.59	1.99	0.73	1.4
ATIC	95.47	89.59	90.85	97.02	95.51	92.37
ATL1	1	1.31	1.43	0.82	0.5	1.21
ATL2	11.11	10.59	8.6	8.34	9.43	9.34
ATL3	10.66	10.07	8.39	9.49	8	12.76
ATM	0.95	1.18	0.61	0.63	0.51	1.12
ATMIN	3.57	2.45	2.5	2.29	1.9	3
ATN1	5.69	2.83	3.25	3.3	4.71	3.52
ATOH1	0	0.51	0	0.15	0	0
ATOH7	0	0	0	0	0	0
ATOH8	0	0	0	0	0	0
ATOX1	125.63	182.87	161.13	152.12	166.18	137.04
ATP10A	0.03	0.64	0.35	0.75	0.57	0.68
ATP10B	0	0	0	0	0	0
ATP10D	0.91	0.56	0.64	0.14	0.27	0.48
ATP11A	2.85	2.77	1.28	1.55	1.74	1.98
ATP11B	1.18	0.49	0.71	0.48	1.07	0.23
ATP11C	7.29	5.56	4.15	5.05	6.34	5.68
ATP12A	0.1	0	0	0	0	0
ATP13A1	11.14	11.58	13.15	11.97	10.22	11.41
ATP13A2	4.82	3.73	4.14	3.99	5.08	2.63
ATP13A3	2.64	2.18	1.13	0.84	1.61	2.38

ATP13A4	0	0	0	0	0	0
ATP13A5	0	0	0	0	0	0
ATP1A1	31.4	31.28	31.63	29.85	30.89	35.59
ATP1A1OS	0.29	0.53	0.87	0.78	0.79	1.97
ATP1A2	0.08	0.09	0.15	0.04	0.09	0.02
ATP1A3	4.09	9.04	8.63	7.36	5.22	8.32
ATP1A4	0	0	0	0	0	0
ATP1B1	12.87	3.48	7.12	5.67	7.4	4.77
ATP1B2	0.55	0.4	0.49	0.39	0.39	0.34
ATP1B3	237.47	165.8	152.97	151.94	159.92	154.14
ATP1B4	1.45	0.61	0.43	0.46	0.68	0.34
ATP2A1	0.02	0	0	0.03	0.12	0.04
ATP2A2	4.04	3.63	2.28	2.81	1.74	2.7
ATP2A3	3.82	9.61	7.78	8.97	6.3	9.16
ATP2B1	2.61	2.98	1.63	2.11	3.09	2.16
ATP2B2	0	0	0	0	0	0
ATP2B3	0	0	0	0	0	0.06
ATP2B4	1.37	4.09	3.24	1.64	2.05	4.69
ATP2C1	18.55	16.44	21.85	18.54	16.78	23.85
ATP2C2	0.06	0	0	0	0	0.02
ATP4A	0	0	0	0	0	0
ATP4B	0	0	0	0	0	0
ATP5A1	447.79	480.72	413.1	459.96	480.72	467.77
ATP5B	808.88	743.6	721.01	784.99	771.75	767.86
ATP5C1	301.65	307.63	284.78	303.87	319.83	291.38
ATP5D	339.25	332.83	407.11	403.89	376.88	318.62
ATP5E	647.95	541.92	415.6	449.15	624.2	436.65
ATP5EP2	15.16	8	5.11	7.16	10.03	6.54
ATP5F1	175.07	174.65	173.73	166.42	180.41	167.59
ATP5G1	360.99	492.6	469.06	497.1	487.77	445.58
ATP5G2	651.89	690.58	703.94	847.26	811.96	746.6
ATP5G3	274.82	290.32	262.79	261.04	307.65	289.58
ATP5H	232.98	241.33	229.95	232.74	267.14	238.06
ATP5I	532.62	430.55	373.23	290.02	557.82	213.28
ATP5J	274.72	254.84	240.73	231.68	278.12	253.81
ATP5J2	410.6	420.87	326.21	353.23	444.07	364.45
ATP5J2-PTCD	0	0	0	0	0	0
ATP5L	184.68	197.07	154.62	154.43	193.76	171.1
ATP5L2	0.2	0.33	0.69	0	0.19	0.21
ATP5O	396.72	418.44	424.54	466.87	485.2	436.06
ATP5S	6.91	5.49	6.17	5.52	6.95	4.12
ATP5SL	31.76	35.71	32.29	34.19	34.29	32.41
ATP6AP1	161.84	134.36	115.15	126.01	145.75	129.61

ATP6AP1L	0.1	0.66	0.57	0.07	0	0
ATP6AP2	105.17	107.75	90.99	79.28	88.85	88.81
ATP6V0A1	25.17	12.08	11.13	15.25	17.12	12.42
ATP6V0A2	3.41	1.82	1.72	2.53	2.95	2.3
ATP6V0A4	0	0	0	0	0	0
ATP6V0B	390.85	436.28	371.86	327.23	391.29	373.85
ATP6V0C	45.84	39.32	52.06	54.25	56.42	50.33
ATP6V0CP3	0.13	0	0	0	0	0
ATP6V0D1	135.46	111.69	112.12	109.76	130.93	94.17
ATP6V0D2	0.29	0.32	0.35	0.19	0.27	0.19
ATP6V0E1	205.35	168.63	156.43	143.55	191.7	147.69
ATP6V0E2	24.82	27.09	23.97	25.4	26.24	24.33
ATP6V0E2-A:	0.24	0.09	0.17	0.2	0.13	0.29
ATP6V1A	25.01	13.8	14.29	14.33	15.7	13.11
ATP6V1B1	0	0.15	0	0.13	0.07	0
ATP6V1B2	131.34	99.28	76.21	75.65	90.44	81.53
ATP6V1C1	26.28	18.14	16.65	15.35	18.01	15.25
ATP6V1C2	0.08	0.11	0	0.34	0.03	0.14
ATP6V1D	24.49	16.77	14.52	15.11	22.7	12.92
ATP6V1E1	116.95	60.58	52.2	62.22	73.67	47.49
ATP6V1E2	0.26	0.29	0.34	0.13	0.21	0.49
ATP6V1F	264.95	236.99	216.32	232.93	276.69	234.87
ATP6V1G1	82.68	54.9	36.12	39.44	53.88	38.17
ATP6V1G2	0.52	0.76	0.47	0.42	0	0.1
ATP6V1G2-D	0	0	0	0	0	0
ATP6V1G3	0	0	0	0	0	0
ATP6V1H	86.88	68.13	66.68	57.14	68.65	59.15
ATP7A	6.11	0.87	0.73	1.99	2.9	1.18
ATP7B	2.46	0.99	1.28	1.81	1.96	1.4
ATP8A1	0.62	0.87	0.92	0.67	0.53	1.36
ATP8A2	2.02	0.01	0	0.76	0.69	0.15
ATP8B1	0.61	0.24	0.16	0.12	0.15	0.2
ATP8B2	1.66	1.47	1.85	1.96	2.3	2.35
ATP8B3	0.48	1.57	1.67	0.66	0.88	1.48
ATP8B4	1.21	6.98	4.99	3.54	2.71	6.13
ATP8B5P	0.06	0	0.03	0	0.08	0.08
ATP9A	0.77	0.86	0.37	0.76	0.63	0.96
ATP9B	3.4	3.42	2.89	3.23	3.18	3.54
ATPAF1	3.9	3.57	3.26	3.4	4.66	3.24
ATPAF2	14.98	21.79	22.53	21.37	18.16	23.77
ATPBD4	11.3	7.74	6.38	10.37	11.79	8.31
ATPBD4-AS1	0	0	0	0	0	0
ATPIF1	366.48	343.98	298.64	343.59	407.67	295.63

ATR	2	3.3	3.13	2.21	2.06	3.25
ATRAID	57.56	99.96	88.35	86.36	93.47	80.84
ATRIP	2.42	3.67	2.6	1.85	2.61	3.14
ATRN	2.61	3.4	2.88	3.12	2.94	2.12
ATRNL1	0.18	0	0	0.09	0	0
ATRX	10.14	9.3	7.72	7.8	9.28	9.08
ATXN1	1.08	0.35	0.35	0.48	0.53	0.18
ATXN10	47.81	49.75	46.96	46.96	48.69	43.18
ATXN1L	2.85	2.18	2.26	1.71	2.35	2.37
ATXN2	16.61	11.77	11.38	14.14	13.95	10.43
ATXN2L	27.54	23.15	21.36	21.06	22.13	17.88
ATXN3	2.88	2.87	2.93	2.38	2.58	2.41
ATXN3L	0	0	0	0	0	0
ATXN7	1.26	0.87	0.94	0.78	1.01	1.1
ATXN7L1	4.02	3.84	3.24	3.47	3.97	3.33
ATXN7L2	5.27	5.68	3.81	4.79	3.69	4.74
ATXN7L3	4.38	3.8	2.96	5.45	4.61	4.84
ATXN7L3B	26.83	23.44	23.21	27.77	29.55	25.29
ATXN8OS	0	0.08	0	0.04	0	0
AUH	3.34	3.74	3.74	4.02	3.78	4.51
AUNIP	12.96	14.11	13.14	15.47	12.27	13.36
AUP1	144.35	166.16	187.08	155.75	162.51	160.97
AURKA	57.89	61.24	49.91	47.05	57.45	50.75
AURKAIP1	218.74	219.59	209.3	219.51	234.41	195.64
AURKAPS1	0	0	0.49	0.07	0.1	0.66
AURKB	81.84	95.84	90.96	95.23	94.28	82.28
AURKC	0	0	0	0	0	0
AUTS2	0	0.01	0.01	0.02	0.04	0
AVEN	1.32	1.27	0.04	1.72	0.83	0.44
AVIL	0.27	0	0.11	0.07	0.05	0.02
AVL9	19.98	16.35	13.35	13.84	15.89	16.88
AVP	0.52	0.21	0	0.09	0.26	0.26
AVPI1	16.21	10.61	10.24	10.99	13.64	7.48
AVPR1A	0.04	0.08	0.01	0.02	0.02	0.05
AVPR1B	0.08	0.13	0.42	0.69	0	0.17
AVPR2	0.56	0.55	0.36	0.21	0.33	0.05
AWAT1	0	0	0	0	0	0
AWAT2	0	0	0	0	0	0
AXDND1	0	0	0	0	0	0
AXIN1	4.94	3.68	2.55	3.64	4.46	3.87
AXIN2	0.03	0.09	0.17	0.01	0.06	0.73
AXL	16.72	1	1.68	13.35	11.26	1.66
AZGP1	0	0	0	0	0	0

AZGP1P1	0	0	0	0	0	0
AZI1	2.18	2.98	2.4	2.9	3	4.99
AZI2	12.55	11.16	8.1	6.59	9.72	11.27
AZIN1	46.12	37.06	37.68	34.21	40.64	45.18
AZU1	3.92	51.25	9.96	0	4.6	85.5
B2M	577.81	810.52	706.5	646.59	691.55	711.51
B3GALNT1	0.02	0.05	0	0.02	0	0.07
B3GALNT2	5.32	9.33	6.95	5.65	5.91	6.1
B3GALT1	0	0	0	0	0	0
B3GALT2	0.08	0.03	0.13	0	0.06	0.08
B3GALT4	0.75	0.21	0.4	0.79	0.46	1.03
B3GALT5	0	0	0	0	0	0
B3GALT6	3.95	3.48	3.71	4.53	3.88	4.58
B3GALTL	1.2	1.07	0.74	0.73	1.34	1.15
B3GAT1	0	0	0	0	0.02	0.02
B3GAT2	0	0.03	0	0	0	0
B3GAT3	31.76	28.07	20.54	25.41	27.6	21.91
B3GNT1	7.02	14.88	9.23	9.79	11.78	12.66
B3GNT2	12.69	15.26	18.37	15.56	16.83	10.73
B3GNT3	0	0	0.02	0	0.13	0.11
B3GNT4	0	0.35	1	0.07	0.19	0.1
B3GNT5	3.79	3.58	3.52	3.32	2.93	4.73
B3GNT6	0.27	0.36	0.2	0.39	0.22	0.52
B3GNT7	0.19	0.08	0.07	0.07	0.05	0.1
B3GNT8	1.82	4.08	4.56	5.01	3.29	7.27
B3GNT9	0.14	0.11	0	0.02	0	0.46
B3GNTL1	16.59	39.07	33.16	26.78	30.92	26.84
B4GALNT1	6.64	8.04	7.32	4.26	6.47	8.41
B4GALNT2	0	0	0	0	0	0
B4GALNT3	0.3	0.15	0.15	0.04	0.14	0.62
B4GALNT4	0.04	0.15	0	0.32	0.5	0.55
B4GALT1	25.93	14.61	14.18	17.2	17.78	14.99
B4GALT2	6.38	5.84	7.81	5.48	5.05	5.48
B4GALT3	27.21	21.94	19.95	22.03	22.56	21.95
B4GALT4	4.05	4.68	2.77	2.18	3.09	2.47
B4GALT5	2.86	3.72	3.21	3.18	2.49	3.78
B4GALT6	0.22	0.08	0.51	0.26	0.14	0.38
B4GALT7	11.84	12.11	11.82	15.3	10.13	12.24
B9D1	19.9	10.32	9.78	21.9	19.29	10.89
B9D2	10.7	7.39	7.53	7.41	9.31	10.69
BAALC	0.72	4.1	5.46	2.28	1.63	0.83
BAAT	0.09	0.02	0	0	0	0.02
BABAM1	112.94	92.36	86.07	89.28	94.4	81.52

BACE1	2.19	2.36	2.34	1.41	2.57	3.31
BACE1-AS	0.5	0.9	1.93	0.96	2.02	1.83
BACE2	0	0	0	0.15	0.07	0
BACH1	5.13	4.55	3.96	4.76	3.5	4.53
BACH2	0.02	0	0	0	0	0
BAD	21.09	17.33	18.95	23.49	20.74	20.09
BAG1	6.87	7.82	6.24	7.57	5.84	8.02
BAG2	19.93	20.86	24.2	17.9	19.04	16.3
BAG3	16.53	5.43	6.76	6.86	9.39	5.25
BAG4	25.38	24.51	26	24.06	24.62	27.84
BAG5	9.21	7.79	8.39	6.55	7.6	7.85
BAG6	28	19.39	21.53	23.33	23.7	23.83
BAGE	0	0	0	0	0	0
BAGE2	0	0	0	0	0	0
BAGE3	0	0	0	0	0	0
BAGE4	0	0	0	0	0	0
BAGE5	0	0	0	0	0	0
BAHCC1	0.06	0.13	0.21	0.11	0.07	0.08
BAHD1	5.09	5.53	5.54	6.63	4.03	5.38
BAI1	0.13	1.79	1.37	1.04	0.92	1.56
BAI2	0	0.07	0.04	0.21	0.05	0.46
BAI3	0	0	0	0.05	0.01	0
BAIAP2	14.02	11.96	17.38	19.05	13.76	12.83
BAIAP2-AS1	1.18	1.85	2.39	1.78	1.48	3.07
BAIAP2L1	1.96	0.78	0.24	0.65	2.67	1.08
BAIAP2L2	0	0.03	0	0	0	0
BAIAP3	0.42	0.21	0	0.04	0.18	0.21
BAK1	31.4	36.51	41.88	36.2	37.93	32.5
BAMBI	1.68	0.78	1.97	1.22	1.63	0.57
BANCR	0	0	0	0	0.11	0
BANF1	192.54	220.04	196.76	219.47	226.96	190.02
BANF2	0	0	0	0	0	0
BANK1	0	0	0.14	0	0	0
BANP	5.99	5.84	8.54	8.05	6.67	6.18
BAP1	9.17	9.58	7.01	7.25	8.82	7.83
BARD1	3.44	5.82	7.65	5.34	5.81	6.74
BARHL1	0	0	0	0	0	0
BARHL2	0	0	0	0	0	0
BARX1	0	0.24	0.19	0.03	0	0.51
BARX2	0	0	0	0	0	0
BASP1	0	0	1.19	0	0.08	1.98
BASP1P1	0	0	0	0	0	0
BATF	13.55	43.8	39.65	30.4	27.04	32.18

BATF2	0.14	0.05	0.06	0.12	0	0
BATF3	1.73	2.61	1.29	2.72	1.34	1.03
BAX	19.55	19.36	15.14	21.29	22.32	18.48
BAZ1A	6.25	5.55	5.62	4.43	5.84	4.41
BAZ1B	10.76	9.42	8.46	8.92	8.97	9.06
BAZ2A	8.7	8.13	6.59	6.81	8.07	8.05
BAZ2B	2.74	1.52	1.22	1.51	1.51	1.58
BBC3	1.95	0.44	0.26	0.81	1.06	0.56
BBIP1	4.07	3.06	3.78	2.96	3.31	1.95
BBOX1	0	0	0	0	0	0
BBS1	3.9	2.07	1.66	1.81	2.2	1.55
BBS10	2.09	2.43	1.37	1.99	2.14	2.09
BBS12	2.74	1.2	1.33	1.02	2.22	0.52
BBS2	6.85	3.82	4.54	3.64	4.63	2.36
BBS4	10.36	8.6	7.48	7.43	8.65	5.88
BBS5	0.97	1.8	0.61	0.93	0.86	0.97
BBS7	4.14	4.93	3.2	5.27	6.69	4.22
BBS9	0.81	0.95	0.62	0.34	0.58	0.38
BBX	5.38	3.69	4.07	3.02	3.93	3.52
BCAM	5.65	10.53	12.06	12.73	10.42	15.97
BCAN	1	0.18	0.23	0.13	0.24	0.1
BCAP29	4.76	4.92	4.84	3.94	5	3.19
BCAP31	429.41	435.38	438.49	429.4	402.7	412.42
BCAR1	3.95	0.08	0	0.42	2.05	0.58
BCAR3	0.09	0.02	0.08	0	0.62	0.15
BCAR4	0	0	0	0	0	0
BCAS1	0.43	0.21	0.33	0.12	0.18	0.25
BCAS2	42.09	40.96	34.9	30.93	38.16	34.97
BCAS3	2.22	1.15	1.81	2.38	2.37	2.49
BCAS4	11.44	6.95	8.96	10.56	11.63	4.23
BCAT1	13.82	21.69	17.39	16.45	17.63	22.45
BCAT2	49.71	50.12	44.14	50.47	45.15	46.82
BCCIP	97.58	106.54	87.62	74.73	92.5	74.3
BCDIN3D	3.49	2.1	3.1	2.39	2.78	2.81
BCDIN3D-AS	0.44	0.24	0.36	0.24	0.41	0.5
BCHE	0	0	0	0	0	0
BCKDHA	43.61	59.66	53.94	57.95	54.57	64.53
BCKDHB	10.02	10.52	9.25	10.25	9.22	10.38
BCKDK	41.43	30.56	28.66	33.13	39.65	32.54
BCL10	10.51	9.04	7.8	7.84	8.09	8.12
BCL11A	6.25	19.37	19.94	21.75	15.25	19.78
BCL11B	0.02	0	0.01	0.03	0.01	0.04
BCL2	0.28	1.6	1.37	1.19	1.32	1.74

BCL2A1	0.09	0	0	0.17	0.25	0.27
BCL2L1	54.75	13.45	16.5	26.63	38.01	14.42
BCL2L10	0	0	0	0	0	0
BCL2L11	25.85	7.11	8.51	12.14	15.41	6.95
BCL2L12	23.99	27.4	27.37	22.57	27.07	18.82
BCL2L13	13.75	8.78	9.56	10.57	9.68	12.24
BCL2L14	0	0	0	0	0.04	0
BCL2L15	0.34	0.4	0.43	0.4	0.12	0.32
BCL2L2	1.69	1.58	0.76	0.58	1.13	1.16
BCL2L2-PABI	28.99	35.86	27.93	26.95	36.09	28.01
BCL3	1.47	0.69	0.29	0.38	0.23	0.24
BCL6	0.5	0.09	0.19	0	0.38	0.69
BCL6B	0.02	0.06	0.25	0	0.17	0
BCL7A	5.18	7.1	6.36	6.66	6.54	8.27
BCL7B	7.35	7.32	7.73	9.38	10.09	6.23
BCL7C	6.09	6.39	8.66	7.05	5.09	4.41
BCL9	1.87	1.6	1.23	1.14	1.59	0.99
BCL9L	0.95	1.4	1.93	1.41	1.44	1.64
BCLAF1	40.49	39.02	35.21	35.01	38.93	35.03
BCMO1	0	0	0	0	0	0
BCO2	0.03	0.02	0	0.04	0	0
BCOR	14.25	14.53	11.31	12.14	11.04	12.63
BCORL1	11.71	10.05	12.68	11.17	10.97	11.23
BCORP1	0	0	0	0	0	0
BCR	2.15	1.65	2.1	1.5	1.72	1.88
BCRP2	0.07	0.55	0.1	0.27	0.16	0.34
BCRP3	0.59	1.77	1.32	2.5	2.13	2.58
BCS1L	54.8	57.86	60.92	58.84	52.67	55.42
BCYRN1	391.9	53.6	255.54	181.13	200.75	72.01
BDH1	13.75	21.35	16.89	18.42	19.13	19.29
BDH2	2.32	2.91	1.17	4.21	3.04	2.91
BDKRB1	0	0.04	0.05	0	0	0
BDKRB2	0	0	0	0	0	0
BDNF	0	0	0	0	0	0.06
BDNF-AS	0	0	0	0	0.19	0
BDP1	3.97	4.62	3.69	3.22	4.06	3.52
BEAN1	0	0	0	0	0	0
BECN1	32.43	20.5	19.09	15.56	21.73	18.5
BEGAIN	0.57	0.62	0.73	0.56	0.93	0.66
BEND2	0.11	0.19	0.23	0.14	0.16	0.37
BEND3	2.06	1.63	1.72	1.41	1.76	2.01
BEND3P3	0.16	0.2	0.35	0.14	0.38	0.19
BEND4	1.36	2.9	2.19	1.65	1.86	2.35

BEND5	0.4	0.45	1.51	0.78	1.18	0.86
BEND6	0.05	0.28	0.02	0.11	0.13	0.11
BEND7	0.72	1.08	0.57	0.94	0.86	0.94
BEST1	0.08	0	0.02	0.02	0	0.16
BEST2	0	0	0	0	0	0
BEST3	0.02	0.04	0	0.02	0.02	0
BEST4	0	0	0	0	0	0.04
BET1	6.21	4.07	2.92	3.55	3.82	2.84
BET1L	6.96	8.63	6.98	5.7	6.73	7.24
BET3L	0	0	0	0	0	0
BEX1	0	0.14	0	0	0	0
BEX2	0.17	0	0	0.12	0	0
BEX4	241.75	53.98	44.57	96.04	180.07	60.47
BEX5	8.49	13.01	10.51	11.04	7.91	9.76
BFAR	19.81	21.52	21.79	17.3	18.98	19.05
BFSP1	0.07	0	0.21	0	0.03	0.18
BFSP2	0	0	0	0	0	0
BGLAP	0	0	0	0	0	0
BGN	0	0	0	0	0	0
BHLHA15	0	0	0	0	0	0
BHLHA9	0	0	0	0	0	0
BHLHB9	0.58	1.3	0.91	0.67	0.74	0.88
BHLHE22	0	0	0	0	0	0
BHLHE23	0	0	0	0	0	0
BHLHE40	32.26	4.16	4.49	6.72	8.56	2.48
BHLHE40-AS	0.08	0.06	0.5	0.22	0	0.08
BHLHE41	0.02	0.02	0.16	0.13	0.09	0.21
BHMT	0	0.02	0	0	0	0
BHMT2	1.05	1.32	1.71	0.61	0.91	1.29
BICC1	0.19	0.02	0.02	0.14	0.15	0.14
BICD1	1.24	2.46	2.71	2.01	2.46	1.21
BICD2	2.65	3.4	2.74	2.64	2.58	2.74
BID	49.68	58.75	56.75	46.82	53.68	52.99
BIK	29.13	83.8	61.3	49.29	50.89	58.17
BIN1	2.54	3.14	2.52	2.68	4.58	2.43
BIN2	18.88	22.94	14.96	17.89	19.51	12.26
BIN3	25.61	35.26	33.91	31.64	27.82	29.6
BIRC2	4.96	3.28	3.1	2.85	4.01	2.84
BIRC3	0	0.11	0.03	0	0	0
BIRC5	98.47	96.22	98.41	106.68	108.59	96.2
BIRC6	3.96	3.19	3.16	2.73	3.12	3.48
BIRC7	0	0	0	0	0	0
BIRC8	0.07	0	0	0	0	0

BIVM	4.29	7.02	4.64	4.46	4.04	3.99
BIVM-ERCC5	0	0	0	0	0	0
BLCAP	27.9	31.83	29.04	26.14	23.92	22.55
BLID	0	0	0	0	0	0
BLK	0	0	0	0	0	0
BLM	7.63	10.79	10.26	10.28	10.12	11.3
BLMH	46.69	46.65	45.9	41.48	45.67	48.2
BLNK	0	0	0	0.04	0	0
BLOC1S1	85.47	86.44	78.11	90.01	90.23	67.11
BLOC1S1-RD	0.87	1.21	0.59	2.14	0.37	1.53
BLOC1S2	18.96	17.63	13.52	13.65	14.9	16.89
BLOC1S3	7.01	6.18	4.11	6.91	4.83	3.95
BLOC1S4	2.08	1.37	1.64	1.11	3.7	0.29
BLOC1S5	6.06	5.06	3.02	8.64	7.39	7.68
BLOC1S5-TXI	8.96	5.35	8.63	4.56	3.88	8.3
BLOC1S6	13.38	10.59	8.44	8	9.45	9.85
BLVRA	123.53	33.61	38.61	63.42	83.27	41.86
BLVRB	221.22	83.47	91.88	153.44	189.23	74.69
BLZF1	6.25	5.46	3.89	5.72	6.5	3.77
BMF	4.03	0.32	0.48	1.29	1.32	0.32
BMI1	3.96	0.4	1.08	1.82	1.56	1.36
BMP1	10.63	12.11	13.26	12.18	9.14	14.83
BMP10	0	0	0	0	0	0
BMP15	0	0	0	0	0	0
BMP2	0	0	0	0	0	0
BMP2K	9	1.4	1.42	2.31	5	1.6
BMP3	0.03	0.06	0.02	0.05	0.01	0.02
BMP4	0.77	4.63	3.77	3.01	2.63	4.58
BMP5	0	0	0	0	0	0
BMP6	3.68	0.98	1.15	1.65	1.98	0.44
BMP7	0.33	0.5	0.41	0.32	0.64	0.33
BMP8A	0.29	0.37	0.32	0.26	0.17	0.3
BMP8B	0.73	0.44	0.88	0.72	0.53	0.42
BMPER	0	0	0	0	0	0
BMPR1A	4.74	0.92	1.12	2.63	4.66	3.27
BMPR1B	0	0	0	0	0	0
BMPR2	1.17	0.89	0.79	0.67	0.7	0.84
BMS1	13.16	13.63	11.51	12.45	13.56	11.56
BMS1P1	0.63	0.89	1.37	0.64	0.84	1.07
BMS1P2	1.08	0.75	0.6	0.47	0.73	0.69
BMS1P4	1.24	1.35	0.57	0.94	0.59	1.43
BMS1P5	0.99	0.89	0.41	0.38	0.83	1.08
BMS1P6	1.29	0.62	0.5	0.52	0.7	0.7

BMX	0.93	0.78	0.51	1.97	3.77	4.36
BNC1	0	0	0	0	0	0
BNC2	0.02	0	0.01	0.02	0.01	0
BNIP1	24.55	21.79	15.85	17.52	19.99	15.99
BNIP2	32.4	27.63	21.34	14.18	21.24	23.27
BNIP3	50.45	35.97	32.33	32.73	42.59	42.45
BNIP3L	69.53	41.79	35.5	56.75	71.61	50.87
BNIPL	1.29	1.02	0.9	0.31	1.02	0.9
BOC	0	0	0	0.01	0.1	0
BOD1	1.94	2.19	2.77	1.76	2.24	3.37
BOD1L1	2.2	2.07	1.65	1.56	1.87	1.88
BOD1L2	0	0.02	0	0	0	0
BOK	0	0	0	0	0	0
BOK-AS1	0	0	0	0	0	0
BOLA1	0.09	0	0	0	0	0
BOLA2	48.84	59.41	50.83	45.17	54.96	43.97
BOLA2B	48.84	59.41	50.83	45.17	54.96	43.97
BOLA3	114.01	90.18	93.68	72.1	91.51	68.03
BOLA3-AS1	0.53	0.52	0.37	0.4	0.37	0.38
BOLL	0	0	0	0	0	0
BOP1	31.37	36.41	43.5	39.15	33.08	34.09
BORA	8.49	12.66	9.16	8.9	7.45	9.75
BPESC1	0	0.03	0	0	0	0.02
BPGM	12.33	8.31	6.07	10.34	7.94	6.54
BPHL	11.48	10.78	10.29	12.94	10.52	12.12
BPI	0	0.06	0	0	0	0.12
BPIFA1	0	0	0	0	0	0
BPIFA2	0	0	0	0	0	0
BPIFA3	0	0	0	0	0	0
BPIFA4P	0	0	0	0	0	0
BPIFB1	0	0	0	0	0	0
BPIFB2	0	0	0	0	0	0
BPIFB3	0	0	0	0	0	0
BPIFB4	0	0	0	0	0	0
BPIFB6	0	0	0	0	0	0
BPIFC	0	0	0	0	0	0
BPNT1	7.79	10.73	10.05	7.92	8.21	10.34
BPTF	4.69	4.68	4.78	4.1	4.46	5.84
BPY2	0	0	0	0	0	0
BPY2B	0	0	0	0	0	0
BPY2C	0	0	0	0	0	0
BRAF	1.73	2.2	2.18	1.97	1.75	2
BRAP	8.53	10.83	7.31	8.08	9.11	9.47

BRAT1	26.18	23.68	23.59	27.44	25.76	25.88
BRCA1	7.9	11.68	11.84	9.34	10.71	10.66
BRCA2	1.39	1.23	1.04	0.82	1.25	1.15
BRCC3	26.8	36.76	26.69	23.62	25.11	24.96
BRD1	1.46	1.58	1.48	1.26	2.23	1.46
BRD2	22.75	22.53	18.26	18.76	18.87	20.23
BRD3	14.05	16.77	13.18	14.55	17.07	17.14
BRD4	6.14	3.87	3.9	4.58	4.66	4.96
BRD7	8.24	10.14	9.13	8.69	9.22	9.56
BRD7P3	0.12	0.12	0.03	0.04	0.08	0
BRD8	21.18	18.63	16.04	14.18	16.06	16.19
BRD9	5.54	7.41	7.92	6.72	7.41	4.71
BRDT	0.13	0.09	0.04	0.09	0.03	0.06
BRE	26.62	25.61	21.91	26.72	24.53	26.67
BRE-AS1	0	0.04	0.04	0.13	0.09	0.05
BREA2	0.32	0.07	0.28	0.02	0.07	0.26
BRF1	1.9	2.24	3.86	2.95	2.48	2.99
BRF2	24.2	23.68	20.65	20.93	21.86	25.06
BRI3	3.38	2.67	2.37	2.04	1.61	1.1
BRI3BP	4.46	10.78	7.02	5.25	6.36	6.07
BRICD5	0.31	0.97	0.14	0.05	0	0.16
BRIP1	2.35	2.56	3.02	1.81	2.92	3.02
BRIX1	54.31	40.62	44.04	36.13	49.91	38.23
BRK1	199.66	151.95	149.22	161.13	183.21	163.22
BRMS1	53.23	56.86	60.64	54.89	50.08	54.25
BRMS1L	2.83	1.74	1.4	1.4	1.66	1.89
BROX	11.38	9.56	8.8	10.19	8.29	9.14
BRPF1	2.73	2.61	2.93	2.45	2.34	2.84
BRPF3	2.27	2.1	1.47	1.85	1.32	1.26
BRS3	0	0	0	0	0	0
BRSK1	0	0	0	0.02	0	0.07
BRSK2	0	0	0	0.01	0.1	0
BRWD1	5.84	5.73	5.21	5.15	5.01	4.11
BRWD1-AS1	0	0	0	0	0	0
BRWD1-IT2	0.3	0.06	0.07	0.21	0.28	0
BRWD3	1.05	0.75	0.59	0.48	0.91	0.87
BSCL2	22.7	19.35	16.04	14.97	11.57	12.03
BSDC1	31.79	10.76	8.64	9.57	17.89	10.07
BSG	394.05	284.93	262.07	290.35	295.74	293.03
BSN	0.12	0.07	0.09	0.19	0.16	0.1
BSN-AS2	0.49	0.94	0.7	0.6	0.37	0.43
BSND	0	0	0	0	0	0
BSPH1	0	0	0	0	0	0

BSPRY	0.56	0.96	1.86	0.82	1.3	1.68
BST1	7.04	12.49	18.35	11.66	10.39	12.08
BST2	128.12	124.14	116.3	109.88	139.24	77.68
BSX	0	0	0	0	0	0
BTAF1	4.64	2.72	3.6	2.52	3.58	2.61
BTBD1	8.4	7.78	7.61	5.42	8.01	4.99
BTBD10	7.19	6.93	4.95	4.68	4.69	4.2
BTBD11	0	0	0	0	0.08	0
BTBD16	0	0	0	0	0	0
BTBD17	0	0	0	0	0	0
BTBD18	0.36	0.43	0.33	0.02	0.16	0.31
BTBD19	1.19	0	0.13	0.23	0.76	0.43
BTBD2	3.76	4.71	4.45	4.84	2.95	5.12
BTBD3	2.61	2.38	2.01	2.81	2.31	2.31
BTBD6	2.72	2.26	2.55	2.15	3.27	2.6
BTBD7	1.82	2.17	1.72	1.94	2.77	2.88
BTBD8	0.47	0.45	0.09	0.39	0.59	0.31
BTBD9	1.24	0.64	1.45	0.79	1.27	0.91
BTC	0	0	0	0	0	0
BTD	9.45	6	8.18	5.09	6.41	5.89
BTF3	707.73	684.18	644.97	707.22	754.28	755.04
BTF3L4	19.83	17.17	16.69	15.91	17.8	16.9
BTF3P11	0.63	0.4	0.92	0.27	1.68	1.4
BTG1	2.22	1.51	0.55	1.05	1.08	0.52
BTG2	22.04	1.87	3.18	6.05	4.22	2.54
BTG3	9.11	7.36	8.48	7.09	8.26	6.76
BTG4	0	0	0	0	0	0
BTK	101.05	71.15	64.82	83.42	86.2	75.82
BTLA	0.02	0	0	0	0	0
BTN1A1	0	0.06	0	0	0	0.16
BTN2A1	2.61	3.01	3.27	2.17	2.83	3
BTN2A2	1.11	1.23	0.38	1.9	1.63	1.58
BTN2A3P	0.38	0.7	0.27	0.58	0.5	0.48
BTN3A1	0.59	0.55	0.87	1.96	0.84	1.44
BTN3A2	2.81	4.68	4.14	4.73	4.65	2.95
BTN3A3	0.87	0.57	0.63	0.89	0.43	0.85
BTNL2	0	0	0	0	0	0
BTNL3	0	0	0	0	0	0
BTNL8	0	0	0	0	0	0
BTNL9	0.39	0.26	0.28	0.67	0.27	0.32
BTRC	5.61	3.23	3.3	3.59	4.16	2.97
BUB1	39.38	32.41	32.51	34.91	34.22	31.13
BUB1B	29.27	29.45	26.59	25.94	27.7	25.93

BUB3	163.63	176.84	145.13	146.46	160.07	150.16
BUD13	9.11	14.59	9.07	12.33	9.34	10.67
BUD31	144.84	118.52	100.63	110.99	128.16	97.03
BVES	0.81	1.14	1.43	0.38	0.96	1.12
BVES-AS1	0	0	0	0	0	0
BYSL	27.45	19.4	28.61	28.98	29.84	27.65
BZRAP1	1.49	6.04	3.25	0.75	1.07	3.83
BZRAP1-AS1	1.33	1.04	1.38	1.64	1.76	0.95
BZW1	95.91	78.27	61.62	60.49	77.4	74.62
BZW2	112.31	130.65	124.53	106.12	103.54	120.01
C10orf10	9.64	0.73	0.69	1.43	2.3	0.22
C10orf105	0	0	0.08	0.16	0	0
C10orf107	0.06	0	0	0	0	0
C10orf11	0.35	2.55	1.2	0.3	2.65	2.47
C10orf111	0.74	0.57	0.94	0.12	0.17	0.81
C10orf113	0	0	0	0	0	0
C10orf114	0	0.15	0.1	0	0.04	0.04
C10orf118	1.7	0.89	0.67	0.56	0.78	0.78
C10orf12	3.07	3.13	3.04	2.71	2.89	3.07
C10orf120	0	0	0	0	0	0
C10orf128	6.02	0.37	1.48	0.65	3.11	0
C10orf129	0	0	0	0	0	0
C10orf131	0.06	0	0	0	0.24	0
C10orf137	3.51	4	4.43	3.81	3.98	3.93
C10orf2	10.7	12.3	13.1	14.41	11.23	12.4
C10orf25	0.02	0.09	0.23	0.22	0.11	0.05
C10orf32	7.93	4.53	4.16	5.34	5.8	5.41
C10orf32-AS:	1.2	1.54	0.67	0	0	1.26
C10orf35	0.07	0	0	0	0	0
C10orf40	0	0	0	0	0	0
C10orf53	0	0	0	0	0	0
C10orf54	7.15	18.61	19.68	14.67	9.38	10.82
C10orf55	0	0.12	0	0.05	0.13	0.07
C10orf62	0	0	0	0	0	0
C10orf67	0.02	0	0.02	0.02	0	0.05
C10orf68	0	0.04	0	0	0	0.05
C10orf71	0	0	0	0	0	0
C10orf76	7.98	6.87	7.09	7.82	7.47	7.74
C10orf82	0	0	0	0	0	0
C10orf88	4.37	3.73	4.28	2.12	3.27	3.85
C10orf90	0	0	0	0	0	0
C10orf91	0	0.42	0.1	0.22	0	0.22
C10orf95	0.06	0.22	0.15	0.11	0.21	0.23

C10orf99	0	0	0	0	0	0
C11orf1	17.26	15.41	19.45	24.72	19.25	19.65
C11orf16	0.04	0.06	0.07	0	0	0
C11orf21	2.44	2.05	0.87	1.61	2.24	1.33
C11orf24	25.92	20.01	17.15	18.23	18.02	18.17
C11orf30	3.52	3.65	3.96	2.99	2.83	3.2
C11orf31	152.72	151.93	122.74	150.48	162.89	132.31
C11orf34	0	0	0	0	0	0.15
C11orf35	0.18	0.17	0.13	0	0.33	0.25
C11orf40	0	0	0	0	0	0
C11orf42	0	0	0	0	0	0
C11orf44	0.07	0.1	0.11	0.12	0.08	0.02
C11orf45	0.98	0.79	1.08	0.29	0.6	2.21
C11orf48	44.26	43.26	38.86	40.18	40.15	36.06
C11orf49	12.99	11.69	10.46	10.05	14.26	10.83
C11orf52	0	0.05	0	0.09	0	0
C11orf53	0	0	0	0	0	0
C11orf54	3.34	4.09	3.77	3.42	3.22	5.53
C11orf57	9.42	8.93	7.94	9.22	10.05	10.14
C11orf58	53.21	52.16	43.2	45.79	54.38	50
C11orf63	1.02	1.46	0.9	0.44	0.58	1.04
C11orf65	0.37	0.18	0.41	0	0.35	0
C11orf68	2.83	2.08	2.82	4.7	3.41	2.67
C11orf70	0.24	0.14	0.17	0.16	0.33	0.35
C11orf71	3.71	3.58	2.95	2.58	6.81	3.01
C11orf73	55.41	51.69	54.87	51.22	53.44	47.73
C11orf74	28.36	19.48	18.2	21.32	20.42	18.05
C11orf75	12.82	23.67	18.03	13.91	21.16	15.59
C11orf80	1.26	2.54	3.56	3.06	2.28	4.3
C11orf82	6.99	6.79	4.84	5.84	6.38	5.73
C11orf83	23.29	30.74	26.06	27.57	21.7	25.67
C11orf84	0.57	0.68	0.41	0.65	0.57	0.53
C11orf85	0	0	0	0	0	0
C11orf86	0	0	0	0	0	0
C11orf87	0.08	0	0	0	0	0
C11orf88	0	0	0	0	0	0
C11orf91	0	0	0.08	0	0	0
C11orf92	0.14	0.18	0.25	0.05	0.12	0.03
C11orf93	0	0	0	0	0	0
C11orf94	0	0.19	0.44	0	0	0
C11orf95	0.36	0.21	0.48	0.21	0.4	0.38
C11orf96	0.23	0.54	0	0.04	0.21	0
C12orf10	50.64	52.35	52.4	59.27	55.66	52.82

C12orf23	0.91	0	0.04	0.03	0.41	0
C12orf29	9.99	10.5	9.49	7.28	9.72	10.3
C12orf36	0	0.16	0	0	0	0
C12orf39	0	0	0	0	0	0
C12orf4	6.57	4.29	6.62	4.83	5.19	4.02
C12orf40	0	0	0	0	0	0
C12orf42	0.34	1.26	1.77	1.3	1.62	0.4
C12orf43	16.76	15.94	14.13	15.37	12.17	14.85
C12orf44	34.59	30.92	36.09	33.22	31.85	28.03
C12orf45	32.85	44.53	37.43	46.72	38.81	33.64
C12orf49	3.34	2.76	2.12	2.05	3.03	1.73
C12orf5	1.22	2	1.69	1.12	0.92	1.83
C12orf50	0.04	0	0.28	0	0	0
C12orf52	30.58	32.37	34.39	33.62	26.8	28.78
C12orf54	0	0	0	0	0	0
C12orf56	0	0	0	0	0	0
C12orf57	266.29	302.98	279.11	321.96	352.77	364.33
C12orf60	2.57	1.02	1.74	1.59	1.03	1.57
C12orf61	0	0	0	0.33	0.04	0.18
C12orf65	21.52	25.44	19.92	20.42	23.97	16.33
C12orf66	3.66	2.79	6.42	3.93	4.94	6.33
C12orf68	0	0	0	0	0	0
C12orf69	0	0.03	0	0.02	0	0
C12orf70	0	0	0	0.08	0	0
C12orf71	0.16	0	0	0.17	0	0.08
C12orf73	4.92	6.37	4.06	3.91	3.82	4.62
C12orf74	0	0	0	0	0	0
C12orf75	1.93	0.56	0.86	0.84	0.36	0.5
C12orf76	4.22	1.71	2.36	2.17	3.95	2.58
C12orf77	0.57	0.72	1.07	0.58	0.71	0.7
C13orf35	0	0	0	0	0	0
C13orf45	0	0	0	0	0	0
C14orf1	42.21	39.31	34.67	34.97	44.43	38.5
C14orf101	1.71	1.22	1.2	1.14	1.28	1
C14orf105	0.35	0.34	0.28	0.07	0.28	0.36
C14orf119	40.27	37.23	26.61	24.14	31.76	35.92
C14orf132	1.15	0.23	0.52	0.21	1.21	0.43
C14orf142	16.55	19.78	13.02	9.46	19.03	18.95
C14orf159	9	19.29	18.36	16.22	15.07	18.27
C14orf166	174.45	170.37	159.97	161.65	170.07	154.47
C14orf166B	0	0	0	0	0	0
C14orf169	2.23	4.46	2.62	3.56	3.23	3.74
C14orf177	0	0	0	0	0	0

C14orf178	1.14	2.13	1.53	0.68	0.91	1.54
C14orf180	0	0	0	0	0	0
C14orf182	0.13	0.14	0.32	0.12	0.24	0.21
C14orf183	0	0	0	0	0.07	0
C14orf2	518.55	580.57	558.77	580.05	733.03	527.02
C14orf23	0.23	0.14	0.2	0.15	0.33	0.06
C14orf28	0	0.43	0.09	0.24	0.4	0.13
C14orf37	0	0.17	0.06	0.39	0.11	0.12
C14orf39	0	0	0	0	0.05	0
C14orf64	0.15	0.12	0.11	0.11	0	0
C14orf79	0.43	0.49	1.54	1	0.81	0.99
C14orf80	2.44	2.06	2.18	2.58	2.41	2.76
C14orf93	3.51	4.71	3.22	3.83	3.07	3.14
C15orf26	1.61	0	0	0.2	0.18	0.05
C15orf27	0.15	0.12	0	0.04	0.23	0
C15orf32	0	0	0	0	0	0
C15orf37	1.36	0.95	1.63	0.9	1.09	1.02
C15orf38	0	0	0.06	0.02	0.1	0.06
C15orf38-AP:	0	0	0	0	0	0
C15orf39	28.2	31.18	37.51	29.5	26.18	29.98
C15orf40	21.21	22.78	18.55	17.49	21.86	17.44
C15orf41	4.68	5.4	4.92	3.92	3.89	4.91
C15orf43	0	0	0	0	0	0
C15orf48	0	0	0	0	0	0
C15orf52	0.1	0.12	0.09	0.24	0.16	0.05
C15orf53	0	0	0	0.02	0	0
C15orf54	0	0	0	0	0	0
C15orf55	0	0	0	0.03	0	0
C15orf56	0	0	0	0	0	0
C15orf57	22.4	12.28	14.92	16.11	13.58	12.75
C15orf59	0	0	0	0	0	0
C15orf60	0	0.13	0	0	0	0.08
C15orf61	44.39	52.44	52.23	53.16	62.22	53.23
C15orf62	0.03	0.11	0.4	0.1	0	0.06
C16orf11	0	0	0	0	0	0
C16orf13	155.23	210.4	201.36	187.73	194.19	217.35
C16orf3	0.16	0	0	0	0	0
C16orf45	0.79	0	0	0	0	0
C16orf46	0.67	0.33	1.28	0.5	0.94	0.67
C16orf52	0.02	0.19	0.22	0.12	0.21	0.12
C16orf54	2.46	9.26	9.08	6.02	4.61	6.02
C16orf55	2.08	2.29	5.92	2.9	3.42	4.33
C16orf58	8.02	13.53	11.87	13.19	9.48	9.73

C16orf59	8.02	12.42	11.74	13.22	9.91	9.13
C16orf62	17.79	12.74	15.46	15.34	15.89	16.52
C16orf70	13.66	10.5	11.01	10.19	12.49	9.26
C16orf71	0.19	0.04	0.02	0	0.03	0.03
C16orf72	1.4	1.6	1.53	0.91	1.82	1.18
C16orf74	0.95	8.1	4.23	2.99	2.63	6.74
C16orf78	0	0	0	0	0	0
C16orf80	53.87	59.07	41.35	42.52	47.5	42.59
C16orf82	0	0	0	0	0	0
C16orf86	1.55	0.66	1.85	2.14	1.64	1.21
C16orf87	7.35	6.48	3.44	5.48	6.59	4.84
C16orf88	16.01	17.96	19.44	18.35	18.05	16.05
C16orf89	0.2	0.03	0.27	0.16	0.15	0.04
C16orf90	0	0	0	0	0	0
C16orf91	34.71	47.63	38.89	37.44	40.65	41.91
C16orf92	0	0	0	0	0	0
C16orf93	0.79	1.26	2.25	0.96	0.82	1.62
C16orf95	8.03	4.14	5.15	4.32	5.65	3.75
C16orf96	0	0	0	0	0	0
C16orf97	0	0	0	0	0	0
C17orf100	0.43	0.04	0.35	0.36	0	0.19
C17orf102	0	0.03	0	0.04	0	0
C17orf103	1.94	0.74	0.86	1.11	1.54	0.97
C17orf104	0.11	0.38	0.14	0.17	0.28	0.95
C17orf105	0	0	0	0	0	0.19
C17orf107	0	0.02	0	0.03	0	0.07
C17orf112	0	0	0	0	0	0
C17orf47	0	0	0	0	0	0
C17orf49	65.02	94.59	80.4	93.55	88.7	76.96
C17orf50	0	0	0	0	0	0
C17orf51	2.73	3.7	2.79	2.81	3	3.88
C17orf53	7.57	8.21	9.32	10.12	8.18	10.06
C17orf58	10.41	13.72	12.54	12.33	14.09	11.87
C17orf59	4.06	6.34	7.58	6.44	4.02	4.11
C17orf61-PLS	2.09	4.91	1.35	0.81	2.69	2.41
C17orf62	31.02	46	42.11	39.91	35.4	42.04
C17orf64	0.32	0.83	0.96	1	1.6	0.98
C17orf66	0	0	0	0	0	0
C17orf67	0.33	0.2	0.4	0.53	0.49	0.52
C17orf70	13.25	15.45	14.53	15.47	12.66	14.78
C17orf72	0.77	0.42	0.49	0.55	0.99	0.36
C17orf74	0	0	0	0	0	0
C17orf75	3.54	4.37	4.37	4.07	5.26	4.23

C17orf76-AS	804.38	936.24	935.1	1190.82	1136.89	998.9
C17orf77	0.24	0.42	0.36	0.24	0.2	0.03
C17orf78	0	0.22	0.07	0	0.11	0.16
C17orf80	6.19	7.29	7.18	4.98	5.21	5.85
C17orf82	0	0	0	0	0	0
C17orf85	3.21	2.92	3.3	2.82	3.92	3.14
C17orf89	79.27	105.53	97.08	96.06	87.69	73.64
C17orf96	1.22	1.05	0.89	1.08	1.53	1.57
C17orf97	0.65	0.29	0.59	0.25	1.07	0.69
C17orf98	0	0.11	0.53	0.1	0	0
C17orf99	0	0	0	0.1	0.13	0
C18orf21	17.96	18.2	18.81	14.83	16.94	16.35
C18orf25	5.67	4.35	5.22	3.76	3.87	3.71
C18orf32	8.97	5.57	2.34	5.11	6.46	4.01
C18orf42	0	0	0	0	0	0
C18orf54	1.59	2.58	1.56	1.65	2.06	1.92
C18orf56	5.31	5.3	4.45	4.46	5.83	4.18
C18orf61	0.26	0.24	0.23	0.22	0.12	0.22
C18orf62	0	0	0	0	0	0
C18orf63	0	0	0	0	0	0
C18orf8	14.27	11.2	9.71	11.08	13.02	10.66
C19orf10	161.54	185.68	188.54	147.04	165.77	179.51
C19orf12	2.07	2	1.93	1.55	2.09	1.6
C19orf18	0	0	0	0	0	0
C19orf21	0	0.1	0	0	0	0
C19orf24	79.33	71.52	75.98	65.61	69.85	65.26
C19orf25	25.55	22.77	20.61	23.36	24.98	22.12
C19orf26	0.4	0	0.05	0.04	0.03	0.03
C19orf33	0.21	0	0	0	0	0
C19orf35	0.21	0.47	0.34	0.1	0.06	0.53
C19orf38	1.97	5.32	3.41	5.72	5.45	5.32
C19orf40	5.03	8.19	5.42	4.32	4.94	5.87
C19orf43	53.92	57.3	57.29	62.77	67.87	67.97
C19orf44	0.7	0.91	1.01	0.45	1.01	1
C19orf45	0	0	0	0	0.08	0
C19orf47	5.47	5.96	6.79	5.1	5.58	4.28
C19orf48	174.88	187.02	163.37	198.78	204.47	173.25
C19orf52	4.9	5.1	7	7.35	8.05	3.59
C19orf53	133	112.64	86.17	111.1	125.59	102.38
C19orf54	2.22	5.12	4.29	3.59	3.37	4.44
C19orf55	2.88	3.14	3.08	3.24	2.98	2.22
C19orf57	0.06	0.64	0.5	0.23	0.06	0.25
C19orf59	0.12	1.96	0.74	0.84	0.27	3.15

C19orf60	70.95	108.09	121.99	107.14	109.11	110.06
C19orf66	5.65	6.04	4.6	5.71	3.42	2.54
C19orf69	0	0	0	0	0	0
C19orf70	66.67	68.75	68.41	69.33	74.17	52.4
C19orf71	0.12	0.09	0.33	0.41	0	0.12
C19orf73	1.92	1.01	1.75	2.29	0.3	1.64
C19orf77	2.57	12.15	15.02	22.12	16.62	11.4
C19orf80	0.09	0	0	0	0	0
C19orf81	0	0	0	0	0	0
C1D	32.65	28.72	19.63	17.47	24.63	29.52
C1GALT1	2.64	2.84	2.18	1.43	2.11	2.12
C1GALT1C1	29.36	36.63	25.19	20.13	24.57	26.53
C1QA	0	0	0	0	0	0
C1QB	0	0	0	0	0	0
C1QBP	622.27	737.57	713.13	691.7	694.65	721.96
C1QC	0	0	0	0	0	0
C1QL1	0	0	0	0	0	0
C1QL2	0	0	0	0	0	0
C1QL3	0.09	0.21	0.11	0.02	0.03	0.06
C1QL4	0.07	0.03	0.29	0.17	0.17	0.22
C1QTNF1	0.38	0.02	0.05	0.13	0	0.18
C1QTNF2	0.03	0.14	0.1	0.15	0.05	0.05
C1QTNF3	0.11	0.23	0.05	0.04	0.01	0.04
C1QTNF3-AN	0	0	0	0	0.05	0
C1QTNF4	0	0	0	0	0	0
C1QTNF5	0	0	0.03	0	0	0
C1QTNF6	2.96	2.07	3.01	3.13	2.94	2.7
C1QTNF7	0	0	0.02	0	0	0.08
C1QTNF8	0	0	0	0	0	0
C1QTNF9	0	0.16	0.24	0.09	0.04	0
C1QTNF9B	0	0	0	0	0	0
C1QTNF9B-A	2.15	4.23	2.64	1.59	2.18	2.86
C1R	0.13	0	0.09	0.2	0.15	0.33
C1RL	2.11	2.99	2.76	2.45	1.64	2.88
C1RL-AS1	0.27	0.64	0.39	0.29	0.42	0.33
C1S	0.31	0.38	0.42	0.06	0.25	0.92
C1orf100	0	0	0	0	0	0
C1orf101	0.02	0	0	0	0	0
C1orf105	0	0	0	0	0	0
C1orf106	0.06	0.03	0.08	0.03	0	0.1
C1orf109	7.42	7.66	6.51	4.78	6.35	6.31
C1orf110	0	0	0	0	0	0
C1orf111	0	0	0	0	0	0

C1orf112	4.26	6.25	6.57	6.33	5.77	7.7
C1orf114	0	0	0	0	0	0
C1orf115	0.7	0.21	0.61	0.05	0.23	1.22
C1orf116	0.3	0	0	0	0.03	0
C1orf122	3.91	2.53	2.35	2.03	4.14	3.63
C1orf123	33.31	36.17	34.12	30.6	36.75	29.07
C1orf127	0	0	0	0	0	0
C1orf131	20.3	22.36	19.35	16.26	18.55	16.4
C1orf140	0.06	0.12	0.15	0.23	0.14	0.14
C1orf141	0.2	0.03	0.2	0.24	0.42	0.09
C1orf145	0.03	0.05	0	0	0	0.11
C1orf146	0	0	0	0	0	0
C1orf151-NB	4.08	1.24	0	6.33	5.47	4.61
C1orf158	0	0	0	0	0	0
C1orf159	10.48	9.77	10.55	11.24	11.85	9.11
C1orf162	0	0.27	0	0.41	0	0.26
C1orf168	0	0	0	0	0	0
C1orf170	0	0	0	0	0	0
C1orf172	0.04	0	0	0	0	0
C1orf173	0	0	0	0	0	0
C1orf174	36.93	39.76	35.64	33.82	39.57	31.91
C1orf177	0	0.07	0.04	0	0.12	0.04
C1orf180	0	0	0.02	0	0	0
C1orf185	0	0	0	0	0	0
C1orf186	38.06	152.63	140.64	101.86	110.61	175.39
C1orf189	0	0.63	0	0	0	0
C1orf192	0	0.08	0.18	0	0.19	0.2
C1orf194	0	0	0	0	0	0
C1orf198	13.28	9.91	8.83	9.21	12.22	8.3
C1orf200	0	0	0.11	0.28	0.54	0.08
C1orf204	0	0	0	0	0	0.03
C1orf21	0.05	3.45	0.18	0.53	1.61	0.11
C1orf210	0	0.96	0.98	0.36	0.69	1.21
C1orf213	2.02	0.06	0.58	0.15	0.23	0.49
C1orf216	7.71	6.2	6.69	7.83	7.69	3.22
C1orf220	0.53	1.06	0.75	0.31	0.4	0.75
C1orf226	0.12	0.08	0	0.01	0	0
C1orf227	0	0	0	0.35	0	0
C1orf228	1.94	14.21	7.68	6.6	5.97	11.08
C1orf229	0.41	0.75	0.82	0.41	0.84	0.74
C1orf233	1.02	2.09	1.2	2.62	1.53	3.36
C1orf27	3.29	2.64	2.88	2.34	3.26	2.68
C1orf35	22.96	23.37	23.21	22.95	23.49	19.95

C1orf43	121.42	122.99	122.32	132.37	142.8	119.06
C1orf50	11.14	10.82	13.43	11.34	10.08	10.75
C1orf51	0	0	0	0	0	0
C1orf52	8.34	8.59	6.01	6.82	9.11	5.38
C1orf53	9.38	9.45	10.19	11.14	10.25	8.4
C1orf54	0	1.75	1.73	2.86	2.71	2.7
C1orf56	3.29	3.63	3.74	2.61	3.68	2.72
C1orf61	0	0	0	0	0.08	0.36
C1orf63	8.19	4.52	5.43	4.48	6.24	2.9
C1orf64	0	0	0	0	0	0
C1orf65	0	0	0	0	0	0
C1orf68	0	0	0	0	0	0
C1orf74	7.52	6.83	6.97	7.09	7.04	6.58
C1orf85	0.06	0	0	0	0.1	0
C1orf86	9.28	6.47	10.13	10.75	13.32	8.07
C1orf87	0	0	0	0	0	0
C1orf94	0	0	0	0	0	0
C1orf95	0	0.04	0	0.01	0	0.08
C2	0	0	0.03	0.02	0	0.22
C20orf111	11.81	14.72	17.69	15.46	14.49	13.71
C20orf112	4.23	4.05	4.58	5.48	5.28	3.46
C20orf118	0.04	0.15	0	0.21	0	0.04
C20orf141	0	0	0	0	0	0
C20orf144	0	0	0	0.1	0	0
C20orf166	0	0	0	0	0	0
C20orf166-A:	0.06	0.03	0.03	0	0	0.1
C20orf173	0	0.08	0	0	0	0
C20orf194	1.37	0.82	0.61	0.91	0.79	0.76
C20orf195	0	0	0	0	0	0
C20orf196	5.47	5.46	8.2	4	4.99	6.66
C20orf197	0.32	1.39	0.73	0.11	0.58	0.68
C20orf201	0.18	0.78	1.23	2.24	0.17	1.81
C20orf202	0	0	0	0	0	0
C20orf203	0.07	0.09	0.15	0.03	0.09	0.2
C20orf24	26.26	44.68	40.84	29.18	35.76	29.85
C20orf26	0.23	0	0	0.05	0	0
C20orf27	100.32	109.09	119.52	135.27	108.11	117.81
C20orf78	0	0	0	0	0	0
C20orf85	0	0	0	0	0	0
C20orf96	2.51	3.52	3.15	5.35	2.29	3.91
C21orf119	4.56	2.1	3.04	4.73	7.84	2.49
C21orf128	0	0	0	0	0	0
C21orf15	0.46	0	0	0.12	0	0

C21orf2	3.26	5.66	6.1	5.36	5.36	5.76
C21orf33	75.9	84.61	87.88	87.52	77.65	77.46
C21orf37	0	0	0.1	0	0	0.11
C21orf49	0.57	0.5	0.06	0.76	0.12	0.26
C21orf54	0	0	0	0	0	0
C21orf58	3.2	2.09	2.23	2.95	3.02	2.98
C21orf59	49.58	49.03	38.86	38.45	45.38	46.9
C21orf62	1.67	2.23	2.29	1.85	1.84	2.12
C21orf67	3.65	1.75	2.14	2.24	2.47	1.92
C21orf7	0	0	0	0	0	0
C21orf88	0.05	0	0.1	0.11	0.1	0
C21orf90	0	0	0	0	0	0
C21orf91	2.69	2.57	2.1	2.02	2.25	2.11
C21orf91-OT	0	0.13	0	0	0	0
C22orf15	0.09	0	0	0.12	0	0
C22orf23	0.08	0.3	0.08	0.03	0.08	0.25
C22orf24	0	0.24	0	0	0.29	0
C22orf26	0.08	0.41	0.11	0.09	0.04	0.29
C22orf28	93.73	87.77	86.54	88.5	84.41	76.86
C22orf29	6.21	6.96	8.73	8.64	7.93	8.24
C22orf31	0.29	0	0	0	0	0.07
C22orf32	21.93	21.93	25.81	26.18	24.63	23.32
C22orf34	1.97	0.93	1.77	1.13	1.15	2.08
C22orf39	21.46	25.46	24.7	22.75	26.12	22.87
C22orf42	0	0	0	0	0	0
C22orf43	0	0.09	0.27	0.08	0.05	0.06
C22orf46	1.16	0.99	1.04	0.96	1.07	0.87
C2CD2	0.8	0.52	0.56	0.67	0.5	0.82
C2CD2L	1.72	1.11	0.3	0.31	0.74	0.33
C2CD3	2.3	2.47	2.34	1.79	2.56	2.46
C2CD4A	0.06	0.02	0.11	0.01	0.04	0.06
C2CD4B	0	0	0	0	0	0
C2CD4C	0.02	0	0	0.02	0	0
C2CD4D	0	0	0	0	0	0
C2CD5	3.74	5.58	4.69	3.9	3.82	4.05
C2orf15	0.53	0.13	0.62	0.48	0.15	0.31
C2orf16	0.1	0	0.16	0.06	0.14	0
C2orf27A	1.37	0.49	0.22	0.47	0	0.25
C2orf27B	0	0	0	0	0	0
C2orf40	0	0	0	0	0	0
C2orf42	4.06	2.54	2.66	2.52	2.71	3.94
C2orf43	6.77	5.76	5.22	6.99	5.88	5.51
C2orf44	7.72	8.01	9.05	9.37	10.51	9.64

C2orf47	36.16	34.75	27.07	24	27.7	28.17
C2orf48	0.44	0.43	0.6	0.45	0.21	0.88
C2orf49	13.42	14.4	11.16	8.5	11.15	10.19
C2orf50	0	0.17	0	0	0	0.37
C2orf53	0	0	0	0	0	0
C2orf54	0.08	0	0.03	0.03	0	0.01
C2orf57	0	0	0	0	0	0
C2orf61	0.03	0	0	0.17	0	0
C2orf62	0.06	0	0.05	0.04	0	0.12
C2orf66	0.26	0	0.03	0.54	0.46	1.06
C2orf68	15.82	16.24	13.84	15.52	14.84	15.13
C2orf69	6.14	8.07	5.49	4.79	5.27	7.54
C2orf70	0	0	0.32	0	0	0
C2orf71	0	0	0	0	0	0
C2orf72	0.06	0	0	0	0.04	0
C2orf73	0	0	0	0	0	0
C2orf74	0	0	0	0	0	0
C2orf76	2.69	1.03	1.79	2.81	2.24	1.89
C2orf78	0	0	0	0	0	0
C2orf80	0	0	0.06	0	0.12	0.25
C2orf81	0.73	0	0	0.14	0.03	0
C2orf82	0.37	0.58	0.84	0.89	0.52	1.47
C2orf83	0.24	0.25	0.45	0.41	0.41	0.34
C2orf88	2.76	1.29	2.3	2.29	2.55	2.09
C2orf91	0.36	0.55	0.67	0.27	0.16	0.61
C3	0.16	0.04	0.06	0	0.04	0.14
C3AR1	0.57	0.86	0.8	0.86	1.11	1.07
C3P1	0	0	0	0	0	0
C3orf14	0	0	0	0	0	0
C3orf17	8.56	6.19	6.52	5.24	6.88	6.04
C3orf18	0	0	0	0	0	0
C3orf20	0.03	0	0	0	0	0
C3orf22	0	0	0	0	0	0
C3orf27	0.03	0.16	0.16	0.06	0.11	0.06
C3orf30	0	0	0	0	0	0
C3orf33	3.88	5.5	4.93	4.07	4.96	3.98
C3orf35	0.15	0.14	0	0	0	0
C3orf36	0	0	0	0	0	0
C3orf37	20.63	16.81	18.91	17.9	23.97	15.55
C3orf38	11.26	9.77	8.66	6.48	7.74	8.18
C3orf43	0	0.06	0.04	0	0	0
C3orf45	0.59	0.59	0.25	0.57	0.27	0.97
C3orf49	0	0.09	0.05	0.12	0.11	0.06

C3orf52	0	0	0	0	0	0
C3orf55	0	0	0	0	0.08	0
C3orf58	6.22	3.25	4.61	4.94	5.2	2.5
C3orf62	1.27	2.2	1.17	1.19	1.32	1.76
C3orf65	0	0.27	0.21	0	0.18	0.04
C3orf67	0	0	0	0	0	0
C3orf70	0.13	0.05	0.06	0.02	0.01	0.08
C3orf72	0.32	0.56	0.67	0.55	0.55	0.71
C3orf79	0	0	0	0	0	0
C3orf80	0.09	0.85	0.23	0.47	0.62	0.34
C4A	0.03	0.07	0.06	0.04	0.01	0.01
C4B	0	0	0	0.02	0	0
C4BPA	0	0	0	0	0	0
C4BPB	0	0	0	0	0	0
C4B_2	0	0	0	0.02	0	0
C4orf17	0	0	0	0.03	0	0
C4orf19	0.73	1.03	1.26	0.37	0.46	0.79
C4orf21	1.09	1.33	0.89	0.94	1.11	1.5
C4orf22	0	0	0	0	0	0.09
C4orf26	0.87	1.28	1.05	0.73	1.12	0.84
C4orf27	27.82	24.89	23.01	20.66	28.16	20.99
C4orf29	5.25	6.24	5.12	5.33	4.67	5.79
C4orf3	17	14.03	8.08	10.28	15.27	13.64
C4orf32	0.94	1.86	1.96	0.87	1.67	2.52
C4orf33	4.57	3.55	2.34	3.83	3.48	3.92
C4orf36	0.51	0.07	0.31	0.12	0.16	0.09
C4orf40	0.07	0	0.06	0.04	0.03	0.1
C4orf45	0	0	0	0	0	0
C4orf46	4.16	9.75	7.96	6.5	6.88	9.19
C4orf47	0.08	0	0	0	0	0
C4orf48	181.28	171.86	177.47	180.5	200.59	143.86
C4orf51	0	0	0	0	0	0
C4orf52	32.46	35.87	31.36	32.43	33.41	28.34
C4orf6	0	0	0	0	0	0
C5	0.15	0.39	0.29	0.22	0.22	0.09
C5AR1	0.38	0.03	0.06	0.02	0	0.06
C5AR2	0	0.3	0.15	0.08	0	0.17
C5orf15	20.73	20.42	15.81	13.79	16.64	12.42
C5orf20	0	0	0	0	0	0
C5orf22	8.1	7.37	5.33	5.19	6.88	6.94
C5orf24	5.24	2.62	3.25	3.91	4.15	2.67
C5orf27	0.3	0.25	0.28	0.26	0.15	0.85
C5orf28	4.06	2.74	3.06	2.5	2.68	1.75

C5orf30	6.05	7.34	6.72	4.74	5.3	6.52
C5orf34	3.01	2.73	2.28	2.33	4.07	4.3
C5orf38	0	0	0	0	0	0
C5orf4	4.84	0.13	0.09	1.09	1.58	0.1
C5orf42	0.22	0.15	0.32	0.21	0.22	0.18
C5orf45	1.72	2.18	1.57	2.31	2.03	1.66
C5orf46	0	0	0	0	0	0
C5orf47	0	0	0	0	0	0
C5orf48	0	0	0	0	0	0
C5orf49	0	0	0	0	0	0
C5orf51	11.13	8.63	7.86	9.02	11.26	9.35
C5orf52	0	0	0	0	0	0
C5orf54	0.7	1.14	1.31	1.02	0.95	0.97
C5orf55	4.24	3.66	3.47	2.67	2.03	2.92
C5orf56	0.78	0.62	0.27	1.15	1.2	0.3
C5orf58	0	0	0	0	0	0
C5orf60	0.18	0.18	0.42	0.13	0.25	0.15
C5orf63	0	0	0	0	0	0
C5orf64	0.17	0.37	0.21	0.28	0.18	0.18
C6	0.02	0	0	0	0	0
C6orf1	40.15	26.67	25.11	20.89	26.88	20.32
C6orf10	0	0	0	0	0	0
C6orf106	13.34	6.56	7.41	9.25	11.67	7.47
C6orf118	0	0	0	0	0	0
C6orf120	2.67	3.16	1.83	3.33	3.42	2.49
C6orf123	0.64	0.66	0.61	0.71	0.63	0.83
C6orf132	0.11	0.14	0.17	0.05	0.05	0.08
C6orf136	7.05	5.42	6.24	6.47	5.6	5.17
C6orf141	0.22	1.24	0.72	0.82	0.46	1.2
C6orf147	0.84	2.08	0.8	0.75	1.28	0.97
C6orf15	0	0	0	0	0	0
C6orf163	0.12	0	0.11	0.12	0.05	0.18
C6orf164	0	0.04	0.04	0	0.14	0
C6orf165	0.43	0.13	0	0	0.09	0
C6orf170	0.91	0.35	0.54	0.47	0.47	0.39
C6orf195	0	0	0	0	0	0
C6orf201	0	0	0	0	0	0
C6orf203	16.08	13.59	13.72	12.1	20.11	14.73
C6orf211	11.84	16.55	10.53	7.28	8.92	13.6
C6orf222	0	0	0	0	0	0
C6orf223	0	0	0	0	0.11	0
C6orf226	2.39	2.84	1.37	1.98	4.28	2.71
C6orf25	0.46	0.45	0.28	0.45	0.19	0.55

C6orf47	6.49	7.56	5.78	6.05	6.04	7.04
C6orf48	75.33	76.85	80.89	80.93	82.09	79.52
C6orf52	2.35	0.73	1.12	1.51	0	1.36
C6orf57	7.93	8.66	7.1	9.38	9.07	5.91
C6orf58	0	0	0	0	0	0
C6orf62	140.28	127.6	95.13	98.42	121.54	92.79
C6orf7	0	0	0	0	0	0
C6orf70	3.69	4.67	6.27	4.98	5.26	4.38
C6orf89	11.72	8	5.24	5.68	7.93	5.68
C6orf99	0	0.5	0	0.09	0.12	0
C7	0.24	0.02	0.1	0	0	0.05
C7orf10	0.05	0.15	0.23	0.23	0.09	0
C7orf13	0	0	0	0	0	0
C7orf25	6.02	4.9	5.3	5.04	5.76	5.14
C7orf26	1.74	1.4	1.95	1.97	1.46	1.06
C7orf29	0	0	0	0	0	0
C7orf31	0.2	0.38	0.53	0.1	0.09	0.28
C7orf33	0	0	0	0	0	0
C7orf34	0.1	0	0	0	0	0
C7orf41	3.07	0.53	1.07	1.15	1.63	1.64
C7orf43	0.44	0.37	0.53	0.56	0.5	0.03
C7orf45	0	0	0	0	0	0
C7orf49	41.13	46	35.69	38.39	36.79	32.66
C7orf50	112.22	133.46	112.43	130.01	117.68	111.54
C7orf53	0.05	0	0.05	0.11	0.05	0
C7orf55	31.81	29.26	26.04	28.26	35.87	22.51
C7orf55-LUC	10.22	6.36	3.56	5.64	3.33	5.32
C7orf57	0	0	0	0	0	0
C7orf60	2.89	2.1	2.88	2.06	2.43	2.53
C7orf61	0.15	0.91	0.28	0.21	0.44	0.31
C7orf62	0	0	0	0	0	0
C7orf63	0	0	0.09	0.11	0	0
C7orf65	0	0.16	0.22	0.13	0.18	0.14
C7orf66	0	0	0	0	0	0
C7orf69	0	0	0	0	0	0
C7orf71	0	0.03	0	0	0	0
C7orf72	0	0	0	0	0	0
C7orf73	50.07	37.62	37.15	39.23	50.09	42.52
C7orf76	0	0	0	0	0	0
C8A	0	0	0	0	0	0
C8B	0	0	0	0	0	0
C8G	0.27	0.77	0.08	0.06	0.76	0
C8orf12	0	0	0	0	0	0

C8orf22	0	0	0	0	0	0
C8orf31	0.05	0.2	0.18	0.03	0.04	0
C8orf33	74.78	83.57	73.08	66.81	76.23	70.42
C8orf34	0	0	0	0	0	0
C8orf37	1	1.12	0.71	0.86	0.93	1.23
C8orf4	0	0	0	0	0	0
C8orf40	47.47	36.95	34.53	39.05	43.61	47.99
C8orf42	0.06	0	0	0	0	0
C8orf44	1.64	2.92	2.11	2.65	1.92	2.48
C8orf44-SGK	1.12	0.11	0.12	0.41	0.61	1.65
C8orf46	0	0	0	0	0	0
C8orf47	0.05	1.26	1.85	0.18	0.36	0.68
C8orf48	0.26	0	0	0	0	0
C8orf56	0.17	0.27	0.31	0.12	0.16	0
C8orf58	0.93	0.73	0.46	0.69	0.71	0.66
C8orf59	121.5	114.39	98.56	97.34	133.31	94.76
C8orf69	0	0	0	0	0	0
C8orf74	0	0	0	0	0	0
C8orf76	40.06	44.7	43.76	37.67	43.5	43.5
C8orf82	1.19	0.3	0.49	0.55	1.59	0.7
C8orf86	0.42	0.41	0.33	0.34	0.38	0.25
C8orf87	0	0	0	0	0	0
C9	0	0	0	0	0	0
C9orf106	0	0	0	0	0	0
C9orf114	14.17	16.98	15.93	15.9	16.01	16.7
C9orf116	14.35	10.26	8.09	10.92	10.88	6.78
C9orf117	0.56	0.34	0.28	0.27	0.29	0.31
C9orf123	34.59	30.45	28.37	30.07	36.09	32.46
C9orf129	0	0	0	0	0	0.29
C9orf131	0.32	0.14	0.52	0.14	0.02	0.14
C9orf135	0	0	0	0	0	0
C9orf139	0	0.23	0.32	0.07	0.07	0.21
C9orf142	56.95	98.4	79.15	90.09	80	79.63
C9orf152	0	0	0	0	0	0
C9orf153	0	0	0	0	0	0
C9orf156	12.91	9.99	8.74	11.79	10.18	11.81
C9orf16	109.84	87.22	81.86	122.79	105.39	98.74
C9orf163	0.06	0.05	0.08	0.06	0.06	0.15
C9orf169	0.32	0.34	0.59	0	0.2	0.11
C9orf170	0	0	0	0	0	0
C9orf171	0	0	0	0	0	0
C9orf172	0.12	0	0.05	0	0	0
C9orf173	0	0	0	0	0	0.25

C9orf174	0	0	0	0	0	0
C9orf24	0	0	0	0	0	0
C9orf3	2.05	3.64	3.35	2.59	2.41	2.51
C9orf37	10.34	11.26	10.5	10.8	14.68	11.13
C9orf40	1.11	1.17	0.98	0.67	1.49	1.34
C9orf41	2.22	2.51	1.97	1.55	1.77	1.53
C9orf43	0.58	0.65	0.13	0.35	0.41	0.18
C9orf47	0	0	0	0	0	0
C9orf50	0	0	0	0	0	0
C9orf53	0.4	0.21	0.24	0.18	0.38	0
C9orf57	0	0	0	0	0	0
C9orf62	0	0	0	0	0	0
C9orf64	0.34	0.55	0.56	0.35	0.22	0.14
C9orf66	0.91	0.42	0.61	0.53	0.73	0.64
C9orf69	3.05	3.8	4.37	3.37	3.78	3.4
C9orf72	2.09	0.42	1.14	0.77	1.9	1.63
C9orf78	52.21	53.33	51.08	46.41	48.58	43.93
C9orf84	0	0	0.04	0	0	0.06
C9orf85	6.52	6.34	5.12	4.86	8.29	8.53
C9orf89	18.35	14.21	13.18	10.97	17.07	13.68
C9orf9	5.87	3.67	3.34	5.06	4.84	2.23
C9orf91	1.37	2.17	1.93	2.29	1.82	1.69
C9orf92	0	0	0	0	0	0
C9orf96	0.11	0	0.1	0.04	0.02	0.05
CA1	2.01	0	0.12	0.04	0.12	0
CA10	0	0	0	0	0	0
CA11	1.53	1.81	2.76	4.1	1.7	1.82
CA12	0	0	0	0	0	0.03
CA13	5.69	2.96	2.71	2.26	3.27	2.16
CA14	0	0	0.04	0.06	0	0
CA2	45.34	16.45	13.75	13.09	19.14	11.21
CA3	0	0	0.08	0.12	0	0
CA4	0	0	0	0	0	0
CA5A	0	0	0	0	0	0
CA5B	2.14	1.43	2.07	1.52	1.35	1.73
CA5BP1	17.88	24.97	15.2	17.54	17.99	21.46
CA6	0	0	0	0	0.07	0
CA7	0	0	0	0	0	0
CA8	34.32	46.47	36.69	24.96	24.34	40.3
CA9	0	0	0	0	0	0
CAAP1	12.45	9.87	6.97	7.62	7.53	8.39
CAB39	7.34	2.67	2.93	2.52	4.41	2.94
CAB39L	1.79	1.46	1.01	1.15	0.68	0.84

CABIN1	4.88	8.97	7.73	5.45	5.37	7.71
CABLES1	1.1	1.21	1.41	0.9	1.15	1.64
CABLES2	0.73	1.37	0.82	0.93	0.92	0.72
CABP1	0.07	0	0	0.1	0	0.58
CABP2	0	0	0	0	0	0
CABP4	1.32	1.95	1.98	0.83	1.31	1.29
CABP5	0	0	0.07	0	0	0
CABP7	0.22	0	0.05	0.27	0.1	0.06
CABS1	0	0	0	0	0	0
CABYR	0.07	0.05	0	0.09	0	0
CACFD1	0.93	0.96	3.42	3.14	1.75	2.58
CACHD1	0.08	0.32	0.66	0.34	0.58	0.33
CACNA1A	0	0.01	0.02	0	0.02	0.01
CACNA1B	0	0	0	0	0	0
CACNA1C	0	0	0	0.02	0	0
CACNA1C-AS	0	0	0.06	0.16	0.12	0.37
CACNA1C-AS	0	0	0	0	0	0
CACNA1C-IT	0	0	0	0	0	0
CACNA1D	0	0	0	0.02	0.02	0.01
CACNA1E	0	0	0	0	0	0
CACNA1F	0	0	0	0	0	0
CACNA1G	0	0.01	0	0.01	0.01	0
CACNA1G-A	0	0	0	0	0	0
CACNA1H	0.67	0.5	0.93	1.19	0.79	1.11
CACNA1I	0	0	0	0	0	0
CACNA1S	0	0	0	0	0	0
CACNA2D1	0	0	0.1	0	0	0
CACNA2D2	0	0.1	0	0.01	0.05	0.08
CACNA2D3	0	0	0.09	0	0	0
CACNA2D3-A	0	0	0.03	0	0	0
CACNA2D4	2.12	1.52	1.21	1.69	1.26	1.37
CACNB1	0.41	0.47	0.23	1.06	0.68	0.61
CACNB2	0.27	0.29	0.24	0.08	0.26	0.27
CACNB3	1.43	2.46	2.9	3.01	2.04	1.82
CACNB4	0	0.11	0.05	0.06	0.05	0.27
CACNG1	0	0	0	0	0	0
CACNG2	0	0	0	0	0	0
CACNG3	0	0	0	0	0	0
CACNG4	0	0	0	0	0	0
CACNG5	0	0	0	0	0	0
CACNG6	0	0	0	0	0	0
CACNG7	0	0	0	0	0	0
CACNG8	0.47	0.55	0.72	0.39	0.34	0.4

CACTIN	1.7	1.42	0.66	0.94	1.13	0.84
CACTIN-AS1	0	0	0	0	0	0
CACUL1	11.22	10.51	8.9	8.83	10.4	9.81
CACYBP	78.3	78.95	73.65	69.51	81.36	69.49
CAD	19.85	22.56	22.43	25.53	23.34	30.27
CADM1	0	0	0	0	0	0
CADM2	0.02	0	0.02	0	0	0
CADM2-AS2	0.21	0.08	0	0	0.1	0
CADM3	0	0	0	0	0	0
CADM4	0.41	0.16	0.25	0.09	0.19	0.07
CADPS	0	0	0	0	0	0
CADPS2	0.07	1.02	0.92	0.26	0.54	0.45
CAGE1	0.11	0.04	0	0	0.02	0.05
CAHM	0.09	0.35	0.08	0.12	0.08	0
CALB1	596.11	0.16	0.64	36.61	69.82	0.29
CALB2	0	0	0	0	0	0
CALCA	0	0	0	0	0	0
CALCB	0	0	0	0	0	0
CALCOCO1	10.53	3.96	4.86	6.49	7.31	4.19
CALCOCO2	10.58	10.2	8.36	9.26	8.15	9.83
CALCR	0	0	0	0	0	0
CALCRL	0.07	0.04	0.31	1.22	0.27	0.73
CALD1	0.02	0	0	0	0	0
CALHM1	0.02	0.11	0.02	0	0	0
CALHM2	11.5	7.02	10.45	7.88	10.56	7.92
CALHM3	0	0	0	0	0	0
CALM1	39.55	36.48	34.97	30.1	34.54	32.22
CALM2	410.5	379.81	310.24	328.92	380.67	369.36
CALM3	70.62	50.49	50.13	57.77	65.7	58.4
CALML3	0	0	0	0	0	0
CALML4	1.17	2.05	1.01	1.34	1.51	1.27
CALML5	0	0	0	0	0	0
CALML6	0	0	0	0	0	0
CALN1	0.06	0.1	0.14	0.22	0.11	0.06
CALR	508.16	847.22	747.59	591.48	641	731.76
CALR3	0	0	0	0	0	0
CALU	27.74	24.43	18.47	18.89	24.09	17.74
CALY	0	0	0	0	0	0
CAMK1	0.05	0.2	0.14	0.03	0	0
CAMK1D	1.9	1.77	3.08	2.15	2.2	2.45
CAMK1G	0	0	0	0	0	0
CAMK2A	0	0	0	0	0	0
CAMK2B	0.04	0	0	0.01	0.12	0

CAMK2D	0	0	0	0	0	0
CAMK2G	9.13	10.03	5.84	7.37	7.72	7.4
CAMK2N1	0	0	0	0	0	0
CAMK2N2	0.22	0.52	0.15	0.38	0.26	0.45
CAMK4	0.03	0	0	0.05	0.06	0.1
CAMKK1	0.85	0.39	0.15	0.34	0.6	0.17
CAMKK2	6.57	12.11	10.76	8.47	7.01	11.46
CAMKMT	10	9.4	7.88	7.02	7.91	8.02
CAMKV	0.46	1.44	0.9	0.71	1.02	1.22
CAMLG	20.58	23.78	24.48	20.82	27.94	24.65
CAMP	0	0	0	0	0	0
CAMSAP1	1.98	1.38	2.09	1.5	2.27	1.88
CAMSAP2	0.14	0.02	0	0.03	0.05	0
CAMSAP3	0.12	0.43	0.57	0.23	0.15	0.43
CAMTA1	57.07	48.75	44.7	45.21	60.77	48.85
CAMTA2	2.97	1.54	1.41	1.99	2.38	1.92
CAND1	9.03	8.91	10.66	10.64	10.9	10.73
CAND2	0.26	0.23	0.28	0.22	0.24	0.52
CANT1	5.38	5.38	6.08	5.99	5.03	7.47
CANX	107.21	117.75	125.71	96.73	107.7	125.57
CAP1	108.86	64.41	67.09	68.56	75.54	57.64
CAP2	0.6	0.08	0	0	0.09	0
CAPG	16.79	21.35	16.56	11.14	16.05	7.55
CAPN1	76.04	49.08	64.92	67.84	58.96	61.39
CAPN10	0.66	1.29	0.1	1.03	0.57	0.7
CAPN11	0.16	0.27	0.49	0.63	0.71	0.43
CAPN12	0.07	0	0.02	0.03	0	0
CAPN13	0	0	0	0	0	0
CAPN14	0	0	0	0	0	0
CAPN2	17.41	13.95	14.03	13.3	14.69	14.56
CAPN3	0.42	0.19	0.02	0	0.17	0.36
CAPN5	0.74	2.19	0.71	1.92	0.68	0.88
CAPN6	0	0	0	0	0	0
CAPN7	3.74	3.91	3.06	3.29	3.25	2.81
CAPN8	0	0	0	0	0	0
CAPN9	0	0	0	0	0	0
CAPNS1	41.88	30.85	27.72	37.97	32.81	28.42
CAPNS2	0	0.06	0	0	0.14	0.15
CAPRIN1	66.11	65.42	70.15	72.74	61.64	73.75
CAPRIN2	0.69	0.25	0.14	0.54	0.43	0.6
CAPS	0.2	0.26	0.34	0.03	0.22	0.75
CAPS2	0.07	0	0	0	0.03	0.03
CAPSL	0	0	0	0	0	0

CAPZA1	134.02	131.78	113.77	94.87	122.37	111.07
CAPZA2	70.16	45.49	38.41	41.96	47.27	40.55
CAPZA3	0	0	0	0	0	0
CAPZB	82.65	84.42	72	84.57	85.29	76.04
CARD10	0.69	0.51	0.53	0.24	0.56	0.02
CARD11	1.52	0.95	0.18	1.17	0.58	0.29
CARD14	0	0	0	0	0	0
CARD16	0	0.58	0	0	0	0
CARD17	0	0	0	0	0	0
CARD18	0	0	0	0	0	0
CARD6	1.85	1.1	1.78	0.81	1.27	1.37
CARD8	12.45	12.57	10.77	7.66	10.93	12.53
CARD9	0.5	0.27	1.13	0.2	0.39	0
CARHSP1	11.62	11.09	11.83	13.62	13.21	10.4
CARKD	14.39	12.9	8.57	10.21	11.93	14.19
CARM1	4.59	4.86	5.38	4.9	4.54	6.04
CARNS1	0.04	0	0.03	0.13	0.05	0.07
CARS	22.06	15.74	18.61	14.5	17.21	14.54
CARS2	3.46	3.36	5.03	5.18	3.8	4.63
CARTPT	0	0	0	0	0	0
CASC1	0	0	0	0	0	0.1
CASC2	0	0.14	0	0.23	0	0
CASC3	25.67	21.34	18.8	20.98	22.23	22.96
CASC4	15.74	14.58	11.12	9.7	11.54	12.46
CASC5	5.98	5.44	5.74	5.73	6	5.66
CASD1	0.95	0.51	0.32	0.14	0.49	0.36
CASK	0.47	0.63	0.67	0.51	0.45	0.57
CASKIN1	0.18	0.08	0.04	0.17	0.31	0.07
CASKIN2	0.9	0.84	0.55	1.04	0.79	0.78
CASP1	0	0.23	0.26	0	0	0.44
CASP10	7.73	4.65	3.76	2.41	3.87	3.58
CASP12	0	0	0	0	0	0
CASP14	0.05	0.13	0.09	0.02	0.06	0.2
CASP2	25.24	37.57	35.41	30.65	26.52	34.14
CASP3	29.06	21.07	18.91	18.76	24.22	21.91
CASP4	24.95	47.77	32.3	37.92	36.36	24.23
CASP5	0	0	0	0	0	0
CASP6	14.63	17.61	16.58	13.38	14.54	10.8
CASP7	10.43	11.27	9.75	5.17	10.67	10.19
CASP8	11.04	11.56	12.55	9.7	10.09	11.68
CASP8AP2	3.59	3.47	3.47	2.72	3.84	3.7
CASP9	21.65	4.64	5.79	6.09	9.76	4.16
CASQ1	0	0	0	0	0	0

CASQ2	0	0	0	0	0	0
CASR	0.16	0	0	0	0	0
CASS4	3.76	0.9	1.9	1.5	2.65	1.61
CAST	5.09	13.44	12.36	8.69	8.42	11.23
CASZ1	0.44	0.63	1.05	0.8	0.88	0.62
CAT	54.74	178.48	138.1	55.84	61.2	152.93
CATSPER1	0.34	0	0	0	0.11	0
CATSPER2	0.3	0.18	0.22	0.04	0.14	0.09
CATSPER2P1	0.3	0.22	0.38	0.27	0.05	0.42
CATSPER3	0	0.43	0.5	1.03	0.1	0.33
CATSPER4	0	0	0	0	0	0
CATSPERB	0	0	0	0	0	0
CATSPERD	0	0	0	0.02	0	0
CATSPERG	0.02	0.03	0.02	0.01	0.04	0.02
CAV1	6.21	0.83	0.31	4.73	0.09	0
CAV2	0.14	0.32	0.55	0.22	0.18	0.5
CAV3	0	0	0	0	0	0
CBFA2T2	1.46	1.96	1.71	1.92	1.59	1.6
CBFA2T3	14.69	7.57	8.85	12.64	15.52	9.06
CBFB	16.3	8.19	6.82	5.94	7.31	6.7
CBL	2.55	3.36	2.76	2.2	2.4	3.82
CBLB	5.39	1.96	1.4	2.39	1.32	1.71
CBLC	0	0	0	0	0	0
CBLL1	24.06	19.13	17.37	22.66	24.51	16.9
CBLN1	0	0	0.11	0	0	0
CBLN2	0	0	0	0	0	0
CBLN3	0	0	0	0	0.15	0
CBLN4	0	0	0	0	0	0
CBR1	73.4	91.02	83.29	86.24	74.75	63.97
CBR3	2.48	7.51	7.73	7.49	5.46	6.09
CBR3-AS1	0.32	0.9	0.83	0.15	0.1	0.44
CBR4	6	4.23	4.73	4.79	5.32	6.28
CBS	61.31	50.96	61.21	58.04	63.82	68.3
CBWD1	31.05	21	19.67	17.87	29.43	19.11
CBWD2	20.95	17.39	16.14	12.47	14.59	13.24
CBWD3	46.17	34.52	34.97	26.68	38.54	34.57
CBWD5	19.8	33.87	20.68	21.36	26.47	23.4
CBWD6	7.63	5.47	3.57	0.54	3.09	2.55
CBX1	13.42	14.01	11.23	8.92	15.28	12.05
CBX2	5.58	6.05	6.25	5.98	5.23	5
CBX3	99.68	81.65	58.53	61.2	81.54	68.48
CBX3P2	0	0	0.14	0.09	0	0
CBX4	0.72	0.7	0.56	0.42	0.7	0.56

CBX5	30.68	36.74	36.67	39.04	32.79	40.93
CBX6	5.68	3.45	3.26	4.77	5.31	3.63
CBX7	0.07	0.01	0	0	0	0
CBX8	1.69	1.26	1.33	3.23	1.37	2.1
CBY1	15.49	8.74	9.94	10.83	10.05	6.98
CBY3	0.1	0	0.27	0	0	0
CC2D1A	2.35	2.56	3.85	4.1	2.37	3.29
CC2D1B	5.31	3.71	2.93	2.69	3.65	3.03
CC2D2A	6.45	2.4	3.31	2.31	3.52	3.05
CC2D2B	0	0	0	0	0	0
CCAR1	27.39	28.49	23.94	21.92	28.78	27.17
CCBE1	0.61	0.55	0.5	0.44	0.33	0.42
CCBL1	3.23	8.79	5.56	5.35	6.51	4.51
CCBL2	10.4	10.45	9.17	9.83	10.61	9.5
CCBP2	0.64	0.49	0.52	0.35	0.44	0.55
CCDC101	23.49	26.82	25.97	28.45	26.55	21.96
CCDC102A	0.54	1.09	0.75	1.29	1	1.62
CCDC102B	5.54	0	0.02	0.7	1.88	0.16
CCDC103	1.38	0.82	0.67	0.81	0.98	0.44
CCDC104	22.39	18.98	15.27	17.08	20.96	17.51
CCDC105	0	0	0	0	0	0
CCDC106	3.49	2.83	3.53	4.8	5	3.6
CCDC107	19.55	16.98	15.86	18.99	16.1	12.49
CCDC108	0.01	0	0	0	0	0.04
CCDC109B	19.71	18.21	19.12	18.43	26.17	19.14
CCDC11	0	0	0.07	0.03	0	0.04
CCDC110	0.15	0	0	0.07	0.05	0
CCDC111	5.15	2.65	2.98	3.05	3.77	3.97
CCDC112	3.44	2.56	1.85	1.63	3.1	1.84
CCDC113	1.29	0.79	1.03	0.98	0.72	0.62
CCDC114	0.4	0.11	0.12	0.08	0.05	0.06
CCDC115	27.66	22.8	18.76	18.71	19.37	18.77
CCDC116	0	0.21	0.15	0	0	0
CCDC117	7.65	12.36	12.93	12.51	11.11	10.12
CCDC12	64.08	66.15	56.35	68.04	71.15	70.8
CCDC120	1.18	1.19	0.77	0.62	1.32	1.09
CCDC121	0.47	0.54	0.65	0.46	0.9	0.94
CCDC122	3.19	2.6	0.7	1.37	0.99	2.05
CCDC124	126.29	148.25	122.6	139.98	135.03	116.66
CCDC125	4	5.46	4.78	3.72	5.32	6.13
CCDC126	0.89	0.98	1.09	0.55	1.09	0.48
CCDC127	17.72	10.46	12	15.72	15.75	14.23
CCDC129	0	0	0.01	0.01	0	0.02

CCDC13	0.05	0.14	0.02	0.09	0.17	0.08
CCDC13-AS1	0	0	0	0	0	0
CCDC130	8.82	7.99	6.4	6.33	7.47	4.2
CCDC132	5.06	3.89	3.34	2.92	2.89	2.35
CCDC134	8.3	9.96	8.74	6.95	8.92	11.09
CCDC135	0	0.06	0.23	0.17	0.02	0.05
CCDC136	0.58	1.04	1.1	0.67	0.85	1.06
CCDC137	24.66	30.23	25.07	28.75	26.05	19.46
CCDC138	6.67	5.73	4.42	4.5	4.5	5.6
CCDC14	3.75	5.2	3.33	3.87	3.52	3.59
CCDC140	0	0	0	0	0	0
CCDC141	0	0.02	0.01	0	0.01	0
CCDC142	2.11	4	5.84	2.12	3.6	4.07
CCDC144A	0.3	0.35	0.24	0.33	0.23	0.32
CCDC144B	3.32	3.76	3.28	2.43	3.27	2.62
CCDC144C	0.07	0.02	0	0.01	0.09	0
CCDC144NL	0	0	0	0	0	0
CCDC146	0	0	0.02	0.03	0	0.02
CCDC147	0	0	0	0.09	0.02	0
CCDC148	0.26	0.29	0.38	0.29	0.19	0
CCDC148-AS1	0	0	0	0	0	0
CCDC149	0	0.03	0	0	0	0.02
CCDC15	0.67	2.04	0.83	1.06	1.33	1.45
CCDC150	0.3	0.42	0.43	0.14	0.17	0.06
CCDC151	0.14	0.03	0	0.14	0.03	0.21
CCDC152	0	0	0	0.02	0.06	0
CCDC153	0	0.49	0.47	0.78	0.2	0.42
CCDC154	0.07	0.5	0	0.02	0.22	0.17
CCDC155	0	0.17	0	0	0	0
CCDC157	0.25	0.59	0.36	0.48	0.21	0.56
CCDC158	0	0	0	0	0	0
CCDC159	2.38	1.99	0.92	1.14	3.28	3.64
CCDC160	0	0	0	0	0	0.06
CCDC162P	0	0	0	0	0	0
CCDC163P	2.96	2.84	3.05	2.34	2.37	2.7
CCDC164	0	0	0	0	0	0
CCDC166	0	0	0	0	0	0
CCDC167	58.75	73.66	71.19	66.89	67.24	57.64
CCDC168	0.02	0.08	0.05	0.02	0.06	0.03
CCDC169	3.68	2.94	3.07	3.29	3.84	4.22
CCDC169-SO	0.1	0	0	0	0	0
CCDC17	0.2	0.1	0.03	0.02	0	0
CCDC170	0.15	0.4	0.23	0.06	0.07	0.31

CCDC171	0.58	0.6	0.55	0.84	0.31	0.86
CCDC172	0	0.03	0	0	0.04	0
CCDC173	0.31	0.16	0.06	0.3	0.45	0.38
CCDC174	4.51	3.72	2.75	3.34	3.37	2.52
CCDC175	0	0	0	0	0	0
CCDC176	0.39	0.45	0.22	0.38	0.34	0.63
CCDC177	0	0	0	0	0	0
CCDC178	0	0	0	0	0	0
CCDC179	0	0	0	0	0	0
CCDC18	2.68	2.88	2.7	2.1	2.64	2.12
CCDC19	0.21	0.33	0.15	0.26	0.31	0.17
CCDC22	22.25	31.96	30.54	30.83	26.24	28.76
CCDC23	11.73	10.66	13.88	12.08	12.16	14.32
CCDC24	1.08	0.16	0.94	0.92	0.34	0.62
CCDC25	31	25.95	23.45	23.28	27.09	24.46
CCDC27	0	0	0	0	0	0
CCDC28A	15.17	11.41	11.55	10.01	13.68	10.63
CCDC28B	32.51	36.81	32.78	41.78	44.9	31.57
CCDC3	0.27	0.59	0.98	0.9	0.5	0.27
CCDC30	0.58	0.36	0.64	0.29	0.28	0.41
CCDC33	0	0	0	0	0	0
CCDC34	12.52	13.2	9.12	10.99	12.13	9.89
CCDC36	0	0.06	0.07	0.02	0.08	0
CCDC37	0	0	0	0	0	0
CCDC38	0.08	0	0.07	0	0	0.04
CCDC39	0	0	0	0.01	0	0.08
CCDC40	0.02	0.05	0	0.01	0.04	0.08
CCDC41	4.71	8.17	6.77	5.72	6.28	7.37
CCDC42	0	0	0	0.19	0	0
CCDC42B	0	0.25	0.19	0	0	0.07
CCDC43	11.86	14.67	10	12.16	12.39	12.97
CCDC47	40.21	35.22	30.99	33.17	37.02	36.66
CCDC50	5.35	4.09	4.3	4.42	4.57	4.09
CCDC51	28.77	27.01	35.98	28.65	35.14	30.27
CCDC53	28.73	34.48	30.56	27.39	31.02	28.93
CCDC54	0	0	0	0	0	0
CCDC57	1.26	2.66	2.16	1.67	1.74	3.22
CCDC58	24.83	29.69	26.77	27.81	30.65	31.43
CCDC59	28.32	27.27	20.92	21.95	25.96	20.26
CCDC6	6.26	6.44	7.6	6.3	6.44	5.39
CCDC60	0	0	0	0	0	0
CCDC61	0.33	0.67	0.6	0.87	0.54	0.54
CCDC62	0.09	0.25	0.3	0.26	0.22	0.27

CCDC63	0	0	0	0	0	0
CCDC64	0.24	0.02	0	0	0.07	0.12
CCDC64B	0	0	0	0	0	0
CCDC65	0.25	0.03	0.6	0.23	0.19	0
CCDC66	1.22	1.91	0.45	1.07	0.62	0.3
CCDC67	0	0	0	0	0	0
CCDC68	0.05	0	0	0	0	0
CCDC69	3.35	3.85	4.09	2.35	2.57	2.55
CCDC7	0.23	0.53	0.65	0.25	0.32	0.6
CCDC70	0	0	0	0	0	0
CCDC71	7.82	5.16	7.08	8.32	9.48	6.79
CCDC71L	0.78	0.44	0.03	0.43	0.2	0.02
CCDC73	0.36	0.17	0.2	0.14	0.12	0.24
CCDC74A	0	0	0	0.2	0	0
CCDC74B	0.28	0	0	0	0	0
CCDC74B-AS	0.31	0.76	0.91	0.3	0.62	0.56
CCDC77	5.37	6.92	5.5	6.68	8.04	5.28
CCDC78	0.19	0.07	0	0.06	0	0.09
CCDC79	0	0	0	0	0	0
CCDC8	0.1	0.05	0.12	0.08	0.08	0.01
CCDC80	0	0	0	0	0	0
CCDC81	0	0.02	0	0	0.11	0
CCDC82	3.72	3.85	3.41	2.54	3.66	2
CCDC83	0	0	0	0	0	0
CCDC84	3.6	1.84	0.69	2.7	3.17	4.15
CCDC85A	0	0	0	0	0	0
CCDC85B	12	15.44	21.17	20.76	17.46	18.78
CCDC85C	1.92	3.77	2.7	2.36	1.9	2.88
CCDC86	65.29	71.49	79.59	73.77	64.96	64.04
CCDC87	0.13	0.04	0.32	0.31	0	0
CCDC88A	3.76	4.55	3.69	3.3	3.45	4.77
CCDC88B	0.28	0.58	0.13	0.33	0.57	0.43
CCDC88C	1.6	2.35	2.04	1.89	1.6	2.66
CCDC89	0	0	0	0	0	0
CCDC9	1.03	1.52	0.85	1.2	1.01	1.01
CCDC90A	5.31	5.19	6.03	4.34	4.79	3.72
CCDC90B	17.56	13.73	15.6	14.09	16.19	12.83
CCDC91	6.07	3.64	3	2.99	3.8	2.32
CCDC92	2.53	1.3	0.72	2	0.66	0.67
CCDC93	2.96	3.38	2.85	2.1	2.83	3.15
CCDC94	25.14	26.22	24.88	23.08	26.21	25.03
CCDC96	0.07	0.13	0.25	0.07	0	0.07
CCDC97	9.94	12.94	13.09	12.95	9.75	14.03

CCER1	0	0	0	0	0	0
CCHCR1	4.15	4.3	5.99	5.5	7.29	4.35
CCIN	0	0	0	0	0	0
CCK	0	0	0	0	0	0
CCKAR	0	0	0	0	0	0
CCKBR	0	0.16	0	0.07	0	0
CCL1	0	0	0	0	0	0
CCL11	0	0	0	0	0	0
CCL13	0	0	0	0	0	0
CCL14	0	0	0	0	0	0
CCL15	0	0	0	0	0	0
CCL15-CCL14	0	0	0	0	0	0
CCL16	0.51	0.78	0.84	0.49	0.65	0.57
CCL17	0	0	0	0	0	0
CCL18	0.21	0	0	0	0.97	0
CCL19	0	0	0	0	0	0
CCL2	34.36	0	0	0.73	2.12	0.64
CCL20	0	0	0	0	0	0
CCL21	0	0	0	0	0	0
CCL22	0.95	0.55	1.15	0.72	0.92	0.81
CCL23	0	0	0	0	0.12	0
CCL24	0.5	0	0	0	0	0
CCL25	0	0	0	0	0.07	0.08
CCL26	0	0	0	0	0	0
CCL27	0	0	0	0	0	0
CCL28	0.13	0.14	0.54	0.07	0.51	0.19
CCL3	0.2	0	0	0	0	0.2
CCL3L1	0	0	0	0	0	0
CCL3L3	0.31	0	0	0.14	0	0
CCL4	0.34	0	0	0.33	0.35	0
CCL4L1	0.44	0	0	0.17	0.06	0
CCL4L2	0.44	0	0	0.17	0.06	0
CCL5	11.03	11.67	13.21	10.61	13.23	14.72
CCL7	0	0	0	0	0	0
CCL8	0	0	0	0	0	0
CCM2	18.26	34.1	35	32.76	24.12	31.38
CCM2L	0	0	0	0	0.08	0
CCNA1	8.28	12.3	12.76	2.07	4.18	13.93
CCNA2	79.99	87.67	78.83	73.93	82.55	72.25
CCNB1	172.82	153.01	140.02	143.66	156.87	132.22
CCNB1IP1	29.05	32.33	28.7	42.62	31.42	32.95
CCNB2	107.55	135.53	114.5	127.71	132.69	116.31
CCNB3	0.06	0.03	0	0	0	0.05

CCNC	32.65	34.9	28.13	23.4	36.95	38.41
CCND1	0.29	0.01	0.22	0	0.37	0
CCND2	0.03	0.06	0.65	0.09	0.12	0.3
CCND3	92.01	58.75	65.01	59.55	58.26	51.58
CCNDBP1	23.03	18.07	12.74	14.46	18.5	14.25
CCNE1	3.27	2.33	2.05	2.17	3.55	2.96
CCNE2	13.85	7.6	5.58	7.12	10.4	7.8
CCNF	36.7	39.03	45.5	44.64	38.1	39.86
CCNG1	95.55	97.78	104.51	91.71	98.33	103.22
CCNG2	0.66	0.11	0.11	0.07	0.26	0.29
CCNH	50.03	38.54	33.12	26.4	41.1	25.64
CCNI	28.6	26.09	39.62	45.72	41.02	28.13
CCNI2	0.03	0	0	0.13	0	0
CCNJ	1.68	2.22	3.05	2.82	3.79	2.79
CCNJL	1.22	0.73	1.1	0.6	0.94	0.52
CCNK	27.01	22	16.95	17.89	23.55	20.17
CCNL1	5.69	8.21	5.18	5.02	5.58	5.47
CCNL2	7.22	7.19	7.26	7.96	8.81	5.91
CCNO	5.45	12.07	6.53	6.36	7.78	9.38
CCNT1	7.23	6.66	7.52	6.33	7.28	6.9
CCNT2	2.89	2.56	2.17	1.87	2.19	2.21
CCNY	3.88	3.81	3.92	3.55	3.3	2.67
CCNYL1	1.49	0.86	1.15	1.16	1.55	0.76
CCP110	4.14	6.08	3.47	3.81	3.68	4.82
CCPG1	6.99	6.1	4.01	2.88	4.48	3.02
CCR1	0	0.15	0.05	0	0	0.03
CCR10	0.18	0.53	1.67	0.55	1.27	0.68
CCR2	0	0	0	0	0	0
CCR3	0	0	0	0	0	0
CCR4	0.09	0	0	0.28	0.34	0
CCR5	0.02	0	0	0	0	0
CCR6	0.79	0.56	0.79	0.39	0.42	0.37
CCR7	0	0.08	0	0.02	0.16	0
CCR8	0	0	0	0	0.1	0
CCR9	0	0.11	0.03	0	0	0
CCRL1	0.1	0	0	0.02	0	0.03
CCRL2	5.62	0.71	2.01	2.61	3.9	2.22
CCRN4L	5.9	4.49	6.93	7.59	4.46	3.09
CCS	14.09	21.96	22.26	24.97	16.14	19.77
CCSAP	1.22	1.21	1.04	0.85	0.89	0.86
CCSER1	0	0	0.16	0.02	0	0.07
CCSER2	1.21	1.65	1.65	1.37	1.54	2.26
CCT2	247.48	231.56	207.74	203.83	232.4	200.65

CCT3	379.11	306.74	278.71	335.97	321.98	293.79
CCT4	185.45	173.96	178.63	177.91	181.73	175.69
CCT5	181.84	185.81	182.83	163.35	182.33	174.47
CCT6A	254.17	254.68	234.8	214.88	233.62	232.58
CCT6B	0.77	0.33	0.58	0.58	0.92	1.03
CCT6P1	1.04	1.4	0.71	0.14	1.03	0.79
CCT6P3	2.08	1.93	0.5	0.82	1.48	0.96
CCT7	404.54	421.77	449.62	461.81	445.31	463.35
CCT8	252.06	233.25	207.64	208.76	256.27	222.1
CCT8L2	0.04	0	0	0	0	0
CCZ1	2.5	1.77	0.3	1.8	2.32	1.64
CCZ1B	3.61	5.58	4.84	5.46	5.69	5.52
CD101	0.04	0.21	0.06	0	0.08	0.38
CD109	1.33	1.03	1.1	1.16	1.17	0.91
CD14	0	0	0	0	0	0
CD151	33.58	18.68	18.29	20.18	24.09	24.76
CD160	0.19	0.11	0	0	0	0.24
CD163	0	0	0	0	0	0
CD163L1	0.44	0.98	0	0.02	0	0.03
CD164	60.2	52.97	46.24	34.89	49.9	51.28
CD164L2	0	0	0	0	0	0
CD177	0	0	0	0	0	0
CD180	0	0.83	0.39	0.11	0	0
CD19	0.11	0.39	0.55	0.47	0.43	0.23
CD1A	0	0	0	0	0	0
CD1B	0	0	0	0	0	0
CD1C	0	0	0	0	0	0
CD1D	0.04	0.03	0.07	0.01	0.04	0.03
CD1E	0	0	0	0	0	0
CD2	0	0	0	0	0	0.1
CD200	0	0.1	0.67	2.31	0.03	0
CD200R1	0	0.11	0	0	0	0
CD200R1L	0	0	0	0	0	0
CD207	1.74	0	0.33	0.16	0.56	0
CD209	0.37	0.45	0.53	0.1	0.32	0.31
CD22	0	0	0	0	0.02	0
CD226	0.08	0	0.03	0.02	0.18	0
CD24	128.24	1.01	2.2	3.53	19.34	1.34
CD244	4.82	5.3	6.08	3.88	3	5.97
CD247	0	0	0	0	0	0.13
CD248	0	0	0	0	0	0
CD27	0	0.09	0	0	0.11	0.06
CD27-AS1	0.33	0.64	0.3	0.11	0.15	0.19

CD274	0	0.02	0	0	0	0.07
CD276	35.76	15.76	10.97	16.2	19.66	14.58
CD28	0.03	0.01	0.03	0.01	0.02	0.02
CD2AP	2.65	1.42	1.16	1.78	1.83	1.56
CD2BP2	18.59	13.73	13.07	13.84	14.74	15.69
CD300A	1.25	4.18	4.15	2.24	1.92	1.67
CD300C	0	0.01	0.61	0	0	0.04
CD300E	0.26	0.17	0.32	0.11	0.25	0.49
CD300LB	0	0	0	0	0	0
CD300LD	0	0	0	0	0	0
CD300LF	0	0	0	0	0.04	0
CD300LG	0.46	0.18	0.37	0.18	0.28	0.47
CD302	4.53	7.29	6.72	6.48	5.64	9.66
CD320	1.09	1.39	0.5	0.84	1.48	1.16
CD33	38.78	49.84	35.56	26.49	33.77	45.75
CD34	0.76	0.09	6.61	26.54	2.53	14.82
CD36	40	0.08	0.05	9.12	4.34	0.08
CD37	19.05	54.13	70.96	58.84	57.67	63.12
CD38	0.25	0	0	0	0	0
CD3D	2.35	4.31	3.06	5.12	4.57	5.09
CD3E	0	0	0	0.03	0.61	0
CD3EAP	15.8	14.79	12.9	13.19	15.75	11.39
CD3G	0.36	0.36	0.44	0	0.06	0.75
CD4	40.87	47.86	54.91	50.71	40.13	64.22
CD40	2.79	4.69	3.8	3.24	2.39	2.43
CD40LG	0	0	0	0	0	0
CD44	37.11	42.04	50	27.69	27.07	46.09
CD46	16.07	14.96	13.68	8.65	11.17	11.7
CD47	24.87	36.73	27.23	22.56	27.01	30.2
CD48	1.44	1.76	1.36	0.28	1.86	2.05
CD5	0	0.07	0	0	0	0
CD52	13.94	9.59	18.35	24.05	21.53	5.85
CD53	39.42	22.51	21.12	14.98	24.37	16.83
CD55	15.4	10.09	5.56	10.3	13.01	8.4
CD58	23.91	34.22	11.81	8.52	19.82	17.54
CD59	23.83	9.62	9.34	12.62	15.6	11.25
CD5L	0.03	0	0	0	0	0
CD6	0	0	0	0	0	0
CD63	421.96	187.5	190.83	246.82	314.41	175.94
CD68	5.05	6.46	8.85	9.27	6.51	8.93
CD69	9.6	17.72	24.28	6.12	13.73	17.98
CD7	0	0	0	0.37	0.11	0
CD70	0	0	0	0	0	0

CD72	0.58	0.34	0.49	0.4	0.23	0.49
CD74	111.23	297.25	570.39	639.95	374.59	417.56
CD79A	0.81	2.75	3.58	3.24	2.5	1.93
CD79B	0	0	0.66	1.02	0.45	0.84
CD80	0.11	0	0	0	0.08	0
CD81	11.46	12.38	14.38	14	16.13	15.08
CD82	4.73	7.56	3.43	2.86	6.02	3.36
CD83	3.25	9.82	7.56	7.55	5.84	9.16
CD84	4.28	2.23	1.39	1.58	3.68	2.52
CD86	2.5	18.01	8.24	2.02	3.21	9.04
CD8A	0	0	0.02	0	0.22	0
CD8B	64.76	56.75	87.89	128.33	94.91	97.6
CD9	18.56	0.32	0	1.11	0.49	0.99
CD93	0	0	0.02	0	0	0
CD96	0.2	0.12	0.12	0.07	0.23	0.05
CD97	14.12	9.68	9.88	7.14	9.68	9.85
CD99	125	115.11	101.3	116.97	113.07	120.47
CD99L2	12.81	13.19	14.79	14.35	14.01	16.23
CD99P1	3.76	1.85	2.34	4.38	4.02	2.27
CDA	0	0.38	1.03	0.22	0.23	4.39
CDADC1	2.71	3.24	2.62	3.03	3.63	3.19
CDAN1	1.01	0.75	1.4	1.08	0.76	1.85
CDC123	94.77	117.75	106.78	103.47	106.27	107.81
CDC14A	2.82	1.75	1.58	1.51	1.98	2.06
CDC14B	0.46	0.96	0.62	0.74	0.8	0.45
CDC14C	0.12	0.1	0.22	0.08	0.15	0.18
CDC16	9.61	7.17	7.43	7.67	8.1	4.98
CDC20	130.54	197.83	192.27	161.25	148.09	163.89
CDC20B	0	0	0	0	0	0
CDC23	22.49	21.02	20.7	18.26	16.69	19.57
CDC25A	3.56	3.78	4.47	3.38	3.44	4.55
CDC25B	6.35	8.55	8.82	8.16	7.76	10.23
CDC25C	8.99	11.54	9.96	11.23	8.34	11.16
CDC26	34.41	57.76	54.22	48.05	45.98	58.48
CDC27	12.2	8.58	8.44	8.11	11.3	8.69
CDC34	4.67	3.44	3.66	4.58	4.17	1.05
CDC37	144.82	128.48	108.21	111.84	124.57	112.3
CDC37L1	8.72	4.87	3.09	3.26	6.05	4.03
CDC40	5.21	5.92	4.88	3.13	5.18	5.53
CDC42	144.8	130.93	134.3	124.09	150.66	152.64
CDC42BPA	2.17	0.74	0.46	0.74	1.02	0.75
CDC42BPB	1.56	0.78	0.8	0.86	1.18	0.91
CDC42BPG	0.03	0.24	0.09	0.12	0.03	0.13

CDC42EP1	8.65	0	0	1.5	2.54	0.07
CDC42EP2	8.87	6.18	8.42	9.74	6	4.65
CDC42EP3	1.66	0.45	0.57	0.06	0.55	1.07
CDC42EP4	13.51	5.14	4.92	8.28	11.74	4.52
CDC42EP5	0	0	0	0	0	0
CDC42SE1	45.55	35.51	40.8	37.71	42.65	39.28
CDC42SE2	24.39	23.95	16.99	16.31	16	20.68
CDC45	44.36	61.32	55.58	56.92	50.29	53.18
CDC5L	9.03	9.22	8.66	8.43	8.98	9.62
CDC6	28.41	31.37	23.6	30.04	27.2	25.29
CDC7	7.47	9.14	10.37	9.74	9.97	9.41
CDC73	5.4	5.25	5.01	3.33	4.52	3.54
CDCA2	32.09	41.31	44.94	34.16	36.56	35.6
CDCA3	42.59	35.74	36.88	38.14	48.24	30.02
CDCA4	32.23	33.58	33.94	34.42	40.26	33.04
CDCA5	50.96	56.12	51.69	53.75	48.55	57.79
CDCA7	27.32	88.03	72.09	56.63	47.15	85.55
CDCA7L	9.2	37.45	37.34	27.06	22.67	36.4
CDCA8	45.32	55.92	52.52	46.94	54.11	41.96
CDCP1	2.9	0.17	0.24	0.17	0.22	0.21
CDCP2	0	0	0	0	0	0
CDH1	0.14	0.2	0.25	0.09	0.12	0.12
CDH10	0	0	0	0	0	0
CDH11	0	0	0	0	0	0
CDH12	0	0.01	0.02	0	0.02	0.03
CDH13	0	0	0	0.04	0	0
CDH15	0	0	0	0.3	0	0
CDH16	0	0	0	0	0	0
CDH17	0.07	0.05	0.04	0	0.02	0.05
CDH18	0	0	0	0	0	0
CDH19	0	0	0	0	0	0
CDH2	0	0.12	0	0	0.05	0
CDH20	0	0	0	0	0	0
CDH22	0	0.03	0.08	0.08	0.05	0.09
CDH23	0.56	1.04	1.14	0.41	0.39	0.79
CDH24	1.2	1.33	1.06	0.62	0.62	1.2
CDH26	0.02	0	3.87	0.56	0	0.09
CDH3	0	0	0	0	0	0.02
CDH4	0	0	0	0	0	0
CDH5	0.04	0	0	0	0	0
CDH6	0.06	0.01	0.05	0.06	0.02	0.02
CDH7	0	0	0	0	0	0
CDH8	0	0	0	0	0	0

CDH9	0	0	0	0	0	0
CDHR1	0.03	0	0	0.02	0	0
CDHR2	0	0	0	0	0	0
CDHR3	0.14	0.16	0.36	0.16	0.11	0.09
CDHR4	0	0	0	0	0	0
CDHR5	0	0	0	0	0	0.05
CDIP1	11.45	8.27	11.66	10.51	10.54	11.22
CDIPT	7.71	5.95	7.09	5.94	5	6.24
CDK1	103.77	77.24	70.38	75.32	86.71	72.34
CDK10	8.71	18.98	14.46	12.52	13.87	12.6
CDK11A	9.02	7.19	4.57	5.06	6.44	3.92
CDK11B	7.03	5.47	5.79	5.22	5.68	6.65
CDK12	7.72	7.23	6.17	5.18	7.07	7.64
CDK13	1.08	1.5	1.72	1.31	1.09	1.48
CDK14	0.07	0.01	0	0.04	0.11	0
CDK15	0.04	0	0	0	0.13	0.02
CDK16	21	22.04	23.09	22.88	22.37	13.58
CDK17	3.78	2.96	3.65	3.5	3.12	3.44
CDK18	0	0.17	0.28	0.15	0	0.05
CDK19	0.88	1.05	0.63	0.59	0.56	1.39
CDK2	19.13	14.82	14.32	19.26	17.65	13.78
CDK20	0	0	0	0	0	0
CDK2AP1	39.98	29.48	26.68	33.89	37.92	26.67
CDK2AP2	68.66	81.21	71.37	69.7	70.37	62.12
CDK3	1.28	1.62	1.44	1.61	0.9	2.17
CDK4	177.46	184.5	173.81	183.65	204.36	188.21
CDK5	24.07	35.69	34.93	25.6	29.01	33.06
CDK5R1	0.56	1.04	0.62	1.06	0.73	0.59
CDK5R2	0	0	0	0.06	0	0
CDK5RAP1	31.57	29.38	27.72	28.19	30.09	24.07
CDK5RAP2	5.1	3.88	4.22	3.19	4.68	3.73
CDK5RAP3	19.46	28.56	29.11	30.56	25.4	30.58
CDK6	3.79	15.1	14.37	9.6	8.08	9.08
CDK7	33.21	29.44	23.44	18.77	25.6	27.64
CDK8	1.39	1.19	1.08	0.93	1.19	0.64
CDK9	6.47	9.65	9.31	8.3	7.67	9
CDKAL1	15.99	11.07	13.36	17.39	13.14	17.15
CDKL1	0	0.56	0.41	0.45	0.73	0.33
CDKL2	0	0	0	0	0	0
CDKL3	0.81	0.25	0.22	0.38	0.09	0.09
CDKL4	0.72	1.47	0.52	0.82	1.04	0.68
CDKL5	0.49	0.27	0.87	0.23	0.16	0.99
CDKN1A	39.84	26.8	14.51	16.21	10.72	16.82

CDKN1B	5.05	4.67	4.45	5.46	4.45	6.86
CDKN1C	1.83	1.42	1.11	2.55	2.43	1.18
CDKN2A	419.84	251.71	203.59	263	345.16	230.32
CDKN2AIP	10.92	12.71	10.9	11	16.81	12.89
CDKN2AIPNL	58.67	61.41	58.67	68.76	74.83	55.87
CDKN2B	36	0.83	0.73	3.4	6.85	0.79
CDKN2B-AS1	2.15	1.25	1.23	0.76	1.29	1.2
CDKN2C	12.34	12.12	16.74	14.54	13.12	9.26
CDKN2D	4.39	1.63	3.04	2.99	2.08	2.69
CDKN3	136.14	148.43	133.66	135.74	158.04	129.19
CDNF	0.28	0.54	0.88	0.04	1.07	0.11
CDO1	6.1	7.76	3.34	5	7.73	4.39
CDON	0.27	0.11	0.16	0.19	0.12	0.07
CDPF1	10.6	9.06	7.21	12.63	10.77	7.67
CDR1	0	0	0	0	0	0
CDR2	1.43	1.07	0.92	1.17	1.99	1.01
CDR2L	3.82	4.37	3.2	3.83	5.5	4.12
CDRT1	0	0	0	0	0	0
CDRT15	0	0	0.12	0	0	0.29
CDRT15L2	0	0	0	0	0.07	0
CDRT15P1	0	0.22	0.2	0.1	0.2	0.42
CDRT15P2	0	0	0	0	0	0
CDRT4	0	0	0	0	0	0
CDRT7	0	0	0	0	0	0
CDS1	0.18	0.46	0.26	0.07	0.06	0.06
CDS2	11.11	7.22	8.61	8.41	9.65	8.8
CDSN	0	0	0	0	0	0
CDT1	9.27	13.01	12.68	12.68	11.76	11.97
CDV3	24.2	20.45	20.69	16.02	22.51	22.82
CDX1	0	0	0	0	0	0
CDX2	10.03	16.85	13.84	8.75	11.65	13.37
CDX4	0	0	0	0	0	0
CDY1	0	0	0	0	0	0
CDY1B	0	0	0	0	0	0
CDY2A	0	0	0	0	0	0
CDY2B	0	0	0	0	0	0
CDYL	3.46	2.54	2.3	1.83	3.09	2.53
CDYL2	0.1	0.05	0.9	0.2	0	0.13
CEACAM1	0	0	0	0	0	0.02
CEACAM16	0	0	0	0	0	0
CEACAM18	0	0	0	0	0	0
CEACAM19	0.23	0.44	0.36	0.25	0.34	0.83
CEACAM20	0	0	0	0	0	0

CEACAM21	0.06	0.74	0.66	0.27	0.84	0.45
CEACAM22P	1.63	1.06	1.67	1.28	1.11	1.65
CEACAM3	0	0	0	0	0	0
CEACAM4	0	0	0	0	0	0
CEACAM5	0.24	0.38	0.45	0.16	0.17	0.14
CEACAM6	0	0	0	0	0	0
CEACAM7	0	0	0	0	0	0
CEACAM8	0.26	0.16	0.21	0.19	0.06	0.38
CEBPA	0.68	6.61	5.48	3.17	1.49	6.23
CEBPA-AS1	0.03	0.11	0.7	0.16	0.13	0.2
CEBPB	0.31	0.52	0.06	0.12	0.2	0.14
CEBPD	1.39	2.63	1.11	0.88	1.43	2.32
CEBPE	0.48	1.1	0.31	0.13	0.27	1.61
CEBPG	8.82	11.55	11.96	9.49	7.24	12.32
CEBPZ	22.93	24.84	21.37	19.37	20.73	21.24
CECR1	0.44	1.2	0.37	0.82	0.77	0.82
CECR2	0.22	0.22	0.01	0.46	0.18	0.03
CECR3	0	0	0	0	0	0
CECR5	28.27	30.59	26.78	37.75	36.86	35.84
CECR5-AS1	0	0	0	0	0	0
CECR6	0.04	0.03	0	0.11	0.02	0.02
CECR7	0	0.02	0	0	0.05	0
CEL	0	0	0	0	0	0
CELA1	0	0.06	0.22	0.17	0	0.08
CELA2A	120.33	0	0.59	5.9	14.71	0.85
CELA2B	64.99	0.13	0.34	7.25	14.32	0.68
CELA3A	0	0	0	0	0	0
CELA3B	0	0	0	0	0	0
CELF1	14.03	15.84	15.84	14.58	13.38	13.91
CELF2	17.53	15.63	13.58	14.98	14.19	15.62
CELF2-AS2	0	0	0.15	0.02	0	0.23
CELF3	0	0.05	0.03	0.01	0.04	0
CELF4	0	0.04	0.02	0	0	0
CELF5	0	0	0	0	0.04	0
CELF6	0	0	0	0	0.02	0
CELP	0	0	0	0	0	0
CELSR1	0.14	0.09	0.01	0.06	0.12	0.03
CELSR2	3.37	2.31	1.64	2.4	2.31	2.55
CELSR3	0.05	0.04	0.09	0	0	0.13
CEMP1	5.53	4.78	3.96	1.98	4.51	4.87
CEND1	0	0	0	0	0.09	0
CENPA	6.41	8.87	5.92	5.79	7.91	6.43
CENPB	1.11	2.04	1.65	2.07	1.82	2.37

CENPBD1	1.93	3.62	4.34	3.9	3.35	3.77
CENPC1	5.17	6.24	5.9	5.65	5.81	5.76
CENPE	4.7	3.87	3.7	2.78	3.39	3.69
CENPF	13.49	12.87	11.82	12.24	13.09	12.95
CENPH	18.68	26.49	26.7	28.65	22.55	23.7
CENPI	5.06	6.19	4.42	5.64	6.79	5.96
CENPJ	5.07	4.64	4.56	2.84	4.26	4.11
CENPK	9.92	9.85	6.1	4.93	6.91	6.57
CENPL	16.77	14.81	16.63	11.05	12.87	13.48
CENPM	90.49	135.42	136.56	133.61	121.8	118.36
CENPN	19.63	19.68	18.18	20.29	22.51	17.97
CENPO	6.43	6.97	8.83	7.53	7.96	7.67
CENPP	5.01	6.62	5.19	4.12	6.81	6.27
CENPQ	5.98	4.78	4.02	4.3	4.19	3.92
CENPT	12.47	14.81	12.9	14.34	12.92	11.03
CENPV	12.16	9.85	8.02	10.11	13.8	7.53
CENPVP1	0	0	0	0	0	0
CENPVP2	0	0	0	0	0	0
CENPW	114.31	131.42	108.87	102.19	126.38	107.11
CEP104	1.85	1.81	1.11	1.41	1.48	1.68
CEP112	0.17	0.13	0	0	0.06	0
CEP120	0.95	0.83	0.61	0.28	0.52	0.96
CEP128	2.08	2.2	1.94	1.57	2.25	1.72
CEP135	1.52	1.55	1.16	1.42	1.49	1.12
CEP152	3.47	4.18	2.5	2.66	3.46	4.36
CEP164	1.93	2.48	1.97	1.56	1.66	1.9
CEP170	6.82	12.24	10.18	7.47	8.4	10.78
CEP170B	0.6	0.36	0.33	0.49	0.35	0.48
CEP170P1	0	0	0.15	0	0	0
CEP19	1.95	1.53	1.84	1.19	1.49	1.42
CEP192	2.08	2.48	2.8	2.06	2.41	3.5
CEP250	2.76	3.58	3.05	3.36	2.75	3.81
CEP290	0.67	0.65	0.48	0.55	0.54	0.68
CEP350	1.95	2.48	2.2	1.52	2.02	2.16
CEP41	6.87	7.39	8.66	6.06	7.91	5.46
CEP44	4.02	2.23	3.13	2.07	3.2	3.76
CEP55	2.77	4.24	3.5	3.53	3.11	5.28
CEP57	9.95	12.82	11.06	11.37	11.67	10.8
CEP57L1	2.22	2.52	2.13	2.5	3.16	2.58
CEP63	5.49	4.93	4.73	3.23	5	4.81
CEP68	5.28	4.16	3.47	3.47	3.7	3.21
CEP70	5.66	12.2	13.56	13.95	12.55	10.35
CEP72	4.92	7.45	7.32	6.9	7.09	8.67

CEP76	6.57	4.76	3.84	4.27	4.47	6.32
CEP78	7.87	11.23	7.88	11.22	9.08	10.42
CEP85	11.6	11.99	8.85	8.54	9.39	9.67
CEP85L	2.36	3.28	3.07	2.55	2.2	2.17
CEP89	2.61	2.07	2.03	2.42	2.31	2.42
CEP95	4.76	6.55	3.63	4.47	5.44	4.93
CEP97	2.28	0.98	1.29	1.9	1.53	1.87
CEPT1	9.09	16.04	11.05	12.66	15	14.5
CER1	0	0	0	0	0	0
CERCAM	9.82	22.04	17.31	14.83	11.91	16.9
CERK	2.55	1.76	3.05	1.72	2.54	1.4
CERKL	0.74	2.27	1.35	0.95	0.9	2.06
CERS1	1.89	3.57	2.51	1.64	1.97	3.4
CERS2	40.17	33.21	38.3	42.4	39.05	38.82
CERS3	0	0	0	0	0	0
CERS4	7.93	9.43	6.39	9.31	6.75	6.98
CERS5	18.61	26.75	17.84	24.8	20.78	18.02
CERS6	2.83	2.88	2.44	2.41	3.07	2.34
CERS6-AS1	0.75	2.85	1.33	1.38	1.21	3.24
CES1	0	0	0	0	0	0
CES1P1	0.03	0	0.03	0	0.03	0
CES1P2	0	0	0	0	0	0
CES2	10.44	10.89	7.64	11.52	9.15	7.91
CES3	1.07	1.39	1.17	1.61	1.78	1.74
CES4A	0	0	0	0	0	0
CES5A	0	0	0	0	0	0
CES5AP1	0	0	0	0	0	0
CETN1	0	0	0	0	0	0
CETN2	30.68	27.85	25.54	25.04	22.34	28.04
CETN3	17.3	16.81	12.66	10.05	13.56	12.62
CETN4P	0.39	0	0.35	0.18	0	0.13
CETP	0	0.1	0	0	0	0.04
CFB	0	0	0	0.04	0	0
CFC1	0	0	0	0	0	0
CFC1B	0	0	0	0	0	0
CFD	57.6	406.29	74.41	27.42	64.84	199.45
CFDP1	26.19	24.88	26.78	23.24	23.49	19.73
CFH	20.11	15.65	14.94	14.63	16.12	12.25
CFHR1	0	0	0	0	0.07	0
CFHR2	0	0	0	0	0	0
CFHR3	0.14	0	0.24	0	0	0
CFHR4	0	0	0	0	0	0
CFHR5	0	0	0	0	0	0

CFI	0	0	0	0	0	0
CFL1	805.99	743.73	644.68	685.48	724.4	656.02
CFL1P1	0.07	0.09	0.03	0	0.1	0.21
CFL2	6.34	6.27	5.75	6.21	6.01	6.38
CFLAR	15.1	9.29	8.52	7.72	8.22	7.93
CFLAR-AS1	0.07	0.12	0.07	0.1	0	0
CFP	18.97	20.87	32.11	16.67	12.93	27.25
CFTR	0	0	0	0	0	0
CGA	0	0	0	0	0	0
CGB	0.25	0	0	0	0	0
CGB1	0	0	0	0	0	0
CGB2	0	0	0	0	0	0
CGB5	0	0	0	0	0	0
CGB7	0	0	0	0	0	0
CGB8	0	0.07	0	0	0.08	0
CGGBP1	8.64	9.59	8.25	8.19	10.18	10.04
CGN	1.12	1.79	1.44	2.51	1.37	2.83
CGNL1	0.05	0.09	0.12	0.08	0.08	0.03
CGREF1	0.69	0.34	0.97	0.54	0.32	0.19
CGRRF1	9.4	6.56	5.06	5.35	6.9	5.76
CH25H	0	0	0	0	0	0
CHAC1	1.05	0	0	0.04	0	0.11
CHAC2	10.02	10.2	7.71	5.48	8.47	9.29
CHAD	0	0	0	0	0	0
CHADL	0	0.02	0	0	0	0
CHAF1A	8.83	10.14	13.2	11.58	11.75	13.21
CHAF1B	17.33	22.79	22.8	20.13	19.4	19.16
CHAMP1	8.42	7.24	4.72	5.64	6.5	6.86
CHAT	0	0	0	0	0	0
CHCHD1	60.97	61.01	57.04	53.41	69.87	56.59
CHCHD10	5.87	6.13	6.55	6.03	7.37	6.99
CHCHD2	995.99	1039.52	919.94	942.43	1070.83	1007.39
CHCHD3	97.17	98.48	92.58	82.43	89.42	85.76
CHCHD4	24.27	30.79	24.85	25.24	26.3	20.27
CHCHD5	39.47	44.1	32.93	43.92	43.3	36.07
CHCHD6	35.12	24.02	27.86	34.72	30.77	26.64
CHCHD7	35.67	54.53	44.72	29.39	34.99	47.02
CHD1	4.74	4.4	4.27	4.17	4.27	4.81
CHD1L	22.87	24.57	22.22	21.83	24.11	24.5
CHD2	5.65	4.62	3.72	3.15	3.63	3.75
CHD3	5.91	4.75	6.07	7.28	6.42	6.36
CHD4	32.74	27.52	29.25	31.75	33.51	29.45
CHD5	0	0.02	0.02	0	0.01	0

CHD6	2.39	1.61	2.02	1.37	1.55	1.71
CHD7	8.9	8.2	8.35	7.97	8.37	8
CHD8	5.88	4.55	5.19	5.69	5.87	5
CHD9	2.4	2	1.82	1.81	1.86	2.2
CHDC2	0	0	0.03	0	0	0
CHDH	1.89	5.09	6.18	3.05	3.3	2.83
CHEK1	16.17	18.12	21.52	16.01	15.91	19.37
CHEK2	17.23	22.45	20.98	25.42	22.79	19.35
CHEK2P2	0.28	0.25	0.29	0.13	0.33	0.58
CHERP	10.34	12.69	12.48	11.98	12.74	13.36
CHFR	0.07	0	0.19	0	0.17	0
CHGA	0	0.17	0	0	0	0.26
CHGB	0.06	0.13	0.08	0.36	0.13	0.17
CHI3L1	0	0.32	0	0	0	0.37
CHI3L2	0	0	0	0	0	0
CHIA	0	0	0	0	0	0
CHIAP2	0.08	0.06	0.07	0	0.04	0.07
CHIC1	1.23	0	0.42	0.08	0	0.17
CHIC2	19.41	13.95	12.54	17.83	19.18	18.34
CHID1	23.01	31.88	31.61	26.34	29.9	31.68
CHIT1	0	0	0	0	0	0
CHKA	3.75	2.83	2.58	1.81	3.48	3.06
CHKB	3.05	5.56	4.65	2.52	3.99	2.51
CHKB-CPT1B	0.12	0	0	0	0.14	0.19
CHL1	0.04	0	0	0	0	0
CHM	4.23	4.59	4.07	2.76	5.18	4.41
CHML	1.55	1.13	1.03	0.67	1.15	1.68
CHMP1A	50.28	55.06	56.24	47.07	50.23	45.75
CHMP1B	12.21	9.68	7.22	7.97	9.32	8.52
CHMP2A	65.42	74.96	76.31	77.17	87.59	81.09
CHMP2B	8.34	9.14	5.07	5.51	7.51	6.82
CHMP3	27.26	20.24	18.74	20.21	20.35	20.05
CHMP4A	52	49.45	35.79	42.06	43.6	37.23
CHMP4B	0.95	1.03	1.62	1.56	1.71	2.61
CHMP4C	0.22	0.2	0.08	0.1	0.1	0.13
CHMP5	28.73	21.34	15.74	17.15	20.7	15.11
CHMP6	18	18.13	17.09	18.84	16.41	13.54
CHMP7	27.7	25.1	26.08	27.86	28.05	24.93
CHN1	0	0.21	0.14	0	0.38	0
CHN2	0	0	0	0	0	0
CHODL	0	0	0	0	0	0
CHODL-AS1	0	0	0	0	0	0
CHORDC1	15.91	18.4	14.02	10.55	13.1	13.5

CHP1	117.69	105.4	95.44	86.52	102.22	98.56
CHP2	0.4	0.28	0.4	0.32	0.45	0.69
CHPF	13.87	6.08	4.24	7.19	7.5	4.56
CHPF2	3.45	3.17	2.1	3.33	3.25	3.92
CHPT1	22.11	17.46	17.01	12.25	22.71	12.15
CHRAC1	24.95	33.68	30.9	32.76	34.41	33.45
CHRD	0	0	0	0	0	0
CHRDL1	4.69	30.67	21.58	13.31	10.96	27.48
CHRDL2	0	0	0	0	0	0
CHRFAM7A	3.14	3.22	2.9	2.01	3.3	2.61
CHRM1	0	0	0	0	0	0
CHRM2	0	0	0.12	0	0	0.11
CHRM3	1.8	2.41	3.1	1.39	1.51	1.96
CHRM4	0.56	0.52	0.65	0.35	0.77	0.57
CHRM5	0	0	0	0	0.02	0
CHRNA1	0	0	0	0	0	0
CHRNA10	0.3	0.27	0.17	0.08	0.29	0.15
CHRNA2	0	0.03	0.03	0	0	0.02
CHRNA3	1.34	1.52	0.59	6.84	1.1	4.7
CHRNA4	0	0	0	0	0	0
CHRNA5	1.04	1.52	1.95	2.07	1.67	0.91
CHRNA6	0.15	0	0	0	0	0.09
CHRNA7	3.89	3.7	3.16	1.98	3.68	2.14
CHRNA9	0	0	0	0	0	0
CHRNB1	3.26	1.91	2.6	2.49	1.61	1.97
CHRNB2	0	0.02	0.14	0.04	0	0
CHRNB3	0	0	0	0	0	0
CHRNB4	0	0.03	0	0	0.2	0
CHRND	0	0.09	0.05	0	0	0
CHRNE	0	0	0	0	0	0
CHRNG	0.17	0	0.03	0.14	0.13	0.07
CHST1	0.04	0.06	0.14	0.04	0.1	0.08
CHST10	2.92	2.68	4	1.62	2.22	3.9
CHST11	2.66	2.35	1.66	1.7	2.74	2.3
CHST12	6.03	6.46	7.34	3.69	6.45	4.18
CHST13	0.54	3.25	2.78	2.38	3.85	2.44
CHST14	1.24	3.46	2.91	1.64	2.62	3.08
CHST15	0.08	0	0	0	0	0
CHST2	0.24	0	0	0.05	0.07	0.09
CHST3	1.36	1.5	2.36	1.33	1.19	1.64
CHST4	0	0.63	0	0	0.03	0.07
CHST5	0	0.1	0.32	0.15	0.21	0.12
CHST6	0.64	1.1	0.97	0.51	0.91	0.94

CHST7	1.08	2.83	4.25	1.86	2.1	3.7
CHST8	0	0	0	0	0	0
CHST9	0	0	0	0	0	0
CHSY1	3.21	2.19	1.77	1.89	1.98	2.11
CHSY3	0.06	0	0	0	0	0
CHTF18	3.56	6.65	6.67	6.01	5.65	5.8
CHTF8	49.09	41.73	54.7	47.69	52.11	56.84
CHTOP	59.25	63.69	58.75	53.01	56.05	59.51
CHUK	3.03	1.87	1.28	0.5	1.48	1.9
CHURC1	18.18	18.17	12.42	14.05	18.64	15.14
CHURC1-FNT	6.23	10.98	11.78	9.91	8.43	8.45
CIAO1	25.69	25.39	27.89	24.81	24.09	25.36
CIAPIN1	59.24	51.27	53.07	48.84	51.99	51.42
CIB1	103.6	102.99	97.35	90.37	89.8	88.93
CIB2	1.64	0.41	0.65	1.26	0.71	0.79
CIB3	0.49	2.21	2	0.84	1.04	1.96
CIB4	0	0	0	0	0	0
CIC	2.4	2	1.46	1.77	2.03	2.13
CIDEA	0	0	0	0	0	0
CIDEB	1.39	1.95	1.19	1.34	1.07	0.91
CIDEC	0.19	0.1	0.47	0	0.12	0.06
CIDECP	6.08	6.26	3.12	2.98	4.09	3.75
CIITA	3.56	8.63	14.94	15.08	9.21	11.06
CILP	0.11	0	0	0.02	0	0
CILP2	0	0.03	0	0	0	0.02
CINP	62.39	56.07	53.34	58.22	65.17	60.11
CIR1	13.29	9.17	8.44	8.95	9.58	8.26
CIRBP	44.61	39.65	34.29	54.42	43.69	47.51
CIRBP-AS1	0	0	0.1	0.23	0.26	0
CIRH1A	62.98	71.01	50.18	64.67	57.2	62.99
CISD1	19.24	16.72	19.44	17.21	17.32	16.28
CISD2	13.36	11.71	11.06	9.59	13.27	9.93
CISD3	13.71	18	17.66	15.34	14.53	15.75
CISH	66.94	64.79	75.21	58.86	60.11	56.36
CIT	5.5	5.89	4.52	4.36	6.15	5.64
CITED1	5.02	6.94	6.74	5.13	5.76	8.3
CITED2	17.62	11.35	8.05	10.5	12.49	9.93
CITED4	0	0	0	0.04	0	0
CIZ1	10.27	10.87	10.57	9.98	10.95	12.85
CKAP2	26.65	23.23	23.74	20.9	25.86	21.33
CKAP2L	12.56	11.39	8.44	8.02	9.81	10.62
CKAP4	0.65	0.59	3.01	1.49	1.13	2.45
CKAP5	19.46	18.08	18.46	16.47	19.31	19.15

CKB	239.31	86.38	110.7	199.52	176.27	112.65
CKLF	30.3	38.88	33.5	24.71	26.73	23.31
CKLF-CMTM1	0.49	0	0.26	0.24	0	0.74
CKM	0.76	3.79	1.03	1.24	1.15	0.73
CKMT1A	1.91	12.77	3.04	8.01	7.87	17.27
CKMT1B	1.93	12.98	13.47	8.55	1.31	13
CKMT2	0	0.08	0.08	0	0	0
CKS1B	254.39	295.46	232.29	268.14	278.12	261.7
CKS2	464.76	297.77	263.98	248.17	361.3	254.67
CLASP1	5.97	4.57	4.36	5.18	5.11	5.14
CLASP2	4.88	5.65	4.83	3.96	4.94	3.99
CLASRP	7.28	7.81	9.41	7.27	7.18	9.1
CLC	4.86	0	0	0	0.36	0.64
CLCA1	0	0	0	0	0	0
CLCA2	0	0	0	0	0	0
CLCA3P	0	0	0	0	0	0
CLCA4	0	0	0	0	0	0
CLCC1	12.84	9.91	9.75	9.58	12.22	9.48
CLCF1	3.35	1	3.63	2.82	1.48	1.45
CLCN1	0	0	0	0	0	0
CLCN2	0.92	1.02	0.55	0.54	0.37	0.5
CLCN3	3.97	2.62	1.82	2.28	2.58	1.87
CLCN4	11.68	2.37	2.81	4.93	5.48	1.61
CLCN5	5.93	5.13	4.72	4.19	4.82	4.4
CLCN6	1.98	2.3	1.28	1.61	2.31	1.23
CLCN7	11.57	13.11	15.24	12.31	10.44	10.49
CLCNKA	0	0	0	0.06	0	0
CLCNKB	0	0	0	0	0	0
CLDN1	0	0	0	0	0	0
CLDN10	0	0	0	0.02	0	0.06
CLDN10-AS1	0.52	0.07	0	0	0	0
CLDN11	0.37	0.49	0.36	0.12	0.22	0.23
CLDN12	2.08	2.64	1.05	1.59	1.57	1.93
CLDN14	0	0	0	0	0	0
CLDN15	1.57	1.53	0.2	0.76	0.59	0.52
CLDN16	0.16	0.27	0.18	0.07	0.21	0.29
CLDN17	0	0	0	0	0	0
CLDN18	0.02	0.07	0.23	0.15	0.04	0.01
CLDN19	0.06	0.22	0.11	0.07	0.06	0.01
CLDN2	0	0	0	0	0	0
CLDN20	0	0.4	0.52	0.04	0.12	0.06
CLDN22	0	0	0	0	0	0
CLDN23	0.38	0.06	0.03	0.05	0.14	0.42

CLDN24	0	0	0	0	0.13	0.38
CLDN25	0	0	0	0	0	0
CLDN3	1.31	0.23	0	0.08	0.17	0
CLDN4	0	0.06	0	0	0	0
CLDN5	0	0.13	0.29	0	0	0.1
CLDN6	0	0	0	0	0	0
CLDN7	0	0.33	0.62	0.18	0.2	0.05
CLDN8	0	0	0	0	0	0
CLDN9	0	0	0	0	0	0
CLDND1	67.26	29.09	21.61	26.81	38.73	25.77
CLDND2	0.32	0.31	0.8	0.6	0.23	0.48
CLEC10A	0	0	0	0	0	0
CLEC11A	26.76	16.55	12.32	56.46	8.44	163.48
CLEC12A	0.26	1.1	0	0	0.05	1.42
CLEC12B	0	0	0	0	0	0.02
CLEC14A	0	0	0	0	0	0
CLEC16A	10.19	9.2	8.99	6.94	7.64	7.33
CLEC17A	0	0.05	0.07	0	0.04	0
CLEC18A	0	0	0	0	0	0
CLEC18B	0	0	0	0	0	0
CLEC18C	0	0	0.03	0	0	0
CLEC19A	0.4	0.2	0.34	0.08	0.35	0.19
CLEC1A	0	0	0	0	0	0
CLEC1B	1.99	0	0	0.19	0.08	0
CLEC2A	0	0	0	0	0	0
CLEC2B	9.42	19.92	15.32	12.59	12.45	18.54
CLEC2D	0.51	0.29	0.29	0.47	0.35	0.34
CLEC2L	0	0	0	0	0	0
CLEC3A	0	0	0	0	0	0
CLEC3B	0	0	0	0	0	0
CLEC4A	0.07	0.11	0	0	0	0
CLEC4C	0	0	0	0	0	0
CLEC4D	0.04	0	0	0	0	0
CLEC4E	0.1	0.05	0.22	0.05	0.19	0.21
CLEC4F	0.21	0	0	0	0	0
CLEC4G	0	0.09	0	0	0	0
CLEC4GP1	0.71	0.62	0.49	0.18	1.31	0.45
CLEC4M	0.21	0	0	0	0	0
CLEC5A	0	0.16	0	0	0	0.15
CLEC6A	0	0	0	0	0	0
CLEC7A	0.48	0.98	0.86	0.57	0.83	0.59
CLEC9A	0	0	0	0	0	0
CLECL1	10.29	33.29	29.13	23.33	23.64	36.55

CLGN	3.55	7.59	6.46	6.08	5.92	6.88
CLHC1	0.44	0.18	0.27	0.17	0.25	0.18
CLIC1	477.14	394.7	379.18	404.45	468.11	356.14
CLIC2	15.17	0.49	0.2	2.64	4.26	0.3
CLIC3	0	0	0	0	0	0.58
CLIC4	12.18	9.21	6.69	7.21	8.27	11.12
CLIC5	0	0	0	0	0	0
CLIC6	0	0.01	0	0	0	0
CLINT1	24.9	33.33	28.75	22.42	25.76	30.41
CLIP1	2.47	2.84	1.59	1.92	2.14	2.14
CLIP2	3.47	1.8	1.86	2.26	1.57	1.61
CLIP3	0.13	0.2	0.32	0.16	0.26	0.07
CLIP4	0.49	0.43	0.12	0.23	0.16	0
CLK1	13.47	11.16	10.53	8.72	12.24	11.48
CLK2	2.17	2.77	1.14	2.42	1.79	1.33
CLK2P	0	0	0	0	0	0
CLK3	31.41	41.83	37.48	32.38	33.61	41.03
CLK4	2.95	2.74	1.66	2.25	2.48	1.89
CLLU1	0	0.04	0	0	0	0
CLLU1OS	0	0	0	0	0	0
CLMN	0.04	0.11	0.08	0.1	0.05	0.06
CLMP	0.03	0.02	0.1	0	0	0.17
CLN3	47.78	48.16	46.02	52.26	52.23	41.68
CLN5	6.97	4.7	4.8	5.05	5.73	6.57
CLN6	28.58	44.9	52.29	58.42	46.64	31.27
CLN8	5.66	5.37	4.09	3.84	4.79	5.05
CLNK	0	0	0	0	0	0
CLNS1A	149.3	177.13	146.96	161.93	160.41	141.72
CLOCK	1.55	0.81	0.52	0.72	0.7	0.94
CLP1	20.74	20.57	27.33	25.33	24.69	23.87
CLPB	26.46	32.56	32.64	27.64	28.11	42.76
CLPP	99.19	111.5	110.07	104.27	113.04	102.22
CLPS	0	0	0	0	0	0
CLPSL1	0.18	0	0.16	0	0	0
CLPSL2	0	0.51	0	0	1.43	0.64
CLPTM1	31.47	31.28	37.01	35.44	30.3	22.12
CLPTM1L	11.22	9.84	9.86	8.69	9.17	9.62
CLPX	28.56	25.56	25.19	28.66	24.32	31.44
CLRN1	0	0	0	0	0	0
CLRN1-AS1	0	0	0	0	0	0
CLRN2	0	0	0	0	0	0
CLRN3	0	0	0	0	0	0
CLSPN	7.82	8.8	7.6	6.93	8.5	6.25

CLSTN1	2.8	2.42	2.12	2.18	2.12	2.58
CLSTN2	0	0	0	0	0	0
CLSTN3	2.73	1.78	2.57	2.34	2.73	2.73
CLTA	302.38	242.6	232.11	281.42	290.23	215.27
CLTB	35.57	36.12	42.92	41.13	35.52	34.78
CLTC	32.5	25.08	26.37	24.7	27.08	30.23
CLTCL1	0.58	1.35	1.05	0.6	0.91	0.93
CLU	1.1	0.46	0.33	0.14	0.36	0.91
CLUAP1	2.62	2.72	2.95	2.15	2.63	3.34
CLUH	11.19	11.27	11.74	12.24	13.03	14.19
CLUHP3	0.71	0	0	0.29	0.4	0
CLUL1	0	0	0	0	0	0
CLVS1	0	0	0	0	0	0
CLVS2	0	0	0	0	0	0
CLYBL	0.37	0.51	0.29	0.47	0.47	0.54
CMA1	0	0	0	0	0	0
CMAHP	0.06	0.59	1.51	0.61	0.57	0.17
CMAS	30.42	13.43	15.6	12.31	21.85	15.53
CMBL	14.18	14.54	15.98	13.05	16.63	16.71
CMC1	47.46	36.08	32.25	30.43	39.11	36.21
CMC2	55.5	56.68	41.59	40.76	62.07	44.23
CMC4	12.88	18.17	18.31	18.5	18.06	16.16
CMIP	7.53	1.54	1.35	2.56	2.27	1.85
CMKLR1	0	0	0	0	0	0
CMPK1	19.07	15.72	13.98	13.47	12.67	15.51
CMPK2	12.51	5.39	7.69	10.75	8.33	6.1
CMSS1	52.86	56.18	55.1	59.7	72.33	54.42
CMTM1	0	0.52	0.38	0.34	0.07	0.1
CMTM2	0	0	0	0	0	0
CMTM3	8.82	14.33	17.5	15.83	15.72	13.73
CMTM4	0.7	0.5	0.9	0.4	0.4	0.6
CMTM5	0.44	0.11	0	0	0.07	0.26
CMTM6	18.23	14.57	8.9	10.23	11.77	9.46
CMTM7	38.6	30.85	35	29.05	35.32	27.06
CMTM8	0.13	0.51	0.71	0.36	0.62	1.12
CMYA5	0.01	0.02	0	0	0.02	0
CN5H6.4	0.1	0.19	1.17	0.31	0.56	0.2
CNBD1	0	0	0	0	0	0
CNBD2	0.08	0.3	0.04	0.12	0	0
CNBP	175.89	184.35	150.04	154.2	173.78	175.4
CNDP1	0	0	0	0	0	0
CNDP2	31.92	29.77	22.64	24.13	22.55	25.98
CNEP1R1	3.68	6.1	3.97	4.66	5.83	6.69

CNFN	0.79	0.5	1.58	0.66	0.3	1.27
CNGA1	0.08	0.05	0.07	0	0.02	0.02
CNGA2	0	0	0	0	0	0
CNGA3	0	0	0	0	0	0
CNGA4	0	0	0	0	0	0
CNGB1	0.03	0	0.13	0.13	0.01	0.03
CNGB3	0	0	0	0	0	0
CNIH	79.56	65.37	61.93	61.13	74.02	69.38
CNIH2	0	0	0	0.04	0	0.06
CNIH3	0.12	0	0	0	0	0
CNIH4	71.71	60.6	50.09	53.95	57.9	46.18
CNKSR1	0.2	0.02	0	0.04	0.11	0
CNKSR2	3.35	2.8	2.6	2.34	2.95	3.51
CNKSR3	0.81	0.57	0.41	0.65	0.4	0.66
CNN1	0.05	0	0	0	0	0
CNN2	61.25	87.28	85.44	70.98	73.12	76.59
CNN3	0.21	0.42	0.43	0.02	0.81	0.18
CNNM1	1.43	2.41	1.76	1.14	2.04	2.18
CNNM2	0.71	0.63	0.38	0.62	0.76	0.27
CNNM3	3.3	3.32	2.02	2.31	2.96	2.54
CNNM4	1.38	0.69	1.14	1.47	1.46	1.2
CNOT1	36.71	43.48	39	35.68	37.21	42.48
CNOT10	21.11	14.84	16.32	15.48	16.17	16.19
CNOT11	2.91	3.19	2.48	3.02	2.97	1.83
CNOT2	22.96	18.7	20.31	16.71	17.61	17.37
CNOT3	5.08	6.91	5.37	5.77	5.89	6.66
CNOT4	8.91	7.68	11.05	10.34	9.9	10.6
CNOT6	4.41	2.92	3.12	2.53	3.26	3.29
CNOT6L	1.29	1.62	1.6	1.21	1.17	1.92
CNOT7	116.73	113.15	98.76	99.07	112.07	102.9
CNOT8	29.05	31.62	24.76	25.24	26.94	30.74
CNP	27.99	19.81	21.72	24.31	19.71	20.98
CNPPD1	32.8	25.61	27.87	28.82	24.15	26.37
CNPY1	0	0	0	0	0	0
CNPY2	119.49	144.32	127.28	111.59	116.97	118.76
CNPY3	29.51	54.06	49.88	42.38	45.92	52.21
CNPY4	3.12	5.6	4.4	2.25	3.48	1.09
CNR1	0	0	0	0	0	0
CNR2	0	0.36	0.88	1.73	0.59	0.63
CNRIP1	71.48	5.19	6.53	20.06	48.91	9.35
CNST	7.15	6.9	9.34	6.82	6.9	7.27
CNTD1	0	0.04	0.19	0.03	0.07	0.05
CNTD2	0	0	0	0	0	0

CNTF	0.12	0	0	0	0	0.2
CNTFR	0	0	0	0	0	0
CNTLN	3.28	3	2.36	2.66	2.62	2.38
CNTN1	0	0	0	0	0	0
CNTN2	0	0.04	0.05	0.01	0.02	0.02
CNTN3	0	0	0	0	0	0
CNTN4	0.18	0.01	0.03	0.1	0.02	0.05
CNTN4-AS2	0	0	0	0	0	0
CNTN5	0	0	0	0	0	0
CNTN6	0	0	0	0	0	0
CNTNAP1	0.26	0.15	0.06	0.54	0.17	0.27
CNTNAP2	0.45	0	0.03	0	0.08	0
CNTNAP3	0	0	0	0	0	0
CNTNAP3B	0	0.01	0	0	0	0
CNTNAP4	0.06	0	0	0	0	0.11
CNTNAP5	0.01	0	0	0	0	0
CNTRL	1.63	3.87	2.73	1.8	2.72	2.34
CNTROB	4.64	3.66	3.1	3.64	3.31	3.41
COA1	98.57	71.6	62.74	77.46	88.64	60.93
COA3	73.31	78.86	79.32	82.91	76.93	72.51
COA4	166.47	148.98	149.36	155.17	173.82	135.01
COA5	13.33	11.92	13.54	11.35	10.17	10.12
COA6	76.91	87.01	79.37	56.18	89.47	75.8
COASY	32.16	35.14	35.62	28.92	29.24	35.36
COBL	0	0	0	0	0	0
COBLL1	0.1	0.13	0	0	0	0.01
COCH	0.13	0	0.04	0.12	0	0
COG1	9.81	9.97	9.51	8.54	7.37	9.52
COG2	8.38	12.84	11.51	6.93	8.58	9.92
COG3	1.94	2.99	2.37	1.23	1.91	2.1
COG4	15.7	13.65	18.1	19.67	19.24	15.24
COG5	6.74	6.19	6.42	6.99	6.41	7.98
COG6	6.08	3.91	3.56	3.06	3.66	2.73
COG7	4.07	8.57	3.92	4.87	4.08	3.3
COG8	20.56	23.87	24.93	21.95	18.24	17.34
COIL	14.04	16.22	16.36	12.8	18.33	14.65
COL10A1	0	0.14	0.71	0.06	0.15	0.58
COL11A1	0	0	0	0	0	0
COL11A2	0.06	0	0	0	0.03	0
COL12A1	0	0	0	0	0	0
COL13A1	0	0	0	0	0	0
COL14A1	0	0	0	0	0.03	0
COL15A1	9.5	0	0.58	2.66	3.55	0

COL16A1	0.03	0	0	0	0	0
COL17A1	0	0	0.04	0	0	0
COL18A1	0.91	0.79	1.34	0.8	1.06	1.14
COL18A1-AS	0	0	0	0	0	0
COL18A1-AS	0	0	0	0	0	0
COL19A1	0.01	0	0	0	0.02	0
COL1A1	0.11	0	0.02	0.11	0	0
COL1A2	0.07	0	0	0	0	0
COL20A1	0	0	0	0	0	0
COL21A1	0	0	0	0	0	0
COL22A1	0	0	0	0	0	0
COL23A1	0	0.04	0	0	0	0
COL24A1	0.22	0.53	0.9	0.65	0.71	0.58
COL25A1	0	0.11	0.25	0	0.11	0.08
COL26A1	0.43	0.34	0.04	0.05	0.46	0.02
COL27A1	0.14	0.78	0.61	0.51	0.63	0.67
COL28A1	0	0	0	0	0	0
COL2A1	0	9.7	3.21	12.44	8.11	6.78
COL3A1	0	0	0	0	0	0
COL4A1	0	0	0	0	0	0
COL4A2	0	0	0	0	0	0
COL4A2-AS1	0.15	0	0	0	0	0
COL4A3	0	0	0.02	0	0	0
COL4A3BP	5.22	3.45	3.71	3.61	3.07	4.12
COL4A4	0	0	0	0	0	0
COL4A5	0	0	0	0	0	0
COL4A6	0	0	0	0	0	0
COL5A1	0	0	0	0	0	0
COL5A2	0.08	0.04	0	0	0.02	0.05
COL5A3	0	0	0	0	0	0
COL6A1	1.47	1.42	1.44	1.71	1.53	1.92
COL6A2	1.3	0.94	1.32	2.41	1.22	2.08
COL6A3	0.02	0	0.23	0	0	0
COL6A4P1	0	0	0	0	0	0
COL6A4P2	0.01	0.08	0.08	0.02	0.03	0
COL6A5	0.01	0	0	0	0	0
COL6A6	0.05	0	0.02	0	0	0.02
COL7A1	0	0	0.01	0	0	0
COL8A1	0	0	0	0	0	0
COL8A2	0	0	0	0	0	0
COL9A1	0	0	0	0	0	0
COL9A2	0	0	0.05	0.21	0.02	0.05
COL9A3	0.18	0.6	1.49	0.34	1.31	0.6

COLEC10	0	0	0	0	0	0
COLEC11	0	0	0	0.04	0	0
COLEC12	0	0	0	0	0	0
COLQ	3.34	0.06	0.23	0.72	1.42	0.54
COMMD1	55.76	38.84	42.41	45	43.74	41.14
COMMD10	12.71	12.27	12.25	12.76	14.61	10.67
COMMD2	10.12	11.25	6.08	5.99	7.75	8.43
COMMD3	52.34	55.03	55.73	59.17	66.79	44.09
COMMD3-BN	0	2.2	1.13	0.44	2.12	1.2
COMMD4	271.85	151.97	145.73	205.77	245.3	147.73
COMMD5	82.62	65.41	46.68	57.81	67.33	47.96
COMMD6	35.52	31.32	26.68	28.15	33.71	26.25
COMMD7	29.35	45.22	40.29	40.91	40.8	35.82
COMMD8	15.82	15.66	15.45	10.24	12.24	11.25
COMMD9	27.99	41.3	33.26	34.05	28.89	31.68
COMP	0.06	0	0.11	0.1	0	0.82
COMT	53.69	89.79	63.14	63.73	63.57	71.94
COMTD1	19.15	27.35	27.87	26.09	22.12	27.33
COPA	49.99	41.15	37.6	35.2	38.71	37.26
COPB1	35.05	32.05	32.72	26.16	32.37	31.4
COPB2	35.04	35.76	29.6	29.72	33.87	31.07
COPE	234.3	272.02	249.07	267.46	256.82	279.98
COPG1	47.83	47.62	37.6	44.48	41.25	42.74
COPG2	6.09	7.53	6.04	7.8	5.71	6.59
COPRS	26.94	21	20.49	22.51	30	22.2
COPS2	18.08	18.55	15.8	17.4	18.06	18.21
COPS3	138.95	117.46	117.61	121.81	132.81	115.01
COPS4	52.34	52.63	42.93	40.93	44.25	45.8
COPS5	140.2	140.2	138.34	120.96	147.03	117
COPS6	119.05	125.88	117.98	131.2	132.19	102.47
COPS7A	37.46	30.16	25.47	30.08	26.78	23.83
COPS7B	34.02	35.55	31.08	30.81	32.66	32
COPS8	34.21	25.58	21.49	19.41	25.06	18.88
COPZ1	99.47	84.96	76.73	94.86	87.24	70.71
COPZ2	0	0	0	0	0	0
COQ10A	4.07	5.37	5.17	2.84	4.64	2.99
COQ10B	13.17	7.19	5.29	6.43	10.52	7.86
COQ2	14.86	22.12	17.98	14.05	19.4	8.82
COQ3	29.49	35.94	24.63	28.01	33.84	22.43
COQ4	27.52	31.61	32.58	29.44	29.5	29.75
COQ5	26.05	30.05	33.56	29.92	24.24	26.44
COQ6	19.65	16.21	14.91	10.52	15.93	12.93
COQ7	4.44	6.52	5.13	5.82	7.08	4.28

COQ9	65.1	55.6	50.22	57.92	56.51	51.39
CORIN	0.01	0.01	0	0	0	0
CORO1A	60.44	242.93	220.11	164.9	135.35	209.28
CORO1B	43.66	32.34	30.3	34.96	32.07	39.56
CORO1C	88.12	35.78	35.82	47.17	58.36	35.14
CORO2A	2.15	2.79	2.08	2.27	4.15	2.2
CORO2B	0	0.05	0	0	0	0
CORO6	0.64	1.74	0.56	0.63	0.9	1.64
CORO7	31.1	38.77	41.07	40.61	33.36	46.44
CORO7-PAM	5.82	9.65	5.54	4.94	8.42	1.36
CORT	0.26	0	0.42	0.16	0.69	0.6
COTL1	70.07	129.01	111.15	161.04	125.15	44.44
COX10	18.94	16.83	16.25	12.49	16.33	14.03
COX10-AS1	1.83	2.4	2.93	1.71	2.59	3.15
COX11	20.41	14.8	13.4	11.41	14.21	15.17
COX14	69.58	67.15	59.72	63.29	70.46	56.35
COX15	10.44	10.18	8.68	9.04	8.42	9.94
COX16	28.36	25.41	23.11	18.64	26.59	25.03
COX17	87.73	82.88	69.86	68.67	86.72	66.16
COX18	2.6	4.37	4.38	2.79	3.67	5.01
COX19	2.6	3.59	1.88	2.42	2.68	1.78
COX20	11.95	12.17	10.31	8.43	11.44	9.38
COX4I1	907.07	850.91	862.22	928.32	994.57	914.28
COX4I2	0	0	0	0	0	0
COX5A	1030.08	1144.59	1088.69	1103.11	1127.55	1124.8
COX5B	769.68	836.23	753.83	806.9	858.42	743.68
COX6A1	559.01	534.59	442.23	498.98	581.53	497.26
COX6A2	0	0	0	0	0	0
COX6B1	541.65	507.99	433.93	490.78	549.89	461.14
COX6B2	1.33	1.51	1.7	1.57	1.16	1.83
COX6C	486.01	448.56	350.18	359.14	463.33	392.22
COX7A1	0	0.08	0	0	0	0.31
COX7A2	386.93	294.47	277.24	333.94	375.13	281.84
COX7A2L	132.86	127.07	124.58	116.21	138.97	124
COX7B	1271.67	1232.1	992.03	1021.38	1319.06	1020.72
COX7B2	0	0	0	0	0	0
COX7C	1207.81	1068.43	960.35	996.92	1330.17	957.8
COX8A	934.02	912.31	824.32	947.7	974.17	876.44
COX8C	0	0	0	0	0.3	0
CP	0	0	0	0	0	0.1
CPA1	0	0	0	0	0	0
CPA2	0	0.04	0.26	0	0	0
CPA3	0.97	0	0	0	0.25	1.92

CPA4	0.24	0.26	0.27	0.18	0.16	0.35
CPA5	0	0	0	0	0	0
CPA6	0	0	0	0	0	0
CPAMD8	0	0	0.08	0.07	0.09	0.12
CPB1	0	0	0	0	0	0
CPB2	0.04	0	0	0.09	0	0
CPB2-AS1	0.32	0.73	0.28	0.32	0.27	0.57
CPD	2.95	4.82	2.88	2.83	2.56	3.93
CPE	0.15	0	0	0.02	0.2	0
CPEB1	0.75	0.37	0.36	0.34	0.5	0
CPEB2	0.06	0.09	0.12	0.02	0.05	0.24
CPEB3	0.16	0.25	0.51	0.38	0.18	0.32
CPEB4	2.75	0.41	0.25	0.52	0.73	0.21
CPED1	6.76	0	0	0.59	0.93	0
CPLX1	0.03	0	0	0	0	0
CPLX2	0	0.05	0	0	0	0
CPLX3	0	0	0	0	0	0
CPLX4	0	0	0	0	0	0
CPM	0.57	0.74	0.62	0.46	0.44	0.46
CPN1	0	0	0	0	0	0
CPN2	0	0.19	0.13	0.1	0.02	0.1
CPNE1	50.92	61.22	48.93	54.6	56.65	48.6
CPNE2	3.48	3.52	3.28	3.26	3.51	4.86
CPNE3	21.48	24.19	23.41	15.84	16.44	21.58
CPNE4	0	0	0	0	0	0
CPNE5	0.02	0	0.02	0	0	0
CPNE6	0	0	0	0	0	0
CPNE7	3.84	3.1	3.62	3.17	3.6	3.66
CPNE8	4.93	3.16	3.42	2.5	2.99	2.74
CPNE9	0.04	0.06	0.07	0.07	0.58	0.15
CPO	0	0	0	0	0	0
CPOX	18.4	1.7	1.18	4.32	9.04	1.56
CPPED1	7.56	4.09	4.27	3.84	3.73	4.55
CPQ	26.95	0.8	1.14	8.09	13.39	2.49
CPS1	0.19	0	0	0	0.28	0.02
CPS1-IT1	0	0	0	0	0	0
CPSF1	27.37	44.75	45.36	40.15	37.04	46.46
CPSF2	15.05	13.03	12.98	14.48	14.42	14.14
CPSF3	42.17	53.65	46.19	48.06	52.42	50.64
CPSF3L	43.24	42.76	41.93	42.46	43.07	40.22
CPSF4	3.99	6.93	5.58	6.55	7.86	4.74
CPSF4L	0	0	0	0	0	0
CPSF6	19.47	21.98	19.4	16.05	19.12	20.22

CPSF7	15.44	16.76	16.73	16.43	17.54	17.01
CPT1A	21.66	31.8	34.79	29.8	30.48	29.68
CPT1B	1.07	1.28	0.56	0.21	0.03	0.31
CPT1C	0	0.02	0.02	0	0	0
CPT2	10.11	11.1	10.68	10.79	8.39	8.09
CPVL	0	0	0	0	0	0
CPXCR1	0	0	0	0	0	0
CPXM1	1.28	4.52	4.56	5.53	3.46	2.44
CPXM2	0.1	0	0	0	0	0
CPZ	0.23	0	0.12	0	0.06	0.17
CR1	0.01	0.07	0.07	0	0.04	0.04
CR1L	0	0	0	0	0	0
CR2	0	0	0	0	0	0
CRABP1	0	0	0	0	0	0
CRABP2	0.59	0	0.07	0	0	0.15
CRADD	19.76	16.54	13.17	10.86	13.38	13.19
CRAMP1L	1.35	1.26	1.57	1.1	1.81	1.82
CRAT	9.34	8.39	8.26	11.9	7.86	12.95
CRB1	0	0.03	0	0	0	0
CRB2	0.01	0	0.06	0	0.07	0
CRB3	0.1	0.27	0.57	0.14	0.83	0.1
CRBN	11.39	8.99	8.43	8.84	9.46	7.06
CRCP	12.17	12.33	9.47	10.29	12.8	12.84
CRCT1	0	0	0	0	0	0
CREB1	3.46	3.08	2.55	2.97	3.18	2.66
CREB3	15.37	12.08	10.62	12.81	14.48	9.61
CREB3L1	0.6	0	0.3	0.02	0	0.14
CREB3L2	14.33	21.66	23.68	21.37	18.97	22.94
CREB3L3	0.12	0	0	0	0	0
CREB3L4	3.84	6.58	6.07	8.86	4.25	7.76
CREB5	0.07	0.01	0	0	0	0.01
CREBBP	4.84	3.84	4.11	4.21	4.81	4.6
CREBL2	1.2	0.55	0.32	0.37	0.62	0.33
CREBRF	0.73	0.35	0.71	0.46	0.47	0.67
CREBZF	2.22	2.55	2.05	1.44	1.7	1.66
CREG1	41.36	37.36	29.91	38.85	39.06	23.24
CREG2	0	0	0	0.01	0	0
CRELD1	2.65	1.32	0.71	1.41	2.56	1.77
CRELD2	30.81	36.61	32.87	18.93	32.17	20.72
CREM	12.39	11.13	5.78	7.68	6.84	7.73
CRH	0	0	0	0	0	0
CRHBP	0	0	0.04	0	0	0
CRHR1	0	0	0	0	0	0

CRHR1-IT1	1.49	0.62	1.58	1.18	0.49	1.19
CRHR2	0.05	0	0.07	0	0	0
CRIM1	2.87	0.75	0.47	0.81	1.38	0.81
CRIP1	0	0.14	0	0	0	0
CRIP2	2.33	0.63	0.27	1.66	3.54	1.37
CRIP3	0	0	0	0	0	0
CRIPAK	0.46	0.09	0.05	0.31	0.28	0.16
CRIPT	17.37	9.52	8.26	9.07	11.38	10.11
CRISP1	0	0	0	0	0	0
CRISP2	0.06	0	0	0.15	1.27	0.11
CRISP3	0.43	0.16	0	0	0.97	0.44
CRISPLD1	2.59	7.11	5.96	5.85	4.31	4.33
CRISPLD2	0.05	0.04	0.06	0.03	0.04	0.09
CRK	6.67	4.71	5.83	5.81	6.15	4.85
CRKL	28.49	30.23	36.56	34.54	31.28	31.12
CRLF1	0.37	0.1	0	0.06	0.16	0.33
CRLF2	4.05	0.32	0	0	2.63	0.36
CRLF3	6.44	11.92	10.83	8.07	9.12	9.14
CRLS1	3.98	3.29	2.89	2.59	3.2	2.67
CRMP1	0.58	0.28	0.17	0.03	0.14	0.12
CRNDE	11.74	19.79	16.53	16.97	20.38	17.51
CRNKL1	5.74	5.55	6.44	6.11	9.42	5.16
CRNN	0	0	0	0	0	0
CROCC	0.22	0.08	0.3	0.21	0.39	0.15
CROCCP2	1.74	1.58	1.12	1.23	0.95	0.68
CROCCP3	0.69	0.45	0.68	0.42	0.6	0.74
CROT	0.02	0.02	0	0	0	0.02
CRP	0	0	0	0	0	0.04
CRSP8P	0	0	0	0	0	0
CRTAC1	0	0	0	0	0	0
CRTAM	0.19	0.11	0.37	0.14	0.18	0.42
CRTAP	21.84	18.92	17.43	18.63	17.19	20.38
CRTC1	1.84	1.43	1.85	2.26	1.66	1.57
CRTC2	3.52	3.83	3.17	3.46	3.99	3.76
CRTC3	5.33	5.67	4.95	5.24	5.56	5.27
CRX	1.32	1.14	1.65	1.04	1.34	1.37
CRY1	0	0	0	0	0.19	0
CRY2	5.41	4.66	4.79	5.36	3.93	4.69
CRYAA	0	0	0	0	0	0
CRYAB	0.12	0	0	0.08	0	0
CRYBA1	0.1	0	0	0	0	0
CRYBA2	0	0	0	0.96	0.22	0.35
CRYBA4	0	0	0.18	0	0	0.39

CRYBB1	0.42	1.59	1	0.82	1.12	2.98
CRYBB2	0	0.43	0.54	0.38	0.31	0.35
CRYBB2P1	8.54	9.98	7.98	6.74	10.87	8.85
CRYBB3	0.09	0	0	0	0	0
CRYBG3	1.46	1.94	1.86	1.93	1.48	2.12
CRYGA	0	0	0	0	0	0
CRYGB	0	0	0	0	0	0
CRYGC	0	0	0	0	0	0
CRYGD	0	0	0	0	0	0
CRYGN	0	0	0	0	0	0
CRYGS	0	0.44	0.68	0.84	0.44	0.47
CRYL1	7.14	8.81	6.58	5.57	7.22	7.97
CRYM	33.98	0.15	1.13	7.31	9.84	1.18
CRYM-AS1	0.37	0.6	0.55	0.35	0.63	0.48
CRYZ	11.86	10.34	8.36	5.91	8.53	10
CRYZL1	8.44	11.33	9.17	8.09	9.08	10.65
CS	143.96	140.55	139.54	155.39	157.38	149.53
CSAD	0.65	1.04	1.23	0.67	0.87	0.84
CSAG1	0	0	0	0.34	0	0.37
CSAG2	0	0	0	0.67	0	0
CSAG3	0	0	0	1.13	0	0
CSAG4	0	0	0	0.44	0	0
CSDA	15.99	12.64	15.54	16.94	17.65	16.56
CSDAP1	0	0.03	0.04	0	0.04	0
CSDC2	0	0	0	0	0	0
CSDE1	97.04	86.52	90.25	90.55	86.95	92.05
CSE1L	58.75	69.39	63.13	60.3	66.09	71.44
CSF1	1.4	0.12	0.08	0.42	0.52	0.35
CSF1R	0.09	0.5	1.38	0.37	0.36	1.65
CSF2	0	0	0	0	0	0
CSF2RA	2.19	7.25	5.13	3.22	2.95	5.68
CSF2RB	1.5	0.27	0.78	0.58	1.25	0.5
CSF3	0	0.03	0.12	0	0	0
CSF3R	0	0.35	0.12	0	0.1	1.14
CSGALNACT1	2.1	0.03	0.1	0.48	1.2	0.09
CSGALNACT2	1.44	0.44	0.49	0.32	0.74	0.41
CSH1	0	0	0	0	0	0
CSH2	0	0	0	0	0	0
CSHL1	0	0	0	0	0	0
CSK	28.1	39.26	35.37	38.08	33.37	39.47
CSMD1	0	0	0.01	0	0.01	0.04
CSMD2	0	0	0	0	0	0
CSMD3	0.07	0.59	1.1	0.89	0.54	0.06

CSN1S1	0	0	0	0	0	0
CSN1S2AP	0	0	0	0	0	0
CSN1S2BP	0	0	0	0	0	0
CSN2	0	0	0	0	0	0
CSN3	0	0	0	0	0	0
CSNK1A1	64.82	53.08	48.1	47.78	48.83	48.22
CSNK1A1L	0.12	0	0	0	0.09	0
CSNK1A1P1	0	0	0	0	0	0
CSNK1D	11.3	7.23	7.64	8.31	10.38	8.43
CSNK1E	7.74	7.86	6.72	6.78	9.54	8.97
CSNK1G1	8.2	6.75	7.13	5.72	5.99	7.05
CSNK1G2	4.1	4.02	4.28	5.37	3.98	4.14
CSNK1G2-AS	0	0	0.05	0	0	0
CSNK1G3	7.75	5.76	3.94	4.51	6.98	4.94
CSNK2A1	77.39	55.3	55.7	56.33	61.79	60.8
CSNK2A2	3.06	2.02	4.03	2.85	3.4	2.91
CSNK2A3	0	0	0.34	0.25	0	0.4
CSNK2B	289.75	252.96	257.67	296.6	298.64	275.1
CSPG4	0.13	0.12	0.11	0.23	0.12	0.03
CSPG4P1Y	0.31	0.16	0	0.13	0.19	0
CSPG5	0	0.11	0.31	0.21	0.07	0.35
CSPP1	6.63	8.54	5.81	6.73	6.76	6.65
CSRNP1	4.27	1.06	1.42	2.48	2.82	2.84
CSRNP2	0.87	0.64	0.49	1.03	0.89	0.32
CSRNP3	0	0	0	0	0	0
CSRP1	46.97	61.87	62.56	55.69	51.3	65.77
CSRP2	4.83	9.46	6	10.44	10.47	8.53
CSRP2BP	5.45	5.4	5.34	8.51	4.82	9.08
CSRP3	0	0	0	0	0	0
CST1	0	0	0	0	0	0
CST11	0	0	0	0	0	0
CST13P	0	0	0	0	0	0
CST2	0	0	0	0	0	0
CST3	126.05	38.27	51.98	68.75	86.5	45
CST4	0	0	0	0	0	0
CST5	0.1	0	0	0	0	0
CST6	0	0	0	0	0	0
CST7	33.5	16.96	17.53	8.06	17.61	11.02
CST8	0	0	0	0	0	0
CST9	0	0	0	0	0	0
CST9L	0	0	0	0	0	0
CSTA	0.09	0	0	0.26	0	0.1
CSTB	206.33	169.55	159.64	168.15	200.02	160.08

CSTF1	31.87	36.28	40.3	43.74	40.82	40.99
CSTF2	47.8	62.55	51.04	45.62	47.95	48.14
CSTF2T	9.28	9.85	12.81	11.63	7.16	10.25
CSTF3	24.97	24.85	29.3	20.79	20.61	21.02
CSTF3-AS1	0.47	1.02	1.25	0.62	0.62	0.46
CSTL1	0	0	0	0	0	0
CT45A1	0	0	0	0	0	0
CT45A2	0	0	0	0	0	0
CT45A3	0	0	0	0	0	0
CT45A4	0	0	0	0	0	0
CT45A5	0	0	0	0	0	0
CT45A6	0	0	0	0	0	0
CT47A1	0	0	0	0	0	0
CT47A10	0	0	0	0	0	0
CT47A11	0	0	0	0	0	0
CT47A12	0	0	0	0	0	0
CT47A2	0	0	0	0	0	0
CT47A3	0	0	0	0	0	0
CT47A4	0	0	0	0	0	0
CT47A5	0	0	0	0	0	0
CT47A6	0	0	0	0	0	0
CT47A7	0	0	0	0	0	0
CT47A8	0	0	0	0	0	0
CT47A9	0	0	0	0	0	0
CT47B1	0	0	0	0	0	0
CT49	0	0	0.1	0.09	0	0
CT60	0	0	0	0	0	0
CT62	0	0	0	0	0	0
CT64	0.1	0.16	0.18	0.03	0	0.15
CTAG1A	0	0	0	0	0	0
CTAG1B	0	0	0	0	0	0
CTAG2	0	0	0	0	0	0
CTAGE1	0	0	0	0	0	0
CTAGE10P	0	0	0	0	0	0
CTAGE11P	0.03	0	0	0.04	0	0
CTAGE15P	0	0	0	0	0	0
CTAGE4	0	0	0	0	0	0
CTAGE5	3.93	4.75	3.89	2.6	3.34	4.18
CTAGE6P	0	0	0	0	0	0
CTAGE7P	0.13	0.04	0.1	0.04	0.05	0
CTAGE9	0.06	0.02	0	0	0	0
CTBP1	23.84	23.04	22.47	31.31	27.1	17.91
CTBP1-AS1	1.88	1.17	0.71	0.82	1.26	0.55

CTBP2	9.8	7.72	8.59	8.02	8.72	8.72
CTBS	4.42	3.66	2.84	1.74	2.3	3
CTC1	2.99	2.65	1.98	1.78	2.28	2.1
CTCF	10.31	15.14	12.71	12.96	12.36	12.71
CTCFL	0.43	0.24	0.17	0.19	0.25	0.55
CTDNEP1	7.15	9.69	7.92	9.18	10.63	12.74
CTDP1	1.38	0.98	0.57	0.66	0.83	1.26
CTDSP1	14.27	14.23	12.16	14.9	13.52	10.21
CTDSP2	14.31	12.11	10.79	9.85	12.04	12.06
CTDSPL	2.28	3.99	4.24	2.54	2.98	3.2
CTDSPL2	6.45	8.83	7.41	6.22	7.23	6.73
CTF1	0.09	0.14	0.2	0	0.13	0.05
CTGF	0	0	0	0	0	0
CTH	7.92	6.88	6.02	4.74	7.41	6.86
CTHRC1	0	0	0	0	0.18	0.19
CTIF	0.57	0.89	0.87	0.46	0.69	0.8
CTLA4	0	0	0	0	0	0
CTNNA1	44.69	22.1	19.91	26.64	27.34	26.42
CTNNA2	0	0	0	0	0	0
CTNNA3	0	0	0	0	0	0
CTNNAL1	13.89	20.72	15.65	18.65	16.5	16.72
CTNNB1	79.14	56.36	77.39	83.4	82.77	80.44
CTNNBIP1	1.18	3.14	1.98	0.95	1.76	1.89
CTNNBL1	43.15	44.17	41.72	43.72	55.68	36.58
CTNND1	1.24	0	0.14	0.1	0.35	0.21
CTNND2	0.03	0	0	0	0	0
CTNS	14.11	11.42	9.5	8.81	6.98	9.43
CTPS1	22.75	30.9	29.2	27.43	26.41	27.1
CTPS2	7.51	7.92	8.04	8.67	7.66	8.6
CTR9	22.19	16.96	13.06	15.59	15.77	12.34
CTRB1	0	0	0	0	0	0
CTRB2	0	0	0	0	0	0
CTRC	0.52	0	0	0	0	0
CTRL	0.58	2.48	0.89	1.06	0.85	0.26
CTSA	123.99	92.59	80.09	85.96	94.1	68.18
CTSB	146.77	83.09	81.87	87.18	93.52	77.37
CTSC	64.71	128.47	114.17	81.16	79.27	102.78
CTSD	152.2	134.4	146.68	141.62	121.51	107.63
CTSE	0.28	0.21	0.03	0.02	0.17	0.29
CTSF	3.3	3.99	3.93	5.07	5.44	2.34
CTSG	0	0.07	0	0	0	0.25
CTSH	54.91	25.34	9.98	17.98	22.52	10.66
CTSK	0.53	0.03	0	0.08	0.08	0.25

CTSL1	83.91	22.63	23.91	42	38.89	38.28
CTSL1P2	0.06	0.14	0.13	0.1	0.05	0.07
CTSL1P8	0	0	0	0	0	0
CTSL2	3.12	3.7	3.4	2.65	2.58	2.58
CTSL3P	0	0	0	0	0	0
CTSO	0.32	0.22	0.23	0.34	0	0.18
CTSS	5.59	14.61	13.19	9.63	9.79	11.74
CTSW	0.06	0.46	1.12	2.53	0.28	0.18
CTSZ	0.8	0.71	0.27	0.45	1.12	0.71
CTTN	57.97	0.16	1.2	15.81	29.37	0.46
CTTNBP2	3.27	0.57	0.83	2.11	1.86	1.49
CTTNBP2NL	2.98	1.8	1.76	1.85	1.95	2.25
CTU1	0.03	0.08	0.13	0.02	0	0
CTU2	53.99	70.03	62.14	58.54	58.05	58.94
CTXN1	1.02	0.33	0.55	0.99	0.4	0.06
CTXN2	0.08	0	0	0	0.05	0
CTXN3	0	0	0	0	0	0
CUBN	0.08	0.02	0.04	0	0.01	0.01
CUEDC1	0.1	0.08	0.04	0.04	0.04	0
CUEDC2	93.86	112.61	122.2	121.6	116.94	103.32
CUL1	25.83	20.59	18.29	19.78	18.74	18.72
CUL2	10.66	9.88	10.41	10.24	11.39	10.16
CUL3	3.78	2.27	2.95	2.55	3.9	3.15
CUL4A	15.01	16.76	17.82	18.48	16.91	17.67
CUL4B	10.24	9.06	8.46	7.71	10.53	10.19
CUL5	5.49	5.7	4.55	3.82	4.47	4.72
CUL7	1.75	1.78	1.21	2.04	1.75	1.88
CUL9	1.17	0.85	0.67	0.84	0.61	0.95
CUTA	241.61	249.14	267.32	294.31	301.03	267.01
CUTC	29.6	43.17	37.59	34.06	31.92	34.27
CUX1	9.43	7.71	7.7	7.74	9.96	8.86
CUX2	0	0	0	0	0	0
CUZD1	0	0	0	0	0	0
CWC15	38.7	38.91	34.12	30.42	37.07	31.08
CWC22	9.56	12.35	14.91	11.31	13.56	13.63
CWC25	10.04	9.26	9.63	7.58	8.29	7.45
CWC27	13.58	12.77	13.03	11.55	13.22	11.84
CWF19L1	23.93	25.59	26.93	24.92	26.89	28.01
CWF19L2	2.57	3.69	3.01	2.76	2.24	2.02
CWH43	0	0	0	0	0	0
CX3CL1	0	0	0	0	0	0
CX3CR1	0	0.09	0	0	0	0
CXADR	2.76	2.67	1.6	1.41	2.25	2.16

CXADRP2	0	0	0	0	0	0
CXADRP3	0	0	0	0	0	0
CXCL1	0	0	0	0	0	0
CXCL10	0.81	0.3	0.06	0.34	0.83	0.44
CXCL11	1.35	0	0.26	0.22	0.48	0.28
CXCL12	0	0	0	0	0	0
CXCL13	0	0	0	0	0	0
CXCL14	0	0	0	0	0	0
CXCL16	1.77	0.46	0.86	0.58	0.95	0.96
CXCL17	0	0	0	0	0	0
CXCL2	1.2	1.34	0.98	0.48	0.54	0.51
CXCL3	0.26	0	0.18	0	0.06	0
CXCL5	0	0.05	0	0.02	0.03	0
CXCL6	0	0	0	0	0	0
CXCL9	0	0	0	0	0	0
CXCR1	0	0	0	0	0	0
CXCR2	0.18	0.28	0.16	0.47	0.24	0.24
CXCR2P1	0	0	0	0	0	0
CXCR3	1.19	0.29	0.08	0.13	0.56	0.09
CXCR4	0.67	0.04	0	0.21	0	0.18
CXCR5	0	0	0	0	0	0
CXCR6	0.49	0	0.42	0.31	0.36	0.38
CXCR7	0	0	0	0	0	0
CXXC1	27.83	34.54	33.2	32.5	27.41	33.55
CXXC11	0	0	0	0	0	0
CXXC1P1	0	0	0	0	0	0
CXXC4	0	0.03	0	0	0.02	0
CXXC5	26.61	25.8	21.55	24.37	24.03	22.33
CXorf21	6.92	26.32	25.13	8.43	8.48	15.32
CXorf22	0	0	0	0	0.04	0
CXorf23	0.38	0.21	0.31	0.23	0.38	0.16
CXorf27	0	0	0	0	0	0
CXorf28	0	0	0	0	0	0
CXorf30	0	0	0	0	0	0
CXorf31	0.32	0.26	0.44	0.45	0	0
CXorf36	0.47	0.43	0.37	0.35	0.29	0.25
CXorf38	7.87	12	11.28	7.5	9.53	10.83
CXorf40A	16.5	21.17	19.59	16.73	14.55	15.83
CXorf40B	42.52	40.78	37.5	30.33	38.88	30.94
CXorf48	0	0	0	0	0	0
CXorf49	0	0	0	0	0	0
CXorf49B	0	0	0	0	0	0
CXorf51A	0	0	0	0	0	0

CXorf51B	0	0	0	0	0	0
CXorf56	22.73	21.43	24.18	22.99	22.41	22.96
CXorf57	3.26	6.27	3.79	3.72	4.44	4.98
CXorf58	0.05	0.11	0.04	0.13	0.05	0
CXorf61	0	0	0	0	0	0
CXorf64	0	0	0	0	0	0
CXorf65	0.27	0.78	0	0.13	0.6	0.64
CXorf66	0	0	0	0	0	0
CYB561	22.95	6.96	5.24	8.99	11.32	4.33
CYB561D1	1.81	2.25	2.2	1.87	2.32	2.82
CYB561D2	28	41.98	39.74	25.71	31.2	28.4
CYB5A	0	0	0	0	0	0
CYB5B	15.16	18.95	16.56	17.46	20.29	18.44
CYB5D1	6.58	5.03	6.29	5.33	4.78	6.82
CYB5D2	9.81	7.37	5.35	8.04	5.81	6.76
CYB5R1	16.45	17.66	15.48	12.53	15.6	13.8
CYB5R2	0	0	0	0	0	0
CYB5R3	74.08	51.73	50.26	57.64	59	52.85
CYB5R4	20.49	15.16	16.03	12	16.5	17.59
CYB5RL	3.21	4.05	3.26	3.1	5.34	4.47
CYBA	90.43	133.57	216.89	178.38	147.55	229.5
CYBASC3	37.31	23.46	22.12	23.82	23.93	20.8
CYBB	0.64	0.08	0.09	0.17	0.24	0
CYBRD1	6.36	6.15	4.74	4.73	6.95	5.55
CYC1	743.3	805.62	842.34	916.05	887.48	664.11
CYCS	56.36	48.25	41.71	35.99	49.89	45.41
CYCSP52	0	0	0.28	0	0	0
CYFIP1	24.3	25.38	27.11	26.29	22.13	29.32
CYFIP2	5.8	9.69	11	9.39	9.71	10.14
CYGB	0	0.05	0.06	0	0	0
CYHR1	28.43	35.91	38.01	35.28	30.21	38.69
CYLC1	0	0	0	0	0	0
CYLC2	0	0	0	0	0	0
CYLD	1.2	1.26	0.71	1.15	0.96	1.13
CYMP	0	0	0	0	0	0
CYP11A1	0	0	0	0	0	0
CYP11B1	0	0	0	0	0	0
CYP11B2	0	0	0	0	0	0
CYP17A1	0	0	0	0	0.19	0
CYP19A1	0	0	0	0	0	0
CYP1A1	23.25	7.76	2.83	15.55	5.88	2.5
CYP1A2	1.21	1.17	0.97	0.92	0.73	1.22
CYP1B1	33.86	22.74	26.19	16.56	12.72	14.9

CYP1B1-AS1	0	0.13	0	0.35	0	0.21
CYP20A1	4.36	4.56	4.39	3.45	3.98	3.94
CYP21A1P	0.03	0.02	0	0.02	0	0
CYP21A2	0	0	0	0	0	0
CYP24A1	0	0	0	0	0	0
CYP26A1	0.38	1.09	1.24	1.87	0.92	1.76
CYP26B1	0.13	0	0	0	0	0
CYP26C1	0	0	0	0	0	0
CYP27A1	0	0	0.03	0	0	0
CYP27B1	0.29	1.42	0.24	0.28	0.22	0.09
CYP27C1	0.14	0.47	0.34	0.14	0.2	0.15
CYP2A13	0	0	0	0	0	0
CYP2A6	0	0	0.04	0	0	0
CYP2A7	0	0	0	0.12	0	0
CYP2B6	0.19	0.31	0.29	0.12	0.39	0.28
CYP2B7P1	0.72	0.39	0.46	0.27	0.45	0.41
CYP2C18	0	0	0	0	0	0
CYP2C19	0	0	0	0	0	0
CYP2C8	0	0	0	0	0	0
CYP2C9	0	0	0	0	0	0
CYP2D6	0.25	0.15	0.14	0	0.16	0.32
CYP2D7P1	0	0.32	0.24	0	0.27	0.08
CYP2E1	0	0.14	0.49	0.28	0.34	0.09
CYP2F1	0.12	0	0	0.28	0	0.25
CYP2G1P	0	0	0	0	0	0
CYP2J2	0	0	0	0	0	0
CYP2R1	1.57	1.93	1.52	1.75	1.79	2.28
CYP2S1	3.8	14.93	19.04	12.81	9.22	15.06
CYP2U1	0.32	1.03	0.28	0.73	0.4	0.86
CYP2W1	0.16	0.05	0.12	0.08	0	0.06
CYP39A1	0	0	0	0	0	0.07
CYP3A4	0.03	0	0	0.04	0	0.05
CYP3A43	0	0	0	0	0	0
CYP3A5	0.15	0.22	0.27	0.19	0.1	0.03
CYP3A7	0	0	0	0	0	0
CYP3A7-CYP3	0	0	0	0	0	0
CYP46A1	0	0	0	0	0	0
CYP4A11	0.03	0.23	0.11	0.06	0	0.1
CYP4A22	0	0	0	0	0	0
CYP4B1	0	0	0	0	0	0
CYP4F11	0	0.14	0	0.03	0.22	0.13
CYP4F12	0	0	0	0	0	0
CYP4F2	0	0	0	0.02	0	0

CYP4F22	0.06	0	0	0	0.16	0.09
CYP4F24P	0	0	0	0	0	0
CYP4F3	0	0	0	0	0	0
CYP4F30P	0	0.15	0	0.05	0	0.06
CYP4F35P	0.13	0.11	0	0.1	0.17	0.05
CYP4F8	0	0	0	0	0	0
CYP4V2	2.35	2.57	2.49	1.73	1.66	1.96
CYP4X1	0	0	0	0	0	0
CYP4Z1	0	0	0	0	0	0
CYP4Z2P	0	0	0	0	0	0
CYP51A1	38.89	27.54	31.62	24.68	38.39	30.88
CYP7A1	0	0	0	0	0	0
CYP7B1	0	0	0	0	0	0
CYP8B1	0.13	0.15	0.15	0.24	0.08	0.06
CYR61	7.47	3.43	3.06	3.35	4.21	2.37
CYS1	0.05	0	0	0.06	0	0
CYSLTR1	2.17	16.68	19.01	8.52	7.76	23.95
CYSLTR2	0.14	0	0	0	0.05	0.15
CYSTM1	92.23	23.9	19.43	31.82	58.33	21.45
CYTH1	4.81	4.58	4.1	4.2	3.73	3.22
CYTH2	6.08	3.7	3.13	4.68	5.62	3.64
CYTH3	0.7	0.56	0.68	0.4	0.53	0.54
CYTH4	0.74	0.4	1.76	1.04	0.28	0.35
CYTIP	1.97	3.75	2.47	2.38	1.57	3.61
CYTL1	2.54	4.92	2.89	26.28	0.22	10.65
CYYR1	0	0	0	0	0	0
D21S2088E	0	0	0.04	0.03	0.04	0.04
D2HGDH	0.65	1.05	1.09	0.88	0.64	0.6
DAAM1	3.28	1.64	1.43	1.66	1.84	1.19
DAAM2	0.08	0	0	0	0	0
DAB1	0	0	0	0	0	0
DAB2	23.26	4.83	5.98	9.07	11.11	5.17
DAB2IP	0.04	0.1	0.02	0.07	0	0.2
DACH1	0	0	0	0	0	0
DACH2	0	0	0	0	0	0
DACT1	0.04	0.09	0	0	0.02	0
DACT2	0	0	0	0	0	0
DACT3	0.46	0.06	0.17	0.12	0.34	0.23
DACT3-AS1	0	0	0	0	0.32	0
DAD1	348.32	254.09	266.61	265.45	320.54	239.65
DAG1	9.42	4.86	4.15	7.31	8.31	6.04
DAGLA	0.33	0.33	0.52	0.27	0.46	0.58
DAGLB	11.26	15.52	15.59	17.18	11.91	17.36

DAK	7.96	6.25	7.78	6.26	8.24	5.85
DALRD3	13.41	15.85	15.14	17.27	11.95	15.04
DANCR	295.7	259.71	274.07	304.86	301.95	266.13
DAND5	1.89	2	2.49	1.62	1.95	2.2
DAO	0	0	0	0	0	0
DAOA	0	0	0	0	0	0
DAOA-AS1	0	0	0	0	0.03	0
DAP	30.58	34.08	35.43	34.5	30.17	28.84
DAP3	68.15	61.86	55.21	60.24	64.35	52.55
DAPK1	20.43	9.8	10.66	10.61	11.86	9.58
DAPK2	1.28	0.16	0.23	0.29	0.74	0.09
DAPK3	20.21	20.78	18.53	19.07	16.9	12.86
DAPL1	0	0	0	0	0	0
DAPP1	1.35	0.24	0.28	0.13	0.25	0.05
DARC	0.24	0	0	0	0	0
DARS	50.69	49.82	57.47	51.97	51.61	55.26
DARS2	15.96	16.93	15.96	13.74	17.88	17.06
DAW1	0	0	0	0	0	0
DAXX	30.5	30.4	29.86	29.34	27.03	28.76
DAZ1	0	0	0	0	0	0
DAZ2	0	0	0	0	0	0
DAZ3	0	0	0	0	0	0
DAZ4	0	0	0	0	0	0
DAZAP1	7.42	6.5	7.7	8.57	8.32	7.64
DAZAP2	95.65	85.12	66.76	72.63	75.82	59.1
DAZL	0	0	0	0	0	0
DBC1	0	0	0	0	0	0
DBF4	10.07	9.36	9.14	7.56	9.78	7.6
DBF4B	4.36	5.26	4.14	3.9	3.92	5.4
DBH	0	0	0	0	0	0
DBI	417.55	357.48	273.43	246.06	377.38	258.89
DBIL5P	0.05	0.11	0.02	0.42	0.05	0.17
DBIL5P2	0.02	0	0.47	0.13	0.08	0
DBN1	26.28	21.06	20.49	30.33	27.15	27.28
DBNDD1	6.16	1.18	1.82	3.79	5.03	3.04
DBNDD2	44.05	25.92	15.95	20.64	43.28	14.16
DBNL	33.35	43.68	36.32	38.91	35.58	39.84
DBP	3.27	5.11	5.19	4.56	5.4	4.4
DBR1	8.28	11.46	8.28	8.38	12.72	12.35
DBT	2.07	1.75	1.44	1.26	1.6	1.68
DBX1	0	0	0	0	0	0
DBX2	0	0	0	0	0	0
DCAF10	2.33	2.04	2.43	1.66	2.24	2.05

DCAF11	19.94	21.5	20.87	17.41	18.1	21.33
DCAF12	21.62	17.85	19.43	17.83	19.19	19.36
DCAF12L1	0	0	0	0	0	0
DCAF12L2	0	0	0	0	0	0
DCAF13	98.75	104.11	95.9	90.04	105.83	96.01
DCAF13P3	0.03	0.25	0.11	0.12	0.17	0
DCAF15	0.88	0.77	0.74	0.99	0.74	0.66
DCAF16	15.58	14.28	15.64	16.96	14.36	18.53
DCAF17	1.57	1.4	1.11	1.29	1.04	1.92
DCAF4	9.89	1.61	13.62	13.5	11.15	15.52
DCAF4L1	0.12	0.08	0	0	0.07	0.02
DCAF4L2	0	0	0	0	0	0
DCAF5	5.24	3.39	3.15	3.18	3.34	2.92
DCAF6	4.81	6.04	5.51	6.08	6.23	5.91
DCAF7	70.22	68.61	61.22	68.24	69.38	68.17
DCAF8	16.78	10.26	7.75	7.47	10.07	9.67
DCAF8L1	0	0	0	0	0	0
DCAF8L2	0	0	0	0	0	0
DCAKD	15.31	23.66	22.68	16.88	18.3	21.43
DCBLD1	0.78	0.97	0.82	0.53	0.47	0.61
DCBLD2	3.59	1.93	1.52	2.05	2.9	1.31
DCC	0.08	0	0	0	0	0
DCD	0	0	0	0	0	0
DCDC1	0	0	0	0.03	0	0.13
DCDC2	0.1	0.21	0.06	0.06	0.14	0.02
DCDC2B	0	0.31	0.26	0.12	0	0.23
DCDC5	0.09	0.16	0.12	0.07	0.02	0.02
DCHS1	0.03	0.21	0.04	0.13	0.03	0.07
DCHS2	0	0	0	0	0	0
DCK	13.46	14.94	12.99	11.5	13.12	16.97
DCLK1	0	0	0	0	0	0
DCLK2	0	0	0.02	0.01	0.05	0.05
DCLK3	0	0	0	0	0	0
DCLRE1A	3.42	4.12	3.57	2.72	3.55	3.6
DCLRE1B	6.64	11.08	9.84	7.73	10.18	9.68
DCLRE1C	5.71	8.07	7.13	5.19	5.04	6.31
DCN	0	0	0	0	0	0
DCP1A	8.69	6.24	6.8	6.3	6.63	5.8
DCP1B	5.32	8.47	7.07	9.12	5.13	7.38
DCP2	6.78	5.65	5.59	4.6	5.28	5.97
DCPS	15.41	28.64	21.93	18.79	23.04	23.62
DCST1	0	0	0	0	0	0
DCST2	0	0	0	0	0	0.12

DCSTAMP	0	0	0	0	0	0
DCT	0	0	0	0	0	0
DCTD	41.2	29.66	29.78	26.99	37.58	33.27
DCTN1	31.44	31.07	28.51	30.12	29.89	32.38
DCTN1-AS1	0.03	0	0	0	0	0
DCTN2	80.35	60.39	55.81	64.76	73.11	56.57
DCTN3	100.35	98.59	70.61	90.38	91.81	69.93
DCTN4	20.75	27.8	22.79	18.31	17.64	18.18
DCTN5	17.71	18.49	18.5	16.55	17.1	16.57
DCTN6	84.34	51.77	44.63	50.32	63.55	39.91
DCTPP1	171.9	240.84	219.96	222.99	215.1	223.36
DCUN1D1	27.19	14.02	12.69	14.37	17.52	11.78
DCUN1D2	1.99	2.85	2.24	2.11	2.13	2.53
DCUN1D3	2.39	2.36	3.17	2.57	2.83	3.63
DCUN1D4	9.82	6.29	7.14	4.93	7.82	7.44
DCUN1D5	9.43	8.76	8.26	7.47	9.04	8.44
DCX	0.05	0.03	0.02	0.03	0.05	0
DCXR	216	263.01	303.86	304.57	287.14	259.08
DDA1	7.13	6.06	6.01	6.56	6.37	6.57
DDAH1	4.95	7.01	7.09	6.51	6.29	4.54
DDAH2	28.73	14.7	17.84	29.7	34.46	19.36
DDB1	67.17	66.14	66.19	72.33	67.2	82.8
DDB2	9.79	14.44	12.08	10.19	11.14	14.36
DDC	0.05	0.18	0.04	0.28	0.04	0
DDHD1	1.46	1.29	1.47	1.19	1.14	0.84
DDHD2	11.32	8.92	7.22	9.41	8.55	6.61
DDI1	0	0	0	0	0	0
DDI2	7.06	7.71	9.48	8.7	8.17	7.23
DDIT3	16.92	6.32	4.1	2.92	7.99	2.19
DDIT4	161.25	21.49	19.41	47.23	44.8	19.64
DDIT4L	0	0	0	0	0	0
DDN	2.56	1.69	1.56	2.38	3.6	2.16
DDO	0	0	0	0	0	0
DDOST	212.01	256.59	226.77	217.23	218.29	234.34
DDR1	2.62	0	0	0.25	0.63	0
DDR2	0.07	0	0	0	0	0
DDRGK1	18.54	31.32	27.84	25.7	24.96	14.58
DDT	140.82	150.38	160.52	154.53	164.1	156.26
DDTL	1.81	3.71	2.15	2.85	2.52	2.31
DDX1	83.44	65.86	59.22	60.6	80.57	59.28
DDX10	25.71	23.27	24.31	23.39	25.66	22.67
DDX11	5.33	9.31	8.24	8.3	7.83	12.21
DDX11-AS1	0.15	0.92	1.16	0.6	1	0.44

DDX11L1	0	0.11	0	0.31	0.19	0.11
DDX11L10	0	0	0	0	0	0
DDX11L2	0.61	0.89	0.72	0.77	0.24	0.23
DDX11L5	0	0	0	0	0	0
DDX11L9	0.18	0.09	0	0	0	0
DDX12P	1.03	2.23	1.77	2	1.71	2.35
DDX17	38.98	44.88	41.56	36.12	39.19	44.4
DDX18	25.95	28.69	24.49	18.67	25.51	19.65
DDX19A	20.56	27.39	23.47	24.81	25.31	27.03
DDX19B	0.05	0	0.05	0.08	0	0
DDX20	11.54	12.87	15.4	13.36	9.13	11.45
DDX21	28.79	26.49	20.14	21.57	24.76	22.19
DDX23	28.93	35.69	29.45	31.88	29.59	42.48
DDX24	48.93	47.78	37.26	41.2	41.48	42.74
DDX25	0	0	0	0	0	0
DDX26B	3.52	3.71	2.26	3.55	3.21	2.59
DDX27	27.84	30.9	25.43	23.43	23.93	25.29
DDX28	15.45	22.66	18.4	17.77	16.33	18.55
DDX31	6.33	6.42	6.89	6.16	5.12	5.51
DDX39A	212.04	231.17	239.38	236.85	255.29	205.45
DDX39B	111.8	150.74	154.99	155.87	148.39	166.16
DDX3X	30.52	26.13	18.56	18.17	27.62	22.87
DDX3Y	0.03	0	0	0	0	0.05
DDX4	0	0	0	0	0	0
DDX41	37.14	48.34	42.28	45.44	42.26	41.66
DDX42	6.41	5.74	3.53	4.25	4.16	4.06
DDX43	0	0.02	0	0	0	0
DDX46	15.07	15.79	13.8	14.18	17.81	16.37
DDX47	41.77	45.04	39.95	39.82	50.99	42.29
DDX49	63.94	69.94	70.91	74.05	62.18	57.55
DDX5	142.23	123.25	102.04	109.23	125.62	126.23
DDX50	10.99	11.27	9.42	9.41	11.25	11.57
DDX51	3.16	2.54	3.89	2.91	2.71	3.19
DDX52	11.18	9.73	8.73	8.78	8.87	9.46
DDX53	0	0	0	0	0	0
DDX54	30.47	36.01	34.16	32.48	30.98	31.16
DDX55	10.34	13.81	10.23	10.13	11.47	11.76
DDX56	51.95	50.44	46.95	50.72	50.85	48.8
DDX58	1.26	1	1.75	0.84	0.99	1.11
DDX59	4.62	5.84	4.06	2.35	3.5	4.26
DDX6	48.38	42.72	42.73	43.98	47.55	52.5
DDX60	0.45	0.62	0.72	0.62	0.7	0.76
DDX60L	0.63	1.18	1.25	0.72	0.6	0.96

DEAF1	4.74	5.02	3.73	4.68	5.05	5.15
1-Dec	0	0	0	0	0	0
DECR1	253.96	100.76	77.76	125.18	163.5	91.15
DECR2	10.28	16.15	12.63	14.43	15.07	13.07
DEDD	27.54	19.22	14.24	20.64	21.98	15.36
DEDD2	20.64	14.75	11.88	15.83	20.42	11.91
DEF6	1.09	7.89	6.95	6.63	5.35	4.79
DEF8	62.94	54.72	42.81	54.33	59.73	41.38
DEFA1	0	0	0	0	0	0
DEFA10P	0	0	0	0	0	0
DEFA11P	0	0	0	0	0	0
DEFA1B	0	0	0	0	0	0
DEFA3	0	0	0	0	0	0
DEFA4	0	0	0	0	0	0
DEFA5	0	0	0	0	0	0
DEFA6	0	0	0	0	0	0
DEFA8P	0	0	0	0	0	0
DEFA9P	0	0	0	0	0	0
DEFB1	0	0	0	0	0	0
DEFB103A	0	0	0	0	0	0
DEFB103B	0	0	0	0	0	0
DEFB104A	0	0	0	0	0	0
DEFB104B	0	0	0	0	0	0
DEFB105A	0	0	0	0	0	0
DEFB105B	0	0	0	0	0	0
DEFB106A	0	0	0	0	0	0
DEFB106B	0	0	0	0	0	0
DEFB107A	0	0	0	0	0	0
DEFB107B	0	0	0	0	0	0
DEFB108B	0	0	0	0	0	0
DEFB109P1	0	0	0	0	0	0
DEFB109P1B	0	0	0	0	0	0
DEFB110	0	0	0	0	0	0
DEFB112	0	0	0	0	0	0
DEFB113	0	0	0	0	0	0
DEFB114	0	0	0	0	0	0
DEFB115	0	0	0	0	0	0
DEFB116	0	0	0	0	0	0
DEFB118	0	0	0	0	0	0
DEFB119	0	0	0	0	0	0
DEFB121	0	0	0	0	0	0
DEFB122	0	0	0	0	0	0
DEFB123	0	0	0	0	0	0

DEFB124	0	0	0	0	0	0
DEFB125	0	0	0	0	0	0
DEFB126	0	0	0	0	0	0
DEFB127	0	0	0	0	0	0
DEFB128	0	0	0	0	0	0
DEFB129	0	0	0	0	0	0
DEFB130	0	0	0	0	0	0
DEFB131	0	0	0	0	0	0
DEFB132	0	0	0	0	0	0
DEFB133	0	0	0	0	0	0
DEFB134	0	0	0	0	0	0
DEFB135	0	0	0	0	0	0
DEFB136	0	0	0	0	0	0
DEFB4A	0	0	0	0	0	0
DEFB4B	0	0	0	0	0	0
DEFT1P	0	0	0	0	0	0
DEFT1P2	0	0	0	0	0	0
DEGS1	55.37	44.65	34.4	30.98	41.22	41.35
DEGS2	15.89	6.2	5.19	10.99	10.07	4.38
DEK	51.06	52.08	44.06	39.45	50.71	43.76
DENND1A	5.13	2.17	2.53	2.7	1.97	1.9
DENND1B	0.35	0.31	0.44	0.32	0.43	0.45
DENND1C	0.97	2.28	2.01	1.31	1.23	1.82
DENND2A	0	0	0	0	0	0
DENND2C	1.85	1.03	1.02	0.77	0.91	0.64
DENND2D	0.54	0.09	0.19	0.77	0.17	0.27
DENND3	1.24	3.99	3.25	0.99	1.35	3.64
DENND4A	6.74	4.01	3.81	3.33	3.93	3.26
DENND4B	5.01	3.47	2.96	2.3	3.92	2.76
DENND4C	2.37	2.44	3.13	1.96	2.45	2.15
DENND5A	4.77	4.61	2.79	2.66	3.92	4.15
DENND5B	0.19	0.22	0.34	0.24	0.18	0.3
DENND5B-AS	0.16	0.03	0	0	0.14	0
DENND6A	1.89	2.06	2.69	2.24	3.02	2.65
DENND6B	1.58	2.47	2.19	1.95	1.25	1.6
DENR	41.43	37.56	38.39	34.6	40.71	37.49
DEPDC1	7.59	6.22	5.04	5.66	4.3	5.57
DEPDC1B	15.84	21.86	18.27	16.67	19.72	18.06
DEPDC4	0.66	1.2	1.27	0.77	1.87	0.8
DEPDC5	1.64	5	4.15	4.44	4.49	4.01
DEPDC7	5.29	5.1	5.97	5.48	6.28	7.32
DEPTOR	9.29	30.29	29.59	26.34	26.36	37.27
DERA	25.41	43.13	37.97	37.1	34.88	40.7

DERL1	47.08	45.23	41.06	37.24	43.08	41.94
DERL2	21.18	21.73	16.08	17.43	20.82	20.23
DERL3	4.27	7.49	9.77	7.41	8.94	6.98
DES	0.65	0	0	0.18	0.53	0
DESI1	15.09	11.74	13.39	13.31	15.21	16.13
DESI2	3.92	2.9	1.67	2.19	3.45	2.57
DET1	5.21	6.07	6.44	7.27	4.5	3.19
DEXI	2.95	4.01	3.14	2.98	2.68	3.12
DFFA	32.15	38.35	33.76	29.57	31.95	32.67
DFFB	5.53	9.09	7.29	8.31	7.45	6.31
DFNA5	0.07	0.28	0.57	0.22	0.25	0.26
DFNB31	0	0.03	0.07	0	0.03	0
DFNB59	0.1	0.04	0	0	0	0
DGAT1	2.03	2.23	1.41	2.4	1.93	1.67
DGAT2	0.75	1.32	2.16	1.52	1.21	1.18
DGAT2L6	0	0	0	0	0	0
DGCR10	0	0	0	0	0	0
DGCR11	1.91	0.96	0.82	0.44	0.56	1.66
DGCR14	4.96	5.25	4.3	5.85	3.85	5.7
DGCR2	15.91	15.38	13.99	14.72	14.82	19.38
DGCR5	0.05	0.11	0	0	0	0
DGCR6	18.57	11.95	11.56	17.65	17.05	12.33
DGCR6L	57.42	78.3	86.18	85.57	67.28	85.78
DGCR8	5.7	6.99	5.98	4.6	6.51	6.62
DGCR9	0.09	0	0	0	0	0
DGKA	4.67	4.93	4.33	5.89	5.39	4.45
DGKB	0.22	0.26	0.42	0.22	0.27	0.34
DGKD	2.04	2.27	1.3	2.26	2.29	1.17
DGKE	0.92	1.2	1.21	0.99	0.7	1.19
DGKG	1.77	0.69	0.63	0.52	0.55	0.4
DGKH	0.28	0.18	0.29	0.29	0.26	0.25
DGKI	0	0	0	0	0	0
DGKK	0	0	0	0	0	0
DGKQ	1.2	1.1	0.5	0.88	1.01	0.72
DGKZ	5.83	8.17	7.84	5.43	4.75	9.04
DGUOK	97.35	85.98	71.37	80.83	95.33	89.8
DHCR24	64.2	84.44	88.1	80.27	81.09	108.9
DHCR7	65.29	56.85	58.38	53.55	73.59	66.59
DHDDS	10.4	10.48	9.74	10.03	9.69	8.55
DHDH	0.42	0	0	0.34	0	0.36
DHFR	24.22	30.43	28.78	28.21	27.54	32.5
DHFRL1	1.48	1.14	0.63	1.03	1.22	1.68
DHH	0	0	0	0	0	0

DHODH	8.08	10	8.41	8.49	8.44	9.41
DHPS	51.55	52.2	49.44	47.67	55.26	52.49
DHRS1	7.8	9.36	6.45	11.06	8.67	8.81
DHRS11	3.92	3.43	0.98	3.15	3.13	2.6
DHRS12	1.14	1.74	0.75	2.17	2.01	0.85
DHRS13	3.7	7.77	8.64	7.72	6	7.25
DHRS2	3.87	0.84	0.29	3.15	0.84	0.31
DHRS3	49.07	0.03	0.23	11.66	5.98	0.29
DHRS4	52.25	36.17	37.62	38.79	35.65	31.22
DHRS4-AS1	7.48	5.42	3.76	4.42	4.86	3.82
DHRS4L1	0	0	0.5	0	0.08	0
DHRS4L2	16.21	16.64	14.47	19.2	17.93	16.79
DHRS7	51.54	59.13	48.81	45.89	56.73	61.15
DHRS7B	16.11	9.23	13.75	12.31	11.68	11.28
DHRS7C	0	0	0	0	0	0
DHRS9	1.49	0	0	0	0.77	0
DHRSX	8.91	8.62	8.68	5.96	7.45	9.77
DHTKD1	13.76	5.93	5.5	9.98	8.92	7
DHX15	65.04	63.39	61.77	55.27	73.2	77.22
DHX16	10.83	13.99	10.91	10.54	11.37	11.34
DHX29	7.14	7.16	6.06	5.33	6.15	4.79
DHX30	30.76	29.75	31.77	32.58	27.67	24.63
DHX32	2.29	2.32	1.62	2.08	2.58	2.2
DHX33	7.62	9.08	6.71	7.06	7.88	8.95
DHX34	5.99	5.15	6.6	6.43	6.2	5.88
DHX35	4.6	4.68	5.2	5.07	4.45	5.43
DHX36	8.58	8.07	7.84	6.45	9.03	7.09
DHX37	8.22	7.2	9.77	8.2	6.7	8.68
DHX38	13.64	10.74	11.97	11.66	12.66	10.32
DHX40	8.53	6.69	7.26	6.99	7.54	9.65
DHX57	4.01	3.28	4.12	4.38	4.23	4.36
DHX58	0.03	0.04	0.49	0.13	0	0
DHX8	11.54	14.21	10.74	13.17	12.05	14.87
DHX9	52.44	57.48	54.78	51.36	54.12	65.13
DIABLO	35.48	41.34	36.34	37.62	38.09	35.71
DIAPH1	14.85	17.66	17.64	18.12	18.01	20.13
DIAPH2	1.92	2.31	2.97	1.65	1.98	3.3
DIAPH3	7.46	5.25	6.61	5.8	7.33	5.89
DIAPH3-AS1	0	0	0	0	0	0
DIAPH3-AS2	0	0	0	0	0	0
DICER1	3.54	4.36	2.84	2.67	3.06	3.81
DICER1-AS1	0.59	0.15	0.09	0.54	0.28	0
DIDO1	9.56	12.35	11.26	10.02	8.11	9.07

DIEXF	4.25	3.78	3.14	2.53	3.12	3.14
DIMT1	51.37	46.02	39.23	35.04	37.34	39.03
DIO1	0	0	0	0	0	0
DIO2	0.01	0	0.02	0.02	0	0
DIO2-AS1	0	0.15	0.05	0	0	0
DIO3	0	0	0	0	0	0
DIO3OS	0	0	0	0	0	0
DIP2A	4.7	3.46	3.98	3.84	2.43	3.39
DIP2A-IT1	0	0.16	0	0.43	0	0
DIP2B	3.35	4.41	3.01	2.8	2.95	3.49
DIP2C	0.1	0.01	0	0.07	0.07	0
DIRAS1	0.09	0.03	0	0.26	0.22	0.09
DIRAS2	0	0	0	0	0	0
DIRAS3	0.23	0	0	0	0	0
DIRC1	0.05	0.11	0.09	0.04	0.1	0.1
DIRC2	0.33	0.22	0.06	0.57	0.25	0.29
DIRC3	0	0	0	0	0.02	0
DIS3	6.86	7.36	7.22	5.97	7.07	6.67
DIS3L	18.19	28.61	29.05	26.64	20.62	30.48
DIS3L2	8.29	8.01	6.83	8.38	6.45	7.58
DISC1	0.52	1.23	0.68	0.5	1.15	1.26
DISC2	0.1	0.16	0.15	0.03	0.08	0.11
DISP1	0.44	0.32	0.35	0.43	0.44	0.37
DISP2	0.09	0.34	0.09	0.32	0.2	0.52
DIXDC1	0.71	1.6	3.13	1.07	1.47	2.3
DKC1	156.27	162.71	150.24	138.1	157.84	128.52
DKFZP434A0	0	0	0	0	0	0
DKFZP434H1	0	0	0	0	0	0
DKFZP434I07	0.68	0.63	0.67	0.58	0.16	0.59
DKFZP434K0	0	0	0	0	0	0
DKFZP434L1	0.16	0.29	0.47	0.29	0.34	0.51
DKFZP586I14	0.35	0.38	0.61	0.18	0.18	0.3
DKFZP686I15	1.59	1.02	0.19	1.1	1.71	0.34
DKFZp434J0	0.27	0	0	0	0.04	0
DKFZp434L1	0	0	0	0	0	0
DKFZp451B0	0	0	0	0	0	0
DKFZp566F0	0	0	0	0	0	0
DKFZp686D0	0	0	0	0	0	0
DKFZp686K1	0	0.11	0	0	0	0
DKFZp686O1	0.05	0.01	0.03	0	0.03	0.02
DKFZp779M	0	0	0	0	0	0
DKK1	0	0	0	0	0	0
DKK2	0	0	0.05	0	0	0

DKK3	0.2	0	0	0.02	0	0.03
DKK4	0	0	0	0	0	0
DKKL1	0	0	0	0	0	0
DLAT	19.48	22.92	16.82	16.8	19.74	18.74
DLC1	19.96	6.53	5.5	5.26	14.52	3.08
DLD	42.03	39.53	33.76	27	29.85	35.33
DLEC1	0	0.02	0	0.04	0	0
DLEU1	25.77	28.58	29.36	23.68	26.38	20.52
DLEU2	3.12	3.57	2.55	2.1	2.1	3.27
DLEU2L	2.49	4.72	2.6	2.91	2.91	2.63
DLEU7	0	0	0	0	0.06	0.26
DLEU7-AS1	0.16	0.06	0.04	0.08	0.27	0.08
DLG1	2.84	2.69	3.44	2.1	3.35	2.65
DLG1-AS1	0.04	0.07	0.32	0	0.13	0
DLG2	0	0	0	0	0.01	0.02
DLG3	1.88	1.68	2.03	1.44	2.04	2.23
DLG4	0.25	0.7	0.49	0.58	0.22	0.56
DLG5	1.83	1.34	1.03	1.27	1.74	1.32
DLG5-AS1	0.1	0.09	0.09	0.09	0.11	0.12
DLGAP1	0	0	0.04	0	0	0
DLGAP1-AS1	2.17	0.43	0.28	0.96	0.96	0.47
DLGAP1-AS3	0	0	0	0	0	0
DLGAP1-AS5	0	0	0	0	0	0
DLGAP2	0	0	0	0	0	0
DLGAP3	0.04	0.05	0.63	0.18	0.15	0.23
DLGAP4	6.91	4.86	3.86	4.85	5.96	3.49
DLGAP5	20.35	21.64	18.12	15.17	19.02	17.44
DLK1	0	0.44	1.2	0.97	1.07	0.66
DLK2	0	0.37	0.53	0.82	0.05	0.05
DLL1	0.11	0.21	0.95	0.53	0.54	0.58
DLL3	0.2	0.51	1.49	2.06	3.21	1.52
DLL4	0.06	0.03	0.21	0.01	0.02	0
DLST	21.75	18.05	13.35	13.06	14.44	14.91
DLX1	4.86	7.79	8.35	7.21	7.39	10.54
DLX2	2.55	4.22	3.88	1.71	2.86	3.23
DLX3	1.55	5.03	6.93	5.07	3.5	8.11
DLX4	4.31	7.96	8.09	7.73	7.15	8.6
DLX5	0	0	0	0	0	0
DLX6	0.08	0.19	0.43	0.11	0.3	1.49
DLX6-AS1	0.44	0.4	0.86	0.23	0.4	0.53
DMAP1	24.89	23.6	20.69	21.35	21.77	19.11
DMBT1	0	0	0	0	0	0
DMBX1	0.51	0.6	0.79	0.49	0.54	0.26

DMC1	1.13	1.76	1.73	0.94	1.35	1.48
DMD	0.29	4.38	4.1	0.4	2.23	2.04
DMGDH	0.17	0	0	0	0	0
DMKN	0	0	0	0	0	0
DMP1	0	0	0	0	0	0
DMPK	0.84	0.14	0.09	0.47	0.38	0.08
DMRT1	0	0	0	0	0	0
DMRT2	0	0	0	0	0	0
DMRT3	0	0	0	0	0	0
DMRTA1	1.05	1.21	1.87	1.12	0.99	0.97
DMRTA2	0	0	0	0	0	0
DMRTB1	0	0	0	0	0	0
DMRTC1	0	0	0	0	0	0
DMRTC1B	0	0	0	0	0	0
DMRTC2	0	0	0	0	0	0
DMTF1	5.3	3.68	3.02	4.71	3.14	3.02
DMWD	0.99	0.07	0.24	0.38	0.17	0.24
DMXL1	2.21	1.11	0.89	1.43	1.06	0.86
DMXL2	1.85	3.84	1.58	1.5	1.28	3.67
DNA2	1.93	2.79	2.07	2.26	2.46	2.85
DNAAF1	0	0	0	0	0	0
DNAAF2	5.81	7.23	6.77	5.45	5.25	5.21
DNAAF3	0.9	0.36	1.06	1.11	3.17	2.87
DNAH1	0.11	0.22	0.15	0.1	0.02	0.07
DNAH10	0.01	0	0	0	0	0.01
DNAH11	0	0.03	0.01	0	0.01	0.03
DNAH12	0	0.03	0	0	0.03	0
DNAH14	3.3	3.64	4.59	3.94	3.96	3.51
DNAH17	0	0	0.01	0	0	0.02
DNAH2	0	0	0	0	0	0
DNAH3	0.02	0.03	0.01	0.03	0.03	0.05
DNAH5	0	0.03	0.01	0	0	0
DNAH6	0	0	0	0	0	0
DNAH7	0.02	0	0	0	0	0.01
DNAH8	0	0	0	0	0	0.01
DNAH9	0.23	0	0	0	0	0
DNAI1	0	0	0	0	0	0
DNAI2	0	0	0	0	0	0
DNAJA1	227.84	224.49	204.83	181.67	211.06	180.06
DNAJA1P5	0	0	0	0	0	0
DNAJA2	40.12	36.2	38.48	32.33	38.38	36.08
DNAJA3	41.53	47.48	44.62	45.99	41.93	50.47
DNAJA4	0.03	0	0	0.09	0	0.26

DNAJB1	65.8	63.87	61.8	64.72	61.73	51.9
DNAJB11	60.56	67.02	59.45	48.57	63.17	45.64
DNAJB12	19.32	19.61	17.59	15.91	15.46	16.33
DNAJB13	0.13	0.09	0.04	0.08	0.11	0.08
DNAJB14	7	6.26	4.23	2.8	3.89	5.67
DNAJB2	0.6	0.3	0.24	0.38	0.04	0.05
DNAJB3	0	0.09	0	0	0	0
DNAJB4	3.55	2.18	1.57	1.22	2.61	2.5
DNAJB5	1.71	5.55	2.36	3.23	3.74	4.13
DNAJB6	59.24	41.76	38.5	42.3	46	38.49
DNAJB7	0.43	0.29	0.38	0.14	0.25	0.71
DNAJB8	0	0	0	0	0	0
DNAJB8-AS1	0	0	0	0	0	0
DNAJB9	6.93	3.68	3.62	3.59	3.62	4.88
DNAJC1	11.89	9.42	8.3	7.09	9.12	5.6
DNAJC10	7.66	6.81	6.08	5.92	4.97	5.66
DNAJC11	36.05	32.1	37.56	33.69	35.46	37.98
DNAJC12	3.01	0	0	0.49	0	0
DNAJC13	1.83	1.8	2.32	1.05	0.92	2.26
DNAJC14	12.91	13.85	12.69	10	11.15	14.15
DNAJC15	0	0	0	0	0	0
DNAJC16	5.94	2.76	2.68	2.83	3.47	3.32
DNAJC17	12.63	15.48	15.41	14.08	16.76	13.74
DNAJC18	2.03	1.52	1.61	1.85	1.71	1.53
DNAJC19	50.64	50.21	44.15	43.78	51.6	45.91
DNAJC2	26.25	24.62	22.01	19.93	20.71	20.93
DNAJC21	2.2	2.69	2.49	1.72	2.21	2.4
DNAJC22	4.05	5.47	1.9	3.5	3.78	3.45
DNAJC24	3.8	2.46	1.78	1.4	1.74	2.06
DNAJC25	1.29	1.15	0.91	0.67	1.02	0.63
DNAJC25-GN	0	0	0	0	0	0.08
DNAJC27	0.35	0.33	0.15	0.24	0.41	0.39
DNAJC27-AS	0.48	0.53	0.61	0.43	0.46	0.69
DNAJC28	0.51	0.19	0	0.15	0.22	0.16
DNAJC3	11.75	13.1	11.52	9.94	11.76	12.21
DNAJC30	5.69	4.41	5.35	5.86	4.85	4.4
DNAJC4	13.24	12.95	13.63	16.27	16.97	17.6
DNAJC5	4.55	4.3	4.24	4.53	3.46	3.6
DNAJC5B	0	0	0	0	0	0
DNAJC5G	0	0.03	0.07	0	0	0.04
DNAJC6	8.25	0.15	0.34	2.37	4.13	0.43
DNAJC7	65.44	62.14	47.8	58.71	62.42	64.02
DNAJC8	113.78	127.45	96.83	98.92	110.16	90.33

DNAJC9	114.84	111.61	104.76	104.54	110.15	81.24
DNAJC9-AS1	0.48	0.32	0.8	0.21	0.2	0.1
DNAL1	1.53	1.71	1.46	1.14	1.54	1.36
DNAL4	7.06	3.26	4.66	5.04	5.55	4.99
DNALI1	0	0.07	0	0	0.1	0
DNASE1	1.51	1.8	1.6	1.02	1.36	1.12
DNASE1L1	2.56	2.48	3.8	3.81	2.43	2.57
DNASE1L2	0.11	0.04	0.21	0	0.11	0.4
DNASE1L3	0	0	0	0	0	0
DNASE2	68.37	42.52	31.6	38.59	44.96	31.52
DNASE2B	0	0	0	0	0	0
DND1	3.38	3.32	2.23	3.02	2.11	3.72
DNER	0	0	0	0	0	0
DNHD1	0.28	1.04	0.84	0.49	0.44	0.54
DNLZ	3.82	4.36	5.15	3.31	4.54	2.57
DNM1	0.63	2.16	1.8	1.96	1.54	2.66
DNM1L	13.82	17.85	13.49	12.48	13.53	16.24
DNM1P35	0.16	0.31	0.55	0.14	0.46	0.29
DNM1P41	3.61	2.99	4.39	3.52	2.92	3.17
DNM1P46	0.16	0.19	0.25	0.15	0.02	0.22
DNM2	9.91	6.07	6.43	6.63	6.6	4.94
DNM3	0.92	0.19	0.09	0.21	0.19	0.23
DNM3OS	0	0.02	0	0	0.03	0.01
DNMBP	1.11	1.31	0.78	1.44	1.05	1.29
DNMBP-AS1	0.55	0.77	0.77	0.63	0.57	0.42
DNMT1	24.54	30.32	21.34	22.69	26.58	27.81
DNMT3A	1.81	1.55	1.53	1.44	1.03	1.95
DNMT3B	4.45	6	6.09	6.64	5.49	5.15
DNMT3L	0	1.1	0.92	0.03	0.04	1.1
DNPEP	15.45	13.48	15.25	17.62	15.85	14.54
DNPH1	176.64	199.54	191.16	211.42	227.8	188.35
DNTT	0	0	0	0	0	0
DNTTIP1	7.5	8.45	12.65	6.63	5.6	7.82
DNTTIP2	41.33	33.67	34.89	34.62	37.19	38.18
DOC2A	0.32	1	0.49	0.95	0.42	1.06
DOC2B	0	0	0	0	0	0
DOC2GP	0	0	0	0	0	0
DOCK1	2.28	3.12	2.43	3.55	3.43	3.49
DOCK10	0.7	0.01	0.11	0.42	0.32	0.21
DOCK11	3.69	2.85	3.52	2.52	2.65	4.07
DOCK2	10.38	17.51	12.68	13.32	11.85	20.25
DOCK3	0.11	0.3	0.48	0.2	0.16	0.44
DOCK4	0.13	0.07	0.09	0.06	0.05	0.1

DOCK5	4.43	5.44	4.84	3.29	4.5	6.25
DOCK6	1.37	0.32	0.19	0.72	0.77	0.34
DOCK7	1.92	1.62	1.88	2.75	2.14	1.37
DOCK8	14.98	7.3	7.82	7.96	10.93	8
DOCK9	0.38	0	0	0.16	0.12	0
DOHH	17.31	16.69	21.46	21.8	25.67	9.61
DOK1	29.7	37.77	48.9	34.76	33.85	43.07
DOK2	7.05	6.74	11.58	8.55	6.54	8.87
DOK3	4.28	3.54	4.87	3.85	3.37	1.05
DOK4	0.93	1.53	1.24	1.61	1.6	1.4
DOK5	0	0	0	0	0	0
DOK6	0	0	0	0.01	0	0
DOK7	0	0	0.03	0	0	0
DOLK	15.2	10.94	15.65	14.58	11	11.22
DOLPP1	21.71	16.12	19.67	16.55	16.71	14.14
DOM3Z	9.83	10.78	10.42	12.16	12.28	12.69
DONSON	1.05	1.14	1.92	1.04	1.94	1.85
DOPEY1	0.69	0.8	0.91	0.61	0.83	1.4
DOPEY2	1.7	3.4	3.58	2.15	2.08	3.17
DOT1L	4.27	4.48	3.58	3.6	3.56	5.18
DPAGT1	34.44	34.98	37.68	32.46	34.7	34.66
DPCD	49.32	26.82	28.45	35.23	30.08	24.93
DPCR1	0	0	0	0	0	0
DPEP1	0.27	0.04	0.08	0.12	0	0.59
DPEP2	0.08	0.84	0.58	0.96	0.12	0.94
DPEP3	0	0	0	0	0	0
DPF1	1.07	1.62	1.09	1.73	1.14	1.21
DPF2	46.74	53.45	49.78	49.55	42.36	48.9
DPF3	2.72	0.28	0.33	0.31	1.22	0.03
DPH1	20.01	23.57	22.97	33.48	29.57	22.37
DPH2	28.33	34.16	24.62	25.25	28.04	27.56
DPH3	6.8	5.37	3.77	4.53	4.67	4.85
DPH3P1	1.36	1.22	1.52	0.81	0.81	1.37
DPH5	22.23	23.79	24.22	25.58	23.07	23.41
DPM1	46.52	45.02	38.07	28.84	36.7	39.08
DPM2	49.29	46.28	41.7	42.29	47.18	36.19
DPM3	58.58	73.59	65.45	59.21	80.48	59.53
DPP10	0	0	0	0	0	0
DPP3	25.37	28.57	33.27	30.29	27.36	28.67
DPP4	0.46	3.58	0.14	2.09	3.03	0
DPP6	0	0	0	0	0	0
DPP7	61.15	80.38	95.1	103.93	84.23	82.16
DPP8	11.78	11.81	11.01	9.96	11.59	12.5

DPP9	23.33	20.58	16.77	13.63	17.22	16.87
DPPA2	0	0	0	0	0	0
DPPA2P3	0	0	0	0	0	0
DPPA3	0	0	0	0.15	0	0
DPPA4	0.13	0.11	0.12	0.11	0.21	0.12
DPPA5	0	0	0	0	0	0
DPRX	0	0	0	0	0	0
DPRXP4	0.24	0.19	0.11	0	0.11	0
DPT	0	0	0	0	0	0
DPY19L1	1.88	1.14	0.83	0.67	1.47	1.07
DPY19L1P1	0.51	0.53	0.6	0.47	0.45	0.98
DPY19L2	1.07	1.95	1.21	1.17	1.04	1.62
DPY19L2P1	2.88	0.03	0.06	0.67	1.22	0
DPY19L2P2	1.89	2.1	2.33	1.73	2.18	2.17
DPY19L2P3	0	0	0	0.14	0	0
DPY19L2P4	0.09	0	0	0.03	0.03	0
DPY19L3	1.66	1.84	2.9	1.88	2.24	2.15
DPY19L4	4.13	3.96	4.49	3.49	4.08	5.46
DPY30	80.98	60.38	55.11	59.38	76.02	59.3
DPYD	0	0.14	0	0.2	0.12	0
DPYD-AS1	0	0	0	0	0	0
DPYS	0	0	0	0	0	0
DPYSL2	21.86	38.14	34.92	33.75	31.32	41.66
DPYSL3	0.03	0	0	0	0	0
DPYSL4	1.18	0.38	0.76	1.37	1.55	0.65
DPYSL5	0.15	0.13	0.21	0.16	0.17	0.07
DQX1	0.08	0.04	0	0	0.1	0
DR1	25.95	21.62	19.53	14.47	20	17.79
DRAM1	1.86	6.82	5.03	5.37	3.48	6.73
DRAM2	19.28	18.94	17.15	17.01	20.33	19.23
DRAP1	28.18	31.93	27.67	39.15	35	28
DRAXIN	0	0	0	0	0	0
DRD1	0	0	0	0	0	0
DRD2	0	0	0	0	0	0
DRD3	0	0	0	0	0	0
DRD4	0	0	0	0	0	0
DRD5	0	0	0	0	0	0
DRG1	135.37	116.21	116.65	112.68	116.98	105.9
DRG2	35.1	31.13	36.81	39.52	32.77	30.05
DRGX	0	0	0	0	0	0
DROSHA	7.66	6.59	6.73	7.06	6.88	6.9
DRP2	0.09	0.03	0.06	0	0	0.05
DSC1	0	0.03	0	0	0	0.01

DSC2	7.41	8.11	7.31	6.93	6.05	8.57
DSC3	0.15	0.12	0.1	0.11	0.07	0.2
DSCAM	0	0	0	0	0	0
DSCAM-AS1	0.07	0.06	0.26	0.1	0	0.07
DSCAM-IT1	0	0	0	0	0	0
DSCAML1	0	0	0	0	0	0
DSCC1	19.22	21.52	21.57	17.37	22.76	21.87
DSCR10	0	0	0	0	0	0
DSCR3	17.01	15.51	15.72	11.94	13.04	17.03
DSCR4	0	0	0	0	0	0
DSCR6	0.42	0.5	0.9	0.34	0.56	0.46
DSCR8	0	0.1	0	0	0	0
DSCR9	0.09	0.14	0.04	0.12	0.21	0.14
DSE	0.49	0.73	0.25	0.1	0.6	0.59
DSEL	0.31	0.35	0.26	0.15	0.2	0.32
DSG1	0	0	0	0	0	0
DSG2	6.5	8.8	6.16	5.03	6.61	8.45
DSG3	0.13	0.05	0.15	0.14	0.05	0.25
DSG4	0	0	0	0	0	0
DSN1	16.72	18.78	13.48	16.44	16.89	15.46
DSP	0.03	0	0	0	0.01	0.01
DSPP	0	0	0	0	0	0
DST	0.63	1.53	1.27	0.82	0.82	1.27
DSTN	134.31	58.4	61.06	62.01	78.68	63.65
DSTNP2	2.02	4.41	2.72	2.09	2.9	1.5
DSTYK	5.26	1.99	2.13	2.94	3.83	3.21
DTD1	72.6	79.72	83.04	82.21	89.49	84.92
DTD2	7.16	8.39	6.45	3.89	6.32	7.36
DTHD1	0	0.05	0	0	0	0
DTL	21.77	29.48	31.9	26.89	23.14	32.39
DTNA	7.29	0	0	0.32	1.16	0
DTNB	5.9	5.21	4.79	5.16	3.4	5.77
DTNBP1	8.73	8.67	12.1	9.03	9.87	8.34
DTWD1	5.02	6.17	4.57	4.54	5.31	4.41
DTWD2	0.81	0.81	0.76	0.44	1.01	0.8
DTX1	0.02	0.02	0	0.03	0.1	0
DTX2	8.56	9.33	11.57	13.61	9.88	11.21
DTX2P1-UPK	0.35	0.93	0.81	0.64	0.34	1.08
DTX3	0	0	0	0	0	0
DTX3L	10.08	10.41	11.87	9.86	9.15	9.02
DTX4	0.53	0.88	1.43	1.19	0.55	0.83
DTYMK	109.42	120.94	103.62	106.37	122.8	103.91
DUOX1	0.03	0	0.04	0.02	0	0.03

DUOX2	0.26	0	0	0	0.03	0.19
DUOXA1	0.11	0	0	0.04	0	0
DUOXA2	0.62	0	0	0	0.3	0
DUPD1	0	0	0	0	0	0
DUS1L	5.27	6.2	6.19	5.87	5.05	6.16
DUS2L	15.71	27.95	26.47	24.56	23.54	26.31
DUS3L	23.82	43.22	38.24	35.46	31.32	31.2
DUS4L	5.28	2.37	2.99	3.09	3.71	3.23
DUSP1	12.59	12.36	9.22	13.16	12.99	2.47
DUSP10	3.23	5.47	5.3	2.94	2.55	2.96
DUSP11	20.7	19.5	16.44	13.88	18.05	20.65
DUSP12	25.1	27.71	18.14	22	17.78	19.55
DUSP13	0.17	0	0	0	0	0
DUSP14	25.97	25.52	18.63	19.64	19.99	18.78
DUSP15	0	0.09	0	0.66	0.21	0.19
DUSP16	0.96	0.9	1.03	0.77	0.49	1.13
DUSP18	2.75	4.19	2.16	1.62	2.86	3.1
DUSP19	1.35	0.75	0.99	0.61	0.9	0.72
DUSP2	13.34	12.74	11.77	21.39	13.23	2.95
DUSP21	0	0	0	0	0	0
DUSP22	3.49	3.27	4.4	5.58	4.45	4.55
DUSP23	6.9	11.08	4.92	7.86	11.33	0.35
DUSP26	0	0	0	0	0	0
DUSP27	0	0.42	0	0	0	0.33
DUSP28	3.08	3.33	3.48	2.48	3.09	2.53
DUSP3	11.06	16.53	12.64	10.46	11.52	11.93
DUSP4	2.26	1.46	1.24	0.92	1.52	0.87
DUSP5	0.49	0.71	0.45	0.46	0.79	0.59
DUSP5P1	0.03	0.64	0.41	0	0.22	0.37
DUSP6	20.12	10.11	5.56	5.36	5.88	7.89
DUSP7	5.87	8.09	7.95	6.79	4.82	7.47
DUSP8	0	0	0.01	0	0	0
DUSP9	3.12	7.46	7.05	4.97	6.23	7.74
DUT	99.11	141.02	130.39	134.86	133.82	145.27
DUX2	0	0	0	0	0	0.05
DUX4	0	0	0	0	0	0
DUX4L2	0	0	0	0	0	0
DUX4L3	0	0	0	0	0	0
DUX4L4	0	0	0	0	0	0
DUX4L5	0	0	0	0	0	0
DUX4L6	0	0	0	0	0	0
DUX4L7	0	0	0	0	0	0
DUXA	1.06	1.49	1.14	0.76	0.74	1.67

DVL1	1.15	1.1	1.44	1.66	0.99	0.86
DVL2	6.21	7.52	9.4	10.65	11.35	7.84
DVL3	3.63	3.56	3.13	3.14	3.17	4.41
DYDC1	0.31	0.46	0.94	0.52	0.23	0.4
DYDC2	0.09	0.14	0	0.03	0.16	0.09
DYM	19.77	20.44	22.66	23.27	15.34	20.03
DYNAP	0.36	0.4	0.07	0.13	0.07	0.41
DYNC1H1	11.24	9.11	11.51	9.51	9.63	11.72
DYNC1I1	0.5	0.97	0.91	0.52	0.23	1.33
DYNC1I2	14.96	13.74	11.52	12.29	12.34	11.43
DYNC1LI1	29.86	24.66	20.09	18.54	25.38	18.42
DYNC1LI2	5.27	4.55	2.5	3.91	3.49	2.89
DYNC2H1	0.42	0.26	0.26	0.28	0.28	0.49
DYNC2LI1	8.22	3.87	3.63	2.55	4.2	5.1
DYNLL1	314.2	287.98	272.34	273.11	341.9	260.06
DYNLL2	2.51	0.74	0.95	1.15	1.21	0.99
DYNLRB1	236.45	164.76	165.46	191.96	217.49	186.25
DYNLRB2	0	0	0	0	0	0
DYNLT1	86.72	49.12	54.15	58.19	56.5	42.22
DYNLT3	13.4	6.41	3.16	3.77	6.52	3.96
DYRK1A	4.41	3.91	2.71	3.05	4.1	3.48
DYRK1B	0.21	0.27	0.14	0.52	0.06	0.12
DYRK2	2.3	2.95	1.99	2.21	2.94	2.73
DYRK3	6.57	2.36	2.68	2.78	1.89	1.63
DYRK4	11.05	7.85	8.24	8.27	10.17	7.88
DYSF	0.01	0	0	0	0	0.05
DYTN	0	0	0	0	0	0
DYX1C1	0.7	1.27	0.63	0.75	1.04	1.22
DYX1C1-CCP(	0	0	0	0	0	0
DZANK1	0.57	1.41	0.16	0.21	0.31	0.22
DZIP1	1.77	1.41	1.45	1.16	1.69	1.27
DZIP1L	1.02	1.7	0.93	1	0.9	1.71
DZIP3	0.95	0.7	0.91	0.98	0.87	1.27
E2F1	2.11	2.26	1.56	3.02	3.86	2.8
E2F2	1.65	3.55	3.41	3.43	3.21	3.58
E2F3	6.21	4.84	5.75	6.92	5.51	6.67
E2F4	15	21.58	17.23	13.8	15.45	17.42
E2F5	1.84	1.35	0.56	0.26	0.86	1.85
E2F6	5.39	4.5	3.58	3.6	4.11	1.95
E2F7	1.52	1.74	1.42	1.15	1.39	1.23
E2F8	4.17	6.46	6.15	4.22	4.69	5
E4F1	11.81	13	11.96	9.12	10.28	9.56
EAF1	10.72	5.54	3.82	5.87	4.81	4.81

EAF2	8.06	26.16	23.79	11.61	13.21	14.84
EAPP	20.45	23.33	22.26	21.59	22.84	19.5
EARS2	9.23	12.47	12.37	15.03	14.68	14.23
EBAG9	29.43	25.23	21.11	18.07	21.58	23.7
EBF1	0	0.05	0.03	0	0.01	0
EBF2	0	0	0	0	0	0
EBF3	0	0.03	1.06	0.71	0.19	0.8
EBF4	0	0.1	0	0.05	0.05	0.13
EBI3	0	0	0	0	0	0.41
EBLN1	0	0	0	0	0	0
EBLN2	0.31	0.18	0.62	0.74	0.08	0.54
EBNA1BP2	167.51	202.42	180.2	171.26	182.88	173.69
EBP	291.63	409.72	439.42	391.91	385.6	375.65
EBPL	22.09	32.42	37.45	35.92	33.49	28.58
ECD	28.62	21.77	20.85	19.86	22.8	17.85
ECE1	6.3	4.42	3.88	3.79	4.3	3.7
ECE2	41.97	70.39	58.25	56.36	68.35	63.62
ECEL1	0	0	0	0	0	0
ECEL1P2	0.12	0	0	0	0	0
ECH1	211.69	200.93	154.83	222.55	193.18	198.77
ECHDC1	0	0	0	0.07	0	0
ECHDC2	0	0	0	0	0	0
ECHDC3	3.56	2.73	4.16	4.13	3.66	4.05
ECHS1	199.22	125.51	117.48	125.71	152.12	130.22
ECI1	15.4	20.36	15.69	21.72	23.22	5.97
ECI2	68.68	56.46	56.54	54.88	57.48	50.94
ECM1	0.45	0	0.42	0.21	0.08	0.34
ECM2	0	0.08	0.05	0	0.08	0.08
ECRP	1.35	6.48	0.51	0	0.28	12.09
ECSCR	0	0	0	0	0	0
ECSIT	40.98	47.86	44.73	44.56	46.72	52.24
ECT2	10.01	7.34	5.58	7.22	7.09	9.01
ECT2L	0.09	0	0.15	0.07	0.06	0.08
EDA	0.01	0.38	0.65	0.02	0.03	0.28
EDA2R	0	0	0	0	0	0
EDAR	0	0	0	0.01	0	0
EDARADD	2.05	2.33	1.47	2.72	2.31	1.91
EDC3	55.68	45.5	39.61	48.15	37.94	43.67
EDC4	16.34	18.44	19.71	20.32	14.96	17.95
EDDM3A	0	0	0	0	0	0
EDDM3B	0	0	0	0	0	0
EDEM1	3.37	2.66	2.35	1.46	2.22	1.45
EDEM2	21.98	22.39	17.89	15.27	18.93	19.03

EDEM3	2.13	4.31	3.61	2.58	2.94	4.03
EDF1	352.14	324.54	314.89	378.5	388.5	348.74
EDIL3	0	0	0	0	0	0
EDN1	0	0	0	0	0	0
EDN2	0	0	0	0	0	0
EDN3	0	0	0	0	0	0
EDNRA	0	0	0	0	0	0
EDNRB	0.05	0.08	0.03	0.06	0.06	0.07
EEA1	3.17	3.38	3.29	2.6	3.46	2.4
EED	8.09	10.28	10.2	7.49	9.92	11.95
EEF1A1	4168.44	3721.28	4218.52	4898.79	4407.39	4701.11
EEF1A2	9.46	0.14	0.4	2.05	5.42	1.71
EEF1B2	980.96	1275.06	1328.06	1459.43	1259.04	1446.74
EEF1D	552.1	571.54	668.06	745.88	662.5	655.39
EEF1DP3	0	0.13	0.26	0	0.81	0.13
EEF1E1	72.22	73.41	62.84	54.21	71.09	61.8
EEF1E1-MUT	1.28	2.44	3.16	0.48	0	1.33
EEF1G	1361.09	1422.41	1718.72	2004.65	1726.76	1899.68
EEF2	773.71	660.21	864.23	984.49	774.27	1049.77
EEF2K	9.17	8.84	9.9	9.36	8.57	11.35
EEFSEC	12.29	16.14	15.78	20.89	17.43	17.14
EEPD1	1.9	2.79	3.36	2.94	1.77	3.04
EFCAB1	0.16	0.05	0	0.02	0	0
EFCAB10	0	0	0	0	0	0
EFCAB11	7.93	3.73	2.1	3.56	4.7	4.06
EFCAB12	0.21	0.19	0	0	0	0
EFCAB13	0.06	0.05	0.02	0	0	0.1
EFCAB14	9.16	10.16	8.39	7.56	6.48	8.07
EFCAB14-AS	0	0	0	0	0	0.17
EFCAB2	2.74	1.88	2.36	1.03	1.76	1.47
EFCAB3	0.05	0.19	0.04	0.13	0.23	0.24
EFCAB4A	0	0	0.37	0	0	0.25
EFCAB4B	4.79	7.71	5.03	5.18	6.29	7.25
EFCAB5	0.43	0.09	0.09	0.07	0.14	0.04
EFCAB6	0	0	0	0	0	0
EFCAB6-AS1	0	0	0	0	0	0
EFCAB7	0.93	0.29	0.77	0.41	1.06	1.45
EFCAB9	0	0	0.1	0	0	0
EFCC1	0	0	0	0	0	0
EFEMP1	0	0	0	0	0	0
EFEMP2	0	0.37	0.03	0.17	0	0
EFHA1	22.43	17	13.38	13.4	17.41	15.61
EFHA2	0.07	0.06	0	0.19	0	0.13

EFHB	0.15	0	0.05	0	0	0
EFHC1	0.42	0.43	0.29	0.42	0.29	0.22
EFHC2	1.45	0.51	0.49	1.38	1.48	1
EFHD1	0	0.12	0.48	0.19	0.04	0.19
EFHD2	6.79	5.37	5.86	5.13	5.27	5.06
EFNA1	2	2.97	3.28	4.45	6.54	3.85
EFNA2	0.03	0.05	0.09	0.28	0.39	0.07
EFNA3	1.38	2.79	3.74	2.67	3.03	2.49
EFNA4	6.45	8.82	13.99	15.09	11.14	11.59
EFNA5	0	0	0	0	0	0
EFNB1	2.36	1.34	2.36	1.17	1.38	1.77
EFNB2	0.28	0.4	0.2	0.29	0.33	0.05
EFNB3	0	0.43	0.54	0.79	1.07	0.78
EFR3A	4.99	4	4.43	3.33	3.52	3.58
EFR3B	0.03	0.04	0.19	0.15	0.06	0.04
EFS	0	0	0	0	0	0
EFTUD1	15.05	17.49	17.77	12.99	14.2	15.96
EFTUD1P1	0	0	0	0	0	0
EFTUD2	57.98	69.54	70.15	73.41	67.89	74.03
EGF	0.07	0.03	0.33	0.02	0.03	0
EGFEM1P	0.13	0.21	0.16	0.14	0.22	0.08
EGFL6	0	0	0	0	0	0
EGFL7	167.74	238.31	354.3	349.36	197.17	278.93
EGFL8	0.29	0.13	0	0.1	0	0.13
EGFLAM	0	0	0	0	0	0
EGFLAM-AS4	0.04	0.03	0.05	0.03	0.14	0
EGFR	0	0	0	0	0	0
EGFR-AS1	0	0	0	0	0	0
EGLN1	0.91	1.23	0.76	0.53	0.71	1.13
EGLN2	53.32	64.35	56.79	63.7	58.38	63.67
EGLN3	4.62	0.04	0.22	0.59	0.73	0.24
EGOT	0.15	0	0.09	0.04	0	0.16
EGR1	38.92	1.48	1.47	16.26	26.78	1.25
EGR2	0.52	0.23	0	0.27	0.14	0
EGR3	0.85	0	0.16	0.14	0.02	0.02
EGR4	0.19	0	0.03	0	0.55	0
EHBP1	3.9	4.2	3.31	2.27	4.3	3
EHBP1L1	1.13	0.5	1.22	1.27	1.08	0.56
EHD1	19.94	14.48	17.75	15.6	16.7	18.14
EHD2	37.52	16.38	25.15	34.4	29.4	29.1
EHD3	0	0.03	0	0	0	0
EHD4	8.83	6.81	4.71	4.98	6.35	4.85
EHF	0	0	0	0	0	0

EHHADH	0.05	0.07	0.1	0.03	0.06	0.09
EHHADH-AS1	0	0	0.05	0	0.15	0.05
EHMT1	10.28	11.52	11.52	9.79	10.27	11.47
EHMT2	6.15	9.19	9.91	9.73	7.91	8.29
EI24	23.66	17.14	21.67	24.69	27.11	17.14
EID1	65.83	40.26	33.03	42.12	47.29	35.04
EID2	1.95	1.19	1.83	1.01	1.84	1.53
EID2B	1.68	1.37	1.16	1.04	1.26	0.56
EID3	0.31	0.24	0.28	0.21	0.14	0.21
EIF1	561.26	348.26	357.92	383.28	456.76	357.45
EIF1AD	19.42	16.58	24.25	20.89	18.91	21.61
EIF1AX	48.72	47.9	44.08	33.95	47.44	44.32
EIF1AY	0	0	0	0	0	0
EIF1B	62.17	52.16	43.33	43.52	49.2	43.05
EIF1B-AS1	0	0	0.13	0.12	0.03	0
EIF2A	27.12	22.98	20.58	24.1	24.78	24.18
EIF2AK1	15.08	13.96	10.85	13.47	11.87	12.85
EIF2AK2	10	9.02	9.18	8.5	8.83	8.54
EIF2AK3	0.76	0.53	0.13	0.76	0.32	0.31
EIF2AK4	5.07	6.3	6.28	4.83	5.08	5.77
EIF2B1	52.38	58.5	47.54	49.56	49.78	51.86
EIF2B2	36.79	39.63	33.49	33.91	37.98	30.92
EIF2B3	45.61	36.86	35.17	44.44	44.59	32.22
EIF2B4	30.78	40.46	34.59	37.58	32.09	31.89
EIF2B5	7.18	8.57	7.81	6.89	7.01	7.77
EIF2D	31.98	35.93	38.14	33.37	34.87	38.05
EIF2S1	54.85	45.94	50.55	40.36	49.48	44.65
EIF2S2	62.14	50.34	46.3	47.26	55.17	38.24
EIF2S3	314.14	297.2	296.48	340.27	323.31	324.52
EIF3A	65.37	69.76	55.11	59.63	60.47	67.11
EIF3B	36.98	28.49	37.45	37.58	34.99	38.14
EIF3C	179.41	171.56	184.13	191.84	176.05	190.57
EIF3CL	0	0	0	2.03	0	0
EIF3D	172.72	159.17	159.54	182.74	153.06	157.68
EIF3E	647.81	649.48	669.02	791.95	714.65	743.19
EIF3F	355.54	361.08	417.92	491.29	439.01	413.55
EIF3G	249.83	274.49	295.94	308.29	281.08	278.18
EIF3H	745.25	682.45	686.72	745.94	747.99	734.96
EIF3I	319.37	284.77	281.39	318.46	299.7	240.52
EIF3IP1	0	0	0	0	0	0
EIF3J	20.81	20.23	21.28	19.81	24.85	23.38
EIF3K	358.44	391.13	400.68	412.12	408.74	389.79
EIF3L	311.43	335.37	381.59	420.74	396.41	402.13

EIF3M	230.47	232.56	224.77	235.5	244.02	229.88
EIF4A1	666.32	734.09	622.19	673.95	669.99	712.94
EIF4A2	75.91	44.15	38.62	41.65	49.92	34.96
EIF4A3	122.03	125.87	116.82	120.44	122.69	124.25
EIF4B	118.83	133.07	156.15	158.46	156.76	186.72
EIF4E	62.15	60.78	52.61	48.11	63.11	54.95
EIF4E1B	0	0	0	0	0	0
EIF4E2	79.38	62.78	60.62	59.11	81.25	62.92
EIF4E3	0.12	0.03	0	0.02	0.11	0.01
EIF4EBP1	593.66	718.99	669.74	764.41	776.22	703.5
EIF4EBP2	11.55	13.04	10.49	10.44	11.58	10.98
EIF4EBP3	2.01	0	0	0	0	0
EIF4ENIF1	3.63	3.1	2.2	3.19	3.42	2.14
EIF4G1	62.22	64.01	57.12	62.18	63.78	59.62
EIF4G2	179.28	154.83	146.75	148.07	166.47	158.02
EIF4G3	3.54	3.2	2.88	2.33	2.57	3.23
EIF4H	197.1	220.32	206.77	215.12	215.58	222.19
EIF5	39.46	28.3	23.48	24.37	29.04	24.65
EIF5A	764.81	764.88	807.46	850.76	908.65	702.68
EIF5A2	3.55	2.88	2.5	2	2.96	2.69
EIF5AL1	1.04	1.14	0.94	0.8	0.77	0.73
EIF5B	28.91	30.59	30.37	26.6	32.13	27.82
EIF6	247.5	233.1	214.44	226.07	232.5	216.41
ELAC1	4.38	4.65	3.26	2.63	3.92	4
ELAC2	46.56	51.15	46.95	44.16	41.01	49.26
ELANE	2.6	7.95	0.92	0.12	2.94	4.74
ELAVL1	7.6	10.06	8.96	9.54	9.26	4.46
ELAVL2	0.02	0	0	0	0	0
ELAVL3	0	0	0	0	0	0
ELAVL4	0	0	0	0	0	0
ELF1	17.75	21.15	18.73	15.89	21.94	19.84
ELF2	5.96	7.08	7.67	6.82	7.18	5.92
ELF3	0	0	0	0	0	0
ELF4	7.53	10.91	11.31	7.88	8.27	8.91
ELF5	0	0	0.06	0	0	0
ELFN1	0.14	0.27	0.41	0.25	0.31	0.31
ELFN2	0	0	0	0	0	0
ELK1	69.46	40.37	39.02	49.09	51.31	44.24
ELK2AP	0	0	0	0	0	0
ELK3	3.22	5.96	5.55	4.6	4.79	4.78
ELK4	4.5	1.74	2.03	2.64	2.43	2.88
ELL	7.66	7.46	6.54	6.15	6.14	7.44
ELL2	0.77	0.37	0.51	0.16	0.15	0.11

ELL3	1.61	1	1.41	1.36	1.99	2.01
ELMO1	18.02	13.62	11.98	12.63	12.83	13.16
ELMO2	8.26	8.76	6.49	6.02	4.51	4.17
ELMO3	0.38	0.27	0.11	0	0.19	0.06
ELMOD1	0.67	1.34	1.2	0.98	0.94	0.72
ELMOD2	2.71	2.39	2.4	2.18	1.98	1.97
ELMOD3	2.9	3.22	3.16	2.04	2.05	3.11
ELMSAN1	6.12	5.69	6.83	6.52	6.12	4.86
ELN	0	0	0	0	0	0
ELOF1	83.67	61.01	67.5	73.46	75.12	51.15
ELOVL1	82.35	89.5	92.95	84.58	80.01	87.94
ELOVL2	0.2	2.03	2.28	1.47	1.69	1.75
ELOVL2-AS1	0.05	0.4	0.17	0.13	0.13	0.61
ELOVL3	0.4	0.27	0	0.08	0.11	0.23
ELOVL4	1.88	0.28	0.48	0.31	0.54	0.02
ELOVL5	75.67	55.71	48.58	57.7	70.67	58.08
ELOVL6	34.52	11.73	12.65	14.56	22.8	11.36
ELOVL7	7.03	2.9	2.89	4.27	5.89	3.34
ELP2	27.43	26.36	26.69	26.43	26.72	32.61
ELP3	27.54	26.97	29.66	30.15	27.01	30.99
ELP4	20.74	23.61	17.85	20.8	21.81	23.87
ELP5	53.08	85.34	81.22	72.27	73.88	69.42
ELP6	32.69	27.74	28.47	33.21	30.64	33.15
ELSPBP1	0.29	0	0	0	0.27	0
ELTD1	0	0	0	0	0	0
EMB	8.49	12.08	12.16	7.57	9.04	11.49
EMBP1	0.02	0.14	0.27	0.06	0.07	0.24
EMC1	17.1	19.79	17.85	15.72	16.17	20.98
EMC10	0	0	0	0.18	0	0.19
EMC2	37.58	31.16	28.34	26.14	38.74	20.6
EMC3	91.64	74.38	68.58	69.71	74.73	62.02
EMC4	151.65	149.93	122.99	132.54	159.17	120.13
EMC6	97.35	85.57	70.48	80.16	98.1	72.16
EMC7	150.8	121.6	102.44	106	129.77	106.58
EMC8	20.4	20.05	22.45	14.68	23.22	11.34
EMC9	7.78	12.21	13.04	11.22	12.81	12.97
EMCN	0	0.03	0.03	0.05	0	0.07
EMCN-IT3	0	0	0	0	0	0
EMD	71.62	60.97	66.42	78.12	71.56	61.93
EME1	5.46	4.64	4.63	4.77	5.34	3.79
EME2	0.6	0.42	0.18	0	0.32	0.07
EMG1	73.7	69.1	62.54	68.03	65.6	56.9
EMID1	0.84	0.74	0.48	0.6	0.69	0.18

EMILIN1	2.98	3.68	7.49	4.11	3.39	4.79
EMILIN2	0.18	0.03	0.15	0	0.07	0.04
EMILIN3	0	0	0	0	0	0
EML1	0.02	0	0	0	0	0
EML2	0.98	1.05	0.61	0.42	0.96	0.87
EML3	4.2	7.04	7.04	7.37	6.09	6.63
EML4	4.17	5.21	4.92	4.68	3.8	4.84
EML5	0.06	0.1	0.08	0.07	0.06	0.05
EML6	0.04	0.01	0	0	0	0.01
EMP1	0.6	0.43	0.86	0.2	0.07	0.63
EMP2	7.63	6.62	8.54	8.65	9.01	7.7
EMP3	168.05	86.18	105.73	146.57	176.55	80.41
EMR1	0	0.04	0	0	0	0
EMR2	2.95	0.18	0.19	0.47	1.41	0.02
EMR3	0	0	0	0	0	0
EMR4P	0	0.09	0.03	0	0	0
EMX1	0	0	0	0	0	0
EMX2	0	0	0.01	0	0	0
EMX2OS	0.34	0.48	0.83	0.37	0.47	0.4
EN1	0	0	0	0	0	0
EN2	0	0	0	0	0	0
ENAH	1.62	1.27	1.48	1.21	1.35	1.62
ENAM	0	0	0	0	0	0
ENC1	0.14	0.17	0.1	0.21	0	0.13
ENDOD1	3.85	3.91	6.73	4.54	4.03	3.1
ENDOG	3.96	3.45	5.83	3.17	3.85	3.06
ENDOU	0	0	0	0.09	0	0
ENDOV	2.65	6.45	3.03	2.84	2.29	1.86
ENG	33.78	72.37	85.46	71.27	54.53	60.83
ENGASE	0.5	0.57	0.52	0.74	0.67	0.62
ENHO	0	0.06	0	0.44	0.13	0
ENKD1	3.31	2.76	2.67	1.78	3.4	2.31
ENKUR	0.27	0.02	0	0	0	0
ENO1	1095.31	1303.36	1322.79	1257.99	1183.79	1321.8
ENO1-AS1	0	0.3	0.53	0.54	0.55	0.19
ENO2	12.32	2.24	3.46	6.62	4.63	4.81
ENO3	3.84	0.84	0.65	1.01	1	1.03
ENO4	0	0	0	0	0.1	0
ENOPH1	23.18	31.61	27.86	27.04	25.07	26.11
ENOSF1	7.24	9.59	9.21	7.48	7.47	7.9
ENOX1	3.02	0.1	0.13	0.33	0.75	0.78
ENOX2	9.08	7.27	6.17	6.33	6.71	5.11
ENPEP	0	0	0	0	0	0

ENPP1	0.74	0.28	0.4	0.27	0.52	0.32
ENPP2	0	0.09	0	0	0	0
ENPP3	9.48	0	0	0.44	0.65	0
ENPP4	3.16	0.94	1.34	1	1.54	1.12
ENPP5	0	0	0	0	0	0
ENPP6	0.17	0.05	0.11	0.03	0.12	0.09
ENPP7	0	0	0	0	0	0
ENSA	46.38	55.8	53.29	49.23	57.49	53.66
ENTHD1	1.16	0	0	0.39	1.02	0
ENTHD2	1.35	0.81	1.44	0.97	0.91	0.81
ENTPD1	2.3	2.78	2.34	2.18	2.5	2.27
ENTPD2	0	0	0	0	0	0
ENTPD3	0.08	0	0	0	0.05	0
ENTPD3-AS1	0.47	0.14	0.35	0.09	0.42	0.51
ENTPD4	6.75	6.02	7.1	4.66	5.32	6.4
ENTPD5	8.43	5.42	4.22	5.9	5.44	4.25
ENTPD6	25.1	23.75	27.82	25.54	24.27	23.21
ENTPD7	2.09	2.19	2.79	1.43	1.85	3.01
ENTPD8	0	0	0	0.05	0.03	0
ENY2	115.54	72.99	56.57	58.73	82.81	67.19
EOGT	2.82	4.3	3.59	3.13	3.37	3.14
EOMES	0.03	0.11	0	0	0.28	0.05
EP300	5.28	6.07	4.16	4.38	4.2	4.55
EP400	2.8	2.93	3.01	2.71	2.63	3.75
EP400NL	0.49	0.27	0.44	0.41	0.51	0.65
EPAS1	6.89	0.1	0.19	0.9	2.2	0.18
EPB41	47.94	29.12	27.08	30.1	32.36	28.39
EPB41L1	0.23	0.27	0.46	0.43	0.42	0.38
EPB41L2	10.73	17.34	13.83	10.53	10.73	15.71
EPB41L3	1.02	3.02	2.31	2.24	1.53	3.85
EPB41L4A	3.27	1.19	0.93	1.27	1.71	0.73
EPB41L4A-AS	31.75	30.95	26.78	39.12	35.94	44.1
EPB41L4B	0.19	0	0.07	0	0.02	0
EPB41L5	2.36	2.22	1.97	1.69	1.94	3.2
EPB42	2.22	0	0	2.19	1.24	0
EPB49	14.54	0.53	0.31	2.18	1.77	0.68
EPC1	5.21	5.24	5.68	5.33	5.35	4.56
EPC2	2.63	1.68	1.48	2.26	1.77	2.38
EPCAM	0.04	0	0	0	0.12	0
EPDR1	9.67	5.7	3.74	4.63	6.96	2.79
EPG5	1.62	1.12	1.23	1.09	0.97	1.36
EPGN	0.42	0.28	0.36	0.48	0.35	0.53
EPHA1	0	0.1	0	0	0.18	0.59

EPHA1-AS1	0	0.11	0.11	0.03	0.05	0.2
EPHA10	0.79	0.74	0.77	0.87	0.84	1.27
EPHA2	0.4	0.04	0.1	0.09	0.09	0
EPHA3	0.02	0	0	0	0.02	0
EPHA4	0.4	0	0	0.03	0	0
EPHA5	0	0	0	0	0	0
EPHA6	0	0	0	0	0	0
EPHA7	0	0	0	0	0	0
EPHA8	0.04	0	0	0	0	0
EPHB1	0.37	0.01	0.11	0	0.1	0
EPHB2	0	0	0	0.03	0.04	0.02
EPHB3	0	0	0.13	0	0	0
EPHB4	1.61	2.05	3.67	1.77	2.35	2.61
EPHB6	1.15	2.89	2.72	1.35	1.28	1
EPHX1	3.59	3.6	2.3	1.73	1.61	4.17
EPHX2	5.53	0	0.06	1.35	1.16	0
EPHX3	0	0.1	0	0	0.2	0.04
EPHX4	0.1	0.86	0.1	0.79	0.54	0.95
EPM2A	0.08	0.5	0.83	0.45	0.49	0.38
EPM2AIP1	3.37	2.52	0.8	1.56	1.39	1.35
EPN1	4.43	5.65	7.27	6.07	5.74	4.63
EPN2	3.27	1.5	0.35	1.65	1.63	1.33
EPN2-AS1	0	0	0	0	0.12	0.06
EPN2-IT1	0.09	0	0	0	0.27	0
EPN3	0	0	0.03	0	0	0
EPO	0	0.22	0	0	0	0.11
EPOR	2.82	1.23	0.96	1.19	1.79	1.16
EPPIN	0.99	1.2	1.49	0.72	0.52	1
EPPIN-WFDC	0	0	0	0	0	0
EPPK1	0	0	0	0	0	0.04
EPRS	40.7	47.69	40.36	38.9	41.34	44.35
EPS15	13.19	11.7	13.65	10.9	13.78	11.33
EPS15L1	9.38	11.26	7.58	9.48	8.68	10.25
EPS8	4.97	3.81	2.83	3.18	3.71	3.79
EPS8L1	0.34	0.47	0.58	0.48	0.42	0.34
EPS8L2	0.28	0.46	0.11	0.08	0.04	0.07
EPS8L3	0	0	0.03	0	0	0
EPSTI1	0	0	0	0	0	0
EPT1	4.07	3.58	3.59	2.8	3.58	4.78
EPX	1.8	0.21	0	0.02	1.09	1.88
EPYC	0	0	0	0.07	0	0
EQTN	0	0	0	0	0	0
ERAL1	72.27	71.57	60.19	65.9	72.3	65.82

ERAP1	11.03	12.81	13.04	12.81	9.13	12.32
ERAP2	0.08	0.61	0.43	0.21	0.24	0.43
ERAS	0.19	0	0	0	0	0
ERBB2	0.21	0.17	0.03	0.04	0.14	0.23
ERBB2IP	3.88	2.28	1.82	1.44	2.5	2.29
ERBB3	0.48	0.47	0.01	0.13	0.01	0.17
ERBB4	0	0	0	0	0	0
ERC1	2.36	3.23	2.6	3.07	2.48	3.19
ERC2	0.16	0.05	0.06	0.1	0.04	0.02
ERC2-IT1	0.03	0.08	0.06	0.04	0	0.13
ERCC1	36.65	39.46	34.63	43.14	37.16	38.69
ERCC2	7.33	11.38	10.02	7.8	6.37	7.53
ERCC3	14.44	12.56	12.48	11.91	15.34	11.57
ERCC4	2.28	2.05	1.64	2.38	1.45	2.11
ERCC5	3.99	5.4	5.03	5.64	4.06	5.2
ERCC6	1.47	0.93	1.1	0.83	0.94	0.86
ERCC6L	8.2	7.73	6.82	6.76	7.28	8.9
ERCC6L2	1.73	2.08	1.63	1.48	1.36	1.82
ERCC8	9.43	10.27	7.8	6.44	7.13	10.89
EREG	0.39	0.36	0.15	0.29	0.2	0.19
ERF	5.54	5.56	5.84	7.01	6.17	6.51
ERG	1.55	9.71	15.35	13.3	7.59	17.39
ERGIC1	12.65	15.41	19.51	16.06	16.26	20.44
ERGIC2	32.68	26.69	26.28	20.66	24.32	31.07
ERGIC3	164.71	158.55	168.62	193.05	188.02	169.89
ERH	134.76	89.68	86.64	87.44	135.9	112.04
ERI1	27.19	18.78	16.69	13.77	17.31	15.56
ERI2	4.31	4.32	1.91	2.79	2.83	3.87
ERI3	36.42	46.17	50.86	52.39	50.72	49.77
ERICH1	18.76	22.21	13.89	13.36	19.79	17.14
ERICH1-AS1	0	0	0	0	0	0
ERLEC1	16.4	14.38	8.31	8.99	10.82	7.44
ERLIN1	11.61	25.69	16.89	14.36	16.08	21.49
ERLIN2	5.36	4.75	4.38	4.32	4.86	4.82
ERMAP	4.11	5.66	2.43	3.52	3.27	2.48
ERMN	0	0.02	0	0	0.02	0
ERMP1	0.47	1.47	0.8	0.88	0.81	1.32
ERN1	0.6	0.48	0.48	0.45	0.56	0.97
ERN2	0.04	0.19	0.17	0.04	0.15	0.21
ERO1L	1.85	3.32	1.61	1.16	1.52	3.4
ERO1LB	0.36	0.83	0.68	0.25	0.48	0.9
ERP27	0.29	0.92	0.53	0.67	0.64	0.94
ERP29	140.83	138.07	124.04	121.22	147.55	124.9

ERP44	22.94	25.61	21.38	17.71	19.75	20.76
ERRFI1	0.16	0.04	0	0.22	0.02	0
ERV3-1	10.83	7.64	5.93	6.81	4.95	6.94
ERVFRD-1	0.04	0.19	0.1	0.08	0.37	0.07
ERVK13-1	1.36	1.29	0.8	0.92	0.77	0.89
ERVMER34-1	0	0	0	0	0	0
ERVV-1	0.59	0.59	0.78	0.57	0.74	1.05
ERVV-2	0	0	0	0	0	0
ESAM	5	0	0.18	4.86	0.11	1.6
ESCO1	4.16	3.16	2.66	2.95	3.29	2.68
ESCO2	11.17	14.59	13.04	10.01	11.38	13.43
ESD	154.94	135.25	125.85	143.84	141.18	128.29
ESF1	10.11	7.85	10.25	8.58	10.15	7.77
ESM1	0	0	0	0	0	0
ESPL1	8.17	12.17	11.15	9.53	8	11.44
ESPN	0.35	0.02	0.21	0.24	0.02	0
ESPNL	0	0	0	0	0	0
ESPNP	0	0	0	0	0	0
ESR1	0	0	0	0	0	0
ESR2	0.77	0.02	0.03	0.1	0.06	0.09
ESRG	1.27	1.16	0.88	0.93	1.6	1.07
ESRP1	2.1	1.61	1.81	2.2	1.83	1.92
ESRP2	0	0.05	0.13	0.12	0	0
ESRRA	4.71	6.84	5.55	3.42	5.44	4.42
ESRRB	0	0	0	0	0.11	0
ESRRG	0	0	0	0	0	0
ESX1	0	0	0	0	0	0
ESYT1	56.72	37.24	40.23	46.66	55.56	43.97
ESYT2	3.74	3.7	3.39	2.96	3.46	4.11
ESYT3	0.7	0.99	1.24	0.98	0.95	2.19
ETAA1	2.9	2.81	3.22	2.56	3.28	2.98
ETF1	44.42	36.23	33.89	36.31	38.88	41.31
ETFA	357.72	341.9	312.77	301.58	324.86	328.36
ETFB	187.37	152.95	137.12	183.9	175.12	137.7
ETFDH	11.11	12.74	10.57	8.95	10.3	10.29
ETHE1	27.03	57.89	52.76	44.68	46.97	53.09
ETNK1	12.17	15.14	11.2	8.74	13.22	12.68
ETNK2	0	0.51	0.36	0	0.08	0
ETS1	0	0.02	0	0	0	0
ETS2	16.32	21.7	23.26	29.74	18.14	25.09
ETV1	0.11	0.01	0.02	0.04	0.01	0.01
ETV2	0.4	0.16	0.55	0.38	0.29	0.41
ETV3	6.72	3.07	3.88	2.58	3.33	4.18

ETV3L	0	0	0	0	0	0
ETV4	16.7	13.75	13.13	14.69	17.02	14.33
ETV5	4.29	3.22	1.47	2.53	3.73	1.05
ETV6	2.83	7.85	8.82	6.86	6.87	9.7
ETV7	0	0	0.22	0	0	0.43
EVA1A	0	0	0	0	0	0
EVA1B	0.36	1.55	1.53	2.26	2.41	2.28
EVA1C	0.04	0.78	0	0.24	0.51	0.62
EVC	0.67	0.84	1.03	0.88	1.15	1.12
EVC2	0	0	0	0	0	0
EVI2A	2.11	5.89	6.27	3.18	3.24	5.42
EVI2B	4.04	11.73	6.14	2.98	2.9	7.87
EVI5	2.37	0.89	0.82	0.84	1.61	0.86
EVI5L	0.27	0.08	0.11	0.31	0.15	0.25
EVL	3.62	3.96	3.64	6.37	2.81	2.81
EVPL	0.17	0	0	0	0	0
EVPLL	0	0	0	0	0	0
EVX1	0	0	0	0	0	0
EVX2	0	0	0	0	0	0
EWSR1	152.29	172.14	158.91	172.12	172.65	165.74
EXD1	0.79	0.89	0.93	0.67	0.79	0.77
EXD2	3.89	3.97	3.64	2.56	2.92	3.27
EXD3	0.18	0.48	0.67	0.61	0.22	0.15
EXO1	13.98	13.52	13.28	12.85	13.86	13.84
EXO5	4.81	8	8.11	6.74	7.41	5.21
EXOC1	8.18	7.61	6.84	6.68	7.45	6.56
EXOC2	10.08	8	6.26	6.62	6.93	7.61
EXOC3	10.95	13.31	14.63	14.84	15.18	12.9
EXOC3L1	0	0	0.05	0	0	0.36
EXOC3L2	0.74	0.67	0.9	0.91	0.58	0.71
EXOC3L4	0	0	0	0	0	0
EXOC4	23.08	19.85	22.26	17.95	19.56	17.23
EXOC5	4.72	3.23	4.07	2.75	4.35	3.09
EXOC6	11.97	8.7	10.41	8.64	9.19	7.24
EXOC6B	4.97	2.8	2.09	3.07	3.25	2.94
EXOC7	14.13	12.49	15.27	11.5	9.79	15.79
EXOC8	1.81	1.44	2.32	1.58	1.6	1.66
EXOG	3.14	4.5	4.6	3.99	4.03	5.24
EXOSC1	37.35	40.06	33.94	39.08	39.87	31.06
EXOSC10	45.45	46.99	41.63	35.74	41.41	42.61
EXOSC2	26.36	34.47	29.58	29.75	28.29	31.97
EXOSC3	40.18	45.01	37.7	33.41	40.93	41.48
EXOSC4	198.28	172.12	156.89	193.17	194.8	153.33

EXOSC5	75.02	108.1	89.29	88.82	85.11	87.68
EXOSC6	5.99	7.02	4.98	6.99	5.43	5.39
EXOSC7	68.28	84.63	57.49	65.97	76.93	67.87
EXOSC8	53.9	54.27	52.01	47.79	53.67	53.28
EXOSC9	36.38	34.55	27.69	33.32	37.99	28.46
EXPH5	2.31	1.3	1.67	1.68	1.53	1.77
EXT1	1.34	0.39	0.69	0.79	1.06	0.28
EXT2	20.36	17.86	16.5	14.65	19.83	17.72
EXTL1	0.02	0.01	0	0.02	0.02	0
EXTL2	6.11	4.98	3.86	3.73	4.01	3.93
EXTL3	10.26	6.48	6.38	8.17	8.31	7.54
EYA1	0	0	0	0	0	0
EYA2	0	0	0	0	0	0
EYA3	12.58	17.58	16.32	14.75	14.37	14.76
EYA4	0	0	0	0	0	0
EYS	0.02	0	0.03	0.01	0	0.02
EZH1	3.23	2.29	1.05	2.22	1.74	2.09
EZH2	20.24	21.93	13.63	19.15	21.88	19.11
EZR	72.78	49.23	43.21	47.82	48.1	48.63
F10	0	0	0.09	0.07	0	0
F11	0	0	0	0	0	0
F11R	16.28	10.46	8.95	7.45	9.76	9.47
F12	4.28	2.32	5.34	6.84	5.95	5.24
F13A1	0	0	0	0	0	0
F13B	0	0	0	0	0	0
F2	0	0.03	0.1	0	0.07	0
F2R	23.78	12	13.39	19.05	21.38	13.6
F2RL1	3.03	11.41	15.46	14.17	9	13.84
F2RL2	0.4	0.22	0.07	0.57	0.56	0.35
F2RL3	7.52	0.9	1.91	2.56	3.49	1.11
F3	0.52	2.75	2.19	0.86	1.11	1.71
F5	0.1	0.02	0.09	0.04	0.04	0.04
F7	0	0	0	0	0	0
F8	0.73	0.47	0.23	0.66	0.5	0.11
F8A1	1.79	2.03	2.96	2.05	2.19	2.52
F8A2	0	0	0	0	0	0
F8A3	0	0	0	0	0	0
F9	0	0	0	0	0	0
FA2H	0	0	0	0.02	0	0
FAAH	0.56	0.55	0.1	0	0.23	0
FAAH2	0	0.12	0.1	0.1	0.1	0
FABP1	0	0	0	0	0	0
FABP12	0	0	0	0	0	0

FABP2	0.19	0	0.12	0	0.06	0.03
FABP3	0.07	0.71	0.19	0.38	0	0.35
FABP4	0	0	0	0	0	0
FABP5	272.62	302.58	221.46	212.81	244.04	310.73
FABP5P3	0	0.07	0	0.19	0	0
FABP6	0	0	0	0	0	0
FABP7	0	0	0	0	0	0
FABP9	0	0	0	0	0	0
FADD	16.16	15.85	22.33	19.8	16.72	13.07
FADS1	30.81	41.35	37.76	31.87	47.97	48.51
FADS2	68.44	68.06	83.27	73.2	112.79	73.81
FADS3	4.22	0.79	0.76	2.99	2.37	1.39
FADS6	0.44	0.34	0.4	0.13	0.3	0.12
FAF1	4.54	4.78	5.71	5.13	3.84	5.33
FAF2	15.85	16.5	12.72	13.23	13.49	10.03
FAH	143.38	154.33	179.42	156.27	136.74	143.74
FAHD1	17.09	13.01	8.61	10.19	12.75	11.15
FAHD2A	33.77	29.11	30.18	36.91	32.5	28.18
FAHD2B	18.9	27.11	24.07	26.66	23.03	23.83
FAHD2CP	2.06	5.2	3.86	4.19	2.83	3.31
FAIM	21.24	33.58	39.03	43.38	36.03	41.41
FAIM2	0.11	0	0	0.05	0	0
FAIM3	0	0	0	0.1	0.05	0
FAM101A	0	0	0	0	0	0
FAM101B	5.81	10.1	8.4	8.14	6.84	10.72
FAM102A	2.3	1.36	1.02	1.13	0.84	1.11
FAM102B	0.24	0.82	0.8	0.44	0.69	0.53
FAM103A1	77.82	67.49	50.06	55.23	71	52.18
FAM104A	19.73	16.49	14.25	12.96	14.9	12.76
FAM104B	24.19	21.65	22.31	20.11	21.96	20.82
FAM105A	0.96	1.29	0.94	0.87	1.65	0.97
FAM105B	2.49	1.67	1.47	1.31	1.4	1.49
FAM106A	0.45	0.52	0.61	0.44	0.66	0.72
FAM106CP	0.15	0.46	0	0	0.17	0.12
FAM107A	0	0	0	0	0	0
FAM107B	1.36	1.09	1.6	0.63	1.44	0.79
FAM108A1	2.06	1.73	3.37	1.98	2.53	3.05
FAM108B1	2.53	2.38	3.35	2.98	2.18	2.19
FAM108C1	1.87	2.21	2.42	1.78	1.97	2.46
FAM109A	2.6	1.11	1.39	1.97	1.28	1.83
FAM109B	0.16	0	0	0	0	0.03
FAM110A	2.48	1.81	4.24	3.58	5.07	2.07
FAM110B	3.51	2.05	1.52	4.02	2.51	4.84

FAM110C	0.02	0	0	0.01	0	0
FAM110D	0	0.08	0	0.14	0	0
FAM111A	7.35	13.54	12.7	11.03	10.32	12.16
FAM111B	0.07	0.06	0.16	0.18	0.04	0.18
FAM114A1	0.92	0.83	1.24	0.36	0.65	0.75
FAM114A2	8.15	5.83	5.59	6.31	5.21	4
FAM115A	3.77	7.33	4.91	7.53	5.44	8.3
FAM115C	0.36	0.55	0.39	0.39	0.49	0.38
FAM117A	4.11	2.55	1.37	2.63	2.44	2.05
FAM117B	1.81	2.17	1.92	1.92	2.09	3.16
FAM118A	9.41	7.19	5.1	7.37	7.44	8.68
FAM118B	12.74	8.34	7.55	8.6	12.46	7
FAM120A	10.95	11.42	9.28	8.01	9.65	7.99
FAM120AOS	7.12	5.39	3.97	5.14	4.46	3.46
FAM120B	20.51	19.95	15.55	15.94	16.25	15.57
FAM120C	13.79	17.21	16.5	10.29	13.98	14.34
FAM122A	1.48	1.25	1.09	1.57	1.2	1.25
FAM122B	2.53	2.82	2.94	2.27	2.67	4.75
FAM122C	4.29	2.77	3.44	2.91	3.3	2.71
FAM124A	0.27	1.2	0.64	0.38	0.11	0.48
FAM124B	3.97	5.22	3.48	4.87	4.75	6.1
FAM126A	4.11	5.07	4.33	3.6	4.06	5.27
FAM126B	2.89	2.14	2.53	2.21	2.48	1.68
FAM127A	0.55	0	0	0	0	0
FAM127B	0	0	0	0	0	0
FAM127C	0	0	0	0	0	0
FAM129A	0.18	0.46	0.63	0.22	0.4	0.44
FAM129B	0.79	0.82	1.48	0.76	1.02	1.5
FAM129C	0.07	0	0.21	0.09	0.06	0.2
FAM131A	0.09	0.07	0	0	0	0
FAM131B	0	0	0.02	0	0	0
FAM131C	0.18	0	0	0.12	0	0
FAM132A	0	0.17	0.47	0	0.07	0.07
FAM133A	0	0	0	0	0	0
FAM133B	1.52	3.5	3.33	2.9	3.31	2.2
FAM133CP	0.13	0.03	0.04	0.12	0.2	0.14
FAM133DP	12.01	8.72	6.5	6.15	7.91	7.61
FAM134A	7.07	6.76	6.81	8.79	7.81	6.06
FAM134B	0.02	0	0	0.03	0.02	0
FAM134C	19.69	14.51	14.06	17.36	15.54	19.29
FAM135A	1.14	1.52	1.62	1.57	1.01	1.46
FAM135B	0	0	0	0	0	0
FAM136A	74.38	93.45	70.75	66.59	81.16	79.51

FAM138A	0.08	0.06	0.17	0.01	0.07	0.13
FAM138B	0.15	0.05	0	0	0.06	0.27
FAM138C	0.04	0.03	0.08	0	0.04	0.06
FAM138D	0	0.25	0.07	0.05	0.5	0.3
FAM138E	0	0	0	0	0.04	0
FAM138F	0.08	0.06	0.17	0.01	0.07	0.13
FAM13A	1.58	1.57	1.78	1.35	1.11	2.01
FAM13A-AS1	0.02	0.04	0.13	0.03	0	0.05
FAM13B	4.88	5.49	3.07	2.76	4.22	4.86
FAM13C	0	0	0	0	0	0
FAM149A	0	0	0.2	0	0.09	0.03
FAM149B1	1.12	0.9	0.72	1.13	0.7	0.73
FAM150A	0.26	2.31	2.44	0.27	1.29	1.18
FAM150B	0	0	0	0	0	0
FAM151A	0	0	0.1	0.04	0	0
FAM151B	0.96	0.65	0.88	0.84	0.58	1.28
FAM153A	0	0	0	0	0	0
FAM153B	0	0	0	0	0	0
FAM153C	1.65	1.81	1.09	0.64	1.2	1.71
FAM154A	0	0	0	0	0	0
FAM154B	0.05	0.04	0.04	0	0.02	0
FAM155A	0	0	0	0	0	0
FAM155B	0	0	0	0	0	0
FAM156A	8.05	19.22	9.03	11.42	12.23	14.8
FAM156B	2.52	0.02	0	1.25	0	0.1
FAM157A	0	0	0	0	0	0
FAM157B	0	0	0	0	0	0
FAM159A	0	0.09	0	0	0	0
FAM159B	0	0	0	0	0	0
FAM160A1	0	0	0	0	0	0.02
FAM160A2	2.74	2.96	2.32	1.66	1.79	1.88
FAM160B1	1.12	1.45	1.63	1.56	1.49	0.65
FAM160B2	16.72	11.47	12.03	11.16	13.65	10.14
FAM161A	1.13	1.57	1.27	1.48	1.28	1.44
FAM161B	0.26	1.18	0.55	0.28	0.49	0.36
FAM162A	104.88	109.26	106.55	89.12	101.17	99.56
FAM162B	0	0	0	0	0	0
FAM163A	0	0	0	0	0	0
FAM163B	0	0	0	0	0	0
FAM166A	0	0	0	0.05	0.07	0.07
FAM166B	0	0.19	0	0.17	0.08	0
FAM167A	0	0	0.13	0	0.07	0.02
FAM167B	0	0	0	0	0	0

FAM168A	4.41	2.19	3.66	4.66	4.53	5.23
FAM168B	7.89	7.55	5.77	5.73	7.76	6.99
FAM169A	1.35	0.62	1.07	0.74	0.82	0.84
FAM169B	0.02	0.07	0	0.02	0.03	0.02
FAM170A	0	0	0	0	0	0
FAM170B	0	0	0	0	0	0
FAM170B-AS	0	0	0	0	0	0
FAM171A1	12.05	3.12	4.1	9.54	7	6.52
FAM171A2	0.33	0.22	0.25	0.08	0.08	0.28
FAM171B	1.84	4	3.48	2.74	2.29	4.69
FAM172A	1.96	2.66	2.05	2.04	2.3	3.22
FAM172BP	0.55	0.35	0.29	0.17	0.23	0.12
FAM173A	7.8	6.24	5.29	6.44	8.67	5.39
FAM173B	9.62	14.68	11.58	10.23	12.34	9.37
FAM174A	7.3	4.88	3.82	3.17	4.29	3.41
FAM174B	0.22	0.24	1.25	0.27	2.44	0.03
FAM175A	5.59	6.28	7.21	8.02	4.44	9.23
FAM175B	10.71	7.94	6.78	7.94	7.84	9.67
FAM177A1	5.7	3.76	3.66	3.38	4.3	4.08
FAM177B	0	0	0	0	0	0
FAM178A	2.79	4.16	3.62	3.19	4.49	2.77
FAM178B	0	0	0	0.06	0.33	0
FAM179A	0.02	0	0	0	0	0
FAM179B	2.18	1.7	1.33	1.5	1.23	1.57
FAM180A	0	0	0	0	0	0
FAM180B	0	0.42	0	0	0.1	0
FAM181A	0	0	0	0	0	0
FAM181A-AS	0	0	0	0	0	0
FAM181B	0	0	0	0	0	0
FAM182A	0.03	0	0	0	0	0.01
FAM182B	0	0	0	0	0	0
FAM183A	0	0	0	0	0	0
FAM183B	0	0	0	0	0	0
FAM184A	0.61	0.24	0.13	0.58	0.25	0.22
FAM184B	0.62	0.45	0.35	0.55	0.56	0.95
FAM185A	2.52	4.63	3.46	4.02	3.91	3.48
FAM186A	0.01	0.05	0	0.01	0	0
FAM186B	0.24	0.04	0.36	0.05	0.09	0.05
FAM187B	0	0	0	0	0	0
FAM188A	10.11	6.31	4.3	3.93	6.33	3.36
FAM188B	1.23	0	0.54	1.07	0	0
FAM189A1	0	0	0	0	0	0.02
FAM189A2	0.07	0	0	0	0	0

FAM189B	1.63	1.92	1.88	1.89	1.9	2.75
FAM192A	31.81	29.87	29.38	23.78	26.97	25.5
FAM193A	2.78	3.13	2.78	2.73	3.14	3.2
FAM193B	1.29	1.58	0.7	0.95	1.04	0.97
FAM194A	0	0	0	0	0	0
FAM194B	0	0	0	0	0	0
FAM195A	39.67	37.04	40.43	40.17	38.82	37.43
FAM195B	50.55	44.29	44.97	66.41	55.07	38.69
FAM196A	0.28	0.1	0.03	0.02	0	0
FAM196B	0	0.19	0.43	0.03	0.04	0.1
FAM197Y2	0	0	0	0	0	0
FAM197Y5	0	0	0	0	0	0
FAM198A	0.13	0.47	1.03	0.33	0.6	0.77
FAM198B	4.46	0.19	0.22	0.07	1.39	6.7
FAM199X	2.85	2	1.89	1.22	2	1.05
FAM19A1	0	0.03	0	0	0	0
FAM19A2	0.21	0	0.03	0.06	0.03	0.44
FAM19A3	0	0.11	0.07	0.15	0	0
FAM19A4	0	0	0	0	0	0
FAM19A5	0	0.04	0	0	0	0
FAM200A	3.37	2.81	4.67	3.65	3.96	4.63
FAM200B	1.94	1.32	1.66	0.91	1.2	1.77
FAM201A	0	0.08	0.08	0.03	0.04	0.39
FAM203A	13.37	16.08	19.86	16.64	15.74	16.18
FAM204A	16.06	15.98	13.37	12.69	12.13	12.45
FAM205A	0	0	0	0	0	0
FAM205B	0	0	0	0	0	0
FAM206A	10.65	9.34	6.24	6.4	8.45	7.56
FAM207A	17.41	13.88	15.36	18.89	15.76	15.17
FAM208A	3.1	3.35	3.18	3.02	3.28	3.74
FAM208B	2.25	2.88	3.95	2.18	2.38	2.87
FAM209A	0	0	0	0	0.11	0
FAM209B	0.12	0	0.21	0.16	0.55	0.23
FAM20A	0	0	0	0	0	0
FAM20B	7.12	4.53	3.06	4.46	4.85	3.84
FAM20C	0.13	0.04	0	0.05	0.3	0.37
FAM210A	7.7	11.06	8.58	8.36	8.65	10.18
FAM210B	4.61	2.35	3.14	3.37	3.94	2.35
FAM211A	2	1.66	2.2	1.75	1.79	2.52
FAM211B	0.18	0.34	0.11	0.25	0.98	0.43
FAM212A	24.65	43.43	54.08	47.2	42.28	39.58
FAM212B	1.27	0.6	0.95	1.18	1.01	0.75
FAM213A	2.16	0.49	1.1	0.66	0.8	0.31

FAM213B	17.31	10.3	13.77	15.49	15.44	13.62
FAM214A	1.32	0.93	0.83	0.9	1.28	0.73
FAM214B	2.98	0.34	0.51	0.91	1.41	0.49
FAM215A	0.71	2.63	1.11	1.75	0.38	2.55
FAM216A	29.38	29.9	26.12	26.11	30.97	26.07
FAM216B	0	0	0	0	0	0
FAM217A	0	0	0	0	0	0
FAM217B	5.89	11.71	11.03	8.47	8.38	11.53
FAM218A	0	0	0	0	0	0
FAM219A	0.63	0.69	0.85	0.53	0.55	0.91
FAM219B	4.11	4.31	4.49	4.03	3.97	2.73
FAM21A	14.59	12.72	10.09	12.32	11.74	11.51
FAM21B	0	0	0	0	0.03	0
FAM21C	0.18	0.18	0.06	0.14	0.11	0.21
FAM220A	3.22	2.02	3.01	4.07	3.42	2.29
FAM221A	0	0	0	0	0	0
FAM221B	0.02	0	0	0	0	0
FAM222A	0.16	0.11	0.47	0.46	0.81	0.19
FAM222A-AS	3.86	8.21	5.62	2.98	5.78	5.26
FAM222B	5.81	4.69	5.14	6.72	7.45	5.36
FAM223A	0.45	0.49	0.11	0.08	0	0
FAM223B	0.45	0.49	0.11	0.08	0	0
FAM224A	0	0	0	0	0	0
FAM224B	0	0	0	0	0	0
FAM225A	0.96	0.61	0.53	0	0	0.76
FAM225B	0.17	0.09	0.06	0.54	0.53	0.11
FAM226A	0	0.01	0	0	0.03	0
FAM226B	0	0.01	0	0	0.03	0
FAM227A	0.42	0.42	0.53	0.33	0.24	0.3
FAM227B	0.19	0.21	0.14	0.1	0.07	0.15
FAM228A	0	0	0.34	0	0	0
FAM228B	0.94	1.4	0.52	0.65	1.57	0.34
FAM229A	0.34	0.27	0.31	0.08	0	0.35
FAM229B	1.51	1.79	1.44	1.66	2.8	1.57
FAM22A	0	0.04	0.07	0.11	0	0.13
FAM22D	0.16	0.03	0.02	0.02	0.17	0
FAM22F	0	0	0	0	0	0
FAM22G	0.18	0.48	0.46	0.29	0.45	0.91
FAM24A	0	0	0	0	0	0
FAM24B	0	0	0	0	0	0
FAM24B-CU	0	0	0	0	0	0
FAM25A	0	0	0	0	0	0
FAM25B	0	0	0	0	0	0

FAM25C	0	0	0	0	0	0
FAM25G	0	0	0	0	0	0
FAM26D	0	0	0	0	0	0
FAM26E	0	0	0	0.01	0.02	0.08
FAM26F	0	0	0	0	0	0
FAM27A	0.96	1.13	2.13	0.87	3.24	3.32
FAM27B	0.96	2.9	1.3	0	0	0
FAM27C	0	0	0	0	0	0
FAM27L	0	0	0	0	0	0
FAM32A	101.14	119.36	108.49	95.22	104.38	103.41
FAM35A	12.11	11.12	9.87	8.13	13.23	9.61
FAM35BP	0	0	0	0	0	0
FAM35DP	1.4	0.53	0.83	0.91	1	1.06
FAM3A	16.41	16.47	14.92	15.81	15.74	13.04
FAM3B	0	0	0	0	0	0
FAM3C	4.44	2.88	2.12	1.68	3.04	2.7
FAM3D	0	0	0	0	0	0
FAM41AY1	0	0	0	0	0	0
FAM41AY2	0	0	0	0	0	0
FAM41C	1.26	0.8	0.52	0.81	0.37	0.99
FAM43A	0.58	0.93	1.31	1.11	0.76	0.8
FAM43B	0	0	0	0	0	0
FAM45A	5.34	12.41	6.16	7.81	5.81	8.76
FAM45B	1.2	1.83	2.49	0.27	1.69	1.93
FAM46A	2.58	8.21	5.87	3.61	3.1	8.74
FAM46B	0.06	0	0	0.13	0.12	0.66
FAM46C	5.31	7.16	3.96	3.8	4.95	5.67
FAM46D	0	0	0	0	0	0
FAM47A	0	0	0	0	0	0
FAM47B	0	0	0	0	0	0
FAM47C	0	0.02	0	0	0	0
FAM47E	0.63	1.53	0.24	1.72	0.31	0.86
FAM47E-STB	0.83	1.52	0.32	0	1.17	0
FAM49A	1.15	2.18	2.14	1.22	1.38	1.2
FAM49B	55.85	67.28	57.17	53.18	63.46	62.8
FAM50A	56.01	34.62	30.35	56.68	41	16.45
FAM50B	0	0	0	0	0	0
FAM53A	0.03	0	0	0	0.05	0
FAM53B	2.6	2.8	2.93	2.94	2.43	3.28
FAM53C	14.63	12.47	11.26	10.84	12.22	10.66
FAM57A	14.26	11.06	11.23	10.39	13.76	7.39
FAM57B	0	0	0	0	0	0
FAM58A	9.14	6.54	7.52	5	6.64	9.22

FAM5B	0	0	0	0	0	0
FAM5C	0.35	0	0	7.48	0.33	0.44
FAM60A	25.96	22.29	18.33	17.56	19.89	21.31
FAM63A	3.4	3.97	3.92	5.29	4.72	3.78
FAM63B	5.3	4.55	5.44	3.61	5.18	3.11
FAM64A	4.58	1.87	2.39	1.98	5.04	9.32
FAM65A	15.27	11.45	11.93	12.86	11.14	10.52
FAM65B	0.48	0.12	0.05	0.28	0.38	0.21
FAM65C	2.21	0.01	0	0.58	0.81	0.02
FAM66A	0	0	0	0	0	0
FAM66B	0	0	0	0.18	0.25	0
FAM66C	0.02	0.02	0.04	0.08	0	0.16
FAM66D	0	0	0	0	0.13	0
FAM66E	0	0	0	0	0	0
FAM69A	1.63	0.94	1.07	1.49	2.67	0.87
FAM69B	1.45	1.56	1.04	4.31	1.6	3.03
FAM69C	0	0	0	0	0	0
FAM71A	0	0	0	0	0	0
FAM71B	0	0	0	0	0	0
FAM71C	0	0	0	0	0	0
FAM71D	0.08	0	0	0	0	0
FAM71E1	0	0	0	0.3	0	0
FAM71E2	0	0	0	0	0	0
FAM71F1	0	0	0	0	0	0
FAM71F2	0	0	0	0	0	0
FAM72A	8.79	10.24	6.28	5.08	8.4	7.35
FAM72B	7.82	9.04	9.65	5.39	9.54	8.37
FAM72D	12.54	11.19	11.52	9.8	10.45	10.66
FAM73A	1.24	2.27	1.32	1.1	1.37	1.77
FAM73B	4.65	3.54	3.95	2.49	4.58	3.69
FAM74A1	0.99	1.13	0.99	0.08	0.75	0.97
FAM74A2	0.22	0.24	0.57	0.16	0.09	0.52
FAM74A3	0	0	0	0.46	0	0
FAM74A4	0	0	0	0	0.18	0
FAM76A	3.78	4.54	3.8	4.24	4.07	4.55
FAM76B	1.36	2.03	1.43	1.27	1.49	1.6
FAM78A	3.75	6.95	6.54	5.63	5.76	6.82
FAM78B	0	0	0	0	0	0
FAM81A	0	0	0	0	0	0.21
FAM81B	0	0	0	0	0	0
FAM83A	64.07	0.22	0.4	9.17	11.26	0.21
FAM83B	0.12	0.16	0.13	0.13	0.08	0.15
FAM83C	0	0	0	0	0	0.02

FAM83D	16.64	14.01	13.96	14.98	17.36	13.38
FAM83E	0.49	0.2	0.29	0.1	0.19	0.05
FAM83F	0	0	0	0	0	0
FAM83G	1.59	1.16	1.21	1.68	1.34	1.31
FAM83H	0.16	0.08	0.12	0.14	0.04	0.05
FAM84A	0.6	0.04	0.01	0	0.05	0.06
FAM84B	1.52	1.32	2.07	2.16	1.79	2.43
FAM86A	9.36	13.24	9.94	12.2	9.73	13.64
FAM86B1	2.97	3.22	3.98	2.99	2.31	3.32
FAM86B2	1.8	1.4	1.13	0.77	1.26	0.66
FAM86B3P	3.42	6.09	4.92	2.48	4.07	5.58
FAM86C1	7.59	9.45	10.11	9.09	6.54	9.95
FAM86C2P	0.44	1.95	1.37	1.87	0.42	0.89
FAM86DP	2.62	5.85	3.48	4.83	4.72	4.9
FAM86EP	2.29	3.05	2.88	2.3	1.89	2.74
FAM86FP	1.03	1.15	1.36	1.4	1.32	1.63
FAM86HP	0.13	0.51	0.21	0.9	0.17	0.46
FAM86JP	0.73	1.03	2.26	1.73	1.77	0.87
FAM89A	3.35	1.53	0.32	1.24	3.25	1.21
FAM89B	0.93	0.59	0.44	1.21	1.01	1.73
FAM8A1	1.43	1.23	0.79	0.84	1.25	1.09
FAM90A1	0.67	0.23	0	0.38	0.24	0
FAM90A10P	0	0	0	0	0	0
FAM90A25P	0	0	0	0	0	0
FAM90A27P	0	0	0	0	0	0
FAM90A2P	0	0	0	0	0	0
FAM90A7P	0	0	0	0	0	0
FAM91A1	8.04	6.06	4.49	4.32	5.04	6.03
FAM91A2	1.2	0.76	0.32	0.53	1.25	0.96
FAM92A1	39.56	67.84	55.47	48.02	56.57	65.56
FAM92A1P2	0.42	0.64	0.59	0.39	0.37	0.24
FAM92B	0	0	0.04	0.03	0	0.08
FAM95B1	0.1	0.27	0.09	0.13	0.23	0.2
FAM96A	172.9	246.67	206.05	148.3	187.11	184.97
FAM96B	196.51	150.06	134.65	144.13	175.2	126.53
FAM98A	18.88	19.52	16	16.38	17.18	16.49
FAM98B	6.25	8.76	9.35	8.02	8.68	11.85
FAM98C	1.66	1.45	2.23	2.33	1.85	1.02
FAM99A	0	0	0	0	0	0
FAM99B	0	0	0	0	0	0
FAM9A	0	0.04	0	0.1	0	0.15
FAM9B	0	0	0.07	0	0	0
FAM9C	0.84	0.51	0.39	0.33	0.23	0.24

FAN1	1.53	2.59	0.94	1.2	0.82	0.87
FANCA	10.9	12.09	9.03	8.54	10.79	13.27
FANCB	5.04	6.15	3.85	3.68	4.9	5.41
FANCC	4.04	3.45	2.87	2.8	4.36	3.02
FANCD2	10.07	13.83	13.24	11.42	12.02	12.44
FANCD2OS	0	0	0	0	0	0
FANCE	0.4	1.13	0.63	0.53	0.41	0.32
FANCF	4.83	5.15	4.83	5.98	3.61	5.27
FANCG	16.44	24.59	19.2	19.43	19.84	21.59
FANCI	33.49	35.69	32.5	31.9	36.32	40.99
FANCL	13.37	14.73	8.43	11.72	12.76	11.85
FANCM	1.46	1.89	1.57	1.18	1.65	1.89
FANK1	0.35	0.09	0.16	0	0.55	0.47
FAP	0	0	0	0	0	0
FAR1	6.05	6.39	4.3	3.9	5.13	5.03
FAR2	12.17	19.69	28.2	21.63	18.03	20.29
FARP1	1.26	2.72	0.4	1.51	0.95	0.55
FARP2	3.3	2.82	3.36	1.94	2.7	3.28
FARS2	13.09	12.72	16.73	16.03	14.45	12.26
FARSA	204.68	273.79	257.7	277.42	265.16	255.34
FARSB	68.48	61.72	59.72	69.47	74.29	65.32
FAS	3.13	7.6	3.16	2.85	4.48	3.81
FAS-AS1	0.34	0.49	0.88	0.07	0	0.34
FASLG	0	0	0	0	0	0
FASN	38.91	41.65	46.81	41.56	42.8	61.08
FASTK	23.27	22.15	24.24	15.73	20.97	15.17
FASTKD1	13	15.5	12.84	10.68	14.12	13.93
FASTKD2	9.8	10.16	10.31	9.06	8.74	10.44
FASTKD3	12.07	12.19	15.98	12.23	12.97	12.37
FASTKD5	23.6	19.91	20.65	19.12	22.29	20.69
FAT1	2.83	2.62	2.89	2.3	2.04	2.58
FAT2	0	0	0	0	0	0
FAT3	0.07	0.09	0.05	0.05	0.04	0.06
FAT4	0	0.03	0.02	0	0	0.09
FATE1	0	0	0	0.05	0	0
FAU	1786.35	1726.79	1850.24	1988.13	1963.24	1885.14
FAXC	0.13	0.2	0.01	0.01	0.04	0.06
FBF1	0.06	0.28	0.29	0.24	0.44	0.28
FBL	276.53	339.42	357.97	385.62	402.43	393.55
FBLIM1	7.65	10.18	9.55	6.51	8.49	7.17
FBLL1	0	0.29	0.2	0.05	0.07	0.15
FBLN1	0.09	0.28	0.14	0.18	0.2	0.34
FBLN2	0	0	0	0.01	0	0

FBLN5	0	0	0	0.44	0	0.2
FBLN7	0	0	0	0	0	0
FBN1	2.1	0.06	0.02	0.2	0.32	0.01
FBN2	0.88	4.15	3.56	1.84	1.94	6.26
FBN3	0	0	0	0.03	0	0
FBP1	0	0	0	0	0	0
FBP2	0.29	0	0	0	0	0
FBRS	4.18	3.68	4	2.95	4.61	4.55
FBRSL1	0.21	0.28	0.57	0.35	0.25	0.23
FBXL12	11.49	18.98	8.87	10.14	13.16	14.45
FBXL13	2.64	2.54	2.14	1.26	1.9	1.09
FBXL14	1.19	0.74	2.08	1.15	2.51	1.63
FBXL15	12.57	12.41	12.09	12.34	13.14	13.56
FBXL16	0	0	0.06	0.01	0	0.06
FBXL17	0.17	0.16	0.34	0.23	0.13	0.1
FBXL18	3.73	3.63	3.97	3.33	3.24	3.78
FBXL19	0.08	0.47	0.42	0.11	0.43	0.66
FBXL19-AS1	0.42	1.32	0.81	0.67	0.57	1.3
FBXL2	0.78	0.48	0.18	0.43	0.44	0
FBXL20	4.21	3.18	3.21	3.71	3.54	4.1
FBXL21	0.08	0	0	0	0	0
FBXL22	0.1	0.27	0.41	0.41	0.51	0.45
FBXL3	1.62	0.93	0.75	1.12	1.2	0.64
FBXL4	5	3.63	3.82	3.42	4.36	4.29
FBXL5	5.2	5.71	5.83	4.99	4	7.53
FBXL6	3.21	2.93	1.6	2.81	3.27	2.67
FBXL7	0	0	0	0	0	0
FBXL8	2.97	1.7	1.62	1.71	0.73	1.94
FBXO10	2.97	5.39	4.34	3.81	3.27	4.27
FBXO11	2.72	2.37	2.35	2.44	3.5	2.98
FBXO15	0.3	0	0	0.06	0.08	0.09
FBXO16	2.69	1.82	2.74	1.98	1.85	2.72
FBXO17	0.13	0.31	0.19	0.07	0.27	0.1
FBXO18	8.95	4.8	5.97	4.38	5.35	4.32
FBXO2	0	0	0	0	0	0
FBXO21	5.82	6.56	6.1	5.24	9	7.35
FBXO22	15.1	20.7	16.89	14.89	16.94	15.67
FBXO22-AS1	24.96	15.79	27.31	15.78	16.15	18.96
FBXO24	0.23	0	0.27	0.36	0.65	0
FBXO25	13.87	11.04	8.17	9.23	10.75	9.05
FBXO27	1.64	1.32	2.93	1.27	1.84	2.32
FBXO28	5.2	4.52	3.74	3.12	5.34	4.05
FBXO3	8.64	7.31	7.11	6.73	8.88	7.56

FBXO30	3.46	2.44	2.49	2.29	2.86	2.36
FBXO31	1.32	2.42	1.52	1.49	1.95	1.03
FBXO32	0.11	0.05	0.16	0.04	0.01	0.06
FBXO33	0.5	0.84	0.56	0.81	0.99	0.47
FBXO34	7.2	9.77	8.51	7.52	8.09	8.82
FBXO36	0.79	0.39	0.83	0.57	0.31	0.24
FBXO38	5.53	5.26	2.79	4.37	4.19	4.59
FBXO39	0	0	0	0	0	0
FBXO4	12.26	11.7	9.53	10.13	13.43	9.77
FBXO40	0.04	0.05	0.13	0.1	0.02	0.16
FBXO41	1.5	2.58	1.99	1.22	2.26	3.69
FBXO42	8.45	12.12	9.29	9.05	8.87	11.62
FBXO43	0.24	0.59	0.89	0.57	0.7	0.78
FBXO44	1.19	0.24	0.95	1.12	0.59	0.59
FBXO45	2.69	3.35	3.96	2.99	3.19	3.89
FBXO46	1.95	2.36	2.28	1.91	1.89	2.07
FBXO47	0	0	0	0	0	0
FBXO48	0.46	0.93	0.52	0.31	0.36	0.65
FBXO5	26.6	21.48	20.82	22.6	25.04	22.72
FBXO6	7.41	5.54	6.94	6.61	9.74	5.8
FBXO7	112.78	66.72	66.63	69.19	75.47	59.51
FBXO8	2.69	2.04	1.71	2.2	2.39	1.49
FBXO9	5.3	3.11	3	3.25	5.32	3.99
FBXW10	0	0	0	0	0	0
FBXW11	7.17	5.45	5.49	5.49	5.07	5.85
FBXW12	0	0	0	0	0	0.26
FBXW2	3.37	4.8	4.64	4.27	4.19	4.72
FBXW4	0.95	1.13	0.7	0.94	2.01	1.98
FBXW4P1	0	0.62	0.06	0.05	0.16	0.17
FBXW5	27.86	34.5	33.18	29.51	29.02	30.92
FBXW7	1.93	2.13	1.77	1.2	1.33	1.87
FBXW8	2.33	3.78	3.19	3.45	2.96	2.94
FBXW9	12.58	12.95	7.52	12.2	9.91	10.69
FCAMR	0	0	0	0	0	0
FCAR	1.64	2.1	2.39	1.15	1.73	2.26
FCER1A	0.91	0	0	0	0.12	0
FCER1G	1.38	10.93	4.93	1.25	4.61	3.48
FCER2	0	0	0	0	0	0
FCF1	9.69	7.27	8.24	5.93	7.33	9.19
FCGBP	0.01	0	0	0	0	0
FCGR1A	0.08	2.6	1.14	0.16	0	1.81
FCGR1B	0.22	1.95	0.89	0.05	0.79	1.94
FCGR1C	0.1	1.41	0.1	0.17	0.45	3.28

FCGR2A	30.7	1.67	4.11	9.4	16.95	5
FCGR2B	2.14	0.03	0	0	0.09	0.37
FCGR2C	1.24	0	0.04	0.05	0.28	0.26
FCGR3A	0	0	0	0	0	0
FCGR3B	0	0	0	0	0	0
FCGRT	0.16	0.32	1.3	0.89	1.56	3.98
FCHO1	1.45	3.93	3.78	1.58	3.45	4.15
FCHO2	1.25	1.28	0.76	0.98	0.92	0.7
FCHSD1	2.27	0.89	0.79	0.91	1.41	1.15
FCHSD2	1.72	3.43	4.57	3.31	2.97	2.18
FCN1	0	0	0	0	0	0
FCN2	0	0	0	0	0	0
FCN3	0	0	0	0	0	0
FCRL1	0	0.02	0.06	0.07	0	0.05
FCRL2	0.06	0	0	0	0	0
FCRL3	0	0	0	0	0	0
FCRL4	0	0	0	0	0	0
FCRL5	0	0	0	0	0	0
FCRL6	0	0	0	0	0	0
FCRLA	0	0	0	0	0	0
FCRLB	1.3	0.23	0.58	0.58	0.12	0.47
FDCSP	0	0	0	0	0	0
FDFT1	226.16	255.82	226.64	211.17	262.49	272.37
FDPS	154.47	154.52	149.62	155.52	190.75	170.07
FDPSL2A	1.23	1.8	1.56	1	1.15	1.53
FDX1	3.97	4.49	4.14	4.74	4.55	4.02
FDX1L	35.42	27.8	28.64	26.93	29.43	15.57
FDXACB1	3.33	4.3	2.66	4.44	5.6	4.48
FDXR	29.73	19.91	18.01	25.4	24.6	17.45
FECH	10.92	6.8	5.02	8.25	9.62	5.84
FEM1A	22.76	20.54	18.39	21.73	18.47	22.75
FEM1B	8.85	9.6	13.49	10.63	8.42	7.86
FEM1C	2.87	3.9	3.59	3.54	3.08	3.54
FEN1	88.07	105.22	99.2	96.22	107.85	102.8
FENDRR	0	0	0.09	0.07	0.08	0.1
FER	2	1.01	1	0.97	2.47	0.4
FER1L5	0	0	0	0	0	0
FER1L6	0.17	0.07	0.1	0.09	0.1	0.14
FER1L6-AS1	0	0	0	0	0	0
FERD3L	0	0	0	0	0	0
FERMT1	3.97	5.57	7.63	5.57	4.85	6.73
FERMT2	0.22	0	0	0	0	0
FERMT3	92.15	23.34	39.82	34.64	55.84	13.72

FES	11.89	21.13	18.12	16.68	14.52	24.92
FETUB	0	0	0	0	0	0
FEV	3.27	0.16	0.25	3.75	4.24	0.48
FEZ1	0.39	0.77	1.13	0.41	0.73	0.88
FEZ2	2.78	2.23	0.91	2.04	2.57	2.29
FEZF1	0	0	0	0	0	0
FEZF1-AS1	0	0	0	0	0	0
FEZF2	0	0	0	0	0	0
FFAR1	0	0	0	0	0	0
FFAR2	0.29	0.44	0.33	0.18	0.29	0.59
FFAR3	0	0	0	0	0	0
FFAR4	0.27	1.49	1.05	0.87	0.47	2.01
FGA	0	0	0	0	0	0
FGB	0.07	0.03	0.04	0.03	0.14	0.03
FGD1	2.13	1.94	3.1	2.3	2.7	2.04
FGD2	0.26	0.17	0.03	0.05	0.07	0
FGD3	7.39	6.42	10.06	11.35	8.91	8.73
FGD4	0.19	0.28	0.32	0.27	0.39	0.17
FGD5	0	0	0	0	0	0
FGD5-AS1	15.8	14.11	10.3	13.63	14.4	12.43
FGD5P1	0.35	0.12	0.32	0.15	0.12	0.31
FGD6	0.32	0.47	0.2	0.14	0.16	0.2
FGF1	0	0	0	0	0	0
FGF10	0.26	0	0	0	0	0
FGF11	0.38	0.45	0.25	0.63	0.38	0.03
FGF12	0	0	0	0	0	0
FGF13	19.38	3.94	3.65	5.56	6.88	3.55
FGF13-AS1	0.34	0	0	0	0	0.09
FGF14	0	0	0	0	0	0
FGF14-AS2	0	0	0	0	0	0
FGF14-IT1	0	0	0	0	0	0
FGF16	0	0	0	0	0	0
FGF17	0	0	0	0	0	0
FGF18	0	0	0	0	0	0
FGF19	0	0	0	0	0	0
FGF2	0.05	0.04	0.01	0.01	0	0.01
FGF20	0	0	0	0	0	0
FGF21	0	0	0	0	0	0
FGF22	0	0	0	0	0	0
FGF23	0	0	0	0	0	0
FGF3	0	0	0	0	0	0
FGF4	0	0	0	0	0	0
FGF5	0.91	0.83	0.84	0.95	0.65	0.71

FGF6	0	0.17	0.1	0.07	0.3	0
FGF7	0.1	0	0	0	0	0
FGF8	0	0	0	0	0	0
FGF9	0.26	0.44	0.28	0.71	0.96	0.9
FGFBP1	0	0	0	0	0	0
FGFBP2	0	0	0	0	0	0
FGFBP3	0.62	0.54	0.73	0.29	0.67	0.26
FGFR1	8.86	4.97	3.39	3.68	5.21	3.56
FGFR1OP	9.99	9.03	8.49	6.37	8.6	7.16
FGFR1OP2	10.19	15.85	10.35	11.34	11.88	10.07
FGFR2	0.06	0.15	0.48	0.59	0.2	0.88
FGFR3	0.42	0	0	0.08	0.19	0.31
FGFR4	1.59	2.44	3.74	2.99	1.98	4.07
FGFRL1	3.56	2.69	2.07	3.18	4.24	2.27
FGG	0	0	0	0	0	0
FGGY	1.06	1.32	2.11	1.38	1.39	1.06
FGL1	0	0	0	0	0	0
FGL2	0.13	0.13	0.16	0.08	0.11	0
FGR	0	0	0	0	0.11	0.15
FH	84.12	89.87	84.34	96.24	92.77	104.07
FHAD1	0.04	0.13	0.11	0.1	0.08	0.02
FHDC1	0.23	0.1	0.21	0.16	0.1	0.1
FHIT	0	0	0	0	0	0
FHL1	0.41	0.9	1.82	2.16	1.02	1.19
FHL2	35.63	15.99	15.18	19.15	21.93	12.26
FHL3	26.44	19.84	20.17	32.39	22.22	12.37
FHL5	0	0	0	0	0	0
FHOD1	3.29	4.85	3.98	4.9	3.35	3.1
FHOD3	0	0.16	0.2	0.06	0.06	0.15
FIBCD1	0.05	0	0	0	0	0
FIBIN	0	0	0	0	0	0
FIBP	59.67	71.27	61.11	70.95	72.09	72.37
FICD	1.4	1.32	2.27	1.71	1.49	2.78
FIG4	6.92	3.1	1.91	3.16	4.34	3.16
FIGF	0.07	0	0	0	0	0
FIGLA	0	0	0	0	0	0
FIGN	0	0	0	0	0	0
FIGNL1	9.45	7.47	9.38	8.85	7.87	7.54
FIGNL2	0	0	0	0	0	0
FILIP1	0	0	0	0	0	0.03
FILIP1L	4.58	0.21	0.69	1.22	1.54	0.36
FIP1L1	23.5	29.45	22.66	26.51	22.46	24.02
FIS1	156.2	108.45	109.86	134	128.25	100.39

FITM1	0	0.36	0.7	0.74	0	0.16
FITM2	2.74	2.16	2.8	3.9	3.59	2.53
FIZ1	0.17	0.45	0.45	0.28	0.49	0.75
FJX1	0.63	0.26	0.32	1.01	0.59	0.63
FKBP10	12	12.46	14.28	16.29	19.48	17.34
FKBP11	29.57	68.53	66.87	78.16	57.5	68.86
FKBP14	2.01	2.16	1.97	1.57	1.85	2.11
FKBP15	5.22	6.15	5.63	5.44	5.49	6.16
FKBP1A	278.75	178.38	150.9	196.92	201.8	174.72
FKBP1A-SDC	0.26	0	0	0	0	0.14
FKBP1AP1	0.81	1.77	0.75	0.53	1	1.35
FKBP1B	4.61	3.19	2.69	2.4	4.17	3.49
FKBP2	97.16	88.67	80.33	92.74	89.35	83.34
FKBP3	55.45	70.53	65.9	58.49	69.84	60.23
FKBP4	75.47	70.55	80.84	78.11	73.22	81.41
FKBP5	18.8	35.24	44.41	35.77	33.8	47.77
FKBP6	0.05	0.13	0.37	0	0.12	0.13
FKBP7	1.76	2.73	2.19	1.76	1.61	3
FKBP8	29.45	22.5	26.68	32.79	27.62	26.67
FKBP9	9.5	11.61	14.7	10.56	12.2	11.56
FKBP9L	0.16	0.24	0.24	0.27	0.61	0.4
FKBPL	6.9	5.85	6.83	5.61	5.83	5.9
FKRP	1.96	1.23	1.84	1.23	1.84	1.48
FKSG29	0.27	0	0.37	0	0.13	0
FKTN	1.59	2.22	1.88	1.52	2.33	2.24
FLAD1	34.7	43.85	38.15	40.11	33.91	34.81
FLCN	33.9	22.36	19.21	23.46	27.31	16.77
FLG	0	0	0	0	0	0
FLG2	0.01	0.07	0.12	0.07	0.1	0.09
FLI1	2.48	7.94	10.69	7.75	4.81	9.11
FLI1-AS1	1.45	3.26	4.53	3.05	1.87	4.06
FLII	27.94	38.67	35.3	32.93	29.46	36.43
FLJ10038	0.29	0.42	0.38	0.36	0.13	0.12
FLJ11235	0.86	0	0	0.3	0.51	0
FLJ12334	0	0.02	0	0	0	0
FLJ12825	0.18	0.27	0.14	0.08	0.44	0.19
FLJ13197	0.74	0.67	0.6	0.49	0.46	0.7
FLJ13224	0.23	0.07	0.29	0.06	0.3	0.14
FLJ14107	0.21	0.39	0.61	0.25	0.35	0.29
FLJ14186	1.53	1.9	2.05	1.97	0.84	2.41
FLJ16171	0	0.08	0.13	0	0	0
FLJ16341	0	0	0	0	0	0
FLJ16779	0	0	0	0	0	0

FLJ20021	4.86	3.37	2.45	1.99	2.54	1.8
FLJ20518	0	0	0	0	0	0
FLJ21408	0	0	0	0	0	0
FLJ22184	0	0.01	0.12	0.26	0.11	0.08
FLJ22447	0	0	0	0.04	0.16	0.17
FLJ22763	0	0	0	0	0	0
FLJ23867	0.22	0.13	0.2	0	0.2	0.05
FLJ25328	0	0	0	0	0	0
FLJ25363	0.08	0.07	0.25	0.04	0.09	0.06
FLJ25758	0	0	0	0	0	0
FLJ26245	0	0	0	0	0	0
FLJ26850	0	0	0	0	0	0
FLJ27352	0.11	0.08	0.1	0	0.1	0
FLJ27354	0.4	0.24	0.73	0.07	0.09	0.6
FLJ30403	3.16	1.45	2.37	1.54	2.12	1.82
FLJ30679	0.07	0.18	0.1	0.05	0	0.3
FLJ30838	0.12	0.14	0.18	0.16	0.13	0.12
FLJ31306	5.59	5.25	3.44	3.59	5.76	4.68
FLJ31485	0	0	0.1	0.1	0.02	0.02
FLJ31662	1.23	1.15	1.57	1.04	1.05	1.18
FLJ31813	0.8	1.36	0.72	0.53	0.69	0.55
FLJ33360	0.03	0.06	0.21	0.03	0.13	0.14
FLJ33534	0.05	0.07	0	0.03	0	0.15
FLJ33581	0	0	0	0	0	0
FLJ33630	0.42	0.33	0.47	0.25	0.35	0.3
FLJ34208	0.34	0.96	0.5	0.52	0.32	1.03
FLJ34503	0	0	0	0	0	0
FLJ35024	0	0.14	0	0.06	0.08	0.36
FLJ35282	0.18	0.34	0.2	0.22	0.4	0.19
FLJ35390	0.05	0	0	0	0	0
FLJ35424	0	0	0	0	0	0
FLJ35946	0	0	0	0	0	0
FLJ36000	0	0.03	0.02	0.01	0	0.02
FLJ36777	0	0.53	0.14	0.11	0.11	0.25
FLJ37035	0.2	0.25	0.05	0.14	0.33	0.31
FLJ37201	0.04	0	0.04	0.07	0.02	0
FLJ37453	1.01	1.62	0.7	1.29	1.26	1.37
FLJ37505	0	0	0	0	0	0
FLJ38109	0.58	0.05	0.21	0.32	0.36	0.29
FLJ38576	0	0	0	0.12	0	0.09
FLJ39051	4.95	7.07	6.07	5.76	6.1	3.61
FLJ39080	0	0	0	0	0	0
FLJ39639	0.4	0.65	0.57	0.34	0.44	0.19

FLJ39739	0	1.21	3.29	0	0.76	1.2
FLJ40194	0.11	0.09	0.1	0	0	0
FLJ40288	0	0	0	0	0	0
FLJ40292	0	0.07	0	0.2	0	0.13
FLJ40852	0.33	0.05	0.18	0.07	0.12	0.13
FLJ41200	0.13	0.06	0.08	0.12	0.05	0.05
FLJ41278	0	0	0	0	0	0
FLJ41350	0	0	0	0	0	0
FLJ41649	0	0	0	0	0	0
FLJ41941	0.1	0.07	0.27	0.18	0.19	0.12
FLJ42102	1.38	1.67	2.25	1.07	0.72	1.44
FLJ42289	0.02	0	0	0	0.02	0
FLJ42351	0	0	0	0.34	0.31	0
FLJ42393	0.22	0.19	0.47	0.31	0.03	0.15
FLJ42627	1.17	1.84	1.01	1.47	1.34	1.56
FLJ42709	0.88	2.1	2.38	1.56	2.95	2.62
FLJ42875	0	0	0	0	0	0
FLJ42969	0	0.09	0	0.08	0	0
FLJ43663	0.59	0.86	0.77	0.71	0.42	0.82
FLJ43681	0.43	1.11	0.98	0.68	0.91	1.71
FLJ43879	0.1	0.28	0.17	0.18	0.08	0.15
FLJ44313	0	0.03	0	0.07	0	0.04
FLJ44511	0	0	0.13	0	0.12	0
FLJ44635	0.16	0.23	0.24	0.25	0.21	0.46
FLJ45079	0	0	0	0	0	0
FLJ45340	1.95	1.79	1.47	1.27	1.52	2.33
FLJ45445	0	0	0	0.03	0.05	0.04
FLJ45513	0.04	0.08	0.36	0.14	0.17	0.14
FLJ45974	0	0	0	0	0	0
FLJ46066	0	0	0	0	0	0
FLJ46257	0	0	0	0	0	0
FLJ46284	0	0	0	0	0	0
FLJ46300	0	0	0	0	0	0
FLJ46361	0.1	0.2	0.42	0.26	0.1	0.09
FLJ46446	4.61	1.68	0.79	2.35	2.35	0.47
FLJ46906	1.31	3.7	2.32	2.55	2.62	2.64
FLNA	21.81	14.51	18.72	19.63	15.12	21.86
FLNB	10.57	6.4	7.88	7.17	7.01	9.21
FLNC	9.52	7.62	9.16	8.72	7.29	8.55
FLOT1	54.38	8.24	7.27	18.16	23.85	3.98
FLOT2	44.09	49.39	42.8	51.8	48.14	45.4
FLRT1	0.22	0.35	0.35	0.08	0	0.11
FLRT2	0	0	0	0	0	0

FLRT3	0	0	0	0	0	0
FLT1	0.32	0.16	0.19	0.22	0	0.15
FLT3	0	0.73	0.43	0.14	0.46	0.23
FLT3LG	4.41	1.6	2.06	2.19	4.2	2.71
FLT4	1.08	0.1	0.13	0.25	0.22	0.22
FLVCR1	2.08	2.51	2.01	1.15	1.67	1.65
FLVCR1-AS1	1.63	1.1	0.64	0.32	0.97	0.12
FLVCR2	0.71	3.52	5.11	2.78	1.7	2.17
FLYWCH1	0.92	0.91	1.61	1.44	1.42	1.54
FLYWCH2	38.24	44.74	45.86	41.15	45.8	40.51
FMN1	0.06	0.16	0.1	0.08	0.12	0.05
FMN2	0	0	0	0	0	0
FMNL1	0.42	1.49	2.5	0.94	0.74	2.04
FMNL2	0.65	0.27	0.51	0.25	0.38	0.24
FMNL3	1.85	1.11	1.42	1.45	1.7	1.01
FMO1	0.97	0	0	0	0.29	0
FMO2	0	0	0	0	0	0
FMO3	0	0	0	0	0	0
FMO4	0.45	0.41	0.73	1.07	0.46	0.14
FMO5	0.34	0.06	0.24	0.25	0.74	1.04
FMO6P	0	0	0	0	0	0
FMO9P	0	0	0	0	0	0
FMOD	0.78	0	0	0	0	0
FMR1	3.17	2.72	2.34	2.74	3.32	3.94
FMR1-AS1	0	0	0	0	0	0
FMR1NB	0	0	0	0	0	0
FN1	0.03	0.03	0.02	0.07	0.02	0
FN3K	5.24	1.98	1.49	2.82	2.82	1.32
FN3KRP	18.89	15.52	15.34	16.86	15.88	15.49
FNBP1	2.16	7.25	7.17	5.95	4.5	6.41
FNBP1L	4.79	1.4	1.63	2.3	2.71	3.36
FNBP4	7.83	8.85	7.28	6.81	7.09	7.5
FNDC1	0	0	0	0	0	0
FNDC3A	6.22	5.85	4.66	4.72	4.35	3.85
FNDC3B	2.25	2.64	3.63	1.22	1.59	2.83
FNDC4	0.71	0.32	0.04	0.4	0.04	0.68
FNDC5	0.39	0.1	0.32	0.29	0.57	0.19
FNDC7	0	0	0	0	0.07	0
FNDC8	0.39	0.57	0.05	0.04	0.16	0.45
FNDC9	0	0.25	0	0	0	0.07
FNIP1	9.01	6.86	7.16	7.48	8.64	8.24
FNIP2	1.59	1.12	0.97	0.85	1.14	1.85
FNTA	22.21	27.28	27.05	25.89	30.53	24.13

FNTB	4.39	5.77	5.04	3.18	5.43	6.99
FOCAD	5.79	5.67	7.71	7.35	5.07	8.43
FOLH1	0.06	0.22	0.13	0.07	0	0.09
FOLH1B	0	0	0	0	0	0
FOLR1	0	0	0	0	0	0
FOLR2	0	0	0.25	0	0.06	0
FOLR3	0	0	0	0	0	0
FOLR4	0	0	0	0	0	0
FONG	0.41	1.03	1.19	0.79	0.23	0.41
FOPNL	29.44	21.2	21.94	18.8	25.31	23.3
FOS	29.39	13.15	8.56	10.03	7.57	13.81
FOSB	1.19	0.92	0.74	0.82	0.54	0.89
FOSL1	26.35	17.63	17.08	19.29	20.04	14.68
FOSL2	0.29	1.51	0.66	1.08	0.29	1.18
FOXA1	0	0.07	0.04	0	0.1	0
FOXA2	0.43	0	0	0.1	0.03	0
FOXA3	0	0	0	0	0	0.08
FOXB1	0.72	0	0	0.43	0	0.16
FOXB2	0	0	0	0	0	0
FOXC1	0.74	0.58	0.64	0.51	0.42	0.28
FOXC2	0	0	0	0	0	0
FOXD1	0	0.05	0	0	0.06	0
FOXD2	0.34	0.81	0.83	1.06	0.87	0.63
FOXD2-AS1	3.42	4.86	4.09	4.2	4.31	5.15
FOXD3	0	0.06	0	0	0	0
FOXD4	0.13	0.24	0.06	0.14	0.48	0.34
FOXD4L1	0	0.08	0	0	0	0.1
FOXD4L2	0	0	0	0.01	0.03	0.04
FOXD4L3	0	0.13	0	0	0	0
FOXD4L4	0	0	0	0.01	0.03	0.04
FOXD4L5	0.09	0	0	0	0	0
FOXD4L6	0	0	0	0	0.02	0
FOXE1	0	0	0	0	0	0
FOXE3	0	0	0	0	0	0
FOXF1	0	0	0	0	0	0.06
FOXF2	0	0	0	0	0	0
FOXG1	0	0	0	0	0	0
FOXH1	0	0	0	0	0.05	0.3
FOXI1	0	0	0	0	0	0
FOXI2	0	0.05	0.02	0	0	0
FOXI3	0	0	0	0	0	0
FOXJ1	0	0	0	0	0	0
FOXJ2	3.8	2.65	2.35	2.37	2.55	1.97

FOXJ3	10.22	9.94	8.87	10.32	10.4	8.74
FOXK1	10.54	10.8	10.5	10.8	10.34	13.67
FOXK2	5.38	4.68	4.82	4.5	5.48	3.95
FOXL1	0	0	0	0	0	0
FOXL2	0	0	0	0	0	0
FOXM1	12.17	13.79	12.42	11.58	11.37	10.57
FOXN1	0	0	0	0	0	0
FOXN2	5.43	3.3	3.89	2.91	4.18	4.1
FOXN3	2.07	3.17	3.36	2.8	2.93	3.77
FOXN3-AS1	0	0	0	0.15	0	0.07
FOXN3-AS2	0	0	0.27	0	0	0
FOXN4	0	0	0	0	0	0
FOXO1	1.64	0.5	0.47	0.9	0.53	0.29
FOXO3	5.14	2.79	3.2	3.01	3.26	4.1
FOXO3B	0.71	0.33	0.52	0.23	0.33	0.34
FOXO4	2.65	3.9	5.27	4.85	2.81	4.62
FOXP1	5.18	6.98	7.54	5.42	5.68	7.71
FOXP2	1.95	3.74	5.03	3.2	4.39	5.67
FOXP3	0.1	0.08	0.03	0.04	0	0.07
FOXP4	1.1	1.61	1.01	0.78	1.04	1.28
FOXQ1	0	0	0	0	0	0
FOXR1	0.13	0.1	0	0.05	0.13	0.4
FOXR2	0	0	0	0	0	0
FOXRED1	29.91	36.25	34	27.21	32.56	36.8
FOXRED2	15.34	16.03	17.38	17.08	16.07	15.29
FOXS1	0	0	0	0	0	0
FP588	0.03	0	0.05	0	0	0.01
FPGS	37.58	56.79	53.08	55.12	45.59	56.31
FPGT	2.21	1.78	1.53	1.84	2.28	1.86
FPGT-TNNI3I	0	0	0	0	0	0
FPR1	0	0	0	0	0	0
FPR2	0.27	0	0	0	0	0
FPR3	1.78	0	0	0.28	0.71	0
FRA10AC1	2.98	3.72	2.8	3.48	3.9	3.55
FRAS1	2.3	0.06	0.05	0.45	0.52	0.37
FRAT1	0.19	0.13	0.2	0.13	0.39	0.47
FRAT2	0.73	1.87	1.22	1.13	1.32	1.28
FREM1	0	0	0	0	0	0
FREM2	0.01	0.03	0	0	0	0.02
FREM3	0	0	0	0	0	0
FRG1	39.11	41.34	34.63	30.05	40.54	35.33
FRG1B	8.01	8.42	7.26	6.9	7.76	6.89
FRG2	0	0.03	0.03	0	0.03	0

FRG2B	0	0	0	0	0	0
FRG2C	0.18	0.02	0	0	0.03	0.11
FRK	0	0	0.05	0	0.02	0
FRMD1	0.15	0	0	0	0	0
FRMD3	0	0	0	0	0	0
FRMD4A	0.04	0.02	0.02	0.04	0.07	0.03
FRMD4B	0.14	0.5	0.6	0.59	0.28	0.65
FRMD5	0	0.08	0.09	0.14	0	0.07
FRMD6	0.03	0.02	0.06	0.07	0	0.08
FRMD6-AS1	0.26	0.49	0.45	0.32	0.14	0.41
FRMD6-AS2	0	0	0	0	0	0
FRMD7	0	0	0.04	0	0	0
FRMD8	10.49	7.22	13.01	13.48	10.32	7.48
FRMD8P1	0	0	0	0	0	0
FRMPD1	0	0	0	0	0.07	0
FRMPD2	0	0	0	0	0	0
FRMPD2P1	0	0	0	0	0	0
FRMPD4	0	0	0	0	0	0
FRRS1	4.68	0.88	0.89	2.14	3.85	1.22
FRRS1L	0	0	0	0	0	0
FRS2	1.41	1.28	1.1	1.25	1.31	1.1
FRS3	0.24	0.11	0	0.02	0.26	0.21
FRY	0.66	0.88	0.68	0.37	0.83	2.1
FRYL	3.38	4.07	2.73	2.42	3.03	3.26
FRZB	0	0	0	0	0	0
FSBP	0	0	0	0	0	0
FSCB	0	0	0	0	0	0
FSCN1	29.94	26.11	32.8	32.73	26.32	30.43
FSCN2	0.13	0.18	0.04	0.09	0	0
FSCN3	0	0	0	0	0	0
FSD1	0.72	0.13	1.18	0.55	2.5	0.65
FSD1L	1.38	0.87	1.23	0.37	1.13	0.49
FSD2	0	0	0	0	0.03	0
FSHB	0	0	0	0	0	0
FSHR	0	0	0	0	0	0
FSIP1	0.05	0	0	0.18	0.05	0.05
FSIP2	0	0	0	0	0	0
FST	0.53	0.26	0.52	0.19	0.46	0.2
FSTL1	5.66	7.84	7.22	8.16	5.89	9.09
FSTL3	4.32	2.18	1.68	1.53	2.64	1.92
FSTL4	0	0	0	0	0	0
FSTL5	1.92	5.55	0.01	4.2	3.98	3.71
FTCD	0	0	0	0	0	0

FTH1	3610.28	667.8	735.54	1379.85	1531.67	560.94
FTH1P18	0	0	0	0	0	0.09
FTH1P3	2.66	0.7	0.55	0.78	0.9	0.35
FTHL17	0	0	0	0	0	0
FTL	6230.28	4354.03	4132.02	4291.2	4322.45	3136.65
FTLP10	0	0.15	0	0.05	0.28	0
FTMT	0	0	0	0	0	0
FTO	14.38	14.32	13.19	13.57	14.18	13.53
FTSJ1	60.6	61.09	68.87	57.25	62.53	73.54
FTSJ2	36.69	34.79	31.66	35.75	34.1	37.28
FTSJ3	17.08	21.46	19.93	20.02	16.05	21.43
FTSJD1	5.46	4.49	3.17	3.16	3.8	3.94
FTSJD2	10.28	8.96	8.53	9.8	9.78	10.08
FTX	1.68	1.95	1.54	0.63	1.2	0.87
FUBP1	45.25	51.3	45.23	44.02	49.07	52.96
FUBP3	3	1.29	2.41	2.04	2.41	2.19
FUCA1	20.51	17.17	25.66	23.36	14.3	19.94
FUCA2	53.13	28.15	19.71	22.59	34.88	23.71
FUK	1.58	2.45	1.56	1.41	1.71	2.35
FUNDC1	29.71	19.23	18.14	16.96	28.61	19.99
FUNDC2	77.23	63.4	53.28	68.1	73.3	59.53
FUNDC2P2	0.05	0.08	0	0.03	0	0
FUOM	44.33	47.29	27.3	36.9	40.01	32.31
FURIN	20.46	3.8	4.03	6.98	9.76	4.54
FUS	76.36	67.72	63.01	70.49	76.85	79.23
FUT1	0.99	2.02	1.76	1.14	1.22	1.53
FUT10	16.01	15.72	14.66	14.67	13.22	17.76
FUT11	2.77	3.37	4.8	3.36	3.05	3.45
FUT2	0.61	0.73	1.24	0.64	0.69	0.76
FUT3	0	0	0	0	0	0
FUT4	0.92	0.95	0.67	0.59	1.09	1.4
FUT5	0	0	0	0	0	0
FUT6	0.61	0.68	0.75	0.47	0.81	0.46
FUT7	0.18	2.14	0.55	0.26	0.29	2.11
FUT8	8.37	3.31	2.83	3.69	6.15	2.97
FUT8-AS1	0	0.15	0.07	0	0	0.26
FUT9	0.03	0.07	0.06	0.02	0.05	0.02
FUZ	0	0	0	0	0.04	0
FXN	20.49	18.95	21.07	20.6	20.97	21.19
FXR1	9.31	8.42	6.94	8.33	7.81	7.68
FXR2	3.2	2.34	2.16	3.29	2.21	2.49
FXYD1	0	0	0	0	0	0
FXYD2	0	0	0	0	0	0

FXYD3	0	0	0	0	0	0
FXYD4	0	0	0	0	0	0
FXYD5	146.1	83	112.78	94.89	101.34	90.5
FXYD6	0	0	0	0	0	0
FXYD6-FXYD2	0	0	0	0	0	0
FXYD7	0	0	0	0	0	0
FYB	0.9	0.73	1.15	0.34	0.48	1.44
FYCO1	3.47	3.47	3.52	1.92	2.65	3.65
FYN	0.29	0	0.02	0.02	0	0
FYTTD1	22.98	18.23	13.62	15.71	15.75	15.79
FZD1	0.3	0.29	0.09	0.48	0.45	0.25
FZD10	0	0	0	0	0	0
FZD2	0.68	0.44	1.08	0.86	0.97	0.79
FZD3	6.4	6.43	5.78	6.19	6.96	6.13
FZD4	0.97	0.55	0.43	0.39	0.68	0.57
FZD5	1.6	1.9	1.48	1.88	2.17	2.26
FZD6	7.51	8.14	5.7	5.16	7.8	7.06
FZD7	0.68	0.94	1.07	0.69	0.66	1.41
FZD8	0	0.02	0.04	0	0	0
FZD9	0.03	0.12	0.09	0.02	0.12	0.16
FZR1	2.95	2.45	3.73	2.54	2.18	2.52
G0S2	0.64	0	0	0.17	0	0
G2E3	6.09	3.52	4.55	3.27	5.35	2.83
G3BP1	109.05	105.71	106.9	111.91	110.2	116.96
G3BP2	29.13	31.59	30.91	27.84	30.88	34.72
G6PC	0.11	0.18	0.2	0.2	0.14	0.07
G6PC2	0	0.02	0	0.02	0	0
G6PC3	51.1	55.91	58.3	68.44	60.89	64
G6PD	74.25	51.23	58.67	62.13	60.82	61.82
GAA	5.41	10.68	6.5	8.66	7.25	10.87
GAB1	2.38	1.38	1.05	1.06	1.6	1.09
GAB2	11.29	9.57	8.49	7.9	9.31	7.14
GAB3	6.99	2.67	2.55	3.45	4.02	2.59
GAB4	0	0	0	0	0	0
GABARAP	308.66	228.56	214.13	274.83	308.42	246.92
GABARAPL1	7.22	1.06	0.76	2.13	2.02	0.32
GABARAPL2	89.74	57.58	42.81	49.21	62.3	53.21
GABARAPL3	0	0	0	0	0	0
GABBR1	0.47	0.25	0.09	0.19	0.35	0.29
GABBR2	0	0	0	0	0	0
GABPA	4.17	3.8	2.79	2.48	4.68	3.49
GABPB1	16.37	17.62	15.9	18.07	16.47	18.17
GABPB1-AS1	2.3	2.24	1.31	1.04	1.83	2.17

GABPB2	1.44	1.76	0.97	0.78	1.32	0.73
GABRA1	0	0	0	0	0	0
GABRA2	0	0	0	0.01	0	0.03
GABRA3	0	0.04	0.05	0.01	0.05	0.03
GABRA4	0	0	0	0	0	0
GABRA5	0	0	0	0	0	0
GABRA6	0	0	0	0	0	0
GABRB1	0	0	0	0	0	0
GABRB2	0	0.29	0.56	0.34	0.23	0.7
GABRB3	0	0	0	0	0	0
GABRD	0	0	0.91	0.08	0.15	0.08
GABRE	0	0	0	0	0	0
GABRG1	0	0	0	0	0	0
GABRG2	0	0	0	0	0	0
GABRG3	0	0	0	0	0	0
GABRP	0	0	0	0	0	0
GABRQ	0.44	0	0	0	0	0
GABRR1	0.03	0	0	0	0	0.03
GABRR2	0	0	0	0	0	0
GABRR3	0	0	0	0	0	0
GAD1	2.3	0	0	0.23	0.52	0
GAD2	0	0	0	0	0	0
GADD45A	10.03	4.84	2.45	1.77	5.54	3.2
GADD45B	3.5	4.66	1.45	2	2.69	4.81
GADD45G	0	0	0	0	0	0
GADD45GIP1	175.5	194.89	267.86	206.25	244.59	193.28
GADL1	0	0	0	0	0	0
GAGE1	0	0	0.04	0	0	0
GAGE10	0	0	0	0	0	0
GAGE12B	0	0	0	0	0	0
GAGE12C	0	0	0	0	0	0
GAGE12D	0	0	0	0	0	0
GAGE12E	0	0	0	0	0	0
GAGE12F	0	0	0	0	0	0
GAGE12G	0	0	0	0	0	0
GAGE12H	0	0	0	0	0	0
GAGE12I	0	0	0	0	0	0
GAGE12J	0	0	0	0	0	0
GAGE13	0	0	0	0	0	0
GAGE2A	0	0	0	0	0	0
GAGE2B	0	0	0	0	0	0
GAGE2C	0	0	0	0	0	0
GAGE2D	0	0	0	0	0	0

GAGE2E	0	0	0	0	0	0
GAGE4	0	0	0	0	0	0
GAGE5	0	0	0	0	0	0
GAGE6	0	0	0	0	0	0
GAGE7	0	0	0	0	0	0
GAGE8	0	0	0	0	0	0
GAK	14.86	18.34	16.09	13.46	12.47	14.82
GAL	0	0	0	0	0	0
GAL3ST1	0	0	0	0	0	0.04
GAL3ST2	0	0	0	0	0	0
GAL3ST3	0	0.03	0	0	0	0
GAL3ST4	0.29	0.1	0.25	0.3	0.26	0.45
GALC	0	0	0	0	0	0
GALE	21.93	24.77	23.35	20.71	20.76	17.54
GALK1	40.17	108.86	116.23	112.64	82.89	89.73
GALK2	16.88	15.88	16.47	14.26	14.54	16.69
GALM	0.63	0.84	0.59	0.33	0.44	0.79
GALNS	4.56	6.68	6.49	5.43	6.33	7.01
GALNT1	7.08	4.97	4.55	2.84	5.68	4.3
GALNT10	2.13	0.81	1.16	1.39	0.97	0.99
GALNT11	8.3	9.64	6.83	5.96	7.2	8.97
GALNT12	0.21	0.19	0.37	0.24	0.23	0.59
GALNT13	0	0	0	0	0	0
GALNT14	15.2	23.98	25.84	20.51	20.03	23.83
GALNT15	0.19	0.23	0.14	0.08	0.26	0.24
GALNT16	1.42	3.74	1.08	1.19	1.66	4.34
GALNT18	8.92	14.41	19.6	16.41	10.78	19.13
GALNT2	28.61	28.53	31.67	35.11	24.81	30.57
GALNT3	0.18	1.35	1.18	0.44	0.52	0.86
GALNT4	1.52	1.32	0.89	1.31	1.84	0.92
GALNT5	0.28	0	0	0	0	0
GALNT6	2.82	3.36	4.85	3.24	3.65	5.37
GALNT7	4.42	6.22	4.55	3.97	5.13	4.35
GALNT8	0	0	0	0	0	0
GALNT9	0	0	0	0.15	0.1	0
GALNTL5	0	0	0	0	0	0
GALNTL6	0	0.02	0	0	0	0
GALP	0	0	0	0	0	0
GALR1	0	0	0	0	0	0
GALR2	0.47	0	0.32	0.12	0.33	0.29
GALR3	0	0	0.12	0	0	0
GALT	8.54	8.81	12.68	8.19	5.62	10.53
GAMT	34.71	59.89	73.21	71.86	60.41	70.79

GAN	0.59	0.58	0.76	0.91	0.75	0.68
GANAB	128.3	142.87	131.71	139.86	124.09	140.78
GANC	1.58	3.04	1.66	1.22	1.72	2.49
GAP43	0	0	0	0	0	0
GAPDH	4631.25	4486.25	4745.28	4924.79	4388.44	4711.88
GAPDHS	0.8	0.67	0.69	0.31	0.47	0.2
GAPT	1.56	5.26	3.37	2.88	6.24	10.82
GAPVD1	16.6	11.39	9.66	11.36	12.55	10.79
GAR1	49.05	46.92	43.8	42.84	47.46	44.72
GAREM	0.02	0.04	0.08	0.1	0.01	0.13
GAREML	3.93	3.97	3.61	2.75	2.91	2.73
GARNL3	0	0.08	0.12	0.12	0.07	0
GARS	136.37	102.05	103.47	103.3	103.08	101.02
GART	58.16	55.17	52.54	57.27	52.06	56.73
GAS1	0	0.02	0.09	0	0	0
GAS2	0.07	0	0.03	0	0	0
GAS2L1	2.09	0.44	0.9	0.7	1.59	0.82
GAS2L2	0	0	0	0	0	0.02
GAS2L3	5.08	3.95	5.34	5.12	5.63	4.02
GAS5	333.25	262.17	180.28	284.18	307.75	262.65
GAS5-AS1	1.73	0.09	1.05	0.32	0.22	2.32
GAS6	0.77	0.07	0.18	0.49	0.44	0.27
GAS6-AS1	2.13	1.51	1.27	2.33	1.57	1.49
GAS7	0.16	0.87	1.12	0.23	0.37	0.31
GAS8	1.71	1.21	1.66	1.36	1.65	1.77
GAST	0	0	0	0	0	0
GATA1	251.69	3.95	4.78	102.05	154.78	8.56
GATA2	24.38	6.97	8.8	14.9	22.48	13.33
GATA3	0.31	0.43	0.54	0.67	0.54	0.48
GATA3-AS1	0	0	0	0	0	0
GATA4	0	0	0	0	0	0
GATA5	0.71	0	0	0.19	0.05	0.14
GATA6	0.15	0.55	0.07	0.08	0.4	0.27
GATAD1	2.59	2.37	1.91	1.65	1.86	2.48
GATAD2A	16.02	12.48	17.4	14.71	13.89	17.83
GATAD2B	6.8	6.07	5.95	5.7	5.98	7.04
GATC	10.43	9.41	11.8	11.02	9.87	10.78
GATM	0.08	1.89	2.38	0.18	3.93	2.12
GATM-AS1	3.11	5.54	5.13	3.31	4.59	4.21
GATS	0.04	0.18	0.11	0.1	0.12	0.03
GATSL1	0	0.36	0.57	0.21	0.18	0.04
GATSL2	1.01	0.36	0.57	0.21	0.18	0.04
GATSL3	2.81	1.45	2.36	1.68	3.13	3.26

GBA	33.14	29.27	22.46	25.53	26.3	27.5
GBA2	3.08	1.44	0.87	3.22	2.43	1.99
GBA3	0	0	0	0	0	0
GBAP1	0.33	0.82	1.44	0.39	0.8	0.7
GBAS	41.34	56.83	50.13	49.46	56.33	57.76
GBE1	15.94	13.93	10.18	10.43	11.22	14.34
GBF1	15.17	13.9	12.78	10.54	14.93	10.7
GBGT1	21.97	45.6	40.3	34.24	24.55	36.16
GBP1	0.1	0.07	0	0.1	0	0.01
GBP1P1	0	0	0	0	0	0
GBP2	7.76	0.29	0.36	2	1.38	0.13
GBP3	0.36	0.19	0.81	1.22	0.63	0.19
GBP4	10.83	1.63	1.43	5.7	5.14	1.67
GBP5	11.11	1.59	0.22	6	6.9	0.24
GBP6	0.28	0.46	0.44	0.21	0.26	0.42
GBP7	0.09	0	0.05	0.1	0.11	0
GBX1	0	0	0	0	0	0
GBX2	0.79	0.04	0	0.66	0.53	0.29
GC	0	0	0	0	0	0
GCA	5.46	7.25	6.34	4.25	5.4	7.44
GCAT	56.95	48.26	46.19	60.98	49.75	49.64
GCC1	4.05	4.74	5.95	6.43	3.42	4.91
GCC2	2.06	1.57	0.92	1.05	1.38	1.1
GCDH	22.88	29.49	21.46	25.91	21.84	19.13
GCFC2	6.58	6.1	7.06	6.2	7.08	6.26
GCG	0	0	0	0	0	0
GCGR	0	0	0	0	0	0
GCH1	0.56	0.82	0.26	0.23	0.61	0.82
GCHFR	37.21	26.75	35.77	57.16	58.92	22.43
GCK	0	0	0	0	0	0
GCKR	0	0	0	0	0	0
GCLC	2.99	2.75	2.35	1.49	2.9	1.94
GCLM	2.57	2.43	2.91	1.57	2.11	1.79
GCM1	0.11	0.03	0.06	0.07	0.04	0.08
GCM2	0	0.02	0	0.06	0	0
GCN1L1	25.38	23.29	27.39	26.06	21.6	27.09
GCNT1	2.1	4.23	5.34	3.1	3.28	4.63
GCNT2	0.8	1.54	1.62	1.45	0.93	1.51
GCNT3	0	0	0	0	0	0
GCNT4	0	0	0	0	0	0
GCNT7	0	0.08	0.13	0.1	0.07	0.11
GCOM1	0	0	0	0	0	0.02
GCSAM	0	0	0	0	0	0

GCSAML	19.06	4.34	3.15	6.47	9.51	2.15
GCSAML-AS1	0	0	0	0	0	0
GCSH	66.06	76.97	73.2	68.6	76.42	69.66
GCSHP3	2.93	2.82	1.36	2.04	1.58	2.79
GDA	0	0	0	0	0	0.03
GDAP1	3.41	2.3	1.57	1.86	2.24	0.79
GDAP1L1	0	0.1	0	0	0	0.03
GDAP2	6.67	6.16	3.73	3.15	4.44	3.51
GDE1	33.18	28.29	29.53	27.26	31.04	27.87
GDEP	0	0	0	0	0	0
GDF1	0	0.11	0.06	0.17	0.13	0.08
GDF10	0	0	0	0	0	0
GDF11	1.42	1.16	1.54	1.31	1.37	1.04
GDF15	121.11	0.25	0	17.6	15.02	0.38
GDF2	2.1	0	0	1	1.08	0
GDF3	0.31	0.05	1.19	1.8	0.47	1.69
GDF5	0	0	0	0	0	0
GDF6	0.02	0	0.05	0	0	0
GDF7	0	0	0.07	0.08	0	0
GDF9	0	0	0	0	0	0
GDI1	35.77	17.78	14.34	23.6	21.94	17.06
GDI2	173.48	184.01	208.3	202.11	195.57	202.82
GDNF	0.06	0.02	0.06	0.04	0.12	0.14
GDPD1	5.16	2.9	3.67	4.35	3.7	2.84
GDPD2	0	0.04	0.06	0	0.03	0
GDPD3	0.21	0	0.25	0	0	0
GDPD4	0	0	0	0	0	0
GDPD5	1.28	1.59	2.14	1.61	0.87	2
GDPGP1	1.62	3.65	4.8	2.92	2.27	2.82
GEM	0.04	0	0	0	0.17	0
GEMIN2	18.82	22.31	14.55	16.22	19.68	15.3
GEMIN4	22.62	29.87	29.18	28.58	27.3	19.48
GEMIN5	8.13	8.75	10.44	9.37	8.74	10.15
GEMIN6	38.59	46.56	42.69	35.5	42.39	43.7
GEMIN7	29.4	34.24	32.81	34.38	40.87	29.49
GEMIN8	9.58	11.83	9.35	10.12	11.27	10.17
GEMIN8P4	0.52	0.62	0.32	0.3	0.41	0.75
GEN1	1.89	1.68	1.33	2.09	2.07	2.61
GET4	17.98	20.26	20.27	21.08	19.11	14.83
GFAP	0.07	0	0	0	0	0
GFER	4.02	5.01	4.52	3.25	4.89	3.45
GFI1	3.39	4.82	5.81	3.61	2.71	4.66
GFI1B	107.51	0.56	5.15	44.2	54.77	5.74

GFM1	20.09	23.87	20.52	14.9	19.4	21.66
GFM2	14.65	13.09	13.76	9.42	15.66	14.2
GFOD1	3.84	2.26	2.85	2.32	2.93	2.61
GFOD2	9.41	11.29	9.68	8.59	7.91	8.37
GFPT1	2.75	2.1	2.08	1.42	1.67	2.04
GFPT2	0.07	0.08	0.4	0.63	0.34	0
GFRA1	0	0.27	5.51	3.99	2.71	0
GFRA2	0	0	0	0	0.05	0
GFRA3	0	0	0	0	0	0
GFRA4	0	0	0	0	0	0
GFRAL	0.2	0.28	0.07	0.22	0.23	0.16
GGA1	15.81	14.55	11.47	11.57	12.18	10.43
GGA2	6.54	7.17	8.23	7.62	7.64	7.59
GGA3	6.85	8.79	7.82	8.23	6.72	8.63
GGACT	1.55	0.94	0.49	0.64	1.42	0.79
GGCT	64.44	72	55.83	53.54	68.94	59.6
GGCX	1.64	2.35	2.09	1.47	1.18	2.14
GGH	115.55	126.85	109.63	94.96	137.52	119.1
GGN	0	0.24	0	0.21	0	0.03
GGNBP1	0	0	0	0	0	0
GGNBP2	22.38	21.48	22.25	17.97	24.72	15.69
GGPS1	8.41	10.92	7.75	8.52	9.91	9.06
GGT1	1.13	4.44	5.39	2.95	2.05	2.02
GGT3P	0	0.03	0.21	0	0	0
GGT5	0.96	3.97	4.89	1.47	2.51	2.77
GGT6	0.82	0.92	0.77	0.7	0.58	0.72
GGT7	0	0.07	0.36	0.02	0.08	0.03
GGT8P	0.03	0.18	0.37	0.44	0.28	0.39
GGTA1P	0	0.16	0	0.03	0	0
GGTLC1	0	0	0	0	0	0
GGTLC2	0	0	0	0	0	0
GH1	0	0	0	0	0	0
GH2	0	0	0	0	0	0
GHDC	3.45	2.82	4.98	3.4	4.68	4.93
GHITM	166.53	148.25	117.16	104.89	137.92	134.03
GHR	0.23	0	0.1	0.06	0.03	0.26
GHRH	0	0	0	0	0	0
GHRHR	0	0	0	0	0	0
GHRL	0.15	0.32	0.51	0.11	0	0.47
GHRLOS	0.28	0.81	0.46	0.41	0.12	0.64
GHSR	0	0	0	0	0.32	0
GID4	1.16	1.82	2.13	1.57	1.27	2.2
GID8	12.52	11.72	9.93	10.05	10.85	10.41

GIF	0	0	0	0.07	0	0
GIGYF1	2.28	2.4	1.88	2.4	2.41	2.36
GIGYF2	9.86	9.5	9.54	11.29	10.75	11.69
GIMAP1	0.04	0.33	0.26	0.05	0.27	0.1
GIMAP1-GIM	0	0	0.56	0.06	0.08	0.06
GIMAP2	2.81	8	6.62	8.86	6.99	11.95
GIMAP4	0	0	0	0	0	0
GIMAP5	2.84	3.3	2.77	0.87	1.01	1.54
GIMAP6	1.23	2.79	1.51	0.99	2.03	2.86
GIMAP7	0	0	0	0	0	0
GIMAP8	0.07	0	0.02	0	0.02	0
GIMD1	0	0	0	0	0	0
GIN1	1.32	0.67	1.18	1.02	1.08	0.81
GINM1	9.78	10.81	9.39	8.46	9	10.57
GINS1	14.49	19.18	14.68	13.76	17.72	22.57
GINS2	74.41	125.73	116.54	114.46	103.71	116.32
GINS3	12.53	15.15	9.67	11.92	9.43	14.27
GINS4	14.46	16.84	17.38	18.1	18.11	17.67
GIP	0	0	0	0	0	0
GIPC1	5.5	2.67	4.44	4.04	4.2	4.34
GIPC2	0	0	0.05	0	0	0
GIPC3	3.58	3.85	5.06	3.82	4.58	3.92
GIPR	0.58	0.14	0.13	0.2	0.48	0.11
GIT1	10.42	9.82	8.66	9.05	10.35	10.3
GIT2	14.43	19.25	15.59	14.24	13.26	17.33
GJA1	0	0.05	0.11	0	0	0.02
GJA10	0	0	0	0	0	0
GJA3	0.42	0.55	0.75	0.32	0.9	1.04
GJA4	0	0	0	0	0	0
GJA5	0	0	0	0	0	0
GJA8	0	0	0	0	0	0
GJA9	0	0	0	0	0	0.16
GJA9-MYCBF	0.17	0	0.2	0	0	0
GJB1	0	0	0	0	0	0
GJB2	0.66	0.5	0.06	0.5	2.44	0.16
GJB3	0	0	0	0	0	0
GJB4	0	0	0	0	0	0
GJB5	0	0	0	0	0	0
GJB6	0	0	0.22	0.18	0	0
GJB7	0.03	0.03	0.12	0	0.03	0
GJC1	1.68	2.7	3.33	2.14	2.98	3.59
GJC2	0	0	0	0	0	0
GJC3	0.14	0	0	0	0.26	0

GJD2	0	0	0	0	0	0
GJD3	0.05	0.06	0.11	0.07	0.2	0.05
GJD4	0	0.18	0.17	0.18	0.04	0
GK	6.7	14.03	8.26	8.66	8.22	11.09
GK2	0	0	0	0	0	0
GK3P	0	0.16	0.12	0	0	0.05
GK5	1.5	1.52	1.74	1.15	1.55	1.68
GKAP1	0.59	0.48	0.71	0.33	0.57	0.39
GKN1	0	0	0	0	0	0
GKN2	0	0	0	0	0	0
GLA	105.98	140.75	135.97	103.95	116.63	120.2
GLB1	43.43	39.7	38.86	36.01	42.77	49.79
GLB1L	0.14	0.89	1.01	1.21	0.83	0.87
GLB1L2	17.12	17.35	21.8	15.62	17.98	19.39
GLB1L3	0.22	0.4	0.17	0.21	0.22	0.22
GLCCI1	0.56	1.09	0.76	0.68	0.82	0.75
GLCE	4.46	2.58	3.82	3.74	2.97	4.13
GLDC	0	0.03	0	0	0	0
GLDN	0.01	0	0	0	0	0
GLE1	19.19	20.4	25.87	23.11	17.66	25.58
GLG1	8.78	6.32	5.87	8.18	7.75	5.86
GLI1	0	0.08	0	0.13	0	0.15
GLI2	0	0	0.01	0	0.03	0
GLI3	0	0.01	0	0	0	0.02
GLI4	1.57	2.12	2.47	1.86	1.01	1.87
GLIPR1	1.79	7.84	6.95	5.34	3.64	8.24
GLIPR1L1	0	0	0	0	0	0
GLIPR1L2	2.67	3.1	3.24	1.87	2.9	2.45
GLIPR2	2.71	4.56	4.79	7.42	7.76	6.49
GLIS1	0	0.04	0	0	0	0
GLIS2	0.84	0.79	0.66	0.9	1.48	1.17
GLIS3	0	0	0	0	0	0
GLIS3-AS1	0	0	0	0	0	0
GLMN	8.07	8.56	10.38	9.35	10.5	10.88
GLO1	88	59.4	60.6	66.57	73.68	68.58
GLOD4	46.5	48.83	40.68	39.52	51.26	32.54
GLOD5	3.12	0	0	0.82	2.66	0.22
GLP1R	0	0	0	0	0	0
GLP2R	0	0	0	0	0	0
GLRA1	0	0.03	0	0	0	0
GLRA2	0	0	0	0	0	0
GLRA3	0	0	0	0	0	0
GLRA4	0	0	0	0	0	0

GLRB	2.92	4.37	1.53	3	2.93	4.38
GLRX	11.2	24.34	25.08	16.22	19.55	21.65
GLRX2	34.49	34.99	27.01	21.62	27.93	24.55
GLRX3	117.82	96.38	79.1	95.97	100.8	79.55
GLRX5	6.95	9.37	7.85	8.61	6.58	6.17
GLS	1.73	1.05	0.74	0.76	1.5	1.5
GLS2	0.06	0.09	0.03	0.15	0.05	0.34
GLT1D1	0	0	0	0	0	0
GLT25D1	35.09	42	51.62	42.37	37.83	32.82
GLT25D2	0	0.01	0	0	0	0
GLT6D1	0	0	0	0	0	0
GLT8D1	7.78	13.39	9.62	6.79	11.16	7.15
GLT8D2	0.04	0	0	0.22	0.04	0
GLTP	12.98	12.07	13.01	11.72	13.81	11.72
GLTPD1	1.24	0.95	0.98	0.99	1.71	0.98
GLTPD2	0	0.1	0	0.04	0	0
GLTSCR1	0.6	0.43	0.72	0.59	0.73	0.32
GLTSCR1L	1.31	1.37	0.98	1.05	1.27	0.88
GLTSCR2	26.55	35.19	47.03	58.45	45.58	48.37
GLUD1	10.34	9.91	11.48	10.6	10.03	9.36
GLUD1P3	0.37	0.55	0.26	0.18	0.22	0.25
GLUD1P7	1.47	1.15	1.46	0.77	0.91	1.31
GLUD2	0.46	0	0	0.14	0	0.04
GLUL	112.56	36.34	32.48	51.45	113.2	36.6
GLYAT	0	0	0	0.05	0	0
GLYATL1	0	0	0	0	0	0
GLYATL2	0	0	0.15	0.72	0	0
GLYATL3	0	0	0	0	0	0
GLYCAM1	0	0	0	0	0	0
GLYCTK	5.81	4.36	5.22	4.75	7.4	6.08
GLYR1	15.24	18.63	15.3	12.73	15.15	15.44
GM140	0	0	0	0	0	0
GM2A	11.2	11.27	16.73	13.24	14.13	13.86
GMCL1	6.26	8.89	7.22	6.29	6.65	8.26
GMCL1P1	0.15	0.4	0.12	0.1	0.05	0.3
GMDS	13.83	28.22	29.05	28.25	25.19	29.4
GMEB1	8.85	9.93	8.66	9.25	10.24	9.26
GMEB2	2.23	3.4	2.58	2.69	3.3	3.84
GMFB	18.35	7.68	6.22	7.58	11.88	8.13
GMFG	88.09	259.5	228.42	232.32	191.76	244.72
GMIP	2.48	2.18	3.55	2.16	1.81	2.13
GML	0	0	0	0	0	0
GMNC	0	0	0	0	0	0

GMNN	103.93	135.71	123.84	118.96	121.18	100.67
GMPPA	27.13	29.15	26.25	26.82	22.73	29.51
GMPPB	4.6	8.11	8.33	11.22	11.93	5.62
GMPR	117.01	22.66	25.28	66.19	95.46	23.18
GMPR2	31.53	41.21	41.01	42.65	32.2	42.62
GMPS	73.67	78.22	73.88	77.85	72.09	85.31
GNA11	1.73	1.07	1.41	1.09	1.9	1.94
GNA12	2.97	1.96	2.26	2.61	2.16	2.57
GNA13	4.94	2.93	3.44	2.94	3.32	3.4
GNA14	0.06	0.09	0	0.02	0.03	0
GNA15	12.27	34.15	35.25	33.59	33.19	27.56
GNAI1	3.2	11.2	13.95	11.09	7.63	13.38
GNAI2	12.75	10.28	14.82	12.48	8.37	13.64
GNAI3	37.37	26.88	26.59	25.98	27.84	30.1
GNAL	0.33	0.31	0.54	0.21	0.23	0.32
GNAO1	0	0.02	0	0	0	0
GNAQ	3.76	2.72	1.96	2.01	3.1	3.26
GNAS	75.4	58.44	59.69	67.82	71.21	81.82
GNAS-AS1	0	0	0	0	0	0
GNAT1	0	0	0	0	0	0
GNAT2	0.44	0.09	0.35	0.11	0.16	0.06
GNAT3	0	0	0	0	0	0
GNAZ	0.75	0.07	0	0.22	0.39	0.02
GNB1	18.78	16.42	14.33	14.05	13.18	15.7
GNB1L	15.92	20.52	15.41	13.45	16.07	17.75
GNB2	9.85	12.88	11.27	10.62	17.4	9.55
GNB2L1	1823.24	1982.06	2266.44	2448.66	2216.52	2307.35
GNB3	0.04	0.64	0.35	0.24	0.29	0.47
GNB4	7.96	14.09	10.64	8.73	9.7	14.38
GNB5	7.01	10.86	8.81	8.7	7.36	10.96
GNE	5.1	4.62	4.51	3.44	4.57	4.62
GNG10	43.5	37.12	32.36	28.61	41.69	39.04
GNG11	17.1	2.29	9.63	17.14	2.07	2.13
GNG12	0.21	0	0	0	0.05	0
GNG12-AS1	0	0	0	0	0	0
GNG13	0	0	0	0.11	0	0.16
GNG2	5.89	0.47	0.2	1.88	1.53	0.08
GNG3	0	0.13	0.22	0	0.31	0.33
GNG4	1.37	5.07	1.35	1.78	1.11	1.64
GNG5	315.18	295.22	237.99	257.39	312.4	261.17
GNG7	1.74	3.14	5.05	3.96	2.56	3.45
GNG8	0.5	0	0	0	0	0
GNGT1	0	0	0	0	0	0

GNGT2	0.09	0.37	0.08	0.82	0	0.39
GNL1	4.36	2.58	2.54	3.54	4.74	2.87
GNL2	55.47	39.24	39.88	45.91	47.52	42.33
GNL3	55.93	70.6	55.85	55.48	64.95	63.72
GNL3L	19.41	20.27	19.05	20.03	18.82	24.23
GNLY	0	0	0	0	0	0
GNMT	0.07	0.28	0.46	0.05	0.07	0.36
GNN	0	0.04	0.01	0.03	0	0
GNPAT	23.72	29.53	29.53	29.8	31.02	28.83
GNPDA1	37.03	0.03	16.73	12.06	24.01	7.93
GNPDA2	5.18	3.28	4.96	2.87	4.06	3.73
GNPNAT1	14.89	16.48	12.37	12.7	15.46	16.77
GNPTAB	5.56	9.86	7.48	6.5	6.05	5.07
GNPTG	45.64	37.25	35.55	28.87	40.96	39.45
GNRH1	0.29	0.22	0.43	0.04	0.57	0.12
GNRH2	0	0.16	0	0.15	0	0
GNRHR	0.01	0.02	0	0	0	0
GNRHR2	0.64	0.7	0.85	0.68	0.67	0.69
GNS	20.6	11.49	13.24	9.53	14.57	13.67
GOLGA1	1.43	1.26	1.46	1.15	1.68	2.2
GOLGA2	6.04	3.83	4.04	2.69	4.58	3.41
GOLGA2P2Y	0.07	0	0	0.03	0	0
GOLGA2P3Y	0.07	0	0	0.03	0	0
GOLGA2P5	0.03	0.46	0.31	0.21	0.03	0.4
GOLGA3	5.3	3.46	3.97	3.03	3.32	3.71
GOLGA4	4.9	4.35	3.68	3.29	3.93	4.22
GOLGA5	9.47	6.46	7.23	5.88	6.9	5.91
GOLGA6A	0	0	0	0	0.06	0.01
GOLGA6B	0	0.02	0	0	0	0
GOLGA6C	0	0.04	0.01	0.01	0	0.03
GOLGA6D	0	0	0	0	0	0
GOLGA6L1	0.15	0	0.53	0	0	0
GOLGA6L10	0.21	0	0.2	0.02	0.04	0
GOLGA6L4	0.49	0.28	0.11	0.04	0.23	0.05
GOLGA6L5	0.12	0	0	0.02	0.03	0.21
GOLGA6L6	0.1	0.25	0.07	0.07	0	0.04
GOLGA6L7P	0.2	0.26	0	0	0.25	0.07
GOLGA6L9	0.35	0.37	0.11	0.04	0.28	0.54
GOLGA7	74.55	56.44	51.7	48.45	54.01	52.13
GOLGA7B	0	0	0	0	0	0.08
GOLGA8A	0.58	0.4	0.71	0.4	0.74	0.16
GOLGA8B	0.79	0.8	0.38	0.42	0.66	0.75
GOLGA8CP	0.02	0	0	0	0	0

GOLGA8DP	0	0.03	0.15	0.02	0.05	0.09
GOLGA8EP	0.07	0.36	0.15	0.12	0.08	0.39
GOLGA8F	0	0	0	0	0.01	0
GOLGA8G	0	0	0	0	0	0
GOLGA8I	0.15	0.98	0.87	0.17	0.67	0.89
GOLGA8S	0.16	2.16	1.82	0.73	0.97	2.15
GOLGA8T	0.85	4.75	3.74	1.57	2.14	3.43
GOLGB1	2.47	2.46	1.88	2.93	2.55	2.19
GOLIM4	14.67	8.59	9.47	10.47	10.75	12.31
GOLM1	8.04	16.23	18.1	11.12	13.59	15.61
GOLPH3	8.58	10.14	9.31	6.78	7.94	8.21
GOLPH3L	15.13	14.88	15.24	15.97	14.23	14.98
GOLT1A	0	0	0	0	0	0
GOLT1B	7.85	8.69	7.61	6.11	6.63	5.85
GON4L	5.18	4.18	4.27	3.87	4.31	4.3
GOPC	3.93	4.06	3.51	2.79	2.84	4.22
GORAB	0.94	1.81	1.63	1.13	2.73	2.06
GORASP1	12.66	10.49	10.82	8.82	11.89	11.36
GORASP2	52.5	43.59	47.2	45.33	48.75	47.85
GOSR1	11.79	12.05	12.36	11.17	11.85	13.31
GOSR2	14.02	15.48	15.06	18.39	17.83	16.07
GOT1	28.35	24.65	17.73	18.08	20.15	17.56
GOT1L1	0	0	0	0	0	0
GOT2	116.65	127.04	109.78	93.89	100.99	102.05
GP1BA	0.15	0.14	0.11	0.02	0.03	0.06
GP1BB	0	0	0.07	0.02	0	0
GP2	0.39	0.51	0.55	0.2	0.33	0.54
GP5	0	0.03	0.04	0	0.06	0
GP6	3.77	0.47	0.57	0.42	0.98	0.63
GP9	0	0	0	0	0	0
GPA33	0	0.19	0.17	0	0.07	0.69
GPAA1	111.32	137.25	122.21	156.73	130.09	129.79
GPAM	3.33	3.52	2.76	2.12	2.59	2.15
GPANK1	10.7	9.07	10.32	10.44	9.76	9.94
GPAT2	0.08	0.14	0.14	0.11	0.07	0.13
GPATCH1	3.91	4.97	3.82	3.61	4.24	3.22
GPATCH11	6.32	6.08	6.34	4.65	5.98	5.11
GPATCH2	3.21	2.75	3.97	2.31	3.24	3.72
GPATCH2L	7.08	6.49	9.08	5.34	5.76	7.65
GPATCH3	11.25	13.2	11.66	12.48	9.38	7.43
GPATCH4	32.27	42.41	37.21	33.03	40.67	37.62
GPATCH8	6.68	6.19	5.07	6.55	5.96	5.55
GPBAR1	0	0.18	0	0.14	0.07	0

GPBP1	13.93	12.8	11.2	12.64	12.09	11.26
GPBP1L1	8.05	6.35	7	6.17	6.38	9.72
GPC1	1.65	1.25	1.82	1.79	1.54	2.67
GPC2	0.17	0.11	0.03	0.12	0	0.29
GPC3	0	0	0	0	0	0
GPC4	0	0	0	0	0	0
GPC5	0	0	0.02	0	0	0
GPC5-AS1	0	0	0	0	0	0
GPC6	0.34	1.28	1.09	1.64	1.21	1.57
GPC6-AS2	0	0	0	0	0	0
GPCPD1	1.9	2.13	2.48	1.67	1.85	2.06
GPD1	0	0.02	0	0	0	0
GPD1L	6.88	5.02	6.53	4.79	4.84	7.02
GPD2	5.05	3.72	3.21	3.33	3.79	4.37
GPER	0	0	0	0	0.05	0.03
GPHA2	0	0	0	0	0	0
GPHB5	0	0	0	0	0	0
GPHN	2.93	5.12	5.94	4	4.02	4.53
GPI	167.8	184.37	198.15	203.05	175.44	207.99
GPIHBP1	0	0	0	0	0	0
GPKOW	26.08	28.13	24.77	27.15	40.26	27.25
GPLD1	0.62	0.5	0.78	1.31	0.88	0.88
GPM6A	0	0	0	0	0	0
GPM6B	9.68	14.24	16.28	11.85	13.43	12.56
GPN1	51.79	50.45	44.75	48.44	45.56	48.71
GPN2	27.57	32.44	34.12	33.35	33.93	32.84
GPN3	25.39	30.22	25.37	25.02	29.25	25.27
GPNMB	0.16	0.13	0	0	0	0.03
GPR1	0.13	0.23	0.27	0.12	0.19	0.33
GPR101	0	0	0	0	0	0
GPR107	2.26	2.16	1.9	1.89	2.36	2.72
GPR108	20.93	18.42	15.19	15.73	17.85	13.59
GPR110	0.02	0.04	0.05	0.15	0.05	0.11
GPR111	0.04	0	0	0	0	0
GPR112	0.01	0	0	0	0	0.01
GPR113	0.05	0.07	0.16	0.17	0.12	0.13
GPR114	0.66	11.05	7.98	4.56	3.74	9.06
GPR115	0	0	0	0	0	0
GPR116	0	0	0	0	0	0
GPR119	0	0	0	0	0	0
GPR12	0.14	0.11	0.27	0.06	0.22	0.14
GPR123	0	0	0	0	0	0
GPR124	1.24	3.42	3.55	2.24	2.31	3.42

GPR125	1.2	1.28	1.04	1.12	0.73	0.88
GPR126	0	0	0	0	0	0
GPR128	0.16	0	0	0	0	0
GPR132	0.07	0.08	0.11	0.06	0.03	0.1
GPR133	0.13	0	0	0	0	0
GPR135	0.12	0	0.04	0.17	0.15	0.21
GPR137	1.75	1.52	2.26	1.88	1.92	1.37
GPR137B	0.9	0.23	0.33	0.72	0.65	0.29
GPR137C	0.45	0	0	0.01	0.04	0
GPR139	0	0	0	0	0	0
GPR141	0	0	0.08	0	0.16	0.17
GPR142	0	0	0	0	0.14	0
GPR143	0	0.03	0.6	0.06	0	0.04
GPR144	0	0	0	0	0	0
GPR146	0.85	8.63	2.01	1.69	2	1.37
GPR148	0	0	0	0	0	0
GPR149	0	0	0	0	0	0
GPR15	0.48	0.24	0.06	0.29	0.46	0.12
GPR150	0	0	0	0	0	0
GPR151	0	0	0.38	0	0	0
GPR152	0	0	0	0.04	0	0
GPR153	0.05	0.13	0.21	0.27	0.25	0.31
GPR155	1.53	1.5	1.59	1.31	1.48	1.62
GPR156	0	0	0.02	0	0	0
GPR157	0.33	1.61	1.57	0.36	0.94	0.93
GPR158	0	0	0	0	0	0
GPR158-AS1	0	0	0	0	0	0
GPR160	1.87	2.86	1.81	1.64	2.53	1.63
GPR161	1.04	1.29	1.57	1.05	1.31	1.66
GPR162	1.34	1.75	1.86	2.33	1.14	1.05
GPR17	0	0	0	0	0	0
GPR171	0.08	0.26	0.45	0.4	0.66	0.04
GPR173	0.06	0.04	0.07	0.02	0.03	0.01
GPR174	0.42	0.62	0.28	0.12	0.17	0.36
GPR176	1.62	0.42	0.16	0.23	0.27	0.23
GPR179	0.04	0.05	0.08	0.01	0.01	0.02
GPR18	0	0.12	0	0.25	0.19	0.15
GPR180	1.44	1.92	2.23	1.14	2.09	1.43
GPR182	0	0	0	0	0	0
GPR183	0.42	0.13	0.08	0.11	0.47	0
GPR19	0	0.1	0.12	0.56	0.48	0.13
GPR20	0	0	0	0	0	0
GPR21	0	0.15	0.17	0	0	0

GPR22	0.2	0.1	0.27	0	0.14	0.1
GPR25	0	0	0	0	0	0
GPR26	0	0	0	0	0	0
GPR27	0	0	0	0	0	0
GPR3	3.07	2.44	3.16	2.28	3.79	2.15
GPR31	0	0	0	0	0	0
GPR32	0	0	0	0	0	0.06
GPR33	0	0	0	0	0	0
GPR34	0.24	0	0.22	0	0	0
GPR35	0.14	0.29	0.63	0.22	0	0
GPR37	0	0	0	0	0	0
GPR37L1	0.34	0.34	0.39	0.47	0.5	0.43
GPR39	0	0	0	0	0	0
GPR4	0.02	0.1	0.02	0.04	0.05	0
GPR45	0	0	0	0	0	0
GPR50	0	0	0	0	0	0
GPR52	0	0.04	0	0.18	0	0
GPR55	0.32	0.01	0	0.16	0.02	0.04
GPR56	0.29	0.97	0.97	0.55	0.64	0.42
GPR6	0	0	0	0	0	0
GPR61	0.03	0	0	0	0	0
GPR62	0	0	0	0	0.03	0
GPR63	1.67	2.14	1.4	1.22	1.17	1.7
GPR64	0	0	0	0	0	0.03
GPR65	0.41	0.22	0.25	0.07	0.06	0.17
GPR68	0.23	0	0	0.04	0	0.03
GPR75	0.78	0.67	1.03	0.14	1	1.28
GPR75-ASB3	2.84	2.74	5.31	5.03	3.7	1.98
GPR78	0.34	0.15	0.23	0.18	0.08	0.34
GPR82	1.37	1.66	2.16	1.5	1.91	1.27
GPR83	0.04	0.07	0.16	0.05	0.24	0.06
GPR84	0.05	0	0.36	0.07	0.09	0.85
GPR85	1.53	3.1	3.1	2.34	3.37	5.7
GPR87	0	0	0.05	0.14	0	0
GPR88	0	0	0	0	0	0
GPR89A	14.13	18.64	13.03	14.03	15.36	12.74
GPR89B	6.26	5.06	7.6	7.84	6.39	3.88
GPR89C	0	0	0	0	0	0
GPR97	0.05	0.63	0.33	0.17	0.08	0.17
GPR98	0.02	0	0	0	0	0
GPRASP1	0	0.32	0.16	0.03	0.12	0.19
GPRASP2	2.07	2.42	3.11	2.78	2.58	3.59
GPRC5A	0	0	0	0	0	0

GPRC5B	6.16	8.24	6.23	5.61	7.53	7.71
GPRC5C	14.47	0.9	0.6	4.27	8.86	0.95
GPRC5D	0	0	0	0	0	0
GPRC6A	0	0	0	0	0	0
GPRIN1	0.5	0.51	1.26	0.71	0.66	0.44
GPRIN2	0	0	0	0	0	0
GPRIN3	0	0.05	0.06	0	0	0
GPS1	80.42	91.94	88.91	80.4	74.55	74.08
GPS2	26.58	36.01	33.96	36.47	26.96	28.59
GPSM1	0.65	0.42	0.73	1.48	1.1	2.25
GPSM2	0.9	2.33	2.2	2.42	2.96	2.38
GPSM3	4.95	8.09	6.93	9.77	5.72	11.77
GPT	0	0	0	0	0	0.04
GPT2	2.05	3.64	3.6	3.32	3.23	2.74
GPX1	609.51	878.7	934.87	873.4	680.77	830.59
GPX2	0.28	0.28	0	0.05	0	0.28
GPX3	0.38	0.23	0.19	0.23	0	0.34
GPX4	478.42	411.36	392.81	427.33	478.29	372.21
GPX5	0	0	0	0	0	0
GPX6	0	0	0	0	0	0
GPX7	35.59	68.15	76.42	65.7	61.49	74.37
GPX8	0.99	0.26	0.22	0.4	0.21	1.35
GRAMD1A	8.09	6.15	4.53	7.26	5.18	4.41
GRAMD1B	0	0.29	0.34	0.13	0.27	0.51
GRAMD1C	0.02	0	0.02	0	0.02	0
GRAMD2	0	0	0.02	0	0	0
GRAMD3	0.05	1.13	1.47	1.49	1.15	1.66
GRAMD4	0.65	1.03	0.54	0.61	0.6	1.26
GRAP	1.82	6.55	5.98	4.9	5.65	4.56
GRAP2	14.89	0	0	4.65	5.92	0.29
GRAPL	0	0	0	0	0.08	0
GRASP	0	0	0	0.06	0	0
GRB10	4.27	3.36	2.63	2.7	2.79	4.56
GRB14	0	0	0	0	0	0
GRB2	79.96	88.98	79.72	70.75	79.82	70.39
GRB7	0	0	0	0	0	0
GREB1	0.55	0.89	1.11	0.7	1.52	0.99
GREB1L	0.02	0	0.03	0	0	0
GREM1	0	0	0	0	0	0
GREM2	0	0	0	0	0	0
GRHL1	0.47	0.57	0.35	0.54	0.74	0.31
GRHL2	0	0	0	0	0	0
GRHL3	0	0	0	0	0	0

GRHPR	99.6	82.3	79.49	87.76	82.66	88.44
GRIA1	0	0	0	0	0	0
GRIA2	0	0	0	0	0	0
GRIA3	0	0	0	0	0	0
GRIA4	0	0	0	0	0	0
GRID1	0	0	0.06	0	0	0
GRID1-AS1	0	0	0	0	0	0
GRID2	0	0	0	0	0	0
GRID2IP	0	0	0	0	0	0
GRIK1	0.07	0	0.04	0	0	0
GRIK1-AS1	0	0	0	0	0	0
GRIK1-AS2	0	0.06	0	0.05	0	0
GRIK2	0	0	0	0	0	0
GRIK3	0	0	0	0	0	0
GRIK4	0	0	0	0	0	0
GRIK5	1.29	3.33	2.7	2.39	1.67	1.93
GRIN1	0	0	0	0	0	0
GRIN2A	0.05	0	0	0	0	0
GRIN2B	0	0	0	0	0	0
GRIN2C	0	0	0.02	0	0.02	0
GRIN2D	0.61	1.44	0.95	0.37	1.03	0.83
GRIN3A	0	0	0	0.01	0.02	0
GRIN3B	0	0	0	0	0.15	0
GRINA	34.11	10.92	14.4	15.32	19.99	12.06
GRIP1	0.09	0.03	0.01	0.02	0.04	0.23
GRIP2	0	0.04	0.01	0	0	0
GRIPAP1	19.94	14.51	15.72	14.45	13.78	14.03
GRK1	0	0	0	0	0	0
GRK4	0.03	0.19	0.18	0.05	0.13	0.1
GRK5	0	0.23	0.73	0	0.24	0.17
GRK6	4	5.64	3.69	4.64	4.31	4.1
GRK7	0	0	0.04	0.09	0	0
GRM1	0	0	0	0	0	0
GRM2	0	0	0	0.01	0.07	0
GRM3	0	0	0	0	0	0
GRM4	0	0	0.13	0	0.02	0
GRM5	0	0	0	0	0	0
GRM5-AS1	0	0	0	0	0	0
GRM6	0.31	0.32	0.26	0.13	0.29	0.37
GRM7	0	0	0	0	0	0
GRM8	0.12	0.04	0.06	0	0.08	0.06
GRN	169.04	94.9	102.28	137.58	134.88	97.84
GRP	0	0	0	0	0	0

GRPEL1	77.18	66.63	59.42	69.38	74.03	64.54
GRPEL2	6.82	4.18	5.05	4.66	5.42	6.19
GRPR	0.22	0.06	0.25	0	0	0
GRSF1	19.72	22.49	22.56	22.07	23.77	13.52
GRTP1	0.43	0.21	0.1	0.81	0.35	0
GRWD1	64.15	70.88	79.75	79.85	65.72	69.15
GRXCR1	0	0	0	0	0	0
GRXCR2	0	0	0	0	0	0
GSC	0	0	0	0	0	0
GSC2	0	0	0	0	0	0
GSDMA	1.44	0.91	0.91	0.74	0.93	1.01
GSDMB	0	0.19	0	0.2	0.17	0.15
GSDMC	0.11	0.31	0.07	0.11	0.24	0.51
GSDMD	65.95	115.77	124.27	108.76	95.62	106.94
GSE1	6.55	4.55	4.1	4.88	5.56	4.49
GSG1	0.66	1.01	1.04	0.47	0.74	0.83
GSG1L	0	0	0	0.02	0	0
GSG2	12.12	17.29	12.39	12.63	12.98	17.77
GSK3A	2.33	1.33	1.55	2	1.31	1.98
GSK3B	1.76	1.79	1.81	1.45	2.3	2.19
GSKIP	3.38	4.03	3.36	2.63	3.1	4.31
GSN	11.61	9.91	10.72	6.41	4.57	6.45
GSPT1	10.79	9.22	11.05	9.38	10.22	8.98
GSPT2	0	0	0.01	0	0	0
GSR	27.39	13.29	15.16	15.96	18.87	11.88
GSS	99.38	81.36	71.22	86.22	84.32	69.8
GSTA1	0	0	0	0	0	0
GSTA2	0	0	0	0	0	0
GSTA3	0	0	0	0	0	0
GSTA4	0.96	0.18	0.57	1.12	0.96	0.57
GSTA5	0	0	0	0	0	0
GSTA7P	0	0	0	0	0	0
GSTCD	3.78	4.68	4.3	3.16	3.92	4
GSTK1	84.22	99.94	93.6	91.04	88.64	89.7
GSTM1	0	0	0	0	0	0
GSTM2	0.13	0.68	0.05	1.1	0.37	0.86
GSTM2P1	0	0	0	0	0	0
GSTM3	1.13	1.15	1.49	1	1.12	0.81
GSTM4	19.19	22	32.09	26.89	23.4	29.27
GSTM5	0	0	0	0	0	0
GSTO1	345.81	198.12	189.09	230.93	261.67	182.91
GSTO2	5.46	0.72	1.53	0.22	1.89	0.37
GSTP1	571.4	622.84	642.85	848.12	777.52	696.67

GSTT1	0	0	0	0	0	0
GSTT2	0	0	0	0	0	0
GSTT2B	0	0	0	0	0	0
GSTTP1	0	0	0	0	0	0
GSTTP2	0.72	0.58	0.5	0.66	0.6	0.76
GSTZ1	28.7	28.67	30.3	28.62	28.97	31.22
GSX1	0	0	0	0	0	0
GSX2	0	0	0	0	0	0
GTDC1	3.5	1.7	1.74	2.33	2.22	2.81
GTDC2	8.02	9.95	9.65	10.33	9.22	7.94
GTF2A1	3.93	3.28	2.75	2.75	2.94	3.09
GTF2A1L	0	0	0	0	0	0
GTF2A2	81.04	49.52	36.11	40.37	63.38	38.62
GTF2B	28.55	22.71	21.19	23.85	27.71	23.96
GTF2E1	12.1	13.92	13.08	12.05	11.9	14.3
GTF2E2	19.14	20.99	19.48	13.05	18.27	18.81
GTF2F1	47.26	39.77	41.67	41.63	39.66	32.25
GTF2F2	47.67	40.84	36.53	31.12	42.38	29.81
GTF2H1	17.36	14.65	15.55	15.35	17.08	16.15
GTF2H2	7.45	11.67	8.92	6.87	8.69	11.15
GTF2H2B	2.85	3.48	4.77	2.75	3.51	3.66
GTF2H2C	14.43	7.33	11.98	8.4	8.52	15.57
GTF2H2D	16.33	20.55	13.04	15.31	19.22	15.07
GTF2H3	16.28	12.14	11.39	8.31	11.14	11.09
GTF2H4	10.01	8.68	5.04	7.79	8.15	9.29
GTF2H5	3.93	3.24	2.7	3.55	4.54	3.07
GTF2I	19.19	27.17	26.87	24.49	26.97	23.44
GTF2IP1	6.58	1.16	1.79	1.42	1.9	2.33
GTF2IRD1	1.02	1.52	1.31	0.94	1.3	0.86
GTF2IRD1P1	0	0.04	0	0	0.05	0.05
GTF2IRD2	0	0.32	0.68	0.27	0.49	0.11
GTF2IRD2B	0.58	0.51	0.7	0.26	0.59	0.79
GTF2IRD2P1	0	0.2	0	0	0.05	0.07
GTF3A	65.97	55.42	58.27	70.02	57.76	57.22
GTF3C1	11.31	12.17	10.91	12.55	9.89	13.01
GTF3C2	26.93	25.78	25.57	24.46	26.42	24.64
GTF3C3	12.7	12.57	11.58	11.17	10.76	11.63
GTF3C4	2.93	3.37	3.35	1.71	2.26	2.68
GTF3C5	56.03	48.94	50.05	67.07	62.48	58.99
GTF3C6	135.38	120.77	104.34	113.91	134.93	109.09
GTPBP1	16.58	12.48	13.64	15.77	15.13	16.1
GTPBP10	3.32	4.29	3.52	2.86	3.54	4.01
GTPBP2	4.01	1.93	0.78	1.43	2.61	0.99

GTPBP3	9.3	12.41	10.36	12.58	10.93	12.52
GTPBP4	72.07	77.76	64.65	63.65	71.79	58.22
GTPBP5	7.82	6.15	6.15	7.1	6	6.94
GTPBP6	8.38	5.76	6.96	7.82	6.65	8.79
GTPBP8	14.79	8.4	7.93	7.77	11.47	7.68
GTSE1	12.36	12.93	11.76	9.48	13.47	11.75
GTSF1	190.68	194.07	172.37	182.08	204.5	183.71
GTSF1L	0	0	0	0	0	0
GUCA1A	0	0	0	0	0	0
GUCA1B	0.44	0.96	0.98	0.46	0.72	0.64
GUCA1C	0	0	0	0	0	0
GUCA2A	0	0	0	0	0	0
GUCA2B	0	0	0	0	0	0
GUCD1	16.87	23.4	22	20.73	19.92	17.64
GUCY1A2	0.15	0	0.01	0.01	0.01	0
GUCY1A3	0.87	6.97	5.54	3.6	2.74	6.46
GUCY1B2	0	0	0	0	0	0
GUCY1B3	1.3	7.07	5.45	3.52	3.17	4.83
GUCY2C	0	0	0	0	0	0
GUCY2D	0	0	0	0	0	0
GUCY2EP	0.18	0	0	0	0	0
GUCY2F	0	0	0	0	0	0
GUCY2GP	0	0	0	0	0	0
GUF1	9.75	8.13	7.52	8.68	7.53	10.4
GUK1	186.79	182.97	200.99	218.64	206.92	148.39
GULP1	0	0	0	0	0	0
GUSB	25.2	27.83	23.72	25.13	29.07	24.17
GUSBP1	8.96	9.22	6.89	8.56	9.87	9.43
GUSBP10	0	0	0	0	0	0
GUSBP11	1.49	1.91	1.83	2.09	1.16	1.64
GUSBP2	0.58	2.04	1.29	1.15	1.31	1.18
GUSBP3	1.71	2.56	3.23	2.1	3.18	2.25
GUSBP4	0.79	1.29	1.47	1.81	1.15	1.73
GUSBP5	0	0	0	0.08	0	0
GUSBP9	0	0.34	0	0.69	0	0
GVINP1	0.11	0.02	0.16	0.02	0.04	0.07
GXYLT1	0.53	0.64	0.64	0.53	0.58	0.36
GXYLT2	0.24	0.5	0.84	0.5	0.92	0.49
GYG1	53.85	37.32	42.69	43.97	46.6	40.58
GYG2	5.64	6.19	0	4.47	5.38	8.3
GYG2P1	0	0	0.08	0.24	0.19	0.1
GYLTL1B	0	0	0	0	0	0
GYPA	0	0	0	0	0.1	0

GYPB	0	0.25	0	1.1	1.05	0
GYPC	0	0	0	0	0	0
GYPE	0.13	0	0.08	0.12	0	0
GYS1	10.41	12.99	16.82	12.39	9.81	12.86
GYS2	0	0	0	0	0	0
GZF1	2.15	2.29	2.77	1.91	2.67	1.85
GZMA	0	0	0	0	0	0
GZMB	0	0	0	0	0	0
GZMH	0	0	0	0	0	0
GZMK	0	0	0	0	0	0
GZMM	0.08	0	0	0	0	0.08
H19	0	0.33	0.09	0.11	0.18	0.1
H1F0	0	0	0	0	0	0
H1FNT	0	0	0	0	0	0
H1FOO	0	0	0	0	0	0
H1FX	4.07	2.39	3.27	5.75	5.35	3.87
H1FX-AS1	0.17	0.05	0.23	0.23	0.07	0
H2AFB1	0	0.13	0	0	0	0
H2AFB2	0	0	0	0	0	0
H2AFB3	0	0.13	0	0	0	0
H2AFJ	7.2	0	0	1.29	1.96	0
H2AFV	73.93	79.35	76.02	76.37	89.34	79.95
H2AFX	50.52	67.18	89.22	78.14	70.33	75.05
H2AFY	39.75	60.83	64.96	53.07	60.01	54.51
H2AFY2	0	0	0	0	0	0
H2AFZ	954.26	1100.5	1016.91	1046.66	1155.05	1050.44
H2BFM	0	0	0	0	0	0
H2BFWT	0	0	0	0	0	0
H2BFXP	0	0	0	0	0	0
H3F3A	362.54	304.8	303.51	402.39	354.53	330.77
H3F3AP4	304.23	275.54	192.64	199.61	341.29	268.84
H3F3B	187.66	222.49	177.28	140.52	201.74	181.88
H3F3C	0.54	0.63	0.35	0.13	0.42	0.42
H6PD	2.23	1.3	1.7	1.14	1.69	1.18
HAAO	8.18	20.38	16.54	14.86	15.99	16.33
HABP2	0	0	0	0	0	0
HABP4	0.79	0.41	0.52	0.39	0.87	0.5
HACE1	0.96	1.81	0.71	0.36	0.62	0.99
HACL1	17.54	17.37	14.76	19.34	18.28	17.64
HADH	42.03	69.45	62.16	59.5	57.05	68.35
HADHA	94.16	85.82	82.69	76.84	86.96	88.49
HADHB	88.29	62.32	62.12	52.15	57.5	56.55
HAGH	16.21	11.94	8.73	12.09	15.07	9.17

HAGHL	9.14	7.56	7.96	6.72	10.65	8.22
HAL	0	0.06	0	0	0	0
HAMP	0	0	0	0	0	0
HAND1	0	0	0	0	0	0
HAND2	0	0	0	0	0	0
HAO1	0	0	0	0	0	0
HAO2	0	0	0	0	0	0
HAP1	0.45	0	0.07	0.34	0.35	0.04
HAPLN1	0	0	0	0	0	0
HAPLN2	0	0	0	0	0	0
HAPLN3	12.13	23.58	24.13	28.17	27.19	32.76
HAPLN4	0	0	0.12	0.13	0.06	0.02
HAR1A	0	0.02	0	0.05	0	0.19
HAR1B	0	0	0.08	0.12	0	0.09
HARBI1	3.79	3.29	3.11	5.76	2.83	2.93
HARS	64.33	42.37	44.12	43.44	46.74	36.61
HARS2	12.55	17.26	13.07	12.88	12.96	20.7
HAS1	0	0	0	0	0	0
HAS2	0	0.07	0	0	0	0
HAS2-AS1	0	0.05	0	0	0	0
HAS3	0	0.42	0.36	0.45	0.43	0.39
HAT1	67.1	68.12	51.85	55.18	60.24	57.55
HAUS1	44.03	61.66	59.4	53.34	52.15	56.08
HAUS2	6.63	6.77	5.86	4.9	5.3	6.76
HAUS3	3.13	3.24	2.35	2.81	3.06	3.54
HAUS4	33.69	55.86	57.31	53.03	39.98	54.7
HAUS5	9.19	12.09	10.06	10.86	8.82	10.03
HAUS6	8.91	6.94	6.25	4.59	5.86	6.98
HAUS7	52.52	86.29	83.31	82.75	70.29	76.65
HAUS8	20.04	17.18	15.8	12.93	19.19	17.87
HAVCR1	0	0	0	0	0.04	0.16
HAVCR1P1	0	0	0.22	0	0.29	0
HAVCR2	0.78	0.53	0.46	0.44	0.27	0.49
HAX1	134.52	118.25	128.53	117.97	138.62	105.27
HBA1	93.42	0.79	0.91	33.19	15.5	1.86
HBA2	12.76	0	0	7.07	1.75	0
HBB	45.11	0	0	0.9	4.8	0
HBBP1	37.34	0.1	0	3.3	1.51	0
HBD	750.7	0.56	7.65	192.85	226.29	1.65
HBE1	1793.46	0.38	4.84	159.77	67.79	0.39
HBEGF	1.56	3.14	2.51	2.27	3.06	2.58
HBG1	634.41	0.39	5.89	136.38	82.66	0
HBG2	990.43	0.39	23.88	513.73	357.74	4.39

HBM	0	0	0	0.11	0	0
HBP1	12.61	6.6	6.73	7.2	9.96	9.18
HBQ1	44.78	47.78	53.7	59.24	52.67	57.1
HBS1L	20.41	16.3	14.67	14.51	17.65	15.8
HBZ	3.33	0.11	0.13	1.16	0.79	0
HCAR1	0.6	0.65	0.48	0.24	0.49	0.68
HCAR2	0	0	0	0	0	0
HCAR3	0	0	0	0	0	0
HCCS	41.47	28.26	28.27	29.88	33.53	29.49
HCFC1	41.32	47.48	46.13	46.16	42.85	52.17
HCFC1R1	14.29	15.21	19.29	19.6	16.42	20.41
HCFC2	0.92	0.89	1.07	0.23	0.46	0.72
HCG11	1.64	1.79	1.42	1.47	2.35	1.38
HCG17	0	0	0.2	0	0.1	0.44
HCG18	3.37	2.97	2.52	2.57	2.22	2.18
HCG22	0	0	0	0.02	0	0
HCG23	0	0	0	0	0	0
HCG25	0.04	0.2	0	0.24	0.43	0.35
HCG26	0	0	0	0	0	0
HCG27	0.09	0	0.16	0.03	0	0
HCG4	0.17	0.25	0.2	0.5	0	0.19
HCG4B	0.07	0.17	0.33	0	0.14	0
HCG9	0.66	0.31	0.34	0.17	0	0
HCK	2.6	12.16	12.81	9.54	7.17	9.56
HCLS1	70.46	76.02	81.31	75.39	70.26	75.39
HCN1	0	0.01	0	0	0.02	0.03
HCN2	0	0	0.02	0	0	0
HCN3	0.48	1.16	0.34	0.36	0.31	0.89
HCN4	0	0	0	0	0	0
HCP5	0	0	0	0.04	0	0
HCRT	0	0.11	0	0	0.14	0.57
HCRTR1	0	0	0	0	0.07	0.04
HCRTR2	0	0	0	0	0	0
HCST	5.65	17.44	9.07	6.68	7.72	7.67
HDAC1	97.24	103.96	84.04	86.89	92.01	98.38
HDAC10	1.98	1.49	1.67	1.67	1.55	3.03
HDAC11	2.03	0.82	1.81	1.31	0.85	1.19
HDAC2	26.74	25.81	22.07	23.04	27.3	28.57
HDAC3	40.2	49.59	41.25	48.59	43.42	45.82
HDAC4	0.63	0.62	0.48	0.77	0.51	0.82
HDAC5	6.82	4.38	6.72	7.06	6.63	6.34
HDAC6	11.98	11.26	11.24	14.87	12.17	10.1
HDAC7	3.48	3.66	4.64	2.87	3.77	3.34

HDAC8	31.94	35.59	32.15	33.88	34.53	33.87
HDAC9	0.95	0	0	0	0	0
HDC	1.52	0.24	0.53	0.82	5.38	1.82
HDDC2	40.95	38.01	36.19	40.19	40.38	39.01
HDDC3	20.77	22.64	22.64	17.39	24.89	14.31
HDGF	33.04	29.11	28.92	25.32	27.26	30.69
HDGFL1	0	0	0	0	0	0
HDGFRP2	1.72	1.68	1.34	1.62	2.19	1.26
HDGFRP3	6.81	6.95	7.15	5.37	6.46	6.07
HDHD1	18.87	20.94	19.46	18.03	17.57	19.89
HDHD2	14.49	16.22	11.91	12.64	13.87	13.64
HDHD3	6.66	13.79	13.45	11.93	13.64	13.85
HDLBP	51.43	44.54	58.44	50.96	46.92	50.59
HDX	0	0	0	0	0	0
HEATR1	7.2	7.9	7.56	7.37	6.76	8.99
HEATR2	7.3	4.69	5.6	5.6	5.45	5.26
HEATR3	0.95	4.29	4.63	4.91	3.34	4.29
HEATR4	0	0	0	0	0	0
HEATR5A	1.48	1.32	1.12	0.71	1.21	1.32
HEATR5B	3.44	3.63	2.98	3.11	4.23	3.11
HEATR6	4.58	3.54	3.67	3.46	5.28	4.58
HEATR8-TTC	0.03	0.11	0	0	0.03	0.02
HEBP1	27.08	33.24	31.63	30.74	26.46	39.56
HEBP2	9.55	8.55	9.34	8.74	11.77	7.09
HECA	0.74	0.86	0.67	0.77	0.82	0.91
HECTD1	5	4.28	4.62	3.72	4.73	5.2
HECTD2	0	0.1	0	0.15	0	0.04
HECTD3	11.17	10.39	10	9.55	10.75	6.23
HECTD4	2.07	1.56	1.02	1	1	1.6
HECW1	0	0	0	0	0	0
HECW2	0	0.01	0	0	0	0
HEG1	0.13	0.28	0.17	0.23	0.18	0.22
HEIH	0	0	0	0	0	0
HELB	1.63	1.46	1.75	1.02	0.58	2.64
HELLS	6.89	11.62	10	9.74	9.38	12.16
HELQ	4.65	2.48	1.37	2.08	2.32	1.96
HELT	0	0	0	0	0	0
HELZ	8.98	7.07	6.49	5.78	6.48	7.11
HELZ2	1.02	0.09	0.27	0.55	0.29	0.28
HEMGN	4.03	0	0.09	1.81	1.39	0
HEMK1	2.86	2.88	2.84	2.6	2.64	3.7
HENMT1	0	0	0	0	0	0
HEPACAM	0.04	0	0	0	0	0

HEPACAM2	0.04	0	0	0	0.03	0
HEPH	0.54	0	0	0.09	0.25	0.09
HEPHL1	0.02	0	0	0	0	0
HEPN1	0	0	0	0	0	0
HERC1	8.48	4.22	3.24	4.16	5.55	4.6
HERC2	4.73	3.06	3.8	3.17	3.28	4.32
HERC2P10	0.97	1.33	1.05	1.21	0.96	0.39
HERC2P2	3.79	0.8	0.89	0.73	1.04	0.77
HERC2P3	6.34	0.52	0.39	1.41	1.52	0.31
HERC2P4	0.88	2	1.6	0.94	1.15	0.67
HERC2P7	1.17	1.18	0.87	0.44	0.93	0.77
HERC2P9	4.14	1.56	1.1	1.1	1.04	0.73
HERC3	2.73	4.99	3.51	2.03	2.84	4.14
HERC4	3.26	3.94	2.58	4.64	4.14	2.75
HERC5	4.17	5.66	5.27	5.28	4.86	2.61
HERC6	4.01	3.27	3.13	3.74	2.86	5.39
HERPUD1	28.07	22.81	16.87	15.04	21.85	13.63
HERPUD2	2.94	2.73	2.79	3.64	2.27	2.39
HES1	1.03	0.08	0.38	0.49	1.36	0.1
HES2	0.42	0.41	0.69	0.48	0.34	0.24
HES3	0	0	0	0	0	0
HES4	0	0	0.07	0.22	0.23	0
HES5	0	0	0	0	0	0
HES6	2.87	4.16	4.32	3.75	2.3	4.63
HES7	0.49	0.38	0.65	0.18	0.46	0.81
HESX1	0.19	0.61	0.42	0.49	0.31	0.39
HEXA	86.87	33.69	54.34	67.49	79.66	55.91
HEXA-AS1	0.44	0.35	0.25	0.19	0.36	0.53
HEXB	49.84	44.24	40.64	35.02	47.62	35.06
HEXDC	0.81	0.32	1.57	0.96	0.89	1.16
HEXIM1	2.38	1.28	1.41	1.4	2.25	2.43
HEXIM2	1.25	2.64	2.23	2.15	1.45	2.52
HEY1	8.43	0	0.03	3.35	2.63	0
HEY2	0	0	0	0	0	0
HEYL	1	0.93	0.13	0.99	1.48	1.1
HFE	0	0	0	0	0	0
HFE2	0	0	0	0	0	0
HFM1	0	0	0	0	0	0
HGC6.3	0	0	0	0	0	0
HGD	0.96	0.03	0	0.18	0.07	0.15
HGF	3.43	8.55	0.71	0	0.42	9.12
HGFAC	0	0	0	0	0	0
HGS	54.34	51.35	48.75	50.4	57.98	49.01

HGSNAT	5.81	6.26	5.84	5.01	5.37	4.67
HHAT	0.05	0.14	0.28	0.3	0.23	0.49
HHATL	0	0	0	0	0	0
HHEX	15.97	12.95	15.12	12.51	10.74	11.41
HHIP	0	0	0	0	0	0
HHIP-AS1	0	0	0	0	0	0
HHIPL1	0	0	0	0	0	0
HHIPL2	0	0	0	0.12	0	0
HHLA1	0	0	0	0	0	0
HHLA2	0.34	0.49	0.28	0.26	0.41	0.16
HHLA3	10.91	3.84	3.97	4.35	6.27	3.16
HIAT1	23.7	21.41	20.37	17.4	20.89	19.94
HIATL1	3.65	2.51	1.97	1.82	3.08	2.5
HIATL2	0.35	0.25	0.24	0.07	0.46	0.45
HIBADH	16.9	17.08	13.3	11.05	17.56	12.69
HIBCH	25.38	24.53	20.81	18.55	24.24	18.19
HIC1	0.14	0.07	0.09	0.1	0.09	0.1
HIC2	1.2	0.85	1.18	1.37	1.57	1.29
HID1	1.11	0.53	0.7	0.81	1.09	0.53
HIF1A	26.82	20.27	13.95	15.98	19.4	16.25
HIF1A-AS2	0	0.03	0.04	0	0	0
HIF1AN	12.21	12	10.68	9.99	9.49	10.8
HIF3A	0.41	0.51	0.5	0.31	0.34	0.32
HIGD1A	47.46	27.58	19.94	20.03	28.7	20.69
HIGD1B	0	0	0.58	0.46	0.64	0.3
HIGD1C	0	0	0	0	0	0
HIGD2A	183.75	185.27	203.01	191.82	228.81	217.5
HIGD2B	0.07	0.32	0	0.05	0.06	0
HILPDA	20.41	23.66	22.26	19.84	20.86	19.51
HILS1	0	0	0	0	0	0
HINFP	7.45	11.13	6.34	6.89	11.16	11.43
HINT1	506.4	537.6	472.17	502.18	520.4	594.1
HINT2	50.79	59.77	43.73	55.92	63.93	41.87
HINT3	2.25	3.26	1.79	1.89	2.91	2.07
HIP1	1.85	2.94	2.23	2.81	2.62	2.76
HIP1R	2.23	3.42	2.31	4.01	3.24	4.99
HIPK1	4.38	4.31	3.56	1.98	2.86	4.27
HIPK2	3.81	1.88	1.57	1.88	2.29	2.04
HIPK3	4.11	5.32	5.07	3.38	3.37	4.88
HIPK4	0	0	0	0	0	0
HIRA	4.87	6.28	4.28	4.04	4.75	4.75
HIRIP3	12.69	18.85	12.09	12.85	11.99	14.04
HIST1H1A	0	0	0.09	0	0.1	0.1

HIST1H1B	0	0	0	0	0	0
HIST1H1C	205.07	278.66	406.83	335.34	284.05	611.69
HIST1H1D	22.53	47.63	58.22	49.85	45.08	78.17
HIST1H1E	862.95	1487.44	2337.06	1810.66	1503.29	3440.35
HIST1H1T	0	0	0	0	0	0
HIST1H2AA	0	0	0	0	0	0
HIST1H2AB	2.84	1.97	3.4	2.97	4.44	4.82
HIST1H2AC	29.13	23.97	40.15	27.93	29.23	43.86
HIST1H2AD	1.13	1.1	0.61	0.67	0.61	0.49
HIST1H2AE	28.3	40.62	69.88	56.5	43.36	84.95
HIST1H2AG	17.54	27.91	55.41	42.56	33.48	81.2
HIST1H2AH	8.61	14.34	23.54	19.12	16.78	20.02
HIST1H2AI	0	0	0	0	0	0
HIST1H2AJ	0	0.16	0	0	0	0
HIST1H2AK	3.53	1.92	4.91	3.37	3.37	5.44
HIST1H2AL	0	0	0	0	0	0
HIST1H2AM	30.04	41.55	56.1	28.86	46.73	35.08
HIST1H2APS1	0	0	0	0	0	0
HIST1H2BA	0	0	0	0	0	0
HIST1H2BB	0	0.16	0	0	0	0
HIST1H2BC	44.21	56.93	72.38	53.2	63.25	54.92
HIST1H2BD	47.86	77.04	110.09	82.17	90.3	109.27
HIST1H2BE	0	0	0	0	0	0
HIST1H2BF	138.26	209.8	305.37	239.23	275.35	309.72
HIST1H2BG	5.98	5.96	9.34	4.45	8.3	7.95
HIST1H2BH	0	0	0	0	0.2	0
HIST1H2BI	2.75	10.58	12.24	12.71	10.79	16.92
HIST1H2BJ	17.75	14.51	23.78	11.07	12.84	21.92
HIST1H2BK	532.94	786.25	1025.73	832.39	775.07	1062.81
HIST1H2BL	0.09	0	0	0.09	0	0
HIST1H2BM	0	0	0	0	0	0
HIST1H2BN	17.54	27.37	35.93	28.19	35.14	37.88
HIST1H2BO	2.46	1.31	3.29	1.45	2.69	1.53
HIST1H3A	25.17	30.12	48.82	40.94	36.33	51.01
HIST1H3B	270.82	445.75	712.83	513.01	510.4	641.08
HIST1H3C	0	0	0	0.13	0.18	0.19
HIST1H3D	23.67	32.2	62.06	42.33	40	60.2
HIST1H3E	0	0	0	0	0	0
HIST1H3F	0	0	0	0	0	0
HIST1H3G	139.33	192.39	351.95	243.26	285.63	230.97
HIST1H3H	23.66	20.02	47.51	33.15	29.45	42.39
HIST1H3I	0.35	0	0	0	0	0
HIST1H3J	1.24	0	0.16	0.25	0.33	0

HIST1H4A	0.48	1.52	0.22	1.01	0.23	0.72
HIST1H4B	1.77	1.88	3.46	2.66	1.94	2.79
HIST1H4C	1780.38	3384.08	3868.02	3020.46	3643.07	2727.25
HIST1H4D	0.24	0.39	1.78	0.69	2.81	3.19
HIST1H4E	115.63	168.97	285.93	219.73	243.18	242.03
HIST1H4F	0	0	0	0	0	0
HIST1H4G	0	0	0	0	0	0
HIST1H4H	9.29	2.27	3.05	2.17	2.74	4.07
HIST1H4I	276.98	510.62	907.14	645.38	681.58	624.53
HIST1H4J	0	0	0	0	0	0
HIST1H4K	18.92	18.44	24.17	24.21	24.27	29.95
HIST1H4L	0	0	0	0	0	0
HIST2H2AA3	235.22	328.84	480.7	388.03	364.06	518.33
HIST2H2AA4	235.22	328.84	480.7	388.03	364.06	518.33
HIST2H2AB	18.85	34.54	40.26	24.21	29.05	37.32
HIST2H2AC	11.6	10.79	26.49	14.67	15.52	25.15
HIST2H2BA	0	0	0	0	0	0
HIST2H2BC	3.64	2.52	0.89	1.21	1.46	2.47
HIST2H2BE	4.41	1.43	2.76	0.8	3.2	1.56
HIST2H2BF	25.14	39.75	63.41	43.34	42.45	54.99
HIST2H3A	14.78	26.64	44.53	28.7	29.2	57.9
HIST2H3C	14.78	26.64	44.53	28.7	29.2	57.9
HIST2H3D	6.54	7.48	11.77	9.54	7.92	12.52
HIST2H4A	37.43	55.16	95.09	55.64	62.17	71.21
HIST2H4B	37.43	55.16	95.09	55.64	62.17	71.21
HIST3H2A	20	12.21	20.91	19.61	18.76	18.76
HIST3H2BB	0.77	1.99	3.26	1.47	3.82	3.63
HIST3H3	0	0	0	0	0	0
HIST4H4	0.42	0	0.39	0.3	0	0
HIVEP1	0.88	0.64	0.56	0.77	0.56	0.69
HIVEP2	0.6	0.96	0.75	0.8	0.4	0.65
HIVEP3	0.44	1.46	1.4	0.68	0.86	1.13
HJURP	32.32	20.26	21.8	26.35	26.67	22.98
HK1	47.96	7.27	7.14	15.27	21.74	11.02
HK2	3.41	6.32	7.18	4.99	4.23	5.64
HK3	0	0	0	0	0	0
HKDC1	0	0	0	0	0	0
HKR1	2.07	2.84	1.96	2.59	2.44	2.19
HLA-A	187.58	243.71	227.7	260.3	205.7	256.6
HLA-B	214.66	256.22	285.62	306.16	249.14	274.04
HLA-C	86.77	103.87	108.45	99.75	96.23	99
HLA-DMA	5.71	9.76	22.71	22.67	14.81	15.39
HLA-DMB	0	0	0.05	0.04	0	0

HLA-DOA	0.15	0.23	0.32	0.11	0.06	0.23
HLA-DOB	1.43	1.69	1	0.72	0.99	0.11
HLA-DPA1	1.9	0.17	6.08	12.66	3.22	0.49
HLA-DPB1	0.62	0.27	5.12	11.22	1.48	0.67
HLA-DPB2	0	0	0	0.04	0.1	0
HLA-DQA1	0	0	0.13	0.03	0	0.05
HLA-DQA2	0	0	0	0	0	0
HLA-DQB1	3.77	20.02	26.17	24.35	20.66	20.23
HLA-DQB2	0	0	0	0	0.06	0
HLA-DRA	67.79	183.5	345.67	409.24	251.13	205.99
HLA-DRB1	16.76	42.31	96.33	117.28	78.25	53.09
HLA-DRB5	2.26	6.32	15	17.42	9.98	9.04
HLA-DRB6	1.18	2.07	5.09	6.36	3.81	2.45
HLA-E	99.94	48.3	51.85	64.15	75.49	58.79
HLA-F	0.18	0.29	0.05	0.12	0.07	0.38
HLA-F-AS1	1.83	1.44	1.95	0.35	2.35	2.2
HLA-G	1.21	0.87	1.16	1.18	1.42	0.51
HLA-H	5.69	7.55	8.83	8.75	6.87	7.56
HLA-J	0.2	0.44	0.5	0.59	0.35	0.53
HLA-L	0.34	0.27	0.37	0.4	0.44	0.45
HLCS	2.82	3.21	1.49	1.78	2.41	2.95
HLF	0.03	0	0	0	0.19	0
HLTF	16.18	7.01	6.4	6.26	10.4	7.75
HLTF-AS1	0	0	0	0	0	0
HLX	4.91	5.01	5.94	4.09	4.87	5.15
HM13	125.84	120.48	121.54	128.84	122.43	124.01
HM13-AS1	0	0.14	1.26	0.36	0.16	0
HMBOX1	11.07	4.28	4.82	6.32	8.19	5.21
HMBS	32.8	43.93	39.83	39.14	38.22	38.07
HMCN1	0.38	0	0.02	0.13	0.13	0
HMG20A	28.49	27.14	25.36	27.82	26.29	34.95
HMG20B	30.08	33.55	38.45	46.25	41.21	25.98
HMGA1	194.35	251.58	337.11	327.32	287.14	330.47
HMGA1P7	0	0	0	0	0	0
HMGA2	3.93	1.15	1.22	2.17	2.69	1.64
HMGB1	265.7	311.35	277.02	282.79	336.63	288.23
HMGB2	235.99	271.42	216.33	222.22	253.75	260.7
HMGB3	40.41	37.98	30.87	36.9	37.65	35.65
HMGB3P1	1.49	1.11	1.88	1.64	0.65	1.4
HMGB4	0	0	0	0	0	0
HMGCL	25.46	18.2	14.46	22.27	20.46	16.96
HMGCLL1	0	0	0	0	0	0
HMGCR	16.29	17.4	12.51	15.1	19.85	19.42

HMGCS1	12.36	5.24	5.19	5.35	10.24	7.61
HMGCS2	0	0	0	0	0	0
HMGN1	199.91	235.19	190.09	173.73	218.39	212.71
HMGN2	702.71	1073.52	860.3	952.98	966.46	932.99
HMGN2P46	0.45	0.67	0.18	0.29	0.58	0.39
HMGN3	85.43	98.68	81.84	72.28	88.8	84.63
HMGN4	24.35	29.09	19.81	19.16	28.1	24.85
HMGN5	34.64	45.01	44.08	34.19	40.44	44.76
HMGXB3	16.37	15.1	14.79	15.93	13.02	12.49
HMGXB4	8.96	7.49	8.6	8.72	8.71	7.15
HMHA1	4.8	9.12	10.39	11.1	7.86	9.35
HMHB1	0	0	0	0	0	0
HMMR	18.14	15.93	13.23	10.77	16.15	14.6
HMOX1	27.24	3.37	4.77	3.43	5.95	3.32
HMOX2	61.65	77.33	75.58	67.27	71.37	69.25
HMP19	0	0	0	0	0	0
HMSD	0	0	0	0	0	0
HMX1	0	0	0	0	0	0
HMX2	0.5	1.88	4.19	2.71	1.99	2.18
HMX3	1.21	2.08	2.48	2.66	2.5	1.37
HN1	161.63	126.75	124.12	118.42	145.44	125.55
HN1L	12.92	13.24	14.07	12.59	14.34	12.82
HNF1A	0.48	0.12	0.04	0.23	0.23	0.11
HNF1A-AS1	1.13	1.45	1.51	1.31	1.08	1.48
HNF1B	0	0	0	0	0	0
HNF4A	0	0	0	0.01	0	0
HNF4G	0	0	0	0	0	0
HNMT	0.6	7.4	4.84	4.5	2.48	8.52
HNRNPA0	17.05	15.7	17.19	19.68	21.21	14.08
HNRNPA1	951.34	926.06	944.48	1228.39	1124.01	1001.93
HNRNPA1L2	3.92	5.14	4.48	4.18	4.44	3.36
HNRNPA1P1(	551.42	726.65	596.34	732.15	694.8	902.44
HNRNPA1P3:	0.17	0.13	0.29	0.55	0.15	0
HNRNPA2B1	234.76	256.7	215.35	212.72	268.23	280.28
HNRNPA3	43.5	54.58	48.99	42.69	50.63	56.35
HNRNPA3P1	0.06	0.03	0.04	0.06	0.03	0
HNRNPAB	37.81	38.43	37.43	31.1	43.17	37.45
HNRNPC	353.78	329.29	298.45	308.26	352.16	327.81
HNRNPCL1	0	0	0	0	0	0
HNRNPD	83.51	92.73	101.48	97.79	111.44	110.64
HNRNPF	244.81	232.01	211.25	222.48	244.23	233.72
HNRNPH1	165.2	186.98	167.46	152.83	200.79	143.67
HNRNPH2	35.32	25.36	21.28	24.1	30.41	21.78

HNRNPH3	83.42	91.74	74.52	78.47	90.07	79.32
HNRNPK	334.23	345.64	298.78	301.8	343.82	346.05
HNRNPKP3	0.34	0.74	0.26	0.38	0.42	0.45
HNRNPL	28.09	24.27	29.21	27.14	31.62	30.71
HNRNPM	219.19	216.29	223.09	223.84	230.41	248.11
HNRNPR	99.79	110.39	93.41	86.06	120.69	101.15
HNRNPU	24.92	25.25	25.46	24.27	28.61	25.01
HNRNPU-AS	0.86	0.63	0.78	0.29	0.94	0.41
HNRNPUL1	27.54	32.52	33.46	35.37	34.89	38.11
HNRNPUL2	1.55	2.11	2.08	1.76	2.17	2.17
HNRNPUL2-I	0.18	0.02	0	0.64	0.64	0
HNRPDL	76.75	97.97	75.2	75.18	85.01	82.67
HNRPLL	10.83	5.9	6.12	6.97	7.07	8.78
HOGA1	0.67	0.88	0.37	0.71	0.56	1.02
HOMER1	0.93	1.04	2.09	1.06	1.93	1.51
HOMER2	7.51	9	11.08	9.01	12.71	9.37
HOMER3	6.35	14.04	6.03	5.6	6.64	11.65
HOMEZ	3.79	1.46	2.26	2.34	2.36	2.36
HOOK1	0	0	0	0	0	0
HOOK2	5.9	6.66	4.75	4.7	3.61	6.49
HOOK3	3.36	3.08	2.56	2.08	2.6	2.63
HOPX	0	0	0	0	0	0
HORMAD1	0	0	0	0	0	0
HORMAD2	0	0	0	0	0	0
HOTAIR	0	0	0	0	0.18	0
HOTAIRM1	4.92	9.39	6.52	5.38	6.47	4.43
HOTTIP	0.4	1.17	1	0.41	0.89	0.7
HOXA-AS3	0.03	0.02	0.03	0	0	0
HOXA-AS4	2.75	4.52	3.47	3.73	7.45	4.75
HOXA1	0.83	2.88	2.89	1.19	0.99	1.97
HOXA10	2.74	4.21	2.84	2.99	4.08	2.97
HOXA10-HO)	0	0	0	0	0	0
HOXA11	0.19	0.61	0.4	0.4	0.94	0.5
HOXA11-AS	4.29	4.03	2.96	2.13	3.56	2
HOXA13	0.29	0.48	0.21	0.22	0.39	0.09
HOXA2	0	0	0.04	0	0	0.04
HOXA3	0	0	0.02	0.02	0.08	0.16
HOXA4	0	0	0.2	0.09	0	0.13
HOXA5	0	0.46	0.86	0.62	0.17	0.91
HOXA6	0.49	0.85	1.52	1.76	0.84	1.48
HOXA7	2.18	6.94	6.49	4.42	5.64	6.04
HOXA9	8.36	10.15	9.43	11.11	9.78	11.61
HOXB-AS3	0	0	0	0	0	0

HOXB-AS5	0	0	0	0	0	0
HOXB1	0	0	0	0	0	0
HOXB13	0	0	0	0	0	0
HOXB2	1.49	0.44	0.94	0.61	1.06	0.38
HOXB3	0.22	0.16	0.06	0	0.06	0.24
HOXB4	1.63	0.74	1.23	0.85	2.3	0.85
HOXB5	0.16	0.19	0.15	0.11	0.12	0.7
HOXB6	0.27	0.25	2.64	1.35	0.55	3.59
HOXB7	0	0	0	0	0	0
HOXB8	0	0	0	0	0	0
HOXB9	0	0	0	0	0	0.11
HOXC-AS5	0	0	0	0	0	0
HOXC10	0.68	2.83	2.94	3.5	2.63	4.73
HOXC11	0.11	0.51	0.1	0.95	0.65	0.92
HOXC12	0	0	0	0	0	0
HOXC13	0	0	0	0	0	0
HOXC4	0	0	0	0	0	0
HOXC5	0	0	0	0	0	0.05
HOXC6	0.8	0.91	0.49	0.5	0.38	0.63
HOXC8	1.09	1.04	1.5	1.17	0.88	0.81
HOXC9	0.29	0.19	0.36	1.04	0.46	1.08
HOXD-AS1	0	0	0	0	0	0
HOXD-AS2	0	0	0	0	0	0
HOXD1	0	0	0	0	0	0
HOXD10	0	0	0	0	0	0
HOXD11	0	0	0	0	0	0
HOXD12	0	0.08	0.35	0	0	0
HOXD13	3.28	8.48	4.94	4.04	5.3	6.87
HOXD3	0	0	0	0	0	0
HOXD4	0	0	0	0	0	0
HOXD8	0	0	0	0.03	0	0
HOXD9	0	0	0	0	0	0
HP	0	0	0	0	0.06	0
HP07349	1.58	1.41	0.36	1.52	3.02	2.97
HP1BP3	21.32	18.33	16.42	16.84	18.88	16.82
HPCA	0	0	0	0	0	0
HPCAL1	25.27	23.79	24.39	13.82	21.37	12.91
HPCAL4	0.51	0	0	0.06	0	0
HPD	0.04	0	0	0	0	0
HPDL	0	0.13	0.19	0.17	0.12	0.29
HPGD	4.75	0	0	0.28	0.5	0
HPGDS	0.97	0.04	0.38	0.45	2.49	1.31
HPN	0	0	0	0	0	0

HPR	0	0.14	0	0.08	0	0.37
HPRT1	116.44	139.14	116.83	108.4	132.75	116.99
HPS1	63.57	44.21	33.85	45.44	46.88	44
HPS3	6.33	5.66	3.06	3.98	4.36	4.54
HPS4	6.37	4.53	4.7	6.15	6.45	5.53
HPS5	4.28	3.54	3.41	2.74	3.17	4.51
HPS6	1.67	2.09	3.71	3.61	2.56	5.26
HPSE	4.55	1.38	1.92	2.03	1.53	2.12
HPSE2	0.02	0	0	0	0	0
HPVC1	0	0	0	0	0	0
HPX	0	0	0	0.03	0	0
HPYR1	0	0	0	0	0	0
HR	0.3	0.26	0.05	0.25	0.37	0.46
HRAS	19.64	17.54	16.13	15.18	22.59	18.64
HRASLS	0	0	0	0	0	0
HRASLS2	0	0	0	0	0	0
HRASLS5	0	0	0	0	0	0
HRC	0	0.05	0	0.02	0	0
HRCT1	0	0	0	0	0	0
HRG	0	0	0	0	0	0
HRH1	0.19	0.02	0.09	0.04	0.13	0.12
HRH2	0.37	2.03	1.74	1.23	1.11	1.74
HRH3	0	0.02	0.2	0	0	0
HRH4	0.39	0.12	0.35	0.07	0.09	0.42
HRK	0	0	0.23	0.03	0	0.05
HRNR	0	0	0	0	0	0
HRSP12	81.49	89.86	95.86	66.55	81.98	90.15
HS1BP3	15	13.18	15.35	11.09	11.79	9.73
HS2ST1	5.22	4.82	4.22	3.94	4.91	5.24
HS3ST1	0	0	0	0	0	0
HS3ST2	0	0	0	0	0	0
HS3ST3A1	2.05	0.5	0.52	0.35	1.48	0.25
HS3ST3B1	2.27	3.27	5.23	4.03	4.8	3.5
HS3ST4	0	0	0	0	0	0
HS3ST5	0	0	0	0	0	0
HS3ST6	0	0	0	0	0	0
HS6ST1	6.38	6.98	7.92	6.09	8.29	6.96
HS6ST2	0	0	0	0	0.05	0
HS6ST2-AS1	0	0	0	0	0	0
HS6ST3	0	0	0	0	0	0
HSBP1	57.63	70.24	56.66	54.41	69.95	61.86
HSBP1L1	31.14	30.94	26.17	18.73	31.98	24.42
HSCB	9.74	10.5	14.81	8.04	9.47	12.07

HSD11B1	0	0	0	0	0	0
HSD11B1L	0	0.23	0.38	0.7	0.35	0.93
HSD11B2	0.91	2.02	2.52	1.63	1.93	0.92
HSD17B1	2.35	2.7	3.2	4.05	3.27	3.87
HSD17B10	307.52	227.75	212.88	268.3	272.55	204.49
HSD17B11	43.24	55.93	50.32	48.75	49.16	62.9
HSD17B12	43.52	15.33	17.51	17.4	19.72	17.38
HSD17B13	0.27	0.93	0.8	0.31	0.72	0.46
HSD17B14	0	0.72	0.39	0.51	0.23	0.31
HSD17B2	0	0	0	0	0	0
HSD17B3	0	0	0	0	0	0
HSD17B4	36.42	27.45	23.36	23.55	24.23	17.86
HSD17B6	0.64	0.39	0.36	0.89	0.19	0.9
HSD17B7	7.88	9.21	6.8	6.82	7.36	8.46
HSD17B7P2	0.19	0.16	0.13	0.05	0.13	0
HSD17B8	10.22	14.23	14.59	24.55	17.31	14.78
HSD3B1	0	0	0	0	0	0
HSD3B2	0	0	0	0	0	0
HSD3B7	3.66	1.07	2.59	0.82	1.59	0.97
HSD3BP4	0	0	0.01	0.01	0.02	0.01
HSD52	0	0	0	0	0	0
HSDL1	6.44	4.36	4.05	4.89	7.88	3.67
HSDL2	18.06	11.16	15.07	17.09	17.21	15.9
HSF1	61.4	64.57	85.16	83.26	74.1	58.35
HSF2	8.68	9.14	7.59	7.15	8.93	11.25
HSF2BP	0.27	0.06	0.11	0	0.07	0
HSF4	0.45	0.2	0.14	0.33	0.06	0.45
HSF5	0	0	0	0	0	0
HSFX1	0.15	0.03	0.21	0.1	0.18	0.11
HSFX2	0	0	0	0	0	0
HSFY1	0	0	0	0	0	0
HSFY1P1	0	0	0	0	0	0
HSFY2	0	0	0	0	0	0
HSH2D	4.48	25.09	22.43	11.98	13.34	20.14
HSP90AA1	541.4	484.99	471.61	401.33	436.15	394.05
HSP90AB1	940.28	938.57	793.03	841.05	867.58	845.83
HSP90AB4P	0.49	0.78	0.48	0.62	0.9	0.57
HSP90B1	201.82	193.02	147.02	136.57	165.14	155.24
HSP90B2P	0.13	0.11	0.18	0.06	0.09	0.06
HSPA12A	1.16	1.5	1.68	0.63	1.19	0.83
HSPA12B	0.07	0.36	0.21	0.3	0.31	0.19
HSPA13	5.94	4.83	5.71	4.25	6.1	5.09
HSPA14	26.87	31.28	23.58	21.5	28.95	31.42

HSPA1A	52.13	48.17	57.16	43.67	45.89	45.12
HSPA1B	22.54	19.39	20.27	18.98	25.68	20.18
HSPA1L	0.35	0.14	0.45	0.02	0.19	0.15
HSPA2	0	0	0	0	0	0
HSPA4	57.92	47.65	53.07	47.7	49.91	57.04
HSPA4L	10.72	11.23	12.34	8.78	7.8	10.47
HSPA5	153.89	169.57	142	113.28	141.04	135.55
HSPA6	0.72	0.17	0.15	0.34	0.18	0
HSPA7	0	0	0	0	0	0
HSPA8	1865.54	1835.69	1879.8	1767.89	1679	1660.43
HSPA9	172.88	196.42	168.75	152.49	170.26	187.66
HSPB1	305.6	211.3	250.16	236.56	268.03	258.23
HSPB11	42.58	38.71	33.35	30.62	37.99	38.62
HSPB2	0	0	0	0	0	0
HSPB2-C11or	0	0	0	0	0	0
HSPB3	0	0	0	0	0.1	0
HSPB6	0	0.04	0.14	0	0	0
HSPB7	0	0	0	0	0	0
HSPB8	0	0	0	0	0	0
HSPB9	1.05	1.67	1.33	1.28	2.26	1.73
HSPBAP1	5.26	4.62	3.5	4.22	3.82	2.57
HSPBP1	34.5	33.32	39.65	43.61	37.81	24.79
HSPD1	478.33	525.95	441.83	428.61	442.85	412.6
HSPE1	502.15	572.37	467.46	415.18	537.99	481.62
HSPE1-MOB4	0.14	0.19	0.09	0.13	0	0.43
HSPG2	2.98	0.95	0.96	1.12	1.59	2.5
HSPH1	22.49	33.5	30.79	25.94	28.22	20.19
HTA	0	0	0	0	0	0
HTATIP2	0	0	0	0	0	0
HTATSF1	26.58	27.84	24.42	26.65	27.3	27.99
HTATSF1P2	1.67	1.08	0.94	0.82	1.07	0.7
HTN1	0	0	0	0	0	0
HTN3	0	0	0	0	0	0
HTR1A	0	0	0	0	0	0
HTR1B	0	0	0	0	0	0
HTR1D	0.14	0.14	0.03	0.25	0.15	0.07
HTR1E	0	0	0	0	0	0
HTR1F	0	0	0	0	0	0
HTR2A	0	0	0	0.01	0.03	0.05
HTR2B	0.69	0.03	0.21	0.18	0.16	0.07
HTR2C	0	0	0	0	0	0
HTR3A	0	0	0	0	0	0
HTR3B	0.31	0.26	0.27	0.06	0.26	0.08

HTR3C	0	0	0	0	0.04	0
HTR3D	0	0	0	0	0	0
HTR3E	0	0	0	0	0	0
HTR4	0	0	0	0	0	0
HTR5A	0	0	0	0	0	0
HTR6	0	0.15	0.17	0.21	0.07	0.41
HTR7	0	0	0	0.02	0	0
HTR7P1	0.14	0.22	0.1	0.17	0.13	0.3
HTRA1	0	0	0	0	0	0
HTRA2	17.68	13.38	15.4	14.2	18.4	12.95
HTRA3	0.37	0	0	0.08	0	0
HTRA4	0.96	0.39	1.03	0.75	0.94	0.94
HTT	1.9	3.05	3.49	3.61	2.6	3.5
HTT-AS1	0	0	0	0	0	0
HULC	0	0	0	0	0	0
HUNK	0.22	0.54	0.38	0.45	0.4	0.36
HUS1	9.7	8.61	7.08	7.49	7.92	7.95
HUS1B	0.97	0.18	0.07	0	0.21	0.53
HUWE1	32.94	31.67	30.54	26.34	30.12	32.12
HVCN1	3.31	6	3.15	4.09	6.39	5.76
HYAL1	0.35	0.14	0.05	0.28	0.04	0.58
HYAL2	8.33	7.64	7.72	6.72	10.58	5.26
HYAL3	12.37	12.01	8.13	8.31	11.53	11.64
HYAL4	0.17	0.03	0.11	0.02	0.09	0.02
HYALP1	0	0	0	0	0	0
HYDIN	0.02	0	0	0	0	0
HYDIN2	0.48	0.6	0.62	0.37	0.41	0.55
HYI	0	0	0	0	0.04	0
HYLS1	6.42	11.49	7.79	8.05	4.97	7.02
HYMAI	0.05	0.17	0	0.04	0.11	0.05
HYOU1	9.7	11.89	10.73	8.49	11.43	12.22
HYPK	68.38	68.8	59.52	56.48	66.93	54.1
IAH1	0	0	0	0	0	0
IAPP	1.15	1.88	1.98	1.02	1.58	0.98
IARS	65.31	71.36	63.65	71.62	68.02	73.21
IARS2	23.79	26.06	21.18	18.28	22.03	23.77
IBA57	3.25	2.81	3.37	2.26	2.82	2.59
IBSP	0	0	0	0	0	0
IBTK	5.43	4.98	4.68	3.58	3.68	5.37
ICA1	4.04	3.94	6.14	3.25	5.6	4.63
ICA1L	0.71	1.12	1.17	0.89	1.29	1.1
ICAM1	6.17	2.15	3.57	3.81	5.23	2.65
ICAM2	79.27	36.71	28.7	51.7	70.67	34.85

ICAM3	7.43	22.94	42.42	20.5	20.26	38.39
ICAM4	22.09	31.92	24.45	28.91	29.7	22.71
ICAM5	0.73	0.5	0.98	1.27	1.12	1.55
ICK	1.59	2.43	2.3	1.93	1.83	2.45
ICMT	19.23	24.42	26.29	22.62	20.89	16.02
ICOS	0	0	0	0	0	0
ICOSLG	0.85	4.55	2.55	2.15	2.2	3.8
ICT1	58.51	59.94	58.77	52.34	54.62	51.8
ID1	57.69	32.17	34.99	25.83	23.15	25.27
ID2	239.46	12.93	7.58	39.93	106.28	5.47
ID3	46.35	33.22	22.89	32.95	30.78	28.57
ID4	0	0	0	0	0	0
IDE	12.84	12.1	10.19	8.6	9.76	11.64
IDH1	40	31.93	27.46	18.47	25.75	24.67
IDH1-AS1	1.03	0.54	0.94	1.43	2.13	0.17
IDH2	90.52	145.69	143.6	180.41	157.41	202.87
IDH3A	37.52	63.25	53.02	33.99	36.88	55.6
IDH3B	144.55	144.97	144.4	145.17	154.33	137.92
IDH3G	110.42	147.18	147.11	152.27	120.25	117.4
IDI1	40.95	37.61	35.69	28.99	46.18	43.03
IDI2	1.16	3.5	2.54	2.1	4.3	1.9
IDI2-AS1	0.17	0.2	0	0.06	0.25	0.43
IDNK	1.81	3.7	2.2	3.68	0.84	1.25
IDO1	1.19	1.63	2.11	0.9	1.32	2.04
IDO2	0	0	0	0	0	0
IDS	44.57	9.84	9.85	18	16.69	11.37
IDUA	0.13	0.5	0.98	0.42	0.32	0
IER2	83.91	37.29	30.58	38.84	60.36	31.5
IER3	121.23	0.63	0.5	10.37	28.61	1.17
IER3IP1	48.7	50.58	35.85	30.9	43.86	42.38
IER5	1.05	0.37	0.46	0.22	0.44	0.73
IER5L	0.03	0	0.12	0.02	0.13	0.05
IFFO1	1.14	2.68	2.99	3.47	2.33	1.8
IFFO2	0.11	0.3	0.25	0.19	0.2	0.55
IFI16	1.74	7.01	4.26	7.26	1.87	1.89
IFI27	0	0.1	0.46	0	0	0
IFI27L1	9.65	10.41	10.26	9.34	8.88	9.56
IFI27L2	30.39	13.7	11.48	5.72	26.95	17.58
IFI30	76.81	129.38	169.14	87.22	89.1	107.5
IFI35	10.84	10.08	10.2	12.45	9.97	7.41
IFI44	1.38	3.8	3.31	3.26	2.08	0.22
IFI44L	0	0	0.03	0	0	0
IFI6	12.9	16.92	25.55	20.39	16.83	20.28

IFIH1	1.65	2.36	1.77	1.36	1.82	2
IFIT1	3.21	0.81	1.43	3.86	2.26	1.74
IFIT1B	0	0.15	0	0	0	0
IFIT2	6.05	1.21	1.22	4.33	3.08	4.29
IFIT3	14.4	4.21	3.04	10.46	8.03	5.74
IFIT5	1.6	3.17	2.37	4.04	3	3.51
IFITM1	394.29	524.98	551.07	617.81	891.84	602.18
IFITM10	0.29	0.37	0.48	0.3	0.22	0.32
IFITM2	1223.57	906.83	1055.76	1324.78	1835.68	1097.71
IFITM3	2.52	5.23	13.31	15.31	7.54	21.88
IFITM4P	1.07	0.42	0	0	0.26	0.27
IFITM5	0	0	0	0	0	0
IFLTD1	0.16	0.22	0.45	0.15	0.09	0.16
IFNA1	0	0	0	0	0	0
IFNA10	0	0	0	0	0	0
IFNA13	0	0	0	0	0	0
IFNA14	0	0	0	0	0	0
IFNA16	0	0	0	0	0	0
IFNA17	0	0	0	0	0	0
IFNA2	0	0	0	0	0	0
IFNA21	0	0	0	0	0	0
IFNA22P	0	0	0	0	0	0
IFNA4	0	0	0	0	0	0
IFNA5	0	0	0	0	0	0
IFNA6	0	0	0	0	0	0
IFNA7	0	0	0	0.15	0	0
IFNA8	0	0	0	0	0	0
IFNAR1	7.52	6.44	4.56	4.38	5.61	6.47
IFNAR2	17.26	20.94	16.54	17.45	21.67	22.09
IFNB1	0	0	0	0	0	0
IFNE	0	0	0	0	0	0
IFNG	0	0	0	0	0	0
IFNGR1	14.15	13.81	9.45	9.81	14.44	13.65
IFNGR2	27.3	24.87	23.5	29.38	31.62	20.01
IFNK	0	0	0	0	0	0
IFNL1	0	0	0.17	0	0	0
IFNL2	0	0	0	0	0	0
IFNL3	0	0.22	0	0	0	0
IFNL4	0	0	0	0	0	0
IFNLR1	1.8	2.48	2.28	1.49	1.86	1.97
IFNW1	0	0.16	0.09	0.03	0.23	0.3
IFRD1	9.08	5.01	1.86	1.83	2.84	2.91
IFRD2	109.74	123.49	109.58	123.6	112.14	96.82

IFT122	3.28	6.3	6.09	4.49	4.96	6.48
IFT140	1.11	1.57	1.09	2.02	1.6	1.11
IFT172	2.12	2.64	3.08	2.28	1.22	3.32
IFT20	9.89	10.54	10.5	10.27	7.51	7.65
IFT27	12.83	11.34	11.39	11.51	12.32	7.89
IFT43	16.56	13.44	7.59	12.3	11.26	10.85
IFT46	14.71	20.96	15.99	18.42	15.31	14.33
IFT52	16.98	16.5	14.93	16.32	18.15	15.78
IFT57	8.91	11	9.96	9.82	8.59	11.31
IFT74	6.92	4	4.21	3.67	5.58	3.47
IFT80	1.35	1.11	1.2	1.21	1.23	1.01
IFT81	1.66	2.4	2.29	2.23	2.96	2.09
IFT88	3.18	2.04	1.69	2.98	3.31	1.74
IGBP1	78.64	74.17	79.18	83.36	78.82	89.78
IGBP1P1	0	0	0	0	0	0
IGDCC3	0.19	1.08	1.67	0.69	0.52	0.77
IGDCC4	0.85	0.71	0.7	0.27	0.69	1.01
IGF1	0.09	0.14	0.07	0.1	0.11	0.19
IGF1R	1.14	1.19	0.67	1.3	1.28	1.6
IGF2	0.04	0	0	0	0	0
IGF2-AS	0	0	0	0	0	0
IGF2BP1	0.1	0.25	0.5	5.25	0.95	0.02
IGF2BP2	5.08	5.15	7.26	4.86	4.41	7.57
IGF2BP3	4.94	3.53	5.08	4.78	4.17	4.21
IGF2R	4.59	2.8	1.73	2.07	2.28	2.47
IGFALS	0	0	0	0	0	0
IGFBP1	0	0	0	0	0	0
IGFBP2	6.94	12.18	12.22	5.87	5.22	12.8
IGFBP3	0	0	0.05	0	0	0
IGFBP4	10.13	16.33	30	36.77	27.73	23.83
IGFBP5	2.21	0.01	0	0.15	0.11	0
IGFBP6	0.16	0.57	0.29	2.49	1.97	0.32
IGFBP7	0.15	1.13	0.62	0.19	0.07	0.15
IGFBPL1	0.06	0.38	0.21	0.11	0.25	0.32
IGFL1	0	0	0	0	0	0
IGFL2	112.12	0.82	0.63	4.6	54.08	1.36
IGFL3	0.24	0	0	0	0	0
IGFL4	0	0	0	0	0	0
IGFLR1	7.11	8.71	5.81	6.09	6.73	4.56
IGFN1	0	0	0	0	0	0
IGHMBP2	7.47	7.08	5.47	4.56	6.77	4.65
IGIP	0.25	0.02	0	0.14	0.1	0
IGJ	0	0	0	0	0	0

IGLL1	12.08	110.26	26.04	24.75	58.11	30.27
IGLL3P	0	0	0	0	0	0
IGLL5	0	0	0	0	0	0
IGLON5	0.02	0.02	0	0	0	0
IGSF1	0	0	0	0	0	0
IGSF10	1.8	6.25	5.49	5.23	3.69	6.18
IGSF11	0	0.02	0	0	0	0
IGSF11-AS1	0	0	0	0	0	0
IGSF21	0	0	0	0	0	0
IGSF22	0	0	0	0	0	0
IGSF23	0	0	0	0	0	0
IGSF3	1.04	1.01	0.95	1.13	1.18	0.94
IGSF5	0.04	0	0.03	0	0	0.04
IGSF6	0	0.06	0	0.2	0	0
IGSF8	6.32	9.03	9.06	9.84	5.96	6.8
IGSF9	0.05	0.04	0	0.01	0.05	0
IGSF9B	0.54	0.21	0.32	0.2	0.19	0.34
IHH	0	0	0	0	0	0
IK	72.61	67.69	48.44	49.27	56.86	42.47
IKBIP	4.22	3.66	4.24	3.2	2.54	3.07
IKBKAP	12.18	10.24	9.98	9.58	11.39	10.52
IKBKB	4.37	6.72	4.46	5.18	4.95	6.46
IKBKE	5.29	11.93	13.49	8.78	8.33	14.83
IKBKG	14.26	10.65	9.74	11.15	13.11	10.63
IKZF1	1.49	0.8	1.48	1.56	1.32	1.11
IKZF2	1.86	4.42	4.44	4.13	4.04	5.85
IKZF3	1.39	1.49	1.54	1.07	1.17	1.37
IKZF4	3.04	0.79	0.71	1.31	1.59	0.95
IKZF5	5.41	5.5	4.67	4.33	3.96	5.33
IL10	0.68	1.07	0.77	0.72	0.95	0.9
IL10RA	0.63	0.3	0.78	0.05	0	0.12
IL10RB	17.56	19.06	16.24	17.45	16.38	17.37
IL11	0.4	0.75	0.5	0.3	0.39	0.55
IL11RA	0.3	0.34	0.63	0.27	0.2	0.39
IL12A	0.47	1.31	0.71	0.72	1.18	1.89
IL12B	0	0	0	0	0.03	0
IL12RB1	1.67	2.75	1.42	1.62	1.52	1.91
IL12RB2	0.06	0	0	0	0	0.04
IL13	0	0	0	0	0	0
IL13RA1	2.07	4.28	2.81	2.84	3.04	4.3
IL13RA2	0	0	0	0	0	0
IL15	1.22	0.26	0.06	0.18	0.35	0.26
IL15RA	11.16	11.07	6.14	8.08	6.89	7.64

IL16	0.41	0.58	0.68	0.57	0.78	0.25
IL17A	0	0	0	0	0	0
IL17B	0.92	0	0	0	0	0
IL17C	0.29	0	0	0	0	0
IL17D	0.08	0.6	0.47	0.32	0.98	0.89
IL17F	0	0	0	0	0	0
IL17RA	1.31	0.94	0.79	0.78	1.01	0.42
IL17RB	2.03	4.02	4.8	2.71	2.43	3.37
IL17RC	3	1.68	2.18	2.1	2.6	2.16
IL17RD	0.42	0.71	0.64	0.4	0.36	0.51
IL17RE	0.03	0.11	0.05	0.19	0.1	0
IL17REL	0	0	0	0	0	0
IL18	2.26	4.08	5.88	4.61	6.76	4.88
IL18BP	1.37	1.64	2.93	0.73	1.13	2
IL18R1	0.06	0	0	0.01	0.29	0.4
IL18RAP	0.3	0.5	0	0.08	0.28	0.11
IL19	0	0	0	0	0	0
IL1A	0	0	0	0	0	0
IL1B	3.85	3.11	2.01	1	1.28	2.17
IL1F10	0	0	0	0	0	0
IL1R1	0.16	0.05	0.03	0.01	0.1	0.08
IL1R2	0	0	0	0	0	0
IL1RAP	7.78	9.5	8.02	6.03	10.48	6.94
IL1RAPL1	0.44	0	0	0.06	0	0
IL1RAPL2	0	0	0	0	0	0
IL1RL1	1.66	1.17	0.61	1.08	4.03	3.81
IL1RL2	0	0	0	0	0	0
IL1RN	0.38	0.07	0	0	0.08	0.09
IL2	0	0	0	0	0	0
IL20	0	0	0	0	0	0
IL20RA	0	0	0	0	0	0
IL20RB	0.04	0.4	0.2	0.1	0.14	0.22
IL21	0	0	0	0	0	0
IL21R	0.59	0.62	1.51	0.81	0.72	0.86
IL21R-AS1	0.24	0.39	0.9	0.18	0.14	0.4
IL22	0	0	0	0	0	0
IL22RA1	0	0	0	0	0	0
IL22RA2	0	0	0	0	0	0
IL23A	0.15	0.29	0	0.05	0.84	0.07
IL23R	2.61	0.38	3.36	0.29	2.14	0.38
IL24	0.26	0	0	0.08	0	0.14
IL25	0	0	0	0	0	0
IL26	0	0	0	0	0	0

IL27	0.15	0.06	0	0	0	0
IL27RA	12.64	19.2	23.47	12.06	16.39	12.97
IL2RA	1.14	3.71	2.24	0.23	1.77	0.49
IL2RB	0.04	0.07	0.16	0.07	0.24	0.25
IL2RG	37.55	122.76	109.55	64.62	89.97	91.12
IL3	0	0	0	0	0	0
IL31	0	0	0	0	0	0
IL31RA	0.01	0.05	0.02	0.06	0.03	0.04
IL32	0.27	0	0	0	0	0.09
IL33	0	0	0	0	0	0
IL34	0.13	0.03	0	0.12	0	0
IL36A	0	0	0	0	0	0
IL36B	0	0	0	0	0	0
IL36G	0	0	0	0	0	0
IL36RN	0	0	0	0	0	0
IL37	0.12	0	0	0	0	0
IL3RA	1.58	6.48	4.98	5.6	1.85	5.54
IL4	0	0	0	0	0	0
IL4I1	0.16	0.05	0.22	0.11	0.12	0.03
IL4R	4.92	3.74	2.1	2.64	3.13	2.9
IL5	0	0	0	0	0	0
IL5RA	0.13	0	0	0	0	0
IL6	0.96	0	0	0	0	0
IL6R	1.19	2.51	3.59	1.78	1.83	2.89
IL6ST	3.92	0.55	1.32	0.82	1.3	0.57
IL7	0.46	0	0	0.15	0.24	0
IL7R	0.06	0	0	0	0	0
IL8	8.6	0.17	0.08	0.36	1.24	0.93
IL9	0	0	0	0	0	0
IL9R	11.97	8.19	18.94	29.84	18.53	13.74
ILDR1	0	0.08	0.03	0.02	0	0.24
ILDR2	0.04	0.93	1.66	0.29	0.13	0.63
ILF2	184.15	237.65	214.54	205.26	213.87	218.67
ILF3	130.86	143.71	131.16	133.39	137.23	146.38
ILF3-AS1	6.23	6.97	7.14	4.74	6.63	6.29
ILK	38.47	43.03	34.28	34.19	37.12	31.8
ILKAP	27.88	32.29	22.32	25.31	23.01	26.24
ILVBL	24.34	32.75	37.68	38.88	28.8	35.66
IMMP1L	17	16.44	14.42	15.17	20.03	14.78
IMMP2L	22.84	6.24	7.98	9.75	10.75	14.22
IMMT	71.97	62.64	57.72	59.4	64.28	59.12
IMP3	156.86	205.36	154.83	159.93	186.01	170.82
IMP4	100.53	117.46	101.48	111.88	101.09	100.95

IMPA1	13.7	10.77	8.49	5.7	8.72	8.28
IMPA2	0.97	5.02	6.59	3.55	3.59	5.56
IMPACT	0.02	0.02	0.02	0.01	0.03	0.04
IMPAD1	4.43	4.99	3.83	3.25	4.64	4.79
IMPDH1	57.13	67.92	67.28	61.02	61.68	60.14
IMPDH2	266.64	339.21	382.47	407.23	350.29	390.58
IMPG1	0	0	0	0	0	0
IMPG2	0.08	0.03	0.06	0.03	0.18	0.07
INA	0.05	0.18	0.23	0.73	0.28	0.16
INADL	0.58	0.62	0.78	0.97	0.41	0.76
INCA1	0.19	0.11	0.04	0.18	0.12	0.25
INCENP	9.43	12.26	10.87	9.02	10.41	15.78
INE1	2.56	1.36	1.12	1.76	1.52	1.9
INE2	0	0.33	0	0	0	1.2
INF2	15.27	17.86	16.96	20.94	19.6	18.22
ING1	3.93	3.67	2.34	2.34	4.14	2.86
ING2	4.45	8.2	4.19	5.85	6.6	5.53
ING3	16.73	12.95	11.87	10.31	12.33	12.81
ING4	18.44	9.02	6.5	9.31	11.84	9.53
ING5	1.69	2.58	3.4	2.07	2.45	2.09
INGX	1.28	2.75	2.6	0.85	1.58	1.93
INHA	1.42	0	0	0.43	0.3	0.21
INHBA	0.47	0	0.56	0	0.26	0.1
INHBA-AS1	0.4	0.5	0.75	0.62	0.47	0.64
INHBB	0	0	0	0	0	0
INHBC	0	0.09	0	0	0.04	0.3
INHBE	0.12	0.01	0.02	0.1	0.09	0.13
INIP	19.62	14.7	16.3	13.51	11.98	11.81
INMT	1.69	1.96	1.92	1.41	1.43	2.24
INMT-FAM18	0	0	0	0	0	0
INO80	2.23	2.18	3.23	2.23	2.32	2.51
INO80B	1.15	0	0.77	0.85	0.37	1.03
INO80B-WBI	1.11	1.62	0	0.39	0.57	0
INO80C	21.33	9.45	8.46	12.25	14.81	10.76
INO80D	2.1	2.96	1.85	1.8	2.01	2.07
INO80E	3.21	5.69	7.03	6.3	6.79	5.83
INPP1	15.91	4.9	4.1	5.03	5.8	4.4
INPP4A	1.55	0.81	0.65	1.29	1.45	1.09
INPP4B	0	0.62	1.37	0.85	0.36	0.05
INPP5A	2.21	3.38	3.98	3.52	3.34	3.49
INPP5B	4.77	6.7	7.42	7.75	7.55	8.33
INPP5D	28.72	25.7	22.69	27.72	26.21	24.38
INPP5E	0.39	0.64	0.89	0.53	0.93	1.44

INPP5F	9.99	2.47	4.95	3.09	4.22	3.03
INPP5J	0.92	0	0	0.07	0.31	0.2
INPP5K	8.61	9.48	7.89	8.59	7.34	9.3
INPPL1	2.79	2.28	1.99	1.76	2.05	2.62
INS	0	0	0	0	0	0
INS-IGF2	0	0	0	0	0	0
INSC	0	0	0	0	0	0
INSIG1	99.08	60.55	69.32	61.7	93.27	62.67
INSIG2	4.03	3.59	2.96	2.44	2.97	3.01
INSL3	0.2	0.31	0	0	0	0.3
INSL4	0	0	0	0	0	0
INSL5	0	0	0	0	0	0
INSL6	0	0	0	0	0	0
INSM1	0	0	0	0	0	0
INSM2	0	0	0	0	0	0
INSR	2.15	1.55	1.98	1.89	2.64	2.04
INSRR	0.29	0	0	0	0.02	0
INTS1	7.46	9.82	12.58	10.1	7.29	9.53
INTS10	64.71	63.78	54.97	47.85	55.54	54.59
INTS12	15.11	17.5	13.62	14.4	13.92	13.28
INTS2	2.89	2.39	2.28	2.65	1.91	3.25
INTS3	5.63	7.5	8.12	5.54	6.34	9.24
INTS4	8.48	9.65	9.58	9.72	8.98	10.88
INTS4L2	0.78	0.84	0.08	0.19	0	0.52
INTS5	21.59	16.51	21.02	23.86	20.83	21.89
INTS6	1.92	1.47	1.32	1.34	1.33	1.06
INTS7	10.88	12.42	12.7	12.02	11.46	15.39
INTS8	8.34	10.13	10.46	9.09	8.4	9.04
INTS9	49.3	41.06	39.55	45.53	44.14	43.46
INTU	1.73	1.73	1.58	1.18	1.21	0.92
INVS	1.75	4.5	2.25	3.09	2.07	3.31
IP6K1	13.47	11.18	12.44	12.6	16.22	9.94
IP6K2	46.75	28.67	21.31	26.96	31.52	26.03
IP6K3	0	0	0	0	0	0
IPCEF1	0.03	0.1	0.1	0.05	0.02	0.31
IPMK	0.27	0.12	0.23	0.09	0.29	0.25
IPO11	18.02	15.79	14.4	14.82	15.04	16.64
IPO11-LRRC7	0	0	0	0	0	0
IPO13	2.59	2.89	3.23	2.63	2.76	1.96
IPO4	34.84	40.57	42.41	40.58	36.69	36.03
IPO5	27.37	28.22	30.95	32.86	26.74	35.48
IPO7	23.76	24.87	25.36	19.23	21.99	30.47
IPO8	3.33	4.23	3.84	3.71	4.26	4.38

IPO9	7.43	7.16	8.02	8.16	8.01	8.45
IPP	4.9	1.05	0.78	1.49	2.01	0.66
IPPK	7.66	4.65	4.67	4.77	5.48	3.67
IPW	0	0	0	0	0	0
IQCA1	0	0	0	0	0	0
IQCB1	10.12	10.52	8.44	8.04	8.55	8.98
IQCC	4.93	3.32	4.05	4.91	4.82	4.22
IQCD	0.11	0	0.72	0.04	0.21	0.06
IQCE	0.49	0.5	0.8	0.39	0.63	0.81
IQCF1	0	0	0	0	0	0
IQCF2	0	0	0	0	0	0
IQCF3	0	0	0.08	0	0	0
IQCF4	0	0	0	0	0	0
IQCF5	0	0	0	0	0	0
IQCF6	0	0	0	0	0	0
IQCG	0.46	1.06	0.93	1.21	0.88	0.64
IQCH	0.27	0.09	0.05	0.48	0.24	0.19
IQCH-AS1	14.32	19.58	21.53	16.65	16.41	17.16
IQCJ	0	0	0	0	0	0
IQCJ-SCHIP1	0	0	0.48	0.16	0.09	0.22
IQCK	1.32	0.95	0.89	2.03	0.92	1.22
IQGAP1	12.71	13.82	11.53	11.34	12.19	15.06
IQGAP2	0.76	1.75	1.27	2.13	1.25	1.88
IQGAP3	3.82	7.01	5.65	4.35	3.71	5.64
IQSEC1	0.96	1.51	1.33	0.93	1.32	1.42
IQSEC2	1.37	0.87	0.85	0.86	0.69	1.19
IQSEC3	0	0	0	0	0	0
IQUB	0.02	0	0	0	0	0
IRAK1	22.74	23.73	20.67	24.03	22.8	18.86
IRAK1BP1	1.25	0.26	0.24	0.95	1	1.2
IRAK2	1.84	2.06	0.81	0.37	1.47	1.94
IRAK3	1.78	5.05	3.44	2.55	2.36	4.79
IRAK4	3.59	5.3	2.91	2	3.27	2.91
IREB2	14.33	12.9	13.85	11.31	13.35	14.54
IRF1	9.49	9.63	12.73	10.04	10.43	9.58
IRF2	11.58	6.97	10.33	8.67	8.92	9.6
IRF2BP1	0.9	1.77	2.13	1.77	1.76	0.97
IRF2BP2	16.41	14.11	12.16	11.43	14.09	10.53
IRF2BPL	0.56	0.32	0.43	0.14	0.33	0.48
IRF3	29.61	34.6	30.36	28.29	33.97	21.85
IRF4	0.07	0.02	0.04	0.1	0.03	0.04
IRF5	6.05	16.52	13	7.75	9.34	10.52
IRF6	7.39	0.05	0.05	1.6	0.72	0.04

IRF7	4.18	6.72	4.99	3.79	4.74	4.25
IRF8	0	0.06	0	0	0	0
IRF9	3.55	1.96	1.1	4.31	1.93	1.21
IRG1	0	0	0	0	0	0
IRGC	0	0	0	0	0	0
IRGM	0.68	1.08	1.14	0.78	1.03	0.32
IRGQ	4.84	4.62	4.81	3.37	4.38	5.32
IRS1	0.02	0	0	0	0	0
IRS2	0.58	0.48	0.44	0.39	0.75	0.28
IRS4	0	0	0	0	0	0
IRX1	0	0	0	0	0	0
IRX2	0	0	0	0	0	0
IRX3	2.2	4.56	5.97	3.68	3.13	5.6
IRX4	0	0	0	0	0	0.03
IRX5	0	0.37	0.13	0.1	0.14	0.22
IRX6	0	0	0	0	0	0
ISCA1	20.17	17.85	14.85	13.88	18.23	14.11
ISCA2	37.33	39.81	36.86	40.57	41.46	33.02
ISCU	62.93	69.93	63	53.66	53.56	50.82
ISG15	109.07	50.25	77.78	126.14	105.64	81.51
ISG20	2.51	1.4	1.18	2.4	2.99	0.9
ISG20L2	20.36	24.56	23.69	21.37	22.42	23.6
ISL1	0.11	0.97	0.49	1.41	0.58	1.33
ISL2	3.06	3.5	2.49	3.7	2.85	2.05
ISLR	0	0	0	0	0	0
ISLR2	0.05	0.06	0.04	0.02	0.02	0.04
ISM1	0	0	0	0	0	0.03
ISM1-AS1	0	0	0	0	0	0
ISM2	2.05	4.45	2.81	3.33	1.82	6.34
ISOC1	27.72	27.76	31.44	19.8	24.92	26.16
ISOC2	44.86	40.4	35	57	50.71	56.57
ISPD	0.48	0.23	0.44	0.19	0.3	0.23
IST1	32.68	27.85	32.44	31.49	33.28	28.8
ISX	0	0	0	0	0	0
ISY1	19.33	19.53	20.6	17.03	17.18	15.06
ISY1-RAB43	1.93	0.84	0.78	0.74	0.29	1.06
ISYNA1	29.62	3.15	0.13	36.06	1.99	2.42
ITCH	5.89	6.14	6.3	5.92	5.59	7.41
ITFG1	16.4	15.56	11.81	10.31	11.59	10.85
ITFG2	10.82	19.2	13	11.48	12.53	12.46
ITFG3	28.19	18.29	25.04	29.72	28.67	24.9
ITGA1	0	0.01	0.01	0.02	0	0.18
ITGA10	0.03	0.03	0	0.01	0	0.06

ITGA11	0.03	0.28	0.07	0.27	0.14	0.35
ITGA2	0.18	1.13	0.97	0.43	0.45	0.87
ITGA2B	5.54	1.01	1.79	6.01	7.67	1.83
ITGA3	0.15	0.02	0.14	0.06	0	0.18
ITGA4	8.02	23.25	16.28	10.03	14.89	21.02
ITGA5	14.52	26.89	27.46	20.04	13.22	22.19
ITGA6	1.25	1.21	1.31	0.94	0.66	2.61
ITGA7	0.56	0.86	1.16	0.78	0.58	1.14
ITGA8	0.94	0.02	0.04	1	1.41	0.07
ITGA9	0.02	0.33	0.6	0.64	0.21	1.09
ITGAD	0	0	0	0	0	0
ITGAE	5.82	8.64	6.41	6.44	6.99	7.92
ITGAL	4.79	18.89	20.47	9.15	7.39	17.01
ITGAM	0	0.16	0	0.08	0.1	0
ITGAV	0.61	0.6	0.5	0.36	0.27	0.57
ITGAX	0.06	0.04	0.07	0.02	0.11	0.09
ITGB1	36.12	17.6	14.7	19.55	28.34	14.3
ITGB1BP1	41.16	48.36	49.68	40.47	43.3	41.09
ITGB1BP2	0.8	1.4	0.39	0.72	0.29	0.68
ITGB2	8.19	25.35	32.93	20.98	20.51	23.83
ITGB2-AS1	0.16	0.26	0.06	0	0.06	0.03
ITGB3	0.13	0	0	0.06	0.15	0
ITGB3BP	20.56	25.74	22.6	21.29	25	18.23
ITGB4	0.05	0.1	0.23	0.11	0.07	0.05
ITGB5	0	0	0	0	0	0
ITGB6	0	0	0	0	0	0
ITGB7	0	0.19	0.17	0.07	0	0
ITGB8	0.06	0.02	0.03	0.03	0.11	0
ITGBL1	0	0	0	0	0	0
ITIH1	0	0	0	0.02	0	0
ITIH2	0	0	0	0	0.02	0
ITIH3	0	0	0	0	0	0
ITIH4	0	0.31	0.02	0.11	0.02	0
ITIH5	0.44	0.54	0.41	0.25	0.42	0.35
ITIH6	0	0	0	0	0	0
ITK	0	0	0	0	0	0
ITLN1	74.38	0	0.41	0.93	1.46	0
ITLN2	4.66	0	0	0.05	0.06	0
ITM2A	78.57	275.81	167.19	140.32	173.33	207.99
ITM2B	275.3	108.5	102.42	84.94	122.61	92.24
ITM2C	54.27	83.86	88.3	77.82	68.65	76.05
ITPA	136.23	110.64	103	125.86	122.29	124.66
ITPK1	12.66	13.14	14.96	13.54	13.18	15.12

ITPK1-AS1	0.11	0	0.28	0	0.06	0.07
ITPKA	0.04	0.54	0.88	0.3	0.8	1.01
ITPKB	1.48	2.34	1.89	2.15	1.73	2.34
ITPKC	0.26	0.85	0.26	0.32	0.4	0.5
ITPR1	3.69	2.47	2.42	1.57	2.04	2.29
ITPR2	2.54	2.38	2.42	1.79	1.72	2.73
ITPR3	0.4	1.47	1.8	1.34	0.81	1.61
ITPRIP	4.33	0.54	1.75	2.88	2.5	0.87
ITPRIPL1	5.55	10.99	11.02	10.6	8.41	9.47
ITPRIPL2	2.77	2.46	3.48	2.96	2.57	3.33
ITSN1	2.24	2.65	3.32	2.98	3.56	4.28
ITSN2	4.38	4.52	3.89	4.65	4.29	3.79
IVD	14.8	17.71	15.02	14.72	15.05	13.34
IVL	0	0	0	0	0	0
IVNS1ABP	10.91	14.49	12.39	10.72	13.16	13.28
IWS1	21.05	18.14	15.17	17.66	19.44	16.26
IYD	0.03	0.03	0.09	0.05	0.01	0
IZUMO1	0	0	0	0	0	0
IZUMO2	0	0	0	0	0	0
IZUMO3	0	0	0	0	0	0
IZUMO4	0.81	0.81	1.25	0.56	0.15	0.16
JAG1	0.38	1.6	0.74	0.97	0.77	1.25
JAG2	2.34	1.43	2.47	1.95	1.29	2.34
JAGN1	53.39	53.44	48.12	59.34	62.16	50.63
JAK1	11.43	10.04	10	10.7	11.14	9.9
JAK2	2.71	0.84	1.26	1.15	1.39	1.05
JAK3	1.47	5.45	6.12	5.44	4.37	4.63
JAKMIP1	0	0	0	0	0	0
JAKMIP2	0.05	0.09	0.12	0.05	0.03	0.12
JAKMIP2-AS1	0	0	0	0	0	0
JAKMIP3	0	0	0	0	0	0
JAM2	0.33	0.47	0.22	0.33	0.42	0.51
JAM3	13.7	17.24	24.23	20.73	18.41	21.98
JARID2	12.6	7.18	6.73	8.69	7.47	6.94
JAZF1	1.91	1.43	1.65	0.55	1.82	1.54
JAZF1-AS1	0	0	0	0	0	0
JDP2	0.31	0.41	0.83	0.49	0.36	0.56
JHDM1D	0.45	0.05	0.04	0.13	0.1	0.08
JKAMP	21.54	15.94	14.1	14.56	18.08	13.7
JMJD1C	4.2	4.69	3.97	3.09	3.45	4.29
JMJD1C-AS1	0.12	0.71	0.06	0.65	0.29	0.06
JMJD4	15.61	17.88	18.68	20.78	19.14	18.79
JMJD6	30.07	20.46	15.75	17.95	22.82	13.49

JMJD7	6.84	11.04	9.28	11.64	7.36	12.03
JMJD7-PLA2(	0.97	0.71	0.71	0.9	0.1	0.83
JMJD8	14.35	17.47	10.06	11.12	15.19	13.61
JMY	1.1	0.68	0.49	0.75	0.83	0.85
JOSD1	5.32	4.59	5.41	5.01	5.07	5.31
JOSD2	35.78	15.73	11.75	20.34	25.07	13.76
JPH1	1.5	2.8	1.7	1.32	2.04	3.56
JPH2	0	0	0	0	0	0
JPH3	0.04	0.1	0.08	0.1	0	0
JPH4	0	0	0.05	0	0.03	0
JPX	4.79	5.9	6.07	5.27	5.93	5.99
JRK	4.27	6.34	7.11	7.7	6.27	8.4
JRKL	0.62	1.06	0.59	0.71	0.97	0.73
JRKL-AS1	0	0	0	0	0.12	0
JSRP1	0	0	0	0	0	0
JTB	58.52	67.44	81.53	71.43	66.32	48.18
JUN	4.86	1.69	1.78	1.77	1.75	2.17
JUNB	34.2	10.72	14.17	20.06	22.07	8.76
JUND	1.66	1.12	1.23	1.83	2.39	1.32
JUP	168.74	31.07	39.26	73.74	84.67	37.02
KAAG1	0	0	0	0	0	0
KAL1	0	0	0	0	0	0
KALRN	0	0.03	0.01	0	0	0.13
KANK1	1.82	3.83	3.69	3.9	2.98	2.9
KANK2	5.66	3.21	3.72	4.8	4.09	3.45
KANK3	0.81	0.54	0.62	0.65	1.09	0.45
KANK4	0.01	0.08	0.15	0.13	0.05	0.06
KANSL1	6.08	5.25	4.08	3.86	4.55	6.28
KANSL1-AS1	0.96	4.07	0.88	1.44	0.61	0.96
KANSL1L	0.67	0.57	0.66	0.54	0.65	1.05
KANSL2	12.37	15.6	13.29	11.39	13.55	13.63
KANSL3	10.97	13.37	10.25	9.88	10.31	13.27
KARS	131.05	123.28	152.37	132.73	142.08	136.94
KAT2A	2.62	1.92	3.02	2.94	3.16	2.75
KAT2B	0.43	0.13	0.12	0.21	0.35	0.12
KAT5	18.36	19.86	17.35	16.25	20.49	15.48
KAT6A	7.21	7.98	8.81	7.83	8.2	8.44
KAT6B	2.81	4.17	2.81	2.44	2.44	2.99
KAT7	26.11	25.45	24.07	27.68	21.42	27.73
KAT8	4.62	6.9	5.96	3.62	5.75	7.04
KATNA1	11.24	18.44	13.35	14.55	15.22	17.02
KATNAL1	1.44	0.82	0.63	0.76	0.63	0.94
KATNAL2	0.13	1.87	0.59	0.37	0.46	0.63

KATNB1	25.47	28.76	26.4	26.11	23.69	32.43
KATNBL1	8.96	8.07	5.23	6.09	8.09	5.07
KAZALD1	1.11	4.87	6.63	5.81	3.3	3.78
KAZN	0.5	1.24	1.35	0.6	1.16	0.95
KBTBD11	0.44	1.16	1.15	0.77	1.4	1.51
KBTBD12	0.61	0.72	0.58	0.28	0.52	0.78
KBTBD13	0.02	0	0	0	0	0.28
KBTBD2	6.83	5.42	4.69	4.05	6.3	4.06
KBTBD3	0.7	0.28	0.29	0.39	0.57	0.28
KBTBD4	5.32	7.13	7.08	9.06	7.23	6.83
KBTBD6	2.99	4.39	3.33	4.86	3.78	3.25
KBTBD7	0	0.08	1.68	0.83	1.51	0.83
KBTBD8	4.1	5.49	5.83	2.74	4.13	4.82
KC6	0	0	0	0	0	0
KCMF1	1.74	1.37	1.54	1.42	1.66	1.23
KCNA1	0	0	0	0	0	0
KCNA10	0	0	0	0	0	0
KCNA2	0	0	0	0	0	0
KCNA3	0	0	0	0	0	0
KCNA4	0	0	0	0	0	0
KCNA5	0	0	0	0	0	0
KCNA6	0.02	0	0	0	0	0
KCNA7	0.8	1.05	1.06	0.75	0.61	0.65
KCNAB1	0.02	0.09	0.07	0	0.24	0.16
KCNAB1-AS1	0	0	0	0	0	0
KCNAB1-AS2	0	0	0	0	0	0
KCNAB2	15.58	56.93	43.94	32.43	25.57	51
KCNAB3	0.24	0.26	0.05	0.02	0.06	0.03
KCNB1	0	0	0	0	0	0
KCNB2	0	0	0	0	0	0
KCNC1	0.01	0.04	0	0.01	0	0
KCNC2	0	0	0	0	0	0
KCNC3	0	0	0	0	0	0
KCNC4	0.2	0.61	0.27	0.3	0.51	1.1
KCND1	0.21	0.33	0.15	0.21	0.06	0.42
KCND2	0	0	0	0	0	0
KCND3	0.11	0	0	0	0	0
KCNE1	0	0	0	0	0	0
KCNE1L	0.26	0.04	0.05	0.14	0	0.05
KCNE2	0.1	0.08	0	0	0.09	0
KCNE3	5.84	8.87	7.52	5.17	4.59	7.15
KCNE4	0.71	0.78	0.76	0.72	0.84	0.68
KCNF1	0	0	0	0	0	0

KCNG1	0	0	0	0	0	0
KCNG2	0.21	0	0.05	0	0	0.11
KCNG3	0	0.03	0	0.03	0.16	0.02
KCNG4	0	0	0	0	0	0
KCNH1	0	0.01	0	0	0	0
KCNH2	36.41	0.54	0.33	12.12	18.05	1.23
KCNH3	0	0.03	0	0.05	0.02	0.02
KCNH4	0.15	0.04	0	0.15	0.12	0.02
KCNH5	0	0	0	0	0	0
KCNH6	0.17	0.14	0.1	0.1	0.1	0.1
KCNH7	0.02	0	0	0	0	0
KCNH8	0	0	0	0	0	0
KCNIP1	0	0	0	0	0	0
KCNIP2	0.21	0.36	0.44	0.05	0.2	0.04
KCNIP3	0	0.15	0.17	0	0	0.14
KCNIP4	0.32	0.31	0.03	0.07	0.07	0
KCNIP4-IT1	0	0	0	0	0	0
KCNJ1	0	0.17	0	0.04	0	0.03
KCNJ10	0	0	0	0	0.01	0.03
KCNJ11	0.98	1.16	1.49	1.23	1.25	1.87
KCNJ12	0	0.02	0.01	0	0.01	0
KCNJ13	0.19	0.17	0	0.04	0	0.04
KCNJ14	0.27	0.07	0.58	0.33	0.15	0.21
KCNJ15	0	0	0	0	0	0
KCNJ16	0	0	0	0	0	0
KCNJ18	0	0	0	0	0	0
KCNJ2	0	0	0	0	0	0
KCNJ2-AS1	0	0	0	0	0	0
KCNJ3	0.03	0.03	0.13	0.07	0.05	0.18
KCNJ4	0	0.18	0	0.11	0	0
KCNJ5	0.43	0.69	0.62	0.6	0.5	0.78
KCNJ6	0	0	0	0	0	0
KCNJ8	0	0	0	0	0	0
KCNJ9	0	0.02	0	0.12	0	0.07
KCNK1	3.68	0	0	0.42	1.24	0
KCNK10	0	0	0.01	0.01	0	0
KCNK12	0	0	0	0	0	0
KCNK13	0	0	0	0	0	0.03
KCNK15	0	0	0	0	0	0
KCNK16	0	0	0	0	0	0
KCNK17	0	0	0	0	0	0
KCNK18	0	0	0	0	0	0
KCNK2	0	0	0	0	0	0

KCNK3	0.11	0.03	0.14	0.03	0	0.05
KCNK4	0	0.03	0	0	0	0
KCNK5	8.53	6.52	5.65	7.43	5.91	8.01
KCNK6	7.74	5.25	6.35	7.96	11.46	4.49
KCNK7	0	0.05	0	0	0	0
KCNK9	0	0	0	0	0	0
KCNMA1	0	0	0	0	0	0
KCNMB1	0	0	0	0	0	0
KCNMB2	0	0	0	0	0	0
KCNMB2-IT1	0	0	0	0	0	0
KCNMB3	0.42	0.05	0.85	0.86	2.38	0.66
KCNMB4	0.16	0.29	0.2	0.34	0.18	0.2
KCNN1	0	0	0	0	0	0
KCNN2	0.66	0.21	0.14	0.12	0.14	0.11
KCNN3	0.11	0.15	0.17	0.09	0.1	0.13
KCNN4	4.11	14.8	21.27	16.04	11.56	16.81
KCNQ1	0.05	0.38	0.53	0.26	0.43	0.71
KCNQ1DN	0	0	0	0	0	0
KCNQ1OT1	2.58	2.83	2.9	1.89	2.18	2.45
KCNQ2	0	0.04	0.1	0	0.05	0.11
KCNQ3	0.02	0	0	0	0	0
KCNQ4	0.57	0.45	0.24	0.67	0.29	0.97
KCNQ5	0.72	1.2	1.01	0.59	0.62	1.1
KCNQ5-AS1	0	0.22	0.25	0.24	0	0
KCNRG	0.07	0.4	0.46	0.14	0.25	0.75
KCNS1	0.03	0.21	0.06	0.02	0.02	0
KCNS2	0	0	0	0.03	0.07	0
KCNS3	0.16	1.09	1.96	0.35	1.1	0.77
KCNT1	0	0	0	0	0	0
KCNT2	0	0	0	0	0.15	0
KCNU1	0	0	0	0	0	0
KCNV1	0	0	0	0	0	0
KCNV2	0.03	0.19	0.37	0.16	0	0.03
KCP	0.04	0.07	0	0.03	0.07	0.03
KCTD1	0.23	1.15	0.93	0.64	0.82	1.29
KCTD10	19.4	8.2	10.99	9.46	10.81	9.3
KCTD11	0	0	0	0	0	0
KCTD12	0.01	0.06	0	0.02	0.04	0.06
KCTD13	6.88	5.09	4.16	6.39	5.07	1.97
KCTD14	0	0	0	0	0	0
KCTD15	4.16	3.65	3.33	4.1	3.86	3.08
KCTD16	0	0	0	0	0	0
KCTD17	1.58	0.83	0.85	0.82	0.83	0.36

KCTD18	1.47	2.25	2.91	1.46	1.76	1.58
KCTD19	0	0	0	0	0	0.03
KCTD2	1.14	0.59	0.97	1.56	1.26	1.17
KCTD20	15.58	13.71	12.74	12.6	12.42	12.63
KCTD21	2.13	2	2.96	2.13	1.91	1.82
KCTD3	2.5	1.51	1.42	1.35	1.95	2.13
KCTD4	0.07	0.11	0	0	0	0.25
KCTD5	5.64	2.7	3.38	3.09	3.67	2.11
KCTD6	6.37	7.37	6.49	5.22	6.89	7.47
KCTD7	0.94	0.94	0.52	1.1	1.51	0.59
KCTD8	0	0	0	0	0	0
KCTD9	9.25	7.1	5.48	4.24	6.93	4.09
KDELC1	9.2	13.62	11.69	8.65	9.09	13.52
KDELC2	3.52	4.85	3.78	2.24	3.19	3.54
KDELR1	60.44	66.36	74.52	72.37	77.32	76.89
KDELR2	72.37	62.05	53.65	58.27	67.22	60.06
KDELR3	7.08	7.31	11.43	9.47	8.43	14.8
KDM1A	8.86	8.75	8.25	8.23	6.74	8.04
KDM1B	1.74	2.76	1.9	0.87	1.6	1.67
KDM2A	8.83	7.16	6.83	7.21	5.58	6.49
KDM2B	4.87	6.99	7.21	6.02	5.49	7.92
KDM3A	4.97	3.63	2.86	3.81	3.43	3.53
KDM3B	3.58	3.44	4.39	3.03	4.82	5.09
KDM4A	7.03	4.54	5.27	5.57	6.03	4.99
KDM4A-AS1	1.42	3.41	3.13	2	1.85	2.9
KDM4B	1.13	0.8	0.76	0.73	1.29	0.76
KDM4C	4.65	3.54	4.16	2.31	3.75	3.67
KDM4D	0.44	0.35	0.2	0.62	0.05	0.02
KDM4E	0	0	0	0	0	0
KDM5A	4.16	4.44	5.4	4.68	5.66	5.61
KDM5B	9.75	9.19	9.59	8.71	8.14	9.28
KDM5B-AS1	0.5	0.16	0.28	0.43	1.86	0.52
KDM5C	19.13	15.46	14.23	14.3	15.85	16.57
KDM5D	0	0	0	0	0.02	0.03
KDM6A	6.51	4.39	4.96	5.14	5.83	4.79
KDM6B	1.31	1.07	0.93	0.57	0.96	1.11
KDM8	2.05	2.43	3.94	4.08	2.46	3.18
KDR	0	0	0	0	0	0
KDSR	6.15	7.32	7.61	5.7	6.76	6.12
KEAP1	74.57	61.99	64.49	71.47	68.37	59.64
KEL	12.82	0	0	2.71	4.52	0
KERA	0	0	0	0	0	0
KGFLP1	0.5	0.5	0.7	0.38	0.39	0.27

KGFLP2	0.11	0.16	0	0.02	0.02	0.02
KHDC1	2.81	3.98	3.74	4.5	2.62	3.9
KHDC1L	0.26	0	0	0	0	0
KHDC3L	0	0	0	0	0	0
KHDRBS1	20.49	17.3	19.85	19.27	25.09	18.41
KHDRBS2	0	0.86	1.84	0.62	0.18	0.13
KHDRBS3	0.95	3.72	1.56	1.38	2.36	2.22
KHK	9.94	8.59	11.73	14.01	9.21	11.98
KHNYN	1.85	1.63	1.23	1.37	2.27	2.19
KHSRP	6.49	6.17	8.03	6.46	5.78	6.94
KIAA0020	28.99	32.65	30.39	26.33	31.56	24.78
KIAA0040	3.58	5.04	3.51	2.81	2.21	4.76
KIAA0087	0.08	0.09	0.09	0.07	0.22	0.13
KIAA0100	9.96	15.82	15.42	13.14	12.43	13.74
KIAA0101	279.32	364.72	316.67	291.81	327.48	398.94
KIAA0125	1.77	1.13	3.28	2.87	3.27	1.54
KIAA0141	10.44	11.62	8.23	8.24	10.59	8.71
KIAA0146	18.67	20.94	18	19.31	18.31	19.85
KIAA0195	8.36	6.3	6.79	6.48	6.91	6.67
KIAA0196	38.8	28.69	27.34	27.43	28.64	27.65
KIAA0226	6.54	3.4	3.56	4.02	4.25	3.29
KIAA0226L	0	0	0	0	0	0
KIAA0232	3	1.55	2.48	2.22	1.6	1.45
KIAA0247	6.51	4.97	4.47	3.78	5.7	4.19
KIAA0317	12.03	8.83	9.11	6.79	8.02	7.24
KIAA0319	0	0	0	0	0	0
KIAA0319L	5.73	5.88	4.37	4.32	3.81	4
KIAA0355	1.59	0.83	0.73	0.96	1.01	0.7
KIAA0368	6.65	7.42	9.01	10.02	7.92	7.83
KIAA0391	16.41	14.21	14.61	14.6	16	14.19
KIAA0408	0.05	0.07	0.03	0.02	0.05	0.04
KIAA0430	1.92	1.45	1.63	1.35	1.23	1.28
KIAA0513	3.93	0.31	0.09	0.93	2.1	0.1
KIAA0556	1.3	1.28	1.12	0.84	0.63	1.32
KIAA0586	4.59	5.75	4.88	3.86	6.1	4.56
KIAA0753	1.31	1.56	1.37	1.42	1.5	1.44
KIAA0754	1.02	0.63	0.86	0.9	1.17	0.98
KIAA0825	0.26	0.74	0.35	0.52	0.25	0.83
KIAA0895	0.21	0.52	0.68	0.46	0.7	0.89
KIAA0895L	1.57	0.84	0.96	1.45	1.61	0.72
KIAA0907	1.98	2.65	2.9	1.82	2.13	3.08
KIAA0922	6.48	7.84	7.03	5.36	6.87	8.02
KIAA0930	1.81	2.89	2.56	1.67	1.83	2.61

KIAA0947	2.59	2.55	2.24	1.53	2.24	2.05
KIAA1009	1.32	1.35	0.94	1.35	1.68	0.84
KIAA1024	0.07	0.01	0.09	0.11	0	0.11
KIAA1024L	0	0	0	0	0	0
KIAA1033	3.19	4.35	4.47	2.78	3.83	4.6
KIAA1045	0	0.25	0.18	0.02	0.03	0.06
KIAA1107	0.11	0.09	0.03	0.14	0.18	0.13
KIAA1109	0.86	0.66	0.63	0.42	0.69	0.84
KIAA1143	10.38	9.46	10.66	9.18	10.73	9.21
KIAA1147	0.51	0.94	0.92	0.76	0.67	1.37
KIAA1161	1.17	2.94	1.8	2.26	2.33	3.43
KIAA1191	17.09	15.6	13.58	14.34	18.45	17.37
KIAA1199	0.02	0.02	0.03	0	0.03	0.01
KIAA1210	0.04	0.06	0.02	0.02	0.03	0.05
KIAA1211	0.3	0.31	0.24	0.09	0.12	0.55
KIAA1211L	0	0	0	0	0	0
KIAA1217	0	0	0	0	0	0
KIAA1239	0	0	0	0	0	0
KIAA1244	0	0.04	0.03	0.02	0.03	0.02
KIAA1257	0	0	0	0	0	0
KIAA1279	9.76	4.83	6.9	5.86	11.4	5.97
KIAA1324	0.59	0.59	0.84	0.54	0.69	0.62
KIAA1324L	0.03	0.06	0.14	0.07	0.06	0.05
KIAA1328	0.8	0.32	0.94	0.48	0.74	0.77
KIAA1377	0.23	0.22	0.16	0.15	0.1	0.12
KIAA1407	0.29	0.04	0.12	0.08	0.1	0.28
KIAA1429	15.81	15.45	15.56	16.75	14.7	17.35
KIAA1430	8.18	6.19	7.26	6.94	7.35	7.91
KIAA1432	1.19	1.82	1.19	0.59	0.91	1.3
KIAA1456	0.25	0.37	0.24	0.21	0.22	0.24
KIAA1462	0.08	0.21	0.4	0.3	0.22	0.09
KIAA1467	6.15	7.53	6.77	4.37	5.87	8.38
KIAA1468	1.23	1.47	1.26	1.69	1.22	0.98
KIAA1522	1.65	1.33	1.04	0.78	0.77	1.31
KIAA1524	2.4	2.18	2.34	2.45	2.14	2.99
KIAA1549	1.32	1.19	1.23	1	0.74	1.22
KIAA1549L	0	0	0	0	0	0
KIAA1551	11.9	10.1	11.93	10.59	14.21	10.76
KIAA1586	8.9	8.27	6.35	5.87	7.43	7.49
KIAA1598	5.39	4.27	3.53	2.55	3.73	4.04
KIAA1609	3.15	1.68	1.84	2.73	2.37	2.18
KIAA1614	0.14	0.29	0.2	0.16	0.11	0.29
KIAA1644	0	0	0	0	0	0

KIAA1656	0.32	0.41	0.2	0.14	0.18	0.14
KIAA1671	1.06	0.17	0.19	0.68	0.43	0.18
KIAA1683	0.15	0	0.03	0	0	0.02
KIAA1704	10.43	9.5	12.41	7.82	9.81	12.4
KIAA1715	4.18	3.84	3.72	2.52	3.79	3.29
KIAA1731	1.98	1.91	1.9	1.23	1.75	1.69
KIAA1737	5.36	4.05	5.79	5.24	5.88	5.37
KIAA1751	0.15	0.2	0.38	0.26	0.28	0.35
KIAA1755	0.01	0.04	0.05	0.01	0.03	0.06
KIAA1804	1.05	1.59	1.28	1.09	1.02	1.08
KIAA1841	1.4	1.84	2.03	1	1.08	0.8
KIAA1875	0.31	0.35	0.49	0.36	0.26	0.32
KIAA1919	2.79	3.56	3.57	2.28	2.67	2.57
KIAA1958	1.25	2.58	3.32	2.77	2.52	2.34
KIAA1967	39.23	47.77	40.92	42.94	37.35	51.43
KIAA1984	0.09	0.07	0.12	0.15	0	0.05
KIAA1984-AS	0.83	1.28	1.26	0.76	1.02	1.74
KIAA2013	5.95	9	8.97	5.66	7.39	7.77
KIAA2018	1.86	2.22	1.59	1.31	1.68	1.82
KIAA2022	0.13	1.25	1.2	0.98	0.53	1.12
KIAA2026	1.08	1.22	1.2	0.72	0.72	0.64
KIDINS220	6.95	4.47	3.43	3.53	5.65	6.33
KIF11	20.85	19.73	17.72	17.36	19.42	22.29
KIF12	0	0	0	0	0	0
KIF13A	2.57	1.85	1.57	1.09	1.55	1.73
KIF13B	3.61	1.79	1.83	1.4	1.93	1.92
KIF14	3.92	3.33	2.89	2.96	3.16	2.43
KIF15	3.46	4.84	3.67	3.45	3.63	4.91
KIF16B	2.76	2.41	1.86	1.89	2.09	2.61
KIF17	0	0.03	0.04	0.04	0.43	0.17
KIF18A	9.02	8.08	6.98	6.79	7.67	8.66
KIF18B	14.36	20.86	19.47	18.3	16.42	18.71
KIF19	0	0	0	0	0	0
KIF1A	0	0	0	0	0	0
KIF1B	3.29	3.58	3.75	4.02	3.57	3.23
KIF1C	2.39	0.69	1.11	0.91	1.52	1.02
KIF20A	12.95	16.16	15.15	15.17	15.43	11.14
KIF20B	7.91	10.45	6.48	7.47	7.8	7.51
KIF21A	3.98	4.5	4.16	3.16	3.03	4.07
KIF21B	0.08	0.62	0.62	0.6	0.58	0.83
KIF22	71.78	98.93	79.27	82.41	88.22	85.39
KIF23	29.68	27.7	20.52	18.92	26.01	24.92
KIF24	2.4	3.22	3.12	2.2	1.98	1.9

KIF25	0	0.29	0	0	0	0
KIF26A	0.18	0.58	0.5	0.47	0.44	0.74
KIF26B	0.09	1.24	1.23	1.3	1.35	1.58
KIF27	0.45	0.32	0.35	0.52	0.15	0.22
KIF2A	10.7	11.55	11.22	12.77	11.8	11.81
KIF2B	0	0	0	0	0	0
KIF2C	46.05	44.46	45.63	47.09	43.62	44.37
KIF3A	2.48	1.52	1.74	1.26	2.13	1.31
KIF3B	2.75	2.13	1.23	1.37	2.85	1.41
KIF3C	2.63	1.46	1.74	2.37	1.99	2.75
KIF4A	19.51	18.32	14.54	16.62	16.45	15.02
KIF4B	0.16	0.01	0	0	0	0
KIF5A	0	0	0	0	0	0
KIF5B	25.55	21.44	17.07	16.55	22.7	18.35
KIF5C	0	0	0.09	0	0	0
KIF6	0	0	0	0	0	0
KIF7	0.19	0.32	0.28	0.58	0.43	0.22
KIF9	4.77	4.77	3.82	4.48	3.88	4.79
KIF9-AS1	0.33	0.7	0.26	0.17	0.33	0.47
KIFAP3	6.57	1.98	2.78	2.73	3.44	1.96
KIFC1	23.48	26.38	25.21	23.69	25.28	28.13
KIFC2	0.18	0.12	0.16	0.03	0.2	0.14
KIFC3	2.51	0.5	0.36	0.86	1.64	0.68
KIN	4.41	4.3	3.22	2.73	3.86	3.22
KIR2DL1	0	0	0	0	0	0
KIR2DL3	0	0	0	0	0	0
KIR2DL4	0	0	0	0	0	0
KIR2DS4	0	0	0	0	0	0
KIR3DL1	0	0	0	0	0	0
KIR3DL2	0	0.03	0	0	0	0
KIR3DL3	0	0	0	0	0	0
KIR3DX1	0.09	0	0	0	0	0
KIRREL	4.22	3.62	4.53	3.89	3.66	5.51
KIRREL2	0.09	0.75	1.32	0.87	0.62	0.82
KIRREL3	0	0.07	0.08	0.12	0.06	0.05
KIRREL3-AS2	0	0	0	0	0	0
KIRREL3-AS3	0	0	0	0	0	0
KISS1	0	0	0	0	0	0
KISS1R	0.05	0	0.17	0.35	0.22	0.42
KIT	50.96	6.26	7.75	9.53	18.8	6.09
KITLG	1.44	0.87	1.05	0.65	1.15	0.87
KL	0	0	0	0	0	0
KLB	0.35	0.19	0.79	0.38	0.42	0.23

KLC1	8.09	7.16	5.58	7.97	7.16	3.73
KLC2	4.2	1.79	2.84	3.22	3.56	0.84
KLC3	1.12	0.59	0.61	0.51	0.59	1.47
KLC4	2.09	3.38	1.45	2.71	2.94	2.08
KLF1	12.34	0	0.17	6.24	7.08	0.05
KLF10	25.77	6.55	8.66	10.41	14.07	5.53
KLF11	0.25	0.88	0.48	0.37	0.09	1.13
KLF12	0.11	0.03	0.14	0.06	0.04	0
KLF13	1.69	2.07	2.09	1.38	1.63	1.84
KLF14	0	0	0	0	0	0
KLF15	0.15	0	0	0.02	0.14	0.15
KLF16	2.17	3.03	3.15	3.06	3.17	3.5
KLF17	0	0.05	0	0.02	0.04	0.07
KLF2	0	0	0	0	0	0
KLF3	2.23	1.04	1.15	1.18	1.39	1.07
KLF4	0.35	0	0.02	0.03	0.05	0.03
KLF5	0.22	0.02	0.14	0.09	0.06	0
KLF6	24.28	7.13	7.34	8.01	9.58	4.97
KLF7	0.27	0.54	0.64	0.37	0.43	0.8
KLF8	0.15	0.16	0.33	0.13	0.17	0.24
KLF9	1.13	1.24	0.99	1.09	1.06	0.99
KLHDC1	0	0.11	0.05	0.02	0.08	0
KLHDC10	1.57	1.53	1.35	1.08	1.31	1.4
KLHDC2	14.92	15.6	14.03	17.23	14.28	14.92
KLHDC3	140.15	119	134.22	148.33	139.86	124.55
KLHDC4	22.59	17.73	17.71	20.29	19.52	18.21
KLHDC7A	0	0	0	0	0	0
KLHDC7B	0	0	0	0	0	0
KLHDC8A	1.21	0	0	0.56	0.53	0.05
KLHDC8B	44.24	13.43	13.87	28.07	35.4	13.26
KLHDC9	0	0.05	0	0	0	0
KLHL1	0	0	0	0	0	0
KLHL10	0.04	0	0	0.1	0	0
KLHL11	22.02	22.99	29.01	28.38	27.87	24.57
KLHL12	12.39	12.36	10.17	13.38	12	12.61
KLHL13	7.47	8.23	9.26	7.54	9.64	10.66
KLHL14	0	0	0	0	0	0
KLHL15	2.05	1.67	1.32	0.72	0.63	1.06
KLHL17	0.2	0.63	0.52	0.16	0.35	0.92
KLHL18	12.53	8.92	8.46	9.19	11.89	8.02
KLHL2	0.28	0.38	0.51	0.19	0.36	0.84
KLHL20	3.63	2.92	2.34	2.2	2.12	2.74
KLHL21	7.4	6.93	7.08	7.49	6.91	4.94

KLHL22	4.22	6.37	6	8.98	5.9	8.1
KLHL23	4.76	6.31	4.83	4.76	4.5	7.35
KLHL24	2.05	1.46	0.81	1.13	1.17	1.4
KLHL25	3.31	3.85	3.27	2.6	3.53	3.17
KLHL26	5.86	4.59	5.79	5.48	2.97	5.1
KLHL28	2.25	2.25	1.91	1.34	1.75	2.2
KLHL29	0.06	0	0.03	0	0	0.01
KLHL3	0.15	0.06	0.17	0.02	0.04	0.06
KLHL30	0	0.17	0.16	0.11	0.19	0.09
KLHL31	0.09	0.11	0.16	0	0.2	0.01
KLHL32	0.38	0	0.03	0.03	0.02	0
KLHL33	0	0	0	0	0	0
KLHL34	0.05	0.06	0.24	0.12	0.1	0.05
KLHL35	0.27	0	0	0.84	0.07	0.04
KLHL36	3.19	2.65	1.31	2.13	2.08	1.27
KLHL38	0.04	0	0	0	0	0
KLHL4	0.11	0.23	0.08	0.26	0.24	0.26
KLHL40	0	0	0	0	0	0
KLHL41	0	0	0	0	0	0
KLHL42	2.03	1.76	1.1	1.02	1.34	1.49
KLHL5	0.9	1.19	0.91	0.75	0.81	1.01
KLHL6	2.76	3.48	3.44	3.61	3.25	2.79
KLHL7	17	17.29	12.54	12.98	17.59	15.08
KLHL7-AS1	0.14	0.36	0.08	0.14	0.19	0.04
KLHL8	3.67	4.04	5.78	4.53	3.17	4.43
KLHL9	8.52	6.83	4.71	5.57	4.66	5.37
KLK1	0	0.14	0.41	0.74	0.25	0.45
KLK10	0.06	0.06	0.07	0.07	0	0.06
KLK11	0	0	0	0	0	0
KLK12	0	0	0	0	0	0
KLK13	0.12	0	0	0	0	0
KLK14	0	0	0	0	0	0
KLK15	0	0	0	0	0	0
KLK2	0	0	0.03	0	0	0
KLK3	0	0	0	0	0	0
KLK4	0	0	0	0	0	0
KLK5	0	0	0	0	0	0
KLK6	0	0	0	0	0	0
KLK7	0.35	0.43	0.45	0.02	0.14	0.46
KLK8	0	0	0	0	0	0
KLK9	0	0	0	0	0	0
KLKB1	0	0	0	0	0	0
KLKP1	0	0	0	0	0	0

KLLN	0.44	0.79	0.31	0.48	0.58	0.52
KLRAP1	0.1	0.21	0	0.16	0	0.03
KLRB1	0	0	0	0	0	0
KLRC1	0.05	0	0	0	0	0
KLRC2	0.75	0	0	0.22	0.24	0
KLRC3	0.84	0	0	0.06	0	0
KLRC4	0	0	0	0	0	0
KLRC4-KLRK1	0	0	0	0	0	0
KLRD1	1.8	1.4	1.87	1.03	1.33	1.72
KLRF1	0	0.1	0	0.08	0.23	0.12
KLRF2	0	0	0	0	0	0
KLRG1	0.29	0.53	0.72	0.37	0.27	0.92
KLRG2	0.19	0	0	0	0	0
KLRK1	0.8	0	0	0	0	0
KMO	0	0.24	0.18	0.04	0	0.02
KNCN	0	0	0	0	0	0
KNDC1	0	0	0	0	0	0
KNG1	0	0	0.02	0.02	0	0
KNSTRN	39.69	40.07	42.18	36.22	51.26	41.32
KNTC1	2.87	2.64	3.16	3.55	3.37	3.4
KPNA1	9.55	7.78	7.35	8.57	10.16	8.13
KPNA2	231.89	221.52	202.68	215.25	219.78	207.54
KPNA3	11.45	12.13	8.56	9.31	9.7	9.65
KPNA4	4.45	3.18	2.98	2.51	4.05	3.56
KPNA5	1.09	0.94	1.06	0.66	0.61	1.59
KPNA6	30.03	16.94	17.62	18.7	19.09	15.87
KPNA7	0	0	0	0	0	0
KPNB1	35.64	34.99	44.73	35.82	42.67	42.36
KPRP	0	0	0	0	0	0
KPTN	6.75	7.82	7.47	8.11	10.58	10.03
KRAS	4.98	4.5	3.36	3.82	4.24	4.21
KRBA1	0.65	1.22	1.73	1.56	0.9	1.95
KRBA2	0.69	0.63	0.6	0.82	0.22	0.39
KRBOX1	0	0	0	0	0	0
KRBOX4	7.82	8.79	6.47	5.87	5.5	7.13
KRCC1	4.07	4.28	4.54	4.03	3.44	3
KREMEN1	5.69	1.33	1.55	1.55	1.59	1.52
KREMEN2	0.34	0.99	0.55	1.98	1.25	0.4
KRI1	13.51	13.85	11.68	12.11	11.73	12.06
KRIT1	5.43	3.79	4.15	2.32	3.87	3.87
KRR1	13.33	11.11	13.69	9.24	12.33	10.74
KRT1	0	0	0	0	0.2	0
KRT10	3.27	3.58	2.55	1.59	4.19	1.99

KRT12	0	0	0	0	0	0
KRT13	0	0	0	0	0	0
KRT14	0	0	0	0	0	0
KRT15	0	0	0	0	0	0
KRT16	0	0	0	0	0	0
KRT16P1	0	0	0	0	0	0
KRT16P2	0	0	0	0	0	0
KRT16P3	0	0	0	0	0	0
KRT17	0	0.07	0	0	0	0.19
KRT18	253.48	131.6	147.18	144.73	160.52	132.5
KRT18P55	0.49	0.12	0.03	0.05	0.07	0.04
KRT19	0	0.08	0	0	0.05	0
KRT19P2	0.14	0.34	1.57	0.1	0.68	0.14
KRT2	0	0	0.03	0.08	0	0.12
KRT20	0	0	0	0	0	0
KRT222	0	0	0	0	0	0
KRT23	0	0	0	0	0	0
KRT24	0	0	0	0	0	0
KRT25	0	0	0	0	0	0
KRT26	0	0	0	0	0	0
KRT27	0	0	0	0	0	0
KRT28	0	0	0	0	0	0
KRT3	0	0	0	0	0	0
KRT31	0	0	0	0	0	0
KRT32	0	0	0	0	0	0
KRT33A	0	0	0	0	0	0
KRT33B	0	0	0	0	0	0
KRT34	0	0	0	0	0	0
KRT35	0	0	0	0	0	0
KRT36	0	0	0	0	0	0
KRT37	0	0	0	0	0	0
KRT38	0	0	0	0	0	0
KRT39	0	0	0	0	0	0
KRT4	0	0	0	0	0	0
KRT40	0	0	0	0	0	0
KRT42P	0.11	0.1	0.03	0.1	0.06	0.21
KRT5	0	0	0	0	0	0
KRT6A	0	0	0	0	0	0
KRT6B	0	0	0	0	0	0
KRT6C	0	0	0	0	0	0
KRT7	0	0	0	0.03	0	0
KRT71	0	0	0	0	0	0
KRT72	0.08	0	0	0	0	0

KRT73	0	0	0	0	0	0
KRT74	0	0	0	0	0	0
KRT75	0	0	0	0	0	0
KRT76	0	0	0	0	0	0
KRT77	0	0	0	0	0	0
KRT78	0	0	0	0	0	0
KRT79	0.64	0	0	0.02	0.34	0.11
KRT8	88.49	12.41	10.31	14.4	21.02	12.07
KRT80	0	0	0	0	0	0
KRT81	0	0	0	0	0	0
KRT82	0	0	0	0	0	0
KRT83	0	0	0	0	0	0
KRT84	0	0	0	0	0	0
KRT85	0	0	0	0	0	0
KRT86	0	0	0	0	0	0
KRT8P41	0.16	0	0	0	0.04	0
KRT9	0	0	0	0	0	0
KRTAP1-1	0	0	0	0	0	0
KRTAP1-3	0	0	0	0	0	0
KRTAP1-4	0	0	0	0	0	0
KRTAP1-5	0	0	0	0.04	0	0
KRTAP10-1	0	0	0	0	0	0
KRTAP10-10	0	0	0	0	0	0.07
KRTAP10-11	0	0	0	0	0	0
KRTAP10-12	0	0	0	0	0	0
KRTAP10-2	0	0	0	0	0	0
KRTAP10-3	0	0	0	0	0	0
KRTAP10-4	0	0	0	0	0	0
KRTAP10-5	0	0	0	0	0	0
KRTAP10-6	0	0	0	0	0	0
KRTAP10-7	0	0	0	0	0	0
KRTAP10-8	0	0	0	0	0	0
KRTAP10-9	0	0	0	0	0	0
KRTAP11-1	0	0	0	0	0	0
KRTAP12-1	0	0	0	0	0	0
KRTAP12-2	0	0	0	0	0	0
KRTAP12-3	0	0	0	0	0	0
KRTAP12-4	0	0	0	0	0	0
KRTAP13-1	0	0	0	0	0	0
KRTAP13-2	0	0	0	0	0	0
KRTAP13-3	0	0	0	0	0	0
KRTAP13-4	0	0	0	0	0	0
KRTAP15-1	0	0	0	0	0	0

KRTAP16-1	0	0	0	0	0	0
KRTAP17-1	0	0	0	0	0	0
KRTAP19-1	0	0	0	0	0	0
KRTAP19-2	0	0	0	0	0	0
KRTAP19-3	0	0	0	0	0	0
KRTAP19-4	0	0	0	0	0	0
KRTAP19-5	0	0	0	0	0	0
KRTAP19-6	0	0	0	0	0	0
KRTAP19-7	0	0	0	0	0	0
KRTAP19-8	0	0	0	0	0	0
KRTAP2-1	0	0	0	0	0	0
KRTAP2-2	0	0	0	0	0	0
KRTAP2-3	0	0	0	0	0	0
KRTAP2-4	0	0	0	0	0	0
KRTAP20-1	0	0	0	0	0	0
KRTAP20-2	0	0	0	0	0	0
KRTAP20-3	0	0	0	0	0	0
KRTAP20-4	0	0	0	0	0	0
KRTAP21-1	0	0	0	0	0	0
KRTAP21-2	0	0	0	0	0	0
KRTAP21-3	0	0	0	0	0	0
KRTAP22-1	0	0	0	0	0	0
KRTAP22-2	0	0	0	0	0	0
KRTAP23-1	0	0	0	0	0	0
KRTAP24-1	0	0	0	0	0	0
KRTAP25-1	0	0	0	0	0	0
KRTAP26-1	0	0	0	0	0	0
KRTAP27-1	0	0	0	0	0	0
KRTAP29-1	0	0	0	0	0	0
KRTAP3-1	0	0	0	0	0	0
KRTAP3-2	0	0	0	0	0	0
KRTAP3-3	0	0	0	0	0	0
KRTAP4-1	0	0	0	0	0	0
KRTAP4-11	0	0	0	0	0	0
KRTAP4-12	0	0	0	0	0	0
KRTAP4-2	0	0	0	0	0	0
KRTAP4-3	0	0	0	0	0	0
KRTAP4-4	0	0	0	0	0	0
KRTAP4-5	0	0	0	0	0	0
KRTAP4-6	0	0	0	0	0	0
KRTAP4-7	0	0	0	0	0	0
KRTAP4-8	0	0	0	0	0	0
KRTAP4-9	0	0	0	0	0	0

KRTAP5-1	0	0	0	0	0	0
KRTAP5-10	0	0	0	0	0.14	0
KRTAP5-11	0	0	0	0	0	0.08
KRTAP5-2	0	0	0	0	0	0
KRTAP5-3	0	0	0	0	0	0
KRTAP5-4	0	0	0	0	0	0
KRTAP5-5	0	0	0	0	0	0
KRTAP5-6	0	0	0	0	0	0
KRTAP5-7	0.17	0	0.16	0	0	0
KRTAP5-8	0	0	0	0	0.12	0
KRTAP5-9	0	0.2	0	0	0	0
KRTAP6-1	0	0	0	0	0	0
KRTAP6-2	0	0	0	0	0	0
KRTAP6-3	0	0	0	0	0	0
KRTAP7-1	0	0	0	0	0	0
KRTAP8-1	0	0	0	0	0	0
KRTAP9-1	0	0	0	0	0	0
KRTAP9-2	0	0	0	0	0	0
KRTAP9-3	0	0	0	0	0	0
KRTAP9-4	0	0	0	0	0	0
KRTAP9-8	0	0	0	0	0	0
KRTAP9-9	0	0	0	0	0	0
KRTCAP2	303.02	298.96	279.22	288.58	313.59	249.82
KRTCAP3	0.35	0	0	0	0	0
KRTDAP	0	0	0	0	0	0
KSR1	1.01	0.3	0.5	0.7	0.8	0.94
KSR2	0	0.02	0	0	0.01	0
KTI12	18.55	25.53	25.38	26.67	19.93	24.45
KTN1	7.58	8.07	6.3	6.92	5.98	7.63
KTN1-AS1	1.39	1.44	1.99	1.57	2.25	2.35
KXD1	94.86	77.84	83.55	79.43	85.21	74.56
KY	0	0	0.03	0.02	0.02	0
KYNU	0	0	0.41	0.06	0	0
L1CAM	0.42	0	0.03	0	0.08	0
L1TD1	1.11	1.21	1.43	0.72	1.21	1.44
L2HGDH	5.34	5.87	6.73	4.3	5.35	4.63
L3HYPDH	6.65	5.97	4.24	4.88	6.39	5.95
L3MBTL1	0	0	0	0	0	0
L3MBTL2	19.25	18.96	17.39	18.95	16.22	15.81
L3MBTL3	1.32	0.61	0.61	0.67	0.95	1.71
L3MBTL4	0	0	0	0	0	0
LACC1	9.52	2.24	1.1	2.89	2.47	1.39
LACE1	2.48	2.57	2.51	1.64	2.31	2.11

LACRT	0	0	0	0	0	0
LACTB	5.41	2.66	4.05	2.01	2.94	2.84
LACTB2	15.78	18.06	12.37	12.87	15.96	12.93
LAD1	0	0	0	0	0	0
LAG3	0	0	0	0	0.11	0
LAGE3	26.99	30.16	28.49	31.82	31.53	19.13
LAIR1	5.69	33.43	22.01	18.07	14.37	29.85
LAIR2	0.12	0.1	0	0.16	0.59	0.53
LALBA	0	0	0	0	0	0
LAMA1	0	0	0	0	0	0
LAMA2	0.01	0	0	0	0.05	0
LAMA3	0	0.03	0.01	0.02	0	0.03
LAMA4	0.01	0.01	0.01	0	0.03	0.02
LAMA5	3.39	1.99	0.88	1.92	1.3	1.72
LAMB1	5.87	2.26	0.28	3.43	3.17	2.37
LAMB2	0.04	0	0	0.04	0	0.11
LAMB2P1	0.15	0.38	0.75	0	0.09	0.49
LAMB3	0.13	0	0	0	0.08	0
LAMB4	0.01	0	0	0	0.01	0
LAMC1	3.23	2.56	2.88	2.56	2.73	4.03
LAMC2	0.11	0.08	0.02	0.1	0.05	0.1
LAMC3	0	0	0	0	0	0
LAMP1	7.67	7.09	8.75	6.64	9.33	5.19
LAMP2	64.44	86.4	69.91	56.65	67.6	83.77
LAMP3	0.43	0.16	0.16	0.1	0.13	0.19
LAMP5	0	0	0	0	0	0
LAMTOR1	149.35	132.22	119.13	135.86	158.66	131.89
LAMTOR2	115.1	123.11	88.92	85.08	96.82	81.56
LAMTOR3	15.64	8.77	9.34	8.07	9	6.26
LAMTOR4	282.91	323.7	288.22	301.1	340.16	275.9
LAMTOR5	155.6	142.49	109.11	119.01	140.24	117.61
LANCL1	5.35	5.6	4.67	5.58	5.56	6.26
LANCL2	4.68	5.6	4.3	4.82	5.44	4.66
LANCL3	4.09	0.28	0.2	2.46	2.65	0.07
LAP3	60.76	54.69	46.9	51.5	46.58	45.51
LAPTM4A	34.07	24.17	21.66	24.4	25.64	27.33
LAPTM4B	167.26	63.7	72.31	103.12	133.04	84.14
LAPTM5	295.19	104.74	107.63	142.42	182.87	102.04
LARGE	0.47	0.28	0.05	0.2	0.2	0.19
LARP1	32.4	27.25	20.93	21.78	27.09	23.15
LARP1B	4.44	5.3	4.85	2.62	4.02	4.31
LARP4	19.36	18.5	18.65	12.45	16.1	14.41
LARP4B	13.89	11.78	11.18	10.62	12.76	10.18

LARP6	0.15	0	0.67	0.2	0	0
LARP7	11.12	13.39	7.9	8.24	10.13	11.13
LARS	34.01	35.3	32.88	31.94	35.12	31.27
LARS2	12.22	10.43	10.54	11.22	10.01	12.08
LARS2-AS1	0.86	1.2	1.29	0.83	1	1.32
LAS1L	115.46	129.76	126.81	131.5	129.03	132.91
LASP1	21.81	19.26	21.21	18.95	18.68	16.69
LAT	3.38	0.75	1.87	4.74	14.36	4.35
LAT2	8.7	64.35	78.74	46.91	41.18	56.11
LATS1	12.45	10.08	10.62	10.39	9.83	7.92
LATS2	0.77	0.76	0.46	0.41	1.18	0.28
LAX1	0.13	0.14	0.22	0.29	0.12	0.39
LAYN	0	0.52	0.13	0.62	0.27	0.17
LBH	6.88	2.93	2.25	4.46	3.35	3.59
LBP	0.04	0	0.18	0	0	0.28
LBR	38.01	50.42	34.5	29.07	37.71	46.01
LBX1	0	0	0	0	0	0
LBX2	0	0	0.14	0.04	0	0
LBX2-AS1	0	0.09	0	0	0.03	0
LCA5	3.22	0.04	0.34	0.39	0.59	0
LCA5L	0	0.07	0.09	0.02	0.15	0
LCAT	0.22	0.66	0.31	0.31	0.42	0.34
LCE1A	0	0	0	0	0	0
LCE1B	0	0	0	0	0	0
LCE1C	0	0	0	0	0	0
LCE1D	0	0	0	0	0	0
LCE1E	0	0	0	0	0	0
LCE1F	0	0	0	0	0	0
LCE2A	0	0	0	0	0	0
LCE2B	0	0	0	0	0	0
LCE2C	0	0	0	0	0	0
LCE2D	0	0	0	0	0	0
LCE3A	0	0	0	0	0	0
LCE3B	0	0	0	0	0	0
LCE3C	0	0	0	0	0	0
LCE3D	0	0	0	0	0	0
LCE3E	0	0	0	0	0	0
LCE4A	0	0	0	0	0	0
LCE5A	0	0	0	0	0	0
LCE6A	0	0	0	0	0	0
LCK	0	0	0	0	0	0
LCLAT1	11.14	9.3	8.61	7.17	10.68	9.44
LCMT1	42.15	33.22	34.7	34.02	38.39	31.02

LCMT2	0	0	0	0	0	0
LCN1	0	0	0	0	0	0
LCN10	0	0	0	0.1	0	0
LCN12	0	0	0	0	0	0
LCN15	0	0	0	0.22	0	0
LCN2	0.1	0	0	0	0	0
LCN6	0	0	0	0	0	0
LCN8	0	0	0	0	0	0
LCN9	0	0	0	0	0	0
LCNL1	0	0	0	0	0	0
LCOR	2.2	2.27	1.6	1.44	1.95	1.56
LCORL	2.31	3.55	2.09	2.44	2.05	2.3
LCP1	26.73	129.01	112.27	69.97	59.53	113.83
LCP2	11.69	9.64	9.43	8.95	11.41	8.43
LCT	0.16	0.12	0.01	0.02	0.23	0.08
LCTL	0	0	0	0.03	0.03	0
LDB1	13.04	8.23	5.58	8.5	9.3	6.64
LDB2	0	0	0	0	0	0
LDB3	0	0.09	0.07	0.09	0.04	0
LDHA	551.64	599.12	644.7	553.57	590.55	620.2
LDHAL6A	0.33	0.15	0.03	0.03	0.1	0.15
LDHAL6B	0	0	0	0	0	0
LDHB	1220.88	1200.14	1105.03	1180.92	1337.99	1181.27
LDHC	0	0	0	0	0	0
LDHD	0.41	0.03	0.07	0.03	0.63	0
LDLR	17.1	12.25	13.05	9.16	15.25	13.45
LDLRAD1	0.31	0.08	0.13	0.09	0.13	0.22
LDLRAD2	0.04	0	0.02	0.09	0	0.04
LDLRAD3	10.56	4.62	3.96	6.06	8.71	3.95
LDLRAD4	0.05	0.38	0.34	0.17	0.08	0.21
LDLRAP1	6.61	8.36	7.9	5.88	7.5	6.89
LDOC1	12.54	0.21	0.25	1.17	1.96	0.94
LDOC1L	2.44	1.14	0.89	1.38	0.1	0
LEAP2	0.34	1.96	1.34	0.47	2.68	0.46
LECT1	0	0	0	0	0	0
LECT2	0	0	0	0	0	0
LEF1	0	0	0	0.01	0	0
LEF1-AS1	0	0	0	0	0	0
LEFTY1	0.05	0	0	0.03	0	0
LEFTY2	0	0	0	0	0	0
LEKR1	0	0	0	0	0	0
LELP1	0	0	0	0	0	0
LEMD1	0	0	0	0	0	0

LEMD1-AS1	0.7	0.86	0.79	0.5	0.49	0.62
LEMD2	1.93	0.65	1.28	1.52	1.82	1.07
LEMD3	2.3	1.98	3.11	1.92	2.28	2.06
LENEP	0	0	0	0	0.24	0
LENG1	10.87	8.23	11.3	7.94	9.97	7.7
LENG8	6.88	5.77	6.18	5.16	5.38	5.82
LENG9	0.04	0.19	0.25	0.05	0	0.16
LEO1	26.17	27.8	25.34	22.85	30.21	30.54
LEP	0	0.05	0.07	0.02	0	0.02
LEPR	2.63	0.86	1.19	0.85	2	1.06
LEPRE1	16.01	25.66	16.63	18.65	14.76	14.91
LEPREL1	0	0	0	0	0	0
LEPREL2	2.5	4.37	3.86	3.95	2.51	6.4
LEPREL4	24.06	31.64	25.53	26.21	24.51	24.15
LEPROT	22.94	4.54	4.04	9.22	13.81	4.57
LEPROTL1	47.88	37.57	31.59	27.58	33.1	30.87
LETM1	16.21	15.44	18.36	17.79	17.96	13.92
LETM2	1.87	2.75	3.24	2.89	2.99	2.51
LETMD1	18.67	22	23.15	24.8	23.4	22.49
LEUTX	0	0	0	0	0	0
LFNG	0.07	0.14	0.06	0.15	0.2	0
LGALS1	156.84	64.85	59.26	32.16	72.19	36.01
LGALS12	0.09	0.24	1.41	0.06	0.46	0.04
LGALS13	0	0	0	0	0	0
LGALS14	0	0	0	0	0	0
LGALS16	0	0	0	0	0	0
LGALS17A	0	0	0	0	0	0
LGALS2	0	0	0	0	0	0
LGALS3	152.61	13.33	12.6	27.45	71.26	8.32
LGALS3BP	55.1	6.05	8.84	15.18	18.52	9.28
LGALS4	0.12	0	0	0	0.17	0.06
LGALS7	0	0	0	0	0	0
LGALS7B	0	0	0	0	0	0
LGALS8	6.05	4.22	3.16	3.94	4.61	3.54
LGALS8-AS1	0.38	0	0.24	0	0.39	0.11
LGALS9	37.36	97.91	98.84	80.03	52.85	104.51
LGALS9B	0	0	0	0.38	0	0
LGALS9C	0	0	0	0	0.1	0
LGALSL	1.38	0.94	1.01	0.87	1.16	0.48
LGI1	0	0	0	0	0	0
LGI2	0	0	0.03	0.01	0.05	0
LGI3	0	0	0	0	0	0.02
LGI4	0.03	0	0	0	0	0

LGMN	0.67	0.74	0.72	0.52	0.49	1.96
LGR4	1.1	1.05	0.29	0.1	0.66	0.5
LGR5	0	0	0	0	0	0
LGR6	0	0	0	0	0	0
LGSN	0.12	0.06	0.13	0.04	0.08	0.02
LHB	0.8	0	0	0.33	0	0.32
LHCGR	0	0	0	0	0	0
LHFP	8.5	9.28	10.07	9.74	9.7	9.95
LHFPL1	0	0	0	0	0	0
LHFPL2	1.4	1.68	1.18	1.05	1.67	1.01
LHFPL3	0	0	0	0	0	0
LHFPL3-AS1	0	0	0	0	0	0
LHFPL3-AS2	0	0	0	0	0	0
LHFPL4	0	0.21	0.05	0.09	0.06	0.01
LHFPL5	0.11	0.25	0.1	0.02	0.18	0.07
LHPP	2.02	2.16	3.05	2.98	2.5	2.01
LHX1	0	0	0.43	0.03	0.04	0.43
LHX2	0	0	0	0	0	0
LHX3	0	0	0	0	0	0
LHX4	0.2	0.58	0.32	0.22	0.04	0.28
LHX5	0	0	0	0	0	0
LHX6	1.25	1.28	0.87	2.14	0.44	0.65
LHX8	0	0	0	0	0	0
LHX9	0.09	0	0	0	0	0
LIAS	11.03	12.61	9.91	9.22	7.92	10.78
LIF	2.26	0.14	0.23	0.82	1.47	0.95
LIFR	0.11	0.02	0.01	0.02	0	0.01
LIG1	24.5	40.13	34.68	36.95	35.95	37.59
LIG3	8.19	13.81	8.59	12.87	10.51	14.14
LIG4	0.75	0.76	0.43	0.6	0.45	0.47
LILRA1	0.05	0.02	0.36	0.29	0.04	0.32
LILRA2	2.75	2.3	1.26	0.31	1.69	1.59
LILRA3	0	0	0	0	0	0
LILRA4	0	0.07	0	0	0.04	0
LILRA5	0	0	0	0	0.07	0
LILRA6	0.02	0.02	0.13	0.06	0.06	0
LILRB1	0.08	0.28	0.25	0.23	0.31	0.24
LILRB2	0	0.04	0.05	0.37	0.07	0.87
LILRB3	0.69	0.93	1.14	0.78	0.99	0.99
LILRB4	0	0	0	0	0	0
LILRB5	0	0	0	0	0	0
LILRP2	0	0	0	0	0	0
LIM2	0	0	0	0	0	0

LIMA1	2.68	2.13	1.87	2.48	2.39	1.59
LIMCH1	0	0	0	0	0	0
LIMD1	4.34	7.04	7.74	6.96	5.55	7.28
LIMD1-AS1	0.21	0.28	0	0	0	0
LIMD2	17.23	14.71	20.53	20.53	20.3	17.36
LIME1	1.14	0.95	0.17	0.74	0.48	0.26
LIMK1	2.49	2.76	3.18	2.55	2.89	2.96
LIMK2	6.35	4.67	5.6	5.36	4.53	5.76
LIMS1	45.42	15.94	18.2	22.59	29.55	14.79
LIMS2	0.07	0	0.03	0.56	0.04	0.36
LIMS3	0.68	0.18	0.31	0.09	0.54	0.19
LIMS3-LOC4	0	0	0	0	0	0
LIMS3L	0.68	0.09	0.3	0.16	0.54	0.18
LIN28A	0.32	0.48	0.38	0.15	0.27	0.38
LIN28B	3.66	5.98	5.97	4.21	4.78	6.73
LIN37	7.55	5.9	6.73	7.63	7.46	6.96
LIN52	4.85	6.04	5.15	7.32	4.18	3.56
LIN54	4.62	4.16	3.75	4	4.06	4.32
LIN7A	1.05	0.19	0.28	0.04	0.06	6.1
LIN7B	4.4	3.06	2.4	4.65	3.09	3.88
LIN7C	9.03	7.89	6.51	6.27	7.07	8.28
LIN9	4.56	3.83	3.9	3.2	4.59	3.84
LINC-ROR	0.28	0.02	0.08	0.17	0.11	0.09
LINC00028	0	0	0	0	0	0
LINC00029	0	0	0	0	0	0
LINC00032	0.03	0	0	0	0	0
LINC00035	0	0	0	0	0	0
LINC00051	0	0	0	0	0.07	0.07
LINC00052	0	0	0	0	0	0
LINC00085	0.98	0.39	0.62	0.62	0.64	0.63
LINC00086	0	0	0	0	0.04	0
LINC00087	0	0.14	0	0	0.1	0.03
LINC00092	0.42	0.43	0.37	0.15	0.48	0.2
LINC00094	6.67	5.48	5.98	7.19	6.42	6.47
LINC00102	0.06	0.12	0.04	0.1	0.05	0.11
LINC00111	0	0	0	0	0	0
LINC00112	0	0	0	0	0	0
LINC00113	0	0.13	0.14	0	0	0.16
LINC00114	0.36	0.19	0.36	0.1	0.12	0
LINC00115	2.19	1.84	1.29	1.55	1.61	2.14
LINC00116	22.07	18.47	18.12	15.33	16.24	15.7
LINC00152	79.41	58.25	56.05	63.02	73.26	32.76
LINC00158	0	0	0	0	0	0

LINC00159	0	0	0	0	0	0
LINC00160	0	0	0	0	0	0
LINC00161	0	0	0	0	0	0
LINC00162	0	0	0	0	0.21	0
LINC00163	0	0	0	0	0	0
LINC00167	0.06	0.13	0.19	0.11	0.21	0.08
LINC00173	0	0.31	0.49	0.03	0	0
LINC00174	0.2	0.54	0.45	0.31	0.23	0.46
LINC00176	0.01	0.07	0.03	0	0	0
LINC00184	0	0	0	0	0	0
LINC00189	0.09	0.13	0.4	0	0.07	0.25
LINC00200	0	0	0	0	0	0
LINC00202-1	0	0	0	0	0	0
LINC00202-2	0	0	0.01	0	0	0
LINC00207	0	0	0	0	0	0
LINC00208	0	0	0	0	0	0
LINC00210	0	0	0	0	0	0
LINC00221	0.06	0	0	0	0.02	0.05
LINC00222	0.04	0	0	0	0	0
LINC00226	0	0	0	0	0	0
LINC00229	0	0	0	0	0	0
LINC00235	0.44	0.93	0	0.41	0.42	0.59
LINC00238	0	0	0	0	0	0
LINC00239	0	0	0	0	0	0
LINC00240	0	0	0.14	0	0	0
LINC00242	0	0.07	0	0.15	0	0
LINC00244	0	0	0	0	0	0
LINC00251	0	0	0	0	0	0
LINC00254	0	0	0	0	0	0
LINC00260	0.21	0.36	0.07	0.48	0.06	0.56
LINC00261	0.47	0	0	0.03	0.14	0
LINC00263	3.43	2.8	6.77	3.34	3.18	5.28
LINC00264	0	0	0	0	0.07	0
LINC00265	1.21	1.47	0.93	1.18	1.06	1.7
LINC00266-1	0	0	0.11	0	0	0.4
LINC00271	0	0.04	0	0	0.04	0
LINC00272	0	0	0	0	0	0
LINC00273	0.19	0.04	0.14	0.04	0.05	0.47
LINC00277	0	0	0	0	0	0
LINC00278	0	0	0	0	0	0
LINC00282	0	0.01	0.02	0	0	0
LINC00284	0	0	0	0	0	0
LINC00290	0	0	0	0	0	0

LINC00293	0	0	0	0.14	0	0.07
LINC00294	5.43	4.4	4.59	3.77	4.76	4.89
LINC00297	0	0	0	0	0	0
LINC00299	0	0	0	0	0	0
LINC00301	0	0	0	0	0	0
LINC00303	0	0	0	0	0	0
LINC00304	0	0	0	0	0	0
LINC00305	0	0	0	0	0	0
LINC00307	0	0	0	0	0	0
LINC00308	0	0	0	0	0	0
LINC00309	0	0	0	0	0	0
LINC00310	0	0.56	0.33	0.04	0	0.37
LINC00311	1.27	0.78	1.11	0.67	0.86	1.11
LINC00312	0	0	0	0	0	0
LINC00313	0	0	0	0	0	0
LINC00314	0.14	0.56	0.26	0.2	0	0
LINC00317	0	0	0	0	0	0
LINC00319	0	0	0	0	0	0
LINC00320	0	0.06	0.03	0	0.07	0.04
LINC00323	0	0	0	0	0	0
LINC00324	0.42	0.53	0.23	0.12	0.03	0.36
LINC00326	0	0	0	0	0	0
LINC00327	0.27	0.47	0.88	0.36	0.82	0.71
LINC00330	0.29	0.43	0.71	0.41	0.25	0.34
LINC00331	0.14	0.23	0	0.1	0	0
LINC00332	0	0	0	0	0	0
LINC00333	0	0	0	0	0	0
LINC00336	0	0.02	0	0	0.1	0
LINC00338	5.19	6.92	4.08	3.75	7.31	3.98
LINC00339	17.15	25.69	21.65	22.66	24.21	18.8
LINC00340	0	0.12	0	0	0	0
LINC00341	0.26	1.16	1.18	1.24	1.02	1.78
LINC00343	0	0	0	0	0	0
LINC00346	0.39	0.31	0.26	0.21	0.3	0.37
LINC00347	0	0	0	0	0	0
LINC00348	0	0.13	0.07	0	0.08	0.16
LINC00351	0.15	0	0	0	0	0.08
LINC00353	0	0	0	0	0.12	0
LINC00359	0	0	0	0	0	0
LINC00366	0	0	0	0	0	0
LINC00379	0	0	0.4	0.3	0	0
LINC00381	0.11	0	0.2	0.04	0.21	0
LINC00398	0	0	0	0	0	0

LINC00410	0.09	0.08	0.14	0.09	0	0.26
LINC00421	0	0	0	0	0	0
LINC00423	0	0	0	0	0	0
LINC00424	0	0	0	0	0	0
LINC00426	0	0.15	0	0	0	0.05
LINC00433	0	0	0	0	0	0
LINC00439	1.01	0.44	0.6	1.11	1.04	0.61
LINC00441	0.07	0	0	0	0.06	0.07
LINC00442	0	0	0	0	0	0
LINC00443	0	0	0	0	0	0
LINC00446	0	0	0	0	0	0
LINC00457	0	0	0	0	0	0
LINC00460	0	0	0	0	0	0
LINC00461	0	0	0.1	0	0	0
LINC00462	0	0	0	0.26	0	0
LINC00466	0.17	0	0.28	0	0.04	0.1
LINC00467	1.77	1.07	0.57	0.65	0.99	1.05
LINC00469	0	0	0	0	0	0
LINC00470	0.04	0	0.04	0	0	0.06
LINC00471	0.8	0.6	0.21	0.33	0.17	0.52
LINC00472	0	0	0	0	0	0
LINC00473	0	0	0	0	0	0
LINC00474	0	0	0	0	0	0
LINC00475	0	0	0	0	0	0
LINC00476	1.3	2.37	1.43	1.36	2.64	2.41
LINC00477	0.39	0.21	0.42	0.13	0.24	0.23
LINC00478	0.2	0	0.04	0.03	0	0.07
LINC00479	0	0	0	0	0	0
LINC00482	0	0.04	0	0	0	0.12
LINC00483	0.79	0.58	1.53	1.23	0.69	0.75
LINC00485	0.43	0.31	0.35	0.09	0.53	0.23
LINC00486	0	0	0	0	0	0
LINC00487	0	0	0	0	0	0
LINC00488	0.08	0.04	0.18	0.1	0.12	0.03
LINC00489	0	0	0	0	0	0
LINC00493	96.49	61.91	55.83	72.05	94.89	80.43
LINC00494	0	0	0	0	0	0
LINC00499	0	0	0	0	0	0
LINC00502	0	0	0	0	0	0
LINC00507	0.57	0.91	1.05	0.78	0.4	1
LINC00511	0	0	0	0	0	0
LINC00514	0.58	0.95	0.68	0.8	0.58	0.69
LINC00515	0.57	0.9	0.52	0.4	0.91	1.53

LINC00516	1.48	2.38	1.7	1.14	1.32	2.1
LINC00518	0.02	0	0	0	0	0
LINC00520	1.38	0	0.07	0.41	0.24	0
LINC00521	0	0	0	0.08	0	0.12
LINC00523	0	0.22	0.08	0	0	0.05
LINC00525	0	0	0	0	0	0
LINC00526	0	0	0	0	0	0
LINC00534	4.13	0	0	0.43	1.28	0.27
LINC00535	0.08	0.24	0.53	0.24	0.4	0.43
LINC00536	0.06	0	0.02	0	0.05	0
LINC00538	0	0	0	0	0	0
LINC00544	0	0	0	0	0	0
LINC00547	0.89	0.98	0.98	0.69	0.65	0.62
LINC00548	0	0	0	0	0	0
LINC00550	0.02	0	0	0.03	0	0
LINC00551	0	0	0	0	0	0
LINC00552	0	0	0	0	0	0
LINC00558	0	0	0	0	0	0
LINC00559	0	0.02	0	0.03	0.02	0.09
LINC00563	0	0	0	0	0	0
LINC00565	0	0	0	0	0	0.06
LINC00568	0.29	0	0.27	0	0.28	0.52
LINC00570	0	0	0	0	0	0
LINC00571	0	0	0	0	0	0
LINC00574	0.45	0.69	0.8	0.48	0.35	1.11
LINC00575	0	0	0	0	0	0.09
LINC00577	0	0	0	0	0	0
LINC00578	0.15	0.16	0.4	0.16	0.26	0.43
LINC00582	0	0	0	0	0.13	0
LINC00583	0	0	0	0	0	0
LINC00588	0	0	0	0	0	0
LINC00589	0	0	0	0	0	0
LINC00592	0	0	0	0	0	0
LINC00593	0	0	0	0	0	0
LINC00595	0	0	0	0	0	0
LINC00597	0	0	0	0.07	0	0
LINC00598	0.22	0.15	0.32	0.18	0.26	0.17
LINC00599	0	0	0	0	0	0
LINC00601	0	0	0	0	0	0
LINC00602	0	0	0	0	0	0
LINC00605	0	0.04	0.05	0.06	0.13	0
LINC00606	0	0	0	0	0	0
LINC00607	0	0	0	0	0.02	0

LINC00608	0	0	0	0	0.05	0
LINC00609	0	0	0	0	0	0
LINC00612	0	0	0	0	0	0
LINC00615	0	0	0	0.04	0	0.11
LINC00616	0	0	0	0	0	0
LINC00617	0.09	0.11	0.15	0.11	0.3	0.11
LINC00619	0	0	0	0	0	0
LINC00620	0.28	0.06	0.62	0.11	0.39	0.31
LINC00622	0	0	0	0	0	0
LINC00623	17.86	15.49	4.51	11.94	13	17.42
LINC00624	0.02	0	0	0	0	0
LINC00626	0	0	0	0	0	0
LINC00628	0	0	0	0	0	0
LINC00629	0.57	0.65	0.31	0.51	0.14	0.06
LINC00630	2.02	2.85	2.55	2.13	2.33	1.83
LINC00633	0	0	0	0	0	0
LINC00634	0	0	0	0.03	0	0.15
LINC00635	0	0	0	0	0	0
LINC00636	0	0	0	0	0	0
LINC00637	0.23	0.39	0.43	0.17	0.24	0.12
LINC00638	0.24	0.52	0.78	0.8	0.27	0.44
LINC00639	0	0	0	0.01	0.02	0.05
LINC00640	0	0	0	0	0	0
LINC00641	1.3	0.84	0.65	0.51	1.28	1
LINC00642	0	0	0	0	0	0
LINC00643	0.01	0.02	0.08	0.01	0	0.03
LINC00645	0	0	0	0	0	0
LINC00648	0.05	0.14	0.05	0.02	0	0.28
LINC00649	1.42	1.77	1.9	1.86	1.78	1.67
LINC00651	0	0	0	0	0	0
LINC00652	0.54	0.51	0.35	0.11	0.26	0.33
LINC00654	0	0	0	0	0	0
LINC00656	0	0	0	0	0	0
LINC00657	9.37	6.79	7.57	7.63	8.3	7.54
LINC00658	0	0	0	0	0	0
LINC00659	0	0	0	0	0	0
LINC00661	0	0	0.02	0	0	0
LINC00662	1.22	2.9	2.59	1.94	2.11	2.04
LINC00663	0.75	0.59	0.82	0.57	0.44	0.64
LINC00665	1.36	1.49	1.39	1.2	1.6	1.47
LINC00667	0.03	0.04	0.05	0.01	0	0.04
LINC00668	0	0.03	0.02	0	0.04	0.04
LINC00669	0.79	1.27	0.53	0.26	0.68	0.97

LINC00670	1.28	1.32	1.1	0.69	1.07	0.62
LINC00671	0.3	0.16	0.29	0.14	0.24	0.11
LINC00672	0.62	0.92	0.88	0.28	0.84	0.57
LINC00673	0	0.05	0.12	0.2	0.03	0.12
LINC00674	2.54	3.47	2.23	2.85	2.5	2.86
LINC00675	0	0	0	0	0	0
LINC00684	0	0	0	0	0	0
LINC00685	1.69	0.46	1.02	0.67	1.15	0.84
LINC00689	0	0	0	0	0.03	0.02
LINC00691	0	0	0.06	0	0.03	0.03
LINC00692	0	0	0	0	0	0
LINC00693	0	0	0	0	0	0
LINC00696	0.02	0.02	0.2	0.08	0.04	0.08
LINC00698	0	0	0	0	0	0
LINC00700	0.06	0.2	0.37	0.03	0.25	0
LINC00701	0	0	0	0	0	0
LINC00704	0	0	0	0	0	0
LINC00705	0	0	0	0	0	0
LINC00707	0.02	0	0	0	0.07	0
LINC00710	0	0	0	0	0	0
LINC00837	0	0	0	0	0	0
LINC00838	0	0	0	0	0	0
LINC00839	0.03	0.08	0.13	0.02	0.12	0.06
LINC00840	0	0	0	0	0	0
LINC00841	0	0	0	0	0	0
LINC00842	0.04	0	0	0	0	0
LINC00847	0	0.07	0.03	0	0.01	0
LINC00851	0	0	0	0	0	0
LINC00852	1.19	1.3	0.78	0.47	0.64	0.86
LINC00853	0	0.19	0.11	0.25	0.23	0.37
LINC00854	0.32	0.57	0.44	0.44	0.23	0.81
LINC00856	0	0.06	0	0.05	0	0
LINC00857	0.28	0.12	0.06	0.07	0	0.13
LINC00858	0	0	0	0	0	0
LINGO1	0.27	0.92	1.43	1.2	0.68	2.67
LINGO2	0.35	0.84	0.4	0.27	0.44	0.75
LINGO3	0.03	0.03	0.1	0.55	0	0.04
LINGO4	0	0	0	0	0	0
LINS	2.13	3.79	3.34	3.61	2.2	2.1
LIPA	18.04	17.86	16.82	14.72	18.72	13.78
LIPC	0.05	0	0.13	0.29	0.26	0.14
LIPE	0.13	0.03	0.02	0.05	0.02	0
LIPF	0	0	0	0	0	0

LIPG	0.03	0	0.13	0	0.05	0.21
LIPH	0.47	0	0	0	0	0.03
LIPI	0	0	0	0	0	0
LIPJ	0	0	0	0	0	0
LIPK	0	0	0	0	0	0
LIPM	0	0	0	0	0	0
LIPN	0	0	0	0	0	0
LIPT1	4.19	4.07	5.34	3.69	3.63	3.87
LIPT2	1.69	3.81	4.05	4.23	2.44	2.93
LITAF	23.73	47.65	37.7	41.64	35.83	32.65
LIX1	0	0	0.03	0.01	0	0.02
LIX1L	4.19	3.68	3.09	2.4	3.3	2.6
LLGL1	5.96	7.36	9.44	7.93	6.61	8.14
LLGL2	3.81	2.22	2.77	1.99	2.85	2.83
LLPH	48.22	55.16	53.01	33.95	51.41	43.52
LMAN1	16.08	16.1	14.61	12.12	13.24	17.07
LMAN1L	0	0	0	0	0	0
LMAN2	123.6	154.4	154.59	140.89	143.11	141.99
LMAN2L	9.11	10.53	12.03	11.41	10.08	16.46
LMBR1	3.36	4.66	3.05	3.47	4.42	3.15
LMBR1L	8.37	7.68	5.48	6.06	7.07	8.97
LMBRD1	12.2	8.58	7.49	7.75	9.88	7.31
LMBRD2	2	2.34	1.46	1.73	1.53	1.32
LMCD1	0	0	0	0	0	0
LMCD1-AS1	0.04	0.03	0	0	0.12	0
LMF1	6.59	6	8.32	6.61	9.18	6.96
LMF2	11.97	13.12	14.03	17.26	17.27	13.76
LMLN	1.13	0.64	0.54	0.42	0.44	0.57
LMNA	87.54	18	22.41	33.77	50.09	13.93
LMNB1	10.4	12.92	14.81	9.89	10.97	17.49
LMNB2	18.43	25.32	20.47	19.53	20.67	21.59
LMO1	0	0.13	0.07	0.77	0.15	0.32
LMO2	7.72	4.83	5.74	5.24	6.28	3.17
LMO3	0.02	0.7	0.96	0.55	0.72	0.15
LMO4	0.88	1.05	0.79	0.98	0.78	1.23
LMO7	1.47	0.8	1.06	1.31	1.21	0.82
LMOD1	0	0.03	0	0	0	0
LMOD2	0	0	0.03	0	0	0
LMOD3	0.38	0.29	0.39	0.22	0.45	0.4
LMTK2	1.75	1.74	1.3	1.63	2.06	1.5
LMTK3	0	0	0.01	0	0	0
LMX1A	0	0	0	0	0	0
LMX1B	0.25	0.21	0.3	0.1	0.19	0.32

LNP1	0.33	0.03	0.28	0.32	0.19	0.38
LNPEP	5.33	9.34	8.11	6.62	5.92	8.3
LNX1	0.05	0.04	0.16	0.05	0.07	0
LNX1-AS1	0	0	0	0	0	0
LNX1-AS2	0	0	0	0	0	0
LNX2	3.26	2.05	2.15	1.98	1.65	1.67
LOC1000096	2.81	1.51	2.25	1.61	2.28	1.57
LOC1000936	0	0	0	0	0	0
LOC1000936	0	0	0	0	0	0
LOC1001267	0.02	0.15	0.04	0	0	0.15
LOC1001278	0.16	0.04	0	0	0	0
LOC1001279	16.9	14.54	13.69	17.29	19.79	12.95
LOC1001280	0	0	0	0	0	0
LOC1001280	0	0	0	0	0	0.07
LOC1001281	0.04	0.17	0.08	0.01	0.09	0.11
LOC1001281	0	0.17	0	0	0	0.11
LOC1001282	0.03	0.03	0.26	0.09	0.06	0.23
LOC1001282	0	0	0	0	0	0
LOC1001282	0.09	0.09	0.14	0	0.08	0
LOC1001282	1.64	2.26	2.48	1.13	1.84	1.5
LOC1001283	0.99	0.6	1.4	0.87	0.42	1.11
LOC1001283	0.44	2.82	1.08	1.38	1.09	1.94
LOC1001283	0.57	0.38	0.53	0.59	0.37	0.63
LOC1001284	0.12	0	0	0	0	0
LOC1001285	0	0	0.04	0	0	0
LOC1001285	0.44	2.15	1.6	1.7	1.33	2.1
LOC1001285	0	0	0	0	0	0
LOC1001285	0.25	0	0	0	0.12	0
LOC1001285	0.65	0.47	0.37	0.69	0.34	0.9
LOC1001285	0.1	0.13	0.14	0.05	0.2	0.26
LOC1001286	0.37	0.81	0.46	0.2	0.22	0.32
LOC1001286	0.72	0.9	1	0.33	0.89	0.75
LOC1001287	0.04	0	0	0	0	0
LOC1001287	0	0.2	0	0.95	0	0
LOC1001287	0	0	0	0.02	0	0
LOC1001287	0	0	0	0	0	0
LOC1001288	0	0.29	0.05	0.33	0.85	0.37
LOC1001288	9.31	7.45	7.19	7.26	6.23	6.84
LOC1001289	0	0	0	0	0	0
LOC1001289	0	0	0	0	0	0
LOC1001290	0	0	0	0	0	0
LOC1001290	0.87	1.39	0.92	1.01	1.39	1.12
LOC1001290	0	0	0	0	0	0

LOC1001290	0	0	0	0	0	0
LOC1001290	0	0	0	0	0.12	0
LOC1001291	0.23	0.06	0.32	0	0.28	0
LOC1001291	0	0.31	0	0	0	0
LOC1001291	0	0	0	0	0	0
LOC1001292	0	0	0	0	0	0
LOC1001292	0	0	0	0	0	0
LOC1001292	9.77	9.35	6.62	8.93	12.38	7.98
LOC1001292	0.61	1.48	1.42	0.91	0.87	0.77
LOC1001293	0	0	0	0	0	0
LOC1001293	0	0	0	0	0	0
LOC1001293	8.51	7.19	6.15	7.09	7.23	6.21
LOC1001294	0	0	0	0	0	0
LOC1001294	0.33	0.87	1.02	0.58	0.42	0.61
LOC1001295	0.41	0.24	0.12	0.37	0.49	0
LOC1001295	0.15	0	0	0	0	0
LOC1001295	0	0	0.03	0	0	0
LOC1001295	0.27	0.47	0.51	0.39	0.33	0.5
LOC1001296	0.19	0	0	0	0.11	0.08
LOC1001296	0	0	0.03	0	0	0
LOC1001296	0	0	0	0	0	0
LOC1001297	0.09	0	0	0	0	0
LOC1001297	0	0.07	0	0.09	0.17	0.14
LOC1001297	0	0	0	0	0	0
LOC1001298	0.15	0.29	0.23	0.28	0.28	0.15
LOC1001299	0.81	0.59	2.12	0.64	1.6	1.96
LOC1001299	0	0	0	0	0	0
LOC1001299	0.34	0.44	0.04	0	0.08	0.05
LOC1001299	0.24	0.53	0.13	0.21	0.15	0.13
LOC1001299	1.23	1.22	0.93	1	0.4	1.19
LOC1001300	0.24	0.41	0.4	0	0.41	0.33
LOC1001300	0	0	0.05	0	0.17	0.12
LOC1001300	0	0	0	0	0	0
LOC1001300	1.03	1.22	2.22	2.14	1.25	0.94
LOC1001301	0	0	0	0	0	0
LOC1001302	0.13	0.06	0.1	0.42	0	0.02
LOC1001302	0	0	0	0	0	0
LOC1001302	0	0	0	0	0	0
LOC1001302	0.29	0.64	0.85	1.44	0.71	0.23
LOC1001302	0	0	0	0	0	0
LOC1001303	0	0	0	0	0	0
LOC1001303	0	0.02	0	0	0	0
LOC1001303	0	0	0	0	0.19	0

LOC1001303	0.73	0.15	0.24	0.17	0	0.23
LOC1001303	0	0	0	0	0	0
LOC1001304	0	0	0	0	0	0.17
LOC1001304	1.59	1.68	2.52	1.26	1.56	1.74
LOC1001304	0	0	0	0	0	0.33
LOC1001304	0.04	0	0	0.03	0	0
LOC1001304	0	0	0	0	0	0
LOC1001305	0	0	0	0	0	0
LOC1001305	1.22	0.77	0.6	0.27	1.04	0.92
LOC1001305	0.63	0.99	0.83	0.82	0.76	1.02
LOC1001306	0	0	0	0	0	0
LOC1001306	0.21	0.1	0.27	0.24	0.13	0.36
LOC1001307	0	0	0	0	0	0
LOC1001307	0.04	0	0.09	0.07	0.09	0
LOC1001307	0.25	0.58	0.37	0.54	0.27	0.31
LOC1001307	0	0	0	0.04	0	0.1
LOC1001308	0	0	0	0	0	0
LOC1001308	0.71	0.48	0.44	0.39	0.74	1.01
LOC1001308	0.36	0.42	0.11	0.1	0.65	0.29
LOC1001308	0	0	0	0	0	0
LOC1001308	0.16	0.21	0.23	0.48	0.35	0.1
LOC1001308	0.19	0.39	0.26	0.23	0.39	0.35
LOC1001309	0.1	0.2	0.16	0.06	0.08	0.26
LOC1001309	0.57	1.02	1.67	0.62	1.41	1.29
LOC1001309	0	0	0	0	0	0
LOC1001309	0.37	0.8	0.42	0.55	0.96	1.21
LOC1001309	0	0	0	0.01	0.02	0
LOC1001310	0	0	0	0	0	0
LOC1001310	0.08	0	0	0	0	0
LOC1001310	2.95	3.89	2.74	2.34	3.69	2.89
LOC1001310	2.82	3.6	3.24	1.92	3.51	3.2
LOC1001310	0.02	0.11	0.02	0.07	0	0.02
LOC1001310	1.25	1.61	1.62	0.93	1.22	1.03
LOC1001311	0	0	0	0	0	0
LOC1001312	0.81	1.81	2.47	1.78	0.64	1.22
LOC1001312	2.52	3.06	3.01	1.8	2.34	2.56
LOC1001312	0	0	0	0	0	0
LOC1001313	0	0	0	0.05	0	0
LOC1001313	0.07	0.18	0.25	0.04	0.2	0.3
LOC1001314	0.05	0.06	0.26	0.06	0	0
LOC1001314	0.23	0.34	0.13	0.12	0.25	0.24
LOC1001315	0.12	0.12	0.08	0.19	0.06	0.1
LOC1001315	1.4	0.98	1.22	0.6	0.9	1.04

LOC1001316	0.32	0.76	0.46	0.32	0.6	0
LOC1001316	0	0	0	0	0	0
LOC1001316	0.13	0.33	0.63	0.15	0.17	0.47
LOC1001316	0.57	0.36	0.25	0.22	0.14	0.77
LOC1001317	3.02	0	0	0.18	0	0
LOC1001318	0	0	0	0	0	0
LOC1001320	0	0	0.02	0	0	0
LOC1001320	0.33	1.09	1.71	0.54	0.45	0.53
LOC1001320	0.08	0.05	0	0.06	0	0.11
LOC1001321	0.1	0	0	0.14	0.1	0.21
LOC1001321	0	0	0	0	0	0
LOC1001322	0.27	0.5	0.83	0.27	0.04	0.16
LOC1001322	7.18	9.22	5.13	4.31	5.88	7.75
LOC1001322	1.57	1.13	0.74	1.7	1.06	1.45
LOC1001322	0	0	0.02	0	0	0
LOC1001323	10.92	11.86	9.27	8.07	11.98	10.28
LOC1001323	0	0	0	0	0	0
LOC1001323	0.62	1.48	1.1	1.35	1.23	0.64
LOC1001327	0	0.05	0	0	0.06	0.07
LOC1001327	0	0	0	0	0	0
LOC1001327	4.61	1.68	0.79	2.35	2.35	0.47
LOC1001327	0	0.11	0.03	0.09	0.07	0
LOC1001328	0	0	0	0	0	0
LOC1001328	1.68	0.86	1.37	1.39	0.54	1.77
LOC1001328	0.02	0	0.06	0	0	0.02
LOC1001330	0.03	0.1	0	0.08	0	0
LOC1001330	0.59	0.2	0.53	0.55	0.48	0.2
LOC1001331	0	0	0	0	0	0
LOC1001331	0.1	0.11	0	0.08	0.05	0.15
LOC1001332	0	0	0	0	0	0
LOC1001332	0.46	0.66	0.5	0.05	0.07	0.54
LOC1001333	1.76	2.01	2	2.34	1.78	2.03
LOC1001333	0.96	1	0.82	0.87	0.61	1.26
LOC1001334	0.27	0	0.15	0.29	0.45	0
LOC1001334	0	0	0	0	0	0
LOC1001336	0.24	0.37	0.36	0.12	0.11	0.21
LOC1001336	0.11	0	0.1	0	0.54	0.11
LOC1001339	0.05	0	0.3	0.1	0.27	0.17
LOC1001339	1.2	0.17	0.1	0.46	0.62	0
LOC1001339	0	0	0.11	0.42	0.12	0
LOC1001342	0.63	0.28	0.22	0.37	0.65	0.35
LOC1001342	4.14	0	0	0.69	0.06	0
LOC1001343	0	0	0	0	0	0

LOC1001343	0.19	0.04	0.09	0.04	0	0
LOC1001348	7.35	4.7	4.23	4.4	5.18	4.55
LOC1001445	0.25	0.28	0.47	0.28	0.18	0.22
LOC1001445	0	0	0	0	0	0
LOC1001446	0	0	0	0	0	0
LOC1001446	1.68	0.31	0.47	1.17	1.72	0.52
LOC1001446	0	0	0	0	0	0
LOC1001697	0	0	0	0	0	0
LOC1001889	1.86	0.97	1.03	0.81	1.19	0.63
LOC1001909	0.14	0.24	0.18	0.1	0.19	0.27
LOC1001909	6.67	12.04	11.91	6.68	7.68	9.61
LOC1001924	0	0	0	0	0	0
LOC1002164	0.3	0.38	0.33	0.27	0.26	0.34
LOC1002165	0.92	1.08	1.29	0.82	0.51	1.41
LOC1002165	0.32	0.31	0.07	0.13	0.28	0.44
LOC1002407	0	0	0	0	0.16	0.17
LOC1002407	0	0.07	0.94	0.06	0	0
LOC1002681	1.2	1.01	1.39	0.55	0.87	1.39
LOC1002706	0	0	0	0	0	0
LOC1002707	0	0	0	0	0	0
LOC1002708	0	0	0	0	0	0
LOC1002717	0	0	0	0.02	0	0
LOC1002717	0.28	0.62	0.13	0.51	0.22	0.09
LOC1002718	0.05	0	0	0	0	0
LOC1002718	1.48	2.05	1.67	2.11	1.77	2.05
LOC1002722	1.24	1.51	3.94	1.54	1.91	1.46
LOC1002722	0.17	0.27	0.07	0.2	0.49	0.21
LOC1002722	0.12	0.28	0	0.19	0.11	0.32
LOC1002867	3.57	1.11	0.06	1.64	1	1.86
LOC1002869	0	0	0	0	0	0
LOC1002870	0	0	0	0	0	0
LOC1002870	3.45	3.68	3.51	4.13	4.03	3.43
LOC1002870	0.19	0.25	0.06	0.17	0	0.19
LOC1002870	1.08	1.1	1.23	0.73	1.37	1.49
LOC1002870	0	0	0	0	0.15	0.08
LOC1002871	1.56	1.12	0.44	0.8	1.89	1.87
LOC1002872	0.12	0.06	0.2	0.12	0.13	0.19
LOC1002873	1.78	1.15	0.88	0.94	0.98	1.08
LOC1002875	0	0	0	0	0	0
LOC1002876	0	0	0	0	0	0
LOC1002877	0	0	0	0	0	0
LOC1002877	1.28	0.8	1	0.78	0.73	0.77
LOC1002878	0.12	0	0	0.09	0.12	0

LOC1002878	0.33	0.38	0.58	0.14	0.51	0.56
LOC1002878	0	0	0	0	0	0
LOC1002879	0	0	0	0	0	0
LOC1002880	8.02	10.02	6.06	6.11	7.54	7.56
LOC1002880	0	0	0	0	0	0
LOC1002881	0	0	0	0	0	0.03
LOC1002881	0.22	0.57	0.2	0.03	0.29	0.22
LOC1002881	0	0	0.1	0	0	0
LOC1002881	0.27	0.15	0.74	0.32	0.39	0.74
LOC1002882	0	0.03	0.03	0	0.03	0
LOC1002883	0	0	0	0	0	0
LOC1002884	1.81	0.86	0.33	0.88	0.17	0.73
LOC1002885	0	0	0	0	0	0
LOC1002885	0	0	0	0	0	0
LOC1002886	1.01	1.44	0.63	1.02	0.91	1.05
LOC1002887	0.33	0.33	0.26	0.4	0.1	0.56
LOC1002887	165.18	77.13	134.29	213.8	181.93	100.87
LOC1002888	0	0	0	0	0	0
LOC1002888	5.9	3.22	2.7	2.83	4.55	3.68
LOC1002888	0.22	0.44	0.17	0.42	0.05	0.17
LOC1002889	0	0	0	0	0.21	0
LOC1002889	0	0	0	0	0	0
LOC1002890	1.61	2.2	2.11	1.31	1.09	1.73
LOC1002890	3.86	4.22	4.18	5.34	4.74	3.96
LOC1002891	0.27	0.35	0.57	0.36	0.47	0.58
LOC1002891	0	0.05	0.13	0.28	0.06	0
LOC1002892	0.04	0.4	0.33	0.11	0.07	0
LOC1002893	1.34	1.86	0.98	1.07	1.44	0.92
LOC1002893	0.39	0.41	1.19	0.99	0.37	0.66
LOC1002894	0	0	0.1	0	0	0
LOC1002894	0.39	0.27	0.31	0.36	0.19	0.22
LOC1002895	0.88	0.19	0	0.27	0.75	0.24
LOC1002895	0.06	0.42	0.57	0.04	0.37	0.71
LOC1002895	0.75	1.03	1.33	0.81	0.44	0.88
LOC1002896	0	0	0	0	0	0
LOC1002896	0.34	0.5	0.43	0.21	0.17	0.19
LOC1002896	0	0	0	0	0	0
LOC1002926	0	0	0.03	0	0.03	0.03
LOC1002941	5.91	5.25	3.53	5.12	5.08	5.24
LOC1002943	0.21	0.18	0.43	0.16	0.21	0.3
LOC1003026	0	0	0	0	0	0
LOC1003037	0.51	0.97	0.28	0	0	0.1
LOC1003350	0.36	0.74	0.74	0.44	0.56	0.74

LOC1003792	0.58	0.57	0.4	0.58	0.63	0.25
LOC1004227	0	0	0	0	0	0
LOC1004988	0.9	0	0	0.21	0	0
LOC1004991	0	0.12	0.14	0	0	0.16
LOC1004991	0	0	0	0	0	0
LOC1004992	2.44	1.56	1.57	2.86	1.37	0.93
LOC1004994	0.36	0.24	0.35	0.53	0.23	0.33
LOC1004994	0.24	0.35	0.09	0.44	0.16	0
LOC1004994	0.6	0.28	0.28	0.26	0.19	0.25
LOC1004994	0.03	0	0.03	0	0	0
LOC1005007	0	0	0	0.08	0	0
LOC1005054	0	0	0	0	0	0
LOC1005054	0	0	0	0	0	0
LOC1005054	0.16	0.34	0.45	0.25	0.3	0.35
LOC1005055	0	0	0	0	0	0
LOC1005055	0	0	0	0	0	0
LOC1005055	0.73	2.72	2.68	2.14	2.35	1.94
LOC1005056	0	0	0	0	0	0
LOC1005056	0	0	0	0	0	0
LOC1005056	0	0	0	0	0	0.22
LOC1005056	0	0	0	0	0	0
LOC1005056	1.41	1.18	0.68	0.69	0.11	0.92
LOC1005056	0.18	0.02	0.03	0.06	0.14	0
LOC1005056	0.1	0.24	0.23	0.17	0.19	0.15
LOC1005056	0.89	0.77	2.66	1.07	0.84	1.37
LOC1005056	0.08	0.14	0.19	0.07	0.1	0.1
LOC1005056	4.4	2.13	3.7	2.59	2.9	0.51
LOC1005056	4.88	3.7	2.72	1.32	1.75	2.03
LOC1005056	1.34	0.28	0.19	0.05	0.4	0.78
LOC1005057	0.68	0.14	0.07	0.17	0.19	0.04
LOC1005057	0	0	0	0	0	0
LOC1005057	0.21	0.85	0.42	0.53	0.56	0.21
LOC1005057	0	0	0	0	0	0
LOC1005057	0	0	0	0	0	0
LOC1005057	12.64	10.83	10.51	7.46	12.41	9.61
LOC1005057	0	0	0	0	0	0
LOC1005057	0.14	0.23	0.37	0.17	0.2	0.11
LOC1005057	0	0	0	0	0	0
LOC1005057	1.02	1.51	0.61	0.74	0.85	1.53
LOC1005057	0	0	0	0	0	0
LOC1005058	0	0	0	0	0	0
LOC1005058	3.78	4.4	3.68	2.71	2.76	3.05
LOC1005058	0	0.2	0	0	0.06	0

LOC1005058	0	0	0	0	0	0
LOC1005058	0	0.03	0.03	0.13	0	0
LOC1005058	0	0	0	0	0	0
LOC1005058	0	0.07	0	0.18	0.08	0.09
LOC1005058	0.68	4.68	3.13	1.79	4.47	0.61
LOC1005058	9.35	9.29	9.7	8.39	8.24	10.03
LOC1005059	0.18	0	0	0	0	0
LOC1005059	0	0	0	0	0	0
LOC1005059	0	0	0.16	0.29	0.11	0
LOC1005059	0	0	0	0	0	0
LOC1005059	0	0	0.17	0	0	0
LOC1005059	0.37	0.53	0.65	0.35	0.61	0.58
LOC1005060	0	0.04	0	0.02	0	0
LOC1005060	0.42	0.51	0.42	0.22	0.44	0.52
LOC1005060	0.32	0	0	0	0.07	0
LOC1005060	0.14	0.19	0.07	0.13	0.12	0.14
LOC1005060	0	0.06	0.02	0.02	0.05	0.03
LOC1005060	7.98	7.12	6.29	3.77	8.37	5.96
LOC1005060	1.36	0.81	1.44	0.75	1.46	1.18
LOC1005060	0.09	0.18	0.05	0.23	0.11	0
LOC1005060	0.77	1.36	0	2	0.37	0.58
LOC1005060	1.66	2.36	1.51	0.88	1.69	1.15
LOC1005060	0.34	0.26	0.39	0.13	0.18	0.25
LOC1005061	1.14	1.35	1.82	0.99	2.03	1.15
LOC1005061	0	0	0	0	0	0
LOC1005061	0.69	0.81	0.85	0.37	0.69	0.79
LOC1005061	0.23	0	0.13	0	0	0.71
LOC1005061	0.11	0	0.21	0	0.11	0
LOC1005061	0.05	0	0	0.07	0.04	0
LOC1005061	0	0	0	0	0	0
LOC1005061	0.78	0	0	0	0.23	0
LOC1005061	5.96	8.13	7.67	6.26	6.38	5.39
LOC1005061	0	0.06	0	0	0	0
LOC1005062	0	0.07	0.06	0.05	0.06	0
LOC1005062	0	0	0	0	0	0
LOC1005062	6.08	7.39	4.47	6.53	8.27	5.77
LOC1005062	0.43	0.65	1.57	0.43	0.58	1.6
LOC1005062	0	0	0	0	0	0
LOC1005063	1.04	1	0.7	0.44	0.53	0.65
LOC1005063	0	0	0	0	0	0.3
LOC1005063	0	0	0.33	0.06	0.17	0
LOC1005063	0	0	0.03	0	0	0
LOC1005063	0.06	0	0	0	0	0

LOC1005063	0	0	0	0	0	0
LOC1005063	1.12	2.47	2.02	2.3	2.18	2.23
LOC1005063	0	0	0	0	0	0
LOC1005063	0	0	0	0	0	0
LOC1005063	0.35	0.39	0.51	0.22	0.26	0.8
LOC1005064	0	0	0	0	0	0
LOC1005064	0.15	1.14	0.73	0.54	0.38	0.46
LOC1005064	0.86	0	0	0.37	1.05	0
LOC1005064	0.08	0	0.07	0	0	0.33
LOC1005064	4.36	3.24	4.06	3.19	2.58	3.41
LOC1005064	0.43	0.58	0.54	0.24	0.26	0.55
LOC1005064	0	0	0	0	0	0
LOC1005064	0	0	0	0	0	0
LOC1005065	5.89	4.11	4.78	6.41	5.08	7.42
LOC1005065	0	0	0	0	0	0.17
LOC1005065	0.35	0.11	0.44	0.26	0.24	0.13
LOC1005066	0	0.04	0.03	0.08	0.08	0.06
LOC1005066	5.39	3.26	2.64	3.65	5.2	2.92
LOC1005066	1.93	2.8	2.04	0.99	1.98	1.68
LOC1005067	7.63	7.23	7.41	5.72	5.61	7.23
LOC1005067	3.71	2.45	1.7	1.46	3.06	1.75
LOC1005067	3.36	3.33	3.89	2.64	3.21	3.59
LOC1005067	0.49	0.88	0.97	0.3	0.5	0.41
LOC1005067	1.44	1.57	1.58	1.11	1.62	1.89
LOC1005067	2.79	2.99	2.36	1.24	2.05	3.84
LOC1005067	0	0.02	0	0	0	0
LOC1005068	0	0	0	0.12	0	0.06
LOC1005068	0.06	0.23	0.11	0.48	0.28	0.53
LOC1005068	0.17	0.17	0.14	0.09	0.14	0.04
LOC1005068	0.16	0.15	0.17	0.2	0.26	0.31
LOC1005068	24.91	29.79	27.58	33.03	32.68	20.63
LOC1005068	0.22	0.03	0	0.07	0.15	0.03
LOC1005068	0	0	0	0	0	0
LOC1005069	0.75	1.35	2.5	1.32	2.45	1.15
LOC1005069	3.38	4.43	4.18	3.64	2.99	4.03
LOC1005070	0	0	0	0	0	0
LOC1005070	2.21	2.7	2.73	2.5	2.24	1.62
LOC1005070	0.1	0.06	0.1	0.02	0.18	0.03
LOC1005070	0	0	0	0	0	0
LOC1005070	0.1	0.19	0	0.21	0.1	0.03
LOC1005071	0.7	0.67	0.54	0.63	0.49	0.24
LOC1005071	0.16	0	0.2	0.04	0	0
LOC1005071	0	0	0	0	0	0

LOC1005071	0.21	0.23	0.17	0.06	0.08	0.2
LOC1005071	1.1	1.75	1.62	1.01	1.47	0.83
LOC1005071	0.16	0	0	0	0	0
LOC1005072	0	0	0	0	0	0
LOC1005072	0	0	0	0	0	0
LOC1005072	3.85	3.59	1.81	1.55	2.69	1.52
LOC1005072	0	0	0	0	0	0
LOC1005072	0.04	0.3	0.67	0.33	0.69	0.3
LOC1005072	0	0	0	0	0	0
LOC1005072	0.18	0.82	0.38	0.67	0.36	0.9
LOC1005072	0.52	0.41	0.07	0.05	0.63	0.3
LOC1005073	0	0.09	0.14	0	0	0
LOC1005073	0	0	0	0	0	0
LOC1005073	0	0	0	0.04	0.04	0.08
LOC1005073	0	0	0	0.01	0	0
LOC1005073	0	0	0	0	0	0
LOC1005073	0.73	1.02	0.69	0.8	0.46	1.38
LOC1005073	0	0	0	0.07	0	0
LOC1005073	0	0	0	0	0	0
LOC1005073	0	0	0	0	0	0
LOC1005073	0	0	0	0	0	0
LOC1005073	0	0	0	0	0	0
LOC1005074	0	0	0	0	0	0
LOC1005074	0	0.24	0.29	0.07	0	0.21
LOC1005074	0	0	0	0.09	0	0
LOC1005074	0	0	0	0	0	0
LOC1005074	0.12	0	0.07	0.12	0.33	0.14
LOC1005074	1.93	3.63	3.24	2.68	3.41	2.22
LOC1005074	0	0	0	0	0	0
LOC1005074	0	0	0	0	0	0
LOC1005075	1.9	2.12	1.6	1.32	1.27	1.1
LOC1005075	0	0	0	0	0	0
LOC1005075	1.11	0.56	0.15	1.08	0.72	0.61
LOC1005075	0.1	0.06	0.06	0.1	0.06	0.1
LOC1005075	0.62	0.28	0	0.56	0.24	1.06
LOC1005075	1.6	0.59	0.66	0.47	0.57	0.31
LOC1005075	0.05	0.11	0.08	0.06	0.09	0.14
LOC1005076	0	0.19	0.07	0	0.03	0.04
LOC1005076	0	0.09	0	0	0	0
LOC1005076	0.11	0	0.03	0.12	0.1	0
LOC1005076	0	0.03	0	0	0	0.06
LOC1005081	0.52	0.44	0.34	0.87	0.36	0.52
LOC1005279	0.28	0.27	0.35	0.06	0.39	0.5

LOC1006165:	0.27	0.32	0	0.09	0	0
LOC1006309	0.53	0.71	0.73	0.47	0.35	0.05
LOC1006309:	0.18	0.55	0.25	0.3	0.56	0.23
LOC1006313	0	0	0	0	0	0
LOC1006527:	0	0	0.31	0	0.3	0
LOC1006527(	0	0	0.07	0	0	0
LOC1006527	0.07	0.06	0.03	0	0.04	0.11
LOC1006527	2.38	3.28	3.53	2.25	2.84	2.94
LOC1006527!	0	0	0	0	0	0
LOC1006529(	0.18	0	0	0	0	0
LOC1006529!	0	0	0.74	0.43	0	0.32
LOC1006535	0	0	0.03	0	0	0
LOC1008626	1.16	1.02	0.8	0.51	1.06	1.49
LOC1008730(	0	0	0	0.1	0	0
LOC1009962!	0.75	0.51	0.55	0.41	0.23	0.42
LOC1009963(	0.03	0	0	0	0	0
LOC1009964!	0.06	0.56	0.42	0.18	0.11	0.43
LOC1010545:	0	0	0	0	0	0
LOC1010556:	0	0	0	0.05	0	0
LOC1010599	0	0	0.16	0	0	0
LOC1011017	1.48	2.38	1.7	1.14	1.32	2.1
LOC113230	0.7	0.55	0.85	0.36	0.88	0.12
LOC115110	0.03	0	0	0.02	0	0
LOC116437	0.15	0.16	0.22	0.37	0.25	0.09
LOC143666	0.58	1.17	1.21	0.9	0.96	0.74
LOC144486	0.2	0.16	0.07	0.26	0.08	0.43
LOC144742	0.04	0.21	0.11	0.12	0.03	0.11
LOC145474	0.04	0	0	0	0.15	0.16
LOC145783	1.4	1.31	1.5	1.41	1.09	0.81
LOC145820	0.86	0.37	0.76	0.29	0.59	0.56
LOC145837	0	0	0	0	0	0
LOC145845	0	0	0	0	0	0
LOC146481	0	0	0	0	0	0
LOC146513	0	0	0	0	0	0
LOC146880	0.4	0.37	0.26	0.46	0.19	0.14
LOC147093	0	0	0	0	0	0
LOC147646	0	0	0	0	0	0
LOC148145	0	0	0	0	0	0
LOC148413	14.39	17.6	22.22	15.78	22.19	21.22
LOC148696	0	0.04	0.02	0.02	0	0
LOC148709	0.59	1.47	0.49	0.13	0.49	0.39
LOC149086	0	0	0	0	0	0
LOC149134	0	0	0	0	0	0

LOC149373	0	0	0	0	0	0
LOC149950	0	0	0	0	0	0
LOC150185	0	0	0	0	0	0
LOC150197	0	0.03	0.12	0.11	0	0
LOC150381	2.51	1.4	1.75	0.91	2.89	1.01
LOC150568	0	0	0	0	0	0
LOC150622	0.1	0.04	0.04	0.03	0.04	0.05
LOC150776	1.76	3.15	1.25	1.19	1.78	1.69
LOC150935	0	0.12	0.07	0.05	0.25	0.19
LOC151009	0	0.31	0.18	0.13	0.12	0
LOC151171	0	0	0	0	0	0
LOC151174	0	0	0	0	0	0
LOC151475	0.69	1.31	1.22	0.6	0.65	0.8
LOC151484	0	0	0	0	0	0
LOC152217	49.54	59.2	45.82	46.9	56.23	48
LOC152225	0.04	0	0	0	0	0.07
LOC152578	0	0	0	0	0	0
LOC152742	0	0	0	0	0	0
LOC153684	1.14	1.33	0.57	0.47	0.43	0.26
LOC153910	0	0	0	0	0	0
LOC154092	0.46	0.29	0.2	0.15	0.2	0.1
LOC154449	0	0	0	0	0	0
LOC154761	0.48	0.52	0.22	0.4	0	0.19
LOC154872	0	0	0	0	0	0
LOC155060	0.14	0.21	0	0.02	0	0.02
LOC157273	0.04	0.14	0.11	0.06	0.04	0.02
LOC157381	0	0	0	0	0.03	0.05
LOC158376	0	0	0	0	0	0
LOC158434	0	0	0	0	0	0
LOC158435	0.06	0	0.11	0.04	0	0.13
LOC158572	0.55	0.2	0.06	0.57	0.03	0.19
LOC158696	0.26	0.67	0.51	0.31	0.21	0.21
LOC1720	0	0	0	0	0	0
LOC200726	0	0	0	0	0	0
LOC200772	0	0	0	0	0	0
LOC201617	0	0	0	0	0	0
LOC201651	0	0	0	0	0	0
LOC202181	2.3	8.26	6.94	4.8	5.06	5.74
LOC202781	2.06	2.1	2.07	1.18	1.87	1.25
LOC220729	5.3	5.82	6.79	6.02	6.41	4.19
LOC221122	0	0	0	0	0	0
LOC253039	0.27	0.42	0.3	0.27	0.2	0.24
LOC253044	0	0	0	0	0	0

LOC253573	0	0	0	0	0	0
LOC254099	0	0	0	0	0	0
LOC254100	0.06	0.4	0.12	0	0.12	0.06
LOC254128	2.57	3.61	2.57	2.92	2.82	2.91
LOC254559	0	0	0	0	0	0
LOC254896	0.61	0.07	0.26	0.31	0.06	0.67
LOC255025	0	0	0	0	0	0
LOC255130	0	0	0	0	0	0
LOC255167	0.07	0.04	0.04	0.02	0	0
LOC255411	0	0	0	0	0	0
LOC255512	0.62	0	0.35	0.05	0	0
LOC255654	0	0	0	0	0	0
LOC256021	0	0	0	0	0	0
LOC256880	0.14	0.49	0.31	0.42	0.48	0.39
LOC257358	0.63	0.53	0	0.32	0.98	0.03
LOC257396	2.09	1.44	1.09	1.87	1.16	1.61
LOC282997	0.72	0.99	1.13	0.67	1.01	0.99
LOC283038	0.14	0.09	0.11	0.16	0.11	0.2
LOC283070	3.44	0.69	0.89	2.95	1.23	0.98
LOC283143	0	0	0	0	0.15	0.03
LOC283174	0.12	0.04	0.16	0.02	0.47	0.23
LOC283177	0	0.06	0.02	0	0	0.03
LOC283194	0	0	0	0	0	0
LOC283214	0	0	0	0	0	0
LOC283299	1.01	0.75	0.56	0.38	0.62	0.45
LOC283332	0	0	0	0	0	0
LOC283335	0.59	0.82	0.95	0.42	0.7	0.69
LOC283403	0	0	0	0	0	0
LOC283440	0	0	0	0	0	0
LOC283585	0.17	0.07	0.12	0.03	0	0.04
LOC283587	0	0.15	0	0	0	0
LOC283663	0.19	0.6	0.75	0.08	0.17	0.37
LOC283683	5.26	7.75	6.79	5.15	5.52	6.33
LOC283688	0.06	0.13	0	0.08	0.03	0.07
LOC283692	0	0	0	0	0.07	0
LOC283693	1.2	0.94	1.46	1.04	0.85	1.51
LOC283710	0	0	0	0.05	0	0
LOC283731	0.28	0.3	0.26	0.24	0.21	0.29
LOC283761	0	0	0	0	0	0
LOC283856	0.03	0.09	0.05	0.03	0	0
LOC283867	0	0	0	0	0	0
LOC283914	0.05	0.26	0.39	0.27	0.05	0.33
LOC283922	4.63	6.06	5.17	4.66	3.6	6.25

LOC284009	0	0.79	0.13	0.1	0	0
LOC284023	3.33	3.3	4.01	3.57	4.29	3.25
LOC284080	0	0	0	0	0	0
LOC284100	0	0.02	0	0.04	0	0
LOC284260	0.5	0.43	0.56	0.48	0.48	0.36
LOC284276	0.08	0.16	0.42	0.44	0.22	0.38
LOC284294	0	0	0	0	0	0
LOC284344	0	0	0	0	0	0
LOC284379	1.83	1.05	1.55	0.61	0.79	0.84
LOC284385	2.47	1.31	1.33	2.62	1.53	0.94
LOC284395	0	0	0.03	0.01	0.03	0.03
LOC284412	0.94	1.14	0.98	0.62	0.74	0.6
LOC284454	5.73	5.31	4.06	2.21	3.15	4.43
LOC284551	0.51	0.5	0.47	0.47	0.43	0.41
LOC284578	0.06	0.15	0.03	0.06	0.03	0
LOC284581	1.4	0.97	1.05	1.12	1.22	0.99
LOC284632	0	0.06	0	0	0.08	0.08
LOC284648	0	0	0.14	0	0	0.15
LOC284661	0	0	0	0	0	0
LOC284688	0	0	0	0	0	0
LOC284751	0.03	0	0	0	0	0
LOC284757	0.11	0.09	0.37	0.2	0.04	0.29
LOC284788	0	0	0	0	0	0
LOC284798	0	0	0	0	0	0
LOC284801	0.19	0.2	0.57	0.52	0.53	0.19
LOC284837	0.22	0.23	0.24	0.04	0.15	0.1
LOC284865	0.59	0.85	0.6	0.35	0.25	0.53
LOC284889	1.64	2.17	2.21	2.19	2.26	1.42
LOC284933	0	0	0	0	0	0
LOC284950	1.1	1.16	1.25	0.67	0.95	0.92
LOC284998	0.06	0.52	0.52	0.25	0.06	0.3
LOC285000	0.05	0	0	0	0	0
LOC285033	1.4	1.84	1.51	1.07	1.33	2.5
LOC285074	1.21	1.7	1.52	1.36	1.36	2.13
LOC285084	0	0	0	0	0	0
LOC285419	0	0	0	0	0	0
LOC285441	0	0	0	0	0	0
LOC285484	0	0	0	0	0	0
LOC285501	0	0.06	0	0	0	0
LOC285540	0.41	0	0.81	0.1	0.38	0.48
LOC285547	0.03	0.82	0.45	0.02	0.2	0.11
LOC285548	0	0	0.43	0.03	0.12	0.47
LOC285577	0	0	0	0	0	0

LOC285593	0	0	0	0	0	0
LOC285626	0	0	0	0	0	0
LOC285627	0	0	0	0	0	0
LOC285629	0	0	0	0.02	0	0.03
LOC285692	0	0	0.04	0	0	0
LOC285696	0.31	0.48	0.34	0.24	0.41	0.61
LOC285740	0	0.12	0.28	0.4	0.44	0.2
LOC285758	0.05	0.19	0	0.08	0	0
LOC285762	0.02	0	0.06	0.01	0.08	0.08
LOC285768	0	0	0	0	0	0
LOC285819	0	0	0	0	0.03	0.03
LOC285847	0	0	0.12	0	0	0
LOC285878	0	0	0	0	0	0
LOC285889	0	0	0	0	0	0
LOC285972	0.47	0.25	0.31	0.46	0.39	0.18
LOC286059	0	0	0	0	0	0.03
LOC286083	0	0	0	0	0	0
LOC286094	0	0	0	0	0	0
LOC286114	0	0	0	0	0	0
LOC286135	0	0	0	0	0	0
LOC286177	0	0.02	0.07	0	0.05	0
LOC286184	0.01	0.05	0.07	0.03	0.08	0.04
LOC286186	1.18	1.24	1.39	0.82	1.33	1.2
LOC286189	0.08	0.11	0.18	0.03	0.04	0.17
LOC286190	0.44	0.41	0.29	0.26	0.41	0.43
LOC286238	0	0	0	0	0	0
LOC286297	0.13	0	0.08	0	0.08	0.21
LOC286359	0	0.2	0.03	0.13	0.06	0.04
LOC286367	0.34	0.25	0.16	0.25	0.04	0.39
LOC286370	0.03	0	0	0	0	0
LOC286437	5.51	6.95	6.55	5.19	5.01	6.03
LOC286442	0.04	0	0	0	0	0
LOC286467	2.39	1.9	2.92	2.23	1.9	2.94
LOC338651	0	0	0.09	0	0	0
LOC338758	0.15	0.08	0.04	0.03	0.09	0.02
LOC338799	0.44	1.09	0.5	0.68	0.28	0.35
LOC338817	0.31	0.31	0.28	0.38	0.32	0.38
LOC338963	0	0	0.1	0	0.1	0
LOC339166	0.71	0.32	0.59	0.38	0.48	0.78
LOC339240	0	0	0	0	0	0
LOC339298	0	0	0	0	0.02	0
LOC339442	0	0	0	0	0	0
LOC339505	0.02	0.03	0	0	0	0

LOC339524	0.32	0.02	0	0.06	0	0.02
LOC339529	0	0	0	0	0	0
LOC339535	0	0	0	0	0	0
LOC339568	0	0	0	0	0	0
LOC339593	0	0	0	0	0	0
LOC339622	0.04	0	0	0	0	0
LOC339666	0.05	0	0.09	0	0.05	0.34
LOC339788	0	0	0	0	0	0.07
LOC339803	0.69	0.48	0.57	0.36	0.26	0.37
LOC339807	0	0	0	0	0	0
LOC339822	0	0	0	0	0	0
LOC339862	0	0.42	0.06	0.07	0.21	0.45
LOC339874	0	0	0	0	0	0
LOC339894	0.1	0.11	0.1	0	0.07	0.04
LOC339975	0.12	0	0	0	0.03	0.06
LOC340017	0	0.09	0.19	0	0	0
LOC340073	0	0	0	0	0	0.07
LOC340074	0.18	0	0	0	0.14	0
LOC340094	0	0	0	0	0	0
LOC340107	0	0	0	0	0	0
LOC340113	0	0	0	0	0	0
LOC340357	0	0	0	0	0	0
LOC340508	0	0	0	0	0	0
LOC340515	0.36	0.16	0.32	0.19	0.14	0.15
LOC341056	0	0.03	0	0	0.07	0
LOC344595	1.12	0.56	0.94	0.86	0.41	0.65
LOC344887	0.1	0.03	0	0	0	0
LOC344967	0.31	0.76	0.63	0.33	0.26	0.2
LOC348761	0.23	0.03	0.08	0.13	0.14	0.34
LOC349160	0	0	0	0	0	0
LOC349196	0.41	0.4	0.72	0.47	0.53	0.44
LOC374443	1.98	2.13	3.48	2.04	2.02	2.84
LOC375196	0	0	0	0	0	0
LOC375295	0	0.07	0	0	0	0
LOC387723	1.12	0.87	0.65	0.2	0.72	0.46
LOC387895	0	0	0	0	0	0
LOC388152	3.23	2.66	3.98	3.53	3.25	3.58
LOC388242	0.1	0	0.05	0.04	0.02	0.05
LOC388499	0	0.24	0	0	0.1	0
LOC388553	0	0	0	0	0	0
LOC388692	0.43	0.38	0.32	0.16	0.24	0.3
LOC388796	18.57	22.57	18.71	20.09	17.07	19.55
LOC388813	0	0	0	0	0	0

LOC388849	0	0	0	0	0	0
LOC388906	0	0	0	0	0	0
LOC388942	0	0	0	0	0	0
LOC388948	0.07	0	0	0	0	0
LOC389023	0	0	0.1	0	0	0.11
LOC389033	0	0	0	0	0	0
LOC389043	0	0	0	0	0	0
LOC389247	0.2	0.08	0	0.21	0.19	0
LOC389332	0	0	0	0	0	0
LOC389458	0	0	0.13	0	0	0
LOC389634	2.43	1.98	2.18	2.29	5	0.64
LOC389641	14.88	13.66	16.47	12.83	16.32	12.87
LOC389705	0	0	0	0.06	0	0.09
LOC389765	0.29	0.07	0.15	0.14	0	0.37
LOC389791	2.07	2.16	1.81	1.73	1.3	2.51
LOC389895	0.43	0	0	0	0	0
LOC389906	0.79	1.53	0.99	1.08	1.02	0.84
LOC390660	0	0	0	0	0	0
LOC390705	0	0	0	0	0	0
LOC391322	0.91	0.12	0.28	0.1	0	0
LOC392196	0	0	0	0	0	0
LOC392232	0	0	0	0	0	0
LOC392364	0	0.06	0	0.05	0.22	0.15
LOC399715	0.03	0.21	0.14	0.12	0.11	0.13
LOC399744	0.93	0.5	0.92	0.97	0.65	1
LOC399753	0	0	0	0	0	0
LOC399815	0	0	0.03	0	0.07	0
LOC399829	0	0.01	0	0	0	0
LOC400027	3.32	1.61	1.68	1.45	1.99	2.47
LOC400043	0	0	0	0	0	0
LOC400084	0	0	0.01	0	0.04	0
LOC400456	0	0	0	0	0	0
LOC400548	0.58	0.43	0.17	0.24	0.09	0.59
LOC400558	0	0	0	0	0	0
LOC400620	0	0	0	0	0	0
LOC400654	0	0	0	0	0	0
LOC400655	0	0.05	0	0.18	0.04	0.09
LOC400657	1.38	0.99	0.74	1.06	0.38	1.21
LOC400680	0.04	0.02	0.02	0.02	0	0
LOC400684	0.41	0.43	0.52	0.56	0.15	0.49
LOC400685	0	0.03	0	0	0	0
LOC400752	0.17	0.21	0.32	0.15	0.29	0.18
LOC400794	0.08	0.03	0	0.06	0.08	0.07

LOC400891	0.34	0.46	0.45	0.32	0.45	0.37
LOC400927	0.87	1.61	0.62	1.66	1.46	0.18
LOC400940	0	0	0	0	0	0
LOC400958	0.44	0.21	0.4	0.39	0.45	0.71
LOC401010	0.07	0.09	0.15	0.15	0.06	0.11
LOC401052	1.51	1.19	1.78	1.11	1.4	1.63
LOC401074	0	0.05	0.11	0.12	0.06	0.24
LOC401109	0	0	0	0	0	0
LOC401127	0	0	0	0	0	0.1
LOC401134	0	0	0	0	0	0
LOC401164	0	0	0	0	0	0
LOC401177	0	0	0	0	0	0
LOC401242	0.05	0.12	0.07	0.05	0.17	0.03
LOC401320	0.47	0.44	0.29	0.4	0.34	0.25
LOC401321	1.28	0.7	0.71	0.79	0.74	0.45
LOC401324	0.26	0.13	0.11	0.12	0.02	0.08
LOC401397	30.2	41.86	33.86	25	34.81	31.35
LOC401463	0	0	0	0	0	0
LOC401497	0	0	0	0	0	0
LOC401557	0.06	0.02	0.11	0.07	0.07	0.1
LOC401980	0	0	0	0.03	0.04	0
LOC402160	0	0	0.03	0	0.03	0
LOC402779	0	0	0	0	0	0
LOC407835	0	0	0	0	0	0
LOC415056	0	0	0	0	0	0
LOC439994	1.24	1.86	0.83	1.09	1.7	1.49
LOC440028	0.4	0.21	0.16	0.03	0.09	0.84
LOC440040	0	0	0	0	0	0
LOC440117	0	0	0	0	0	0
LOC440173	0.3	0.5	0.49	0.06	0.73	0.28
LOC440243	0	0.04	0	0.24	0.1	0.41
LOC440288	11.22	13.56	12.32	12.29	11.25	11.93
LOC440297	2.98	1.21	2.31	1.79	1.86	1.76
LOC440300	1.34	1.71	1.49	1.08	1.46	1.33
LOC440311	0	0	0	0	0.1	0
LOC440335	0.13	0	0	0.28	0	0
LOC440354	11.02	11.68	10.32	10.03	10.74	10.85
LOC440356	0	0	0	0	0.24	0
LOC440434	4.39	6.16	5.8	3.52	4.57	5.21
LOC440461	0	0	0	0	0.04	0
LOC440518	0	0	0	0	0	0
LOC440563	0.07	0.02	0	0	0	0
LOC440600	0.4	0.47	0.9	0.41	0.4	0.51

LOC440700	0.05	0.25	0.11	0	0.06	0.13
LOC440704	0	0.06	0.1	0.02	0.08	0.03
LOC440894	0.69	2.42	2.23	2.07	1.47	1.7
LOC440895	0	0	0	0	0	0
LOC440896	0.42	0.53	0.2	0.14	0.46	0.07
LOC440900	0	0	0	0	0	0
LOC440905	0	0	0	0	0	0
LOC440910	0	0	0.03	0	0	0
LOC440925	0	0	0	0	0	0
LOC440970	0.1	0.07	0.07	0.06	0.1	0.18
LOC441009	0	0	0	0	0	0
LOC441025	0	0	0	0	0	0
LOC441081	0.93	1.69	2.06	0.94	1.12	1.03
LOC441155	0.36	0.23	0.29	0.26	0.42	0.5
LOC441204	0	0.18	0	0.09	0.09	0
LOC441242	0.11	0.15	0.07	0.05	0.14	0.15
LOC441454	0.74	0.8	0.62	0.8	0.9	0.8
LOC441455	0.05	0	0	0	0	0
LOC441461	0	1.06	0.27	0.21	0	0.15
LOC441601	0.08	0.06	0	0.05	0	0
LOC441666	0.16	0.08	0.24	0.24	0.1	0.12
LOC442028	0.09	0.25	0.06	0.06	0	0.03
LOC442132	0.11	0.28	0.38	0.17	0.1	0.32
LOC442459	0	0	0	0.02	0	0
LOC442497	0	0	0	0	0	0
LOC493754	2.76	3.24	2.28	2.95	3.62	1.74
LOC494127	0	0	0	0	0	0
LOC494141	0.87	0.69	0.14	0.35	0.58	0.55
LOC494558	0	0	0	0	0	0
LOC503519	0	0	0	0	0	0
LOC541471	95.48	46.18	53.08	46.84	70.92	35.41
LOC541473	0.47	0.16	0.48	0.22	0.03	0.03
LOC550112	6.2	4.58	2.29	4.31	3.48	2.42
LOC550113	0	0	0	0	0	0
LOC550643	85.23	79.99	81.94	73.59	94.13	82.48
LOC553103	0.13	0.07	0.1	0	0	0.09
LOC554206	0.89	0.58	0.2	0.26	0.14	0.75
LOC554223	0.91	1.08	1.78	1.49	1.06	1.29
LOC574538	0	0	0	0	0	0
LOC595101	0.89	1.6	1.33	0.91	0.67	0.38
LOC606724	0	1.38	0.74	0.11	0.73	0.98
LOC613037	3.58	4.44	2.93	2.66	3.5	2.68
LOC613038	0.1	0	0.05	0.04	0.02	0.05

LOC619207	0	0	0	0.05	0	0.01
LOC63930	0	0	0	0	0	0
LOC641367	0.32	0.11	0.15	0.22	0.16	0.14
LOC641515	0	0	0	0	0	0
LOC641746	0	0	0	0	0	0
LOC642236	1.49	2.16	1.23	1.75	1.94	2.97
LOC642361	1.5	2.11	1.78	1.35	1.82	1.31
LOC642366	0	0.03	0.05	0.03	0.02	0.14
LOC642423	0	0	0	0	0	0
LOC642426	0	0	0.05	0	0	0
LOC642846	1.24	1.26	0.67	1.07	0.68	0.84
LOC642852	0.96	0.2	0.25	0.24	0.28	0.27
LOC642929	0.15	0.04	0.21	0.05	0	0
LOC643037	0	0	0	0	0	0
LOC643201	0.07	0.01	0.07	0.02	0.1	0.03
LOC643339	0.43	0	0	0	0	0
LOC643355	0	0	0	0	0	0
LOC643387	0.07	0	0	0.06	0	0
LOC643401	1.7	2.9	0.56	1.12	1.75	0.4
LOC643406	6.37	8.59	10	6.05	6.03	8.44
LOC643441	0	0	0	0	0	0
LOC643486	0	0	0	0	0	0
LOC643529	0	0	0	0	0	0
LOC643542	0.02	0	0	0	0	0
LOC643623	0.09	0.25	0.52	0.21	0.28	0.36
LOC643648	0	0	0	0	0	0.16
LOC643669	0	0.23	0.4	0.05	0.07	0
LOC643714	0	0	0	0	0	0
LOC643723	0	0.04	0	0	0	0
LOC643733	0.38	0	0.12	0	0	0
LOC643770	0.18	0.28	0.08	0.03	0.26	0.18
LOC643802	0.06	0.31	0.15	0.12	0.05	0.17
LOC643837	6.12	7.01	6.22	4.36	6.04	4.56
LOC643923	0	0	0	0	0.68	0.24
LOC644145	0	0	0	0	0	0
LOC644172	0	0	0	0	0	0.04
LOC644189	0	0	0	0	0	0
LOC644248	0	0	0	0	0	0
LOC644554	0.07	0	0.2	0.14	0	0.15
LOC644649	0	0	0	0	0	0
LOC644656	0.93	1.16	0.73	0.69	0.69	0.47
LOC644669	0.28	0.19	0.11	0.05	0.13	0.23
LOC644838	0	0	0	0	0	0

LOC644936	2.74	1.56	1.95	2.55	2.26	1.28
LOC644961	1.82	2.28	1.69	1.84	1.89	1.89
LOC645166	4.68	7.85	3.61	4.41	4.97	1.51
LOC645212	5.6	4.06	2.74	3.41	5.11	5.05
LOC645249	3.28	5.05	7.29	6.15	5.48	5.05
LOC645355	0	0	0	0	0	0
LOC645434	0	0	0	0	0	0
LOC645513	2.63	6.14	6.86	4.98	4.27	6.45
LOC645638	0	0	0.24	0	2.11	0.13
LOC645752	0	0	0.02	0.03	0	0
LOC645949	0	0	0	0	0	0
LOC646168	0	0	0	0	0	0
LOC646214	4.69	5.25	5.23	3.14	3.93	5.05
LOC646268	0	0	0	0	0	0
LOC646278	2.05	2.09	1.91	1.23	2.03	1.63
LOC646329	0.26	0.65	0.24	0.18	0.12	0.27
LOC646471	0.07	0.53	0.55	0.35	0.32	0.31
LOC646498	0	0	0	0	0	0
LOC646626	0.73	0.3	0	0	0	0
LOC646719	1.45	1.4	1.52	1.31	1.38	1.16
LOC646736	0.35	0.11	0	0.06	0	0
LOC646743	0	0	0	0	0	0
LOC646762	8.63	3.79	5.1	4.18	4.9	4.78
LOC646813	0	0	0	0	0.13	0
LOC646862	0	0	0	0	0	0
LOC646903	0	0	0	0	0	0
LOC646938	0	0.14	0	0	0	0.18
LOC646999	0	0.07	0.12	0.04	0.11	0.03
LOC647323	0	0	0	0	0	0
LOC647859	0	0	0	0	0	0
LOC648691	0.21	0.37	0.41	0.28	0.34	0.33
LOC648987	2.95	3.08	2.41	3.98	3.07	3.97
LOC649133	0	0	0	0	0	0
LOC649330	0	0	0.07	0	0	0
LOC649352	0	0	0	0	0	0
LOC649395	0.45	0.22	0.29	0.27	0.47	0.3
LOC650226	0	0	0	0	0	0
LOC650293	0	0.1	0	0.17	0.12	0
LOC650368	0.23	0.32	0.29	0.11	0	0.06
LOC652276	2.5	3.55	3.34	2.7	3.45	3.39
LOC653160	0.16	0.15	0	0.14	0.26	0
LOC653486	0	0	0	0	0	0
LOC653501	0.22	0.2	0	0.44	0.09	0.44

LOC653513	1.07	0.32	0	0.3	0.42	0
LOC653653	0.64	0.19	0.38	0.24	0.16	0.59
LOC653712	0.08	0.06	0.14	0	0	0
LOC653786	0	0	0	0	0	0.06
LOC654342	4.83	6.52	4.29	2.79	6.94	0.99
LOC654433	11.76	17.55	13.55	10.28	14.23	14.61
LOC727677	0	0	0	0	0	0
LOC727849	0.78	1.05	1.37	0.75	1.29	0.46
LOC727896	0.7	0.89	0.99	0.28	0.54	0.44
LOC727915	0	0	0	0	0	0
LOC727924	0	0	0	0	0	0
LOC727982	0	0	0	0	0	0
LOC728012	0	0	0	0	0	0
LOC728024	0.48	0.71	0.94	0.52	0.43	0.43
LOC728040	0	0	0	0	0	0
LOC728084	0	0	0	0	0	0
LOC728175	0.04	0	0	0	0	0
LOC728190	4.4	3.77	3.32	2.11	3.19	3.89
LOC728218	0.26	0.09	0.33	0	0.11	0.08
LOC728228	0.82	0	0	0	0	0
LOC728323	1.85	0.6	0.77	1.11	1.05	0.89
LOC728342	0.33	0.17	0.15	0.19	0.03	0.16
LOC728377	0	0.04	0	0.04	0.03	0.15
LOC728392	0	0	0	0.14	0	0
LOC728407	0.77	0.69	0.76	0.81	0.75	1.55
LOC728431	0.37	1.66	1.05	0.36	0.18	1.76
LOC728463	0	0	0	0	0	0
LOC728537	0.16	0	0	0.04	0	0
LOC728554	26.07	30.92	21.62	26.5	25.55	20.91
LOC728558	0.97	1.38	1.73	0.76	0.84	0.84
LOC728606	0.96	0.77	0.64	0.44	0.43	0.83
LOC728613	0.19	0.22	0.06	0.22	0.23	0.03
LOC728716	0	0	0	0	0	0
LOC728724	2.51	0.16	0	0	0.25	0.27
LOC728730	0.82	0.9	0.44	0.77	0.59	0.78
LOC728739	0	0	0	0	0	0
LOC728743	0.6	0.66	1.34	0.64	0.96	0.46
LOC728752	1.67	2.42	1.5	1.4	1.36	1.78
LOC728819	0	0	0.48	0	0	0
LOC728875	0	0	4.88	0	0.64	0.54
LOC728989	0	0	0	0	0	0
LOC729013	6.04	6.6	5.52	3.98	6.1	7.07
LOC729020	0.04	0.22	0.22	0.11	0	0.08

LOC729041	0.96	0.52	0.49	0.62	0.56	0.18
LOC729059	0	0	0	0	0	0
LOC729080	0	0.13	0.13	0	0	0.08
LOC729121	0	0	0	0	0	0
LOC729176	0	0	0	0	0.09	0
LOC729177	0	0	0	0	0	0
LOC729444	0.05	0.05	0.15	0.05	0.03	0.05
LOC729506	0	0	0	0	0	0
LOC729603	1.04	0.94	1.3	0.59	0.62	0.34
LOC729609	0.52	0.13	0.12	0.44	0.32	0.91
LOC729683	0.17	0.13	0.11	0	0.04	0.06
LOC729732	1.74	3.06	3.34	2.08	2.08	2.57
LOC729737	0.23	0.22	0.2	0.05	0.23	0.46
LOC729739	1.02	0.17	0	0.6	0.5	0.17
LOC729852	3.84	5.68	6.06	3.7	4.08	3.87
LOC729911	0	0	0	0	0	0
LOC729950	0	0	0	0	0.03	0
LOC729966	0	0	0	0	0	0
LOC729970	0	0	0	0	0	0
LOC729987	0.21	0.48	0.55	0.19	0.49	0.36
LOC730091	0	0.02	0.12	0	0.09	0.08
LOC730101	0.71	0.19	0	0.34	0.23	0.21
LOC730102	1.45	3.46	2.49	1.55	1.69	3.77
LOC730159	0	0	0	0	0	0
LOC730183	3.95	1.67	2.06	1.86	0.94	1.84
LOC730227	0.3	0.3	0.2	0.16	0	0.16
LOC730441	0	0	0	0	0	0
LOC730668	0.04	0.16	0.09	0.14	0.02	0.14
LOC730811	0	0	0	0	0	0
LOC731223	0	0	0	0	0.16	0
LOC731275	4.14	4.36	2.9	3.99	3.66	4.09
LOC731424	1.79	0.8	0.95	0.94	1.39	0.82
LOC731779	0	0	0	0	0	0
LOC732275	0	0	0	0	0	0
LOC79015	0	0	0	0.05	0	0
LOC81691	13.85	5.36	7.3	11.35	12.94	6.57
LOC84931	0	0	0	0	0	0
LOC90246	0.05	0	0.1	0.22	0.2	0.03
LOC90499	0.34	0.28	0.04	0.02	0.06	0.06
LOC90784	3.38	3.42	4.35	3.02	4.9	4.86
LOC90834	3.32	3.1	4.08	2.25	2.83	3.75
LOC91450	0.08	0.18	0.35	0	0	0.16
LOC91948	3.48	3.28	3.83	2.37	2.6	4.45

LOC92249	8.09	7.49	5.01	6.29	5.84	6.63
LOC93432	0	0	0	0.1	0	0
LOC93622	4.2	3.34	3.6	3.54	4.4	1.76
LOC96610	4.21	6.12	3.61	4.12	5.82	5.21
LOH12CR1	4.78	4.99	4.63	3.6	2.84	4.29
LOH12CR2	1.82	2.71	2.47	1.56	1.73	1.79
LONP1	19.18	16.94	11.31	14.22	13.36	13.56
LONP2	14.23	14.22	11.96	13.52	13.88	14.7
LONRF1	0.99	0.48	0.69	0.64	1.2	0.88
LONRF2	0.27	0.06	0.09	0.06	0.06	0.1
LONRF3	0.03	0	0	0	0	0
LOR	0.06	0	0	0	0	0
LOX	0.14	0.12	0.33	0.18	0.36	0.28
LOXHD1	0	0	0	0	0	0
LOXL1	0.06	0.22	0.64	0.76	0.21	0.26
LOXL1-AS1	2.97	6.35	7.54	8.23	6.47	8.72
LOXL2	0.63	0.1	0.07	0.3	0.28	0.29
LOXL3	2.99	14.49	15.8	11.62	8.49	10.78
LOXL4	0.28	0.23	0.13	0.04	0	0.04
LPA	0	0	0	0	0	0
LPAL2	0.74	0.81	1.03	0.67	0.95	0.94
LPAR1	0	0	0	0	0.02	0
LPAR2	1.32	1.7	1.33	2.56	0.78	1.3
LPAR3	0	0	0	0	0	0
LPAR4	0	0.42	0.18	0.06	0	0.28
LPAR5	17.59	0.34	0.02	3.02	9.38	0.75
LPAR6	1.25	1.47	1.06	0.58	0.59	0.69
LPCAT1	2.24	2.98	3.02	3.52	2.41	2.77
LPCAT2	5.01	4.34	3.35	2.71	3.38	4.73
LPCAT3	29.74	44.31	48.42	37.08	35.82	40.84
LPCAT4	1.36	2.48	2.27	1.34	1.8	2.32
LPGAT1	1.32	1.21	1.53	1.53	1.3	2.17
LPHN1	1.74	1.81	2.33	1.68	2.16	2.1
LPHN2	0	0	0	0	0	0
LPHN3	0	0	0	0	0	0
LPIN1	2.33	1.96	1.46	1.76	1.96	2.16
LPIN2	2.65	2.23	1.8	2.12	2.25	2.5
LPIN3	0.8	0.79	0.79	0.47	0.75	0.74
LPL	0.02	0	0	0	0	0
LPO	0	0	0	0	0	0
LPP	1.87	1.62	1.98	1.37	1.56	1.72
LPP-AS2	0.25	0.1	0.08	0.14	0.05	0.14
LPPR1	0	0	0	0	0	0

LPPR2	7.04	5.37	5.05	6.5	6.67	9.98
LPPR3	0.19	2.06	1.67	0.36	0.69	2.58
LPPR4	0	0	0	0	0	0
LPPR5	0	0	0	0	0	0
LPXN	7.1	23.53	14.21	6.56	9	9.4
LRAT	0.07	0.02	0.07	0.01	0.03	0.06
LRBA	4	2.24	3.24	2.53	2.36	3.34
LRCH1	0.73	0.86	0.82	0.58	0.41	0.63
LRCH2	0	0	0	0	0	0
LRCH3	9.77	10.7	9.84	8.58	10.91	8.66
LRCH4	2.25	3.24	2.83	2.41	2.23	2.42
LRCOL1	0	0	0	0	0	0
LRFN1	0.33	0.62	0.72	0.69	0.68	1.13
LRFN2	0	0	0	0	0	0
LRFN3	0.15	0.55	1.04	0.6	0.19	0.62
LRFN4	3.19	3.83	5.44	2.92	3.09	2.76
LRFN5	0	0	0.04	0	0	0
LRG1	4.38	15.52	10.09	4.67	4	12.1
LRGUK	0.03	0.06	0.02	0.09	0	0.03
LRIF1	13.17	11.91	10.39	8.23	10.76	9.42
LRIG1	1.33	0.99	0.84	0.65	1.21	1.04
LRIG2	1.89	2.94	2.74	2.32	2.82	2.8
LRIG3	0	0	0	0	0	0
LRIT1	0	0	0	0	0	0
LRIT2	0	0	0	0	0	0
LRIT3	0	0.05	0	0	0	0
LRMP	3	6.89	5.48	4.31	4.39	7.43
LRP1	0	0	0	0.01	0.13	0.06
LRP10	7.63	4.39	5.06	4.28	4.56	3.65
LRP11	0.3	1.11	0.92	0.21	0.55	0.63
LRP12	12.56	1.71	2.06	4	8.98	2.24
LRP1B	0	0	0	0	0	0
LRP2	0	0	0	0	0	0
LRP2BP	0.18	0.15	0.08	0.05	0.12	0.08
LRP3	1.35	1.36	1.24	2.25	2.63	3.06
LRP4	0.54	0.68	0.7	0.57	0.86	0.82
LRP4-AS1	0.65	0.14	0.25	0.14	0.13	0.44
LRP5	3.81	4.69	3.81	5.26	2.96	4.51
LRP5L	0.85	0.51	0.55	0.37	0.68	1.15
LRP6	1.43	1.03	0.97	0.8	1.23	1.19
LRP8	0.87	1.55	1.36	0.96	0.88	1.25
LRPAP1	14.04	20.1	17.18	15.14	15.51	20.17
LRPPRC	34.14	35.14	35.7	34.37	29.93	43.11

LRR1	28.14	39.8	35.29	26.71	35.96	32.25
LRRC1	0.41	0.25	0.5	0.68	0.3	0.07
LRRC10	0	0.07	0	0.02	0	0
LRRC10B	0	0	0	0	0	0
LRRC14	17	16.85	14.76	14.92	16.95	13.9
LRRC14B	0	0	0	0	0	0
LRRC15	0	0	0	0	0	0
LRRC16A	3.17	0.18	0.16	0.48	1.51	0.34
LRRC16B	0.05	0.01	0.2	0	0	0
LRRC17	0	0	0	0.05	0	0.03
LRRC18	0	0.18	0.17	0.03	0.04	0.19
LRRC19	0.12	0.05	0.15	0.08	0.19	0.21
LRRC2	0.21	0.45	0.47	0.12	0.26	0.34
LRRC2-AS1	0	0	0	0	0	0
LRRC20	1.99	3.5	3.38	4.02	2.85	2.91
LRRC23	0.46	1.39	0.67	1.97	2.27	1.02
LRRC24	0.05	0	0	0	0	0
LRRC25	0.51	0.45	0.75	0.56	0.68	0.22
LRRC26	0.13	0.6	0.64	0.61	0.66	1.73
LRRC27	1.4	3.17	2.31	0.8	1.21	1.97
LRRC28	5.54	7.06	6.39	6.78	5.04	6.59
LRRC29	0.35	0.32	0.7	1.08	0.27	0.35
LRRC3	0.38	1.01	0.81	0.32	1.51	0.34
LRRC3-AS1	0	0	0	0	0	0
LRRC30	0	0	0	0	0	0
LRRC31	0	0	0	0	0	0
LRRC32	0.55	0.05	0.2	0.18	0.15	0
LRRC33	5.03	8.91	13.7	8.6	9.24	9.46
LRRC34	8	8.54	3.45	4.5	6.61	3.61
LRRC36	0	0.03	0	0	0	0
LRRC37A	0.27	0.34	0.3	0.11	0.22	0.55
LRRC37A11P	0	0.04	0.02	0.01	0	0.02
LRRC37A2	0.46	1.36	1.14	0.38	0.53	1.2
LRRC37A3	1.05	0.99	0.98	0.69	0.89	0.86
LRRC37A4P	1.34	4.46	2.7	2.09	2.12	2.99
LRRC37A5P	0	0	0	0	0	0
LRRC37A6P	0.02	0.08	0.01	0.07	0.07	0.06
LRRC37B	0.92	0.95	0.76	0.25	0.8	2.04
LRRC37BP1	1.54	3.56	2.99	2.17	2.44	2.91
LRRC38	0	0	0	0	0	0
LRRC39	0.17	0.03	0	0	0	0.04
LRRC3B	0	0	0	0	0	0
LRRC3C	0	0	0	0	0	0

LRRC4	0.1	0.12	0.05	0	0.09	0.2
LRRC40	9.21	8.46	7.21	6.16	9.11	9.12
LRRC41	43.91	34.54	35.14	37.87	32.56	38.41
LRRC42	17.11	13.42	11.06	10.41	12.91	11.38
LRRC43	0	0.03	0.04	0.13	0.04	0.2
LRRC45	2.88	2.6	3.35	3.53	2.47	2.14
LRRC46	1.42	0.64	0.28	0.21	0.33	0.35
LRRC47	13.43	13.06	14.51	13.08	14.98	9.92
LRRC48	0.46	0.23	0.26	0.17	0.3	0.44
LRRC49	0.18	0.36	0.33	0.23	0.1	0.08
LRRC4B	0.1	0	0	0	0	0.05
LRRC4C	0	0	0	0	0	0
LRRC52	0	0	0	0	0	0
LRRC55	0	0	0	0	0.03	0
LRRC56	0.12	0.08	0.07	0.06	0.08	0.18
LRRC57	13.28	10.72	9.45	8.97	9.27	10.22
LRRC58	9.3	12.45	12.74	8.88	10.38	9.87
LRRC59	51.65	60.22	66.91	63.61	57.37	62.84
LRRC6	0.32	0.03	0.07	0.21	0.04	0.08
LRRC61	0	0	0	0	0	0
LRRC66	0	0	0	0	0	0
LRRC69	0.06	0.18	0	0.12	0.32	0
LRRC7	0.05	0	0	0	0	0
LRRC70	0	0.03	0.09	0	0.12	0.1
LRRC71	0	0	0	0	0	0
LRRC72	0	0	0	0	0	0
LRRC73	0.14	0	0	0	0.07	0
LRRC8A	4.05	2.56	3.31	4.47	4.96	3.07
LRRC8B	2.37	0.98	1.17	1.8	2.1	1.01
LRRC8C	4.66	4.61	3.2	3.7	3.25	3.7
LRRC8D	15.93	14.4	12.15	14.37	12.69	11.37
LRRC8E	1.93	0.02	0.32	1.21	0.98	1.05
LRRC9	0	0	0	0	0	0
LRRCC1	4.97	9.36	7.7	5.9	6.22	8.38
LRRD1	0	0	0	0	0	0
LRRFIP1	22.07	24.52	23.49	14.19	18.86	16.87
LRRFIP2	8.31	7.51	5.72	6.35	7.29	5.58
LRRIQ1	0	0	0	0	0	0
LRRIQ3	0.43	0.68	0.12	0.25	0.43	0.41
LRRIQ4	0	0.19	0	0.03	0.08	0.04
LRRK1	0.6	1.38	1.71	1.19	1.2	1.65
LRRK2	0.1	0	0.01	0.02	0	0.04
LRRN1	0	0	0	0.03	0	0

LRRN2	0	0	0	0	0	0
LRRN3	0.79	0.03	0	0.12	0.2	0
LRRN4	0	0.08	0.05	0	0	0
LRRN4CL	1.13	1.17	1.71	0.62	0.98	1.02
LRRTM1	0	0	0	0	0	0
LRRTM2	0.03	0.01	0	0	0.01	0.05
LRRTM3	0	0	0	0	0	0
LRRTM4	0	0	0	0	0	0
LRSAM1	3.82	3.93	5.09	5.31	3.77	3.8
LRTM1	0	0	0.05	0	0	0.06
LRTM2	0	0	0	0	0	0
LRTOMT	5.96	5.83	7.87	4.22	4.61	5.53
LRWD1	18.19	13.51	11.22	14.24	14.8	13.59
LSAMP	1.1	2.88	4.62	5.33	2.75	0.21
LSAMP-AS3	0	0	0	0	0	0
LSG1	27.43	32.66	25.39	25.44	25.71	25.33
LSM1	122.23	108.1	93.07	75.95	115.07	88.7
LSM10	50.38	58.72	59.87	46.88	55.68	47.98
LSM11	0.31	0.56	0.13	0.35	0.32	0.14
LSM12	87.6	79.07	73.4	65.16	75.75	75.56
LSM14A	20.38	23.18	19.77	18.27	21.17	21.57
LSM14B	1.24	0.98	1.4	0.87	1.38	0.49
LSM2	111.52	142.59	130.37	144.36	154.16	134.12
LSM3	237.09	203.68	189.7	204.37	256.03	203.43
LSM4	142.06	134.63	122.99	144.79	145.55	139.93
LSM5	28.37	30.36	24.14	26.02	28.06	26.06
LSM6	34.23	65.61	40.56	37.01	43.13	51.31
LSM7	327.52	408.62	332.65	380.75	398.44	373.55
LSMD1	65.36	101.13	98.85	92.42	102.89	94.07
LSP1	0.13	0.32	0.25	0.89	0.41	0.05
LSP1P3	0	0	0	0	0	0
LSR	0.38	0.21	0.17	0.44	0.29	0.27
LSS	14.81	14.62	14.9	12.34	20.01	20.84
LST1	12.51	28.43	31.6	24.43	18.68	33.3
LTA	0	0	0	0	0	0.05
LTA4H	42.02	48.89	42.07	63.76	44.79	55.98
LTB	0.73	0.89	2.7	1.92	0.53	0.37
LTB4R	0.56	1.29	1.13	0.39	0.57	1
LTB4R2	0.12	0.1	0.24	0.22	0.21	0.28
LTBP1	6.46	0.07	0.01	1.28	2.12	0.12
LTBP2	0.01	0.03	0.13	0.03	0	0
LTBP3	2.51	2.29	2.59	2.33	1.71	1.61
LTBP4	0.8	0.8	1.11	1.65	1.7	0.56

LTBR	37.06	46.96	36.85	37.94	37.21	36.87
LTC4S	0.72	0.38	1.21	0.08	0.23	0
LTF	1.08	0	0.05	0.12	0.14	0
LTK	0.03	0.02	0	0	0	0
LTN1	3.17	1.88	1.73	2.19	2.12	2.92
LTV1	44.99	36.41	26.8	26.73	32.64	28.21
LUC7L	27.18	25.93	24.42	24.01	24.07	22.32
LUC7L2	10.47	13.91	19.41	13.13	15.7	17.1
LUC7L3	39.63	35.59	33.72	29.07	32.44	33.67
LUM	0	0	0	0	0	0
LURAP1	1.91	4.56	3.83	3	1.68	2.65
LURAP1L	0	0	0	0	0	0
LUST	1.96	1.15	0.88	0.54	0.61	0.78
LUZP1	5.85	7.06	7.54	6.05	6.05	6.77
LUZP2	0.11	0.03	0.13	0.06	0.12	0.13
LUZP4	0	0	0	0	0	0
LUZP6	23.6	17.42	18.28	15.51	16.2	19.45
LXN	420.35	13.7	22.4	171.49	291.85	19.56
LY6D	0	0	0	0	0	0
LY6E	259.49	297.27	423.94	391.32	369.16	382.22
LY6G5B	9.07	7.73	5.6	7.13	7.84	6.89
LY6G5C	3.61	3.72	2.4	3.04	3.02	2.76
LY6G6C	0	0	0	0	0.25	0
LY6G6D	1.09	0	0	0.31	1.25	0
LY6G6E	0	0	0	0	0	0
LY6G6F	0.61	0	0	1.5	1.23	0
LY6H	0	0	0	0	0	0
LY6K	0	0	0	0	0	0
LY75	0.16	0.27	0.09	0.22	0.29	0.14
LY75-CD302	0	0	0	0	0	0
LY86	0.35	3.15	3.08	2.82	1.93	2.59
LY86-AS1	1.61	1.32	1	1.36	1.14	1.35
LY9	0	0	0.03	0	0	0
LY96	2.91	3.03	2.42	0.73	1.51	1.06
LYAR	34.69	38.27	32.03	26.4	28.37	35.08
LYG1	0	0.06	0	0.11	0	0.16
LYG2	0	1.13	0.16	0.12	0	0.54
LYL1	18.97	11.55	16.04	19.18	18.06	4.2
LYN	13.7	10.45	10.78	10.37	13.99	9.54
LYNX1	0	0	0	0	0.04	0
LYPD1	0	0	0	0	0	0
LYPD2	0	0	0	0	0	0
LYPD3	0.09	0	0	0.06	0.08	0.18

LYPD4	0	0	0	0	0	0
LYPD5	0	0	0	0	0	0
LYPD6	0	0.01	0	0	0	0
LYPD6B	0	0	0	0	0	0
LYPD8	0	0	0	0	0	0
LYPLA1	158.09	74.86	70.99	73.54	107.32	83.24
LYPLA2	49.24	53.88	49.81	52.89	50.42	50.67
LYPLAL1	8.84	8.45	5.12	6.76	7.76	5.62
LYRM1	11.9	19.09	13.91	10.35	12.87	15.81
LYRM2	7.2	5.33	4.95	5.31	5.36	4.18
LYRM4	60.09	57.37	58.44	61.37	66.72	53.06
LYRM5	10.68	10.28	6.15	4.73	8.39	5.97
LYRM7	5.38	6.19	5.46	3.72	4.96	5.54
LYRM9	1.92	1.72	0.37	3.84	1.44	1.69
LYSMD1	0.73	0.51	0.74	0.92	1.21	0.57
LYSMD2	6.5	15.12	9.38	8.86	9.7	6.18
LYSMD3	4.87	3.66	4.3	3.03	3.31	3.68
LYSMD4	2.84	2.17	1.39	2.44	1.58	1.05
LYST	0.6	1.98	0.41	0.28	0.43	3.01
LYVE1	0.73	0	0	0	0	0
LYZ	17.78	16.17	11.86	8.14	11.38	16.03
LYZL1	0	0	0	0	0	0
LYZL2	0	0	0	0	0	0
LYZL4	0	0	0	0	0	0
LYZL6	0	0	0	0	0	0
LZIC	22.07	25.35	23.24	21.84	24.55	23.94
LZTFL1	2.65	3.23	3.26	2.52	2.87	3.93
LZTR1	3.17	3.56	2.22	2.01	1.72	1.61
LZTS1	0.39	1.62	1.46	0.85	0.94	1.51
LZTS1-AS1	0	0.07	0	0	0	0
LZTS2	12.82	7.95	9.9	11.44	7.69	7.5
M1	0	0	0	0	0	0
M1AP	0.23	0.11	0.84	0.53	0.19	0.7
M6PR	141.3	118.89	110.63	99.57	129.6	101.76
MAATS1	0	0	0	0	0	0
MAB21L1	4.47	2.53	4.96	7.87	6.56	2.06
MAB21L2	0.03	0.02	0	0	0.02	0
MAB21L3	2.11	1.87	2.14	1.25	1.37	1.99
MACC1	0.2	0.23	0.16	0.13	0.18	0.23
MACC1-AS1	0	0	0	0	0	0
MACF1	2.58	3.71	4.3	3.82	3.45	4.43
MACROD1	1.51	3.32	2.76	2.16	1.37	3.23
MACROD2	0	0	0	0	0	0

MACROD2-A	0	0	0	0	0	0
MAD1L1	17.82	18.64	22.67	17.87	19.02	15.92
MAD2L1	84.7	93.59	73.88	67.03	91.8	97.36
MAD2L1BP	54.77	27.36	32.65	33.79	46.96	33.41
MAD2L2	74.75	45.88	46.99	74.6	75.92	41.8
MADCAM1	0	0	0	0	0	0
MADD	10.12	7.44	7.53	7.05	6.75	8.19
MAEA	33.75	35.43	30.52	32.22	34.01	28.24
MAEL	0	0	0	0	0	0
MAF	0	0	0	0	0	0
MAF1	66.47	68.74	85.25	89.61	78.77	44.98
MAFA	0.06	0.1	0	0.06	0	0
MAFB	0.06	0	0	0.03	0	0.04
MAFF	4.33	1.75	1.04	1.44	2.5	1.01
MAFG	3.5	2.56	2.7	2.29	3.46	2.38
MAFG-AS1	0.07	0.19	0.11	0.16	0.18	0
MAFK	1.26	0.77	0.32	0.93	0.94	0.79
MAG	0	0	0	0	0	0
MAGEA1	0	0	0	0	0	0
MAGEA10	0.2	0.36	0.47	0.24	0.73	0.51
MAGEA10-M	0	0	0	0	0	0
MAGEA11	0	0	0	0	0	0
MAGEA12	0	0	0	0	0	0
MAGEA2	0	0	0	0	0	0
MAGEA2B	0	0	0	0	0	0
MAGEA3	0	0	0	0	0	0
MAGEA4	0	0	0	0	0	0
MAGEA5	0	0	0	0	0	0
MAGEA6	0	0	0	0	0	0
MAGEA8	0	0	0	0	0	0
MAGEA9	0	0	0	0	0	0
MAGEA9B	0	0	0	0	0	0
MAGEB1	0	0	0	0	0	0
MAGEB10	0.24	0.32	0.27	0.17	0.33	0.06
MAGEB16	0	0	0	0	0	0
MAGEB18	0	0	0	0	0	0
MAGEB2	0	0	0	0	0	0
MAGEB3	0	0	0	0	0	0
MAGEB4	0	0	0	0	0	0
MAGEB5	0	0	0	0	0	0
MAGEB6	0	0	0	0	0	0
MAGEC1	0	0	0	0	0	0
MAGEC2	0	0	0	0	0	0

MAGEC3	0	0	0	0	0	0
MAGED1	39.24	37.25	30.2	44.19	49.16	36.08
MAGED2	61.87	35.07	25.65	43.55	44.76	31.38
MAGED4	1.02	1.09	2.27	3.28	3	0.43
MAGED4B	1.96	2.15	4.41	6.2	5.78	0.82
MAGEE1	1.1	0.14	0.25	0.43	0.78	0.42
MAGEE2	0	0	0	0	0	0
MAGEF1	2.04	0.84	0.2	0.91	0.97	0.22
MAGEH1	3.44	4.81	3.47	5.76	7.08	2.96
MAGEL2	0	0	0	0	0	0
MAGI1	5.23	0.3	0.44	1.3	2.78	0.26
MAGI1-AS1	0	0	0	0	0	0
MAGI2	0	0	0	0	0	0
MAGI2-AS2	0	0	0	0	0	0
MAGI2-AS3	0.14	0.12	0.2	0.02	0.1	0.09
MAGI3	0.9	1.03	0.98	0.6	0.81	0.99
MAGIX	7.55	2.06	1.25	4.52	10.59	2.1
MAGOH	28.16	36.21	38.19	29.87	44.52	38.12
MAGOH2	0	0.1	0	0	0.24	0
MAGOHB	11.53	10.54	7.94	8.49	12.59	9.19
MAGT1	33.26	37.76	36.07	32.16	37.05	35.85
MAK	0.1	0.27	0.29	0.14	0.22	0.12
MAK16	44.76	39.93	40.1	33.64	37.68	34.85
MAL	0	0	0	0	0	0
MAL2	0	0	0	0	0	0
MALAT1	161.32	61.18	64.55	49.83	79.15	56.89
MALL	0.06	0.05	0.29	0.11	0.12	0.11
MALSU1	83.75	93.22	91.11	77.4	90.67	81.12
MALT1	0.75	0.22	0.77	0.43	1.01	0.25
MAMDC2	0.04	0	0	0	0	0
MAMDC4	0.14	0.34	0.05	0.03	0.04	0.12
MAML1	4.42	4.75	3.64	4.68	4.27	4.9
MAML2	0.19	0.23	0.19	0.27	0.18	0.03
MAML3	1.35	0.92	1.19	0.9	1.25	1.59
MAMLD1	0.05	1.95	1.03	0.56	0.76	0.89
MAMSTR	0.14	0.37	0.22	0.07	0.18	0.48
MAN1A1	4.15	0.86	0.43	0.77	1.24	0.28
MAN1A2	2.14	1.74	2.53	2.01	1.9	2.29
MAN1B1	14.67	18.08	15.1	17.1	16.23	18.01
MAN1C1	0	0.11	0.04	0	0.04	0.07
MAN2A1	1.18	2.43	2.15	1.4	1.19	1.28
MAN2A2	4.51	3.7	2.4	2.27	3.75	2.11
MAN2B1	18.76	37.49	37.44	35.97	21.46	49.82

MAN2B2	0.65	1.14	0.59	0.36	0.79	0.85
MAN2C1	12.55	19.03	14.49	17.47	18.66	14.86
MANBA	7.8	8.49	8.77	6.82	5.74	8.18
MANBAL	13.86	10.34	11.23	12.34	11.7	8.78
MANEA	2.02	2.99	2.38	1.66	2.56	3.63
MANEAL	1.84	2.26	3.02	1.92	1.95	2.29
MANF	16.41	20.1	20.89	13.96	18.6	13.4
MANSC1	7.77	0.96	1.11	2.39	3.95	2
MANSC4	0	0	0	0	0	0.07
MAOA	10.79	2.51	1.66	4.37	5.27	0.85
MAOB	0	0	0	0	0	0
MAP10	0	0	0	0	0	0
MAP1A	4.2	4.2	6.59	6.89	4.8	6.23
MAP1B	0.21	0.11	0.25	0.17	0.08	0.13
MAP1LC3A	0	0.07	0	0	0	0
MAP1LC3B	92.66	31.56	31.62	38.82	56.94	33.07
MAP1LC3B2	10	3.11	2.3	5.12	7.45	2.72
MAP1LC3C	2.83	3.75	3.72	2.05	3.46	4.47
MAP1S	19.56	17.99	17.6	21.61	20.68	17.1
MAP2	0.13	0	0	0	0	0
MAP2K1	7.69	3.71	4.5	4.41	4.95	6.68
MAP2K2	20.21	13.42	18.53	15.63	17.14	18.74
MAP2K3	35.96	31.08	35.52	25.97	32.57	33.66
MAP2K4	2.78	2.85	2.34	1.85	2.58	2.73
MAP2K4P1	0.15	0	0.32	0	0	0
MAP2K5	14.56	18.93	18.84	16.63	14.17	18.94
MAP2K6	0.04	0.87	0.4	0.46	0.82	0.68
MAP2K7	1.38	1.32	1.56	1.66	2.4	1.13
MAP3K1	1.68	3.54	2.45	1.99	2.62	3.33
MAP3K10	0.04	0	0.04	0.07	0.02	0.04
MAP3K11	3.72	4.88	6.58	5.33	3.98	5.61
MAP3K12	0.76	0.15	0.6	0.28	0.67	0.15
MAP3K13	1.08	0.86	1.2	0.67	0.95	1.42
MAP3K14	0.18	0.24	0.34	0.2	0.38	0.29
MAP3K14-AS	0.51	0.07	0.08	0.06	0	0.09
MAP3K15	0.69	0.49	1	0.55	0.8	0.65
MAP3K19	0	0	0	0	0	0
MAP3K2	1.24	1	0.99	0.44	0.68	1.05
MAP3K3	1.79	1.88	1.59	1.11	1.83	0.92
MAP3K4	4.7	4.8	4.47	4.7	3.27	3.86
MAP3K5	0.4	0.49	0.33	0.77	0.84	0.37
MAP3K6	0.55	0.28	0.83	0.34	0.52	0.56
MAP3K7	9.39	6.99	5.63	6	8.34	6.67

MAP3K8	1.4	0.77	1.02	0.45	0.97	1.1
MAP3K9	0.52	0.43	0.51	0.14	0.21	0.41
MAP4	35.92	34.3	30.13	31.35	30.52	31.58
MAP4K1	0.1	0.09	0.62	0.02	0.08	0.08
MAP4K2	2.94	6.41	8.72	8.6	6.4	9.64
MAP4K3	1.01	0.66	0.18	0.5	0.8	0.64
MAP4K4	7.6	3.45	3.02	3.45	6.48	4.04
MAP4K5	3.16	3.17	1.83	1.88	1.82	2.34
MAP6	0.12	0	0	0	0	0
MAP6D1	0.93	2.36	1.74	1.61	2.44	0.77
MAP7	1.1	2.87	2.71	1.6	1.24	1.62
MAP7D1	3.19	2.65	2.12	2.83	4.11	3.01
MAP7D2	0	0.03	0	0	0	0
MAP7D3	14.8	13.47	12.98	11.89	13.02	11.79
MAP9	0.24	0.08	0	0.06	0.06	0
MAPK1	4.82	2.38	3.21	3.5	2.38	1.99
MAPK10	0	0	0	0	0	0
MAPK11	0.18	0.63	1.04	0.21	0.15	0.37
MAPK12	10.21	10.5	8.83	9.83	9.93	12.72
MAPK13	0.61	0.99	0.89	0.55	0.76	0.46
MAPK14	5.27	6.37	5.99	5.76	5	6.13
MAPK15	0	0	0	0	0	0
MAPK1IP1L	39.08	32.26	30.44	31.11	35.33	29.47
MAPK3	3.87	4.14	4.13	4.89	5.48	3.5
MAPK4	0	0	0	0	0.01	0
MAPK6	13.1	16.39	12.88	10.5	15.5	17.51
MAPK7	2.36	1.75	1.24	2.32	1.71	1.55
MAPK8	3.94	3.27	3.25	1.8	4.23	4.14
MAPK8IP1	0.27	0.07	0.15	0	0.34	0.23
MAPK8IP2	0	0.04	0.02	0.17	0.3	0.04
MAPK8IP3	0.36	0.86	0.24	0.27	0.62	0.68
MAPK9	2.73	1.75	2.31	1.19	1.92	1.61
MAPKAP1	6.15	5.97	5.81	6.04	6.03	5.3
MAPKAPK2	2.32	1.7	1.3	1.43	2.2	1.93
MAPKAPK3	43.32	78.99	84.52	83.54	71.69	63.78
MAPKAPK5	2.3	2.61	3.6	1.46	2.93	2.17
MAPKAPK5-A	8.77	13.82	12.52	9.98	13.53	13.09
MAPKBP1	1.64	2.47	2.32	1.51	1.87	2.67
MAPRE1	60.49	47.96	46.13	45.32	57.79	56.09
MAPRE2	28.16	15.31	17.08	15.99	13.93	16.36
MAPRE3	8	0.06	0.15	1.09	1.38	0.12
MAPT	0.11	0.13	0.42	0.1	0.15	0.34
MAPT-AS1	0	0	0	0	0	0

MAPT-IT1	0	0	0	0	0	0
1-Mar	0	0	0	0	0	0
2-Mar	0	0	0	0	0	0
1-Mar	0.32	0.34	0.21	0.24	0.29	0.27
10-Mar	0	0	0	0	0	0
11-Mar	0	0	0	0	0	0
2-Mar	2.72	1.54	1.2	1.49	1.78	1.58
3-Mar	9.41	3.55	2.6	2.61	4.73	2.99
4-Mar	0.08	0	0	0	0	0
5-Mar	9.6	9.31	10.41	8.01	8.98	10.31
6-Mar	19.88	14.48	12.97	12.42	14.51	13.73
7-Mar	18.89	13.97	12.64	12.18	14.79	12.17
8-Mar	2.13	1.5	1.05	2.49	2.78	0.49
9-Mar	0.78	0.58	0.4	0.42	0.48	0.96
MARCKS	0.15	1.72	2.42	0.27	0.17	0.38
MARCKSL1	32.37	15.38	12.7	25.77	28.91	14.88
MARCO	0	0	0	0	0	0
MARK1	0	0	0	0	0	0.01
MARK2	1.73	1.37	1.68	1.12	1.55	1.61
MARK2P9	0	0	0	0	0.08	0
MARK3	5.2	3.85	3.6	4.44	4.39	4.48
MARK4	0.71	0.81	0.7	0.48	0.66	0.65
MARS	75.18	79.22	66.47	63.45	66.74	65.59
MARS2	17.7	15.64	14.24	15.33	12.76	16.34
MARVELD1	3.64	5.17	4.22	5.55	4.28	4.19
MARVELD2	0.18	0.17	0.14	0.03	0.05	0.16
MARVELD3	0.99	0.94	1.56	1.02	0.87	1.02
MAS1	0	0	0	0	0	0
MAS1L	0.07	0	0	0	0	0
MASP1	0	0	0	0	0	0
MASP2	0	0.09	0.08	0.12	0.25	0.06
MAST1	1.63	1.64	1.58	1.92	1.62	1.85
MAST2	2.87	2.98	2.25	2.33	1.89	1.69
MAST3	5.73	3.82	4.87	4.39	4.07	4.57
MAST4	2.03	1.2	1.12	1.53	1.9	1.1
MASTL	6.2	7.3	6.49	4.58	5.65	4.89
MAT1A	0	0	0	0	0	0
MAT2A	135.08	166.58	130.41	118.83	130.28	146.44
MAT2B	48.42	64.77	50.45	39.83	57.95	59.15
MATK	13.1	22.78	25.82	23.37	22.31	27
MATN1	0	0	0	0.11	0	0.04
MATN1-AS1	0.07	0.03	0.1	0.06	0.1	0.15
MATN2	0.63	1.29	1.09	0.8	2.11	2

MATN3	0	0	0	0	0	0
MATN4	0	0	0	0	0	0
MATR3	61.22	65.01	53.5	55.42	60.74	65.26
MAU2	7.52	7.68	7.52	7.83	5.68	8.2
MAVS	6.63	14.04	15.31	10.1	9.08	13.67
MAX	41.95	56.18	60.46	48.71	54.18	71.57
MAZ	65.06	60.6	72.77	61.36	65.61	54.82
MB	0	0	0	0	0	0
MB21D1	10.96	10.29	10.93	7.23	9.84	7.41
MB21D2	1.4	1.05	1.86	2.31	1	1.62
MBD1	14.82	15.5	14.07	13.09	14.67	11.75
MBD2	2.14	2.49	2.58	2.06	1.99	2.97
MBD3	13.88	15.1	12.05	14.43	13.34	11.21
MBD3L1	0	0	0	0	0	0
MBD3L2	0	0	0	0	0	0
MBD3L3	0	0	0	0	0	0
MBD3L4	0	0	0	0	0	0
MBD3L5	0	0	0	0	0	0
MBD4	17.15	15.15	11.59	9.55	12.85	12.46
MBD5	1.05	0.95	0.93	0.48	0.54	0.58
MBD6	3.91	2.53	2.84	3.44	3.44	2.7
MBIP	9.64	8.66	8.32	7.82	9.79	8.83
MBL1P	0	0	0	0	0	0
MBL2	0	0	0	0	0	0
MBLAC1	0.35	0.2	0.18	0.14	0.19	0.25
MBLAC2	2.82	1.81	2.24	1.3	1.94	3.39
MBNL1	32.89	22.19	20.77	18.74	22.72	19.85
MBNL1-AS1	0.13	0.1	0.13	0.01	0.01	0.16
MBNL2	7.91	0.77	1.33	1.88	2.69	1.13
MBNL3	3.16	3.02	2.49	3.23	3.06	3.66
MBOAT1	14.12	19.23	18.66	14.41	19.03	15.05
MBOAT2	6.98	2.64	4.61	5.49	4.66	4.57
MBOAT4	0.09	0.07	0.04	0.09	0.04	0
MBOAT7	46.88	31.03	32.94	36.74	37.23	33.2
MBP	1.59	3.22	4.7	4.69	3.07	4.09
MBTD1	0.71	0.99	0.65	0.28	0.72	0.72
MBTPS1	14.34	13.44	14.49	10.89	14.49	18.91
MBTPS2	8.13	7.32	7.34	5.95	8.53	7.65
MC1R	1.03	0.46	1.25	1.39	0.62	0.64
MC2R	0.01	0.02	0.07	0.03	0.02	0
MC3R	0	0	0	0	0	0
MC4R	0	0	0	0	0	0
MC5R	0	0	0	0	0	0

MCAM	3.17	6.63	6.62	7.19	5.95	10.41
MCAT	15.71	22.7	23.67	22.74	21.61	14.6
MCC	0.16	0.14	0.15	0.04	0.25	0.1
MCCC1	12.69	12.11	12.29	15.43	10.88	13.1
MCCC2	26.13	31.33	28.3	29.47	27.21	31.92
MCCD1	0	0	0	0	0	0
MCEE	13.17	10.75	8.77	6.08	10.54	8.14
MCF2	0	0	0	0	0	0.14
MCF2L	0	0.18	0.38	0.5	0.21	0.29
MCF2L-AS1	0	1.66	1.03	0.55	1.12	1.13
MCF2L2	0.37	0.24	0.38	0.26	0.15	0.27
MCFD2	31.33	25.57	23.87	23.28	26.13	24.54
MCHR1	0	0	0	0	0	0
MCHR2	0	0.06	0	0	0.03	0.1
MCIN	2.17	1.93	3.14	1.35	1.82	1.49
MCL1	9.21	5.48	4.49	4	5.67	4.31
MCM10	11.63	17.72	14.92	16.75	14.27	18.71
MCM2	92.45	119.81	130.13	137.87	116.31	140.5
MCM3	96.33	115.29	106.25	107.66	97.19	111.55
MCM3AP	7.31	7.91	7.33	7.05	7.89	7.16
MCM3AP-AS	2.21	1.49	1.42	1.48	1.61	1.58
MCM4	88.87	121.14	112.8	114.04	99.4	124.01
MCM5	152.42	176.95	173.68	196.05	160.37	171.55
MCM6	51.19	61.7	59.05	53.7	56.64	58.68
MCM7	217.8	291.96	267.04	265.1	262.01	287
MCM8	1.59	1.28	1.44	1.2	1.17	1.08
MCM9	2.73	3.46	2.88	3.43	3.01	3.08
MCMBP	8.24	5.87	4.53	4.95	6.78	4.45
MCMDC2	0.35	0.25	0.45	0.31	0.47	0.82
MCOLN1	11.25	5.21	6.72	8.97	9.89	7.86
MCOLN2	1.89	2.19	3.08	2.99	2.24	2.86
MCOLN3	1.42	1.24	0.89	1.41	1.76	0.41
MCPH1	15.02	13.99	12.45	13.01	14.02	15.55
MCRS1	47.84	54.61	56.05	54.7	54.9	53.3
MCTP1	0.06	0.19	0	0	0.02	0.2
MCTP2	0.7	1.78	1.25	0.71	1.11	1.26
MCTS1	10.02	12.3	11.13	9.76	10.93	11.32
MCU	10.57	13.96	13.25	10.58	11.48	10.52
MDC1	7.74	8.53	8.9	7.82	8.53	9.69
MDFI	0.74	4.4	5.02	3.05	3.23	4.62
MDFIC	0.64	1.19	0.51	0.4	0.67	1.09
MDGA1	0	0.13	0.11	0.04	0.06	0.03
MDGA2	0	0	0	0	0	0

MDH1	175.73	177.93	158.24	170.15	198.98	173.98
MDH1B	0.09	0	0.09	0.02	0.09	0
MDH2	358.56	353.51	359.6	384.13	379.67	379.57
MDK	145.83	77.04	96	164.13	141.27	100.44
MDM1	1.36	2.23	3.09	1.5	2.04	2.37
MDM2	8.01	8.9	7.7	8.04	6.83	9.9
MDM4	8.12	7.41	6.71	7.62	7.81	8.24
MDN1	3.47	4.03	3.76	3.23	3.41	4.36
MDP1	11.31	20.4	17.73	18.44	22.11	13.5
MDS2	0	0	0	0	0	0
ME1	6.38	12.81	16.71	14.86	8.9	15.4
ME2	7.31	9.84	8.25	6.56	6.62	6.55
ME3	0.6	0	0	0	0	0
MEA1	96.8	94.68	76.9	95.78	104.57	67.45
MEAF6	16.09	11.28	12.36	10.12	13.3	11.9
MECOM	1.43	2.16	3.76	1.88	2.05	2.86
MECP2	8	6.2	6.7	6.92	7.61	6.92
MECR	19.22	19.58	16.51	23.19	22.92	17.13
MED1	12.88	14.8	11.48	11.78	12.54	11.26
MED10	58.67	45.1	29.54	35.72	39.44	35.63
MED11	13.4	19.3	13.12	14.76	13.16	9.14
MED12	14.3	18.96	19.69	23.28	17.31	22.95
MED12L	0.92	0.61	0.85	0.78	1.09	1.08
MED13	4.13	2.3	1.93	1.97	2.58	2.62
MED13L	3.66	7.35	5.7	3.28	4.61	6.01
MED14	4.09	3.51	2.76	3.24	3.5	3.37
MED15	18.38	16.04	16.58	18.17	15.58	16.8
MED16	31.46	15.21	21.86	29.54	21.68	16.82
MED17	7.98	7.25	7.72	8.98	9.89	7.81
MED18	7.87	10.38	10.9	11.69	8.97	11.2
MED19	12.18	10.21	7.82	8.67	10.56	8.6
MED20	10.15	11.28	10.83	11.48	8.91	10.51
MED21	22.5	18.22	17.48	15.31	21.97	19.57
MED22	8.44	8.78	5.84	6.65	9.35	5.36
MED23	6.72	3.27	2.89	4.05	3.71	4.03
MED24	35.01	34.4	33.91	33.23	31.12	30.07
MED25	5.42	3.48	3.41	4.59	4.73	3.33
MED26	0.55	0.68	1.05	0.72	1.08	0.69
MED27	23.95	29.42	21.48	27.4	26.76	20.57
MED28	58.01	72.28	64.48	59.35	63.36	63.05
MED29	12.23	14.08	12.99	13.43	13.66	12.73
MED30	29.78	46.01	28.98	31.99	38.07	33.79
MED31	7.54	6.98	5.39	5.02	7.51	6.78

MED4	31.03	24.5	20.41	20.93	25.46	20.18
MED4-AS1	0	0.15	0	0	0	0
MED6	31.88	38.56	36.31	31.71	42.57	37.03
MED7	9.19	9.12	7.95	7.26	6.27	8.89
MED8	23.72	26.02	25.72	23.48	24.78	23.46
MED9	12.15	11.45	8	9.66	8.85	11.53
MEDAG	0	0	0	0	0	0
MEF2A	3.11	2.3	1.65	1.74	2.04	2.56
MEF2B	0	0	0.24	0.18	0	0.05
MEF2BNB	15.46	15.36	11.33	11.79	13.12	11.4
MEF2BNB-M	0.44	0.49	0	0.46	0.25	0.16
MEF2C	3.71	8.42	9.74	8.09	5.6	7.18
MEF2D	3	3.05	3.77	2.69	2.29	3.78
MEFV	1.95	2.96	2.57	1.85	2.2	2.93
MEG3	0.05	0.04	0.06	0.05	0.03	0.12
MEG8	0	0	0	0	0	0
MEG9	0	0	0	0	0	0
MEGF10	0	0.06	0.53	0.1	0.07	0.29
MEGF11	0.17	0.17	0.5	0.25	0.15	0.31
MEGF6	0.13	0.75	0.99	0.42	0.32	1.07
MEGF8	3.04	2.99	3.73	2.96	2.79	4.17
MEGF9	0.9	0.67	0.46	0.46	1.01	0.83
MEI1	0.02	0.09	0	0	0.1	0.16
MEIG1	0.65	0.51	0.83	0.27	0.12	0.52
MEIOB	0	0	0	0	0	0
MEIS1	8.24	8.9	9.38	8.76	8.69	7.62
MEIS1-AS3	0	0	0	0	0	0.09
MEIS2	4.35	2.78	4.85	3.71	3.41	4.2
MEIS3	0.37	0	0	0.08	0.39	0.42
MEIS3P1	0	0.02	0	0	0	0
MELK	30.57	28.6	19.32	22.37	29.45	24.32
MEMO1	42.88	36.63	28.86	31.87	38.67	30.03
MEN1	5.42	6.06	4.97	4.23	5.52	4.8
MEOX1	0	0	0	0	0	0
MEOX2	0	0	0	0	0	0
MEP1A	0	0	0	0	0	0
MEP1B	0	0	0	0	0	0
MEPCE	7.1	10.26	10.79	9.41	11.9	9.68
MEPE	0	0.03	0	0	0	0
MERTK	0.88	1.13	1.54	2.12	1.55	1.22
MESDC1	0.93	0.87	0.68	0.69	0.79	0.73
MESDC2	28.13	26.85	25.74	20.06	27.33	25.31
MESP1	0.46	0.98	0.24	1.08	1.18	1.13

MESP2	0	0.07	0	0	0	0
MEST	0.29	0.81	2.2	1.07	1.77	1.5
MESTIT1	0.08	0.03	0.21	0.02	0.21	0.11
MET	0	0.03	0.05	0.08	0.06	0.06
METAP1	33.66	30.22	24.75	26.61	30.58	33.29
METAP1D	3.85	3.15	2.77	3.53	2.86	4.44
METAP2	45.17	33.94	33.47	36.91	40.69	33.61
METRN	2.73	7.29	8.01	6.08	4.11	8.91
METRNL	4.42	3.08	3.42	2.23	3.59	2.52
METTL1	15.24	22.42	20.47	17.38	21.03	12.41
METTL10	7.49	6.95	5.74	5.23	6.24	7.5
METTL11B	0	0	0	0	0	0
METTL12	2.99	1.8	3.54	4.28	2.37	3.25
METTL13	18.5	18.31	22.95	26.67	19.66	25.63
METTL14	8.54	6.01	6.37	6.6	5.12	5.66
METTL15	5.21	3.73	4.03	2.87	2.97	4.48
METTL16	7.32	8.13	7.59	7.3	8.28	8.73
METTL17	32.97	55.37	31.53	40.84	40.32	36.68
METTL18	6.1	4.85	6.23	3.71	7.4	4.37
METTL20	1.67	1.76	0.96	2.24	1.42	1.41
METTL21A	18.04	24.93	24.87	18.17	21	19.41
METTL21B	8.7	12.15	10.49	10.32	8.91	9.94
METTL21C	0	0	0.06	0	0	0
METTL21D	2.36	3.32	3.6	2.66	3.2	4
METTL21EP	0	0	0	0	0	0.03
METTL22	8.99	11.96	11.51	14.92	12.82	10.87
METTL23	21.34	23.86	24.7	30.48	36.22	29.68
METTL24	0	0.11	0	0	0	0
METTL25	2.94	2.64	2.14	2.17	2.29	3.28
METTL2A	19.89	18.74	20.49	20.13	17.51	21.21
METTL2B	23.54	25.62	23.6	25.56	23.53	24.67
METTL3	20.17	19.2	15.27	19.11	20.38	17.56
METTL4	3.95	1.99	1.4	1.42	3.22	1.36
METTL5	76.82	78.2	69.04	62.27	80.28	63.67
METTL6	5.15	5.7	3.25	3.72	4.52	5.03
METTL7A	3.17	1.94	2.55	2.62	3.08	2.06
METTL7B	7.46	12.31	13.11	7.27	6.87	13.03
METTL8	3.46	4.92	4.68	3.57	3.64	4.98
METTL9	9.16	5.83	7.3	8.65	9.97	3.22
MEX3A	0.81	0.31	0.73	0.83	0.8	0.54
MEX3B	1.6	1.21	0.98	1.87	2.54	1.69
MEX3C	0.21	1.35	0.8	0.46	0.46	0.96
MEX3D	0	0.02	0.03	0	0	0.06

MFAP1	32.77	27.9	28.65	27.92	29.99	25.51
MFAP2	0	0	0	0	0	0
MFAP3	5.42	5.4	3.86	3.25	4.67	4.69
MFAP3L	0.01	0	0.1	0	0.05	0
MFAP4	0	0.98	0	0.55	0.3	0.16
MFAP5	0.46	0.36	0.41	0.21	0.29	0.16
MFF	33.7	35.73	31.1	34.43	34.41	33.07
MFGE8	18.84	7.37	8.3	10.84	21.71	9.17
MFHAS1	4.91	3.35	2.83	3.59	3.07	3.59
MFI2	2.87	1.76	2.19	3.13	3.02	0.89
MFI2-AS1	0.08	0.51	0.22	0	0.39	0.66
MFN1	8.14	5.76	4.58	5.45	5.22	5.77
MFN2	23.37	15.08	16.91	19.7	16.77	18.87
MFNG	114.11	112.04	126.16	132.33	117.67	113.95
MFRP	0	0	0.03	0	0	0
MFSD1	55.19	67.21	60.78	50.4	61.57	75.65
MFSD10	61.79	65.6	63.79	58.28	59.67	66.98
MFSD11	5.14	4.28	5.44	3.29	5.26	4.86
MFSD12	9.08	7.26	6.79	7.79	6.46	5.43
MFSD2A	3.17	4.56	5.6	4.68	5.15	4.89
MFSD2B	0.05	0	0	0	0	0
MFSD3	15.73	24.68	25.03	22.57	26.2	18.36
MFSD4	0	0.08	0.18	0.12	0.18	0.41
MFSD5	13.84	16.94	23.03	18	17.05	12.51
MFSD6	3.57	0.95	1.32	1.63	2.11	1.08
MFSD6L	0	0	0	0	0	0
MFSD7	0	0	0.04	0.03	0	0
MFSD8	1.32	2.72	1.4	1.45	2.31	3.31
MFSD9	4.47	4.09	2.7	4.04	3.2	5.82
MGA	5.37	6.2	6.72	5.32	4.85	6.82
MGAM	0	0	0	0	0	0.08
MGARP	0.06	0.18	0.31	0	0.05	0
MGAT1	44.88	37.7	36.74	36.97	41.89	46.42
MGAT2	14.19	17.86	16.79	14.99	15.06	15.61
MGAT3	1.19	0.09	0.08	0.18	0.36	0.32
MGAT4A	0.65	1.19	1.15	0.71	0.96	1.37
MGAT4B	8.31	5.28	5.01	7.45	8.65	9.52
MGAT4C	0	0	0	0	0	0
MGAT5	2.72	2.66	2.46	2.16	2.11	3.46
MGAT5B	0	0.01	0	0	0	0
MGC12916	0.48	1.04	0.44	0.25	0.7	0.71
MGC15885	0	0	0.03	0	0	0
MGC16025	0	0.03	0	0.1	0	0

MGC16121	0.6	0.55	0	0.14	0	0
MGC16142	0.47	0.76	0.12	0.44	0.34	0.43
MGC16275	2.53	2.28	1.38	2.25	1.75	1.28
MGC21881	1.39	1.36	1.09	1.26	1.19	1.98
MGC27345	3.17	4.55	4.53	3.52	3.22	4.45
MGC27382	0	0	0	0	0	0
MGC2752	11.88	4.2	5.89	4.8	8.75	4.57
MGC2889	0	0.05	0	0	0	0.03
MGC32805	0.11	0.04	0.13	0	0.12	0
MGC34034	0.02	0.04	0.11	0.03	0.02	0.09
MGC34796	0	0	0.07	0.05	0	0
MGC39372	0	0	0	0	0	0
MGC45800	0	0	0	0	0	0
MGC45922	0.08	0	0	0.34	0	0
MGC57346	7.99	6.62	9.78	7.46	6.3	6.71
MGC72080	8.38	6.67	7.65	7.13	10.22	5.87
MGEA5	5.42	4.57	3.62	3.62	4.4	3.86
MGLL	0	0	0	0	0	0
MGME1	20.07	15.84	9.2	15.6	16.78	14.69
MGMT	0	0	0	0	0	0
MGP	0.48	0	0	0	0.2	0
MGRN1	14.26	12.96	13.46	12.27	10.9	9.95
MGST1	211.96	315.43	234.27	201.68	254.97	273.01
MGST2	78.61	61.41	59.22	50.61	56.49	73.15
MGST3	42.7	34.93	22.35	32.82	42.49	27.02
MIA	0.16	0	0	0	0	0
MIA-RAB4B	0	0	0	0	0.17	0
MIA2	0	0	0	0	0	0
MIA3	4.06	3.81	2.72	3.4	3.24	3.73
MIAT	0.04	0.02	0.05	0.1	0.03	0
MIB1	1.64	0.95	0.8	0.89	1.07	1.3
MIB2	0.35	1.09	0.68	0.55	0.53	1.3
MICA	18.81	5.77	4.59	7.71	12.34	6.38
MICAL1	2.59	3.29	3.26	4.1	2.69	2.76
MICAL2	5.44	0.82	0.85	1.64	2.74	1.78
MICAL3	2.68	3.02	2.39	2.45	3.27	3.57
MICALCL	0.17	0	0	0	0.23	0.05
MICALL1	0.87	2.05	1.43	1.23	1.21	1.25
MICALL2	6.63	1.65	1.76	1.97	3.24	1.78
MICB	8.27	8.71	11.35	7.36	8.93	7.49
MICU1	33.19	22.11	15.94	21.95	25.46	18.19
MID1	0.06	0	0	0.03	0.16	0.06
MID1IP1	7.07	3.95	3.2	5.35	5.11	5.03

MID2	0.1	0.09	0.11	0.04	0.1	0.03
MIDN	2.5	0.74	1.29	1.52	1.3	1.92
MIEN1	103.07	98.91	85.45	97.23	128.38	89.71
MIER1	13.06	7.95	6.28	7.72	12.27	9.79
MIER2	4.58	3.31	4.41	4.49	4.17	2.89
MIER3	5.82	4.61	3.15	4.03	3.03	4.24
MIF	2050.33	2017.17	1823.32	2161.62	2422.51	2190.83
MIF4GD	16.81	18.59	14.59	16.44	18.11	15.73
MIIP	53.16	29.09	24.89	31.92	34.84	25.22
MILR1	0.97	2.49	1.53	0.4	0.86	0.83
MIMT1	0	0	0	0	0	0
MINA	9.61	11.29	9.76	9.88	9.98	9.26
MINK1	3.21	1.01	1.31	1.58	1.87	0.88
MINOS1	41.85	42.1	38.22	35.73	40.72	34.47
MINOS1P1	0.12	0.39	0.21	0.02	0.18	0.39
MINPP1	30.36	8.35	5.59	10.39	16.77	9.02
MIOS	3.7	4.69	4.75	6.62	3.86	4.83
MIOX	0	0	0	0	0	0
MIP	0.08	0.27	0	0.15	0.03	0
MIPEP	12.56	13.56	12.08	7.83	10.15	13.78
MIPEPP3	1.75	1.83	1.18	1.46	1.33	1.93
MIPOL1	0	0	0.02	0	0	0
MIR1-1	0	0	0	0	0	0
MIR1-2	0	0	0	0	0	0
MIR100	0	0	0	0	0	0
MIR100HG	0	0	0	0	0	0
MIR101-1	0	0	0	0	0	0
MIR101-2	0	0	0	0	0	0
MIR103A1	0	0	0	0	0	0
MIR103A2	0	0	0	0	0	0
MIR103B1	0	0	0	0	0	0
MIR103B2	0	0	0	0	0	0
MIR105-1	0	0	0	0	0	0
MIR105-2	0	0	0	0	0	0
MIR106A	0	0	0	0	0	0
MIR106B	0	0	0	0	0	0
MIR107	0	0	0	0	0	0
MIR10A	0	0	0	0	0	0
MIR10B	0	0	0	0	0	0
MIR1178	0	0	0	0	0	0
MIR1179	0	0	0	0	0	0
MIR1180	0	0	0	0	0	0
MIR1181	0	0	0	0	0	0

MIR1182	0	0	0	0	0.48	0
MIR1184-1	0	0	0	0	0	0
MIR1184-2	0	0	0	0	0	0
MIR1184-3	0	0	0	0	0	0
MIR1185-1	0	0	0	0	0	0
MIR1185-2	0	0	0	0	0	0
MIR1193	0	0	0	0	0	0
MIR1197	0	0	0	0	0	0
MIR1200	0	0	0	0	0	0
MIR1203	0	0	0	0	0	0
MIR1204	0	0	5.17	0	0	0
MIR1205	0	0	0	0	0	0
MIR1206	0	0	0	2.61	0	0
MIR1207	0	0	0	0	0	0
MIR1208	0	0	0	0	0	0
MIR122	0	0	0	0	0	0
MIR1224	0	0	0	0	0	0
MIR1225	0	0	0	0	0	0
MIR1226	0	0	0	0	0	0
MIR1227	0	0	0	0	0	0
MIR1228	0	0	0	0	0	0
MIR1229	0	0	0	0	0	0
MIR1231	0	0	0	0	0	0
MIR1233-1	0	0	0	0	0	0
MIR1233-2	0	0	0	0	0	0
MIR1234	0	0	0	0	0	0
MIR1236	0	0	0	0	0	0
MIR1237	0	0	0	0	0	0
MIR1238	0	0	0	0	0	0
MIR124-1	0	0	0	0	0	0
MIR124-2	0	0	0	0	0	0
MIR124-3	0	0	0	0	0	0
MIR1243	0	0	0	0	0	0
MIR1244-1	0	0	0.76	0	0	0
MIR1244-2	0	0	0.76	0	0	0
MIR1244-3	0	0	0.76	0	0	0
MIR1245A	0	0	0	0	0	0
MIR1245B	0	0	0	0	0	0
MIR1246	0	0	0	0	0	0
MIR1247	0	0	0	0	0	0
MIR1248	0	2.19	0	0	0	0
MIR1249	0	0	0	0	0	0
MIR1250	0	0	0	0	0	0

MIR1251	0	0	0	0	0	0
MIR1252	0	0	0	0	0	0
MIR1253	0	0	0	0	0	0
MIR1256	0	0	0	0	0	0
MIR1257	0	0	0	0	0	0
MIR1258	0	0	0	0	0	0
MIR125A	0	0	0	0	0	0
MIR125B1	0	0	0	0	0	0
MIR125B2	0	0	0	0	0	0
MIR126	0	0	0	0	0	0
MIR1260A	0	0	0	0	0	0
MIR1260B	0	0	0	0	0	0
MIR1262	0	0	0	0	0	0
MIR1264	0	0	0	0	0	0
MIR1265	0	0	0	0	0	0
MIR1266	0	0	0	0	0	0
MIR127	0	0	0	0	0	0
MIR1270-1	0	0	0	0	0	0
MIR1270-2	0	0	0	0	0	0
MIR1272	0	0	0	0	0	0
MIR1275	0	0	0	0	0	0
MIR1276	0	0	0	0	0	0
MIR1277	0	0	0	0	0	0
MIR1278	0	0	0	0	0	0
MIR1279	0	0	0	0	0	0
MIR128-1	0	0	0	0	0	0
MIR128-2	0	0	0	0	0	0
MIR1281	0	0	0	0	0	0
MIR1282	0	0	0	0	0	0
MIR1283-1	0	0	0	0	0	0
MIR1283-2	0	0	0	0	0	0
MIR1284	0	0	0	0	0	0
MIR1286	0	0	0	0	0	0
MIR1287	0	0	0	0	0	0
MIR1288	0	0	0	0	0	0
MIR1289-2	0	0	0	0	0	0
MIR129-1	0	0	0	0	0	0
MIR129-2	0	0	0	0	0	0
MIR1291	0	0	0	0	0	0
MIR1292	0	0	0	0	0	0
MIR1293	0	0	0	0	0	0
MIR1295A	0	0	0	0	0	0
MIR1295B	0	0	0	0	0	0

MIR1296	0	0	0	0	0	0
MIR1297	0	0	0	0	0	0
MIR1298	0	0	0	0	0	0
MIR1301	0	0	0	0	0	0
MIR1304	0	0	0	0	0	0
MIR1305	0	0	0	0	0	0
MIR1306	0	0	0	0	0	0
MIR1307	0	0	0	0	0	0
MIR130A	0	0	0	0	0	0
MIR130B	0	0	0	0	0	0
MIR132	0	0	0	0	0	0
MIR1322	0	0	0	0	0	0
MIR1323	0	0	0	0	0	0
MIR1324	0	0	0	0	0	0
MIR133A1	0	0	0	0	0	0
MIR133A2	0	0	0	0	0	0
MIR133B	0	0	0	0	0	0
MIR134	0	0	0	0	0	0
MIR1343	0	0	0	0	0	0
MIR135A1	0	0	0	0	0	0
MIR135A2	0	0	0	0	0	0
MIR135B	0	0	0	0	0	0
MIR136	0	0	0	0	0	0
MIR137	0	0	0	0	0	0
MIR137HG	0	0	0	0	0	0
MIR138-1	0	0	0	0	0	0
MIR138-2	0	0	0	0	0	0
MIR139	0	0	0	0	0	0
MIR140	0	0	0	0	0	0
MIR141	0	0	0	0	0	0
MIR142	0	1.49	0	0	0	0
MIR143	0	0	0	0	0	0
MIR143HG	0.53	0.8	0.73	0.57	0.54	0.55
MIR144	0	0	0	0	0	0
MIR145	0	0	0	0	0	0
MIR1468	0	0	0	0	0	0
MIR1469	0	0	0	0	0	0
MIR146A	0	0	0	0	0	0
MIR146B	0	0	0	0	0	0
MIR1470	0	0	0	0	0	0
MIR1471	0	0	0	0	0	0
MIR147A	0	0	0	0	0	0
MIR147B	0	0	0	0	0	0

MIR148A	0	0	0	0	0	0
MIR148B	0	0	0	0	0	0
MIR149	0	0	0	0	0	0
MIR150	0	0	0	0	0	0
MIR152	0	0	0	0	0	0
MIR153-1	0	0	0	0	0	0
MIR153-2	0	0	0	0	0	0
MIR1537	0	0	0	0	0	0
MIR1538	0	0	0	0	0	0
MIR1539	0	0	0	0	0	0
MIR154	0	0	0	0	0	0
MIR155	0	0	0	0	0	0
MIR155HG	0.31	0.44	0.51	0.11	0.48	0.36
MIR15A	0	0	0	0	0	0
MIR15B	0	0	0	0	0	0
MIR16-1	0	0	0	0	0	0
MIR16-2	0	0	0	0	0	0
MIR17	0	0	0	0	0	0
MIR17HG	0.88	1.33	1.65	0.5	0.76	0.95
MIR181A1	0	0	0	0	0	0
MIR181A2	0	0	0	0	0	1.27
MIR181A2HG	0.39	0.31	0	0.27	0.63	0.13
MIR181B1	0	0	0	0	0	0
MIR181B2	0	0	0	0	0	0
MIR181C	0	0	0	0	0	0
MIR181D	0	0	0	0	0	0
MIR182	0	0	0	0	0	0
MIR1827	0	0	0	0	0	0
MIR183	0	0	0	0	0	0
MIR184	0	0	0	0	0	0
MIR185	0	0	0	0	0	0
MIR186	0	0	0	0	0	0
MIR187	0	0	0	0	0	0
MIR188	0	0	0	0	0	0
MIR18A	0	0	0	0	0	0
MIR18B	0	0	0	0	0	0
MIR1908	0	0	0	0	0	0
MIR1909	0	1.7	0	0	0	0
MIR190A	0	0	0	0	0	0
MIR190B	0	0	0	0	0	0
MIR191	0	0	0	0	0	0
MIR1910	0	0	0	0	0	0
MIR1911	0	0	0	0	0	0

MIR1912	0	0	0	0	0	0
MIR1913	0	0	0	0	0	0
MIR1914	0	0	0	0	0	0
MIR1915	0	0	0	0	0	0
MIR192	0	1.03	0	0	0	0
MIR193A	0	0	0	0	0	0
MIR193B	0	0	0	0	0	0
MIR194-1	0	0	0	0	0	0
MIR194-2	0	0	0	0	0	0
MIR195	0	0	0	0	0	0
MIR196A1	0	0	0	0	0	0
MIR196A2	0	0	0	0	0	0
MIR196B	0	0	0	0	0	0
MIR197	0	1.89	0	0	0	0
MIR1972-1	0	0	0	0	0	0
MIR1972-2	0	0	0	0	0	0
MIR1973	0	0	0	0	0	0
MIR1976	0	0	0	0	0	0
MIR198	0	0	0	0	0	0
MIR199A1	0	0	0	0	0	0
MIR199A2	0	0	0	0	0	0
MIR199B	0	0	0	0	0	0
MIR19A	0	0	0	0	0	0
MIR19B1	0	0	0	0	0	0
MIR19B2	0	0	0	0	0	0
MIR200A	0	0	0	0	0	0
MIR200B	0	0	0	0	0	0
MIR200C	0	0	0	0	0	0
MIR202	0	0	0	0	0	0
MIR203	0	0	0	0	0	0
MIR204	0	0	0	0	0	0
MIR205	0	0	0	0	0	0
MIR2052	0	0	0	0	0	0
MIR2053	0	0	0	0	0	0
MIR2054	0	0	0	0	0	0
MIR205HG	0	0.17	0	0	0.09	0.09
MIR206	0	0	0	0	0	0
MIR208A	0	0	0	0	0	0
MIR208B	0	0	0	0	0	0
MIR20A	0	0	0	0	0	0
MIR20B	0	0	0	0	0	0
MIR21	0	0	0	0	0	0
MIR210	0	0	0	0	0	0

MIR210HG	0.47	0.43	0.32	0.24	0.31	0.07
MIR211	0	0	0	0	0	0
MIR2110	0	0	0	0	1.3	0
MIR2113	0	0	0	0	0	0
MIR2114	0	0	0	0	0	0
MIR2116	0	0	0	0	0	0
MIR2117	0	0	0	0	0	0
MIR212	0	0	0	0	0	0
MIR214	0	0	0	0	0	0
MIR215	0	0	0	0	0	0
MIR216A	0	0	0	0	0	0
MIR216B	0	0	0	0	0	0
MIR217	0	0	0	0	0	0
MIR218-1	0	0	0	0	0	0
MIR218-2	0	0	0	0	0	0
MIR219-1	0	0	0	0	0	0
MIR219-2	0	0	0	0	0	0
MIR22	0	0	0	0	0	0
MIR221	0	1.03	0	0	0	0
MIR222	0	0	0	0	0	0
MIR223	1.27	0	0	0	0	0
MIR2276	0	0	0	0	0	0
MIR2277	0	0	0	0	0	0
MIR2278	0	0	0	0	0	0
MIR22HG	0.76	0.45	0.26	0.43	0.36	0.33
MIR2355	0	0	0	0	0	0
MIR2392	0	0	0	0	0	0
MIR23A	0	0	0	0	0	0
MIR23B	0	0	0	0	0	0
MIR23C	0	0	0	0	0	0
MIR24-1	0	0	0	0	0	0
MIR24-2	0	0	0	0	0	0
MIR2467	0	0	0	0	0	0
MIR25	0	0	0	0	1.92	0
MIR2681	0	0	0	0	0	0
MIR2682	0	0	0	0	0	0
MIR26A1	0	0	0	0	0	0
MIR26A2	0	0	0	0	0	0
MIR26B	0	0	0	0	0	0
MIR27A	0	0	0	0	0	0
MIR27B	0	0	0	0	0	0
MIR2861	0	0	0	0	0	0
MIR2909	0	0	0	0	0	0

MIR296	0	0	0	0	0	0
MIR2964A	0	0	0	0	0	0
MIR298	0	0	0	0	0	0
MIR299	0	0	0	0	0	0
MIR29A	0	0	0	0	0	0
MIR29B1	0	0	0	0	0	0
MIR29B2	0	0	0	0	0	0
MIR29C	0	0	0	0	0	0
MIR300	0	0	0	0	0	0
MIR301A	0	0	0	0	0	0
MIR301B	0	0	0	0	0	0
MIR302A	0	0	0	0	0	0
MIR302B	0	0	0	0	0	0
MIR302C	0	0	0	0	0	0
MIR302D	0	0	0	0	0	0
MIR302F	0	0	0	0	0	0
MIR3064	0	0	0	0	0	0
MIR3065	0	0	0	0	0	0
MIR3074	0	0	0	0	0	0
MIR30A	0	0	0	0	0	0
MIR30B	0	0	0	0	0	0
MIR30C1	0	0	0	0	0	0
MIR30C2	0	0	0	0	0	0
MIR30D	0	0	0	0	0	0
MIR30E	0	0	0	0	0	0
MIR31	0	0	0	0	0	0
MIR3115	0	0	0	0	0	0
MIR3117	0	0	0	0	0	0
MIR3119-1	0	0	0	0	0	0
MIR3119-2	0	0	0	0	0	0
MIR3120	0	0	0	0	0	0
MIR3121	0	0	0	0	0	0
MIR3122	0	0	0	0	0	0
MIR3123	0	0	0	0	0	0
MIR3124	0	0	0	0	0	0
MIR3125	0	0	0	0	0	0
MIR3126	0	0	0	0	0	0
MIR3127	0	0	0	0	0	0
MIR3128	0	0	0	0	0	0
MIR3129	0	0	0	0	0	0
MIR3130-1	0	0	0	0	0	0
MIR3130-2	0	0	0	0	0	0
MIR3131	0	0	0	0	0	0

MIR3132	0	0	0	0	0	0
MIR3134	0	0	0	0	0	0
MIR3136	0	0	0	0	0	0
MIR3138	0	0	0	0	0	0
MIR3140	0	0	0	0	0	0
MIR3141	0	0	0	0	0	0
MIR3142	0	0	0	0	0	0
MIR3143	0	0	0	0	0	0
MIR3145	0	0	0	0	0	0
MIR3146	0	0	0	0	0	0
MIR3147	0	0	0	0	0	0
MIR3148	0	0	0	0	0	0
MIR3150A	0	0	0	0	0	0
MIR3150B	0	0	0	0	0	0
MIR3151	0	0	0	0	0	0
MIR3152	0	0	0	0	0	0
MIR3153	0	0	0	0	0	0
MIR3154	0	0	0	0	0	0
MIR3155A	0	0	0	0	0	0
MIR3155B	0	0	0	0	0	0
MIR3156-1	0	0	0	0	0	0
MIR3156-2	0	0	0	0	0	0
MIR3156-3	0	0	0	0	0	0
MIR3157	0	0	0	0	0	0
MIR3158-1	0	0	0	0	0	0
MIR3158-2	0	0	0	0	0	0
MIR3160-1	0	0	0	0	0	0
MIR3160-2	0	0	0	0	0	0
MIR3162	0	0	0	0	0	0
MIR3165	0	0	0	0	0	0
MIR3167	0	0	0	0	0	0
MIR3169	0	0	0	0	0	0
MIR3170	0	0	0	0	0	0
MIR3173	0	0	0	0	0	0
MIR3175	0	0	0	0	0	0
MIR3176	0	0	0	0	0	0
MIR3177	0	0	0	0	0	0
MIR3178	0	0	0	0	0	0
MIR3179-1	0	0	0	0	0	0
MIR3179-2	0	0	0	0	0	0
MIR3179-3	0	0	0	0	0	0
MIR3180-1	0	0	0	0	0	0
MIR3180-2	0	0	0	0	0	0

MIR3180-3	0	0	0	0	0	0
MIR3180-4	0	0	0	0	0	0
MIR3180-5	0	0	0	0	0	0
MIR3182	0	0	0	0	0	0
MIR3183	0	0	0	0	0	0
MIR3184	0	0	0	0	0	0
MIR3185	0	0	0	0	0	0
MIR3186	0	0	0	0	0	0
MIR3187	0	0	0	0	0	0
MIR3188	0	0	0	0	0	0
MIR3189	2.41	0	0	0	0	0
MIR3190	0	0	0	0	0	0
MIR3191	0	0	0	0	0	0
MIR3192	0	0	0	0	0	0
MIR3193	0	0	0	0	0	0
MIR3194	0	0	0	0	0	0
MIR3196	0	0	0	0	0	0
MIR3197	0	0	0	0	0	0
MIR3198-1	0	0	0	0	0	0
MIR3198-2	0	0	0	0	0	0
MIR3199-1	0	0	0	0	0	0
MIR3199-2	0	0	0	0	0	0
MIR31HG	0	0	0	0	0	0
MIR32	0	0	0	0	0	0
MIR3200	0	0	0	0	0	0
MIR3201	0	0	0	0	0	0
MIR3202-1	0	0	0	0	0	0
MIR3202-2	0	0	0	0	0	0
MIR320A	2	0	0	0	0	0
MIR320B1	0	0	0	0	0	0
MIR320B2	0	0	0	0	0	0
MIR320C1	0	0	0	0	0	0
MIR320C2	0	0	0	0	0	0
MIR320D1	0	0	0	0	0	0
MIR320D2	0	0	0	0	0	0
MIR320E	0	0	0	0	0	0
MIR323A	0	0	0	0	0	0
MIR323B	0	0	0	0	0	0
MIR324	0	3.2	0	0	0	0
MIR326	0	0	0	0	0	0
MIR328	0	0	0	0	0	0
MIR329-1	0	0	0	0	0	0
MIR329-2	0	0	0	0	0	0

MIR330	0	0	0	0	0	0
MIR331	0	0	0	0	0	0
MIR335	0	0	0	0	0	0
MIR337	0	0	0	0	0	0
MIR338	0	0	0	0	0	0
MIR339	0	0	0	0	0	0
MIR33A	0	0	0	0	0	0
MIR33B	0	0	0	0	0	0
MIR340	0	0	0	0	0	0
MIR345	0	0	0	0	0	0
MIR346	0	0	0	0	0	0
MIR34A	0	0	0	0	0	0
MIR34B	0	0	0	0	0	0
MIR34C	0	0	0	0	0	0
MIR3529	0	0	0	0	0	0
MIR3545	0	0	0	0	0	0
MIR3591	0	0	0	0	0	0
MIR3605	0	0	0	0	0	1.47
MIR3606	0	0	0	0	0	0
MIR3607	0	0	0	0	0	0
MIR3609	0	0	0	0	0	0
MIR3610	0	0	0	0	0	0
MIR3612	0	0	0	0	0	0
MIR3613	0	0	0	0	0	0
MIR3614	0	0	0	0	0	0
MIR3615	0	0	0	0	0	0
MIR3616	0	0	0	0	0	0
MIR3618	0	0	0	0	0	0
MIR3619	0	0	0	0	0	0
MIR362	0	0	0	0	0	0
MIR3620	0	0	0	0	0	0
MIR3621	0	0	0	0	0	0
MIR3622A	0	0	0	0	0	0
MIR3622B	0	0	0	0	0	0
MIR363	0	0	0	0	0	0
MIR3646	0	0	0	0	0	0
MIR3648	0	0	1.15	0	0.61	0.63
MIR3649	0	0	0	0	0	0
MIR3650	0	0	0	0	0	0
MIR3651	0	0	0	0	0	0
MIR3652	0	0	0	0	0	1.19
MIR3653	0	0	0	0	0	0
MIR3654	0	0	0	0	7.75	0

MIR3655	0	0	0	0	0	0
MIR3656	0	0	0	0	0	0
MIR3658	0	0	0	0	0	0
MIR3659	0	0	0	0	0	0
MIR365A	0	0	0	0	0	0
MIR365B	0	0	0	0	0	0
MIR3660	0	0	0	0	0	0
MIR3661	0	0	0	0	0	0
MIR3662	0	0	0	0	0	0
MIR3663	0	0	0	0	0	0
MIR3664	0	0	0	0	0	0
MIR3665	0	0	0	0	0	0
MIR3666	0	0	0	0	0	0
MIR3668	0	0	0	0	0	0
MIR367	0	2.23	0	0	0	0
MIR3671	0	0	0	0	0	0
MIR3675	0	0	0	0	0	0
MIR3676	0	0	0	0	0	0
MIR3677	0	0	0	0	0	0
MIR3678	0	0	0	0	0	0
MIR3679	0	0	0	0	0	0
MIR3680-1	0	0	0	0	0	0
MIR3680-2	0	0	0	0	0	0
MIR3682	0	0	0	0	0	0
MIR3684	0	0	0	0	0	0
MIR3685	0	0	0	0	0	0
MIR3687	0	0	0	0	0	0
MIR3688-1	0	0	0	0	0	0
MIR3688-2	0	0	0	0	0	0
MIR3689A	0	0	0	0	0	0
MIR3689B	0	0	0	0	0	0
MIR3689C	0	0	0	0	0	0
MIR3689D1	0	0	0	0	0	0
MIR3689D2	0	0	0	0	0	0
MIR3689E	0	0	0	0	0	0
MIR3689F	0	0	0	0	0	0
MIR369	0	0	0	0	0	0
MIR3690	0	0	0	0	0	0
MIR3691	0	0	0	0	0	0
MIR3692	0	0	0	0	0	0
MIR3714	0	0	0	0	0	0
MIR371A	0	0	0	0	0	0
MIR371B	0	0	0	0	0	0

MIR372	0	0	0	0	0	0
MIR373	0	0	0	0	0	0
MIR374A	0	0	0	0	0	0
MIR374B	0	0	0	0	0	0
MIR374C	0	0	0	0	0	0
MIR375	0	0	0	0	0	0
MIR376A1	0	0	0	0	0	0
MIR376A2	0	0	0	0	0	0
MIR376B	0	0	0	0	0	0
MIR376C	0	0	0	0	0	0
MIR377	0	0	0	0	0	0
MIR378C	0	0	0	0	0	0
MIR378D1	0	0	0	0	0	0
MIR378D2	0	0	0	0	0	0
MIR378E	0	0	0	0	0	0
MIR378F	0	0	0	0	0	0
MIR379	0	0	0	0	0	0
MIR380	0	0	0	0	0	0
MIR381	0	0	0	0	0	0
MIR382	0	0	0	0	0	0
MIR383	0	0	0	0	0	0
MIR384	0	0	0	0	0	0
MIR3907	0	0	0	0	0	0
MIR3908	0	0	0	0	0	0
MIR3909	0	0	0	0	0	0
MIR3910-1	0	0	0	0	0	0
MIR3910-2	0	0	0	0	0	0
MIR3911	0	1.05	0	0	0	2.58
MIR3912	0	0	0	0	0	0
MIR3913-1	0	0	0	0	0	0
MIR3913-2	0	0	0	0	0	0
MIR3914-1	0	0	0	0	0	0
MIR3914-2	0	0	0	0	0	0
MIR3916	0	1.32	0	0	0	0
MIR3917	0	0	0	0	0	0
MIR3918	0	0	0	0	0	0
MIR3919	0	0	0	0	0	0
MIR3920	0	0	0	0	0	0
MIR3921	0	0	0	0	0	0
MIR3922	0	0	0	0	0	0
MIR3924	0	0	0	0	0	0
MIR3925	0	0	0	0	0	0
MIR3926-1	0	0	0	0	0	0

MIR3926-2	0	0	0	0	0	0
MIR3928	0	0	0	0	0	0
MIR3935	0	0	0	0	0	0
MIR3936	0	0	0	0	0	0
MIR3938	0	0	0	0	0	0
MIR3939	0	0	0	0	0	0
MIR3940	0	0	0	0	0	0
MIR3941	0	0	0	0	0	0
MIR3942	0	0	0	0	0	0
MIR3943	0	0	0	0	0	0
MIR3944	0	0	0	0	0	0
MIR3945	0	0	0	0	0	0
MIR3960	0	0	0	0	0	0
MIR3973	0	0	0	0	0	0
MIR3974	0	0	0	0	0	0
MIR3975	0	0	0	0	0	0
MIR3976	0	0	0	0	0	0
MIR3977	0	0	0	0	0	0
MIR3978	0	0	0	0	0	0
MIR409	0	0	0	0	0	0
MIR410	0	0	0	0	0	0
MIR411	0	0	0	0	0	0
MIR412	0	0	0	0	0	0
MIR421	0	0	0	0	0	0
MIR423	0	0	0	0	0	0
MIR424	0	0	0	0	0	0
MIR425	0	0	0	0	0	0
MIR4251	0	0	0	0	0	0
MIR4252	0	0	0	0	0	0
MIR4253	0	0	0	0	0	0
MIR4254	0	0	0	0	0	0
MIR4255	0	0	0	0	0	0
MIR4256	0	0	0	0	0	0
MIR4257	0	0	0	0	0	0
MIR4258	0	0	0	0	0	0
MIR4260	0	0	0	0	0	0
MIR4261	0	0	0	0	0	0
MIR4262	0	0	0	0	0	0
MIR4263	0	0	0	0	0	0
MIR4264	0	0	0	0	0	0
MIR4265	0	0	0	0	0	0
MIR4266	0	0	0	0	0	0
MIR4267	0	0	0	0	0	0

MIR4268	0	0	0	0	0	0
MIR4269	0	0	0	0	0	0
MIR4270	0	0	0	0	0	0
MIR4271	0	0	0	0	0	0
MIR4272	0	0	0	0	0	0
MIR4273	0	0	0	0	0	0
MIR4274	0	0	0	0	0	0
MIR4275	0	0	0	0	0	0
MIR4276	0	0	0	0	0	0
MIR4277	0	0	0	0	0	0
MIR4278	0	0	0	0	0	0
MIR4279	0	0	0	0	0	0
MIR4280	0	0	0	0	0	0
MIR4281	0	0	0	0	0	0
MIR4282	0	0	0	0	0	0
MIR4283-1	0	0	0	0	0	0
MIR4283-2	0	0	0	0	0	0
MIR4284	0	0	0	0	0	0
MIR4285	0	0	0	0	0	0
MIR4287	0	0	0	0	0	0
MIR4288	0	0	0	0	0	0
MIR4289	0	0	0	0	0	0
MIR429	0	0	0	0	0	0
MIR4290	0	0	0	0	0	0
MIR4291	0	0	0	0	0	0
MIR4292	0	0	0	0	0	0
MIR4294	0	0	0	0	0	0
MIR4295	0	0	0	0	0	0
MIR4296	0	0	0	0	0	0
MIR4297	0	0	0	0	0	0
MIR4298	0	0	0	0	0	0
MIR4299	0	0	0	0	0	0
MIR4300	0	0	0	0	0	0
MIR4301	0	0	0	0	0	0
MIR4302	0	0	0	0	0	0
MIR4303	0	0	0	0	0	0
MIR4304	0	0	0	0	0	0
MIR4305	0	0	0	0	0	0
MIR4306	0	0	0	0	0	0
MIR4307	0	0	0	0	0	0
MIR4308	0	0	0	0	0	0
MIR4309	0	0	0	0	0	0
MIR431	0	0	0	0	0	0

MIR4310	0	0	0	0	0	0
MIR4311	0	0	0	0	0	0
MIR4312	0	0	0	0	0	0
MIR4313	0	0	0	0	0	0
MIR4314	0	0	0	0	0	0
MIR4315-1	0	0	0	0	0	0
MIR4315-2	0	0	0	0	0	0
MIR4316	0	0	0	0	0	0
MIR4317	0	0	0	0	0	0
MIR4318	0	0	0	0	0	0
MIR4319	0	0	0	0	0	0
MIR432	0	0	0	0	0	0
MIR4320	0	0	0	0	0	0
MIR4321	0	0	0	0	0	0
MIR4322	0	0	0	0	0	0
MIR4323	0	0	0	0	0	0
MIR4324	0	0	0	0	0	0
MIR4325	0	0	0	0	0	0
MIR4326	0	0	0	0	0	0
MIR4327	0	0	0	0	0	0
MIR4328	0	0	0	0	0	0
MIR4329	0	0	0	0	0	0
MIR433	0	0	0	0	0	0
MIR4330	0	0	0	0	0	0
MIR4417	0	0	0	0	0	0
MIR4420	0	0	0	0	0	0
MIR4422	0	0	0	0	0	0
MIR4423	0	0	0	0	0	0
MIR4424	0	0	0	0	0	0
MIR4426	3.5	0	0	2.59	0	0
MIR4427	0	0	0	0	0	0
MIR4429	0	0	0	0	0	0
MIR4432	0	0	0	0	0	0
MIR4434	0	0	0	0	0	0
MIR4435-1	0	0	0	0	0	0
MIR4435-2	0	0	0	0	0	0
MIR4436A	0	0	0	0	0	0
MIR4436B1	0	0	0	0	0	0
MIR4436B2	0	0	0	0	0	0
MIR4437	0	0	0	0	0	0
MIR4439	0	0	0	0	0	0
MIR4440	0	0	0	0	0	0
MIR4441	0	0	0	0	0	0

MIR4442	0	0	0	0	0	0
MIR4443	0	0	0	0	0	0
MIR4444-1	0	0	0	0	0	0
MIR4446	0	0	0	0	0	0
MIR4449	0	0	0	0	0	0
MIR4450	0	0	0	0	0	0
MIR4451	0	0	0	0	0	0
MIR4453	0	0	0	0	0	0
MIR4454	0	0	0	0	0	0
MIR4456	0	0	0	0	0	0
MIR4457	0	0	0	0	0	0
MIR4458	0	0	0	0	0	0
MIR4460	0	0	0	0	0	0
MIR4461	403.51	457.81	435.47	266.28	297.36	271.48
MIR4462	0	0	0	0	0	0
MIR4464	0	0	0	0	0	0
MIR4465	0	0	0	0	0	0
MIR4466	0	0	0	0	0	0
MIR4467	0	0	0	0	0	0
MIR4468	0	0	0	0	0	0
MIR4469	0	0	0	0	0	0
MIR4470	0	0	0	0	0	0
MIR4471	0	0	0	0	0	0
MIR4472-1	0	0	0	0	0	0
MIR4472-2	0	0	0	0	0	0
MIR4473	0	0	0	0	0	0
MIR4474	0	0	0	0	0	0
MIR4475	0	0	0	0	0	0
MIR4476	0	0	0	0	0	0
MIR4478	0	0	0	0	0	0
MIR4479	0	0	0	0	0	0
MIR448	0	0	0	0	0	0
MIR4480	0	0	0	0	0	0
MIR4482-1	0	0	0	0	0	0
MIR4483	0	0	0	0	0	0
MIR4484	0	0	0	0	0	0
MIR4485	0	0	0	0	0	0
MIR4486	0	0	0	0	0	0
MIR4488	0	0	0	0	0	0
MIR4489	0	0	0	0	0	0
MIR4490	0	0	0	0	0	0
MIR4491	0	0	0	0	0	0
MIR4492	0	0	0	0	0	0

MIR4493	0	0	0	0	0	0
MIR4497	0	0	0	0	0	0
MIR4498	0	0	0	0	0	0
MIR4499	0	0	0	0	0	0
MIR449A	0	0	0	0	0	0
MIR449B	0	0	0	0	0	0
MIR449C	0	0	0	0	0	0
MIR4500	0	0	0	0	0	0
MIR4500HG	0	0	0	0	0	0
MIR4503	0	0	0	0	0	0
MIR4505	0	0	0	0	0	0
MIR4508	0	0	0	0	0	0
MIR4509-1	0	0	0	0	0	0
MIR4509-2	0	0	0	0	0	0
MIR4509-3	0	0	0	0	0	0
MIR450A1	0	0	0	0	0	0
MIR450A2	0	0	0	0	0	0
MIR450B	0	0	0	0	0	0
MIR4510	0	0	0	0	0	0
MIR4511	0	0	0	0	0	0
MIR4513	0	0	0	0	0	0
MIR4514	0	0	0	0	0	0
MIR4515	0	0	0	0	0	0
MIR4516	0	0	0	0	0	0
MIR4517	0	0	0	0	0	0
MIR4518	0	0	0	0	0	0
MIR4519	0	0	0	0	0	0
MIR451A	0	0	0	0	0	0
MIR451B	0	0	0	0	0	0
MIR452	0	0	0	0	0	0
MIR4520A	0	0	0	0	0	0
MIR4520B	0	0	0	0	0	0
MIR4521	0	0	0	0	0	0
MIR4522	0	0	0	0	0	0
MIR4523	0	0	0	0	0	0
MIR4524A	0	0	0	0	0	0
MIR4524B	0	0	0	0	0	0
MIR4526	0	0	0	0	0	0
MIR4529	0	0	0	0	0	0
MIR4530	0	0	0	0	0	0
MIR4531	0	0	0	0	0	0
MIR4532	0	0	0	0	0	0
MIR4533	0	0	0	0	0	0

MIR4534	0	0	0	0	0	0
MIR4535	0	0	0	0	0	0
MIR4536-1	0	0	0	0	0	0
MIR454	0	0	0	0	0	0
MIR4540	0	0	0	0	0	0
MIR455	0	0	0	0	0	0
MIR4632	0	0	0	0	0	0
MIR4633	0	0	0	0	0	0
MIR4634	0	0	0	0	0	0
MIR4635	0	0	0	0	0	0
MIR4636	0	0	0	0	0	0
MIR4637	0	0	0	0	0	0
MIR4638	0	0	0	0	0	0
MIR4639	0	0	0	0	0	0
MIR4640	0	0	0	0	0	0
MIR4641	0	0	0	0	0	0
MIR4642	0	0	0	0	0	0
MIR4643	0	0	0	0	0	0
MIR4644	0	0	0	0	0	0
MIR4645	0	0	0	0	0	0
MIR4646	0	0	0	0	0	0
MIR4647	0	0	0	0	0	0
MIR4648	0	0	0	0	0	0
MIR4649	0	0	0	0	0	0
MIR4650-1	0	0	0	0	0	0
MIR4650-2	0	0	0	0	0	0
MIR4651	0	0	0	0	0	0
MIR4652	0	0	0	0	0	0
MIR4653	0	0	0	0	0	0
MIR4654	0	0	0	0	0	0
MIR4655	0	0	0	0	0	0
MIR4656	0	0	0	0	0	0
MIR4657	0	0	0	0	0	0
MIR4658	0	0	0	0	0	0
MIR4659A	0	0	0	0	0	0
MIR4659B	0	0	0	0	0	0
MIR4660	0	0	0	0	0	0
MIR4661	0	0	0	0	0	0
MIR4663	0	0	0	0	0	0
MIR4664	0	0	0	0	0	0
MIR4665	0	0	0	0	0	0
MIR4666A	0	0	0	0	0	0
MIR4667	0	0	0	0	0	0

MIR4668	0	0	0	0	0	0
MIR4669	0	0	0	0	0	0
MIR4670	0	0	0	0	0	0
MIR4671	0	0	0	0	0	0
MIR4672	0	0	0	0	0	0
MIR4673	0	0	0	0	0	0
MIR4674	0	0	0	0	0	0
MIR4675	0	0	0	0	0	0
MIR4676	0	0	0	0	0	0
MIR4677	0	0	0	0	0	0
MIR4678	0	0	0	0	0	0
MIR4679-1	0	0	0	0	0	0
MIR4679-2	0	0	0	0	0	0
MIR4680	0	0	0	0	0	0
MIR4681	0	0	0	0	0	0
MIR4682	0	0	0	0	0	0
MIR4683	0	0	0	0	0	0
MIR4684	0	0	0	0	0	0
MIR4685	0	0	0	0	0	0
MIR4686	0	0	0	0	0	0
MIR4687	0	0	0	0	0	0
MIR4688	0	0	0	0	0	0
MIR4689	0	0	0	0	0	0
MIR4690	0	0	0	0	0	0
MIR4691	0	0	0	4.22	0	0
MIR4692	0	0	0	0	0	0
MIR4693	0	0	0	0	0	0
MIR4694	0	0	0	0	0	0
MIR4695	0	0	0	0	0	0
MIR4696	0	0	0	0	0	0
MIR4697	0	0	0	0	0	0
MIR4698	0	0	0	0	0	0
MIR4699	0	0	0	0	0	0
MIR4700	0	0	0	0	0.67	0
MIR4701	0	0	0	0	0	0
MIR4703	0	0	0	0	0	0
MIR4705	0	0	0	0	0	0
MIR4706	0	0	0	0	0	0
MIR4707	0	0	0	0	0	0
MIR4708	0	0	0	0	0	0
MIR4709	0	0	0	0	0	0
MIR4710	0	0	0	0	0	0
MIR4711	0	0	0	0	0	0

MIR4712	0	0	0	0	0	0
MIR4713	0	0	0	0	0	0
MIR4714	0	0	0	0	0	0
MIR4715	0	0	0	0	0	0
MIR4716	0	0	0	0	0	0
MIR4717	0	0	0	0	0	0
MIR4718	0	0	0	0	0	0
MIR4719	0	0	0	0	0	0
MIR4720	0	0	0	0	0	0
MIR4721	0	0	0	0	0	0
MIR4722	0	0	0	0	0	0
MIR4723	0	0	0	0	0	0
MIR4724	0	0	0	0	0	0
MIR4725	0	0	0	0	0	0
MIR4726	0	0	0	0	0	0
MIR4727	0	0	0	0	0	0
MIR4728	0	0	0	0	0	0
MIR4729	0	0	0	0	0	0
MIR4730	0	0	0	0	0	0
MIR4731	0	0	0	0	0	0
MIR4732	0	0	0	0	0	0
MIR4733	0	0	0	0	0	0
MIR4734	0	0	0	0	0	0
MIR4735	0	0	0	0	0	0
MIR4736	0	0	0	0	0	0
MIR4737	2.04	0	1.14	3.04	0	4.08
MIR4738	0	0	0	0	0	0
MIR4739	0	0	0	0	0	0
MIR4740	0	0	0	0	0	0
MIR4741	0	0	0	0	0	0
MIR4742	0	0	0	0	0	0
MIR4743	0	0	0	0	0	0
MIR4744	0	0	0	0	0	0
MIR4745	0	0	0	0	0	0
MIR4746	0	0	0	0	0	0
MIR4747	0	0	0	0	0	0
MIR4748	0	0	0	0	0	0
MIR4749	0	0	0	0	0	0
MIR4750	0	0	0	0	0	0
MIR4751	0	0	0	0	0	3.47
MIR4752	0	0	0	0	0	0
MIR4753	0	0	0	0	0	0
MIR4754	0	0	0	0	0	0

MIR4755	0	0	0	0	0	0
MIR4756	0	0	0	0	0	0
MIR4757	0	0	0	0	0	0
MIR4758	0	0	0	0	0	0
MIR4759	0	0	0	0	0	0
MIR4760	0	0	0	0	0	0
MIR4761	0	0	0	0	0	0
MIR4762	0	0	0	0	0	0
MIR4763	0	0	0	0	0	0
MIR4764	0	0	0	0	0	0
MIR4765	0	0	0	0	0	0
MIR4766	0	0	0	0	0	0
MIR4767	0	0	0	0	0	0
MIR4768	0	0	0	0	0	0
MIR4769	0	0	0	0	0	0
MIR4770	0	0	0	0	0	0
MIR4772	0	0	0	0	0	0
MIR4773-1	0	0	0	0	0	0
MIR4773-2	0	0	0	0	0	0
MIR4774	0	0	0	0	0	0
MIR4775	0	0	0	0	0	0
MIR4776-1	0	0	0	0	0	0
MIR4776-2	0	0	0	0	0	0
MIR4777	0	0	0	0	0	0
MIR4778	0	0	0	0	0	0
MIR4779	0	0	0	0	0	0
MIR4780	0	0	0	0	0	0
MIR4781	0	0	0	0	0	0
MIR4782	0	0	0	0	0	0
MIR4783	0	0	0	0	0	0
MIR4784	0	0	0	0	0	0
MIR4785	0	0	0	0	0	0
MIR4786	0	0	0	0	0	0
MIR4787	0	0	0	0	0	0
MIR4788	0	0	0	0	0	0
MIR4789	0	0	0	0	0	0
MIR4790	0	0	0	0	0	0
MIR4791	0	0	0	0	0	0
MIR4792	0	0	0	0	0	0
MIR4793	0	0	0	0	0	0
MIR4794	0	0	0	0	0	0
MIR4795	0	0	0	0	0	0
MIR4796	0	0	0	0	0	0

MIR4797	0	0	0	0	0	0
MIR4798	0	0	0	0	0	0
MIR4799	0	0	0	0	0	0
MIR4800	0	0	0	0	0	0
MIR4801	0	0	0	0	0	0
MIR4802	0	0	0	0	0	0
MIR4803	0	0	0	0	0	0
MIR4804	0	0	0	0	0	0
MIR483	0	0	0	0	0	0
MIR484	0	0	0	0	0	0
MIR485	0	0	0	0	0	0
MIR486	0	0	0	0	0	0
MIR487A	0	0	0	0	0	0
MIR487B	0	0	0	0	0	0
MIR488	0	0	0	0	0	0
MIR489	0	0	0	0	0	0
MIR490	0	0	0	0	0	0
MIR491	0	0	0	0	0	0
MIR492	0	0	0	0	0	0
MIR493	0	0	0	0	0	0
MIR494	0	0	0	0	0	0
MIR495	0	0	0	0	0	0
MIR496	0	0	0	0	0	0
MIR497	0	0	0	0	0	0
MIR497HG	0.21	0	0	0	0	0
MIR498	0	0	0	0	0	0
MIR4999	0	0	0	0	0	0
MIR499A	0	0	0	0	0	0
MIR499B	0	0	0	0	0	0
MIR5000	0	0	0	0	0	0
MIR5001	0	0	0	2.17	0	0
MIR5002	0	0	0	0	0	0
MIR5003	0	0	0	0	0	0
MIR5004	0	0	0	0	0	0
MIR5006	0	0	0	0	0	0
MIR5007	0	0	0	0	0	0
MIR5008	0	0	0	0	0	0
MIR5009	0	0	0	0	0	0
MIR500A	0	0	0	0	0	0
MIR500B	0	0	0	0	0	0
MIR501	0	0	0	0	0	0
MIR5010	0	0	0	0	0	0
MIR5011	0	0	0	0	0	0

MIR502	0	0	0	0	0	0
MIR503	0	0	0	0	0	0
MIR504	0	0	0	0	0	0
MIR5047	0	0	0	0	0	0
MIR505	0	0	0	0	0	0
MIR506	0	0	0	0	0	0
MIR507	0	0	0	0	0	0
MIR508	0	0	0	0	0	0
MIR5087	0	0	0	0	0	0
MIR5088	0	0	0	0	0	0
MIR5089	0	0	0	0	0	0
MIR509-1	0	0	0	0	0	0
MIR509-2	0	0	0	0	0	0
MIR509-3	0	0	0	0	0	0
MIR5090	0	0	0	0	0	0
MIR5091	0	0	0	0	0	0
MIR5092	0	0	0	0	0	0
MIR5093	0	0	0	0	0	0
MIR5094	0	0	0	0	0	0
MIR5095	0	0	0	0	0	0
MIR510	0	0	0	0	0	0
MIR5100	0	0	0	0	0	0
MIR511-1	0	0	0	0	0	0
MIR511-2	0	0	0	0	0	0
MIR512-1	0	0	0	0	0	0
MIR512-2	0	0	0	0	0	0
MIR514A1	0	0	0	0	0	0
MIR514A2	0	0	0	0	0	0
MIR514A3	0	0	0	0	0	0
MIR514B	0	0	0	0	0	0
MIR515-1	0	0	0	0	0	0
MIR515-2	0	0	0	0	0	0
MIR516A1	0	0	0	0	0	0
MIR516A2	0	0	0	0	0	0
MIR516B1	0	0	0	0	0	0
MIR516B2	0	0	0	0	0	0
MIR517A	0	0	0	0	0	0
MIR517B	0	0	0	0	0	0
MIR517C	0	0	0	0	0	0
MIR5186	0	0	0	0	0	0
MIR5187	0	0	0	0	0	0
MIR5188	0	0	0	0	0	0
MIR5189	0	0	0	0	0	0

MIR518A1	0	0	0	0	0	0
MIR518A2	0	0	0	0	0	0
MIR518B	0	0	0	0	0	0
MIR518C	0	0	0	0	0	0
MIR518D	0	0	0	0	0	0
MIR518E	0	0	0	0	0	0
MIR518F	0	0	0	0	0	0
MIR5190	0	0	0	0	0	0
MIR5191	0	0	0	0	0	0
MIR5192	0	0	0	0	0	0
MIR5193	0	0	0	0	0	0
MIR5194	0	0.91	0	0	0	0
MIR5196	0	0	0	0	0	0
MIR5197	0	0	0	0	0	0
MIR519A1	0	0	0	0	0	0
MIR519A2	0	0	0	0	0	0
MIR519B	0	0	0	0	0	0
MIR519C	0	0	0	0	0	0
MIR519D	0	0	0	0	0	0
MIR519E	0	0	0	0	0	0
MIR520A	0	0	0	0	0	0
MIR520B	0	0	0	0	0	0
MIR520C	0	0	0	0	0	0
MIR520D	0	0	0	0	0	0
MIR520E	0	0	0	0	0	0
MIR520F	0	0	0	0	0	0
MIR520G	0	0	0	0	0	0
MIR520H	0	0	0	0	0	0
MIR521-1	0	0	0	0	0	0
MIR521-2	0	0	0	0	0	0
MIR522	0	0	0	0	0	0
MIR523	0	0	0	0	0	0
MIR524	0	0	0	0	0	0
MIR525	0	0	0	0	0	0
MIR526A1	0	0	0	0	0	0
MIR526A2	0	0	0	0	0	0
MIR526B	0	0	0	0	0	0
MIR527	0	0	0	0	0	0
MIR532	0	0	0	0	0	0
MIR539	0	0	0	0	0	0
MIR541	0	0	0	0	0	0
MIR542	0	0	0	0	0	0
MIR543	0	0	0	0	0	0

MIR545	0	0	0	0	0	0
MIR548A1	0	0	0	0	0	0
MIR548A2	0	0	0	0	0	0
MIR548A3	0	0	0	0	0	0
MIR548AA1	0	0	0	0	0	0
MIR548AA2	0	0	0	0	0	0
MIR548AC	0	0	0	0	0	0
MIR548AD	0	0	0	0	0	0
MIR548AE2	0	0	0	0	0	0
MIR548AI	0	0	0	0	0	0
MIR548AJ2	0	0	0	0	0	0
MIR548AL	0	0	0	0	0	0
MIR548AN	0	0	0	0	0	0
MIR548AO	0	0	0	0	0	0
MIR548AP	0	0	0	0	0	0
MIR548AQ	0	0	0	0	0	0
MIR548AR	0	0	0	0	0	0
MIR548AS	0	0	0	0	0	0
MIR548AU	0	0	0	0	0	0
MIR548AV	0	0	0	0	0	0
MIR548AX	0	0	0	0	0	0
MIR548B	0	0	0	0	0	0
MIR548C	0	0	0	0	0	0
MIR548D1	0	0	0	0	0	0
MIR548D2	0	0	0	0	0	0
MIR548F1	0	0	0	0	0	0
MIR548F2	0	0	0	0	0	0
MIR548F3	0	0	0	0	0	0
MIR548F4	0	0	0	0	0	0
MIR548F5	0	0	0	0	0	0
MIR548G	0	0	0	0	0	0
MIR548H2	0	0	0	0	0	0
MIR548H3	0	0	0	0	0	0
MIR548H4	0	0	0	0	0	0
MIR548I1	0	0	0	0	0	0
MIR548I2	0	0	0	0	0	0
MIR548I3	0	0	0	0	0	0
MIR548I4	0	0	0	0	0	0
MIR548J	0	0	0	0	0	0
MIR548K	0	0	0	0	0	0
MIR548M	0	0	0	0	0	0
MIR548N	0	0	0	0	0	0
MIR548O2	0	0	0	0	0	0

MIR548Q	0	0	0	0	0	0
MIR548T	0	0	0	0	0	0
MIR548W	0	0	0	0	0	0
MIR548X	0	0	0	0	0	0
MIR548Y	0	0	0	0	0	0
MIR548Z	0	0	0	0	0	0
MIR549	0	0	0	0	0	0
MIR550A1	0	0	0	0	0	0
MIR550A2	0	0	0	0	0	0
MIR550A3	0	0	0	0	0	0
MIR550B1	0	0	0	0	0	0
MIR550B2	0	0	0	0	0	0
MIR551A	0	0	0	0	0	0
MIR551B	0	0	0	0	0	0
MIR553	0	0	0	0	0	0
MIR554	0	0	0	0	0	0
MIR555	0	0	0	0	0	0
MIR556	0	0	0	0	0	0
MIR557	0	0	0	0	0	0
MIR5579	0	0	0	0	0	0
MIR558	0	0	0	0	0	0
MIR5580	0	0	0	0	0	0
MIR5581	0	0	0	0	0	0
MIR5582	0	0	0	0	0	0
MIR5583-1	0	0	0	0	0	0
MIR5583-2	0	0	0	0	0	0
MIR5584	0	0	0	0	0	0
MIR5586	0	0	0	0	0	0
MIR5587	0	0	0	0	0	0
MIR559	0	0	0	0	0	0
MIR561	0	0	0	0	0	0
MIR563	0	0	0	0	0	0
MIR564	0	0	0	0	0	0
MIR567	0	0	0	0	0	0
MIR568	0	0	0	0	0	0
MIR5680	0	0	0	0	0	0
MIR5681A	0	0	0	0	0	0
MIR5681B	0	0	0	0	0	0
MIR5684	0	0	0	0	0	0
MIR5685	0	0	0	0	0	0
MIR5687	0	0	0	0	0	0
MIR5688	0	0	0	0	0	0
MIR5689	0	0	0	0	0	0

MIR569	0	0	0	0	0	0
MIR5690	0	0	0	0	0	0
MIR5691	0	0	0	0	0	0
MIR5692A1	0	0	0	0	0	0
MIR5692B	0	0	0	0	0	0
MIR5692C1	0	0	0	0	0	0
MIR5692C2	0	0	0	0	0	0
MIR5693	0	0	0	0	0	0
MIR5694	0	0	0	0	0	0
MIR5695	0	0	0	0	0	0
MIR5696	0	0	0	0	0	0
MIR5698	0	0	0	0	0	0
MIR5699	0	0	0	0	0	0
MIR570	0	0	0	0	0	0
MIR5700	0	0	0	0	0	0
MIR5702	0	0	0	0	0	0
MIR5703	0	0	0	0	0	0
MIR5704	0	0	0	0	0	0
MIR5705	0	0	0	0	0	0
MIR5707	0	0	0	0	0	0
MIR5708	0	0	0	0	0	0
MIR572	0	0	0	0	0	0
MIR573	0	1.21	1.38	0	0	0
MIR574	0	0	0	0	0	0
MIR575	0	0	0	0	0	0
MIR577	0	0	0	0	0	0
MIR578	0	0	0	0	0	0
MIR580	0	0	0	0	0	0
MIR581	0	0	0	0	0	0
MIR583	0	0	0	0	0	0
MIR585	0	0	0	0	0	0
MIR586	0	0	0	0	0	0
MIR589	0	0	1.38	0	0	0
MIR590	0	0	0	0	0	0
MIR591	0	0	0	0	0	0
MIR592	0	0	0	0	0	0
MIR593	0	0	0	0	0	0
MIR595	0	0	0	0	0	0
MIR596	0	0	0	0	0	0
MIR597	3.08	0	0	1.14	0	0
MIR598	0	0	0	0	0	0
MIR599	0	0	0	0	0	0
MIR600	0	0	0	0	0	0

MIR600HG	0.95	0.6	1.25	0.97	0.44	1.13
MIR601	0	0	0	0	0	0
MIR602	0	0	0	0	0	0
MIR603	0	0	0	0	0	0
MIR604	0	0	0	0	0	0
MIR605	0	0	0	0	0	0
MIR608	0	0	0	0	0	0
MIR609	0	0	0	0	0	0
MIR610	0	0	0	0	0	0
MIR611	0	0	0	0	0	0
MIR612	0	0	0	0	0	0
MIR613	0	0	0	0	0	0
MIR614	0	0	0	0	0	0
MIR615	0	0	0	0	0	0
MIR617	0	0	0	0	0	0
MIR618	0	0	0	0	0	0
MIR620	0	0	0	0	0	0
MIR621	0	0	0	0	0	0
MIR622	0	0	0	0	0	0
MIR623	0	0	0	0	0	0
MIR624	0	0	0	0	0	0
MIR626	0	0	0	0	0	0
MIR627	0	0	0	0	0	0
MIR628	0	0	0	0	0	0
MIR629	0	0	0	0	0	0
MIR630	0	0	0	0	0	0
MIR631	0	0	0	0	0	0
MIR632	0	0	0	0	0	1.62
MIR634	0	0	0	0	0	0
MIR635	0	0	0	0	0	0
MIR636	0	0	0	0	0	0
MIR637	0	0	0	0	0	0
MIR638	0	0	0	0	0	0
MIR639	0	0	2.81	0	0	0
MIR641	0	0	0	0	0	0
MIR642A	0	0	0	0	0	0
MIR642B	0	0	0	0	0	0
MIR643	0	0	0	0	0	0
MIR644A	0	0	0	0	0	0
MIR644B	0	0	0	0	0	0
MIR645	0	0	0	0	0	0
MIR647	0	0	0	0	0	0
MIR648	0	0	0	0	0	0

MIR650	0	0	0	0	0	0
MIR651	0	0	0	0	0	0
MIR653	0	0	0	0	0	0
MIR654	0	0	0	0	0	0
MIR655	0	0	0	0	0	0
MIR656	0	0	0	0	0	0
MIR657	0	0	0	0	0	0
MIR658	0	0	1.36	0	1.46	0
MIR659	0	0	0	0	0	0
MIR660	0	0	0	0	0	0
MIR661	0	0	0	0	0	0
MIR662	0	0	0	0	0	0
MIR663A	1.64	0	1.52	0	0	0
MIR663B	2.38	0	0	0	0	0
MIR664	0	0	0	0	0	0
MIR665	0	0	0	0	0	0
MIR668	0	0	0	0	0	0
MIR670	0	0	0	0	0	0
MIR671	0	0	0	0	0	0
MIR675	0	0	0	0	0	0
MIR676	0	0	0	0	0	0
MIR7-1	0	0	0	0	0	0
MIR7-2	0	0	0	0	0	0
MIR7-3	0	0	0	0	0	0
MIR7-3HG	0.1	0	0	0	0	0
MIR708	0	0	0	0	0	0
MIR711	0	0	0	0	0	0
MIR718	0	0	0	0	0	0
MIR744	0	0	0	0	0	0
MIR758	0	0	0	0	0	0
MIR759	0	0	0	0	0	0
MIR760	0	0	0	0	0	0
MIR761	0	0	0	0	0	0
MIR762	0	0	0	0	0	0
MIR764	0	0	0	0	0	0
MIR765	0	0	0	0	0	0
MIR766	0	0	0	0	0	0
MIR767	0	0	0	0	0	0
MIR769	0	0	0	0	0	0
MIR770	0	0	0	0	0	0
MIR802	0	0	0	0	0	0
MIR873	0	0	0	0	0	0
MIR874	0	0	0	0	0	0

MIR875	0	0	0	0	0	0
MIR876	0	0	0	0	0	0
MIR877	0	0	0	0	0	0
MIR885	0	0	0	0	0	0
MIR888	0	0	0	0	0	0
MIR889	0	0	0	0	0	0
MIR890	0	0	0	0	0	0
MIR891A	0	0	0	0	0	0
MIR891B	0	0	0	0	0	0
MIR892A	0	0	0	0	0	0
MIR892B	0	0	0	0	0	0
MIR9-1	0	0	0	0	0	0
MIR9-2	0	0	0	0	0	0
MIR9-3	0	0	0	0	0	0
MIR920	0	0	0	0	0	0
MIR921	0	0	0	0	0	0
MIR922	0	0	0	0	0	0
MIR92A1	0	0	0	0	0	0
MIR92A2	0	0	0	0	0	0
MIR92B	0	0	0	0	0	0
MIR93	0	0	0	0	0	0
MIR933	0	0	2.05	0	0	0
MIR934	0	0	0	0	0	0
MIR935	0	0	0	0	0	0
MIR936	0	0	0	0	0	0
MIR937	0	0	0	0	0	0
MIR938	0	0	0	0	0	0
MIR939	0	0	0	0	0	0
MIR940	0	0	0	0	0	0
MIR941-1	0	0	0	0.85	0	0
MIR941-2	0	0	0	0	0	0
MIR941-3	0	0.49	0	0	0	0
MIR941-4	0	0.49	0	0	0	0
MIR942	0	0	0	0	0	0
MIR943	0	0	0	0	0	0
MIR944	0	0	0	0	0	0
MIR96	0	0	0	0	0	0
MIR98	0	0.92	0	0	0	0
MIR99A	0	0	0	0	0	0
MIR99B	0	0	0	0	0	0
MIRLET7A1	0	0	0	0	0	0
MIRLET7A2	0	0	0	0	0	0
MIRLET7A3	0	0	0	0	0	0

MIRLET7B	0	0	0	0	0	0
MIRLET7BHG	0.35	1.08	1.15	0.58	0.57	0.56
MIRLET7C	0	0	0	0	0	0
MIRLET7D	0	0	0	0	0	0
MIRLET7DHG	1	1.39	2.04	1.13	1.55	2.78
MIRLET7E	0	0	0	0	0	0
MIRLET7F1	0	0	0	0	0	0
MIRLET7F2	0	0	0	0	0	0
MIRLET7G	0	0	0	0	0	0
MIRLET7I	0	0	0	0	0	0
MIS12	13.61	8.82	8.02	10.8	9.71	9.77
MIS18A	25.16	39.67	27.46	27.5	29.3	30.39
MIS18BP1	9.54	10.88	8.88	7.99	7.83	9.61
MITD1	16.76	17.78	15.22	14.01	19.25	10.34
MITF	3.74	0.51	0.14	1.38	2.94	0.38
MIXL1	0	0	0	0	0	0
MKI67	20.53	21.05	20.03	18.77	19.92	20.3
MKI67IP	60.61	70.27	59.75	61.89	74.47	68.78
MKKS	28.45	22.33	19.2	18.01	20.57	24.17
MKL1	9.03	10.73	10.92	11.85	10.86	12.15
MKL2	2.96	2.76	2.36	1.94	1.44	2.83
MKLN1	4.49	3.09	2.68	2.61	2.86	2.56
MKNK1	11.96	15.53	12.48	11.81	8.94	12.74
MKNK1-AS1	0.06	0.02	0	0	0	0
MKNK2	5.87	7.53	6.98	5.2	5.5	6.68
MKRN1	9.14	7.85	7.33	8.7	6.89	7.24
MKRN2	15.33	18.5	17.68	19.98	15.85	17.82
MKRN3	0	0	0	0	0	0
MKRN7P	0	0	0	0	0	0
MKRN9P	0	0	0	0	0	0
MKS1	7.77	11.04	11.07	8.88	9.24	10.1
MKX	0	0	0	0	0	0
MLANA	1.01	1.06	1.42	0.59	0.68	0.45
MLC1	72.72	109.23	88.44	75.67	72.93	89.7
MLEC	12.01	21.74	18.69	15.82	14.41	19.13
MLF1	0	0	0	0	0	0
MLF1IP	34.42	35.25	32.46	35.45	36.63	38.44
MLF2	270.37	187.41	145.29	190.46	211.75	175.78
MLH1	22.61	26.18	25.46	19.1	22.98	23.81
MLH3	0	0.02	0	0	0.01	0.05
MLIP	0	0	0	0	0	0
MLIP-IT1	0	0	0	0	0	0
MLK7-AS1	0.25	0.18	0.08	0.12	0.49	0.06

MLKL	4.04	8.04	10.15	5.05	4.93	8.2
MLL	2.68	2.54	2.61	2.36	2.58	2.45
MLL2	3.09	3.43	4	2.86	3.06	3.55
MLL3	2.65	2.14	2.16	2.34	2.51	2.51
MLL4	0.98	1.15	1.31	0.91	1.04	1.34
MLL5	8.53	8.17	7.97	6.82	7.55	6.6
MLLT1	5.15	4.61	3.63	4.14	4.08	4.77
MLLT10	6.74	7.4	6.57	5.41	5.35	5.92
MLLT10P1	0	0.35	0	0	0.14	0.3
MLLT11	3.86	5.81	6.5	4.56	4.77	3.32
MLLT3	8.79	4.57	4.99	7.66	7.25	5.57
MLLT4	1.8	0.52	0.71	0.68	0.65	0.87
MLLT4-AS1	0.56	0.12	0.32	0.25	0.12	0.2
MLLT6	3.35	4.03	3.64	2.87	3.65	3.96
MLN	0	0	0	0	0	0
MLNR	0	0	0	0	0	0.06
MLPH	0.44	0	0.03	0	0.07	0.02
MLST8	47.55	57.18	53.66	61.16	57.18	50.84
MLX	38.63	32.17	32.97	29.76	35.57	22.07
MLXIP	5.47	3.39	2.43	3.42	3.32	3.13
MLXIPL	0	0	0	0	0	0
MLYCD	1.71	1.93	1.07	1.11	1.87	0.92
MMAA	0.35	0.56	0.7	0.4	0.35	0.36
MMAB	9.79	13.39	12.69	12.57	13.63	13
MMACHC	4.97	5.05	5.96	6.71	5.72	6.31
MMADHC	119.05	99.81	86.43	93.65	97.16	95
MMD	1.8	2.54	2.68	1.81	1.92	1.66
MMD2	0.78	0.71	0.49	0.23	0.66	0.57
MME	0.83	1.09	0	0.14	0.25	0.13
MMEL1	0	0	0	0	0	0
MMGT1	21.14	17.1	20.56	16.55	21.86	21.2
MMP1	0.29	0	0	0	0	0
MMP10	0	0	0	0	0	0
MMP11	0.13	0	0.09	0.49	0.25	0.07
MMP12	0	0	0	0	0	0
MMP13	0	0	0	0	0	0
MMP14	0	0.02	0	0.03	0.02	0
MMP15	0.08	0.79	0.31	0.35	0.86	0.64
MMP16	0	0	0	0.01	0	0.01
MMP17	0.98	2.45	2.04	1.32	1.9	1.76
MMP19	0.12	0.1	0.03	0	0.03	0.06
MMP2	13.56	6.32	6.99	6.91	5.46	5.57
MMP20	0	0	0	0	0	0

MMP21	0	0	0	0	0	0
MMP23A	0.17	0	0	0	0	0.09
MMP23B	0	0	0	0	0	0
MMP24	0.4	0.28	0.73	0.38	0.31	0.2
MMP25	0.06	0.39	0.28	0.55	0.25	0.39
MMP26	0	0	0	0	0	0
MMP27	0	0	0	0	0	0
MMP28	0.08	0.06	0.49	0.69	0.29	0
MMP3	0	0	0	0	0	0
MMP7	0	0	0	0	0	0
MMP8	0	0	0	0	0	0
MMP9	0	0.02	0.09	0	0	0
MMRN1	0.01	0	0	0	0	0
MMRN2	0.07	0.03	0.05	0.04	0.02	0.03
MMS19	18.7	19.56	14.63	22.07	17.77	17.99
MMS22L	5.03	5.79	5.09	3.57	4.73	5.12
MN1	0.03	0	0	0	0	0
MNAT1	16.03	18.38	11	9.56	16.44	10.68
MND1	24.56	33.19	32.81	26.83	28.59	33.68
MNDA	0	0	0	0	0	0.59
MNF1	53.53	56.54	51.57	57.2	61.28	42.14
MNS1	1.97	1.98	1.4	2.46	2.52	2.84
MNT	1.8	1.08	1.41	1.35	1.46	1.61
MNX1	0.23	0.17	0.17	0.1	0.39	0.17
MOAP1	11.01	8.01	6.68	7.71	8.44	8.38
MOB1A	49.04	55.26	49.03	47.28	51.13	56.57
MOB1B	8.76	3.93	3.52	3.8	4.59	3.38
MOB2	3.26	3.54	3.59	4.35	3.62	2.77
MOB3A	18.95	29.53	27.74	20.89	21.52	27.2
MOB3B	16.79	3.26	2.75	4.73	7.31	3.12
MOB3C	2.51	1.14	1.78	2.21	1.29	2.72
MOB4	10.69	13.61	9.24	7.33	11.89	9.51
MOBP	0.02	0	0	0	0	0
MOCOS	1.8	4.88	4.44	2.3	4	5.91
MOCS1	3.34	0	0.1	0	0	0
MOCS2	27.4	18.72	18.07	17.45	21.44	19.64
MOCS3	6.41	6.92	6.91	6.34	7.57	5.11
MOG	0.91	2.46	2.55	1.41	1.76	2.03
MOGAT1	0	0	0	0	0	0
MOGAT2	0	0	0	0	0	0
MOGAT3	0.3	0.1	0.21	0.06	0.36	0.22
MOGS	5.86	6.59	7.43	6.12	6.69	7.67
MOK	1.24	1.21	0.61	1.08	1.75	1.42

MON1A	11.26	11.99	11.92	14.59	10.2	11.27
MON1B	20.57	19.14	17.55	16.37	18.2	22.49
MON2	2.61	2.17	1.93	2.1	1.91	2.53
MORC1	0	0	0	0	0	0
MORC2	2.18	1.61	1.96	1.7	2.3	1.79
MORC2-AS1	6.67	4.82	5.03	6.47	7.01	3.08
MORC3	4.6	4.76	3.56	2.72	4.85	4.98
MORC4	1.22	3.03	2.07	1.31	1.37	1.77
MORF4L1	240.53	202.45	153.84	190.19	228.43	170.93
MORF4L2	202.03	138.49	129.92	142.23	168.01	147.04
MORF4L2-AS	0.13	0.28	0.26	0.09	0.65	0.49
MORN1	0.32	0.5	0.5	0.44	0.82	0.51
MORN2	21.94	16.15	16.15	15.87	14.54	17.71
MORN3	0.92	0.16	0	0	0.81	0.33
MORN4	0.32	0.62	0.55	0.46	0.59	0.52
MORN5	0	0	0	0	0	0
MOS	0	0	0	0	0	0
MOSPD1	11.03	5.15	6.33	5.78	6.27	5.61
MOSPD2	2.86	2.07	2.59	2.48	3.14	1.79
MOSPD3	10.19	9.41	10.46	10.77	10.12	8.67
MOV10	12.5	17.89	18.22	13.76	13.85	15.05
MOV10L1	0	0	0	0	0	0
MOXD1	0.17	0	0	0	0	0
MOXD2P	0	0	0	0	0	0
MPC1	60.86	68.36	58.99	45.73	63.45	59.77
MPC1L	0	0	0	0	0	0
MPC2	27.66	21.73	22.08	23.72	26.78	20.12
MPDU1	56.67	69.04	78.95	82.27	71.06	65.33
MPDZ	0	0.01	0	0	0	0
MPEG1	0	0	0	0	0.02	0
MPG	75.47	78.65	71.9	74.24	68.1	71.91
MPHOSPH10	19.77	21.14	16.93	13.09	17.81	18.21
MPHOSPH6	37.2	39.63	38.48	31.29	41.33	37.65
MPHOSPH8	10.74	11.19	10.67	9.41	11.49	8.87
MPHOSPH9	4.34	6.03	6	4.2	5.74	6.22
MPI	24.62	35.33	29.6	31.02	32.92	33.81
MPL	0.44	0.47	0.66	0.25	0.39	0.55
MPLKIP	47.54	52.48	48.16	42.53	53.05	48.76
MPND	1.3	1.49	1.75	2.3	2.01	1.71
MPO	1.49	22.53	3.83	1.09	1.58	38.05
MPP1	185.75	44.63	49.64	84.43	117.52	44.57
MPP2	1.7	3.62	4.99	5.36	2.25	5.78
MPP3	0.29	0.47	0.12	0.52	0.37	0.47

MPP4	0	0	0	0	0	0
MPP5	1.93	2.04	2.18	2.5	1.67	2.07
MPP6	2.66	2.44	4.35	3.47	3.69	5.04
MPP7	0.65	0.35	0.21	0.19	0.37	0.24
MPPE1	3.5	6.83	6.05	4.31	5.14	5.51
MPPED1	0	0	0	0.02	0	0
MPPED2	0.01	1.14	0.09	0.93	0.68	0.3
MPRIP	2.48	2.48	2.51	2.59	2.85	2.91
MPST	44.78	43.95	42.16	55.99	50.35	51.13
MPV17	99.71	98.81	81.52	113.03	103.94	80.89
MPV17L	0.94	0.46	0.82	0.27	0.78	0.34
MPV17L2	25.15	24.4	19.19	19.35	19.2	20.79
MPZ	0.08	0.15	0.24	0.08	0.21	0.31
MPZL1	7.73	14.73	9.63	8.8	9.99	10.22
MPZL2	0	0	0	0	0	0
MPZL3	6.35	1.3	0.72	1.46	2.01	1.75
MR1	3.26	3.26	3.24	2.04	2.2	2.48
MRAP	0	0	0	0	0	0
MRAP2	10.61	3.33	2.95	6.59	6.88	3.06
MRAS	0.04	0.07	0.01	0.05	0	0.03
MRC1	0.01	0	0	0	0	0
MRC2	4.36	6.59	6.41	4.87	4.23	5.83
MRE11A	7.34	8.46	7.7	7.28	7.41	7.33
MREG	1.26	1.55	1.45	1.92	1.31	2.39
MRFAP1	128.57	129.77	141.72	138.5	142.74	132.48
MRFAP1L1	38.4	37.89	37.03	33.8	39.68	32.79
MRGBP	3.01	2.77	3.71	3.04	2.89	3.36
MRGPRD	0	0	0	0	0	0
MRGPRE	0	0	0	0	0	0
MRGPRF	0	0	0	0	0	0
MRGPRG	0	0	0	0	0	0
MRGPRG-AS	0	0	0	0	0	0
MRGPRX1	0	0	0	0	0	0
MRGPRX2	0	0	0	0	0	0
MRGPRX3	0.05	0	0.12	0	0.24	0.07
MRGPRX4	0	0	0	0	0	0
MRI1	18.84	24.25	27.88	23.66	19.51	30.91
MRM1	19.26	26.18	21.66	16.1	30.23	21.44
MRO	0.1	0.36	0.29	0.21	0.23	0.32
MROH1	15.75	11.5	13.6	11.06	15.01	13.4
MROH2B	0	0	0	0	0	0
MROH5	0	0	0	0	0	0
MROH6	0.25	0.49	0.47	0.45	0.21	0.36

MROH7	0	0	0	0	0	0
MROH8	0.11	0	0.38	0.26	0.28	0.19
MROH9	0	0	0	0	0	0
MRP63	43.08	47.28	39.18	43.11	43.35	36.26
MRPL1	31.04	44.48	38.26	31.41	37.63	34.68
MRPL10	38.31	39.37	38.84	46.37	40.17	40.55
MRPL11	70.33	93.08	82.6	88.9	88.82	85.06
MRPL12	121.11	158.7	169.82	160.73	164.55	150.68
MRPL13	205.68	225.56	198.84	178.23	208.4	197.63
MRPL14	208.97	166.2	131.49	175.26	194.84	158.56
MRPL15	180.31	173.49	169.66	174.93	184.61	158.88
MRPL16	290.33	139.66	133.69	273.63	307.76	219.4
MRPL17	44.21	44.31	46.5	43.96	45.55	43.78
MRPL18	100.7	121.62	126.2	106.15	115.64	96.52
MRPL19	10.45	10.06	8.38	8.33	9.21	10.23
MRPL2	58.84	52.02	51.2	62.18	57.6	45.39
MRPL20	230.04	224.59	192.25	171.95	204.9	188.86
MRPL21	181.75	159	177.23	167.23	193.44	163.72
MRPL22	31.8	36.51	32.65	31.06	37.82	30.2
MRPL23	50.86	69.72	74.48	83.3	73.48	88.95
MRPL23-AS1	0	0	0	0	0	0
MRPL24	61.34	57.87	60.86	69.89	76.03	72.06
MRPL27	113.86	138.93	114.27	116.72	134.29	117.25
MRPL28	137.68	127.64	110.29	136.91	132.37	113.81
MRPL3	138.65	147.2	136.45	140.83	139.4	143.45
MRPL30	17.75	15.78	15	16.04	16.26	19.09
MRPL32	97.73	88.47	84.06	77.48	86.96	82.85
MRPL33	114.62	119.18	82.05	68.58	107.97	85.55
MRPL34	63.96	63.81	52.32	52.75	61.22	51.23
MRPL35	17.29	13.61	12.04	12.02	15.08	12.6
MRPL36	180.31	161.76	152.97	160.92	178.87	134.97
MRPL37	230.9	237.04	243.27	244.97	247.6	238.91
MRPL38	76.51	92.96	89.99	86.04	86.87	76.11
MRPL39	92.07	97.16	81.84	66.99	89.25	84.08
MRPL4	129.15	166.85	146.82	155.52	140.22	149.49
MRPL40	84.43	95.34	86.92	82.97	101.05	90.34
MRPL41	161.79	137.08	144.47	137.69	159.77	99.58
MRPL42	26.79	19.52	17.59	17.7	22.81	21.02
MRPL42P5	0.12	0.48	0	0.08	0.57	0
MRPL43	86.62	82.78	80.51	75.3	75.71	79.93
MRPL44	45.12	47.53	37.42	39.56	43.73	40.34
MRPL45	60.33	57.33	56.69	60.58	53.62	56.38
MRPL45P2	1.47	0.76	0.44	0.3	0.36	0.33

MRPL46	83.54	89.12	83.61	81.29	86.8	76.13
MRPL47	75.52	80.21	74.6	63.92	86.86	79.43
MRPL48	83.58	72.53	77.26	74.18	83.66	67.98
MRPL49	54.3	37.83	37.95	35.53	38.76	37.97
MRPL50	50.67	35.1	27.14	27.3	40.14	35.59
MRPL51	289.61	261.87	245.2	253.04	303.51	232.79
MRPL52	94.96	94.76	84.5	90.14	96.88	78.24
MRPL53	42.29	42.75	35.3	41.04	43.02	34.71
MRPL54	107.6	109.26	101.02	107.27	116.93	101.54
MRPL55	109.89	118.11	84.77	110.63	109.91	89
MRPL9	85.3	107.64	107.17	91.68	92.07	90.66
MRPS10	43.84	43.92	40.45	35.84	46.56	37.85
MRPS11	112.58	108.99	101.07	101.5	108.13	93.41
MRPS12	169.61	172.97	162.68	158.43	171.39	153.58
MRPS14	21.59	26.53	20.23	18.6	24.59	20.09
MRPS15	94.59	77.74	76.88	80.53	101.92	74.02
MRPS16	87.73	91.43	86.47	85.52	84.22	91.52
MRPS17	93.56	71.9	65.72	62.89	88.56	68.92
MRPS18A	82.88	72.41	75.12	79.07	77.52	63.87
MRPS18B	78.18	95.73	89.99	94.76	86.31	76.41
MRPS18C	65.76	68.56	61.19	56.43	70.13	52.1
MRPS2	169.3	144.87	143.93	155.73	155.71	158.61
MRPS21	298.15	273.46	227.53	262.75	314.33	203.76
MRPS22	87.86	79.11	78.46	77.88	84.06	77.35
MRPS23	74.37	59.64	45.08	51.49	59.33	43.56
MRPS24	266.73	216.44	235.48	228.09	255.71	211.07
MRPS25	22.92	25.98	26.28	24.19	26.2	27.68
MRPS26	123.41	155.26	124.91	124.69	122.58	124.92
MRPS27	37.2	54.61	54.51	58.48	53.64	60.19
MRPS28	112.15	92.63	79.89	85.89	98.23	90.62
MRPS30	61.34	38.23	42.16	44.76	44.27	40.23
MRPS31	24.83	30.4	24.99	24.85	28.91	24.07
MRPS31P5	0.89	1.86	0.92	1.03	1.04	1.18
MRPS33	81.73	74.98	57.04	67.51	64.62	68.28
MRPS34	186.65	242.56	236.34	211.56	215.92	230.3
MRPS35	63.94	63.36	54.55	50.69	60.7	63.31
MRPS36	37.74	22.64	20.78	18.94	27.59	20.56
MRPS5	43.87	43.86	38.51	35.31	42.55	32.3
MRPS6	67.37	69.03	75.24	75.22	59.78	61.77
MRPS7	94.27	100.01	83.74	90.27	99.69	75.3
MRPS9	47.77	46.76	47.04	44.26	46.4	43.95
MRRF	38.7	33.74	27.31	35.3	30.69	31.33
MRS2	12.42	12.14	12.6	9.8	9.26	14.11

MRS2P2	0.43	0.63	0.62	0.5	0.28	0.47
MRTO4	53.41	59.73	58.56	60.64	54.07	58.3
MRVI1	0	0.1	0	0	0	0.01
MRVI1-AS1	0	0	0	0.09	0	0
MS4A1	0	0	0	0	0	0
MS4A10	1.2	2.32	1.83	1.31	1.3	1.13
MS4A12	0	0	0	0	0	0
MS4A13	0	0	0	0	0	0
MS4A14	0	0	0	0	0	0
MS4A15	0.09	0	0	0	0	0
MS4A2	4.62	0.53	0.79	1.18	12.69	1.02
MS4A3	73.56	21.62	25.28	77.65	68.9	123.71
MS4A4A	24.8	3.13	3.47	30.25	28.84	20.65
MS4A5	0	0	0	0	0	0
MS4A6A	18.62	2.7	2.59	13.54	15.16	10.02
MS4A6E	0.24	0	0	0	0	0
MS4A7	0.57	1.26	0.53	0.52	0.82	0.84
MS4A8B	0	0	0	0	0.21	0
MSANTD1	0	0.04	0	0	0	0
MSANTD2	0.13	0.31	0.51	0.05	0.09	0.47
MSANTD3	7.48	5.8	3.63	5	5.71	2.45
MSANTD3-TI	0.78	0.36	0.31	0.31	0.39	0
MSANTD4	5.69	5.6	5.52	4.37	4.44	4.14
MSC	0.47	0	0	0	0	0
MSGN1	0	0	0	0	0	0
MSH2	18.26	17.57	13.1	18.38	16.81	15.75
MSH3	2.97	4.59	4.26	4.04	4.02	3.45
MSH4	0.02	0.03	0	0	0	0
MSH5	2.3	4.86	3.04	3.72	3.64	4.5
MSH5-SAPCD	0.32	0.54	0	0	0.41	0.13
MSH6	19.46	26.04	30.55	28.5	25.66	24.54
MSI1	0.15	0	0	0.05	0	0.22
MSI2	3.7	2.45	1.51	2.95	2.4	1.67
MSL1	2.92	2.48	2.19	1.26	2.02	1.46
MSL2	4.21	4.21	6.08	5.14	3.39	7.1
MSL3	34.05	14.14	12.18	21.07	21.47	11.47
MSL3P1	0	0	0	0	0	0
MSLN	0	0	0	0	0	0
MSMB	0	0	0	0	0	0
MSMO1	17.58	15.58	13.45	9.36	16.17	16.61
MSMP	2.68	1.47	0.45	0.86	1.04	1.94
MSN	352.04	458.77	431.62	404.06	355.67	432.82
MSR1	0	0	0	0	0	0

MSRA	1.46	2.56	1.89	3.61	2.1	2.56
MSRB1	28.64	42.56	32.16	28.14	34.64	38.53
MSRB2	1.56	2.36	1.84	2.64	1.63	1.62
MSRB3	0.5	1.22	0.31	0.32	0.26	0.17
MSS51	0.32	0.43	0.35	0.3	0.3	0.46
MST1	0	0.1	0.06	0	0	0.1
MST1L	0	0.01	0	0	0	0
MST1P2	0	0.02	0.05	0	0.03	0
MST1R	0	0	0	0	0	0
MST4	62.62	40.5	37.7	29.71	40.24	42.98
MSTN	0	0	0	0	0	0
MSTO1	24.78	28.59	21.95	21.68	28.17	23.54
MSTO2P	0.89	2.17	0	1.33	0.82	0.68
MSX1	0.53	0.39	0.52	0.79	0.72	0.42
MSX2	0.5	0.29	0.21	0.57	0.47	0.37
MSX2P1	0	0	0	0	0	0
MT1A	0	0	0	0	0	0
MT1B	0	0	0	0	0	0
MT1DP	0	0	0	0	0	0
MT1E	387.42	356.38	379.96	311.65	389.43	318.12
MT1F	110.17	149.93	105.39	105.38	134.41	112.16
MT1G	0	0	0	0	0	0
MT1H	0	0	0	0.12	0	0
MT1IP	0.25	0	0.08	0.12	0.05	0.08
MT1JP	0.41	0.72	1.6	1.4	1.45	0.37
MT1L	0	0	0	0	0	0
MT1M	73.93	119.22	110.16	68.33	99.5	96.78
MT1P2	0	0	0	0	0	0
MT1X	259.24	305.26	246.75	225.98	268.83	217.8
MT2A	994.55	1241.27	1134.51	597.27	855.37	852.8
MT3	0	0	0	0	0	0
MT4	0	0.51	0	0	0	0
MTA1	2.97	2.83	4.35	2.47	3.27	4.66
MTA2	24.41	28.74	35.69	38.27	28.91	36.02
MTA3	7.58	4.65	6.76	5.11	5.88	5.84
MTAP	10.19	13.01	12.86	12.5	11.61	11.85
MTBP	4.71	4.6	4	3.55	4.38	3.2
MTCH1	18.48	17.02	15.94	17.85	19.27	11.99
MTCH2	90.52	116.56	95.53	100.18	107.97	95.7
MTCP1	0.36	1.5	0.93	1.57	0.91	1
MTDH	24.86	32.04	29.02	22.11	27.63	23.2
MTERF	9.26	7.78	6.42	6.47	8.36	7.06
MTERFD1	28.18	40.04	35.08	27.24	33.31	27.94

MTERFD2	19.54	18.53	15.93	15.54	20.01	15.47
MTERFD3	0	0	0	0	0	0
MTF1	3.4	3.66	3	2.15	2.75	2.39
MTF2	16.43	21.14	16.59	16.22	18.61	16.59
MTFMT	23.57	20.49	21.19	18.77	21.12	21.95
MTFP1	102.52	117.9	139.3	121.28	134.36	133.47
MTFR1	6.1	3.77	5.16	5.27	5.27	6.24
MTFR1L	27.68	22.84	23.01	22.38	20.17	19.06
MTFR2	12.5	17.15	12.53	8.65	14.15	13.96
MTG1	33.93	44.01	36.05	48.12	52.33	39.87
MTHFD1	54.82	71.72	74.57	71.96	64.2	80.74
MTHFD1L	4.14	8.32	8.44	5.62	7.61	7.54
MTHFD2	173.66	114.12	113.34	107.5	130.83	123.71
MTHFD2L	0.9	0.93	0.94	0.92	0.59	0.88
MTHFR	3.61	1.03	0.97	1.33	2.37	0.86
MTHFS	34.24	24.63	24.84	25.99	29.58	27.32
MTHFSD	4.68	4.68	3.4	4.1	4.91	3.71
MTIF2	17.34	21.63	20.25	16.08	19.1	19.15
MTIF3	34.49	25.86	21.99	21.42	24.76	20.32
MTL5	0.61	0.6	1.08	0.82	0.83	0.46
MTM1	4.4	6.42	5.36	3.69	4.15	4.15
MTMR1	1.98	1.92	1.37	1.1	1.21	1.19
MTMR10	0.51	1.23	1.89	0.56	0.72	1.35
MTMR11	0.11	0	0	0.09	0.02	0
MTMR12	4.17	3.39	2.47	2.95	2.74	3.46
MTMR14	22.51	31.94	36.44	31.75	26.78	36.82
MTMR2	14.74	12.96	14.77	12.56	15.09	13.17
MTMR3	3.29	1.96	1.77	2.38	3	2.25
MTMR4	4.32	4.76	4.52	5.27	5.26	3.98
MTMR6	4.72	3.37	3.25	2.81	3.01	3.32
MTMR7	0.25	0.19	0.04	0.06	0.01	0.22
MTMR8	3.44	4.87	2.29	2.48	4.41	3.09
MTMR9	2.83	2.83	2.54	1.95	2.25	2.05
MTMR9LP	0.06	0.09	0	0	0	0
MTNR1A	0	0	0	0	0	0
MTNR1B	0	0	0	0	0	0
MTO1	13.37	12.09	12.37	8.88	12.78	10.87
MTOR	7.98	8.41	7.57	6.05	6.33	7.49
MTOR-AS1	0	0	0	0	0	0
MTPAP	18.41	22.03	21.54	20.25	20.03	18.5
MTPN	23.6	17.42	18.28	15.51	16.2	19.45
MTR	3.51	3.78	4.33	3.09	3.68	3.87
MTRF1	2.68	4.48	2.67	3.22	2.81	3.02

MTRF1L	9.67	6.1	6.47	5.5	9.11	6.24
MTRNR2L1	5284.21	5952.43	6919.32	6287.36	4860.5	5656.67
MTRNR2L10	2621.77	2875.49	3539.45	3076.86	2304.29	2723.54
MTRNR2L2	91965.14	106734.4	114695.22	103474.28	84485.74	96631.08
MTRNR2L3	195.04	204.93	263.02	221.29	173.43	208.44
MTRNR2L4	177.76	185.06	217.07	188.32	144.01	190.96
MTRNR2L5	14.63	16.64	17.16	17.54	13.19	14.34
MTRNR2L6	1136.41	1261.58	1496.54	1364.44	1006.03	1196.85
MTRNR2L7	3.45	3.55	4.34	4.32	2.66	3.69
MTRNR2L8	28321.6	32907.95	38088.66	34413.77	26444.32	31337.46
MTRR	8.23	8.59	9.06	6.34	7.55	9.34
MTSS1	12.53	0.37	0.7	6.05	6.75	0.44
MTSS1L	2.1	1.51	1.42	1.59	1.56	1.78
MTTP	0	0	0	0	0	0
MTUS1	0	0.2	0.06	0	0	0.35
MTUS2	0.05	0	0	0	0	0
MTUS2-AS1	0	0	0	0	0	0
MTVR2	0	0.76	0	0.33	0.37	0.29
MTX1	30.74	44.98	32.73	36.28	35.81	33.28
MTX2	32.13	31.88	23.13	22.53	31.2	21.02
MTX3	0.22	0.24	0.25	0.25	0.18	0.2
MUC1	1.49	2.45	0.68	1.12	2.32	0.84
MUC12	0.09	0.02	0	0.01	0.05	0.03
MUC13	0	0	0	0	0	0
MUC15	0	0	0	0	0	0
MUC16	0	0	0	0	0	0
MUC17	0	0.02	0.04	0.01	0	0.01
MUC2	0	0	0	0	0	0
MUC20	0.48	0.36	0.17	0.45	0.31	0.37
MUC21	0	0.02	0	0.01	0	0
MUC22	0.01	0	0.01	0.01	0	0
MUC4	0	0.03	0	0.28	0.02	0.01
MUC5B	0	0	0	0	0	0
MUC6	0	0	0	0	0	0
MUC7	0	0	0	0	0	0
MUCL1	0	0	0	0	0	0
MUL1	31.86	21.97	26.35	22.33	28.63	23.67
MUM1	6.74	6.22	6.39	6.9	5.77	6.54
MUM1L1	0	0	0	0	0	0
MURC	0.05	0.05	0.03	0.06	0.21	0.23
MUS81	10.7	12.91	14.11	11.51	13.15	13.52
MUSK	0	0	0	0	0	0
MUSTN1	0.22	1.64	0	0.21	0	0.54

MUT	10.05	7.53	5.92	7.51	7.91	7.34
MUTYH	5.06	10.74	10	11.46	7.01	12.22
MVB12A	17.26	21.33	22.37	25.04	23.56	16.73
MVB12B	0.69	0.04	0.17	0.15	0.74	0
MVD	60.5	44.78	45.08	52.63	73.72	54.28
MVK	16.3	10.85	9.77	10.36	16.6	9.57
MVP	6.98	0.92	1.55	1.55	1.65	0.77
MX1	0.16	0.13	0.4	0.1	0	0.12
MX2	0	0	0.09	0.05	0.09	0.02
MXD1	6.03	1.81	1.56	2.39	4.07	2.57
MXD3	6.14	7.38	9.05	8.27	9.22	5.61
MXD4	4.57	1.15	0.63	1.62	1.85	0.78
MXI1	2.7	1.9	2.94	1.66	1.96	1.74
MXRA5	0	0	0	0	0	0
MXRA7	4.5	3.55	5.37	4.75	5.28	4.05
MXRA8	0.1	0.03	0.03	0.24	0	0.16
MYADM	31.54	31.5	27.75	22.86	27.3	26.22
MYADML	0	0	0	0	0	0
MYADML2	0	0	0	0	0	0
MYB	18.65	29.65	22.13	15.24	19.81	22.96
MYBBP1A	37.04	34.34	42.69	41.9	29.39	47.86
MYBL1	0	0.34	0.08	0.07	0.05	0.09
MYBL2	28.92	22.07	26.11	30.33	25.42	18.11
MYBPC1	0	0	0	0	0	0
MYBPC2	0	0	0	0	0	0
MYBPC3	0	0	0	0	0	0
MYBPH	0	0	0	0	0	0
MYBPHL	0.46	0	0	0.04	0	0
MYC	190.2	250.34	278.8	209.83	197.09	297.19
MYCBP	23.32	31.09	25.62	22.31	24.6	26.2
MYCBP2	2.14	2.5	2.18	1.85	2.77	2.39
MYCBP2-AS1	0	0	0	0	0	0
MYCBPAP	0.02	0	0	0.08	0.09	0
MYCL1	1.49	1.23	0.8	0.78	1.45	0.92
MYCN	0.34	0	0	0	0	0.14
MYCNOS	0	0	0	0	0	0
MYCT1	0.19	0	0	0.08	0.02	0
MYD88	29.46	12.92	16.48	18.9	26.36	14.31
MYEF2	0.57	0.66	0.43	0.94	0.99	0.41
MYEOV	0.06	0	0.15	0	0	0
MYEOV2	146.71	125.11	101.39	111.5	142.14	99.53
MYF5	0	0	0	0	0	0
MYF6	0	0	0	0	0	0

MYH1	0	0	0	0	0	0
MYH10	10.5	2.37	2.24	5.78	7.62	2.69
MYH11	0.13	0.12	0.2	0.14	0.14	0.07
MYH13	0	0	0	0	0	0
MYH14	0	0	0	0	0	0
MYH15	0	0	0	0	0	0
MYH16	0	0.03	0	0	0	0
MYH2	0	0	0	0	0	0
MYH3	0	0.08	0.05	0.01	0.02	0
MYH4	0	0	0	0	0	0
MYH6	0	0	0	0.01	0	0
MYH7	0	0	0	0	0	0
MYH7B	0	0.04	0.04	0.02	0	0.01
MYH8	0	0	0	0	0	0
MYH9	33.15	25.67	28.27	23.48	22.47	35.85
MYL1	0	0	0	0	0	0
MYL10	0.22	0.34	0.31	0.11	0.22	0.2
MYL12A	208.28	116.28	90.42	90.69	150.26	91.4
MYL12B	380.47	255.86	194.5	223.53	261.23	223.05
MYL2	0	0	0	0	0	0
MYL3	0	0	0	0.06	0	0
MYL4	5.12	0	1.25	8.22	12.25	0.18
MYL5	3.04	2.31	3.34	1.1	1.74	0.74
MYL6	815.12	636.29	546.61	575.13	719.38	584.1
MYL6B	32.49	35.85	37.68	41.45	50.96	42.05
MYL7	0	0	0	0	0	0
MYL9	0	0	0	0	0	0
MYLIP	3.65	1.51	1.89	1.4	1.83	2.28
MYLK	0.52	0.38	0.32	0.48	0.38	0.64
MYLK-AS1	0.52	1.15	1.05	0.86	1.09	0.21
MYLK2	0	0	0	0	0.07	0
MYLK3	0.55	0.81	0.76	0.45	0.61	0.65
MYLK4	0	0.1	0.05	0.01	0	0.02
MYLPF	0.61	1.26	0.9	0.34	0.94	0.99
MYNN	7.17	4.64	4.54	6.23	5.6	4.57
MYO10	0.19	0.17	0.19	0.09	0.11	0.11
MYO15A	0	0	0	0	0	0
MYO15B	0.01	0.01	0.04	0.01	0	0.05
MYO16	0	0	0	0	0.09	0
MYO16-AS1	0	0	0	0	0	0
MYO18A	5.15	7.74	10.79	7.23	6.01	11.42
MYO18B	0	0	0	0	0	0
MYO19	15.4	16.2	14.25	16.5	13.17	16.38

MYO1A	0.02	0	0	0	0.08	0
MYO1B	0.28	1.82	1.95	1.22	0.52	2.12
MYO1C	8.84	1.69	3.64	5.64	4.48	2.82
MYO1D	7.43	2.81	2.92	5.22	7.16	4.08
MYO1E	0.54	0.36	0.03	0.29	0.11	0.1
MYO1F	4.11	17.35	14.55	9.18	7.4	15.49
MYO1G	2.62	15	16.39	12.25	10.71	12.91
MYO1H	0	0.03	0	0	0	0
MYO3A	0	0	0	0	0	0
MYO3B	0.44	0.06	0.09	0.03	0.09	0.01
MYO5A	2.01	1.48	1.11	1.04	1.03	1.53
MYO5B	0.02	0.01	0.01	0.03	0.03	0.02
MYO5C	0.14	0.13	0.03	0.05	0.07	0.06
MYO6	3.35	1.76	0.09	0.56	0.49	0.49
MYO7A	0	0	0	0	0	0
MYO7B	0	0	0	0	0	0
MYO9A	1.75	2.39	2.29	2.5	2.2	2.39
MYO9B	2.72	2.91	3.66	3.19	3.24	4.01
MYOC	0	0	0	0	0	0
MYOCD	0	0	0	0	0	0
MYOD1	0	0	0	0	0	0
MYOF	0.29	1.27	0.5	0.14	0.02	0.75
MYOG	0	0	0	0	0	0
MYOM1	0.22	0	0	0	0	0.01
MYOM2	0.1	0.28	0.14	0.16	0.27	0.17
MYOM3	0	0.02	0.04	0.01	0.02	0.03
MYOT	0.17	0.03	0.04	0.2	0.04	0.04
MYOZ1	0.24	0.07	0.13	0.23	0.31	0.1
MYOZ2	0	0	0	0	0	0
MYOZ3	1.04	1.7	2.98	2.79	2.57	2.65
MYPN	0	0	0	0	0	0.03
MYPOP	0.2	0.77	0.47	0.14	0.82	0.36
MYRF	0.76	0.26	0.42	0.47	0.47	0.25
MYRIP	0.73	0.02	0.04	0.02	0.2	0
MYSM1	1.54	1.43	0.76	0.53	1.02	1.84
MYT1	0	0.04	0	0.13	0.01	0.08
MYT1L	0	0	0	0	0	0
MYZAP	0	0	0	0	0	0
MZB1	0.38	0.97	1.56	0.33	0	0.86
MZF1	4.51	2.23	1.57	1.85	2.64	1.85
MZT1	18.44	15.43	11.3	8.58	16.17	13.36
MZT2A	71.94	65.4	54.07	83.72	71.26	63.81
MZT2B	40.35	46.13	45.76	54.29	53.36	52.23

N4BP1	1.3	1.45	1.12	1.13	0.89	1.2
N4BP2	1.69	1.69	1.17	1.06	1.6	1.33
N4BP2L1	2.16	2.41	1.18	1.1	1.08	1.74
N4BP2L2	15.48	18.88	13.26	13.48	13.15	12.37
N4BP2L2-IT2	0.15	0.05	0.46	0.09	0.38	0.25
N4BP3	0.34	0.16	0.3	0.18	0.08	0.26
N6AMT1	8.92	4.26	3.39	3.73	5.5	5.03
N6AMT2	10.82	18.91	13.41	8.72	16.19	12.36
NAA10	228.69	216.92	207.62	224.78	239.08	199.17
NAA11	0	0	0	0	0	0
NAA15	27.03	29.98	29.88	27.59	29.57	28.11
NAA16	2.38	4.52	1.75	1.96	4.05	3.13
NAA20	100.11	106.13	91.47	69.31	94.6	93.15
NAA25	8.17	9.05	8.74	8.48	9.67	8.88
NAA30	1.11	0.6	0.58	0.89	0.46	0.87
NAA35	6.63	5.85	5.54	5.04	5.87	5.71
NAA38	3.55	3.31	2.73	2.41	3.43	2.37
NAA40	7	6.13	6.41	7.26	7.96	6.09
NAA50	30.82	24.57	23.33	18.45	22.04	25.46
NAA60	23.02	17.84	16.05	16.17	20.76	18.6
NAAA	35.77	42.88	38.93	33.95	37.8	43.33
NAALAD2	0	0	0.04	0.05	0	0.07
NAALADL1	0	0.09	0	0	0	0
NAALADL2	0	0	0	0	0	0
NAALADL2-A	0	0	0	0	0	0
NAB1	1.39	1.16	0.65	1.29	1.46	1.18
NAB2	3.33	1.32	0.91	2.37	3.33	1.42
NABP1	5.71	5.26	5.74	2.78	6.17	3.02
NABP2	43.36	44.86	46.95	35.63	50.06	40.29
NACA	932.54	952.73	898.38	973.43	1054.46	976.69
NACA2	0.21	0	0	0.08	0.21	0
NACAD	0	0	0	0	0	0.02
NACAP1	0.39	0.87	0.49	0.83	0.34	1.08
NACC1	1.65	2.17	2.13	1.76	1.95	1.51
NACC2	0.53	0.43	0.45	0.5	0.66	0.7
NADK	3.29	3.87	3.36	2.82	2.83	4.07
NADKD1	4.13	4.3	3.1	3.55	4.01	3.14
NADSYN1	20.74	29.6	23.93	19.94	23.28	26.12
NAE1	53.86	68.84	56.31	52.27	66.49	59.24
NAF1	4.97	7.2	4.78	5.81	5.87	6.38
NAGA	18.16	19.61	18.19	17.55	16.74	19.11
NAGK	14.3	9.22	8.3	9.02	8.06	9.13
NAGLU	2.1	2.09	1.06	2.69	1.88	1.65

NAGPA	19.83	26.99	20.1	14.56	16.8	17.39
NAGPA-AS1	0.25	0.68	0.28	0.13	0.41	0.12
NAGS	0.07	0	0	0	0.03	0.04
NAIF1	0.84	1.47	0.68	1.58	1.26	1.22
NAIP	0.71	1.1	1.16	0.47	0.5	0.81
NALCN	0	0	0	0	0	0
NALCN-AS1	0	0	0	0	0	0
NAMPT	11.78	9.28	8.68	7.83	10.37	9.98
NANOG	0.49	0.46	0.24	0.56	0.27	0.62
NANOGNB	0	0	0	0	0	0
NANOS1	0.33	0.57	0.32	0.5	0.34	0.12
NANOS2	0	0	0	0	0	0
NANOS3	0.38	0	0.26	0.33	0.18	0
NANP	5.73	5.6	4.98	4.65	6.66	7.22
NANS	91.71	96.59	101.25	93.4	105.16	98.49
NAP1L1	95.74	109.41	110.95	120.99	103.66	120.76
NAP1L2	0	0	0	0	0	0
NAP1L3	0.21	0.19	0.97	1.33	0.77	0.48
NAP1L4	54.57	53.86	44.89	53.64	52.53	50.88
NAP1L5	7.71	3.6	4.39	3.15	4.6	3.36
NAP1L6	0.26	0.09	0.69	0.4	0.5	0.22
NAPA	56.56	36.82	36.23	44.32	52.61	37.16
NAPA-AS1	0.12	0.39	0.79	1.19	0.93	0.74
NAPB	1.12	0.69	0.58	0.42	0.71	0.28
NAPEPLD	0.87	1.35	1.2	0.86	1.1	1.41
NAPG	7.52	3.92	3.95	3.29	4.4	3.85
NAPRT1	49.75	68.78	60.89	67.23	59.45	59.09
NAPSA	0	0	0	0	0	0
NAPSB	0.11	0	0	0	0	0
NARF	62.37	43.89	42.6	44.72	51.4	50.42
NARFL	18.8	23.2	23.83	21.53	16.85	18.99
NARG2	5.68	4.7	4.72	4.88	4.42	6.94
NARR	0	0	0	0	0	0
NARS	69.58	53.67	52.3	45.14	50.86	51.12
NARS2	14.94	20.3	21.41	17.57	15.43	19.45
NASP	87.46	107.97	81.28	87.49	100.59	90.06
NAT1	8.25	7.63	7.84	5.26	7.18	5.35
NAT10	25.97	32.2	39.17	35.78	31.78	43.34
NAT14	3.74	1.67	3.52	2.89	3.33	2.71
NAT16	0.02	0	0	0	0	0
NAT2	0	0	0	0	0	0
NAT6	6.87	6.12	7.31	7.26	7.44	11.07
NAT8	0	0.11	0	0	0	0

NAT8B	0	0	0	0	0.54	0
NAT8L	0.89	0.84	0.89	1.76	1.46	1.03
NAT9	20.39	11.33	10.75	13.14	15.87	14.89
NAV1	0.48	0.34	0.45	0.29	0.47	0.52
NAV2	0.01	0.14	0.15	0	0	0.04
NAV2-AS4	0	0	0	0	0	0
NAV2-AS5	0	0	0	0	0	0
NAV3	0.04	0.01	0	0	0	0
NBAS	12.1	5.16	6.42	8.38	8.44	6.34
NBEA	0.28	0.24	0.16	0.26	0.47	0.32
NBEAL1	1.3	1.08	0.87	0.7	1.03	0.9
NBEAL2	3.55	3.61	4.57	3.05	4.86	4.89
NBEAP1	0	0	0	0	0	0.3
NBL1	0	4.02	6.24	0	1.76	1.34
NBLA00301	0	0	0	0	0	0
NBN	24.82	28.88	23.82	19.8	21.86	21.87
NBPF1	1.94	2.15	1.72	1.81	2.51	2.61
NBPF10	2.84	2.95	1.27	1.69	1.89	1.65
NBPF11	0	0	0.59	0.3	0.31	0.64
NBPF14	5.22	5.83	2.03	3.02	3.99	3.55
NBPF15	2.06	2.5	1.63	2.24	2.61	3.91
NBPF16	2.35	1.3	0.41	0.7	1.49	0.81
NBPF22P	0	0	0	0	0	0
NBPF24	1.82	2.05	0.52	1.12	1.16	2.06
NBPF3	1.42	1.05	1.43	1.04	1.05	1.69
NBPF4	0	0	0	0	0	0
NBPF6	0	0	0	0	0	0
NBPF7	0.2	0.27	0.36	0.19	0	0.08
NBPF9	1.28	2.14	1.37	0.78	1.77	1.53
NBR1	11.34	4.82	4.82	6.2	7.96	3.25
NBR2	3.19	1.91	2.55	1.95	1.48	2.45
NCALD	1.2	5.72	3.88	2.18	1	5.38
NCAM1	0.14	0	0	0.22	0.01	0.05
NCAM2	0.01	0.07	0	0	0	0.13
NCAN	0	0	0	0	0	0
NCAPD2	36.96	47.58	40.08	46.27	38.67	48.27
NCAPD3	11.56	12.41	8.22	11.1	12.58	9.99
NCAPG	13.18	14.42	13.96	11.54	14.7	13.21
NCAPG2	13.87	15.86	9.85	9.11	13.95	14.06
NCAPH	16.82	21.04	16.66	18.84	20.53	20.28
NCAPH2	28.46	32.5	33.18	41.21	34.71	29.26
NCBP1	20.72	22.62	19.73	18.16	21.26	20.08
NCBP2	38.88	52.59	37.68	41.38	48.96	44.24

NCCRP1	0.19	0.13	0.18	0.12	0.08	0
NCDN	12.64	7.02	6.65	10.01	8.54	12
NCEH1	2.75	1.71	1.77	0.9	2.03	1.5
NCF1	2.3	4.01	4.09	4.09	2.32	1.31
NCF1B	0.11	0.1	0.06	0.25	0	0.18
NCF1C	0.51	0.92	0.78	1.18	1.04	1.48
NCF2	0	0.29	0.24	0.19	0.22	0.65
NCF4	24.88	62.03	71.77	57.55	55.39	54.52
NCK1	13.01	11.61	11.58	11.04	10.75	11.01
NCK2	5.24	3.4	4.13	3.56	3.99	3.06
NCKAP1	2.99	0.31	0.16	1.04	1.71	0.21
NCKAP1L	20.55	47.97	43.78	32.56	29.59	46.98
NCKAP5	0	0	0	0.02	0.01	0
NCKAP5L	3.43	2.25	3.64	2.41	2.83	3.64
NCKIPSD	13.27	8.55	9.72	10.59	9.13	8.4
NCL	439.13	471.67	391.96	402.14	430.24	444.12
NCLN	43.66	48.42	48.54	42.55	45.41	43.12
NCMAP	0.8	0.9	0.81	0.38	0.78	0.74
NCOA1	1.34	0.89	0.38	1.25	1.15	1.06
NCOA2	10.66	9.29	8.8	10.46	8.08	7.77
NCOA3	17.88	5.61	5.07	7.92	9.45	6.02
NCOA4	53.89	44.05	37.95	43.25	42.59	55.32
NCOA5	8.71	9.79	9.91	9.89	9.31	10.66
NCOA6	2.35	2.94	2.26	2.28	2.69	2.65
NCOA7	14.02	5.61	4.77	3.96	6.54	6.19
NCOR1	3.53	4.55	3.15	2.71	3.03	2.97
NCOR1P1	0	0	0	0	0.2	0
NCOR2	3.11	3.13	3.43	3.42	3.22	3.84
NCR1	0	0	0	0	0	0
NCR2	0	0	0	0	0	0
NCR3	0.09	0.34	0.32	0.15	0.76	0.77
NCR3LG1	3.61	7.55	5.85	5.17	4.26	7.6
NCRNA0018!	0.14	0	0	0	0	0.03
NCRUPAR	4.56	5.94	5.23	5.22	2.52	4.06
NCS1	3.15	2.42	3.62	2.37	2.6	2.98
NCSTN	30.39	36.14	23.96	20.79	27.5	29.66
NDC80	21.13	13.73	11.83	12.23	14.31	11.25
NDE1	6.98	7.14	7.2	6.34	6.23	5.86
NDEL1	19.36	12.13	11.62	12.43	16.7	9.04
NDFIP1	9.73	7.05	5.71	4.66	6.79	6.72
NDFIP2	7.4	0.22	0.23	1.19	2.89	0.19
NDFIP2-AS1	0	0	0	0	0	0
NDN	0	0	0	0	0	0

NDNF	0	0	0	0	0	0
NDNL2	2.05	0.56	0.57	0.46	1.22	2.26
NDOR1	4.01	4.68	2.89	3.09	3.43	4.22
NDP	0	0	0	0	0.03	0
NDRG1	2.24	1.73	1.98	1.51	1.96	1.94
NDRG2	26.69	11.82	10.11	17.5	18.9	12.08
NDRG3	18.87	20.58	18.61	19.45	20.86	17.07
NDRG4	0.13	0	0.04	0.03	0.02	0
NDST1	10.07	11.93	12.05	13.2	11.52	11.13
NDST2	1.67	1.4	1.59	1.48	2.26	1.34
NDST3	0.08	0.11	0.14	0.07	0.16	0.22
NDST4	0	0	0	0	0	0
NDUFA1	1010.33	975.76	922.23	752.24	1102.68	743.78
NDUFA10	47.47	39	29.72	37.4	44.04	33.21
NDUFA11	248.14	270.33	250.67	292.32	280.39	289.25
NDUFA12	235.34	228.58	215.37	225	261.54	220.33
NDUFA13	567.59	595.3	536.43	592.42	614.91	522.21
NDUFA2	150.45	138.54	119.83	136.94	146.61	119.24
NDUFA3	173.48	177.19	160.21	160.7	201.42	115.83
NDUFA4	178.26	133.03	112.5	124.85	164.97	125.2
NDUFA4L2	0.96	0.1	0.59	0.31	0.48	0.9
NDUFA5	32.46	28.25	24.59	21.77	32.87	28.71
NDUFA6	127.95	107.12	103.14	101.18	132.6	96.56
NDUFA7	93.2	65.13	60.61	64.36	89.23	59.47
NDUFA8	162.93	149.79	134.45	148.55	157.35	115.85
NDUFA9	111.54	108.99	101.11	98.4	107.59	93.51
NDUFAB1	372.53	340.1	291.12	309.97	339.62	331.57
NDUFAF1	12.06	15.51	13.44	12.1	17.78	10.29
NDUFAF2	93.74	104.85	96.97	92.98	98.42	95.02
NDUFAF3	76.38	84.53	75.27	90.04	83.01	75.08
NDUFAF4	22.68	21.05	19.22	16.31	18.33	18.29
NDUFAF4P1	0	0.1	0.2	0.11	0.13	0.03
NDUFAF5	7.5	6.1	5.4	4.35	6.42	4.58
NDUFAF6	45.81	31.86	30.43	32.86	39.43	31
NDUFAF7	5.85	6.7	6.28	4.48	3.38	4.94
NDUFB1	128.76	125.94	106.86	92.84	145.31	88.37
NDUFB10	234.26	283.46	278.49	251.48	271.53	273.41
NDUFB11	319.64	299.52	303.38	360.13	359.14	327.47
NDUFB2	248.25	257.03	217.51	248.96	277.58	228.94
NDUFB2-AS1	0.37	0.79	0.42	1.01	0.3	1.01
NDUFB3	147.29	150.74	125.4	100.25	138.55	102.27
NDUFB4	253.86	254.67	228.29	228.18	283.56	249.69
NDUFB5	72.36	60.09	70.03	55.1	60.24	73.31

NDUFB6	193.49	156.85	123.69	123.7	174.92	139.25
NDUFB7	329.42	350.75	341.01	361.92	364.04	295.93
NDUFB8	322.06	346.01	281.6	328.57	343.44	292.94
NDUFB9	1088.09	712.88	712.78	947.19	963.2	684.08
NDUFC1	136.46	158	149.4	130.59	163.2	126.24
NDUFC2	119.37	88.2	85.65	82.88	91.51	92.5
NDUFC2-KCT	0.24	0.96	0.22	0	0	0
NDUFS1	26.21	28.14	20.43	18.05	26.07	23.21
NDUFS2	93.53	102.12	76.57	92.78	91.35	92.6
NDUFS3	160.19	258.71	223.43	220.19	199.2	220.03
NDUFS4	74.98	83.9	90.87	80.07	98.29	78.72
NDUFS5	658.5	627.53	542.19	646.78	708.39	606.56
NDUFS6	293.5	286.54	274.77	276.16	293.97	244.3
NDUFS7	264.33	319.55	281.09	295.26	327.04	267.84
NDUFS8	276.55	261.98	244.72	309.07	295.92	265.39
NDUFV1	170.93	189.2	178.34	214.97	187.29	174.13
NDUFV2	275.61	278.39	232.35	227.57	265.82	224.03
NDUFV3	38.66	41.43	39.63	40.97	39.96	40.71
NEAT1	11.61	8.54	5.01	3.14	5.09	6.12
NEB	0	0	0	0	0.01	0
NEBL	0	0	0	0	0	0
NEBL-AS1	0	0	0	0	0	0
NECAB1	1.23	0.83	1.01	0.8	2.07	1.07
NECAB2	0.14	0	0	0	0	0
NECAB3	4.48	2.28	2.5	2.77	2.95	2.94
NECAP1	10.93	5.92	4.6	4.41	4.85	5.5
NECAP2	32.25	21.75	21.64	19.13	21.94	18.77
NEDD1	4.97	4	3.42	4.39	5.46	4.21
NEDD4	2.99	2.94	2.02	2.46	3.06	2.67
NEDD4L	0.48	0.14	0.3	0.24	0.3	0.21
NEDD8	296.8	322.49	250.63	288.13	326.82	266.06
NEDD8-MDP	0.38	0	0	0	0.76	0.41
NEDD9	4.2	0.83	2.14	1.14	0.58	0.83
NEFH	0	0	0	0.04	0	0
NEFL	0	0	0.1	0	0	0
NEFM	0	0	0	0	0	0
NEGR1	1.75	2.24	2.55	1.12	0.95	2.07
NEGR1-IT1	0	0	0	0	0	0
NEIL1	0.24	0.03	0.29	0.03	0.15	0.12
NEIL2	23.79	19.74	15.16	24.04	27.29	14.08
NEIL3	9.38	9.26	6.49	8.05	7.29	7.88
NEK1	2.52	1.73	1.74	1.7	2.69	1.57
NEK10	0.25	0.13	0.26	0	0.05	0

NEK11	0.4	0.43	0.46	0.13	0.62	0.82
NEK2	11.86	14.27	10.44	8.71	8.95	12.82
NEK3	3.77	4.89	3.23	2.45	2.57	4.25
NEK4	5.65	8.07	1.74	4.45	5.49	6.87
NEK5	3.66	4.89	5.27	3.69	4.1	4.06
NEK6	12.09	12.53	17.25	13	13.17	7.08
NEK7	1.1	1.01	0.43	0.83	0.53	0.78
NEK8	1.96	1.53	1.36	0.79	1.11	1.43
NEK9	6.66	7.99	9.02	6.86	7.55	11.16
NELFA	28.17	26.44	25.8	25.91	26.1	26.09
NELFB	5.28	4.43	3.55	4.12	4.2	4.36
NELFCD	72.41	66.17	58.45	77.56	71.98	72.26
NELFE	72.87	80.25	77.68	78.84	70.12	63.37
NELL1	0	0	0	0	0	0
NELL2	0.11	0	0	0	0.02	0
NEMF	10.19	11.41	6.95	8.76	10.46	8.69
NENF	12.42	23.38	22.89	23.15	26.54	6.01
NEO1	17.62	0.42	0.37	2.64	3.54	0.39
NES	0	0	0	0.04	0	0
NET1	95.74	17.76	17.77	29.32	64.27	15.58
NETO1	0	0	0	0	0	0
NETO2	2.29	2.07	2.15	1.82	3.1	2.42
NEU1	48.71	33.2	35.71	38.22	52.84	31.64
NEU2	0	0	0	0	0	0
NEU3	7.66	8.94	8.63	7.88	7.73	10.98
NEU4	0.07	0	0	0	0	0
NEURL	0.2	0.03	0.02	0	0.25	0
NEURL1B	0	0.06	0.15	0.21	0	0.16
NEURL2	0.9	1.23	0.29	0.58	0.34	0.06
NEURL3	0	0	0	0	0	0
NEURL4	0.75	0.63	0.97	0.67	1.4	0.66
NEUROD1	0	0	0	0	0	0
NEUROD2	0	0	0	0	0	0
NEUROD4	0	0	0	0	0	0
NEUROD6	0	0	0	0	0	0
NEUROG1	0	0	0	0	0	0
NEUROG2	0	0	0	0	0	0
NEUROG3	0	0	0	0	0	0
NEXN	3.55	1.67	1.87	1.18	2.26	2.08
NF1	2.18	2.24	1.9	1.85	1.98	3.16
NF1P2	0	0	0	0	0	0
NF2	28.42	34.48	33.8	32.18	29.88	29.93
NFAM1	0.03	0.1	0.15	0.07	0.01	0.07

NFASC	0.04	0	0	0	0.01	0
NFAT5	2.82	2.57	2.25	2.56	2.61	2.14
NFATC1	12.81	3.4	3.4	5.36	7.87	3.3
NFATC2	1.19	2.16	2.59	1.99	1.94	1.58
NFATC2IP	2.69	3.55	2.48	3.06	3.32	3.34
NFATC3	13.72	9.75	10.86	9.7	11.32	11.59
NFATC4	0	0	0	0	0	0.02
NFE2	158.1	65.08	60.73	97.94	118.98	67.48
NFE2L1	5.43	5.16	4.76	4.75	5.95	7.52
NFE2L2	25.78	12.67	16.74	16.04	15.6	9.93
NFE2L3	0.7	0.83	0.66	1.16	0.9	0.97
NFIA	2.4	0.31	0.69	0.93	1.2	0.72
NFIB	0	0.24	0.39	0.06	0.16	0.29
NFIC	10.79	8.84	9.46	9.26	8.78	8.11
NFIL3	0.64	1.03	0.78	0.39	0.97	1.04
NFIX	1.4	1.27	1.43	1.1	0.84	1.21
NFKB1	2.18	1.76	1.79	1.74	2.22	1.49
NFKB2	0.83	0.28	0.28	0.59	0.47	0.65
NFKBIA	33.81	32.35	27.67	29.54	23.7	25.91
NFKBIB	12.64	13.79	11.01	16.48	14.12	12.12
NFKBID	2.39	3.03	0.78	1.95	1.99	2.09
NFKBIE	1.7	3.31	4	1.4	1.47	2.64
NFKBIL1	12.52	9.02	9.73	7.24	13.52	4.77
NFKBIZ	2.27	0.68	0.29	1.62	0.5	0.67
NFRKB	9.26	10.16	10.25	9.26	8.06	10.08
NFS1	14.31	17.04	12.89	11.95	13.18	14.96
NFU1	27.32	31.8	31	29.62	31.4	33.21
NFX1	26.03	26.46	25.93	25.63	23.94	24.14
NFXL1	4.01	7.14	4.79	4.84	4.35	6.14
NFYA	7.84	7.57	9.15	8.29	7.79	9.02
NFYB	5.35	8.4	7.16	4.39	5.37	6.29
NFYC	33.21	30.18	36	30.42	35.89	27.99
NGB	0	0	0	0	0	0
NGDN	56.52	58.16	41.59	59.3	55.57	48.63
NGEF	0	0	0	0	0	0
NGF	0	0	0	0	0	0
NGFR	0	0	0	0.1	0	0.19
NGFRAP1	279.97	169.86	159.64	221.43	251.88	191.25
NGLY1	25.22	25.21	20.69	27.63	24.65	20.69
NGRN	45.52	32.95	34.72	34.91	39.81	29.36
NHEG1	0	0	0.25	0	0.06	0
NHEJ1	21.13	14.69	16.34	21.23	18.7	15.05
NHLH1	0.14	0	0	0	0	0.03

NHLH2	0	0	0	0	0	0
NHLRC1	0.17	0.03	0.07	0.16	0.02	0
NHLRC2	3.74	3.22	3.79	3.15	3.66	3.46
NHLRC3	1.22	2.46	1.54	1.66	1.18	1.62
NHLRC4	0.29	0.26	0.6	0.05	0.31	0.4
NHP2	410.18	443.16	458.89	493.83	495.25	437.22
NHP2L1	257.08	286.99	283.61	268.4	279.18	284.38
NHS	0	0	0	0	0	0
NHSL1	0.78	4.43	3.13	2.79	2.35	4.7
NHSL2	0.59	0	0.23	0	0.32	0.1
NICN1	4.3	4.56	5.2	6.21	6.3	5.62
NID1	11.97	15.64	16.03	11.97	12.51	20.86
NID2	1.76	0.07	0.03	0.02	0.42	0
NIF3L1	21.84	31.8	19.37	21.56	23.08	20.56
NIM1	0.02	0.04	0	0.03	0	0.03
NIN	1.75	2.75	1.61	1.34	1.28	2.23
NINJ1	24.43	44.21	37.43	32.44	32.1	33.46
NINJ2	4.83	3.13	3.56	3.44	2.25	2.26
NINL	0	0.02	0	0	0.03	0
NIP7	24.95	35.43	30.54	24.88	30.26	26.41
NIPA1	1.09	1.5	1.45	1.27	1.81	1.33
NIPA2	36.79	40.39	43.32	33.51	38.31	33.6
NIPAL1	0.1	0	0.28	0.07	0	0.03
NIPAL2	3.03	5.32	4.84	4.24	3.2	3.93
NIPAL3	6.02	4.83	5.4	6.7	4.88	5.98
NIPAL4	0	0	0	0	0	0
NIPBL	6.83	5.67	4.55	5.03	5.65	5.52
NIPSNAP1	37.47	24.35	28.19	33.04	39.85	27.83
NIPSNAP3A	50.41	36.91	25.52	28.04	35.2	30.27
NIPSNAP3B	2.25	0.77	1.64	0.79	1.33	1.55
NISCH	8.82	7.43	7.1	6.86	7.99	9.4
NIT1	9.93	8.26	8.91	8.89	8.47	7.5
NIT2	36.36	48.24	38.07	40.69	38.39	39.33
NKAIN1	0.1	0.08	0.23	0.1	0.09	0.28
NKAIN2	0	0	0	0	0	0
NKAIN3	0	0	0	0	0	0
NKAIN4	0	0	0	0	0	0
NKAP	29.12	29.33	30.16	26.03	28.67	34.35
NKAPL	0	0	0	0	0	0
NKAPP1	0.94	1.65	1.36	0.94	1.92	1.14
NKD1	0.19	0.29	0.34	0.18	0.16	0.19
NKD2	2.13	2.37	0.65	1.27	0.84	0.51
NKG7	1.42	16.11	11.28	6.96	3.87	10.25

NKIRAS1	1.93	4.49	2.95	2.45	3.36	3.11
NKIRAS2	30.31	21.42	15.08	18.61	23.57	16.57
NKPD1	0.18	0.4	0.1	0.19	0.13	0.34
NKRF	4.63	4	3.95	3.22	4.01	4.91
NKTR	3.97	3.41	2.9	2.02	3.05	2.74
NKX1-2	0	0	0	0	0	0
NKX2-1	0	0	0	0.02	0	0
NKX2-2	0.04	0	0.26	0.15	0	0.07
NKX2-3	0	0	0	0	0	0
NKX2-4	0	0	0	0	0	0
NKX2-5	0	0	0	0	0	0
NKX2-6	0	0	0	0	0	0
NKX2-8	0	0	0	0	0	0
NKX3-1	3.81	0.7	0.98	2.27	2.19	1.44
NKX3-2	0.13	0.41	0.48	0.29	0.15	0.63
NKX6-1	0.07	1.19	0.63	0.52	1.62	0.35
NKX6-2	0	0	0	0	0	0
NKX6-3	0	0	0	0	0	0
NLE1	17.7	25.54	25.09	27.14	23.76	24.51
NLGN1	0	0	0	0	0	0
NLGN2	0.59	0.64	0.58	0.56	0.87	0.43
NLGN3	0.24	0.1	0.21	0.1	0.27	0.4
NLGN4X	0	0	0	0	0	0
NLGN4Y	0	0	0	0	0	0
NLGN4Y-AS1	0	0	0	0	0.15	0
NLK	4.86	1.84	1.65	2.68	3.45	2.41
NLN	6.29	6.63	5.87	5.78	5.74	6.44
NLRC3	0.65	2.12	3.08	2.17	1.94	4.1
NLRC4	0	0.8	1.34	0.77	0.53	1.27
NLRC5	0.95	2.04	1.76	1.74	1.13	2.21
NLRP1	0.1	0.1	0.61	0.5	0.3	0.7
NLRP10	0	0	0	0	0	0
NLRP11	0.02	0.25	0.34	0.16	0.1	0.31
NLRP12	2.35	3.09	2.98	1.97	2.53	2.87
NLRP13	0	0	0	0	0	0
NLRP14	0	0	0.02	0	0	0.02
NLRP2	0	0.16	0.49	0.09	0	0.06
NLRP3	0.08	1.46	0.86	0.67	0.21	1.14
NLRP4	0	0	0	0	0	0
NLRP5	0	0	0	0	0	0
NLRP6	0	0	0	0	0	0
NLRP7	0	0	0	0	0	0
NLRP8	0	0.19	0.19	0.07	0.02	0.13

NLRP9	0.06	0.11	0	0	0.06	0
NLRX1	3.45	4.35	4.36	3.95	4.76	5.27
NMB	33.76	56.74	64.64	61.06	50.53	57.61
NMBR	0	0	0	0	0	0
NMD3	22.48	21.18	19.62	17.54	17.67	18.14
NME1	782.26	841.32	754.84	841.59	935.1	778.97
NME1-NME2	27.47	51.21	39.94	35.44	31.74	51.41
NME2	1060.21	1126.23	1082.63	1204.17	1195.18	1118.29
NME3	1.67	1.56	1.18	0.47	1.22	0.69
NME4	55.63	77.23	87.39	88.16	85.27	66.48
NME5	0	0	0	0	0	0
NME6	19.44	24.22	22.11	17.43	21.11	18.08
NME7	13.62	7.86	6	11.42	7.84	6.42
NME8	0	0	0	0	0	0
NME9	0.21	0.22	0.13	0.21	0.1	0
NMI	36.64	51.73	43.97	36.46	43.74	43.91
NMNAT1	4.47	5.42	5.18	3.86	4.46	4.07
NMNAT2	0	0	0	0	0	0
NMNAT3	3.44	0.89	1.75	2.48	2.7	0.88
NMRAL1	41.25	39.32	39.37	46.32	39.27	38.51
NMRK1	9.35	4.74	2.84	2.89	5.41	2.33
NMRK2	0	0	0	0	0	0
NMS	0	0	0	0	0	0
NMT1	19.03	19.54	19.14	17.24	17.67	19.29
NMT2	6.12	2.97	4.05	4.4	4.94	4.35
NMU	88.67	0.15	0.09	4.86	10.31	0.29
NMUR1	0.22	0.31	0.24	0.18	0.08	0.11
NMUR2	0	0	0	0	0	0
NNAT	0.06	0.15	0	0.09	0	0.25
NNMT	0	0	0	0	0	0
NNT	16.07	25.93	22.94	17.55	19.39	24.92
NOA1	25.47	26.31	29.06	26.72	27.03	31.27
NOB1	61.39	62.57	62.24	71.2	60.59	73.22
NOBOX	0	0	0	0	0	0
NOC2L	110.83	119.75	137.64	129.69	112.62	138.69
NOC3L	8.69	7.74	9.33	7.37	9.57	8.86
NOC4L	50.9	80.11	70.88	69.31	57.81	68.8
NOD1	2.39	3.52	2.77	2.65	1.85	2.44
NOD2	0.23	0.89	1	0.51	0.47	0.89
NODAL	0	0	0	0	0	0
NOG	0.04	0.34	0.54	0.67	0.74	0.28
NOL10	20.13	22.13	19.35	17.13	20.45	15.94
NOL11	35.64	41.02	35.18	33.38	33.36	37.92

NOL12	11.62	15.3	11.6	13.11	13.56	13.28
NOL3	9.05	3.59	3.13	3.55	3.66	2.66
NOL4	0	0	0	0	0	0
NOL6	23.42	27.24	28.43	25.69	24.08	24.86
NOL7	121.81	128	119.09	122.99	129.29	96.06
NOL8	13.72	13.89	11.45	12.02	13.58	13.7
NOL9	5.9	6.61	6.69	7.97	5.59	7.64
NOLC1	71.27	74.6	66.42	66.02	70.05	72.26
NOM1	1.9	3.07	3.14	1.85	2.55	2.58
NOMO1	5.25	7.09	7.6	7.45	6.81	7.2
NOMO2	5.11	9.72	11.14	7.84	8.13	8.7
NOMO3	1.98	2.86	4.71	2.21	3.05	4.07
NONO	413.32	417.66	397.98	425.55	441.33	404.03
NOP10	410.96	303.75	249.07	288.35	395.81	244.76
NOP14	8.86	11.89	11.6	10.32	7.57	11.58
NOP14-AS1	0.94	1.21	1.76	1.24	1.01	1.19
NOP16	172.23	219.05	188.9	171.55	190.21	177.98
NOP2	41.2	45.14	43.42	40.77	33.84	43.86
NOP56	96.83	117.32	95.38	101.89	103.38	101.63
NOP58	102.09	102	98.26	94.17	104.75	100.2
NOP9	2.88	3.3	3.56	4.42	3.06	4.92
NOS1	0.13	0.14	0.15	0.07	0.14	0.19
NOS1AP	0	0	0	0.01	0	0.03
NOS2	0	0.01	0	0	0	0
NOS3	0	0.21	0.15	0.23	0.07	0.03
NOSIP	80.51	85.88	86.51	91.34	83.2	72.51
NOSTRIN	12.75	0.31	0.77	4.95	5.27	0.28
NOTCH1	1.41	2.96	2.09	1.7	1.48	3.35
NOTCH2	9.62	10.05	13.37	9.7	9.23	9.32
NOTCH2NL	4.63	4.76	3.55	2.99	4.5	3.69
NOTCH3	0.42	0.31	0.25	0.38	0.64	0.39
NOTCH4	0.2	0.02	0.03	0.01	0.05	0.03
NOTO	0	0	0	0	0	0
NOTUM	1.79	0.42	1.91	0.71	1	0.71
NOV	0.08	0.27	0.33	0.02	0.13	0
NOVA1	0	0	0	0	0	0
NOVA2	0	0	0	0	0	0
NOX1	0.06	0	0	0	0	0.06
NOX3	0.04	0	0	0	0.07	0.58
NOX4	0.02	0.13	0.11	0.05	0.06	0.08
NOX5	0	0	0	0	0	0
NOXA1	0.2	0.48	0.19	0.72	0.31	0
NOXO1	0.18	0.36	0.13	0.03	0.09	0.05

NOXRED1	0	0.03	0.11	0	0.04	0
NPAP1	0.22	0.36	0.28	0.1	0.23	0.24
NPAS1	0.69	1.76	1.79	1.34	0.61	0.84
NPAS2	0.07	0	0	0	0	0
NPAS3	0	0	0	0	0	0
NPAS4	0	0	0	0	0	0
NPAT	5.71	3.43	5.23	4.13	4.74	3.83
NPB	0	0.31	0	0	0	0
NPBWR1	0	0	0	0	0	0
NPBWR2	0	0	0	0	0	0
NPC1	3.24	2.7	4.34	3.94	3.45	5.32
NPC1L1	0	0	0.01	0	0	0
NPC2	93.69	85.98	79.12	99.01	99	72.28
NPDC1	25.22	53.64	63.14	53.68	40.03	40.25
NPEPL1	3.72	3.5	5.2	3.45	2.55	2.65
NPEPPS	16.5	17.2	19.14	16.65	16.82	17.79
NPFF	0.28	0.55	0	0.38	0	0
NPFFR1	0	0	0	0	0	0
NPFFR2	0.23	0.3	0.39	0.22	0.36	0.45
NPHP1	0	0	0	0	0.03	0
NPHP3	0.31	0.32	0.16	0.22	0.13	0.17
NPHP3-ACAD	0	0.02	0.07	0	0	0.06
NPHP3-AS1	0	0	0	0	0	0
NPHP4	1.38	1.24	1.07	1.28	0.96	1.39
NPHS1	0.33	0.37	0.62	0.21	0.43	0.55
NPHS2	0	0	0	0	0	0
NPIP	10.23	13.42	3.41	4.41	9.47	10.47
NPIPL3	1.85	2.09	2.16	2.23	1.2	2.64
NPL	7.25	1.35	1.6	2.42	5.13	1.27
NPLOC4	8.8	6.94	5.83	5.82	5.71	6.05
NPM1	1411.54	1429.27	1393.65	1421.76	1485.1	1485.04
NPM2	0.97	1.31	0.13	0.53	1.12	0.84
NPM3	43.44	69.14	68.99	62.49	57.79	44.84
NPNT	0	0	0	0	0	0
NPPA	0.18	0.07	0.17	0.51	0.17	0
NPPA-AS1	0.23	0.05	0.06	0	0.21	0.18
NPPB	0.23	0	0	0	0	0
NPPC	0	0	0	0	0	0
NPR1	0	0	0	0	0	0
NPR2	0.15	0.7	0.54	0.6	0.26	0.77
NPR3	7.18	3.22	7.4	7.97	7.95	5.36
NPRL2	22.41	27.28	19.82	23.94	23.34	20.23
NPRL3	10.84	7.41	8.88	9.46	8.81	7.89

NPS	0	0	0	0	0	0
NPSR1	0	0	0	0	0	0
NPSR1-AS1	0	0.02	0	0	0	0
NPTN	26.75	21.85	17.58	16.51	19.43	18.53
NPTX1	0	0	0.01	0.04	0.01	0.25
NPTX2	0	0	0	0	0	0
NPTXR	0.05	0.51	0.33	0.17	0.28	0.48
NPVF	0	0	0	0	0	0
NPW	51.24	84.9	132.32	157.58	97.49	98.78
NPY	0	0	0	0	0	0
NPY1R	0.52	0	0	0.1	0.14	0
NPY2R	0	0	0	0	0	0
NPY5R	0	0	0	0	0	0
NPY6R	0	0.06	0	0	0.05	0
NQO1	24.28	24.63	15.26	14.43	11.57	11.9
NQO2	43.29	26.7	17.08	27.99	32.11	17.24
NR0B1	0	0	0	0	0	0
NR0B2	0	0	0	0	0	0
NR1D1	4.41	4.23	5.23	4.09	4.71	6.26
NR1D2	0.73	0.38	0.3	0.25	0.23	0.16
NR1H2	25.6	13.93	15.19	14.28	17.5	15.34
NR1H3	5.65	8.82	6.96	5.51	5.25	10
NR1H4	0.12	0	0	0	0.06	0
NR1I2	0	0	0	0	0	0
NR1I3	0.16	0	0	0	0	0
NR2C1	5.43	3.38	4.63	5.69	4.14	4.14
NR2C2	1.1	1.18	1.46	1.19	1.44	1.49
NR2C2AP	19.38	18.23	21.63	24.54	20.66	22.46
NR2E1	0.02	0	0	0	0	0
NR2E3	0	0	0.07	0.08	0.07	0
NR2F1	0.41	0.36	0.38	0.33	0.39	0.09
NR2F2	0.12	0	0	0	0	0
NR2F6	20.16	21.3	23.16	19.47	21.57	10.19
NR3C1	3.6	4.17	4.16	3.32	3.09	3.43
NR3C2	0.16	0.37	0.13	0.15	0.04	0.21
NR4A1	5.5	0.34	0.82	1.28	2.79	0.82
NR4A2	1.42	0.37	0.15	0.28	0.52	0.62
NR4A3	0.06	0	0	0	0.13	0
NR5A1	0	0	0	0.08	0	0
NR5A2	0.39	0.21	0.22	0.8	0.23	0.24
NR6A1	0.39	0.52	0.57	0.78	0.44	1.65
NRADDP	0.08	0	0	0.16	0	0.23
NRAP	0	0	0	0	0	0

NRARP	0.98	0.68	1.23	0.79	1.4	1.98
NRAS	16.57	15.04	12.85	12.59	14.73	12.48
NRBF2	9.22	13.26	10.94	9.87	10.18	9.68
NRBP1	56.74	50.58	51.06	52.7	50.78	40
NRBP2	1.42	0.35	0.37	0.51	0.42	0.57
NRCAM	0.07	0	0.03	0.02	0	0
NRD1	36.46	34.05	33.97	31.33	31.73	33.51
NRDE2	1.88	3.82	1.95	2.8	2.57	3.4
NREP	11.29	28.5	21.47	17.48	21.52	24.15
NREP-AS1	0	0	0	0	0	0
NRF1	3.19	7.31	7.31	6.43	5.75	5.24
NRG1	0	0	0	0	0	0
NRG1-IT3	0	0	0	0	0	0
NRG2	0	0.08	0.04	0.04	0.02	0.04
NRG3	0	0	0	0	0	0
NRG4	0.27	0.69	0.28	0.37	0.1	0.31
NRGN	0.75	0.15	0.06	0.86	0.71	0.38
NRIP1	2.24	2.05	2.44	3.72	2.82	2.8
NRIP2	0.76	1	0.58	0.31	0.4	0.34
NRIP3	2.55	3.79	5.15	2.72	5.1	1.72
NRK	0	0	0	0	0	0
NRL	0.07	0.21	0.21	0.44	0.46	0.04
NRM	26.16	37.83	37.49	44.33	39.97	37.59
NRN1	8.4	15.47	13.07	12.82	12.53	12.67
NRN1L	0.51	0.51	0	0.62	0.12	0
NRON	0	0	0	0	0.05	0.03
NRP1	1.82	8.91	10.05	4.35	1.86	6.57
NRP2	0	0	0.14	0.06	0	0.17
NRSN1	0.18	0.38	0.44	0.14	0.26	0.24
NRSN2	14.23	6.05	0.65	9.63	10.07	10.58
NRTN	0.28	0.16	0.25	0.19	0.26	0.28
NRXN1	0	0	0	0	0.01	0
NRXN2	0.14	0.31	0.96	0.61	1.05	0.73
NRXN3	0.03	0.08	0.1	0.03	0.06	0.06
NS3BP	0.23	0.46	0	0.68	0.27	0.47
NSA2	63.54	64.42	64.47	60.74	83.53	57.32
NSD1	4.82	4.8	4.76	4.45	4.7	5
NSDHL	83.57	58.35	55.65	61.15	71.75	63.85
NSF	12.4	12.36	11.71	12.72	7.76	11.1
NSFL1C	27.47	31.64	29.73	27.36	30.46	22.4
NSFP1	0	0	0	0	0	0
NSG1	0	0	0.1	0	0.03	0.11
NSL1	2.62	2.42	2.06	2.3	2.44	2.34

NSMAF	23.35	32.51	25.29	23.24	28.38	32.51
NSMCE1	84.6	71.59	60.54	94.07	72.62	59.25
NSMCE2	52.09	40.09	36.85	36.71	42.71	31.5
NSMCE4A	19.96	32.64	26.98	33.53	28.64	21.15
NSMF	3.2	3.7	4.39	4.35	4.34	4
NSRP1	18.36	19.26	17.26	16.69	19.89	14.21
NSUN2	35.16	32.87	29.57	32.2	34.24	25.63
NSUN3	6.12	4.91	5.41	7.97	7.17	6.51
NSUN4	6.6	6.71	6.02	5.7	5.64	7.55
NSUN5	32.99	32.99	28.12	30.16	27.42	27.21
NSUN5P1	3.66	2.8	6.11	7.5	4.12	4.91
NSUN5P2	6.01	3.98	3.88	4.77	5.42	3.65
NSUN6	3.96	4.98	6.56	4.58	6.39	3.64
NSUN7	0	0.05	0	0	0	0
NT5C	57.67	58.54	52.07	60.73	71.15	56.78
NT5C1A	0	0	0	0.14	0	0.21
NT5C1B	0	0.08	0	0	0	0
NT5C1B-RDH	0	0	0.06	0	0	0
NT5C2	28.54	18.88	15.82	17.63	20.56	16.38
NT5C3	26.25	26.96	25.24	20.85	26.9	25.39
NT5C3B	24.83	30.34	26.12	26.89	33.78	25.27
NT5DC1	12.29	11.34	11.57	6.96	9.86	10.48
NT5DC2	19.09	13.46	15.34	23.5	26.37	19.72
NT5DC3	8.15	5.87	5.78	6.28	5.91	5.97
NT5E	0	0	0.02	0	0	0
NT5M	1.18	0.04	1.03	1.3	1.51	0.38
NTAN1	7.93	9.3	13.74	12.27	10.52	7.36
NTF3	0	0	0	0	0	0
NTF4	0	0	0	0	0	0
NTHL1	36.61	82.92	72.76	62.56	63.66	69.98
NTM	0	0	0	0	0	0
NTMT1	93.21	99.99	99.09	115.39	120.29	101.05
NTN1	0.18	0.25	0.35	0.33	0.34	0.17
NTN3	0	0	0	0	0	0
NTN4	0	0	0	0	0	0
NTN5	0	0	0	0	0	0
NTNG1	0	0	0	0	0	0
NTNG2	0.09	0.04	0.09	0.08	0.13	0.07
NTPCR	29.55	37.39	35.7	35.92	34.52	41.4
NTRK1	17.68	0.02	1.3	3.17	12.12	3.29
NTRK2	0.01	0	0	0	0	0
NTRK3	0	0	0	0	0	0
NTRK3-AS1	0	0	0	0	0	0

NTS	63.11	0.63	0.28	8.77	19.57	0.56
NTSR1	0	0.07	1.73	0.05	0	0.02
NTSR2	0	0	0	0	0	0
NUAK1	0	0	0.01	0	0	0.03
NUAK2	0.5	1.01	0.7	0.35	0.75	0.82
NUB1	10.2	7.06	6.13	7.64	9.08	9.69
NUBP1	28.12	38.67	28.25	39.77	36.32	31.58
NUBP2	103.14	129.96	116.23	141.26	127.11	119.61
NUBPL	3.3	4.95	3.95	4.02	4.63	3.43
NUCB1	52.41	28.69	34.96	41.75	36.49	34.93
NUCB2	29.12	94.29	60.66	44.55	46.36	73.48
NUCKS1	28.74	24.98	23.62	24.49	29.99	31.7
NUDC	196.33	205.85	193.7	182.65	180.97	177.74
NUDCD1	31.23	25.01	19.01	17.11	25.75	22.72
NUDCD2	49.45	41.01	42.1	43.26	41.91	40.54
NUDCD3	18.74	14.71	13.97	14.63	15.35	15.24
NUDT1	66.8	97.84	98.56	115.74	101.79	108.86
NUDT10	0.13	0.46	0.04	0.45	0.35	0.51
NUDT11	12.21	19.54	15.53	17.44	16.68	20.16
NUDT12	2.71	2.9	2.65	2.2	2.4	1.62
NUDT13	0.91	0.39	0.8	0.68	0.73	1.06
NUDT14	31.67	14.49	19.09	20.6	22.52	12.41
NUDT15	15.44	17.86	14.27	15.93	14.3	14.51
NUDT16	12.02	9.66	8.28	7.02	10.24	9.34
NUDT16L1	27.55	31.63	31.69	31.56	30.16	29
NUDT16P1	0	0.03	0	0	0	0
NUDT17	2.45	3.65	3.4	2.47	3.52	1.63
NUDT18	0.69	2.45	4.33	1.36	3.4	1.44
NUDT19	23.48	18.08	20.62	19.02	21.4	17.71
NUDT2	25.13	17.92	18.59	21.91	18.83	18
NUDT21	30.21	32.1	27.17	28.92	32.1	32.59
NUDT22	31.86	24.82	22.56	22.61	22.37	22.21
NUDT3	3.67	2.81	3.41	5.38	4.05	6.05
NUDT4	8.79	1.25	2.34	4.29	2.73	2.08
NUDT4P1	10.34	3.41	3.47	2.72	4.38	3.19
NUDT5	101.51	118.52	106.65	109.79	115.36	110.43
NUDT6	5.4	5.55	4.48	3.32	4.19	6.75
NUDT7	1.33	5.51	2.57	3.62	2.32	2.17
NUDT8	15.73	16.26	20.36	22.53	15.32	16.35
NUDT9	5.66	12.46	8.68	7.24	7.3	9.34
NUDT9P1	0	0	0	0.1	0	0.22
NUF2	14.32	13.14	10.38	10.8	16.34	12.26
NUFIP1	6.02	6.49	6.43	6.26	6.29	7.08

NUFIP2	5.32	5.84	4.9	3.84	4.27	6.01
NUGGC	0.4	0.94	0.74	0.41	0.44	0.61
NUMA1	9.74	12.46	11.85	14.01	12.11	14.97
NUMB	10.68	10.21	7.54	8.84	8.56	8.25
NUMBL	1.68	0.56	0.96	0.92	0.81	1.13
NUP107	40.19	35.39	24.93	28.3	29.14	27.51
NUP133	12.39	13.96	13.92	11.19	16.05	16.01
NUP153	7.86	7.54	8.21	6.68	8.51	6.47
NUP155	13.12	17.83	19.58	17.34	18.85	19.71
NUP160	13.47	18.05	15.31	13.91	13.38	20.46
NUP188	31.44	37.53	34.72	29.96	32.54	38.1
NUP205	13.84	16.48	15.93	14.43	14.71	17.97
NUP210	11.47	14.22	12.82	13.4	10.45	13.74
NUP210L	0	0.03	0	0.01	0	0.01
NUP210P1	0	0	0	0	0	0
NUP214	15.32	19.82	18.47	16.37	14.18	18.82
NUP35	28.81	17.27	19.07	25.21	25.76	20.78
NUP37	36.29	42.87	36.78	28.04	34.31	29.67
NUP43	20.52	18.05	17.52	18.16	20.5	17.18
NUP50	23.64	23.61	23.54	19.64	24.3	24.42
NUP54	25.92	28.91	25.79	24.92	26.98	25.03
NUP62	44.85	50.47	46.43	46.91	47.01	43.82
NUP62CL	3.66	3.2	2.91	3.02	4.59	4.08
NUP85	24.74	29.33	21.6	23.14	26.49	26.47
NUP88	41.25	42.34	33	39.18	40.43	39.8
NUP93	49.12	62.27	50.94	55.24	58.38	59.07
NUP98	23.16	21.42	21.95	21.23	22.34	19.5
NUPL1	16.4	10.72	8.77	8.8	13.74	10.86
NUPL2	15.24	16.11	9.75	9.07	12.99	12.95
NUPR1	4.71	0	0	0	0	0
NUPR1L	0.14	0.64	0	0.26	0.39	0.27
NUS1	3.84	3.62	2.97	2.43	3.74	3.65
NUSAP1	63.57	66.66	54.37	60.82	67.01	53.04
NUTF2	275.32	270	259.48	283.51	306.85	276.72
NVL	8.99	13.66	12.17	8.19	7.71	9.08
NWD1	0.26	0.42	0.32	0.28	0.36	0.43
NXF1	25.75	20.13	19.54	20.88	21.43	20.79
NXF2	0.02	0	0	0	0	0
NXF2B	0.02	0	0	0	0	0
NXF3	96.01	49.6	31.12	68.95	68.27	46.6
NXF4	0	0	0	0	0	0.02
NXF5	0	0	0	0	0	0
NXN	14.14	12.81	12.66	13.22	16.6	5.92

NXNL1	0	0	0	0	0	0
NXNL2	0.21	0.41	0.69	0.25	0.45	0.19
NXPE1	0	0	0	0	0	0
NXPE2	0	0	0	0	0	0
NXPE3	4.1	2.79	3.03	2.19	3.02	3.82
NXPE4	0	0	0	0	0	0
NXPH1	0	0	0	0	0	0
NXPH2	0	0	0	0	0	0
NXPH3	0	0	0	0.02	0	0.08
NXPH4	0.41	0.16	0.79	0.17	0.31	0.17
NXT1	44.21	74.6	54.94	58.58	58.33	54.96
NXT2	10.57	12.31	8.7	11.35	10.48	9.22
NYAP1	0	0	0	0	0	0
NYAP2	0.09	0.27	0.33	0.22	0.32	0.23
NYNRIN	0.4	0.04	0	0.69	0.78	0.23
NYX	0	0	0	0	0	0
OACYLP	0	0	0	0	0	0
OAF	1.11	2.01	1.24	1.32	0.64	1.13
OARD1	18.2	17.88	14.88	15.3	16.56	16.76
OAS1	0	0.74	0.98	0.49	0	0.5
OAS2	0.07	0.07	0.06	0.27	0	0.04
OAS3	0.39	0.45	0.39	0.45	0.3	0.31
OASL	3.11	0.72	0.2	1.94	1.1	0.71
OAT	159.58	65.77	61.07	74.68	102.11	67.17
OAZ1	1044.59	964.5	829.07	775.38	892.5	838.79
OAZ2	87.66	62.7	61.67	64.59	83.33	67.72
OAZ3	2.07	2	1.8	0.57	0.63	0.68
OBFC1	2.27	3.47	3.95	3.39	2.87	3.87
OBP2A	0	0	0	0	0	0
OBP2B	0	0	0	0	0	0
OBSCN	0.12	0.01	0	0.09	0.03	0.01
OBSL1	3.92	4.19	3.51	5.35	3.89	5.52
OC90	0	0	0	0	0	0
OCA2	0.21	0.04	1.51	0.29	0.11	0.23
OCEL1	6.24	5.36	9.81	8.81	7.91	3.46
OCIAD1	58.6	43.53	38.45	40.97	44.14	40.23
OCIAD2	97.56	79.16	73.52	73.95	89.05	90.49
OCLM	0.88	0.21	0	0.18	1.09	0.53
OCLN	2.03	4.79	3.64	3.76	2.97	4.55
OCM	0	0	0	0	0	0
OCM2	0	0	0	0	0	0
OCRL	5.34	3.8	4.18	4.64	4.86	4.27
OCSTAMP	0	0	0	0	0	0

ODAM	0.06	0	0	0	0	0
ODC1	57.91	64.1	63.78	66.55	64.56	52.06
ODF1	0	0	0	0	0	0
ODF2	11.73	15.34	12.86	11.77	10.21	13.43
ODF2L	6.59	7.7	7.26	4.09	5.52	6.57
ODF3	0	0	0	0	0	0
ODF3B	0	0	0	0	0	0
ODF3L1	0	0	0	0	0	0
ODF3L2	0	0	0.04	0	0	0
ODF4	0	0	0	0	0	0
OFD1	4.11	3.44	3.26	4.15	4.07	3.42
OGDH	33.54	25.45	23.33	24.15	27.26	25.27
OGDHL	0.75	2.28	0.89	1.67	1.56	1.06
OGFOD1	2.77	2.82	2.85	1.51	2.08	2.65
OGFOD2	10.57	16.09	13.08	11.76	13.63	13.15
OGFOD3	3.83	4.18	3.39	5.77	5.13	3.26
OGFR	3.34	3.87	4.38	4.41	5.01	4.97
OGFRL1	0	0.5	0.12	0.21	0.4	0.17
OGG1	10.8	11.01	10.94	11.41	10.52	9.36
OGN	0	0.1	0.1	0.02	0.1	0.16
OGT	17.67	18.15	12.43	16.05	15.92	18.49
OIP5	29.82	41.48	31.43	33.08	34.39	31.6
OIP5-AS1	25.24	25.95	24.83	22.25	26.75	24.52
OIT3	0.03	0	0	0	0	0
OLA1	38.14	31.15	31.24	39.1	37.57	38.71
OLAH	0.45	0.05	0.76	0.34	0.26	0.43
OLFM1	0	0.2	0.05	0	0	0.13
OLFM2	0.16	0.09	0.22	0.16	0.3	0.28
OLFM3	0	0	0	0	0	0
OLFM4	0	0	0	0	0	0
OLFML1	0.13	0.04	0.05	0.02	0	0.02
OLFML2A	0.22	0.45	0.45	0.29	0.37	0.31
OLFML2B	0.92	0	0.04	0.92	0.89	0.42
OLFML3	0	0	0	0	0	0
OLIG1	0.03	0.2	0.18	0	0	0.2
OLIG2	0.29	0.73	1.41	0.26	0.52	1.27
OLIG3	0	0	0	0	0	0
OLR1	0	0	0	0	0	0
OMA1	5.78	9.14	7.99	8.81	10.11	10.24
OMD	0.23	0.11	0.34	0.06	0.19	0.26
OMG	0	0.12	0	0.08	0	0
OMP	0	0	0	0	0	0
ONECUT1	0	0	0	0.02	0	0

ONECUT2	0.38	1.09	0.66	0.54	0.46	0.7
ONECUT3	0	0	0	0	0	0
OOEP	0	0	0	0	0	0
OPA1	10.96	8.34	8.36	7.72	8.06	10.13
OPA1-AS1	0	0	0.31	0	0	0
OPA3	5.76	5.54	6	3.88	6.02	5.28
OPALIN	0	0	0	0	0	0
OPCML	0	0	0	0	0	0
OPHN1	4.72	5.14	3.89	3.37	4.5	4.79
OPLAH	0	0	0	0	0	0
OPN1LW	0	0	0	0	0	0
OPN1MW	0	0	0	0	0	0
OPN1MW2	0	0	0	0	0	0
OPN1SW	0	0	0	0.14	0	0.07
OPN3	0.19	0.62	1.11	0.82	0.47	1.45
OPN4	0	0	0	0	0	0
OPN5	0	0	0	0	0	0
OPRD1	0	0	0	0	0	0
OPRK1	0.07	0.04	0.1	0.07	0.02	0.03
OPRL1	0.11	0.6	1.41	1.1	0.3	0.38
OPRM1	0.03	0.02	0.25	0.15	0.18	0.06
OPTC	0	0	0	0	0	0
OPTN	10.29	3.26	2.94	3.46	4.46	2.54
OR10A2	0	0	0	0	0	0
OR10A3	0	0	0	0	0	0
OR10A4	0.08	0.06	0	0	0	0.47
OR10A5	0	0	0	0.06	0	0
OR10A6	0	0	0	0	0	0
OR10A7	0	0	0	0	0	0
OR10AD1	0	0	0	0	0	0
OR10AG1	0	0	0	0	0	0
OR10C1	0	0	0	0	0	0
OR10G2	0	0	0	0	0	0
OR10G3	0	0	0	0	0	0
OR10G4	0	0	0	0	0	0
OR10G7	0	0	0	0	0	0
OR10G8	0	0	0	0	0	0
OR10G9	0	0	0	0	0	0
OR10H1	0	0	0	0	0	0
OR10H2	0	0	0	0	0	0
OR10H3	0	0	0	0	0	0
OR10H4	0	0	0	0	0	0
OR10H5	0	0	0	0	0	0

OR10J1	0	0	0	0	0	0
OR10J3	0	0	0	0	0	0
OR10J5	0	0	0	0	0	0
OR10K1	0	0	0	0	0	0
OR10K2	0	0	0	0	0	0
OR10P1	0	0	0	0	0	0
OR10Q1	0	0	0	0	0	0
OR10R2	0	0	0	0	0	0
OR10S1	0	0	0	0	0	0
OR10T2	0	0	0	0	0	0
OR10V1	0	0	0	0	0	0
OR10V2P	0	0	0	0	0	0
OR10W1	0	0	0	0	0	0
OR10X1	0	0	0	0	0	0
OR10Z1	0	0	0	0	0	0
OR11A1	0.1	0.1	0.24	0.18	0.12	0
OR11G2	0	0	0	0	0	0
OR11H1	0	0	0	0	0	0
OR11H12	0	0	0	0	0	0
OR11H2	0	0	0	0	0	0
OR11H4	0	0	0	0	0	0
OR11H6	0	0	0	0	0	0
OR11L1	0	0.31	0	0	0	0
OR12D2	0	0	0	0	0	0
OR12D3	0	0	0	0	0	0
OR13A1	0	0	0	0	0	0
OR13C2	0	0	0	0	0	0
OR13C3	0	0	0	0	0	0
OR13C4	0	0	0	0	0	0
OR13C5	0	0	0	0	0	0
OR13C8	0	0	0	0	0	0
OR13C9	0	0	0	0	0	0
OR13D1	0	0	0.13	0	0	0
OR13F1	0	0	0	0	0	0
OR13G1	0	0	0	0	0	0
OR13H1	0	0	0	0	0	0
OR13J1	0	0	0	0	0	0
OR14A16	0	0	0	0	0	0
OR14C36	0	0	0	0	0	0
OR14I1	0	0	0	0	0	0
OR14J1	0	0	0	0	0	0
OR1A1	0	0	0	0	0	0
OR1A2	0	0	0	0	0	0

OR1B1	0	0	0	0	0	0
OR1C1	0	0	0	0	0	0
OR1D2	0	0	0	0	0	0
OR1D4	0	0	0	0	0	0
OR1D5	0	0	0	0	0	0
OR1E1	0	0	0	0	0	0
OR1E2	0	0	0	0	0	0
OR1F1	0	0	0	0	0	0
OR1F2P	0	0	0	0	0	0
OR1G1	0	0	0	0	0	0
OR1I1	0	0	0	0	0	0
OR1J1	0	0	0	0	0	0
OR1J2	0	0	0	0	0	0
OR1J4	0	0	0	0	0	0
OR1K1	0	0	0	0	0	0
OR1L1	0.08	0	0	0	0	0
OR1L3	0	0	0	0	0	0
OR1L4	0	0	0	0	0	0
OR1L6	0	0	0	0	0	0
OR1L8	0	0	0	0	0	0
OR1M1	0	0	0	0	0	0
OR1N1	0	0	0	0	0	0
OR1N2	0	0	0	0	0	0
OR1Q1	0	0	0	0	0	0
OR1S1	0	0	0	0	0	0
OR1S2	0	0	0	0	0	0
OR2A1	0	0.11	0	0	0	0
OR2A12	0	0	0	0	0	0
OR2A14	0	0	0	0	0	0
OR2A2	0	0	0	0	0	0
OR2A20P	0	0.37	0.53	0	0.32	0.17
OR2A25	0	0	0	0	0	0
OR2A4	0.33	0	0	0	0	0
OR2A42	0	0.11	0	0	0	0
OR2A5	0	0	0	0	0	0
OR2A7	0	0	0	0	0	0
OR2A9P	0	0	0	0	0	0
OR2AE1	0	0	0	0	0	0
OR2AG1	0	0.22	0	0	0	0
OR2AG2	0.16	0.54	0.37	0	0.15	0.16
OR2AK2	0	0	0	0	0.29	0
OR2AP1	0	0	0	0	0	0
OR2AT4	1.12	0	0	0.61	0.68	0

OR2B11	0.08	0	0	0	0	0
OR2B2	0	0	0	0.22	0	0
OR2B3	0	0	0	0	0	0
OR2B6	0	0	0.08	0	0	0.08
OR2C1	0	0	0	0	0	0
OR2C3	0	0	0	0	0	0
OR2D2	0	0	0	0	0	0
OR2D3	0	0.12	0.14	0	0	0.16
OR2F1	0	0	0	0	0	0
OR2F2	0	0	0	0	0	0
OR2G2	0	0	0	0	0	0
OR2G3	0	0	0	0	0	0
OR2G6	0	0	0	0	0	0
OR2H1	0	0	0	0	0	0
OR2H2	0	0	0	0	0	0
OR2J2	0	0	0	0	0	0
OR2J3	0	0.2	0	0	0	0
OR2K2	0	0	0	0	0	0
OR2L13	0.04	0	0	0	0	0
OR2L1P	0	0	0.15	0.06	0	0
OR2L2	0	0	0	0	0	0
OR2L3	0	0.2	0	0	0	0
OR2L5	0	0	0	0	0	0
OR2L8	0	0	0	0	0	0
OR2M1P	0	0	0	0	0	0
OR2M2	0	0	0	0	0	0
OR2M3	0	0	0	0	0	0
OR2M4	0	0	0	0	0	0
OR2M5	0	0	0	0	0	0
OR2M7	0	0	0	0	0	0
OR2S2	0	0	0	0	0	0
OR2T1	0	0	0	0	0	0
OR2T10	0	0.07	0	0	0	0
OR2T11	0	0	0	0	0	0
OR2T12	0	0	0	0.17	0	0
OR2T2	0	0	0	0	0	0
OR2T27	0	0	0	0	0	0
OR2T29	0	0	0	0	0	0
OR2T3	0	0	0	0	0	0
OR2T33	0	0.06	0.22	0	0	0
OR2T34	0	0	0	0	0	0
OR2T35	0	0	0	0	0	0
OR2T4	0	0	0	0	0	0

OR2T5	0	0	0	0	0	0
OR2T6	0	0	0	0	0	0
OR2T8	0.16	0	0	0	0	0.08
OR2V1	0	0	0	0	0	0
OR2V2	0	0	0	0	0	0
OR2W1	0	0	0	0	0	0
OR2W3	0.74	0.52	0.82	1.3	0.7	0.74
OR2W5	0	0	0	0	0	0
OR2Y1	0	0	0	0	0	0
OR2Z1	0	0	0	0	0	0
OR3A1	0	0	0	0	0	0
OR3A2	0	0	0	0	0	0
OR3A3	0	0	0	0	0	0
OR3A4P	0	0	0	0	0	0
OR4A15	0	0	0	0	0	0
OR4A16	0	0	0	0	0	0
OR4A47	0	0	0	0	0	0
OR4A5	0	0	0	0	0	0
OR4B1	0	0	0	0	0	0
OR4C11	0	0	0	0	0	0
OR4C12	0	0	0	0	0	0
OR4C13	0	0	0	0	0	0
OR4C15	0	0	0	0	0	0
OR4C16	0	0	0	0	0	0
OR4C3	0	0	0	0	0	0
OR4C45	0	0	0	0	0	0
OR4C46	0	0	0	0	0	0
OR4C6	0	0	0	0	0	0
OR4D1	0	0	0	0	0	0
OR4D10	0	0	0	0	0	0
OR4D11	0	0	0	0	0	0
OR4D2	0	0	0	0	0	0
OR4D5	0	0	0	0	0	0
OR4D6	0	0	0	0	0	0
OR4D9	0	0	0	0	0	0
OR4E2	0	0	0	0	0	0
OR4F13P	0.04	0.03	0.03	0	0	0.07
OR4F15	0	0	0	0	0	0
OR4F16	0	0	0	0	0.02	0
OR4F17	0	0	0	0	0	0
OR4F21	0	0	0	0	0.01	0
OR4F29	0	0	0	0	0.02	0
OR4F3	0	0	0	0	0.02	0

OR4F4	0	0	0	0	0	0
OR4F5	0	0	0	0	0	0
OR4F6	0	0	0	0	0	0
OR4K1	0	0	0	0	0	0
OR4K13	0	0	0	0	0	0
OR4K14	0	0	0	0	0	0
OR4K15	0	0	0	0	0	0
OR4K17	0	0	0	0	0	0
OR4K2	0	0	0	0	0	0
OR4K5	0	0	0	0	0	0
OR4L1	0	0	0	0	0	0
OR4M1	0	0	0	0	0	0
OR4M2	0	0	0	0	0	0
OR4N2	0	0	0	0	0	0
OR4N3P	0	0	0	0	0	0
OR4N4	0	0	0	0	0	0
OR4N5	0	0	0	0	0	0
OR4P4	0	0	0	0	0	0
OR4Q3	0	0	0	0	0	0
OR4S1	0	0	0	0	0	0
OR4S2	0	0	0	0	0	0
OR4X1	0	0	0	0	0	0
OR4X2	0	0	0	0	0	0
OR51A2	0	0	0	0	0	0
OR51A4	0	0	0	0	0	0
OR51A7	0	0	0	0	0	0
OR51B2	0	0	0	0	0	0
OR51B4	0	0	0	0	0	0
OR51B5	0.1	0	0	0	0	0
OR51B6	0	0	0	0	0	0
OR51D1	0	0	0	0	0	0
OR51E1	0	0	0	0	0	0
OR51E2	0.27	0.34	0.38	0.45	0.34	0.18
OR51F1	0	0	0	0	0	0
OR51F2	0	0	0	0	0	0
OR51G1	0	0	0	0	0	0
OR51G2	0	0	0	0	0	0
OR51I1	0	0	0	0	0	0
OR51I2	0	0	0	0	0	0
OR51L1	0	0	0	0	0	0
OR51M1	0	0	0	0	0	0
OR51Q1	0	0	0	0	0	0
OR51S1	0	0	0	0	0	0

OR51T1	0	0	0	0	0	0
OR51V1	0	0	0	0	0	0
OR52A1	0	0	0	0	0	0
OR52A5	0	0	0	0	0	0
OR52B2	0	0	0	0	0	0
OR52B4	0	0	0	0	0	0
OR52B6	0	0	0	0	0.07	0
OR52D1	0	0	0	0	0	0
OR52E2	0	0	0	0	0	0
OR52E4	0	0	0	0	0	0
OR52E6	0	0	0	0	0	0
OR52E8	0	0	0	0	0	0
OR52H1	0	0	0	0	0	0
OR52I1	0	0	0.07	0	0	0
OR52I2	0	0	0	0	0	0
OR52J3	0	0	0	0	0	0
OR52K1	0	0	0	0	0	0
OR52K2	0	0	0	0	0	0
OR52L1	0	0	0	0	0	0
OR52M1	0	0	0	0	0	0
OR52N1	0	0	0	0	0	0
OR52N2	0	0	0	0	0	0
OR52N4	0	0.06	0	0	0	0
OR52N5	0	0	0	0	0	0
OR52R1	0	0.06	0	0	0	0
OR52W1	0	0	0	0	0	0
OR56A1	0	0	0	0	0	0
OR56A3	0	0	0	0	0	0
OR56A4	0	0	0	0	0	0
OR56A5	0	0	0	0	0	0
OR56B1	0	0	0	0	0	0
OR56B4	0	0	0	0	0	0
OR5A1	0	0	0	0	0	0
OR5A2	0	0	0	0	0	0
OR5AC2	0	0	0	0	0	0
OR5AK2	0	0	0	0	0	0
OR5AK4P	0	0.07	0	0	0	0
OR5AN1	0	0	0	0	0	0
OR5AP2	0	0	0	0	0	0
OR5AR1	0	0	0	0	0	0
OR5AS1	0	0	0	0	0	0
OR5AU1	0	0	0	0	0	0
OR5B12	0	0	0	0	0	0

OR5B17	0	0	0	0	0	0
OR5B2	0	0	0	0	0	0
OR5B21	0	0	0	0	0	0
OR5B3	0	0	0	0	0	0
OR5C1	0	0	0	0	0	0
OR5D13	0	0	0	0	0	0
OR5D14	0	0	0	0	0	0
OR5D16	0	0	0	0	0	0
OR5D18	0	0	0	0	0	0
OR5E1P	0	0	0	0	0	0
OR5F1	0	0	0	0	0	0
OR5H1	0	0	0	0	0	0
OR5H14	0	0	0	0	0	0
OR5H15	0	0	0	0	0	0
OR5H2	0	0	0	0	0	0
OR5H6	0	0	0	0	0	0
OR5I1	0	0	0	0	0	0
OR5J2	0	0	0	0	0	0
OR5K1	0	0	0	0	0	0
OR5K2	0	0	0	0	0	0
OR5K3	0	0	0	0	0	0
OR5K4	0	0	0	0	0	0
OR5L1	0	0	0	0	0	0
OR5L2	0	0	0	0	0	0
OR5M1	0	0	0	0	0	0
OR5M10	0	0	0	0	0	0
OR5M11	0	0	0	0	0	0
OR5M3	0	0	0	0	0	0
OR5M8	0	0	0	0	0	0
OR5M9	0	0	0	0	0	0
OR5P2	0	0	0	0	0	0
OR5P3	0	0	0	0	0	0
OR5R1	0	0	0	0	0	0
OR5T1	0	0	0	0	0	0
OR5T2	0	0	0	0	0	0
OR5T3	0	0	0	0	0	0
OR5V1	0	0	0	0	0	0
OR5W2	0	0	0	0	0	0
OR6A2	0	0.04	0	0	0	0
OR6B1	0	0	0	0	0	0
OR6B2	0	0	0	0	0	0
OR6B3	0	0	0	0	0	0
OR6C1	0	0	0	0	0	0

OR6C2	0	0	0	0	0	0
OR6C3	0	0	0	0	0	0
OR6C4	0	0	0	0	0	0
OR6C6	0	0	0	0	0	0
OR6C65	0	0	0	0	0	0
OR6C68	0	0	0	0	0	0
OR6C70	0	0	0	0	0	0
OR6C74	0	0	0	0	0	0
OR6C75	0	0	0	0	0	0
OR6C76	0	0	0	0	0	0
OR6F1	0	0	0	0	0	0
OR6K2	0	0	0	0	0	0
OR6K3	0	0	0	0	0	0
OR6K6	0	0	0	0	0	0
OR6M1	0	0	0	0	0	0
OR6N1	0	0	0	0	0	0
OR6N2	0	0	0	0	0	0
OR6P1	0	0	0	0	0	0
OR6Q1	0	0	0	0	0	0
OR6S1	0	0	0	0	0	0
OR6T1	0	0	0	0	0	0
OR6V1	0	0	0	0	0	0
OR6W1P	0	0	0	0.03	0	0
OR6X1	0	0	0	0	0	0
OR6Y1	0	0	0	0	0	0
OR7A10	0	0	0	0	0	0
OR7A17	0	0	0	0	0	0
OR7A5	0	0	0	0	0	0
OR7C1	0	0	0	0	0	0
OR7C2	0	0	0	0	0	0
OR7D2	1.5	1.55	2	0.75	1.07	1.36
OR7D4	0	0	0	0	0	0
OR7E12P	0.22	0.1	0.36	0.33	0.07	0.28
OR7E14P	0.23	0.52	1.46	0.59	0.75	0.93
OR7E156P	0.03	0.02	0.03	0.13	0.05	0.03
OR7E24	0	0	0	0	0	0
OR7E2P	0.23	0.18	0	0	0	0
OR7E37P	0.74	0.16	0.36	0.21	0.32	0.56
OR7E5P	0	0.03	0.07	0	0.03	0.06
OR7E91P	0	0.1	0	0.04	0.12	0
OR7G1	0	0	0	0	0	0
OR7G2	0	0	0	0	0	0
OR7G3	0	0	0	0	0	0

OR8A1	0	0	0	0	0	0
OR8B12	0	0	0	0	0	0
OR8B2	0	0	0	0	0	0
OR8B3	0	0	0	0	0	0
OR8B4	0	0	0	0	0	0
OR8B8	0	0	0	0	0	0
OR8D1	0	0	0	0	0	0
OR8D2	0	0	0	0	0	0
OR8D4	0	0	0	0	0	0
OR8G1	0	0	0	0	0	0
OR8G2	0	0	0	0	0	0
OR8G5	0.07	0	0	0	0	0
OR8H1	0	0	0	0	0	0
OR8H2	0	0	0	0	0	0
OR8H3	0	0	0	0	0	0
OR8I2	0	0	0	0	0	0
OR8J1	0	0	0	0	0	0
OR8J3	0	0	0	0	0	0
OR8K1	0	0	0	0	0	0
OR8K3	0	0	0	0	0	0
OR8K5	0	0	0	0	0	0
OR8S1	0	0	0	0	0	0
OR8U1	0	0	0	0	0	0
OR8U8	0	0	0	0	0	0
OR9A2	0	0	0	0	0	0
OR9A4	0	0	0	0	0	0
OR9G1	0	0	0	0	0	0
OR9G4	0	0	0	0	0	0
OR9G9	0	0	0	0	0	0
OR9I1	0	0	0	0	0	0
OR9K2	0	0	0	0	0	0
OR9Q1	0	0	0	0	0	0
OR9Q2	0	0	0	0	0	0
ORAI1	6.48	9.35	10.4	7.87	7.4	8.97
ORAI2	5.93	6.19	6.4	5.82	5.78	5.39
ORAI3	1.48	1.96	2.68	1.99	2	3.61
ORAOV1	1.51	3.15	1.88	1.52	1.78	2.99
ORC1	29.77	30.77	26.87	29.62	25.35	32.72
ORC2	10.13	10.52	8.74	8.54	9.87	10.35
ORC3	22.54	19.83	18.82	20.67	21.05	17.24
ORC4	16.5	14.96	15.13	12.43	14.72	15.35
ORC5	23.42	26.04	26.53	24.13	24.07	26.25
ORC6	33.87	35.84	28.33	28.81	32.78	29.6

ORM1	0	0	0	0	0	0
ORM2	0.09	0	0	0	0	0
ORMDL1	21.95	23.51	20.92	13.87	21.1	18.08
ORMDL2	19.87	22.34	15.91	18.69	24.06	20.57
ORMDL3	7.13	5.21	3.6	6.5	5.2	5.3
OS9	37.8	42.08	39.53	36.86	34.65	47.4
OSBP	3.13	2.68	2.51	2.47	2.82	2.28
OSBP2	4.28	1.12	1.28	1.84	2.58	1.47
OSBPL10	0.23	0.01	0.02	0	0.02	0.04
OSBPL10-AS	0	0	0	0	0	0
OSBPL11	1.59	1.05	1.28	1.17	1.01	0.74
OSBPL1A	3.92	5.71	3.57	4.77	3.31	5.57
OSBPL2	6.92	5.12	4.8	5.54	5.31	5.62
OSBPL3	4.92	2.48	2.28	2.75	4.17	3.42
OSBPL5	1.98	2.57	2.1	2.63	2.36	2.55
OSBPL6	6.12	2.61	4.05	2.77	3.07	3.71
OSBPL7	2.12	2.42	2.91	2.7	2.41	2.21
OSBPL8	11.23	6.82	5.77	5.25	8.8	7.13
OSBPL9	27.7	19.44	15.73	16.47	16.18	15.75
OSCAR	0.49	0.21	0.55	0.29	0.36	0.27
OSCP1	0	0.08	0	0	0	0
OSGEP	28.41	42.14	39.23	34.37	32.79	38.57
OSGEPL1	2.82	4.72	4.15	4.9	3.52	5.01
OSGIN1	7.25	3.91	2.02	2.07	3.28	0.77
OSGIN2	1.82	1.7	1.26	1.58	1.61	1.67
OSM	0.6	4.57	1.72	4.08	3.1	3.64
OSMR	0.69	0.33	0.47	0.48	0.39	0.51
OSR1	0.51	0	0	0.35	0.33	0
OSR2	12.99	20.84	18.79	16.91	15.18	17.47
OST4	357.53	286.96	263.8	325.98	364.84	271.1
OSTC	156.79	166.82	160.46	156.86	157.35	151.47
OSTCP1	0.07	0.13	0.06	0.09	0	0.07
OSTF1	46.28	27.78	31.25	22.68	29.85	25.97
OSTM1	6.56	3.01	2.29	2.59	3.38	2.44
OSTN	0	0	0	0	0	0
OTC	0	0	0	0	0	0
OTOA	0	0	0	0	0	0
OTOF	0	0	0	0	0	0
OTOGL	0	0	0	0	0	0
OTOL1	0	0	0	0	0	0
OTOP1	0	0	0	0	0	0
OTOP2	0	0	0	0	0	0
OTOP3	0	0	0	0	0	0

OTOR	0	0	0	0	0	0
OTOS	0	0	0	0	0	0
OTP	0	0	0	0	0	0.06
OTUB1	64.72	59.87	57.69	57.18	62.31	54.27
OTUB2	1.29	0.84	0.6	0.71	0.6	0.81
OTUD1	0.09	0.24	0.32	0.16	0.24	0.14
OTUD3	0.58	0.74	0.29	0.44	0.6	0.47
OTUD4	1	1.33	1.08	0.57	1.22	0.88
OTUD5	20.07	10.67	11.24	14.14	16.05	11.81
OTUD6A	0.99	0.76	0.85	0.69	0.22	1.3
OTUD6B	8.36	7.71	6.2	4.41	6.43	6.38
OTUD7A	0.02	0	0.09	0.1	0.02	0.05
OTUD7B	0.82	0.56	0.47	0.48	0.29	0.78
OTX1	4.91	6.92	7.44	6.92	6.1	6.6
OTX2	0	0	0	0	0	0
OTX2-AS1	0.14	0.23	0.26	0.07	0.04	0.16
OVCA2	50.18	41.68	32.53	35.45	41.56	33.18
OVCH1	0	0	0	0	0	0
OVCH2	0	0	0	0	0	0
OVGP1	0	0.21	0	0.05	0	0.5
OVOL1	0.05	0	0	0	0	0
OVOL2	0	0	0	0	0.23	0.58
OVOL3	0.69	0	0.32	0.24	0	0.35
OXA1L	101.85	120.55	106.46	120.49	127.29	123.71
OXCT1	24.45	29.09	21.05	20.35	21.43	22.33
OXCT2	0	0.1	0.26	0.08	0.23	0.21
OXER1	0.59	1.33	1.81	1.6	1.39	2.64
OXGR1	0	0	0	0	0	0
OXLD1	16.75	17.48	14.36	22.33	18.3	13.98
OXNAD1	6.48	7.24	6.87	5.32	5.3	4.27
OXR1	6.92	7.4	5.07	4.49	6.65	5.29
OXSM	22.44	18.52	16.51	15.43	17.14	17.41
OXSR1	4.54	2.89	1.89	4.29	4.54	3.49
OXT	1.63	5.94	3.43	4.32	4.66	6.58
OXTR	0.03	0.25	0.17	0.06	0.17	0.29
P2RX1	7.29	5.7	7.89	5.84	6.56	4.8
P2RX2	0.05	0	0	0	0	0
P2RX3	0	0	0	0.04	0	0
P2RX4	8.78	9.99	9.79	8.77	9.35	9.39
P2RX5	4.6	4.07	4.26	4.5	5.46	5.02
P2RX5-TAX1I	2.33	2.49	1.9	1.96	2.06	1.74
P2RX6	0.05	0	0	0	0	0
P2RX6P	0	0	0	0	0	0

P2RX7	0.19	0.2	0.71	0.45	0.31	0.26
P2RY1	7.87	3.03	2.9	3.88	4.73	2.51
P2RY10	0	0	0	0	0	0
P2RY11	0	0	1.78	0.47	0	0.45
P2RY12	0	0.04	0	0	0	0
P2RY13	0	0	0	0	0	0.05
P2RY14	0.97	0	0.08	0.04	0.47	0.14
P2RY2	1.67	13.51	12	4.14	4.74	10.19
P2RY4	0	0	0	0	0	0
P2RY6	2.5	14	16.73	8.64	4.93	11.91
P2RY8	6.68	35.7	29.81	16.83	16.72	37.57
P4HA1	17.61	18.78	19	15.19	15.22	17.19
P4HA2	13.33	5.78	8.57	5.79	6.11	7.12
P4HA3	0	0	0	0	0	0
P4HB	264.13	263.12	222.12	224	240.37	308.76
P4HTM	4.74	5.03	2.98	3.53	3.57	4.65
PA2G4	163.69	186.8	177.92	184.68	189.15	168.28
PA2G4P4	0	0.11	0.18	0	0.03	0
PAAF1	24.23	24.69	25.14	27.97	26.37	27.63
PABPC1	1051.44	1169.37	1338.55	1389.46	1228.59	1486.76
PABPC1L	0.62	1.45	0.19	0.76	0.56	0.88
PABPC1L2A	0	0	0	0	0	0
PABPC1L2B	0	0	0	0	0	0
PABPC1P2	0.67	0.94	1.1	0.45	0.74	1.07
PABPC3	0.31	0.7	0.5	0.7	0.52	0.82
PABPC4	18.38	16.35	22.97	23.48	19.76	25.57
PABPC4L	0	0.17	0.13	0.1	0.01	0.13
PABPC5	0	0	0	0	0	0
PABPN1	1	1.45	0.76	1.73	1.78	0.69
PABPN1L	0	0.05	0.11	0	0	0
PACRG	0	0	0.04	0	0	0.05
PACRG-AS1	0	0	0	0	0	0
PACRGL	1.94	2.25	2.32	2.09	1.34	2.14
PACS1	3.92	3.92	3.93	3.78	4.67	3.78
PACS2	2.19	2.85	2.92	2.32	2.39	2.42
PACSIN1	0	0	0	0	0	0
PACSIN2	13.12	8.34	8.09	8.5	12.87	11.32
PACSIN3	3.17	7.37	7.95	7.9	7.22	9.04
PADI1	0	0	0	0	0	0
PADI2	0	0	0	0	0	0
PADI3	0	0	0	0	0	0
PADI4	0	0	0	0	0	0
PADI6	0	0	0	0	0	0

PAEP	0	0	0	0	0	0
PAF1	26.65	26.82	22.34	27.59	21.93	20.56
PAFAH1B1	7.86	5.57	5.86	5.64	7.43	8.14
PAFAH1B2	48.78	45.42	49.03	38.72	48.61	44.71
PAFAH1B3	110.61	105.8	103.83	128.76	123.17	94.36
PAFAH2	2.22	1.96	2.96	3.43	3.52	2.54
PAG1	0.22	0.13	0.16	0.1	0.1	0.09
PAGE1	0	0	0	0	0	0
PAGE2	0	0	0.16	0	0.21	0
PAGE2B	4.12	0	0	1.61	2.03	0
PAGE3	0	0	0	0	0	0
PAGE4	0	0	0	0	0	0
PAGE5	0.15	0	0	0	0	0
PAGR1	11.83	19.1	19.99	16.6	15.74	12.1
PAH	0	0	0	0	0	0
PAICS	120.7	135.42	121.14	123.37	132.84	129.25
PAIP1	11.83	11.64	12.77	9.91	12.44	10.36
PAIP2	114.4	126.04	129.83	132.91	131.22	146.32
PAIP2B	1.22	0.48	0.2	0.34	0.59	0.63
PAK1	6.37	11.41	10.59	11.38	10.93	10.96
PAK1IP1	39.34	47.04	32.42	35.39	36.19	32.27
PAK2	17.7	19.06	17.15	16.01	15.73	18.05
PAK3	0	0	0	0	0	0
PAK4	0.94	0.78	1.51	1.42	1.33	0.77
PAK6	0.02	0.15	0.18	0.05	0	0.04
PAK7	0	0	0	0	0	0
PALB2	4.32	4.13	5.7	4.84	4.07	5.36
PALD1	3.33	3.53	3.79	3.45	5.39	4.51
PALLD	0.02	0.27	0.01	0.15	0.09	0.03
PALM	1.79	2.17	2.54	3.15	2.46	2.24
PALM2	0.05	0.05	0.07	0.05	0.11	0.09
PALM2-AKAF	0	0	0	0	0	0
PALM3	0.03	0.33	1.4	1.25	0.61	1.68
PALMD	0.51	0	0	0	0	0
PAM	18.16	5.07	5.16	7.56	6.62	6.84
PAM16	72	55.97	49.46	53.42	64.2	48.64
PAMR1	0	0	0	0	0	0
PAN2	2.6	3.32	2.8	3.37	3.05	2.59
PAN3	4.74	3.91	2.8	3.27	3.83	3.77
PAN3-AS1	0.07	0	0.25	0	0.06	0.17
PANK1	0.65	1.04	1.31	0.8	0.7	0.99
PANK2	7.11	6.37	4.58	4.97	6.9	5.77
PANK3	8.71	5.81	5.03	3.73	6.6	4.5

PANK4	8.61	9.25	7.38	7.48	9.59	6.7
PANX1	2.16	2.04	1.67	2.32	1.84	1.18
PANX2	1.01	0.21	0.32	0.81	0.63	0.77
PANX3	0	0	0	0	0	0
PAOX	0.22	0.66	1.1	1.07	0.75	0.5
PAPD4	18.8	12.69	7.75	9.37	10.96	9.27
PAPD5	2.1	1.47	1.73	1.52	1.58	1.91
PAPD7	1.22	1.16	1.33	0.88	1.06	1.04
PAPL	0.47	0.58	1.19	0.27	0.34	0.59
PAPLN	0.25	0.61	0.62	0.72	0.55	0.51
PAPOLA	5.75	6.2	4.83	4.44	5.7	4.89
PAPOLB	0	0	0	0	0	0
PAPOLG	3.24	3.2	3.82	3.1	3.63	3.22
PAPPA	0.08	0.03	0.02	0.08	0.08	0.03
PAPPA2	0	0	0	0.06	0	0
PAPSS1	24.67	19.56	13.93	12.23	12.83	12.83
PAPSS2	0.04	0	0.02	0	0	0
PAQR3	0.76	0.67	0.66	0.73	0.91	1.2
PAQR4	3.49	6.49	8.29	7.14	7.52	7.13
PAQR5	0.08	0.1	0.17	0.11	0.01	0.17
PAQR6	0.05	0.07	0.13	0.22	0.1	0
PAQR7	0.93	0.29	0.36	0.88	0.87	0.47
PAQR8	2.09	1.04	1.08	2.33	1.26	1.26
PAQR9	0.07	0.22	0.19	0.14	0.26	0.14
PAR-SN	0.55	0.54	1.22	0.77	0.55	0.3
PAR1	0	0	0	0	0	0
PAR4	0	0	0	0	0	0
PAR5	0	0	0	0	0	0
PARD3	3.61	2.78	3.33	2.85	2.69	2.28
PARD3B	0.9	0.01	0	0.03	0.19	0
PARD6A	1.08	2.03	2.09	2.11	1.19	2.55
PARD6B	3.07	2.86	3.74	2.41	3.65	3.41
PARD6G	0.96	1.27	2.41	1	1.11	1.47
PARD6G-AS1	0.3	0.05	0.3	0	0	0.33
PARG	4.51	4.18	4.08	5.22	3.45	6.18
PARK2	0.43	0.35	0.68	0.42	0.25	0.61
PARK7	447.97	422.67	384.93	409.24	453.91	403.76
PARL	52.38	66.07	55.99	56.83	54.93	59.76
PARM1	0	0.01	0.12	0	0.04	0
PARN	19.74	16.67	16.19	15.47	18.78	17.17
PARP1	48.82	53.59	43.23	51.86	44.29	43.54
PARP10	7.49	4.39	5.54	6.09	3.48	3.41
PARP11	0.65	1.21	1.63	1.23	1.04	1.2

PARP12	2.62	1.6	1.33	1.29	1.86	1.43
PARP14	6.83	9.43	8.2	7.37	7.17	6.03
PARP15	0	0	0	0	0	0
PARP16	7.92	14	15.02	14.69	11.98	17.52
PARP2	22.12	20.5	19.9	18.68	20.73	21.62
PARP3	0.81	0.93	0.69	0.75	1.91	0.88
PARP4	12.45	11.22	10.17	9.46	7.73	11.75
PARP6	5.55	4.52	4.92	3.79	4.08	3.47
PARP8	2.12	3.24	2.01	1.6	1.46	3.19
PARP9	4.86	5.1	4.1	3.23	3.38	3.99
PARPBP	9.02	10.59	7.68	7.18	9.84	10.43
PARS2	3.82	6.83	8.48	5.84	4.56	5.88
PART1	0.41	0.51	0.33	0.1	0.03	0.47
PARVA	0.03	0.05	0.02	0.02	0.02	0.04
PARVB	10.42	7.91	9.57	10.88	11.11	9.98
PARVG	5.34	32.6	27.37	18.55	15.68	30.19
PASD1	0	0	0	0	0	0
PASK	3.86	5.02	4.34	3.96	4.73	5.52
PATE1	0	0	0	0	0	0
PATE2	0.06	0	0	0	0.07	0.1
PATE3	0	0	0	0	0	0
PATE4	0.08	0.36	0.22	0.17	0.19	0.3
PATL1	4.51	5.55	4.07	3.28	3.89	4.67
PATL2	0	0.15	0.1	0.08	0	0.19
PATZ1	3.31	3.36	4.46	4.81	3.39	3.13
PAWR	1.36	2.06	3.19	1.83	2.08	1.15
PAX1	0	0	0	0	0	0
PAX2	0	0	0	0.01	0	0.07
PAX3	0	0	0	0	0	0
PAX4	0	0	0	0	0	0
PAX5	3.02	7.42	7.22	5.34	5.83	9.88
PAX6	1.14	1.41	1.68	1.34	0.94	2.02
PAX7	0	0	0	0	0	0
PAX8	0.98	1.37	0.95	1.05	0.73	0.87
PAX9	1.2	1.53	2.32	1.85	1.66	1.21
PAXBP1	1.1	1.31	0.93	1.15	1.1	0.95
PAXBP1-AS1	1.05	0.99	1.18	0.68	1.02	1.19
PAXIP1	2.7	2.85	3.36	3.92	3.94	4.39
PBDC1	62.2	64.46	46.47	47.1	58.58	27.3
PBK	44.58	22.5	17.45	22.9	33.95	22.73
PBLD	0.91	0.5	0.89	0.44	0.69	1.36
PBOV1	0.3	0.29	0.42	0.37	0.3	0.36
PBRM1	3.93	3.59	3.32	3.84	4.52	3.78

PBX1	0	0	0	0	0	0
PBX2	2.08	2.14	1.82	1.74	2.05	3.09
PBX3	0.44	2	1.63	1.2	1.77	1.53
PBX4	0.09	0.2	0.21	0.44	0.24	0.09
PBXIP1	4.96	5.02	3.9	4.48	3.57	4.15
PC	1.5	2.13	3.23	5.05	3.27	4.84
PCA3	0.1	0.18	0.23	0.04	0.15	0.08
PCAT1	0.42	0.41	0.29	0.37	0.13	0.53
PCBD1	68.78	54.99	55.15	56.29	65.87	48.91
PCBD2	10.57	8.65	6.61	6.22	6.75	7.64
PCBP1	137.69	87.96	76.52	101.53	125.32	77.82
PCBP1-AS1	0.12	0.25	0.28	0.21	0.32	0.19
PCBP2	96.14	100.62	117.56	119.04	114.52	126.56
PCBP3	0	0	0	0	0	0.12
PCBP4	6.51	6.27	5.25	6.2	4.98	4.76
PCCA	9.54	8.35	9.21	9.84	9.02	8.86
PCCA-AS1	0.18	0	0	0	0	0.19
PCCB	77.51	77.21	60.35	62.62	86.53	70.38
PCDH1	0.04	0	0	0.05	0.11	0
PCDH10	0	0	0	0	0	0
PCDH11X	1.58	3.61	3.63	3.84	3.32	2.77
PCDH11Y	0.88	1.1	1.53	0.58	0.97	1.1
PCDH12	0	0	0	0	0	0
PCDH15	0	0	0	0	0	0
PCDH17	0	0	0	0	0	0
PCDH18	0	0	0	0	0	0
PCDH19	0	0	0	0	0	0
PCDH20	0	0	0	0	0	0
PCDH7	0	0.01	0	0	0	0
PCDH8	0	0	0	0	0	0
PCDH9	0.05	0	0.06	0	0.02	0.04
PCDH9-AS2	0	0	0	0	0	0
PCDH9-AS3	0	0	0	0	0	0
PCDHA1	0.03	0	0	0	0	0
PCDHA10	0	0.02	0	0	0	0
PCDHA11	0	0	0	0	0	0
PCDHA12	0	0	0	0	0	0
PCDHA13	0	0	0	0	0	0
PCDHA2	0	0.07	0.13	0	0	0
PCDHA3	0.24	0.3	0.3	0.59	0.11	0.3
PCDHA4	0	0	0.16	0	0	0
PCDHA5	0	0	0	0	0	0
PCDHA6	0	0	0	0	0	0

PCDHA7	0	0	0	0	0	0
PCDHA8	0	0	0	0	0	0
PCDHA9	0.25	0.47	0.19	0.25	0.27	0.21
PCDHAC1	0	0	0	0	0	0
PCDHAC2	0	0	0	0	0	0
PCDHB1	0	0	0	0	0	0
PCDHB10	0.02	0.04	0	0.11	0.02	0
PCDHB11	0.04	0	0.14	0.02	0.04	0.08
PCDHB12	0	0	0	0	0	0
PCDHB13	0.07	0.09	0	0	0	0
PCDHB14	0	0	0	0	0	0.08
PCDHB15	0	0.02	0	0	0	0
PCDHB16	0	0.04	0.07	0.02	0.12	0.02
PCDHB17	0	0	0	0	0	0
PCDHB18	0	0	0	0	0	0
PCDHB19P	0.08	0.08	0.13	0.07	0.06	0.02
PCDHB2	0.03	0	0	0	0	0
PCDHB3	0	0	0	0	0	0.09
PCDHB4	0	0	0	0	0	0
PCDHB5	0	0	0	0	0	0
PCDHB6	0	0	0	0	0	0
PCDHB7	0	0	0	0	0	0
PCDHB8	0	0.42	0.11	0	0	0
PCDHB9	0.54	0.5	0.67	0.22	0.64	0.5
PCDHGA1	0	0	0	0	0	0
PCDHGA10	1.43	1.65	1.55	1.81	0.84	1.13
PCDHGA11	0.1	0.35	0	0.06	0.12	0
PCDHGA12	0	0	0.15	0	0	0
PCDHGA2	0	0	0	0	0	0
PCDHGA3	0	0	0	0	0	0
PCDHGA4	0.12	0	0	0	0	0
PCDHGA5	0	0.09	0	0	0	0.06
PCDHGA6	0	0.05	0	0	0	0
PCDHGA7	0.03	0	0	0	0	0
PCDHGA8	0	0	0	0	0.02	0
PCDHGA9	0.03	0.15	0	0	0	0.03
PCDHGB1	0	0.07	0	0	0	0
PCDHGB2	0	0.14	0	0	0.11	0.49
PCDHGB3	0	0.05	0.11	0	0	0
PCDHGB4	0	0	0	0	0	0
PCDHGB5	0	0	0	0	0	0.03
PCDHGB6	0	0	0	0	0	0
PCDHGB7	0	0.06	0	0	0.03	0

PCDHGB8P	0	0	0	0	0.07	0
PCDHGC3	0	0	0	0	0	0
PCDHGC4	0	0	0.08	0	0	0
PCDHGC5	0	0	0.03	0	0	0.03
PCDP1	0	0	0	0	0.13	0
PCED1A	2.46	2.75	3.09	2.28	2.89	2.9
PCED1B	3.48	0.31	0.78	0.51	0.94	0
PCED1B-AS1	1.03	0.39	0.53	0.35	0.63	0.36
PCF11	4.35	5.16	4.59	4.38	4.3	5.59
PCGEM1	0	0	0	0	0	0
PCGF1	13.12	13.6	17.49	12.8	13	10.13
PCGF2	0.73	0.3	0.77	0.64	1.06	0.89
PCGF3	2.73	2.36	1.83	1.97	1.8	2.57
PCGF5	20.97	13.47	14.82	12.42	20.99	18.16
PCGF6	9.44	11.51	5.47	8.8	10.46	9.35
PCID2	33.79	44.02	37.02	31.78	36.24	32.89
PCIF1	18.57	27.03	25.37	25.19	24.33	26.01
PCK1	0	0	0	0	0	0
PCK2	29.03	34.56	27.62	20.93	27.55	29.3
PCLO	0	0.01	0	0	0	0.01
PCM1	15.41	16	12.26	11.36	12.35	13.96
PCMT1	166.99	86.42	93.19	100.8	133.06	81.15
PCMTD1	4.32	1.96	1.74	1.75	1.96	2.26
PCMTD2	5.44	2.72	2.46	2.91	2.89	3.09
PCNA	201.21	249.43	210.07	219.04	219.36	238.11
PCNA-AS1	0	0	0	0	0	0
PCNAP1	0	0	0	0	0	0
PCNP	50.73	29.27	21.84	22.15	39.27	32.91
PCNT	3.39	2.44	2.65	2.96	3.3	3.18
PCNX	0.69	1.19	1.06	0.66	0.85	1.47
PCNXL2	0.17	0.01	0.06	0.06	0	0.06
PCNXL3	4.04	4.19	3	3.19	3.02	3.7
PCNXL4	6.43	6.48	5.23	4.32	5.96	6.06
PCOLCE	2.3	1.35	1.39	1.27	1.09	1.68
PCOLCE-AS1	0	0	0	0.04	0	0
PCOLCE2	2.58	10.89	5.83	5.81	4.19	7.51
PCP2	0	0	0	0	0	0
PCP4	0	0	0	0	0	0
PCP4L1	0	0.58	0	0.15	0.85	0.8
PCSK1	0	0	0	0	0	0
PCSK1N	0	0	0	0.2	0	0
PCSK2	0	0	0	0	0	0
PCSK4	0.36	0.09	0.1	0	0	0.03

PCSK5	0.33	0.06	0.1	0.12	0.24	0.04
PCSK6	1.07	0.62	1.28	0.79	0.6	0.79
PCSK7	6.5	4.66	3.57	5.11	4.46	4.83
PCSK9	0.18	0	0	0	0	0
PCTP	12.88	4.59	6.49	8.27	8.23	6.53
PCYOX1	8.5	9.45	9.26	7.1	7.17	9.68
PCYOX1L	6.95	6.2	6.86	5.23	5.01	6.3
PCYT1A	15.66	11.77	12.81	10.79	12.3	12.5
PCYT1B	0.97	0.13	0.17	0.75	0.77	0.28
PCYT2	5.65	4.46	5.13	4.82	5.08	5.96
PDAP1	34.86	32.58	28.67	30.92	32.47	30.52
PDC	0.13	0	0	0	0	0
PDCD1	0	0	0	0	0	0
PDCD10	58.16	37.26	34	33.38	46.98	35.64
PDCD11	20.84	19.27	22.51	17.09	19.07	19.75
PDCD1LG2	0.03	0	0	0	0	0.03
PDCD2	30.43	28.92	27.41	25.17	34.39	24.07
PDCD2L	25.86	24.37	21.91	17.48	22.85	21.08
PDCD4	68.82	22.2	17.41	26.87	48.84	21.03
PDCD5	252.11	246.1	200.94	182.84	251.52	223.92
PDCD6	128.98	139.48	103.97	114.13	122.86	121.19
PDCD6IP	28.11	27.81	24.67	23.21	25.38	25.74
PDCD7	12.76	21.83	18.9	16.07	19.2	16.39
PDCL	9.94	7.93	7.86	7.93	10.28	8.15
PDCL2	0	0	0	0	0	0
PDCL3	37.89	45.16	34.33	37.51	37.55	30.64
PDCL3P4	0.46	0	0	0	0.08	0
PDDC1	12.74	14.69	14.82	12.45	13.42	15.31
PDE10A	0	0	0	0	0	0
PDE11A	0.17	0.15	0.16	0.18	0.13	0.15
PDE12	18.18	17.33	18.54	17.37	14.84	21.7
PDE1A	0	0	0	0	0	0
PDE1B	0.17	0.43	0.35	0.13	0.58	0.1
PDE1C	0	0	0	0	0	0
PDE2A	0	0.04	0	0	0	0.19
PDE3A	0.77	0.26	0.14	0.29	0.95	0.38
PDE3B	0.76	0.24	0.32	0.15	0.43	0.54
PDE4A	1.48	1.33	1.96	1.64	2.75	1.92
PDE4B	0.16	1.94	0.68	0.03	0.05	0.81
PDE4C	1.23	1.77	1.91	1.16	1.32	1.52
PDE4D	0.98	2.81	4.28	1.43	1.84	2.77
PDE4DIP	5.95	6.13	3.14	2.31	3.4	2.71
PDE5A	0.07	0.13	0.08	0.04	0.04	0.1

PDE6A	1.14	1.52	1.46	0.86	1.1	1.05
PDE6B	0.19	0.27	0.23	0.14	0.44	0.3
PDE6C	0	0	0.02	0	0	0
PDE6D	13.17	15.51	17.74	12.77	13.18	14.76
PDE6G	0	0.65	1.24	0	0	0
PDE6H	0	0	0	0	0.1	0
PDE7A	1.16	1.87	2.65	1.51	1.87	2.01
PDE7B	0.12	0.11	0.07	0.02	0.04	0.02
PDE8A	1.82	2.7	1.51	1.3	1.62	1.5
PDE8B	0.08	0.07	0.02	0.05	0.13	0.11
PDE9A	0	0.08	0	0	0	0
PDF	1.03	1.79	1.8	3.75	2.75	0.58
PDGFA	1.74	1.91	1.66	2.35	3.99	1.13
PDGFB	0	0	0	0	0	0
PDGFC	2.75	4.49	2.85	2.75	3.44	3.99
PDGFD	0	0	0	0	0	0
PDGFRA	0.79	0.08	0	0.08	0.13	0.11
PDGFRB	0	0	0.03	0	0	0.1
PDGFRL	0.16	2.45	1.81	0.75	0.55	2.08
PDHA1	94.22	118.38	107.04	98.36	99.82	104.91
PDHA2	0	0	0	0	0	0
PDHB	86.34	75.71	66.92	62.5	92.17	69.02
PDHX	15.63	10.8	10.81	11	14.08	10.84
PDIA2	0.82	0.07	0	0	0.04	0
PDIA3	160.63	158.78	133.11	139.4	145.94	150.19
PDIA3P	0.16	0	0.87	0.32	0.26	0.36
PDIA4	45.14	54.2	43.67	39.52	49.64	52.47
PDIA5	4.61	4.48	3.86	4.6	4.13	4.53
PDIA6	123.79	162.83	153.87	132.61	148.85	151.94
PDIK1L	3.27	2.33	2.2	2.74	2.27	3.91
PDILT	0	0	0	0	0	0
PDK1	8.81	13.33	14.58	11.8	9.09	14.58
PDK2	11.9	11.58	14.11	15.5	12.08	14.84
PDK3	26.08	26.45	22.76	24.71	23.76	26.27
PDK4	0	0	0	0	0	0
PDLIM1	114.11	108.26	129.75	115.42	130.22	106.47
PDLIM2	3.86	17.06	20.07	14.11	11.16	17.78
PDLIM3	0	0	0	0	0	0
PDLIM4	0	0	0	0	0	0
PDLIM5	14.12	8.7	8.22	5.29	9.42	6.94
PDLIM7	32.77	27.79	35.36	33.12	30.67	29.48
PDP1	3.68	6.88	6.6	4.81	5.19	7.64
PDP2	2.87	2.74	3.97	2.94	2.92	3.84

PDPK1	3.96	3.65	2.73	2.4	3.78	2.73
PDPN	0	0.03	0.09	0.04	0	0
PDPR	6.64	8.85	6.69	6.75	5.37	8.86
PDRG1	43.11	31.9	29.4	29.26	37.07	35.8
PDS5A	7.33	6.13	6.04	4.7	5.82	5.4
PDS5B	9.69	9.51	7.43	7.8	9.19	8.91
PDSS1	7.57	5.24	7.38	4.78	4.93	8.3
PDSS2	3.46	6.83	6.6	4.34	4.66	6.21
PDX1	0	0	0	0.02	0	0
PDXDC1	15.3	18.05	17.45	16.94	18.52	19.99
PDXDC2P	1.63	1.43	1.3	1.25	0.93	1.16
PDXK	5.4	4.08	4.79	4	4.95	6.05
PDXP	7.55	4.62	4.28	4.46	6.23	5.03
PDYN	0	0	0	0	0	0
PDZD11	55.07	29.19	37.6	40.93	45.15	25.31
PDZD2	0.13	0.01	0.02	0.03	0.03	0.1
PDZD3	0	0	0	0	0	0
PDZD4	0	0	0.02	0.01	0.04	0
PDZD7	0	0.2	0.1	0.86	0.07	0.14
PDZD8	4.02	1.49	1.31	2.1	2.89	1.74
PDZD9	0	0.11	0.06	0	0	0
PDZK1	0	0.08	0.03	0.09	0.15	0.06
PDZK1IP1	0.17	0	0	0.06	0.25	0
PDZK1P1	0.02	0	0.04	0	0	0
PDZRN3	0	0	0	0	0	0
PDZRN4	0	0	0	0	0	0
PEA15	7.57	3.88	4.08	4.62	6.17	3.13
PEAK1	6.24	4.16	5.31	4.86	4.36	5.29
PEAR1	0.13	0.05	0.01	0.43	0.5	0.05
PEBP1	228.4	298.08	250.5	264.11	262.03	265.17
PEBP4	0	0	0	0	0	0
PECAM1	0.61	0.29	0.5	0.16	0.64	0.19
PECR	8.95	6.32	7.59	5.39	7.01	5.9
PEF1	66.28	72.18	60.36	69.98	67.31	56.31
PEG10	3.38	0.87	1.62	1.88	1.83	3.45
PEG3	0	0	0	0	0	0
PEG3-AS1	0	0	0	0	0	0
PELI1	2.16	0.71	0.74	0.98	1.45	1.42
PELI2	2.65	1.6	1.23	1.34	1.58	1.75
PELI3	1.08	0.78	1.1	0.95	1.54	1.26
PELO	11.89	9.56	10.2	7.68	11.66	9
PELP1	10.58	11.87	10.95	12.71	12.75	14.5
PEMT	63.62	74.28	84.32	81.64	91.86	79.63

PENK	0	0	0	0	0	0
PEPD	54.76	45.25	39.29	44.85	51.88	36.58
PER1	2.25	2.09	2.27	2.19	2.67	3.6
PER2	6.25	6.83	5.83	6.41	6.16	5.13
PER3	5.58	3.12	3.17	3.74	4.1	3.44
PER4	0	0	0	0	0	0
PERP	0.37	0.07	0.28	0.05	0.14	0.14
PES1	31.02	28.84	28.56	34.35	29.71	33.31
PET100	159.9	171.82	150.58	109.88	197.06	120.13
PET112	24.96	28.15	32.23	25.36	28.86	26.01
PET117	13	18.03	11.52	15.57	13.51	16.04
PEX1	1.3	1.22	1.3	0.95	2.54	2.35
PEX10	7.21	5.94	7.5	9.87	7.56	6.16
PEX11A	0.11	0.29	0.14	0.11	0.1	0.09
PEX11B	17.43	20.56	17.03	17.35	12.84	16.6
PEX11G	0	0.27	0	0	0.16	0
PEX12	3.35	2.1	2.37	2.68	1.8	2.65
PEX13	5.88	5.88	4.45	4.43	5.03	3.95
PEX14	6.8	4.75	6.44	5.92	6.1	7.42
PEX16	17.03	18.94	21.1	14.29	15.61	17.78
PEX19	18.09	10.88	8.36	10.93	13.82	10.13
PEX2	8.81	11.5	8.9	9.69	9.46	9.3
PEX26	5.25	8.01	7.23	6.54	7.11	5.96
PEX3	9.65	10.48	7.55	6.58	6.05	5.49
PEX5	5	5.85	4.41	4.61	4.09	9.75
PEX5L	0.02	0.09	0.07	0.04	0.05	0.11
PEX6	3.45	3.19	2.85	2.73	2.25	2.94
PEX7	2.68	1.87	0.93	1.99	1.29	0.82
PF4	0	0	0	0	0	0
PF4V1	0	0	0	0	0	0
PFAS	19.12	23.92	28.62	32.16	25.95	29.21
PFDN1	72.6	56.02	56.94	59.45	65.32	57.19
PFDN2	133.06	97.92	88.55	83.11	109.09	82.93
PFDN4	24.53	15.58	13.63	14.16	17.05	13.02
PFDN5	422.18	376.13	354.3	413.4	414.99	364.18
PFDN6	15.16	22.19	20.81	16.58	20.79	21.25
PFKFB1	0	0.18	0	0.13	0.04	0
PFKFB2	0	0	0.01	0	0	0
PFKFB3	4.57	4.65	4.81	3.81	4.59	4.73
PFKFB4	7.12	0.88	0.91	2.38	3.51	1.77
PFKL	56.45	79.93	84.3	78.11	63.89	89.7
PFKM	9.16	0.45	1.71	4.2	2.25	2.31
PFKP	103.03	68.58	76.55	84.46	72.66	80.77

PFN1	879.7	796.47	708.07	792.34	855.44	762.54
PFN1P2	0.71	1.03	0.46	0.4	0.62	0.59
PFN2	4.76	5.03	3.62	7.12	2.29	5.54
PFN3	0	0	0	0	0	0
PFN4	0.1	0.24	0.09	0.71	0.1	0.1
PGA3	0	0	0	0	0	0
PGA4	0	0	0	0	0	0
PGA5	0	0	0	0	0	0
PGAM1	525.64	557.88	505.93	471.29	527.58	498.05
PGAM1P5	0.07	0	0	0.14	0.16	0
PGAM2	0.27	0.14	0.17	0	0.09	0.18
PGAM4	0.12	0.6	0	0.08	0.22	0.06
PGAM5	12.31	11.94	11.78	15.48	13.44	7.73
PGAP1	0.35	0.4	0.21	0.25	0.19	0.64
PGAP2	20.6	24.68	25.47	27.78	27.37	23.36
PGAP3	1.58	0.99	1.05	1.97	1.23	0.8
PGBD1	6.64	3.52	5.13	4.31	5.3	4.4
PGBD2	5.28	1.64	1.88	1.78	3.45	1.6
PGBD3	0.85	0.94	0.82	0.9	1.01	0.74
PGBD4	0.73	1.15	0.41	0.07	0.51	0.83
PGBD5	0.04	0	0.02	0.1	0.06	0.07
PGC	0	0	0	0	0	0.06
PGCP1	0	0	0	0	0	0
PGD	217.8	204.32	223.37	207.49	211.97	215.21
PGF	0.2	0.92	0.62	0.41	0.38	0.12
PGGT1B	3.67	3.19	1.69	2.6	4.12	2.44
PGK1	741.6	839.23	756.43	716.03	730.9	850.19
PGK2	0	0	0	0	0	0
PGLS	116.59	168.81	175.34	164.9	151.42	164.72
PGLYRP1	0.73	0.38	0	0	0.15	0.08
PGLYRP2	0	0	0	0	0	0
PGLYRP3	2.59	0	0	0.09	0	0
PGLYRP4	9.87	0	0.04	1.11	0.99	0
PGM1	13.22	26.28	25.13	24.76	21.61	29.08
PGM2	14.76	20.24	16.58	12.72	14.75	17.65
PGM2L1	1.3	1.32	1.19	0.79	1.13	1.3
PGM3	5.78	7.87	6.14	5.26	6.9	5.57
PGM5	0	0	0	0	0	0
PGM5-AS1	0	0	0	0	0	0
PGM5P2	2.82	2.99	4.92	1.94	2.91	3.88
PGP	2.18	2.68	2.83	1.85	2.3	1.29
PGPEP1	4.44	3.15	3.26	2.79	4.01	3.85
PGPEP1L	0	0	0	0	0	0

PGR	0	0	0	0	0	0
PGRMC1	73.35	98.97	102.62	95.27	92.48	111.8
PGRMC2	3.19	2.2	2.15	2.43	3.01	2.81
PGS1	11.66	16.86	12.15	13.46	11.74	13.22
PHACTR1	7.69	6.61	8.56	7.4	7.08	7.64
PHACTR2	5.37	1.17	0.85	1.72	2.49	1.25
PHACTR3	0	0	0	0	0	0
PHACTR4	10.6	10.08	8.24	8.51	7.4	8.98
PHAX	13.4	14.2	14.4	13.37	14.14	11.85
PHB	251.82	258.57	246.09	250.53	261.19	241.62
PHB2	311.94	346.26	317.22	360.66	330.08	335.72
PHC1	3.4	2.93	1.93	3.53	3.29	2.59
PHC2	2.71	2.63	1.93	2.75	1.86	3
PHC3	2.91	3.34	2.94	2.47	2.78	2.43
PHEX	0	0.04	0	0	0	0
PHEX-AS1	0	0	0	0	0	0
PHF1	2	1.44	1.08	1.07	0.97	1.12
PHF10	2.34	3.01	2.43	1.77	2.36	3.18
PHF11	7.76	8.73	8.45	9.52	6.95	7.66
PHF12	3.58	2.99	2.19	2.21	2.44	2.83
PHF13	4.53	2.74	3.8	3.57	5.29	4.06
PHF14	5.61	5.46	6.58	5.86	6.14	7.48
PHF15	1.37	2.14	2.71	2.2	1.93	2.65
PHF16	4.14	4.48	4.57	4.51	4	4.63
PHF17	15.07	14.02	14.97	16.32	13.21	14.71
PHF19	34.42	69.13	57.59	50.11	39.85	57.04
PHF2	1.68	1.49	0.54	1.23	1.49	1.53
PHF20	5.84	5.57	5.53	5.29	5.85	6.27
PHF20L1	4.9	3.77	4.3	6.36	4.23	4.13
PHF21A	1.5	1.92	1.1	1.07	1.85	1.93
PHF21B	0	0	0	0	0	0
PHF23	7.14	6.71	6.57	7.21	7.49	6.64
PHF3	7.74	9.19	10.13	7.07	7.99	9.7
PHF5A	30.43	45.39	39.99	41.33	46.93	53.26
PHF6	29.98	27.85	27.14	25.09	27.33	27.32
PHF7	1.74	3.15	2.59	2.77	1.96	1.42
PHF8	11.21	11.49	11.08	11.64	10.73	13.83
PHGDH	53.74	100.26	93.31	94.97	77.97	112.13
PHGR1	0	0	0	0	0	0
PHIP	4.4	3.85	4.16	2.9	4.15	2.98
PHKA1	3.19	1.83	2.17	1.47	2.34	1.93
PHKA2	3.51	7.1	7.8	6.78	4.84	7.16
PHKA2-AS1	0.19	0	0.11	0.39	0.45	0.25

PHKB	8.53	8.92	7.65	7.37	5.66	8.75
PHKG1	0.88	1.65	1.66	1.63	1.38	1.21
PHKG2	10.2	9.14	7.51	10.01	9.89	10.03
PHLDA1	13.09	0.72	1.11	3.14	2.01	0.32
PHLDA2	13.32	6.92	8.71	10.26	24.11	11.66
PHLDA3	0.2	0.23	0.41	0.14	0.23	0.15
PHLDB1	3.58	1.65	2.28	2.3	1.14	1.21
PHLDB2	0	0	0	0	0	0
PHLDB3	1.8	1.28	1.97	3.99	1.14	1.91
PHLPP1	0.84	1.2	1.66	1.21	1.52	1.68
PHLPP2	1.16	0.78	0.79	1.09	0.59	1.17
PHOSPHO1	0.18	0.34	0.76	0.37	0.31	0.26
PHOSPHO2	4.97	2.67	2.81	3.46	5.18	2.95
PHOSPHO2-K	0.16	0	0	0	0	0
PHOX2A	0	0	0	0	0	0
PHOX2B	0	0	0	0	0	0
PHPT1	109.31	127.56	116.76	136.12	125.54	145.34
PHRF1	6.35	6.73	6.92	6.06	7.33	6.32
PHTF1	2.13	1.2	1.66	0.89	1.63	1.02
PHTF2	8.65	4.82	3.44	3.51	4.96	4.44
PHYH	18.31	19.57	18.41	18.85	18.53	13.97
PHYHD1	0.62	0.6	0.69	0.52	0.47	0.69
PHYHIP	0.02	0.07	0	0	0	0
PHYHIPL	0	0	0	0	0	0
PI15	0	0	0	0	0	0
PI16	0	0	0	0	0	0
PI3	0	0	0	0	0	0
PI4K2A	5.66	0.97	0.89	1.31	2.97	0.98
PI4K2B	0.77	0.73	0.85	0.54	0.61	0.49
PI4KA	6.62	6.97	6.2	6.95	6.52	7.8
PI4KAP1	0.66	0.67	0.29	0.32	0.31	0.66
PI4KAP2	0.42	1.19	0.6	0.39	1.08	1.4
PI4KB	15.9	17.13	14.59	17.14	15.97	15.95
PIANP	0.03	0	0.03	0	0	0
PIAS1	47.73	62.45	58.27	53.73	55.15	62.28
PIAS2	4.49	4.51	4.26	4.09	4.22	2.75
PIAS3	14.28	8.3	7.85	10.75	12.21	8.68
PIAS4	22.56	25.19	24.21	23.17	22.28	18.83
PIBF1	1.9	3.17	3.11	3.06	2.29	3.27
PICALM	8.81	2.76	1.95	3.02	3.27	2.74
PICK1	1.49	1.52	1.46	1.48	1.05	2.01
PID1	0	0	0.05	0	0	0
PIDD	3.34	3.32	3.97	3.45	4.29	4.67

PIEZO1	7.6	9.88	7.37	6.78	7.02	8.05
PIEZO2	0	0	0	0	0	0
PIF1	1.46	3.12	3.33	2.42	3.61	1.7
PIFO	0.06	0	0.17	0.71	0.47	0.19
PIGA	5.66	5.47	4.69	4.45	5.7	3.7
PIGB	4.57	6.5	6.59	5.27	4.92	6.15
PIGC	17.78	19.64	21.63	19.13	18.84	21.36
PIGF	34.22	37.71	31.02	31.56	39.12	31.62
PIGG	7.11	6.4	5.96	4.85	6.35	6.7
PIGH	13.21	21.51	22.16	22.31	19.14	19.31
PIGK	4.73	7.06	5.42	3.79	4.16	5.42
PIGL	9.72	8.64	8.83	6.81	11.54	9.79
PIGM	5.76	7.59	5.65	6.6	6.13	6.78
PIGN	5.61	5.59	5.19	4.54	4.84	5.9
PIGO	8.78	11.84	10.99	8.09	9.2	10.56
PIGP	15.01	15.11	12.51	13.02	14.93	15.51
PIGQ	6.42	11.05	7.72	8.1	8.96	6.31
PIGR	0.39	0.44	0.48	0.27	0.27	0.37
PIGS	33.59	41.2	30.49	32.26	31.9	34.36
PIGT	57.85	66.5	56.84	65.61	68.33	57.47
PIGU	28.91	40.09	32.53	33.53	35.6	33.16
PIGV	4.41	4.24	3.8	3.93	4.56	5.56
PIGW	12.35	11.81	10.25	10.12	7.83	13.07
PIGX	2.48	4.15	3.92	2.1	3.1	2.71
PIGY	62.86	55.27	40.56	44.42	54.68	43.01
PIGZ	0.7	0.15	1.04	0.45	0.2	0.99
PIH1D1	47.3	44.24	42.55	45.83	41.52	40.36
PIH1D2	0.43	0.34	0.33	0.5	0.29	0.62
PIH1D3	0.14	0.08	0.15	0.12	0.05	0
PIK3AP1	1.7	4.87	1.95	1.3	2.26	3.8
PIK3C2A	2.45	2.19	2.02	1.4	2	2.22
PIK3C2B	3.67	3.8	3.63	2.93	2.72	2.63
PIK3C2G	0	0	0	0	0	0
PIK3C3	11.51	10.23	9.31	8.07	8.87	6.66
PIK3CA	1.68	1.14	0.63	0.79	1.39	0.99
PIK3CB	4.85	3.83	3.83	3.99	4.07	4.13
PIK3CD	4.49	14.28	14.44	11.92	9.36	14.33
PIK3CG	1.55	2.46	2.35	1.56	2.27	2.47
PIK3IP1	2.13	2.04	1.01	1.22	0.98	0.25
PIK3R1	0.69	1.03	1.41	0.62	0.74	1.13
PIK3R2	3.82	2.25	2.35	2.28	3.4	2.45
PIK3R3	4.66	0.19	0.37	0.62	0.81	0.53
PIK3R4	8.57	7.67	9.17	7.8	7.19	7.9

PIK3R5	1.2	1.64	1.81	1.24	1.4	3.51
PIK3R6	0.36	0.13	0.99	0.54	0.57	0.24
PIKFYVE	1.56	2.1	2.68	1.9	1.75	2.37
PILRA	0	0	0.13	0	0.14	0.07
PILRB	2.72	2.57	1.11	1.47	1.55	1.73
PIM1	191.76	55.08	64.06	107.05	133.27	55.25
PIM2	163.43	30.42	27.95	48.35	85.81	37.91
PIM3	4.47	3.69	3.9	2.84	3.99	1.81
PIN1	102.68	87.51	93.58	99.81	106.04	77.04
PIN1P1	0	0	0.07	0	0	0
PIN4	49.61	40.75	35.5	35.77	46.71	31.87
PIN4P1	1.26	1.43	1.87	1.14	2.03	1.49
PINK1	4.87	2.62	2.19	2.19	2.93	3.03
PINLYP	0	0	0	0	0	0
PINX1	43.05	45.76	42.63	39.26	46.07	45.43
PION	0.16	0.21	0.16	0.26	0.02	0.16
PIP	0	0	0	0	0	0
PIP4K2A	27.72	11.57	14.08	14.43	19.5	12.73
PIP4K2B	4.92	5.78	5.02	5.59	5.66	6.3
PIP4K2C	15.25	7.38	7.5	6.19	9.82	5.78
PIP5K1A	18.84	15.6	0.02	11.6	0.53	15.13
PIP5K1B	0.96	0	0	0.28	0.49	0
PIP5K1C	1.99	1.86	2.41	1.49	2.21	2.35
PIP5K1P1	0	0	0	0	0	0
PIP5KL1	0.53	0.05	0	0.07	0.07	0.05
PIPOX	0.27	0.33	0.35	0.13	0.33	0.41
PIPSL	0.34	0.27	0.02	0.16	0	0.16
PIR	30.45	48.51	45.97	63.07	54.16	54.42
PIR-FIGF	0.09	0	0	0	0.12	0.08
PIRT	0	0	0	0	0	0
PISD	34.04	27.94	26.56	26.17	25.96	23.59
PISRT1	0	0	0	0	0	0
PITHD1	42.45	37.75	42.64	44.38	41.63	30.75
PITPNA	2.97	4.24	2.76	2.17	3.18	2.51
PITPNA-AS1	10.99	15.89	12.09	16.11	19.42	15.51
PITPNB	30.78	31.09	31.1	30.59	31.27	28.95
PITPNC1	0.35	0.14	0.51	0.65	0.32	0.59
PITPNM1	1.71	1.52	0.78	1.07	1.07	1.47
PITPNM2	1.07	0.43	0.42	0.63	0.65	0.49
PITPNM3	0	0	0.02	0	0	0.02
PITRM1	40.16	29.71	27.41	32.4	34.72	30.78
PITRM1-AS1	0.02	0.07	0.08	0.26	0.07	0
PITX1	0.92	3.35	0.73	1.91	2.24	1.5

PITX2	0	0	0	0	0	0
PITX3	0	0	0.05	0	0	0
PIWIL1	0	0	0	0	0	0
PIWIL2	0.6	0.73	0.66	0.42	0.38	0.36
PIWIL3	0.69	0.19	0.19	0.16	0.72	0.56
PIWIL4	0	0.68	0	0	0	0.07
PJA1	10.25	12.84	10.08	15.92	10.45	10.27
PJA2	10.97	11.46	9.06	8.08	11.37	8.06
PKD1	1.89	1.74	0.83	1.08	1.28	1.52
PKD1L1	0	0	0	0	0	0
PKD1L2	0	0	0	0	0	0
PKD1L3	0	0	0	0	0	0
PKD1P1	2.94	3.05	1.4	1.61	2.34	2.9
PKD2	0.48	0.34	0.52	0.35	0.43	0.43
PKD2L1	0	0	0	0	0	0
PKD2L2	0	0	0.17	0	0	0
PKDCC	0.03	0.05	0	0.12	0.47	0.15
PKDREJ	0	0	0.02	0	0	0.01
PKHD1	0.01	0	0	0	0	0.01
PKHD1L1	0.57	0	0	0.02	0.05	0
PKI55	2.84	1.89	3.33	2.53	2.59	3.5
PKIA	0.6	1.56	1.61	0.91	0.9	1.24
PKIB	0.36	0.15	0.04	0.03	0	0.47
PKIG	13.61	3.93	2.92	8.36	12.95	6.58
PKLR	0	0.04	0	0	0.03	0
PKM	324.06	349.99	392.09	322.22	284.49	414.77
PKMYT1	58.18	71.95	71.41	79	72.77	70.07
PKN1	13.99	13.36	14.27	13.32	8.87	14.25
PKN2	0.87	1.18	1.31	0.88	1.33	1.1
PKN3	3.1	4.52	4	4.45	4.32	4.4
PKNOX1	5.81	4.23	4.51	3.42	3.89	4.22
PKNOX2	0	0	0	0	0	0
PKP1	0	0	0	0	0	0
PKP2	2.7	3.3	2.7	2.61	2.4	3.68
PKP3	0	0	0	0	0	0
PKP4	7.6	6.41	6.3	5.68	6.55	6.34
PLA1A	0	0	0	0	0	0
PLA2G10	0	0.12	0	0.05	0	0
PLA2G12A	6.72	7.44	6.66	7.31	7.05	8.44
PLA2G12B	0	0	0	0	0	0
PLA2G15	17.67	10.59	11.16	9.6	13.35	8.94
PLA2G16	0.83	0.17	0	0.05	0	0.16
PLA2G1B	0	0	0	0	0	0

PLA2G2A	0	0	0	0	0	0
PLA2G2C	0	0	0	0	0	0
PLA2G2D	0	0	0	0	0	0
PLA2G2E	0	0	0	0	0	0
PLA2G2F	0	0	0	0	0	0
PLA2G3	0	0	0	0	0	0
PLA2G4A	3.76	3.67	3.56	3.39	4.07	1.39
PLA2G4B	0	0.33	0	0	0.41	0
PLA2G4C	0	0	0	0.02	0.03	0
PLA2G4D	0	0	0	0	0	0
PLA2G4E	0.37	0.32	0.53	0.25	0.41	0.22
PLA2G4F	0	0	0	0	0	0
PLA2G5	0	0	0	0	0	0
PLA2G6	1.17	1.08	1.93	0.68	0.68	1.05
PLA2G7	0	0.07	0	0.06	0	0
PLA2R1	0	0	0	0	0	0
PLAA	10.09	11.52	12.97	8.92	11.1	12.22
PLAC1	0.2	0	0	0	0	0
PLAC1L	0.09	0	0	0.25	0.39	0
PLAC4	0.04	0.01	0.01	0.01	0.02	0.04
PLAC8	6.24	28.24	14.76	11.86	7.89	22.34
PLAC8L1	0.11	0.09	0	0.08	0.1	0.11
PLAC9	0	0	0	0	0	0
PLAG1	0.33	0.18	0.07	0.09	0.41	0.13
PLAGL1	0.56	5.92	3.47	2.89	2.95	4.33
PLAGL2	6.14	9.14	8.26	7.11	5.65	7.29
PLAT	0.48	0.08	0.02	0.28	0.51	0.05
PLAU	3.43	13.82	7.21	4.3	2.92	8.07
PLAUR	6.23	14.81	12.34	6.19	9.74	13.93
PLB1	0	0.1	0.05	0	0	0
PLBD1	0	0	0	0	0	0
PLBD2	8.17	5.42	6.49	7.08	5.47	5.4
PLCB1	0	0	0	0	0	0
PLCB2	1.96	7.89	6.41	3.78	3.96	4.69
PLCB3	1.15	1.04	1.21	0.54	0.88	0.94
PLCB4	0	0	0	0	0	0
PLCD1	1.8	1.41	0.9	0.94	0.54	0.83
PLCD3	1.49	1.27	1.17	1.57	2.28	1.63
PLCD4	0.18	0.46	0.37	0.2	0.28	0.36
PLCE1	0.07	0.02	0.01	0.02	0.03	0.01
PLCG1	4.32	2.4	2.13	4.46	2.29	2.92
PLCG2	3.79	6.73	6.99	5.59	4.48	4.85
PLCH1	2.39	5.18	3.46	4.24	3.13	7.85

PLCH2	0	0	0	0	0	0
PLCL1	0.38	0	0	0	0	0
PLCL2	1.47	0.38	0.79	0.64	0.79	0.82
PLCXD1	9.27	16.67	17.11	16.89	18.38	18.01
PLCXD2	0.21	0	0	0.12	0.17	0.11
PLCXD3	0.01	0.01	0.01	0	0	0.01
PLCZ1	0	0	0	0	0	0
PLD1	1.9	1.68	1.11	0.84	1.54	1.61
PLD2	1.32	2.05	1.84	1.89	3.13	2.18
PLD3	27.66	15.07	18.94	25.18	22.91	19.9
PLD4	0.96	4.28	5.32	5.66	2.42	3.06
PLD5	0	0	0	0	0	0
PLD6	48.09	43.77	39.47	46.27	48.09	40.21
PLEC	6.2	1.75	2.55	2.64	3.56	1.95
PLEK	11.7	40.84	25.73	15.29	16.91	27.87
PLEK2	0	0	0	0	0	0
PLEKHA1	1.83	0.76	0.96	0.87	1.1	1.27
PLEKHA2	3.48	7.02	7.37	4.21	4.96	5.54
PLEKHA3	1.07	1.17	0.71	0.43	1.54	1.2
PLEKHA4	2.31	0.3	0.82	1.92	1.57	0.91
PLEKHA5	5.05	5.1	6.2	3.96	5.12	6.02
PLEKHA6	0.3	0	0	0.01	0	0.01
PLEKHA7	1.29	1.76	2.23	1.7	1.68	2.43
PLEKHA8	0.51	0.45	0.53	0.6	0.49	0.72
PLEKHA8P1	0.81	1.84	1.64	1.66	2.02	2.17
PLEKHB1	0.33	0	0	0	0	0
PLEKHB2	38.07	28.68	29.27	23.36	30.24	24.17
PLEKHD1	0	0	0	0	0	0
PLEKHF1	5.17	5.15	7	7.18	5.49	3.85
PLEKHF2	14.32	11.29	10.65	9.47	18.54	11.97
PLEKHG1	0.01	0.02	0.04	0.03	0.02	0.07
PLEKHG2	3.06	3.1	3.42	2.73	2.43	2.96
PLEKHG3	2.61	6.21	6.92	4.59	3.76	4.14
PLEKHG4	1.08	0.75	1.33	1.52	1.13	1.29
PLEKHG4B	0.26	0.09	0.4	0.53	0.42	0.06
PLEKHG5	0.83	0.28	0.12	0.56	0.27	0.2
PLEKHG6	0.27	0.19	0.11	0.04	0.07	0.09
PLEKHG7	0	0	0	0	0	0
PLEKHH1	0.34	0.37	0.56	0.1	0.28	0.45
PLEKHH2	0.33	0.41	0.42	0.5	0.4	0.61
PLEKHH3	1.38	0.6	0.51	0.38	0.34	0.63
PLEKHJ1	101.28	100.94	80.39	93.14	99.61	96.74
PLEKHM1	5.3	4.94	5.37	4.58	4.46	5.09

PLEKHM1P	4.45	3.09	2.48	3.29	1.66	2.57
PLEKHM2	5.71	4.27	3.28	4.96	4.87	4.02
PLEKHM3	0.59	0.78	0.85	0.59	0.58	0.64
PLEKHN1	0.28	0	0	0	0	0
PLEKHO1	2.86	7.83	9.26	10.12	6.9	8.69
PLEKHO2	6.92	10.05	14.88	10.57	8.91	13.34
PLEKHS1	0	0	0	0	0	0
PLG	0	0	0	0	0	0
PLGLA	0	0	0	0	0	0
PLGLB1	0.29	0.22	0.15	0.22	0.13	0.46
PLGLB2	0	0	0	0	0	0
PLGRKT	20.39	28.09	24.17	30.15	26.42	27.38
PLIN1	0	0	0	0	0	0
PLIN2	63.87	27.9	25.89	36.73	50.85	22.56
PLIN3	54.11	53.76	52.5	52.31	53.67	45.25
PLIN4	0.07	0.14	0.04	0.18	0.04	0.09
PLIN5	0.09	0.02	0.21	0.15	0.15	0.08
PLK1	169.98	156.19	154.27	154.68	155.15	147.63
PLK1S1	7.04	7.13	7.8	7.37	7.04	5.57
PLK2	1.42	3.28	2.31	1.27	0.97	2.92
PLK3	1.59	2.11	3.22	1.89	2.53	1.61
PLK4	5.9	8.16	4.79	5.87	7.35	9.79
PLK5	0	0	0.04	0	0	0
PLLP	0	0	0	0	0	0
PLN	0	0.03	0	0	0	0.04
PLOD1	33.29	27.93	30.6	27.19	28.78	29.93
PLOD2	0.07	0	0	0	0	0
PLOD3	32.36	25.36	24.27	28	26.92	26.72
PLP1	0	0	0	0	0	0
PLP2	535.43	345.27	335.82	406.06	645.95	377.34
PLRG1	29.77	21.85	18.65	17	21.09	17.42
PLS1	3.03	5.08	3.67	3.12	3.45	4.56
PLS3	17.19	21.09	19.31	11.65	11.86	19.68
PLSCR1	18.39	11.07	11.86	8.78	14.08	6.97
PLSCR2	0	0.11	0	0.03	0	0.05
PLSCR3	7.58	1.54	5.44	6.48	4.98	4.57
PLSCR4	0.17	0.05	0	0	0	0
PLSCR5	0	0	0	0	0	0
PLTP	44.47	51.91	67.83	55.7	44.23	57.68
PLVAP	0.55	0.46	0.59	0.73	0.37	0.42
PLXDC1	0.22	0.2	0.1	0.15	0.03	0.11
PLXDC2	5.08	1.27	6.51	3.98	4.37	6.11
PLXNA1	11.52	2.7	1.93	3.33	3.35	2.26

PLXNA2	0.01	0.02	0.09	0	0	0.01
PLXNA3	2.66	0.72	1.36	0.97	0.93	1.69
PLXNA4	0	0	0	0	0.07	0.02
PLXNB1	0.84	0.71	0.52	0.86	0.92	1.24
PLXNB2	4.71	8.14	10.63	8.83	6.8	10.18
PLXNB3	1.92	0.02	0.04	0.11	0.31	0
PLXNC1	0.37	0.47	0.56	0.28	0.42	0.49
PLXND1	1.47	4.65	4.65	3.65	3.07	5.98
PM20D1	0	0.11	0.15	0	0.06	0
PM20D2	2.07	7.34	7.02	4.04	4.07	6.15
PMAIP1	16.62	9.93	8.33	6.02	9.81	10.32
PMCH	0	0.09	0	0	0	0
PMCHL1	0	0	0	0	0	0
PMCHL2	0	0.08	0.26	0.14	0.04	0.15
PMEL	0	0.03	0.09	0.35	0.37	0.07
PMEPA1	0	0	0	0	0	0
PMF1	51.11	59.92	58.86	58.87	63.98	58.37
PMF1-BGLAI	2.08	4.24	1.79	2.49	2.78	2.21
PMFBP1	0.04	0.02	0.1	0	0	0.41
PML	15.13	19.71	23.82	18.18	15.43	22.63
PMM1	17.38	13.75	13.96	14.14	13.71	10.66
PMM2	12.97	17.61	17.78	14.75	15.55	16.86
PMP2	0	0	0	0	0	0.01
PMP22	41.77	10.62	8.82	16.6	40.27	7.27
PMPCA	110.65	96.05	102.57	110.04	103.89	95.58
PMPCB	27.21	25.47	19.53	20.97	24.27	20.9
PMS1	7.69	6.87	6.04	5.82	7.34	6.03
PMS2	6.54	5.71	4.67	4.08	5.04	6.6
PMS2CL	0.56	0.33	0.36	0.07	0	0.24
PMS2L2	2.98	5.09	2.86	2.04	4.59	1.42
PMS2P1	11.5	11.39	11.71	9.9	9.77	8.46
PMS2P3	0.48	0.65	0.84	1.2	0.68	0.63
PMS2P4	4.77	7.47	3.17	4.82	4.56	3.75
PMS2P5	7.41	9.92	6.18	7.21	6.98	6.25
PMVK	32.91	52.77	52.47	41.19	43.89	52.25
PNCK	0	0	0	0	0	0
PNISR	10.86	14.73	11.29	10.35	11.29	10.9
PNKD	79.62	108.98	93.96	81.97	82.87	81.53
PNKP	1.16	3.17	3.09	2.5	2.9	1.92
PNLDC1	0	0.06	0.11	0	0.07	0
PNLIP	4.3	0	0	0.48	0.24	0
PNLIPRP1	0	0	0	0	0	0
PNLIPRP2	0	0	0	0	0	0

PNLIPRP3	0	0	0	0	0	0
PNMA1	14.59	8.79	4.49	11.92	8.72	2.8
PNMA2	3.66	5.58	6.7	7.78	7.55	8.58
PNMA3	0	0	0	0	0	0
PNMA5	0	0	0	0	0	0
PNMA6A	0	0	0	0	0	0
PNMA6C	0	0	0	0	0	0
PNMA6D	0	0	0	0	0	0
PNMAL1	2.88	0.19	1.42	1.22	1.02	0.23
PNMAL2	0	0	0	0	0	0
PNMT	0	0	0	0	0	0
PNN	20.47	22.63	16.14	16.2	19.24	18.48
PNO1	25.54	32.06	26.95	25.14	28.22	24.33
PNOC	0.51	0	0.12	0.13	0	0.19
PNP	57.07	53.37	51.88	47.11	48.05	43.48
PNPLA1	0.11	0.18	0.32	0.16	0.12	0.17
PNPLA2	6.83	4.64	5.31	5.74	5.68	6.24
PNPLA3	0.25	0.87	0.63	0.32	0.76	1.03
PNPLA4	0.22	0.27	0.1	0.2	0.04	0.28
PNPLA5	0.03	0.08	0	0.02	0	0
PNPLA6	3.58	4.29	7.16	4.03	6.6	7.21
PNPLA7	0.02	0.06	0.01	0.01	0.03	0.11
PNPLA8	3.93	1.36	1.94	1.37	1.92	1.09
PNPO	21.67	23.87	23.46	22.07	24.11	24.83
PNPT1	17.37	17.69	13.27	12.51	15.21	15.86
PNRC1	3.84	2.29	1.8	2.83	2.4	4.72
PNRC2	53.53	55.32	45.86	44.14	58.15	58.34
POC1A	21.51	25.88	23.99	32.06	23.34	22.64
POC1B	5.54	4.69	4.04	3.43	4.93	5.38
POC1B-GALN	0	0.28	0.41	0.12	0	0
POC5	8.18	6.55	3.83	5.42	4.31	5.1
PODN	0	0	0	0	0	0
PODNL1	0.43	0.43	0.2	0.1	0.07	0.07
PODXL	1.98	8.18	7.97	6.57	5.87	8.13
PODXL2	2	1.21	5.28	4.03	2.67	6.07
POF1B	0	0.06	0	0	0	0.04
POFUT1	10.08	13.44	10.92	9.52	9.29	12.19
POFUT2	1.26	1.04	0.56	0.62	1.06	0.75
POGK	13.43	14.66	16.5	15.02	13.32	16.05
POGLUT1	7.52	8	6.74	6.1	8.59	8.57
POGZ	6.7	5.73	4.95	7.11	5.87	6.67
POLA1	15.33	16.49	16.23	15.1	17.96	18.3
POLA2	25.19	33.54	30.35	37.23	33.18	35.31

POLB	28.6	18.05	21.01	22.26	24.23	26.46
POLD1	32.31	36.22	39.54	40.47	36.95	43.08
POLD2	143.45	185.45	185.06	192.77	169.24	176.68
POLD3	6.33	9.44	8.64	7.26	7.15	9.05
POLD4	25.77	19.17	20.74	19.56	20.5	12.52
POLDIP2	43	49.27	45.74	45.92	38.77	39.87
POLDIP3	31.96	30.22	31.03	32.7	31.54	31.56
POLE	8.52	8.98	7.72	8.68	8.69	8.92
POLE2	14.05	18.02	19.79	16.01	16.97	18.57
POLE3	66.57	90.99	91.3	89.23	74.75	83.35
POLE4	79.78	70.57	75.7	84.22	86.73	59.29
POLG	13.44	16.13	16.36	14.6	12.59	11.68
POLG2	6.18	6.27	9.17	4.61	6.73	6.82
POLH	4.18	4.35	3.91	3.99	3.91	4.18
POLI	0.65	0.6	1.04	0.91	0.7	0.73
POLK	2.32	1.71	1.26	1.02	2.16	1.52
POLL	9.13	7.92	6.98	8.66	8.56	7.25
POLM	1.76	3.17	3.73	4.17	2.39	3.36
POLN	0.2	0.18	0.09	0.21	0.36	0.48
POLQ	1.66	2.85	2.21	1.84	1.46	2.46
POLR1A	20.05	25.02	23.51	21.16	21.82	24.76
POLR1B	15.2	18.78	17.69	19.21	17.53	19.59
POLR1C	46.54	43.58	50.31	50.42	45.74	33.46
POLR1D	187.9	166.79	157.56	182.43	199.97	171.22
POLR1E	33.03	37.09	37.04	38.1	27.36	29.4
POLR2A	35.96	33.47	35.47	36.33	30.43	40.6
POLR2B	30.53	27.19	25.67	24.02	27.96	26.68
POLR2C	25.47	31.02	29.48	26.31	26.54	33.27
POLR2D	58.12	70.91	58.23	58.22	62.57	70.59
POLR2E	121.07	123.97	121.34	127.03	122.34	123.12
POLR2F	84.76	77.9	73.99	75.66	77.67	71.75
POLR2G	123.44	112.21	98.62	96.29	119.22	93.16
POLR2H	133.16	121.6	105.89	120.12	128.42	102.73
POLR2I	98.22	108.35	103.04	112.29	120.13	82.47
POLR2J	129.33	149.02	129.83	146.58	152.83	140.31
POLR2J2	0	0.79	1.06	0.82	1.73	1.26
POLR2J3	6.15	9.85	8.31	9.46	7.66	6.21
POLR2J4	0.46	0.44	0.66	0.62	0.86	0.63
POLR2K	116.54	94.56	73.29	78	98.35	82.53
POLR2L	229.08	201.54	168.39	184.88	240.74	185.21
POLR2M	14.56	13.7	11.15	10.06	11.67	12.74
POLR3A	8.7	8.07	8.21	7.28	8.62	8.65
POLR3B	6.62	4.49	5.04	5.63	6.38	5.59

POLR3C	31.52	30.07	30.9	30.5	23.64	30.58
POLR3D	30.15	29.19	39.01	42.49	39.87	26.04
POLR3E	22.59	18.27	16.94	18.86	17.89	20.3
POLR3F	7.46	5.15	2.76	4.9	5.59	6.09
POLR3G	2.83	2.16	2.74	2.14	2.46	1.93
POLR3GL	19.4	18.54	19.24	19.29	18.48	14.85
POLR3H	17.93	18.12	16.72	19.75	18.9	15.71
POLR3K	98.09	111.54	94.33	103.86	130.52	101.88
POLRMT	10.62	15.81	16.66	19.65	13.94	9.11
POM121	15.21	16.69	16.63	13.62	14.77	14.85
POM121C	8.03	9.14	9.26	8.67	10.57	9.19
POM121L10I	0.24	0.32	0.33	0.23	0.24	0.2
POM121L12	0	0	0	0	0	0
POM121L1P	0	0	0	0	0	0
POM121L2	0	0	0	0	0	0
POM121L4P	0	0	0	0.09	0.03	0.08
POM121L8P	0.06	0.07	0.08	0.06	0.08	0.12
POM121L9P	0	0	0	0	0.01	0
POMC	0	0	0	0	0	0
POMGNT1	5.09	3.92	3.7	5.54	4.51	4.68
POMP	201.88	106.66	105.9	115.28	148.95	94.93
POMT1	12.39	13.05	15.77	13.99	11.14	14.09
POMT2	1.36	1.13	0.85	1.11	0.46	0.43
POMZP3	3.16	3.87	2.91	2.73	4.7	2.17
PON1	0	0	0	0	0	0
PON2	35.81	18.09	15.19	15.78	20.63	13.91
PON3	0	0	0	0	0	0
POP1	8.74	10.8	11.14	10.42	8.55	9.2
POP4	21.39	24.44	22.31	19.24	20.72	19.73
POP5	48.43	60.76	51.75	50.04	53.53	54.37
POP7	94.92	95.24	86.61	89.87	98.87	87.96
POPDC2	0	0.14	0	0.03	0.04	0.05
POPDC3	10.35	18.65	15.29	10.01	11.63	11.23
POR	51.21	32.35	29.83	34.67	37.67	31.04
PORCN	10.45	8.35	10.4	10.93	10.21	8.19
POSTN	0	0	0	0	0	0
POT1	2.71	4.5	3.19	3.33	4.24	3.63
POTEA	0	0	0	0	0	0
POTEB	0	0	0	0	0	0
POTEC	0	0	0	0	0	0
POTED	0	0	0	0	0	0
POTEE	0.24	0.13	0.08	0.02	0.01	0.09
POTEF	0.06	0.13	0.15	0.11	0.09	0.04

POTEG	0	0	0	0	0	0
POTEH	0	0	0	0	0	0
POTEM	0.14	0.15	0.13	0.02	0.03	0.12
POU1F1	0	0	0	0	0	0
POU2AF1	0.66	0.59	0.41	0.37	0.36	0.65
POU2F1	1.81	2.72	2.7	1.92	1.66	2.42
POU2F2	0.01	0.07	0.32	0.6	0.37	0.48
POU2F3	0.03	0.09	0.37	0.04	0.08	0.17
POU3F1	0	0	0	0	0	0
POU3F2	0.12	0	0	0.02	0.05	0
POU3F3	0	0	0	0	0	0
POU3F4	0	0	0	0	0	0
POU4F1	1.81	1	0.61	0.21	0.72	0.46
POU4F1-AS1	0	0	0.13	0.04	0	0
POU4F2	0	0	0	0	0	0
POU4F3	0	0	0	0	0	0
POU5F1	3.96	4.05	4.61	2.26	3.79	2.78
POU5F1B	0	0	0	0	0	0
POU5F1P3	0	0	0.06	0	0	0
POU5F1P4	0.21	0.41	0.53	0.03	0	0.24
POU5F2	0	0.05	0.05	0	0	0
POU6F1	0.03	0.16	0.37	0.17	0.04	0.02
POU6F2	0	0	0	0	0	0
POU6F2-AS1	0	0	0	0	0	0
PP12613	0	0	0	0	0	0
PP14571	0	0	0	0	0	0
PP2D1	0.03	0	0.13	0.17	0	0.04
PP7080	4.44	7.05	4.18	3.64	6	6.99
PPA1	251.77	310.26	338.77	326.21	320.94	350.1
PPA2	62.36	59.81	48.26	47.63	55.88	55.34
PPAN	9.9	14.78	17.88	18.19	10.05	14.38
PPAN-P2RY1	3	1.94	0.94	1.3	2.84	4.45
PPAP2A	2.76	4.39	1.98	2.74	2.43	2.94
PPAP2B	0.2	0.19	0.42	0.53	0.31	0.27
PPAP2C	0	0	0	0	0	0
PPAPDC1A	1.03	0	0	0	0	0
PPAPDC1B	31.72	32.25	29.23	27.58	33.61	26.48
PPAPDC2	2.23	1.64	1.32	0.91	2.45	2.11
PPAPDC3	0.07	0	0	0.05	0	0
PPARA	1.21	0.96	0.98	0.76	0.98	1.15
PPARD	3.44	1.08	0.67	3.11	1.82	1.48
PPARG	0	0	0	0	0	0
PPARGC1A	0	0	0	0	0	0

PPARGC1B	1.71	2.82	2.39	1.6	1.69	2.8
PPAT	12.8	14.33	13	11.75	11.68	12.55
PPBP	0.35	0	0	0	0	0
PPBPP2	0	0	0	0	0	0
PPCDC	11.24	13.25	13.27	9.41	12.24	9.63
PPCS	16.28	7.51	11.96	9.9	13.12	9.95
PPDPF	10.96	8.5	12.07	12.66	11.95	11.27
PPEF1	0.15	0	0	0	0	0
PPEF2	0.16	0.12	0.36	0.18	0.19	0.23
PPFIA1	2.11	2.03	1.7	1.42	1.82	1.86
PPFIA2	0	0	0	0	0	0
PPFIA3	0.52	0.76	0.62	0.71	0.92	1.04
PPFIA4	0	0	0	0.05	0	0
PPFIBP1	8.8	2.75	3.63	3.43	3.56	4.24
PPFIBP2	2.92	3.07	3.61	2.58	2.28	2.7
PPHLN1	45.66	35.14	26.37	34.37	42.26	33.24
PPIA	978.9	871.74	727.38	805.58	952.8	808.58
PPIAL4A	0.2	0	0	0.09	0	0.14
PPIAL4B	0.03	0	0	0	0	0
PPIAL4C	0.01	0	0	0	0	0
PPIAL4D	0	0.13	0	0	0	0
PPIAL4E	0	0	0	0	0	0
PPIAL4F	0	0.13	0	0	0	0
PPIAL4G	0.28	0.32	0.35	0.3	0.8	0.5
PPIAP30	0.66	0.72	0	0.27	0.13	0.13
PPIB	843.77	1029.98	873.07	851.96	885.78	905.98
PPIC	40.71	43.36	29.9	14.59	20.42	34.39
PPID	46.86	37.56	33.42	28.44	37.3	33.83
PPIE	36.76	36.08	31.79	27.78	34.06	28.15
PPIEL	0.89	1.27	1.88	0.83	1.22	1.3
PPIF	77.95	77.57	83.15	72.96	75.28	83.17
PPIG	28.34	33.33	28.89	26.75	33.7	27.7
PPIH	104.69	145.41	136.3	109.91	142.69	135.11
PPIL1	48.48	50.75	45.59	41.44	35.93	47.17
PPIL2	14.02	13.39	14.28	14.78	12	13.3
PPIL3	11.21	8.69	5.65	11.08	9.01	7.51
PPIL4	13.5	10.54	10.07	10.42	9.69	11.41
PPIL6	1.05	0.96	1.17	0.73	1.02	1.33
PPIP5K1	3.56	5.53	5.23	3.45	3.16	5.03
PPIP5K2	23.49	11.13	8.53	7.64	17.63	12.12
PPL	0	0.04	0.15	0	0.13	0
PPM1A	2.09	1.62	1.29	1.79	1.58	1.28
PPM1B	3.13	2.71	2.42	2.26	3.69	2.45

PPM1D	5.45	5.57	4.47	6.66	5.01	5.07
PPM1E	0.2	0.46	0.18	0.12	0.2	0.09
PPM1F	5.14	5.07	7.04	6.04	5.25	4.87
PPM1G	119.28	126.69	116.39	137.47	125.83	123.46
PPM1H	1.18	1.45	1.15	1.77	1.51	1.56
PPM1J	2.86	2.26	1.99	3.2	3.61	1.1
PPM1K	2.95	3.81	4.33	3.26	3.33	3.41
PPM1L	1.17	1.46	1.57	2.28	3.35	2.94
PPM1M	1.1	2.31	3.81	1.91	1.8	0.25
PPM1N	0.26	0.27	0.56	0.03	1.15	0.4
PPME1	31.5	22.25	25.38	24.7	26.69	19.22
PPOX	9.58	9.37	8.88	10.21	8.4	8.57
PPP1CA	202.07	277.95	269.92	278.18	256.23	252.01
PPP1CB	31.57	30.6	24.16	20.47	26.53	24.32
PPP1CC	30.67	44.13	40.55	41.08	34.66	32.93
PPP1R10	19.1	14.48	16.2	18.32	14.49	18.96
PPP1R11	26.94	21.02	24.29	23.27	22.66	19.63
PPP1R12A	16.51	10.12	9.29	8.88	13.51	9.22
PPP1R12B	2.36	1.86	1.23	1.17	2.72	2.13
PPP1R12C	1.13	1.05	1.08	0.54	1.02	1.22
PPP1R13B	0.58	0.98	0.75	0.7	0.36	0.8
PPP1R13L	0.21	0.42	0.53	0.61	0.68	0.78
PPP1R14A	36.35	39.74	32.69	37.48	50.87	26.59
PPP1R14B	11.43	12.48	10.35	11.47	10.45	9.15
PPP1R14C	5.39	0.97	1.52	0.3	3.05	0.98
PPP1R14D	0	0	0.1	0	0	0
PPP1R15A	64.17	14.41	11	23.29	30.47	8.66
PPP1R15B	11.89	8.24	10.13	8.26	10.23	11.93
PPP1R16A	6.63	8.54	9.29	9.05	8.21	8.77
PPP1R16B	0.77	0.41	0.37	0.32	0.59	0.69
PPP1R17	0	0	0	0	0	0
PPP1R18	3.65	4.41	6.2	5.48	3.75	3.86
PPP1R1A	0.53	0.06	0	0.08	0.08	0.16
PPP1R1B	0.58	0	0	0	0	0
PPP1R1C	0	0	0	0	0	0
PPP1R2	10.2	8.89	8.27	8.77	8	6.83
PPP1R21	0.76	1.36	0.87	0.8	1.11	0.73
PPP1R26	1.58	1.59	1.53	0.97	2.07	1.41
PPP1R27	0.1	0	0	0	0	0
PPP1R2P3	0	0	0	0	0	0.03
PPP1R2P9	0	0	0	0	0	0
PPP1R32	0.15	0.2	0.22	0	0	0
PPP1R35	1.67	2.7	2.52	2.71	3.08	0.93

PPP1R36	0	0	0	0	0	0
PPP1R37	0.49	0.16	0.11	0.22	0.29	0.28
PPP1R3A	0	0	0	0	0	0
PPP1R3B	5.34	2.42	2.45	2.17	2.08	1.36
PPP1R3C	0	0	0	0	0	0
PPP1R3D	0.29	0.66	0.44	0.26	0.32	0.7
PPP1R3E	0.42	0.48	0.33	0.55	0.55	0.33
PPP1R3F	0.15	0.21	0.35	0.03	0	0
PPP1R3G	0.34	0	0	0.06	0	0
PPP1R42	0	0	0	0	0	0
PPP1R7	51.25	53.63	55.88	60.34	54.24	51.39
PPP1R8	7.43	6.75	7.19	7.91	7.43	14
PPP1R9A	0.04	0	0.01	0	0	0.04
PPP1R9B	0.86	0.75	0.54	0.37	0.56	0.62
PPP2CA	50.39	63.53	62.95	54.56	60.47	42.27
PPP2CB	11.33	6.89	4.88	5.32	6.63	4.33
PPP2R1A	154.52	161.57	153.34	171.38	148.57	161.84
PPP2R1B	11.87	15.42	15.41	13.59	14.41	19.04
PPP2R2A	10.02	7.23	7.26	7.33	9.26	8.48
PPP2R2B	0	0.09	0.03	0	0	0
PPP2R2B-IT1	0	0	0	0	0	0
PPP2R2C	0	0	0	0	0	0
PPP2R2D	10.07	12.08	11.67	10.19	10.75	12.25
PPP2R3A	0.68	0.32	0.35	0.45	0.44	0.67
PPP2R3B	1	0.81	1.16	1.1	1.28	1.29
PPP2R3C	12.66	14.62	10.82	11.02	11.4	11.12
PPP2R4	42.22	38.9	42.63	45.07	44.67	42.94
PPP2R5A	3.52	2.08	3.12	1.7	2.73	3.28
PPP2R5B	0.5	0.21	0.15	0.09	0.05	0.22
PPP2R5C	48.49	38.49	37.29	38.92	45.15	39.74
PPP2R5D	33.01	29.3	30.08	29.44	28.02	24.37
PPP2R5E	2.4	2.13	2.2	0.96	2.14	1.99
PPP3CA	6.92	7.81	8.8	6.76	7.86	6.12
PPP3CB	1.73	1.06	1.21	0.89	1.23	1.19
PPP3CC	4.87	5.35	4.95	5.13	5.12	3.82
PPP3R1	8.18	5.81	4.06	4.51	7.15	6.76
PPP3R2	0	0	0	0	0	0
PPP4C	43.41	53.32	50.4	53.26	49.23	42.77
PPP4R1	8.56	7.89	9.98	6.94	6.49	7.39
PPP4R1L	0.31	0.05	0.08	0.04	0	0.03
PPP4R2	4.87	4.89	3.38	4.49	3.01	3.27
PPP4R4	0	0	0.16	0	0	0
PPP5C	45.69	51.77	46.31	53.14	55.11	45.69

PPP5D1	0	0.12	0.17	0	0.35	0.14
PPP6C	27.25	22.92	20.24	22.12	21.8	23.22
PPP6R1	0.78	1.66	1.21	0.45	0.53	1.33
PPP6R2	7.88	6.09	6.45	7.22	7.08	5.82
PPP6R3	19.98	16.93	14.37	15.53	16.37	18.8
PPRC1	14.44	16.82	17.51	17.52	16.61	13.68
PPT1	57.68	66	62.57	62.07	61.58	57.21
PPT2	19.66	14.18	11.49	17.02	18.6	19.49
PPT2-EGFL8	0	0.24	0.04	0.1	0.4	0.4
PPTC7	1.59	1.84	1.08	0.72	1.35	0.9
PPWD1	13.12	17	19.01	17.19	15.34	20.71
PPY	0.19	0	0	0.13	0	0
PPY2	0	0	0	0	0	0
PPYR1	0	0	0	0	0	0
PQBP1	145.5	119.83	113.24	133.71	128.47	107.06
PQLC1	4.01	6.17	6.1	5.85	5.34	5.96
PQLC2	11.46	7.84	9.17	7.55	11.08	5.48
PQLC3	5.61	10.81	10.43	10.05	8.53	7.78
PRAC	0	0	0	0	0	0
PRADC1	25.5	34.71	35.1	26.88	28.76	24.41
PRAF2	2.04	0.9	0.55	0.54	1.42	1.09
PRAM1	1.43	9.82	7.5	1.74	2.45	11.06
PRAME	0.37	0.85	1.48	0.64	0.51	1.63
PRAMEF1	0	0	0	0	0	0
PRAMEF10	0	0	0.05	0.16	0.07	0.12
PRAMEF11	0	0	0	0	0	0
PRAMEF12	0	0	0	0	0	0
PRAMEF13	0	0	0	0	0	0
PRAMEF14	0	0	0	0	0	0
PRAMEF15	0	0	0	0	0	0
PRAMEF16	0	0.22	0.16	0	0.2	0
PRAMEF17	0.1	0	0.17	0.13	0	0.12
PRAMEF18	0	0	0	0	0	0
PRAMEF19	0	0	0	0	0	0
PRAMEF2	0	0	0	0	0	0
PRAMEF20	0	0	0	0	0	0
PRAMEF21	0	0	0	0	0	0
PRAMEF22	0	0	0	0	0	0
PRAMEF3	0	0	0	0	0	0
PRAMEF4	0	0	0	0	0	0
PRAMEF5	0	0	0	0	0	0
PRAMEF6	0	0	0	0	0	0
PRAMEF7	0	0	0	0	0	0

PRAMEF8	0	0	0	0	0	0
PRAMEF9	0	0	0	0	0	0
PRAP1	0.11	0.17	0.33	0	0	0
PRB1	0	0	0	0	0	0
PRB2	0	0	0	0	0	0
PRB3	0	0	0	0	0	0
PRB4	0	0	0	0	0	0
PRC1	38.02	27.57	23.96	23.83	28.12	21.8
PRCC	13.19	13.32	13.54	14.93	13.21	12.29
PRCD	0	0	0	0	0	0
PRCP	25.55	30.69	27.1	22.59	28.66	28.98
PRDM1	0.03	0.44	0.14	0.2	0.2	0.2
PRDM10	2.35	2.04	2.22	1.5	1.2	1.2
PRDM11	0.16	0.06	0.09	0.08	0.05	0.16
PRDM12	0	0.02	0	0	0	0
PRDM13	0	0	0	0	0	0
PRDM14	0.06	0.08	0.03	0.01	0	0.03
PRDM15	1.12	1.25	1.61	1.41	0.86	1.54
PRDM16	0	0	0	0	0.02	0
PRDM2	4.13	4.9	5.36	3.95	5.21	5.18
PRDM4	1.76	2.73	2.26	1.85	1.97	2.17
PRDM5	0	0	0	0	0	0
PRDM6	0.07	0	0.13	0	0	0
PRDM7	0.21	0.09	0.16	0.1	0.22	0.15
PRDM8	1.93	0.22	0.53	0.46	1.7	0.57
PRDM9	0	0	0	0	0	0
PRDX1	715.05	785.91	724.33	739.27	748.7	660.77
PRDX2	452.97	473.84	429.32	490.76	503.19	514.41
PRDX3	218.1	247.37	214.32	168.66	197.3	207.77
PRDX4	544.55	608.57	605.65	640.4	654.7	628.84
PRDX5	154.89	224	204.88	230.43	216.97	200.58
PRDX6	242.28	236.88	234.4	241.02	243.92	250.54
PREB	34.15	36.44	43.19	38.62	37.67	20.9
PRELID1	372.78	373.45	399	404.4	434.16	297.93
PRELID2	8.58	9.44	8.49	7.89	11.25	9.42
PRELP	0.41	0.45	0.4	0.27	0.32	0.24
PREP	19.11	23.83	23.94	18.08	20.17	13.19
PREPL	6.52	4.07	3.52	3.15	4.54	5.26
PREX1	0.17	0.78	0.81	0.45	0.44	1.11
PREX2	0	0.02	0	0	0	0
PRF1	0.03	0	0	0	0.03	0.03
PRG1	0	0	0	0	0	0
PRG2	25.56	3.09	0.67	1.39	5.27	6.45

PRG3	0	0	0	0	0	0
PRG4	0	0	0.01	0.01	0	0
PRH1	0.49	0	0	0	0	0.25
PRH1-PRR4	2.79	2.2	1.22	1.92	1.33	1.8
PRH2	0.05	0.01	0.06	0.02	0.02	0.1
PRHOXNB	0	0	0	0	0	0
PRICKLE1	7.29	0	0.17	2.29	2.81	0
PRICKLE2	0	0	0	0	0	0
PRICKLE2-AS	0.01	0.01	0.01	0.02	0.03	0.02
PRICKLE2-AS	0	0	0	0	0	0
PRICKLE2-AS	2.43	3.11	3.97	1.81	2.61	2.52
PRICKLE3	14.45	17.64	17.94	16.87	16.41	18.86
PRICKLE4	0.32	0.29	0.58	0.72	0.43	0.78
PRIM1	29.6	32.63	37.8	33.41	36.32	29.98
PRIM2	14.85	12.33	17.46	15.23	12.71	13.59
PRIMA1	0	0	0	0	0	0
PRKAA1	7.37	4.65	3.28	3.94	5.02	4.51
PRKAA2	0	0.02	0	0.01	0	0.01
PRKAB1	28.4	16.9	12.14	18.44	28.1	18.23
PRKAB2	1.2	1.64	1.39	1.38	0.88	1.45
PRKACA	1.27	0.61	0.9	1.14	0.95	1.38
PRKACB	8.98	10.36	6.32	8.94	10.16	8.65
PRKACG	0	0	0	0	0	0
PRKAG1	22.65	18.81	16.76	23.76	22.39	20.28
PRKAG2	2.15	4.12	2.92	2	2.93	4.07
PRKAG2-AS1	1.49	2.2	1.82	1.59	2.74	1.81
PRKAG3	0	0	0	0	0	0
PRKAR1A	62.58	35.56	29.26	31.9	27.98	30.44
PRKAR1B	5.35	5.32	3.56	6.71	5.83	5.91
PRKAR2A	11.93	18.26	20.93	18.04	15.48	23.4
PRKAR2B	16.86	0.51	0.65	3.45	8.26	0.86
PRKCA	1.74	1.01	1.06	1.06	1.39	1.13
PRKCB	3.92	1.2	1.16	1.73	1.72	1.8
PRKCD	1.83	5.02	3	1.28	2.05	2.42
PRKCDBP	0	0	0	0	0	0
PRKCE	0.77	0.83	1.09	1.4	0.86	1.33
PRKCG	0	0	0	0	0	0
PRKCH	1.08	3.67	3.96	2.87	3.59	3.08
PRKCI	3.13	1.85	1.85	2.3	3.97	2.95
PRKCQ	4.84	6.16	7.2	8.67	7.27	8.05
PRKCQ-AS1	1.81	2.23	1.8	1.92	1.33	2.66
PRKCSH	41.98	39.98	47.12	49.46	47.28	51.48
PRKCZ	1.13	1.08	0.53	1.87	0.97	1.09

PRKD1	0.28	0.02	0	0	0.04	0
PRKD2	2.1	3.56	3.48	2.94	2.32	2.63
PRKD3	0.84	2.85	2.34	1.77	2.07	2.91
PRKDC	50.15	54.93	47.23	50.85	51.45	58.59
PRKG1	0	0	0	0	0	0
PRKG1-AS1	0	0	0	0	0	0
PRKG2	0	0	0	0	0	0
PRKRA	26.41	23.54	20.68	20.99	22.94	16.95
PRKRIP1	5.53	5.45	6.35	5	4.5	5.03
PRKRIR	3.19	3.75	3.57	4.2	4.17	4.39
PRKX	2.93	3.51	3.81	2.86	3.38	4.09
PRKXP1	1.09	1	1	0.71	0.87	0.91
PRKY	0.05	0.08	0.11	0.04	0.03	0.04
PRL	0	0	0	0	0	0
PRLH	0	0	0	0	0	0
PRLHR	0	0	0	0	0	0
PRLR	0.03	0.01	0.01	0.01	0.01	0.01
PRM1	0	0	0	0	0	0
PRM2	0	0	0	0	0	0
PRM3	0	0	0	0	0	0
PRMT1	178.2	180.32	185.42	216.89	199.06	201.11
PRMT10	6.25	5.77	4.56	7.63	5.21	5.98
PRMT2	28.72	19.69	16.46	19.67	19.41	21.33
PRMT3	10.27	8.12	8.76	8.72	10.84	10.18
PRMT5	76.89	84.32	85.88	89.62	83.02	87.11
PRMT6	21.81	17.98	18.94	14.31	16.59	21.06
PRMT7	24.7	34.69	33.23	32.08	28.23	29.73
PRMT8	0	0	0	0	0	0
PRND	0.13	0.25	0.17	0.25	0.07	0.28
PRNP	22.11	17.2	18.22	14.87	16.74	15.95
PRNT	0	0	0	0	0	0
PROB1	0.29	0.33	0.27	0.31	0.65	0.27
PROC	0	0.3	0.15	0.32	0	0.51
PROCA1	0.18	0.81	1.17	0.04	0.75	0.49
PROCR	5.85	3.3	3.32	5.35	3.86	6.07
PRODH	0	0	0.34	0.08	0.14	0.04
PRODH2	0	0	0	0	0	0
PROK1	0	0	0	0	0	0
PROK2	0	0	0	0	0	0
PROKR1	0	0	0	0	0.12	0
PROKR2	0	0	0	0	0	0
PROL1	0	0	0	0	0	0
PROM1	0	0	0	0	0	0

PROM2	0.22	0.09	0.38	0.17	0.2	0.14
PROP1	0	0	0	0	0	0
PRORSD1P	0.45	0.5	0.48	0.16	0.45	0.68
PROS1	21.47	4.65	4.64	5.11	10.65	4.93
PROSAPIP1	0.19	0.31	0.29	0.33	0.17	0.38
PROSC	38.45	53.42	43.96	44.78	37.12	43.96
PROSER1	7.78	8.35	5.26	6.86	8.43	6.1
PROSER2	0.92	1.17	1.1	0.88	1.01	1.15
PROSER2-AS	0.11	0.09	0.25	0.11	0.13	0.05
PROX1	1.4	0.12	0	0.29	0.21	0
PROX2	0	0	0.06	0.06	0.02	0.06
PROZ	0	0.04	0	0.07	0	0
PRPF18	13.44	11.28	9.11	8.39	13.43	7.99
PRPF19	16.89	17.88	20.49	15.92	18.52	22.45
PRPF3	25.58	19.23	23.28	21.24	26.02	20.27
PRPF31	57.47	67.31	62.33	65.08	52.08	58.47
PRPF38A	27.18	28.03	22.27	20.86	28.67	23.05
PRPF38B	14.68	20.17	17.41	14.93	17.71	17.76
PRPF39	4.89	5.01	3.52	4.44	6.53	6.72
PRPF4	32.75	35.04	28.57	31.76	35.13	33.17
PRPF40A	20.54	22.38	19.02	18.75	22.63	21.77
PRPF40B	0.35	0.16	0	0.14	0.14	0.05
PRPF4B	4.63	6.38	5.25	3.61	6.04	3.64
PRPF6	43.32	39.23	42.98	45.74	37.24	32.83
PRPF8	57.56	68.37	63.55	60.5	62.19	80.27
PRPH	0.12	0.06	0.15	0.25	0.42	0.25
PRPH2	0.02	0.06	0.08	0.03	0	0.15
PRPS1	36.5	36.82	33.15	40.94	41.16	41.66
PRPS1L1	0	0	0	0	0	0
PRPS2	73.68	72.64	73.76	71.08	83	66
PRPSAP1	10.34	17.73	14.22	14.58	12.4	13.29
PRPSAP2	22.57	18.6	15.02	17.35	24.4	19.67
PRR11	15.25	18.79	19.59	15.84	18.12	17.28
PRR12	0.54	0.78	0.81	0.62	0.64	0.77
PRR13	127.46	154.83	159.07	150.31	151.02	147.67
PRR14	6.4	3.67	4.43	5.08	5.22	4.08
PRR14L	6.84	6.97	7.46	6.79	6.9	7.67
PRR15	0.49	0.38	0.24	0.18	0.34	0.31
PRR15L	0	0	0	0	0	0
PRR16	0	0.09	0	0	0	0
PRR18	0	0	0	0	0	0
PRR19	2.24	0.25	1.39	2.71	1.89	1.02
PRR20A	0	0	0	0	0	0

PRR20B	0	0	0	0	0	0
PRR20C	0	0	0	0	0	0
PRR20D	0	0	0	0	0	0
PRR20E	0	0	0	0	0	0
PRR21	0	0	0	0	0	0
PRR22	0.32	0.3	0.05	0.37	0.41	0.44
PRR23A	0.07	0.2	0.16	0.18	0.28	0.28
PRR23B	0	0	0	0	0	0
PRR23C	0.27	0.12	0.39	0.16	0.35	0.42
PRR24	7.18	2.98	3.92	4.89	4.03	2.49
PRR25	0	0	0	0.17	0	0.06
PRR26	0	0	0	0	0	0
PRR3	11.49	9.43	6.33	10.34	9.99	8.95
PRR4	0	0	0	0	0	0
PRR5	14.69	3.54	3.17	7.25	6	1.89
PRR5-ARHGA	0	0	0.07	0	0	0
PRR5L	0.24	0.34	0.52	0.63	0	0
PRR7	2.25	3.44	3.96	2.99	3.71	2.1
PRR7-AS1	0.46	0.49	0.73	0.45	0.47	0.82
PRR9	0.06	0	0	0	0	0
PRRC1	8.99	9.92	8.15	5.94	8.33	7.73
PRRC2A	19.71	23.52	24.6	23.68	22.2	23.15
PRRC2B	6.73	5.71	7.12	7.89	6.18	7.65
PRRC2C	26.93	26.93	27.02	23.71	26.23	25.83
PRRG1	8.07	4.75	4.79	4.04	6.24	4.76
PRRG2	0.54	0.43	0.3	0.89	0.36	0.76
PRRG3	0	0	0	0	0	0
PRRG4	1.39	2.68	2.21	1.19	2	2.39
PRRT1	0.04	0.09	0.04	0.16	0.07	0.04
PRRT2	0	0.02	0	0.04	0.15	0.05
PRRT3	0.47	0.78	0.96	1	1.15	0.55
PRRT3-AS1	0	0.42	0	0	0.41	0.13
PRRT4	0.21	1.72	1.29	0.61	0.52	0.84
PRRX1	0	0	0	0	0	0
PRRX2	0	0	0	0	0	0
PRSS1	0	0	0	0	0	0
PRSS12	0.11	0.3	0.29	0.1	0.3	0.19
PRSS16	0.61	0.9	1.03	0.91	0.75	0.98
PRSS2	0	0	0	0	0	0
PRSS21	0	0	0	0	0	0
PRSS22	0	0	0	0	0	0
PRSS23	24.41	0.08	0.07	3.18	5.79	0.23
PRSS27	0	0	0	0	0	0

PRSS3	0	0	0	0	0	0
PRSS30P	0	0	0	0	0.02	0
PRSS33	0	0	0	0	0	0
PRSS35	0	0	0.13	0	0	0
PRSS36	0	0.16	0	0.04	0.16	0.13
PRSS37	0	0	0	0	0	0
PRSS38	0	0	0	0	0	0
PRSS3P2	0	0	0	0	0	0
PRSS41	0	0	0	0	0	0
PRSS42	0	0	0	0	0	0
PRSS45	0	0	0	0	0	0
PRSS46	0	0	0	0	0	0
PRSS48	0	0	0	0	0	0
PRSS50	0	0	0	0	0	0
PRSS53	0.33	0.77	0.87	0.9	0.55	0.41
PRSS54	0	0	0	0	0	0
PRSS55	0	0	0	0	0	0
PRSS56	0	0	0	0	0	0
PRSS57	76.67	428.21	362.05	294.87	251.2	457.31
PRSS58	0	0	0	0	0	0
PRSS8	0.11	0	0	0	0	0.04
PRTFDC1	12.03	15.23	11.43	14.94	12.26	16.79
PRTG	0.49	0.5	0.42	0.41	0.31	0.48
PRTN3	91.68	1754.06	149.6	3.83	63.68	1930.6
PRUNE	12.64	9.82	9.64	13.29	10.83	9.74
PRUNE2	0.01	0	0	0	0	0
PRX	0.1	0.09	0.05	0.23	0.05	0.18
PRY	0	0	0	0	0	0
PRY2	0	0	0	0	0	0
PSAP	204.36	124.63	101.82	108.16	121.2	107.37
PSAPL1	0	0	0	0	0	0
PSAT1	71.74	75.66	69.97	48.53	50.21	63.59
PSCA	0.26	0.18	0.41	0	0	0
PSD	0.04	0	0	0.03	0.04	0.08
PSD2	0	0	0	0	0	0
PSD3	2.42	4.37	3.25	2.29	2.81	3.99
PSD4	6.18	5.7	6.7	6.44	6.73	10.8
PSEN1	9.94	11	9.16	9.51	7.19	9.84
PSEN2	10.72	7.46	6.83	6.85	8.54	7.67
PSENEN	78.92	63.71	60.78	59.78	63.65	58.86
PSG1	0	0	0	0	0	0
PSG10P	0	0	0	0	0	0
PSG11	0	0	0	0	0	0

PSG2	0	0	0	0	0	0
PSG3	0	0	0	0	0	0
PSG4	0	0	0	0	0	0
PSG5	0	0	0	0	0	0
PSG6	0	0	0	0	0	0
PSG7	0	0	0	0	0	0.09
PSG8	0	0	0	0	0	0
PSG9	0	0	0	0	0	0
PSIMCT-1	0	0.16	0	0.12	0.19	0.7
PSIP1	11.88	8.66	7.41	11.53	12.97	7.51
PSKH1	23.32	20.99	23.04	23.83	21.05	27.14
PSKH2	0	0	0	0	0	0
PSMA1	225.77	216.24	198.51	196.46	207.91	201.25
PSMA2	212.65	218.76	198.78	183.97	225.19	192.17
PSMA3	289.72	287.77	270.27	261.59	285.31	236.31
PSMA4	495.32	543.54	447.04	413.99	504.63	445.74
PSMA5	76.61	70.32	62.34	67.53	66.84	56.08
PSMA6	308.23	375.49	351.28	356.06	349.47	344.28
PSMA7	527.6	481.12	448.19	454.8	537.35	438.59
PSMA8	0	0	0	0	0	0
PSMB1	478.36	447.18	406.93	432.34	501.93	420.65
PSMB10	63.88	111.77	134.43	120.92	82.97	93.96
PSMB11	0	0	0	0	0	0
PSMB2	71.14	73.52	66.89	67.53	72.85	70.18
PSMB3	449.06	468.03	396.78	390.3	450.92	394.11
PSMB4	499.22	465.39	453.24	457.2	499.09	376.16
PSMB5	293.29	245.07	252.51	275.01	289.94	227.22
PSMB6	417.2	393.42	361.11	343.85	389.02	318.72
PSMB7	314.49	275.49	259.81	272.09	298.71	231.31
PSMB8	70.38	142.54	152.8	155.02	128.44	124.64
PSMB9	5.05	21.48	28.12	25.68	13.65	15.6
PSMC1	141.07	134.73	120.44	111.62	122.45	100.09
PSMC2	47.3	48.22	41.48	42.77	43.05	35.13
PSMC3	166.15	178.05	190.49	170.87	179.05	145.28
PSMC3IP	8.99	10.69	6.12	5.73	7.09	8.09
PSMC4	75.69	59.69	43.76	52.1	65.26	46.12
PSMC5	148.44	164.71	144.39	152.85	150.28	141.98
PSMC6	78.65	73.42	74.16	69.77	76.9	60.28
PSMD1	68.95	71.32	72.21	70.26	74.14	60.49
PSMD10	98.99	85.5	75.59	77.27	101.85	68.22
PSMD11	76.53	77.48	69.73	65.96	68.81	59.12
PSMD12	67.62	55.33	48.85	46.34	61.25	54.77
PSMD13	143.02	128.52	118.01	115.71	121.08	110.9

PSMD14	158.06	126.69	117.96	127.87	131.38	110.76
PSMD2	67.39	67.72	70.42	66.44	66.63	47.55
PSMD3	37.04	45.03	61.27	51.53	55.43	31.82
PSMD4	180.54	170.74	143.95	152.29	156.98	140.95
PSMD5	0.19	0.09	0.08	0.06	0.08	0.1
PSMD6	104.15	103.37	100.11	94.38	97.89	95.58
PSMD6-AS2	0.29	1	0.48	0.18	0.35	0.52
PSMD7	103.21	92.05	81.03	85.53	93.19	68.42
PSMD8	201.81	213.01	187.46	215.61	227.69	183.73
PSMD9	47.11	43.67	35.65	37.75	42.57	34.34
PSME1	278.94	366.72	382.87	377.08	347.2	325.71
PSME2	165.86	247.39	265.56	237.56	193.84	203.85
PSME3	64.87	64.18	66.97	62.33	59.95	63.59
PSME4	5.54	4.9	3.58	3.51	4.51	4.25
PSMF1	67.3	53.37	61.91	63.02	58.16	60.31
PSMG1	122.98	130.73	134.33	107.8	120.87	114.57
PSMG2	94.41	97.94	90.36	78.43	93.77	101.29
PSMG3	76.79	81.98	68.48	70.12	72.8	58.06
PSMG3-AS1	0.88	1.63	1.97	1.04	1.41	1.37
PSMG4	40.26	39.13	38.91	40.37	39.72	33.11
PSORS1C1	0	0	0	0	0	0
PSORS1C2	0	0	0	0	0	0
PSORS1C3	0	0	0	0	0	0
PSPC1	33.12	27.48	26.46	28.7	26.78	33.26
PSPH	16.24	24.03	23.34	17.46	18.86	23.02
PSPN	0.72	0	0.66	0.13	0.17	0.73
PSRC1	15.93	12.86	17.82	12.7	13.55	14.65
PSTK	3.66	3.02	4.59	2.01	3.17	2.69
PSTPIP1	0	1.76	1.17	0.17	0.49	3.6
PSTPIP2	18.49	12.97	11.85	11.59	14.38	12.05
PTAFR	0.82	1	1.39	0.61	0.47	0.84
PTAR1	1.82	1.36	1.79	1.25	1.64	2.06
PTBP1	153.89	167.99	164.86	159	165.53	170.06
PTBP2	1.96	1.18	1.14	1.1	0.98	2.4
PTBP3	16.28	15.53	14.06	10.22	14.42	14.49
PTCD1	8.71	7.47	7.22	8.67	8.75	8.42
PTCD2	6	7.1	6.64	5.36	5.4	7.63
PTCD3	15.7	18.74	14.96	16.11	14.91	16.22
PTCH1	0.67	0.04	0.06	0.61	0.17	0.18
PTCH2	0	0.09	0.42	0.01	0.2	0.45
PTCHD1	0	0	0	0	0	0
PTCHD2	0	0.03	0.03	0	0.01	0.01
PTCHD3	0	0	0	0	0	0

PTCHD3P1	9.42	10.5	8.19	8.87	8.76	6.5
PTCHD4	0.48	0.61	0.7	0.16	0.3	0.36
PTCRA	0	0	0	0	0	0
PTCSC3	1.05	1.18	0.6	1.14	0.81	0.54
PTDSS1	115.56	165.97	187.18	151.13	136.89	164.53
PTDSS2	3.66	4.28	4.94	4.31	3.99	3.95
PTEN	9.44	14.17	11.69	10.2	11.9	12.99
PTENP1	0.04	0.03	0	0	0.05	0
PTER	2.93	4.21	2.63	2.68	3.28	4.52
PTF1A	0	0	0	0	0	0
PTGDR	0	0	0	0	0	0
PTGDR2	0.23	0.04	0.14	0.61	0.74	0.2
PTGDS	0	0	0	0	0.09	0.1
PTGER1	0	0	0.05	0	0	0
PTGER2	0	0	0	0	0	0
PTGER3	0.05	0	0	0	0.54	0.22
PTGER4	0.32	0.87	0.45	0.45	0.5	0.75
PTGER4P2-C	0.49	0.67	0.47	0.42	0.52	0.41
PTGES	0.1	0.03	0.36	0	0.25	0.43
PTGES2	20.42	19.95	20.55	26.31	19.19	24.21
PTGES3	312.41	310.02	297.23	267.19	300.84	323.97
PTGES3L	2.17	0.96	1.28	1.36	0.7	0.8
PTGES3L-AA	0.97	0	0.21	0	0.37	0
PTGFR	0	0	0	0	0	0
PTGFRN	6.14	3.96	3.29	3.21	2.9	3.87
PTGIR	0.88	0.17	0.91	1.63	0.43	0.32
PTGIS	0.81	0.64	0.52	0.41	0.39	0.57
PTGR1	0	0	0	0	0	0
PTGR2	5.12	6.16	4.14	6.01	6.57	6.65
PTGS1	19.02	2.46	4.24	7.11	6.95	3.44
PTGS2	0	0	0.06	0	0	0
PTH	0	0	0	0	0	0
PTH1R	0	0	0	0	0	0
PTH2	0	0	0	0	0	0
PTH2R	0	0	0	0	0	0
PTHLH	7.72	0	0	0.24	2.6	0.15
PTK2	15.01	0.15	0.01	3.55	3.55	0.1
PTK2B	15.25	24.38	24.59	15.54	13.44	20.83
PTK6	0.5	0.68	1.07	0.32	0.31	0.29
PTK7	4.38	7.6	7.07	8.44	6.49	7.64
PTMA	1296.07	1577.39	1439.54	1405.31	1606.95	1610.81
PTMS	14.7	6.58	10.82	8.36	13.18	8.43
PTN	0	0	0	0	0	0

PTOV1	12.11	8.74	12.16	10.56	11.32	9.44
PTOV1-AS1	2.19	2.52	1.72	2.5	2.18	2.59
PTP4A1	38.24	35.16	29.89	26.93	35.6	32.9
PTP4A2	22.77	24.43	20.01	15.7	21.81	16.98
PTP4A3	25.71	12.53	13.99	23.15	29.81	15.02
PTPDC1	0.45	0.24	0.29	0.49	0.56	0.39
PTPLA	21.2	12.75	18.82	21.59	31.36	13.02
PTPLAD1	70.69	80.01	67.51	62.76	72.08	70.4
PTPLAD2	10.67	18.82	20.38	18.44	13.72	21.51
PTPLB	4.22	6.06	6.21	4.24	3.33	9.57
PTPMT1	37.17	48.05	42.87	39.58	37.43	40.7
PTPN1	16.07	13.76	14.74	14.74	13.71	14.36
PTPN11	17.93	19.69	17.18	14.43	20.38	14.24
PTPN12	4.29	2.86	2.58	2.14	3.47	3.85
PTPN13	1.55	1.71	1.51	1.04	1.25	1.22
PTPN14	3.51	3.81	2.78	2.8	2.38	2.41
PTPN18	4.55	4.8	5.11	5.37	5.88	7.22
PTPN2	31.01	29.96	29.57	27.71	33.16	27.36
PTPN20A	0	0	0	0	0	0
PTPN20B	0	0	0	0	0	0
PTPN21	0.01	0	0	0.02	0.01	0
PTPN22	0.08	2.5	2.05	0.53	0.79	1.69
PTPN23	5.04	4.89	5.04	5.29	3.76	4.33
PTPN3	0.03	0.2	0.06	0.02	0.07	0.11
PTPN4	0.26	0.31	0.52	0.33	0.29	0.29
PTPN5	0	0	0	0	0	0
PTPN6	61.19	79.51	71.09	67.39	75.79	66.56
PTPN7	85.9	65.25	60.05	56.61	77.99	62.01
PTPN9	4.1	3.59	3.6	2.54	3.11	2.22
PTPRA	4.02	3.6	5.16	3.37	4.83	3.45
PTPRB	0.23	0.08	0.07	0.05	0.06	0.13
PTPRC	17.45	11.51	10.14	9.38	10.66	11.24
PTPRCAP	5.74	110.89	131.12	143.11	116.97	40.72
PTPRD	0	0	0	0.01	0	0
PTPRE	0.47	0.9	0.41	0.58	0.34	0.29
PTPRF	3.52	7.52	7.24	7.53	6.6	9.6
PTPRG	1.32	1.56	1.55	0.96	1.06	0.97
PTPRG-AS1	0.9	1.05	1.04	0.63	0.76	0.68
PTPRH	0.15	0	0	0	0	0
PTPRJ	2.17	0.74	0.4	0.45	0.71	0.74
PTPRK	0.46	3.07	2.41	3.58	2.86	3.63
PTPRM	0.09	0.05	0.05	0	0.02	0.01
PTPRN	0	0	0	0	0	0

PTPRN2	0	0	0	0	0.01	0
PTPRO	0	0	0	0	0	0
PTPRQ	0	0	0	0	0	0
PTPRR	0.11	0	0	0	0.03	0
PTPRS	12.5	7.31	10.74	10.79	11.88	10.48
PTPRT	0	0	0	0	0	0
PTPRU	1.77	3.89	3.78	2.4	2.99	6.46
PTPRVP	0.09	0.07	0.02	0.1	0.08	0.16
PTPRZ1	0	0	0	0	0	0.01
PTRF	46.67	10.96	16.82	30.85	36.85	12.96
PTRH1	21.86	20.15	19.11	23.6	26.29	13.9
PTRH2	86.05	75.78	62.66	75.25	80.35	74.01
PTRHD1	96.45	83.33	81.33	88.64	98.15	83.29
PTS	22.97	9.69	7.36	6.25	11.79	9.13
PTTG1	218.12	203.18	196.4	216.35	240.08	202.18
PTTG1IP	36.02	41.3	40.93	36.12	40	42.32
PTTG2	0	0.11	0	0	0.14	0
PTTG3P	0	0	0.12	0	0	0.27
PTX3	0	0	0	0	0	0
PTX4	0.05	0.21	0.1	0.04	0.2	0.11
PUF60	349.21	329.81	310.23	375.16	349.32	311.3
PUM1	14.04	13.88	13.67	14.13	13.16	14.85
PUM2	4.72	3.69	4.13	3.37	3.65	4.96
PURA	2.82	2.54	3.65	3.17	2.77	2.8
PURB	0.87	0.88	0.63	0.76	0.95	0.9
PURG	0.57	0	0	0	0.07	0
PUS1	6.84	7.44	8.35	6.88	7.36	6.35
PUS10	1.66	0.69	1.17	0.92	0.65	0.77
PUS3	11.71	11.3	11.66	10.67	12.59	7.33
PUS7	13.12	13.06	12.72	10.19	12.77	12.5
PUS7L	5.01	6.29	6.31	4.07	4.77	5.55
PUSL1	22.34	28.93	29.96	25.9	31.76	26.42
PVALB	0	0	0	0	0	0
PVR	13.92	10.02	11	7.51	8.41	10.09
PVRIG	0.11	0.14	0.21	0.11	0.1	0.06
PVRL1	0.69	1.56	1.45	1.43	1.64	2.16
PVRL2	14.17	8.8	11.27	10.9	12.1	7.58
PVRL3	0	0	0	0	0.06	0
PVRL3-AS1	0	0	0	0	0	0
PVRL4	0	0	0.04	0.05	0	0.04
PVT1	5.02	2.33	2.23	2.71	2.95	2.19
PWP1	56.01	48.06	44.74	50.65	50.38	43.58
PWP2	19.44	19.77	21.06	22.15	18.53	20.41

PWRN1	0	0	0	0	0	0
PWRN2	0	0	0	0	0	0
PWWP2A	1.11	0.98	1.2	1.07	1.17	1.81
PWWP2B	1.18	1.46	1.23	1.14	1.68	1.64
PXDC1	0	0	0.03	0	0	0
PXDN	0	0	0	0	0	0
PXDNL	0	0.04	0	0	0	0
PXK	13.91	43.8	34.68	19.6	18.37	38.42
PXMP2	11.31	12.43	15.07	16.02	14.54	7.17
PXMP4	6.17	5.46	6.52	4.61	6.48	5.51
PXN	11.87	5.73	4.91	7	8.66	6.32
PXN-AS1	12.25	18.15	12.93	14.77	15.35	13.7
PXT1	0	0	0	0	0.03	0
PYCARD	106.85	197.27	196.38	157.99	134.59	157.83
PYCR1	88.61	82.96	71.62	84.36	87.88	84.85
PYCR2	34.54	22.49	22.22	29.01	26.62	20.06
PYCRL	35.37	30.96	30.07	33.4	29.16	32.75
PYDC1	0	0	0	0	0	0
PYDC2	0	0	0	0	0	0
PYGB	13.33	16.51	12.82	10.89	10.85	19.08
PYGL	35.83	67.09	50.32	45.05	38.75	59.69
PYGM	0.07	0.14	0.06	0	0.04	0.07
PYGO1	0.96	0.57	1.37	0.5	0.51	0.73
PYGO2	7.3	7.35	10.46	9.44	8.66	8.48
PYHIN1	0	0	0	0	0	0
PYROXD1	4.21	3.44	2.05	2.74	1.88	1.74
PYROXD2	0	0	0.16	0	0	0
PYURF	62.86	55.27	40.56	44.42	54.68	43.01
PYY	0	0	0	0	0	0
PYY2	0	0	0	0	0	0
PZP	0	0	0	0	0	0
QARS	74.52	95.01	89.36	115.24	93.25	114.44
QDPR	30.75	43.9	46.04	39.83	35.18	35.19
QKI	1.78	2.01	1.63	1.13	1.58	1.98
QPCT	0	0	0	0	0	0
QPCTL	8.74	10.21	12.56	10.21	8.24	13.34
QPRT	27.25	34.94	41.51	40.61	26.67	32.74
QRFP	1.06	0	0	0.3	0.41	0
QRFPR	0.16	0	0.2	0.14	0.18	0
QRICH1	21.14	25.49	23.71	25.76	26.41	19.52
QRICH2	0.35	0.17	0.22	0.31	0.22	0.27
QRSL1	6.02	9.79	7.34	7.74	8.41	10.76
QSER1	3.06	1.76	2.73	1.68	2.63	2.29

QSOX1	37.33	44.71	31.03	25.88	27.47	51.39
QSOX2	0.66	0.53	0.44	0.44	0.35	0.31
QTRT1	28.11	32.76	26.59	34.18	31.01	32.06
QTRTD1	8.27	10.16	9.13	10.26	9.9	10.56
R3HCC1	71.56	74.67	66.37	76.79	80.66	67.79
R3HCC1L	4.51	4.56	5.08	6.24	7.68	4.23
R3HDM1	13.96	12.34	11.1	9.94	13.14	12.82
R3HDM2	7.6	7.12	5.79	7.75	9	5.8
R3HDM4	14.36	10.18	11.04	15.76	14.76	11.7
R3HDML	0	0	0	0	0	0
RAB10	39.21	35.37	32.44	28.97	34.11	30.04
RAB11A	40.22	34.32	31.88	30	38.23	33.26
RAB11B	16.17	16.49	18.51	17.95	18.09	14.31
RAB11B-AS1	2.64	1.67	2.05	1.57	1.15	2.28
RAB11FIP1	16.32	10.03	7.95	7.09	10.87	9.61
RAB11FIP2	0.33	0.59	0.38	0.19	0.22	0.49
RAB11FIP3	0.52	0.84	0.56	0.48	0.47	0.51
RAB11FIP4	0.57	0.84	1	0.77	0.88	0.85
RAB11FIP5	1.22	1.64	1.6	1.5	1.41	1.85
RAB12	0.28	0.5	0.47	0.19	0.21	0.17
RAB13	62.35	51.76	50.03	56.2	57.69	49.66
RAB14	12.76	11.4	11.88	10.35	11.92	12.4
RAB15	0.69	2.75	3.37	2.31	2.18	3.51
RAB17	0	0	0	0	0	0
RAB18	9.26	8.13	5.75	5.89	7.26	7.43
RAB19	0	0	0	0	0	0
RAB1A	51.99	48.08	48.22	38.3	48.56	41.55
RAB1B	99.76	103.71	106.02	99.39	91.7	81.05
RAB20	13.15	8.64	10.85	7.32	14.49	8.03
RAB21	5.24	4.96	4.63	4.04	5.22	3.6
RAB22A	1.04	1.27	0.9	1.08	1.09	1.31
RAB23	0.93	1	0.62	0.68	0.96	0.54
RAB24	19.27	13.4	10.72	14.74	16.52	10.48
RAB25	0	0	0	0	0	0
RAB26	1.52	1.05	1.89	1.11	1.04	1.16
RAB27A	3.5	6.18	2.12	1.02	2.56	6.78
RAB27B	0	0	0	0	0	0
RAB28	7.19	6.17	4.02	3.54	6.2	5.03
RAB2A	22.18	19.87	23.02	20.37	25.05	16.88
RAB2B	4.91	3.13	2.97	3.56	3.45	3.98
RAB30	2.05	2.09	1.02	0.96	1.58	1.79
RAB31	9.16	5.92	4.96	3.13	8.55	3.49
RAB32	0	0	0	0	0	0

RAB33A	2.41	21.53	17.47	14.18	8.09	12.74
RAB33B	1.38	1.72	1.58	1.58	1.17	1.6
RAB34	0	0.32	0	0	0	0
RAB35	10.03	8.55	10.11	9.98	9.28	10.23
RAB36	0.36	0.47	0.19	0.28	0.2	0.23
RAB37	0.2	3.24	1.74	2.62	3.1	2.63
RAB38	19.11	21.46	19.7	13.99	27.05	19.11
RAB39A	0.46	0.07	0.53	0.23	0.16	0.25
RAB39B	1.38	1.05	1.34	1	0.75	0.68
RAB3A	3.09	3.62	7.31	5.09	2.92	5.15
RAB3B	1.15	0.69	1.29	1.19	0.89	0.94
RAB3C	0.08	0.12	0.21	0.32	2.46	5.01
RAB3D	2.98	5.03	6.09	4.45	4.24	6.18
RAB3GAP1	9.49	8.55	6.96	9.2	7.58	9.25
RAB3GAP2	5.19	3.81	4.38	3.05	4.06	4.04
RAB3IL1	0.54	0.71	0.28	0.36	0.44	0.39
RAB3IP	1.84	1.63	1.66	1.17	1.39	1.38
RAB40A	0.45	0	0	0	0	0
RAB40AL	0.07	0	0	0.05	0	0
RAB40B	4.67	3.36	2.01	2.85	3.03	2.82
RAB40C	2.96	5.57	4.09	3.86	2.97	5.26
RAB41	0	0	0	0	0.14	0.07
RAB42	2.24	4.17	5.06	3.07	3.16	3.65
RAB43	0.34	0	0.14	0.37	0.37	0.26
RAB44	0.14	1.41	0.41	0.2	0.29	1.14
RAB4A	4.55	6.24	8.01	6.11	6.05	6.85
RAB4B	6.9	10.33	8.75	8.6	6.39	9.43
RAB4B-EGLN	0.58	0	1.18	0	0	0
RAB5A	17.02	9.72	10.32	9.18	11.23	11.81
RAB5B	5.46	2.97	2.34	3.11	3.54	2.86
RAB5C	91.29	84.54	69.92	73.82	77.1	80.77
RAB6A	33.83	23.1	19.19	18.74	25.2	21.23
RAB6B	1.27	0.68	0.42	1.08	1.04	1.01
RAB6C	0.03	0	0.34	0.14	0	0.05
RAB7A	133.06	110.78	98.15	86.5	112.89	87.97
RAB7L1	10.72	9.29	6.47	6.21	7.86	8.17
RAB8A	54.2	96.38	94.72	62.24	65.75	84.6
RAB8B	5.25	3.48	3.5	3.84	4.66	4.69
RAB9A	36.77	30.02	22.59	28.73	31.53	24.23
RAB9B	0	0.02	0.03	0.01	0	0.04
RAB9BP1	0	0	0	0	0	0
RABAC1	54.82	53.5	63.05	71.73	70.95	62.49
RABEP1	13.15	11.55	10.92	11.81	14.09	11.62

RABEP2	2.39	2.59	2.39	2.99	3.65	3.49
RABEPK	37.36	43.6	49.58	47.24	45.49	44.02
RABGAP1	2.41	1.96	1.89	1.81	1.69	1.81
RABGAP1L	10.86	6.48	4.54	9.7	7.05	5.35
RABGEF1	4.6	4.22	5.19	4.2	3.73	5.28
RABGGTA	13.84	15.01	11.14	15.56	13.57	15.75
RABGGTB	35.41	34.52	29.02	25.68	33.57	23.99
RABIF	8.57	9.21	5.83	7.24	7.07	6.82
RABL2A	1.92	2.77	1.79	1.8	2.82	1.65
RABL2B	7.01	9.14	8.97	6.66	6.81	7.17
RABL3	10.49	12.56	8.15	8.57	9.71	9.12
RABL5	10.7	10.75	10.23	9.92	9.45	7.36
RABL6	6.36	8.47	8.72	6.84	8.96	8.56
RAC1	27.14	26.05	24.96	32.39	25.82	12.6
RAC2	293.32	97.96	105.56	123.78	151.13	83.3
RAC3	3.25	3.54	6.28	5.79	4.18	3.72
RACGAP1	36.82	40.14	36.17	37.19	37.63	38.41
RACGAP1P	0.05	0.19	0.24	0.2	0.15	0.19
RAD1	11.29	9.89	9.16	8.43	9.7	10.79
RAD17	6.72	8.02	3.81	5.2	7.9	5.66
RAD18	5.07	6.34	5.71	5.94	4.66	4.81
RAD21	38.58	40.26	33.87	28.27	39.54	42.44
RAD21-AS1	0	0.21	0.17	0.04	0.07	0.11
RAD21L1	0	0	0	0	0	0
RAD23A	51.41	45.76	44.07	37.79	41.72	35.53
RAD23B	5.68	4.04	4.94	3.16	4.03	3.65
RAD50	5.27	6.18	4.05	4.72	4.84	5.92
RAD51	29.14	34.45	34.13	40.74	32.62	31.75
RAD51AP1	9.85	16.62	8.09	8.45	9.77	10.14
RAD51AP2	0	0	0	0	0	0
RAD51B	3.29	2.82	2.24	3.64	1.69	2.91
RAD51C	47.85	57.24	41.41	43.1	53.86	47.01
RAD51D	7.82	13.01	10.98	7.45	9.99	10.99
RAD51L3-RF	0	0	0.18	0.07	0.24	0
RAD52	0.22	1.03	0.7	0.72	0.77	0.44
RAD54B	6.83	9.55	7.44	6.37	7.74	7.63
RAD54L	6.18	8.06	8.93	7.59	7.74	9.25
RAD54L2	6.68	7.49	10.65	9.28	7.55	7.78
RAD9A	1.97	1.94	2.29	1.98	2.49	2.46
RAD9B	0.2	0.37	0.25	0	0.37	0.28
RADIL	0.14	1.24	1.34	0.92	1.12	0.8
RAE1	48.08	40.16	30.96	36.06	42.53	41.65
RAET1E	0.04	0.47	0.74	0.17	0.33	0.01

RAET1G	0.34	0.6	0	0.65	0.82	0.35
RAET1K	0.46	1.94	1.04	0.56	0.97	1.28
RAET1L	0	0	0	0	0	0
RAF1	33.75	32.63	27.41	29.4	30.05	29.84
RAG1	0	0	0	0	0.01	0
RAG2	0.25	0.06	0.33	0	0.2	0.61
RAI1	1.9	2.14	2.11	1.29	1.99	2.34
RAI14	7.4	1.78	1.1	2.58	3.75	2.87
RAI2	0.43	0	0	0.27	0.31	0
RALA	23.24	16.12	15.23	15.74	14.96	14.6
RALB	25.24	14.6	15.33	11.28	16.64	13.57
RALBP1	10.66	13.32	15.7	11.87	13.13	13.41
RALGAPA1	1.1	0.46	0.71	0.62	0.58	0.51
RALGAPA2	1.05	2.04	1.59	0.92	1.05	2.72
RALGAPB	1.94	1.51	1.22	1.87	1	1.5
RALGDS	3.6	2.73	1.82	1.75	2.31	2.2
RALGPS1	0.05	0.09	0.15	0.03	0	0
RALGPS2	1.64	1.67	1.71	1.34	1.51	0.87
RALY	18.39	20.93	22.89	24.76	20.4	14.58
RALYL	0	0	0	0	0	0
RAMP1	52.96	7.15	19.38	22.21	30.77	12.85
RAMP2	0.5	0	0	0.07	0.76	0.3
RAMP2-AS1	1.76	2.07	4.55	1.28	2.47	2.58
RAMP3	0	0	0	0	0	0
RAN	876.46	907.81	805.86	826.11	889.98	829.31
RANBP1	196.12	218.75	210.1	214.27	225.37	205.56
RANBP10	2.21	3.42	3.12	2.38	2.85	2.7
RANBP17	0	0	0	0	0	0
RANBP2	8.25	7.68	7.27	6.9	6.9	8.4
RANBP3	15.83	15.38	13.59	16.73	15.26	17.75
RANBP3L	0	0	0	0	0	0
RANBP6	2.67	4.19	2.89	2.79	2.91	5
RANBP9	1.87	1.53	1.05	0.96	1.54	1.7
RANGAP1	64.11	61.27	56.6	64.72	58.28	62.43
RANGRF	54.56	73.47	64.27	71.69	74.55	54.77
RAP1A	9.09	7.42	6.49	5.71	6.15	6.81
RAP1B	112.47	81.84	80.37	75.42	96.82	86.85
RAP1GAP	10.78	0.42	0.02	3.41	3.81	0.31
RAP1GAP2	0.48	1.38	1.65	1.28	0.45	1.8
RAP1GDS1	21.19	18.48	17.14	18.09	21.17	19.93
RAP2A	1.53	0.71	1.16	0.7	1.27	1.18
RAP2B	14.85	16.69	17.44	17.41	15.99	11.96
RAP2C	10.68	5.43	5.86	4.59	6.51	7.3

RAPGEF1	1.78	1.88	1.54	2.17	2.28	1.84
RAPGEF2	5.08	1.43	0.89	1.96	2.92	1.14
RAPGEF3	0	0.03	0.06	0.06	0.03	0.03
RAPGEF4	0.4	0.61	0.21	0.27	0.52	0.3
RAPGEF4-AS	0	0	0	0.04	0.03	0
RAPGEF5	0	0	0	0	0	0
RAPGEF6	4.95	6.14	5.98	4.16	3.88	5.39
RAPGEFL1	0.6	0.77	0.68	0.48	0.61	0.28
RAPH1	0.04	0.03	0.03	0.08	0.18	0
RAPSN	0	0	0	0	0	0
RARA	4.09	4.16	6.79	3.62	4.22	5.2
RARB	0.13	0.06	0	0.02	0.17	0.08
RARG	0.88	1.55	2.91	2.08	2.03	1.39
RARRES1	0.15	0.21	0.16	0	0	0.36
RARRES2	0	0	0	0	0	0
RARRES3	0.62	0.16	0.76	2.29	0.88	0
RARS	56.68	53.6	50.86	48.69	44.32	40.66
RARS2	23.16	22.66	21.68	21.68	23.68	19.25
RASA1	2.4	0.95	0.88	1.09	1.64	0.25
RASA2	2.4	4.63	3.13	2.54	3.09	2.86
RASA3	8.06	5.41	5.58	4.51	7.31	5.3
RASA4	0.25	0.2	0.44	0.29	0.28	0.24
RASA4CP	0.43	0.14	0.28	0.28	0	0.84
RASAL1	1.15	2.4	1.04	2.38	1.67	3.1
RASAL2	0.52	1.19	0.93	0.79	0.63	1.09
RASAL2-AS1	1.12	0.44	0.79	0.14	0.35	0.69
RASAL3	0.33	1.44	1.56	1.84	0.83	0.94
RASD1	0	0	0.08	0.31	0.12	0
RASD2	0	0	0	0	0.16	0
RASEF	0.06	0	0	0	0	0
RASGEF1A	0	0	0	0	0.02	0
RASGEF1B	0.03	0.15	0	0.17	0.24	0
RASGEF1C	0.1	0	0.21	0.02	0.25	0
RASGRF1	0.82	0.31	0.1	0.2	0.46	0.45
RASGRF2	0	0.03	0.06	0.02	0.05	0.04
RASGRP1	0.29	0.4	0.55	0.59	0.69	0.83
RASGRP2	2.42	10.84	8.25	5.16	4.93	8.64
RASGRP3	0.18	0.05	0.08	0.01	0	0
RASGRP4	0.78	2.24	2.25	1.15	1.59	2.55
RASIP1	0	0	0.1	0.09	0.08	0
RASL10A	0.15	0	0.09	0.24	0.52	0.25
RASL10B	4.28	5.9	6.24	6.31	5.63	5.65
RASL11A	8.29	9.36	5.46	5.08	6.8	9.61

RASL11B	0.19	0.51	0.31	0.18	0.57	0.5
RASL12	0	0	0	0	0	0
RASSF1	37.81	35.98	21.9	23.58	34.89	27.9
RASSF10	0	0	0	0	0	0
RASSF2	2.78	8.73	10.47	7.11	6.36	12.55
RASSF3	0.79	1.74	1.12	1.82	2.14	1.96
RASSF4	0.09	2.06	3.09	0.36	1.25	2.17
RASSF5	8.69	8.46	8.12	5.91	6.49	8.62
RASSF6	0.05	0.06	0.02	0.01	0.04	0.25
RASSF7	16.5	18.52	15.17	19.59	15.89	16.14
RASSF8	0.32	0.2	0.29	0.13	0.16	0.12
RASSF9	0	0	0	0	0	0
RAVER1	15.58	17.39	15.82	13.99	16.21	15.09
RAVER2	1.77	1.23	0.74	0.91	1.75	1.7
RAX	0	0	0	0	0	0
RAX2	0.07	0.11	0	0.02	0	0.1
RB1	13.3	10.43	9.96	7.85	9.38	9.65
RB1CC1	21.79	11.67	11.01	10.56	13.46	12.24
RBAK	1.33	0.79	0.92	0.78	1.02	1.2
RBAK-LOC38	0	0	0	0.1	0	0
RBBP4	42.65	48.53	42.06	46.86	48.01	43.82
RBBP5	14.37	9.88	12.37	11.08	12.09	11.96
RBBP6	5.05	4.75	3.73	3.73	4.66	4.61
RBBP7	162.42	224.66	195.84	190.84	193.87	194.18
RBBP8	9.5	10.72	10.11	7.99	9.1	6.73
RBBP8NL	0	0	0	0	0	0
RBBP9	2.97	3.01	2.89	3.11	1.91	3.31
RBCK1	14.81	13.38	12.87	13.52	11.77	13.43
RBFA	9.56	11.97	8.58	11.94	10.45	10.94
RBFOX1	0	0	0	0	0	0
RBFOX2	1.5	1.92	1.55	2.15	2.28	1.86
RBFOX3	0	0	0	0	0.05	0
RBKS	2.29	1.45	1.34	0.47	1.32	0.83
RBL1	7.32	6.88	5.97	5.76	5.72	5.58
RBL2	4.72	6.79	6.11	4.83	6.12	5.14
RBM10	46.3	50.86	48.33	52.18	43.94	41.23
RBM11	0.49	0	0	0	0	0
RBM12	15.32	18.7	17.81	16.22	17.25	18.26
RBM12B	5.74	5.45	6.1	4.96	6.39	6.03
RBM12B-AS:	3.04	2.4	3.39	2.34	2.56	2.27
RBM14	29.76	35.57	35.61	33.86	33.47	36.96
RBM14-RBM	8.38	12.36	10	7.36	8.96	16.42
RBM15	11.56	13.82	13.06	11.79	13.04	15.79

RBM15B	2.51	2.24	1.91	2.19	2.79	2.85
RBM17	27.79	38.75	33.19	27.34	31.23	26.1
RBM18	13.99	12.25	9.8	9.21	11.43	9.18
RBM19	24.98	21.79	21.87	17.4	19.77	20.63
RBM20	0.14	0.01	0.01	0	0.2	0
RBM22	23.76	27.94	28.11	24.66	30.76	28.4
RBM23	29.88	38.45	39.08	36.61	32.02	32.29
RBM24	0	0	0	0	0	0
RBM25	27.46	29.58	25.8	21.81	27.8	25.77
RBM26	3.31	4.31	3.92	2.65	3.83	3.23
RBM26-AS1	1.16	0.38	0.71	0.39	0.81	0.51
RBM27	9.76	10.67	9.79	9.32	7.3	8.62
RBM28	17.42	22.97	19.25	19.47	17.24	21.31
RBM3	238.46	227.03	212.57	241.57	256.89	254.12
RBM33	3.22	2.8	2.51	2.47	2.76	2.68
RBM34	32.86	34.13	32.3	26.61	29.43	31.32
RBM38	6.57	5.18	5.19	6.58	7.37	4.98
RBM39	50.38	51.02	40.72	40.16	47.17	39.59
RBM4	122.19	121.74	111.76	116.36	125.98	114.61
RBM41	9.17	7.49	6.16	4.45	5.58	6.99
RBM42	67.84	61.9	67.92	58.6	64.57	43.71
RBM43	2	1.96	2.23	1.26	1.26	1.35
RBM44	0	0	0	0	0	0
RBM45	8.03	5.77	6.7	5.59	5.82	4.17
RBM46	0	0	0	0	0	0
RBM47	0	0.53	0.16	0	0	0.23
RBM48	10.54	9.95	11.09	10.7	11.27	7.73
RBM4B	10	14.75	12.34	11.05	13.04	10.34
RBM5	20.09	16.78	13.97	17.51	16.49	19.6
RBM6	10.02	11.35	7.74	9.23	10.09	11.03
RBM7	15.29	13.51	10.53	10.95	13.78	13.28
RBM8A	50.85	44.76	38.05	40.26	50.42	40.59
RBMS1	1.93	1.38	1.15	1.43	1.27	1.76
RBMS2	2.07	2.71	2.66	2.54	1.87	3.33
RBMS3	0.13	0	0	0	0	0
RBMX	231.04	233.58	228.15	242.87	239.75	246.6
RBMX2	27.33	23.19	20.88	21.53	28.74	17.03
RBMXL1	5.72	6.12	4.18	5.73	5.35	5.17
RBMXL2	0	0	0	0	0	0
RBMXL3	0	0	0	0	0	0
RBMY1A1	0	0	0	0	0	0
RBMY1A3P	0	0	0	0	0	0
RBMY1B	0	0	0	0	0	0

RBMY1D	0	0	0	0	0	0
RBMY1E	0	0	0	0	0	0
RBMY1F	0	0	0	0	0	0
RBMY1J	0	0	0	0	0	0
RBMY2EP	0	0	0	0	0	0
RBMY2FP	0.04	0	0	0	0	0
RBMY3AP	0	0	0	0	0	0
RBP1	0.39	0.48	0.45	0.16	0.16	0.25
RBP2	0	0	0	0	0	0
RBP3	0	0	0	0	0	0
RBP4	0	0	0	0	0	0
RBP5	0.08	0.13	0.45	0.17	0	0
RBP7	0	0	0	0	0	0
RBPJ	16.39	18.74	18.26	16.1	16.62	18.66
RBPJL	0	0	0	0	0	0
RBPMS	0.37	2.41	2.3	1.87	1.71	2.06
RBPMS2	5.41	2.33	3.36	6.07	5.56	2.7
RBX1	135.23	167.26	128.38	143.1	154.82	139.19
RC3H1	3.56	4.41	3.63	3.04	3.39	4.57
RC3H2	9.89	7.01	8.9	6.53	7.11	6.47
RCAN1	3.08	1.45	0.59	1.11	1.28	0.92
RCAN2	0	0	0	0	0	0
RCAN3	2.57	2.44	2.65	2.15	3.08	1.93
RCAN3AS	0	0	0	0	0	0.61
RCBTB1	9.53	10.48	9.09	9.38	5.74	9.07
RCBTB2	7.85	6.16	2.92	4.42	4.37	3.36
RCC1	57.05	76.68	81.67	76.56	68.23	87.91
RCC2	29.16	28.8	28.32	29.27	33.01	34.32
RCCD1	2.78	3.69	3.47	2.78	2.84	2.1
RCE1	19.74	20.04	17.25	17.54	16.57	10.41
RCHY1	9.58	9.2	7.17	7.77	8.29	7.73
RCL1	55.69	22.64	24.68	25.77	33.98	23.91
RCN1	40.3	38.99	44.36	42.64	38.56	44.35
RCN2	14.93	19.2	16.77	15.85	15.32	10.97
RCN3	0.96	0.16	0.29	0.33	0.72	0
RCOR1	3.51	2.14	1.76	1.22	2.04	2.76
RCOR2	0	0	0	0	0	0.03
RCOR3	6.58	5.24	5.86	4.61	5.03	5.07
RCSD1	1.85	8.73	10.44	8.53	6.58	7.47
RCVRN	0	0	0	0	0	0
RD3	0.07	0.03	0.2	0.05	0.13	0
RD3L	0	0.7	0.35	0.13	0	0.45
RDH10	8.62	6.73	5.26	3.89	6.22	5.32

RDH11	29.74	18.8	22.78	21.53	26.63	25.44
RDH12	0	0.06	0	0.03	0.04	0
RDH13	18.36	24.93	27.34	23.3	24.5	22.44
RDH14	3.31	2.02	1.48	1.77	2.82	2.97
RDH16	0	0	0.08	0	0	0
RDH5	0	0.38	0	0.16	0.12	0
RDH8	0	0	0	0	0	0
RDM1	4.19	2.13	1.53	3.76	3.86	2.43
RDX	11.34	10.04	9.78	9.82	11.14	8.33
REC8	0.16	0.57	0.03	0.05	0.19	0.13
RECK	1.15	0.77	1.39	1.08	0.79	1.06
RECQL	7.83	5.33	5.15	5.16	6.19	5.34
RECQL4	11.17	11.55	8.33	11.06	10.67	10.36
RECQL5	4.52	4.03	4.62	4.18	3.97	3.88
REEP1	0.09	0	0.02	0.14	0.05	0.04
REEP2	1.48	0.64	0.74	1.2	1.94	0.38
REEP3	1.9	1.42	1.29	1.49	1.44	1.59
REEP4	109.04	117.37	115.04	111.79	101.66	95.31
REEP5	44.58	25.12	20.07	20.07	27.58	22.92
REEP6	13.65	15.52	11.59	12.29	13.17	12.92
REG1A	0	0	0	0	0	0
REG1B	0	0	0	0	0	0
REG1P	0	0	0	0	0	0
REG3A	0	0	0	0	0	0
REG3G	0	0	0	0	0	0
REG4	0	0	0	0	0	0
REL	4.47	4.2	3.44	4.95	3.79	4.63
RELA	18.3	16.4	16	17.23	15.76	14.43
RELB	0.94	0.56	1.15	0.73	0.43	0.62
RELL1	6.05	9.31	9.23	7.3	6.36	7.63
RELL2	1.96	2.23	1.59	2.77	2.38	2.76
RELN	0	0	0	0	0	0
RELT	3.99	6.12	5.33	5.27	3.66	3.21
REM1	0	0	0	0	0	0
REM2	0	0	0	0	0	0.08
REN	0	0	0	0	0	0
RENBP	3.26	1.17	1.4	1.19	1.01	0.62
REP15	0.67	0.26	0.61	0	0.06	0.14
REPIN1	4.34	2.85	2.32	2.67	4.12	4.11
REPS1	2.3	3.15	2.24	2.2	1.82	1.98
REPS2	0.25	0.17	0.07	0.06	0.07	0.04
RER1	46.09	40.53	40.1	41.8	44.77	31.39
RERE	3.43	1.11	0.94	1.22	1.44	0.86

REREP3	0	0	0	0.07	0	0
RERG	0	0	0	0	0	0
RERGL	0	0	0	0	0	0
RESP18	0	0	0	0	0	0
REST	7.67	5.12	4.42	4.36	6.24	5.06
RET	0.8	1.7	0.24	0.13	0.36	7.77
RETN	0	0	0	0	0	0
RETNLB	0	0	0	0	0	0
RETSAT	5.27	6.71	6.96	5.72	4.87	4.02
REV1	1.6	1.56	1.62	0.97	1.48	1.45
REV3L	1.37	1.49	1.6	1.07	1.09	1.25
REXO1	1.51	0.99	1.49	1.09	1.08	0.83
REXO1L1	1.13	1.79	1.57	0.87	1.35	1.58
REXO1L2P	0	0	0	0	0	0
REXO2	106.36	74.62	51.94	50.36	69.12	54.09
REXO4	8.09	7.38	7.21	6.77	5.26	6.06
RFC1	10	12.46	11.67	11.94	10.21	13.58
RFC2	40.1	53.84	52.92	61.58	49.26	55.77
RFC3	33.63	30.47	26.4	27.32	29.83	28.23
RFC4	35.1	44.11	34.78	38.72	41.02	35.19
RFC5	30.45	31.78	25.5	29.62	28.35	30.73
RFESD	12.73	0.61	1.2	5.89	8.64	1.42
RFFL	2.1	5.2	4.64	2.54	3.82	4.38
RFK	53.97	14.36	12.89	16.63	19.34	13.63
RFNG	2.27	1.95	2.93	1.56	3.76	1.93
RFPL1	0	0	0	0	0	0
RFPL1-AS1	0.23	0.27	0.27	0.23	0.23	0.21
RFPL2	0	0	0	0	0	0
RFPL3	0	0	0	0	0	0
RFPL3S	0.07	0	0	0	0	0.18
RFPL4A	0	0	0	0	0	0
RFPL4B	0	0	0	0	0	0
RFT1	11.38	19.13	18.52	18.19	15.54	17.71
RFTN1	3.81	5.11	5.22	3.26	3.6	4.18
RFTN2	0.16	0	0.04	0.06	0	0
RFWD2	3.15	2.67	3.18	3.17	1.61	1.38
RFWD3	21.85	23.35	25.56	22.63	23.33	26.79
RFX1	1.09	0.66	1.59	1.09	0.67	0.77
RFX2	0.86	2.5	1.35	1.59	1.18	0.87
RFX3	3.58	1.55	1.34	1.72	1.65	1.18
RFX4	0	0	0	0	0	0
RFX5	9.26	8.51	8.26	8.28	8.64	9.89
RFX6	0	0	0	0	0	0

RFX7	4.12	5.07	4.52	3.62	3.38	4.26
RFX8	0	0	0	0	0	0.26
RFXANK	47	56.35	66.36	63.61	69.55	58.56
RFXAP	0.36	0.57	1.11	0.44	0.56	0.65
RGAG1	0.12	0	0	0.1	0	0
RGAG4	0.62	0.09	0.02	0.14	0.61	0.24
RGCC	1.03	0.38	0.5	0.38	1.37	0.14
RGL1	0.03	0.02	0	0	0.09	0.11
RGL2	1.62	2.59	2.52	3.82	2.88	3.51
RGL3	0	0	0	0	0	0
RGL4	0.38	0.18	0.3	0.21	0.12	0.13
RGMA	0.07	0	0.48	0.46	0.22	0.14
RGMB	0	0	0	0	0	0
RGN	0	0	0	0	0.04	0.1
RGP1	7.21	7.17	6.3	3.78	4.98	4.2
RGPD1	0.03	0	0.02	0.02	0	0
RGPD2	0	0	0	0	0	0
RGPD3	0.13	0.4	0.22	0.28	0	0
RGPD4	0	0.05	0	0.03	0.04	0
RGPD5	1.37	4.14	3.45	2.15	3.74	2.75
RGPD6	1.19	2.05	1.59	0.73	1.89	1.43
RGPD8	2.75	0.07	0	0	0	0
RGR	0	0.04	0	0	0	0
RGS1	0	0.17	0	0	0	0
RGS10	94.76	56.16	72.72	72.14	66.82	68.55
RGS11	0	0	0	0	0	0
RGS12	1.61	1.46	1.34	1.89	1.9	1.7
RGS13	0.1	0	0	0.03	0	0
RGS14	2.04	8.67	10.27	9.89	5.58	10.36
RGS16	5.46	6.96	5.91	4.27	4.95	5.13
RGS17	0.56	0.52	1.26	0.67	0.63	1.24
RGS18	1.55	8.24	9.75	11.09	5.58	15.26
RGS19	20.23	56.42	62.62	46.47	44.78	54.48
RGS2	7.37	0.88	2.86	2.39	2.11	1.41
RGS20	20.56	4.06	3.28	5.29	9	4.33
RGS21	0	0	0	0	0	0
RGS22	0.02	0	0	0	0	0
RGS3	0.32	0.08	0.78	0.57	0.1	0.03
RGS4	0	0	0	0	0	0
RGS5	1.79	0.36	0.24	0.74	1.03	1.04
RGS6	0.94	0	0	0.1	0.46	0
RGS7	0	0	0	0	0	0
RGS7BP	0	0	0	0	0	0

RGS8	0	0	0	0	0	0
RGS9	0.32	0	0.12	0.12	0.47	0.05
RGS9BP	0.3	0.56	0.62	0.68	0.93	0.54
RGSL1	0	0	0	0	0	0
RHAG	91.77	2.06	1.51	21.03	68.32	2.99
RHBDD1	0	0	0	0	0	0
RHBDD2	40.95	20.58	20.18	17.71	22.83	23.13
RHBDD3	13.77	10.79	15.78	15.73	15.11	12.24
RHBDF1	0.46	0.52	0.29	0.13	0.05	0.1
RHBDF2	2.12	5.25	5.39	5.06	3.14	4.64
RHBDL1	0.05	0	0	0.13	0.14	0
RHBDL2	0.34	0.75	0.54	0.62	0.61	0.86
RHBDL3	0	0	0	0	0	0
RHBG	0.13	0.39	0.34	0.04	0.21	0.29
RHCE	4.75	0	0.13	1.72	0.68	0
RHCG	0.38	0	0	0.21	0	0
RHD	12.18	0.76	0.96	5.65	2.52	0.73
RHEB	37.74	32.6	29	24.47	34.54	34.67
RHEBL1	3.94	7.44	2.31	4.59	8.26	2.22
RHNO1	16.27	25.71	20.71	19.78	19.43	20.58
RHO	0	0	0	0	0	0
RHOA	394.91	368.38	379.26	388.52	379.77	388.61
RHOB	0	0	0	0	0	0
RHOBTB1	1.4	0.09	0.2	0.44	0.31	0
RHOBTB2	2.8	3.33	3.21	1.99	1.83	1.86
RHOBTB3	3.05	1.38	1.11	1.1	1.34	0.89
RHOC	125.03	47.99	51.92	61.89	87.53	54.7
RHOD	0	0	0	0	0	0
RHOF	16.36	37.23	42.14	33.15	27.7	42.74
RHOG	83.41	98.19	121.11	97.73	87.93	92.41
RHOH	17.82	15.17	8.12	7.53	8.59	8.9
RHOJ	0.05	0.04	0	0	0	0.1
RHOQ	1.83	1.04	1.03	1.11	1.05	1.5
RHOT1	3.63	3.18	2.19	2.35	2.63	3.31
RHOT2	27.81	29.32	24.74	28.48	30.61	26.8
RHOU	0.21	1.97	1.2	0.93	0.8	1.45
RHOV	0.17	0.03	0.84	1.05	1.53	0.18
RHOXF1	0	0.08	0	0	0	0
RHOXF2	0.23	2.11	0	0	0.06	0
RHOXF2B	0	0	0	0	0	2.86
RHPN1	0.66	1.72	1.66	1.02	0.68	0.97
RHPN1-AS1	1.07	1.94	4.61	1.76	0.97	2.93
RHPN2	3.17	5	3.08	2.6	4.44	3.04

RIBC1	0.77	0.06	0.45	0.21	0	0
RIBC2	0	0	0	0	0	0
RIC3	0.03	0.05	0	0.07	0	0.01
RIC8A	23.52	20.57	22.12	25.5	23.1	18.97
RIC8B	1.5	2.72	1.28	1.62	1.32	2.14
RICTOR	5.48	1.39	1.47	2	4.16	2.2
RIF1	8.78	7.66	6.88	6.58	7.58	9.6
RIIAD1	0	0	0	0	0	0
RILP	3.77	5.58	4.89	3.23	4.74	3.48
RILPL1	0.84	1.12	0.84	1.02	1.53	0.62
RILPL2	32.81	56.21	55.12	33.4	39.68	54.48
RIMBP2	0	0	0	0	0	0
RIMBP3	0	0.15	0	0.09	0.14	0.31
RIMBP3B	0.12	0.17	0.26	0.12	0.26	0.27
RIMBP3C	0	0	0	0	0	0
RIMKLA	0.03	0.05	0.37	0.07	0.16	0.36
RIMKLB	1.69	2.81	2.32	2.59	2.31	2.56
RIMS1	0	0.07	0	0	0	0
RIMS2	0.03	0.9	0.54	0.63	0.65	0.59
RIMS3	0.11	0.24	0.35	0.36	0.26	0.52
RIMS4	0	0	0	0	0	0
RIN1	0	0.04	0.42	0.54	0	1.63
RIN2	0.02	0	0	0.04	0.02	0
RIN3	3.43	5.61	9.85	7.11	6.66	6.71
RING1	2.61	2.46	1.84	1.86	2.02	2.08
RINL	1.06	0.37	0.83	1.81	0.47	0.76
RINT1	10.85	9.84	10.12	9.67	9.22	11.38
RIOK1	22.51	22.34	16.99	20.19	19.94	19.74
RIOK2	15.39	14.14	11.39	12.76	10.85	11.81
RIOK3	13.17	9.94	7.78	7.01	9.6	6.26
RIPK1	3.69	3.03	1.77	2.54	5.55	2.66
RIPK2	16.06	22.3	19.11	16.42	18.2	15.85
RIPK3	1.7	3.52	4.37	3.34	2.87	0.94
RIPK4	0	0	0	0	0	0
RIPPLY1	0	0	0	0	0	0
RIPPLY2	0	0	0	0	0	0
RIT1	5.18	1.81	1.56	2.89	1.65	2.14
RIT2	0	0	0	0	0	0
RLBP1	0	0	0	0	0.04	0
RLF	5.33	4.04	4.1	4.97	4.87	4.65
RLIM	20.55	18.88	18.83	16.35	17.91	18.97
RLN1	0	0	0	0	0	0
RLN2	0	0	0	0	0	0

RLN3	0	0	0	0	0	0
RLTPR	0.24	0.63	0.38	0.74	0.35	0.64
RMDN1	18.36	18.72	16.47	15.44	16.34	17.38
RMDN2	0	0	0	0	0	0
RMDN3	19.93	9.88	9.42	12.84	14.22	12.89
RMI1	7.48	8.9	8.41	7.69	6.96	7.93
RMI2	2.05	2.52	2.54	2.42	3.07	1.91
RMND1	19.4	14.39	18.57	20.33	16.18	21.59
RMND5A	9.4	6.64	5.48	6.45	8.68	6.63
RMND5B	11.37	8.6	6.91	8.65	9.85	7.39
RMRP	9.48	22.1	33.01	14.74	15.87	18.27
RMST	0	0	0	0	0	0
RNA5SP244	0	0	0	0	0	0
RNA5SP411	0	0	0	0	0	0
RNASE1	5.65	0	0	0.99	2.74	0
RNASE10	0	0	0	0	0	0
RNASE11	0	0	0	0	0	0
RNASE12	0	0	0	0	0	0
RNASE13	0	0	0	0	0	0
RNASE2	4.32	29.53	3.04	0	4.8	80.7
RNASE3	0.89	4.74	0	0	0.32	10.46
RNASE4	0	0	0.31	0.23	0	0
RNASE6	1.03	0	0	0.35	0.34	0
RNASE7	0	0	0	0	0	0
RNASE8	0	0	0	0	0	0
RNASE9	0	0	0	0	0	0
RNASEH1	37.39	38.85	33.89	33.93	29.99	31.77
RNASEH2A	85.54	85.88	88.34	100.63	103.43	82.25
RNASEH2B	16.74	29.02	26.05	26.45	28.26	23.86
RNASEH2B-A	0.13	0.62	0.12	0.18	0.49	0.92
RNASEH2C	13.61	15.57	14.55	16.9	13.78	15.57
RNASEK	359.03	262.41	297.05	313.93	396.96	275.3
RNASEK-C17	2.34	0.2	0	2.55	1.69	0.53
RNASEL	2	1.61	1.97	2.06	2.56	1.88
RNASET2	58.58	41.03	42.46	33.1	71.4	37.96
RND1	0.49	0.43	0.94	0.15	0.93	0.09
RND2	0.2	0.15	0.26	0.05	0.14	0.05
RND3	0	0	0	0	0	0
RNF10	27.66	30.49	35.1	31.02	26.75	26.58
RNF103	3.93	2.41	1.75	1.67	1.68	1.84
RNF103-CHM	0.36	0	0	0	0	0.25
RNF11	2.82	0.8	1.43	0.94	1.52	1.08
RNF111	1.41	1.66	1.14	0.98	1.47	1.35

RNF112	0	0	0	0	0	0
RNF113A	35.82	42.65	37.26	35.35	39.5	37.56
RNF113B	0	0.09	0	0	0	0
RNF114	36.56	35.23	34.57	34.67	37.89	36.75
RNF115	5.13	3.82	2.89	4.29	4.62	3.24
RNF121	6.73	4.93	3.05	4.4	5.03	3.52
RNF122	1.51	1.63	2.72	3	2.77	2.49
RNF123	9.56	5.85	5.86	6.5	7.68	6.87
RNF125	2.31	3.07	2.47	2.21	2.86	3.88
RNF126	13.87	11.78	9.63	11.79	12.29	11.44
RNF126P1	0	0	0	0	0	0.08
RNF128	0	0	0	0	0	0
RNF13	9.16	10.64	7.67	9.2	8.08	9.06
RNF130	41.64	44.08	32	36.42	46.82	40.44
RNF133	0	0	0.05	0	0	0
RNF135	7.5	12.47	16.42	15.75	12.76	15.92
RNF138	14.4	14.84	14.39	12.37	13.56	15.93
RNF138P1	0.35	0.43	0.42	0.35	0.3	0.36
RNF139	10.96	7	7.71	9.46	8.97	6.6
RNF14	15.12	10.06	10.06	8.49	9.41	9.17
RNF141	6.97	5.89	5.39	5.62	7.07	5.71
RNF144A	16.54	8.3	12.07	15.58	12.18	10.24
RNF144A-AS	4.57	3.08	3.42	4.1	4.45	1.88
RNF144B	0.09	0.12	0.13	0.1	0.07	0.03
RNF145	3.71	1.2	2.09	2.43	2.89	1.27
RNF146	5.17	3.7	4.23	3.4	5.13	5.75
RNF148	0.06	0	0.16	0	0.11	0
RNF149	7.82	7.64	6.57	8.22	8.2	5.1
RNF150	0.03	0.09	0.1	0.19	0.03	0.05
RNF151	0.2	0	0	0	0.09	0
RNF152	0.16	0.03	0	0	0	0
RNF157	1.3	3.65	2.03	2.14	2.06	2.31
RNF157-AS1	0	0.08	0	0	0	0
RNF165	0	0	0	0	0	0
RNF166	5.1	7.97	6.46	6.23	6.64	6.69
RNF167	29.01	27.94	26.15	28.91	28.83	24.45
RNF168	10.47	8.68	10.05	12.38	10.34	10.75
RNF169	1.3	1.77	1.57	1.19	1.21	1.72
RNF17	0	0	0	0	0	0
RNF170	4.83	6.96	5.03	2.91	3.79	5.54
RNF175	0	0.26	0	0	0	0
RNF180	0	0	0	0	0	0
RNF181	89.97	89.71	89.1	79.27	94.12	72.99

RNF182	0	0.19	0.02	0	0	0
RNF183	0.38	0	0	0	0	0
RNF185	17.27	13.13	12.55	12.16	14.44	12.84
RNF186	0	0	0	0	0	0
RNF187	4.93	3.18	5.21	3.23	4.06	4.49
RNF19A	5.91	6.12	5.72	5.91	5.35	5.54
RNF19B	0.49	0.11	0.39	0.49	0.32	0.35
RNF2	15.5	13.89	12.99	13.89	14.29	10.99
RNF20	9.98	11.38	9.49	9.55	8.77	10.46
RNF207	1.63	3.08	2.19	2.22	1.42	2.37
RNF208	3.84	2.19	3.58	1.86	2.22	3.37
RNF212	0	0	0	0	0	0.03
RNF213	2.65	1.98	3.14	2.47	2.41	2.63
RNF214	5.43	4.72	5.57	5.15	5.81	5.27
RNF215	0.31	0	0.21	0.24	0.36	0.23
RNF216	11.01	13.24	12.88	11.92	10.12	10.88
RNF216-IT1	0.27	0	0	0	0	0
RNF216P1	13.33	13.8	15.56	12.09	14.89	14.79
RNF217	1.75	0.95	0.27	0.34	0.2	0.55
RNF219	10.11	8.21	10.9	5.44	7.21	6.88
RNF220	5.23	5.32	5.56	6.01	6.74	6.08
RNF222	0.43	0.34	0.72	0.41	0.5	0.68
RNF223	0	0	0	0	0	0
RNF224	0	0	0	0	0	0
RNF24	25.34	17.62	19.47	20.12	19.99	19.64
RNF25	17.24	13.81	12.97	12.93	10.31	12.49
RNF26	27.34	34.05	29.5	32.27	32.43	30.45
RNF31	1.92	2.25	2.49	2.13	2.37	3
RNF32	0	0.03	0.04	0	0	0.09
RNF34	20.64	20.29	20.53	19.1	16.78	16.51
RNF38	5.41	5.14	4.16	4.31	4.17	4.24
RNF39	0.17	0	0.06	0	0.13	0.1
RNF4	42.83	33.53	30.42	29.12	34.76	30.52
RNF40	15.16	15.6	14.87	13.49	14.64	17.37
RNF41	10.3	12.07	12.29	9.18	10.76	11.29
RNF43	0.03	0.06	0.17	0	0	0
RNF44	1.79	2.03	1.16	0.99	1.56	1.45
RNF5	1.95	0.76	0.88	3.29	5.44	0.98
RNF5P1	92.02	63.12	59.4	69.8	70.65	58.23
RNF6	9.85	7.96	7.7	6.82	8.75	7.42
RNF7	53.73	47.28	43.57	42.43	47.91	41.95
RNF8	5.38	5.79	6.48	5.84	5.59	6.03
RNFT1	10.33	15.38	11.5	9.45	13.28	14.27

RNFT2	9.12	8.76	13.62	10.72	10.59	11.37
RNGTT	7.37	7.75	5.19	4.64	5.95	5.9
RNH1	135.92	112.38	112.76	124.26	117.99	106.97
RNLS	0	0	0	0	0	0
RNMT	4.15	5.72	4.63	4.23	5.4	5
RNMTL1	30.99	31.66	29.99	29.78	29.23	26.34
RNPC3	4.24	2.79	2.45	3.46	2.52	2.94
RNPEP	4.93	5.63	3.81	2.89	5.6	5.57
RNPEPL1	10.36	8.54	8.16	7.94	6.4	6.5
RNPS1	141.99	129.43	119.66	128.33	135.57	121.53
RNU11	0	0	0	0	0	0
RNU12	3.26	0.66	0.75	1.19	0.8	1.63
RNU4ATAC	0	1.61	3.66	4.36	0.98	5.96
RNU5D-1	0	0	0	0	0	0
RNU5E-1	0	0	0	0	0	0
RNU5F-1	0	0	0	0	0	0
RNU6-16	0	0	0	0	0	0
RNU6-19	0	0	0	0	0	0
RNU6-28	6.65	1.15	0	2.51	0	4.97
RNU6-33	0	0	0	0	0	0
RNU6-34	0	0	0	0	0	0
RNU6-35	0	0	0	0	0	0
RNU6-52	0	0	0	0	0	0
RNU6-53	0	0	0	0	0	0
RNU6-57	0	0	0	0	0	0
RNU6-6	0	0	0	0	0	0
RNU6-64	0	0	0	0	0	0
RNU6-66	0	1.77	0	0	0	0
RNU6-67	0	0	0	0	0	0
RNU6-69	0	0	0	0	0	0
RNU6-71	0	0	0	0	0	0
RNU6-72	0	0	0	0	0	0
RNU6-76	0	0	0	0	0	0
RNU6-78	0	0	0	0	0	0
RNU6-79	0	0	0	0	0	0
RNU6-81	0	0	0	0	0	0
RNU6-83	0	0	0	0	0	0
RNU6ATAC	2.1	0	0	1.54	0	0
RNU86	0	0	0	0	0	0
RNY4	0	0	0	0	0	0
RNY5	0	0	0	0	0	0
ROBO1	12.7	2.56	2.69	3.59	4.54	2.97
ROBO2	0	0	0	0	0	0

ROBO3	0.25	0.22	0.41	0.58	0.3	0.19
ROBO4	0	0	0	0	0	0
ROCK1	8.09	8.19	7.06	5	8.61	8.33
ROCK1P1	0.07	0.1	0	0.12	0.05	0.18
ROCK2	2.05	1.97	1.87	1.35	1.87	2.14
ROGDI	4.58	8.14	4.9	6.92	5.99	6.61
ROM1	0.43	0.12	0.58	0.54	0.82	0.32
ROMO1	254.27	277.73	217.78	224.44	299.36	225.58
ROPN1	0	0	0	0	0	0
ROPN1B	0	0	0	0	0	0
ROPN1L	0.16	2.13	0.78	1.51	0.47	0
ROR1	0	0	0.03	0	0	0.04
ROR2	0.04	0.06	0.08	0.13	0.08	0
RORA	0.03	0	0.05	0	0.01	0.01
RORB	0.02	0.17	0	0.08	0	0.06
RORC	0	0	0	0	0	0
ROS1	0	0	0	0	0	0
RP1	0	0	0	0	0	0
RP1-177G6.2	0.46	0.37	0.66	0.16	0.16	0.25
RP1L1	0	0.01	0	0	0.09	0.1
RP2	5.2	8.95	7.34	6.62	5.92	7.49
RP9	16.6	19.04	18.06	20.35	24.38	15.39
RP9P	3.05	3.98	4.35	2.5	4.79	3.08
RPA1	43.55	40.39	32.21	37.77	40.66	38
RPA2	89.42	91.76	78.09	80.16	81.15	78.45
RPA3	36.34	35.39	34.37	32.17	33.02	32.5
RPA4	0.29	0	0.35	0.2	0.05	0
RPAIN	48.08	31.05	22.39	30.65	33.96	27.93
RPAP1	21.5	30.08	30.01	27.95	24.32	30.05
RPAP2	5.55	7.33	5.59	6.05	5.23	6.92
RPAP3	10.66	8.73	6.36	6.07	7.64	7.28
RPE	21.23	23.33	20.12	17.91	20.31	24.36
RPE65	0	0	0	0	0	0
RPF1	30.99	23.32	20.49	22.57	23.92	20.67
RPF2	52.98	46.95	49.96	47.83	58.54	48.06
RPGR	1.95	3.99	1.58	1.86	2.33	1.66
RPGRIP1	0.04	0.03	0	0	0.02	0
RPGRIP1L	0.52	0.78	0.49	1.22	0.94	0.65
RPH3A	0	0	0	0.01	0	0
RPH3AL	0.54	0.8	1.19	1.12	0.92	0.75
RPIA	13.02	12.39	9.14	7.88	12.42	9.65
RPL10	2053.64	2251.32	2430.59	2841.05	2772.61	2777.41
RPL10A	2095.62	2295.94	2424.77	2746.56	2627.71	2682.13

RPL10L	0.1	0.23	0	0	0.09	0.79
RPL11	2287.62	2929.29	2941.85	3049.35	3113.04	3343.55
RPL12	2140.11	2057.35	2339.93	2857.46	2558.31	2585.67
RPL13	519.33	589.97	618.41	760.33	662.03	728.81
RPL13A	1340.32	1346.8	1487.66	1909.99	1700.33	1748
RPL13AP17	0	0	0	0	0	0
RPL13AP20	0.89	0.23	1.8	0.25	1.69	0.86
RPL13AP3	2.03	2.46	2.72	2.19	2.28	2.74
RPL13AP5	2300.96	2570.48	2743.05	3126.59	2960.97	3094.11
RPL13AP6	0.73	0.6	1.75	0.57	0.6	1.5
RPL13P5	2.86	3.9	3.33	2.8	3.58	3.27
RPL14	1428.73	1380.54	1479.07	1605.08	1559.01	1619.21
RPL15	979.61	1076.06	1128.68	1186.7	1188.77	1259.98
RPL17	2772.03	3029.26	3205.31	3575.52	3504.33	3479.34
RPL17-C18or	7.9	2.02	3.53	2.06	4.52	3.96
RPL18	1624.87	1694.84	1996.2	2234.79	2147.46	2045.91
RPL18A	3279.01	3954.79	4316.75	4999.92	4400.62	4711.08
RPL19	3907.89	4119.24	4189.87	4809.07	4855.4	4579.48
RPL19P12	0.31	0.54	0.25	0.89	0.32	0.96
RPL21	1425.4	1232.6	1002.2	1261.08	1577.03	2170.24
RPL21P28	1166.26	1707.11	1714.24	1758.1	1740.99	1046.82
RPL21P44	0	0.29	0.04	0.19	0.2	0.13
RPL22	190.21	191.18	199.63	215.05	227.62	231.06
RPL22L1	83.33	56.98	64.65	64.26	72.83	67.18
RPL23	2484.08	2613.27	2952.78	3302.82	3202.97	2799.48
RPL23A	1831.75	2187.37	2242.17	2421.46	2455.98	2431.92
RPL23AP32	0.27	0.66	0.36	0.27	0	0
RPL23AP53	5.56	3.65	4.48	3.4	4.68	5.64
RPL23AP64	0.51	0.41	1.17	1.44	0.49	0.16
RPL23AP7	16.25	21.35	12.45	11.73	16.23	16.15
RPL23AP82	12.17	11.24	11.14	8.74	10.92	10.04
RPL23P8	0	0.3	0.35	0.94	0.77	0.63
RPL24	1488.58	1105.19	1350.29	1596.03	1551.14	1387.98
RPL26	2395.83	2814.01	2839.9	3229.29	3281.19	3223.19
RPL26L1	87.79	94.82	90.66	86.47	95.57	85.39
RPL27	2497.61	2633.51	2802.95	2802.94	2965.15	2745.45
RPL27A	224.75	208.5	218.37	256.99	238.73	242.35
RPL28	810.02	964.42	1089.92	1285.18	1081.69	1224.15
RPL29	2663.69	3194.86	3450.29	3898.71	3648.83	3826.83
RPL29P2	0.38	0.5	1.17	1.01	1.53	0.96
RPL3	2587.16	2628.18	2822.54	3353.43	3236.81	3224.8
RPL30	3745.65	4274.7	4148.67	4611.45	4715.89	4679.15
RPL31	2763.16	3148.72	3219.08	3570.3	3744.99	3388.41

RPL31P11	0.23	0.29	0.35	0.13	0.27	0.39
RPL32	727	760.5	813.66	929.8	900.34	967.38
RPL32P3	1.06	0.71	0.39	0.95	1.23	0.77
RPL34	1015.23	1021.08	1090.91	1138.09	1357.61	1003.34
RPL34-AS1	0.1	0.01	0.04	0.04	0.04	0.02
RPL35	2390.07	2762.59	2845.63	2947.17	3048.12	2803.97
RPL35A	2027.38	1936.03	2082.02	2253.3	2408.56	2176.58
RPL36	2044.93	2435.47	2341.17	2640.81	2793.27	2356.76
RPL36A	1140.16	1239.68	1204.59	1314.78	1558.84	1305.02
RPL36A-HNR	59.19	56.07	48.02	57.61	69.16	59.21
RPL36AL	485.87	490.56	422.93	459.36	557.44	441.31
RPL37	501.57	520.97	568.95	559.56	638.89	574.46
RPL37A	4452.41	4703.33	5260.73	5348.89	6150.15	5165
RPL38	2494.98	2686.2	3031.16	2914.43	3448.94	2566.71
RPL39	4194.39	5013.45	5426.27	5325.1	5840.67	5595.79
RPL39L	50.92	72.91	68.92	67.08	69.74	74.63
RPL3L	0	0	0	0	0	0
RPL4	5054.68	5092.59	5393.89	6302.65	5764.23	5669.05
RPL41	6788.16	7919.84	8243.19	9102.69	10035.62	8410.35
RPL5	2507.11	2783.94	3225.2	3161.16	3394.9	3456.57
RPL6	1870.4	1997.44	2062.46	2174.12	2245.13	2281.81
RPL7	5211.81	6293.92	6493.75	6588.05	7025.52	7255.3
RPL7A	2075.32	2363.06	2605.89	2919.51	2753.12	2875.53
RPL7L1	73.31	85.61	89.21	77.68	77.48	78.54
RPL8	4058.66	4572	4871.83	5242.97	4958.72	5105.44
RPL9	2434.45	2876.57	3053.44	3303.62	3258.36	3442.03
RPLP0	3530.79	3633.02	4326.39	4905.24	4451.74	4784.87
RPLP0P2	0.64	0.69	0.77	0.56	0.62	0.67
RPLP1	10219.37	10572.72	10441.35	12677.23	11421.92	12104.54
RPLP2	3268.2	3187.63	3408.86	4051.09	3794.35	3734.3
RPN1	164.41	196.11	206.62	197.58	180.78	204.98
RPN2	168.74	211.16	170.9	160.14	173.46	188.07
RPP14	11.72	11.65	11.94	9.41	10.83	11.79
RPP21	107.67	78.71	70.7	94.33	106.66	65.94
RPP25	0.76	4.74	7.49	7.69	2.67	2.68
RPP25L	38.09	35.58	38.33	42.43	41.74	39.64
RPP30	24.39	22.73	18.49	19.57	22.45	20.37
RPP38	19.88	19.48	16.61	21.03	17.86	17.38
RPP40	21.81	25.95	28.54	25.11	24.55	21.17
RPPH1	35.57	21.04	74.3	41.78	36.78	79.21
RPRD1A	8.73	6.47	6.05	5.25	6.44	8.06
RPRD1B	8.05	7.01	9.39	6.96	9.15	8.52
RPRD2	8.83	7.18	6.96	6.85	7.57	7.97

RPRM	0	0	0	0	0	0
RPRML	0	0	0	0	0	0
RPS10	2467.17	2592.85	2779.4	2981.92	2951.59	3080.48
RPS10-NUDT	5.77	7.95	8.73	4.1	7.41	7.11
RPS10P7	0.76	0.84	0.33	0.75	0.72	0.58
RPS11	2778.52	2760.41	3006.27	3753.59	3533.62	3297.31
RPS12	4193.19	4663.58	4936.37	5764.73	5323.08	5537.99
RPS13	2852.74	3228.18	3235.05	3486.97	3467.75	3599.99
RPS14	3726.8	4359.04	4485.3	5079.16	5067.34	5260.62
RPS15	2729.19	3508.18	3547.06	4003.18	3886.33	3799.66
RPS15A	2417.97	2711.03	2927.99	3070.31	3148.17	3109.81
RPS15AP10	0.37	0.61	0.69	0.42	1.2	0.83
RPS16	3237.55	3589.4	3761.26	4341.46	4157.77	4121.04
RPS16P5	0.09	0.22	0.48	0.19	0.1	0.23
RPS17	2800.65	3149.13	3178.08	3350.54	3483.37	3559.7
RPS17L	2800.65	3149.13	3178.08	3350.54	3483.37	3559.7
RPS18	5776.2	6520.65	7425.18	8772.71	8015.51	8368.68
RPS19	1992.69	1964.78	2100.49	2474.81	2444.39	2485.88
RPS19BP1	159.95	131.23	118.86	117.28	142.57	111.95
RPS2	2067.31	2096.12	2738.99	3195.55	2996.93	2944.5
RPS20	3236.29	3454.78	3910.43	4127.7	4120.49	4330.97
RPS21	2426.8	2465.48	2506.71	2487.02	2955.64	2428.33
RPS23	583.16	602.53	650.08	759.31	757.44	743.81
RPS24	1965.28	2306.51	2187.41	2407.35	2504.24	2497.81
RPS25	3182.93	3482.78	3915.55	4075.41	4476.15	3758.93
RPS26	1370.18	1428.12	1566.4	1723.45	1763.98	1638.69
RPS26P11	0.46	0	0.84	0.58	0	0.17
RPS27	3177.9	3614.55	3398.25	3346.45	4202.39	3191.48
RPS27A	1862.02	1976.1	1929.02	2137.39	2195.92	2239.5
RPS27L	111.34	117.35	88.36	95.58	99.13	103.24
RPS28	4015.08	3699.8	3877.14	5018.91	5536.92	4226
RPS29	3382.59	4167.4	4705.01	3696.34	5424.01	3829.97
RPS2P32	4.8	4.02	4.06	2.23	3.05	3.37
RPS3	1993.58	2624	2844.51	3106.48	2949.98	3083.1
RPS3A	2378.12	2786.01	2860.33	3018.3	3008.67	3329.87
RPS4X	3434.39	3878.99	4005.39	4797.13	4610.4	4603.58
RPS4Y1	0	0	0	0	0	0
RPS4Y2	0	0	0	0	0	0
RPS5	3602.95	3985.04	4578.97	5193.98	4698.29	4358.4
RPS6	2737.32	3059.14	3019.52	3457.35	3518.08	3668.65
RPS6KA1	23.41	29.66	26.23	26.63	24.07	26.31
RPS6KA2	0.7	0.17	0.22	0.45	0.48	0.09
RPS6KA2-IT1	0	0	0	0	0	0

RPS6KA3	5.2	3.64	3.22	2.78	4.14	4.13
RPS6KA4	3.23	3.45	3.23	2.44	1.95	4.32
RPS6KA5	2.73	2.57	1.47	1.14	1.95	2.24
RPS6KA6	0.09	0.18	0.2	0.1	0.18	0.12
RPS6KB1	5.91	6.58	4.83	4.09	5.43	7.39
RPS6KB2	36.43	36.75	42.79	41.2	36.95	45.99
RPS6KC1	6.32	3.4	3.18	4.33	3.28	2.95
RPS6KL1	0.42	0.49	0.72	0.83	0.48	0.63
RPS7	1113.38	1231.11	1220.24	1295.5	1346.12	1405.89
RPS7P5	0	0.04	0	0.06	0	0.08
RPS8	3217.66	3788.6	3805.52	4392.27	4179.87	4296.2
RPS9	1519.72	1497.04	1577.73	1876.43	1843.48	1799.03
RPSA	2576.86	2994.76	3405.13	3778.71	3393.85	3357.1
RPSAP52	0.28	0.41	0.35	0.77	0.89	0.54
RPSAP58	105.94	111.71	131.94	137.31	136.75	127.39
RPSAP9	1.59	1.34	1.55	1.3	2.08	2.46
RPTN	0	0	0	0	0	0
RPTOR	5	5.34	5.47	4.25	4.58	4.83
RPUSD1	26.5	41.95	41.26	37.93	29.13	24.77
RPUSD2	18.02	24.33	19.34	22.35	23.04	19.49
RPUSD3	70.48	91.6	98.04	95.05	101.25	88.99
RPUSD4	31.54	32.07	28.09	33.77	28.22	28.28
RQCD1	68.05	79.78	82.19	78.85	85.78	70.33
RRAD	0	0	0	0	0	0
RRAGA	37.94	28.81	27.9	29.8	30.98	30.37
RRAGB	6.76	5.12	4.64	3.9	6.29	6.08
RRAGC	4.56	2.83	3.23	2.03	3.78	2.5
RRAGD	1.98	2.61	1.6	1.78	1.81	2.7
RRAS	3.14	2.66	1.08	3.63	4.46	3.17
RRAS2	1.99	1.17	1.06	1	2.08	0.87
RRBP1	7.32	6.59	7.32	5.29	6.59	5.87
RREB1	7.72	9.95	10.52	8.45	9.65	8.23
RRH	0	0	0	0.03	0.05	0.1
RRM1	56.11	55.19	50.37	59.78	54.68	57.09
RRM2	93.01	90.92	84.69	74.27	93.37	79.48
RRM2B	5.23	2.9	2.7	2.03	2.98	2.54
RRN3	23.23	16.64	14.16	15.99	17.42	14.44
RRN3P1	0.06	0.55	0	0	0.1	0.06
RRN3P2	1.48	2.31	1.75	1.34	1.01	1.02
RRN3P3	1.15	1.24	1.58	1.58	0.88	0.71
RRNAD1	2.81	3.33	3.27	2.4	3.44	4.25
RRP1	52.77	65.68	59.88	59.3	62.44	41.12
RRP12	26.08	28.34	29.07	25.04	21.78	26.8

RRP15	5.08	5.22	4.33	3.41	4.76	4.1
RRP1B	18.2	28.54	28.63	26.63	21.64	34.97
RRP36	8.67	5.59	5.59	6.75	5.91	5.91
RRP7A	29.75	36.35	36.03	30.26	33.24	28
RRP7B	9.21	6.61	8.24	4.68	5.54	6.13
RRP8	20.67	19.66	18.84	22.37	20.37	15.88
RRP9	66.23	70.16	73.66	71.1	69.73	56.76
RRS1	54.68	63.06	59.24	65.84	58.51	65.72
RS1	0	0	0	0	0	0
RSAD1	4.99	12.06	9.42	7.14	6.45	7.12
RSAD2	15.19	3.64	6.76	13.34	8.1	6.82
RSBN1	3.04	2.31	2.83	2.46	2.79	3.35
RSBN1L	2.56	3.2	3.8	3.17	3.41	2.94
RSBN1L-AS1	8.18	7.49	7.33	5.61	7.34	6.93
RSC1A1	5.53	6.11	5	5.76	5.09	2.8
RSF1	9.99	6.38	7.73	7.98	10.24	7.15
RSG1	1.05	1.85	0.7	1.06	1.09	2.47
RSL1D1	58.51	64.82	65.45	66.44	63.91	63.77
RSL24D1	102.82	107.71	91.39	82.82	101.64	117.56
RSPH1	0	0	0	0	0	0.05
RSPH10B	0.04	0	0	0.01	0.03	0.02
RSPH10B2	0.04	0	0	0.01	0.03	0.02
RSPH3	2.23	2.37	1.92	0.93	2.01	2.36
RSPH4A	0	0	0	0	0.03	0.03
RSPH6A	0.06	0	0	0	0	0
RSPH9	0	0	0.07	0	0	0
RSPO1	0	0	0	0	0	0
RSPO2	0	0	0	0	0	0
RSPO3	0	0	0	0	0	0
RSPO4	0	0	0	0	0	0
RSPRY1	13.65	12.53	14.23	13.25	10.77	12.52
RSRC1	29.02	31.1	31.17	30.13	28.37	31.59
RSRC2	18.98	17.96	12.75	11.39	16.88	11.14
RSU1	23.05	26.29	21.83	20.18	20.84	21.15
RSU1P2	0	0.05	0.08	0	0	0
RTBDN	0.24	0.26	0.11	0.1	0.11	0.16
RTCA	18.57	16.79	13.83	11.2	15.54	14.38
RTDR1	0	0	0	0	0	0
RTEL1	2.56	3.04	4.52	4.22	4.01	3.35
RTEL1-TNFR:	0.78	0.62	0.21	0.39	1.05	0.91
RTF1	12.18	12.34	10.58	9.51	11.7	11.44
RTFDC1	126.56	108.11	95.89	107.31	100.83	93.51
RTKN	4.34	2.07	1.97	3.99	2.7	2.54

RTKN2	0.81	1.04	0.88	1.05	1.13	1.4
RTL1	0.02	0	0	0	0.05	0
RTN1	0	0	0	0	0	0
RTN2	2.89	2.01	1.95	3.32	2.02	2.58
RTN3	76.8	80.15	84.46	67.68	69.99	88.45
RTN4	9.68	7.2	5.81	4.6	6.59	5.4
RTN4IP1	8.4	6.6	8.1	6.46	6.33	7.47
RTN4R	0.66	1.85	1.98	1.94	1.78	2.11
RTN4RL1	0.16	0.47	0.86	0.47	0.26	0.84
RTN4RL2	0.78	0.33	0.6	0.95	0.96	0.42
RTP1	0	0	0	0	0	0
RTP2	0	0	0	0	0	0
RTP3	0	0	0	0	0	0
RTP4	0	0	0.49	0.32	0	0
RTTN	2	2.29	1.59	1.59	1.75	1.76
RUFY1	6.75	5.85	5.71	5.99	5.95	6.07
RUFY2	0.75	0.68	0.72	0.4	0.66	0.47
RUFY3	1.9	2.53	2.24	1.14	1.94	1.19
RUFY4	0	0	0	0	0	0
RUNDC1	3.95	4.73	3.85	3.23	3.54	3.1
RUNDC3A	0.42	0.37	0.03	0	0.34	0
RUNDC3B	0.09	0.61	0.36	0.25	0.32	0.13
RUNX1	7.48	14.81	14.61	12.29	10.2	14.2
RUNX1-IT1	0.74	1.49	1.08	1.21	1.53	0.2
RUNX1T1	0.34	0	0	0.12	0.17	0
RUNX2	0.3	0.58	0.01	0.02	0.06	0.03
RUNX3	0.83	1.35	1.95	1.34	1.53	1.41
RUSC1	19.16	23.22	20.53	21.42	21.68	20.82
RUSC1-AS1	0	0.1	0.17	0.08	0	0
RUSC2	10.21	4.67	5.09	5.78	6.96	6.43
RUVBL1	94.62	87.36	73.61	80.01	99.42	81.63
RUVBL2	236.58	265.23	258.86	281.92	239.24	240.51
RWDD1	36.55	40.32	36.97	30.78	35.92	39.08
RWDD2A	3.08	3.56	4.18	1.94	1.76	4.62
RWDD2B	17.35	15.34	13.02	15.37	16.29	11.87
RWDD3	5.18	9.85	6.63	7.28	5.44	6.61
RWDD4	14.77	14.63	11.35	8.45	12.79	10.94
RXFP1	0.08	0.02	0.02	0	0	0
RXFP2	0	0	0	0	0.05	0
RXFP3	0	0	0	0	0	0
RXFP4	0.27	1.15	0	0	0	0.67
RXRA	1.42	1.43	1.18	0.72	1.11	0.91
RXRB	2.04	3.34	2.56	2.6	2.14	4.01

RXRG	0	0	0	0	0	0
RYBP	3.62	0.98	1.25	1.12	2.8	0.62
RYK	1.84	3.38	2.91	1.64	2.44	2.69
RYR1	0	0	0	0	0	0
RYR2	0	0	0	0	0	0
RYR3	0.02	0.01	0	0	0	0.03
S100A1	0	0	0	0.1	0	0
S100A10	19.4	64.66	67.75	64.74	57.32	70.98
S100A11	180.77	222.92	179.63	120.43	148.18	153.85
S100A12	0	0	0	0	0	0
S100A13	113.42	39.99	39.6	48.68	64.27	54.78
S100A14	0	0	0	0	0	0
S100A16	0.5	0	0	0	0	0
S100A2	0.48	0.32	0.15	0.17	0.61	0.32
S100A3	0.32	0	0.2	0	0	0
S100A4	56.86	64.67	153.73	93.3	87.29	119.66
S100A5	0.22	0	0	0	0.11	0
S100A6	0	0.19	0	0.65	0	0
S100A7	0	0	0	0	0	0
S100A7A	0	0	0	0	0	0
S100A7L2	0	0	0	0	0	0
S100A8	0	0	0	0	0	0
S100A9	0	0	0	0	0	0
S100B	0	0	0	0	0	0
S100G	0	0	0	0	0	0
S100P	0	0.13	0.45	1.26	0.31	0.33
S100PBP	6.72	5.36	5.41	5.27	5.75	6.1
S100Z	0.27	1.05	1.7	1.01	0.31	0.34
S1PR1	1.84	0.11	0	0	0.07	0.15
S1PR2	4.7	8.13	8.87	5.97	6.6	8.11
S1PR3	0.16	0.12	0.17	0.02	0.06	0.15
S1PR4	4.31	10.11	14.39	6.58	8.45	9.7
S1PR5	0.07	0.1	0.15	0.8	0.09	0.32
SAA1	0	0	0	0	0	0
SAA2	0.14	0.18	0.17	0.05	0.24	0.26
SAA2-SAA4	0	0	0	0	0	0
SAA3P	0	0	0	0	0	0
SAA4	0	0	0	0	0	0
SAAL1	29.08	26.38	22.14	21.15	25.77	30.25
SAC3D1	2.77	2.03	3.45	1.08	2.3	1.21
SACM1L	6.5	8.61	6.47	4.88	5	8.36
SACS	3.01	3.35	3.61	2.69	3.01	3.46
SAE1	99.86	113	104.59	107.8	117.7	102.65

SAFB	53.02	65.45	59.88	56.08	55.58	58.3
SAFB2	12.68	17.02	13.57	12.43	13.49	16.82
SAG	0	0	0	0.06	0	0
SAGE1	0	0	0	0	0	0
SALL1	0	0	0	0	0	0.01
SALL2	1.01	0.05	0.16	0.75	0.37	0.36
SALL3	0	0	0.02	0	0.01	0
SALL4	0	0	0	0	0	0
SAMD1	2.33	1.97	3.58	3.15	2.48	4.35
SAMD10	2.13	2.9	2.13	2.63	2.8	4.92
SAMD11	0.13	0.2	0.06	0.09	0.15	0.23
SAMD12	0	0.03	0.13	0.01	0	0.01
SAMD12-AS1	0	0	0	0	0	0
SAMD13	0.96	2.66	1.92	1.02	1.92	1.84
SAMD14	0	0	0.06	0	0.21	0
SAMD15	0.29	0.17	0.2	0.05	0.03	0.11
SAMD3	0.18	0	0	0	0	0.03
SAMD4A	0.08	0.19	0.17	0.09	0.13	0.13
SAMD4B	3.07	2.58	2.02	1.88	2.09	1.94
SAMD5	0.14	0.09	0.09	0.09	0.07	0.14
SAMD7	0.03	0	0	0	0	0
SAMD8	1.76	0.85	0.71	0.85	0.65	0.72
SAMD9	1.87	1.64	1.59	1.04	1.11	1.67
SAMD9L	0.17	0.05	0	0.08	0	0.07
SAMHD1	6.35	4.72	3.13	5.33	2.73	1.98
SAMM50	68.34	79.6	77.65	63.58	65.99	73.69
SAMSN1	171.51	60.73	60.33	78.23	115.49	45.95
SAMSN1-AS1	0.93	0.51	0.17	0.19	0.27	0.19
SAP130	10.61	12.86	12.51	13.27	12.47	9.28
SAP18	62.51	53.2	46.87	49.79	60.24	51.61
SAP25	0.28	0	0.26	0.13	0	0.09
SAP30	1.31	0.98	0.95	0.57	1.43	0.83
SAP30BP	24.47	23.05	21.97	22.29	24.22	20.34
SAP30L	0.44	0.45	0.52	0.47	0.56	0.78
SAPCD1	0.64	0.23	0.31	0.06	0.09	0
SAPCD2	7.92	14.24	14.56	9.02	10.71	13.25
SAR1A	43.58	44.9	36.08	34.89	40.55	32.32
SAR1B	11.1	11.21	9.23	8.56	10.16	8.57
SARDH	0	0	0	0	0	0
SARM1	0.11	0.08	0.01	0.01	0.03	0.06
SARNP	115.35	106.95	108.18	99.26	125.76	100.07
SARS	108.22	71.6	66.4	72.3	69.99	65.12
SARS2	26.8	25.21	21.43	24.14	18.95	20.85

SART1	4.7	4.73	4.1	4.21	3.95	4.04
SART3	12.74	15.11	13.9	15.76	17.48	15.58
SASH1	0.09	0.12	0.27	0.1	0.07	0.18
SASH3	10.92	56.6	58.36	41.62	27.92	42.73
SASS6	3.76	4.02	2.79	3.19	3.35	3.35
SAT1	181.67	41.66	34.1	41.13	76.11	27.64
SAT2	9.55	14.68	17.1	12.42	9.05	10.45
SATB1	5.47	23.28	15.86	23.35	15.11	25.56
SATB2	3.44	3.04	3.16	3.42	3.33	3.04
SATB2-AS1	0.02	0	0	0.03	0.08	0
SATL1	0	0	0	0	0	0
SAV1	2.09	0.99	1.16	1.15	2.16	1.76
SAYSD1	3.89	4.1	3.49	2.76	4.51	4.13
SBDS	31.43	21.83	21.65	18.85	21.63	17.41
SBDSP1	7.54	11.05	8.37	6.37	5.93	7.56
SBF1	2.28	2.16	2.76	2.68	2.02	2.96
SBF1P1	0	0	0	0	0	0
SBF2	0.84	0.55	0.33	0.47	0.56	0.52
SBF2-AS1	1.61	1.07	1.38	1.69	1.25	2.38
SBK1	0.05	0.02	0.11	0.21	0.19	0.03
SBK2	0	0	0	0	0	0
SBNO1	12.48	13.12	11.39	9.69	12.38	11.98
SBNO2	0.69	0.78	1.16	0.67	1.35	1.15
SBSN	0	0	0	0	0	0
SBSPON	0.35	0.34	0.29	0.09	0.19	0.27
SC5DL	4.15	3.78	4.49	3.48	4.79	4.07
SCAF1	1.31	1.66	2.55	1.85	1.04	1.7
SCAF11	11.33	12.73	11.12	8.69	10.4	10.65
SCAF4	20.72	22.97	20.09	18.03	21.75	21.74
SCAF8	3.91	4.21	2.96	3.64	3.3	4.12
SCAI	1.87	1.67	1.7	1.37	1.66	1.52
SCAMP1	5.24	6.38	6.11	3.82	5.08	7.12
SCAMP2	121.64	136.41	131.44	124.05	123.19	120.81
SCAMP3	88.09	76.82	88.21	84.54	82.19	75.43
SCAMP4	13.11	14.17	13.02	11.64	14.02	15.48
SCAMP5	1.25	2.41	2.57	1.96	1.17	1.77
SCAND1	83.7	115.21	109.74	122.4	113.26	111.1
SCAND2P	1.86	1.65	2.33	2.3	1.96	1.91
SCAND3	0	0	0	0	0	0
SCAP	22.12	19.1	21.62	21.13	17.9	19.94
SCAPER	3.73	5.55	5.5	4.82	3.28	4.02
SCARA3	0.04	0.05	0.07	0.1	0	0.02
SCARA5	0	0	0	0	0	0

SCARB1	50.94	64.93	60.11	55.64	50.03	60.97
SCARB2	4.8	5.85	6.84	4.96	5.3	5.73
SCARF1	1.34	2.34	3.57	3.01	2.42	1.57
SCARF2	0.02	0.33	0	0.24	0.31	0.02
SCARNA1	0	0.56	0	0.51	0.69	0
SCARNA10	1.12	0.89	0	0	0	0.28
SCARNA11	0	0	0	0	0	0
SCARNA12	4.69	4.32	15.85	4.61	4.89	3.98
SCARNA13	0	0	0.12	1.18	2.45	0
SCARNA14	0	0	0	0	1.82	0
SCARNA15	1.03	0	0	0	0	0
SCARNA16	0	1.43	1.63	1.28	0.58	1.19
SCARNA17	4.54	0.65	1.32	1.44	0.79	1.04
SCARNA18	0.95	0	0	0	0	0
SCARNA2	4.13	4.59	6.42	4.92	5.94	3.95
SCARNA20	0	0	0	0	2.94	0.99
SCARNA21	0	1.47	0.84	0.67	2.7	0.91
SCARNA22	24.21	15.35	57.23	47.83	23.93	38.95
SCARNA23	0.99	0	0	0	0	0
SCARNA27	0	0	0	0	0	0
SCARNA3	0	0	0	0	0	0
SCARNA4	0	0	0	0	0.99	0
SCARNA5	2.43	0.55	0.32	0.49	1.68	1.05
SCARNA6	0	0	0	0.26	0.36	0
SCARNA7	0	0.67	0.25	0	0	0
SCARNA8	0	0	0	0	0	0
SCARNA9	10.5	5.7	4.68	6.64	4.18	4.63
SCARNA9L	2.35	0	0.72	0.94	1	0.26
SCCPDH	37.24	43.74	45.5	40.18	41.12	41.79
SCD	154.85	74.7	85.52	74.51	130.61	108.9
SCD5	6.05	11.69	11.29	10.47	10.78	17.29
SCEL	0	0	0	0	0	0
SCFD1	17.66	15.31	16.48	13.7	17.23	15.06
SCFD2	8.17	10.82	11.88	11.65	6.97	9.38
SCG2	0.06	0	0	0	0.11	0.09
SCG3	0.07	0.16	0.04	0.02	0.06	0.13
SCG5	0	0.1	0.06	0	0	0.12
SCGB1A1	0	0	0	0	0	0
SCGB1B2P	0	0	0	0	0	0
SCGB1C1	0	0	0	0	0	0
SCGB1D1	0	0	0	0	0	0
SCGB1D2	0	0	0	0	0	0
SCGB1D4	0	0	0	0	0	0

SCGB2A1	0	0	0	0	0	0
SCGB2A2	0	0	0	0	0	0
SCGB2B2	0	0	0	0	0	0
SCGB2B3P	0	0	0	0	0	0
SCGB3A1	0	0	0	0	0	0
SCGB3A2	0	0	0	0	0	0
SCGN	0.05	0	0	0	0	0
SCHIP1	0	0.53	0	0.24	0.42	0.28
SCIMP	0.13	0.09	0.06	0	0.13	0.03
SCIN	0.07	0.16	0.03	0.02	0.06	0.03
SCLT1	5.01	6.86	4.89	4.95	4.64	4.95
SCLY	11.57	10.83	13.62	16.26	10.81	10.55
SCMH1	6.95	10.26	11.57	9.9	8.8	9.81
SCML1	0	0	0	0	0	0
SCML2	10.39	8.95	7.78	8.66	8.07	7.07
SCML4	0.15	0.11	0.08	0.08	0.14	0.05
SCN10A	0	0	0	0	0	0
SCN11A	0.21	0.42	0.38	0.31	0.44	0.6
SCN1A	0	0	0	0	0	0
SCN1B	0.16	0.29	0.05	0.04	0.34	0.31
SCN2A	0.14	0	0	0	0	0
SCN2B	0.02	0.09	0.05	0.06	0.1	0.03
SCN3A	0	0	0	0	0	0
SCN3B	0.09	0.49	0.16	0.31	0.3	0.32
SCN4A	0	0	0	0	0	0
SCN4B	0.03	0	0.1	0	0	0
SCN5A	0.72	0.79	1.06	0.63	0.83	0.75
SCN7A	0	0	0	0	0	0
SCN8A	0	0	0.01	0	0.01	0.05
SCN9A	0.39	0.02	0.07	0.03	0.02	0.02
SCNM1	20.52	24.77	23.11	23	25.65	23.49
SCNN1A	0	0	0.02	0.05	0	0
SCNN1B	0	0	0	0	0	0
SCNN1D	0	0.06	0	0.14	0	0.02
SCNN1G	0.02	0.02	0	0.01	0	0
SCO1	35.8	38.69	29.28	29.58	38.98	34.95
SCO2	59.06	43.17	45.59	42.59	42.9	39.64
SCOC	23.04	13.13	9.47	9.75	14.83	14.2
SCP2	32.87	32.58	29.41	21.85	32.79	36.35
SCP2D1	0	0	0	0	0	0
SCPEP1	2.66	14.86	14.1	6.91	4.31	6.35
SCRG1	0	0.14	0	0	0	0
SCRIB	2.68	2.29	2.13	2.8	2.19	2.75

SCRN1	8.48	11.87	9.92	10.98	11.39	9.57
SCRN2	16.36	13.13	15.29	18.22	15.89	16.07
SCRN3	4.68	3.11	1.88	2.72	3	2.49
SCRT1	0	0	0	0	0	0
SCRT2	0	0	0	0	0	0
SCT	0	0	0	0	0	0
SCTR	0	0.03	0	0	0	0
SCUBE1	0	0	0	0	0.05	0
SCUBE2	0	0	0	0	0	0
SCUBE3	0.04	0.02	0.13	0.14	0	0.17
SCXA	0	0	0	0	0	0
SCXB	0	0	0	0	0	0
SCYL1	31.13	25.65	21.17	23.81	24.77	21.47
SCYL2	7.43	7.38	6	4.26	6.9	4.11
SCYL3	1.54	1.77	1.37	2.21	1.49	1.82
SDAD1	19.37	17.91	13.58	13.7	17.13	16.82
SDC1	2.24	12.34	10.7	6.58	6.85	12.28
SDC2	8.15	0	0.02	1.88	1.16	0
SDC3	0.7	0.02	0	0.12	0.38	0
SDC4	0	0	0	0	0	0
SDC4P	0	0	0	0	0	0
SDCBP	188.62	98.82	84.94	88.93	126.83	104.96
SDCBP2	0.57	0	0	0.11	0.39	0
SDCBP2-AS1	0.35	0.55	0.82	0.91	1.21	1.48
SDCCAG3	3.31	3.73	3.51	4.49	3.25	3.21
SDCCAG8	2.99	5.33	5.12	3.53	4	6.64
SDE2	10.76	7.22	6.32	6.14	7.28	5.35
SDF2	37.46	27.98	23.39	30.07	33.79	26.5
SDF2L1	78.65	115.53	110.03	80.63	102.57	89.39
SDF4	29.26	35.14	34.59	30.83	30.08	34.97
SDHA	109.05	115.17	99.97	97.2	105.82	115.3
SDHAF1	35.33	33.09	33.76	38.44	34.42	32.02
SDHAF2	60.24	50.1	41.85	39.62	50.57	41.13
SDHAP1	1.76	2.07	1.75	1.06	1.4	1.54
SDHAP2	2.98	3.56	2.22	1.46	2.32	3.24
SDHAP3	0.17	0	0.12	0.31	0.37	0.14
SDHB	213.38	231.18	191.84	182.24	206.15	172.99
SDHC	45.09	47.46	38.77	40.27	42.79	41.12
SDHD	128.95	149.56	138.13	97.49	125.83	142.43
SDK1	0	0.04	0	0	0	0
SDK2	0	0	0	0	0	0
SDPR	0.85	0	0.08	0.31	0	0
SDR16C5	0	0	0	0	0	0

SDR39U1	39.35	45.46	35.8	37.68	37.29	35.26
SDR42E1	0	0	0	0	0.05	0
SDR9C7	0	0	0	0	0	0
SDS	0.14	0.29	0.04	0	0	0.09
SDSL	8.07	6.15	6.66	4.93	2.9	2.59
SEBOX	0	0	0.22	0	0	0
SEC11A	255.29	217.15	198.98	184.23	239.36	217.04
SEC11C	65.62	72.94	63.89	55.37	57.68	50.98
SEC13	144.41	147.84	140.54	133.13	139.01	134.84
SEC14L1	23.85	4.69	5.8	7.54	9.61	5.22
SEC14L1P1	0.69	0.43	0.13	0.25	0.26	0.61
SEC14L2	3.38	0.53	0.55	1.58	2.05	0.55
SEC14L3	0	0.02	0	0	0.09	0
SEC14L4	0.92	1.06	1.92	0.67	0.91	1.03
SEC14L5	0.07	0.05	0	0.03	0.06	0.09
SEC14L6	0	0	0	0	0	0
SEC16A	11.99	10.84	12.95	11.17	10	15.72
SEC16B	0	0	0	0	0	0.02
SEC1P	0	0	0.03	0.04	0.03	0
SEC22A	5.59	6.3	5.16	4.87	5.31	4.22
SEC22B	39.89	31.24	24.92	27.19	31.17	24.51
SEC22C	13.05	7.3	9.24	6.38	7.6	6.42
SEC23A	13.28	10.13	7.03	10.29	10.14	10.89
SEC23B	26.09	22.62	17.02	16.28	20.21	17.65
SEC23IP	7.79	7.48	7.38	5.61	7.07	7.16
SEC24A	4.79	4.4	4.34	4.3	3.94	4.35
SEC24B	10.91	9.6	10.68	9.25	8.6	7.44
SEC24B-AS1	0.21	0.53	1.75	1.04	1.48	1.67
SEC24C	23.97	27	24.93	24.69	23.67	22.08
SEC24D	1.27	3.89	3.02	1.98	2.4	2.6
SEC31A	38.38	35.08	34.63	34.79	33.64	35.91
SEC31B	0.3	0.46	0.57	0.42	0.55	0.82
SEC61A1	98.35	105.71	107.05	92.17	90.75	103.89
SEC61A2	2.45	1.93	1.92	1.38	2.38	1.87
SEC61B	258.82	219.58	188.81	196.41	235	186.52
SEC61G	159.48	147.81	121.52	123.05	150.45	120.22
SEC62	11.74	9.79	8.41	7.76	9.95	8.05
SEC63	7.43	6.76	6.49	5.9	7.66	7.1
SECISBP2	4.66	7.84	5.44	6.38	7.07	8.02
SECISBP2L	3.58	2.37	2.1	2.27	3.24	2.57
SECTM1	0	0	0	0	0	0
SEH1L	52.55	51.81	48.29	50.96	50.69	50.7
SEL1L	5.47	5.55	5.13	3.87	3.83	7.24

SEL1L2	0	0	0	0	0	0
SEL1L3	1.79	1.14	1.28	0.49	0.79	1.27
SELE	0	0	0	0	0	0
SELENBP1	0	0	0	0	0	0
SELK	43.74	34.75	28.95	25.43	37.22	24.49
SELL	2.37	4.39	5.44	4.55	6.26	4.43
SELM	15.86	3.02	4.34	8.43	8.25	3.74
SELO	2.06	1.75	2.32	1.86	1.2	2.5
SELP	0	0.22	0.06	0.49	0.04	0.12
SELPLG	19.94	15.16	11.58	13.2	17.41	10.63
SELRC1	21.91	27.14	28.92	24.16	24.29	29.83
SELT	44.54	41.17	28.12	27.65	33.88	30.54
SELV	0	0	0	0	0	0
SEMA3A	0	0.4	0.09	0	0	0
SEMA3B	0	0.29	0.16	0.41	0.61	0
SEMA3C	0	0	0	0	0.05	0
SEMA3D	0	0.27	0.16	0.09	0	0.03
SEMA3E	0.09	0.02	0.04	0.02	0.09	0.02
SEMA3F	0.06	0.57	0.23	0.18	0.1	0.5
SEMA3G	0	0.04	0.07	0.08	0.01	0.12
SEMA4A	0	0.04	0.29	0	0	0
SEMA4B	0.48	0.75	0.25	0.63	0.51	0.62
SEMA4C	3.62	1.87	1.61	2.67	2.72	3.31
SEMA4D	1.31	1.95	3.4	2.31	2.25	2.74
SEMA4F	1.44	1.97	2.36	2.43	2.45	2.1
SEMA4G	0.23	0.21	0.3	0.19	0.17	0.75
SEMA5A	0.02	0.04	0.04	0.06	0.01	0.02
SEMA5B	0	0	0	0	0	0
SEMA6A	0.1	0.02	0.03	0.1	0.06	0
SEMA6B	0.09	0.38	0.32	0.23	0.12	0.54
SEMA6C	0.83	0.18	0.24	0.68	0.75	0.26
SEMA6D	0	0	0	0	0	0
SEMA7A	37.75	5.5	9.34	13.27	16.5	10.57
SEMG1	0	0	0	0	0	0
SEMG2	0	0	0	0	0	0
SENP1	6.9	6.18	6.85	5.66	7.07	5.67
SENP2	10.55	7.72	9.35	8.4	10.83	6.83
SENP3	2.79	1.24	1.94	2.37	1.52	2.17
SENP3-EIF4A	0.18	0.4	0	0	0.36	0
SENP5	17.66	14.99	16.96	15.14	16.3	15.98
SENP6	3.94	3.49	2.75	2.42	3.17	2
SENP7	1.47	0.99	1.44	1.03	1.03	1.41
SENP8	4.81	5.42	5.24	4.27	2.43	4.86

15-Sep	74.46	77.6	63.55	55.85	74.81	70.46
SEPHS1	12.6	16.77	13.62	12.79	16.26	14.04
SEPHS2	14.43	19	17.83	14.8	21.13	13.19
SEPN1	4.66	4.98	4.94	4.94	3.68	6.06
SEPP1	0	0	0	0	0	0
SEPSECS	1.33	1.09	1.3	1.35	1.02	1.02
1-Sep	0.28	0.86	0.64	0.66	0.89	1.71
10-Sep	5.55	0	0	0.03	0.51	0
11-Sep	14.63	12.69	12.79	10.61	14.66	10.8
12-Sep	0	0	0	0	0	0
14-Sep	0.34	0.58	0.4	0.15	0.25	0.31
2-Sep	61.46	45.72	41.77	35.51	42.23	40.33
3-Sep	0	0	0	0.08	0	0
4-Sep	0.13	0	0.16	0	0	0.13
5-Sep	8.1	4.47	7.62	12.62	9.52	9.69
SEPT5-GP1B	0	0.05	0	0.42	0	0.17
6-Sep	17.07	37.82	37.6	38.61	28.33	43.52
7-Sep	27.91	27.81	22.51	23.11	25.58	23.29
SEPT7L	0	0	0	0.84	0.19	0.01
SEPT7P2	1.29	3.21	1.4	1.09	1.42	1.34
8-Sep	17.02	11.45	10.42	12.89	21.74	14.12
9-Sep	7.24	8.56	7.71	9.19	7.13	11.09
SEPW1	200.81	146.39	148.89	162.72	177.49	131.62
SERAC1	2.41	2.22	1.72	0.99	1.61	1.69
SERBP1	61.74	66.84	64.55	59.57	65.26	62.19
SERF1A	0	0	0	0	0	0
SERF1B	19.64	18.47	13.28	18.17	17.97	15.57
SERF2	136.38	122.02	89.08	109.95	123.1	109.57
SERF2-C15O	0	0	0	0	0	0.27
SERGEF	27.42	34.01	27.16	27.54	26.11	28.07
SERHL	0	0.05	0	0	0	0
SERHL2	0	0	0	0	0.5	0
SERINC1	28.08	16.53	13.81	14.25	19.09	15.6
SERINC2	0.81	1.58	3.25	2.78	1.68	1.61
SERINC3	61.4	38.47	32.93	35.21	45.2	39.06
SERINC4	0.1	0.08	0.12	0.04	0	0.1
SERINC5	4.04	12.82	9.45	13.33	6.59	16.41
SERP1	64.73	68.32	72.59	69.12	70.7	71.42
SERP2	0.3	0	0	0	0	0
SERPINA1	0	0	0	0	0	0
SERPINA10	0.03	0.03	0	0	0	0.04
SERPINA11	0	0	0	0	0	0
SERPINA12	0	0	0	0	0	0

SERPINA13P	0	0	0	0	0	0
SERPINA3	0	0	0	0	0	0
SERPINA4	0	0	0	0	0	0
SERPINA5	0	0	0	0	0	0
SERPINA6	0	0	0	0	0	0
SERPINA7	0	0	0	0	0	0
SERPINA9	0	0	0	0	0	0
SERPINB1	61.06	24.94	27.42	24.33	35.32	30.57
SERPINB10	0	0	0	0	0	0
SERPINB11	0	0	0	0	0	0
SERPINB12	0	0	0	0	0	0
SERPINB13	0	0	0	0	0	0
SERPINB2	0.08	0	0	0	0	0
SERPINB3	0.08	0	0	0	0	0
SERPINB4	0	0	0	0	0	0
SERPINB5	0	0	0	0	0	0
SERPINB6	114.09	51.53	53.52	77.1	83.23	51.79
SERPINB7	0	0	0	0	0	0
SERPINB8	0	0.04	0	0.05	0	0.12
SERPINB9	0.75	0.5	0.92	0.52	0.41	0.59
SERPINC1	0	0	0	0	0	0
SERPIND1	0.03	0	0	0	0.03	0.03
SERPINE1	25.01	0	0.02	0.94	0.89	0.09
SERPINE2	7.76	30.45	27.21	25.84	21.04	31.84
SERPINE3	0.05	0	0	0	0	0
SERPINF1	4.15	2.08	1.47	2.79	3.92	1.68
SERPINF2	0.04	0.03	0.03	0.07	0.2	0.14
SERPING1	0	0.03	0.3	0.2	0.08	0.04
SERPINH1	172	58.44	43.8	93.75	92.11	51.31
SERPINI1	3.51	0.14	0.25	0.58	0.72	0
SERPINI2	0	0	0	0	0	0
SERTAD1	29.45	10.89	9.69	14.53	13.13	6.5
SERTAD2	4.49	4.37	3.73	3.55	4.33	4.38
SERTAD3	11.49	8.62	5.13	7.09	8.16	4.89
SERTAD4	0	0	0	0	0	0
SERTAD4-AS	0	0	0	0	0	0
SERTM1	0	0	0	0	0	0
SESN1	4.97	4.34	4.86	3.85	5.19	7.05
SESN2	9.16	6.35	6.93	7.1	5.9	5.48
SESN3	0.18	0	0.01	0.01	0	0
SESTD1	0.46	0.94	0.61	0.7	0.68	0.95
SET	174.54	230.17	225.79	194.26	219.8	199.53
SETBP1	0.03	0	0	0	0.03	0

SETD1A	3.22	3.17	3.53	2.63	3.16	2.88
SETD1B	3.21	3.15	3.4	3.04	2.93	3.06
SETD2	5.84	6.37	5.42	6.13	5.97	5.76
SETD3	11.94	16.66	16.24	12.67	15.24	16.87
SETD4	1.89	5.95	6.32	5.03	4.04	4.34
SETD5	6.27	6.32	6.54	6.08	5.74	6.36
SETD5-AS1	1.83	2.57	1.39	2.14	1.51	1.17
SETD6	0.03	0.34	0.33	0.14	0.3	0.15
SETD7	2.42	2.76	3.31	2.34	2.04	3.71
SETD8	9.4	7.38	6.01	7.69	6.79	4.44
SETD9	4.61	5.84	5.2	7.39	4.87	6.06
SETDB1	21.77	22.65	21.04	18.92	17.38	20.57
SETDB2	1.93	4.64	4.65	4.59	3.18	5.04
SETMAR	7.5	10.87	10.95	11.49	7.33	10.85
SETX	5.35	3.91	4.31	3.82	4.14	3.89
SEZ6	0	0.03	0	0.05	0	0.12
SEZ6L	0	0.01	0.13	0	0	0
SEZ6L2	1.09	0.52	1.05	0.83	0.5	0.44
SF1	41.01	49.13	42.47	40.81	39.33	46.83
SF3A1	25.52	25.91	26.64	25.5	24.71	27.31
SF3A2	11.11	16.44	12.11	15.28	13.23	13.66
SF3A3	78.62	90.29	81.43	83.06	79.2	86.74
SF3B1	49.72	47.81	48.18	42.51	49.29	53.87
SF3B14	235.51	178.32	165.49	158.79	199.33	179.18
SF3B2	45.99	52.21	52.61	51.86	52.38	52.29
SF3B3	33.51	33.9	34.59	38.3	34.15	40.28
SF3B4	42.38	45.26	47.03	47.34	45.04	45.5
SF3B5	228.06	216.55	204.73	210.62	260.63	212.48
SFI1	1.14	2.72	3.06	2.1	2.06	1.77
SFMBT1	1.2	0.9	1.36	1.61	1.55	1.85
SFMBT2	0.87	0.65	0.97	0.64	1.23	1.19
SFN	0	0	0	0	0.05	0
SFPQ	18.78	18.65	19.55	19.43	24.41	17.32
SFR1	6.59	9.62	7.69	7.3	7.97	6.94
SFRP1	0	0	0	0	0.08	0
SFRP2	0	0	0	0	0	0
SFRP4	0.07	0	0	0	0	0
SFRP5	0	0	0	0.05	0.19	0.04
SFSWAP	8.49	9.11	8.59	8.42	7.91	8.73
SFT2D1	130.42	97.01	93.61	85.3	103.12	81.38
SFT2D2	61.89	67.4	70.97	54.36	64.4	66.86
SFT2D3	0.47	0.51	0.29	0.7	0.22	0.34
SFTA1P	0	0	0	0	0	0

SFTA2	0	0	0	0	0	0
SFTA3	0	0	0	0	0	0
SFTPA1	0	0	0	0	0	0
SFTPA2	0	0	0	0	0	0
SFTPB	0.32	0.44	0.58	0.25	0.42	0.31
SFTPC	0	0	0	0	0.06	0
SFTPD	0	0	0	0	0	0
SFXN1	75.75	54.5	50.72	47.25	55.35	51.76
SFXN2	2.75	5.43	4.67	2.81	4.53	4.35
SFXN3	15.8	5.94	7.59	5.87	6.9	7.87
SFXN4	45.94	58.75	55.8	51.75	47.39	54.24
SFXN5	2.38	1.52	1.28	1.97	1.91	1.59
SGCA	0	0	0	0.05	0	0
SGCB	2.67	2.26	2.21	2.3	1.75	2.45
SGCD	0	0	0	0	0	0
SGCE	1.73	1.28	2.24	2.23	3.35	7.53
SGCG	0	0	0	0	0	0
SGCZ	0	0	0	0	0	0
SGIP1	0	0.15	0	0.04	0	0
SGK1	9.79	1.22	0.2	0.92	4.7	0.8
SGK110	0	0	0	0	0	0
SGK196	124.49	97	100.06	95.72	112.95	92.09
SGK2	0	0	0	0	0	0
SGK223	5.26	4.25	4.8	4.28	2.66	5.96
SGK3	1.14	9.05	6.69	3.05	2.18	4.51
SGK494	1	1.76	1.43	0.71	0.87	0.6
SGMS1	7.21	4.12	2.62	4.82	4.83	2.5
SGMS2	0.04	0	0.01	0	0	0
SGOL1	9.65	8.75	8.72	8.12	11.29	8.16
SGOL1-AS1	0.77	0.33	0	0.47	0.17	0.18
SGOL2	6.13	6.78	6.21	5	5.35	5.67
SGPL1	7.69	8.62	7.46	5.96	7.87	5.73
SGPP1	1.76	0.24	0.54	0.39	0.54	0.22
SGPP2	0.06	0	0	0	0	0
SGSH	0.9	0	0.12	0.31	0.6	0.35
SGSM1	0.27	0.53	0.63	0.25	0.42	0.34
SGSM2	2.01	1.27	1.08	1.66	1.38	0.73
SGSM3	16.92	16.81	16.51	14.91	18.34	12.96
SGTA	70.11	63.3	59.01	61.24	60.17	57.23
SGTB	3.14	2.34	2.24	1.87	2.42	2.31
SH2B1	0.29	0.38	0.23	0.34	0.7	0.63
SH2B2	0.08	0.86	0.39	0.08	0.37	0.04
SH2B3	4.45	3.17	2.99	2.84	3.54	2.48

SH2D1A	0	0	0	0	0	0
SH2D1B	0	0	0	0	0	0
SH2D2A	1.6	0	0	0.36	0.69	0.21
SH2D3A	0.53	1.42	0.8	0.96	0.38	1.29
SH2D3C	0.78	1.73	3.97	1.33	1.89	0.81
SH2D4A	0.02	0	0	0	0	0
SH2D4B	0	0	0	0	0	0
SH2D5	0.22	0.79	0.59	0.39	0.89	0.5
SH2D6	0	0.07	0	0	0	0
SH2D7	0	0	0	0	0	0.04
SH3BGR	1.54	1.22	0	1.17	1.02	0.7
SH3BGRL	98.1	85.7	75.9	75.76	92.65	98.61
SH3BGRL2	1.24	0.05	0.09	0.25	0.18	0
SH3BGRL3	558.6	177.43	219.31	279.46	349.13	242.65
SH3BP1	0.43	0.69	0.99	0.41	0.62	0.66
SH3BP2	2.13	2.22	2	1.38	1.97	2.03
SH3BP4	1.02	0.32	1.34	0.91	0.55	1.18
SH3BP5	2.65	1.64	0.15	1.16	0.93	1.31
SH3BP5-AS1	0.61	0.86	0.61	0.23	0.52	0.69
SH3BP5L	1.1	0.58	1.4	0.99	1.06	0.74
SH3D19	0.02	0.16	0.09	0.04	0	0.14
SH3D21	0.09	0.6	0.06	0.13	0.33	0.1
SH3GL1	7.48	5.93	5.02	4.86	6.06	4.05
SH3GL1P1	0.09	0.21	0.1	0.14	0.11	0.18
SH3GL1P2	0.09	0	0.06	0	0	0
SH3GL2	0.24	0.4	0	0.02	0	0
SH3GL3	1.99	0	0	1.12	0.42	0
SH3GLB1	9	7.72	7.77	6.97	6.82	7.08
SH3GLB2	1.12	0.7	0.75	0.31	0.91	1.47
SH3KBP1	9.9	16.67	17.2	12.73	14.33	10.04
SH3PXD2A	1.28	0.43	0.14	0.22	0.33	0.23
SH3PXD2B	0.76	0.25	0.33	0.7	0.75	0.68
SH3RF1	2.19	4.22	3.22	2.04	2.09	2.72
SH3RF2	0	0.09	0	0.2	0.14	0.15
SH3RF3	0.53	0	0	0.06	0.16	0
SH3RF3-AS1	0	0	0.05	0.02	0	0
SH3TC1	1.17	0.59	0.44	0.42	0.75	0.46
SH3TC2	0.03	0.01	0.04	0	0.02	0.01
SH3YL1	2	0.98	1.81	2.07	1.54	1.17
SHANK1	0.05	0.03	0	0.09	0.06	0.11
SHANK2	0.03	0	0	0	0	0
SHANK2-AS1	0.2	0.07	0.12	0.19	0	0.27
SHANK2-AS3	1.14	1.12	2.33	1.12	2.01	1.11

SHANK3	0.13	0.3	0.27	0.41	0.23	0.42
SHARPIN	21.02	25.83	25.17	28.52	26.84	16.69
SHB	0.42	0.15	0.19	0.39	0.34	0.07
SHBG	0	0	0.08	0	0	0
SHC1	17.59	17.82	18.77	18.71	16.33	19.93
SHC2	0	0	0.03	0	0.11	0.03
SHC3	0	0	0	0	0	0
SHC4	0.1	0	0	0.12	0	0.02
SHCBP1	16.22	13.08	10.28	9.19	14.22	8.91
SHCBP1L	0	0	0	0	0	0
SHD	0	0	0	0.08	0.08	0.14
SHE	0.04	0.08	0.09	0.05	0.05	0.09
SHF	0.29	0.3	0.24	0.5	0.44	1.31
SHFM1	339.69	313.69	266.85	259.93	345.63	273.7
SHH	0	0.07	0.09	0	0	0
SHISA2	0.12	0.11	0.04	0.04	0.17	0.16
SHISA3	0.34	4.08	3.5	1.55	1.24	3.49
SHISA4	0.05	0.13	0	0	0	0
SHISA5	119.03	81.75	106.28	122.7	119.1	92.56
SHISA6	0	0	0	0	0	0
SHISA7	0.02	0	0	0	0	0
SHISA8	0	0	0	0	0.05	0.11
SHISA9	5.49	7.12	7.43	4.39	5.29	6.71
SHKBP1	30.43	45.46	39.7	41.52	41.34	42.94
SHMT1	17.06	25.11	18.67	19.28	19.03	25.04
SHMT2	87.31	104.21	94.66	92.55	96.82	108.51
SHOC2	8.48	7.48	8.7	5.85	6.78	6.84
SHOX	2.13	2.4	2.12	1.8	1.34	1.91
SHOX2	2.43	3.12	3.56	2.26	2.95	2.85
SHPK	11.86	10.74	12.3	10.56	11.38	13.5
SHPRH	3.14	3.47	2.43	2.9	2.72	2.61
SHQ1	18.94	19.07	16.22	16.11	18.55	15.59
SHROOM1	0.52	0.41	0.67	0.24	0.47	0.4
SHROOM2	0.09	0.01	0.03	0.11	0.11	0.1
SHROOM3	0.05	0.01	0.01	0.01	0	0
SHROOM4	0.04	0.09	0.09	0.06	0.03	0.04
SI	0	0	0	0	0	0
SIAE	0.35	0.14	0.28	0.14	0.26	0.25
SIAH1	3.31	3.78	3.75	4.2	4.25	4.78
SIAH2	2.01	2.88	2.48	3.02	2.06	2.94
SIAH3	0.03	0.01	0.02	0	0	0.02
SIDT1	0	0.09	0.07	0.01	0.03	0.07
SIDT2	2.64	1.46	0.72	1.07	1.17	0.86

SIGIRR	20.73	17.26	17.65	17.86	21.69	15.92
SIGLEC1	0	0	0	0	0	0
SIGLEC10	2.28	3.47	7.03	2.68	3.5	2.82
SIGLEC11	0.07	0.1	0.38	0.13	0.17	0.13
SIGLEC12	23.65	34.56	39.42	33.09	28.25	50.1
SIGLEC14	0	0.04	0.39	0	0	0.21
SIGLEC15	0.05	0.08	0	0	0	0
SIGLEC16	0.24	0.2	0.23	0.07	0.15	0.18
SIGLEC17P	0.25	0.71	0.09	0.07	0.43	0.1
SIGLEC5	0.29	1.68	2.51	2	1.95	1.02
SIGLEC6	174.98	51.74	53.71	61.03	106.67	18.68
SIGLEC7	0	0.1	0	0	0.05	0.36
SIGLEC8	0.13	0.21	0.25	0.07	0.2	0.22
SIGLEC9	0.22	0.2	0	0	0	0
SIGLECL1	0	0	0	0	0	0
SIGMAR1	140.03	156.32	156.8	169.59	160.03	151.44
SIK1	0.75	0.27	0.37	0.14	0.14	0.12
SIK2	2.99	3.9	3.01	2.93	3.15	4.36
SIK3	0.81	1.11	1.32	1.02	0.96	1.26
SIKE1	8.26	7.33	6.54	5.13	6.51	6.03
SIL1	22.6	24.94	29.44	29.26	22.97	25.31
SIM1	0	0	0	0	0	0
SIM2	0	0.01	0.06	0	0	0
SIMC1	1.01	3.6	5.27	3.52	2.82	3.67
SIN3A	13.32	14.35	13.73	15	13.94	15.26
SIN3B	3.06	1.95	1.41	1.72	1.3	1.7
SIPA1	1.64	5.99	6.79	6.76	3.56	4.28
SIPA1L1	1.94	1.53	1.69	2.39	3.8	0.85
SIPA1L2	0	0	0.01	0	0	0
SIPA1L3	2.59	3.02	2.83	2.55	2.78	3.34
SIRPA	2	2.5	2.38	1.98	2.72	2.29
SIRPB1	0	0.37	0.08	0	0	0.06
SIRPB2	0.14	0.38	0.24	0.16	0.22	0.39
SIRPD	0	0	0	0	0	0
SIRPG	0	0	0	0	0	0
SIRT1	0.87	0.91	0.56	0.77	1.42	1.21
SIRT2	24.57	23.04	24.85	27.15	23.68	23.84
SIRT3	6.05	6.83	4.73	4.26	6.22	4.65
SIRT4	1.44	1.24	0.52	0.4	0.42	0.32
SIRT5	5.73	5.58	4.9	4.03	4.46	4.43
SIRT6	6.52	7.87	9.38	7.82	7.54	9.03
SIRT7	2.32	2.74	3.34	3.19	2.56	2.22
SIT1	0	0	0.06	0	0.29	0

SIVA1	198.27	338.46	273.53	265.57	294.97	299.58
SIX1	0.9	5.34	4.96	4.49	4.11	5.36
SIX2	0	0	0.03	0.19	0.19	0.07
SIX3	2.12	3.22	2.95	2.64	2.85	3
SIX4	1.76	2.02	2.08	1.64	1.27	2.15
SIX5	0.48	0.77	0.58	0.54	0.47	0.64
SIX6	0	0	0	0	0	0
SKA1	13.76	8.46	11.75	9.54	10.76	8.37
SKA2	24	27.74	24.34	26.14	31.15	26.98
SKA3	18.26	21.74	20.49	18.45	21.38	18.46
SKAP1	0	0	0	0	0	0
SKAP2	5.2	4.53	3.23	2.36	4.65	3.88
SKI	2.11	1.54	1.66	1.15	1.64	1.7
SKIDA1	0.1	0.18	0.22	0.18	0.15	0.25
SKIL	3.56	1.04	0.87	1.03	1.97	0.93
SKINTL	0	0	0	0	0	0
SKIV2L	6.96	7.12	7.61	7.77	6.4	8.27
SKIV2L2	22.24	21.13	21.96	21.14	21.5	23.53
SKOR1	0	0	0	0	0	0.03
SKP1	205.9	154.58	145.56	161.61	186.95	160.53
SKP1P2	0.41	0	0.25	0.07	0.08	0.22
SKP2	20.77	49.71	47.02	35.9	33.09	42.18
SLA	0.26	2.14	1.92	1.94	1.96	3.98
SLA2	0.19	0.6	0.92	0.7	0.85	0.55
SLAIN1	1.61	2.07	1.51	1.29	2.16	1.14
SLAIN2	2.29	2.15	1.7	1.63	1.97	1.35
SLAMF1	0.07	0.06	0	0	0.17	0.52
SLAMF6	1.34	1.07	1.81	1.49	1.46	1.59
SLAMF7	0.7	0.77	1.15	0.3	0.43	0.62
SLAMF8	0	0	0	0	0	0
SLAMF9	0	0	0	0	0	0
SLBP	56.58	65.05	65.7	66.54	66.68	48.57
SLC10A1	0.05	0.11	0.17	0.06	0.26	0.14
SLC10A2	0	0	0	0.03	0	0
SLC10A3	7.7	6.92	10.08	7	8.25	11.19
SLC10A4	2.82	2.71	3.25	1.77	2.3	3.13
SLC10A5	0.17	0.27	0	0.04	0	0.06
SLC10A6	0	0	0	0	0	0
SLC10A7	1.82	3.73	3.44	1.29	2.96	3.16
SLC11A1	0.12	0.15	0.05	0.13	0.37	0.17
SLC11A2	5.54	6.04	6.26	7.2	5.75	4.9
SLC12A1	0	0	0	0	0	0
SLC12A2	2.45	2.09	1.91	1.83	1.62	1.41

SLC12A3	0.04	0.01	0.02	0.05	0	0.03
SLC12A4	0.67	0.6	0.63	1.03	0.66	1.01
SLC12A5	0.06	0.16	0.2	0.13	0.1	0.17
SLC12A6	2.32	2.23	2.12	2.08	1.93	3.17
SLC12A7	3.36	3.27	4.06	2.89	2.62	2.83
SLC12A8	0.35	0.3	0.19	0.19	0.09	0.29
SLC12A9	2.41	2.86	3.19	2.46	1.99	3.72
SLC13A1	0	0.05	0	0	0	0
SLC13A2	0	0	0	0	0	0
SLC13A3	0	0	0	0	0	0
SLC13A4	0	0.06	0.09	0.02	0	0.03
SLC13A5	0	0	0	0	0	0
SLC14A1	0.27	0.16	0.25	0.09	0.1	0.08
SLC14A2	0.56	0.86	0.82	0.6	0.39	0.51
SLC15A1	0.42	0.42	0.28	0.22	0.2	0.21
SLC15A2	0.57	0.62	0.35	0.27	0.36	0.24
SLC15A3	0	0.25	0.03	0.17	0.26	0.27
SLC15A4	1.83	2.08	1.49	1.66	1.83	1.93
SLC15A5	0	0	0	0	0	0
SLC16A1	50.36	35.54	37.01	41.68	41.17	36.16
SLC16A10	1.01	0.85	1.29	1.02	0.76	1.79
SLC16A11	0	0	0	0	0	0
SLC16A12	0.01	0.05	0.04	0.06	0.1	0.07
SLC16A13	2.76	4.19	5.02	4.42	3.23	3.83
SLC16A14	3.11	4.85	4.53	2.48	4.14	4.44
SLC16A2	0.12	1.62	2.56	2.99	1.2	0.55
SLC16A3	34.73	33.82	30.15	24.47	23.94	31.05
SLC16A4	0.36	1.03	0.83	0.5	0.51	0.72
SLC16A5	2.12	0.57	1.63	2.45	1.9	1.08
SLC16A6	0.12	0.07	0.03	0.12	0.09	0.08
SLC16A7	1.23	0.8	0.85	0.76	0.81	1.15
SLC16A8	0	0	0	0.05	0.04	0
SLC16A9	1.53	1.07	1.06	1.41	1.25	1.06
SLC17A1	0	0	0	0	0	0
SLC17A2	0	0	0	0	0	0
SLC17A3	0	0	0	0	0	0
SLC17A4	0	0	0	0	0	0
SLC17A5	5.19	5.01	4.21	3.53	4.37	3.32
SLC17A6	0	0	0	0	0	0
SLC17A7	0.02	0.02	0	0.15	0	0.02
SLC17A8	0	0	0	0	0	0
SLC17A9	16.81	27.8	30.66	20.26	21.61	21.66
SLC18A1	0.08	0	0	0.07	0	0.05

SLC18A2	2.17	0.47	0.74	1.14	1.45	0.85
SLC18A3	0	0	0	0	0	0
SLC18B1	4.53	4.36	4.4	5.58	5.48	5.48
SLC19A1	37.48	42.79	47.21	55.29	45.23	49.8
SLC19A2	4.11	4.48	4.21	4.44	4.2	2.48
SLC19A3	0.04	0.04	0	0.07	0.09	0.12
SLC1A1	0	0	0	0.01	0	0
SLC1A2	0.06	0.03	0.06	0.03	0.03	0.07
SLC1A3	0.8	3.65	2.79	0.93	0.84	2.01
SLC1A4	3.57	4.4	2.79	2.79	2.63	2.31
SLC1A5	116.98	104.63	127.33	115.74	107.8	116.02
SLC1A6	0	0.04	0.02	0.02	0	0
SLC1A7	0	0	0	0	0	0
SLC20A1	85.57	40.16	50.98	49.03	57.3	34.81
SLC20A2	11.42	8.64	7.86	6.48	8.36	7.76
SLC22A1	0	0	0	0	0	0
SLC22A10	0	0	0	0	0	0
SLC22A11	0	0	0	0	0	0
SLC22A12	0	0	0	0	0	0
SLC22A13	0	0.02	0	0	0	0
SLC22A14	0	0	0	0	0	0
SLC22A15	0.09	0.93	0.22	0.04	0.04	0.51
SLC22A16	0	0	0	0	0	0
SLC22A17	0.09	0	0	0.23	0.09	0
SLC22A18	9.09	3.29	1.07	5.39	5.92	1.87
SLC22A18AS	0	0.15	0.18	0	0.09	0.1
SLC22A2	0	0	0	0	0	0
SLC22A20	0.06	0.32	0.06	0.01	0.09	0.16
SLC22A23	0.26	0.21	0.03	0.14	0.09	0.19
SLC22A24	0.07	0	0	0	0	0
SLC22A25	0	0	0	0	0	0
SLC22A3	0	0	0	0	0	0
SLC22A31	0	0	0	0	0.04	0
SLC22A4	0.91	2.2	1.53	1.34	0.9	2.98
SLC22A5	3.57	7.11	5.93	4.27	3.67	7.05
SLC22A6	0	0	0	0	0	0
SLC22A7	0	0	0	0	0	0
SLC22A8	0	0	0	0	0	0
SLC22A9	0	0.2	0.07	0.02	0.06	0
SLC23A1	0	0	0.06	0	0	0.03
SLC23A2	3.24	3.2	2.99	3.11	2.84	3.04
SLC23A3	0.18	0	0	0	0	0.11
SLC24A1	0.86	1.43	2.3	1.16	1.14	1.92

SLC24A2	0.01	0.03	0.07	0.02	0.03	0.07
SLC24A3	0	0	0	0	0	0
SLC24A4	0.33	0.54	0.5	0.24	0.28	0.32
SLC24A5	0	0	0	0	0.04	0
SLC24A6	3.89	7.12	5.92	4.01	4.27	8.58
SLC25A1	34.25	46.2	38.23	44.59	44.98	29.29
SLC25A10	12.12	19.81	17.4	20.55	15.28	20.15
SLC25A11	64.07	57.52	50.81	62.06	61.15	45.34
SLC25A12	4.57	5.19	5.26	4.27	4.67	5.79
SLC25A13	17.06	13.73	15.44	11.11	14.59	14.72
SLC25A14	14.64	12.86	13.2	11.26	12.86	10.42
SLC25A15	11.53	13.96	15.96	11.22	11.87	13.5
SLC25A16	2.32	3.46	2.01	1.96	1.7	1.79
SLC25A17	31.4	22.6	23.59	26.18	33.39	24.72
SLC25A18	0	0	0.03	0	0.1	0
SLC25A19	43.13	55.5	49.2	45.42	50.33	45.73
SLC25A2	0	0	0	0	0.1	0
SLC25A20	12.99	13.44	13.4	15.28	11.05	11.44
SLC25A21	0.07	0	0.15	0.14	0.47	0.06
SLC25A22	6.07	7.15	7.19	8.45	6.54	2.96
SLC25A23	7.73	5.77	5.63	5.81	7.17	5.5
SLC25A24	4.15	6.4	6.52	5.4	6.15	6.12
SLC25A25	7.6	3.87	4.88	4.67	5.08	3.28
SLC25A26	10.37	10.53	8.05	10.19	9.53	9.46
SLC25A27	0	0.16	0	0.02	0	0.27
SLC25A28	2.26	2.31	0.66	0.83	1.73	1.07
SLC25A29	1.27	3.78	4.74	4.13	4.24	3.42
SLC25A3	654.42	628.23	587.87	676.08	688.06	683.13
SLC25A30	1.7	1.6	2.5	1.01	1.75	2.5
SLC25A31	0	0	0	0	0	0
SLC25A32	33.31	41.89	39.18	32.35	32.34	40.21
SLC25A33	3.49	6.43	2.44	3.26	4.03	4.2
SLC25A34	0.53	0.55	0.88	0.71	0.41	1.04
SLC25A35	0.99	0.89	1.12	0.67	0.56	0.74
SLC25A36	5.5	5.41	4.28	3.37	5.49	5.47
SLC25A37	145.99	25.7	20.33	65.1	75.62	22.14
SLC25A38	29.05	24.27	23.29	26.08	25.23	24.05
SLC25A39	281.23	193.73	193.45	238.57	240.58	194.23
SLC25A3P1	0	0	0	0	0	0
SLC25A4	12.43	3.3	6.77	7.04	5.92	5.33
SLC25A40	2.18	3.15	2.86	2.56	3.71	3.53
SLC25A41	0	0.19	0	0	0.05	0.15
SLC25A42	0.96	0.88	0.59	0.9	1.09	1.1

SLC25A43	3.4	4.46	4.26	4.34	3.07	3.02
SLC25A44	14.17	11.42	11.51	10.57	13.24	11.4
SLC25A45	2.35	2.21	2.3	1.82	1.5	1.5
SLC25A46	16.49	15.17	13.97	12.93	12.13	16.2
SLC25A47	0	0	0	0	0	0
SLC25A48	0.19	0	0.12	0.04	0	0
SLC25A5	1140.98	1259.32	1136.46	1200.39	1311.19	1163.97
SLC25A5-AS1	0.33	0.55	0.9	0.85	0.61	0.53
SLC25A51	7.49	6.6	7.79	5.55	8.29	5.5
SLC25A51P1	0	0.04	0	0	0.05	0
SLC25A52	0.15	0	0	0	0.14	0
SLC25A53	0.37	0.7	0.79	0.38	0.93	0.35
SLC25A6	864.55	886.29	1010.2	1261.44	1147.48	1096.21
SLC26A1	0.13	0.62	0.2	0.4	0	0.22
SLC26A10	0.52	0.74	0.43	0.3	0.44	0.86
SLC26A11	0.05	0	0	0.04	0	0
SLC26A2	2.25	4.71	3.71	2.55	3.59	6.33
SLC26A3	0	0	0	0	0	0
SLC26A4	0.07	0.12	0.22	0.1	0.09	0.33
SLC26A4-AS1	0.24	0.04	0.07	0	0	0
SLC26A5	0	0	0	0	0	0
SLC26A6	7.54	9.57	10.13	10.75	9.33	9.16
SLC26A7	0	0	0	0	0	0
SLC26A8	0	0.12	0	0	0.09	0.09
SLC26A9	0	0	0.03	0.04	0	0.02
SLC27A1	2.44	2.56	1.8	2.78	2.09	2.65
SLC27A2	48.85	29.39	23.91	41.58	50.43	37.22
SLC27A3	0.09	1.7	2.12	0.77	1.18	1.63
SLC27A4	7.01	9.38	8.57	6.97	5.35	6.27
SLC27A5	1.58	2.18	0.97	1.22	2.08	1.23
SLC27A6	0.66	0.04	0	0.06	0.08	0
SLC28A1	0	0	0	0	0	0
SLC28A2	0.75	0.72	0.74	0.35	0.44	0.37
SLC28A3	0	0	0	0	0	0
SLC29A1	62.21	112.76	115.59	102.55	80.48	111.12
SLC29A2	8.19	12.92	11.02	10.39	11.03	15.42
SLC29A3	7.1	8.61	10.33	9.84	10.65	9.08
SLC29A4	1.04	0.68	1.25	2.4	1	1.71
SLC2A1	24.55	12.93	13.95	14.59	11.8	11.23
SLC2A1-AS1	0.14	0.05	0.12	0	0	0.21
SLC2A10	0.02	0.05	0.02	0	0.01	0.07
SLC2A11	1.66	1.82	1.81	2.15	1.76	1.35
SLC2A12	0.58	0.11	0.28	0.25	0.33	0.2

SLC2A13	0.13	0.29	0.18	0.18	0.02	0.05
SLC2A14	0	0.07	0	0	0	0.04
SLC2A2	0	0	0	0	0	0
SLC2A3	26.13	4.29	3.25	9.28	19.01	3.7
SLC2A4	0.9	0.63	1.12	2	1.37	1.69
SLC2A4RG	1.26	1.53	2.03	1.44	1.92	2.22
SLC2A5	1.2	3.6	5.44	3.99	4.26	6.91
SLC2A6	3.22	2.64	5.21	4.25	4.57	4.28
SLC2A7	0	0	0	0	0	0
SLC2A8	4.8	2.77	4.49	3.93	4.47	2.34
SLC2A9	0.6	0.72	0.37	1.07	0.11	0
SLC30A1	3.07	2.85	1.44	1.34	1.17	1.04
SLC30A10	0	0	0	0	0	0
SLC30A2	0	0	0	0	0	0
SLC30A3	7.16	9.95	12.55	8.59	6.89	10.8
SLC30A4	0.05	0.1	0.03	0.01	0.18	0.02
SLC30A5	6.14	5.47	5.2	4.18	4.91	4.31
SLC30A6	7.05	5.55	6.78	5.65	6.67	5.74
SLC30A7	5.02	4.69	3.97	3.33	3.44	4.06
SLC30A8	0.05	0.01	0.01	0	0	0.03
SLC30A9	19.88	17.85	15.36	16.44	19.29	14.79
SLC31A1	12.72	14.34	11.87	9.79	10.73	12.42
SLC31A2	4.61	6.33	6.71	6.4	7.33	7.45
SLC32A1	0	0	0	0	0.03	0
SLC33A1	8.85	8.72	9.25	6.75	6.74	10.73
SLC34A1	0	0	0	0	0	0
SLC34A2	0	0	0	0.02	0	0.02
SLC34A3	0	0	0.03	0	0	0
SLC35A1	10.55	13.52	10.51	7.23	12.87	10.02
SLC35A2	26.53	24.07	26.24	20.8	24.2	21.18
SLC35A3	2.51	2.66	2.58	1.77	2.55	2
SLC35A4	0	0	0	0	0	0
SLC35A5	6.25	6.36	5.37	3.27	5.81	4.07
SLC35B1	58.7	65.79	64.96	58.3	72.34	55.55
SLC35B2	64.58	54.53	50.93	56.71	70.22	55.06
SLC35B3	7.98	7.18	6.9	4.58	8.69	10.03
SLC35B4	7.66	8.81	9.43	7.7	7.8	9.63
SLC35C1	8.32	8.89	9.41	8.56	8.73	6.89
SLC35C2	31.55	32.09	28.9	34.22	28.82	32.35
SLC35D1	3.94	5.25	3.4	2.54	5.05	4.29
SLC35D2	1.97	0.9	1.34	1.61	1.52	1.21
SLC35D3	0.03	0.05	0.06	0.02	0.35	0
SLC35E1	5.72	5.65	5.87	5.59	4.7	5.98

SLC35E2	4.65	4.75	5.35	4.02	4.44	5.32
SLC35E2B	9.56	8.88	8.74	8.78	9.23	11.69
SLC35E3	7.14	8.83	7.45	5.61	5.34	7.2
SLC35E4	1.74	2.88	4.13	3.2	3.46	2.95
SLC35F1	0.11	0.21	0.15	0.11	0.13	0.15
SLC35F2	23.1	33.47	33.76	31.39	35.36	36.9
SLC35F3	0	0	0	0	0	0
SLC35F4	0	0	0	0	0	0
SLC35F5	10.56	9.55	8.64	7.88	8.99	8.22
SLC35F6	51.89	55.55	55.44	58.38	62.98	49.32
SLC35G1	2.57	6.3	3.55	3.05	4.35	4.64
SLC35G2	2.33	4.73	4.73	4.33	1.98	2.8
SLC35G3	0.08	0.31	0.04	0.08	0	0.05
SLC35G5	0	0	0	0	0	0.25
SLC35G6	0	0.05	0	0	0.06	0
SLC36A1	4.63	2.53	2.34	1.84	3.01	1.53
SLC36A2	0.49	0.6	0.91	0.4	0.63	0.56
SLC36A3	0	0.06	0.07	0.01	0	0.05
SLC36A4	2.02	1.34	2.29	1.74	3	2.8
SLC37A1	1.86	3.52	1.85	3.54	1.71	2.48
SLC37A2	1.6	3.42	5.51	1.77	1.81	1.71
SLC37A3	2.47	0.45	0.6	1.54	1.12	0.64
SLC37A4	26.1	36.83	29.05	36.23	28.6	42.39
SLC38A1	25.51	20.95	16.81	14.94	19.72	18.4
SLC38A10	5.8	10.53	11.47	7.65	9.05	9.93
SLC38A11	0	0	0	0	0	0
SLC38A2	31.65	15.1	15.17	15.66	19	18.07
SLC38A3	0	0	0	0.27	0.06	0.33
SLC38A4	0	0	0	0	0	0
SLC38A5	216.96	211.63	246.13	263.6	250.12	225.77
SLC38A6	24.42	5.87	6.46	7.73	16.68	5.8
SLC38A7	11.59	10.08	6.1	7.55	9.22	4.19
SLC38A8	0.38	0	0	0	0	0
SLC38A9	5.58	10.08	8.93	7.69	5.86	10.04
SLC39A1	44.59	39.08	33.81	28.62	40.16	38.32
SLC39A10	5.52	8.18	7.35	5.64	8.01	7.92
SLC39A11	16.56	17.1	14.72	13.56	18.61	16.29
SLC39A12	0	0	0	0	0	0
SLC39A13	6.95	2.74	2.29	5.61	2.99	3.61
SLC39A14	40.53	35.64	33.3	31.24	33.85	34.24
SLC39A2	0	0.13	0	0	0	0
SLC39A3	145.73	132.27	140.77	150.59	146.89	143.66
SLC39A4	35.1	33.37	32.62	36.65	44.16	37.46

SLC39A5	0	0.06	0	0	0	0
SLC39A6	8.45	9.94	6.66	5.17	8.76	10
SLC39A7	10.86	9.4	9.99	8.83	8.32	12.84
SLC39A8	11.12	5.63	8.37	9.05	6.81	6.01
SLC39A9	18.66	20.67	18.55	12.63	18.3	16.65
SLC3A1	0	0	0	0	0	0
SLC3A2	165.83	156.25	136.23	138.64	143.51	122.97
SLC40A1	81.89	0.12	1.33	9.05	6.66	0
SLC41A1	10.1	5.24	4.63	4.55	5.94	5.81
SLC41A2	1.24	0.37	0.22	1.04	0.52	0.89
SLC41A3	12.26	9.82	7.15	11.94	9.06	10.38
SLC43A1	27.11	38.2	37.4	37.07	28.32	32.67
SLC43A2	5.95	4.51	7.84	3.11	5.38	6.59
SLC43A3	126.78	110.76	106.72	107.57	125.94	107.08
SLC44A1	8.48	1.05	1.09	1.74	1.26	1.55
SLC44A2	17.57	5.58	7.53	12.39	13.75	4.62
SLC44A3	0.07	0	0	0	0	0
SLC44A4	0.22	0.73	0.7	0.41	0.41	0.52
SLC44A5	0	0	0	0	0	0
SLC45A1	0	0	0	0	0	0
SLC45A2	0	0.07	0	0	0	0
SLC45A3	0.54	0.73	0.45	0.47	0.16	0.76
SLC45A4	0.75	0.31	0.2	0.19	0.35	0.33
SLC46A1	0	0	0	0	0	0
SLC46A2	0	0.05	0	0	0.19	0.03
SLC46A3	3.28	2.02	1.6	1.63	1.52	2.79
SLC47A1	0.16	0.05	0.17	0.11	0.1	0.22
SLC47A2	0	0	0	0	0	0
SLC48A1	36.1	19.7	16.31	19.06	19.09	15.37
SLC4A1	1.87	0.29	0.27	0.38	0.13	0.31
SLC4A10	0	0	0	0	0	0
SLC4A11	7.69	9.95	9.18	10.12	7.58	10.76
SLC4A1AP	13.86	15.59	12.71	9.69	13.41	11.03
SLC4A2	10.8	5.11	5.62	6.7	7.5	5.49
SLC4A3	0.31	0.13	0.24	0.35	0.58	0.17
SLC4A4	0	0	0	0	0	0
SLC4A5	3.72	2.13	2.73	2.49	2.88	2.11
SLC4A7	0.69	0.29	0.43	0.25	0.35	0.23
SLC4A8	0.34	0.32	0.55	0.37	0.59	0.44
SLC4A9	0	0	0	0	0	0
SLC50A1	29.52	32.39	33.29	25.52	31.41	24.13
SLC51A	0	0.04	0	0.07	0	0.05
SLC51B	0	0	0	0	0	0

SLC52A1	0.11	0.13	0.23	0.07	0.17	0.08
SLC52A2	27.7	26.88	26.72	27.14	23.41	26.32
SLC52A3	0.03	0	0.12	0	0	0
SLC5A1	0	0	0	0	0	0
SLC5A10	0	0.03	0.07	0.07	0.03	0.07
SLC5A11	0	0	0	0	0	0
SLC5A12	0	0	0	0	0	0
SLC5A2	0	0.13	0	0.12	0	0.08
SLC5A3	1.73	0.84	1.37	1.09	0.88	0.99
SLC5A4	0	0	0	0	0	0
SLC5A5	0.68	0.93	1.18	1.11	0.62	0.77
SLC5A6	36.18	48.81	49.31	47.58	42.47	48.1
SLC5A7	0	0	0	0	0.03	0
SLC5A8	0	0	0	0	0	0
SLC5A9	0	0	0	0	0	0
SLC6A1	0	0	0	0	0	0
SLC6A1-AS1	0	0	0	0	0	0
SLC6A10P	0.1	0.07	0.09	0.03	0.07	0.1
SLC6A11	0	0	0	0	0	0
SLC6A12	0	0	0	0	0	0
SLC6A13	0	0	0	0	0	0.07
SLC6A14	0.07	0.14	0.09	0.05	0.04	0.11
SLC6A15	0	0	0	0	0	0
SLC6A16	0	0	0	0	0	0
SLC6A17	0	0	0	0	0	0
SLC6A18	0.14	0	0	0	0	0
SLC6A19	41.25	0.19	0.52	12.49	23.07	0.66
SLC6A2	0	0	0	0	0	0
SLC6A20	0.22	0.29	0.27	0.29	0.2	0.32
SLC6A3	0	0	0	0	0	0
SLC6A4	0.67	0.63	0.67	0.52	0.36	0.49
SLC6A5	0	0	0	0	0	0
SLC6A6	6.38	5.19	4.1	4.75	4.24	6.04
SLC6A7	0	0	0	0	0	0
SLC6A8	5.03	2.63	3.3	4.61	3.11	3.62
SLC6A9	1.69	0.62	0.2	1.78	0.76	1.03
SLC7A1	13.76	12.69	12.54	10.46	11.71	14.16
SLC7A10	0	0	0	0	0	0
SLC7A11	1.25	0.6	0.58	0.37	0.49	0.62
SLC7A11-AS1	0.03	0.1	0.09	0	0.06	0.06
SLC7A13	0	0	0	0	0	0
SLC7A14	0.25	0.31	0.29	0.15	0.23	0.28
SLC7A2	1.17	8.15	7.21	5.42	3.01	6.96

SLC7A3	0	0	0	0.02	0	0
SLC7A4	0	0	0	0	0	0
SLC7A5	28.07	39.28	41.8	35.75	31.95	37.83
SLC7A5P1	0.67	0.42	1.84	2.32	0.89	2.46
SLC7A5P2	1.38	2.74	2.03	1.67	1.91	2.34
SLC7A6	7.67	9.46	6.89	6.68	8.08	7.06
SLC7A6OS	6.16	4.99	4.23	3.73	5.51	5.77
SLC7A7	0.34	0.4	0.42	0.47	0.37	0.89
SLC7A8	6.19	0	0	0.25	0.11	0.17
SLC7A9	0	0	0	0	0	0
SLC8A1	0	0	0	0	0	0
SLC8A1-AS1	0	0	0	0	0	0
SLC8A2	0	0	0	0	0	0
SLC8A3	0.32	0.16	0	0	0.03	0.04
SLC9A1	1.87	0.82	1.51	0.85	0.53	1.26
SLC9A2	0.05	0	0	0	0	0
SLC9A3	0.03	0	0.02	0	0	0.03
SLC9A3R1	25.32	41.22	41.82	36.39	32.97	36.91
SLC9A3R2	0.26	0.58	0.34	0.23	0.22	0.46
SLC9A4	0.27	0.36	0.65	0.18	0.25	0.18
SLC9A5	0	0	0.02	0	0	0.06
SLC9A6	3.56	3.71	2.99	3	3.47	3.83
SLC9A7	1.65	2.25	2.05	1.7	1.98	2.39
SLC9A7P1	0.19	0.02	0.07	0.03	0.05	0.09
SLC9A8	2.3	3.3	2.13	2.24	2.32	2.87
SLC9A9	0.12	0.02	0	0	0.08	0
SLC9A9-AS1	0	0	0	0	0	0
SLC9B1	0.1	0.04	0	0.51	0.34	0.2
SLC9B2	6.01	7.53	6.4	6.27	5.87	7.6
SLC9C1	0	0	0	0	0	0
SLC9C2	0	0	0.03	0	0	0
SLCO1A2	0.15	0.16	0.07	0.07	0.12	0.1
SLCO1B1	0	0	0	0	0	0
SLCO1B3	0	0.02	0	0.07	0	0
SLCO1B7	0	0	0	0	0	0
SLCO1C1	0	0	0	0	0	0
SLCO2A1	0	0	0.22	0	0	0
SLCO2B1	1.47	0	0.02	0.75	1.06	0.04
SLCO3A1	0.62	0.48	0.61	0.18	0.44	0.46
SLCO4A1	5.66	14.18	15.64	10.31	8.49	12.84
SLCO4C1	0.12	1.11	0.77	0.58	0.37	0.41
SLCO5A1	0	0	0.02	0.26	0	0.41
SLCO6A1	0	0	0	0	0	0

SLED1	0	0	0	0	0	0
SLFN11	5.56	8.77	8.21	9.12	9.42	11.08
SLFN12	0	0	0	0	0	0
SLFN12L	0	0.07	0.38	0.12	0.16	0.17
SLFN13	0.18	0.33	0.3	0.2	0.23	0.14
SLFN14	6.69	0.08	0.05	2.46	2.01	0.26
SLFN5	0.53	0.11	0.03	0.27	0.34	0.14
SLFNL1	0	0.11	0.2	0	0.25	0.14
SLFNL1-AS1	1.74	2.97	2.29	1.33	1.11	2.43
SLIRP	522.73	475.1	417.49	387.1	542.55	365.1
SLIT1	0	0	0	0	0	0
SLIT2	0	0	0	0	0	0
SLIT2-IT1	0	0	0	0	0	0
SLIT3	0.03	0	0.01	0	0	0
SLITRK1	0	0	0	0	0	0
SLITRK2	0	0	0	0	0	0
SLITRK3	0	0	0	0	0	0
SLITRK4	0.01	0.01	0	0	0	0.01
SLITRK5	0	1.13	0.91	0.25	0.7	0.61
SLITRK6	0	0	0	0	0	0
SLK	3.51	3.46	2.53	2.42	2.86	3.12
SLMAP	4.06	5.92	3.13	2.58	3.81	3.29
SLMO1	1.04	1.94	1.62	1.37	1.11	1.18
SLMO2	37.26	26.92	23.35	17.17	26.87	23.41
SLMO2-ATP5	6.79	3.12	7.17	10.27	6.57	6.9
SLN	0	0	0	0	0.21	0
SLPI	0	2.05	0.12	0	0	0.82
SLTM	15.78	17	9.88	11.93	16.36	15.1
SLU7	14.64	13.7	12.63	13.01	15.15	13.79
SLURP1	0	0	0	0	0	0
SLX1A	35.38	49.51	42.17	39.47	43.66	50.1
SLX1A-SULT1	0.66	2.05	0.47	0.36	0.95	0.25
SLX1B	35.38	49.51	42.17	39.47	43.66	50.1
SLX1B-SULT1	0.66	2.05	0.47	0.36	0.95	0.25
SLX4	2.43	2.35	1.83	2.13	1.63	2.3
SLX4IP	2.67	5.22	3.47	3.02	4.14	4.37
SMA4	1.21	4.71	4.27	4.44	2.36	4.98
SMA5	6.01	9.62	7.89	4.38	7.25	8.58
SMAD1	3.17	4.36	3.44	2.72	3.44	3.33
SMAD2	6.88	6.14	7.11	5.43	5.38	7.72
SMAD3	1.24	0.5	0.97	0.91	1.22	1.37
SMAD4	1.36	1.27	1.36	1.74	1.01	1.4
SMAD5	5.24	5.61	4.78	5.24	5.11	5.63

SMAD5-AS1	0.08	0.19	0.08	0	0.23	0.08
SMAD6	0.77	0.32	0.26	0.48	0.48	0.33
SMAD7	1.15	0.25	0.04	0.13	0.12	0.03
SMAD9	0.88	1.36	1.81	1.19	0.94	1.74
SMAGP	5.98	13.38	23.43	19.05	15.26	15.15
SMAP1	9.76	7.51	8.33	8.86	9.06	8.22
SMAP2	7.75	5.76	4.07	4.32	4.64	5.4
SMARCA1	0.36	0.31	0.18	0.14	0.1	0.43
SMARCA2	4.19	5.13	4.97	4.36	4.81	5.79
SMARCA4	26.07	26.92	29.73	32.23	31.44	31.35
SMARCA5	13.9	12.48	11.43	12.09	12.77	11.85
SMARCAD1	7.03	5.11	5.08	4.87	5.69	6.66
SMARCAL1	9.86	6.27	7.61	5.55	8.17	7.53
SMARCB1	53.55	67.23	61.72	66.01	60.89	61.88
SMARCC1	14.87	14.3	16.14	14.23	17.11	17.08
SMARCC2	11.39	9.61	8.58	7.68	10.7	9.64
SMARCD1	6.12	7.27	5.96	7.11	5.75	5.53
SMARCD2	6.07	6.1	6.45	4.71	3.96	5.1
SMARCD3	0.18	0.14	0.68	0.31	0.33	0.32
SMARCE1	46.35	48.62	48.07	48.2	51.12	47.22
SMC1A	21.77	26.36	22.87	20.83	23.88	24.45
SMC1B	0	0	0	0.01	0.06	0
SMC2	12.39	16.77	14.38	12.13	14.99	14.72
SMC3	20.63	24.44	16.2	15.05	23.78	16.39
SMC4	25.14	27.21	21.77	22.48	23.7	24.47
SMC5	4.11	6.7	4.61	4.01	4.36	4.57
SMC6	7.64	6.16	4.93	5.33	5.75	3.14
SMCHD1	2.31	1.9	1.25	2.03	2.1	1.5
SMCP	0	0	0	0	0	0
SMCR5	0.08	0.16	0.16	0.44	0.27	0.65
SMCR7	2.83	4.64	3.33	4.03	4.4	4.56
SMCR7L	19.43	17.32	16.03	19.01	15.47	17.43
SMCR8	5.53	5.69	7.35	5.87	6.98	7.09
SMCR9	0	0	0	0	0	0
SMEK1	4.62	4.17	3.54	2.99	4.16	4.63
SMEK2	10.51	12.81	9.34	9.13	10.97	14.01
SMEK3P	0	0	0	0	0	0
SMG1	2.45	2.37	1.64	1.46	2.15	2.43
SMG1P1	4.31	5.74	3.7	1.67	3.21	4.04
SMG5	12.6	13.44	14.97	14.68	14.55	14.54
SMG6	2.97	2.83	3.31	2.47	3.49	3.41
SMG7	11.31	11.39	10.38	9	9.99	9.91
SMG7-AS1	0.1	0	0.05	0.04	0.09	0.24

SMG8	13.79	13.92	15.42	14.36	13.13	13.28
SMG9	6.18	5.46	5.03	4.77	5.86	6.66
SMIM1	10.45	0.24	0	2.11	2.17	0.46
SMIM10	3.36	5.64	3.65	4.19	5.75	4.63
SMIM11	21.25	18.2	22.85	22.22	30.42	22.55
SMIM12	13.57	13.35	13.12	11.73	14.46	12.32
SMIM13	0.46	1.2	1.09	0.84	1.15	1.09
SMIM14	2.48	0.92	2.79	1.16	1.36	1.7
SMIM15	0	0.08	0.09	0.02	0.1	0.08
SMIM16	0	0.08	0	0	0.15	0.05
SMIM17	0.39	0.69	1.08	0.74	0.4	0.35
SMIM18	0.3	0.39	0.27	0.27	0.47	0.2
SMIM2-IT1	0	0	0	0	0	0
SMIM3	17.53	40.58	38.06	25.2	28.01	35.7
SMIM4	46.35	31.14	33.47	28.87	34.29	18.61
SMIM5	0.23	0.63	0.18	0.17	0.05	0.13
SMIM6	0	0.22	0	0	0	0
SMIM7	48.28	43.68	38.13	36.76	45.3	40.96
SMIM8	4.04	3.78	3.2	4.12	4.42	3.88
SMIM9	0	0	0	0	0	0
SMKR1	0.89	1.17	0.68	0.87	1.05	0.97
SMLR1	0	0.07	0	0	0	0
SMN1	52.59	73.41	55.4	48.22	57.07	54.28
SMN2	21.4	22.1	21.16	13.73	20.9	22.08
SMNDC1	16.01	13.91	15.02	10.9	16.43	13.47
SMO	0.6	1.12	1.65	0.84	1.12	0.94
SMOC1	0	0	0	0	0	0
SMOC2	0	0	0	0	0	0
SMOX	7.52	3.54	2.39	2.11	3.22	2.37
SMPD1	1.75	0.84	0.71	1.16	0.81	0.42
SMPD2	3.4	7.86	7.41	5.54	2.91	5.33
SMPD3	0.04	0	0.05	0	0.04	0.11
SMPD4	30.85	31.22	24.78	29.78	28.63	30.48
SMPDL3A	1.81	0.63	0.15	0.62	0.63	0.2
SMPDL3B	0.51	1.62	3.54	1.44	1.76	5.77
SMPX	0	0	0	0	0	0
SMR3A	0	0	0	0	0	0
SMR3B	0	0	0	0	0	0
SMS	153.18	146.35	140.66	143.86	160.15	140.3
SMTN	6.03	5.87	4.65	6.7	5.25	4.52
SMTNL1	0	0.11	0	0.19	0.09	0.05
SMTNL2	0.65	0.18	0.56	0.37	0.11	0.5
SMU1	23.85	22.39	26.19	23.53	26.05	23.77

SMUG1	18.03	18.84	12.69	17.35	13.34	13.13
SMURF1	0.67	0.64	0.39	0.43	0.5	0.42
SMURF2	0.39	0.65	0.76	0.55	0.65	0.32
SMYD1	0	0	0	0	0	0
SMYD2	14.67	17.58	13.99	6.85	11.12	9.95
SMYD3	53.86	94.73	96.33	82.52	82.82	77.71
SMYD4	5.48	5.41	5.18	5.38	5.03	4.67
SMYD5	15.38	13.75	10.52	13.6	14.75	13.65
SNAI1	2.31	2.17	1.8	2.28	1.61	1.32
SNAI2	0	0	0	0	0	0
SNAI3	4.78	8.32	11.37	6.67	5.17	8.34
SNAI3-AS1	3.59	3.02	4.39	2.57	2.44	3.6
SNAP23	56.17	27.08	27.04	27.35	30.78	28.7
SNAP25	0	0	0	0	0	0
SNAP25-AS1	0	0	0	0.02	0	0
SNAP29	11.63	10.18	11.01	8.96	10.09	8.89
SNAP47	23.4	16.62	15.66	17.73	19.68	18.85
SNAP91	0.2	0	0	0	0	0
SNAPC1	4.73	3.34	2.96	2.5	2.83	4.01
SNAPC2	21.45	12.24	20.58	16.54	17.19	7.42
SNAPC3	3.83	2.37	2.76	3.46	2.95	2.76
SNAPC4	3.85	3.97	2.96	3.36	2.78	4.26
SNAPC5	15.4	15.68	9.86	9.99	9.96	14.97
SNAPIN	44.39	40.52	36.74	37.97	41.41	27.72
SNAR-A1	0	0	0	0	0	0
SNAR-A10	4.68	0.27	5.37	2.91	0.51	0
SNAR-A11	1.56	1.36	2.69	1.25	0.57	2.36
SNAR-A12	0	0	0	0	0	0
SNAR-A13	0	0	0	0	0	0
SNAR-A14	1.56	1.36	1.34	1.25	0.51	4.71
SNAR-A2	0	0	0	0	0	0
SNAR-A3	0	0	2.69	1.66	0.44	14.13
SNAR-A4	3.12	1.36	5.37	2.49	0.64	2.36
SNAR-A5	7.79	1.36	1.34	2.49	0.64	0
SNAR-A6	2.34	1.09	1.34	0	0.64	0
SNAR-A7	5.45	0.68	0	1.25	0.64	2.36
SNAR-A8	0	0.95	1.34	0.42	0.38	9.42
SNAR-A9	0	0.54	12.09	2.49	0.51	0
SNAR-B1	72.54	32.18	62.84	50.06	45.79	77.13
SNAR-B2	72.54	32.18	62.84	50.06	45.79	77.13
SNAR-C1	0	0	0	0	0	0
SNAR-C2	0	0	0	0	0	0
SNAR-C3	1.34	0	0	0	1.36	0

SNAR-C4	0	0	0	0	0	0
SNAR-C5	0	0	0	0	0	0
SNAR-D	0	0	0	0	0	0
SNAR-E	0	0	1.07	0	0	0
SNAR-F	0	0	0	0	0	0
SNAR-G1	0	0	0	0	0	0
SNAR-G2	0	0	0	0	0	0
SNAR-H	0	0	0	0	0	0
SNAR-I	0	0	0	0	0	0
SNCA	1.09	0	0.19	0.33	0.11	0.02
SNCAIP	0	0	0	0	0	0
SNCB	0	0.09	0	0	0	0.3
SNCG	1.99	0	0	0.33	0.99	0.48
SND1	73.12	71.75	81.25	84.79	69.53	99.17
SND1-IT1	0.14	0.27	0.42	0.14	0.4	0.4
SNED1	0.05	0.04	0.1	0.14	0.05	0
SNF8	15.76	12.48	13.74	12.3	12.84	10.54
SNHG1	65.4	59.71	30.98	36.75	55.83	36.9
SNHG10	9.07	11.17	10.94	7.74	10.79	10.38
SNHG11	8.16	9.49	7.66	7.92	8.53	10.88
SNHG12	4.29	4.58	3.63	3.27	2.34	2.92
SNHG15	34.26	60.68	50.59	51.17	59.17	52.66
SNHG16	156.93	151.29	155.1	167.45	162.99	172.68
SNHG3	33.05	45.77	27.56	24.16	31.6	35.58
SNHG4	7.05	7.39	7.92	7.59	7.5	8.04
SNHG5	64.57	70.86	43.62	50.29	59.71	65.79
SNHG6	270.28	375.63	296.04	297.62	353.19	349.57
SNHG7	8.8	11.35	6.17	8.34	9.52	9.21
SNHG8	234.5	232.37	252.47	198.18	295.4	227.09
SNHG9	26.34	28.79	19.65	17.54	27.29	18.03
SNIP1	6.37	6.11	7.93	5	4.8	4.03
SNN	2.87	3.55	4.35	3.99	3.21	2.41
SNORA1	0	0	0	0	0	0.99
SNORA10	3.85	1.56	2.66	2.11	6.64	1.92
SNORA11	0	0	0	0	0	0
SNORA11B	0	0.83	0	0	1.02	1.03
SNORA11C	0	0	0	0	0	0
SNORA11D	0	0	0	0.38	0	0
SNORA11E	0	0	0	0.38	0	0
SNORA12	4.16	0	1.53	2.42	0	4.16
SNORA13	0	0	0	0	0	0.96
SNORA14A	0	0	0	0	0	0
SNORA14B	0	2.28	0.87	1.37	0	0

SNORA15	0	0	0	0	0.95	0
SNORA16A	0.95	0.77	0	0	0	0
SNORA16B	0	0	0	0	0	0
SNORA17	0	0	0	0	0	0
SNORA18	3.89	3.15	1.79	6.4	0	1.94
SNORA19	0	0	0	0	1.03	0
SNORA20	0	0	0	0	0	0
SNORA21	0	0	0	0.7	1.9	0
SNORA22	0	0	0	0	0	0
SNORA23	0	0	0	0	0.57	1.17
SNORA24	0	2.39	3.62	1.44	0.97	2.95
SNORA25	0	0	0	0	0	1.94
SNORA26	0	0	2.01	0.8	0	1.09
SNORA27	0	0	0	0	0	0
SNORA28	0	0	0	0	0	0
SNORA29	0.89	0.72	0	0	0	0
SNORA2A	2.82	0	0	0	0	0
SNORA2B	0	0	0	0	0	0
SNORA3	0.99	7.24	7.32	2.18	2.94	1.99
SNORA30	0	0	0	0	0	0
SNORA31	0	0	0	1.45	0	0.99
SNORA32	1.1	0	0	0	1.09	0
SNORA33	1.92	0	2.66	0.7	0	1.92
SNORA34	0.92	2.23	0	0	0	0
SNORA35	0	0	0	0	0	0
SNORA36A	0	0	0	0.73	0	0
SNORA36B	0	0	0	0	0	0
SNORA36C	0	0	0	0	0	0
SNORA37	0	0	0	0	0	0
SNORA38	0	0	0	0	0	0
SNORA38B	0	0	0	0	0	0
SNORA39	0	0	0	0	0	0
SNORA4	0	0	1.7	0	0	0
SNORA40	0	1.67	1.89	6.03	3.05	0
SNORA41	0	0	1.79	0	0	0
SNORA42	0.95	0	0.88	0.69	0	0
SNORA43	0	0	0	0	0	0
SNORA44	0	0	0	0	1.92	0.97
SNORA45	1.97	2.39	1.81	0	0.97	0.98
SNORA46	0	0	0.87	0	0	0.94
SNORA47	0	0	0	0	0	0
SNORA48	1.88	3.04	0	0	1.86	0.94
SNORA49	0	0	0	0	0	0

SNORA50	0	2.26	0	0	0	0
SNORA51	0.97	0.79	2.69	0	0	0
SNORA52	0.95	0.77	0	0.69	0.94	0
SNORA53	0	0	0.73	0.57	0	0
SNORA54	0	0	0	0	0	0
SNORA55	0	0	0	0	0	0
SNORA56	1	0	0	0.74	0	0
SNORA57	0.82	1.98	0	1.19	1.61	0.82
SNORA58	1.29	1.38	0.4	1.54	0.84	2.16
SNORA59A	0	0.64	2.19	0.58	1.17	0.4
SNORA59B	0	0.64	2.19	0.58	1.17	0.4
SNORA5A	0.95	0	0.88	0	0	0
SNORA5B	0	0	0	0	0	0
SNORA5C	0.92	0	0.85	0.67	0	2.76
SNORA6	0.82	0.66	4.5	2.97	3.21	1.63
SNORA60	0	0	0	0	0	0
SNORA61	0.99	0.8	1.83	0.73	0.98	0
SNORA62	0	1.26	2.86	2.83	2.3	3.12
SNORA63	0	0	1.73	0.69	1.86	2.82
SNORA64	1.9	1.54	0.88	1.39	1.88	0.95
SNORA65	0	1.51	0	0	0	0
SNORA66	0	1.56	0.89	2.81	2.85	3.85
SNORA67	7.37	2.98	8.48	4.71	6.36	13.82
SNORA68	0	0	0.89	0	0	0
SNORA69	0	0	0	0	0	0
SNORA70	13.17	12.94	2.6	8.25	7.43	5.65
SNORA70B	0	0	0	0	0	0
SNORA70C	0	0	0	0	0	0
SNORA70D	0	0	0	0	0	0
SNORA70E	0	0	0	0	0	0
SNORA70F	0	0	0	0	0	0
SNORA70G	0	0	1.61	0.64	0	0
SNORA71A	0	5.16	5.03	0.67	0	5.47
SNORA71B	0.93	2.26	1.71	0	0	0
SNORA71C	4.56	3.68	12.58	3.99	4.49	9.11
SNORA71D	0	0	2.52	0	1.8	0.91
SNORA72	0	0	0	0	0.96	0.97
SNORA74A	0	0.87	0	0	0	0
SNORA74B	0	0	0	0.38	0.51	0
SNORA75	0	0.74	0	0	0.91	0.92
SNORA76	4.81	3.89	10.62	8.43	6.64	8.65
SNORA77	0	0.85	0.97	0	0	0
SNORA78	0	0	0	0	1.02	5.14

SNORA79	0	0	0	0	0	0
SNORA7A	0	0	0	0	0	0.73
SNORA7B	0	0	0	0.66	0	0
SNORA8	0	0	0	0.66	0	0
SNORA80	0.93	0	0	0	0.92	0
SNORA80B	0	0	0	2.04	0	0.93
SNORA81	0	0	0	0	0	0.64
SNORA84	0	2.33	4.43	2.81	0	0
SNORA9	1.92	0	2.66	0	0	0
SNORD10	23.89	9.98	61.38	44.36	13.79	32.15
SNORD100	0	0	0	0	0	0
SNORD101	0	0	0	0	0	0
SNORD102	0	0	0	1.85	0	4.94
SNORD103A	0	0	0	0	0	0
SNORD103B	0	0	0	0	0	0
SNORD104	0	1.7	0	1.55	0	2.08
SNORD105	0	0	0	0	0	0
SNORD105B	0	0	0	0	0	0
SNORD107	0	0	0	0	0	0
SNORD108	0	0	0	0	0	0
SNORD109A	0	0	0	0	0	0
SNORD109B	0	0	0	0	0	0
SNORD11	0	0	0	0	0	0
SNORD110	0	0	0	0	0	0
SNORD111	0	0	0	0	0	0
SNORD111B	0	0	0	0	0	0
SNORD113-1	0	0	0	0	0	0
SNORD113-2	0	0	0	0	0	0
SNORD113-4	0	0	0	0	0	0
SNORD113-5	0	0	0	0	0	0
SNORD113-6	0	0	0	0	0	0
SNORD113-7	0	0	0	0	0	0
SNORD113-9	0	0	0	0	0	0
SNORD114-1	0	0	0	0	0	0
SNORD114-1	0	0	0	0	0	0
SNORD114-1	0	0	0	0	0	0
SNORD114-1	0	0	0	0	0	0
SNORD114-1	0	0	0	0	0	0
SNORD114-1	0	0	0	0	0	0
SNORD114-1	0	0	0	0	0	0
SNORD114-1	0	0	0	0	0	0
SNORD114-1	0	0	0	0	0	0
SNORD114-1	0	0	0	0	0	0

SNORD114-1	0	0	0	0	0	0
SNORD114-2	0	0	0	0	0	0
SNORD114-2	0	0	0	0	0	0
SNORD114-2	0	0	0	0	0	0
SNORD114-2	0	0	0	0	0	0
SNORD114-2	0	0	0	0	0	0
SNORD114-2	0	0	0	0	0	0
SNORD114-2	0	0	0	0	0	0
SNORD114-2	0	0	0	0	0	0
SNORD114-2	0	0	0	0	0	0
SNORD114-2	0	0	0	0	0	0
SNORD114-2	0	0	0	0	0	0
SNORD114-3	0	0	0	0	0	0
SNORD114-3	0	0	0	0	0	0
SNORD114-3	0	0	0	0	0	0
SNORD114-4	0	0	0	0	0	0
SNORD114-5	0	0	0	0	0	0
SNORD114-6	0	0	0	0	0	0
SNORD114-7	0	0	0	0	0	0
SNORD114-8	0	0	0	0	0	0
SNORD114-9	0	0	0	0	0	0
SNORD115-1	0	0	0	0	0	0
SNORD115-1	0	0	0	0	0	0
SNORD115-1	0	0	0	0	0	0
SNORD115-1	0	0	0	0	0	0
SNORD115-1	0	0	0	0	0	0
SNORD115-1	0	0	0	0	0	0
SNORD115-1	0	0	0	0	0	0
SNORD115-1	0	0	0	0	0	0
SNORD115-1	0	0	0	0	0	0
SNORD115-1	0	0	0	0	0	0
SNORD115-1	0	0	0	0	0	0
SNORD115-2	0	0	0	0	0	0
SNORD115-2	0	0	0	0	0	0
SNORD115-2	0	0	0	0	0	0
SNORD115-2	0	0	0	0	0	0
SNORD115-2	0	0	0	0	0	0
SNORD115-2	0	0	0	0	0	0
SNORD115-2	0	0	0	0	0	0
SNORD115-2	0	0	0	0	0	0
SNORD115-2	0	0	0	0	0	0
SNORD115-2	0	0	0	0	0	0
SNORD115-2	0	0	0	0	0	0

SNORD115-3	0	0	0	0	0	0
SNORD115-3	0	0	0	0	0	0
SNORD115-3	0	0	0	0	0	0
SNORD115-3	0	0	0	0	0	0
SNORD115-3	0	0	0	0	0	0
SNORD115-3	0	0	0	0	0	0
SNORD115-3	0	0	0	0	0	0
SNORD115-3	0	0	0	0	0	0
SNORD115-3	0	0	0	0	0	0
SNORD115-3	0	0	0	0	0	0
SNORD115-3	0	0	0	0	0	0
SNORD115-4	0	0	0	0	0	0
SNORD115-4	0	0	0	0	0	0
SNORD115-4	0	0	0	0	0	0
SNORD115-4	0	0	0	0	0	0
SNORD115-4	0	0	0	0	0	0
SNORD115-4	0	0	0	0	0	0
SNORD115-4	0	0	0	0	0	0
SNORD115-4	0	0	0	0	0	0
SNORD115-4	0	0	0	0	0	0
SNORD115-5	0	0	0	0	0	0
SNORD115-6	0	0	0	0	0	0
SNORD115-7	0	0	0	0	0	0
SNORD115-8	0	0	0	0	0	0
SNORD115-9	0	0	0	0	0	0
SNORD116-1	0	0	0	0	0	0
SNORD116-1	0	0	0	0	0	0
SNORD116-1	0	0	0	0	0	0
SNORD116-1	0	0	0	0	0	0
SNORD116-1	0	0	0	0	0	0
SNORD116-1	0	0	0	0	0	0
SNORD116-1	0	0	0	0	0	0
SNORD116-1	0	0	0	0	0	0
SNORD116-1	0	0	0	0	0	0
SNORD116-1	0	0	0	0	0	0
SNORD116-1	0	0	0	0	0	0
SNORD116-2	0	0	0	0	0	0
SNORD116-2	0	0	0	0	0	0
SNORD116-2	0	0	0	0	0	0
SNORD116-2	0	0	0	0	0	0
SNORD116-2	0	0	0	0	0	0
SNORD116-2	0	0	0	0	0	0
SNORD116-2	0	0	0	0	0	0

SNORD116-2	0	0	0	0	0	0
SNORD116-2	0	0	0	0	0	0
SNORD116-2	0	0	0	0	0	0
SNORD116-2	0	0	0	0	0	0
SNORD116-3	0	0	0	0	0	0
SNORD116-4	0	0	0	0	0	0
SNORD116-5	0	0	0	0	0	0
SNORD116-6	0	0	0	0	0	0
SNORD116-7	0	0	0	0	0	0
SNORD116-8	0	0	0	0	0	0
SNORD116-9	0	0	0	0	0	0
SNORD117	0	0	0	0	0	0
SNORD119	0	0	0	0	0	0
SNORD11B	0	0	0	0	0	0
SNORD12	0	0	0	0	0	0
SNORD121A	0	0	0	0	0	0
SNORD121B	0	0	0	0	0	0
SNORD123	0	0	0	0	0	0
SNORD124	0	0	0	0	0	0
SNORD125	0	0	0	0	0	0
SNORD126	0	0	0	0	0	0
SNORD127	0	0	0	0	0	0
SNORD12B	0	0	0	0	0	0
SNORD12C	0	0	0	0	2.12	0
SNORD15A	0.82	0	0	0	0	0
SNORD15B	0	0.68	2.32	0	0	0.84
SNORD16	1.47	1.2	1.36	0	0	1.47
SNORD17	10.73	9.26	7.46	7.95	7.92	6.88
SNORD18A	0	0	0	1.94	0	0
SNORD18B	0	2.12	0	0	0	0
SNORD18C	0	0	0	0	0	0
SNORD19	0	0	0	0	0	0
SNORD19B	0	0	0	0	0	0
SNORD1A	0	0	2.19	0	0	0
SNORD1B	0	0	1.72	0	0	0
SNORD1C	0	0	0	0	0	0
SNORD2	0	0	0	0	0	0
SNORD20	0	0	0	0	0	0
SNORD21	0	0	1.47	0	0	0
SNORD22	1.04	0.84	0	2.29	0	0
SNORD23	0	0	0	2.81	1.26	1.27
SNORD24	0	0	0	0	0	0
SNORD25	0	0	2.58	2.09	0	0

SNORD26	0	0	0	0	2.31	0
SNORD27	2.47	0	2.29	0	0	4.94
SNORD28	0	0	0	0	0	0
SNORD29	0	0	0	0	0	0
SNORD30	0	0	0	0	0	0
SNORD31	0	0	0	2.03	2.72	2.72
SNORD32A	2	3.27	0	2.98	3.99	4
SNORD32B	0	0	0	0	0	0
SNORD33	0	0	1.82	0	0	0
SNORD34	0	0	0	0	0	2.86
SNORD35A	0	0	1.72	0	0	0
SNORD35B	1.82	0	0	0	0	0
SNORD36A	0	0	0	0	0	0
SNORD36B	2.53	0	0	0	0	0
SNORD36C	0	0	0	0	0	0
SNORD37	0	0	0	0	0	0
SNORD38A	0	0	0	0	0	0
SNORD38B	0	0	0	0	0	0
SNORD41	0	0	0	0	0	0
SNORD42A	0	0	2.87	0	0	0
SNORD42B	0	0	0	0	0	0
SNORD43	0	0	0	0	0	0
SNORD44	0	0	0	0	3.28	0
SNORD45A	0	1.57	0	0	0	0
SNORD45B	0	0	0	0	0	0
SNORD45C	0	0	0	0	0	0
SNORD46	0	0	0	0	0	0
SNORD47	0	0	4.1	0	0	11.06
SNORD48	0	0	0	0	0	0
SNORD49A	2.53	0	0	0	0	0
SNORD49B	0	0	0	0	0	0
SNORD4A	0	0	0	0	0	0
SNORD4B	0	0	0	0	0	0
SNORD5	0	0	0	0	0	0
SNORD50A	0	1.89	0	1.72	0	0
SNORD50B	0	0	0	0	0	0
SNORD51	0	0	0	0	0	0
SNORD52	0	0	0	0	0	0
SNORD53	0	0	0	0	0	0
SNORD54	0	0	2.87	0	3.1	0
SNORD55	7.08	0	0	1.76	0	0
SNORD56	0	0	0	0	0	0
SNORD56B	0	0	0	0	0	0

SNORD57	0	0	0	0	0	0
SNORD58A	0	0	0	0	0	0
SNORD58B	0	0	0	0	0	0
SNORD58C	0	0	0	0	3.44	0
SNORD59A	0	1.89	0	1.72	0	9.24
SNORD59B	0	0	0	0	0	0
SNORD6	0	0	0	0	0	0
SNORD60	0	0	0	1.46	0	0
SNORD61	0	0	0	0	0	0
SNORD62A	0	0	0	0	1.85	0
SNORD62B	0	0	0	0	1.85	0
SNORD63	0	0	2.52	0	0	0
SNORD64	0	0	0	0	0	0
SNORD65	2.41	0	0	0	0	0
SNORD66	0	0	0	0	0	0
SNORD67	0	0	0	0	0	1.26
SNORD68	0	2.02	0	3.69	2.47	0
SNORD69	0	0	0	0	0	0
SNORD7	0	0	0	0	0	0
SNORD70	1.79	0	1.66	0	7.13	0
SNORD71	0	0	0	0	0	0
SNORD72	0	0	0	0	0	0
SNORD73A	0	0	0	0	0	0
SNORD74	0	0	0	0	0	0
SNORD75	0	0	0	0	0	0
SNORD76	6.12	1.67	3.78	10.64	4.07	2.04
SNORD77	0	0	0	0	0	0
SNORD78	0	0	0	0	0	0
SNORD79	0	0	0	0	2.04	0
SNORD8	0	0	1.19	0	0	1.29
SNORD80	0	2.07	2.34	3.78	7.58	5.05
SNORD81	0	0	0	0	0	0
SNORD82	0	0	0	0	0	0
SNORD83A	0	0	0	0	1.58	0
SNORD83B	3.28	0	1.52	0	0	0
SNORD84	0	0	0	0	2.16	0
SNORD85	0	0	0	0	0	0
SNORD86	0	0	0	0	0	0
SNORD87	0	0	0	0	0	0
SNORD88A	0	0	0	0	0	0
SNORD88B	0	0	0	1.14	0	0
SNORD88C	0	0	0	0	0	0
SNORD89	2.41	0	0	0	0	0

SNORD9	0	0	0	0	0	0
SNORD90	0	0	0	0	0	0
SNORD91A	0	0	0	0	1.66	0
SNORD91B	0	0	0	0	0	0
SNORD92	0	0	0	0	0	0
SNORD93	0	0	0	0	0	0
SNORD94	0	0	0.85	0	0	0
SNORD95	0	0	0	0	0	0
SNORD96A	0	0	0	0	0	0
SNORD96B	0	0	0	0	0	0
SNORD97	0.87	0	0.8	0	0	0
SNORD98	2.78	0	0	0	0	0
SNORD99	0	0	0	0	0	0
SNPH	0	0	0	0	0	0
SNRK	2.88	3.01	2.26	3.21	3.52	2.76
SNRK-AS1	0.05	0	0	0	0	0
SNRNP200	45.34	40.84	39.58	40.18	40.99	41.59
SNRNP25	90.3	115.39	119.54	103	109.29	86.81
SNRNP27	31.42	22.91	18.92	16.06	23.94	18.66
SNRNP35	15.83	17.09	19.69	22.21	26.66	21.56
SNRNP40	112.57	119.82	100.8	102.85	115.7	105.64
SNRNP48	2.13	3	2.65	2.79	2.75	4.27
SNRNP70	16.86	12.7	18.53	14.94	19.48	17.21
SNRPA	79.24	82.97	85.73	91.55	88.37	91.29
SNRPA1	118.32	146.3	123.2	104.4	125.68	109.73
SNRPB	521.6	543.58	446.99	530.87	598.26	515.68
SNRPB2	48.23	47.72	39.54	40.36	47.8	36.91
SNRPC	176.47	202.31	195.77	187.51	214.03	176.39
SNRPD1	157.41	199.29	160.97	166.69	203.39	187.75
SNRPD2	780.15	785.35	667	847.89	930.45	774.38
SNRPD2P2	0	0	0.28	0	0	0
SNRPD3	118.15	136.52	123.55	123.21	130.78	122.24
SNRPE	135.61	145.21	117.46	125.55	153.83	132.54
SNRPF	212.11	202.92	187.56	172.42	218.26	188.12
SNRPG	488.99	513.9	428.4	418.81	575.29	418.26
SNRPN	0	0	0	0	0	0
SNTA1	0.91	0.59	0.92	1.27	1.42	0.7
SNTB1	0.25	0.65	0.69	0.57	0.88	1.15
SNTB2	0.77	0.59	0.73	0.43	0.67	0.41
SNTG1	0	0	0	0	0	0
SNTG2	0	0	0	0	0	0
SNTN	0.12	0.62	0.27	0.24	0.38	0.27
SNUPN	64.51	65.32	59.53	57.46	61.6	51.96

SNURF	0	0	0	0	0	0
SNW1	52.54	43.78	35.21	37.06	45.22	40.64
SNX1	20.92	21.8	18.45	19.15	18.64	18.22
SNX10	4.58	6.86	4.89	2.46	4.6	5.2
SNX11	14.05	10	8.76	8.95	8.53	7.59
SNX12	10.99	10.08	8.7	9.07	10.08	9.16
SNX13	1.95	2.26	1.83	2.04	2.28	1.92
SNX14	9.33	8.07	10.15	7.33	6.78	8.26
SNX15	3.34	4.45	4.2	3.63	3.04	3.67
SNX16	1.41	1.27	1.05	0.82	1.21	0.4
SNX17	65.78	77.88	85.86	74.24	73.77	68.18
SNX18	0.52	0.87	0.58	0.69	0.7	0.66
SNX19	0	0	0.01	0	0	0
SNX2	50.56	63.71	57.29	45.06	61.45	56.4
SNX20	1.65	8.36	8.9	3.55	3.77	8.53
SNX21	0.5	1.08	0.13	0.11	0.18	0.78
SNX22	5.48	5.95	6.94	6.05	4.83	5.74
SNX24	2.21	0.6	0.6	0.28	0.55	0.35
SNX25	0.5	0.34	0.08	0.51	0.58	0.09
SNX27	10.18	11.34	9.73	8.22	9.45	9.34
SNX29	1.14	0.93	1.13	1.25	0.4	1.05
SNX29P1	0.89	0.43	0.71	0	0.13	0.35
SNX29P2	0	0.32	0.07	0	0.12	0.34
SNX3	36.32	34.81	31.28	29.4	31.62	30.2
SNX30	1.04	0.95	0.78	0.71	0.67	1
SNX31	0	0	0	0	0	0
SNX32	0.52	0.03	0.04	0	0.04	0
SNX33	2.9	3.38	2.51	2.8	3.78	3.1
SNX4	20.71	18.86	14.87	14.74	16.34	17.49
SNX5	58.13	35.93	35.35	40.76	50.61	40
SNX6	29.17	23.14	22.29	21.64	25.03	21.73
SNX7	0	0	0	0	0	0
SNX8	7.41	5.17	5.16	5.35	6.09	4.95
SNX9	4	2.39	1.58	2.01	3.05	1.41
SOAT1	7.51	9.47	7.32	7.07	7.22	7.76
SOAT2	0	0	0	0	0	0
SOBP	0.45	0.31	0.56	0.71	0.66	0.78
SOCS1	9.42	0.98	1.71	3.1	3.72	0.57
SOCS2	37.04	29.13	25.75	30.08	28.69	15.74
SOCS2-AS1	4.29	2.37	5.1	3.48	2.45	2.02
SOCS3	0.86	0.29	0.4	0.94	1.66	0.84
SOCS4	3.97	3.2	1.98	1.5	2.15	2.36
SOCS5	6.38	2.27	2.55	2.29	4.04	3.37

SOCS6	1.96	2.38	2.04	1.78	1.79	2.07
SOCS7	0.98	1.29	1	1.11	1.21	1.08
SOD1	485.17	430.41	399.98	479.54	487.07	443.16
SOD2	66.61	30.45	25.11	40.44	42.83	31.42
SOD3	0	0	0	0	0	0
SOGA1	3.83	4.7	3.96	3.21	3.52	4.9
SOGA2	0.55	0.2	0.26	0.11	0.2	0.13
SOGA3	0	0	0	0	0	0
SOHLH1	0	0	0	0	0	0
SOHLH2	0	0	0	0	0	0.14
SOLH	1.02	1.32	1.25	1.4	1.2	1.29
SON	28.22	26.34	23.49	22.58	27.39	24.19
SORBS1	3.43	0.9	0.78	0.9	1.31	1.04
SORBS2	0	0	0	0	0	0
SORBS3	15.06	26.28	33.5	32.55	26.17	24.39
SORCS1	0.05	0	0	0	0	0
SORCS2	0	0	0	0	0	0
SORCS3	0	0	0	0	0	0
SORD	32.58	37.47	43.15	37.15	31.75	45.14
SORL1	0.93	4.23	5.72	2.42	3.27	4.84
SORT1	7.05	3.23	2.23	2.61	3.33	2.54
SOS1	12.77	3.52	3.5	5.9	9.78	2.9
SOS2	0.94	1.09	1.13	0.75	1.06	1.06
SOST	0	0	0	0	0	0
SOSTDC1	0	0	0	0	0	0
SOWAHA	0	0	0	0.01	0.02	0
SOWAHB	0	0	0	0	0	0
SOWAHC	0.22	0.07	0.24	0.15	0.14	0.1
SOWAHD	0.19	0.56	0.39	0.26	0.36	1
SOX1	0	0	0	0	0	0
SOX10	0	0.06	0	0.04	0	0
SOX11	0	0.02	0	0	0	0
SOX12	0.68	0.4	0.54	0.6	0.64	0.69
SOX13	0.09	0	0	0	0	0
SOX14	0	0	0	0	0	0
SOX15	0.11	0.09	0	0.04	0	0.05
SOX17	0	0	0	0	0	0
SOX18	0.35	0.07	0.36	0.09	0.16	0.26
SOX2	0	0.05	0.05	0	0	0
SOX2-OT	0.02	0.01	0	0	0.02	0
SOX21	0.03	0	0	0	0	0
SOX3	0	0	0	0	0	0
SOX30	0.07	0	0	0	0	0

SOX4	5.03	6.33	6.6	6.06	8.87	9.69
SOX5	0	0	0	0	0	0
SOX6	0	0	0	0.01	0	0
SOX7	11.48	1.47	0.87	3.08	1.71	1.41
SOX8	0.12	1.15	0.85	1.05	0.45	0.56
SOX9	0	0	0	0	0	0
SP1	7.3	6.66	5.7	5.48	6.36	7.32
SP100	19.95	14.61	11.38	12.41	13.1	10.23
SP110	9.62	9.6	10.77	8.9	9.12	9.27
SP140	0	0	0	0	0	0.05
SP140L	6.72	5.32	6.11	5.64	6.12	5.68
SP2	4.66	5.42	4.11	4.33	5.3	4.05
SP3	3.92	3.57	3.15	4.08	3.75	4.35
SP4	1.97	2.08	1.9	1.82	1.51	2.32
SP5	0	0	0	0	0	0
SP6	0	0	0	0	0	0
SP7	0	0	0.11	0.25	0.05	0.12
SP8	0	0	0	0	0	0
SP9	0	0	0	0	0	0
SPA17	0.08	0.98	0	1.89	1.1	0.5
SPACA1	0	0	0	0	0	0
SPACA3	0.29	0	0	0	0	0
SPACA4	0	0	0	0	0	0
SPACA5	0	0	0	0	0	0.11
SPACA5B	0	0	0	0	0	0
SPACA7	0	0	0	0	0	0
SPAG1	0.1	0.32	0.18	0.05	0	0.23
SPAG11A	0	0	0	0	0	0
SPAG11B	0	0	0	0	0	0
SPAG16	0	0	0	0	0	0
SPAG17	0	0	0	0.01	0	0.01
SPAG4	0.83	1.14	0.28	0.57	1.12	0.63
SPAG5	16.07	16.42	19.99	18.45	16.17	16.69
SPAG5-AS1	0	0	0	0	0	0.11
SPAG6	0.1	0.08	0.03	0.02	0.04	0.06
SPAG7	48.65	57.01	46.71	51.94	57.37	47.78
SPAG8	0.26	0.14	0.71	0.18	0.12	0.04
SPAG9	2.77	1.77	1.73	1.95	2.25	1.93
SPAM1	0	0.06	0	0	0	0
SPANXA1	0	0	0	0	0	0
SPANXA2	0	0	0	0	0	0
SPANXA2-OT	0	0	0.11	0	0	0.04
SPANXB1	0	0	0	0	0	0

SPANXB2	0	0	0	0	0	0
SPANXC	0	0	0	0	0	0
SPANXD	0	0	0	0	0	0
SPANXE	0	0	0	0	0	0
SPANXF1	0	0	0	0	0	0
SPANXN1	0	0	0	0	0	0
SPANXN2	0	0	0	0	0	0
SPANXN3	0	0	0	0	0	0
SPANXN4	0	0	0	0	0	0
SPANXN5	0	0	0	0	0	0
SPARC	3.94	14.74	21.34	16.62	12.22	19.15
SPARCL1	0	0.04	0.05	0.02	0.02	0
SPAST	6.92	5.93	4.97	4.37	6.23	4.81
SPATA12	0.03	0.17	0.28	0	0.09	0.15
SPATA13	2.52	3.29	2.5	2.83	2.77	3.28
SPATA13-AS	0	0	0	0	0	0
SPATA16	0.07	0	0	0	0	0
SPATA17	0.25	0.15	0	0	0	0.25
SPATA18	0	0	0	0	0	0
SPATA19	0	0	0	0	0	0
SPATA2	2.55	2.18	2.17	2.27	1.94	1.19
SPATA20	9.37	12.68	10.88	12.74	13.23	12.55
SPATA21	0.04	0	0	0	0	0
SPATA22	0	0	0	0	0	0
SPATA24	1.66	1.03	1.95	0.74	1.8	1.67
SPATA25	0	0.35	0.1	0	0	0
SPATA2L	10.83	5.84	6.24	7.56	7.26	4.64
SPATA3	0	0	0	0	0	0
SPATA31A1	0	0	0	0	0	0
SPATA31A2	0	0	0	0	0	0
SPATA31A3	0	0	0	0.04	0	0
SPATA31A4	0	0	0	0	0	0
SPATA31A5	0	0	0	0	0	0
SPATA31A6	0	0	0	0	0.03	0
SPATA31A7	0	0	0	0	0	0
SPATA31C1	0	0.08	0	0.03	0.03	0
SPATA31C2	0.29	1.39	0.95	1.06	0.77	0.47
SPATA31D1	0	0	0	0	0	0
SPATA31D3	0	0	0	0	0	0
SPATA31D4	0	0	0	0	0	0
SPATA31D5P	0	0	0	0	0	0
SPATA31E1	0	0	0	0	0	0
SPATA32	0.06	0.05	0.05	0	0	0

SPATA4	0	0	0	0	0	0
SPATA5	3.44	3.95	3.12	2.27	2.72	3.35
SPATA5L1	0.57	1.86	1.51	0.96	1.71	1.18
SPATA6	0	0	0	0	0	0
SPATA6L	0	0	0	0	0.02	0
SPATA7	3.75	2.06	2.86	1.72	1.71	2.27
SPATA8	0	0	0	0	0	0
SPATA9	0.25	0.19	0.23	0.13	0.47	0
SPATC1	0	0	0	0.03	0	0
SPATC1L	0	0	0	0	0	0
SPATS1	0	0	0	0	0	0
SPATS2	6.51	5.24	5.04	5.65	7.38	4.62
SPATS2L	4.45	3.87	4.24	2.83	3.35	2.98
SPC24	20.94	24.33	33.33	30.94	28.11	32.66
SPC25	34.5	29.62	29.19	25.77	33.76	26.59
SPCS1	141.4	143.82	131.4	126.73	149.08	131.54
SPCS2	65.56	63.42	54.42	58.67	64.33	53.45
SPCS3	22.65	19.31	21.64	15.05	17.79	27.18
SPDEF	0	0	0	0	0	0
SPDL1	9.93	10.84	9.84	7.07	10.17	9.82
SPDYA	0	0.07	0.06	0	0.04	0.09
SPDYC	0	0	0	0	0	0
SPDYE1	0.72	0.87	1.12	0.57	0.62	0.73
SPDYE2	0.77	1.11	1.11	0.4	0.66	1.15
SPDYE2L	0.77	1.11	1.11	0.4	0.66	1.15
SPDYE3	0.16	0.28	0.32	0.13	0.37	0.15
SPDYE4	0	0	0	0	0	0
SPDYE5	0.61	1.64	1.73	1.02	1.09	1.42
SPDYE6	0	0.13	0	0	0.16	0.4
SPDYE7P	3.74	4.1	4.19	3.37	2.88	3.98
SPDYE8P	1.78	1.89	1.22	1.53	0.77	1.37
SPECC1	9.86	5.5	5.38	7.43	7.42	5.51
SPECC1L	4.78	4.64	6.52	4.94	4.81	6.38
SPEF1	0.19	0	0	0	0.04	0
SPEF2	0.29	0.25	0.2	0.67	0.3	0.22
SPEG	0.05	0	0	0.11	0.45	0
SPEM1	0	0	0	0	0	0
SPEN	5.14	4.78	3.92	3.61	4.16	4.89
SPERT	0	0	0	0	0	0
SPESP1	0	0	0	0	0	0
SPG11	4.3	3.3	4.33	3.1	3.62	3.68
SPG20	15.37	26.84	22.42	17.94	18.48	29.65
SPG20OS	0	1.6	0.89	0.63	0.55	0.82

SPG21	67.9	68.48	62.26	60.57	68.59	65.19
SPG7	6.28	6.41	5.93	7.2	6.58	4.69
SPHAR	0	0	0	0	0	0
SPHK1	4.4	5.2	4.75	5.95	7.01	6.84
SPHK2	3.55	6.51	5.55	5.78	6.76	7.39
SPHKAP	0	0	0	0	0	0
SPI1	4.86	16.9	18.4	11.09	9.66	19.5
SPIB	1.06	1.64	1.28	0.97	1.42	1.55
SPIC	0	0	0	0	0	0
SPICE1	2.71	2.05	1.35	2.03	1.68	1.81
SPIN1	12.15	9.63	8.81	10	12.26	10.36
SPIN2A	0.35	0	0	0.22	0	0
SPIN2B	17.71	17.32	11.3	16.61	17.11	17.01
SPIN3	3.54	3.94	0.19	2.12	2.81	2.8
SPIN4	21.21	20.18	14.52	14.45	19.19	13.58
SPINK1	0	0	0	0	0	0
SPINK13	0	0	0	0	0	0
SPINK14	0	0	0	0	0	0
SPINK2	0.34	1.4	4.69	1.65	4.88	3.36
SPINK4	1.46	3.67	10.22	6.66	6.07	9.28
SPINK5	0	0	0	0	0	0
SPINK6	0	0	0	0	0	0
SPINK7	0	0	0	0	0	0
SPINK8	0	0	0	0	0	0
SPINK9	0	0	0	0	0	0
SPINT1	16.62	11.67	15.2	18.15	20.44	8.85
SPINT2	116.85	91.09	74.68	95.8	102.24	97.48
SPINT3	0	0	0	0	0	0
SPINT4	0	0	0	0	0	0
SPIRE1	1.34	1.9	1.94	0.92	1.48	1.76
SPIRE2	0.38	0.39	0.16	0.39	0.19	0.26
SPN	37.44	40.81	39.14	28.6	40.97	42.59
SPNS1	55.02	35.35	41.09	53.32	65.77	32.46
SPNS2	0.02	0.61	1.47	0.07	0.2	0.35
SPNS3	0	0.18	0.39	0.62	0.15	0
SPO11	0	0	0	0	0	0
SPOCD1	0	0	0	0	0	0
SPOCK1	0	0	0	0	0	0
SPOCK2	0	0.17	0.22	0.16	0.06	0.38
SPOCK3	0	0	0	0	0	0
SPON1	0.03	0	0	0	0	0.03
SPON2	0	0.49	0	0.4	0.04	0
SPOP	17.33	22.45	18.94	24.05	19.59	18.23

SPOPL	0.89	0.2	0.34	0.63	0.49	0.54
SPP1	0.1	0	0	0	0	0
SPP2	0	0	0	0	0	0
SPPL2A	11.36	9.12	8.81	10.89	11.25	6.79
SPPL2B	8.37	9.75	7.01	7.29	6.62	8.54
SPPL2C	0	0	0	0	0	0
SPPL3	2.43	1.75	3.15	2.01	2.5	2.76
SPR	56.61	66.24	64.93	50.43	59.9	57.07
SPRED1	1.61	1.05	1.14	0.7	1.01	1.35
SPRED2	6.14	4.26	4.15	3.09	3.24	2.13
SPRED3	0.32	0.24	0.16	0.12	0.06	0.15
SPRN	0.76	0.85	0.55	0.66	1.28	0.79
SPRNP1	0	0	0	0	0	0
SPRR1A	0	0	0	0	0	0
SPRR1B	0	0	0	0	0	0
SPRR2A	0	0	0	0	0	0
SPRR2B	0	0	0	0	0	0
SPRR2C	0	0	0	0	0	0
SPRR2D	0	0	0	0	0	0
SPRR2E	0	0	0	0	0	0
SPRR2F	0	0	0	0	0	0
SPRR2G	0	0	0	0	0	0
SPRR3	0	0	0	0	0	0
SPRR4	0	0	0	0	0	0
SPRTN	4.53	4.95	4.16	3.08	4.37	4.86
SPRY1	1.15	0.18	0.46	0.32	0.93	0.09
SPRY2	5.84	3.14	1.76	1.27	1.75	3.22
SPRY3	0	0.18	0.12	0.23	0.08	0.14
SPRY4	1.28	0.06	0.03	0.18	0.4	0.06
SPRYD3	17.56	13.22	15.51	12.95	13.66	14.68
SPRYD4	14.12	14.21	12.61	12.97	12.33	11.83
SPRYD7	6.28	4.12	4.48	3.14	4.42	3.85
SPSB1	0.4	0	0.26	0.47	0.2	0.17
SPSB2	6.59	5.18	5.34	6.02	4.71	5.66
SPSB3	10.27	10.5	8.2	9.38	7.92	7.54
SPSB4	0	0.02	0.11	0	0.06	0.12
SPTA1	0.67	0	0	0.13	0	0
SPTAN1	1.18	5.48	5.6	3.93	2.47	7.08
SPTB	7.42	0.16	0.08	3.15	2.84	0.02
SPTBN1	2.1	6.28	6.28	4.69	3.43	9.13
SPTBN2	1.39	0.97	1.46	1.02	0.76	1.74
SPTBN4	0.02	0	0.08	0.01	0	0
SPTBN5	0	0	0	0	0	0

SPTLC1	24.7	21.24	24.01	18.11	22.38	18.37
SPTLC2	19.76	20.81	20.86	20.95	23.84	24.48
SPTLC3	0	0	0	0	0	0
SPTSSA	4.75	8.93	7.62	3.43	4.65	10.1
SPTSSB	0	0	0	0	0	0
SPTY2D1	6.18	4.29	4.09	3.89	4.08	3.36
SPTY2D1-AS:	0.11	0.5	0.14	0	0.41	0
SPZ1	0	0	0	0	0	0
SQLE	219.59	37.2	37.98	93.57	142.9	40.14
SQRDL	8.78	43.52	34.86	19.32	13.16	31.27
SQSTM1	128.8	41.64	44.04	57.45	65.82	41.73
SRA1	49.02	23.09	16.78	23.97	29.22	19.04
SRBD1	8.15	6.35	5.09	6.81	7.48	6.56
SRC	2.35	2.11	1.72	2.46	1.79	1.71
SRCAP	13.43	14.29	13.99	11.88	12.23	15.29
SRCIN1	0	0	0.02	0	0	0
SRCRB4D	0.16	0.08	0.1	0.23	0.05	0.13
SRD5A1	9.65	7.42	6.9	6.97	9.78	7.66
SRD5A1P1	0	0	0.03	0	0	0
SRD5A2	0	0	0	0	0	0
SRD5A3	9.41	15.58	14.72	12.82	12.35	13.02
SRD5A3-AS1	0	0	0	0	0	0.08
SREBF1	5.49	4.92	6.6	6.24	8.37	6.01
SREBF2	21.4	18.47	17.61	22.9	25.66	21.18
SREK1	7.38	7.93	4.6	4.58	6.39	5.1
SREK1IP1	3.3	3.47	2.9	2.56	3	2.64
SRF	2.06	2.58	1.92	2.07	2.21	2.02
SRFBP1	8.81	7.9	7.97	7.68	7.39	7.84
SRG7	0	0	0	0	0	0
SRGAP1	0.79	0.48	0.28	0.35	0.61	0.55
SRGAP2	4.81	6.57	5.95	3.52	4.22	5.79
SRGAP2B	1.12	0	0.25	2.44	0	1.87
SRGAP2C	4.53	2.3	2.85	1.93	3.67	1.68
SRGAP2D	2.98	2.69	2.92	0.98	1.83	1.24
SRGAP3	0.11	0.26	0.48	0.18	0.11	0.35
SRGN	60.62	97.83	43.92	26.9	30.02	74.19
SRI	95.76	92.45	85.13	84.71	102.26	79.66
SRL	0.15	0.16	0.11	0.21	0.11	0.12
SRM	50.68	51.83	64.81	72.58	66.71	55.61
SRMS	0	0	0	0	0	0
SRP14	434.04	293.96	248.39	274.41	327.75	252.47
SRP19	35.74	31.93	22.83	22.3	29.99	20.98
SRP54	46.6	37.11	30.11	29.36	41.39	34.76

SRP68	15.4	19.94	18.69	21.07	18.2	10.99
SRP72	46.66	55.49	48.97	46.53	51.31	49.63
SRP9	179.7	179.17	142.57	124.96	174.97	167.36
SRPK1	30.92	32.77	26.94	27.57	29.83	32.26
SRPK2	7.47	8.35	8.86	6.43	7.4	7.42
SRPK3	0	0	0	0	0	0
SRPR	47.82	44.26	35.92	40.77	38.57	39.26
SRPRB	56.34	78.37	70.93	63.96	64.45	64.71
SRPX	1.49	0.21	0	0.92	0.04	0
SRPX2	0.17	0	0.03	0.02	0	0
SRR	6.23	6.08	6.35	5.7	5.58	9.15
SRRD	5.61	3.36	3.53	4.07	4.15	3.72
SRRM1	38.03	45.76	32.05	31.26	41.69	34.98
SRRM2	36.99	37.69	33.28	32.96	35.8	43.28
SRRM2-AS1	2.05	1.19	1.16	0.51	0.65	0.88
SRRM3	0.36	0.54	0.7	0.57	0.59	1.1
SRRM4	0.04	0.03	0.06	0.02	0.01	0.01
SRRM5	0.13	0.08	0.26	0	0.29	0.56
SRRT	47.44	47.47	45.01	47.66	49.81	57.87
SRSF1	52.76	67.42	64.61	59.52	65.7	67.54
SRSF10	45.94	61.45	46.17	50.61	51.77	51.76
SRSF11	37.54	35.47	30.22	30.73	37.71	30.69
SRSF12	0.17	0.59	1.19	0.62	0.81	1.06
SRSF2	188.45	248.13	228.18	224.91	247.44	201.55
SRSF3	184.48	212.19	184.54	184.87	203.01	200.51
SRSF4	36.43	36.51	25.95	31.67	34.43	32.99
SRSF5	78.99	77.63	51.47	57.36	74.66	75.71
SRSF6	68.52	66.91	68.61	58.52	71.09	79.59
SRSF7	86.63	110.96	101.82	100.49	103.69	108.91
SRSF8	2.83	2.8	2.38	2.21	3.05	2.33
SRSF9	32.4	25.73	28.62	27.14	32.37	21.59
SRXN1	4.81	1.54	1.12	1.72	3.46	3.33
SRY	0	0	0	0	0	0
SS18	18.81	24.77	19.49	19.16	18.66	19.67
SS18L1	0.79	1.04	1.09	1.23	0.81	0.74
SS18L2	40.73	66	54.96	40.79	52.9	57.15
SSB	109.08	103.43	84.37	76.77	104.65	87.07
SSBP1	320.63	347.93	351.35	353.06	370.02	349.01
SSBP2	8.28	8.99	8.01	9.31	11.49	10.08
SSBP3	1.9	3.48	2.92	2.9	2.18	2.03
SSBP4	3.83	3.31	3.84	3.65	4.21	2.8
SSC5D	0.15	0.01	0.04	0.23	0.08	0
SSFA2	3.01	4.94	3.51	3.33	3.59	3.35

SSH1	4.1	1.51	1.49	1.8	1.7	1.99
SSH2	2.93	4.01	2.7	2.45	3.06	4.15
SSH3	3.65	6.52	4.32	5.49	4.33	7.06
SSNA1	63.25	80.69	75.77	67.49	67.82	64.67
SSPN	0	0	0	0.06	0	0
SSPO	0.03	0.01	0.03	0	0	0.01
SSR1	17.66	22.95	24.1	18.43	21.2	22.38
SSR2	198.78	250.17	228.6	226.93	231.62	213.53
SSR3	63.15	73.14	62.58	61.1	61.24	64.87
SSR4	645.02	748.89	811.72	856.03	789.06	781.5
SSR4P1	0	0	0.09	0.02	0.06	0.03
SSRP1	27.95	31.39	33.49	37.87	33.75	33.24
SSSCA1	70.57	79.04	60.25	81.28	76.82	61.34
SST	0	0	0	0	0	0
SSTR1	0	0	0	0	0	0
SSTR2	0.65	0.65	0.23	0.51	0.91	0.4
SSTR3	0	0	0	0	0	0
SSTR4	0	0	0	0	0	0
SSTR5	0	0	0	0	0	0
SSTR5-AS1	0.21	0.08	0.11	0	0.23	0.35
SSU72	58	58.77	64.06	61.64	59.32	64.38
SSUH2	0	0	0	0	0	0
SSX1	0	0	0	0	0	0
SSX2	0	0	0	0	0	0
SSX2B	0	0	0	0	0	0
SSX2IP	3.52	1.42	1.73	1.43	2.54	1.34
SSX3	0	0	0	0	0	0
SSX4	0	0	0	0	0	0
SSX4B	0	0	0	0	0	0
SSX5	0	0	0	0	0	0
SSX6	0	0	0	0	0	0
SSX7	0	0	0	0.12	0	0
SSX8	0	0	0	0	0	0
SSX9	0	0	0	0	0	0
ST13	113.17	105.33	91.35	102.22	116.07	102.59
ST13P4	0	0	0.12	0	0.16	0.07
ST14	0	0	0	0	0.12	0
ST18	0	0	0	0	0	0
ST20	22.25	19.34	18.32	17.31	14.33	18.16
ST20-MTHFS	0.47	0	0	0	0.29	0.15
ST3GAL1	13.8	6.36	8.75	8.82	10.11	6.74
ST3GAL2	3.76	2.6	3.49	2.72	2.72	2.49
ST3GAL3	5.49	4.61	6.19	5.83	5.43	5.17

ST3GAL4	11.61	27.64	16.91	23.41	20.64	24.44
ST3GAL5	1.88	1.31	0.65	1.21	2.11	1.4
ST3GAL6	1.46	3.05	2.51	2.01	1.5	4.27
ST3GAL6-AS	0.51	0.65	1.13	0.21	0	0.31
ST5	0.05	0.13	0.18	0.05	0.16	0.02
ST6GAL1	13.06	11.73	15.82	17.55	11.08	16.1
ST6GAL2	0.02	0.04	0.02	0.05	0.08	0.08
ST6GALNAC1	2.27	0.07	0	0.1	0.33	0
ST6GALNAC2	0.28	0.39	0.1	0.51	0	0.46
ST6GALNAC3	0	0	0	0	0	0
ST6GALNAC4	16.46	20.42	25.54	23.69	19.73	21.64
ST6GALNAC5	0	0	0	0	0	0
ST6GALNAC6	3.8	7.05	6.3	5.79	4.19	5.91
ST7	15.09	6.34	6.01	6.49	8.53	4.05
ST7-AS1	0	0	0	0	0	0
ST7-AS2	0	0	0	0	0	0
ST7-OT3	0.16	0.12	0.14	0.23	0.14	0
ST7-OT4	0	0.22	0.09	0	0.18	0.1
ST7L	3.46	3.17	3.92	2.91	3.42	3.01
ST8SIA1	0.11	0.16	0.19	0.08	0.06	0.2
ST8SIA2	0	0	0	0	0	0
ST8SIA3	0	0	0	0	0	0
ST8SIA4	1.23	3.76	2.27	2.21	1.9	3.18
ST8SIA5	0.08	0	0	0.12	0	0.28
ST8SIA6	3.07	0.51	2.51	1.89	2.95	1.25
ST8SIA6-AS1	0	0	0	0	0	0
STAB1	0	0	0	0	0	0
STAB2	0	0	0.02	0	0	0
STAC	0.64	0	0	0	0	0
STAC2	0.26	0.2	0.3	0.21	0.3	0.45
STAC3	0.64	1.28	2.7	1.09	1.33	2.34
STAG1	3.41	4.82	4.55	3.3	3.49	4.89
STAG2	25.22	19.62	17.25	15.31	21.8	21.56
STAG3	0.11	0.1	0.23	0.17	0.12	0.12
STAG3L1	4.41	3.05	2.58	2.76	4	3.4
STAG3L2	7.13	15.08	12.13	9.34	9.54	11.65
STAG3L3	5.28	7.54	5.03	3.92	5.02	3.58
STAG3L4	3.27	4.55	4.69	4	5.01	4.6
STAM	24.48	6.92	8.12	11.63	18.56	8.73
STAM2	3.43	3.44	2.34	2.17	2.72	3.17
STAMBP	5.43	4.87	4.25	3.95	4.43	3.96
STAMBPL1	2.54	6.1	4.8	2.16	3.36	4.42
STAP1	1.49	0.51	0.05	0.07	0.7	0.35

STAP2	0.1	0.26	0.44	0.66	0.32	0.87
STAR	7.09	34.76	14.52	11.1	17.6	19.23
STARD10	3.68	3.37	5.27	5.45	4.88	4.1
STARD13	1.48	1.36	1.55	1.71	1.49	0.84
STARD13-AS	0.25	0.69	0.11	0	0	0
STARD3	13.98	12.41	13.49	12.98	13.62	18.59
STARD3NL	23.1	28.54	28.4	18.67	21.58	19.13
STARD4	4.58	3.98	2.77	2.33	3.98	2.23
STARD4-AS1	0.18	0.37	0.25	0.13	0.14	0.4
STARD5	2.41	2.8	2.31	1.15	1.19	1.33
STARD6	0.12	0	0	0	0	0
STARD7	50.72	48.74	53.48	52.44	55.11	39.49
STARD8	17.85	2.64	5.32	7.74	10.64	4.33
STARD9	0.4	0.8	0.94	0.63	0.49	0.4
STAT1	13.76	13.31	8.26	10.48	9.86	11.49
STAT2	3.77	3.4	3.94	3.5	3.32	1.79
STAT3	28.34	18.73	16.72	18.01	21.3	20.49
STAT4	0.78	7.39	2.55	1.31	1.8	2.72
STAT5A	17.76	11.44	10.85	15.44	15.56	11.46
STAT5B	4	4.52	3.47	5.15	4.84	3.55
STAT6	15.25	22.9	20.6	18.15	19.84	22.92
STATH	0.14	0	0	0	0	0
STAU1	31.58	22.18	22.25	25.45	27.3	21.81
STAU2	13.16	14.56	12.38	11.41	12.77	12.9
STAU2-AS1	0.53	0.32	0.69	0.19	0.41	0.37
STBD1	6.73	4.04	4.81	3.3	4.12	5.03
STC1	0	0	0	0	0	0.02
STC2	2.81	3.03	2.86	2.25	2.41	3.82
STEAP1	5.46	11.18	9.36	8.76	7.01	9.12
STEAP1B	0	0	0	0	0	0
STEAP2	0.33	0.43	0.31	0.21	0.17	0.18
STEAP3	13.26	9.79	13.72	10.44	11.93	12.93
STEAP4	0.19	0.12	0.13	0.02	0.14	0.09
STH	0	0	0	0	0	0
STIL	7.88	6.61	7.65	7.3	6.9	6.64
STIM1	13.84	10.46	8.73	12.53	9.57	10.18
STIM2	0.63	0.45	0.61	0.54	0.51	0.51
STIP1	193.86	189.94	181.03	186.75	185.29	160.12
STK10	9.16	15.1	17.28	14.58	9.89	16.88
STK11	0.73	0.75	0.69	0.29	0.46	0.43
STK11IP	5.1	6.35	7.03	5.01	8.25	7.61
STK16	2.13	3.12	2.91	3.09	3.29	4.72
STK17A	3.96	1.32	1.01	1.11	1.44	0.67

STK17B	3.46	7.59	4.38	2.59	2.53	5.5
STK19	14.81	8.47	7.16	8.26	10.23	7.65
STK24	3.92	2.31	2.64	1.91	2.78	2.28
STK25	20.18	17.91	22.32	16.12	17.7	19.62
STK3	15.74	10.41	7.79	10.19	13.39	12.31
STK31	0	0	0	0	0	0
STK32A	0	0	0	0	0	0
STK32B	3.63	0.64	1.84	2.64	0.75	2.1
STK32C	2.23	3.4	3.76	2.27	1.58	3.55
STK33	0	0	0	0	0	0
STK35	4.01	3.58	3.21	3.47	3.19	3.8
STK36	2.47	1.61	0.89	1.25	2.26	1.29
STK38	11.73	14.42	12.38	11.55	13.09	11.94
STK38L	2.39	1.94	2.53	2.34	2.35	2.87
STK39	0.13	0.79	0.18	0.65	0.23	0.29
STK4	7.81	10.33	10.73	8.77	9.04	12
STK4-AS1	0	0.11	0.19	0.14	0	0.04
STK40	4.71	3.6	4.8	3.51	5.24	3.57
STL	0.1	0.06	0.07	0.1	0.07	0.13
STMN1	228.08	237.06	220.27	277.57	285.86	262.5
STMN2	0	0.13	0	0	0	0
STMN3	2.16	4.84	8.91	8.45	5.72	8.5
STMN4	0	0	0	0	0	0
STMND1	0	0	0	0	0	0
STOM	47.4	47.83	51.03	37.54	45.42	37.05
STOML1	6.8	6.02	5.37	6.37	6.18	3.04
STOML2	247.55	279.36	268.72	250.81	254.97	254.91
STOML3	0	0	0	0	0	0
STON1	1.01	0.66	0.2	0.44	0.78	0.19
STON1-GTF2	0.17	0	0.05	0	0	0.26
STON2	24.85	10.1	14.71	14.46	21.19	13.57
STOX1	0.35	1.4	1.19	0.92	1.08	1.37
STOX2	0.02	0	0	0	0	0
STPG1	1.64	1.94	1.64	1.76	3.53	1.44
STPG2	0	0	0	0	0	0
STRA13	166.27	152.63	140.6	162.01	157.76	133.27
STRA6	0	0	0	0	0	0
STRA8	0	0	0	0	0	0
STRADA	6.54	7.29	8.74	6.94	7.48	9.34
STRADB	22.9	35.04	37.86	29.21	28.95	42.52
STRAP	144.97	128.78	124.58	121.12	143.47	124.5
STRBP	1.59	2.74	3.43	1.99	3.55	2.73
STRC	0	0.04	0	0	0.04	0

STRIP1	10.06	12.45	14.07	10.26	11.16	10.35
STRIP2	2.34	2.14	1.22	1.53	2.06	1.74
STRN	1.3	0.63	0.75	0.57	0.6	0.92
STRN3	7.54	2.25	2.01	2.55	3.44	2.84
STRN4	6.8	5.43	5.27	5.36	6.97	3.9
STS	0.39	0.2	0.06	0.1	0.25	0.15
STT3A	92.35	93.82	88.02	91.98	105.01	87.39
STT3B	2.9	4.47	4.03	3.47	4.49	3.68
STUB1	15.56	8.44	13.48	10.17	9.62	8.58
STX10	36.73	55.7	55.83	57.39	57.73	52.2
STX11	0.62	0.44	0.58	0.67	0.82	0.36
STX12	7.47	6.76	4.25	5.99	6.85	4.74
STX16	4.42	4.97	3.16	3.07	3.57	3.68
STX16-NPEPI	0.18	0	0.47	0.86	0.11	0.02
STX17	5.82	4.52	4.01	4.18	3.31	3.87
STX18	16.79	16.33	12.32	11.46	13.25	13.54
STX19	0	0	0	0	0	0
STX1A	0.21	0.03	0.03	0.15	0.36	0.04
STX1B	0.09	0.16	0.16	0.12	0.11	0.16
STX2	2.4	2.42	1.72	0.88	1.44	1.28
STX3	3.43	1.35	1.21	2.05	1.14	1.68
STX4	12.48	10.04	11.21	7.78	10.27	12.43
STX5	1.94	1.94	2.44	2.03	1.91	2.17
STX6	1.67	1.07	1.34	1.81	1.28	1.77
STX7	7.47	7.03	6.51	4.39	5.97	6.86
STX8	25.44	31.35	40.37	32.52	33.21	24.89
STXBP1	6.25	4.35	4.34	5.22	5.59	4.68
STXBP2	0	0	0.87	0.41	0.41	0.16
STXBP3	10.07	11.44	6.92	6.63	7.87	6.96
STXBP4	1.18	1.16	1.09	1	0.93	0.99
STXBP5	3.5	0.37	0.09	0.46	1.4	0.4
STXBP5-AS1	0.07	0	0	0	0.02	0
STXBP5L	0.08	0.05	0.03	0	0.12	0.03
STXBP6	0.23	0	0	0	0.11	0
STYK1	0.32	0	0.33	0	0	0
STYX	5.72	6.06	5.8	4.48	5.86	6.34
STYXL1	23.72	22.38	18.79	18.63	17.16	16.84
SUB1	71.74	63.94	62.66	60.56	74.01	71.13
SUCLA2	30.06	30.16	25.27	20.63	31.1	23.21
SUCLG1	115.65	109.9	87.41	97.27	113.12	101.98
SUCLG2	25.58	22.03	18.62	19.74	25.88	28.47
SUCNR1	16.58	19.93	15.58	2.05	3.07	12.1
SUCO	1.97	1.04	1.18	1.22	1.7	1.03

SUDS3	5.6	6.49	5.45	5.22	6	4.24
SUFU	1.82	1.5	2.71	3.09	2.07	2.08
SUGP1	22.56	20.83	20.68	22.65	21.03	17.92
SUGP2	6.9	6.39	6.63	6.86	6.79	5.88
SUGT1	40.76	34.11	33.72	26.83	35.53	26.94
SUGT1P1	1.71	0.65	1.34	0.71	0.71	0.73
SUGT1P3	0.14	0.55	0.08	0.25	0	0.16
SULF1	0.22	0	0	0.02	0.13	0
SULF2	0.09	0.03	0	0.14	0.07	0.09
SULT1A1	8.05	13.86	12	14.32	11.32	10.88
SULT1A2	2.8	3.36	2.66	1.81	2.27	2.95
SULT1A3	3.12	4.71	5.03	2.77	3.1	7.92
SULT1A4	3.12	4.71	5.03	2.77	3.1	7.92
SULT1B1	0.12	0	0	0	0	0.06
SULT1C2	0.05	0.02	0.13	0	0.02	0.08
SULT1C2P1	0.04	0.16	0.07	0.06	0.22	0.05
SULT1C3	0	0	0	0	0	0
SULT1C4	0	0	0	0	0	0
SULT1E1	0	0.03	0	0	0	0.04
SULT2A1	0.52	0.67	1.01	0.69	0.47	0.58
SULT2B1	0.57	0.3	0.41	0.48	1.14	0.38
SULT4A1	0	0	0	0	0	0
SULT6B1	0	0	0	0	0	0
SUMF1	10.65	9.83	11.66	7.3	12.39	11.48
SUMF2	53.84	68.02	69.92	76.21	56.32	73.7
SUMO1	107.61	94.22	77.78	82.21	95.44	77.82
SUMO1P1	0.5	0.05	0.24	0.17	0.65	0.19
SUMO1P3	0.76	0.87	0.75	1.22	0.46	0.97
SUMO2	341.56	310.19	291.7	289.63	345.72	307.34
SUMO3	73.6	80.5	71.68	76.06	75.83	76.88
SUMO4	0.12	0.28	0.74	0.26	0	0
SUN1	8.38	2.87	3.1	3.07	5.13	3.05
SUN2	2.48	2.71	3.08	1.85	2.14	2.16
SUN3	0	0	0	0	0.16	0
SUN5	0	0	0	0	0	0
SUOX	15.53	10.1	9.51	11.31	13.49	8.42
SUPT16H	53.82	53.99	45.97	47.81	52.76	49.64
SUPT20H	11.41	11.5	10.01	10.99	10.66	10.59
SUPT20HL1	0	0	0	0	0	0
SUPT20HL2	0	0	0	0	0	0
SUPT3H	3.29	1.18	1.18	1.15	1.42	1.04
SUPT4H1	42.13	35.07	29.91	33.01	38.6	54.59
SUPT5H	37.67	33.53	32.15	32.57	37.04	32.32

SUPT6H	11.72	8.92	11.05	10.74	10.9	11.46
SUPT7L	13.3	8.18	6.89	8.63	8.79	7.72
SUPV3L1	24.98	21.99	21.65	23.81	21.09	20.3
SURF1	17.98	20.32	20.25	18.92	20.05	20.04
SURF2	35.4	33.23	31.36	37.96	36.74	30.63
SURF4	84.33	85.28	76.77	76.29	78.1	82.77
SURF6	39.87	36.52	37.95	40.99	39.77	38.79
SUSD1	5.55	2.38	2.56	4.9	4.73	3.5
SUSD2	0	0.09	0	0	0	0
SUSD3	6.51	19.43	27.7	17.36	18.06	15.23
SUSD4	0.02	0	0	0	0	0
SUSD5	0.04	0.03	0.04	0	0.03	0.07
SUV39H1	31.71	50.67	48.94	43.58	34.49	50.04
SUV39H2	8.23	12.17	11.52	9.08	12.88	15.74
SUV420H1	4.17	1.38	1.94	2.23	2.2	1.66
SUV420H2	0.38	0.78	0.76	0.85	0.84	0.93
SUZ12	5.92	5.2	3.89	3.3	5.31	5.19
SUZ12P1	1.22	2.42	1.86	1.35	2.17	0.69
SV2A	0.08	0.04	0.07	0	0	0
SV2B	0.1	0.1	0.16	0.09	0.12	0.1
SV2C	0	0	0	0	0	0
SVEP1	0.03	0	0	0.04	0	0
SVIL	0.15	1.89	0.98	0.71	0.61	1.71
SVIP	16.52	11.23	13.08	10.83	11.78	11.66
SVOP	0.19	0.3	0.21	0.15	0.3	0.12
SVOPL	0	0.17	0.97	0.55	0.75	0.37
SWAP70	16.71	11.7	11.02	12.32	13.3	9.57
SWI5	20.2	22.45	23.16	21.26	21	17.26
SWSAP1	6.87	5.97	7.12	5.61	5.46	4.82
SWT1	0.4	0.21	0.59	0.39	0.72	0.21
SYAP1	36.06	20.66	18.75	17.51	22.9	19.45
SYBU	11.33	0	0.02	0.75	0.91	0
SYCE1	0	0	0	0	0	0
SYCE1L	0.18	0.42	0.98	0.68	1.68	0.45
SYCE2	0.5	0.79	0.38	0.23	0.24	0
SYCE3	0	0.15	0	0	0.19	0.2
SYCN	0	0	0	0	0	0
SYCP1	0	0	0	0	0	0
SYCP2	0.04	0	0	0	0	0
SYCP2L	1.44	2.25	1.64	1.22	1.43	1.48
SYCP3	0	0	0	0	0	0
SYDE1	0.34	0.14	0.27	0.22	0.36	0.27
SYDE2	0.73	0.58	0.77	0.97	0.6	0.44

SYF2	25.29	17.92	14.96	15.71	20.87	15.1
SYK	13.72	15.05	12.73	13.16	15.25	12.2
SYMPK	14.06	14.05	11.35	11.43	12.3	11.05
SYN1	0.25	0	0.13	0.02	0.17	0
SYN2	0.34	0	0	0	0.07	0.04
SYN3	0.31	0	0	0.02	0.13	0
SYNC	0	0.16	0.03	0	0.07	0.07
SYNCRIP	36.48	40.92	41.69	35.51	43.2	46.01
SYNDIG1	0	0	0	0	0	0
SYNDIG1L	0	0	0	0	0	0
SYNE1	0.12	0.14	0.15	0.09	0.05	0.02
SYNE2	0.5	0.02	0.11	0.1	0.01	0.13
SYNE3	1.31	4.04	4.78	2.87	2.33	4.2
SYNE4	0	0	0.09	0	0.05	0
SYNGAP1	0.2	0.26	0.12	0.1	0.18	0.28
SYNGR1	18.9	31.74	38.3	27.03	28.14	30.86
SYNGR2	165.96	154.74	134.24	140.9	142.58	138.9
SYNGR3	0.36	0	0	0	0.1	0
SYNGR4	0.3	0	0.14	0.1	0	0.23
SYNJ1	1.07	0.59	1.05	0.62	0.71	0.68
SYNJ2	3.24	0.69	1.15	1.44	1.67	0.93
SYNJ2-IT1	0	0	0.5	0.11	0.31	0.41
SYNJ2BP	2.85	3.1	3.19	2.38	2.86	2.65
SYNJ2BP-CO	0.4	0.5	0.14	0.41	0.71	0.91
SYNM	0	0	0	0	0	0
SYNPO	0.06	0.21	0.04	0.11	0.16	0.42
SYNPO2	0.27	0.09	0.11	0.1	0.11	0.14
SYNPO2L	0.19	0.29	0.28	0.2	0.2	0.36
SYNPR	0	0	0	0	0	0
SYNPR-AS1	0	0	0	0	0	0
SYNRG	2.4	2.28	2.59	1.95	2.6	2.02
SYP	0.24	0.38	0.47	1.1	0.63	0.73
SYPL1	53.31	72.79	61.03	47.64	61.9	66.24
SYPL2	1.17	0.29	0.49	0.24	0.49	0.47
SYS1	10.52	10.51	7.33	10.83	11.86	6.47
SYS1-DBNDD	0	2.5	0	0	0.96	2.35
SYT1	0	0	0	0	0.03	0
SYT10	0	0	0	0	0	0
SYT11	0.48	0.3	0.27	0.37	0.26	0.23
SYT12	0	0	0	0	0	0
SYT13	0.09	0.2	0.14	0.06	0.1	0.11
SYT14	0	0	0	0	0	0
SYT14L	0	0	0	0	0	0

SYT15	0.33	0.27	0.37	0.3	0.41	0.35
SYT16	0	0	0	0	0	0
SYT17	0	0	0	0	0	0
SYT2	0.02	0	0.01	0	0	0
SYT3	0.78	0.16	0	0.18	0.2	0.12
SYT4	0	0	0	0	0	0
SYT5	0.04	0	0	0	0	0
SYT6	0	0	0	0.48	0	0
SYT7	0.01	0.04	0.1	0.02	0.04	0.04
SYT8	0	0	0	0	0	0
SYT9	0	0	0	0	0	0
SYTL1	3.32	11.56	7.1	8.65	9.49	7.04
SYTL2	0.1	0	0.12	0	0.02	0
SYTL3	0.03	0.23	0.03	0	0.06	0
SYTL4	9.35	3.47	3.42	3.7	4.26	4.3
SYTL5	0	0	0	0	0	0
SYVN1	13.81	8.15	7.2	8.07	8.9	6.68
SZRD1	28.53	27.81	30.35	32.2	29.98	32.31
SZT2	1.3	1.47	1.5	1.42	1.2	2.07
T	0	0	0	0	0	0
TAAR1	0	0	0	0	0	0
TAAR2	0	0	0	0	0	0
TAAR3	0	0	0	0	0	0
TAAR5	0	0	0	0	0	0
TAAR6	0	0	0	0	0	0
TAAR8	0	0	0.14	0	0	0
TAAR9	0	0	0	0	0	0
TAB1	9.24	7.44	8.97	9.64	8.09	7.38
TAB2	3.6	2.18	1.43	1.63	2.25	2.86
TAB3	1.06	1.12	1.24	0.82	0.96	1.02
TAC1	0	0	0	0	0	0
TAC3	0.4	0	0	0	0	0
TAC4	0	0.35	0.13	0.1	0	0
TACC1	9.24	8.28	6.74	5	7.38	5.99
TACC2	0.42	0	0	0.24	0.25	0
TACC3	40.85	51.34	52.48	53.23	43.48	46.05
TACO1	25.63	27	27.58	30.75	29.34	17.65
TACR1	0	0.01	0	0.02	0	0.08
TACR2	0	0	0.05	0	0.17	0
TACR3	0	0	0	0	0	0
TACSTD2	0	0	0	0	0	0
TADA1	20.17	19.62	19.13	18.56	19.44	19.04
TADA2A	9.19	9.85	10.66	7.53	8.7	9.92

TADA2B	3.17	2.55	2.24	2.02	2.37	1.91
TADA3	26.41	19.99	20.56	26.73	30.72	26.34
TAF1	3.68	3.4	4.44	3.08	3.56	5.56
TAF10	4.04	4.93	4.22	7.51	6.01	2.33
TAF11	18.09	25.08	16.14	18.86	25.48	19.3
TAF12	29.25	34.77	34.87	28.71	32.35	29.93
TAF13	6.96	5.06	2.21	5.24	6.5	1.86
TAF15	6.09	7.21	5	5.34	6.83	10.51
TAF1A	2.19	5.39	3.12	2.73	3.54	2.43
TAF1B	4.57	6.05	4.92	4.9	4.77	6.43
TAF1C	4.37	5.71	4.32	6.46	4.18	5.85
TAF1D	25.62	31.84	27.5	22.99	26.95	23.42
TAF1L	0.07	0.13	0.08	0.11	0.05	0.16
TAF2	15.61	15.88	13.02	9.3	14.66	12.22
TAF3	7.23	6.23	6.76	5.23	6.72	5.31
TAF4	0.36	1.01	0.8	0.51	0.65	0.84
TAF4B	1.63	3.22	2.25	2.54	1.93	1.51
TAF5	1.49	2.61	2.46	2.08	1.7	2.75
TAF5L	1.7	2.02	2.47	1.05	0.81	2.35
TAF6	22.69	16.88	17.71	17.68	17.93	17.82
TAF6L	5.47	3.51	4.15	5.97	4.97	3.6
TAF7	41.19	25.23	21.02	26.02	30.8	18.5
TAF7L	0.1	0	0	0	0.13	0
TAF8	6.68	5.15	7.33	6.36	6.87	7.52
TAF9	188.58	164.72	140.12	147.4	186.22	140.73
TAF9B	39.35	36.92	34.07	32.37	35.36	35.95
TAGAP	0.87	7.09	7.57	4.58	2.88	8.04
TAGLN	0.46	0.1	0.54	0.05	0.06	0.33
TAGLN2	513.58	443.48	421.8	441.86	473.24	436.06
TAGLN3	0	0	0	0	0	0
TAL1	9.76	4.88	5.64	7.84	7.76	5.93
TAL2	0	0.3	0	0	0	0
TALDO1	391.39	369.05	341.64	361.89	365.57	361.29
TAMM41	16.68	17.14	19.06	21.25	18.58	18.01
TANC1	9.86	1.67	2.3	3.1	4.27	1.37
TANC2	0.86	0.93	0.99	0.52	0.64	0.74
TANGO2	13.02	12.91	14.64	11.54	11.99	14.62
TANGO6	4.79	4.45	4.4	5.7	4.97	4.03
TANK	8.14	9.38	5.6	5.72	6.98	5.52
TAOK1	2.44	2.17	1.44	1.72	2.01	2.07
TAOK2	5	3.79	3.59	3.99	2.97	3.61
TAOK3	30.72	51.1	48.58	42.32	40.46	43.03
TAP1	9.85	14.76	19.13	13.62	13.95	19.18

TAP2	5.49	5.36	5.62	4.08	4.35	5.86
TAPBP	67.15	74.52	84.94	88.23	65.63	81.05
TAPBPL	3.43	3.67	5.71	3.57	3.24	3.18
TAPT1	0.28	0.22	0.12	0.29	0.58	0.65
TAPT1-AS1	0	0.37	0.19	0.26	0.06	0.26
TARBP1	0.74	1.16	0.91	0.5	0.61	0.64
TARBP2	33.42	27.72	35.91	36.62	36.99	37.08
TARDBP	45.25	50.72	46.05	42.81	52.14	50.88
TARM1	0	0	0	0	0	0
TARP	23.79	100.68	42.16	20.62	16.58	132.16
TARS	105.98	84.71	80.54	80.66	84.72	80.51
TARS2	17.38	19.73	17.74	17.52	16.04	14.37
TARSL2	2.05	2.17	0.95	1.52	1.6	0.89
TAS1R1	0	0	0	0	0	0
TAS1R2	0	0	0	0	0	0
TAS1R3	0	0	0	0.08	0	0
TAS2R1	0	0	0	0	0	0
TAS2R10	0	0.07	0	0	0	0
TAS2R13	0	0	0.04	0	0	0.05
TAS2R14	0	0	0	0.39	0.23	0.16
TAS2R16	0	0	0	0	0	0
TAS2R19	0.26	0	0	0.06	0.08	0.26
TAS2R20	0.93	0.37	0.44	0.45	0.4	0.2
TAS2R3	0.07	0.22	0.13	0	0	0
TAS2R30	0.16	0	0	0.06	0	0
TAS2R31	0.3	0.06	0.21	0	0	0.09
TAS2R38	0	0	0	0	0	0
TAS2R39	0	0	0	0	0	0
TAS2R4	0	0	0	0	0	0
TAS2R40	0	0	0	0	0	0
TAS2R41	0	0	0	0	0	0
TAS2R42	0	0	0	0	0	0
TAS2R43	0	0	0	0	0	0.15
TAS2R46	0	0	0	0	0	0.16
TAS2R5	0	0.73	0.12	0	0.19	0.33
TAS2R50	0	0.12	0	0.11	0	0
TAS2R60	0	0	0	0	0	0
TAS2R7	0	0.17	0	0	0	0
TAS2R8	0	0	0	0	0	0
TAS2R9	0	0	0	0	0	0
TASP1	2.62	2.68	2.77	2.99	3.69	3.15
TAT	0.32	0.45	0.58	0.28	0.29	0.18
TATDN1	51.49	55.98	42.51	44.36	44.23	44.62

TATDN2	1.75	1.81	2.95	1.66	1.77	2.49
TATDN3	9.14	11.98	10.3	8.45	10.06	9.94
TAX1BP1	15.75	18.22	10.48	9.87	15.58	13.43
TAX1BP3	43.7	21.38	35.25	34.43	24.83	32.69
TAZ	6.54	8.62	7.5	10.82	8.36	5.68
TBATA	0	0	0	0	0	0
TBC1D1	2.31	4.21	5.54	4.18	2.66	5.33
TBC1D10A	3.96	2.36	2.71	3.18	3.84	2.88
TBC1D10B	4.11	5.85	4.94	4.44	5.03	3.78
TBC1D10C	0.73	6.55	9.07	6.3	4.33	6.61
TBC1D12	0	0.01	0.01	0	0	0
TBC1D13	4.75	7.51	8.09	6.4	5.21	6.56
TBC1D14	19	15.37	16.3	15.7	18.13	17.61
TBC1D15	7.33	12.26	11.55	11.78	9.07	13.01
TBC1D16	1.29	1.59	1.91	1.58	1.59	2.75
TBC1D17	2.69	1.68	1.25	2.34	2.31	1.24
TBC1D19	2.71	1.16	1.53	0.97	0.95	1.02
TBC1D2	1.87	1.2	1.28	1.01	1.99	2.63
TBC1D20	3.11	2.82	2.37	2.6	2.68	2.74
TBC1D21	0	0	0	0	0	0
TBC1D22A	7.44	5.43	7.23	6.17	4.9	4.7
TBC1D22B	5.58	2.95	3.49	3.95	4.03	2.74
TBC1D23	20.36	4.5	4.19	6.51	12.62	4.69
TBC1D24	3.67	3.96	5.32	3.99	3.03	5.22
TBC1D25	5.93	4.64	7.17	7.24	4.41	5.58
TBC1D26	0	0	0	0	0	0
TBC1D28	0	0	0	0	0	0
TBC1D29	0	0	0.11	0.08	0	0
TBC1D2B	2.68	4.48	3.83	2.36	2.74	3.29
TBC1D3	0	0	0	0	0	0
TBC1D30	0.08	0.01	0.03	0.01	0.03	0.22
TBC1D3B	0	0	0.19	0	0	0
TBC1D3C	0	0	0	0.26	0	0
TBC1D3F	0.45	0.99	0	0	0.56	0.39
TBC1D3G	0	0	0	0	0	0
TBC1D3H	0	0	0	0	0	0
TBC1D3P1-D	0	0.07	0	0	0	0
TBC1D3P2	0	0	0	0	0	0
TBC1D3P5	0	0	0.04	0.02	0.02	0
TBC1D4	2.91	2.21	2.61	2.1	2.38	3.19
TBC1D5	4.04	4.67	4.81	4.5	2.93	5.07
TBC1D7	42	21.79	22.06	30.27	31.07	19.64
TBC1D8	2.41	3.34	2.81	2.41	3.03	2.21

TBC1D8B	0.93	0.6	0.91	1.13	0.44	0.47
TBC1D9	0.53	0.37	0.16	0.37	0.66	0.17
TBC1D9B	24.9	22.17	23.27	21.19	20.53	25.44
TBCA	155.52	155.47	115.07	131.65	147.56	135.18
TBCB	67.85	60.66	50.42	69.92	68.74	49.13
TBCC	18.03	17.48	16.19	19.68	15.34	15.29
TBCCD1	16.57	14.6	17.41	15.51	13.5	16.32
TBCD	12.09	13.7	12.24	15.32	13.15	15.77
TBCE	18.82	20.3	15.4	14.18	21.03	14.05
TBCEL	1.13	0.48	0.31	0.42	0.46	0.66
TBCK	1.77	1.97	1.55	2.12	3.24	1.95
TBK1	8.97	8.42	6.5	4.46	7.12	6.95
TBKBP1	1.05	0.61	0.58	0.58	1.09	0.44
TBL1X	10.97	8.67	8.07	6.41	9.37	8.8
TBL1XR1	23.99	26.86	23.05	18.02	21.49	23.18
TBL1Y	0	0	0	0	0	0
TBL2	14.13	15.43	16.36	13.06	16.43	11.9
TBL3	24.36	24.67	21.88	29.09	24.37	25.7
TBP	10.54	13.9	13.37	15.24	12.38	11.57
TBPL1	74.22	14.37	18.04	32.16	49.23	14.81
TBPL2	0	0	0	0	0	0
TBR1	0	0	0	0	0.02	0
TBRG1	5.05	4.89	4.31	4.04	3.77	3.69
TBRG4	66.89	69.82	67.87	63	67.53	64.05
TBX1	0	0	0.05	0.17	0.09	0.15
TBX10	0	0	0	0	0	0
TBX15	0	0	0	0.06	0.14	0
TBX18	0	0	0	0	0	0
TBX19	0.54	0.31	0.07	0	0	0.26
TBX2	0.06	0.3	0.16	0.09	0.16	0.33
TBX20	3.84	3.33	4.32	4.4	3.43	4.93
TBX21	0	0	0	0	0	0
TBX22	0	0	0	0	0	0
TBX3	0.52	0.75	0.62	0.47	0.57	0.46
TBX4	0	0	0	0	0.03	0
TBX5	0	0	0	0	0	0
TBX5-AS1	0	0	0	0	0	0
TBX6	0.08	0	0	0	0	0.08
TBXA2R	24.05	10.86	16.22	15.48	16.09	10.25
TBXAS1	10.53	30.81	33.12	24.2	21.38	31.18
TC2N	0	0	0	0	0	0
TCAIM	6.51	10.56	7.76	9.75	7.03	11.32
TCAM1P	0.18	0.21	0.05	0.13	0.17	0.3

TCAP	0.08	0.06	0.29	0	0.15	0.08
TCEA1	105.19	91.64	91.99	77.7	97.32	96.23
TCEA2	1.14	0.71	0.84	1.22	1.39	2.75
TCEA3	0.94	0.96	0.3	0.45	0.27	0.14
TCEAL1	14.73	19.56	16.95	15.28	15.01	17.71
TCEAL2	0	0	0	0	0	0
TCEAL3	9.01	4.9	0	7.51	2.59	3.86
TCEAL4	72.97	80.29	57.01	59.46	72	56.05
TCEAL5	0	0	0	0	0	0
TCEAL6	0	0	0	0	0	0
TCEAL7	0	0	0	0	0	0
TCEAL8	91.71	44.73	45.81	52.78	87.02	0
TCEANC	1.92	2.12	1.81	2.49	1.82	1.38
TCEANC2	2.9	4.26	3.71	2.67	3.29	4.54
TCEB1	116.95	99.05	80.1	76.46	97.89	84.91
TCEB2	253.83	227.9	204.34	242.25	245.06	209.6
TCEB3	15.89	13.55	11.64	11.94	11.92	12.94
TCEB3B	0	0	0	0	0	0
TCEB3C	0	0.05	0	0	0.04	0
TCEB3CL	0	0	0.08	0	0	0.09
TCEB3CL2	0	0.05	0	0	0.04	0
TCERG1	26.71	36.05	26.07	24.27	28.39	25.71
TCERG1L	0	0	0	0	0	0
TCF12	18.35	12.6	11.38	10.48	11.32	11.41
TCF15	0	0	0	0.26	0	0
TCF19	11.46	18.34	15.26	12.38	15.06	19.35
TCF20	2.91	2.45	3.05	2.26	2.55	3.56
TCF21	0	0	0	0	0	0
TCF23	0	0	0	0	0	0
TCF24	0.54	0.59	0.92	0.64	0.76	1.18
TCF25	67.95	55.08	63.06	74.05	64.45	49.57
TCF3	7.6	8.07	6.11	7.57	7.76	7.24
TCF4	2.68	1.43	1.42	1.54	1.68	1.83
TCF7	1.91	3.22	3	2.93	3.77	2.12
TCF7L1	0.25	0.55	1.1	1.53	1.96	0.63
TCF7L2	2.26	1.58	0.89	1.39	1.69	1.13
TCFL5	0.3	0.54	0.25	0.12	0.68	0.51
TCHH	0.04	0.03	0.08	0.06	0	0
TCHHL1	0	0	0	0	0	0
TCHP	7.32	7.59	6.66	5.33	7.9	7.38
TCIRG1	2.39	11.95	9.84	7.93	4.88	5
TCL1A	0	0	0	0	0	0.05
TCL1B	0	0	0	0	0	0

TCL6	0	0.02	0.04	0.02	0.04	0.09
TCN1	12.1	1.13	0.92	1.08	4.42	5.3
TCN2	0.68	1.17	2.83	1.31	0.3	1.8
TCOF1	21.13	30.27	30.74	26.63	26.08	28.22
TCP1	233.48	207.16	213.63	226.12	213.15	220.75
TCP10	0	0	0	0	0	0
TCP10L	0.05	0.09	0	0.07	0.03	0
TCP10L2	0	0	0	0	0	0
TCP11	0	0	0	0	0	0
TCP11L1	3.8	3.76	2.85	2.64	3.3	3
TCP11L2	2.52	0.76	0.58	1.71	1.53	0.77
TCTA	17.44	14.52	8.85	10.93	10.47	12.1
TCTE1	0	0	0	0	0	0
TCTE3	0.11	0	0.29	0	0	0.11
TCTEX1D1	3.74	2.3	1.69	0.56	2.94	2.04
TCTEX1D2	26.21	33.86	24.5	27.5	31.3	32.62
TCTEX1D4	0.12	0.24	0.05	0.08	0.11	0.06
TCTN1	4.7	4.44	2.23	3.55	2.87	2.99
TCTN2	1.37	3.38	3.37	2.56	2.49	2.02
TCTN3	18.22	20.43	17.61	19.58	15.76	20.69
TDG	19.91	15.68	12.42	11.42	19.29	15.07
TDGF1	0.24	0.19	0.33	0.04	0.16	0.28
TDGF1P3	0.25	0.06	0.1	0.13	0.05	0.03
TDH	0.06	0	0	0	0.05	0
TDO2	0	0	0.2	0	0	0
TDP1	5.3	5.92	4.81	4.93	3.61	5.7
TDP2	11.03	11.49	13.02	8.84	10.31	10.89
TDRD1	0	0.04	0.03	0	0	0.02
TDRD10	0	0	0	0	0	0
TDRD12	0	0	0	0	0	0
TDRD3	0.82	2.6	2.68	2.8	1.8	2.71
TDRD5	0	0	0	0	0	0
TDRD6	0	0	0	0	0	0
TDRD7	0.35	0.65	0.44	0.61	0.65	0.74
TDRD9	2.38	11.98	10.35	5.85	5.88	11
TDRG1	0	0	0	0	0	0
TDRKH	3.01	3.71	1.94	2.4	1.97	1.08
TEAD1	0.03	0.05	0.01	0.1	0.06	0.02
TEAD2	0	0	0	0	0	0
TEAD3	0.02	0.12	0.18	0.28	0.02	0.2
TEAD4	13.49	26.41	32.11	21.92	19.83	31.18
TEC	2	3.5	4.01	3.09	2.99	3.71
TECPR1	2.82	2.55	2.39	2.46	2.15	2.67

TECPR2	2.06	1.01	0.92	0.7	1.2	1.58
TECR	259.43	288.76	333.34	357.98	314.66	329.76
TECRL	0.05	0.08	0.02	0	0	0.03
TECTA	0.03	0	0	0	0	0
TECTB	0	0	0	0	0	0
TEDDM1	0.03	0	0.02	0	0.03	0
TEF	0.69	1.23	1.05	0.88	0.37	1.25
TEFM	17.18	16.59	15.61	15.1	16.93	15.31
TEK	0.65	0	0	0	0.59	0.11
TEKT1	0	0	0	0	0	0
TEKT2	0	0	0	0	0	0
TEKT3	0	0	0	0	0	0
TEKT4	0.3	0.2	0.1	0.21	0.05	0.36
TEKT4P2	26.27	27.46	23.79	27.51	25.81	23.55
TEKT5	0	0	0	0	0	0
TELO2	19.27	20.72	19.7	20.28	19.2	23.97
TEN1	26.12	17.46	19.63	18.19	27.41	18.85
TEN1-CDK3	0	0.07	0	0	0	0
TENC1	1.02	0.1	0.08	0.3	0.14	0.27
TENM1	0.01	0	0	0	0	0
TENM2	0	0	0	0	0	0
TENM3	0	0	0	0	0	0
TENM4	0	0	0	0	0	0.02
TEP1	0.87	0.93	1.12	0.55	0.8	0.8
TEPP	0	0.08	0	0	0	0
TERC	3.23	2.56	2.43	2.52	1.63	2.29
TERF1	9.95	10.92	11.06	11.46	11.81	9.36
TERF2	5.42	6.27	4.36	4.55	4.13	4.15
TERF2IP	14.98	13	11.24	11.27	12.96	8.38
TERT	0.02	0.37	0.32	0.09	0.2	0.27
TES	3.3	10.79	5.96	7.3	5	7.78
TESC	68.73	26.52	29.52	36.16	53.94	16.64
TESK1	1.69	2.5	0.99	1.86	1.53	1.95
TESK2	4.78	2.52	2.02	1.19	2.12	1.41
TESPA1	4.71	2.46	3.01	4.78	5.51	2.24
TET1	1.87	0.53	0.64	0.97	1.81	0.41
TET2	3.32	2.78	2.5	2.95	2.48	2.46
TET3	2.81	3.21	2.94	2.25	2.81	3.32
TEX10	32.06	42.07	40.39	41.62	39.23	36.55
TEX101	0.64	0.31	0.68	0.22	0.3	0.67
TEX11	0.1	0.06	0.07	0.05	0.01	0.06
TEX12	0	0	0	0.05	0	0
TEX13A	0	0	0	0	0	0

TEX13B	0	0	0	0	0	0
TEX14	0.02	0	0	0.03	0	0
TEX15	1.37	0.81	0.52	0.54	0.97	1.02
TEX19	0	0.3	0.31	0.13	0.04	0.23
TEX2	5.9	5.13	3.92	6.12	5.8	3.93
TEX21P	0.22	0	0	0	0	0
TEX22	0.09	0	0.03	0.16	0.05	0.03
TEX26	0	0	0	0	0	0
TEX26-AS1	0	0	0	0	0	0
TEX261	14.96	11.93	8.5	10.03	7.6	11.58
TEX264	45.31	57.92	61.72	60.48	52.32	50.88
TEX28	0	0	0	0	0	0
TEX29	0	0	0	0	0	0
TEX30	40.8	69.31	51.81	37.99	40.43	54.07
TEX33	0	0	0	0	0	0
TEX35	0.09	0.07	0.16	0	0	0.18
TEX36	0	0	0	0	0	0
TEX37	0	0	0	0	0	0
TEX38	0	0	0	0.06	0.09	0
TEX40	1.17	2.78	2.34	4.58	4.45	2.9
TEX9	1.75	1.59	1.07	0.84	1.36	1.5
TF	0	0	0	0	0	0
TFAM	13.9	13.79	11.45	9.93	12.87	14.19
TFAMP1	0.3	0.32	0.21	0.36	0.25	0.23
TFAP2A	0.4	0.55	0.51	0.24	0.16	0.4
TFAP2B	0	0	0	0	0	0
TFAP2C	0	0	0	0	0	0
TFAP2D	0	0	0	0	0	0
TFAP2E	0.03	0.56	0.65	0.51	0.29	0.31
TFAP4	1.44	2.04	2.78	2.63	2.28	3.9
TFB1M	5.84	5.03	5.42	6.27	4.64	3.9
TFB2M	31.26	38.75	37.33	30.94	36.48	44.29
TFCP2	5.58	3.2	4.14	3.94	3.57	3.87
TFCP2L1	0.09	0.58	0.97	0.11	1.4	2.62
TFDP1	11.84	12.72	8.75	10.63	9.44	9.24
TFDP2	13.86	7.3	7.13	9.66	10.58	8.56
TFDP3	0	0	0	0	0	0
TFE3	4.05	3.22	4.96	5.18	5.92	4.16
TFEB	0.42	0.64	0.93	1.17	0.46	1.24
TFEC	0.32	1.35	1.23	0.55	0.2	0.64
TFF1	0	0	0	0	0	0
TFF2	0	0	0	0	0	0
TFF3	0	0	0	0	0	0

TFG	73.82	42.86	41.35	45.14	53.52	43.04
TFIP11	19.47	20.87	23.82	21.91	18.17	21.55
TFPI	2.74	4.69	2.85	2.15	3.83	3.32
TFPI2	0	0	0	0	0	0
TFPT	13.76	14.54	18.99	12.22	18.39	10.72
TFR2	4.9	4.84	6.97	4.42	5.93	4.95
TFRC	134.17	88.94	77.86	82.89	94.18	90.67
TG	0.02	0.02	0	0	0	0
TGDS	6.65	10.27	9.48	6.21	7.98	9.65
TGFA	0.02	0	0.02	0	0	0
TGFB1	9.84	5.9	6.86	5.94	5.69	4.94
TGFB1I1	1.28	1.17	0.5	3.34	1.46	2.92
TGFB2	0	0.01	0	0	0	0
TGFB3	0.55	0.86	1.38	1.59	0.84	0.9
TGFBI	0	0	0	0	0	0
TGFBR1	2.49	2.03	1.53	1.71	2.79	1.58
TGFBR2	1.65	0.77	1.15	1	0.49	0.79
TGFBR3	0.66	0.23	0.08	0.26	0.34	0.03
TGFBR3L	0.18	0	0.77	0.17	0	0.06
TGFBRAP1	3.24	3.02	3.31	4.04	3.06	2.86
TGIF1	19.4	14.56	8.51	10.18	12.96	9.55
TGIF2	8.75	9.65	8.9	11.43	9.59	12.11
TGIF2-C20or	4.72	0	0	2.27	0.81	1.63
TGIF2LX	0	0	0	0	0	0
TGIF2LY	0	0	0	0	0	0
TGM1	0.08	0.04	0.02	0.11	0.2	0.08
TGM2	3.84	0.12	1.37	0.49	0.3	0.2
TGM3	0	0	0	0	0	0
TGM4	0.02	0	0	0	0	0
TGM5	0.06	0.39	0.43	0	0.05	0.15
TGM6	0	0	0	0	0	0
TGM7	0	0	0	0	0	0
TGOLN2	15.77	17.52	10.72	9.44	11.32	15.07
TGS1	15.54	21.72	21.72	19.54	20.04	21.19
TH	0	0	0.04	0	0	0
THADA	5.53	6.72	6.09	7.07	4.82	5.98
THAP1	10.95	8.72	6.76	6.94	8.62	6.69
THAP10	3.43	1.22	1.49	1.24	1.65	1.02
THAP11	4.63	6.78	7.37	5.43	3.26	5.12
THAP2	0.45	0.55	0.37	0.46	0.97	1.03
THAP3	11.63	13.06	12.07	12.83	11.39	13.59
THAP4	21.46	25.39	21.62	26.18	25.31	25.73
THAP5	5.04	4.42	5.58	4.1	5.16	3.88

THAP6	1.77	1.47	1.7	1.14	1.57	1.81
THAP7	18.2	21.82	23.83	22.72	23.37	18.72
THAP7-AS1	3.33	1.21	1.65	1.12	1.67	2.36
THAP8	2.44	1.06	1.75	1.37	1.3	0.62
THAP9	1.53	2.1	1.1	1.22	1.26	1.38
THAP9-AS1	7.55	6.85	5.6	6.82	6.29	7.8
THBD	0.11	0	0.08	0.12	0	0
THBS1	0.3	0.01	0.02	0	0.25	0
THBS2	0	0	0.02	0	0	0
THBS3	0.83	0.52	0.89	0.57	0.66	0.21
THBS4	0	0	0	0	0	0
THEG	0	0	0	0	0	0
THEG5	0	0	0	0	0	0
THEGL	0	0	0	0	0	0
THEM4	4.81	5.99	4.64	4.8	5.2	6.76
THEM5	0	0	0	0	0	0
THEM6	24.93	53.42	58.32	45.76	47.03	60.46
THEMIS	0.02	0	0	0	0	0
THEMIS2	6.83	5.58	3.96	5.41	5.22	5.85
THG1L	28.47	20.98	16.97	24.62	22.17	20.41
THNSL1	3.41	3.31	2.91	3.57	2.85	3.58
THNSL2	0	0	0	0	0	0
THOC1	10.5	8.16	7.29	7.66	9.95	10.03
THOC2	27.29	27.18	21.92	19.8	25.84	26.64
THOC3	39.8	48.41	40.85	36.42	44.78	48.75
THOC5	19.31	17.88	19.12	16.93	18.57	23.27
THOC6	42.89	62.74	60.93	62.85	60.61	55.11
THOC7	84.27	86.93	70.75	66.26	84.8	73.64
THOP1	27.58	33.1	31.12	31.49	32.82	27.06
THPO	0	0	0	0	0	0
THRA	4.34	3.8	3.62	3.48	4.36	4.35
THRAP3	67.08	73.51	63.85	70.46	69.11	60.32
THRB	1.12	0	0	0.24	0.06	0
THRB-AS1	0.26	0	0	0	0	0
THRSP	0.06	0	0	0	0	0.07
THSD1	0	0	0	0	0.07	0
THSD4	0	0	0	0	0	0
THSD7A	0.14	0.53	0.3	0	0.02	0.47
THSD7B	0	0	0	0	0	0
THTPA	7.72	10.93	9.72	9.93	6.16	5.47
THUMPD1	37.84	17.44	17.89	21.5	32.3	14.77
THUMPD2	3.59	6.17	3.38	3.91	2.92	3.91
THUMPD3	10.36	8.71	8.21	8.44	10.93	8.18

THY1	0.03	0	0	0.17	0	0.06
THYN1	50.71	57.36	52.29	59.03	56.16	38.92
TIA1	9.46	9.45	8.71	7.37	12.23	9.15
TIAF1	0	0.21	0.76	0	0.15	0.1
TIAL1	5.68	4.71	4.15	3.54	5.06	6.05
TIAM1	0.25	0	0	0	0.08	0.1
TIAM2	0.38	0	0	0.09	0.07	0
TICAM1	7.2	6.83	7.2	5.61	6.82	5.36
TICAM2	0.25	0.33	0.15	0.53	0.75	0.34
TICRR	3.4	4.71	5.18	5.52	4.25	4.69
TIE1	0.19	0.76	0.33	0.37	0.58	0.64
TIFA	3.06	6.96	6.75	5.23	5.76	7.73
TIFAB	0	0	0	0	0	0
TIGD1	7.09	7.44	7.02	6.39	5.85	5.88
TIGD2	3.18	4.06	4.57	2.34	3.27	3.51
TIGD3	0	0.39	0.62	0.08	0.75	0.15
TIGD4	0.09	0.16	0.08	0.1	0	0
TIGD5	2.56	3	4.23	2.93	3.13	2.15
TIGD6	1.07	1.01	0.71	1.06	1.12	0.84
TIGD7	0.3	0.4	0.48	0.58	0.46	0.54
TIGIT	0	0	0	0	0	0
TIMD4	0	0	0	0	0	0
TIMELESS	12.65	15.38	16.3	14.16	16.08	18.09
TIMM10	174.34	178.29	166.21	185.14	211.36	149.78
TIMM10B	26.55	26.28	22.41	23.81	26.24	23.37
TIMM13	165.39	188.67	179.51	189.37	191.69	182
TIMM17A	73.43	74.42	61.86	50.95	65.43	55.91
TIMM17B	136.03	158.76	161.24	159.47	168.03	171.13
TIMM21	34.58	37.47	35.35	32.69	34.98	37.94
TIMM22	48.46	46.85	40.5	42.31	42.35	36.69
TIMM23	146.39	122.59	128.67	124.72	150.55	121.55
TIMM44	70.04	67.18	58.11	67.1	65.29	53.95
TIMM50	64.58	68.02	73	69.14	67.79	67.49
TIMM8A	57.55	46.05	47.22	52.8	67.57	47.89
TIMM8B	120.1	130.24	95.28	108.27	128.83	88.12
TIMM9	44.58	41.44	41.38	43.64	50.6	34.64
TIMMDC1	72.04	63.68	62.02	60.6	63.77	60.71
TIMP1	1633.1	130.79	168.46	298.25	396.6	173.54
TIMP2	0.08	1.41	2.42	1.08	0.84	1.11
TIMP3	24.51	0.03	0.13	4.48	11.09	0.65
TIMP4	0	0	0	0	0	0.09
TINAG	0	0	0	0	0	0
TINAGL1	0.17	0.35	0.27	0.22	0.12	0.13

TINCR	0.08	0.21	0.29	0.02	0.04	0.02
TINF2	16.85	18.28	16.81	15.64	19.42	17.4
TIPARP	4.5	2.61	3.19	3.51	2.75	3.01
TIPARP-AS1	0.15	0.09	0.06	0.22	0	0.21
TIPIN	61.91	63.99	60.1	55.2	56.22	58.21
TIPRL	31.73	32.14	32.8	29.68	32.5	30.03
TIRAP	2.92	4.92	2.19	2.87	3.36	3.91
TISP43	0	0	0	0	0	0
TJAP1	8.39	6.94	5.43	6.81	7.05	6.24
TJP1	0.86	0.05	0.01	0.16	0.24	0.06
TJP2	7.7	2.95	2.59	2.24	5.13	2.6
TJP3	0	0.04	0	0.02	0	0.09
TK1	130.13	124.54	116.3	138.88	134.15	104.64
TK2	1.37	0.92	0.83	0.86	1.12	1.01
TKT	411.55	367.93	372.98	472.82	434.17	381.54
TKTL1	0	0	0	0	0	0
TKTL2	0	0	0	0	0	0
TLCD1	4.37	5.88	8.61	14.72	8.98	7.6
TLCD2	2.39	2.26	2.54	1.63	2.07	2.12
TLE1	0	0.04	0	0	0.04	0
TLE2	0.06	0	0.15	0.04	0.09	0
TLE3	9.91	8.94	9.18	9.37	10.5	8.48
TLE4	0.49	0.67	0.13	0.16	0.52	0.48
TLE6	0	0	0.07	0.15	0	0
TLK1	12.45	8.17	5.51	6.25	9.56	7.2
TLK2	1.97	2.26	1.97	1.97	1.58	1.9
TLL1	0	0	0	0	0	0
TLL2	0.03	0.08	0.1	0.02	0.08	0.02
TLN1	21.95	10.14	12.35	14.51	13.86	13.55
TLN2	0.58	0.7	0.69	0.76	0.46	0.74
TLR1	0.38	1.12	0.82	1.45	0.46	1.52
TLR10	0.38	0.65	0.4	0.47	0.19	0.43
TLR2	0.04	0	0	0	0	0
TLR3	0.22	0	0	0.03	0.02	0
TLR4	0.18	2.15	1.1	0.22	0.11	1.75
TLR5	0.02	0	0	0	0.03	0
TLR6	0.3	0.61	0.59	0.29	0.46	0.43
TLR7	0.35	0.16	0.19	0.15	0.19	0.21
TLR8	0	0	0	0	0	0
TLR8-AS1	0.11	0.15	0.22	0.13	0.04	0.1
TLR9	0.04	0.15	0.05	0.01	0.12	0.51
TLX1	0	0	0	0	0	0
TLX1NB	0	0	0	0	0	0

TLX2	0	0	0	0	0	0
TLX3	0	0	0	0	0	0
TM2D1	11.33	11.37	9.48	7.69	13.01	8.2
TM2D2	17.32	17.66	20.35	16.21	16.16	17.49
TM2D3	22.58	21.67	16.63	20.57	24.46	18.85
TM4SF1	11.14	0	0	0.21	0.21	0.04
TM4SF18	0.06	0	0	0	0	0
TM4SF19	0.08	0	0.07	0.05	0	0
TM4SF19-TC	0	0	0	0	0	0
TM4SF20	0.06	0.18	0.18	0.26	0.12	0
TM4SF4	0	0	0	0	0	0
TM4SF5	0.23	0	0	0	0	0
TM6SF1	17.61	2.75	2.52	5.26	8.24	2.25
TM6SF2	0	0	0.14	0	0	0
TM7SF2	16.34	13.51	18.31	18.29	16.42	12.24
TM7SF3	4.75	9.38	8.27	4.55	6.44	8.91
TM9SF1	25.75	26.29	25.7	23.01	22.65	27.6
TM9SF2	50.06	51.92	49.11	39.72	48.74	48.56
TM9SF3	5.73	4.48	4.02	3.14	6.06	3.38
TM9SF4	13.44	10.32	10.3	11.95	10.87	10.43
TMA16	13.47	10.88	10.17	11.61	14.14	11.79
TMA7	615.24	688.31	575.4	489.05	685.82	496.87
TMBIM1	57.56	26.3	30.8	43.02	51.23	29.26
TMBIM4	39.06	52.91	43.83	39.55	38.46	43.11
TMBIM6	151.72	126.67	126.94	128.73	137.63	135.66
TMC1	0.02	0.06	0	0	0	0.06
TMC2	0	0	0	0	0.02	0
TMC3	0	0	0	0.02	0.06	0
TMC4	0.18	0.12	0.14	0.06	0	0.09
TMC5	1.99	2.14	2.02	1.65	1.48	1.72
TMC6	29.26	13.7	12.61	17.1	22.86	13.67
TMC7	0.36	0.61	0.74	0.28	0.39	0.29
TMC8	1.54	3.34	2.91	2.51	2.54	2.86
TMCC1	1.19	1.13	0.75	0.41	1.06	0.8
TMCC2	1.98	0.33	0.08	0.64	0.47	0.46
TMCC3	1.39	0.7	1.97	2.06	1.95	1.7
TMCO1	28.62	29.23	25.9	20.27	25.85	21.07
TMCO2	0	0	0	0	0	0
TMCO3	4.25	2.7	1.84	1.82	2.56	1.12
TMCO4	1.42	4.87	3.03	2.7	2.89	2.93
TMCO5A	0	0	0	0	0	0
TMCO5B	0	0	0	0	0	0
TMCO6	7.76	9.33	6.89	9.28	4.96	10.47

TMED1	37.08	33.13	34.09	30.2	43.27	33.56
TMED10	43.58	47.93	49.24	42.97	45.62	50.63
TMED10P1	0.48	0.22	0.48	0.29	0.26	0.09
TMED11P	0	0	0	0	0	0
TMED2	122.64	141.24	142.78	127.62	126.7	141.61
TMED3	190.35	242.13	252.56	202.75	210.84	239.1
TMED4	31.28	27.83	21.81	27.32	27.21	32.75
TMED5	7.69	9.11	8.5	5.48	7.81	8.69
TMED6	0.08	0.58	0.08	0.11	0.08	0.33
TMED7	12.75	11.52	11.2	7.8	11.03	12.25
TMED7-TICAI	0.82	0.36	1.05	0.54	0.21	0
TMED8	6.78	6.29	7.14	6.65	7.4	5.66
TMED9	62.32	38.52	64.53	58.83	85.27	68.83
TMEFF1	0.16	0.24	0	0	0	0.09
TMEFF2	0	0	0	0	0	0
TMEM100	0.55	0	0	0	0	0
TMEM101	38.78	40.49	43.63	40.25	49.01	36.05
TMEM102	1.76	2.33	1.2	2.35	1.24	1.55
TMEM104	5.05	6.2	5.01	4.92	3.94	3.48
TMEM105	0.23	0.23	0.58	0.34	0.36	0.28
TMEM106A	1.52	2.78	2.58	2.03	2.09	2.17
TMEM106B	3.63	3.05	2.01	1.82	1.94	2.62
TMEM106C	71.87	92.48	82.16	77.63	89.59	85.11
TMEM107	6.97	6.85	6.94	5.47	5.95	6.11
TMEM108	0	0	0	0	0	0
TMEM109	93.88	98.91	111.42	92.43	94.85	103.64
TMEM11	52.97	50.67	45.43	44.34	55.26	46.47
TMEM110	1.1	1.4	1.88	1.45	1.15	1.62
TMEM110-M	0	0	0	0.25	0.59	0.82
TMEM114	0	0	0	0	0	0
TMEM115	36.33	33.42	38.93	34.93	30.91	30.56
TMEM116	1.73	2.85	2.44	2.67	1.45	3.08
TMEM117	2.36	1.58	1.46	1.84	2.01	0.82
TMEM119	0.15	0.48	0.56	0.05	0.15	0.13
TMEM120A	8.77	10.21	10.68	7.48	10.36	6.03
TMEM120B	3.45	4.51	4.14	4.26	4	3.19
TMEM121	0.49	0.66	1.13	1.26	0.65	1.05
TMEM123	23.68	35.4	24.32	22.68	23.79	25.99
TMEM125	0	0	0	0	0	0
TMEM126A	52.43	56.51	52.13	45.79	58.79	48.54
TMEM126B	50.85	50.38	43.15	31.66	45.87	35.3
TMEM127	5.62	5.43	6.33	3.75	3.8	4.7
TMEM128	7.04	9.71	4.76	5.17	4.38	5.69

TMEM129	3.62	2.81	2.89	3.62	3	3.05
TMEM130	0.52	0.75	0.61	0.4	0.46	0.39
TMEM131	1.59	1.65	1.57	1.42	1.29	1.46
TMEM132A	1.36	0.91	0.36	0.64	0.58	0.9
TMEM132B	0	0	0	0	0	0
TMEM132C	0	0	0	0	0	0
TMEM132D	0	0	0	0	0	0
TMEM132E	0.15	0	0.05	0.36	0.45	0.13
TMEM133	0	0	0	0	0	0
TMEM134	16.89	11.16	10.76	17.55	15.43	12.77
TMEM135	4.95	2.04	2.16	2.05	3.52	1.76
TMEM136	0.78	0.88	1.15	0.62	0.57	1.06
TMEM138	18.33	18.25	17.14	13.21	13.92	14.63
TMEM139	0.05	0.04	0.25	0	0	0.32
TMEM140	7.31	5.95	4.16	3.88	5.34	5.94
TMEM141	46.59	33.61	33.05	45.45	45.05	30.71
TMEM143	2.29	3.56	2.99	5.04	2.1	2.95
TMEM144	1.27	3.59	2.77	1.13	2.17	2.66
TMEM145	0.33	0.03	0	0	0	0
TMEM147	149.96	191.04	169.69	183.17	175.75	150.61
TMEM14A	11.98	6.04	0.07	4.09	7.47	13.02
TMEM14B	141.09	93.6	104.85	113.3	126.29	97.31
TMEM14C	109.37	63.37	65.43	74.63	72.34	63.38
TMEM14E	0	0	0	0.04	0	0
TMEM150A	8.13	7.86	10.45	8.03	5.36	7.18
TMEM150B	0	0	0.24	0	0	0
TMEM150C	0.86	1.14	0.97	1.7	1.33	0.99
TMEM151A	0	0	0.03	0.04	0	0.06
TMEM151B	0	0	0	0	0	0
TMEM154	0.41	1.19	1.2	0.86	1.05	1.15
TMEM155	0	0	0	0	0	0
TMEM156	0.25	1.22	2.05	1.3	1.21	1.6
TMEM158	1.32	0.22	0.33	0.44	0.68	0.32
TMEM159	12.36	11.8	15.28	14.99	14.46	18.45
TMEM160	60.61	80.61	103.37	93.85	97.85	89.62
TMEM161A	46.08	62.22	61.66	80.64	55.75	65.68
TMEM161B	3.08	2.36	2.25	1.84	2.5	1.48
TMEM161B-/	2.71	1.21	1.64	0.83	1.85	1.16
TMEM163	0.67	1.94	1.18	2.56	1.33	2.41
TMEM164	10.31	9.57	6.58	6.94	9.07	8.32
TMEM165	31.23	35.14	30.7	28.74	34.87	23.33
TMEM167A	21.48	18.7	17.08	16.66	21.72	19.52
TMEM167B	4.06	4.69	4.06	2.52	3.38	7.72

TMEM168	0	0	0	0	0	0
TMEM169	0.17	0.1	0.22	0.2	0.13	0.49
TMEM17	1.02	2.67	2.98	1.72	2.34	1.69
TMEM170A	7.64	9.24	6.59	5.93	7.03	11.02
TMEM170B	0.33	0.67	0.43	0.36	0.43	0.44
TMEM171	0.99	0	0	0.21	0.34	0
TMEM173	27.73	30.91	39.12	29.26	45.42	34.8
TMEM174	0	0	0	0	0	0
TMEM175	7.04	6.41	5.98	4.76	4.19	2.58
TMEM176A	0	0.12	0.34	0	0	0
TMEM176B	0.26	0	0	0	0	0
TMEM177	33.87	36.75	34.84	50.52	41.77	39.08
TMEM178A	0	0.04	0	0	0	0.06
TMEM178B	0	0	0	0	0.06	0
TMEM179	0	0	0	0	0	0
TMEM179B	74.93	76.6	79.83	84.7	81.92	66.49
TMEM18	15.83	17.63	15.47	18.17	17.27	18.4
TMEM180	10.35	5.58	6.72	11.89	7.55	8.98
TMEM181	2.24	1.79	1.63	2.37	1.81	2.07
TMEM182	0.73	0.67	0.72	0.28	0.88	0.41
TMEM183A	2.12	3.36	2.31	1.1	2.38	1.89
TMEM183B	41.65	49.19	55.54	59.46	47.59	36.22
TMEM184A	0.25	0.27	0.22	0.07	0	0.08
TMEM184B	7.98	9.48	9.83	9.54	10.31	8.81
TMEM184C	15.16	16.46	16.69	14.05	14.46	15.03
TMEM185A	5.95	6.4	7.6	5.5	5.03	6.73
TMEM185B	1.52	1.16	1.11	1.3	0.95	0.78
TMEM186	14.51	17.81	12.52	13.47	15.58	12.99
TMEM187	10.99	7.64	9.82	11.36	9.03	9.08
TMEM189	17.69	14.56	14.34	15.6	16.59	9.78
TMEM189-U	2.26	8.23	1.2	3.07	4.72	2.63
TMEM19	4.64	3.35	3.55	3.07	3.37	3.82
TMEM190	0	0	0	0	0	0
TMEM191A	0.59	0.58	0.52	0.57	0.69	0.84
TMEM191B	0.58	0.78	1.18	0.43	0.67	0.92
TMEM191C	0	0.68	0	0	0	0
TMEM192	7.42	10.47	9.04	8.11	7.41	8.2
TMEM194A	9.11	8.89	7.71	6.76	9.07	6.71
TMEM194B	1.86	1.99	2.63	1.88	2.84	3.19
TMEM196	0	0	0	0	0	0
TMEM198	0.03	0.29	0.09	0.39	0.22	0.2
TMEM198B	1.48	1.25	1.16	1.28	1.49	1.9
TMEM199	25.64	23.27	18.25	16.03	19.83	18.01

TMEM2	4.66	0.06	0.29	0.93	0.82	0
TMEM200A	0.15	0	0.12	0	0.02	0
TMEM200B	0	0	0	0	0	0
TMEM200C	0	0	0	0	0	0
TMEM201	11.91	11.79	12.15	10.3	9.43	13.59
TMEM202	0	0	0	0	0	0
TMEM203	3.27	4.05	1.63	3.14	4	3.35
TMEM204	0	0	0	0	0	0
TMEM205	41.98	51.31	48.67	52.75	51.88	53.03
TMEM206	15.4	11.6	9.28	6.85	14.73	9.19
TMEM207	0	0	0	0	0	0
TMEM208	94.48	99.67	88.94	86.6	85.2	88.43
TMEM209	15.24	14.92	11.71	10.77	15.64	11.92
TMEM211	0	0	0	0	0	0
TMEM212	9.1	11.01	11.13	7.92	8.23	9.51
TMEM213	0.68	1.16	1.12	0.52	0.67	0.97
TMEM214	8.01	6.73	7.28	7.66	6.78	7.19
TMEM215	0	0	0	0	0	0
TMEM216	16.07	24.6	24.11	14.82	19.5	23.15
TMEM217	0.79	0.2	0.14	0.29	0.59	0.04
TMEM218	4.25	5.08	4.9	5.24	5.77	4.18
TMEM219	74.07	84.8	98.88	103.07	96.02	100.44
TMEM220	2.76	4.58	3.55	4.64	3.23	4.2
TMEM220-A	0.94	0.96	0.97	0.54	1.24	0.99
TMEM221	0.42	0.21	0.46	0.48	0.47	0.35
TMEM222	49.65	38.52	40.56	47.24	40.31	37.12
TMEM223	26.16	34.37	34.85	31.85	26.94	30.99
TMEM225	0	0	0	0	0	0
TMEM229A	0	0	0	0	0	0
TMEM229B	0	0	0.2	0.07	0.13	0.09
TMEM230	97.56	85.52	80.09	85.97	88.56	77.11
TMEM231	2.51	2.23	1.95	2.52	1.43	2.61
TMEM232	0	0	0	0	0	0
TMEM233	17.76	0.04	0	2.91	5.52	0.19
TMEM234	7.64	6.41	5.69	5.32	4.28	6.43
TMEM235	0	0	0	0	0	0
TMEM236	0.25	0.28	0.33	0.14	0.34	0.3
TMEM237	3.55	2.66	2.21	2.98	2.59	2.54
TMEM238	0	0	0.22	0.08	0	0.12
TMEM239	0	0	0	0	0	0
TMEM240	0	0	0	0	0	0
TMEM241	7.07	8.33	6.58	7.86	6.55	6.87
TMEM242	5.21	4.52	3.02	3.67	4.14	3.97

TMEM243	4.07	6.84	5.38	4.42	4.11	7.19
TMEM244	0	0	0	0	0	0
TMEM245	2.16	1.82	0.72	0.89	1.76	1.24
TMEM246	0	0	0	0	0	0
TMEM247	0	0	0	0	0	0
TMEM248	6.95	6.33	5.42	6.04	6.51	5.21
TMEM249	0.24	0.44	0.16	0.24	0.25	0
TMEM25	0.1	0.04	0.09	0.24	0.06	0.05
TMEM251	10.77	6.47	7.27	5.53	10.24	5.26
TMEM252	0	0	0	0	0	0
TMEM253	0.34	0.19	0.04	0.2	0.09	0
TMEM254	5.01	7.62	5.74	7.96	7.39	6.79
TMEM254-AS	0.2	0.02	0.12	0.1	0.23	0.17
TMEM255A	0.2	2.79	4.1	2.19	2.46	5.94
TMEM255B	0	0.06	0.27	0	0	0.08
TMEM256	74.58	80.56	73.14	82.69	93.32	63.31
TMEM257	0	0	0	0	0	0
TMEM258	308.49	414.13	370.46	364.9	455.03	349.74
TMEM259	7.16	7.54	6.45	7.02	5.81	6.26
TMEM26	0	0	0	0	0	0
TMEM27	0.47	0	0.04	0.07	0	0.57
TMEM30A	17.78	10.3	10.42	7.96	10.53	11.53
TMEM30B	0	0.01	0	0	0.02	0
TMEM30C	0	0	0	0	0	0.3
TMEM31	0	0.09	0.1	0	0.21	0
TMEM33	18.62	19.09	19.2	12.61	16.06	17.52
TMEM35	0	0	0	0	0	0.04
TMEM37	14.79	10.22	7.99	10.89	13.16	5.22
TMEM38A	3.79	1.87	2.77	1.71	1.06	2.17
TMEM38B	11.19	7.29	9.19	5.9	8	7.16
TMEM39A	5.97	6.71	6.27	4.65	5.1	4.42
TMEM39B	23.06	26.01	28.14	26.05	22.47	23.07
TMEM40	0.06	0.04	0.08	0.12	0	0.09
TMEM41A	14.4	12.75	12.59	11.99	14.86	11.37
TMEM41B	12.01	8.7	9.86	7.06	10.11	8.99
TMEM42	0.08	0.06	0.07	0.16	0	0
TMEM43	17.83	14.18	14.32	19.27	12.21	15.17
TMEM44	0.72	0.66	0.86	0.42	0.33	0.79
TMEM44-AS	1.13	0.92	0.99	0.97	0.38	0.92
TMEM45A	1.67	0.15	0.22	0.56	0.41	0.68
TMEM45B	0.2	0.28	0.08	0.11	0.1	0.24
TMEM47	0	0	0	0.04	0	0
TMEM48	19.46	31.86	29.53	24.09	27.74	33.58

TMEM5	4.91	8.97	6.29	6.52	8.12	8.1
TMEM50A	73.58	48.36	38.37	40.48	49.03	38.48
TMEM50B	16.27	18.17	15.48	11.05	10.92	14.52
TMEM51	2.79	1.55	1.17	2.05	2.16	2.94
TMEM51-AS	0.14	0.04	0	0.01	0.01	0
TMEM52	1.05	1.46	2.88	4.51	2.29	1.96
TMEM52B	0.03	0.08	0	0.03	0	0.03
TMEM53	0.05	0.53	0.64	0.31	0.32	0.4
TMEM54	5.2	2.39	1.21	5.47	4.48	4.15
TMEM55A	4.8	3.66	3.77	2.18	4.01	2.94
TMEM55B	2.86	3.4	3.06	1.92	3.32	2.28
TMEM56	0.4	0.18	0.4	0.28	0.33	0.38
TMEM56-RW	0.44	0.14	0.13	0.08	0	0
TMEM57	4.76	2.06	1.23	1.86	2.32	1.6
TMEM59	68.38	65.25	62.1	56.01	67.29	61.24
TMEM59L	0.65	0	0	0.73	0	0.09
TMEM60	35	29.11	20.5	18.72	33.72	22.26
TMEM61	0	0	0	0	0	0
TMEM62	2	2.07	1.35	2.01	2.12	1.02
TMEM63A	3.87	3.85	3.48	4.54	3.46	4.37
TMEM63B	6.73	2.31	2.77	3.83	5.02	3.09
TMEM63C	0.59	0.39	0.56	0.63	0.26	1.63
TMEM64	2.31	1.51	1.62	1.33	1.36	1.45
TMEM65	0.12	0.63	0.45	0.44	0.59	0.85
TMEM66	195.08	164.66	148.33	147.1	149.69	138.05
TMEM67	1.79	2.96	2.43	1.47	1.54	2.25
TMEM68	7.25	7.56	5.52	5.15	6.94	7.54
TMEM69	30.17	28.48	22.67	24.61	27.66	23.29
TMEM70	53.12	59.6	52.72	46.54	53.04	51.49
TMEM71	0	0	0.23	0.08	0	0.36
TMEM72	0	0	0.45	0	0	0.07
TMEM72-AS	0	0	0.02	0	0	0
TMEM74	0.95	2.09	3.11	1.75	1.6	2.57
TMEM74B	1.04	0.17	0	0.77	0.82	0
TMEM79	0.69	1.93	1.66	1.61	1.19	1.24
TMEM80	16.87	18.82	17.57	18.52	19.21	17.68
TMEM81	0.45	1.38	0.57	0.98	0.86	0.4
TMEM82	0	0	0	0	0	0
TMEM86A	0.83	0.11	0.32	0.7	0.25	0.04
TMEM86B	0.25	0.67	0.16	0.56	0.32	0.73
TMEM87A	12.9	18.76	13.37	10.04	12.96	14.07
TMEM87B	5.36	1.15	0.98	1.4	3.09	1.74
TMEM88	0	0	0.16	0	0.08	0

TMEM88B	0	0	0	0	0	0
TMEM89	0	0.14	0	0	0	0.18
TMEM8A	1.57	1.17	1.45	1.39	1.2	1.19
TMEM8B	1.24	0.03	0.16	0.07	0.66	0.02
TMEM8C	0	0	0	0	0	0
TMEM9	56.96	53.47	44.52	51.17	51.67	56.85
TMEM91	4.14	2.19	4.21	7.88	6.87	4.82
TMEM92	0.11	1.1	0.91	0.83	0.91	1.9
TMEM95	0	0	0	0	0	0
TMEM97	37.25	49.58	49.09	63.61	69.74	64.88
TMEM98	47.29	31.45	36.15	46.6	45.19	31.86
TMEM99	3	2.63	2.42	3.25	2.5	2.46
TMEM9B	24.5	23.58	19.9	13.43	20.42	15.22
TMEM9B-AS	0.44	0.69	1.27	0.7	0.62	0.44
TMF1	5.88	6.39	6.21	4.54	4.63	4.29
TMIE	0	0.13	0.47	0.6	0	0.08
TMIGD1	0	0	0	0	0	0
TMIGD2	0.43	0.13	1.42	1.08	1.36	0.69
TMLHE	16.81	17.61	15.75	15.51	15.3	17.45
TMLHE-AS1	0.22	0.1	0.16	0.09	0.25	0.22
TMOD1	18.3	0.62	0.92	4.81	6.89	0.17
TMOD2	0.75	0.67	0.9	0.66	0.74	0.98
TMOD3	12.73	7.64	7.61	7.26	8.71	8.01
TMOD4	0	0.18	0.42	0.12	0.32	0.23
TMPO	49.35	59.23	54.87	54.96	54.97	60.85
TMPO-AS1	0.59	1.19	0.82	1.36	0.65	0.61
TMPPE	3.62	4.22	3.67	3.16	3.32	3.43
TMPRSS11A	0	0	0	0	0.06	0
TMPRSS11B	0.09	0.09	0.19	0.13	0.17	0.12
TMPRSS11BI	0.07	0.03	0.1	0.03	0	0.02
TMPRSS11D	0	0	0	0	0	0
TMPRSS11E	0	0	0	0	0	0
TMPRSS11F	0	0	0	0	0	0
TMPRSS11GI	0	0	0	0	0	0
TMPRSS12	0	0	0	0	0	0
TMPRSS13	0	0	0	0	0	0
TMPRSS15	0	0	0	0	0	0
TMPRSS2	0	0	0	0	0	0
TMPRSS3	0.03	0.03	0.11	0.08	0	0.13
TMPRSS4	0.04	0.26	0.04	0.13	0	0
TMPRSS4-AS	0	0	0	0	0	0
TMPRSS5	0	0.05	0	0	0	0
TMPRSS6	0	0	0.04	0	0	0

TMPRSS7	0	0.02	0	0.02	0	0.03
TMPRSS9	0	0.04	0	0	0.04	0.05
TMSB10	1366.09	736.21	714.68	763.14	1015.78	801.87
TMSB15A	35.95	49.68	45.12	70.81	72.04	67.28
TMSB15B	0	0.1	0	1.17	0.46	0.37
TMSB4X	1010.6	1471.41	1104.97	836.37	1107.39	976.8
TMSB4Y	0.04	0	0.04	0	0	0
TMTC1	0.01	0.04	0.04	0.01	0.01	0.1
TMTC2	0.1	0.15	0.01	0	0.18	0
TMTC3	1.23	1.04	1.32	1.26	1.12	1.12
TMTC4	2.26	3.54	1.89	2.42	2.36	3.18
TMUB1	13.74	17.28	16.98	14.86	14.86	14.62
TMUB2	12.23	9.71	9.42	7.73	10.11	6.24
TMX1	29.24	39.06	32.39	23.43	27.52	34.84
TMX2	119.54	122.29	125	110.1	115.55	102.59
TMX2-CTNNI	0.15	0.46	0	0.24	0	0
TMX3	7.58	5.31	4.46	3.87	6.02	4.9
TMX4	7.23	12.2	8.84	6.17	8	6.91
TNC	0.02	0.02	0.03	0.06	0.03	0.03
TNF	0.13	0	0	0	0.08	0.27
TNFAIP1	8.59	5.19	5.4	5.38	5.62	6.78
TNFAIP2	1.7	2.07	1.82	0.89	1.7	1
TNFAIP3	0.49	0.31	0.34	0.22	0.69	0.48
TNFAIP6	0	0	0	0	0	0
TNFAIP8	12.86	7.06	5.26	5.48	9.61	5.43
TNFAIP8L1	9.16	9.14	8.98	9.3	9.75	8.74
TNFAIP8L2	12.83	29.78	30.83	33.04	25.76	33.78
TNFAIP8L2-S	4.73	5.53	7.29	5.16	4.5	5.34
TNFAIP8L3	1.51	0.35	0.57	0.79	0.53	0.76
TNFRSF10A	13.94	9.46	8.61	11.24	14.01	7.57
TNFRSF10B	18.93	17.51	15.77	13.09	16.61	21.2
TNFRSF10C	0.6	1.01	0.1	0.37	1.32	0.05
TNFRSF10D	4.33	3.05	0.41	0.85	1.74	2.58
TNFRSF11A	0.84	0.7	0.37	0.8	0.46	1.17
TNFRSF11B	0	0	0	0	0	0
TNFRSF12A	20.46	3.46	3.61	5.65	7.12	3.91
TNFRSF13B	0	0	0.1	0.04	0.01	0
TNFRSF13C	0	0	0	0	0.31	0
TNFRSF14	3.6	0.79	2.91	4.44	4.78	1.46
TNFRSF17	0	0	0	0	0	0
TNFRSF18	0	0	0	0	0	0
TNFRSF19	0	0.05	0	0.01	0	0
TNFRSF1A	18.68	56.55	51.26	46.06	32.74	53.75

TNFRSF1B	5.33	13.43	15.91	12.56	8.54	10.57
TNFRSF21	6.09	4.09	5.22	6.23	6.34	5.75
TNFRSF25	0.44	0.29	0.26	0.13	0.25	0
TNFRSF4	0.62	0.49	0	0	0.66	0
TNFRSF6B	0	0.16	0	0	0	0.18
TNFRSF8	2.24	0.29	7.3	4.33	4.84	6.63
TNFRSF9	0.41	0.36	0.52	0.35	0.42	0.37
TNFSF10	0.91	4.34	1.59	1.2	2.9	3.98
TNFSF11	0	0	0	0.02	0	0.03
TNFSF12	0.49	0.43	0.7	0.3	0.31	0.27
TNFSF12-TN	0	0	0	0.1	0	0
TNFSF13	4.31	5.1	8.92	3.57	5.73	5.62
TNFSF13B	0.36	2.56	1.16	0.04	1.03	2.85
TNFSF14	0.91	2.23	3.05	1.06	1.48	2.75
TNFSF15	0.1	0.08	0.15	0.08	0.09	0.18
TNFSF18	0	0	0	0	0	0
TNFSF4	0	0.2	0.19	0.07	0.1	0.17
TNFSF8	0	0	0	0	0	0
TNFSF9	4.7	2.26	3.22	2.77	4.44	4.61
TNIK	4.7	1.9	1.56	2.82	2.7	2.31
TNIP1	6.36	5.84	6.12	4.4	5.09	6.78
TNIP2	11.08	7.66	9.33	6.72	7.13	7.67
TNIP3	0.11	0.2	0.06	0.1	0.03	0.22
TNK1	0.03	0	0.02	0	0	0
TNK2	0.33	0.61	0.47	0.31	0.2	0.31
TNKS	3.14	2.42	3.01	2.82	2.44	2.48
TNKS1BP1	1.88	1.62	2.9	2.34	1.86	2.02
TNKS2	2.99	3.05	2.69	1.35	2.44	2.58
TNMD	0	0	0	0	0	0
TNN	0	0	0	0	0	0
TNNC1	0	0	0	0.08	0.33	0
TNNC2	0	0	0	0	0	0
TNNI1	0.1	0.14	0.16	0.06	0.18	0.07
TNNI2	0	0	0.21	0	0	0
TNNI3	2.23	0	0.09	1.03	2.03	0.47
TNNI3K	0	0	0	0	0	0
TNNT1	13.11	7.84	24.96	20.3	39.58	23.82
TNNT2	0	0	0	0	0	0
TNNT3	0	0	0	0.18	0	0.07
TNP1	0	0	0	0	0	0
TNP2	0	0	0	0	0	0
TNPO1	13.06	11.92	12.2	9.94	11.8	13.13
TNPO2	18	13.96	12.47	10.92	14.55	18.1

TNPO3	17.49	24.4	24.69	24.01	20.88	21.59
TNR	0	0	0	0	0	0
TNRC18	1.2	1.53	1.65	1.14	0.98	1.85
TNRC18P1	0.08	0	0.14	0.08	0.04	0.08
TNRC6A	2.7	1.87	2.17	1.98	1.96	2.19
TNRC6B	4.16	2.42	3.45	2.9	3.62	2.81
TNRC6C	1.54	1.8	1.38	1.19	1.81	1.28
TNS1	0.24	0.73	1.41	0.44	0.65	1.12
TNS3	0.09	0	0	0.03	0.05	0
TNS4	0.12	0.43	0.17	0.73	0.3	0.23
TNXA	0	0	0	0	0	0
TNXB	0	0	0	0.01	0	0
TOB1	22.25	16.65	11.15	11.72	14.67	11.72
TOB1-AS1	0.11	0.25	0.1	0.04	0.4	0.27
TOB2	5.27	3.67	5.09	5.26	5.43	5.91
TOB2P1	0.93	0.13	0.13	0.07	0.29	0.15
TOE1	10.81	12.93	11.34	13.51	13.43	10.06
TOLLIP	17.09	9.38	9.64	13.19	12.78	8.61
TOM1	26.17	15.78	14.37	15.13	14.26	11.3
TOM1L1	12.65	8	5.03	5.6	8.11	6.2
TOM1L2	2.5	2	1.34	2.27	0.92	2.25
TOMM20	86.46	82.21	79.21	93.36	89.95	97.49
TOMM20L	0.53	0.21	0.49	0.09	0.25	0
TOMM22	169.85	175.02	180.65	169.96	175.16	152.38
TOMM34	52.31	50.61	44.55	59.21	52.09	59.07
TOMM40	21.12	24.66	24.32	22.92	23.92	18.98
TOMM40L	3.17	3.68	4.33	2.61	3.79	3.87
TOMM5	171.78	191.74	172.27	157.54	206.41	161.05
TOMM6	270.63	269.25	247.47	249.5	276.16	253.94
TOMM7	260.48	255.1	221.95	228.69	271.85	198.9
TOMM70A	7.47	4.66	4.6	4.3	4.46	4.02
TONSL	7.16	14.49	11.94	12.16	12.85	11.22
TOP1	24.95	13.24	13.66	15.82	20.2	15.24
TOP1MT	114.44	227.07	267.5	220.21	182.12	253.59
TOP1P1	0	0	0	0	0	0
TOP1P2	0	0	0.13	0	0	0
TOP2A	52.92	55.14	46.19	47.82	49.77	53.17
TOP2B	6.95	7.63	6.21	6.38	7.7	10.05
TOP3A	11.77	12.48	12.04	14.97	13.26	16.92
TOP3B	4.81	6.11	3.84	3.74	5.68	6.64
TOPAZ1	0	0	0	0	0	0
TOPBP1	4.52	5.43	5.27	5.11	6.11	4.16
TOPORS	10.61	8.4	9.65	9.98	11.88	10.34

TOR1A	35.08	30.12	21.98	23.93	25.72	25.79
TOR1AIP1	10.97	10.07	10.73	8.84	11.73	8.76
TOR1AIP2	23.48	18.26	19.51	20.63	21.57	15.76
TOR1B	3.11	5.36	5.58	4.1	5.56	4.7
TOR2A	12.62	31.22	22.96	21.4	28.6	25.93
TOR3A	39.69	49.26	42.86	44.18	37.51	29.59
TOR4A	1	1.07	1.41	1.23	1.92	1.32
TOX	1.31	4.4	4.62	3.36	4.43	1.45
TOX2	0	0	0	0	0	0
TOX3	0	0	0	0	0	0
TOX4	20.4	16.67	16.86	14.88	14.4	15.04
TP53	45.1	54.67	55.95	70.7	60.83	78.64
TP53AIP1	0	0	0.13	0	0.18	0
TP53BP1	7.69	7.77	5.55	6.59	7.18	9.04
TP53BP2	3	4.59	3.01	2.51	3.03	3.81
TP53I11	4.37	7.88	7.04	7.59	7.17	6.42
TP53I13	12.6	25.56	20.51	23.6	24.32	16.76
TP53I3	22.78	9.41	5.58	12.64	12.58	6.53
TP53INP1	2.82	0.41	0.36	0.41	1.23	0.19
TP53INP2	2.35	0.79	0.93	1.3	1.13	0.36
TP53RK	20.07	16.28	19.41	17.25	19.78	14.08
TP53TG1	0	0	0	0	0	0
TP53TG3	0	0	0	0	0	0
TP53TG3B	0	0	0	0	0	0
TP53TG3C	0	0.1	0	0	0	0
TP53TG3D	0	0	0	0	0	0
TP53TG5	0	0	0	0	0	0
TP63	0	0	0	0	0	0
TP73	0.33	0.13	0.01	0.56	0.22	0.05
TP73-AS1	1.63	0.95	1.41	1.37	0.99	0.92
TPBG	3.18	2.33	2.25	2.16	3.69	3.05
TPBGL	0	0	0	0	0.05	0
TPCN1	6.47	4.46	5.54	3.98	5.83	4.71
TPCN2	1.88	1.98	1.63	1.49	2.06	1.53
TPD52	16.97	17.82	16.02	14.14	17.88	18.4
TPD52L1	5.7	3.66	3.97	5.2	9.34	5.41
TPD52L2	61.87	49.13	48.44	42.34	45.61	46.43
TPD52L3	0	0	0	0	0	0
TPGS1	0.62	0.7	1.44	1.7	0.71	0.21
TPGS2	49.29	54.1	50.2	53.21	55.44	52.38
TPH1	0.04	0.06	0	0	0	0
TPH2	0	0	0	0	0	0
TPI1	1252.82	1164.63	1094.3	1263.61	1223.84	1098.08

TPI1P2	0.07	0.23	0.67	0.51	0.66	0
TPI1P3	0.23	0.26	0.24	0.13	0.43	0.2
TPK1	1.02	4.61	4.43	1.37	2.16	3.7
TPM1	18.89	4.98	7.61	9.27	11.69	4.02
TPM2	0.06	0	0.06	0.13	0	0.06
TPM3	175.77	188.93	179.7	147.79	153.72	166.56
TPM3P9	5.36	7.43	7.33	6.32	7.38	6.82
TPM4	55.16	45.55	41.78	33.85	39.46	26.64
TPMT	8.86	7.18	6.17	6.47	7.45	5.94
TPO	0	0	0	0	0	0
TPP1	81.39	62.05	47.51	57.21	63.35	50.32
TPP2	11.9	14.24	13.09	12.34	12.72	14.28
TPPP	0.17	0.04	0.02	0.02	0.03	0.21
TPPP2	0	0	0	0	0	0
TPPP3	0	0	0	0	0	0
TPR	9.68	9.67	8.57	8.18	8.5	7.57
TPRA1	13.05	11.62	11.76	8.57	10.48	9.05
TPRG1	0	0	0	0	0	0
TPRG1-AS2	0.18	0	0	0	0	0.01
TPRG1L	4.71	2.1	2.19	1.93	2.78	2.09
TPRKB	41.27	31.35	35.24	26.91	36.09	33.46
TPRN	0.86	1.73	0.92	1.03	0.94	1.44
TPRX1	0	0	0	0	0	0
TPRXL	0	0.05	0	0	0.02	0
TPSAB1	1031.37	224.89	90.54	223.3	787.09	71.87
TPSB2	1871.03	433.22	169.66	402.93	1385.74	135.79
TPSD1	841.31	193.38	70.73	177.53	639.91	50.97
TPSG1	0.55	0.18	0	0	0	0
TPST1	3.89	3.18	3.39	2.8	2.99	2.33
TPST2	76.44	22.24	38.84	41.05	64.73	29.94
TPT1	2105.19	1926.7	2122.47	2297.25	2206.05	2456.59
TPT1-AS1	1.7	1.45	1.03	1.9	0.75	0.71
TPTE	0.04	0	0	0.12	0.1	0
TPTE2	0.08	0.06	0	0.05	0	0
TPTE2P1	0.15	0.25	0.39	0.2	0.36	0.3
TPTE2P3	0.03	0.26	0.08	0.06	0.03	0.23
TPTE2P5	0.14	0.27	0.32	0.09	0.13	0.19
TPTE2P6	0	0	0	0	0.03	0.02
TPTEP1	7.77	9.13	8.1	9.76	7.81	5.9
TPX2	66.36	65.05	63.83	65.26	66.87	58.52
TRA2A	49.89	52.74	39.12	42.05	56.89	52.48
TRA2B	38.02	47.06	42.55	38.89	42.03	41.7
TRABD	40.92	40.23	35.74	38.89	33.85	39.24

TRABD2A	4.5	5.26	4.19	3.76	3.67	2.21
TRABD2B	0	0	0	0	0	0
TRADD	7.79	8.41	7.73	7.53	6.07	7.41
TRAF1	0.04	0.05	0.17	0.2	0.22	0.02
TRAF2	15.6	17.07	17.71	14.88	16.51	18.59
TRAF3	1.96	2.63	2.99	2.02	1.66	2.49
TRAF3IP1	1.87	2.28	2.51	1.51	2.5	1.68
TRAF3IP2	2.07	4.53	4.54	3.72	3.84	4.65
TRAF3IP2-AS	2.5	4.1	3.23	3.36	2.23	2.69
TRAF3IP3	0.13	1.78	1.44	1.69	1.7	2.18
TRAF4	4.43	2.87	3.8	4.34	3.84	3.66
TRAF5	1.99	5.01	4.45	3.28	3.49	4.01
TRAF6	3.77	3.48	3.75	3.11	3.21	3.4
TRAF7	17.58	19.46	18.41	18.24	18.88	14.81
TRAFD1	23.99	20.23	14.81	20.62	19.67	16.86
TRAIP	7.99	9.44	11.16	13.81	9.96	12.14
TRAK1	3.27	3.35	3.45	2.43	2.35	3.22
TRAK2	8.58	8.44	6.39	5.16	5.81	7.96
TRAM1	43.18	60.13	60.49	39.71	44.14	37.3
TRAM1L1	0.18	0	0.03	0.45	0	0.18
TRAM2	3.34	4.55	3.05	3.16	3.36	3.55
TRANK1	2.16	2.19	2.64	2.97	2.91	2.77
TRAP1	158.26	151.5	161.04	145.88	142.38	157.43
TRAPPC1	51.35	43.17	42.1	49.46	46.77	48.55
TRAPPC10	2.35	1.22	1.49	1.03	1.58	1.25
TRAPPC11	7.6	6.77	5.19	4.67	4.94	6.07
TRAPPC12	2.73	2.56	1.81	2.32	2.88	2.17
TRAPPC13	6.93	5.56	4.58	4.71	5.03	5.31
TRAPPC2	24.79	26.44	20.86	16.85	21.92	22.4
TRAPPC2L	179.1	215.16	196.67	219.36	215.49	182.81
TRAPPC2P1	16.3	14.52	7.06	9.59	11.89	9.23
TRAPPC3	50.95	45.56	36.47	35.59	51.4	39.23
TRAPPC4	62.09	45.42	44.37	43.67	52.58	40.19
TRAPPC5	211.15	155.25	136.58	177.53	184.26	138.43
TRAPPC6A	25.34	27.51	23.19	30.45	25.39	29.06
TRAPPC6B	7.13	4.72	3.39	4.88	4.37	3.48
TRAPPC8	1.3	2.22	2.01	1.22	1.49	1.59
TRAPPC9	6.86	9.44	11.92	10.07	7.45	12.7
TRAT1	0	0	0	0	0	0
TRDMT1	1.16	1.23	0.85	1.15	1.53	1.23
TRDN	1.55	0	0	0.08	0.09	0
TREH	0	0	0	0	0	0
TREM1	0	0	0	0	0	0

TREM2	0	0	0.08	0	0	0
TREML1	0	0	0	0	0	0
TREML2	0.02	0.03	0.04	0	0.09	0.06
TREML3P	0	0	0	0	0	0
TREML4	0	0	0	0	0	0
TREML5P	0	0	0	0	0	0
TRERF1	0.88	2.3	1.6	1.31	1.95	3.8
TREX1	0.86	0.53	0.13	0.42	0.81	0.29
TREX2	0.18	0.48	1.21	0.17	0.06	0.49
TRH	0	0	0.2	0	0.03	0
TRHDE	0	0	0	0	0	0
TRHDE-AS1	0	0	0	0	0	0
TRHR	0	0	0	0	0	0
TRIAP1	59.68	66.59	59.67	47.4	64.51	52.25
TRIB1	3.64	1.15	0.97	1	1.28	0.57
TRIB2	4.17	1.14	0.78	1.19	1.98	1.13
TRIB3	19.41	24.26	26.45	22.19	22.04	18.19
TRIL	0	0	0	0	0	0.03
TRIM10	0	0	0	0	0.11	0
TRIM11	5.98	7.38	6.57	5.9	4.38	4.15
TRIM13	5.34	6.76	6.01	5.33	5.02	5.87
TRIM14	12.26	15.15	11.92	10.74	14.51	14.78
TRIM15	0.26	0.16	0	0.05	0.16	0.2
TRIM16	4.49	2.85	2.93	1.97	2.1	1.42
TRIM16L	4.48	3.18	5.3	3.34	2.34	2.95
TRIM17	0.07	0	0.2	0	0.03	0.15
TRIM2	0.2	0.15	0.13	0.18	0	0.06
TRIM21	21.02	13.27	13.8	16.48	13.29	12.26
TRIM22	1.37	5.38	5.78	3.97	2.08	3.87
TRIM23	1.84	1.9	1.87	1.4	1.42	1.74
TRIM24	13.46	5.79	5.44	9.59	10.52	7.69
TRIM25	15.07	15	14.64	14.28	13.34	15.68
TRIM26	4.64	8.44	7.73	7.2	6.91	8.96
TRIM27	101.39	65.49	64.31	67.83	68.83	57.02
TRIM28	34.43	30	31.23	39.83	32.9	30.6
TRIM29	0	0	0	0	0	0
TRIM3	1.49	0.66	1.53	0.61	1.22	0.62
TRIM31	0	0	0	0	0	0
TRIM32	10.01	10.04	9.11	9.64	11.81	10.91
TRIM33	2.33	2.15	1.62	1.46	2	2.07
TRIM34	0.35	0.65	0.84	1.75	0.48	0.79
TRIM35	12.49	11.41	9.5	8.82	8.71	12.39
TRIM36	0.64	2.74	2.12	1	1.53	1.34

TRIM37	7.43	5.41	4.03	5.26	6.27	5.43
TRIM38	2.77	2.72	1.82	1.33	2.31	2.05
TRIM39	0.9	1.12	1.27	0.48	0.88	0.57
TRIM39-RPP2	0.15	0.6	0.65	0	0.95	0.58
TRIM4	10.64	7.86	9.24	6.87	7.81	11.66
TRIM40	0	0	0	0	0	0
TRIM41	1.44	1.26	2.07	1.1	0.84	1.24
TRIM42	0	0	0	0	0	0
TRIM43	0	0	0.04	0	0	0
TRIM43B	0	0	0	0	0	0
TRIM44	3.7	2.01	2.55	2.19	2.61	3.05
TRIM45	1.05	1	1.44	1.13	1.2	0.82
TRIM46	0	0	0.02	0.03	0	0.05
TRIM47	3.31	8.11	12.23	7.35	7.51	8.2
TRIM48	0.05	0	0	0	0	0
TRIM49	0	0	0	0	0	0
TRIM49B	0	0	0	0	0	0
TRIM49C	0	0	0	0	0	0
TRIM49D1P	0	0	0	0	0	0
TRIM49D2P	0	0	0	0	0	0
TRIM5	13.47	9.43	8.09	8.74	11.75	11.13
TRIM50	0	0	0	0	0	0
TRIM51	0	0	0	0	0	0
TRIM51HP	0	0	0	0	0	0
TRIM52	1.98	1.4	1.16	1.23	1.25	1.37
TRIM53AP	0	0	0	0	0	0
TRIM54	0	0.03	0.11	0	0	0
TRIM55	0	0	0	0	0	0
TRIM56	2.98	2.79	3.6	2.57	2.16	2.69
TRIM58	4.14	2.64	3.21	2.71	3.37	2.86
TRIM59	0.23	0.22	0.46	0.39	0.53	0.43
TRIM6	4.67	5.85	7.11	3.77	4.8	4.56
TRIM6-TRIM	0.38	1.5	1.28	0.37	1.34	2.39
TRIM60	0	0	0	0	0	0
TRIM61	0.47	0.63	0.22	0.26	0.36	0.57
TRIM62	0.87	0.59	0.57	0.49	0.44	0.78
TRIM63	0.76	0	0	0.32	0.48	0
TRIM64	0	0	0	0	0	0
TRIM64B	0	0	0	0	0	0
TRIM64C	0	0	0	0	0	0
TRIM65	1.99	3.14	3.98	3.1	2.73	2.35
TRIM66	0.6	0.49	0.33	0.32	0.48	0.33
TRIM67	0.41	0	0	0.02	0.12	0

TRIM68	3.7	4	3.2	3.53	4.13	3.12
TRIM69	0.16	0	0	0	0	0
TRIM7	6.84	6.2	5.97	5.92	7.96	2.9
TRIM71	0.02	0.37	0.59	0.02	0.04	0.35
TRIM72	0.09	0.12	0.21	0.18	0.22	0.26
TRIM73	0.03	0	0.03	0	0.16	0
TRIM74	0.03	0	0.03	0	0.16	0
TRIM77	0	0	0	0	0	0
TRIM8	4.79	2.89	2.54	2.89	2.06	1.16
TRIM9	0	2.34	0.9	0.08	0.35	0.37
TRIML1	0	0	0	0	0	0
TRIML2	0	0	0	0	0	0
TRIO	0.64	0.96	0.68	0.62	0.71	1.03
TRIOBP	2.58	1.86	1.74	1.2	1.73	1.74
TRIP10	14.33	6.6	4.1	8.24	8.01	5.31
TRIP11	3.31	2.64	2.5	1.93	2.72	2.89
TRIP12	12.11	7.63	8.96	8.86	11.1	9.99
TRIP13	42.25	39.56	42	38.19	38.07	32.24
TRIP4	18.63	17.37	14.44	16.37	17.48	11.94
TRIP6	7.93	6.58	8.46	10.05	10.86	7.29
TRIQK	2.82	1.11	2.1	1.57	2.79	1.44
TRIT1	13.16	15.12	12.37	10.56	14.19	13.6
TRMT1	41.35	39.35	41.58	39.26	36.17	35.04
TRMT10A	1.18	1.33	1.03	0.8	0.59	1.21
TRMT10B	1.79	1.93	2.08	1.41	2.07	1.77
TRMT10C	54.17	40.65	28.25	33.73	50.28	35.07
TRMT11	6.33	8.6	7.63	5.48	6.23	8.21
TRMT112	369.4	337.37	308.38	354.27	406.52	324.45
TRMT12	16.58	6.61	7.17	9.38	10.89	8.66
TRMT13	2.76	2.53	3.24	2.66	3.74	3.91
TRMT1L	4.89	4.53	3.72	3.19	4.31	4.52
TRMT2A	21.78	23.13	29.41	20.74	22.46	21.32
TRMT2B	16.51	16.69	15.54	15.48	13.69	17.87
TRMT44	1.44	1.15	0.94	1.02	0.85	0.75
TRMT5	7.09	3.64	5.91	7.05	6.3	6.29
TRMT6	10.27	9.27	7.13	7.68	8.1	8.72
TRMT61A	24	23.65	21.25	21.06	25	17.53
TRMT61B	11.02	13.53	14.8	14.07	11.58	13.89
TRMU	24.71	21.9	20.2	17.39	16.96	19.08
TRNAU1AP	15.46	14.08	9.88	11.94	14.19	12.71
TRNP1	0	0.3	0.1	0.1	0.22	0.38
TRNT1	9.89	8.6	7.72	5.78	9.29	7.97
TRO	0	0	0	0	0	0

TROAP	29.29	28.34	24	24.19	26.22	22.3
TROVE2	26.78	25.03	24.63	28.75	25.41	21
TRPA1	0	0	0	0	0	0
TRPC1	0.2	0.45	0.21	0.18	0.4	0.05
TRPC2	0.06	0.59	0.4	0.56	0.24	1.01
TRPC3	0	0	0	0	0	0
TRPC4	0	0	0	0	0	0
TRPC4AP	16.5	20	21.92	23.32	19.08	14.01
TRPC5	0	0	0.07	0.03	0	0
TRPC5OS	0	0	0	0	0.08	0
TRPC6	0	0	0	0	0.01	0
TRPC7	0	0	0	0	0	0
TRPM1	0.08	0.09	0.03	0	0.05	0.16
TRPM2	0.07	1.83	0.7	0.2	0.33	0.37
TRPM3	0	0	0	0	0	0
TRPM4	0.74	0.36	0.31	0.75	0.42	0.74
TRPM5	0	0	0	0.01	0	0
TRPM6	0.36	0.13	0.13	0.07	0.04	0.14
TRPM7	4.51	4.47	4.15	3.53	3.95	4.42
TRPM8	0.04	0.02	0.03	0.05	0.06	0.08
TRPS1	0	0	0.01	0	0	0
TRPT1	19.14	26.34	22.34	25.81	22.13	30.99
TRPV1	1.11	1.4	1.33	0.84	1.06	1.04
TRPV2	52.76	12.53	22.02	19.97	18.38	9.54
TRPV3	0.11	0.02	0	0	0.04	0.01
TRPV4	0.05	0.49	0.07	0.28	0.37	0.1
TRPV5	0	0	0	0	0	0.15
TRPV6	0	0	0.02	0.05	0	0
TRRAP	3.69	3.44	3.79	3.11	3.94	3.53
TRUB1	5.64	4.41	4.5	3.12	4.18	2.67
TRUB2	68.4	74.18	73.91	76.82	70.13	74.58
TSACC	0.95	0.75	1.26	1.02	0.3	0.96
TSC1	4.28	3.02	3.68	3.02	3.61	2.47
TSC2	7.5	6.35	7.13	7.93	6.03	7.38
TSC22D1	67.15	5.74	5.37	16.57	26.95	5.48
TSC22D1-AS:	0.03	0.12	0.15	0.02	0.02	0.12
TSC22D2	10.56	5.09	4.93	7.74	8.79	4.15
TSC22D3	6.61	10.02	8.59	6.87	9.34	11.95
TSC22D4	3.57	3.49	5.22	3.64	4.54	3.96
TSEN15	33.57	36.5	28.08	26.44	31.26	29.46
TSEN2	7.78	9.7	8.22	7.21	5.85	6
TSEN34	6.74	5.17	4.25	6.05	4.14	2.92
TSEN54	11.16	15.04	15.97	20.46	14.29	13.07

TSFM	34.6	35.14	27.28	26.28	37.3	27.07
TSG1	0.53	0.88	0.43	0.27	0.53	0.63
TSG101	58.34	26.3	18.08	30.82	40.81	22.71
TSGA10	0.18	0.27	0.2	0.03	0.17	0.2
TSGA10IP	0	0	0	0.06	0	0
TSGA13	0	0	0	0	0	0
TSHB	0	0	0	0	0	0
TSHR	0	0	0.02	0	0	0
TSHZ1	0.55	0.89	0.86	1.27	0.84	1.08
TSHZ2	0.1	0.06	0.1	0.06	0.07	0.02
TSHZ3	0.07	0.27	0.16	0.03	0.08	0.07
TSIX	0.3	0.24	0.33	0.23	0.3	0.28
TSKS	0	0	0	0	0	0
TSKU	11.46	5.8	8.4	7.31	8.47	10.21
TSLP	1.13	0.77	0.67	0.21	0.98	1.08
TSN	46.98	44.57	39.85	38.8	41.83	43.97
TSNARE1	8.4	9.82	6.87	6.29	5.06	9.12
TSNAX	13.2	9.78	8.46	8.94	13.22	8.98
TSNAX-DISC1	0	0.09	0	0	0	0.01
TSNAXIP1	0	0.14	0	0.06	0.25	0
TSPAN1	0	0.18	0	0	0.09	0
TSPAN10	0	0.07	0.04	0.16	0.04	0
TSPAN11	0	0	0	0	0	0
TSPAN12	0.03	0	0.08	0.02	0.11	0
TSPAN13	19.62	16.19	14.07	13.94	15.77	17.44
TSPAN14	10.85	16.37	15.97	13.37	11.47	19.86
TSPAN15	2.93	22.47	33.2	28.42	14.53	17.61
TSPAN16	0	0	0	0	0	0
TSPAN17	4.66	2.57	3.56	4.64	5.12	3.71
TSPAN18	0.24	0.16	0.55	0.69	0.21	0.47
TSPAN19	0	0	0	0	0	0
TSPAN2	0.18	0.2	0.31	0.4	0.11	0.11
TSPAN3	56.08	67.98	62.54	65.3	55.01	62.16
TSPAN31	6.56	4.25	4.5	3.65	4.88	3.57
TSPAN32	3.99	2.5	3.5	2.04	2.84	1.44
TSPAN33	0.39	0.64	0.89	0.85	0.82	1.33
TSPAN4	40.73	45.53	40.85	34.96	36.91	37.63
TSPAN5	3.08	1.87	1.72	2.26	2.78	1.8
TSPAN6	0	0.2	0	0	0	0
TSPAN7	1.73	0.26	0	5.69	4.15	9.85
TSPAN8	1.01	0	0.56	1.4	3.77	0
TSPAN9	0.58	0.03	0.2	0.24	0.44	0.29
TSPEAR	0	0	0	0	0	0

TSPO	346.77	332.19	321.31	329.79	335.49	346.39
TSPO2	0	0	0	0	0	0
TSPY1	0	0	0	0	0	0
TSPY2	0	0	0	0	0	0
TSPY26P	0	0	0	0.04	0.06	0.13
TSPY3	0	0	0	0	0	0
TSPY4	0	0	0	0	0	0
TSPY8	0	0	0	0	0	0
TSPYL1	18.67	11.23	11.03	10.38	14.9	13.01
TSPYL2	9	3.21	3.97	3.74	5.85	4.21
TSPYL4	3.54	3.79	2.08	3.53	3.62	2.06
TSPYL5	0	0	0	0	0	0
TSPYL6	0	0	0	0	0	0
TSR1	24.21	25.29	24.01	26.18	22.99	24.94
TSR2	108.99	94.41	90.02	92.94	110.99	86.26
TSR3	11.84	15.13	15.35	13.24	11.26	10.64
TSSC1	26.23	30.4	23.38	28.45	24.86	25.87
TSSC4	41.17	41.16	36.01	37.9	33.56	22.82
TSSK1B	0	0	0	0	0	0
TSSK2	0	0	0	0	0	0
TSSK3	0.92	0.18	0.47	0.47	0.11	0.23
TSSK4	0	0.32	0.1	0.28	0.28	0.35
TSSK6	0.47	0.53	0.71	0.14	0	1.32
TST	23.65	21.76	18.77	18.62	17.74	21.79
TSTA3	133.33	107.18	100.1	118.45	118.48	98.63
TSTD1	0.12	0.29	0	0	0	0
TSTD2	3.11	2.97	4.37	3.91	2.71	5.12
TSTD3	3.34	2.4	2.49	1.81	3.68	2.58
TTBK1	0	0	0	0	0	0
TTBK2	0.14	0.25	0.53	0.33	0.25	0.26
TTC1	56.9	52.05	45.08	40.99	54.66	41.64
TTC12	0	0	0	0	0	0
TTC13	2.17	1.89	1.55	1.09	2.15	1.4
TTC14	5.83	3.74	4.6	3.72	4.98	2.92
TTC16	0	0	0	0	0	0
TTC17	5.96	8.31	5.75	5.9	5.49	6.45
TTC18	0.24	0.49	0.11	0.08	0.14	0.43
TTC19	3.58	3.47	2.85	2.18	3.4	3.84
TTC21A	0.07	0	0.03	0.06	0.02	0
TTC21B	2.3	2.32	1.81	1.52	1.18	2.49
TTC22	0	0.03	0.06	0	0	0.03
TTC23	0.09	0.1	0.08	0.05	0.09	0.05
TTC23L	0.11	0	0	0	0	0

TTC24	0	0	0	0	0	0
TTC25	0	0.28	0	0.05	0.03	0
TTC26	3.84	3.29	2.76	3.51	2.91	2.91
TTC27	15.25	13.71	17.24	13.63	12.98	13.81
TTC28	1.08	0.17	0.38	0.38	0.62	0.12
TTC28-AS1	7.9	5.42	3.17	2.83	4.57	2.03
TTC29	0	0	0.15	0	0.08	0.25
TTC3	8.77	13	13.39	15.59	11.35	14
TTC30A	0.25	0.4	0.15	0.57	0.08	0
TTC30B	1.22	0.33	0.55	0.69	0.25	0.34
TTC31	8.68	7.92	9.37	10.18	9.55	10.15
TTC32	8.52	7.53	3.85	4.18	8.91	5.32
TTC33	1.45	1.55	2.06	1.34	1.76	1.48
TTC34	0	0	0	0	0	0
TTC36	0	0	0.11	0	0	0
TTC37	7.23	5.57	6.18	5.31	6.21	6.03
TTC38	12.37	7.16	8.93	8.06	9.92	7.55
TTC39A	0.35	0.68	0.25	0.78	0.38	0.87
TTC39B	0.32	0.07	0.23	0.43	0.45	0.07
TTC39C	3.4	5.5	3.73	3.08	4.17	5.23
TTC3P1	0.27	0.54	0.58	0.36	0.51	0.78
TTC4	17.02	24.65	21.05	24.26	21.19	18.7
TTC40	0	0	0	0	0	0
TTC5	21.42	21.26	17.68	15.89	17.64	15.63
TTC7A	1.29	4.73	2.26	2.03	1.06	2.86
TTC7B	2.33	1.47	1.03	1.5	1.28	1.78
TTC8	6.12	3.84	1.49	1.63	3.99	2.25
TTC9	0.03	0.16	0.06	0.11	0.18	0.1
TTC9B	0.09	0	0	0	0	0
TTC9C	12.71	8.55	10.84	11.08	12.71	9.2
TTF1	4.26	4.47	3.05	4.25	4.87	3.72
TTF2	11.01	14.65	11.08	10.53	11.65	14.34
TTI1	11.44	10.62	13.32	13.1	10.54	15.38
TTI2	23.83	27.18	30.87	25.38	28.91	28.86
TTK	13.44	14.45	14.74	14.8	15.1	16.68
TTL	6.19	2.94	3.69	4.08	4.05	4.26
TTLL1	3.58	5.24	6.39	4.12	3.96	4.73
TTLL10	0	0	0	0	0	0
TTLL11	0.04	0.26	0.03	0.28	0.42	0.2
TTLL12	18.33	23.78	28.74	26.52	24.52	28.18
TTLL13	0.12	0.03	0	0.06	0	0
TTLL2	0	0	0	0	0	0
TTLL3	2.59	3.07	1.46	1.16	1.05	1.36

TTLL4	11.93	15.78	12.21	12.92	12.66	10.89
TTLL5	7.38	6.97	6.93	7.91	6.57	8.03
TTLL6	0	0	0	0	0	0
TTLL7	1.27	0.45	0.27	0.27	0.69	0.16
TTLL9	0.09	0	0	0	0	0.05
TTN	0	0	0	0	0	0
TTN-AS1	0	0	0	0	0	0
TTPA	0.06	0.41	0.91	0.14	0.66	0.79
TTPAL	3.27	2.34	1.93	2.58	2.53	2.56
TTR	0	0	0	0	0	0
TTTY1	0	0	0	0	0	0
TTTY10	0	0	0	0	0	0
TTTY11	0	0	0	0	0	0
TTTY12	0	0	0	0	0	0
TTTY13	0	0	0	0	0	0
TTTY14	0	0	0	0	0	0
TTTY15	0.05	0.12	0.11	0.1	0.11	0.27
TTTY16	0	0	0	0	0	0
TTTY17A	0	0	0	0	0	0
TTTY17B	0	0	0	0	0	0
TTTY17C	0	0	0	0	0	0
TTTY18	0	0	0	0	0	0
TTTY19	0	0	0	0	0	0
TTTY1B	0	0	0	0	0	0
TTTY2	0	0	0	0	0	0
TTTY20	0	0	0	0	0	0
TTTY21	0	0	0	0	0	0
TTTY21B	0	0	0	0	0	0
TTTY22	0	0	0	0	0	0
TTTY23	0	0	0	0	0	0
TTTY23B	0	0	0	0	0	0
TTTY2B	0	0	0	0	0	0
TTTY3	0	0	0	0	0	0
TTTY3B	0	0	0	0	0	0
TTTY4	0	0	0	0	0	0
TTTY4B	0	0	0	0	0	0
TTTY4C	0	0	0	0	0	0
TTTY5	0	0	0	0	0	0
TTTY6	0	0	0	0	0	0
TTTY6B	0	0	0	0	0	0
TTTY7	0	0	0	0	0	0
TTTY7B	0	0	0	0	0	0
TTTY8	0	0	0	0	0	0

TTTY8B	0	0	0	0	0	0
TTTY9A	0	0	0	0	0	0
TTTY9B	0	0	0	0	0	0
TTYH1	0.19	1.31	1.14	2.04	1.54	1.3
TTYH2	7.62	4.77	3.07	3.58	3.96	7.09
TTYH3	3.19	3.25	4.06	2.87	3.37	3.99
TUB	0.97	1.09	1.68	1.86	1.31	2.04
TUBA1A	142.03	52.81	50.32	74.91	111.07	79.04
TUBA1B	1770.25	2267.3	2240.27	2234.29	2212.83	2253.92
TUBA1C	469.33	341.67	306.25	365.28	390.75	364
TUBA3C	0	0	0	0	0.09	0
TUBA3D	0.05	0.11	0.04	0.09	0	0.05
TUBA3E	0	0	0	0	0	0
TUBA3FP	1.28	0.83	0.97	0.66	0.59	0.94
TUBA4A	42.81	64.91	94.82	62.41	48.95	80.14
TUBA4B	0	0.22	0	0	0	0
TUBA8	1.31	1.34	2.45	3.53	1.8	2.15
TUBAL3	1.32	0	0.11	0.15	0.38	0
TUBB	974.63	993.52	954.86	1105.07	1052.64	1026.45
TUBB1	0.62	0.29	0.19	0.47	0.43	0.44
TUBB2A	95.09	26.85	31.32	46.29	62.6	19.49
TUBB2B	7.99	1.66	1.44	4.42	4.65	1.7
TUBB3	17.03	5.22	7.18	6.8	6.8	7.17
TUBB4A	40.38	43.41	53.15	54.09	43.92	44.39
TUBB4B	571.88	559.08	572.65	565.54	629.8	556.54
TUBB6	77.98	78.05	70.82	75.34	82.23	69.93
TUBB8	0.05	0.01	0	0.03	0.05	0
TUBBP5	0	0	0	0	0	0
TUBD1	9.92	7.85	6.94	5.28	7.73	7.3
TUBE1	3.71	4.81	3.49	4.6	3.02	2.69
TUBG1	70.93	57.18	45.11	62.87	66.67	46.09
TUBG2	0.55	0.56	0.6	0.55	0.4	0.37
TUBGCP2	19.91	15.64	18.68	18.27	19.54	18.33
TUBGCP3	8.38	10	12.16	8.64	9.82	7.01
TUBGCP4	10.76	12.71	11.6	12.65	13.65	10.45
TUBGCP5	5.28	5.52	5.54	4.7	6.19	4.81
TUBGCP6	3.57	4.38	3.27	3.89	3.06	5.69
TUFM	315.31	357.14	313.43	331.28	310.03	328.95
TUFT1	6.26	4.12	4.24	3.12	3.81	3.36
TUG1	5.94	4.08	3.49	3.11	4.47	4.5
TULP1	0	0	0	0	0	0
TULP2	0.13	0	0	0.03	0	0.04
TULP3	2.81	3.53	5.24	3.42	4.39	3.36

TULP4	2.77	3.15	3.17	2.86	2.28	2.83
TUSC1	1.16	1.52	1.93	1.82	1.32	1.47
TUSC2	18.42	18.56	19.73	18.72	18.78	18
TUSC3	0.02	0	0	0	0	0
TUSC5	0	0	0	0	0	0
TUT1	18.24	11.38	13.91	18.16	12.56	15.56
TVP23A	0.2	0.11	0.37	0.17	0.13	0.32
TVP23B	19.5	12.67	9.13	7.46	10.28	7.06
TVP23C	4.14	4.08	3.62	2.85	3.84	3.68
TVP23C-CDR	1.82	0.19	0.46	0.68	1.06	0.4
TWF1	8.39	4.34	1.64	2.87	3.7	2.93
TWF2	81.56	83.4	101.15	70.92	63.79	79.04
TWIST1	1.92	0.25	0.33	0.43	0.97	0.36
TWIST2	0	0	0.18	0	0	0
TWISTNB	16.46	14.49	15.12	14.22	16.8	18.05
TWSG1	6.87	6.06	4.79	4.84	5	5.26
TXK	0.3	0.02	0	0.03	0.05	0
TXLNA	36.95	37.29	30.59	34.08	33.18	35.76
TXLNB	0.03	0.19	0.1	0.12	0.1	0.02
TXLNG	13.24	14.45	12.24	14.1	13.92	14
TXLNG2P	0	0.04	0.01	0.02	0	0.02
TXN	619.24	353.39	302.04	382.75	437.45	345.01
TXN2	171.64	142.25	137.32	157.69	160.38	142.46
TXNDC11	2.73	3.6	2.39	2.1	2.32	1.84
TXNDC12	31.56	56.32	49.91	37.82	36.68	51.21
TXNDC15	10.81	8.3	9.83	8.35	11.01	10.46
TXNDC16	3.08	1.72	2.52	1.72	2.33	2.27
TXNDC17	61.53	65.43	59.4	53.84	66.78	57.85
TXNDC2	0.08	0	0	0.13	0.18	0
TXNDC5	11.39	5.14	0	4.88	5.41	1.54
TXNDC8	0	0	0	0	0	0
TXNDC9	31.74	23.16	20.45	16.07	27.22	25.96
TXNIP	13.93	7.54	9.49	8.44	10.2	10.16
TXNL1	108.63	88.11	85.51	103.33	106.81	89.48
TXNL4A	37.16	39.94	40.6	33.21	49.76	42.27
TXNL4B	10.88	12.31	7.78	7.56	9.05	13.9
TXNRD1	28.71	36.06	28.1	23.8	22.48	29.82
TXNRD2	5.86	8.1	7.89	10.45	8.92	4.61
TXNRD3	0.57	1.06	0.85	0.77	1.06	0.99
TXNRD3NB	0	0	0	0	0	0
TYK2	9.08	10.95	8.78	15.09	11.03	12.46
TYMP	0.74	0.86	0.7	1.24	0.7	1.1
TYMS	171.65	271.85	219.86	217.87	232.01	244.28

TYR	0	0	0	0	0	0
TYRO3	4.16	7.09	7.55	7.26	5.62	5.43
TYRO3P	0.81	1.57	0.74	1.43	0.69	0.91
TYROBP	6.63	17.51	14.06	6.16	5.93	11.04
TYRP1	0	0.02	0	0	0	0
TYSND1	2.52	4.19	5.3	3.47	3.16	4.33
TYW1	12.41	11.74	10.79	10.3	8.05	9.45
TYW1B	0.67	1.85	0.79	0.62	0.52	1.28
TYW3	9.73	7.87	8.42	5.89	8.74	7.58
TYW5	1.56	2.23	1.74	1.15	1.26	1.48
U2AF1	204.44	228.67	204.12	205.23	217.07	215.28
U2AF1L4	9.84	5.37	11.37	8.8	9.2	7.53
U2AF2	6.53	6.77	7.08	5.79	6.43	7.65
U2SURP	15.56	17.7	16.39	15.53	16.85	17.76
UACA	0.08	0.07	0.29	0.03	0.01	0.04
UAP1	13.01	22.53	20.64	15.08	15.68	16.96
UAP1L1	2.77	2.5	2.2	1.81	2.94	1.7
UBA1	165.88	198.54	210.35	209.6	198.18	220.07
UBA2	9.88	9.14	11.71	9.64	9.56	9.58
UBA3	26.58	28.47	16.85	20.3	25.27	21.3
UBA5	6.52	9.73	8.41	8.81	9.62	5.55
UBA52	310.27	292.2	301.04	354.13	344.69	326.7
UBA6	4.04	3.22	2.08	2.25	2.61	3.23
UBA7	2.48	3.11	2.62	3.8	1.84	1.13
UBAC1	56.2	20.54	21.46	29.78	36.17	20.31
UBAC2	37.1	31.14	28.54	31.71	39.17	32.72
UBAC2-AS1	0.24	0.9	0.69	1.89	0.92	0.44
UBALD1	2.5	1.3	1.56	1.54	1.41	1.08
UBALD2	1.32	1.26	1.2	2.07	2.04	0.95
UBAP1	18.12	19.26	21.31	19.94	21.48	17.47
UBAP1L	0.27	0.39	0.45	0.04	0.31	0.22
UBAP2	18.42	16.22	15.63	18.27	18.25	15.64
UBAP2L	100.29	89.98	90.74	96.74	93.42	92.81
UBASH3A	0	0	0	0.25	0	0
UBASH3B	0.6	1.36	0.47	0.41	0.35	0.32
UBB	0.4	1.01	0.86	0.12	0.63	0.49
UBC	739.66	613.48	593.28	646.81	678.92	576.58
UBD	0	0	0	0	0	0
UBE2A	88.96	70.32	65.28	63.09	66.82	78.13
UBE2B	24	22.01	21.19	15.62	25.77	16.16
UBE2C	0.44	0.62	0.44	0.76	1.29	0.22
UBE2D1	3.81	6.19	5.88	5.42	5.88	4.15
UBE2D2	17.02	17.8	21.85	20.22	18.81	15.86

UBE2D3	161.8	139.45	137.03	138.41	170.45	128.11
UBE2D4	3.82	3.31	7.34	1.41	1.86	0.22
UBE2DNL	0	0	0	0	0	0
UBE2E1	4.25	2.9	3.35	2.96	4.28	4.76
UBE2E2	17.19	12.27	15.96	14.65	14.79	13.62
UBE2E3	29.77	33.91	30.31	34.67	37.7	32.27
UBE2F	55.09	21.13	26.56	31.35	38.47	22.48
UBE2F-SCLY	0	0.81	0.29	0.34	1.72	0
UBE2G1	16.57	17.66	20.56	18.31	16.71	14.78
UBE2G2	35.51	32.86	30.71	26.88	32.09	35.43
UBE2H	3.67	1.95	1.4	1.53	2.38	2.18
UBE2I	74.53	68.73	74.71	73.28	82.6	71.64
UBE2J1	8.54	10.44	8.28	4.45	6.17	8.4
UBE2J2	17.45	17.53	15.74	16.16	20.2	13.02
UBE2K	19.8	14.15	14.81	13.05	15.83	15.27
UBE2L3	75.29	78.44	68	65.82	78.25	76.58
UBE2L6	98.35	65.14	66.61	94.89	79.34	50.1
UBE2M	10.46	3.06	4.72	3.99	4.64	3.33
UBE2MP1	0	0	0	0	0	0
UBE2N	74.48	76.34	66.84	56.65	73.65	68.71
UBE2NL	0	0.18	0.07	0	0	0
UBE2O	3.94	4.2	3.86	4.64	4.03	4.74
UBE2Q1	2.03	1.28	1.34	2.13	2.19	1.53
UBE2Q2	6.29	4.02	4.27	2.95	4.34	4.56
UBE2Q2P1	3.28	2.64	2.8	2.53	2.48	1.79
UBE2Q2P2	0.76	0.35	0	0.12	0.12	0.06
UBE2Q2P3	2.53	1.93	2.21	1.49	2.82	1.5
UBE2QL1	0	0	0	0	0.02	0.04
UBE2R2	5.16	3.77	3.55	3.22	3.88	4.04
UBE2S	17.67	7.85	16.11	13.73	14.06	11.93
UBE2T	87.4	81.4	73.2	79.54	97.04	75.38
UBE2U	0	0	0	0	0	0
UBE2V1	80.9	37.37	38.74	51.94	55.41	45.39
UBE2V2	122.96	109.1	98.15	76.44	112.31	104.36
UBE2W	7.67	5.18	4.89	4.49	5.17	6.22
UBE2Z	8.39	6.55	6.87	6.6	8.5	6.15
UBE3A	8.14	7.84	10.01	6.97	9.3	7.12
UBE3B	22.92	15.82	14.9	13.99	15.65	12.96
UBE3C	5.75	3.36	3.63	3.15	4.18	4.11
UBE3D	6.27	5.23	5.78	4.87	5.77	7
UBE4A	19.36	14.01	15.12	11.88	13.35	13.77
UBE4B	10.37	9.79	10.06	9.14	9.83	11.11
UBFD1	7.37	5.18	5.08	5.12	6.65	3.93

UBIAD1	18.17	24.54	19.82	20.8	20.23	21.06
UBL3	0.43	1.22	0.9	0.33	0.8	0.94
UBL4A	63.27	61.58	61.12	65.5	60.84	64.67
UBL4B	0	0	0	0	0	0
UBL5	536.65	478.69	422.57	383.05	580.83	423.74
UBL7	114.36	135.75	134.77	155.64	136.01	148.15
UBLCP1	13.96	0.34	3.82	1.75	4.26	0.16
UBN1	4.48	5.14	3.85	3.65	2.91	3.68
UBN2	1.58	1.54	1.45	1.04	1.48	1.43
UBOX5	4.76	5.35	5.18	5.42	4.28	4.98
UBOX5-AS1	0	0	0	0	0	0
UBP1	4.19	3.08	3.27	2.91	2.87	3.71
UBQLN1	37	33.31	33.19	29.96	33.5	38.59
UBQLN2	23.09	28.57	28.3	25.51	25.83	30.57
UBQLN3	0	0	0	0	0	0
UBQLN4	7.56	6.33	4.82	6.93	7.26	5.81
UBQLNL	0	0	0	0	0	0
UBR1	6.02	2.74	2.14	3.19	3.48	2.5
UBR2	9.17	9.33	7.81	5.77	7.08	9.58
UBR3	2.11	0.61	0.69	0.61	1.03	1.01
UBR4	5.75	4.14	4.99	4.54	4.71	4.95
UBR5	7.37	5.84	4.92	5.93	6.22	6.68
UBR7	22.5	27.55	22.15	23.57	26.48	23.14
UBTD1	0.67	1.06	1.23	0.96	1.91	1.12
UBTD2	0.53	2.36	1.57	1.55	1.62	2.59
UBTF	16.19	15.85	14.99	15.21	18.71	17.37
UBTFL1	0	0	0	0	0	0
UBXN1	105.18	79.73	81.28	86.21	90.25	83.99
UBXN10	3.63	0	0	0.22	0.62	0
UBXN11	5.45	6.76	8.54	9.67	5.72	8.6
UBXN2A	10.12	11.31	8.86	8.07	10.44	11.29
UBXN2B	11.81	12.99	10.56	7.59	10.3	12.68
UBXN4	14.29	14.46	11.63	10.27	11.41	11.87
UBXN6	5.2	3.77	3.41	4.34	4.02	4.06
UBXN7	9.61	6.35	8.25	7.5	8.56	7.85
UBXN8	0.31	0.04	0.05	0.07	0.35	0
UCA1	3.4	0.13	0.17	0.31	0.18	0.35
UCHL1	43.17	5.83	1.06	189.71	56.68	138.52
UCHL3	68.64	67.96	66.75	69.73	69.44	63.44
UCHL5	54.75	57.21	51.33	45.81	52.85	53.44
UCK1	5.76	9.58	6.42	6.86	7.59	8.29
UCK2	14.87	16.88	15.95	15.03	20.36	16.17
UCKL1	16.97	12.05	13.24	10.22	14.27	10.49

UCKL1-AS1	1.17	1.11	1.34	0.71	0.72	0.71
UCMA	0	0	0	0	0	0
UCN	0.66	1.15	0.48	1.56	3.14	0.8
UCN2	0.05	0	0	0	0	0
UCN3	0	0	0	0	0	0
UCP1	0	0	0	0	0	0
UCP2	57.69	46.35	43.81	48.25	42.74	48.91
UCP3	0	0.05	0	0.28	0.06	0
UEVLD	3.5	2.26	1.15	1.39	1.75	1.62
UFC1	90.83	101.48	102.42	105.95	108.01	94.89
UFD1L	45.22	40.03	39.56	40.13	40.89	37.99
UFL1	6.25	8.41	6.65	5.15	7.1	6.29
UFM1	14.7	15.26	11.28	8.67	12.18	11.45
UFSP1	0.62	1.16	0.92	0.48	1.76	1.25
UFSP2	13.09	12.39	10.65	9.52	11.29	9.3
UG0898H09	0.27	0.21	0.49	0.33	0.26	0.21
UGCG	2.88	4.68	3.05	4.79	3.46	3.61
UGDH	7.8	11.82	10.98	9.03	10.32	12.2
UGDH-AS1	47.79	53.29	56.37	33.31	41.7	50.32
UGGT1	8.59	7.63	7.77	6.59	7.04	9.16
UGGT2	2.47	1.22	1.01	0.94	0.77	1.13
UGP2	29.46	30.6	25.83	22.5	31.62	27.92
UGT1A1	0	0	0	0	0	0
UGT1A10	0	0	0	0	0	0
UGT1A3	0	0	0	0	0	0
UGT1A4	0	0	0	0	0	0
UGT1A5	0	0	0	0	0	0
UGT1A6	0.4	0	0	0	0.08	0.4
UGT1A7	0	0	0	0	0	0
UGT1A8	0	0	0	0	0	0
UGT1A9	0	0	0	0	0	0
UGT2A1	0	0.04	0.08	0	0	0
UGT2A2	0	0	0	0	0	0
UGT2A3	0.02	0	0.05	0.17	0.25	0.25
UGT2B10	0	0	0	0	0	0
UGT2B11	0.04	0	0	0	0	0
UGT2B15	0	0	0	0.03	0	0.03
UGT2B17	0	0	0	0	0	0
UGT2B28	0	0	0.07	0.07	0.09	0
UGT2B4	0	0	0	0	0	0
UGT2B7	0	0	0	0	0	0
UGT3A1	0	0	0	0	0	0
UGT3A2	0.08	2.22	0	0	0.23	0.51

UGT8	1.45	2.01	1.54	1.32	1.62	1.74
UHMK1	4.72	3.32	2.42	2.26	2.96	2.87
UHRF1	22.61	41.93	30.84	34.11	30.26	41.27
UHRF1BP1	3.57	3.64	3.93	3.14	3.7	3.87
UHRF1BP1L	5.62	5.59	4.31	4.68	4.39	5.76
UHRF2	1.25	1.15	1.23	0.88	1.25	0.99
UIMC1	11.34	9.69	10.2	9.47	8.72	8.18
ULBP1	2.92	5.7	3.46	3.6	4.04	5.13
ULBP2	2.13	4.15	2.92	2.06	2.25	4.3
ULBP3	1.84	8.1	5.63	6.28	7.28	9.7
ULK1	1.31	0.86	0.7	0.98	0.87	0.91
ULK2	0.3	0.22	0.36	0.23	0.29	0.27
ULK3	23.58	25.59	20.94	26.36	22.69	20.23
ULK4	1.29	1.6	1.73	1.96	1.41	2.32
ULK4P1	1.96	1	0.94	0.6	2.31	0
ULK4P2	1.96	1	0.94	0.6	2.31	0
ULK4P3	2.95	4.34	4.61	5.83	4.36	7.13
UMOD	0	0	0	0	0	0
UMODL1	0	0	0	0	0	0
UMPS	13.84	14.64	16.11	15.2	13.37	15.04
UNC119	4.31	6.78	6.62	5.51	8.36	3.65
UNC119B	1.31	1.27	0.26	0.8	1.32	0.85
UNC13A	0.18	0.03	0	0.01	0.05	0.03
UNC13B	4.69	5.88	4.36	3.96	4.11	5.43
UNC13C	0	0	0	0	0	0
UNC13D	17.75	12.96	12.97	13.27	16.96	12.1
UNC45A	27.6	15.93	22.03	22.66	22.12	20.69
UNC45B	0	0	0.01	0	0	0
UNC50	27.29	25.47	18.93	19.21	24.32	16.45
UNC5A	0.08	0.05	0.05	0.16	0	0.24
UNC5B	0.41	0.02	0	0.06	0.06	0.08
UNC5B-AS1	0	0	0	0.26	0	0
UNC5C	0	0	0	0	0	0
UNC5CL	0	0	0.02	0	0	0
UNC5D	0	0	0	0	0	0
UNC79	0	0	0	0.01	0.01	0.01
UNC80	0	0	0	0	0	0.05
UNC93A	0	0	0	0	0	0
UNC93B1	1.94	5.2	6.68	4.15	3.24	5.01
UNCX	0	0	0	0	0	0
UNG	37.57	57.07	48.83	62.43	46.37	45.78
UNK	12.66	16.2	13.23	12.52	14.17	12.73
UNKL	2.47	3.3	1.88	2.18	3.14	2.54

UNQ6494	0	0	0	0	0	0
UNQ6975	0	0	0	0	0	0
UOX	0.29	0	0	0	0	0
UPB1	0.21	0	0.07	0.02	0.24	0
UPF1	3.41	4.5	3.74	3.11	4.06	4.6
UPF2	7.46	4.01	5.08	4.34	7.02	3.72
UPF3A	11.47	9.47	7.45	7.34	9.46	7
UPF3B	19.06	22.1	13.75	14.74	19.63	20.23
UPK1A	0	0	0	0.56	0	0
UPK1A-AS1	0	0	0	0.41	0	0
UPK1B	0.42	0.55	0.49	0.22	0.46	0.59
UPK2	0	0	0	0	0.16	0
UPK3A	0	0.46	0.97	0.33	0.67	1.89
UPK3B	0.14	0.46	0.78	0.54	1.05	0.8
UPK3BL	5.54	4.46	4.38	4.13	3	6.03
UPP1	8.19	6.38	2.87	1.36	3.75	1.56
UPP2	0	0	0	0	0	0
UPRT	9.91	6.4	4.9	10.03	9.52	7.38
UQCC	15.46	15.01	16.37	14.48	18.48	14.5
UQCR10	251.12	250.89	225.69	238.56	282.7	226.39
UQCR11	229.61	191.63	153.57	174.65	225.59	149.52
UQCRB	66.82	53.47	50.26	46.43	60.37	49.37
UQCRBP1	0.1	0.26	0	0	0.56	0
UQCRC1	365.06	419.7	411.1	427.45	406.84	370.18
UQCRC2	117.5	127.26	115.53	121.28	128.57	124.98
UQCRFS1	230.71	223.42	191.84	197.13	228.67	189.71
UQCRH	953.3	792.45	766.6	854.79	971.75	784.19
UQCRHL	0.79	1.24	0.87	0.8	1.03	0.58
UQCRQ	158.94	145.14	138.73	126.97	173.21	117.05
URB1	6.51	6.38	6.77	5.08	6.21	7.6
URB2	7.94	9.33	10.18	10.5	7.39	11.77
URGCP	8.94	7.61	9.63	7.27	8.02	10.66
URGCP-MRP	0	0.31	0	0	0	0.75
URI1	2.94	2.9	2.43	2.12	2.82	2.95
URM1	21.96	22.25	21.26	20.26	24.41	22.45
UROC1	0	0	0	0	0	0
UROD	196.34	115.15	100	132.96	147.08	84
UROS	53.48	52.24	51.38	59.27	45.15	45.67
USB1	31.58	33.39	34.96	31.54	32.37	29.03
USE1	26.27	31.89	26.2	38.94	28.73	28.27
USF1	18.75	21.24	25.46	25.62	20.07	22.41
USF2	10.38	4.59	5.84	8.38	5.79	4.15
USH1C	0.46	0	0	0	0.09	0

USH1G	0.24	0	0	0.14	0.04	0
USH2A	0	0.01	0	0	0	0.02
USHBP1	0	0.05	0	0	0	0
USMG5	485.83	358.26	296.7	285.76	419.22	268.09
USO1	14.89	11.14	11.5	11.09	12.5	13.35
USP1	15.82	16.07	14.31	11.93	17.88	17.79
USP10	62.09	58.47	57.11	57.77	58.37	53.23
USP11	39.55	36.51	33.19	46.08	36.53	35.91
USP12	3.76	1.29	0.98	0.95	1.59	1.06
USP13	2.34	3.2	3.45	3.05	2.74	3.39
USP14	29.64	27.18	25.24	23.81	25.93	24.42
USP15	21.47	12.13	10.38	11.15	12.47	7.32
USP16	13.1	9.82	10.11	8.69	9.91	11.74
USP17L10	0	0	0	0	0	0
USP17L11	0	0	0	0	0	0
USP17L12	0	0	0	0	0	0
USP17L13	0	0	0	0	0	0
USP17L15	0	0.04	0	0	0	0
USP17L17	0	0	0	0	0	0
USP17L18	0	0	0	0	0	0
USP17L19	0	0	0	0	0	0
USP17L1P	0	0	0	0	0	0
USP17L2	0	0	0	0	0	0
USP17L20	0	0	0	0	0	0
USP17L21	0	0	0	0	0	0
USP17L22	0	0	0	0	0	0
USP17L24	0	0	0	0	0	0
USP17L25	0	0	0	0	0	0
USP17L26	0	0	0	0	0	0
USP17L27	0	0	0	0	0	0
USP17L28	0	0	0	0	0	0
USP17L29	0	0	0	0	0	0
USP17L3	0	0	0	0	0	0
USP17L30	0	0	0	0	0	0
USP17L4	0	0	0	0	0	0
USP17L5	0	0	0	0	0	0
USP17L6P	0	0.19	0	0	0	0
USP17L7	0	0	0	0	0	0
USP17L8	0	0	0	0	0	0
USP17L9P	0	0	0	0	0	0
USP18	4.17	7.69	4.85	5.82	6.58	8.52
USP19	4.12	5.18	4.86	5.68	5.18	3.1
USP2	6.73	10.56	10.5	8.39	7.7	6.68

USP20	5.66	6.23	4.77	5.02	5.21	5.29
USP21	3.74	6.43	6.87	7.28	4.41	4.75
USP22	24.01	20.5	18.67	21.19	23.2	17.06
USP24	3.41	1.79	1.26	1.3	2.41	2.35
USP25	0.83	0.86	1.09	0.46	0.57	0.59
USP26	0	0	0	0	0	0
USP27X	0.49	0.32	0.38	0.2	0.41	0.8
USP28	3.49	5.36	4.6	3.79	3.45	4.79
USP29	0	0.03	0.08	0.02	0.08	0
USP3	17.82	15.78	17.42	17.52	17.25	15.37
USP30	2.63	3.24	2.16	2.71	2.71	3.5
USP30-AS1	0	0.13	0.08	0.33	0.15	0.33
USP31	0.85	0.92	0.75	0.32	0.71	0.7
USP32	3.22	3.29	2.05	2.07	2.93	2.7
USP32P1	0.45	0.21	0.15	0.25	0.36	0.74
USP32P2	0.35	0.3	0.26	0.07	0.27	0.02
USP33	14.37	13.76	11.23	13.54	13.49	11.64
USP34	2.91	2.78	2.06	2.35	2.79	2.67
USP35	0.35	0.28	0.12	0.13	0.05	0.08
USP36	11.72	16.64	16.68	11.05	10.9	14.7
USP37	2.94	3.78	2.93	3.21	2.63	3.73
USP38	7.51	4.89	5.79	6.2	5.58	5.11
USP39	56.26	70.7	59.72	57.79	57.82	61.88
USP4	9.43	9.43	8.61	10.69	7.37	9.23
USP40	4.06	2.9	3.37	2.2	2.74	4.27
USP42	3.51	3.25	3.38	4.57	3.58	3.21
USP43	0.16	0.21	0.56	0.02	0.18	0.14
USP44	0.04	0.24	0.08	0.09	0.12	0.13
USP45	1.18	0.64	1.11	1.01	1.32	1.05
USP46	2.12	2.15	2.4	1.46	1.92	2.21
USP47	4.71	4.79	4.33	4.53	5.28	4.69
USP48	14.08	11.86	9.7	11.17	13.83	7.23
USP49	0.84	1.39	0.59	1.08	0.89	1.06
USP5	28.32	24.55	27.95	30.87	27.64	29.84
USP50	0	0	0	0	0	0
USP51	0	0	0	0	0	0
USP53	0.63	1.01	0.84	0.67	0.84	1.31
USP54	3.07	2.25	3.41	1.59	2.67	2.43
USP6	0.1	0.08	0	0.03	0.01	0
USP6NL	2.45	1.31	2.41	1.64	2.23	1.57
USP7	7.04	7.3	7.56	6.79	6.3	6.95
USP8	8.09	7.57	5.24	4.87	5.85	5.05
USP9X	22.9	14.95	13.69	15.15	16.96	13.45

USP9Y	0	0	0	0	0	0
USPL1	5.43	5.93	6.01	5.05	5.55	4.55
UST	1.15	0.47	0.71	0.49	0.41	0.56
UTF1	0	0	0	0	0	0
UTP11L	54.47	48.74	50.33	44.29	49.6	47.06
UTP14A	44.77	38.7	35.25	44.9	40.81	42.94
UTP14C	3.66	2.12	1.48	2.12	1.91	2.42
UTP15	12.06	10.17	9.02	7.72	11.31	10.8
UTP18	61.38	63.19	65.96	59.33	62.49	61.45
UTP20	3.81	3.99	3.72	3.91	3.59	3.32
UTP23	17.34	22.26	20.08	13.85	18.24	19.2
UTP3	14.66	11.38	10.98	8.6	11.16	10.19
UTP6	36.05	41.86	30.19	27.86	34.95	31.59
UTRN	0.97	1.67	1.54	1.18	1.24	1.98
UTS2	0	0	0	0	0	0
UTS2D	0.37	0.43	0.36	0.25	0.11	0.68
UTS2R	0	0	0	0	0	0
UTY	0.13	0.12	0.18	0.13	0.12	0.05
UVRAG	2.39	2.52	1.69	2.77	2.06	1.6
UVSSA	1.24	1.35	0.77	0.7	1.47	0.82
UXS1	18.8	19.89	19.89	22.02	18.66	20.27
UXT	343.51	359.17	374.26	419.14	408.26	362.32
VAC14	5.41	7.98	7.82	8.99	6.44	7
VAMP1	5.39	2.44	3.39	3.25	2.2	3.56
VAMP2	6.39	2.37	2.5	4.28	4.52	3.15
VAMP3	38.16	22.56	26.46	23.73	27.6	20.53
VAMP4	0.77	0.7	1.01	1.69	0.72	0.96
VAMP5	1.57	1.62	2.76	1.17	0.69	1.22
VAMP7	45.73	42.87	37.62	32.35	38.76	40.03
VAMP8	214.79	494.03	380.97	282.5	299.08	374.48
VANGL1	2	4.09	3.07	2.69	1.86	3.11
VANGL2	2.11	1.74	1.16	0.92	1.78	1.13
VAPA	53.62	29.42	24.47	28.5	41.26	25.79
VAPB	4.44	3.44	2.89	3.98	3.79	3.24
VARS	6.99	9.22	9.53	12.39	11.88	9.9
VARS2	2.41	1.55	2.76	2.35	1.8	2.04
VASH1	5.58	0.85	1.41	1.88	3.26	1.28
VASH2	0.49	0.37	0.17	0.46	0.45	0.75
VASN	0	0.02	0.05	0.14	0.12	0
VASP	2.79	2.88	2.2	2.08	2.33	2.27
VAT1	217.65	87.69	78.47	102.49	115	92.15
VAT1L	0	0	0	0	0	0
VAV1	7.9	8.6	12.67	9.57	6.79	11.94

VAV2	2.1	2.35	2.56	2.08	1.93	1.95
VAV3	0.35	1.8	1.44	0.63	0.88	1.42
VAV3-AS1	0	0.21	0	0.55	0	0.4
VAX1	0	0	0	0	0	0
VAX2	1.13	2.36	2.37	1.56	0.94	1.68
VBP1	112.33	87.61	86.33	76.92	94.7	82.08
VCAM1	0	0	0	0	0	0
VCAN	5.77	2.84	6.08	8.79	6	10.8
VCL	27.99	13.21	11.9	14.44	15.91	13.76
VCP	126.33	117.79	131.01	123.91	124.49	133.48
VCPIP1	6.34	6.1	7.51	4.81	5.35	6.12
VCX	0	0	0	0	0	0
VCX2	0	0	0	0	0	0
VCX3A	0	0	0	0	0	0
VCX3B	0	0	0	0	0	0
VCY	0	0	0	0	0	0
VCY1B	0	0	0	0	0	0
VDAC1	174.62	189.24	199.9	171.15	188.94	184.24
VDAC2	245.16	214.6	226.26	210.68	247.4	214.93
VDAC3	303.16	339.59	295.01	305.97	308.13	295.67
VDR	1.71	3.72	3.35	2.32	2.32	3.29
VEGFA	0.62	0.27	0.67	0.44	0.43	0.35
VEGFB	2.64	3.66	2.27	3.93	3.07	2.91
VEGFC	0	0	0	0	0	0
VENTX	0.37	0.42	0.5	0.41	0.45	0.31
VENTXP1	0	0	0	0	0.03	0
VENTXP7	0	0	0	0	0	0
VEPH1	0	0	0	0	0	0
VEZF1	10.46	8.74	8.12	9.04	11.55	8.68
VEZT	7.74	7.1	7.88	5.77	6.04	5.77
VGF	0	0	0	0.02	0	0
VGLL1	0	0	0	0	0	0
VGLL2	0	0	0	0	0	0
VGLL3	0.01	0.11	0.02	0.03	0.02	0.03
VGLL4	5.28	5.25	4.84	3.7	4.89	5.07
VHL	4.19	4.38	4.45	3.69	4.84	3.91
VHLL	0	0	0	0	0	0
VIL1	0	0	0	0	0	0
VILL	1	1.75	0.84	1.24	1.16	0.53
VIM	333.22	176.71	238.2	244.44	216.75	169.77
VIMP	33.07	27.63	19.69	19.35	26.59	16.86
VIP	0	0	0	0	0	0
VIPAS39	12.02	9.47	6.92	10.75	8.94	9.22

VIPR1	0	0.02	0.24	0.52	0.32	0.06
VIPR2	0.08	0.15	0.18	0.17	0.15	0.06
VIT	0	0	0	0	0	0
VKORC1	100.8	113.73	117.79	104.21	113.44	113.43
VKORC1L1	2.33	1.73	1.22	2.14	2.71	2.33
VLDLR	0.2	1.67	0.65	1.18	0.58	0.89
VMA21	35.73	31.99	30.29	27.36	34.58	36.26
VMAC	0.78	1.15	1.74	1.29	1.01	1.32
VMO1	0.53	0	0.19	0.22	0.35	0
VMP1	33.42	34.69	34.2	28.18	35.59	33.24
VN1R1	0	0.08	0.26	0.26	0.05	0
VN1R10P	0	0	0	0	0	0
VN1R2	0.23	0.27	0.5	0.3	0.46	0.18
VN1R4	0	0	0	0	0	0
VN1R5	0	0	0	0	0	0
VNN1	0.17	0.65	3	2.68	0.75	2.12
VNN2	0.11	0.04	0.89	0.44	0.22	0.37
VNN3	0	0	0	0	0	0
VOPP1	8.7	11.86	10.43	9.81	10.61	11.98
VPRBP	10.72	13.44	13.06	13.36	14.06	14.31
VPREB1	0	0	0	0	0	0
VPREB3	0	0.55	0.26	0	0	0
VPS11	14.73	15.27	13.73	13.94	14.72	13.23
VPS13A	1.78	1.76	1.58	1.14	1.45	1.79
VPS13A-AS1	0	0	0.54	0.14	1.25	1.25
VPS13B	8.16	8.31	8.34	6.61	7.23	10.74
VPS13C	5.78	6.51	5.24	4.21	5.24	7.52
VPS13D	2.19	1.63	1.49	1.58	1.95	1.97
VPS16	14.08	15.18	16.52	14.79	13.43	14
VPS18	16.63	14.72	17.42	17.46	14.86	12.05
VPS25	111.11	107.05	94.36	98.59	101.49	86.17
VPS26A	44.89	39.63	36.33	30.04	47.74	42.26
VPS26B	4.81	4.83	4.71	4.9	3.19	3.36
VPS28	206.71	184.34	175.98	201.29	214.52	184.53
VPS29	95.05	105.86	86.25	72.82	96.14	85.59
VPS33A	25.88	28.5	29.65	22.23	23.73	24.04
VPS33B	7.46	7.38	9.31	6.83	7.56	6.87
VPS35	41.98	47.45	46.58	39.61	47.92	50.67
VPS36	5.7	7.98	7.66	6.45	7.15	7.2
VPS37A	10.07	10.24	7.49	7.13	8.8	10.57
VPS37B	9.28	5.9	6.47	8.31	10.21	5.7
VPS37C	1.01	1.6	0.87	1.36	1.56	1.48
VPS37D	0.19	0	0	0	0.09	0

VPS39	8.13	8.04	7.29	8.75	8.36	9.69
VPS41	11.72	9.55	7.95	8.18	7.62	8.84
VPS45	9.26	8.78	4.7	5.89	9.88	6.82
VPS4A	42.51	51.45	58.77	56.72	61.21	36.57
VPS4B	14.46	13.05	9.45	10.43	11.23	11.35
VPS51	18.6	26.26	31.93	35.65	24.47	25.45
VPS52	19.29	15.78	15.43	19.54	17.7	16.63
VPS53	9.19	11.01	11.38	10.71	9.68	11.91
VPS54	0.94	0.74	0.7	0.65	0.79	0.93
VPS72	58.62	60.21	58.9	63.29	55.53	58.05
VPS8	3.36	3.2	2.76	3.1	2.09	1.91
VPS9D1	0.25	0.09	0.31	0.18	0.11	0.17
VRK1	50.68	46.72	39.71	46.37	54.35	38.93
VRK2	20.91	24.68	23.42	19.06	20.61	18.08
VRK3	17.17	16.76	19.09	18.03	18.61	14.75
VRTN	0.09	0.02	0	0	0	0
VSIG1	1.51	1.73	2.26	0.97	1.43	1.48
VSIG10	2.54	3.56	3.87	3.61	3.45	4.2
VSIG10L	1.3	0.44	0.69	0.81	0.76	0.5
VSIG2	0	0.11	0.06	0	0.13	0.07
VSIG4	0	1.8	2.38	0.63	0.19	3.68
VSIG8	0	0	0	0	0	0
VSNL1	0	0.32	0	0	0	0
VSTM1	0	0	0	0	0	0
VSTM2A	0	0	0	0	0	0
VSTM2B	0	0	0.11	0	0	0
VSTM2L	0.04	0.15	0.55	0.13	0	0.11
VSTM4	0.59	1.32	1.72	0.7	0.86	0.96
VSTM5	0	0	0	0.09	0	0
VSX1	0	0.03	0	0	0	0
VSX2	0	0	0	0	0	0
VTA1	38.94	36.76	31.23	30.52	30.42	30.08
VTCN1	0	0	0	0	0	0
VTI1A	5.98	3.94	3.98	4.08	4.89	5.03
VTI1B	72.51	69.75	65.39	58.37	72.45	54.58
VTN	0.98	0.1	0.04	0.03	0.04	0
VTRNA1-1	0	2.47	1.4	0	0	0
VTRNA1-2	0	0	0	0	0	0
VTRNA1-3	0	0	0	0	0	0
VTRNA2-1	0	0	0	0	0	0
VWA1	0.42	0.37	0.59	0.36	0.38	1.07
VWA2	0.03	0.07	0.08	0.1	0.1	0.08
VWA3A	0	0.01	0	0	0	0

VWA3B	0	0.02	0	0	0	0
VWA5A	0.28	0.25	0.33	0.11	0	0.68
VWA5B1	0	0	0	0	0	0
VWA5B2	0.02	0.11	0.02	0.02	0	0
VWA7	0.25	0.02	0	0.03	0.19	0.03
VWA8	1.91	1.51	1.08	0.74	1.34	1.12
VWA8-AS1	0	0	0.03	0	0	0
VWA9	51.88	56.44	44.18	48.85	52.63	48.91
VWC2	0	0	0	0	0	0
VWC2L	0	0	0	0	0	0
VWC2L-IT1	0	0	0	0	0	0
VWCE	0.1	0.02	0	0.1	0.21	0.33
VWDE	0.64	0.87	1.1	0.4	0.3	0.67
VWF	0	0	0.04	0.01	0	0.01
WAC	12.01	10.14	9.93	9.4	11.36	9.61
WAC-AS1	10.09	10.67	8.96	9.83	10.23	10.19
WAPAL	4.88	5.19	5	4.25	4.74	4.41
WARS	114.55	80.01	76.46	69.87	77.95	60.85
WARS2	8.82	10.47	7.98	6.31	7.71	9.28
WAS	3.16	5.27	7.34	3.29	4.17	6.48
WASF1	11.2	11.09	6.72	9.02	10.2	6.48
WASF2	34.86	34	30.14	28.03	32.31	28.05
WASF3	1.28	1.26	0.72	1.06	0.93	0.89
WASH1	15.79	11.37	11.08	18.37	16.57	15.58
WASH2P	19.75	14.01	15.71	23.63	21.57	11.69
WASH3P	23.56	14.61	19.24	32.6	29.3	16.48
WASH5P	2.56	1.91	1.54	1.6	1.74	1.22
WASH7P	8.27	8.88	4.74	4.61	4.73	5.22
WASL	2.16	1.49	1.26	1.43	1.28	1.24
WBP1	14.58	12.65	16.51	17.58	14.85	12.3
WBP11	33.73	33.04	30.62	31.81	30.48	30.64
WBP11P1	0.02	0.03	0	0	0.04	0
WBP1L	15.92	8.9	10.08	11.03	11.77	6.87
WBP2	47.04	26.86	23.2	33.87	41.07	27.98
WBP2NL	0	0	0	0	0	0
WBP4	6.58	3.87	4.78	5.04	5.65	3.76
WBP5	0	0	0	0	0	0
WBSCR16	7.5	5.84	5.65	4.82	4.55	4.94
WBSCR17	0	0	0	0	0	0
WBSCR22	101.9	76.91	77.25	85.18	80.91	66.26
WBSCR27	0	0	0	0.06	0	0
WBSCR28	0	0	0	0	0	0
WDFY1	7.43	5.6	4.21	4.65	5.57	6.67

WDFY2	8.22	5.12	6.34	6.11	6.19	5.13
WDFY3	1.01	0.53	0.63	0.41	0.85	0.99
WDFY3-AS2	0.38	0	0	0.1	0.18	0
WDFY4	1.39	5.66	6.52	3.67	3.11	8.03
WDHD1	6.93	7.71	8.13	7.59	6.96	8.66
WDPCP	1.87	1.86	0.71	2.04	1.62	0.76
WDR1	79.34	56.81	48.92	63.17	58.13	52.65
WDR11	6.71	5.63	3.97	4.39	4.86	6.64
WDR11-AS1	0.3	0.9	1.14	0.41	0.32	0.58
WDR12	40.4	37.99	34.92	34.51	40.11	38.4
WDR13	43.6	24.99	30.26	37.29	35.09	31.28
WDR16	0.07	0	0.07	0	0	0
WDR17	0.13	0.16	0.21	0.29	0.24	0.52
WDR18	88.26	117.02	120.74	135.69	116.94	119.64
WDR19	2.01	2.48	1.36	1.32	0.83	1.07
WDR20	9.93	8.83	9.3	6.93	8.16	5.5
WDR24	7.38	8.77	11.2	9.01	7.01	8.15
WDR25	7.82	8.52	8.95	7.82	6.99	9.45
WDR26	2.03	0.56	1.05	1.34	1.38	1.28
WDR27	0.62	0.74	0.85	0.97	1	0.76
WDR3	20.86	23.78	23.74	20.98	21.94	23.18
WDR31	0.76	0.58	1.96	1.71	0.75	0.68
WDR33	15.14	16.68	18.03	14.33	17.31	16.97
WDR34	53.39	71.94	62.34	71.6	56.93	65.86
WDR35	2.37	2.19	0.91	1.58	2.08	1.21
WDR36	14.9	20.31	16.13	12.79	14.84	18.15
WDR37	1.91	2.33	2.02	2.3	2.24	2.67
WDR38	0.18	0.05	0.05	0	0.11	0.3
WDR4	39.7	45.71	43.62	39.01	37.67	45.54
WDR41	18.96	15.99	16.33	12.01	17.86	14.66
WDR43	31.77	40.91	35.66	34.63	35.37	36.37
WDR44	14.15	4.61	3.78	6.81	7.55	2.84
WDR45	42.38	19.25	15.23	23.46	22.1	17.41
WDR45B	25.21	15.71	12.36	16.89	16.08	20.62
WDR46	37.63	44.57	37.75	37.1	39.46	38.17
WDR47	2.46	1.86	1.69	1.67	2.41	1.9
WDR48	10.91	5.19	4.62	8.47	8.75	6.72
WDR49	0	0	0	0	0	0
WDR5	16.19	21.58	19.82	20.54	19.56	13.85
WDR52	0.24	0.11	0.22	0.11	0.13	0.36
WDR52-AS1	0	0	0	0	0	0
WDR53	4.95	4.65	6.83	4.55	4.49	5.67
WDR54	47.33	53.68	47.63	55.88	50.09	44.51

WDR55	22.61	22.44	21.6	19.7	21.22	20.38
WDR59	3.07	2.71	2.09	3.51	3.21	3.87
WDR5B	0.93	1.16	0.64	0.64	0.78	0.63
WDR6	20.24	22.72	23.84	27.2	27.75	28.82
WDR60	2.88	3.53	3.66	3.3	3.55	3.29
WDR61	148	160.96	144.57	127.85	154.9	139.81
WDR62	3.68	5.89	3.84	4.76	3.22	3.05
WDR63	0.2	0	0.13	0.07	0	0
WDR64	0	0	0	0	0	0
WDR65	0	0	0	0	0.05	0
WDR66	0.21	0.1	0.23	0.1	0.11	0.35
WDR67	6.26	5.04	4.16	5.46	4.64	5.01
WDR7	2.33	1.71	1.66	1.23	1.69	1.6
WDR70	14.68	16.57	16.5	16.75	16.97	14
WDR72	0	0	0	0	0.02	0
WDR73	27.11	25.09	23.28	24.14	21.49	23.04
WDR74	80.54	84.65	87.31	84.38	87.47	92.94
WDR75	28.17	22.99	21.13	23.1	25.96	24.55
WDR76	8.63	11.09	13.11	11.12	12.89	9.63
WDR77	42.18	40	46.1	45.54	39.8	35.56
WDR78	0.51	0.5	0.12	0.12	0.16	0.3
WDR81	3.66	3.35	1.28	3.13	2.07	1.92
WDR82	9.24	9.32	9.63	9.34	7.38	10.95
WDR83	12.71	12.48	11.16	16.77	11.09	10.84
WDR83OS	189.43	152.19	137.66	161.15	184.22	142.65
WDR85	19.46	18.09	14.18	18.54	16.83	19.04
WDR86	0	0.48	0.46	0.17	0.27	0.29
WDR86-AS1	1.54	2.34	1.46	1.47	1.08	1.92
WDR87	0	0	0	0	0	0
WDR88	0.04	0.03	0	0.03	0	0
WDR89	13.14	10.37	12.85	10.39	13.38	8.37
WDR90	0.61	1.21	0.26	0.85	0.69	0.66
WDR91	4.58	3.89	4.09	3.15	3.46	3.85
WDR92	9.78	10.08	10.1	6.62	7.29	10.85
WDR93	0	0	0	0	0	0
WDR96	0.11	0	0.01	0.06	0	0
WDSUB1	10.72	10.84	9.25	8.14	10.4	8.85
WDTC1	5.35	2.99	2.81	3.44	3.73	2.97
WDYHV1	13.19	12.25	14.07	11.87	15.91	15.82
WEE1	4.75	2.96	4.18	3.3	4.09	3.73
WEE2	0	0	0	0	0	0
WFDC1	0.16	0.86	2.9	1.13	0.26	0.5
WFDC10A	0.18	0	0	0	0	0

WFDC10B	0	0	0	0	0	0
WFDC11	0	0	0	0	0	0
WFDC12	0	0	0	0	0	0
WFDC13	0	0	0	0	0	0
WFDC2	0	0.46	0	0	0.55	0
WFDC3	0	0	0	0	0	0
WFDC5	0	0	0	0	0	0
WFDC6	0	0	0	0	0	0
WFDC8	0.6	0.36	1.14	0.24	0.09	1.38
WFDC9	0	0	0	0	0	0
WFIKKN1	0	0.06	0	0.08	0.04	0
WFIKKN2	0	0	0	0	0	0
WFS1	0.02	0	0.18	0	0	0.06
WHAMM	2.27	2.79	2.16	2.26	2.13	2.38
WHAMMP1	0.38	0.89	1.07	0.45	0.22	0.41
WHAMMP2	0.09	0.37	0.22	0.06	0.19	0.23
WHAMMP3	0.64	0.25	0.37	0.47	0.2	0.29
WHSC1	4.07	5.8	4.13	4.6	4.22	5.19
WHSC1L1	10.82	13.11	12.5	12.23	12.74	14.24
WIBG	29.69	24.89	23.37	18.63	25.97	18.28
WIF1	0	0	0	0	0	0
WIPF1	3	5.25	5.24	3.15	4.04	4.55
WIPF2	3.52	3.25	3.38	3.41	4.31	2.64
WIPF3	3.52	0.13	0.13	0.12	0.07	0.07
WIPI1	5.46	1.79	0.56	2.59	1.2	0.54
WIPI2	5.6	4.07	5.24	5.06	5.53	4.12
WISP1	0	0	0	0	0	0
WISP2	0	0	0	0	0	0
WISP3	0	0	0	0	0	0
WIZ	1.84	1.89	1.41	2.12	1.1	1.34
WLS	0	0	0	0	0	0
WNK1	11.12	7.07	6.05	6.21	7.26	6.45
WNK2	0.71	0.68	0.69	0.79	0.85	0.64
WNK3	0.01	0.03	0.01	0.04	0.01	0.03
WNK4	0.03	0.03	0	0	0.02	0.04
WNT1	0	0	0	0	0	0
WNT10A	0	0	0	0.11	0	0
WNT10B	2.08	3.12	3.4	3.24	2.69	3.83
WNT11	0.23	0.45	0.21	0.87	0.15	0.62
WNT16	0	0	0	0	0	0
WNT2	0	0	0	0	0	0
WNT2B	0	0.09	0.3	0.25	0.14	0
WNT3	0.15	0.16	0.18	0.07	0.24	0.05

WNT3A	0	0	0	0	0	0
WNT4	0	0	0	0	0.1	0
WNT5A	0	0	0	0	0	0
WNT5B	0.2	0	0.43	0.26	0.03	0.31
WNT6	0.04	0.1	0	0.03	0	0
WNT7A	0	0	0	0	0	0
WNT7B	0.23	0.53	0.49	0.19	0.19	0.35
WNT8A	0	0	0	0	0	0
WNT8B	0	0.06	0	0.2	0	0.34
WNT9A	0	0	0.28	0.08	0	0.56
WNT9B	0	0	0	0	0	0
WRAP53	16.69	13.87	15.54	17.05	21.64	15.48
WRAP73	14.67	19.19	10.57	12.09	17.13	12.49
WRB	26.26	24.34	17.2	14.75	20.83	17.89
WRN	7.44	4.81	4.12	4.56	5.81	4.07
WRNIP1	1.44	0.94	0.88	0.81	1.97	1.01
WSB1	11.36	9.64	6.6	8.73	11.75	10.05
WSB2	19.92	5.62	5.16	6.58	12.48	3.06
WSCD1	0.49	1.16	0.87	0.38	0.44	0.78
WSCD2	0	0	0	0	0	0
WT1	2.09	3.93	4.92	2.9	4.65	2.8
WT1-AS	3.17	2.33	1.96	2.64	3.2	1.91
WTAP	41.85	44.06	48.35	49.19	55.18	41.94
WTAPP1	0	0	0	0.02	0	0.03
WTH3DI	0.07	0.05	0	0.05	0.01	0
WTIP	0.03	0	0	0	0.16	0.03
WWC1	2.14	1.86	1.92	1.03	2.09	2.06
WWC2	1.34	1.09	1.17	0.86	1.29	0.87
WWC2-AS2	0	0	0	0.23	0.16	0
WWC3	3.2	2.5	2.2	3.52	3.1	2.16
WWOX	5.56	7.91	6.17	6.63	4.28	6.59
WWP1	3.63	2.26	2.66	1.64	1.5	2.34
WWP2	19.21	19.78	15.3	13.84	16.66	15.85
WWTR1	7.49	0.72	0.86	3.28	3.56	0.64
WWTR1-AS1	0.23	0.24	0.25	0.08	0.3	0.26
XAB2	6.42	9.89	7.49	8.22	6.99	8
XAF1	0.81	0.73	0.77	0.53	0.64	0.99
XAGE1A	0	0	0	0	0	0
XAGE1B	0	0	0	0	0	0
XAGE1C	0	0	0	0	0	0
XAGE1D	0	0	0	0	0	0
XAGE1E	0	0	0	0	0	0
XAGE2	0	0	0	0	0	0

XAGE2B	0	0	0	0	0	0
XAGE3	0	0	0	0	0	0
XAGE5	0	0	0	0	0	0
XBP1	25.94	79.34	85.38	50.64	40.79	66.2
XCL1	0	0	0	0	0	0
XCL2	0	0	0.13	0	0	0
XCR1	0	0.04	0	0	0	0
XDH	0.07	0.02	0.09	0.02	0.08	0.07
XG	0	0	0	0	0	0
XGPY2	0	0	0	0	0	0
XIAP	8.29	6.81	6.82	6.24	6.42	6.92
XIRP1	0.01	0	0	0.01	0.07	0
XIRP2	0	0	0	0	0	0
XIST	0	0.01	0	0	0	0
XK	10.9	1.71	0.43	4.66	5.08	0.23
XKR3	0	0	0	0	0	0
XKR4	0	0	0	0	0	0
XKR5	0.12	0.18	0.28	0.1	0.2	0.17
XKR6	0.14	0.24	0.07	0.09	0.05	0.04
XKR7	0	0	0	0	0	0
XKR8	5.01	10.42	10.12	9.72	8.18	7.81
XKR9	0.53	0.99	1.36	0.67	0.67	0.89
XKRX	0	0	0	0	0	0
XKRY	0	0	0	0	0	0
XKRY2	0	0	0	0	0	0
XPA	6.07	8.79	7.24	9.07	6.53	5.28
XPC	7.43	5	4.32	6.26	4.5	5.37
XPNPEP1	35.03	28.1	23.06	31.7	27.02	26.77
XPNPEP2	0	0	1.02	0.45	0.25	0.33
XPNPEP3	10.35	12.3	10.54	10.43	11.21	13.47
XPO1	17.51	17.17	18.52	16.14	18.35	19.39
XPO4	4.77	4.8	5.07	5.1	4.86	5.45
XPO5	14.64	16.94	17.8	16.51	15.77	20.01
XPO6	11.5	9.85	10.32	9.78	9.75	9.09
XPO7	29.19	24.76	27.68	25.28	27.89	30.38
XPOT	28.67	30.35	24.87	21.38	26.23	24.65
XPR1	13.78	5.61	5.43	5.93	8.28	5.07
XRCC1	15.12	18.9	16.27	18.35	14.41	16.25
XRCC2	4.7	6.82	7.25	6.35	7.24	7.21
XRCC3	4.75	5.96	9.18	6.06	5.5	6.66
XRCC4	5.62	6.95	4.98	3.44	6.2	6.59
XRCC5	110.68	94.58	98.98	104.47	113.16	107.18
XRCC6	362.39	392.69	360.56	380.69	358.93	355.56

XRCC6BP1	11.55	18.42	15.61	10.82	14.47	11.21
XRN1	2.34	2.14	1.39	1.56	2.09	2.79
XRN2	38.09	35.81	29.61	32.85	32.37	32.18
XRRA1	5.87	4.62	5.16	4.61	4.36	4.8
XXYLT1	2.71	4.59	3	4.53	4.28	2.31
XYLB	4.81	5.12	5.45	4.91	5.74	5.64
XYLT1	0.17	0.59	0.81	0.39	0.56	0.65
XYLT2	5.39	6.23	7.64	6.18	5.07	5.85
YAE1D1	17.63	15.05	16.29	14.24	20.61	12.11
YAF2	4.31	3.45	3.48	2.28	3.26	3.11
YAP1	0.04	0	0	0	0	0
YARS	85.03	94.99	95.19	84.85	84.2	88.75
YARS2	24.37	21.39	22.69	22.98	23.66	24.67
YBEY	6.47	3.14	1.85	5.39	7.43	2.61
YBX1	95.89	88.02	96.9	85.61	95.22	89.25
YBX2	0.05	0.11	0.04	0.2	0.18	0.24
YDJC	57.93	75.83	70.64	63.58	72.66	70.73
YEATS2	7.42	8.85	8.36	6.4	6.16	7.79
YEATS4	36.07	33.15	35.5	27.68	35.91	38.99
YES1	6.63	3.84	3.87	3.62	6.05	5.56
YIF1A	95.26	101.09	91.18	99.33	101.03	88.92
YIF1B	45.46	41.95	38.44	43.63	43.62	37.15
YIPF1	11.57	16.08	15.48	10.22	9.91	13.34
YIPF2	11.55	12.65	13.12	13.79	10.32	12.59
YIPF3	67.41	61.75	50.71	54.99	62.85	60.69
YIPF4	24.4	15.38	15.81	16.38	19.18	16.88
YIPF5	15.13	14.29	14.03	10.89	12.21	11.12
YIPF6	8.97	4.45	3.09	3.47	6.42	4.35
YIPF7	0.08	0	0	0	0	0
YJEFN3	0.6	0.84	0.89	1.47	1.93	0.71
YKT6	9.59	9.36	10.33	7.1	8.57	7.51
YLPM1	6.59	6.67	6.36	7.12	6.24	6.87
YME1L1	30.72	25.56	24.13	21.69	27.59	24.83
YOD1	0	0	0	0	0	0
YPEL1	0.46	0.81	0.65	0.5	0.51	0.48
YPEL2	0.77	0.36	0.09	0.23	0.31	0.1
YPEL3	5.27	1.3	0.6	1.48	2.19	2.74
YPEL4	0.13	0.14	0.36	0.24	0	0.13
YPEL5	12.64	4.39	1.4	3.79	4.32	3.01
YRDC	4.04	2.48	2.48	3.06	1.95	1.84
YTHDC1	6.02	4.88	4.04	4.64	5.35	4.97
YTHDC2	2.02	2.41	2.15	2.03	2.41	2.53
YTHDF1	4.68	4.3	6.43	4.94	4.47	2.92

YTHDF2	64.25	59.05	65.9	65.19	63.76	60.33
YTHDF3	30.56	23.9	28.75	25.79	23.8	26.46
YWHAB	155.22	155.58	163.68	146.14	153.11	154.14
YWHAE	358.77	322.98	351.59	369.18	389.13	350.93
YWHAG	73.22	53.77	55	52.79	59.8	63.8
YWHAH	11.76	15.23	12.13	12.06	9.71	8.5
YWHAQ	165.42	178.98	183.56	189.44	185.7	199.43
YWHAZ	596.57	378.88	333.97	360.81	437.65	352.42
YY1	8.87	7.33	5.74	5.11	8.37	7.55
YY1AP1	26.65	26.36	25.8	22.74	24.71	22.52
YY1P2	0.28	0.32	0.11	0.08	0.45	0.28
YY2	1.15	1.28	0.49	0.18	0.33	0.46
ZACN	0	0.1	0	0	0	0
ZADH2	2.82	4.04	3.02	2.43	2.41	3.93
ZAK	15.71	10.86	9.66	11.64	12.93	11.42
ZAN	0	0	0	0	0	0
ZAP70	0.1	0.19	0.29	0	0	0
ZAR1	0	0	0	0	0	0
ZAR1L	0	0	0	0	0	0
ZBBX	0	0	0	0	0.17	0
ZBED1	12.79	13.81	11.16	12.54	12.07	11.77
ZBED2	0	0	0	0	0	0
ZBED3	1.16	1.13	1.16	0.84	1.05	1.18
ZBED3-AS1	0.33	0.63	0.24	1.01	1.07	1.01
ZBED4	2.29	3.21	3.34	3.07	3.37	3.65
ZBED5	4.91	7.08	4.92	6.17	5.65	4.8
ZBED6	2.36	1.48	1.5	1.67	1.38	1.23
ZBP1	0.09	0.05	0.2	0.04	0.14	0.24
ZBTB1	5.34	5.23	4.76	5.73	6.12	5.28
ZBTB10	0.56	0.61	0.33	0.37	0.57	0.83
ZBTB11	7.83	4.62	4.39	5.39	6.58	5.28
ZBTB12	0	0	0.07	0.03	0	0.04
ZBTB14	2.01	2.91	2.35	2.59	2.33	2.64
ZBTB16	1.96	9.65	8.44	4.18	3.49	8.17
ZBTB17	5.95	6.51	6.81	5.07	5.8	7.15
ZBTB18	0	0.31	0.24	0.09	0.02	0.28
ZBTB2	3.49	6.36	4.99	4.46	3.43	3.96
ZBTB20	1.93	0.2	0.68	0.39	0.6	0.18
ZBTB20-AS1	0	0.03	0.2	0.15	0	0
ZBTB21	1.98	2.4	2.3	2.37	2.74	2.76
ZBTB22	0.3	0.2	0.1	0.46	0.21	0.45
ZBTB24	2.38	2.26	2.7	3.32	2.33	2.66
ZBTB25	1.47	2.2	2.17	1.27	2.73	1.82

ZBTB26	2.29	1.25	1.84	2.27	1.68	1.22
ZBTB3	4.66	4.93	5.52	5	4.26	5.9
ZBTB32	0.26	0	0.03	0	0	0.08
ZBTB33	14.3	13.26	15.39	12.51	12.65	18.16
ZBTB34	0.55	1.03	0.5	0.76	0.63	0.69
ZBTB37	1.15	0.54	0.55	1.21	1.19	0.94
ZBTB38	1.59	1.14	1.03	0.84	0.95	0.76
ZBTB39	2.49	2.27	2.61	1.78	2.25	2.15
ZBTB4	2.3	1.25	1.62	1.39	1.72	1.42
ZBTB40	3.32	2.77	2.77	2.19	2.18	2.23
ZBTB41	0.22	0.26	0.24	0.24	0.43	0.34
ZBTB42	0.2	0.22	0.49	0.55	0.23	0.67
ZBTB43	2.56	1.53	2.36	2.05	1.72	2.29
ZBTB44	1.84	1.72	1.85	2.02	2.37	2.43
ZBTB45	3.81	2.04	3.06	3.2	2.89	3.22
ZBTB46	0.12	0.18	0.21	0.05	0.16	0.25
ZBTB47	0.22	0.24	0.44	0.39	0.26	0.3
ZBTB48	11.43	13.56	12.91	12.86	12.63	13.38
ZBTB49	1.96	1.78	0.25	0.82	1.24	1
ZBTB5	2.3	2.16	2.22	2.28	2.08	2.25
ZBTB6	2.36	1.78	2.02	1.33	1.6	1.66
ZBTB7A	0.83	0.73	0.6	0.69	1.07	0.81
ZBTB7B	1.47	1.73	1.32	1.33	1.4	1.9
ZBTB7C	0.03	0.11	0.05	0	0	0.07
ZBTB8A	2.22	3.13	3.26	1.93	2.23	2.31
ZBTB8B	0	0.03	0.05	0.02	0	0.02
ZBTB8OS	34.2	30.8	25.98	19.28	25.71	27.08
ZBTB9	10.22	7.1	10.94	10.4	7.9	7.83
ZC2HC1A	1.98	0.46	0.46	0.3	0.66	0.7
ZC2HC1B	0	0	0	0	0	0
ZC2HC1C	0	0.15	0.26	0	0.05	0
ZC3H10	2.64	4.52	4.84	3.73	3.74	3.87
ZC3H11A	5.86	5.19	5.15	4.79	5.95	7.5
ZC3H12A	1.78	1.91	1.06	1.87	0.61	0.9
ZC3H12B	0.25	0.53	0.34	0.3	0.5	0.25
ZC3H12C	0.37	0.58	0.37	0.36	0.27	0.56
ZC3H12D	0.81	1.23	1.58	0.89	0.82	1.42
ZC3H13	15.87	17.11	16.95	14.25	17.01	17.47
ZC3H14	16.18	13.53	12.86	13.76	13.89	13.24
ZC3H15	65.2	69.17	61.2	51.77	64.42	58.72
ZC3H18	4.09	4.83	6.16	3.32	3.38	4.25
ZC3H3	24.84	31.58	29.97	28.56	23.02	32.96
ZC3H4	3.64	4.97	5.82	4.46	5.4	4.92

ZC3H6	0.4	0.41	0.49	0.37	0.32	0.37
ZC3H7A	6.88	9.56	3.79	5	5.82	5.5
ZC3H7B	6.25	6.83	7.36	7.12	6.06	6.65
ZC3H8	5.16	5.43	4.3	3.27	4.06	4.8
ZC3HAV1	20.02	17.45	15.41	20.02	17.82	19.42
ZC3HAV1L	5.37	5	5.58	4.55	5.55	4.17
ZC3HC1	26.07	27.21	30.02	22.74	22.9	22.41
ZC4H2	7.08	10.5	7.82	11.72	10.27	11.8
ZCCHC10	8.97	10.95	6.46	6.39	9.55	7.95
ZCCHC11	2.3	3.14	3.19	2.31	2.47	2.68
ZCCHC12	1.11	0.26	0.06	0.44	0.66	0.71
ZCCHC13	0	0	0	0	0	0
ZCCHC14	1.57	0.39	0.57	0.47	0.55	0.57
ZCCHC16	0	0	0.02	0	0	0
ZCCHC17	54.89	57.42	55.52	60.09	54.7	47.35
ZCCHC18	1.01	1.23	1.76	1.29	0.88	0.95
ZCCHC2	1.57	2.15	1.64	1.6	1.44	2.1
ZCCHC24	0.19	0.24	0.17	0.34	0.14	0.19
ZCCHC3	3.15	3.17	1.96	2.69	4.27	2.92
ZCCHC4	3.29	3.81	4.09	2.78	4.37	5.04
ZCCHC5	0	0	0	0	0	0.28
ZCCHC6	3.88	3.47	3.09	2.5	4.27	3.6
ZCCHC7	17.46	15.16	14.51	14.78	15.88	15.62
ZCCHC8	2.8	5.75	7.76	5.51	6.31	6.47
ZCCHC9	8.13	16.61	14.92	11.88	13.76	12.27
ZCRB1	19.26	25.06	20.13	17.66	23.62	24.03
ZCWPW1	0.48	0.9	0.57	0.54	0.49	0.53
ZCWPW2	0.27	0.18	0.16	0.25	0.08	0.05
ZDBF2	0.74	0.26	0.25	0.14	0.29	0.02
ZDHHC1	0	0.17	0	0.25	0	0
ZDHHC11	0.51	0.82	0.54	0.25	0.37	0.82
ZDHHC12	50.88	47.12	50.21	49.18	42.15	48.6
ZDHHC13	7.47	7.76	7.61	5.17	7.96	7.5
ZDHHC14	2.52	1.33	1.25	0.93	1.56	1
ZDHHC15	3.51	4.58	4.7	3.16	3.96	3.77
ZDHHC16	13.58	16.41	26.2	21.07	15.67	11.73
ZDHHC17	1.29	1.58	1.32	1.66	2.08	2.3
ZDHHC18	1.86	2.32	1.47	1.08	1.38	2.02
ZDHHC19	0	0	0	0	0	0
ZDHHC2	4.66	3.35	3.22	2.36	4.07	2.59
ZDHHC20	4.33	5.18	3.89	2.26	3.5	4.92
ZDHHC21	0.53	0.48	0.56	0.16	0.34	0.69
ZDHHC22	0.04	0.05	0.35	0.28	0	0

ZDHHC23	0.25	0.02	0.12	0.07	0	0.14
ZDHHC24	13.52	22.63	21.08	20.35	19.13	20.04
ZDHHC3	2.43	4.87	4.45	4.1	3.63	2.96
ZDHHC4	28.55	29.02	28.16	27.86	31.63	21.87
ZDHHC5	12.61	14.81	15.24	11.4	12.33	13.87
ZDHHC6	22.2	25.05	20.75	17.89	25.22	20.47
ZDHHC7	4.13	3.19	2.13	3.48	2.78	2.07
ZDHHC8	0.79	0.93	1.2	0.68	0.87	0.8
ZDHHC8P1	0.46	0.27	0.18	0.19	0.17	0.13
ZDHHC9	15.04	13.77	13.17	12.23	12	12.88
ZEB1	0.53	1.84	1.08	1.04	0.49	0.8
ZEB1-AS1	0.7	0.34	0.27	0.02	0.19	0.17
ZEB2	12.9	10.11	9.55	12.31	12.06	10.91
ZEB2-AS1	0.24	0	0	0	0.21	0.85
ZER1	5.01	3.59	3.59	6.77	5.42	4.02
ZFAND1	15.54	31.19	28.98	21	24.53	32.16
ZFAND2A	5.52	5.7	2.6	4.29	4.66	3.92
ZFAND2B	18.41	17.31	15.74	16.4	16.6	11.98
ZFAND3	1.7	1.12	0.54	0.93	0.93	1.41
ZFAND4	0.56	0.03	0	0.13	0.09	0.16
ZFAND5	10.07	8.84	7.16	7.89	9.71	5.64
ZFAND6	97.85	64.24	47.39	55.63	93.46	55.22
ZFAS1	266.31	294.4	236.59	254.87	329.37	314.6
ZFAT	5.15	5.34	5.53	5.27	4.98	7.29
ZFAT-AS1	0.18	0.07	0	0	0	0
ZFC3H1	3.96	2.6	2.87	2.39	3.17	3.06
ZFHX2	1.09	0.27	0.71	0.51	0.58	0.54
ZFHX3	0.24	0.23	0.61	0.26	0.49	0.22
ZFHX4	0.01	0	0	0	0	0
ZFHX4-AS1	0	0	0	0	0	0
ZFP1	5.49	4.39	5.35	4.32	5.58	4.97
ZFP106	7.57	7.93	6.58	5.82	6.53	6.3
ZFP112	0.18	0.33	0	0.3	0.24	0
ZFP14	1.13	1.2	1.44	0.52	1.14	1.03
ZFP2	0	0	0	0	0	0
ZFP28	2.07	1.06	0.96	0.96	0.94	0.48
ZFP3	0.03	0	0.01	0.01	0	0
ZFP30	1.35	1.49	1.44	1.06	1.02	1.49
ZFP36	14.85	10.69	12.85	9.7	11.1	11.32
ZFP36L1	17.69	5.81	5.07	7.97	9.26	4.61
ZFP36L2	10.71	25.07	26.29	21.2	17.52	17.32
ZFP37	0.02	0	0	0	0	0
ZFP41	3.21	1.25	2.32	1.78	2.07	2.81

ZFP42	0.48	0.69	1.54	0.61	0.92	0.95
ZFP57	0	0	0	0	0	0
ZFP62	3.32	3.38	2.9	3.97	2.61	3.92
ZFP64	4.2	5.96	5.9	5.09	4.32	6.16
ZFP69	0.48	0.46	0.31	0.31	1.42	0.66
ZFP69B	1.38	0.43	1.06	0.51	2.4	0.63
ZFP82	0	0	0	0	0	0
ZFP90	1.09	2.12	1.31	1.52	1.87	1.25
ZFP91	7.7	5.26	5.53	4.83	5.33	7.91
ZFP91-CNTF	0	0	0.21	0	0	0.12
ZFP92	0.12	0.05	0.05	0.25	0.23	0
ZFPL1	16.77	20.71	22.5	22.04	20.48	21.02
ZFPM1	0.58	0.09	0.06	0.37	0.3	0.16
ZFPM2	0.02	0	0	0	0	0
ZFR	30.1	27.88	24.28	24.78	29.3	24.34
ZFR2	0.05	0.05	0.08	0.21	0.05	0.1
ZFX	6.98	7.49	7.57	7.18	6.62	5.88
ZFX-AS1	0.21	0	0	0	0	0
ZFY	0	0	0.01	0	0	0
ZFYVE1	4.72	2.41	3.02	3.7	3.46	1.96
ZFYVE16	1.43	1.26	1.02	1.27	0.72	0.86
ZFYVE19	7.04	5.67	6.46	6.11	6.57	5.6
ZFYVE20	2.7	1.95	2.18	2.43	2.77	2.14
ZFYVE21	30.93	23.06	17.81	20.95	25.75	14.9
ZFYVE26	3.46	2.92	3.49	2.87	3.53	3.07
ZFYVE27	12.18	11.72	10.02	9.05	11.27	8.29
ZFYVE28	0.66	0	0.1	0	0.03	0.17
ZFYVE9	1.73	2.71	3.09	2.55	2.06	3.2
ZG16	0	0.19	0	0.07	0.09	0.15
ZG16B	0.66	0	2.68	0	0.27	0.67
ZGLP1	0.11	1	0.27	0.75	0.42	0.3
ZGPAT	7.43	6.25	5.83	7.1	5.25	5.64
ZHX1	14.19	11.46	11.11	8.91	10.78	8.93
ZHX1-C8ORF	1.62	2.16	3.18	2.05	2.83	2.26
ZHX2	1.26	2.52	2.2	1.47	1.61	1.96
ZHX3	1.13	1.31	1.47	1.3	1.24	1.58
ZIC1	0	0	0	0	0	0
ZIC2	0.76	1.25	1.1	1.53	1.69	1.44
ZIC3	0	0	0	0	0	0
ZIC4	0	0	0	0	0	0
ZIC5	0.06	0.15	0.27	0.11	0.12	0.16
ZIK1	0	0	0	0	0	0
ZIM2	0	0	0	0	0	0

ZIM3	0.08	0	0.03	0.12	0.11	0.12
ZKSCAN1	9.93	7.7	7.89	8.03	7.67	9.39
ZKSCAN2	0.61	0.63	0.37	0.62	0.45	0.77
ZKSCAN3	6.28	6.74	6.65	5.23	5.72	5.83
ZKSCAN4	3.6	5.2	5.69	4.97	2.42	3.49
ZKSCAN5	3.06	3.76	3.5	3.13	2.68	3.25
ZKSCAN7	1.23	1.86	2.3	1.24	1.6	2.56
ZKSCAN8	10.63	7.06	6.86	6.52	7.07	7.66
ZMAT1	0.04	0.08	0.27	0.14	0.11	0.16
ZMAT2	29.91	28.42	27.37	29.04	29.39	30.69
ZMAT3	1.16	0.77	0.61	0.53	0.93	0.72
ZMAT4	0.07	0	0	0	0	0
ZMAT5	28.17	26.4	26.29	32.89	31.98	24.44
ZMIZ1	3.58	4.5	3.6	3.71	4.48	4.33
ZMIZ1-AS1	0	0	0	0	0	0
ZMIZ2	3.36	2.25	2.35	2.89	2.4	3.13
ZMPSTE24	31.45	26.51	25.28	16.08	24.84	24.26
ZMYM1	3.85	3.41	4.69	2.75	3.68	4.6
ZMYM2	2.13	1.74	1.32	1.66	1.64	2.1
ZMYM3	19.75	18.91	20.94	21.58	18.65	17.13
ZMYM4	5.12	5.53	4.15	4.38	3.54	5.21
ZMYM5	5.08	4.51	2.64	3.55	4.4	4.87
ZMYM6	3.2	2.29	2.74	2.25	2.61	2.61
ZMYM6NB	20.7	28.11	27.6	24.51	27.96	26.57
ZMYND10	0	0.56	0.54	0.03	0.51	0.21
ZMYND11	2.24	3.11	3	4.13	2.71	3.16
ZMYND12	0.14	0	0	0	0	0
ZMYND15	0	0	0	0	0	0
ZMYND19	3.68	3.21	4.53	3.52	3.64	4.33
ZMYND8	22.36	8.5	10.4	14.29	19.92	9.73
ZNF10	1.26	0.75	1.56	0.61	1.05	1.01
ZNF100	4.44	4.63	4.76	3.12	4.28	4.53
ZNF101	5.64	9.66	9.19	8.56	9.32	12.71
ZNF107	1.27	3.12	3.32	3.6	2.4	1.83
ZNF114	6.39	9.37	9.38	10.24	8.65	9.21
ZNF117	2.09	1.51	1.77	1.22	1.53	1.42
ZNF12	1.08	0.8	1.26	1.05	1.33	0.82
ZNF121	26.67	28.26	30.58	29.94	28.21	43.36
ZNF124	10.72	12.13	11.77	13.7	11.75	14.72
ZNF131	7.61	6.95	5.96	5.55	6.31	6.92
ZNF132	0.41	0.08	0	0.15	0.23	0.19
ZNF133	1.86	1.41	1.52	4.33	2.81	1.04
ZNF134	10.24	7.89	9.05	9.31	5.9	6.79

ZNF135	0.02	0.06	0	0	0	0
ZNF136	2.53	3.55	5.21	3.18	2.77	2.82
ZNF137P	0.22	0.09	0	0.1	0.03	0.06
ZNF138	4.49	4.97	5.16	4.6	4.16	4.5
ZNF14	2.68	4.29	4.04	4.89	2.92	2.88
ZNF140	7.04	5.56	3.96	4.84	3.72	4.88
ZNF141	0.08	0.04	0.03	0.1	0	0.05
ZNF142	7.98	9.39	8.47	8.54	8.09	8.64
ZNF143	6.58	6.83	5.13	4.73	6.98	6.29
ZNF146	30.46	29.09	25.34	22.32	29.04	25.55
ZNF148	3.38	3.47	3.41	2.88	2.89	3.43
ZNF154	0.03	0.84	0.32	0.05	0.03	0.05
ZNF155	0	0	0.03	0	0.11	0.08
ZNF157	0.44	0.14	0.16	0	0.17	0.06
ZNF16	9.48	8.48	8.69	10.24	11.48	10.87
ZNF160	5	4.84	4.38	3.85	2.81	5.38
ZNF165	1.39	2.09	1.47	1.51	0.99	3.06
ZNF169	1.82	1.77	3.1	2.92	1.29	1.96
ZNF17	3.3	4.09	2.72	3.75	2.52	3.84
ZNF174	7.25	6.68	6.26	5.11	5.99	7.06
ZNF175	1.92	0.86	0.89	0.86	2.11	0.58
ZNF177	0	0.12	0.63	0.13	0.33	0.4
ZNF18	1.09	3.58	2.97	3.14	2.49	3.29
ZNF180	2.12	3.72	2.73	3.21	4.27	4.13
ZNF181	1.6	1.58	1.02	1.85	2.29	1.66
ZNF182	3.1	3.99	3.99	3.87	2.48	3.23
ZNF184	0	0	0	0	0	0
ZNF185	1.06	1.08	0.72	1.32	0.68	0.91
ZNF189	1.25	1.7	1.25	0.93	0.91	0.99
ZNF19	1.89	0.36	0.32	0.53	0.85	0.46
ZNF195	14.09	10.79	12.06	9.63	11.41	11.9
ZNF197	2.35	1.81	2.03	2.84	2.65	2.38
ZNF2	2.3	2.58	3.56	2.6	2.76	3.11
ZNF20	1.83	1.06	1.62	1.82	2.04	2.05
ZNF200	4.6	4.69	5.38	6.04	3.78	4.6
ZNF202	0.86	1.61	1.36	2.56	3.79	1.95
ZNF204P	0.02	0.1	0.06	0.04	0.12	0.12
ZNF205	0	0	0.2	0	0	0
ZNF205-AS1	0.9	0.7	1.12	0.27	0.39	0.04
ZNF207	150.38	124.46	122.08	133.72	143.05	139.7
ZNF208	0.04	0.01	0.04	0.01	0	0.02
ZNF211	2.97	4.07	3.24	2.56	3.61	3.56
ZNF212	3.82	4.71	4.47	4.28	4.98	4.91

ZNF213	1.04	1.26	0.71	0.99	1.34	1.38
ZNF214	0.38	0	0.03	0	0.1	0
ZNF215	3.15	3.92	3.1	1.92	1.96	3.53
ZNF217	10.54	8.2	9.65	9.44	9.45	7.81
ZNF219	0.77	0.31	0.91	0.77	0.49	0.43
ZNF22	9.75	17.36	17.4	22.68	6.53	11.29
ZNF221	1.06	1.06	1.59	0.61	1.03	1.37
ZNF222	2.94	3.65	2.69	2.55	2.31	3.06
ZNF223	0	0	0	0	0	0
ZNF224	2.51	2.14	3.16	1.69	2.88	2.95
ZNF225	2.09	1.59	1.5	1.4	2.27	1.84
ZNF226	4.54	4.05	4.67	2.44	5.96	2.43
ZNF227	4.02	3.47	4.14	4.77	5.17	3.96
ZNF229	0	0	0	0	0	0
ZNF23	2.31	2.25	2.18	3.27	2.5	1.84
ZNF230	0.79	1.11	1.07	1.18	1.02	1.19
ZNF232	3.66	4.31	4.74	4.89	3.15	4.85
ZNF233	0.24	0	0	0	0.17	0.35
ZNF234	2.72	2.96	1.64	2.37	0.67	1.66
ZNF235	0.82	1.42	1.01	1.07	1.12	0.62
ZNF236	1.25	1.39	1.4	1.46	1.19	1.16
ZNF239	0	0	0	0	0	0
ZNF24	8.45	6.2	6.85	6.47	6.86	6.46
ZNF248	1.05	4.09	3.27	3.4	1.66	4.47
ZNF25	1.61	0.75	0.3	0.4	1.5	0.57
ZNF250	2.27	3.28	3.34	3.31	3.51	2.79
ZNF251	1.6	1.53	0.44	1.02	1.1	1.3
ZNF252P	2.63	2.58	3.31	2.42	2.69	2
ZNF252P-AS	0.18	0.14	0.25	0.03	0.1	0.05
ZNF253	0.05	0.3	0.2	0.2	0.11	0.16
ZNF254	3.28	3.76	4	3.17	3.23	3.57
ZNF256	0	0	0	0	0.02	0
ZNF257	0.09	0.08	0.06	0.04	0	0.17
ZNF259	25.52	28.37	30.77	28.71	24.07	17.53
ZNF26	5.68	3.97	3.86	3.92	3.9	3.1
ZNF260	4.31	3.5	3.6	2.91	3.5	3.74
ZNF263	0.73	0.4	0.16	0.47	0.42	1.97
ZNF264	0.54	0.77	1.07	0.6	0.64	0.74
ZNF266	5.17	7.43	4.87	4.45	5.3	5.74
ZNF267	7.5	7.15	6.03	5.4	7.04	6.16
ZNF268	2.96	3.88	3.3	2.95	2.44	3.13
ZNF271	5.76	5.98	4.6	4.69	6.39	4.11
ZNF273	2.45	3.11	2.33	2.31	1.97	2.35

ZNF274	8.83	6.93	7.82	7.87	6.42	5.78
ZNF275	5.24	4.85	3.98	5.1	4.81	5.48
ZNF276	1.49	2.03	1.35	1.3	1.14	0.92
ZNF277	6.59	5.65	5.83	5.38	6.72	5.68
ZNF28	0.03	0.06	0.04	0.05	0.09	0.01
ZNF280A	0	0	0	0	0	0
ZNF280B	1.86	2.28	1.6	1.87	1.42	1.41
ZNF280C	4.02	6.61	5.42	4.36	4.16	5.27
ZNF280D	3.65	1.19	1.56	3.36	2.2	3.93
ZNF281	9.22	9.89	11.6	11.95	10.11	9.98
ZNF282	9.41	9.47	9.43	8.48	9.57	10.81
ZNF283	3.84	3.57	2.37	1.72	2.27	3.12
ZNF284	0.13	0.32	0	0.44	0.22	0
ZNF285	0.08	0.02	0	0	0	0
ZNF286A	4.83	4.44	5.59	4.75	5.15	5.94
ZNF286B	0.59	0.73	1.11	1.02	1.03	0.72
ZNF287	0	0	0.47	0.56	0.03	0.33
ZNF292	3.79	4.86	5.38	4.35	4.8	4.78
ZNF295-AS1	0	0	0	0	0	0
ZNF296	14.22	17.55	22.36	18.23	14.64	14.66
ZNF3	10.07	10.78	10.67	10.74	10.62	11.54
ZNF30	1.79	2.86	1.85	1.51	3.21	2.84
ZNF300	0	0	0.1	0	0.02	0.14
ZNF300P1	0	0	0	0	0	0
ZNF302	3.58	3.55	3.31	3.14	2.72	3.32
ZNF304	2.32	1.44	1.89	1.92	1.57	0
ZNF311	0	0	0	0	0	0
ZNF317	9.29	9.31	10.69	11.2	10.31	12.41
ZNF318	1.25	1.21	1.09	1.28	1.21	1.13
ZNF319	1.71	2.13	1.96	2.28	1.08	2.17
ZNF32	11.66	9.84	10.99	11.35	7.47	14.67
ZNF32-AS1	0	0	0	0	0	0
ZNF32-AS2	0.18	0	0.16	0	0.15	0
ZNF32-AS3	0	0	0	0	0	0
ZNF320	0.17	0.3	0.27	0.18	0.2	0.17
ZNF321P	0.65	0.5	0	0.43	0.5	0.4
ZNF322	4.3	3.55	2.95	3.09	3.72	3.13
ZNF324	4.31	4.82	4.47	4.92	4.07	4.1
ZNF324B	2.14	1.25	1.56	2.3	1.61	2.27
ZNF326	17.32	18.13	15.97	17.35	15.48	16.4
ZNF329	0.35	0.69	0.64	0.36	0.49	0.48
ZNF330	35.86	34.52	29.91	25.9	29.29	28.19
ZNF331	0.42	0.99	0.45	0.63	0.89	0.52

ZNF333	3.22	2.59	2.5	1.68	3.61	2.38
ZNF334	0.02	0	0	0	0	0.04
ZNF335	5.7	5.03	5.31	4.95	4.25	4.6
ZNF337	3.3	4.36	3.79	3.83	2.6	3.59
ZNF33A	3.96	4.6	4.85	3.28	4.11	5.77
ZNF33B	2.65	3.29	3.19	3.28	3.37	4.12
ZNF33BP1	0	0	0	0	0	0
ZNF34	2.49	4.01	4.02	3.5	2.96	5.03
ZNF341	1.23	1.81	1.95	0.89	2.21	1.69
ZNF343	2.12	4.36	4.41	6.23	4.92	5.05
ZNF345	1.82	0.28	2.28	1.45	0.8	0.88
ZNF346	9.63	7.9	7.49	8.64	8.94	9.88
ZNF347	0.48	0.4	0.29	0.28	0.4	0.62
ZNF35	0.3	0.37	0.05	0	0	0
ZNF350	0	0	0	0	0	0
ZNF354A	0.38	0.72	0.35	0.51	0.58	0.63
ZNF354B	1.23	1.54	1.03	1.23	1.47	1.54
ZNF354C	0	0	0	0	0	0
ZNF358	1.25	0.75	2.22	1.49	1.82	1.84
ZNF362	2.7	4.51	5.73	5.32	3.54	3.89
ZNF365	0.05	0.04	0	0	0	0
ZNF366	0	0	0	0	0	0
ZNF367	0.53	0.31	0.34	0.24	0.5	0.18
ZNF37A	5.32	3.77	3.73	3.1	3.53	4.34
ZNF37BP	2.34	1.78	1.55	1.35	1.43	1.87
ZNF382	0	0	0.17	0	0	0.05
ZNF383	2.07	2.8	3.06	2.81	1.91	4.67
ZNF384	11.3	10.94	12.32	11.63	9.72	10.19
ZNF385A	12.35	12.97	12.48	16.6	15.58	12.8
ZNF385B	0	0	0	0	0	0
ZNF385C	0.06	0.04	0.08	0.14	0	0.03
ZNF385D	0	0	0	0	0	0
ZNF391	1.73	1.36	1.58	0.68	1.41	1.23
ZNF394	13.67	11.14	12.14	14.9	13.26	11.41
ZNF395	4.62	4.51	5.36	5.3	5.41	5.69
ZNF396	0	0.02	0.38	0.5	0.15	0.29
ZNF397	3.43	2.9	1.34	1.8	2.55	1.72
ZNF398	3.89	3.29	4.07	3.08	3.54	4.21
ZNF404	0	0	0	0.05	0	0
ZNF407	1.33	1.58	2.97	1.87	1.81	1.95
ZNF408	11.08	7.47	9.08	11.26	10.84	7.31
ZNF41	2.11	3.86	4.41	3.36	3.19	4.26
ZNF410	24.38	17.85	14.66	16.48	21.91	17.43

ZNF414	7.55	6.52	7.27	7.66	6.66	7
ZNF415	0.03	0	0	0.05	0.03	0.05
ZNF416	5.89	3.31	4.14	4.24	3.61	3.72
ZNF417	0.98	1.81	2.41	1.57	1.07	1.36
ZNF418	0.02	0	0	0.01	0	0
ZNF419	3.05	4.28	4.05	1.79	2.64	3.67
ZNF420	3.95	3.1	2.94	3.26	2.47	2.93
ZNF423	0	0	0	0	0	0
ZNF425	0.68	0.43	0.64	0.54	0.73	0.53
ZNF426	0.45	0.24	0.35	0.18	0.27	0.29
ZNF428	3.06	3.77	2.65	4.35	5.25	2.07
ZNF429	2.13	5.18	3.69	2.14	3.48	3.18
ZNF43	0.25	0.48	0.46	0.27	0.31	0.48
ZNF430	0.55	0.81	1.14	0.9	0.73	0.77
ZNF431	2.73	3.01	2.66	1.92	2.58	2.44
ZNF432	0.6	0.58	1.2	0.88	0.67	0.49
ZNF433	0.1	0.27	0.25	0.22	0.21	0.23
ZNF436	2.4	2.61	2.99	2.32	3.1	2.56
ZNF438	0.78	1.63	1.06	1.15	1.57	1.27
ZNF439	0.03	0.26	0.29	0.12	0.25	0
ZNF44	4.28	2.51	1.6	3.76	2.39	2.72
ZNF440	4.06	3.45	3.8	4.79	3.94	3.02
ZNF441	1.53	1.63	1.81	1.48	1.68	1.68
ZNF442	2.19	3.65	3.36	2.38	1.97	1.97
ZNF443	3.69	4.66	4.83	4.15	3.75	3.09
ZNF444	3.04	2.57	3.7	4.15	2.47	2.28
ZNF445	4.83	7.49	7.25	7.38	6.46	10.14
ZNF446	2.32	1.68	1.58	2.24	2.48	1.78
ZNF449	1.56	0.52	0.67	1.43	1.46	0.58
ZNF45	3.94	4.08	3.59	2.58	1.98	3.01
ZNF451	9.49	5.55	6.33	6.36	6.35	5.88
ZNF454	0	0	0	0	0	0
ZNF460	30.02	22.38	24	22.63	23.76	24.68
ZNF461	1.19	0.66	1.25	0.74	1.25	1.14
ZNF462	2.82	1.14	1.8	2.44	2.25	2.28
ZNF467	3.39	2.49	3.16	2.44	3.59	1.8
ZNF468	1.78	2.97	0.6	2.45	1.74	0.12
ZNF469	0.25	0.01	0.08	0.16	0.11	0.14
ZNF470	1.09	1.08	0.4	0.51	0.67	0.63
ZNF471	1.24	1.31	1.81	1.19	1.23	1.47
ZNF473	4.45	4.53	3.76	5.58	5.39	4.85
ZNF474	0	0	0	0	0.11	0
ZNF479	0.11	0	0	0	0	0

ZNF48	1.57	1.45	1.09	1.01	1.96	0.72
ZNF480	5.3	5.22	4.98	6.21	4.68	5.84
ZNF483	2.32	4.33	4.12	2.93	3.27	4.02
ZNF484	1.3	0.94	1.33	0.66	1.19	1.08
ZNF485	0.12	0.08	0.1	0.24	0.2	0
ZNF486	0.43	0.57	0.42	0.17	0.25	0.27
ZNF487P	0.75	0.51	0.09	0.48	0.71	0
ZNF488	0.54	0.56	0.15	0.83	0.82	0.93
ZNF490	5.89	6.25	7.81	5.52	4.82	7.55
ZNF491	0.21	0.36	0.36	0.3	0.17	0.27
ZNF492	2.25	3.3	3.96	2.12	3.11	3.26
ZNF493	1.78	2.82	2.29	1.18	1.91	2.07
ZNF496	7.28	7.95	6.83	7.42	6.44	5.5
ZNF497	0.1	0.32	0.38	0.44	0.31	0.22
ZNF500	3.39	3.81	3.77	4.18	4	4.19
ZNF501	0	0	0	0	0	0.08
ZNF502	0	0	0	0	0.02	0
ZNF503	0	0.31	0.37	0.18	0.16	0.32
ZNF503-AS1	0.06	0	0.34	0	0.04	0
ZNF503-AS2	0.12	0.35	1.27	0.53	0.44	0.15
ZNF506	2.01	2.66	2.96	2.45	1.49	1.96
ZNF507	2.4	2.39	2.19	1.68	1.67	1.99
ZNF510	0.28	0.13	0.36	0.18	0.25	0.25
ZNF511	41.12	50.13	47.27	55.33	61.67	40.69
ZNF512	12.64	16.35	12.94	13.05	13.71	14.87
ZNF512B	1.37	1.1	1.35	1.43	1.42	1.53
ZNF513	0.36	0.45	0.66	0.61	0.7	0.47
ZNF514	0.21	0.09	0.32	0.06	0.59	0.06
ZNF516	0.47	0.86	0.61	0.4	0.32	0.59
ZNF517	0.76	0.43	0.33	0.37	0.32	0.19
ZNF518A	1.61	1.22	1.3	1.12	1.13	1.39
ZNF518B	0.18	0	0	0.01	0	0
ZNF519	0.52	1.44	1.29	0.84	2.11	1.1
ZNF521	3.13	4.97	4.89	9.13	7.13	8.93
ZNF524	13.08	8.2	9.26	12.6	9.13	11.9
ZNF525	0.07	0.08	0.03	0.07	0.04	0.1
ZNF526	6.93	7.35	11.92	8.07	7.77	8.28
ZNF527	1.77	1.71	1.34	1.6	1.68	2.08
ZNF528	0.28	0.4	0.41	0.22	0.38	0.6
ZNF529	2.24	3.05	2.94	1.4	1.92	1.9
ZNF530	2.7	2.49	2.05	1.52	2.5	2.38
ZNF532	0	0	0	0.01	0.03	0
ZNF534	0	0	0	0	0	0.11

ZNF536	0	0	0	0	0	0
ZNF540	0.07	0.21	0.45	0.15	0.61	0
ZNF541	0.03	0	0	0	0.01	0
ZNF542	0.23	0.65	0.43	0.23	0.33	0.31
ZNF543	4.32	2.59	3.91	5.09	4.68	4.23
ZNF544	0.04	0.18	0.18	0.16	0.17	0.23
ZNF546	0.29	0.38	0.31	0.33	0.12	0.28
ZNF547	1.78	0.84	0.96	1.62	1.11	0.4
ZNF548	3.35	3.59	2.81	2.42	2.79	2.4
ZNF549	3.15	3.71	3.45	3.14	3.76	3.29
ZNF550	2.73	1.78	2.31	3.49	1.95	2.75
ZNF551	3.64	5.81	4.28	3.54	4.62	3.9
ZNF552	3.37	3.83	6.55	4.13	4.02	5.96
ZNF554	0.86	0.86	0.75	0.57	0.47	0.93
ZNF555	1.19	1.09	1.18	1.38	1.37	1.1
ZNF556	0	0.18	0.3	0.15	0	0.24
ZNF557	3.21	3.16	3.76	3.69	3.39	3.69
ZNF558	1.61	0.32	0.84	1.11	0.73	0.91
ZNF559	1.02	0.92	2.04	0.93	0.57	1.5
ZNF559-ZNF	0.11	0	0.16	0.08	0	0
ZNF560	0	0	0	0	0.02	0
ZNF561	3.91	2.99	4.53	2.23	2.82	2.96
ZNF562	10.96	11.1	11.67	9.95	10.58	9.66
ZNF563	1.12	2.38	1.94	1.89	1.91	2.14
ZNF564	3.27	4.08	4.6	6.55	3.8	3.95
ZNF565	2.78	2.65	1.98	1.66	2.35	2.1
ZNF566	1.71	2.3	2.03	1.33	1.54	1.64
ZNF567	3.67	2.59	2.39	2.47	2.62	2.04
ZNF568	0.04	0.02	0.07	0.03	0.03	0.02
ZNF569	1.66	1.71	1.36	2.28	1.95	2.04
ZNF57	4.68	5.88	7.34	6.55	5.08	5.48
ZNF570	0.52	1.83	1.41	1.1	0.68	1.06
ZNF571	2.84	0.92	2.69	2.13	0.88	1.08
ZNF572	2.49	1.17	0.69	2.7	1.17	1.89
ZNF573	2.48	2.87	3.37	2.55	3.69	1.81
ZNF574	23.08	32.39	32.6	35.6	25.31	28.55
ZNF575	0.04	0.03	0.36	0.24	0.21	0
ZNF576	6.6	6.82	6.94	4.64	5.89	5.87
ZNF577	0.05	0.21	0.33	0.11	0.05	0.12
ZNF578	0.82	0.64	0.86	0.72	0.77	0.71
ZNF579	0.34	0.11	0.06	0.07	0.1	0.03
ZNF580	4.94	3.54	4.73	4.91	4.27	3.47
ZNF581	51.15	46.13	51.16	64.91	50.03	45.13

ZNF582	0.45	0	0.12	0	0.09	0
ZNF582-AS1	0.13	0.04	0	0	0	0
ZNF583	1.35	2.85	3.49	1.92	1.55	1.78
ZNF584	12.23	12.11	11.18	15.05	13.03	8.37
ZNF585A	2.9	1.66	2.11	1.91	1.74	1.58
ZNF585B	0.04	0.16	0.06	0.05	0.07	0.09
ZNF586	0.46	0	0.71	0.1	0	0
ZNF587	4.93	4.76	4.43	4.01	4.02	4.13
ZNF587B	5.53	6.56	7.08	6.68	5.95	6.53
ZNF589	2.07	4.85	3.3	2.7	4.3	3.54
ZNF592	8.91	10.69	11.13	9.19	8.83	10.59
ZNF593	141.62	176.16	152.67	162.42	163.15	144.92
ZNF594	0.42	0.71	0.65	0.42	0.25	1.13
ZNF595	0.47	0.69	0.58	0.78	0.48	0.59
ZNF596	3.1	1.12	1.91	1.49	1.23	1.47
ZNF597	8.8	5.45	5.64	5.08	5.66	3.86
ZNF598	1.25	1.1	1.2	0.56	0.96	1.73
ZNF599	0.19	0	0.3	0.16	0.2	0.26
ZNF600	0	0	0.07	0.12	0.03	0.15
ZNF605	1.55	0.93	0.91	0.74	0.79	0.45
ZNF606	0	0	0	0	0	0
ZNF607	0.38	1.53	1.19	1.38	2.65	1.1
ZNF608	0	0	0.01	0	0	0
ZNF609	7.28	6.67	4.55	5.68	5.86	7.67
ZNF610	0	0.11	0	0.02	0	0
ZNF611	2.03	1.97	1.67	2.23	2.67	2.23
ZNF613	0.91	2.03	1.15	0.63	1.96	0.55
ZNF614	5.25	5.53	4.17	3.81	4.15	4.67
ZNF615	1.08	0.5	0.08	1.14	0.4	0.75
ZNF616	1.79	2.67	3.55	1.68	2.19	3.92
ZNF618	0.68	0.36	0.51	0.2	0.22	0.18
ZNF619	3.87	2.4	3.62	4.51	2.41	5.51
ZNF620	4.27	4.28	4.2	2.97	3.82	4.17
ZNF621	1.81	1.44	1.38	1.51	2.14	1.48
ZNF622	49.81	44.75	45.1	47.24	40.8	42
ZNF623	7.22	4.65	3.46	5.7	5.25	6.06
ZNF624	1.18	1.02	0.5	0.63	0.85	1.54
ZNF625	0.08	0.17	0.19	0.28	0.12	0.06
ZNF625-ZNF	0	0.14	0.18	0	0	0.23
ZNF626	0.32	0.52	0.57	0.29	0.29	0.55
ZNF627	8.94	5.48	7.14	5.48	7.5	3.91
ZNF628	0.56	0.4	0.78	0.63	0.6	0.38
ZNF629	2.45	4.05	5.08	4.59	3.73	5.36

ZNF630	0	0	0	0	0	0
ZNF638	13.18	12.33	9.89	12.15	12.67	12.64
ZNF639	4.13	2.73	2.88	2.55	2.43	2.19
ZNF641	0.84	0.56	0.2	0.47	0.3	0.24
ZNF644	8.5	7.84	7.04	7.59	8.08	6.59
ZNF645	0	0	0	0	0	0
ZNF646	3.82	4.11	4.66	4.07	3.51	4.26
ZNF648	0	0	0	0	0	0
ZNF649	0.65	2.5	1.16	0.22	1.53	1.57
ZNF652	2.81	3.44	4.06	3.5	3.21	4.98
ZNF653	0.43	0.05	0.09	0.16	0.03	0.33
ZNF654	1.3	1.35	1.84	1.16	1.18	1.81
ZNF655	16.94	11.3	13.39	11.58	12.49	9.81
ZNF658	0.84	0.82	0.72	0.86	0.79	0.62
ZNF658B	0	0	0.13	0	0	0.03
ZNF660	0	0.03	0	0	0	0
ZNF662	0.11	0.16	0.52	0.26	0.35	0.38
ZNF663	0	0	0	0	0	0
ZNF664	32.35	28.65	20.23	30.72	30.6	29.43
ZNF664-FAM	0	0	0	0	0	0
ZNF665	0.65	0.4	0.94	0.58	0.38	0.6
ZNF667	0.38	0.22	0.33	0.15	0.16	0.2
ZNF668	7.52	11.6	9.79	9.57	11.78	8.16
ZNF669	6.53	10.14	7.25	8.49	7.31	8.95
ZNF670	2.75	3.76	4.01	3.43	4.06	3.34
ZNF670-ZNF	0.56	2.26	1.86	1.23	1.32	1.92
ZNF671	2.62	1.83	2.57	2.78	2.36	2.2
ZNF672	25.69	13.15	14.24	21.22	24.68	9.66
ZNF674	1.62	1.18	1.41	1.02	1.78	1.21
ZNF674-AS1	3.96	1.95	2.91	1.65	2.16	2.17
ZNF675	5.59	6.76	5.62	4.82	5.56	5.38
ZNF676	0	0	0	0	0.04	0
ZNF677	0.45	0.58	0.49	0.38	0.51	0.47
ZNF678	0.53	0.79	0.67	0.34	0.61	0.63
ZNF679	0	0	0.05	0.07	0	0
ZNF680	2.44	2.69	2.63	3.43	1.58	2.72
ZNF681	1.63	1.67	2.14	1.39	1.7	1.59
ZNF682	0.43	0.44	0.22	0.24	0.21	0.21
ZNF683	0	0	0	0	0	0
ZNF684	1.06	2.22	1.51	1.77	2.09	0.53
ZNF687	7.39	6.38	7.95	8.41	6.93	8.91
ZNF688	1.77	1.53	3.74	1.81	2.62	3.31
ZNF689	9.63	10.03	8.37	13.19	10.81	10.01

ZNF69	1.71	2.56	2.46	1.56	0.23	0.56
ZNF691	6.93	8.61	9.88	9.88	7.5	9.23
ZNF692	7.78	6.1	4.06	4.72	5.26	2.02
ZNF695	11.43	13.31	15.39	15.41	15.55	16.18
ZNF696	2.66	2.12	2.98	2.36	2.69	1.93
ZNF697	0.3	0.37	0.58	0.22	0.08	0.36
ZNF699	0.46	0.56	0.68	0.34	0.96	0.29
ZNF7	19.54	19.95	16.18	22.12	19.78	16.38
ZNF70	1.56	1.62	2.47	1.88	2.12	2.4
ZNF700	3.97	4.29	5.07	4.06	3.08	3.34
ZNF701	0.03	0.09	0.06	0.2	0.05	0.07
ZNF702P	0.42	0.43	0.35	0.29	0.8	0.25
ZNF703	0.17	0.4	0.5	0.19	0.32	0.24
ZNF704	0.03	0.12	0.05	0.1	0.04	0.1
ZNF705A	0	0	0	0	0	0
ZNF705B	0	0	0	0	0	0
ZNF705D	0	0	0	0	0	0
ZNF705G	0	0	0	0	0	0
ZNF706	76.36	96.43	85.75	78.91	88.55	89.15
ZNF707	3.83	4.5	2.49	2.98	3.32	4.54
ZNF708	2.82	4.17	3.72	2.94	2.38	3.21
ZNF709	2.21	2.28	2.11	1.49	1.61	1.99
ZNF71	2.98	3.12	3.56	3.7	2.75	2.88
ZNF710	1.42	2.2	2.4	1.78	2.12	1.66
ZNF711	2.8	3.08	1.61	2.41	1.93	2.92
ZNF713	1.47	1.25	1.73	1.1	1.58	1.88
ZNF714	0.4	0.56	0.43	0.32	0.41	0.37
ZNF716	0.5	0.31	0.47	0.27	0.33	0.26
ZNF717	1.02	1.36	2.06	1.62	1.39	1.71
ZNF718	4.17	3.01	3.32	2.53	2.17	2.2
ZNF720	1.9	1	1.11	0.69	0.99	0.77
ZNF721	5.6	4.45	3.47	3.8	3.88	3.83
ZNF724P	14.51	17.59	12.01	15.69	11.03	15.73
ZNF726	0.81	1.22	1.34	2.32	1.75	1.79
ZNF727	0	0	0	0	0	0
ZNF728	0.11	0.09	0.08	0.02	0.14	0.08
ZNF729	0	0	0.02	0.04	0	0
ZNF732	0.07	0.03	0	0	0.14	0.09
ZNF733P	0	0	0	0	0	0
ZNF735	0	0.04	0	0.04	0	0
ZNF736	1.69	2.26	3.54	3.15	3.02	5.44
ZNF737	0.26	0.29	0.26	0.16	0.32	0.31
ZNF738	0.84	1.37	1.47	0.88	0.83	0.87

ZNF74	5.5	5.08	4.02	5.34	6.49	6.48
ZNF740	1.14	1.09	1.54	1.2	1.54	1.67
ZNF746	1.02	1.2	1.25	1.26	0.74	0.75
ZNF747	7.85	6.21	5.51	5.27	5.14	4.32
ZNF749	3.46	3.08	2.37	1.57	3.16	4.11
ZNF750	0	0	0	0	0	0
ZNF75A	3.3	1.23	1.08	2.24	1.64	0.18
ZNF75D	1.65	1.98	1.87	1.36	1.58	2.27
ZNF76	7	10.51	11.74	7.09	8.59	8.67
ZNF761	2.97	2.98	2.78	1.9	2.11	2.09
ZNF763	0.23	0.31	0.61	0.58	0.12	0.03
ZNF764	6.11	4.79	5.87	7.47	7.54	4.76
ZNF765	4.54	4.01	4.83	3.87	3.86	3.3
ZNF766	8.35	8.27	8.02	8.94	9.72	6.87
ZNF767	0.92	0.71	0.87	0.62	0.62	1.05
ZNF768	8.86	5.3	6.82	7	8.54	7.87
ZNF77	2.75	1.63	2.99	1.69	3.18	2.56
ZNF770	10.97	10.85	10.87	10.37	11.17	13.89
ZNF771	11.13	7.44	6.29	9.37	9.78	3.89
ZNF772	0.69	0.38	0.57	0.34	0.54	0.69
ZNF773	4.29	4.64	6.71	4.99	5.17	3.45
ZNF774	0.44	0.7	1.26	1.58	0.57	0.86
ZNF775	0.32	0.23	0.03	0.33	0.09	0.03
ZNF776	1.09	1.12	1.18	1.81	1.57	1.26
ZNF777	1.14	1.51	1.04	1.12	1.43	1.15
ZNF778	1.48	1.07	1.1	1.81	1.35	1.4
ZNF780A	2.13	2.9	2.77	1.93	1.86	1.8
ZNF780B	0.91	1.31	0.98	0.66	0.72	1.44
ZNF781	0	0	0	0	0	0
ZNF782	0.44	0.62	0.32	0.56	0.43	0.53
ZNF783	0.69	0.35	0.21	0.13	0.13	0.21
ZNF784	0.37	0.67	0.37	0.36	0.45	0.71
ZNF785	1.08	1.39	1.7	1.01	1.66	1.13
ZNF786	2.13	2.56	2.08	3.07	4.35	3.28
ZNF787	0.42	0.76	0.56	0.63	0.54	0.97
ZNF788	3.7	9.79	0.67	9.31	6.96	9.29
ZNF789	4.59	3.44	2.86	2.11	3.23	2.77
ZNF79	1.8	3.15	2.27	3.38	2.3	3.61
ZNF790	2.71	2.71	2.6	1.27	2.32	2.1
ZNF790-AS1	0.07	0.17	0.27	0.26	0.14	0.37
ZNF791	6.47	4.6	3.26	3.41	3.79	3.98
ZNF792	1.27	0.15	0.1	0.24	0.77	0.32
ZNF793	1.49	2.23	2.45	1.55	1.36	1.96

ZNF799	3.51	4.03	5.09	4.63	4.33	3.76
ZNF8	8.03	7.45	7.83	7.17	6.54	7.18
ZNF80	0.9	0.24	0.5	0.37	0.44	0.33
ZNF800	5.45	5.98	7.64	5.13	6.33	4.61
ZNF804A	0.46	1.33	0.55	0.74	0.64	1.28
ZNF804B	0	0	0	0	0	0
ZNF805	2	1.71	1.64	1.45	2.26	1.55
ZNF808	2.37	3.14	3.52	3.52	2.65	2.15
ZNF81	1.3	1.32	1.11	1.33	1.38	1.5
ZNF812	0.11	0.03	0.03	0	0	0
ZNF813	1.84	2.18	2.87	2.34	2.99	3.42
ZNF814	1.2	1.48	1.91	0.86	1.07	1.69
ZNF815P	0.87	1.19	0.8	0.38	0.44	0.42
ZNF816	0.14	0	0.09	0.1	0.05	0
ZNF816-ZNF	0	0	0.49	0	0.18	0
ZNF818P	0.69	1.96	1.61	0.91	1.06	0.93
ZNF821	0.72	1.15	1.27	0.52	1.19	0.52
ZNF823	4.87	5.68	5.32	4.41	4.68	5.58
ZNF826P	0	0	0	0	0	0.13
ZNF827	1.47	1.18	1.63	1.03	0.83	1.49
ZNF829	0.35	0.32	0.46	0.24	0.29	0.37
ZNF83	3.11	2.73	2.01	2.04	3.62	2.94
ZNF830	9.13	9.85	10.25	7.37	10.54	9.28
ZNF831	0	0	0	0	0	0
ZNF833P	0.33	0.51	0.14	0.28	0.31	0.71
ZNF835	0.32	0.49	0.51	0.4	0.25	0.19
ZNF836	0.2	1.28	0.54	1.23	1	0.84
ZNF837	0	0.41	0.32	0	0.36	0
ZNF839	1.51	0.86	0.93	0.96	1.35	1.09
ZNF84	3.93	3.24	3.07	2.85	2.44	3.02
ZNF841	0.07	0.12	0.23	0.19	0.17	0.06
ZNF843	0	0	0	0	0	0.19
ZNF844	0.03	0.06	0.05	0.04	0.05	0
ZNF845	2.5	1.93	2.68	2.49	2.08	3.12
ZNF846	0.28	0.11	0.44	0.59	0.36	0.56
ZNF847P	0	0	0	0	0	0
ZNF85	0.19	0.33	0.51	0.17	0.24	0.1
ZNF850	2.49	2.83	3.09	2.35	1.88	2.3
ZNF853	0.02	0.1	0.04	0.14	0.1	0.41
ZNF860	0.06	0.04	0	0	0	0
ZNF862	0.21	0.16	0.53	0.52	0.26	0.28
ZNF865	1.04	1.19	0.87	0.6	0.64	0.82
ZNF876P	0.9	0.92	1.59	0.85	1.57	1.35

ZNF878	0.04	0.07	0.09	0.44	0	0
ZNF879	0	0.02	0	0	0	0
ZNF880	0	0	0	0	0	0
ZNF883	0	0	0	0	0	0
ZNF890P	0	0	0	0.03	0	0
ZNF90	0.01	0.02	0.04	0	0	0.07
ZNF91	3.31	3.25	4.9	3.45	3.02	3.45
ZNF92	7.46	10.08	10.41	6.14	9.43	9.67
ZNF93	0.08	0.15	0	0	0.02	0.11
ZNF98	0.38	0.74	0.84	0.6	0.48	0.23
ZNF99	0.1	0.16	0.15	0.08	0.1	0.23
ZNFX1	5.56	3.67	5.42	4.74	3.48	4.49
ZNHIT1	103.91	111.73	118.23	117.84	116.89	97.96
ZNHIT2	16.41	11.72	12.16	14.11	14.39	8.6
ZNHIT3	55.37	61.71	59.17	63.69	63.53	58.82
ZNHIT6	5.88	5.5	4.97	3.52	4.62	5.23
ZNRD1	72.82	96.77	91.71	84.2	76.06	79.39
ZNRD1-AS1	0.51	0.29	1.44	0.77	0.71	1.56
ZNRF1	2.68	3.28	2.18	2.53	3.89	2.38
ZNRF2	0.36	0.21	0.12	0.1	0.22	0.23
ZNRF2P1	0.04	0.09	0.17	0.5	0.18	0.29
ZNRF2P2	0	0	0	0.28	0.39	0
ZNRF3	1.26	1.14	1.01	1.05	1.3	1.14
ZNRF3-AS1	1.71	2.42	2.27	1.39	1.65	1.91
ZNRF4	0	0	0	0	0	0
ZP1	0	0	0	0.05	0	0
ZP2	0	0	0	0	0	0
ZP3	0.08	0	0	0	0	0.91
ZP4	0	0	0	0	0	0
ZPBP	0	0	0	0	0	0
ZPBP2	0	0	0	0	0	0
ZPLD1	1.12	0	0	0.01	0.04	0
ZRANB1	1.44	0.67	0.91	0.91	1.18	1.45
ZRANB2	16.01	19.69	16.52	15.25	18.01	16.93
ZRANB2-AS1	0.09	0.07	0	0.01	0.09	0.09
ZRANB2-AS2	0.4	0.16	0.74	0.42	0.1	0.81
ZRANB3	1.81	2.1	2.41	2.07	2.5	4.03
ZRSR2	3.51	3.03	4.8	2.79	2.89	1.97
ZSCAN1	0	0	0	0	0	0
ZSCAN10	0	0	0	0	0	0
ZSCAN12	0.2	0.5	0.28	0.3	0.3	0.27
ZSCAN12P1	0.37	0.31	0.46	0.37	0.27	0.7
ZSCAN16	3.38	4.35	2.39	3.27	3.7	2.82

ZSCAN18	0	0.29	0.05	0.1	0.19	0.1
ZSCAN2	14.43	16.86	17.66	17.71	17.55	16.42
ZSCAN20	1.46	0.85	1.21	0.68	0.37	0.82
ZSCAN21	4.23	4.53	1.95	3.6	3.33	2.82
ZSCAN22	7.46	6.17	7.2	4.63	4.76	4.81
ZSCAN23	0.02	0.07	0	0.05	0.07	0.05
ZSCAN25	5.62	5.26	4.14	3.08	4.45	5.62
ZSCAN26	3.79	2.59	2.27	3.24	4.24	3.95
ZSCAN29	2.9	3.65	2.85	1.81	2.71	3.27
ZSCAN30	0.93	0.48	1.19	0.7	0.87	0.67
ZSCAN31	3.12	2.63	2.36	0.92	2.39	1.88
ZSCAN32	3.59	3.75	4.74	4.68	4.93	3.48
ZSCAN4	0	0.03	0	0	0	0.03
ZSCAN5A	2.15	1.21	2.4	1.01	2.11	1.91
ZSCAN5B	0.15	0.07	0	0	0	0
ZSCAN9	4.87	3.03	4.31	3.64	5.28	4.67
ZSWIM1	13.72	10.99	11.69	11.61	12.94	9.32
ZSWIM2	0	0	0	0	0	0
ZSWIM3	2.88	2.39	4.3	1.85	2.67	1.92
ZSWIM4	0.32	0.05	0.11	0.29	0.17	0.12
ZSWIM5	0.01	0.01	0.1	0.01	0.01	0.01
ZSWIM6	1.22	0.65	0.5	0.83	0.52	0.38
ZSWIM7	8.89	10.61	8.99	7	11.23	7.99
ZSWIM8	1.24	0.65	0.67	0.61	0.65	0.66
ZSWIM8-AS1	0.42	0.27	0.31	0.39	0	0
ZUFSP	9.32	7.51	5.55	5.66	6.28	6.97
ZW10	13.31	15.68	14.68	13.59	12.7	16.5
ZWILCH	27.96	29.3	25.02	21.89	26.04	23.27
ZWINT	85.38	100.95	81.02	77.95	98.24	82.91
ZXDA	0.26	0.33	1.03	0.41	0.44	0.53
ZXDB	1.28	1.76	1.97	1.39	1.9	1.53
ZXDC	2.09	1.72	1.27	1.39	1.8	1.98
ZYG11A	0.76	0.8	1.2	0.76	0.73	0.47
ZYG11B	2.24	2.42	2.19	2.29	1.99	1.93
ZYX	23.57	24.17	28.82	23.36	25.64	20.02
ZZEF1	3.17	2.56	2.95	2.51	2.65	3.67
ZZZ3	6.41	7.76	6.88	7.81	6.72	6.42
