# Supplementary material for: Integrated multi-omics analyses reveal homology-directed repair pathway as a unique dependency in near-haploid leukemia
Source: Blood Cancer J. 2023 Jun 8;13(1):92. doi: 10.1038/s41408-023-00863-1 (PMC10247733; doi:10.1038/s41408-023-00863-1)
Supplement: Supplementary file 5 — Supplementary Table 4 [file 41408_2023_863_MOESM5_ESM.pdf]

Signature genes	Intersection of signature genes and cluster 2 genes
UBE2J2	TYMS
AURKAIP1	PCNA
MRPL20	RRM2
SSU72	TUBB4B
CDK11A	H2AFZ
GNB1	SPC25
RER1	H2AFX
FAM213B	HMGN2
ICMT	CENPW
PARK7	CKS1B
SLC25A33	CDKN3
TARDBP	ANP32E
SRM	DTYMK
UBIAD1	MAD2L1
MAD2L2	NUCKS1
EFHD2	BUB3
SDHB	TSPO
MRTO4	ARL6IP1
AKR7A2	HSP90AA1
CAPZB	RAN
EIF4G3	CALM2
CDC42	
TCEB3	
PITHD1	
SRRM1	
TMEM50A	
ZNF593	
HMGN2	
NUDC	
TMEM222	
RPA2	
DNAJC8	
ATPIF1	
RCC1	
SNHG12	
TAF12	
MECR	
ZCCHC17	
EIF3I	
HDAC1	
ZBTB8OS	
AK2	
PSMB2	
CLSPN	
TRAPPC3	
MRPS15	
C1orf109	
YRDC	
C1orf122	
UTP11L	
AKIRIN1	
PPT1	
ZMPSTE24	
CTPS1	
PPIH	
EBNA1BP2	
CDC20	
MED8	
DPH2	
ATP6V0B	
UROD	
TXNDC12	
KTI12	
BTF3L4	
MAGOH	
YIPF1	
HSPB11	
TMEM59	
TM2D1	
AK4	
SERBP1	
SRSF11	
ACADM	
NEXN	
SSX2IP	
SH3GLB1	
15-Sep	
LRRC8D	
ZNF326	
MTF2	
TMED5	
DR1	
ALG14	
PTBP2	
RTCA	
TMEM167B	
PSRC1	
PSMA5	
LAMTOR5	
WDR77	
ATP5F1	
RAP1A	
CAPZA1	
BCAS2	
SIKE1	
VANGL1	
CD58	
GDAP2	
WDR3	
RBM8A	
ANP32E	
MRPS21	
MCL1	
TNFAIP8L2	
SCNM1	
PSMB4	
RP1-178F15.4	
SLC39A1	
TPM3	
C1orf43	
CKS1B	
FLAD1	
SLC50A1	
KRTCAP2	
MTX1	
SCAMP3	
DAP3	
GPATCH4	
MRPL24	
HDGF	
PRCC	
NTRK1	
PIGM	
F11R	
PFDN2	
SDHC	
FCGR2A	
TMCO1	
UCK2	
TIPRL	
NME7	
PRRC2C	
CACYBP	
MRPS14	
TOR3A	
ACBD6	
KIAA1614-AS1	
GLUL	
RGS16	
DHX9	
RNF2	
UCHL5	
TROVE2	
GLRX2	
TIMM17A	
RNPEP	
NUCKS1	
BATF3	
TATDN3	
ANGEL2	
CNIH4	
PYCR2	
ACBD3	
PARP1	
PSEN2	
ARF1	
C1orf35	
MRPL55	
ARV1	
FAM89A	
C1orf131	
COA6	
GGPS1	
CEP170	
COX20	
CNST	
AHCTF1	
ACP1	
ADI1	
RRM2	
HPCAL1	
ODC1	
PDIA6	
E2F6	
SDC1	
SF3B6	
RAB10	
EPT1	
PREB	
ATRAID	
EIF2B4	
SNX17	
PPM1G	
GPN1	
SUPT7L	
MRPL33	
RBKS	
WDR43	
DPY30	
FAM98A	
CEBPZOS	
CEBPZ	
CYP1B1-AS1	
SRSF7	
GEMIN6	
PIGF	
MCFD2	
CALM2	
CHAC2	
RTN4	
CFAP36	
VRK2	
COMMD1	
RAB1A	
C1D	
PNO1	
PPP3R1	
NFU1	
PCBP1	
SNRPG	
FAM136A	
MPHOSPH10	
SPR	
SMYD5	
TPRKB	
BOLA3	
MOB1A	
PCGF1	
AUP1	
HTRA2	
POLE4	
MRPL19	
TRABD2A	
RNF181	
TMEM150A	
USP39	
RPIA	
MRPS5	
CIAO1	
ANKRD39	
UNC50	
MITD1	
TXNDC9	
EIF5B	
PDCL3	
C2orf49	
UXS1	
NPHP1	
RP11-65I12.1	
ACTR3	
DDX18	
TFCP2L1	
NIFK	
TSN	
POLR2D	
IMP4	
PLEKHB2	
UBXN4	
MMADHC	
PRPF40A	
PSMD14	
SPC25	
SSB	
METTL5	
GORASP2	
DYNC1I2	
HAT1	
MTX2	
ITGA4	
ZNF804A	
ZC3H15	
ORMDL1	
PMS1	
COQ10B	
HSPE1	
MOB4	
C2orf47	
BZW1	
NDUFB3	
WDR12	
FASTKD2	
CCNYL1	
IDH1	
RPE	
ARPC2	
AAMP	
TMBIM1	
BCS1L	
TUBA4A	
DNPEP	
MRPL44	
NCL	
PTMA	
COPS7B	
EIF4E2	
COPS8	
LRRFIP1	
SCLY	
ILKAP	
HES6	
TRAF3IP1	
MYEOV2	
PPP1R7	
2-Sep	
DTYMK	
CRBN	
ARL8B	
ARPC4	
EMC3	
SEC13	
CHCHD4	
MRPS25	
DPH3	
OXNAD1	
RAB5A	
UBE2E1	
NGLY1	
CMC1	
GPD1L	
CMTM6	
DYNC1LI1	
EIF1B	
HIGD1A	
KIAA1143	
EXOSC7	
LZTFL1	
CCDC12	
ELP6	
MAP4	
NME6	
PLXNB1	
UQCRC1	
P4HTM	
NDUFAF3	
RHOA	
GNAI2	
IFRD2	
HYAL3	
HYAL2	
TUSC2	
RASSF1	
CYB561D2	
CYB561D2	
MANF	
TEX264	
RRP9	
ALAS1	
SMIM4	
SPCS1	
SELK	
ARF4	
PDHB	
THOC7	
PSMD6	
LRIG1	
TMF1	
UBA3	
PPP4R2	
CHMP2B	
ARL13B	
NIT2	
TFG	
TRMT10C	
PCNP	
CD47	
ATG3	
NAA50	
TMEM39A	
TIMMDC1	
COX17	
RP11-18H7.1	
NDUFB4	
EAF2	
FAM162A	
HACD2	
UMPS	
RAB7A	
ISY1	
CNBP	
MBD4	
CDV3	
SRPRB	
MRPS22	
RNF7	
ATP1B3	
PCOLCE2	
U2SURP	
COMMD2	
SELT	
SUCNR1	
CCNL1	
SHOX2	
MFSD1	
PDCD10	
LRRC34	
SEC62	
GNB4	
ACTL6A	
MRPL47	
PARL	
ALG3	
ECE2	
PSMD2	
EIF4G1	
POLR2H	
EPHB3	
TRA2B	
DNAJB11	
ATP13A3	
PPP1R2	
TFRC	
TCTEX1D2	
PCGF3	
FGFRL1	
SLBP	
LETM1	
TNIP2	
ADD1	
NOP14	
LRPAP1	
LYAR	
STX18	
STX18-AS1	
MSX1	
MRFAP1	
BLOC1S4	
TADA2B	
GRPEL1	
QDPR	
RBPJ	
UBE2K	
RHOH	
TMEM33	
COMMD8	
SLC30A4	
USP46	
SCFD2	
RCHY1	
THAP6	
11-Sep	
MRPL1	
ENOPH1	
THAP9	
MRPS18C	
PTPN13	
KLHL8	
NUDT9	
NAP1L5	
SMARCAD1	
PDLIM5	
RAP1GDS1	
EIF4E	
ADH5	
H2AFZ	
SLC39A8	
UBE2D3	
CISD2	
PPA2	
AIMP1	
GAR1	
C4orf32	
C4orf3	
MAD2L1	
EXOSC9	

BBS7
KIAA1109
PGRMC2
NDUFC1
NAA15
MGST2
SCOC
ZNF330
ANAPC10
LSM6
ARHGAP10
KIAA0922
PPID
TMA16
RP11-218F10.3
TMEM192
NEK1
SAP30
SPCS3
DCTD
PDCD6
CLPTM1L
MRPL36
NDUFS6
MED10
NSUN2
FAM173B
DAP
OTULIN
SUB1
BRIX1
DNAJC21
MRPS30
PELO
MOCS2
NDUFS4
CCNO
DHX29
SREK1IP1
CWC27
SGTB
NLN
SREK1
CDK7
AK6
SCAMP1
HOMER1
ZCCHC9
TMEM167A
CCNH
POLR3G
NR2F1
CHD1
FAM174A
SRP19
REEP5
ATG12
CTC-546K23.1
ISOC1
SLC22A4
LEAP2
ZCCHC10
PPP2CA
UBE2B
SAR1B
DDX46
ETF1
HSPA9
PAIP2
UBE2D2
PURA
CYSTM1
SRA1
GNPDA1
NDFIP1
GEMIN5
MRPL22
CLINT1
TTC1
PTTG1
NUDCD2
PANK3
RPL26L1
ATP6V0E1
BNIP1
SFXN1
SIMC1
NOP16
CLTB
FAF2
LMAN2
GRK6
PRR7
TMED9
HNRNPAB
CANX
TRIM7
NQO2
TUBB2A
SNRNP48
EEF1E1
NOL7
DEK
ACOT13
GMNN
U91328.19
ABT1
ZNRD1
ABCF1
PPP1R10
DHX16
CSNK2B
CLIC1
BRD2
SLC39A7
PFDN6
UQCC2
NUDT3
SNRPC
TAF11
SRPK1
SRSF3
MTCH1
CCDC167
CCND3
MRPS10
MEA1
KLHDC3
RRP36
POLR1C
YIPF3
MRPS18A
MRPL14
SLC29A1
NFKBIE
CD2AP
ICK
FBXO9
BAG2
FKBP1C
SDHAF4
SYNCRIP
UBE2J1
NDUFAF4
COQ3
ATG5
RTN4IP1
CISTM1
CD164
ZBTB24
CDC40
GTF3C6
RPF2
SLC16A10
TUBE1
RWDD1
GOPC
ASF1A
HDDC2
CENPW
MTFR2
NHSL1
ABRACL
CITED2
LTV1
SF3B5
PPIL4
GINM1
PCMT1
TFB1M
TMEM242
DYNLT1
WTAP
MRPL18
SFT2D1
MPC1
PHF10
PSMB1
PDCD2
ZFAND2A
PSMG3
FTSJ2
BRAT1
AIMP2
RAC1
KDELR2
NDUFA4
PHF14
TWISTNB
NUPL2
MALSU1
CYCS
NFE2L3
HNRNPA2B1
HOXA11
TAX1BP1
LSM5
RP9
7-Sep
RALA
MPLKIP
PSMA2
MRPL32
STK17A
BLVRA
DBNL
YKT6
PPIA
HUS1
SEC61G
VOPP1
MRPS17
CCT6A
CHCHD2
ASL
TMEM248
SBDS
FZD9
VPS37D
DNAJC30
WBSCR22
ABHD11
EIF4H
STYXL1
GSAP
SRI
STEAP1
KRIT1
BET1
PON2
SHFM1
PDAP1
BUD31
ATP5J2
ZSCAN21
COPS6
GNB2
POP7
SH2B2
PRKRIP1
POLR2J
DNAJC2
PSMC2
SRPK2
BCAP29
LINC00998
CAPZA2
ST7
FAM3C
NDUFA5
RBM28
HILPDA
CALU
STRIP2
BPGM
TTC26
C7orf55
LUC7L2
SSBP1
GSTK1
CDK5
ABCF2
RHEB
DNAJB6
HCCS
RAB9A
CTPS2
SYAP1
PDHA1
MBTPS2
PRDX4
MID1IP1
FUNDC1
FTSJ1
EBP
PRAF2
NUDT11
CXorf65
PBDC1
TIMM8A
GLA
ARMCX6
PRPS1
UBE2A
UPF3B
MCTS1
RAB33A
HPRT1
HTATSF1
BCAP31
NAA10
ATP6AP1
UBL4A
FBA1
VBP1
FBXO25
ERICH1
AGPAT5
PINX1
NEIL2
CTSB
AC145124.2
VPS37A
SLC7A2
INTS10
REEP4
SLC39A14
CCDC25
LEPROTL1
MAK16
ERLIN2
BRF2
LSM1
PLPP5
TM2D2
UBE2V2
LYPLA1
TGS1
LYN
IMPAD1
SDCBP
CA8
RAB2A
GGH
ARMC1
MTFR1
CTD-2532N20.1
RRS1
COPS5
CSPP1
TRAM1
TERF1
UBE2W
TCEB1
TMEM70
MRPS28
TPD52
FABP5
IMPA1
CA2
RIPK2
OSGIN2
C8orf37
PTDSS1
MTDH
LAPTM4B
POP1
OSR2
POLR2K
ZNF706
SLC25A32
UTP23
MRPL13
DERL1
C8orf76
NDUFB9
MYC
FAM49B
PYCRL
TSTA3
GRINA
EXOSC4
CYC1
HGH1
SLC52A2
SLC39A4
COMMD5
C8orf33
PUM3
MTAP
NDUFB6
BAG1
CHMP5
UBAP1
DCTN3
SIGMAR1
GBA2
CLTA
TOMM5
EXOSC3
FXN
C9orf40
OSTF1
RFK
PSAT1
ISCA1
SPTLC1
NOL8
CENPP
FAM120AOS
AAED1
ANP32B
NANS
SEC61B
MRPL50
SMC2
RAD23B
TXN
KIAA0368
DNAJC25
SLC31A1
CDC26
PRPF4
POLE3
ATP6V1G1
PHF19
RAB14
STOM
NDUFA8
RBM18
PSMB7
PPP6C
RABEPK
HSPA5
SLC27A4
URM1
SET
ENDOG
DOLPP1
NTMT1
ASB6
TOR1A
C9orf78
FAM78A
MED27
GTF3C4
SURF2
SURF4
WDR5
MRPS2
QSOX2
GPSM1
AGPAT2
EDF1
TRAF2
C9orf142
SSNA1
TUBB4B
NELFB
MRPL41
DPH7
ARRDC1
PSMD13
RNH1
TALDO1
CD151
CHID1
TOLLIP
CD81
TSSC4
PHLDA2
RHOG
MRPL17
PPFIBP2
AKIP1

TMEM9B
TMEM41B
COPB1
PSMA1
C11orf58
NUCB2
LDHA
TSG101
SPTY2D1
ARL14EP
RCN1
CSTF3
CD59
APIP
COMMD9
API5
PEX16
SPI1
PTPMT1
MTCH2
SLC43A3
TMX2
CTNND1
CCDC86
TMEM109
TMEM138
TMEM216
FADS1
GANAB
POLR2G
TMEM179B
SLC3A2
ATL3
RTN3
NAA40
OTUB1
FKBP2
MRPL49
SSSCA1
RNASEH2C
CFL1
FOSL1
EIF1AD
BANF1
KLC2
RAB1B
YIF1A
BRMS1
CORO1B
MRPL21
FAM86C1
LAMTOR1
P2RY2
MRPL48
COA4
UCP2
SPCS2
RSF1
NDUFC2
RAB30-AS1
PCF11
TMEM126B
TMEM126A
C11orf73
CTSC
CHORDC1
CWC15
TMEM123
DCUN1D5
AASDHPPT
SLC35F2
DIXDC1
TIMM8B
SDHD
PTS
RBM7
REXO2
ZPR1
CEP164
ARCN1
TRAPPC4
VPS11
H2AFX
SC5D
TBRG1
DCPS
VPS26B
GTPBP4
ANKRD16
RBM17
ATP5C1
TAF3
PROSER2
NUDT5
CDC123
PRPF18
MLLT10
RAB18
ZEB1
KIF5B
HNRNPF
TIMM23
ZWINT
CISD1
UBE2D1
TFAM
REEP3
HNRNPH3
RUFY2
DDX21
SUPV3L1
AIFM2
SAR1A
ASCC1
DNAJC9
MRPS16
ANXA7
CHCHD1
SAMD8
VDAC2
PPIF
GHITM
RPP30
5-Mar
IDE
TCTN3
PGAM1
EXOSC1
UBTD1
AVPI1
CUTC
BLOC1S2
NDUFB8
MRPL43
NPM3
TAF5
GSTO1
SMNDC1
SMC3
GPAM
ZDHHC6
FAM204A
FAM45A
SFXN4
PRDX3
BUB3
BCCIP
PTPRE
GLRX3
BNIP3
ZNF511
FUOM
ECHS1
TIGAR
MRPL51
COPS7A
TPI1
EMG1
LPCAT3
M6PR
MAGOHB
YBX3
SMIM10L1
STRAP
MGST1
GOLT1B
CMAS
KRAS
MRPS35
CCDC91
ERGIC2
FAM60A
ZCRB1
RPAP3
ARF3
MCRS1
TMBIM6
ATF1
ATG101
KRT18
C12orf10
PRR13
COPZ1
GTSF1
CD63
ORMDL2
PYM1
NABP2
CNPY2
PTGES3
NDUFA4L2
TSFM
GNS
LLPH
TMBIM4
CNOT2
RAB21
KRR1
PPP1R12A
CCDC59
C12orf29
EEA1
NUDT4
UBE2N
MRPL42
RP11-1105G2.3
METAP2
TMPO
IKBIP
ACTR6
GAS2L3
ARL1
CHPT1
NUP37
HSP90B1
TDG
TXNRD1
C12orf45
PRDM4
ISCU
ALKBH2
TCHP
ATP2A2
ARPC3
GPN3
FAM216A
VPS29
HVCN1
BRAP
C12orf49
TESC
PEBP1
SUDS3
PRKAB1
RAB35
SRSF9
DYNLL1
POP5
MLEC
C12orf43
VPS33A
C12orf65
RILPL2
EIF2B1
GTF2H3
BRI3BP
RAN
PUS1
EP400NL
NOC4L
PGAM5
N6AMT2
SAP18
MRPL57
PARP4
MTMR6
MTIF3
POLR1D
POMP
CCNA1
ALG5
EXOSC8
GTF2F2
ZC3H13
SUCLA2
NUDT15
ITM2B
DLEU2
VPS36
SUGT1
DIS3
UCHL3
FBXL3
RBM26
TGDS
DNAJC3
PCCA
TEX30
LAMP1
ADPRHL1
TFDP1
OSGEP
TMEM55B
PNP
METTL17
HNRNPC
DAD1
MRPL52
PSMB5
EMC9
CHMP4A
NEDD8
BAZ1A
GEMIN2
LRR1
RPL36AL
TMX1
C14orf166
ERO1A
PSMC6
GNPNAT1
CDKN3
SOCS4
PSMA3
TIMM9
JKAMP
DHRS7
MNAT1
ATP6V1D
PIGH
RAD51B
ERH
MED6
EIF2B2
TMED10
AHSA1
SLIRP
PSMC1
CALM1
NDUFB1
DDX24
YY1
HSP90AA1
CINP
EIF5
TRMT61A
APOPT1
JAG2
NIPA1
NIPA2
KLF13
AVEN
EMC7
KATNBL1
EMC4
NOP10
DPH6
MEIS2
SRP14
KNSTRN
C15orf57
RAD51
TMEM87A
SNAP23
TGM5
EIF3J
SORD
GALK2
USP8
LYSMD2
CCPG1
MNS1
GTF2A2
BNIP2
FAM96A
PPIB
MTFMT
HACD3
RAB11A
ANP32A
ADPGK
SCAMP2
MPI
COX5A
PPCDC
COMMD4
SNUPN
IMP3
UBE2Q2
ETFA
IDH3A
WDR61
PSMA4
MORF4L1
TMED3
MTHFS
MESDC2
FAM103A1
WDR73
MRPL46
MRPS11
CIB1
VIMP
SNRPA1
POLR3K
SNRNP25
LUC7L
MRPL28
FAM195A
STUB1
WDR24
UBE2I
MRPS34
HAGH
FAHD1
NDUFB10
ZNF598
NTHL1
PGP
ECI1
SRRM2
DNAJA3
HMOX2
ALG1
EEF2KMT
NUBP1
DEXI
CLEC16A
GSPT1
RP11-166B2.1
ARL6IP1
COQ7
NDUFAB1
TAOK2
PPP4C
BOLA2B
SEPHS2
ZNF747
SRCAP
STX4
ORC6
NETO2
NUDT21
HERPUD1
FAM192A
ARL2BP
CIAPIN1
COQ9
POLR2C
CFAP20
GOT2
DYNC1LI2
FAM96B
KIAA0895L
E2F4
TMEM208
ATP6V0D1
NIP7
NQO1
AARS
PSMD7
GABARAPL2
TERF2IP
CMC2
ATMIN
GCSH
MPHOSPH6
RP11-505K9.1
GINS2
EMC8
COX4I1
MAP1LC3B
CYBA
PIEZO1
APRT

TRAPPC2L
CHMP1A
DEF8
FAM101B
FAM57A
GLOD4
PITPNA
TSR1
EMC6
NCBP3
UBE2G1
PSMB6
KIF1C
RABEP1
RPAIN
CTC-524C5.2
C1QBP
DERL2
TXNDC17
RNASEK-C17orf49
ELP5
EIF5A
MPDU1
TRAPPC1
HES7
PER1
STX8
SCO1
HS3ST3B1
CENPV
COPS3
MED9
DRG2
NLK
TMEM199
ERAL1
DHRS13
TEFM
COPRS
PSMD11
DUSP14
DDX52
CISD3
PSMB3
KRT10
EIF1
DNAJC7
RAB5C
TUBG1
VPS25
COA3
PSME3
RND2
G6PC3
CCDC43
EFTUD2
NMT1
PNPO
CBX1
SNX11
SNF8
PHB
SLC35B1
MRPL27
LRRC59
LUC7L3
NME1
UTP18
COX11
COIL
MRPS23
DYNLL2
SUPT4H1
RAD51C
SKA2
CTD-2319I12.2
STRADA
PSMC5
ICT1
NT5C
HN1
NUP85
MRPS7
SLC25A19
GRB2
GALK1
H3F3B
SPHK1
SRSF2
SYNGR2
ARL16
HGS
MRPL12
SLC25A10
ARHGDIA
ALYREF
PCYT2
PYCR1
STRA13
DCXR
GPS1
SLC16A3
CSNK1D
TYMS
METTL4
MYL12A
MYL12B
NDUFV2
VAPA
TUBB6
SEH1L
FAM210A
SNRPD1
ZNF24
SLC39A6
HAUS1
IER3IP1
ELAC1
TXNL1
SEC11C
LMAN1
TMX3
TIMM21
CNDP2
CTDP1
TXNL4A
ZCCHC3
TRIB3
PSMF1
NSFL1C
SNRPB
NOP56
IDH3B
MRPS26
ITPA
TMEM230
PCNA
TRMT6
MKKS
ESF1
NDUFAF5
SNRPB2
RRBP1
NAA20
NXT1
APMAP
ABHD12
MAPRE1
CHMP4B
EIF2S2
DYNLRB1
NCOA6
EIF6
SPAG4
NFS1
ROMO1
RBM39
SCAND1
C20orf24
SAMHD1
SRSF6
YWHAB
TOMM34
TP53RK
RNF114
DPM1
PFDN4
RTFDC1
RAE1
CTSZ
PRELID3B
TAF4
PSMA7
ADRM1
SAMD10
TPGS1
CDC34
BSG
RNF126
WDR18
TMEM259
POLR2E
NDUFS7
SCAMP4
MKNK2
PLEKHJ1
TIMM13
S1PR4
DOHH
MFSD12
MRPL54
SH3GL1
MYDGF
C19orf70
CLPP
ELAVL1
NDUFA7
PPAN
DNMT1
MRPL4
KEAP1
TMED1
YIPF2
LDLR
TMEM205
PLPPR2
C19orf43
ASNA1
PRDX2
RNASEH2A
GCDH
CALR
GADD45GIP1
C19orf53
ASF1B
ADGRL1
GIPC1
ADGRE2
RAB8A
FAM32A
SMIM7
SIN3B
BABAM1
CERS1
COPE
DDX49
TMEM161A
ZNF738
UQCRFS1
POP4
URI1
PDCD5
CEBPG
PEPD
KCTD15
PDCD2L
TMEM147
PSENEN
THAP8
TBCB
CAPNS1
ZNF527
PSMD8
EIF3K
ACTN4
NFKBIB
TIMM50
EID2
PSMC4
AKT2
B9D2
GSK3A
PLAUR
ZNF283
ZNF180
PVRL2
TOMM40
GEMIN7
CD3EAP
VASP
OPA3
FKRP
AP2S1
SAE1
NAPA
CARD8
EMP3
GRWD1
BAX
NOSIP
BCL2L12
PRMT1
CTU1
CD33
SIGLEC6
ZNF614
ZNF480
TFPT
RDH13
HSPBP1
UBE2S
ISOC2
ZNF274
ERVK3-1
CHMP2A
UBE2M
ATP6V1E1
BCL2L13
BID
USP18
MRPL40
UFD1L
GNB1L
COMT
TANGO2
TRMT2A
RANBP1
SNAP29
UBE2L3
SDF2L1
CHCHD10
SNRPD3
SRRD
PITPNB
HSCB
UQCR10
MTFP1
SLC35E4
YWHAH
RBFOX2
TXN2
IFT27
KCTD17
GGA1
ANKRD54
POLR2F
DDX17
CBX6
RPS19BP1
ADSL
RBX1
RANGAP1
PHF5A
POLR3H
DESI1
SNU13
NDUFA6
RRP7A
BIK
MCAT
TSPO
PARVG
FAM118A
TTC38
TRMU
CRELD2
SCO2
BTG3
MRPL39
ATP5J
USP16
MIS18A
C21orf59
IL10RB
IFNAR1
ITSN1
ATP5O
CBR1
PIGP
DSCR3
PSMG1
WDR4
NDUFV3
PDXK
CSTB
UBE2G2
SUMO3
FAM207A
