# Supplementary material for: Integrated multi-omics analyses reveal homology-directed repair pathway as a unique dependency in near-haploid leukemia
Source: Blood Cancer J. 2023 Jun 8;13(1):92. doi: 10.1038/s41408-023-00863-1 (PMC10247733; doi:10.1038/s41408-023-00863-1)
Supplement: Supplementary file 6 — Supplementary Table 5 [file 41408_2023_863_MOESM6_ESM.pdf]

	p_val	avg_log2FC	pct.1	pct.2	p_val_adj	cluster	gene
hg19-TUBA1	9.53E-303	-2.2916453	0.758	0.876	5.92E-300	0	TUBA1B
hg19-TUBB	6.55E-265	-1.6271158	0.839	0.924	4.07E-262	0	TUBB
hg19-HMGB2	8.03E-228	-1.762902	0.659	0.83	4.99E-225	0	HMGB2
hg19-TK1	4.16E-213	-1.1798272	0.359	0.601	2.58E-210	0	TK1
hg19-TUBB4	3.31E-212	-1.554458	0.491	0.704	2.06E-209	0	TUBB4B
hg19-KIAA01	6.06E-211	-1.4908336	0.424	0.679	3.76E-208	0	KIAA0101
hg19-TYMS	5.60E-194	-1.1523295	0.439	0.675	3.48E-191	0	TYMS
hg19-MKI67	5.55E-189	-0.944651	0.25	0.501	3.44E-186	0	MKI67
hg19-H2AFZ	5.00E-184	-1.1786821	0.768	0.88	3.10E-181	0	H2AFZ
hg19-PCNA	3.15E-181	-0.9740013	0.44	0.687	1.96E-178	0	PCNA
hg19-CKS1B	7.15E-176	-0.9977808	0.441	0.668	4.44E-173	0	CKS1B
hg19-HIST1H	2.82E-161	-2.642777	0.542	0.73	1.75E-158	0	HIST1H4C
hg19-CDK1	3.77E-159	-0.9147801	0.281	0.498	2.34E-156	0	CDK1
hg19-NUSAP	3.33E-147	-1.0390811	0.352	0.55	2.07E-144	0	NUSAP1
hg19-UBE2C	3.74E-145	-1.5548171	0.328	0.526	2.32E-142	0	UBE2C
hg19-MAD2L	4.18E-141	-0.6372783	0.365	0.572	2.60E-138	0	MAD2L1
hg19-RRM2	4.87E-138	-0.4050381	0.268	0.457	3.03E-135	0	RRM2
hg19-CENPW	9.34E-138	-0.7099663	0.48	0.655	5.80E-135	0	CENPW
hg19-SPC25	7.62E-134	-0.3343545	0.212	0.413	4.73E-131	0	SPC25
hg19-DUT	9.20E-133	-1.0641268	0.723	0.836	5.71E-130	0	DUT
hg19-CDCA3	4.05E-131	-0.3501289	0.225	0.414	2.52E-128	0	CDCA3
hg19-UBE2T	3.05E-127	-0.6010802	0.423	0.598	1.89E-124	0	UBE2T
hg19-HMGB1	1.46E-125	-0.6559986	0.969	0.977	9.09E-123	0	HMGB1
hg19-C21orf	7.11E-123	-0.5082554	0.313	0.494	4.42E-120	0	C21orf58
hg19-TUBA1	3.13E-118	-0.7782783	0.45	0.633	1.94E-115	0	TUBA1A
hg19-H2AFX	2.35E-116	-0.5352938	0.382	0.537	1.46E-113	0	H2AFX
hg19-BIRC5	2.51E-114	-0.8662854	0.32	0.5	1.56E-111	0	BIRC5
hg19-AURKB	2.25E-113	-0.7474852	0.31	0.483	1.40E-110	0	AURKB
hg19-MCM7	2.74E-113	-0.7279896	0.492	0.664	1.70E-110	0	MCM7
hg19-TPX2	1.20E-111	-0.429402	0.295	0.462	7.42E-109	0	TPX2
hg19-DHFR	1.75E-111	-0.658357	0.429	0.605	1.09E-108	0	DHFR
hg19-STMN1	3.12E-111	-0.6915018	0.951	0.957	1.93E-108	0	STMN1
hg19-MND1	1.24E-106	-0.442253	0.355	0.523	7.70E-104	0	MND1
hg19-GTSE1	2.86E-106	-0.7107222	0.283	0.458	1.78E-103	0	GTSE1
hg19-HMMR	6.79E-106	-0.4199445	0.213	0.392	4.22E-103	0	HMMR
hg19-CDKN3	6.51E-104	-0.9033498	0.359	0.526	4.04E-101	0	CDKN3
hg19-CORO1	3.53E-103	-0.7148691	0.61	0.771	2.19E-100	0	CORO1A
hg19-CENPF	4.60E-100	-0.8848439	0.313	0.478	2.86E-97	0	CENPF
hg19-CALM2	1.36E-97	-0.6168552	0.853	0.936	8.42E-95	0	CALM2
hg19-HSP90	1.51E-95	-0.4636195	0.966	0.984	9.36E-93	0	HSP90AA1
hg19-DTYMK	2.04E-95	-0.4516128	0.386	0.548	1.27E-92	0	DTYMK
hg19-ARL6IP	1.32E-94	-0.941135	0.517	0.67	8.23E-92	0	ARL6IP1
hg19-TOP2A	4.58E-94	-1.3540561	0.323	0.492	2.84E-91	0	TOP2A
hg19-CKS2	2.86E-92	-1.0040575	0.549	0.695	1.78E-89	0	CKS2
hg19-CCNA2	4.18E-92	-0.3213008	0.196	0.369	2.60E-89	0	CCNA2
hg19-ACTB	8.71E-92	-0.4149923	0.998	0.996	5.41E-89	0	ACTB
hg19-KIFC1	1.91E-91	-0.3457874	0.303	0.451	1.19E-88	0	KIFC1
hg19-HMGN	7.55E-90	-0.7715057	0.797	0.852	4.69E-87	0	HMGN2
hg19-MCM5	2.37E-89	-0.5397853	0.524	0.673	1.47E-86	0	MCM5
hg19-NUCKS	2.86E-88	-0.7438054	0.772	0.864	1.78E-85	0	NUCKS1
hg19-CDC20	9.36E-88	-0.8241238	0.328	0.469	5.82E-85	0	CDC20
hg19-TUBA1	1.80E-85	-0.6939432	0.35	0.502	1.12E-82	0	TUBA1C
hg19-CKAP2L	3.42E-85	-0.3077392	0.187	0.355	2.12E-82	0	CKAP2L
hg19-CCNB2	1.02E-84	-0.6100004	0.338	0.489	6.34E-82	0	CCNB2
hg19-SMC4	3.58E-84	-0.6361802	0.432	0.605	2.23E-81	0	SMC4
hg19-KIF22	6.57E-83	-0.3687819	0.372	0.526	4.08E-80	0	KIF22
hg19-TACC3	7.69E-83	-0.3404748	0.308	0.451	4.78E-80	0	TACC3
hg19-ANP32	5.06E-82	-0.4780106	0.39	0.552	3.15E-79	0	ANP32E
hg19-DLGAP	1.20E-80	-0.5356784	0.281	0.426	7.42E-78	0	DLGAP5
hg19-PTTG1	8.94E-80	-1.2105632	0.414	0.551	5.55E-77	0	PTTG1
hg19-ASPM	3.03E-79	-0.4270994	0.248	0.402	1.88E-76	0	ASPM
hg19-HMGB3	5.26E-78	-0.7915226	0.643	0.764	3.27E-75	0	HMGB3
hg19-PLK1	4.40E-76	-0.4918052	0.246	0.395	2.73E-73	0	PLK1
hg19-MXD3	3.58E-73	-0.5265025	0.417	0.545	2.23E-70	0	MXD3
hg19-KIF4A	3.45E-71	-0.2840176	0.216	0.365	2.14E-68	0	KIF4A
hg19-PLP2	5.35E-68	-0.4611321	0.691	0.819	3.32E-65	0	PLP2
hg19-SPC24	1.46E-65	-0.2800813	0.264	0.389	9.04E-63	0	SPC24
hg19-NKG7	1.04E-64	-0.4917379	0.563	0.709	6.48E-62	0	NKG7
hg19-KPNA2	1.58E-64	-0.8377473	0.423	0.556	9.84E-62	0	KPNA2
hg19-VIM	1.68E-64	-0.5354567	0.896	0.944	1.04E-61	0	VIM
hg19-BUB3	1.48E-61	-0.5554476	0.577	0.71	9.21E-59	0	BUB3
hg19-PKM	6.55E-61	-0.3990249	0.804	0.875	4.07E-58	0	PKM
hg19-FOS	2.34E-60	-0.8949727	0.541	0.692	1.45E-57	0	FOS
hg19-PRC1	2.73E-60	-0.3489204	0.322	0.437	1.69E-57	0	PRC1
hg19-TAGLN	7.63E-59	-0.4009232	0.445	0.581	4.74E-56	0	TAGLN2
hg19-CKAP2	3.82E-58	-0.3567118	0.33	0.463	2.38E-55	0	CKAP2
hg19-HIST2H	4.49E-56	-0.3706813	0.355	0.461	2.79E-53	0	HIST2H2AC
hg19-HSPA8	1.51E-54	-0.4493003	0.854	0.915	9.37E-52	0	HSPA8
hg19-CENPA	3.62E-54	-0.4574764	0.192	0.338	2.25E-51	0	CENPA
hg19-EMP3	2.29E-53	-0.7548267	0.402	0.556	1.42E-50	0	EMP3
hg19-CDCA8	4.74E-53	-0.2951392	0.264	0.38	2.94E-50	0	CDCA8
hg19-MCM1	5.43E-51	-0.2593327	0.344	0.443	3.37E-48	0	MCM10
hg19-RAN	3.48E-49	-0.3897772	0.867	0.915	2.16E-46	0	RAN
hg19-NUF2	4.62E-48	-0.3247755	0.31	0.409	2.87E-45	0	NUF2
hg19-EGR1	6.36E-48	-0.5495942	0.517	0.63	3.95E-45	0	EGR1
hg19-HN1	6.84E-48	-0.5462315	0.534	0.656	4.25E-45	0	HN1
hg19-IER2	3.81E-46	-0.5048045	0.709	0.818	2.36E-43	0	IER2
hg19-PHGDH	6.19E-46	-0.3264425	0.414	0.519	3.84E-43	0	PHGDH
hg19-CENPE	1.18E-44	-0.4026893	0.284	0.396	7.34E-42	0	CENPE
hg19-JUNB	9.34E-43	-0.4668809	0.357	0.479	5.80E-40	0	JUNB
hg19-CCNB1	2.05E-42	-0.5693396	0.35	0.44	1.27E-39	0	CCNB1
hg19-UBB	1.70E-39	-0.4837663	0.959	0.973	1.05E-36	0	UBB
hg19-SGOL2	3.28E-39	-0.2678663	0.369	0.444	2.04E-36	0	SGOL2
hg19-PFN1	1.12E-38	-0.303331	0.984	0.985	6.95E-36	0	PFN1
hg19-TROAP	2.55E-37	-0.3212046	0.346	0.428	1.58E-34	0	TROAP
hg19-UBE2S	7.09E-36	-0.4548641	0.373	0.475	4.40E-33	0	UBE2S
hg19-FLNA	2.53E-33	-0.2517418	0.535	0.646	1.57E-30	0	FLNA
hg19-MX1	1.37E-32	-0.4354352	0.586	0.675	8.50E-30	0	MX1
hg19-UNG	1.72E-30	-0.2546414	0.367	0.458	1.07E-27	0	UNG
hg19-SLC25A	1.88E-30	-0.3790224	0.951	0.955	1.17E-27	0	SLC25A5
hg19-VPREB	5.50E-30	0.29685766	0.915	0.882	3.41E-27	0	VPREB3
hg19-FXYD2	5.14E-28	-0.3066041	0.538	0.619	3.19E-25	0	FXYD2
hg19-CD69	1.24E-25	-0.31286	0.392	0.479	7.70E-23	0	CD69
hg19-KLF6	2.04E-25	-0.4315461	0.602	0.717	1.27E-22	0	KLF6
hg19-LTB	3.20E-24	-0.4444112	0.673	0.774	1.99E-21	0	LTB
hg19-IFI6	5.36E-23	-0.2962398	0.516	0.581	3.33E-20	0	IFI6
hg19-S100A	7.68E-23	-0.4096473	0.53	0.639	4.77E-20	0	S100A6
hg19-DDIT4	1.40E-22	-0.3898584	0.367	0.462	8.72E-20	0	DDIT4
hg19-S100A1	1.89E-22	-0.3647895	0.369	0.43	1.18E-19	0	S100A10
hg19-LSP1	5.63E-20	-0.441875	0.457	0.55	3.50E-17	0	LSP1
hg19-C1QTN	1.60E-18	0.46345092	0.57	0.534	9.92E-16	0	C1QTNF4
hg19-GSN	6.46E-14	-0.2746291	0.369	0.444	4.01E-11	0	GSN
hg19-TIMP1	4.18E-11	-0.351499	0.438	0.516	2.59E-08	0	TIMP1
hg19-IGLL1	0.00015392	-0.2583164	0.643	0.673	0.09558361	0	IGLL1
hg19-HCST	0.00163944	-0.2851556	0.412	0.461	1	0	HCST
hg19-MCM7.	2.15E-240	0.85214286	0.842	0.574	1.34E-237	1	MCM7
hg19-PCNA.1	1.20E-228	0.80763907	0.854	0.578	7.45E-226	1	PCNA
hg19-DUT.1	5.19E-225	0.75715627	0.952	0.777	3.22E-222	1	DUT
hg19-MCM5.	3.67E-214	0.80735222	0.839	0.592	2.28E-211	1	MCM5
hg19-TYMS.1	8.47E-198	0.63968249	0.839	0.57	5.26E-195	1	TYMS
hg19-UNG.1	9.82E-185	0.72133485	0.624	0.393	6.10E-182	1	UNG
hg19-KIAA01	3.60E-156	0.54455716	0.836	0.57	2.24E-153	1	KIAA0101
hg19-PKM.1	3.99E-101	0.51811328	0.952	0.837	2.48E-98	1	PKM
hg19-DHFR.1	1.63E-95	0.62500488	0.713	0.531	1.01E-92	1	DHFR
hg19-TK1.1	1.40E-94	0.55568324	0.688	0.512	8.67E-92	1	TK1
hg19-STMN1	7.20E-74	0.25409046	0.998	0.946	4.47E-71	1	STMN1
hg19-HSP90	3.41E-64	0.27446464	0.995	0.976	2.12E-61	1	HSP90AA1
hg19-TAGLN	4.57E-64	0.41127068	0.655	0.525	2.84E-61	1	TAGLN2
hg19-RAN.1	3.41E-60	0.30044394	0.966	0.89	2.11E-57	1	RAN
hg19-PRDX2	3.82E-52	0.45228626	0.616	0.499	2.38E-49	1	PRDX2
hg19-AURKB	1.20E-46	-0.5164221	0.339	0.468	7.44E-44	1	AURKB
hg19-MCM1	1.38E-45	0.31587426	0.504	0.401	8.54E-43	1	MCM10
hg19-IGLL1.1	8.28E-44	0.30451879	0.783	0.639	5.14E-41	1	IGLL1
hg19-PLIN3	5.22E-35	-0.4017206	0.466	0.552	3.24E-32	1	PLIN3
hg19-HIST1H	4.65E-32	-0.4938769	0.473	0.554	2.89E-29	1	HIST1H1C
hg19-KLF6.1	9.40E-32	-0.5468376	0.634	0.705	5.84E-29	1	KLF6
hg19-EMP3.1	6.53E-31	-0.6631589	0.452	0.538	4.05E-28	1	EMP3
hg19-PLK1.1	1.06E-25	-0.4628198	0.299	0.376	6.60E-23	1	PLK1
hg19-TMEM	3.47E-25	-0.3204943	0.368	0.443	2.15E-22	1	TMEM173
hg19-LSP1.1	1.86E-24	-0.4675087	0.487	0.539	1.15E-21	1	LSP1
hg19-S100A	4.63E-24	-0.833023	0.804	0.823	2.87E-21	1	S100A4
hg19-DDIT4.	2.38E-21	-0.4321317	0.381	0.455	1.48E-18	1	DDIT4
hg19-HMGB	1.33E-20	-0.4458858	0.93	0.759	8.27E-18	1	HMGB2
hg19-CENPE.	3.97E-19	-0.3341646	0.316	0.383	2.46E-16	1	CENPE
hg19-NKG7.1	4.10E-18	-0.4824833	0.639	0.684	2.54E-15	1	NKG7
hg19-IRX1	8.63E-18	-0.3378401	0.489	0.548	5.36E-15	1	IRX1
hg19-H1FX	1.35E-17	-0.4391471	0.651	0.693	8.37E-15	1	H1FX
hg19-DLGAP	2.42E-17	-0.4565368	0.337	0.406	1.50E-14	1	DLGAP5
hg19-TIMP1.	4.42E-16	-0.4914258	0.458	0.508	2.74E-13	1	TIMP1
hg19-HCST.1	4.68E-16	-0.3466536	0.41	0.459	2.91E-13	1	HCST
hg19-CCNB1.	8.99E-16	-0.4890794	0.371	0.431	5.58E-13	1	CCNB1
hg19-TSPO	9.13E-16	-0.4934761	0.617	0.67	5.67E-13	1	TSPO
hg19-CKAP2.	7.19E-14	-0.3303942	0.388	0.443	4.47E-11	1	CKAP2
hg19-UBE2C.	1.93E-13	-1.1880051	0.436	0.492	1.20E-10	1	UBE2C
hg19-PTTG1.	2.30E-13	-1.0000222	0.477	0.53	1.43E-10	1	PTTG1
hg19-MXD3.1	5.60E-13	-0.3720418	0.481	0.524	3.48E-10	1	MXD3
hg19-S100A	1.42E-12	-0.6086035	0.587	0.621	8.79E-10	1	S100A6
hg19-HMMR	2.15E-12	-0.35607	0.315	0.359	1.34E-09	1	HMMR
hg19-KPNA2.	8.02E-12	-0.6138485	0.494	0.533	4.98E-09	1	KPNA2
hg19-HIST2H	1.83E-11	-0.3090063	0.389	0.448	1.14E-08	1	HIST2H2AC
hg19-ARL6IP	3.22E-11	-0.816651	0.627	0.637	2.00E-08	1	ARL6IP1
hg19-TUBA1.	3.22E-10	-0.4285159	0.566	0.598	2.00E-07	1	TUBA1A
hg19-TOP2A.	4.00E-10	-0.9632693	0.418	0.462	2.49E-07	1	TOP2A
hg19-LTB.1	1.37E-09	-0.4526792	0.741	0.754	8.53E-07	1	LTB
hg19-CD37	1.97E-09	-0.4632011	0.555	0.586	1.22E-06	1	CD37
hg19-HN1.1	3.61E-09	-0.5402589	0.618	0.631	2.24E-06	1	HN1
hg19-MX1.1	5.09E-09	-0.3651439	0.635	0.659	3.16E-06	1	MX1
hg19-BIRC5.1	6.55E-09	-0.6065855	0.416	0.469	4.07E-06	1	BIRC5
hg19-CCNB2.	1.33E-08	-0.3637335	0.416	0.464	8.28E-06	1	CCNB2
hg19-CENPF.	5.89E-08	-0.65448	0.412	0.447	3.66E-05	1	CENPF
hg19-IGJ	7.26E-08	-0.259404	0.468	0.504	4.51E-05	1	IGJ
hg19-ACTG1	1.49E-07	-0.2556257	0.995	0.982	9.24E-05	1	ACTG1
hg19-TUBA1	1.75E-07	-0.5031112	0.441	0.474	0.00010869	1	TUBA1C
hg19-HIST1H	2.50E-07	-1.4925883	0.705	0.683	0.00015534	1	HIST1H4C
hg19-MKI67.	4.51E-07	-0.5774043	0.409	0.452	0.00027977	1	MKI67
hg19-CD52	7.71E-07	-0.473531	0.358	0.398	0.00047879	1	CD52
hg19-CENPA.	1.09E-06	-0.4104151	0.274	0.312	0.00067518	1	CENPA
hg19-FXYD2.	7.34E-06	-0.357046	0.585	0.604	0.00455762	1	FXYD2
hg19-FOS.1	1.48E-05	-0.590255	0.648	0.66	0.00920911	1	FOS
hg19-PLP2.1	1.89E-05	-0.3143629	0.803	0.787	0.01174159	1	PLP2
hg19-TROAP	4.63E-05	-0.263415	0.391	0.413	0.02876794	1	TROAP
hg19-S100A1	0.000201	-0.2923845	0.403	0.419	0.12481873	1	S100A10
hg19-CDKN3.	0.00029042	-0.4344629	0.469	0.493	0.18035019	1	CDKN3
hg19-FLNA.1	0.0004649	-0.2850345	0.615	0.623	0.28870169	1	FLNA
hg19-CDK1.1	0.00048159	-0.3652928	0.481	0.441	0.29906602	1	CDK1
hg19-UBB.1	0.00144995	-0.3110655	0.991	0.964	0.900417	1	UBB
hg19-IER2.1	0.00168154	-0.2511386	0.806	0.79	1	1	IER2
hg19-ASPM.1	0.00223334	-0.2629552	0.392	0.361	1	1	ASPM
hg19-NUCKS	0.00458301	-0.2871949	0.92	0.825	1	1	NUCKS1
hg19-HIST1H	0	2.72223629	0.981	0.636	0	2	HIST1H4C
hg19-KIAA01	0	1.85677403	0.99	0.557	0	2	KIAA0101
hg19-TUBA1	0	1.71178143	0.999	0.823	0	2	TUBA1B
hg19-TUBB.1	0	1.57833336	0.999	0.888	0	2	TUBB
hg19-DUT.2	0	1.50871608	0.996	0.778	0	2	DUT
hg19-TK1.2	0	1.47685921	0.949	0.476	0	2	TK1
hg19-TYMS.2	0	1.40150763	0.969	0.562	0	2	TYMS
hg19-PCNA.2	0	1.34848236	0.978	0.571	0	2	PCNA
hg19-HMGB	0	1.14472127	0.993	0.756	0	2	HMGB2
hg19-MCM7.	0	1.04328382	0.925	0.573	0	2	MCM7
hg19-DHFR.2	0	1.00825377	0.898	0.508	0	2	DHFR
hg19-RRM2.	0	0.93904863	0.76	0.354	0	2	RRM2
hg19-MKI67.	0	0.86999524	0.813	0.38	0	2	MKI67
hg19-UBE2T.	0	0.86554367	0.86	0.506	0	2	UBE2T
hg19-AURKB	0	0.81006007	0.778	0.386	0	2	AURKB
hg19-TUBB4	0	0.72049178	0.957	0.604	0	2	TUBB4B
hg19-HIST2H	1.56E-304	0.95915151	0.734	0.385	9.66E-302	2	HIST2H2AC
hg19-H2AFZ.	2.04E-297	1.16953332	0.998	0.829	1.26E-294	2	H2AFZ
hg19-UBE2C.	1.24E-295	0.30184707	0.832	0.421	7.69E-293	2	UBE2C
hg19-NUSAP	3.62E-294	0.74319728	0.838	0.448	2.25E-291	2	NUSAP1
hg19-SPC25.	8.12E-293	0.60109231	0.685	0.313	5.04E-290	2	SPC25
hg19-CDK1.2	1.46E-284	0.65220217	0.785	0.391	9.06E-282	2	CDK1
hg19-C21orf	3.48E-271	0.85940764	0.748	0.402	2.16E-268	2	C21orf58
hg19-STMN1	1.54E-260	0.90367355	0.999	0.948	9.58E-258	2	STMN1
hg19-TOP2A.	9.79E-250	0.73168361	0.795	0.394	6.08E-247	2	TOP2A
hg19-MXD3.	4.78E-239	0.7705618	0.781	0.47	2.97E-236	2	MXD3
hg19-H2AFX.	2.32E-237	0.62619794	0.763	0.456	1.44E-234	2	H2AFX
hg19-MCM5.	2.13E-236	0.86400438	0.897	0.595	1.32E-233	2	MCM5
hg19-MND1.	9.22E-235	0.74508074	0.749	0.439	5.72E-232	2	MND1
hg19-HMGN	1.01E-230	0.91717229	0.991	0.813	6.25E-228	2	HMGN2
hg19-CENPW	1.38E-221	0.70381882	0.877	0.57	8.54E-219	2	CENPW
hg19-CKS1B.	9.86E-217	0.66930793	0.905	0.567	6.12E-214	2	CKS1B
hg19-GTSE1.	1.09E-203	0.56352203	0.7	0.37	6.79E-201	2	GTSE1
hg19-HMGB1	1.69E-203	0.68160779	0.997	0.971	1.05E-200	2	HMGB1
hg19-BIRC5.	3.65E-189	0.58857477	0.735	0.411	2.26E-186	2	BIRC5
hg19-CDKN3.	1.06E-182	0.35184478	0.756	0.442	6.60E-180	2	CDKN3
hg19-SPC24.	3.93E-182	0.56351608	0.588	0.321	2.44E-179	2	SPC24
hg19-HIST1H	3.04E-171	0.5560045	0.775	0.497	1.89E-168	2	HIST1H1C
hg19-MCM1	2.08E-168	0.39607267	0.631	0.384	1.29E-165	2	MCM10
hg19-CDCA3.	8.55E-161	0.37589285	0.599	0.332	5.31E-158	2	CDCA3
hg19-KIFC1.1	1.99E-154	0.40953346	0.633	0.38	1.23E-151	2	KIFC1
hg19-ANP32	1.14E-147	0.63026994	0.758	0.474	7.07E-145	2	ANP32E
hg19-SMC4.1	1.52E-141	0.50746406	0.833	0.52	9.41E-139	2	SMC4
hg19-DTYMK	1.02E-139	0.51695018	0.737	0.473	6.33E-137	2	DTYMK
hg19-KIF22.1	4.00E-135	0.46561808	0.707	0.453	2.48E-132	2	KIF22
hg19-MAD2L	4.87E-131	0.45611533	0.752	0.486	3.02E-128	2	MAD2L1
hg19-PHGDH	3.16E-130	0.49436181	0.7	0.46	1.96E-127	2	PHGDH
hg19-CKS2.1	1.74E-126	0.28052977	0.892	0.622	1.08E-123	2	CKS2
hg19-TUBA1.	6.72E-119	0.46590284	0.824	0.551	4.17E-116	2	TUBA1A
hg19-TACC3.	5.49E-115	0.32992944	0.616	0.384	3.41E-112	2	TACC3
hg19-NUCKS	1.50E-113	0.56081115	0.98	0.819	9.33E-111	2	NUCKS1
hg19-CKAP2.	3.46E-108	0.40837005	0.649	0.396	2.15E-105	2	CKAP2
hg19-HMGB3	2.49E-97	0.41262883	0.929	0.704	1.55E-94	2	HMGB3
hg19-PKM.2	2.63E-95	0.49271603	0.968	0.84	1.63E-92	2	PKM
hg19-CKAP2L	1.06E-93	0.41080337	0.515	0.283	6.58E-91	2	CKAP2L
hg19-IGLL1.2	6.44E-90	0.89801022	0.836	0.637	4.00E-87	2	IGLL1
hg19-S100A	1.10E-81	-1.173026	0.814	0.821	6.84E-79	2	S100A4
hg19-SLC25A	1.27E-79	0.60057974	0.997	0.947	7.90E-77	2	SLC25A5
hg19-BUB3.1	1.77E-74	0.33358578	0.867	0.647	1.10E-71	2	BUB3
hg19-MDK	2.56E-55	0.25557475	0.698	0.532	1.59E-52	2	MDK
hg19-ACTB.1	2.77E-54	0.30657347	1	0.996	1.72E-51	2	ACTB
hg19-UBB.2	3.08E-52	0.50886016	0.996	0.965	1.91E-49	2	UBB
hg19-LTB.2	3.75E-49	-0.9737782	0.749	0.752	2.33E-46	2	LTB
hg19-TSPO.1	3.63E-47	-0.7608405	0.637	0.664	2.25E-44	2	TSPO
hg19-ARL6IP	6.51E-46	-0.2526825	0.813	0.604	4.04E-43	2	ARL6IP1
hg19-HSP90	1.48E-45	0.3979895	0.998	0.976	9.17E-43	2	HSP90AA1
hg19-PFN1.1	9.79E-36	0.3406338	0.999	0.982	6.08E-33	2	PFN1
hg19-S100A	2.11E-35	-0.7640674	0.593	0.618	1.31E-32	2	S100A6
hg19-RAN.2	2.16E-31	0.32837885	0.983	0.891	1.34E-28	2	RAN
hg19-CD37.1	2.46E-26	-0.7479257	0.573	0.581	1.53E-23	2	CD37
hg19-H1FX.1	3.59E-26	0.2755688	0.824	0.661	2.23E-23	2	H1FX
hg19-PRDX1	5.31E-23	0.31690641	0.999	0.986	3.30E-20	2	PRDX1
hg19-CALM2	6.73E-23	0.26548556	0.988	0.904	4.18E-20	2	CALM2
hg19-HCST.2	2.68E-22	-0.3581115	0.438	0.452	1.67E-19	2	HCST
hg19-MZT2B	3.39E-21	0.3398974	0.99	0.915	2.10E-18	2	MZT2B
hg19-CORO1	1.80E-18	0.25058842	0.837	0.717	1.12E-15	2	CORO1A
hg19-JUNB.1	2.12E-18	-0.2595228	0.565	0.432	1.31E-15	2	JUNB
hg19-CD52.1	6.79E-12	-0.5168845	0.362	0.395	4.22E-09	2	CD52
hg19-AK5	7.85E-10	-0.513694	0.776	0.739	4.88E-07	2	AK5
hg19-TIMP1.	2.59E-06	-0.4799059	0.523	0.494	0.00161069	2	TIMP1
hg19-KLF6.2	1.79E-05	-0.3605614	0.748	0.681	0.01112066	2	KLF6
hg19-PLIN3.1	2.64E-05	-0.3459259	0.567	0.531	0.01638826	2	PLIN3
hg19-FOS.2	0.00088205	-0.6280214	0.698	0.65	0.54775362	2	FOS
hg19-LSP1.2	0.00311515	-0.5083937	0.567	0.522	1	2	LSP1
hg19-S100A	0	1.78965342	0.954	0.8	0	3	S100A4
hg19-LSP1.3	6.10E-223	1.50002296	0.722	0.501	3.79E-220	3	LSP1
hg19-S100A	2.05E-199	1.50409529	0.784	0.589	1.28E-196	3	S100A6
hg19-EMP3.2	6.75E-195	1.71673371	0.72	0.492	4.19E-192	3	EMP3
hg19-CD37.2	1.59E-171	1.42612008	0.732	0.558	9.88E-169	3	CD37
hg19-TSPO.2	1.38E-170	1.12136313	0.816	0.637	8.56E-168	3	TSPO
hg19-STMN1	6.75E-163	-1.2567426	0.881	0.967	4.19E-160	3	STMN1
hg19-IGLL1.3	3.20E-162	-2.4239781	0.398	0.706	1.98E-159	3	IGLL1
hg19-TUBA1	1.31E-157	-2.1223659	0.73	0.867	8.14E-155	3	TUBA1B
hg19-TIMP1.	5.80E-155	1.31460186	0.665	0.474	3.60E-152	3	TIMP1
hg19-KIAA01	6.07E-155	-1.5855515	0.338	0.662	3.77E-152	3	KIAA0101
hg19-HMGN	2.38E-150	-1.3479377	0.67	0.864	1.48E-147	3	HMGN2
hg19-TYMS.3	1.30E-144	-1.2639618	0.355	0.661	8.08E-142	3	TYMS
hg19-NKG7.2	1.23E-143	0.9954314	0.803	0.657	7.66E-141	3	NKG7
hg19-TK1.3	9.46E-135	-1.0034592	0.284	0.584	5.88E-132	3	TK1
hg19-HMGB1	1.93E-133	-0.9084311	0.951	0.979	1.20E-130	3	HMGB1
hg19-H2AFZ.	2.83E-124	-1.3168518	0.716	0.874	1.76E-121	3	H2AFZ
hg19-CENPW	7.66E-121	-0.8108749	0.38	0.649	4.76E-118	3	CENPW
hg19-HMGB	5.83E-118	-1.7504295	0.629	0.815	3.62E-115	3	HMGB2
hg19-DUT.3	2.48E-117	-1.3356039	0.63	0.837	1.54E-114	3	DUT
hg19-UBE2T.	4.94E-117	-0.616172	0.318	0.593	3.07E-114	3	UBE2T
hg19-MCM7.	1.15E-114	-0.8890227	0.38	0.661	7.15E-112	3	MCM7
hg19-CKS1B.	7.01E-102	-0.9318174	0.383	0.651	4.35E-99	3	CKS1B
hg19-CDK1.3	3.59E-101	-0.8084084	0.214	0.483	2.23E-98	3	CDK1
hg19-C21orf	2.93E-100	-0.4895225	0.223	0.487	1.82E-97	3	C21orf58
hg19-GTSE1.	7.78E-100	-0.6216886	0.182	0.453	4.83E-97	3	GTSE1
hg19-DTYMK	8.33E-97	-0.4729161	0.274	0.546	5.17E-94	3	DTYMK
hg19-C1QTN	8.61E-94	-0.4896731	0.35	0.571	5.35E-91	3	C1QTNF4
hg19-PCNA.3	4.92E-87	-1.0229491	0.422	0.661	3.06E-84	3	PCNA
hg19-MDK.1	1.17E-86	-0.527107	0.366	0.584	7.27E-84	3	MDK
hg19-PRDX1.	6.86E-86	-0.679444	0.97	0.99	4.26E-83	3	PRDX1
hg19-NUSAP	7.40E-85	-0.9496636	0.293	0.536	4.60E-82	3	NUSAP1
hg19-TUBB.2	2.81E-84	-1.4356587	0.849	0.913	1.74E-81	3	TUBB
hg19-RRM2.	3.12E-84	-0.3997123	0.206	0.444	1.94E-81	3	RRM2
hg19-HCST.3	4.38E-84	0.87638203	0.541	0.436	2.72E-81	3	HCST
hg19-H2AFX.	1.05E-82	-0.4825481	0.303	0.531	6.50E-80	3	H2AFX
hg19-CDC20.	1.40E-82	-0.6893906	0.238	0.467	8.71E-80	3	CDC20
hg19-HMGB3	2.77E-82	-0.959266	0.544	0.765	1.72E-79	3	HMGB3
hg19-PLP2.2	3.81E-82	0.80226027	0.845	0.782	2.37E-79	3	PLP2
hg19-MND1.	2.04E-81	-0.5097635	0.289	0.513	1.26E-78	3	MND1
hg19-CENPF.	2.41E-81	-0.7771359	0.226	0.471	1.50E-78	3	CENPF
hg19-PTTG1.	1.61E-80	-1.2237374	0.313	0.55	9.97E-78	3	PTTG1
hg19-CDCA3.	4.22E-79	-0.3239926	0.17	0.4	2.62E-76	3	CDCA3
hg19-UBE2C.	2.10E-78	-1.4483335	0.278	0.511	1.31E-75	3	UBE2C
hg19-MKI67.	8.81E-78	-0.8286371	0.231	0.475	5.47E-75	3	MKI67
hg19-MAD2L	1.46E-77	-0.6380174	0.319	0.555	9.06E-75	3	MAD2L1
hg19-CD52.2	1.14E-76	1.02501278	0.538	0.369	7.07E-74	3	CD52
hg19-LMNA	4.01E-73	0.84021965	0.397	0.276	2.49E-70	3	LMNA
hg19-CDKN3.	6.62E-73	-0.7877114	0.296	0.517	4.11E-70	3	CDKN3
hg19-FOS.3	3.74E-72	1.14331958	0.728	0.647	2.32E-69	3	FOS
hg19-DHFR.3	6.60E-69	-0.6420742	0.359	0.595	4.10E-66	3	DHFR
hg19-KLF6.3	8.00E-69	0.84032219	0.752	0.682	4.97E-66	3	KLF6
hg19-MCM1	2.47E-66	-0.2565486	0.245	0.446	1.54E-63	3	MCM10
hg19-CKS2.2	1.24E-65	-0.9796031	0.477	0.689	7.71E-63	3	CKS2
hg19-PHGDH	1.90E-65	-0.3852433	0.306	0.523	1.18E-62	3	PHGDH
hg19-HIST2H	1.05E-64	-0.2895483	0.252	0.464	6.55E-62	3	HIST2H2AC
hg19-BIRC5.	3.72E-64	-0.8297045	0.272	0.486	2.31E-61	3	BIRC5
hg19-SMC4.2	3.96E-64	-0.6208438	0.354	0.597	2.46E-61	3	SMC4
hg19-TROAP	9.86E-64	-0.3375993	0.228	0.436	6.12E-61	3	TROAP
hg19-CCNB2.	2.32E-63	-0.5923627	0.275	0.481	1.44E-60	3	CCNB2
hg19-MXD3.	3.37E-63	-0.4364232	0.325	0.544	2.09E-60	3	MXD3
hg19-KIF22.2	8.48E-63	-0.3867775	0.308	0.517	5.27E-60	3	KIF22
hg19-MCM5.	3.47E-61	-0.6627956	0.46	0.665	2.15E-58	3	MCM5
hg19-AURKB	8.62E-60	-0.6167486	0.262	0.47	5.35E-57	3	AURKB
hg19-TMSB4	9.15E-60	-0.2730257	1	1	5.68E-57	3	TMSB4X
hg19-NUCKS	1.02E-59	-0.9417274	0.723	0.86	6.32E-57	3	NUCKS1
hg19-ANP32	4.85E-59	-0.6489079	0.324	0.544	3.01E-56	3	ANP32E
hg19-TUBB4	2.85E-58	-1.0730558	0.494	0.68	1.77E-55	3	TUBB4B
hg19-ASPM.2	9.84E-57	-0.3815593	0.182	0.394	6.11E-54	3	ASPM
hg19-CMTM	1.84E-55	-0.2753909	0.337	0.506	1.14E-52	3	CMTM8
hg19-DLGAP	1.36E-54	-0.4916841	0.216	0.419	8.47E-52	3	DLGAP5
hg19-GCHFR	1.88E-52	-0.2698238	0.354	0.551	1.16E-49	3	GCHFR
hg19-LGALS1	1.85E-50	0.67712181	0.332	0.252	1.15E-47	3	LGALS1
hg19-PRC1.1	3.42E-48	-0.3299598	0.25	0.434	2.12E-45	3	PRC1
hg19-AHNAK	3.79E-48	0.35918398	0.339	0.246	2.35E-45	3	AHNAK
hg19-TOP2A.	9.96E-48	-1.2477544	0.279	0.479	6.19E-45	3	TOP2A
hg19-NUF2.1	2.80E-47	-0.2771732	0.223	0.41	1.74E-44	3	NUF2
hg19-SPC25.	6.81E-47	-0.2953137	0.2	0.392	4.23E-44	3	SPC25
hg19-HIST1H	6.71E-45	-2.3453635	0.592	0.701	4.17E-42	3	HIST1H4C
hg19-TPX2.1	1.87E-43	-0.3800378	0.278	0.446	1.16E-40	3	TPX2
hg19-BUB3.2	3.28E-42	-0.6025534	0.516	0.703	2.03E-39	3	BUB3
hg19-HMMR	3.37E-42	-0.3699928	0.187	0.375	2.09E-39	3	HMMR
hg19-MZT2B	1.30E-41	-0.6461281	0.844	0.938	8.05E-39	3	MZT2B
hg19-IER2.2	1.94E-41	0.4689823	0.822	0.789	1.21E-38	3	IER2
hg19-UNG.2	1.33E-38	-0.266408	0.288	0.459	8.29E-36	3	UNG
hg19-HN1.2	1.68E-38	-0.6168176	0.473	0.651	1.05E-35	3	HN1
hg19-CCNB1.	1.38E-36	-0.5284873	0.275	0.441	8.56E-34	3	CCNB1
hg19-CKAP2L	2.68E-35	-0.2625152	0.161	0.34	1.67E-32	3	CKAP2L
hg19-CENPE.	7.65E-35	-0.392067	0.226	0.391	4.75E-32	3	CENPE
hg19-PRDX2.	9.46E-34	-0.2539175	0.371	0.543	5.87E-31	3	PRDX2
hg19-CDCA8.	2.70E-33	-0.2757414	0.212	0.374	1.68E-30	3	CDCA8
hg19-KIFC1.2	3.75E-33	-0.2762928	0.286	0.437	2.33E-30	3	KIFC1
hg19-PLK1.2	2.00E-32	-0.4187993	0.219	0.382	1.24E-29	3	PLK1
hg19-RAN.3	4.36E-32	-0.5788246	0.835	0.914	2.71E-29	3	RAN
hg19-ARL6IP	2.52E-30	-0.7922217	0.493	0.656	1.56E-27	3	ARL6IP1
hg19-KPNA2.	1.83E-28	-0.6296719	0.388	0.546	1.14E-25	3	KPNA2
hg19-CENPA.	1.02E-27	-0.425664	0.153	0.327	6.35E-25	3	CENPA
hg19-SLC25A	3.68E-27	-0.5074258	0.941	0.956	2.29E-24	3	SLC25A5
hg19-KIF4A.1	4.27E-27	-0.2715744	0.194	0.351	2.65E-24	3	KIF4A
hg19-CKAP2.	7.53E-27	-0.4087062	0.289	0.454	4.68E-24	3	CKAP2
hg19-ACTB.2	2.75E-26	-0.3208492	0.998	0.996	1.71E-23	3	ACTB
hg19-DDIT4.	5.07E-26	0.64681536	0.489	0.434	3.15E-23	3	DDIT4
hg19-PHPT1	2.08E-25	-0.2784195	0.522	0.689	1.29E-22	3	PHPT1
hg19-LTB.3	5.10E-24	0.46131643	0.75	0.752	3.17E-21	3	LTB
hg19-CCNA2.	1.45E-23	-0.2574053	0.196	0.349	8.98E-21	3	CCNA2
hg19-FLNA.2	1.35E-22	0.52787094	0.622	0.621	8.40E-20	3	FLNA
hg19-UBE2S.	3.04E-22	-0.5361108	0.318	0.472	1.89E-19	3	UBE2S
hg19-TUBA1	6.23E-22	-0.5084827	0.346	0.485	3.87E-19	3	TUBA1C
hg19-JUNB.2	3.01E-21	0.83076126	0.481	0.447	1.87E-18	3	JUNB
hg19-S100A1	1.69E-20	1.21967404	0.434	0.413	1.05E-17	3	S100A10
hg19-SLC9A3	2.75E-15	0.65062709	0.331	0.302	1.71E-12	3	SLC9A3R2
hg19-HSP90	7.74E-15	-0.378177	0.983	0.979	4.81E-12	3	HSP90AA1
hg19-RETN	9.57E-14	0.27899483	0.323	0.328	5.94E-11	3	RETN
hg19-CRIP2	7.51E-13	0.52227021	0.33	0.294	4.66E-10	3	CRIP2
hg19-ISG15	2.07E-12	-0.2742795	0.641	0.772	1.28E-09	3	ISG15
hg19-ACTG1.	2.19E-12	-0.3995356	0.975	0.986	1.36E-09	3	ACTG1
hg19-UBB.3	4.42E-10	-0.4898298	0.949	0.973	2.75E-07	3	UBB
hg19-EEF1B	1.57E-09	-0.3046408	0.965	0.978	9.74E-07	3	EEF1B2
hg19-HSPA8.	3.79E-09	-0.39121	0.858	0.908	2.35E-06	3	HSPA8
hg19-HNRN	2.51E-08	-0.363533	0.545	0.636	1.56E-05	3	HNRNPH1
hg19-PFN1.2	1.18E-07	-0.3113925	0.978	0.986	7.35E-05	3	PFN1
hg19-GSN.1	1.62E-07	0.48006064	0.425	0.427	0.00010078	3	GSN
hg19-TYROB	5.54E-06	0.55208444	0.344	0.369	0.00344272	3	TYROBP
hg19-IGJ.1	7.98E-06	0.46615202	0.48	0.5	0.00495462	3	IGJ
hg19-TMEM	2.84E-05	0.40956047	0.415	0.431	0.01761898	3	TMEM173
hg19-SRGN	0.00032093	0.2655837	0.377	0.415	0.19930028	3	SRGN
hg19-CRIP1	0.0007592	0.3363177	0.263	0.284	0.47146115	3	CRIP1
hg19-TAGLN	0.00101374	0.61698207	0.523	0.554	0.62953449	3	TAGLN2
hg19-CFP	0.00273172	0.28197947	0.306	0.344	1	3	CFP
hg19-NUSAP	3.81E-104	-0.9569113	0.238	0.54	2.36E-101	4	NUSAP1
hg19-UBE2C.	5.64E-103	-1.383295	0.213	0.516	3.50E-100	4	UBE2C
hg19-TUBA1	1.25E-100	-1.9552067	0.761	0.861	7.74E-98	4	TUBA1B
hg19-HMGN	2.56E-98	-1.174902	0.703	0.857	1.59E-95	4	HMGN2
hg19-KIAA01	6.82E-94	-1.3497073	0.383	0.652	4.24E-91	4	KIAA0101
hg19-TCL1A	9.02E-94	-0.9676228	0.751	0.896	5.60E-91	4	TCL1A
hg19-TK1.4	1.01E-93	-0.9196927	0.295	0.579	6.29E-91	4	TK1
hg19-HMGB	3.39E-88	-1.5560314	0.641	0.811	2.11E-85	4	HMGB2
hg19-FOS.4	1.33E-82	1.05390393	0.759	0.644	8.27E-80	4	FOS
hg19-CENPW	8.43E-82	-0.6870705	0.388	0.645	5.23E-79	4	CENPW
hg19-BIRC5.	6.24E-81	-0.8029557	0.216	0.491	3.88E-78	4	BIRC5

hg19-CDK1.4	4.62E-77	-0.7772354	0.207	0.48	2.87E-74	4	CDK1
hg19-CDC20.3	2.42E-76	-0.7443201	0.212	0.467	1.50E-73	4	CDC20
hg19-MKI67.4	5.66E-75	-0.8435234	0.205	0.475	3.51E-72	4	MKI67
hg19-TYMS.4	5.96E-75	-0.9178229	0.398	0.651	3.70E-72	4	TYMS
hg19-CDKN3.	4.71E-72	-0.7868868	0.261	0.518	2.93E-69	4	CDKN3
hg19-C1QTN	9.07E-65	-0.3835063	0.352	0.567	5.63E-62	4	C1QTNF4
hg19-H2AFX.	4.88E-63	-0.431362	0.295	0.529	3.03E-60	4	H2AFX
hg19-PRC1.2	1.81E-62	-0.3522315	0.201	0.438	1.13E-59	4	PRC1
hg19-ACTG1.	9.67E-62	0.29384103	0.997	0.983	6.00E-59	4	ACTG1
hg19-H2AFZ.	1.05E-61	-0.9823465	0.769	0.865	6.52E-59	4	H2AFZ
hg19-UBE2T.	1.27E-61	-0.4156597	0.352	0.585	7.88E-59	4	UBE2T
hg19-CENPF.	1.72E-61	-0.79676	0.225	0.468	1.07E-58	4	CENPF
hg19-CORO1	3.74E-60	0.5828878	0.806	0.725	2.32E-57	4	CORO1A
hg19-DUT.4	4.81E-60	-0.9848251	0.68	0.827	2.99E-57	4	DUT
hg19-TPX2.2	4.42E-59	-0.3510613	0.226	0.45	2.75E-56	4	TPX2
hg19-MND1.	1.07E-58	-0.451197	0.284	0.511	6.62E-56	4	MND1
hg19-DHFR.4	1.87E-58	-0.6873776	0.356	0.593	1.16E-55	4	DHFR
hg19-TROAP.	2.56E-58	-0.3105382	0.204	0.436	1.59E-55	4	TROAP
hg19-TUBB.3	7.41E-58	-1.1965793	0.855	0.911	4.60E-55	4	TUBB
hg19-TUBB4	1.95E-57	-1.1075117	0.47	0.68	1.21E-54	4	TUBB4B
hg19-SPC25.3	5.25E-57	-0.3021501	0.159	0.395	3.26E-54	4	SPC25
hg19-AK5.1	2.06E-56	0.65188157	0.799	0.737	1.28E-53	4	AK5
hg19-AURKB	2.89E-56	-0.654311	0.237	0.47	1.79E-53	4	AURKB
hg19-PTTG1.	4.74E-55	-1.1099371	0.315	0.547	2.94E-52	4	PTTG1
hg19-CKS1B.	7.90E-55	-0.7410622	0.423	0.642	4.90E-52	4	CKS1B
hg19-MCM7.	1.00E-54	-0.7392641	0.427	0.65	6.22E-52	4	MCM7
hg19-CCNB2.	1.10E-54	-0.5642425	0.257	0.481	6.85E-52	4	CCNB2
hg19-CCNB1.	4.14E-52	-0.5258307	0.228	0.445	2.57E-49	4	CCNB1
hg19-RRM2.3	4.33E-52	-0.3981924	0.22	0.439	2.69E-49	4	RRM2
hg19-TOP2A.	5.16E-52	-1.2512448	0.243	0.481	3.20E-49	4	TOP2A
hg19-VPREB3	2.32E-50	-0.7643284	0.766	0.905	1.44E-47	4	VPREB3
hg19-MAD2L	2.58E-50	-0.4873993	0.323	0.551	1.60E-47	4	MAD2L1
hg19-DLGAP	2.73E-50	-0.4850249	0.196	0.419	1.70E-47	4	DLGAP5
hg19-LTB.4	8.21E-49	0.70216264	0.799	0.745	5.10E-46	4	LTB
hg19-STMN1	3.20E-48	-0.7849938	0.943	0.958	1.99E-45	4	STMN1
hg19-CDCA3.	3.59E-45	-0.3083225	0.189	0.395	2.23E-42	4	CDCA3
hg19-GTSE1.	1.05E-44	-0.5624069	0.227	0.443	6.53E-42	4	GTSE1
hg19-KIF22.3	1.55E-43	-0.2803929	0.309	0.514	9.64E-41	4	KIF22
hg19-CENPE.	1.64E-43	-0.2917733	0.181	0.395	1.02E-40	4	CENPE
hg19-DTYMK	2.38E-43	-0.4005569	0.327	0.536	1.48E-40	4	DTYMK
hg19-ANP32	1.10E-42	-0.4777183	0.32	0.541	6.85E-40	4	ANP32E
hg19-KIFC1.3	1.20E-41	-0.2971678	0.242	0.44	7.47E-39	4	KIFC1
hg19-CKAP2.	3.95E-41	-0.4980775	0.244	0.458	2.45E-38	4	CKAP2
hg19-C21orf	1.24E-40	-0.460008	0.276	0.476	7.72E-38	4	C21orf58
hg19-HMGB3	7.72E-39	-0.764414	0.588	0.756	4.80E-36	4	HMGB3
hg19-ASPM.3	1.46E-38	-0.3922133	0.186	0.39	9.09E-36	4	ASPM
hg19-KLF6.4	5.10E-38	0.59691256	0.718	0.688	3.17E-35	4	KLF6
hg19-HIST2H	1.18E-36	-0.2817869	0.27	0.459	7.30E-34	4	HIST2H2AC
hg19-MXD3.4	1.74E-36	-0.37681	0.345	0.538	1.08E-33	4	MXD3
hg19-CKS2.3	9.15E-36	-0.8863284	0.504	0.682	5.68E-33	4	CKS2
hg19-PCNA.4	4.01E-35	-0.7257868	0.48	0.651	2.49E-32	4	PCNA
hg19-IGLL1.4	4.06E-34	-1.1405925	0.533	0.684	2.52E-31	4	IGLL1
hg19-SMC4.3	3.04E-32	-0.5348386	0.395	0.588	1.89E-29	4	SMC4
hg19-PHGDH	3.14E-32	-0.2734067	0.33	0.517	1.95E-29	4	PHGDH
hg19-PRDX2.	4.09E-32	-0.3110058	0.352	0.543	2.54E-29	4	PRDX2
hg19-PLK1.3	1.22E-31	-0.4439117	0.2	0.383	7.57E-29	4	PLK1
hg19-UBE2S.	3.33E-31	-0.4967998	0.276	0.475	2.07E-28	4	UBE2S
hg19-NUF2.2	3.75E-31	-0.2804592	0.227	0.407	2.33E-28	4	NUF2
hg19-CDCA8.	4.29E-31	-0.2588974	0.188	0.375	2.66E-28	4	CDCA8
hg19-HMMR	2.04E-29	-0.3143072	0.188	0.372	1.27E-26	4	HMMR
hg19-CD69.1	2.90E-29	0.92687992	0.502	0.454	1.80E-26	4	CD69
hg19-MCM5.	6.91E-29	-0.4409598	0.484	0.659	4.29E-26	4	MCM5
hg19-NUCKS	8.43E-28	-0.7426022	0.751	0.855	5.23E-25	4	NUCKS1
hg19-KCNMB	7.72E-26	0.63043389	0.505	0.484	4.80E-23	4	KCNMB1
hg19-HIST1H	1.19E-25	-2.2440982	0.623	0.695	7.37E-23	4	HIST1H4C
hg19-NKG7.3	2.78E-25	0.50192535	0.695	0.673	1.73E-22	4	NKG7
hg19-MS4A1	6.27E-24	0.72346251	0.478	0.452	3.89E-21	4	MS4A1
hg19-CCNA2.	9.59E-24	-0.2559572	0.176	0.35	5.95E-21	4	CCNA2
hg19-LSP1.4	9.03E-23	-0.2766885	0.362	0.55	5.61E-20	4	LSP1
hg19-ARL6IP.	6.24E-21	-0.6974921	0.498	0.653	3.88E-18	4	ARL6IP1
hg19-HMGB1	3.58E-20	-0.4223147	0.984	0.974	2.22E-17	4	HMGB1
hg19-KPNA2.	2.25E-19	-0.5365335	0.393	0.543	1.40E-16	4	KPNA2
hg19-TUBA1	5.45E-19	-0.513393	0.329	0.486	3.38E-16	4	TUBA1C
hg19-BUB3.3	1.17E-17	-0.4350357	0.55	0.697	7.26E-15	4	BUB3
hg19-MZT2B	1.70E-17	-0.4873233	0.883	0.931	1.05E-14	4	MZT2B
hg19-CENPA.	7.98E-16	-0.3965212	0.16	0.324	4.96E-13	4	CENPA
hg19-UBB.4	9.36E-16	-0.5673239	0.96	0.971	5.81E-13	4	UBB
hg19-ISG15.3	2.13E-15	0.8618755	0.731	0.758	1.32E-12	4	ISG15
hg19-HN1.3	2.98E-14	-0.4249971	0.509	0.644	1.85E-11	4	HN1
hg19-FLNA.3	1.64E-13	0.40880836	0.61	0.623	1.02E-10	4	FLNA
hg19-GSN.2	6.27E-13	0.47976747	0.431	0.426	3.89E-10	4	GSN
hg19-CKAP2L	2.49E-12	-0.2506301	0.19	0.333	1.55E-09	4	CKAP2L
hg19-PKM.3	7.31E-12	-0.4035098	0.784	0.869	4.54E-09	4	PKM
hg19-SLC25A	1.54E-10	-0.3330996	0.939	0.956	9.57E-08	4	SLC25A5
hg19-RAN.4	3.54E-09	-0.4311869	0.876	0.908	2.20E-06	4	RAN
hg19-HNRNP	1.36E-07	-0.3886999	0.534	0.636	8.42E-05	4	HNRNPH1
hg19-DDIT4.3	1.35E-06	0.47024697	0.423	0.443	0.00083973	4	DDIT4
hg19-HCST.4	3.35E-06	0.36028381	0.412	0.454	0.00208249	4	HCST
hg19-LY6E	6.86E-05	0.46994798	0.432	0.467	0.04259746	4	LY6E
hg19-EEPD1	0.00048764	0.2579143	0.296	0.41	0.30282182	4	EEPD1
hg19-PREX1	0.00120852	0.49999454	0.31	0.341	0.75049232	4	PREX1
hg19-IGJ.2	0.00327131	0.54539825	0.467	0.501	1	4	IGJ
hg19-NR4A1	0.0032784	0.34077346	0.307	0.402	1	4	NR4A1
hg19-MX1.2	0.00919466	0.68275958	0.595	0.662	1	4	MX1
hg19-RAN.5	1.68E-139	-0.6610228	0.695	0.919	1.04E-136	5	RAN
hg19-SLC25A	1.05E-136	-0.5637849	0.743	0.969	6.55E-134	5	SLC25A5
hg19-PLP2.3	3.13E-115	-1.0326869	0.608	0.803	1.94E-112	5	PLP2
hg19-PKM.4	4.57E-115	-0.5107297	0.666	0.873	2.84E-112	5	PKM
hg19-TUBA1	3.77E-114	-1.6112305	0.593	0.868	2.34E-111	5	TUBA1B
hg19-EEF1B2	3.51E-111	-0.2930011	0.821	0.987	2.18E-108	5	EEF1B2
hg19-HSPA8.	2.01E-101	-0.4303127	0.721	0.914	1.25E-98	5	HSPA8
hg19-RPS2	1.27E-96	-0.4670884	0.999	1	7.90E-94	5	RPS2
hg19-TUBB.4	6.01E-94	-1.0453526	0.741	0.916	3.73E-91	5	TUBB
hg19-VIM.1	3.70E-93	-0.4515547	0.765	0.945	2.30E-90	5	VIM
hg19-KIAA01	1.63E-92	-1.0820465	0.394	0.637	1.01E-89	5	KIAA0101
hg19-H2AFZ.	7.06E-90	-0.7173962	0.663	0.868	4.38E-87	5	H2AFZ
hg19-PCNA.5	2.30E-79	-0.4855752	0.406	0.647	1.43E-76	5	PCNA
hg19-ACTB.3	1.47E-76	-0.6028816	0.961	0.999	9.16E-74	5	ACTB
hg19-HMGB2	3.54E-72	-0.965857	0.616	0.804	2.20E-69	5	HMGB2
hg19-HIST1H	3.30E-67	-1.7530263	0.499	0.701	2.05E-64	5	HIST1H4C
hg19-PFN1.3	4.82E-67	-0.3032917	0.906	0.99	2.99E-64	5	PFN1
hg19-STMN1	5.20E-66	-0.3612198	0.84	0.964	3.23E-63	5	STMN1
hg19-IGLL1.5	1.22E-65	-1.211307	0.48	0.68	7.60E-63	5	IGLL1
hg19-DUT.5	3.73E-63	-0.4828773	0.673	0.82	2.32E-60	5	DUT
hg19-TYMS.5	1.33E-58	-0.8499925	0.497	0.631	8.23E-56	5	TYMS
hg19-MCM7.	1.36E-58	-0.4704021	0.5	0.634	8.45E-56	5	MCM7
hg19-EMR2	1.46E-55	0.28812454	0.577	0.401	9.06E-53	5	EMR2
hg19-HMGB3	2.83E-54	-0.4723988	0.615	0.746	1.75E-51	5	HMGB3
hg19-SMC4.4	1.30E-52	-0.2826205	0.403	0.577	8.08E-50	5	SMC4
hg19-DHFR.5	2.83E-51	-0.3395898	0.435	0.575	1.76E-48	5	DHFR
hg19-HSP90A	1.01E-50	-0.2635181	0.887	0.986	6.29E-48	5	HSP90AA1
hg19-TOP2A.	1.82E-50	-0.9467751	0.306	0.464	1.13E-47	5	TOP2A
hg19-NCF1	8.77E-45	0.38345579	0.586	0.394	5.45E-42	5	NCF1
hg19-SPC25.4	2.52E-43	-0.2930845	0.257	0.376	1.56E-40	5	SPC25
hg19-HMGB1	3.50E-42	-0.4028848	0.883	0.982	2.17E-39	5	HMGB1
hg19-PTTG1.	1.30E-39	-1.0841413	0.426	0.527	8.05E-37	5	PTTG1
hg19-CKS1B.	1.61E-39	-0.5262021	0.528	0.623	1.00E-36	5	CKS1B
hg19-MCM5.	3.16E-39	-0.4900228	0.557	0.645	1.96E-36	5	MCM5
hg19-TSHZ2	7.36E-39	0.31682096	0.516	0.321	4.57E-36	5	TSHZ2
hg19-TUBB4	4.62E-37	-0.7566622	0.597	0.66	2.87E-34	5	TUBB4B
hg19-CKS2.4	1.74E-34	-0.6141069	0.59	0.667	1.08E-31	5	CKS2
hg19-MYC	8.05E-33	-0.4677516	0.58	0.63	5.00E-30	5	MYC
hg19-TMSB4	2.37E-31	-0.2645893	1	1	1.47E-28	5	TMSB4X
hg19-BUB3.4	6.42E-31	-0.2577291	0.617	0.684	3.99E-28	5	BUB3
hg19-PHPT1.	7.69E-31	-0.3573996	0.617	0.671	4.77E-28	5	PHPT1
hg19-C10orf1	6.54E-29	0.59410213	0.616	0.448	4.06E-26	5	C10orf10
hg19-NUSAP	4.05E-28	-0.5781905	0.447	0.509	2.51E-25	5	NUSAP1
hg19-S100A4	1.20E-27	-0.3052105	0.698	0.828	7.44E-25	5	S100A4
hg19-CENPW	3.16E-27	-0.3723194	0.571	0.618	1.97E-24	5	CENPW
hg19-CDK1.5	2.07E-26	-0.6667968	0.395	0.453	1.29E-23	5	CDK1
hg19-PDGFA	7.30E-26	0.58502081	0.546	0.37	4.53E-23	5	PDGFA
hg19-RRM2.4	1.03E-25	-0.2995722	0.36	0.418	6.41E-23	5	RRM2
hg19-UBE2T.	1.39E-25	-0.4923183	0.546	0.559	8.66E-23	5	UBE2T
hg19-CDKN3.	3.81E-25	-0.4899001	0.439	0.492	2.36E-22	5	CDKN3
hg19-C21orf	4.52E-25	-0.30333	0.404	0.457	2.81E-22	5	C21orf58
hg19-TK1.5	1.29E-24	-0.4236299	0.505	0.549	8.00E-22	5	TK1
hg19-MAD2L	3.00E-24	-0.4508771	0.491	0.527	1.86E-21	5	MAD2L1
hg19-ARL6IP.	1.26E-23	-0.7638174	0.577	0.639	7.80E-21	5	ARL6IP1
hg19-TMEM1	1.34E-22	0.5335782	0.574	0.418	8.35E-20	5	TMEM173
hg19-ASPM.4	7.42E-22	-0.272725	0.314	0.371	4.61E-19	5	ASPM
hg19-KIF4A.2	1.26E-21	-0.2787545	0.282	0.335	7.83E-19	5	KIF4A
hg19-HGS	6.45E-20	0.27078023	0.57	0.43	4.01E-17	5	HGS
hg19-TUBA1.	1.01E-17	-0.2548094	0.547	0.595	6.27E-15	5	TUBA1A
hg19-CDC20.	1.99E-17	-0.5486877	0.422	0.439	1.23E-14	5	CDC20
hg19-PREX1.	4.31E-17	0.33541581	0.465	0.328	2.68E-14	5	PREX1
hg19-H2AFX.	5.77E-17	-0.304955	0.495	0.502	3.59E-14	5	H2AFX
hg19-UBE2S.	7.06E-16	-0.3836088	0.414	0.455	4.38E-13	5	UBE2S
hg19-CENPE.	4.23E-15	-0.3420348	0.342	0.372	2.62E-12	5	CENPE
hg19-BIRC5.5	7.29E-15	-0.6758391	0.447	0.46	4.53E-12	5	BIRC5
hg19-ANP32	1.43E-14	-0.4123107	0.503	0.517	8.88E-12	5	ANP32E
hg19-GTSE1.	3.59E-13	-0.4309704	0.392	0.42	2.23E-10	5	GTSE1
hg19-HMMR	3.86E-13	-0.3592124	0.334	0.352	2.40E-10	5	HMMR
hg19-UBE2C.	1.07E-12	-1.0398318	0.48	0.481	6.67E-10	5	UBE2C
hg19-C19orf	1.21E-12	0.30628303	0.553	0.439	7.49E-10	5	C19orf66
hg19-MND1.	7.69E-12	-0.4561538	0.493	0.484	4.77E-09	5	MND1
hg19-WASH4	8.37E-12	0.35690203	0.577	0.454	5.20E-09	5	WASH4P
hg19-KIFC1.4	1.10E-11	-0.2901617	0.423	0.417	6.83E-09	5	KIFC1
hg19-CCNB2.	2.93E-11	-0.5930707	0.47	0.454	1.82E-08	5	CCNB2
hg19-MKI67.	4.86E-10	-0.6807282	0.456	0.443	3.02E-07	5	MKI67
hg19-KPNA2.	5.56E-10	-0.5601713	0.512	0.527	3.45E-07	5	KPNA2
hg19-HNRNP	6.29E-09	0.46127582	0.469	0.636	3.91E-06	5	HNRNPH1
hg19-AURKB	7.76E-09	-0.3795018	0.457	0.443	4.82E-06	5	AURKB
hg19-CD37.3	8.41E-09	0.44078248	0.646	0.575	5.22E-06	5	CD37
hg19-TAGLN.	1.18E-08	-0.3036189	0.55	0.55	7.33E-06	5	TAGLN2
hg19-KCNQ1	2.18E-07	0.50919804	0.454	0.329	0.00013548	5	KCNQ1OT1
hg19-POU4F	6.54E-07	0.26464284	0.395	0.266	0.00040598	5	POU4F1
hg19-TUBA1	2.34E-06	-0.6056144	0.496	0.466	0.00145584	5	TUBA1C
hg19-C1QTN	2.76E-06	-0.4126419	0.562	0.541	0.00171187	5	C1QTNF4
hg19-TPX2.3	1.05E-05	-0.3224941	0.46	0.422	0.00650006	5	TPX2
hg19-FOS.5	1.62E-05	0.33918585	0.598	0.662	0.01005022	5	FOS
hg19-CENPF.	0.00012094	-0.3914453	0.462	0.439	0.07510322	5	CENPF
hg19-KIF22.4	0.00012678	-0.2633593	0.532	0.488	0.07872743	5	KIF22
hg19-HIST2H	0.00014121	-0.3534291	0.474	0.434	0.0876919	5	HIST2H2AC
hg19-H1FX.2	0.00057915	0.56657921	0.631	0.689	0.35965017	5	H1FX
hg19-EGR1.1	0.00062364	0.30988149	0.627	0.603	0.38728088	5	EGR1
hg19-CCNB1.	0.00078419	-0.3286432	0.468	0.416	0.48698398	5	CCNB1
hg19-IRX1.1	0.00174609	0.53804298	0.608	0.531	1	5	IRX1
hg19-DDIT4.4	0.00797009	0.36583285	0.518	0.435	1	5	DDIT4
hg19-DLGAP	0.00980803	-0.4383186	0.447	0.389	1	5	DLGAP5
hg19-UBE2C.	0	3.08566711	0.993	0.446	0	6	UBE2C
hg19-PTTG1.	0	2.76087212	0.99	0.488	0	6	PTTG1
hg19-TOP2A.	0	2.64775325	0.955	0.419	0	6	TOP2A
hg19-CKS2.5	0	2.51889505	0.999	0.638	0	6	CKS2
hg19-TUBB4	0	2.4455092	0.997	0.633	0	6	TUBB4B
hg19-KPNA2.	0	2.41370139	0.972	0.495	0	6	KPNA2
hg19-CDC20.	0	2.35617125	0.966	0.401	0	6	CDC20
hg19-CENPF.	0	2.313437	0.966	0.404	0	6	CENPF
hg19-HMGB2	0	2.26234851	0.999	0.777	0	6	HMGB2
hg19-NUSAP	0	2.14283249	0.973	0.473	0	6	NUSAP1
hg19-CDKN3.	0	2.14018346	0.975	0.455	0	6	CDKN3
hg19-HN1.4	0	2.12715748	0.982	0.604	0	6	HN1
hg19-CDK1.6	0	2.03159283	0.965	0.413	0	6	CDK1
hg19-MKI67.	0	1.99664316	0.966	0.408	0	6	MKI67
hg19-PLK1.4	0	1.9716282	0.926	0.323	0	6	PLK1
hg19-CCNB1.	0	1.95817685	0.919	0.386	0	6	CCNB1
hg19-TUBA1	0	1.93912312	0.937	0.435	0	6	TUBA1C
hg19-DLGAP	0	1.88669962	0.937	0.356	0	6	DLGAP5
hg19-CCNB2.	0	1.82086918	0.954	0.42	0	6	CCNB2
hg19-BIRC5.6	0	1.81690059	0.962	0.425	0	6	BIRC5
hg19-CENPA.	0	1.81599929	0.916	0.263	0	6	CENPA
hg19-AURKB	0	1.77967589	0.959	0.408	0	6	AURKB
hg19-GTSE1.	0	1.64586409	0.926	0.384	0	6	GTSE1
hg19-CENPE.	0	1.58368138	0.889	0.335	0	6	CENPE
hg19-HMMR	0	1.52714007	0.878	0.315	0	6	HMMR
hg19-CDCA3.	0	1.20702915	0.863	0.337	0	6	CDCA3
hg19-ARL6IP.	4.66E-306	2.2827833	0.992	0.611	2.89E-303	6	ARL6IP1
hg19-TUBA1	5.89E-304	2.00521963	1	0.839	3.66E-301	6	TUBA1B
hg19-TPX2.4	2.55E-300	1.46114847	0.884	0.393	1.58E-297	6	TPX2
hg19-H2AFX.	5.30E-287	1.24849785	0.928	0.472	3.29E-284	6	H2AFX
hg19-PIF1	6.02E-282	1.12645159	0.757	0.235	3.74E-279	6	PIF1
hg19-CKS1B.	3.58E-276	1.59836815	0.992	0.591	2.23E-273	6	CKS1B
hg19-UBE2S.	1.89E-272	1.58477891	0.921	0.42	1.17E-269	6	UBE2S
hg19-KIF23	4.77E-272	1.09340937	0.769	0.239	2.96E-269	6	KIF23
hg19-KIF20A	2.26E-266	1.01819924	0.763	0.262	1.40E-263	6	KIF20A
hg19-MXD3.5	3.57E-259	1.26656518	0.944	0.486	2.22E-256	6	MXD3
hg19-CKAP2.	2.47E-258	1.33623621	0.895	0.401	1.53E-255	6	CKAP2
hg19-ASPM.5	1.39E-253	1.4739086	0.846	0.334	8.64E-251	6	ASPM
hg19-PSRC1	2.96E-252	0.61553098	0.715	0.244	1.84E-249	6	PSRC1
hg19-TROAP.	1.32E-245	1.06863541	0.825	0.381	8.17E-243	6	TROAP
hg19-KIFC1.5	7.81E-245	1.09009795	0.835	0.389	4.85E-242	6	KIFC1
hg19-CDCA8.	1.70E-242	1.14913715	0.794	0.323	1.06E-239	6	CDCA8
hg19-DEPDC1	4.89E-240	0.89849567	0.749	0.249	3.04E-237	6	DEPDC1
hg19-CCNA2.	2.77E-239	1.03964072	0.79	0.298	1.72E-236	6	CCNA2
hg19-PRC1.3	6.98E-239	1.17889141	0.83	0.382	4.33E-236	6	PRC1
hg19-SGOL2.	1.33E-238	1.07550955	0.819	0.4	8.23E-236	6	SGOL2
hg19-AURKA	4.05E-236	1.2423867	0.794	0.367	2.52E-233	6	AURKA
hg19-SMC4.5	4.61E-235	1.41169923	0.959	0.539	2.86E-232	6	SMC4
hg19-BUB3.5	5.76E-225	1.59641246	0.986	0.659	3.58E-222	6	BUB3
hg19-FAM64	5.86E-225	1.00375568	0.759	0.332	3.64E-222	6	FAM64A
hg19-NUF2.3	1.04E-220	0.97496858	0.788	0.359	6.44E-218	6	NUF2
hg19-TUBB.5	3.64E-217	1.51368616	0.999	0.898	2.26E-214	6	TUBB
hg19-KIF4A.3	1.08E-216	1.09183143	0.756	0.302	6.73E-214	6	KIF4A
hg19-HMGB3	1.46E-216	1.5317946	0.992	0.719	9.06E-214	6	HMGB3
hg19-CKAP2L	3.39E-215	0.90805579	0.755	0.287	2.10E-212	6	CKAP2L
hg19-TACC3.	6.87E-209	0.96197038	0.816	0.391	4.26E-206	6	TACC3
hg19-HIST1H	8.33E-205	2.15611113	0.975	0.667	5.17E-202	6	HIST1H4C
hg19-SPC25.5	2.88E-203	0.73803356	0.781	0.339	1.79E-200	6	SPC25
hg19-H2AFZ.	2.90E-203	1.36066305	1	0.844	1.80E-200	6	H2AFZ
hg19-NEK2	1.54E-195	0.73573382	0.708	0.301	9.57E-193	6	NEK2
hg19-TUBA1.	8.81E-194	1.28187721	0.955	0.567	5.47E-191	6	TUBA1A
hg19-KIF22.5	1.87E-193	0.8879666	0.871	0.465	1.16E-190	6	KIF22
hg19-C21orf	2.27E-190	0.93533667	0.847	0.426	1.41E-187	6	C21orf58
hg19-CDC25B	3.03E-186	0.72125621	0.735	0.364	1.88E-183	6	CDC25B
hg19-ANP32	2.21E-184	1.09889387	0.91	0.488	1.37E-181	6	ANP32E
hg19-CENPW	1.49E-183	1.00349978	0.94	0.593	9.25E-181	6	CENPW
hg19-UBE2T.	1.32E-170	0.90480677	0.899	0.535	8.21E-168	6	UBE2T
hg19-HMGN2	2.33E-168	1.16648222	0.975	0.83	1.45E-165	6	HMGN2
hg19-RACGA	1.03E-166	0.80561768	0.708	0.333	6.40E-164	6	RACGAP1
hg19-MAD2L	1.28E-158	0.97526209	0.878	0.501	7.96E-156	6	MAD2L1
hg19-NUCKS	1.81E-157	1.31940499	0.999	0.832	1.12E-154	6	NUCKS1
hg19-HMGB1	7.55E-156	0.89827378	0.999	0.974	4.69E-153	6	HMGB1
hg19-DTYMK	6.14E-147	0.74194847	0.854	0.488	3.81E-144	6	DTYMK
hg19-CALM2	7.17E-146	1.10019526	0.999	0.911	4.45E-143	6	CALM2
hg19-CBR3	2.84E-139	0.86590389	0.78	0.454	1.76E-136	6	CBR3
hg19-MND1.	2.28E-114	0.48703722	0.783	0.464	1.42E-111	6	MND1
hg19-HIST2H	2.73E-113	0.50649624	0.731	0.417	1.69E-110	6	HIST2H2AC
hg19-STMN1	4.99E-111	0.93217374	1	0.953	3.10E-108	6	STMN1
hg19-PLIN3.2	3.59E-105	0.68298134	0.825	0.516	2.23E-102	6	PLIN3
hg19-TMSB4	3.63E-95	0.39766817	1	1	2.25E-92	6	TMSB4X
hg19-PHGDH	1.52E-93	0.65686375	0.766	0.477	9.47E-91	6	PHGDH
hg19-UBB.5	3.67E-91	0.86313879	1	0.967	2.28E-88	6	UBB
hg19-KIAA01	4.32E-86	0.53140839	0.952	0.598	2.68E-83	6	KIAA0101
hg19-SPC24.	6.86E-85	0.36506991	0.62	0.343	4.26E-82	6	SPC24
hg19-TYMS.6	1.19E-84	0.72954918	0.906	0.602	7.42E-82	6	TYMS
hg19-VIM.2	1.55E-80	0.76715679	0.999	0.929	9.62E-78	6	VIM
hg19-RAN.6	1.12E-64	0.59028234	0.999	0.898	6.94E-62	6	RAN
hg19-ACTB.4	1.72E-63	0.53669286	1	0.996	1.07E-60	6	ACTB
hg19-RPS18	3.27E-61	-0.3246671	1	1	2.03E-58	6	RPS18
hg19-IGLL1.6	9.05E-60	1.11003334	0.868	0.653	5.62E-57	6	IGLL1
hg19-HIST1H	9.96E-54	0.30609156	0.771	0.522	6.18E-51	6	HIST1H1C
hg19-HSPA8.	2.35E-49	0.60946563	0.985	0.896	1.46E-46	6	HSPA8
hg19-RPS2.1	7.53E-49	-0.256993	1	1	4.68E-46	6	RPS2
hg19-IRX1.2	7.89E-43	0.39839043	0.749	0.522	4.90E-40	6	IRX1
hg19-PLP2.4	4.06E-41	0.52218869	0.926	0.781	2.52E-38	6	PLP2
hg19-UNG.3	2.71E-39	-0.3429853	0.323	0.445	1.69E-36	6	UNG
hg19-MX1.3	6.56E-36	0.25509386	0.825	0.643	4.07E-33	6	MX1
hg19-SLC25A	5.27E-26	0.51940606	0.997	0.951	3.27E-23	6	SLC25A5
hg19-H1FX.3	1.92E-24	0.36672307	0.9	0.67	1.19E-21	6	H1FX
hg19-MZT2B	3.42E-23	0.26739877	0.994	0.921	2.12E-20	6	MZT2B
hg19-ACTG1.	3.68E-22	0.33551403	1	0.984	2.29E-19	6	ACTG1
hg19-RP11-3	9.41E-22	0.3484627	0.689	0.529	5.85E-19	6	RP11-386I14.4
hg19-CORO1	4.31E-21	0.3307372	0.867	0.726	2.68E-18	6	CORO1A
hg19-IER2.3	1.08E-19	0.36408678	0.93	0.784	6.71E-17	6	IER2
hg19-KLF6.5	3.11E-18	0.25070542	0.868	0.679	1.93E-15	6	KLF6
hg19-UGT3A	8.84E-16	0.33697478	0.453	0.308	5.49E-13	6	UGT3A2
hg19-PFN1.4	1.30E-13	0.35644468	1	0.984	8.10E-11	6	PFN1
hg19-TIMP1.	2.74E-09	-0.4611023	0.484	0.499	1.70E-06	6	TIMP1
hg19-CD37.4	5.52E-09	-0.5711642	0.59	0.579	3.43E-06	6	CD37
hg19-HCST.5	1.43E-07	-0.2729582	0.461	0.449	8.87E-05	6	HCST
hg19-S100A4	1.88E-07	-0.5948856	0.858	0.817	0.00011696	6	S100A4
hg19-LY6E.1	2.68E-06	-0.2835007	0.463	0.463	0.00166486	6	LY6E
hg19-MCM5.	1.02E-05	-0.5745958	0.656	0.638	0.00635212	6	MCM5
hg19-TAGLN.	0.00275251	-0.3906084	0.574	0.548	1	6	TAGLN2
hg19-EMP3.3	0.00283058	-0.3617883	0.62	0.514	1	6	EMP3
hg19-IGJ.3	0.00703255	-0.2672036	0.593	0.491	1	6	IGJ
hg19-PTTG1.	2.12E-233	1.76487117	0.934	0.493	1.32E-230	7	PTTG1
hg19-TMSB4	2.23E-214	0.70571996	1	1	1.39E-211	7	TMSB4X
hg19-HMGB1	1.04E-166	1.01511638	0.999	0.974	6.47E-164	7	HMGB1
hg19-CDC20.	8.62E-161	1.28862129	0.785	0.415	5.35E-158	7	CDC20
hg19-ARL6IP.	9.81E-154	1.7398543	0.912	0.617	6.09E-151	7	ARL6IP1
hg19-HMGB3	2.79E-152	1.51906157	0.987	0.721	1.73E-149	7	HMGB3
hg19-TROAP.	5.59E-151	0.97086931	0.743	0.387	3.47E-148	7	TROAP
hg19-HMMR	1.52E-130	0.89479654	0.693	0.329	9.42E-128	7	HMMR
hg19-CCNB1.	7.66E-122	1.11611876	0.722	0.4	4.76E-119	7	CCNB1
hg19-CCNB2.	7.98E-120	1.01668995	0.769	0.434	4.95E-117	7	CCNB2
hg19-HMGN2	1.89E-116	0.98828947	0.95	0.832	1.17E-113	7	HMGN2
hg19-BIRC5.7	6.32E-115	1.28807391	0.773	0.438	3.92E-112	7	BIRC5
hg19-HMGB2	1.14E-113	1.18032327	0.958	0.78	7.07E-111	7	HMGB2
hg19-NUCKS	3.10E-108	1.28140381	0.956	0.835	1.92E-105	7	NUCKS1
hg19-CDC25B	5.76E-100	0.54705431	0.654	0.37	3.58E-97	7	CDC25B
hg19-TUBA1	3.06E-99	1.35731225	0.738	0.45	1.90E-96	7	TUBA1C
hg19-PLK1.5	3.15E-98	0.77146322	0.658	0.342	1.96E-95	7	PLK1
hg19-PTMS	4.36E-92	0.37337245	0.594	0.353	2.71E-89	7	PTMS
hg19-CENPF.	2.04E-90	0.93577345	0.732	0.421	1.27E-87	7	CENPF
hg19-ACTB.5	2.06E-89	0.66879375	1	0.996	1.28E-86	7	ACTB
hg19-UBE2C.	1.64E-88	1.30657085	0.74	0.464	1.02E-85	7	UBE2C
hg19-CDKN3.	3.45E-84	0.85839138	0.757	0.471	2.14E-81	7	CDKN3
hg19-TUBA1	4.59E-78	0.87420064	0.981	0.841	2.85E-75	7	TUBA1B
hg19-NUSAP	3.91E-75	0.77759173	0.756	0.489	2.43E-72	7	NUSAP1
hg19-PLIN3.3	4.07E-70	0.54948823	0.776	0.52	2.53E-67	7	PLIN3
hg19-CCNA2.	7.74E-70	0.44608636	0.585	0.313	4.81E-67	7	CCNA2
hg19-FAM64	7.71E-69	0.51744548	0.585	0.345	4.79E-66	7	FAM64A
hg19-CENPW	3.38E-67	0.75474362	0.833	0.601	2.10E-64	7	CENPW
hg19-MKI67.	8.38E-67	0.59173419	0.712	0.426	5.20E-64	7	MKI67
hg19-ASPM.6	5.36E-65	0.67385164	0.618	0.35	3.33E-62	7	ASPM
hg19-CENPE.	9.06E-65	0.4720262	0.608	0.355	5.63E-62	7	CENPE
hg19-ANP32	1.95E-63	0.75940677	0.765	0.499	1.21E-60	7	ANP32E
hg19-TUBB4	1.98E-60	1.18866765	0.82	0.645	1.23E-57	7	TUBB4B
hg19-SGOL2.	2.19E-60	0.34025142	0.627	0.414	1.36E-57	7	SGOL2
hg19-HN1.5	9.79E-60	0.59663801	0.852	0.614	6.08E-57	7	HN1
hg19-CALM2	4.35E-58	0.86802002	0.987	0.912	2.70E-55	7	CALM2
hg19-DLGAP	2.45E-57	0.60240052	0.62	0.378	1.52E-54	7	DLGAP5
hg19-CKS1B.	1.27E-56	1.04428644	0.819	0.603	7.86E-54	7	CKS1B
hg19-KPNA2.	3.69E-56	0.91679821	0.737	0.512	2.29E-53	7	KPNA2
hg19-NEK2.1	1.03E-54	0.27743127	0.548	0.313	6.43E-52	7	NEK2
hg19-PRC1.4	1.04E-54	0.37301652	0.626	0.397	6.47E-52	7	PRC1
hg19-MAD2L	4.74E-54	0.66663081	0.751	0.51	2.94E-51	7	MAD2L1
hg19-IFI6.1	2.68E-52	0.50624002	0.74	0.555	1.66E-49	7	IFI6
hg19-TPX2.5	1.46E-51	0.60970136	0.624	0.411	9.04E-49	7	TPX2
hg19-LTB.5	3.41E-50	0.99166301	0.914	0.741	2.12E-47	7	LTB
hg19-RAN.7	2.71E-49	0.75782096	0.984	0.899	1.68E-46	7	RAN
hg19-CDCA8.	7.30E-49	0.37205754	0.553	0.34	4.53E-46	7	CDCA8
hg19-CKAP2.	9.84E-49	0.37658325	0.655	0.418	6.11E-46	7	CKAP2
hg19-DEPDC1	3.97E-48	0.37128476	0.501	0.266	2.47E-45	7	DEPDC1
hg19-TUBB.6	3.80E-47	0.7365249	0.978	0.9	2.36E-44	7	TUBB
hg19-GTSE1.	5.46E-47	0.73529933	0.637	0.404	3.39E-44	7	GTSE1
hg19-UBB.6	1.29E-46	0.89757539	0.994	0.968	8.01E-44	7	UBB
hg19-GCHFR	4.78E-46	0.49218801	0.708	0.514	2.97E-43	7	GCHFR
hg19-CKS2.6	8.12E-44	0.89218928	0.817	0.651	5.04E-41	7	CKS2
hg19-DTYMK	1.14E-43	0.40966782	0.715	0.498	7.05E-41	7	DTYMK
hg19-VIM.3	2.68E-43	0.83140041	0.984	0.93	1.67E-40	7	VIM
hg19-TUBA1.	1.05E-42	0.74070219	0.778	0.579	6.52E-40	7	TUBA1A
hg19-NUF2.4	1.58E-38	0.50142496	0.569	0.375	9.79E-36	7	NUF2
hg19-ACTG1.	8.69E-38	0.56069436	0.999	0.984	5.40E-35	7	ACTG1
hg19-TACC3.	1.06E-37	0.39277861	0.605	0.406	6.56E-35	7	TACC3
hg19-KIF4A.4	2.22E-36	0.49349064	0.528	0.318	1.38E-33	7	KIF4A
hg19-SMC4.6	2.52E-36	0.65576915	0.776	0.552	1.57E-33	7	SMC4
hg19-APLP2	6.12E-36	0.35973513	0.703	0.486	3.80E-33	7	APLP2
hg19-RAB30-	1.13E-34	0.32049778	0.614	0.436	7.02E-32	7	RAB30-AS1
hg19-CENPA.	9.49E-34	0.46431876	0.529	0.29	5.90E-31	7	CENPA
hg19-HSP90A	2.24E-33	0.51524589	0.997	0.978	1.39E-30	7	HSP90AA1
hg19-CDK1.7	6.11E-33	0.54421268	0.624	0.437	3.79E-30	7	CDK1
hg19-HSPA8.	2.47E-32	0.91024097	0.968	0.897	1.53E-29	7	HSPA8
hg19-H2AFX.	4.26E-32	0.25103341	0.662	0.491	2.64E-29	7	H2AFX
hg19-MX1.4	9.28E-31	0.55861249	0.813	0.644	5.76E-28	7	MX1
hg19-TOP2A.	1.02E-30	1.05341915	0.618	0.443	6.33E-28	7	TOP2A
hg19-PPP1R1	1.73E-30	0.29378156	0.675	0.474	1.08E-27	7	PPP1R14B
hg19-UBE2S.	3.87E-27	0.63041312	0.629	0.441	2.40E-24	7	UBE2S
hg19-KIF22.6	4.42E-27	0.298959	0.648	0.48	2.75E-24	7	KIF22
hg19-STMN1	2.78E-26	0.52556238	0.997	0.953	1.73E-23	7	STMN1
hg19-HNRNP	9.06E-26	0.66962182	0.782	0.614	5.63E-23	7	HNRNPH1
hg19-H2AFZ.	9.07E-25	0.49933341	0.974	0.846	5.63E-22	7	H2AFZ
hg19-PDGFA	8.43E-21	0.35914863	0.525	0.372	5.23E-18	7	PDGFA
hg19-IGLL1.7	2.20E-20	0.64350427	0.784	0.659	1.36E-17	7	IGLL1
hg19-MCM5.	3.91E-18	-0.7890677	0.564	0.644	2.43E-15	7	MCM5
hg19-MZT2B	4.10E-18	0.49438555	0.994	0.921	2.54E-15	7	MZT2B
hg19-BUB3.6	2.00E-17	0.50818412	0.794	0.672	1.24E-14	7	BUB3
hg19-FXYD2.	2.07E-15	0.33077518	0.715	0.593	1.28E-12	7	FXYD2
hg19-FOS.6	8.83E-14	-0.6930684	0.646	0.658	5.48E-11	7	FOS
hg19-TYMS.7	2.79E-11	-0.673508	0.602	0.623	1.73E-08	7	TYMS
hg19-PRDX1.	5.77E-11	0.27570848	0.996	0.987	3.58E-08	7	PRDX1
hg19-CDC37	8.54E-11	0.2507849	0.756	0.63	5.30E-08	7	CDC37
hg19-DUT.6	3.60E-10	-0.6861077	0.852	0.808	2.24E-07	7	DUT
hg19-ISG15.2	2.80E-09	0.26643786	0.861	0.748	1.74E-06	7	ISG15
hg19-CD37.5	7.09E-08	-0.548872	0.604	0.578	4.40E-05	7	CD37
hg19-HIST1H	6.83E-07	-0.8364071	0.681	0.687	0.0004241	7	HIST1H4C
hg19-PCNA.6	1.51E-05	-0.7086098	0.652	0.63	0.00934887	7	PCNA
hg19-UNG.4	5.85E-05	-0.2937971	0.433	0.437	0.03630177	7	UNG
hg19-HCST.6	0.00020977	-0.2742791	0.472	0.448	0.13026667	7	HCST
hg19-MCM7.	0.00075644	-0.4435351	0.649	0.623	0.46974757	7	MCM7
hg19-RRM2.5	0.00727564	-0.3000118	0.493	0.409	1	7	RRM2
hg19-EGR1.2	0.00933585	-0.4418654	0.633	0.602	1	7	EGR1
hg19-IGJ.4	0.00977958	-0.3085506	0.567	0.493	1	7	IGJ
