# Supplementary material for: Integrated multi-omics analyses reveal homology-directed repair pathway as a unique dependency in near-haploid leukemia
Source: Blood Cancer J. 2023 Jun 8;13(1):92. doi: 10.1038/s41408-023-00863-1 (PMC10247733; doi:10.1038/s41408-023-00863-1)
Supplement: Supplementary file 7 — Supplementary Table 6 [file 41408_2023_863_MOESM7_ESM.pdf]

Enriched Reactome Pathways

Reactome pathways	Homo sapier	Client Text B	Client Text B	Client Text B	Client Text B	Client Text B	Client Text Box Input (FDR)
Manipulation of host energy metabolism (R-HSA-9636667)	2	2	0.01	+	> 100	9.74E-05	1.39E-03
Formation of annular gap junctions (R-HSA-196025)	11	3	0.04	+	66.85	2.28E-05	3.49E-04
Gap junction degradation (R-HSA-190873)	12	3	0.05	+	61.28	2.84E-05	4.27E-04
Vpu mediated degradation of CD4 (R-HSA-180534)	51	12	0.21	+	57.67	2.02E-17	7.18E-15
Negative regulation of NOTCH4 signaling (R-HSA-9604323)	52	12	0.21	+	56.56	2.47E-17	7.71E-15
Cross-presentation of soluble exogenous antigens (endosomes) (R-HSA-12	49	11	0.2	+	55.02	7.37E-16	7.07E-14
Regulation of activated PAK-2p34 by proteasome mediated degradation (F	49	11	0.2	+	55.02	7.37E-16	6.81E-14
FBXL7 down-regulates AURKA during mitotic entry and in early mitosis (R-	54	12	0.22	+	54.47	3.69E-17	1.02E-14
SCF-beta-TrCP mediated degradation of Emi1 (R-HSA-174113)	54	12	0.22	+	54.47	3.69E-17	9.19E-15
Regulation of ornithine decarboxylase (ODC) (R-HSA-350562)	50	11	0.2	+	53.92	8.97E-16	7.71E-14
Autodegradation of the E3 ubiquitin ligase COP1 (R-HSA-349425)	51	11	0.21	+	52.87	1.09E-15	9.03E-14
Ubiquitin-dependent degradation of Cyclin D (R-HSA-75815)	51	11	0.21	+	52.87	1.09E-15	8.74E-14
p53-Independent G1/S DNA damage checkpoint (R-HSA-69613)	51	11	0.21	+	52.87	1.09E-15	8.46E-14
p53-Independent DNA Damage Response (R-HSA-69610)	51	11	0.21	+	52.87	1.09E-15	8.21E-14
Ubiquitin Mediated Degradation of Phosphorylated Cdc25A (R-HSA-69601)	51	11	0.21	+	52.87	1.09E-15	7.97E-14
Vif-mediated degradation of APOBEC3G (R-HSA-180585)	52	11	0.21	+	51.85	1.31E-15	9.09E-14
Regulation of Apoptosis (R-HSA-169911)	52	11	0.21	+	51.85	1.31E-15	8.84E-14
AUF1 (hnRNP D0) binds and destabilizes mRNA (R-HSA-450408)	53	11	0.22	+	50.87	1.58E-15	1.04E-13
Regulation of RUNX3 expression and activity (R-HSA-8941858)	53	11	0.22	+	50.87	1.58E-15	1.01E-13
NIK-->noncanonical NF-kB signaling (R-HSA-5676590)	58	12	0.24	+	50.71	7.86E-17	1.51E-14
Degradation of AXIN (R-HSA-4641257)	54	11	0.22	+	49.93	1.89E-15	1.18E-13
SCF(Skp2)-mediated degradation of p27/p21 (R-HSA-187577)	59	12	0.24	+	49.85	9.43E-17	1.68E-14
GLI3 is processed to GLI3R by the proteasome (R-HSA-5610785)	59	12	0.24	+	49.85	9.43E-17	1.57E-14
Degradation of GLI2 by the proteasome (R-HSA-5610783)	59	12	0.24	+	49.85	9.43E-17	1.47E-14
Degradation of GLI1 by the proteasome (R-HSA-5610780)	59	12	0.24	+	49.85	9.43E-17	1.38E-14
Dectin-1 mediated noncanonical NF-kB signaling (R-HSA-5607761)	59	12	0.24	+	49.85	9.43E-17	1.31E-14
Folding of actin by CCT/TriC (R-HSA-390450)	10	2	0.04	+	49.02	1.05E-03	1.29E-02
Hh mutants are degraded by ERAD (R-HSA-5362768)	55	11	0.22	+	49.02	2.27E-15	1.38E-13
Stabilization of p53 (R-HSA-69541)	55	11	0.22	+	49.02	2.27E-15	1.35E-13
ROS sensing by NFE2L2 (R-HSA-8932339)	56	11	0.23	+	48.15	2.70E-15	1.46E-13
Degradation of DVL (R-HSA-4641258)	56	11	0.23	+	48.15	2.70E-15	1.43E-13
Metabolism of polyamines (R-HSA-351202)	57	11	0.23	+	47.3	3.21E-15	1.67E-13
CDT1 association with the CDC6:ORC:origin complex (R-HSA-68827)	58	11	0.24	+	46.49	3.81E-15	1.83E-13
Hh mutants abrogate ligand secretion (R-HSA-5387390)	58	11	0.24	+	46.49	3.81E-15	1.79E-13
Regulation of HMOX1 expression and activity (R-HSA-9707587)	64	12	0.26	+	45.96	2.25E-16	2.67E-14
Defective CFTR causes cystic fibrosis (R-HSA-5678895)	60	11	0.24	+	44.94	5.32E-15	2.41E-13
Gluconeogenesis (R-HSA-70263)	33	6	0.13	+	44.56	1.10E-08	1.90E-07
Activation of NF-kappaB in B cells (R-HSA-1169091)	66	12	0.27	+	44.56	3.13E-16	3.54E-14
Asymmetric localization of PCP proteins (R-HSA-4608870)	63	11	0.26	+	42.8	8.59E-15	3.46E-13
Autodegradation of Cdh1 by Cdh1:APC/C (R-HSA-174084)	63	11	0.26	+	42.8	8.59E-15	3.40E-13
Hedgehog ligand biogenesis (R-HSA-5358346)	64	11	0.26	+	42.13	1.00E-14	3.85E-13
p53-Dependent G1/S DNA damage checkpoint (R-HSA-69580)	64	11	0.26	+	42.13	1.00E-14	3.79E-13
p53-Dependent G1 DNA Damage Response (R-HSA-69563)	64	11	0.26	+	42.13	1.00E-14	3.73E-13
Orc1 removal from chromatin (R-HSA-68949)	70	12	0.29	+	42.02	5.89E-16	6.12E-14
Regulation of RUNX2 expression and activity (R-HSA-8939902)	70	12	0.29	+	42.02	5.89E-16	5.87E-14
Oxygen-dependent proline hydroxylation of Hypoxia-inducible Factor Alpha	65	11	0.27	+	41.48	1.17E-14	4.23E-13
The role of GTSE1 in G2/M progression after G2 checkpoint (R-HSA-88522	78	13	0.32	+	40.85	4.64E-17	1.05E-14
Endosomal/Vacuolar pathway (R-HSA-1236977)	12	2	0.05	+	40.85	1.44E-03	1.74E-02
Regulation of RAS by GAPs (R-HSA-5658442)	66	11	0.27	+	40.85	1.36E-14	4.84E-13
G1/S DNA Damage Checkpoints (R-HSA-69615)	66	11	0.27	+	40.85	1.36E-14	4.78E-13
Downstream signaling events of B Cell Receptor (BCR) (R-HSA-1168372)	79	13	0.32	+	40.33	5.38E-17	1.12E-14
APC/C:Cdc20 mediated degradation of Securin (R-HSA-174154)	67	11	0.27	+	40.24	1.58E-14	5.47E-13
Regulation of PTEN stability and activity (R-HSA-8948751)	68	11	0.28	+	39.65	1.83E-14	6.08E-13
ER-Phagosome pathway (R-HSA-1236974)	90	14	0.37	+	38.13	6.38E-18	3.18E-15
Apoptosis induced DNA fragmentation (R-HSA-140342)	13	2	0.05	+	37.71	1.66E-03	1.98E-02
CDK-mediated phosphorylation and removal of Cdc6 (R-HSA-69017)	72	11	0.29	+	37.45	3.22E-14	1.03E-12
Cdc20:Phospho-APC/C mediated degradation of Cyclin A (R-HSA-174184)	72	11	0.29	+	37.45	3.22E-14	1.02E-12
APC:Cdc20 mediated degradation of cell cycle proteins prior to satisfation	73	11	0.3	+	36.93	3.70E-14	1.14E-12
APC/C:Cdh1 mediated degradation of Cdc20 and other APC/C:Cdh1 targete	73	11	0.3	+	36.93	3.70E-14	1.12E-12
Regulation of APC/C activators between G1/S and early anaphase (R-HSA-	80	12	0.33	+	36.77	2.50E-15	1.45E-13
Signaling by NOTCH4 (R-HSA-9013694)	80	12	0.33	+	36.77	2.50E-15	1.42E-13
Cellular response to hypoxia (R-HSA-1234174)	74	11	0.3	+	36.43	4.23E-14	1.26E-12
Infection with Mycobacterium tuberculosis (R-HSA-9635486)	27	4	0.11	+	36.31	7.34E-06	1.17E-04
APC/C:Cdc20 mediated degradation of mitotic proteins (R-HSA-176409)	75	11	0.31	+	35.95	4.84E-14	1.42E-12
Degradation of beta-catenin by the destruction complex (R-HSA-195253)	82	12	0.33	+	35.87	3.27E-15	1.66E-13
Cyclin E associated events during G1/S transition (R-HSA-69202)	82	12	0.33	+	35.87	3.27E-15	1.63E-13
Activation of APC/C and APC/C:Cdc20 mediated degradation of mitotic pro	76	11	0.31	+	35.48	5.52E-14	1.60E-12
ABC transporter disorders (R-HSA-5619084)	77	11	0.31	+	35.02	6.29E-14	1.80E-12
Cyclin A:Cdk2-associated events at S phase entry (R-HSA-69656)	84	12	0.34	+	35.02	4.25E-15	1.96E-13
Antigen processing-Cross presentation (R-HSA-1236975)	106	15	0.43	+	34.68	1.40E-18	8.72E-16
Regulation of mitotic cell cycle (R-HSA-453276)	87	12	0.35	+	33.81	6.22E-15	2.67E-13
APC/C-mediated degradation of cell cycle proteins (R-HSA-174143)	87	12	0.35	+	33.81	6.22E-15	2.63E-13
Switching of origins to a post-replicative state (R-HSA-69052)	90	12	0.37	+	32.68	9.01E-15	3.51E-13
PCP/CE pathway (R-HSA-4086400)	91	12	0.37	+	32.32	1.02E-14	3.72E-13
Hedgehog 'on' state (R-HSA-5632684)	84	11	0.34	+	32.1	1.50E-13	4.11E-12
Response of Mtb to phagocytosis (R-HSA-9637690)	23	3	0.09	+	31.97	1.57E-04	2.20E-03
Defective Intrinsic Pathway for Apoptosis (R-HSA-9734009)	23	3	0.09	+	31.97	1.57E-04	2.19E-03
Interleukin-1 signaling (R-HSA-9020702)	100	13	0.41	+	31.86	8.66E-16	7.71E-14
Regulation of mRNA stability by proteins that bind AU-rich elements (R-H:	85	11	0.35	+	31.72	1.69E-13	4.52E-12
Host Interactions of HIV factors (R-HSA-162909)	124	16	0.51	+	31.63	3.51E-19	2.92E-16
UCH proteinases (R-HSA-5689603)	95	12	0.39	+	30.96	1.63E-14	5.55E-13
MAPK6/MAPK4 signaling (R-HSA-5687128)	88	11	0.36	+	30.64	2.39E-13	6.20E-12
CLEC7A (Dectin-1) signaling (R-HSA-5607764)	96	12	0.39	+	30.64	1.82E-14	6.14E-13
Sema3A PAK dependent Axon repulsion (R-HSA-399954)	16	2	0.07	+	30.64	2.39E-03	2.85E-02
TNFR2 non-canonical NF-kB pathway (R-HSA-5668541)	98	12	0.4	+	30.01	2.28E-14	7.49E-13
Transcriptional regulation by RUNX3 (R-HSA-8878159)	93	11	0.38	+	28.99	4.16E-13	1.05E-11
Downstream TCR signaling (R-HSA-202424)	102	12	0.42	+	28.84	3.54E-14	1.10E-12
Hedgehog 'off' state (R-HSA-5610787)	112	13	0.46	+	28.45	3.32E-15	1.62E-13
Antigen Presentation: Folding, assembly and peptide loading of class I MH	26	3	0.11	+	28.28	2.19E-04	2.98E-03
Detoxification of Reactive Oxygen Species (R-HSA-3299685)	35	4	0.14	+	28.01	1.87E-05	2.92E-04
RUNX1 regulates transcription of genes involved in differentiation of HSCs	98	11	0.4	+	27.51	7.04E-13	1.72E-11
Assembly of the pre-replicative complex (R-HSA-68867)	99	11	0.4	+	27.23	7.80E-13	1.87E-11
Cell-extracellular matrix interactions (R-HSA-446353)	18	2	0.07	+	27.23	2.96E-03	3.48E-02
Cellular response to chemical stress (R-HSA-9711123)	154	17	0.63	+	27.06	2.80E-19	3.48E-16
Cytoprotection by HMOX1 (R-HSA-9707564)	120	13	0.49	+	26.55	7.55E-15	3.09E-13
Gene and protein expression by JAK-STAT signaling after Interleukin-12 sti	37	4	0.15	+	26.5	2.29E-05	3.48E-04
ABC-family proteins mediated transport (R-HSA-382556)	102	11	0.42	+	26.43	1.05E-12	2.48E-11
Prefoldin mediated transfer of substrate to CCT/TriC (R-HSA-389957)	28	3	0.11	+	26.26	2.68E-04	3.63E-03
RHO GTPases Activate ROCKs (R-HSA-5627117)	19	2	0.08	+	25.8	3.26E-03	3.76E-02
Transcriptional regulation by RUNX2 (R-HSA-8878166)	117	12	0.48	+	25.14	1.60E-13	4.33E-12
Synthesis of DNA (R-HSA-69239)	119	12	0.49	+	24.72	1.93E-13	5.11E-12
Pyruvate metabolism (R-HSA-70268)	30	3	0.12	+	24.51	3.23E-04	4.36E-03
EPHB-mediated forward signaling (R-HSA-3928662)	41	4	0.17	+	23.91	3.33E-05	4.97E-04
TCR signaling (R-HSA-202403)	124	12	0.51	+	23.72	3.03E-13	7.79E-12
Deregulated CDK5 triggers multiple neurodegenerative pathways in Alzhei	21	2	0.09	+	23.34	3.90E-03	4.42E-02
Neurodegenerative Diseases (R-HSA-8863678)	21	2	0.09	+	23.34	3.90E-03	4.40E-02
DNA Replication Pre-Initiation (R-HSA-69002)	116	11	0.47	+	23.24	3.85E-12	8.72E-11
Interleukin-1 family signaling (R-HSA-446652)	138	13	0.56	+	23.09	4.00E-14	1.20E-12
RHO GTPases activate IQGAPs (R-HSA-5626467)	32	3	0.13	+	22.98	3.86E-04	5.17E-03
G1/S Transition (R-HSA-69206)	130	12	0.53	+	22.63	5.10E-13	1.26E-11
Cooperation of Prefoldin and TriC/CCT in actin and tubulin folding (R-HSA-	33	3	0.13	+	22.28	4.19E-04	5.56E-03

Chaperone Mediated Autophagy (R-HSA-9613829)	22	2	0.09	+	22.28	4.25E-03	4.77E-02
Signaling by Hedgehog (R-HSA-5358351)	148	13	0.6	+	21.53	9.22E-14	2.61E-12
Interleukin-12 signaling (R-HSA-9020591)	46	4	0.19	+	21.31	5.07E-05	7.52E-04
C-type lectin receptors (CLRs) (R-HSA-5621481)	138	12	0.56	+	21.31	9.84E-13	2.34E-11
Apoptosis (R-HSA-109581)	173	15	0.71	+	21.25	1.17E-15	8.34E-14
Signaling by high-kinase activity BRAF mutants (R-HSA-6802948)	35	3	0.14	+	21.01	4.93E-04	6.43E-03
RHO GTPases Activate WASPs and WAVEs (R-HSA-5663213)	35	3	0.14	+	21.01	4.93E-04	6.40E-03
FCERI mediated NF-kB activation (R-HSA-2871837)	141	12	0.58	+	20.86	1.25E-12	2.91E-11
Beta-catenin independent WNT signaling (R-HSA-3858494)	142	12	0.58	+	20.71	1.35E-12	3.11E-11
Recycling pathway of L1 (R-HSA-437239)	48	4	0.2	+	20.43	5.92E-05	8.73E-04
Mitotic G1 phase and G1/S transition (R-HSA-453279)	147	12	0.6	+	20.01	1.97E-12	4.52E-11
Gap junction trafficking (R-HSA-190828)	49	4	0.2	+	20.01	6.38E-05	9.36E-04
PTEN Regulation (R-HSA-6807070)	137	11	0.56	+	19.68	2.06E-11	4.22E-10
Gap junction trafficking and regulation (R-HSA-157858)	50	4	0.2	+	19.61	6.87E-05	1.00E-03
Programmed Cell Death (R-HSA-5357801)	202	16	0.82	+	19.41	4.56E-16	4.94E-14
Signaling by the B Cell Receptor (BCR) (R-HSA-983705)	169	13	0.69	+	18.85	4.50E-13	1.12E-11
MAP2K and MAPK activation (R-HSA-5674135)	39	3	0.16	+	18.85	6.63E-04	8.51E-03
G2/M Transition (R-HSA-69275)	195	15	0.8	+	18.85	6.11E-15	2.67E-13
Mitotic G2-G2/M phases (R-HSA-453274)	197	15	0.8	+	18.66	7.04E-15	2.92E-13
DNA Replication (R-HSA-69306)	159	12	0.65	+	18.5	4.69E-12	1.04E-10
Glycolysis (R-HSA-70171)	67	5	0.27	+	18.29	1.11E-05	1.76E-04
S Phase (R-HSA-69242)	161	12	0.66	+	18.27	5.38E-12	1.19E-10
Pyruvate metabolism and Citric Acid (TCA) cycle (R-HSA-71406)	54	4	0.22	+	18.16	9.10E-05	1.30E-03
G2/M Checkpoints (R-HSA-69481)	150	11	0.61	+	17.97	5.16E-11	1.01E-09
Interleukin-12 family signaling (R-HSA-447115)	56	4	0.23	+	17.51	1.04E-04	1.46E-03
Regulation of expression of SLITs and ROBOs (R-HSA-9010553)	169	12	0.69	+	17.4	9.18E-12	1.97E-10
HIV Infection (R-HSA-162906)	227	16	0.93	+	17.28	2.54E-15	1.41E-13
Glucose metabolism (R-HSA-70326)	86	6	0.35	+	17.1	2.03E-06	3.30E-05
Aggrephagy (R-HSA-9646399)	44	3	0.18	+	16.71	9.22E-04	1.17E-02
Signaling downstream of RAS mutants (R-HSA-9649948)	45	3	0.18	+	16.34	9.81E-04	1.24E-02
Paradoxical activation of RAF signaling by kinase inactive BRAF (R-HSA-68	45	3	0.18	+	16.34	9.81E-04	1.23E-02
Signaling by RAS mutants (R-HSA-6802949)	45	3	0.18	+	16.34	9.81E-04	1.22E-02
Signaling by moderate kinase activity BRAF mutants (R-HSA-6802946)	45	3	0.18	+	16.34	9.81E-04	1.22E-02
EPH-Ephrin signaling (R-HSA-2682334)	91	6	0.37	+	16.16	2.76E-06	4.47E-05
Fc epsilon receptor (FCERI) signaling (R-HSA-2454202)	187	12	0.76	+	15.73	2.80E-11	5.67E-10
Separation of Sister Chromatids (R-HSA-2467813)	187	12	0.76	+	15.73	2.80E-11	5.62E-10
Ub-specific processing proteases (R-HSA-5689880)	204	13	0.83	+	15.62	4.25E-12	9.55E-11
Disorders of transmembrane transporters (R-HSA-5619115)	173	11	0.71	+	15.58	2.17E-10	4.07E-09
EPH-ephrin mediated repulsion of cells (R-HSA-3928665)	50	3	0.2	+	14.71	1.31E-03	1.60E-02
Signaling by NOTCH (R-HSA-157118)	201	12	0.82	+	14.63	6.18E-11	1.20E-09
Apoptotic execution phase (R-HSA-75153)	51	3	0.21	+	14.42	1.38E-03	1.68E-02
Mitotic Anaphase (R-HSA-68882)	227	13	0.93	+	14.04	1.52E-11	3.18E-10
Mitotic Metaphase and Anaphase (R-HSA-2555396)	228	13	0.93	+	13.98	1.60E-11	3.32E-10
The citric acid (TCA) cycle and respiratory electron transport (R-HSA-1428!	176	10	0.72	+	13.93	4.23E-09	7.59E-08
Signaling by ROBO receptors (R-HSA-376176)	215	12	0.88	+	13.68	1.29E-10	2.44E-09
TCF dependent signaling in response to WNT (R-HSA-201681)	201	11	0.82	+	13.41	9.82E-10	1.79E-08
Transcriptional regulation by RUNX1 (R-HSA-8878171)	204	11	0.83	+	13.22	1.14E-09	2.06E-08
RAF/MAP kinase cascade (R-HSA-5673001)	264	14	1.08	+	13	6.24E-12	1.36E-10
MAPK1/MAPK3 signaling (R-HSA-5684996)	269	14	1.1	+	12.76	7.93E-12	1.72E-10
Neddylation (R-HSA-8951664)	233	12	0.95	+	12.62	3.12E-10	5.80E-09
Deubiquitination (R-HSA-5688426)	282	14	1.15	+	12.17	1.45E-11	3.06E-10
Respiratory electron transport (R-HSA-611105)	102	5	0.42	+	12.02	7.54E-05	1.09E-03
Respiratory electron transport, ATP synthesis by chemiosmotic coupling, a	126	6	0.51	+	11.67	1.63E-05	2.58E-04
Platelet degranulation (R-HSA-114608)	127	6	0.52	+	11.58	1.71E-05	2.67E-04
Regulation of PLK1 Activity at G2/M Transition (R-HSA-2565942)	85	4	0.35	+	11.53	4.76E-04	6.25E-03
Signaling by BRAF and RAF1 fusions (R-HSA-6802952)	64	3	0.26	+	11.49	2.57E-03	3.05E-02
Semaphorin interactions (R-HSA-373755)	64	3	0.26	+	11.49	2.57E-03	3.04E-02
MAPK family signaling cascades (R-HSA-5683057)	308	14	1.26	+	11.14	4.44E-11	8.86E-10
Response to elevated platelet cytosolic Ca2+ (R-HSA-76005)	132	6	0.54	+	11.14	2.10E-05	3.24E-04
Class I MHC mediated antigen processing & presentation (R-HSA-983169)	378	17	1.54	+	11.02	3.40E-13	8.65E-12
Loss of proteins required for interphase microtubule organization from the	68	3	0.28	+	10.81	3.03E-03	3.55E-02
Loss of Nlp from mitotic centrosomes (R-HSA-380259)	68	3	0.28	+	10.81	3.03E-03	3.53E-02
PIP3 activates AKT signaling (R-HSA-1257604)	251	11	1.02	+	10.74	9.02E-09	1.57E-07
Signaling by WNT (R-HSA-195721)	297	13	1.21	+	10.73	3.64E-10	6.72E-09
Diseases of programmed cell death (R-HSA-9645723)	70	3	0.29	+	10.5	3.28E-03	3.77E-02
VEGFA-VEGFR2 Pathway (R-HSA-4420097)	94	4	0.38	+	10.43	6.85E-04	8.76E-03
Signaling by Interleukins (R-HSA-449147)	448	19	1.83	+	10.4	3.15E-14	1.02E-12
AURKA Activation by TPX2 (R-HSA-8854518)	71	3	0.29	+	10.36	3.41E-03	3.90E-02
Translocation of SLC2A4 (GLUT4) to the plasma membrane (R-HSA-14451	72	3	0.29	+	10.21	3.54E-03	4.03E-02
Regulation of actin dynamics for phagocytic cup formation (R-HSA-202948	123	5	0.5	+	9.96	1.76E-04	2.43E-03
Cell Cycle Checkpoints (R-HSA-69620)	271	11	1.11	+	9.95	1.93E-08	3.29E-07
M Phase (R-HSA-68886)	375	15	1.53	+	9.8	4.74E-11	9.37E-10
Neutrophil degranulation (R-HSA-6798695)	477	19	1.95	+	9.76	9.26E-14	2.59E-12
Antigen processing: Ubiquitination & Proteasome degradation (R-HSA-983	308	12	1.26	+	9.55	6.43E-09	1.14E-07
Signaling by VEGF (R-HSA-194138)	104	4	0.42	+	9.43	9.86E-04	1.22E-02
Diseases of signal transduction by growth factor receptors and second me	418	16	1.71	+	9.38	1.87E-11	3.86E-10
Intracellular signaling by second messengers (R-HSA-9006925)	291	11	1.19	+	9.27	3.89E-08	6.50E-07
Axon guidance (R-HSA-422475)	549	20	2.24	+	8.93	9.33E-14	2.58E-12
Metabolism of amino acids and derivatives (R-HSA-71291)	369	13	1.51	+	8.64	4.57E-09	8.14E-08
Nervous system development (R-HSA-9675108)	574	20	2.34	+	8.54	2.07E-13	5.44E-12
Fcgamma receptor (FCGR) dependent phagocytosis (R-HSA-2029480)	147	5	0.6	+	8.34	3.89E-04	5.19E-03
L1CAM interactions (R-HSA-373760)	119	4	0.49	+	8.24	1.60E-03	1.92E-02
Cell Cycle, Mitotic (R-HSA-69278)	517	16	2.11	+	7.59	3.87E-10	7.09E-09
Infectious disease (R-HSA-5663205)	895	27	3.65	+	7.39	1.55E-16	1.94E-14
Cytokine Signaling in Immune system (R-HSA-1280215)	697	21	2.84	+	7.38	7.09E-13	1.72E-11
Innate Immune System (R-HSA-168249)	1107	33	4.52	+	7.31	3.05E-20	7.61E-17
Clathrin-mediated endocytosis (R-HSA-8856828)	144	4	0.59	+	6.81	3.12E-03	3.62E-02
Cellular responses to stress (R-HSA-2262752)	718	19	2.93	+	6.49	9.19E-11	1.78E-09
Cellular responses to stimuli (R-HSA-8953897)	732	19	2.99	+	6.36	1.26E-10	2.41E-09
Cell Cycle (R-HSA-1640170)	648	16	2.64	+	6.05	8.99E-09	1.58E-07
Metabolism of carbohydrates (R-HSA-71387)	286	7	1.17	+	6	1.91E-04	2.63E-03
RHO GTPase Effectors (R-HSA-195258)	288	7	1.17	+	5.96	1.99E-04	2.72E-03
Adaptive Immune System (R-HSA-1280218)	826	20	3.37	+	5.93	1.25E-10	2.40E-09
Platelet activation, signaling and aggregation (R-HSA-76002)	260	6	1.06	+	5.66	7.54E-04	9.58E-03
Metabolism of RNA (R-HSA-8953854)	661	15	2.7	+	5.56	8.18E-08	1.36E-06
Transport of small molecules (R-HSA-382551)	719	16	2.93	+	5.45	3.71E-08	6.25E-07
Disease (R-HSA-1643685)	1679	33	6.85	+	4.82	5.55E-15	2.47E-13
Immune System (R-HSA-168256)	2055	38	8.38	+	4.53	1.25E-16	1.63E-14
Developmental Biology (R-HSA-1266738)	1090	20	4.45	+	4.5	1.35E-08	2.31E-07
Signaling by Rho GTPases (R-HSA-194315)	670	12	2.73	+	4.39	1.94E-05	3.00E-04
RHO GTPase cycle (R-HSA-9012999)	448	8	1.83	+	4.38	5.31E-04	6.86E-03
Signaling by Rho GTPases, Miro GTPases and RHOBTB3 (R-HSA-9716542)	685	12	2.79	+	4.29	2.40E-05	3.63E-04
Metabolism (R-HSA-1430728)	2087	32	8.51	+	3.76	1.31E-11	2.80E-10
Hemostasis (R-HSA-109582)	670	10	2.73	+	3.66	4.28E-04	5.64E-03
Post-translational protein modification (R-HSA-597592)	1396	20	5.7	+	3.51	7.05E-07	1.16E-05
Metabolism of proteins (R-HSA-392499)	1914	26	7.81	+	3.33	2.58E-08	4.38E-07
Gene expression (Transcription) (R-HSA-74160)	1454	19	5.93	+	3.2	5.39E-06	8.67E-05
Generic Transcription Pathway (R-HSA-212436)	1197	15	4.88	+	3.07	9.89E-05	1.40E-03
RNA Polymerase II Transcription (R-HSA-73857)	1319	16	5.38	+	2.97	8.19E-05	1.18E-03
Signal Transduction (R-HSA-162582)	2500	28	10.2	+	2.75	3.65E-07	6.02E-06
Unclassified (UNCLASSIFIED)	10082	5	41.13	-	0.12	1.22E-17	5.06E-15

Gene List
CYBA
TMSB4X
PRDX1
TPI1
CFL1
ALDOA
ENO1
ITM2A
GSTP1
HMGB1
CYC1
ACTB
TIMP1
ACTG1
PPIB
B2M
PRDX2
RAN
COX4I1
CALM2
ARHGDIB
PSMD8
PSMA4
MYL6
PGK1
STMN1
UQCRB
PPIA
ARPC3
ATP5B
PSMB3
PSMB1
MDH2
SLC25A5
PKM
COPE
CORO1A
SKP1
AIF1
PGAM1
C1QBP
EIF3I
CLIC1
PRTN3

LDHA
PSMB5
MRPL13
LDHB
PDHA1
PEBP1
PARK7
TUBB4B
VDAC3
PSMA7
ATP5O
CLTA
TUBB
PSMA1
RBM8A
AZU1
PSMB2
TUFM
HLA-A
NCL
HSP90AA1
PRDX4
PSME2
HMGB2
ETFA
CACYBP
TALDO1
EBP
PSMA3
VDAC2
S100A11
BCAP31
PSMB7
BASP1
ILF2
EIF3K
UQCRFS1
HSD17B10
CD63
CCT5
