# Supplementary material for: Integrated multi-omics analyses reveal homology-directed repair pathway as a unique dependency in near-haploid leukemia
Source: Blood Cancer J. 2023 Jun 8;13(1):92. doi: 10.1038/s41408-023-00863-1 (PMC10247733; doi:10.1038/s41408-023-00863-1)
Supplement: Supplementary file 3 — Supplementary Table 2 [file 41408_2023_863_MOESM3_ESM.pdf]

MG1

gene	ratio	p_value	BH_adjusted_p_value
RP11-34P13.	NA	NA	NA
FAM138A	NA	NA	NA
OR4F5	NA	NA	NA
RP11-34P13.	NA	2.27E-101	1.26E-100
RP11-34P13.	NA	NA	NA
RP11-34P13.	NA	NA	NA
RP11-34P13.	NA	NA	NA
FO538757.3	NA	NA	NA
FO538757.2	2.36381323	1.26E-116	1.01E-115
AP006222.2	1.1385214	1.13E-112	8.17E-112
RP5-857K21.	NA	NA	NA
RP4-669L17.	NA	NA	NA
RP4-669L17.	NA	8.05E-111	5.54E-110
OR4F29	NA	NA	NA
RP5-857K21.	NA	2.17E-38	4.41E-38
RP5-857K21.	NA	NA	NA
OR4F16	NA	NA	NA
RP11-206L10	NA	NA	NA
RP11-206L10	1.49805447	9.63E-14	1.36E-13
FAM87B	NA	NA	NA
LINC00115	6.36673152	3.80E-93	1.80E-92
FAM41C	0.74902724	3.35E-38	6.78E-38
RP11-54O7.1	NA	2.86E-126	3.23E-125
RP11-54O7.1	2.99610895	2.19E-95	1.08E-94
RP11-54O7.2	1.49805447	4.65E-16	6.83E-16
RP11-54O7.3	Inf	9.14E-59	2.46E-58
SAMD11	Inf	5.18E-89	2.26E-88
NOC2L	2.0043414	3.14E-40	6.53E-40
KLHL17	2.99610895	0.043506	0.04707657
PLEKHN1	NA	8.94E-55	2.28E-54
PERM1	NA	3.28E-107	2.06E-106
RP11-54O7.1	Inf	6.08E-47	1.39E-46
HES4	2.14721141	4.34E-20	6.77E-20
ISG15	2.28778971	0.39404545	0.40682749
RP11-54O7.1	NA	NA	NA
AGRN	1.5729572	6.37E-10	8.38E-10
RP11-54O7.1	NA	NA	NA
RNF223	NA	NA	NA
C1orf159	2.46108949	1.28E-33	2.42E-33
LINC01342	NA	NA	NA
RP11-465B2	NA	NA	NA
TTLL10-AS1	NA	NA	NA
TTLL10	NA	NA	NA

TNFRSF18	NA	NA	NA
TNFRSF4	1.49805447	0.87939432	0.88485701
SDF4	1.62053692	3.31E-48	7.67E-48
B3GALT6	2.33926968	5.25E-18	7.95E-18
FAM132A	0.99870298	0.20998677	0.22053838
RP5-902P8.1	NA	8.67E-105	5.15E-104
UBE2J2	1.55410413	2.69E-20	4.21E-20
RP5-902P8.1	0	3.70E-06	4.50E-06
SCNN1D	Inf	3.82E-70	1.23E-69
ACAP3	2.73394942	1.17E-37	2.34E-37
PUSL1	2.49675746	2.20E-15	3.19E-15
CPSF3L	1.97192885	2.12E-10	2.81E-10
CPTP	2.70802155	2.26E-09	2.95E-09
TAS1R3	NA	NA	NA
DVL1	3.54085603	6.17E-93	2.90E-92
MXRA8	NA	2.28E-87	9.63E-87
AURKAIP1	1.62992547	1.45E-67	4.48E-67
CCNL2	1.98492218	0.14855717	0.15711345
MRPL20	2.00970263	6.25E-55	1.60E-54
RP4-758J18.:	1.99740597	4.02E-43	8.69E-43
ANKRD65	NA	4.22E-12	5.78E-12
RP4-758J18.:	NA	NA	NA
TMEM88B	NA	NA	NA
RP4-758J18.:	NA	2.53E-11	3.41E-11
VWA1	1.46238651	0.00026681	0.00030954
ATAD3C	3.74513619	8.77E-83	3.42E-82
ATAD3B	1.51802853	4.92E-26	8.39E-26
ATAD3A	1.5096673	1.18E-34	2.27E-34
TMEM240	Inf	1.66E-121	1.55E-120
SSU72	2.05003796	2.00E-84	8.03E-84
RP5-832C2.5	1.21716926	0.48835548	0.50162751
AL645728.1	NA	NA	NA
C1orf233	1.71652075	6.38E-26	1.09E-25
RP11-345P4.	3.74513619	1.84E-25	3.11E-25
MIB2	2.01661179	7.42E-57	1.95E-56
MMP23B	0	8.99E-05	1.06E-04
CDK11B	1.61328943	6.99E-23	1.14E-22
RP11-345P4.	Inf	1.50E-21	2.40E-21
SLC35E2B	1.90661479	0.00061852	0.00071006
RP11-345P4.	NA	NA	NA
CDK11A	1.44811933	1.90E-73	6.41E-73
SLC35E2	1.49805447	2.06E-14	2.95E-14
NADK	2.59038586	1.34E-103	7.76E-103
GNB1	2.19958242	2.16E-109	1.43E-108
RP1-140A9.1	Inf	1.58E-72	5.24E-72

CALML6	NA	NA	NA
TMEM52	3.78674881	2.28E-107	1.44E-106
CFAP74	NA	0.02258698	0.02469405
RP11-547D2	NA	NA	NA
GABRD	0	6.50E-66	1.96E-65
RP11-547D2	NA	1.68E-137	3.62E-136
PRKCZ	1.81014916	3.60E-40	7.49E-40
RP5-892K4.1	Inf	1.35E-67	4.17E-67
RP11-181G1	Inf	0.31715645	0.32977145
FAAP20	2.32368677	6.09E-110	4.09E-109
RP11-181G1	NA	NA	NA
SKI	2.37191958	7.38E-108	4.71E-107
MORN1	1.68531128	3.58E-05	4.25E-05
RER1	1.49805447	1.12E-58	3.00E-58
PEX10	1.82850767	4.51E-38	9.10E-38
PLCH2	NA	NA	NA
RP3-395M20	NA	NA	NA
RP3-395M20	NA	NA	NA
PANK4	Inf	1.20E-46	2.73E-46
HES5	NA	NA	NA
RP3-395M20	2.24708171	5.37E-20	8.37E-20
RP3-395M20	Inf	7.49E-24	1.24E-23
TNFRSF14	1.49805447	1.33E-38	2.70E-38
RP3-395M20	NA	NA	NA
FAM213B	2.3540856	1.71E-11	2.31E-11
MMEL1	NA	5.92E-18	8.95E-18
RP13-436F1	NA	NA	NA
TTC34	NA	1.44E-13	2.02E-13
RP11-740P5.	NA	NA	NA
RP11-740P5.	NA	NA	NA
ACTRT2	NA	NA	NA
LINC00982	Inf	4.41E-46	9.93E-46
PRDM16	Inf	2.65E-93	1.26E-92
RP1-163G9.2	NA	NA	NA
RP11-22L13.	NA	NA	NA
ARHGEF16	0	4.23E-85	1.72E-84
RP11-168F9.	NA	NA	NA
MEGF6	1.49805447	0.32861948	0.34135784
RP11-46F15.	NA	NA	NA
TPRG1L	1.08949416	2.49E-15	3.61E-15
WRAP73	1.49805447	4.62E-80	1.72E-79
TP73	NA	9.88E-134	1.64E-132
RP5-1092A1	NA	NA	NA
RP5-1092A1	NA	NA	NA
TP73-AS1	3.37062257	1.55E-07	1.94E-07

CCDC27	NA	NA	NA
SMIM1	0.93628405	8.72E-113	6.33E-112
LRRC47	1.95899431	9.16E-50	2.18E-49
RP1-286D6.5	NA	5.25E-101	2.89E-100
CEP104	1.88643897	1.64E-15	2.39E-15
DFFB	2.77140078	0.00430856	0.00482803
C1orf174	2.01738003	0.00027061	0.0003139
LINC01134	0.74902724	7.71E-06	9.31E-06
LINC01346	NA	NA	NA
RP13-614K1	NA	NA	NA
RP5-1166F1(	NA	NA	NA
RP1-37J18.1	NA	NA	NA
RP1-37J18.2	NA	NA	NA
AJAP1	NA	NA	NA
RP11-542C1(	NA	NA	NA
RP1-58B11.1	NA	NA	NA
RP11-154H1	NA	NA	NA
NPHP4	5.24319066	2.56E-37	5.12E-37
KCNAB2	2.09727626	6.05E-42	1.29E-41
CHD5	NA	2.92E-121	2.71E-120
RPL22	2.00330994	3.23E-87	1.36E-86
RP1-120G22	1.34824903	0.16870424	0.17801619
RNF207	2.43433852	3.57E-83	1.40E-82
ICMT	1.47602426	1.49E-40	3.12E-40
LINC00337	4.49416342	1.56E-26	2.67E-26
HES3	NA	NA	NA
GPR153	Inf	0.00033256	0.00038487
ACOT7	1.01580406	1.19E-132	1.86E-131
RP1-202O8.3	0.74902724	0.75413305	0.76344899
HES2	NA	9.18E-05	1.08E-04
ESPN	0	3.20E-51	7.75E-51
RP1-202O8.2	NA	NA	NA
TNFRSF25	Inf	6.16E-97	3.13E-96
PLEKHG5	NA	5.39E-73	1.80E-72
NOL9	1.97820014	1.26E-52	3.12E-52
TAS1R1	NA	NA	NA
ZBTB48	1.10725766	1.89E-97	9.72E-97
KLHL21	3.07101167	6.81E-101	3.73E-100
PHF13	4.94357977	1.68E-99	8.96E-99
THAP3	2.38975357	0.1323582	0.14041766
DNAJC11	3.95914397	1.19E-72	3.97E-72
RP11-242F2	NA	7.05E-52	1.73E-51
RP11-312B8.	Inf	7.70E-101	4.22E-100
CAMTA1	2.20595865	1.49E-68	4.66E-68
RP11-334N1	NA	NA	NA

RP3-453P22.	NA	NA	NA
RP4-549F15.	NA	NA	NA
RP11-338N1(	NA	8.37E-60	2.29E-59
RP11-338N1(	NA	NA	NA
RP11-338N1(	NA	0.00245985	0.00277391
VAMP3	3.95319931	1.50E-72	4.99E-72
PER3	3.932393	2.97E-113	2.18E-112
RP3-467L1.4	2.99610895	2.79E-81	1.06E-80
UTS2	NA	5.28E-72	1.74E-71
TNFRSF9	NA	5.09E-106	3.10E-105
PARK7	1.43149635	8.29E-122	7.89E-121
ERRFI1	NA	6.70E-37	1.33E-36
RP11-431K24	NA	NA	NA
RP11-431K24	NA	NA	NA
RP11-431K24	NA	NA	NA
SLC45A1	NA	NA	NA
RERE	2.67509728	1.25E-110	8.54E-110
RP5-1115A1!	NA	NA	NA
RP4-633I8.4	NA	0.22131413	0.23213927
ENO1	0.87405284	1.88E-140	5.57E-139
ENO1-AS1	2.24708171	0.01088812	0.01204392
CA6	NA	NA	NA
SLC2A7	NA	NA	NA
SLC2A5	0.93628405	1.12E-31	2.06E-31
GPR157	0.96303502	0.00020541	0.00023898
MIR34AHG	NA	NA	NA
RP3-510D11.	NA	NA	NA
RP3-510D11.	NA	NA	NA
H6PD	0.24967575	1.96E-30	3.56E-30
SPSB1	Inf	1.54E-26	2.65E-26
RP13-392I16	NA	NA	NA
SLC25A33	2.26078343	5.17E-24	8.55E-24
TMEM201	3.31712062	6.78E-86	2.79E-85
PIK3CD	2.37191958	1.35E-07	1.70E-07
PIK3CD-AS1	NA	0.45283752	0.46580361
PIK3CD-AS2	2.92801556	8.39E-139	2.06E-137
CLSTN1	1.89753567	1.22E-05	1.46E-05
AL357140.1	NA	NA	NA
CTNNBIP1	1.38649723	1.69E-138	4.02E-137
RP11-84A14.	NA	NA	NA
LZIC	1.68531128	1.12E-20	1.77E-20
NMNAT1	1.78614187	6.41E-09	8.26E-09
RBP7	NA	6.92E-16	1.01E-15
UBE4B	1.69779507	0.00020332	0.00023658
KIF1B	2.24708171	2.76E-53	6.90E-53

PGD	1.61487524	7.90E-107	4.91E-106
RP4-736L20.:	NA	9.39E-135	1.66E-133
APITD1-COR	Inf	4.11E-10	5.43E-10
APITD1	1.8398436	3.67E-21	5.83E-21
CORT	Inf	4.66E-107	2.90E-106
DFFA	1.62289235	0.12383575	0.13160495
RP5-1113E3.	NA	NA	NA
PEX14	1.32520204	5.05E-94	2.42E-93
CASZ1	2.99610895	2.91E-33	5.48E-33
RP4-734G22.	NA	5.39E-67	1.65E-66
C1orf127	NA	NA	NA
TARDBP	1.82237555	5.35E-59	1.45E-58
RP4-635E18.	NA	NA	NA
MASP2	NA	4.62E-92	2.14E-91
RP4-635E18.	NA	9.84E-176	3.57E-172
SRM	2.05225387	2.45E-77	8.73E-77
EXOSC10	1.30644286	2.60E-77	9.25E-77
RP4-635E18.	Inf	3.87E-123	3.86E-122
RP4-635E18.	1.02140078	0.82790337	0.83517699
MTOR	2.07084001	6.78E-49	1.59E-48
MTOR-AS1	NA	NA	NA
ANGPTL7	NA	NA	NA
UBIAD1	1.82116426	1.05E-10	1.40E-10
PTCHD2	NA	5.69E-85	2.31E-84
RP1-69M21.:	NA	NA	NA
FBXO2	NA	NA	NA
FBXO44	Inf	1.90E-24	3.16E-24
FBXO6	0.58257674	2.45E-111	1.70E-110
MAD2L2	1.61290532	2.06E-48	4.80E-48
DRAXIN	NA	7.75E-127	8.99E-126
AGTRAP	Inf	5.69E-44	1.24E-43
C1orf167	NA	NA	NA
RP11-56N19.	NA	NA	NA
MTHFR	3.18336576	1.66E-52	4.11E-52
CLCN6	1.62289235	3.59E-26	6.13E-26
NPPA-AS1	1.24837873	1.54E-09	2.02E-09
NPPA	NA	NA	NA
NPPB	NA	NA	NA
KIAA2013	1.91240997	3.15E-59	8.56E-59
PLOD1	1.81495061	6.50E-18	9.83E-18
MFN2	2.71076524	3.61E-135	6.52E-134
MIIP	2.03523562	1.37E-07	1.72E-07
TNFRSF8	2.0130107	4.18E-73	1.40E-72
TNFRSF1B	2.44127396	0.13396768	0.14198993
VPS13D	5.43044747	5.82E-82	2.24E-81

RP5-888M10	NA	NA	NA
DHRS3	NA	1.91E-32	3.56E-32
RP11-474O2	NA	NA	NA
AADACL4	NA	NA	NA
AADACL3	NA	NA	NA
C1orf158	NA	NA	NA
PRAMEF12	NA	NA	NA
PRAMEF1	NA	NA	NA
RP5-845O24	NA	NA	NA
PRAMEF11	NA	NA	NA
HNRNPCL1	NA	NA	NA
PRAMEF2	NA	NA	NA
PRAMEF4	NA	NA	NA
PRAMEF10	NA	NA	NA
PRAMEF7	NA	NA	NA
PRAMEF6	NA	NA	NA
PRAMEF27	NA	NA	NA
PRAMEF26	NA	NA	NA
PRAMEF25	NA	NA	NA
HNRNPCL3	NA	8.82E-29	1.56E-28
HNRNPCL2	NA	NA	NA
RP13-221M1	NA	NA	NA
HNRNPCL4	NA	NA	NA
PRAMEF9	NA	NA	NA
PRAMEF18	NA	NA	NA
PRAMEF5	NA	NA	NA
PRAMEF8	NA	NA	NA
PRAMEF15	NA	NA	NA
RP11-219C24	NA	NA	NA
PRAMEF14	NA	NA	NA
PRAMEF19	NA	NA	NA
PRAMEF17	NA	NA	NA
PRAMEF20	NA	NA	NA
LRRC38	NA	NA	NA
RP4-597A16.	NA	NA	NA
PDPN	NA	NA	NA
CTA-520D8.2	NA	NA	NA
PRDM2	3.12637456	1.40E-86	5.83E-86
RP11-344F13	NA	NA	NA
RP4-704D23.	NA	NA	NA
KAZN	2.2250515	0.00408177	0.00457616
TMEM51-AS	NA	NA	NA
TMEM51	2.64130657	2.27E-35	4.41E-35
C1orf195	NA	NA	NA
FHAD1	0	0.00356101	0.00400098

RP3-467K16.	NA	NA	NA
RP3-467K16.	NA	NA	NA
RP3-467K16.	NA	3.71E-43	8.02E-43
EFHD2	1.89314576	5.32E-28	9.32E-28
CTRC	NA	NA	NA
CELA2A	NA	NA	NA
CELA2B	NA	6.39E-68	1.98E-67
CASP9	2.0598249	9.04E-13	1.25E-12
DNAJC16	4.24448768	4.16E-12	5.72E-12
RP4-680D5.8	NA	NA	NA
AGMAT	1.49805447	0.00077641	0.00088874
RP4-680D5.2	NA	NA	NA
DDI2	2.66653696	4.23E-95	2.07E-94
RSC1A1	NA	NA	NA
RP4-680D5.9	NA	0.38801892	0.40076511
PLEKHM2	2.36231667	1.71E-29	3.07E-29
RP11-288I21	NA	NA	NA
SLC25A34	NA	4.92E-47	1.12E-46
RP11-169K16	NA	NA	NA
TMEM82	NA	NA	NA
FBLIM1	2.71522374	5.21E-32	9.65E-32
UQCRHL	1.02991245	1.23E-05	1.48E-05
FLJ37453	1.87256809	5.40E-11	7.24E-11
SPEN	1.95282101	6.07E-21	9.60E-21
ZBTB17	2.33030696	4.88E-06	5.92E-06
C1orf64	NA	NA	NA
RP11-5P18.5	NA	NA	NA
HSPB7	NA	NA	NA
CLCNKA	NA	NA	NA
CLCNKB	NA	NA	NA
FAM131C	0	2.12E-57	5.60E-57
EPHA2	NA	5.00E-153	1.03E-150
RP11-276H7.	NA	NA	NA
RP11-276H7.	NA	NA	NA
ARHGEF19-A	NA	NA	NA
ARHGEF19	1.2733463	0.89088561	0.89592277
RSG1	1.24837873	0.27728192	0.28920596
FBXO42	2.41993415	1.16E-34	2.23E-34
SZRD1	2.32092947	9.98E-72	3.27E-71
SPATA21	Inf	1.07E-75	3.71E-75
NECAP2	1.83971602	9.28E-06	1.12E-05
RP4-798A10.	1.49805447	2.03E-51	4.93E-51
RP4-798A10.	0	1.05E-08	1.35E-08
RP4-798A10.	NA	NA	NA
RP5-875O13.	NA	NA	NA

FAM231C	NA	NA	NA
NBPF1	1.90661479	4.79E-15	6.92E-15
RP5-1182A14	NA	1.37E-30	2.49E-30
RP1-163M9.8	NA	NA	NA
FAM231A	NA	NA	NA
RP1-163M9.7	NA	NA	NA
FAM231C	NA	NA	NA
RP5-1182A14	NA	NA	NA
CROCC	2.62159533	6.49E-05	7.64E-05
RP11-108M9	NA	NA	NA
RP11-108M9	NA	NA	NA
RP11-108M9	NA	3.97E-33	7.46E-33
RP11-108M9	NA	NA	NA
RP11-108M9	NA	2.38E-139	6.22E-138
MFAP2	NA	NA	NA
RP1-37C10.3	NA	NA	NA
ATP13A2	2.0598249	3.03E-60	8.36E-60
SDHB	1.09531119	8.72E-141	2.71E-139
PADI2	NA	NA	NA
RP11-380J14	NA	NA	NA
RP11-380J14	NA	NA	NA
PADI1	NA	4.72E-63	1.35E-62
PADI3	Inf	1.62E-79	5.97E-79
PADI4	NA	6.24E-09	8.05E-09
AC004824.2	NA	NA	NA
PADI6	NA	NA	NA
RP1-20B21.4	NA	NA	NA
RCC2	2.55784833	9.56E-139	2.33E-137
ARHGEF10L	NA	NA	NA
RP11-473A10	NA	NA	NA
ACTL8	2.99610895	1.86E-65	5.55E-65
RP11-174G17	NA	NA	NA
IGSF21	NA	NA	NA
RP11-174G17	NA	NA	NA
RP11-422P22	NA	NA	NA
KLHDC7A	NA	2.56E-85	1.04E-84
PAX7	NA	NA	NA
TAS1R2	NA	NA	NA
RP13-279N2	NA	NA	NA
ALDH4A1	5.74254215	4.55E-99	2.41E-98
IFFO2	1.24837873	1.84E-10	2.45E-10
UBR4	2.07084001	1.75E-18	2.67E-18
RP1-43E13.2	4.49416342	8.03E-57	2.11E-56
EMC1	1.44811933	7.89E-64	2.30E-63
MRTO4	1.577914	7.77E-19	1.19E-18

AKR7A3	0	0.00138213	0.0015704
AKR7A2	1.18627664	1.17E-144	7.19E-143
PQLC2	1.02140078	1.88E-90	8.40E-90
CAPZB	1.09399907	4.61E-131	6.59E-130
RP5-1056L3.	NA	NA	NA
MINOS1	2.06984459	1.67E-117	1.37E-116
MINOS1-NBI	NA	NA	NA
NBL1	1.83655717	0.45589707	0.46884443
HTR6	NA	NA	NA
TMCO4	1.19844358	7.67E-68	2.38E-67
RNF186	NA	NA	NA
RP11-91K11.	NA	NA	NA
OTUD3	2.12224384	4.09E-18	6.20E-18
PLA2G2E	NA	NA	NA
PLA2G2A	NA	NA	NA
PLA2G5	NA	NA	NA
PLA2G2D	NA	NA	NA
PLA2G2F	NA	NA	NA
RP3-340N1.2	NA	NA	NA
PLA2G2C	NA	NA	NA
UBXN10-AS1	0	9.87E-14	1.39E-13
UBXN10	0	4.36E-16	6.41E-16
RP3-340N1.6	NA	NA	NA
VWA5B1	NA	NA	NA
RP4-745E8.2	NA	NA	NA
LINC01141	NA	NA	NA
RP4-749H3.2	NA	NA	NA
CAMK2N1	NA	2.21E-45	4.93E-45
MUL1	1.36778887	3.47E-64	1.01E-63
FAM43B	NA	NA	NA
CDA	3.74513619	6.19E-53	1.54E-52
PINK1	1.49805447	2.89E-19	4.45E-19
PINK1-AS	1.49805447	0.03119508	0.03394967
DDOST	0.87806134	1.03E-140	3.16E-139
KIF17	0	6.67E-52	1.64E-51
SH2D5	2.24708171	0.72106114	0.73097291
RP5-930J4.2	NA	NA	NA
HP1BP3	1.86845256	0.04742562	0.05124453
EIF4G3	1.98492218	0.00455781	0.00510324
ECE1	2.99610895	2.26E-44	4.95E-44
RP3-329E20.	NA	NA	NA
RP5-1071N3.	NA	2.32E-52	5.73E-52
RP11-293F5.	NA	NA	NA
NBPF3	1.49805447	2.52E-17	3.77E-17
ALPL	NA	3.70E-131	5.31E-130

RP11-63N8.3	NA	NA	NA
RAP1GAP	0.74902724	0.00022298	0.00025904
USP48	1.73212549	1.44E-37	2.88E-37
LDLRAD2	Inf	2.22E-16	3.28E-16
HSPG2	2.62159533	1.39E-22	2.25E-22
CELA3B	NA	NA	NA
CELA3A	NA	NA	NA
RP1-224A6.3	Inf	1.22E-51	2.97E-51
LINC00339	1.99740597	5.35E-12	7.33E-12
CDC42	1.53907263	9.42E-116	7.35E-115
RP1-224A6.9	NA	3.45E-80	1.29E-79
WNT4	NA	NA	NA
ZBTB40	3.10311284	1.00E-100	5.48E-100
EPHA8	NA	NA	NA
C1QA	NA	3.14E-121	2.90E-120
C1QC	NA	2.64E-77	9.38E-77
C1QB	NA	4.11E-78	1.48E-77
EPHB2	0.37451362	0.00317834	0.00357524
RP11-69E9.1	NA	NA	NA
LACTBL1	NA	NA	NA
C1orf234	0	9.67E-14	1.36E-13
KDM1A	1.63715953	9.18E-27	1.58E-26
RP1-184J9.2	NA	NA	NA
LUZP1	1.6168027	1.86E-65	5.55E-65
HTR1D	NA	4.92E-47	1.12E-46
LINC01355	2.37191958	2.19E-20	3.44E-20
HNRNPR	1.69677599	6.33E-21	1.00E-20
ZNF436	5.24319066	3.79E-100	2.04E-99
ZNF436-AS1	2.24708171	8.69E-52	2.13E-51
RP5-1057J7.1	NA	1.34E-124	1.41E-123
TCEA3	2.49675746	1.46E-05	1.75E-05
ASAP3	Inf	0.24672573	0.2582414
E2F2	2.99610895	5.09E-75	1.75E-74
RP1-150O5.3	NA	8.68E-109	5.65E-108
ID3	4.28711524	1.43E-139	3.84E-138
MDS2	NA	NA	NA
RPL11	1.99530889	1.44E-65	4.32E-65
TCEB3	1.79574479	1.13E-14	1.63E-14
TCEB3-AS1	1.31079767	0.0929421	0.09932584
PITHD1	1.82396709	0.41600704	0.42881816
LYPLA2	1.79372312	4.42E-71	1.44E-70
GALE	1.19232907	2.23E-82	8.63E-82
HMGCL	1.12354086	1.47E-118	1.24E-117
FUCA1	1.92931258	0.49999525	0.51320588
CNR2	Inf	2.52E-15	3.66E-15

RP11-4M23.3	NA	NA	NA
PNRC2	1.88731272	1.92E-05	2.30E-05
SRSF10	1.53876248	2.52E-40	5.25E-40
RP11-293P2(	NA	NA	NA
MYOM3	NA	1.62E-101	9.00E-101
RP11-293P2(	NA	NA	NA
IL22RA1	NA	NA	NA
IFNLR1	NA	NA	NA
RP11-10N16.	NA	NA	NA
RP11-10N16.	NA	NA	NA
GRHL3	NA	NA	NA
STPG1	1.81906615	9.42E-35	1.82E-34
NIPAL3	2.62159533	6.62E-52	1.62E-51
RCAN3	3.44552529	2.59E-41	5.47E-41
RP4-594I10.3	Inf	6.38E-141	2.01E-139
NCMAP	NA	NA	NA
SRRM1	1.78410031	2.17E-11	2.93E-11
CLIC4	2.33696498	0.06581011	0.07072608
RUNX3	3.83325704	3.49E-134	5.93E-133
RP11-84D1.1	NA	NA	NA
RP11-84D1.2	NA	NA	NA
RP4-799D16.	NA	NA	NA
RP4-706G24.	NA	3.70E-131	5.31E-130
SYF2	1.16848249	2.75E-133	4.47E-132
RSRP1	2.02577821	2.61E-09	3.40E-09
RP3-465N24.	Inf	1.72E-140	5.14E-139
RHD	NA	NA	NA
TMEM50A	1.52512775	6.20E-68	1.93E-67
RHCE	NA	1.38E-47	3.18E-47
TMEM57	1.64072633	4.06E-05	4.81E-05
LDLRAP1	3.49546044	1.18E-118	1.00E-117
RP11-70P17.	Inf	3.78E-58	1.01E-57
MAN1C1	Inf	2.23E-08	2.85E-08
RP1-187B23.	2.24708171	9.55E-47	2.17E-46
SEPN1	2.47178988	1.61E-71	5.24E-71
RP1-317E23.	NA	NA	NA
RP1-317E23.	0.74902724	1.35E-14	1.94E-14
MTFR1L	2.20867006	0.60737623	0.61988184
RP1-317E23.	NA	NA	NA
AUNIP	1.29561468	2.25E-60	6.23E-60
RP1-125I3.2	NA	NA	NA
PAQR7	Inf	3.52E-08	4.47E-08
STMN1	1.12463486	4.40E-145	3.06E-143
PAFAH2	2.62159533	1.86E-45	4.16E-45
EXTL1	NA	3.85E-14	5.47E-14

SLC30A2	NA	3.28E-107	2.06E-106
TRIM63	NA	NA	NA
PDIK1L	6.74124514	6.31E-33	1.18E-32
FAM110D	1.12354086	2.68E-24	4.46E-24
RP11-96L14.8	NA	NA	NA
RP11-96L14.7	0.37451362	0.00403433	0.00452353
ZNF593	1.88848227	1.01E-08	1.30E-08
CNKSR1	NA	1.04E-22	1.69E-22
CATSPER4	NA	NA	NA
CEP85	1.93865873	6.21E-07	7.68E-07
SH3BGRL3	1.56657747	2.70E-110	1.83E-109
UBXN11	1.52302205	7.67E-31	1.40E-30
CD52	1.14557107	6.58E-48	1.52E-47
AIM1L	Inf	1.50E-20	2.35E-20
ZNF683	NA	NA	NA
LIN28A	NA	NA	NA
DHDDS	1.87256809	1.09E-15	1.59E-15
RP3-476K8.3	NA	3.48E-118	2.91E-117
HMGN2	1.77260247	1.35E-112	9.76E-112
RPS6KA1	1.5396671	9.41E-25	1.57E-24
RP5-968P14.	Inf	3.22E-153	6.71E-151
ARID1A	2.18763511	8.00E-127	9.27E-126
PIGV	0.99870298	5.36E-14	7.59E-14
ZDHHC18	1.68531128	0.00014274	0.00016666
SFN	NA	8.18E-59	2.20E-58
GPN2	0.91185925	2.20E-90	9.82E-90
RP1-50O24.6	NA	NA	NA
GPATCH3	2.72015155	1.03E-78	3.73E-78
NUDC	0.86157537	9.01E-124	9.25E-123
NR0B2	NA	NA	NA
KDF1	NA	NA	NA
TRNP1	0.66260102	3.37E-111	2.34E-110
FAM46B	NA	8.46E-43	1.82E-42
AL137860.1	NA	NA	NA
SLC9A1	2.7464332	7.67E-31	1.40E-30
RP11-40H20.	NA	NA	NA
WDTC1	1.90137683	0.92853989	0.93187866
TMEM222	1.9051345	6.69E-15	9.64E-15
SYTL1	1.03314102	3.20E-95	1.57E-94
MAP3K6	2.62159533	3.43E-91	1.56E-90
FCN3	NA	NA	NA
CD164L2	NA	NA	NA
GPR3	1.79766537	6.05E-07	7.48E-07
WASF2	2.10837296	8.71E-63	2.50E-62
RP4-752I6.1	NA	NA	NA

RP1-159A19.	0.74902724	2.78E-09	3.61E-09
AHDC1	1.87256809	0.04188578	0.0453748
FGR	NA	NA	NA
RP11-288L9.:	0	3.88E-11	5.22E-11
IFI6	1.36429961	1.67E-96	8.44E-96
RP11-288L9.4	NA	1.29E-43	2.80E-43
FAM76A	1.31079767	1.52E-27	2.65E-27
STX12	1.39105058	3.49E-29	6.21E-29
PPP1R8	1.88679013	6.88E-25	1.16E-24
AL109927.1	NA	3.65E-49	8.58E-49
THEMIS2	0.71646084	2.72E-66	8.23E-66
RPA2	1.03579767	2.89E-135	5.24E-134
SMPDL3B	1.49805447	3.08E-09	4.00E-09
RP11-460I13	NA	4.45E-89	1.94E-88
XKR8	1.66450497	2.19E-51	5.33E-51
EYA3	2.80885214	1.96E-82	7.59E-82
PTAFR	Inf	2.08E-52	5.14E-52
DNAJC8	1.08689394	3.08E-120	2.77E-119
ATPIF1	1.51975456	5.32E-85	2.16E-84
RP5-1092A3.	Inf	2.10E-91	9.57E-91
RP5-1092A3.	NA	NA	NA
SESN2	2.49675746	1.07E-57	2.85E-57
MED18	1.87256809	8.04E-40	1.66E-39
PHACTR4	2.18466278	1.83E-23	3.00E-23
RCC1	1.51209874	1.00E-61	2.83E-61
TRNAU1AP	1.30188067	1.08E-74	3.71E-74
SNHG12	1.83272622	2.85E-34	5.46E-34
TAF12	1.50593897	1.74E-69	5.54E-69
RAB42	2.49675746	3.18E-95	1.56E-94
RP11-442N2	NA	1.87E-112	1.34E-111
RNU11	Inf	1.46E-82	5.68E-82
GMEB1	2.24708171	3.53E-53	8.83E-53
YTHDF2	1.71605494	0.74156172	0.75112572
OPRD1	NA	1.53E-150	2.36E-148
RP1-212P9.3	NA	4.68E-103	2.68E-102
EPB41	2.28391912	3.06E-66	9.24E-66
TMEM200B	Inf	2.43E-148	2.94E-146
RP11-242O2	NA	NA	NA
SRSF4	1.78614187	3.22E-62	9.16E-62
MECR	2.00682769	1.48E-21	2.36E-21
RP11-467D1	NA	NA	NA
PTPRU	1.73212549	9.83E-52	2.40E-51
RP3-437I16.1	NA	NA	NA
RP4-656G21.	NA	NA	NA
RP11-111I12	NA	NA	NA

RP5-893G23.	NA	NA	NA
RP4-591L5.1	NA	NA	NA
RP4-591L5.2	NA	NA	NA
MATN1	NA	8.67E-19	1.33E-18
MATN1-AS1	NA	1.27E-46	2.89E-46
LAPTM5	1.58722438	2.19E-66	6.66E-66
RP5-1125N1	NA	NA	NA
RP5-1125N1	NA	NA	NA
RP1-65J11.5	NA	NA	NA
SDC3	NA	1.63E-05	1.95E-05
PUM1	2.01821228	2.51E-108	1.62E-107
NKAIN1	Inf	1.68E-59	4.57E-59
SNRNP40	1.15258477	1.67E-119	1.46E-118
ZCCHC17	1.74498653	2.44E-34	4.67E-34
RP11-266K2	NA	NA	NA
FABP3	NA	2.67E-20	4.18E-20
SERINC2	0.94876783	7.86E-139	1.94E-137
LINC01226	NA	3.68E-151	6.13E-149
RP11-73M7.	NA	NA	NA
TINAGL1	NA	1.02E-20	1.61E-20
HCRTR1	NA	5.03E-145	3.36E-143
PEF1	1.91305605	5.32E-09	6.88E-09
RP11-73M7.	NA	NA	NA
RP11-73M7.	NA	NA	NA
COL16A1	NA	NA	NA
ADGRB2	Inf	2.75E-125	2.98E-124
RP11-84A19.	NA	NA	NA
SPOCD1	NA	NA	NA
RP11-84A19.	NA	0.05832705	0.06281428
RP11-84A19.	Inf	2.15E-19	3.33E-19
PTP4A2	2.16735852	3.02E-94	1.46E-93
RP4-534N18.	NA	NA	NA
KHDRBS1	1.50659851	4.89E-40	1.01E-39
RP11-277A4.	NA	2.10E-147	2.37E-145
TMEM39B	2.83225924	0.00509179	0.00569374
KPNA6	1.96442992	2.07E-36	4.08E-36
TXLNA	2.14586182	5.94E-06	7.19E-06
CCDC28B	1.38436284	9.92E-126	1.10E-124
RP4-622L5.7	0	3.07E-10	4.07E-10
IQCC	1.49805447	0.0102263	0.01131805
DCDC2B	NA	NA	NA
TMEM234	2.30469919	1.32E-83	5.22E-83
EIF3I	1.06379355	1.03E-123	1.06E-122
FAM167B	Inf	1.09E-115	8.47E-115
LCK	NA	NA	NA

HDAC1	1.02681217	2.25E-126	2.57E-125
MARCKSL1	1.60081736	3.40E-31	6.22E-31
RP4-811H24.	NA	NA	NA
TSSK3	0.74902724	3.28E-06	4.00E-06
FAM229A	6.74124514	8.87E-71	2.87E-70
BSDC1	2.04542053	5.75E-45	1.27E-44
RP1-27O5.3	NA	NA	NA
ZBTB8B	NA	1.09E-14	1.56E-14
ZBTB8A	1.62289235	4.64E-11	6.23E-11
ZBTB8OS	1.67484351	4.31E-14	6.11E-14
RBBP4	1.59330649	3.27E-18	4.96E-18
SYNC	2.67509728	2.17E-77	7.74E-77
RP11-114B7.	NA	NA	NA
KIAA1522	2.24708171	0.60393218	0.616575
YARS	1.66560004	1.84E-35	3.58E-35
S100PBP	2.08939177	5.35E-08	6.77E-08
FNDC5	1.49805447	2.19E-08	2.79E-08
HPCA	NA	NA	NA
TMEM54	2.82697377	1.37E-05	1.64E-05
RNF19B	1.99740597	1.20E-17	1.81E-17
RP1-117O3.2	NA	NA	NA
AK2	1.46400778	1.89E-60	5.24E-60
AZIN2	0.37451362	1.74E-35	3.38E-35
TRIM62	2.62159533	0.01234389	0.0136268
ZNF362	1.49805447	0.00022476	0.0002611
RP11-415J8.:	Inf	1.64E-144	9.77E-143
A3GALT2	NA	NA	NA
RP11-415J8.:	NA	NA	NA
PHC2	1.77893969	1.16E-19	1.80E-19
RP11-415J8.!	NA	NA	NA
ZSCAN20	Inf	0.00068868	0.00078966
CSMD2	NA	2.35E-95	1.16E-94
HMGB4	NA	NA	NA
CSMD2-AS1	NA	NA	NA
C1orf94	NA	NA	NA
SMIM12	1.7639813	0.00690289	0.00768862
GJB5	NA	NA	NA
GJB4	NA	NA	NA
RP1-34M23.!	NA	NA	NA
GJB3	NA	NA	NA
GJA4	NA	NA	NA
RP5-997D16.	NA	NA	NA
DLGAP3	4.49416342	2.85E-24	4.74E-24
RP11-244H3.	Inf	0.66587925	0.67734126
ZMYM6NB	NA	NA	NA

ZMYM6	11.9844358	1.60E-102	9.08E-102
ZMYM1	1.87256809	0.00301958	0.00339834
SFPQ	1.44001847	1.64E-110	1.12E-109
ZMYM4	2.32198444	1.06E-68	3.32E-68
ZMYM4-AS1	NA	NA	NA
KIAA0319L	0.96303502	6.10E-16	8.94E-16
NCDN	2.38326848	5.04E-88	2.16E-87
RP4-728D4.2	1.8385214	3.79E-09	4.92E-09
TFAP2E	1.87256809	3.42E-46	7.72E-46
PSMB2	1.24503486	8.73E-137	1.78E-135
C1orf216	2.14256628	2.39E-18	3.64E-18
CLSPN	1.11918605	3.35E-64	9.80E-64
RP11-435D7.	NA	NA	NA
AGO4	1.07003891	1.59E-31	2.93E-31
AGO1	2.7464332	1.03E-76	3.63E-76
RP4-789D17.	0.74902724	0.86149343	0.86761485
AGO3	3.51466627	7.31E-62	2.07E-61
RP4-665N4.8	NA	NA	NA
TEKT2	NA	2.39E-56	6.23E-56
ADPRHL2	1.65629967	1.96E-91	8.97E-91
COL8A2	NA	NA	NA
TRAPPC3	1.5734599	4.95E-32	9.16E-32
MAP7D1	1.88193093	1.12E-09	1.47E-09
THRAP3	1.50114963	3.55E-73	1.19E-72
SH3D21	NA	6.55E-22	1.05E-21
EVA1B	2.0598249	8.71E-10	1.14E-09
RP11-268J15	0	1.10E-106	6.83E-106
STK40	2.24708171	1.16E-42	2.50E-42
LSM10	2.46335014	9.59E-115	7.27E-114
OSCP1	NA	4.71E-24	7.80E-24
MRPS15	1.41414705	3.27E-43	7.07E-43
CSF3R	NA	1.93E-178	3.51E-174
RP4-614N24.	NA	NA	NA
GRIK3	NA	NA	NA
RP5-1180C18	NA	NA	NA
LINC01137	6.74124514	2.46E-15	3.56E-15
ZC3H12A	2.24708171	1.13E-05	1.35E-05
MEAF6	1.7857603	5.88E-24	9.73E-24
SNIP1	2.8463035	7.47E-11	9.99E-11
DNALI1	NA	0.05255704	0.05667775
GNL2	2.04585051	1.33E-07	1.67E-07
RSPO1	NA	NA	NA
C1orf109	2.62159533	0.49604428	0.50932341
CDCA8	1.6879487	6.95E-91	3.14E-90
EPHA10	NA	NA	NA

MANEAL	1.63023575	2.05E-25	3.47E-25
RP11-109P14	NA	NA	NA
YRDC	1.62023622	0.00564562	0.00630333
C1orf122	1.80832099	0.1072293	0.11417923
MTF1	3.932393	4.40E-126	4.94E-125
INPP5B	2.99610895	8.37E-102	4.68E-101
RP11-109P14	NA	3.31E-36	6.52E-36
SF3A3	1.14888388	7.92E-137	1.62E-135
FHL3	1.43860787	4.59E-41	9.65E-41
UTP11L	1.05745022	8.51E-138	1.88E-136
POU3F1	Inf	0.01160288	0.0128236
RP5-884C9.2	NA	7.48E-69	2.35E-68
LINC01343	NA	NA	NA
RP11-329N2	NA	NA	NA
RRAGC	1.47222595	8.99E-50	2.13E-49
RP5-864K19.	0.37451362	1.28E-32	2.39E-32
MYCBP	1.51678016	8.66E-20	1.34E-19
RP5-864K19.	NA	NA	NA
GJA9	NA	NA	NA
RHBDL2	NA	1.35E-88	5.87E-88
AKIRIN1	1.68706135	1.48E-74	5.06E-74
NDUFS5	1.63891235	1.59E-32	2.95E-32
MACF1	2.36854559	6.69E-22	1.07E-21
RP11-420K8.	NA	NA	NA
BMP8A	NA	NA	NA
RP11-69E11.	0.74902724	0.84391531	0.85071465
PABPC4	1.68772232	0.05923599	0.06378178
RP11-69E11.	NA	NA	NA
HEYL	2.24708171	4.56E-15	6.59E-15
RP1-144F13.	NA	NA	NA
NT5C1A	NA	5.41E-108	3.46E-107
HPCAL4	Inf	3.20E-59	8.68E-59
PPIE	1.55302895	1.09E-133	1.80E-132
BMP8B	1.79766537	0.94051159	0.94347587
OXCT2	2.24708171	6.00E-12	8.21E-12
RP1-118J21.2	1.12354086	9.53E-24	1.57E-23
TRIT1	1.71206226	0.16774246	0.17702191
MYCL	0.59922179	0.79789789	0.80611815
RP1-118J21.5	NA	1.94E-111	1.35E-110
MFSD2A	0.83225249	5.20E-27	9.00E-27
CAP1	1.00889383	1.73E-113	1.28E-112
PPT1	1.02461273	1.45E-139	3.88E-138
RLF	1.87256809	1.61E-63	4.67E-63
TMCO2	0	2.41E-141	8.10E-140
RP1-39G22.7	4.94357977	9.19E-81	3.46E-80

ZMPSTE24	1.25347415	1.14E-59	3.12E-59
COL9A2	0.37451362	4.97E-06	6.03E-06
SMAP2	1.43072618	3.33E-20	5.20E-20
ZFP69B	0.37451362	2.96E-47	6.78E-47
ZFP69	2.99610895	0.01459882	0.01607793
RP11-656D1(	1.49805447	1.32E-48	3.08E-48
EXO5	2.24708171	2.77E-08	3.53E-08
RP11-656D1(	NA	NA	NA
RP11-656D1(	NA	NA	NA
ZNF684	1.81906615	1.01E-25	1.72E-25
RIMS3	1.12354086	1.93E-05	2.31E-05
RP4-739H11.	NA	NA	NA
NFYC-AS1	4.86867704	5.06E-18	7.67E-18
NFYC	1.45996834	1.40E-44	3.08E-44
KCNQ4	6.74124514	2.27E-56	5.91E-56
CITED4	2.90248054	0.72319974	0.73309995
RP5-1066H1:	NA	NA	NA
CTPS1	1.4366588	3.67E-50	8.76E-50
SLFNL1-AS1	NA	9.32E-78	3.34E-77
SLFNL1	Inf	7.60E-15	1.09E-14
SCMH1	2.29389591	5.82E-12	7.96E-12
RP11-399E6.	NA	NA	NA
FOXO6	2.99610895	2.28E-30	4.13E-30
RP11-399E6.	NA	NA	NA
EDN2	NA	NA	NA
HIVEP3	0.21400778	1.86E-141	6.36E-140
RP11-486B1(	NA	NA	NA
GUCA2B	NA	NA	NA
GUCA2A	NA	NA	NA
FOXJ3	2.62159533	1.53E-90	6.86E-90
RIMKLA	3.49546044	1.87E-138	4.42E-137
ZMYND12	NA	NA	NA
PPCS	1.5571882	1.34E-90	6.02E-90
CCDC30	Inf	1.02E-37	2.06E-37
PPIH	1.27718747	6.04E-133	9.60E-132
RP5-994D16.	0.37451362	2.64E-67	8.10E-67
YBX1	1.96371042	1.43E-34	2.75E-34
CLDN19	NA	NA	NA
P3H1	1.71206226	5.20E-50	1.24E-49
C1orf50	1.19636295	1.54E-67	4.75E-67
RP5-994D16.	NA	NA	NA
RP5-994D16.	NA	NA	NA
SVBP	1.45944482	0.00152565	0.00173119
ERMAP	Inf	0.0001037	0.00012151
RP11-342M1	1.12354086	3.28E-13	4.58E-13

ZNF691	1.08949416	1.84E-89	8.10E-89
SLC2A1	1.49805447	0.00147598	0.00167557
SLC2A1-AS1	Inf	0.53076753	0.54408321
FAM183A	NA	NA	NA
EBNA1BP2	1.24492472	1.38E-95	6.82E-95
CFAP57	NA	3.89E-102	2.19E-101
TMEM125	NA	NA	NA
C1orf210	NA	1.05E-125	1.16E-124
TIE1	Inf	5.34E-30	9.64E-30
MPL	NA	NA	NA
RP1-92O14.3	2.49675746	0.13363113	0.14172431
CDC20	0.85192874	5.19E-134	8.72E-133
ELOVL1	1.55797665	6.22E-34	1.18E-33
MED8	1.56135255	2.35E-53	5.88E-53
RP1-92O14.6	NA	6.97E-94	3.33E-93
SZT2	5.24319066	4.04E-123	4.03E-122
SZT2-AS1	NA	NA	NA
HYI	NA	2.85E-61	8.01E-61
HYI-AS1	0.74902724	5.43E-30	9.78E-30
PTPRF	1.98129785	8.13E-63	2.33E-62
KDM4A	1.21716926	0.58702384	0.59981901
KDM4A-AS1	1.56614786	5.51E-25	9.28E-25
ST3GAL3	0.49935149	6.14E-44	1.34E-43
RP11-184I16	NA	NA	NA
ARTN	Inf	0.14367676	0.15205832
RP11-7O11.3	2.99610895	6.12E-28	1.07E-27
IPO13	2.01661179	9.06E-12	1.24E-11
DPH2	1.56755185	1.16E-15	1.70E-15
ATP6V0B	1.56533594	2.30E-54	5.84E-54
B4GALT2	2.08185512	6.51E-102	3.65E-101
CCDC24	2.62159533	4.36E-05	5.16E-05
SLC6A9	5.24319066	3.28E-113	2.41E-112
RP5-1198O2(	NA	2.36E-64	6.91E-64
KLF17	NA	NA	NA
KLF18	NA	NA	NA
DMAP1	1.19439478	1.06E-85	4.34E-85
ERI3	1.8003727	1.55E-18	2.37E-18
RNF220	2.43433852	2.53E-96	1.27E-95
TMEM53	NA	3.43E-51	8.30E-51
C1orf228	1.79418152	6.60E-37	1.31E-36
KIF2C	2.04280156	9.59E-109	6.23E-108
RP11-269F19	NA	0.00901092	0.00999426
RPS8	1.9863275	4.98E-73	1.66E-72
BEST4	NA	7.65E-06	9.24E-06
PLK3	1.5729572	0.57298549	0.58596972

TCTEX1D4	Inf	9.94E-56	2.57E-55
BTBD19	NA	2.95E-29	5.26E-29
PTCH2	Inf	8.74E-24	1.44E-23
EIF2B3	1.3618677	8.94E-88	3.82E-87
HECTD3	1.74773022	3.41E-15	4.94E-15
UROD	1.05243068	6.80E-125	7.26E-124
ZSWIM5	NA	NA	NA
LINC01144	Inf	5.08E-78	1.83E-77
HPDL	NA	NA	NA
MUTYH	1.71206226	7.78E-18	1.17E-17
TOE1	1.43732254	1.15E-55	2.98E-55
TESK2	1.49805447	0.20791751	0.21845367
CCDC163P	0.44941634	1.96E-05	2.34E-05
MMACHC	2.09727626	4.49E-52	1.10E-51
PRDX1	0.9456066	5.29E-123	5.24E-122
AKR1A1	1.63347183	2.11E-95	1.04E-94
NASP	1.04133055	5.42E-139	1.36E-137
CCDC17	Inf	3.58E-139	9.19E-138
GPBP1L1	2.79444777	2.69E-39	5.53E-39
TMEM69	1.39255768	1.56E-35	3.03E-35
IPP	Inf	5.98E-35	1.16E-34
MAST2	1.97470817	1.63E-58	4.38E-58
PIK3R3	5.9922179	1.42E-17	2.13E-17
PIK3R3	NA	NA	NA
RP4-533D7.4	NA	NA	NA
RP4-533D7.5	0	5.68E-12	7.78E-12
TSPAN1	NA	NA	NA
RP11-322N2	NA	NA	NA
POMGNT1	2.31841764	7.89E-08	9.94E-08
LURAP1	1.59168288	0.01232635	0.01360827
RAD54L	2.08063121	7.21E-09	9.29E-09
LRRC41	1.73967616	1.03E-28	1.82E-28
UQCRH	1.85683495	3.63E-50	8.66E-50
NSUN4	1.23976922	6.31E-06	7.63E-06
FAAH	NA	2.36E-20	3.70E-20
DMBX1	NA	NA	NA
RP11-49P4.7	NA	NA	NA
MKNK1-AS1	NA	NA	NA
KNCN	NA	NA	NA
MKNK1	1.46348399	3.39E-16	5.00E-16
MOB3C	2.62159533	0.17095933	0.18036428
ATPAF1	1.4900861	5.60E-77	1.98E-76
TEX38	NA	NA	NA
EFCAB14-AS	NA	NA	NA
EFCAB14	1.92250324	8.49E-81	3.20E-80

CYP4B1	NA	NA	NA
CYP4A11	NA	NA	NA
CYP4X1	NA	NA	NA
CYP4Z1	NA	NA	NA
CYP4A22-AS1	NA	1.48E-16	2.19E-16
CYP4A22	NA	NA	NA
LINC00853	1.99740597	1.11E-20	1.75E-20
PDZK1IP1	NA	1.28E-39	2.63E-39
TAL1	1.70233463	1.95E-13	2.73E-13
RP1-18D14.7	NA	3.48E-118	2.91E-117
STIL	1.49805447	1.87E-07	2.34E-07
CMPK1	1.91750973	0.87490576	0.88053591
LINC01389	NA	3.56E-81	1.35E-80
FOXE3	NA	5.14E-08	6.50E-08
FOXD2-AS1	1.64785992	0.00019857	0.00023109
FOXD2	1.59168288	9.90E-10	1.30E-09
RP11-543D5.	NA	NA	NA
TRABD2B	NA	NA	NA
RP11-543D5.	NA	NA	NA
RP11-330M1	NA	NA	NA
RP4-683M8.:	NA	NA	NA
RP11-193P11	NA	NA	NA
AL356289.1	NA	NA	NA
RP5-1024N4.	NA	NA	NA
SLC5A9	NA	NA	NA
RP5-1024N4.	NA	NA	NA
SPATA6	NA	NA	NA
AGBL4	NA	NA	NA
BEND5	Inf	1.66E-143	7.91E-142
RP11-141A19	NA	NA	NA
RP11-141A19	NA	NA	NA
ELAVL4	NA	NA	NA
RP11-567C20	NA	NA	NA
RP11-567C20	NA	NA	NA
DMRTA2	NA	NA	NA
FAF1	1.60019455	5.17E-107	3.22E-106
RP5-850O15.	NA	NA	NA
CDKN2C	2.33578231	2.52E-06	3.08E-06
C1orf185	NA	NA	NA
LINC01562	NA	2.34E-18	3.56E-18
RNF11	1.49805447	5.67E-52	1.39E-51
RP11-296A18	NA	5.28E-27	9.12E-27
TTC39A	2.24708171	5.29E-31	9.66E-31
TTC39A-AS1	NA	8.75E-122	8.30E-121
EPS15	3.09597925	4.83E-100	2.60E-99

RP11-253A2(	NA	1.09E-129	1.45E-128
RP11-191G2	Inf	1.38E-25	2.34E-25
OSBPL9	1.49805447	9.13E-10	1.20E-09
NRDC	1.61127952	6.70E-18	1.01E-17
RP4-657D16.	NA	3.84E-125	4.13E-124
RP4-657D16.	NA	3.52E-48	8.15E-48
RAB3B	0.85603113	2.19E-99	1.16E-98
TXNDC12	1.15434501	3.15E-145	2.34E-143
KTI12	1.23488274	1.64E-97	8.45E-97
RP11-91A18.	NA	NA	NA
TXNDC12-AS	NA	NA	NA
BTF3L4	1.4192095	4.58E-132	6.94E-131
ZFYVE9	1.34824903	0.00022869	0.00026562
CC2D1B	1.24837873	3.37E-21	5.35E-21
RP11-155O1	Inf	7.68E-127	8.94E-126
ORC1	1.81343436	1.12E-05	1.35E-05
PRPF38A	1.91157993	4.68E-54	1.18E-53
ZCCHC11	1.88391699	4.56E-27	7.89E-27
RP11-25O10.	NA	8.60E-05	1.01E-04
GPX7	1.62231706	6.52E-28	1.14E-27
FAM159A	0	2.88E-95	1.42E-94
COA7	1.14899324	4.09E-38	8.26E-38
ZYG11B	1.99740597	5.73E-74	1.94E-73
ZYG11A	NA	1.68E-06	2.05E-06
RP4-631H13.	NA	NA	NA
ECHDC2	NA	4.95E-32	9.17E-32
SCP2	1.223857	7.22E-124	7.44E-123
AL445183.1	NA	NA	NA
PODN	NA	NA	NA
RP11-334A1	NA	NA	NA
SLC1A7	NA	NA	NA
RP11-334A1	NA	NA	NA
CPT2	1.95899431	0.00038178	0.00044119
RP5-1024G6.	Inf	7.75E-07	9.56E-07
C1orf123	1.41647725	4.84E-118	4.03E-117
RP5-1024G6.	NA	1.88E-114	1.42E-113
MAGOH	1.48296073	1.37E-95	6.79E-95
RP5-1024G6.	NA	1.58E-09	2.07E-09
LRP8	7.11575875	6.57E-110	4.41E-109
RP4-784A16.	NA	NA	NA
RP4-784A16.	NA	1.76E-22	2.85E-22
RP4-784A16.	NA	NA	NA
RP4-784A16.	NA	2.88E-130	3.94E-129
RP11-117D2	NA	NA	NA
RP11-117D2	NA	NA	NA

DMRTB1	NA	NA	NA
GLIS1	NA	8.44E-67	2.57E-66
NDC1	1.94192247	1.38E-83	5.45E-83
YIPF1	1.62289235	9.71E-52	2.37E-51
DIO1	NA	NA	NA
HSPB11	1.76465739	1.78E-06	2.18E-06
LRRC42	0.81144617	1.61E-132	2.50E-131
LDLRAD1	NA	NA	NA
TMEM59	0.80847384	6.19E-145	4.02E-143
TCEANC2	1.83095547	3.55E-14	5.04E-14
CDCP2	NA	NA	NA
RP11-446E24	NA	1.96E-21	3.13E-21
CYB5RL	1.5421149	0.00015633	0.00018235
MRPL37	1.44058942	5.94E-105	3.55E-104
SSBP3	1.80554987	0.36877666	0.38176013
SSBP3-AS1	NA	9.79E-108	6.23E-107
RP5-997D24.	NA	NA	NA
RP4-705F19.	NA	NA	NA
RP5-866L20.	NA	NA	NA
ACOT11	3.74513619	8.09E-75	2.78E-74
FAM151A	NA	NA	NA
MROH7	NA	NA	NA
MROH7-TTC4	NA	NA	NA
TTC4	0.93628405	0.00010227	0.00011985
PARS2	1.71206226	0.27271988	0.28467687
TTC22	NA	NA	NA
RP11-67L3.2	NA	NA	NA
LEXM	NA	NA	NA
DHCR24	2.24708171	1.07E-97	5.53E-97
RP11-67L3.5	NA	1.19E-63	3.44E-63
RP11-67L3.4	0.37451362	2.63E-66	7.94E-66
TMEM61	NA	NA	NA
RP11-12C17.	NA	NA	NA
BSND	NA	NA	NA
PCSK9	NA	NA	NA
USP24	1.61040856	0.51127972	0.52452142
RP11-101C11	NA	NA	NA
RP11-90C4.1	NA	NA	NA
RP11-90C4.2	NA	NA	NA
RP11-90C4.3	NA	NA	NA
RP1-158P9.1	NA	NA	NA
RP11-504A18	NA	NA	NA
RP4-710M16	NA	NA	NA
PLPP3	0	1.15E-18	1.76E-18
PRKAA2	NA	NA	NA

C1orf168	NA	NA	NA
RP11-378I13	NA	NA	NA
C8A	NA	NA	NA
C8B	Inf	8.09E-05	9.51E-05
RP5-1103B4.	NA	NA	NA
DAB1	NA	NA	NA
RP6-102O10.	NA	NA	NA
DAB1-AS1	NA	NA	NA
RP11-393I23	NA	NA	NA
OMA1	1.55567195	1.02E-11	1.39E-11
TACSTD2	NA	2.67E-09	3.48E-09
MYSM1	2.87127108	8.86E-21	1.40E-20
JUN	1.12354086	4.23E-28	7.42E-28
LINC01135	NA	NA	NA
RP11-63G10.	NA	4.60E-56	1.19E-55
RP11-63G10.	NA	NA	NA
RP4-794H19.	NA	0.00241914	0.00272885
LINC01358	NA	NA	NA
RP11-145M4	NA	NA	NA
RP11-145M4	NA	NA	NA
RP11-467C18	NA	2.58E-134	4.43E-133
FGGY	1.99740597	2.05E-29	3.67E-29
RP4-782L23.1	NA	NA	NA
HOOK1	NA	6.39E-68	1.98E-67
CYP2J2	NA	NA	NA
C1orf87	NA	NA	NA
RP11-575B7.	NA	NA	NA
RP11-776H12	NA	NA	NA
RP11-436K8.	NA	NA	NA
NFIA	4.49416342	1.04E-21	1.67E-21
NFIA-AS2	NA	NA	NA
TM2D1	1.46400778	3.21E-32	5.96E-32
RP11-430G17	Inf	3.04E-128	3.76E-127
INADL	4.11964981	4.56E-133	7.34E-132
L1TD1	NA	0.99471457	0.99526316
KANK4	NA	5.51E-95	2.69E-94
USP1	1.5864813	1.69E-40	3.53E-40
DOCK7	1.64785992	1.00E-07	1.26E-07
ANGPTL3	NA	NA	NA
RP11-230B22	NA	3.35E-45	7.44E-45
ATG4C	1.72852439	2.82E-71	9.17E-71
RP4-771M4.3	NA	NA	NA
RP11-5P4.1	NA	NA	NA
RP11-5P4.2	NA	NA	NA
RP11-5P4.3	NA	NA	NA

RP11-335E6.	NA	NA	NA
RP11-335E6.	NA	NA	NA
FOXD3-AS1	NA	NA	NA
FOXD3	Inf	4.62E-92	2.14E-91
ALG6	1.7061176	0.94299026	0.94585776
ITGB3BP	1.51925336	2.14E-113	1.57E-112
EFCAB7	1.49805447	7.24E-05	8.53E-05
PGM1	1.07552629	9.84E-100	5.26E-99
ROR1	NA	1.72E-110	1.17E-109
ROR1-AS1	NA	NA	NA
UBE2U	NA	NA	NA
CACHD1	1.68531128	9.41E-37	1.87E-36
RAVER2	2.99610895	3.26E-77	1.16E-76
JAK1	1.57830739	2.55E-23	4.18E-23
RP4-535B20.	NA	NA	NA
RP4-535B20.	NA	0.13377061	0.14180592
AK4	2.24708171	4.18E-28	7.33E-28
DNAJC6	Inf	2.39E-13	3.34E-13
RP5-1044H5.	NA	NA	NA
LEPROT	1.70233463	0.47317262	0.48625228
LEPR	0.74902724	2.09E-10	2.77E-10
PDE4B	0	3.77E-142	1.39E-140
RP11-397C1[illegible]	NA	NA	NA
SGIP1	NA	4.32E-143	1.89E-141
RP4-598P13.	NA	NA	NA
TCTEX1D1	1.24837873	9.44E-85	3.82E-84
INSL5	NA	NA	NA
WDR78	2.24708171	2.49E-23	4.09E-23
RP11-342H2[illegible]	NA	NA	NA
MIER1	1.76333495	1.94E-08	2.47E-08
SLC35D1	2.92120623	1.31E-40	2.74E-40
C1orf141	NA	NA	NA
IL23R	1.87256809	8.66E-31	1.58E-30
IL12RB2	NA	0.00033669	0.0003896
SERBP1	1.40193653	7.50E-74	2.54E-73
RP11-393N2	NA	NA	NA
GADD45A	2.02677958	0.98560675	0.98658562
GNG12	Inf	4.04E-64	1.18E-63
GNG12-AS1	NA	NA	NA
DIRAS3	NA	NA	NA
WLS	NA	NA	NA
RPE65	NA	NA	NA
DEPDC1	1.55797665	5.91E-40	1.22E-39
RP4-694A7.2	NA	3.00E-94	1.44E-93
DEPDC1-AS1	1.12354086	0.00262932	0.00296262

RP11-424D14	NA	NA	NA
RP11-74K19.	NA	NA	NA
RP3-380B4.1	NA	NA	NA
LRRC7	1.12354086	6.87E-11	9.19E-11
RP11-181B18	NA	NA	NA
LRRC40	1.22568093	4.06E-07	5.04E-07
SRSF11	1.34978944	2.69E-87	1.13E-86
RP4-677H15.	NA	NA	NA
ANKRD13C	1.64785992	0.00700049	0.00779637
HHLA3	1.28404669	1.46E-13	2.04E-13
RP11-180O5.	NA	NA	NA
AL158839.1	NA	NA	NA
CTH	0.37451362	1.13E-137	2.48E-136
RP11-42O15.	0.37451362	0.64433342	0.65616036
RP5-952N6.1	NA	NA	NA
RP11-99H8.1	NA	NA	NA
PTGER3	NA	1.09E-25	1.84E-25
RP3-333A15.	NA	NA	NA
ZRANB2-AS1	NA	0.00021029	0.00024448
ZRANB2	2.15573693	4.18E-49	9.82E-49
NEGR1	0.74902724	3.17E-90	1.41E-89
RP11-82L20.	NA	NA	NA
RP4-660H19.	NA	NA	NA
RP11-262K1.	NA	NA	NA
LINC01360	NA	NA	NA
RP4-788P17.	NA	NA	NA
LRRIQ3	2.24708171	1.81E-65	5.41E-65
FPGT	12.733463	3.32E-10	4.39E-10
FPGT-TNNI3	NA	4.72E-63	1.35E-62
TNNI3K	NA	NA	NA
RP11-439H8.	NA	NA	NA
RP4-650F12.	NA	NA	NA
LRRC53	NA	NA	NA
ERICH3	NA	8.24E-21	1.30E-20
ERICH3-AS1	NA	NA	NA
RP11-17E13.	NA	5.14E-08	6.50E-08
CRYZ	1.40442607	5.39E-95	2.64E-94
TYW3	2.03936828	2.00E-36	3.95E-36
RP11-93N20.	NA	NA	NA
RP11-510C10	NA	NA	NA
RP11-510C10	NA	NA	NA
LHX8	NA	NA	NA
RP11-510C10	NA	NA	NA
SLC44A5	NA	NA	NA
RP4-682C21.	NA	NA	NA

ACADM	1.13936538	2.16E-127	2.58E-126
RABGGTB	2.18466278	7.30E-130	9.77E-129
MSH4	NA	NA	NA
ASB17	NA	NA	NA
RP11-550H2.	NA	NA	NA
RP11-550H2.	NA	NA	NA
ST6GALNAC3	NA	NA	NA
RP5-1102E8.	NA	NA	NA
RP11-415A2(	NA	NA	NA
ST6GALNAC5	NA	NA	NA
RP4-564M11	NA	NA	NA
PIGK	1.26151956	1.12E-88	4.87E-88
AK5	0	3.31E-90	1.48E-89
RP11-375A5.	NA	NA	NA
ZZZ3	2.11490043	0.34693127	0.35967925
USP33	2.36231667	2.70E-89	1.19E-88
FAM73A	3.44552529	1.72E-114	1.30E-113
NEXN-AS1	NA	1.04E-93	4.96E-93
NEXN	1.93498703	1.13E-41	2.39E-41
FUBP1	1.80171417	0.36988401	0.38288461
DNAJB4	Inf	6.09E-47	1.39E-46
GIPC2	NA	NA	NA
RP11-386I14	Inf	2.89E-144	1.63E-142
PTGFR	NA	NA	NA
IFI44L	NA	0.02164078	0.0236767
IFI44	Inf	9.66E-108	6.14E-107
ADGRL4	NA	NA	NA
RP4-726F1.1	NA	NA	NA
RP4-726F1.2	NA	NA	NA
RP11-339A1	NA	NA	NA
RP11-339D2	NA	NA	NA
RP5-933B4.1	NA	NA	NA
RP5-887A10.	NA	NA	NA
ADGRL2	NA	NA	NA
RP5-837I24.2	NA	NA	NA
RP5-837I24.4	NA	NA	NA
RP11-147G1(	NA	NA	NA
LINC01362	NA	NA	NA
LINC01361	NA	NA	NA
RP11-413G15	NA	NA	NA
RP11-475O6.	NA	NA	NA
RP5-836J3.1	NA	NA	NA
TTLL7	0.82392996	0.12498073	0.13279065
RP11-486G15	2.7464332	8.81E-14	1.24E-13
PRKACB	1.71206226	6.26E-09	8.07E-09

SAMD13	0.62418936	2.08E-47	4.77E-47
DNASE2B	NA	5.68E-50	1.35E-49
RPF1	1.0965141	6.11E-93	2.87E-92
GNG5	1.95366966	5.41E-28	9.48E-28
CTBS	1.63424125	3.83E-37	7.63E-37
LINC01555	NA	NA	NA
SSX2IP	0.93628405	5.34E-16	7.84E-16
LPAR3	NA	6.07E-05	7.16E-05
MCOLN2	0.79896239	8.21E-11	1.10E-10
WDR63	NA	NA	NA
MCOLN3	8.98832685	1.45E-63	4.19E-63
SYDE2	19.4747082	1.02E-124	1.08E-123
C1orf52	1.64275292	1.09E-20	1.71E-20
BCL10	1.04863813	1.25E-58	3.36E-58
RP11-131L23	Inf	6.90E-77	2.44E-76
DDAH1	2.99610895	1.22E-119	1.07E-118
RP11-131L23	NA	NA	NA
RP4-621F18.	Inf	1.06E-154	2.70E-152
RP11-290M5	NA	0.59019793	0.6028925
CYR61	0.37451362	0.0473676	0.05118793
RP11-290M5	NA	NA	NA
ZNHIT6	1.7077821	2.34E-11	3.15E-11
COL24A1	1.49805447	0.29582646	0.30808747
ODF2L	4.49416342	2.44E-124	2.55E-123
CLCA2	NA	NA	NA
CLCA1	NA	NA	NA
CLCA4	NA	NA	NA
RP4-651E10.	NA	NA	NA
RP4-612B15.	1.87256809	0.43897777	0.45193138
SH3GLB1	1.76957685	3.33E-06	4.05E-06
15-Sep	1-Jan	3.74E-113	2.74E-112
HS2ST1	1.80321372	1.01E-36	2.00E-36
RP4-604K5.3	NA	NA	NA
RP5-1052I5.2	NA	NA	NA
LINC01140	NA	1.30E-67	4.02E-67
LMO4	2.41150233	0.89786548	0.90273303
LINC01364	NA	NA	NA
RP5-1027O1	NA	NA	NA
PKN2-AS1	Inf	2.36E-33	4.45E-33
PKN2	2.33030696	7.17E-07	8.85E-07
GTF2B	0.73238219	2.37E-106	1.46E-105
CCBL2	0.98309825	8.32E-91	3.75E-90
RBMXL1	2.16682879	0.00032157	0.0003723
GBP3	NA	1.50E-27	2.61E-27
GBP1	NA	NA	NA

GBP2	NA	NA	NA
GBP7	NA	NA	NA
GBP4	Inf	2.87E-05	3.41E-05
GBP5	NA	3.14E-08	3.99E-08
RP4-620F22.	NA	NA	NA
GBP6	NA	NA	NA
LRRC8B	2.47755163	1.34E-43	2.90E-43
FLJ27354	3.37062257	7.34E-30	1.32E-29
LRRC8C	2.57998271	2.49E-32	4.62E-32
RP11-302M6	NA	NA	NA
RP5-943J3.2	2.7464332	4.97E-69	1.57E-68
LRRC8D	2.58414397	3.43E-71	1.11E-70
RP11-302M6	NA	NA	NA
ZNF326	1.11016537	4.94E-128	6.06E-127
RP5-827O9.1	NA	NA	NA
BARHL2	NA	5.60E-91	2.53E-90
RP4-665J23.:	2.3151751	8.91E-93	4.18E-92
RP4-665J23.:	NA	5.44E-132	8.17E-131
RP4-665J23.4	NA	NA	NA
ZNF644	1.85473411	0.60628769	0.61884052
HFM1	NA	NA	NA
CDC7	1.74773022	5.72E-05	6.76E-05
TGFBR3	Inf	3.68E-16	5.42E-16
BRDT	NA	2.21E-66	6.71E-66
EPHX4	4.49416342	4.17E-18	6.33E-18
SETSIP	Inf	1.21E-06	1.49E-06
BTBD8	0	0.00314506	0.00353824
KIAA1107	0	5.69E-14	8.05E-14
RP4-621B10.	NA	NA	NA
C1orf146	NA	NA	NA
GLMN	1.07003891	2.34E-107	1.47E-106
RPAP2	2.37926299	2.42E-07	3.01E-07
GFI1	1.54798962	0.56629008	0.57931852
EVI5	1.68531128	1.38E-46	3.14E-46
RPL5	1.63755811	6.08E-37	1.21E-36
FAM69A	Inf	3.46E-22	5.58E-22
MTF2	1.71976654	3.77E-45	8.37E-45
TMED5	1.4029399	4.00E-40	8.30E-40
CCDC18	1.39474037	1.53E-87	6.49E-87
DR1	1.40633685	1.80E-36	3.55E-36
FNBP1L	1.21716926	1.39E-38	2.82E-38
BCAR3	NA	1.19E-153	2.59E-151
RP5-1033H2:	NA	NA	NA
RP4-561L24.:	NA	0.00048233	0.00055541
DNTTIP2	1.20379377	7.55E-64	2.20E-63

GCLM	2.55702402	2.46E-52	6.07E-52
ABCA4	NA	NA	NA
RP11-78O9.1	NA	NA	NA
ARHGAP29	NA	3.71E-88	1.60E-87
RP11-148B18	NA	NA	NA
RP11-148B18	NA	NA	NA
ABCD3	1.56318728	1.19E-83	4.72E-83
F3	1.24837873	4.96E-52	1.22E-51
RP11-86H7.6	NA	NA	NA
SLC44A3	NA	1.82E-65	5.45E-65
CNN3	0.74902724	0.62086046	0.63314505
RP4-639F20.	NA	NA	NA
ALG14	1.29377432	4.19E-34	8.01E-34
RP11-313A24	NA	NA	NA
TMEM56	NA	2.35E-40	4.90E-40
TMEM56-RW	0.74902724	3.77E-70	1.21E-69
RP11-57H12.	NA	NA	NA
RWDD3	2.99610895	1.00E-07	1.26E-07
RP11-57H12.	NA	NA	NA
RP4-586O15.	NA	NA	NA
RP11-14O19.	NA	NA	NA
RP11-14O19.	NA	NA	NA
FLJ31662	NA	NA	NA
RP11-286B14	NA	NA	NA
RP11-286B14	NA	NA	NA
RP11-147C23	NA	NA	NA
RP5-898J17.1	NA	NA	NA
PTBP2	0.93628405	1.44E-35	2.80E-35
DPYD	0.74902724	4.87E-21	7.72E-21
DPYD-AS1	NA	NA	NA
DPYD-AS2	NA	NA	NA
RP11-272L13	NA	NA	NA
RP11-490G2.	NA	NA	NA
MIR137HG	NA	NA	NA
RP5-1070A16	NA	NA	NA
SNX7	NA	1.17E-37	2.36E-37
PLPPR5	NA	NA	NA
RP5-896L10.1	NA	NA	NA
PLPPR4	NA	NA	NA
RP11-413P11	NA	NA	NA
PALMD	NA	NA	NA
FRRS1	Inf	4.69E-88	2.02E-87
AGL	3.37062257	1.87E-07	2.33E-07
RP5-884G6.2	1.49805447	0.01271048	0.01402551
SLC35A3	2.20546909	3.34E-45	7.41E-45

MFSD14A	1.40817121	5.08E-07	6.29E-07
RP4-714D9.2	NA	NA	NA
SASS6	1.38649723	5.77E-14	8.17E-14
TRMT13	1.81014916	0.41081905	0.42363891
LRRC39	NA	NA	NA
DBT	1.56614786	3.54E-06	4.31E-06
RTCA-AS1	Inf	2.25E-37	4.51E-37
RTCA	1.55233181	6.34E-66	1.91E-65
RP5-837M10	NA	NA	NA
CDC14A	0.99870298	1.99E-07	2.49E-07
RP5-837M10	NA	NA	NA
GPR88	NA	NA	NA
LINC01349	NA	NA	NA
VCAM1	NA	NA	NA
EXTL2	2.12224384	0.00014683	0.00017138
RP4-549L20.	Inf	3.31E-119	2.87E-118
SLC30A7	2.29701686	1.65E-40	3.43E-40
DPH5	1.51053826	2.41E-80	9.02E-80
RP11-421L21	0.74902724	1.00E-86	4.20E-86
RP11-421L21	1.8385214	2.37E-28	4.18E-28
RP4-575N6.4	NA	NA	NA
S1PR1	NA	NA	NA
LINC01307	NA	NA	NA
RP11-411H5.	Inf	3.55E-70	1.14E-69
OLFM3	NA	NA	NA
RP11-202K23	NA	NA	NA
RP5-936J12.	NA	NA	NA
COL11A1	NA	NA	NA
RP11-153F1.	NA	NA	NA
RP11-153F1.	NA	NA	NA
RNPC3	1.89753567	1.04E-19	1.61E-19
AMY2B	NA	5.10E-28	8.93E-28
AMY2A	NA	NA	NA
AMY1A	NA	NA	NA
AMY1B	NA	NA	NA
AMY1C	NA	NA	NA
RP11-251P6.	NA	NA	NA
RP11-24P14.	NA	NA	NA
RP5-947P14.	NA	NA	NA
RP11-233E12	NA	NA	NA
PRMT6	2.10303801	2.76E-87	1.16E-86
NTNG1	NA	NA	NA
VAV3	1.92607004	9.72E-13	1.35E-12
VAV3-AS1	Inf	7.29E-37	1.45E-36
RP11-356N1.	NA	5.11E-124	5.29E-123

SLC25A24	0.96932937	1.40E-11	1.90E-11
RP11-483I13	2.24708171	3.17E-91	1.44E-90
NBPF4	NA	NA	NA
RP11-483I13	NA	NA	NA
NBPF6	NA	NA	NA
RP11-131J3.1	NA	NA	NA
FAM102B	5.24319066	2.40E-112	1.71E-111
HENMT1	NA	NA	NA
PRPF38B	1.69543327	1.57E-28	2.77E-28
FNDC7	NA	NA	NA
RP11-293A1(	NA	NA	NA
STXBP3	1.70233463	0.40070456	0.41353787
AKNAD1	NA	NA	NA
RP11-475E11	NA	1.93E-109	1.28E-108
SPATA42	NA	NA	NA
GPSM2	1.2963933	1.31E-74	4.48E-74
CLCC1	1.85285685	2.40E-15	3.48E-15
WDR47	2.24708171	5.18E-71	1.68E-70
TAF13	2.34071012	4.88E-52	1.20E-51
RP5-1065J22	2.62159533	7.59E-100	4.06E-99
TMEM167B	1.93978849	0.50280392	0.51591362
SCARNA2	NA	NA	NA
C1orf194	NA	NA	NA
KIAA1324	Inf	4.32E-143	1.89E-141
SARS	0.89883268	2.51E-111	1.74E-110
CELSR2	2.39688716	0.9050589	0.90967194
PSRC1	0.91245136	3.32E-108	2.13E-107
MYBPHL	NA	1.34E-111	9.40E-111
SORT1	13.2328145	2.75E-142	1.04E-140
PSMA5	1.22433588	7.66E-109	5.00E-108
SYPL2	NA	0.79529053	0.80352867
ATXN7L2	2.62159533	0.01006686	0.01114295
CYB561D1	6.36673152	6.70E-58	1.78E-57
AMIGO1	NA	4.89E-144	2.59E-142
GPR61	NA	7.97E-25	1.34E-24
RP5-1160K1.	NA	NA	NA
GNAI3	1.26894026	2.59E-83	1.02E-82
GNAT2	0	1.94E-56	5.06E-56
AMPD2	1.77042802	0.5440175	0.55722479
RP5-1160K1.	NA	NA	NA
GSTM4	1.06225681	3.83E-90	1.70E-89
GSTM2	NA	NA	NA
GSTM1	NA	NA	NA
AC000032.2	NA	NA	NA
GSTM5	NA	NA	NA

RP4-735C1.4	NA	NA	NA
GSTM3	NA	1.28E-39	2.63E-39
EPS8L3	NA	NA	NA
RP4-735C1.6	NA	NA	NA
RP11-195M1	NA	NA	NA
RP11-195M1	NA	NA	NA
CSF1	Inf	2.31E-64	6.77E-64
AHCYL1	1.75110422	9.36E-07	1.15E-06
STRIP1	1.85053788	3.39E-05	4.03E-05
RP4-773N10.	3.74513619	2.03E-109	1.35E-108
ALX3	NA	NA	NA
LINC01397	NA	NA	NA
UBL4B	NA	NA	NA
SLC6A17	NA	NA	NA
RP5-1028L1C	NA	NA	NA
RP5-1028L1C	NA	NA	NA
KCNC4-AS1	NA	2.62E-19	4.05E-19
KCNC4	2.3540856	7.95E-16	1.16E-15
RP5-1074L1.	1.49805447	2.73E-20	4.28E-20
RBM15	3.94941634	1.29E-59	3.52E-59
SLC16A4	2.54669261	0.08318249	0.0890165
RP5-1074L1.	1.42996109	5.43E-05	6.41E-05
LAMTOR5	1.80213427	0.00171411	0.00194189
PROK1	NA	NA	NA
RP11-470L1S	NA	NA	NA
RP11-470L1S	NA	NA	NA
RP11-470L1S	NA	NA	NA
KCNA10	NA	NA	NA
KCNA2	NA	NA	NA
RP11-284N8.	NA	NA	NA
KCNA3	NA	NA	NA
CD53	1.71206226	2.35E-42	5.02E-42
RP11-96K19.	NA	NA	NA
LRIF1	2.47504652	2.72E-14	3.88E-14
RP11-96K19.	Inf	1.45E-128	1.83E-127
DRAM2	1.27420725	1.00E-135	1.89E-134
CEPT1	2.24708171	4.12E-08	5.22E-08
RP5-1180E21	NA	NA	NA
RP5-1180E21	1.49805447	3.69E-40	7.68E-40
DENND2D	NA	3.62E-49	8.51E-49
CHI3L2	NA	NA	NA
CHIA	NA	NA	NA
RP5-1125M8	NA	NA	NA
PIFO	0	8.67E-151	1.43E-148
OVGP1	NA	4.53E-29	8.06E-29

WDR77	1.3917111	1.49E-43	3.24E-43
ATP5F1	1.17867758	7.82E-129	9.98E-128
C1orf162	2.24708171	1.64E-08	2.10E-08
TMIGD3	0.74902724	1.10E-30	2.00E-30
ADORA3	0	3.04E-11	4.10E-11
RAP1A	1.55680171	9.67E-65	2.86E-64
LINC01160	NA	9.57E-141	2.95E-139
FAM212B	Inf	0.0010717	0.0012229
FAM212B-AS	NA	3.64E-106	2.23E-105
DDX20	1.31079767	6.59E-05	7.76E-05
KCND3	NA	1.99E-78	7.21E-78
KCND3-AS1	NA	NA	NA
RP11-88H9.2	NA	NA	NA
RP5-965F6.2	NA	NA	NA
CTTNBP2NL	1.31079767	0.2813227	0.29335306
WNT2B	0.74902724	2.07E-17	3.11E-17
RP4-671G15.	0	1.16E-63	3.37E-63
ST7L	2.80885214	0.15773827	0.16659997
CAPZA1	1.37138075	7.83E-99	4.13E-98
MOV10	2.14721141	0.09673958	0.10332937
RP11-426L16	NA	0.02457123	0.02683591
RHOC	1.30627455	9.65E-44	2.10E-43
RP11-426L16	NA	NA	NA
PPM1J	2.24708171	3.29E-95	1.61E-94
RP11-426L16	NA	NA	NA
FAM19A3	Inf	1.49E-131	2.19E-130
LINC01356	0	1.27E-45	2.85E-45
RP3-522D1.1	6.74124514	6.87E-26	1.17E-25
SLC16A1	3.04292315	7.08E-104	4.14E-103
SLC16A1-AS1	2.60286965	2.04E-91	9.31E-91
RP11-31F15.	Inf	1.14E-25	1.93E-25
LRIG2	3.74513619	4.11E-118	3.43E-117
MAGI3	2.39688716	3.39E-82	1.31E-81
PHTF1	2.13472763	5.60E-07	6.93E-07
RSBN1	1.80234679	0.70075908	0.71142536
RP5-1073O3.	NA	NA	NA
PTPN22	1.94747082	6.46E-15	9.31E-15
AP4B1-AS1	NA	1.82E-22	2.94E-22
BCL2L15	Inf	0.00014764	0.00017231
AP4B1	0.61284047	0.12613395	0.13400024
DCLRE1B	2.24708171	0.00041427	0.00047815
HIPK1-AS1	0	6.47E-51	1.56E-50
HIPK1	1.75858569	6.54E-13	9.09E-13
OLFML3	NA	NA	NA
SYT6	NA	NA	NA

RP4-543J13.1	NA	NA	NA
TRIM33	2.29701686	5.01E-139	1.26E-137
BCAS2	1.24011132	6.08E-125	6.50E-124
DENND2C	3.74513619	1.10E-05	1.33E-05
AMPD1	NA	NA	NA
NRAS	2.24708171	6.28E-12	8.59E-12
CSDE1	2.16671398	2.62E-118	2.20E-117
SIKE1	1.68828361	1.02E-12	1.42E-12
SYCP1	NA	NA	NA
TSHB	NA	NA	NA
TSPAN2	Inf	9.16E-60	2.51E-59
RP4-666F24.	NA	NA	NA
RP4-663N10.	NA	NA	NA
NGF	NA	NA	NA
VANGL1	1.89753567	0.4519854	0.4649621
CASQ2	NA	NA	NA
NHLH2	NA	NA	NA
RP11-12L8.1	NA	NA	NA
SLC22A15	3.49546044	9.05E-12	1.23E-11
RP11-159M1	NA	NA	NA
MAB21L3	NA	1.09E-25	1.84E-25
RP5-1185H1	NA	NA	NA
ATP1A1	2.00045079	1.33E-22	2.16E-22
RP4-655J12.4	NA	4.23E-95	2.07E-94
RP4-655J12.5	NA	NA	NA
RP5-1086K13	Inf	0.37022748	0.38321829
CD58	0.69312968	8.13E-145	5.14E-143
IGSF3	1.87256809	0.00774782	0.00861597
C1orf137	NA	NA	NA
CD2	NA	5.77E-98	2.99E-97
RP4-753F5.1	2.99610895	0.00049916	0.00057457
PTGFRN	3.30820363	5.01E-88	2.15E-87
CD101	0.74902724	0.00753328	0.00838253
RP11-27K13.	NA	6.01E-07	7.43E-07
TTF2	1.91131088	0.70191362	0.712438
TRIM45	0.74902724	3.34E-40	6.94E-40
VTCN1	NA	NA	NA
LINC01525	NA	NA	NA
RP11-188D8.	Inf	7.38E-148	8.47E-146
MAN1A2	1.7726978	3.58E-13	5.00E-13
AL157902.3	NA	7.72E-97	3.92E-96
FAM46C	3.74513619	2.39E-55	6.14E-55
GDAP2	2.03307393	8.59E-42	1.83E-41
WDR3	1.43117704	2.23E-38	4.51E-38
SPAG17	NA	NA	NA

TBX15	NA	NA	NA
RP4-712E4.1	NA	NA	NA
RP4-712E4.2	NA	NA	NA
WARS2	1.34036453	2.37E-103	1.37E-102
WARS2-IT1	NA	NA	NA
RP11-418J17	2.24708171	1.36E-82	5.31E-82
RP11-418J17	NA	NA	NA
RP5-834N19.	NA	NA	NA
HAO2	NA	NA	NA
HSD3B2	NA	NA	NA
HSD3B1	NA	NA	NA
ZNF697	4.49416342	6.07E-08	7.66E-08
PHGDH	0.97303538	3.73E-135	6.72E-134
HMGCS2	NA	NA	NA
REG4	NA	NA	NA
ADAM30	NA	NA	NA
NOTCH2	1.87256809	6.63E-16	9.70E-16
RP11-114O1	NA	2.40E-11	3.24E-11
SEC22B	2.44419414	4.24E-63	1.22E-62
AC253572.1	1.24837873	2.94E-08	3.74E-08
NBPF26	Inf	6.52E-96	3.24E-95
PPIAL4A	NA	NA	NA
AC241377.2	NA	NA	NA
LINC00623	7.49027237	1.21E-05	1.45E-05
CH17-118O6.	NA	NA	NA
FCGR1B	0.93628405	8.25E-119	7.03E-118
RP11-439A1	NA	NA	NA
RP11-439A1	Inf	6.97E-94	3.33E-93
RP11-439A1	NA	NA	NA
FAM72B	4.86867704	2.94E-47	6.73E-47
SRGAP2C	1.41482923	7.48E-05	8.80E-05
SRGAP2-AS1	NA	NA	NA
CH17-437K3.	NA	NA	NA
RP11-343N1	NA	NA	NA
RP11-344P1	NA	4.78E-09	6.18E-09
RP11-344P1	Inf	2.40E-40	4.99E-40
RP11-344P1	NA	NA	NA
RP11-763B2	NA	NA	NA
RP11-403I13	NA	1.49E-17	2.24E-17
RP11-403I13	NA	2.28E-20	3.58E-20
RP5-998N21.	NA	NA	NA
RP5-998N21.	NA	NA	NA
HIST2H3PS2	NA	NA	NA
FAM72C	2.17898833	4.65E-19	7.16E-19
IGKV1OR1-1	NA	NA	NA

RP11-14N7.2	2.38752432	1.21E-74	4.16E-74
PPIAL4E	NA	NA	NA
RP11-763B22	0.74902724	1.82E-25	3.08E-25
NBPF15	5.9922179	3.84E-91	1.74E-90
PPIAL4F	NA	NA	NA
CH17-125A10	NA	NA	NA
RP11-289H16	NA	0.02164078	0.0236767
SRGAP2B	1.1770428	0.04259651	0.04611995
FAM72D	1.37978702	4.30E-21	6.83E-21
AC241585.2	NA	NA	NA
PPIAL4D	NA	NA	NA
CH17-373J23	1.49805447	1.75E-29	3.14E-29
RP11-89F3.2	0	7.77E-27	1.34E-26
NBPF20	0.49935149	0.37475291	0.38770343
GPR89A	1.28999135	7.99E-15	1.15E-14
PDZK1	NA	NA	NA
CD160	NA	7.85E-117	6.31E-116
RNF115	1.68531128	2.09E-26	3.59E-26
POLR3C	1.31079767	1.54E-96	7.79E-96
NUDT17	1.62289235	3.27E-12	4.49E-12
PIAS3	2.67509728	7.13E-59	1.92E-58
ANKRD35	1.75553259	2.91E-08	3.70E-08
ITGA10	2.99610895	1.43E-74	4.90E-74
RP11-315I20	NA	NA	NA
PEX11B	1.78339818	4.75E-09	6.15E-09
RBM8A	1.29095017	2.19E-97	1.13E-96
RP11-315I20	0	1.54E-95	7.62E-95
CH17-270A2.	NA	NA	NA
LIX1L	1.99027237	3.99E-75	1.38E-74
ANKRD34A	0	6.31E-10	8.30E-10
CH17-270A2.	NA	3.18E-27	5.51E-27
POLR3GL	2.17641877	7.06E-15	1.02E-14
TXNIP	1.40993362	6.01E-125	6.42E-124
HFE2	NA	NA	NA
RP11-458D21	NA	1.11E-29	1.99E-29
NBPF10	0.74902724	8.80E-86	3.62E-85
RP11-458D21	NA	NA	NA
NOTCH2NL	NA	1.20E-24	2.00E-24
CH17-353B19	Inf	4.44E-108	2.84E-107
AC243756.1	NA	NA	NA
CH17-408M7	NA	NA	NA
NBPF12	Inf	3.02E-40	6.30E-40
RP4-704D21.	NA	NA	NA
PRKAB2	7.86478599	2.50E-73	8.42E-73
RP11-337C18	0	1.27E-40	2.65E-40

FMO5	0	3.90E-86	1.61E-85
CHD1L	1.40442607	1.00E-05	1.20E-05
LINC00624	NA	1.55E-80	5.81E-80
BCL9	2.99610895	0.23670696	0.24796961
ACP6	1.72276265	5.00E-83	1.96E-82
RP11-533N1	NA	NA	NA
GJA5	NA	NA	NA
RP11-433J22	NA	NA	NA
RP11-433J22	NA	NA	NA
GJA8	NA	NA	NA
GPR89B	1.24837873	0.03302879	0.03591515
RP6-74O6.2	NA	NA	NA
XXyac-YX155	Inf	0.74626995	0.75568787
RP11-94I2.4	Inf	6.27E-27	1.08E-26
NBPF11	1.49805447	0.00046582	0.00053691
RP4-565E6.1	NA	NA	NA
XXyac-YX155	NA	8.37E-60	2.29E-59
RP11-495P1(	NA	NA	NA
RP11-495P1(	NA	NA	NA
RP11-495P1(	NA	NA	NA
RP6-74O6.6	NA	NA	NA
LINC01138	1.94747082	0.00038329	0.00044288
RP11-495P1(	Inf	3.72E-13	5.19E-13
AC245100.1	Inf	1.44E-13	2.02E-13
PPIAL4G	NA	NA	NA
NBPF14	3.99481193	0.86959012	0.87552606
PDE4DIP	6.59143969	2.16E-144	1.25E-142
RP4-791M13	NA	NA	NA
RP4-791M13	NA	NA	NA
RP4-791M13	NA	NA	NA
NBPF9	Inf	3.37E-78	1.22E-77
CH17-189H2(	3.45979248	5.35E-123	5.30E-122
RP11-326G2	0	1.02E-23	1.68E-23
NBPF19	1.71206226	2.26E-50	5.41E-50
PPIAL4C	NA	NA	NA
LINC00869	0.92526894	3.95E-103	2.27E-102
RP11-353N4.	NA	NA	NA
RP11-353N4.	NA	NA	NA
FAM231D	1.49805447	2.80E-69	8.89E-69
FCGR1A	0.37451362	4.77E-144	2.56E-142
HIST2H2BF	Inf	7.19E-114	5.35E-113
RP11-196G1	NA	NA	NA
HIST2H3D	0	0.85132871	0.85785411
HIST2H4A	NA	NA	NA
HIST2H3C	NA	NA	NA

HIST2H2AA3	NA	NA	NA
HIST2H2BE	Inf	2.42E-55	6.23E-55
RP11-196G1‹	2.99610895	6.06E-17	9.01E-17
HIST2H2AA4	NA	2.40E-83	9.44E-83
HIST2H3A	NA	NA	NA
HIST2H4B	NA	2.94E-81	1.12E-80
RP11-196G1‹	2.24708171	2.37E-85	9.66E-85
HIST2H2AC	0.99870298	2.28E-25	3.86E-25
HIST2H2AB	NA	0.15802963	0.16689799
BOLA1	NA	NA	NA
SV2A	NA	1.16E-48	2.72E-48
SF3B4	1.0052734	2.72E-135	4.96E-134
MTMR11	Inf	7.46E-05	8.78E-05
OTUD7B	1.68531128	9.79E-06	1.18E-05
VPS45	2.24708171	9.81E-12	1.34E-11
PLEKHO1	2.54971898	3.26E-08	4.15E-08
ANP32E	1.30867778	3.88E-65	1.15E-64
RP4-790G17.	NA	0.00048233	0.00055541
CA14	NA	4.72E-63	1.35E-62
APH1A	1.63563091	0.99161227	0.99232334
C1orf54	1.87256809	7.13E-52	1.75E-51
CIART	NA	7.54E-79	2.75E-78
MRPS21	1.80034784	2.40E-07	3.00E-07
AC242988.1	NA	NA	NA
PRPF3	1.49805447	6.99E-22	1.12E-21
RPRD2	1.31079767	0.54816022	0.56134132
TARS2	1.87256809	0.00758321	0.00843705
ECM1	0.37451362	0.00011128	0.0001303
FALEC	0	3.31E-07	4.12E-07
RP11-54A4.2	NA	2.65E-67	8.14E-67
ADAMTSL4	2.49675746	1.91E-18	2.92E-18
MCL1	1.75157139	9.33E-14	1.32E-13
ENSA	1.62155575	7.96E-65	2.36E-64
GOLPH3L	1.54798962	1.26E-90	5.67E-90
HORMAD1	NA	8.24E-21	1.30E-20
CTSS	0.70741461	7.30E-141	2.29E-139
CTSK	0.74902724	2.66E-64	7.80E-64
ARNT	1.8385214	9.20E-11	1.23E-10
SETDB1	2.39688716	0.06082615	0.06545513
CERS2	1.97743191	2.62E-89	1.15E-88
RP11-316M1	Inf	4.71E-132	7.13E-131
RP11-316M1	NA	3.74E-52	9.22E-52
ANXA9	Inf	1.01E-05	1.22E-05
FAM63A	2.24708171	0.02200211	0.02406187
PRUNE	2.78210117	2.54E-26	4.35E-26

BNIPL	Inf	4.18E-42	8.91E-42
C1orf56	1.12354086	4.41E-21	7.00E-21
CDC42SE1	2.25860521	1.45E-14	2.07E-14
MLLT11	1.99173152	4.24E-18	6.43E-18
GABPB2	1.44043699	4.50E-20	7.02E-20
SEMA6C	0.74902724	4.40E-42	9.38E-42
TNFAIP8L2	1.05475264	2.75E-138	6.35E-137
SCNM1	1.55961836	1.94E-67	5.97E-67
LYSMD1	Inf	0.00585895	0.0065383
TMOD4	NA	9.82E-60	2.68E-59
VPS72	1.29761057	2.47E-122	2.39E-121
PIP5K1A	0.64915694	1.89E-106	1.17E-105
PSMD4	1.16735188	2.11E-128	2.63E-127
RP11-126K1.	3.37062257	9.09E-34	1.73E-33
ZNF687	3.12094682	5.66E-10	7.45E-10
PI4KB	2.30058366	7.17E-77	2.53E-76
RP11-126K1.	NA	NA	NA
RFX5	1.77893969	8.38E-06	1.01E-05
RP11-126K1.	NA	4.99E-61	1.39E-60
RP11-126K1.	2.85992218	2.50E-92	1.16E-91
SELENBP1	NA	NA	NA
PSMB4	1.44187743	2.09E-86	8.68E-86
POGZ	1.84760052	1.68E-76	5.89E-76
CGN	1.74773022	2.89E-28	5.08E-28
TUFT1	4.49416342	1.85E-39	3.81E-39
RP11-404E16	NA	NA	NA
SNX27	1.87256809	0.00021204	0.0002465
RP11-98D18.	NA	NA	NA
CELF3	NA	NA	NA
RIIAD1	NA	NA	NA
RP11-98D18.	NA	9.03E-88	3.85E-87
MRPL9	1.30726452	2.54E-92	1.18E-91
OAZ3	3.1459144	2.71E-13	3.80E-13
RP11-98D18.	NA	NA	NA
RP11-98D18.	NA	NA	NA
TDRKH	1.24837873	1.69E-05	2.02E-05
RP11-98D18.	1.49805447	1.06E-10	1.41E-10
RP11-98D18.	NA	9.25E-135	1.64E-133
LINGO4	NA	NA	NA
RORC	NA	NA	NA
C2CD4D	NA	NA	NA
AL450992.2	NA	NA	NA
THEM5	NA	NA	NA
THEM4	2.11919901	1.34E-99	7.15E-99
S100A10	1.4662936	1.17E-83	4.63E-83

AL591893.1	NA	NA	NA
S100A11	1.25512672	9.00E-102	5.03E-101
TCHHL1	NA	NA	NA
TCHH	NA	1.82E-43	3.94E-43
AL589986.2	NA	NA	NA
RPTN	NA	NA	NA
FLG-AS1	NA	NA	NA
RP11-107M1	NA	NA	NA
HRNR	NA	NA	NA
FLG	NA	NA	NA
FLG2	NA	9.03E-88	3.85E-87
CRNN	NA	NA	NA
LCE5A	NA	NA	NA
LCE3C	NA	NA	NA
AL135842.1	NA	NA	NA
CRCT1	NA	NA	NA
LCE3E	NA	NA	NA
LCE3D	NA	NA	NA
LCE3B	NA	NA	NA
LCE3A	NA	NA	NA
LINC00302	NA	NA	NA
LCE2D	NA	NA	NA
LCE2C	NA	NA	NA
LCE2B	NA	NA	NA
LCE2A	NA	NA	NA
LCE4A	NA	NA	NA
C1orf68	NA	NA	NA
KPRP	NA	NA	NA
LCE1F	NA	NA	NA
LCE1E	NA	NA	NA
LCE1D	NA	NA	NA
LCE1C	NA	NA	NA
LCE1B	NA	NA	NA
LCE1A	NA	NA	NA
LCE6A	NA	NA	NA
SMCP	NA	NA	NA
IVL	NA	NA	NA
LINC01527	NA	NA	NA
SPRR4	NA	NA	NA
SPRR1A	NA	NA	NA
SPRR3	NA	NA	NA
SPRR1B	NA	NA	NA
SPRR2D	NA	NA	NA
SPRR2A	NA	NA	NA
SPRR2B	NA	NA	NA

SPRR2E	NA	NA	NA
SPRR2F	NA	NA	NA
SPRR2G	NA	NA	NA
RP1-140J1.1	NA	NA	NA
LELP1	NA	NA	NA
PRR9	NA	NA	NA
LOR	NA	NA	NA
PGLYRP3	NA	NA	NA
PGLYRP4	NA	NA	NA
S100A9	0	1.44E-134	2.52E-133
S100A12	NA	NA	NA
S100A8	NA	6.23E-43	1.34E-42
S100A7A	NA	NA	NA
S100A7L2	NA	NA	NA
S100A7	NA	NA	NA
BX470102.3	NA	NA	NA
S100A6	NA	1.49E-86	6.22E-86
S100A5	0.74902724	1.73E-09	2.26E-09
S100A4	1.06961089	3.17E-139	8.19E-138
S100A3	NA	1.83E-92	8.55E-92
S100A2	3.74513619	5.08E-38	1.02E-37
S100A16	NA	NA	NA
S100A14	NA	0.08618533	0.09219188
S100A13	1.58950547	3.62E-112	2.58E-111
S100A1	NA	1.16E-09	1.52E-09
RP1-178F15.	1.49805447	7.59E-06	9.17E-06
CHTOP	1.82665997	4.31E-98	2.24E-97
SNAPIN	1.86476573	3.12E-08	3.96E-08
ILF2	0.93126183	4.01E-110	2.71E-109
NPR1	NA	NA	NA
INTS3	2.76203794	3.80E-44	8.31E-44
RP11-216N1	NA	NA	NA
RP11-216N1	NA	NA	NA
AL513523.2	NA	1.89E-21	3.01E-21
SLC27A3	0.74902724	1.83E-74	6.26E-74
GATAD2B	2.19714656	2.07E-126	2.37E-125
DENND4B	1.71206226	3.25E-29	5.79E-29
CRTC2	0.49935149	6.60E-05	7.78E-05
SLC39A1	1.86492496	0.35281803	0.36565694
RP11-422P2	2.24708171	0.00045254	0.00052173
RP11-422P2	NA	3.96E-112	2.82E-111
CREB3L4	0.79896239	2.96E-130	4.03E-129
JTB	2.2709144	4.08E-142	1.50E-140
RP11-422P2	1.87256809	6.65E-07	8.22E-07
RAB13	1.25198415	7.33E-143	3.10E-141

RPS27	2.47558781	1.74E-144	1.03E-142
NUP210L	NA	1.29E-84	5.22E-84
TPM3	1.56916466	1.86E-54	4.74E-54
C1orf189	NA	NA	NA
C1orf43	1.9189954	0.76719832	0.77605307
UBAP2L	1.61951835	0.25649351	0.26814022
HAX1	1.56463467	6.01E-30	1.08E-29
AQP10	NA	4.09E-129	5.28E-128
ATP8B2	1.12354086	7.65E-05	9.00E-05
RP11-350G8.	NA	1.35E-33	2.56E-33
IL6R	1.02991245	1.87E-41	3.97E-41
SHE	NA	NA	NA
RP11-350G8.	NA	NA	NA
TDRD10	NA	NA	NA
UBE2Q1	2.44037906	1.02E-11	1.39E-11
UBE2Q1-AS1	NA	NA	NA
CHRNB2	NA	NA	NA
RP11-61L14.	NA	NA	NA
ADAR	2.27291024	3.83E-117	3.11E-116
KCNN3	NA	2.30E-86	9.54E-86
PMVK	1.88769996	1.36E-50	3.25E-50
RP11-307C12	NA	6.70E-38	1.35E-37
PBXIP1	0.56177043	8.48E-51	2.04E-50
PYGO2	1.87256809	1.69E-05	2.02E-05
RP11-307C12	1.31079767	0.0036604	0.00411061
SHC1	2.8463035	2.44E-110	1.65E-109
CKS1B	1.37207392	1.08E-107	6.85E-107
FLAD1	1.80515564	2.34E-18	3.56E-18
LENEP	0	2.21E-06	2.70E-06
ZBTB7B	1.04031561	3.73E-22	6.01E-22
DCST2	NA	1.44E-124	1.51E-123
DCST1	NA	NA	NA
RP11-307C12	0.58257674	4.54E-62	1.29E-61
ADAM15	1.67039702	3.12E-68	9.75E-68
EFNA4	1.10273454	1.53E-130	2.13E-129
RP11-540D14	NA	NA	NA
EFNA3	1.61886532	2.97E-60	8.22E-60
EFNA1	2.49675746	0.30283606	0.31517073
SLC50A1	1.18468594	2.30E-31	4.21E-31
DPM3	1.88347626	1.18E-84	4.76E-84
KRTCAP2	1.16249027	1.28E-141	4.45E-140
RP11-201K10	NA	NA	NA
TRIM46	NA	8.68E-72	2.85E-71
MUC1	1.87256809	6.71E-18	1.01E-17
AC234582.2	NA	NA	NA

AC234582.1	NA	NA	NA
RP11-263K19	0	0.00370187	0.00415642
THBS3	1.87256809	5.26E-74	1.79E-73
MTX1	1.11472877	2.80E-140	8.11E-139
RP11-263K19	NA	8.59E-25	1.44E-24
GBA	1.46060311	7.04E-09	9.08E-09
FAM189B	2.08745296	5.10E-08	6.45E-08
SCAMP3	1.36812118	6.71E-72	2.21E-71
CLK2	2.43433852	0.00017563	0.00020456
HCN3	3.74513619	1.35E-42	2.90E-42
PKLR	NA	NA	NA
FDPS	0.87510113	3.78E-143	1.68E-141
RUSC1-AS1	NA	1.69E-29	3.03E-29
RUSC1	1.74773022	8.05E-06	9.72E-06
ASH1L	2.62159533	1.12E-78	4.07E-78
ASH1L-AS1	6.17947471	7.94E-127	9.22E-126
RP11-29H23.	NA	0.05255704	0.05667775
MSTO1	1.02991245	3.68E-61	1.03E-60
YY1AP1	1.64785992	2.25E-09	2.93E-09
DAP3	1.13524441	3.68E-141	1.19E-139
RP11-243J18	NA	NA	NA
GON4L	1.46684501	2.23E-20	3.50E-20
SYT11	Inf	5.50E-145	3.61E-143
RIT1	0.74902724	1.66E-40	3.47E-40
KIAA0907	2.23147698	1.99E-103	1.15E-102
RXFP4	NA	NA	NA
ARHGEF2	1.77150886	2.08E-65	6.22E-65
RP11-336K24	2.24708171	3.61E-57	9.50E-57
RP11-336K24	NA	1.13E-120	1.03E-119
SSR2	1.3959144	3.67E-107	2.30E-106
UBQLN4	2.36789256	7.72E-41	1.62E-40
LAMTOR2	1.86616615	0.36012851	0.37293518
RAB25	Inf	6.49E-21	1.03E-20
MEX3A	1.60505837	8.14E-90	3.60E-89
LMNA	0.82151374	3.70E-106	2.26E-105
SEMA4A	NA	NA	NA
SLC25A44	1.42996109	1.78E-05	2.13E-05
PMF1-BGLAP	NA	2.54E-21	4.05E-21
PMF1	1.43117704	1.86E-105	1.12E-104
BGLAP	NA	6.16E-08	7.77E-08
PAQR6	1.24837873	1.57E-64	4.63E-64
SMG5	1.58617533	2.48E-49	5.84E-49
TMEM79	4.11964981	2.82E-33	5.31E-33
GLMP	NA	3.65E-49	8.58E-49
VHLL	Inf	8.37E-60	2.29E-59

CCT3	1.05310481	5.92E-126	6.63E-125
TSACC	1.99740597	1.25E-06	1.53E-06
RHBG	NA	NA	NA
RP11-139I14	NA	NA	NA
C1orf61	0.84265564	3.43E-33	6.44E-33
RP11-98G7.1	0	0.76472162	0.77363406
MEF2D	2.07422927	1.29E-30	2.35E-30
RP11-284F21	NA	2.64E-126	3.00E-125
IQGAP3	3.74513619	1.19E-14	1.70E-14
TTC24	NA	NA	NA
APOA1BP	1.36524823	1.54E-81	5.90E-81
GPATCH4	1.85186634	2.07E-13	2.90E-13
AL365181.2	0.37451362	0.04587993	0.04960391
RP11-284F21	NA	NA	NA
HAPLN2	NA	NA	NA
RP11-284F21	NA	5.51E-06	6.67E-06
BCAN	Inf	9.72E-57	2.55E-56
RP11-284F21	1.49805447	4.45E-25	7.49E-25
RP11-284F21	NA	2.89E-57	7.64E-57
NES	NA	1.73E-48	4.02E-48
RP11-66D17.	NA	1.22E-58	3.26E-58
CRABP2	NA	0.00335483	0.00377236
RP11-66D17.	NA	NA	NA
ISG20L2	2.2559987	6.11E-29	1.09E-28
RRNAD1	1.32181277	1.57E-55	4.05E-55
MRPL24	1.36090864	2.35E-113	1.73E-112
HDGF	1.66269573	0.27237955	0.28433798
PRCC	1.77769131	3.17E-32	5.89E-32
SH2D2A	Inf	2.49E-18	3.79E-18
NTRK1	1.49805447	0.00402033	0.0045084
INSRR	NA	NA	NA
PEAR1	NA	1.32E-73	4.47E-73
LRRC71	NA	NA	NA
ARHGEF11	1.87256809	0.21532113	0.2260361
ETV3L	NA	NA	NA
ETV3	2.24708171	2.04E-35	3.96E-35
RP11-71G12.	NA	NA	NA
RP11-85G21.	NA	NA	NA
RP11-85G21.	NA	NA	NA
RP11-85G21.	NA	NA	NA
FCRL5	NA	NA	NA
FCRL4	NA	NA	NA
FCRL3	NA	NA	NA
RP11-367J7.3	NA	NA	NA
FCRL2	NA	NA	NA

FCRL1	NA	NA	NA
CD5L	NA	NA	NA
KIRREL	2.99610895	2.17E-21	3.46E-21
RP11-404O1:	NA	NA	NA
CD1D	NA	2.36E-17	3.54E-17
RP11-404O1:	NA	NA	NA
CD1A	NA	NA	NA
CD1C	NA	NA	NA
CD1B	NA	NA	NA
CD1E	NA	NA	NA
OR10T2	NA	NA	NA
OR10K2	NA	NA	NA
OR10K1	NA	NA	NA
RP11-144L1.4	NA	NA	NA
OR10R2	NA	NA	NA
OR6Y1	NA	NA	NA
OR6P1	NA	NA	NA
OR10X1	NA	NA	NA
OR10Z1	NA	NA	NA
SPTA1	Inf	7.30E-81	2.75E-80
OR6K2	NA	NA	NA
OR6K3	NA	NA	NA
OR6K6	NA	NA	NA
OR6N1	NA	NA	NA
OR6N2	NA	NA	NA
MNDA	0	5.10E-146	5.29E-144
PYHIN1	NA	2.02E-07	2.52E-07
IFI16	1.28059495	5.15E-22	8.28E-22
AIM2	NA	NA	NA
CADM3	NA	NA	NA
CADM3-AS1	NA	NA	NA
ACKR1	NA	NA	NA
FCER1A	NA	NA	NA
OR10J3	NA	NA	NA
RP11-550P17	NA	NA	NA
OR10J1	NA	NA	NA
RP11-180D2:	NA	NA	NA
OR10J5	NA	NA	NA
APCS	NA	NA	NA
CRP	NA	NA	NA
DUSP23	1.56614786	7.91E-55	2.02E-54
FCRL6	NA	NA	NA
SLAMF8	NA	NA	NA
C1orf204	NA	NA	NA
VSIG8	NA	NA	NA

RP11-190A1?	NA	NA	NA
RP11-190A1?	NA	NA	NA
CFAP45	0	1.43E-10	1.91E-10
TAGLN2	1.33206506	5.31E-125	5.69E-124
IGSF9	NA	5.26E-29	9.35E-29
SLAMF9	NA	NA	NA
LINC01133	NA	7.60E-105	4.52E-104
PIGM	2.04542053	4.26E-107	2.66E-106
KCNJ10	Inf	1.30E-67	4.02E-67
RP11-536C5.	NA	NA	NA
KCNJ9	NA	NA	NA
IGSF8	1.64785992	2.45E-23	4.02E-23
ATP1A2	NA	4.85E-25	8.16E-25
ATP1A4	NA	NA	NA
CASQ1	NA	NA	NA
RP11-536C5.	Inf	0.07458713	0.08000708
PEA15	1.84135863	1.66E-37	3.32E-37
DCAF8	1.28999135	1.78E-08	2.27E-08
RP11-574F21	NA	1.99E-52	4.91E-52
RP11-574F21	NA	7.64E-09	9.84E-09
PEX19	1.7183566	0.00109455	0.00124873
COPA	1.22267681	3.01E-69	9.53E-69
NCSTN	1.25972763	1.38E-40	2.89E-40
NHLH1	Inf	9.29E-32	1.71E-31
VANGL2	1.74773022	5.02E-52	1.23E-51
SLAMF6	Inf	0.01861601	0.02042032
RP11-528G1.	NA	NA	NA
CD84	2.99610895	1.95E-68	6.09E-68
SLAMF1	Inf	3.74E-62	1.06E-61
RP11-404F1(	NA	6.92E-139	1.72E-137
CD48	1.91418072	0.75474064	0.76396277
SLAMF7	NA	NA	NA
LY9	NA	NA	NA
CD244	1.94747082	1.51E-07	1.89E-07
ITLN1	NA	2.40E-65	7.15E-65
RP11-312J18	NA	1.77E-93	8.42E-93
RP11-544M2	NA	NA	NA
ITLN2	NA	NA	NA
F11R	1.55567195	1.29E-09	1.69E-09
RP11-544M2	NA	NA	NA
TSTD1	Inf	7.75E-16	1.13E-15
USF1	1.49805447	5.26E-07	6.51E-07
ARHGAP30	1.73881323	8.37E-06	1.01E-05
AL591806.1	NA	NA	NA
PVRL4	NA	NA	NA

RP11-544M2	NA	NA	NA
KLHDC9	NA	NA	NA
PFDN2	1.70532686	0.74352403	0.75307129
NIT1	1.08192823	2.48E-119	2.16E-118
DEDD	2.17574578	0.07757079	0.0831633
UFC1	1.55788801	1.10E-69	3.51E-69
RP11-297K8.	Inf	6.94E-07	8.56E-07
USP21	4.49416342	0.02731344	0.02978066
PPOX	1.60505837	8.85E-76	3.08E-75
B4GALT3	1.66574714	6.13E-13	8.52E-13
ADAMTS4	NA	1.26E-105	7.59E-105
NDUFS2	0.71335927	3.63E-144	2.00E-142
FCER1G	1.19844358	4.59E-114	3.43E-113
APOA2	NA	0.3365742	0.34926095
TOMM40L	2.12224384	0.00130833	0.00148775
NR1I3	Inf	8.00E-130	1.07E-128
PCP4L1	NA	1.26E-13	1.77E-13
MPZ	1.56614786	2.27E-63	6.55E-63
SDHC	1.17620683	2.38E-145	1.87E-143
CFAP126	NA	NA	NA
FCGR2A	1.92607004	9.61E-59	2.59E-58
HSPA6	0	5.73E-09	7.41E-09
FCGR3A	NA	3.03E-46	6.83E-46
RP11-5K23.5	NA	NA	NA
FCGR3B	NA	NA	NA
FCGR2B	0.49935149	5.73E-74	1.94E-73
RP11-25K21.	NA	NA	NA
FCRLA	NA	NA	NA
FCRLB	1.24837873	0.00038252	0.00044202
DUSP12	1.2733463	2.49E-47	5.71E-47
RP11-474I16	NA	2.17E-108	1.40E-107
ATF6	1.42996109	3.87E-15	5.59E-15
OLFML2B	NA	2.32E-16	3.42E-16
RP11-227F8.	NA	NA	NA
NOS1AP	NA	2.62E-42	5.60E-42
RP11-565P22	NA	NA	NA
RP11-565P22	NA	NA	NA
C1orf226	NA	4.58E-34	8.74E-34
C1orf111	NA	NA	NA
SH2D1B	NA	NA	NA
UHMK1	1.65693904	1.27E-12	1.76E-12
RP11-359K18	NA	NA	NA
UAP1	1.80145791	0.06471201	0.06957481
DDR2	NA	8.12E-79	2.96E-78
HSD17B7	0.79896239	8.46E-138	1.87E-136

CCDC190	NA	NA	NA
RGS4	NA	NA	NA
RGS5	0.74902724	5.30E-72	1.75E-71
RP11-267N1:	NA	NA	NA
RGS5	1.19844358	3.67E-25	6.19E-25
RP11-77M5.:	NA	NA	NA
NUF2	0.76110832	5.35E-116	4.20E-115
PBX1	Inf	2.44E-91	1.11E-90
RP11-506O2	NA	NA	NA
LMX1A	NA	NA	NA
RP11-38C18.	NA	NA	NA
RP11-38C18.	NA	NA	NA
RXRG	NA	NA	NA
RP11-280O1	NA	NA	NA
LRRC52	NA	NA	NA
RP11-306I1.2	NA	7.60E-123	7.49E-122
MGST3	1.30315453	1.52E-137	3.29E-136
ALDH9A1	1.49805447	1.73E-58	4.64E-58
RP11-466F5.	Inf	1.03E-130	1.44E-129
TMCO1	1.17194738	1.64E-71	5.37E-71
RP11-466F5.	NA	1.11E-06	1.37E-06
UCK2	2.15226814	0.0017447	0.00197605
RP11-525G1:	NA	NA	NA
FAM78B	NA	NA	NA
RP11-375H1!	NA	NA	NA
RP11-479J7.2	NA	NA	NA
RP11-479J7.:	NA	NA	NA
RP11-276E17	NA	NA	NA
POGK	2.51945525	9.43E-49	2.21E-48
TADA1	1.49805447	2.54E-14	3.62E-14
ILDR2	0.89883268	0.64918153	0.66087488
MAEL	NA	1.32E-73	4.47E-73
RP11-102C16	NA	NA	NA
GPA33	NA	0.00110931	0.0012651
DUSP27	Inf	1.16E-62	3.32E-62
LINC01363	NA	NA	NA
RP11-277B1!	0.62418936	2.18E-16	3.22E-16
POU2F1	1.94747082	0.02129061	0.02331046
CD247	NA	3.50E-79	1.28E-78
RP11-104L21	NA	NA	NA
CREG1	1.27833982	4.15E-143	1.83E-141
RP3-455J7.4	0	6.23E-06	7.54E-06
RCSD1	1.62645914	4.06E-23	6.63E-23
MPZL1	1.79766537	2.92E-08	3.72E-08
ADCY10	NA	5.09E-119	4.37E-118

RP1-313L4.3	NA	5.83E-98	3.02E-97
MPC2	1.91212939	1.33E-07	1.67E-07
DCAF6	1.89597519	0.87899856	0.88450784
GPR161	1.67090691	4.07E-41	8.57E-41
TIPRL	1.34061563	1.15E-76	4.03E-76
SFT2D2	2.09727626	7.27E-67	2.22E-66
TBX19	Inf	3.48E-106	2.13E-105
RP5-968D22.	NA	2.92E-130	3.97E-129
RP5-968D22.	NA	4.51E-80	1.68E-79
XCL2	NA	6.95E-73	2.32E-72
XCL1	NA	1.64E-144	9.77E-143
DPT	NA	NA	NA
RP1-10C16.1	Inf	6.20E-84	2.47E-83
LINC00626	NA	NA	NA
LINC00970	NA	NA	NA
RP5-1018K9.	NA	NA	NA
ATP1B1	1.21716926	0.06603921	0.07096809
NME7	0.79063986	3.17E-94	1.53E-93
RP4-800F24.	NA	NA	NA
BLZF1	1.44043699	7.83E-62	2.21E-61
CCDC181	NA	2.17E-38	4.41E-38
SLC19A2	1.49805447	2.59E-23	4.24E-23
RP1-206D15.	NA	1.96E-125	2.13E-124
F5	NA	NA	NA
SELP	NA	NA	NA
C1orf112	0.65539883	1.75E-63	5.05E-63
SELL	0.18725681	1.84E-82	7.15E-82
SELE	NA	NA	NA
RP1-117P20.	NA	NA	NA
METTL18	1.67090691	1.21E-85	4.97E-85
SCYL3	1.87256809	4.23E-10	5.58E-10
KIFAP3	2.99610895	7.30E-39	1.49E-38
RP11-332H1	NA	NA	NA
METTL11B	NA	NA	NA
RP11-297H3.	NA	NA	NA
LINC01142	NA	NA	NA
RP11-576I22	NA	NA	NA
GORAB	3.2457847	4.77E-46	1.07E-45
RP11-545I10	NA	NA	NA
RP1-79C4.1	NA	NA	NA
PRRX1	NA	NA	NA
RP1-79C4.4	NA	NA	NA
MROH9	NA	NA	NA
FMO3	NA	NA	NA
FMO2	NA	NA	NA

RP1-127D3.4	NA	NA	NA
RP1-45C12.1	NA	NA	NA
FMO1	NA	NA	NA
FMO4	NA	3.52E-09	4.57E-09
PRRC2C	1.69810523	1.62E-59	4.43E-59
MYOC	NA	NA	NA
VAMP4	1.1770428	1.18E-05	1.42E-05
METTL13	2.24708171	0.01180314	0.01304255
DNM3	0.37451362	3.88E-14	5.51E-14
DNM3OS	NA	NA	NA
PIGC	1.9683739	4.45E-14	6.31E-14
C1orf105	NA	NA	NA
SUCO	1.59168288	0.0043795	0.00490662
FASLG	NA	NA	NA
RP1-15D23.2	NA	NA	NA
RP3-471M13	NA	NA	NA
TNFSF18	NA	NA	NA
RP3-395P12.	NA	NA	NA
TNFSF4	Inf	1.17E-100	6.35E-100
RP11-296O1	3.74513619	0.01515377	0.01667797
PRDX6	1.08752044	1.19E-141	4.16E-140
SLC9C2	NA	NA	NA
RP3-436N22.	NA	NA	NA
ANKRD45	0	3.76E-15	5.44E-15
RP11-360D2.	NA	NA	NA
KLHL20	1.3732166	0.00042663	0.00049221
CENPL	2.11089494	5.82E-07	7.19E-07
DARS2	1.99740597	1.94E-84	7.80E-84
GAS5-AS1	NA	3.92E-14	5.57E-14
ZBTB37	1.12354086	8.65E-29	1.53E-28
SERPINC1	NA	2.00E-44	4.40E-44
RC3H1	1.57689945	1.09E-60	3.03E-60
RP11-160H2.	NA	NA	NA
RP11-160H2.	Inf	9.70E-127	1.12E-125
RABGAP1L	2.3540856	8.01E-05	9.42E-05
GPR52	NA	NA	NA
RP1-102G20.	NA	NA	NA
CACYBP	0.92809796	1.87E-129	2.45E-128
MRPS14	1.56493191	5.18E-60	1.42E-59
TNN	NA	NA	NA
KIAA0040	3.74513619	7.86E-34	1.49E-33
RP5-1114G2.	NA	NA	NA
RP3-518E13.	NA	NA	NA
TNR	NA	NA	NA
RP11-222A5.	NA	NA	NA

RP11-318C24	NA	2.59E-13	3.63E-13
RFWD2	1.61154345	0.00977616	0.01082581
RP11-492I21	NA	NA	NA
RP11-195C7.	NA	NA	NA
PAPPA2	NA	NA	NA
ASTN1	NA	NA	NA
BRINP2	NA	NA	NA
RP1-35C21.1	NA	NA	NA
RP1-35C21.2	NA	NA	NA
RP11-63B19.	NA	NA	NA
SEC16B	NA	5.81E-73	1.94E-72
RASAL2-AS1	Inf	3.81E-47	8.70E-47
RASAL2	2.87127108	2.02E-12	2.78E-12
RP4-593C16.	NA	NA	NA
LINC00083	NA	NA	NA
TEX35	NA	7.20E-126	8.01E-125
C1orf220	Inf	2.54E-81	9.66E-81
RP5-1098D14	0	2.72E-45	6.06E-45
RP11-428K3.	NA	NA	NA
RALGPS2	1.66450497	0.00398231	0.00446687
ANGPTL1	NA	NA	NA
FAM20B	2.62159533	4.14E-116	3.26E-115
TOR3A	1.52146158	1.46E-53	3.67E-53
ABL2	2.72645914	1.51E-111	1.06E-110
SOAT1	1.83095547	3.02E-22	4.88E-22
AXDND1	NA	9.95E-170	1.50E-166
RP11-545A1(	NA	NA	NA
NPHS2	NA	NA	NA
TDRD5	NA	NA	NA
RP11-12M5.:	NA	NA	NA
FAM163A	NA	1.89E-61	5.30E-61
RP11-12M5.:	NA	6.16E-82	2.37E-81
RP11-12M5.4	NA	NA	NA
TOR1AIP2	2.17898833	3.99E-25	6.73E-25
RP11-533E19	1.49805447	8.28E-95	4.03E-94
TOR1AIP1	1.17239046	4.01E-17	5.99E-17
RP11-533E19	NA	NA	NA
RP11-533E19	1.49805447	2.57E-43	5.56E-43
CEP350	1.94747082	3.57E-08	4.53E-08
RP11-502H18	NA	NA	NA
QSOX1	1.2291729	1.05E-122	1.03E-121
LHX4	3.74513619	6.26E-103	3.57E-102
ACBD6	1.83355626	3.71E-30	6.71E-30
OVAAL	NA	0.64647372	0.6582291
XPR1	3.55787938	2.86E-32	5.31E-32

RP11-46A10.	NA	NA	NA
KIAA1614	NA	4.23E-20	6.60E-20
RP11-46A10.	Inf	4.28E-40	8.89E-40
KIAA1614-AS	0.99870298	4.64E-17	6.92E-17
STX6	5.05593385	7.44E-130	9.95E-129
MR1	1.99740597	0.01586221	0.01744814
IER5	1.4192095	2.15E-125	2.33E-124
RP11-540K16	NA	NA	NA
RP11-540K16	NA	NA	NA
GM140	NA	NA	NA
CACNA1E	NA	NA	NA
RP11-33M22	NA	NA	NA
ZNF648	NA	NA	NA
RP11-538D16	NA	1.55E-53	3.89E-53
RP11-538D16	NA	NA	NA
GS1-122H1.1	NA	NA	NA
LINC01344	NA	NA	NA
GLUL	1.382008	1.33E-127	1.60E-126
TEDDM1	NA	NA	NA
LINC00272	NA	NA	NA
RGSL1	NA	NA	NA
RNASEL	Inf	5.80E-25	9.76E-25
RGS16	1.12354086	8.28E-88	3.54E-87
RP11-317P15	NA	NA	NA
RGS8	NA	NA	NA
RP11-317P15	NA	NA	NA
NPL	1.74773022	9.40E-06	1.13E-05
DHX9	2.19227484	4.34E-86	1.80E-85
SHCBP1L	NA	NA	NA
LAMC1	2.55917639	2.99E-14	4.25E-14
RP11-181K3.	NA	NA	NA
LAMC2	NA	NA	NA
NMNAT2	NA	NA	NA
SMG7	2.80885214	7.23E-125	7.72E-124
NCF2	NA	3.42E-82	1.32E-81
ARPC5	1.60642437	6.27E-44	1.37E-43
RGL1	NA	NA	NA
APOBEC4	NA	NA	NA
RP11-444D13	NA	NA	NA
COLGALT2	Inf	3.67E-65	1.09E-64
TSEN15	1.39272252	2.56E-34	4.90E-34
RP11-382D12	NA	NA	NA
RP11-382D12	0	4.91E-68	1.53E-67
C1orf21	0.37451362	4.36E-102	2.45E-101
EDEM3	1.87256809	0.01186417	0.01310759

FAM129A	0	8.69E-125	9.24E-124
GS1-120K12.	NA	NA	NA
RNF2	2.03735409	4.41E-46	9.93E-46
TRMT1L	0.99870298	7.04E-78	2.53E-77
SWT1	0	0.0035822	0.00402355
IVNS1ABP	2.01821228	2.56E-17	3.83E-17
GS1-279B7.2	NA	2.26E-59	6.16E-59
GS1-279B7.1	NA	NA	NA
LINC01350	NA	NA	NA
GS1-204I12.4	NA	NA	NA
HMCN1	NA	NA	NA
GS1-174L6.4	NA	NA	NA
PRG4	NA	5.01E-117	4.06E-116
TPR	1.68010971	1.23E-43	2.67E-43
C1orf27	1.61328943	0.00010696	0.00012529
AL596220.1	NA	NA	NA
OCLM	Inf	1.32E-100	7.17E-100
RP11-295K2.	NA	0.00216643	0.00244652
PDC	NA	NA	NA
GS1-304P7.3	NA	NA	NA
PTGS2	NA	NA	NA
PACERR	NA	1.65E-46	3.75E-46
PLA2G4A	2.62159533	0.76997501	0.77881839
LINC01036	NA	NA	NA
LINC01037	NA	NA	NA
RP11-445P19	NA	NA	NA
ERVMER61-1	NA	NA	NA
RP11-201A3.	NA	NA	NA
RP11-669M2	NA	NA	NA
RP11-316I3.1	NA	NA	NA
RP11-316I3.2	NA	NA	NA
LINC01035	NA	NA	NA
RP11-398M1	NA	NA	NA
RP11-445J9.1	NA	NA	NA
BRINP3	NA	1.70E-13	2.39E-13
RP11-547I7.1	NA	NA	NA
LINC01351	NA	4.97E-05	5.88E-05
RP11-161I10	NA	NA	NA
RP11-463J7.2	NA	NA	NA
RP11-309H21	NA	NA	NA
RP11-309H21	NA	NA	NA
RP11-541F9.	NA	NA	NA
RP11-541F9.	NA	NA	NA
RGS18	1.46684501	9.90E-07	1.22E-06
RGS21	NA	NA	NA

RP5-1011O1.	NA	NA	NA
RP5-1011O1.	NA	NA	NA
RGS1	NA	5.44E-132	8.17E-131
RGS13	NA	NA	NA
RGS2	1.87256809	1.79E-05	2.14E-05
LINC01032	NA	0.0003184	0.00036865
UCHL5	1.29324234	7.72E-64	2.25E-63
TROVE2	1.70233463	0.71601496	0.7263038
RP11-101E1:	2.24708171	4.66E-95	2.28E-94
GLRX2	1.61478599	0.21695964	0.22771662
CDC73	1.55567195	1.85E-24	3.09E-24
B3GALT2	NA	NA	NA
LINC01031	NA	NA	NA
RP11-21J7.1	NA	NA	NA
RP11-98G13.	NA	NA	NA
RP11-563D1(	NA	NA	NA
RP11-476H2(	NA	NA	NA
RP11-476B1.	NA	NA	NA
KCNT2	NA	NA	NA
CFH	2.24708171	1.71E-09	2.23E-09
CFHR3	NA	9.81E-71	3.17E-70
RP4-608O15.	NA	NA	NA
CFHR1	NA	3.39E-157	1.43E-154
CFHR4	NA	NA	NA
CFHR2	NA	NA	NA
CFHR5	NA	NA	NA
F13B	NA	NA	NA
ASPM	1.47389231	1.57E-33	2.97E-33
ZBTB41	Inf	0.02400066	0.02621906
CRB1	NA	NA	NA
RP11-75C23.	NA	NA	NA
DENND1B	2.8463035	1.52E-60	4.22E-60
RP11-448G4.	NA	NA	NA
C1orf53	3.18336576	8.05E-108	5.12E-107
LHX9	NA	5.76E-32	1.06E-31
NEK7	1.77893969	4.89E-21	7.76E-21
ATP6V1G3	NA	NA	NA
RP11-553K8.	NA	3.33E-76	1.16E-75
PTPRC	1.4200308	3.37E-20	5.28E-20
RP11-553K8.	NA	NA	NA
MIR181A1H(	2.11089494	1.48E-64	4.36E-64
RP11-16L9.2	NA	6.59E-30	1.19E-29
LINC01222	NA	1.69E-07	2.11E-07
LINC01221	NA	NA	NA
RP11-382E9.	NA	NA	NA

NR5A2	NA	0.64017694	0.65218385
RP11-532L16	NA	NA	NA
LINC00862	NA	NA	NA
ZNF281	1.8268957	6.01E-18	9.08E-18
RP11-469A15	0	4.35E-104	2.55E-103
RP11-121P12	NA	NA	NA
KIF14	1.69779507	1.99E-14	2.84E-14
DDX59	1.24837873	2.66E-08	3.39E-08
RP11-92G12.	Inf	4.35E-08	5.52E-08
CAMSAP2	Inf	1.29E-14	1.85E-14
GPR25	NA	NA	NA
C1orf106	NA	6.78E-33	1.27E-32
KIF21B	2.99610895	0.04451431	0.04815903
RP11-168O1(	NA	1.47E-39	3.03E-39
RP11-168O1(	NA	NA	NA
CACNA1S	NA	NA	NA
ASCL5	NA	NA	NA
TMEM9	1.82424376	0.15463131	0.16338505
IGFN1	NA	NA	NA
RP11-567E21	NA	NA	NA
PKP1	NA	NA	NA
TNNT2	NA	NA	NA
LAD1	NA	NA	NA
RP11-27G14.	NA	NA	NA
TNNI1	NA	NA	NA
PHLDA3	Inf	3.24E-30	5.87E-30
CSRP1	1.75680934	1.65E-33	3.11E-33
RP11-134G8.	1.49805447	2.77E-30	5.01E-30
RP11-134G8.	5.24319066	1.69E-117	1.39E-116
NAV1	0.74902724	2.39E-19	3.70E-19
RP11-25B7.1	NA	NA	NA
IPO9-AS1	2.49675746	5.66E-54	1.43E-53
RP11-90L20.:	NA	NA	NA
IPO9	2.10440986	1.36E-22	2.21E-22
SHISA4	0.74902724	4.93E-09	6.38E-09
RP11-307B6.	NA	NA	NA
LMOD1	NA	NA	NA
TIMM17A	1.69380026	0.3028363	0.31517073
RNPEP	1.25418514	1.27E-142	5.08E-141
RP11-465N4.	NA	1.82E-94	8.79E-94
RP11-465N4.	NA	NA	NA
ELF3	NA	2.46E-98	1.29E-97
GPR37L1	0	1.43E-119	1.26E-118
ARL8A	2.95865759	0.0334366	0.03634553
PTPN7	2.39018854	4.42E-133	7.11E-132

LGR6	NA	NA	NA
UBE2T	1.29156588	1.14E-79	4.23E-79
PPP1R12B	1.71206226	2.21E-11	2.99E-11
SYT2	NA	NA	NA
RP11-569A1:	Inf	1.09E-14	1.57E-14
RP11-569A1:	NA	NA	NA
KDM5B	1.77676228	3.83E-13	5.34E-13
RP11-480I12	Inf	7.59E-100	4.06E-99
PCAT6	2.99610895	2.14E-48	4.98E-48
RABIF	1.80399518	2.25E-16	3.33E-16
KLHL12	1.38570039	2.54E-11	3.43E-11
ADIPOR1	2.06968052	1.01E-78	3.68E-78
CYB5R1	1.57689945	2.97E-15	4.30E-15
TMEM183A	2.12053373	3.86E-65	1.15E-64
PPFIA4	Inf	1.84E-113	1.36E-112
MYOG	NA	NA	NA
ADORA1	NA	NA	NA
RP11-335O1:	NA	NA	NA
RP11-335O1:	NA	NA	NA
MYBPH	NA	6.24E-09	8.05E-09
CHI3L1	0	7.24E-65	2.15E-64
CHIT1	NA	NA	NA
LINC01353	NA	NA	NA
LINC01136	NA	2.21E-66	6.71E-66
BTG2	2.62159533	1.94E-18	2.96E-18
FMOD	NA	NA	NA
PRELP	NA	NA	NA
OPTC	NA	NA	NA
ATP2B4	1.05745022	7.02E-05	8.27E-05
LAX1	NA	3.07E-05	3.65E-05
ZC3H11A	1.39105058	0.42063424	0.43341548
ZBED6	2.99610895	3.31E-13	4.62E-13
SNRPE	1.85793303	5.06E-17	7.54E-17
LINC00303	NA	NA	NA
SOX13	NA	NA	NA
RP11-74C13.	NA	NA	NA
ETNK2	Inf	2.18E-52	5.39E-52
RP11-74C13.	NA	NA	NA
REN	NA	NA	NA
KISS1	0	4.68E-103	2.68E-102
GOLT1A	NA	NA	NA
PLEKHA6	NA	NA	NA
RP11-203F1(	NA	NA	NA
LINC00628	NA	NA	NA
RP11-739N2(	NA	NA	NA

PPP1R15B	3.21641108	1.42E-91	6.48E-91
PIK3C2B	1.18595979	8.50E-17	1.26E-16
MDM4	2.46108949	1.89E-32	3.52E-32
RP11-430C7.	NA	NA	NA
LRRN2	NA	NA	NA
RP11-430C7.	NA	NA	NA
RP11-23I7.1	NA	NA	NA
NFASC	Inf	3.70E-131	5.31E-130
CNTN2	NA	5.04E-119	4.33E-118
TMEM81	Inf	2.80E-141	9.28E-140
RBBP5	2.28869434	8.28E-65	2.45E-64
DSTYK	1.63849708	8.99E-80	3.33E-79
TMCC2	3.74513619	0.00010107	0.00011847
RP11-383G1(	NA	NA	NA
NUAK2	NA	NA	NA
KLHDC8A	NA	NA	NA
LEMD1-AS1	NA	NA	NA
LEMD1	NA	NA	NA
BLACAT1	NA	NA	NA
RP11-576D8.	NA	2.22E-08	2.83E-08
CDK18	NA	1.68E-132	2.59E-131
MFSD4	NA	6.14E-129	7.88E-128
ELK4	1.65856031	0.80291352	0.81100479
SLC45A3	1.49805447	0.00336794	0.00378688
NUCKS1	1.33387293	2.35E-87	9.92E-87
RAB29	1.8268957	1.30E-50	3.12E-50
SLC41A1	1.60505837	0.70075908	0.71142536
PM20D1	NA	NA	NA
SLC26A9	0.74902724	9.07E-24	1.50E-23
RP4-681L3.2	NA	NA	NA
RAB7B	0.61744137	4.89E-140	1.38E-138
CTSE	0.37451362	5.55E-48	1.28E-47
C1orf186	1.09416724	3.41E-102	1.92E-101
AVPR1B	NA	1.16E-32	2.17E-32
RP11-38J22.3	Inf	3.26E-141	1.07E-139
RP11-38J22.1	NA	NA	NA
FAM72A	0.81712062	0.21350042	0.22415073
SRGAP2	1.53210117	3.08E-38	6.23E-38
IKBKE	2.44127396	0.00044539	0.00051362
C1orf147	NA	1.97E-49	4.64E-49
RP11-534L2(	NA	7.75E-131	1.09E-129
RASSF5	3.54085603	4.19E-89	1.83E-88
EIF2D	1.58692211	2.38E-19	3.67E-19
RP11-343H5.	0	9.81E-52	2.40E-51
DYRK3	4.49416342	1.35E-08	1.73E-08

MAPKAPK2	2.49675746	0.00243834	0.00275034
IL10	NA	NA	NA
IL19	NA	NA	NA
IL20	NA	NA	NA
IL24	NA	NA	NA
FCMR	NA	5.84E-96	2.91E-95
PIGR	NA	NA	NA
FCAMR	NA	NA	NA
C1orf116	NA	NA	NA
PFKFB2	NA	NA	NA
YOD1	NA	NA	NA
C4BPB	NA	NA	NA
C4BPA	NA	NA	NA
RP11-164O2	NA	NA	NA
RP11-164O2	NA	NA	NA
RP11-6J21.2	NA	NA	NA
CD55	2.9128837	0.04238487	0.04589902
RP11-357P1	NA	NA	NA
RP11-357P1	NA	NA	NA
CR2	NA	NA	NA
CR1	NA	NA	NA
RP11-78B10.	NA	NA	NA
CR1L	NA	NA	NA
CD46	1.45644185	2.50E-15	3.63E-15
C1orf132	3.37062257	1.29E-09	1.69E-09
RP11-328D5.	NA	NA	NA
CD34	1.01916821	4.00E-47	9.14E-47
PLXNA2	0.37451362	0.00069627	0.00079821
RP11-565N2	NA	NA	NA
RP11-565N2	NA	NA	NA
RP11-459K2	NA	NA	NA
RP5-1051D1	NA	NA	NA
RP11-385M4	NA	NA	NA
RP11-372M1	NA	NA	NA
RP11-372M1	NA	NA	NA
RP1-272L16.	NA	NA	NA
CAMK1G	NA	NA	NA
LAMB3	NA	NA	NA
RP1-28O10.1	NA	NA	NA
G0S2	NA	NA	NA
HSD11B1	NA	NA	NA
TRAF3IP3	1.21716926	6.70E-35	1.29E-34
C1orf74	3.74513619	6.97E-33	1.31E-32
IRF6	NA	NA	NA
DIEXF	1.72852439	1.30E-06	1.60E-06

SYT14	NA	NA	NA
SERTAD4-AS	NA	NA	NA
SERTAD4	NA	NA	NA
HHAT	NA	8.05E-59	2.17E-58
KCNH1	NA	NA	NA
RP11-543B1(	NA	NA	NA
RCOR3	2.33520256	0.00163688	0.00185613
TRAF5	3.37062257	1.18E-64	3.48E-64
LINC00467	3.49546044	1.42E-57	3.75E-57
RD3	NA	NA	NA
RP11-359E8.	NA	NA	NA
SLC30A1	1.7061176	5.32E-34	1.01E-33
RP11-354K1.	NA	NA	NA
RP11-354K1.	NA	NA	NA
NEK2	0.81144617	7.86E-118	6.51E-117
RP11-122M1	NA	6.73E-60	1.85E-59
RP11-122M1	NA	NA	NA
LPGAT1	2.19714656	8.84E-62	2.50E-61
RP11-552D8.	NA	5.58E-56	1.45E-55
INTS7	1.61632193	1.11E-08	1.43E-08
DTL	3.09597925	1.08E-79	3.99E-79
RP11-15I11.2	NA	NA	NA
RP11-15I11.3	Inf	2.95E-05	3.50E-05
PPP2R5A	1.5897721	1.34E-69	4.27E-69
RP11-384C4.	NA	4.12E-28	7.24E-28
TMEM206	1.55155642	6.08E-51	1.47E-50
NENF	2.22646628	5.84E-96	2.91E-95
RP11-61J19.2	NA	NA	NA
RP11-61J19.3	NA	NA	NA
RP11-61J19.5	5.9922179	1.70E-95	8.41E-95
ATF3	1.87256809	1.49E-31	2.75E-31
RP11-338C15	NA	NA	NA
FAM71A	NA	NA	NA
RP11-338C15	NA	NA	NA
BATF3	3.25145914	2.24E-120	2.03E-119
NSL1	1.04085603	6.95E-139	1.72E-137
TATDN3	1.90661479	5.13E-31	9.38E-31
SPATA45	Inf	8.76E-05	1.03E-04
FLVCR1-AS1	0.99870298	0.00017615	0.00020516
FLVCR1	4.49416342	2.32E-58	6.22E-58
RP11-348H3.	NA	NA	NA
VASH2	2.7464332	0.21264187	0.22328812
ANGEL2	1.53747696	0.19224765	0.20235303
RPS6KC1	1.40442607	0.06111252	0.06575549
RP11-554K11	NA	NA	NA

RP11-323I1.1	NA	NA	NA
LINC00538	NA	NA	NA
PROX1	NA	3.27E-97	1.67E-96
RP11-478J18	NA	NA	NA
PROX1-AS1	NA	NA	NA
SMYD2	2.30469919	3.96E-24	6.57E-24
PTPN14	3.74513619	1.12E-52	2.77E-52
CENPF	1.41215686	8.17E-134	1.37E-132
KCNK2	NA	NA	NA
RP11-199H2.	NA	NA	NA
KCTD3	1.92607004	3.46E-59	9.39E-59
USH2A	NA	NA	NA
RP5-1111A8.	NA	NA	NA
RP11-22M7.2	NA	NA	NA
RP5-1099E6.	NA	NA	NA
ESRRG	NA	1.03E-98	5.44E-98
GPATCH2	1.56047341	0.19260192	0.20271416
SPATA17	Inf	5.12E-12	7.02E-12
SPATA17-AS	NA	NA	NA
LINC00210	NA	NA	NA
RP11-152L7.	NA	NA	NA
RP11-152L7.	NA	NA	NA
RRP15	2.00929529	0.17738044	0.18693209
TGFB2-AS1	NA	1.55E-11	2.10E-11
TGFB2	Inf	1.03E-130	1.44E-129
C1orf143	NA	NA	NA
RP11-412H9.	NA	NA	NA
LYPLAL1-AS1	NA	0.22131413	0.23213927
LYPLAL1	1.28404669	6.62E-105	3.95E-104
RP11-135J2.3	NA	NA	NA
RP11-392O1	Inf	9.13E-51	2.20E-50
RP11-392O1	NA	NA	NA
RP11-95P13.	NA	NA	NA
RP11-95P13.	NA	NA	NA
SLC30A10	NA	NA	NA
EPRS	1.1593639	4.26E-123	4.25E-122
BPNT1	0.88120851	2.75E-90	1.23E-89
IARS2	2.18137757	7.04E-24	1.16E-23
RAB3GAP2	2.99610895	4.34E-33	8.14E-33
RP11-302I18	NA	NA	NA
RP11-410C4.	NA	NA	NA
RP11-410C4.	NA	NA	NA
MARK1	NA	NA	NA
C1orf115	2.99610895	2.34E-45	5.22E-45
2-Mar	NA	4.76E-19	7.32E-19

1-Mar	NA	NA	NA
RNU6ATAC3!	NA	NA	NA
RP11-295M1	NA	NA	NA
LINC01352	NA	NA	NA
HLX-AS1	0.37451362	2.76E-24	4.59E-24
HLX	1.54486868	8.11E-34	1.54E-33
C1orf140	NA	NA	NA
RP11-103C3.	NA	NA	NA
DUSP10	2.62159533	3.39E-23	5.55E-23
RP11-191N8.	NA	NA	NA
RP11-815M8	NA	7.72E-97	3.92E-96
RP11-400N1:	NA	NA	NA
RP11-400N1:	NA	NA	NA
RP11-400N1:	NA	NA	NA
RP11-358H9.	NA	NA	NA
RP11-378J18	NA	1.03E-130	1.44E-129
HHIPL2	NA	NA	NA
TAF1A	2.99610895	0.01188166	0.01312611
TAF1A-AS1	NA	1.34E-06	1.64E-06
MIA3	1.55155642	0.91988065	0.92364815
RP11-378J18	0	0.01552656	0.01708307
AIDA	1.70973609	8.01E-05	9.42E-05
BROX	2.30700389	7.59E-27	1.31E-26
FAM177B	NA	NA	NA
RP11-452F1!	3.20417207	8.44E-42	1.80E-41
RP11-455P21	NA	NA	NA
DISP1	Inf	0.02777457	0.03027253
TLR5	NA	NA	NA
RP11-239E1(	NA	NA	NA
RP11-239E1(	NA	NA	NA
SUSD4	NA	1.12E-25	1.90E-25
CCDC185	NA	NA	NA
CAPN8	NA	NA	NA
CAPN2	1.69591073	2.49E-69	7.91E-69
TP53BP2	2.09727626	0.22104615	0.23195206
RP11-504P24	NA	NA	NA
FBXO28	2.65040407	0.18286266	0.19259764
DEGS1	1.41293774	2.67E-73	8.99E-73
RP11-365O1(	NA	8.17E-54	2.06E-53
NVL	1.09233139	2.00E-41	4.22E-41
CNIH4	1.34985122	2.69E-96	1.35E-95
WDR26	2.60188409	7.51E-63	2.15E-62
CNIH3	0.93628405	3.54E-52	8.73E-52
RP11-3L21.2	NA	NA	NA
RP11-449J1.:	NA	NA	NA

DNAH14	1.3732166	1.45E-40	3.03E-40
LBR	2.1593578	1.19E-112	8.60E-112
RP11-496N1:	NA	NA	NA
ENAH	5.01272074	3.18E-87	1.34E-86
RP11-145A3.	NA	NA	NA
SRP9	1.753582	1.12E-15	1.64E-15
EPHX1	1.28404669	1.76E-67	5.41E-67
RP11-285F7.	1.49805447	1.79E-24	2.99E-24
TMEM63A	2.24708171	7.40E-68	2.29E-67
LEFTY1	NA	3.03E-112	2.17E-111
RP4-559A3.7	NA	NA	NA
PYCR2	1.12354086	3.97E-128	4.89E-127
LEFTY2	NA	NA	NA
SDE2	1.41482923	6.13E-10	8.07E-10
RP11-396C2:	NA	3.27E-97	1.67E-96
H3F3A	1.48748468	1.16E-143	5.73E-142
RP11-396C2:	Inf	1.08E-86	4.51E-86
ACBD3	1.00554341	1.00E-115	7.78E-115
RP11-275I14	NA	NA	NA
MIXL1	NA	2.38E-139	6.22E-138
LIN9	Inf	1.05E-42	2.26E-42
PARP1	1.56653696	2.12E-45	4.73E-45
C1orf95	NA	1.18E-59	3.21E-59
ITPKB	1.71206226	1.07E-37	2.16E-37
ITPKB-AS1	NA	1.84E-36	3.63E-36
PSEN2	2.01661179	0.30724234	0.31968287
ADCK3	1.29831388	8.03E-54	2.03E-53
CDC42BPA	0.97699205	6.77E-47	1.54E-46
RP5-1087E8.	NA	NA	NA
RP11-1B20.1	NA	NA	NA
CTD-2090I13	Inf	8.25E-123	8.10E-122
ZNF678	1.99740597	1.45E-59	3.97E-59
SNAP47	2.12670233	1.13E-06	1.39E-06
JMJD4	1.81343436	7.08E-10	9.32E-10
SNAP47-AS1	NA	NA	NA
PRSS38	NA	NA	NA
WNT9A	NA	6.27E-37	1.25E-36
WNT3A	NA	NA	NA
RP5-881P19.	NA	NA	NA
ARF1	1.54027522	1.50E-38	3.06E-38
C1orf35	1.73947648	1.09E-14	1.57E-14
MRPL55	2.10334946	9.26E-109	6.02E-108
GUK1	2.34756098	1.05E-128	1.33E-127
GJC2	Inf	3.47E-50	8.28E-50
IBA57-AS1	NA	2.45E-12	3.37E-12

IBA57	1.61328943	1.20E-33	2.27E-33
C1orf145	NA	NA	NA
OBSCN	Inf	2.91E-75	1.01E-74
RP5-1139B1:	NA	NA	NA
RP5-1139B1:	NA	NA	NA
RP5-1139B1:	NA	NA	NA
RP11-245P1(	NA	NA	NA
TRIM11	1.58365759	3.13E-50	7.48E-50
RP11-245P1(	NA	NA	NA
RP11-245P1(	NA	1.75E-09	2.28E-09
TRIM17	NA	NA	NA
HIST3H3	NA	NA	NA
HIST3H2A	1.62832008	4.03E-11	5.42E-11
HIST3H2BB	Inf	0.00274107	0.00308662
RNF187	1.66804647	3.67E-30	6.63E-30
RHOU	1.49805447	1.41E-09	1.85E-09
RP5-1065P1<	NA	NA	NA
RP5-1065P1<	NA	NA	NA
RP5-1061H2(	NA	NA	NA
TMEM78	NA	5.14E-86	2.12E-85
RP5-1061H2(	NA	1.07E-80	4.04E-80
RAB4A	2.01536825	2.02E-18	3.08E-18
SPHAR	NA	NA	NA
RP4-803J11.:	Inf	0.00402423	0.00451249
CCSAP	2.24708171	0.08504618	0.09098943
ACTA1	NA	NA	NA
NUP133	1.62832008	1.71E-09	2.23E-09
RP4-613A2.1	Inf	3.20E-26	5.47E-26
ABCB10	3.12094682	3.48E-37	6.94E-37
TAF5L	2.34071012	3.49E-13	4.87E-13
URB2	1.68531128	2.22E-20	3.48E-20
RP11-286M1	NA	NA	NA
RP11-552D4.	NA	NA	NA
GALNT2	2.31366191	5.32E-130	7.19E-129
RP5-956O18.	NA	3.34E-06	4.06E-06
RP5-956O18.	NA	5.65E-99	2.99E-98
PGBD5	0	5.79E-22	9.31E-22
RP4-553F17.	NA	NA	NA
RP11-543E8.	NA	NA	NA
COG2	2.24708171	1.34E-19	2.07E-19
AGT	NA	NA	NA
RP11-99J16_	NA	NA	NA
CAPN9	NA	NA	NA
RP5-858B6.1	NA	NA	NA
C1orf198	1.32181277	1.13E-25	1.92E-25

RP5-858B6.3	NA	NA	NA
TTC13	1.49805447	0.91308358	0.9171278
ARV1	0.91451	6.23E-136	1.19E-134
FAM89A	1.40334988	1.51E-60	4.20E-60
TRIM67	NA	NA	NA
RP5-1097F14	NA	NA	NA
C1orf131	1.26715134	9.84E-58	2.61E-57
GNPAT	1.86729325	7.47E-09	9.62E-09
EXOC8	1.68531128	0.27591033	0.28785814
SPRTN	1.8385214	3.73E-29	6.64E-29
EGLN1	1.80447471	1.10E-119	9.74E-119
RP11-295G20	1.58127972	0.58363301	0.59655586
TSNAX-DISC1	Inf	0.000139	0.00016234
TSNAX	1.27775235	6.14E-111	4.24E-110
LINC00582	NA	NA	NA
DISC1	1.87256809	1.32E-23	2.17E-23
RP5-865N13.	NA	NA	NA
RP5-865N13.	NA	NA	NA
SIPA1L2	NA	8.95E-82	3.43E-81
RP11-66A2.1	NA	NA	NA
RP11-66A2.2	NA	NA	NA
MAP10	NA	NA	NA
NTPCR	1.47414935	8.04E-06	9.71E-06
PCNXL2	1.49805447	2.11E-18	3.22E-18
RP5-862P8.2	1.90661479	7.61E-23	1.24E-22
KCNK1	Inf	1.53E-81	5.86E-81
SLC35F3	Inf	3.39E-157	1.43E-154
RP4-799P18.	NA	NA	NA
RP4-799P18.	NA	NA	NA
RP5-827C21.	NA	NA	NA
RP5-827C21.	NA	NA	NA
RP5-827C21.	4.99351492	8.84E-106	5.37E-105
COA6	1.6954968	1.76E-06	2.15E-06
TARBP1	2.93369001	4.46E-41	9.39E-41
LINC01354	NA	2.21E-66	6.71E-66
RP5-855F14.	NA	NA	NA
IRF2BP2	2.64130657	1.35E-74	4.61E-74
RP4-781K5.2	NA	4.41E-58	1.18E-57
LINC00184	NA	NA	NA
RP4-781K5.9	NA	NA	NA
RP4-781K5.4	NA	NA	NA
RP4-781K5.5	NA	NA	NA
RP4-781K5.6	NA	8.48E-157	3.42E-154
RP4-781K5.7	NA	NA	NA
LINC01132	Inf	0.00060646	0.00069635

AL360294.1	NA	NA	NA
RP11-443B7.	NA	1.45E-06	1.78E-06
RP11-443B7.	7.49027237	5.71E-22	9.17E-22
RP11-443B7.	NA	2.10E-25	3.56E-25
LINC01348	NA	NA	NA
RP4-597N16.	Inf	8.59E-14	1.21E-13
TOMM20	1.91269455	0.01788727	0.0196352
RBM34	1.9661965	1.66E-56	4.33E-56
ARID4B	2.03682845	0.82992666	0.8371715
GGPS1	1.70091602	8.67E-97	4.40E-96
TBCE	1.45399405	5.63E-103	3.22E-102
B3GALNT2	2.15345331	0.00177579	0.00201076
GNG4	0.69140976	2.19E-135	4.01E-134
LYST	0.55778624	8.69E-145	5.44E-143
LYST-AS1	NA	NA	NA
RP5-940F7.2	NA	NA	NA
RP4-580N22.	NA	NA	NA
NID1	1.09473212	1.54E-07	1.93E-07
GPR137B	1.79766537	4.73E-39	9.69E-39
ERO1B	1.39105058	2.38E-21	3.80E-21
EDARADD	0.37451362	0.01203961	0.01329708
LGALS8	2.99610895	9.66E-15	1.39E-14
LGALS8-AS1	NA	1.41E-19	2.19E-19
RP11-385F5.	Inf	5.87E-68	1.82E-67
RP11-385F5.	0.37451362	5.29E-09	6.84E-09
HEATR1	1.99740597	8.55E-08	1.08E-07
ACTN2	Inf	6.35E-99	3.35E-98
MTR	1.89459831	6.35E-46	1.43E-45
MT1HL1	NA	1.28E-33	2.43E-33
RYR2	NA	1.58E-23	2.60E-23
RP11-193H5.	NA	NA	NA
ZP4	NA	NA	NA
MTRNR2L11	NA	3.27E-97	1.67E-96
RP11-136B18	NA	NA	NA
LINC01139	NA	NA	NA
RP11-177F15	NA	0.05255704	0.05667775
RP11-544D2	NA	4.22E-17	6.29E-17
CHRM3	1.64785992	3.97E-06	4.83E-06
CHRM3-AS2	0	1.66E-86	6.90E-86
CHRM3-AS1	NA	NA	NA
RP11-35L17.	NA	NA	NA
FMN2	NA	1.28E-39	2.63E-39
RP11-567G2	NA	NA	NA
RP11-177F1	NA	NA	NA
GREM2	NA	NA	NA

RP11-467I20	NA	NA	NA
RP11-80B9.4	NA	NA	NA
RP11-80B9.1	NA	NA	NA
RGS7	NA	NA	NA
RP11-527D7.	NA	9.05E-34	1.72E-33
FH	0.69552529	7.66E-127	8.92E-126
KMO	0.37451362	4.91E-07	6.09E-07
OPN3	1.49805447	3.22E-16	4.74E-16
CHML	1.58448069	1.59E-17	2.40E-17
WDR64	NA	9.41E-24	1.55E-23
EXO1	1.87256809	1.56E-36	3.09E-36
BECN2	NA	NA	NA
MAP1LC3C	NA	NA	NA
PLD5	NA	NA	NA
RP11-561I11	NA	NA	NA
RP11-561I11	NA	NA	NA
RP11-261C1(	NA	NA	NA
LINC01347	NA	NA	NA
CEP170	2.30950065	1.41E-05	1.68E-05
RP11-261C1(	NA	NA	NA
RP11-261C1(	NA	NA	NA
SDCCAG8	1.9865505	1.61E-37	3.24E-37
AKT3	Inf	1.79E-34	3.43E-34
RP11-269F2(	NA	NA	NA
RP11-278H7.	NA	NA	NA
ZBTB18	Inf	1.10E-93	5.25E-93
RP11-278H7.	NA	NA	NA
RP11-278H7.	NA	NA	NA
C1orf100	NA	NA	NA
RP11-518L1(	Inf	3.53E-127	4.18E-126
ADSS	1.38349737	1.17E-120	1.07E-119
C1orf101	NA	0.40171797	0.41453657
DESI2	2.54669261	2.85E-126	3.23E-125
RP11-173A6.	NA	NA	NA
COX20	1.97094264	2.59E-68	8.10E-68
HNRNPU-AS:	1.79766537	2.87E-05	3.41E-05
RP11-11N7.5	NA	NA	NA
HNRNPU	1.86100903	8.44E-16	1.23E-15
RP11-11N7.4	NA	5.41E-10	7.14E-10
RP11-156E8.	2.24708171	0.0244592	0.02671516
EFCAB2	4.11964981	0.00546834	0.0061084
KIF26B	2.24708171	0.00010095	0.00011833
RP11-62I21.1	NA	NA	NA
RP11-522M2	NA	NA	NA
RP11-522M2	0.74902724	0.02950072	0.03212112

SMYD3	1.95246433	3.48E-19	5.37E-19
RP11-83A16.	NA	NA	NA
RP11-36N20.	NA	1.31E-33	2.48E-33
RP11-690C23	NA	3.48E-60	9.59E-60
TFB2M	1.52302205	2.45E-18	3.73E-18
CNST	2.78210117	5.57E-98	2.90E-97
RP11-439E19	NA	2.82E-115	2.17E-114
RP11-439E19	NA	0.10239071	0.10914055
SCCPDH	1.28696498	5.45E-109	3.57E-108
RP11-439E19	Inf	1.07E-134	1.89E-133
AHCTF1	1.74773022	2.67E-12	3.68E-12
ZNF695	2.0598249	3.89E-103	2.23E-102
ZNF670-ZNF	3.74513619	2.40E-16	3.55E-16
ZNF670	1.09473212	2.11E-38	4.28E-38
ZNF669	0.56177043	0.38248324	0.39549792
C1orf229	NA	NA	NA
ZNF124	4.04474708	0.02904548	0.03163493
RP11-488L18	2.24708171	2.51E-66	7.60E-66
RP11-488L18	NA	1.56E-22	2.53E-22
ZNF496	1.62289235	3.22E-43	6.97E-43
NLRP3	1.49805447	4.00E-09	5.18E-09
OR2B11	NA	NA	NA
GCSAML	14.2315175	3.63E-145	2.58E-143
GCSAML-AS1	NA	NA	NA
OR2C3	NA	NA	NA
RP11-978I15	NA	NA	NA
OR2G2	NA	NA	NA
OR2G3	NA	NA	NA
RP11-634B7.	NA	NA	NA
OR13G1	NA	NA	NA
OR6F1	NA	NA	NA
OR14A2	NA	NA	NA
OR14K1	NA	NA	NA
OR1C1	NA	NA	NA
OR14A16	NA	NA	NA
OR11L1	NA	NA	NA
TRIM58	1.68531128	6.88E-08	8.68E-08
OR2W3	NA	0.00596164	0.00664881
OR2T8	NA	2.39E-56	6.23E-56
OR2AJ1	NA	NA	NA
OR2L13	NA	NA	NA
OR2L8	NA	NA	NA
OR2AK2	NA	NA	NA
OR2L5	NA	NA	NA
OR2L2	NA	NA	NA

OR2L3	NA	NA	NA
OR2M5	NA	NA	NA
OR2M2	NA	NA	NA
OR2M3	NA	NA	NA
OR2M4	NA	NA	NA
OR2T33	NA	NA	NA
OR2T12	NA	NA	NA
OR2M7	NA	NA	NA
OR14C36	NA	NA	NA
OR2T4	NA	NA	NA
OR2T6	NA	NA	NA
OR2T1	NA	NA	NA
OR2T2	NA	NA	NA
OR2T3	NA	NA	NA
RP11-407H1[illegible]	NA	NA	NA
OR2T5	NA	NA	NA
OR2G6	NA	NA	NA
RP11-438F1[illegible]	NA	NA	NA
OR2T29	NA	NA	NA
OR2T34	NA	NA	NA
OR2T10	NA	NA	NA
OR2T11	NA	NA	NA
OR2T35	NA	NA	NA
OR2T27	NA	NA	NA
OR14I1	NA	NA	NA
XX-CR54.1	NA	NA	NA
LYPD8	NA	NA	NA
SH3BP5L	1.42996109	5.43E-35	1.05E-34
ZNF672	1.47932879	9.00E-89	3.92E-88
ZNF692	1.59575368	9.02E-20	1.40E-19
AL672294.1	NA	NA	NA
PGBD2	3.74513619	7.32E-23	1.19E-22
FAM110C	NA	NA	NA
AC079779.7	NA	NA	NA
SH3YL1	1.56047341	0.22717899	0.23816707
ACP1	1.88448444	0.2835311	0.29557095
FAM150B	NA	NA	NA
AC079779.5	NA	NA	NA
RP11-356M6	NA	NA	NA
AC079779.6	NA	NA	NA
AC105393.1	NA	NA	NA
AC105393.2	NA	NA	NA
AC093326.1	NA	NA	NA
AC093326.2	NA	NA	NA
AC093326.3	NA	NA	NA

TMEM18	1.46500916	2.97E-84	1.19E-83
AC092159.2	Inf	1.10E-09	1.45E-09
AC092159.1	NA	NA	NA
AC092159.3	NA	NA	NA
AC116609.1	NA	NA	NA
RP13-539J13	NA	NA	NA
AC116609.3	NA	NA	NA
AC116609.2	NA	NA	NA
LINC01115	NA	NA	NA
AC113607.2	NA	NA	NA
AC113607.3	NA	NA	NA
AC116614.1	NA	NA	NA
SNTG2	NA	NA	NA
AC114808.3	NA	NA	NA
AC114808.2	NA	NA	NA
AC108462.1	NA	NA	NA
TPO	NA	NA	NA
AC144450.1	NA	NA	NA
AC141930.2	NA	NA	NA
AC144450.2	NA	5.39E-67	1.65E-66
PXDN	NA	NA	NA
MYT1L	NA	1.08E-44	2.37E-44
AC093390.1	NA	NA	NA
MYT1L-AS1	NA	NA	NA
AC018685.1	NA	NA	NA
AC018685.2	NA	NA	NA
AC011995.1	NA	NA	NA
AC011995.3	NA	NA	NA
LINC01250	NA	NA	NA
AC019118.3	NA	NA	NA
AC019118.4	NA	NA	NA
TSSC1	1.80260401	1.02E-62	2.92E-62
TRAPPC12	1.45399405	3.54E-52	8.73E-52
TRAPPC12-A:	Inf	1.19E-63	3.44E-63
RP11-1293J1	NA	1.11E-114	8.44E-114
ADI1	1.39390402	2.42E-99	1.28E-98
AC142528.1	1.34824903	4.41E-34	8.42E-34
AC108488.4	1.39105058	0.58577352	0.59860887
RP13-512J5.:	NA	4.12E-28	7.24E-28
RNASEH1	1.9935648	3.01E-71	9.78E-71
RNASEH1-AS	2.40991372	1.73E-44	3.80E-44
RPS7	1.74438291	1.44E-15	2.09E-15
COLEC11	NA	NA	NA
AC010907.2	NA	NA	NA
ALLC	NA	NA	NA

AC010907.5	NA	NA	NA
DCDC2C	NA	NA	NA
AC019172.2	NA	NA	NA
LINC01304	NA	NA	NA
AC012445.1	NA	NA	NA
LINC01249	NA	NA	NA
AC107057.1	NA	NA	NA
LINC01248	NA	NA	NA
SOX11	NA	NA	NA
AC010729.2	NA	NA	NA
AC010729.3	NA	NA	NA
AC017053.1	NA	NA	NA
LINC01247	NA	NA	NA
AC021021.2	NA	NA	NA
MIR7515HG	NA	NA	NA
LINC00487	NA	NA	NA
NRIR	NA	9.20E-54	2.32E-53
CMPK2	4.30690661	2.74E-145	2.09E-143
RSAD2	5.9922179	2.51E-140	7.31E-139
RP11-439M1	Inf	3.32E-126	3.74E-125
RNF144A	4.54766537	1.94E-145	1.62E-143
AC019048.1	NA	NA	NA
RP11-557L19	0	6.48E-15	9.34E-15
AC092580.3	NA	NA	NA
AC092580.4	NA	NA	NA
AC007463.2	NA	NA	NA
LINC00298	NA	NA	NA
LINC00299	NA	NA	NA
U91324.1	NA	NA	NA
AC011747.3	NA	NA	NA
AC011747.4	NA	1.97E-57	5.21E-57
AC011747.6	NA	NA	NA
ID2-AS1	NA	NA	NA
ID2	1.61886532	1.38E-05	1.65E-05
RP11-434B12	Inf	2.07E-112	1.48E-111
KIDINS220	1.83095547	7.56E-26	1.29E-25
MBOAT2	1.59792477	2.73E-93	1.29E-92
RP11-734K21	NA	NA	NA
RP11-734K21	NA	NA	NA
RP11-734K21	NA	NA	NA
ASAP2	2.24708171	8.28E-72	2.72E-71
ITGB1BP1	2.27813894	7.90E-131	1.11E-129
CPSF3	1.16296334	1.71E-87	7.27E-87
IAH1	2.78210117	5.40E-07	6.69E-07
ADAM17	1.71206226	0.00145367	0.00165055

RP11-400L8.:	NA	NA	NA
RP11-214N9.	7.49027237	3.32E-62	9.44E-62
YWHAQ	1.32864831	2.73E-67	8.40E-67
RP11-521D1:	NA	NA	NA
RP11-521D1:	NA	NA	NA
RP11-521D1:	NA	NA	NA
TAF1B	0.88521401	7.50E-118	6.21E-117
RP11-95D17.	Inf	0.20234935	0.21276352
GRHL1	0.49935149	0.1886422	0.19859263
AC010969.1	NA	NA	NA
RP11-254F7.	NA	NA	NA
RP11-254F7.	NA	0.22955634	0.2406177
KLF11	0.62418936	0.00837414	0.0092988
RP11-254F7.	NA	NA	NA
CYS1	Inf	1.01E-06	1.24E-06
AC104794.4	NA	NA	NA
RP11-254F7.	1.34824903	2.04E-14	2.92E-14
RRM2	1.22856319	9.10E-120	8.06E-119
C2orf48	5.9922179	0.00050394	0.00057989
HPCAL1	1.72852439	0.13750679	0.14564737
ODC1	2.18148643	4.15E-106	2.53E-105
RP11-320M2	NA	0.0038068	0.00427344
AC007249.3	NA	NA	NA
NOL10	1.37864434	2.96E-58	7.92E-58
AC092687.5	NA	NA	NA
RN7SL832P	5.9922179	4.95E-69	1.56E-68
AC092687.3	NA	NA	NA
ATP6V1C2	Inf	1.54E-14	2.20E-14
RP11-791G1!	NA	4.62E-92	2.14E-91
PDIA6	0.96382181	9.56E-142	3.38E-140
AC092687.4	NA	NA	NA
RP11-245G1:	NA	NA	NA
KCNF1	NA	NA	NA
AC062028.1	NA	0.24882742	0.26035107
C2orf50	NA	3.67E-81	1.39E-80
PQLC3	1.42783317	6.36E-10	8.37E-10
ROCK2	2.81986725	1.61E-102	9.12E-102
AC099344.2	NA	NA	NA
AC099344.3	NA	NA	NA
LINC00570	NA	2.36E-58	6.33E-58
E2F6	2.39688716	2.60E-18	3.96E-18
GREB1	NA	0.66511293	0.67667557
NTSR2	NA	NA	NA
RP11-494G9.	NA	NA	NA
LPIN1	3.18336576	0.35483924	0.36764669

AC012456.3	NA	NA	NA
AC012456.4	NA	NA	NA
MIR3681HG	0	2.08E-176	1.26E-172
AC096559.2	NA	NA	NA
RP11-333O1.	NA	NA	NA
TRIB2	1.79766537	0.00370313	0.00415758
AC092635.1	NA	NA	NA
LINC00276	NA	NA	NA
AC016730.1	NA	NA	NA
AC011897.2	NA	NA	NA
FAM84A	NA	7.93E-26	1.35E-25
AC068286.1	NA	NA	NA
NBAS	2.04280156	1.69E-05	2.02E-05
AC008278.2	NA	NA	NA
DDX1	1.58127972	3.81E-32	7.06E-32
AC008278.3	NA	NA	NA
AC008271.1	NA	NA	NA
AC113608.1	NA	NA	NA
MYCNUT	NA	NA	NA
MYCNOS	NA	NA	NA
MYCN	NA	NA	NA
AC010145.4	NA	NA	NA
GACAT3	NA	NA	NA
AC142119.1	NA	NA	NA
AC010745.1	NA	NA	NA
AC010745.2	NA	NA	NA
AC010745.3	NA	NA	NA
AC010745.4	NA	NA	NA
AC010880.1	NA	NA	NA
AC104623.2	NA	NA	NA
FAM49A	1.24837873	3.97E-79	1.45E-78
AC008069.1	NA	NA	NA
AC080094.1	NA	NA	NA
RAD51AP2	NA	NA	NA
VSNL1	NA	NA	NA
SMC6	2.09727626	0.1719401	0.1813779
GEN1	3.59533074	6.62E-83	2.59E-82
MSGN1	NA	NA	NA
KCNS3	2.0598249	0.00012903	0.00015086
RP11-111J6.:	NA	5.94E-64	1.73E-63
RDH14	1.93978849	9.11E-07	1.12E-06
NT5C1B-RDH	NA	NA	NA
NT5C1B	NA	NA	NA
AC106053.1	NA	NA	NA
LINC01376	NA	7.29E-115	5.55E-114

OSR1	NA	NA	NA
AC010096.1	NA	2.47E-28	4.35E-28
AC010096.2	NA	NA	NA
AC019055.1	NA	NA	NA
LINC00954	Inf	0.0061396	0.00684559
TTC32	1.26559775	0.27397025	0.28591626
RP11-79O8.1	Inf	4.89E-144	2.59E-142
WDR35	1.3732166	0.71310932	0.7234373
AC079145.4	NA	NA	NA
MATN3	NA	NA	NA
LAPTM4A	1.02896671	1.58E-77	5.66E-77
RP11-644K8.	NA	1.50E-75	5.20E-75
AC098828.2	NA	NA	NA
SDC1	1.27107652	2.31E-06	2.82E-06
PUM2	1.8612192	3.80E-64	1.11E-63
RHOB	NA	NA	NA
AC023137.2	NA	NA	NA
AC012065.4	NA	NA	NA
HS1BP3	1.63424125	1.04E-57	2.76E-57
RP11-130L8.:	NA	NA	NA
GDF7	7.49027237	7.99E-64	2.33E-63
RP11-130L8.	Inf	0.00246054	0.00277451
LDAH	2.30950065	3.12E-34	5.96E-34
AC012361.1	NA	NA	NA
RP11-116D2.	NA	NA	NA
APOB	NA	NA	NA
TDRD15	0	6.79E-146	6.85E-144
AC067959.1	NA	NA	NA
AC011752.1	NA	NA	NA
AC009411.2	NA	NA	NA
AC009411.1	NA	NA	NA
AC018742.1	NA	NA	NA
AC068490.2	NA	NA	NA
AC068490.1	NA	NA	NA
AC096570.2	NA	NA	NA
AC096570.1	NA	NA	NA
AC016768.1	NA	NA	NA
AC012506.1	NA	NA	NA
AC012506.2	NA	NA	NA
AC012506.3	NA	NA	NA
AC012506.4	NA	NA	NA
KLHL29	NA	NA	NA
AC011239.1	NA	NA	NA
RP11-557N2	NA	7.15E-24	1.18E-23
ATAD2B	1.19844358	5.52E-50	1.31E-49

UBXN2A	2.62159533	4.92E-83	1.93E-82
MFSD2B	3.2457847	1.12E-53	2.81E-53
C2orf44	0.97949716	0.00059587	0.00068429
FKBP1B	1.76770428	0.00236598	0.00266955
SF3B6	1.41703387	2.47E-122	2.39E-121
FAM228B	1.40442607	3.32E-71	1.08E-70
TP53I3	0.97949716	1.13E-113	8.35E-113
PFN4	NA	1.57E-27	2.73E-27
RP11-507M3	NA	NA	NA
AC008073.7	NA	NA	NA
FAM228A	NA	0.03199769	0.03481271
AC008073.9	NA	1.35E-14	1.94E-14
ITSN2	1.62913424	0.18586615	0.19571557
AC009228.1	NA	8.75E-11	1.17E-10
NCOA1	1.55567195	0.07524385	0.08069721
PTRHD1	1.87256809	0.29070018	0.30283564
CENPO	3.2457847	2.90E-53	7.26E-53
ADCY3	2.58754864	5.19E-23	8.47E-23
RP11-443B2(	1.99740597	2.40E-14	3.42E-14
DNAJC27	0.74902724	2.61E-29	4.66E-29
DNAJC27-AS	Inf	1.68E-26	2.89E-26
EFR3B	NA	3.74E-17	5.59E-17
POMC	NA	NA	NA
LINC01381	NA	NA	NA
DNMT3A	1.77893969	3.61E-12	4.96E-12
AC012074.2	NA	1.76E-12	2.43E-12
DTNB	1.28404669	2.70E-91	1.23E-90
AC104699.1	NA	NA	NA
ASXL2	2.27964811	3.07E-14	4.37E-14
KIF3C	4.11964981	1.52E-26	2.62E-26
RAB10	1.45124027	1.26E-118	1.07E-117
GAREM2	2.07684825	1.05E-51	2.56E-51
HADHA	1.24353066	1.64E-123	1.66E-122
HADHB	1.30475712	1.73E-138	4.11E-137
ADGRF3	NA	7.30E-81	2.75E-80
EPT1	1.98271916	1.15E-13	1.62E-13
DRC1	NA	NA	NA
OTOF	NA	NA	NA
C2orf70	NA	NA	NA
CIB4	NA	NA	NA
AC015977.6	NA	NA	NA
KCNK3	NA	NA	NA
SLC35F6	1.4205689	1.36E-14	1.95E-14
CENPA	1.84944997	1.48E-92	6.90E-92
DPYSL5	NA	3.17E-68	9.89E-68

AC013472.4	NA	NA	NA
MAPRE3	Inf	6.68E-95	3.26E-94
TMEM214	2.92120623	3.31E-13	4.62E-13
AGBL5	1.84760052	3.15E-10	4.16E-10
AGBL5-AS1	Inf	0.00250079	0.00281955
RP11-503P1(	NA	2.73E-120	2.46E-119
RP11-195B1:	NA	5.54E-80	2.06E-79
OST4	2.38052909	4.06E-142	1.49E-140
EMILIN1	1.48719901	1.04E-06	1.28E-06
KHK	1.1770428	5.26E-93	2.48E-92
CGREF1	NA	NA	NA
ABHD1	0.37451362	5.66E-98	2.94E-97
PREB	1.98313878	0.35917552	0.37201201
PRR30	NA	NA	NA
TCF23	NA	5.06E-06	6.13E-06
SLC5A6	1.93498703	1.23E-84	4.96E-84
ATRAID	1.47734404	1.71E-120	1.55E-119
CAD	1.39105058	3.83E-25	6.45E-25
SLC30A3	2.62159533	1.02E-64	3.00E-64
DNAJC5G	NA	0.00110931	0.0012651
TRIM54	NA	NA	NA
UCN	1.60505837	7.66E-13	1.06E-12
MPV17	1.62554847	5.09E-75	1.75E-74
GTF3C2	2.0028337	1.06E-106	6.58E-106
GTF3C2-AS1	NA	1.84E-64	5.42E-64
AC074117.1(	3.99481193	1.24E-119	1.09E-118
EIF2B4	0.82104909	3.57E-125	3.86E-124
SNX17	1.23877582	8.88E-143	3.73E-141
ZNF513	8.98832685	2.98E-41	6.28E-41
PPM1G	1.65331502	9.61E-46	2.15E-45
NRBP1	1.39105058	1.53E-126	1.75E-125
KRTCAP3	2.24708171	1.17E-57	3.09E-57
IFT172	2.99610895	0.06086159	0.06548938
FNDC4	2.69649805	0.02555118	0.02788771
GCKR	NA	NA	NA
AC109829.1	NA	NA	NA
AC109829.2	NA	NA	NA
C2orf16	NA	NA	NA
ZNF512	2.14007782	0.60574375	0.61835489
CCDC121	Inf	0.52964152	0.54295962
GPN1	1.29063155	3.21E-73	1.08E-72
SUPT7L	1.55353797	9.07E-86	3.73E-85
SLC4A1AP	1.18595979	2.02E-73	6.82E-73
LINC01460	NA	2.06E-37	4.11E-37
MRPL33	1.74694754	2.39E-13	3.35E-13

RBKS	0.18725681	1.78E-140	5.30E-139
BRE	1.1523496	2.57E-132	3.93E-131
AC093690.1	NA	NA	NA
FLJ31356	NA	NA	NA
FOSL2	1.49805447	8.14E-61	2.27E-60
RP11-373D2:	NA	NA	NA
AC104695.4	NA	NA	NA
PLB1	NA	1.50E-23	2.47E-23
AC074011.2	NA	NA	NA
AC097724.3	1.49805447	4.31E-66	1.30E-65
PPP1CB	1.9947778	0.00221942	0.00250573
SPDYA	NA	1.35E-12	1.86E-12
RP11-713D1!	NA	5.71E-52	1.40E-51
TRMT61B	1.12354086	1.70E-108	1.10E-107
WDR43	1.31234524	4.64E-36	9.11E-36
FAM179A	NA	NA	NA
C2orf71	NA	NA	NA
AC105398.3	NA	NA	NA
CLIP4	0	2.54E-96	1.28E-95
ALK	NA	NA	NA
AC106870.1	NA	NA	NA
AC106870.2	NA	NA	NA
AC016907.3	0	4.23E-82	1.63E-81
YPEL5	1.81906615	1.16E-09	1.53E-09
LBH	1.68531128	2.92E-16	4.31E-16
AC109642.1	NA	NA	NA
LCLAT1	1.99740597	9.26E-17	1.37E-16
CAPN13	NA	NA	NA
GALNT14	1.83095547	9.53E-95	4.64E-94
AC009305.1	NA	NA	NA
CAPN14	NA	NA	NA
EHD3	NA	NA	NA
XDH	NA	NA	NA
SRD5A2	NA	9.14E-85	3.70E-84
AL133247.2	NA	NA	NA
AL133249.1	NA	NA	NA
RP11-1057B(	NA	1.25E-21	1.99E-21
MEMO1	1.99740597	0.00573619	0.00640288
DPY30	1.64031159	5.26E-17	7.84E-17
CTC-336P14.:	1.49805447	2.71E-11	3.66E-11
SPAST	2.43433852	1.24E-26	2.13E-26
RP11-563N4.	1.72852439	2.23E-49	5.25E-49
SLC30A6	2.36231667	5.25E-86	2.16E-85
NLRC4	0.74902724	3.85E-13	5.37E-13
YIPF4	2.02514772	0.00138358	0.00157196

AL133245.2	2.62159533	0.02677407	0.0292031
BIRC6	1.65170109	0.83438184	0.8415252
BIRC6-AS1	NA	NA	NA
BIRC6-AS2	NA	NA	NA
TTC27	2.30950065	0.58881214	0.60151077
LINC00486	NA	NA	NA
AL121656.5	NA	NA	NA
LTBP1	Inf	1.05E-34	2.03E-34
RASGRP3	Inf	7.27E-07	8.98E-07
AC020594.5	Inf	1.44E-124	1.51E-123
FAM98A	2.12791829	4.29E-28	7.52E-28
LINC01317	NA	NA	NA
LINC01318	NA	NA	NA
AC073218.1	NA	NA	NA
LINC01320	NA	NA	NA
RP11-529E15	NA	NA	NA
AC073218.3	NA	NA	NA
AC012593.1	NA	0.0091276	0.0101181
AC019064.1	NA	NA	NA
RP11-490M8	0.74902724	5.14E-51	1.24E-50
CRIM1	2.24708171	8.60E-15	1.24E-14
RP11-78I14.1	NA	9.18E-05	1.08E-04
FEZ2	1.22209707	1.01E-63	2.93E-63
VIT	NA	NA	NA
STRN	2.36231667	0.85653105	0.86276089
HEATR5B	1.94747082	0.00012085	0.00014144
GPATCH11	1.58127972	2.07E-13	2.91E-13
EIF2AK2	2.04422017	4.31E-56	1.12E-55
SULT6B1	NA	NA	NA
CEBPZOS	1.76295435	2.09E-46	4.72E-46
CEBPZ	1.92710891	1.71E-63	4.94E-63
NDUFAF7	1.99740597	1.97E-59	5.37E-59
PRKD3	1.49805447	3.71E-05	4.40E-05
AC007391.2	Inf	1.03E-153	2.34E-151
QPCT	NA	0.0091276	0.0101181
AC006369.2	NA	NA	NA
CDC42EP3	1.08949416	4.96E-39	1.02E-38
AC006369.3	NA	NA	NA
LINC00211	Inf	5.39E-09	6.97E-09
RMDN2	NA	NA	NA
RMDN2-AS1	NA	4.18E-70	1.34E-69
CYP1B1	5.09338521	1.98E-145	1.63E-143
CYP1B1-AS1	1.12354086	6.16E-10	8.11E-10
AC009229.6	NA	NA	NA
RP11-314C9.	NA	NA	NA

AC009229.5	NA	NA	NA
ATL2	1.24837873	1.46E-11	1.98E-11
AC016995.3	NA	NA	NA
AC093822.1	NA	NA	NA
HNRNPLL	1.04276341	1.40E-120	1.27E-119
AC011247.3	NA	NA	NA
GALM	Inf	0.015975	0.01756582
AC074366.3	NA	6.78E-49	1.59E-48
SRSF7	1.41466075	1.73E-74	5.92E-74
GEMIN6	1.34440786	2.04E-54	5.18E-54
DHX57	2.24708171	4.10E-36	8.05E-36
AC018693.6	NA	NA	NA
MORN2	1.78339818	5.77E-09	7.45E-09
ARHGEF33	NA	NA	NA
RP11-173C1.	NA	NA	NA
SOS1	2.58754864	9.19E-81	3.46E-80
CDKL4	0.74902724	0.34172932	0.35446845
MAP4K3	5.24319066	7.24E-21	1.15E-20
AC007246.3	2.88087399	3.31E-63	9.52E-63
TMEM178A	0	7.40E-138	1.66E-136
THUMPD2	1.62645914	1.37E-11	1.86E-11
SLC8A1-AS1	NA	NA	NA
SLC8A1	NA	1.06E-158	7.11E-156
AC007317.1	NA	NA	NA
AC104654.1	NA	NA	NA
AC104654.2	NA	NA	NA
C2orf91	NA	NA	NA
AC013480.2	NA	NA	NA
PKDCC	2.0598249	8.64E-08	1.09E-07
AC083949.1	NA	NA	NA
EML4	2.12514705	3.30E-49	7.75E-49
COX7A2L	1.95211872	6.61E-32	1.22E-31
KCNG3	Inf	5.81E-44	1.27E-43
MTA3	1.7183566	4.74E-15	6.84E-15
OXER1	3.74513619	3.49E-103	2.01E-102
HAAO	1.1385214	1.05E-107	6.70E-107
AC016735.1	NA	NA	NA
AC016735.2	NA	NA	NA
AC016735.3	NA	NA	NA
AC093609.1	NA	5.19E-23	8.46E-23
ZFP36L2	2.05336777	2.36E-09	3.07E-09
LINC01126	NA	3.00E-09	3.89E-09
AC010883.5	0.74902724	2.55E-06	3.12E-06
THADA	1.95899431	1.15E-67	3.57E-67
PLEKHH2	0.99870298	8.02E-60	2.20E-59

C1GALT1C1L	NA	NA	NA
DYNC2LI1	1.1770428	1.45E-91	6.62E-91
ABCG5	NA	NA	NA
ABCG8	NA	NA	NA
LRPPRC	2.33030696	1.60E-68	5.01E-68
RP11-559M2	NA	0.21325368	0.22390464
PPM1B	1.49805447	1.65E-15	2.41E-15
SLC3A1	NA	3.13E-68	9.76E-68
PREPL	2.15345331	2.46E-24	4.10E-24
CAMKMT	1.13982406	6.54E-60	1.80E-59
RP11-89K21.	3.37062257	1.16E-34	2.23E-34
SIX3-AS1	2.37191958	0.20660844	0.21711598
SIX3	2.4830766	1.54E-46	3.48E-46
AC012354.6	2.12224384	2.77E-51	6.71E-51
SIX2	Inf	4.77E-98	2.48E-97
AC093702.1	Inf	1.61E-102	9.08E-102
AC009236.2	Inf	5.75E-13	8.00E-13
SRBD1	1.58127972	3.16E-10	4.18E-10
PRKCE	1.68531128	0.0089825	0.00996395
U51244.2	NA	NA	NA
AC017006.2	NA	NA	NA
AC017006.3	NA	NA	NA
EPAS1	NA	5.46E-42	1.16E-41
AC016912.3	NA	5.14E-08	6.50E-08
TMEM247	NA	1.32E-104	7.81E-104
ATP6V1E2	2.92801556	1.57E-105	9.47E-105
RP11-417F21	NA	NA	NA
RHOQ	1.97957198	6.10E-48	1.41E-47
RP11-417F21	Inf	3.34E-57	8.79E-57
PIGF	1.97708352	9.71E-10	1.27E-09
CRIPT	1.51205498	3.34E-21	5.31E-21
SOCS5	2.54669261	4.51E-08	5.71E-08
RP11-333I13	Inf	3.25E-135	5.86E-134
LINC01118	Inf	1.39E-136	2.77E-135
LINC01119	0.74902724	5.85E-10	7.71E-10
AC016722.3	NA	2.26E-48	5.26E-48
AC016722.4	NA	1.70E-48	3.96E-48
MCFD2	1.44567495	2.13E-20	3.35E-20
TTC7A	3.6381323	1.33E-78	4.85E-78
RP11-15I20.1	NA	NA	NA
AC093732.1	NA	NA	NA
C2orf61	NA	NA	NA
RP11-761B3.	NA	NA	NA
AC073283.7	NA	4.58E-158	2.86E-155
CALM2	1.27960907	2.78E-141	9.23E-140

AC073283.4	NA	NA	NA
AC106869.2	NA	NA	NA
BCYRN1	13.4824903	4.56E-133	7.34E-132
EPCAM	NA	NA	NA
MSH2	1.18115834	9.76E-94	4.65E-93
KCNK12	Inf	1.99E-131	2.91E-130
HCG2040054	NA	4.58E-53	1.14E-52
MSH6	1.5421149	1.26E-44	2.77E-44
FBXO11	2.41993415	0.55980716	0.57288028
AC079807.2	NA	3.77E-142	1.39E-140
AC079807.4	NA	NA	NA
FOXN2	2.01661179	2.26E-06	2.77E-06
RP11-191L17	Inf	5.33E-27	9.21E-27
PPP1R21	1.60505837	3.34E-22	5.39E-22
STON1	Inf	7.55E-96	3.75E-95
STON1-GTF2	NA	NA	NA
GTF2A1L	NA	NA	NA
RP11-310N1	NA	NA	NA
LHCGR	NA	NA	NA
RP11-460M2	NA	NA	NA
FSHR	NA	NA	NA
RP11-152D8.	NA	NA	NA
RP11-443F4.	NA	NA	NA
NRXN1	NA	NA	NA
RP11-358H1	NA	NA	NA
AC007682.1	NA	NA	NA
AC087073.1	NA	NA	NA
AC139712.4	NA	NA	NA
AC010967.2	NA	NA	NA
AC010967.3	NA	NA	NA
ASB3	4.49416342	7.40E-69	2.33E-68
GPR75-ASB3	NA	NA	NA
CHAC2	1.34100038	2.21E-51	5.37E-51
ERLEC1	2.30058366	4.93E-87	2.07E-86
GPR75	NA	5.28E-27	9.12E-27
PSME4	2.12881425	1.55E-88	6.73E-88
ACYP2	1.92607004	4.32E-32	8.01E-32
RP11-477N3.	0	2.08E-09	2.71E-09
TSPYL6	NA	NA	NA
C2orf73	NA	NA	NA
SPTBN1	0.97547733	1.02E-107	6.47E-107
AC092839.3	NA	NA	NA
AC092839.4	NA	NA	NA
AC093110.3	NA	NA	NA
EML6	NA	5.87E-32	1.08E-31

AC104781.1	NA	NA	NA
RTN4	1.09573853	1.27E-138	3.07E-137
CLHC1	2.8463035	1.12E-62	3.19E-62
AC012358.7	NA	NA	NA
RPS27A	1.85883593	0.17489187	0.18436306
MTIF2	1.32614658	4.82E-54	1.22E-53
AC012358.8	NA	1.57E-101	8.75E-101
CCDC88A	1.88817283	3.74E-101	2.06E-100
CFAP36	1.25606106	1.14E-95	5.62E-95
PPP4R3B	1.67224686	1.46E-16	2.17E-16
RP11-554J4.1	7.86478599	1.58E-68	4.94E-68
PNPT1	1.93297352	0.00026628	0.00030893
EFEMP1	NA	NA	NA
RP11-481J13	NA	NA	NA
MIR217HG	NA	NA	NA
AC011306.1	NA	NA	NA
AC007743.1	NA	NA	NA
CCDC85A	NA	NA	NA
VRK2	1.3732166	3.85E-59	1.04E-58
FANCL	1.26398346	2.26E-127	2.70E-126
RP11-334G2	5.9922179	9.80E-65	2.90E-64
AC007250.4	NA	NA	NA
LINC01122	Inf	0.62282721	0.63504363
AC007131.1	NA	NA	NA
RP11-444A2	NA	1.98E-32	3.69E-32
AC007131.2	NA	NA	NA
AC007179.1	NA	NA	NA
AC007381.3	NA	NA	NA
MIR4432HG	NA	NA	NA
BCL11A	2.54215305	1.29E-59	3.51E-59
AC009970.1	NA	NA	NA
PAPOLG	0.87386511	2.01E-26	3.44E-26
LINC01185	NA	NA	NA
REL	2.63546621	2.26E-10	2.99E-10
PUS10	3.74513619	3.35E-19	5.16E-19
PEX13	2.14007782	0.0175883	0.01931636
KIAA1841	2.24708171	0.38220085	0.39522845
C2orf74	NA	3.76E-110	2.54E-109
RP11-493E1	NA	NA	NA
AHSA2	1.79115209	3.16E-30	5.71E-30
USP34	2.20867006	4.97E-17	7.41E-17
RP11-355B1	0.74902724	4.50E-19	6.93E-19
XPO1	2.21770809	1.40E-75	4.86E-75
FAM161A	1.68531128	6.70E-13	9.31E-13
CCT4	1.30467816	8.52E-98	4.40E-97

AC107081.5	NA	5.09E-45	1.13E-44
COMMD1	1.42538765	5.03E-108	3.22E-107
AC107081.1	NA	NA	NA
AC018462.2	NA	NA	NA
B3GNT2	1.12354086	8.23E-29	1.46E-28
AC093159.1	0.74902724	4.18E-82	1.61E-81
TMEM17	1.87256809	6.58E-49	1.54E-48
AC092155.4	NA	NA	NA
EHBP1	2.49675746	6.47E-47	1.47E-46
AC007098.1	NA	NA	NA
AC009501.4	1.87256809	0.00172629	0.00195544
OTX1	0.96303502	0.89499043	0.90000091
WDPCP	2.62159533	2.32E-06	2.84E-06
MDH1	0.82112612	9.09E-144	4.55E-142
AC016734.1	NA	NA	NA
UGP2	1.30188067	3.63E-86	1.50E-85
VPS54	2.32198444	1.28E-05	1.54E-05
AC012368.1	NA	NA	NA
PELI1	1.49805447	3.93E-34	7.50E-34
AC074289.1	0.74902724	2.21E-62	6.29E-62
LINC00309	NA	NA	NA
AC114752.1	NA	NA	NA
AC114752.3	NA	NA	NA
AC008074.3	NA	NA	NA
LGALSL	1.59168288	1.94E-06	2.38E-06
AC008074.4	NA	NA	NA
RP11-568N6.	NA	0.28666397	0.29879468
AFTPH	1.68531128	0.01524077	0.01677271
AC008074.1	NA	NA	NA
AC007365.1	NA	NA	NA
SERTAD2	3.6770428	7.00E-26	1.19E-25
AC007365.3	Inf	3.10E-116	2.45E-115
AC007880.1	NA	1.48E-64	4.36E-64
AC007386.2	NA	NA	NA
SLC1A4	1.39105058	2.20E-17	3.30E-17
AC007386.4	NA	NA	NA
CEP68	2.32198444	0.00087604	0.0010019
RAB1A	1.33433267	1.66E-54	4.23E-54
ACTR2	1.91369965	4.41E-76	1.54E-75
SPRED2	1.60505837	0.00793625	0.00881794
AC074391.1	NA	2.25E-110	1.52E-109
AC012370.3	NA	0.03359203	0.03649917
AC007389.3	NA	9.81E-71	3.17E-70
RP11-418H1(	NA	3.74E-19	5.76E-19
RP11-340F1(	NA	NA	NA

AC118345.1	NA	NA	NA
AC092669.6	NA	NA	NA
AC092669.3	NA	1.00E-95	4.98E-95
MEIS1-AS3	NA	NA	NA
MEIS1	1.49805447	1.10E-50	2.64E-50
MEIS1-AS2	1.49805447	1.01E-19	1.57E-19
AC007392.3	NA	NA	NA
AC007392.4	NA	NA	NA
AC007403.2	NA	NA	NA
LINC01628	NA	NA	NA
AC007403.3	NA	NA	NA
AC023115.2	NA	NA	NA
AC078941.1	NA	NA	NA
AC023115.4	NA	NA	NA
ETAA1	4.49416342	7.35E-28	1.29E-27
AC007422.1	NA	NA	NA
AC010987.5	NA	NA	NA
AC010987.6	NA	NA	NA
C1D	1.25659175	9.29E-127	1.07E-125
WDR92	0.80664472	6.67E-98	3.45E-97
RP11-474G2:	NA	6.88E-28	1.20E-27
PNO1	1.17347601	1.26E-111	8.84E-111
PPP3R1	1.79766537	0.61556794	0.62788899
RP11-474G2:	Inf	2.36E-64	6.94E-64
RP11-474G2:	1.49805447	0.00018072	0.00021045
CNRIP1	2.33520256	3.66E-35	7.10E-35
AC015969.3	NA	NA	NA
PLEK	1.66450497	7.96E-64	2.32E-63
FBXO48	6.74124514	9.96E-26	1.69E-25
APLF	1.49805447	1.82E-57	4.81E-57
PROKR1	NA	1.48E-51	3.60E-51
ARHGAP25	1.07003891	8.22E-23	1.34E-22
AC097495.2	0	0.6774439	0.68860305
AC097495.3	NA	NA	NA
BMP10	NA	NA	NA
GKN2	NA	NA	NA
GKN1	NA	NA	NA
ANTXR1	0.56177043	2.91E-58	7.79E-58
GFPT1	3.62029831	0.5464322	0.55963492
NFU1	1.38524917	3.97E-120	3.56E-119
AAK1	2.7464332	2.11E-23	3.46E-23
ANXA4	0.79735158	4.38E-119	3.78E-118
AC092431.3	NA	NA	NA
GMCL1	2.17217899	1.58E-54	4.03E-54
SNRNP27	1.77626459	1.04E-14	1.49E-14

MXD1	1.1770428	9.45E-59	2.54E-58
AC019206.1	NA	NA	NA
ASPRV1	0.74902724	0.00939161	0.01040632
PCBP1	1.45074749	6.51E-86	2.68E-85
AC016700.2	NA	NA	NA
AC016700.5	2.24708171	0.07186057	0.07711887
C2orf42	1.62289235	7.95E-15	1.14E-14
TIA1	1.28832685	4.32E-35	8.37E-35
PCYOX1	1.70233463	0.3374341	0.35011321
SNRPG	1.92822166	2.31E-21	3.68E-21
FAM136A	2.13842653	4.72E-65	1.40E-64
AC022201.5	NA	2.13E-22	3.45E-22
AC022201.4	NA	NA	NA
TGFA	NA	NA	NA
ADD2	4.86867704	1.53E-122	1.49E-121
AC007395.3	NA	NA	NA
FIGLA	NA	6.70E-38	1.35E-37
CLEC4F	Inf	6.31E-15	9.09E-15
CD207	NA	8.09E-14	1.14E-13
LINC01143	NA	7.59E-100	4.06E-99
VAX2	2.24708171	1.35E-66	4.09E-66
ATP6V1B1	Inf	0.04768331	0.05151683
AC007040.11	2.24708171	3.10E-72	1.02E-71
ATP6V1B1-A	1.87256809	1.14E-81	4.36E-81
ANKRD53	NA	NA	NA
TEX261	2.03990397	0.02427848	0.02652096
AC007040.6	NA	NA	NA
AC007040.8	NA	NA	NA
NAGK	0.90671718	1.53E-113	1.13E-112
RP11-467P9.	Inf	8.71E-38	1.75E-37
MCEE	1.77893969	0.01179445	0.01303373
MPHOSPH10	1.2533228	1.50E-66	4.56E-66
PAIP2B	5.74254215	1.08E-46	2.47E-46
ZNF638	1.77547197	4.96E-32	9.19E-32
DYSF	NA	NA	NA
CYP26B1	NA	1.45E-06	1.78E-06
EXOC6B	5.9922179	2.75E-31	5.04E-31
SPR	1.19095331	9.53E-134	1.59E-132
EMX1	NA	NA	NA
RP11-466M2	NA	NA	NA
SFXN5	2.62159533	9.49E-77	3.35E-76
RAB11FIP5	1.97470817	4.11E-28	7.21E-28
NOTO	NA	NA	NA
SMYD5	2.47178988	4.99E-20	7.78E-20
PRADC1	2.25763139	3.00E-80	1.12E-79

CCT7	1.01935286	1.75E-132	2.70E-131
FBXO41	4.68142023	6.57E-94	3.14E-93
EGR4	1.49805447	0.87353212	0.87920221
ALMS1	2.24708171	0.33287836	0.34562349
NAT8	NA	NA	NA
TPRKB	1.63740838	3.37E-36	6.62E-36
RP11-434P11	Inf	2.63E-134	4.50E-133
DUSP11	1.06440713	1.91E-139	5.04E-138
C2orf78	NA	NA	NA
STAMBP	2.58535208	0.00793625	0.00881794
ACTG2	NA	NA	NA
DGUOK	2.24708171	8.12E-124	8.35E-123
DGUOK-AS1	3.21011673	1.64E-139	4.39E-138
AC073046.25	Inf	2.54E-72	8.39E-72
TET3	1.97470817	3.60E-73	1.21E-72
RP11-287D1.	NA	NA	NA
BOLA3	2.43621109	6.70E-145	4.30E-143
BOLA3-AS1	Inf	0.0175882	0.01931636
MOB1A	1.59970817	9.37E-36	1.83E-35
RP11-287D1.	Inf	3.39E-157	1.43E-154
MTHFD2	1.69761283	6.92E-47	1.58E-46
RP11-287D1.	NA	NA	NA
SLC4A5	NA	0.0011667	0.00132971
DCTN1	2.03735409	5.06E-11	6.80E-11
DCTN1-AS1	NA	NA	NA
C2orf81	NA	1.00E-128	1.27E-127
WDR54	1.30143482	2.84E-117	2.31E-116
RTKN	1.49805447	0.00126294	0.00143696
INO80B	7.86478599	5.28E-122	5.05E-121
INO80B-WBI	NA	NA	NA
WBP1	Inf	3.50E-39	7.18E-39
MOGS	1.6031811	4.34E-06	5.27E-06
MRPL53	1.99740597	3.18E-24	5.28E-24
CCDC142	2.71522374	1.19E-07	1.49E-07
TTC31	1.78614187	0.1473935	0.15592821
LBX2	NA	NA	NA
RP11-523H2(	NA	2.07E-112	1.48E-111
LBX2-AS1	NA	6.66E-74	2.26E-73
PCGF1	1.77042802	3.06E-71	9.96E-71
TLX2	NA	NA	NA
DQX1	Inf	0.14891611	0.15748389
AUP1	1.59888506	2.59E-126	2.95E-125
HTRA2	1.45597429	8.02E-38	1.61E-37
LOXL3	1.34824903	3.19E-06	3.88E-06
DOK1	1.91418072	9.45E-39	1.93E-38

M1AP	0.74902724	0.09349446	0.09991026
SEMA4F	3.74513619	4.97E-77	1.76E-76
RP11-259N1!	3.74513619	1.16E-58	3.11E-58
HK2	3.1459144	2.27E-118	1.90E-117
LINC01291	NA	6.70E-38	1.35E-37
AC104135.2	Inf	6.12E-146	6.20E-144
LINC01293	NA	NA	NA
POLE4	1.58053163	3.61E-52	8.90E-52
TACR1	NA	NA	NA
RP11-355F16	NA	5.37E-05	6.34E-05
EVA1A	NA	NA	NA
AC007099.2	NA	NA	NA
AC007099.1	NA	NA	NA
MRPL19	1.1797179	2.28E-81	8.67E-81
GCFC2	2.19090467	1.21E-82	4.71E-82
RP11-342K6.	1.12354086	3.72E-10	4.92E-10
RP11-342K6.	NA	NA	NA
RP11-342K6.	0	2.00E-89	8.79E-89
LRRTM4	NA	NA	NA
AC079117.1	NA	NA	NA
AC084149.2	NA	NA	NA
AC084149.1	NA	5.79E-95	2.83E-94
AC073628.1	NA	NA	NA
AC012494.1	NA	NA	NA
AC092660.1	NA	NA	NA
REG3G	NA	NA	NA
REG1B	NA	NA	NA
REG1A	NA	NA	NA
REG3A	NA	NA	NA
CTNNA2	0	3.01E-137	6.36E-136
AC010975.1	NA	NA	NA
AC016716.2	NA	NA	NA
LRRTM1	NA	NA	NA
AC008067.2	NA	NA	NA
AC084193.1	NA	NA	NA
AC012075.2	NA	NA	NA
AC079896.1	NA	NA	NA
AC010105.1	NA	NA	NA
AC010744.1	NA	NA	NA
AC106874.1	NA	NA	NA
SUCLG1	1.0712191	2.23E-138	5.19E-137
DNAH6	NA	NA	NA
TRABD2A	1.39818418	3.42E-24	5.69E-24
AC022210.1	NA	NA	NA
TMSB10	2.08529805	2.54E-21	4.05E-21

KCMF1	2.24708171	2.99E-72	9.89E-72
RP11-60H5.1	NA	NA	NA
TCF7L1	1.74773022	5.31E-24	8.78E-24
TGOLN2	1.83971602	0.00453194	0.00507522
RETSAT	1.22568093	5.58E-06	6.76E-06
ELMOD3	2.07684825	2.84E-91	1.29E-90
RP11-717A5.	NA	NA	NA
CAPG	1.12354086	1.13E-46	2.58E-46
SH2D6	NA	NA	NA
MAT2A	2.25059827	2.51E-143	1.16E-141
GGCX	1.42996109	1.44E-54	3.68E-54
VAMP8	1.63628492	3.40E-18	5.16E-18
AC016753.2	NA	NA	NA
VAMP5	1.1770428	1.50E-92	7.01E-92
RNF181	1.26055803	3.34E-139	8.62E-138
TMEM150A	1.33301457	8.10E-11	1.08E-10
USP39	2.20546909	0.14797446	0.15652456
C2orf68	1.98780305	2.37E-07	2.96E-07
SFTPB	NA	NA	NA
GNLY	NA	0.03359203	0.03649917
ATOH8	NA	8.57E-135	1.52E-133
AC105053.3	NA	NA	NA
ST3GAL5	1.93498703	0.02170318	0.02374354
ST3GAL5-AS	NA	NA	NA
RP11-548P2.	Inf	2.02E-05	2.41E-05
POLR1A	2.13004621	2.17E-49	5.13E-49
PTCD3	1.86048701	1.11E-11	1.51E-11
IMMT	1.07229162	7.65E-133	1.21E-131
RP11-301O1	Inf	3.98E-28	6.99E-28
MRPL35	1.06736381	1.78E-117	1.46E-116
REEP1	0	1.77E-50	4.25E-50
KDM3A	1.49805447	0.56418357	0.57722865
CHMP3	2.27431907	1.63E-53	4.09E-53
RNF103-CHN	NA	9.79E-32	1.81E-31
AC015971.2	NA	4.01E-05	4.75E-05
RNF103	2.99610895	1.64E-25	2.77E-25
RMND5A	1.6665856	1.02E-08	1.30E-08
CD8A	NA	NA	NA
CD8B	1.61789883	1.08E-20	1.70E-20
RGPD1	NA	NA	NA
PLGLB1	0	7.17E-11	9.59E-11
RP11-223J6.	NA	NA	NA
IGKV3OR2-2	NA	NA	NA
AC133644.2	Inf	0.00036958	0.00042723
LINC00152	1.9758822	1.04E-106	6.43E-106

RP11-1399P1	NA	NA	NA
PLGLB2	NA	NA	NA
RGPD2	NA	NA	NA
KRCC1	1.85473411	7.13E-47	1.62E-46
SMYD1	NA	NA	NA
FABP1	NA	NA	NA
THNSL2	NA	NA	NA
FOXI3	NA	NA	NA
TEX37	NA	NA	NA
AC104134.2	NA	NA	NA
EIF2AK3	6.74124514	7.89E-08	9.94E-08
AC062029.1	0	7.69E-47	1.75E-46
RPIA	1.63053548	9.01E-81	3.39E-80
AC096579.13	NA	NA	NA
IGKC	Inf	1.85E-84	7.46E-84
IGKV1-12	NA	NA	NA
IGKV1-8	NA	NA	NA
IGKJ5	NA	NA	NA
IGKJ4	NA	NA	NA
IGKJ3	NA	NA	NA
IGKJ2	NA	NA	NA
IGKJ1	NA	NA	NA
IGKV4-1	NA	NA	NA
IGKV5-2	NA	NA	NA
IGKV7-3	NA	NA	NA
IGKV2-4	NA	NA	NA
IGKV1-5	NA	NA	NA
IGKV1-6	NA	NA	NA
IGKV3-7	NA	NA	NA
IGKV1-9	NA	NA	NA
IGKV2-10	NA	NA	NA
IGKV3-11	NA	NA	NA
IGKV1-13	NA	NA	NA
IGKV2-14	NA	NA	NA
IGKV3-15	NA	NA	NA
IGKV1-16	NA	NA	NA
IGKV1-17	NA	NA	NA
IGKV2-18	NA	NA	NA
IGKV2-19	NA	NA	NA
IGKV3-20	NA	NA	NA
IGKV6-21	NA	NA	NA
IGKV1-22	NA	NA	NA
IGKV2-23	NA	NA	NA
IGKV2-24	NA	NA	NA
IGKV3-25	NA	NA	NA

IGKV2-26	NA	NA	NA
IGKV1-27	NA	NA	NA
IGKV2-28	NA	NA	NA
IGKV2-29	NA	NA	NA
IGKV2-30	NA	NA	NA
IGKV3-31	NA	NA	NA
IGKV1-32	NA	NA	NA
IGKV1-33	NA	NA	NA
IGKV3-34	NA	NA	NA
IGKV1-35	NA	NA	NA
IGKV2-36	NA	NA	NA
IGKV1-37	NA	NA	NA
IGKV2-38	NA	NA	NA
IGKV1-39	NA	NA	NA
IGKV2-40	NA	NA	NA
IGKV2D-40	NA	NA	NA
IGKV1D-39	NA	NA	NA
IGKV2D-38	NA	NA	NA
IGKV1D-37	NA	NA	NA
IGKV2D-36	NA	NA	NA
IGKV1D-35	NA	NA	NA
IGKV3D-34	NA	NA	NA
IGKV1D-33	NA	NA	NA
IGKV1D-32	NA	NA	NA
IGKV3D-31	NA	NA	NA
IGKV2D-30	NA	NA	NA
IGKV2D-29	NA	NA	NA
IGKV2D-28	NA	NA	NA
IGKV1D-27	NA	NA	NA
IGKV2D-26	NA	NA	NA
IGKV3D-25	NA	NA	NA
IGKV2D-24	NA	NA	NA
IGKV2D-23	NA	NA	NA
IGKV1D-22	NA	NA	NA
IGKV6D-21	NA	NA	NA
IGKV3D-20	NA	NA	NA
IGKV2D-19	NA	NA	NA
IGKV2D-18	NA	NA	NA
IGKV6D-41	NA	NA	NA
IGKV1D-17	NA	NA	NA
IGKV1D-16	NA	NA	NA
IGKV3D-15	NA	NA	NA
IGKV3D-11	NA	NA	NA
IGKV2D-14	NA	NA	NA
IGKV1D-13	NA	NA	NA

IGKV1D-12	NA	NA	NA
IGKV2D-10	NA	NA	NA
IGKV1D-42	NA	NA	NA
IGKV1D-43	NA	NA	NA
IGKV1D-8	NA	NA	NA
IGKV3D-7	NA	NA	NA
IGKV1OR2-1	NA	NA	NA
CH17-132F21	NA	NA	NA
RP11-575H3.	NA	8.05E-14	1.14E-13
IGKV1OR2-1	NA	NA	NA
RP11-407P15	NA	NA	NA
TEKT4	NA	NA	NA
AC103563.8	NA	NA	NA
MAL	NA	NA	NA
AC103563.9	NA	NA	NA
MRPS5	1.3732166	1.73E-53	4.34E-53
ZNF514	1.12354086	9.54E-48	2.20E-47
ZNF2	1.49805447	2.41E-39	4.96E-39
AC092835.2	NA	NA	NA
PROM2	NA	NA	NA
KCNIP3	NA	4.74E-71	1.53E-70
FAHD2A	1.37978702	2.90E-45	6.46E-45
RP11-440D17	NA	NA	NA
RP11-440D17	NA	NA	NA
TRIM43	NA	NA	NA
AC008268.1	NA	NA	NA
LINC00342	4.86867704	0.54919833	0.56227743
ANKRD36C	1.81906615	0.23578267	0.24702986
GPAT2	NA	NA	NA
ADRA2B	NA	6.50E-06	7.86E-06
ASTL	NA	NA	NA
DUSP2	1.65482762	1.03E-30	1.88E-30
AC012307.2	NA	NA	NA
STARD7	1.66322458	0.0316863	0.03447807
TMEM127	1.55797665	3.25E-07	4.04E-07
CIAO1	1.52545791	8.04E-36	1.57E-35
SNRNP200	1.78147019	6.69E-80	2.48E-79
AC021188.4	0.74902724	0.36539818	0.37832746
ITPRIPL1	2.29389591	5.54E-24	9.16E-24
NCAPH	1.59927437	3.08E-81	1.17E-80
NEURL3	NA	NA	NA
RP11-363D14	Inf	1.50E-75	5.20E-75
ARID5A	1.60505837	4.69E-109	3.08E-108
KANSL3	2.18946423	1.15E-102	6.55E-102
FER1L5	NA	NA	NA

LMAN2L	2.14007782	3.22E-50	7.68E-50
CNNM4	1.74773022	2.14E-32	3.99E-32
RP11-353K11	0.74902724	9.70E-10	1.27E-09
CNNM3	1.33160398	0.00439382	0.00492205
ANKRD23	2.99610895	2.21E-58	5.92E-58
ANKRD39	1.85053788	1.42E-06	1.74E-06
SEMA4C	1.49805447	1.28E-13	1.79E-13
FAM178B	NA	NA	NA
IGKV2OR2-1	NA	NA	NA
IGKV2OR2-2	NA	NA	NA
IGKV1OR2-3	NA	NA	NA
FAHD2B	1.00583658	8.22E-124	8.44E-123
ANKRD36	1.62289235	0.02159938	0.02364424
RP11-734K23	NA	NA	NA
AC159540.3	NA	NA	NA
IGKV1OR2-9	NA	NA	NA
IGKV2OR2-1(	NA	NA	NA
IGKV2OR2-7I	NA	NA	NA
IGKV3OR2-5	NA	NA	NA
IGKV1OR2-6	NA	NA	NA
IGKV2OR2-7	NA	NA	NA
IGKV2OR2-8	NA	NA	NA
IGKV1OR2-1	NA	NA	NA
AC159540.1	2.99610895	9.54E-57	2.50E-56
AC159540.2	NA	NA	NA
ANKRD36B	2.62159533	1.32E-57	3.51E-57
COX5B	1.85237929	0.03538203	0.03840498
ACTR1B	1.72503243	3.11E-29	5.55E-29
LINC01125	Inf	2.57E-60	7.09E-60
ZAP70	NA	NA	NA
TMEM131	2.33030696	3.23E-60	8.91E-60
VWA3B	NA	NA	NA
AC092675.3	NA	NA	NA
CNGA3	NA	NA	NA
INPP4A	2.71522374	6.90E-35	1.33E-34
COA5	2.0575688	5.68E-24	9.40E-24
UNC50	1.46748193	9.98E-49	2.33E-48
MGAT4A	3.27699416	2.71E-83	1.07E-82
AC109826.1	1.49805447	1.14E-60	3.15E-60
KIAA1211L	NA	2.54E-98	1.33E-97
TSGA10	1.74773022	8.34E-13	1.16E-12
C2orf15	Inf	0.00518819	0.00579904
RP11-111H13	Inf	1.01E-21	1.62E-21
LIPT1	1.33160398	3.66E-37	7.29E-37
RP11-38C17.	1.12354086	0.00812116	0.00901954

MITD1	1.01790881	7.30E-144	3.75E-142
MRPL30	1.80862674	5.42E-06	6.57E-06
LYG2	1.99740597	2.73E-40	5.69E-40
LYG1	0	2.43E-22	3.93E-22
TXNDC9	0.86328563	4.61E-143	2.01E-141
EIF5B	1.57456801	5.65E-73	1.89E-72
REV1	1.73346304	0.04318315	0.04673838
RP11-527J8.:	NA	0.91173019	0.91586982
AFF3	NA	NA	NA
AC092667.2	NA	NA	NA
LINC01104	NA	NA	NA
LONRF2	NA	NA	NA
CHST10	1.30094204	0.35013444	0.36293789
NMS	NA	NA	NA
PDCL3	0.91439689	3.75E-100	2.02E-99
AC068538.4	NA	NA	NA
AC092168.3	NA	NA	NA
AC092168.4	NA	NA	NA
NPAS2	NA	NA	NA
AC016738.3	NA	NA	NA
AC016738.4	NA	7.22E-27	1.24E-26
RPL31	2.10308048	6.51E-73	2.17E-72
TBC1D8	2.24708171	6.39E-28	1.12E-27
RP11-299H2:	NA	2.40E-50	5.74E-50
CNOT11	1.26953769	4.37E-116	3.44E-115
RNF149	1.3618677	0.65348261	0.66510416
CREG2	NA	5.53E-46	1.24E-45
RFX8	NA	1.12E-40	2.35E-40
AC092570.2	NA	NA	NA
MAP4K4	1.78216825	0.11610888	0.1234946
LINC01127	NA	NA	NA
IL1R2	NA	NA	NA
IL1R1	NA	6.32E-07	7.81E-07
AC007271.3	NA	NA	NA
IL1RL2	NA	NA	NA
IL1RL1	2.0598249	2.82E-24	4.70E-24
IL18R1	Inf	3.19E-143	1.44E-141
IL18RAP	Inf	9.24E-09	1.19E-08
SLC9A4	NA	NA	NA
SLC9A2	NA	NA	NA
MFSD9	1.78614187	5.17E-16	7.60E-16
TMEM182	Inf	4.70E-52	1.16E-51
AC007251.2	NA	NA	NA
AC108051.1	NA	NA	NA
AC108051.2	NA	1.35E-88	5.87E-88

AC108051.3	NA	NA	NA
AC073987.1	NA	NA	NA
AC013727.1	NA	NA	NA
AC013727.2	NA	NA	NA
RP11-76I14.1	NA	NA	NA
AC096554.1	NA	NA	NA
AC068535.2	NA	NA	NA
AC068535.3	NA	NA	NA
LINC01102	NA	NA	NA
LINC01103	NA	NA	NA
AC013402.4	NA	NA	NA
AC013402.3	NA	NA	NA
AC013402.5	NA	NA	NA
AC068057.1	NA	NA	NA
LINC01114	0.88521401	1.21E-10	1.61E-10
POU3F3	2.45136187	1.25E-60	3.47E-60
AC018730.4	NA	1.19E-43	2.58E-43
AC010884.1	NA	NA	NA
MRPS9	1.04683325	2.21E-134	3.83E-133
UTAT33	NA	NA	NA
AC104655.3	NA	NA	NA
GPR45	NA	NA	NA
RP11-332H14	NA	9.58E-05	1.12E-04
TGFBRAP1	2.17574578	3.50E-30	6.32E-30
AC012360.4	NA	2.43E-148	2.94E-146
RP11-332H14	1.7655642	6.01E-24	9.93E-24
C2orf49	1.36697471	1.80E-21	2.87E-21
FHL2	1.24837873	1.31E-77	4.69E-77
AC012360.6	NA	NA	NA
NCK2	3.28605498	4.23E-141	1.36E-139
AC009505.2	NA	NA	NA
AC009505.4	NA	NA	NA
C2orf40	NA	NA	NA
UXS1	1.46924573	2.00E-83	7.89E-83
AC018878.3	NA	NA	NA
RGPD3	Inf	1.94E-06	2.37E-06
AC108868.5	NA	5.78E-19	8.88E-19
AC108868.6	2.24708171	1.74E-20	2.73E-20
ST6GAL2	NA	NA	NA
AC016994.2	NA	NA	NA
AC006227.1	NA	NA	NA
AC096669.1	NA	NA	NA
AC096669.2	NA	NA	NA
AC096669.3	NA	NA	NA
GACAT1	NA	NA	NA

RGPD4-AS1	Inf	5.35E-13	7.44E-13
RGPD4	NA	NA	NA
SLC5A7	NA	NA	NA
LINC01593	NA	NA	NA
LINC01594	NA	NA	NA
SULT1C3	NA	NA	NA
SULT1C2	NA	NA	NA
SULT1C4	NA	1.86E-31	3.41E-31
GCC2	1.30094204	2.70E-41	5.70E-41
GCC2-AS1	Inf	1.17E-42	2.52E-42
LIMS1	2.29838495	4.48E-57	1.18E-56
LIMS1-AS1	NA	NA	NA
RANBP2	1.60505837	1.25E-40	2.62E-40
CCDC138	2.32198444	5.31E-26	9.06E-26
EDAR	NA	NA	NA
SH3RF3-AS1	NA	NA	NA
SH3RF3	NA	NA	NA
AC109344.1	NA	NA	NA
10-Sep	NA	6.97E-94	3.33E-93
SOWAHC	NA	3.59E-37	7.16E-37
RP11-506F3.	NA	NA	NA
RGPD5	NA	1.70E-62	4.86E-62
LIMS3	NA	NA	NA
LINC01123	0.99870298	5.26E-18	7.97E-18
MALL	NA	NA	NA
NPHP1	NA	1.54E-06	1.89E-06
LINC00116	2.23449302	5.29E-109	3.47E-108
RP11-1223D1	NA	NA	NA
LINC01106	1.49805447	4.48E-22	7.21E-22
RP13-1039J1	NA	NA	NA
AC108938.5	NA	NA	NA
LIMS3L	NA	NA	NA
RP13-1039J1	NA	NA	NA
RGPD6	NA	NA	NA
AC123886.2	NA	NA	NA
BUB1	0.90953307	1.23E-82	4.80E-82
AC114776.3	NA	NA	NA
ACOXL	NA	NA	NA
AC096670.3	NA	NA	NA
BCL2L11	2.28453307	1.65E-17	2.48E-17
AC108463.1	NA	NA	NA
MIR4435-2H	3.27699416	1.29E-138	3.10E-137
RP11-181E1(	NA	5.68E-50	1.35E-49
AC068491.2	NA	NA	NA
RP11-803D5.	2.24708171	4.95E-06	6.00E-06

RP11-68E19.	NA	NA	NA
AC017002.1	NA	8.69E-42	1.85E-41
AC017002.2	NA	1.87E-29	3.34E-29
AC017002.4	NA	NA	NA
ANAPC1	1.9865505	2.94E-13	4.11E-13
MERTK	1.87256809	7.64E-14	1.08E-13
TMEM87B	1.24837873	1.31E-08	1.67E-08
FBLN7	Inf	3.35E-81	1.27E-80
ZC3H8	1.44043699	0.00770127	0.00856473
ZC3H6	0.46814202	1.55E-119	1.36E-118
RGPD8	NA	9.29E-07	1.14E-06
TTL	2.49675746	3.61E-59	9.79E-59
POLR1B	1.74773022	3.64E-40	7.57E-40
CHCHD5	1.89532859	4.59E-22	7.39E-22
AC012442.6	NA	8.89E-76	3.09E-75
AC079922.3	1.68531128	0.00041143	0.00047494
SLC20A1	2.1352866	1.66E-09	2.17E-09
NT5DC4	NA	8.76E-27	1.51E-26
CKAP2L	1.85053788	1.21E-89	5.35E-89
IL1A	NA	NA	NA
IL1B	0.37451362	5.23E-93	2.47E-92
IL37	NA	2.22E-08	2.83E-08
IL36G	NA	1.01E-11	1.37E-11
IL36A	NA	NA	NA
IL36B	NA	NA	NA
IL36RN	NA	NA	NA
IL1F10	NA	NA	NA
IL1RN	NA	1.95E-83	7.70E-83
PSD4	2.24708171	5.62E-74	1.91E-73
AC016683.5	NA	NA	NA
PAX8	0.93628405	1.07E-32	2.00E-32
RP11-65I12.1	2.16566571	3.83E-23	6.27E-23
IGKV1OR2-1(	NA	NA	NA
AC016745.3	NA	NA	NA
RP11-480C16	NA	1.64E-05	1.97E-05
CBWD2	1.31079767	9.11E-97	4.62E-96
FOXD4L1	Inf	9.80E-59	2.64E-58
RP11-395L14	NA	4.22E-12	5.78E-12
PGM5P4-AS1	NA	NA	NA
FAM138B	NA	NA	NA
RABL2A	5.24319066	2.77E-08	3.52E-08
AC017074.1	NA	NA	NA
AC017074.2	NA	NA	NA
SLC35F5	1.28404669	0.00639767	0.00712939
AC024704.2	NA	1.37E-44	3.02E-44

AC104653.1	Inf	4.78E-20	7.45E-20
RP11-141B14	0.49935149	1.57E-38	3.19E-38
ACTR3	1.15663741	2.31E-123	2.33E-122
LINC01191	0	1.32E-140	4.00E-139
AC110769.3	NA	NA	NA
AC010982.2	NA	NA	NA
DPP10	NA	3.18E-78	1.15E-77
DPP10-AS3	NA	NA	NA
DPP10-AS2	NA	NA	NA
DPP10-AS1	NA	6.87E-74	2.33E-73
AC009312.1	NA	NA	NA
DDX18	1.71111111	0.00167212	0.0018955
AC009404.2	2.99610895	2.51E-104	1.48E-103
CCDC93	1.77893969	0.49637289	0.50963198
AC009303.1	NA	4.66E-51	1.13E-50
RP11-98C1.2	NA	NA	NA
INSIG2	2.82965845	9.52E-07	1.17E-06
AC093901.1	NA	NA	NA
RP11-19E11.	NA	NA	NA
EN1	NA	NA	NA
MARCO	NA	NA	NA
RP11-77A13.	NA	NA	NA
C1QL2	NA	NA	NA
STEAP3	1.06440713	0.00225623	0.00254682
STEAP3-AS1	NA	4.81E-113	3.52E-112
C2orf76	1.55567195	1.58E-107	1.00E-106
DBI	1.68662999	7.98E-47	1.82E-46
TMEM37	0.59922179	1.89E-50	4.52E-50
SCTR	NA	NA	NA
AC013275.2	NA	NA	NA
CFAP221	NA	NA	NA
TMEM177	1.90030984	1.90E-11	2.57E-11
PTPN4	4.86867704	7.66E-112	5.40E-111
EPB41L5	4.49416342	9.44E-11	1.26E-10
AC012363.4	NA	NA	NA
TMEM185B	0.83545346	7.90E-77	2.79E-76
RALB	1.35870057	1.77E-84	7.13E-84
AC012363.2	NA	NA	NA
AC012363.13	NA	NA	NA
INHBB	NA	NA	NA
AC073257.2	NA	NA	NA
AC073257.1	NA	NA	NA
AC018866.1	NA	NA	NA
GLI2	Inf	2.04E-92	9.51E-92
RP11-297J22	NA	NA	NA

AC067960.1	NA	NA	NA
AC079988.3	NA	NA	NA
TFCP2L1	3.18336576	5.10E-116	4.01E-115
CLASP1	1.28404669	0.0135032	0.01488848
RP11-204L24	Inf	2.80E-140	8.11E-139
NIFK-AS1	1.66450497	1.91E-05	2.28E-05
NIFK	1.06047843	2.20E-121	2.05E-120
TSN	1.16982906	2.93E-136	5.71E-135
AC018737.3	NA	9.20E-86	3.78E-85
AC062020.2	NA	NA	NA
AC062020.1	NA	NA	NA
AC073409.1	NA	NA	NA
AC079154.1	NA	NA	NA
CNTNAP5	NA	NA	NA
AC097499.1	NA	NA	NA
AC114813.1	NA	NA	NA
AC023347.1	NA	NA	NA
GYPC	NA	NA	NA
RP11-521O1	NA	NA	NA
RP11-521O1	NA	NA	NA
BIN1	1.13225048	8.40E-65	2.48E-64
CYP27C1	NA	3.34E-19	5.15E-19
ERCC3	1.13982406	3.86E-42	8.23E-42
MAP3K2	1.23752326	1.11E-27	1.94E-27
AC068282.3	NA	5.56E-53	1.39E-52
PROC	1.49805447	1.57E-12	2.16E-12
IWS1	1.42603262	1.48E-50	3.55E-50
AC010976.2	NA	NA	NA
MYO7B	NA	NA	NA
RP11-286H1	NA	NA	NA
LIMS2	NA	2.16E-76	7.58E-76
GPR17	NA	NA	NA
WDR33	1.63424125	7.24E-67	2.21E-66
SFT2D3	2.08063121	5.03E-38	1.01E-37
POLR2D	2.01221724	5.18E-25	8.72E-25
AMMECR1L	1.63424125	1.21E-41	2.57E-41
RP11-395A1	5.61770428	4.55E-65	1.35E-64
SAP130	3.29571984	3.79E-77	1.35E-76
UGGT1	2.33520256	2.04E-110	1.38E-109
HS6ST1	2.4887034	2.54E-45	5.66E-45
AC012451.1	NA	NA	NA
AC068483.1	NA	NA	NA
AC079586.1	NA	NA	NA
AC079776.1	NA	NA	NA
AC079776.3	NA	NA	NA

RAB6C	NA	NA	NA
POTEF	NA	NA	NA
AC018804.3	NA	NA	NA
CCDC74B	Inf	1.23E-49	2.91E-49
SMPD4	2.71869145	1.71E-83	6.76E-83
MZT2B	2.00217408	1.06E-34	2.04E-34
TUBA3E	NA	NA	NA
CCDC115	1.68531128	6.28E-05	7.40E-05
IMP4	1.79766537	2.19E-14	3.12E-14
PTPN18	1.94967384	0.00070056	0.00080303
POTEI	NA	NA	NA
AC013269.3	NA	NA	NA
CFC1B	NA	NA	NA
AC013269.4	NA	NA	NA
AC013269.5	NA	NA	NA
TISP43	NA	NA	NA
AC140481.4	NA	NA	NA
CFC1	NA	NA	NA
POTEJ	NA	NA	NA
GPR148	NA	NA	NA
AC140481.8	NA	NA	NA
AMER3	NA	NA	NA
AC133785.1	NA	NA	NA
ARHGEF4	NA	5.65E-99	2.99E-98
FAM168B	2.08771421	3.15E-109	2.08E-108
PLEKHB2	1.71595331	1.84E-29	3.29E-29
POTEE	NA	NA	NA
LINC01120	NA	NA	NA
RP11-109E12	NA	NA	NA
MZT2A	2.01469466	4.96E-47	1.13E-46
TUBA3D	NA	NA	NA
CCDC74A	1.87256809	0.00129186	0.0014694
LINC01087	NA	NA	NA
C2orf27B	NA	0.00727894	0.0081025
AC103564.7	NA	NA	NA
AJ239322.3	NA	NA	NA
AJ239322.1	NA	NA	NA
RP1-156L9.1	NA	NA	NA
ANKRD30BL	NA	NA	NA
AC097532.2	NA	NA	NA
RP11-725P16	NA	NA	NA
GPR39	NA	NA	NA
LYPD1	NA	NA	NA
AC010974.3	NA	NA	NA
NCKAP5	NA	6.73E-123	6.65E-122

AC016909.1	NA	NA	NA
AC010890.1	NA	NA	NA
AC011243.1	NA	NA	NA
MGAT5	2.58754864	6.09E-81	2.30E-80
TMEM163	0.61284047	2.92E-48	6.77E-48
CCNT2-AS1	1.74773022	1.07E-22	1.74E-22
ACMSD	NA	NA	NA
CCNT2	3.49546044	1.98E-39	4.07E-39
MAP3K19	NA	3.75E-93	1.77E-92
RAB3GAP1	1.83095547	7.53E-07	9.30E-07
ZRANB3	1.49805447	9.59E-18	1.45E-17
R3HDM1	2.70971618	1.76E-125	1.92E-124
UBXN4	1.60713611	6.94E-25	1.17E-24
LCT	NA	2.78E-134	4.75E-133
AC011893.3	NA	NA	NA
MCM6	1.95282101	1.18E-08	1.51E-08
DARS	1.80447471	6.51E-38	1.31E-37
DARS-AS1	0.74902724	1.71E-115	1.32E-114
AC068492.1	NA	NA	NA
CXCR4	NA	0.04216748	0.04567724
THSD7B	NA	NA	NA
HNMT	1.11219196	2.75E-100	1.49E-99
AC069394.1	NA	NA	NA
AC097721.2	2.24708171	7.33E-22	1.18E-21
AC114763.1	NA	NA	NA
SPOPL	3.37062257	7.65E-15	1.10E-14
RP11-231E19	NA	1.08E-22	1.76E-22
AC092620.3	NA	5.19E-35	1.00E-34
NXPH2	NA	NA	NA
AC062021.1	NA	NA	NA
AC016710.1	NA	NA	NA
AC078851.1	NA	NA	NA
AC073928.2	NA	NA	NA
LRP1B	NA	NA	NA
KYNU	NA	2.34E-18	3.56E-18
ARHGAP15	1.49805447	1.16E-34	2.23E-34
RP11-190J23	NA	NA	NA
AC096558.1	NA	NA	NA
RP11-570L15	NA	NA	NA
RP11-570L15	NA	NA	NA
AC092652.1	NA	NA	NA
RP11-434H14	NA	NA	NA
CTD-2252P21	NA	NA	NA
AC079584.2	NA	NA	NA
AC016910.1	NA	NA	NA

GTDC1	0.64202335	7.02E-30	1.26E-29
ZEB2	2.37816148	1.79E-106	1.10E-105
RP11-107E5.	NA	NA	NA
ZEB2-AS1	0.74902724	1.58E-27	2.74E-27
LINC01412	NA	1.97E-49	4.64E-49
TEX41	NA	NA	NA
AC023128.1	NA	NA	NA
RP11-514A9.	NA	NA	NA
AC064865.1	NA	NA	NA
AC092484.1	NA	NA	NA
AC079163.1	NA	NA	NA
AC062031.1	NA	NA	NA
AC103881.1	NA	NA	NA
AC062032.1	NA	NA	NA
ACVR2A	0.44941634	1.20E-90	5.38E-90
AC009480.3	NA	NA	NA
ORC4	1.62289235	1.44E-107	9.13E-107
MBD5	0.89883268	9.65E-10	1.27E-09
EPC2	2.34071012	3.95E-80	1.47E-79
AC105402.4	NA	NA	NA
KIF5C	0.74902724	4.81E-13	6.69E-13
LYPD6B	NA	NA	NA
LYPD6	Inf	9.12E-118	7.52E-117
MMADHC	1.49805447	4.37E-55	1.12E-54
AC144449.1	NA	3.18E-27	5.51E-27
AC007364.1	NA	NA	NA
AC016682.1	NA	NA	NA
AC113610.1	NA	NA	NA
RND3	NA	NA	NA
AC104777.2	NA	NA	NA
AC104777.3	NA	NA	NA
AC104777.1	NA	NA	NA
AC104777.4	NA	NA	NA
AC023469.2	NA	NA	NA
AC023469.1	NA	NA	NA
AC018731.3	NA	NA	NA
RBM43	1.49805447	0.03446394	0.03742188
NMI	1.06214518	4.72E-130	6.39E-129
TNFAIP6	NA	NA	NA
RIF1	1.6079118	2.65E-23	4.34E-23
NEB	NA	1.62E-66	4.91E-66
ARL5A	2.01828794	1.60E-65	4.78E-65
CACNB4	0.89883268	1.52E-10	2.02E-10
AC079790.2	NA	NA	NA
STAM2	2.24708171	3.37E-60	9.31E-60

FMNL2	3.49546044	7.79E-45	1.72E-44
PRPF40A	1.15322791	1.02E-128	1.29E-127
ARL6IP6	1.41293774	9.80E-62	2.77E-61
AC011901.2	NA	NA	NA
AC079150.3	NA	NA	NA
RPRM	NA	NA	NA
GALNT13	NA	NA	NA
AC009227.2	NA	NA	NA
AC009227.3	NA	NA	NA
AC061961.2	NA	NA	NA
KCNJ3	NA	1.55E-11	2.10E-11
AC092625.1	NA	NA	NA
AC093375.1	NA	NA	NA
RP11-605B1(	NA	NA	NA
NR4A2	Inf	4.24E-15	6.12E-15
GPD2	2.78210117	0.04710726	0.05090962
GALNT5	NA	NA	NA
ERMN	NA	5.84E-97	2.97E-96
CYTIP	0.59922179	1.79E-79	6.60E-79
ACVR1C	NA	1.70E-100	9.21E-100
AC019186.1	NA	2.83E-123	2.84E-122
ACVR1	1.74773022	0.75020624	0.75962726
UPP2	NA	NA	NA
RP11-546J1.:	Inf	6.87E-141	2.16E-139
RP11-1152H:	NA	NA	NA
CCDC148-AS:	NA	NA	NA
CCDC148	NA	NA	NA
PKP4	2.13184675	2.12E-77	7.56E-77
AC005042.1	NA	NA	NA
AC005042.4	NA	NA	NA
AC005042.5	NA	NA	NA
DAPL1	4.68142023	3.11E-116	2.46E-115
TANC1	1.94747082	1.18E-68	3.72E-68
WDSUB1	2.7464332	1.64E-16	2.42E-16
BAZ2B	5.09338521	1.69E-127	2.03E-126
AC008277.1	NA	NA	NA
AC009506.1	2.3540856	4.04E-22	6.50E-22
AC009961.3	3.74513619	4.97E-129	6.40E-128
7-Mar	1-Jan	5.18E-10	6.84E-10
CD302	2.45514483	1.14E-85	4.66E-85
LY75-CD302	NA	NA	NA
LY75	0	1.32E-104	7.81E-104
PLA2R1	NA	NA	NA
ITGB6	NA	NA	NA
RBMS1	1.33916991	7.24E-88	3.10E-87

AC009313.1	NA	NA	NA
TANK	1.12354086	1.19E-18	1.82E-18
AC009299.2	NA	NA	NA
AC009299.3	NA	2.13E-20	3.35E-20
PSMD14	0.96585091	1.99E-133	3.25E-132
TBR1	0	0.64301944	0.65493255
AC009487.4	NA	NA	NA
SLC4A10	NA	NA	NA
DPP4	6.74124514	2.02E-145	1.64E-143
AC008063.2	NA	NA	NA
AC008063.3	NA	NA	NA
GCG	NA	NA	NA
AC007750.5	NA	NA	NA
FAP	NA	NA	NA
IFIH1	0.93628405	9.73E-08	1.22E-07
GCA	1.6692607	8.16E-81	3.08E-80
KCNH7	NA	NA	NA
AC007740.1	NA	NA	NA
FIGN	Inf	2.38E-139	6.22E-138
AC092684.1	NA	NA	NA
GRB14	NA	NA	NA
COBLL1	0.74902724	1.51E-25	2.55E-25
AC019181.2	NA	NA	NA
SLC38A11	NA	NA	NA
AC013463.2	NA	NA	NA
SCN3A	NA	NA	NA
SCN2A	NA	5.79E-16	8.49E-16
CSRNP3	NA	NA	NA
GALNT3	NA	1.01E-10	1.34E-10
AC009495.2	NA	NA	NA
AC009495.4	NA	NA	NA
AC009495.3	NA	2.57E-109	1.70E-108
TTC21B	7.49027237	0.31480458	0.32736356
AC010127.3	NA	NA	NA
SCN1A	NA	NA	NA
SCN9A	3.74513619	4.68E-121	4.30E-120
SCN7A	NA	NA	NA
XIRP2	NA	NA	NA
XIRP2-AS1	NA	NA	NA
AC074363.1	NA	NA	NA
AC016723.4	NA	NA	NA
B3GALT1	NA	NA	NA
STK39	1.34824903	0.0012025	0.00136939
CERS6	2.10093006	5.92E-54	1.49E-53
NOSTRIN	1.49805447	0.90526261	0.90982628

SPC25	0.75708129	2.64E-142	1.00E-140
G6PC2	NA	NA	NA
ABCB11	NA	NA	NA
DHRS9	0	1.21E-175	3.66E-172
AC007556.3	NA	NA	NA
LRP2	NA	NA	NA
BBS5	Inf	7.99E-149	1.03E-146
RP11-724O1(	NA	NA	NA
KLHL41	NA	NA	NA
FASTKD1	1.68531128	0.4316492	0.44448744
PPIG	1.55294009	3.99E-69	1.26E-68
CCDC173	1.12354086	0.05489059	0.05916261
PHOSPHO2	1.23054475	1.27E-19	1.97E-19
KLHL23	2.63072981	5.71E-120	5.09E-119
SSB	0.94695671	2.67E-118	2.24E-117
METTL5	1.62160536	1.59E-41	3.37E-41
UBR3	1.7655642	0.32058748	0.33326255
MYO3B	NA	3.17E-21	5.03E-21
AC012594.1	NA	NA	NA
AC007277.3	NA	NA	NA
LINC01124	NA	NA	NA
SP5	0	1.12E-66	3.40E-66
AC007405.4	0	3.38E-134	5.77E-133
AC007405.6	0.14980545	2.30E-60	6.36E-60
ERICH2	0	2.07E-168	2.69E-165
GAD1	0	3.72E-119	3.22E-118
AC007405.8	NA	NA	NA
GORASP2	1.25462062	9.09E-126	1.01E-124
TLK1	2.60951425	3.86E-98	2.01E-97
METTL8	1.9688716	4.56E-35	8.84E-35
DCAF17	1.12354086	2.10E-08	2.68E-08
CYBRD1	1.81495061	1.46E-19	2.27E-19
DYNC1I2	0.91474123	4.22E-138	9.65E-137
SLC25A12	1.9661965	3.42E-17	5.11E-17
HAT1	0.66848667	1.64E-140	4.90E-139
METAP1D	1.49805447	0.00164855	0.00186913
DLX1	1.67090691	0.02113041	0.02313925
DLX2	2.88910506	8.80E-49	2.06E-48
DLX2-AS1	NA	0.03625915	0.03934056
AC104088.1	NA	NA	NA
ITGA6	0.1997406	5.26E-141	1.67E-139
AC078883.4	NA	NA	NA
AC078883.3	0.59922179	3.22E-67	9.87E-67
AC093818.1	1.81906615	0.32887659	0.34160536
PDK1	2.6483463	5.69E-33	1.07E-32

RAPGEF4-AS	NA	NA	NA
RAPGEF4	2.24708171	4.28E-31	7.83E-31
AC013461.1	1.34824903	1.22E-14	1.74E-14
MLK7-AS1	NA	NA	NA
CDCA7	1.99891004	5.36E-99	2.84E-98
RP11-796E1(	NA	NA	NA
AC106900.6	NA	NA	NA
SP3	1.77893969	2.36E-70	7.60E-70
RP11-394I13	NA	0.60957199	0.62198285
RP11-394I13	NA	NA	NA
RP11-394I13	NA	NA	NA
OLA1	1.68376371	6.11E-60	1.68E-59
LINC01305	NA	NA	NA
SP9	1.49805447	1.37E-05	1.64E-05
CIR1	1.53646613	1.09E-35	2.13E-35
SCRN3	2.43433852	1.17E-34	2.25E-34
GPR155	1.79766537	1.87E-05	2.23E-05
AC010894.3	Inf	0.21302131	0.22367361
AC018890.6	NA	3.84E-125	4.13E-124
WIPF1	2.87127108	6.36E-107	3.95E-106
AC010894.5	NA	NA	NA
CHRNA1	NA	NA	NA
AC018890.4	NA	NA	NA
CHN1	Inf	2.90E-09	3.76E-09
ATF2	2.51459144	1.16E-58	3.12E-58
AC096649.2	NA	NA	NA
ATP5G3	1.91844784	1.00E-33	1.90E-33
AC096649.3	NA	NA	NA
AC016751.3	NA	NA	NA
AC016751.2	NA	NA	NA
KIAA1715	2.6528048	1.77E-79	6.54E-79
EVX2	NA	4.63E-22	7.45E-22
HOXD13	1.71206226	0.00040821	0.00047128
HOXD12	NA	NA	NA
HOXD11	NA	NA	NA
HOXD10	NA	NA	NA
HOXD-AS2	NA	NA	NA
HOXD9	NA	NA	NA
HOXD8	NA	NA	NA
HOXD3	NA	NA	NA
AC009336.19	NA	NA	NA
HOXD4	NA	NA	NA
RP11-387A1.	NA	NA	NA
HAGLR	NA	4.11E-11	5.52E-11
HAGLROS	1.49805447	4.46E-52	1.10E-51

HOXD1	NA	NA	NA
MTX2	1.45181823	1.90E-105	1.15E-104
AC017048.1	NA	NA	NA
AC017048.2	NA	NA	NA
RP11-324L17	NA	NA	NA
LINC01116	Inf	1.95E-80	7.29E-80
LINC01117	NA	NA	NA
AC092162.1	NA	NA	NA
AC073636.1	NA	NA	NA
AC079305.11	NA	NA	NA
AC079305.8	NA	NA	NA
HNRNPA3	1.49805447	1.41E-72	4.69E-72
NFE2L2	1.46748193	2.38E-83	9.36E-83
AC079305.10	Inf	0.91221764	0.91630876
RP11-337N6.	NA	NA	NA
RP11-337N6.	NA	NA	NA
AGPS	1.65574442	2.26E-19	3.49E-19
TTC30B	Inf	7.91E-38	1.59E-37
AC073834.3	NA	NA	NA
TTC30A	Inf	6.15E-156	2.11E-153
PDE11A	NA	NA	NA
AC012499.1	NA	NA	NA
RBM45	0.74902724	2.13E-69	6.78E-69
OSBPL6	1.3658732	1.51E-05	1.81E-05
AC009948.5	2.14007782	1.46E-05	1.75E-05
PRKRA	1.85336227	7.77E-19	1.19E-18
DFNB59	NA	7.60E-123	7.49E-122
FKBP7	1.44455253	4.91E-91	2.23E-90
PLEKHA3	2.06398617	8.57E-33	1.60E-32
TTN-AS1	NA	1.18E-62	3.36E-62
TTN	NA	NA	NA
RP11-65L3.4	NA	NA	NA
RP11-65L3.2	NA	NA	NA
RP11-171I2.5	NA	NA	NA
RP11-171I2.2	NA	NA	NA
RP11-171I2.4	NA	NA	NA
RP11-171I2.3	NA	NA	NA
RP11-171I2.1	NA	NA	NA
CCDC141	NA	NA	NA
SESTD1	1.42315175	5.68E-12	7.78E-12
AC093911.1	NA	NA	NA
ZNF385B	NA	NA	NA
CWC22	1.26758456	3.31E-31	6.06E-31
AC009478.1	NA	NA	NA
SCHLAP1	NA	NA	NA

UBE2E3	1.95038911	1.79E-65	5.36E-65
AC104076.3	NA	NA	NA
AC068196.1	NA	NA	NA
AC104820.2	NA	NA	NA
AC020595.1	NA	NA	NA
ITGA4	1.92250324	7.15E-13	9.93E-13
CERKL	NA	1.25E-07	1.57E-07
NEUROD1	NA	8.61E-111	5.90E-110
AC013733.3	NA	NA	NA
AC013733.4	NA	NA	NA
RP11-366L5.	Inf	1.95E-22	3.16E-22
SSFA2	3.13655156	3.01E-75	1.04E-74
PPP1R1C	NA	3.00E-94	1.44E-93
PDE1A	NA	NA	NA
DNAJC10	2.63942931	1.15E-88	5.00E-88
FRZB	NA	NA	NA
NCKAP1	2.24708171	7.70E-08	9.70E-08
DUSP19	4.49416342	1.55E-72	5.15E-72
AC064871.3	NA	NA	NA
NUP35	1.31079767	1.49E-16	2.21E-16
RP11-438L19	0.74902724	0.05101772	0.05507995
AC093639.1	NA	NA	NA
ZNF804A	0.74902724	2.60E-67	8.00E-67
AC007966.1	NA	NA	NA
FSIP2	NA	NA	NA
FSIP2-AS1	NA	2.95E-33	5.55E-33
LINC01473	Inf	1.10E-27	1.91E-27
RP11-410E4.	0	2.84E-19	4.39E-19
ZC3H15	1.3924773	3.06E-70	9.83E-70
AC018867.1	NA	NA	NA
ITGAV	1.68531128	0.25900423	0.27067136
AC017101.10	NA	0.02164078	0.0236767
FAM171B	2.14007782	6.18E-05	7.29E-05
ZSWIM2	NA	NA	NA
AC007319.1	NA	NA	NA
CALCRL	Inf	2.52E-05	3.01E-05
TFPI	0.93628405	0.00512053	0.00572482
LINC01090	NA	NA	NA
GULP1	NA	0.66541104	0.67690295
DIRC1	NA	NA	NA
COL3A1	NA	NA	NA
COL5A2	Inf	7.99E-149	1.03E-146
AC133106.2	NA	NA	NA
WDR75	1.49805447	1.13E-57	3.01E-57
SLC40A1	Inf	3.88E-20	6.05E-20

ASNSD1	1.81749256	2.87E-09	3.72E-09
ANKAR	2.62159533	2.72E-41	5.74E-41
OSGEPL1	1.1770428	3.33E-58	8.90E-58
OSGEPL1-AS	0.74902724	4.33E-74	1.47E-73
RP11-455J20	NA	1.18E-64	3.48E-64
ORMDL1	1.89071633	1.25E-26	2.14E-26
PMS1	1.9214177	5.60E-27	9.67E-27
C2orf88	3.55787938	1.85E-116	1.47E-115
MSTN	NA	NA	NA
HIBCH	1.22124006	1.27E-78	4.62E-78
INPP1	0.82392996	6.47E-136	1.23E-134
MFSD6	1.49805447	4.98E-127	5.84E-126
RP11-647K16	NA	0.00150565	0.00170893
NEMP2	5.9922179	0.04797978	0.05183096
AC093388.3	1.49805447	0.01100092	0.01216498
NAB1	1.59168288	7.70E-08	9.70E-08
AC006460.2	NA	NA	NA
AC005540.3	NA	NA	NA
GLS	2.78210117	3.14E-10	4.15E-10
STAT1	3.52042802	1.50E-86	6.23E-86
AC067945.2	NA	NA	NA
STAT4	5.61770428	4.36E-141	1.40E-139
AC092614.2	NA	1.56E-76	5.50E-76
MYO1B	3.13229572	5.24E-124	5.42E-123
NABP1	1.73212549	2.52E-09	3.27E-09
AC098872.3	NA	NA	NA
SDPR	NA	NA	NA
AC098617.1	NA	NA	NA
TMEFF2	NA	NA	NA
AC013401.1	NA	NA	NA
AC013401.2	NA	NA	NA
PCGEM1	NA	NA	NA
RP11-764E7.	NA	NA	NA
AC018799.1	NA	NA	NA
AC006196.1	NA	NA	NA
AC010983.1	NA	6.20E-55	1.59E-54
AC064834.1	0.74902724	0.84979462	0.85649856
RP11-762E8.	NA	NA	NA
AC064834.2	NA	NA	NA
AC064834.3	Inf	3.97E-95	1.95E-94
SLC39A10	1.51303502	4.55E-52	1.12E-51
DNAH7	NA	3.63E-44	7.94E-44
STK17B	2.37191958	2.69E-76	9.41E-76
RP11-347P5.	1.19844358	8.72E-51	2.10E-50
HECW2	NA	1.29E-139	3.49E-138

AC020571.3	Inf	8.74E-31	1.59E-30
RP11-369J9.:	NA	4.71E-13	6.56E-13
CCDC150	0.96303502	1.03E-15	1.51E-15
GTF3C3	1.49805447	0.00258918	0.00291812
C2orf66	NA	1.97E-49	4.64E-49
PGAP1	3.18336576	6.67E-41	1.40E-40
ANKRD44	1.19844358	5.89E-17	8.76E-17
AC013264.2	NA	NA	NA
AC010746.3	NA	NA	NA
SF3B1	1.01619619	1.22E-129	1.61E-128
COQ10B	1.13645512	1.12E-89	4.95E-89
HSPD1	0.97122362	1.21E-136	2.46E-135
HSPE1	1.74519997	2.19E-06	2.68E-06
HSPE1-MOB4	NA	NA	NA
MOB4	1.25836576	2.21E-84	8.89E-84
RFTN2	NA	2.13E-20	3.35E-20
AC011997.1	Inf	1.00E-55	2.59E-55
MARS2	1.26398346	0.1358645	0.14395828
BOLL	NA	NA	NA
PLCL1	NA	NA	NA
AC109589.1	NA	NA	NA
AC005235.1	NA	NA	NA
AC019330.1	NA	NA	NA
AC018717.1	NA	NA	NA
SATB2	2.04280156	0.0172572	0.01895732
RP11-486F17	NA	NA	NA
SATB2-AS1	0	2.58E-31	4.74E-31
AC093590.1	NA	NA	NA
FTCDNL1	2.7464332	2.97E-54	7.55E-54
C2orf69	1.79312581	0.98850589	0.9892693
TYW5	2.20546909	4.75E-18	7.20E-18
C2orf47	0.77345204	1.50E-118	1.27E-117
SPATS2L	1.95713568	7.59E-10	9.98E-10
KCTD18	2.69649805	3.97E-29	7.06E-29
SGOL2	1.12867118	3.36E-90	1.50E-89
AOX1	Inf	1.64E-59	4.46E-59
AC007163.2	NA	NA	NA
AC007163.3	NA	NA	NA
AC007163.6	NA	NA	NA
BZW1	1.44390793	2.03E-126	2.33E-125
CLK1	1.71206226	2.12E-29	3.79E-29
PPIL3	1.82371849	2.85E-05	3.39E-05
NIF3L1	1.27775235	6.48E-96	3.22E-95
ORC2	2.48115272	2.19E-10	2.90E-10
AC005037.3	2.7464332	0.01065554	0.01178881

FAM126B	1.84135863	3.09E-61	8.65E-61
NDUFB3	1.78339818	0.15105323	0.15968811
RP11-13J8.1	NA	NA	NA
CFLAR	1.29831388	1.53E-23	2.52E-23
CFLAR-AS1	NA	NA	NA
CASP10	1.49805447	6.35E-46	1.43E-45
CASP8	1.49805447	2.87E-30	5.20E-30
ALS2CR12	NA	0.02104074	0.02304244
TRAK2	2.77580682	2.16E-138	5.03E-137
STRADB	2.13573983	9.42E-05	1.10E-04
ALS2CR11	NA	NA	NA
TMEM237	1.90377756	3.87E-15	5.60E-15
MPP4	Inf	9.37E-102	5.23E-101
ALS2	2.29114214	4.44E-23	7.26E-23
CDK15	NA	3.06E-09	3.97E-09
RP11-107N1!	Inf	6.15E-05	7.25E-05
FZD7	1.79766537	0.07039717	0.07557074
KIAA2012	Inf	5.64E-69	1.78E-68
AC079354.3	NA	NA	NA
AC079354.5	5.9922179	4.33E-45	9.61E-45
SUMO1	1.93946785	0.43439069	0.4472597
NOP58	1.07069538	4.25E-90	1.89E-89
RP11-686O6.	Inf	3.46E-05	4.10E-05
RP11-686O6.	1.49805447	0.2323135	0.24345146
BMPR2	4.49416342	6.18E-84	2.46E-83
FAM117B	2.99610895	5.71E-113	4.16E-112
ICA1L	7.86478599	1.47E-115	1.14E-114
WDR12	1.45565671	4.43E-67	1.36E-66
CARF	Inf	5.47E-76	1.91E-75
NBEAL1	1.97201521	2.54E-27	4.41E-27
CYP20A1	2.27964811	5.22E-29	9.28E-29
ABI2	2.72373541	2.92E-81	1.11E-80
RP11-363J17	NA	NA	NA
RAPH1	NA	3.17E-10	4.20E-10
CD28	NA	NA	NA
CTLA4	NA	NA	NA
ICOS	NA	NA	NA
AC009965.2	NA	NA	NA
AC009498.1	NA	NA	NA
AC016903.1	NA	NA	NA
AC016903.2	NA	NA	NA
PARD3B	NA	NA	NA
NRP2	2.0598249	2.15E-16	3.17E-16
AC007362.3	NA	NA	NA
AC007679.3	NA	NA	NA

INO80D	2.99610895	4.97E-17	7.41E-17
AC007383.3	NA	3.53E-127	4.18E-126
NDUFS1	1.64420613	4.18E-05	4.95E-05
AC007383.4	NA	6.07E-07	7.50E-07
EEF1B2	1.75810728	1.27E-12	1.76E-12
GPR1	NA	NA	NA
ZDBF2	NA	5.55E-102	3.11E-101
AC017081.1	NA	NA	NA
ADAM23	NA	NA	NA
AC010731.2	NA	NA	NA
AC010731.3	NA	NA	NA
AC010731.4	NA	NA	NA
DYTN	NA	NA	NA
MDH1B	NA	NA	NA
FASTKD2	1.33160398	5.81E-69	1.83E-68
RP11-95H11.	NA	NA	NA
AC008269.2	NA	NA	NA
CPO	NA	NA	NA
KLF7	5.9922179	0.29046321	0.30260616
AC007879.1	NA	NA	NA
AC007879.2	NA	NA	NA
AC007879.4	NA	0.00073116	0.00083747
AC007879.3	NA	NA	NA
AC007879.7	Inf	9.38E-63	2.68E-62
AC007879.5	NA	NA	NA
AC007879.6	NA	NA	NA
CREB1	1.33160398	1.38E-12	1.91E-12
METTL21A	2.00710211	1.95E-41	4.12E-41
AC079767.4	NA	NA	NA
CCNYL1	1.22568093	0.77872081	0.78735735
RP11-801F7.	NA	0.03083857	0.03357176
FZD5	1.49805447	2.55E-103	1.47E-102
AC096772.6	Inf	1.12E-152	2.14E-150
PLEKHM3	NA	1.66E-103	9.60E-103
CRYGD	NA	NA	NA
CRYGC	NA	NA	NA
CRYGB	NA	NA	NA
CRYGA	NA	NA	NA
C2orf80	Inf	1.66E-67	5.10E-67
IDH1	1.31585866	3.35E-73	1.12E-72
IDH1-AS1	1.24837873	5.90E-25	9.92E-25
PIKFYVE	1.35761187	0.48933344	0.50260359
PTH2R	NA	NA	NA
MAP2	NA	7.65E-06	9.24E-06
UNC80	0.74902724	2.62E-09	3.41E-09

RPE	1.41482923	6.33E-59	1.71E-58
KANSL1L	1.49805447	9.98E-13	1.38E-12
RP11-260M2	Inf	2.19E-69	6.96E-69
AC007038.7	1.49805447	0.35134041	0.36414635
AC006994.2	NA	2.11E-133	3.43E-132
ACADL	NA	NA	NA
AC006994.3	NA	NA	NA
MYL1	NA	NA	NA
LANCL1-AS1	NA	NA	NA
LANCL1	2.88910506	3.20E-12	4.39E-12
CPS1	NA	2.32E-48	5.39E-48
ERBB4	NA	NA	NA
AC093381.2	NA	NA	NA
AC108066.1	NA	NA	NA
IKZF2	3.95914397	2.20E-119	1.92E-118
RP11-105N1	5.24319066	5.18E-31	9.46E-31
RP11-105N1	NA	NA	NA
RP11-105N1	NA	NA	NA
AC079610.2	NA	NA	NA
SPAG16	NA	1.49E-17	2.24E-17
AC107218.3	NA	NA	NA
VWC2L	NA	NA	NA
BARD1	1.97243839	5.52E-11	7.41E-11
AC072062.1	NA	5.60E-91	2.53E-90
ABCA12	NA	NA	NA
AC072062.3	NA	NA	NA
AC073284.4	NA	NA	NA
ATIC	1.01580406	7.61E-109	4.97E-108
FN1	NA	5.09E-91	2.30E-90
AC012462.1	NA	NA	NA
AC012462.2	NA	NA	NA
AC012462.3	NA	NA	NA
AC012668.1	NA	NA	NA
AC012668.2	NA	NA	NA
AC012668.3	NA	NA	NA
LINC00607	NA	NA	NA
LINC01614	NA	NA	NA
AC122136.2	NA	NA	NA
AC093382.1	Inf	1.42E-87	6.02E-87
MREG	1.1770428	1.61E-10	2.15E-10
PECR	1.46060311	8.05E-131	1.13E-129
TMEM169	NA	7.00E-116	5.48E-115
XRCC5	1.03022351	1.69E-111	1.19E-110
PKI55	1.71206226	8.43E-08	1.06E-07
4-Mar	NA	NA	NA

AC012513.6	NA	NA	NA
AC069155.1	NA	NA	NA
AC098820.2	NA	NA	NA
SMARCAL1	1.39105058	1.70E-76	5.97E-76
AC098820.4	Inf	9.90E-21	1.56E-20
RPL37A	2.55957801	4.12E-145	2.89E-143
LINC01280	NA	NA	NA
AC073321.4	NA	3.48E-60	9.59E-60
IGFBP2	1.36372774	1.22E-137	2.67E-136
IGFBP5	NA	NA	NA
AC007563.5	NA	NA	NA
AC007563.3	NA	NA	NA
AC007557.4	NA	NA	NA
TNP1	NA	NA	NA
AC007557.5	NA	NA	NA
AC007557.2	NA	NA	NA
AC007557.3	NA	NA	NA
AC007563.1	NA	NA	NA
AC007557.1	NA	NA	NA
RP11-574O1(	NA	NA	NA
DIRC3-AS1	NA	NA	NA
DIRC3	NA	NA	NA
TNS1	Inf	6.68E-06	8.08E-06
AC010136.2	NA	NA	NA
RUFY4	NA	NA	NA
CXCR2	0	3.46E-42	7.39E-42
CXCR1	NA	NA	NA
ARPC2	1.76301649	8.01E-07	9.88E-07
GPBAR1	NA	5.67E-112	4.01E-111
AAMP	0.91254727	8.51E-127	9.86E-126
PNKD	1.53271383	1.34E-54	3.41E-54
TMBIM1	0.96070885	1.56E-21	2.50E-21
CATIP-AS2	NA	NA	NA
CATIP	Inf	4.14E-59	1.12E-58
SLC11A1	0.34046693	4.40E-64	1.28E-63
CTDSP1	1.35840533	5.76E-16	8.44E-16
RP11-378A1:	0.74902724	1.22E-06	1.50E-06
VIL1	NA	7.97E-25	1.34E-24
USP37	1.87256809	3.86E-05	4.57E-05
RQCD1	2.2865042	1.28E-116	1.02E-115
PLCD4	NA	NA	NA
RP11-548H3.	1.31079767	0.60375116	0.61642487
ZNF142	5.9922179	7.25E-90	3.21E-89
BCS1L	1.53627015	5.12E-54	1.29E-53
RNF25	0.74902724	2.92E-126	3.30E-125

STK36	1.99740597	7.46E-80	2.77E-79
TTLL4	3.16255945	5.00E-58	1.33E-57
CYP27A1	Inf	3.52E-24	5.84E-24
RP11-459I19	NA	NA	NA
PRKAG3	NA	NA	NA
WNT6	0	3.79E-111	2.63E-110
WNT10A	NA	2.99E-74	1.02E-73
LINC01494	NA	NA	NA
AC097468.7	NA	NA	NA
CDK5R2	NA	8.88E-06	1.07E-05
LINC00608	NA	NA	NA
FEV	Inf	0.18714035	0.19703443
CRYBA2	NA	NA	NA
AC097468.4	NA	NA	NA
CFAP65	NA	NA	NA
IHH	NA	NA	NA
RP11-33O4.1	2.24708171	5.84E-06	7.07E-06
NHEJ1	Inf	5.43E-15	7.83E-15
RP11-33O4.2	NA	NA	NA
SLC23A3	1.49805447	3.57E-15	5.16E-15
CNPPD1	1.16675397	4.60E-89	2.01E-88
FAM134A	2.17065036	3.12E-49	7.35E-49
ZFAND2B	2.09473719	1.29E-63	3.74E-63
ABCB6	NA	NA	NA
ATG9A	2.20302129	1.06E-46	2.41E-46
ANKZF1	1.16099222	1.96E-130	2.71E-129
GLB1L	Inf	7.62E-106	4.63E-105
STK16	1.75858569	0.08848582	0.0946248
TUBA4A	0.86208795	3.31E-142	1.24E-140
TUBA4B	NA	6.14E-129	7.88E-128
DNAJB2	1.68531128	3.13E-28	5.51E-28
PTPRN	0	8.75E-122	8.30E-121
AC114803.3	NA	NA	NA
RESP18	NA	NA	NA
DNPEP	2.0366808	3.45E-23	5.64E-23
AC053503.4	NA	8.25E-13	1.14E-12
DES	NA	7.97E-25	1.34E-24
AC053503.6	NA	NA	NA
SPEG	Inf	1.38E-92	6.45E-92
AC053503.11	NA	NA	NA
RP11-316O1	0	0.64451469	0.65630812
GMPPA	0.94442565	1.30E-101	7.23E-101
ASIC4	Inf	2.40E-09	3.12E-09
CHPF	1.19844358	2.57E-100	1.39E-99
TMEM198	2.99610895	9.43E-09	1.21E-08

RP11-256I23	NA	NA	NA
OBSL1	1.18179853	5.41E-46	1.22E-45
RP11-256I23	NA	2.38E-117	1.94E-116
INHA	NA	5.40E-133	8.63E-132
STK11IP	1.39105058	6.65E-29	1.18E-28
AC009955.8	NA	2.43E-122	2.35E-121
SLC4A3	Inf	2.15E-08	2.74E-08
RP11-256I23	NA	5.60E-91	2.53E-90
AC009502.4	NA	NA	NA
AC008281.1	NA	NA	NA
AC114765.1	NA	NA	NA
AC114765.2	NA	NA	NA
AC067956.1	NA	NA	NA
AC093843.1	NA	NA	NA
EPHA4	NA	NA	NA
CTD-2308L22	NA	NA	NA
AC068489.1	NA	NA	NA
PAX3	NA	NA	NA
CCDC140	NA	NA	NA
RP11-384O8.	NA	7.60E-105	4.52E-104
SGPP2	0.24967575	4.38E-80	1.63E-79
RP11-16P6.1	2.24708171	0.00210308	0.00237616
FARSB	1.34085123	4.32E-43	9.34E-43
MOGAT1	NA	NA	NA
ACSL3	1.62913424	3.94E-48	9.12E-48
AC097461.4	NA	NA	NA
KCNE4	NA	NA	NA
AC013448.1	NA	NA	NA
AC013448.2	NA	NA	NA
SCG2	NA	1.15E-85	4.70E-85
AP1S3	2.43433852	0.50082047	0.51396566
WDFY1	1.59168288	0.00097831	0.00111752
MRPL44	1.07003891	9.97E-110	6.66E-109
AC073641.2	NA	NA	NA
SERPINE2	1.1523496	1.11E-87	4.71E-87
FAM124B	0.99870298	0.07186057	0.07711887
RP11-92F20.	NA	1.61E-102	9.08E-102
CUL3	1.5729572	3.92E-24	6.51E-24
DOCK10	NA	3.00E-35	5.82E-35
AC067961.1	NA	NA	NA
NYAP2	NA	0.80486567	0.81293135
RP11-314B1.	NA	NA	NA
AC068138.1	NA	NA	NA
IRS1	NA	NA	NA
RP11-395N3.	NA	NA	NA

RHBDD1	NA	NA	NA
COL4A4	NA	NA	NA
COL4A3	NA	NA	NA
AC097662.2	Inf	4.85E-08	6.14E-08
MFF	1.85073546	1.04E-27	1.81E-27
TM4SF20	NA	NA	NA
AGFG1	2.17686041	3.56E-79	1.31E-78
C2orf83	NA	NA	NA
SLC19A3	NA	NA	NA
CCL20	NA	3.03E-46	6.83E-46
DAW1	NA	NA	NA
SPHKAP	NA	NA	NA
AC009410.1	NA	NA	NA
AC074019.1	NA	NA	NA
PID1	NA	NA	NA
DNER	NA	NA	NA
TRIP12	1.8385214	5.60E-11	7.51E-11
FBXO36	2.99610895	1.94E-10	2.58E-10
SLC16A14	1.97470817	3.84E-06	4.66E-06
AC009950.2	NA	2.77E-73	9.33E-73
SP110	1.87256809	0.66306079	0.67462559
AC009950.1	NA	NA	NA
SP140	NA	4.01E-22	6.46E-22
SP140L	2.18466278	9.45E-10	1.24E-09
SP100	1.81906615	0.00108257	0.00123522
AC010149.4	NA	NA	NA
AC098823.3	NA	NA	NA
CAB39	1.51365921	3.49E-29	6.21E-29
ITM2C	1.34745641	2.29E-80	8.59E-80
AC012507.3	NA	NA	NA
GPR55	3.74513619	9.50E-122	8.99E-121
AC012507.4	NA	NA	NA
SPATA3-AS1	1.49805447	2.47E-11	3.33E-11
SPATA3	NA	NA	NA
C2orf72	NA	5.79E-16	8.49E-16
PSMD1	2.18816946	4.48E-49	1.05E-48
HTR2B	NA	NA	NA
ARMC9	1.3732166	0.07986663	0.08557408
B3GNT7	NA	3.64E-106	2.23E-105
AC017104.2	NA	7.50E-22	1.20E-21
NCL	1.00832685	1.57E-140	4.73E-139
LINC00471	2.99610895	4.62E-14	6.55E-14
NMUR1	NA	NA	NA
C2orf57	NA	NA	NA
PTMA	1.69263998	0.01549336	0.01704759

PDE6D	2.24708171	5.67E-09	7.33E-09
COPS7B	1.62667531	3.73E-06	4.54E-06
NPPC	NA	1.73E-80	6.47E-80
DIS3L2	1.34824903	2.63E-17	3.94E-17
AC105461.1	NA	NA	NA
ALPP	NA	NA	NA
AC068134.8	NA	NA	NA
ALPPL2	NA	NA	NA
AC068134.6	NA	NA	NA
ALPI	NA	NA	NA
ECEL1	NA	NA	NA
PRSS56	NA	1.76E-65	5.27E-65
CHRND	NA	NA	NA
CHRNG	NA	NA	NA
TIGD1	2.52796693	7.48E-14	1.06E-13
EIF4E2	1.40716832	2.07E-105	1.25E-104
AC073254.1	NA	NA	NA
EFHD1	0.89883268	0.03085109	0.03358337
GIGYF2	1.81639105	4.11E-12	5.65E-12
KCNJ13	NA	NA	NA
C2orf82	3.06744488	1.34E-143	6.57E-142
NGEF	NA	NA	NA
NEU2	NA	NA	NA
INPP5D	2.39688716	9.25E-96	4.59E-95
ATG16L1	1.99740597	3.04E-18	4.62E-18
SAG	NA	1.42E-07	1.78E-07
RP11-400N9.	Inf	0.01182278	0.01306266
DGKD	2.40758755	1.88E-66	5.70E-66
USP40	1.31079767	1.24E-09	1.62E-09
UGT1A8	NA	NA	NA
UGT1A10	NA	NA	NA
UGT1A9	NA	NA	NA
UGT1A7	NA	NA	NA
UGT1A6	NA	NA	NA
AC114812.8	NA	NA	NA
UGT1A5	NA	NA	NA
UGT1A4	NA	NA	NA
UGT1A3	NA	NA	NA
UGT1A1	NA	1.19E-43	2.58E-43
MROH2A	NA	NA	NA
HJURP	2.03901859	3.42E-30	6.19E-30
TRPM8	NA	NA	NA
AC005538.5	NA	NA	NA
SPP2	NA	NA	NA
AC006037.2	NA	NA	NA

RP11-309M7	NA	NA	NA
AC097713.3	NA	NA	NA
AC097713.4	NA	NA	NA
ARL4C	NA	9.99E-22	1.60E-21
LINC01173	NA	NA	NA
SH3BP4	1.62289235	0.02279529	0.02492029
AC114814.4	NA	NA	NA
AC114814.3	NA	NA	NA
AGAP1	4.6190013	9.47E-88	4.04E-87
AC064874.1	NA	NA	NA
GBX2	4.49416342	1.53E-73	5.16E-73
AC079135.1	Inf	2.51E-46	5.66E-46
ASB18	Inf	1.62E-116	1.29E-115
IQCA1	NA	NA	NA
RP11-785G1	Inf	3.40E-111	2.36E-110
IQCA1-AS1	NA	NA	NA
ACKR3	NA	NA	NA
AC011286.1	NA	NA	NA
AC105760.3	NA	NA	NA
AC105760.2	Inf	8.59E-12	1.17E-11
COPS8	1.51776572	1.22E-24	2.04E-24
AC107079.1	NA	NA	NA
AC112715.2	NA	NA	NA
COL6A3	NA	8.07E-07	9.96E-07
AC112721.1	NA	7.74E-138	1.72E-136
AC112721.2	NA	NA	NA
MLPH	NA	1.32E-73	4.47E-73
PRLH	NA	3.01E-137	6.36E-136
RAB17	0.74902724	1.87E-86	7.77E-86
AC104667.3	NA	NA	NA
AC096574.5	NA	NA	NA
LRRFIP1	1.63945247	0.05064855	0.05469114
RBM44	NA	1.01E-45	2.26E-45
RAMP1	0.56177043	3.23E-54	8.19E-54
UBE2F	2.08306115	0.00195437	0.00221062
UBE2F-SCLY	0	1.13E-69	3.59E-69
SCLY	1.16848249	1.89E-25	3.20E-25
ESPNL	NA	2.39E-56	6.23E-56
KLHL30	NA	NA	NA
FAM132B	1.77893969	2.98E-41	6.28E-41
ILKAP	1.39757521	2.71E-90	1.21E-89
HES6	1.4766537	6.11E-23	9.96E-23
PER2	1.99740597	0.76185173	0.77081669
AC012485.2	NA	NA	NA
TRAF3IP1	1.70233463	2.02E-48	4.71E-48

ASB1	2.04280156	0.44793748	0.46094621
AC016999.2	NA	NA	NA
LINC01107	NA	NA	NA
AC113618.1	NA	NA	NA
AC144525.1	NA	NA	NA
TWIST2	NA	NA	NA
FLJ43879	NA	NA	NA
HDAC4	0.24967575	2.54E-128	3.16E-127
AC017028.1C	NA	NA	NA
AC062017.1	1.31079767	7.66E-55	1.96E-54
AC079612.1	NA	NA	NA
AC079612.2	NA	NA	NA
RP11-315F2:	NA	NA	NA
AC093802.1	NA	NA	NA
NDUFA10	0.95471865	1.73E-138	4.11E-137
OR6B2	NA	NA	NA
PRR21	NA	NA	NA
OR6B3	NA	NA	NA
MYEOV2	2.36319729	5.47E-141	1.73E-139
OTOS	NA	9.79E-32	1.81E-31
AC124861.1	NA	NA	NA
GPC1	3.74513619	9.97E-88	4.25E-87
ANKMY1	5.05593385	0.00092888	0.00106179
DUSP28	3.1459144	6.04E-10	7.96E-10
RNPEPL1	2.28549337	0.00974678	0.01079458
CAPN10-AS1	4.04474708	5.84E-18	8.84E-18
CAPN10	1.75553259	0.04001423	0.04337066
GPR35	Inf	6.09E-45	1.35E-44
AQP12B	NA	NA	NA
AC011298.2	NA	NA	NA
AQP12A	NA	NA	NA
KIF1A	NA	NA	NA
AGXT	NA	NA	NA
C2orf54	NA	NA	NA
CROCC2	NA	NA	NA
AC104809.2	NA	NA	NA
SNED1	NA	NA	NA
AC093585.6	NA	NA	NA
AC005237.4	NA	NA	NA
MTERF4	2.32198444	3.69E-05	4.38E-05
PASK	1.99740597	7.38E-42	1.57E-41
PPP1R7	0.9353138	1.64E-134	2.85E-133
ANO7	3.37062257	2.90E-13	4.05E-13
HDLBP	2.07277978	2.88E-42	6.16E-42
2-Sep	1-Jan	4.43E-11	5.95E-11

AC005104.3	NA	NA	NA
FARP2	3.57868569	1.31E-44	2.87E-44
STK25	1.74773022	9.72E-34	1.84E-33
BOK-AS1	NA	2.04E-122	1.98E-121
BOK	NA	NA	NA
RP11-367H1.	NA	NA	NA
THAP4	2.23911334	5.94E-131	8.44E-130
ATG4B	1.26953769	2.24E-86	9.32E-86
DTYMK	1.66481903	3.90E-55	1.00E-54
AC133528.2	0	2.34E-64	6.87E-64
ING5	1.18394628	1.72E-73	5.81E-73
AC114730.11	NA	NA	NA
D2HGDH	2.13184675	0.00010616	0.00012438
AC114730.8	NA	4.22E-18	6.40E-18
AC114730.7	NA	NA	NA
GAL3ST2	NA	NA	NA
AC114730.5	NA	NA	NA
AC114730.3	NA	NA	NA
NEU4	NA	1.38E-32	2.57E-32
AC114730.2	NA	NA	NA
PDCD1	NA	NA	NA
RTP5	NA	NA	NA
AC131097.4	NA	NA	NA
AC093642.3	Inf	0.72852067	0.7383287
AC093642.4	NA	NA	NA
AC093642.6	NA	NA	NA
RP11-341N2.	Inf	3.40E-156	1.21E-153
AY269186.1	NA	NA	NA
AY269186.2	NA	NA	NA
CHL1	NA	NA	NA
CHL1-AS1	NA	NA	NA
LINC01266	NA	NA	NA
AC090044.2	NA	NA	NA
AC087430.1	NA	NA	NA
CNTN6	NA	NA	NA
RP11-204C23	NA	NA	NA
CNTN4	NA	8.68E-93	4.07E-92
CNTN4-AS2	NA	NA	NA
CNTN4-AS1	NA	NA	NA
IL5RA	NA	NA	NA
TRNT1	1.72852439	6.17E-26	1.05E-25
AC024060.1	NA	NA	NA
CRBN	1.52075227	5.04E-21	7.99E-21
RP11-97C16.	3.74513619	4.20E-96	2.10E-95
AC026188.1	NA	NA	NA

SUMF1	1.19844358	4.79E-31	8.75E-31
LRRN1	NA	NA	NA
SETMAR	1.14850843	0.77732752	0.78599241
ITPR1-AS1	NA	1.05E-52	2.61E-52
ITPR1	0.93628405	4.57E-81	1.73E-80
EGOT	NA	NA	NA
AC018816.3	Inf	1.54E-24	2.57E-24
BHLHE40-AS	NA	7.99E-149	1.03E-146
BHLHE40	Inf	2.49E-118	2.09E-117
ARL8B	1.74382904	3.34E-11	4.49E-11
AC026202.3	NA	NA	NA
RP11-572B2.	NA	2.58E-134	4.43E-133
EDEM1	2.41353221	4.25E-49	9.97E-49
AC026202.5	NA	NA	NA
AC027119.1	NA	NA	NA
AC026167.1	NA	NA	NA
AC069277.2	NA	NA	NA
GRM7-AS3	NA	NA	NA
GRM7	NA	7.12E-36	1.39E-35
GRM7-AS2	NA	NA	NA
GRM7-AS1	NA	NA	NA
RP11-157E16	NA	NA	NA
LMCD1-AS1	NA	NA	NA
AC018832.1	NA	NA	NA
AC023481.1	NA	NA	NA
LMCD1	NA	2.44E-85	9.96E-85
AC034187.2	NA	NA	NA
SSUH2	NA	2.74E-07	3.42E-07
CAV3	NA	NA	NA
OXTR	4.49416342	1.59E-137	3.44E-136
RAD18	1.85473411	2.92E-39	6.00E-39
SRGAP3	1.49805447	1.10E-58	2.96E-58
SRGAP3-AS1	NA	NA	NA
SRGAP3-AS2	NA	NA	NA
SRGAP3-AS3	NA	NA	NA
SRGAP3-AS4	NA	4.18E-70	1.34E-69
RP11-380O2	NA	5.32E-125	5.69E-124
THUMPD3-A	2.20059037	2.64E-82	1.02E-81
THUMPD3	1.36697471	1.10E-51	2.69E-51
SETD5	2.21770809	1.44E-06	1.76E-06
LHFPL4	Inf	5.67E-06	6.87E-06
MTMR14	1.64785992	9.59E-63	2.74E-62
CPNE9	NA	3.10E-23	5.07E-23
BRPF1	1.36429961	0.19851681	0.20880635
OGG1	1.49805447	1.38E-06	1.69E-06

CAMK1	NA	7.16E-42	1.53E-41
TADA3	1.89894229	1.62E-36	3.21E-36
ARPC4	1.69779507	0.40949124	0.42231767
ARPC4-TTLL3	NA	2.67E-07	3.33E-07
TTLL3	2.44419414	2.47E-83	9.74E-83
RP11-266J6.2	NA	5.20E-22	8.36E-22
RPUSD3	1.39941714	5.79E-93	2.73E-92
CIDEC	NA	NA	NA
JAGN1	1.54610528	8.77E-64	2.55E-63
IL17RE	1.49805447	0.05829855	0.06278732
IL17RC	2.62159533	8.34E-35	1.61E-34
CRELD1	1.87256809	2.43E-96	1.22E-95
PRRT3	2.62159533	7.47E-09	9.62E-09
PRRT3-AS1	Inf	2.76E-22	4.46E-22
RP11-1020A1	0	0.00059475	0.00068303
EMC3	0.99370947	3.98E-92	1.84E-91
AC022007.5	NA	5.03E-145	3.36E-143
FANCD2	1.69345288	2.22E-06	2.71E-06
FANCD2OS	NA	NA	NA
BRK1	1.85000703	1.20E-09	1.57E-09
VHL	1.49805447	0.08504618	0.09098943
IRAK2	1.12354086	0.03324798	0.03614699
TATDN2	2.16823674	3.20E-122	3.08E-121
RP11-438J1.1	NA	NA	NA
LINC00852	Inf	1.49E-29	2.68E-29
GHRL	2.99610895	6.51E-38	1.31E-37
GHRLOS	1.49805447	1.52E-11	2.06E-11
SEC13	1.03437095	5.80E-141	1.84E-139
ATP2B2	NA	NA	NA
LINC00606	NA	NA	NA
AC018495.3	NA	NA	NA
SLC6A11	NA	NA	NA
SLC6A1	NA	NA	NA
SLC6A1-AS1	NA	NA	NA
HRH1	NA	4.64E-87	1.95E-86
ATG7	1.42315175	1.24E-19	1.92E-19
VGLL4	1.53854243	5.06E-07	6.27E-07
RP11-169K17	NA	NA	NA
RP11-169K17	NA	NA	NA
TAMM41	1.01653696	1.23E-123	1.26E-122
SYN2	0	1.03E-82	4.01E-82
TIMP4	NA	NA	NA
PPARG	NA	NA	NA
TSEN2	1.73458939	4.60E-77	1.63E-76
MKRN2OS	NA	1.37E-83	5.42E-83

MKRN2	1.52008469	8.84E-21	1.40E-20
RAF1	2.47755163	7.99E-68	2.48E-67
TMEM40	NA	NA	NA
CAND2	NA	5.25E-101	2.89E-100
RP11-767C1.	1.49805447	7.46E-34	1.42E-33
RPL32	2.16336607	2.35E-87	9.92E-87
RP11-767C1.	NA	NA	NA
IQSEC1	1.67429618	6.69E-06	8.08E-06
NUP210	2.34922179	1.79E-110	1.22E-109
HDAC11-AS1	NA	NA	NA
HDAC11	0.59922179	1.63E-23	2.68E-23
FBLN2	NA	NA	NA
LINC00620	NA	NA	NA
WNT7A	NA	NA	NA
TPRXL	NA	NA	NA
CHCHD4	2.12224384	0.20866825	0.21920437
TMEM43	1.87256809	1.05E-15	1.53E-15
RP11-434D1:	NA	3.18E-27	5.51E-27
AC093495.4	Inf	0.14344331	0.15182897
XPC	1.9661965	2.44E-05	2.91E-05
LSM3	1.79811211	1.99E-15	2.90E-15
RP11-536I6.2	NA	NA	NA
LINC01267	NA	NA	NA
SLC6A6	1.3732166	2.42E-17	3.63E-17
GRIP2	NA	NA	NA
AC090952.5	NA	NA	NA
CCDC174	1.34824903	1.04E-88	4.53E-88
C3orf20	Inf	3.40E-111	2.36E-110
AC090957.2	NA	NA	NA
RP11-95M5.:	NA	NA	NA
FGD5	NA	NA	NA
FGD5-AS1	1.90823606	2.86E-22	4.63E-22
NR2C2	2.7464332	6.95E-22	1.12E-21
MRPS25	1.37803231	2.30E-132	3.53E-131
RBSN	3.83876459	1.43E-40	2.98E-40
CAPN7	2.7464332	1.05E-69	3.35E-69
SH3BP5-AS1	Inf	2.33E-29	4.17E-29
SH3BP5	0.65539883	8.94E-79	3.26E-78
METTL6	1.45399405	8.03E-57	2.11E-56
EAF1	1.44455253	9.01E-34	1.71E-33
EAF1-AS1	1.12354086	0.95270349	0.95501971
COLQ	NA	4.38E-29	7.79E-29
HACL1	0.92187968	2.06E-50	4.95E-50
BTD	1.42315175	7.43E-92	3.42E-91
ANKRD28	1.60766822	4.48E-75	1.54E-74

GALNT15	NA	NA	NA
DPH3	1.84711571	6.58E-14	9.30E-14
OXNAD1	1.63180934	1.65E-55	4.25E-55
RFTN1	2.17898833	0.07464996	0.08006974
RP11-415F2:	NA	3.95E-112	2.82E-111
RP11-415F2:	NA	1.03E-86	4.28E-86
RP11-415F2:	Inf	9.59E-43	2.06E-42
RP11-194G1(	NA	NA	NA
LINC00690	NA	NA	NA
DAZL	NA	NA	NA
AC091493.2	NA	NA	NA
PLCL2	2.24708171	0.40684328	0.41965833
PLCL2-AS1	NA	NA	NA
TBC1D5	2.24708171	9.61E-48	2.21E-47
AC132807.1	NA	NA	NA
RP11-320G1(	NA	9.22E-19	1.41E-18
SATB1	1.67315175	0.00451722	0.00505904
KCNH8	NA	NA	NA
EFHB	0	0.00522059	0.0058349
RAB5A	1.54912451	1.03E-39	2.13E-39
PP2D1	NA	NA	NA
KAT2B	0	0.62802015	0.64005065
SGOL1	1.47266372	4.97E-84	1.98E-83
RP11-669C1!	NA	NA	NA
RP11-901H1:	NA	NA	NA
ZNF385D	NA	3.38E-32	6.27E-32
ZNF385D-AS	NA	NA	NA
ZNF385D-AS	NA	NA	NA
UBE2E2-AS1	Inf	1.46E-121	1.36E-120
UBE2E2	1.50753583	4.83E-36	9.48E-36
UBE2E1-AS1	NA	6.57E-21	1.04E-20
UBE2E1	1.25766899	7.32E-118	6.06E-117
NKIRAS1	0.87386511	4.94E-38	9.96E-38
RPL15	1.83349513	0.1450871	0.15353302
NR1D2	11.9844358	8.95E-109	5.82E-108
LINC00691	NA	NA	NA
THRB	NA	NA	NA
RP11-317L1(	NA	NA	NA
AC133680.1	NA	NA	NA
RARB	NA	NA	NA
TOP2B	1.85934997	1.20E-83	4.77E-83
NGLY1	1.79949227	0.00357032	0.00401095
OXSM	1.87256809	4.39E-59	1.19E-58
AC099754.1	NA	NA	NA
LRRC3B	NA	NA	NA

AC114877.3	NA	NA	NA
NEK10	Inf	0.70765066	0.71806019
SLC4A7	2.24708171	0.01822893	0.01999935
RP11-222K16	NA	NA	NA
RP11-222K16	NA	3.00E-35	5.82E-35
EOMES	NA	4.06E-19	6.25E-19
AC098973.2	NA	NA	NA
AC098973.1	NA	NA	NA
AC092415.1	NA	NA	NA
CMC1	1.28404669	2.02E-85	8.25E-85
AZI2	1.33016906	2.87E-21	4.57E-21
ZCWPW2	0.74902724	1.44E-05	1.72E-05
LINC00693	NA	NA	NA
RBMS3-AS3	NA	NA	NA
RBMS3	NA	NA	NA
RBMS3-AS2	NA	NA	NA
RBMS3-AS1	NA	NA	NA
AC116035.1	NA	NA	NA
AC116035.2	NA	NA	NA
TGFBR2	1.59168288	4.62E-20	7.20E-20
GADL1	NA	NA	NA
STT3B	1.86680635	0.12619826	0.13406071
OSBPL10	Inf	4.18E-38	8.43E-38
OSBPL10-AS	NA	NA	NA
ZNF860	NA	NA	NA
GPD1L	1.49805447	0.60121941	0.61390908
CMTM8	1.36429961	1.45E-11	1.97E-11
CMTM7	1.88857295	1.14E-09	1.49E-09
CMTM6	1.77735277	0.00047221	0.00054414
DYNC1LI1	1.47602426	8.72E-76	3.03E-75
CNOT10	2.9461738	3.39E-64	9.92E-64
CNOT10-AS1	NA	5.77E-98	2.99E-97
TRIM71	3.89494163	8.50E-78	3.06E-77
CCR4	NA	6.17E-76	2.15E-75
GLB1	1.71206226	3.18E-61	8.92E-61
TMPPE	1.49805447	6.60E-50	1.57E-49
CRTAP	2.1579118	2.01E-42	4.31E-42
RP11-627J17	NA	NA	NA
SUSD5	NA	NA	NA
FBXL2	0.74902724	0.00442151	0.00495246
UBP1	2.0598249	3.22E-87	1.35E-86
CLASP2	1.99740597	9.73E-09	1.25E-08
RP11-10C24.	NA	1.52E-09	1.99E-09
RP11-10C24.	Inf	1.30E-15	1.89E-15
RP11-10C24.	Inf	2.66E-10	3.52E-10

PDCD6IP	1.3732166	1.45E-61	4.09E-61
AC018359.1	NA	NA	NA
AC123023.1	NA	NA	NA
AC018359.3	NA	NA	NA
ARPP21	NA	NA	NA
ARPP21-AS1	NA	NA	NA
STAC	NA	NA	NA
DCLK3	NA	NA	NA
RP11-640L9.1	NA	NA	NA
TRANK1	2.62159533	0.20152358	0.21191982
RP11-129K12	NA	1.10E-39	2.27E-39
EPM2AIP1	3.34181383	2.39E-51	5.81E-51
MLH1	2.04734112	7.69E-37	1.53E-36
LRRFIP2	1.85965383	2.46E-17	3.69E-17
RP11-285J16	1.49805447	1.34E-16	1.99E-16
RP11-259K5.	NA	NA	NA
RP11-259K5.	1.49805447	1.96E-05	2.34E-05
GOLGA4	2.19227484	6.16E-19	9.45E-19
C3orf35	Inf	0.00356154	0.00400133
ITGA9	5.24319066	7.77E-16	1.14E-15
CTDSPL	2.66840953	3.93E-55	1.01E-54
VILL	5.24319066	2.33E-109	1.54E-108
PLCD1	0.37451362	2.09E-72	6.91E-72
DLEC1	NA	1.33E-118	1.12E-117
ACAA1	1.04643511	2.98E-135	5.41E-134
MYD88	4.19455253	4.20E-08	5.33E-08
OXSR1	1.60505837	9.07E-36	1.77E-35
SLC22A13	NA	0.00021029	0.00024448
SLC22A14	NA	6.24E-09	8.05E-09
XYLB	2.7464332	4.71E-06	5.71E-06
ACVR2B-AS1	Inf	2.75E-33	5.17E-33
ACVR2B	2.02237354	4.03E-09	5.23E-09
EXOG	2.99610895	4.34E-83	1.70E-82
SCN5A	1.49805447	2.88E-14	4.10E-14
SCN10A	NA	NA	NA
RP11-134J21	NA	NA	NA
SCN11A	NA	NA	NA
WDR48	1.74773022	0.00151343	0.00171765
GORASP1	1.33160398	2.83E-07	3.52E-07
TTC21A	Inf	6.53E-16	9.57E-16
CSRNP1	1.74773022	1.34E-18	2.04E-18
XIRP1	NA	NA	NA
CX3CR1	NA	NA	NA
CCR8	NA	NA	NA
SLC25A38	1.39722389	2.07E-31	3.80E-31

RPSA	2.14579146	1.59E-127	1.91E-126
MOBP	Inf	5.04E-119	4.33E-118
MYRIP	NA	1.25E-48	2.93E-48
EIF1B	1.13150923	1.15E-131	1.70E-130
ENTPD3-AS1	1.21716926	0.00461745	0.00516906
ENTPD3	NA	NA	NA
RPL14	1.89488721	8.93E-42	1.90E-41
ZNF619	3.74513619	2.01E-28	3.55E-28
ZNF620	8.23929961	1.61E-09	2.10E-09
ZNF621	4.74383917	1.09E-35	2.13E-35
RP11-528N2	NA	NA	NA
RP11-761N2	NA	NA	NA
RP11-520A2	NA	NA	NA
RP11-372H2.	NA	NA	NA
CTNNB1	2.41561284	1.01E-24	1.69E-24
ULK4	0.99870298	0.29022638	0.30237678
TRAK1	2.64362554	3.60E-34	6.88E-34
CCK	NA	NA	NA
RP11-333B1	NA	NA	NA
LYZL4	NA	NA	NA
VIPR1	Inf	7.51E-13	1.04E-12
VIPR1-AS1	NA	0.0003184	0.00036865
SEC22C	2.41093142	2.11E-46	4.77E-46
SS18L2	2.14790737	1.10E-53	2.76E-53
NKTR	1.70502253	0.53389997	0.5472633
RP4-613B23.	Inf	1.92E-41	4.06E-41
ZBTB47	2.24708171	5.55E-23	9.05E-23
KLHL40	NA	NA	NA
CCDC13	0.37451362	2.30E-127	2.75E-126
HHATL	NA	NA	NA
HHATL-AS1	NA	NA	NA
CCDC13-AS1	NA	NA	NA
RP4-613B23.	NA	NA	NA
RP11-70C1.1	0.37451362	0.01660844	0.0182557
HIGD1A	1.91476681	2.30E-46	5.20E-46
RP11-70C1.3	NA	NA	NA
ACKR2	NA	0.9584947	0.96061268
RP11-136C2	NA	NA	NA
KRBOX1	NA	NA	NA
CYP8B1	NA	NA	NA
ZNF662	NA	NA	NA
KRBOX1-AS1	NA	NA	NA
FAM198A	2.37191958	0.13122724	0.13924809
POMGNT2	3.02285992	7.79E-94	3.72E-93
RP11-136C2	NA	NA	NA

SNRK	1.33160398	1.38E-81	5.30E-81
RP11-188P2(	NA	5.83E-98	3.02E-97
SNRK-AS1	NA	NA	NA
ANO10	1.64785992	9.61E-25	1.61E-24
ABHD5	2.0130107	1.75E-06	2.15E-06
RP4-672N11.	NA	NA	NA
RP4-555D20.	NA	NA	NA
RP4-555D20.	3.74513619	1.80E-06	2.20E-06
RP4-555D20.	4.49416342	3.30E-06	4.01E-06
TOPAZ1	NA	NA	NA
RP11-424N2	Inf	1.22E-68	3.81E-68
TCAIM	1.46684501	1.30E-30	2.36E-30
LINC00694	2.78210117	3.39E-34	6.47E-34
RP11-101C21	NA	NA	NA
ZNF445	2.3540856	7.40E-119	6.32E-118
ZNF852	0.93628405	2.45E-18	3.73E-18
ZKSCAN7	NA	0.00216643	0.00244652
RP11-944L7.	NA	NA	NA
ZNF660	NA	NA	NA
ZNF197	2.69649805	1.82E-15	2.64E-15
ZNF197-AS1	NA	NA	NA
ZNF35	0	4.77E-36	9.37E-36
RP11-348P1(	Inf	0.49716449	0.51041584
ZNF502	NA	NA	NA
ZNF501	NA	NA	NA
KIAA1143	1.68531128	0.01475704	0.01624725
KIF15	1.32520204	9.36E-23	1.52E-22
TMEM42	1.49805447	5.39E-42	1.15E-41
TGM4	NA	NA	NA
RP11-272D2(	NA	NA	NA
ZDHHC3	1.9994694	5.60E-44	1.22E-43
EXOSC7	1.03799206	1.50E-143	7.24E-142
CLEC3B	NA	2.65E-67	8.14E-67
CDCP1	NA	NA	NA
TMEM158	1.42315175	0.63227976	0.64431946
LARS2	4.04474708	3.99E-136	7.65E-135
LARS2-AS1	NA	NA	NA
LIMD1	2.7464332	1.19E-71	3.90E-71
LIMD1-AS1	Inf	1.57E-81	6.00E-81
SACM1L	2.88087399	6.62E-113	4.82E-112
SLC6A20	NA	NA	NA
LZTFL1	1.2733463	3.65E-48	8.46E-48
CCR9	NA	NA	NA
FYCO1	4.49416342	5.33E-90	2.36E-89
CXCR6	NA	9.18E-05	1.08E-04

XCR1	NA	NA	NA
CCR3	NA	NA	NA
CCR1	NA	7.60E-105	4.52E-104
CCR2	NA	NA	NA
RP11-24F11.	NA	2.09E-82	8.11E-82
CCR5	NA	NA	NA
CCRL2	0.37451362	1.31E-124	1.38E-123
LTF	NA	NA	NA
RTP3	NA	NA	NA
LRRC2	NA	NA	NA
LRRC2-AS1	NA	NA	NA
TDGF1	NA	NA	NA
ALS2CL	3.74513619	1.58E-88	6.83E-88
TMIE	1.12354086	2.29E-32	4.27E-32
PRSS50	NA	NA	NA
PRSS46	NA	NA	NA
PRSS45	NA	NA	NA
PRSS42	NA	NA	NA
MYL3	NA	NA	NA
PTH1R	0	4.35E-104	2.55E-103
CCDC12	1.31748541	2.94E-144	1.65E-142
NBEAL2	1.34824903	0.78989234	0.79829686
SETD2	1.42315175	4.33E-34	8.26E-34
KIF9-AS1	2.99610895	6.55E-41	1.38E-40
KIF9	2.10388533	1.96E-112	1.40E-111
KLHL18	2.60772446	8.27E-141	2.58E-139
RP11-708J19	4.68142023	4.66E-109	3.06E-108
PTPN23	2.56809339	8.17E-111	5.62E-110
SCAP	1.84220212	5.26E-116	4.13E-115
ELP6	1.33754864	3.22E-93	1.53E-92
CSPG5	2.24708171	4.62E-08	5.85E-08
SMARCC1	1.56199582	8.63E-28	1.51E-27
DHX30	1.85579883	1.98E-88	8.56E-88
MAP4	2.37191958	7.50E-56	1.94E-55
CDC25A	1.8624461	3.11E-65	9.26E-65
CAMP	NA	1.40E-113	1.03E-112
ZNF589	1.8385214	0.01815251	0.01991672
NME6	1.36891185	1.49E-27	2.60E-27
SPINK8	NA	NA	NA
FBXW12	Inf	2.27E-101	1.26E-100
PLXNB1	0.42801556	1.97E-89	8.66E-89
CCDC51	1.62832008	3.17E-45	7.04E-45
TMA7	2.42964929	1.85E-145	1.58E-143
RP11-24C3.2	NA	0.10239071	0.10914055
ATRIP	NA	NA	NA

ATRIP	NA	3.76E-54	9.52E-54
TREX1	NA	NA	NA
SHISA5	2.52702118	2.07E-83	8.17E-83
PFKFB4	Inf	4.25E-23	6.95E-23
UCN2	NA	1.21E-21	1.94E-21
COL7A1	0	5.72E-32	1.06E-31
UQCRC1	1.07713139	5.13E-145	3.42E-143
TMEM89	NA	4.76E-08	6.03E-08
SLC26A6	1.72276265	1.60E-83	6.30E-83
CELSR3	NA	1.12E-124	1.19E-123
CELSR3-AS1	NA	NA	NA
RP11-572O6.	NA	NA	NA
NCKIPSD	2.54669261	1.59E-16	2.35E-16
IP6K2	1.32328145	7.81E-63	2.24E-62
PRKAR2A	1.79900292	0.00012674	0.00014823
PRKAR2A-AS	Inf	2.76E-33	5.19E-33
SLC25A20	1.21716926	1.90E-53	4.76E-53
ARIH2OS	4.49416342	5.68E-05	6.71E-05
ARIH2	2.05767253	3.16E-89	1.39E-88
RP13-131K1!	2.99610895	9.04E-19	1.39E-18
RP13-131K1!	NA	9.37E-83	3.65E-82
RP13-131K1!	Inf	1.68E-125	1.84E-124
P4HTM	0.99870298	2.64E-103	1.52E-102
WDR6	2.30058366	0.00099747	0.00113905
DALRD3	1.28999135	3.74E-94	1.80E-93
NDUFAF3	1.531019	1.16E-87	4.93E-87
IMPDH2	1.07066835	1.75E-128	2.18E-127
RP13-131K1!	Inf	1.25E-102	7.10E-102
QRICH1	2.99610895	0.1933119	0.20343781
QARS	1.7798667	5.39E-29	9.58E-29
USP19	2.57998271	0.00542047	0.0060568
LAMB2	Inf	1.27E-05	1.52E-05
CCDC71	2.2262754	0.94815591	0.95082883
KLHDC8B	1.23877582	3.32E-123	3.32E-122
C3orf84	NA	NA	NA
CCDC36	NA	NA	NA
RP11-3B7.1	NA	NA	NA
C3orf62	5.74254215	1.56E-125	1.70E-124
USP4	1.59168288	0.60792083	0.62040275
GPX1	2.55377683	3.33E-145	2.42E-143
RHOA	1.21092737	8.63E-145	5.42E-143
TCTA	1.79115209	4.40E-25	7.42E-25
AMT	Inf	3.43E-14	4.88E-14
NICN1	2.24708171	2.14E-14	3.05E-14
DAG1	1.29831388	0.00341896	0.00384329

BSN-AS2	NA	3.50E-79	1.28E-78
BSN	NA	1.50E-14	2.14E-14
BSN-AS1	NA	NA	NA
APEH	1.57190223	6.73E-80	2.49E-79
MST1	NA	2.47E-26	4.23E-26
RNF123	2.0598249	5.70E-09	7.36E-09
AMIGO3	NA	8.61E-111	5.90E-110
GMPPB	1.23054475	2.33E-88	1.00E-87
IP6K1	2.8463035	3.72E-22	6.00E-22
CDHR4	NA	5.56E-53	1.39E-52
FAM212A	1.24604531	3.29E-63	9.48E-63
UBA7	2.99610895	1.09E-13	1.53E-13
TRAIP	1.07003891	1.22E-67	3.76E-67
CAMKV	4.49416342	6.58E-68	2.04E-67
MST1R	NA	2.06E-108	1.33E-107
CTD-2330K9.	NA	NA	NA
CTD-2330K9.	NA	NA	NA
MON1A	1.52480545	0.00022986	0.00026696
RBM6	1.76241703	0.63857287	0.65058623
RBM5	2.007393	1.35E-28	2.39E-28
RBM5-AS1	NA	1.02E-07	1.28E-07
SEMA3F-AS1	NA	NA	NA
SEMA3F	Inf	1.60E-118	1.35E-117
GNAT1	NA	NA	NA
SLC38A3	NA	2.57E-109	1.70E-108
GNAI2	1.50856714	1.35E-132	2.11E-131
U73166.2	Inf	2.94E-75	1.02E-74
SEMA3B-AS1	NA	NA	NA
SEMA3B	Inf	0.00791185	0.00879192
LSMEM2	Inf	1.95E-123	1.98E-122
IFRD2	1.77326524	4.30E-17	6.42E-17
HYAL3	0.96749351	2.50E-141	8.37E-140
NAT6	1.34824903	2.38E-35	4.63E-35
HYAL1	NA	2.82E-31	5.18E-31
HYAL2	2.01856493	2.14E-13	2.99E-13
TUSC2	2.03999771	9.95E-100	5.32E-99
RASSF1	1.1385214	1.20E-113	8.91E-113
RASSF1-AS1	4.49416342	1.44E-53	3.62E-53
ZMYND10-AS	NA	NA	NA
ZMYND10	2.99610895	1.87E-52	4.63E-52
NPRL2	1.22568093	3.89E-96	1.95E-95
CYB561D2	1.11417802	3.19E-121	2.95E-120
XXcos-LUCA1	NA	NA	NA
TMEM115	1.53459239	6.59E-24	1.09E-23
CACNA2D2	NA	7.24E-120	6.43E-119

CYB561D2	1.49805447	9.37E-45	2.07E-44
C3orf18	NA	1.57E-28	2.78E-28
HEMK1	1.78216825	1.29E-18	1.97E-18
RP11-804H8.	NA	NA	NA
CISH	2.11629918	3.85E-11	5.18E-11
MAPKAPK3	1.63079348	2.42E-16	3.57E-16
RP11-804H8.	Inf	5.59E-09	7.22E-09
DOCK3	2.24708171	3.03E-14	4.31E-14
MANF	1.22909875	5.54E-95	2.71E-94
RBM15B	1.93616475	4.69E-07	5.82E-07
VPRBP	2.38326848	2.40E-72	7.95E-72
RAD54L2	3.34565499	1.58E-128	1.98E-127
TEX264	1.4192095	5.00E-123	4.97E-122
GRM2	NA	4.19E-70	1.34E-69
IQCF6	NA	NA	NA
IQCF3	NA	NA	NA
IQCF2	NA	NA	NA
IQCF5-AS1	NA	NA	NA
IQCF5	NA	NA	NA
IQCF1	NA	NA	NA
RRP9	1.22982175	1.69E-55	4.37E-55
PARP3	1.49805447	4.88E-69	1.54E-68
GPR62	NA	NA	NA
PCBP4	1.63180934	3.72E-47	8.52E-47
ABHD14B	1.71776913	1.10E-30	2.00E-30
ABHD14A	1.68177814	2.60E-86	1.08E-85
ABHD14A-AC	NA	NA	NA
ACY1	Inf	0.00154621	0.0017543
RPL29	2.14454275	2.33E-132	3.57E-131
DUSP7	2.45136187	0.02912205	0.03171452
POC1A	1.24837873	4.22E-125	4.54E-124
ALAS1	1.42497865	2.29E-126	2.61E-125
TLR9	NA	NA	NA
RP11-330H6.	NA	NA	NA
TWF2	0.94644527	1.06E-142	4.36E-141
RP5-1157M2	0	1.39E-23	2.29E-23
PPM1M	2.88910506	0.00101043	0.00115356
WDR82	1.80234679	5.34E-26	9.11E-26
GLYCTK	1.3732166	7.30E-57	1.92E-56
DNAH1	3.74513619	8.51E-36	1.67E-35
BAP1	1.88326848	3.30E-43	7.14E-43
PHF7	1.97743191	9.99E-11	1.33E-10
SEMA3G	Inf	8.30E-59	2.24E-58
TNNC1	NA	6.93E-43	1.49E-42
NISCH	2.30469919	2.07E-52	5.13E-52

STAB1	Inf	1.00E-152	1.93E-150
NT5DC2	2.66439689	2.81E-105	1.69E-104
SMIM4	1.97470817	1.72E-82	6.67E-82
PBRM1	2.4012932	1.84E-08	2.35E-08
GNL3	1.11765844	3.79E-66	1.14E-65
GLT8D1	1.81495061	4.18E-60	1.15E-59
SPCS1	1.52224786	6.52E-34	1.24E-33
NEK4	2.85992218	7.61E-29	1.35E-28
ITIH1	NA	NA	NA
ITIH3	NA	NA	NA
ITIH4	NA	2.49E-51	6.04E-51
RP5-966M1.6	Inf	2.82E-31	5.18E-31
ITIH4-AS1	NA	NA	NA
MUSTN1	NA	NA	NA
TMEM110-M	NA	3.23E-05	3.83E-05
TMEM110	0.88521401	5.35E-25	9.01E-25
SFMBT1	2.04734112	0.32160025	0.33420046
RP11-894J14	NA	2.00E-44	4.40E-44
RFT1	2.05584072	1.21E-68	3.78E-68
PRKCD	1.02140078	5.54E-60	1.52E-59
TKT	1.28305592	5.01E-105	3.00E-104
DCP1A	1.90293406	5.91E-05	6.98E-05
CACNA1D	NA	1.28E-110	8.76E-110
RP11-884K10	NA	NA	NA
CHDH	4.49416342	1.91E-107	1.21E-106
IL17RB	1.34824903	1.35E-11	1.83E-11
RP11-884K10	0.28088521	6.01E-32	1.11E-31
ACTR8	1.74773022	0.01324091	0.01460372
SELK	1.7026081	1.71E-23	2.81E-23
CACNA2D3	Inf	1.46E-67	4.51E-67
CACNA2D3-A	NA	NA	NA
LRTM1	Inf	4.23E-38	8.54E-38
RP11-889D3.	NA	NA	NA
RP11-889D3.	NA	NA	NA
RP11-64C1.1	NA	NA	NA
RP11-875H7.	NA	NA	NA
RP11-875H7.	NA	NA	NA
WNT5A	NA	NA	NA
WNT5A-AS1	NA	NA	NA
RP11-875H7.	NA	NA	NA
ERC2	Inf	1.89E-43	4.09E-43
RP11-58O15.	NA	NA	NA
CCDC66	2.09727626	2.84E-48	6.59E-48
FAM208A	1.97470817	0.68829632	0.69920331
ARHGEF3	1.64785992	2.56E-79	9.43E-79

ARHGEF3-AS	NA	NA	NA
SPATA12	NA	8.01E-62	2.26E-61
RP11-157F2(	NA	NA	NA
IL17RD	NA	7.65E-06	9.24E-06
HESX1	0.56177043	0.27970307	0.2916977
APPL1	2.32929202	9.25E-142	3.29E-140
ASB14	NA	NA	NA
DNAH12	NA	NA	NA
PDE12	2.15345331	7.34E-66	2.21E-65
ARF4	1.05939764	4.12E-133	6.64E-132
ARF4-AS1	1.39105058	0.07035714	0.07553223
DENND6A	1.87256809	1.42E-14	2.03E-14
DENND6A-AS	NA	NA	NA
RP11-755B1(	NA	1.28E-142	5.08E-141
SLMAP	1.44043699	6.79E-25	1.14E-24
FLNB	3.08973735	3.73E-125	4.03E-124
FLNB-AS1	NA	3.14E-121	2.90E-120
DNASE1L3	NA	NA	NA
ABHD6	1.68531128	1.08E-28	1.91E-28
RPP14	1.57554005	6.33E-12	8.65E-12
RP11-80H18.	NA	1.56E-76	5.50E-76
RP11-80H18.	NA	NA	NA
PXK	1.05182548	4.91E-99	2.60E-98
PDHB	0.76055073	1.38E-143	6.71E-142
RP11-802O2	2.0598249	0.00057566	0.00066141
RP11-359I18	NA	NA	NA
KCTD6	1.2067661	0.25944262	0.27111387
ACOX2	NA	1.94E-111	1.35E-110
FAM107A	NA	NA	NA
RP11-475O2	NA	NA	NA
FAM3D-AS1	NA	NA	NA
FAM3D	NA	NA	NA
C3orf67	NA	1.12E-29	2.01E-29
C3orf67-AS1	NA	NA	NA
CTD-2185K1(	NA	NA	NA
RP11-285B2	NA	NA	NA
RP11-719N2	NA	NA	NA
FHIT	NA	NA	NA
PTPRG	2.43433852	2.91E-05	3.46E-05
C3orf14	NA	NA	NA
FEZF2	NA	NA	NA
CADPS	NA	3.09E-105	1.86E-104
LINC00698	NA	NA	NA
SYNPR	NA	NA	NA
SYNPR-AS1	NA	NA	NA

SNTN	NA	NA	NA
C3orf49	0	2.39E-37	4.79E-37
THOC7	1.5553679	4.35E-66	1.31E-65
THOC7-AS1	NA	NA	NA
ATXN7	3.67023346	2.41E-119	2.10E-118
SCAANT1	NA	NA	NA
PSMD6-AS2	NA	0.69149503	0.70237405
RP11-245J9.5	0	9.83E-34	1.86E-33
PSMD6	0.88241565	1.88E-126	2.15E-125
RP11-245J9.4	Inf	3.39E-157	1.43E-154
RP11-245J9.6	Inf	9.11E-151	1.45E-148
PRICKLE2-AS	NA	NA	NA
LINC00994	NA	NA	NA
PRICKLE2	NA	NA	NA
PRICKLE2-AS	NA	NA	NA
PRICKLE2-AS	NA	NA	NA
PRICKLE2-AS	NA	NA	NA
RP11-14D22.	NA	NA	NA
RP11-14D22.	NA	NA	NA
ADAMTS9	NA	3.34E-06	4.06E-06
ADAMTS9-AS	NA	NA	NA
ADAMTS9-AS	NA	NA	NA
RP11-543A18	NA	NA	NA
MAGI1	2.80885214	4.97E-06	6.03E-06
MAGI1-AS1	NA	NA	NA
SLC25A26	1.6332955	1.54E-23	2.53E-23
LRIG1	1.74773022	3.20E-24	5.31E-24
KBTBD8	6.36673152	1.07E-40	2.25E-40
RP11-85I21.1	NA	NA	NA
SUCLG2	0.69140976	7.56E-131	1.07E-129
SUCLG2-AS1	NA	2.43E-15	3.53E-15
FAM19A1	NA	NA	NA
FAM19A4	NA	NA	NA
EOGT	2.49675746	3.22E-18	4.90E-18
CTD-2013N24	Inf	5.95E-97	3.03E-96
TMF1	1.9214177	0.00552981	0.00617593
UBA3	1.41162826	1.95E-83	7.70E-83
ARL6IP5	1.33672553	1.11E-60	3.08E-60
LMOD3	1.12354086	1.42E-09	1.86E-09
FRMD4B	Inf	6.61E-44	1.44E-43
RP11-62G11.	NA	NA	NA
RP11-444P10	NA	NA	NA
MITF	1.99740597	3.26E-82	1.26E-81
LINC01212	NA	5.60E-91	2.53E-90
RP11-231I13	NA	NA	NA

FOXP1	1.26055803	2.56E-16	3.77E-16
FOXP1-AS1	NA	NA	NA
RP11-154H2:	NA	NA	NA
RP11-154H2:	NA	9.88E-134	1.64E-132
RP11-154H2:	0.14980545	4.95E-57	1.30E-56
EIF4E3	NA	8.81E-43	1.90E-42
GPR27	NA	NA	NA
PROK2	NA	NA	NA
LINC00877	NA	NA	NA
RP11-398A8.	NA	NA	NA
RP11-398A8.	NA	NA	NA
LINC00870	NA	NA	NA
RP11-433A1(	NA	NA	NA
RP11-433A1(	NA	NA	NA
RP11-447M4	NA	NA	NA
RYBP	6.74124514	6.35E-142	2.30E-140
RP11-654C2:	NA	NA	NA
SHQ1	2.53516911	6.37E-69	2.01E-68
GXYLT2	1.99740597	1.94E-30	3.53E-30
PPP4R2	1.45196049	3.12E-41	6.58E-41
EBLN2	2.87127108	2.47E-62	7.04E-62
PDZRN3	NA	NA	NA
PDZRN3-AS1	NA	NA	NA
RP11-20B7.1	NA	NA	NA
RP11-508O1:	NA	NA	NA
CNTN3	NA	NA	NA
RP11-803B1.	Inf	1.10E-56	2.88E-56
CTD-2026G6.	0.37451362	0.75256903	0.76197719
FRG2C	NA	NA	NA
LINC00960	NA	NA	NA
ZNF717	3.07101167	6.29E-19	9.65E-19
ROBO2	NA	NA	NA
RP11-354H2:	NA	NA	NA
RP11-354H2:	NA	NA	NA
RP11-314M2	NA	NA	NA
ROBO1	3.2457847	3.36E-81	1.27E-80
RP11-47P18.	NA	NA	NA
RP11-47P18.	NA	NA	NA
RP11-6B4.1	NA	NA	NA
RP11-520D1!	NA	NA	NA
RP11-520D1!	NA	NA	NA
GBE1	1.64785992	1.45E-38	2.94E-38
RP11-142L1.	NA	NA	NA
RP11-359D2.	NA	NA	NA
RP11-260O1:	NA	NA	NA

LINC00971	NA	NA	NA
RP11-735B13	NA	NA	NA
CADM2	NA	4.57E-111	3.17E-110
CADM2-AS2	NA	NA	NA
CADM2-AS1	NA	NA	NA
RP11-789F5.	NA	NA	NA
RP11-331K15	NA	NA	NA
VGLL3	NA	NA	NA
LINC00506	NA	NA	NA
CHMP2B	1.30094204	5.71E-130	7.70E-129
POU1F1	NA	NA	NA
RP11-451B8.	NA	NA	NA
HTR1F	NA	NA	NA
CGGBP1	2.36937187	4.99E-91	2.26E-90
RP11-159G9.	2.24708171	7.50E-70	2.40E-69
ZNF654	2.03307393	1.14E-19	1.77E-19
C3orf38	1.45863199	3.84E-60	1.06E-59
EPHA3	NA	NA	NA
PROS1	0.93628405	1.94E-30	3.52E-30
ARL13B	1.31971466	3.28E-94	1.58E-93
STX19	NA	NA	NA
DHFRL1	10.4863813	4.50E-79	1.65E-78
NSUN3	1.1523496	1.51E-25	2.56E-25
LINC00879	NA	NA	NA
MTRNR2L12	0.93628405	2.97E-08	3.78E-08
EPHA6	NA	NA	NA
RP11-389G6.	NA	NA	NA
ARL6	0.99870298	8.42E-52	2.06E-51
RP11-389G6.	NA	1.15E-85	4.70E-85
CRYBG3	1.02991245	2.57E-06	3.14E-06
RP11-389G6.	NA	NA	NA
MINA	1.71206226	0.32401393	0.33665086
GABRR3	NA	NA	NA
OR5AC2	NA	NA	NA
RP11-343D2.	NA	NA	NA
OR5H1	NA	NA	NA
OR5H14	NA	NA	NA
OR5H15	NA	NA	NA
RP11-325B23	NA	NA	NA
OR5H6	NA	NA	NA
OR5H2	NA	NA	NA
OR5K4	NA	NA	NA
OR5K3	NA	NA	NA
OR5K1	NA	NA	NA
OR5K2	NA	NA	NA

CLDND1	1.98721512	1.77E-43	3.84E-43
CPOX	3.12829023	7.44E-46	1.67E-45
RP11-227H4.	NA	NA	NA
GPR15	NA	NA	NA
ST3GAL6-AS	Inf	2.81E-14	4.01E-14
ST3GAL6	1.87256809	7.36E-37	1.46E-36
DCBLD2	1.33160398	5.26E-36	1.03E-35
CTD-2021J15	NA	NA	NA
LINC00973	NA	NA	NA
RP11-779P15	NA	NA	NA
RP11-779P15	NA	NA	NA
RP11-201E8.	NA	NA	NA
COL8A1	NA	3.37E-08	4.28E-08
RP11-383I23	Inf	5.16E-97	2.63E-96
CMSS1	1.62888893	1.91E-45	4.27E-45
FILIP1L	Inf	8.27E-55	2.11E-54
TMEM30C	NA	NA	NA
TBC1D23	2.16385646	3.49E-15	5.06E-15
NIT2	1.02872728	1.33E-141	4.61E-140
TOMM70A	1.87256809	6.59E-12	9.00E-12
LNP1	0.74902724	2.75E-14	3.91E-14
TMEM45A	Inf	6.10E-140	1.71E-138
ADGRG7	NA	NA	NA
TFG	1.47161822	1.45E-113	1.07E-112
ABI3BP	NA	NA	NA
IMPG2	NA	2.66E-34	5.09E-34
SENP7	2.99610895	5.52E-26	9.41E-26
TRMT10C	1.12106063	6.35E-71	2.06E-70
PCNP	1.71588382	6.46E-28	1.13E-27
ZBTB11	3.59533074	7.78E-86	3.20E-85
ZBTB11-AS1	3.49546044	8.35E-35	1.61E-34
RPL24	2.20480435	2.51E-140	7.31E-139
CEP97	5.9922179	6.05E-76	2.11E-75
NXPE3	3.21011673	9.70E-15	1.39E-14
RP11-49I4.3	NA	NA	NA
NFKBIZ	0.93628405	1.96E-11	2.65E-11
RP11-221J22	NA	NA	NA
RP11-221J22	NA	NA	NA
ZPLD1	NA	NA	NA
RP11-280H2	NA	NA	NA
ALCAM	1.69345288	3.05E-24	5.08E-24
CBLB	1.87256809	6.48E-08	8.18E-08
LINC00882	NA	5.29E-18	8.01E-18
DUBR	Inf	7.06E-45	1.56E-44
RP11-446H1	NA	NA	NA

RP11-446H18	NA	NA	NA
CCDC54	NA	NA	NA
RP11-115H18	NA	4.36E-16	6.41E-16
BBX	1.76309488	1.69E-08	2.17E-08
LINC00635	NA	NA	NA
LINC00636	NA	5.41E-108	3.46E-107
CD47	1.13235294	1.26E-127	1.53E-126
LINC01215	1.9661965	0.0411336	0.04457061
IFT57	1.48806744	1.07E-51	2.61E-51
HHLA2	NA	3.65E-49	8.58E-49
MYH15	Inf	1.23E-38	2.51E-38
KIAA1524	0.63379228	4.49E-95	2.20E-94
AC069499.1	NA	NA	NA
DZIP3	1.71206226	0.00062803	0.00072076
RETNLB	NA	NA	NA
TRAT1	NA	NA	NA
GUCA1C	NA	NA	NA
MORC1	NA	NA	NA
MORC1-AS1	NA	NA	NA
FLJ22763	NA	NA	NA
RP11-59E19.	NA	NA	NA
LINC00488	NA	NA	NA
DPPA2	NA	NA	NA
DPPA4	NA	NA	NA
LINC01205	NA	NA	NA
RP11-457K10	NA	NA	NA
RP11-119D18	NA	NA	NA
RP11-553A10	NA	NA	NA
PVRL3-AS1	NA	NA	NA
PVRL3	NA	5.70E-20	8.88E-20
CD96	Inf	8.74E-63	2.50E-62
RP11-615J4.3	NA	NA	NA
ZBED2	NA	NA	NA
PLCXD2	NA	1.51E-70	4.88E-70
PLCXD2-AS1	NA	NA	NA
PHLDB2	NA	NA	NA
ABHD10	1.23589494	3.49E-113	2.55E-112
TAGLN3	NA	NA	NA
TMPRSS7	NA	NA	NA
C3orf52	NA	NA	NA
GCSAM	NA	NA	NA
RP11-757F18	NA	NA	NA
SLC9C1	NA	NA	NA
RP11-90K6.1	NA	NA	NA
CD200	NA	1.70E-66	5.18E-66

RP11-231E6.	NA	5.69E-11	7.62E-11
BTLA	NA	NA	NA
ATG3	1.45124027	1.01E-66	3.08E-66
SLC35A5	2.02237354	3.19E-74	1.09E-73
CCDC80	NA	7.88E-117	6.32E-116
RP11-572C1!	NA	NA	NA
CD200R1L	NA	NA	NA
CD200R1	NA	NA	NA
RP11-484K9.	NA	2.15E-35	4.17E-35
GTPBP8	1.38790341	8.35E-76	2.91E-75
C3orf17	1.94747082	0.20042639	0.21079047
RP11-572M1	NA	1.19E-07	1.49E-07
RP11-572M1	NA	NA	NA
RP11-572M1	NA	NA	NA
BOC	0	1.77E-06	2.16E-06
CFAP44	7.49027237	5.18E-94	2.48E-93
CFAP44-AS1	NA	1.86E-31	3.41E-31
SPICE1	0.59922179	1.13E-23	1.85E-23
SIDT1	NA	4.98E-101	2.74E-100
SIDT1-AS1	NA	6.73E-123	6.65E-122
USF3	1.99740597	1.49E-23	2.46E-23
NAA50	1.71256462	0.00030559	0.00035409
RP11-271C2	NA	0.05253411	0.05667775
ATP6V1A	2.13184675	7.13E-97	3.63E-96
GRAMD1C	NA	0.92789068	0.93127864
RP11-255E6.	NA	1.35E-10	1.79E-10
ZDHHC23	0.56177043	0.0515757	0.05567573
CCDC191	0	0.00098901	0.00112946
QTRTD1	1.77401188	1.24E-13	1.74E-13
DRD3	NA	NA	NA
RP11-553L6.	NA	NA	NA
ZNF80	NA	NA	NA
TIGIT	NA	NA	NA
RP11-553L6.	NA	4.12E-28	7.24E-28
ZBTB20	0.74902724	0.976289	0.97758225
ZBTB20-AS1	NA	NA	NA
RP11-197K3.	NA	NA	NA
ZBTB20-AS2	NA	0.00013894	0.00016228
ZBTB20-AS3	NA	NA	NA
ZBTB20-AS4	NA	NA	NA
AC069063.1	NA	NA	NA
AC069063.2	NA	NA	NA
RP11-190P1	NA	NA	NA
GAP43	NA	NA	NA
LSAMP	13.1079767	2.64E-135	4.82E-134

LSAMP-AS1	NA	NA	NA
LINC00903	NA	NA	NA
TUSC7	NA	NA	NA
LINC00901	NA	NA	NA
RP11-384F7.	NA	NA	NA
RP11-768G7.	NA	NA	NA
RP11-768G7.	NA	NA	NA
RP11-384F7.	NA	NA	NA
IGSF11	NA	NA	NA
IGSF11-AS1	NA	NA	NA
C3orf30	NA	NA	NA
RP11-484M3	NA	NA	NA
UPK1B	NA	NA	NA
B4GALT4	0.68093385	5.62E-53	1.40E-52
B4GALT4-AS	NA	NA	NA
ARHGAP31	2.24708171	1.53E-133	2.51E-132
ARHGAP31-A	NA	NA	NA
TMEM39A	0.94613967	1.15E-40	2.41E-40
POGLUT1	1.67639429	1.48E-09	1.94E-09
RP11-190C22	Inf	5.75E-139	1.44E-137
TIMMDC1	1.40351706	1.79E-68	5.61E-68
CD80	NA	7.26E-41	1.52E-40
RP11-190C22	NA	3.46E-109	2.28E-108
ADPRH	NA	2.83E-123	2.84E-122
PLA1A	NA	NA	NA
POPDC2	0.74902724	4.77E-20	7.44E-20
COX17	1.96379577	1.50E-43	3.26E-43
MAATS1	NA	NA	NA
RP11-169N1	NA	NA	NA
NR1I2	NA	NA	NA
GSK3B	1.38708748	1.41E-18	2.15E-18
RP11-18H7.1	0.74902724	5.48E-40	1.14E-39
GPR156	NA	NA	NA
LRRC58	2.62718509	3.04E-130	4.13E-129
RP11-174O3.	NA	NA	NA
FSTL1	4.68142023	5.80E-08	7.33E-08
NDUFB4	1.92563682	9.90E-46	2.22E-45
HGD	NA	NA	NA
RABL3	2.11089494	0.09916206	0.10584209
GTF2E1	1.99740597	1.87E-18	2.85E-18
RP11-500K19	NA	NA	NA
STXBP5L	Inf	3.71E-73	1.24E-72
POLQ	1.1523496	0.63283397	0.64484801
ARGFX	NA	NA	NA
FBXO40	NA	NA	NA

HCLS1	1.48022049	3.33E-107	2.09E-106
GOLGB1	1.08949416	3.57E-15	5.17E-15
IQCB1	1.71206226	6.69E-08	8.45E-08
EAF2	1.29267604	5.02E-64	1.47E-63
SLC15A2	1.49805447	1.82E-08	2.32E-08
ILDR1	NA	NA	NA
CD86	2.7464332	1.70E-63	4.92E-63
CASR	NA	NA	NA
CSTA	0.18725681	1.86E-121	1.74E-120
CCDC58	1.33610264	1.86E-13	2.60E-13
FAM162A	1.56698336	2.66E-30	4.83E-30
WDR5B	1.31079767	3.94E-47	9.01E-47
RP11-299J3.8	0.74902724	2.81E-24	4.68E-24
KPNA1	3.29571984	1.01E-125	1.12E-124
PARP9	2.24708171	1.36E-38	2.77E-38
DTX3L	2.8463035	7.18E-112	5.07E-111
PARP15	NA	NA	NA
PARP14	2.01462498	7.52E-24	1.24E-23
HSPBAP1	1.58127972	2.38E-13	3.33E-13
DIRC2	1.87256809	1.94E-35	3.77E-35
RP11-67L2.2	Inf	4.16E-21	6.60E-21
SEMA5B	NA	NA	NA
PDIA5	1.72852439	3.55E-26	6.06E-26
SEC22A	1.08608949	2.55E-97	1.31E-96
RP11-797D24	NA	NA	NA
ADCY5	NA	NA	NA
RP11-797D24	NA	1.53E-68	4.80E-68
HACD2	1.47834323	0.00019036	0.00022161
MYLK-AS1	1.49805447	5.78E-12	7.91E-12
MYLK	1.87256809	0.13518464	0.14325466
MYLK-AS2	NA	NA	NA
RP11-9N20.3	NA	NA	NA
CCDC14	1.73774319	1.03E-29	1.85E-29
ROPN1	NA	NA	NA
KALRN	Inf	1.01E-10	1.35E-10
UMPS	1.12354086	1.72E-139	4.57E-138
ITGB5	Inf	8.06E-60	2.21E-59
ITGB5-AS1	NA	NA	NA
MUC13	NA	NA	NA
HEG1	Inf	4.70E-31	8.60E-31
SLC12A8	NA	0.10239071	0.10914055
ZNF148	1.61554894	4.33E-07	5.37E-07
SNX4	1.04469588	1.95E-84	7.84E-84
OSBPL11	2.24708171	3.40E-66	1.03E-65
RP11-379B18	NA	0.00042058	0.00048531

RP11-666A2(	NA	NA	NA
ALG1L	NA	NA	NA
ROPN1B	NA	NA	NA
SLC41A3	1.55567195	7.99E-08	1.01E-07
RP11-158I23	NA	NA	NA
RP11-124N2.	NA	NA	NA
ALDH1L1	NA	5.66E-14	8.02E-14
ALDH1L1-AS:	NA	NA	NA
ALDH1L1-AS:	NA	NA	NA
RP11-71E19.	NA	NA	NA
RP11-71E19.	NA	NA	NA
KLF15	0	1.19E-38	2.42E-38
CCDC37-AS1	NA	NA	NA
CFAP100	NA	NA	NA
ZXDC	1.5729572	1.05E-15	1.54E-15
UROC1	NA	NA	NA
CHST13	0.18725681	9.78E-76	3.40E-75
C3orf22	NA	NA	NA
TXNRD3	2.24708171	1.79E-66	5.42E-66
TXNRD3NB	NA	NA	NA
CHCHD6	1.715514	2.58E-20	4.04E-20
PLXNA1	1.42996109	5.16E-12	7.07E-12
C3orf56	NA	NA	NA
RP11-88I21.2	NA	NA	NA
LINC01471	NA	NA	NA
RP11-59J16.2	NA	NA	NA
RP11-59J16.3	NA	NA	NA
RP11-153F1(	Inf	3.95E-07	4.91E-07
TPRA1	2.32805763	1.58E-101	8.78E-101
MCM2	2.54320876	2.54E-53	6.37E-53
PODXL2	1.38570039	4.15E-77	1.47E-76
ABTB1	0.74902724	2.09E-95	1.03E-94
MGLL	NA	1.76E-12	2.43E-12
KBTBD12	NA	2.99E-74	1.02E-73
SEC61A1	2.2038686	1.61E-116	1.28E-115
RUVBL1	0.94442565	6.14E-95	2.99E-94
RUVBL1-AS1	NA	NA	NA
EEFSEC	1.31971466	9.59E-84	3.81E-83
DNAJB8	NA	NA	NA
DNAJB8-AS1	NA	NA	NA
GATA2	1.79766537	0.00244884	0.00276184
GATA2-AS1	1.41482923	0.00478897	0.00535909
LINC01565	NA	3.00E-94	1.44E-93
RPN1	1.64662186	2.04E-29	3.64E-29
RAB7A	1.10966998	7.92E-130	1.06E-128

RP11-723O4.	Inf	1.11E-32	2.08E-32
RP11-723O4.	NA	3.13E-29	5.59E-29
ACAD9	1.49805447	1.56E-117	1.28E-116
KIAA1257	NA	0.58474496	0.59765877
RP11-723O4.	NA	NA	NA
EFCC1	NA	NA	NA
GP9	NA	NA	NA
RAB43	NA	9.80E-08	1.23E-07
ISY1-RAB43	NA	NA	NA
RP11-434H6.	Inf	1.00E-152	1.93E-150
ISY1	0.98652368	1.19E-127	1.45E-126
RP11-434H6.	Inf	3.57E-52	8.80E-52
CNBP	1.70379586	4.90E-11	6.58E-11
COPG1	1.45863199	1.67E-53	4.19E-53
HMCES	1.57903039	4.16E-76	1.45E-75
H1FX	1.24007597	1.80E-145	1.58E-143
H1FX-AS1	0	1.65E-90	7.38E-90
EFCAB12	0	1.04E-13	1.47E-13
MBD4	1.57587549	1.69E-94	8.20E-94
IFT122	2.24708171	0.000389	0.00044936
RHO	NA	NA	NA
H1FOO	NA	NA	NA
PLXND1	3.44552529	6.54E-124	6.75E-123
TMCC1	1.39105058	1.30E-16	1.93E-16
TMCC1-AS1	2.45136187	2.42E-38	4.89E-38
RP11-93K22.	0	8.61E-07	1.06E-06
TRH	NA	NA	NA
ALG1L2	NA	9.32E-135	1.65E-133
RP11-93K22.	NA	8.11E-110	5.43E-109
RP11-77P16.	NA	9.18E-31	1.67E-30
COL6A5	NA	NA	NA
COL6A6	NA	NA	NA
PIK3R4	3.74513619	5.05E-99	2.67E-98
ATP2C1	2.27964811	3.07E-15	4.45E-15
AC055733.1	NA	NA	NA
RP11-39E3.3	NA	NA	NA
ASTE1	1.41482923	0.00029417	0.00034099
NEK11	Inf	1.87E-08	2.39E-08
RP11-265F1‹	NA	6.85E-69	2.16E-68
NUDT16	1.42996109	2.28E-09	2.97E-09
MRPL3	1.36746749	2.26E-82	8.76E-82
RP11-517B1‹	Inf	0.91637724	0.92028319
CPNE4	NA	1.94E-111	1.35E-110
ACPP	1.24837873	0.70172115	0.7122825
DNAJC13	1.60505837	0.02290493	0.02503713

ACAD11	Inf	1.66E-17	2.50E-17
NPHP3-ACAD	Inf	5.42E-143	2.34E-141
ACKR4	NA	NA	NA
UBA5	1.59168288	8.85E-76	3.08E-75
NPHP3	4.68142023	4.21E-89	1.84E-88
NPHP3-AS1	NA	NA	NA
RP11-469L4.	NA	NA	NA
TMEM108	NA	NA	NA
TMEM108-A	NA	NA	NA
BFSP2	NA	NA	NA
BFSP2-AS1	NA	NA	NA
RP11-91K8.5	NA	NA	NA
CDV3	2.06109876	2.40E-94	1.16E-93
TOPBP1	2.30469919	8.21E-75	2.82E-74
TF	NA	NA	NA
SRPRB	1.22049616	1.51E-127	1.82E-126
RAB6B	0.48466468	2.09E-139	5.49E-138
C3orf36	NA	NA	NA
SLCO2A1	NA	7.11E-62	2.01E-61
RP11-202A1	NA	NA	NA
RYK	2.88375486	5.21E-106	3.17E-105
RP11-200A1.	NA	NA	NA
RP11-375I20	NA	NA	NA
AMOTL2	0.24967575	0.01467208	0.01615665
ANAPC13	1.40310736	8.98E-122	8.51E-121
CEP63	0.86983808	3.45E-29	6.16E-29
EPHB1	NA	3.05E-146	3.18E-144
KY	NA	NA	NA
PPP2R3A	1.49805447	0.00178993	0.00202629
RP11-305O4.	NA	6.94E-13	9.64E-13
MSL2	3.07101167	1.51E-79	5.60E-79
PCCB	0.68910506	4.58E-139	1.16E-137
STAG1	3.2457847	7.37E-95	3.59E-94
RP11-102M1	Inf	0.03719461	0.04033864
SLC35G2	16.4785992	3.91E-90	1.74E-89
RP11-731C1	2.24708171	0.46917402	0.48225241
NCK1-AS1	1.24837873	0.86795572	0.87402601
NCK1	1.04581161	1.15E-107	7.33E-107
IL20RB	0.74902724	2.78E-29	4.96E-29
IL20RB-AS1	NA	NA	NA
SOX14	NA	NA	NA
LINC01210	NA	NA	NA
RP11-2A4.4	NA	NA	NA
CLDN18	NA	NA	NA
RP11-461M2	NA	1.29E-40	2.70E-40

DZIP1L	0.58257674	6.85E-87	2.87E-86
A4GNT	NA	NA	NA
DBR1	1.38281952	2.78E-73	9.33E-73
ARMC8	1.32065329	1.84E-29	3.29E-29
NME9	NA	NA	NA
MRAS	NA	NA	NA
ESYT3	5.9922179	7.51E-56	1.95E-55
CEP70	1.75934305	0.00990195	0.01096242
FAIM	1.80953115	1.75E-20	2.75E-20
PIK3CB	2.2811284	1.03E-27	1.80E-27
LINC01391	NA	1.92E-166	1.94E-163
FOXL2	NA	NA	NA
FOXL2NB	NA	NA	NA
PRR23A	NA	NA	NA
MRPS22	0.7946202	6.70E-135	1.20E-133
PRR23B	NA	NA	NA
PRR23C	NA	NA	NA
BPESC1	NA	NA	NA
PISRT1	Inf	1.37E-22	2.22E-22
RP11-219D1!	NA	6.75E-147	7.29E-145
COPB2	1.715514	6.07E-59	1.64E-58
RP11-319G6.	NA	0.00102231	0.00116705
RBP2	NA	NA	NA
RP11-319G6.	NA	NA	NA
RBP1	NA	NA	NA
NMNAT3	2.46108949	1.21E-109	8.05E-109
RP11-442N1.	NA	NA	NA
RP11-442N1.	NA	NA	NA
RP11-166D1ł	NA	NA	NA
CLSTN2	NA	NA	NA
RP11-68L1.2	NA	NA	NA
RP11-68L1.1	NA	NA	NA
CLSTN2-AS1	NA	NA	NA
TRIM42	NA	NA	NA
RP11-691G1	NA	NA	NA
SLC25A36	1.78491597	2.06E-27	3.58E-27
SPSB4	NA	1.48E-28	2.61E-28
RP11-231L11	NA	NA	NA
PXYLP1	2.13184675	2.34E-55	6.02E-55
RP11-438D8.	NA	NA	NA
ZBTB38	1.12354086	1.17E-05	1.40E-05
RASA2	4.49416342	4.76E-64	1.39E-63
AC010184.1	NA	NA	NA
RP11-340E6.	NA	NA	NA
RNF7	1.78422129	0.00018437	0.00021468

GRK7	NA	NA	NA
ATP1B3	1.1492708	1.16E-134	2.05E-133
ATP1B3-AS1	NA	1.91E-27	3.32E-27
TFDP2	2.00364786	3.65E-27	6.32E-27
GK5	1.90137683	8.19E-59	2.21E-58
XRN1	1.91418072	0.00679588	0.00757036
ATR	0.81144617	4.43E-14	6.28E-14
PLS1	1.49805447	0.14132807	0.14963372
PLS1-AS1	NA	NA	NA
TRPC1	1.49805447	9.53E-68	2.95E-67
PCOLCE2	1.49805447	5.20E-52	1.28E-51
RP11-372E1.	NA	NA	NA
PAQR9	0.99870298	3.95E-36	7.77E-36
PAQR9-AS1	Inf	1.16E-142	4.75E-141
U2SURP	1.29326426	9.02E-84	3.58E-83
RP11-91G21.	1.19844358	9.45E-23	1.54E-22
RP11-80H8.3	NA	NA	NA
CHST2	0.74902724	1.22E-34	2.34E-34
SLC9A9	Inf	1.25E-100	6.83E-100
SLC9A9-AS1	NA	NA	NA
SLC9A9-AS2	NA	NA	NA
C3orf58	1.74773022	2.69E-05	3.20E-05
RP11-88H10.	NA	NA	NA
RP11-88H10.	NA	NA	NA
RP11-274H2.	NA	NA	NA
RP11-274H2.	NA	NA	NA
PLOD2	Inf	2.20E-46	4.97E-46
PLSCR4	0	5.59E-77	1.98E-76
PLSCR2	NA	NA	NA
PLSCR1	1.25643279	2.19E-100	1.19E-99
PLSCR5	NA	NA	NA
PLSCR5-AS1	NA	NA	NA
RP11-649A1(	NA	NA	NA
RP11-232M2	NA	NA	NA
ZIC4	NA	NA	NA
ZIC4-AS1	NA	NA	NA
ZIC1	NA	NA	NA
RP11-78O22.	NA	NA	NA
RP11-71N10.	NA	4.30E-54	1.09E-53
RP11-639B1.	NA	NA	NA
RP11-501O2.	NA	NA	NA
RP11-501O2.	NA	NA	NA
RP11-501O2.	NA	NA	NA
RP11-501O2.	NA	NA	NA
AGTR1	NA	NA	NA

CPB1	NA	NA	NA
RP11-680B3.	NA	NA	NA
CPA3	NA	NA	NA
GYG1	1.31559912	3.46E-136	6.71E-135
HLTF	1.89931907	7.86E-06	9.49E-06
HLTF-AS1	NA	NA	NA
HPS3	1.26151956	7.67E-68	2.38E-67
CP	NA	NA	NA
RP11-206M1	0	9.37E-65	2.77E-64
TM4SF18	NA	NA	NA
TM4SF1	0.49935149	8.02E-42	1.71E-41
TM4SF1-AS1	NA	NA	NA
RP11-278L1!	NA	NA	NA
TM4SF4	NA	NA	NA
WWTR1	Inf	0.07848333	0.08410187
WWTR1-AS1	0	6.01E-09	7.76E-09
COMMD2	1.71652075	0.04456938	0.04821572
ANKUB1	NA	NA	NA
RNF13	1.21342412	1.20E-26	2.07E-26
PFN2	1.49805447	3.59E-49	8.44E-49
RP11-651P2:	NA	NA	NA
RP11-167H9.	NA	NA	NA
RP11-167H9.	NA	NA	NA
RP11-167H9.	NA	NA	NA
LINC01213	NA	NA	NA
LINC01214	NA	NA	NA
TSC22D2	1.38281952	3.61E-08	4.59E-08
SERP1	2.06592761	2.19E-119	1.91E-118
EIF2A	1.45049719	1.01E-65	3.03E-65
SELT	1.72160136	9.26E-19	1.42E-18
ERICH6	NA	NA	NA
ERICH6-AS1	Inf	9.74E-08	1.23E-07
SIAH2	1.63976233	1.22E-42	2.62E-42
SIAH2-AS1	NA	5.67E-112	4.01E-111
AC016142.1	NA	NA	NA
CLRN1-AS1	NA	NA	NA
RP11-166N6.	NA	NA	NA
RP11-166N6.	NA	NA	NA
RP11-373I8.1	NA	NA	NA
CLRN1	NA	NA	NA
MED12L	1.99740597	2.88E-39	5.92E-39
GPR171	3.74513619	9.28E-42	1.97E-41
P2RY14	NA	NA	NA
GPR87	NA	NA	NA
P2RY13	NA	9.64E-116	7.50E-115

P2RY12	NA	NA	NA
IGSF10	2.51459144	3.10E-70	9.96E-70
RP11-454C1ε	NA	NA	NA
AADACL2	NA	NA	NA
AADACL2-AS	NA	NA	NA
RP11-64D22.	NA	NA	NA
AADAC	NA	NA	NA
SUCNR1	1.22840467	6.11E-25	1.03E-24
RP11-246A1(	Inf	1.14E-52	2.83E-52
MBNL1	2.40029183	3.38E-122	3.24E-121
MBNL1-AS1	Inf	2.37E-47	5.43E-47
TMEM14EP	NA	NA	NA
RP11-362A9.	NA	NA	NA
RP11-788A4.	NA	NA	NA
P2RY1	4.74383917	3.15E-74	1.07E-73
RP11-529G2	NA	1.18E-54	3.02E-54
RAP2B	3.29571984	2.47E-84	9.93E-84
RP11-292E2.	NA	0.08314998	0.08898696
RP11-292E2.	NA	NA	NA
RP11-292E2.	NA	NA	NA
C3orf79	NA	NA	NA
RP11-217E22	NA	NA	NA
ARHGEF26	1.91418072	0.73216625	0.74185758
DHX36	1.89366745	4.74E-06	5.75E-06
GPR149	NA	NA	NA
RP11-656A1!	NA	NA	NA
RP11-439C8.	NA	NA	NA
RP11-439C8.	NA	NA	NA
MME	0.74902724	0.17470792	0.18419056
MME-AS1	NA	NA	NA
LINC01487	NA	NA	NA
RP11-451G4.	NA	1.39E-136	2.77E-135
RP11-451G4.	NA	NA	NA
PLCH1	4.86867704	7.91E-68	2.45E-67
PLCH1-AS1	NA	NA	NA
PLCH1-AS2	NA	NA	NA
C3orf33	1.26758456	5.91E-06	7.15E-06
SLC33A1	1.87256809	1.78E-27	3.10E-27
GMPS	1.62964034	6.01E-16	8.81E-16
KCNAB1	NA	4.22E-12	5.78E-12
KCNAB1-AS2	NA	NA	NA
KCNAB1-AS1	NA	NA	NA
RP11-305K5.	Inf	3.75E-42	8.01E-42
SSR3	1.6271971	1.25E-63	3.63E-63
TIPARP-AS1	2.24708171	0.03938981	0.04269897

TIPARP	2.99610895	5.98E-45	1.33E-44
LINC00886	NA	1.28E-142	5.08E-141
LEKR1	NA	NA	NA
LINC00880	NA	NA	NA
RP11-6F2.5	NA	NA	NA
LINC00881	Inf	7.45E-134	1.25E-132
CCNL1	2.01778766	0.90135841	0.90610319
RP11-555M1	NA	NA	NA
VEPH1	NA	NA	NA
PTX3	NA	6.24E-59	1.68E-58
PQLC2L	NA	3.45E-89	1.51E-88
RP11-290K4.	NA	NA	NA
RP11-290K4.	NA	NA	NA
SHOX2	1.83095547	0.36615498	0.37906778
RSRC1	1.62084583	5.27E-29	9.36E-29
RP11-538P18	4.41093818	4.19E-72	1.38E-71
MLF1	NA	9.36E-51	2.25E-50
GFM1	2.57478113	3.47E-85	1.41E-84
LXN	0.82392996	8.77E-115	6.66E-114
RP11-379F4.	NA	NA	NA
RP11-379F4.	NA	3.44E-08	4.37E-08
RARRES1	NA	NA	NA
MFSD1	1.15691336	2.03E-145	1.64E-143
RP11-379F4.	NA	4.48E-12	6.14E-12
RP11-379F4.	0	4.57E-62	1.30E-61
IQCJ-SCHIP1	NA	5.05E-57	1.33E-56
IQCJ	NA	NA	NA
IQCJ-SCHIP1-	NA	NA	NA
SCHIP1	Inf	6.58E-105	3.93E-104
IL12A-AS1	4.49416342	8.82E-21	1.39E-20
IL12A	1.49805447	7.83E-33	1.47E-32
LINC01100	NA	1.11E-60	3.08E-60
C3orf80	1.98271916	4.74E-94	2.27E-93
RP11-432B6.	NA	NA	NA
IFT80	3.2457847	4.09E-73	1.37E-72
SMC4	1.25173008	8.54E-126	9.47E-125
TRIM59	0.74902724	2.55E-05	3.04E-05
KPNA4	2.41353221	8.90E-123	8.74E-122
ARL14	NA	NA	NA
RP11-16N11.	Inf	1.13E-137	2.49E-136
PPM1L	0.18725681	2.30E-29	4.12E-29
B3GALNT1	NA	NA	NA
NMD3	1.31079767	4.32E-50	1.03E-49
SPTSSB	NA	NA	NA
RP11-3P17.4	Inf	0.00026191	0.00030396

RP11-3P17.5	NA	2.32E-48	5.39E-48
OTOL1	NA	NA	NA
RP11-774I5.1	NA	NA	NA
RP11-10O22.	NA	NA	NA
LINC01192	NA	NA	NA
RP11-418B1.	NA	NA	NA
LINC01323	NA	NA	NA
LINC01324	NA	NA	NA
SI	NA	NA	NA
RP11-747D1	NA	NA	NA
SLITRK3	NA	NA	NA
LINC01322	NA	NA	NA
BCHE	NA	7.99E-149	1.03E-146
LINC01326	NA	NA	NA
RP11-12K11.	NA	0.0003184	0.00036865
ZBBX	NA	NA	NA
LINC01327	NA	NA	NA
SERPINI2	NA	NA	NA
WDR49	NA	NA	NA
PDCD10	1.26901309	3.19E-113	2.34E-112
SERPINI1	NA	1.40E-13	1.96E-13
RP11-298O2	NA	NA	NA
RP11-298O2	NA	NA	NA
RP11-298O2	NA	NA	NA
RP11-298O2	NA	NA	NA
LINC01330	NA	NA	NA
GOLIM4	2.05502345	5.24E-17	7.81E-17
RP11-637O1	NA	NA	NA
RP11-368I23	NA	1.91E-27	3.32E-27
RP11-368I23	NA	NA	NA
RP11-368I23	NA	NA	NA
RP11-152C1	NA	NA	NA
MECOM	1.64785992	3.67E-28	6.45E-28
RP11-641D5.	NA	NA	NA
AC074033.1	NA	NA	NA
TERC	NA	NA	NA
ACTRT3	0.74902724	0.03018987	0.03286754
RP11-816J6.3	Inf	4.68E-128	5.75E-127
MYNN	2.7464332	0.10859445	0.11561061
RP11-362K14	Inf	2.61E-32	4.85E-32
LRRC34	1.75858569	5.95E-09	7.68E-09
RP11-362K14	NA	1.64E-58	4.40E-58
RP11-362K14	0.74902724	4.85E-13	6.75E-13
LRRIQ4	0	6.77E-08	8.54E-08
LRRC31	NA	NA	NA

SAMD7	NA	NA	NA
RP11-379K17	NA	3.90E-124	4.05E-123
SEC62	1.42266537	2.40E-68	7.50E-68
SEC62-AS1	NA	NA	NA
GPR160	2.90248054	4.75E-73	1.59E-72
PHC3	2.24708171	3.54E-60	9.77E-60
RP11-379K17	NA	0.8126495	0.82015394
PRKCI	2.08939177	2.27E-15	3.29E-15
RP11-543D1(	NA	NA	NA
SKIL	1.49805447	1.33E-45	2.97E-45
RP11-469J4.3	NA	NA	NA
CLDN11	NA	5.16E-14	7.31E-14
SLC7A14	NA	2.41E-26	4.12E-26
RP11-373E16	NA	NA	NA
RPL22L1	2.0856097	5.66E-143	2.43E-141
EIF5A2	2.33520256	1.29E-20	2.03E-20
SLC2A2	NA	NA	NA
TNIK	1.24837873	1.20E-14	1.73E-14
RP11-185E8.	0	8.76E-138	1.93E-136
PLD1	0.85603113	5.77E-107	3.59E-106
TMEM212	NA	NA	NA
TMEM212-A:	NA	NA	NA
FNDC3B	1.3618677	1.46E-08	1.87E-08
GHSR	NA	NA	NA
TNFSF10	1.19844358	1.94E-06	2.37E-06
RP11-408H1.	NA	4.79E-31	8.75E-31
NCEH1	5.24319066	7.35E-35	1.42E-34
ECT2	2.65564202	6.23E-78	2.24E-77
SPATA16	NA	NA	NA
RP11-324C1(	NA	NA	NA
NLGN1	NA	NA	NA
NLGN1-AS1	NA	NA	NA
NAALADL2	NA	NA	NA
NAALADL2-A	NA	NA	NA
NAALADL2-A	NA	NA	NA
RP11-809F4.	NA	NA	NA
LINC01208	NA	NA	NA
RP11-644C3.	NA	NA	NA
LINC01209	NA	3.20E-59	8.68E-59
TBL1XR1	1.76612738	5.76E-11	7.72E-11
TBL1XR1-AS:	NA	NA	NA
LINC00501	NA	NA	NA
RP11-706D8.	Inf	1.42E-74	4.86E-74
LINC00578	NA	NA	NA
RP11-114M1	NA	NA	NA

RP11-114M1	NA	7.77E-80	2.88E-79
KCCAT211	NA	NA	NA
RP11-2L8.1	NA	NA	NA
RP11-2L8.2	NA	NA	NA
RP11-33A14.	NA	NA	NA
KCNMB2	NA	NA	NA
RP11-385J1.3	NA	NA	NA
KCNMB2-AS1	NA	NA	NA
ZMAT3	8.98832685	5.99E-82	2.31E-81
RP11-360P21	NA	NA	NA
PIK3CA	2.69649805	1.27E-15	1.85E-15
KCNMB3	2.52796693	1.43E-67	4.39E-67
ZNF639	2.04734112	6.60E-23	1.08E-22
RP11-255C15	NA	1.83E-67	5.64E-67
MFN1	1.63849708	3.86E-10	5.10E-10
GNB4	1.94952295	1.48E-54	3.77E-54
AC007620.3	Inf	1.62E-116	1.29E-115
RP11-145M9	NA	3.65E-78	1.32E-77
ACTL6A	1.03365759	1.84E-128	2.29E-127
RP11-15L13.4	NA	NA	NA
RP11-145M9	Inf	3.54E-132	5.39E-131
MRPL47	1.25900323	9.36E-116	7.31E-115
NDUFB5	1.33773636	9.03E-110	6.04E-109
USP13	2.14007782	0.15561137	0.16439187
PEX5L	NA	NA	NA
RP11-494H4.	Inf	3.37E-155	9.85E-153
PEX5L-AS1	NA	NA	NA
PEX5L-AS2	NA	NA	NA
RP11-420J11	NA	NA	NA
RP11-496B10	NA	NA	NA
TTC14	1.53062088	1.01E-33	1.91E-33
CCDC39	0	3.36E-115	2.57E-114
CCDC39-AS1	NA	NA	NA
FXR1	1.35169283	2.07E-109	1.37E-108
DNAJC19	2.26557621	3.38E-130	4.59E-129
RP11-275H4.	NA	NA	NA
RP11-4B14.3	NA	NA	NA
SOX2	NA	NA	NA
LINC01206	NA	NA	NA
RP11-416O18	NA	NA	NA
RP11-338L18	NA	NA	NA
FLJ46066	NA	NA	NA
RP11-139K4.	NA	NA	NA
RP11-646E18	NA	NA	NA
RP11-646E18	NA	NA	NA

RP11-225N1(	NA	NA	NA
ATP11B	7.49027237	0.00765385	0.00851356
RP11-531F1(	NA	2.14E-43	4.63E-43
DCUN1D1	2.43433852	1.19E-17	1.80E-17
MCCC1	1.76241703	4.75E-05	5.62E-05
MCCC1-AS1	NA	2.00E-92	9.33E-92
LAMP3	NA	NA	NA
MCF2L2	0	2.46E-127	2.94E-126
B3GNT5	1.74773022	1.39E-10	1.85E-10
KLHL6	2.54669261	1.53E-05	1.83E-05
KLHL6-AS1	NA	NA	NA
KLHL24	1.49805447	5.91E-39	1.21E-38
YEATS2	2.24708171	0.59617035	0.60889053
YEATS2-AS1	NA	0.05917316	0.06371791
MAP6D1	3.17587549	2.56E-118	2.15E-117
PARL	1.43102091	3.04E-97	1.56E-96
ABCC5	1.77893969	1.00E-16	1.49E-16
ABCC5-AS1	NA	NA	NA
HTR3D	NA	NA	NA
HTR3C	NA	NA	NA
HTR3E-AS1	NA	NA	NA
HTR3E	NA	NA	NA
RP11-778D9.	Inf	4.17E-95	2.04E-94
RP11-778D9.	0.74902724	2.86E-34	5.47E-34
EIF2B5	2.0598249	5.00E-51	1.21E-50
DVL3	2.18715953	1.12E-10	1.49E-10
AP2M1	1.45378848	4.38E-125	4.70E-124
ABCF3	1.54486868	3.80E-22	6.12E-22
VWA5B2	NA	NA	NA
ALG3	1.47041509	4.64E-132	7.02E-131
ECE2	1.67235641	1.66E-40	3.47E-40
CAMK2N2	2.99610895	0.84838678	0.85512711
PSMD2	1.09857328	1.29E-120	1.18E-119
EIF4G1	1.5300035	1.85E-32	3.44E-32
FAM131A	Inf	4.19E-131	5.99E-130
CLCN2	1.19844358	0.02788056	0.03038622
POLR2H	1.49310496	8.96E-45	1.98E-44
THPO	NA	NA	NA
CHRD	0.74902724	3.84E-11	5.17E-11
RP11-433C9.	NA	NA	NA
EIF2B5-AS1	NA	NA	NA
EPHB3	0.56177043	1.38E-52	3.42E-52
MAGEF1	1.04863813	9.35E-127	1.08E-125
RP11-329B9.	NA	2.69E-71	8.75E-71
RP11-329B9.	NA	NA	NA

RP11-329B9.	NA	NA	NA
RP11-329B9.	NA	NA	NA
VPS8	1.63023575	0.15358112	0.16231321
C3orf70	NA	NA	NA
EHHADH-AS1	NA	NA	NA
EHHADH	NA	NA	NA
MAP3K13	NA	4.68E-58	1.25E-57
TMEM41A	1.8799115	0.13383298	0.14186374
LIPH	NA	NA	NA
SENP2	2.28274968	2.19E-07	2.73E-07
IGF2BP2	1.88464918	3.84E-11	5.17E-11
IGF2BP2-AS1	NA	NA	NA
TRA2B	1.60438326	8.17E-14	1.15E-13
ETV5	3.05372643	0.05927094	0.06381564
ETV5-AS1	NA	NA	NA
DGKG	2.7464332	6.98E-79	2.55E-78
RP11-78H24.	NA	NA	NA
RP11-48F14.	NA	NA	NA
RP11-48F14.	NA	NA	NA
CRYGS	Inf	0.03324289	0.03614362
TBCCD1	1.31079767	2.01E-42	4.30E-42
DNAJB11	0.98205793	3.82E-103	2.19E-102
RP11-573D15	NA	1.70E-100	9.21E-100
AHSG	NA	3.13E-29	5.59E-29
FETUB	NA	NA	NA
HRG	NA	NA	NA
KNG1	NA	3.02E-83	1.19E-82
RP11-573D15	Inf	5.53E-56	1.43E-55
EIF4A2	1.39614601	5.21E-93	2.45E-92
RFC4	1.11521833	2.18E-112	1.56E-111
RP11-573D15	NA	3.57E-115	2.73E-114
RP11-573D15	4.11964981	3.54E-14	5.03E-14
ADIPOQ	NA	NA	NA
ADIPOQ-AS1	NA	NA	NA
ST6GAL1	1.69779507	2.90E-24	4.83E-24
RPL39L	2.24708171	2.15E-70	6.91E-70
RP11-208N14	NA	NA	NA
RTP1	NA	NA	NA
MASP1	NA	NA	NA
RTP4	NA	9.26E-42	1.97E-41
RP11-567G1	NA	NA	NA
SST	NA	NA	NA
RTP2	NA	NA	NA
RP11-211G3.	NA	NA	NA
BCL6	0.74902724	2.48E-54	6.29E-54

RP11-211G3.	NA	NA	NA
RP11-30O15.	NA	NA	NA
RP11-44H4.1	NA	NA	NA
RP11-132N1!	NA	NA	NA
RP11-132N1!	NA	NA	NA
RP11-132N1!	NA	NA	NA
RP11-430L16	NA	NA	NA
LPP-AS2	Inf	1.18E-102	6.72E-102
LPP	1.83095547	6.62E-10	8.72E-10
LPP-AS1	NA	NA	NA
TPRG1-AS1	NA	NA	NA
TPRG1	NA	NA	NA
TPRG1-AS2	NA	NA	NA
TP63	NA	NA	NA
P3H2	NA	NA	NA
P3H2-AS1	NA	NA	NA
CLDN1	NA	NA	NA
CLDN16	NA	NA	NA
TMEM207	NA	NA	NA
IL1RAP	2.46108949	1.52E-94	7.37E-94
RP11-268E23	NA	NA	NA
RP11-95L3.2	NA	NA	NA
GMNC	NA	NA	NA
OSTN	NA	NA	NA
OSTN-AS1	NA	NA	NA
UTS2B	NA	NA	NA
CCDC50	1.44727297	2.01E-10	2.67E-10
PYDC2	NA	NA	NA
RP11-197K6.	NA	NA	NA
RP11-655G2.	NA	NA	NA
FGF12	NA	NA	NA
FGF12-AS1	NA	NA	NA
FGF12-AS2	NA	NA	NA
FGF12-AS3	NA	NA	NA
MB21D2	2.99610895	0.01470319	0.01618895
RP11-143P4.	NA	NA	NA
HRASLS	0	1.02E-171	2.06E-168
ATP13A5	NA	NA	NA
ATP13A5-AS	NA	NA	NA
ATP13A4	NA	7.76E-67	2.37E-66
ATP13A4-AS	NA	NA	NA
OPA1	2.86696632	2.10E-69	6.68E-69
OPA1-AS1	NA	NA	NA
RP11-528A4.	NA	NA	NA
RP11-699L21	NA	NA	NA

RP11-699L21	NA	NA	NA
RP11-407B7.	NA	NA	NA
RP11-135A1.	NA	NA	NA
RP11-135A1.	NA	1.80E-118	1.52E-117
HES1	0.37451362	8.13E-65	2.40E-64
RP11-513G1	NA	2.79E-13	3.90E-13
RP11-513G1	NA	NA	NA
RP11-513G1	NA	NA	NA
LINC00887	1.49805447	2.61E-34	4.99E-34
CPN2	NA	NA	NA
LRRC15	NA	NA	NA
GP5	NA	4.04E-114	3.02E-113
ATP13A3	2.30469919	5.72E-98	2.97E-97
LINC00884	0.93628405	5.30E-61	1.48E-60
TMEM44-AS	2.0598249	3.00E-07	3.74E-07
TMEM44	2.49675746	9.61E-09	1.24E-08
AC046143.3	Inf	5.77E-05	6.81E-05
RP11-534C1	Inf	1.22E-127	1.48E-126
LSG1	1.28404669	2.43E-16	3.59E-16
FAM43A	1.39105058	1.29E-08	1.65E-08
AC106706.1	NA	NA	NA
AC090505.6	NA	8.66E-15	1.24E-14
AC090505.4	NA	3.97E-33	7.46E-33
AC090505.1	NA	NA	NA
XXYLT1	1.83511673	7.19E-27	1.24E-26
XXYLT1-AS1	NA	NA	NA
XXYLT1-AS2	Inf	7.88E-47	1.79E-46
ACAP2	1.71444012	0.04046899	0.04385833
PPP1R2	1.35051881	1.14E-71	3.74E-71
AC091633.3	NA	NA	NA
APOD	NA	NA	NA
AC069513.4	2.99610895	2.11E-17	3.17E-17
LINC00969	2.2770428	3.70E-119	3.20E-118
MUC20	0.74902724	8.10E-91	3.65E-90
MUC4	Inf	9.78E-105	5.81E-104
AC124944.5	NA	NA	NA
TNK2	2.8463035	0.14987466	0.15847911
TNK2-AS1	NA	3.85E-43	8.32E-43
AC124944.3	NA	NA	NA
RP11-352G9.	NA	5.02E-38	1.01E-37
RP11-480A1	Inf	6.19E-23	1.01E-22
TFRC	2.68913057	7.48E-137	1.53E-135
LINC00885	NA	5.70E-29	1.01E-28
ZDHHC19	NA	2.26E-20	3.54E-20
SLC51A	NA	2.84E-06	3.46E-06

PCYT1A	1.64369866	0.00165735	0.00187899
RP11-447L10	NA	NA	NA
AC069257.8	NA	NA	NA
TCTEX1D2	2.91680018	8.30E-83	3.24E-82
TM4SF19-TC	NA	5.95E-133	9.47E-132
TM4SF19-AS	NA	0.94669673	0.94947051
TM4SF19	NA	NA	NA
UBXN7	3.18336576	9.77E-55	2.50E-54
UBXN7-AS1	NA	NA	NA
RNF168	2.05447471	1.29E-50	3.10E-50
CTD-2002J20	NA	NA	NA
SMCO1	NA	NA	NA
WDR53	2.24708171	8.20E-25	1.37E-24
FBXO45	2.63942931	1.16E-67	3.60E-67
LINC01063	NA	1.82E-96	9.16E-96
NRROS	1.32520204	1.88E-72	6.24E-72
PIGX	2.00700888	3.73E-15	5.40E-15
CEP19	1.55567195	1.81E-05	2.17E-05
PAK2	1.84564228	2.97E-44	6.50E-44
SENP5	2.06628203	4.99E-57	1.31E-56
NCBP2	1.77004761	1.96E-07	2.45E-07
NCBP2-AS1	NA	NA	NA
NCBP2-AS2	1.99138968	3.55E-47	8.11E-47
PIGZ	1.49805447	7.84E-15	1.13E-14
MFI2	1.29831388	2.51E-30	4.55E-30
MFI2-AS1	1.55155642	9.06E-14	1.28E-13
DLG1	2.87127108	1.39E-57	3.67E-57
DLG1-AS1	0	4.56E-45	1.01E-44
AC128709.3	NA	NA	NA
AC128709.4	NA	NA	NA
AC128709.2	NA	NA	NA
RP13-616I3.1	NA	0.72049369	0.73047929
BDH1	2.05288947	4.83E-20	7.54E-20
RP11-496H1.	Inf	1.36E-71	4.44E-71
AC024560.2	NA	2.04E-54	5.18E-54
RUBCN	2.62159533	1.63E-55	4.20E-55
FYTTD1	1.83435242	0.06333298	0.0681083
RP11-803P9.	NA	8.32E-107	5.16E-106
LRCH3	4.68142023	1.32E-115	1.03E-114
AC055764.1	NA	NA	NA
IQCG	Inf	0.04307573	0.04662768
RPL35A	2.20146048	1.83E-141	6.29E-140
LMLN	3.74513619	0.00085171	0.0009742
LMLN-AS1	NA	2.04E-122	1.98E-121
ZNF595	6.36673152	6.35E-22	1.02E-21

ZNF718	2.62159533	1.51E-20	2.37E-20
ZNF732	NA	NA	NA
ZNF141	NA	NA	NA
RP11-2H3.7	NA	NA	NA
RP11-2H3.6	Inf	3.98E-136	7.64E-135
ZNF721	1.87256809	1.88E-27	3.27E-27
PIGG	1.49805447	3.72E-15	5.38E-15
RP11-1263C1	Inf	1.11E-103	6.47E-103
PDE6B	NA	NA	NA
RP11-1191J2	NA	NA	NA
RP11-1191J2	NA	NA	NA
ATP5I	2.0914159	3.66E-123	3.66E-122
MYL5	2.09727626	2.09E-15	3.03E-15
MFSD7	NA	NA	NA
PCGF3	3.15661479	9.56E-52	2.34E-51
RP11-1191J2	NA	NA	NA
RP11-440L14	NA	0.52245019	0.53573883
RP11-440L14	3.74513619	3.39E-06	4.13E-06
AC139887.4	NA	NA	NA
CPLX1	Inf	1.73E-25	2.93E-25
RP11-440L14	NA	NA	NA
GAK	2.24708171	9.06E-63	2.59E-62
TMEM175	1.3732166	0.24980983	0.26133375
DGKQ	1.60505837	0.53903258	0.55246196
SLC26A1	Inf	0.00091289	0.00104384
IDUA	NA	3.82E-18	5.80E-18
FGFRL1	1.71206226	6.07E-42	1.29E-41
RP11-460I19	NA	NA	NA
RNF212	NA	NA	NA
RP11-20I20.2	NA	NA	NA
AC092535.3	NA	NA	NA
SPON2	1.12354086	3.63E-06	4.41E-06
RP11-20I20.4	NA	NA	NA
CTBP1-AS	NA	1.44E-45	3.21E-45
CTBP1	2.81391313	1.31E-143	6.42E-142
CTBP1-AS2	2.80885214	5.74E-80	2.13E-79
MAEA	1.04551719	2.43E-76	8.51E-76
UVSSA	1.12354086	0.00122777	0.00139756
CRIPAK	1.49805447	5.95E-21	9.42E-21
NKX1-1	NA	NA	NA
RP13-1016M	NA	NA	NA
RP11-1398P2	1.24837873	6.52E-08	8.23E-08
FAM53A	Inf	1.40E-32	2.61E-32
SLBP	1.41269525	4.49E-56	1.17E-55
RP11-572O1	1.66450497	4.21E-59	1.14E-58

TMEM129	1.84135863	5.02E-91	2.27E-90
TACC3	0.92121741	3.95E-142	1.45E-140
FGFR3	1.49805447	1.36E-11	1.84E-11
LETM1	1.68531128	1.06E-08	1.36E-08
WHSC1	2.40552978	5.65E-57	1.48E-56
NELFA	2.12224384	7.86E-64	2.29E-63
C4orf48	2.13617099	1.07E-60	2.97E-60
NAT8L	2.47755163	1.17E-54	3.00E-54
POLN	Inf	6.57E-13	9.14E-13
RP11-317B7.	NA	NA	NA
HAUS3	2.12224384	8.16E-10	1.07E-09
MXD4	1.39818418	4.21E-16	6.20E-16
ZFYVE28	3.74513619	0.0463619	0.05011903
RP11-478C1.	NA	NA	NA
CFAP99	NA	NA	NA
CFAP99	NA	NA	NA
RNF4	2.38506041	1.50E-34	2.87E-34
FAM193A	2.32198444	4.35E-50	1.04E-49
TNIP2	1.70183394	0.13484578	0.14290392
SH3BP2	2.80885214	1.94E-93	9.22E-93
ADD1	2.49675746	4.96E-07	6.15E-07
MFSD10	1.00916843	1.66E-143	7.91E-142
NOP14-AS1	2.09727626	2.33E-14	3.32E-14
NOP14	1.79766537	4.05E-43	8.76E-43
GRK4	2.09727626	3.19E-25	5.38E-25
HTT-AS	NA	NA	NA
HTT	3.39943131	1.24E-104	7.34E-104
MSANTD1	Inf	2.96E-105	1.78E-104
RGS12	1.49805447	0.00062564	0.00071815
HGFAC	NA	NA	NA
DOK7	NA	NA	NA
LRPAP1	0.8688716	5.78E-144	3.03E-142
AL590235.1	NA	NA	NA
RP11-529E1(	0	3.14E-121	2.90E-120
RP3-368B9.2	NA	NA	NA
RP3-513G18.	NA	NA	NA
AC141928.1	Inf	0.00506002	0.00565856
ADRA2C	4.24448768	6.02E-120	5.36E-119
OTOP1	NA	NA	NA
TMEM128	1.45399405	1.70E-26	2.92E-26
LYAR	1.22624681	2.62E-51	6.35E-51
ZBTB49	5.24319066	1.54E-05	1.85E-05
RP11-265O1.	NA	NA	NA
NSG1	NA	NA	NA
STX18	0.86251621	2.58E-122	2.49E-121

STX18-AS1	2.99610895	0.04845065	0.05233651
LINC01396	NA	NA	NA
MSX1	1.64072633	3.59E-43	7.76E-43
CYTL1	1.29377432	2.76E-44	6.05E-44
STK32B	1.09473212	5.98E-16	8.77E-16
EVC2	NA	NA	NA
EVC	3.37062257	1.78E-05	2.12E-05
CRMP1	NA	1.84E-95	9.09E-95
C4orf50	NA	NA	NA
JAKMIP1	NA	NA	NA
RP11-586D1!	NA	NA	NA
RP11-586D1!	NA	NA	NA
RP11-1406H:	NA	NA	NA
WFS1	NA	5.56E-54	1.41E-53
PPP2R2C	NA	NA	NA
MAN2B2	1.49805447	0.0020257	0.00228988
MRFAP1	1.58463558	6.70E-78	2.41E-77
RP11-539L1C	Inf	2.34E-07	2.93E-07
AC093323.3	1.74124514	2.02E-61	5.69E-61
RP11-539L1C	Inf	2.59E-116	2.05E-115
S100P	2.99610895	1.13E-97	5.81E-97
MRFAP1L1	2.36534917	2.95E-14	4.20E-14
BLOC1S4	1.48244974	1.89E-36	3.73E-36
KIAA0232	1.91418072	2.39E-64	7.02E-64
TBC1D14	2.57998271	1.14E-45	2.56E-45
RP11-367J11	NA	1.62E-93	7.69E-93
CCDC96	Inf	0.0001073	0.00012568
TADA2B	2.37191958	4.19E-18	6.36E-18
GRPEL1	1.0936501	3.00E-103	1.73E-102
RP11-367J11	Inf	4.72E-119	4.07E-118
SORCS2	NA	NA	NA
PSAPL1	NA	NA	NA
AFAP1-AS1	NA	NA	NA
AFAP1	1.24837873	2.21E-13	3.10E-13
AC097381.1	NA	NA	NA
ABLIM2	Inf	1.10E-36	2.18E-36
RP11-338K1:	NA	NA	NA
RP11-1258F1	NA	NA	NA
SH3TC1	0	3.68E-23	6.02E-23
AC104650.2	NA	NA	NA
HTRA3	Inf	3.18E-62	9.04E-62
RP11-774O3.	NA	NA	NA
RP11-774O3.	NA	NA	NA
RP11-774O3.	Inf	0.00241286	0.00272193
ACOX3	1.40442607	6.94E-69	2.19E-68

TRMT44	1.91418072	0.2955867	0.30785544
RP11-689P11	NA	NA	NA
RP11-689P11	NA	9.38E-63	2.68E-62
GPR78	NA	NA	NA
CPZ	NA	NA	NA
RP11-1338A2	NA	NA	NA
HMX1	NA	NA	NA
FAM90A26	NA	NA	NA
USP17L10	NA	NA	NA
USP17L11	NA	NA	NA
USP17L12	NA	NA	NA
USP17L13	NA	NA	NA
USP17L15	NA	NA	NA
USP17L17	NA	NA	NA
USP17L18	NA	NA	NA
USP17L19	NA	NA	NA
USP17L20	NA	NA	NA
USP17L21	NA	NA	NA
USP17L22	NA	NA	NA
USP17L23	NA	NA	NA
USP17L24	NA	NA	NA
USP17L25	NA	NA	NA
USP17L26	NA	NA	NA
USP17L5	NA	NA	NA
USP17L27	NA	NA	NA
USP17L28	NA	NA	NA
USP17L29	NA	NA	NA
USP17L30	NA	NA	NA
DEFB131	NA	NA	NA
SLC2A9	2.62159533	5.61E-15	8.09E-15
DRD5	NA	NA	NA
RP13-560N1	NA	NA	NA
RP11-448G1	NA	NA	NA
WDR1	1.51632343	6.83E-116	5.35E-115
ZNF518B	NA	3.24E-55	8.32E-55
CLNK	NA	1.86E-62	5.31E-62
RP11-61G19.	NA	NA	NA
RP11-61G19.	NA	NA	NA
HS3ST1	NA	NA	NA
RP11-281P23	NA	NA	NA
RP11-281P23	NA	NA	NA
RP11-74M11	NA	NA	NA
RP11-1J7.1	NA	NA	NA
RP11-168E17	NA	NA	NA
RP11-22A3.2	Inf	1.79E-14	2.56E-14

RP11-22A3.1	NA	5.83E-98	3.02E-97
RAB28	1.4778105	0.00049812	0.00057344
LINC01097	0	2.69E-117	2.19E-116
NKX3-2	1.34824903	2.37E-41	5.01E-41
LINC01096	Inf	9.54E-92	4.38E-91
BOD1L1	2.7464332	2.15E-16	3.18E-16
AC006445.1	NA	NA	NA
LINC01182	NA	NA	NA
AC007126.1	NA	NA	NA
LINC01085	NA	NA	NA
RP11-669M1	NA	NA	NA
RP11-669M1	NA	NA	NA
AC006296.1	NA	NA	NA
AC006296.2	NA	NA	NA
LINC00504	NA	2.90E-121	2.70E-120
CPEB2-AS1	NA	NA	NA
CPEB2	Inf	7.81E-97	3.97E-96
RP11-665G4.	NA	NA	NA
C1QTNF7	NA	NA	NA
RP11-484O2.	NA	NA	NA
CC2D2A	1.68531128	1.50E-19	2.33E-19
RP11-799M1	NA	2.72E-103	1.57E-102
FBXL5	5.24319066	4.13E-83	1.62E-82
FAM200B	1.9661965	4.91E-96	2.45E-95
BST1	1.15758755	3.01E-109	1.98E-108
CD38	NA	NA	NA
FGFBP1	NA	NA	NA
FGFBP2	NA	NA	NA
PROM1	NA	NA	NA
TAPT1	14.2315175	5.77E-22	9.27E-22
RP11-452J21	NA	NA	NA
TAPT1-AS1	NA	5.45E-09	7.04E-09
RP11-446J8.:	NA	NA	NA
LDB2	NA	NA	NA
RP11-141E1:	NA	NA	NA
RP11-576E2(	NA	NA	NA
QDPR	1.1797179	1.71E-138	4.07E-137
CLRN2	NA	NA	NA
LAP3	1.01118677	3.47E-141	1.13E-139
AC006160.5	NA	1.95E-49	4.61E-49
MED28	1.6237474	2.35E-08	2.99E-08
FAM184B	1.73638132	5.70E-29	1.01E-28
DCAF16	1.57830739	0.30004343	0.31233578
NCAPG	0.97373541	2.32E-99	1.24E-98
LCORL	1.39818418	2.50E-20	3.91E-20

RP11-608B3.	NA	NA	NA
RP11-3J1.1	NA	NA	NA
RP11-608O2	NA	NA	NA
RP11-94H6.1	NA	NA	NA
SLIT2	NA	NA	NA
PACRGL	3.18336576	1.35E-42	2.91E-42
KCNIP4	NA	NA	NA
RP11-362J17	NA	NA	NA
RP11-120A1.	NA	NA	NA
RP11-556G2:	NA	NA	NA
RP11-556G2:	NA	NA	NA
KCNIP4-IT1	NA	NA	NA
RP11-17E2.2	NA	NA	NA
ADGRA3	2.83560311	4.24E-126	4.76E-125
RP11-412P1:	NA	NA	NA
RP11-552M1	NA	NA	NA
RP11-453O5.	NA	NA	NA
RP11-380P1:	NA	NA	NA
ERVH-1	NA	NA	NA
PPARGC1A	NA	NA	NA
RP13-497K6.	NA	NA	NA
DHX15	1.74211953	0.00195779	0.00221436
RP11-496D2	NA	NA	NA
SOD3	NA	NA	NA
CCDC149	NA	0.03914439	0.04244054
LGI2	0	0.11281669	0.12002817
SEPSECS	1.74773022	1.30E-28	2.30E-28
SEPSECS-AS1	2.08063121	1.67E-18	2.55E-18
RP11-717K1:	NA	NA	NA
RP11-660M5	NA	0.00048233	0.00055541
PI4K2B	1.74773022	2.81E-48	6.52E-48
ZCCHC4	0.74902724	2.68E-10	3.55E-10
ANAPC4	1.97957198	7.42E-16	1.09E-15
RP11-302F1:	NA	NA	NA
SLC34A2	NA	NA	NA
SEL1L3	15.729572	1.90E-134	3.29E-133
RP11-302F1:	NA	NA	NA
SMIM20	2.52648076	8.77E-97	4.45E-96
RP13-494C2:	NA	NA	NA
RBPJ	1.57126766	1.40E-124	1.48E-123
CCKAR	NA	NA	NA
TBC1D19	1.12354086	3.59E-32	6.66E-32
STIM2	3.20038911	1.62E-65	4.84E-65
RP11-293A2:	NA	5.63E-61	1.57E-60
RP11-415C1!	NA	NA	NA

AC024132.1	NA	NA	NA
RP11-415C15	NA	NA	NA
RP11-415C15	NA	NA	NA
AC007106.1	NA	NA	NA
RP11-180C1.	NA	NA	NA
RP11-123O2:	NA	NA	NA
RP11-292B1.	NA	NA	NA
RP11-769N2:	NA	3.62E-19	5.57E-19
RP11-472K22	NA	NA	NA
RP11-472K22	NA	NA	NA
PCDH7	NA	7.88E-117	6.32E-116
RP11-619J20	NA	NA	NA
RP11-617I14	NA	NA	NA
RP11-315A1:	NA	NA	NA
RP11-665I14	NA	NA	NA
RP11-734I18	NA	NA	NA
RP11-240A1(	NA	NA	NA
RP11-802H3.	NA	NA	NA
RP11-79E3.3	NA	NA	NA
RP11-79E3.2	NA	NA	NA
RP11-548L2C	NA	NA	NA
RP11-725M2	NA	NA	NA
ARAP2	Inf	8.61E-85	3.49E-84
RP11-431M7	NA	1.24E-09	1.62E-09
DTHD1	NA	NA	NA
RP11-431M7	NA	NA	NA
RP11-722M1	NA	NA	NA
RP11-103J17	NA	NA	NA
NWD2	NA	NA	NA
C4orf19	NA	NA	NA
RP11-36B15.	NA	2.00E-06	2.45E-06
RELL1	1.87256809	1.84E-05	2.20E-05
PGM2	0.81870419	4.87E-114	3.63E-113
AC021106.1	NA	NA	NA
TBC1D1	1.74342547	0.06680763	0.07177261
PTTG2	0.74902724	1.25E-47	2.87E-47
RP11-600L4.	NA	NA	NA
RP11-83C7.1	NA	NA	NA
LINC01258	NA	NA	NA
LINC01259	NA	NA	NA
RP11-213G2	NA	NA	NA
RP11-617D2(	NA	NA	NA
KLF3	1.97470817	5.79E-05	6.84E-05
TLR10	NA	4.99E-61	1.39E-60
TLR1	NA	6.93E-60	1.90E-59

TLR6	NA	6.73E-08	8.49E-08
FAM114A1	NA	NA	NA
TMEM156	2.99610895	0.01776216	0.01950258
KLHL5	2.30950065	8.16E-58	2.17E-57
RP11-360F5.	Inf	5.81E-73	1.94E-72
RP11-360F5.	NA	NA	NA
WDR19	6.74124514	2.81E-60	7.78E-60
RFC1	1.68385968	2.43E-19	3.76E-19
KLB	NA	1.30E-67	4.02E-67
RPL9	1.9235146	2.28E-24	3.80E-24
LIAS	1.74230249	9.84E-08	1.24E-07
UGDH	1.85053788	0.17447966	0.18396059
UGDH-AS1	Inf	0.44806796	0.46105433
SMIM14	0.24967575	2.03E-127	2.43E-126
RP11-539G1	Inf	1.72E-24	2.87E-24
UBE2K	1.63584722	0.33638928	0.34908902
PDS5A	2.47892348	6.62E-135	1.18E-133
N4BP2	3.21011673	5.45E-15	7.86E-15
RHOH	2.15719844	7.74E-13	1.07E-12
RP11-395I6.2	NA	NA	NA
AC195454.1	0.74902724	0.51170707	0.52493014
CHRNA9	NA	NA	NA
RBM47	1.49805447	1.02E-25	1.74E-25
RP11-588L15	NA	NA	NA
NSUN7	NA	NA	NA
APBB2	2.46108949	3.30E-74	1.12E-73
RP11-632F7.	NA	NA	NA
UCHL1-AS1	NA	NA	NA
UCHL1	Inf	1.44E-55	3.71E-55
LIMCH1	NA	6.80E-112	4.80E-111
RP11-227F19	NA	NA	NA
PHOX2B	NA	NA	NA
RP11-227F19	NA	NA	NA
RP11-227F19	NA	NA	NA
LINC00682	NA	5.14E-86	2.12E-85
RP11-457P14	NA	NA	NA
TMEM33	1.42151155	6.62E-47	1.51E-46
DCAF4L1	2.24708171	8.81E-32	1.63E-31
RP11-814H16	NA	5.51E-95	2.69E-94
SLC30A9	2.28549337	2.16E-17	3.24E-17
BEND4	1.21716926	3.31E-11	4.46E-11
RP11-63A11.	NA	NA	NA
SHISA3	2.41353221	8.92E-33	1.67E-32
ATP8A1	2.43433852	4.68E-13	6.52E-13
RP11-109E24	NA	NA	NA

RP11-109E24	NA	NA	NA
GRXCR1	NA	NA	NA
RP11-395F4.	NA	NA	NA
RP11-324J13	NA	NA	NA
RP11-663P9.	NA	NA	NA
LVCAT1	NA	NA	NA
KCTD8	0	2.04E-139	5.38E-138
YIPF7	NA	NA	NA
GUF1	2.02861543	2.12E-31	3.89E-31
GNPDA2	0.91547773	8.46E-12	1.15E-11
RP11-700J17	NA	3.98E-26	6.79E-26
RP11-700J17	Inf	1.80E-120	1.63E-119
RP11-362I1.1	NA	NA	NA
GABRG1	NA	NA	NA
GABRA2	NA	NA	NA
RP11-436F23	NA	NA	NA
COX7B2	NA	NA	NA
GABRA4	NA	NA	NA
GABRB1	NA	NA	NA
COMMD8	1.010156	8.17E-107	5.07E-106
ATP10D	1.49805447	2.73E-49	6.43E-49
AC092597.3	NA	NA	NA
CORIN	NA	4.67E-112	3.32E-111
RP11-121C2.	NA	NA	NA
RP11-121C2.	1.99740597	2.04E-28	3.60E-28
NFXL1	1.28404669	3.70E-06	4.50E-06
NIPAL1	NA	0.00037433	0.00043266
CNGA1	NA	NA	NA
TXK	Inf	1.51E-115	1.17E-114
TEC	1.68531128	3.31E-08	4.21E-08
SLAIN2	2.78804583	4.54E-34	8.66E-34
SLC10A4	1.31971466	8.81E-112	6.20E-111
ZAR1	0.74902724	1.55E-26	2.66E-26
FRYL	2.65040407	1.24E-30	2.25E-30
OCIAD1	1.32101167	8.75E-132	1.30E-130
OCIAD1-AS1	NA	NA	NA
OCIAD2	1.92997529	1.38E-12	1.90E-12
CWH43	NA	NA	NA
DCUN1D4	2.29389591	2.64E-26	4.51E-26
RP11-365H22	NA	0.00286694	0.00322735
LRRC66	NA	6.07E-05	7.16E-05
SGCB	1.49805447	0.12645575	0.13431852
RP11-535C7.	NA	NA	NA
SPATA18	NA	NA	NA
USP46	2.24708171	2.26E-05	2.69E-05

USP46-AS1	6.74124514	1.32E-116	1.05E-115
RP11-177B4.	NA	NA	NA
ERVMER34-1	NA	0.0003184	0.00036865
LINC01618	NA	NA	NA
RASL11B	0	2.24E-90	1.00E-89
SCFD2	1.07003891	3.81E-48	8.82E-48
RP11-752D24	NA	NA	NA
FIP1L1	1.21206226	1.38E-94	6.70E-94
RP11-231C18	NA	NA	NA
LNX1	Inf	1.91E-51	4.64E-51
LNX1-AS1	NA	NA	NA
RP11-571I18	NA	6.21E-12	8.49E-12
CHIC2	1.66828794	3.01E-87	1.27E-86
RP11-571I18	NA	NA	NA
GSX2	NA	NA	NA
PDGFRA	NA	1.02E-85	4.20E-85
RP11-231C18	NA	NA	NA
RP11-231C18	NA	NA	NA
RP11-545H22	NA	NA	NA
AC006552.1	NA	NA	NA
KIT	0.24967575	2.42E-143	1.12E-141
RP11-273B19	NA	NA	NA
RP11-273B19	NA	NA	NA
RP11-463H12	NA	NA	NA
RP11-530I17	NA	NA	NA
KDR	NA	NA	NA
SRD5A3	1.84135863	2.50E-15	3.63E-15
RP11-177J6.1	0.74902724	0.04483026	0.04849216
TMEM165	1.97318369	6.04E-25	1.02E-24
CLOCK	4.11964981	2.61E-50	6.24E-50
RP11-528I4.2	NA	1.26E-10	1.67E-10
PDCL2	NA	NA	NA
NMU	0.37451362	3.86E-13	5.39E-13
RP11-345F18	NA	1.38E-109	9.17E-109
EXOC1	0.91547773	3.65E-14	5.18E-14
CEP135	3.37062257	0.03857827	0.04182925
KIAA1211	Inf	1.40E-29	2.52E-29
AASDH	1.03542	9.60E-37	1.90E-36
RP11-646I6.5	2.99610895	0.00170051	0.00192672
PPAT	1.32417315	1.68E-08	2.15E-08
RP11-646I6.6	NA	2.01E-138	4.69E-137
PAICS	1.66029864	2.01E-22	3.25E-22
SRP72	1.84780686	5.72E-07	7.07E-07
ARL9	0	3.48E-60	9.59E-60
THEGL	NA	NA	NA

HOPX	NA	1.08E-43	2.35E-43
SPINK2	0.74902724	2.13E-104	1.26E-103
REST	1.47602426	1.34E-13	1.88E-13
RP11-738E22	Inf	5.54E-10	7.31E-10
NOA1	2.21141375	7.81E-12	1.07E-11
POLR2B	1.69868677	2.47E-77	8.81E-77
IGFBP7	0.64202335	1.63E-121	1.53E-120
IGFBP7-AS1	NA	NA	NA
RP11-300M6	NA	NA	NA
RP11-319E12	NA	NA	NA
RP11-319E12	NA	NA	NA
RP11-685F15	NA	NA	NA
RP11-4O3.1	NA	NA	NA
RP11-4O3.2	NA	NA	NA
RP11-577G20	NA	NA	NA
RP11-577G20	NA	NA	NA
RP11-506N2.	NA	NA	NA
RP11-340A13	NA	NA	NA
RP11-340A13	NA	NA	NA
RP11-622J8.1	NA	NA	NA
RP11-725D20	NA	NA	NA
RP11-525J21	NA	NA	NA
RP11-593F5.	NA	NA	NA
RP11-16N2.1	NA	0.00021029	0.00024448
ADGRL3	NA	2.32E-52	5.73E-52
RP11-798L4.	Inf	1.99E-108	1.29E-107
ADGRL3-AS1	NA	NA	NA
RP11-257A22	NA	NA	NA
RP11-12K22.	NA	NA	NA
TECRL	NA	NA	NA
RP11-707A18	NA	NA	NA
EPHA5	NA	NA	NA
EPHA5-AS1	NA	NA	NA
RP11-807H7.	NA	NA	NA
RP11-51P8.1	NA	NA	NA
RP11-25H12.	NA	NA	NA
RP11-19C24.	NA	NA	NA
RP11-584P21	NA	2.22E-129	2.90E-128
CENPC	2.37191958	1.26E-11	1.71E-11
STAP1	2.24708171	0.00041437	0.00047823
UBA6	1.78339818	0.01385003	0.01526533
UBA6-AS1	1.81906615	0.75750363	0.76658844
GNRHR	NA	NA	NA
TMPRSS11D	NA	NA	NA
TMPRSS11A	NA	NA	NA

TMPRSS11F	NA	NA	NA
TMPRSS11B	NA	NA	NA
YTHDC1	1.83745744	3.28E-40	6.83E-40
TMPRSS11E	NA	NA	NA
UGT2B17	NA	NA	NA
UGT2B15	NA	NA	NA
UGT2B10	NA	NA	NA
RP11-468N1	NA	NA	NA
UGT2A3	NA	NA	NA
UGT2B11	NA	5.66E-128	6.91E-127
UGT2B7	NA	NA	NA
RP11-704M1	0	5.27E-129	6.78E-128
UGT2B28	NA	NA	NA
UGT2B4	NA	NA	NA
UGT2A1	NA	NA	NA
UGT2A2	NA	NA	NA
SULT1B1	NA	NA	NA
SULT1E1	NA	NA	NA
CSN1S1	NA	NA	NA
CSN2	NA	NA	NA
STATH	NA	NA	NA
AC063956.1	NA	NA	NA
HTN3	NA	NA	NA
HTN1	NA	NA	NA
PRR27	NA	NA	NA
ODAM	NA	NA	NA
FDCSP	NA	NA	NA
CSN3	NA	NA	NA
CABS1	NA	NA	NA
SMR3A	NA	NA	NA
SMR3B	NA	NA	NA
PROL1	NA	NA	NA
MUC7	NA	NA	NA
AMTN	NA	NA	NA
AMBN	NA	NA	NA
ENAM	NA	NA	NA
JCHAIN	NA	3.18E-84	1.27E-83
UTP3	1.54971153	4.08E-15	5.90E-15
RP11-46J23.	NA	0.00135704	0.00154238
RUFY3	2.16385646	0.68104916	0.69207381
GRSF1	1.16319524	3.22E-115	2.47E-114
MOB1B	5.05593385	5.22E-117	4.22E-116
DCK	1.59367497	0.01331815	0.01468623
SLC4A4	Inf	2.17E-131	3.15E-130
GC	NA	NA	NA

RP11-545L5.:	NA	NA	NA
NPFFR2	NA	NA	NA
ADAMTS3	2.24708171	3.12E-05	3.71E-05
RP11-373J21	NA	NA	NA
COX18	1.59168288	4.34E-15	6.27E-15
ANKRD17	1.98492218	9.80E-26	1.66E-25
RP11-692D1:	NA	1.29E-138	3.10E-137
ALB	NA	NA	NA
AFP	NA	NA	NA
AFM	NA	NA	NA
RP11-622A1.	NA	NA	NA
RASSF6	NA	NA	NA
AC112518.3	NA	NA	NA
CXCL8	Inf	9.16E-07	1.13E-06
CXCL6	NA	NA	NA
PF4V1	NA	NA	NA
CXCL1	NA	NA	NA
PF4	NA	NA	NA
PPBP	NA	NA	NA
CXCL5	NA	0.0091276	0.0101181
CXCL3	1.87256809	0.19471487	0.20487863
CXCL2	Inf	3.11E-84	1.24E-83
MTHFD2L	2.2595655	7.50E-20	1.17E-19
RP11-629B1:	Inf	0.58866726	0.60139664
EPGN	NA	NA	NA
EREG	0.74902724	0.62307156	0.63525707
RP11-727M1	NA	NA	NA
AREG	NA	2.94E-05	3.49E-05
AC142293.3	NA	NA	NA
BTC	NA	NA	NA
PARM1	NA	3.34E-141	1.09E-139
RP11-44F21.	NA	NA	NA
RP11-44F21.	NA	NA	NA
RP11-44F21.	NA	NA	NA
RP11-542G1.	NA	NA	NA
RP11-542G1.	NA	NA	NA
RP11-542G1.	NA	NA	NA
RP11-567N4.	NA	NA	NA
RP11-567N4.	NA	NA	NA
RCHY1	1.99740597	1.48E-32	2.75E-32
THAP6	1.68531128	1.19E-13	1.67E-13
C4orf26	NA	9.63E-31	1.75E-30
CDKL2	NA	NA	NA
G3BP2	2.27829118	6.28E-35	1.21E-34
USO1	1.36489408	3.51E-59	9.50E-59

RP11-556N4.	Inf	6.52E-102	3.65E-101
PPEF2	NA	NA	NA
NAAA	1.49111904	4.62E-113	3.38E-112
SDAD1	1.58414956	0.09218712	0.09853062
AC110615.1	NA	5.44E-15	7.85E-15
RP11-630D6.	NA	NA	NA
AC112719.2	NA	NA	NA
CXCL9	NA	NA	NA
ART3	NA	NA	NA
CXCL10	Inf	1.28E-142	5.08E-141
CXCL11	NA	4.79E-31	8.75E-31
NUP54	1.51588846	1.57E-09	2.05E-09
SCARB2	3.05852789	6.83E-67	2.08E-66
FAM47E	3.74513619	4.97E-118	4.14E-117
FAM47E-STB	NA	NA	NA
STBD1	0	8.75E-14	1.24E-13
CCDC158	NA	NA	NA
SHROOM3	NA	NA	NA
RP11-359D1	NA	NA	NA
RP11-359D1	NA	NA	NA
SOWAHB	NA	NA	NA
11-Sep	1-Jan	9.11E-143	3.82E-141
CCNI	1.69071765	1.23E-20	1.94E-20
CCNG2	1.49805447	5.16E-24	8.55E-24
RP11-625I7.1	NA	NA	NA
CXCL13	NA	NA	NA
CNOT6L	2.08063121	5.41E-11	7.25E-11
MRPL1	1.1632937	1.37E-140	4.15E-139
FRAS1	NA	1.56E-96	7.89E-96
ANXA3	0.21400778	3.65E-122	3.50E-121
LINC01094	0	4.86E-13	6.76E-13
RP11-109G2	Inf	1.62E-15	2.35E-15
BMP2K	1.23369192	2.91E-05	3.46E-05
PAQR3	1.59792477	5.56E-59	1.50E-58
LINC01088	3.32900994	5.89E-33	1.10E-32
NAA11	NA	NA	NA
GK2	NA	NA	NA
LINC00989	0	9.88E-134	1.64E-132
RP11-234K1	NA	NA	NA
RP11-452C8.	Inf	2.82E-31	5.18E-31
PCAT4	NA	NA	NA
ANTXR2	1.31079767	1.93E-24	3.22E-24
RP11-377G1	NA	NA	NA
PRDM8	1.42315175	9.33E-06	1.12E-05
FGF5	Inf	3.69E-17	5.51E-17

C4orf22	NA	1.53E-81	5.86E-81
BMP3	NA	NA	NA
PRKG2	NA	NA	NA
RP11-100N2(	NA	NA	NA
RASGEF1B	NA	1.65E-112	1.19E-111
RP11-689K5.	NA	NA	NA
RP11-127B2(	1.12354086	3.71E-10	4.90E-10
HNRNPD	1.27577121	3.45E-110	2.33E-109
RP11-127B2(	3.29571984	1.60E-68	5.01E-68
HNRNPDL	1.3873557	3.50E-108	2.25E-107
ENOPH1	1.23187124	3.61E-63	1.04E-62
TMEM150C	Inf	5.72E-05	6.76E-05
LINC00575	NA	NA	NA
SCD5	1.23589494	1.78E-74	6.09E-74
SEC31A	2.05547009	2.25E-36	4.45E-36
THAP9-AS1	1.90881135	1.23E-23	2.03E-23
THAP9	0.44941634	0.66061395	0.67228689
LIN54	1.87256809	1.65E-13	2.31E-13
COPS4	1.11062659	2.62E-142	1.00E-140
PLAC8	0.5599524	3.01E-54	7.65E-54
RP11-576N1	NA	4.87E-96	2.43E-95
COQ2	0.85163371	1.90E-91	8.70E-91
HPSE	1.19844358	3.66E-54	9.27E-54
HELQ	2.24708171	0.00016606	0.00019358
MRPS18C	1.67608879	0.45967011	0.47264425
FAM175A	1.1523496	3.00E-59	8.14E-59
GPAT3	1.98492218	0.11707624	0.12450889
RP11-767N1	NA	NA	NA
RP11-42A4.1	NA	NA	NA
NKX6-1	2.03901859	0.00770127	0.00856473
CDS1	4.49416342	0.33132558	0.34405063
WDFY3	1.49805447	3.35E-08	4.26E-08
WDFY3-AS1	NA	0.0091276	0.0101181
ARHGAP24	NA	3.11E-33	5.86E-33
RP13-514E2	NA	NA	NA
MAPK10	NA	NA	NA
RP11-778J15	NA	NA	NA
PTPN13	3.2457847	2.67E-06	3.26E-06
SLC10A6	NA	NA	NA
C4orf36	2.24708171	5.42E-31	9.90E-31
AFF1	2.99610895	0.2225189	0.2333625
KLHL8	1.75733313	2.10E-33	3.96E-33
RP11-529H2.	NA	NA	NA
HSD17B13	Inf	7.30E-76	2.54E-75
HSD17B11	0.91237892	1.42E-139	3.82E-138

NUDT9	0.89467142	2.04E-90	9.13E-90
RP11-710E1.	NA	NA	NA
SPARCL1	NA	NA	NA
RP11-742B1ٰ	NA	NA	NA
DSPP	NA	7.29E-115	5.55E-114
DMP1	NA	NA	NA
IBSP	NA	NA	NA
MEPE	NA	NA	NA
SPP1	NA	0.00188436	0.00213223
PKD2	1.31079767	2.10E-32	3.90E-32
ABCG2	0	7.12E-92	3.28E-91
PPM1K	1.55567195	3.51E-59	9.50E-59
RP11-10L7.1	NA	3.01E-137	6.36E-136
HERC6	3.2457847	2.08E-55	5.35E-55
HERC5	3.0460441	0.00259361	0.00292293
PYURF	1.89157326	5.18E-17	7.72E-17
HERC3	2.54669261	1.53E-23	2.52E-23
PIGY	NA	NA	NA
NAP1L5	1.45124027	4.46E-06	5.42E-06
FAM13A-AS1	Inf	8.97E-144	4.51E-142
FAM13A	2.45136187	2.93E-36	5.78E-36
TIGD2	2.46108949	0.00206496	0.00233382
RP11-84C13.	NA	0.49372788	0.5069737
GPRIN3	NA	2.22E-129	2.90E-128
RP11-115D1	NA	NA	NA
SNCA	18.3511673	1.80E-145	1.58E-143
RP11-115D1	NA	NA	NA
RP11-115D1	NA	NA	NA
RP11-115D1	NA	NA	NA
SNCA-AS1	NA	5.76E-32	1.06E-31
MMRN1	NA	4.12E-28	7.24E-28
CCSER1	NA	2.29E-41	4.83E-41
AC004054.1	NA	NA	NA
RP11-763F8.	NA	NA	NA
RP11-354O2	NA	NA	NA
RP11-562F9.	NA	NA	NA
RP11-9B6.1	NA	NA	NA
GRID2	NA	NA	NA
RP11-428L21	NA	NA	NA
RP11-703C1	NA	NA	NA
ATOH1	NA	2.56E-19	3.95E-19
RP11-363G1	NA	NA	NA
SMARCAD1	1.74773022	3.95E-57	1.04E-56
HPGDS	NA	0.33974986	0.35247567
PDLIM5	1.11284047	3.91E-06	4.75E-06

RP11-554D13	NA	NA	NA
BMPR1B-AS1	NA	NA	NA
BMPR1B	NA	NA	NA
UNC5C	NA	NA	NA
RP11-710C12	NA	NA	NA
PDHA2	NA	NA	NA
RP11-145G20	NA	1.00E-95	4.98E-95
RP11-431J17	NA	NA	NA
STPG2	NA	NA	NA
STPG2-AS1	NA	NA	NA
RP11-323J4.1	0.37451362	8.23E-20	1.28E-19
RAP1GDS1	1.49805447	1.01E-41	2.15E-41
TSPAN5	1.83095547	8.85E-43	1.91E-42
RP11-724M2	NA	NA	NA
RP11-1299A1	NA	NA	NA
EIF4E	1.51274128	7.49E-47	1.71E-46
RP11-571L19	NA	NA	NA
METAP1	1.93498703	2.03E-24	3.38E-24
RP11-571L19	Inf	0.09256089	0.09892428
ADH5	1.12068197	2.23E-126	2.55E-125
RP11-696N14	Inf	1.16E-38	2.37E-38
ADH4	NA	NA	NA
ADH6	NA	5.68E-94	2.72E-93
ADH1A	NA	NA	NA
ADH1B	NA	NA	NA
ADH1C	NA	NA	NA
ADH7	NA	NA	NA
C4orf17	NA	NA	NA
TRMT10A	2.88910506	1.34E-14	1.92E-14
MTTP	NA	NA	NA
RP11-766F14	NA	NA	NA
RP11-766F14	NA	NA	NA
DAPP1	NA	NA	NA
LAMTOR3	1.57148852	3.05E-20	4.77E-20
DNAJB14	2.3594358	1.01E-36	1.99E-36
H2AFZ	1.70909177	6.43E-18	9.72E-18
RP11-15B17.	0	2.86E-126	3.23E-125
DDIT4L	NA	NA	NA
RP11-588P8.	NA	NA	NA
EMCN	NA	1.32E-100	7.17E-100
PPP3CA	1.68929547	3.77E-37	7.51E-37
FLJ20021	1.31079767	0.0228738	0.02500461
BANK1	Inf	2.31E-11	3.11E-11
RP11-498M5	NA	NA	NA
SLC39A8	2.0598249	3.04E-46	6.86E-46

RP11-499E18	1.49805447	0.33016407	0.34286414
AF213884.2	1.49805447	2.23E-107	1.41E-106
NFKB1	2.43433852	1.63E-36	3.22E-36
MANBA	4.64396887	9.46E-16	1.38E-15
UBE2D3	1.75946904	2.73E-14	3.89E-14
RP11-10L12.4	1.99740597	0.00955046	0.01057974
CISD2	1.82161386	2.64E-36	5.20E-36
SLC9B1	Inf	4.82E-08	6.10E-08
SLC9B2	2.14494163	3.04E-56	7.90E-56
BDH2	2.3540856	1.71E-13	2.40E-13
CENPE	1.20335523	3.25E-72	1.07E-71
RP11-328K4.	NA	NA	NA
RP11-297P16	NA	NA	NA
RP11-297P16	NA	NA	NA
TACR3	NA	NA	NA
RP11-703G6.	NA	NA	NA
RP11-729M2	NA	NA	NA
CXXC4	NA	2.06E-106	1.27E-105
AC004053.1	NA	NA	NA
AC004063.1	NA	NA	NA
RP11-556I14	NA	NA	NA
RP11-556I14	NA	NA	NA
AC004069.2	1.12354086	2.02E-07	2.52E-07
TET2	10.4863813	4.84E-130	6.54E-129
TET2-AS1	NA	NA	NA
PPA2	0.92593317	3.91E-143	1.73E-141
AC004066.3	NA	NA	NA
ARHGEF38	NA	1.97E-49	4.64E-49
INTS12	1.02498464	4.85E-123	4.82E-122
GSTCD	3.65150778	1.95E-75	6.75E-75
RP11-45L9.1	NA	NA	NA
NPNT	NA	1.61E-26	2.76E-26
RP11-710F7.	NA	NA	NA
RP11-710F7.	NA	8.16E-98	4.22E-97
TBCK	1.49805447	0.10459518	0.11143142
AIMP1	1.03063825	7.97E-123	7.84E-122
GIMD1	NA	NA	NA
RP13-612N2	NA	NA	NA
DKK2	NA	1.24E-38	2.51E-38
RP11-713M6	NA	NA	NA
PAPSS1	2.0598249	6.12E-45	1.35E-44
SGMS2	Inf	3.21E-48	7.45E-48
RP11-286E11	NA	8.67E-109	5.65E-108
CYP2U1	1.19844358	4.71E-17	7.03E-17
RP11-286E11	NA	6.97E-11	9.33E-11

HADH	0.7846952	2.68E-144	1.52E-142
LEF1	NA	7.29E-77	2.58E-76
RPL34-AS1	NA	1.15E-22	1.87E-22
RPL34	2.28001101	1.41E-112	1.02E-111
OSTC	1.85951534	0.00098648	0.00112664
ETNPPL	NA	NA	NA
COL25A1	NA	NA	NA
AC004051.2	NA	NA	NA
SEC24B-AS1	0.99870298	1.30E-23	2.14E-23
SEC24B	2.24708171	0.00329272	0.00370275
CCDC109B	2.16550449	3.77E-77	1.34E-76
CASP6	1.09233139	7.10E-59	1.91E-58
AC004067.5	NA	3.17E-21	5.03E-21
PLA2G12A	2.03049552	2.87E-59	7.80E-59
CFI	NA	NA	NA
RP11-602N24	Inf	2.39E-31	4.39E-31
GAR1	1.13715953	1.08E-134	1.91E-133
RRH	NA	1.66E-31	3.06E-31
LRIT3	NA	3.21E-82	1.24E-81
EGF	NA	7.54E-79	2.75E-78
ELOVL6	1.49805447	2.01E-33	3.80E-33
ENPEP	NA	NA	NA
RP11-380D23	NA	NA	NA
RP11-380D23	NA	NA	NA
PITX2	NA	NA	NA
LINC01438	NA	NA	NA
RP11-255I10	NA	NA	NA
RP11-269F21	NA	NA	NA
RP11-269F21	NA	NA	NA
RP11-269F21	NA	NA	NA
C4orf32	2.31190138	5.58E-140	1.57E-138
RP11-73K9.2	Inf	6.87E-156	2.31E-153
AP1AR	4.11964981	6.17E-78	2.22E-77
RP11-73K9.3	NA	8.67E-109	5.65E-108
TIFA	1.19844358	3.17E-66	9.58E-66
ALPK1	0.99870298	5.43E-12	7.43E-12
NEUROG2	Inf	0.00088635	0.00101356
RP11-402J6.1	0	0.00010218	0.00011975
ZGRF1	2.99610895	2.20E-52	5.44E-52
LARP7	1.09233139	4.53E-138	1.04E-136
RP11-148B6.	NA	NA	NA
RP11-148B6.	NA	6.59E-30	1.19E-29
ANK2	NA	8.05E-65	2.38E-64
RP11-119H12	NA	NA	NA
RP11-650J17	NA	NA	NA

RP11-650J17	NA	NA	NA
CAMK2D	NA	NA	NA
ARSJ	NA	NA	NA
RP11-26P13.	NA	NA	NA
UGT8	NA	2.77E-73	9.33E-73
NDST4	NA	NA	NA
RP11-659O3.	3.932393	7.27E-08	9.16E-08
MTRNR2L13	NA	NA	NA
RP11-55L3.1	NA	NA	NA
TRAM1L1	Inf	6.97E-65	2.07E-64
AC107399.2	NA	NA	NA
AC113617.1	NA	NA	NA
LINC01378	NA	NA	NA
AC108056.1	NA	NA	NA
NDST3	2.24708171	1.62E-45	3.62E-45
SNHG8	2.24421736	1.13E-130	1.57E-129
PRSS12	NA	9.92E-124	1.02E-122
RP11-384K6.	0	5.49E-147	6.00E-145
METTL14	1.84375935	7.47E-05	8.79E-05
SEC24D	1.07003891	2.64E-18	4.02E-18
SYNPO2	NA	4.18E-08	5.30E-08
MYOZ2	NA	2.85E-61	8.01E-61
USP53	1.12354086	6.97E-79	2.55E-78
C4orf3	1.75824288	2.01E-44	4.41E-44
FABP2	NA	NA	NA
RP11-33B1.4	NA	2.95E-25	4.97E-25
RP11-33B1.3	NA	NA	NA
PDE5A	0	1.40E-141	4.85E-140
LINC01365	NA	NA	NA
RP11-700N1.	NA	NA	NA
RP11-96A1.5	NA	NA	NA
MAD2L1	0.97677201	5.00E-110	3.37E-109
RP11-679C8.	NA	2.06E-106	1.27E-105
RP11-501E14	NA	NA	NA
PRDM5	NA	NA	NA
NDNF	NA	NA	NA
RP11-647P12	NA	NA	NA
TNIP3	NA	NA	NA
QRFPR	NA	NA	NA
ANXA5	0.68093385	3.14E-144	1.74E-142
TMEM155	NA	NA	NA
PP12613	NA	NA	NA
EXOSC9	0.93968872	1.03E-122	1.01E-121
CCNA2	1.4679212	3.00E-65	8.94E-65
BBS7	1.82575389	0.00316541	0.00356091

RP11-63B13.	NA	NA	NA
TRPC3	NA	NA	NA
KIAA1109	1.60505837	0.90670422	0.91117421
ADAD1	NA	NA	NA
IL2	NA	NA	NA
IL21	NA	NA	NA
IL21-AS1	NA	NA	NA
BBS12	2.24708171	3.67E-71	1.19E-70
FGF2	NA	5.56E-54	1.41E-53
RP11-170N1(	NA	1.24E-42	2.67E-42
NUDT6	0.7864786	1.95E-112	1.40E-111
SPATA5	1.04863813	0.0002927	0.00033933
SPRY1	0.74902724	1.29E-14	1.85E-14
RP11-381N2(	NA	NA	NA
RP11-381N2(	NA	NA	NA
LINC01091	NA	NA	NA
AC096732.2	NA	NA	NA
CTD-2325B1:	NA	NA	NA
RP11-93I21.3	NA	NA	NA
ANKRD50	2.49675746	9.59E-10	1.26E-09
FAT4	NA	NA	NA
RP11-399F2.	NA	NA	NA
RP11-318I4.1	NA	NA	NA
RP11-810P8.	NA	NA	NA
RP11-125O1	NA	NA	NA
INTU	5.61770428	5.61E-97	2.86E-96
SLC25A31	NA	4.31E-28	7.56E-28
HSPA4L	1.61328943	2.74E-31	5.02E-31
RP11-50D9.3	NA	7.99E-149	1.03E-146
PLK4	1.63023575	2.64E-29	4.71E-29
MFSD8	0.97949716	2.03E-77	7.24E-77
ABHD18	3.55787938	3.56E-92	1.65E-91
LARP1B	1.84220212	0.13129356	0.13931032
PGRMC2	1.56135255	3.52E-95	1.73E-94
RP11-420A2:	Inf	1.83E-18	2.79E-18
RP11-130C6.	NA	NA	NA
RP11-184M1	NA	NA	NA
RP11-184M1	NA	NA	NA
JADRR	NA	NA	NA
JADE1	1.38649723	1.48E-15	2.15E-15
SCLT1	1.09473212	1.84E-44	4.04E-44
C4orf33	0.84265564	2.35E-95	1.16E-94
RP11-419L4.	NA	NA	NA
RP11-519M1	NA	NA	NA
RP11-422J15	NA	NA	NA

RP11-521E5.	NA	NA	NA
RP11-401I19	NA	NA	NA
RP11-314N1	NA	NA	NA
RP11-614F1	NA	NA	NA
RP11-745L13	NA	NA	NA
RP11-789C2.	NA	NA	NA
RP11-1E22.1	NA	NA	NA
RP11-62N21.	NA	NA	NA
LINC01256	NA	NA	NA
RP11-404I7.2	NA	NA	NA
RP11-404I7.1	NA	NA	NA
RP11-9G1.3	NA	NA	NA
PCDH10	NA	NA	NA
RP11-427M2	NA	NA	NA
PABPC4L	0.74902724	0.0001563	0.00018234
RP11-400D2.	NA	NA	NA
RP11-400D2.	NA	NA	NA
CTD-2012I17	NA	NA	NA
RP11-192C21	NA	NA	NA
RP11-553P9.	NA	NA	NA
RP11-553P9.	NA	NA	NA
RP11-780O1	NA	NA	NA
LINC00613	NA	NA	NA
RP11-775H9.	NA	NA	NA
RP11-775H9.	NA	NA	NA
RP11-775H9.	NA	NA	NA
RP11-138I17	NA	2.16E-76	7.58E-76
RP11-785F11	NA	NA	NA
PCDH18	NA	NA	NA
RP11-714L20	NA	2.74E-07	3.42E-07
RP13-884E18	NA	NA	NA
RP13-884E18	NA	NA	NA
RP11-793B23	NA	NA	NA
LINC00616	NA	NA	NA
SLC7A11	1.31079767	2.36E-06	2.88E-06
RP11-733C7.	NA	NA	NA
LINC00498	NA	NA	NA
LINC00499	NA	NA	NA
LINC00500	NA	NA	NA
RP11-173E2.	NA	NA	NA
RP11-98O2.1	NA	NA	NA
RP11-371F15	NA	NA	NA
RP11-577B7.	NA	NA	NA
NOCT	4.49416342	5.81E-51	1.40E-50
ELF2	2.6909497	7.95E-47	1.81E-46

MGARP	NA	8.75E-32	1.62E-31
NDUFC1	2.18756598	1.97E-121	1.84E-120
NAA15	1.53134457	6.49E-17	9.65E-17
RP11-83A24.	2.82965845	1.77E-33	3.33E-33
RAB33B	8.23929961	1.62E-89	7.15E-89
SETD7	1.79766537	3.83E-54	9.69E-54
RP11-342I1.2	0	0.00201259	0.00227562
RP11-308D13	NA	NA	NA
MGST2	0.98020848	1.69E-102	9.53E-102
MAML3	1.24837873	2.24E-14	3.19E-14
RP11-392B6.	NA	NA	NA
SCOC	1.95579334	0.08176101	0.08757798
SCOC-AS1	2.0598249	1.81E-47	4.15E-47
CLGN	1.60505837	2.13E-87	9.01E-87
MGAT4D	NA	NA	NA
ELMOD2	1.42315175	0.0026564	0.00299257
UCP1	NA	NA	NA
TBC1D9	1.49805447	2.83E-94	1.37E-93
RP11-102N1	NA	NA	NA
RP11-102N1	1.49805447	5.20E-11	6.97E-11
RNF150	0	5.50E-111	3.80E-110
ZNF330	1.74444501	1.62E-29	2.91E-29
RP11-586L23	NA	NA	NA
RP11-362F19	NA	NA	NA
RP11-362F19	NA	NA	NA
RP11-208N20	NA	NA	NA
IL15	NA	4.29E-48	9.91E-48
INPP4B	1.87256809	8.40E-48	1.93E-47
RP11-223C24	NA	NA	NA
RP11-223C24	NA	NA	NA
RP11-284M1	NA	2.17E-38	4.41E-38
USP38	2.99610895	4.17E-59	1.13E-58
RP11-364L4.	NA	NA	NA
GAB1	2.62159533	9.48E-10	1.24E-09
SMARCA5	2.24708171	1.09E-10	1.46E-10
SMARCA5-AS	NA	3.04E-123	3.05E-122
FREM3	NA	NA	NA
RP13-578N3.	NA	NA	NA
GYPE	NA	1.24E-42	2.67E-42
RP11-673E1.	Inf	1.11E-116	8.91E-116
RP11-673E1.	NA	NA	NA
GYPB	Inf	1.53E-81	5.86E-81
GYPA	NA	NA	NA
RP11-361D14	NA	NA	NA
HHIP-AS1	NA	5.19E-35	1.00E-34

HHIP	NA	NA	NA
RP13-539F13	NA	NA	NA
ANAPC10	1.32520204	1.29E-30	2.34E-30
ABCE1	1.54798962	6.45E-62	1.83E-61
OTUD4	3.4330415	0.00017472	0.00020353
RP11-142A22	NA	NA	NA
SMAD1	1.9661965	0.00030224	0.00035024
SMAD1-AS2	NA	NA	NA
SMAD1-AS1	NA	4.71E-13	6.56E-13
RP11-557J10	NA	NA	NA
MMAA	Inf	7.24E-11	9.69E-11
RP11-557J10	NA	NA	NA
C4orf51	NA	NA	NA
ZNF827	1.19844358	2.60E-71	8.48E-71
RP11-181K12	NA	NA	NA
RP11-203B7.	NA	NA	NA
RP11-203B7.	NA	NA	NA
LINC01095	NA	NA	NA
LSM6	1.94859717	3.54E-35	6.86E-35
RP11-6L6.2	NA	NA	NA
RP11-6L6.7	NA	NA	NA
SLC10A7	1.07003891	0.00591658	0.00660179
POU4F2	NA	NA	NA
TTC29	1.49805447	0.06202808	0.06672083
RP11-292D4.	NA	NA	NA
RP11-292D4.	NA	1.42E-74	4.86E-74
RP11-292D4.	NA	NA	NA
EDNRA	NA	NA	NA
RP11-752L20	NA	NA	NA
RP11-425A23	NA	4.58E-34	8.74E-34
TMEM184C	1.38281952	2.19E-31	4.03E-31
PRMT9	4.6190013	1.07E-66	3.26E-66
ARHGAP10	2.24708171	0.00105316	0.00120182
NR3C2	0	0.00804583	0.0089386
RP11-76G10.	NA	NA	NA
RP11-503L19	NA	NA	NA
RP11-563M4	NA	NA	NA
RP11-342A1.	NA	NA	NA
RP11-24I21.1	NA	NA	NA
RP11-526A4.	NA	NA	NA
RP11-707F2.	NA	NA	NA
RP11-423J7.1	NA	NA	NA
DCLK2	NA	8.52E-09	1.10E-08
LRBA	1.64785992	4.72E-11	6.34E-11
RP11-1336O1	NA	1.82E-96	9.16E-96

MAB21L2	NA	NA	NA
RPS3A	1.66978873	6.51E-35	1.26E-34
SH3D19	0.0936284	9.36E-143	3.91E-141
RP11-372K14	Inf	1.19E-122	1.16E-121
PRSS48	NA	NA	NA
RP11-731D1.	NA	NA	NA
RP11-610P16	NA	NA	NA
FAM160A1	NA	NA	NA
RP11-164P12	NA	NA	NA
GATB	1.13488975	7.46E-110	5.01E-109
RP11-164P12	NA	NA	NA
RP11-424M2	NA	NA	NA
RP11-503L23	NA	NA	NA
RP11-73G16.	NA	NA	NA
RP11-73G16.	NA	NA	NA
RP11-18H21.	NA	NA	NA
RP11-18H21.	NA	NA	NA
RP11-18H21.	NA	NA	NA
RP11-461L13	NA	NA	NA
RP11-461L13	NA	NA	NA
RP11-461L13	NA	5.11E-124	5.29E-123
FBXW7	1.38281952	6.36E-19	9.77E-19
DEAR	NA	NA	NA
DKFZP434I07	5.24319066	3.79E-128	4.68E-127
TMEM154	1.49805447	0.00340757	0.00383095
RP11-768B22	NA	NA	NA
ARFIP1	1.42996109	0.03097212	0.03371108
TIGD4	NA	4.49E-106	2.74E-105
FHDC1	NA	NA	NA
RP11-285C1.	NA	NA	NA
TRIM2	1.49805447	2.80E-27	4.85E-27
MND1	1.47743905	2.08E-113	1.53E-112
KIAA0922	1.77893969	8.81E-06	1.06E-05
TLR2	NA	NA	NA
RNF175	0	5.11E-124	5.29E-123
RP11-153M7	NA	NA	NA
SFRP2	NA	1.29E-139	3.49E-138
RP11-290O1	NA	NA	NA
DCHS2	NA	NA	NA
RP11-9N12.2	NA	NA	NA
RP11-9N12.3	NA	NA	NA
PLRG1	1.38281952	1.61E-49	3.81E-49
FGB	NA	NA	NA
FGA	NA	NA	NA
FGG	NA	NA	NA

LRAT	Inf	2.53E-14	3.61E-14
RP11-21G20.	NA	NA	NA
RBM46	NA	NA	NA
AC097467.2	NA	NA	NA
RP11-92A5.2	NA	NA	NA
NPY2R	NA	NA	NA
RP11-27G13.	NA	NA	NA
MAP9	0.24967575	7.04E-21	1.11E-20
RP13-487K5.	NA	NA	NA
GUCY1A3	1.56614786	0.39031142	0.40308702
GUCY1B3	2.90248054	2.63E-12	3.62E-12
ASIC5	NA	NA	NA
TDO2	NA	1.81E-07	2.26E-07
CTSO	0.74902724	3.66E-20	5.72E-20
RP11-171N4.	NA	NA	NA
RP11-171N4.	NA	NA	NA
RP11-171N4.	NA	NA	NA
RP11-171N4.	NA	NA	NA
PDGFC	0.89169909	2.06E-62	5.87E-62
RP11-154F14	NA	NA	NA
GLRB	1.3732166	8.10E-67	2.47E-66
GRIA2	NA	NA	NA
RP11-364P22	NA	NA	NA
RP11-364P22	NA	NA	NA
FAM198B	0.74902724	4.39E-86	1.81E-85
RP11-597D13	Inf	2.01E-05	2.40E-05
RP11-597D13	NA	NA	NA
TMEM144	3.74513619	1.71E-39	3.53E-39
RXFP1	Inf	1.03E-153	2.34E-151
RP11-781M1	NA	NA	NA
C4orf46	1.3732166	3.78E-53	9.45E-53
ETFDH	1.73458939	6.39E-17	9.50E-17
PPID	0.8591783	1.42E-117	1.16E-116
FNIP2	2.0598249	0.00019135	0.00022274
C4orf45	NA	NA	NA
RAPGEF2	0.74902724	0.54246797	0.55573177
RP11-138A23	NA	NA	NA
RP11-6C14.1	NA	NA	NA
RP11-502M1	NA	NA	NA
RP11-234O6.	NA	NA	NA
FSTL5	2.99610895	9.32E-76	3.24E-75
RP11-497K21	NA	NA	NA
AC005150.1	NA	NA	NA
RP11-563E2.	NA	NA	NA
NAF1	1.66450497	1.19E-29	2.13E-29

NPY1R	NA	NA	NA
NPY5R	NA	NA	NA
TKTL2	NA	NA	NA
TMA16	1.69971565	1.76E-14	2.52E-14
1-Mar	0-Jan	0.00081667	0.00093447
RP11-218F1(	Inf	1.02E-44	2.25E-44
LINC01207	NA	1.14E-122	1.11E-121
APELA	NA	NA	NA
TRIM61	NA	NA	NA
FAM218A	NA	NA	NA
RP11-366M4	NA	NA	NA
TRIM60	NA	1.19E-07	1.49E-07
TMEM192	1.75110422	0.11502812	0.12235943
KLHL2	1.1770428	4.04E-11	5.43E-11
MSMO1	1.12354086	1.35E-54	3.43E-54
CPE	NA	3.05E-54	7.73E-54
LINC01179	NA	NA	NA
TLL1	NA	NA	NA
RP11-217C7.	NA	NA	NA
SPOCK3	NA	NA	NA
RP11-436A7.	NA	NA	NA
RP11-310I9.1	NA	NA	NA
ANXA10	NA	NA	NA
DDX60	Inf	7.73E-10	1.02E-09
DDX60L	2.24708171	4.18E-67	1.28E-66
PALLD	Inf	1.93E-136	3.82E-135
RP11-610J23	NA	NA	NA
RP11-635L1.	NA	NA	NA
CBR4	0.91547773	5.15E-112	3.66E-111
RP11-483A2(	NA	NA	NA
SH3RF1	1.28404669	0.000174	0.0002027
RP11-327O1	NA	NA	NA
NEK1	1.16515348	5.63E-95	2.75E-94
CLCN3	1.04863813	3.25E-06	3.95E-06
C4orf27	0.95101211	1.13E-126	1.30E-125
RP11-205M3	NA	NA	NA
RP11-6E9.5	NA	NA	NA
RP11-6E9.4	NA	NA	NA
MFAP3L	0.93628405	3.90E-27	6.75E-27
AADAT	5.61770428	5.41E-27	9.35E-27
LINC01612	NA	1.42E-74	4.86E-74
RP11-789C1.	NA	NA	NA
RP11-322J23	NA	NA	NA
RP11-344G1	NA	NA	NA
RP11-717H1	NA	NA	NA

RP11-97E7.1	NA	NA	NA
RP11-97E7.2	NA	NA	NA
GALNTL6	NA	NA	NA
RP11-10K16.	0.74902724	4.25E-71	1.38E-70
GALNT7	2.55917639	1.93E-14	2.75E-14
RP11-798M1	NA	0.23111347	0.24222187
HMGB2	1.29996046	1.50E-144	9.07E-143
RP11-798M1	3.74513619	0.00118943	0.00135502
SAP30	1.37181393	2.24E-107	1.41E-106
SCRG1	Inf	3.32E-34	6.34E-34
RP11-489G1	NA	NA	NA
HAND2	NA	NA	NA
HAND2-AS1	NA	6.65E-83	2.60E-82
RP11-475B2.	NA	NA	NA
RP11-161D1!	NA	NA	NA
RP11-161D1!	NA	NA	NA
RP11-161D1!	NA	NA	NA
RP11-148L24	NA	NA	NA
RP11-248N2:	NA	NA	NA
RP11-248N2:	NA	NA	NA
FBXO8	2.49675746	0.03365296	0.03656318
CEP44	1.94747082	1.03E-06	1.27E-06
RP11-51M24	NA	NA	NA
HPGD	Inf	1.07E-09	1.40E-09
RP11-440I14	NA	NA	NA
RP11-440I14	NA	NA	NA
GLRA3	NA	NA	NA
ADAM29	NA	NA	NA
RP13-577H1:	NA	NA	NA
RP11-287F9.	NA	NA	NA
RP11-598D1·	NA	NA	NA
GPM6A	NA	NA	NA
RP11-806K1!	NA	NA	NA
WDR17	0.74902724	0.85315943	0.85960337
SPATA4	NA	NA	NA
ASB5	NA	NA	NA
RP11-87F15.	NA	NA	NA
SPCS3	1.79853633	0.51730726	0.53055505
RP11-313E1!	NA	NA	NA
VEGFC	NA	NA	NA
RP11-624A4.	NA	NA	NA
RP11-765K1·	NA	NA	NA
RP11-487E1:	NA	NA	NA
NEIL3	0.74902724	2.71E-80	1.02E-79
RP11-376O6.	NA	NA	NA

AGA	0.62418936	1.14E-125	1.26E-124
RP11-130F1(	NA	1.80E-118	1.52E-117
RP11-140M1	NA	NA	NA
LINC01098	NA	NA	NA
LINC01099	NA	NA	NA
RP11-404J23	NA	1.53E-68	4.80E-68
RP11-460H9.	NA	NA	NA
LINC00290	NA	NA	NA
RP11-665C1	NA	NA	NA
RP11-540E1(	NA	NA	NA
AC108142.1	NA	NA	NA
TENM3	NA	NA	NA
RP11-18D7.3	NA	NA	NA
RP11-18D7.2	NA	NA	NA
RP11-188P1	Inf	3.06E-14	4.36E-14
DCTD	1.37190252	9.23E-88	3.94E-87
RP11-335L23	NA	NA	NA
RP11-335L23	NA	NA	NA
WWC2-AS2	Inf	1.27E-142	5.08E-141
WWC2	2.99610895	5.37E-63	1.54E-62
WWC2-AS1	NA	NA	NA
CLDN22	NA	NA	NA
CLDN24	NA	NA	NA
CDKN2AIP	1.30094204	2.05E-21	3.26E-21
RP11-367N1	NA	2.02E-65	6.04E-65
ING2	1.99337894	1.34E-64	3.94E-64
RP11-367N1	Inf	3.23E-140	9.31E-139
RWDD4	2.25414801	1.05E-62	3.01E-62
TRAPPC11	2.07084001	1.95E-28	3.44E-28
STOX2	NA	2.80E-84	1.12E-83
ENPP6	NA	NA	NA
RP11-290F5.	0	1.19E-08	1.52E-08
RP11-290F5.	NA	NA	NA
IRF2	1.49805447	7.94E-77	2.80E-76
RP11-326I11	2.99610895	3.69E-05	4.37E-05
RP11-326I11	NA	NA	NA
RP11-242J7.	NA	NA	NA
CASP3	1.78444724	0.01936898	0.02123857
PRIMPOL	0.61284047	1.33E-15	1.94E-15
CENPU	1.52008469	2.83E-137	6.04E-136
ACSL1	1.10570687	0.16918669	0.17851488
RP11-701P1(	NA	NA	NA
MIR3945HG	3.74513619	4.79E-22	7.71E-22
LINC01093	NA	NA	NA
RP11-386B1	NA	NA	NA

HELT	NA	NA	NA
RP11-386B13	NA	NA	NA
SLC25A4	0.37451362	5.51E-69	1.74E-68
CFAP97	1.49805447	6.87E-58	1.83E-57
SNX25	2.99610895	0.18057037	0.19026064
LRP2BP	Inf	2.66E-138	6.17E-137
RP11-714G18	NA	NA	NA
ANKRD37	1.99740597	9.03E-30	1.62E-29
UFSP2	1.95899431	1.97E-55	5.08E-55
C4orf47	NA	1.44E-124	1.51E-123
CCDC110	NA	NA	NA
RP11-279O9.	NA	1.19E-07	1.49E-07
PDLIM3	NA	NA	NA
SORBS2	NA	NA	NA
RP11-301L8.1	NA	NA	NA
RP11-626E13	NA	NA	NA
RP11-45I20.1	NA	NA	NA
TLR3	NA	2.49E-51	6.04E-51
FAM149A	NA	7.14E-13	9.91E-13
CYP4V2	4.49416342	1.07E-88	4.65E-88
KLKB1	NA	NA	NA
F11	NA	NA	NA
F11-AS1	NA	NA	NA
RP11-215A19	NA	NA	NA
MTNR1A	NA	NA	NA
FAT1	1.92607004	0.22202721	0.23286031
RP11-11N5.1	NA	NA	NA
RP11-11N5.3	NA	NA	NA
RP11-217E13	NA	NA	NA
RP11-696F12	NA	NA	NA
RP11-308K2.	NA	NA	NA
RP11-138B4.	NA	NA	NA
RP11-91J3.2	NA	NA	NA
RP11-91J3.3	NA	NA	NA
RP11-237D3.	NA	NA	NA
RP11-565A3.	NA	NA	NA
RP11-565A3.	NA	NA	NA
ZFP42	NA	1.62E-79	5.97E-79
TRIML2	NA	NA	NA
TRIML1	NA	NA	NA
RP11-366H4.	NA	NA	NA
RP11-366H4.	NA	NA	NA
LINC01060	NA	NA	NA
RP11-756P10	NA	NA	NA
RP11-756P10	NA	NA	NA

RP11-756P1(	NA	NA	NA
RP11-818C3.	NA	NA	NA
RP11-706F1.	NA	NA	NA
RP11-706F1.	NA	NA	NA
LINC01262	0.74902724	3.85E-17	5.75E-17
RP11-462G2:	NA	NA	NA
AF146191.4	NA	0.00390444	0.0043806
LINC01596	NA	NA	NA
FRG1	0.81494163	8.85E-144	4.46E-142
FRG2	NA	NA	NA
DBET	NA	NA	NA
DUX4	NA	NA	NA
CTD-2231H1(	NA	NA	NA
PLEKHG4B	1.49805447	5.33E-06	6.46E-06
LRRC14B	NA	NA	NA
CCDC127	1.14850843	9.35E-62	2.64E-61
SDHA	0.94321948	2.50E-104	1.47E-103
CTD-2083E4.	NA	0.13377061	0.14180592
PDCD6	1.59224353	3.34E-74	1.14E-73
AHRR	6.52723735	4.14E-135	7.44E-134
EXOC3-AS1	2.11089494	1.43E-52	3.55E-52
EXOC3	1.22209707	1.07E-15	1.57E-15
PP7080	1.58884565	1.40E-55	3.61E-55
SLC9A3	NA	4.45E-89	1.94E-88
RP11-310P5.	NA	NA	NA
RP11-310P5.	NA	NA	NA
CEP72	1.02140078	1.72E-43	3.73E-43
TPPP	NA	2.73E-120	2.46E-119
CTD-2589H1!	Inf	1.18E-69	3.76E-69
ZDHHC11B	1.49805447	0.10503997	0.11189872
ZDHHC11	1.49805447	4.34E-57	1.14E-56
BRD9	2.62159533	2.11E-71	6.88E-71
TRIP13	1.16296334	2.86E-48	6.64E-48
RP11-661C8.	NA	NA	NA
RP11-661C8.	NA	1.62E-79	5.97E-79
RP11-43F13.	Inf	1.89E-34	3.62E-34
RP11-43F13.	0.74902724	9.09E-23	1.48E-22
NKD2	3.90190933	4.29E-145	2.99E-143
SLC12A7	2.49675746	3.28E-07	4.08E-07
CTD-3080P1:	NA	NA	NA
SLC6A19	NA	NA	NA
SLC6A18	NA	NA	NA
TERT	Inf	5.54E-34	1.05E-33
CLPTM1L	2.03242147	5.56E-51	1.34E-50
LINC01511	NA	NA	NA

SLC6A3	NA	NA	NA
LPCAT1	1.53289295	1.26E-29	2.26E-29
CTD-2245E15	NA	NA	NA
CTD-2012J19	NA	NA	NA
CTD-2587M2	NA	NA	NA
MRPL36	1.76748873	0.00015104	0.00017625
NDUFS6	1.87119625	2.29E-27	3.97E-27
CTD-2194D22	NA	NA	NA
CTD-2194D22	NA	NA	NA
IRX4	NA	NA	NA
CTD-2194D22	NA	NA	NA
CTD-2194D22	NA	NA	NA
RP11-259O2.	NA	NA	NA
RP11-259O2.	NA	NA	NA
RP11-259O2.	NA	NA	NA
LSINCT5	NA	1.45E-16	2.14E-16
RP11-129I19	NA	NA	NA
IRX2	NA	NA	NA
C5orf38	NA	NA	NA
RP11-468D1	NA	NA	NA
RP11-35O7.1	NA	NA	NA
LINC01377	NA	NA	NA
LINC01019	NA	NA	NA
RP11-121L11	NA	NA	NA
LINC01017	NA	NA	NA
IRX1	NA	NA	NA
CTD-2012M1	NA	NA	NA
CTD-2287N1	NA	NA	NA
CTD-2383I20	NA	NA	NA
CTD-2008N3.	NA	NA	NA
RP11-445O3.	NA	NA	NA
RP11-445O3.	NA	NA	NA
RP11-445O3.	NA	NA	NA
CTD-2318H2	NA	NA	NA
CTD-2161F6.	NA	NA	NA
CTD-2161F6.	NA	NA	NA
CTD-2161F6.	NA	NA	NA
CTD-2046I8.1	NA	NA	NA
CTD-2247C11	NA	NA	NA
CTD-2247C11	NA	NA	NA
LINC01020	NA	NA	NA
CTD-2247C11	NA	NA	NA
CTD-2247C11	NA	NA	NA
CTD-2297D1(	NA	NA	NA
ADAMTS16	NA	NA	NA

CTD-2297D1(	NA	NA	NA
ICE1	2.20026751	1.30E-39	2.67E-39
CTC-471C19.:	NA	NA	NA
CTC-471C19.:	NA	NA	NA
FLJ33360	NA	NA	NA
MED10	1.57390533	0.05696627	0.06136338
UBE2QL1	NA	2.37E-63	6.84E-63
LINC01018	NA	NA	NA
NSUN2	1.47932879	2.69E-06	3.28E-06
SRD5A1	1.78339818	1.43E-06	1.76E-06
CTD-2044J15	NA	1.96E-125	2.13E-124
PAPD7	2.18946423	0.76284099	0.77177455
RP11-332J15	NA	NA	NA
RP11-332J15	NA	NA	NA
RP11-332J15	NA	NA	NA
RP11-332J15	NA	NA	NA
RP11-122F24	NA	NA	NA
RP11-404K5.	NA	NA	NA
CTD-2296D1.	NA	NA	NA
CTD-2296D1.	NA	NA	NA
CTD-2296D1.	NA	NA	NA
CTD-2296D1.	NA	NA	NA
CTD-2296D1.	NA	NA	NA
ADCY2	NA	NA	NA
RP11-711G1(	NA	NA	NA
C5orf49	NA	NA	NA
MTRR	2.08657588	1.56E-78	5.66E-78
FASTKD3	1.77676228	8.33E-16	1.22E-15
CTD-2072I24	NA	NA	NA
RP11-480D4.	NA	NA	NA
RP11-480D4.	NA	NA	NA
RP11-480D4.	NA	NA	NA
MIR4458HG	NA	1.45E-06	1.78E-06
RP11-417J1.:	NA	NA	NA
RP11-315A1(	NA	NA	NA
RP11-143A1:	NA	NA	NA
CTD-2215L1C	NA	NA	NA
SEMA5A	NA	NA	NA
CTD-2201E9.	NA	NA	NA
CTD-2201E9.	NA	NA	NA
SNHG18	NA	NA	NA
TAS2R1	0	2.92E-108	1.88E-107
CTD-2143L24	NA	NA	NA
RP11-447B1:	NA	NA	NA
CTD-2199O4.	NA	2.34E-18	3.56E-18

CTD-2199O4.	NA	NA	NA
CTD-2199O4.	NA	NA	NA
CTD-2199O4.	0.74902724	5.69E-15	8.21E-15
FAM173B	1.34824903	1.58E-132	2.46E-131
CTD-2256P15	Inf	1.43E-107	9.05E-107
CCT5	0.94038456	8.23E-139	2.03E-137
CTD-2256P15	0	4.67E-08	5.92E-08
CTD-2256P15	NA	NA	NA
CMBL	1.04863813	1.42E-121	1.34E-120
CTD-2256P15	1.12354086	4.65E-46	1.05E-45
6-Mar	1-Jan	1.02E-06	1.26E-06
ROPN1L-AS1	NA	NA	NA
ROPN1L	1.60505837	0.44793748	0.46094621
LINC01513	NA	1.31E-44	2.88E-44
RP11-1C1.6	NA	NA	NA
RP11-1C1.4	NA	4.78E-09	6.18E-09
RP11-1C1.5	NA	NA	NA
ANKRD33B	1.29831388	0.07435443	0.07976219
ANKRD33B-A	NA	NA	NA
DAP	1.76819545	1.49E-05	1.79E-05
CTD-2154B17	NA	5.48E-110	3.68E-109
CTD-2154B17	NA	NA	NA
CTNND2	NA	NA	NA
RP11-308B16	NA	NA	NA
LINC01194	NA	NA	NA
RP11-419C19	NA	NA	NA
CTC-305H11.	NA	NA	NA
DNAH5	NA	1.86E-104	1.10E-103
CTB-51A17.1	NA	NA	NA
TRIO	1.59168288	6.20E-08	7.82E-08
AC016549.1	3.74513619	1.63E-07	2.04E-07
FAM105A	1.12354086	1.63E-105	9.85E-105
CTD-2165H16	Inf	6.19E-126	6.92E-125
OTULIN	2.60661479	1.50E-39	3.10E-39
ANKH	2.16385646	3.60E-73	1.21E-72
CTB-40H15.4	Inf	3.92E-141	1.27E-139
RP1-251I12.1	NA	NA	NA
RP1-137K24.	NA	NA	NA
FBXL7	NA	NA	NA
CTD-2350J17	NA	NA	NA
11-Mar	NA	NA	NA
RP11-19O2.1	NA	NA	NA
RP11-19O2.2	NA	NA	NA
RP1-167G20.	NA	NA	NA
RP1-167G20.	NA	NA	NA

ZNF622	1.30712596	1.84E-84	7.42E-84
FAM134B	NA	5.76E-36	1.13E-35
RP11-260E18	NA	NA	NA
CTC-461F20.1	NA	NA	NA
MYO10	1.49805447	0.0120933	0.01335423
BASP1	0.19066148	1.80E-145	1.58E-143
CTD-2228A4.	NA	NA	NA
AC091878.1	NA	NA	NA
CTD-2139B15	NA	0.50830229	0.52152586
CTD-2139B15	NA	NA	NA
CTD-2139B15	NA	NA	NA
RP11-321E2.	NA	4.56E-09	5.90E-09
RP11-321E2.	NA	NA	NA
RP11-454P21	NA	NA	NA
CTD-2023M8	NA	NA	NA
RP11-124N3.	NA	NA	NA
RP11-124N3.	NA	NA	NA
CDH18	NA	2.51E-23	4.11E-23
RP11-420O10	NA	NA	NA
RP11-774D14	NA	NA	NA
CTD-2333M2	NA	NA	NA
RP11-374A4.	NA	NA	NA
RP11-360I2.1	NA	1.28E-90	5.75E-90
RP11-804N13	NA	NA	NA
CDH12	NA	0.0091276	0.0101181
CTD-2234B20	NA	NA	NA
PRDM9	NA	NA	NA
C5orf17	NA	NA	NA
CTD-2074D8.	NA	NA	NA
CDH10	NA	NA	NA
RP11-116O17	NA	NA	NA
RP11-730N24	NA	NA	NA
RP11-730N24	NA	NA	NA
RP11-549K20	NA	4.03E-19	6.20E-19
RP11-184E9.	NA	NA	NA
RP11-192H6.	NA	NA	NA
RP11-184E9.	NA	NA	NA
CTD-2306M5	NA	1.66E-31	3.06E-31
RP11-351N6.	NA	NA	NA
CTD-2533K21	NA	NA	NA
CDH9	NA	NA	NA
CTD-3007L5.1	NA	NA	NA
LINC01021	2.99610895	1.44E-11	1.96E-11
CTD-2061E9.	NA	NA	NA
CTD-2134P3.	NA	NA	NA

CTD-2118P12	NA	6.76E-40	1.40E-39
CTD-2010I22	NA	NA	NA
RP11-152K4.	NA	NA	NA
CDH6	NA	NA	NA
DROSHA	2.00875486	0.7578986	0.76694536
C5orf22	4.11964981	5.01E-33	9.39E-33
PDZD2	NA	8.76E-27	1.51E-26
RP11-5N11.4	NA	NA	NA
RP11-5N11.5	NA	NA	NA
RP11-5N11.6	NA	NA	NA
CTD-2152M2	NA	NA	NA
GOLPH3	1.62289235	0.0663457	0.07128901
CTD-2186M1	2.37191958	0.15659614	0.16541293
MTMR12	2.54669261	1.33E-92	6.25E-92
ZFR	1.45500693	4.24E-66	1.28E-65
SUB1	1.5292969	3.32E-73	1.11E-72
CTD-2004A9.	NA	NA	NA
NPR3	2.24708171	0.05516348	0.05945321
CTD-2066L21	NA	NA	NA
CTD-2218G2(	NA	NA	NA
CTD-2066L21	NA	NA	NA
CTD-2066L21	NA	NA	NA
RP11-113I22	NA	NA	NA
CTD-2203K17	NA	3.32E-104	1.95E-103
TARS	1.63757916	6.42E-23	1.05E-22
CTD-2233C11	NA	NA	NA
ADAMTS12	NA	NA	NA
RXFP3	NA	NA	NA
SLC45A2	NA	NA	NA
AMACR	0.74902724	4.14E-25	6.98E-25
C1QTNF3-AN	NA	NA	NA
C1QTNF3	0.74902724	4.83E-58	1.29E-57
RP11-1250I1	NA	NA	NA
CTD-2024P1(	NA	NA	NA
RAI14	0.59922179	5.05E-80	1.88E-79
CTD-2024P1(	NA	NA	NA
CTD-2517O1(	NA	4.31E-07	5.34E-07
TTC23L	NA	1.49E-17	2.24E-17
RAD1	1.75907912	0.00213812	0.00241545
BRIX1	0.9625	2.49E-131	3.61E-130
DNAJC21	2.49256123	8.25E-61	2.30E-60
AGXT2	NA	NA	NA
PRLR	NA	NA	NA
SPEF2	0.29961089	1.65E-84	6.64E-84
CTD-2113L7.	NA	NA	NA

IL7R	Inf	1.84E-145	1.58E-143
CAPSL	NA	NA	NA
RP11-79C6.3	NA	NA	NA
UGT3A1	NA	NA	NA
UGT3A2	Inf	7.80E-114	5.80E-113
LMBRD2	1.94747082	1.24E-62	3.54E-62
SKP2	1.88754864	1.13E-58	3.04E-58
NADK2	1.93865873	6.18E-08	7.80E-08
NADK2-AS1	NA	8.75E-11	1.17E-10
RANBP3L	NA	3.85E-11	5.17E-11
SLC1A3	0.62418936	1.13E-16	1.68E-16
CTD-2282P23	NA	NA	NA
CTD-2353F22	NA	1.09E-36	2.17E-36
CTD-2653M2	NA	NA	NA
NIPBL	1.92363813	2.08E-18	3.18E-18
C5orf42	1.49805447	6.03E-11	8.08E-11
NUP155	1.94747082	3.94E-09	5.11E-09
WDR70	1.52480545	1.08E-29	1.94E-29
GDNF	NA	NA	NA
GDNF-AS1	NA	NA	NA
CTD-2194L12	NA	NA	NA
CTD-2194L12	NA	NA	NA
CTD-2130F23	NA	NA	NA
CTD-2130F23	NA	NA	NA
CTD-2116N24	NA	NA	NA
CTD-2207A17	NA	NA	NA
EGFLAM	NA	NA	NA
EGFLAM-AS4	NA	NA	NA
EGFLAM-AS3	NA	NA	NA
EGFLAM-AS2	NA	NA	NA
EGFLAM-AS1	NA	NA	NA
CTD-2263F21	NA	NA	NA
LIFR	NA	1.17E-136	2.37E-135
LIFR-AS1	0	6.00E-08	7.58E-08
CTD-2196P11	NA	NA	NA
LINC01265	NA	NA	NA
OSMR-AS1	NA	NA	NA
RP11-122C5.	NA	NA	NA
OSMR	NA	0.21812387	0.22891209
RICTOR	1.49805447	5.16E-16	7.58E-16
FYB	2.24708171	3.79E-42	8.09E-42
C9	NA	NA	NA
DAB2	1.5421149	0.01063967	0.01177196
CTD-2078B5.	Inf	5.51E-95	2.69E-94
LINC00603	NA	NA	NA

LINC00604	NA	NA	NA
PTGER4	NA	1.50E-42	3.21E-42
TTC33	0.54928664	3.20E-84	1.28E-83
PRKAA1	1.69516691	1.63E-57	4.31E-57
AC008810.1	NA	NA	NA
RPL37	2.61746652	2.30E-144	1.32E-142
CARD6	2.7464332	4.45E-91	2.02E-90
C7	NA	NA	NA
MROH2B	NA	NA	NA
C6	NA	NA	NA
PLCXD3	NA	NA	NA
OXCT1	1.73346304	1.60E-37	3.20E-37
C5orf51	1.49805447	3.98E-39	8.15E-39
FBXO4	1.49805447	0.00113432	0.00129346
RP11-112L7.1	NA	NA	NA
GHR	Inf	2.15E-30	3.89E-30
CCDC152	NA	5.31E-92	2.45E-91
SEPP1	NA	NA	NA
CTD-2325A15	NA	NA	NA
CTD-2201E18	Inf	4.93E-48	1.14E-47
CTD-2035E11	0.74902724	5.76E-08	7.29E-08
CTD-2035E11	NA	2.15E-46	4.86E-46
ANXA2R	2.43433852	9.78E-15	1.41E-14
AC025171.1	Inf	9.66E-50	2.29E-49
CTD-2035E11	Inf	6.95E-79	2.54E-78
ZNF131	2.15105258	0.00073585	0.00084279
NIM1K	NA	1.33E-55	3.43E-55
CTD-2636A23	NA	5.30E-16	7.78E-16
HMGCS1	1.32684825	2.68E-26	4.59E-26
CCL28	NA	NA	NA
C5orf28	1.38570039	0.15463131	0.16338505
C5orf34	2.24708171	1.26E-14	1.80E-14
RP11-159F24	NA	1.56E-76	5.50E-76
RP11-159F24	NA	3.53E-87	1.48E-86
PAIP1	1.74400372	4.87E-95	2.38E-94
NNT-AS1	2.01167315	0.00053147	0.00061117
NNT	1.74773022	3.93E-15	5.69E-15
RP11-8L21.1	NA	NA	NA
FGF10	NA	NA	NA
FGF10-AS1	NA	NA	NA
RP11-473L15	NA	NA	NA
RP11-503D12	NA	NA	NA
RP11-53O19.	Inf	7.84E-135	1.40E-133
RP11-53O19.	NA	NA	NA
MRPS30	1.24837873	3.19E-133	5.17E-132

RP11-53O19.	3.2457847	6.47E-47	1.47E-46
RP11-357F12	NA	NA	NA
HCN1	NA	NA	NA
CTD-2013M1	NA	NA	NA
EMB	1.84375935	0.02524465	0.02756144
RP11-269M2	NA	NA	NA
PARP8	1.70233463	0.00019273	0.00022434
RP11-317O2	NA	NA	NA
RP11-317O2	NA	NA	NA
CTD-2089N3.	NA	NA	NA
CTD-2089N3.	NA	NA	NA
CTD-2089N3.	NA	NA	NA
CTD-2314G2	Inf	2.96E-42	6.33E-42
ISL1	1.74773022	4.02E-08	5.09E-08
CTD-2335O3.	NA	NA	NA
CTD-2203A3.	NA	NA	NA
CTD-2288O8.	NA	NA	NA
ITGA1	0	9.73E-31	1.77E-30
PELO	1.00955845	2.45E-141	8.22E-140
CTD-2175A2	NA	NA	NA
ITGA2	3.37062257	5.40E-07	6.68E-07
CTD-2366F13	0	4.04E-17	6.03E-17
MOCS2	1.89931907	6.39E-23	1.04E-22
CTD-2366F13	0.74902724	0.08542283	0.09138702
FST	3.74513619	1.34E-05	1.61E-05
NDUFS4	1.37279908	1.09E-131	1.62E-130
CTD-2081C1(	NA	NA	NA
CTD-2081C1(	NA	1.54E-79	5.68E-79
ARL15	0.74902724	3.69E-75	1.27E-74
LINC01033	Inf	1.96E-125	2.13E-124
HSPB3	NA	NA	NA
SNX18	1.77893969	1.44E-11	1.95E-11
RP11-45H22.	NA	NA	NA
ESM1	NA	NA	NA
GZMK	NA	NA	NA
CTD-2313F11	NA	NA	NA
CTD-2313F11	NA	NA	NA
GZMA	NA	NA	NA
CDC20B	NA	NA	NA
GPX8	1.31079767	4.41E-14	6.26E-14
MCIDAS	0	2.51E-109	1.66E-108
CCNO	1.81343436	3.68E-68	1.15E-67
RP11-506H2(	Inf	0.28750855	0.29959679
DHX29	1.51131159	3.64E-83	1.43E-82
SKIV2L2	1.8053477	0.03406735	0.03700232

PLPP1	0.0956205	1.80E-145	1.58E-143
SLC38A9	1.2733463	1.17E-79	4.34E-79
DDX4	NA	NA	NA
IL31RA	NA	NA	NA
AC008914.1	NA	NA	NA
IL6ST	1.22568093	6.00E-09	7.74E-09
CTD-2031P19	NA	NA	NA
FLJ31104	0	0.07418576	0.07958596
CTD-2227I18	NA	NA	NA
ANKRD55	1.74773022	3.55E-107	2.22E-106
RP11-155L15	NA	NA	NA
CTC-236F12.4	NA	NA	NA
C5orf67	NA	NA	NA
AC008940.1	NA	NA	NA
MAP3K1	1.49805447	4.81E-47	1.10E-46
AC008937.2	NA	NA	NA
AC008937.3	NA	NA	NA
SETD9	1.93865873	1.05E-06	1.30E-06
MIER3	2.43433852	7.60E-10	9.99E-10
CTD-2310F14	Inf	3.39E-157	1.43E-154
AC016644.1	NA	NA	NA
GPBP1	1.65667201	5.48E-30	9.89E-30
CTD-2023N9.	NA	NA	NA
ACTBL2	NA	NA	NA
CTD-2023N9.	NA	NA	NA
CTD-2023N9.	NA	NA	NA
RP11-772C9.	NA	NA	NA
RP11-478P10	NA	NA	NA
CTD-2037L6.1	NA	NA	NA
PLK2	0.87386511	5.85E-30	1.05E-29
GAPT	0.70960475	2.77E-132	4.23E-131
CTD-2117L12	NA	NA	NA
RAB3C	0	2.75E-145	2.09E-143
RP11-479O10	NA	NA	NA
CTD-2176I21	NA	NA	NA
PDE4D	2.24708171	4.97E-16	7.30E-16
RP11-266N13	Inf	4.29E-24	7.10E-24
CTD-2254N19	NA	NA	NA
AC109486.1	NA	NA	NA
PART1	NA	0.01534287	0.01688302
DEPDC1B	1.61998914	7.82E-58	2.08E-57
ELOVL7	1.49805447	0.00572707	0.00639309
RP11-231G3.	0.74902724	0.00114979	0.00131077
ERCC8	1.59168288	5.41E-12	7.41E-12
AC104113.3	NA	NA	NA

NDUFAF2	1.75833421	0.00028949	0.00033565
SMIM15	NA	NA	NA
CTC-436P18.:	NA	NA	NA
CTC-436P18.:	NA	NA	NA
ZSWIM6	2.99610895	3.31E-07	4.11E-07
RP11-2O17.2	NA	NA	NA
CTD-2170G1.	NA	NA	NA
CTD-2244C2(	NA	NA	NA
KIF2A	1.87937743	2.72E-21	4.33E-21
DIMT1	1.34075875	2.60E-55	6.69E-55
IPO11	2.45514483	3.90E-59	1.06E-58
LRRC70	NA	NA	NA
HTR1A	NA	NA	NA
RNF180	NA	NA	NA
RGS7BP	NA	NA	NA
FAM159B	NA	NA	NA
SREK1IP1	1.36429961	2.82E-67	8.66E-67
CWC27	1.30623043	1.82E-71	5.94E-71
RP11-307L14	NA	2.90E-73	9.75E-73
RP11-307L14	0.74902724	3.22E-142	1.21E-140
ADAMTS6	2.62159533	3.55E-110	2.40E-109
CTD-2116N2(	0	6.19E-78	2.23E-77
CENPK	1.15390683	5.57E-81	2.11E-80
PPWD1	2.03307393	0.00326251	0.00366924
TRIM23	2.24708171	0.3254167	0.33805026
TRAPPC13	1.12354086	2.32E-92	1.08E-91
CTC-534A2.2	1.19844358	2.30E-50	5.51E-50
SGTB	1.27775235	6.45E-21	1.02E-20
NLN	2.38975357	6.83E-09	8.80E-09
CTD-2033C11	Inf	2.40E-24	3.99E-24
ERBB2IP	1.95579334	1.51E-49	3.57E-49
SREK1	1.09203971	4.68E-126	5.25E-125
AC025442.3	NA	NA	NA
CTD-2016O1	NA	NA	NA
RP11-305P14	NA	NA	NA
RP11-5P22.3	NA	NA	NA
MAST4	2.49675746	1.59E-05	1.90E-05
MAST4-AS1	1.71206226	4.52E-22	7.28E-22
CD180	Inf	4.67E-13	6.50E-13
CTD-2187J20	NA	NA	NA
RP11-434D9.	NA	NA	NA
RP11-83M16	NA	1.06E-91	4.87E-91
RP11-83M16	NA	NA	NA
RP11-434D9.	NA	NA	NA
RP11-404L6.:	NA	NA	NA

PIK3R1	1.49805447	0.7167905	0.72700918
CTD-2582M2	NA	NA	NA
CTC-537E7.3	NA	NA	NA
CTC-537E7.1	NA	NA	NA
CTC-537E7.2	NA	NA	NA
CTC-340D7.1	NA	NA	NA
RP11-141O1	NA	NA	NA
RP11-141O1	NA	NA	NA
CTC-498J12.1	NA	NA	NA
SLC30A5	2.04812135	1.11E-74	3.80E-74
CTC-498J12.3	Inf	3.42E-60	9.44E-60
CCNB1	0.71850262	9.94E-139	2.42E-137
CENPH	1.13444902	4.71E-96	2.35E-95
MRPS36	1.89384728	8.99E-08	1.13E-07
CDK7	0.74902724	1.84E-105	1.11E-104
CCDC125	1.61328943	3.00E-23	4.92E-23
AK6	1.54499624	3.41E-22	5.50E-22
TAF9	1.6822415	4.92E-21	7.80E-21
RAD17	0.83891051	1.55E-68	4.85E-68
MARVELD2	NA	1.52E-13	2.14E-13
RP11-241G9.	NA	NA	NA
OCLN	1.91418072	3.69E-19	5.69E-19
GTF2H2C	2.24708171	3.89E-07	4.82E-07
SERF1B	NA	1.76E-12	2.43E-12
SMN2	1.49805447	8.73E-83	3.41E-82
RP11-497H1(	1.49805447	7.59E-16	1.11E-15
SERF1A	1.74773022	0.03699768	0.04012746
SMN1	0.99870298	1.92E-33	3.63E-33
NAIP	2.99610895	1.72E-53	4.31E-53
GTF2H2	1.41095828	0.18414543	0.19393744
RP11-136K7.	NA	NA	NA
RP11-136K7.	NA	NA	NA
RP11-136K7.	NA	NA	NA
BDP1	2.86915518	1.22E-07	1.53E-07
MCCC2	1.87256809	0.03387052	0.03679515
CARTPT	NA	NA	NA
MAP1B	NA	1.63E-26	2.80E-26
MRPS27	1.59300159	0.00047084	0.00054259
PTCD2	1.59792477	0.02340792	0.0255777
ZNF366	NA	NA	NA
CTC-347C20.:	NA	4.79E-31	8.75E-31
CTC-347C20.	NA	NA	NA
CTD-2631K1(	NA	1.90E-24	3.16E-24
TNPO1	2.53617994	1.44E-118	1.21E-117
CTD-2376I4.1	0	9.21E-12	1.26E-11

CTD-2376I4.2	NA	3.70E-39	7.59E-39
FCHO2	1.49805447	6.12E-30	1.10E-29
TMEM171	NA	4.04E-88	1.74E-87
RP11-232L2.1	NA	NA	NA
TMEM174	NA	NA	NA
RP11-60A8.1	NA	NA	NA
RP11-60A8.2	NA	NA	NA
FOXD1	1.68531128	0.14544564	0.15390346
FOXD1-AS1	NA	NA	NA
LINC01385	NA	NA	NA
LINC01386	NA	NA	NA
RP11-79P5.9	2.69649805	1.84E-83	7.28E-83
BTF3	1.53478275	6.96E-137	1.43E-135
ANKRA2	2.46108949	5.69E-11	7.62E-11
UTP15	1.66828794	2.70E-10	3.57E-10
ARHGEF28	NA	NA	NA
CTC-575I10.1	NA	NA	NA
RP11-428C6.	NA	NA	NA
CTD-2292M1	NA	NA	NA
LINC01335	NA	NA	NA
CTD-2275D24	NA	NA	NA
LINC01333	NA	NA	NA
LINC01332	NA	NA	NA
LINC01331	NA	NA	NA
ENC1	NA	4.35E-69	1.38E-68
HEXB	1.10512215	9.98E-140	2.74E-138
GFM2	1.10382961	1.03E-09	1.36E-09
NSA2	1.73881323	1.04E-63	3.02E-63
FAM169A	2.0598249	1.51E-36	2.98E-36
CTD-2377O17	NA	0.02164078	0.0236767
CTD-2503O10	NA	NA	NA
GCNT4	Inf	3.40E-156	1.21E-153
LINC01336	NA	NA	NA
ANKRD31	Inf	1.17E-37	2.36E-37
CTD-2235C13	NA	NA	NA
HMGCR	1.90377756	3.66E-22	5.91E-22
CTD-2235C13	Inf	8.00E-130	1.07E-128
COL4A3BP	1.22840467	1.74E-120	1.58E-119
POLK	1.7183566	1.00E-59	2.74E-59
CTC-366B18.	NA	7.82E-89	3.41E-88
CTC-366B18.	Inf	1.89E-36	3.73E-36
ANKDD1B	Inf	2.44E-136	4.79E-135
POC5	0.78844972	6.01E-121	5.52E-120
CTC-235G5.3	NA	NA	NA
SV2C	NA	NA	NA

AC113404.1	NA	6.61E-155	1.84E-152
RP11-466P24	NA	NA	NA
IQGAP2	1.32101167	5.40E-56	1.40E-55
CTD-2236F14	NA	NA	NA
F2RL2	NA	7.97E-25	1.34E-24
CTD-2384B11	NA	NA	NA
F2R	3.32900994	1.29E-133	2.12E-132
F2RL1	2.43433852	2.69E-41	5.68E-41
S100Z	0.92526894	2.46E-25	4.16E-25
CRHBP	0	3.72E-84	1.49E-83
AGGF1	1.99740597	3.29E-98	1.72E-97
ZBED3	2.76563903	6.52E-50	1.55E-49
CTC-564N23.	NA	NA	NA
ZBED3-AS1	2.99610895	3.12E-07	3.88E-07
PDE8B	Inf	3.77E-142	1.39E-140
WDR41	1.47222595	1.46E-92	6.83E-92
OTP	NA	1.68E-137	3.62E-136
TBCA	1.84867936	3.24E-24	5.38E-24
RP11-107N7.	NA	NA	NA
AP3B1	1.45124027	2.14E-23	3.52E-23
SCAMP1-AS1	2.08063121	4.60E-29	8.19E-29
SCAMP1	2.49675746	2.50E-06	3.05E-06
LHFPL2	1.08192823	0.04551423	0.0492144
ARSB	2.56809339	2.67E-45	5.96E-45
DMGDH	0	5.50E-30	9.92E-30
BHMT2	NA	NA	NA
BHMT	NA	NA	NA
JMY	1.32520204	2.34E-26	4.01E-26
HOMER1	2.51459144	2.48E-07	3.09E-07
PAPD4	1.90661479	2.56E-37	5.12E-37
CMYA5	Inf	4.72E-83	1.85E-82
CTC-431G16.	NA	NA	NA
LINC01455	NA	NA	NA
MTX3	NA	7.88E-117	6.32E-116
THBS4	NA	NA	NA
CTD-2201I18	NA	0.0091276	0.0101181
SERINC5	1.72852439	1.05E-38	2.13E-38
CTC-458I2.2	NA	NA	NA
SPZ1	NA	NA	NA
ZFYVE16	2.8463035	2.79E-60	7.71E-60
CTD-2015H6.	1.49805447	9.87E-36	1.93E-35
FAM151B	0.74902724	1.57E-10	2.08E-10
ANKRD34B	NA	NA	NA
LINC01337	NA	2.43E-07	3.03E-07
DHFR	1.98403048	2.23E-07	2.78E-07

CTC-325J23.2	NA	0.00075478	0.00086436
MSH3	1.55567195	7.74E-29	1.37E-28
RASGRF2-AS	NA	NA	NA
RASGRF2	NA	NA	NA
CTD-2193P3.	NA	NA	NA
CKMT2-AS1	1.14850843	0.02310813	0.0252562
CKMT2	NA	NA	NA
ZCCHC9	1.44973014	2.32E-08	2.96E-08
ACOT12	0	3.37E-66	1.02E-65
SSBP2	2.33696498	2.95E-14	4.20E-14
CTD-2249K22	Inf	0.02477267	0.02705103
ATG10	1.41162826	2.04E-12	2.82E-12
ATG10-AS1	NA	NA	NA
RPS23	2.36664549	1.11E-140	3.40E-139
ATP6AP1L	0.74902724	1.47E-17	2.21E-17
CTD-2015A6.	NA	NA	NA
CTD-2015A6.	NA	NA	NA
CTD-2218K11	NA	NA	NA
LINC01338	NA	NA	NA
RP11-78C3.1	NA	NA	NA
CTD-2227C6.	NA	NA	NA
CTD-2227C6.	NA	NA	NA
RP11-343L5.1	1.49805447	2.98E-13	4.17E-13
TMEM167A	2.36545659	2.05E-130	2.83E-129
XRCC4	1.40442607	2.14E-88	9.25E-88
VCAN	7.77115759	1.95E-134	3.37E-133
VCAN-AS1	NA	NA	NA
HAPLN1	NA	NA	NA
EDIL3	NA	3.74E-17	5.59E-17
CTD-2269F5.	NA	2.31E-09	3.01E-09
CTC-384G19.	NA	NA	NA
RP11-1072N1	NA	NA	NA
CTD-2331D1	NA	NA	NA
CTD-2331D1	NA	NA	NA
CTD-2331D1	NA	NA	NA
CTC-480C2.1	NA	NA	NA
CTD-2036A18	NA	NA	NA
CTC-261N6.3	NA	NA	NA
CTC-261N6.1	NA	NA	NA
CTC-261N6.2	NA	NA	NA
COX7C	2.0249398	8.97E-119	7.63E-118
CTC-493L21.1	NA	NA	NA
CTC-493L21.2	NA	NA	NA
RP11-72L22.1	NA	NA	NA
CTC-242N15.	NA	NA	NA

RASA1	3.18336576	5.31E-80	1.97E-79
CTC-428H11.	3.74513619	6.10E-63	1.75E-62
CCNH	0.72384985	1.28E-110	8.73E-110
CTD-2316B1.	NA	NA	NA
CTD-2316B1.	NA	NA	NA
TMEM161B	1.71206226	5.28E-12	7.24E-12
TMEM161B-/	1.78339818	9.63E-14	1.36E-13
CTC-498M16	NA	NA	NA
CTC-498M16	NA	NA	NA
LINC00461	NA	NA	NA
CTC-467M3.1	NA	NA	NA
CTC-467M3.3	NA	NA	NA
CTC-467M3.2	NA	NA	NA
MEF2C	2.17109344	2.88E-20	4.51E-20
MEF2C-AS1	1.93498703	2.28E-58	6.09E-58
CTD-2232E5.	NA	NA	NA
RP11-116A1.	NA	NA	NA
AC113167.1	NA	NA	NA
AC113167.2	NA	NA	NA
RP11-61G23.	NA	6.37E-118	5.29E-117
CETN3	1.12354086	2.23E-102	1.26E-101
MBLAC2	1.99740597	0.0680524	0.0730969
POLR3G	2.32390502	1.80E-77	6.44E-77
LYSMD3	2.78210117	5.91E-35	1.14E-34
ADGRV1	NA	NA	NA
RP11-414H23	NA	NA	NA
RP11-414H23	NA	NA	NA
RP11-213H15	NA	NA	NA
LUCAT1	NA	0.44274034	0.45564983
RP11-213H15	NA	NA	NA
CTD-2061E19	NA	NA	NA
ARRDC3	13.4824903	2.41E-56	6.28E-56
ARRDC3-AS1	Inf	0.53954529	0.5529562
RP11-348J24	NA	NA	NA
RP11-348J24	NA	NA	NA
RP11-133F8.	NA	NA	NA
CTC-529L17.1	NA	NA	NA
CTC-529L17.2	NA	NA	NA
CTC-458G6.4	NA	NA	NA
CTC-458G6.2	NA	NA	NA
CTD-2091N2	NA	NA	NA
NR2F1-AS1	1.32520204	8.07E-54	2.03E-53
NR2F1	1.29377432	2.71E-51	6.58E-51
FAM172A	2.0598249	5.68E-16	8.34E-16
POU5F2	2.24708171	9.75E-09	1.25E-08

RP11-185E12	NA	NA	NA
KIAA0825	Inf	1.79E-80	6.71E-80
SLF1	0.84758345	1.63E-89	7.18E-89
MCTP1	0	1.07E-125	1.18E-124
CTC-484P3.3	NA	NA	NA
CTC-558O19.	NA	NA	NA
FAM81B	NA	NA	NA
TTC37	2.0598249	1.07E-12	1.48E-12
ARSK	1.39105058	1.68E-36	3.32E-36
GPR150	NA	NA	NA
RFESD	1.74773022	1.55E-09	2.03E-09
SPATA9	NA	1.54E-19	2.39E-19
CTD-2154I11	0	8.48E-98	4.38E-97
RHOBTB3	2.08657588	0.001887	0.00213509
GLRX	2.35137664	0.00455041	0.0050955
AC008592.4	NA	NA	NA
AC008592.8	NA	1.89E-61	5.30E-61
LINC01554	NA	2.80E-84	1.12E-83
ELL2	3.74513619	8.80E-08	1.11E-07
CTD-2337A12	NA	NA	NA
AC008592.7	NA	NA	NA
RP11-254I22	NA	NA	NA
RP11-254I22	NA	NA	NA
RP11-254I22	NA	NA	NA
PCSK1	NA	NA	NA
CAST	1.59904691	6.13E-18	9.27E-18
CTC-506B8.1	NA	NA	NA
ERAP1	1.93498703	4.56E-75	1.57E-74
CTD-2260A17	NA	1.64E-59	4.46E-59
CTD-2260A17	NA	NA	NA
CTD-2260A17	Inf	2.01E-13	2.81E-13
ERAP2	2.80885214	0.00313083	0.00352245
LNPEP	2.99610895	2.66E-46	6.00E-46
CTD-2215E18	NA	NA	NA
LIX1	NA	NA	NA
RIOK2	0.90953307	1.76E-126	2.02E-125
CTD-2215E18	NA	NA	NA
CTD-2215E18	NA	NA	NA
LINC01340	NA	NA	NA
RP11-455B3.	NA	NA	NA
AC008834.1	NA	NA	NA
RGMB	0.74902724	3.29E-77	1.17E-76
RGMB-AS1	NA	NA	NA
AC008522.1	NA	2.82E-31	5.18E-31
CHD1	1.97877345	4.80E-16	7.05E-16

CTD-2007H1:	NA	8.68E-109	5.65E-108
CTD-2151A2.	NA	NA	NA
RP11-346J10	NA	NA	NA
CTD-2001C1:	NA	9.55E-41	2.00E-40
FAM174A	1.37978702	6.76E-68	2.10E-67
CTC-293G12.	NA	NA	NA
ST8SIA4	1.49805447	7.73E-10	1.02E-09
CTD-2333K2.	NA	NA	NA
SLCO4C1	0.37451362	7.72E-90	3.42E-89
SLCO6A1	NA	NA	NA
RP11-58B2.1	NA	NA	NA
LINC00492	NA	NA	NA
CTD-2340E1.	NA	NA	NA
LINC00491	NA	NA	NA
PAM	2.88910506	1.81E-18	2.76E-18
GIN1	1.24837873	1.52E-06	1.86E-06
PPIP5K2	1.61328943	3.63E-24	6.03E-24
CTC-503K11.:	NA	NA	NA
C5orf30	1.34824903	1.85E-08	2.37E-08
CTD-2154H6.	NA	NA	NA
CTD-2193G5.	NA	NA	NA
NUDT12	2.03307393	7.51E-31	1.37E-30
RP11-138J23	NA	NA	NA
RP11-6N13.1	NA	NA	NA
RP11-6N13.4	NA	NA	NA
CTD-2374C24	NA	NA	NA
CTC-254B4.1	NA	NA	NA
EFNA5	NA	1.89E-61	5.30E-61
RP11-252I13	NA	NA	NA
FBXL17	0.62418936	0.00194924	0.0022051
LINC01023	1.49805447	4.10E-95	2.01E-94
FER	3.74513619	3.92E-79	1.44E-78
CTD-2197I11	NA	NA	NA
CTD-2587M2	NA	2.39E-121	2.23E-120
PJA2	2.52796693	5.63E-39	1.15E-38
RP11-231G1!	NA	NA	NA
RP11-231G1!	NA	NA	NA
RP11-231G1!	NA	NA	NA
CTC-332L22.:	Inf	2.09E-138	4.86E-137
MAN2A1	1.15758755	0.01788727	0.0196352
AC011366.3	NA	NA	NA
TMEM232	NA	1.59E-80	5.96E-80
SLC25A46	1.45525292	7.47E-08	9.42E-08
CTC-551A13.	NA	NA	NA
TSLP	1.71206226	1.29E-74	4.42E-74

CTC-551A13.	NA	NA	NA
WDR36	2.04280156	0.00029893	0.00034647
CAMK4	NA	1.57E-10	2.09E-10
AC010468.2	NA	NA	NA
CTC-499J9.1	NA	NA	NA
STARD4	1.62289235	0.03137852	0.03414726
STARD4-AS1	5.24319066	1.25E-114	9.46E-114
NREP	1.49805447	6.92E-22	1.11E-21
NREP-AS1	NA	NA	NA
EPB41L4A	Inf	0.90843308	0.91270937
EPB41L4A-A!	2.57098538	1.33E-129	1.75E-128
CTC-459M5.1	NA	NA	NA
CTC-459M5.2	NA	NA	NA
RP11-526F3.	NA	NA	NA
EPB41L4A-A!	NA	NA	NA
RP11-159K7.	NA	NA	NA
APC	1.7655642	6.73E-62	1.91E-61
CTC-554D6.1	NA	NA	NA
CTC-487M23	NA	2.46E-10	3.26E-10
SRP19	1.55779284	3.39E-25	5.71E-25
REEP5	1.14557107	1.25E-136	2.52E-135
ZRSR1	NA	4.40E-10	5.81E-10
CTC-487M23	NA	1.20E-41	2.54E-41
CTC-487M23	NA	NA	NA
DCP2	1.78339818	0.00035449	0.00040991
MCC	NA	1.64E-58	4.40E-58
CTD-2201G3.	NA	NA	NA
TSSK1B	NA	NA	NA
YTHDC2	2.06874189	1.11E-18	1.69E-18
RP11-371M2	NA	NA	NA
KCNN2	NA	NA	NA
RP11-492A1(	NA	NA	NA
CTC-313D10.	NA	NA	NA
RP11-438C1!	NA	NA	NA
RP11-438C1!	NA	NA	NA
TRIM36	2.0598249	6.30E-54	1.59E-53
CTC-428G20.	NA	NA	NA
PGGT1B	1.74773022	7.20E-26	1.22E-25
CTC-428G20.	1.49805447	2.44E-30	4.43E-30
CCDC112	1.77626459	5.23E-07	6.48E-07
FEM1C	1.60505837	0.0002266	0.00026321
TMED7-TICA	NA	3.65E-49	8.58E-49
TICAM2	NA	NA	NA
AC010226.4	1.49805447	4.76E-06	5.77E-06
TMED7	2.24708171	0.6971076	0.70783713

CTC-505O3.3	NA	NA	NA
CTC-505O3.2	NA	NA	NA
CDO1	1.27775235	6.13E-20	9.54E-20
ATG12	1.41482923	4.20E-60	1.16E-59
AP3S1	2.01923077	4.97E-69	1.57E-68
AC026449.1	NA	NA	NA
LVRN	NA	1.34E-05	1.60E-05
CTD-2287O1(	NA	NA	NA
ARL14EPL	NA	NA	NA
CTD-2287O1(	NA	NA	NA
CTD-2287O1(	1.49805447	3.11E-77	1.11E-76
COMMD10	0.96101608	5.49E-74	1.86E-73
CTC-339F2.2	NA	NA	NA
SEMA6A	0	6.05E-77	2.14E-76
SEMA6A-AS1	NA	1.96E-125	2.13E-124
CTB-118N6.2	NA	NA	NA
RP11-249M1	NA	NA	NA
CTC-472C24.	NA	NA	NA
RP11-492L8.:	NA	NA	NA
LINC00992	NA	NA	NA
CTD-3179P9.	NA	NA	NA
HNCAT21	NA	NA	NA
CTD-2281M2	NA	NA	NA
RP11-2N5.2	NA	NA	NA
RP11-2N5.1	NA	NA	NA
CTC-448D22.	NA	NA	NA
DTWD2	0.24967575	1.46E-36	2.90E-36
CTB-161M19	NA	NA	NA
DMXL1	2.99610895	5.78E-16	8.48E-16
TNFAIP8	0.35110652	1.66E-141	5.72E-140
HSD17B4	0.74902724	9.43E-142	3.34E-140
FAM170A	NA	NA	NA
CTC-552D5.1	NA	NA	NA
RP11-574H6.	NA	NA	NA
PRR16	NA	1.02E-07	1.28E-07
CTD-2334D1!	NA	NA	NA
CTC-546K23.	1.87256809	1.39E-27	2.42E-27
FTMT	NA	NA	NA
SRFBP1	1.34824903	1.77E-39	3.64E-39
LOX	0	1.11E-42	2.39E-42
CTC-441N14.	NA	NA	NA
ZNF474	NA	4.09E-09	5.30E-09
CTC-441N14.	NA	NA	NA
CTD-2544H1	NA	NA	NA
SNCAIP	NA	NA	NA

CTD-2544H1.	NA	NA	NA
CTD-2280E9.	NA	NA	NA
MGC32805	NA	NA	NA
RP11-166A1.	NA	NA	NA
SNX2	0.71094111	3.46E-139	8.89E-138
SNX24	1.31079767	1.08E-46	2.47E-46
PPIC	0.84349914	7.06E-144	3.65E-142
RP11-359P5.	NA	7.54E-79	2.75E-78
AC106786.1	Inf	1.35E-129	1.78E-128
PRDM6	NA	4.85E-114	3.61E-113
CEP120	2.39688716	1.77E-36	3.49E-36
CSNK1G3	1.94066148	2.81E-42	6.01E-42
LINC01170	NA	NA	NA
CTC-369A16.	NA	NA	NA
CTD-2308B1	NA	NA	NA
CTD-2308B1	NA	NA	NA
CTD-2308B1	NA	NA	NA
CTD-2308B1	NA	NA	NA
ZNF608	NA	NA	NA
RP11-43D2.2	NA	NA	NA
RP11-284A2	NA	NA	NA
RP11-284A2	NA	NA	NA
RP11-284A2	NA	NA	NA
RP11-257I8.2	NA	NA	NA
RP11-508M8	NA	NA	NA
RP11-395P1	NA	NA	NA
RP11-395P1	NA	NA	NA
RP11-756H2	NA	NA	NA
RP11-114J13	NA	NA	NA
CTC-339D2.1	NA	NA	NA
GRAMD3	Inf	1.59E-112	1.14E-111
RP11-517I3.1	NA	NA	NA
ALDH7A1	NA	NA	NA
PHAX	1.0830529	2.05E-77	7.31E-77
TEX43	NA	NA	NA
RP11-434D1.	NA	1.15E-85	4.70E-85
LMNB1	1.45399405	1.06E-33	2.01E-33
3-Mar	1-Jan	6.80E-31	1.24E-30
C5orf63	NA	NA	NA
MEGF10	0.37451362	9.46E-41	1.98E-40
CTD-2195M1	NA	NA	NA
PRRC1	1.49805447	0.24398343	0.25541532
CTD-2195M1	NA	NA	NA
CTXN3	NA	NA	NA
CTC-548H10.	NA	NA	NA

LINC01183	NA	NA	NA
CTC-228N24.	NA	NA	NA
LINC01184	2.7464332	3.48E-12	4.79E-12
SLC12A2	1.45644185	1.44E-22	2.33E-22
FBN2	1.09473212	0.0229205	0.02505264
SLC27A6	NA	0.0091276	0.0101181
ISOC1	1.39912635	1.77E-92	8.25E-92
ADAMTS19-/	NA	NA	NA
ADAMTS19	NA	NA	NA
CTC-575N7.1	NA	NA	NA
KIAA1024L	Inf	1.06E-91	4.87E-91
CHSY3	NA	3.33E-76	1.16E-75
HINT1	1.86780549	1.44E-21	2.30E-21
LYRM7	2.18466278	1.36E-49	3.23E-49
CDC42SE2	2.12224384	3.08E-88	1.33E-87
RAPGEF6	1.74773022	7.57E-73	2.52E-72
CTC-432M15	NA	NA	NA
FNIP1	3.02285992	2.48E-139	6.44E-138
MEIKIN	NA	NA	NA
CTB-127M13	NA	NA	NA
AC034228.4	NA	NA	NA
ACSL6	Inf	2.04E-149	2.87E-147
AC034228.3	NA	NA	NA
AC034228.2	NA	NA	NA
IL3	NA	NA	NA
CSF2	NA	NA	NA
AC063976.3	NA	NA	NA
P4HA2-AS1	NA	NA	NA
P4HA2	0.74902724	1.30E-42	2.79E-42
PDLIM4	NA	NA	NA
SLC22A4	1.49805447	7.09E-41	1.49E-40
SLC22A5	1.06112192	8.21E-55	2.10E-54
C5orf56	3.74513619	1.39E-53	3.49E-53
AC116366.5	NA	1.60E-23	2.63E-23
AC116366.6	NA	NA	NA
IRF1	2.17217899	0.83681419	0.84383765
RP11-89G4.1	NA	NA	NA
IL5	NA	NA	NA
RAD50	1.8053477	3.62E-58	9.68E-58
TH2LCRR	NA	NA	NA
IL13	NA	NA	NA
IL4	NA	NA	NA
AC004237.1	NA	NA	NA
KIF3A	2.03307393	0.83843663	0.84542671
CCNI2	NA	6.43E-129	8.23E-128

8-Sep	1-Jan	2.27E-90	1.01E-89
SOWAHA	NA	NA	NA
AC004775.5	NA	5.76E-32	1.06E-31
SHROOM1	2.24708171	2.88E-08	3.66E-08
GDF9	NA	1.64E-59	4.46E-59
UQCRQ	2.15533946	1.10E-87	4.68E-87
LEAP2	1.12354086	1.34E-16	1.98E-16
AFF4	2.66320796	1.10E-29	1.98E-29
CTC-350I8.1	NA	2.43E-148	2.94E-146
ZCCHC10	1.68531128	1.10E-21	1.75E-21
HSPA4	1.62451362	3.88E-37	7.73E-37
RP11-485M7	NA	NA	NA
CTB-49A3.2	NA	NA	NA
FSTL4	NA	NA	NA
CTB-49A3.5	NA	NA	NA
CTB-49A3.4	NA	NA	NA
CTB-3M24.3	NA	NA	NA
WSPAR	NA	NA	NA
C5orf15	1.20266345	6.21E-53	1.55E-52
VDAC1	1.29426186	3.45E-91	1.57E-90
CTB-113I20.2	Inf	8.98E-56	2.32E-55
TCF7	1.97957198	7.32E-05	8.62E-05
SKP1	1.19728678	6.31E-127	7.38E-126
CTD-2410N1	NA	NA	NA
PPP2CA	1.02174998	2.74E-141	9.16E-140
CTD-2410N1	NA	3.33E-76	1.16E-75
CDKL3	3.74513619	6.53E-57	1.71E-56
UBE2B	1.54119217	1.12E-117	9.24E-117
RP11-215P8.	Inf	1.06E-98	5.60E-98
CDKN2AIPNL	1.8624461	3.28E-08	4.17E-08
RP11-215P8.	NA	NA	NA
AC005355.1	NA	NA	NA
AC005355.2	NA	NA	NA
JADE2	2.02677958	0.14273768	0.1510909
SAR1B	1.14360409	3.02E-35	5.86E-35
SEC24A	2.57998271	1.27E-17	1.91E-17
CAMLG	1.80819857	1.62E-90	7.27E-90
DDX46	1.599697	1.51E-38	3.07E-38
C5orf24	2.02861543	1.04E-21	1.67E-21
TXNDC15	1.57903039	6.55E-35	1.27E-34
PCBD2	2.12514705	4.82E-50	1.15E-49
CATSPER3	1.12354086	1.58E-25	2.67E-25
PITX1	1.40804262	2.90E-108	1.86E-107
C5orf66	0	2.26E-22	3.66E-22
CTC-276P9.4	NA	3.67E-65	1.09E-64

C5orf66-AS1	NA	NA	NA
CTC-276P9.2	NA	NA	NA
CTC-276P9.3	NA	NA	NA
C5orf66-AS2	NA	NA	NA
H2AFY	1.17441818	6.33E-100	3.40E-99
CTC-203F4.2	0	1.28E-07	1.60E-07
DCANP1	NA	NA	NA
TIFAB	NA	NA	NA
CTB-138E5.1	NA	5.68E-94	2.72E-93
NEUROG1	NA	NA	NA
CTC-321K16.1	NA	5.69E-11	7.62E-11
CXCL14	NA	NA	NA
CTC-321K16.4	NA	NA	NA
RP11-119J18	NA	NA	NA
SLC25A48	Inf	7.38E-148	8.47E-146
IL9	NA	NA	NA
GS1-39E22.2	NA	NA	NA
GS1-39E22.1	NA	NA	NA
LECT2	NA	NA	NA
TGFBI	NA	NA	NA
SMAD5-AS1	NA	2.49E-05	2.97E-05
SMAD5	2.53242542	4.69E-125	5.03E-124
AC009014.3	NA	NA	NA
TRPC7	NA	NA	NA
TRPC7-AS1	NA	NA	NA
TRPC7-AS2	NA	NA	NA
AC063980.3	NA	NA	NA
RP11-114H2	NA	NA	NA
CTB-1I21.1	NA	NA	NA
SPOCK1	NA	1.03E-98	5.44E-98
KLHL3	NA	2.22E-84	8.93E-84
HNRNPA0	1.42603262	1.47E-117	1.21E-116
RP11-381K2(	NA	NA	NA
RP11-381K2(	4.24448768	8.87E-125	9.42E-124
MYOT	NA	NA	NA
PKD2L2	NA	1.51E-53	3.80E-53
FAM13B	3.89494163	1.39E-58	3.73E-58
RP11-325L7.	NA	0.34229471	0.35503462
RP11-325L7.	NA	NA	NA
WNT8A	NA	NA	NA
NME5	NA	NA	NA
BRD8	1.22209707	2.75E-68	8.59E-68
KIF20A	1.54798962	1.99E-57	5.26E-57
CDC23	1.4381323	1.01E-31	1.85E-31
GFRA3	NA	NA	NA

CDC25C	1.49805447	5.22E-27	9.02E-27
FAM53C	1.79372312	0.0003623	0.00041886
RP11-256P1.	NA	NA	NA
KDM3B	2.15043304	1.54E-14	2.20E-14
REEP2	Inf	6.96E-58	1.85E-57
EGR1	1.49805447	0.31477992	0.32735668
ETF1	1.95490797	5.20E-16	7.63E-16
HSPA9	1.38243282	3.89E-58	1.04E-57
CTNNA1	1.71206226	2.10E-05	2.51E-05
AC034243.1	NA	NA	NA
LRRTM2	NA	2.92E-130	3.97E-129
SIL1	0.59538062	2.76E-141	9.18E-140
CTB-46B19.2	NA	NA	NA
MATR3	Inf	4.59E-13	6.39E-13
MATR3	2.24708171	1.34E-50	3.22E-50
PAIP2	1.63537613	1.03E-40	2.16E-40
CTB-43P18.1	Inf	2.04E-85	8.32E-85
SLC23A1	NA	2.93E-90	1.30E-89
MZB1	0.83225249	4.36E-21	6.91E-21
PROB1	2.24708171	0.86938471	0.87541641
SPATA24	1.59168288	3.22E-38	6.51E-38
DNAJC18	1.31079767	1.96E-30	3.56E-30
ECSCR	NA	NA	NA
ECSCR	Inf	3.97E-41	8.35E-41
TMEM173	0.84265564	1.69E-140	5.05E-139
UBE2D2	1.86239109	0.6234406	0.63559761
CTD-3224K1!	Inf	2.67E-40	5.56E-40
CXXC5	1.90881135	1.67E-09	2.18E-09
CTD-3224K1!	NA	NA	NA
CTB-35F21.1	NA	2.01E-138	4.69E-137
CTB-35F21.2	NA	NA	NA
CTB-35F21.3	NA	NA	NA
CTB-35F21.5	NA	NA	NA
PSD2	NA	NA	NA
NRG2	1.79766537	0.00039557	0.00045683
CTB-35F21.4	NA	NA	NA
LINC01024	0.74902724	7.16E-06	8.65E-06
PURA	1.97339868	2.14E-08	2.72E-08
IGIP	0.74902724	9.64E-93	4.52E-92
CYSTM1	1.63507165	3.09E-29	5.51E-29
PFDN1	1.50890994	1.17E-14	1.69E-14
AC011380.1	NA	NA	NA
HBEGF	1.63424125	0.25443222	0.26604665
CTC-329D1.3	NA	NA	NA
SLC4A9	NA	NA	NA

CTC-329D1.2	Inf	0.00017234	0.0002008
ANKHD1	1.87256809	0.88186546	0.88709749
ANKHD1-EIF4	0.74902724	1.31E-78	4.78E-78
SRA1	1.42023346	2.06E-61	5.79E-61
EIF4EBP3	Inf	6.55E-143	2.80E-141
APBB3	NA	NA	NA
SLC35A4	NA	NA	NA
CD14	NA	1.70E-100	9.21E-100
NDUFA2	2.02469012	2.57E-46	5.81E-46
TMCO6	2.20302129	2.00E-84	8.03E-84
IK	1.19163424	6.96E-135	1.24E-133
WDR55	2.27964811	0.28680682	0.2988999
DND1	0.37451362	1.25E-53	3.13E-53
HARS	0.88521401	3.13E-115	2.40E-114
HARS2	1.49805447	8.33E-22	1.33E-21
ZMAT2	1.43327374	6.28E-129	8.04E-128
PCDHA1	NA	NA	NA
AC005609.16	NA	NA	NA
PCDHA2	NA	NA	NA
PCDHA3	NA	0.17298661	0.18242882
PCDHA4	NA	NA	NA
PCDHA5	0	3.01E-137	6.36E-136
PCDHA6	NA	3.05E-50	7.28E-50
PCDHA7	NA	2.34E-18	3.56E-18
PCDHA8	NA	NA	NA
PCDHA9	Inf	8.62E-70	2.75E-69
AC005609.19	NA	NA	NA
PCDHA10	NA	1.95E-64	5.73E-64
AC005609.20	NA	NA	NA
PCDHA11	NA	NA	NA
PCDHA12	NA	NA	NA
AC005609.18	NA	NA	NA
AC005609.17	NA	NA	NA
PCDHA13	NA	NA	NA
PCDHAC1	NA	1.30E-67	4.02E-67
CTC-270D5.2	NA	NA	NA
PCDHAC2	NA	NA	NA
CTC-270D5.1	NA	NA	NA
PCDHB1	NA	NA	NA
CH17-140K24	NA	NA	NA
PCDHB2	NA	NA	NA
AC005754.7	NA	NA	NA
PCDHB3	NA	NA	NA
AC005754.8	NA	NA	NA
PCDHB4	NA	NA	NA

PCDHB5	NA	NA	NA
CH17-140K24	NA	NA	NA
PCDHB6	NA	NA	NA
CH17-140K24	NA	NA	NA
PCDHB7	NA	NA	NA
PCDHB8	NA	NA	NA
PCDHB16	0	7.24E-65	2.15E-64
CH17-140K24	NA	NA	NA
PCDHB10	NA	1.30E-67	4.02E-67
CH17-140K24	NA	NA	NA
PCDHB9	NA	NA	NA
CH17-140K24	NA	NA	NA
PCDHB11	NA	NA	NA
PCDHB12	NA	NA	NA
PCDHB13	NA	1.22E-58	3.26E-58
PCDHB14	NA	NA	NA
CH17-140K24	NA	NA	NA
PCDHB15	NA	NA	NA
TAF7	1.99373426	3.18E-52	7.85E-52
SLC25A2	NA	NA	NA
AC005618.6	Inf	3.35E-107	2.10E-106
PCDHGA1	NA	NA	NA
PCDHGA2	NA	NA	NA
PCDHGA3	NA	NA	NA
PCDHGB1	NA	1.22E-58	3.26E-58
AC005618.8	NA	NA	NA
PCDHGA4	NA	NA	NA
PCDHGB2	NA	NA	NA
PCDHGA5	NA	1.91E-119	1.67E-118
PCDHGB3	NA	NA	NA
PCDHGA6	0.74902724	0.33416817	0.34686342
PCDHGA7	NA	NA	NA
PCDHGB4	NA	NA	NA
PCDHGA8	NA	3.26E-17	4.88E-17
PCDHGB5	NA	NA	NA
PCDHGA9	NA	NA	NA
PCDHGB6	NA	NA	NA
PCDHGA10	Inf	7.23E-22	1.16E-21
PCDHGB7	NA	8.73E-51	2.10E-50
PCDHGA11	0	5.32E-113	3.88E-112
AC005618.9	NA	NA	NA
PCDHGA12	NA	NA	NA
PCDHGC3	NA	NA	NA
PCDHGC4	NA	NA	NA
PCDHGC5	NA	NA	NA

DIAPH1	2.17630748	4.21E-108	2.70E-107
CTD-2024I7.1	NA	NA	NA
AC008781.7	NA	NA	NA
HDAC3	2.09056856	7.11E-12	9.71E-12
RELL2	2.24708171	0.31756171	0.33017391
FCHSD1	1.12354086	0.00602534	0.00671944
ARAP3	0	4.68E-30	8.44E-30
PCDH1	NA	0.35771936	0.37054612
KIAA0141	1.64785992	5.67E-09	7.33E-09
PCDH12	NA	2.85E-61	8.01E-61
AC005740.6	NA	2.94E-109	1.94E-108
RNF14	1.77042802	2.95E-05	3.50E-05
AC005740.5	NA	NA	NA
GNPDA1	0.24967575	1.85E-94	8.93E-94
NDFIP1	1.52529183	4.48E-123	4.46E-122
SPRY4	NA	0.38801892	0.40076511
SPRY4-IT1	Inf	3.10E-152	5.51E-150
AC005592.2	NA	NA	NA
FGF1	NA	NA	NA
AC005592.3	NA	NA	NA
AC005592.1	NA	NA	NA
ARHGAP26	0.74902724	6.78E-53	1.69E-52
ARHGAP26-A	NA	NA	NA
NR3C1	1.66000631	0.05639351	0.06075002
CTD-2050E21	NA	NA	NA
CTD-2050E21	NA	NA	NA
HMHB1	NA	NA	NA
YIPF5	1.72276265	3.47E-80	1.30E-79
KCTD16	NA	2.28E-71	7.43E-71
CTC-367F4.1	NA	NA	NA
CTC-806A22.	NA	1.37E-83	5.42E-83
CTB-73N10.1	NA	NA	NA
PRELID2	2.29389591	3.09E-31	5.67E-31
GRXCR2	NA	NA	NA
SH3RF2	NA	NA	NA
PLAC8L1	2.24708171	3.75E-05	4.44E-05
RP11-118M9	NA	NA	NA
LARS	1.66370473	6.82E-81	2.58E-80
RP11-449H3.	NA	1.76E-12	2.43E-12
RBM27	2.68072906	1.52E-97	7.83E-97
CTC-359M8.1	NA	NA	NA
POU4F3	NA	NA	NA
CTB-1H10.1	NA	NA	NA
TCERG1	1.40872095	5.12E-106	3.12E-105
GPR151	NA	NA	NA

CTB-99A3.1	NA	NA	NA
PPP2R2B	NA	3.31E-154	8.11E-152
CTC-255N20.	NA	NA	NA
STK32A	NA	NA	NA
DPYSL3	NA	1.19E-07	1.49E-07
CTB-108O6.2	NA	NA	NA
JAKMIP2-AS1	NA	NA	NA
JAKMIP2	NA	NA	NA
SPINK1	NA	NA	NA
SCGB3A2	NA	NA	NA
C5orf46	NA	NA	NA
CTC-327F10.!	NA	NA	NA
CTC-327F10.4	NA	NA	NA
SPINK5	NA	NA	NA
SPINK14	NA	NA	NA
SPINK6	NA	NA	NA
CTC-295J13.3	NA	NA	NA
SPINK13	NA	NA	NA
RP11-373N2	NA	1.01E-11	1.37E-11
SPINK7	NA	NA	NA
SPINK9	NA	NA	NA
FBXO38	2.24708171	2.84E-34	5.44E-34
CTD-2283N1!	NA	4.07E-06	4.94E-06
HTR4	NA	NA	NA
ADRB2	Inf	5.96E-66	1.79E-65
SH3TC2	Inf	0.19290473	0.2030211
RP11-44B19.	NA	NA	NA
CTC-529P8.1	NA	1.24E-11	1.69E-11
ABLIM3	NA	6.07E-05	7.16E-05
RP11-331K21	NA	NA	NA
AC012613.2	NA	NA	NA
AFAP1L1	NA	9.11E-49	2.13E-48
GRPEL2	2.48115272	1.55E-50	3.71E-50
GRPEL2-AS1	NA	NA	NA
PCYOX1L	4.74383917	0.00048713	0.00056083
IL17B	NA	NA	NA
RP11-394O4.	NA	8.50E-11	1.14E-10
CARMN	NA	NA	NA
RP11-394O4.	NA	NA	NA
RP11-394O4.	NA	NA	NA
CSNK1A1	1.71368353	1.86E-21	2.96E-21
ARHGEF37	5.24319066	2.93E-73	9.83E-73
PPARGC1B	3.6770428	1.09E-89	4.82E-89
PDE6A	0.74902724	6.03E-16	8.83E-16
SLC26A2	0.96749351	1.33E-44	2.94E-44

TIGD6	2.24708171	7.98E-17	1.18E-16
HMGXB3	1.64785992	7.05E-05	8.31E-05
CSF1R	NA	2.87E-61	8.06E-61
PDGFRB	NA	3.05E-146	3.18E-144
CDX1	NA	NA	NA
SLC6A7	NA	NA	NA
CAMK2A	NA	NA	NA
ARSI	NA	NA	NA
TCOF1	1.63121487	1.59E-08	2.03E-08
CD74	1.44072153	3.75E-39	7.69E-39
RPS14	2.12212311	1.86E-119	1.63E-118
CTC-367J11.1	NA	2.99E-07	3.72E-07
NDST1	2.3151751	9.32E-88	3.98E-87
SYNPO	1.49805447	6.27E-78	2.26E-77
CTB-140J7.2	NA	NA	NA
MYOZ3	1.49805447	3.71E-10	4.91E-10
CTC-345K18.:	NA	NA	NA
RBM22	1.56614786	3.20E-50	7.65E-50
DCTN4	2.84006161	7.92E-137	1.62E-135
SMIM3	2.32929202	1.63E-90	7.30E-90
IRGM	2.99610895	4.12E-137	8.61E-136
ZNF300	NA	1.61E-102	9.08E-102
AC008641.1	NA	NA	NA
GPX3	NA	NA	NA
TNIP1	1.76636274	2.88E-05	3.42E-05
ANXA6	0.59922179	5.85E-130	7.87E-129
CTB-17P3.4	NA	3.07E-72	1.01E-71
CCDC69	1.49805447	1.13E-25	1.91E-25
GM2A	2.46108949	1.78E-10	2.37E-10
SLC36A3	NA	NA	NA
SLC36A2	NA	NA	NA
SLC36A1	1.68531128	0.45182933	0.46484563
FAT2	NA	NA	NA
CTC-251D13.	Inf	1.07E-155	3.41E-153
CTB-113P19.	NA	NA	NA
SPARC	1.05331955	7.41E-105	4.42E-104
CTB-113P19.	NA	NA	NA
CTB-113P19.	Inf	2.79E-124	2.92E-123
ATOX1	1.96007128	2.85E-63	8.21E-63
CTB-113P19.	NA	NA	NA
RP11-54C4.3	NA	1.45E-19	2.24E-19
G3BP1	1.36952317	1.16E-91	5.34E-91
GLRA1	NA	NA	NA
CTB-12O2.1	NA	NA	NA
NMUR2	NA	NA	NA

LINC01470	NA	NA	NA
GRIA1	NA	NA	NA
AC091962.3	NA	NA	NA
RP11-461L18	NA	NA	NA
FAM114A2	2.62159533	0.15410554	0.16284848
MFAP3	2.62159533	1.16E-44	2.55E-44
GALNT10	1.70233463	1.41E-23	2.33E-23
SAP30L-AS1	NA	1.58E-109	1.05E-108
SAP30L	1.95664258	4.08E-94	1.96E-93
HAND1	NA	NA	NA
CTB-158E9.1	NA	NA	NA
LARP1	2.10466104	4.09E-116	3.22E-115
FAXDC2	NA	4.06E-116	3.20E-115
CNOT8	1.51218706	5.98E-46	1.34E-45
GEMIN5	2.02237354	4.67E-07	5.79E-07
MRPL22	1.40480209	3.97E-81	1.51E-80
KIF4B	NA	0.46372044	0.47670083
SGCD	NA	NA	NA
AC016577.1	NA	NA	NA
CTC-340I23.2	NA	NA	NA
TIMD4	NA	NA	NA
HAVCR1	NA	NA	NA
HAVCR2	NA	8.92E-45	1.97E-44
CTB-120L21.	NA	NA	NA
MED7	1.26758456	5.14E-88	2.21E-87
ITK	NA	1.62E-79	5.97E-79
FAM71B	NA	NA	NA
CTB-4E7.1	NA	NA	NA
CTC-248O19.	NA	NA	NA
CYFIP2	1.76241703	0.07276693	0.07807307
FNDC9	NA	NA	NA
CTB-47B11.3	NA	NA	NA
ADAM19	NA	5.39E-67	1.65E-66
NIPAL4	NA	NA	NA
AC106801.1	NA	NA	NA
SOX30	NA	NA	NA
C5orf52	NA	NA	NA
AC008694.3	NA	NA	NA
THG1L	1.41095828	2.03E-62	5.77E-62
LSM11	2.3151751	2.48E-72	8.21E-72
CLINT1	1.60104572	2.51E-08	3.20E-08
CTC-436K13.	NA	NA	NA
CTC-436K13.	NA	NA	NA
CTC-436K13.	NA	NA	NA
CTC-436K13.	NA	NA	NA

CTC-436K13.1	NA	NA	NA
RP11-524N5.	NA	NA	NA
RP11-542A14	NA	NA	NA
RP11-542A14	NA	NA	NA
RP11-32D16.	NA	NA	NA
EBF1	Inf	3.10E-152	5.51E-150
CTD-2363C16	NA	NA	NA
CTD-2363C16	NA	NA	NA
RP11-175K6.	NA	8.66E-69	2.72E-68
RNF145	2.00803047	3.88E-65	1.15E-64
CTB-11I22.1	NA	2.26E-39	4.64E-39
CTB-11I22.2	NA	NA	NA
UBLCP1	Inf	2.53E-111	1.76E-110
AC008697.1	NA	0.00110931	0.0012651
IL12B	NA	NA	NA
AC008703.1	NA	NA	NA
AC008691.1	NA	NA	NA
AC011343.1	NA	NA	NA
ADRA1B	NA	NA	NA
TTC1	1.52146158	2.28E-63	6.58E-63
PWWP2A	1.8385214	3.18E-55	8.16E-55
FABP6	NA	NA	NA
CTB-127C13.	NA	NA	NA
CCNJL	1.49805447	2.62E-72	8.68E-72
C1QTNF2	NA	0.19684326	0.20708208
ZBED8	3.74513619	1.11E-08	1.43E-08
SLU7	1.24306648	2.06E-88	8.88E-88
PTTG1	1.29457857	1.24E-141	4.34E-140
MIR3142HG	3.74513619	1.33E-88	5.77E-88
ATP10B	NA	1.18E-37	2.37E-37
CTC-348L5.1	NA	NA	NA
CTC-529G1.1	NA	NA	NA
RP11-109J4.1	NA	NA	NA
GABRB2	0.74902724	4.24E-31	7.76E-31
GABRA6	NA	NA	NA
RP11-348M1	NA	NA	NA
GABRA1	NA	NA	NA
LINC01202	NA	NA	NA
GABRG2	NA	NA	NA
RP11-96G10.	NA	NA	NA
RP11-167P20	NA	NA	NA
RP11-541P9.	NA	1.56E-76	5.50E-76
CCNG1	1.83923442	4.48E-18	6.79E-18
NUDCD2	1.84035187	4.60E-06	5.58E-06
HMMR	0.93037067	2.37E-127	2.83E-126

HMMR-AS1	NA	NA	NA
MAT2B	1.52183312	3.10E-51	7.52E-51
CTC-207P7.1	NA	NA	NA
CTB-32P11.1	NA	NA	NA
CTC-340A15.	0	6.16E-16	9.02E-16
CTD-2313P7.	NA	NA	NA
CTB-181F24.	NA	NA	NA
CTC-535M15	NA	NA	NA
RP11-67M9.:	NA	NA	NA
CTB-7E3.1	NA	NA	NA
CTC-264O10.	NA	NA	NA
CTC-264O10.	NA	NA	NA
TENM2	NA	NA	NA
CTB-180C19.	NA	NA	NA
CTC-286N12.	NA	NA	NA
CTB-105L4.2	NA	NA	NA
CTB-78F1.1	NA	NA	NA
CTC-353G13.	NA	NA	NA
CTB-77H17.1	NA	NA	NA
CTB-178M22	NA	NA	NA
CTB-178M22	Inf	1.76E-77	6.28E-77
WWC1	2.46108949	3.62E-100	1.95E-99
RARS	1.19602736	2.80E-101	1.55E-100
FBLL1	Inf	8.44E-50	2.01E-49
PANK3	1.80085272	3.49E-12	4.80E-12
CTC-558O2.2	NA	NA	NA
SLIT3	NA	NA	NA
CTC-558O2.1	NA	NA	NA
CTB-174D11.	NA	NA	NA
CTB-174D11.	NA	NA	NA
SPDL1	1.66450497	6.13E-12	8.38E-12
DOCK2	1.67429618	0.67610743	0.68732158
CTB-37A13.1	NA	NA	NA
FAM196B	NA	NA	NA
FOXI1	NA	NA	NA
LINC01187	NA	NA	NA
LCP2	3.19584955	2.10E-24	3.50E-24
CTB-114C7.4	NA	NA	NA
LINC01366	NA	1.69E-13	2.37E-13
KCNIP1	NA	NA	NA
KCNMB1	Inf	6.62E-11	8.86E-11
CTD-2270F17	NA	NA	NA
CTB-147C13.	NA	NA	NA
CTC-265N9.1	NA	NA	NA
CTC-455F18.:	NA	NA	NA

GABRP	NA	NA	NA
RANBP17	NA	NA	NA
RP11-546B8.	NA	NA	NA
TLX3	NA	NA	NA
NPM1	1.04904016	3.65E-107	2.29E-106
FGF18	NA	NA	NA
CTB-78H18.1	NA	NA	NA
SMIM23	NA	NA	NA
FBXW11	2.93369001	1.64E-78	5.94E-78
STK10	2.60868107	4.29E-18	6.50E-18
EFCAB9	0.74902724	1.84E-63	5.31E-63
UBTD2	2.28453307	2.95E-45	6.58E-45
SH3PXD2B	2.99610895	9.34E-53	2.32E-52
CTB-54I1.1	NA	NA	NA
NEURL1B	0.37451362	0.92523571	0.92876812
CTB-79E8.2	NA	NA	NA
RP11-779O1	NA	NA	NA
RP11-779O1	NA	NA	NA
DUSP1	2.99610895	0.17250797	0.18195579
RP11-536N1	NA	NA	NA
ERGIC1	2.93369001	2.70E-94	1.30E-93
CTC-308K20.	Inf	5.11E-117	4.13E-116
CTC-308K20.	NA	NA	NA
RPL26L1	1.95807349	5.54E-43	1.19E-42
ATP6V0E1	1.78075185	4.95E-24	8.20E-24
CREBRF	4.49416342	1.43E-75	4.97E-75
CTC-209H22.	NA	NA	NA
BNIP1	1.35934573	7.62E-45	1.69E-44
NKX2-5	NA	NA	NA
STC2	1.52926394	4.01E-26	6.84E-26
CTB-32H22.1	NA	NA	NA
CTB-164N12.	NA	NA	NA
CTB-33O18.1	NA	NA	NA
CTB-33O18.2	NA	NA	NA
CTB-33O18.3	NA	NA	NA
BOD1	1.93865873	1.14E-13	1.61E-13
CTB-43E15.1	NA	NA	NA
CTB-43E15.2	NA	NA	NA
LINC01484	NA	NA	NA
LINC01485	NA	NA	NA
CPEB4	1.12354086	0.27993407	0.29192182
C5orf47	NA	NA	NA
HMP19	NA	NA	NA
RP11-619L12	NA	NA	NA
RP11-619L12	NA	NA	NA

CTC-430J12.2	NA	NA	NA
LINC01411	NA	1.46E-82	5.68E-82
RP11-267A15	NA	NA	NA
MSX2	Inf	2.77E-22	4.48E-22
CTD-2532K18	NA	NA	NA
CTD-2532K18	NA	NA	NA
CTC-281M20	NA	NA	NA
CTC-281M20	NA	NA	NA
DRD1	NA	NA	NA
SFXN1	1.84760052	0.82083094	0.82831878
HRH2	0.95875486	7.64E-49	1.79E-48
CPLX2	NA	NA	NA
THOC3	1.35241029	3.97E-95	1.95E-94
RP11-91H12.	NA	NA	NA
RP11-826N14	NA	NA	NA
RP11-826N14	NA	NA	NA
FAM153B	NA	NA	NA
RP11-844P9.	NA	NA	NA
RP11-826N14	NA	NA	NA
RP11-844P9.	NA	7.61E-110	5.11E-109
SIMC1	3.59533074	1.49E-08	1.90E-08
KIAA1191	2.24708171	2.44E-10	3.23E-10
RP11-843P14	NA	NA	NA
RP11-843P14	NA	5.50E-20	8.57E-20
ARL10	0	2.54E-52	6.27E-52
NOP16	1.49953185	3.01E-44	6.59E-44
HIGD2A	1.7674091	1.24E-37	2.48E-37
CLTB	1.77620783	2.00E-26	3.42E-26
FAF2	1.63424125	7.18E-26	1.22E-25
RNF44	2.18466278	1.60E-18	2.44E-18
CDHR2	NA	1.23E-33	2.32E-33
GPRIN1	2.46108949	1.85E-10	2.46E-10
SNCB	NA	8.46E-31	1.54E-30
EIF4E1B	NA	NA	NA
TSPAN17	2.79899652	3.00E-77	1.07E-76
RP11-375B1.	NA	NA	NA
RP11-375B1.	NA	NA	NA
LINC01574	NA	NA	NA
UNC5A	Inf	1.17E-20	1.84E-20
HK3	NA	NA	NA
UIMC1	1.07469125	5.78E-07	7.15E-07
ZNF346	1.08949416	1.24E-27	2.17E-27
FGFR4	1.83095547	0.01772478	0.01946389
NSD1	2.31221452	3.44E-27	5.96E-27
RAB24	3.74513619	1.57E-88	6.80E-88

MXD3	1.49805447	2.59E-42	5.54E-42
PRELID1	2.40746269	4.01E-127	4.72E-126
LMAN2	1.47529922	4.50E-131	6.43E-130
RGS14	2.59278659	1.89E-09	2.46E-09
SLC34A1	NA	3.31E-14	4.70E-14
PFN3	NA	NA	NA
F12	1.76179646	6.52E-39	1.33E-38
GRK6	1.51023378	3.37E-29	6.02E-29
PRR7-AS1	0.74902724	0.00226405	0.00255549
PRR7	2.0598249	0.40479083	0.41761247
DBN1	1.49805447	3.17E-29	5.66E-29
PDLIM7	1.27612048	3.65E-126	4.11E-125
RP11-1334A2	NA	NA	NA
DOK3	2.99610895	4.17E-82	1.61E-81
DDX41	1.2443517	4.52E-94	2.17E-93
FAM193B	2.62159533	5.48E-11	7.36E-11
RP11-1277A3	Inf	4.80E-131	6.84E-130
TMED9	1.20824593	1.24E-142	5.02E-141
B4GALT7	2.06311011	8.18E-12	1.12E-11
RP11-1277A3	0.74902724	0.04710651	0.05090962
RP11-1101H1	NA	NA	NA
FAM153A	NA	NA	NA
RP11-1026M	NA	NA	NA
RP11-1026M	NA	NA	NA
RP11-423H2.	NA	NA	NA
RP11-1252I4	NA	NA	NA
RP11-423H2.	2.99610895	1.20E-33	2.27E-33
RP11-423H2.	NA	0.10268633	0.10944924
PROP1	NA	NA	NA
FAM153C	NA	NA	NA
N4BP3	1.49805447	1.79E-06	2.19E-06
RMND5B	1.74773022	0.00010662	0.0001249
NHP2	1.94778978	8.57E-09	1.10E-08
HNRNPAB	1.81565671	2.86E-07	3.57E-07
PHYKPL	2.13184675	0.01599824	0.01758924
COL23A1	NA	1.30E-118	1.10E-117
RP11-1259L2	NA	NA	NA
CLK4	3.29571984	2.68E-05	3.19E-05
ZNF354A	2.99610895	1.41E-11	1.91E-11
ZNF354B	1.87256809	8.50E-10	1.12E-09
ZFP2	NA	9.23E-23	1.50E-22
RP11-281O1	NA	NA	NA
ZNF454	NA	1.23E-07	1.55E-07
RP11-281O1	NA	NA	NA
GRM6	NA	1.31E-142	5.18E-141

ZNF879	NA	NA	NA
ZNF354C	NA	5.50E-20	8.57E-20
ADAMTS2	1.59168288	1.01E-15	1.47E-15
RP11-443C1(	NA	NA	NA
RP11-443C1(	NA	NA	NA
RP11-798K2:	NA	NA	NA
RP11-798K2:	NA	2.42E-68	7.56E-68
RUFY1	1.23877582	6.17E-24	1.02E-23
RP11-1379J2	NA	1.86E-62	5.31E-62
HNRNPH1	1.80003384	1.59E-08	2.03E-08
RP11-1379J2	0	4.34E-42	9.25E-42
CBY3	NA	NA	NA
CANX	2.04711516	7.27E-119	6.21E-118
MAML1	2.29389591	4.26E-71	1.38E-70
LTC4S	NA	4.28E-58	1.14E-57
MGAT4B	2.24708171	1.92E-88	8.30E-88
SQSTM1	1.7426348	3.69E-23	6.03E-23
C5orf45	1.19844358	2.75E-12	3.78E-12
CTC-241N9.1	0.37451362	0.12269825	0.13042665
TBC1D9B	2.47595115	3.01E-105	1.81E-104
RNF130	2.30700389	8.71E-53	2.17E-52
CTC-563A5.2	NA	NA	NA
RASGEF1C	0.74902724	5.47E-32	1.01E-31
MAPK9	2.24708171	1.95E-77	6.96E-77
CTB-129O4.1	1.99740597	2.35E-27	4.07E-27
GFPT2	Inf	0.95020716	0.9527253
CTC-573N18.	NA	NA	NA
CNOT6	1.84916099	1.09E-82	4.26E-82
SCGB3A1	NA	NA	NA
FLT4	NA	9.12E-118	7.52E-117
RP11-451H2:	NA	NA	NA
OR2Y1	NA	NA	NA
MGAT1	1.50753583	1.54E-17	2.31E-17
LINC00847	NA	NA	NA
ZFP62	2.17898833	1.51E-20	2.38E-20
BTNL8	NA	NA	NA
BTNL3	NA	NA	NA
BTNL9	1.49805447	4.71E-88	2.02E-87
OR2V1	NA	NA	NA
OR2V2	NA	NA	NA
CTC-338M12	NA	6.06E-40	1.25E-39
CTC-338M12	5.61770428	1.36E-75	4.72E-75
TRIM7	2.11089494	4.04E-45	8.97E-45
CTC-338M12	0	2.58E-45	5.76E-45
CTC-338M12	NA	NA	NA

TRIM41	2.62159533	3.27E-40	6.80E-40
CTC-338M12	NA	NA	NA
GNB2L1	1.43180354	1.57E-108	1.02E-107
TRIM52	1.79766537	2.70E-06	3.29E-06
CTC-338M12	NA	NA	NA
AC138035.2	NA	NA	NA
AC138035.1	NA	NA	NA
OR4F3	NA	NA	NA
LINC00266-3	NA	NA	NA
RP3-416J7.4	NA	NA	NA
RP3-416J7.5	NA	NA	NA
DUSP22	1.84135863	0.03433131	0.03728456
IRF4	NA	2.25E-19	3.47E-19
EXOC2	3.06420233	4.97E-12	6.82E-12
RP1-20B11.2	NA	NA	NA
HUS1B	0.74902724	3.30E-06	4.01E-06
RP11-532F6.	NA	6.69E-49	1.57E-48
RP11-532F6.	NA	NA	NA
RP11-532F6.	NA	NA	NA
RP11-284J1.:	NA	NA	NA
RP5-1077H2:	NA	NA	NA
RP5-1077H2:	NA	NA	NA
RP5-856G1.1	NA	NA	NA
RP5-856G1.2	NA	NA	NA
FOXQ1	NA	NA	NA
LINC01394	NA	NA	NA
RP4-668J24.:	NA	NA	NA
FOXF2	NA	NA	NA
RP11-157J24	NA	NA	NA
FOXCUT	NA	NA	NA
FOXC1	2.16823674	9.30E-69	2.92E-68
GMDS	1.04031561	3.94E-140	1.12E-138
GMDS-AS1	9.73735409	1.04E-94	5.06E-94
RP1-136B1.1	NA	3.27E-66	9.89E-66
LINC01600	NA	NA	NA
RP11-145H9.	NA	NA	NA
MYLK4	NA	0.0003184	0.00036865
WRNIP1	2.09727626	6.78E-52	1.66E-51
SERPINB1	1.06873399	7.14E-87	2.99E-86
SERPINB9P1	NA	NA	NA
SERPINB9	NA	7.21E-40	1.49E-39
RP1-90J20.2	NA	NA	NA
SERPINB6	0.81756568	2.64E-142	1.00E-140
LINC01011	Inf	3.21E-51	7.77E-51
NQO2	2.16641724	8.42E-09	1.08E-08

RP1-90J20.8	3.74513619	1.00E-125	1.11E-124
RP1-40E16.2	NA	NA	NA
RIPK1	2.88910506	3.98E-10	5.26E-10
RP1-40E16.1	2.24708171	1.81E-11	2.45E-11
BPHL	0.80252918	6.21E-132	9.28E-131
RP1-40E16.1	NA	1.40E-124	1.48E-123
TUBB2A	0.94163424	3.83E-90	1.70E-89
RP1-40E16.9	NA	2.40E-11	3.23E-11
TUBB2B	Inf	1.22E-11	1.66E-11
PSMG4	1.96659492	5.13E-83	2.01E-82
SLC22A23	Inf	7.55E-28	1.32E-27
RP11-506K6.	NA	1.53E-150	2.36E-148
RP1-223B1.1	NA	NA	NA
PXDC1	NA	1.86E-31	3.41E-31
RP11-420L9.	NA	NA	NA
FAM50B	NA	NA	NA
RP1-140K8.1	NA	NA	NA
RP1-140K8.5	NA	NA	NA
RP3-406P24.	NA	5.78E-19	8.88E-19
RP3-406P24.	NA	NA	NA
PRPF4B	1.72601929	2.47E-13	3.46E-13
FAM217A	NA	NA	NA
C6orf201	NA	2.75E-05	3.27E-05
ECI2	1.70904806	1.07E-23	1.77E-23
RP3-400B16.	NA	NA	NA
RP3-400B16.	NA	NA	NA
RP3-400B16.	NA	NA	NA
RP3-527G5.1	NA	NA	NA
RP11-274H2	NA	7.75E-131	1.09E-129
RP4-529N6.2	NA	NA	NA
RP4-529N6.1	NA	NA	NA
CDYL	2.24708171	2.00E-13	2.80E-13
RP3-430A16.	NA	NA	NA
RPP40	0.67192149	6.88E-62	1.95E-61
RP11-428J1.	1.87256809	5.67E-64	1.66E-63
RP11-428J1.	Inf	2.28E-81	8.67E-81
PPP1R3G	NA	4.54E-73	1.52E-72
LYRM4	2.04170328	2.62E-114	1.97E-113
FARS2	1.07360571	9.66E-105	5.74E-104
RP1-232P20.	NA	NA	NA
RP1-256G22.	NA	NA	NA
RP3-380B8.4	NA	3.21E-82	1.24E-81
NRN1	1.51776572	5.07E-93	2.39E-92
F13A1	NA	NA	NA
LY86-AS1	0	1.72E-117	1.41E-116

LY86	1.52926394	3.35E-06	4.07E-06
RP1-80N2.3	Inf	3.56E-05	4.23E-05
RP1-80N2.2	Inf	1.34E-22	2.17E-22
RP3-470L22.1	NA	NA	NA
RP3-429O6.1	NA	NA	NA
RP11-556O1!	NA	NA	NA
RREB1	2.34922179	1.28E-10	1.70E-10
SSR1	1.91611619	1.44E-10	1.92E-10
RP11-69L16.4	NA	NA	NA
CAGE1	Inf	3.52E-09	4.56E-09
RIOK1	1.16731518	6.57E-25	1.10E-24
RP3-512B11.	NA	2.89E-10	3.82E-10
DSP	NA	7.31E-13	1.01E-12
SNRNP48	1.19844358	1.17E-128	1.48E-127
BMP6	2.24708171	0.00010684	0.00012516
TXNDC5	Inf	1.92E-129	2.51E-128
BLOC1S5-TXI	NA	NA	NA
BLOC1S5	1.56614786	1.04E-13	1.46E-13
EEF1E1-BLO(	NA	NA	NA
BLOC1S5	NA	NA	NA
EEF1E1	1.56165113	6.24E-34	1.19E-33
RP11-203H2.	NA	NA	NA
RP11-203H2.	NA	NA	NA
SLC35B3	2.71522374	3.99E-14	5.66E-14
HULC	NA	NA	NA
RP11-314C16	NA	NA	NA
RP11-354I10	NA	NA	NA
OFCC1	NA	NA	NA
TFAP2A	1.74773022	0.70827584	0.71865438
TFAP2A-AS1	Inf	1.29E-57	3.41E-57
RP1-290I10.3	NA	NA	NA
LINC00518	NA	NA	NA
MIR5689HG	NA	2.41E-25	4.07E-25
RP1-290I10.5	NA	NA	NA
GCNT2	1.49805447	7.71E-25	1.29E-24
RP11-360O1!	NA	1.90E-38	3.86E-38
C6orf52	1.79766537	7.78E-08	9.80E-08
PAK1IP1	1.09016836	5.98E-90	2.65E-89
TMEM14C	1.27040894	1.13E-110	7.74E-110
RP11-421M1	0.74902724	0.0563238	0.06067853
TMEM14B	1.98605707	1.04E-98	5.48E-98
RP11-637O1!	NA	NA	NA
MAK	0	0.02476712	0.0270466
GCM2	NA	NA	NA
RP11-637O1!	NA	NA	NA

SYCP2L	2.24708171	0.00308391	0.00347031
ELOVL2	2.15345331	0.10974254	0.11679862
ELOVL2-AS1	NA	NA	NA
SMIM13	1.66620345	0.01077528	0.01191983
ERVFRD-1	NA	4.77E-66	1.43E-65
RP3-510L9.1	NA	NA	NA
NEDD9	1.49805447	7.30E-41	1.53E-40
RP11-716O2	NA	NA	NA
RP11-716O2	NA	NA	NA
TMEM170B	1.56047341	1.10E-22	1.79E-22
RP11-679B1	NA	NA	NA
ADTRP	NA	NA	NA
RP3-413H6.2	NA	NA	NA
RP11-456H1	NA	NA	NA
RP11-456H1	NA	NA	NA
HIVEP1	0.56177043	3.37E-21	5.35E-21
EDN1	NA	NA	NA
RP11-125M1	NA	7.61E-110	5.11E-109
PHACTR1	1.53854243	1.81E-25	3.07E-25
RP1-257A7.4	Inf	4.96E-18	7.51E-18
TBC1D7	1.17239046	1.13E-97	5.83E-97
RP1-257A7.5	2.24708171	1.19E-05	1.43E-05
GFOD1	1.75244108	0.18116261	0.19087357
GFOD1-AS1	NA	NA	NA
SIRT5	1.60019455	2.60E-100	1.41E-99
RP1-223E5.4	2.80885214	1.33E-70	4.30E-70
NOL7	1.13424125	3.20E-129	4.15E-128
RANBP9	3.18336576	1.11E-71	3.64E-71
MCUR1	2.23061957	2.95E-106	1.81E-105
RP3-500L14.	NA	3.68E-82	1.42E-81
RNF182	NA	1.70E-100	9.21E-100
RP3-380E11.	NA	NA	NA
CD83	2.30058366	4.50E-05	5.33E-05
RP11-359N1	NA	NA	NA
LINC01108	NA	NA	NA
RP3-448I9.2	NA	NA	NA
RP3-448I9.1	NA	NA	NA
RP11-330A1	NA	NA	NA
RP11-146I2.	NA	NA	NA
RP11-560J1.	0.49935149	6.04E-51	1.46E-50
JARID2	1.4192095	1.76E-46	3.98E-46
JARID2-AS1	NA	0.16385858	0.17295336
DTNBP1	1.01563015	1.08E-128	1.36E-127
RP11-401E1	NA	NA	NA
MYLIP	0.95875486	5.67E-134	9.53E-133

GMPR	0.68876068	6.85E-130	9.18E-129
RP3-467D16.	NA	NA	NA
ATXN1	0.74902724	7.23E-17	1.07E-16
RP1-151F17.	NA	NA	NA
RP1-151F17.	NA	1.32E-73	4.47E-73
STMND1	NA	NA	NA
RBM24	NA	NA	NA
CAP2	NA	NA	NA
FAM8A1	1.29377432	6.05E-18	9.15E-18
NUP153	2.80885214	2.29E-119	2.00E-118
RP11-500C11	2.64656291	1.00E-73	3.39E-73
KIF13A	1.94747082	5.15E-58	1.37E-57
NHLRC1	NA	NA	NA
TPMT	2.10837296	0.14069724	0.14897452
KDM1B	1.60505837	1.62E-08	2.07E-08
DEK	1.56096106	1.03E-34	1.98E-34
RNF144B	0.74902724	1.20E-10	1.61E-10
RP11-686D16	NA	NA	NA
RP1-167F1.2	NA	NA	NA
RP3-471C18.	NA	NA	NA
RP3-471C18.	NA	NA	NA
RP4-625H18.	NA	NA	NA
ID4	NA	NA	NA
MBOAT1	NA	3.81E-26	6.51E-26
RP11-239H6.	NA	NA	NA
E2F3	1.85869722	1.63E-15	2.38E-15
CDKAL1	1.41482923	1.41E-05	1.69E-05
RP3-348I23.2	NA	NA	NA
RP1-135L22.	NA	NA	NA
LINC00581	NA	NA	NA
RP11-204E9.	NA	NA	NA
SOX4	2.79012646	1.35E-69	4.31E-69
CASC15	0	8.48E-157	3.42E-154
NBAT1	NA	NA	NA
RP11-524C21	NA	NA	NA
RP3-404K8.2	NA	NA	NA
PRL	NA	NA	NA
HDGFL1	NA	NA	NA
RP1-309H15.	NA	NA	NA
RP1-209A6.1	NA	NA	NA
RP11-439H9.	NA	NA	NA
NRSN1	NA	NA	NA
DCDC2	NA	NA	NA
KAAG1	NA	NA	NA
MRS2	2.39323337	1.29E-87	5.48E-87

GPLD1	Inf	1.26E-142	5.08E-141
ALDH5A1	2.20546909	1.22E-74	4.17E-74
KIAA0319	NA	3.65E-49	8.58E-49
TDP2	1.87256809	1.13E-65	3.38E-65
ACOT13	1.62289235	2.67E-09	3.47E-09
RP1-30M3.5	Inf	1.14E-48	2.66E-48
C6orf62	2.14536196	2.51E-66	7.60E-66
RP1-30M3.6	NA	2.14E-37	4.29E-37
RP3-369A17.	NA	NA	NA
GMNN	1.11171411	6.25E-116	4.90E-115
C6orf229	NA	NA	NA
RP3-369A17.	NA	NA	NA
FAM65B	NA	9.11E-07	1.12E-06
RP11-367G6.	Inf	3.08E-108	1.98E-107
RP3-425P12.	NA	NA	NA
RP3-425P12.	NA	NA	NA
RP3-425P12.	NA	NA	NA
RP3-522P13.	NA	NA	NA
LRRC16A	0	7.24E-72	2.38E-71
SCGN	NA	NA	NA
HIST1H2AA	NA	NA	NA
HIST1H2BA	NA	NA	NA
SLC17A4	NA	NA	NA
SLC17A1	NA	NA	NA
SLC17A3	NA	NA	NA
SLC17A2	NA	NA	NA
TRIM38	0.81712062	3.07E-82	1.19E-81
U91328.21	NA	NA	NA
U91328.19	3.74513619	8.98E-09	1.16E-08
U91328.22	Inf	7.29E-115	5.55E-114
HIST1H1A	NA	3.00E-94	1.44E-93
HIST1H3A	NA	1.31E-13	1.85E-13
HIST1H4A	Inf	4.58E-94	2.20E-93
HIST1H4B	NA	1.67E-89	7.34E-89
HIST1H3B	1.49805447	9.09E-42	1.93E-41
HIST1H2AB	Inf	7.80E-09	1.00E-08
HIST1H2BB	NA	NA	NA
HIST1H3C	NA	NA	NA
HIST1H1C	2.2811284	1.25E-51	3.06E-51
HFE	NA	NA	NA
HIST1H4C	2.5485195	2.83E-99	1.51E-98
HIST1H1T	NA	NA	NA
HIST1H2BC	4.49416342	8.55E-40	1.77E-39
HIST1H2AC	4.49416342	7.96E-10	1.05E-09
HIST1H1E	1.49805447	7.88E-76	2.74E-75

HIST1H2BD	2.62159533	1.96E-07	2.45E-07
RP1-221C16.	NA	NA	NA
HIST1H2BE	NA	0.01251177	0.01380961
HIST1H4D	Inf	0.22023831	0.23111774
RP1-34B20.2	1.49805447	1.19E-25	2.02E-25
HIST1H3D	NA	NA	NA
HIST1H2AD	NA	NA	NA
HIST1H2BF	0	3.44E-47	7.87E-47
HIST1H4E	1.49805447	0.00806539	0.00895979
HIST1H2BG	1.49805447	2.40E-48	5.58E-48
HIST1H2AE	0.74902724	3.65E-06	4.43E-06
HIST1H3E	NA	NA	NA
HIST1H1D	8.98832685	1.17E-117	9.63E-117
HIST1H4F	NA	NA	NA
HIST1H4G	NA	NA	NA
HIST1H3F	NA	NA	NA
HIST1H2BH	NA	NA	NA
HIST1H3G	0.74902724	0.12288783	0.13061287
HIST1H2BI	Inf	1.69E-87	7.18E-87
HIST1H4H	Inf	1.44E-19	2.23E-19
BTN3A2	2.08063121	1.67E-08	2.13E-08
BTN2A2	1.12354086	3.67E-80	1.37E-79
BTN3A1	4.49416342	6.48E-116	5.08E-115
BTN3A3	Inf	3.17E-141	1.05E-139
BTN2A1	1.42315175	5.02E-09	6.50E-09
BTN1A1	NA	2.22E-08	2.83E-08
HCG11	1.63849708	7.06E-15	1.02E-14
CTA-14H9.5	0.74902724	0.00197153	0.00222975
HMGN4	0.74902724	1.29E-25	2.18E-25
ABT1	1.49805447	3.53E-13	4.92E-13
RP11-457M1	NA	NA	NA
ZNF322	3.22081712	1.46E-92	6.83E-92
RP11-457M1	Inf	2.21E-50	5.30E-50
LINC00240	NA	0.99823134	0.9983414
RP11-239L2(	NA	NA	NA
RP1-86C11.7	NA	NA	NA
HIST1H2BJ	2.99610895	3.66E-25	6.17E-25
HIST1H2AG	Inf	1.46E-138	3.48E-137
HIST1H4I	4.49416342	2.77E-06	3.38E-06
HIST1H2BK	0.62418936	0.27090814	0.28286707
HIST1H2AH	Inf	1.16E-48	2.72E-48
PRSS16	2.99610895	2.83E-07	3.52E-07
POM121L2	NA	NA	NA
ZNF391	Inf	1.07E-157	5.54E-155
RP1-153G14.	NA	NA	NA

ZNF184	NA	NA	NA
LINC01012	NA	NA	NA
HIST1H2BL	NA	NA	NA
HIST1H2AI	NA	NA	NA
HIST1H3H	Inf	0.00011365	0.00013305
HIST1H2AJ	NA	NA	NA
HIST1H2BM	NA	NA	NA
HIST1H4J	NA	4.28E-96	2.14E-95
HIST1H4K	NA	NA	NA
HIST1H2BN	1.98271916	4.48E-05	5.30E-05
HIST1H2AK	NA	5.48E-18	8.29E-18
HIST1H2AL	NA	NA	NA
HIST1H1B	NA	NA	NA
HIST1H3I	NA	NA	NA
HIST1H4L	NA	NA	NA
HIST1H3J	NA	1.11E-29	1.99E-29
HIST1H2AM	NA	1.14E-135	2.15E-134
HIST1H2BO	0	0.01284399	0.01417026
OR2B2	NA	NA	NA
OR2B6	NA	1.69E-46	3.84E-46
RP1-313I6.12	1.34824903	2.07E-06	2.53E-06
ZNF165	1.87256809	0.01525093	0.01678287
ZSCAN16-AS	2.15345331	4.57E-52	1.12E-51
ZSCAN16	0.49935149	4.02E-84	1.61E-83
RP1-265C24.	NA	1.09E-115	8.47E-115
ZKSCAN8	4.49416342	1.96E-48	4.57E-48
ZSCAN9	0.85603113	4.49E-50	1.07E-49
ZKSCAN4	0.85603113	2.98E-09	3.87E-09
NKAPL	NA	1.00E-144	6.19E-143
ZSCAN26	0.99870298	2.73E-06	3.33E-06
RP5-874C20.	NA	NA	NA
PGBD1	2.24708171	1.33E-15	1.94E-15
ZSCAN31	0.99870298	1.82E-33	3.43E-33
ZKSCAN3	8.23929961	5.35E-35	1.03E-34
ZSCAN12	NA	NA	NA
ZSCAN23	Inf	2.00E-92	9.33E-92
GPX6	NA	NA	NA
GPX5	NA	NA	NA
ZBED9	NA	NA	NA
RP5-1186N2	Inf	6.49E-130	8.70E-129
LINC00533	NA	NA	NA
XXbac-BPG3	0.53501946	3.12E-40	6.48E-40
LINC01623	NA	2.77E-73	9.33E-73
HCG14	Inf	0.54280389	0.5560445
TRIM27	1.97279005	7.51E-12	1.02E-11

LINC01556	1.24837873	1.37E-67	4.22E-67
HCG15	NA	NA	NA
HCG16	NA	NA	NA
ZNF311	NA	NA	NA
XXbac-BPG2!	NA	NA	NA
OR2W1	NA	NA	NA
OR2B3	NA	NA	NA
OR2J3	NA	NA	NA
XXbac-BCX1!	NA	NA	NA
OR2J2	NA	NA	NA
XXbac-BPG3(	NA	NA	NA
OR14J1	NA	NA	NA
OR5V1	NA	NA	NA
OR12D3	NA	NA	NA
OR12D2	NA	NA	NA
OR11A1	NA	NA	NA
OR10C1	NA	NA	NA
OR2H1	NA	NA	NA
MAS1L	NA	NA	NA
XXbac-BPG1:	NA	7.01E-72	2.30E-71
LINC01015	NA	NA	NA
UBD	NA	NA	NA
GABBR1	2.24708171	2.30E-22	3.71E-22
OR2H2	NA	NA	NA
MOG	NA	1.22E-58	3.26E-58
ZFP57	NA	NA	NA
HLA-F	NA	NA	NA
XXbac-BPG1:	Inf	3.58E-51	8.66E-51
HLA-G	NA	NA	NA
HCP5B	NA	NA	NA
HLA-A	0.71431622	3.96E-143	1.75E-141
HCG9	0	1.89E-57	4.99E-57
ZNRD1-AS1	2.99610895	1.80E-61	5.05E-61
ZNRD1	1.53710329	8.27E-56	2.14E-55
PPP1R11	1.51382347	1.30E-11	1.76E-11
RNF39	NA	NA	NA
TRIM31	NA	1.10E-14	1.57E-14
TRIM31-AS1	NA	NA	NA
TRIM40	NA	NA	NA
TRIM10	NA	NA	NA
TRIM15	NA	5.66E-128	6.91E-127
TRIM26	2.24708171	5.38E-36	1.06E-35
HCG17	Inf	2.80E-150	4.16E-148
HCG18	1.42996109	1.47E-30	2.67E-30
TRIM39	1.31079767	3.06E-30	5.53E-30

TRIM39-RPP:	NA	NA	NA
RPP21	1.89040208	3.96E-05	4.69E-05
HLA-E	0.97767766	2.43E-139	6.33E-138
XXbac-BPG2	NA	1.29E-94	6.24E-94
GNL1	1.14137484	7.35E-94	3.51E-93
PRR3	2.00875486	7.65E-07	9.44E-07
ABCF1	1.42188221	3.48E-82	1.34E-81
PPP1R10	1.29243915	8.80E-102	4.92E-101
MRPS18B	0.99315463	1.89E-140	5.59E-139
ATAT1	2.01661179	4.14E-48	9.58E-48
C6orf136	2.15719844	1.82E-20	2.85E-20
DHX16	1.35308146	2.77E-59	7.52E-59
PPP1R18	2.213643	0.90732132	0.91169335
NRM	1.59168288	1.40E-08	1.79E-08
MDC1	2.14494163	8.73E-46	1.96E-45
MDC1-AS1	NA	NA	NA
TUBB	1.1357565	1.83E-136	3.62E-135
XXbac-BPG2!	Inf	1.26E-130	1.75E-129
FLOT1	2.46554799	1.41E-60	3.92E-60
XXbac-BPG2!	NA	1.01E-90	4.54E-90
IER3	0.99870298	1.33E-11	1.81E-11
HCG20	NA	NA	NA
LINC00243	NA	6.32E-07	7.81E-07
XXbac-BPG2:	NA	NA	NA
DDR1-AS1	NA	9.99E-36	1.95E-35
DDR1	0.74902724	8.38E-05	9.84E-05
GTF2H4	NA	8.82E-29	1.56E-28
VARS2	3.18336576	1.07E-07	1.34E-07
SFTA2	NA	NA	NA
DPCR1	NA	NA	NA
HCG21	NA	NA	NA
MUC21	NA	NA	NA
MUC22	NA	NA	NA
HCG22	NA	NA	NA
C6orf15	NA	NA	NA
PSORS1C1	NA	4.90E-89	2.14E-88
CDSN	NA	NA	NA
PSORS1C2	NA	NA	NA
CCHCR1	0.92526894	2.86E-28	5.04E-28
TCF19	1.48140942	0.00192041	0.00217262
POU5F1	Inf	2.04E-122	1.98E-121
XXbac-BPG2!	1.68531128	8.54E-06	1.03E-05
HCG27	0	0.99637562	0.99670525
XXbac-BPG2!	NA	NA	NA
HLA-C	0.85197411	6.15E-142	2.23E-140

XXbac-BPG24	NA	NA	NA
HLA-B	0.99870298	2.24E-142	8.57E-141
XXbac-BPG18	4.11964981	4.88E-17	7.28E-17
MICA	2.15345331	3.00E-08	3.82E-08
LINC01149	NA	8.14E-32	1.50E-31
MICB	1.12354086	2.06E-11	2.78E-11
MCCD1	NA	NA	NA
ATP6V1G2-D	NA	NA	NA
DDX39B	1.94747082	2.45E-16	3.62E-16
DDX39B-AS1	NA	1.36E-148	1.71E-146
ATP6V1G2	5.24319066	2.45E-61	6.87E-61
NFKBIL1	1.87256809	1.68E-31	3.08E-31
LTA	NA	5.50E-145	3.61E-143
TNF	NA	6.93E-48	1.60E-47
LTB	0.67935029	3.51E-44	7.67E-44
LST1	1.04181587	2.18E-123	2.21E-122
NCR3	0.49935149	8.56E-62	2.42E-61
AIF1	1.1094614	1.80E-145	1.58E-143
PRRC2A	2.18523543	1.80E-97	9.26E-97
BAG6	1.3618677	5.59E-48	1.29E-47
APOM	1.3732166	1.21E-49	2.87E-49
C6orf47	1.71206226	0.01590744	0.01749471
C6orf47-AS1	Inf	7.02E-82	2.69E-81
GPANK1	1.3569334	8.23E-21	1.30E-20
CSNK2B	1.40665532	6.76E-13	9.38E-13
XXbac-BPG32	NA	NA	NA
LY6G5B	2.99610895	0.00118835	0.00135387
LY6G5C	1.94747082	0.06626897	0.07121078
ABHD16A	2.24708171	4.06E-13	5.66E-13
XXbac-BPG32	NA	NA	NA
LY6G6F	NA	NA	NA
XXbac-BPG32	NA	0.00079475	0.0009095
LY6G6E	NA	NA	NA
LY6G6D	NA	NA	NA
C6orf25	NA	NA	NA
LY6G6C	NA	5.76E-24	9.52E-24
DDAH2	1.77735277	4.27E-24	7.08E-24
CLIC1	1.12669244	4.61E-110	3.11E-109
MSH5	0.74902724	7.15E-19	1.10E-18
MSH5-SAPCD	NA	NA	NA
SAPCD1	NA	NA	NA
SAPCD1-AS1	NA	0.08314998	0.08898696
VWA7	NA	NA	NA
VARS	1.42188221	3.16E-70	1.01E-69
LSM2	1.89348002	2.84E-87	1.20E-86

HSPA1L	NA	0.0190627	0.02090904
HSPA1A	0.70560537	2.36E-144	1.35E-142
HSPA1B	0.97373541	1.64E-128	2.05E-127
C6orf48	2.19520104	4.20E-91	1.90E-90
NEU1	0.86138132	1.89E-113	1.39E-112
SLC44A4	Inf	2.78E-79	1.02E-78
EHMT2	1.7426348	9.37E-08	1.18E-07
EHMT2-AS1	NA	NA	NA
C2	NA	4.95E-43	1.07E-42
ZBTB12	NA	2.77E-53	6.93E-53
XXbac-BPG1:	NA	NA	NA
C2-AS1	NA	NA	NA
CFB	NA	NA	NA
NELFE	1.20761534	4.94E-33	9.28E-33
SKIV2L	1.99740597	1.63E-37	3.27E-37
DXO	2.40758755	1.71E-34	3.28E-34
STK19	2.12224384	2.76E-12	3.79E-12
C4A	NA	NA	NA
C4A-AS1	NA	NA	NA
AL645922.1	NA	NA	NA
C4B	NA	NA	NA
C4B-AS1	NA	NA	NA
CYP21A2	NA	NA	NA
TNXB	NA	NA	NA
ATF6B	1.78289582	2.54E-30	4.60E-30
FKBPL	1.31079767	1.35E-67	4.17E-67
PRRT1	0.42801556	1.46E-07	1.83E-07
PPT2	2.37191958	6.66E-46	1.50E-45
PPT2-EGFL8	NA	2.81E-16	4.14E-16
EGFL8	1.87256809	8.56E-25	1.43E-24
AGPAT1	2.65564202	3.04E-75	1.05E-74
RNF5	1.5077821	5.70E-69	1.80E-68
AGER	8.98832685	1.23E-57	3.25E-57
PBX2	2.66101782	2.69E-35	5.22E-35
GPSM3	2.09619853	7.21E-10	9.49E-10
NOTCH4	Inf	6.64E-80	2.47E-79
XXbac-BPG1!	NA	NA	NA
XXbac-BPG1!	NA	4.99E-41	1.05E-40
C6orf10	NA	NA	NA
HCG23	NA	NA	NA
BTNL2	NA	NA	NA
HLA-DRA	1.40202534	3.41E-29	6.09E-29
HLA-DRB5	Inf	1.63E-21	2.60E-21
HLA-DRB1	1.43292167	1.57E-22	2.54E-22
HLA-DQA1	NA	4.56E-73	1.53E-72

HLA-DQB1	1.18595979	5.65E-36	1.11E-35
HLA-DQB1-A	NA	NA	NA
HLA-DQA2	NA	NA	NA
HLA-DQB2	NA	NA	NA
HLA-DOB	2.24708171	5.67E-10	7.48E-10
XXbac-BPG2	NA	NA	NA
TAP2	2.02237354	0.09451811	0.10099821
PSMB8	1.24124514	1.35E-130	1.88E-129
PSMB8-AS1	1.81906615	0.00012034	0.00014086
PSMB9	1.95141307	8.90E-15	1.28E-14
TAP1	2.45514483	0.58256412	0.59549686
HLA-DMB	NA	1.19E-11	1.62E-11
XXbac-BPG1	NA	NA	NA
HLA-DMA	1.87256809	9.70E-28	1.69E-27
BRD2	1.78370046	2.16E-32	4.02E-32
XXbac-BPG1	NA	NA	NA
HLA-DOA	NA	NA	NA
HLA-DPA1	Inf	6.48E-32	1.20E-31
HLA-DPB1	0.56177043	6.96E-28	1.22E-27
HCG24	NA	NA	NA
COL11A2	Inf	6.22E-117	5.02E-116
RXRB	1.68531128	0.77434433	0.78310691
SLC39A7	1.73774319	1.57E-19	2.44E-19
HSD17B8	1.09143969	3.50E-117	2.84E-116
RING1	1.41707856	2.95E-107	1.86E-106
XXbac-BPG1	0.37451362	1.02E-33	1.93E-33
HCG25	1.49805447	3.77E-24	6.25E-24
VPS52	1.89753567	1.50E-12	2.08E-12
RPS18	2.17599834	8.13E-107	5.05E-106
B3GALT4	1.12354086	3.40E-07	4.22E-07
WDR46	1.36822309	2.14E-89	9.40E-89
PFDN6	1.44618278	3.36E-61	9.42E-61
RGL2	1.34824903	9.83E-09	1.26E-08
TAPBP	1.88715953	1.02E-61	2.89E-61
ZBTB22	2.99610895	2.33E-46	5.27E-46
DAXX	1.72473377	1.23E-05	1.48E-05
KIFC1	1.08479807	1.18E-122	1.15E-121
PHF1	1.3658732	4.73E-19	7.27E-19
CUTA	1.48274868	4.23E-109	2.78E-108
SYNGAP1	2.99610895	9.80E-31	1.78E-30
XXbac-BPG2	Inf	2.91E-82	1.13E-81
ZBTB9	NA	0.00595068	0.00663943
BAK1	1.67429618	1.36E-08	1.74E-08
LINC00336	NA	NA	NA
ITPR3	1.68531128	0.02570565	0.02804956

UQCC2	1.6875949	2.62E-23	4.30E-23
IP6K3	NA	NA	NA
LEMD2	2.99610895	1.13E-16	1.68E-16
MLN	NA	NA	NA
LINC01016	NA	NA	NA
RP3-468B3.2	NA	9.22E-19	1.41E-18
GRM4	NA	9.99E-22	1.60E-21
HMGA1	2.23847741	1.24E-130	1.72E-129
C6orf1	1.90051687	3.71E-30	6.71E-30
NUDT3	1.61209322	4.82E-12	6.61E-12
RPS10-NUDT	NA	3.80E-10	5.02E-10
RPS10	2.70650024	1.80E-145	1.58E-143
PACSIN1	NA	0.00044227	0.00051011
SPDEF	NA	NA	NA
C6orf106	2.47178988	1.61E-13	2.26E-13
RP11-140K17	2.09727626	0.0075832	0.00843705
SNRPC	1.42687994	2.33E-135	4.26E-134
UHRF1BP1	1.74773022	2.13E-14	3.04E-14
TAF11	1.65385214	1.79E-22	2.90E-22
ANKS1A	1.55567195	0.12951205	0.13748436
TCP11	NA	4.95E-67	1.51E-66
SCUBE3	NA	1.00E-33	1.90E-33
RP3-329A5.8	Inf	1.45E-65	4.35E-65
ZNF76	2.72373541	1.01E-05	1.21E-05
DEF6	1.11145977	9.66E-105	5.74E-104
PPARD	0.74902724	0.0299559	0.03261478
FANCE	2.78210117	4.47E-100	2.41E-99
RPL10A	1.7598415	3.18E-21	5.06E-21
TEAD3	1.49805447	4.67E-05	5.52E-05
TULP1	NA	NA	NA
RP3-340B19.	NA	1.09E-25	1.84E-25
FKBP5	2.75186094	1.07E-70	3.45E-70
RP3-510O8.4	NA	NA	NA
ARMC12	NA	6.14E-145	4.01E-143
CLPSL2	4.49416342	2.51E-11	3.39E-11
CLPSL1	0	6.98E-08	8.81E-08
CLPS	NA	NA	NA
LHFPL5	NA	NA	NA
SRPK1	1.1251907	1.86E-128	2.32E-127
SLC26A8	NA	3.43E-100	1.85E-99
MAPK14	1.57607815	0.03315711	0.03605252
MAPK13	NA	6.97E-81	2.63E-80
RP1-179N16.	NA	0.00096725	0.00110502
BRPF3	2.0598249	1.97E-48	4.59E-48
PNPLA1	NA	NA	NA

C6orf222	NA	NA	NA
ETV7	NA	3.32E-101	1.83E-100
RP1-50J22.4	NA	NA	NA
PXT1	NA	NA	NA
KCTD20	4.10466926	7.56E-136	1.44E-134
STK38	2.82325651	1.48E-59	4.05E-59
SRSF3	1.44685644	2.57E-89	1.13E-88
PANDAR	NA	5.60E-160	4.24E-157
CDKN1A	1.28404669	9.58E-21	1.51E-20
RAB44	0.74902724	1.50E-49	3.55E-49
CPNE5	NA	NA	NA
PPIL1	1.14724425	2.68E-62	7.61E-62
C6orf89	2.37191958	1.63E-15	2.37E-15
RP1-90K10.4	NA	NA	NA
PI16	Inf	9.12E-118	7.52E-117
MTCH1	1.4432476	4.39E-129	5.66E-128
FGD2	NA	2.06E-37	4.11E-37
PIM1	3.25723771	5.67E-119	4.87E-118
TMEM217	0.74902724	0.00083871	0.00095945
TBC1D22B	2.14007782	2.49E-10	3.30E-10
RNF8	1.9758822	3.21E-08	4.08E-08
CMTR1	2.87127108	2.12E-62	6.03E-62
CCDC167	1.79593017	2.97E-17	4.45E-17
RP1-153P14.	NA	NA	NA
RP1-153P14.	NA	NA	NA
MDGA1	0.74902724	0.00554306	0.00619035
RP3-441A12.	NA	9.22E-19	1.41E-18
ZFAND3	2.17217899	7.29E-17	1.08E-16
BTBD9	2.24708171	2.17E-07	2.71E-07
BTBD9-AS1	NA	NA	NA
GLO1	1.21893583	2.64E-65	7.87E-65
RP3-503A6.2	NA	NA	NA
DNAH8	NA	NA	NA
RP1-207H1.3	NA	NA	NA
GLP1R	NA	NA	NA
SAYSD1	1.39105058	0.40085007	0.41366451
KCNK5	1.3732166	0.00306835	0.00345302
KCNK17	NA	NA	NA
KCNK16	NA	NA	NA
KIF6	NA	7.46E-10	9.81E-10
DAAM2	0	1.27E-84	5.12E-84
RP11-61I13.3	NA	NA	NA
MOCS1	NA	NA	NA
RP11-552E2(	NA	NA	NA
TDRG1	NA	NA	NA

LINC00951	NA	NA	NA
RP11-552E2(	NA	NA	NA
LRFN2	NA	NA	NA
RP11-570K4.	NA	NA	NA
UNC5CL	Inf	1.56E-73	5.27E-73
OARD1	1.6332955	1.80E-42	3.85E-42
TSPO2	NA	NA	NA
APOBEC2	NA	NA	NA
NFYA	4.34435798	4.87E-92	2.25E-91
TREML1	NA	NA	NA
TREM2	NA	5.31E-92	2.45E-91
TREML2	Inf	2.78E-70	8.92E-70
TREML4	NA	NA	NA
TREM1	NA	NA	NA
NCR2	NA	NA	NA
FOXP4-AS1	2.99610895	1.19E-80	4.49E-80
LINC01276	NA	NA	NA
FOXP4	2.04045351	1.68E-60	4.65E-60
MDFI	2.20731035	1.50E-20	2.36E-20
TFEB	1.74773022	6.21E-08	7.84E-08
RP11-298J23	NA	NA	NA
PGC	NA	NA	NA
RP11-298J23	NA	NA	NA
FRS3	NA	2.66E-05	3.17E-05
PRICKLE4	1.74773022	4.71E-07	5.84E-07
PRICKLE4	NA	NA	NA
TOMM6	NA	NA	NA
USP49	6.74124514	1.26E-98	6.60E-98
RP11-298J23	NA	NA	NA
RP5-973N23.	NA	1.11E-44	2.44E-44
MED20	2.03307393	2.26E-07	2.82E-07
BYSL	1.0872976	2.72E-117	2.22E-116
CCND3	1.51104339	4.97E-49	1.17E-48
RP11-533O2(	NA	NA	NA
TAF8	1.66450497	1.03E-07	1.29E-07
RP11-7K24.3	NA	4.00E-88	1.72E-87
C6orf132	1.24837873	0.03978848	0.04312855
GUCA1A	NA	NA	NA
RP1-139D8.6	NA	NA	NA
GUCA1B	Inf	8.37E-60	2.29E-59
MRPS10	1.47362967	1.02E-28	1.80E-28
TRERF1	0.56177043	3.98E-47	9.11E-47
UBR2	1.14850843	3.90E-40	8.11E-40
PRPH2	NA	NA	NA
TBCC	1.18394628	1.76E-81	6.70E-81

GLTSCR1L	3.74513619	2.27E-47	5.21E-47
RPL7L1	1.88741665	9.21E-19	1.41E-18
C6orf226	1.39105058	8.04E-07	9.92E-07
PTCRA	NA	NA	NA
CNPY3	1.36333735	1.13E-115	8.74E-115
RP3-475N16.	0.74902724	2.00E-26	3.42E-26
GNMT	0.74902724	0.08975208	0.09595629
PEX6	0.98309825	2.23E-40	4.64E-40
PPP2R5D	1.43196384	0.10756931	0.11453268
MEA1	1.62869876	1.09E-38	2.22E-38
KLHDC3	1.67744335	9.03E-53	2.24E-52
RRP36	1.77448119	3.31E-22	5.34E-22
RP1-20C7.6	NA	1.03E-86	4.28E-86
CUL7	3.74513619	3.18E-42	6.80E-42
KLC4	0.83225249	7.51E-31	1.37E-30
MRPL2	1.39155771	6.85E-102	3.83E-101
RP11-387M2	NA	0.07532475	0.08077442
PTK7	1.72276265	0.00924508	0.01024646
SRF	2.29389591	4.84E-09	6.26E-09
CUL9	1.49805447	2.34E-55	6.02E-55
RP3-330M21	NA	5.02E-38	1.01E-37
DNPH1	1.97296219	3.79E-37	7.55E-37
TTBK1	NA	NA	NA
SLC22A7	NA	NA	NA
CRIP3	NA	NA	NA
ZNF318	1.9661965	0.00044539	0.00051362
ABCC10	Inf	8.55E-102	4.78E-101
DLK2	0.74902724	7.30E-117	5.88E-116
TJAP1	2.19357977	2.31E-23	3.79E-23
LRRC73	0.74902724	2.67E-23	4.37E-23
POLR1C	0.8271866	5.61E-92	2.59E-91
YIPF3	1.26205959	8.23E-135	1.46E-133
RP3-337H4.9	0.74902724	3.35E-64	9.80E-64
XPO5	3.95914397	1.78E-76	6.27E-76
RP3-337H4.1	NA	NA	NA
POLH	5.24319066	3.31E-06	4.03E-06
RP3-337H4.8	NA	3.68E-151	6.13E-149
GTPBP2	Inf	4.88E-17	7.28E-17
MAD2L1BP	0.7202185	1.01E-128	1.29E-127
RSPH9	NA	2.80E-75	9.67E-75
MRPS18A	1.32264936	2.06E-116	1.64E-115
RP1-261G23.	NA	NA	NA
VEGFA	2.99610895	3.31E-68	1.03E-67
RP1-261G23.	NA	7.08E-143	3.00E-141
RP11-344J7.:	NA	NA	NA

RP5-1120P11	NA	NA	NA
C6orf223	0	4.23E-121	3.89E-120
RP5-1120P11	NA	1.55E-11	2.10E-11
MRPL14	1.91724691	0.00283148	0.00318783
TMEM63B	1.99740597	2.30E-26	3.94E-26
CAPN11	Inf	2.39E-15	3.47E-15
RP1-302G2.5	NA	NA	NA
SLC29A1	1.38613086	8.30E-69	2.61E-68
HSP90AB1	1.26443642	1.94E-96	9.74E-96
SLC35B2	1.43251459	5.28E-88	2.26E-87
NFKBIE	1.51192535	2.66E-30	4.81E-30
TMEM151B	NA	NA	NA
RP11-444E17	NA	NA	NA
TCTE1	NA	NA	NA
AARS2	4.99351492	2.33E-130	3.20E-129
SPATS1	NA	NA	NA
CDC5L	1.26953769	2.57E-122	2.48E-121
RP3-449H6.1	NA	NA	NA
SUPT3H	2.24708171	1.96E-119	1.71E-118
RUNX2	Inf	2.14E-43	4.63E-43
RP1-244F24.	NA	0.05619008	0.06053807
RP1-166H4.2	NA	NA	NA
CLIC5	NA	NA	NA
RP1-8B1.4	Inf	1.74E-123	1.77E-122
ENPP4	1.87256809	3.44E-08	4.36E-08
ENPP5	NA	NA	NA
RCAN2	NA	NA	NA
RP11-795J1.1	NA	NA	NA
CYP39A1	NA	2.03E-87	8.61E-87
SLC25A27	NA	0.00061	0.00070037
RP11-446F17	NA	NA	NA
TDRD6	NA	NA	NA
PLA2G7	NA	NA	NA
ANKRD66	NA	NA	NA
RP11-268F1.	NA	NA	NA
MEP1A	NA	NA	NA
ADGRF5	NA	NA	NA
RP3-365O12.	NA	NA	NA
ADGRF1	NA	1.09E-25	1.84E-25
TNFRSF21	1.87256809	2.14E-71	6.98E-71
RP11-385F7.	Inf	1.10E-39	2.27E-39
CD2AP	1.07003891	6.31E-48	1.46E-47
ADGRF2	NA	NA	NA
ADGRF4	NA	NA	NA
RP11-550C4.	NA	NA	NA

OPN5	NA	NA	NA
PTCHD4	NA	NA	NA
MUT	2.87127108	1.04E-08	1.33E-08
CENPQ	0.66580199	1.72E-107	1.09E-106
GLYATL3	NA	NA	NA
C6orf141	3.08973735	8.70E-34	1.65E-33
RHAG	Inf	1.28E-90	5.75E-90
CRISP2	NA	5.14E-86	2.12E-85
CRISP3	NA	NA	NA
PGK2	NA	NA	NA
RP3-417L20.4	NA	NA	NA
RP11-719J20	NA	NA	NA
CRISP1	NA	NA	NA
DEFB133	NA	NA	NA
DEFB114	NA	NA	NA
DEFB113	NA	NA	NA
DEFB110	NA	NA	NA
DEFB112	NA	NA	NA
RP11-397G1	NA	NA	NA
RP11-524K22	NA	NA	NA
RP1-28O17.1	NA	NA	NA
TFAP2D	NA	NA	NA
TFAP2B	NA	NA	NA
RP3-335N17.	NA	NA	NA
PKHD1	NA	NA	NA
LINCMD1	NA	NA	NA
IL17A	NA	NA	NA
IL17F	NA	NA	NA
MCM3	1.907187	0.02702462	0.02947106
PAQR8	0.64202335	2.75E-09	3.57E-09
EFHC1	2.69649805	4.93E-61	1.38E-60
TRAM2	2.62159533	0.07169679	0.0769522
TRAM2-AS1	2.49675746	4.89E-81	1.85E-80
TMEM14A	1.67509728	2.06E-05	2.46E-05
GSTA2	NA	NA	NA
GSTA1	NA	NA	NA
GSTA5	NA	NA	NA
GSTA3	NA	NA	NA
GSTA4	0.74902724	3.78E-38	7.64E-38
ICK	1.87256809	0.00044149	0.00050925
FBXO9	1.88297125	4.24E-15	6.13E-15
GCM1	NA	NA	NA
ELOVL5	2.09317201	2.43E-05	2.89E-05
RP3-483K16.	NA	2.27E-101	1.26E-100
GCLC	2.8463035	6.47E-105	3.86E-104

RP1-27K12.4	NA	NA	NA
RP1-27K12.2	NA	NA	NA
LINC01564	NA	NA	NA
KLHL31	NA	5.28E-72	1.74E-71
RP11-79N23.	NA	NA	NA
RP13-476E2(	NA	5.56E-53	1.39E-52
LRRC1	3.74513619	6.12E-74	2.08E-73
RP3-523E19.	NA	NA	NA
MLIP	NA	NA	NA
RP11-411K7.	NA	NA	NA
MLIP-AS1	NA	NA	NA
TINAG	NA	NA	NA
RP11-124I4.2	NA	NA	NA
RP11-524H1!	NA	NA	NA
FAM83B	NA	NA	NA
HCRTR2	NA	NA	NA
GFRAL	NA	NA	NA
HMGCLL1	NA	NA	NA
BMP5	NA	NA	NA
COL21A1	NA	NA	NA
DST	1.74773022	1.06E-15	1.55E-15
RP11-472M1	NA	NA	NA
BEND6	2.24708171	0.0146514	0.01613486
KIAA1586	1.55567195	0.00459874	0.00514843
ZNF451	2.38083658	2.85E-114	2.13E-113
RP11-203B9.	NA	0.18948651	0.1994699
BAG2	1.4804303	3.23E-45	7.17E-45
RAB23	2.62159533	8.35E-128	1.02E-126
PRIM2	1.30475712	5.42E-12	7.42E-12
RP1-71H19.2	NA	NA	NA
XXbac-BPGB	NA	7.91E-05	9.30E-05
XXbac-BPGB	NA	3.03E-46	6.83E-46
XXbac-BPGB	1.87256809	3.06E-08	3.89E-08
LINC00680	2.24708171	0.4675865	0.48064787
XXbac-BPG5!	NA	NA	NA
RP11-143A2.	NA	NA	NA
RP11-343D2	NA	NA	NA
AL356135.1	NA	NA	NA
KHDRBS2	0.74902724	5.23E-10	6.89E-10
FKBP1C	0.99870298	5.97E-08	7.54E-08
LGSN	NA	NA	NA
PTP4A1	1.98492218	3.88E-08	4.93E-08
RP5-1148A2.	NA	6.60E-109	4.32E-108
PHF3	1.82149805	0.5848812	0.59776434
EYS	NA	2.78E-43	6.02E-43

RP11-59D5__	Inf	1.07E-155	3.41E-153
RP11-349P19	NA	NA	NA
RP11-301G19	3.80755512	1.07E-70	3.46E-70
RP11-542F9.	NA	NA	NA
RP11-406O1	NA	NA	NA
RP3-525N10.	Inf	1.75E-138	4.15E-137
ADGRB3	Inf	0.89790638	0.90273303
LMBRD1	2.16385646	9.75E-43	2.10E-42
COL19A1	NA	0.01247871	0.0137748
COL9A1	NA	NA	NA
RP1-149L1.1	NA	NA	NA
RP11-462G2.	NA	1.19E-153	2.59E-151
RP11-462G2.	1.49805447	8.22E-33	1.54E-32
FAM135A	2.24708171	0.04254352	0.04606532
SDHAF4	1.38281952	1.31E-08	1.68E-08
RP11-134K13	NA	5.51E-95	2.69E-94
SMAP1	1.85734396	1.13E-18	1.73E-18
B3GAT2	2.14007782	6.77E-84	2.69E-83
OGFRL1	1.49805447	2.88E-15	4.18E-15
RP3-331H24.	NA	NA	NA
RP3-331H24.	NA	NA	NA
LINC00472	NA	NA	NA
LINC01626	NA	NA	NA
RIMS1	NA	NA	NA
KCNQ5	2.02237354	1.42E-08	1.82E-08
KCNQ5-AS1	NA	NA	NA
RP11-257K9.	NA	NA	NA
KHDC1L	0	4.61E-46	1.04E-45
KHDC1	2.9079881	3.88E-71	1.26E-70
RP11-398K22	3.37062257	1.23E-114	9.31E-114
DPPA5	NA	NA	NA
KHDC3L	NA	NA	NA
OOEP	NA	9.25E-135	1.64E-133
OOEP-AS1	NA	NA	NA
DDX43	NA	NA	NA
MB21D1	2.31841764	1.78E-14	2.55E-14
MTO1	2.76203794	1.89E-81	7.18E-81
RP11-505P4.	NA	NA	NA
EEF1A1	2.04187507	1.17E-06	1.44E-06
RP11-505P4.	NA	1.69E-55	4.37E-55
SLC17A5	1.16515348	3.23E-52	7.97E-52
RP11-553A21	Inf	2.51E-139	6.52E-138
CD109	1.12354086	4.78E-104	2.80E-103
RP11-554D15	NA	NA	NA
RP11-554D15	NA	NA	NA

RP11-554D15	NA	NA	NA
COL12A1	NA	NA	NA
COX7A2	2.02761248	1.81E-113	1.34E-112
TMEM30A	2.24708171	6.85E-11	9.17E-11
RP1-234P15.	1.49805447	3.11E-56	8.09E-56
FILIP1	NA	6.22E-117	5.02E-116
RP11-415D17	NA	NA	NA
RP11-415D17	NA	NA	NA
SENP6	1.90823606	1.16E-23	1.90E-23
MYO6	1.68531128	0.00965909	0.01069812
IMPG1	NA	NA	NA
RP11-35J1.1	NA	NA	NA
RP11-35J1.2	NA	NA	NA
RP11-354K4.	NA	NA	NA
HTR1B	NA	NA	NA
MEI4	NA	NA	NA
RP3-390M24	NA	NA	NA
RP11-173D14	NA	NA	NA
IRAK1BP1	Inf	6.68E-122	6.36E-121
PHIP	1.90789957	1.75E-20	2.75E-20
HMGN3	1.98298026	1.01E-93	4.82E-93
HMGN3-AS1	0.74902724	5.58E-07	6.90E-07
RP1-232L24.3	NA	NA	NA
LCA5	1.49805447	2.18E-19	3.37E-19
RP11-177G23	NA	7.31E-62	2.07E-61
SH3BGRL2	Inf	9.11E-151	1.45E-148
LINC01621	NA	NA	NA
RP1-92C4.2	NA	NA	NA
ELOVL4	0.74902724	5.79E-57	1.52E-56
AL133475.1	NA	NA	NA
TTK	0.96932937	1.71E-81	6.51E-81
BCKDHB	0.99870298	1.06E-84	4.26E-84
RP11-250B2.	Inf	1.27E-17	1.91E-17
RP11-250B2.	NA	1.80E-76	6.33E-76
RP11-250B2.	NA	2.57E-44	5.63E-44
FAM46A	0.64007782	2.31E-116	1.83E-115
RP11-244G12	NA	NA	NA
LINC01526	NA	NA	NA
RP11-379B8.	NA	6.56E-44	1.43E-43
RP11-801I18	NA	NA	NA
IBTK	3.13655156	3.32E-73	1.11E-72
TPBG	1.56614786	5.31E-40	1.10E-39
UBE3D	1.22341115	2.48E-66	7.50E-66
RP11-445L13	NA	NA	NA
DOPEY1	0	1.32E-23	2.18E-23

AL139333.1	NA	NA	NA
PGM3	1.24837873	7.85E-67	2.39E-66
RWDD2A	1.87256809	8.70E-05	1.02E-04
ME1	1.48177127	6.50E-14	9.19E-14
PRSS35	NA	6.74E-117	5.44E-116
SNAP91	NA	NA	NA
RP3-393K13.	NA	NA	NA
RIPPLY2	NA	NA	NA
CYB5R4	2.1614786	1.70E-09	2.21E-09
RP4-676J13.:	NA	NA	NA
RP11-51G5.1	NA	NA	NA
MRAP2	0.80894942	3.32E-62	9.44E-62
CEP162	1.60505837	2.92E-07	3.64E-07
LINC01611	NA	NA	NA
TBX18	NA	NA	NA
TBX18-AS1	NA	NA	NA
RP11-30P6.6	NA	NA	NA
NT5E	NA	NA	NA
SNX14	1.24837873	2.19E-19	3.39E-19
RP11-321N4.	0.74902724	2.49E-05	2.97E-05
SYNCRIP	1.58770638	1.54E-05	1.85E-05
HTR1E	NA	NA	NA
CGA	NA	NA	NA
RP11-393I2.4	Inf	4.80E-42	1.02E-41
ZNF292	1.89997153	5.25E-18	7.95E-18
GJB7	NA	NA	NA
SMIM8	1.42556797	0.56190578	0.57493061
C6orf163	Inf	3.87E-10	5.11E-10
CFAP206	NA	NA	NA
RP3-382I10.7	NA	0.05255704	0.05667775
SLC35A1	1.32520204	2.37E-48	5.50E-48
RARS2	1.37889105	6.10E-10	8.04E-10
ORC3	3.67380026	8.54E-45	1.89E-44
AKIRIN2	0.96932937	1.31E-115	1.01E-114
SPACA1	NA	NA	NA
CNR1	NA	NA	NA
RP11-459O1.	NA	NA	NA
RNGTT	3.86997406	1.20E-95	5.93E-95
RP11-63L7.5	NA	NA	NA
PNRC1	0.80009728	8.67E-144	4.38E-142
SRSF12	1.42315175	0.00838056	0.00930535
PM20D2	2.22438392	8.89E-95	4.33E-94
GABRR1	NA	NA	NA
GABRR2	NA	NA	NA
UBE2J1	1.58205753	0.00072073	0.00082588

RRAGD	2.7668149	3.82E-67	1.17E-66
ANKRD6	3.89494163	2.87E-91	1.31E-90
RP11-16C18.	0	7.75E-131	1.09E-129
LYRM2	1.83095547	0.21638032	0.22712172
MDN1	1.58884565	1.75E-17	2.63E-17
RP1-122O8.7	NA	NA	NA
CASP8AP2	1.68531128	1.82E-10	2.41E-10
GJA10	NA	NA	NA
RP11-63K6.7	NA	NA	NA
BACH2	NA	NA	NA
RP3-512E2.2	NA	NA	NA
RP1-45N11.1	NA	NA	NA
MAP3K7	1.70403696	1.92E-09	2.51E-09
RP1-177I10.1	NA	NA	NA
CASC6	NA	NA	NA
RP1-149C7.1	NA	NA	NA
RP11-127B1(	NA	NA	NA
RP1-23E21.2	Inf	5.38E-83	2.10E-82
EPHA7	NA	NA	NA
RP11-524K1(	NA	NA	NA
RP3-463P15.	NA	NA	NA
MANEA-AS1	1.49805447	5.15E-12	7.06E-12
MANEA	3.1459144	7.47E-61	2.08E-60
FUT9	NA	NA	NA
UFL1-AS1	NA	NA	NA
UFL1	1.18595979	1.73E-88	7.50E-88
FHL5	NA	NA	NA
RP3-417O22.	NA	NA	NA
GPR63	2.09727626	1.30E-54	3.33E-54
NDUFAF4	1.40770489	2.08E-47	4.78E-47
KLHL32	NA	NA	NA
MMS22L	3.70946822	3.49E-120	3.14E-119
RP11-436D2:	NA	NA	NA
RP1-104O17.	NA	NA	NA
RP11-111D3.	NA	NA	NA
RP1-273N12.	NA	NA	NA
POU3F2	1.49805447	1.53E-08	1.96E-08
FBXL4	2.24708171	9.34E-44	2.03E-43
FAXC	1.49805447	5.97E-07	7.38E-07
COQ3	1.39105058	5.81E-62	1.65E-61
PNISR	1.69779507	1.20E-57	3.19E-57
RP11-98I9.4	Inf	1.90E-06	2.32E-06
USP45	0.98309825	1.86E-88	8.05E-88
TSTD3	4.11964981	8.59E-42	1.83E-41
CCNC	1.67202209	1.24E-45	2.77E-45

RP1-199J3.7	Inf	2.19E-49	5.18E-49
PRDM13	NA	NA	NA
MCHR2	NA	NA	NA
MCHR2-AS1	NA	NA	NA
SIM1	NA	NA	NA
RP1-121G13.	NA	NA	NA
ASCC3	1.39402291	0.62988056	0.64191063
RP3-467N11.	1.12354086	2.98E-12	4.10E-12
GRIK2	NA	NA	NA
HACE1	5.24319066	2.29E-37	4.57E-37
LINC00577	NA	5.92E-17	8.81E-17
LIN28B	1.62645914	0.06223276	0.06693305
BVES	3.37062257	1.07E-69	3.40E-69
BVES-AS1	NA	0.24099508	0.25234519
POPDC3	2.15719844	2.21E-117	1.80E-116
RP3-355L5.5	NA	1.76E-159	1.28E-156
PREP	1.44973014	1.06E-18	1.63E-18
RP3-355L5.4	NA	NA	NA
RP3-359N14.	NA	NA	NA
PRDM1	0	1.85E-135	3.42E-134
ATG5	1.29761057	1.69E-57	4.46E-57
RP11-404H1	NA	NA	NA
AIM1	NA	NA	NA
RP3-335E1.1	NA	NA	NA
RTN4IP1	1.03711464	3.32E-103	1.91E-102
QRSL1	1.39683458	3.53E-55	9.07E-55
RP1-60O19.2	NA	0.03359203	0.03649917
RP1-60O19.1	NA	NA	NA
CD24	4.49416342	1.79E-26	3.07E-26
C6orf203	1.38101897	5.78E-129	7.43E-128
BEND3	Inf	2.79E-94	1.35E-93
PDSS2	1.04863813	4.82E-105	2.89E-104
SOBP	2.7464332	1.99E-05	2.38E-05
RP1-67A8.3	NA	NA	NA
SCML4	NA	1.62E-116	1.29E-115
SEC63	1.81906615	3.31E-18	5.02E-18
RP1-191J18.	1.49805447	3.68E-08	4.67E-08
RP1-111B22.	NA	3.90E-124	4.05E-123
OSTM1	1.61328943	5.58E-20	8.70E-20
OSTM1-AS1	NA	3.43E-20	5.36E-20
NR2E1	NA	NA	NA
SNX3	1.83074424	9.59E-54	2.42E-53
RP1-128O3.4	NA	NA	NA
LACE1	0.74902724	1.77E-36	3.50E-36
FOXO3	2.52796693	1.40E-29	2.50E-29

LINC00222	NA	NA	NA
ARMC2	1.12354086	0.00810867	0.00900621
ARMC2-AS1	NA	NA	NA
SESN1	2.24708171	9.43E-28	1.65E-27
RP11-787I22	NA	NA	NA
CEP57L1	3.2457847	0.0049863	0.00557647
CD164	1.12354086	3.80E-59	1.03E-58
RP11-425D1(	Inf	8.89E-75	3.05E-74
PPIL6	0.74902724	8.01E-06	9.66E-06
SMPD2	1.61328943	6.02E-06	7.28E-06
MICAL1	2.24708171	0.61538537	0.62773806
ZBTB24	1.61328943	3.34E-15	4.83E-15
RP5-919F19.	NA	NA	NA
AK9	1.49805447	1.26E-32	2.35E-32
FIG4	Inf	7.96E-35	1.53E-34
RP1-249I4.2	NA	1.95E-13	2.74E-13
GPR6	NA	NA	NA
WASF1	3.57228375	9.73E-35	1.87E-34
CDC40	1.38708748	1.30E-64	3.82E-64
METTL24	NA	NA	NA
DDO	NA	NA	NA
SLC22A16	NA	NA	NA
CTA-331P3.1	NA	1.87E-10	2.49E-10
CDK19	1.62289235	0.00158671	0.00179991
AMD1	2.04622525	4.21E-06	5.12E-06
GTF3C6	1.90446673	0.71194822	0.72229978
RPF2	1.01653696	2.70E-110	1.83E-109
SLC16A10	1.44043699	1.62E-53	4.08E-53
RP11-428F8.	NA	NA	NA
KIAA1919	2.41353221	9.36E-48	2.15E-47
RP5-1112D6.	1.87256809	6.57E-30	1.18E-29
RP5-1112D6.	Inf	3.96E-15	5.72E-15
REV3L	1.24837873	0.01374019	0.0151461
RP5-1112D6.	Inf	3.16E-75	1.09E-74
TRAF3IP2-AS	1.77042802	4.13E-59	1.12E-58
TRAF3IP2	0.74902724	3.43E-86	1.42E-85
C6orf3	Inf	2.44E-126	2.79E-125
FYN	0.37451362	3.97E-40	8.25E-40
RP1-97J1.2	NA	NA	NA
WISP3	NA	NA	NA
TUBE1	1.9661965	0.57883022	0.59184681
FAM229B	2.39688716	3.11E-120	2.80E-119
LAMA4	NA	NA	NA
RP1-142L7.5	NA	NA	NA
RP1-142L7.9	NA	NA	NA

RP1-142L7.8	NA	NA	NA
RP11-506B6.	NA	NA	NA
RP11-506B6.	NA	NA	NA
RFPL4B	NA	NA	NA
RP1-105O18.	NA	NA	NA
RP11-437J19	NA	NA	NA
RP1-124C6.1	NA	NA	NA
RP11-346D19	NA	NA	NA
RP11-367G18	NA	NA	NA
RP1-249H1.2	NA	NA	NA
MARCKS	8.23929961	1.99E-145	1.63E-143
LINC01268	NA	NA	NA
RP11-544L8_	NA	NA	NA
HDAC2	1.54669261	4.93E-97	2.52E-96
RP3-399L15.:	Inf	5.95E-135	1.07E-133
HS3ST5	NA	NA	NA
RP3-399L15.:	NA	NA	NA
RP3-399L15.:	NA	NA	NA
RP11-282C5.	NA	NA	NA
FRK	NA	NA	NA
NT5DC1	1.28999135	1.11E-76	3.92E-76
COL10A1	NA	NA	NA
RP3-486I3.5	NA	NA	NA
TSPYL4	2.62159533	2.40E-98	1.26E-97
DSE	4.49416342	1.62E-25	2.74E-25
RP3-486I3.7	NA	5.63E-61	1.57E-60
TSPYL1	1.77893969	5.15E-59	1.39E-58
RP1-93H18.1	NA	NA	NA
RP1-93H18.6	NA	NA	NA
FAM26F	NA	5.24E-64	1.53E-63
RP11-259P20	NA	NA	NA
TRAPPC3L	NA	NA	NA
FAM26E	NA	NA	NA
FAM26D	NA	NA	NA
RWDD1	2.21910081	6.96E-132	1.04E-130
RSPH4A	NA	4.32E-06	5.25E-06
ZUFSP	1.04863813	1.04E-60	2.90E-60
KPNA5	2.99610895	8.75E-104	5.10E-103
FAM162B	NA	NA	NA
GPRC6A	NA	NA	NA
RFX6	NA	NA	NA
VGLL2	NA	NA	NA
ROS1	NA	NA	NA
RP1-179P9.3	NA	NA	NA
RP1-92C8.3	NA	NA	NA

DCBLD1	2.24708171	4.88E-83	1.91E-82
GOPC	1.64651032	0.02084183	0.02282874
NUS1	2.01871975	6.91E-68	2.14E-67
SLC35F1	NA	NA	NA
CEP85L	2.14007782	8.76E-12	1.19E-11
PLN	0	4.39E-177	3.98E-173
MCM9	8.98832685	9.16E-114	6.80E-113
ASF1A	1.29933296	2.21E-87	9.34E-87
RP11-351A11	0	4.08E-107	2.55E-106
FAM184A	0.96303502	3.90E-84	1.56E-83
MAN1A1	4.49416342	9.82E-54	2.47E-53
TBC1D32	3.74513619	1.22E-06	1.49E-06
GJA1	NA	7.74E-138	1.72E-136
HSF2	2.78210117	0.12396256	0.131732
SERINC1	2.54669261	5.60E-96	2.79E-95
PKIB	0.42801556	7.08E-60	1.94E-59
FABP7	NA	NA	NA
SMPDL3A	Inf	4.31E-17	6.44E-17
CLVS2	NA	NA	NA
TRDN	NA	NA	NA
RP11-532N4.	NA	NA	NA
NKAIN2	NA	7.97E-25	1.34E-24
RNF217-AS1	NA	2.92E-130	3.97E-129
RNF217	2.39688716	5.24E-10	6.91E-10
TPD52L1	2.99610895	1.49E-130	2.06E-129
HDDC2	2.06398617	8.67E-100	4.64E-99
RP11-735G4.	NA	NA	NA
RP11-138M1	NA	NA	NA
RP11-624M8	NA	NA	NA
RP1-293L8.2	NA	NA	NA
HEY2	NA	5.59E-32	1.03E-31
NCOA7	1.66088648	0.27294693	0.28489748
NCOA7-AS1	NA	NA	NA
HINT3	2.52796693	1.54E-06	1.89E-06
TRMT11	1.07948043	4.37E-60	1.20E-59
RP11-527F13	Inf	5.94E-64	1.73E-63
CENPW	1.65930339	8.25E-39	1.68E-38
RSPO3	NA	NA	NA
RNF146	0.99870298	1.17E-15	1.70E-15
ECHDC1	NA	NA	NA
RP3-403A15.	NA	NA	NA
KIAA0408	NA	NA	NA
SOGA3	NA	NA	NA
C6orf58	NA	NA	NA
RP11-325O24	NA	NA	NA

THEMIS	NA	NA	NA
PTPRK	2.09727626	3.19E-14	4.53E-14
RP11-103C16	NA	NA	NA
RP1-86D1.5	NA	NA	NA
LAMA2	NA	5.56E-34	1.06E-33
RP1-69D17.3	NA	NA	NA
RP1-69D17.4	NA	NA	NA
ARHGAP18	0.90953307	5.02E-53	1.25E-52
TMEM244	NA	NA	NA
L3MBTL3	3.74513619	7.30E-09	9.40E-09
RP11-73O6.3	NA	NA	NA
SAMD3	NA	NA	NA
TMEM200A	Inf	0.00474383	0.00530923
RP11-102N1	NA	NA	NA
SMLR1	NA	NA	NA
EPB41L2	1.56704383	1.88E-40	3.92E-40
AKAP7	1.14850843	1.06E-25	1.80E-25
ARG1	NA	NA	NA
MED23	2.09727626	3.55E-24	5.90E-24
ENPP3	NA	8.11E-31	1.48E-30
OR2A4	NA	NA	NA
CTAGE9	NA	NA	NA
ENPP1	NA	7.38E-148	8.47E-146
RP11-69I8.2	NA	NA	NA
CTGF	NA	1.77E-88	7.66E-88
RP11-69I8.3	NA	NA	NA
RP3-523C21.	NA	NA	NA
LINC01013	NA	NA	NA
MOXD1	NA	NA	NA
STX7	1.72852439	2.14E-37	4.28E-37
TAAR9	NA	NA	NA
TAAR8	NA	NA	NA
TAAR6	NA	NA	NA
TAAR5	NA	NA	NA
TAAR2	NA	NA	NA
TAAR1	NA	NA	NA
VNN1	1.68531128	0.00245516	0.00276879
VNN3	NA	8.53E-72	2.80E-71
VNN2	0.99870298	2.70E-29	4.83E-29
RP1-55C23.7	NA	NA	NA
SLC18B1	0.95709036	3.50E-16	5.16E-16
RPS12	2.64297882	2.00E-145	1.64E-143
LINC00326	NA	NA	NA
EYA4	NA	NA	NA
RP1-283K11.	NA	NA	NA

RP1-283K11.	NA	NA	NA
TARID	NA	NA	NA
RP11-704J17	NA	NA	NA
LINC01312	NA	NA	NA
TCF21	NA	NA	NA
TBPL1	3.3402566	2.39E-54	6.08E-54
SLC2A12	NA	NA	NA
SGK1	NA	3.00E-125	3.25E-124
RP11-557H1!	NA	NA	NA
LINC01010	NA	NA	NA
RP11-557H1!	NA	3.67E-65	1.09E-64
RP11-557H1!	NA	5.28E-72	1.74E-71
CTA-31J9.2	NA	NA	NA
RP1-287H17.	NA	NA	NA
ALDH8A1	NA	2.11E-89	9.27E-89
RP11-349J5.:	NA	NA	NA
HBS1L	1.39105058	6.04E-129	7.76E-128
CTA-212D2.2	NA	6.58E-106	4.01E-105
MYB	2.24083982	6.00E-13	8.35E-13
MYB-AS1	NA	NA	NA
RP1-32B1.4	NA	NA	NA
AHI1	1.21716926	9.55E-33	1.79E-32
RP3-388E23.	0	4.13E-69	1.31E-68
LINC00271	NA	9.98E-105	5.93E-104
RP1-38C16.2	NA	NA	NA
RP11-394G3.	NA	NA	NA
PDE7B	NA	NA	NA
RP13-143G1!	NA	NA	NA
MTFR2	1.2012701	2.90E-143	1.32E-141
BCLAF1	2.07794653	0.02031699	0.02226597
RP3-406A7.7	Inf	1.51E-80	5.67E-80
MAP7	1.87256809	0.10141594	0.10817779
RP3-325F22.	Inf	8.97E-138	1.98E-136
MAP3K5	1.3732166	2.22E-50	5.32E-50
RP3-325F22.	NA	NA	NA
PEX7	1.41482923	1.51E-50	3.61E-50
SLC35D3	NA	7.05E-52	1.73E-51
NHEG1	NA	NA	NA
RP11-204P2.	NA	NA	NA
IL20RA	NA	NA	NA
IL22RA2	NA	NA	NA
IFNGR1	1.07003891	5.88E-103	3.36E-102
OLIG3	NA	NA	NA
RP11-95M15	NA	NA	NA
RP11-356I2.1	NA	NA	NA

RP11-356I2.4	Inf	2.52E-58	6.73E-58
TNFAIP3	Inf	2.33E-78	8.45E-78
RP11-10J5.1	NA	NA	NA
RP11-240M1	NA	NA	NA
PERP	Inf	0.22199943	0.23284463
ARFGEF3	0	2.72E-10	3.61E-10
PBOV1	NA	2.97E-64	8.70E-64
RP3-422G23.	NA	NA	NA
HEBP2	2.03399638	3.20E-92	1.49E-91
NHSL1	1.39105058	2.29E-13	3.21E-13
RP11-390P2.	1.49805447	5.69E-15	8.21E-15
GVQW2	Inf	3.53E-17	5.28E-17
CCDC28A	1.43732254	3.54E-11	4.76E-11
ECT2L	NA	NA	NA
REPS1	2.39688716	1.98E-11	2.68E-11
ABRACL	1.84182912	1.75E-50	4.20E-50
HECA	4.86867704	2.19E-49	5.17E-49
RP1-225E12.	NA	NA	NA
TXLNB	Inf	1.51E-40	3.15E-40
RP11-445F6.	NA	NA	NA
CITED2	1.09143969	5.77E-137	1.19E-135
LINC01625	NA	NA	NA
RP5-899B16.	NA	1.09E-148	1.38E-146
RP5-899B16.	NA	NA	NA
RP3-332B22.	NA	NA	NA
RP3-460G2.2	3.08220403	7.81E-145	4.97E-143
RP11-63E9.1	NA	NA	NA
NMBR	NA	NA	NA
RP11-137J7.2	NA	NA	NA
GJE1	NA	NA	NA
VTA1	1.42188221	3.20E-24	5.31E-24
RP11-753B14	NA	NA	NA
ADGRG6	NA	NA	NA
RP11-440G9.	NA	NA	NA
RP1-67K17.3	NA	NA	NA
HIVEP2	Inf	2.41E-05	2.88E-05
RP1-67K17.4	NA	NA	NA
RP11-439L18	NA	NA	NA
RP11-439L18	NA	NA	NA
LINC01277	NA	NA	NA
RP1-45I4.3	NA	NA	NA
AIG1	1.02991245	5.60E-07	6.92E-07
RP1-45I4.2	NA	NA	NA
ADAT2	2.30058366	0.91328946	0.9172838
PEX3	1.20996708	3.78E-37	7.53E-37

FUCA2	0.64202335	3.65E-145	2.59E-143
PHACTR2	1.45644185	8.55E-68	2.65E-67
PHACTR2-AS	NA	2.08E-69	6.61E-69
RP3-468K18.	NA	NA	NA
LTV1	0.78195151	4.51E-126	5.06E-125
RP3-468K18.	NA	NA	NA
ZC2HC1B	NA	NA	NA
PLAGL1	1.74773022	2.90E-12	3.99E-12
SF3B5	1.88282312	8.25E-27	1.42E-26
STX11	0.74902724	7.30E-29	1.30E-28
UTRN	1.07003891	1.34E-36	2.64E-36
RP1-91J24.3	NA	NA	NA
EPM2A	4.49416342	5.40E-26	9.21E-26
RP3-466P17.	Inf	4.18E-09	5.42E-09
RP3-466P17.	NA	NA	NA
AL023806.1	NA	5.35E-68	1.66E-67
FBXO30	1.8385214	1.30E-18	1.98E-18
RP11-545I5.3	0.74902724	0.1019892	0.10875731
SHPRH	1.81495061	6.93E-108	4.42E-107
GRM1	NA	NA	NA
RAB32	0	2.44E-163	2.21E-160
RP11-715G15	NA	NA	NA
ADGB	NA	NA	NA
STXBP5-AS1	NA	NA	NA
RP11-497D6.	NA	NA	NA
RP11-497D6.	NA	NA	NA
LUADT1	NA	NA	NA
STXBP5	1.33160398	0.54023374	0.55356795
SAMD5	NA	NA	NA
RP11-307P5.	NA	NA	NA
RP11-307P5.	NA	NA	NA
RP11-242F11	NA	NA	NA
RP11-631F7.	NA	NA	NA
RP11-631F7.	NA	NA	NA
SASH1	NA	1.09E-129	1.45E-128
UST	2.99610895	2.08E-114	1.56E-113
UST-AS1	NA	NA	NA
RP11-162J8.3	NA	NA	NA
RP1-111D6.3	NA	NA	NA
TAB2	1.20996708	2.21E-05	2.64E-05
RP1-111D6.2	NA	NA	NA
SUMO4	Inf	3.09E-14	4.40E-14
ZC3H12D	NA	5.57E-111	3.85E-110
PPIL4	1.10966998	2.60E-109	1.72E-108
RP1-12G14.5	NA	NA	NA

GINM1	1.74773022	2.13E-07	2.65E-07
RP1-12G14.9	NA	NA	NA
RP1-12G14.7	Inf	4.21E-37	8.38E-37
KATNA1	2.02514772	3.02E-10	4.00E-10
LATS1	2.24708171	4.25E-88	1.82E-87
AL358852.1	0	0.49178294	0.50500516
NUP43	1.47389231	2.20E-16	3.26E-16
PCMT1	1.11631086	4.92E-83	1.93E-82
RP11-350J20	Inf	4.42E-82	1.70E-81
LRP11	5.24319066	0.00997613	0.0110432
RP11-350J20	NA	NA	NA
RAET1E-AS1	NA	NA	NA
RAET1E	NA	2.42E-44	5.31E-44
RAET1E-AS1	NA	NA	NA
RAET1G	0.74902724	8.63E-24	1.42E-23
ULBP2	0.81144617	1.41E-75	4.91E-75
ULBP1	2.24708171	5.82E-42	1.24E-41
RAET1L	NA	NA	NA
RP11-472G2	NA	NA	NA
ULBP3	NA	1.10E-71	3.60E-71
PPP1R14C	3.49546044	9.88E-27	1.70E-26
IYD	NA	3.45E-89	1.51E-88
PLEKHG1	NA	NA	NA
RP11-136K14	NA	NA	NA
MTHFD1L	1.75976279	0.02071309	0.02269321
RP1-292B18.	NA	8.17E-54	2.06E-53
RP1-292B18.	NA	NA	NA
RP1-297M16	NA	NA	NA
AKAP12	0.74902724	2.07E-20	3.25E-20
ZBTB2	1.55567195	0.13819557	0.14635984
RMND1	1.93498703	8.65E-125	9.21E-124
ARMT1	1.76853653	0.00152428	0.00172975
CCDC170	1.19844358	6.89E-70	2.20E-69
ESR1	NA	NA	NA
RP3-443C4.2	NA	NA	NA
SYNE1	Inf	1.88E-139	4.98E-138
SYNE1-AS1	NA	NA	NA
RP3-398G3.5	NA	NA	NA
NANOGP11	NA	NA	NA
MYCT1	NA	NA	NA
VIP	NA	NA	NA
RP1-200K18.	NA	NA	NA
FBXO5	2.10303801	4.28E-27	7.41E-27
RP1-101K10.	NA	1.43E-15	2.08E-15
MTRF1L	2.29593131	9.01E-13	1.25E-12

RGS17	2.09727626	0.05408537	0.05830857
RP11-306O1:	NA	1.28E-39	2.63E-39
RP11-475C16	NA	NA	NA
OPRM1	NA	6.80E-33	1.27E-32
IPCEF1	0.37451362	3.01E-54	7.63E-54
CNKSR3	1.59168288	4.31E-09	5.58E-09
SCAF8	1.76241703	2.59E-19	3.99E-19
TIAM2	0.99870298	1.28E-13	1.80E-13
RP11-477D1!	Inf	9.89E-45	2.18E-44
TFB1M	1.02498464	1.81E-115	1.40E-114
CLDN20	Inf	1.80E-15	2.62E-15
RP1-257I9.2	NA	NA	NA
NOX3	NA	NA	NA
RP11-518I13	NA	NA	NA
RP11-230C9.	NA	7.38E-108	4.71E-107
RP11-230C9.	Inf	1.07E-156	3.98E-154
ARID1B	2.40758755	1.29E-27	2.24E-27
RP11-230C9.	NA	NA	NA
RP1-137K2.2	NA	NA	NA
TMEM242	1.53316513	1.56E-101	8.69E-101
RP5-933K21.	NA	2.32E-33	4.37E-33
ZDHHC14	2.39688716	0.0142047	0.01564674
SNX9	1.40993362	3.39E-08	4.31E-08
RP11-52J3.3	NA	NA	NA
RP11-52J3.2	NA	NA	NA
RP3-403L10.:	NA	NA	NA
SYNJ2	1.49805447	1.33E-40	2.77E-40
SERAC1	2.24708171	4.06E-17	6.05E-17
GTF2H5	2.11400581	5.53E-79	2.03E-78
TULP4	3.40466926	3.07E-87	1.29E-86
RP11-732M1	NA	NA	NA
TMEM181	2.44682231	1.19E-87	5.08E-87
DYNLT1	1.7061176	1.46E-13	2.06E-13
SYTL3	NA	4.95E-27	8.56E-27
EZR	1.46060311	1.25E-48	2.91E-48
EZR-AS1	NA	NA	NA
C6orf99	Inf	4.62E-144	2.49E-142
RSPH3	1.74773022	0.10283152	0.10959754
RP1-111C20.	NA	1.24E-38	2.51E-38
TAGAP	1.94747082	3.24E-51	7.84E-51
RP1-111C20.	NA	NA	NA
RP11-13P5.1	NA	NA	NA
RP11-13P5.2	NA	NA	NA
FNDC1	NA	NA	NA
RP11-125D1:	NA	NA	NA

RP11-125D12	NA	NA	NA
RP3-393E18.	NA	1.32E-51	3.23E-51
SOD2	1.73555092	3.75E-64	1.10E-63
WTAP	1.5711303	9.42E-35	1.82E-34
ACAT2	0.69603003	7.87E-137	1.61E-135
TCP1	1.48758902	1.41E-28	2.50E-28
MRPL18	1.89902073	8.87E-07	1.09E-06
PNLDC1	0.74902724	0.82394663	0.83137039
RP1-249F5.3	NA	NA	NA
MAS1	NA	1.33E-36	2.62E-36
IGF2R	2.03307393	0.26932964	0.28123509
AIRN	NA	NA	NA
SLC22A1	NA	3.67E-65	1.09E-64
SLC22A2	NA	NA	NA
RP1-276N6.2	NA	NA	NA
SLC22A3	NA	NA	NA
LPA	NA	NA	NA
RP1-81D8.3	NA	NA	NA
PLG	NA	NA	NA
RP11-235G24	NA	NA	NA
RP11-235G24	NA	NA	NA
RP3-428L16.	NA	NA	NA
RP3-428L16.	0.24967575	0.57900776	0.59199498
MAP3K4	2.99610895	2.52E-75	8.73E-75
AGPAT4	1.49805447	0.64247105	0.65441074
PARK2	0	4.97E-69	1.57E-68
PACRG	NA	1.04E-73	3.52E-73
PACRG-AS2	NA	NA	NA
PACRG-AS3	NA	NA	NA
PACRG-AS1	NA	NA	NA
CAHM	1.49805447	0.00011411	0.00013358
QKI	2.93190661	1.01E-132	1.58E-131
RP5-826L7.1	NA	NA	NA
RP1-230L10.	NA	NA	NA
RP1-155D22.	NA	NA	NA
RP1-155D22.	NA	NA	NA
RP11-347L18	NA	NA	NA
RP11-300M2	NA	NA	NA
C6orf118	NA	NA	NA
PDE10A	NA	NA	NA
RP11-252P19	NA	NA	NA
LINC00473	NA	NA	NA
LINC00602	NA	NA	NA
RP11-459F1.	NA	NA	NA
T	NA	NA	NA

RP4-655C5.4	NA	NA	NA
RP4-655C5.9	NA	NA	NA
PRR18	NA	3.98E-39	8.16E-39
SFT2D1	1.69940588	1.05E-05	1.27E-05
MPC1	1.50492628	6.10E-48	1.41E-47
RP1-168L15.!	Inf	4.66E-54	1.18E-53
RPS6KA2	1.49805447	3.42E-78	1.24E-77
RP11-514O1:	1.79766537	2.81E-23	4.60E-23
RPS6KA2-AS	NA	NA	NA
RNASET2	1.40481457	2.27E-116	1.80E-115
RP1-167A14.	NA	0.00062118	0.00071307
FGFR1OP	2.33030696	0.04702074	0.05082218
CCR6	NA	5.95E-133	9.47E-132
TCP10L2	NA	NA	NA
GPR31	NA	NA	NA
RP11-568A7.	NA	NA	NA
RP11-568A7.	NA	NA	NA
RP11-568A7.	NA	NA	NA
UNC93A	NA	NA	NA
TTLL2	NA	NA	NA
TCP10	NA	NA	NA
RP11-351J23	NA	NA	NA
RP11-351J23	NA	NA	NA
LINC01558	NA	NA	NA
RP3-431P23.	NA	NA	NA
MLLT4-AS1	0.74902724	1.14E-64	3.36E-64
MLLT4	1.89161116	0.00130007	0.00147855
RP3-470B24.	NA	NA	NA
KIF25-AS1	NA	NA	NA
KIF25	NA	0.10239071	0.10914055
FRMD1	NA	NA	NA
RP11-503C2‹	NA	NA	NA
RP11-503C2‹	NA	NA	NA
RP11-503C2‹	NA	NA	NA
RP11-503C2‹	NA	NA	NA
DACT2	NA	NA	NA
RP1-39J2.1	NA	NA	NA
SMOC2	NA	NA	NA
RP1-125N5.2	NA	NA	NA
RP3-495K2.3	NA	NA	NA
RP3-495K2.2	NA	NA	NA
RP3-495K2.1	NA	NA	NA
LINC01615	NA	NA	NA
RP11-417E7.	NA	NA	NA
XXyac-YX65C	NA	NA	NA

THBS2	NA	NA	NA
XXyac-YX65C	NA	NA	NA
RP1-137D17.	NA	NA	NA
RP1-137D17.	NA	1.11E-44	2.44E-44
WDR27	1.99740597	4.15E-62	1.18E-61
C6orf120	3.22081712	3.56E-54	9.02E-54
PHF10	1.54126758	0.32210741	0.33470833
RP1-266L20.:	NA	0.00603539	0.00673022
RP1-266L20.4	NA	NA	NA
TCTE3	2.99610895	1.16E-14	1.66E-14
ERMARD	3.37062257	3.58E-07	4.45E-07
LINC00242	NA	NA	NA
LINC00574	NA	9.92E-66	2.98E-65
RP1-182D15.	NA	NA	NA
RP11-302L19	NA	NA	NA
RP11-302L19	NA	NA	NA
RP5-1086L22	NA	NA	NA
RP5-894D12.	NA	NA	NA
LINC01624	NA	NA	NA
DLL1	0.93628405	5.57E-06	6.74E-06
FAM120B	2.99610895	1.06E-33	2.01E-33
RP5-894D12.	NA	2.40E-11	3.23E-11
PSMB1	1.05999522	1.72E-142	6.68E-141
TBP	2.79182879	0.07579877	0.08127312
PDCD2	1.961738	7.97E-41	1.67E-40
XX-C2158C12	NA	NA	NA
XX-C2158C12	1.74773022	5.59E-26	9.52E-26
XX-C2158C6.:	NA	NA	NA
XX-C2158C6.:	NA	NA	NA
ABC7-48172:	NA	NA	NA
AC093627.7	NA	NA	NA
AC093627.8	NA	NA	NA
AC093627.9	0	1.08E-27	1.89E-27
AC093627.10	NA	0.25013448	0.2616432
RP11-90P13.	NA	NA	NA
AC093627.11	NA	NA	NA
AC093627.12	NA	NA	NA
FAM20C	0	1.81E-20	2.85E-20
WI2-2373I1.:	NA	NA	NA
AC226118.1	NA	NA	NA
AC147651.1	NA	NA	NA
PDGFA	Inf	2.50E-34	4.79E-34
HRAT92	NA	0.01774706	0.01948718
PRKAR1B	3.31712062	6.28E-15	9.05E-15
AC147651.5	NA	NA	NA

AC147651.4	1.87256809	0.0071091	0.00791636
DNAAF5	1.7655642	1.24E-112	8.95E-112
SUN1	2.42542153	1.29E-56	3.37E-56
GET4	0.37451362	1.09E-121	1.03E-120
RP11-449P15	Inf	3.99E-29	7.11E-29
ADAP1	2.06731518	2.43E-08	3.10E-08
COX19	2.16385646	8.90E-08	1.12E-07
CYP2W1	NA	NA	NA
C7orf50	2.11626569	1.60E-122	1.56E-121
AC073957.15	NA	NA	NA
GPR146	1.56614786	1.15E-06	1.41E-06
RP11-449P15	NA	4.91E-46	1.11E-45
AC091729.7	NA	NA	NA
AC091729.8	NA	NA	NA
GPER1	NA	1.82E-99	9.66E-99
ZFAND2A	1.55567195	0.00037654	0.00043519
AC091729.9	2.49675746	0.93122188	0.93441523
UNCX	0	2.64E-136	5.18E-135
AC073094.4	NA	NA	NA
MICALL2	1.12354086	2.17E-11	2.93E-11
AC102953.4	NA	NA	NA
RP11-1246C1	1.74773022	1.21E-22	1.96E-22
INTS1	1.52008469	3.51E-10	4.65E-10
AC102953.6	NA	NA	NA
MAFK	1.2963933	0.04684811	0.05063861
TMEM184A	NA	6.60E-111	4.55E-110
PSMG3	1.89028158	7.89E-35	1.52E-34
PSMG3-AS1	2.33030696	1.57E-35	3.06E-35
AC074389.5	NA	NA	NA
ELFN1	5.9922179	8.85E-107	5.49E-106
AC074389.7	NA	NA	NA
AC074389.6	NA	NA	NA
ELFN1-AS1	1.89337441	1.16E-22	1.88E-22
MAD1L1	2.75152863	4.41E-89	1.93E-88
AC110781.3	NA	3.62E-39	7.42E-39
FTSJ2	1.6798572	1.15E-05	1.38E-05
NUDT1	1.68590575	7.90E-25	1.32E-24
SNX8	2.24708171	0.91926249	0.92307854
EIF3B	1.68785323	1.77E-06	2.17E-06
AC004840.8	NA	NA	NA
CHST12	1.17035506	9.41E-136	1.78E-134
GRIFIN	NA	NA	NA
LFNG	Inf	3.02E-120	2.71E-119
BRAT1	1.57054098	9.94E-06	1.20E-05
IQCE	4.06614786	2.72E-65	8.11E-65

TTYH3	2.0598249	4.78E-106	2.92E-105
AMZ1	NA	NA	NA
GNA12	1.66450497	3.96E-33	7.44E-33
CARD11	NA	5.53E-62	1.57E-61
AC004906.3	NA	NA	NA
AC024028.1	NA	NA	NA
AC091801.1	NA	NA	NA
AC073316.2	NA	NA	NA
AC073316.1	NA	NA	NA
SDK1	NA	NA	NA
AC011284.3	NA	NA	NA
FOXK1	1.78147019	1.22E-12	1.69E-12
AP5Z1	1.81906615	0.00244044	0.00275253
RADIL	3.99481193	0.99803247	0.99819754
PAPOLB	NA	0.09736952	0.10399611
MMD2	NA	NA	NA
RBAK-RBAKD	1.715514	7.82E-52	1.91E-51
RBAK	4.99351492	3.34E-73	1.12E-72
RBAKDN	NA	NA	NA
WIPI2	1.73291895	4.99E-77	1.77E-76
SLC29A4	2.62159533	3.21E-72	1.06E-71
TNRC18	1.87256809	6.68E-13	9.28E-13
AC093620.5	NA	NA	NA
AC092171.2	NA	1.91E-27	3.32E-27
RP11-1275H	0.49935149	1.17E-10	1.56E-10
RP11-1275H	4.49416342	2.02E-35	3.93E-35
RP11-1275H	1.49805447	9.77E-87	4.08E-86
FBXL18	2.43433852	3.22E-16	4.74E-16
AC092171.4	NA	NA	NA
ACTB	0.9285154	1.05E-140	3.22E-139
CTB-161C1.1	0	7.85E-79	2.86E-78
FSCN1	1.39964214	2.87E-30	5.20E-30
RNF216	1.93170182	5.75E-14	8.13E-14
OCM	NA	4.47E-93	2.11E-92
CCZ1	1.72601929	0.00176018	0.00199346
RSPH10B	NA	5.66E-128	6.91E-127
PMS2	1.28404669	6.91E-48	1.59E-47
AIMP2	0.8642622	2.67E-100	1.45E-99
EIF2AK1	1.7061176	1.39E-25	2.36E-25
ANKRD61	NA	1.77E-88	7.66E-88
AC004895.4	NA	9.41E-24	1.55E-23
USP42	1.85053788	6.18E-81	2.34E-80
CYTH3	1.49805447	0.00013992	0.0001634
FAM220A	1.2733463	4.13E-42	8.80E-42
RAC1	1.5399432	6.32E-47	1.44E-46

DAGLB	1.87256809	7.55E-12	1.03E-11
KDELR2	1.33250128	1.40E-129	1.84E-128
GRID2IP	NA	NA	NA
ZDHHC4	1.56047341	1.60E-36	3.15E-36
AC079742.4	Inf	5.03E-10	6.63E-10
C7orf26	1.89753567	0.00039596	0.00045722
ZNF853	Inf	2.92E-95	1.44E-94
ZNF316	3.99481193	4.95E-62	1.40E-61
AC073343.13	NA	NA	NA
ZNF12	1.74773022	1.73E-15	2.51E-15
RSPH10B2	NA	NA	NA
CCZ1B	1.76383833	3.61E-38	7.30E-38
C1GALT1	2.17217899	2.03E-17	3.04E-17
COL28A1	NA	NA	NA
RP5-1159O4.	Inf	2.06E-106	1.27E-105
RP5-1159O4.	Inf	3.76E-61	1.05E-60
MIOS	1.77401188	0.13295999	0.14103726
RP5-1007H1(	Inf	3.25E-135	5.86E-134
RPA3	1.62772314	1.64E-45	3.67E-45
UMAD1	1.49805447	2.95E-08	3.75E-08
AC007161.5	NA	2.76E-116	2.18E-115
AC009473.1	Inf	0.02180709	0.02385291
AC006042.7	NA	NA	NA
GLCCI1	1.87256809	1.69E-07	2.11E-07
ICA1	1.01653696	0.14387312	0.15225726
AC006042.6	NA	NA	NA
AC007009.1	NA	NA	NA
AC007128.1	NA	1.96E-21	3.13E-21
RP4-594A5.1	NA	NA	NA
NXPH1	NA	NA	NA
RP4-668E10.	NA	NA	NA
AC060834.3	NA	NA	NA
AC006373.1	NA	NA	NA
CTD-2060L22	NA	1.75E-135	3.23E-134
AC004535.2	NA	NA	NA
NDUFA4	1.90421713	6.60E-19	1.01E-18
RP5-855F16.	NA	3.69E-11	4.97E-11
PHF14	1.57871895	9.16E-27	1.58E-26
THSD7A	1.12354086	2.79E-44	6.11E-44
AC004160.4	NA	NA	NA
TMEM106B	0.85603113	8.72E-11	1.17E-10
VWDE	1.74773022	2.72E-06	3.31E-06
AC005281.1	NA	0.00596164	0.00664881
AC005281.2	NA	NA	NA
SCIN	NA	NA	NA

ARL4A	1.64396887	2.62E-53	6.55E-53
AC011288.2	NA	NA	NA
AC006000.5	NA	NA	NA
AC005019.3	NA	NA	NA
ETV1	2.99610895	0.09482468	0.10131983
DGKB	NA	3.62E-19	5.57E-19
AC006150.1	NA	NA	NA
AC006458.3	NA	NA	NA
AGMO	NA	NA	NA
MEOX2	NA	NA	NA
AC005550.3	NA	NA	NA
MEOX2-AS1	NA	NA	NA
AC005550.5	NA	NA	NA
AC006041.1	NA	NA	NA
ISPD	1.49805447	0.40479083	0.41761247
ISPD-AS1	NA	NA	NA
SOSTDC1	NA	NA	NA
GS1-166A23.	NA	NA	NA
LRRC72	NA	NA	NA
GS1-166A23.	NA	2.80E-140	8.11E-139
ANKMY2	0.70960475	3.44E-68	1.07E-67
BZW2	1.22047379	1.10E-119	9.74E-119
AC073333.8	NA	1.87E-29	3.34E-29
TSPAN13	2.14144967	0.37952047	0.3925462
AC073333.1	NA	NA	NA
AGR2	NA	2.06E-131	2.99E-130
AGR3	NA	7.43E-45	1.64E-44
AC003075.4	2.24708171	2.64E-92	1.23E-91
AHR	1.60505837	7.63E-87	3.20E-86
AC019117.1	NA	NA	NA
AC019117.2	NA	NA	NA
AC017060.1	NA	NA	NA
AC006482.1	NA	NA	NA
SNX13	1.87256809	2.52E-10	3.34E-10
PRPS1L1	NA	NA	NA
HDAC9	NA	NA	NA
AC010082.2	NA	NA	NA
AC004744.3	NA	NA	NA
TWIST1	2.30950065	7.17E-28	1.25E-27
AC003986.6	NA	NA	NA
AC003986.7	NA	NA	NA
AC003986.5	NA	NA	NA
FERD3L	NA	NA	NA
AC007091.1	NA	NA	NA
TWISTNB	1.29221493	1.42E-96	7.19E-96

TMEM196	NA	NA	NA
AC004543.2	NA	NA	NA
RP11-486P11	NA	NA	NA
MACC1	2.24708171	4.61E-10	6.09E-10
MACC1-AS1	NA	NA	NA
AC099342.1	NA	NA	NA
CTA-293F17.	NA	2.37E-63	6.83E-63
ITGB8	NA	5.85E-09	7.55E-09
ABCB5	NA	NA	NA
SP8	0	6.93E-33	1.30E-32
LINC01162	NA	NA	NA
SP4	0.99870298	0.31119428	0.32372057
DNAH11	NA	3.25E-135	5.86E-134
CDCA7L	1.83632484	8.61E-28	1.50E-27
RAPGEF5	NA	NA	NA
STEAP1B	NA	NA	NA
AC002480.3	NA	NA	NA
AC002480.4	NA	NA	NA
AC002480.2	NA	NA	NA
AC073072.5	NA	NA	NA
IL6	NA	NA	NA
AC073072.1	NA	NA	NA
TOMM7	2.46368353	2.46E-145	1.92E-143
FAM126A	1.67429618	1.82E-73	6.15E-73
KLHL7-AS1	0	7.84E-35	1.51E-34
KLHL7	2.08063121	3.94E-09	5.11E-09
NUPL2	1.56871742	4.38E-125	4.70E-124
AC005082.12	NA	7.30E-81	2.75E-80
GPNMB	0.74902724	4.27E-45	9.49E-45
MALSU1	1.599408	7.50E-24	1.24E-23
IGF2BP3	1.08949416	1.94E-44	4.27E-44
TRA2A	1.3153649	2.95E-121	2.74E-120
CCDC126	0.83225249	4.11E-21	6.52E-21
AC006026.2	NA	NA	NA
FAM221A	Inf	2.63E-134	4.50E-133
AC006026.13	NA	NA	NA
STK31	NA	NA	NA
AC009508.1	NA	NA	NA
AC004485.3	NA	NA	NA
NPY	NA	NA	NA
MPP6	1.48392189	0.16598988	0.17518257
DFNA5	NA	5.28E-27	9.12E-27
OSBPL3	4.49416342	1.82E-29	3.27E-29
CYCS	1.7106753	0.05723748	0.06165186
C7orf31	1.87256809	3.91E-15	5.66E-15

NPVF	NA	NA	NA
AC003985.1	NA	NA	NA
RP5-978I12.1	NA	NA	NA
AC091705.1	NA	NA	NA
AC003090.1	NA	NA	NA
CTD-2227E11	Inf	3.18E-52	7.84E-52
NFE2L3	0.84265564	7.50E-75	2.57E-74
CTB-119C2.1	2.24708171	0.14581385	0.15427508
HNRNPA2B1	1.2770031	2.96E-136	5.78E-135
CBX3	2.49836827	3.64E-140	1.04E-138
SNX10	2.86861495	3.47E-72	1.15E-71
AC004540.4	Inf	8.21E-134	1.37E-132
AC004540.5	Inf	2.50E-67	7.68E-67
KIAA0087	NA	NA	NA
AC004947.2	NA	NA	NA
C7orf71	NA	NA	NA
SKAP2	1.62289235	7.21E-23	1.17E-22
HOXA1	4.11964981	9.58E-42	2.04E-41
HOTAIRM1	2.1875	1.60E-34	3.08E-34
HOXA2	NA	1.11E-29	1.99E-29
HOXA3	Inf	1.88E-28	3.32E-28
HOXA-AS2	NA	NA	NA
HOXA4	Inf	1.55E-116	1.24E-115
HOXA-AS3	Inf	0.90400192	0.90871026
HOXA5	3.74513619	3.46E-15	5.00E-15
HOXA6	2.62159533	5.48E-113	4.00E-112
HOXA7	1.67963684	0.00028287	0.00032802
HOXA9	1.53679726	1.57E-13	2.20E-13
HOXA10-HOX	NA	NA	NA
HOXA10-AS	2.14938251	9.75E-40	2.01E-39
HOXA10	1.61843385	0.00768939	0.00855257
HOXA11	1.07003891	3.38E-07	4.21E-07
HOXA11-AS	1.15475032	2.32E-119	2.02E-118
RP1-170O19.	NA	NA	NA
HOXA13	1.94747082	5.48E-20	8.54E-20
HOTTIP	2.80885214	1.22E-94	5.93E-94
RP1-170O19.	NA	NA	NA
EVX1-AS	NA	NA	NA
EVX1	NA	NA	NA
AC004009.3	NA	NA	NA
HIBADH	1.70233463	7.47E-86	3.07E-85
AC004549.6	NA	4.21E-116	3.31E-115
TAX1BP1	1.43411313	7.84E-112	5.52E-111
JAZF1	2.49675746	4.02E-09	5.21E-09
JAZF1-AS1	NA	2.26E-39	4.64E-39

CREB5	NA	NA	NA
TRIL	NA	NA	NA
AC005013.5	NA	NA	NA
CTB-113D17.	NA	4.30E-54	1.09E-53
AC005162.5	NA	NA	NA
CPVL	NA	7.43E-45	1.64E-44
AC005162.4	NA	NA	NA
CHN2	NA	8.17E-54	2.06E-53
AC004593.3	NA	NA	NA
AC007255.8	NA	NA	NA
PRR15	Inf	7.12E-06	8.60E-06
WIPF3	NA	4.47E-148	5.34E-146
SCRN1	3.05372643	1.93E-57	5.11E-57
AC007285.6	NA	NA	NA
FKBP14	0.89883268	9.43E-22	1.51E-21
PLEKHA8	3.42412451	1.34E-26	2.30E-26
AC007285.7	NA	NA	NA
MTURN	1.89459831	3.49E-103	2.01E-102
AC007036.6	NA	NA	NA
ZNRF2	2.99610895	4.31E-40	8.95E-40
GS1-114I9.3	NA	NA	NA
LINC01176	NA	1.13E-11	1.53E-11
NOD1	3.2457847	1.15E-55	2.97E-55
GS1-114I9.1	NA	1.25E-13	1.76E-13
GGCT	1.84101566	0.00057181	0.0006571
RP4-777O23.	NA	1.11E-60	3.08E-60
RP4-777O23.	NA	NA	NA
RP4-777O23.	NA	NA	NA
GARS	1.97422179	0.0003489	0.00040356
CRHR2	NA	NA	NA
INMT	NA	NA	NA
INMT-FAM18	NA	NA	NA
FAM188B	NA	NA	NA
AC004691.5	NA	NA	NA
RP5-877J2.1	NA	NA	NA
AQP1	NA	NA	NA
GHRHR	NA	NA	NA
ADCYAP1R1	NA	NA	NA
NEUROD6	NA	NA	NA
AC006380.3	NA	NA	NA
CCDC129	NA	NA	NA
PPP1R17	NA	NA	NA
PDE1C	NA	NA	NA
LSM5	2.05829538	1.85E-126	2.12E-125
AVL9	2.09727626	3.47E-05	4.12E-05

RP11-225B1	0.74902724	0.94319684	0.94601266
LINC00997	Inf	0.00779383	0.00866554
RP11-265E1	NA	NA	NA
KBTBD2	4.68142023	0.7864963	0.79499754
FKBP9	1.55155642	1.85E-16	2.74E-16
NT5C3A	1.22290161	9.36E-83	3.65E-82
RP9	1.35190282	1.46E-53	3.67E-53
BBS9	0.74902724	3.96E-71	1.29E-70
RP11-89N17	NA	NA	NA
RP11-89N17	NA	NA	NA
RP11-89N17	NA	NA	NA
RP11-89N17	NA	NA	NA
BMPER	NA	NA	NA
AC009262.2	NA	NA	NA
NPSR1	NA	NA	NA
DPY19L1	1.49805447	0.00161807	0.00183504
TBX20	NA	5.32E-10	7.02E-10
AC009531.2	NA	NA	NA
AC007652.1	NA	NA	NA
HERPUD2	1.68531128	0.00065367	0.00075
RP11-379H1	1.79766537	2.59E-37	5.18E-37
AC018647.3	NA	NA	NA
SEPT7-AS1	Inf	2.31E-115	1.78E-114
7-Sep	1-Jan	9.16E-109	5.95E-108
AC083864.3	NA	NA	NA
AC083864.4	NA	NA	NA
EEPD1	2.49675746	0.00069265	0.00079411
AC007327.5	NA	NA	NA
RP11-182J23	NA	4.01E-05	4.75E-05
KIAA0895	3.74513619	2.09E-39	4.30E-39
ANLN	1.59168288	8.02E-88	3.43E-87
AC006960.7	NA	NA	NA
AOAH	NA	5.69E-11	7.62E-11
RP11-85E16.	NA	0.05255704	0.05667775
AC007349.4	NA	NA	NA
AC007349.5	NA	NA	NA
AC007349.7	NA	NA	NA
ELMO1	2.99610895	1.63E-46	3.70E-46
ELMO1-AS1	NA	NA	NA
GPR141	NA	2.86E-22	4.61E-22
EPDR1	1.9214177	1.72E-48	4.02E-48
NME8	NA	NA	NA
SFRP4	NA	1.46E-121	1.36E-120
STARD3NL	1.79231518	1.34E-12	1.86E-12
TRGC2	1.03711464	7.26E-35	1.40E-34

TRGJ2	NA	NA	NA
TRGJP2	NA	NA	NA
TRGC1	1.48036092	6.07E-36	1.19E-35
TRGJ1	NA	NA	NA
TRGJP	NA	NA	NA
TRGJP1	NA	NA	NA
TRGV11	NA	NA	NA
TRGVB	NA	NA	NA
TRGV10	NA	NA	NA
TRGV9	0	1.21E-77	4.32E-77
TRGVA	NA	NA	NA
RP11-121A8.	Inf	1.08E-123	1.10E-122
TRGV8	NA	NA	NA
TRGV7	NA	NA	NA
TRGV6	NA	NA	NA
TRG-AS1	2.32733463	2.60E-115	2.00E-114
TRGV5	2.99610895	0.12813766	0.13605721
TRGV5P	NA	NA	NA
TRGV4	NA	NA	NA
TRGV3	NA	3.33E-76	1.16E-75
TRGV2	NA	NA	NA
TRGV1	NA	NA	NA
AMPH	NA	3.55E-36	6.99E-36
VPS41	2.03307393	0.03395788	0.03688784
POU6F2	NA	NA	NA
POU6F2-AS2	NA	NA	NA
POU6F2-AS1	NA	NA	NA
AC011290.4	NA	NA	NA
YAE1D1	1.35401078	2.10E-31	3.86E-31
AC004837.4	NA	NA	NA
RALA	1.36309461	8.94E-99	4.71E-98
AC004837.5	NA	NA	NA
LINC00265	NA	6.16E-70	1.97E-69
RP11-467D6.	Inf	0.00031052	0.00035974
CDK13	2.24708171	1.56E-06	1.91E-06
MPLKIP	1.81820321	5.10E-29	9.07E-29
SUGCT	Inf	0.02190318	0.02395512
AC004988.1	NA	NA	NA
LINC01450	NA	NA	NA
LINC01449	NA	NA	NA
INHBA	NA	3.73E-25	6.29E-25
INHBA-AS1	NA	NA	NA
GLI3	NA	NA	NA
LINC01448	NA	NA	NA
AC010132.1C	NA	NA	NA

RP11-111K18	NA	2.67E-09	3.48E-09
C7orf25	1.49805447	8.66E-08	1.09E-07
RP11-111K18	NA	NA	NA
PSMA2	1.72954192	9.41E-13	1.30E-12
MRPL32	1.19417866	1.20E-89	5.32E-89
AC005537.2	NA	NA	NA
HECW1	NA	NA	NA
AC004692.4	NA	NA	NA
AC011738.4	NA	NA	NA
STK17A	1.0083059	1.68E-98	8.82E-98
COA1	1.78590378	1.81E-11	2.45E-11
BLVRA	0.83981842	1.34E-126	1.54E-125
MRPS24	0.89883268	2.67E-26	4.57E-26
URGCP-MRP	NA	NA	NA
URGCP	1.29377432	7.33E-18	1.11E-17
UBE2D4	2.49675746	9.87E-67	3.01E-66
SPDYE1	NA	NA	NA
AC004951.6	1.91418072	0.44871432	0.46169324
LINC00957	NA	0.01196134	0.01321334
DBNL	1.42720055	2.85E-105	1.71E-104
PGAM2	NA	7.88E-117	6.32E-116
POLM	1.35761187	0.41794381	0.43076563
AEBP1	NA	NA	NA
POLD2	1.86140373	0.0027485	0.0030948
MYL7	NA	NA	NA
GCK	NA	NA	NA
YKT6	2.20899558	7.83E-57	2.05E-56
CAMK2B	0	7.54E-64	2.20E-63
NUDCD3	1.96966422	2.01E-39	4.13E-39
NPC1L1	NA	NA	NA
DDX56	1.04671755	3.72E-83	1.46E-82
TMED4	1.70233463	2.20E-15	3.20E-15
OGDH	1.83511673	1.56E-06	1.91E-06
ZMIZ2	2.58754864	2.51E-39	5.17E-39
AC013436.6	NA	NA	NA
PPIA	1.32571419	3.73E-145	2.63E-143
H2AFV	1.81381206	2.43E-25	4.10E-25
AC004854.4	NA	1.20E-11	1.63E-11
PURB	2.88375486	2.97E-103	1.71E-102
RP4-673M15	NA	7.03E-36	1.38E-35
MYO1G	2.22835603	3.09E-90	1.38E-89
SNHG15	2.04240566	1.33E-87	5.65E-87
CCM2	1.82277727	0.00021709	0.00025232
NACAD	NA	NA	NA
TBRG4	1.25462062	2.04E-51	4.97E-51

RAMP3	NA	NA	NA
AC073325.1	NA	NA	NA
ADCY1	NA	2.20E-06	2.69E-06
IGFBP1	NA	NA	NA
IGFBP3	NA	NA	NA
AC073115.6	NA	NA	NA
AC073115.7	NA	1.49E-42	3.20E-42
AC023669.1	NA	NA	NA
AC092657.2	NA	NA	NA
AC004869.3	NA	NA	NA
AC004869.2	NA	NA	NA
AC011294.3	NA	NA	NA
AC004901.1	NA	NA	NA
AC004870.3	NA	NA	NA
TNS3	NA	NA	NA
LINC01447	NA	NA	NA
C7orf65	NA	8.18E-140	2.25E-138
HUS1	1.45399405	0.20604924	0.21655344
LINC00525	NA	NA	NA
PKD1L1	NA	NA	NA
C7orf69	NA	NA	NA
SUN3	Inf	0.00016902	0.00019698
C7orf57	NA	1.24E-38	2.51E-38
UPP1	1.24837873	4.85E-56	1.26E-55
ABCA13	NA	NA	NA
AC091770.3	NA	NA	NA
AC010971.1	NA	NA	NA
VWC2	NA	NA	NA
ZPBP	NA	NA	NA
C7orf72	NA	NA	NA
AC020743.2	NA	NA	NA
AC020743.3	NA	NA	NA
AC020743.4	NA	NA	NA
IKZF1	2.34071012	4.06E-10	5.37E-10
FIGNL1	2.24708171	2.39E-69	7.59E-69
DDC	Inf	2.21E-11	2.98E-11
DDC-AS1	NA	NA	NA
GRB10	1.49805447	5.89E-09	7.60E-09
AC004920.3	NA	NA	NA
RP4-724E13.	NA	NA	NA
COBL	NA	NA	NA
RP4-718N17.	NA	NA	NA
RP11-153N1	NA	NA	NA
POM121L12	NA	NA	NA
GS1-278J22.	NA	NA	NA

GS1-278J22.1	NA	NA	NA
LINC01446	NA	NA	NA
RP11-806J6.1	NA	NA	NA
HPVC1	NA	NA	NA
LINC01445	NA	NA	NA
VSTM2A	NA	NA	NA
GS1-18A18.2	NA	NA	NA
SEC61G	1.95682007	8.94E-56	2.31E-55
RP11-745C15	NA	NA	NA
EGFR	NA	NA	NA
EGFR-AS1	NA	NA	NA
ELDR	NA	NA	NA
LANCL2	0.74902724	0.00043762	0.00050483
VOPP1	2.28453307	1.56E-129	2.06E-128
RP11-310H4.	NA	NA	NA
RP11-310H4.	NA	NA	NA
RP11-310H4.	NA	NA	NA
14-Sep	NA	1.39E-136	2.77E-135
RP11-15K19.	NA	NA	NA
ZNF713	0.74902724	0.57121526	0.58425817
GBAS	1.70889177	4.85E-23	7.92E-23
MRPS17	2.58318368	1.88E-109	1.25E-108
PSPH	1.72144856	0.67990753	0.69099112
CCT6A	1.12970569	5.57E-101	3.06E-100
SUMF2	1.48892	1.67E-70	5.38E-70
PHKG1	2.49675746	0.02731344	0.02978066
CHCHD2	1.87129741	8.23E-45	1.82E-44
NUPR2	NA	NA	NA
RP11-760D2.	NA	NA	NA
RP11-760D2.	NA	NA	NA
RP11-760D2.	NA	NA	NA
AC118758.1	NA	NA	NA
RP13-580B18	NA	NA	NA
ZNF479	NA	NA	NA
RP11-1217F2	NA	NA	NA
RP11-114G1	NA	NA	NA
ZNF716	NA	NA	NA
RP11-196D18	NA	NA	NA
RP5-905H7.9	NA	NA	NA
RP5-905H7.8	NA	NA	NA
RP11-73B2.2	NA	NA	NA
RP11-73B2.4	NA	NA	NA
RP11-340I6.3	NA	NA	NA
RP11-340I6.1	NA	NA	NA
AC079355.1	NA	NA	NA

RP11-340I6.7	NA	7.05E-52	1.73E-51
RP11-340I6.8	NA	1.64E-59	4.46E-59
LINC01005	NA	NA	NA
ZNF727	NA	NA	NA
RP11-321E8.	NA	NA	NA
ZNF735	NA	NA	NA
ZNF679	NA	NA	NA
ZNF736	2.7464332	2.41E-46	5.45E-46
ZNF680	1.84375935	3.00E-18	4.55E-18
ZNF107	2.24708171	3.86E-41	8.13E-41
ZNF138	1.44811933	2.37E-43	5.14E-43
ZNF273	2.67509728	3.05E-14	4.35E-14
RP11-797H7.	NA	NA	NA
ZNF117	2.52796693	7.15E-21	1.13E-20
ERV3-1	2.68256266	5.83E-132	8.73E-131
AC104073.2	NA	NA	NA
RP11-328P23	NA	7.44E-119	6.35E-118
ZNF92	2.04280156	9.79E-41	2.05E-40
RP11-667F9.	NA	NA	NA
AC104057.1	NA	NA	NA
RP11-479O9.	NA	1.32E-74	4.54E-74
VKORC1L1	2.12767157	1.22E-77	4.36E-77
GUSB	1.29377432	8.41E-20	1.31E-19
ASL	1.10966998	1.74E-139	4.62E-138
AC068533.7	NA	NA	NA
CRCP	1.92607004	6.63E-05	7.81E-05
RP5-1132H15	NA	NA	NA
TPST1	1.74773022	2.76E-33	5.20E-33
LINC00174	Inf	7.86E-139	1.94E-137
GS1-124K5.3	Inf	0.02441846	0.02667227
GS1-124K5.4	2.0598249	5.46E-39	1.12E-38
GS1-124K5.1	1.34824903	7.90E-18	1.19E-17
KCTD7	Inf	7.15E-06	8.64E-06
RABGEF1	1.94747082	5.08E-28	8.90E-28
RP11-792A8.	3.17088197	1.21E-85	4.94E-85
RP11-458F8.	2.99610895	0.43149719	0.44435614
TMEM248	1.85934997	9.19E-24	1.51E-23
SBDS	1.31079767	1.40E-111	9.79E-111
TYW1	1.79231518	0.67952715	0.69064323
RP11-166O4.	1.87256809	0.11090021	0.11801687
LINC01372	Inf	3.70E-85	1.50E-84
RP4-736H5.3	NA	NA	NA
RP11-358M3	NA	NA	NA
RP5-945F2.2	NA	NA	NA
RP5-945F2.3	NA	NA	NA

RP11-123O1.	NA	NA	NA
RP11-3P22.1	NA	NA	NA
RP11-3P22.2	NA	NA	NA
RP5-942I16.1	NA	0.22131413	0.23213927
AUTS2	Inf	1.64E-08	2.09E-08
WBSCR17	NA	NA	NA
CALN1	NA	1.97E-49	4.64E-49
TYW1B	1.12354086	3.02E-52	7.46E-52
POM121	3.20779056	1.36E-123	1.39E-122
RP11-313P13	0.74902724	4.06E-34	7.76E-34
TRIM74	NA	NA	NA
NSUN5	1.16133581	7.46E-79	2.73E-78
TRIM50	NA	NA	NA
FKBP6	NA	NA	NA
FZD9	1.49805447	0.75336032	0.76273581
BAZ1B	1.64917401	0.00115946	0.00132162
BCL7B	2.08987846	6.19E-99	3.27E-98
TBL2	1.15390683	2.16E-98	1.13E-97
MLXIPL	NA	0.00177283	0.00200753
VPS37D	2.24708171	7.13E-25	1.20E-24
DNAJC30	1.25572213	2.25E-114	1.69E-113
WBSCR22	1.30094204	2.28E-106	1.40E-105
STX1A	Inf	3.38E-15	4.89E-15
ABHD11-AS1	NA	3.35E-45	7.44E-45
ABHD11	1.19844358	4.19E-107	2.61E-106
CLDN3	NA	0.00279162	0.00314315
CLDN4	NA	NA	NA
WBSCR27	NA	2.97E-96	1.49E-95
WBSCR28	NA	NA	NA
RP11-731K22	NA	NA	NA
ELN	NA	3.06E-85	1.25E-84
CTB-51J22.1	NA	NA	NA
LIMK1	1.5421149	3.54E-08	4.49E-08
EIF4H	1.68188167	3.14E-10	4.16E-10
LAT2	1.35587801	2.23E-41	4.72E-41
RFC2	1.22290161	1.78E-134	3.09E-133
CLIP2	1.60505837	8.35E-38	1.68E-37
GTF2IRD1	2.99610895	1.21E-80	4.56E-80
RP5-1186P1(	NA	2.64E-126	3.00E-125
GTF2I	2.34458822	2.05E-144	1.18E-142
NCF1	0.32101167	2.72E-129	3.53E-128
GTF2IRD2	0.89883268	1.79E-28	3.16E-28
GATSL2	NA	9.90E-12	1.35E-11
WBSCR16	1.86642853	8.29E-52	2.03E-51
GTF2IRD2B	1.49805447	1.19E-17	1.79E-17

AC004878.2	NA	1.22E-20	1.91E-20
AC004878.8	NA	NA	NA
TRIM73	4.49416342	2.62E-06	3.19E-06
POM121C	2.40385485	3.51E-103	2.02E-102
SPDYE5	NA	NA	NA
HIP1	1.83511673	0.46124736	0.47423914
CCL26	NA	NA	NA
CCL24	NA	NA	NA
RHBDD2	1.33754864	6.27E-73	2.09E-72
POR	2.17574578	0.1013618	0.10812641
TMEM120A	1.59792477	2.94E-96	1.47E-95
STYXL1	1.26151956	2.27E-83	8.95E-83
MDH2	1.14443647	7.84E-140	2.17E-138
SRRM3	1.49805447	8.28E-35	1.60E-34
HSPB1	1.44692803	4.20E-124	4.36E-123
YWHAG	3.28368191	1.35E-134	2.36E-133
SSC4D	2.99610895	0.00202314	0.00228713
ZP3	NA	1.56E-76	5.50E-76
DTX2	2.99610895	3.21E-10	4.24E-10
AC007078.4	NA	NA	NA
UPK3B	NA	NA	NA
SPDYE16	NA	1.07E-112	7.72E-112
POMZP3	1.19844358	1.63E-05	1.95E-05
AC005522.7	NA	NA	NA
CCDC146	Inf	1.91E-30	3.47E-30
FGL2	NA	NA	NA
AC073635.5	NA	NA	NA
GSAP	Inf	1.21E-05	1.45E-05
RP5-899E9.1	NA	NA	NA
PTPN12	1.63976233	4.46E-13	6.21E-13
APTR	1.3658732	2.06E-30	3.75E-30
RSBN1L	2.03735409	0.00198887	0.00224909
TMEM60	1.78767834	0.00116264	0.00132516
PHTF2	1.95899431	9.04E-07	1.11E-06
RP5-1185I7.1	Inf	0.65328582	0.66494116
MAGI2	NA	NA	NA
MAGI2-AS1	NA	NA	NA
MAGI2-AS2	NA	NA	NA
RP5-1093G2	NA	NA	NA
GNAI1	1.27775235	2.16E-38	4.38E-38
AC003988.1	NA	NA	NA
AC004862.6	NA	NA	NA
CD36	Inf	1.06E-49	2.51E-49
GNAT3	NA	NA	NA
SEMA3C	NA	NA	NA

AC005008.2	NA	NA	NA
AC008163.4	NA	NA	NA
AC010091.1	NA	NA	NA
HGF	0.05851775	1.80E-145	1.58E-143
CACNA2D1	NA	3.78E-14	5.36E-14
AC006145.4	NA	NA	NA
PCLO	NA	1.48E-28	2.61E-28
AC079799.2	NA	NA	NA
SEMA3E	NA	7.13E-55	1.82E-54
SEMA3A	Inf	4.77E-141	1.52E-139
AC003984.1	NA	NA	NA
AC074183.4	NA	NA	NA
SEMA3D	1.49805447	3.74E-43	8.09E-43
LINC00972	NA	7.82E-08	9.85E-08
GRM3	NA	NA	NA
AC005009.1	NA	NA	NA
AC005009.2	NA	NA	NA
KIAA1324L	Inf	3.39E-157	1.43E-154
CTA-254O6.1	NA	NA	NA
AC005076.5	3.44552529	1.38E-68	4.34E-68
DMTF1	1.97470817	1.12E-22	1.82E-22
TMEM243	2.87127108	1.72E-80	6.46E-80
TP53TG1	NA	NA	NA
CROT	NA	NA	NA
ABCB4	NA	NA	NA
ABCB1	NA	5.48E-101	3.01E-100
RUNDC3B	0	1.17E-57	3.09E-57
SLC25A40	2.09727626	1.00E-23	1.65E-23
DBF4	1.90293406	3.25E-29	5.79E-29
ADAM22	11.2354086	6.74E-135	1.20E-133
SRI	1.59540815	2.60E-12	3.58E-12
CTB-167B5.1	NA	NA	NA
AC003991.3	NA	NA	NA
STEAP4	Inf	2.23E-147	2.47E-145
AC002127.4	NA	NA	NA
ZNF804B	NA	NA	NA
C7orf62	NA	NA	NA
AC002383.2	NA	NA	NA
RP5-1121E1(	NA	NA	NA
STEAP1	1.16099222	7.23E-24	1.19E-23
STEAP2	NA	2.11E-133	3.43E-132
CFAP69	NA	8.12E-113	5.91E-112
AC002064.4	NA	NA	NA
AC002064.5	NA	NA	NA
GTPBP10	2.08063121	9.75E-13	1.35E-12

CLDN12	0.96303502	1.36E-22	2.21E-22
CDK14	NA	2.21E-49	5.22E-49
AC002456.2	NA	NA	NA
FZD1	6.74124514	1.88E-45	4.20E-45
RP11-115N4.	NA	NA	NA
RP11-142A5.	NA	NA	NA
CTB-111F10.	NA	NA	NA
MTERF1	1.17035506	1.04E-29	1.87E-29
AKAP9	1.93795936	6.88E-11	9.22E-11
CYP51A1	3.74513619	6.51E-24	1.08E-23
LRRD1	NA	1.68E-132	2.59E-131
CYP51A1-AS:	NA	4.77E-20	7.44E-20
KRIT1	1.85628489	4.62E-15	6.67E-15
ANKIB1	2.62159533	3.46E-39	7.09E-39
GATAD1	1.04863813	2.15E-07	2.68E-07
AC007566.1C	NA	4.55E-09	5.89E-09
ERVW-1	NA	NA	NA
PEX1	2.24708171	1.05E-55	2.70E-55
RBM48	1.12354086	3.03E-75	1.05E-74
FAM133B	1.66342412	7.18E-18	1.09E-17
CDK6	3.07413262	2.71E-145	2.08E-143
AC002454.1	4.49416342	5.13E-99	2.72E-98
SAMD9	1.64785992	3.32E-06	4.04E-06
SAMD9L	NA	4.32E-143	1.89E-141
HEPACAM2	NA	NA	NA
VPS50	2.24708171	2.46E-10	3.26E-10
CALCR	NA	NA	NA
GNGT1	NA	NA	NA
TFPI2	NA	NA	NA
AC002076.1C	NA	NA	NA
GNG11	2.3151751	4.26E-51	1.03E-50
BET1	1.82149805	4.25E-35	8.24E-35
AC006378.2	NA	NA	NA
AC003092.1	NA	NA	NA
AC003092.2	NA	NA	NA
COL1A2	NA	0.22131413	0.23213927
CASD1	4.99351492	4.55E-68	1.42E-67
SGCE	0.65539883	1.27E-143	6.27E-142
PEG10	1.77893969	9.51E-05	1.12E-04
PPP1R9A	NA	6.73E-114	5.01E-113
AC002429.5	NA	NA	NA
PON1	NA	NA	NA
PON3	NA	NA	NA
PON2	1.37086117	2.50E-67	7.68E-67
GS1-293C5.1	NA	NA	NA

AC004012.1	NA	2.27E-101	1.26E-100
ASB4	NA	NA	NA
PDK4	NA	NA	NA
AC002451.3	NA	NA	NA
DYNC1I1	2.99610895	4.21E-10	5.55E-10
SLC25A13	1.9865505	1.51E-32	2.82E-32
SHFM1	1.90034101	2.09E-28	3.69E-28
DLX6-AS1	Inf	2.22E-79	8.18E-79
DLX6	1.12354086	6.11E-17	9.08E-17
DLX5	NA	NA	NA
SDHAF3	1.672977	3.87E-05	4.59E-05
TAC1	NA	NA	NA
ASNS	1.97470817	5.47E-27	9.46E-27
OCM2	NA	NA	NA
LMTK2	Inf	1.11E-58	2.98E-58
BHLHA15	NA	NA	NA
TECPR1	2.67509728	3.88E-17	5.79E-17
BRI3	1.83569888	6.94E-05	8.17E-05
BAIAP2L1	Inf	2.66E-93	1.26E-92
RP11-307C18	NA	8.17E-05	9.60E-05
NPTX2	NA	NA	NA
TMEM130	NA	7.50E-22	1.20E-21
TRRAP	1.32181277	0.14972783	0.15833308
AC004893.11	5.24319066	1.44E-33	2.72E-33
SMURF1	2.62159533	2.07E-58	5.53E-58
KPNA7	NA	NA	NA
ARPC1A	2.63342208	2.63E-86	1.09E-85
ARPC1B	2.05483743	5.32E-47	1.21E-46
PDAP1	1.60505837	1.16E-37	2.33E-37
BUD31	1.41952743	1.17E-95	5.81E-95
PTCD1	2.47755163	0.13410249	0.14212451
ATP5J2-PTCD	NA	6.25E-82	2.40E-81
CPSF4	2.3151751	0.50894563	0.5221564
ATP5J2	1.6559279	0.00048336	0.00055656
ZNF789	2.46108949	1.01E-10	1.35E-10
ZNF394	1.65693904	2.49E-23	4.09E-23
ZKSCAN5	2.24708171	1.05E-96	5.34E-96
FAM200A	2.0598249	2.64E-65	7.87E-65
ZNF655	1.72276265	0.98043023	0.9816206
GS1-259H13.	NA	NA	NA
GS1-259H13.	1.49805447	3.19E-15	4.61E-15
ZSCAN25	5.49286641	1.34E-89	5.91E-89
CYP3A5	1.12354086	0.63616357	0.648168
CYP3A7-CYP3	NA	NA	NA
CYP3A7	NA	NA	NA

CYP3A4	NA	NA	NA
RP11-757A1:	NA	NA	NA
CYP3A43	NA	NA	NA
OR2AE1	NA	NA	NA
TRIM4	2.90248054	7.98E-19	1.22E-18
GJC3	NA	1.03E-16	1.52E-16
RP4-604G5.1	NA	NA	NA
AZGP1	NA	NA	NA
ZKSCAN1	1.9599546	1.14E-17	1.72E-17
ZSCAN21	1.07003891	1.97E-23	3.24E-23
ZNF3	3.34181383	3.33E-28	5.85E-28
COPS6	1.01534803	3.06E-123	3.07E-122
MCM7	0.95282617	4.20E-139	1.07E-137
AP4M1	1.49805447	2.87E-13	4.01E-13
TAF6	1.55252918	1.82E-28	3.22E-28
RP11-506M1	NA	6.61E-103	3.77E-102
CNPY4	2.99610895	2.37E-06	2.90E-06
MBLAC1	0.83225249	8.13E-06	9.81E-06
AC073842.19	NA	NA	NA
LAMTOR4	1.88034188	2.22E-10	2.95E-10
C7orf43	3.18336576	8.72E-11	1.17E-10
GAL3ST4	Inf	0.0066531	0.00741313
GPC2	1.24837873	4.43E-78	1.60E-77
STAG3	0.56177043	6.70E-14	9.47E-14
GATS	1.87256809	6.16E-31	1.12E-30
PVRIG	NA	NA	NA
SPDYE3	Inf	5.92E-18	8.95E-18
PILRB	2.838419	1.22E-49	2.90E-49
PILRA	0	6.59E-27	1.14E-26
ZCWPW1	4.24448768	6.90E-25	1.16E-24
MEPCE	2.20026751	1.37E-05	1.64E-05
RP11-758P17	NA	7.34E-77	2.59E-76
PPP1R35	1.66450497	9.82E-10	1.29E-09
RP11-758P17	NA	0.58204523	0.59499998
C7orf61	1.04863813	0.01388937	0.01530775
TSC22D4	1.99905945	3.93E-08	4.98E-08
NYAP1	NA	1.98E-32	3.69E-32
RP11-44M6.:	NA	NA	NA
AGFG2	Inf	0.02691699	0.02935722
SAP25	2.24708171	5.31E-05	6.27E-05
LRCH4	1.37821012	1.38E-95	6.82E-95
FBXO24	0	0.12915858	0.13711714
PCOLCE-AS1	NA	NA	NA
PCOLCE	1.31079767	2.38E-57	6.28E-57
MOSPD3	2.03581762	1.01E-12	1.40E-12

TFR2	1.81343436	1.77E-12	2.45E-12
ACTL6B	NA	NA	NA
GNB2	1.45964282	1.30E-67	4.01E-67
GIGYF1	2.87127108	1.20E-51	2.93E-51
POP7	2.12872128	3.00E-29	5.36E-29
EPO	NA	0.38801892	0.40076511
ZAN	NA	NA	NA
EPHB4	2.29701686	8.32E-47	1.89E-46
SLC12A9	2.007393	1.00E-07	1.26E-07
RP11-126L15	0.74902724	2.07E-15	3.00E-15
TRIP6	1.28999135	6.31E-144	3.29E-142
SRRT	1.56122545	3.13E-40	6.51E-40
UFSP1	1.74773022	3.91E-20	6.11E-20
ACHE	1.24837873	8.86E-10	1.16E-09
MUC3A	NA	2.91E-101	1.61E-100
RP11-395B7.	NA	NA	NA
MUC12	0.74902724	4.64E-15	6.70E-15
RP11-395B7.	Inf	0.05327863	0.05744909
MUC17	NA	NA	NA
TRIM56	2.0354001	9.03E-118	7.46E-117
SERPINE1	NA	3.96E-16	5.82E-16
AP1S1	1.91548111	2.62E-14	3.73E-14
VGF	1.07672665	1.00E-118	8.50E-118
NAT16	NA	9.42E-102	5.25E-101
MOGAT3	Inf	9.67E-127	1.11E-125
PLOD3	1.60820554	5.03E-42	1.07E-41
ZNHIT1	1.86138858	6.89E-33	1.29E-32
CLDN15	3.21011673	1.95E-43	4.23E-43
FIS1	2.22307443	3.01E-39	6.19E-39
RP11-132A1.	NA	NA	NA
RP11-132A1.	NA	NA	NA
IFT22	2.61246085	4.54E-127	5.34E-126
COL26A1	Inf	2.10E-135	3.85E-134
LINC01007	NA	NA	NA
MYL10	NA	NA	NA
AC004953.1	NA	NA	NA
CUX1	3.06744488	3.08E-65	9.18E-65
CTA-339C12.	NA	NA	NA
CTA-305I2.1	NA	NA	NA
SH2B2	1.47389231	2.08E-36	4.11E-36
SPDYE6	NA	NA	NA
PRKRIP1	1.3732166	1.61E-71	5.27E-71
RP11-514P8.	NA	NA	NA
ORAI2	1.23877582	1.59E-74	5.44E-74
ALKBH4	2.2250515	5.70E-29	1.01E-28

LRWD1	1.76021401	1.83E-56	4.77E-56
POLR2J	1.99314081	4.98E-85	2.02E-84
RASA4B	Inf	1.05E-123	1.07E-122
POLR2J3	1.78339818	0.01354244	0.01493084
RP11-514P8.	NA	NA	NA
SPDYE2	NA	NA	NA
RASA4	0	6.99E-17	1.04E-16
RP11-514P8.	NA	9.10E-16	1.33E-15
UPK3BL	Inf	7.30E-81	2.75E-80
RP11-577H5.	NA	NA	NA
SPDYE2B	NA	NA	NA
POLR2J2	2.99610895	3.57E-40	7.41E-40
AC105052.2	NA	NA	NA
FAM185A	1.19844358	1.45E-08	1.85E-08
FBXL13	2.99610895	2.28E-35	4.43E-35
LRRC17	NA	1.98E-118	1.67E-117
NFE4	2.92120623	1.49E-135	2.77E-134
RP11-645N1	0	1.40E-113	1.03E-112
ARMC10	2.07138397	1.22E-64	3.61E-64
NAPEPLD	1.02991245	1.63E-15	2.38E-15
PMPCB	1.2067661	1.21E-109	8.09E-109
DNAJC2	1.54888132	6.58E-31	1.20E-30
PSMC2	0.88773599	9.44E-137	1.92E-135
SLC26A5	NA	NA	NA
CTB-107G13.	NA	NA	NA
RELN	NA	NA	NA
ORC5	1.10570687	3.20E-55	8.23E-55
LHFPL3	NA	NA	NA
RP11-325F22	NA	NA	NA
LINC01004	Inf	1.57E-09	2.05E-09
KMT2E-AS1	1.19844358	1.39E-06	1.70E-06
KMT2E	2.18568604	1.28E-37	2.57E-37
CTB-152G17.	0.37451362	0.00020518	0.00023872
SRPK2	1.39524681	1.07E-110	7.31E-110
RP4-778K6.3	NA	NA	NA
PUS7	2.37734732	2.72E-47	6.24E-47
RINT1	1.23369192	1.16E-42	2.49E-42
EFCAB10	NA	NA	NA
RP11-251G2.	1.49805447	4.65E-38	9.39E-38
ATXN7L1	2.3151751	1.56E-48	3.62E-48
CDHR3	1.49805447	1.69E-11	2.28E-11
SYPL1	1.14226654	5.79E-82	2.23E-81
NAMPT	2.67044493	0.07831536	0.08392683
RP11-22N19.	Inf	5.46E-105	3.26E-104
CTB-30L5.1	NA	NA	NA

CCDC71L	1.97112431	9.22E-72	3.02E-71
RP5-884M6.1	Inf	2.51E-39	5.16E-39
PIK3CG	5.24319066	6.66E-83	2.60E-82
PRKAR2B	4.49416342	3.28E-126	3.71E-125
HBP1	0.91547773	1.51E-48	3.52E-48
CTA-363E19.	NA	6.20E-84	2.47E-83
COG5	1.66450497	1.07E-22	1.74E-22
GPR22	NA	1.40E-113	1.03E-112
DUS4L	1.5421149	0.41719827	0.43002164
RP4-593H12.	Inf	1.45E-71	4.74E-71
BCAP29	1.80862674	0.348664	0.36145499
SLC26A4-AS1	Inf	3.22E-22	5.19E-22
SLC26A4	NA	4.83E-114	3.61E-113
AC002467.7	1.42315175	0.35349096	0.3663125
CBLL1	2.28453307	1.26E-53	3.16E-53
SLC26A3	NA	NA	NA
DLD	1.27420725	3.63E-79	1.33E-78
LAMB1	2.66320796	1.22E-80	4.58E-80
CTB-13F3.1	NA	1.60E-24	2.68E-24
LAMB4	NA	1.53E-68	4.80E-68
NRCAM	NA	2.07E-65	6.19E-65
PNPLA8	1.87256809	2.99E-41	6.30E-41
THAP5	2.39688716	1.04E-05	1.25E-05
DNAJB9	1.33754864	1.55E-51	3.78E-51
AC005487.2	NA	NA	NA
C7orf66	NA	NA	NA
AC004014.3	NA	NA	NA
AC002386.1	NA	NA	NA
AC073071.1	NA	NA	NA
AC003088.1	NA	NA	NA
IMMP2L	2.52796693	1.26E-13	1.78E-13
LRRN3	NA	NA	NA
DOCK4	NA	1.87E-16	2.76E-16
DOCK4-AS1	NA	NA	NA
ZNF277	1.94747082	9.83E-20	1.53E-19
AC004112.4	NA	NA	NA
IFRD1	1.70931857	2.39E-30	4.34E-30
LSMEM1	NA	9.43E-42	2.00E-41
AC002463.3	NA	NA	NA
TMEM168	NA	8.82E-29	1.56E-28
C7orf60	1.87256809	4.31E-12	5.92E-12
HRAT17	NA	NA	NA
GPR85	1.67639429	7.12E-07	8.79E-07
LINC00998	1.69743583	1.17E-09	1.53E-09
PPP1R3A	NA	NA	NA

FOXP2	0.81144617	2.97E-22	4.80E-22
AC073626.2	NA	NA	NA
MDFIC	1.69868677	4.77E-10	6.29E-10
LINC01392	NA	NA	NA
LINC01393	NA	7.05E-18	1.07E-17
RP11-458K1(	NA	NA	NA
RP11-458K1(	NA	NA	NA
RP11-458K1(	NA	NA	NA
TFEC	2.37191958	4.39E-05	5.19E-05
TES	2.12224384	1.02E-48	2.39E-48
AC073130.3	NA	NA	NA
AC002066.1	NA	NA	NA
AC073130.2	NA	NA	NA
CAV2	NA	NA	NA
CAV1	0.74902724	2.97E-05	3.53E-05
AC006159.5	NA	NA	NA
AC006159.4	NA	NA	NA
LINC01510	NA	NA	NA
MET	Inf	1.01E-07	1.27E-07
CAPZA2	1.23127765	1.87E-129	2.45E-128
ST7-AS1	0.74902724	2.79E-20	4.36E-20
ST7	1.61328943	4.99E-27	8.63E-27
AC106873.4	NA	NA	NA
ST7-AS2	NA	NA	NA
WNT2	NA	NA	NA
AC002465.2	NA	NA	NA
ASZ1	NA	NA	NA
CFTR	NA	NA	NA
AC000111.3	NA	NA	NA
AC000111.6	NA	NA	NA
CTTNBP2	1.39105058	2.98E-45	6.62E-45
AC003084.2	NA	NA	NA
LSM8	1.8198197	0.73021384	0.74000333
ANKRD7	NA	NA	NA
RP11-328J2.:	NA	NA	NA
RP4-742N3.1	NA	NA	NA
RP5-1006K1:	NA	NA	NA
KCND2	NA	NA	NA
RP4-797C5.2	NA	NA	NA
TSPAN12	Inf	5.27E-26	8.98E-26
ING3	1.47031273	8.35E-51	2.01E-50
CPED1	Inf	1.06E-154	2.70E-152
WNT16	NA	NA	NA
FAM3C	1.83244163	8.87E-05	1.04E-04
RP4-745K6.1	NA	NA	NA

PTPRZ1	NA	NA	NA
AASS	2.24708171	6.30E-67	1.92E-66
RP11-560I19	NA	NA	NA
FEZF1	NA	NA	NA
FEZF1-AS1	NA	NA	NA
CADPS2	1.49805447	0.00178198	0.00201752
RP5-1101C3.	NA	NA	NA
RNF133	NA	NA	NA
RNF148	NA	3.34E-53	8.35E-53
TAS2R16	NA	NA	NA
RP11-61D3.1	NA	NA	NA
SLC13A1	NA	NA	NA
IQUB	NA	NA	NA
RP11-332K1!	NA	NA	NA
NDUFA5	1.71368353	0.00207036	0.00233978
ASB15	NA	NA	NA
RP11-390E2:	NA	NA	NA
LMOD2	NA	NA	NA
WASL	1.3732166	2.33E-25	3.94E-25
RP11-390E2:	2.24708171	6.96E-16	1.02E-15
HYAL4	NA	NA	NA
SPAM1	NA	NA	NA
RP4-630C24.	NA	NA	NA
TMEM229A	NA	NA	NA
RP5-921G16.	NA	NA	NA
RP11-264K2:	NA	NA	NA
RP5-921G16.	NA	NA	NA
GPR37	NA	NA	NA
C7orf77	NA	NA	NA
POT1	1.74773022	0.56857639	0.58159184
POT1-AS1	Inf	2.96E-151	5.06E-149
RP11-3B12.5	NA	NA	NA
RP11-3B12.2	NA	NA	NA
RP11-3B12.3	NA	NA	NA
RP11-807H1	NA	NA	NA
AC005276.1	NA	NA	NA
AC000370.2	NA	NA	NA
GRM8	0.74902724	1.10E-32	2.06E-32
AC000099.1	NA	NA	NA
ZNF800	2.30950065	2.19E-60	6.05E-60
AC000123.4	NA	NA	NA
AC000124.1	NA	NA	NA
GCC1	1.40442607	0.09958826	0.10627824
ARF5	1.09097445	4.72E-117	3.82E-116
FSCN3	NA	5.86E-12	8.02E-12

PAX4	NA	NA	NA
AC073934.6	NA	NA	NA
SND1	1.78070626	8.02E-30	1.44E-29
SND1-IT1	4.24448768	1.61E-82	6.25E-82
LRRC4	Inf	1.29E-127	1.55E-126
LEP	NA	NA	NA
RP11-62J1.4	NA	1.82E-65	5.45E-65
RBM28	1.23324687	7.09E-107	4.41E-106
PRRT4	1.74773022	8.86E-11	1.18E-10
IMPDH1	1.77042802	0.9870563	0.98798209
AC010655.1	NA	NA	NA
HILPDA	1.64170353	0.92565248	0.92913508
METTL2B	1.52075227	3.00E-18	4.56E-18
RP11-212P7.	3.74513619	8.15E-15	1.17E-14
RP11-274B2:	NA	2.81E-38	5.70E-38
FAM71F2	NA	9.25E-135	1.64E-133
RP11-274B2:	NA	9.36E-17	1.39E-16
FAM71F1	NA	NA	NA
CALU	1.42585908	4.68E-70	1.50E-69
OPN1SW	NA	NA	NA
CCDC136	1.83095547	9.33E-11	1.24E-10
FLNC	2.47178988	2.21E-55	5.69E-55
RP11-309L24	NA	NA	NA
KCP	Inf	8.12E-06	9.80E-06
ATP6V1F	1.91651102	6.23E-13	8.65E-13
RP11-309L24	Inf	4.84E-16	7.11E-16
IRF5	4.01750973	7.62E-88	3.26E-87
RP11-309L24	NA	9.63E-05	1.13E-04
TNPO3	2.24708171	4.74E-51	1.14E-50
TSPAN33	1.37978702	0.13056548	0.1385783
SMO	2.38752432	5.10E-98	2.65E-97
RP11-286H1	NA	NA	NA
AHCYL2	0.24967575	1.07E-30	1.94E-30
STRIP2	2.24708171	9.27E-16	1.35E-15
SMKR1	5.61770428	3.66E-143	1.63E-141
RP11-448A1	3.2457847	2.67E-62	7.60E-62
NRF1	2.24708171	3.52E-23	5.76E-23
RP11-738B7.	NA	NA	NA
UBE2H	1.49805447	6.43E-28	1.13E-27
RP11-306G2	Inf	1.69E-13	2.37E-13
ZC3HC1	1.2753707	1.08E-51	2.63E-51
KLHDC10	2.09727626	9.69E-25	1.62E-24
RP11-775D2:	NA	NA	NA
TMEM209	2.92801556	5.76E-50	1.37E-49
RP11-775D2:	NA	NA	NA

SSMEM1	NA	NA	NA
CPA2	NA	9.40E-104	5.48E-103
CPA4	NA	4.02E-120	3.60E-119
CPA5	NA	NA	NA
CPA1	NA	NA	NA
CEP41	1.86296518	1.73E-43	3.75E-43
RP11-2E11.5	NA	NA	NA
MESTIT1	NA	NA	NA
MEST	0.74902724	9.32E-16	1.36E-15
RP11-2E11.6	Inf	3.16E-05	3.76E-05
COPG2	0.64202335	3.46E-41	7.30E-41
RP11-2E11.9	NA	1.11E-29	1.99E-29
TSGA13	NA	2.08E-141	7.05E-140
KLF14	NA	NA	NA
RP11-36B6.1	NA	NA	NA
AC016831.7	0.74902724	3.90E-07	4.84E-07
AC058791.1	Inf	1.64E-144	9.77E-143
RP11-138A9.	4.11964981	8.71E-125	9.25E-124
RP11-138A9.	2.99610895	3.73E-58	9.96E-58
LINC-PINT	2.80885214	6.88E-41	1.45E-40
MKLN1	2.24708171	1.21E-14	1.73E-14
RP11-180C1(	Inf	1.20E-118	1.02E-117
PODXL	2.33520256	1.89E-31	3.47E-31
AC009518.4	NA	NA	NA
PLXNA4	NA	7.75E-127	8.99E-126
AC018643.4	NA	NA	NA
AC011625.1	NA	NA	NA
FLJ40288	0	3.76E-20	5.88E-20
AC009365.4	NA	NA	NA
CHCHD3	1.52302205	8.12E-58	2.16E-57
AC009518.8	NA	NA	NA
EXOC4	1.72852439	1.15E-20	1.81E-20
LRGUK	NA	0.00073116	0.00083747
AC008154.4	NA	NA	NA
SLC35B4	1.81582361	6.95E-31	1.27E-30
AC008154.5	NA	NA	NA
RP11-38M8.:	Inf	0.02488837	0.02717573
AKR1B1	1.28356031	3.80E-125	4.10E-124
AKR1B10	NA	NA	NA
AKR1B15	NA	NA	NA
BPGM	1.93170182	6.07E-13	8.44E-13
AC009276.4	NA	NA	NA
CALD1	NA	8.28E-143	3.48E-141
AGBL3	0.74902724	3.00E-05	3.56E-05
C7orf49	1.7219017	2.54E-06	3.10E-06

TMEM140	0.74902724	1.32E-56	3.45E-56
RP11-134L1C	NA	NA	NA
WDR91	1.12354086	1.98E-35	3.85E-35
AC009542.2	NA	NA	NA
STRA8	NA	NA	NA
CNOT4	2.20765923	0.08166998	0.08748564
NUP205	1.68531128	0.95642539	0.95864472
C7orf73	2.17467575	2.72E-121	2.53E-120
SLC13A4	NA	NA	NA
RP11-644N4.	Inf	1.60E-132	2.48E-131
FAM180A	NA	NA	NA
AC015987.1	NA	4.04E-114	3.02E-113
MTPN	2.18734334	1.39E-83	5.51E-83
LUZP6	NA	NA	NA
AC024084.1	NA	NA	NA
AC009784.3	NA	NA	NA
AC009264.1	NA	NA	NA
CHRM2	NA	1.52E-128	1.91E-127
PTN	NA	NA	NA
AC078842.4	NA	NA	NA
AC078842.3	NA	NA	NA
DGKI	NA	NA	NA
AC090498.1	1.53550584	3.77E-142	1.39E-140
CREB3L2	2.34071012	3.10E-96	1.55E-95
AC022173.2	NA	NA	NA
AKR1D1	NA	1.25E-48	2.93E-48
AC083867.4	NA	NA	NA
TRIM24	1.75430063	4.39E-26	7.49E-26
SVOPL	1.49805447	6.08E-92	2.80E-91
ATP6V0A4	NA	NA	NA
TMEM213	NA	NA	NA
KIAA1549	2.15345331	0.62417515	0.63627497
ZC3HAV1L	Inf	1.63E-19	2.52E-19
ZC3HAV1	2.97639771	3.82E-93	1.81E-92
TTC26	1.64785992	5.83E-08	7.37E-08
UBN2	5.74254215	3.40E-29	6.07E-29
C7orf55	1.57638412	2.62E-15	3.79E-15
LUC7L2	1.22421656	7.03E-90	3.11E-89
C7orf55-LUC	NA	8.24E-21	1.30E-20
RP11-634H2.	0.74902724	2.81E-24	4.68E-24
KLRG2	NA	3.65E-49	8.58E-49
CLEC2L	NA	NA	NA
HIPK2	4.24448768	4.77E-45	1.06E-44
TBXAS1	1.40817121	4.14E-28	7.27E-28
PARP12	1.68531128	3.74E-08	4.75E-08

KDM7A	2.24708171	2.15E-63	6.22E-63
JHDM1D-AS1	Inf	1.39E-86	5.80E-86
SLC37A3	3.37062257	2.59E-06	3.16E-06
RAB19	NA	NA	NA
MKRN1	1.59614137	6.66E-39	1.36E-38
DENND2A	Inf	3.52E-112	2.51E-111
ADCK2	2.0598249	2.07E-14	2.96E-14
NDUFB2	2.16709765	8.61E-116	6.73E-115
NDUFB2-AS1	0.49935149	1.90E-20	2.98E-20
BRAF	1.99740597	3.06E-53	7.65E-53
MRPS33	1.87412209	0.00621389	0.00692587
TMEM178B	NA	NA	NA
RP5-842K16.	NA	NA	NA
RP5-842K16.	NA	NA	NA
RP11-744I24	NA	NA	NA
RP11-744I24	NA	NA	NA
AGK	2.07422927	1.94E-30	3.52E-30
RP5-894A10.	2.99610895	6.17E-30	1.11E-29
KIAA1147	1.56047341	0.55771235	0.57080094
WEE2-AS1	Inf	3.56E-137	7.49E-136
WEE2	NA	NA	NA
SSBP1	1.28169694	9.96E-106	6.04E-105
TAS2R3	NA	NA	NA
TAS2R4	1.74773022	3.23E-36	6.35E-36
TAS2R5	NA	5.50E-20	8.57E-20
PRSS37	NA	NA	NA
MGAM	NA	1.36E-15	1.98E-15
OR9A4	NA	NA	NA
CLEC5A	0	8.13E-145	5.14E-143
TAS2R38	NA	NA	NA
MGAM2	NA	NA	NA
PRSS58	NA	NA	NA
U66059.58	NA	NA	NA
TRBV1	NA	NA	NA
TRBV2	NA	NA	NA
TRBV3-1	NA	NA	NA
TRBV4-1	NA	NA	NA
TRBV5-1	NA	NA	NA
TRBV6-1	NA	NA	NA
TRBV7-1	NA	NA	NA
TRBV4-2	NA	NA	NA
TRBV6-2	NA	NA	NA
TRBV7-2	NA	NA	NA
TRBV8-1	NA	NA	NA
TRBV5-2	NA	NA	NA

TRBV6-4	NA	NA	NA
TRBV7-3	NA	NA	NA
TRBV8-2	NA	NA	NA
TRBV5-3	NA	NA	NA
TRBV9	NA	NA	NA
TRBV10-1	NA	NA	NA
TRBV11-1	NA	NA	NA
TRBV12-1	NA	NA	NA
TRBV10-2	NA	NA	NA
TRBV11-2	NA	NA	NA
TRBV12-2	NA	NA	NA
TRBV6-5	NA	NA	NA
TRBV7-4	NA	NA	NA
TRBV5-4	NA	NA	NA
TRBV6-6	NA	NA	NA
TRBV7-5	NA	NA	NA
TRBV5-5	NA	NA	NA
TRBV6-7	NA	NA	NA
TRBV7-6	NA	NA	NA
TRBV5-6	NA	NA	NA
TRBV6-8	NA	NA	NA
TRBV7-7	NA	NA	NA
TRBV5-7	NA	NA	NA
TRBV7-9	NA	NA	NA
TRBV13	NA	NA	NA
TRBV10-3	NA	NA	NA
TRBV11-3	NA	NA	NA
TRBV12-3	NA	NA	NA
TRBV12-4	NA	NA	NA
TRBV12-5	NA	NA	NA
TRBV14	NA	NA	NA
TRBV15	NA	NA	NA
TRBV16	NA	NA	NA
TRBV17	NA	NA	NA
TRBV18	NA	NA	NA
TRBV19	NA	NA	NA
TRBV20-1	NA	NA	NA
TRBV21-1	NA	NA	NA
TRBV22-1	NA	NA	NA
TRBV23-1	NA	NA	NA
TRBV24-1	NA	NA	NA
MTRNR2L6	NA	6.27E-37	1.25E-36
TRBV25-1	NA	NA	NA
TRBVA	NA	NA	NA
TRBV26	NA	NA	NA

TRBVB	NA	NA	NA
TRBV27	NA	NA	NA
TRBV28	NA	NA	NA
TRBC1	NA	3.85E-43	8.32E-43
TRBV29-1	NA	NA	NA
PRSS2	NA	5.83E-98	3.02E-97
PRSS1	NA	NA	NA
TRBD1	NA	NA	NA
TRBJ1-1	NA	NA	NA
TRBJ1-2	NA	NA	NA
TRBJ1-3	NA	NA	NA
TRBJ1-4	NA	NA	NA
TRBJ1-5	NA	NA	NA
TRBJ1-6	NA	NA	NA
TRBJ2-1	NA	NA	NA
TRBJ2-2	NA	NA	NA
TRBJ2-2P	NA	NA	NA
TRBJ2-3	NA	NA	NA
TRBJ2-4	NA	NA	NA
TRBJ2-5	NA	NA	NA
TRBJ2-6	NA	NA	NA
TRBJ2-7	NA	NA	NA
TRBC2	NA	NA	NA
TRBV30	NA	NA	NA
EPHB6	1.87256809	1.83E-23	3.00E-23
TRPV6	NA	NA	NA
RP11-114L10	NA	NA	NA
TRPV5	NA	2.11E-22	3.41E-22
C7orf34	NA	NA	NA
KEL	NA	NA	NA
OR9A2	NA	NA	NA
OR6V1	NA	NA	NA
PIP	NA	NA	NA
TAS2R39	NA	NA	NA
RP11-556I13	NA	NA	NA
TAS2R40	NA	NA	NA
GSTK1	1.26419529	7.28E-135	1.30E-133
AC073342.12	NA	5.53E-46	1.24E-45
TMEM139	NA	NA	NA
CASP2	1.90377756	0.01067144	0.01180567
CLCN1	NA	NA	NA
FAM131B	0.74902724	1.26E-89	5.59E-89
RP11-563K23	NA	NA	NA
AC093673.5	1.12354086	2.83E-17	4.24E-17
ZYX	1.73086024	3.19E-91	1.45E-90

EPHA1	0	1.15E-46	2.61E-46
EPHA1-AS1	Inf	7.29E-45	1.61E-44
TAS2R60	NA	NA	NA
TAS2R41	NA	NA	NA
CTAGE15	NA	NA	NA
TCAF2	NA	2.29E-67	7.05E-67
CTAGE6	NA	NA	NA
TCAF1	2.54669261	8.16E-89	3.56E-88
OR2F2	NA	NA	NA
RP4-669B10.	NA	NA	NA
AC004853.1	NA	NA	NA
OR6B1	NA	NA	NA
OR2A5	NA	NA	NA
OR2A25	NA	NA	NA
OR2A12	NA	NA	NA
OR2A2	NA	NA	NA
OR2A14	NA	NA	NA
CTAGE4	NA	NA	NA
ARHGEF35	0	1.77E-18	2.69E-18
RP4-545C24.	0	8.48E-09	1.09E-08
OR2A42	NA	NA	NA
OR2A1-AS1	Inf	1.06E-08	1.35E-08
OR2A7	NA	NA	NA
CTAGE8	NA	NA	NA
OR2A1	NA	NA	NA
ARHGEF5	NA	1.88E-134	3.27E-133
NOBOX	NA	NA	NA
TPK1	1.76770428	5.63E-27	9.72E-27
AC006007.1	NA	NA	NA
CNTNAP2	NA	NA	NA
AC006004.1	NA	NA	NA
C7orf33	NA	NA	NA
RP5-1136G1:	1.12354086	3.28E-36	6.45E-36
CUL1	1.59888506	0.10049878	0.10723732
EZH2	1.92397192	0.00348808	0.00392001
GHET1	NA	0.00087797	0.00100404
PDIA4	1.06592338	6.08E-122	5.80E-121
ZNF786	2.43433852	0.10398631	0.11078926
ZNF425	NA	1.06E-11	1.44E-11
ZNF398	1.26758456	1.70E-15	2.47E-15
ZNF282	2.80885214	2.69E-123	2.71E-122
ZNF212	1.43563554	0.01152769	0.01274127
ZNF783	Inf	1.26E-68	3.96E-68
AC004941.3	NA	NA	NA
RP11-143I21	NA	NA	NA

ZNF777	1.60019455	0.85164381	0.85812397
ZNF746	1.90661479	4.46E-20	6.97E-20
KRBA1	1.28404669	2.30E-20	3.61E-20
ZNF467	3.09597925	0.04217411	0.0456817
SSPO	0	9.95E-170	1.50E-166
ZNF862	0.74902724	1.82E-38	3.69E-38
RP4-751H13.	NA	2.97E-64	8.70E-64
ATP6V0E2-A:	Inf	2.28E-57	6.01E-57
ATP6V0E2	2.18856396	1.38E-05	1.66E-05
RP11-445N2(	NA	NA	NA
RP11-728K2(	Inf	6.97E-94	3.33E-93
RP11-728K2(	NA	NA	NA
RP11-545G3.	NA	NA	NA
ACTR3C	NA	9.36E-51	2.25E-50
LRRC61	NA	NA	NA
ZBED6CL	NA	NA	NA
RARRES2	NA	NA	NA
RP4-584D14.	NA	NA	NA
REPIN1	2.09103437	5.88E-08	7.43E-08
ZNF775	3.29571984	9.30E-12	1.27E-11
RP4-584D14.	NA	NA	NA
RP11-511P7.	3.74513619	1.22E-20	1.93E-20
LINC00996	Inf	2.69E-28	4.73E-28
GIMAP8	NA	1.95E-15	2.84E-15
GIMAP7	NA	NA	NA
GIMAP4	NA	NA	NA
GIMAP6	1.34824903	5.37E-07	6.64E-07
GIMAP2	0.99870298	2.00E-08	2.56E-08
GIMAP1	1.49805447	6.89E-12	9.42E-12
GIMAP1-GIM	NA	NA	NA
GIMAP5	NA	0.16508868	0.1742416
TMEM176B	NA	3.06E-40	6.38E-40
TMEM176A	NA	2.25E-49	5.31E-49
AOC1	NA	6.11E-35	1.18E-34
KCNH2	3.49546044	0.68085863	0.69191896
NOS3	Inf	3.48E-54	8.81E-54
ATG9B	NA	1.49E-114	1.13E-113
ABCB8	2.9128837	0.00045284	0.00052205
RP11-148K1.	NA	NA	NA
ASIC3	0	0.96966566	0.97116453
CDK5	1.07152508	3.96E-134	6.72E-133
SLC4A2	1.75486381	0.00147334	0.00167268
RP11-148K1.	NA	2.04E-146	2.15E-144
FASTK	1.67479124	1.30E-20	2.05E-20
TMUB1	1.65432634	0.37363655	0.38661463

AGAP3	1.98780305	1.79E-50	4.30E-50
GBX1	NA	NA	NA
ASB10	NA	NA	NA
IQCA1L	NA	NA	NA
ABCF2	1.84375935	1.65E-06	2.02E-06
CHPF2	1.79766537	3.19E-07	3.96E-07
SMARCD3	0.56177043	2.50E-144	1.42E-142
RP4-548D19.	2.24708171	2.02E-09	2.63E-09
RP5-1070G2	NA	NA	NA
NUB1	1.35190282	8.24E-56	2.13E-55
WDR86	1.74773022	0.41958676	0.43240985
RP4-555L14.	NA	NA	NA
CRYGN	NA	NA	NA
RHEB	1.91712438	1.39E-32	2.60E-32
PRKAG2	1.90377756	2.74E-08	3.49E-08
PRKAG2-AS1	2.00546002	0.00031114	0.00036043
RP11-796I2.1	NA	NA	NA
GALNTL5	NA	NA	NA
GALNT11	2.68401427	3.40E-05	4.04E-05
RP5-981O7.2	NA	NA	NA
KMT2C	2.49675746	1.71E-41	3.62E-41
LINC01003	2.29701686	2.78E-19	4.29E-19
XRCC2	2.36692607	9.99E-86	4.10E-85
ACTR3B	1.56047341	5.25E-66	1.58E-65
LINC01287	NA	NA	NA
DPP6	NA	1.97E-49	4.64E-49
RP5-1048B1	NA	NA	NA
AC006019.3	NA	NA	NA
AC006019.4	NA	NA	NA
RP11-476H2	NA	NA	NA
PAXIP1-AS2	NA	8.43E-38	1.69E-37
PAXIP1	2.20765923	2.32E-50	5.55E-50
RP11-5C23.1	NA	NA	NA
RP11-5C23.2	NA	NA	NA
PAXIP1-AS1	1.78216825	3.36E-50	8.02E-50
HTR5A-AS1	NA	NA	NA
HTR5A	NA	NA	NA
AC099552.4	NA	NA	NA
AC099552.3	NA	NA	NA
AC099552.2	NA	NA	NA
AC144652.1	2.99610895	1.87E-105	1.13E-104
INSIG1	1.33960641	1.00E-86	4.19E-86
BLACE	NA	NA	NA
AC008060.7	NA	NA	NA
AC008060.8	NA	NA	NA

EN2	NA	2.06E-131	2.99E-130
CNPY1	NA	NA	NA
AC008060.5	NA	NA	NA
AC009403.2	1.87256809	1.10E-49	2.60E-49
RBM33	1.7305112	1.77E-11	2.39E-11
SHH	0.99870298	4.56E-20	7.11E-20
AC021218.2	NA	NA	NA
AC073133.2	NA	NA	NA
AC073133.1	NA	NA	NA
LINC01006	NA	NA	NA
LINC00244	NA	NA	NA
RNF32	NA	NA	NA
AC005534.8	NA	9.99E-36	1.95E-35
LMBR1	3.69163424	1.39E-83	5.48E-83
RP5-1121A1!	NA	NA	NA
NOM1	1.61632193	0.80552446	0.81355146
MNX1	2.90248054	0.00345913	0.00388796
MNX1-AS2	NA	0.15149489	0.16012703
MNX1-AS1	1.38101897	1.40E-42	3.01E-42
RP4-639J15.:	NA	NA	NA
RP4-639J15.:	NA	NA	NA
UBE3C	2.47595115	5.52E-112	3.91E-111
RP5-1142J19	NA	NA	NA
DNAJB6	1.57766123	2.00E-88	8.65E-88
AC006372.4	NA	NA	NA
AC006372.5	NA	NA	NA
AC006372.6	NA	NA	NA
PTPRN2	NA	1.98E-32	3.69E-32
AC005481.5	NA	NA	NA
AC011899.9	NA	1.32E-73	4.47E-73
RP11-452C1:	NA	NA	NA
AC011899.1C	NA	NA	NA
AC078942.1	NA	NA	NA
LINC01022	NA	NA	NA
NCAPG2	1.80647745	2.92E-38	5.91E-38
ESYT2	1.66088648	6.59E-22	1.06E-21
WDR60	1.01338979	1.53E-28	2.71E-28
VIPR2	NA	NA	NA
PLCXD1	1.61456982	3.80E-89	1.66E-88
GTPBP6	1.5204135	2.79E-139	7.21E-138
LINC00685	2.29389591	3.66E-13	5.10E-13
PPP2R3B	1.76241703	0.05305677	0.05721327
RP13-465B1	NA	2.05E-05	2.44E-05
RP13-465B1	NA	0.00129646	0.00147454
SHOX	NA	NA	NA

RP11-309M2	NA	NA	NA
CRLF2	NA	8.94E-73	2.98E-72
CSF2RA	1.1770428	5.26E-57	1.38E-56
IL3RA	0.18725681	2.44E-129	3.18E-128
SLC25A6	1.78590494	8.85E-10	1.16E-09
LINC00106	0.49935149	3.36E-90	1.50E-89
ASMTL-AS1	Inf	9.20E-128	1.12E-126
ASMTL	1.02832553	3.90E-110	2.63E-109
P2RY8	2.02962219	9.00E-30	1.62E-29
AKAP17A	1.61111519	3.35E-58	8.94E-58
ASMT	NA	NA	NA
RP13-297E16	NA	NA	NA
RP13-297E16	NA	NA	NA
DHRSX	2.28274968	1.92E-15	2.78E-15
ZBED1	2.18715953	0.01200471	0.01325964
LINC00102	NA	NA	NA
CD99	1.56116542	1.99E-124	2.09E-123
XG	NA	NA	NA
GYG2	3.29571984	1.48E-74	5.06E-74
GYG2-AS1	NA	NA	NA
ARSD	1.68531128	2.78E-06	3.38E-06
ARSD-AS1	NA	NA	NA
ARSE	NA	NA	NA
ARSH	NA	NA	NA
ARSF	NA	NA	NA
LINC01546	NA	NA	NA
MXRA5	NA	NA	NA
PRKX	1.80200756	5.16E-06	6.25E-06
PRKX-AS1	NA	NA	NA
RP11-706O15	2.0598249	0.00124249	0.00141395
RP11-706O15	NA	2.11E-133	3.43E-132
RP11-706O15	NA	NA	NA
RP11-706O15	NA	NA	NA
RP11-707P20	NA	NA	NA
RP11-733O18	NA	NA	NA
NLGN4X	NA	NA	NA
VCX3A	NA	NA	NA
RP11-1M18.1	NA	NA	NA
PUDP	1.04102091	2.95E-129	3.83E-128
STS	NA	2.52E-62	7.16E-62
VCX	NA	NA	NA
PNPLA4	NA	NA	NA
RP11-692P14	NA	NA	NA
VCX2	NA	NA	NA
VCX3B	NA	NA	NA

ANOS1	NA	NA	NA
FAM9A	NA	NA	NA
GS1-542M4.4	NA	NA	NA
FAM9B	NA	NA	NA
GS1-519E5.1	NA	NA	NA
TBL1X	2.34621767	2.26E-69	7.19E-69
GPR143	NA	3.54E-14	5.03E-14
SHROOM2	0	1.18E-118	1.00E-117
CLDN34	NA	NA	NA
WWC3	2.19714656	1.06E-11	1.45E-11
WWC3-AS1	NA	5.11E-21	8.10E-21
CLCN4	1.49805447	5.85E-08	7.39E-08
RP6-1O2.1	NA	NA	NA
MID1	NA	1.53E-150	2.36E-148
RP11-120D5.	1.49805447	0.01289669	0.01422753
HCCS	1.17214186	2.26E-66	6.86E-66
ARHGAP6	NA	NA	NA
AMELX	NA	NA	NA
GS1-590J6.3	NA	2.19E-30	3.96E-30
MSL3	2.26491569	1.60E-05	1.91E-05
FRMPD4	NA	NA	NA
FRMPD4-AS1	NA	NA	NA
PRPS2	1.65076877	5.66E-08	7.16E-08
TLR7	NA	1.35E-33	2.56E-33
TLR8-AS1	NA	NA	NA
TLR8	NA	NA	NA
TMSB4X	1.10185849	7.20E-144	3.71E-142
FAM9C	NA	0.38801892	0.40076511
RP11-791M2	NA	NA	NA
GS1-600G8.5	NA	NA	NA
GS1-600G8.3	NA	NA	NA
ATXN3L	NA	NA	NA
LINC01203	NA	NA	NA
RP11-1L9.1	NA	NA	NA
EGFL6	NA	NA	NA
TCEANC	1.87256809	2.13E-80	7.98E-80
RAB9A	1.48765132	1.26E-75	4.38E-75
TRAPPC2	1.49805447	6.76E-35	1.31E-34
OFD1	1.28059495	5.20E-41	1.09E-40
GPM6B	1.56047341	9.42E-14	1.33E-13
RP1-122K4.2	NA	NA	NA
RP1-122K4.3	NA	NA	NA
GEMIN8	1.56047341	1.85E-106	1.14E-105
GLRA2	NA	NA	NA
FANCB	2.52796693	7.43E-36	1.46E-35

MOSPD2	3.932393	5.30E-32	9.80E-32
ASB9	3.1459144	1.43E-85	5.87E-85
ASB11	NA	2.46E-98	1.29E-97
PIGA	1.78614187	1.55E-64	4.55E-64
FIGF	Inf	2.01E-66	6.11E-66
PIR	1.22974621	3.37E-48	7.82E-48
BMX	0.74902724	4.22E-48	9.76E-48
ACE2	NA	1.48E-64	4.36E-64
GS1-594A7.3	NA	NA	NA
TMEM27	1.49805447	1.66E-119	1.46E-118
CA5B	1.99740597	0.23486076	0.24609241
INE2	NA	0.08314998	0.08898696
ZRSR2	1.23877582	8.57E-53	2.13E-52
AP1S2	1.42497865	1.48E-19	2.30E-19
GRPR	NA	NA	NA
RP11-431J24	NA	NA	NA
MAGEB17	NA	NA	NA
CTPS2	1.39818418	1.12E-62	3.21E-62
S100G	NA	NA	NA
SYAP1	1.38165159	2.41E-69	7.66E-69
TXLNG	1.42585908	7.64E-61	2.13E-60
RBBP7	0.9619614	2.29E-136	4.51E-135
REPS2	NA	2.85E-33	5.37E-33
NHS	NA	NA	NA
NHS-AS1	NA	NA	NA
SCML1	NA	NA	NA
RAI2	NA	NA	NA
LINC01456	NA	NA	NA
BEND2	NA	NA	NA
SCML2	1.41482923	7.00E-12	9.55E-12
CDKL5	0.74902724	0.34339494	0.35611472
RS1	NA	NA	NA
PPEF1	NA	NA	NA
PPEF1-AS1	NA	NA	NA
PHKA2-AS1	NA	NA	NA
PHKA2	2.3594358	1.05E-34	2.03E-34
ADGRG2	NA	NA	NA
PDHA1	0.88927063	3.37E-137	7.09E-136
MAP3K15	2.49675746	1.48E-89	6.51E-89
SH3KBP1	1.29108642	4.80E-64	1.40E-63
CXorf23	2.12224384	0.00399545	0.00448132
MAP7D2	NA	2.51E-72	8.30E-72
EIF1AX	1.90998089	4.57E-57	1.20E-56
EIF1AX-AS1	NA	7.20E-126	8.01E-125
RPS6KA3	3.7151751	4.12E-90	1.83E-89

CNKSR2	2.12224384	6.08E-12	8.32E-12
KLHL34	NA	1.40E-11	1.90E-11
SMPX	NA	NA	NA
MBTPS2	2.20546909	1.73E-07	2.17E-07
YY2	2.0598249	3.78E-28	6.64E-28
SMS	1.48804839	1.65E-61	4.65E-61
PHEX	NA	8.32E-107	5.16E-106
PHEX-AS1	NA	NA	NA
PTCHD1-AS	NA	NA	NA
ZNF645	NA	NA	NA
RP11-494I9.2	NA	NA	NA
GS1-433O24	NA	NA	NA
DDX53	NA	NA	NA
PTCHD1	0	1.41E-108	9.15E-108
PRDX4	1.25940194	2.78E-114	2.09E-113
ACOT9	1.2795882	1.00E-59	2.75E-59
RP13-314C10	NA	NA	NA
SAT1	1.34241245	8.55E-134	1.43E-132
APOO	1.66088648	6.83E-22	1.10E-21
CXorf58	NA	2.80E-150	4.16E-148
KLHL15	3.37062257	9.26E-109	6.02E-108
EIF2S3	2.16091044	5.26E-33	9.87E-33
ZFX-AS1	NA	NA	NA
ZFX	1.79312581	0.35999227	0.37281538
PDK3	1.5396671	1.36E-22	2.21E-22
PCYT1B	NA	NA	NA
PCYT1B-AS1	NA	NA	NA
POLA1	2.21303502	1.26E-34	2.42E-34
ARX	1.12354086	3.95E-29	7.04E-29
RP11-86A5.1	NA	NA	NA
MAGEB18	NA	NA	NA
MAGEB6P1	NA	NA	NA
MAGEB6	NA	NA	NA
MAGEB5	NA	NA	NA
VENTXP1	NA	NA	NA
RP11-268G1	NA	NA	NA
RP11-268G1	NA	5.06E-06	6.13E-06
DCAF8L2	NA	NA	NA
MAGEB10	NA	NA	NA
DCAF8L1	NA	NA	NA
RP11-149B9.	NA	NA	NA
IL1RAPL1	NA	NA	NA
MAGEB2	NA	3.98E-136	7.64E-135
MAGEB3	NA	NA	NA
MAGEB4	NA	NA	NA

MAGEB1	NA	NA	NA
NR0B1	NA	NA	NA
CXorf21	0.37451362	4.89E-107	3.04E-106
GK	1.47222595	7.91E-89	3.45E-88
GK-AS1	NA	2.91E-18	4.42E-18
TAB3	1.49805447	0.07783292	0.08341968
TAB3-AS1	NA	NA	NA
TAB3-AS2	NA	NA	NA
FTHL17	NA	NA	NA
DMD	2.69649805	1.76E-40	3.68E-40
DMD-AS3	NA	NA	NA
RP11-305F18	NA	NA	NA
FAM47A	NA	NA	NA
RP11-545D19	NA	NA	NA
TMEM47	NA	NA	NA
FAM47B	NA	NA	NA
MAGEB16	NA	NA	NA
CFAP47	NA	NA	NA
RP11-87M18	NA	NA	NA
FAM47C	NA	NA	NA
PRRG1	1.54798962	3.36E-27	5.83E-27
RP5-972B16.	NA	NA	NA
LANCL3	0.12483787	1.64E-132	2.54E-131
XK	1.3732166	4.98E-23	8.13E-23
CYBB	0	2.31E-136	4.54E-135
DYNLT3	1.34824903	5.86E-139	1.47E-137
AL121578.2	NA	NA	NA
HYPM	NA	NA	NA
SYTL5	NA	NA	NA
SRPX	NA	2.95E-70	9.48E-70
RP13-43E11.	NA	NA	NA
RPGR	1.87256809	9.22E-05	1.08E-04
OTC	NA	NA	NA
TSPAN7	NA	3.54E-56	9.22E-56
RP4-646N3.1	NA	NA	NA
MID1IP1	1.35462373	3.39E-87	1.42E-86
MID1IP1-AS1	0	4.48E-35	8.68E-35
RP11-265P11	NA	NA	NA
LINC01281	NA	NA	NA
LINC01282	NA	NA	NA
RP11-157D23	NA	NA	NA
RP13-126P21	NA	NA	NA
AC092198.1	NA	NA	NA
BCOR	1.34824903	0.00080161	0.00091729
RP11-320G24	NA	NA	NA

ATP6AP2	0.81560744	8.02E-139	1.98E-137
CXorf38	2.01167315	1.00E-27	1.75E-27
MED14	2.40758755	0.0009533	0.00108936
MED14OS	8.23929961	1.45E-32	2.71E-32
USP9X	2.39363052	1.43E-76	5.03E-76
RP5-1172N1(	Inf	8.10E-138	1.80E-136
DDX3X	1.89997153	0.00367866	0.00413086
NYX	NA	NA	NA
CASK	1.60505837	3.25E-06	3.96E-06
CASK-AS1	NA	NA	NA
GPR34	NA	5.90E-07	7.29E-07
GPR82	Inf	5.24E-119	4.50E-118
RP11-524P6.	NA	NA	NA
RP1-154K9.2	NA	NA	NA
AL023875.1	NA	NA	NA
RP3-326I13.1	NA	NA	NA
MAOA	2.24708171	9.35E-06	1.13E-05
MAOB	NA	NA	NA
NDP	NA	NA	NA
NDP-AS1	NA	NA	NA
EFHC2	0.74902724	1.53E-19	2.38E-19
FUNDC1	1.17289536	1.44E-107	9.13E-107
DUSP21	NA	NA	NA
KDM6A	1.41482923	0.3911712	0.40392896
CXorf36	NA	0.00021029	0.00024448
RP11-342D14	NA	NA	NA
LINC01204	NA	NA	NA
MIR222HG	2.16385646	1.38E-28	2.44E-28
RP6-99M1.3	NA	7.00E-68	2.17E-67
RP5-1158E12	0.74902724	0.02086926	0.02285741
RP5-1158E12	NA	NA	NA
LINC01186	NA	NA	NA
KRBOX4	1.94747082	5.14E-20	8.02E-20
ZNF674	1.31079767	2.15E-26	3.69E-26
ZNF674-AS1	1.73212549	0.16143551	0.17044539
CHST7	1.77893969	0.00119486	0.00136094
SLC9A7	1.87256809	4.69E-52	1.15E-51
RP2	2.62159533	5.46E-18	8.26E-18
LINC01545	0	3.75E-26	6.41E-26
JADE3	1.49805447	1.08E-38	2.20E-38
RGN	0	9.44E-49	2.21E-48
NDUFB11	1.53485028	3.59E-38	7.25E-38
RBM10	1.67782101	5.46E-33	1.02E-32
UBA1	1.31483918	5.74E-130	7.74E-129
CDK16	2.72950603	5.86E-18	8.86E-18

USP11	1.73774319	5.88E-75	2.02E-74
RP11-571E6.	NA	NA	NA
ZNF157	NA	5.04E-119	4.33E-118
ZNF41	2.62159533	1.33E-87	5.63E-87
LINC01560	1.02991245	3.23E-77	1.15E-76
ARAF	1.71464066	1.60E-22	2.60E-22
SYN1	Inf	2.95E-14	4.21E-14
TIMP1	1.0633616	1.19E-129	1.57E-128
CFP	1.00401523	4.85E-139	1.23E-137
ELK1	2.54431474	8.18E-93	3.85E-92
UXT	1.55820221	1.44E-123	1.46E-122
UXT-AS1	NA	NA	NA
AL022578.1	NA	2.08E-06	2.55E-06
ZNF81	2.88910506	1.29E-05	1.55E-05
ZNF182	2.56809339	2.99E-59	8.11E-59
ZNF630	NA	NA	NA
SPACA5	NA	NA	NA
ZNF630-AS1	NA	NA	NA
SPACA5B	NA	NA	NA
SSX5	NA	NA	NA
SSX1	NA	NA	NA
RP11-344N1	NA	NA	NA
SSX3	NA	NA	NA
SSX4	NA	NA	NA
SSX4B	NA	NA	NA
SLC38A5	0.962535	2.05E-127	2.46E-126
FTSJ1	1.64260359	1.83E-21	2.91E-21
AF196972.9	0.74902724	1.06E-77	3.79E-77
PORCN	1.90661479	1.08E-42	2.32E-42
EBP	1.15346334	3.60E-137	7.55E-136
TBC1D25	1.53062088	1.37E-11	1.85E-11
RP11-1148L6	Inf	5.66E-91	2.56E-90
RBM3	1.89354086	4.96E-60	1.36E-59
RP11-1148L6	NA	NA	NA
WDR13	2.38840761	1.16E-16	1.73E-16
WAS	1.35449092	1.07E-103	6.21E-103
SUV39H1	2.75642023	1.26E-05	1.51E-05
AF196970.3	NA	2.57E-109	1.70E-108
GLOD5	NA	2.68E-65	8.01E-65
GATA1	3.74513619	0.03775571	0.04094471
HDAC6	2.55917639	2.89E-30	5.23E-30
ERAS	NA	1.82E-96	9.16E-96
PCSK1N	NA	NA	NA
TIMM17B	1.68565113	3.25E-40	6.76E-40
PQBP1	1.02559114	8.20E-131	1.15E-129

SLC35A2	1.20156453	1.12E-60	3.12E-60
PIM2	1.11589772	1.80E-42	3.85E-42
OTUD5	2.10772781	0.00016867	0.00019658
KCND1	0.74902724	0.8730625	0.87877829
GRIPAP1	2.64656291	0.04251704	0.0460394
TFE3	2.49675746	2.74E-123	2.76E-122
CCDC120	4.49416342	1.14E-82	4.44E-82
PRAF2	5.24319066	3.84E-24	6.37E-24
WDR45	1.88021123	6.08E-37	1.21E-36
GPKOW	0.7367481	6.92E-126	7.73E-125
AC231657.1	NA	NA	NA
MAGIX	0.79308766	8.82E-113	6.40E-112
PLP2	1.6567026	7.66E-83	2.99E-82
PRICKLE3	1.06440713	4.94E-47	1.13E-46
SYP	3.1459144	2.11E-14	3.02E-14
SYP-AS1	NA	NA	NA
CACNA1F	NA	2.19E-30	3.96E-30
CCDC22	0.94232459	8.18E-126	9.08E-125
FOXP3	NA	8.68E-93	4.07E-92
PPP1R3F	Inf	2.30E-126	2.63E-125
LL0XNC01-7F	0.74902724	3.62E-08	4.59E-08
GAGE10	NA	NA	NA
GAGE12J	NA	NA	NA
GAGE13	NA	NA	NA
GAGE2E	NA	NA	NA
GAGE12B	NA	NA	NA
GAGE12C	NA	NA	NA
GAGE12D	NA	NA	NA
GAGE12E	NA	NA	NA
GAGE12F	NA	NA	NA
GAGE12G	NA	NA	NA
GAGE12H	NA	NA	NA
GAGE1	NA	NA	NA
GAGE2A	NA	NA	NA
PAGE1	NA	NA	NA
PAGE4	NA	NA	NA
USP27X-AS1	Inf	7.96E-19	1.22E-18
USP27X	2.24708171	0.00026387	0.00030618
CLCN5	1.76241703	6.17E-29	1.09E-28
AKAP4	NA	NA	NA
CCNB3	NA	8.96E-35	1.73E-34
DGKK	NA	NA	NA
SHROOM4	Inf	9.57E-141	2.95E-139
BMP15	NA	NA	NA
LINC01284	NA	NA	NA

NUDT10	NA	0.00073116	0.00083747
RP11-348F1.	NA	NA	NA
RP11-348F1.	NA	NA	NA
CXorf67	NA	5.48E-110	3.68E-109
NUDT11	1.3299055	1.01E-49	2.40E-49
LINC01496	NA	NA	NA
CENPVP3	NA	NA	NA
RP11-141O1!	NA	NA	NA
CENPVP1	NA	NA	NA
GSPT2	NA	NA	NA
MAGED1	1.94747082	2.46E-07	3.06E-07
RP11-114H2(	NA	NA	NA
RP11-33H15.	NA	NA	NA
MAGED4B	NA	NA	NA
MAGED4	NA	NA	NA
RP11-363G1(	NA	NA	NA
XAGE2	NA	NA	NA
XAGE1A	NA	NA	NA
XAGE1B	NA	NA	NA
SSX7	NA	NA	NA
SSX2	NA	NA	NA
SSX2B	NA	NA	NA
SPANXN5	NA	NA	NA
XAGE5	NA	NA	NA
XAGE3	NA	NA	NA
FAM156B	0	4.35E-104	2.55E-103
FAM156A	NA	3.53E-127	4.18E-126
GPR173	NA	NA	NA
TSPYL2	1.62289235	6.83E-10	8.99E-10
RP11-236P2⁄	NA	NA	NA
KANTR	Inf	8.19E-124	8.42E-123
RP11-258C1!	2.47178988	7.12E-46	1.60E-45
KDM5C	2.24708171	0.00032837	0.0003801
IQSEC2	4.11964981	3.32E-09	4.31E-09
SMC1A	1.53142698	2.75E-110	1.86E-109
RIBC1	0.37451362	6.79E-35	1.31E-34
HSD17B10	1.44891015	9.00E-114	6.69E-113
RP3-339A18.	NA	0.00013894	0.00016228
HUWE1	2.03101616	0.43317101	0.4460292
PHF8	1.76770428	1.79E-45	3.99E-45
FAM120C	0.95969115	3.67E-79	1.35E-78
WNK3	NA	NA	NA
TSR2	1.38904112	6.01E-109	3.93E-108
FGD1	1.79766537	0.00488673	0.00546714
GNL3L	1.59470315	0.53731896	0.55073676

ITIH6	NA	NA	NA
MAGED2	0.91344785	1.31E-135	2.44E-134
TRO	NA	NA	NA
PFKFB1	NA	1.09E-88	4.74E-88
APEX2	1.52529183	6.72E-39	1.37E-38
ALAS2	NA	NA	NA
PAGE2B	NA	1.01E-45	2.26E-45
PAGE2	NA	NA	NA
FAM104B	2.08063121	1.05E-92	4.92E-92
MTRNR2L10	NA	7.02E-41	1.47E-40
PAGE5	NA	1.03E-51	2.51E-51
PAGE3	NA	NA	NA
MAGEH1	1.49805447	8.07E-43	1.74E-42
USP51	NA	NA	NA
FOXR2	NA	NA	NA
RRAGB	1.7183566	0.01481011	0.0163037
RP13-188A5.	NA	3.69E-62	1.05E-61
KLF8	NA	NA	NA
UBQLN2	1.69971565	0.3432179	0.35595147
RP11-431N1!	NA	NA	NA
LINC01420	2.18360483	2.55E-60	7.05E-60
RP11-445O1(	NA	NA	NA
SPIN3	6.36673152	5.80E-50	1.38E-49
SPIN2B	1.97743191	1.80E-29	3.23E-29
RP3-323P24.	NA	5.51E-16	8.09E-16
SPIN2A	Inf	2.68E-29	4.80E-29
FAAH2	NA	2.68E-94	1.30E-93
ZXDB	1.58127972	1.42E-48	3.31E-48
NLRP2P	NA	NA	NA
ZXDA	1.12354086	4.77E-18	7.23E-18
SPIN4	3.08973735	1.04E-125	1.14E-124
SPIN4-AS1	NA	0.02859177	0.03115387
ARHGEF9	1.66828794	1.95E-33	3.67E-33
AMER1	2.69649805	2.23E-109	1.47E-108
ASB12	0	2.52E-14	3.60E-14
MTMR8	Inf	0.20544416	0.21596754
ZC4H2	2.3540856	7.29E-11	9.75E-11
ZC3H12B	Inf	0.0034849	0.00391668
LAS1L	1.08892195	1.84E-121	1.72E-120
MSN	2.10891164	6.05E-77	2.14E-76
RP6-159A1.3	NA	2.10E-06	2.57E-06
RP6-159A1.4	2.23411154	6.36E-38	1.28E-37
VSIG4	0	1.80E-145	1.58E-143
HEPH	NA	NA	NA
EDA2R	NA	NA	NA

AR	2.34695201	1.71E-05	2.04E-05
RP6-201G10.	NA	3.01E-86	1.25E-85
OPHN1	Inf	9.71E-31	1.77E-30
YIPF6	1.44043699	2.61E-36	5.15E-36
STARD8	Inf	1.61E-89	7.10E-89
EFNB1	1.45124027	0.02337621	0.02554458
PJA1	1.19844358	7.86E-36	1.54E-35
LINC00269	NA	5.94E-64	1.73E-63
FAM155B	NA	NA	NA
RP13-57D9.3	NA	NA	NA
EDA	2.24708171	2.07E-11	2.80E-11
AWAT2	NA	NA	NA
OTUD6A	NA	NA	NA
IGBP1	1.17759437	3.92E-110	2.65E-109
IGBP1-AS2	NA	NA	NA
IGBP1-AS1	NA	NA	NA
DGAT2L6	NA	NA	NA
AWAT1	NA	NA	NA
P2RY4	NA	NA	NA
ARR3	NA	NA	NA
RAB41	NA	NA	NA
PDZD11	1.85402782	2.05E-21	3.27E-21
KIF4A	1.37086117	5.33E-115	4.08E-114
GDPD2	NA	1.76E-12	2.43E-12
DLG3	2.49675746	4.12E-20	6.44E-20
DLG3-AS1	NA	NA	NA
TEX11	NA	NA	NA
SLC7A3	NA	NA	NA
SNX12	1.49805447	1.22E-32	2.28E-32
FOXO4	1.54798962	0.00034787	0.00040239
CXorf65	0.87386511	1.71E-44	3.76E-44
IL2RG	1.20525292	3.07E-28	5.40E-28
MED12	2.6528048	8.20E-104	4.78E-103
NLGN3	0.74902724	1.10E-14	1.58E-14
RP5-1091N2.	Inf	0.00022173	0.00025763
GJB1	NA	NA	NA
ZMYM3	1.97304736	4.54E-11	6.10E-11
NONO	1.75757659	0.00025572	0.00029686
ITGB1BP2	1.99740597	0.17283804	0.18228274
TAF1	2.838419	6.36E-74	2.16E-73
OGT	1.8799115	4.33E-76	1.51E-75
ACRC	1.49805447	8.66E-36	1.69E-35
CXCR3	Inf	0.01442598	0.01588855
LINC00891	0.74902724	4.48E-127	5.28E-126
CXorf49	NA	NA	NA

RP11-402P6.	NA	NA	NA
CXorf49B	NA	NA	NA
NHSL2	1.68531128	7.85E-10	1.03E-09
RGAG4	0.24967575	3.97E-130	5.39E-129
PIN4	1.876004	8.94E-40	1.85E-39
ERCC6L	2.17217899	0.03376159	0.03667901
RPS4X	1.79937325	3.48E-14	4.95E-14
CITED1	1.43994029	1.64E-39	3.39E-39
HDAC8	1.99740597	1.44E-46	3.26E-46
PHKA1	3.07101167	8.60E-54	2.17E-53
PHKA1-AS1	NA	NA	NA
DMRTC1B	NA	NA	NA
RP1-172N19.	NA	NA	NA
RP11-69A21.	NA	NA	NA
DMRTC1	NA	NA	NA
LINC00684	NA	NA	NA
PABPC1L2B-/	NA	NA	NA
PABPC1L2B	NA	NA	NA
PABPC1L2A	NA	NA	NA
PABPC1L2B-/	NA	NA	NA
NAP1L6	NA	4.56E-104	2.67E-103
NAP1L2	NA	NA	NA
CDX4	NA	NA	NA
CHIC1	Inf	3.27E-29	5.82E-29
TSIX	NA	5.84E-97	2.97E-96
XIST	NA	NA	NA
JPX	2.10129789	4.08E-50	9.73E-50
RP13-216E22	0	5.86E-111	4.05E-110
FTX	5.24319066	1.31E-24	2.19E-24
ZCCHC13	NA	NA	NA
SLC16A2	Inf	0.69573347	0.70652094
RLIM	2.11567342	7.38E-33	1.38E-32
KIAA2022	2.7464332	2.50E-55	6.42E-55
ABCB7	0.79308766	7.05E-115	5.38E-114
UPRT	1.94747082	7.57E-28	1.32E-27
ZDHHC15	Inf	4.77E-110	3.22E-109
MAGEE2	NA	NA	NA
RP11-212D3.	NA	NA	NA
PBDC1	0.92670347	1.14E-121	1.07E-120
MAGEE1	Inf	2.29E-25	3.87E-25
MIR325HG	NA	NA	NA
FGF16	NA	NA	NA
ATRX	1.43403505	6.09E-19	9.35E-19
MAGT1	2.0191169	4.25E-36	8.35E-36
COX7B	1.74653967	0.00035519	0.0004107

ATP7A	0.99870298	0.32096702	0.33359976
PGAM4	NA	NA	NA
PGK1	0.74161724	4.86E-140	1.37E-138
TAF9B	1.72276265	1.59E-36	3.13E-36
CYSLTR1	1.10151064	7.61E-109	4.97E-108
ZCCHC5	0	2.92E-130	3.97E-129
LPAR4	0.12483787	2.98E-115	2.29E-114
P2RY10	NA	NA	NA
GPR174	NA	NA	NA
ITM2A	0.56728289	1.71E-139	4.55E-138
TBX22	NA	NA	NA
FAM46D	NA	NA	NA
BRWD3	3.21011673	1.39E-10	1.85E-10
HMGN5	1.02595045	2.44E-134	4.21E-133
SH3BGRL	1.38773263	2.00E-139	5.26E-138
POU3F4	NA	NA	NA
CYLC1	NA	NA	NA
RPS6KA6	NA	2.17E-110	1.48E-109
HDX	NA	NA	NA
APOOL	2.15043304	5.55E-64	1.62E-63
SATL1	NA	NA	NA
RP5-928E24.	NA	NA	NA
ZNF711	5.11835279	4.54E-97	2.32E-96
POF1B	NA	NA	NA
CHM	1.63849708	0.00845135	0.0093828
DACH2	NA	NA	NA
KLHL4	NA	1.65E-77	5.89E-77
RP1-138A5.3	NA	NA	NA
CPXCR1	NA	NA	NA
RP13-348B1:	NA	NA	NA
TGIF2LX	NA	NA	NA
RP13-212L9.	NA	NA	NA
PABPC5-AS1	NA	NA	NA
PABPC5	NA	NA	NA
PCDH11X	2.24708171	1.72E-29	3.08E-29
NAP1L3	2.99610895	0.01714506	0.01883641
FAM133A	Inf	6.30E-31	1.15E-30
DIAPH2	1.31828794	1.78E-118	1.50E-117
RPA4	3.2457847	0.00010776	0.00012621
DIAPH2-AS1	NA	NA	NA
RP11-485F1:	NA	NA	NA
PCDH19	NA	NA	NA
TNMD	NA	NA	NA
TSPAN6	NA	2.22E-129	2.90E-128
SRPX2	NA	NA	NA

RP11-524D16	NA	NA	NA
SYTL4	1.55567195	1.86E-19	2.87E-19
CSTF2	1.2012701	6.70E-140	1.87E-138
NOX1	NA	NA	NA
XKRX	NA	NA	NA
ARL13A	NA	NA	NA
TRMT2B	1.87256809	0.20782381	0.21836787
TMEM35	NA	NA	NA
CENPI	5.24319066	0.00765371	0.00851356
DRP2	NA	1.14E-109	7.61E-109
TAF7L	Inf	1.08E-132	1.70E-131
TIMM8A	1.79231518	1.35E-09	1.77E-09
BTK	1.88347626	0.00033967	0.00039303
RPL36A	2.7163428	1.34E-138	3.21E-137
RPL36A-HNR	NA	NA	NA
GLA	0.83624274	2.86E-140	8.26E-139
HNRNPH2	1.08114309	1.35E-139	3.64E-138
ARMCX4	NA	NA	NA
ARMCX1	NA	NA	NA
ARMCX6	1.78830253	0.7559244	0.76507562
ARMCX3	2.01661179	0.00091689	0.00104828
ARMCX3-AS1	NA	NA	NA
ARMCX2	NA	NA	NA
NXF5	NA	NA	NA
ZMAT1	NA	0.0012513	0.00142389
TCEAL2	NA	3.38E-08	4.29E-08
TCEAL6	NA	NA	NA
BEX5	1.42437966	2.91E-49	6.86E-49
NXF2	NA	NA	NA
NXF2B	NA	NA	NA
TCP11X2	NA	NA	NA
TMSB15A	1.32045008	1.44E-112	1.04E-111
ARMCX5	1.92607004	4.73E-11	6.35E-11
RP4-769N13.	Inf	1.04E-05	1.25E-05
GPRASP1	2.99610895	2.76E-28	4.86E-28
GPRASP2	3.99481193	3.30E-34	6.31E-34
BHLHB9	1.12354086	7.57E-08	9.54E-08
LINC00630	4.49416342	5.89E-17	8.76E-17
LL0XNC01-23	0.93628405	9.73E-06	1.17E-05
RAB40AL	Inf	0.24105417	0.2523925
LL0XNC01-23	NA	NA	NA
BEX1	NA	3.18E-75	1.10E-74
NXF3	0.99870298	1.40E-61	3.94E-61
BEX4	1.64785992	7.62E-104	4.45E-103
TCEAL8	1.50329243	5.60E-25	9.43E-25

TCEAL5	NA	NA	NA
BEX2	NA	NA	NA
TCEAL7	NA	NA	NA
WBP5	NA	NA	NA
NGFRAP1	1.83220442	1.73E-42	3.70E-42
RP13-349O2(	NA	NA	NA
LL0XNC01-25	NA	NA	NA
RAB40A	NA	1.21E-21	1.94E-21
TCEAL4	1.28789575	6.12E-127	7.15E-126
TCEAL3	3.42412451	3.70E-54	9.39E-54
TCEAL3-AS1	NA	NA	NA
TCEAL1	1.59927437	4.06E-58	1.08E-57
MORF4L2	1.26055803	4.39E-86	1.81E-85
MORF4L2-AS	3.74513619	5.66E-08	7.16E-08
GLRA4	NA	NA	NA
TMEM31	2.24708171	7.66E-11	1.02E-10
PLP1	NA	NA	NA
RAB9B	NA	1.53E-82	5.96E-82
LL0XNC01-11	Inf	2.91E-85	1.19E-84
TMSB15B	0	4.00E-08	5.07E-08
H2BFWT	NA	NA	NA
H2BFM	NA	NA	NA
RP11-722G7.	NA	4.41E-07	5.47E-07
SLC25A53	4.86867704	9.40E-108	5.98E-107
ZCCHC18	1.3732166	9.95E-49	2.33E-48
FAM199X	1.93498703	0.60665044	0.61917594
ESX1	NA	NA	NA
IL1RAPL2	NA	NA	NA
TEX13A	NA	NA	NA
NRK	NA	NA	NA
SERPINA7	NA	NA	NA
MUM1L1	NA	NA	NA
CXorf57	2.7464332	1.77E-14	2.53E-14
RNF128	NA	NA	NA
TBC1D8B	1.99740597	0.12440721	0.13218903
MORC4	1.62289235	1.40E-61	3.94E-61
RIPPLY1	NA	8.61E-08	1.08E-07
CLDN2	NA	NA	NA
RBM41	1.57689945	1.19E-18	1.81E-18
NUP62CL	0.93628405	1.70E-56	4.43E-56
PIH1D3	NA	0.00048233	0.00055541
FRMPD3-AS1	NA	NA	NA
FRMPD3	NA	3.98E-39	8.16E-39
PRPS1	1.08491389	5.04E-86	2.08E-85
TSC22D3	0.9079118	2.60E-106	1.60E-105

NCBP2L	NA	NA	NA
MID2	0	4.09E-77	1.45E-76
RP6-191P20.	NA	NA	NA
TEX13B	NA	NA	NA
VSIG1	0	1.77E-17	2.65E-17
PSMD10	1.99740597	2.08E-47	4.78E-47
ATG4A	2.39688716	4.81E-09	6.22E-09
COL4A6	NA	NA	NA
COL4A5	NA	NA	NA
RP6-24A23.6	NA	NA	NA
IRS4	NA	NA	NA
RP6-24A23.3	NA	NA	NA
GUCY2F	NA	1.50E-42	3.23E-42
NXT2	1.29831388	1.68E-07	2.10E-07
KCNE5	NA	4.39E-77	1.56E-76
ACSL4	1.7183566	2.26E-08	2.88E-08
TMEM164	1.42556797	6.32E-16	9.26E-16
AMMECR1	2.38326848	0.14855717	0.15711345
RGAG1	NA	NA	NA
CHRDL1	1.60820554	3.01E-06	3.66E-06
PAK3	NA	NA	NA
CAPN6	NA	NA	NA
DCX	NA	NA	NA
LINC00890	NA	NA	NA
ALG13	2.14938251	9.31E-23	1.51E-22
ALG13-AS1	NA	NA	NA
TRPC5	NA	NA	NA
TRPC5OS	Inf	3.37E-155	9.85E-153
ZCCHC16	NA	NA	NA
LHFPL1	NA	NA	NA
AMOT	NA	0.10576102	0.11264039
XACT	1.74773022	8.54E-30	1.54E-29
HTR2C	NA	NA	NA
IL13RA2	NA	NA	NA
LRCH2	NA	NA	NA
RBMXL3	NA	NA	NA
LUZP4	NA	NA	NA
PLS3-AS1	NA	0.00168489	0.00190938
PLS3	2.13472763	7.13E-18	1.08E-17
DANT1	NA	NA	NA
DANT2	2.99610895	9.99E-97	5.06E-96
AGTR2	NA	NA	NA
SLC6A14	NA	NA	NA
CT83	NA	NA	NA
RP11-232D9.	NA	NA	NA

KLHL13	0.94876783	2.32E-96	1.16E-95
WDR44	2.49675746	4.98E-17	7.42E-17
DOCK11	2.07422927	1.86E-37	3.73E-37
IL13RA1	1.04212485	2.86E-50	6.85E-50
ZCCHC12	NA	1.59E-28	2.81E-28
LINC01285	0	0.1196492	0.12722283
LONRF3	NA	1.58E-20	2.48E-20
RP13-347D8.	NA	NA	NA
KIAA1210	NA	NA	NA
PGRMC1	1.24601213	2.09E-59	5.69E-59
RP5-1139I1.1	NA	NA	NA
RP5-1139I1.2	0	0.00173328	0.00196324
SLC25A43	1.60505837	5.96E-58	1.59E-57
SLC25A5	1.07509833	4.94E-121	4.54E-120
CXorf56	1.58127972	2.62E-47	6.01E-47
UBE2A	1.59624157	3.60E-43	7.78E-43
NKRF	1.70049427	1.26E-22	2.04E-22
6-Sep	1-Jan	5.25E-22	8.44E-22
SOWAHD	0.65539883	2.99E-91	1.36E-90
RPL39	2.18157835	9.40E-94	4.48E-93
UPF3B	1.41810213	1.66E-115	1.28E-114
RNF113A	1.25836576	1.14E-107	7.25E-107
NDUFA1	2.00757088	2.59E-79	9.52E-79
AKAP14	0	3.86E-43	8.33E-43
NKAP	1.17444562	3.42E-130	4.65E-129
RHOXF1-AS1	NA	4.18E-08	5.30E-08
RHOXF2B	NA	NA	NA
RHOXF1	NA	NA	NA
LINC01402	NA	NA	NA
RHOXF2	NA	NA	NA
ZBTB33	1.68531128	2.06E-45	4.59E-45
TMEM255A	2.24708171	9.77E-26	1.66E-25
ATP1B4	NA	NA	NA
LAMP2	1.27404633	6.09E-135	1.09E-133
CUL4B	3.11134391	0.00013402	0.00015663
MCTS1	1.04722487	3.84E-137	8.04E-136
C1GALT1C1	0.72632944	2.99E-134	5.10E-133
CT47B1	NA	NA	NA
CT47A8	NA	NA	NA
RP1-321E8.5	NA	NA	NA
CT47A12	NA	NA	NA
CT47A11	NA	NA	NA
CT47A10	NA	NA	NA
CT47A9	NA	NA	NA
CT47A6	NA	NA	NA

CT47A7	NA	NA	NA
CT47A5	NA	NA	NA
CT47A4	NA	NA	NA
CT47A3	NA	NA	NA
CT47A2	NA	NA	NA
CT47A1	NA	NA	NA
GLUD2	NA	5.28E-112	3.74E-111
RP13-192B19	NA	NA	NA
GRIA3	NA	NA	NA
THOC2	1.42622995	4.16E-57	1.09E-56
XIAP	3.10707595	1.83E-104	1.08E-103
XIAP-AS1	0	1.52E-128	1.91E-127
RP1-315G1.3	1.19844358	1.20E-47	2.76E-47
STAG2	2.27918288	6.83E-35	1.32E-34
SH2D1A	NA	6.78E-49	1.59E-48
TEX13D	NA	NA	NA
TENM1	NA	NA	NA
TEX13C	NA	NA	NA
RP11-13E5.2	NA	NA	NA
DCAF12L2	NA	NA	NA
DCAF12L1	NA	NA	NA
PRR32	NA	NA	NA
CTD-2076M1	NA	NA	NA
ACTRT1	NA	NA	NA
RP1-30E17.2	NA	NA	NA
SMARCA1	0.74902724	2.24E-46	5.06E-46
OCRL	3.74513619	1.31E-62	3.73E-62
APLN	2.99610895	0.21225894	0.22289892
XPNPEP2	0.24967575	2.85E-114	2.13E-113
SASH3	2.22835603	0.8087409	0.81648177
ZDHHC9	2.13611471	8.73E-45	1.93E-44
RP4-537K23.	NA	NA	NA
UTP14A	1.13314377	2.26E-73	7.60E-73
BCORL1	1.64369866	0.71756632	0.72775538
ELF4	2.46738384	1.38E-82	5.39E-82
AIFM1	0.93043227	2.53E-115	1.94E-114
RAB33A	0.79063986	8.16E-141	2.56E-139
ZNF280C	1.74773022	0.00027335	0.00031704
SLC25A14	1.67429618	7.87E-19	1.21E-18
GPR119	NA	NA	NA
RBMX2	1.39329542	6.34E-84	2.52E-83
RP1-274L7.1	NA	NA	NA
ENOX2	1.4381323	1.38E-108	8.94E-108
LINC01201	NA	NA	NA
ARHGAP36	NA	NA	NA

IGSF1	Inf	1.26E-84	5.10E-84
OR13H1	NA	NA	NA
FIRRE	2.99610895	8.69E-44	1.89E-43
STK26	1.88053647	3.89E-11	5.23E-11
FRMD7	NA	NA	NA
RAP2C	1.91418072	2.57E-12	3.54E-12
RAP2C-AS1	1.12354086	0.29841214	0.31065548
MBNL3	4.49416342	4.66E-05	5.51E-05
HS6ST2	NA	1.21E-72	4.04E-72
HS6ST2-AS1	NA	NA	NA
USP26	NA	NA	NA
TFDP3	NA	NA	NA
GPC4	NA	NA	NA
GPC3	NA	NA	NA
CCDC160	NA	2.32E-48	5.39E-48
PHF6	1.59168288	5.30E-19	8.15E-19
HPRT1	1.13366285	7.41E-104	4.33E-103
MIR503HG	1.56614786	3.36E-05	3.99E-05
LINC00629	0	1.15E-10	1.53E-10
PLAC1	NA	3.01E-137	6.36E-136
RP11-308B5.	NA	NA	NA
FAM122B	2.00682769	5.02E-27	8.68E-27
FAM122C	1.24837873	4.23E-49	9.93E-49
MOSPD1	1.78934284	7.73E-36	1.51E-35
RP3-473B4.3	NA	NA	NA
SMIM10	2.31221452	6.81E-49	1.59E-48
FAM127C	NA	NA	NA
FAM127A	NA	2.07E-10	2.74E-10
FAM127B	NA	NA	NA
RP11-85L21.[illegible]	NA	2.99E-74	1.02E-73
SMIM10L2B	Inf	1.10E-06	1.35E-06
LINC00633	NA	NA	NA
RP11-85L21.[illegible]	NA	NA	NA
CT55	0	1.86E-123	1.88E-122
ZNF75D	0.65539883	2.59E-08	3.29E-08
RP13-210D1[illegible]	NA	NA	NA
ZNF449	2.24708171	1.56E-46	3.53E-46
RP11-265D1[illegible]	NA	NA	NA
SMIM10L2A	NA	1.55E-34	2.97E-34
DDX26B-AS1	Inf	0.52833752	0.54165344
DDX26B	2.09727626	1.53E-09	2.00E-09
CT45A1	NA	NA	NA
CT45A3	NA	NA	NA
CT45A5	NA	NA	NA
CT45A6	NA	NA	NA

CT45A2	NA	NA	NA
CT45A7	NA	NA	NA
CT45A8	NA	NA	NA
CT45A9	NA	NA	NA
CT45A10	NA	NA	NA
SAGE1	NA	NA	NA
MMGT1	2.15629053	1.23E-59	3.36E-59
SLC9A6	1.87256809	1.33E-07	1.67E-07
FHL1	1.24837873	1.48E-47	3.41E-47
MAP7D3	1.90928511	0.00132618	0.00150768
ADGRG4	NA	NA	NA
BRS3	NA	NA	NA
HTATSF1	1.47948355	3.31E-47	7.59E-47
VGLL1	NA	NA	NA
CD40LG	NA	NA	NA
ARHGEF6	2.06874189	4.04E-35	7.82E-35
RP11-1114A5	2.99610895	2.29E-48	5.34E-48
RBMX	1.54174773	1.66E-79	6.10E-79
GPR101	NA	NA	NA
RP1-137H15.	NA	NA	NA
ZIC3	NA	NA	NA
FGF13	5.24319066	1.99E-51	4.84E-51
RP6-27P15.2	NA	NA	NA
FGF13-AS1	NA	NA	NA
F9	NA	NA	NA
MCF2	0	3.68E-151	6.13E-149
ATP11C	2.39688716	1.77E-60	4.92E-60
CXorf66	NA	NA	NA
RP11-364B14	NA	NA	NA
AC004070.1	NA	NA	NA
SOX3	NA	NA	NA
XXyac-YR29II	NA	NA	NA
LINC00632	NA	NA	NA
CDR1	NA	NA	NA
CDR1-AS	NA	NA	NA
RP11-298A8.	NA	NA	NA
SPANXB1	NA	NA	NA
RP3-507I15.2	NA	NA	NA
LDOC1	0.74902724	7.59E-61	2.12E-60
SPANXC	NA	NA	NA
SPANXA2-OT	NA	NA	NA
SPANXA1	NA	NA	NA
SPANXA2	NA	NA	NA
RP1-171K16.	NA	NA	NA
SPANXD	NA	NA	NA

MAGEC3	NA	NA	NA
MAGEC1	NA	NA	NA
MAGEC2	NA	NA	NA
SPANXN4	NA	NA	NA
GS1-256O22	NA	NA	NA
SPANXN3	NA	NA	NA
SLITRK4	NA	NA	NA
SPANXN2	NA	NA	NA
RP3-526F5.2	NA	NA	NA
RP11-143G3.	NA	NA	NA
SPANXN1	NA	NA	NA
SLITRK2	NA	NA	NA
TMEM257	NA	NA	NA
CXorf51B	NA	NA	NA
CXorf51A	NA	NA	NA
L29074.3	NA	NA	NA
FMR1	3.09824903	1.50E-108	9.74E-108
FMR1NB	NA	NA	NA
AC002368.4	NA	NA	NA
AFF2	NA	NA	NA
IDS	1.99740597	1.79E-77	6.38E-77
AF011889.5	NA	NA	NA
AF011889.2	NA	NA	NA
LINC00893	Inf	0.87653374	0.88212543
CXorf40A	1.33160398	6.51E-58	1.73E-57
RP5-937E21.	NA	NA	NA
MAGEA9B	NA	NA	NA
HSFX2	NA	NA	NA
TMEM185A	2.17574578	8.29E-74	2.81E-73
MAGEA11	NA	NA	NA
HSFX1	NA	NA	NA
MAGEA9	NA	NA	NA
LINC00850	NA	NA	NA
MAGEA8-AS	NA	NA	NA
MAGEA8	NA	NA	NA
CXorf40B	1.14137484	1.18E-107	7.50E-107
XX-FW81066	NA	NA	NA
LINC00894	Inf	5.81E-138	1.31E-136
MAMLD1	1.87256809	6.80E-64	1.98E-63
MTM1	2.37191958	0.00437236	0.00489892
MTMR1	2.24708171	1.83E-29	3.28E-29
CD99L2	1.39105058	0.59797136	0.61069559
AF003626.1	NA	NA	NA
HMGB3	1.25999155	8.41E-116	6.58E-115
GPR50-AS1	NA	NA	NA

GPR50	NA	NA	NA
AF003625.3	NA	NA	NA
VMA21	2.29579893	4.76E-132	7.18E-131
WI2-80269A(	NA	1.03E-21	1.64E-21
XX-FW80269	NA	NA	NA
PASD1	NA	NA	NA
PRRG3	NA	NA	NA
FATE1	NA	NA	NA
CNGA2	NA	NA	NA
RP11-366F6.	NA	NA	NA
MAGEA4	NA	NA	NA
GABRE	NA	NA	NA
MAGEA10	NA	NA	NA
RP11-329E24	NA	NA	NA
GABRA3	NA	NA	NA
GABRQ	NA	NA	NA
MAGEA3	NA	NA	NA
MAGEA2B	NA	NA	NA
CSAG1	Inf	3.19E-39	6.55E-39
MAGEA12	NA	NA	NA
RP1-14D6.7	NA	NA	NA
MAGEA2	NA	NA	NA
MAGEA6	NA	NA	NA
CETN2	1.29227776	1.49E-76	5.25E-76
NSDHL	0.64047257	3.04E-130	4.13E-129
ZNF185	Inf	3.75E-125	4.04E-124
PNMA5	NA	NA	NA
PNMA3	NA	3.13E-29	5.59E-29
PNMA6A	NA	NA	NA
MAGEA1	NA	NA	NA
RP11-635O1(	NA	NA	NA
LL0XNC01-16	NA	NA	NA
ZNF275	3.49546044	3.19E-94	1.53E-93
U82695.5	NA	NA	NA
ZFP92	2.49675746	0.02650785	0.02891968
TREX2	NA	NA	NA
HAUS7	0.92934861	9.15E-126	1.01E-124
U82695.9	NA	NA	NA
BGN	NA	NA	NA
ATP2B3	NA	NA	NA
FAM58A	2.29055204	2.92E-13	4.08E-13
RP11-66N11.	1.83095547	1.22E-17	1.83E-17
RP11-66N11.	NA	NA	NA
DUSP9	1.60006208	3.67E-48	8.49E-48
PNCK	NA	8.43E-29	1.49E-28

SLC6A8	3.07636187	1.19E-67	3.68E-67
BCAP31	1.03659152	1.36E-137	2.96E-136
ABCD1	1.23589494	3.77E-18	5.71E-18
U52111.14	NA	NA	NA
PLXNB3	NA	NA	NA
SRPK3	NA	NA	NA
IDH3G	0.95565544	1.75E-135	3.23E-134
SSR4	1.58396274	4.20E-78	1.52E-77
PDZD4	NA	2.92E-47	6.70E-47
L1CAM	1.49805447	9.43E-07	1.16E-06
AVPR2	NA	NA	NA
ARHGAP4	1.64785992	2.75E-19	4.25E-19
NAA10	1.53120426	1.31E-55	3.40E-55
RENBP	0.67412451	5.14E-69	1.62E-68
HCFC1	2.41587658	9.29E-111	6.36E-110
HCFC1-AS1	Inf	2.06E-89	9.05E-89
TMEM187	1.08949416	6.04E-93	2.84E-92
IRAK1	2.15978906	6.04E-88	2.59E-87
MECP2	1.56704383	1.19E-17	1.80E-17
OPN1LW	NA	NA	NA
OPN1MW	NA	NA	NA
OPN1MW2	NA	NA	NA
OPN1MW3	NA	NA	NA
TEX28	NA	NA	NA
TKTL1	NA	NA	NA
XX-FW83128	NA	7.38E-65	2.18E-64
FLNA	1.2733463	7.84E-34	1.49E-33
EMD	2.25332361	1.46E-77	5.21E-77
RPL10	1.91180333	0.00015215	0.00017753
XX-FW83563	3.74513619	0.71601496	0.7263038
DNASE1L1	2.0598249	2.58E-38	5.22E-38
TAZ	1.02991245	7.09E-123	7.00E-122
CH17-340M2	1.65410182	0.00126523	0.00143947
ATP6AP1	1.08312572	9.08E-139	2.23E-137
GDI1	1.28404669	1.32E-17	1.99E-17
FAM50A	1.24837873	4.02E-129	5.20E-128
PLXNA3	1.44043699	2.15E-06	2.63E-06
LAGE3	2.08751014	4.58E-102	2.57E-101
UBL4A	1.7153888	4.27E-30	7.71E-30
SLC10A3	2.1025326	0.16854366	0.17785709
FAM3A	2.01661179	0.21159592	0.22221552
G6PD	1.13205253	9.18E-124	9.41E-123
IKBKG	1.8612192	1.38E-08	1.77E-08
FAM223A	NA	NA	NA
CTAG1A	NA	NA	NA

CTAG1B	NA	NA	NA
FAM223B	NA	NA	NA
CTAG2	NA	NA	NA
GAB3	1.49805447	4.35E-28	7.63E-28
DKC1	1.23016709	9.76E-115	7.40E-114
MPP1	0.93093385	1.65E-133	2.70E-132
SMIM9	NA	NA	NA
F8	0.37451362	1.55E-90	6.96E-90
H2AFB1	NA	NA	NA
F8A1	1.14694796	1.61E-110	1.10E-109
FUNDC2	1.49146479	3.83E-127	4.53E-126
MTCP1	2.62159533	1.80E-56	4.69E-56
CMC4	1.49805447	6.28E-23	1.02E-22
BRCC3	1.64559014	7.41E-09	9.55E-09
VBP1	1.10342236	5.11E-113	3.73E-112
RAB39B	1.41482923	1.10E-12	1.53E-12
CLIC2	Inf	4.78E-58	1.27E-57
RP13-228J13	NA	NA	NA
H2AFB2	NA	NA	NA
F8A2	NA	NA	NA
F8A3	0.74902724	0.01595278	0.01754245
H2AFB3	NA	0.00110931	0.0012651
TMLHE-AS1	Inf	5.08E-98	2.64E-97
RP11-218L14	NA	NA	NA
TMLHE	0.79308766	2.86E-94	1.38E-93
SPRY3	0	7.53E-43	1.62E-42
VAMP7	1.91169638	1.47E-33	2.78E-33
IL9R	2.24708171	0.7703715	0.77917599
WASIR1	NA	NA	NA
AC144568.2	NA	NA	NA
OR4F21	NA	NA	NA
ZNF596	1.49805447	1.45E-17	2.18E-17
RP5-855D21.	NA	NA	NA
RP5-855D21.	Inf	2.91E-76	1.02E-75
RP11-63E5.6	NA	NA	NA
FAM87A	NA	NA	NA
FBXO25	0.731608	1.59E-136	3.17E-135
RP11-91J19.3	NA	NA	NA
TDRP	NA	NA	NA
ERICH1	1.45990957	4.63E-90	2.05E-89
ERICH1-AS1	NA	1.18E-54	3.02E-54
RP11-43A14.	NA	NA	NA
RP11-555E9.	NA	NA	NA
CTD-3212A4.	NA	NA	NA
CTD-2281E23	NA	NA	NA

CTD-2281E23	NA	NA	NA
AF067845.1	NA	NA	NA
AF067845.2	NA	NA	NA
AF067845.3	NA	NA	NA
DLGAP2	NA	NA	NA
DLGAP2-AS1	NA	NA	NA
CLN8	1.52579622	3.75E-45	8.34E-45
CTD-2336O2	Inf	3.03E-17	4.53E-17
CTD-2336O2	2.54987996	9.30E-86	3.82E-85
ARHGEF10	1.96296793	1.23E-44	2.70E-44
KBTBD11-OT	0.99870298	2.43E-05	2.90E-05
RP11-439C15	NA	NA	NA
KBTBD11	1.77893969	1.77E-43	3.84E-43
RP11-439C15	Inf	2.42E-66	7.33E-66
RP11-1049H7	NA	NA	NA
MYOM2	2.62159533	0.00202217	0.00228617
AC133633.2	NA	NA	NA
CH17-31A6.1	NA	NA	NA
CH17-174L20	NA	NA	NA
AC133633.1	NA	NA	NA
RP11-378A12	NA	NA	NA
RP11-134O2	1.98271916	6.92E-05	8.15E-05
GS1-57L11.1	NA	NA	NA
CSMD1	NA	NA	NA
RP5-991O23	NA	NA	NA
RP11-281H11	NA	NA	NA
RP11-158A14	NA	NA	NA
RP11-124B13	NA	4.63E-22	7.45E-22
RP11-115C21	2.33268482	3.07E-05	3.65E-05
MCPH1	1.71206226	0.04034223	0.04372356
ANGPT2	3.29571984	1.22E-107	7.71E-107
CTD-2541M1	NA	NA	NA
MCPH1-AS1	Inf	5.50E-145	3.61E-143
AGPAT5	1.96759394	4.13E-18	6.26E-18
XKR5	NA	NA	NA
GS1-24F4.2	NA	9.22E-19	1.41E-18
DEFB1	NA	NA	NA
DEFA6	NA	NA	NA
GS1-24F4.3	NA	NA	NA
DEFA4	0	5.93E-131	8.42E-130
DEFA1	NA	3.55E-36	6.99E-36
DEFA1B	NA	1.02E-20	1.61E-20
DEFA3	0	3.34E-101	1.84E-100
DEFA5	NA	NA	NA
FAM66B	NA	7.31E-62	2.07E-61

USP17L1	NA	NA	NA
USP17L4	NA	NA	NA
ZNF705G	NA	NA	NA
DEFB4B	NA	NA	NA
DEFB103B	NA	NA	NA
SPAG11B	NA	NA	NA
DEFB104B	NA	NA	NA
DEFB106B	NA	NA	NA
DEFB105B	NA	NA	NA
DEFB107B	NA	NA	NA
PRR23D1	NA	NA	NA
PRR23D2	NA	NA	NA
DEFB107A	NA	NA	NA
DEFB105A	NA	NA	NA
DEFB106A	NA	NA	NA
DEFB104A	NA	NA	NA
SPAG11A	NA	NA	NA
DEFB103A	NA	NA	NA
DEFB4A	NA	NA	NA
ZNF705B	NA	NA	NA
FAM66E	NA	NA	NA
USP17L8	NA	NA	NA
USP17L3	NA	NA	NA
FAM85B	NA	1.04E-13	1.46E-13
RP11-556O5.	NA	NA	NA
SGK223	8.61381323	1.16E-138	2.79E-137
CTA-398F10.	NA	NA	NA
CTA-398F10.	NA	4.58E-34	8.74E-34
CTD-3023L14	NA	NA	NA
CTD-3023L14	NA	NA	NA
CTD-3023L14	NA	NA	NA
CLDN23	0.74902724	7.82E-129	9.98E-128
RP11-211C9.	NA	NA	NA
MFHAS1	2.53516911	3.23E-27	5.60E-27
ERI1	2.17686041	5.77E-12	7.89E-12
PPP1R3B	1.24837873	2.85E-14	4.06E-14
RP11-10A14.	NA	2.12E-18	3.23E-18
RP11-10A14.	NA	NA	NA
RP11-10A14.	NA	NA	NA
RP11-10A14.	NA	NA	NA
RP11-10A14.	NA	NA	NA
RP11-115J16	NA	NA	NA
RP11-10A14.	NA	NA	NA
RP11-115J16	NA	NA	NA
RP11-115J16	NA	NA	NA

RP11-375N1!	Inf	1.09E-81	4.17E-81
TNKS	1.5729572	0.01691963	0.01859437
LINC00599	NA	6.66E-23	1.08E-22
RP11-1E4.1	Inf	6.30E-53	1.57E-52
MSRA	0.79308766	2.07E-123	2.09E-122
LINCR-0001	NA	NA	NA
RP11-981G7.	NA	NA	NA
PRSS51	NA	NA	NA
RP11-981G7.	NA	0.01402324	0.01544966
PRSS55	NA	NA	NA
RP1L1	NA	1.26E-08	1.62E-08
C8orf74	NA	NA	NA
SOX7	5.74254215	5.69E-09	7.35E-09
CTD-2135J3.4	NA	NA	NA
CTD-2135J3.3	NA	NA	NA
PINX1	1.18705486	7.95E-96	3.95E-95
RP11-177H2.	NA	2.46E-95	1.21E-94
XKR6	2.62159533	0.0554606	0.05976633
AF131215.6	NA	9.11E-49	2.13E-48
AF131215.8	2.24708171	1.26E-63	3.66E-63
LINC00529	NA	NA	NA
AF131216.1	0.37451362	7.18E-27	1.24E-26
MTMR9	1.93297352	3.55E-17	5.31E-17
AF131216.6	NA	0.10239071	0.10914055
SLC35G5	Inf	0.92440844	0.92799595
AF131216.5	Inf	7.59E-100	4.06E-99
FAM167A	0	1.58E-80	5.93E-80
BLK	NA	NA	NA
RP11-148O2	NA	4.57E-87	1.92E-86
RP11-148O2	NA	NA	NA
RP11-148O2	NA	NA	NA
RP11-148O2	NA	NA	NA
LINC00208	NA	NA	NA
GATA4	NA	NA	NA
C8orf49	NA	NA	NA
NEIL2	1.61328943	1.59E-93	7.54E-93
FDFT1	0.83506415	6.09E-143	2.60E-141
RP11-297N6.	1.64785992	3.60E-08	4.57E-08
CTSB	1.5571882	6.84E-128	8.34E-127
DEFB136	NA	0.02676509	0.02919506
DEFB135	NA	4.02E-120	3.60E-119
DEFB134	NA	NA	NA
RP11-1236K1	NA	NA	NA
ZNF705D	NA	NA	NA
FAM66D	NA	NA	NA

USP17L7	NA	NA	NA
USP17L2	NA	NA	NA
FAM86B1	2.69649805	0.74116947	0.75077032
AC145124.2	2.99610895	4.65E-60	1.28E-59
DEFB130	NA	NA	NA
FAM66A	NA	NA	NA
RP11-351I21	NA	5.56E-53	1.39E-52
FAM86B2	NA	2.72E-103	1.57E-102
RP11-419I17	NA	1.95E-13	2.74E-13
LONRF1	2.42332341	8.38E-05	9.85E-05
RP11-252C15	NA	NA	NA
LINC00681	NA	NA	NA
KIAA1456	NA	4.91E-108	3.14E-107
DLC1	3.2457847	1.05E-102	5.99E-102
RP11-10C8.2	NA	NA	NA
C8orf48	Inf	3.70E-131	5.31E-130
RP11-480O10	NA	NA	NA
SGCZ	NA	NA	NA
RP11-3G21.1	NA	NA	NA
TUSC3	NA	NA	NA
RP11-77B22.	NA	NA	NA
MSR1	NA	NA	NA
RP11-13N12.	NA	NA	NA
RP11-13N12.	NA	NA	NA
FGF20	NA	NA	NA
MICU3	2.24708171	2.37E-08	3.02E-08
RP11-468H14	NA	2.15E-24	3.58E-24
ZDHHC2	1.33960641	8.18E-05	9.61E-05
CNOT7	1.93207026	1.09E-12	1.51E-12
VPS37A	1.9688716	8.76E-57	2.30E-56
MTMR7	NA	NA	NA
SLC7A2	1.75486381	2.85E-53	7.12E-53
PDGFRL	1.12354086	2.47E-56	6.43E-56
MTUS1	1.68531128	3.11E-13	4.34E-13
RP11-326L2.	NA	NA	NA
RP11-156K13	NA	NA	NA
FGL1	NA	NA	NA
RP11-156K13	NA	NA	NA
RP11-156K13	NA	NA	NA
PCM1	1.66088648	2.27E-107	1.43E-106
ASAH1	1.41528903	2.25E-47	5.16E-47
CTD-2547L16	Inf	8.98E-61	2.50E-60
NAT1	0.58257674	5.45E-67	1.67E-66
NAT2	NA	NA	NA
PSD3	2.82965845	3.25E-45	7.23E-45

RP11-161I2.1	NA	NA	NA
RP11-420B22	NA	NA	NA
RP11-1080G	NA	NA	NA
RP11-1080G	NA	3.76E-63	1.08E-62
RP11-618M2	NA	NA	NA
RP11-618M2	NA	NA	NA
SH2D4A	NA	5.04E-119	4.33E-118
CSGALNACT1	NA	NA	NA
RP11-1105O	NA	NA	NA
INTS10	1.7309453	2.41E-12	3.33E-12
LPL	NA	NA	NA
AC100802.3	NA	NA	NA
SLC18A1	Inf	1.25E-79	4.62E-79
ATP6V1B2	1.05877534	9.07E-123	8.89E-122
LZTS1	Inf	1.41E-132	2.21E-131
LZTS1-AS1	NA	NA	NA
RP11-563N1	NA	NA	NA
RP11-108E14	NA	NA	NA
RP11-369E15	NA	NA	NA
RP11-369E15	NA	NA	NA
RP11-369E15	NA	NA	NA
RP11-369E15	NA	NA	NA
RP11-421P23	NA	NA	NA
RP11-421P23	NA	NA	NA
RP11-24P4.1	NA	NA	NA
GFRA2	0	8.68E-109	5.65E-108
DOK2	1.29142627	4.35E-39	8.90E-39
XPO7	1.75591631	1.83E-52	4.52E-52
NPM2	1.04863813	1.35E-96	6.85E-96
FGF17	NA	6.05E-18	9.15E-18
DMTN	Inf	4.30E-14	6.10E-14
FAM160B2	2.57998271	6.54E-46	1.47E-45
NUDT18	1.60505837	2.95E-06	3.59E-06
HR	0.99870298	0.25714337	0.2687886
REEP4	1.13412034	1.74E-64	5.12E-64
LGI3	Inf	2.51E-70	8.08E-70
SFTPC	NA	NA	NA
BMP1	2.20963035	1.88E-59	5.13E-59
PHYHIP	NA	NA	NA
POLR3D	1.86805588	5.53E-33	1.04E-32
CTD-2530N2	NA	NA	NA
PIWIL2	NA	3.67E-65	1.09E-64
SLC39A14	1.78404669	3.42E-52	8.43E-52
PPP3CC	1.89753567	3.58E-33	6.73E-33
RP11-582J16	0	1.80E-24	3.01E-24

SORBS3	2.4402271	5.12E-110	3.45E-109
RP11-582J16	NA	7.13E-13	9.90E-13
PDLIM2	1.91030978	8.97E-18	1.35E-17
AC037459.4	NA	NA	NA
C8orf58	1.81906615	3.45E-57	9.08E-57
CCAR2	2.12224384	4.52E-19	6.96E-19
RP11-582J16	2.24708171	3.76E-78	1.36E-77
BIN3	1.48261061	4.39E-38	8.86E-38
EGR3	Inf	2.86E-37	5.71E-37
PEBP4	NA	1.51E-43	3.28E-43
RP11-87E22.	NA	NA	NA
RP11-875O1	0	1.70E-19	2.63E-19
RHOBTB2	1.71206226	0.26109112	0.27280511
TNFRSF10B	1.46809339	1.63E-17	2.44E-17
RP11-875O1	NA	3.33E-06	4.05E-06
RP11-875O1	NA	NA	NA
TNFRSF10C	Inf	0.0010089	0.00115188
TNFRSF10D	0.93628405	1.60E-53	4.01E-53
RP11-1149O	NA	NA	NA
TNFRSF10A	2.0130107	0.17505696	0.18452637
RP11-1149O	NA	2.64E-126	3.00E-125
CHMP7	1.61746461	3.76E-65	1.12E-64
R3HCC1	1.58245191	2.42E-81	9.20E-81
LOXL2	0	9.41E-168	1.14E-164
ENTPD4	2.93369001	9.61E-64	2.79E-63
CTC-756D1.2	NA	3.27E-97	1.67E-96
CTC-756D1.3	NA	NA	NA
SLC25A37	2.42664304	1.39E-125	1.53E-124
NKX3-1	Inf	3.86E-33	7.25E-33
NKX2-6	NA	NA	NA
RP11-175E9.	NA	NA	NA
RP11-213G6.	NA	NA	NA
STC1	NA	NA	NA
RP11-203E8.	NA	NA	NA
ADAM28	Inf	1.37E-14	1.97E-14
RP11-624C2	NA	NA	NA
ADAMDEC1	NA	NA	NA
ADAM7	NA	NA	NA
RP11-561E1.	NA	NA	NA
GS1-72M22.	NA	NA	NA
NEFM	NA	NA	NA
NEFL	NA	NA	NA
CTD-2168K2	NA	NA	NA
CTD-2168K2	NA	NA	NA
DOCK5	2.62159533	6.37E-118	5.29E-117

GNRH1	0.74902724	1.83E-24	3.06E-24
RP11-395I14	NA	NA	NA
KCTD9	1.30386223	5.48E-20	8.54E-20
CDCA2	1.33754864	8.09E-125	8.61E-124
RP11-219J21	NA	NA	NA
RP11-219J21	NA	NA	NA
RP11-299D14	NA	NA	NA
EBF2	NA	1.47E-18	2.24E-18
PPP2R2A	1.54404738	1.80E-71	5.86E-71
BNIP3L	2.13026095	1.29E-34	2.48E-34
PNMA2	3.1459144	4.67E-10	6.16E-10
DPYSL2	2.43433852	4.71E-37	9.36E-37
ADRA1A	NA	NA	NA
RP11-521M1	NA	NA	NA
RP11-521M1	NA	NA	NA
STMN4	NA	NA	NA
TRIM35	1.68531128	0.90979028	0.91397172
PTK2B	1.73346304	2.56E-101	1.42E-100
CHRNA2	NA	NA	NA
EPHX2	NA	1.94E-111	1.35E-110
CLU	2.18946423	1.24E-08	1.59E-08
SCARA3	Inf	2.23E-87	9.42E-87
CCDC25	1.47670453	1.02E-104	6.03E-104
RP11-16P20.	NA	NA	NA
ESCO2	1.68025029	8.38E-42	1.78E-41
PBK	1.06180784	1.43E-102	8.13E-102
SCARA5	NA	NA	NA
RP11-597M1	NA	NA	NA
NUGGC	NA	4.04E-114	3.02E-113
ELP3	1.64369866	3.63E-46	8.19E-46
RP11-380I10	NA	NA	NA
PNOC	0.74902724	3.11E-23	5.10E-23
ZNF395	1.63424125	1.61E-08	2.06E-08
FBXO16	1.49805447	4.88E-63	1.40E-62
RP11-181B1	NA	NA	NA
FZD3	2.10743257	2.28E-84	9.16E-84
EXTL3	1.80447471	1.40E-30	2.54E-30
EXTL3-AS1	NA	3.99E-21	6.33E-21
INTS9	1.1770428	1.51E-44	3.32E-44
RP11-662B1	NA	NA	NA
HMBOX1	0.85603113	1.82E-28	3.21E-28
CTD-2647L4.	NA	6.56E-44	1.43E-43
KIF13B	2.69649805	1.61E-77	5.77E-77
CTD-2647L4.	NA	NA	NA
DUSP4	1.64785992	1.21E-07	1.52E-07

RP11-486M2	NA	NA	NA
RP11-486M2	NA	NA	NA
LINC00589	NA	NA	NA
AC145110.1	NA	1.35E-10	1.79E-10
RP11-94H18.	NA	NA	NA
RP11-94H18.	NA	NA	NA
RP11-489E7.	NA	NA	NA
FAM183CP	NA	NA	NA
SARAF	1.53023226	5.82E-71	1.88E-70
RP11-489E7.	0.37451362	4.19E-16	6.16E-16
LEPROTL1	1.45748217	1.71E-102	9.69E-102
MBOAT4	NA	NA	NA
RP11-51J9.5	3.65150778	2.90E-45	6.46E-45
DCTN6	1.38281952	2.75E-63	7.93E-63
RP11-51J9.4	NA	NA	NA
RP11-51J9.6	0	6.56E-05	7.73E-05
RBPMS-AS1	Inf	5.94E-69	1.87E-68
RBPMS	1.19844358	3.66E-92	1.69E-91
GTF2E2	1.51053826	7.44E-55	1.90E-54
CTD-2373N4.	NA	NA	NA
SMIM18	NA	NA	NA
GSR	2.12224384	1.79E-71	5.83E-71
UBXN8	NA	NA	NA
PPP2CB	2.34202883	8.31E-26	1.41E-25
TEX15	2.02237354	1.02E-09	1.34E-09
PURG	NA	9.81E-71	3.17E-70
WRN	2.67509728	2.91E-24	4.84E-24
RP11-363L24	NA	NA	NA
RP11-566H8.	NA	NA	NA
RP11-566H8.	NA	NA	NA
NRG1	NA	NA	NA
RP11-11N9.4	1.82243635	0.39780103	0.41061142
RP11-722E23	10.4863813	3.38E-104	1.99E-103
FUT10	1.63849708	9.07E-23	1.48E-22
TTI2	1.68531128	1.63E-35	3.18E-35
MAK16	1.93338654	6.39E-05	7.53E-05
RNF122	0.74902724	1.62E-72	5.39E-72
DUSP26	NA	NA	NA
RP11-317N1.	NA	NA	NA
RP1-273G13.	NA	NA	NA
RP11-431M3	NA	NA	NA
RP1-84O15.2	NA	NA	NA
RP11-431M3	NA	NA	NA
LINC01288	NA	NA	NA
UNC5D	NA	NA	NA

RP11-473J6.1	NA	NA	NA
RP11-89M20	NA	NA	NA
RP11-962G1!	NA	NA	NA
KCNU1	NA	NA	NA
RP11-419C2:	NA	NA	NA
RP11-527N2:	NA	NA	NA
LINC01605	NA	NA	NA
RP11-150O1:	NA	NA	NA
RP11-150O1:	NA	NA	NA
RP11-150O1:	NA	NA	NA
RP11-150O1:	NA	NA	NA
RP11-346L1.:	NA	NA	NA
ZNF703	1.49805447	2.69E-15	3.90E-15
RP11-863K1(	NA	NA	NA
RP11-863K1(	0	1.58E-11	2.14E-11
ERLIN2	1.961738	4.52E-19	6.96E-19
PROSC	1.69374628	6.28E-64	1.83E-63
ADGRA2	1.59575368	3.77E-06	4.58E-06
BRF2	1.17347601	4.49E-94	2.16E-93
RAB11FIP1	1.65226596	5.64E-09	7.29E-09
GOT1L1	NA	NA	NA
ADRB3	NA	NA	NA
EIF4EBP1	2.40623551	2.04E-144	1.18E-142
KB-1836B5.4	0	5.26E-25	8.85E-25
ASH2L	1.0872976	1.86E-64	5.47E-64
RP11-90P5.5	NA	2.72E-84	1.09E-83
STAR	0.80350195	1.13E-140	3.46E-139
RP11-90P5.2	0	8.52E-92	3.92E-91
LSM1	1.95268144	0.004735	0.00529967
BAG4	2.07422927	0.37238309	0.38536159
DDHD2	2.56809339	1.19E-77	4.26E-77
PLPP5	2.19159821	1.30E-14	1.86E-14
WHSC1L1	2.28132296	1.35E-71	4.42E-71
RP11-513D5.	NA	NA	NA
RP11-513D5.	NA	NA	NA
RP11-350N1!	0.93628405	3.19E-26	5.46E-26
LETM2	1.04863813	0.00028145	0.00032642
RP11-350N1!	NA	NA	NA
RP11-350N1!	Inf	6.06E-36	1.19E-35
FGFR1	1.88959144	5.30E-22	8.53E-22
RP11-350N1!	NA	NA	NA
C8orf86	NA	NA	NA
RP11-675F6.	NA	NA	NA
RP11-675F6.	NA	NA	NA
RP11-495O1(	NA	NA	NA

TACC1	2.12012794	8.39E-13	1.16E-12
RP11-723D2:	Inf	2.22E-129	2.90E-128
PLEKHA2	1.69224672	1.97E-14	2.82E-14
CTD-2544N1	Inf	1.37E-83	5.42E-83
HTRA4	NA	1.09E-66	3.33E-66
TM2D2	1.65804087	1.45E-41	3.08E-41
ADAM9	4.04474708	1.67E-24	2.78E-24
ADAM32	NA	0.00596164	0.00664881
RP11-122L4.	NA	NA	NA
CTD-2024D2:	NA	NA	NA
ADAM18	Inf	0.27671459	0.28864743
ADAM2	NA	NA	NA
IDO1	Inf	1.32E-131	1.95E-130
RP11-44K6.3	NA	NA	NA
RP11-44K6.4	NA	NA	NA
IDO2	NA	NA	NA
RP11-359E1!	NA	NA	NA
RP11-359E1!	NA	NA	NA
C8orf4	NA	NA	NA
RP11-470M1	NA	NA	NA
CTA-392C11.	NA	NA	NA
CTA-392C11.	NA	NA	NA
RP11-301G7.	NA	NA	NA
ZMAT4	0.74902724	1.72E-45	3.85E-45
RP11-465K1(	NA	NA	NA
SFRP1	NA	NA	NA
RP11-15G16.	NA	NA	NA
GOLGA7	2.18833448	7.42E-25	1.25E-24
GINS4	2.2591628	1.24E-23	2.05E-23
RP11-360L9.	0	9.63E-132	1.43E-130
RP11-360L9.	1.49805447	0.00742794	0.00826633
GPAT4	1.89568622	0.00160375	0.00181902
RP11-360L9.	Inf	5.95E-133	9.47E-132
NKX6-3	NA	NA	NA
ANK1	Inf	1.27E-13	1.78E-13
RP11-930P1	NA	NA	NA
RP11-930P1	Inf	1.73E-11	2.35E-11
KAT6A	2.33696498	6.83E-21	1.08E-20
RP11-589C2	2.24708171	2.92E-22	4.72E-22
RP11-589C2	NA	NA	NA
AP3M2	1.16921325	0.11677327	0.12419396
PLAT	NA	5.16E-43	1.11E-42
RP11-231D2(	0	8.56E-69	2.69E-68
IKBKB	2.63171732	2.97E-116	2.34E-115
POLB	2.0191169	8.25E-24	1.36E-23

DKK4	NA	NA	NA
VDAC3	1.14705047	3.91E-122	3.74E-121
SLC20A2	2.57098538	1.16E-51	2.83E-51
RP11-503E24	0	6.39E-36	1.25E-35
SMIM19	1.91587919	5.41E-65	1.61E-64
CHRNB3	NA	NA	NA
RP11-412B14	NA	NA	NA
CHRNA6	NA	NA	NA
THAP1	1.91750973	9.78E-39	1.99E-38
RNF170	2.41993415	2.09E-09	2.72E-09
HOOK3	1.74319066	1.30E-10	1.73E-10
RP11-598P20	NA	NA	NA
FNTA	1.02870694	3.43E-141	1.12E-139
POMK	0.74902724	0.96386921	0.96573232
HGSNAT	1.18595979	9.64E-08	1.21E-07
LINC00293	NA	NA	NA
RP11-350F16	NA	NA	NA
RP11-350F16	NA	NA	NA
RP11-1134I1	Inf	3.06E-38	6.19E-38
IGLV8OR8-1	NA	NA	NA
SPIDR	1.73638132	0.00574989	0.00641738
CEBPD	1.30810977	4.71E-112	3.35E-111
PRKDC	2.01411655	4.33E-21	6.86E-21
MCM4	2.67833982	4.76E-143	2.06E-141
UBE2V2	1.67335872	1.16E-16	1.72E-16
RP11-567J20	NA	9.04E-66	2.72E-65
RP11-770E5.	NA	NA	NA
RP11-567J20	NA	NA	NA
AC026904.1	NA	NA	NA
RP11-567J20	NA	NA	NA
RP11-770E5.	NA	NA	NA
RP11-770E5.	NA	NA	NA
EFCAB1	NA	NA	NA
SNAI2	NA	NA	NA
C8orf22	NA	NA	NA
RP11-10H3.1	NA	NA	NA
RP11-738G5.	NA	NA	NA
RP11-738G5.	NA	NA	NA
RP11-109P6.	NA	NA	NA
RP11-401N13	NA	NA	NA
SNTG1	NA	NA	NA
RP11-401H2.	NA	NA	NA
PXDNL	NA	NA	NA
RP11-11C20.	NA	NA	NA
PCMTD1	2.11089494	1.19E-26	2.05E-26

RP11-110G2	1.4192095	5.26E-15	7.59E-15
RP11-546K22	0	4.14E-78	1.49E-77
RP11-546K22	NA	2.06E-11	2.78E-11
RP11-546K22	NA	NA	NA
ST18	NA	6.23E-43	1.34E-42
RP11-26M5.3	NA	NA	NA
RP11-26M5.2	NA	NA	NA
RP11-1023P1	NA	NA	NA
FAM150A	0.6866083	5.21E-120	4.65E-119
RB1CC1	2.29540605	1.01E-53	2.54E-53
NPBWR1	NA	NA	NA
RP11-162D9.	NA	NA	NA
OPRK1	NA	NA	NA
RP11-1081M	NA	NA	NA
RP11-1081M	NA	NA	NA
RP11-400K9.	NA	NA	NA
RP11-400K9.	NA	NA	NA
ATP6V1H	1.27864246	7.27E-112	5.12E-111
RGS20	1.73710572	6.34E-05	7.47E-05
RP11-1070A2	NA	2.57E-116	2.04E-115
TCEA1	1.86612683	0.00091604	0.00104738
AC100821.2	NA	NA	NA
RP11-30L15.(	NA	6.41E-92	2.95E-91
LYPLA1	1.79629415	0.87204277	0.8778493
MRPL15	1.17905038	9.32E-120	8.26E-119
SOX17	NA	NA	NA
RP11-53M11	NA	NA	NA
RP1	NA	NA	NA
XKR4	NA	1.48E-16	2.19E-16
RP11-386G2	NA	NA	NA
RP11-386G2	NA	NA	NA
RP11-628E19	NA	NA	NA
TMEM68	1.45863199	1.76E-07	2.20E-07
TGS1	1.59961749	2.14E-14	3.05E-14
LYN	2.1751751	0.01582836	0.01741197
RPS20	2.4027182	1.84E-145	1.58E-143
CTA-397H3.3	NA	3.52E-24	5.84E-24
MOS	NA	NA	NA
PLAG1	1.87256809	0.07822745	0.08383758
CHCHD7	2.10970554	8.15E-104	4.75E-103
RP11-140I16	1.49805447	0.39751137	0.41033578
SDR16C5	NA	NA	NA
PENK	NA	NA	NA
RP11-17A4.2	NA	NA	NA
LINC00968	NA	NA	NA

RP11-17A4.3	NA	NA	NA
RP11-17A4.4	NA	NA	NA
IMPAD1	2.34478092	2.08E-80	7.79E-80
LINC01606	NA	NA	NA
RP11-513O1	NA	NA	NA
LINC00588	NA	NA	NA
RP11-756K1	NA	NA	NA
RP11-246K1	1.87256809	0.00117329	0.00133688
RP11-388G2	NA	NA	NA
CTD-2339F6.	0.74902724	2.04E-11	2.76E-11
RP11-44D19.	NA	NA	NA
LINC01602	8.98832685	3.60E-87	1.51E-86
FAM110B	1.31079767	0.00981301	0.01086528
RP11-1112C	NA	NA	NA
RP11-648L3.	NA	NA	NA
UBXN2B	1.58127972	0.12428004	0.13206165
CYP7A1	NA	NA	NA
SDCBP	0.70694706	2.09E-145	1.67E-143
NSMAF	1.97694074	3.49E-26	5.96E-26
TOX	0.79308766	2.22E-07	2.77E-07
RP11-25K19.	2.42788139	2.29E-23	3.76E-23
RP11-379I19	NA	NA	NA
RP11-379I19	NA	NA	NA
RP11-27P7.1	NA	NA	NA
CA8	0.98011011	7.71E-77	2.72E-76
LINC01301	Inf	5.79E-85	2.34E-84
RAB2A	1.46114589	1.31E-69	4.18E-69
RP11-91I20.4	NA	3.44E-77	1.22E-76
RP11-91I20.3	NA	NA	NA
RP11-91I20.2	NA	NA	NA
RP11-91I20.1	NA	NA	NA
CHD7	2.19055135	1.74E-37	3.48E-37
RP11-33I11.2	NA	NA	NA
AC022182.1	NA	NA	NA
AC022182.3	NA	1.10E-71	3.60E-71
CLVS1	NA	NA	NA
RP11-787D1	NA	NA	NA
RP11-787D1	NA	NA	NA
ASPH	2.39688716	9.60E-83	3.74E-82
RP11-280G9.	NA	NA	NA
RP11-705O2	NA	9.85E-09	1.27E-08
RP11-705O2	NA	NA	NA
RP11-705O2	NA	NA	NA
NKAIN3	NA	NA	NA
RP11-577N1.	NA	NA	NA

UG0898H09	NA	NA	NA
RP11-659E9.	NA	3.18E-27	5.51E-27
GGH	0.91035618	2.75E-138	6.35E-137
RP11-659E9.	NA	NA	NA
TTPA	1.49805447	4.12E-100	2.22E-99
YTHDF3-AS1	1.80447471	1.38E-06	1.69E-06
YTHDF3	2.7317464	1.25E-136	2.52E-135
RP11-16E18.	2.24708171	2.13E-33	4.02E-33
CTD-3046C4.	NA	NA	NA
RP11-45K10.	NA	NA	NA
RP11-573J24	NA	NA	NA
RP11-32K4.1	NA	NA	NA
RP11-579E24	NA	NA	NA
LINC01289	NA	NA	NA
MIR124-2HG	NA	NA	NA
RP11-21C4.1	NA	NA	NA
BHLHE22	NA	4.30E-54	1.09E-53
CYP7B1	NA	NA	NA
RP11-1D12.2	NA	NA	NA
LINC00251	NA	NA	NA
LINC01299	NA	NA	NA
CTD-3025N20	1.49805447	9.39E-29	1.66E-28
ARMC1	1.3765906	3.06E-116	2.41E-115
MTFR1	1.53109979	1.51E-84	6.10E-84
RP11-707M3	0.37451362	2.12E-41	4.48E-41
PDE7A	1.8954975	3.10E-24	5.16E-24
CTD-2532N20	2.24708171	0.02173648	0.0237771
DNAJC5B	NA	0.19550059	0.20568149
AC084082.3	NA	3.38E-32	6.27E-32
TRIM55	NA	4.09E-23	6.68E-23
CRH	NA	NA	NA
LINC00967	NA	NA	NA
RRS1-AS1	1.49805447	1.05E-14	1.51E-14
RRS1	1.39206005	3.63E-98	1.90E-97
ADHFE1	NA	1.78E-56	4.65E-56
C8orf46	NA	NA	NA
MYBL1	11.2354086	4.74E-127	5.57E-126
VCPIP1	1.47464737	0.89581173	0.90077688
C8orf44-SGK	0	5.71E-37	1.14E-36
C8orf44	4.1536965	1.22E-05	1.47E-05
AC090154.1	NA	NA	NA
SGK3	2.33520256	8.90E-42	1.89E-41
MCMDC2	Inf	2.22E-129	2.90E-128
TCF24	1.53210117	1.62E-27	2.81E-27
PPP1R42	NA	1.94E-111	1.35E-110

COPS5	1.15436118	5.25E-139	1.32E-137
CSPP1	1.20102643	2.56E-88	1.10E-87
ARFGEF1	1.59367497	5.76E-69	1.82E-68
RP11-7F18.2	4.49416342	6.51E-09	8.39E-09
CPA6	NA	NA	NA
RP11-396J6.1	NA	NA	NA
PREX2	NA	NA	NA
RP11-403D15	NA	NA	NA
C8orf34-AS1	NA	NA	NA
C8orf34	NA	NA	NA
RP11-865I6.2	NA	NA	NA
LINC01592	NA	NA	NA
RP11-21C17.	NA	NA	NA
LINC01603	NA	NA	NA
SULF1	NA	NA	NA
SLCO5A1	0.24967575	4.58E-67	1.40E-66
RP11-102F4.	NA	NA	NA
RP11-159H10	Inf	0.00720538	0.00802259
PRDM14	NA	NA	NA
NCOA2	2.49675746	5.56E-60	1.53E-59
RP11-333A23	NA	NA	NA
RP11-333A23	NA	NA	NA
TRAM1	1.93353543	3.26E-18	4.95E-18
LACTB2-AS1	Inf	3.51E-38	7.09E-38
LACTB2	1.03144734	4.63E-105	2.77E-104
XKR9	NA	3.21E-60	8.87E-60
RP11-326E22	NA	NA	NA
EYA1	Inf	1.07E-156	3.98E-154
RP11-1102P1	NA	3.26E-141	1.07E-139
RP11-1144P2	NA	NA	NA
RP11-281N10	NA	NA	NA
MSC-AS1	NA	NA	NA
MSC	NA	NA	NA
TRPA1	NA	NA	NA
RP11-142A23	NA	NA	NA
RP11-351E7.	NA	NA	NA
KCNB2	NA	0.38801892	0.40076511
RP11-1145L2	NA	NA	NA
RP11-531A24	NA	NA	NA
TERF1	1.04937974	1.02E-111	7.18E-111
RP11-531A24	NA	NA	NA
RP11-531A24	NA	0.07565295	0.08112157
SBSPON	Inf	4.28E-96	2.14E-95
C8orf89	NA	NA	NA
RPL7	1.67714984	6.76E-41	1.42E-40

RDH10	2.76203794	2.67E-128	3.32E-127
RDH10-AS1	NA	1.35E-107	8.54E-107
RP11-434I12	NA	NA	NA
RP11-434I12	NA	NA	NA
STAU2-AS1	NA	5.63E-50	1.34E-49
STAU2	1.45399405	0.3195768	0.33223095
RP11-463D1!	NA	NA	NA
RP11-463D1!	NA	NA	NA
UBE2W	1.20996708	9.78E-85	3.95E-84
TCEB1	1.77215502	0.95911402	0.96118028
TMEM70	1.189245	1.61E-85	6.57E-85
LY96	0.59922179	7.70E-11	1.03E-10
RP11-6I2.3	NA	NA	NA
RP11-6I2.4	NA	NA	NA
RP1-16A9.1	NA	NA	NA
JPH1	8.80107004	2.26E-145	1.79E-143
GDAP1	1.77893969	1.33E-15	1.94E-15
CTD-2320G1	NA	NA	NA
MIR2052HG	NA	NA	NA
RP11-730G2	NA	NA	NA
RP11-758M4	NA	NA	NA
PI15	NA	NA	NA
RP11-3N13.2	NA	NA	NA
CRISPLD1	2.24708171	7.51E-41	1.58E-40
RP11-300E4.	NA	NA	NA
RP11-697M1	NA	1.28E-28	2.27E-28
CASC9	NA	NA	NA
HNF4G	NA	NA	NA
RP11-706J10	NA	NA	NA
LINC01109	NA	NA	NA
LINC01111	NA	NA	NA
ZFHX4-AS1	NA	NA	NA
ZFHX4	NA	NA	NA
RP11-48D4.2	NA	NA	NA
PEX2	1.75140192	1.34E-26	2.31E-26
RP11-38H17.	NA	NA	NA
RP11-91P17.	NA	NA	NA
PKIA-AS1	NA	NA	NA
PKIA	2.7464332	4.12E-38	8.31E-38
ZC2HC1A	0.37451362	5.04E-08	6.38E-08
IL7	NA	NA	NA
RP11-79H23.	NA	NA	NA
RP11-1114I9	NA	NA	NA
STMN2	NA	NA	NA
HEY1	Inf	2.88E-130	3.94E-129

LINC01607	NA	NA	NA
RP11-26J3.1	Inf	4.83E-137	1.00E-135
MRPS28	1.04328794	3.29E-117	2.68E-116
RP11-26J3.4	NA	NA	NA
RP11-92K15.	NA	1.81E-28	3.19E-28
TPD52	1.83735741	6.20E-16	9.09E-16
RP11-92K15.	NA	NA	NA
RP11-941H1!	NA	NA	NA
RP11-48B3.5	2.99610895	2.73E-63	7.88E-63
ZBTB10	1.71206226	1.02E-17	1.54E-17
RP11-48B3.3	NA	8.97E-07	1.11E-06
RP11-48B3.4	1.12354086	7.64E-14	1.08E-13
ZNF704	0.74902724	2.56E-62	7.29E-62
PAG1	NA	9.41E-56	2.43E-55
RP11-172E1(	NA	NA	NA
RP11-1149M	NA	NA	NA
RP11-1149M	NA	0.00021029	0.00024448
RP11-363E6.	Inf	1.95E-10	2.59E-10
RP11-363E6.	0	2.96E-08	3.76E-08
FABP5	1.5742753	3.55E-14	5.04E-14
RP11-157I4.4	NA	NA	NA
PMP2	NA	NA	NA
FABP9	NA	NA	NA
FABP4	NA	NA	NA
RP11-257P3.	NA	NA	NA
FABP12	NA	NA	NA
IMPA1	1.24837873	5.60E-67	1.71E-66
SLC10A5	Inf	2.91E-98	1.52E-97
ZFAND1	1.55657223	2.33E-91	1.06E-90
CHMP4C	NA	NA	NA
RP13-923O2:	NA	NA	NA
SNX16	1.64785992	2.17E-23	3.56E-23
RP11-354A14	NA	8.68E-109	5.65E-108
RP11-99H20.	NA	NA	NA
RP11-653B1(	NA	NA	NA
RP11-731N1(	NA	NA	NA
CTD-2272D1!	NA	NA	NA
LINC01419	NA	NA	NA
RP11-120I21	NA	NA	NA
RALYL	NA	NA	NA
LRRCC1	1.55061779	2.02E-16	2.98E-16
RP11-219B4.	Inf	4.01E-38	8.09E-38
E2F5	2.01167315	0.00858143	0.00952548
RP11-219B4.	NA	NA	NA
C8orf59	1.94817413	1.16E-10	1.54E-10

CA13	2.24708171	2.31E-14	3.30E-14
RP11-219B4.	NA	1.18E-16	1.74E-16
RP11-219B4.	NA	NA	NA
CA1	NA	NA	NA
CA3	Inf	0.00054324	0.00062455
CA3-AS1	2.24708171	5.31E-14	7.52E-14
CA2	0.73588641	2.21E-143	1.03E-141
RP11-317J10	NA	NA	NA
CTA-392E5.1	NA	NA	NA
ATP6V0D2	NA	NA	NA
PSKH2	NA	NA	NA
CTD-3118D1:	NA	NA	NA
CTD-3118D1:	NA	NA	NA
SLC7A13	NA	NA	NA
WWP1	5.9922179	2.84E-78	1.03E-77
RMDN1	1.27082149	4.88E-112	3.47E-111
CPNE3	1.49805447	5.73E-79	2.10E-78
CNGB3	NA	NA	NA
RP11-386D6.	NA	NA	NA
RP11-386D6.	NA	NA	NA
CNBD1	NA	NA	NA
AF121898.3	NA	0.38801892	0.40076511
DCAF4L2	NA	NA	NA
CTB-118P15.	NA	NA	NA
MMP16	NA	2.73E-132	4.17E-131
RP11-586K2.	NA	NA	NA
RP11-642C5.	NA	NA	NA
RP11-37B2.1	5.24319066	1.44E-40	3.01E-40
RIPK2	1.54586472	3.26E-32	6.06E-32
OSGIN2	2.16566571	4.49E-67	1.38E-66
NBN	1.77343214	2.14E-55	5.51E-55
DECR1	0.86507371	1.42E-140	4.27E-139
CALB1	Inf	1.53E-81	5.86E-81
LINC00534	NA	NA	NA
AB015752.3	NA	NA	NA
LINC01030	NA	1.76E-22	2.85E-22
TMEM64	2.24708171	0.00128719	0.00146428
RP11-68L18.:	0	1.33E-77	4.75E-77
NECAB1	0.74902724	1.80E-15	2.61E-15
RP11-662G2:	NA	NA	NA
C8orf88	2.29593131	4.14E-50	9.88E-50
TMEM55A	1.3172548	4.58E-36	9.00E-36
GS1-251I9.3	NA	NA	NA
OTUD6B-AS:	1.62369775	1.72E-48	4.02E-48
OTUD6B	2.60188409	5.91E-40	1.22E-39

LRRC69	1.31079767	5.85E-11	7.84E-11
SLC26A7	NA	2.32E-52	5.73E-52
RP11-122C21	NA	NA	NA
RUNX1T1	NA	9.99E-36	1.95E-35
GS1-5L10.1	NA	NA	NA
RP11-700E23	NA	NA	NA
RP11-700E23	NA	NA	NA
RP11-587H10	NA	NA	NA
RP11-587H10	NA	NA	NA
RP11-100L22	NA	NA	NA
RP11-100L22	NA	NA	NA
RP11-100L22	NA	NA	NA
CTD-3239E11	NA	NA	NA
TRIQK	0.74902724	0.790692	0.799016
C8orf87	NA	NA	NA
LINC00535	Inf	3.10E-152	5.51E-150
RP11-388K12	NA	NA	NA
RP11-388K12	NA	NA	NA
FAM92A1	2.05351288	4.26E-63	1.23E-62
CTD-2006H14	NA	1.46E-61	4.11E-61
RP11-10N23.	Inf	0.42144557	0.43415283
RBM12B	3.30820363	1.63E-31	3.00E-31
RP11-10N23.	NA	NA	NA
RP11-10N23.	Inf	0.00807712	0.00897227
TMEM67	2.3540856	2.51E-65	7.50E-65
RP3-388N13.	NA	NA	NA
PDP1	2.24708171	8.23E-93	3.87E-92
RP3-388N13.	Inf	7.16E-48	1.65E-47
KB-1184D12.	NA	NA	NA
CDH17	NA	NA	NA
GEM	NA	NA	NA
RAD54B	1.12354086	0.24842399	0.25995893
FSBP	NA	NA	NA
KIAA1429	1.52075227	3.07E-14	4.37E-14
RP11-267M2	NA	NA	NA
RP11-267M2	0	4.35E-07	5.39E-07
RP11-22C11.	0	1.10E-09	1.44E-09
ESRP1	1.74773022	4.04E-37	8.04E-37
DPY19L4	2.37191958	0.18124733	0.19095175
KB-1608C10.	NA	NA	NA
INTS8	1.64785992	5.26E-06	6.38E-06
CCNE2	2.0598249	0.03330736	0.03620721
RP11-347C18	0.74902724	7.39E-57	1.94E-56
NDUFAF6	1.79580443	1.06E-07	1.33E-07
TP53INP1	NA	2.14E-47	4.92E-47

RP11-320N2	NA	NA	NA
RP11-320N2	NA	NA	NA
PLEKHF2	2.14938251	4.69E-05	5.55E-05
C8orf37-AS1	NA	1.29E-40	2.70E-40
LINC01298	NA	NA	NA
C8orf37	0.59922179	5.46E-26	9.31E-26
RP11-90D11.	NA	3.28E-143	1.47E-141
RP11-31K23.	NA	NA	NA
KB-1043D8.8	NA	NA	NA
GDF6	Inf	2.99E-74	1.02E-73
UQCRB	1.43628521	1.73E-142	6.71E-141
KB-1043D8.6	Inf	0.20406845	0.21454623
MTERF3	0.731608	3.07E-132	4.67E-131
PTDSS1	1.86747267	1.58E-32	2.94E-32
KB-1448A5.1	NA	NA	NA
SDC2	NA	1.05E-141	3.71E-140
CPQ	0	3.83E-140	1.09E-138
KB-1958F4.2	NA	NA	NA
KB-1000E4.2	NA	NA	NA
TSPYL5	NA	3.24E-55	8.32E-55
MTDH	2.17556124	1.78E-120	1.62E-119
LAPTM4B	1.72455672	0.00898932	0.00997091
MATN2	0.37451362	0.00479865	0.00536959
RPL30	2.05978946	1.92E-144	1.13E-142
KB-1208A12.	1.24837873	0.04792057	0.05177008
ERICH5	2.62159533	6.56E-38	1.32E-37
HRSP12	1.67415028	3.93E-05	4.65E-05
POP1	1.53550584	1.24E-17	1.87E-17
NIPAL2	2.80885214	3.66E-54	9.27E-54
KB-1458E12.	NA	7.75E-127	8.99E-126
STK3	3.74513619	1.33E-38	2.70E-38
KCNS2	NA	NA	NA
KB-1517D11.	NA	NA	NA
KB-1205A7.2	NA	NA	NA
RP11-44N12.	1.12354086	1.24E-51	3.04E-51
OSR2	1.54798962	2.68E-138	6.20E-137
RP11-44N12.	NA	NA	NA
RP11-410L14	3.29571984	5.82E-89	2.54E-88
VPS13B	2.37191958	3.83E-20	5.99E-20
CTD-2340D6.	NA	NA	NA
RP11-402L5.	NA	NA	NA
COX6C	1.79137152	0.55162435	0.5646975
RGS22	NA	NA	NA
FBXO43	2.46108949	9.61E-05	1.13E-04
POLR2K	1.58767023	6.66E-24	1.10E-23

SPAG1	1.99740597	2.05E-11	2.77E-11
RNF19A	2.99610895	7.67E-22	1.23E-21
KB-1991G8.1	NA	NA	NA
KB-173C10.2	NA	NA	NA
KB-173C10.1	NA	NA	NA
KB-1615E4.2	NA	NA	NA
KB-1615E4.3	NA	NA	NA
ANKRD46	1.5172603	1.57E-46	3.55E-46
SNX31	NA	NA	NA
KB-1083B1.1	NA	NA	NA
PABPC1	2.2873869	3.33E-144	1.84E-142
YWHAZ	2.10650797	6.16E-56	1.60E-55
FLJ42969	NA	2.56E-85	1.04E-84
KB-1460A1.1	NA	NA	NA
KB-1460A1.3	NA	NA	NA
KB-1460A1.5	0	8.50E-36	1.66E-35
ZNF706	1.87926062	5.18E-23	8.45E-23
KB-1410C5.5	Inf	3.47E-48	8.04E-48
KB-1410C5.2	NA	NA	NA
KB-1410C5.3	NA	NA	NA
KB-1930G5.3	NA	NA	NA
KB-1930G5.4	NA	NA	NA
KB-1562D12.	NA	NA	NA
GRHL2	NA	NA	NA
NCALD	0.99870298	0.01599824	0.01758924
KB-1107E3.1	0	8.26E-72	2.71E-71
RRM2B	5.05593385	7.56E-91	3.41E-90
UBR5-AS1	5.24319066	2.78E-56	7.25E-56
UBR5	2.09450209	3.21E-20	5.03E-20
KB-431C1.5	NA	1.23E-32	2.29E-32
KB-1980E6.3	1.87256809	5.77E-05	6.81E-05
ODF1	NA	1.24E-38	2.51E-38
KLF10	1.49805447	1.63E-67	5.03E-67
KB-1732A1.1	0.74902724	0.07528202	0.08073336
AZIN1	1.97820014	1.63E-42	3.50E-42
KB-1254G8.1	NA	7.75E-131	1.09E-129
AZIN1-AS1	2.09727626	1.67E-21	2.66E-21
KB-1507C5.3	NA	5.76E-24	9.52E-24
KB-1507C5.4	NA	1.79E-05	2.14E-05
KB-1639H6.2	NA	NA	NA
ATP6V1C1	1.30386223	5.28E-120	4.71E-119
LINC01181	NA	NA	NA
BAALC-AS2	NA	1.62E-56	4.24E-56
BAALC	1.9820413	0.18543486	0.19527276
BAALC-AS1	1.49805447	7.43E-24	1.23E-23

RP11-318M2	NA	NA	NA
FZD6	2.62159533	3.06E-30	5.53E-30
CTHRC1	NA	NA	NA
RP11-1C8.6	Inf	0.00615021	0.00685644
SLC25A32	1.85285685	5.29E-05	6.25E-05
DCAF13	0.90541754	1.18E-103	6.86E-103
RP11-1C8.5	NA	1.03E-130	1.44E-129
RP11-1C8.7	0	2.56E-14	3.65E-14
RP11-1C8.4	NA	9.63E-05	1.13E-04
RIMS2	1.49805447	0.09564951	0.10218912
DPYS	NA	7.65E-06	9.24E-06
DCSTAMP	NA	NA	NA
LRP12	1.74773022	0.40012284	0.41296101
ZFPM2	NA	6.08E-11	8.15E-11
RP11-574O7.	NA	NA	NA
RP11-642D2:	NA	NA	NA
RP11-152P17	NA	NA	NA
RP11-778D1:	NA	NA	NA
RP11-395G2:	1.96803235	5.30E-13	7.38E-13
OXR1	1.72852439	0.00264057	0.00297511
RP11-649G1!	0	1.64E-82	6.39E-82
ABRA	NA	NA	NA
ANGPT1	0.37451362	7.45E-103	4.24E-102
RSPO2	NA	NA	NA
EIF3E	1.32181277	2.95E-108	1.89E-107
RP11-35G22.	NA	NA	NA
EMC2	0.62918288	3.28E-131	4.73E-130
RP11-790J24	NA	NA	NA
TMEM74	1.99740597	0.1054303	0.11230134
RP11-1084E!	NA	9.10E-16	1.33E-15
TRHR	NA	NA	NA
NUDCD1	1.33160398	4.26E-92	1.97E-91
RP11-122A2:	NA	1.54E-23	2.53E-23
ENY2	1.65511627	2.53E-45	5.64E-45
PKHD1L1	NA	NA	NA
EBAG9	1.77142208	0.00866034	0.0096113
SYBU	Inf	1.62E-122	1.58E-121
RP11-422N1(	NA	NA	NA
KCNV1	NA	NA	NA
RP11-696P8.	NA	NA	NA
RP11-211F2.	NA	NA	NA
RP11-238I10	NA	NA	NA
KB-1299A7.2	NA	NA	NA
LINC01608	NA	NA	NA
LINC01609	NA	NA	NA

RP11-114O8.	NA	NA	NA
RP11-1101K5	NA	NA	NA
RP11-58O3.2	NA	NA	NA
CSMD3	5.9922179	6.32E-12	8.64E-12
RP11-67H2.1	NA	NA	NA
RP11-267L5.	NA	NA	NA
TRPS1	0	4.36E-16	6.41E-16
AF178030.2	NA	NA	NA
LINC00536	NA	NA	NA
RP11-536K17	NA	NA	NA
EIF3H	1.44204217	4.81E-93	2.27E-92
UTP23	1.58062441	2.83E-55	7.29E-55
RAD21	1.93186608	5.25E-25	8.84E-25
AC087350.1	NA	NA	NA
RAD21-AS1	NA	5.59E-06	6.77E-06
AARD	NA	NA	NA
SLC30A8	NA	NA	NA
MED30	2.05629176	1.68E-24	2.81E-24
EXT1	4.49416342	2.14E-82	8.28E-82
SAMD12	NA	5.33E-10	7.03E-10
AC023590.1	Inf	1.26E-146	1.35E-144
SAMD12-AS1	NA	NA	NA
TNFRSF11B	NA	NA	NA
COLEC10	NA	NA	NA
RP11-278I4.2	NA	NA	NA
MAL2	NA	NA	NA
RP11-4K16.2	NA	NA	NA
NOV	0	7.61E-20	1.18E-19
RP11-775B15	NA	NA	NA
ENPP2	NA	NA	NA
TAF2	1.66828794	1.34E-47	3.07E-47
DSCC1	2.36359706	0.00157966	0.00179203
KB-1471A8.1	Inf	1.17E-124	1.23E-123
DEPTOR	1.54345006	1.75E-68	5.48E-68
RP11-760H22	6.74124514	1.43E-88	6.20E-88
COL14A1	Inf	3.35E-107	2.10E-106
MRPL13	1.10326586	1.63E-141	5.62E-140
MTBP	1.31079767	8.59E-13	1.19E-12
SNTB1	1.32520204	1.22E-18	1.87E-18
RP11-713M1	NA	NA	NA
RP11-713M1	Inf	4.44E-44	9.70E-44
RP11-369K17	Inf	1.36E-113	1.01E-112
HAS2	NA	NA	NA
HAS2-AS1	NA	NA	NA
RP11-3G20.1	NA	NA	NA

RP11-3G20.2	NA	NA	NA
RP11-785H2(	NA	NA	NA
RP11-398G2	NA	NA	NA
RP11-94A24.	NA	NA	NA
RP11-96B2.1	NA	NA	NA
LINC01151	NA	NA	NA
RP11-973F15	NA	NA	NA
RP11-44N11.	NA	8.21E-34	1.56E-33
RP11-44N11.	0	3.77E-133	6.08E-132
ZHX2	2.62159533	0.58827539	0.60103013
RP11-557C18	Inf	1.45E-11	1.96E-11
RP11-557C18	NA	NA	NA
DERL1	1.01157287	4.70E-111	3.26E-110
TBC1D31	2.80885214	0.16166875	0.17067177
FAM83A	Inf	5.95E-133	9.47E-132
RP11-539E17	NA	NA	NA
FAM83A-AS1	NA	4.59E-58	1.22E-57
C8orf76	1.10636133	7.58E-104	4.42E-103
ZHX1-C8orf7(	0.99870298	2.05E-15	2.98E-15
ZHX1	2.77821012	1.65E-97	8.49E-97
ATAD2	1.82879377	2.70E-07	3.36E-07
WDYHV1	1.95768482	0.00066243	0.0007599
FBXO32	0.62418936	1.85E-38	3.75E-38
RP11-174I12	NA	NA	NA
CTD-2552K11	NA	NA	NA
KLHL38	NA	NA	NA
ANXA13	NA	NA	NA
FAM91A1	2.9461738	6.84E-101	3.75E-100
FER1L6	NA	2.42E-28	4.27E-28
RP11-245A18	NA	NA	NA
FER1L6-AS1	NA	NA	NA
RP11-959I15	NA	NA	NA
FER1L6-AS2	NA	NA	NA
RP11-37N22.	3.74513619	4.27E-39	8.75E-39
RP11-383J24	NA	NA	NA
TMEM65	2.14007782	5.12E-06	6.21E-06
TRMT12	2.14007782	4.32E-43	9.34E-43
RNF139-AS1	Inf	0.37405495	0.38702548
RNF139	1.88464918	3.50E-09	4.54E-09
TATDN1	1.21020516	1.34E-127	1.62E-126
RP11-158K1.	0	1.04E-16	1.54E-16
NDUFB9	1.52837445	2.55E-110	1.73E-109
MTSS1	Inf	0.00026755	0.00031037
RP11-532M2	NA	8.57E-150	1.22E-147
RP11-1082L8	NA	NA	NA

RP11-1082L8	NA	2.47E-28	4.35E-28
LINC00964	NA	NA	NA
RP11-1082L8	NA	NA	NA
ZNF572	1.12354086	3.81E-17	5.69E-17
RP11-6D1.3	NA	NA	NA
SQLE	2.18175957	5.41E-92	2.50E-91
KIAA0196	1.76897922	1.79E-05	2.14E-05
KIAA0196-AS	NA	NA	NA
NSMCE2	1.06042058	2.06E-116	1.64E-115
RP11-550A5.	NA	NA	NA
TRIB1	2.49675746	3.80E-50	9.07E-50
RP11-136O1	NA	NA	NA
RP11-697B2	NA	NA	NA
LINC00861	NA	NA	NA
RP11-622O1	NA	NA	NA
RP11-65D17.	NA	NA	NA
RP11-103H7.	NA	NA	NA
RP11-103H7.	NA	NA	NA
RP11-103H7.	NA	NA	NA
FAM84B	Inf	2.75E-71	8.95E-71
RP11-103H7.	NA	NA	NA
RP11-89K10.	NA	NA	NA
RP11-351C8.	NA	NA	NA
PCAT1	0	2.63E-149	3.57E-147
PCAT2	NA	NA	NA
PRNCR1	0.74902724	1.08E-20	1.70E-20
CASC19	NA	NA	NA
CCAT1	NA	NA	NA
CASC8	NA	NA	NA
CASC21	NA	NA	NA
CCAT2	NA	NA	NA
POU5F1B	NA	8.61E-08	1.08E-07
AC108925.1	NA	NA	NA
CASC11	1.49805447	0.00180898	0.00204757
MYC	1.51585585	5.73E-63	1.65E-62
PVT1	2.12224384	2.07E-09	2.70E-09
AC026248.1	NA	NA	NA
LINC00976	NA	1.40E-124	1.48E-123
LINC00977	Inf	0.00114681	0.00130745
CCDC26	1.87256809	0.02013416	0.02206694
RP11-26E5.1	NA	NA	NA
RP11-419K1	NA	1.31E-142	5.18E-141
GSDMC	NA	6.97E-94	3.33E-93
RP11-473O4.	NA	NA	NA
FAM49B	1.73998834	2.31E-26	3.96E-26

RP11-473O4.	0	0.06436961	0.06921898
RP11-473O4.	NA	NA	NA
ASAP1	2.00958527	4.88E-05	5.78E-05
KB-1568E2.1	NA	NA	NA
ADCY8	NA	NA	NA
RP11-737F9.	NA	NA	NA
RP11-381I15	NA	NA	NA
CTD-2501M5	NA	NA	NA
EFR3A	0.68093385	1.58E-133	2.59E-132
OC90	NA	NA	NA
RP11-240B1:	NA	NA	NA
HHLA1	NA	NA	NA
KCNQ3	NA	NA	NA
HPYR1	NA	NA	NA
LRRC6	Inf	8.10E-41	1.70E-40
TMEM71	NA	NA	NA
PHF20L1	1.45484136	1.55E-64	4.57E-64
AF230666.2	NA	0.00256266	0.00288858
TG	NA	NA	NA
SLA	0.64202335	2.72E-101	1.50E-100
WISP1	NA	NA	NA
NDRG1	1.12354086	0.71892472	0.72905156
ST3GAL1	2.36057069	2.58E-16	3.81E-16
RP11-629O1.	1.49805447	0.35740991	0.3702467
CTD-2588J1.:	NA	NA	NA
CTD-2309H9.	NA	NA	NA
RP11-157E21	NA	NA	NA
RP11-513H8.	NA	NA	NA
ZFAT	1.79766537	0.00423866	0.00475117
ZFAT-AS1	NA	NA	NA
CTD-2342N2:	0.49935149	6.97E-40	1.44E-39
AC083843.1	4.11964981	8.28E-66	2.49E-65
AC083843.2	NA	NA	NA
NCRNA0025(	NA	NA	NA
RP11-1057B8	NA	NA	NA
LINC01591	NA	NA	NA
RP11-343P9.	NA	NA	NA
KHDRBS3	1.11539926	4.46E-141	1.43E-139
RP11-149P2	NA	NA	NA
RP11-431D1:	NA	NA	NA
RP11-30J20.:	Inf	6.85E-150	1.00E-147
RP11-238K6.	NA	NA	NA
RP11-238K6.	NA	NA	NA
RP11-489O1	NA	NA	NA
FAM135B	NA	NA	NA

COL22A1	NA	NA	NA
RP11-324F11	NA	NA	NA
CTD-2534J5.1	NA	NA	NA
RP11-398H6.	NA	NA	NA
KCNK9	NA	NA	NA
TRAPPC9	1.49805447	0.89642778	0.90134639
RP11-284H18	NA	NA	NA
CTA-204B4.2	2.54669261	5.05E-80	1.88E-79
CHRAC1	1.89931907	9.07E-14	1.28E-13
AGO2	2.77140078	4.80E-84	1.92E-83
ERICD	Inf	0.00030117	0.00034902
PTK2	NA	0.0050972	0.00569944
AC105009.1	NA	NA	NA
RP11-128L5.1	NA	NA	NA
DENND3	1.12354086	1.23E-09	1.62E-09
RP11-809O17	0.74902724	0.00285631	0.00321559
SLC45A4	1.68531128	9.10E-10	1.19E-09
RP11-10J21.4	NA	NA	NA
RP11-10J21.3	NA	NA	NA
RP11-10J21.5	NA	NA	NA
RP11-10J21.6	NA	NA	NA
LINC01300	NA	NA	NA
GPR20	NA	NA	NA
CTD-3064M3	NA	NA	NA
PTP4A3	1.05220493	7.48E-43	1.61E-42
CTD-3064M3	NA	0.13377061	0.14180592
AC138647.1	NA	NA	NA
RP11-953B20	NA	NA	NA
LINC00051	NA	NA	NA
TSNARE1	0.79896239	2.18E-103	1.26E-102
RP13-467H17	NA	0.0020007	0.00226232
ADGRB1	1.87256809	5.21E-69	1.64E-68
ARC	1.12354086	1.68E-31	3.10E-31
AP006547.3	NA	NA	NA
AC145123.2	NA	NA	NA
JRK	1.650399	1.98E-12	2.74E-12
PSCA	1.12354086	0.3925988	0.40538005
LY6K	Inf	4.55E-24	7.54E-24
CTD-2292P10	NA	3.81E-39	7.80E-39
THEM6	1.11619745	4.54E-78	1.64E-77
CTD-2292P10	NA	NA	NA
SLURP1	NA	NA	NA
LYPD2	NA	NA	NA
RP11-706C16	NA	NA	NA
LYNX1	NA	NA	NA

LY6D	NA	NA	NA
RP11-706C16	NA	NA	NA
RP11-706C16	NA	NA	NA
GML	NA	NA	NA
CYP11B1	NA	NA	NA
CYP11B2	NA	NA	NA
RP11-273G15	0.74902724	2.65E-16	3.90E-16
LY6E	1.75874533	2.48E-44	5.44E-44
RP11-520P18	NA	NA	NA
LY6H	NA	NA	NA
GPIHBP1	NA	NA	NA
ZFP41	1.74773022	0.09900261	0.10568434
RP13-582O9.	NA	NA	NA
GLI4	2.24708171	1.99E-56	5.20E-56
MINCR	1.961738	4.04E-21	6.41E-21
ZNF696	1.01118677	1.39E-113	1.03E-112
RP13-582O9.	2.24708171	0.00068028	0.00078013
TOP1MT	1.6976886	2.50E-72	8.29E-72
RHPN1-AS1	5.9922179	1.33E-87	5.65E-87
RHPN1	1.38708748	2.90E-13	4.06E-13
RP11-909N17	NA	NA	NA
MAFA-AS1	NA	NA	NA
MAFA	NA	9.98E-35	1.92E-34
ZC3H3	1.88170257	1.46E-17	2.20E-17
RP11-661A12	0.74902724	6.23E-06	7.53E-06
RP11-661A12	NA	1.53E-81	5.86E-81
GSDMD	1.08425621	1.22E-140	3.71E-139
MROH6	1.58617533	0.00984998	0.01090555
RP11-661A12	NA	2.34E-18	3.56E-18
NAPRT	1.35430177	2.19E-145	1.74E-143
RP11-661A12	0.37451362	1.10E-12	1.52E-12
EEF1D	1.74652239	4.18E-66	1.26E-65
TIGD5	1.9539841	0.01121631	0.01240166
PYCRL	1.39722389	4.21E-111	2.92E-110
TSTA3	1.35777729	4.17E-91	1.89E-90
RP11-661A12	0	1.42E-83	5.62E-83
ZNF623	1.31079767	1.06E-49	2.52E-49
ZNF707	2.32198444	2.18E-09	2.84E-09
RP11-429J17	2.99610895	3.41E-09	4.43E-09
CCDC166	NA	NA	NA
RP11-429J17	NA	NA	NA
MAPK15	NA	NA	NA
RP11-429J17	NA	NA	NA
FAM83H	Inf	4.12E-15	5.96E-15
FAM83H-AS1	NA	9.18E-05	1.08E-04

RP11-429J17	NA	NA	NA
RP11-429J17	NA	NA	NA
SCRIB	1.23145156	1.91E-47	4.39E-47
PUF60	1.03130989	4.62E-97	2.36E-96
NRBP2	2.43433852	4.53E-09	5.87E-09
EPPK1	NA	NA	NA
PLEC	1.56614786	4.16E-62	1.18E-61
PARP10	2.09727626	1.97E-12	2.71E-12
GRINA	1.45074749	2.40E-141	8.09E-140
SPATC1	NA	NA	NA
SMPD5	NA	1.01E-11	1.37E-11
OPLAH	NA	NA	NA
CTD-3065J16	5.24319066	0.0001167	0.0001366
EXOSC4	1.28974512	3.02E-136	5.88E-135
GPAA1	1.58168373	7.48E-122	7.12E-121
CYC1	1.30487182	9.04E-132	1.34E-130
SHARPIN	1.87582473	7.48E-109	4.89E-108
MAF1	1.86765322	7.39E-11	9.89E-11
WDR97	0	6.20E-33	1.16E-32
HGH1	1.37978702	5.42E-30	9.77E-30
MROH1	1.68531128	2.29E-33	4.32E-33
BOP1	1.07401839	7.36E-133	1.17E-131
SCX	Inf	7.64E-98	3.95E-97
HSF1	1.62094176	1.62E-46	3.68E-46
DGAT1	1.49805447	8.20E-87	3.43E-86
GS1-393G12	NA	NA	NA
SCRT1	Inf	1.82E-96	9.16E-96
TMEM249	Inf	2.60E-84	1.04E-83
GS1-393G12	NA	4.33E-99	2.30E-98
SLC52A2	1.45238208	1.34E-75	4.64E-75
FBXL6	1.24338521	2.13E-102	1.20E-101
ADCK5	1.07003891	1.57E-70	5.07E-70
CPSF1	1.8835832	5.65E-59	1.53E-58
GS1-393G12	Inf	0.11107468	0.11819561
SLC39A4	1.81249253	6.91E-20	1.08E-19
VPS28	1.01305934	2.45E-145	1.91E-143
TONSL	2.19714656	1.06E-49	2.51E-49
TONSL-AS1	NA	NA	NA
CYHR1	2.03434025	1.09E-13	1.54E-13
CTD-2517M2	NA	8.61E-111	5.90E-110
KIFC2	2.38326848	1.57E-41	3.33E-41
FOXH1	0	0.25703564	0.26869148
PPP1R16A	1.64785992	1.89E-07	2.36E-07
GPT	0	0.43884356	0.45181885
MFSD3	1.09284302	2.68E-89	1.17E-88

RECQL4	1.36891185	0.00030866	0.00035763
CTD-2517M2	NA	6.11E-144	3.19E-142
LRRC14	2.12591554	9.52E-27	1.64E-26
LRRC24	0.37451362	4.52E-24	7.49E-24
C8orf82	1.59732314	1.28E-117	1.05E-116
ARHGAP39	NA	NA	NA
CTD-2517M2	NA	NA	NA
AF186192.1	NA	NA	NA
AF186192.6	NA	NA	NA
ZNF251	2.71522374	0.00034001	0.0003934
ZNF34	1.12354086	5.16E-52	1.27E-51
RPL8	1.78359905	2.68E-42	5.74E-42
ZNF517	Inf	0.03277343	0.03564602
ZNF7	2.03901859	3.85E-29	6.86E-29
COMMD5	1.62809393	1.64E-86	6.83E-86
ZNF250	2.24708171	4.03E-61	1.13E-60
ZNF16	1.79115209	1.30E-102	7.35E-102
RP5-1047A1!	NA	NA	NA
ZNF252P-AS	Inf	1.19E-07	1.50E-07
C8orf33	1.80738726	4.38E-09	5.67E-09
WASH1	0.81924854	1.46E-28	2.57E-28
XXyac-YRM2(	0.74902724	0.03092589	0.03366277
FAM138C	NA	NA	NA
PGM5P3-AS1	NA	NA	NA
RP11-143M1	1.34824903	0.00061322	0.00070403
LINC01388	4.49416342	1.24E-06	1.52E-06
FOXD4	2.24708171	7.34E-12	1.00E-11
CBWD1	1.81906615	0.45417359	0.46709849
C9orf66	4.49416342	1.13E-17	1.70E-17
DOCK8	1.30475712	2.96E-45	6.60E-45
RP11-59O6.3	NA	NA	NA
RP11-165F24	NA	NA	NA
KANK1	1.7061176	2.13E-19	3.30E-19
RP11-130C1!	NA	NA	NA
DMRT1	NA	NA	NA
DMRT3	NA	NA	NA
LINC01230	NA	NA	NA
DMRT2	NA	NA	NA
SMARCA2	1.40202534	2.28E-25	3.86E-25
RP11-264I13	NA	NA	NA
VLDLR-AS1	NA	9.72E-25	1.63E-24
RP11-91N2.3	NA	NA	NA
VLDLR	1.60505837	0.81175926	0.81934664
KCNV2	NA	NA	NA
PUM3	1.39633473	6.35E-19	9.74E-19

LINC01231	NA	NA	NA
RFX3	1.12354086	1.04E-08	1.34E-08
AL365202.1	NA	NA	NA
RFX3-AS1	NA	3.70E-117	3.01E-116
RP11-509J21	NA	1.91E-119	1.67E-118
RP11-509J21	NA	NA	NA
GLIS3	NA	1.61E-102	9.08E-102
RP11-252M1	NA	NA	NA
GLIS3-AS1	NA	NA	NA
RP11-358M1	NA	NA	NA
SLC1A1	NA	NA	NA
SPATA6L	NA	NA	NA
PLPP6	1.64785992	0.01852446	0.02032235
CDC37L1-AS1	0.74902724	7.25E-05	8.53E-05
CDC37L1	1.45863199	2.13E-88	9.20E-88
AK3	1.4696104	1.52E-27	2.64E-27
RCL1	1.04551719	4.05E-75	1.40E-74
RP11-125K10	NA	NA	NA
JAK2	Inf	2.87E-08	3.65E-08
IGHEP2	NA	7.76E-80	2.88E-79
INSL6	NA	NA	NA
INSL4	NA	NA	NA
RLN2	NA	NA	NA
RLN1	NA	NA	NA
RP11-12D24.	NA	NA	NA
PLGRKT	1.61231287	8.04E-110	5.39E-109
CD274	NA	NA	NA
PDCD1LG2	NA	NA	NA
RP11-574F11	NA	NA	NA
RIC1	0.97373541	1.45E-54	3.69E-54
RP11-207C16	NA	NA	NA
ERMP1	1.79766537	3.18E-50	7.60E-50
KIAA2026	1.79115209	0.09741934	0.10404319
MLANA	NA	NA	NA
RANBP6	2.8463035	2.58E-27	4.48E-27
RP11-218I7.2	NA	3.17E-21	5.03E-21
IL33	NA	NA	NA
TPD52L3	NA	NA	NA
UHRF2	2.04734112	5.88E-91	2.66E-90
RP11-307L3.4	NA	NA	NA
GLDC	NA	NA	NA
RP11-390F4.	NA	NA	NA
RP11-390F4.	NA	NA	NA
RP11-390F4.	NA	6.06E-61	1.69E-60
RP11-146B14	NA	NA	NA

AL513412.1	NA	NA	NA
KDM4C	1.79766537	8.88E-64	2.58E-63
RP11-77E14.	0	0.00172452	0.00195356
TMEM261	1.72199045	4.08E-11	5.48E-11
RP11-29B9.1	NA	NA	NA
RP11-29B9.2	NA	NA	NA
PTPRD	NA	NA	NA
PTPRD-AS1	NA	1.82E-65	5.45E-65
RP11-75C9.2	NA	NA	NA
RP11-527D1!	NA	NA	NA
PTPRD-AS2	NA	NA	NA
RP11-87N24.	NA	NA	NA
RP11-109M1	NA	NA	NA
RP11-23D5.1	NA	NA	NA
RP11-74C3.1	NA	NA	NA
TYRP1	NA	1.97E-49	4.64E-49
LURAP1L-AS	NA	NA	NA
LURAP1L	NA	2.34E-13	3.28E-13
MPDZ	NA	NA	NA
LINC01235	Inf	1.88E-114	1.42E-113
RP11-536O1	NA	NA	NA
LINC00583	NA	NA	NA
RP11-284P2(	NA	NA	NA
NFIB	1.60505837	0.00160517	0.00182052
RP11-120J1.:	NA	NA	NA
RP11-408A1:	Inf	3.10E-152	5.51E-150
RP11-408A1:	Inf	5.93E-42	1.26E-41
ZDHHC21	1.99740597	1.22E-29	2.19E-29
CER1	NA	NA	NA
FREM1	NA	NA	NA
TTC39B	Inf	6.02E-75	2.07E-74
SNAPC3	1.34824903	1.28E-48	2.99E-48
PSIP1	1.41210053	3.16E-17	4.73E-17
CCDC171	Inf	3.23E-76	1.13E-75
C9orf92	NA	NA	NA
BNC2	NA	2.85E-61	8.01E-61
RP11-183I6.2	NA	NA	NA
RP11-62F24.	NA	NA	NA
RP11-62F24.	NA	NA	NA
RP11-132E11	NA	NA	NA
CNTLN	2.0598249	1.96E-61	5.50E-61
SH3GL2	NA	8.09E-14	1.14E-13
RP11-570H1!	NA	NA	NA
ADAMTSL1	NA	NA	NA
SAXO1	NA	NA	NA

RRAGA	1.01118677	8.60E-122	8.17E-121
HAUS6	2.15629053	5.90E-73	1.97E-72
PLIN2	1.27446425	1.71E-130	2.36E-129
DENND4C	1.71206226	0.14139829	0.14969934
RP11-146N2:	NA	NA	NA
RP11-513M1	2.99610895	1.00E-78	3.65E-78
RP11-513M1	0	0.29702723	0.309267
RPS6	1.85161604	0.85775373	0.86389653
ACER2	NA	NA	NA
SLC24A2	NA	NA	NA
RP11-378I6.1	NA	NA	NA
MLLT3	1.55567195	1.56E-19	2.41E-19
FOCAD	1.03711464	6.28E-21	9.94E-21
FOCAD-AS1	Inf	1.63E-96	8.22E-96
HACD4	1.6454041	1.58E-78	5.72E-78
IFNB1	NA	7.29E-115	5.55E-114
IFNW1	NA	NA	NA
IFNA21	NA	NA	NA
IFNA4	NA	NA	NA
IFNA7	NA	NA	NA
IFNA10	NA	NA	NA
IFNA16	NA	NA	NA
IFNA17	NA	NA	NA
IFNA14	NA	NA	NA
IFNA5	NA	NA	NA
KLHL9	3.44552529	4.41E-65	1.31E-64
IFNA6	NA	NA	NA
IFNA13	NA	NA	NA
IFNA2	NA	NA	NA
RP11-354P17	NA	NA	NA
IFNA8	NA	NA	NA
IFNA1	NA	NA	NA
IFNE	NA	NA	NA
MTAP	1.07859922	5.87E-105	3.51E-104
RP11-145E5.	NA	NA	NA
RP11-70L8.4	0.74902724	7.95E-33	1.49E-32
CDKN2A-AS1	NA	NA	NA
CDKN2A	1.73841945	8.00E-12	1.09E-11
CDKN2B-AS1	2.24708171	0.00622254	0.00693508
CDKN2B	0.99870298	7.16E-89	3.12E-88
RP11-408N14	NA	8.82E-29	1.56E-28
DMRTA1	1.49805447	0.85062635	0.85724159
LINC01239	NA	NA	NA
RP11-370B1:	NA	NA	NA
RP11-315I14	NA	NA	NA

ELAVL2	NA	1.24E-145	1.23E-143
RP11-321L2.1	NA	NA	NA
RP11-321L2.1	NA	NA	NA
IZUMO3	NA	NA	NA
RP11-20A20.	NA	NA	NA
TUSC1	1.67384658	5.28E-09	6.83E-09
LINC01241	NA	2.30E-116	1.82E-115
RP11-80I3.1	NA	NA	NA
RP11-18A15.	NA	NA	NA
RP11-337A23	NA	NA	NA
CAAP1	1.64785992	3.53E-70	1.13E-69
PLAA	1.81906615	1.01E-14	1.45E-14
IFT74	0.91547773	6.50E-72	2.14E-71
IFT74-AS1	0	4.19E-27	7.26E-27
LRRC19	NA	NA	NA
RP11-56F10.	NA	NA	NA
TEK	NA	NA	NA
LINC00032	NA	NA	NA
EQTN	NA	NA	NA
MOB3B	1.12354086	3.76E-118	3.14E-117
RP11-298E2.	NA	NA	NA
IFNK	NA	NA	NA
C9orf72	0.74902724	6.63E-12	9.07E-12
RP11-575I8.1	NA	NA	NA
LINGO2	2.99610895	6.01E-92	2.77E-91
LINC01242	NA	NA	NA
LINC01243	NA	NA	NA
ACO1	2.15896086	2.02E-29	3.61E-29
DDX58	3.74513619	5.37E-62	1.52E-61
TOPORS	1.89040208	0.27169976	0.28364466
TOPORS-AS1	1.64785992	2.12E-08	2.71E-08
NDUFB6	1.54504668	1.17E-83	4.63E-83
GVQW1	0	3.97E-10	5.25E-10
TAF1L	NA	NA	NA
RP11-555J4.4	NA	NA	NA
RP11-555J4.3	NA	NA	NA
TMEM215	NA	NA	NA
RP11-462B18	NA	NA	NA
APTX	1.59300159	5.71E-15	8.24E-15
DNAJA1	0.8583573	7.60E-136	1.44E-134
SMU1	1.52686321	6.14E-32	1.13E-31
B4GALT1	3.20038911	1.00E-84	4.06E-84
B4GALT1-AS	2.24708171	0.28329158	0.2953552
SPINK4	1.49805447	1.42E-89	6.28E-89
BAG1	1.85053788	1.99E-17	2.99E-17

CHMP5	1.16593862	1.47E-132	2.28E-131
NFX1	1.85285685	1.47E-136	2.92E-135
AQP7	NA	3.18E-73	1.07E-72
RP11-311H1(	NA	NA	NA
AQP3	1.08949416	5.28E-45	1.17E-44
NOL6	1.89753567	5.27E-40	1.09E-39
RP11-255A1:	NA	7.02E-82	2.69E-81
ANKRD18B	1.82371849	0.8538831	0.86023696
CYP4F26P	Inf	1.06E-139	2.90E-138
TRBV20OR9-	NA	NA	NA
TRBV21OR9-	NA	NA	NA
TRBV22OR9-	NA	NA	NA
TRBV23OR9-	NA	NA	NA
TRBV24OR9-	NA	NA	NA
PTENP1-AS	NA	NA	NA
TRBV26OR9-	NA	NA	NA
RP11-384P7.	NA	NA	NA
RP11-384P7.	NA	NA	NA
LINC01251	NA	1.34E-05	1.60E-05
PRSS3	NA	NA	NA
UBE2R2-AS1	NA	NA	NA
TRBV29OR9-	NA	NA	NA
UBE2R2	2.06527898	2.16E-09	2.81E-09
UBAP2	1.64988432	0.0006713	0.00076988
RP11-537H1!	NA	NA	NA
DCAF12	2.27383268	2.10E-64	6.16E-64
UBAP1	1.74038682	1.11E-54	2.84E-54
KIF24	0.37451362	0.00021335	0.000248
NUDT2	1.4014058	1.56E-52	3.88E-52
KIAA1161	0.99870298	1.36E-43	2.96E-43
C9orf24	NA	NA	NA
FAM219A	1.29377432	2.55E-09	3.31E-09
DNAI1	NA	NA	NA
ENHO	2.24708171	3.07E-13	4.29E-13
RP11-296L22	NA	NA	NA
CNTFR	NA	NA	NA
CNTFR-AS1	NA	NA	NA
RPP25L	1.39506323	6.93E-118	5.75E-117
DCTN3	1.28767954	9.12E-124	9.35E-123
ARID3C	NA	4.17E-07	5.17E-07
SIGMAR1	1.66524805	1.37E-53	3.43E-53
GALT	1.35538262	1.58E-92	7.39E-92
RP11-195F1!	NA	NA	NA
IL11RA	0.74902724	7.20E-41	1.51E-40
CCL27	NA	2.80E-84	1.12E-83

RP11-195F19	1.12354086	0.43050981	0.4433645
RP11-195F19	Inf	2.62E-18	3.99E-18
CCL19	NA	NA	NA
CCL21	NA	NA	NA
FAM205A	NA	NA	NA
FAM205C	NA	NA	NA
PHF24	NA	1.84E-09	2.40E-09
DNAJB5-AS1	NA	NA	NA
DNAJB5	2.7464332	5.24E-21	8.31E-21
C9orf131	NA	NA	NA
VCP	1.49805447	1.81E-42	3.88E-42
FANCG	1.03711464	2.91E-64	8.52E-64
PIGO	0.64202335	3.56E-41	7.51E-41
RP11-182N2	NA	8.83E-56	2.29E-55
STOML2	1.71114672	5.46E-28	9.56E-28
FAM214B	0.74902724	0.0001731	0.00020168
UNC13B	2.99610895	1.27E-08	1.63E-08
RUSC2	1.12354086	0.03797296	0.04117539
FAM166B	NA	1.79E-18	2.73E-18
TESK1	4.11964981	4.42E-103	2.53E-102
CD72	1.12354086	1.25E-12	1.73E-12
RP11-331F9.	NA	1.05E-52	2.61E-52
RP11-331F9.	NA	NA	NA
SIT1	NA	0.36547661	0.37838707
RMRP	NA	1.81E-21	2.89E-21
CCDC107	1.82762646	1.98E-29	3.55E-29
ARHGEF39	1.24837873	1.38E-144	8.38E-143
CA9	NA	NA	NA
TPM2	1.12354086	2.02E-13	2.83E-13
TLN1	1.99740597	1.54E-44	3.38E-44
CREB3	1.93713941	8.01E-11	1.07E-10
GBA2	1.2067661	0.00272289	0.00306652
RGP1	2.59662776	1.58E-99	8.43E-99
MSMP	0.37451362	0.12026082	0.12785817
RP11-112J3.	NA	NA	NA
RP11-112J3.	3.37062257	4.33E-46	9.75E-46
NPR2	Inf	8.51E-20	1.32E-19
SPAG8	Inf	2.74E-108	1.77E-107
HINT2	1.49805447	4.85E-123	4.82E-122
TMEM8B	4.49416342	1.68E-08	2.15E-08
FAM221B	NA	NA	NA
LINC00950	NA	NA	NA
OR13J1	NA	NA	NA
HRCT1	NA	NA	NA
LINC00961	NA	NA	NA

RP11-327L3.:	NA	NA	NA
OR2S2	NA	NA	NA
RECK	1.12354086	0.27102113	0.28296877
GLIPR2	1.54798962	3.03E-14	4.31E-14
CCIN	NA	4.85E-25	8.16E-25
CLTA	1.11640726	4.20E-119	3.63E-118
GNE	0	2.40E-55	6.17E-55
RNF38	2.49675746	1.02E-20	1.61E-20
MELK	1.40442607	5.18E-27	8.95E-27
PAX5	3.32900994	4.43E-109	2.91E-108
RP11-344B2:	NA	NA	NA
RP11-297B1:	NA	NA	NA
LINC01400	NA	1.17E-136	2.37E-135
RP11-220I1.!	Inf	2.80E-150	4.16E-148
EBLN3	3.37062257	1.22E-137	2.68E-136
RP11-220I1.:	NA	1.86E-123	1.88E-122
ZCCHC7	1.53854243	6.16E-27	1.06E-26
LINC01627	NA	NA	NA
GRHPR	1.32235673	2.66E-126	3.02E-125
ZBTB5	3.18336576	3.85E-83	1.51E-82
POLR1E	1.26398346	1.07E-78	3.90E-78
RP11-613M1	NA	5.53E-46	1.24E-45
FBXO10	2.09727626	1.13E-09	1.48E-09
RP11-613M1	NA	1.45E-26	2.50E-26
TOMM5	1.60019455	0.05431257	0.05854309
RP11-613M1	NA	NA	NA
FRMPD1	NA	1.45E-61	4.07E-61
TRMT10B	Inf	1.56E-118	1.32E-117
EXOSC3	1.40211995	5.92E-80	2.20E-79
DCAF10	1.3732166	0.00139358	0.00158311
SLC25A51	1.39818418	2.11E-27	3.66E-27
SHB	2.77140078	3.04E-48	7.06E-48
RP11-113O2.	NA	NA	NA
ALDH1B1	2.31317235	4.18E-05	4.95E-05
IGFBPL1	NA	NA	NA
FAM95C	Inf	1.52E-45	3.40E-45
ANKRD18A	2.24708171	6.93E-94	3.31E-93
FAM201A	NA	6.56E-101	3.60E-100
RP11-402N8.	NA	NA	NA
CNTNAP3	NA	NA	NA
SPATA31A1	NA	NA	NA
FAM74A1	NA	NA	NA
RP11-347J14	NA	NA	NA
RP11-347J14	NA	NA	NA
GLIDR	Inf	0.32892937	0.34164061

RP11-216M2	NA	NA	NA
ANKRD20A2	Inf	0.99417797	0.99478113
FAM95B1	8.23929961	1.03E-05	1.24E-05
AC129778.2	NA	5.77E-98	2.99E-97
BMS1P14	NA	4.25E-27	7.36E-27
CH17-80A12.	NA	NA	NA
IGKV1OR9-2	NA	NA	NA
RP11-318K12	NA	NA	NA
PGM5P2	NA	NA	NA
PGM5-AS1	NA	NA	NA
BX255923.3	NA	1.37E-62	3.90E-62
FOXD4L6	NA	NA	NA
CBWD7	0.44941634	7.93E-126	8.81E-125
RP11-4L24.3	Inf	7.79E-09	1.00E-08
RP11-187C18	NA	NA	NA
RP11-157L3.	NA	NA	NA
CNTNAP3B	NA	1.43E-15	2.08E-15
SPATA31A6	NA	NA	NA
FAM74A7	NA	NA	NA
RP11-286O1.	NA	NA	NA
RP11-475I24	NA	NA	NA
RP11-475I24	NA	NA	NA
RP11-175I6.5	NA	NA	NA
RP11-459O1(	NA	NA	NA
RP11-459O1(	NA	NA	NA
FP325317.1	NA	NA	NA
SPATA31A5	NA	NA	NA
RP11-144A1(	NA	NA	NA
FAM74A6	NA	2.33E-16	3.44E-16
RP11-144A1(	NA	NA	NA
SPATA31A7	NA	NA	NA
RP11-101E5.	NA	NA	NA
RP11-101E5.	NA	NA	NA
RP11-160N1.	NA	NA	NA
RP11-160N1.	NA	NA	NA
FAM27C	2.16385646	1.02E-49	2.41E-49
RP11-374M1	NA	NA	NA
RP11-374M1	0	0.18626698	0.19612626
RP11-374M1	NA	NA	NA
RP11-449H15	NA	NA	NA
RP11-111F5.	NA	NA	NA
RP11-111F5.	0.99870298	9.49E-18	1.43E-17
RP11-111F5.	NA	NA	NA
LINC01189	NA	NA	NA
RP11-211N8.	NA	NA	NA

LINC01410	2.99610895	4.35E-74	1.48E-73
RP11-262H14	Inf	2.12E-111	1.48E-110
RP11-262H14	Inf	0.05618954	0.06053807
RP11-381O7.	NA	NA	NA
RP11-149F8.	NA	NA	NA
RP11-764K9.	NA	1.19E-153	2.59E-151
ANKRD20A4	NA	0.00034389	0.00039787
IGKV1OR-2	NA	NA	NA
IGKV1OR9-1	NA	NA	NA
FOXD4L5	NA	NA	NA
CBWD5	2.36231667	1.74E-30	3.16E-30
FOXD4L4	NA	NA	NA
IGKV1OR-3	NA	NA	NA
RP11-15E1.5	NA	5.05E-91	2.29E-90
ANKRD20A3	1.49805447	1.42E-25	2.41E-25
RP11-327I22	NA	NA	NA
ZNF658	1.12354086	2.86E-06	3.49E-06
RP11-395E19	NA	NA	NA
RP11-395E19	NA	NA	NA
SPATA31A3	NA	NA	NA
RP11-118H15	NA	NA	NA
ANKRD20A1	NA	1.70E-100	9.21E-100
RP11-561O23	NA	1.28E-67	3.97E-67
CBWD3	1.26151956	6.79E-23	1.11E-22
FOXD4L3	NA	NA	NA
RP11-561O23	NA	NA	NA
RP11-561O23	NA	NA	NA
PGM5	NA	NA	NA
PGM5-AS1	NA	NA	NA
RP11-88I18.2	1.49805447	0.09208192	0.09842398
TMEM252	NA	NA	NA
RP11-274B18	NA	NA	NA
LINC01506	NA	NA	NA
PIP5K1B	NA	2.72E-101	1.50E-100
FAM122A	3.85214008	2.79E-88	1.20E-87
RP11-203L2.4	NA	NA	NA
PRKACG	NA	NA	NA
FXN	1.83160567	0.26418767	0.275977
TJP2	2.03307393	4.77E-13	6.63E-13
BANCR	NA	NA	NA
FAM189A2	NA	NA	NA
APBA1	0	1.13E-120	1.03E-119
RP11-470P21	NA	NA	NA
RP11-109D9.	NA	NA	NA
PTAR1	1.61789883	1.87E-50	4.47E-50

C9orf135-AS1	NA	NA	NA
C9orf135	NA	NA	NA
MAMDC2-AS	0	8.17E-144	4.16E-142
MAMDC2	NA	NA	NA
SMC5-AS1	0.74902724	8.66E-20	1.34E-19
SMC5	1.72450457	2.44E-20	3.83E-20
KLF9	2.04280156	5.79E-40	1.20E-39
TRPM3	NA	5.57E-07	6.89E-07
RP11-141J10	NA	NA	NA
RP11-141J10	NA	NA	NA
TMEM2	Inf	4.98E-36	9.77E-36
ABHD17B	2.24708171	0.32913383	0.3418334
AL138751.1	NA	NA	NA
C9orf85	1.73212549	0.00722745	0.00804568
C9orf57	NA	NA	NA
GDA	NA	NA	NA
RP11-63P12.	NA	NA	NA
LINC01504	Inf	0.00327409	0.00368203
ZFAND5	1.66450497	1.02E-05	1.23E-05
TMC1	NA	NA	NA
LINC01474	NA	NA	NA
ALDH1A1	NA	NA	NA
ANXA1	0.49935149	4.15E-121	3.82E-120
RP11-128I7.1	NA	NA	NA
RP11-171A24	NA	NA	NA
RORB-AS1	NA	NA	NA
RORB	NA	4.98E-09	6.44E-09
TRPM6	NA	7.75E-131	1.09E-129
C9orf40	1.39506323	2.37E-70	7.64E-70
C9orf41-AS1	NA	7.45E-42	1.59E-41
CARNMT1	1.52686321	8.47E-05	9.95E-05
NMRK1	1.91418072	4.58E-28	8.03E-28
OSTF1	1.20575116	1.13E-138	2.73E-137
RP11-470F18	NA	NA	NA
PCSK5	0.74902724	1.15E-68	3.60E-68
RFK	2.34311085	1.37E-93	6.53E-93
GCNT1	2.3540856	0.29702724	0.309267
PRUNE2	NA	0.00168489	0.00190938
PCA3	NA	0.00110931	0.0012651
FOXB2	NA	NA	NA
VPS13A-AS1	NA	4.71E-13	6.56E-13
VPS13A	3.74513619	7.20E-19	1.10E-18
GNA14	NA	7.65E-06	9.24E-06
GNA14-AS1	NA	NA	NA
GNAQ	2.93369001	9.28E-64	2.70E-63

CEP78	1.63180934	9.75E-23	1.58E-22
PSAT1	1.42497865	1.56E-107	9.89E-107
RP11-165H2:	NA	NA	NA
RP11-375O1:	NA	NA	NA
TLE4	2.09727626	1.38E-20	2.17E-20
RP11-403N1(	NA	NA	NA
RP11-403N1(	NA	NA	NA
RP11-327L9.:	NA	NA	NA
LINC01507	NA	NA	NA
RP11-289F5.	NA	NA	NA
TLE1	2.24708171	1.76E-44	3.86E-44
RP11-154D1:	NA	NA	NA
RP11-383M4	NA	NA	NA
SPATA31D4	NA	NA	NA
RP11-383M4	NA	NA	NA
SPATA31D3	NA	NA	NA
SPATA31D1	NA	NA	NA
RP11-15B24.	NA	NA	NA
RP11-15B24.	NA	NA	NA
RP11-15B24.	NA	NA	NA
RASEF	NA	NA	NA
RP11-439K3.	NA	NA	NA
FRMD3	NA	5.48E-101	3.01E-100
RP11-439K3.	NA	NA	NA
IDNK	1.07003891	4.39E-25	7.40E-25
UBQLN1	1.99439782	2.18E-57	5.76E-57
RP11-522I20	2.24708171	0.09857841	0.10524389
GKAP1	2.7464332	1.92E-13	2.70E-13
RP11-575L7.:	NA	NA	NA
RP11-575L7.4	NA	NA	NA
KIF27	Inf	3.34E-68	1.04E-67
C9orf64	NA	NA	NA
HNRNPK	1.29389254	7.43E-138	1.67E-136
RP11-575L7.:	NA	0.63371934	0.64571391
RMI1	1.67090691	2.04E-13	2.86E-13
RP11-158D2.	NA	NA	NA
SLC28A3	NA	NA	NA
RP11-380F14	NA	NA	NA
NTRK2	NA	6.16E-82	2.37E-81
AGTPBP1	2.3540856	0.00369088	0.00414433
RP11-202I11	NA	NA	NA
RP11-213G2.	NA	NA	NA
NAA35	1.06592338	6.41E-33	1.20E-32
GOLM1	1.80953115	0.58149614	0.59447217
C9orf153	NA	2.50E-26	4.27E-26

ISCA1	2.20931563	3.24E-119	2.81E-118
ZCCHC6	1.9661965	3.48E-48	8.05E-48
RP11-359J6.1	NA	NA	NA
RP11-395D3.	NA	NA	NA
GAS1	Inf	1.26E-33	2.39E-33
GAS1RR	NA	NA	NA
RP11-276H19	NA	NA	NA
C9orf170	NA	NA	NA
DAPK1	3.6770428	2.22E-132	3.41E-131
CTSL	0.79814378	3.00E-135	5.44E-134
XXyac-YM21(	NA	NA	NA
XXyac-YM21(	NA	NA	NA
XXyac-YM21(	NA	NA	NA
SPATA31E1	NA	NA	NA
CDK20	NA	NA	NA
SPIN1	2.29701686	8.07E-62	2.28E-61
NXNL2	Inf	3.66E-96	1.83E-95
RP13-60M5.2	NA	NA	NA
RP11-791O2	NA	NA	NA
C9orf47	NA	NA	NA
S1PR3	NA	7.78E-111	5.36E-110
SHC3	NA	NA	NA
CKS2	1.72675943	2.36E-51	5.74E-51
SECISBP2	2.04585051	2.69E-42	5.76E-42
SEMA4D	5.43044747	5.33E-98	2.77E-97
GADD45G	NA	NA	NA
UNQ6494	NA	NA	NA
RP5-1050E16	NA	NA	NA
RP5-1050E16	NA	NA	NA
RP11-406A2(	NA	NA	NA
MIR4290HG	NA	NA	NA
LINC01508	NA	NA	NA
LINC01501	NA	NA	NA
DIRAS2	NA	NA	NA
SYK	1.89337441	1.85E-83	7.29E-83
RP11-367F23	NA	NA	NA
RP11-367F23	NA	NA	NA
RP11-305L7.	NA	0.02068765	0.0226667
RP11-305L7.(	NA	8.66E-15	1.24E-14
RP11-305L7.:	Inf	5.81E-138	1.31E-136
LINC00484	NA	3.04E-45	6.77E-45
RP11-305L7.7	0	3.14E-121	2.90E-120
AUH	0.90671718	4.46E-50	1.06E-49
NFIL3	2.7464332	1.43E-45	3.20E-45
RP11-440G5.	NA	NA	NA

ROR2	0	0.00028309	0.00032826
SPTLC1	1.43563554	5.79E-45	1.28E-44
LINC00475	NA	NA	NA
RP11-100G1!	NA	NA	NA
RP11-62C3.6	NA	NA	NA
IARS	1.84503033	1.21E-09	1.58E-09
AL136097.1	NA	NA	NA
NOL8	1.59435798	7.74E-35	1.49E-34
CENPP	1.96966422	1.24E-107	7.88E-107
OGN	NA	NA	NA
OMD	NA	NA	NA
ASPN	NA	2.64E-77	9.38E-77
ECM2	NA	NA	NA
IPPK	1.59168288	0.00025937	0.00030104
BICD2	1.64785992	0.61356104	0.6259475
ZNF484	NA	2.92E-40	6.08E-40
FGD3	0.74902724	3.24E-11	4.37E-11
SUSD3	1.20114278	7.78E-123	7.66E-122
RP11-274J16	NA	NA	NA
CARD19	1.83603018	1.78E-05	2.13E-05
NINJ1	1.85365326	0.00563214	0.00628867
RP11-370F5.	NA	NA	NA
WNK2	1.87256809	1.81E-91	8.30E-91
C9orf129	NA	NA	NA
RP11-165J3.!	NA	NA	NA
RP11-165J3.(	Inf	1.61E-79	5.95E-79
FAM120AOS	2.09056856	3.39E-66	1.02E-65
FAM120A	3.40733959	1.44E-144	8.75E-143
PHF2	2.49675746	0.35470426	0.36752783
RP11-53B5.1	NA	NA	NA
BARX1	2.24708171	5.17E-81	1.96E-80
BARX1-AS1	NA	NA	NA
PTPDC1	4.11964981	0.54919833	0.56227743
RP11-2B6.3	NA	1.87E-29	3.34E-29
RP11-2B6.2	Inf	4.97E-32	9.20E-32
MIRLET7DHC	NA	5.09E-119	4.37E-118
ZNF169	2.52796693	8.86E-66	2.66E-65
NUTM2F	NA	NA	NA
RP11-307E17	0	2.99E-44	6.56E-44
MFSD14B	1.88269008	1.45E-39	2.98E-39
PCAT7	Inf	0.00744687	0.00828689
FBP2	NA	NA	NA
FBP1	NA	NA	NA
C9orf3	2.46108949	0.0001336	0.00015615
RP11-54O15.	NA	NA	NA

FANCC	0.74902724	1.97E-17	2.95E-17
RP11-80I15.4	NA	NA	NA
RP11-435O5.	NA	NA	NA
RP11-435O5.	Inf	3.14E-13	4.39E-13
PTCH1	0	1.76E-88	7.62E-88
RP11-435O5.	NA	NA	NA
RP11-435O5.	0	5.24E-64	1.53E-63
RP11-435O5.	NA	0.4077228	0.42054165
RP11-332M4	NA	NA	NA
RP11-180I4.2	NA	NA	NA
RP11-180I4.4	NA	NA	NA
ERCC6L2	2.24708171	1.33E-41	2.81E-41
LINC00092	NA	NA	NA
RP11-569G1	NA	NA	NA
RP11-569G1	NA	NA	NA
HSD17B3	NA	NA	NA
RP11-240L7.4	NA	NA	NA
SLC35D2	2.02677958	1.65E-34	3.17E-34
ZNF367	2.24708171	1.83E-35	3.57E-35
HABP4	1.5396671	3.43E-08	4.35E-08
CDC14B	1.49805447	0.0848583	0.09079914
AAED1	1.45500693	2.89E-14	4.12E-14
RP11-535M1	NA	NA	NA
ZNF510	Inf	2.58E-53	6.46E-53
ZNF782	0.99870298	3.44E-54	8.73E-54
MFSD14C	0.74902724	0.04227787	0.04578752
NUTM2G	NA	NA	NA
CTSV	0.69909209	2.19E-135	4.01E-134
RP11-498P14	NA	NA	NA
RP11-498P14	Inf	2.58E-90	1.15E-89
RP11-498P14	NA	NA	NA
CCDC180	1.87256809	1.68E-19	2.60E-19
TDRD7	0.99870298	4.47E-21	7.08E-21
TMOD1	0.24967575	5.19E-09	6.72E-09
RP11-244N9.	NA	NA	NA
TSTD2	1.77893969	4.52E-19	6.96E-19
NCBP1	1.54637881	0.00047038	0.00054209
RP11-244N9.	NA	NA	NA
XPA	1.05331955	1.08E-131	1.60E-130
RP11-546O6.	NA	NA	NA
PTCSC2	NA	NA	NA
FOXE1	NA	NA	NA
TRMO	1.49805447	6.89E-118	5.72E-117
HEMGN	NA	2.20E-06	2.69E-06
ANP32B	1.53007939	2.14E-86	8.90E-86

RP11-535C21	NA	NA	NA
NANS	0.89761969	1.33E-141	4.61E-140
TRIM14	2.24708171	3.69E-09	4.78E-09
CORO2A	2.24708171	0.13634193	0.14443884
TBC1D2	0.74902724	1.59E-101	8.83E-101
GABBR2	NA	NA	NA
ANKS6	2.45514483	5.19E-117	4.20E-116
GALNT12	1.64785992	0.00015879	0.0001852
RP11-92C4.3	NA	NA	NA
COL15A1	Inf	2.39E-13	3.35E-13
RP11-92C4.6	NA	NA	NA
TGFBR1	2.14494163	5.60E-31	1.02E-30
RP11-96L7.2	NA	NA	NA
ALG2	1.31079767	4.44E-55	1.14E-54
SEC61B	2.01384667	1.07E-78	3.90E-78
NAMA	NA	NA	NA
RP11-547C13	NA	NA	NA
RP11-554F20	NA	5.26E-29	9.35E-29
NR4A3	NA	9.36E-51	2.25E-50
STX17-AS1	Inf	1.72E-76	6.04E-76
STX17	1.59168288	0.13109468	0.1391237
RP11-60I3.4	NA	NA	NA
ERP44	0.88773599	8.33E-129	1.06E-127
INVS	1.07003891	1.89E-07	2.36E-07
TEX10	1.82850767	0.06463684	0.06949811
MSANTD3	3.23579767	2.64E-135	4.81E-134
MSANTD3-TI	NA	NA	NA
TMEFF1	NA	9.10E-16	1.33E-15
MURC	Inf	1.15E-59	3.14E-59
PLPPR1	NA	NA	NA
BAAT	Inf	8.32E-107	5.16E-106
MRPL50	1.12563311	7.96E-58	2.11E-57
ZNF189	1.2733463	1.59E-08	2.04E-08
ALDOB	NA	NA	NA
TMEM246-AS	NA	NA	NA
TMEM246	NA	NA	NA
RNF20	1.46060311	1.32E-12	1.83E-12
GRIN3A	NA	NA	NA
PPP3R2	NA	NA	NA
LINC00587	NA	NA	NA
CYLC2	NA	NA	NA
LINC01492	NA	NA	NA
RP11-436F21	NA	NA	NA
SMC2-AS1	Inf	0.03490647	0.03789559
SMC2	1.1154868	8.28E-103	4.71E-102

OR13F1	NA	NA	NA
RP11-317C2(	NA	NA	NA
OR13C4	NA	NA	NA
OR13C3	NA	NA	NA
OR13C8	NA	NA	NA
OR13C5	NA	NA	NA
OR13C2	NA	NA	NA
OR13C9	NA	NA	NA
OR13D1	NA	NA	NA
NIPSNAP3A	1.1770428	9.96E-120	8.82E-119
NIPSNAP3B	0.24967575	1.35E-25	2.28E-25
ABCA1	NA	NA	NA
RP11-217B7.	NA	NA	NA
RP11-217B7.	NA	NA	NA
SLC44A1	1.08192823	2.25E-51	5.46E-51
FSD1L	2.99610895	0.0040517	0.00454273
RP11-235C2:	Inf	9.12E-118	7.52E-117
FKTN	4.99351492	4.17E-56	1.08E-55
TAL2	NA	5.51E-16	8.09E-16
TMEM38B	1.16708895	2.40E-63	6.93E-63
RP11-6F6.1	NA	NA	NA
RP11-308N1!	NA	NA	NA
RP11-540H2:	NA	NA	NA
RP11-308N1!	NA	NA	NA
LINC01505	NA	NA	NA
ZNF462	1.34824903	6.10E-08	7.70E-08
RP11-508N1:	NA	NA	NA
RP11-196I18	NA	NA	NA
RAD23B	1.71268801	3.01E-10	3.98E-10
LINC01509	NA	NA	NA
KLF4	0.49935149	2.38E-59	6.47E-59
RP11-505C1:	NA	NA	NA
RP11-339N8.	NA	NA	NA
ACTL7B	NA	NA	NA
ACTL7A	NA	NA	NA
IKBKAP	1.59168288	0.00357627	0.00401713
FAM206A	2.78210117	1.10E-58	2.97E-58
CTNNAL1	1.72852439	7.88E-11	1.05E-10
TMEM245	2.838419	9.19E-113	6.66E-112
FRRS1L	NA	3.70E-131	5.31E-130
EPB41L4B	Inf	5.19E-12	7.11E-12
PTPN3	Inf	0.04624891	0.04999986
PALM2	NA	NA	NA
RP11-406O2:	NA	NA	NA
PALM2-AKAF	NA	NA	NA

AKAP2	NA	NA	NA
C9orf152	NA	NA	NA
TXN	1.69350718	2.55E-18	3.87E-18
TXNDC8	NA	NA	NA
SVEP1	NA	NA	NA
RP11-410K21	NA	NA	NA
MUSK	NA	NA	NA
LPAR1	NA	NA	NA
RP11-202G1	NA	NA	NA
OR2K2	NA	NA	NA
KIAA0368	1.88885129	5.35E-40	1.11E-39
ZNF483	Inf	0.05912373	0.06366847
PTGR1	NA	3.75E-93	1.77E-92
RP11-16L21.	NA	NA	NA
DNAJC25	1.49805447	0.06577199	0.07069349
DNAJC25-GN	0	6.98E-79	2.55E-78
GNG10	2.99610895	4.95E-111	3.43E-110
C9orf84	NA	NA	NA
UGCG	1.55567195	2.45E-12	3.37E-12
SUSD1	1.40442607	9.26E-33	1.73E-32
PTBP3	2.48613296	2.82E-41	5.94E-41
HSDL2	1.25643279	2.57E-51	6.23E-51
C9orf147	0.74902724	2.67E-29	4.77E-29
KIAA1958	1.12354086	3.13E-52	7.73E-52
INIP	1.63740838	1.12E-20	1.77E-20
RP11-276E15	3.74513619	8.62E-52	2.11E-51
SNX30	5.9922179	2.63E-06	3.20E-06
SLC46A2	NA	NA	NA
RP11-408O1	NA	6.61E-103	3.77E-102
ZNF883	NA	NA	NA
ZFP37	NA	NA	NA
FAM225B	1.12354086	0.00918525	0.01018139
FAM225A	1.49805447	1.34E-54	3.43E-54
SLC31A2	1.32520204	8.46E-87	3.54E-86
FKBP15	1.31971466	4.44E-27	7.69E-27
SLC31A1	1.51507782	0.00042206	0.00048699
CDC26	1.91256469	3.95E-80	1.47E-79
PRPF4	1.51074985	1.56E-08	1.99E-08
RNF183	NA	NA	NA
WDR31	1.87256809	5.39E-13	7.49E-13
BSPRY	1.78614187	0.0027044	0.00304627
HDHD3	1.93297352	1.69E-78	6.12E-78
ALAD	1.74773022	2.26E-11	3.06E-11
POLE3	1.78638295	1.80E-13	2.52E-13
C9orf43	0.37451362	0.34058037	0.35329763

RGS3	0.74902724	2.47E-23	4.05E-23
RP11-168K11	NA	NA	NA
RP11-168K11	NA	NA	NA
RP11-18B16.	NA	NA	NA
ZNF618	0.74902724	1.89E-38	3.84E-38
AMBP	NA	NA	NA
KIF12	NA	NA	NA
COL27A1	2.09103437	1.79E-84	7.20E-84
ORM1	NA	NA	NA
ORM2	NA	NA	NA
AKNA	2.24708171	7.54E-61	2.10E-60
RP11-9M16.2	NA	2.07E-10	2.74E-10
DFNB31	0.74902724	1.34E-20	2.11E-20
ATP6V1G1	1.54049935	8.80E-56	2.28E-55
C9orf91	4.49416342	1.92E-101	1.07E-100
RP11-402G3.	NA	NA	NA
RP11-402G3.	NA	NA	NA
TNFSF15	NA	NA	NA
TNFSF8	NA	NA	NA
TNC	NA	NA	NA
RP11-523L1.	NA	NA	NA
1-Dec	NA	NA	NA
RP11-445L6.	NA	NA	NA
RP11-284G1(	NA	NA	NA
LINC00474	NA	NA	NA
RP11-787B4.	NA	NA	NA
PAPPA	NA	0.00048233	0.00055541
PAPPA-AS2	NA	NA	NA
RP11-45A16.	NA	NA	NA
PAPPA-AS1	NA	NA	NA
ASTN2	1.08192823	5.55E-46	1.25E-45
ASTN2-AS1	NA	NA	NA
RP11-67K19.	NA	NA	NA
TRIM32	1.66450497	2.86E-38	5.79E-38
RP11-388N2.	NA	NA	NA
RP11-500B12	NA	NA	NA
TLR4	0.37451362	4.13E-82	1.59E-81
RP11-281A2(	NA	2.40E-59	6.52E-59
RP11-281A2(	NA	2.23E-42	4.78E-42
RP11-349E4.	NA	NA	NA
RP11-127L21	NA	NA	NA
BRINP1	NA	NA	NA
RP11-295D22	NA	NA	NA
LINC01613	NA	NA	NA
RP11-360A18	NA	NA	NA

CDK5RAP2	1.41482923	7.31E-60	2.00E-59
MEGF9	8.23929961	2.67E-96	1.34E-95
FBXW2	1.73135804	2.80E-20	4.38E-20
PSMD5	NA	1.46E-121	1.36E-120
PSMD5-AS1	NA	3.18E-78	1.15E-77
PHF19	1.99879692	7.90E-18	1.19E-17
TRAF1	0.74902724	0.00097623	0.00111522
C5	0	2.28E-11	3.07E-11
CNTRL	1.7061176	3.31E-30	5.99E-30
RAB14	1.42556797	2.38E-42	5.09E-42
GSN	0.14980545	1.01E-144	6.25E-143
RP11-477J21	NA	NA	NA
GSN-AS1	NA	4.71E-13	6.56E-13
STOM	1.15128261	4.75E-48	1.10E-47
RP11-162D1(	NA	NA	NA
DAB2IP	Inf	1.70E-24	2.84E-24
RP11-524G2	NA	NA	NA
TTLL11	1.92607004	3.75E-07	4.66E-07
TTLL11-IT1	Inf	0.03554357	0.03857339
NDUFA8	1.58250362	1.00E-34	1.93E-34
MORN5	NA	NA	NA
LHX6	3.74513619	9.62E-14	1.36E-13
RBM18	1.55567195	2.00E-46	4.52E-46
MRRF	1.50628554	1.08E-63	3.14E-63
PTGS1	1.66450497	1.52E-17	2.29E-17
RP11-542K23	NA	NA	NA
RP11-542K23	NA	4.76E-90	2.11E-89
RP11-542K23	NA	NA	NA
OR1J1	NA	NA	NA
OR1J2	NA	NA	NA
OR1J4	NA	NA	NA
OR1N1	NA	NA	NA
OR1N2	NA	NA	NA
OR1L8	NA	NA	NA
RP11-64P14.	NA	NA	NA
OR1Q1	NA	NA	NA
OR1B1	NA	NA	NA
OR1L1	NA	NA	NA
OR1L3	NA	NA	NA
OR1L4	NA	NA	NA
OR1L6	NA	NA	NA
OR5C1	NA	NA	NA
PDCL	1.1385214	5.10E-98	2.65E-97
OR1K1	NA	NA	NA
RC3H2	1.33522247	1.07E-16	1.58E-16

ZBTB6	8.23929961	1.09E-97	5.62E-97
ZBTB26	1.49805447	3.35E-61	9.38E-61
RABGAP1	6.24189364	7.15E-27	1.23E-26
GPR21	NA	NA	NA
STRBP	1.76333495	6.66E-14	9.41E-14
CRB2	NA	NA	NA
DENND1A	3.10311284	3.81E-06	4.63E-06
RP11-230L22	NA	NA	NA
RP11-417B4.	NA	NA	NA
LHX2	NA	9.60E-35	1.85E-34
RP11-85O21.	NA	NA	NA
RP11-85O21.	NA	NA	NA
NEK6	1.94066148	8.35E-26	1.42E-25
RP11-121A14	NA	NA	NA
PSMB7	1.22428457	3.41E-129	4.41E-128
ADGRD2	NA	7.30E-81	2.75E-80
NR5A1	NA	NA	NA
RP11-10J18.3	NA	NA	NA
NR6A1	2.72859922	5.40E-67	1.65E-66
MIR181A2HG	Inf	9.55E-128	1.16E-126
RP11-175D17	NA	2.37E-63	6.83E-63
OLFML2A	NA	NA	NA
WDR38	NA	NA	NA
RPL35	1.97902585	7.16E-104	4.18E-103
ARPC5L	1.8144138	1.14E-15	1.66E-15
GOLGA1	4.49416342	1.75E-73	5.89E-73
SCAI	1.77042802	0.66099018	0.6725943
PPP6C	1.07924355	8.66E-142	3.10E-140
RABEPK	1.79528751	4.21E-14	5.98E-14
HSPA5	0.93007174	1.27E-137	2.79E-136
RP11-65N13.	NA	NA	NA
GAPVD1	1.99740597	0.30847374	0.32094573
MAPKAP1	1.93018557	2.42E-08	3.09E-08
RP11-12A16.	NA	NA	NA
PBX3	5.74254215	3.46E-05	4.11E-05
RP11-343J18	NA	NA	NA
RP11-343J18	NA	NA	NA
MVB12B	1.49805447	0.00579502	0.00646735
RP11-205K6.	NA	NA	NA
RP11-205K6.	NA	NA	NA
RP11-123K19	0	3.44E-54	8.74E-54
LMX1B	2.24708171	0.04225843	0.0457703
ZBTB43	2.09727626	4.33E-68	1.35E-67
ZBTB34	2.62159533	1.10E-28	1.95E-28
RALGPS1	2.24708171	2.09E-55	5.37E-55

ANGPTL2	NA	NA	NA
RP13-225O2	NA	NA	NA
GARNL3	Inf	2.16E-33	4.07E-33
SLC2A8	0.99870298	9.12E-32	1.68E-31
ZNF79	0.74902724	5.07E-24	8.40E-24
RPL12	2.07491373	1.78E-90	7.97E-90
LRSAM1	1.24837873	5.81E-05	6.86E-05
FAM129B	3.04292315	6.56E-89	2.86E-88
STXBP1	1.49805447	0.12859573	0.13653559
RP11-56D16.	0.37451362	4.63E-08	5.86E-08
PTRH1	2.17065036	1.94E-07	2.42E-07
CFAP157	NA	1.77E-69	5.64E-69
TTC16	NA	1.06E-49	2.51E-49
TOR2A	2.2865042	2.19E-63	6.33E-63
SH2D3C	0	1.02E-46	2.32E-46
AL162426.1	NA	NA	NA
CDK9	1.1770428	5.74E-107	3.57E-106
FPGS	1.28094513	4.64E-32	8.59E-32
ENG	1.72852439	1.05E-19	1.63E-19
RP11-228B1!	NA	1.09E-13	1.53E-13
AK1	2.62159533	0.01766259	0.01939678
ST6GALNAC(	2.45774562	1.07E-34	2.07E-34
ST6GALNAC4	1.74230249	1.83E-10	2.44E-10
PIP5KL1	1.49805447	0.00033014	0.00038213
RP11-203J24	NA	NA	NA
DPM2	1.81431042	1.49E-42	3.20E-42
FAM102A	3.74513619	3.14E-08	3.99E-08
RP11-379C1(	NA	NA	NA
NAIF1	1.39105058	8.47E-36	1.66E-35
SLC25A25	3.29571984	0.00072619	0.0008321
RP11-379C1(	NA	NA	NA
SLC25A25-A!	1.99740597	4.60E-63	1.32E-62
PTGES2	1.64180718	1.62E-55	4.18E-55
PTGES2-AS1	Inf	1.56E-09	2.03E-09
LCN2	NA	NA	NA
C9orf16	1.95653976	1.94E-53	4.86E-53
CIZ1	2.11444147	1.84E-12	2.53E-12
DNM1	2.16385646	0.96200999	0.96397599
GOLGA2	2.29389591	5.17E-20	8.06E-20
SWI5	1.75257829	8.72E-20	1.35E-19
TRUB2	1.16228364	1.54E-101	8.55E-101
RP11-339B2:	Inf	1.43E-07	1.79E-07
COQ4	2.4530642	3.56E-49	8.37E-49
SLC27A4	2.45136187	0.00051579	0.0005934
URM1	1.68635741	4.16E-55	1.07E-54

RP11-339B2:	NA	NA	NA
MIR219A2	NA	2.23E-42	4.78E-42
CERCAM	2.2811284	1.19E-38	2.42E-38
ODF2	1.30599621	5.03E-80	1.87E-79
ODF2-AS1	NA	NA	NA
GLE1	1.67782101	6.72E-53	1.67E-52
RP11-216B9.	NA	0.01686595	0.01853651
SPTAN1	2.71522374	9.26E-80	3.43E-79
RP11-216B9.	NA	NA	NA
WDR34	1.25914061	4.31E-141	1.39E-139
HMGA1P4	Inf	0.5169714	0.53024056
SET	1.68941641	3.90E-18	5.91E-18
PKN3	2.24708171	2.27E-14	3.24E-14
ZDHHC12	1.6718619	3.01E-94	1.45E-93
RP11-545E1	1.41482923	2.66E-12	3.66E-12
ZER1	1.34824903	2.14E-87	9.05E-87
TBC1D13	2.77140078	4.39E-81	1.66E-80
ENDOG	1.10020321	7.14E-118	5.92E-117
C9orf114	1.10214008	7.37E-69	2.32E-68
CCBL1	1.19844358	1.28E-88	5.57E-88
LRRC8A	1.32520204	0.24683163	0.25833734
PHYHD1	NA	NA	NA
RP11-101E3.	NA	NA	NA
DOLK	1.19844358	5.52E-05	6.52E-05
NUP188	1.75430063	0.00012331	0.00014428
RP11-167N5.	Inf	1.09E-140	3.33E-139
SH3GLB2	1.64785992	1.20E-35	2.35E-35
FAM73B	4.34435798	5.71E-53	1.42E-52
DOLPP1	1.3618677	0.00691368	0.00770016
CRAT	1.83095547	3.62E-12	4.97E-12
RP11-247A1:	NA	NA	NA
PPP2R4	1.9804788	4.00E-10	5.29E-10
RP11-247A1:	NA	3.23E-41	6.82E-41
IER5L	2.99610895	3.45E-07	4.29E-07
RP11-247A1:	NA	4.52E-45	1.00E-44
RP11-247A1:	NA	NA	NA
RP11-344B5.	NA	NA	NA
RP11-344B5.	NA	NA	NA
RP11-344B5.	NA	NA	NA
C9orf106	NA	4.51E-95	2.21E-94
RP11-65J3.1!	NA	NA	NA
LINC01503	Inf	0.81886413	0.82637999
RP11-65J3.3	NA	NA	NA
RP11-65J3.2	NA	NA	NA
RP11-65J3.1	NA	0.03359203	0.03649917

RP11-492E3.	Inf	3.98E-136	7.64E-135
RP11-492E3.	NA	NA	NA
NTMT1	1.54938967	5.69E-51	1.37E-50
C9orf50	NA	NA	NA
ASB6	1.60505837	1.79E-35	3.48E-35
RP11-483H2(	NA	NA	NA
PRRX2	NA	NA	NA
PRRX2-AS1	NA	NA	NA
PTGES	NA	5.84E-96	2.91E-95
TOR1B	1.02991245	3.97E-117	3.22E-116
TOR1A	1.67429618	2.08E-54	5.28E-54
C9orf78	1.51457713	1.77E-39	3.64E-39
USP20	2.80885214	2.74E-24	4.56E-24
FNBP1	2.33030696	0.32122022	0.33384379
RP11-409K2(	NA	NA	NA
RP11-138E2.	Inf	2.51E-58	6.71E-58
GPR107	1.94747082	0.2866894	0.29879468
RP11-88G17.	NA	NA	NA
NCS1	2.78210117	7.65E-56	1.98E-55
HMCN2	NA	NA	NA
ASS1	2.24708171	4.60E-36	9.03E-36
FUBP3	1.95899431	4.27E-19	6.58E-19
PRDM12	NA	0.00162101	0.00183826
EXOSC2	1.36678166	1.40E-41	2.97E-41
ABL1	3.05372643	1.32E-83	5.25E-83
QRFP	NA	1.48E-51	3.60E-51
FIBCD1	0	2.50E-62	7.12E-62
RP11-83J21.3	NA	NA	NA
LAMC3	NA	3.75E-93	1.77E-92
AIF1L	1.18595979	5.43E-12	7.43E-12
NUP214	2.27204929	0.00721623	0.00803417
RP11-544A12	Inf	1.38E-109	9.17E-109
RP11-544A12	1.42315175	0.833774	0.84095891
FAM78A	1.88053647	1.31E-06	1.61E-06
PLPP7	NA	9.64E-116	7.50E-115
PRRC2B	3.30252918	8.33E-129	1.06E-127
RP11-334J6.7	NA	0.32076322	0.33342613
POMT1	1.94747082	0.00271132	0.00305368
RP11-334J6.6	NA	1.07E-120	9.73E-120
UCK1	1.40726329	3.81E-34	7.28E-34
RAPGEF1	1.60505837	4.31E-07	5.35E-07
RP11-323H21	NA	NA	NA
MED27	1.15610726	3.83E-53	9.56E-53
NTNG2	2.62159533	4.49E-12	6.16E-12
SETX	2.51459144	0.00136748	0.00155405

TTF1	1.48597339	2.38E-72	7.88E-72
CFAP77	NA	NA	NA
BARHL1	NA	NA	NA
DDX31	2.29701686	0.00289459	0.00325828
GTF3C4	1.85869722	1.14E-27	1.99E-27
AK8	5.9922179	2.49E-91	1.13E-90
C9orf9	1.49805447	8.02E-07	9.89E-07
TSC1	2.24708171	2.26E-39	4.65E-39
GFI1B	0.99870298	1.80E-21	2.87E-21
GTF3C5	1.1741508	4.24E-33	7.96E-33
CEL	Inf	4.61E-17	6.87E-17
RALGDS	2.56809339	9.84E-28	1.72E-27
GBGT1	1.60505837	1.56E-10	2.08E-10
OBP2B	NA	NA	NA
ABO	NA	NA	NA
SURF6	1.51416259	6.90E-31	1.26E-30
MED22	3.5311284	4.85E-65	1.44E-64
RPL7A	1.68236811	7.83E-28	1.37E-27
SURF1	1.44518196	6.92E-123	6.84E-122
SURF2	1.81824932	1.00E-09	1.32E-09
SURF4	1.37760788	5.06E-88	2.17E-87
STKLD1	0	5.26E-17	7.84E-17
REXO4	1.47770047	0.43944279	0.45238445
ADAMTS13	0	1.67E-22	2.70E-22
CACFD1	2.24708171	3.52E-86	1.46E-85
SLC2A6	1.39105058	1.19E-52	2.96E-52
TMEM8C	NA	NA	NA
ADAMTSL2	NA	NA	NA
FAM163B	NA	NA	NA
LL09NC01-25	NA	NA	NA
DBH	NA	NA	NA
DBH-AS1	NA	NA	NA
SARDH	NA	NA	NA
VAV2	2.17217899	0.85021943	0.85687911
LINC00094	2.37926299	1.52E-54	3.87E-54
BRD3	1.95508804	0.11823319	0.12572455
RP11-374P2(	NA	NA	NA
LL09NC01-25	NA	1.42E-31	2.61E-31
WDR5	2.13385666	6.30E-22	1.01E-21
LL09NC01-13	Inf	5.56E-149	7.47E-147
RP11-145E17	NA	NA	NA
RXRA	1.45399405	0.01734252	0.01904989
RP11-473E2.	NA	NA	NA
RP11-473E2.	NA	NA	NA
RP11-473E2.	NA	NA	NA

COL5A1	NA	NA	NA
COL5A1-AS1	NA	NA	NA
FCN2	NA	NA	NA
FCN1	NA	NA	NA
RP11-447M1	NA	NA	NA
OLFM1	0.74902724	1.14E-06	1.40E-06
RP11-399H1:	NA	NA	NA
RP11-399H1:	NA	NA	NA
C9orf62	NA	NA	NA
RP11-555H7.	NA	NA	NA
PPP1R26-AS:	1.12354086	0.00543345	0.00607093
PPP1R26	2.14007782	7.51E-52	1.84E-51
C9orf116	1.9661965	7.95E-14	1.12E-13
MRPS2	1.33022265	5.80E-100	3.11E-99
RP11-426A6.	NA	2.17E-21	3.46E-21
LCN1	NA	NA	NA
OBP2A	NA	NA	NA
PAEP	NA	NA	NA
LINC01502	0	1.77E-93	8.42E-93
RP11-98L5.5	NA	NA	NA
GLT6D1	NA	NA	NA
LCN9	NA	NA	NA
SOHLH1	NA	NA	NA
KCNT1	NA	NA	NA
CAMSAP1	1.94747082	4.53E-32	8.39E-32
RP11-432J22	2.24708171	1.27E-08	1.62E-08
UBAC1	2.54870342	3.17E-112	2.26E-111
NACC2	2.13184675	5.84E-34	1.11E-33
RP11-83N9.5	Inf	4.00E-88	1.72E-87
C9orf69	1.52183312	2.49E-32	4.62E-32
RP11-83N9.6	NA	8.61E-111	5.90E-110
LHX3	NA	NA	NA
QSOX2	1.87256809	5.43E-14	7.68E-14
WI2-1959D1!	NA	NA	NA
CR392000.1	NA	NA	NA
CCDC187	NA	NA	NA
DKFZP434A0	NA	NA	NA
GPSM1	1.81906615	1.26E-32	2.35E-32
DNLZ	5.9922179	3.34E-69	1.06E-68
CARD9	0	6.80E-44	1.48E-43
SNAPC4	4.60116732	9.53E-128	1.16E-126
SDCCAG3	2.58754864	6.18E-56	1.60E-55
PMPCA	1.00795023	2.14E-107	1.35E-106
INPP5E	3.99481193	2.83E-50	6.77E-50
SEC16A	3.55787938	2.31E-129	3.01E-128

C9orf163	Inf	2.48E-15	3.59E-15
NOTCH1	1.59168288	8.14E-06	9.82E-06
RP11-413M3	NA	NA	NA
NALT1	NA	3.31E-154	8.11E-152
LINC01451	NA	NA	NA
RP11-251M1	3.16255945	0.02673844	0.02916774
EGFL7	1.75308807	9.33E-25	1.56E-24
AGPAT2	1.72957198	7.10E-09	9.15E-09
FAM69B	2.16763943	0.00078744	0.0009012
SNHG7	2.02718381	2.05E-131	2.99E-130
LCN10	NA	NA	NA
RP11-216L13	NA	NA	NA
LCN6	NA	NA	NA
LCN8	NA	NA	NA
LCN15	NA	2.27E-101	1.26E-100
TMEM141	1.99740597	5.82E-08	7.36E-08
RP11-216L13	NA	NA	NA
CCDC183	NA	0.00393667	0.00441649
CCDC183-AS1	NA	2.96E-76	1.04E-75
RABL6	2.37898588	7.77E-124	8.01E-123
RABL6	NA	NA	NA
C9orf172	1.49805447	0.0109368	0.01209481
PHPT1	1.61585692	5.55E-26	9.47E-26
MAMDC4	1.74773022	1.42E-35	2.76E-35
EDF1	1.49953185	1.84E-142	7.09E-141
TRAF2	1.4713035	9.40E-28	1.64E-27
FBXW5	1.85554475	1.64E-27	2.86E-27
C8G	Inf	6.84E-127	7.98E-126
LCN12	0	2.29E-26	3.92E-26
PRR31	NA	NA	NA
RP11-229P13	NA	NA	NA
PTGDS	NA	4.62E-92	2.14E-91
LCNL1	NA	NA	NA
C9orf142	1.99161878	4.61E-67	1.41E-66
CLIC3	NA	3.53E-77	1.25E-76
ABCA2	2.27829118	3.06E-57	8.06E-57
C9orf139	0.29961089	6.00E-130	8.06E-129
FUT7	1.56047341	2.56E-33	4.82E-33
RP11-229P13	NA	NA	NA
NPDC1	1.94010334	0.72106114	0.73097291
ENTPD2	NA	NA	NA
RP11-229P13	NA	NA	NA
RP11-229P13	NA	NA	NA
SAPCD2	1.64369866	8.06E-118	6.66E-117
RP11-229P13	NA	1.43E-103	8.27E-103

UAP1L1	1.61328943	5.47E-31	9.99E-31
MAN1B1-AS	1.1770428	0.02615645	0.02853974
MAN1B1	1.14850843	5.03E-90	2.23E-89
DPP7	1.67616193	2.07E-81	7.89E-81
GRIN1	NA	2.43E-122	2.35E-121
LRRC26	0.96303502	8.73E-64	2.54E-63
RP11-350O1	NA	NA	NA
TMEM210	NA	NA	NA
ANAPC2	1.24837873	2.60E-41	5.48E-41
SSNA1	2.29496473	5.14E-90	2.28E-89
TPRN	1.87256809	6.48E-27	1.12E-26
TMEM203	2.45070077	5.45E-26	9.29E-26
NDOR1	3.74513619	1.43E-19	2.22E-19
RNF208	2.63171732	4.80E-79	1.76E-78
CYSRT1	Inf	3.20E-89	1.40E-88
RNF224	NA	NA	NA
SLC34A3	NA	NA	NA
TUBB4B	0.76393325	5.77E-143	2.48E-141
FAM166A	Inf	6.72E-43	1.45E-42
C9orf173-AS	Inf	6.88E-98	3.56E-97
C9orf173	NA	NA	NA
NELFB	1.57736324	2.25E-05	2.68E-05
TOR4A	0.94876783	1.36E-56	3.55E-56
NRARP	0.74902724	2.45E-82	9.49E-82
EXD3	0.59922179	1.05E-06	1.29E-06
NOXA1	0	6.65E-90	2.95E-89
ENTPD8	NA	1.75E-135	3.23E-134
NSMF	2.15668187	3.37E-82	1.30E-81
PNPLA7	1.12354086	4.54E-17	6.78E-17
MRPL41	1.79131452	1.18E-09	1.54E-09
DPH7	1.78491597	3.99E-29	7.10E-29
ZMYND19	2.19159821	5.37E-105	3.21E-104
ARRDC1	1.5969826	3.92E-106	2.39E-105
ARRDC1-AS1	1.88697246	2.89E-88	1.25E-87
EHMT1	1.40442607	4.37E-10	5.77E-10
RP11-188C1	NA	NA	NA
CACNA1B	NA	NA	NA
RP11-885N1	NA	NA	NA
FAM157B	NA	3.34E-57	8.79E-57
RP11-304M2	NA	NA	NA
LINC01001	Inf	0.77632517	0.7850664
RP11-304M2	NA	NA	NA
BET1L	2.37191958	1.45E-54	3.71E-54
SCGB1C1	NA	NA	NA
ODF3	NA	NA	NA

RP11-304M2	NA	7.75E-127	8.99E-126
RIC8A	1.83272622	1.92E-35	3.73E-35
SIRT3	1.29377432	7.10E-18	1.07E-17
PSMD13	0.89330707	1.45E-140	4.38E-139
NLRP6	NA	NA	NA
RP11-326C3.	NA	NA	NA
ATHL1	1.87256809	1.47E-37	2.94E-37
IFITM5	NA	NA	NA
IFITM2	1.44257098	1.27E-121	1.20E-120
RP11-326C3.	NA	NA	NA
IFITM1	1.18595979	1.49E-137	3.23E-136
RP11-326C3.	Inf	5.48E-24	9.08E-24
IFITM3	1.35401078	6.79E-140	1.89E-138
RP11-326C3.	NA	NA	NA
RP11-326C3.	NA	NA	NA
RP11-326C3.	NA	NA	NA
RP11-326C3.	0.74902724	2.56E-44	5.62E-44
RP11-326C3.	Inf	4.52E-25	7.61E-25
B4GALNT4	0.42801556	9.67E-88	4.12E-87
PKP3	NA	6.09E-45	1.35E-44
SIGIRR	1.80447471	7.13E-71	2.31E-70
ANO9	NA	NA	NA
PTDSS2	2.17898833	1.95E-17	2.93E-17
RP13-317D1	NA	0.25773262	0.26938902
RNH1	0.96792104	3.10E-144	1.72E-142
RP13-46H24.	NA	NA	NA
HRAS	2.32822633	5.30E-112	3.75E-111
LRRC56	NA	1.37E-40	2.87E-40
LMNTD2	Inf	9.32E-50	2.21E-49
RP11-496I9.1	NA	9.47E-84	3.76E-83
RASSF7	1.76241703	0.67515868	0.68654946
MIR210HG	1.49805447	2.20E-05	2.63E-05
PHRF1	1.93297352	4.42E-07	5.48E-07
IRF7	1.1523496	4.06E-51	9.84E-51
CDHR5	NA	NA	NA
SCT	NA	1.32E-51	3.23E-51
DRD4	NA	1.45E-21	2.31E-21
DEAF1	1.67588109	1.38E-34	2.64E-34
RP11-754B1	NA	NA	NA
EPS8L2	0.18725681	8.85E-74	3.00E-73
TMEM80	1.43117704	0.0001618	0.00018867
AP006621.9	NA	NA	NA
TALDO1	0.92373332	1.59E-137	3.44E-136
PDDC1	1.84375935	2.78E-32	5.16E-32
AP006621.5	1.73638132	0.00017328	0.00020187

AP006621.6	NA	NA	NA
CEND1	NA	NA	NA
SLC25A22	0.94729915	6.83E-32	1.26E-31
PANO1	0.74902724	1.58E-27	2.76E-27
PIDD1	1.61328943	0.0005218	0.0006001
RPLP2	2.18238121	1.13E-144	6.93E-143
PNPLA2	1.3732166	4.75E-72	1.57E-71
AP006621.8	NA	1.24E-42	2.67E-42
CRACR2B	Inf	4.28E-21	6.79E-21
CD151	1.15856731	9.20E-126	1.02E-124
POLR2L	2.10764292	4.28E-103	2.45E-102
TSPAN4	1.23619942	3.78E-143	1.68E-141
RP11-1391J7	2.43433852	3.45E-05	4.09E-05
CHID1	1.56105677	1.79E-106	1.10E-105
AP2A2	2.13611471	0.00012541	0.00014671
MUC6	NA	NA	NA
RP13-870H1	NA	NA	NA
MUC5AC	NA	NA	NA
MUC5B	NA	NA	NA
RP11-532E4.	NA	NA	NA
TOLLIP	1.14473974	2.62E-114	1.97E-113
TOLLIP-AS1	5.24319066	4.16E-25	7.01E-25
BRSK2	Inf	4.74E-62	1.34E-61
MOB2	1.87256809	6.33E-24	1.05E-23
DUSP8	0	2.14E-86	8.88E-86
KRTAP5-AS1	NA	NA	NA
AP006285.2	NA	NA	NA
KRTAP5-1	NA	NA	NA
KRTAP5-2	NA	NA	NA
KRTAP5-3	NA	NA	NA
KRTAP5-4	NA	NA	NA
KRTAP5-5	NA	NA	NA
AP006285.7	NA	NA	NA
FAM99A	NA	NA	NA
FAM99B	NA	NA	NA
AP006285.6	NA	NA	NA
KRTAP5-6	NA	NA	NA
IFITM10	NA	1.58E-18	2.40E-18
RP11-295K3.	NA	NA	NA
CTSD	1.3264024	3.75E-90	1.67E-89
AC068580.6	NA	NA	NA
AC068580.7	NA	NA	NA
SYT8	NA	NA	NA
TNNI2	NA	2.34E-41	4.95E-41
LSP1	0.37451362	1.61E-43	3.50E-43

PRR33	NA	NA	NA
LINC01150	NA	NA	NA
TNNT3	NA	1.16E-18	1.77E-18
MRPL23	1.62173107	7.39E-117	5.95E-116
MRPL23-AS1	NA	NA	NA
LINC01219	NA	NA	NA
IGF2	NA	NA	NA
INS-IGF2	NA	NA	NA
IGF2-AS	NA	NA	NA
INS	NA	NA	NA
TH	NA	2.28E-71	7.43E-71
ASCL2	0.81144617	7.93E-138	1.76E-136
C11orf21	0.74902724	2.60E-11	3.50E-11
TSPAN32	Inf	6.08E-12	8.32E-12
CD81-AS1	NA	0.0091276	0.0101181
CD81	1.21324472	3.83E-106	2.34E-105
TSSC4	1.52128013	4.82E-72	1.59E-71
AC124057.5	NA	NA	NA
TRPM5	NA	NA	NA
KCNQ1	0.74902724	4.55E-07	5.64E-07
KCNQ1OT1	0.74902724	1.27E-17	1.92E-17
KCNQ1-AS1	NA	NA	NA
KCNQ1DN	NA	NA	NA
CDKN1C	1.28999135	4.10E-59	1.11E-58
SLC22A18AS	0.93628405	8.96E-28	1.56E-27
SLC22A18	0.74902724	1.03E-90	4.64E-90
PHLDA2	1.40442607	3.21E-37	6.41E-37
NAP1L4	2.11154345	7.42E-16	1.09E-15
CTC-343N3.1	NA	NA	NA
CARS	1.12354086	2.56E-12	3.53E-12
CARS-AS1	NA	NA	NA
OSBPL5	0.59922179	5.08E-51	1.23E-50
AC108448.3	NA	NA	NA
MRGPRG	NA	NA	NA
MRGPRG-AS	NA	NA	NA
AC109309.4	NA	NA	NA
MRGPRE	NA	NA	NA
RP5-1173A5.	NA	NA	NA
ZNF195	1.37978702	2.40E-05	2.86E-05
RP13-726E6.	NA	NA	NA
RP13-726E6.	Inf	2.71E-38	5.48E-38
ART5	0.74902724	9.03E-16	1.32E-15
ART1	NA	NA	NA
CHRNA10	Inf	2.43E-87	1.03E-86
NUP98	2.24708171	3.90E-72	1.29E-71

PGAP2	1.7183566	0.70866209	0.71900607
RHOG	1.79766537	0.00020436	0.00023778
AC090587.5	0	0.0364833	0.03957904
STIM1	1.74773022	8.28E-62	2.34E-61
RRM1	1.52631965	2.08E-55	5.35E-55
RRM1-AS1	NA	NA	NA
RP11-23F23.	Inf	3.31E-41	6.99E-41
AC009758.1	NA	NA	NA
TRIM21	0.29961089	3.24E-56	8.44E-56
OR52K2	NA	NA	NA
OR52K1	NA	NA	NA
OR52M1	NA	NA	NA
C11orf40	NA	NA	NA
OR52I2	NA	NA	NA
OR52I1	NA	NA	NA
TRIM68	1.74773022	4.33E-51	1.05E-50
OR51D1	NA	NA	NA
OR51E1	NA	NA	NA
OR51E2	NA	NA	NA
MMP26	NA	NA	NA
OR51F1	NA	NA	NA
OR52R1	NA	NA	NA
OR51F2	NA	NA	NA
OR51S1	NA	NA	NA
OR51H1	NA	NA	NA
OR51T1	NA	NA	NA
OR51A7	NA	NA	NA
OR51G2	NA	NA	NA
OR51G1	NA	NA	NA
OR51A4	NA	NA	NA
OR51A2	NA	NA	NA
OR51L1	NA	NA	NA
OR52J3	NA	NA	NA
OR52E2	NA	NA	NA
AC113331.9	NA	NA	NA
OR52A5	NA	NA	NA
OR52A1	NA	NA	NA
OR51V1	NA	NA	NA
AC104389.16	NA	NA	NA
HBB	NA	3.23E-23	5.29E-23
HBD	Inf	1.77E-88	7.66E-88
HBG1	NA	NA	NA
HBG2	NA	9.79E-32	1.81E-31
AC104389.2	NA	NA	NA
AC104389.1	NA	NA	NA

HBE1	NA	2.22E-08	2.83E-08
OR51B4	NA	NA	NA
OR51B2	NA	NA	NA
OR51B5	NA	NA	NA
OR51B6	NA	NA	NA
OR51M1	NA	NA	NA
OR51J1	NA	NA	NA
OR51Q1	NA	NA	NA
OR51I1	NA	NA	NA
OR51I2	NA	NA	NA
OR52D1	NA	NA	NA
UBQLN3	NA	NA	NA
UBQLNL	NA	NA	NA
OR52H1	NA	NA	NA
AC015691.13	NA	7.21E-40	1.49E-39
OR52B6	NA	NA	NA
TRIM6	1.99740597	6.17E-09	7.96E-09
TRIM6-TRIM	NA	NA	NA
TRIM34	NA	7.88E-117	6.32E-116
TRIM5	2.09727626	0.00309953	0.00348767
TRIM22	4.49416342	1.55E-124	1.63E-123
OR56B1	NA	NA	NA
OR52N4	NA	NA	NA
OR52N5	NA	NA	NA
OR52N1	NA	NA	NA
OR52N2	NA	NA	NA
OR52E6	NA	NA	NA
OR52E8	NA	NA	NA
OR52E4	NA	NA	NA
OR52E5	NA	NA	NA
AC025016.1	NA	NA	NA
OR56A3	NA	NA	NA
OR56A5	NA	NA	NA
OR52L1	NA	NA	NA
OR56A4	NA	NA	NA
OR56A1	NA	NA	NA
OR56B4	NA	NA	NA
OR52B2	NA	NA	NA
OR52W1	NA	NA	NA
RP11-290F24	0.74902724	0.32134378	0.33395308
C11orf42	NA	NA	NA
FAM160A2	2.37191958	1.30E-17	1.96E-17
CNGA4	NA	NA	NA
CCKBR	NA	NA	NA
PRKCDBP	NA	2.72E-84	1.09E-83

RP11-304C12	NA	NA	NA
RP11-304C12	NA	NA	NA
SMPD1	1.99740597	4.25E-24	7.04E-24
APBB1	1.17035506	1.69E-06	2.07E-06
HPX	NA	0.00110931	0.0012651
TRIM3	0.74902724	9.51E-08	1.20E-07
ARFIP2	1.9758822	2.00E-17	3.01E-17
TIMM10B	2.17525718	2.75E-78	9.95E-78
TIMM10B	NA	1.01E-45	2.26E-45
DNHD1	0.37451362	0.25649351	0.26814022
RRP8	1.3264024	3.30E-42	7.05E-42
RP11-732A19	NA	NA	NA
ILK	1.02140078	3.83E-106	2.34E-105
TAF10	2.22033074	7.02E-118	5.82E-117
TPP1	1.78614187	0.00238645	0.00269248
RP11-732A19	Inf	1.60E-18	2.44E-18
DCHS1	0.74902724	7.23E-10	9.51E-10
RP11-732A19	NA	NA	NA
RP11-732A19	NA	NA	NA
MRPL17	1.68104158	5.53E-15	7.97E-15
OR2AG2	NA	NA	NA
OR2AG1	NA	NA	NA
OR6A2	NA	NA	NA
OR10A5	NA	NA	NA
OR10A2	NA	NA	NA
OR10A4	NA	NA	NA
OR2D2	NA	NA	NA
OR2D3	NA	NA	NA
ZNF215	2.62159533	3.06E-05	3.63E-05
ZNF214	NA	NA	NA
NLRP14	NA	NA	NA
RBMXL2	NA	NA	NA
RP11-324J3.1	NA	NA	NA
SYT9	NA	NA	NA
CTD-2516F10	Inf	3.99E-29	7.11E-29
OLFML1	NA	NA	NA
PPFIBP2	0.85603113	3.43E-127	4.07E-126
CTD-2516F10	NA	NA	NA
CYB5R2	NA	NA	NA
OVCH2	NA	NA	NA
RP11-35J10.5	NA	NA	NA
RP11-35J10.4	NA	NA	NA
OR5P2	NA	NA	NA
OR5P3	NA	NA	NA
OR10A6	NA	NA	NA

OR10A3	NA	NA	NA
NLRP10	NA	NA	NA
EIF3F	1.69753086	4.80E-31	8.78E-31
CASC23	NA	NA	NA
TUB	2.14007782	1.24E-50	2.98E-50
RP11-236J17	NA	NA	NA
RIC3	NA	NA	NA
RP11-379P15	NA	NA	NA
LMO1	Inf	1.01E-48	2.36E-48
STK33	NA	NA	NA
TRIM66	1.87256809	0.00383408	0.00430353
RP11-152H18	NA	NA	NA
RPL27A	2.2815581	1.70E-140	5.08E-139
ST5	Inf	6.00E-124	6.20E-123
RP11-152H18	NA	2.95E-25	4.97E-25
RP11-318C2.	NA	NA	NA
AKIP1	2.01462498	5.86E-15	8.44E-15
C11orf16	NA	NA	NA
ASCL3	NA	NA	NA
TMEM9B	1.27244386	3.10E-55	7.97E-55
TMEM9B-AS	NA	4.50E-76	1.57E-75
NRIP3	3.2457847	9.64E-29	1.71E-28
RP11-467K18	NA	NA	NA
SCUBE2	NA	NA	NA
DENND5A	3.22081712	2.35E-48	5.45E-48
TMEM41B	1.64785992	0.34586771	0.35859709
IPO7	2.16566571	5.16E-92	2.38E-91
CTD-2371O3.	NA	0.00051996	0.00059806
CTD-2371O3.	2.43433852	9.11E-30	1.64E-29
ZNF143	1.31079767	1.66E-15	2.42E-15
WEE1	2.92801556	1.62E-31	2.98E-31
SWAP70	2.16682879	3.57E-16	5.26E-16
RP11-540A21	Inf	1.36E-120	1.24E-119
SBF2-AS1	1.71206226	0.05235914	0.05651136
SBF2	1.39105058	5.08E-38	1.02E-37
RP11-1H15.2	NA	3.84E-66	1.16E-65
RP11-748C4.	NA	NA	NA
RP11-351I24	NA	NA	NA
ADM	Inf	3.00E-13	4.19E-13
AMPD3	4.86867704	1.51E-21	2.41E-21
MTRNR2L8	0.74902724	6.13E-131	8.70E-130
RNF141	1.9539841	0.17997957	0.18964915
MRVI1-AS1	NA	2.54E-72	8.39E-72
LYVE1	NA	NA	NA
MRVI1	Inf	2.46E-98	1.29E-97

CTR9	1.40035527	2.39E-09	3.11E-09
RP11-685M7	NA	NA	NA
EIF4G2	2.21785138	1.96E-135	3.61E-134
RP11-685M7	NA	1.48E-14	2.12E-14
ZBED5	1.8385214	1.01E-12	1.40E-12
ZBED5-AS1	0.65132803	1.00E-73	3.39E-73
CTD-2003C8.	NA	NA	NA
RP11-179A1(	NA	NA	NA
CTD-3224I3.3	NA	NA	NA
GALNT18	1.68531128	5.00E-06	6.06E-06
CSNK2A3	Inf	3.49E-44	7.64E-44
RP11-567I13	0	8.09E-14	1.14E-13
RP11-483L5.	NA	8.96E-35	1.73E-34
USP47	1.66718966	4.54E-09	5.88E-09
DKK3	NA	NA	NA
RP13-631K18	NA	NA	NA
RP13-631K18	NA	NA	NA
RP13-631K18	NA	NA	NA
MICAL2	1.49805447	0.60429429	0.61690996
RP11-265D17	NA	NA	NA
MICALCL	Inf	1.65E-46	3.75E-46
PARVA	NA	NA	NA
RP11-51B23.	NA	NA	NA
TEAD1	Inf	3.37E-155	9.85E-153
RP11-47J17.3	NA	NA	NA
RP11-47J17.2	NA	4.02E-120	3.60E-119
LINC00958	0.37451362	1.35E-52	3.35E-52
CTC-497E21.1	NA	NA	NA
RASSF10	NA	NA	NA
CTC-497E21.5	NA	NA	NA
ARNTL	1.72276265	1.54E-73	5.19E-73
BTBD10	1.72276265	3.06E-13	4.27E-13
PTH	NA	NA	NA
FAR1	2.14007782	8.40E-67	2.56E-66
RP11-98J9.1	NA	NA	NA
RP11-98J9.2	NA	NA	NA
RP11-98J9.3	NA	NA	NA
RP11-231N3.	Inf	7.53E-63	2.16E-62
SPON1	0	8.34E-95	4.06E-94
RP11-21L19.	NA	NA	NA
RRAS2	2.18466278	0.40479083	0.41761247
COPB1	1.25643279	8.00E-122	7.61E-121
RP11-140L24	NA	NA	NA
PSMA1	1.2067661	2.73E-116	2.16E-115
PDE3B	0.13618677	4.69E-136	8.97E-135

CYP2R1	0.53501946	4.35E-140	1.23E-138
CALCB	NA	NA	NA
CALCA	NA	NA	NA
INSC	NA	NA	NA
RP11-531H8.	NA	NA	NA
RP11-531H8.	NA	NA	NA
RP11-396O2(	NA	NA	NA
RP11-396O2(	NA	NA	NA
RP11-222N1:	NA	NA	NA
SOX6	NA	1.13E-08	1.44E-08
CTD-3096P4.	NA	NA	NA
C11orf58	1.48129771	1.00E-26	1.72E-26
PLEKHA7	1.49805447	8.80E-06	1.06E-05
RPS13	1.97492382	9.62E-82	3.69E-81
PIK3C2A	1.49805447	2.74E-50	6.56E-50
NUCB2	0.85136526	2.16E-122	2.09E-121
NCR3LG1	2.79636835	8.20E-35	1.58E-34
RP1-239B22.	1.87256809	7.04E-34	1.34E-33
KCNJ11	2.99610895	1.13E-57	3.00E-57
ABCC8	NA	NA	NA
USH1C	NA	NA	NA
OTOG	NA	NA	NA
RP11-358H1{	NA	NA	NA
RP11-358H1{	NA	NA	NA
MYOD1	NA	NA	NA
KCNC1	NA	1.12E-06	1.38E-06
SERGEF	1.60820554	6.02E-08	7.60E-08
TPH1	2.99610895	4.88E-28	8.55E-28
SAAL1	0.9902394	1.27E-114	9.63E-114
MRGPRX3	NA	NA	NA
RP11-113D6.	NA	NA	NA
MRGPRX4	NA	NA	NA
SAA4	NA	NA	NA
SAA2-SAA4	NA	NA	NA
SAA2	NA	NA	NA
SAA1	NA	NA	NA
HPS5	3.18336576	0.57439982	0.58738299
GTF2H1	1.79766537	0.73783798	0.74747915
LDHA	0.95158413	3.05E-124	3.18E-123
LDHC	NA	1.85E-96	9.31E-96
LDHAL6A	NA	NA	NA
TSG101	1.01754644	1.15E-107	7.29E-107
UEVLD	3.74513619	2.44E-34	4.67E-34
SPTY2D1-AS:	Inf	3.01E-57	7.93E-57
SPTY2D1	2.55917639	9.79E-22	1.57E-21

TMEM86A	NA	4.73E-40	9.81E-40
IGSF22	NA	NA	NA
RP11-1081L1	NA	NA	NA
PTPN5	NA	NA	NA
MRGPRX1	NA	NA	NA
RP11-583F24	NA	NA	NA
MRGPRX2	NA	NA	NA
ZDHHC13	1.63023575	0.05934091	0.06388339
CSRP3	NA	NA	NA
RP11-428C19	NA	NA	NA
E2F8	1.92607004	1.89E-29	3.37E-29
RP11-428C19	NA	NA	NA
NAV2	NA	0.00037673	0.00043538
NAV2-AS5	NA	NA	NA
NAV2-AS4	NA	NA	NA
RP11-359E10	Inf	1.53E-81	5.86E-81
NAV2-AS3	NA	2.06E-37	4.11E-37
NAV2-AS2	NA	NA	NA
NAV2-AS1	NA	NA	NA
DBX1	NA	NA	NA
HTATIP2	NA	NA	NA
PRMT3	2.54669261	2.12E-23	3.47E-23
SLC6A5	NA	NA	NA
NELL1	NA	NA	NA
RP11-701I24	NA	NA	NA
RP11-670N15	NA	NA	NA
CTD-2019O4.	NA	NA	NA
ANO5	4.86867704	1.64E-49	3.88E-49
CTD-3064C13	NA	NA	NA
CTD-2140G10	NA	NA	NA
SLC17A6	NA	NA	NA
CTD-2140G10	NA	NA	NA
LINC01495	NA	NA	NA
RP11-34N19.	NA	NA	NA
FANCF	1.10151064	4.00E-26	6.82E-26
GAS2	NA	2.40E-34	4.61E-34
SVIP	2.66840953	3.06E-144	1.71E-142
RP11-17A1.3	NA	1.34E-50	3.22E-50
CCDC179	NA	NA	NA
RP11-266A24	NA	NA	NA
RP11-945A11	NA	NA	NA
RP11-945A11	NA	NA	NA
RP11-2F20.1	NA	NA	NA
LUZP2	NA	NA	NA
RP11-405K6.	NA	NA	NA

RP11-20J1.1	NA	NA	NA
CTD-2507G9.	NA	NA	NA
RP11-430L3.	NA	NA	NA
ANO3	NA	NA	NA
MUC15	NA	NA	NA
SLC5A12	NA	NA	NA
FIBIN	NA	NA	NA
BBOX1	NA	NA	NA
BBOX1-AS1	NA	NA	NA
CCDC34	1.66450497	0.05111036	0.05517668
LGR4	2.69649805	8.47E-44	1.85E-43
RP11-159H2	NA	5.24E-05	6.19E-05
LIN7C	1.24837873	0.004351	0.00487529
BDNF-AS	NA	3.32E-07	4.12E-07
LINC00678	NA	NA	NA
BDNF	Inf	4.48E-66	1.35E-65
RP11-406D1.	NA	9.14E-60	2.50E-59
KIF18A	1.64785992	2.22E-13	3.10E-13
METTL15	2.24708171	6.41E-56	1.66E-55
RP11-22P4.2	NA	NA	NA
RP11-960D2	NA	NA	NA
RP11-115J23	NA	NA	NA
RP11-466I1.1	NA	NA	NA
RP11-460B1	NA	NA	NA
RP11-460B1	NA	NA	NA
CTD-3012A1	NA	NA	NA
RP5-1027O1	NA	NA	NA
CTD-3138F1	NA	NA	NA
LINC01616	NA	NA	NA
KCNA4	NA	NA	NA
RP11-624D1	NA	NA	NA
FSHB	NA	NA	NA
ARL14EP	1.67716968	1.33E-71	4.34E-71
MPPED2	4.86867704	1.05E-113	7.78E-113
RP4-710M3.	NA	NA	NA
RP5-1024C2	NA	5.76E-32	1.06E-31
DCDC1	NA	NA	NA
RP1-296L11.	NA	NA	NA
DNAJC24	2.04734112	3.51E-113	2.57E-112
IMMP1L	2.09179558	3.98E-67	1.22E-66
ELP4	0.96932937	5.09E-134	8.57E-133
Z83001.1	NA	NA	NA
PAX6	1.89753567	9.64E-06	1.16E-05
RCN1	1.3172548	1.06E-110	7.27E-110
PAUPAR	NA	NA	NA

RP1-17K7.2	NA	NA	NA
RP1-17K7.3	NA	NA	NA
RP1-65P5.3	NA	NA	NA
RP1-65P5.5	NA	NA	NA
WT1	1.85628489	6.24E-20	9.70E-20
WT1-AS	1.49805447	2.71E-16	3.99E-16
EIF3M	1.03103509	5.54E-127	6.49E-126
CCDC73	NA	0.02823045	0.03076387
PRRG4	1.9193823	1.14E-08	1.47E-08
QSER1	2.06874189	3.39E-44	7.43E-44
DEPDC7	0.33706226	7.23E-131	1.02E-129
TCP11L1	1.49805447	9.83E-16	1.44E-15
AC131263.1	NA	NA	NA
LINC00294	2.99610895	4.77E-05	5.64E-05
CSTF3	1.80819857	0.0014125	0.00160441
CSTF3-AS1	Inf	6.87E-27	1.19E-26
HIPK3	1.49805447	2.84E-05	3.38E-05
KIAA1549L	NA	2.49E-51	6.04E-51
RP4-541C22.	NA	NA	NA
C11orf91	NA	0.38801892	0.40076511
CD59	1.20598096	2.00E-143	9.45E-142
FBXO3	2.57998271	1.84E-33	3.47E-33
FBXO3-AS1	NA	9.41E-24	1.55E-23
RP11-646J21	NA	NA	NA
RP11-646J21	NA	NA	NA
RP11-646J21	NA	NA	NA
RP11-646J21	NA	NA	NA
LMO2	1.54546126	6.17E-64	1.80E-63
RP13-786C16	NA	NA	NA
CAPRIN1	2.04422017	8.12E-112	5.72E-111
NAT10	1.40888457	0.00680119	0.00757581
ABTB2	1.08608949	1.61E-18	2.46E-18
CAT	1.5606909	4.10E-22	6.60E-22
ELF5	NA	NA	NA
RP4-594L9.2	NA	NA	NA
RP11-350D17	NA	NA	NA
EHF	NA	NA	NA
APIP	1.29561468	6.28E-117	5.07E-116
PDHX	1.53550584	2.53E-68	7.92E-68
RP4-607I7.1	NA	6.65E-83	2.60E-82
CD44	0.95731289	4.19E-107	2.61E-106
RP1-68D18.4	NA	NA	NA
SLC1A2	NA	NA	NA
RP1-68D18.3	NA	NA	NA
RP4-683L5.1	NA	NA	NA

PAMR1	NA	NA	NA
RP5-945I17.2	NA	NA	NA
FJX1	1.72692391	0.01378004	0.01518911
RP1-276E15.	NA	NA	NA
TRIM44	1.7726978	2.94E-17	4.40E-17
RP11-698N1	NA	NA	NA
LDLRAD3	3.49546044	0.35120632	0.36402817
COMMD9	1.44195992	9.66E-20	1.50E-19
PRR5L	0.37451362	7.87E-28	1.37E-27
RP11-514F3.	NA	NA	NA
RP11-219O3.	NA	NA	NA
TRAF6	1.49805447	0.00013444	0.00015712
RAG1	Inf	5.30E-100	2.85E-99
RAG2	4.49416342	1.24E-106	7.69E-106
C11orf74	1.14137484	1.54E-136	3.08E-135
RP11-159D8.	NA	NA	NA
RP11-63D14.	NA	NA	NA
RP11-277K23	NA	NA	NA
LINC01493	NA	NA	NA
LRRC4C	NA	NA	NA
RP11-63C8.1	NA	NA	NA
RP11-124G5.	NA	NA	NA
LINC01499	NA	NA	NA
RP11-375D13	NA	NA	NA
RP11-375D13	NA	NA	NA
RP11-148I19	NA	NA	NA
CTD-2572N1	NA	NA	NA
API5	1.78070626	1.24E-15	1.80E-15
RP11-484D2.	NA	NA	NA
TTC17	1.23877582	7.90E-12	1.08E-11
RP11-484D2.	NA	NA	NA
HSD17B12	1.40993362	6.21E-36	1.22E-35
RP11-613D13	NA	NA	NA
ALKBH3	1.69135183	0.07765809	0.08324213
ALKBH3-AS1	NA	1.00E-62	2.87E-62
C11orf96	1.87256809	0.70673159	0.71716772
RP11-613D13	NA	NA	NA
ACCSL	NA	NA	NA
ACCS	0.56177043	1.01E-85	4.15E-85
EXT2	1.49805447	0.00143939	0.00163465
ALX4	NA	NA	NA
RP11-58K22.	NA	NA	NA
CD82	0.74902724	9.75E-53	2.42E-52
RP11-58K22.	NA	NA	NA
RP11-58K22.	NA	NA	NA

RP11-45A12.	NA	NA	NA
RP11-45A12.	NA	NA	NA
TSPAN18	1.77893969	1.88E-05	2.24E-05
TP53I11	1.9661965	1.20E-12	1.67E-12
AC068858.1	NA	NA	NA
PRDM11	0.74902724	4.21E-07	5.23E-07
CTD-2560E9.	NA	NA	NA
SYT13	NA	NA	NA
CTD-2560E9.	NA	NA	NA
RP11-430H1(	NA	NA	NA
RP11-430H1(	NA	NA	NA
RP11-430H1(	NA	NA	NA
RP11-430H1(	NA	NA	NA
RP11-958J22	NA	NA	NA
CHST1	NA	3.67E-65	1.09E-64
RP11-495O1	NA	NA	NA
CTD-2210P2	NA	NA	NA
CTD-2210P2	NA	NA	NA
DKFZp779M(	NA	2.29E-89	1.01E-88
SLC35C1	1.53459239	4.83E-23	7.89E-23
CTD-2210P2	NA	NA	NA
CRY2	3.62029831	2.09E-112	1.50E-111
MAPK8IP1	Inf	0.06058459	0.06520679
RP11-618K1:	NA	2.06E-16	3.05E-16
C11orf94	Inf	0.03330374	0.03620544
PEX16	1.33160398	6.38E-139	1.59E-137
GYLTL1B	NA	NA	NA
PHF21A	1.81906615	0.93039657	0.93369038
RP11-702F3.	NA	NA	NA
RP11-702F3.	NA	3.88E-121	3.57E-120
RP11-702F3.	NA	NA	NA
CTD-2589M5	NA	NA	NA
CTD-2589M5	NA	NA	NA
CREB3L1	1.49805447	7.79E-20	1.21E-19
DGKZ	1.84628644	0.00035413	0.00040953
MDK	2.22528462	7.22E-19	1.11E-18
CHRM4	NA	1.65E-112	1.19E-111
AMBRA1	1.55919956	1.40E-10	1.86E-10
HARBI1	5.24319066	4.64E-06	5.63E-06
ATG13	2.04280156	8.84E-06	1.07E-05
ARHGAP1	1.71206226	0.13736935	0.14551029
ZNF408	1.3658732	3.79E-48	8.78E-48
F2	NA	NA	NA
CKAP5	1.63676322	3.03E-51	7.34E-51
LRP4-AS1	Inf	5.49E-37	1.09E-36

LRP4	2.62159533	0.00615014	0.00685644
C11orf49	0.62418936	2.61E-90	1.17E-89
RP11-390K5.	NA	1.14E-64	3.37E-64
ARFGAP2	1.94747082	0.29811073	0.31035951
PACSIN3	1.53614061	8.29E-44	1.81E-43
DDB2	0.8827821	1.57E-143	7.51E-142
RP11-17G12.	NA	NA	NA
ACP2	1.5421149	2.09E-76	7.34E-76
NR1H3	1.49805447	3.75E-54	9.50E-54
MADD	2.59278659	1.06E-18	1.62E-18
RP11-17G12.	NA	NA	NA
MYBPC3	0	9.05E-120	8.03E-119
SPI1	1.36137067	1.89E-140	5.59E-139
RP11-750H9.	NA	NA	NA
SLC39A13	1.68531128	1.68E-16	2.48E-16
PSMC3	0.97261746	4.14E-135	7.44E-134
RAPSN	NA	3.53E-15	5.10E-15
CELF1	2.55917639	1.35E-52	3.35E-52
NDUFS3	1.24277591	2.02E-134	3.50E-133
PTPMT1	2.44629108	7.06E-140	1.96E-138
KBTBD4	1.92607004	0.00130714	0.0014865
RP11-76I23.7	Inf	2.69E-28	4.74E-28
FAM180B	Inf	3.45E-41	7.28E-41
C1QTNF4	NA	4.09E-22	6.59E-22
MTCH2	1.50352982	4.42E-38	8.92E-38
AGBL2	NA	7.72E-97	3.92E-96
FNBP4	1.72641644	7.70E-06	9.30E-06
NUP160	2.32592668	6.93E-81	2.62E-80
PTPRJ	2.0598249	0.58791767	0.60069849
PTPRJ-AS1	NA	NA	NA
OR4B1	NA	NA	NA
OR4X2	NA	NA	NA
OR4X1	NA	NA	NA
OR4S1	NA	NA	NA
OR4C3	NA	NA	NA
OR4C5	NA	NA	NA
OR4A47	NA	NA	NA
TRIM49B	NA	NA	NA
TRIM64C	NA	NA	NA
FOLH1	NA	4.25E-22	6.85E-22
OR4C13	NA	NA	NA
OR4C12	NA	NA	NA
RP11-347H1!	NA	NA	NA
OR4C46	NA	NA	NA
OR4A5	NA	NA	NA

TRIM48	NA	NA	NA
OR4A16	NA	NA	NA
OR4A15	NA	NA	NA
OR4C15	NA	NA	NA
OR4C16	NA	NA	NA
OR4C11	NA	NA	NA
OR4P4	NA	NA	NA
OR4S2	NA	NA	NA
OR4C6	NA	NA	NA
RP11-674C21	NA	NA	NA
OR5D13	NA	NA	NA
OR5D14	NA	NA	NA
OR5L1	NA	NA	NA
OR5D18	NA	NA	NA
OR5L2	NA	NA	NA
OR5D16	NA	NA	NA
TRIM51	NA	NA	NA
OR5W2	NA	NA	NA
OR5I1	NA	NA	NA
OR10AG1	NA	NA	NA
OR5F1	NA	NA	NA
OR5AS1	NA	NA	NA
OR8I2	NA	NA	NA
OR8H2	NA	NA	NA
OR8H3	NA	NA	NA
OR8J3	NA	NA	NA
OR8K5	NA	NA	NA
OR5J2	NA	NA	NA
OR5T2	NA	NA	NA
OR5T3	NA	NA	NA
OR5T1	NA	NA	NA
OR8H1	NA	NA	NA
OR8K3	NA	NA	NA
OR8K1	NA	NA	NA
OR8J1	NA	NA	NA
OR8U1	NA	NA	NA
OR5R1	NA	NA	NA
OR5M9	NA	NA	NA
OR5M3	NA	NA	NA
OR5M8	NA	NA	NA
OR5M11	NA	NA	NA
OR5M10	NA	NA	NA
OR5M1	NA	NA	NA
OR5AP2	NA	NA	NA
OR5AR1	NA	NA	NA

OR9G1	NA	NA	NA
OR9G4	NA	NA	NA
AP000479.1	NA	NA	NA
OR5AK2	NA	NA	NA
LRRC55	NA	NA	NA
APLNR	NA	NA	NA
TNKS1BP1	1.92607004	4.57E-37	9.09E-37
RP11-872D1	NA	1.43E-119	1.26E-118
SSRP1	2.14721141	5.73E-54	1.45E-53
P2RX3	NA	NA	NA
PRG3	NA	NA	NA
PRG2	NA	1.56E-32	2.90E-32
RP11-872D1	NA	NA	NA
SLC43A3	1.75733313	5.02E-22	8.07E-22
RTN4RL2	1.26398346	2.02E-73	6.82E-73
RP11-624G1	NA	3.93E-136	7.61E-135
SLC43A1	1.64785992	6.52E-25	1.10E-24
TIMM10	2.31607106	2.63E-122	2.53E-121
SMTNL1	NA	NA	NA
UBE2L6	1.78444724	1.10E-08	1.41E-08
SERPING1	Inf	0.44057486	0.45349839
YPEL4	NA	2.06E-131	2.99E-130
CLP1	0.7846952	1.74E-90	7.77E-90
ZDHHC5	1.76241703	4.08E-17	6.09E-17
MED19	1.13088426	2.28E-39	4.68E-39
TMX2	1.31461923	5.72E-94	2.74E-93
TMX2-CTNNI	NA	NA	NA
C11orf31	1.91516981	2.91E-44	6.38E-44
RP11-691N7	NA	NA	NA
BTBD18	NA	NA	NA
CTNND1	2.24708171	9.22E-10	1.21E-09
OR9Q1	NA	NA	NA
OR6Q1	NA	NA	NA
RP11-659P1	NA	NA	NA
OR9I1	NA	NA	NA
OR9Q2	NA	NA	NA
OR1S2	NA	NA	NA
OR1S1	NA	NA	NA
OR10Q1	NA	NA	NA
OR10W1	NA	NA	NA
OR5B17	NA	NA	NA
OR5B3	NA	NA	NA
OR5B2	NA	NA	NA
OR5B12	NA	NA	NA
RP11-655C2.	NA	NA	NA

OR5B21	NA	NA	NA
LPXN	2.06731518	1.79E-12	2.47E-12
ZFP91	1.92250324	3.31E-07	4.12E-07
ZFP91-CNTF	NA	NA	NA
CMB9-55A18	NA	NA	NA
CNTF	NA	3.09E-105	1.86E-104
GLYAT	NA	NA	NA
GLYATL2	NA	1.82E-96	9.16E-96
GLYATL1	NA	NA	NA
RP11-780O24	NA	NA	NA
RP11-142C4.	NA	NA	NA
GLYATL1P3	NA	NA	NA
FAM111B	NA	NA	NA
AP001258.4	0.37451362	0.85102967	0.85760041
FAM111A	2.17217899	0.00849019	0.00942477
DTX4	1.31079767	6.69E-36	1.31E-35
MPEG1	NA	NA	NA
RP11-1036E2	NA	NA	NA
OR5AN1	NA	NA	NA
OR5A2	NA	NA	NA
OR5A1	NA	NA	NA
OR4D6	NA	NA	NA
OR4D10	NA	NA	NA
OR4D11	NA	NA	NA
OR4D9	NA	NA	NA
AP000442.4	Inf	4.00E-88	1.72E-87
OSBP	1.93170182	3.96E-09	5.13E-09
AP000442.1	0	0.96226325	0.96417651
PATL1	1.12354086	1.19E-11	1.61E-11
OR10V1	NA	NA	NA
STX3	1.24837873	2.46E-101	1.36E-100
AP000640.10	NA	NA	NA
MRPL16	0.95330739	2.88E-143	1.31E-141
GIF	NA	NA	NA
TCN1	NA	8.26E-46	1.85E-45
RP11-736I10	NA	3.21E-82	1.24E-81
OOSP2	NA	5.47E-100	2.94E-99
MS4A3	0.03524834	1.80E-145	1.58E-143
MS4A2	NA	3.54E-42	7.55E-42
AP001257.1	NA	NA	NA
MS4A6A	0.74902724	9.91E-75	3.40E-74
MS4A4E	NA	NA	NA
MS4A4A	0.53501946	6.47E-107	4.02E-106
MS4A6E	NA	NA	NA
MS4A7	0.62418936	3.54E-143	1.58E-141

MS4A14	0	1.17E-136	2.37E-135
MS4A5	NA	NA	NA
MS4A1	NA	NA	NA
MS4A12	NA	NA	NA
MS4A13	NA	NA	NA
LINC00301	NA	NA	NA
MS4A8	NA	NA	NA
MS4A18	NA	NA	NA
MS4A15	NA	NA	NA
MS4A10	NA	NA	NA
RP11-804A2:	NA	NA	NA
RP11-804A2:	NA	4.39E-40	9.11E-40
RP11-804A2:	Inf	5.95E-133	9.47E-132
CCDC86	1.38101897	6.95E-40	1.44E-39
PTGDR2	NA	0.05255704	0.05667775
ZP1	NA	NA	NA
PRPF19	1.22241245	8.45E-45	1.87E-44
RP11-881M1	NA	NA	NA
RP11-881M1	NA	1.76E-12	2.43E-12
TMEM109	1.49215662	3.08E-73	1.03E-72
RP11-881M1	NA	3.97E-33	7.46E-33
TMEM132A	1.92607004	1.10E-09	1.44E-09
SLC15A3	2.99610895	3.06E-40	6.37E-40
CD6	NA	NA	NA
RP11-881M1	NA	NA	NA
CD5	NA	1.24E-113	9.19E-113
VPS37C	1.2733463	4.38E-73	1.47E-72
PGA3	NA	NA	NA
PGA4	NA	NA	NA
PGA5	NA	NA	NA
VWCE	0	4.16E-98	2.17E-97
DDB1	1.93297352	1.54E-14	2.20E-14
TKFC	1.56047341	4.53E-66	1.37E-65
CYB561A3	3.02491769	3.61E-97	1.85E-96
TMEM138	1.63917555	9.28E-21	1.46E-20
TMEM216	1.87256809	3.70E-05	4.38E-05
CPSF7	2.24708171	2.39E-15	3.46E-15
RP11-286N2:	2.24708171	1.12E-96	5.69E-96
SDHAF2	1.39722389	5.14E-51	1.24E-50
PPP1R32	Inf	3.50E-78	1.26E-77
RP11-286N2:	NA	NA	NA
LRRC10B	NA	1.62E-79	5.97E-79
SYT7	Inf	1.04E-07	1.31E-07
RP11-794G2	Inf	1.85E-71	6.04E-71
RP11-855O1	NA	NA	NA

RP11-855O1(	NA	NA	NA
DAGLA	Inf	3.92E-139	1.00E-137
DKFZP434K0:	NA	NA	NA
MYRF	2.24708171	0.00144716	0.00164326
TMEM258	2.01251792	2.94E-33	5.53E-33
FEN1	1.12080718	2.79E-137	5.97E-136
FADS2	1.43839744	9.20E-122	8.71E-121
FADS1	1.14123441	2.53E-145	1.95E-143
FADS3	2.43433852	0.17128924	0.18070183
RAB3IL1	1.09233139	1.76E-27	3.06E-27
BEST1	7.49027237	1.20E-138	2.90E-137
FTH1	5.30904325	1.80E-145	1.58E-143
RP11-810P1:	NA	NA	NA
INCENP	1.99740597	2.95E-83	1.16E-82
SCGB1D1	NA	NA	NA
SCGB2A1	NA	NA	NA
RP11-703H8.	NA	NA	NA
SCGB1D2	NA	NA	NA
SCGB2A2	NA	NA	NA
SCGB1D4	NA	NA	NA
RP11-703H8.	NA	NA	NA
ASRGL1	NA	NA	NA
CTD-2531D1!	NA	NA	NA
SCGB1A1	NA	NA	NA
CTD-2531D1!	NA	NA	NA
AHNAK	2.15719844	4.44E-13	6.19E-13
RP11-864I4.3	NA	NA	NA
RP11-864I4.4	NA	1.22E-58	3.26E-58
EEF1G	2.30469919	1.60E-76	5.61E-76
RP11-864I4.1	NA	9.11E-49	2.13E-48
TUT1	1.56047341	1.38E-64	4.06E-64
MTA2	4.04474708	6.63E-91	3.00E-90
EML3	1.78216825	1.72E-19	2.67E-19
RP11-831H9.	NA	NA	NA
ROM1	2.24708171	7.45E-33	1.39E-32
B3GAT3	1.91565373	1.16E-87	4.93E-87
GANAB	2.46222783	3.54E-126	3.99E-125
INTS5	2.22211414	9.86E-06	1.19E-05
RP11-831H9.	NA	NA	NA
C11orf98	0	8.09E-71	2.62E-70
LBHD1	0	0.00044548	0.00051369
METTL12	2.64772419	1.30E-84	5.24E-84
UQCC3	1.71435111	2.55E-29	4.56E-29
UBXN1	1.46536965	5.57E-40	1.16E-39
LRRN4CL	Inf	9.04E-61	2.51E-60

BSCL2	0	2.55E-07	3.18E-07
HNRNPUL2-E	0	3.06E-93	1.45E-92
GNG3	NA	NA	NA
HNRNPUL2	1.44579676	0.0004986	0.00057396
TTC9C	1.24837873	1.85E-53	4.63E-53
ZBTB3	6.74124514	5.57E-47	1.27E-46
POLR2G	1.18754002	8.30E-138	1.84E-136
RP11-727F15	2.24708171	9.74E-66	2.92E-65
TAF6L	1.39818418	9.06E-17	1.34E-16
TMEM223	1.41305848	2.24E-109	1.48E-108
RP11-727F15	Inf	0.06581011	0.07072608
TMEM179B	1.83410453	1.92E-54	4.88E-54
NXF1	1.94066148	0.23949521	0.25083259
STX5	1.34824903	2.28E-98	1.20E-97
RP11-727F15	0	4.94E-55	1.26E-54
WDR74	1.30981726	1.91E-116	1.52E-115
SLC3A2	1.30702739	1.75E-74	5.97E-74
CHRM1	NA	NA	NA
AP000438.2	NA	NA	NA
SLC22A6	NA	NA	NA
SLC22A8	NA	NA	NA
SLC22A24	NA	NA	NA
SLC22A10	NA	NA	NA
SLC22A25	NA	NA	NA
SLC22A9	NA	NA	NA
HRASLS5	NA	NA	NA
LGALS12	0	3.51E-145	2.53E-143
RARRES3	Inf	0.00077744	0.00088986
HRASLS2	NA	NA	NA
PLA2G16	0.18725681	3.41E-118	2.86E-117
RP11-697H9.	NA	NA	NA
ATL3	1.64302749	6.02E-22	9.67E-22
RP11-697H9.	NA	NA	NA
RTN3	1.20192743	3.18E-145	2.34E-143
C11orf95	2.46108949	0.08258397	0.08843338
C11orf84	2.41093142	2.64E-72	8.71E-72
MARK2	2.48677043	1.57E-66	4.76E-66
RCOR2	0.56177043	1.13E-33	2.14E-33
NAA40	2.09727626	5.80E-20	9.03E-20
COX8A	2.11740248	1.02E-84	4.14E-84
AP000721.4	NA	NA	NA
OTUB1	1.4804303	3.10E-102	1.75E-101
MACROD1	0.89883268	1.75E-144	1.03E-142
RP11-21A7A.	NA	NA	NA
RP11-21A7A.	NA	NA	NA

FLRT1	NA	0.00455062	0.0050955
RP11-21A7A.	1.49805447	6.30E-39	1.29E-38
STIP1	1.51957825	1.23E-42	2.64E-42
FERMT3	1.26758456	1.87E-08	2.38E-08
TRPT1	2.0648859	0.78470003	0.79322606
NUDT22	1.51750973	2.49E-17	3.73E-17
RP11-783K1(	Inf	1.60E-90	7.18E-90
DNAJC4	1.80897144	6.00E-55	1.54E-54
VEGFB	2.52111607	1.76E-116	1.40E-115
FKBP2	1.84284479	9.34E-36	1.83E-35
PPP1R14B	2.95403652	1.80E-145	1.58E-143
RP11-783K1(	3.18336576	0.0070333	0.00783243
RP11-783K1(	NA	0.2711146	0.28305007
PLCB3	1.61328943	0.02896908	0.03155551
BAD	2.32774618	2.44E-77	8.69E-77
GPR137	3.58463035	7.08E-17	1.05E-16
KCNK4	NA	4.74E-71	1.53E-70
TEX40	0.34570488	4.43E-145	3.07E-143
ESRRA	2.12514705	1.60E-16	2.36E-16
TRMT112	1.83202589	7.08E-06	8.56E-06
PRDX5	1.38097963	2.38E-145	1.87E-143
AP003774.1	Inf	1.05E-64	3.09E-64
CCDC88B	4.04474708	8.14E-39	1.66E-38
RPS6KA4	1.91662852	2.74E-41	5.78E-41
AP003774.6	NA	NA	NA
AP003774.5	NA	NA	NA
AP003774.4	NA	6.59E-30	1.19E-29
AP005273.1	NA	NA	NA
SLC22A11	NA	NA	NA
SLC22A12	NA	NA	NA
NRXN2	2.54669261	8.14E-09	1.05E-08
AP001092.4	NA	NA	NA
RASGRP2	1.60856669	7.68E-64	2.24E-63
PYGM	NA	2.02E-36	3.98E-36
SF1	2.11355947	1.08E-49	2.56E-49
AP001462.6	2.24708171	0.02372711	0.02592338
MAP4K2	2.24708171	4.73E-52	1.16E-51
MEN1	2.4012932	6.48E-57	1.70E-56
CDC42BPG	Inf	1.35E-105	8.17E-105
EHD1	1.5729572	2.52E-19	3.88E-19
MIR194-2HG	1.49805447	2.12E-59	5.76E-59
ATG2A	3.2457847	5.58E-23	9.11E-23
PPP2R5B	2.49675746	1.86E-07	2.33E-07
GPHA2	NA	NA	NA
C11orf85	NA	NA	NA

BATF2	NA	4.60E-56	1.19E-55
ARL2	2.1614786	1.00E-14	1.44E-14
ARL2-SNX15	NA	NA	NA
SNX15	NA	3.01E-94	1.45E-93
SAC3D1	1.80905409	1.45E-09	1.90E-09
NAALADL1	NA	NA	NA
CDCA5	2.20603912	2.54E-05	3.02E-05
ZFPL1	1.49805447	6.17E-59	1.67E-58
TMEM262	0	2.18E-174	5.64E-171
VPS51	1.1770428	1.94E-29	3.47E-29
AP003068.9	NA	7.24E-120	6.43E-119
TM7SF2	1.07293091	1.28E-137	2.80E-136
ZNHIT2	2.12881425	5.51E-63	1.58E-62
AP003068.12	NA	NA	NA
FAU	1.56459643	3.43E-113	2.51E-112
SYVN1	1.3732166	1.62E-50	3.89E-50
MRPL49	1.74363718	0.00218126	0.00246311
SPDYC	NA	NA	NA
AP003068.23	1.87256809	1.55E-21	2.48E-21
CAPN1	1.46060311	7.23E-50	1.72E-49
POLA2	1.65856031	6.16E-19	9.45E-19
CDC42EP2	1.87256809	3.68E-60	1.02E-59
DPF2	2.32198444	3.15E-15	4.56E-15
TIGD3	3.99481193	0.80632827	0.81427261
RP11-867O8.	NA	NA	NA
SLC25A45	1.49805447	0.00093579	0.00106956
FRMD8	2.53516911	5.23E-128	6.40E-127
NEAT1	2.26794598	9.77E-97	4.95E-96
CMB9-22P13	1.87256809	5.91E-05	6.98E-05
CMB9-22P13	NA	NA	NA
MALAT1	2.04562202	3.35E-09	4.34E-09
AP000769.7	NA	1.62E-78	5.89E-78
AP000769.1	2.24708171	0.01948992	0.0213673
SCYL1	2.24708171	3.36E-07	4.17E-07
LTBP3	1.87256809	1.15E-50	2.76E-50
SSSCA1	1.62788586	1.74E-15	2.53E-15
FAM89B	1.93865873	5.90E-09	7.62E-09
EHBP1L1	2.99610895	2.26E-46	5.10E-46
SIPA1	2.33350793	9.95E-37	1.97E-36
KCNK7	0.74902724	1.87E-06	2.29E-06
MAP3K11	2.32900657	6.86E-82	2.63E-81
PCNXL3	1.74773022	6.89E-74	2.33E-73
RELA	2.08063121	0.80291352	0.81100479
KAT5	1.38281952	4.70E-128	5.77E-127
RNASEH2C	1.89944706	1.38E-69	4.39E-69

RP11-770G2:	NA	NA	NA
AP5B1	1.94747082	3.76E-16	5.53E-16
OVOL1	NA	0.13377061	0.14180592
OVOL1-AS1	NA	9.03E-88	3.85E-87
RP11-770G2:	NA	NA	NA
CFL1	1.20268644	1.12E-143	5.52E-142
SNX32	NA	7.97E-22	1.28E-21
MUS81	1.53747696	3.71E-16	5.45E-16
EFEMP2	NA	6.52E-62	1.85E-61
CTSW	NA	0.03441288	0.03737091
FIBP	1.42101167	3.42E-134	5.82E-133
CCDC85B	1.89237864	4.51E-21	7.15E-21
FOSL1	1.42315175	1.10E-40	2.30E-40
C11orf68	1.60019455	0.99264776	0.99330478
DRAP1	1.83625162	7.35E-45	1.63E-44
TSGA10IP	NA	4.89E-34	9.31E-34
SART1	1.35401078	1.31E-90	5.90E-90
EIF1AD	1.51274128	1.03E-20	1.63E-20
BANF1	1.56502049	2.23E-54	5.66E-54
CST6	NA	NA	NA
CATSPER1	NA	3.22E-26	5.50E-26
GAL3ST3	NA	NA	NA
SF3B2	1.50865392	1.29E-81	4.94E-81
RP11-1167A:	NA	5.92E-17	8.81E-17
PACS1	2.57478113	8.04E-110	5.39E-109
RP11-755F1(	Inf	1.34E-15	1.96E-15
KLC2	1.30094204	1.71E-44	3.76E-44
RP11-755F1(	NA	NA	NA
RP11-867G2:	NA	NA	NA
RP11-867G2:	NA	8.82E-20	1.37E-19
RAB1B	2.10993588	1.22E-66	3.73E-66
RP11-867G2:	NA	3.73E-51	9.02E-51
RP11-867G2:	Inf	0.00029331	0.00034001
CNIH2	1.87256809	0.00051623	0.00059388
YIF1A	1.55771151	6.36E-111	4.39E-110
TMEM151A	1.49805447	7.93E-31	1.44E-30
RP11-867G2:	NA	NA	NA
CD248	Inf	7.36E-100	3.95E-99
RIN1	1.49805447	7.03E-54	1.78E-53
RP11-867G2:	NA	NA	NA
BRMS1	1.44623498	4.94E-77	1.75E-76
B4GAT1	1.21716926	3.78E-37	7.53E-37
RP11-867G2:	5.24319066	1.28E-10	1.71E-10
SLC29A2	1.93498703	8.58E-31	1.56E-30
NPAS4	NA	NA	NA

MRPL11	1.93331807	4.18E-52	1.03E-51
PELI3	1.87256809	2.70E-12	3.72E-12
CTD-3074O7.	1.31079767	6.67E-28	1.17E-27
DPP3	1.14137484	3.90E-92	1.81E-91
CTD-3074O7.	NA	NA	NA
BBS1	0.74902724	2.07E-33	3.91E-33
ZDHHC24	1.56614786	2.13E-53	5.34E-53
ACTN3	NA	NA	NA
CTD-3074O7.	NA	NA	NA
CTSF	1.49805447	6.42E-16	9.40E-16
CCDC87	NA	1.59E-80	5.96E-80
CCS	0.8688716	5.44E-87	2.28E-86
RBM14	1.36361369	9.89E-68	3.06E-67
RBM14-RBM	NA	2.91E-46	6.56E-46
RBM4	2.27383268	4.66E-94	2.24E-93
RBM4B	1.23369192	3.29E-57	8.68E-57
RP11-658F2.	1.19844358	0.41170568	0.42448082
SPTBN2	2.56809339	9.36E-09	1.20E-08
C11orf80	1.12354086	9.19E-36	1.80E-35
RCE1	0.80350195	5.21E-92	2.41E-91
PC	2.62159533	3.45E-12	4.75E-12
LRFN4	2.30822679	1.12E-124	1.19E-123
C11orf86	NA	NA	NA
SYT12	NA	3.03E-13	4.24E-13
RHOD	NA	NA	NA
KDM2A	3.7057137	1.13E-138	2.74E-137
ADRBK1	1.75231143	5.59E-10	7.37E-10
ANKRD13D	2.80885214	8.79E-71	2.84E-70
SSH3	6.17947471	1.92E-08	2.46E-08
RAD9A	1.88053647	2.46E-36	4.84E-36
POLD4	1.59168288	2.71E-124	2.83E-123
AP003419.11	NA	NA	NA
CTC-1337H24	NA	2.27E-101	1.26E-100
CLCF1	Inf	1.20E-32	2.23E-32
CTC-1337H24	0	1.20E-31	2.21E-31
PPP1CA	1.22197797	4.95E-139	1.25E-137
TBC1D10C	1.16515348	3.72E-85	1.51E-84
CARNS1	Inf	1.68E-21	2.68E-21
RPS6KB2	2.14772096	1.04E-17	1.57E-17
AP003419.16	1.49805447	1.13E-09	1.48E-09
CORO1B	0.99176755	4.83E-145	3.28E-143
PTPRCAP	NA	1.09E-73	3.67E-73
GPR152	NA	NA	NA
CABP4	NA	NA	NA
TMEM134	1.9157812	4.09E-25	6.90E-25

AIP	1.09613742	1.66E-136	3.29E-135
PITPNM1	1.49805447	9.57E-12	1.30E-11
CDK2AP2	1.63295038	6.97E-112	4.92E-111
CABP2	NA	NA	NA
GSTP1	1.35927532	9.56E-133	1.51E-131
C11orf72	NA	8.66E-15	1.24E-14
RP11-655M1	NA	NA	NA
NDUFV1	1.20014591	5.99E-138	1.35E-136
RP11-655M1	Inf	2.06E-139	5.41E-138
NUDT8	1.39319066	4.67E-78	1.68E-77
TBX10	NA	NA	NA
ACY3	NA	1.19E-43	2.58E-43
ALDH3B2	NA	NA	NA
RP11-119D9.	NA	NA	NA
RP5-903G2.2	1.12354086	9.33E-11	1.24E-10
UNC93B1	1.46400778	7.23E-16	1.06E-15
ALDH3B1	0.74902724	1.16E-46	2.63E-46
RP5-901A4.1	0	7.89E-13	1.09E-12
NDUFS8	1.66979276	3.61E-20	5.64E-20
TCIRG1	1.39105058	3.32E-99	1.76E-98
RP11-802E16	2.99610895	2.13E-52	5.27E-52
CHKA	1.98129785	4.26E-36	8.37E-36
CTD-2655K5.	NA	3.44E-77	1.22E-76
KMT5B	1.45399405	5.50E-05	6.50E-05
C11orf24	1.93018557	2.68E-81	1.02E-80
LRP5	2.24708171	8.55E-14	1.21E-13
PPP6R3	2.54134241	6.59E-80	2.45E-79
CTD-2007L18	NA	NA	NA
GAL	NA	NA	NA
MTL5	2.43433852	7.50E-40	1.55E-39
CPT1A	3.27699416	2.50E-41	5.28E-41
RP11-757G1.	NA	8.98E-81	3.38E-80
MRPL21	1.4621331	3.51E-64	1.03E-63
IGHMBP2	1.78614187	0.23794334	0.24923603
RP11-757G1.	NA	NA	NA
MRGPRD	NA	NA	NA
RP11-554A1:	NA	NA	NA
MRGPRF	NA	NA	NA
MRGPRF-AS	NA	NA	NA
TPCN2	2.7464332	0.00214184	0.0024195
RP11-554A1:	0	7.34E-34	1.40E-33
RP11-554A1:	NA	NA	NA
RP11-554A1:	NA	1.52E-128	1.91E-127
MYEOV	Inf	0.94281594	0.9457352
RP11-211G2:	NA	NA	NA

RP11-211G2:	NA	NA	NA
AP000439.1	NA	NA	NA
AP000439.2	NA	NA	NA
AP000439.5	NA	NA	NA
AP000439.3	NA	NA	NA
CCND1	0.74902724	1.04E-42	2.24E-42
ORAOV1	1.42315175	3.18E-10	4.21E-10
AP001888.1	NA	NA	NA
FGF19	NA	NA	NA
FGF4	NA	NA	NA
FGF3	NA	NA	NA
RP11-643C9.	NA	NA	NA
RP11-643C9.	NA	NA	NA
RP11-626H1:	NA	NA	NA
RP11-626H1:	NA	NA	NA
RP11-626H1:	NA	NA	NA
ANO1-AS2	NA	NA	NA
ANO1	NA	NA	NA
ANO1-AS1	NA	NA	NA
FADD	1.16953376	2.69E-82	1.04E-81
RP11-805J14	NA	2.05E-36	4.05E-36
PPFIA1	1.97470817	1.45E-22	2.35E-22
AP000487.6	NA	NA	NA
CTA-797E19.	NA	NA	NA
AP000487.4	NA	NA	NA
AP000487.5	0.74902724	0.50064659	0.51381629
CTTN	Inf	2.88E-70	9.24E-70
SHANK2	NA	NA	NA
SHANK2-AS1	NA	NA	NA
SHANK2-AS2	NA	NA	NA
SHANK2-AS3	NA	NA	NA
DHCR7	1.83690013	6.26E-28	1.10E-27
RP11-660L16	1.69716298	2.05E-22	3.32E-22
NADSYN1	1.90823606	1.02E-11	1.39E-11
KRTAP5-7	NA	3.31E-14	4.70E-14
KRTAP5-8	NA	NA	NA
KRTAP5-9	NA	1.21E-72	4.04E-72
KRTAP5-10	NA	1.57E-64	4.63E-64
KRTAP5-11	NA	NA	NA
RP11-167J8.:	NA	NA	NA
FAM86C1	1.61632193	0.25281305	0.26438404
DEFB108B	NA	NA	NA
RP11-849H4.	2.04280156	1.08E-14	1.55E-14
CTD-2313N1:	NA	NA	NA
RNF121	1.33160398	6.13E-39	1.25E-38

IL18BP	2.99610895	3.99E-06	4.85E-06
NUMA1	1.85053788	1.11E-24	1.85E-24
RP11-849H4.	NA	2.72E-18	4.13E-18
LRTOMT	4.49416342	2.20E-21	3.50E-21
LAMTOR1	1.79244826	7.91E-34	1.50E-33
ANAPC15	2.08379896	1.66E-110	1.13E-109
FOLR3	NA	NA	NA
RP11-807H2	NA	NA	NA
FOLR1	NA	NA	NA
FOLR2	0	3.15E-74	1.07E-73
INPPL1	1.71206226	9.50E-19	1.45E-18
PHOX2A	NA	NA	NA
CLPB	1.34824903	3.50E-106	2.14E-105
AP000593.7	NA	NA	NA
RP11-45F15.	NA	NA	NA
RP11-45F15.	NA	NA	NA
RP11-7N14.1	NA	NA	NA
LINC01537	NA	NA	NA
PDE2A	Inf	0.18198503	0.19169555
RP11-169D4.	Inf	0.72429443	0.73412758
RP11-31L22.	NA	NA	NA
ARAP1	1.9661965	4.83E-67	1.48E-66
ARAP1-AS1	NA	5.87E-14	8.31E-14
ARAP1-AS2	NA	NA	NA
STARD10	2.19542466	1.16E-20	1.82E-20
ATG16L2	3.37062257	8.76E-15	1.26E-14
FCHSD2	1.87256809	0.00273683	0.00308204
RP11-800A3.	NA	NA	NA
RP11-800A3.	NA	1.99E-52	4.91E-52
P2RY2	1.23589494	1.42E-93	6.74E-93
P2RY6	1.41482923	0.75710873	0.76623154
RP11-800A3.	NA	1.70E-11	2.30E-11
ARHGEF17	Inf	1.76E-48	4.09E-48
RELT	2.72373541	0.17267452	0.18212087
RP11-809N8.	NA	NA	NA
FAM168A	0.74902724	2.94E-78	1.06E-77
RP11-809N8.	Inf	6.21E-70	1.99E-69
PLEKHB1	NA	3.53E-127	4.18E-126
RAB6A	2.49095105	7.57E-38	1.52E-37
RP11-456I15	NA	NA	NA
RP11-707G1	NA	4.85E-31	8.87E-31
MRPL48	1.2367659	3.30E-131	4.76E-130
COA4	1.91883668	4.63E-23	7.57E-23
PAAF1	1.09097445	7.71E-67	2.35E-66
DNAJB13	NA	3.63E-44	7.94E-44

RP11-167N4.	NA	NA	NA
UCP2	1.29451446	1.09E-55	2.83E-55
UCP3	NA	1.75E-07	2.18E-07
C2CD3	4.86867704	6.89E-09	8.88E-09
PPME1	1.24837873	1.97E-19	3.05E-19
P4HA3	NA	NA	NA
RP11-632K5.	NA	3.98E-26	6.79E-26
PGM2L1	2.0598249	0.1605825	0.16955464
RP11-702H2:	NA	NA	NA
KCNE3	1.68531128	0.26899222	0.28089891
RP11-702H2:	NA	4.35E-104	2.55E-103
LIPT2	2.7464332	0.18978117	0.19976849
AP001372.2	0.37451362	0.00118154	0.0013462
POLD3	1.30475712	6.34E-44	1.38E-43
CHRDL2	NA	NA	NA
RNF169	2.12723735	1.82E-91	8.34E-91
XRRA1	2.66840953	1.79E-26	3.06E-26
SPCS2	0.857757	2.22E-135	4.05E-134
NEU3	2.20546909	2.18E-96	1.10E-95
OR2AT4	NA	NA	NA
CTD-2562J17	NA	NA	NA
SLCO2B1	NA	NA	NA
CTD-2562J17	NA	NA	NA
TPBGL	NA	3.59E-37	7.16E-37
CTD-2562J17	0	3.60E-55	9.23E-55
ARRB1	1.79231518	2.84E-22	4.59E-22
CTD-2562J17	NA	NA	NA
RPS3	1.8853092	4.09E-17	6.11E-17
KLHL35	Inf	1.59E-71	5.18E-71
GDPD5	1.44455253	1.83E-100	9.90E-100
SERPINH1	1.09233139	5.60E-71	1.81E-70
CTD-2530H1:	NA	NA	NA
MAP6	NA	NA	NA
MOGAT2	NA	NA	NA
CTD-2530H1:	NA	NA	NA
DGAT2	0.74902724	1.64E-110	1.12E-109
RP11-535A1!	NA	NA	NA
RP11-535A1!	NA	NA	NA
UVRAG	2.30469919	0.00345336	0.00388172
CTD-2011F1:	NA	NA	NA
WNT11	Inf	1.27E-19	1.96E-19
RP11-619A14	NA	NA	NA
RP11-619A14	NA	0.00058361	0.00067037
PRKRIR	2.5507414	5.96E-45	1.32E-44
RP11-111M2	4.49416342	5.40E-97	2.76E-96

RP11-111M2	NA	8.63E-27	1.49E-26
RP11-111M2	Inf	1.05E-123	1.07E-122
RP11-111M2	1.78491597	0.00115102	0.00131209
EMSY	2.12224384	0.02558544	0.02792342
RP11-672A2.	NA	NA	NA
RP11-672A2.	NA	NA	NA
RP11-672A2.	NA	NA	NA
AP001189.4	NA	NA	NA
LRRC32	Inf	5.59E-60	1.54E-59
RP11-672A2.	NA	NA	NA
RP11-672A2.	NA	NA	NA
RP11-21L23.4	NA	NA	NA
TSKU	2.02237354	0.60592504	0.61850515
RP11-21L23.1	Inf	2.05E-123	2.07E-122
ACER3	1.46924573	5.61E-20	8.74E-20
CTD-2547H18	NA	NA	NA
B3GNT6	NA	6.78E-49	1.59E-48
CAPN5	1.12354086	1.71E-07	2.14E-07
OMP	NA	NA	NA
MYO7A	NA	3.34E-31	6.11E-31
GDPD4	NA	NA	NA
PAK1	2.00415396	3.60E-16	5.29E-16
RP11-739B23	NA	NA	NA
CLNS1A	1.28807613	8.10E-90	3.58E-89
AQP11	Inf	6.28E-16	9.20E-16
RSF1	1.08359274	8.87E-104	5.17E-103
AAMDC	1.25729572	4.03E-97	2.06E-96
RP11-91P24.	NA	8.32E-107	5.16E-106
RP11-91P24.	NA	NA	NA
RP11-91P24.	NA	NA	NA
INTS4	1.01118677	1.09E-05	1.31E-05
KCTD14	NA	5.17E-51	1.25E-50
RP11-7I15.3	NA	NA	NA
NDUFC2-KCT	NA	NA	NA
RP11-7I15.4	NA	NA	NA
THRSP	NA	NA	NA
NDUFC2	1.64922179	0.4829956	0.49626245
ALG8	1.20335523	1.31E-92	6.15E-92
KCTD21-AS1	2.49675746	1.23E-53	3.10E-53
KCTD21	7.49027237	3.12E-12	4.30E-12
USP35	Inf	4.69E-65	1.39E-64
GAB2	3.18336576	1.03E-73	3.48E-73
RP11-452H21	NA	NA	NA
RP11-452H21	NA	NA	NA
RP11-452H21	Inf	8.40E-118	6.94E-117

NARS2	1.18748221	3.02E-06	3.68E-06
RP11-843A2:	NA	NA	NA
TENM4	NA	NA	NA
RP11-673F1{	NA	NA	NA
CTD-2337I7.1	NA	NA	NA
RP11-802F5.	NA	NA	NA
RP11-258O1:	NA	NA	NA
CTD-2555I5.1	NA	NA	NA
RP11-686G2:	NA	NA	NA
MIR4300HG	NA	0.38801892	0.40076511
RP11-876F1⁄	NA	NA	NA
RP11-179A1(	NA	NA	NA
FAM181B	NA	1.22E-32	2.27E-32
RP11-718B1:	NA	NA	NA
PRCP	1.3618677	3.99E-136	7.65E-135
DDIAS	1.2733463	7.08E-20	1.10E-19
RAB30	0.74902724	4.94E-12	6.76E-12
RAB30-AS1	2.02514772	0.61520283	0.62758715
RP11-113K21	NA	1.09E-66	3.33E-66
PCF11	1.56740885	1.54E-42	3.31E-42
PCF11-AS1	Inf	4.20E-33	7.87E-33
ANKRD42	3.37062257	0.12639063	0.1342572
RP11-727A2:	NA	NA	NA
CCDC90B	1.9896036	1.68E-81	6.42E-81
RP11-727A2:	NA	1.98E-32	3.69E-32
DLG2	0	0.30311673	0.31544448
RP11-160H1:	NA	NA	NA
CTD-2537O9.	NA	NA	NA
AP000857.3	NA	NA	NA
TMEM126B	1.73062914	6.49E-16	9.50E-16
TMEM126A	1.90446673	1.02E-15	1.49E-15
CREBZF	4.11964981	2.42E-32	4.49E-32
CCDC89	NA	NA	NA
SYTL2	NA	8.11E-24	1.34E-23
CCDC83	NA	NA	NA
RP11-90K17.	NA	NA	NA
PICALM	1.52579622	2.62E-24	4.35E-24
RP11-12D16.	NA	0.05255704	0.05667775
EED	1.31079767	1.24E-103	7.17E-103
C11orf73	2.07500789	1.82E-72	6.04E-72
CCDC81	NA	3.65E-69	1.15E-68
RP11-317J19	NA	NA	NA
ME3	NA	NA	NA
CTD-2005H7.	NA	NA	NA
CTD-2005H7.	NA	NA	NA

PRSS23	NA	1.09E-36	2.17E-36
AP000654.4	NA	NA	NA
RP11-736K2(	Inf	2.94E-15	4.27E-15
FZD4	0.89883268	1.92E-23	3.15E-23
RP11-736K2(	NA	NA	NA
TMEM135	2.46108949	0.33887415	0.35158726
RP11-720D4.	NA	NA	NA
RP11-164N3.	NA	NA	NA
RP11-164N3.	NA	NA	NA
RP11-164N3.	NA	NA	NA
RAB38	1.44455253	1.42E-86	5.92E-86
CTSC	1.50093535	0.01303053	0.01437257
GRM5-AS1	NA	NA	NA
GRM5	NA	NA	NA
TYR	NA	NA	NA
NOX4	NA	NA	NA
RP11-643G5.	NA	NA	NA
TRIM77	NA	NA	NA
RP11-313I2.1	NA	NA	NA
TRIM49	NA	NA	NA
TRIM64B	NA	NA	NA
TRIM49D1	NA	NA	NA
TRIM49D2	NA	NA	NA
TRIM64	NA	NA	NA
TRIM49C	NA	NA	NA
UBTFL1	NA	NA	NA
NAALAD2	NA	NA	NA
CHORDC1	1.47450016	2.77E-32	5.15E-32
RP11-699F1(	NA	NA	NA
RP11-201M2	NA	NA	NA
FAT3	NA	NA	NA
RP11-675M1	NA	NA	NA
RP11-203F8.	NA	NA	NA
RP11-676F2(	NA	NA	NA
RP11-676F2(	NA	NA	NA
RP11-676F2(	NA	NA	NA
MTNR1B	NA	NA	NA
SLC36A4	1.12354086	8.96E-45	1.98E-44
RP11-755E2:	NA	NA	NA
CCDC67	NA	NA	NA
SMCO4	2.48843493	1.00E-118	8.50E-118
CEP295	1.74773022	3.63E-35	7.04E-35
SCARNA9	NA	NA	NA
TAF1D	1.74388906	3.07E-30	5.55E-30
C11orf54	1.20996708	9.96E-12	1.36E-11

MED17	1.57054098	3.20E-09	4.15E-09
VSTM5	NA	NA	NA
HEPHL1	NA	NA	NA
PANX1	3.49546044	0.83559782	0.8426579
RP11-680H2(	NA	NA	NA
IZUMO1R	NA	NA	NA
GPR83	0	1.05E-125	1.16E-124
MRE11A	1.18115834	8.40E-122	7.98E-121
RP11-685N1(	NA	1.08E-14	1.55E-14
ANKRD49	1.44811933	4.62E-50	1.10E-49
C11orf97	NA	NA	NA
PIWIL4	0	0.00181919	0.00205901
FUT4	1.82575389	4.13E-06	5.01E-06
RP11-867G2.	NA	9.85E-09	1.27E-08
RP11-867G2.	NA	NA	NA
RP11-867G2.	NA	NA	NA
AMOTL1	2.59662776	1.72E-56	4.49E-56
RP11-856F16	NA	NA	NA
CWC15	1.59551155	3.33E-66	1.01E-65
KDM4D	NA	6.54E-21	1.03E-20
KDM4E	NA	NA	NA
SRSF8	2.03485733	1.70E-74	5.81E-74
ENDOD1	1.64785992	1.75E-103	1.01E-102
RP11-712B9.	NA	NA	NA
SESN3	NA	0.00458856	0.00513735
RP11-338H14	NA	NA	NA
RP11-644L4.	NA	NA	NA
FAM76B	2.24708171	1.16E-63	3.35E-63
CEP57	1.58127972	4.18E-109	2.75E-108
MTMR2	1.87256809	3.65E-05	4.32E-05
MAML2	NA	8.75E-14	1.23E-13
CCDC82	1.26398346	3.16E-83	1.24E-82
JRKL	3.37062257	2.49E-129	3.24E-128
JRKL-AS1	NA	NA	NA
RP11-49K4.2	NA	NA	NA
RP11-360K13	NA	NA	NA
RP11-882G5.	NA	NA	NA
RP11-379J13	NA	NA	NA
RP11-115E19	NA	NA	NA
RP11-99C10.	NA	NA	NA
CNTN5	NA	NA	NA
CTD-2383M3	NA	4.22E-12	5.78E-12
ARHGAP42	Inf	2.62E-12	3.61E-12
TMEM133	NA	3.08E-82	1.19E-81
PGR	NA	NA	NA

TRPC6	NA	NA	NA
ANGPTL5	NA	NA	NA
CEP126	NA	1.03E-120	9.42E-120
C11orf70	NA	5.14E-08	6.50E-08
RP11-732A2:	NA	NA	NA
RP11-732A2:	NA	8.91E-58	2.37E-57
YAP1	NA	NA	NA
RP11-864G5.	NA	NA	NA
BIRC3	Inf	1.69E-122	1.65E-121
BIRC2	2.24708171	3.28E-08	4.17E-08
TMEM123	1.94972919	6.93E-112	4.89E-111
RP11-315O6.	0	7.13E-26	1.21E-25
RP11-315O6.	NA	NA	NA
MMP7	NA	NA	NA
MMP20	NA	NA	NA
RP11-817J15	NA	NA	NA
RP11-817J15	NA	NA	NA
MMP27	NA	NA	NA
MMP8	NA	NA	NA
MMP10	NA	NA	NA
MMP1	NA	NA	NA
MMP3	NA	NA	NA
MMP12	NA	NA	NA
MMP13	NA	NA	NA
DCUN1D5	1.46626552	7.18E-73	2.39E-72
DYNC2H1	0.44941634	0.00326251	0.00366924
RP11-563P16	NA	NA	NA
PDGFD	1.49805447	1.43E-36	2.82E-36
RP11-617B3.	NA	NA	NA
DDI1	NA	NA	NA
RP11-886D15	NA	NA	NA
CASP4	1.40918684	1.81E-60	5.00E-60
CASP5	NA	NA	NA
CASP1	Inf	2.56E-98	1.34E-97
CARD16	0	7.58E-50	1.80E-49
CARD17	NA	NA	NA
CARD18	NA	NA	NA
RP11-94P11.	NA	NA	NA
GRIA4	NA	NA	NA
MSANTD4	2.39688716	3.16E-95	1.56E-94
KBTBD3	0.74902724	7.31E-81	2.76E-80
AASDHPPT	1.01653696	2.76E-128	3.43E-127
RP11-677I18	NA	1.75E-81	6.68E-81
RP11-677I18	NA	NA	NA
GUCY1A2	0.74902724	7.02E-08	8.85E-08

RP11-819C21	3.2457847	8.72E-59	2.35E-58
CWF19L2	0.99870298	3.07E-32	5.70E-32
ALKBH8	2.24708171	7.67E-27	1.32E-26
ELMOD1	2.24708171	6.67E-40	1.38E-39
SLN	NA	0.00300849	0.00338627
SLC35F2	1.14850843	1.91E-44	4.19E-44
RAB39A	0.74902724	1.16E-33	2.20E-33
CUL5	1.53747696	0.02517696	0.02748919
RP11-144G7.	NA	NA	NA
ACAT1	0.83375883	1.01E-143	5.02E-142
NPAT	1.61328943	0.00056308	0.0006472
ATM	2.9079881	9.38E-10	1.23E-09
C11orf65	0	5.89E-06	7.13E-06
KDELC2	3.89494163	1.04E-34	2.00E-34
EXPH5	1.04863813	2.02E-58	5.42E-58
DDX10	1.46924573	5.82E-26	9.92E-26
RP11-25I9.3	NA	NA	NA
RP11-25I9.2	NA	NA	NA
RP11-708B6.	NA	NA	NA
C11orf87	NA	NA	NA
RP11-262A12	NA	NA	NA
ZC3H12C	1.79766537	1.40E-13	1.96E-13
RDX	1.18373054	7.37E-76	2.57E-75
RP11-347E1(	NA	NA	NA
FDX1	2.3002913	3.52E-85	1.43E-84
ARHGAP20	NA	NA	NA
RP11-89C3.3	NA	NA	NA
RP11-89C3.4	NA	NA	NA
C11orf53	NA	NA	NA
COLCA1	NA	1.28E-142	5.08E-141
COLCA2	NA	NA	NA
POU2AF1	NA	NA	NA
RP11-794P6.	NA	NA	NA
RP11-794P6.	NA	NA	NA
BTG4	NA	NA	NA
RP11-794P6.	NA	NA	NA
C11orf88	NA	NA	NA
LAYN	0.74902724	0.00075551	0.00086515
SIK2	5.05593385	2.57E-136	5.05E-135
PPP2R1B	2.58414397	2.78E-12	3.83E-12
RP11-108O1(	NA	NA	NA
ALG9	2.24708171	5.99E-21	9.49E-21
RP11-108O1(	NA	NA	NA
FDXACB1	2.69649805	4.48E-05	5.30E-05
C11orf1	1.67639429	6.32E-14	8.94E-14

CRYAB	NA	2.28E-71	7.43E-71
HSPB2	NA	NA	NA
HSPB2-C11or	NA	NA	NA
C11orf52	NA	NA	NA
DIXDC1	1.06440713	1.02E-59	2.78E-59
DLAT	1.41634241	1.23E-15	1.80E-15
PIH1D2	2.24708171	0.10548615	0.11235424
C11orf57	1.65196418	4.75E-23	7.76E-23
TIMM8B	1.78782414	0.00012701	0.00014852
SDHD	1.3334331	6.00E-33	1.13E-32
AP002884.2	NA	NA	NA
IL18	1.08192823	4.43E-65	1.32E-64
RP11-356J5.4	NA	NA	NA
TEX12	Inf	2.92E-127	3.48E-126
BCO2	1.49805447	0.00047915	0.00055206
PTS	1.50656615	0.0804042	0.08614498
PLET1	NA	NA	NA
RP11-356J5.1	NA	5.77E-85	2.34E-84
RP11-356J5.1	NA	0.15153795	0.16016321
RP11-65M17	NA	1.69E-55	4.37E-55
RP11-65M17	NA	NA	NA
RP11-27G22.	NA	NA	NA
RP11-629G1	NA	NA	NA
NCAM1	NA	NA	NA
NCAM1-AS1	NA	NA	NA
RP11-839D1	NA	NA	NA
TTC12	NA	NA	NA
ANKK1	NA	NA	NA
RP11-159N1	NA	NA	NA
DRD2	NA	NA	NA
TMPRSS5	NA	3.90E-35	7.56E-35
ZW10	1.49805447	2.71E-07	3.37E-07
RP11-667M1	NA	5.28E-112	3.74E-111
CLDN25	NA	NA	NA
USP28	2.38752432	9.33E-25	1.56E-24
RP11-667M1	NA	NA	NA
HTR3B	NA	NA	NA
HTR3A	NA	NA	NA
ZBTB16	0.74902724	9.58E-46	2.15E-45
RP11-64D24.	NA	NA	NA
NNMT	NA	5.28E-27	9.12E-27
RP11-64D24.	NA	NA	NA
C11orf71	2.3540856	3.11E-36	6.13E-36
C11orf71	NA	NA	NA
RBM7	1.08479807	2.44E-110	1.65E-109

RP11-212D1?	NA	NA	NA
REXO2	1.3018237	3.27E-73	1.10E-72
NXPE1	NA	NA	NA
NXPE4	NA	NA	NA
NXPE2	NA	NA	NA
CADM1	NA	NA	NA
AP000462.3	NA	NA	NA
AP000462.2	NA	NA	NA
RP11-136I14	NA	NA	NA
RP11-136I14	NA	NA	NA
AP000997.1	NA	NA	NA
AP000997.2	NA	NA	NA
AP000797.3	NA	NA	NA
LINC00900	NA	NA	NA
AP000797.4	NA	NA	NA
AP001891.1	NA	NA	NA
AP000770.1	NA	NA	NA
BUD13	2.15345331	1.11E-12	1.54E-12
AP006216.1C	NA	1.92E-40	4.00E-40
ZPR1	1.49206226	5.42E-59	1.46E-58
APOA5	NA	NA	NA
AP006216.5	NA	NA	NA
APOA4	NA	NA	NA
APOC3	NA	NA	NA
APOA1	Inf	1.40E-11	1.90E-11
APOA1-AS	NA	NA	NA
SIK3	2.62159533	2.36E-73	7.96E-73
AP000936.4	NA	NA	NA
PAFAH1B2	2.28549337	3.84E-97	1.96E-96
SIDT2	1.99740597	9.98E-15	1.43E-14
TAGLN	0.99870298	0.00195437	0.00221062
PCSK7	2.82965845	2.41E-116	1.91E-115
RNF214	1.37978702	2.09E-108	1.35E-107
BACE1	1.46238651	0.00124249	0.00141395
BACE1-AS	0	1.63E-104	9.60E-104
CEP164	1.79454442	0.04133994	0.04479152
AP000892.4	NA	NA	NA
DSCAML1	NA	NA	NA
RP11-35P15.	NA	NA	NA
FXYD2	NA	NA	NA
RP11-728F11	NA	NA	NA
FXYD6-FXYD2	NA	NA	NA
RP11-728F11	NA	NA	NA
FXYD6	NA	NA	NA
TMPRSS13	NA	1.06E-91	4.87E-91

IL10RA	Inf	7.64E-70	2.44E-69
TMPRSS4-AS	NA	NA	NA
AP002962.1	NA	NA	NA
TMPRSS4	NA	1.18E-37	2.37E-37
SCN4B	NA	0.10090371	0.1076504
SCN2B	NA	NA	NA
JAML	Inf	4.86E-12	6.67E-12
MPZL3	1.87256809	0.00015715	0.00018329
MPZL2	NA	NA	NA
CD3E	Inf	4.90E-17	7.30E-17
CD3D	0.4766537	1.51E-63	4.37E-63
CD3G	NA	NA	NA
UBE4A	1.21342412	0.00362112	0.00406675
RP11-770J1.5	0.74902724	4.18E-15	6.04E-15
ATP5L	1.81536476	0.00862739	0.0095759
RP11-770J1.4	Inf	3.13E-59	8.50E-59
KMT2A	3.72016861	5.67E-79	2.08E-78
RP11-770J1.3	NA	NA	NA
TTC36	NA	0.03359203	0.03649917
TMEM25	NA	0.35428354	0.36711286
IFT46	1.43563554	2.51E-21	4.00E-21
ARCN1	1.57607815	1.05E-15	1.53E-15
PHLDB1	2.39688716	2.93E-14	4.18E-14
AP002954.3	NA	3.17E-21	5.03E-21
TREH	Inf	2.23E-42	4.78E-42
AP002954.4	NA	NA	NA
DDX6	2.73394942	8.22E-113	5.97E-112
RP11-158I9.8	1.31079767	0.0159046	0.01749265
CXCR5	NA	NA	NA
RP11-158I9.5	NA	NA	NA
BCL9L	1.34824903	0.00047498	0.00054729
UPK2	NA	3.13E-12	4.30E-12
FOXR1	Inf	7.86E-24	1.30E-23
RP11-110I1.1	7.49027237	0.00010315	0.00012087
CCDC84	2.34071012	7.58E-17	1.13E-16
RP11-110I1.1	NA	2.59E-25	4.38E-25
RPS25	2.01392894	9.26E-139	2.26E-137
TRAPPC4	1.23769741	5.51E-59	1.49E-58
SLC37A4	1.37017178	7.40E-69	2.33E-68
RP11-110I1.6	NA	8.43E-47	1.92E-46
HYOU1	1.72276265	0.20960944	0.22016758
RP11-110I1.1	1.12354086	2.47E-31	4.53E-31
RP11-110I1.1	3.74513619	2.50E-88	1.08E-87
VPS11	2.3540856	0.0005213	0.00059956
HMBS	1.53249251	1.07E-77	3.85E-77

H2AFX	1.80918887	6.49E-11	8.69E-11
DPAGT1	1.11062659	1.00E-30	1.83E-30
C2CD2L	1.02991245	3.33E-10	4.40E-10
HINFP	2.19357977	1.17E-23	1.93E-23
ABCG4	NA	1.09E-129	1.45E-128
NLRX1	2.17898833	0.14703129	0.1555541
PDZD3	NA	4.22E-12	5.78E-12
CCDC153	3.74513619	9.72E-34	1.84E-33
CBL	1.83095547	1.34E-17	2.01E-17
MCAM	1.3732166	1.15E-05	1.39E-05
RNF26	1.73555092	1.48E-64	4.36E-64
RP11-334E6.	0	1.11E-118	9.41E-118
C1QTNF5	NA	NA	NA
MFRP	NA	NA	NA
USP2	2.54669261	8.65E-19	1.33E-18
RP11-334E6.	NA	4.18E-08	5.30E-08
USP2-AS1	2.69649805	1.23E-41	2.60E-41
THY1	NA	NA	NA
RP11-196E1.	Inf	2.01E-138	4.69E-137
PVRL1	1.10966998	3.76E-89	1.65E-88
CTD-2523D1:	NA	NA	NA
CTD-2523D1:	NA	NA	NA
RP11-831A1(	NA	NA	NA
RP11-831A1(	NA	NA	NA
TRIM29	NA	NA	NA
AP000679.2	NA	NA	NA
OAF	1.69135183	4.12E-13	5.74E-13
POU2F3	NA	0.08314998	0.08898696
RP11-778O1	NA	NA	NA
TMEM136	NA	NA	NA
ARHGEF12	1.49805447	4.13E-10	5.45E-10
GRIK4	NA	NA	NA
RP11-640N1	NA	4.86E-05	5.75E-05
TBCEL	2.24708171	4.72E-16	6.94E-16
TECTA	NA	1.32E-73	4.47E-73
SC5D	1.14557107	6.68E-99	3.53E-98
RP11-730K1	NA	NA	NA
SORL1	0.9192607	5.96E-117	4.81E-116
BLID	NA	NA	NA
MIR100HG	NA	NA	NA
RP11-716H6.	NA	NA	NA
RP11-716H6.	NA	NA	NA
UBASH3B	4.49416342	7.38E-108	4.71E-107
CRTAM	NA	NA	NA
C11orf63	1.49805447	3.71E-26	6.34E-26

BSX	NA	NA	NA
HSPA8	1.3067188	9.38E-32	1.73E-31
CLMP	NA	NA	NA
CTD-2216M2	NA	NA	NA
RP11-109E1(	NA	NA	NA
GRAMD1B	Inf	1.47E-25	2.48E-25
SCN3B	3.74513619	1.30E-25	2.20E-25
RP11-634B2:	NA	NA	NA
ZNF202	3.2457847	4.48E-35	8.67E-35
OR6X1	NA	NA	NA
OR6M1	NA	NA	NA
TMEM225	NA	NA	NA
OR8D4	NA	NA	NA
OR4D5	NA	NA	NA
OR6T1	NA	NA	NA
OR10S1	NA	NA	NA
OR10G6	NA	NA	NA
OR10G4	NA	NA	NA
OR10G9	NA	NA	NA
OR10G8	NA	NA	NA
OR10G7	NA	NA	NA
VWA5A	0.74902724	0.00620898	0.00692125
OR10D3	NA	NA	NA
OR8G1	NA	NA	NA
OR8G5	NA	NA	NA
OR8D1	NA	NA	NA
OR8D2	NA	NA	NA
OR8B2	NA	NA	NA
OR8B3	NA	NA	NA
OR8B4	NA	NA	NA
OR8B8	NA	NA	NA
OR8B12	NA	NA	NA
OR8A1	NA	NA	NA
PANX3	NA	NA	NA
TBRG1	1.59927437	8.18E-11	1.09E-10
SIAE	NA	4.48E-35	8.68E-35
SPA17	0	3.36E-70	1.08E-69
NRGN	1.33160398	2.48E-31	4.56E-31
RP11-677M1	NA	NA	NA
VSIG2	NA	NA	NA
ESAM	6.74124514	2.22E-17	3.33E-17
RP11-677M1	Inf	2.51E-93	1.19E-92
MSANTD2	Inf	8.70E-110	5.82E-109
AP000866.1	0	0.78675664	0.79521639
RP11-677M1	Inf	3.35E-05	3.98E-05

ROBO3	0.29961089	7.70E-131	1.09E-129
RP11-664I21	NA	NA	NA
ROBO4	NA	NA	NA
RP11-664I21	NA	NA	NA
HEPACAM	NA	NA	NA
HEPN1	NA	NA	NA
CCDC15	5.61770428	9.66E-27	1.66E-26
SLC37A2	1.87256809	0.01953054	0.02141054
TMEM218	1.65091718	4.15E-23	6.79E-23
RP11-687M2	NA	NA	NA
PKNOX2-AS1	NA	NA	NA
PKNOX2	NA	NA	NA
RP11-687M2	NA	NA	NA
FEZ1	NA	NA	NA
AP000708.1	NA	NA	NA
EI24	2.0737531	2.17E-51	5.27E-51
STT3A-AS1	NA	2.33E-16	3.44E-16
STT3A	1.26151956	1.95E-101	1.08E-100
CHEK1	1.523883	7.17E-21	1.13E-20
ACRV1	NA	NA	NA
PATE1	NA	NA	NA
PATE2	NA	0.00256266	0.00288858
PATE3	NA	NA	NA
PATE4	NA	NA	NA
RP11-680F2(	NA	NA	NA
HYLS1	1.04863813	5.79E-93	2.73E-92
PUS3	1.12354086	8.76E-56	2.27E-55
RP11-680F2(	NA	NA	NA
DDX25	NA	NA	NA
RP11-680F2(	NA	NA	NA
RP11-680F2(	NA	NA	NA
RP11-680F2(	NA	NA	NA
CDON	0	9.21E-61	2.56E-60
RP11-680F2(	NA	NA	NA
RP11-673E1	NA	NA	NA
RP11-50B3.2	NA	NA	NA
RPUSD4	2.1614786	2.72E-31	4.99E-31
RP11-50B3.4	NA	1.75E-135	3.23E-134
FAM118B	1.08479807	3.45E-138	7.93E-137
SRPRA	1.74773022	7.72E-11	1.03E-10
FOXRED1	1.34824903	1.48E-78	5.37E-78
TIRAP	7.49027237	5.01E-43	1.08E-42
RP11-712L6.	NA	NA	NA
RP11-712L6.	2.24708171	3.47E-92	1.61E-91
DCPS	1.08107024	2.27E-128	2.83E-127

ST3GAL4-AS	1.68531128	0.00177736	0.00201241
ST3GAL4	2.05426282	1.84E-88	7.97E-88
KIRREL3	Inf	5.35E-99	2.83E-98
KIRREL3-AS1	NA	NA	NA
RP11-115C1(	NA	NA	NA
RP11-688I9.4	NA	NA	NA
KIRREL3-AS2	NA	NA	NA
RP11-688I9.2	NA	NA	NA
KIRREL3-AS3	NA	NA	NA
RP11-168K9.	NA	NA	NA
RP11-168K9.	NA	NA	NA
CTD-2234N1	NA	NA	NA
CTD-2234N1	NA	NA	NA
RP11-480C22	NA	NA	NA
RP11-702B1(	NA	NA	NA
RP11-702B1(	NA	NA	NA
ETS1	NA	1.76E-12	2.43E-12
RP11-1007G	NA	NA	NA
RP11-264E2(	NA	NA	NA
RP11-264E2(	NA	NA	NA
RP11-744N1	NA	4.99E-84	1.99E-83
FLI1	1.42315175	0.00359713	0.00404007
SENCR	1.12354086	3.47E-61	9.71E-61
KCNJ1	NA	NA	NA
KCNJ5	NA	8.32E-59	2.24E-58
C11orf45	1.49805447	1.93E-12	2.66E-12
TP53AIP1	NA	NA	NA
ARHGAP32	4.24448768	1.81E-19	2.79E-19
BARX2	NA	9.42E-102	5.25E-101
RP11-237N1	NA	NA	NA
LINC01395	NA	NA	NA
TMEM45B	Inf	6.20E-84	2.47E-83
NFRKB	1.65226596	3.43E-22	5.53E-22
PRDM10	2.99610895	5.55E-08	7.02E-08
LINC00167	NA	7.87E-05	9.25E-05
APLP2	0.98040214	2.01E-143	9.49E-142
ST14	NA	1.53E-102	8.66E-102
ZBTB44	2.11681611	5.41E-54	1.37E-53
RP11-121M2	Inf	7.25E-11	9.70E-11
ADAMTS8	Inf	4.58E-08	5.80E-08
ADAMTS15	Inf	4.84E-78	1.75E-77
C11orf44	NA	NA	NA
RP11-890B1	NA	8.12E-113	5.91E-112
RP11-890B1	NA	NA	NA
SNX19	NA	NA	NA

AP002856.4	NA	NA	NA
AP002856.6	NA	NA	NA
AP002856.7	NA	NA	NA
AP002856.5	NA	NA	NA
NTM	NA	NA	NA
AP003025.2	NA	NA	NA
RP11-217L21	NA	NA	NA
NTM-AS1	NA	NA	NA
AP004372.1	NA	NA	NA
RP11-697E14	NA	NA	NA
OPCML	NA	NA	NA
RP11-448P19	NA	NA	NA
SPATA19	NA	NA	NA
IGSF9B	NA	1.71E-11	2.31E-11
RP11-713P17	NA	NA	NA
RP11-713P17	Inf	4.33E-05	5.13E-05
RP11-713P17	NA	NA	NA
JAM3	2.0598249	6.11E-25	1.03E-24
NCAPD3	1.71046518	0.00301449	0.00339282
RP11-700F16	NA	5.69E-85	2.31E-84
VPS26B	1.82935498	2.20E-12	3.04E-12
THYN1	1.38957467	6.45E-64	1.88E-63
ACAD8	1.56047341	1.41E-52	3.50E-52
GLB1L3	NA	NA	NA
GLB1L2	1.05331955	5.84E-54	1.48E-53
B3GAT1	NA	NA	NA
AP004550.1	NA	NA	NA
RP11-469N6.	NA	NA	NA
RP11-469N6.	NA	NA	NA
RP11-469N6.	NA	NA	NA
RP11-555G19	NA	NA	NA
AP005135.2	NA	NA	NA
RP11-631M2	NA	NA	NA
TUBB8	NA	5.56E-54	1.41E-53
ZMYND11	2.49675746	5.22E-79	1.91E-78
DIP2C	Inf	1.46E-121	1.36E-120
RP11-490E15	NA	NA	NA
RP11-809C18	NA	NA	NA
PRR26	NA	9.64E-116	7.50E-115
RP11-164C1.	NA	NA	NA
LARP4B	2.4583458	1.97E-127	2.36E-126
RP11-363N22	NA	3.34E-06	4.06E-06
GTPBP4	1.56047341	6.92E-64	2.01E-63
IDI2	NA	2.03E-87	8.61E-87
IDI2-AS1	0.37451362	4.10E-22	6.61E-22

IDI1	1.33950126	1.35E-112	9.76E-112
WDR37	1.49805447	0.34586771	0.35859709
LINC00200	NA	NA	NA
ADARB2	NA	NA	NA
ADARB2-AS1	NA	NA	NA
LINC00700	NA	NA	NA
RP11-69C17.	NA	NA	NA
RP11-69C17.	NA	NA	NA
RP11-69C17.	NA	NA	NA
LINC00701	NA	NA	NA
RP11-446F3.	NA	NA	NA
RP11-526P5.	NA	NA	NA
RP11-526P5.	NA	NA	NA
RP11-118K6.	NA	NA	NA
RP11-118K6.	1.49805447	1.47E-52	3.65E-52
PFKP	1.47464737	4.62E-77	1.64E-76
RP11-298E9.	NA	NA	NA
PITRM1	2.24708171	2.01E-14	2.87E-14
PITRM1-AS1	NA	7.20E-126	8.01E-125
RP11-298E9.	NA	NA	NA
RP11-298E9.	NA	NA	NA
RP11-195B3.	NA	NA	NA
RP11-482E14	NA	NA	NA
RP11-482E14	NA	NA	NA
RP11-184A2.	NA	NA	NA
RP11-184A2.	NA	NA	NA
KLF6	1.96296793	8.07E-13	1.12E-12
RP11-464C19	NA	NA	NA
RP11-464C19	NA	NA	NA
RP11-433J20	NA	NA	NA
RP11-433J20	NA	NA	NA
LINC00702	NA	NA	NA
LINC00703	NA	NA	NA
RP11-34C15.	NA	NA	NA
LINC00704	NA	NA	NA
LINC00705	NA	NA	NA
AKR1E2	NA	1.01E-11	1.37E-11
AKR1C2	NA	NA	NA
AKR1C1	NA	NA	NA
RP11-499O7.	NA	NA	NA
AKR1C3	2.99610895	1.58E-39	3.26E-39
AKR1C3	NA	NA	NA
AKR1C4	NA	NA	NA
RP11-445P17	NA	NA	NA
RP11-445P17	NA	NA	NA

UCN3	NA	NA	NA
TUBAL3	NA	NA	NA
NET1	2.37191958	2.91E-66	8.80E-66
CALML5	NA	1.34E-63	3.87E-63
CALML3-AS1	NA	NA	NA
CALML3	NA	NA	NA
RP11-116G8.	NA	NA	NA
RP13-463N1(	NA	5.76E-36	1.13E-35
RP11-336A1(	NA	NA	NA
RP11-336A1(	NA	NA	NA
ASB13	1.72852439	1.39E-07	1.75E-07
FAM208B	2.48540856	1.10E-76	3.88E-76
RP11-336A1(	NA	NA	NA
GDI2	1.32181277	3.16E-100	1.70E-99
RP11-318E3.	0.74902724	1.60E-40	3.34E-40
ANKRD16	1.32328145	1.25E-44	2.75E-44
FBXO18	1.76333495	0.26032092	0.27201602
RP11-536K7.	NA	6.86E-05	8.08E-05
IL15RA	3.37062257	6.87E-84	2.73E-83
IL2RA	4.11964981	4.72E-68	1.47E-67
RP11-536K7.	NA	NA	NA
RBM17	2.19796517	2.54E-79	9.34E-79
PFKFB3	1.60505837	1.51E-08	1.93E-08
RP11-414H1	NA	NA	NA
RP11-563J2.2	Inf	1.03E-153	2.34E-151
RP11-563J2.3	NA	NA	NA
PRKCQ	1.79766537	0.07035714	0.07553223
RP11-554I8.1	NA	NA	NA
RP11-554I8.2	NA	NA	NA
LINC00706	NA	NA	NA
LINC00707	NA	NA	NA
RP11-799O2	NA	NA	NA
RP11-393K12	NA	NA	NA
SFMBT2	1.28404669	1.82E-26	3.12E-26
RP5-1031D4.	NA	NA	NA
RP11-385N2	NA	NA	NA
ITIH5	NA	8.76E-27	1.51E-26
ITIH2	NA	NA	NA
KIN	1.15094429	9.03E-101	4.94E-100
ATP5C1	1.10171338	6.62E-131	9.38E-130
TAF3	1.94135631	3.75E-20	5.86E-20
GATA3-AS1	NA	NA	NA
RP11-379F12	NA	NA	NA
RP11-379F12	NA	NA	NA
GATA3	1.56614786	1.50E-14	2.15E-14

LINC00708	NA	NA	NA
RP5-1119O2	NA	NA	NA
RP11-543F8.	NA	NA	NA
RP11-428L9.	NA	NA	NA
RP11-428L9.	NA	NA	NA
LINC00709	NA	NA	NA
RP5-1051H1	NA	NA	NA
RP5-933E2.1	NA	NA	NA
RP11-271F1	NA	NA	NA
RP11-271F1	NA	NA	NA
SFTA1P	NA	1.97E-49	4.64E-49
RP1-251M9.	NA	NA	NA
CELF2	2.45987354	1.74E-85	7.12E-85
RP1-251M9.	NA	NA	NA
RP11-397O4	NA	NA	NA
CELF2-AS2	NA	NA	NA
RP3-323N1.2	NA	4.19E-23	6.85E-23
CELF2-AS1	NA	NA	NA
USP6NL	1.28832685	0.00064318	0.00073805
RP11-138I18	NA	NA	NA
RP11-138I18	NA	NA	NA
ECHDC3	1.67429618	8.66E-06	1.04E-05
PROSER2	1.45863199	1.94E-35	3.78E-35
PROSER2-AS	NA	2.15E-24	3.58E-24
UPF2	2.06239006	2.28E-115	1.76E-114
DHTKD1	2.7464332	1.36E-20	2.14E-20
SEC61A2	3.2457847	2.10E-29	3.75E-29
NUDT5	1.14306526	6.06E-132	9.06E-131
CDC123	1.02472241	8.27E-94	3.94E-93
RP11-186N1	0	8.05E-57	2.11E-56
CAMK1D	2.24708171	5.02E-141	1.60E-139
CCDC3	4.19455253	1.38E-140	4.17E-139
OPTN	1.81906615	3.34E-14	4.75E-14
MCM10	2.194824	1.79E-11	2.42E-11
UCMA	NA	NA	NA
PHYH	0.93122305	2.76E-136	5.40E-135
SEPHS1	1.70403696	1.81E-12	2.50E-12
RP11-445K1	NA	NA	NA
BEND7	3.08973735	3.64E-122	3.48E-121
RP11-214D1	NA	1.38E-109	9.17E-109
PRPF18	1.84760052	7.12E-24	1.17E-23
RP11-295P9.	Inf	6.83E-78	2.46E-77
FRMD4A	NA	NA	NA
RP11-295P9.	NA	0.08314998	0.08898696
RP11-295P9.	NA	NA	NA

RP11-353M9	NA	NA	NA
RP11-142M1	NA	NA	NA
RP11-397C18	NA	NA	NA
FAM107B	2.03990397	0.0046212	0.00517293
RP11-7C6.1	NA	NA	NA
CDNF	1.49805447	3.38E-12	4.65E-12
HSPA14	1.08762859	1.52E-59	4.15E-59
RP11-398C13	2.62159533	1.53E-10	2.04E-10
SUV39H2	1.83382531	7.58E-46	1.70E-45
DCLRE1C	2.44682231	2.59E-05	3.09E-05
MEIG1	2.24708171	4.83E-07	5.99E-07
OLAH	NA	4.02E-120	3.60E-119
ACBD7	NA	1.55E-13	2.18E-13
C10orf111	1.49805447	7.18E-15	1.03E-14
RPP38	1.01031581	1.51E-129	1.98E-128
NMT2	2.34071012	7.20E-20	1.12E-19
FAM171A1	2.43433852	0.09810292	0.10476091
RP11-25G10.	NA	NA	NA
ITGA8	Inf	0.0982611	0.10492366
FAM188A	2.08063121	0.01254499	0.01384459
RP11-461K13	NA	NA	NA
PTER	1.31079767	1.36E-17	2.04E-17
C1QL3	NA	NA	NA
RSU1	1.03484026	5.69E-98	2.96E-97
CUBN	NA	4.00E-08	5.08E-08
RP11-406H2	Inf	1.63E-85	6.67E-85
TRDMT1	0.65539883	4.17E-16	6.13E-16
VIM-AS1	1.31079767	8.02E-41	1.68E-40
VIM	0.87159533	1.51E-131	2.22E-130
RP11-124N1	NA	0.02035194	0.02230292
ST8SIA6	Inf	8.14E-26	1.38E-25
ST8SIA6-AS1	NA	NA	NA
HACD1	1.85514886	9.59E-05	1.12E-04
STAM-AS1	5.24319066	3.07E-119	2.66E-118
STAM	1.33754864	1.06E-20	1.67E-20
RP11-390B4.	NA	NA	NA
TMEM236	NA	1.24E-42	2.67E-42
MRC1	NA	NA	NA
RP11-457D2.	NA	NA	NA
SLC39A12	NA	NA	NA
SLC39A12-AS	NA	NA	NA
CACNB2	NA	1.03E-130	1.44E-129
RP11-499P20	1.12354086	0.03685684	0.03997709
RP11-383B4.	NA	NA	NA
NSUN6	1.31079767	1.36E-35	2.66E-35

ARL5B	1.90661479	7.31E-46	1.64E-45
RP11-139J15	NA	NA	NA
MALRD1	NA	NA	NA
RP11-49L2.1	NA	NA	NA
RP11-354E11	NA	4.83E-114	3.61E-113
PLXDC2	Inf	1.09E-05	1.32E-05
RP11-575A19	NA	NA	NA
NEBL	NA	NA	NA
C10orf113	NA	NA	NA
NEBL-AS1	NA	NA	NA
RP11-275N1.	NA	NA	NA
CASC10	11.2354086	1.97E-71	6.42E-71
SKIDA1	4.49416342	9.60E-34	1.82E-33
MLLT10	1.59168288	0.0692764	0.0743896
DNAJC1	1.83971602	0.00096484	0.00110241
EBLN1	NA	NA	NA
RP11-108B14	NA	NA	NA
RP11-573G6.	NA	NA	NA
COMMD3	1.20887307	2.45E-131	3.55E-130
COMMD3-BN	NA	NA	NA
BMI1	2.55550469	1.82E-24	3.03E-24
RP11-573G6.	NA	1.44E-124	1.51E-123
RP11-573G6.	NA	NA	NA
RP11-573G6.	Inf	1.48E-62	4.23E-62
SPAG6	NA	NA	NA
PIP4K2A	2.66840953	2.61E-17	3.90E-17
ARMC3	NA	NA	NA
MSRB2	1.48000563	3.88E-52	9.55E-52
PTF1A	NA	NA	NA
LINC01552	NA	NA	NA
C10orf67	NA	NA	NA
RP11-371A19	NA	NA	NA
OTUD1	1.49805447	0.03558633	0.03861749
KIAA1217	NA	NA	NA
ARHGAP21	1.40442607	8.10E-66	2.43E-65
PRTFDC1	1.29978256	3.88E-121	3.57E-120
RP11-165A20	Inf	1.48E-127	1.79E-126
ENKUR	Inf	2.98E-79	1.09E-78
THNSL1	0.33290099	3.54E-35	6.86E-35
LINC01516	NA	NA	NA
GPR158-AS1	NA	NA	NA
GPR158	NA	NA	NA
LINC00836	NA	NA	NA
RP11-435M3	NA	NA	NA
MYO3A	NA	NA	NA

GAD2	NA	NA	NA
APBB1IP	1.2443517	6.53E-21	1.03E-20
RP11-128B1(	NA	NA	NA
LINC00264	NA	NA	NA
RP13-16H11.	NA	NA	NA
LINC00202-2	NA	NA	NA
RP13-16H11.	NA	NA	NA
PDSS1	1.26502378	6.29E-105	3.76E-104
RP13-16H11.	NA	0.77679212	0.78549482
ABI1	1.40817121	0.59995534	0.61268729
LINC00202-1	NA	3.52E-16	5.18E-16
LINC00614	NA	NA	NA
ANKRD26	1.49805447	1.71E-27	2.98E-27
YME1L1	2.13852704	3.34E-07	4.16E-07
MASTL	1.68531128	0.92338449	0.92706372
ACBD5	1.94747082	8.81E-07	1.09E-06
RP11-85G18.	NA	NA	NA
RP11-748L13	NA	NA	NA
PTCHD3	NA	NA	NA
RAB18	1.4778105	0.00025754	0.00029893
MKX	NA	NA	NA
MKX-AS1	NA	NA	NA
ARMC4	NA	0.06801161	0.07305741
RP11-218D6.	NA	NA	NA
MPP7	NA	3.63E-49	8.54E-49
RP11-351M1	NA	NA	NA
RP11-351M1	NA	NA	NA
WAC-AS1	2.20963035	1.55E-05	1.86E-05
WAC	2.09103437	0.00178995	0.00202629
BAMBI	1.4205689	2.07E-24	3.46E-24
RP11-478H13	NA	NA	NA
LINC00837	NA	NA	NA
C10orf126	NA	NA	NA
LYZL1	NA	NA	NA
SVIL-AS1	2.14721141	8.57E-92	3.94E-91
SVIL	3.47762646	8.57E-23	1.39E-22
KIAA1462	0.99870298	5.34E-12	7.31E-12
MTPAP	1.96966422	0.25087954	0.26240741
MAP3K8	3.99481193	3.16E-85	1.29E-84
RP11-253D19	0	7.74E-138	1.72E-136
RP11-253D19	NA	NA	NA
LYZL2	NA	NA	NA
RP11-14C22.	NA	NA	NA
RP11-14C22.	NA	NA	NA
ZNF438	1.24837873	2.06E-95	1.01E-94

RP11-192P3.	NA	1.48E-28	2.61E-28
ZEB1-AS1	2.7464332	1.12E-11	1.52E-11
ZEB1	1.74773022	7.44E-19	1.14E-18
RP11-472N1:	NA	NA	NA
RP11-472N1:	NA	0.29393389	0.3061516
ARHGAP12	2.99610895	1.16E-13	1.64E-13
KIF5B	1.61508998	4.15E-52	1.02E-51
RP11-166N1'	NA	NA	NA
EPC1	1.63849708	1.44E-07	1.80E-07
RP11-166N1'	NA	NA	NA
RP11-135A2·	NA	1.68E-143	8.00E-142
RP11-135A2·	NA	NA	NA
CCDC7	2.24708171	2.80E-05	3.33E-05
CCDC7	NA	3.46E-109	2.28E-108
RP11-479G2:	Inf	3.46E-23	5.66E-23
ITGB1	2.12958724	1.33E-23	2.20E-23
RP11-462L8.	NA	NA	NA
RP11-342D1:	NA	NA	NA
NRP1	1.12354086	4.77E-41	1.00E-40
RP11-342D1:	NA	NA	NA
RP11-90B22.	NA	NA	NA
RP11-476F1·	NA	NA	NA
RP13-259N1:	NA	NA	NA
LINC00838	NA	NA	NA
RP11-490O2·	NA	NA	NA
PARD3	1.99740597	1.29E-11	1.75E-11
PARD3-AS1	0.74902724	0.14351857	0.15189977
RP11-297A1(	NA	NA	NA
CUL2	1.13982406	3.19E-33	6.00E-33
RP11-297A1(	Inf	2.04E-122	1.98E-121
CREM	3.22462573	4.77E-127	5.60E-126
RP11-324I22	NA	4.23E-31	7.75E-31
CCNY	2.85992218	4.43E-127	5.21E-126
RP11-324I22	1.87256809	6.51E-13	9.05E-13
GJD4	NA	4.29E-48	9.91E-48
RP11-425A6.	NA	7.20E-126	8.01E-125
FZD8	NA	9.37E-88	4.00E-87
PCAT5	NA	NA	NA
RP11-309N2·	NA	NA	NA
RP11-810B2:	NA	NA	NA
RP11-92J19.:	NA	NA	NA
RP11-322I2.:	NA	NA	NA
ANKRD30A	NA	NA	NA
RP11-20F24.	NA	NA	NA
LINC00993	NA	NA	NA

MTRNR2L7	NA	NA	NA
RP11-258F22	NA	2.70E-96	1.36E-95
ZNF248	1.34824903	0.00567265	0.00633312
RP11-258F22	NA	NA	NA
RP11-162G10	NA	0.00764615	0.00850656
ZNF25	4.49416342	9.37E-36	1.83E-35
RP11-393J16	NA	NA	NA
ZNF33A	2.21451531	0.00026201	0.00030406
ZNF37A	2.39688716	4.66E-48	1.08E-47
RP11-508N22	NA	0.40939858	0.42224611
RP11-508N22	NA	0.97503184	0.9763773
RP11-508N22	NA	NA	NA
RP11-672F9.	NA	NA	NA
RP11-291L22	NA	NA	NA
IGKV1OR10-	NA	NA	NA
LINC00839	NA	NA	NA
ZNF33B	2.92801556	3.64E-42	7.78E-42
LINC01518	NA	NA	NA
AL022344.5	NA	NA	NA
AL022344.7	NA	NA	NA
BMS1	1.63676322	8.79E-21	1.39E-20
RP11-124O1	NA	NA	NA
LINC01264	NA	NA	NA
RET	0	6.65E-143	2.83E-141
RP11-351D16	0	2.46E-34	4.72E-34
CSGALNACT2	4.11964981	2.29E-56	5.98E-56
RASGEF1A	NA	NA	NA
RP11-168L22	NA	NA	NA
FXYD4	NA	NA	NA
HNRNPF	1.16533925	8.03E-138	1.78E-136
RP11-517P14	2.49675746	9.24E-21	1.46E-20
ZNF487	4.11964981	7.75E-34	1.47E-33
ZNF239	NA	NA	NA
ZNF485	NA	NA	NA
ZNF32-AS3	NA	NA	NA
RP11-402L1.	NA	NA	NA
ZNF32	2.89840974	3.54E-48	8.21E-48
ZNF32-AS1	NA	NA	NA
ZNF32-AS2	NA	NA	NA
RP11-402L1.	NA	NA	NA
RP11-272J7.	NA	NA	NA
LINC00619	NA	NA	NA
LINC00840	NA	NA	NA
RP11-168P8.	NA	NA	NA
RP11-168P8.	NA	NA	NA

LINC00841	NA	NA	NA
RP11-20J15.2	NA	NA	NA
RP11-20J15.3	NA	NA	NA
C10orf142	NA	NA	NA
CXCL12	NA	NA	NA
RP11-733D4.	NA	NA	NA
TMEM72-AS	NA	NA	NA
RP11-285G1	NA	NA	NA
TMEM72	NA	5.68E-94	2.72E-93
RP11-285G1	NA	NA	NA
RASSF4	2.99610895	5.32E-38	1.07E-37
C10orf10	NA	0.00132157	0.00150254
C10orf25	1.12354086	3.04E-06	3.70E-06
ZNF22	1.14090241	5.55E-110	3.73E-109
RP11-445N1	NA	NA	NA
OR13A1	NA	NA	NA
ALOX5	Inf	3.43E-118	2.87E-117
RP11-67C2.2	NA	NA	NA
8-Mar	1-Jan	0.0002191	0.0002546
ZFAND4	NA	1.35E-33	2.56E-33
FAM21C	Inf	2.43E-148	2.94E-146
AGAP4	0.99870298	1.53E-14	2.18E-14
TIMM23	1.2075227	4.48E-69	1.42E-68
NCOA4	1.46924573	0.19734137	0.20759406
MSMB	NA	NA	NA
ANTXRL	NA	NA	NA
AGAP14P	NA	NA	NA
ANXA8L1	NA	NA	NA
NPY4R	NA	NA	NA
GPRIN2	NA	NA	NA
SYT15	NA	NA	NA
RP11-38L15.	NA	NA	NA
RP11-38L15.	NA	NA	NA
RP11-38L15.	NA	NA	NA
PTPN20	NA	NA	NA
GDF10	NA	NA	NA
GDF2	NA	NA	NA
RBP3	NA	NA	NA
ZNF488	Inf	8.62E-119	7.34E-118
ANXA8	NA	NA	NA
FAM25G	NA	NA	NA
RP11-144G6.	NA	NA	NA
AGAP9	3.74513619	4.26E-09	5.52E-09
LINC00864	NA	NA	NA
ABC7-42404	NA	NA	NA

CH17-360D5.	NA	NA	NA
CH17-360D5.	NA	NA	NA
CH17-360D5.	NA	NA	NA
FAM25C	NA	NA	NA
FRMPD2	NA	NA	NA
MAPK8	1.84760052	0.84107455	0.84789812
ARHGAP22	2.12881425	2.80E-66	8.46E-66
RP11-534L6.:	NA	NA	NA
WDFY4	1.71206226	3.33E-31	6.09E-31
LRRC18	NA	NA	NA
RP11-523O1:	NA	NA	NA
VSTM4	Inf	1.29E-106	7.96E-106
FAM170B-AS	NA	NA	NA
FAM170B	NA	NA	NA
C10orf128	Inf	1.05E-126	1.21E-125
C10orf71-AS:	NA	NA	NA
C10orf71	NA	NA	NA
DRGX	NA	NA	NA
RP11-123B3.	NA	NA	NA
ERCC6	0.89883268	0.22380096	0.2346799
PGBD3	NA	NA	NA
ERCC6-PGBD	0	1.84E-95	9.09E-95
CHAT	NA	NA	NA
SLC18A3	NA	NA	NA
C10orf53	NA	NA	NA
OGDHL	0.37451362	5.73E-05	6.77E-05
PARG	2.67509728	8.38E-56	2.17E-55
TIMM23B	3.21011673	1.43E-53	3.59E-53
LINC00843	NA	0.19469686	0.20487156
AGAP6	2.8463035	3.31E-80	1.24E-79
FAM21A	2.13184675	4.88E-46	1.10E-45
ASAH2	0	0.92194038	0.92566508
SGMS1	2.80885214	6.20E-96	3.09E-95
RP11-512N4.	NA	NA	NA
SGMS1-AS1	0.74902724	0.06046855	0.06508962
ASAH2B	Inf	0.98146547	0.98260287
A1CF	NA	NA	NA
RP11-96B5.4	NA	NA	NA
PRKG1	NA	NA	NA
RP11-96B5.3	NA	NA	NA
RP11-40C11.	NA	NA	NA
CSTF2T	1.98271916	0.8538831	0.86023696
PRKG1-AS1	NA	NA	NA
DKK1	Inf	1.28E-142	5.08E-141
LINC01468	NA	NA	NA

RP11-556E13	NA	NA	NA
MBL2	NA	NA	NA
RP11-319F12	0	1.06E-142	4.35E-141
RP11-71J2.1	NA	NA	NA
PCDH15	NA	NA	NA
RP11-257I14	NA	NA	NA
RP11-598C10	NA	NA	NA
RP11-598C10	NA	NA	NA
MTRNR2L5	NA	NA	NA
ZWINT	1.08661698	5.22E-135	9.35E-134
IPMK	1.31079767	3.03E-28	5.32E-28
CISD1	1.5518639	4.64E-14	6.57E-14
RP11-179B15	NA	2.17E-21	3.46E-21
RP11-179B15	NA	NA	NA
UBE2D1	1.69098573	4.02E-31	7.35E-31
TFAM	1.25242206	4.71E-50	1.12E-49
BICC1	Inf	1.44E-78	5.21E-78
LINC00844	NA	NA	NA
PHYHIPL	NA	NA	NA
FAM13C	NA	NA	NA
RP11-443O13	NA	NA	NA
RP11-135D11	NA	NA	NA
SLC16A9	2.80885214	0.3760113	0.38898312
MRLN	NA	NA	NA
CCDC6	1.89337441	3.74E-07	4.65E-07
LINC01553	NA	NA	NA
ANK3	NA	NA	NA
RP11-388P9.	NA	NA	NA
RP11-388P9.	NA	NA	NA
RP11-131N11	NA	NA	NA
CDK1	0.9637833	2.04E-143	9.57E-142
RHOBTB1	Inf	6.15E-20	9.56E-20
LINC00845	NA	NA	NA
TMEM26	NA	NA	NA
TMEM26-AS1	NA	NA	NA
C10orf107	NA	NA	NA
RP11-63A2.2	NA	5.19E-35	1.00E-34
RP11-491H19	NA	NA	NA
ARID5B	2.99610895	0.58505948	0.59791286
RTKN2	2.49675746	7.06E-08	8.90E-08
RP11-120C12	NA	NA	NA
ZNF365	NA	NA	NA
RP11-436D10	NA	1.46E-82	5.68E-82
ADO	1.81582361	0.00086953	0.00099452
EGR2	0.74902724	4.92E-30	8.88E-30

RP11-144G1(	NA	NA	NA
NRBF2	1.57976654	8.22E-78	2.95E-77
JMJD1C	1.68531128	1.63E-27	2.84E-27
JMJD1C-AS1	Inf	3.57E-05	4.24E-05
REEP3	1.61328943	0.80853978	0.81632415
RP11-170M1	NA	NA	NA
RP11-174J11	NA	NA	NA
RP11-179K3.	NA	NA	NA
RP11-252C24	NA	NA	NA
RP11-428G2.	NA	NA	NA
LINC01515	1.42315175	0.00119377	0.00135979
CTNNA3	NA	NA	NA
RP11-210G2.	NA	NA	NA
LRRTM3	NA	NA	NA
RP11-344A5.	NA	NA	NA
DNAJC12	Inf	4.33E-139	1.10E-137
RP11-57G10.	0.74902724	0.27529231	0.28724639
SIRT1	2.24708171	1.62E-62	4.61E-62
HERC4	7.24059663	1.17E-100	6.39E-100
MYPN	NA	NA	NA
ATOH7	NA	NA	NA
RP11-153K11	NA	NA	NA
PBLD	0.74902724	2.72E-43	5.89E-43
HNRNPH3	1.0902746	2.70E-139	6.99E-138
RUFY2	1.68531128	6.07E-09	7.83E-09
DNA2	1.87256809	6.82E-19	1.05E-18
SLC25A16	4.04474708	6.94E-12	9.49E-12
TET1	4.49416342	1.23E-55	3.18E-55
CCAR1	1.74479286	0.28481496	0.29689226
STOX1	1.87256809	0.29702724	0.309267
DDX50	1.47834323	2.86E-44	6.27E-44
DDX21	1.56679432	5.18E-19	7.96E-19
KIF1BP	0.97949716	3.56E-64	1.04E-63
SRGN	0.2807855	3.22E-145	2.35E-143
VPS26A	1.2034932	1.33E-119	1.17E-118
SUPV3L1	1.62289235	2.51E-50	6.00E-50
RP11-227H1!	NA	NA	NA
HKDC1	NA	NA	NA
RP11-227H1!	NA	NA	NA
HK1	1.21716926	8.35E-11	1.12E-10
TACR2	NA	5.92E-17	8.81E-17
TSPAN15	2.60868107	2.37E-24	3.95E-24
NEUROG3	NA	NA	NA
RP11-343J3.:	NA	NA	NA
C10orf35	NA	5.72E-05	6.75E-05

RP11-242G2(	NA	NA	NA
COL13A1	NA	NA	NA
RP11-262I2.2	NA	NA	NA
H2AFY2	NA	3.85E-14	5.47E-14
AIFM2	1.52480545	3.64E-39	7.46E-39
TYSND1	1.5729572	4.45E-06	5.40E-06
SAR1A	1.56450044	2.43E-31	4.46E-31
PPA1	1.12224496	3.66E-138	8.38E-137
NPFFR1	NA	1.91E-27	3.32E-27
LRRC20	3.62029831	0.00018667	0.00021734
EIF4EBP2	1.91240997	4.04E-06	4.91E-06
NODAL	NA	NA	NA
PALD1	1.93498703	2.25E-05	2.68E-05
PRF1	Inf	1.85E-05	2.21E-05
ADAMTS14	Inf	5.04E-13	7.01E-13
TBATA	NA	NA	NA
SGPL1	3.74513619	1.06E-07	1.33E-07
PCBD1	2.13236583	3.81E-102	2.15E-101
RP11-432J9.3	NA	NA	NA
RP11-432J9.(	NA	NA	NA
UNC5B	Inf	2.93E-06	3.57E-06
UNC5B-AS1	NA	NA	NA
SLC29A3	0.64202335	1.10E-119	9.70E-119
CDH23	Inf	0.91472106	0.91867081
CDH23-AS1	NA	NA	NA
C10orf105	NA	3.59E-37	7.16E-37
C10orf54	1.40849687	1.70E-65	5.08E-65
PSAP	1.10234197	6.88E-126	7.69E-125
RP11-472G2	NA	1.96E-108	1.27E-107
CHST3	5.05593385	3.74E-109	2.46E-108
RP11-150D2(	NA	NA	NA
SPOCK2	0.74902724	1.64E-33	3.10E-33
ASCC1	1.56047341	6.75E-116	5.29E-115
ANAPC16	1.85297815	0.04576715	0.04948493
DDIT4	1.74124514	3.46E-33	6.50E-33
RP11-442H2:	NA	6.27E-37	1.25E-36
DNAJB12	1.71998847	7.27E-88	3.11E-87
MICU1	1.65574442	9.35E-10	1.23E-09
RP11-167P22	NA	NA	NA
MCU	3.83876459	3.29E-92	1.53E-91
OIT3	NA	NA	NA
PLA2G12B	NA	NA	NA
P4HA1	1.4381323	1.76E-30	3.20E-30
RP11-344N1(	NA	NA	NA
RP11-344N1(	Inf	0.15635404	0.16516682

NUDT13	0.49935149	5.91E-10	7.79E-10
RP11-152N1:	9.73735409	2.20E-136	4.34E-135
ECD	1.67090691	8.05E-17	1.20E-16
FAM149B1	1.04863813	1.95E-21	3.11E-21
DNAJC9	1.33779748	5.60E-77	1.98E-76
DNAJC9-AS1	NA	0.57279192	0.58580478
MRPS16	2.18194891	2.30E-101	1.28E-100
RP11-152N1:	1.41482923	0.00093493	0.00106864
CFAP70	Inf	0.02532947	0.02765072
ANXA7	0.79408151	3.52E-141	1.14E-139
RP11-537A6.	NA	2.72E-84	1.09E-83
MSS51	1.49805447	4.17E-27	7.22E-27
PPP3CB	1.08369898	1.23E-87	5.21E-87
USP54	1.34824903	9.36E-27	1.61E-26
RP11-464F9.	1.49805447	2.98E-06	3.63E-06
RP11-464F9.	Inf	9.57E-102	5.33E-101
MYOZ1	NA	6.60E-19	1.01E-18
SYNPO2L	Inf	2.72E-85	1.11E-84
RP11-464F9.	NA	NA	NA
AGAP5	Inf	1.00E-104	5.95E-104
RP11-574K11	NA	7.45E-134	1.25E-132
SEC24C	2.79899652	3.04E-90	1.36E-89
FUT11	4.49416342	8.20E-45	1.81E-44
CHCHD1	1.94790129	3.01E-32	5.59E-32
ZSWIM8	1.49805447	1.20E-24	2.01E-24
RP11-574K11	NA	NA	NA
ZSWIM8-AS1	NA	NA	NA
NDST2	Inf	3.51E-136	6.79E-135
CAMK2G	1.89753567	3.99E-24	6.63E-24
RP11-574K11	NA	NA	NA
RP11-574K11	NA	NA	NA
PLAU	1.42996109	3.52E-20	5.49E-20
C10orf55	NA	3.93E-35	7.61E-35
VCL	1.88464918	1.01E-05	1.21E-05
AP3M1	1.99740597	1.45E-24	2.42E-24
ADK	1.10321841	1.98E-124	2.08E-123
RP11-46O21.	NA	NA	NA
RP11-222G7.	NA	1.97E-49	4.64E-49
KAT6B	1.65574442	7.91E-17	1.17E-16
RP11-77G23.	NA	NA	NA
RP11-77G23.	NA	NA	NA
DUPD1	NA	NA	NA
DUSP13	NA	1.87E-29	3.34E-29
SAMD8	5.24319066	0.00070656	0.0008098
VDAC2	1.09997007	3.36E-125	3.64E-124

COMTD1	2.04071707	9.07E-09	1.17E-08
ZNF503-AS1	NA	NA	NA
RP11-399K21	NA	NA	NA
ZNF503	1.74773022	5.38E-38	1.08E-37
ZNF503-AS2	1.49805447	5.38E-06	6.52E-06
RP11-399K21	1.49805447	2.43E-06	2.97E-06
RP11-399K21	NA	2.86E-22	4.61E-22
RP11-399K21	NA	NA	NA
C10orf11	0.93628405	5.02E-91	2.27E-90
AC013286.1	NA	NA	NA
RP11-369F10	NA	NA	NA
RP11-325D15	NA	NA	NA
KCNMA1	NA	NA	NA
KCNMA1-AS1	NA	NA	NA
KCNMA1-AS2	NA	NA	NA
RP11-328K22	NA	NA	NA
KCNMA1-AS3	NA	NA	NA
RP13-39P12.	NA	1.28E-39	2.63E-39
DLG5	4.11964981	3.70E-59	1.00E-58
RP11-126H7.	NA	NA	NA
POLR3A	2.7464332	1.06E-94	5.16E-94
RPS24	1.7976022	0.00485918	0.00543665
RP11-157J13	NA	NA	NA
RP11-90J7.3	NA	NA	NA
LINC00856	NA	NA	NA
RP11-90J7.2	NA	0.3061233	0.31855506
RP11-90J7.4	NA	NA	NA
RP11-17G2.1	NA	NA	NA
RP11-31E13.	NA	NA	NA
ZMIZ1-AS1	NA	NA	NA
RP11-481G8.	NA	NA	NA
ZMIZ1	2.37191958	9.00E-134	1.50E-132
PPIF	1.61448358	6.72E-44	1.47E-43
ZCCHC24	2.24708171	4.71E-06	5.71E-06
RP11-342M3	NA	NA	NA
RP11-342M3	NA	NA	NA
EIF5AL1	2.93849147	3.08E-16	4.54E-16
SFTPA2	NA	NA	NA
SFTPA1	NA	NA	NA
RP11-589B3.	NA	NA	NA
RP11-506M1	NA	1.23E-11	1.67E-11
NUTM2B-AS	2.86392767	9.72E-82	3.72E-81
NUTM2B	NA	NA	NA
RP11-182L21	2.46108949	2.15E-79	7.91E-79
NUTM2E	0	1.03E-63	2.98E-63

SFTPD	NA	NA	NA
SFTPD-AS1	NA	3.23E-27	5.61E-27
TMEM254-A:	NA	6.92E-139	1.72E-137
TMEM254	1.99740597	0.0016975	0.00192343
PLAC9	NA	NA	NA
ANXA11	1.523883	8.59E-73	2.86E-72
LINC00857	NA	NA	NA
MAT1A	NA	1.09E-129	1.45E-128
RP11-36D19.	NA	NA	NA
DYDC1	NA	NA	NA
DYDC2	Inf	1.61E-77	5.74E-77
FAM213A	NA	NA	NA
TSPAN14	1.39949826	1.12E-67	3.47E-67
RP11-137H2.	NA	NA	NA
SH2D4B	NA	NA	NA
RP11-514F8.	NA	NA	NA
NRG3	NA	NA	NA
RP11-202D1ℓ	NA	NA	NA
RP11-344L13	NA	NA	NA
RP11-219F1(	NA	NA	NA
RP11-338I21	Inf	2.00E-92	9.33E-92
GHITM	0.99548136	1.29E-138	3.10E-137
C10orf99	NA	NA	NA
CDHR1	NA	NA	NA
LRIT2	NA	NA	NA
LRIT1	NA	NA	NA
RGR	NA	NA	NA
LINC00858	NA	NA	NA
CCSER2	1.92607004	1.30E-10	1.73E-10
LINC01519	NA	NA	NA
RP11-475D1.	NA	NA	NA
RP11-475D1.	NA	NA	NA
LINC01520	NA	NA	NA
GRID1-AS1	NA	NA	NA
GRID1	NA	NA	NA
RP11-93H12.	NA	NA	NA
WAPL	1.65856031	1.07E-11	1.45E-11
RP11-77P6.2	0.74902724	7.05E-14	9.96E-14
OPN4	NA	NA	NA
LDB3	NA	7.46E-10	9.81E-10
RP11-359E3.	Inf	1.24E-112	8.95E-112
BMPR1A	1.49805447	6.22E-09	8.03E-09
MMRN2	NA	NA	NA
SNCG	0.49935149	0.00785647	0.00873412
ADIRF	NA	NA	NA

RP11-96C23.	NA	NA	NA
RP11-96C23.	NA	NA	NA
FAM25A	NA	NA	NA
GLUD1	1.42585908	0.99492588	0.9954197
FAM35A	1.72852439	1.70E-33	3.21E-33
NUTM2A-AS	0.99870298	0.00888745	0.00986093
NUTM2A	NA	NA	NA
LINC00863	NA	2.58E-134	4.43E-133
NUTM2D	NA	NA	NA
LINC00864	NA	NA	NA
MINPP1	0.87386511	2.16E-41	4.56E-41
RP11-57C13.	NA	NA	NA
RP11-57C13.	NA	NA	NA
RP11-57C13.	NA	NA	NA
PAPSS2	NA	NA	NA
ATAD1	1.24837873	7.70E-05	9.06E-05
KLLN	Inf	3.66E-58	9.76E-58
PTEN	1.9441044	2.91E-07	3.63E-07
RNLS	NA	NA	NA
LIPJ	NA	NA	NA
LIPF	NA	NA	NA
LIPK	NA	NA	NA
LIPN	NA	1.86E-31	3.41E-31
LIPM	NA	NA	NA
ANKRD22	0.37451362	5.25E-08	6.64E-08
STAMBPL1	2.7464332	3.73E-87	1.57E-86
ACTA2-AS1	NA	1.49E-17	2.24E-17
ACTA2	1.12354086	2.85E-32	5.29E-32
FAS	2.49675746	4.20E-39	8.60E-39
CH25H	NA	NA	NA
LIPA	1.34036453	3.18E-71	1.03E-70
RP11-149I23	NA	NA	NA
IFIT2	0.74902724	0.6938464	0.7046835
IFIT3	0.83225249	3.60E-11	4.85E-11
IFIT1B	NA	NA	NA
IFIT1	4.49416342	1.49E-123	1.52E-122
IFIT5	1.77893969	0.04859859	0.05249319
SLC16A12	NA	NA	NA
SLC16A12-AS	NA	NA	NA
PANK1	3.932393	1.43E-38	2.91E-38
RP11-80H5.2	Inf	1.07E-132	1.68E-131
RP11-80H5.6	NA	NA	NA
RP11-80H5.9	NA	1.32E-100	7.17E-100
RP11-80H5.7	NA	NA	NA
KIF20B	1.71487815	1.77E-28	3.12E-28

LINC00865	NA	NA	NA
LINC01374	NA	NA	NA
LINC01375	NA	NA	NA
RP11-15K3.1	NA	NA	NA
RP11-94M14	NA	NA	NA
RP11-94M14	NA	NA	NA
RP11-30G13.	NA	NA	NA
HTR7	NA	7.74E-138	1.72E-136
RPP30	1.08425621	5.55E-118	4.62E-117
ANKRD1	NA	NA	NA
RP11-236B18	NA	NA	NA
RP11-236B18	NA	NA	NA
LINC00502	NA	NA	NA
PCGF5	3.27292336	7.27E-139	1.80E-137
HECTD2	NA	2.53E-07	3.16E-07
PPP1R3C	NA	NA	NA
TNKS2-AS1	0.74902724	0.06923327	0.0743477
TNKS2	2.53516911	2.25E-75	7.79E-75
FGFBP3	2.66320796	1.56E-39	3.22E-39
RP11-402D2	1.87256809	5.32E-109	3.48E-108
BTAF1	2.3540856	7.72E-30	1.39E-29
CPEB3	Inf	8.23E-70	2.63E-69
5-Mar	1-Jan	2.57E-110	1.74E-109
IDE	1.88754864	0.00425603	0.00477036
KIF11	1.32782101	1.31E-69	4.18E-69
HHEX	1.57448583	4.15E-62	1.18E-61
EXOC6	1.87256809	1.40E-35	2.73E-35
RP11-348J12	NA	NA	NA
CYP26C1	NA	NA	NA
RP11-348J12	NA	NA	NA
CYP26A1	0.99870298	0.05031209	0.0543343
MYOF	6.74124514	1.06E-95	5.24E-95
CEP55	2.04109922	0.00023056	0.00026776
FFAR4	3.74513619	3.87E-05	4.59E-05
RBP4	NA	NA	NA
PDE6C	NA	NA	NA
FRA10AC1	1.28832685	1.60E-112	1.15E-111
LGI1	NA	7.30E-81	2.75E-80
RP11-437J2.4	NA	NA	NA
SLC35G1	2.37191958	1.17E-11	1.59E-11
PLCE1	2.24708171	3.77E-21	5.98E-21
PLCE1-AS2	NA	NA	NA
RP11-391J2.3	NA	NA	NA
PLCE1-AS1	NA	NA	NA
RP11-76P2.4	NA	5.46E-113	3.98E-112

NOC3L	1.30094204	7.32E-65	2.17E-64
TBC1D12	NA	1.24E-145	1.23E-143
HELLS	1.9027708	1.46E-81	5.59E-81
CYP2C18	NA	NA	NA
RP11-400G3.	NA	NA	NA
CYP2C19	NA	NA	NA
CYP2C9	NA	NA	NA
CYP2C8	NA	NA	NA
RP11-310E22	NA	5.00E-75	1.72E-74
ACSM6	NA	1.03E-19	1.60E-19
RP11-310E22	NA	NA	NA
PDLIM1	1.14715883	1.02E-105	6.21E-105
SORBS1	0	6.56E-14	9.28E-14
ALDH18A1	1.90661479	2.08E-38	4.22E-38
TCTN3	0.82705091	8.45E-137	1.73E-135
ENTPD1	0.29961089	3.62E-14	5.14E-14
ENTPD1-AS1	2.62159533	4.32E-06	5.25E-06
AL365273.1	NA	NA	NA
RP11-248J23	NA	NA	NA
C10orf131	0	2.72E-11	3.67E-11
RP11-690P14	NA	NA	NA
CC2D2B	NA	NA	NA
CCNJ	2.34071012	3.68E-08	4.67E-08
ZNF518A	2.24708171	2.04E-20	3.20E-20
BLNK	NA	NA	NA
RP11-35J23.:	NA	NA	NA
DNTT	NA	NA	NA
OPALIN	NA	NA	NA
TLL2	NA	0.79177177	0.80006257
TM9SF3	1.8691941	4.37E-34	8.34E-34
PIK3AP1	0.8688716	3.44E-45	7.63E-45
LCOR	1.15758755	2.49E-24	4.15E-24
C10orf12	3.74513619	1.66E-09	2.17E-09
RP11-175O1!	1.16515348	0.00013788	0.00016111
SLIT1	NA	8.73E-51	2.10E-50
SLIT1-AS1	NA	NA	NA
ARHGAP19-S	NA	NA	NA
ARHGAP19	2.47755163	9.49E-68	2.94E-67
FRAT1	2.62159533	0.27671191	0.28864743
FRAT2	2.31165303	2.59E-06	3.16E-06
RP11-452K12	0	1.43E-40	2.98E-40
RRP12	2.99610895	4.54E-134	7.65E-133
RP11-452K12	NA	NA	NA
PGAM1	1.27380263	5.86E-97	2.99E-96
EXOSC1	1.25935349	4.23E-77	1.50E-76

ZDHHC16	2.39477722	6.95E-86	2.86E-85
MMS19	2.40758755	1.36E-13	1.92E-13
UBTD1	1.7077821	2.06E-14	2.94E-14
ANKRD2	NA	NA	NA
HOGA1	NA	NA	NA
RP11-548K23	NA	NA	NA
C10orf62	NA	NA	NA
MORN4	NA	NA	NA
PI4K2A	2.43433852	1.02E-14	1.47E-14
AVPI1	1.85758755	0.00103302	0.00117912
MARVELD1	2.00250139	1.11E-14	1.59E-14
ZFYVE27	2.49675746	1.14E-21	1.82E-21
SFRP5	NA	NA	NA
LINC00866	NA	NA	NA
GOLGA7B	NA	2.10E-127	2.51E-126
CRTAC1	NA	NA	NA
R3HCC1L	1.15758755	2.87E-30	5.20E-30
LOXL4	NA	NA	NA
RP11-34A14.	NA	NA	NA
PYROXD2	Inf	0.0035589	0.00399886
HPS1	1.8154389	7.97E-06	9.61E-06
HPSE2	NA	9.64E-116	7.50E-115
CNNM1	1.49805447	4.00E-11	5.39E-11
GOT1	1.27775235	7.30E-69	2.30E-68
RP11-441O1	1.12354086	1.26E-07	1.58E-07
LINC01475	NA	NA	NA
RP11-129J12	NA	NA	NA
NKX2-3	NA	NA	NA
SLC25A28	1.49805447	1.84E-23	3.03E-23
RP11-85A1.3	0	0.00014582	0.00017021
RP11-483F11	NA	1.38E-109	9.17E-109
ENTPD7	1.74773022	2.03E-09	2.65E-09
CUTC	1.34285063	3.58E-82	1.38E-81
COX15	2.78804583	3.73E-16	5.49E-16
ABCC2	NA	1.55E-11	2.10E-11
DNMBP	4.49416342	5.20E-68	1.62E-67
DNMBP-AS1	NA	NA	NA
CPN1	NA	NA	NA
ERLIN1	1.7655642	3.01E-15	4.36E-15
CHUK	2.93369001	1.15E-100	6.29E-100
RP11-316M2	NA	NA	NA
RP11-316M2	NA	NA	NA
CWF19L1	1.44257098	1.35E-49	3.19E-49
BLOC1S2	2.11681611	2.48E-06	3.02E-06
PKD2L1	NA	NA	NA

RP11-34D15.	NA	0.38801892	0.40076511
SCD	1.72116897	4.93E-14	6.99E-14
OLMALINC	1.87256809	2.30E-16	3.40E-16
RP11-285F16	NA	5.53E-46	1.24E-45
WNT8B	NA	NA	NA
SEC31B	10.4863813	1.37E-37	2.75E-37
RP11-411B6.	NA	NA	NA
NDUFB8	1.64166819	1.20E-06	1.47E-06
HIF1AN	1.61632193	1.19E-51	2.90E-51
PAX2	0.74902724	0.35328767	0.36612275
RP11-179B2.	4.49416342	6.40E-60	1.76E-59
SLF2	1.66450497	8.57E-77	3.02E-76
RP11-108L7.	NA	9.18E-05	1.08E-04
MRPL43	1.79523937	0.67307327	0.68454396
SEMA4G	0.74902724	8.20E-84	3.26E-83
RP11-108L7.	NA	3.44E-77	1.22E-76
C10orf2	1.99740597	9.27E-18	1.40E-17
LZTS2	2.46454123	2.79E-64	8.19E-64
PDZD7	2.24708171	1.48E-57	3.92E-57
SFXN3	1.68531128	4.72E-62	1.34E-61
RP11-108L7.	1.61632193	4.71E-56	1.22E-55
KAZALD1	1.74230249	2.36E-05	2.82E-05
TLX1	Inf	3.66E-86	1.51E-85
LINC01514	NA	NA	NA
LBX1	NA	NA	NA
RP11-107I14	NA	NA	NA
RP11-107I14	NA	NA	NA
RP11-107I14	NA	NA	NA
BTRC	1.68531128	4.08E-10	5.39E-10
DPCD	1.85232411	1.41E-32	2.63E-32
POLL	2.34695201	0.39519872	0.40799495
FBXW4	1.79766537	0.04517885	0.04886048
RP11-190J1.	NA	NA	NA
FGF8	NA	9.70E-153	1.93E-150
NPM3	1.79451315	0.00126294	0.00143696
MGEA5	2.37734732	1.34E-16	1.98E-16
KCNIP2-AS1	Inf	4.54E-16	6.67E-16
KCNIP2	Inf	4.63E-102	2.60E-101
C10orf76	2.69649805	1.34E-50	3.23E-50
HPS6	1.21716926	4.44E-31	8.12E-31
LDB1	1.08949416	5.50E-12	7.53E-12
PPRC1	1.99740597	1.67E-53	4.19E-53
NOLC1	1.47362967	4.20E-18	6.37E-18
ELOVL3	0	4.08E-21	6.48E-21
PITX3	NA	1.43E-132	2.23E-131

GBF1	1.34824903	0.37643134	0.38939545
NFKB2	2.0598249	0.26753339	0.27942377
PSD	NA	0.00065572	0.00075224
FBXL15	1.53034013	8.27E-80	3.07E-79
CUEDC2	1.17276265	1.11E-139	3.03E-138
C10orf95	0.99870298	1.75E-14	2.50E-14
MFSD13A	1.64785992	1.10E-07	1.38E-07
ACTR1A	1.70233463	1.12E-11	1.52E-11
RP11-18I14.1	0	1.84E-62	5.24E-62
SUFU	1.74773022	5.75E-20	8.95E-20
RP11-47A8.5	0.74902724	0.03501637	0.03801263
TRIM8	0.99870298	3.89E-08	4.93E-08
ARL3	1.76657367	0.08561182	0.0915838
SFXN2	3.74513619	1.66E-59	4.52E-59
WBP1L	2.92801556	4.72E-32	8.75E-32
CYP17A1	NA	NA	NA
CYP17A1-AS1	Inf	1.69E-33	3.20E-33
RP11-753C18	NA	1.13E-17	1.71E-17
BORCS7	2.95865759	1.80E-100	9.80E-100
BORCS7-ASN	NA	NA	NA
AS3MT	NA	NA	NA
RP11-724N1.	NA	NA	NA
CNNM2	1.24837873	0.24757383	0.25908424
NT5C2	1.94747082	4.77E-07	5.91E-07
RPEL1	NA	1.32E-11	1.79E-11
INA	0	8.76E-142	3.13E-140
PCGF6	1.88391699	1.45E-14	2.08E-14
TAF5	1.46060311	3.12E-117	2.54E-116
USMG5	2.18825758	2.95E-125	3.20E-124
PDCD11	2.7378237	1.09E-133	1.80E-132
CALHM2	2.09727626	0.04288898	0.04643107
RP11-225H22	1.49805447	2.47E-19	3.81E-19
RP11-225H22	NA	7.01E-64	2.04E-63
CALHM1	NA	NA	NA
CALHM3	NA	1.14E-22	1.86E-22
NEURL1-AS1	NA	NA	NA
NEURL1	0	3.33E-07	4.14E-07
SH3PXD2A	7.49027237	1.36E-37	2.72E-37
RP11-416N2.	NA	NA	NA
SH3PXD2A-A	NA	NA	NA
OBFC1	2.24708171	0.20642192	0.21693254
SLK	1.62289235	0.00186886	0.00211482
COL17A1	NA	NA	NA
SFR1	1.31079767	2.28E-22	3.69E-22
CFAP43	NA	1.21E-72	4.04E-72

GSTO1	0.85989408	8.35E-145	5.26E-143
GSTO2	1.49805447	2.53E-14	3.61E-14
ITPRIP	0.74902724	8.67E-05	1.02E-04
RP11-127L20	NA	NA	NA
RP11-127L20	NA	NA	NA
CFAP58-AS1	NA	NA	NA
CFAP58	NA	NA	NA
RP11-127O4.	NA	NA	NA
RP11-127O4.	NA	NA	NA
SORCS3	NA	NA	NA
SORCS3-AS1	NA	NA	NA
RP11-56I23.1	NA	NA	NA
RP11-298H24	NA	NA	NA
SORCS1	NA	NA	NA
LINC01435	NA	NA	NA
RP11-163F15	NA	NA	NA
RP11-655H13	NA	NA	NA
XPNPEP1	1.24837873	0.68333459	0.69427958
ADD3	2.97639771	4.46E-143	1.94E-141
MXI1	1.44257098	3.52E-11	4.74E-11
RP11-549L6.1	NA	NA	NA
SMNDC1	1.49805447	1.51E-114	1.14E-113
RP11-525A16	NA	NA	NA
DUSP5	2.47755163	0.00058963	0.0006772
SMC3	1.11979572	4.49E-139	1.14E-137
RBM20	Inf	1.25E-48	2.93E-48
RP11-313D6.	NA	3.01E-137	6.36E-136
PDCD4-AS1	0.99870298	2.44E-91	1.11E-90
PDCD4	1.23752326	6.69E-104	3.91E-103
BBIP1	2.22211414	8.55E-14	1.21E-13
RP11-348N5.	NA	6.70E-38	1.35E-37
RP11-348N5.	NA	8.68E-93	4.07E-92
SHOC2	1.89753567	0.00058233	0.00066894
ADRA2A	Inf	1.05E-13	1.48E-13
RP11-381K7.	NA	NA	NA
GPAM	1.38281952	0.33365651	0.34639179
TECTB	NA	NA	NA
ACSL5	1.52075227	4.66E-08	5.90E-08
RP11-324O2.	NA	9.32E-33	1.74E-32
ZDHHC6	1.8076524	5.84E-17	8.69E-17
VTI1A	2.07084001	0.7167905	0.72700918
RP11-25C19.	NA	NA	NA
TCF7L2	2.87127108	8.94E-44	1.95E-43
RP11-57H14.	Inf	3.38E-19	5.21E-19
HABP2	NA	NA	NA

NRAP	NA	NA	NA
CASP7	1.46400778	5.95E-17	8.86E-17
RP11-211N1:	NA	NA	NA
PLEKHS1	NA	NA	NA
DCLRE1A	2.99610895	0.00038555	0.00044546
NHLRC2	1.62289235	3.31E-18	5.03E-18
ADRB1	0.24967575	0.01026899	0.01136461
CCDC186	1.93498703	5.03E-05	5.94E-05
TDRD1	NA	NA	NA
VWA2	NA	NA	NA
CTB-1144G6.	NA	NA	NA
AFAP1L2	NA	4.63E-22	7.45E-22
ABLIM1	2.24708171	1.12E-21	1.79E-21
RP11-106M7	NA	NA	NA
FAM160B1	2.7464332	9.01E-36	1.76E-35
TRUB1	1.87256809	0.52185193	0.5351556
RP11-383C6.	NA	NA	NA
ATRNL1	NA	NA	NA
GFRA1	3.99481193	7.77E-120	6.89E-119
CCDC172	NA	NA	NA
PNLIPRP3	NA	NA	NA
PNLIP	NA	NA	NA
PNLIPRP1	NA	NA	NA
C10orf82	NA	0.01127359	0.01246423
RP11-498B4.	NA	NA	NA
HSPA12A	1.92607004	1.78E-71	5.81E-71
RP11-539I5.1	NA	NA	NA
ENO4	NA	5.56E-34	1.06E-33
SHTN1	2.24708171	3.20E-50	7.65E-50
VAX1	NA	NA	NA
MIR3663HG	NA	NA	NA
KCNK18	NA	NA	NA
RP11-501J20	NA	NA	NA
SLC18A2	NA	NA	NA
RP11-129M1	0.74902724	6.33E-15	9.12E-15
PDZD8	2.12224384	2.03E-05	2.42E-05
CTA-109P11.	NA	NA	NA
EMX2OS	NA	3.76E-87	1.58E-86
EMX2	NA	NA	NA
CTA-109P11.	NA	NA	NA
RP11-328K1!	NA	NA	NA
RP11-355F2:	NA	NA	NA
RAB11FIP2	1.73458939	2.39E-26	4.10E-26
RP11-354M2	NA	NA	NA
CASC2	Inf	4.76E-121	4.38E-120

FAM204A	1.65432634	0.35985607	0.37269559
LINC00867	0.74902724	2.70E-37	5.39E-37
PRLHR	NA	6.67E-162	5.26E-159
CACUL1	2.40205286	7.37E-12	1.01E-11
RP11-498J9.2	NA	NA	NA
NANOS1	1.81906615	2.31E-22	3.74E-22
EIF3A	2.00126196	0.24672573	0.2582414
FAM45A	1.30265606	2.05E-80	7.67E-80
SFXN4	1.60421582	5.72E-46	1.29E-45
PRDX3	1.51394895	2.58E-69	8.20E-69
GRK5	1.04863813	3.29E-22	5.30E-22
RP11-79M19	NA	NA	NA
RGS10	1.85717712	1.82E-52	4.50E-52
TIAL1	2.12345586	0.06201462	0.0667103
BAG3	1.34036453	4.75E-98	2.48E-97
INPP5F	4.06614786	6.96E-102	3.90E-101
MCMBP	2.33787289	2.76E-49	6.50E-49
SEC23IP	2.27482346	1.48E-50	3.56E-50
PLPP4	NA	NA	NA
LINC01561	NA	NA	NA
WDR11-AS1	NA	0.88146125	0.88674004
WDR11	2.07084001	2.90E-93	1.37E-92
RP11-95I16.4	NA	2.47E-28	4.35E-28
RP11-95I16.2	NA	NA	NA
RP11-95I16.6	NA	NA	NA
LINC01153	NA	NA	NA
FGFR2	NA	3.34E-81	1.27E-80
RP11-62L18.3	NA	NA	NA
RP11-78A18.	NA	NA	NA
ATE1	3.07101167	1.54E-16	2.27E-16
ATE1-AS1	0.99870298	1.27E-41	2.69E-41
RP11-500G2	Inf	2.89E-116	2.28E-115
NSMCE4A	1.4398776	8.56E-95	4.17E-94
RP11-500G2	NA	7.77E-44	1.69E-43
TACC2	NA	3.17E-100	1.71E-99
BTBD16	NA	NA	NA
PLEKHA1	1.81014916	1.12E-10	1.50E-10
ARMS2	NA	NA	NA
HTRA1	NA	NA	NA
DMBT1	NA	NA	NA
C10orf120	NA	NA	NA
CUZD1	NA	NA	NA
FAM24B	NA	NA	NA
FAM24A	NA	NA	NA
C10orf88	1.60505837	1.24E-13	1.74E-13

PSTK	1.73638132	0.0693958	0.07451341
IKZF5	2.15345331	7.00E-26	1.19E-25
ACADSB	2.09727626	8.64E-05	1.01E-04
HMX3	Inf	8.40E-22	1.35E-21
HMX2	1.46924573	6.96E-56	1.81E-55
BUB3	0.99510535	2.80E-138	6.46E-137
RP11-282I1.1	NA	NA	NA
RP11-338O1.	NA	NA	NA
RP11-282I1.2	NA	NA	NA
GPR26	NA	NA	NA
CPXM2	NA	NA	NA
RP11-391M7	NA	NA	NA
CHST15	NA	NA	NA
OAT	1.53210117	1.85E-74	6.32E-74
NKX1-2	NA	NA	NA
LHPP	0.99870298	4.58E-50	1.09E-49
RP11-12J10.3	NA	NA	NA
FAM53B	1.5729572	7.53E-08	9.50E-08
RP11-12J10.4	NA	NA	NA
FAM53B-AS1	NA	NA	NA
METTL10	2.13472763	3.32E-23	5.44E-23
FAM175B	1.32520204	4.47E-68	1.39E-67
ZRANB1	2.20026751	2.91E-69	9.23E-69
RP11-298J20	NA	NA	NA
CTBP2	1.53789635	0.5016464	0.51475504
RP11-59C5.3	NA	1.21E-105	7.31E-105
RP11-151A1(	Inf	2.69E-119	2.34E-118
TEX36-AS1	NA	NA	NA
TEX36	NA	NA	NA
FLJ37035	3.37062257	0.99513299	0.995572
RP11-383C5.	NA	NA	NA
EDRF1	2.52796693	3.29E-128	4.07E-127
EDRF1-AS1	NA	NA	NA
MMP21	NA	NA	NA
UROS	2.12881425	4.61E-131	6.59E-130
BCCIP	1.50386089	2.36E-54	6.01E-54
DHX32	1.56047341	5.48E-19	8.42E-19
FANK1	Inf	5.86E-06	7.10E-06
FANK1-AS1	NA	NA	NA
ADAM12	NA	1.07E-156	3.98E-154
LINC00601	NA	NA	NA
C10orf90	NA	NA	NA
DOCK1	1.77893969	7.29E-10	9.58E-10
RP11-223P11	NA	NA	NA
RP11-223P11	NA	NA	NA

FAM196A	NA	2.20E-06	2.69E-06
NPS	NA	NA	NA
FOXI2	NA	NA	NA
CLRN3	NA	NA	NA
PTPRE	0.29961089	5.99E-138	1.35E-136
RP11-4C20.3	NA	NA	NA
MKI67	1.72029333	2.84E-64	8.33E-64
LINC01163	NA	NA	NA
RP11-264E18	NA	NA	NA
RP11-442O1	NA	NA	NA
RP11-442O1	NA	NA	NA
RP11-168C9.	NA	NA	NA
MGMT	NA	2.33E-16	3.44E-16
RP11-109A6.	NA	NA	NA
RP11-109A6.	NA	NA	NA
EBF3	2.11089494	1.91E-07	2.38E-07
RP11-234G1	NA	NA	NA
RP11-234G1	NA	NA	NA
LINC00959	Inf	6.79E-101	3.72E-100
RP11-500G1	Inf	1.64E-144	9.77E-143
GLRX3	0.95493512	2.86E-128	3.55E-127
RP11-540N6.	NA	NA	NA
TCERG1L	NA	NA	NA
TCERG1L-AS	NA	NA	NA
LINC01164	NA	NA	NA
ABC11-4932	NA	NA	NA
PPP2R2D	1.95713568	3.67E-34	7.01E-34
BNIP3	1.62707352	2.31E-07	2.88E-07
RP11-45A17.	2.99610895	0.00051779	0.00059559
RP11-45A17.	Inf	9.32E-32	1.72E-31
JAKMIP3	NA	NA	NA
RP11-140A1	NA	0.00673984	0.00750886
DPYSL4	0.65539883	2.15E-51	5.23E-51
STK32C	1.68190661	3.06E-39	6.28E-39
LRRC27	0.87386511	2.97E-06	3.62E-06
PWWP2B	2.17898833	0.85408673	0.86034656
RP11-432J24	Inf	1.28E-151	2.24E-149
RP11-432J24	Inf	6.62E-26	1.13E-25
C10orf91	3.74513619	3.21E-05	3.81E-05
RP11-432J24	NA	NA	NA
LINC01165	NA	NA	NA
INPP5A	1.26758456	2.66E-05	3.17E-05
NKX6-2	NA	NA	NA
CFAP46	NA	NA	NA
LINC01166	NA	NA	NA

LINC01167	NA	NA	NA
LINC01168	NA	NA	NA
ADGRA1	NA	NA	NA
ADGRA1-AS1	NA	NA	NA
KNDC1	Inf	6.59E-46	1.48E-45
UTF1	NA	0.02068765	0.0226667
VENTX	NA	NA	NA
MIR202HG	NA	NA	NA
RP13-49I15.6	Inf	6.44E-13	8.95E-13
ADAM8	4.49416342	1.52E-14	2.17E-14
TUBGCP2	1.68929547	1.51E-40	3.16E-40
RP11-122K13	NA	6.36E-12	8.69E-12
ZNF511	1.75332833	5.35E-16	7.86E-16
PRAP1	NA	NA	NA
CALY	NA	NA	NA
RP11-122K13	NA	NA	NA
FUOM	1.87256809	1.76E-70	5.66E-70
ECHS1	1.05488003	3.69E-134	6.28E-133
RP11-122K13	NA	NA	NA
PAOX	2.54669261	3.77E-67	1.16E-66
RP11-108K14	NA	NA	NA
MTG1	1.94747082	0.00098466	0.00112463
SPRN	NA	NA	NA
CYP2E1	Inf	2.67E-21	4.24E-21
RP11-108K14	Inf	5.93E-12	8.11E-12
SYCE1	NA	NA	NA
FRG2B	NA	NA	NA
FAM138D	NA	NA	NA
IQSEC3	NA	4.79E-31	8.75E-31
RP11-598F7.	NA	NA	NA
RP11-598F7.	NA	NA	NA
RP11-598F7.	NA	NA	NA
RP11-598F7.	NA	NA	NA
SLC6A12	NA	0.00216643	0.00244652
RP11-283I3.1	NA	NA	NA
RP11-283I3.2	NA	NA	NA
SLC6A13	NA	NA	NA
KDM5A	2.0598249	5.08E-07	6.30E-07
CCDC77	1.09473212	7.54E-29	1.34E-28
B4GALNT3	0.74902724	3.67E-124	3.82E-123
NINJ2	1.49805447	2.67E-44	5.85E-44
RP5-1154L15	NA	NA	NA
RP11-218M2	3.74513619	0.9665618	0.96821624
RP11-218M2	NA	NA	NA
WNK1	1.72144856	3.98E-27	6.89E-27

RAD52	2.24708171	1.47E-85	5.99E-85
RP11-359B1:	9.73735409	1.29E-27	2.24E-27
ERC1	1.12354086	2.21E-117	1.80E-116
RP5-951N9.1	NA	NA	NA
LINC00942	NA	NA	NA
WNT5B	NA	0.00048371	0.00055692
FBXL14	NA	1.65E-130	2.29E-129
ADIPOR2	1.47031273	4.60E-22	7.41E-22
CACNA2D4	1.4014058	9.03E-53	2.24E-52
LRTM2	0	1.82E-94	8.79E-94
RP5-1096D1	NA	NA	NA
LINC00940	NA	3.46E-36	6.82E-36
DCP1B	3.37062257	4.53E-11	6.08E-11
CACNA1C	NA	1.29E-40	2.70E-40
RP5-1096D1	NA	0.22131413	0.23213927
RP1-53O8.2	NA	NA	NA
CACNA1C-AS	NA	NA	NA
CACNA1C-AS	NA	NA	NA
CACNA1C-AS	NA	0.05248562	0.05663776
CACNA1C-AS	NA	2.00E-44	4.40E-44
RP11-986G1	NA	NA	NA
RP11-885B4.	NA	NA	NA
RP11-885B4.	NA	NA	NA
FKBP4	1.1812722	1.44E-78	5.21E-78
RP4-816N1.6	1.74773022	0.00024408	0.00028336
RP4-816N1.7	NA	6.50E-37	1.29E-36
ITFG2	2.41353221	1.39E-75	4.84E-75
NRIP2	NA	5.03E-145	3.36E-143
FOXM1	2.38083658	5.78E-105	3.45E-104
RHNO1	2.07339424	8.77E-19	1.34E-18
TULP3	0.52872511	3.72E-53	9.30E-53
TEAD4	2.10143753	0.06789577	0.07293729
RP11-253E3.	10.4863813	5.70E-72	1.88E-71
TSPAN9	0.18725681	3.07E-101	1.69E-100
RP5-1063M2	NA	NA	NA
RP5-1063M2	NA	NA	NA
RP11-476M1	NA	NA	NA
PRMT8	NA	2.54E-72	8.39E-72
RP11-476M1	NA	NA	NA
CRACR2A	4.49416342	8.15E-49	1.91E-48
PARP11	2.49675746	9.65E-06	1.16E-05
RP11-664D1.	NA	NA	NA
RP11-320N7.	NA	NA	NA
CCND2-AS1	Inf	2.59E-37	5.17E-37
CCND2-AS2	NA	NA	NA

CCND2	4.86867704	1.42E-38	2.89E-38
TIGAR	0.74902724	2.50E-121	2.33E-120
FGF23	NA	NA	NA
FGF6	NA	6.42E-05	7.57E-05
C12orf4	1.35761187	0.99616851	0.99655303
RAD51AP1	1.25684231	4.77E-133	7.65E-132
DYRK4	1.38281952	4.42E-11	5.94E-11
RP11-500M8	NA	NA	NA
AKAP3	Inf	5.39E-103	3.08E-102
NDUFA9	0.70622568	3.75E-142	1.39E-140
RP11-234B2	NA	NA	NA
RP11-234B2	NA	NA	NA
GALNT8	NA	9.25E-08	1.16E-07
RP11-234B2	NA	NA	NA
KCNA6	NA	NA	NA
KCNA1	NA	NA	NA
RP11-429A2	NA	NA	NA
RP11-429A2	NA	NA	NA
KCNA5	NA	NA	NA
RP11-319E1	NA	NA	NA
RP11-1038A	NA	NA	NA
RP11-319E1	NA	NA	NA
RP11-1038A	NA	NA	NA
RP11-1038A	NA	NA	NA
NTF3	NA	NA	NA
ANO2	NA	NA	NA
VWF	NA	NA	NA
RP3-454B23.	NA	NA	NA
CD9	Inf	1.10E-14	1.58E-14
PLEKHG6	NA	3.72E-18	5.65E-18
TNFRSF1A	1.66015738	4.93E-34	9.40E-34
SCNN1A	NA	1.71E-23	2.80E-23
LTBR	2.42185473	2.29E-70	7.36E-70
RP1-102E24.	2.24708171	2.72E-86	1.13E-85
CD27	NA	NA	NA
TAPBPL	4.49416342	4.38E-36	8.60E-36
VAMP1	1.68531128	1.42E-43	3.09E-43
RP1-102E24.	NA	2.43E-148	2.94E-146
MRPL51	1.81197553	0.40610955	0.41892531
NCAPD2	2.56485084	7.94E-125	8.46E-124
RP5-940J5.3	NA	2.03E-08	2.59E-08
GAPDH	1.23770213	1.63E-115	1.26E-114
RP5-940J5.9	4.24448768	1.42E-14	2.04E-14
IFFO1	0.99870298	7.45E-15	1.07E-14
RP5-940J5.8	NA	1.05E-52	2.61E-52

NOP2	1.45996834	2.68E-82	1.04E-81
CHD4	1.68531128	0.01983118	0.02173749
RP5-940J5.6	NA	4.52E-06	5.48E-06
LPAR5	1.49805447	0.00011618	0.000136
ACRBP	NA	1.25E-114	9.44E-114
ING4	1.79766537	1.69E-15	2.46E-15
RP4-761J14.8	NA	2.00E-44	4.40E-44
ZNF384	Inf	1.62E-52	4.01E-52
PIANP	NA	NA	NA
RP4-761J14.9	NA	NA	NA
COPS7A	1.39474037	6.10E-17	9.07E-17
RP4-761J14.1	NA	5.25E-21	8.32E-21
MLF2	1.76894772	9.81E-24	1.62E-23
PTMS	1.66620345	1.36E-106	8.39E-106
LAG3	0	1.91E-52	4.72E-52
CD4	2.31956822	9.73E-71	3.15E-70
GPR162	2.0598249	5.74E-36	1.13E-35
P3H3	2.02237354	7.66E-07	9.45E-07
GNB3	1.19844358	1.25E-13	1.76E-13
CDCA3	1.20413239	1.55E-128	1.94E-127
USP5	1.5711303	1.64E-05	1.96E-05
TPI1	1.23233212	3.62E-127	4.28E-126
SPSB2	1.74773022	1.91E-14	2.73E-14
LRRC23	1.24837873	1.74E-19	2.68E-19
U47924.32	NA	1.82E-99	9.66E-99
ENO2	0.74902724	6.66E-81	2.52E-80
ATN1	2.33696498	1.06E-129	1.42E-128
C12orf57	1.35626331	2.04E-145	1.64E-143
U47924.31	0.37451362	0.02946877	0.03208826
PTPN6	1.25684231	5.48E-94	2.62E-93
U47924.27	NA	4.17E-07	5.17E-07
U47924.29	NA	NA	NA
PHB2	1.46603286	7.33E-46	1.65E-45
EMG1	1.42083517	4.26E-73	1.43E-72
RP3-461F17.	Inf	0.0071433	0.00795395
LPCAT3	1.32181277	3.10E-100	1.68E-99
C1S	Inf	0.9050583	0.90967194
C1R	0	1.03E-13	1.45E-13
C1RL	3.1459144	1.76E-65	5.27E-65
C1RL-AS1	1.08949416	3.31E-30	5.99E-30
ABC12-4924	NA	NA	NA
RBP5	Inf	1.96E-125	2.13E-124
RP11-273B2(	NA	NA	NA
CLSTN3	3.932393	2.15E-72	7.14E-72
RP11-273B2(	NA	NA	NA

PEX5	2.99610895	9.03E-68	2.80E-67
ACSM4	NA	NA	NA
CD163L1	3.09824903	0.00124023	0.00141165
CD163	NA	NA	NA
APOBEC1	NA	NA	NA
GDF3	0.37451362	5.59E-69	1.76E-68
DPPA3	NA	NA	NA
CLEC4C	NA	NA	NA
NANOGNB	NA	NA	NA
NANOG	NA	0.00310902	0.00349813
SLC2A14	NA	NA	NA
SLC2A3	1.49805447	1.26E-15	1.83E-15
RP11-69M1.8	NA	1.30E-91	5.94E-91
FOXJ2	4.30690661	5.55E-55	1.42E-54
C3AR1	0.74902724	6.71E-09	8.65E-09
NECAP1	2.24708171	0.00890097	0.00987533
CLEC4A	0.37451362	2.30E-57	6.06E-57
ZNF705A	NA	1.06E-91	4.87E-91
FAM66C	Inf	0.00016297	0.00019001
RP11-266K4.	NA	NA	NA
RP11-266K4.	NA	NA	NA
FAM90A1	0	1.33E-16	1.97E-16
RP11-266K4.	Inf	4.03E-64	1.18E-63
LINC00937	4.49416342	5.57E-09	7.20E-09
RP11-90D4.3	NA	NA	NA
CLEC6A	NA	NA	NA
CLEC4D	NA	NA	NA
CLEC4E	NA	NA	NA
RP11-561P12	NA	NA	NA
AICDA	NA	NA	NA
MFAP5	NA	1.76E-99	9.38E-99
RP11-20D14.	NA	NA	NA
RIMKLB	4.86867704	5.02E-104	2.94E-103
A2ML1-AS1	NA	NA	NA
RP11-20D14.	NA	NA	NA
A2ML1-AS2	NA	NA	NA
A2ML1	NA	NA	NA
RP11-259O18	NA	NA	NA
PHC1	1.49805447	0.05176282	0.05587441
M6PR	1.39912635	5.68E-60	1.56E-59
KLRG1	8.23929961	0.00041062	0.00047404
RP11-259O18	NA	NA	NA
A2M-AS1	NA	NA	NA
A2M	NA	NA	NA
PZP	NA	NA	NA

LINC00987	NA	1.98E-138	4.65E-137
RP11-118B2:	NA	NA	NA
RP13-735L24	Inf	7.21E-51	1.74E-50
KLRB1	Inf	1.75E-10	2.32E-10
RP11-705C1!	Inf	2.43E-31	4.46E-31
CLEC2D	6.74124514	1.33E-28	2.36E-28
RP11-75L1.1	NA	NA	NA
CLECL1	1.84851676	2.00E-10	2.66E-10
CD69	2.19714656	2.48E-97	1.27E-96
KLRF1	2.24708171	0.00151686	0.00172144
CLEC2B	1.20810845	5.07E-27	8.77E-27
RP11-290C1(	NA	1.84E-75	6.37E-75
KLRF2	NA	NA	NA
CLEC2A	NA	NA	NA
AC091814.2	NA	NA	NA
AC091814.3	NA	NA	NA
CLEC12A	0.10700389	1.76E-134	3.06E-133
CLEC1B	NA	NA	NA
CLEC12B	NA	NA	NA
RP11-133L14	NA	NA	NA
CLEC9A	NA	NA	NA
CLEC1A	NA	NA	NA
CLEC7A	NA	2.21E-20	3.46E-20
OLR1	NA	NA	NA
TMEM52B	NA	NA	NA
GABARAPL1	0.37451362	4.56E-94	2.19E-93
RP11-656E2(	NA	NA	NA
KLRD1	NA	8.37E-60	2.29E-59
RP11-277P1:	NA	NA	NA
RP11-277P1:	NA	NA	NA
RP11-277P1:	0	1.26E-52	3.14E-52
KLRK1	NA	NA	NA
KLRC4-KLRK1	NA	NA	NA
KLRC4	NA	NA	NA
KLRC3	NA	NA	NA
RP11-277P1:	NA	NA	NA
KLRC2	NA	NA	NA
KLRC1	NA	2.22E-08	2.83E-08
EIF2S3L	Inf	1.27E-32	2.37E-32
RP11-291B2:	NA	NA	NA
MAGOHB	1.45644185	1.24E-50	2.98E-50
STYK1	NA	1.43E-44	3.15E-44
YBX3	1.83244163	0.00136995	0.00155675
RP13-81N3.2	NA	NA	NA
TAS2R7	NA	NA	NA

TAS2R8	NA	NA	NA
TAS2R9	NA	NA	NA
PRH1	0.37451362	3.59E-05	4.26E-05
TAS2R10	NA	1.01E-11	1.37E-11
PRR4	2.99610895	1.21E-71	3.97E-71
PRH1-PRR4	NA	NA	NA
TAS2R13	NA	NA	NA
PRH2	NA	NA	NA
TAS2R14	0.37451362	0.05532683	0.05962572
TAS2R50	NA	NA	NA
TAS2R20	NA	NA	NA
TAS2R19	NA	NA	NA
TAS2R31	NA	1.93E-35	3.75E-35
TAS2R46	NA	6.66E-23	1.08E-22
TAS2R43	NA	NA	NA
TAS2R30	NA	9.23E-11	1.23E-10
SMIM10L1	2.47019621	1.65E-93	7.82E-93
TAS2R42	NA	NA	NA
RP11-711K1.	NA	NA	NA
PRB3	NA	NA	NA
PRB4	NA	NA	NA
PRB1	NA	NA	NA
PRB2	NA	NA	NA
RP11-434C1.	NA	NA	NA
LINC01252	NA	4.84E-62	1.37E-61
ETV6	2.76203794	1.15E-48	2.68E-48
BCL2L14	NA	1.62E-79	5.97E-79
LRP6	2.24708171	1.23E-06	1.51E-06
MANSC1	1.49805447	4.60E-06	5.58E-06
LOH12CR2	0.24967575	0.03486142	0.03785121
BORCS5	1.57689945	5.94E-23	9.69E-23
DUSP16	1.87256809	2.79E-44	6.10E-44
RP11-253I19	NA	NA	NA
CREBL2	3.99481193	1.06E-10	1.41E-10
RP11-180M1	NA	NA	NA
GPR19	0	3.09E-47	7.08E-47
RP11-180M1	NA	NA	NA
CDKN1B	1.64072633	0.2971475	0.30937446
RP11-180M1	NA	NA	NA
APOLD1	1.64785992	6.65E-82	2.56E-81
DDX47	Inf	0.64225657	0.65422901
GPRC5A	NA	NA	NA
RP11-392P7.	NA	NA	NA
GPRC5D	NA	NA	NA
RP11-392P7.	NA	NA	NA

HEBP1	1.55155642	4.85E-119	4.19E-118
RP11-377D9.	NA	NA	NA
FAM234B	1.42315175	0.00012687	0.00014838
GSG1	NA	1.06E-128	1.34E-127
EMP1	0.23832685	8.92E-113	6.47E-112
LINC01559	NA	NA	NA
GRIN2B	NA	NA	NA
RP11-4N23.4	NA	NA	NA
RP11-134N1.	NA	NA	NA
ATF7IP	1.29142627	1.16E-66	3.52E-66
PLBD1	NA	NA	NA
RP11-502N1:	NA	NA	NA
PLBD1-AS1	NA	NA	NA
GUCY2C	NA	NA	NA
RP11-174G6.	NA	NA	NA
HIST4H4	Inf	4.89E-144	2.59E-142
H2AFJ	NA	3.24E-55	8.32E-55
WBP11	1.83482641	8.56E-25	1.43E-24
C12orf60	1.74773022	1.78E-12	2.45E-12
SMCO3	NA	NA	NA
ART4	NA	NA	NA
RP11-233G1.	NA	NA	NA
MGP	NA	3.05E-50	7.28E-50
ERP27	1.49805447	0.00012753	0.00014912
ARHGDIB	1.16407907	1.77E-142	6.83E-141
PDE6H	NA	1.84E-95	9.09E-95
LINC01489	NA	NA	NA
RERG	NA	NA	NA
RERG-AS1	NA	NA	NA
PTPRO	NA	NA	NA
EPS8	2.99610895	7.63E-22	1.22E-21
RP11-6B19.1	NA	NA	NA
RP11-6B19.2	NA	NA	NA
STRAP	1.41073265	1.02E-81	3.91E-81
DERA	1.38976138	8.27E-113	6.00E-112
SLC15A5	NA	NA	NA
MGST1	1.32352386	3.19E-27	5.54E-27
LMO3	NA	2.16E-14	3.09E-14
RP11-424M2	NA	NA	NA
RP11-69C13.	NA	NA	NA
RP11-239A1	NA	NA	NA
RP11-871F6.	NA	NA	NA
RP11-606D9.	NA	NA	NA
RERGL	NA	NA	NA
PIK3C2G	NA	NA	NA

PLCZ1	NA	NA	NA
RP11-361I14	NA	NA	NA
CAPZA3	NA	NA	NA
PLEKHA5	1.31079767	9.73E-32	1.79E-31
AEBP2	1.89040208	1.25E-46	2.83E-46
RP11-405A1:	NA	NA	NA
RP11-664H1	NA	NA	NA
CTC-465D4.1	NA	NA	NA
RP11-284H1!	NA	NA	NA
PDE3A	Inf	1.77E-27	3.07E-27
SLCO1C1	NA	NA	NA
SLCO1B3	NA	NA	NA
RP11-545J16	NA	NA	NA
SLCO1B7	NA	NA	NA
RP11-125O5.	NA	NA	NA
SLCO1B1	NA	NA	NA
SLCO1A2	NA	NA	NA
IAPP	NA	1.17E-136	2.37E-135
PYROXD1	2.16385646	0.00213441	0.00241141
RECQL	2.59956512	8.35E-17	1.24E-16
GOLT1B	1.77042802	2.01E-06	2.46E-06
SPX	NA	7.31E-62	2.07E-61
GYS2	NA	NA	NA
LDHB	0.87787691	8.32E-123	8.17E-122
RP11-59N23.	NA	NA	NA
RP11-59N23.	NA	NA	NA
KCNJ8	NA	NA	NA
ABCC9	NA	NA	NA
RP11-729I10	NA	NA	NA
CMAS	1.47266372	2.20E-113	1.62E-112
ST8SIA1	NA	NA	NA
RP11-73M14	NA	NA	NA
C2CD5	6.36673152	6.98E-99	3.68E-98
RP11-359J14	NA	NA	NA
RP11-268P4.	NA	NA	NA
RP11-268P4.	NA	1.28E-90	5.75E-90
ETNK1	2.50636037	8.14E-30	1.46E-29
RP11-114G2:	NA	NA	NA
RP11-449P1.	NA	NA	NA
RP11-153K1(	NA	NA	NA
RP11-153K1(	NA	NA	NA
SOX5	NA	NA	NA
RP11-437F6.	NA	NA	NA
RP11-444D3.	NA	NA	NA
RP11-778H2.	NA	NA	NA

LINC00477	NA	NA	NA
RP11-625L16	NA	NA	NA
BCAT1	2.70522459	9.14E-139	2.24E-137
RP11-625L16	NA	NA	NA
RP11-662I13	NA	NA	NA
C12orf77	NA	NA	NA
RP11-713N1	NA	NA	NA
LRMP	1.78614187	0.67610743	0.68732158
RP11-713N1	NA	NA	NA
RP11-713N1	NA	NA	NA
CASC1	NA	NA	NA
LYRM5	1.89869695	0.07469226	0.08011037
KRAS	1.74456977	1.96E-12	2.71E-12
RP11-295I5.3	NA	5.35E-09	6.91E-09
RP11-295I5.4	NA	NA	NA
RP11-707G1	0.74902724	5.23E-42	1.11E-41
LMNTD1	NA	3.93E-28	6.90E-28
RP11-443N2	NA	NA	NA
RP11-443N2	NA	NA	NA
RP11-443N2	NA	NA	NA
RASSF8-AS1	Inf	2.32E-46	5.26E-46
RASSF8	NA	0.02634015	0.02873845
BHLHE41	0.42801556	2.37E-59	6.43E-59
SSPN	0	7.98E-61	2.22E-60
RP11-283G6.	NA	NA	NA
RP11-283G6.	NA	NA	NA
RP11-283G6.	NA	NA	NA
RP11-283G6.	NA	0.10723105	0.11417923
RP11-612B6.	NA	NA	NA
RP11-513G1	NA	NA	NA
ITPR2	1.84375935	0.24672573	0.2582414
RP11-666F17	NA	9.18E-05	1.08E-04
ASUN	2.04734112	5.31E-06	6.44E-06
FGFR1OP2	1.83435242	2.02E-10	2.68E-10
RP11-421F16	NA	2.67E-05	3.18E-05
TM7SF3	1.60389528	1.41E-74	4.81E-74
MED21	1.92458387	3.52E-19	5.42E-19
RP11-582E3.	1.1770428	0.96945899	0.97101115
C12orf71	NA	NA	NA
RP11-582E3.	NA	NA	NA
STK38L	1.99740597	1.71E-26	2.93E-26
ARNTL2	3.12094682	4.66E-102	2.61E-101
ARNTL2-AS1	NA	NA	NA
SMCO2	1.49805447	1.19E-05	1.43E-05
PPFIBP1	2.43433852	2.17E-11	2.93E-11

RP11-709A2:	NA	NA	NA
RP11-1060J1	NA	NA	NA
REP15	Inf	4.40E-133	7.09E-132
RP11-1060J1	NA	NA	NA
RP11-1060J1	NA	NA	NA
MRPS35	1.17771578	4.64E-108	2.97E-107
MANSC4	NA	NA	NA
RP11-860B1:	NA	NA	NA
KLHL42	2.00875486	0.88452896	0.88967813
RP11-860B1:	NA	NA	NA
PTHLH	Inf	8.57E-150	1.22E-147
RP11-993B2:	NA	NA	NA
CCDC91	1.15475032	3.62E-92	1.68E-91
RP11-967K21	NA	6.09E-32	1.12E-31
RP11-425D1.	NA	NA	NA
RP11-977P2.	NA	NA	NA
RP11-946L16	0	0.00023646	0.00027455
FAR2	0.78159364	3.73E-56	9.70E-56
RP11-946L16	Inf	5.98E-21	9.48E-21
RP11-996F15	Inf	0.67323122	0.68465991
RP11-996F15	Inf	4.50E-59	1.22E-58
RP11-996F15	0	2.40E-34	4.60E-34
RP11-996F15	Inf	1.50E-127	1.80E-126
ERGIC2	1.33710647	1.40E-115	1.09E-114
OVCH1-AS1	NA	NA	NA
OVCH1	NA	NA	NA
TMTC1	NA	NA	NA
RP11-310I24	NA	NA	NA
RP11-776A1:	NA	NA	NA
RP11-776A1:	NA	NA	NA
RP11-155I9.1	NA	NA	NA
IPO8	2.44682231	1.52E-121	1.42E-120
CAPRIN2	4.49416342	4.08E-27	7.07E-27
RP11-77I22.2	NA	NA	NA
LINC00941	NA	7.72E-97	3.92E-96
TSPAN11	NA	NA	NA
RP11-551L14	NA	NA	NA
RP11-551L14	NA	NA	NA
DDX11-AS1	1.49805447	1.38E-07	1.73E-07
DDX11	2.14721141	2.99E-58	7.99E-58
OVOS2	0.87386511	1.39E-09	1.82E-09
FAM60A	1.33240422	1.36E-51	3.32E-51
FLJ13224	Inf	0.00218195	0.00246374
RP11-771K4.	NA	1.08E-35	2.10E-35
DENND5B	1.64785992	0.00204524	0.00231168

DENND5B-A!	NA	NA	NA
METTL20	0.56177043	0.71911835	0.72920715
AMN1	1.71206226	6.92E-105	4.13E-104
RP11-467L13	1.87256809	0.0005133	0.00059062
H3F3C	Inf	1.63E-13	2.28E-13
RP11-428G5.	NA	NA	NA
KIAA1551	1.20996708	6.04E-96	3.01E-95
RP11-50I19.2	1.49805447	0.07326089	0.07859841
RP11-843B15	NA	NA	NA
BICD1	1.60505837	0.03916706	0.04246258
FGD4	Inf	3.61E-122	3.46E-121
DNM1L	2.0130107	0.2520599	0.26361161
RP11-278C7.	0.37451362	0.10773093	0.11469804
YARS2	1.29377432	2.15E-50	5.16E-50
PKP2	1.39105058	0.02173291	0.02377463
RP11-267D19	NA	NA	NA
SYT10	NA	NA	NA
RP11-847H18	NA	NA	NA
ALG10	2.43433852	0.3632709	0.37614638
RP11-847H18	NA	NA	NA
RP11-313F23	NA	NA	NA
RP11-313F23	NA	NA	NA
ALG10B	1.12354086	4.38E-23	7.15E-23
RP11-804F13	NA	NA	NA
CPNE8	1.42996109	2.88E-36	5.66E-36
RP11-396F22	4.99351492	0.00012124	0.00014189
RP11-554L12	NA	NA	NA
KIF21A	1.92363813	3.77E-19	5.81E-19
ABCD2	NA	5.48E-110	3.68E-109
C12orf40	NA	NA	NA
SLC2A13	2.49675746	1.76E-07	2.20E-07
RP11-476D10	NA	NA	NA
AC079630.2	NA	NA	NA
AC079630.4	NA	9.99E-36	1.95E-35
LRRK2	0	4.10E-121	3.77E-120
RP11-115F18	NA	NA	NA
CNTN1	NA	NA	NA
PDZRN4	NA	NA	NA
RP11-413B19	NA	NA	NA
RP11-630C16	NA	NA	NA
RP11-630C16	NA	9.95E-13	1.38E-12
GXYLT1	2.32198444	1.60E-10	2.12E-10
YAF2	2.64202335	5.63E-98	2.93E-97
PPHLN1	1.58859623	5.37E-104	3.15E-103
ZCRB1	2.24076081	6.44E-111	4.44E-110

PRICKLE1	Inf	6.75E-147	7.29E-145
RP11-328C8.	NA	7.05E-52	1.73E-51
RP11-609L23	NA	NA	NA
RP11-25I15.2	NA	NA	NA
RP11-25I15.3	NA	NA	NA
RP11-547C5.	NA	NA	NA
RP11-547C5.	NA	NA	NA
RP11-118A3.	NA	NA	NA
ADAMTS20	NA	NA	NA
PUS7L	1.33576524	3.92E-60	1.08E-59
IRAK4	3.65150778	9.62E-72	3.15E-71
TWF1	1.24837873	7.81E-05	9.19E-05
TMEM117	1.12354086	1.07E-50	2.57E-50
RP11-46I1.1	NA	NA	NA
RP11-499A1(	NA	NA	NA
NELL2	NA	2.72E-103	1.57E-102
DBX2	NA	NA	NA
RP11-478B9.	NA	NA	NA
ANO6	3.16255945	5.23E-67	1.60E-66
RP11-352M1	NA	NA	NA
RP11-352M1	2.56809339	3.27E-26	5.60E-26
ARID2	3.37062257	6.92E-106	4.21E-105
SCAF11	1.95323257	2.43E-06	2.97E-06
SLC38A1	1.93227316	1.36E-32	2.53E-32
SLC38A2	1.51886079	0.20586309	0.21637033
RP11-474P2.	NA	NA	NA
RP11-96H19.	NA	0.00516667	0.00577534
RP11-474P2.	NA	NA	NA
RP11-474P2.	Inf	0.10144888	0.10820657
RP11-446N1!	2.24708171	9.95E-102	5.54E-101
SLC38A4	NA	NA	NA
RP11-618L22	NA	NA	NA
AMIGO2	1.19844358	0.08941196	0.09559829
PCED1B	0.74902724	1.30E-27	2.27E-27
RP11-493L12	NA	NA	NA
RP11-493L12	Inf	2.14E-48	4.99E-48
RP11-493L12	NA	NA	NA
RP11-493L12	NA	NA	NA
RP1-90J4.1	NA	NA	NA
RPAP3	1.28059495	3.30E-31	6.04E-31
RP1-197B17.	4.11964981	1.34E-121	1.26E-120
ENDOU	NA	1.02E-20	1.61E-20
RP1-197B17.	NA	3.90E-124	4.05E-123
RAPGEF3	0.49935149	3.62E-68	1.13E-67
SLC48A1	2.88375486	3.14E-100	1.70E-99

HDAC7	3.54085603	4.51E-93	2.13E-92
RP5-1057I20	NA	2.19E-24	3.65E-24
RP5-1057I20	NA	9.42E-102	5.25E-101
RP5-1057I20	NA	NA	NA
VDR	3.74513619	2.03E-21	3.23E-21
RP11-89H19.	0	2.51E-72	8.30E-72
RP11-89H19.	NA	NA	NA
TMEM106C	2.05107458	8.08E-26	1.37E-25
COL2A1	4.79377432	1.83E-83	7.22E-83
RP1-228P16.	NA	NA	NA
RP1-228P16.	NA	NA	NA
RP1-228P16.	NA	NA	NA
RP1-228P16.	NA	0.02865859	0.03122293
SENP1	2.43433852	4.55E-51	1.10E-50
PFKM	11.2354086	1.44E-84	5.81E-84
ASB8	1.74098223	6.98E-12	9.54E-12
RP11-474C8.	NA	NA	NA
CCDC184	NA	1.52E-128	1.91E-127
RP11-370I10	NA	NA	NA
OR10AD1	NA	NA	NA
RP11-370I10	NA	NA	NA
RP11-370I10	NA	NA	NA
H1FNT	NA	NA	NA
ZNF641	0.93628405	0.99803247	0.99819754
RP11-370I10	NA	NA	NA
RP11-370I10	NA	5.04E-119	4.33E-118
ANP32D	NA	NA	NA
C12orf54	NA	NA	NA
RP11-722P11	NA	NA	NA
OR8S1	NA	NA	NA
LALBA	NA	NA	NA
KANSL2	1.49805447	0.3559202	0.36872455
CCNT1	2.52796693	5.22E-60	1.44E-59
LINC00935	NA	NA	NA
ADCY6	0.93628405	5.08E-16	7.46E-16
RP11-579D7.	NA	NA	NA
RP11-579D7.	Inf	7.59E-10	9.98E-10
CACNB3	2.19714656	0.02124682	0.02326391
DDX23	2.00389105	1.39E-43	3.02E-43
RND1	1.49805447	1.69E-45	3.77E-45
RP11-302B13	0.74902724	0.00559201	0.00624463
CCDC65	NA	7.54E-30	1.36E-29
FKBP11	1.13588746	5.74E-126	6.43E-125
AC073610.5	NA	NA	NA
ARF3	1.65261565	2.79E-28	4.91E-28

WNT10B	1.3732166	0.12761955	0.1355388
WNT1	NA	NA	NA
DDN	1.61632193	1.56E-21	2.49E-21
RP11-386G1	NA	NA	NA
RP11-386G1	1.91418072	0.03554085	0.03857275
PRKAG1	1.8385214	1.80E-137	3.87E-136
KMT2D	2.03307393	7.46E-107	4.64E-106
RHEBL1	2.14007782	0.08198894	0.08781175
DHH	NA	4.79E-31	8.75E-31
RP11-386G1	NA	1.89E-61	5.30E-61
LMBR1L	1.45124027	1.85E-47	4.26E-47
TUBA1B	1.42269503	9.05E-140	2.49E-138
RP11-386G1	0.20806312	4.71E-134	7.94E-133
TUBA1A	0.54928664	2.50E-145	1.94E-143
TUBA1C	1.38211168	1.33E-133	2.18E-132
RP11-977B1	0.74902724	8.15E-79	2.97E-78
RP11-161H2	NA	1.93E-09	2.52E-09
RP11-161H2	NA	7.88E-34	1.50E-33
PRPH	2.24708171	0.11792585	0.12540508
TROAP	2.32198444	0.01801315	0.0197674
C1QL4	0.74902724	4.66E-45	1.03E-44
DNAJC22	1.49805447	0.00415954	0.00466307
SPATS2	2.25809682	3.79E-07	4.71E-07
KCNH3	0	0.02322871	0.02538494
MCRS1	1.9320147	0.16004145	0.16900303
PRPF40B	Inf	6.60E-129	8.44E-128
RP11-133N2	NA	NA	NA
FAM186B	NA	NA	NA
FMNL3	2.24708171	0.17398594	0.18346137
TMBIM6	1.06809926	7.69E-142	2.77E-140
NCKAP5L	1.99740597	0.00039596	0.00045722
BCDIN3D-AS	Inf	0.84736067	0.85414031
BCDIN3D	1.87256809	3.70E-21	5.88E-21
RP11-70F11.	NA	NA	NA
FAIM2	NA	NA	NA
RP11-70F11.	NA	NA	NA
RP11-70F11.	NA	NA	NA
AQP2	NA	NA	NA
RP11-469H8.	Inf	2.63E-134	4.50E-133
RP11-469H8.	NA	NA	NA
AQP5	NA	NA	NA
AQP6	NA	4.16E-55	1.07E-54
RACGAP1	2.11867704	0.32248813	0.33508474
ASIC1	2.01661179	4.37E-06	5.31E-06
SMARCD1	3.41223519	1.28E-144	7.81E-143

GPD1	NA	3.98E-136	7.64E-135
COX14	2.14427405	3.69E-102	2.08E-101
RP4-605O3.4	3.74513619	8.66E-05	1.02E-04
CERS5	1.12354086	8.68E-79	3.16E-78
LIMA1	0.37451362	3.26E-97	1.67E-96
RP3-405J10.4	NA	NA	NA
FAM186A	NA	NA	NA
LARP4	3.4297563	3.29E-136	6.38E-135
DIP2B	2.47178988	2.90E-09	3.77E-09
ATF1	0.95330739	4.29E-107	2.67E-106
TMPRSS12	NA	NA	NA
METTL7A	3.55787938	2.13E-06	2.61E-06
RP11-407N8.	NA	5.41E-108	3.46E-107
HIGD1C	NA	NA	NA
RP11-407N8.	NA	2.21E-66	6.71E-66
SLC11A2	1.43563554	5.25E-38	1.06E-37
LETMD1	1.81906615	4.56E-14	6.47E-14
CSRNP2	2.99610895	2.20E-05	2.62E-05
TFCP2	2.44682231	6.27E-74	2.13E-73
POU6F1	2.24708171	1.24E-47	2.85E-47
DAZAP2	1.66190418	1.06E-38	2.15E-38
SMAGP	1.65856031	2.45E-17	3.68E-17
BIN2	1.25347415	5.73E-39	1.17E-38
CELA1	NA	1.11E-41	2.36E-41
GALNT6	1.74773022	0.00083376	0.00095384
SLC4A8	Inf	0.98829452	0.98911233
RP11-607P23	NA	NA	NA
SCN8A	NA	0.00019792	0.00023035
RP11-923I11	NA	NA	NA
RP11-923I11	NA	NA	NA
RP11-923I11	NA	NA	NA
RP11-923I11	NA	NA	NA
RP11-923I11	NA	NA	NA
ANKRD33	NA	NA	NA
ACVRL1	NA	3.82E-20	5.97E-20
ACVR1B	0.97949716	2.18E-66	6.63E-66
GRASP	0.74902724	0.54187911	0.55515986
NR4A1	1.12354086	0.08285782	0.08871092
RP11-1100L3	NA	NA	NA
ATG101	1.48336767	6.25E-66	1.88E-65
RP11-1100L3	NA	4.89E-93	2.30E-92
RP1-288H2.2	NA	NA	NA
KRT80	NA	NA	NA
C12orf80	NA	NA	NA
LINC00592	NA	NA	NA

KRT7	0.37451362	3.09E-95	1.52E-94
RP3-416H24.	NA	NA	NA
KRT86	NA	3.53E-31	6.46E-31
RP11-845M1	NA	NA	NA
KRT81	0	9.32E-21	1.47E-20
RP11-845M1	NA	NA	NA
KRT83	NA	NA	NA
KRT85	NA	NA	NA
KRT84	NA	NA	NA
RP3-416H24.	NA	NA	NA
KRT82	NA	NA	NA
RP11-1020M	NA	NA	NA
KRT75	NA	NA	NA
KRT6B	NA	NA	NA
KRT6C	NA	NA	NA
KRT6A	NA	NA	NA
KRT5	NA	NA	NA
KRT71	NA	NA	NA
KRT74	NA	NA	NA
KRT72	NA	NA	NA
KRT73-AS1	NA	NA	NA
KRT73	NA	NA	NA
KRT2	NA	1.02E-18	1.56E-18
KRT1	NA	NA	NA
KRT77	NA	NA	NA
RP11-641A6.	NA	NA	NA
KRT76	NA	NA	NA
KRT3	NA	NA	NA
KRT4	NA	NA	NA
KRT79	NA	NA	NA
KRT78	NA	NA	NA
KRT8	1.7183566	1.05E-28	1.87E-28
KRT18	0.84567591	3.00E-143	1.36E-141
EIF4B	2.53242542	2.06E-122	2.00E-121
RP11-983P16	0.99870298	2.47E-66	7.47E-66
TNS2	0.74902724	7.72E-13	1.07E-12
SPRYD3	2.49675746	9.51E-78	3.41E-77
IGFBP6	5.9922179	1.18E-32	2.21E-32
SOAT2	NA	NA	NA
CSAD	2.34071012	4.90E-35	9.49E-35
RP11-1136G	NA	NA	NA
ZNF740	4.04474708	1.46E-137	3.17E-136
ITGB7	NA	3.42E-29	6.10E-29
RARG	10.4863813	4.79E-132	7.22E-131
MFSD5	2.05378436	1.01E-77	3.61E-77

ESPL1	1.56047341	1.01E-07	1.26E-07
PFDN5	1.69904453	3.66E-76	1.28E-75
RP11-680A1	4.49416342	5.13E-19	7.89E-19
C12orf10	1.26328474	7.38E-138	1.66E-136
AAAS	1.06361868	1.06E-111	7.43E-111
SP7	NA	3.32E-106	2.04E-105
SP1	2.54669261	2.30E-111	1.60E-110
AMHR2	NA	1.55E-34	2.97E-34
PRR13	1.92914929	0.01699148	0.01867107
PCBP2	1.40963923	1.89E-109	1.26E-108
MAP3K12	3.2457847	1.03E-17	1.55E-17
RP11-793H1	5.9922179	8.70E-99	4.59E-98
TARBP2	1.64644666	1.84E-05	2.20E-05
RP11-793H1	NA	1.92E-06	2.35E-06
NPFF	NA	NA	NA
ATF7	2.01661179	0.00475806	0.00532483
RP11-793H1	NA	3.20E-69	1.01E-68
ATP5G2	1.86947613	0.00864055	0.00958993
CALCOCO1	1.49805447	1.03E-34	1.99E-34
CISTR	0	5.54E-103	3.16E-102
RP11-686F1	NA	1.13E-69	3.61E-69
HOXC13-AS	NA	NA	NA
HOXC13	NA	NA	NA
HOXC12	NA	NA	NA
HOTAIR	NA	1.14E-09	1.50E-09
HOXC11	1.02991245	1.39E-32	2.59E-32
HOXC10	1.85758755	0.02372714	0.02592338
RP11-834C1	NA	NA	NA
HOXC6	1.38281952	1.79E-17	2.68E-17
HOXC9	3.74513619	0.05331238	0.05748206
HOXC-AS1	NA	NA	NA
HOXC8	1.82371849	0.00495424	0.00554096
HOXC4	NA	9.03E-88	3.85E-87
AC012531.2	NA	NA	NA
HOXC5	NA	0.00390444	0.0043806
FLJ12825	NA	NA	NA
RP11-834C1	NA	NA	NA
RP11-834C1	NA	NA	NA
SMUG1	1.84135863	0.37043846	0.38339292
RP11-834C1	NA	1.64E-59	4.46E-59
RP11-834C1	NA	NA	NA
RP11-834C1	Inf	5.11E-21	8.10E-21
RP11-834C1	NA	NA	NA
RP11-834C1	NA	NA	NA
CBX5	1.42714065	4.93E-24	8.16E-24

RP11-968A1!	NA	NA	NA
HNRNPA1	1.46277211	2.28E-88	9.84E-88
NFE2	1.95029733	2.72E-13	3.80E-13
COPZ1	1.80074356	0.00068546	0.00078602
RP11-753H1(	NA	0.68766998	0.69860616
RP11-753H1(	NA	NA	NA
GPR84	Inf	8.63E-17	1.28E-16
ZNF385A	1.82371849	6.03E-32	1.11E-31
ITGA5	2.8463035	1.05E-97	5.41E-97
LINC01154	NA	NA	NA
GTSF1	1.93512986	0.00100486	0.00114741
NCKAP1L	2.11919901	2.36E-07	2.95E-07
RP11-1049A:	NA	NA	NA
PDE1B	3.74513619	3.32E-20	5.19E-20
PPP1R1A	5.9922179	0.03191134	0.03472086
LACRT	NA	NA	NA
DCD	NA	NA	NA
RP11-850F7.	NA	NA	NA
MUCL1	NA	NA	NA
TESPA1	1.64785992	8.20E-84	3.26E-83
RP11-616L12	NA	NA	NA
NEUROD4	NA	NA	NA
OR9K2	NA	NA	NA
OR10A7	NA	NA	NA
OR6C74	NA	NA	NA
OR6C6	NA	NA	NA
OR6C1	NA	NA	NA
OR6C3	NA	NA	NA
OR6C75	NA	NA	NA
OR6C65	NA	NA	NA
OR6C76	NA	NA	NA
RP11-110A1:	NA	NA	NA
OR6C2	NA	NA	NA
OR6C70	NA	NA	NA
OR6C68	NA	NA	NA
OR6C4	NA	NA	NA
OR2AP1	NA	NA	NA
OR10P1	NA	NA	NA
RP11-644F5.	NA	NA	NA
METTL7B	1.49805447	2.11E-54	5.37E-54
ITGA7	0.74902724	0.02183726	0.02388446
RP11-644F5.	NA	0.68188341	0.69288276
BLOC1S1	1.74683852	0.00044713	0.00051556
RDH5	1.49805447	0.00018361	0.0002138
CD63	1.21862462	2.88E-128	3.57E-127

RP11-644F5.	2.54669261	5.21E-30	9.40E-30
GDF11	2.99610895	2.01E-121	1.87E-120
SARNP	1.80647745	5.38E-18	8.15E-18
RP11-762I7.5	NA	7.05E-62	1.99E-61
ORMDL2	1.28184043	3.05E-65	9.10E-65
DNAJC14	2.41993415	1.67E-79	6.16E-79
MMP19	NA	0.4906801	0.50392971
PYM1	1.36943364	1.48E-103	8.57E-103
DGKA	2.49675746	3.68E-05	4.37E-05
PMEL	0	3.09E-102	1.74E-101
CDK2	1.76241703	0.01788727	0.0196352
RP11-973D8.	NA	NA	NA
RAB5B	2.07084001	0.02881681	0.03139153
SUOX	1.60505837	0.02751354	0.02999343
IKZF4	3.74513619	1.93E-107	1.22E-106
RP11-603J24	NA	3.59E-37	7.16E-37
RP11-603J24	NA	NA	NA
RPS26	2.39575579	1.91E-145	1.61E-143
ERBB3	1.49805447	8.50E-07	1.05E-06
RP11-603J24	NA	NA	NA
PA2G4	1.19140574	3.38E-122	3.24E-121
RP11-603J24	Inf	9.30E-82	3.56E-81
RPL41	2.77681845	4.97E-145	3.36E-143
ZC3H10	2.62159533	8.16E-08	1.03E-07
ESYT1	2.65783858	4.95E-19	7.62E-19
RP11-603J24	NA	NA	NA
RP11-603J24	NA	2.08E-87	8.79E-87
RP11-603J24	NA	3.13E-68	9.76E-68
MYL6B	2.81039972	2.72E-132	4.15E-131
MYL6	1.09225604	1.80E-145	1.58E-143
SMARCC2	1.56225681	4.82E-81	1.83E-80
RNF41	1.4287001	5.97E-51	1.44E-50
NABP2	1.97569503	9.01E-37	1.79E-36
SLC39A5	NA	7.29E-115	5.55E-114
ANKRD52	1.89459831	7.08E-23	1.15E-22
COQ10A	1.08479807	2.49E-140	7.29E-139
RP11-977G19	0	9.64E-116	7.50E-115
CS	2.29798648	3.07E-138	7.06E-137
RP11-977G19	NA	1.10E-34	2.12E-34
RP11-977G19	2.78210117	1.96E-76	6.86E-76
RP11-977G19	2.24708171	1.55E-12	2.15E-12
CNPY2	1.95876567	3.02E-38	6.11E-38
PAN2	1.74773022	2.30E-24	3.83E-24
IL23A	0.74902724	2.44E-21	3.88E-21
STAT2	0.83225249	1.89E-97	9.72E-97

APOF	NA	NA	NA
RP11-121C6.	NA	1.42E-67	4.37E-67
TIMELESS	2.26732569	3.38E-38	6.83E-38
MIP	NA	1.79E-18	2.74E-18
SPRYD4	1.87256809	9.60E-12	1.31E-11
GLS2	NA	NA	NA
RBMS2	1.1770428	7.49E-11	1.00E-10
BAZ2A	1.66034371	1.68E-07	2.10E-07
ATP5B	0.80481951	5.69E-140	1.59E-138
PTGES3	1.52108925	6.16E-37	1.22E-36
NACA	1.52657985	1.08E-119	9.59E-119
PRIM1	0.88218764	5.49E-133	8.77E-132
HSD17B6	0.74902724	2.45E-119	2.13E-118
SDR9C7	NA	NA	NA
RDH16	0.74902724	0.0076517	0.00851221
GPR182	NA	NA	NA
ZBTB39	2.09727626	2.60E-22	4.20E-22
TAC3	NA	NA	NA
MYO1A	NA	5.78E-19	8.88E-19
NEMP1	3.83876459	4.46E-79	1.63E-78
NAB2	1.40442607	1.54E-43	3.35E-43
STAT6	2.22438392	2.49E-60	6.87E-60
LRP1	NA	1.76E-75	6.09E-75
LRP1-AS	NA	NA	NA
NXPH4	1.55797665	2.20E-137	4.70E-136
SHMT2	1.65196418	1.11E-100	6.05E-100
RP11-123K3.	NA	3.81E-123	3.81E-122
NDUFA4L2	1.39105058	0.83235609	0.83957546
STAC3	1.13645512	8.80E-58	2.34E-57
RP11-123K3.	NA	NA	NA
R3HDM2	1.78614187	7.64E-18	1.15E-17
RP11-756H6.	0	2.04E-20	3.21E-20
INHBC	NA	NA	NA
INHBE	NA	1.40E-27	2.45E-27
GLI1	2.99610895	8.00E-51	1.93E-50
ARHGAP9	1.51033361	5.35E-70	1.71E-69
MARS	1.22460009	1.39E-61	3.92E-61
DDIT3	1.22568093	2.22E-98	1.16E-97
MBD6	1.97957198	1.78E-08	2.28E-08
DCTN2	1.01902543	1.21E-134	2.13E-133
KIF5A	Inf	4.28E-96	2.14E-95
PIP4K2C	3.44552529	2.62E-53	6.55E-53
DTX3	NA	NA	NA
ARHGEF25	NA	2.53E-06	3.09E-06
AC025165.8	NA	NA	NA

SLC26A10	NA	0.01812707	0.0198912
B4GALNT1	3.99481193	6.85E-56	1.78E-55
OS9	1.29377432	1.38E-110	9.41E-110
RP11-571M6	NA	3.46E-109	2.28E-108
AGAP2	7.49027237	1.70E-53	4.27E-53
AGAP2-AS1	NA	2.17E-110	1.48E-109
TSPAN31	1.07672665	7.03E-23	1.14E-22
CDK4	1.72550154	1.05E-23	1.73E-23
9-Mar	1-Jan	7.64E-123	7.52E-122
CYP27B1	6.74124514	8.59E-95	4.18E-94
METTL1	1.35934573	7.65E-63	2.19E-62
METTL21B	2.17217899	4.22E-07	5.24E-07
RP11-571M6	NA	NA	NA
TSFM	0.82255138	3.26E-142	1.23E-140
AVIL	2.49675746	2.50E-13	3.49E-13
RP11-571M6	0.74902724	5.56E-19	8.55E-19
RP11-571M6	0	1.34E-22	2.18E-22
CTDSP2	2.92952875	1.25E-100	6.83E-100
RP11-620J15	NA	NA	NA
RP11-620J15	NA	6.76E-12	9.24E-12
RP11-620J15	NA	2.61E-18	3.96E-18
RP11-620J15	2.40177212	8.73E-40	1.80E-39
XRCC6BP1	1.56827578	4.27E-32	7.91E-32
RP11-58A17.	NA	NA	NA
RP11-362K2.	NA	NA	NA
RP11-767I20	NA	NA	NA
LRIG3	NA	NA	NA
RP11-150C16	NA	NA	NA
RP11-272B17	NA	NA	NA
RP11-272B17	NA	NA	NA
SLC16A7	2.09727626	9.22E-77	3.25E-76
RP11-316A16	NA	NA	NA
FAM19A2	2.99610895	1.72E-23	2.83E-23
USP15	1.61554894	5.66E-08	7.15E-08
MON2	1.24837873	0.01301156	0.01435251
RP11-863H1.	NA	2.03E-08	2.59E-08
LINC01465	NA	3.18E-18	4.84E-18
RP11-631N16	1.49805447	8.70E-08	1.10E-07
RP11-631N16	1.12354086	2.73E-32	5.08E-32
PPM1H	Inf	0.34058109	0.35329763
RP11-848D3.	NA	NA	NA
AVPR1A	NA	NA	NA
RP11-1022B3	NA	NA	NA
DPY19L2	0.99870298	5.91E-22	9.50E-22
RP11-415I12	NA	1.56E-22	2.53E-22

TMEM5	1.97065499	1.74E-10	2.32E-10
TMEM5-AS1	NA	NA	NA
SRGAP1	1.12354086	7.15E-60	1.96E-59
RP11-196H14	NA	NA	NA
RP11-196H14	NA	NA	NA
RP11-196H14	NA	NA	NA
C12orf66	1.04863813	2.13E-106	1.31E-105
C12orf56	Inf	1.37E-83	5.42E-83
XPOT	2.65726329	1.35E-94	6.56E-94
RP11-114F3.	Inf	2.07E-11	2.80E-11
TBK1	1.12354086	2.29E-63	6.61E-63
RP11-439H13	NA	NA	NA
RP11-338E21	NA	NA	NA
RASSF3	5.11835279	2.22E-39	4.56E-39
RP11-338E21	NA	NA	NA
GNS	1.79766537	0.00010032	0.00011761
RP11-629N8.	NA	NA	NA
TBC1D30	1.31079767	3.82E-10	5.06E-10
RP11-766N7.	NA	NA	NA
RP11-766N7.	NA	NA	NA
RP11-168J19	NA	NA	NA
WIF1	NA	NA	NA
LEMD3	1.59168288	2.07E-15	3.01E-15
MSRB3	2.24708171	7.64E-24	1.26E-23
RP11-230G5.	NA	NA	NA
RP11-221N13	NA	NA	NA
RP11-221N13	NA	NA	NA
RP11-221N13	NA	NA	NA
HMGA2	1.47535668	1.04E-77	3.72E-77
RP11-366L20	NA	1.42E-17	2.13E-17
RP11-366L20	NA	NA	NA
RP11-366L20	NA	NA	NA
LLPH	1.11883985	1.71E-128	2.14E-127
RP11-745O10	NA	1.62E-93	7.69E-93
LLPH-AS1	NA	NA	NA
TMBIM4	1.15758755	3.30E-114	2.47E-113
TMBIM4	NA	NA	NA
IRAK3	1.31079767	4.27E-58	1.14E-57
RP11-335I12	NA	NA	NA
HELB	1.59168288	7.65E-06	9.24E-06
GRIP1	Inf	6.60E-21	1.04E-20
RP11-123O10	NA	NA	NA
RP11-123O10	NA	NA	NA
CAND1	2.20365984	2.61E-66	7.91E-66
RP11-473M1	NA	NA	NA

RP11-542B15	NA	NA	NA
RP11-335O4.	NA	NA	NA
DYRK2	2.79182879	7.76E-119	6.63E-118
RP11-335O4.	NA	5.66E-05	6.68E-05
RP11-43N5.1	NA	NA	NA
LINC01479	NA	NA	NA
IFNG-AS1	NA	NA	NA
IFNG	NA	NA	NA
IL26	NA	NA	NA
IL22	NA	NA	NA
MDM1	0.69140976	5.67E-100	3.04E-99
RP11-81H14.	NA	0.00168489	0.00190938
RP11-71J4.2	NA	NA	NA
RP11-81H14.	NA	NA	NA
RAP1B	1.96921677	2.81E-32	5.23E-32
RP11-637A17	1.49805447	3.57E-28	6.28E-28
NUP107	2.18333471	2.19E-09	2.86E-09
SLC35E3	1.80321372	1.31E-41	2.77E-41
MDM2	2.99610895	7.44E-25	1.25E-24
RP11-611O2.	NA	3.37E-44	7.37E-44
RP11-611O2.	NA	NA	NA
CPM	NA	2.09E-86	8.69E-86
RP11-324P9.	NA	NA	NA
CPSF6	1.46151656	1.36E-19	2.11E-19
RP11-1143G9	NA	NA	NA
LYZ	0.18309555	1.10E-119	9.70E-119
RP11-1143G9	1.12354086	6.24E-11	8.35E-11
YEATS4	1.11440638	7.72E-134	1.29E-132
RP11-956E11	NA	NA	NA
FRS2	2.24708171	1.12E-33	2.13E-33
CCT2	1.22335011	1.84E-131	2.69E-130
LRRC10	NA	NA	NA
BEST3	NA	1.15E-116	9.21E-116
RP11-588G2	NA	9.10E-16	1.33E-15
RAB3IP	3.22081712	1.84E-31	3.39E-31
AC025263.3	NA	NA	NA
MYRFL	NA	2.28E-34	4.37E-34
LINC01481	2.30950065	3.04E-25	5.12E-25
RP11-611E13	NA	NA	NA
RP11-320P7.	NA	NA	NA
LINC01481	Inf	1.78E-64	5.24E-64
CNOT2	1.83585107	3.05E-11	4.11E-11
AC092881.1	NA	NA	NA
KCNMB4	3.37062257	0.01712389	0.01881429
RP11-588H23	NA	NA	NA

PTPRB	NA	1.91E-141	6.53E-140
RP11-785D18	NA	NA	NA
PTPRR	NA	NA	NA
CTD-2021H9.	NA	NA	NA
CTD-2021H9.	NA	NA	NA
CTD-2021H9.	NA	NA	NA
TSPAN8	NA	NA	NA
LGR5	NA	NA	NA
RP11-186F10	NA	NA	NA
ZFC3H1	1.28832685	3.47E-65	1.03E-64
THAP2	1.92607004	2.73E-09	3.55E-09
RP11-293I14	NA	NA	NA
TMEM19	1.45399405	2.96E-23	4.85E-23
RP11-498M1	NA	NA	NA
RAB21	1.98426514	4.25E-20	6.64E-20
RP11-2H8.2	NA	NA	NA
TBC1D15	1.36964981	0.00786327	0.00874114
TPH2	NA	NA	NA
RP11-89M22	NA	NA	NA
TRHDE	NA	NA	NA
RP11-12K6.2	NA	NA	NA
RP11-314D7.	NA	NA	NA
RP11-314D7.	NA	NA	NA
RP11-295G1	NA	NA	NA
RP11-711C17	NA	NA	NA
RP11-711C17	NA	NA	NA
RP11-81H3.2	NA	NA	NA
RP11-274M1	NA	NA	NA
ATXN7L3B	1.91750973	1.18E-49	2.80E-49
RP11-56G10.	NA	NA	NA
RP11-81K13.	NA	NA	NA
KCNC2	NA	NA	NA
RP11-560G2.	NA	NA	NA
CAPS2	0	1.35E-14	1.94E-14
GLIPR1L1	NA	NA	NA
GLIPR1L2	NA	7.23E-96	3.59E-95
GLIPR1	1.24057636	2.65E-135	4.83E-134
RP11-585P4.	Inf	2.43E-52	6.00E-52
KRR1	1.28502838	1.68E-45	3.75E-45
RP11-114H23	NA	NA	NA
RP11-114H23	NA	NA	NA
RP11-290L1.	NA	NA	NA
PHLDA1	1.59168288	3.69E-37	7.36E-37
RP11-290L1.	NA	NA	NA
RP11-290L1.	NA	0.00546514	0.0061052

NAP1L1	1.76840794	3.67E-07	4.56E-07
RP11-54A9.1	NA	NA	NA
BBS10	0.49935149	7.82E-09	1.01E-08
OSBPL8	2.21863764	6.17E-116	4.84E-115
RP11-20E24.	0	0.5868217	0.59964623
ZDHHC17	2.8463035	5.21E-07	6.45E-07
CSRP2	2.65040407	2.47E-07	3.08E-07
RP11-461F16	NA	NA	NA
E2F7	2.12224384	5.84E-11	7.82E-11
RP1-81P11.1	NA	NA	NA
RP1-34H18.1	NA	NA	NA
RP1-97G4.1	NA	NA	NA
RP11-781A6.	NA	NA	NA
NAV3	0	7.56E-77	2.67E-76
RP11-136F16	NA	NA	NA
RP11-38F22.	NA	NA	NA
RP11-754N2	NA	NA	NA
RP11-90C1.1	NA	NA	NA
RP11-171L9.	NA	NA	NA
RP11-123M2	NA	NA	NA
SYT1	NA	NA	NA
RP11-390N6.	NA	NA	NA
RP1-78O14.1	NA	1.98E-138	4.65E-137
RP11-359M6	NA	NA	NA
PAWR	1.66450497	0.00013054	0.00015261
RP11-530C5.	NA	NA	NA
PPP1R12A	1.32891929	2.04E-136	4.02E-135
RP11-530C5.	NA	NA	NA
RP11-84G21.	4.11964981	1.57E-45	3.50E-45
OTOGL	NA	NA	NA
PTPRQ	NA	NA	NA
RP11-272K23	NA	NA	NA
MYF6	NA	NA	NA
MYF5	NA	NA	NA
LINC01490	NA	NA	NA
LIN7A	0.30842298	1.80E-145	1.58E-143
ACSS3	NA	0.07671805	0.08225394
RP11-543H12	NA	NA	NA
PPFIA2	NA	NA	NA
RP11-121G22	NA	NA	NA
RP11-315E17	NA	NA	NA
RP11-362A1.	NA	NA	NA
CCDC59	1.46798404	4.21E-39	8.63E-39
METTL25	1.49805447	4.35E-97	2.22E-96
RP11-263K4.	NA	NA	NA

RP11-263K4.	NA	NA	NA
RP11-263K4.	NA	2.26E-19	3.49E-19
TMTC2	0.74902724	3.75E-63	1.08E-62
RP11-87P13.	NA	4.68E-103	2.68E-102
RP11-384P14	NA	NA	NA
RP11-788H18	NA	NA	NA
SLC6A15	NA	NA	NA
TSPAN19	NA	NA	NA
LRRIQ1	NA	NA	NA
RP11-193M2	NA	NA	NA
ALX1	0.24967575	1.14E-50	2.75E-50
RP11-408B1	NA	NA	NA
RASSF9	NA	NA	NA
NTS	NA	NA	NA
MGAT4C	NA	NA	NA
RP11-18J9.3	NA	NA	NA
RP11-202H2.	NA	NA	NA
RP11-248E9.	NA	NA	NA
RP11-248E9.	NA	NA	NA
C12orf50	NA	1.69E-55	4.37E-55
C12orf29	1.46868086	2.36E-24	3.94E-24
CEP290	2.14721141	7.82E-50	1.86E-49
TMTC3	2.49675746	1.54E-10	2.05E-10
KITLG	7.49027237	1.35E-66	4.11E-66
RP11-900F13	NA	NA	NA
RP11-900F13	NA	NA	NA
RP11-13A1.1	NA	0.00049939	0.00057479
RP11-13A1.3	NA	NA	NA
DUSP6	1.1432521	7.91E-78	2.84E-77
RP11-823E8.	Inf	0.4519938	0.4649621
RP11-1109F1	NA	NA	NA
RP11-1109F1	NA	NA	NA
POC1B	1.68531128	1.13E-35	2.20E-35
POC1B-GALN	NA	NA	NA
GALNT4	NA	NA	NA
RP11-734K2.	3.74513619	0.00096305	0.00110044
RP11-981P6.	NA	0.13287426	0.14095456
ATP2B1	1.3172548	2.39E-60	6.62E-60
LINC00936	1.49805447	1.98E-43	4.29E-43
RP11-654D1	NA	NA	NA
RP11-654D1	NA	NA	NA
RP11-567C2.	NA	NA	NA
RP11-753N8.	NA	NA	NA
RP11-632B2	NA	NA	NA
RP11-20L19.	NA	NA	NA

CCER1	NA	NA	NA
LINC00615	NA	NA	NA
EPYC	NA	NA	NA
KERA	NA	NA	NA
LUM	Inf	3.70E-131	5.31E-130
DCN	NA	NA	NA
RP11-121E16	NA	NA	NA
RP11-693J15	NA	NA	NA
RP11-1041F2	NA	NA	NA
RP11-587P21	NA	NA	NA
RP11-587P21	NA	NA	NA
AC123512.1	NA	NA	NA
AC123512.2	NA	NA	NA
AC123512.3	NA	NA	NA
BTG1	0.88773599	2.94E-136	5.74E-135
RP11-796E2.	NA	2.84E-48	6.59E-48
RP11-756G2(	NA	NA	NA
CLLU1OS	NA	NA	NA
CLLU1	NA	NA	NA
RP11-693J15	NA	NA	NA
RP11-693J15	NA	1.33E-20	2.09E-20
C12orf74	NA	NA	NA
PLEKHG7	NA	NA	NA
EEA1	1.77547197	0.00147202	0.00167128
RP11-511B2:	0	8.15E-48	1.88E-47
RP11-511B2:	NA	0.01221419	0.0134869
RP11-202G1	NA	NA	NA
RP11-511B2:	NA	NA	NA
RP11-486A1	NA	NA	NA
NUDT4	2.23771887	1.05E-26	1.81E-26
UBE2N	1.47850155	3.67E-23	6.00E-23
MRPL42	1.71264066	8.37E-27	1.44E-26
SOCS2	4.48667315	1.83E-145	1.58E-143
CRADD	2.71522374	2.17E-123	2.19E-122
RP11-887P2.	Inf	4.48E-08	5.67E-08
RP11-1060G	NA	NA	NA
RP11-1060G	NA	2.07E-10	2.74E-10
PLXNC1	1.49805447	3.53E-66	1.07E-65
RP11-74K11.	NA	NA	NA
RP11-1105G	NA	1.14E-12	1.57E-12
RP11-1105G	0.24967575	2.75E-90	1.23E-89
CEP83	1.83511673	2.33E-32	4.33E-32
CEP83-AS1	3.74513619	2.55E-19	3.93E-19
RP11-778J16	NA	NA	NA
TMCC3	2.80885214	4.87E-48	1.13E-47

NDUFA12	1.68131401	2.07E-21	3.30E-21
NR2C1	1.49805447	6.01E-27	1.04E-26
FGD6	2.24708171	5.86E-15	8.44E-15
VEZT	2.69649805	7.93E-46	1.78E-45
RP11-167N2	NA	NA	NA
RP11-167N2	NA	NA	NA
RP11-167N2	NA	NA	NA
METAP2	1.769131	9.19E-23	1.49E-22
USP44	1.49805447	0.00260472	0.00293509
NTN4	NA	NA	NA
RP11-536G4	NA	1.80E-118	1.52E-117
RP11-536G4	NA	NA	NA
SNRPF	1.90706277	3.09E-84	1.24E-83
CCDC38	NA	8.32E-107	5.16E-106
AMDHD1	NA	3.90E-05	4.62E-05
HAL	NA	1.03E-86	4.28E-86
RP11-256L6.	NA	NA	NA
LTA4H	1.52686321	4.85E-53	1.21E-52
RP11-256L6.	NA	NA	NA
ELK3	1.49805447	1.07E-36	2.11E-36
RP11-394J1.	NA	0.0091276	0.0101181
CDK17	1.81906615	0.99989645	0.99989645
RP11-510I5.	NA	NA	NA
CFAP54	Inf	3.89E-102	2.19E-101
NEDD1	5.84241245	2.55E-101	1.41E-100
RP11-541G9.	NA	NA	NA
RP11-397H6.	NA	NA	NA
RP11-541G9.	NA	NA	NA
RP11-690J15	NA	NA	NA
RP11-181C3.	0	8.83E-72	2.89E-71
TMPO-AS1	2.53837008	2.91E-34	5.56E-34
TMPO	1.55401628	8.92E-54	2.25E-53
SLC25A3	1.01306281	2.66E-136	5.20E-135
IKBIP	1.30512322	6.64E-62	1.88E-61
APAF1	3.30820363	6.78E-37	1.35E-36
ANKS1B	NA	0.00596164	0.00664881
RP11-473C1	NA	NA	NA
RP11-406H4.	NA	NA	NA
FAM71C	NA	NA	NA
UHRF1BP1L	1.81906615	2.54E-52	6.27E-52
RP11-175P1	NA	NA	NA
ACTR6	1.15029183	1.84E-60	5.08E-60
DEPDC4	0.53501946	0.57528462	0.58825462
SCYL2	2.12224384	8.83E-46	1.98E-45
SLC17A8	NA	NA	NA

NR1H4	NA	NA	NA
GAS2L3	1.87256809	2.70E-07	3.36E-07
ANO4	NA	NA	NA
RP11-263E1.	NA	NA	NA
RP11-350G2	NA	NA	NA
SLC5A8	NA	NA	NA
UTP20	1.31079767	0.54350076	0.55672694
ARL1	1.49805447	8.61E-17	1.28E-16
RP11-321F8.	NA	NA	NA
SPIC	NA	NA	NA
MYBPC1	NA	NA	NA
RP11-755O1	NA	NA	NA
RP11-285E2	NA	NA	NA
CHPT1	2.0016022	0.0361822	0.03925942
SYCP3	Inf	4.15E-144	2.26E-142
GNPTAB	2.73986279	1.95E-141	6.64E-140
DRAM1	1.23227062	1.51E-118	1.27E-117
RP11-512N2	NA	NA	NA
RP11-554E2	NA	2.08E-141	7.05E-140
CCDC53	1.4197979	3.60E-48	8.33E-48
RP11-554E2	NA	2.90E-109	1.91E-108
NUP37	0.74902724	8.22E-141	2.57E-139
AC079907.1	NA	NA	NA
PARPBP	1.74773022	4.85E-23	7.92E-23
PMCH	NA	1.73E-35	3.37E-35
RP11-210L7.	Inf	9.11E-151	1.45E-148
IGF1	NA	NA	NA
LINC00485	NA	NA	NA
PAH	NA	NA	NA
ASCL1	NA	NA	NA
RP11-328J6.	NA	NA	NA
RP11-552I14	NA	NA	NA
C12orf42	1.74773022	7.06E-16	1.03E-15
RP11-626I20	NA	NA	NA
STAB2	NA	NA	NA
RP11-341G2	NA	NA	NA
RP11-341G2	NA	NA	NA
RP11-341G2	Inf	0.00067049	0.000769
NT5DC3	2.16385646	5.71E-60	1.57E-59
RP11-650K2	NA	NA	NA
RP11-650K2	NA	2.47E-28	4.35E-28
HSP90B1	0.91476943	2.44E-142	9.32E-141
C12orf73	1.98271916	2.34E-66	7.08E-66
TDG	1.81629403	1.77E-16	2.62E-16
GLT8D2	NA	0.9502571	0.9527253

HCFC2	2.62159533	0.95022278	0.9527253
NFYB	1.18595979	9.44E-125	1.00E-123
RP11-818F2(	NA	NA	NA
TXNRD1	2.46602814	1.24E-60	3.44E-60
RP11-818F2(	0	1.30E-70	4.19E-70
EID3	Inf	1.26E-05	1.51E-05
CHST11	3.932393	9.45E-68	2.93E-67
SLC41A2	Inf	0.31045032	0.32296518
C12orf45	1.31495893	3.73E-23	6.11E-23
ALDH1L2	NA	NA	NA
RP11-61E11.	NA	NA	NA
RP11-61E11.	NA	3.26E-73	1.09E-72
KIAA1033	2.20302129	0.00227583	0.00256863
APPL2	1.49805447	1.11E-58	2.98E-58
C12orf75	1.84375935	0.94794924	0.95067413
RP11-474B1(	NA	NA	NA
CASC18	NA	NA	NA
RP11-114F1(	NA	NA	NA
NUAK1	Inf	6.79E-38	1.37E-37
RP11-114F1(	NA	NA	NA
CKAP4	2.99610895	1.41E-21	2.26E-21
RP11-651L5.:	NA	2.92E-130	3.97E-129
TCP11L2	NA	5.42E-34	1.03E-33
POLR3B	1.19844358	3.63E-52	8.94E-52
RP11-144F1!	NA	NA	NA
RFX4	Inf	1.36E-18	2.08E-18
RP11-482D2⁄	NA	1.68E-132	2.59E-131
RP11-482D2⁄	NA	1.06E-49	2.51E-49
RIC8B	3.18336576	2.52E-58	6.73E-58
RP11-412D9.	NA	NA	NA
TMEM263	Inf	1.59E-119	1.39E-118
MTERF2	NA	NA	NA
CRY1	NA	5.76E-24	9.52E-24
RP11-797M1	NA	NA	NA
BTBD11	0	2.63E-149	3.57E-147
RP11-128P1(	NA	NA	NA
PWP1	1.66970655	1.50E-62	4.28E-62
PRDM4	1.82575389	0.06248805	0.06720364
RP11-864J10	NA	4.83E-114	3.61E-113
ASCL4	NA	NA	NA
RP11-554D1⁄	NA	NA	NA
RP11-554D1⁄	NA	NA	NA
RP11-554D1⁄	NA	NA	NA
WSCD2	NA	NA	NA
CMKLR1	NA	NA	NA

LINC01498	NA	NA	NA
FICD	0.66580199	1.57E-91	7.20E-91
SART3	1.54798962	1.60E-74	5.46E-74
ISCU	1.96068894	9.91E-136	1.87E-134
TMEM119	NA	5.99E-16	8.78E-16
SELPLG	2.99610895	3.29E-09	4.27E-09
RP11-689B2:	NA	NA	NA
CORO1C	2.23210117	4.65E-10	6.13E-10
SSH1	1.59168288	0.17922209	0.18886194
DAO	NA	2.92E-40	6.08E-40
SVOP	NA	6.05E-39	1.24E-38
USP30	1.68531128	7.32E-08	9.23E-08
USP30-AS1	NA	0.13532662	0.14339673
ALKBH2	1.86642853	2.07E-71	6.76E-71
UNG	1.46748193	4.53E-14	6.42E-14
ACACB	3.74513619	4.06E-22	6.54E-22
FOXN4	NA	NA	NA
MYO1H	NA	NA	NA
LINC01486	NA	NA	NA
RP11-256L11	NA	8.81E-152	1.55E-149
KCTD10	1.87256809	1.61E-08	2.06E-08
UBE3B	1.95579334	1.98E-08	2.53E-08
MMAB	1.7565683	6.76E-51	1.63E-50
MVK	1.03711464	1.25E-133	2.06E-132
FAM222A	2.24708171	9.80E-62	2.77E-61
FAM222A-AS	Inf	3.70E-126	4.17E-125
TRPV4	NA	2.51E-13	3.51E-13
GLTP	1.44357977	1.30E-15	1.89E-15
RP1-7G5.6	NA	NA	NA
TCHP	1.97957198	0.00098012	0.00111952
GIT2	1.66088648	5.58E-10	7.36E-10
RP11-837J7.4	NA	NA	NA
ANKRD13A	1.55155642	0.10076785	0.10751178
C12orf76	1.71206226	1.32E-08	1.69E-08
IFT81	2.03307393	8.24E-34	1.57E-33
ATP2A2	1.77653896	9.30E-84	3.69E-83
ANAPC7	1.963666	5.37E-07	6.65E-07
ARPC3	1.08419146	4.80E-144	2.57E-142
GPN3	1.21798342	9.86E-77	3.48E-76
FAM216A	1.2975275	6.56E-133	1.04E-131
VPS29	1.47547074	3.65E-118	3.05E-117
RAD9B	1.12354086	0.42461022	0.43738807
PPTC7	2.40758755	5.59E-17	8.32E-17
TCTN1	1.94747082	1.41E-51	3.44E-51
HVCN1	0.89883268	3.74E-139	9.59E-138

PPP1CC	1.72942067	5.72E-31	1.05E-30
RP1-74B13.2	NA	NA	NA
CCDC63	NA	NA	NA
MYL2	NA	NA	NA
LINC01405	NA	1.48E-51	3.60E-51
RP1-46F2.3	NA	NA	NA
CUX2	NA	0.24882742	0.26035107
FAM109A	3.74513619	5.70E-07	7.05E-07
RP3-473L9.4	Inf	9.01E-12	1.23E-11
SH2B3	2.69649805	5.10E-33	9.57E-33
ATXN2	2.99610895	2.28E-124	2.39E-123
RP11-686G8.	NA	NA	NA
BRAP	1.67429618	9.14E-29	1.62E-28
RP11-686G8.	Inf	3.10E-152	5.51E-150
ACAD10	2.14007782	5.51E-13	7.67E-13
RP11-162P2	NA	NA	NA
ALDH2	0	4.01E-52	9.88E-52
MAPKAPK5-A	1.8443789	2.22E-08	2.83E-08
MAPKAPK5	2.6139522	3.19E-81	1.21E-80
TMEM116	1.60505837	0.04470728	0.04836202
ERP29	1.06940324	3.53E-145	2.53E-143
RP1-267L14.	0	3.51E-21	5.57E-21
NAA25	2.62159533	1.85E-132	2.86E-131
RP1-267L14.	NA	NA	NA
TRAFD1	1.49805447	0.12282487	0.1305536
HECTD4	2.78210117	4.89E-05	5.79E-05
RP3-521E19.	NA	NA	NA
RPL6	1.86721214	8.95E-06	1.08E-05
PTPN11	1.74773022	1.63E-64	4.80E-64
RPH3A	NA	NA	NA
OAS1	1.12354086	1.34E-05	1.60E-05
RP1-71H24.1	NA	NA	NA
OAS3	1.60505837	0.05074063	0.05478732
OAS2	NA	8.07E-14	1.14E-13
DTX1	NA	NA	NA
RASAL1	0	2.18E-36	4.31E-36
CFAP73	Inf	3.74E-11	5.04E-11
DDX54	1.48491365	2.47E-46	5.58E-46
RITA1	1.87256809	0.09098941	0.09726768
RP11-545P7.	NA	NA	NA
IQCD	NA	1.86E-08	2.38E-08
TPCN1	4.11964981	3.85E-56	1.00E-55
SLC8B1	0.56177043	1.62E-135	3.01E-134
PLBD2	1.9661965	1.88E-15	2.74E-15
SDS	Inf	2.88E-130	3.94E-129

SDSL	1.56047341	1.56E-114	1.18E-113
LHX5	NA	NA	NA
LHX5-AS1	NA	NA	NA
LINC01234	NA	0.20797298	0.21849929
RBM19	2.65564202	6.00E-86	2.47E-85
RP11-780K2.	NA	NA	NA
RP11-100F1!	NA	NA	NA
RP11-100F1!	NA	NA	NA
RP11-139B1.	NA	NA	NA
TBX5	NA	NA	NA
TBX5-AS1	NA	NA	NA
RP11-162N7.	NA	NA	NA
TBX3	2.62159533	1.04E-49	2.46E-49
RP4-601P9.2	NA	1.91E-36	3.76E-36
RP4-601P9.1	NA	NA	NA
RP11-25E2.1	NA	NA	NA
RP11-116D1	NA	NA	NA
RP11-116D1	NA	NA	NA
RP11-116D1	NA	NA	NA
RP11-116D1	NA	NA	NA
RP11-116D1	NA	NA	NA
RP11-1028N	NA	NA	NA
RP11-1028N	NA	NA	NA
RP11-1028N	NA	NA	NA
RP11-110L1!	NA	NA	NA
RP11-110L1!	NA	NA	NA
MED13L	1.62645914	8.54E-15	1.23E-14
RP11-493P1.	NA	NA	NA
RP11-148B3.	NA	NA	NA
RP11-148B3.	NA	NA	NA
MAP1LC3B2	2.24708171	0.59383272	0.60657132
RP11-497G1!	NA	NA	NA
RP11-497G1!	NA	0.13377061	0.14180592
RP11-497G1!	NA	3.68E-16	5.41E-16
RP11-497G1!	NA	1.69E-24	2.82E-24
C12orf49	2.24708171	8.43E-65	2.49E-64
RNFT2	2.79636835	5.58E-57	1.47E-56
RP11-240G2	NA	6.27E-37	1.25E-36
HRK	4.11964981	0.00357329	0.00401404
FBXW8	1.49805447	3.15E-68	9.83E-68
RP11-240G2	NA	3.74E-17	5.59E-17
RP11-231I16	NA	7.52E-54	1.90E-53
TESC	1.39996757	4.21E-132	6.40E-131
TESC-AS1	NA	NA	NA
FBXO21	2.12881425	1.87E-27	3.26E-27

NOS1	NA	1.19E-07	1.49E-07
RP11-227B2:	NA	NA	NA
KSR2	NA	NA	NA
RP11-465L8.	NA	NA	NA
RFC5	1.68531128	3.40E-41	7.17E-41
RP11-33N14.	NA	NA	NA
WSB2	2.55702402	9.64E-91	4.34E-90
RP11-33N14.	NA	1.30E-67	4.02E-67
VSIG10	1.7880005	0.00788146	0.00875975
RP11-136L23	0	9.79E-48	2.25E-47
PEBP1	1.33013096	6.36E-117	5.13E-116
TAOK3	2.08698429	9.25E-98	4.77E-97
RP11-131L12	NA	6.59E-18	9.96E-18
SUDS3	2.09103437	3.57E-16	5.26E-16
RP11-131L12	0	4.38E-08	5.54E-08
RP11-131L12	NA	1.29E-30	2.34E-30
RP11-121J20	NA	NA	NA
RP11-3L23.2	NA	NA	NA
RP11-357K6.	NA	NA	NA
RP11-357K6.	NA	NA	NA
RP11-357K6.	NA	NA	NA
SRRM4	NA	NA	NA
RP11-364C1:	NA	NA	NA
RP11-64B16.	NA	NA	NA
HSPB8	NA	NA	NA
RP11-64B16.	NA	NA	NA
RP11-64B16.	NA	NA	NA
LINC00934	NA	NA	NA
CCDC60	NA	NA	NA
RP11-768F2:	NA	1.14E-22	1.85E-22
TMEM233	NA	5.19E-35	1.00E-34
PRKAB1	1.20996708	1.86E-73	6.27E-73
CIT	2.19357977	3.91E-11	5.26E-11
RP1-127H14.	NA	NA	NA
AC004813.1	NA	NA	NA
CCDC64	NA	1.28E-05	1.54E-05
RAB35	1.29593601	1.22E-43	2.66E-43
RP1-267D11.	2.16385646	0.00037358	0.00043182
GCN1	2.32456729	2.14E-54	5.43E-54
RPLP0	2.03825228	2.64E-79	9.71E-79
PXN-AS1	2.95371118	4.64E-145	3.17E-143
PXN	2.57998271	8.61E-36	1.68E-35
RP1-278C19.	Inf	0.00082513	0.00094409
SIRT4	0	1.18E-75	4.11E-75
PLA2G1B	NA	1.25E-13	1.76E-13

MSI1	Inf	1.58E-06	1.94E-06
RP1-166H1.2	NA	NA	NA
COX6A1	1.72244468	9.93E-23	1.61E-22
AL021546.6	NA	NA	NA
TRIAP1	1.84720263	0.62197262	0.63420792
GATC	2.7464332	1.60E-86	6.66E-86
SRSF9	1.80495679	0.01370044	0.0151032
DYNLL1	1.70600543	0.0002628	0.00030496
NRAV	2.33030696	2.70E-48	6.28E-48
RP11-18C24.	NA	4.99E-61	1.39E-60
COQ5	1.05745022	5.19E-141	1.65E-139
RNF10	1.89869695	0.20558409	0.21610212
POP5	1.6943721	2.12E-57	5.60E-57
RP11-728G1	1.29377432	1.10E-29	1.97E-29
CABP1	1.24837873	1.18E-14	1.69E-14
MLEC	1.49582523	2.38E-67	7.31E-67
RP11-173P1	NA	0.65223635	0.66391022
RP11-173P1	NA	1.46E-121	1.36E-120
RP11-173P1	0.74902724	1.03E-25	1.75E-25
UNC119B	1.94747082	1.25E-71	4.08E-71
RP11-173P1	NA	NA	NA
ACADS	1.24837873	1.74E-110	1.18E-109
RP11-173P1	NA	NA	NA
SPPL3	2.68200075	2.27E-63	6.55E-63
HNF1A	NA	3.75E-93	1.77E-92
C12orf43	1.62034464	6.83E-53	1.70E-52
OASL	0.74902724	1.77E-12	2.44E-12
P2RX7	0.74902724	7.58E-11	1.01E-10
RP11-340F1	NA	NA	NA
P2RX4	1.23589494	2.14E-44	4.69E-44
CAMKK2	2.15345331	6.79E-62	1.92E-61
ANAPC5	2.0264778	4.35E-57	1.14E-56
RP11-711D1	NA	NA	NA
RNF34	1.35485809	2.28E-05	2.72E-05
KDM2B	1.83511673	0.00809644	0.00899318
RP13-941N1	NA	NA	NA
ORAI1	1.90700613	6.19E-60	1.70E-59
MORN3	0.89883268	5.46E-27	9.43E-27
TMEM120B	1.67963684	4.97E-12	6.80E-12
RHOF	2.48011241	3.45E-06	4.19E-06
LINC01089	1.26151956	0.25889472	0.2705725
RP11-347I19	NA	NA	NA
RP11-347I19	0	0.10583436	0.11271188
SETD1B	2.07422927	1.68E-13	2.36E-13
HPD	NA	3.35E-107	2.10E-106

RP11-7M8.2	NA	NA	NA
RP11-87C12.	NA	NA	NA
PSMD9	1.8954975	2.44E-51	5.92E-51
RP11-87C12.	NA	1.30E-42	2.80E-42
WDR66	Inf	2.20E-113	1.62E-112
RP11-87C12.	2.24708171	1.45E-44	3.19E-44
BCL7A	2.22737047	7.79E-71	2.52E-70
MLXIP	2.27336337	5.24E-75	1.80E-74
LRRC43	NA	2.70E-112	1.93E-111
IL31	NA	NA	NA
B3GNT4	2.24708171	1.02E-43	2.21E-43
DIABLO	2.54669261	3.36E-06	4.08E-06
RP11-512M8	NA	NA	NA
VPS33A	1.26151956	3.27E-101	1.80E-100
CLIP1	1.85628489	9.28E-25	1.55E-24
CLIP1-AS1	Inf	8.00E-130	1.07E-128
ZCCHC8	1.49805447	0.45935503	0.47234704
RP11-15J22.8	Inf	1.94E-09	2.52E-09
RSRC2	1.69135183	3.92E-22	6.33E-22
KNTC1	1.87256809	2.67E-36	5.25E-36
RP11-324E6.	1.87256809	6.04E-53	1.50E-52
RP11-324E6.	NA	NA	NA
HCAR2	NA	NA	NA
HCAR3	NA	NA	NA
HCAR1	NA	NA	NA
DENR	2.86272054	3.58E-127	4.23E-126
CCDC62	0.89883268	3.59E-23	5.88E-23
HIP1R	0.97949716	1.49E-30	2.70E-30
VPS37B	1.77676228	0.16268236	0.17172185
RP11-463O1	NA	1.69E-32	3.15E-32
ABCB9	9.73735409	4.23E-39	8.66E-39
OGFOD2	1.74773022	1.71E-42	3.66E-42
ARL6IP4	2.01438759	1.69E-47	3.89E-47
PITPNM2	7.49027237	2.30E-62	6.54E-62
RP11-197N1	NA	NA	NA
MPHOSPH9	2.62159533	1.34E-06	1.64E-06
C12orf65	1.81660629	1.55E-38	3.15E-38
RP11-282O1	NA	1.70E-128	2.12E-127
CDK2AP1	2.01661179	0.69499678	0.70581234
RP11-282O1	NA	4.10E-18	6.22E-18
SBNO1	2.27589045	9.26E-20	1.44E-19
RP13-942N8.	1.66828794	0.13390032	0.14192683
KMT5A	2.24708171	7.73E-82	2.96E-81
RILPL2	1.43411313	3.21E-72	1.06E-71
SNRNP35	1.17186519	7.92E-123	7.79E-122

RILPL1	2.24708171	0.14191983	0.15024272
RP11-486O1	Inf	3.21E-83	1.26E-82
TMED2	1.57719003	9.95E-14	1.40E-13
DDX55	1.53679726	2.29E-20	3.59E-20
EIF2B1	0.99513619	8.02E-124	8.25E-123
GTF2H3	1.55567195	0.0007079	0.00081128
TCTN2	1.49805447	7.42E-38	1.49E-37
RP11-338K1	NA	5.09E-46	1.14E-45
ATP6V0A2	1.71652075	3.63E-08	4.61E-08
DNAH10	NA	7.72E-97	3.92E-96
CCDC92	1.70233463	0.00046764	0.00053897
RP11-380L11	NA	2.26E-54	5.73E-54
DNAH10OS	NA	NA	NA
ZNF664	4.09761959	3.33E-120	2.99E-119
FAM101A	NA	NA	NA
RP11-214K3.	NA	6.73E-06	8.14E-06
RP11-214K3.	NA	0.31318432	0.32573467
RP11-522N1	NA	NA	NA
NCOR2	1.95899431	2.06E-13	2.89E-13
RP11-83B20.	Inf	1.50E-39	3.10E-39
SCARB1	2.41791249	1.10E-108	7.15E-108
UBC	0.92132164	3.21E-116	2.53E-115
DHX37	2.03025804	1.76E-37	3.53E-37
BRI3BP	1.55733001	0.10195854	0.10873101
THRIL	NA	3.35E-81	1.27E-80
AACS	1.15758755	1.06E-85	4.36E-85
RP11-158L12	NA	NA	NA
TMEM132B	NA	3.50E-79	1.28E-78
LINC00939	NA	NA	NA
RP5-916L7.2	NA	NA	NA
RP3-446N13.	NA	NA	NA
RP3-446N13.	NA	NA	NA
RP1-116K23.	NA	NA	NA
RP4-765H13.	NA	NA	NA
RP4-809F18.	NA	NA	NA
RP4-809F18.	NA	NA	NA
RP5-944M2.	NA	NA	NA
RP5-944M2.	NA	NA	NA
RP5-944M2.	NA	NA	NA
RP11-407A1	NA	NA	NA
RP11-407A1	NA	NA	NA
RP11-407A1	NA	NA	NA
LINC00943	NA	NA	NA
LINC00944	NA	NA	NA
RP11-983C2.	NA	NA	NA

RP11-983C2.	NA	NA	NA
RP11-575F12	NA	NA	NA
RP11-575F12	NA	NA	NA
RP11-575F12	NA	NA	NA
RP11-955H22	NA	NA	NA
RP11-955H22	NA	NA	NA
RP11-351O2.	NA	NA	NA
RP11-526P6.	NA	NA	NA
RP11-749H20	NA	NA	NA
FLJ37505	NA	NA	NA
LINC00507	NA	NA	NA
LINC00508	NA	NA	NA
RP11-349K16	NA	NA	NA
RP13-653N12	NA	NA	NA
RP13-653N12	NA	NA	NA
RP11-417O18	NA	NA	NA
TMEM132C	NA	NA	NA
SLC15A4	1.74773022	1.66E-19	2.57E-19
RP11-21K12.	NA	4.18E-08	5.30E-08
GLT1D1	NA	NA	NA
TMEM132D	NA	NA	NA
RP11-669N7.	NA	NA	NA
RP11-669N7.	NA	NA	NA
RP11-337L12	NA	NA	NA
RP11-174M1	NA	NA	NA
RP11-572C21	NA	NA	NA
RP11-474D1.	NA	NA	NA
RP11-474D1.	NA	NA	NA
RP11-474D1.	NA	NA	NA
RP11-474D1.	NA	NA	NA
RP11-143E21	NA	NA	NA
FZD10	NA	NA	NA
RP11-143E21	NA	NA	NA
PIWIL1	Inf	8.90E-11	1.19E-10
RIMBP2	NA	NA	NA
RP11-117L5.4	NA	NA	NA
RP11-662M2	NA	NA	NA
RP11-662M2	NA	NA	NA
STX2	4.86867704	1.86E-100	1.01E-99
RP11-989F5.	NA	NA	NA
RP11-989F5.	NA	NA	NA
RAN	1.35431665	5.01E-105	3.00E-104
ADGRD1	NA	NA	NA
RP11-243M5	NA	NA	NA
RP11-76C10.	NA	NA	NA

RP11-76C10.	NA	NA	NA
LINC01257	0	9.02E-11	1.20E-10
RP11-495K9.	NA	NA	NA
RP13-507P19	NA	NA	NA
RP13-507P19	NA	NA	NA
RP11-897M7	NA	1.75E-09	2.28E-09
RP11-897M7	NA	NA	NA
RP11-495K9.	NA	NA	NA
RP11-495K9.	NA	NA	NA
RP11-495K9.	NA	NA	NA
SFSWAP	1.6728275	0.82689216	0.83424967
RP11-495K9.	NA	NA	NA
MMP17	1.94747082	1.09E-22	1.76E-22
RP11-417L19	NA	2.03E-137	4.36E-136
ULK1	5.24319066	1.19E-116	9.53E-116
RP11-417L19	NA	1.24E-87	5.29E-87
PUS1	1.95525292	2.76E-54	7.00E-54
EP400	2.89396887	7.17E-28	1.25E-27
RP13-820C6.	NA	NA	NA
RP13-820C6.	NA	2.66E-34	5.09E-34
EP400NL	1.59575368	0.0611484	0.06579019
DDX51	2.90622568	2.52E-26	4.32E-26
NOC4L	0.86652171	3.10E-137	6.53E-136
RP13-977J11	NA	1.31E-24	2.19E-24
RP13-977J11	3.74513619	1.56E-09	2.04E-09
RP13-977J11	NA	NA	NA
GALNT9	0	1.43E-103	8.27E-103
RP13-895J2.2	NA	NA	NA
RP13-895J2.3	NA	2.67E-62	7.61E-62
RP13-895J2.6	NA	NA	NA
RP13-895J2.7	NA	NA	NA
RP13-714J12	NA	1.50E-75	5.20E-75
RP11-503G7.	NA	NA	NA
RP11-503G7.	NA	NA	NA
FBRSL1	1.91418072	1.54E-11	2.08E-11
RP13-554M1	NA	4.18E-70	1.34E-69
LRCOL1	NA	NA	NA
P2RX2	NA	NA	NA
POLE	1.79766537	4.65E-80	1.73E-79
PXMP2	1.91987508	3.66E-68	1.14E-67
RP13-672B3.	NA	4.78E-09	6.18E-09
PGAM5	2.2931757	2.58E-29	4.60E-29
ANKLE2	1.74773022	5.21E-39	1.07E-38
GOLGA3	3.05372643	1.21E-86	5.03E-86
CHFR	NA	0.0127198	0.01403495

RP11-46H11.	NA	NA	NA
RP11-46H11.	Inf	7.32E-06	8.85E-06
ZNF605	2.24708171	3.51E-69	1.11E-68
ZNF26	2.8463035	1.06E-57	2.80E-57
RP11-386I8.6	NA	0.02164078	0.0236767
ZNF84	1.8385214	4.29E-07	5.32E-07
ZNF140	1.40442607	1.13E-18	1.73E-18
ZNF891	2.39688716	1.11E-65	3.34E-65
CTD-2140B24	0	6.63E-119	5.68E-118
ZNF10	Inf	1.05E-51	2.58E-51
CTD-2140B24	NA	NA	NA
ZNF268	1.39105058	1.54E-21	2.45E-21
ANHX	NA	NA	NA
AL356585.2	Inf	2.87E-08	3.65E-08
LINC00349	NA	NA	NA
LINC00388	NA	NA	NA
LINC00387	NA	NA	NA
LINC00408	NA	NA	NA
RP11-442J17	NA	NA	NA
LINC00442	NA	NA	NA
RP11-301J16	NA	NA	NA
TUBA3C	NA	NA	NA
RP11-408E5.	NA	NA	NA
LINC00421	NA	NA	NA
RP11-385E5.	NA	NA	NA
TPTE2	NA	NA	NA
LINC00350	NA	NA	NA
MPHOSPH8	1.22821858	8.86E-132	1.32E-130
RP11-523H24	Inf	1.36E-64	4.02E-64
PSPC1	1.14850843	1.32E-110	9.04E-110
ZMYM5	1.18595979	2.06E-16	3.04E-16
RP11-61K9.2	NA	6.32E-07	7.81E-07
RP11-61K9.3	0.74902724	1.72E-15	2.50E-15
ZMYM2	2.70802155	3.52E-42	7.51E-42
LINC01072	NA	NA	NA
GJA3	4.11964981	6.02E-23	9.81E-23
LINC00556	NA	NA	NA
GJB2	NA	2.40E-39	4.93E-39
GJB6	NA	NA	NA
CRYL1	2.15345331	2.38E-115	1.83E-114
RP11-476H16	NA	NA	NA
IFT88	Inf	5.79E-132	8.68E-131
RP11-172H24	1.94747082	1.23E-52	3.05E-52
IL17D	1.34824903	0.00018041	0.00021011
N6AMT2	1.64302749	7.36E-14	1.04E-13

XPO4	3.2457847	5.62E-88	2.41E-87
LATS2	1.87256809	0.07214795	0.07741812
LATS2-AS1	NA	0.00029653	0.0003437
SAP18	1.59550445	4.67E-59	1.26E-58
SKA3	1.45525292	0.00138857	0.00157753
MRPL57	2.06436053	2.97E-92	1.38E-91
LINC01046	NA	NA	NA
LINC00539	0.74902724	1.24E-20	1.95E-20
ZDHHC20	1.99740597	4.31E-07	5.35E-07
MICU2	1.13366285	7.90E-144	4.05E-142
FGF9	3.74513619	3.43E-28	6.02E-28
LINC00424	NA	NA	NA
LINC00540	NA	NA	NA
AL354828.1	NA	NA	NA
AL354828.2	NA	NA	NA
LINC00621	NA	NA	NA
RP11-124N1!	NA	NA	NA
RP11-124N1!	NA	NA	NA
LINC00362	NA	NA	NA
SGCG	NA	NA	NA
SACS	2.67509728	8.92E-81	3.36E-80
SACS-AS1	NA	NA	NA
LINC00327	NA	NA	NA
LINC00352	NA	NA	NA
TNFRSF19	NA	NA	NA
MIPEP	1.16515348	0.00020732	0.00024116
C1QTNF9B-A	1.91418072	4.75E-34	9.05E-34
C1QTNF9B	NA	2.42E-68	7.56E-68
RP11-307N1(	NA	1.75E-05	2.10E-05
SPATA13	2.34071012	4.02E-49	9.45E-49
SPATA13	NA	NA	NA
SPATA13-AS	NA	NA	NA
C1QTNF9	NA	NA	NA
C1QTNF9-AS	NA	NA	NA
C1QTNF9B-A	NA	NA	NA
LINC00566	NA	NA	NA
PARP4	1.63023575	0.01089622	0.01205141
ATP12A	NA	NA	NA
RNF17	NA	NA	NA
CENPJ	1.87256809	4.57E-07	5.67E-07
PABPC3	1.49805447	6.37E-26	1.09E-25
AMER2	NA	NA	NA
LINC00463	NA	NA	NA
LINC01053	NA	NA	NA
LINC01076	NA	NA	NA

MTMR6	1.8385214	0.00105316	0.00120182
RP11-271M2	NA	1.32E-51	3.23E-51
NUP58	2.20168612	2.10E-50	5.02E-50
ATP8A2	NA	8.62E-44	1.88E-43
SHISA2	NA	NA	NA
LINC00415	NA	NA	NA
RNF6	1.56614786	0.03554085	0.03857275
RP11-380N8.	4.49416342	1.68E-111	1.18E-110
CDK8	1.78934284	3.69E-08	4.69E-08
WASF3	1.5729572	0.00589784	0.00658129
WASF3-AS1	NA	3.46E-11	4.67E-11
GPR12	Inf	1.26E-72	4.18E-72
USP12	2.24708171	1.12E-61	3.16E-61
USP12-AS1	NA	NA	NA
USP12-AS2	NA	2.09E-71	6.83E-71
RP11-428O1	NA	0.73956269	0.74918457
LINC00412	Inf	5.90E-23	9.62E-23
RPL21	1.91666568	1.65E-28	2.92E-28
RASL11A	1.99740597	1.48E-05	1.77E-05
LINC01079	NA	NA	NA
GTF3A	1.93790877	1.03E-29	1.85E-29
MTIF3	1.33853941	9.05E-140	2.49E-138
LNX2	3.99481193	3.63E-43	7.84E-43
POLR1D	1.93923975	7.03E-12	9.61E-12
RP11-87C7.3	NA	NA	NA
GSX1	NA	NA	NA
PDX1-AS1	NA	NA	NA
PDX1	NA	NA	NA
ATP5EP2	2.92801556	2.73E-143	1.25E-141
LINC00543	1.74773022	9.45E-07	1.16E-06
CDX2	1.77150886	0.00075411	0.00086365
URAD	NA	NA	NA
FLT3	NA	3.19E-26	5.45E-26
PAN3-AS1	2.24708171	0.00382143	0.00428959
PAN3	1.9193823	1.37E-06	1.68E-06
FLT1	NA	8.26E-141	2.58E-139
POMP	1.69670952	0.00066432	0.00076198
SLC46A3	1.12354086	0.00384042	0.00431037
MTUS2	NA	2.21E-66	6.71E-66
MTUS2-AS2	NA	NA	NA
MTUS2-AS1	NA	NA	NA
SLC7A1	2.45136187	2.50E-115	1.92E-114
UBL3	1.12354086	5.19E-73	1.74E-72
LINC00297	NA	NA	NA
LINC00572	NA	NA	NA

LINC00544	NA	NA	NA
RP11-629E24	NA	NA	NA
LINC00365	NA	NA	NA
LINC00384	NA	NA	NA
LINC00385	NA	NA	NA
KATNAL1	2.52796693	1.65E-78	5.97E-78
LINC00427	NA	NA	NA
LINC00426	NA	9.94E-06	1.20E-05
LINC01058	NA	NA	NA
HMGB1	1.22428405	8.21E-142	2.95E-140
USPL1	1.02991245	1.38E-14	1.97E-14
ALOX5AP	2.24708171	2.80E-45	6.24E-45
LINC00398	NA	NA	NA
TEX26-AS1	NA	NA	NA
LINC00545	NA	NA	NA
MEDAG	NA	NA	NA
TEX26	NA	NA	NA
RP11-252M2	NA	NA	NA
LINC01066	NA	NA	NA
HSPH1	1.50325605	4.13E-51	9.99E-51
B3GLCT	1.49805447	8.48E-16	1.24E-15
RP11-326L9.	NA	NA	NA
RXFP2	NA	NA	NA
LINC01073	NA	NA	NA
FRY-AS1	NA	9.86E-81	3.71E-80
FRY	0.56177043	0.0027067	0.00304867
ZAR1L	NA	8.82E-22	1.41E-21
BRCA2	1.31079767	0.20198204	0.21238962
N4BP2L1	1.59168288	2.64E-05	3.14E-05
N4BP2L2	1.4844358	4.01E-68	1.25E-67
PDS5B	2.36534917	0.10157848	0.10833843
RP11-380B4.	Inf	5.66E-111	3.91E-110
LINC00423	NA	NA	NA
KL	NA	NA	NA
STARD13	1.05745022	2.56E-17	3.83E-17
RP11-141M1	NA	5.33E-23	8.69E-23
RP11-141M1	NA	NA	NA
RP11-37L2.1	NA	2.32E-52	5.73E-52
RP11-179A7.	NA	NA	NA
RFC3	1.60136858	4.78E-29	8.50E-29
RP11-16D22.	NA	NA	NA
LINC00457	NA	NA	NA
RP11-266E6.	NA	NA	NA
RP11-307O1	NA	NA	NA
NBEA	Inf	0.01145944	0.01266662

MAB21L1	2.24708171	6.38E-06	7.72E-06
LINC00445	NA	NA	NA
DCLK1	NA	NA	NA
SOHLH2	NA	1.09E-36	2.17E-36
CCDC169-SO	NA	NA	NA
CCDC169	0.49935149	2.90E-128	3.59E-127
SPG20	1.32973375	2.22E-65	6.62E-65
SPG20-AS1	2.99610895	1.99E-98	1.04E-97
CCNA1	0.45908121	2.97E-142	1.12E-140
SERTM1	NA	NA	NA
RFXAP	1.32181277	1.82E-08	2.32E-08
SMAD9	1.68531128	1.16E-06	1.42E-06
ALG5	1.17610417	1.04E-142	4.29E-141
EXOSC8	0.75596267	8.99E-136	1.70E-134
SUPT20H	1.31585866	0.00121238	0.0013803
AL138706.2	NA	NA	NA
CSNK1A1L	NA	NA	NA
LINC01048	NA	NA	NA
LINC00547	NA	NA	NA
POSTN	NA	NA	NA
TRPC4	NA	NA	NA
RP11-93G5.1	NA	6.85E-12	9.36E-12
LINC00571	NA	0.01402324	0.01544966
UFM1	2.30621544	1.49E-125	1.63E-124
LINC00437	NA	NA	NA
LINC00366	NA	NA	NA
FREM2	NA	NA	NA
FREM2-AS1	NA	NA	NA
STOML3	NA	NA	NA
PROSER1	2.57478113	2.75E-105	1.66E-104
NHLRC3	2.13611471	4.37E-34	8.34E-34
RP11-50D16.	NA	4.78E-09	6.18E-09
LHFP	1.7077821	6.73E-08	8.49E-08
COG6	1.12354086	1.55E-06	1.89E-06
RP11-427J23	NA	NA	NA
LINC00332	NA	NA	NA
LINC00548	NA	NA	NA
RP11-172E9.	NA	NA	NA
LINC00598	NA	NA	NA
FOXO1	Inf	1.05E-115	8.16E-115
MRPS31	0.9302435	1.86E-107	1.17E-106
SLC25A15	2.30469919	1.63E-38	3.30E-38
ELF1	1.34665535	3.90E-106	2.38E-105
WBP4	1.48819885	1.44E-50	3.45E-50
KBTBD6	2.0598249	0.00979089	0.01084145

RP11-74J13.8	NA	0.07268712	0.07799206
KBTBD7	2.24708171	1.97E-26	3.38E-26
MTRF1	1.92607004	0.3800829	0.39308404
NAA16	1.31079767	4.26E-26	7.27E-26
RGCC	2.20963035	4.38E-13	6.11E-13
VWA8	1.68531128	3.81E-21	6.05E-21
VWA8-AS1	NA	1.46E-47	3.35E-47
DGKH	6.74124514	6.98E-26	1.19E-25
RP11-187A9.	NA	NA	NA
AKAP11	1.98271916	0.83539513	0.84250033
TNFSF11	NA	NA	NA
FAM216B	NA	NA	NA
LINC01050	NA	NA	NA
LINC00428	NA	NA	NA
EPSTI1	NA	2.12E-14	3.02E-14
DNAJC15	NA	5.65E-99	2.99E-98
LINC00400	NA	NA	NA
ENOX1	NA	0.01468115	0.01616566
ENOX1-AS2	NA	NA	NA
ENOX1-AS1	NA	NA	NA
RP11-168P13	NA	1.97E-18	3.00E-18
CCDC122	1.99740597	0.82285018	0.83031025
LACC1	2.24708171	5.48E-85	2.22E-84
RP11-65B7.2	NA	7.02E-39	1.43E-38
LINC00284	NA	NA	NA
LINC00390	NA	NA	NA
SMIM2-AS1	Inf	7.45E-24	1.23E-23
SMIM2	NA	NA	NA
RP11-478K15	NA	NA	NA
RP11-478K15	NA	NA	NA
RP11-269C23	NA	NA	NA
SERP2	NA	NA	NA
TUSC8	Inf	4.58E-29	8.14E-29
TSC22D1	1.9193823	0.0012025	0.00136939
TSC22D1-AS1	Inf	2.55E-86	1.06E-85
LINC00407	NA	NA	NA
RP11-442J21	NA	NA	NA
LINC00330	NA	6.52E-12	8.91E-12
NUFIP1	1.05544747	6.54E-76	2.28E-75
RP11-321C24	NA	NA	NA
GPALPP1	1.39818418	4.33E-10	5.71E-10
GTF2F2	1.10725766	1.06E-130	1.47E-129
KCTD4	NA	NA	NA
TPT1	2.29557384	3.34E-140	9.60E-139
RP11-290D2.	0	1.85E-38	3.74E-38

TPT1-AS1	3.74513619	1.07E-17	1.61E-17
RP11-290D2.	NA	2.37E-63	6.83E-63
SLC25A30	4.11964981	1.09E-30	1.98E-30
SLC25A30-AS	NA	1.32E-104	7.81E-104
COG3	2.78210117	9.05E-45	2.00E-44
ERICH6B	NA	NA	NA
LINC01055	NA	NA	NA
SPERT	NA	NA	NA
SIAH3	NA	NA	NA
ZC3H13	1.33853941	1.48E-89	6.55E-89
CPB2-AS1	1.49805447	0.73079796	0.74055391
CPB2	NA	7.50E-22	1.20E-21
LCP1	1.71360188	2.92E-09	3.79E-09
LRRC63	NA	5.68E-50	1.35E-49
LINC00563	NA	NA	NA
KIAA0226L	NA	NA	NA
LINC01198	NA	NA	NA
RP11-189B4.	NA	NA	NA
LRCH1	2.54669261	6.14E-06	7.43E-06
ESD	1.0195093	3.59E-141	1.16E-139
HTR2A	NA	NA	NA
HTR2A-AS1	NA	NA	NA
RP11-279N8.	NA	NA	NA
LINC00444	NA	NA	NA
LINC00562	2.24708171	6.27E-24	1.04E-23
SUCLA2	1.00731249	4.61E-102	2.59E-101
SUCLA2-AS1	NA	1.87E-67	5.74E-67
NUDT15	1.55750108	5.03E-80	1.87E-79
RP11-90M2.5	Inf	5.67E-45	1.26E-44
MED4	1.62289235	2.14E-63	6.17E-63
MED4-AS1	NA	2.85E-05	3.39E-05
ITM2B	0.85953945	1.09E-143	5.42E-142
LINC00441	Inf	3.26E-141	1.07E-139
RB1	2.36692607	4.31E-59	1.17E-58
RP11-305D15	NA	NA	NA
LPAR6	2.99610895	5.59E-48	1.29E-47
RCBTB2	2.14007782	0.08877744	0.09492547
LINC01077	NA	NA	NA
LINC00462	NA	1.45E-08	1.86E-08
CYSLTR2	NA	NA	NA
RP11-156K23	1.12354086	9.35E-59	2.52E-58
FNDC3A	1.98271916	6.93E-05	8.16E-05
MLNR	NA	NA	NA
CDADC1	1.69779507	8.95E-15	1.29E-14
CAB39L	1.04863813	0.0143591	0.01581584

SETDB2	2.10143753	3.82E-82	1.47E-81
PHF11	1.715514	3.30E-43	7.14E-43
RCBTB1	2.51459144	2.74E-49	6.45E-49
ARL11	1.59168288	0.25152289	0.26306516
EBPL	1.54474708	0.04762915	0.05146139
KPNA3	1.58793774	1.21E-14	1.73E-14
RP11-195L15	NA	1.00E-105	6.09E-105
SPRYD7	1.85628489	2.91E-39	5.98E-39
DLEU2	1.8271725	1.82E-48	4.23E-48
TRIM13	2.07684825	1.86E-09	2.42E-09
KCNRG	1.49805447	0.10184981	0.10862144
DLEU1	2.14815359	2.69E-35	5.23E-35
DLEU1-AS1	NA	NA	NA
DLEU7	NA	2.44E-120	2.20E-119
DLEU7-AS1	NA	NA	NA
RNASEH2B-A	Inf	3.16E-127	3.75E-126
RNASEH2B	1.1545779	3.01E-119	2.61E-118
LINC00371	NA	NA	NA
RP11-457D13	NA	NA	NA
FAM124A	3.37062257	5.55E-16	8.14E-16
SERPINE3	NA	2.56E-85	1.04E-84
INTS6	1.4766537	3.51E-06	4.27E-06
INTS6-AS1	1.68531128	0.01795012	0.0197018
WDFY2	1.39105058	0.00560082	0.00625408
DHRS12	0	1.78E-38	3.60E-38
RP11-327P2.	1.12354086	0.21560671	0.2263228
LINC00282	NA	2.82E-138	6.49E-137
CCDC70	NA	NA	NA
ATP7B	3.37062257	2.47E-123	2.49E-122
ALG11	0.89883268	0.00013332	0.00015583
UTP14C	2.20302129	6.99E-33	1.31E-32
NEK5	1.49805447	1.51E-39	3.11E-39
RP11-248G5.	NA	1.37E-54	3.48E-54
NEK3	1.1523496	2.64E-104	1.55E-103
RP13-444H2.	NA	NA	NA
THSD1	NA	7.87E-29	1.40E-28
VPS36	1.53909706	0.0150888	0.01660748
RP11-245D16	2.0598249	0.03279465	0.03566696
CKAP2	1.45964282	9.43E-95	4.59E-94
LINC00345	NA	2.72E-103	1.57E-102
RP11-78J21.4	NA	NA	NA
HNRNPA1L2	1.68531128	5.28E-111	3.65E-110
RP11-93H24.	1.19844358	2.82E-19	4.35E-19
SUGT1	1.09473212	2.72E-119	2.36E-118
LECT1	NA	NA	NA

PCDH8	NA	NA	NA
OLFM4	NA	NA	NA
LINC01065	NA	NA	NA
RP11-24H2.3	NA	NA	NA
RP11-384G2:	NA	NA	NA
LINC00558	NA	NA	NA
LINC00458	NA	NA	NA
RP11-108H9.	NA	NA	NA
RP11-78L16.	NA	NA	NA
RP11-415G4.	NA	NA	NA
PRR20A	NA	NA	NA
PRR20C	NA	NA	NA
PRR20B	NA	NA	NA
PRR20D	NA	NA	NA
PRR20E	NA	NA	NA
PCDH17	NA	7.51E-45	1.66E-44
RP11-95F22.	NA	NA	NA
RP11-538C21	NA	2.26E-39	4.64E-39
LINC00374	NA	NA	NA
DIAPH3	2.13184675	8.96E-53	2.23E-52
DIAPH3-AS1	NA	NA	NA
DIAPH3-AS2	NA	NA	NA
LINC00434	NA	NA	NA
TDRD3	3.5311284	4.49E-08	5.68E-08
LINC00378	NA	NA	NA
LINC01442	NA	NA	NA
PCDH20	NA	NA	NA
RP11-310K1(	NA	NA	NA
RP11-432J3.4	NA	NA	NA
LINC00358	NA	NA	NA
LINC01075	NA	NA	NA
LINC01074	NA	NA	NA
LINC00459	NA	NA	NA
LINC00448	NA	NA	NA
RP11-527N1:	NA	NA	NA
LINC00376	NA	NA	NA
LINC00395	NA	NA	NA
RP11-473M1	NA	NA	NA
RP11-394A14	NA	NA	NA
RP11-394A14	NA	NA	NA
LINC00355	Inf	2.36E-21	3.76E-21
LINC01052	NA	NA	NA
PCDH9	NA	1.05E-15	1.53E-15
PCDH9-AS1	NA	NA	NA
PCDH9-AS2	NA	NA	NA

PCDH9-AS3	NA	NA	NA
PCDH9-AS4	NA	NA	NA
LINC00364	NA	NA	NA
LINC00550	NA	NA	NA
RP11-411G9.	NA	NA	NA
LINC00383	NA	NA	NA
RP11-459J23	NA	NA	NA
KLHL1	NA	NA	NA
ATXN8OS	NA	NA	NA
LINC00348	NA	NA	NA
DACH1	0	2.88E-83	1.13E-82
MZT1	1.82280754	4.55E-36	8.94E-36
BORA	1.23752326	2.29E-104	1.35E-103
DIS3	1.43430748	4.69E-57	1.23E-56
PIBF1	0.58257674	3.78E-12	5.19E-12
KLF5	Inf	7.50E-12	1.02E-11
LINC00393	NA	NA	NA
LINC00392	NA	NA	NA
KLF12	0	5.98E-85	2.42E-84
LINC00402	NA	NA	NA
RP11-512C24	NA	NA	NA
LINC00381	NA	NA	NA
RP11-285E18	NA	NA	NA
LINC00347	NA	NA	NA
LINC01078	NA	NA	NA
TBC1D4	1.77401188	4.31E-16	6.33E-16
RP11-182M2	NA	5.68E-34	1.08E-33
COMMD6	2.05866661	5.35E-39	1.09E-38
UCHL3	1.46186958	1.79E-84	7.20E-84
LMO7-AS1	NA	NA	NA
LMO7	1.58127972	0.00041264	0.00047631
LMO7DN	NA	NA	NA
LINC00561	NA	NA	NA
LINC01034	NA	NA	NA
KCTD12	0.37451362	1.10E-39	2.26E-39
AC000403.4	NA	7.63E-20	1.19E-19
IRG1	NA	NA	NA
CLN5	0.79735158	3.28E-115	2.51E-114
FBXL3	1.49805447	2.52E-08	3.21E-08
AC001226.7	0	1.81E-11	2.45E-11
MYCBP2	1.90293406	7.73E-13	1.07E-12
MYCBP2-AS1	NA	2.59E-76	9.08E-76
SCEL	NA	NA	NA
SCEL-AS1	NA	NA	NA
SLAIN1	1.52926394	1.55E-22	2.51E-22

EDNRB-AS1	NA	NA	NA
EDNRB	NA	NA	NA
RNF219-AS1	NA	NA	NA
RP11-318G2	NA	NA	NA
LINC01069	NA	NA	NA
LINC00446	NA	NA	NA
RP11-203P2.	NA	NA	NA
RP11-52L5.6	NA	NA	NA
POU4F1	3.62029831	1.13E-72	3.75E-72
RNF219	1.49805447	8.76E-52	2.14E-51
LINC00331	NA	NA	NA
RBM26	1.86770428	2.99E-45	6.65E-45
RBM26-AS1	5.24319066	9.64E-38	1.94E-37
NDFIP2-AS1	NA	NA	NA
NDFIP2	0	3.18E-74	1.08E-73
LINC01068	NA	NA	NA
LINC01038	NA	NA	NA
LINC00382	NA	NA	NA
LINC01080	NA	NA	NA
SPRY2	1.5729572	2.00E-18	3.05E-18
LINC00377	NA	NA	NA
LINC00564	NA	NA	NA
RP11-452B18	NA	NA	NA
SLITRK1	NA	NA	NA
LINC00333	NA	NA	NA
LINC00375	NA	NA	NA
LINC00351	NA	NA	NA
SLITRK6	NA	NA	NA
RP11-30L8.1	NA	NA	NA
LINC00430	NA	NA	NA
RP11-342C20	NA	NA	NA
MIR4500HG	NA	NA	NA
RP11-29P20.	NA	NA	NA
SLITRK5	1.72276265	1.02E-61	2.89E-61
LINC00397	NA	NA	NA
RP11-471M2	NA	NA	NA
RP11-545P6.	NA	NA	NA
LINC00433	NA	NA	NA
LINC00560	NA	NA	NA
LINC01047	NA	NA	NA
LINC00440	NA	NA	NA
LINC01040	NA	NA	NA
LINC00353	NA	NA	NA
RP11-75N6.3	NA	NA	NA
LINC00559	NA	NA	NA

LINC01049	NA	NA	NA
LINC00410	NA	NA	NA
LINC00380	NA	NA	NA
LINC00379	1.49805447	1.09E-17	1.65E-17
GPC5	NA	NA	NA
GPC5-AS2	NA	5.04E-119	4.33E-118
GPC5-AS1	NA	NA	NA
LINC00363	NA	NA	NA
RP11-632L2.:	2.99610895	1.72E-14	2.47E-14
GPC6	0.83225249	1.59E-61	4.48E-61
GPC6-AS2	NA	NA	NA
GPC6-AS1	NA	NA	NA
DCT	NA	7.45E-134	1.25E-132
TGDS	1.07360571	3.74E-134	6.35E-133
GPR180	2.62159533	4.24E-05	5.02E-05
LINC00391	NA	NA	NA
SOX21	NA	NA	NA
SOX21-AS1	NA	NA	NA
LINC00557	NA	NA	NA
ABCC4	1.95899431	2.04E-19	3.15E-19
CLDN10	NA	NA	NA
CLDN10-AS1	NA	1.15E-85	4.70E-85
DZIP1	2.49675746	3.56E-106	2.18E-105
DNAJC3-AS1	2.52796693	2.14E-06	2.62E-06
DNAJC3	1.17997442	1.28E-125	1.40E-124
UGGT2	1.74773022	0.76066514	0.76970199
HS6ST3	NA	NA	NA
LINC00359	NA	NA	NA
RP11-65L19.4	NA	NA	NA
OXGR1	NA	NA	NA
LINC00456	NA	NA	NA
MBNL2	Inf	3.91E-96	1.96E-95
RAP2A	1.49805447	4.59E-28	8.05E-28
RP11-120E13	NA	NA	NA
RP11-383H17	NA	NA	NA
IPO5	2.32198444	6.87E-77	2.43E-76
FARP1	3.81322957	2.78E-140	8.11E-139
RNF113B	NA	NA	NA
RP11-261P24	NA	NA	NA
FARP1-AS1	NA	4.74E-71	1.53E-70
STK24	2.27336337	1.30E-07	1.64E-07
STK24-AS1	0	0.00045216	0.00052133
SLC15A1	NA	NA	NA
DOCK9	NA	NA	NA
DOCK9-AS1	NA	NA	NA

DOCK9-AS2	NA	NA	NA
UBAC2-AS1	1.79766537	0.89930351	0.90408757
UBAC2	1.8799115	1.05E-32	1.95E-32
GPR18	NA	NA	NA
GPR183	NA	7.65E-06	9.24E-06
RP11-214F16	NA	NA	NA
LINC00449	Inf	8.68E-17	1.29E-16
TM9SF2	1.79766537	3.32E-06	4.04E-06
LINC01039	NA	NA	NA
CLYBL	0.53501946	5.82E-88	2.49E-87
CLYBL-AS2	NA	NA	NA
CLYBL-AS1	NA	NA	NA
ZIC5	Inf	7.86E-83	3.07E-82
ZIC2	4.30690661	1.51E-109	1.00E-108
LINC00554	NA	NA	NA
RP11-12G12.	2.8463035	7.52E-117	6.05E-116
PCCA	0.87386511	2.80E-52	6.92E-52
PCCA-AS1	NA	NA	NA
GGACT	1.19844358	0.00013261	0.00015502
RP11-151A6.	NA	7.46E-105	4.44E-104
TMTC4	1.42315175	2.38E-13	3.33E-13
NALCN-AS1	NA	NA	NA
LINC00411	NA	NA	NA
NALCN	NA	NA	NA
ITGBL1	NA	2.82E-31	5.18E-31
FGF14	NA	NA	NA
RP11-397O8.	NA	NA	NA
FGF14-AS1	NA	NA	NA
FGF14-AS2	NA	NA	NA
LINC00555	NA	NA	NA
RP11-29B2.6	NA	2.26E-09	2.94E-09
TPP2	2.07422927	1.16E-28	2.05E-28
METTL21C	NA	NA	NA
CCDC168	Inf	5.26E-49	1.23E-48
LINC00283	NA	NA	NA
TEX30	1.88319259	0.13363113	0.14172431
KDELC1	1.15758755	2.43E-99	1.29E-98
BIVM	2.43433852	3.88E-127	4.58E-126
BIVM-ERCC5	NA	NA	NA
ERCC5	2.62159533	3.54E-14	5.03E-14
RP11-255P5.	NA	NA	NA
RP11-255P5.	NA	NA	NA
SLC10A2	NA	NA	NA
LINC01309	NA	NA	NA
DAOA-AS1	NA	NA	NA

DAOA	NA	NA	NA
RP11-252M2	NA	NA	NA
LINC00460	NA	NA	NA
EFNB2	0.74902724	3.19E-16	4.71E-16
ARGLU1	1.72742167	0.00062744	0.00072012
LINC00551	NA	5.47E-100	2.94E-99
LINC00443	NA	NA	NA
RP11-282A1:	NA	NA	NA
RP11-282A1:	NA	NA	NA
FAM155A	NA	NA	NA
RP11-346C4.	NA	NA	NA
LIG4	Inf	1.38E-89	6.09E-89
ABHD13	2.08063121	0.12076031	0.12838169
TNFSF13B	2.0598249	7.95E-79	2.90E-78
MYO16	NA	NA	NA
MYO16-AS2	NA	NA	NA
MYO16-AS1	NA	NA	NA
RP11-54H7.4	NA	NA	NA
LINC00370	NA	NA	NA
LINC01067	NA	NA	NA
LINC00399	NA	NA	NA
LINC00676	NA	NA	NA
IRS2	2.24708171	3.41E-80	1.27E-79
RP11-40E6.1	NA	NA	NA
RP11-40E6.2	NA	NA	NA
RP11-403A3.	NA	NA	NA
RP11-403A3.	NA	NA	NA
RP11-403A3.	NA	NA	NA
LINC00396	NA	NA	NA
COL4A1	NA	9.17E-78	3.29E-77
COL4A2	NA	NA	NA
COL4A2-AS2	NA	NA	NA
COL4A2-AS1	NA	NA	NA
RAB20	1.30094204	3.32E-92	1.54E-91
RP11-90L1.8	0	2.04E-32	3.79E-32
CARKD	1.13562194	0.00017167	0.00020004
CARS2	1.58489821	7.79E-25	1.31E-24
ING1	1.8385214	5.96E-16	8.73E-16
LINC00567	NA	NA	NA
LINC00346	NA	NA	NA
ANKRD10	1.63976233	0.01674118	0.01840049
RP11-365P1:	1.49805447	4.19E-20	6.54E-20
LINC00431	NA	NA	NA
LINC00368	NA	NA	NA
ARHGEF7-AS	NA	NA	NA

ARHGEF7	2.8463035	9.64E-95	4.68E-94
ARHGEF7-AS	NA	NA	NA
TEX29	NA	6.39E-68	1.98E-67
RP11-65D24.	NA	NA	NA
RP11-65D24.	NA	NA	NA
LINC00354	NA	NA	NA
SOX1	NA	NA	NA
RP11-450H6.	NA	NA	NA
LINC00404	NA	NA	NA
LINC00403	NA	NA	NA
LINC01070	NA	NA	NA
LINC01043	NA	NA	NA
LINC01044	NA	NA	NA
SPACA7	NA	NA	NA
TUBGCP3	1.74773022	1.24E-29	2.22E-29
RP11-88E10.	NA	NA	NA
ATP11AUN	NA	NA	NA
RP11-88E10.	0.74902724	2.46E-06	3.00E-06
ATP11A	3.74513619	1.56E-30	2.84E-30
ATP11A-AS1	NA	NA	NA
MCF2L	0.44941634	2.02E-80	7.56E-80
RP11-120K24	NA	1.32E-100	7.17E-100
MCF2L-AS1	3.37062257	2.20E-34	4.22E-34
F7	NA	NA	NA
F10	NA	NA	NA
F10-AS1	NA	NA	NA
PROZ	NA	NA	NA
RP11-98F14.	NA	5.83E-98	3.02E-97
PCID2	1.23450785	8.41E-47	1.91E-46
CUL4A	2.27048881	4.18E-54	1.06E-53
LAMP1	1.32684825	3.47E-95	1.70E-94
GRTP1	1.49805447	2.56E-12	3.53E-12
GRTP1-AS1	NA	NA	NA
ADPRHL1	0.44941634	6.71E-24	1.11E-23
DCUN1D2	1.74773022	3.12E-45	6.94E-45
DCUN1D2-AS	NA	NA	NA
TMCO3	1.23589494	2.11E-29	3.77E-29
TFDP1	2.35655492	4.07E-107	2.54E-106
ATP4B	NA	NA	NA
GRK1	NA	NA	NA
TMEM255B	0.24967575	1.26E-109	8.42E-109
GAS6-AS1	3.31712062	3.95E-101	2.18E-100
GAS6	0.99870298	1.01E-27	1.76E-27
GAS6-AS2	NA	NA	NA
RP11-199F6.	NA	NA	NA

LINC00454	NA	NA	NA
LINC00453	NA	NA	NA
LINC00452	NA	NA	NA
LINC00565	NA	NA	NA
RASA3	1.74773022	8.31E-21	1.31E-20
CDC16	2.44419414	6.68E-06	8.07E-06
UPF3A	1.77661832	8.51E-45	1.88E-44
CHAMP1	2.3479123	2.91E-61	8.15E-61
LINC01054	NA	NA	NA
RP11-536C1(	NA	NA	NA
OR11H12	NA	NA	NA
RP11-536C1(	NA	NA	NA
RP11-536C1(	NA	NA	NA
CTD-2311B1:	NA	NA	NA
CTD-2311B1:	NA	NA	NA
POTEM	NA	NA	NA
CTD-2311B1:	NA	NA	NA
CTD-2314B2.	NA	NA	NA
RP11-146E1:	NA	NA	NA
RP11-496I2.2	NA	NA	NA
AL589743.1	1.97470817	7.04E-69	2.22E-68
LINC01297	Inf	2.88E-130	3.94E-129
POTEG	NA	NA	NA
RP11-244H1{	NA	NA	NA
RP11-244H1{	NA	NA	NA
RP11-597A1:	NA	NA	NA
RP11-597A1:	NA	NA	NA
OR11H2	NA	NA	NA
OR4N2	NA	NA	NA
OR4Q3	NA	NA	NA
OR4M1	NA	NA	NA
OR4K2	NA	NA	NA
OR4K5	NA	NA	NA
OR4K1	NA	NA	NA
OR4K15	NA	NA	NA
OR4K14	NA	NA	NA
OR4K13	NA	NA	NA
OR4L1	NA	NA	NA
OR4K17	NA	NA	NA
OR4N5	NA	NA	NA
OR11G2	NA	NA	NA
OR11H6	NA	NA	NA
OR11H4	NA	NA	NA
TTC5	1.05418648	1.40E-64	4.13E-64
CTD-2292M1	0	9.54E-65	2.82E-64

CTD-2292M1	NA	NA	NA
CCNB1IP1	1.84760052	4.54E-29	8.07E-29
RPPH1	Inf	1.50E-33	2.84E-33
PARP2	1.42671855	5.56E-16	8.16E-16
RP11-203M5	NA	NA	NA
TEP1	1.49805447	0.00784512	0.00872203
KLHL33	NA	NA	NA
OSGEP	1.26264591	1.16E-126	1.33E-125
RP11-203M5	NA	NA	NA
APEX1	1.06863834	1.47E-136	2.92E-135
TMEM55B	1.46400778	1.68E-139	4.50E-138
PNP	0.80009728	4.01E-144	2.19E-142
RP11-203M5	NA	NA	NA
RNASE10	NA	NA	NA
RNASE9	NA	NA	NA
RP11-14J7.7	NA	NA	NA
RNASE11	NA	NA	NA
RP11-14J7.6	NA	NA	NA
RNASE12	NA	NA	NA
OR6S1	NA	NA	NA
RNASE4	NA	NA	NA
ANG	0.37451362	6.71E-101	3.68E-100
RP11-903H1:	NA	NA	NA
RP11-903H1:	NA	NA	NA
EDDM3A	NA	NA	NA
EDDM3B	NA	NA	NA
RNASE6	NA	3.57E-106	2.18E-105
RP11-219E7.	NA	NA	NA
RNASE1	NA	1.45E-26	2.50E-26
RP11-219E7.	NA	NA	NA
RP11-219E7.	NA	NA	NA
RNASE3	0	1.96E-145	1.63E-143
RP11-219E7.	NA	NA	NA
RP11-84C10.	NA	NA	NA
RP11-84C10.	0	1.02E-145	1.02E-143
RNASE2	0.02618976	1.80E-145	1.58E-143
METTL17	1.36047804	2.22E-131	3.23E-130
RP11-84C10.	NA	NA	NA
SLC39A2	NA	2.30E-86	9.54E-86
NDRG2	2.17217899	1.37E-43	2.97E-43
TPPP2	NA	NA	NA
AL161668.5	NA	NA	NA
RP11-998D1(	NA	NA	NA
RNASE13	NA	NA	NA
RNASE7	NA	NA	NA

RNASE8	NA	NA	NA
ARHGEF40	4.74383917	2.60E-76	9.10E-76
ZNF219	4.24448768	2.06E-07	2.57E-07
TMEM253	NA	2.36E-58	6.33E-58
RP11-998D1(	NA	NA	NA
OR5AU1	NA	NA	NA
HNRNPC	1.50968533	9.34E-108	5.95E-107
RPGRIP1	NA	NA	NA
SUPT16H	1.61632193	5.95E-07	7.35E-07
RP11-524O1.	1.49805447	0.00014498	0.00016925
CHD8	1.94747082	1.00E-53	2.52E-53
RAB2B	3.37062257	1.59E-16	2.35E-16
TOX4	1.23752326	1.47E-61	4.14E-61
METTL3	2.14938251	7.19E-43	1.55E-42
SALL2	NA	1.09E-56	2.86E-56
AE000658.22	NA	NA	NA
OR10G3	NA	NA	NA
TRAV1-1	NA	NA	NA
OR10G2	NA	NA	NA
TRAV1-2	NA	NA	NA
OR4E2	NA	NA	NA
TRAV2	NA	NA	NA
AE000658.31	NA	NA	NA
TRAV3	NA	NA	NA
TRAV4	NA	NA	NA
TRAV5	NA	NA	NA
TRAV6	NA	NA	NA
TRAV7	NA	NA	NA
TRAV8-1	NA	NA	NA
TRAV9-1	NA	NA	NA
TRAV10	NA	NA	NA
TRAV11	NA	NA	NA
TRAV12-1	NA	NA	NA
TRAV8-2	NA	NA	NA
TRAV8-3	NA	NA	NA
TRAV13-1	NA	NA	NA
TRAV12-2	NA	NA	NA
TRAV8-4	NA	NA	NA
TRAV8-5	NA	NA	NA
TRAV13-2	NA	NA	NA
TRAV14DV4	NA	NA	NA
TRAV9-2	NA	NA	NA
TRAV15	NA	NA	NA
TRAV12-3	NA	NA	NA
TRAV8-6	NA	NA	NA

TRAV16	NA	NA	NA
TRAV17	NA	NA	NA
TRAV18	NA	NA	NA
TRAV19	NA	NA	NA
TRAV20	NA	NA	NA
TRAV21	NA	NA	NA
TRAV22	NA	NA	NA
TRAV23DV6	NA	NA	NA
TRDV1	NA	NA	NA
TRAV24	NA	NA	NA
TRAV25	NA	NA	NA
TRAV26-1	NA	NA	NA
TRAV8-7	NA	NA	NA
TRAV27	NA	NA	NA
TRAV28	NA	NA	NA
TRAV29DV5	NA	NA	NA
TRAV30	NA	NA	NA
TRAV31	NA	NA	NA
TRAV32	NA	NA	NA
TRAV33	NA	NA	NA
TRAV26-2	NA	NA	NA
TRAV34	NA	NA	NA
TRAV35	NA	NA	NA
TRAV36DV7	NA	NA	NA
TRAV37	NA	NA	NA
TRAV38-1	NA	NA	NA
TRAV38-2DV	NA	NA	NA
TRAV39	NA	NA	NA
TRAV40	NA	NA	NA
TRAV41	NA	NA	NA
AE000661.5(	0.74902724	7.06E-33	1.32E-32
TRDV2	NA	5.28E-27	9.12E-27
TRDD1	NA	NA	NA
TRDD2	NA	NA	NA
TRDD3	NA	NA	NA
TRDJ1	NA	NA	NA
TRDJ4	NA	NA	NA
TRDJ2	NA	NA	NA
TRDJ3	NA	NA	NA
TRDC	5.63741552	1.81E-145	1.58E-143
TRDV3	NA	NA	NA
TRAJ61	NA	NA	NA
TRAJ60	NA	NA	NA
TRAJ59	NA	NA	NA
TRAJ58	NA	NA	NA

TRAJ57	NA	NA	NA
TRAJ56	NA	NA	NA
TRAJ55	NA	NA	NA
TRAJ54	NA	NA	NA
TRAJ53	NA	NA	NA
TRAJ52	NA	NA	NA
TRAJ51	NA	NA	NA
TRAJ50	NA	NA	NA
TRAJ49	NA	NA	NA
TRAJ48	NA	NA	NA
TRAJ47	NA	NA	NA
TRAJ46	NA	NA	NA
TRAJ45	NA	NA	NA
TRAJ44	NA	NA	NA
TRAJ43	NA	NA	NA
TRAJ42	NA	NA	NA
TRAJ41	NA	NA	NA
TRAJ40	NA	NA	NA
TRAJ39	NA	NA	NA
TRAJ38	NA	NA	NA
TRAJ37	NA	NA	NA
TRAJ36	NA	NA	NA
TRAJ35	NA	NA	NA
TRAJ34	NA	NA	NA
TRAJ33	NA	NA	NA
TRAC	0.24967575	2.80E-102	1.58E-101
TRAJ32	NA	NA	NA
TRAJ31	NA	NA	NA
TRAJ30	NA	NA	NA
TRAJ29	NA	NA	NA
TRAJ28	NA	NA	NA
TRAJ27	NA	NA	NA
TRAJ26	NA	NA	NA
TRAJ25	NA	NA	NA
TRAJ24	NA	NA	NA
TRAJ23	NA	NA	NA
TRAJ22	NA	NA	NA
TRAJ21	NA	NA	NA
TRAJ20	NA	NA	NA
TRAJ19	NA	NA	NA
TRAJ18	NA	NA	NA
TRAJ17	NA	NA	NA
TRAJ16	NA	NA	NA
TRAJ14	NA	NA	NA
TRAJ13	NA	NA	NA

TRAJ12	NA	NA	NA
TRAJ11	NA	NA	NA
TRAJ10	NA	NA	NA
TRAJ9	NA	NA	NA
TRAJ8	NA	NA	NA
TRAJ7	NA	NA	NA
TRAJ6	NA	NA	NA
TRAJ5	NA	NA	NA
TRAJ4	NA	NA	NA
TRAJ3	NA	NA	NA
TRAJ2	NA	NA	NA
TRAJ1	NA	NA	NA
AE000662.93	Inf	6.11E-09	7.88E-09
AE000662.92	NA	NA	NA
DAD1	1.54197891	7.74E-83	3.02E-82
CH17-302M2	NA	NA	NA
ABHD4	5.24319066	3.72E-05	4.42E-05
OR6J1	NA	NA	NA
AC243945.1	NA	NA	NA
OXA1L	1.48261061	5.76E-123	5.71E-122
SLC7A7	1.12354086	3.22E-37	6.43E-37
MRPL52	2.17662107	1.77E-80	6.62E-80
MMP14	Inf	1.60E-104	9.47E-104
LRP10	1.87256809	0.0707609	0.07595221
REM2	NA	2.58E-134	4.43E-133
RBM23	3.69520104	1.68E-130	2.32E-129
PRMT5-AS1	Inf	0.47221157	0.48531964
PRMT5	1.30019822	2.24E-40	4.67E-40
RP11-298I3.1	NA	NA	NA
HAUS4	1.54486868	0.99699694	0.99727179
RP11-298I3.5	NA	NA	NA
AJUBA	0.79584144	2.41E-116	1.91E-115
RP11-298I3.4	Inf	8.39E-10	1.10E-09
C14orf93	1.12354086	2.41E-59	6.54E-59
PSMB5	1.24808007	4.01E-95	1.97E-94
PSMB11	NA	NA	NA
CDH24	1.12354086	0.00341041	0.00383391
ACIN1	2.13669875	1.90E-10	2.52E-10
C14orf119	1.03074102	5.51E-137	1.14E-135
CEBPE	0.11179511	3.44E-145	2.50E-143
SLC7A8	Inf	8.18E-140	2.25E-138
RNF212B	NA	NA	NA
HOMEZ	0.89883268	6.48E-58	1.72E-57
PPP1R3E	1.3618677	4.17E-90	1.85E-89
BCL2L2	1.28404669	0.0874712	0.09355082

BCL2L2-PABI	NA	NA	NA
PABPN1	2.25021572	2.98E-55	7.65E-55
SLC22A17	0	6.61E-155	1.84E-152
EFS	NA	5.44E-132	8.17E-131
RP11-124D2.	NA	NA	NA
IL25	NA	NA	NA
CMTM5	NA	NA	NA
MYH6	NA	NA	NA
MYH7	NA	NA	NA
CTD-2201G1	NA	NA	NA
NGDN	1.15067952	2.18E-114	1.64E-113
ZFHX2	5.24319066	2.82E-80	1.05E-79
RP11-66N24.	NA	NA	NA
THTPA	1.31079767	4.88E-06	5.92E-06
AP1G2	1.16099222	1.90E-79	7.00E-79
RP11-66N24.	Inf	3.32E-25	5.60E-25
JPH4	NA	2.59E-86	1.08E-85
RP11-66N24.	NA	NA	NA
DHRS2	NA	NA	NA
LINC00596	NA	NA	NA
DHRS4-AS1	1.87256809	6.97E-10	9.17E-10
DHRS4	1.38101897	4.44E-57	1.17E-56
DHRS4L2	1.1945693	4.60E-89	2.01E-88
LRRC16B	NA	NA	NA
CPNE6	NA	NA	NA
NRL	1.49805447	0.00739194	0.00822728
PCK2	1.29831388	3.14E-46	7.09E-46
DCAF11	1.48467899	5.27E-73	1.76E-72
RP11-468E2.	NA	NA	NA
FITM1	0.74902724	8.19E-06	9.88E-06
PSME1	0.92746043	4.58E-141	1.46E-139
EMC9	1.61886532	2.19E-110	1.49E-109
RP11-468E2.	1.49805447	2.60E-47	5.97E-47
PSME2	1.2559218	2.60E-99	1.38E-98
RNF31	NA	1.14E-127	1.38E-126
RP11-468E2.	1.87256809	0.94009853	0.94316582
IRF9	2.62159533	0.32490615	0.33753921
REC8	0.56177043	3.75E-16	5.52E-16
IPO4	2.21827297	0.56173075	0.57478394
RP11-468E2.	NA	NA	NA
TM9SF1	2.24708171	1.02E-16	1.51E-16
RP11-468E2.	NA	NA	NA
RP11-468E2.	NA	5.25E-36	1.03E-35
TSSK4	NA	NA	NA
CHMP4A	1.45944482	1.31E-95	6.47E-95

MDP1	2.02237354	2.20E-33	4.15E-33
NEDD8-MDP	NA	NA	NA
NEDD8	1.96399437	9.50E-51	2.29E-50
GMPR2	0.95805809	3.01E-141	9.95E-140
TINF2	1.55423152	0.00824056	0.00915129
TGM1	1.49805447	1.49E-48	3.46E-48
RABGGTA	1.7183566	1.21E-40	2.53E-40
DHRS1	0.81712062	2.38E-139	6.22E-138
NOP9	2.11089494	1.83E-44	4.03E-44
CIDEB	NA	0.48586704	0.49912796
LTB4R2	1.49805447	1.51E-08	1.93E-08
LTB4R	4.24448768	1.32E-32	2.47E-32
ADCY4	0	9.48E-50	2.25E-49
RP11-934B9.	NA	NA	NA
RIPK3	1.99740597	6.70E-50	1.59E-49
NFATC4	NA	3.04E-17	4.54E-17
NYNRIN	NA	2.30E-05	2.74E-05
CBLN3	NA	7.74E-07	9.55E-07
KHNYN	1.81014916	7.94E-56	2.06E-55
SDR39U1	1.6197714	9.46E-123	9.28E-122
RP11-80A15.	NA	2.94E-53	7.35E-53
CMA1	NA	0.38801892	0.40076511
CTSG	0	3.08E-144	1.71E-142
RP11-104E1	NA	NA	NA
GZMH	NA	NA	NA
GZMB	NA	NA	NA
STXBP6	NA	NA	NA
AL137164.1	NA	NA	NA
RP11-89K22.	NA	NA	NA
RP11-314P1	NA	NA	NA
NOVA1	Inf	3.53E-75	1.22E-74
NOVA1-AS1	NA	NA	NA
RP11-626P1	NA	NA	NA
RP11-626P1	NA	NA	NA
RP11-384J4.	NA	NA	NA
MIR4307HG	NA	NA	NA
LINC00645	NA	NA	NA
CTD-2591A6.	NA	NA	NA
RP11-412H8.	NA	NA	NA
FOXG1-AS1	NA	NA	NA
FOXG1	NA	0.52796143	0.54129846
LINC01551	NA	6.58E-117	5.30E-116
RP11-966I7.	NA	NA	NA
RP11-966I7.	NA	NA	NA
RP11-966I7.	NA	NA	NA

CTD-2384A14	NA	NA	NA
RP11-148E17	NA	NA	NA
CTD-2384A14	NA	NA	NA
RP11-260G1	NA	NA	NA
PRKD1	NA	6.12E-114	4.56E-113
CTD-2503I6.1	NA	NA	NA
RP11-269C4.	NA	NA	NA
CTD-2251F13	NA	NA	NA
RP11-1103G	NA	NA	NA
G2E3	1.29831388	8.55E-51	2.06E-50
SCFD1	1.49805447	5.84E-86	2.41E-85
RP11-159L20	NA	NA	NA
COCH	Inf	2.58E-32	4.79E-32
RP11-829H16	NA	NA	NA
STRN3	1.94747082	5.16E-06	6.25E-06
AP4S1	1.39818418	5.20E-07	6.44E-07
HECTD1	1.3845655	1.64E-20	2.58E-20
RP11-596D2	NA	9.17E-78	3.29E-77
HEATR5A	1.1770428	3.94E-26	6.73E-26
RP11-176H8.	2.80885214	1.03E-59	2.80E-59
CTD-2213F21	NA	0.54183127	0.5551422
DTD2	1.94747082	8.72E-58	2.32E-57
GPR33	NA	NA	NA
NUBPL	1.87256809	0.33391616	0.3466415
CTD-2213F21	NA	NA	NA
CTD-2213F21	NA	NA	NA
RP11-159D2	NA	NA	NA
RP11-187E13	NA	2.46E-98	1.29E-97
RP11-187E13	NA	NA	NA
ARHGAP5-AS	2.99610895	6.70E-54	1.69E-53
ARHGAP5	1.92607004	3.06E-13	4.27E-13
AKAP6	0	3.01E-45	6.69E-45
RP11-320M1	NA	NA	NA
NPAS3	NA	1.75E-09	2.28E-09
EGLN3	NA	NA	NA
EGLN3-AS1	NA	NA	NA
SPTSSA	1.71814779	0.00068937	0.00079041
EAPP	1.07293091	3.20E-143	1.44E-141
RP11-671J11	NA	NA	NA
RP11-671J11	NA	NA	NA
SNX6	1.71074122	6.81E-48	1.57E-47
CFL2	1.35538262	8.56E-39	1.75E-38
BAZ1A	1.5568786	4.99E-44	1.09E-43
RP11-73E17.	1.74773022	5.80E-15	8.36E-15
SRP54	0.84101304	1.28E-60	3.54E-60

FAM177A1	2.3769131	4.69E-124	4.87E-123
PPP2R3C	1.91824049	4.69E-33	8.80E-33
KIAA0391	5.9922179	1.65E-90	7.38E-90
RP11-561B1:	NA	1.45E-26	2.50E-26
PSMA6	1.33754864	3.40E-73	1.14E-72
RP11-561B1:	NA	NA	NA
NFKBIA	1.15887233	7.86E-48	1.81E-47
RP11-561B1:	NA	NA	NA
INSM2	NA	7.88E-34	1.50E-33
RALGAPA1	3.2457847	1.01E-07	1.27E-07
RP11-317N8.	NA	1.21E-72	4.04E-72
BRMS1L	1.15758755	4.54E-59	1.23E-58
RP11-317N8.	NA	1.95E-13	2.74E-13
RP11-116N8.	NA	NA	NA
RP11-116N8.	NA	NA	NA
LINC00609	NA	NA	NA
PTCSC3	NA	NA	NA
RP11-259K1!	NA	NA	NA
MBIP	1.29831388	5.86E-65	1.74E-64
SFTA3	NA	NA	NA
NKX2-1	Inf	3.09E-20	4.83E-20
NKX2-1-AS1	NA	NA	NA
NKX2-8	NA	NA	NA
RP11-964E11	NA	NA	NA
PAX9	1.87256809	2.03E-07	2.54E-07
SLC25A21	NA	NA	NA
RP11-81F13.	NA	NA	NA
RP11-81F13.	NA	NA	NA
SLC25A21-AS	NA	NA	NA
MIPOL1	NA	3.05E-50	7.28E-50
RP11-356O9.	NA	NA	NA
RP11-356O9.	NA	NA	NA
FOXA1	NA	3.62E-104	2.13E-103
TTC6	NA	NA	NA
LINC00517	NA	NA	NA
CTD-2058B2	NA	NA	NA
CTD-2058B2	NA	NA	NA
CTD-2142D1	NA	NA	NA
SSTR1	NA	NA	NA
CLEC14A	NA	NA	NA
RP11-96D24.	NA	NA	NA
LINC00639	NA	NA	NA
SEC23A	1.46924573	8.22E-50	1.95E-49
RP11-545M1	0	3.07E-22	4.95E-22
GEMIN2	0.90671718	1.89E-104	1.12E-103

TRAPPC6B	2.02237354	9.82E-32	1.81E-31
RP11-407N1	0.37451362	5.86E-32	1.08E-31
PNN	1.80840839	0.00551218	0.006157
MIA2	NA	NA	NA
RP11-407N1	NA	NA	NA
CTAGE5	1.40442607	3.28E-46	7.38E-46
RP11-407N1	Inf	4.43E-130	6.00E-129
FBXO33	1.78934284	1.30E-09	1.70E-09
RP11-111A2	NA	3.89E-102	2.19E-101
RP11-662J14	NA	NA	NA
RP11-662J14	NA	1.65E-46	3.75E-46
RP11-129M6	NA	NA	NA
CTD-2298J14	NA	NA	NA
LRFN5	NA	NA	NA
RP11-313C4.	NA	NA	NA
RP11-214N1.	NA	NA	NA
RP11-305B6.	NA	NA	NA
RP11-99L13.	NA	NA	NA
RP11-99L13.	NA	NA	NA
FSCB	NA	NA	NA
RP11-163M1	NA	NA	NA
RP11-398E1(	NA	NA	NA
RP11-857B2	NA	NA	NA
C14orf28	1.49805447	1.29E-07	1.61E-07
RP11-857B2	NA	NA	NA
KLHL28	2.24708171	2.29E-19	3.54E-19
FAM179B	7.30301556	1.73E-82	6.73E-82
CTD-3028N1	NA	1.98E-32	3.69E-32
CTD-3028N1	NA	NA	NA
PRPF39	1.38281952	6.77E-39	1.38E-38
FKBP3	1.05647919	7.93E-117	6.36E-116
FANCM	7.49027237	1.67E-38	3.38E-38
MIS18BP1	1.3732166	4.97E-94	2.39E-93
RP11-454K7.	NA	NA	NA
RP11-454K7.	NA	NA	NA
RP11-945F5.	NA	NA	NA
RP11-369C8.	NA	NA	NA
LINC00871	NA	NA	NA
RPL10L	NA	1.43E-103	8.27E-103
MDGA2	NA	NA	NA
LINC00648	NA	NA	NA
RP11-2G1.1	NA	NA	NA
RP11-816J8.	1.49805447	1.05E-13	1.47E-13
RPS29	2.56661421	2.64E-143	1.22E-141
LRR1	1.39177358	5.07E-69	1.60E-68

RPL36AL	1.69563692	2.18E-25	3.69E-25
MGAT2	1.79613674	5.36E-11	7.19E-11
RP11-649E7.	NA	4.88E-39	9.99E-39
DNAAF2	1.35599758	4.57E-67	1.40E-66
POLE2	1.63023575	5.54E-63	1.59E-62
KLHDC1	Inf	3.91E-99	2.07E-98
RP11-831F12	NA	NA	NA
KLHDC2	1.41861219	2.94E-69	9.31E-69
NEMF	1.53330282	0.87838443	0.88393889
RP11-596C23	NA	2.17E-06	2.65E-06
RP11-596C23	0.59922179	6.04E-13	8.39E-13
ARF6	1.93795936	5.46E-21	8.64E-21
LINC01588	1.49805447	3.31E-31	6.06E-31
VCPKMT	1.33754864	0.00026467	0.00030709
SOS2	1.34824903	3.53E-17	5.27E-17
L2HGDH	2.80885214	0.15031579	0.1589363
ATP5S	1.29243915	2.18E-16	3.22E-16
RP11-247L20	NA	6.32E-44	1.38E-43
CDKL1	1.49805447	0.00032831	0.00038005
RP11-247L20	NA	6.37E-118	5.29E-117
MAP4K5	2.43433852	1.55E-73	5.22E-73
RP11-406H23	NA	4.08E-10	5.39E-10
ATL1	Inf	1.57E-10	2.08E-10
SAV1	2.51673152	8.68E-25	1.45E-24
NIN	2.07422927	4.61E-30	8.33E-30
RP11-286O18	NA	NA	NA
RP11-218E20	NA	NA	NA
PYGL	1.23411154	1.30E-134	2.28E-133
ABHD12B	NA	6.23E-43	1.34E-42
RP11-218E20	NA	NA	NA
RP11-218E20	NA	9.40E-18	1.42E-17
TRIM9	0.42801556	1.46E-53	3.67E-53
RP11-1140I5	NA	NA	NA
TMX1	1.28536773	1.82E-111	1.27E-110
LINC00519	NA	NA	NA
LINC00640	NA	1.61E-38	3.26E-38
RP11-255G12	NA	NA	NA
RP11-255G12	NA	NA	NA
FRMD6-AS2	NA	NA	NA
FRMD6	NA	1.41E-66	4.29E-66
RP11-102G14	NA	NA	NA
FRMD6-AS1	1.87256809	4.25E-18	6.44E-18
RP11-280K24	NA	NA	NA
GNG2	0.85603113	2.30E-34	4.41E-34
RP11-463J10	NA	NA	NA

RP11-463J10	NA	NA	NA
RP11-463J10	NA	NA	NA
C14orf166	1.29201138	4.12E-99	2.18E-98
NID2	NA	NA	NA
RP11-1033H1	NA	7.38E-65	2.18E-64
PTGDR	NA	NA	NA
PTGER2	NA	NA	NA
TXNDC16	0.87386511	7.05E-05	8.30E-05
GPR137C	Inf	5.20E-11	6.98E-11
ERO1A	2.29786322	2.10E-87	8.87E-87
RP11-841O2	2.15345331	7.18E-29	1.27E-28
PSMC6	1.05862516	3.76E-115	2.88E-114
STYX	2.95668646	5.74E-85	2.32E-84
GNPNAT1	1.97957198	0.00093753	0.00107148
RP11-589M4	NA	2.03E-144	1.18E-142
RP11-589M4	NA	NA	NA
FERMT2	0.74902724	5.85E-57	1.54E-56
DDHD1	1.60019455	6.56E-05	7.73E-05
RP11-368P1	NA	NA	NA
RP11-547D2	0	5.87E-17	8.73E-17
AL163953.2	NA	NA	NA
AL162759.1	NA	NA	NA
BMP4	2.12224384	6.80E-06	8.22E-06
CDKN3	1.56311828	1.82E-65	5.45E-65
CNIH1	1.7699943	1.10E-36	2.17E-36
RP11-794A8.	NA	NA	NA
GMFB	1.52221664	0.55266569	0.56569967
CGRRF1	1.46238651	1.30E-15	1.90E-15
SAMD4A	1.87256809	6.29E-52	1.54E-51
GCH1	1.34824903	2.54E-08	3.24E-08
WDHD1	1.23369192	4.95E-37	9.84E-37
SOCS4	1.99740597	3.56E-33	6.69E-33
MAPK1IP1L	2.02063162	1.41E-10	1.87E-10
LGALS3	0.97373541	6.66E-122	6.35E-121
DLGAP5	0.98735409	3.11E-139	8.04E-138
RP11-698F2	NA	1.17E-114	8.84E-114
RP11-665C1	NA	NA	NA
FBXO34	1.40035527	0.01325054	0.01461346
RP11-665C1	NA	NA	NA
ATG14	2.15345331	1.85E-31	3.41E-31
TBPL2	NA	NA	NA
KTN1-AS1	5.24319066	7.91E-24	1.30E-23
KTN1	2.6731339	1.81E-145	1.58E-143
RP11-813I20	NA	NA	NA
LINC00520	NA	NA	NA

CTD-2588E21	Inf	3.77E-126	4.24E-125
PELI2	2.99610895	9.98E-13	1.38E-12
RP11-930O1	NA	NA	NA
RP11-930O1	NA	NA	NA
RP11-930O1	NA	NA	NA
TMEM260	0.84265564	0.10338014	0.11016932
RP11-624J12	NA	1.80E-118	1.52E-117
RP11-1085N	NA	NA	NA
RP11-1085N	NA	NA	NA
RP11-1085N	NA	NA	NA
OTX2	NA	NA	NA
OTX2-AS1	NA	NA	NA
RP11-1085N	NA	NA	NA
RP11-108M1	NA	NA	NA
EXOC5	2.18715953	5.08E-94	2.44E-93
AP5M1	1.89597519	0.0016915	0.00191675
RP11-932A1(	NA	NA	NA
NAA30	2.30950065	2.88E-12	3.96E-12
C14orf105	NA	NA	NA
SLC35F4	NA	NA	NA
RP11-409I10	NA	NA	NA
C14orf37	NA	8.99E-05	1.05E-04
ACTR10	0.67559319	1.39E-125	1.52E-124
PSMA3	0.92464286	6.66E-132	9.95E-131
PSMA3-AS1	1.89970676	8.73E-23	1.42E-22
LINC00216	NA	8.12E-113	5.91E-112
ARID4A	1.14850843	4.71E-85	1.91E-84
RP11-517O1	NA	NA	NA
TOMM20L	NA	6.69E-14	9.46E-14
RP11-517O1	NA	NA	NA
TIMM9	1.85884246	1.47E-14	2.11E-14
KIAA0586	1.89040208	0.06441176	0.06926021
DACT1	NA	NA	NA
LINC01500	NA	NA	NA
DAAM1	1.70233463	0.60846565	0.62092383
GPR135	0.74902724	6.97E-08	8.79E-08
L3HYPDH	0.95330739	2.53E-05	3.02E-05
JKAMP	1.23054475	1.86E-114	1.40E-113
CCDC175	NA	4.22E-12	5.78E-12
RTN1	NA	1.22E-58	3.26E-58
RP11-62H20.	NA	NA	NA
LRRC9	NA	NA	NA
RP11-16B13.	NA	NA	NA
PCNXL4	2.54669261	3.50E-53	8.76E-53
DHRS7	0.78103695	2.01E-142	7.74E-141

CTD-2184C24	NA	2.25E-15	3.26E-15
PPM1A	1.70468268	5.96E-05	7.03E-05
CTD-2568P8.	NA	1.15E-85	4.70E-85
C14orf39	NA	NA	NA
SIX6	NA	NA	NA
RP11-1042B1	NA	NA	NA
SALRNA1	NA	NA	NA
SIX1	1.87256809	2.78E-28	4.90E-28
SIX4	1.24837873	2.43E-15	3.53E-15
MNAT1	1.02723735	3.36E-122	3.23E-121
TRMT5	2.37191958	3.93E-55	1.01E-54
SLC38A6	2.43433852	9.01E-37	1.79E-36
PRKCH	1.69779507	3.07E-12	4.22E-12
TMEM30B	NA	NA	NA
RP11-902B17	NA	1.31E-77	4.69E-77
RP11-47I22.4	NA	NA	NA
RP11-47I22.1	NA	NA	NA
RP11-47I22.2	NA	4.83E-114	3.61E-113
FLJ22447	NA	9.43E-18	1.42E-17
HIF1A-AS1	NA	NA	NA
HIF1A	1.55353797	5.19E-11	6.96E-11
HIF1A-AS2	NA	NA	NA
SNAPC1	1.91418072	2.07E-11	2.79E-11
CTD-2277K2.	NA	NA	NA
SYT16	NA	NA	NA
RP11-355I22	NA	NA	NA
RP11-355I22	NA	NA	NA
LINC00643	NA	NA	NA
LINC00644	NA	NA	NA
KCNH5	NA	NA	NA
RP11-1112J2	NA	NA	NA
RHOJ	NA	NA	NA
RP11-696D21	NA	NA	NA
GPHB5	NA	NA	NA
PPP2R5E	2.20659375	2.16E-64	6.35E-64
CTD-2302E22	Inf	5.48E-92	2.53E-91
WDR89	1.77893969	2.64E-14	3.77E-14
CTD-2302E22	NA	NA	NA
CTD-2302E22	0	8.05E-05	9.46E-05
SGPP1	1.31079767	8.11E-19	1.24E-18
SYNE2	0	2.32E-98	1.21E-97
ESR2	NA	NA	NA
RP11-544I20	1.12354086	3.12E-10	4.12E-10
CTD-2555O1(	NA	NA	NA
MTHFD1	1.4875048	6.14E-49	1.44E-48

CTD-2555O1(	NA	NA	NA
CTD-2555O1(	Inf	3.28E-72	1.08E-71
ZBTB25	2.12224384	4.29E-07	5.32E-07
AKAP5	Inf	3.98E-121	3.67E-120
ZBTB1	1.49805447	0.11490852	0.12223938
RP11-973N1:	NA	NA	NA
HSPA2	NA	NA	NA
PPP1R36	NA	3.72E-18	5.65E-18
RP11-973N1:	NA	NA	NA
PLEKHG3	2.47178988	6.67E-05	7.85E-05
SPTB	Inf	1.48E-14	2.12E-14
AL135745.1	NA	NA	NA
CHURC1	1.83435242	0.02084183	0.02282874
CHURC1-FNT	NA	NA	NA
GPX2	NA	6.72E-75	2.31E-74
RAB15	2.09727626	1.34E-34	2.57E-34
FNTB	1.10382961	3.87E-70	1.24E-69
RP11-840I19	NA	NA	NA
MAX	1.99108506	0.90917296	0.91340214
RP11-840I19	NA	NA	NA
CTD-2509G1(	NA	NA	NA
CTD-2509G1(	NA	NA	NA
FUT8	1.70233463	7.10E-40	1.47E-39
FUT8-AS1	Inf	2.95E-147	3.24E-145
CTD-2014B1(	NA	2.00E-92	9.33E-92
RP11-783L4.	NA	NA	NA
RP11-72M17	1.39105058	3.90E-17	5.82E-17
GPHN	1.9661965	0.00126753	0.00144199
RP11-862P1:	NA	NA	NA
FAM71D	0.89883268	1.32E-11	1.79E-11
MPP5	0.79896239	3.82E-10	5.05E-10
ATP6V1D	1.36516255	7.70E-109	5.03E-108
EIF2S1	1.03325632	2.68E-93	1.27E-92
PLEK2	NA	NA	NA
TMEM229B	1.12354086	0.24087395	0.25223291
PLEKHH1	1.49805447	0.71950166	0.72955506
PIGH	1.46348399	6.33E-20	9.84E-20
ARG2	1.16099222	4.17E-57	1.10E-56
VTI1B	2.28095732	1.86E-112	1.33E-111
RP11-1012A:	NA	NA	NA
RDH11	1.59168288	1.03E-62	2.95E-62
RDH12	NA	1.06E-91	4.87E-91
ZFYVE26	2.24708171	2.96E-26	5.07E-26
RAD51B	0.85603113	2.70E-92	1.26E-91
CTD-2566J3.:	NA	4.18E-08	5.30E-08

CTD-2325P2.	NA	NA	NA
CTD-2325P2.	NA	NA	NA
ZFP36L1	2.24708171	3.86E-46	8.69E-46
ACTN1	1.68780804	5.62E-31	1.03E-30
ACTN1-AS1	NA	NA	NA
DCAF5	2.0598249	0.67534839	0.68666539
RP11-817G1:	NA	0.00890851	0.00988309
RP11-363J20	NA	3.61E-63	1.04E-62
EXD2	1.39105058	0.19473762	0.20489067
RP11-363J20	NA	NA	NA
GALNT16	1.49805447	3.14E-20	4.91E-20
ERH	1.52267553	1.07E-41	2.27E-41
SLC39A9	1.52801556	2.55E-14	3.64E-14
PLEKHD1	Inf	1.95E-83	7.71E-83
CCDC177	0	0.25564252	0.26728139
SUSD6	2.49675746	7.31E-66	2.20E-65
SRSF5	1.66391051	2.75E-83	1.08E-82
SLC10A1	NA	NA	NA
SMOC1	NA	NA	NA
SLC8A3	NA	NA	NA
RP11-486O1:	NA	NA	NA
RP11-718G2.	NA	NA	NA
COX16	2.99610895	0.0003944	0.00045551
SYNJ2BP-CO.	NA	2.66E-34	5.09E-34
SYNJ2BP	1.89753567	7.79E-34	1.48E-33
RP11-486O1:	NA	NA	NA
ADAM21	NA	NA	NA
ADAM20P1	NA	7.75E-127	8.99E-126
ADAM20	NA	1.84E-45	4.11E-45
MED6	1.02606471	9.91E-93	4.65E-92
CTD-2540L5.!	NA	NA	NA
CTD-2540L5.(	NA	NA	NA
TTC9	2.3540856	2.23E-115	1.72E-114
LINC01269	NA	NA	NA
MAP3K9	0.37451362	1.18E-12	1.64E-12
RP6-65G23.3	Inf	5.69E-90	2.52E-89
RP6-65G23.5	Inf	0.5526333	0.56569843
RP1-292L20.:	2.49675746	2.59E-07	3.22E-07
PCNX	3.49546044	4.13E-11	5.55E-11
RP6-91H8.3	NA	NA	NA
RP6-91H8.2	NA	NA	NA
RP6-91H8.1	NA	NA	NA
RP6-91H8.5	NA	NA	NA
RP1-261D10.	NA	2.92E-36	5.76E-36
SIPA1L1	1.64785992	2.88E-13	4.02E-13

RP6-114E22.	NA	NA	NA
RGS6	NA	NA	NA
AC005477.1	NA	NA	NA
RP3-514A23.	NA	NA	NA
DPF3	0.74902724	4.33E-86	1.79E-85
DCAF4	1.39105058	8.24E-25	1.38E-24
ZFYVE1	3.37062257	5.48E-74	1.86E-73
RP11-109N2:	Inf	4.53E-44	9.90E-44
RBM25	1.35706111	4.10E-52	1.01E-51
PSEN1	1.20996708	1.55E-65	4.63E-65
PAPLN	NA	0.90812528	0.91245064
RP4-647C14.	Inf	7.38E-148	8.47E-146
RP4-647C14.	0	0.00030055	0.00034832
NUMB	1.64072633	0.0038979	0.00437408
HEATR4	NA	NA	NA
C14orf169	1.31828794	7.22E-20	1.12E-19
RP3-414A15.	NA	NA	NA
ACOT1	NA	NA	NA
ACOT2	NA	NA	NA
ACOT4	NA	NA	NA
ACOT6	NA	NA	NA
RP3-414A15.	NA	NA	NA
DNAL1	1.90137683	7.54E-05	8.87E-05
RP4-693M11	NA	NA	NA
PNMA1	1.81014916	0.10509566	0.11195147
ELMSAN1	3.1459144	1.40E-104	8.26E-104
RP5-1021I20	Inf	7.06E-46	1.59E-45
RP5-1021I20	NA	NA	NA
PTGR2	1.8385214	1.01E-61	2.86E-61
RP5-1021I20	NA	4.12E-28	7.24E-28
RP5-1021I20	NA	6.88E-28	1.20E-27
ZNF410	Inf	3.07E-19	4.74E-19
RP5-1021I20	NA	0.05607447	0.06042068
FAM161B	0.74902724	2.96E-68	9.23E-68
COQ6	1.03314102	1.58E-140	4.75E-139
ENTPD5	2.99610895	4.06E-34	7.76E-34
AC005480.1	NA	8.08E-128	9.83E-127
BBOF1	2.62159533	0.00023483	0.00027269
ALDH6A1	1.12354086	5.18E-17	7.72E-17
LIN52	2.24708171	2.25E-97	1.16E-96
VSX2	NA	NA	NA
ABCD4	1.34824903	0.15720449	0.16604587
VRTN	NA	NA	NA
SYNDIG1L	NA	NA	NA
RP3-449M8.9	NA	NA	NA

RP3-449M8.6	NA	NA	NA
NPC2	1.25643279	4.49E-145	3.09E-143
ISCA2	1.36132728	2.55E-42	5.45E-42
LTBP2	NA	6.87E-58	1.83E-57
CTD-2207P18	NA	NA	NA
CTD-2207P18	NA	NA	NA
AREL1	1.45399405	1.11E-38	2.27E-38
FCF1	2.70482058	3.84E-97	1.96E-96
YLPM1	2.24708171	1.81E-28	3.20E-28
PROX2	NA	NA	NA
DLST	2.0105468	0.00266094	0.0029975
RPS6KL1	1.90661479	0.77991562	0.78852146
PGF	4.49416342	5.31E-88	2.28E-87
EIF2B2	1.20810845	3.98E-126	4.48E-125
RP11-950C14	NA	4.02E-120	3.60E-119
MLH3	NA	1.43E-15	2.08E-15
ACYP1	1.54299611	0.88268486	0.88787252
ZC2HC1C	1.49805447	7.31E-64	2.13E-63
NEK9	1.49805447	0.23970267	0.25103538
RP11-950C14	NA	1.43E-15	2.08E-15
TMED10	1.31205442	6.32E-137	1.30E-135
CTD-3075F15	NA	NA	NA
RP11-293M1	NA	NA	NA
RP11-293M1	NA	NA	NA
FOS	3.99481193	9.99E-92	4.59E-91
LINC01220	0	5.95E-60	1.63E-59
RP11-293M1	NA	2.73E-120	2.46E-119
JDP2	2.90248054	1.95E-140	5.76E-139
BATF	1.82226029	7.37E-39	1.50E-38
AC007182.6	NA	3.71E-23	6.06E-23
FLVCR2	0.99870298	8.65E-05	1.02E-04
TTLL5	3.07101167	1.19E-131	1.76E-130
C14orf1	1.86000052	2.63E-85	1.07E-84
RP11-270M1	NA	NA	NA
IFT43	1.19076125	1.94E-103	1.12E-102
TGFB3	2.7464332	3.69E-21	5.86E-21
RP11-270M1	NA	NA	NA
GPATCH2L	1.92250324	0.00216428	0.00244471
RP11-516J2.1	NA	NA	NA
ESRRB	NA	NA	NA
RP11-187O7.	NA	NA	NA
RP11-99E15.	NA	NA	NA
VASH1	1.49805447	0.11342183	0.12066492
RP11-488C13	NA	NA	NA
RP11-488C13	NA	NA	NA

RP11-488C13	1.24837873	0.09910888	0.10579155
ANGEL1	1.99740597	1.40E-09	1.83E-09
LRRC74A	NA	NA	NA
RP11-488C13	NA	NA	NA
LINC01629	NA	NA	NA
IRF2BPL	5.24319066	2.60E-67	8.00E-67
RP11-7F17.8	5.24319066	1.83E-127	2.20E-126
RP11-7F17.5	NA	NA	NA
RP11-7F17.4	NA	NA	NA
RP11-7F17.3	NA	NA	NA
CIPC	5.24319066	3.53E-91	1.60E-90
RP11-463C8.	NA	NA	NA
TMEM63C	Inf	0.06486257	0.06972841
ZDHHC22	NA	1.64E-144	9.77E-143
RP11-463C8.	NA	2.58E-134	4.43E-133
NGB	NA	NA	NA
POMT2	4.74383917	1.96E-59	5.35E-59
GSTZ1	1.14137484	2.29E-140	6.70E-139
TMED8	1.63849708	1.03E-20	1.62E-20
SAMD15	0	5.37E-40	1.11E-39
NOXRED1	NA	2.35E-30	4.26E-30
AF111168.2	NA	NA	NA
VIPAS39	4.30690661	2.62E-06	3.19E-06
AHSA1	0.90659699	1.11E-132	1.74E-131
ISM2	2.0598249	0.47285243	0.48595076
SPTLC2	1.69779507	2.93E-34	5.59E-34
ALKBH1	1.67090691	3.80E-53	9.48E-53
SLIRP	2.34306936	5.80E-138	1.31E-136
SNW1	1.22323153	5.47E-126	6.12E-125
C14orf178	Inf	1.68E-27	2.93E-27
ADCK1	0.81144617	3.27E-66	9.88E-66
NRXN3	NA	NA	NA
RP11-332E19	NA	NA	NA
RP11-232C2.	NA	NA	NA
RP11-232C2.	NA	NA	NA
RP11-232C2.	NA	NA	NA
CTD-2243E23	NA	NA	NA
RP11-588P7.	NA	NA	NA
RP11-242P2.	NA	NA	NA
RP11-242P2.	NA	NA	NA
RP11-526N18	NA	NA	NA
DIO2	NA	NA	NA
DIO2-AS1	NA	NA	NA
CEP128	0.91547773	6.68E-120	5.95E-119
TSHR	NA	NA	NA

RP11-114N1!	NA	NA	NA
GTF2A1	1.59168288	1.45E-65	4.34E-65
CTD-2506P8.	Inf	1.40E-42	3.01E-42
STON2	2.45136187	2.53E-35	4.92E-35
RP11-299L17	NA	NA	NA
SEL1L	4.66061392	3.69E-58	9.84E-58
LINC01467	NA	NA	NA
RP11-226P1.	NA	NA	NA
RP11-666E17	NA	NA	NA
RP11-406A9.	NA	NA	NA
RP11-11K13.	NA	NA	NA
RP11-452D2:	NA	NA	NA
RP11-452D2:	NA	NA	NA
RP11-353P1!	NA	NA	NA
RP11-789A2:	NA	NA	NA
CTD-2128A3.	NA	NA	NA
CTD-2128A3.	NA	NA	NA
LINC00911	NA	NA	NA
RP11-497E1!	NA	NA	NA
RP11-497E1!	NA	NA	NA
FLRT2	NA	NA	NA
CTD-2341M2	0	5.13E-137	1.06E-135
RP11-1079H!	NA	NA	NA
RP11-26L16.	NA	NA	NA
RP11-322L2(	NA	NA	NA
RP11-736P1(	NA	NA	NA
RP11-1141N	NA	NA	NA
RP11-594C1:	NA	NA	NA
RP11-594C1:	NA	NA	NA
RP11-325D8.	NA	NA	NA
CTD-2007A1(	NA	NA	NA
RP11-1152H:	NA	NA	NA
RP11-804L24	NA	NA	NA
GALC	NA	NA	NA
GPR65	NA	1.75E-135	3.23E-134
RP11-300J18	NA	NA	NA
RP11-300J18	NA	NA	NA
LINC01146	NA	NA	NA
RP11-753D2(	NA	NA	NA
RP11-753D2(	NA	NA	NA
RP11-753D2(	NA	NA	NA
KCNK10	NA	NA	NA
SPATA7	0.93628405	1.10E-46	2.49E-46
PTPN21	NA	NA	NA
RP11-507K2.	NA	NA	NA

RP11-507K2.	NA	NA	NA
ZC3H14	1.73967616	7.99E-21	1.26E-20
EML5	Inf	4.73E-16	6.96E-16
RP11-976B1(	NA	NA	NA
TTC8	0.99870298	0.81014912	0.81785795
FOXN3	1.9688716	0.28178671	0.29382002
RP11-681H1{	NA	1.28E-41	2.72E-41
RP11-356K2:	NA	NA	NA
RP11-356K2:	NA	NA	NA
RP11-33N16.	NA	NA	NA
FOXN3-AS1	4.49416342	1.88E-73	6.32E-73
FOXN3-AS2	NA	NA	NA
RP11-944C7.	NA	NA	NA
EFCAB11	1.06112192	1.12E-106	6.92E-106
TDP1	3.33657588	7.21E-12	9.85E-12
KCNK13	NA	6.86E-51	1.66E-50
PSMC1	1.09545233	9.20E-116	7.19E-115
NRDE2	2.24708171	5.89E-11	7.89E-11
RP11-471B2:	NA	NA	NA
CALM1	1.22818437	7.43E-108	4.73E-107
RP11-471B2:	NA	NA	NA
RP11-1078H!	NA	NA	NA
LINC00642	NA	NA	NA
RP11-1078H!	NA	NA	NA
TTC7B	1.84375935	8.59E-05	1.01E-04
RP11-1078H!	NA	NA	NA
RP11-1078H!	NA	NA	NA
RP11-661G1(	NA	NA	NA
RP11-661G1(	NA	NA	NA
CTD-3035D6.	NA	NA	NA
RPS6KA5	1.74773022	4.63E-12	6.35E-12
C14orf159	1.82371849	2.65E-98	1.38E-97
GPR68	NA	NA	NA
CTD-2547L24	NA	NA	NA
CCDC88C	2.48115272	4.24E-65	1.26E-64
RP11-895M1	NA	7.02E-82	2.69E-81
PPP4R3A	1.84375935	1.13E-44	2.49E-44
CATSPERB	NA	NA	NA
TC2N	NA	NA	NA
FBLN5	NA	NA	NA
TRIP11	1.56318728	2.48E-35	4.81E-35
ATXN3	1.60505837	0.00082677	0.00094591
AL049872.1	NA	NA	NA
NDUFB1	1.9062437	4.76E-20	7.43E-20
CPSF2	1.74773022	0.00121128	0.00137913

RP11-472N1!	NA	NA	NA
SLC24A4	NA	1.22E-16	1.80E-16
RIN3	1.72692391	1.15E-39	2.37E-39
LGMN	0	2.40E-117	1.95E-116
GOLGA5	0.98309825	1.47E-07	1.84E-07
RP11-862G1!	NA	NA	NA
RP11-862G1!	NA	NA	NA
CHGA	NA	NA	NA
ITPK1	1.76437527	0.00240709	0.00271559
ITPK1-AS1	NA	4.90E-22	7.88E-22
MOAP1	1.94747082	2.13E-13	2.99E-13
TMEM251	1.9661965	2.47E-17	3.69E-17
RP11-371E8.	NA	NA	NA
C14orf142	2.04585051	0.01091243	0.0120686
UBR7	1.49805447	1.96E-09	2.55E-09
BTBD7	2.24708171	5.50E-44	1.20E-43
UNC79	NA	NA	NA
COX8C	NA	3.97E-33	7.46E-33
PRIMA1	NA	NA	NA
FAM181A-AS	NA	NA	NA
FAM181A	NA	NA	NA
ASB2	NA	1.34E-08	1.72E-08
RP11-131H2	NA	NA	NA
LINC00521	NA	NA	NA
OTUB2	2.99610895	1.13E-48	2.65E-48
DDX24	1.17978649	1.05E-121	9.89E-121
IFI27L1	2.0488098	8.19E-16	1.20E-15
IFI27	4.49416342	2.22E-92	1.03E-91
IFI27L2	1.49805447	5.02E-27	8.68E-27
PPP4R4	0	1.41E-07	1.76E-07
SERPINA10	NA	NA	NA
SERPINA6	NA	NA	NA
SERPINA1	NA	NA	NA
RP11-349I1.2	NA	NA	NA
SERPINA11	NA	NA	NA
SERPINA9	NA	NA	NA
SERPINA12	NA	NA	NA
SERPINA4	NA	NA	NA
SERPINA5	NA	NA	NA
SERPINA3	NA	NA	NA
SERPINA3	NA	NA	NA
GSC	NA	NA	NA
RP11-179A9.	NA	NA	NA
RP11-991C1.	NA	NA	NA
RP11-991C1.	NA	NA	NA

DICER1	1.96411587	7.17E-72	2.35E-71
DICER1-AS1	1.24837873	1.76E-23	2.89E-23
CLMN	0	2.15E-19	3.33E-19
RP11-725G5.	NA	NA	NA
SYNE3	1.24837873	0.00493828	0.00552345
RP11-1070N	NA	5.17E-53	1.29E-52
SNHG10	2.50284711	1.04E-74	3.55E-74
GLRX5	2.30537605	1.30E-112	9.38E-112
RP11-1070N	NA	NA	NA
RP11-1070N	NA	NA	NA
RP11-1070N	NA	NA	NA
TCL1B	NA	NA	NA
TCL1A	NA	NA	NA
RP11-164H1:	NA	0.36065879	0.373463
RP11-185P18	NA	NA	NA
TUNAR	0.74902724	1.48E-20	2.33E-20
C14orf132	1.49805447	9.38E-21	1.48E-20
BDKRB2	NA	NA	NA
RP11-404P21	NA	NA	NA
RP11-404P21	NA	NA	NA
BDKRB1	NA	NA	NA
RP11-404P21	NA	NA	NA
RP11-404P21	NA	7.65E-06	9.24E-06
ATG2B	2.09727626	0.80632827	0.81427261
GSKIP	1.67090691	2.63E-10	3.48E-10
AK7	NA	0.67742831	0.68860305
RP11-872J21	2.32198444	9.20E-64	2.67E-63
PAPOLA	2.28778971	4.88E-87	2.05E-86
RP11-433J8.1	0	4.72E-171	8.56E-168
RP11-433J8.2	NA	NA	NA
VRK1	0.53309146	6.95E-143	2.95E-141
LINC00618	Inf	4.36E-08	5.53E-08
RP11-907D1.	NA	NA	NA
RP11-907D1.	NA	NA	NA
RP11-359N5.	NA	NA	NA
CTD-2506J14	NA	NA	NA
RP11-76E12.	NA	NA	NA
RP11-204N1	NA	NA	NA
RP11-204N1	NA	NA	NA
LINC01550	Inf	8.61E-111	5.90E-110
RP11-61O1.1	NA	NA	NA
RP11-61O1.2	NA	NA	NA
RP11-1082A:	NA	NA	NA
C14orf177	NA	NA	NA
AL132796.1	NA	NA	NA

RP11-1127D7	NA	NA	NA
AL162151.4	NA	NA	NA
BCL11B	Inf	2.59E-45	5.76E-45
AL109767.1	NA	NA	NA
CTD-2200A1(	NA	NA	NA
SETD3	2.18099107	0.00132858	0.00151031
CCNK	2.07177747	9.25E-82	3.55E-81
CCDC85C	2.46108949	6.89E-83	2.70E-82
RP11-688G1!	NA	NA	NA
RP11-543C4.	NA	1.96E-71	6.38E-71
HHIPL1	NA	NA	NA
CYP46A1	NA	1.82E-96	9.16E-96
RP11-543C4.	NA	NA	NA
EML1	NA	7.38E-148	8.47E-146
EVL	1.91536965	4.14E-12	5.69E-12
RP11-436M1	NA	NA	NA
CTD-2376I20	NA	NA	NA
DEGS2	2.43433852	2.53E-06	3.09E-06
RP11-638I2.4	NA	5.85E-23	9.55E-23
YY1	1.78228356	2.50E-09	3.25E-09
SLC25A29	1.72276265	1.48E-40	3.09E-40
RP11-638I2.6	NA	2.44E-91	1.11E-90
SLC25A47	NA	7.01E-72	2.30E-71
WARS	2.11089494	4.19E-25	7.07E-25
RP11-638I2.8	NA	1.40E-113	1.03E-112
RP11-638I2.9	NA	NA	NA
WDR25	1.3732166	6.23E-44	1.36E-43
BEGAIN	0.87386511	1.86E-33	3.52E-33
CTD-2062F14	NA	NA	NA
LINC00523	NA	0.1382489	0.14640778
RP11-566J3.2	NA	0.03359203	0.03649917
DLK1	1.08949416	1.17E-20	1.84E-20
MEG3	NA	NA	NA
RP11-123M6	NA	NA	NA
RTL1	NA	NA	NA
MEG8	NA	NA	NA
SNHG23	2.24708171	0.044499	0.04814533
AL132709.8	NA	NA	NA
SNHG24	NA	NA	NA
MIR381HG	NA	NA	NA
MEG9	NA	NA	NA
AL132709.1	NA	NA	NA
RP11-8L8.2	Inf	5.38E-125	5.75E-124
CTD-2561F5.	NA	NA	NA
RP11-168L7.	NA	NA	NA

LINC00524	NA	NA	NA
RP11-168L7.:	NA	NA	NA
RP11-1029J1	NA	NA	NA
DIO3OS	NA	NA	NA
DIO3	NA	NA	NA
RP11-1029J1	NA	NA	NA
RP11-1029J1	NA	NA	NA
LINC00239	NA	NA	NA
PPP2R5C	1.34635276	1.07E-114	8.13E-114
CTD-2017C7.	NA	NA	NA
CTD-2017C7.	NA	6.24E-59	1.68E-58
RP11-1017G:	7.49027237	2.78E-27	4.83E-27
RP11-1017G:	0	1.31E-57	3.48E-57
DYNC1H1	1.16025788	6.85E-98	3.54E-97
RP11-1017G:	NA	NA	NA
HSP90AA1	1.02741968	8.09E-121	7.41E-120
WDR20	1.74773022	0.1550077	0.16377321
MOK	11.9844358	9.86E-69	3.10E-68
ZNF839	1.74773022	0.00229361	0.00258821
CINP	1.54620623	1.65E-92	7.70E-92
TECPR2	0.89883268	1.16E-49	2.76E-49
ANKRD9	1.95899431	4.96E-34	9.46E-34
CTD-2555C1(	NA	NA	NA
RCOR1	1.72601929	5.36E-35	1.04E-34
RP11-661D1!	NA	NA	NA
TRAF3	1.98129785	4.83E-22	7.77E-22
AMN	NA	1.03E-120	9.42E-120
CDC42BPB	2.47178988	5.19E-63	1.49E-62
RP11-365N1!	NA	NA	NA
RP11-736N1	NA	NA	NA
EXOC3L4	NA	NA	NA
LINC00677	NA	NA	NA
TNFAIP2	1.42996109	0.02084183	0.02282874
LINC00605	NA	NA	NA
RP11-736N1	NA	NA	NA
RP11-45P15.	NA	8.59E-48	1.98E-47
EIF5	1.79938727	1.67E-07	2.09E-07
MARK3	2.24708171	4.64E-17	6.92E-17
CKB	0.94982068	4.51E-134	7.61E-133
RP11-600F2	NA	5.69E-85	2.31E-84
TRMT61A	1.78471922	3.89E-36	7.65E-36
RP11-894P9.	NA	NA	NA
BAG5	1.9865505	1.10E-18	1.68E-18
KLC1	1.94747082	1.48E-19	2.29E-19
APOPT1	1.71387588	0.03581452	0.03886279

RP11-73M18	NA	NA	NA
RP11-894P9.	5.24319066	5.15E-74	1.75E-73
RP11-73M18	Inf	5.27E-97	2.69E-96
XRCC3	1.98492218	3.84E-07	4.77E-07
ZFYVE21	1.50940337	1.46E-56	3.82E-56
PPP1R13B	4.74383917	2.29E-57	6.05E-57
LINC00637	NA	0.13377061	0.14180592
CTD-2134A5.	NA	NA	NA
CTD-2134A5.	NA	NA	NA
C14orf2	1.90182133	5.35E-41	1.13E-40
TDRD9	0.90953307	2.17E-54	5.52E-54
RD3L	NA	3.53E-127	4.18E-126
ASPG	Inf	8.68E-46	1.95E-45
CTD-2223O1ε	NA	7.01E-72	2.30E-71
KIF26A	1.87256809	4.85E-05	5.73E-05
RP11-260M1	NA	NA	NA
RP11-260M1	NA	NA	NA
TMEM179	NA	NA	NA
C14orf180	NA	NA	NA
RP11-614O9.	NA	NA	NA
RP11-982M1	NA	NA	NA
RP11-982M1	NA	2.22E-08	2.83E-08
RP11-982M1	Inf	5.60E-91	2.53E-90
INF2	1.97470817	3.31E-46	7.47E-46
RP11-982M1	NA	NA	NA
ADSSL1	0.74902724	1.95E-72	6.47E-72
SIVA1	1.99663058	8.31E-94	3.96E-93
AKT1	2.30822679	2.87E-136	5.61E-135
RP11-982M1	NA	0.05411278	0.05833467
ZBTB42	6.74124514	6.14E-66	1.85E-65
LINC00638	1.49805447	1.57E-32	2.93E-32
CTD-3051D2:	NA	NA	NA
CTD-3051D2:	NA	9.28E-27	1.60E-26
CEP170B	2.88910506	3.70E-58	9.88E-58
CTD-3051D2:	NA	NA	NA
PLD4	0.58257674	2.45E-61	6.87E-61
AHNAK2	NA	6.99E-24	1.15E-23
C14orf79	Inf	6.83E-07	8.44E-07
CDCA4	1.6175801	1.55E-54	3.95E-54
GPR132	NA	1.19E-63	3.44E-63
RP11-44N21.	NA	3.93E-19	6.05E-19
JAG2	2.0105468	0.0009203	0.00105205
NUDT14	1.43292167	4.09E-137	8.56E-136
BRF1	1.45124027	3.27E-29	5.83E-29
BTBD6	2.35804871	7.11E-70	2.27E-69

PACS2	2.51945525	2.69E-100	1.45E-99
TEX22	1.12354086	1.30E-54	3.33E-54
RP11-521B24	7.49027237	0.45292247	0.46586458
MTA1	2.14830889	4.15E-75	1.43E-74
RP11-521B24	NA	5.18E-49	1.21E-48
CRIP2	8.23929961	0.00439382	0.00492205
CRIP1	NA	NA	NA
AL928654.7	NA	NA	NA
C14orf80	1.22402012	5.33E-39	1.09E-38
TMEM121	2.39688716	0.10044504	0.10718628
IGHA2	NA	2.92E-108	1.88E-107
AL928742.12	NA	NA	NA
IGHE	NA	NA	NA
RP11-731F5.	NA	NA	NA
IGHG4	NA	1.66E-31	3.06E-31
IGHG2	NA	NA	NA
RP11-731F5.	NA	NA	NA
IGHGP	NA	NA	NA
AL928768.3	NA	NA	NA
IGHA1	Inf	7.99E-06	9.64E-06
IGHEP1	NA	NA	NA
IGHG1	NA	NA	NA
IGHG3	NA	1.31E-21	2.10E-21
IGHD	NA	NA	NA
IGHM	NA	1.42E-27	2.48E-27
IGHJ6	NA	NA	NA
IGHJ3P	NA	NA	NA
IGHJ5	NA	NA	NA
IGHJ4	NA	NA	NA
IGHJ3	NA	NA	NA
IGHJ2P	NA	NA	NA
IGHJ2	NA	NA	NA
IGHJ1	NA	NA	NA
IGHD7-27	NA	NA	NA
IGHJ1P	NA	NA	NA
IGHD1-26	NA	NA	NA
IGHD6-25	NA	NA	NA
IGHD5-24	NA	NA	NA
IGHD4-23	NA	NA	NA
IGHD3-22	NA	NA	NA
IGHD2-21	NA	NA	NA
AL122127.25	NA	NA	NA
IGHD1-20	NA	NA	NA
IGHD6-19	NA	NA	NA
IGHD5-18	NA	NA	NA

IGHD4-17	NA	NA	NA
IGHD3-16	NA	NA	NA
IGHD2-15	NA	NA	NA
IGHD1-14	NA	NA	NA
IGHD6-13	NA	NA	NA
IGHD5-12	NA	NA	NA
IGHD4-11	NA	NA	NA
IGHD3-10	NA	NA	NA
IGHD3-9	NA	NA	NA
IGHD2-8	NA	NA	NA
IGHD1-7	NA	NA	NA
IGHD6-6	NA	NA	NA
IGHD5-5	NA	NA	NA
IGHD4-4	NA	NA	NA
IGHD3-3	NA	NA	NA
IGHD2-2	NA	NA	NA
KIAA0125	0.67242218	5.45E-49	1.28E-48
IGHD1-1	NA	NA	NA
IGHV6-1	NA	NA	NA
IGHVII-1-1	NA	NA	NA
IGHV1-2	NA	NA	NA
IGHVIII-2-1	NA	NA	NA
IGHV1-3	NA	NA	NA
IGHV4-4	NA	NA	NA
CH17-262H1:	NA	NA	NA
IGHV2-5	NA	7.65E-06	9.24E-06
IGHVIII-5-1	NA	NA	NA
IGHVIII-5-2	NA	NA	NA
IGHV3-6	NA	NA	NA
IGHV3-7	NA	NA	NA
CH17-224D4.	NA	NA	NA
CH17-224D4.	NA	NA	NA
IGHV3-11	NA	NA	NA
IGHVIII-11-1	NA	NA	NA
IGHV1-12	NA	NA	NA
IGHV3-13	NA	NA	NA
IGHVIII-13-1	NA	NA	NA
IGHV1-14	NA	NA	NA
IGHV3-15	NA	NA	NA
IGHVII-15-1	NA	NA	NA
IGHV3-16	NA	NA	NA
IGHV1-17	NA	NA	NA
IGHV1-18	NA	NA	NA
IGHV3-19	NA	NA	NA
AB019440.5(	NA	NA	NA

IGHV3-20	NA	NA	NA
IGHV3-21	NA	NA	NA
IGHV3-22	NA	NA	NA
IGHVII-22-1	NA	NA	NA
IGHVIII-22-2	NA	NA	NA
IGHV3-23	NA	NA	NA
IGHV1-24	NA	NA	NA
LINC00226	NA	NA	NA
IGHV3-25	NA	NA	NA
IGHVIII-25-1	NA	NA	NA
IGHV2-26	NA	NA	NA
IGHVIII-26-1	NA	NA	NA
IGHVII-26-2	NA	NA	NA
IGHV7-27	NA	NA	NA
IGHV4-28	NA	NA	NA
IGHV3-29	NA	NA	NA
IGHV3-30	NA	NA	NA
IGHVII-30-1	NA	NA	NA
IGHV3-30-2	NA	NA	NA
IGHV4-31	NA	NA	NA
IGHVII-31-1	NA	NA	NA
IGHV3-32	NA	NA	NA
IGHV3-33	NA	NA	NA
IGHVII-33-1	NA	NA	NA
IGHV3-33-2	NA	NA	NA
IGHV4-34	NA	NA	NA
IGHV7-34-1	NA	NA	NA
IGHV3-35	NA	NA	NA
IGHV3-36	NA	NA	NA
IGHV3-37	NA	NA	NA
IGHV3-38	NA	NA	NA
IGHVIII-38-1	NA	NA	NA
IGHV4-39	NA	NA	NA
IGHV7-40	NA	NA	NA
IGHVII-40-1	NA	NA	NA
IGHV3-41	NA	NA	NA
IGHV3-42	NA	NA	NA
IGHV3-43	NA	NA	NA
IGHVIII-44	NA	NA	NA
LINC00221	NA	NA	NA
IGHVIV-44-1	NA	NA	NA
IGHVII-44-2	NA	NA	NA
IGHV1-45	NA	NA	NA
IGHV1-46	NA	NA	NA
IGHVII-46-1	NA	NA	NA

IGHV3-47	NA	NA	NA
IGHVIII-47-1	NA	NA	NA
IGHV3-48	NA	NA	NA
IGHV3-49	NA	NA	NA
IGHVII-49-1	NA	NA	NA
IGHV3-50	NA	NA	NA
IGHV5-51	NA	NA	NA
IGHVIII-51-1	NA	NA	NA
IGHVII-51-2	NA	NA	NA
IGHV3-52	NA	NA	NA
IGHV3-53	NA	NA	NA
IGHVII-53-1	NA	NA	NA
IGHV3-54	NA	NA	NA
IGHV4-55	NA	NA	NA
IGHV7-56	NA	NA	NA
IGHV3-57	NA	NA	NA
IGHV1-58	NA	NA	NA
IGHV4-59	NA	NA	NA
IGHV3-60	NA	NA	NA
IGHVII-60-1	NA	NA	NA
IGHV4-61	NA	NA	NA
IGHV3-64	NA	NA	NA
IGHV3-62	NA	NA	NA
IGHVII-62-1	NA	NA	NA
IGHV3-63	NA	NA	NA
IGHV3-65	NA	NA	NA
IGHVII-65-1	NA	NA	NA
IGHV3-66	NA	NA	NA
IGHV1-67	NA	NA	NA
IGHVII-67-1	NA	NA	NA
IGHVIII-67-2	NA	NA	NA
IGHVIII-67-3	NA	NA	NA
IGHVIII-67-4	NA	NA	NA
CH17-212P11	NA	NA	NA
IGHV1-68	NA	NA	NA
IGHV1-69	NA	NA	NA
IGHV2-70	NA	NA	NA
CH17-212P11	NA	NA	NA
CH17-212P11	NA	NA	NA
IGHV1-69-2	NA	NA	NA
IGHV2-70	NA	NA	NA
IGHV3-71	NA	NA	NA
IGHV3-72	NA	NA	NA
IGHV3-73	NA	NA	NA
IGHV3-74	NA	NA	NA

IGHVII-74-1	NA	NA	NA
IGHV3-75	NA	NA	NA
IGHV3-76	NA	NA	NA
IGHVIII-76-1	NA	NA	NA
IGHV5-78	NA	NA	NA
IGHVII-78-1	NA	NA	NA
IGHV3-79	NA	NA	NA
IGHV7-81	NA	NA	NA
IGHVIII-82	NA	NA	NA
IGHV1OR15-	NA	NA	NA
IGHV1OR15-	NA	NA	NA
IGHV3OR15-	NA	NA	NA
IGHD5OR15-	NA	NA	NA
IGHD4OR15-	NA	NA	NA
IGHD3OR15-	NA	NA	NA
IGHD2OR15-	NA	NA	NA
IGHD1OR15-	NA	NA	NA
RP11-173D3.	NA	8.37E-60	2.29E-59
RP11-509A1	NA	NA	NA
GOLGA6L6	NA	NA	NA
IGHV1OR15-	NA	NA	NA
RP11-467L1	NA	NA	NA
RP11-403B2.	NA	0.01402324	0.01544966
RP11-403B2.	NA	NA	NA
RP11-403B2.	NA	NA	NA
POTEB2	NA	NA	NA
LINC01193	NA	NA	NA
IGHD5OR15-	NA	NA	NA
IGHD4OR15-	NA	NA	NA
IGHD3OR15-	NA	NA	NA
IGHD2OR15-	NA	NA	NA
IGHD1OR15-	NA	NA	NA
RP11-854K1	NA	NA	NA
RP11-32B5.7	NA	1.25E-13	1.76E-13
RP11-275E1	NA	NA	NA
RP11-275E1	NA	NA	NA
RP11-32B5.8	NA	NA	NA
POTEB3	NA	NA	NA
RP11-11H9.2	NA	NA	NA
RP11-983G1	NA	NA	NA
RP11-294C1	NA	NA	NA
RP11-294C1	NA	NA	NA
RP11-294C1	NA	NA	NA
RP11-294C1	NA	NA	NA
RP11-294C1	NA	NA	NA

RP11-294C11	NA	NA	NA
RP11-361C13	NA	NA	NA
POTEB	NA	NA	NA
OR4M2	NA	NA	NA
OR4N4	NA	NA	NA
RP11-2F9.4	NA	NA	NA
IGHV1OR15-	NA	NA	NA
IGHV1OR15-	NA	NA	NA
IGHV4OR15-	NA	NA	NA
IGHV1OR15-	NA	NA	NA
GOLGA6L22	NA	NA	NA
NIPA1	2.20026751	7.85E-18	1.18E-17
NIPA2	2.03372244	7.15E-17	1.06E-16
CYFIP1	1.68531128	5.08E-07	6.30E-07
TUBGCP5	2.3540856	6.92E-101	3.79E-100
GOLGA6L1	NA	NA	NA
GOLGA8S	NA	NA	NA
GOLGA6L2	NA	NA	NA
MKRN3	NA	NA	NA
AC126407.1	NA	NA	NA
MKRN3-AS1	NA	NA	NA
MAGEL2	NA	NA	NA
NDN	NA	NA	NA
RP11-484P15	NA	NA	NA
PWRN4	NA	NA	NA
PWRN2	NA	NA	NA
RP11-580I1.1	NA	NA	NA
RP11-580I1.2	NA	NA	NA
RP11-350A1.	NA	NA	NA
RP11-107D24	NA	NA	NA
PWRN3	NA	NA	NA
PWRN1	NA	NA	NA
NPAP1	NA	NA	NA
SNRPN	NA	1.92E-166	1.94E-163
SNURF	NA	NA	NA
RP11-701H24	NA	NA	NA
RP11-701H24	NA	NA	NA
PWAR6	NA	NA	NA
RP11-701H24	NA	NA	NA
AC124312.1	NA	NA	NA
UBE3A	1.73162211	2.30E-06	2.81E-06
AC124997.1	NA	NA	NA
ATP10A	3.74513619	1.36E-08	1.74E-08
RP11-2C7.1	NA	NA	NA
RP11-1084I9	NA	NA	NA

RP11-1084I9	NA	NA	NA
LINC00929	NA	NA	NA
AC009878.2	NA	NA	NA
GABRB3	0	2.89E-39	5.93E-39
AC011196.3	NA	NA	NA
GABRA5	NA	NA	NA
GABRG3	NA	NA	NA
GABRG3-AS1	NA	NA	NA
RP11-100M1	NA	NA	NA
RP11-100M1	NA	NA	NA
RP11-30G8.2	NA	NA	NA
RP11-30G8.1	NA	NA	NA
OCA2	0.74902724	2.83E-29	5.06E-29
AC090696.2	NA	NA	NA
HERC2	2.12561784	9.14E-09	1.18E-08
GOLGA8F	NA	NA	NA
GOLGA8G	NA	NA	NA
GOLGA8M	Inf	3.10E-152	5.51E-150
RP11-578F21	NA	NA	NA
APBA2	2.52796693	8.48E-80	3.14E-79
RP13-126C7.	NA	NA	NA
FAM189A1	NA	NA	NA
NSMCE3	1.23877582	1.62E-82	6.28E-82
RP11-300A12	NA	NA	NA
RP11-680F8.	NA	NA	NA
TJP1	0	0.10131734	0.10808533
RP11-680F8.	NA	NA	NA
RP11-143J24	NA	NA	NA
CTD-2022H16	NA	NA	NA
GOLGA8J	NA	NA	NA
GOLGA8T	NA	NA	NA
RP11-932O9.	NA	NA	NA
DKFZP434L18	Inf	5.15E-135	9.25E-134
CHRFAM7A	0.74902724	2.71E-31	4.96E-31
GOLGA8R	Inf	1.26E-86	5.24E-86
AC026150.8	NA	NA	NA
GOLGA8Q	6.74124514	5.45E-112	3.86E-111
GOLGA8H	0.37451362	3.64E-32	6.76E-32
RP11-932O9.	Inf	1.15E-112	8.34E-112
RP11-932O9.	Inf	0.66702621	0.67843188
RP11-932O9.	NA	6.61E-103	3.77E-102
ARHGAP11B	2.27048881	3.24E-16	4.78E-16
RP11-932O9.	2.24708171	0.66530875	0.67683683
FAN1	3.932393	1.20E-44	2.64E-44
MTMR10	1.49805447	4.86E-26	8.30E-26

TRPM1	NA	NA	NA
RP11-348B1:	NA	NA	NA
RP11-16E12.	1.49805447	4.21E-57	1.11E-56
RP11-16E12.	NA	NA	NA
KLF13	2.16682879	6.75E-11	9.04E-11
OTUD7A	Inf	8.18E-140	2.25E-138
CHRNA7	Inf	1.98E-87	8.39E-87
RP11-717I24	NA	NA	NA
GOLGA8K	NA	NA	NA
GOLGA8O	NA	1.32E-104	7.81E-104
RP11-1000B(	4.11964981	2.32E-45	5.16E-45
RP11-1000B(	NA	2.90E-28	5.11E-28
GOLGA8N	Inf	1.75E-138	4.15E-137
RP11-1000B(	NA	3.65E-69	1.15E-68
ARHGAP11A	2.54017933	9.70E-10	1.27E-09
SCG5	Inf	1.75E-59	4.76E-59
RP11-1000B(	NA	NA	NA
RP11-758N1:	NA	NA	NA
GREM1	NA	9.24E-11	1.23E-10
FMN1	NA	3.98E-136	7.64E-135
RP11-489D6.	NA	NA	NA
RYR3	NA	NA	NA
RP11-3D4.4	Inf	2.35E-17	3.52E-17
RP11-3D4.3	Inf	4.74E-109	3.11E-108
RP11-3D4.2	NA	6.97E-94	3.33E-93
AVEN	1.96966422	2.71E-12	3.73E-12
CHRM5	NA	NA	NA
EMC7	1.07264876	2.09E-101	1.16E-100
PGBD4	1.12354086	7.80E-24	1.29E-23
KATNBL1	2.14815359	3.37E-18	5.12E-18
EMC4	1.66251869	3.82E-13	5.33E-13
SLC12A6	5.61770428	0.02751354	0.02999343
RP11-1084A:	NA	NA	NA
NOP10	2.21098401	7.62E-96	3.78E-95
NUTM1	NA	NA	NA
LPCAT4	3.85214008	1.15E-123	1.17E-122
GOLGA8A	8.42655642	2.80E-116	2.21E-115
GOLGA8B	3.37062257	8.32E-111	5.72E-110
CTD-2125J1.:	NA	NA	NA
GJD2	NA	NA	NA
RP11-814P5.	NA	NA	NA
ACTC1	NA	NA	NA
AQR	1.40993362	0.46997606	0.48304945
ZNF770	2.33625162	1.05E-104	6.20E-104
RP11-463I20	NA	NA	NA

RP11-463I20	NA	NA	NA
NANOGP8	NA	NA	NA
RP11-323I15	NA	NA	NA
DPH6	1.57830739	5.98E-66	1.80E-65
DPH6-AS1	NA	NA	NA
RP11-184D1:	NA	NA	NA
RP11-684B2:	NA	NA	NA
RP11-475A1:	NA	NA	NA
RP11-475A1:	NA	NA	NA
C15orf41	3.37062257	2.48E-06	3.03E-06
RP11-16L14.:	NA	NA	NA
MEIS2	1.87256809	0.28692428	0.29900513
RP11-128A1:	2.99610895	1.29E-31	2.38E-31
RP11-128A1:	Inf	6.77E-22	1.09E-21
RP11-720L8.:	NA	NA	NA
TMCO5A	NA	NA	NA
RP11-1008C:	NA	NA	NA
RP11-1008C:	NA	NA	NA
RP11-346D1:	NA	NA	NA
SPRED1	1.49805447	1.35E-08	1.74E-08
FAM98B	2.19159821	1.45E-54	3.69E-54
RASGRP1	3.37062257	4.59E-08	5.81E-08
RP11-102L12	NA	1.46E-121	1.36E-120
RP11-275I4.1	NA	NA	NA
RP11-275I4.2	NA	NA	NA
C15orf53	NA	0.17396314	0.183448
RP11-326N1:	NA	NA	NA
RP11-326N1:	NA	1.47E-18	2.24E-18
RP11-624L4.:	NA	NA	NA
RP11-62C7.2	NA	NA	NA
RP11-265N7.	NA	NA	NA
RP11-265N7.	NA	NA	NA
C15orf54	NA	NA	NA
RP11-462P6.	NA	NA	NA
RP11-624L4.:	NA	NA	NA
RP11-27M9.:	NA	NA	NA
THBS1	NA	NA	NA
CTD-2033D1!	NA	NA	NA
CTD-2033D1!	NA	NA	NA
FSIP1	Inf	1.06E-125	1.16E-124
RP11-37C7.3	Inf	1.18E-09	1.54E-09
GPR176	NA	8.68E-18	1.31E-17
RP11-325N1!	NA	1.26E-27	2.20E-27
EIF2AK4	2.36231667	1.18E-133	1.94E-132
SRP14	1.6502992	7.65E-102	4.28E-101

SRP14-AS1	2.99610895	4.94E-79	1.81E-78
RP11-521C2(	NA	NA	NA
RP11-521C2(	NA	2.93E-90	1.30E-89
BMF	Inf	2.51E-19	3.88E-19
RP11-521C2(	NA	NA	NA
BUB1B	1.58448069	3.78E-67	1.16E-66
BUB1B-PAK6	NA	NA	NA
PAK6	NA	2.72E-12	3.75E-12
PLCB2	1.38281952	1.28E-09	1.67E-09
ANKRD63	NA	NA	NA
PLCB2-AS1	NA	NA	NA
RP11-133K1.	NA	NA	NA
INAFM2	2.57998271	1.45E-37	2.91E-37
C15orf52	0.74902724	0.08780179	0.09389885
PHGR1	NA	NA	NA
DISP2	Inf	9.10E-83	3.55E-82
RP11-64K12.	NA	NA	NA
KNSTRN	1.43083408	9.74E-106	5.91E-105
IVD	1.78147019	1.54E-05	1.85E-05
BAHD1	1.8385214	4.52E-11	6.07E-11
RP11-64K12.	NA	NA	NA
RP11-64K12.	NA	NA	NA
CHST14	1.31079767	4.77E-40	9.90E-40
RP11-111A2.	NA	NA	NA
C15orf57	1.64302749	1.55E-79	5.74E-79
RPUSD2	1.17347601	1.44E-89	6.35E-89
CASC5	2.11490043	3.96E-24	6.57E-24
AC022405.1	NA	NA	NA
RAD51	1.09771233	2.16E-128	2.69E-127
RMDN3	1.52008469	1.16E-22	1.89E-22
GCHFR	1.89040208	2.91E-21	4.63E-21
DNAJC17	0.93628405	4.19E-104	2.46E-103
C15orf62	NA	6.91E-34	1.31E-33
ZFYVE19	1.12354086	2.14E-115	1.65E-114
PPP1R14D	NA	NA	NA
RP11-532F1.	1.12354086	5.73E-11	7.68E-11
SPINT1	1.77401188	1.67E-20	2.63E-20
RHOV	5.74254215	1.29E-90	5.77E-90
CTD-2129N1.	Inf	1.07E-156	3.98E-154
VPS18	2.14007782	1.87E-18	2.85E-18
RP11-540O1	Inf	2.18E-51	5.30E-51
DLL4	NA	2.22E-129	2.90E-128
CHAC1	0	2.92E-130	3.97E-129
INO80	1.62289235	1.65E-18	2.51E-18
RP11-540O1	NA	NA	NA

RP11-540O1:	NA	NA	NA
RP11-540O1:	NA	NA	NA
EXD1	NA	NA	NA
CHP1	2.0598249	1.67E-13	2.35E-13
OIP5	1.3229572	2.69E-75	9.28E-75
NUSAP1	1.15929844	1.35E-128	1.71E-127
NDUFAF1	1.43892075	1.41E-55	3.64E-55
RTF1	1.43930724	4.70E-90	2.09E-89
ITPKA	1.14694796	5.43E-64	1.59E-63
LTK	Inf	2.85E-49	6.71E-49
RPAP1	1.07360571	7.86E-97	3.99E-96
TYRO3	2.21827297	2.13E-29	3.80E-29
RP11-380D1:	NA	NA	NA
MGA	2.48677043	1.63E-27	2.84E-27
RP11-107F6.	Inf	2.08E-05	2.48E-05
MAPKBP1	1.21716926	2.39E-18	3.63E-18
AC073657.1	NA	9.56E-10	1.25E-09
RP11-23P13.	NA	NA	NA
JMJD7	NA	NA	NA
JMJD7-PLA2(	Inf	2.29E-28	4.04E-28
PLA2G4B	NA	NA	NA
SPTBN5	NA	NA	NA
RP11-23P13.	NA	NA	NA
EHD4	1.14850843	1.05E-75	3.65E-75
EHD4-AS1	NA	NA	NA
PLA2G4E-AS:	NA	NA	NA
PLA2G4E	NA	NA	NA
CTD-2382E5.	NA	NA	NA
PLA2G4D	NA	NA	NA
PLA2G4F	NA	NA	NA
VPS39	1.3618677	2.75E-30	4.99E-30
RP11-546B1!	NA	NA	NA
TMEM87A	1.48103113	6.98E-28	1.22E-27
GANC	1.32520204	3.36E-08	4.26E-08
CAPN3	0	1.05E-105	6.36E-105
ZNF106	2.56957955	5.97E-59	1.61E-58
SNAP23	1.73812731	1.21E-25	2.05E-25
RP11-265N6.	NA	3.02E-129	3.91E-128
LRRC57	1.39105058	2.33E-23	3.81E-23
HAUS2	1.34100038	8.42E-13	1.17E-12
RP11-265N6.	NA	1.04E-10	1.39E-10
STARD9	1.74773022	1.49E-07	1.86E-07
CDAN1	1.24837873	0.10343513	0.11022145
CTD-2036P1(	NA	NA	NA
CTD-2036P1(	NA	1.81E-07	2.26E-07

TTBK2	1.40442607	9.17E-39	1.87E-38
CTD-2036P1(	4.49416342	4.23E-46	9.54E-46
UBR1	1.74773022	0.64638276	0.65817343
EPB42	1.49805447	0.0016427	0.00186262
TMEM62	1.49805447	1.03E-06	1.27E-06
RP11-473C1ɛ	NA	0.00073116	0.00083747
CCNDBP1	0.80975918	3.47E-138	7.97E-137
TGM5	0.24967575	3.65E-93	1.73E-92
TGM7	NA	NA	NA
RP11-402F9.	NA	NA	NA
LCMT2	NA	NA	NA
ADAL	Inf	9.38E-63	2.68E-62
ZSCAN29	4.8151751	5.81E-28	1.02E-27
TUBGCP4	2.3540856	1.31E-65	3.93E-65
TP53BP1	1.49805447	0.03285836	0.03573411
MAP1A	2.84189746	1.63E-20	2.57E-20
PPIP5K1	1.49805447	0.64042877	0.65240377
CKMT1B	0.24967575	8.49E-93	3.99E-92
STRC	NA	3.69E-11	4.97E-11
CATSPER2	2.32198444	3.10E-27	5.37E-27
CKMT1A	0.44941634	7.45E-103	4.24E-102
PDIA3	0.9167199	8.83E-142	3.15E-140
ELL3	NA	6.59E-30	1.19E-29
RP11-296A1(	NA	NA	NA
SERF2	2.17315707	2.83E-101	1.56E-100
AC018512.1	NA	NA	NA
SERINC4	NA	9.73E-29	1.72E-28
HYPK	1.02991245	7.25E-59	1.95E-58
MFAP1	1.04671755	6.13E-132	9.17E-131
WDR76	2.29114214	6.75E-24	1.11E-23
FRMD5	NA	0.00073116	0.00083747
CASC4	2.49675746	1.52E-28	2.68E-28
RP11-616K2ː	NA	NA	NA
RP11-516C1.	NA	NA	NA
CTDSPL2	2.64130657	1.82E-33	3.43E-33
CTD-2329K1(	NA	NA	NA
EIF3J-AS1	1.97470817	0.05934091	0.06388339
EIF3J	1.3935983	3.70E-143	1.65E-141
SPG11	1.3618677	0.87224722	0.87800638
PATL2	1.49805447	2.94E-06	3.58E-06
B2M	1.44066822	3.80E-142	1.40E-140
TRIM69	2.66320796	2.21E-99	1.18E-98
CTD-2008A1.	NA	NA	NA
CTD-2008A1.	NA	NA	NA
C15orf43	NA	NA	NA

SORD	1.56278522	5.98E-45	1.33E-44
RP11-109D2(	NA	NA	NA
RP11-109D2(	NA	NA	NA
DUOX2	NA	1.85E-96	9.31E-96
DUOXA2	NA	NA	NA
DUOXA1	NA	NA	NA
DUOX1	NA	6.58E-07	8.13E-07
SHF	1.59168288	1.27E-06	1.56E-06
RP11-519G1(	NA	NA	NA
CTD-2651B2(	NA	9.02E-06	1.09E-05
SLC28A2	1.49805447	0.00159051	0.00180411
GATM	0	4.02E-116	3.17E-115
GATM-AS1	Inf	3.48E-06	4.24E-06
SPATA5L1	1.56047341	8.98E-58	2.38E-57
C15orf48	NA	3.09E-48	7.16E-48
RP11-519G1(	NA	NA	NA
RP11-519G1(	NA	NA	NA
SLC30A4	NA	1.86E-62	5.31E-62
RP11-96O20.	NA	NA	NA
RP11-96O20.	0.24967575	1.11E-11	1.51E-11
BLOC1S6	1.81160076	3.62E-07	4.50E-07
RP11-96O20.	NA	NA	NA
SQRDL	0.84758345	1.04E-91	4.76E-91
CTD-2306A1:	NA	NA	NA
RP11-718O1	NA	NA	NA
RP11-627D1(	NA	NA	NA
AC066615.1	NA	NA	NA
SEMA6D	NA	NA	NA
CTD-2050N2.	NA	NA	NA
RP11-142J21	NA	NA	NA
RP11-552E1(	NA	NA	NA
LINC01491	NA	NA	NA
RP11-208K4.	NA	NA	NA
CTD-2007H1	NA	NA	NA
SLC24A5	NA	NA	NA
MYEF2	0.74902724	1.53E-39	3.16E-39
RP11-605F2:	NA	NA	NA
CTXN2	0	1.90E-34	3.65E-34
SLC12A1	NA	NA	NA
RP11-605F2:	NA	NA	NA
RP11-154J22	Inf	2.25E-141	7.58E-140
DUT	1.53992556	1.64E-51	3.99E-51
FBN1	Inf	0.07208899	0.07735943
RP11-227D1:	NA	NA	NA
RP11-227D1:	NA	7.65E-44	1.67E-43

CEP152	2.838419	3.87E-72	1.28E-71
RP11-227D13	NA	3.01E-119	2.61E-118
RP11-485O10	NA	NA	NA
SHC4	0.74902724	5.39E-06	6.52E-06
EID1	1.67387116	2.68E-14	3.81E-14
RP11-138E16	NA	NA	NA
SECISBP2L	2.37191958	0.49999525	0.51320588
RP11-325E5.	NA	NA	NA
COPS2	1.33576524	1.17E-70	3.78E-70
GALK2	1.01338979	1.40E-125	1.54E-124
FAM227B	2.24708171	5.85E-43	1.26E-42
RP11-295H24	1.49805447	2.56E-99	1.36E-98
FGF7	Inf	6.45E-131	9.15E-130
DTWD1	1.09357977	3.54E-77	1.26E-76
ATP8B4	4.74383917	8.92E-132	1.33E-130
CTD-2647E9.	NA	6.59E-06	7.97E-06
SLC27A2	1.28404669	5.11E-60	1.41E-59
HDC	Inf	2.90E-41	6.12E-41
GABPB1	1.39105058	4.40E-48	1.02E-47
GABPB1-AS1	2.23689087	7.40E-74	2.51E-73
CTD-3110H11	NA	1.28E-90	5.75E-90
USP8	1.84375935	9.85E-09	1.27E-08
RP11-562A8.	NA	NA	NA
RP11-562A8.	NA	NA	NA
USP50	NA	NA	NA
TRPM7	2.41353221	1.89E-42	4.06E-42
RP11-120K9.	NA	1.03E-86	4.28E-86
SPPL2A	1.02991245	8.57E-49	2.01E-48
RP11-507J18	NA	NA	NA
CTD-2378E12	NA	1.34E-111	9.40E-111
AP4E1	5.80496109	8.50E-119	7.24E-118
RP11-108K3.	NA	NA	NA
TNFAIP8L3	NA	NA	NA
CYP19A1	NA	5.44E-132	8.17E-131
RP11-108K3.	NA	NA	NA
GLDN	NA	NA	NA
RP11-707P17	NA	NA	NA
DMXL2	1.87256809	9.84E-30	1.77E-29
RP11-707P17	NA	NA	NA
SCG3	Inf	1.39E-40	2.90E-40
RP11-313P18	NA	4.29E-48	9.91E-48
RP11-313P18	NA	NA	NA
LYSMD2	1.28832685	5.17E-79	1.89E-78
TMOD2	2.99610895	1.42E-11	1.92E-11
TMOD3	2.90007982	6.48E-69	2.04E-68

RP11-56B16.	NA	1.14E-12	1.57E-12
RP11-56B16.	NA	NA	NA
LEO1	1.33420477	1.23E-78	4.47E-78
MAPK6	3.06420233	1.34E-140	4.07E-139
CTD-2650P22	NA	NA	NA
CTD-2184D3.	NA	NA	NA
CTD-2184D3.	NA	NA	NA
BCL2L10	NA	NA	NA
GNB5	1.15758755	5.42E-109	3.55E-108
CTD-2184D3.	NA	4.85E-25	8.16E-25
CTD-2184D3.	NA	NA	NA
RP11-430B1.	NA	1.00E-152	1.93E-150
MYO5C	NA	NA	NA
MYO5A	1.49805447	0.34268949	0.35542378
AC018902.1	NA	NA	NA
ARPP19	2.44641961	1.19E-72	3.95E-72
RP11-23N2.4	NA	NA	NA
FAM214A	2.99610895	2.20E-40	4.59E-40
ONECUT1	NA	0.14576254	0.1542298
RP11-209K1(	NA	NA	NA
RP11-209E8.	NA	NA	NA
WDR72	NA	NA	NA
RP11-643A5.	NA	NA	NA
UNC13C	NA	NA	NA
RP11-548M1	NA	NA	NA
RSL24D1	1.36810628	1.54E-122	1.50E-121
RAB27A	0.75839008	3.80E-145	2.67E-143
PIGBOS1	1.80274352	9.38E-18	1.42E-17
PIGB	1.32520204	4.93E-08	6.23E-08
CCPG1	2.29389591	0.8009066	0.80906774
C15orf65	0	0.01062363	0.01175494
DYX1C1	1.21716926	2.64E-30	4.78E-30
PYGO1	1.60505837	0.16236997	0.17140207
PRTG	0.85603113	1.51E-30	2.75E-30
RP11-420M1	NA	NA	NA
NEDD4	3.932393	1.30E-35	2.54E-35
RFX7	3.18336576	1.07E-80	4.01E-80
TEX9	0.99870298	6.52E-17	9.69E-17
RP11-48G14.	Inf	0.00031741	0.00036763
RP11-48G14.	NA	9.60E-94	4.57E-93
MNS1	1.31079767	1.62E-11	2.19E-11
RP11-48G14.	Inf	2.00E-92	9.33E-92
ZNF280D	3.2457847	2.62E-71	8.51E-71
RP11-323F24	0.74902724	1.08E-10	1.44E-10
TCF12	2.15345331	1.19E-93	5.66E-93

LINC00926	Inf	8.39E-52	2.05E-51
LINC01413	NA	2.28E-132	3.51E-131
CGNL1	NA	4.95E-67	1.51E-66
CTD-2515H2	NA	NA	NA
CTD-2515H2	NA	NA	NA
CTD-2515H2	NA	NA	NA
GCOM1	NA	NA	NA
MYZAP	NA	NA	NA
POLR2M	1.88548236	1.14E-21	1.82E-21
RP11-344A1	NA	7.59E-100	4.06E-99
ALDH1A2	0.24967575	2.51E-05	2.99E-05
CTD-2330J20	NA	NA	NA
AQP9	NA	NA	NA
LIPC	NA	1.64E-07	2.05E-07
RP11-355N1	Inf	3.06E-33	5.76E-33
RP11-50C13.	NA	NA	NA
ADAM10	2.27204929	3.15E-107	1.98E-106
RP11-30K9.6	Inf	4.29E-12	5.89E-12
FAM63B	2.21933996	5.27E-23	8.61E-23
RP11-30K9.5	NA	1.73E-35	3.37E-35
RNF111	1.49805447	8.88E-24	1.46E-23
SLTM	1.52723735	8.50E-36	1.66E-35
AC025918.2	NA	NA	NA
CCNB2	0.61952253	3.37E-140	9.65E-139
RP11-59H7.4	NA	NA	NA
MYO1E	NA	0.10350982	0.11029456
LDHAL6B	NA	NA	NA
FAM81A	NA	2.65E-05	3.16E-05
GCNT3	NA	NA	NA
GTF2A2	2.09089442	1.84E-49	4.36E-49
BNIP2	1.65146969	2.33E-106	1.44E-105
RP11-361D1	NA	NA	NA
FOXB1	NA	6.60E-07	8.15E-07
RP11-82L7.4	NA	NA	NA
ANXA2	0.95745221	2.12E-130	2.92E-129
ICE2	4.59403372	6.00E-130	8.06E-129
RORA-AS1	Inf	3.82E-107	2.39E-106
RORA	1.49805447	2.16E-46	4.88E-46
CTD-2501E1	NA	1.55E-66	4.70E-66
RP11-219B1	NA	NA	NA
RORA-AS2	NA	NA	NA
RP11-554D2	NA	NA	NA
RP11-39M21	NA	NA	NA
RP11-39M21	NA	NA	NA
CTD-2026J24	NA	NA	NA

RP11-507B12	NA	NA	NA
RP11-259A24	NA	NA	NA
RP11-507B12	NA	NA	NA
RP11-162I7.1	NA	NA	NA
RP11-16B9.1	NA	NA	NA
VPS13C	1.65226596	6.44E-05	7.59E-05
RP11-643M1	NA	7.84E-72	2.57E-71
C2CD4A	NA	NA	NA
C2CD4B	NA	NA	NA
RP11-299H22	NA	NA	NA
RP11-1O10.1	NA	NA	NA
RP11-299H22	NA	2.08E-141	7.05E-140
RP11-299H22	NA	NA	NA
RP11-435J9.2	NA	9.82E-91	4.42E-90
TLN2	1.99740597	3.46E-20	5.42E-20
MGC15885	NA	NA	NA
RP11-625H11	NA	NA	NA
RP11-1069G	NA	7.05E-52	1.73E-51
RP11-1069G	NA	NA	NA
TPM1	1.36298399	9.46E-78	3.40E-77
RP11-244F12	Inf	1.32E-15	1.92E-15
RP11-244F12	NA	1.47E-18	2.24E-18
RP11-69G7.1	NA	NA	NA
LACTB	1.10570687	3.14E-84	1.26E-83
RPS27L	1.78949668	8.31E-09	1.07E-08
RAB8B	2.24708171	4.55E-15	6.58E-15
APH1B	0.88946984	1.71E-106	1.05E-105
CA12	NA	NA	NA
RP11-321G1	NA	NA	NA
AC007950.1	NA	NA	NA
AC007950.2	1.12354086	7.23E-29	1.28E-28
USP3	1.67090691	9.61E-26	1.63E-25
USP3-AS1	0.85603113	0.12970907	0.13768544
FBXL22	0.93628405	4.20E-19	6.47E-19
HERC1	1.75553259	0.00058065	0.0006671
RP11-317G6	NA	NA	NA
DAPK2	Inf	4.51E-43	9.74E-43
RP11-111E14	NA	NA	NA
FAM96A	1.79089225	0.00509999	0.0057022
SNX1	1.1770428	3.64E-106	2.23E-105
SNX22	1.9661965	7.58E-14	1.07E-13
PPIB	0.96509279	2.12E-143	9.93E-142
CSNK1G1	1.6728275	7.20E-26	1.22E-25
CTD-2116N1	NA	0.03650938	0.03960495
KIAA0101	1.93308787	1.49E-67	4.58E-67

TRIP4	1.24837873	8.88E-115	6.74E-114
ZNF609	2.47405966	1.04E-31	1.91E-31
OAZ2	1.96061165	1.55E-08	1.98E-08
AC100830.4	Inf	1.13E-133	1.86E-132
AC100830.5	Inf	2.11E-61	5.94E-61
AC100830.3	3.74513619	6.36E-124	6.56E-123
RBPMS2	2.51945525	6.29E-25	1.06E-24
PIF1	2.03307393	2.93E-24	4.88E-24
AC069368.3	NA	NA	NA
PLEKHO2	1.39818418	1.15E-05	1.38E-05
ANKDD1A	0.87386511	0.02561939	0.02795749
CTD-2017F17	NA	0.00010186	0.00011938
CTD-2017F17	2.24708171	8.34E-56	2.16E-55
SPG21	1.97331445	2.75E-08	3.50E-08
MTFMT	1.59408361	2.52E-94	1.22E-93
SLC51B	NA	2.86E-126	3.23E-125
RP11-325L12	NA	NA	NA
RASL12	NA	NA	NA
KBTBD13	NA	NA	NA
RP11-325L12	NA	NA	NA
UBAP1L	2.0598249	0.24895637	0.26047096
PDCD7	1.73930558	2.45E-23	4.02E-23
CLPX	1.8954975	0.00569527	0.00635798
CILP	NA	4.58E-34	8.74E-34
PARP16	2.80885214	5.10E-09	6.60E-09
IGDCC3	2.14376761	0.00023627	0.00027434
IGDCC4	1.62289235	0.56787244	0.58090453
DPP8	1.80321372	0.02814007	0.03066721
HACD3	1.30063644	1.60E-66	4.85E-66
VWA9	1.98018695	1.53E-08	1.96E-08
SLC24A1	1.94747082	2.36E-09	3.08E-09
RP11-16E23.	NA	2.97E-43	6.43E-43
DENND4A	2.20765923	0.54746869	0.5606648
RAB11A	1.23711516	1.36E-139	3.65E-138
MEGF11	1.49805447	2.31E-56	6.03E-56
RP11-653J6.:	NA	NA	NA
DIS3L	2.29489196	2.89E-37	5.76E-37
RP11-352G1:	Inf	3.34E-57	8.79E-57
TIPIN	1.10856031	2.04E-136	4.02E-135
MAP2K1	1.5421149	1.75E-40	3.64E-40
CTD-3185P2.	2.49675746	1.37E-38	2.79E-38
SNAPC5	2.21659805	1.05E-101	5.86E-101
RPL4	1.97616984	7.33E-05	8.63E-05
ZWILCH	1.74773022	1.30E-08	1.67E-08
LCTL	0.37451362	6.55E-53	1.63E-52

LINC01169	Inf	1.29E-35	2.52E-35
SMAD6	4.38715953	3.86E-139	9.89E-138
RP11-622C24	NA	NA	NA
RP11-798K3.	NA	NA	NA
RP11-798K3.	NA	NA	NA
RP11-798K3.	NA	NA	NA
SMAD3	1.72276265	0.11604863	0.12343775
RP11-342M2	NA	NA	NA
AAGAB	1.78102032	7.04E-09	9.08E-09
IQCH	Inf	3.26E-109	2.15E-108
IQCH-AS1	2.80885214	7.23E-16	1.06E-15
C15orf61	2.38409889	1.69E-81	6.45E-81
RP11-502I4.3	1.12354086	0.23118573	0.24228361
MAP2K5	1.7726978	3.61E-29	6.43E-29
SKOR1	NA	2.48E-91	1.13E-90
RP11-34F13.	NA	3.31E-14	4.70E-14
PIAS1	2.30950065	0.24906294	0.26056743
CALML4	1.24837873	0.98498553	0.98607259
RP11-315D16	NA	NA	NA
CLN6	1.65449687	9.06E-11	1.21E-10
RP11-315D16	NA	NA	NA
FEM1B	3.16524413	1.35E-132	2.11E-131
ITGA11	Inf	2.99E-07	3.72E-07
CORO2B	NA	NA	NA
ANP32A	1.10646577	1.52E-128	1.91E-127
SPESP1	NA	9.03E-88	3.85E-87
NOX5	NA	NA	NA
RP11-809H16	NA	NA	NA
EWSAT1	NA	6.09E-45	1.35E-44
RP11-809H16	NA	NA	NA
GLCE	1.88021123	3.46E-16	5.10E-16
RP11-352D13	NA	NA	NA
RP11-352D13	NA	NA	NA
PAQR5	0.74902724	0.13094879	0.138977
RP11-253M7	NA	4.23E-23	6.91E-23
KIF23	1.49805447	1.43E-119	1.26E-118
RPLP1	2.42060121	1.16E-141	4.07E-140
RP11-253M7	NA	1.73E-10	2.30E-10
DRAIC	Inf	0.03257085	0.03542993
RP11-279F6.	NA	NA	NA
PCAT29	NA	NA	NA
RP11-19J5.2	NA	NA	NA
LINC00593	NA	NA	NA
TLE3	2.13359274	0.03664633	0.03975114
AC011648.1	NA	NA	NA

RP11-543G1:	NA	NA	NA
RP11-96C21.	NA	NA	NA
RP11-96C21.	NA	2.74E-85	1.12E-84
SALRNA3	NA	NA	NA
SALRNA2	NA	NA	NA
UACA	NA	1.86E-104	1.10E-103
RP11-138H8.	NA	NA	NA
RP11-138H8.	NA	NA	NA
LARP6	Inf	3.10E-05	3.68E-05
LRRC49	NA	3.40E-81	1.29E-80
THAP10	0.37451362	7.04E-28	1.23E-27
THSD4	NA	NA	NA
CT62	NA	NA	NA
THSD4-AS1	NA	NA	NA
RP11-1123I8	NA	NA	NA
NR2E3	NA	NA	NA
CTD-2524L6.:	Inf	2.18E-60	6.02E-60
MYO9A	1.60505837	3.36E-06	4.09E-06
RP11-390D1:	NA	NA	NA
SENP8	1.99740597	6.50E-39	1.33E-38
RP11-2I17.4	NA	NA	NA
GRAMD2	NA	NA	NA
PKM	0.98735409	1.78E-141	6.10E-140
PARP6	2.24708171	2.62E-05	3.12E-05
RP11-106M3	NA	NA	NA
RP11-106M3	NA	3.21E-82	1.24E-81
CELF6	NA	NA	NA
RP11-106M3	NA	NA	NA
HEXA	1.23145156	1.42E-39	2.92E-39
HEXA-AS1	1.49805447	3.14E-06	3.83E-06
TMEM202	NA	NA	NA
RP11-1007O:	2.49675746	0.03446394	0.03742188
RP11-1007O:	NA	1.77E-14	2.53E-14
ARIH1	1.79766537	1.25E-83	4.94E-83
RP11-1006G	NA	NA	NA
RP11-1006G	NA	6.97E-94	3.33E-93
GOLGA6B	NA	NA	NA
HIGD2B	NA	1.49E-15	2.17E-15
BBS4	4.34435798	1.69E-23	2.78E-23
ADPGK	1.6172179	1.06E-68	3.32E-68
ADPGK-AS1	NA	5.43E-22	8.73E-22
NEO1	0.74902724	2.70E-42	5.78E-42
RP11-272D1:	NA	NA	NA
HCN4	NA	NA	NA
RP11-272D1:	NA	NA	NA

REC114	NA	NA	NA
NPTN	4.74383917	7.78E-68	2.41E-67
CD276	1.97112431	9.92E-08	1.25E-07
RP11-8P11.4	NA	NA	NA
C15orf59	NA	NA	NA
C15orf59-AS	NA	NA	NA
RP11-24D15.	NA	NA	NA
TBC1D21	NA	NA	NA
LOXL1-AS1	3.26848249	3.93E-79	1.44E-78
LOXL1	1.5729572	1.58E-14	2.26E-14
STOML1	1.93498703	0.49178294	0.50500516
PML	1.69677599	4.67E-59	1.26E-58
GOLGA6A	NA	NA	NA
ISLR2	NA	NA	NA
RP11-247C2.	NA	9.36E-17	1.39E-16
RP11-665J16	NA	NA	NA
ISLR	NA	NA	NA
STRA6	NA	NA	NA
RP11-60L3.1	NA	NA	NA
CCDC33	NA	NA	NA
RP11-60L3.6	NA	NA	NA
CYP11A1	NA	NA	NA
CTD-2311M2	NA	NA	NA
CTD-2311M2	NA	1.41E-19	2.19E-19
CTD-2311M2	NA	NA	NA
SEMA7A	1.87256809	0.1580439	0.16690334
UBL7	1.38873699	6.15E-141	1.94E-139
UBL7-AS1	1.48022049	5.68E-43	1.23E-42
RP11-10O17.	NA	2.84E-38	5.75E-38
ARID3B	3.08973735	6.88E-100	3.69E-99
CLK3	1.27008966	6.37E-69	2.01E-68
CTD-3154N5.	NA	4.21E-116	3.31E-115
CTD-3154N5.	NA	NA	NA
EDC3	2.14721141	1.78E-48	4.15E-48
CYP1A1	5.05593385	3.67E-85	1.49E-84
CYP1A2	NA	5.28E-112	3.74E-111
CSK	2.06208101	1.56E-84	6.28E-84
LMAN1L	NA	NA	NA
CPLX3	NA	3.18E-27	5.51E-27
ULK3	2.48115272	5.54E-28	9.70E-28
SCAMP2	1.21790649	6.23E-138	1.40E-136
MPI	0.95528111	1.60E-108	1.04E-107
FAM219B	2.12429036	1.30E-24	2.17E-24
COX5A	1.61457395	7.61E-10	1.00E-09
RPP25	1.74773022	3.20E-11	4.32E-11

SCAMP5	3.0793342	1.61E-21	2.57E-21
PPCDC	1.71759694	7.59E-60	2.08E-59
C15orf39	2.42740308	8.93E-36	1.75E-35
RP11-69H7.3	NA	NA	NA
RP11-69H7.2	NA	NA	NA
GOLGA6D	NA	NA	NA
GOLGA6C	NA	NA	NA
COMMD4	1.14530159	4.23E-144	2.29E-142
RP11-817O1:	0.74902724	2.28E-08	2.91E-08
NEIL1	Inf	1.28E-129	1.69E-128
MAN2C1	1.74038682	4.80E-17	7.15E-17
RP11-817O1:	1.94747082	3.09E-25	5.22E-25
SIN3A	2.42619692	2.98E-99	1.58E-98
PTPN9	2.12224384	0.98560675	0.98658562
SNUPN	1.45897479	1.44E-85	5.87E-85
CTD-2026K11	NA	NA	NA
CTD-2026K11	NA	3.18E-34	6.09E-34
IMP3	1.68077011	2.32E-19	3.59E-19
CTD-2026K11	0	4.28E-08	5.42E-08
CTD-2026K11	NA	NA	NA
SNX33	2.62159533	3.70E-38	7.47E-38
CSPG4	NA	NA	NA
CTD-2026K11	NA	NA	NA
CTD-2026K11	0	3.55E-140	1.02E-138
ODF3L1	NA	NA	NA
DNM1P35	NA	1.83E-53	4.58E-53
RP11-24M17	NA	NA	NA
UBE2Q2	1.60766822	6.26E-08	7.90E-08
FBXO22	1.59948525	1.46E-123	1.48E-122
NRG4	0	1.22E-08	1.57E-08
TMEM266	0	1.95E-119	1.70E-118
RP11-593F2:	NA	6.42E-31	1.17E-30
ETFA	0.98357112	1.00E-142	4.16E-141
ISL2	3.15379889	2.84E-25	4.80E-25
RP11-685G9.	NA	NA	NA
RP11-685G9.	Inf	2.27E-29	4.06E-29
SCAPER	1.98271916	0.00615046	0.00685644
RCN2	2.06850568	2.05E-16	3.03E-16
PSTPIP1	0.93628405	1.67E-113	1.23E-112
TSPAN3	1.76770428	1.03E-13	1.45E-13
RP11-797A1	NA	NA	NA
PEAK1	1.71206226	5.93E-11	7.95E-11
HMG20A	3.27206635	4.50E-74	1.53E-73
RP11-307C1	NA	NA	NA
RP11-307C1	NA	3.85E-14	5.47E-14

LINGO1	1.77486889	2.22E-70	7.13E-70
LINGO1-AS1	NA	1.34E-111	9.40E-111
LINGO1-AS2	NA	NA	NA
CTD-2071N1.	NA	NA	NA
RP11-114H2	NA	NA	NA
RP11-114H2	NA	NA	NA
TBC1D2B	2.79444777	3.13E-60	8.65E-60
RP11-114H2	NA	2.21E-66	6.71E-66
SH2D7	NA	1.10E-71	3.60E-71
CIB2	2.2865042	6.01E-09	7.75E-09
IDH3A	1.14754814	6.57E-84	2.61E-83
RP11-285A1.	NA	9.31E-12	1.27E-11
ACSBG1	0	1.76E-152	3.33E-150
RP11-762H8.	NA	NA	NA
DNAJA4	NA	NA	NA
WDR61	1.13302221	7.75E-136	1.47E-134
RP11-762H8.	NA	NA	NA
RP11-762H8.	NA	NA	NA
CRABP1	NA	NA	NA
IREB2	1.92607004	6.01E-92	2.77E-91
RP11-650L12	NA	2.74E-07	3.42E-07
HYKK	2.37191958	1.27E-47	2.91E-47
PSMA4	0.74146512	9.63E-138	2.12E-136
CHRNA5	1.7305112	0.13316106	0.14124228
RP11-650L12	NA	4.78E-09	6.18E-09
CHRNA3	1.49805447	2.83E-07	3.53E-07
CHRNB4	Inf	1.22E-34	2.34E-34
RP11-335K5.	NA	NA	NA
ADAMTS7	3.2457847	7.61E-63	2.18E-62
MORF4L1	1.51409985	1.74E-102	9.85E-102
CTSH	2.02785423	0.00429801	0.00481651
RASGRF1	2.99610895	3.48E-59	9.43E-59
RP11-16K12.	NA	2.21E-19	3.42E-19
ANKRD34C-A	NA	NA	NA
ANKRD34C	NA	NA	NA
TMED3	1.65643909	3.27E-64	9.59E-64
KIAA1024	1.49805447	1.17E-84	4.70E-84
RP11-38G5.2	NA	NA	NA
MTHFS	1.650399	6.57E-39	1.34E-38
RP11-38G5.4	0.74902724	1.79E-09	2.33E-09
ST20-MTHFS	NA	2.04E-122	1.98E-121
ST20	2.24708171	1.51E-131	2.22E-130
ST20-AS1	1.28404669	7.99E-06	9.64E-06
BCL2A1	NA	1.06E-108	6.86E-108
ZFAND6	1.17208892	1.56E-131	2.28E-130

FAH	1.10788595	2.44E-120	2.21E-119
RP11-2E17.2	NA	1.52E-128	1.91E-127
LINC01314	NA	NA	NA
LINC00927	NA	NA	NA
RP11-210M1	NA	5.83E-98	3.02E-97
ARNT2	Inf	5.94E-64	1.73E-63
RP11-210M1	NA	NA	NA
RP11-379K2	NA	NA	NA
RP11-379K2	NA	NA	NA
ABHD17C	1.3203192	4.86E-48	1.12E-47
RP11-28H5.2	Inf	4.10E-123	4.09E-122
CEMIP	NA	NA	NA
RP11-351M8	NA	NA	NA
RP11-351M8	NA	NA	NA
MESDC2	1.04201408	3.76E-118	3.14E-117
RP11-775C2	NA	NA	NA
RP11-775C2	NA	1.38E-60	3.83E-60
MESDC1	2.47297882	1.98E-63	5.73E-63
CFAP161	NA	2.76E-97	1.42E-96
IL16	0.37451362	2.08E-133	3.39E-132
STARD5	2.49675746	0.15112712	0.15975692
TMC3-AS1	1.49805447	0.0003913	0.00045199
TMC3	NA	NA	NA
RP11-761I4.	NA	3.26E-141	1.07E-139
CTD-2240J17	NA	2.36E-17	3.54E-17
CTD-2240J17	NA	1.41E-11	1.91E-11
CTD-2240J17	NA	5.56E-53	1.39E-52
CTD-2240J17	Inf	0.80088548	0.80906774
CTD-2034I4.1	NA	NA	NA
CTD-2034I4.2	NA	NA	NA
RP11-499F3.	NA	NA	NA
RP11-499F3.	NA	0.10239071	0.10914055
RP11-276M1	NA	NA	NA
MEX3B	2.99610895	1.29E-61	3.63E-61
LINC01583	NA	NA	NA
EFTUD1	1.64785992	3.60E-37	7.17E-37
SAXO2	1.24837873	3.25E-20	5.08E-20
GOLGA6L10	1.49805447	1.43E-38	2.92E-38
GOLGA6L9	0	1.52E-15	2.21E-15
RPS17	2.1414045	3.55E-122	3.40E-121
RP11-152F1	NA	NA	NA
RP11-379H8.	NA	NA	NA
CPEB1	Inf	1.60E-119	1.41E-118
CPEB1-AS1	Inf	1.14E-60	3.17E-60
AP3B2	NA	7.92E-21	1.25E-20

AC105339.1	NA	0.09805185	0.10471254
RP11-752G1!	NA	NA	NA
SNHG21	2.5659745	4.15E-125	4.46E-124
FSD2	Inf	1.06E-17	1.60E-17
WHAMM	1.49805447	4.75E-10	6.27E-10
HOMER2	1.91512646	1.98E-45	4.43E-45
RP11-382A2(	NA	4.00E-88	1.72E-87
FAM103A1	1.59237642	8.86E-21	1.40E-20
C15orf40	2.11600195	8.74E-114	6.49E-113
RP11-382A2(	NA	NA	NA
BTBD1	1.89753567	0.00016451	0.00019179
RP11-90B9.3	NA	1.55E-155	4.86E-153
RP11-382A2(	NA	NA	NA
TM6SF1	1.99740597	0.0012918	0.0014694
RP11-382A2(	2.25693733	2.96E-137	6.30E-136
RP11-382A2(	NA	NA	NA
RP11-382A2(	NA	1.41E-11	1.91E-11
BNC1	NA	NA	NA
SH3GL3	0.74902724	1.86E-05	2.22E-05
ADAMTSL3	NA	NA	NA
RP13-262C2.	NA	NA	NA
UBE2Q2L	2.7464332	1.49E-09	1.94E-09
GOLGA6L4	0.56177043	0.39303802	0.40581046
RP11-182J1.:	NA	NA	NA
RP11-182J1.:	NA	NA	NA
RP11-182J1.:	2.24708171	0.37265162	0.38561748
ZSCAN2	1.5172603	4.59E-29	8.17E-29
RP11-182J1.!	NA	4.22E-12	5.78E-12
RP11-182J1.:	Inf	1.22E-14	1.75E-14
RP11-182J1.:	NA	2.41E-26	4.12E-26
WDR73	1.38361976	1.14E-51	2.79E-51
NMB	2.18957482	8.37E-89	3.65E-88
SEC11A	1.29811192	6.53E-145	4.23E-143
RP11-245C1;	NA	2.32E-48	5.39E-48
ZNF592	2.95668646	1.13E-79	4.19E-79
ALPK3	NA	3.09E-105	1.86E-104
SLC28A1	NA	NA	NA
PDE8A	2.51945525	7.73E-140	2.15E-138
AKAP13	2.07084001	1.23E-67	3.80E-67
RP11-815J21	NA	NA	NA
RP11-815J21	NA	NA	NA
RP11-815J21	NA	NA	NA
RP11-158M2	NA	NA	NA
RP11-158M2	NA	6.82E-108	4.35E-107
RP11-158M2	NA	NA	NA

RP11-158M2	NA	3.98E-72	1.31E-71
KLHL25	3.1459144	5.29E-25	8.91E-25
RP11-553E24	NA	NA	NA
LINC01584	NA	NA	NA
AGBL1	NA	NA	NA
AGBL1-AS1	NA	NA	NA
RP11-182L7.	NA	NA	NA
RP11-133L19	NA	NA	NA
RP11-138H1(	NA	NA	NA
RP11-648K4.	NA	0.38801892	0.40076511
LINC00052	NA	NA	NA
NTRK3	NA	2.49E-09	3.24E-09
NTRK3-AS1	NA	NA	NA
MRPL46	1.10997152	3.16E-104	1.86E-103
MRPS11	1.65366225	2.25E-06	2.75E-06
DET1	1.3732166	1.67E-73	5.62E-73
LINC01586	NA	NA	NA
AEN	2.06773716	2.34E-06	2.86E-06
ISG20	3.59533074	7.36E-36	1.44E-35
RP11-343B18	NA	NA	NA
ACAN	NA	9.30E-07	1.15E-06
HAPLN3	1.59927437	4.01E-27	6.95E-27
MFGE8	2.0130107	0.00070522	0.00080832
RP11-326A19	NA	3.89E-102	2.19E-101
RP11-326A19	0	3.69E-58	9.85E-58
ABHD2	2.2262754	7.53E-119	6.43E-118
RLBP1	NA	NA	NA
FANCI	1.25095271	6.11E-128	7.46E-127
POLG	1.7061176	0.00774896	0.00861671
RP11-217B1.	0.99870298	4.36E-09	5.64E-09
MIR9-3HG	NA	NA	NA
RHCG	NA	NA	NA
LINC00928	NA	NA	NA
RP11-429B14	NA	NA	NA
TICRR	1.72852439	0.87981752	0.88523373
RP11-429B14	NA	NA	NA
KIF7	Inf	1.29E-90	5.80E-90
PLIN1	NA	NA	NA
PEX11A	NA	NA	NA
WDR93	NA	NA	NA
MESP1	2.82965845	7.85E-128	9.56E-127
MESP2	NA	2.27E-101	1.26E-100
ANPEP	1.91418072	0.27476797	0.28673227
AP3S2	4.49416342	2.56E-120	2.32E-119
C15orf38-AP.	NA	NA	NA

ARPIN	NA	NA	NA
ZNF710	1.07003891	2.04E-36	4.03E-36
RP11-617F23	0.29961089	2.08E-81	7.93E-81
IDH2	1.1920793	1.71E-139	4.55E-138
CTD-2315E11	NA	NA	NA
SEMA4B	2.49675746	2.85E-53	7.12E-53
CIB1	1.8039635	0.21724972	0.22800789
GDPGP1	2.39688716	0.00010054	0.00011786
RP11-697E2.	Inf	4.04E-35	7.83E-35
NGRN	1.31079767	3.81E-23	6.23E-23
RP11-697E2.	NA	NA	NA
RP11-697E2.	NA	1.06E-91	4.87E-91
ZNF774	3.74513619	1.72E-52	4.26E-52
IQGAP1	2.10743257	6.32E-12	8.64E-12
RP11-154B1	NA	NA	NA
CRTC3	3.1348177	1.29E-78	4.69E-78
CTD-3065B2	NA	NA	NA
CTD-3065B2	NA	2.09E-82	8.11E-82
CRTC3-AS1	0.49935149	1.48E-71	4.82E-71
RP11-387D1	NA	0.39735263	0.41019526
BLM	1.77042802	9.76E-09	1.25E-08
FURIN	3.97560611	4.67E-99	2.48E-98
FES	1.87256809	3.20E-18	4.86E-18
MAN2A2	1.94747082	1.14E-27	1.99E-27
AC068831.1	NA	1.50E-75	5.20E-75
HDDC3	1.76333495	0.02765987	0.03014932
UNC45A	2.06874189	0.28294883	0.29501481
AC068831.3	NA	NA	NA
AC068831.6	NA	0.0005036	0.00057953
RCCD1	1.324367	2.22E-100	1.20E-99
PRC1	2.09727626	8.55E-14	1.21E-13
PRC1-AS1	NA	1.11E-103	6.47E-103
VPS33B	1.99740597	0.00054025	0.00062116
AC068831.1	3.74513619	1.11E-06	1.37E-06
AC068831.1	1.49805447	8.79E-128	1.07E-126
SV2B	NA	5.56E-34	1.06E-33
CRAT37	NA	3.74E-17	5.59E-17
RP11-692C2	NA	NA	NA
RP11-692C2	NA	NA	NA
SLCO3A1	2.11089494	5.93E-07	7.33E-07
RP11-152L2	NA	NA	NA
RP11-24J19.	NA	NA	NA
ST8SIA2	NA	NA	NA
RP11-763K1	NA	NA	NA
C15orf32	NA	NA	NA

LINC00930	NA	NA	NA
RP11-386M2	NA	NA	NA
FAM174B	0	1.32E-140	4.00E-139
RP11-386M2	NA	NA	NA
CTD-2313J17	NA	6.60E-111	4.55E-110
CTD-2313J17	NA	NA	NA
RP11-437B1(	0.37451362	3.12E-27	5.42E-27
CHD2	1.46060311	0.00057071	0.00065588
RGMA	NA	5.99E-15	8.63E-15
RP11-326A1:	NA	NA	NA
RP11-266O8.	NA	NA	NA
RP11-164C1:	NA	NA	NA
RP11-739G5.	NA	NA	NA
LINC01579	NA	NA	NA
RP11-76E17.	NA	NA	NA
RP11-76E17.	1.49805447	0.22790176	0.238911
RP11-76E17.	NA	NA	NA
LINC01580	NA	NA	NA
LINC01581	NA	NA	NA
MCTP2	2.49675746	5.09E-11	6.83E-11
RP11-57P19.	NA	NA	NA
RP11-255M2	0.24967575	8.72E-54	2.20E-53
CTD-2576F9.	NA	NA	NA
RP11-255M2	NA	NA	NA
RP11-255M2	NA	NA	NA
LINC01197	NA	NA	NA
CTD-2536I1.:	NA	NA	NA
LINC00924	NA	3.04E-45	6.77E-45
RP11-398J10	NA	NA	NA
RP11-61O11.	NA	3.67E-65	1.09E-64
RP11-759A2·	NA	NA	NA
RP11-4G2.1	NA	NA	NA
RP11-236L14	NA	NA	NA
RP11-236L14	NA	NA	NA
NR2F2-AS1	NA	NA	NA
RP11-327J17	NA	NA	NA
RP11-327J17	NA	NA	NA
RP11-327J17	NA	NA	NA
RP11-327J17	NA	NA	NA
NR2F2	NA	1.20E-62	3.42E-62
RP11-262A1(	NA	NA	NA
RP11-522B1!	NA	NA	NA
RP11-522B1!	NA	NA	NA
RP11-522B1!	NA	NA	NA
RP11-522B1!	NA	NA	NA

SPATA8-AS1	NA	NA	NA
SPATA8	NA	NA	NA
RP11-315L6.1	NA	NA	NA
CTD-2147F2.1	NA	NA	NA
CTD-2147F2.1	NA	3.84E-125	4.13E-124
RP11-461F11	NA	NA	NA
LINC00923	NA	NA	NA
RP11-753A21	NA	NA	NA
ARRDC4	2.7464332	0.0094358	0.01045464
RP11-753A21	NA	NA	NA
AC144835.1	NA	NA	NA
CTD-2544M6	NA	NA	NA
LINC01582	NA	NA	NA
RP11-183E24	NA	NA	NA
RP11-167B3.	NA	NA	NA
RP11-167B3.	NA	NA	NA
RP11-167B3.	NA	NA	NA
FAM169B	NA	NA	NA
RP11-35O15.	NA	0.62462798	0.63666503
IGF1R	1.49805447	3.80E-36	7.47E-36
RP11-654A16	NA	NA	NA
RP11-654A16	NA	NA	NA
PGPEP1L	NA	NA	NA
RP11-6O2.2	Inf	2.62E-149	3.57E-147
SYNM	NA	NA	NA
RP11-6O2.4	NA	NA	NA
TTC23	Inf	1.02E-09	1.34E-09
RP11-6O2.3	NA	NA	NA
LRRC28	2.0598249	0.06983507	0.07498063
AC022819.2	NA	NA	NA
RP11-20G13.	NA	NA	NA
RP11-20G13.	NA	NA	NA
RP11-20G13.	NA	NA	NA
RP11-20G13.	NA	NA	NA
MEF2A	4.74383917	2.10E-115	1.62E-114
LYSMD4	0.99870298	0.00119704	0.00136334
CTD-2054N24	Inf	7.94E-121	7.28E-120
CTD-3076O17	NA	NA	NA
ADAMTS17	Inf	6.17E-10	8.12E-10
CTD-3076O17	Inf	1.03E-153	2.34E-151
RP11-90E5.1	NA	7.60E-105	4.52E-104
SPATA41	NA	NA	NA
CERS3-AS1	NA	NA	NA
CERS3	NA	NA	NA
RP11-526I2.5	4.49416342	1.63E-24	2.71E-24

RP11-526I2.1	NA	NA	NA
LINS1	1.62289235	2.87E-14	4.09E-14
ASB7	3.37062257	1.46E-31	2.69E-31
RP11-66B24.	NA	NA	NA
AC087762.1	NA	NA	NA
RP11-66B24.	NA	1.29E-40	2.70E-40
RP11-66B24.	NA	NA	NA
ALDH1A3	NA	7.00E-59	1.89E-58
RP11-66B24.	NA	NA	NA
RP11-66B24.	2.03307393	1.24E-62	3.54E-62
LRRK1	2.51945525	1.68E-33	3.18E-33
RP11-505E24	NA	NA	NA
RP11-505E24	NA	NA	NA
RP11-424I19	NA	NA	NA
RP11-424I19	NA	NA	NA
CHSY1	3.91158668	0.00143681	0.00163182
VIMP	1.27372652	5.35E-92	2.47E-91
SNRPA1	1.21774367	4.45E-116	3.50E-115
RP11-299G2(	NA	2.03E-70	6.53E-70
PCSK6	Inf	0.38544309	0.39853159
PCSK6-AS1	NA	1.45E-143	7.02E-142
RP11-14C10.	NA	NA	NA
RP11-14C10.	NA	NA	NA
RP11-14C10.	NA	NA	NA
TM2D3	1.72775616	9.42E-55	2.41E-54
TARSL2	2.20302129	4.52E-31	8.27E-31
RP11-89K11.	NA	0.24998475	0.26150166
OR4F6	NA	NA	NA
OR4F15	NA	NA	NA
OR4F4	NA	NA	NA
FAM138E	NA	NA	NA
AC140725.4	Inf	3.26E-66	9.86E-66
WASIR2	1.33754864	0.17447966	0.18396059
POLR3K	1.93115537	1.04E-06	1.28E-06
SNRNP25	2.12573093	2.08E-82	8.06E-82
RHBDF1	0.74902724	0.80682846	0.81468703
MPG	1.2542436	2.92E-137	6.23E-136
NPRL3	1.85758755	9.50E-12	1.29E-11
Z69720.2	NA	1.40E-06	1.72E-06
Z69666.2	NA	NA	NA
HBZ	Inf	4.53E-111	3.14E-110
HBM	NA	NA	NA
HBA2	NA	1.92E-74	6.57E-74
HBA1	3.37062257	1.00E-15	1.47E-15
HBQ1	1.90848036	1.36E-48	3.17E-48

LA16c-OS12.	2.24708171	9.03E-09	1.16E-08
LUC7L	1.33868698	8.73E-83	3.41E-82
FAM234A	1.4381323	0.00071937	0.00082438
AC004754.3	NA	0.06485778	0.06972739
RGS11	NA	NA	NA
ARHGDIG	1.33754864	4.01E-27	6.95E-27
PDIA2	0.37451362	1.20E-92	5.62E-92
AXIN1	2.40477166	3.59E-66	1.08E-65
MRPL28	1.25210523	7.98E-100	4.27E-99
TMEM8A	2.54669261	0.00058737	0.00067465
NME4	2.54063353	1.91E-138	4.50E-137
DECR2	1.76770428	0.97297753	0.97437395
LA16c-359F1	Inf	9.16E-80	3.39E-79
RAB11FIP3	3.37062257	3.74E-33	7.03E-33
LINC00235	0	1.53E-18	2.33E-18
CAPN15	3.74513619	3.46E-140	9.89E-139
LA16c-366D1	NA	NA	NA
PRR35	NA	NA	NA
PIGQ	2.80885214	4.12E-102	2.32E-101
NHLRC4	Inf	6.04E-19	9.28E-19
RAB40C	1.89227934	5.37E-09	6.94E-09
WFIKKN1	0	7.60E-05	8.94E-05
C16orf13	2.20012076	1.57E-115	1.22E-114
FAM195A	2.2038686	4.80E-101	2.64E-100
MSLN	NA	NA	NA
AL022341.3	NA	8.12E-113	5.91E-112
WDR90	1.69779507	8.87E-32	1.64E-31
LA16c-349E1	NA	6.16E-82	2.37E-81
RHOT2	1.34384298	8.07E-47	1.84E-46
RHBDL1	6.74124514	6.76E-54	1.71E-53
LA16c-313D1	NA	NA	NA
STUB1	1.80783734	1.06E-46	2.42E-46
JMJD8	1.80321372	1.09E-22	1.77E-22
WDR24	1.59168288	4.10E-29	7.29E-29
FBXL16	7.49027237	2.33E-56	6.08E-56
LA16c-380A1	NA	NA	NA
METRN	2.03154966	4.64E-42	9.89E-42
FAM173A	1.98995296	0.95456431	0.95683219
CCDC78	2.24708171	8.64E-40	1.79E-39
HAGHL	2.30469919	1.18E-36	2.34E-36
NARFL	1.06257352	4.00E-46	9.02E-46
MSLNL	NA	NA	NA
RPUSD1	1.64190739	0.00124136	0.00141284
CHTF18	1.2067661	0.00012162	0.00014233
GNG13	NA	NA	NA

PRR25	NA	0.00216643	0.00244652
LA16c-360A4	NA	1.31E-142	5.18E-141
LMF1	1.12354086	1.52E-24	2.53E-24
LA16c-306A4	NA	NA	NA
LMF1-AS1	NA	NA	NA
RP11-161M6	0.89883268	2.63E-87	1.11E-86
SOX8	1.38281952	7.65E-44	1.67E-43
RP11-161M6	NA	NA	NA
RP11-161M6	NA	NA	NA
SSTR5	NA	NA	NA
C1QTNF8	NA	NA	NA
LA16c-381G6	NA	NA	NA
RP11-616M2	NA	2.23E-147	2.47E-145
CACNA1H	1.87256809	5.71E-09	7.38E-09
RP11-616M2	NA	NA	NA
RP11-616M2	NA	NA	NA
RP11-616M2	NA	NA	NA
TPSG1	NA	NA	NA
RP11-616M2	NA	3.75E-93	1.77E-92
TPSB2	0.86510456	5.47E-23	8.92E-23
TPSAB1	0.39521538	1.77E-50	4.25E-50
TPSD1	NA	1.25E-17	1.88E-17
RP11-616M2	NA	NA	NA
LA16c-358B7	Inf	1.25E-09	1.63E-09
UBE2I	1.85195918	2.64E-14	3.77E-14
LA16c-358B7	1.87256809	3.14E-13	4.38E-13
BAIAP3	0.74902724	1.34E-17	2.01E-17
TSR3	2.18788025	9.10E-67	2.78E-66
GNPTG	0.88946984	1.01E-119	8.92E-119
LA16c-316G1	NA	4.85E-25	8.16E-25
UNKL	3.40807393	2.05E-111	1.43E-110
LA16c-312E8	NA	2.35E-120	2.12E-119
C16orf91	2.06142539	7.86E-58	2.09E-57
CCDC154	Inf	3.99E-58	1.06E-57
CLCN7	1.78102032	0.40978609	0.42259773
LA16c-390E6	NA	4.89E-144	2.59E-142
LA16c-390E6	NA	NA	NA
PTX4	NA	NA	NA
TELO2	1.79115209	1.15E-13	1.62E-13
IFT140	2.99610895	1.79E-07	2.24E-07
LA16c-385E7	NA	9.33E-104	5.44E-103
TMEM204	NA	NA	NA
LA16c-395F1	NA	NA	NA
CRAMP1	3.18336576	0.89027014	0.89535345
LA16c-395F1	NA	NA	NA

LA16c-431H6	1.92607004	1.14E-76	4.01E-76
HN1L	2.53938502	5.70E-100	3.06E-99
MAPK8IP3	2.99610895	0.26786956	0.27975877
LA16c-329F2	NA	NA	NA
LA16c-361A3	NA	NA	NA
NME3	3.31712062	3.84E-61	1.08E-60
MRPS34	1.42961054	1.68E-58	4.51E-58
EME2	3.2457847	4.85E-41	1.02E-40
SPSB3	1.44973014	3.04E-94	1.46E-93
NUBP2	1.7146849	1.34E-26	2.30E-26
IGFALS	NA	NA	NA
HAGH	1.73912071	1.36E-11	1.85E-11
FAHD1	1.52579622	2.57E-78	9.29E-78
MEIOB	NA	NA	NA
LINC00254	NA	NA	NA
XX-DJ76P10_	NA	NA	NA
HS3ST6	NA	NA	NA
MSRB1	1.45555648	4.45E-76	1.55E-75
RPL3L	NA	NA	NA
NDUFB10	1.50325154	5.07E-34	9.67E-34
RPS2	2.17457738	1.87E-115	1.44E-114
SNHG9	2.21520821	1.03E-142	4.27E-141
RNF151	0.74902724	2.09E-08	2.67E-08
AC005363.11	NA	7.83E-119	6.69E-118
TBL3	2.24708171	3.28E-12	4.52E-12
NOXO1	NA	1.55E-34	2.97E-34
GFER	2.05629176	0.9986538	0.99870885
AC005606.14	NA	NA	NA
SYNGR3	NA	2.58E-75	8.92E-75
ZNF598	1.85528285	0.48137772	0.49462815
AC005606.15	NA	0.00019525	0.00022725
NPW	1.85149477	0.51495885	0.52820622
SLC9A3R2	1.30009728	6.33E-49	1.48E-48
NTHL1	1.16747821	1.10E-138	2.68E-137
TSC2	1.97743191	4.81E-59	1.30E-58
PKD1	2.54669261	5.55E-67	1.70E-66
RP11-304L19	NA	NA	NA
RP11-304L19	NA	NA	NA
RAB26	2.7464332	4.67E-15	6.74E-15
SNHG19	2.30155642	7.43E-48	1.71E-47
TRAF7	2.02010376	1.06E-58	2.86E-58
CASKIN1	0.74902724	2.82E-05	3.36E-05
MLST8	2.16555494	0.00975412	0.01080205
BRICD5	2.99610895	1.04E-13	1.46E-13
RP11-304L19	NA	8.83E-56	2.29E-55

PGP	1.86209219	3.87E-12	5.31E-12
E4F1	1.53372244	3.09E-31	5.67E-31
RP11-304L19	NA	9.25E-135	1.64E-133
DNASE1L2	Inf	7.18E-123	7.09E-122
ECI1	1.09829275	2.82E-145	2.13E-143
RP11-304L19	1.49805447	0.8695901	0.87552606
RNPS1	1.70071336	5.73E-34	1.09E-33
AC009065.4	0	1.26E-108	8.19E-108
RP11-304L19	1.81906615	8.75E-06	1.05E-05
ABCA3	1.74773022	3.50E-07	4.35E-07
CCNF	2.3927259	4.20E-08	5.33E-08
RP11-715J22	NA	5.99E-32	1.11E-31
RP11-715J22	NA	NA	NA
C16orf59	1.82371849	9.49E-09	1.22E-08
RP11-715J22	Inf	0.27488195	0.28683472
NTN3	NA	NA	NA
TBC1D24	2.14721141	1.72E-17	2.58E-17
RP11-20I23.1	NA	NA	NA
ATP6V0C	0	8.61E-61	2.40E-60
RP11-20I23.3	NA	NA	NA
AMDHD2	2.70184825	0.96118252	0.96320003
CEMP1	NA	NA	NA
PDPK1	1.56414511	0.61867517	0.63095199
RP11-20I23.1	1.74773022	4.65E-14	6.58E-14
RP11-20I23.8	0.74902724	1.29E-13	1.82E-13
RP11-20I23.6	6.74124514	6.28E-46	1.41E-45
RP11-20I23.7	NA	NA	NA
CTD-3126B10	NA	NA	NA
CTD-3126B10	NA	1.35E-88	5.87E-88
KCTD5	1.91001946	3.14E-11	4.23E-11
PRSS27	Inf	4.50E-37	8.97E-37
SRRM2-AS1	2.24708171	6.57E-26	1.12E-25
SRRM2	1.70282451	5.94E-20	9.25E-20
TCEB2	2.20949925	1.31E-124	1.38E-123
CTD-2270P14	0.74902724	7.36E-25	1.24E-24
PRSS33	NA	NA	NA
PRSS21	NA	6.09E-45	1.35E-44
ZG16B	NA	5.51E-95	2.69E-94
PRSS22	NA	NA	NA
LA16c-325D7	NA	NA	NA
LA16c-325D7	Inf	6.12E-146	6.20E-144
FLYWCH2	1.69363381	0.48721586	0.50048526
FLYWCH1	1.97470817	2.68E-07	3.34E-07
LA16c-321D4	NA	9.45E-96	4.69E-95
KREMEN2	0.90399839	7.86E-110	5.27E-109

PKMYT1	1.83745744	2.36E-68	7.37E-68
PAQR4	1.09143969	6.17E-26	1.05E-25
LA16c-380H5	NA	1.61E-07	2.02E-07
LA16c-380H5	NA	NA	NA
LA16c-380H5	0.89883268	0.60049693	0.61320586
CLDN9	NA	1.63E-11	2.20E-11
CLDN6	NA	NA	NA
TNFRSF12A	1.61040856	0.0104586	0.01157375
HCFC1R1	1.84760052	0.88084139	0.88616561
THOC6	1.5745974	6.07E-79	2.22E-78
CCDC64B	NA	NA	NA
RP11-473M2	NA	NA	NA
MMP25	0	5.98E-37	1.19E-36
MMP25-AS1	3.29571984	0.81236326	0.81991067
IL32	0.24967575	3.72E-15	5.38E-15
RP11-473M2	1.12354086	1.58E-20	2.48E-20
ZSCAN10	NA	3.67E-65	1.09E-64
ZNF213-AS1	1.60505837	7.28E-41	1.53E-40
ZNF205	NA	0.54860514	0.56176523
ZNF213	2.87127108	2.44E-35	4.74E-35
RP11-473M2	1.87256809	0.21407418	0.2247401
AJ003147.8	NA	NA	NA
AJ003147.9	NA	NA	NA
OR1F1	NA	NA	NA
ZNF200	3.932393	9.71E-45	2.14E-44
MEFV	NA	NA	NA
LINC00921	NA	0.31462001	0.32720913
ZNF263	0.91547773	2.81E-66	8.49E-66
TIGD7	1.49805447	3.36E-61	9.43E-61
ZNF75A	1.91418072	2.22E-25	3.75E-25
LA16c-360H6	NA	1.10E-21	1.76E-21
OR2C1	NA	NA	NA
LA16c-306E5	NA	1.11E-16	1.65E-16
MTRNR2L4	NA	2.13E-20	3.35E-20
ZSCAN32	1.12354086	6.22E-16	9.11E-16
ZNF174	2.37191958	8.87E-25	1.48E-24
ZNF597	1.12354086	1.73E-09	2.26E-09
NAA60	2.08771421	2.79E-65	8.33E-65
C16orf90	NA	NA	NA
CLUAP1	0.81712062	1.28E-126	1.47E-125
NLRC3	1.70233463	0.0079607	0.00884458
LA16c-390H2	NA	1.88E-29	3.37E-29
RP11-461A8.	NA	NA	NA
SLX4	2.12224384	8.15E-86	3.35E-85
DNASE1	2.59956512	4.32E-12	5.93E-12

TRAP1	1.24038911	2.30E-132	3.53E-131
RP11-461A8.	NA	3.56E-32	6.61E-32
CREBBP	2.3540856	1.22E-60	3.39E-60
ADCY9	4.99351492	1.59E-86	6.62E-86
RP11-462G1:	NA	NA	NA
RP11-95P2.3	0.99870298	0.92441532	0.92799595
SRL	NA	NA	NA
LINC01569	2.7464332	1.51E-18	2.30E-18
TFAP4	1.05896954	5.04E-11	6.76E-11
GLIS2	2.14007782	5.81E-15	8.37E-15
GLIS2-AS1	0	1.03E-87	4.41E-87
PAM16	3.37062257	8.46E-16	1.24E-15
CORO7-PAM	NA	NA	NA
CORO7	1.4432476	2.59E-08	3.30E-08
VASN	NA	1.09E-129	1.45E-128
DNAJA3	1.73881323	0.51797925	0.53121422
NMRAL1	2.06335805	0.25529872	0.26693732
HMOX2	1.09594512	2.27E-120	2.06E-119
CDIP1	1.80377988	0.00020902	0.00024313
C16orf96	NA	NA	NA
RP11-709D2⁄	NA	3.14E-121	2.90E-120
UBALD1	2.70616292	1.54E-92	7.19E-92
MGRN1	2.24708171	3.16E-17	4.72E-17
RP11-709D2⁄	NA	NA	NA
NUDT16L1	1.22757242	7.32E-75	2.51E-74
ANKS3	1.45265888	0.02092804	0.0229204
C16orf71	0.37451362	2.99E-31	5.48E-31
ZNF500	1.87256809	7.26E-09	9.36E-09
12-Sep	NA	2.29E-33	4.31E-33
SMIM22	NA	NA	NA
RP11-127I20	NA	NA	NA
ROGDI	1.75430063	0.00011031	0.00012918
GLYR1	3.12451362	5.23E-114	3.90E-113
UBN1	1.76503448	2.90E-05	3.45E-05
PPL	0.74902724	3.31E-05	3.93E-05
SEC14L5	0	1.06E-158	7.11E-156
RP11-165E7.	2.99610895	6.28E-21	9.94E-21
NAGPA	1.15326416	3.48E-49	8.18E-49
ALG1	1.03437095	7.01E-92	3.23E-91
C16orf89	NA	NA	NA
EEF2KMT	1.39743888	3.36E-18	5.10E-18
RP11-10K17.	NA	NA	NA
RP11-382N1:	NA	NA	NA
RP11-420N3.	NA	4.27E-60	1.18E-59
LINC01570	NA	NA	NA

RBFOX1	NA	NA	NA
RP11-26O3.1	NA	NA	NA
CTD-2535I10	NA	NA	NA
RP11-279O1	NA	NA	NA
RP11-279O1	NA	NA	NA
RP11-279O1	NA	NA	NA
RP11-568A1	NA	NA	NA
RP11-483K5.	NA	NA	NA
TMEM114	NA	NA	NA
METTL22	2.33696498	9.87E-30	1.77E-29
ABAT	2.24708171	0.70518855	0.71564192
TMEM186	1.4200308	2.21E-33	4.16E-33
PMM2	1.79766537	0.04159911	0.04506963
RP11-152P2	NA	2.23E-42	4.78E-42
RP11-77H9.2	2.99610895	3.66E-30	6.62E-30
CARHSP1	1.93029944	0.2085743	0.21911836
RP11-77H9.5	NA	3.88E-121	3.57E-120
RP11-77H9.6	Inf	2.12E-77	7.55E-77
USP7	2.12723735	4.92E-126	5.51E-125
RP11-77H9.8	Inf	4.58E-73	1.53E-72
RP11-473I1.6	NA	NA	NA
C16orf72	2.6483463	8.41E-111	5.78E-110
RP11-473I1.5	1.49805447	5.28E-11	7.08E-11
RP11-243A1	NA	2.65E-05	3.16E-05
LINC01177	NA	NA	NA
LINC01195	NA	NA	NA
RP11-418I22	NA	NA	NA
RP11-418I22	NA	NA	NA
RP11-297M9	NA	NA	NA
GRIN2A	NA	NA	NA
RP11-895K1	NA	NA	NA
ATF7IP2	2.06462636	2.52E-59	6.85E-59
RP11-609N1	NA	NA	NA
LINC01290	NA	5.81E-17	8.64E-17
EMP2	1.71206226	2.14E-33	4.04E-33
RP11-27M24	NA	1.96E-21	3.13E-21
TEKT5	NA	1.09E-36	2.17E-36
RP11-109M1	NA	NA	NA
NUBP1	1.0469358	5.72E-103	3.27E-102
TVP23A	Inf	0.06905135	0.07415672
RP11-876N2	NA	NA	NA
CIITA	7.49027237	5.43E-138	1.24E-136
DEXI	1.33888619	1.17E-07	1.46E-07
RP11-876N2	Inf	2.64E-09	3.43E-09
CLEC16A	1.26758456	3.13E-15	4.53E-15

RP11-66H6.3	NA	2.71E-09	3.52E-09
RP11-396B1	NA	1.24E-56	3.25E-56
RMI2	1.43385214	6.28E-53	1.56E-52
SOCS1	5.36802853	4.55E-145	3.11E-143
TNP2	NA	NA	NA
PRM3	NA	NA	NA
PRM2	NA	NA	NA
PRM1	NA	NA	NA
RP11-485G7.	Inf	3.51E-60	9.67E-60
AC009121.2	NA	NA	NA
RP11-485G7.	NA	NA	NA
CTD-3088G3.	0.37451362	0.00014185	0.00016563
CTD-3088G3.	NA	NA	NA
LITAF	1.78872176	8.62E-56	2.23E-55
SNN	2.39205473	7.18E-15	1.03E-14
TXNDC11	1.93498703	7.72E-55	1.97E-54
RP11-490O6.	0.74902724	0.76501765	0.77389039
ZC3H7A	2.99610895	1.27E-11	1.73E-11
RP11-486I11	0.37451362	4.68E-06	5.68E-06
BCAR4	NA	1.35E-10	1.79E-10
RSL1D1	1.26935558	1.72E-91	7.87E-91
RP11-166B2.	NA	1.35E-107	8.54E-107
GSPT1	1.38685435	4.34E-80	1.62E-79
RP11-166B2.	Inf	0.00534237	0.00597026
RP11-166B2.	0.37451362	1.92E-142	7.40E-141
TNFRSF17	NA	NA	NA
SNX29	Inf	0.28916221	0.30130267
RP11-166B2.	NA	NA	NA
RP11-276H1.	NA	NA	NA
RP11-276H1.	NA	1.65E-112	1.19E-111
RP11-165M1	NA	NA	NA
CTD-3037G2	NA	4.74E-71	1.53E-70
CTD-3037G2	NA	NA	NA
CTD-3037G2	NA	NA	NA
CPPED1	0.53056096	1.08E-135	2.04E-134
CTD-2583P5.	NA	NA	NA
CTD-2583P5.	NA	3.40E-14	4.84E-14
SHISA9	NA	3.17E-10	4.20E-10
AC009134.1	NA	NA	NA
U91319.1	NA	NA	NA
AC003009.1	NA	NA	NA
U95743.1	NA	NA	NA
ERCC4	1.49805447	4.00E-48	9.26E-48
CTD-2135D7.	NA	0.00256266	0.00288858
CTD-2135D7.	NA	NA	NA

CTD-2135D7.	NA	NA	NA
CTD-2135D7.	NA	NA	NA
MKL2	1.60505837	0.0053859	0.00601854
CTA-276F8.1	Inf	2.88E-17	4.31E-17
CTA-276F8.2	NA	NA	NA
MIR193BHG	0	2.37E-31	4.34E-31
RP11-65J21.:	NA	NA	NA
RP11-65J21.4	NA	6.07E-05	7.16E-05
PARN	1.16848249	1.63E-56	4.26E-56
BFAR	1.63676322	2.50E-05	2.98E-05
PLA2G10	NA	NA	NA
RP11-82O18.	NA	NA	NA
NPIPA3	NA	7.54E-79	2.75E-78
RP11-719K4.	NA	NA	NA
NPIPA2	NA	NA	NA
NPIPA1	2.0598249	9.00E-120	7.99E-119
NOMO1	2.34071012	3.88E-38	7.83E-38
MIR3179-1	NA	1.37E-83	5.42E-83
MIR3180-1	NA	NA	NA
PDXDC1	1.69066148	9.78E-30	1.76E-29
RP11-680G24	NA	NA	NA
NTAN1	1.78830253	0.0013479	0.00153208
RRN3	3.03356031	4.49E-07	5.57E-07
RP11-72I8.1	NA	8.37E-60	2.29E-59
MIR3180-4	Inf	2.03E-27	3.53E-27
NPIPA5	NA	3.50E-79	1.28E-78
RP11-1021N	NA	NA	NA
MPV17L	NA	3.31E-120	2.97E-119
C16orf45	NA	NA	NA
KIAA0430	2.37191958	2.77E-87	1.17E-86
CTB-193M12	NA	NA	NA
NDE1	2.24708171	0.0707609	0.07595221
CTB-193M12	3.21011673	6.38E-14	9.03E-14
MYH11	Inf	3.08E-45	6.85E-45
AF001548.5	0.74902724	3.18E-05	3.78E-05
AF001548.6	NA	NA	NA
FOPNL	1.33420477	1.73E-69	5.49E-69
CTA-972D3.2	NA	NA	NA
ABCC1	1.72276265	1.39E-05	1.67E-05
ABCC6	NA	NA	NA
RP11-517A5.	NA	NA	NA
NOMO3	1.24837873	0.0033877	0.00380886
RP11-517A5.	NA	NA	NA
MIR3180-2	NA	NA	NA
AC138969.4	NA	3.84E-79	1.41E-78

NPIPA7	NA	NA	NA
RP11-14N9.2	NA	NA	NA
XYLT1	2.99610895	8.59E-30	1.54E-29
CTD-2576D5.	NA	4.22E-12	5.78E-12
RP11-916L7.	NA	NA	NA
CTD-3229J4.	NA	NA	NA
CTA-481E9.4	NA	NA	NA
CTA-481E9.3	NA	NA	NA
NPIPA8	NA	NA	NA
RP11-1212A	NA	NA	NA
MIR3180-3	NA	NA	NA
MIR3179-3	Inf	2.39E-71	7.79E-71
NOMO2	NA	1.45E-13	2.04E-13
RP11-457I16	NA	NA	NA
RPS15A	2.20487373	2.24E-113	1.65E-112
RP11-1035H	NA	2.09E-82	8.11E-82
ARL6IP1	1.35077384	1.57E-122	1.53E-121
RP11-1035H	NA	NA	NA
SMG1	3.70107576	2.67E-129	3.47E-128
CTD-2288F12	NA	6.24E-09	8.05E-09
TMC7	0.74902724	0.00208476	0.00235576
RP11-626G1	0	0.15533046	0.16410466
RP11-626G1	NA	7.35E-07	9.08E-07
COQ7	2.19357977	0.00066432	0.00076198
RP11-626G1	NA	1.09E-25	1.84E-25
RP11-626G1	NA	1.21E-29	2.17E-29
ITPRIPL2	2.76563903	4.41E-115	3.37E-114
CTD-2349B8.	NA	NA	NA
RP11-626G1	2.99610895	0.74587943	0.75541476
SYT17	Inf	4.35E-17	6.49E-17
CLEC19A	NA	NA	NA
CTA-363E6.2	NA	NA	NA
TMC5	2.52796693	1.09E-138	2.65E-137
CTA-363E6.5	NA	NA	NA
CTA-363E6.6	NA	NA	NA
CTA-363E6.7	0	0.62351036	0.63563301
GDE1	1.97470817	8.16E-09	1.05E-08
CCP110	1.60505837	2.80E-49	6.60E-49
C16orf62	1.2733463	2.34E-75	8.09E-75
AC002550.6	NA	2.14E-81	8.16E-81
KNOP1	1.40744634	2.19E-51	5.33E-51
AC002550.5	0	4.87E-60	1.34E-59
IQCK	1.9661965	0.00056961	0.00065467
CTD-2380F24	NA	NA	NA
GPRC5B	1.74773022	0.01480977	0.0163037

GPR139	NA	NA	NA
RP11-204E4.	NA	NA	NA
GP2	NA	NA	NA
UMOD	NA	NA	NA
PDILT	NA	NA	NA
RP11-429K17	NA	NA	NA
ACSM5	NA	NA	NA
CTD-2194A8.	NA	NA	NA
ACSM2A	NA	NA	NA
ACSM2B	NA	NA	NA
RP11-143N1	NA	NA	NA
ACSM3	0.74902724	1.66E-130	2.29E-129
ACSM1	NA	4.04E-114	3.02E-113
THUMPD1	1.64785992	0.00013673	0.00015977
AC004381.7	NA	NA	NA
ERI2	2.37191958	1.43E-34	2.75E-34
AC004381.6	1.08192823	4.51E-47	1.03E-46
DCUN1D3	11.2354086	8.32E-38	1.67E-37
LYRM1	2.45269703	4.84E-33	9.08E-33
DNAH3	NA	1.89E-08	2.42E-08
CTC-508F8.1	Inf	3.37E-81	1.28E-80
TMEM159	2.57998271	4.37E-42	9.31E-42
AF001550.7	NA	2.18E-60	6.02E-60
ZP2	NA	NA	NA
ANKS4B	NA	0.00048233	0.00055541
CRYM	Inf	0.05049065	0.05452389
CRYM-AS1	1.99740597	1.44E-36	2.84E-36
NPIPB3	NA	NA	NA
CTB-31N19.5	NA	NA	NA
METTL9	2.34478092	1.26E-33	2.39E-33
IGSF6	NA	6.73E-123	6.65E-122
OTOA	NA	0.00013894	0.00016228
RP11-645C24	NA	0.72327392	0.73313417
NPIPB4	0.74902724	2.09E-23	3.44E-23
UQCRC2	0.99378811	5.37E-131	7.64E-130
PDZD9	NA	NA	NA
CTD-2649C14	Inf	3.22E-153	6.71E-151
C16orf52	3.42412451	1.37E-107	8.68E-107
RP11-101E7.	NA	NA	NA
VWA3A	NA	NA	NA
SDR42E2	0.74902724	8.89E-11	1.19E-10
EEF2K	1.5693904	1.04E-13	1.47E-13
RP11-141O1	NA	NA	NA
POLR3E	2.60531213	5.25E-133	8.41E-132
CDR2	1.97112431	0.02561974	0.02795749

RP11-21M24	NA	NA	NA
RP11-21M24	NA	0.00015263	0.00017809
NPIPB5	0.93628405	1.37E-38	2.79E-38
RP11-105C19	NA	NA	NA
RP11-105C19	Inf	1.13E-99	6.03E-99
HS3ST2	NA	NA	NA
RP11-20G6.2	NA	NA	NA
USP31	1.84375935	4.90E-08	6.20E-08
SCNN1G	NA	NA	NA
CTC-391G2.1	NA	NA	NA
SCNN1B	NA	NA	NA
COG7	1.34824903	0.31131833	0.32383104
CTD-2270L9.4	1.95899431	4.42E-30	7.99E-30
GGA2	1.41780156	1.70E-05	2.04E-05
EARS2	1.61632193	1.57E-50	3.76E-50
UBFD1	1.65226596	0.04057064	0.04396587
NDUFAB1	1.80053311	0.01330848	0.01467645
PALB2	1.81906615	0.74627405	0.75568787
CTD-2196E14	NA	NA	NA
DCTN5	1.99416342	1.40E-52	3.48E-52
PLK1	1.20391592	1.87E-51	4.54E-51
CTD-2196E14	NA	NA	NA
ERN2	NA	NA	NA
CTD-2385L22	NA	NA	NA
CHP2	NA	NA	NA
PRKCB	1.48735409	2.80E-48	6.49E-48
AC004125.3	NA	NA	NA
CACNG3	NA	NA	NA
CTD-2313J23	NA	NA	NA
RBBP6	2.1976953	4.99E-33	9.36E-33
CTD-2540M1	NA	NA	NA
LINC01567	NA	NA	NA
TNRC6A	2.88514195	1.88E-137	4.04E-136
CTD-2515A14	NA	1.35E-14	1.94E-14
SLC5A11	NA	NA	NA
ARHGAP17	2.54669261	2.02E-89	8.88E-89
RP11-266L9.5	NA	1.47E-10	1.95E-10
LCMT1-AS1	2.43433852	4.20E-21	6.66E-21
LCMT1	1.46809339	2.16E-26	3.71E-26
LCMT1-AS2	NA	NA	NA
AQP8	NA	NA	NA
ZKSCAN2	3.37062257	2.23E-22	3.61E-22
CTD-2547G23	NA	NA	NA
CTD-2547G23	1.20996708	3.28E-08	4.17E-08
CTA-249B10.	NA	NA	NA

HS3ST4	NA	NA	NA
AC009158.1	NA	1.16E-05	1.39E-05
AC002331.1	NA	NA	NA
RP11-142A1:	NA	2.29E-41	4.83E-41
C16orf82	NA	NA	NA
RP11-673P17	NA	NA	NA
RP11-673P17	NA	NA	NA
KDM8	1.49805447	4.18E-40	8.68E-40
CTD-3203P2.	NA	0.83949664	0.84640147
NSMCE1	1.2688745	3.84E-128	4.74E-127
FLJ21408	0	2.03E-22	3.28E-22
CTD-3203P2.	NA	3.82E-38	7.71E-38
IL4R	3.20038911	8.80E-26	1.50E-25
IL21R	1.31079767	0.00142895	0.00162298
IL21R-AS1	NA	NA	NA
GTF3C1	1.61789883	2.01E-55	5.18E-55
KIAA0556	2.99610895	2.79E-19	4.31E-19
CTD-2049O4.	NA	NA	NA
GSG1L	NA	NA	NA
XPO6	2.59484436	1.42E-92	6.65E-92
RP11-57A19.	NA	NA	NA
SBK1	0	0.30075728	0.31306091
NPIPB6	NA	4.28E-96	2.14E-95
EIF3CL	Inf	1.08E-24	1.80E-24
RP11-57A19.	NA	NA	NA
NPIPB7	NA	5.30E-58	1.41E-57
RP11-435I10	NA	3.17E-27	5.51E-27
CLN3	1.44455253	4.75E-101	2.61E-100
APOBR	0.85603113	1.60E-70	5.14E-70
IL27	NA	NA	NA
NUPR1	NA	NA	NA
RP11-666O2.	4.74383917	5.88E-61	1.64E-60
SGF29	1.34824903	3.81E-104	2.24E-103
SULT1A2	1.87256809	0.00058118	0.00066766
SULT1A1	2.16610579	1.28E-63	3.70E-63
NPIPB8	NA	NA	NA
EIF3C	2.99610895	1.13E-96	5.70E-96
NPIPB9	NA	NA	NA
RP11-1348G	Inf	1.18E-39	2.43E-39
RP11-1348G	0	1.88E-21	2.99E-21
RP11-1348G	4.49416342	1.68E-98	8.82E-98
ATXN2L	2.99610895	4.59E-134	7.75E-133
RP11-24N18.	NA	0.05255704	0.05667775
TUFM	1.0879407	1.81E-140	5.36E-139
SH2B1	3.62990123	1.15E-121	1.08E-120

RP11-22P6.2	3.74513619	0.02751225	0.02999343
ATP2A1	NA	1.66E-136	3.29E-135
ATP2A1-AS1	1.48063524	7.95E-11	1.06E-10
RABEP2	1.84760052	0.37085464	0.38380176
CD19	0.37451362	5.69E-95	2.78E-94
NFATC2IP	1.98832685	3.75E-11	5.05E-11
RP11-264B1	Inf	1.29E-127	1.55E-126
RP11-264B1	0	9.47E-06	1.14E-05
SPNS1	1.45863199	1.91E-94	9.21E-94
LAT	17.2276265	1.41E-63	4.10E-63
RP11-264B1	NA	NA	NA
RP11-426C2	Inf	0.00113525	0.00129443
RP11-426C2	NA	NA	NA
RP11-426C2	NA	NA	NA
RP11-426C2	NA	NA	NA
RP11-426C2	NA	2.99E-08	3.80E-08
RP11-231C1	1.49805447	0.00180729	0.00204578
NPIPB11	Inf	5.64E-101	3.09E-100
BOLA2	Inf	1.07E-157	5.54E-155
RP11-345J4.	1.65574442	3.44E-45	7.63E-45
SLX1B	0	6.61E-155	1.84E-152
SULT1A4	NA	1.58E-43	3.43E-43
RP11-345J4.	NA	NA	NA
RP11-231C1	NA	NA	NA
SPN	1.92664225	0.4033286	0.41617492
AC009133.22	NA	NA	NA
QPRT	1.63069471	5.28E-39	1.08E-38
C16orf54	0.74902724	1.99E-129	2.61E-128
AC009133.17	NA	NA	NA
ZG16	NA	NA	NA
KIF22	0.84331039	3.58E-145	2.56E-143
AC009133.21	NA	9.03E-88	3.85E-87
MAZ	2.20162052	5.81E-119	4.98E-118
AC009133.1	Inf	2.17E-41	4.60E-41
AC009133.12	0.89883268	0.00228174	0.00257498
PRRT2	0.74902724	4.57E-37	9.10E-37
AC009133.2	NA	NA	NA
PAGR1	2.85992218	7.61E-69	2.39E-68
CTD-2574D2	NA	NA	NA
MVP	0.18725681	5.79E-29	1.03E-28
CDIPT	1.7183566	1.63E-25	2.76E-25
CTD-2574D2	NA	2.55E-21	4.05E-21
CDIPT-AS1	0.74902724	3.33E-22	5.37E-22
SEZ6L2	2.01661179	1.01E-20	1.60E-20
ASPHD1	1.69677599	3.19E-35	6.19E-35

KCTD13	1.44043699	5.31E-09	6.87E-09
CTD-2574D22	NA	0.00109288	0.0012469
TMEM219	1.76122621	1.13E-22	1.83E-22
TAOK2	1.97470817	5.40E-13	7.51E-13
HIRIP3	1.03588873	5.15E-120	4.60E-119
INO80E	2.21712062	5.60E-20	8.72E-20
DOC2A	0.99870298	1.54E-104	9.14E-104
C16orf92	NA	NA	NA
FAM57B	Inf	0.07761441	0.08320022
ALDOA	0.99469535	6.51E-136	1.24E-134
CTD-2515O1(	NA	3.93E-65	1.17E-64
PPP4C	1.4774046	2.33E-138	5.42E-137
TBX6	NA	2.79E-09	3.62E-09
YPEL3	1.24837873	3.19E-61	8.95E-61
RP11-455F5.	0.74902724	7.05E-32	1.30E-31
GDPD3	Inf	4.40E-12	6.04E-12
RP11-455F5.	NA	NA	NA
RP11-455F5.	NA	NA	NA
MAPK3	1.24180831	1.33E-96	6.74E-96
CORO1A	0.61003249	2.89E-145	2.17E-143
RP11-455F5.	2.99610895	7.27E-37	1.44E-36
BOLA2B	2.0598249	8.73E-18	1.32E-17
SLX1A	0.74902724	3.91E-60	1.08E-59
SULT1A3	2.69649805	8.05E-99	4.25E-98
RP11-347C12	NA	NA	NA
RP11-347C12	Inf	1.95E-09	2.54E-09
CD2BP2	1.92874514	2.30E-09	2.99E-09
RP11-347C12	1.31079767	3.46E-13	4.83E-13
TBC1D10B	2.3540856	7.73E-36	1.51E-35
RP11-347C12	Inf	1.28E-129	1.69E-128
MYLPF	1.49805447	2.65E-26	4.54E-26
ZNF48	1.30475712	0.00320554	0.00360561
1-Sep	0-Jan	1.55E-06	1.89E-06
ZNF771	1.42315175	1.21E-12	1.68E-12
DCTPP1	2.12666589	1.46E-24	2.44E-24
SEPHS2	0	1.14E-109	7.60E-109
ITGAL	4.94357977	2.44E-139	6.36E-138
RP11-297C4.	NA	NA	NA
RP11-297C4.	NA	7.21E-40	1.49E-39
RP11-297C4.	NA	9.53E-09	1.22E-08
ZNF768	2.926432	1.41E-116	1.12E-115
AC002310.11	NA	NA	NA
ZNF747	1.7183566	0.00116795	0.00133105
AC002310.13	NA	NA	NA
AC002310.12	1.24837873	3.68E-27	6.37E-27

ZNF764	2.62159533	2.98E-106	1.83E-105
ZNF688	1.69345288	5.78E-10	7.61E-10
AC002310.7	5.9922179	1.08E-21	1.72E-21
ZNF785	Inf	1.00E-152	1.93E-150
RP11-146F11	NA	NA	NA
ZNF689	1.3732166	2.83E-09	3.68E-09
PRR14	1.60505837	1.08E-11	1.47E-11
FBRS	3.95319931	2.93E-108	1.88E-107
RP11-146F11	1.94747082	4.12E-15	5.96E-15
SRCAP	1.07003891	6.27E-09	8.09E-09
RP11-2C24.9	Inf	4.73E-14	6.70E-14
TMEM265	NA	NA	NA
PHKG2	1.21206226	2.99E-83	1.18E-82
CCDC189	1.69971565	0.66099018	0.6725943
RNF40	2.9444519	1.60E-91	7.31E-91
ZNF629	1.9661965	4.61E-63	1.33E-62
RP11-2C24.7	0	0.01296754	0.01430482
BCL7C	2.23983306	4.53E-123	4.51E-122
MIR762HG	3.55787938	2.05E-130	2.83E-129
CTF1	Inf	2.26E-157	1.14E-154
FBXL19-AS1	2.99610895	5.12E-08	6.48E-08
FBXL19	2.09030857	4.38E-53	1.09E-52
ORAI3	0.79224035	4.26E-139	1.09E-137
RP11-1072A3	0.74902724	4.61E-63	1.32E-62
SETD1A	3.05852789	6.35E-130	8.52E-129
HSD3B7	0.24967575	6.91E-53	1.72E-52
STX1B	NA	0.38801892	0.40076511
STX4	1.08858625	4.81E-136	9.19E-135
RP11-196G1	0	0.67592162	0.68720971
RP11-196G1	Inf	2.27E-63	6.55E-63
ZNF668	1.74773022	0.42108363	0.43382925
AC135050.5	NA	0.00013894	0.00016228
ZNF646	2.3540856	2.59E-78	9.38E-78
PRSS53	NA	5.40E-12	7.40E-12
RP11-196G1	NA	NA	NA
VKORC1	1.87126317	6.80E-11	9.11E-11
BCKDK	1.72716869	8.63E-28	1.51E-27
KAT8	1.41482923	2.33E-49	5.50E-49
RP11-388M2	NA	NA	NA
PRSS8	NA	NA	NA
PRSS36	Inf	1.77E-64	5.20E-64
FUS	1.77842806	5.43E-05	6.42E-05
RP11-388M2	NA	NA	NA
RP11-388M2	Inf	5.57E-128	6.82E-127
PYCARD	1.39840319	1.28E-121	1.20E-120

PYCARD-AS1	3.74513619	8.84E-34	1.68E-33
TRIM72	0.74902724	3.09E-22	4.98E-22
PYDC1	NA	NA	NA
ITGAM	Inf	4.32E-106	2.63E-105
ITGAX	0	1.43E-132	2.23E-131
ITGAD	NA	NA	NA
COX6A2	NA	NA	NA
RP11-452L6.8	NA	NA	NA
ZNF843	NA	NA	NA
RP11-452L6.1	NA	4.05E-18	6.15E-18
RP11-452L6.5	NA	3.59E-154	8.68E-152
ARMC5	3.2457847	0.01716014	0.01885184
TGFB1I1	2.62159533	3.74E-94	1.80E-93
SLC5A2	NA	0.05255704	0.05667775
AC026471.6	NA	2.38E-117	1.94E-116
C16orf58	1.99740597	2.52E-07	3.14E-07
RP11-452L6.7	0	1.86E-28	3.28E-28
AHSP	NA	0.00013894	0.00016228
CTD-2014E2.	NA	NA	NA
CTD-2014E2.	NA	NA	NA
CTD-2358C21	NA	NA	NA
CTD-2358C21	NA	NA	NA
CTD-2358C21	NA	NA	NA
ZNF720	3.29571984	1.05E-36	2.07E-36
ZNF267	1.56047341	0.40508366	0.41789081
IGHV1OR16-	NA	NA	NA
IGHV1OR16-	NA	NA	NA
IGHV3OR16-	NA	NA	NA
RP11-17M15	NA	NA	NA
RP11-56L13.1	NA	NA	NA
TP53TG3D	NA	NA	NA
RP11-56L13.1	NA	NA	NA
RP11-626K17	NA	3.06E-85	1.25E-84
RP11-586K12	NA	NA	NA
RP11-586K12	NA	NA	NA
TP53TG3	NA	NA	NA
RP11-586K12	NA	NA	NA
RP11-586K12	NA	NA	NA
RP11-67H24.	NA	NA	NA
IGHV2OR16-	NA	NA	NA
RP11-989E6.	NA	NA	NA
IGHV3OR16-	NA	NA	NA
IGHV3OR16-	NA	NA	NA
IGHV1OR16-	NA	NA	NA
IGHV3OR16-	NA	NA	NA

RP11-989E6.	NA	NA	NA
IGHV1OR16-	NA	NA	NA
IGHV3OR16-	NA	NA	NA
RP11-19N8.4	NA	NA	NA
RP11-19N8.7	NA	NA	NA
RP11-1437A8	NA	NA	NA
TP53TG3C	NA	NA	NA
RP11-1437A8	NA	NA	NA
RP11-1437A8	NA	NA	NA
TP53TG3E	NA	NA	NA
TP53TG3B	NA	NA	NA
RP11-1437A8	NA	NA	NA
TP53TG3F	NA	NA	NA
RP11-23E10.	NA	NA	NA
RP11-989E6.	NA	2.01E-53	5.03E-53
RP11-23E10.	NA	NA	NA
IGHV3OR16-	NA	NA	NA
IGHV3OR16-	NA	NA	NA
RP11-812E19	NA	NA	NA
RP11-812E19	NA	NA	NA
IGHV3OR16-	NA	NA	NA
IGHV3OR16-	NA	NA	NA
RP11-812E19	NA	NA	NA
LINC00273	NA	NA	NA
CTD-2144E22	NA	NA	NA
CTD-2144E22	NA	NA	NA
CTD-2144E22	NA	NA	NA
RP11-244B22	NA	NA	NA
RP11-488I20	NA	NA	NA
RP11-244B22	NA	NA	NA
RP11-488I20	NA	NA	NA
LINC01566	NA	NA	NA
RP11-80F22.	NA	NA	NA
RP11-80F22.	NA	NA	NA
RP11-80F22.	NA	NA	NA
RP11-14K3.7	NA	NA	NA
FLJ26245	NA	NA	NA
RP11-352B15	NA	NA	NA
SHCBP1	2.06549935	0.01208442	0.01334523
RP11-46D6.1	Inf	1.65E-44	3.63E-44
VPS35	1.5359799	2.69E-25	4.55E-25
ORC6	1.6869396	9.19E-78	3.30E-77
MYLK3	NA	1.61E-79	5.93E-79
C16orf87	1.55567195	0.00960102	0.01063445
GPT2	2.41731518	4.23E-09	5.49E-09

DNAJA2	1.04718371	3.25E-58	8.68E-58
RP11-169E6.	NA	0.00043703	0.00050417
RP11-169E6.	NA	NA	NA
NETO2	1.67429618	1.68E-63	4.86E-63
ITFG1-AS1	NA	4.63E-142	1.69E-140
ITFG1	1.07672665	3.46E-116	2.73E-115
RP11-329J18	NA	9.64E-23	1.57E-22
RP11-474B1.	NA	1.13E-120	1.03E-119
PHKB	1.74098223	1.13E-79	4.19E-79
RP11-523L2(	NA	NA	NA
AC140542.2	NA	NA	NA
RP11-523L2(	NA	NA	NA
ABCC12	NA	NA	NA
ABCC11	NA	NA	NA
LONP2	3.29571984	1.27E-45	2.85E-45
SIAH1	1.52801556	4.09E-10	5.40E-10
N4BP1	2.78210117	1.00E-103	5.84E-103
RP11-44I10.3	NA	NA	NA
CTC-527H23.	NA	NA	NA
RP11-42I10.1	Inf	1.41E-26	2.43E-26
CTC-527H23.	Inf	9.88E-53	2.45E-52
CTC-527H23.	NA	NA	NA
RP11-22D3.1	NA	NA	NA
RP11-22D3.2	NA	NA	NA
CBLN1	0	3.06E-33	5.77E-33
RP11-491F9.	NA	4.58E-86	1.89E-85
RP11-491F9.	NA	NA	NA
RP11-491F9.	NA	NA	NA
RP11-491F9.	NA	NA	NA
C16orf78	NA	NA	NA
RP11-189E14	NA	NA	NA
RP11-189E14	NA	NA	NA
RP11-189E14	NA	NA	NA
ZNF423	NA	9.14E-71	2.96E-70
RP11-305A4.	NA	NA	NA
CNEP1R1	1.94747082	0.01394048	0.01536221
RP11-429P3.	NA	NA	NA
HEATR3	1.49805447	5.95E-25	1.00E-24
RP11-429P3.	NA	NA	NA
PAPD5	2.46108949	2.60E-16	3.83E-16
ADCY7	2.90248054	2.06E-63	5.96E-63
BRD7	2.51459144	1.12E-135	2.10E-134
RP11-21B23.	NA	NA	NA
RP11-21B23.	NA	NA	NA
RP11-21B23.	NA	NA	NA

NKD1	2.37191958	3.38E-47	7.73E-47
RP11-401P9.	NA	NA	NA
RP11-401P9.	NA	6.59E-30	1.19E-29
RP11-401P9.	0	1.62E-30	2.94E-30
RP11-401P9.	NA	0.08314998	0.08898696
SNX20	1.49805447	2.90E-30	5.24E-30
NOD2	NA	1.27E-87	5.38E-87
RP11-327F22	NA	NA	NA
RP11-327F22	NA	NA	NA
CYLD	3.12094682	6.11E-82	2.35E-81
RP11-327F22	NA	NA	NA
RP11-327F22	NA	NA	NA
CTD-2034I21	NA	NA	NA
CTD-2034I21	NA	NA	NA
RP11-883G14	NA	NA	NA
RP11-883G14	NA	NA	NA
RP11-883G14	NA	NA	NA
RP11-883G14	NA	NA	NA
SALL1	NA	NA	NA
AC009166.5	NA	NA	NA
RP11-437L7.	NA	NA	NA
LINC01571	NA	NA	NA
RP11-152O14	NA	NA	NA
LINC00919	NA	NA	NA
RP11-152O14	NA	NA	NA
RP11-142G1.	NA	NA	NA
RP11-142G1.	NA	NA	NA
CASC22	NA	NA	NA
TOX3	NA	NA	NA
CASC16	NA	NA	NA
RP11-297L17	NA	NA	NA
RP11-297L17	NA	NA	NA
RP11-297L17	NA	NA	NA
RP11-467J12	NA	NA	NA
RP11-295M3	NA	1.91E-119	1.67E-118
CHD9	1.60505837	6.72E-09	8.67E-09
AC007906.1	NA	NA	NA
RP11-44F14.	NA	NA	NA
RP11-44F14.	NA	NA	NA
RBL2	2.45136187	0.34520404	0.35797037
AKTIP	1.49805447	1.48E-29	2.65E-29
RPGRIP1L	2.58754864	5.30E-16	7.78E-16
FTO	3.74513619	2.84E-123	2.86E-122
RP11-357N1.	NA	NA	NA
RP11-357N1.	NA	NA	NA

RP11-357N1:	NA	NA	NA
RP11-324D1:	NA	NA	NA
RP11-324D1:	NA	NA	NA
RP11-324D1:	NA	1.90E-38	3.85E-38
IRX3	1.49469561	2.11E-117	1.73E-116
RP11-434E6.	Inf	0.00621375	0.00692587
RP11-434E6.	NA	NA	NA
RP11-1136G	NA	NA	NA
RP11-1136G	0	7.85E-31	1.43E-30
CRNDE	2.21069172	1.65E-96	8.31E-96
IRX5	3.74513619	4.26E-18	6.46E-18
CTD-3032H1:	NA	0.70090979	0.71153855
CTD-3032H1:	NA	NA	NA
RP11-26L20.:	NA	NA	NA
IRX6	NA	NA	NA
RP11-26L20.	NA	NA	NA
MMP2	1.39105058	2.44E-105	1.47E-104
RP11-212I21	NA	NA	NA
RP11-212I21	0	2.58E-134	4.43E-133
LPCAT2	0.77577821	2.04E-109	1.35E-108
RP11-212I21	2.62159533	0.00491443	0.00549711
CAPNS2	NA	NA	NA
SLC6A2	NA	NA	NA
CES1	0	1.12E-09	1.47E-09
CES5A	NA	NA	NA
RP11-461O7.	NA	NA	NA
RP11-355E1(	NA	NA	NA
CTD-2050B1:	NA	NA	NA
GNAO1	NA	1.30E-83	5.13E-83
DKFZP434H1	NA	NA	NA
RP11-441F2.	NA	NA	NA
AMFR	1.98879646	2.04E-65	6.09E-65
RP11-413H2:	Inf	2.42E-13	3.39E-13
NUDT21	1.97600519	2.15E-47	4.93E-47
OGFOD1	2.34071012	1.59E-43	3.46E-43
RP11-413H2:	NA	9.55E-52	2.33E-51
BBS2	1.49805447	5.34E-83	2.09E-82
MT4	NA	NA	NA
MT3	NA	2.44E-91	1.11E-90
MT2A	1.53366115	0.37419448	0.38714777
RP11-249C2	NA	NA	NA
MT1E	2.27276265	1.32E-139	3.58E-138
MT1M	1.95542509	8.48E-45	1.87E-44
MT1A	NA	NA	NA
MT1B	NA	5.89E-79	2.15E-78

MT1F	1.90062612	1.67E-10	2.22E-10
MT1G	NA	2.25E-32	4.19E-32
MT1H	NA	NA	NA
RP11-249C24	Inf	1.66E-33	3.13E-33
MT1X	1.82069772	0.4203348	0.43315615
RP11-343H19	NA	1.53E-68	4.80E-68
NUP93	1.31079767	1.28E-54	3.27E-54
SLC12A3	NA	NA	NA
HERPUD1	1.27851201	2.00E-135	3.68E-134
CETP	NA	3.45E-80	1.29E-79
NLRC5	2.49675746	3.59E-39	7.36E-39
RP11-322D14	NA	NA	NA
RP11-407G23	NA	NA	NA
CPNE2	2.19542466	2.22E-51	5.39E-51
FAM192A	1.28564377	1.03E-102	5.86E-102
RSPRY1	1.75633973	4.20E-14	5.96E-14
ARL2BP	1.90575284	1.03E-37	2.08E-37
RP11-407G23	NA	NA	NA
PLLP	NA	2.29E-41	4.83E-41
CCL22	NA	NA	NA
CX3CL1	NA	NA	NA
CCL17	NA	7.88E-117	6.32E-116
CIAPIN1	0.83601105	2.70E-114	2.03E-113
COQ9	1.45024423	1.10E-78	4.00E-78
POLR2C	1.53123923	2.48E-46	5.60E-46
DOK4	1.91418072	0.02457484	0.02683823
CCDC102A	1.41162826	2.62E-87	1.10E-86
ADGRG5	1.91418072	3.38E-08	4.29E-08
ADGRG1	2.62159533	0.12703653	0.1349275
RP11-405F3.	NA	NA	NA
ADGRG3	1.12354086	0.54453389	0.55772222
RP11-405F3.	NA	NA	NA
DRC7	0	0.76085812	0.76985432
KATNB1	1.7655642	0.71989555	0.72991366
KIFC3	3.5311284	2.45E-13	3.43E-13
CTD-2600O9.	1.12354086	3.08E-21	4.90E-21
CTD-2600O9.	NA	NA	NA
CNGB1	NA	NA	NA
TEPP	NA	3.13E-68	9.76E-68
ZNF319	3.37062257	2.23E-16	3.30E-16
USB1	2.25860521	2.67E-18	4.05E-18
MMP15	2.54669261	0.00632258	0.00704615
CFAP20	1.1753029	1.68E-82	6.54E-82
CTB-134F13.	NA	NA	NA
RP11-459F6.	NA	4.72E-63	1.35E-62

CSNK2A2	3.21356847	5.16E-138	1.18E-136
RP11-459F6.	NA	NA	NA
CCDC113	0.37451362	5.01E-54	1.27E-53
PRSS54	NA	NA	NA
GINS3	1.97957198	5.97E-13	8.30E-13
RP11-481J2.:	NA	NA	NA
RP11-481J2.:	5.49286641	2.81E-51	6.82E-51
RP11-481J2.:	NA	NA	NA
NDRG4	NA	NA	NA
SETD6	0.37451362	2.78E-62	7.89E-62
CNOT1	1.77122911	6.83E-40	1.41E-39
RP11-481J2.4	NA	2.36E-17	3.54E-17
AC010287.1	3.74513619	2.44E-40	5.09E-40
SLC38A7	1.8385214	0.00057511	0.00066082
GOT2	1.64785992	0.01735473	0.01906215
RP11-410D1	NA	NA	NA
RP11-700H1:	Inf	3.91E-30	7.06E-30
RP11-430C1.	NA	NA	NA
RP11-457D2(	NA	NA	NA
RP11-354I13	NA	NA	NA
RP11-354I13	NA	NA	NA
CDH8	NA	NA	NA
CTC-420A11.	NA	NA	NA
AC009110.1	NA	NA	NA
RP11-368L12	NA	NA	NA
RP11-2K6.2	NA	NA	NA
RP11-370P15	NA	NA	NA
RP11-21L1.1	NA	7.24E-120	6.43E-119
RP11-744D14	NA	NA	NA
AC012322.1	NA	NA	NA
RP11-467L24	NA	NA	NA
RP11-439I14	NA	NA	NA
CDH11	NA	NA	NA
RP11-95H3.1	NA	NA	NA
RP11-256I9.2	NA	NA	NA
RP11-256I9.3	NA	NA	NA
LINC00922	NA	NA	NA
RP11-351A2(	NA	NA	NA
RP11-351A2(	NA	NA	NA
RP11-513N24	NA	NA	NA
CDH5	NA	NA	NA
LINC00920	0.74902724	0.26388914	0.27569691
BEAN1	NA	NA	NA
BEAN1-AS1	NA	NA	NA
RP11-403P17	NA	NA	NA

TK2	1.99740597	5.90E-20	9.19E-20
RP11-403P17	NA	NA	NA
RP11-403P17	0.74902724	6.51E-49	1.53E-48
CKLF	1.69345288	1.69E-05	2.03E-05
CKLF-CMTM1	NA	0.02164078	0.0236767
RP11-403P17	NA	4.72E-129	6.09E-128
CMTM1	NA	3.20E-69	1.01E-68
CMTM2	NA	1.89E-96	9.53E-96
RP11-403P17	NA	NA	NA
CMTM3	1.82195814	6.84E-16	1.00E-15
CMTM4	4.11964981	1.96E-90	8.76E-90
DYNC1LI2	1.17347601	1.31E-105	7.90E-105
RP11-63M22	NA	0.00209533	0.00236755
RP11-63M22	NA	NA	NA
RP11-61A14.	Inf	9.57E-141	2.95E-139
CCDC79	NA	6.85E-33	1.28E-32
NAE1	1.2963933	1.89E-118	1.59E-117
RP11-61A14.	0.24967575	0.27715984	0.28909526
CA7	NA	3.74E-17	5.59E-17
PDP2	4.36932555	1.90E-47	4.37E-47
CDH16	NA	NA	NA
RRAD	NA	7.01E-72	2.30E-71
FAM96B	1.7873846	2.02E-12	2.78E-12
CES2	2.29873876	1.25E-15	1.83E-15
CES3	3.74513619	4.66E-112	3.32E-111
CES4A	NA	7.30E-81	2.75E-80
CBFB	2.07138397	6.12E-62	1.73E-61
C16orf70	1.49805447	0.03218409	0.03501342
B3GNT9	0.74902724	9.59E-20	1.49E-19
TRADD	1.71206226	1.84E-22	2.97E-22
FBXL8	2.24708171	6.29E-17	9.35E-17
RP11-5A19.5	NA	NA	NA
HSF4	0.93628405	7.78E-32	1.44E-31
NOL3	1.84375935	9.69E-26	1.65E-25
KIAA0895L	1.92607004	1.91E-17	2.87E-17
EXOC3L1	NA	1.71E-21	2.73E-21
E2F4	1.79880026	0.23527027	0.24650726
ELMO3	5.24319066	4.80E-127	5.63E-126
LRRC29	0.29961089	7.98E-98	4.12E-97
TMEM208	1.56448197	2.26E-42	4.83E-42
FHOD1	1.6728275	6.76E-33	1.27E-32
SLC9A5	NA	0.00256266	0.00288858
PLEKHG4	1.34824903	0.18777263	0.19768867
KCTD19	Inf	3.55E-80	1.32E-79
LRRC36	NA	NA	NA

TPPP3	NA	5.28E-112	3.74E-111
ZDHHC1	2.24708171	0.02862528	0.0311885
HSD11B2	1.31079767	2.22E-13	3.11E-13
RP11-297D2:	NA	NA	NA
ATP6V0D1	0.94585191	1.09E-125	1.20E-124
RP11-297D2:	0.87386511	0.04331742	0.04687811
AGRP	NA	NA	NA
CTD-2012K14	Inf	9.22E-28	1.61E-27
FAM65A	2.33030696	1.16E-53	2.91E-53
CTD-2012K14	NA	NA	NA
CTD-2012K14	NA	NA	NA
CTD-2012K14	NA	NA	NA
CTD-2012K14	1.12354086	3.56E-85	1.45E-84
CTD-2012K14	3.74513619	8.47E-84	3.37E-83
CTCF	1.83000973	0.42997879	0.44284276
AC009095.4	NA	NA	NA
RLTPR	9.36284047	3.17E-105	1.90E-104
ACD	1.34241245	2.49E-69	7.91E-69
PARD6A	1.92607004	9.81E-13	1.36E-12
ENKD1	2.40596628	8.23E-46	1.85E-45
C16orf86	1.16515348	3.74E-28	6.58E-28
GFOD2	2.0842497	1.02E-25	1.72E-25
RANBP10	2.12224384	1.10E-103	6.38E-103
TSNAXIP1	0	5.66E-39	1.16E-38
CENPT	1.43147428	9.59E-104	5.59E-103
THAP11	1.70757258	2.77E-11	3.73E-11
NUTF2	1.81997682	6.34E-10	8.34E-10
EDC4	0	2.51E-76	8.78E-76
CTC-479C5.1(	NA	4.28E-96	2.14E-95
NRN1L	NA	2.95E-25	4.97E-25
PSKH1	2.8463035	0.02245723	0.02455367
CTRL	1.49805447	0.01413753	0.01557463
CTC-479C5.1:	1.12354086	7.97E-23	1.30E-22
PSMB10	1.35934573	2.12E-124	2.22E-123
LCAT	2.99610895	3.05E-17	4.56E-17
SLC12A4	1.87256809	0.96449265	0.96630361
DPEP3	NA	NA	NA
DPEP2	0	7.32E-18	1.11E-17
DUS2	0.98735409	1.68E-112	1.21E-111
CTC-479C5.6	NA	NA	NA
DDX28	0.85999424	2.20E-61	6.17E-61
NFATC3	1.76309488	5.69E-76	1.98E-75
RP11-96D1.5	NA	NA	NA
RP11-96D1.1	NA	1.68E-137	3.62E-136
ESRP2	NA	3.97E-33	7.46E-33

PLA2G15	0.93628405	1.18E-105	7.15E-105
RP11-96D1.7	NA	NA	NA
SLC7A6	2.11490043	1.93E-05	2.30E-05
SLC7A6OS	1.73346304	0.01568242	0.01725352
PRMT7	1.3732166	1.43E-15	2.08E-15
SMPD3	0.74902724	7.73E-21	1.22E-20
RP11-71L14.4	NA	NA	NA
ZFP90	4.49416342	7.14E-06	8.63E-06
RP11-615I2.1	NA	NA	NA
RP11-615I2.6	NA	NA	NA
CDH3	NA	8.68E-93	4.07E-92
RP11-615I2.2	NA	NA	NA
CDH1	NA	NA	NA
RP11-354M1	NA	NA	NA
TANGO6	1.06112192	1.95E-41	4.14E-41
RP11-521L9.1	NA	NA	NA
HAS3	0	0.06079044	0.06542446
CHTF8	1.94102757	6.32E-39	1.29E-38
UTP4	1.56135255	2.95E-08	3.75E-08
SNTB2	21.7217899	4.51E-96	2.25E-95
RP11-343C2.	NA	NA	NA
VPS4A	1.69543327	7.57E-22	1.21E-21
COG8	1.80321372	3.03E-17	4.54E-17
PDF	NA	1.83E-07	2.29E-07
RP11-343C2.	NA	NA	NA
RP11-343C2.	NA	NA	NA
RP11-343C2.	NA	NA	NA
NIP7	1.49242269	2.46E-47	5.64E-47
TMED6	Inf	1.43E-124	1.51E-123
TERF2	1.89040208	3.56E-21	5.65E-21
CYB5B	1.17638634	1.89E-47	4.34E-47
NFAT5	2.19714656	1.30E-06	1.59E-06
NQO1	1.69444576	3.54E-05	4.20E-05
CTD-2033A16	NA	NA	NA
CTD-2033A16	Inf	0.41155695	0.42435159
NOB1	1.39023995	5.25E-56	1.36E-55
WWP2	2.14007782	3.29E-13	4.59E-13
CLEC18A	NA	3.80E-07	4.72E-07
AC009022.1	NA	NA	NA
PDPR	1.79115209	2.52E-28	4.44E-28
RP11-296I10	NA	1.97E-42	4.23E-42
CLEC18C	NA	NA	NA
AC009060.2	NA	NA	NA
AC009060.3	NA	NA	NA
EXOSC6	1.68050983	2.21E-37	4.42E-37

AARS	0.87386511	1.72E-131	2.51E-130
DDX19B	NA	3.11E-84	1.24E-83
RP11-529K1.	NA	NA	NA
DDX19A	1.79766537	5.76E-07	7.12E-07
ST3GAL2	1.42556797	0.86509687	0.87119552
RP11-529K1.	NA	1.92E-42	4.12E-42
FUK	1.04863813	1.01E-11	1.37E-11
COG4	1.49805447	1.51E-35	2.94E-35
SF3B3	2.24708171	8.89E-37	1.76E-36
IL34	NA	NA	NA
MTSS1L	1.59168288	0.00075695	0.00086674
VAC14	1.8385214	0.09621926	0.10278572
RP11-394B2.	NA	NA	NA
VAC14-AS1	NA	2.00E-92	9.33E-92
RP11-424M2	NA	NA	NA
HYDIN	NA	NA	NA
RP11-23E19.	NA	NA	NA
RP11-23E19.	NA	NA	NA
CMTR2	3.11134391	7.08E-35	1.37E-34
CTD-2520I13	NA	NA	NA
CALB2	NA	2.72E-120	2.46E-119
RP11-510M2	NA	NA	NA
ZNF23	Inf	2.25E-12	3.10E-12
AC010547.9	NA	NA	NA
RP11-510M2	0	0.10923641	0.11628041
ZNF19	0.12483787	1.32E-100	7.17E-100
RP11-510M2	NA	NA	NA
CHST4	0	2.14E-91	9.77E-91
TAT	NA	NA	NA
TAT-AS1	0.74902724	8.30E-32	1.53E-31
RP11-432I5.2	NA	NA	NA
MARVELD3	NA	1.22E-09	1.60E-09
PHLPP2	2.46108949	2.71E-38	5.49E-38
RP11-432I5.6	NA	NA	NA
RP11-432I5.8	NA	NA	NA
RP11-432I5.4	NA	NA	NA
AP1G1	2.34922179	0.8714295	0.87728061
ATXN1L	1.65856031	4.57E-57	1.20E-56
IST1	2.04280156	2.96E-35	5.75E-35
ZNF821	1.9661965	8.10E-28	1.42E-27
PKD1L3	NA	NA	NA
DHODH	1.42123117	1.48E-44	3.25E-44
TXNL4B	1.97243839	7.24E-10	9.52E-10
HP	NA	1.53E-150	2.36E-148
HPR	NA	NA	NA

RP11-384M1	NA	NA	NA
DHX38	1.61040856	0.05232761	0.0564807
PMFBP1	NA	0.13610002	0.14419942
RP11-328J14	NA	0.00021029	0.00024448
LINC01572	Inf	1.45E-114	1.10E-113
AC004158.3	NA	0.00048233	0.00055541
AC004158.1	NA	NA	NA
RP5-991G20.	5.24319066	5.51E-100	2.96E-99
RP5-991G20.	NA	NA	NA
ZFHX3	2.62159533	8.22E-88	3.51E-87
RP5-991G20.	Inf	9.17E-14	1.29E-13
RP11-346C20	NA	0.00103778	0.00118449
RP11-346C20	NA	7.48E-69	2.35E-68
HCCAT5	NA	NA	NA
C16orf47	NA	NA	NA
AC140912.1	NA	NA	NA
LINC01568	NA	NA	NA
RP11-44L9.1	NA	NA	NA
RP11-44L9.2	NA	NA	NA
RP11-44L9.3	NA	NA	NA
CTD-2009A10	NA	NA	NA
RP11-53L24.	NA	NA	NA
AC009120.4	NA	5.41E-10	7.14E-10
AC009120.10	NA	1.67E-56	4.37E-56
AC009120.11	NA	2.59E-09	3.37E-09
PSMD7	0.85701583	7.47E-132	1.11E-130
AC009120.5	NA	NA	NA
RP11-252A24	NA	1.32E-131	1.95E-130
NPIPB15	NA	1.02E-78	3.73E-78
CLEC18B	NA	NA	NA
RP11-252A24	NA	NA	NA
GLG1	1.62289235	0.96676873	0.96837005
RFWD3	4.38715953	1.14E-139	3.10E-138
MLKL	1.08192823	2.83E-39	5.80E-39
FA2H	0	1.76E-100	9.53E-100
WDR59	1.78934284	6.20E-62	1.76E-61
ZNRF1	2.46838521	2.11E-122	2.04E-121
RP11-252E2.	NA	0.00168489	0.00190938
LDHD	NA	4.71E-132	7.13E-131
RP11-252E2.	NA	NA	NA
ZFP1	3.37062257	0.00096662	0.00110438
CTRB2	NA	NA	NA
CTRB1	NA	NA	NA
BCAR1	0.49935149	2.42E-70	7.77E-70
CFDP1	1.33881246	2.67E-129	3.47E-128

RP11-77K12.	NA	NA	NA
RP11-77K12.	0	1.43E-119	1.26E-118
TMEM170A	1.67782101	0.01946336	0.02133948
CHST6	NA	NA	NA
RP11-77K12.	NA	NA	NA
RP11-77K12.	NA	NA	NA
RP11-77K12.	NA	NA	NA
CHST5	NA	0.00596164	0.00664881
TMEM231	1.12354086	5.35E-05	6.33E-05
RP11-490B18	Inf	3.98E-136	7.64E-135
GABARAPL2	1.52397237	9.63E-77	3.39E-76
RP11-490B18	NA	7.26E-41	1.52E-40
ADAT1	3.2457847	1.25E-43	2.72E-43
KARS	1.36298399	1.61E-81	6.14E-81
TERF2IP	1.01975997	3.72E-131	5.33E-130
RP11-2C15.1	NA	NA	NA
RP11-293N1	NA	NA	NA
RP11-150D5.	NA	NA	NA
CNTNAP4	Inf	9.11E-151	1.45E-148
RP11-58C22.	NA	NA	NA
RP11-58C22.	NA	NA	NA
RP11-96P7.1	NA	NA	NA
CTD-2336H1	NA	NA	NA
MON1B	2.49675746	5.79E-65	1.72E-64
SYCE1L	1.39105058	4.53E-24	7.50E-24
RP11-538I12	NA	NA	NA
RP11-538I12	NA	NA	NA
ADAMTS18	NA	NA	NA
RP11-449J10	NA	NA	NA
RP11-571O6.	NA	NA	NA
NUDT7	0.24967575	2.02E-145	1.64E-143
RP11-264M1	NA	NA	NA
VAT1L	NA	NA	NA
RP11-281J9.:	NA	NA	NA
CLEC3A	NA	NA	NA
WWOX	1.09473212	2.43E-88	1.05E-87
RP11-190D6.	NA	NA	NA
RP11-264L1.	NA	NA	NA
RP11-556H2.	NA	NA	NA
RP11-679B1	NA	NA	NA
RP11-679B1	NA	NA	NA
RP11-467I17	NA	NA	NA
MAF	NA	2.00E-44	4.40E-44
RP11-70D24.	NA	NA	NA
RP11-70D24.	NA	NA	NA

RP11-70D24.	NA	NA	NA
LINC01229	NA	NA	NA
MAFTRR	NA	NA	NA
LINC01228	NA	NA	NA
RP11-148M9	NA	NA	NA
RP11-525K1(	NA	NA	NA
RP11-525K1(	NA	NA	NA
RP11-525K1(	NA	NA	NA
DYNLRB2	NA	NA	NA
RP11-109P1	NA	NA	NA
LINC01227	NA	NA	NA
CDYL2	0.37451362	1.34E-120	1.22E-119
CTD-2055G2	NA	NA	NA
PRCAT47	NA	2.51E-107	1.58E-106
CMC2	1.90094034	3.92E-26	6.70E-26
CENPN	1.4381323	1.52E-41	3.21E-41
RP11-303E1(	Inf	1.32E-131	1.95E-130
ATMIN	1.68531128	2.79E-38	5.66E-38
C16orf46	1.49805447	1.84E-09	2.40E-09
RP11-303E1(	NA	NA	NA
RP11-303E1(	NA	NA	NA
RP11-303E1(	NA	NA	NA
GCSH	1.98721512	2.80E-70	9.01E-70
AC092718.1	NA	NA	NA
BCO1	NA	NA	NA
RP11-55K13.	NA	1.43E-24	2.39E-24
GAN	3.68751871	2.74E-46	6.18E-46
RP11-391L3.	NA	NA	NA
CMIP	3.20038911	1.61E-128	2.02E-127
RP11-960L18	0.74902724	0.3555146	0.36832538
PLCG2	1.40993362	0.04227938	0.04578752
RP11-510J16	NA	NA	NA
SDR42E1	NA	NA	NA
HSD17B2	NA	NA	NA
RP11-510J16	NA	NA	NA
MPHOSPH6	1.46702968	7.64E-35	1.48E-34
CTD-2588J6.	NA	NA	NA
RP11-481E4.	NA	NA	NA
RP11-2L4.1	NA	NA	NA
CDH13	NA	3.23E-36	6.35E-36
CTD-3253I12	NA	NA	NA
RP11-543N1	NA	NA	NA
RP11-298D2	NA	NA	NA
RP11-298D2	NA	NA	NA
RP11-298D2	NA	NA	NA

RP11-483P21	NA	6.73E-123	6.65E-122
HSBP1	1.87441907	4.47E-05	5.29E-05
MLYCD	1.05544747	1.68E-90	7.53E-90
RP11-505K9.	NA	1.35E-54	3.44E-54
RP11-483P21	NA	NA	NA
OSGIN1	1.74773022	0.00933647	0.01034711
NECAB2	NA	NA	NA
SLC38A8	NA	NA	NA
MBTPS1	2.29873876	4.21E-80	1.57E-79
RP11-505K9.	1.42315175	3.03E-25	5.11E-25
HSDL1	1.12354086	3.24E-80	1.21E-79
DNAAF1	Inf	7.55E-25	1.27E-24
TAF1C	1.95899431	0.00059816	0.00068687
ADAD2	NA	NA	NA
RP11-486L19	NA	5.56E-54	1.41E-53
KCNG4	NA	NA	NA
RP11-558A11	NA	NA	NA
WFDC1	2.99610895	7.90E-20	1.23E-19
RP11-558A11	NA	NA	NA
RP11-558A11	NA	NA	NA
ATP2C2	NA	NA	NA
RP11-517C16	NA	NA	NA
TLDC1	3.37062257	2.34E-70	7.53E-70
RP11-517C16	NA	NA	NA
COTL1	1.73761542	2.62E-29	4.68E-29
RP11-61F12.	NA	NA	NA
KLHL36	2.30058366	4.35E-16	6.40E-16
USP10	2.18466278	7.19E-13	9.99E-13
CRISPLD2	NA	5.84E-96	2.91E-95
RP11-254F19	NA	NA	NA
ZDHHC7	2.20765923	1.17E-72	3.88E-72
KIAA0513	NA	6.23E-19	9.57E-19
FAM92B	NA	NA	NA
RP11-442O1	NA	NA	NA
RP11-442O1	NA	NA	NA
CTC-786C10.	NA	4.36E-16	6.41E-16
LINC00311	2.24708171	4.95E-27	8.56E-27
RP11-680G10	NA	1.82E-65	5.45E-65
LA16c-352F7	NA	1.45E-143	7.02E-142
RP11-118F19	NA	NA	NA
CTD-2542L18	NA	1.96E-21	3.13E-21
GSE1	2.65040407	1.29E-55	3.34E-55
GINS2	1.60019455	1.18E-44	2.61E-44
C16orf74	1.74148833	5.55E-47	1.27E-46
RP11-568J23	NA	2.31E-09	3.01E-09

EMC8	1.51179809	1.54E-08	1.97E-08
RP11-568J23	NA	NA	NA
RP11-568J23	NA	NA	NA
COX4I1	1.36552174	8.34E-139	2.05E-137
IRF8	NA	NA	NA
RP11-542M1	NA	NA	NA
RP11-542M1	NA	NA	NA
RP11-805I24	Inf	1.61E-102	9.08E-102
RP11-805I24	NA	NA	NA
RP11-805I24	NA	NA	NA
LINC01082	NA	NA	NA
RP11-514D2:	NA	NA	NA
RP11-514D2:	NA	NA	NA
RP11-514D2:	NA	NA	NA
LINC00917	NA	NA	NA
RP11-158I3.:	NA	NA	NA
FENDRR	NA	NA	NA
FOXF1	NA	NA	NA
RP11-463O9.	NA	3.82E-38	7.71E-38
MTHFSD	1.22568093	3.05E-78	1.10E-77
FOXC2-AS1	NA	NA	NA
FOXC2	NA	5.79E-95	2.83E-94
FOXL1	NA	3.91E-30	7.06E-30
RP11-463O9.	NA	NA	NA
RP11-58A18.	NA	NA	NA
RP11-58A18.	NA	NA	NA
CTD-2015G9.	NA	NA	NA
CTD-2015G9.	NA	NA	NA
RP11-134D3.	NA	NA	NA
RP11-134D3.	NA	NA	NA
C16orf95	2.7464332	3.95E-56	1.03E-55
RP11-899L11	NA	NA	NA
RP11-899L11	NA	NA	NA
RP11-178L8.4	NA	NA	NA
RP11-178L8.7	0	1.04E-47	2.40E-47
RP11-178L8.8	NA	NA	NA
FBXO31	2.66320796	1.43E-40	2.98E-40
RP11-178L8.9	NA	NA	NA
MAP1LC3B	1.44291137	6.68E-19	1.03E-18
RP11-178L8.:	NA	5.28E-112	3.74E-111
ZCCHC14	1.49805447	0.00042453	0.00048982
RP11-482M8	NA	NA	NA
RP11-482M8	NA	NA	NA
JPH3	NA	3.52E-24	5.84E-24
RP11-278A2:	Inf	1.09E-111	7.66E-111

RP11-278A2:	1.49805447	1.98E-19	3.06E-19
KLHDC4	1.19439478	2.77E-58	7.41E-58
RP4-536B24.	NA	0.9070073	0.9114283
RP4-536B24.	NA	NA	NA
SLC7A5	2.79406871	1.58E-142	6.15E-141
RP4-536B24.	NA	NA	NA
CA5A	Inf	3.84E-78	1.39E-77
BANP	1.36891185	0.3453367	0.35808748
RP11-863P1:	NA	NA	NA
RP11-863P1:	NA	NA	NA
RP11-863P1:	NA	NA	NA
RP11-863P1:	NA	NA	NA
RP11-863P1:	Inf	4.57E-87	1.92E-86
RP11-863P1:	NA	NA	NA
LA16c-444G7	NA	NA	NA
LA16c-444G7	NA	NA	NA
ZNF469	Inf	3.10E-63	8.92E-63
ZFPM1	5.9922179	7.10E-48	1.64E-47
RP11-21B21.	NA	NA	NA
ZC3H18	2.8795936	1.86E-64	5.47E-64
IL17C	NA	NA	NA
CYBA	1.12278487	1.94E-144	1.14E-142
MVD	1.1797179	1.37E-78	4.99E-78
SNAI3-AS1	1.15758755	1.51E-19	2.33E-19
SNAI3	4.21327821	8.39E-36	1.64E-35
AC138028.1	NA	NA	NA
RNF166	2.10867451	0.00042041	0.00048515
RP5-1142A6.	NA	NA	NA
CTU2	1.51802853	4.15E-33	7.79E-33
RP5-1142A6.	0.74902724	7.37E-82	2.83E-81
PIEZO1	1.73555092	1.97E-08	2.51E-08
RP5-1142A6.	Inf	2.46E-12	3.38E-12
RP5-1142A6.	Inf	1.06E-154	2.70E-152
RP5-1142A6.	NA	NA	NA
RP5-1142A6.	Inf	2.98E-09	3.87E-09
CDT1	1.27244386	9.53E-95	4.64E-94
APRT	1.60554431	1.84E-117	1.51E-116
GALNS	2.33030696	5.81E-24	9.60E-24
AC092384.1	NA	NA	NA
TRAPPC2L	1.7166547	5.91E-08	7.46E-08
PABPN1L	NA	7.62E-37	1.51E-36
CBFA2T3	2.13611471	1.77E-05	2.11E-05
RP11-830F9.	NA	NA	NA
RP11-830F9.	Inf	2.88E-130	3.94E-129
CTD-2555A7.	NA	NA	NA

CTD-2555A7.	2.24708171	4.29E-09	5.56E-09
ACSF3	2.16385646	0.01484203	0.01633785
CTD-2555A7.	NA	NA	NA
LINC00304	Inf	1.86E-62	5.31E-62
RP11-46C24.	NA	NA	NA
CDH15	Inf	0.00055745	0.00064077
SLC22A31	0.74902724	9.24E-07	1.14E-06
RP11-46C24.	5.24319066	5.52E-07	6.83E-07
ZNF778	1.77893969	7.35E-29	1.30E-28
RP11-46C24.	NA	NA	NA
ANKRD11	1.50686656	1.76E-97	9.06E-97
AC137932.4	1.74773022	0.0316657	0.03445773
AC137932.5	1.12354086	4.30E-28	7.54E-28
AC137932.6	NA	2.63E-134	4.50E-133
SPG7	1.34259599	2.17E-90	9.72E-90
RP11-104N1(	Inf	5.65E-80	2.10E-79
RPL13	2.07618235	7.79E-74	2.64E-73
CPNE7	2.07855058	3.54E-07	4.40E-07
DPEP1	Inf	2.26E-57	5.98E-57
CHMP1A	1.77301384	1.37E-06	1.68E-06
SPATA33	2.17686041	2.65E-83	1.04E-82
CDK10	1.53459239	3.52E-05	4.18E-05
RP11-368I7.4	Inf	2.53E-28	4.46E-28
RP11-368I7.6	NA	4.53E-35	8.78E-35
SPATA2L	1.23054475	3.11E-16	4.58E-16
VPS9D1	Inf	9.06E-07	1.12E-06
VPS9D1-AS1	5.24319066	1.34E-70	4.32E-70
ZNF276	1.64785992	0.29022638	0.30237678
FANCA	2.5502594	1.12E-99	6.00E-99
SPIRE2	1.49805447	2.16E-56	5.62E-56
TCF25	1.48897536	4.81E-71	1.56E-70
MC1R	2.24708171	1.45E-12	2.00E-12
RP11-566K11	NA	NA	NA
RP11-566K11	NA	NA	NA
TUBB3	Inf	0.19468767	0.20487156
DEF8	1.9450546	2.90E-30	5.26E-30
CENPBD1	1.20996708	9.19E-62	2.59E-61
RP11-566K11	NA	3.74E-17	5.59E-17
DBNDD1	2.55917639	0.00185903	0.00210383
GAS8	1.49805447	0.00165882	0.00188054
GAS8-AS1	NA	NA	NA
PRDM7	NA	NA	NA
FAM157C	1.49805447	1.07E-32	2.00E-32
RP11-356C4.	3.74513619	3.02E-09	3.92E-09
RP11-356C4.	NA	NA	NA

AC137934.1	Inf	1.01E-37	2.03E-37
RP11-1228E1	NA	NA	NA
RP11-1228E1	NA	8.68E-109	5.65E-108
SCGB1C2	NA	NA	NA
DOC2B	NA	NA	NA
AC108004.2	NA	NA	NA
RPH3AL	0.65539883	1.34E-05	1.61E-05
RP11-1260E1	NA	NA	NA
RP11-1260E1	NA	NA	NA
RP11-1260E1	NA	NA	NA
AC108004.3	NA	6.12E-146	6.20E-144
C17orf97	1.7183566	7.10E-69	2.24E-68
FAM101B	1.37876495	4.52E-78	1.63E-77
RP5-1029F21	NA	NA	NA
VPS53	2.94929475	5.00E-89	2.18E-88
RP5-1029F21	NA	NA	NA
RP5-1029F21	NA	NA	NA
FAM57A	1.66888525	1.22E-07	1.53E-07
GEMIN4	2.15629053	0.00038594	0.00044589
GLOD4	1.22953528	2.50E-86	1.04E-85
RP11-676J12	Inf	1.07E-71	3.51E-71
AC087392.1	NA	NA	NA
RNMTL1	1.4381323	1.48E-29	2.65E-29
RP11-676J12	NA	NA	NA
RP11-676J12	NA	4.86E-137	1.01E-135
NXN	1.61328943	1.66E-31	3.05E-31
RP11-676J12	0	6.67E-162	5.26E-159
RP11-676J12	0	4.47E-148	5.34E-146
TIMM22	1.9661965	1.64E-06	2.01E-06
ABR	2.81634241	7.85E-111	5.40E-110
AC016292.1	NA	NA	NA
AC144836.1	NA	NA	NA
BHLHA9	NA	NA	NA
TUSC5	NA	NA	NA
YWHAE	1.48515732	8.40E-86	3.45E-85
CRK	1.94747082	1.66E-11	2.24E-11
RP11-818O2	NA	NA	NA
MYO1C	1.90137683	0.00016867	0.00019658
INPP5K	1.60505837	1.86E-12	2.57E-12
PITPNA-AS1	2.43433852	6.33E-100	3.40E-99
PITPNA	1.32586431	7.14E-31	1.30E-30
SLC43A2	2.3540856	2.03E-17	3.04E-17
SCARF1	1.79766537	0.00388502	0.00436016
RILP	2.0737531	2.63E-40	5.48E-40
PRPF8	1.83272622	3.85E-09	4.99E-09

RP11-961A1!	1.49805447	1.29E-19	2.00E-19
TLCD2	1.21716926	3.02E-14	4.30E-14
MIR22HG	0	1.44E-22	2.34E-22
WDR81	2.78210117	9.30E-12	1.27E-11
RP11-961A1!	NA	4.63E-22	7.45E-22
SERPINF2	0.74902724	0.03112672	0.03387731
SERPINF1	0.24967575	1.50E-100	8.18E-100
SMYD4	1.67429618	0.31819053	0.33080874
RPA1	2.1422179	0.18448863	0.19428761
RTN4RL1	0.99870298	6.76E-31	1.23E-30
CTD-2545H1.	NA	NA	NA
AC099684.1	NA	NA	NA
DPH1	0	6.13E-29	1.09E-28
OVCA2	NA	1.05E-125	1.16E-124
RP11-667K14	NA	6.98E-96	3.47E-95
RP11-667K14	NA	NA	NA
HIC1	6.89105058	4.05E-73	1.36E-72
SMG6	1.81014916	1.45E-28	2.57E-28
RP11-667K14	NA	NA	NA
AC090617.1	NA	NA	NA
AL450226.2	NA	NA	NA
SRR	0.51249232	1.10E-103	6.38E-103
TSR1	1.32520204	1.23E-90	5.55E-90
SGSM2	2.14007782	1.61E-10	2.15E-10
RP1-59D14.1	NA	NA	NA
RP1-59D14.5	NA	NA	NA
MNT	2.53516911	4.85E-30	8.76E-30
RP1-59D14.1	Inf	3.67E-65	1.09E-64
METTL16	3.74513619	8.24E-114	6.12E-113
PAFAH1B1	2.02962219	6.51E-40	1.35E-39
RP11-74E22.	NA	NA	NA
RP11-74E22.	NA	NA	NA
CLUH	1.59614137	7.67E-42	1.63E-41
RP11-74E22.	NA	4.42E-07	5.48E-07
RP11-74E22.	NA	7.88E-34	1.50E-33
hsa-mir-125:	NA	NA	NA
RAP1GAP2	0.95330739	0.15127498	0.1599039
CTD-3060P21	NA	NA	NA
OR1D5	NA	NA	NA
OR1D2	NA	NA	NA
OR1G1	NA	NA	NA
CTD-2309O5.	NA	NA	NA
OR1A2	NA	NA	NA
OR1A1	NA	NA	NA
OR3A2	NA	NA	NA

OR3A1	NA	NA	NA
OR1E1	NA	NA	NA
OR3A3	NA	NA	NA
OR1E2	NA	NA	NA
SPATA22	Inf	3.40E-111	2.36E-110
ASPA	NA	NA	NA
TRPV3	NA	5.95E-19	9.14E-19
TRPV1	3.74513619	1.05E-18	1.61E-18
RP11-235E17	0	0.04525679	0.04894186
SHPK	2.3540856	4.21E-77	1.49E-76
CTNS	0.83225249	3.12E-14	4.44E-14
RP11-235E17	NA	NA	NA
TAX1BP3	1.48765132	3.02E-82	1.17E-81
P2RX5-TAX1I	2.24708171	1.47E-10	1.96E-10
EMC6	2.03089905	8.71E-31	1.59E-30
P2RX5	2.52449921	0.0003149	0.00036474
ITGAE	2.16650906	2.41E-114	1.81E-113
CTD-3195I5.3	NA	NA	NA
GSG2	1.31079767	1.18E-06	1.45E-06
CTD-3195I5.4	NA	7.54E-79	2.75E-78
NCBP3	1.61328943	2.75E-08	3.50E-08
CAMKK1	5.24319066	3.51E-75	1.21E-74
P2RX1	1.12354086	3.64E-64	1.06E-63
ATP2A3	1.57689945	2.59E-37	5.18E-37
RP11-459C13	NA	NA	NA
ZZEF1	2.3540856	1.46E-83	5.79E-83
CYB5D2	2.52796693	4.26E-38	8.60E-38
ANKFY1	1.68531128	7.35E-63	2.11E-62
RP11-810M2	NA	NA	NA
RP11-104O19	0	8.67E-23	1.41E-22
UBE2G1	1.41806127	1.47E-82	5.72E-82
SPNS3	Inf	0.64414333	0.65600769
RP13-580F15	Inf	4.68E-128	5.75E-127
AC118754.4	NA	5.79E-12	7.93E-12
SPNS2	0.74902724	2.17E-36	4.27E-36
MYBBP1A	1.72144856	4.17E-61	1.17E-60
GGT6	NA	NA	NA
RP11-141J13	NA	NA	NA
SMTNL2	1.12354086	2.61E-35	5.07E-35
RP11-141J13	NA	NA	NA
ALOX15	NA	NA	NA
PELP1	1.63676322	9.85E-25	1.65E-24
AC091153.4	NA	NA	NA
RP11-314A20	0.37451362	7.18E-41	1.51E-40
ARRB2	1.6940616	1.56E-77	5.58E-77

MED11	1.80862674	0.02902636	0.03161601
RP11-314A2(	NA	NA	NA
CXCL16	0.18725681	3.16E-78	1.14E-77
ZMYND15	NA	0.000313	0.00036257
TM4SF5	NA	NA	NA
VMO1	1.31079767	0.0191146	0.0209647
GLTPD2	NA	NA	NA
PSMB6	1.39818418	1.90E-55	4.90E-55
RP11-81A22.	4.49416342	1.55E-05	1.86E-05
PLD2	8.98832685	0.08276776	0.08861972
MINK1	1.60505837	8.18E-06	9.87E-06
CHRNE	NA	NA	NA
C17orf107	NA	0.28339515	0.29544621
GP1BA	NA	2.92E-130	3.97E-129
SLC25A11	1.46486972	9.82E-41	2.06E-40
RNF167	1.907187	0.85734613	0.86353395
PFN1	1.86899179	0.08392396	0.08980467
ENO3	0.74902724	2.33E-49	5.50E-49
SPAG7	1.0646286	8.56E-144	4.34E-142
CAMTA2	0.89883268	2.07E-41	4.39E-41
RP5-1050D4.	Inf	4.50E-11	6.05E-11
RP5-1050D4.	NA	9.18E-05	1.08E-04
RP5-1050D4.	NA	2.13E-20	3.35E-20
RP5-1050D4.	NA	6.46E-28	1.13E-27
INCA1	Inf	1.96E-86	8.15E-86
KIF1C	2.30950065	5.05E-67	1.54E-66
AC109333.1(	NA	2.16E-76	7.58E-76
SLC52A1	NA	NA	NA
RP11-46I8.3	NA	NA	NA
ZFP3	NA	NA	NA
ZNF232	0.87386511	1.08E-39	2.22E-39
USP6	0.74902724	1.58E-09	2.06E-09
ZNF594	1.87256809	1.54E-05	1.84E-05
RP11-333E1.	1.24837873	0.00184437	0.00208737
SCIMP	NA	2.17E-38	4.41E-38
RP11-333E1.	NA	NA	NA
RABEP1	1.76811191	0.00032707	0.00037864
NUP88	1.12354086	2.54E-48	5.91E-48
RP11-420A6.	0	7.77E-100	4.16E-99
RPAIN	1.78254078	0.87040754	0.87630042
CTC-524C5.2	1.41365704	2.63E-15	3.81E-15
C1QBP	1.3059756	7.57E-121	6.94E-120
DHX33	2.50761293	1.26E-134	2.21E-133
CTC-524C5.5	NA	1.39E-136	2.77E-135
DERL2	1.33610264	1.14E-72	3.79E-72

MIS12	2.10303801	4.87E-30	8.78E-30
NLRP1	Inf	0.01347383	0.014857
WSCD1	2.78210117	9.96E-71	3.22E-70
AIPL1	Inf	6.61E-103	3.77E-102
FAM64A	2.12224384	2.32E-26	3.97E-26
PITPNM3	NA	7.21E-20	1.12E-19
KIAA0753	4.11964981	0.01922146	0.02108063
CTC-281F24.!	NA	1.24E-80	4.64E-80
TXNDC17	1.57699607	2.76E-34	5.28E-34
MED31	1.388646	3.47E-76	1.21E-75
C17orf100	3.74513619	2.48E-101	1.37E-100
C17orf100	NA	NA	NA
CTC-281F24.	NA	NA	NA
SLC13A5	NA	NA	NA
XAF1	NA	3.76E-87	1.58E-86
FBXO39	NA	NA	NA
TEKT1	NA	NA	NA
ALOX12-AS1	1.78339818	0.42048451	0.43328581
RP11-530N7.	NA	NA	NA
RP11-589P1(	NA	NA	NA
RP11-589P1(	0.74902724	1.55E-53	3.89E-53
ALOX12	NA	NA	NA
RNASEK	0.96303502	2.23E-27	3.87E-27
RNASEK-C17	1.49805447	1.90E-18	2.89E-18
C17orf49	1.81906615	5.61E-68	1.74E-67
MIR497HG	0	5.59E-132	8.38E-131
BCL6B	0	3.70E-41	7.79E-41
SLC16A13	0	2.19E-63	6.33E-63
SLC16A11	NA	NA	NA
CLEC10A	NA	NA	NA
ASGR2	Inf	1.31E-16	1.94E-16
ASGR1	1.49805447	3.43E-38	6.93E-38
DLG4	1.24837873	0.62178923	0.63405656
ACADVL	1.08520482	1.60E-142	6.22E-141
DVL2	1.58365759	8.91E-08	1.12E-07
PHF23	1.5584599	4.60E-62	1.31E-61
GABARAP	2.7464332	2.19E-06	2.68E-06
CTD-2545G1	NA	NA	NA
CTDNEP1	2.10570902	1.74E-94	8.42E-94
RP1-4G17.5	NA	NA	NA
ELP5	2.10592764	4.13E-132	6.29E-131
CLDN7	0.59922179	4.84E-103	2.77E-102
SLC2A4	1.74773022	1.62E-11	2.20E-11
RP1-4G17.2	NA	NA	NA
YBX2	1.49805447	3.60E-08	4.57E-08

EIF5A	1.84944997	0.22538612	0.23632846
GPS2	1.30094204	3.11E-20	4.86E-20
RP11-542C1(	NA	5.60E-91	2.53E-90
NEURL4	4.24448768	3.47E-20	5.43E-20
ACAP1	2.45136187	0.08148817	0.08729604
KCTD11	Inf	1.53E-106	9.48E-106
RP11-542C1(	NA	NA	NA
TMEM95	NA	NA	NA
TNK1	NA	NA	NA
TMEM256-PI	Inf	3.86E-05	4.58E-05
TMEM256-PI	NA	4.12E-28	7.24E-28
TMEM256	2.19528728	3.14E-42	6.71E-42
NLGN2	2.7464332	0.00035064	0.00040554
SPEM1	NA	NA	NA
C17orf74	NA	1.82E-65	5.45E-65
TMEM102	0.96303502	0.26697376	0.27885531
RP11-104H1!	NA	NA	NA
FGF11	1.31079767	3.06E-11	4.12E-11
RP11-104H1!	NA	NA	NA
CHRNB1	1.49805447	4.45E-08	5.63E-08
ZBTB4	2.03307393	1.56E-41	3.30E-41
SLC35G6	NA	0.00051496	0.00059249
POLR2A	2.01984873	1.57E-88	6.80E-88
TNFSF12	1.87256809	2.62E-17	3.92E-17
TNFSF12-TN	NA	NA	NA
RP11-186B7.	0	4.73E-16	6.95E-16
TNFSF13	Inf	3.98E-39	8.16E-39
SENP3	3.27699416	1.18E-117	9.73E-117
SENP3-EIF4A	0	6.73E-33	1.26E-32
EIF4A1	1.10616651	1.63E-104	9.60E-104
CD68	1.55353797	1.65E-34	3.16E-34
AC113189.5	2.0598249	1.88E-20	2.95E-20
MPDU1	1.50440216	5.27E-75	1.81E-74
SOX15	Inf	2.11E-133	3.43E-132
FXR2	1.60766822	5.55E-05	6.56E-05
SHBG	NA	1.79E-05	2.14E-05
SAT2	1.90767875	8.37E-05	9.84E-05
ATP1B2	NA	NA	NA
TP53	1.93795936	1.15E-31	2.12E-31
WRAP53	1.21984436	2.39E-123	2.41E-122
EFNB3	0.74902724	1.76E-61	4.94E-61
DNAH2	NA	NA	NA
KDM6B	3.5311284	2.91E-11	3.93E-11
TMEM88	0.74902724	3.86E-20	6.03E-20
NAA38	2.10823142	2.19E-96	1.10E-95

CYB5D1	1.23589494	3.00E-29	5.36E-29
CHD3	2.36300259	7.34E-129	9.38E-128
AC025335.1	Inf	1.34E-11	1.81E-11
AC104581.1	NA	NA	NA
KCNAB3	5.9922179	1.46E-57	3.87E-57
RP11-1099M	NA	NA	NA
TRAPPC1	1.50130405	7.64E-90	3.38E-89
CNTROB	3.32900994	9.47E-69	2.97E-68
GUCY2D	NA	0.00130999	0.00148956
ALOX15B	NA	1.32E-73	4.47E-73
RP11-599B1:	1.49805447	5.27E-40	1.09E-39
ALOX12B	0.56177043	2.19E-05	2.62E-05
AC129492.6	1.68531128	0.8406686	0.84753597
ALOXE3	NA	3.20E-59	8.68E-59
HES7	1.75591631	2.08E-59	5.67E-59
PER1	1.07003891	0.00016451	0.00019179
RP11-599B1:	NA	NA	NA
VAMP2	2.12591554	0.04413049	0.04774947
TMEM107	1.45863199	3.40E-48	7.87E-48
RP11-599B1:	NA	2.58E-07	3.22E-07
BORCS6	1.32782101	4.68E-87	1.96E-86
AC129492.1	NA	1.77E-88	7.66E-88
AURKB	0.90337224	2.16E-140	6.34E-139
LINC00324	Inf	2.57E-15	3.72E-15
CTC1	0.89883268	1.41E-83	5.56E-83
PFAS	1.71935798	6.12E-55	1.57E-54
RP11-849F2.	0.24967575	4.87E-12	6.67E-12
SLC25A35	2.39688716	0.06197833	0.06667522
RANGRF	1.97820014	4.96E-12	6.79E-12
ARHGEF15	NA	1.05E-10	1.40E-10
AC135178.7	NA	2.61E-26	4.46E-26
RP11-849F2.	NA	NA	NA
ODF4	NA	NA	NA
RP11-849F2.	NA	NA	NA
KRBA2	NA	4.02E-51	9.73E-51
RP11-849F2.	NA	4.21E-108	2.70E-107
RPL26	2.02820137	1.10E-63	3.20E-63
RNF222	Inf	1.42E-85	5.83E-85
NDEL1	1.34036453	1.50E-49	3.54E-49
MYH10	1.65856031	3.31E-90	1.47E-89
CCDC42	NA	5.53E-15	7.97E-15
SPDYE4	NA	NA	NA
MFSD6L	NA	NA	NA
PIK3R6	0	5.47E-100	2.94E-99
PIK3R5	1.99740597	1.88E-15	2.73E-15

CTB-41I6.1	NA	NA	NA
CTB-41I6.2	NA	NA	NA
NTN1	3.2457847	2.85E-62	8.11E-62
RP11-85B7.2	NA	NA	NA
RP11-85B7.5	NA	9.55E-18	1.44E-17
STX8	1.19704353	2.67E-128	3.32E-127
RP11-85B7.4	NA	NA	NA
AC087501.1	NA	NA	NA
RP11-565F19	NA	NA	NA
RP11-565F19	NA	NA	NA
CFAP52	NA	5.78E-19	8.88E-19
RP11-55L4.2	NA	NA	NA
USP43	Inf	3.00E-12	4.13E-12
RP11-55L4.1	NA	NA	NA
RP11-477N1	NA	NA	NA
DHRS7C	NA	NA	NA
GSG1L2	NA	NA	NA
RP11-477N1	NA	NA	NA
GLP2R	NA	NA	NA
RCVRN	NA	NA	NA
GAS7	1.49805447	2.76E-40	5.75E-40
RP11-401O9.	NA	NA	NA
MYH13	NA	NA	NA
RP11-401O9.	NA	NA	NA
RP11-799N1	NA	NA	NA
MYHAS	NA	NA	NA
MYH8	NA	NA	NA
MYH4	NA	NA	NA
MYH1	NA	NA	NA
MYH2	NA	NA	NA
CTC-297N7.7	NA	NA	NA
MYH3	0.49935149	2.66E-43	5.76E-43
CTC-297N7.1	NA	1.46E-16	2.17E-16
SCO1	1.3069761	2.49E-112	1.78E-111
CTC-297N7.1	NA	NA	NA
ADPRM	3.74513619	1.29E-35	2.52E-35
TMEM220	1.71652075	0.00674821	0.00751772
TMEM220-A	Inf	1.99E-11	2.70E-11
CTC-297N7.9	NA	NA	NA
RP11-963H4.	NA	2.29E-41	4.83E-41
LINC00675	NA	NA	NA
PIRT	NA	NA	NA
RP11-963H4.	NA	NA	NA
SHISA6	NA	NA	NA
RP11-680C21	NA	NA	NA

DNAH9	NA	NA	NA
RP11-628O1ៈ	NA	NA	NA
RP11-1096G	NA	3.62E-19	5.57E-19
ZNF18	2.24708171	0.18175628	0.19146571
MAP2K4	2.53516911	1.45E-24	2.43E-24
RP11-471L1	NA	NA	NA
LINC00670	NA	NA	NA
MYOCD	NA	NA	NA
AC005358.3	NA	NA	NA
RP11-1090M	NA	1.80E-118	1.52E-117
ARHGAP44	2.37191958	1.36E-08	1.74E-08
RP11-597M1	NA	NA	NA
RP11-597M1	NA	NA	NA
ELAC2	1.23301407	4.43E-86	1.83E-85
RP11-488L1.	NA	NA	NA
AC003664.1	NA	NA	NA
HS3ST3A1	1.99740597	6.97E-55	1.78E-54
RP11-131K5.	NA	NA	NA
AC005304.1	NA	7.65E-13	1.06E-12
RP11-131K5.	NA	2.01E-23	3.30E-23
COX10	2.31841764	3.14E-13	4.39E-13
AC005224.2	Inf	2.27E-41	4.80E-41
CDRT15	NA	NA	NA
RP11-214O1	NA	NA	NA
HS3ST3B1	1.3732166	3.98E-31	7.28E-31
RP11-214O1	Inf	0.14753464	0.15606842
RP11-214O1	1.87256809	8.13E-09	1.05E-08
AC022816.2	NA	NA	NA
AC013248.2	NA	NA	NA
AC005863.1	NA	NA	NA
AC005863.2	NA	NA	NA
AC005772.2	NA	NA	NA
CDRT7	NA	NA	NA
CDRT8	NA	NA	NA
PMP22	1.43563554	2.39E-26	4.10E-26
RP11-849N1	NA	NA	NA
AC005703.3	NA	NA	NA
AC005703.2	NA	NA	NA
TEKT3	NA	NA	NA
RP11-849N1	NA	NA	NA
CDRT4	0	9.05E-10	1.19E-09
TVP23C-CDR	NA	NA	NA
TVP23C	2.24708171	0.00091859	0.00105017
RP11-385D1	4.74383917	7.07E-111	4.87E-110
CDRT1	0	1.21E-49	2.88E-49

RP11-385D13	NA	NA	NA
TRIM16	4.66061392	7.71E-122	7.34E-121
RP11-640I15	NA	NA	NA
ZNF286A	3.86997406	2.53E-34	4.85E-34
TBC1D26	NA	NA	NA
TBC1D26	NA	NA	NA
AC005324.6	NA	NA	NA
AC005324.7	NA	NA	NA
CTD-3157E16	NA	NA	NA
CTD-3157E16	NA	NA	NA
AC015922.6	NA	2.11E-22	3.41E-22
ADORA2B	2.03901859	2.12E-58	5.67E-58
ZSWIM7	1.69779507	5.14E-21	8.15E-21
TTC19	1.9758822	2.99E-11	4.03E-11
CTC-529I10.1	NA	3.36E-30	6.07E-30
NCOR1	1.51192535	9.49E-18	1.43E-17
CTC-529I10.2	0	3.22E-76	1.13E-75
PIGL	1.83632484	6.36E-25	1.07E-24
CENPV	1.7987457	0.27397025	0.28591626
UBB	NA	3.45E-80	1.29E-79
RP11-138I1.4	NA	NA	NA
RP11-138I1.2	Inf	6.47E-48	1.49E-47
TRPV2	1.70233463	3.66E-27	6.34E-27
RP11-138I1.3	NA	2.92E-108	1.88E-107
LRRC75A	1.99027237	7.09E-115	5.41E-114
ZNF287	NA	NA	NA
ZNF624	Inf	3.18E-10	4.21E-10
RP11-92B11.	NA	NA	NA
CCDC144A	1.91418072	1.38E-17	2.08E-17
RP11-219A15	2.12224384	8.37E-41	1.75E-40
RP11-92B11.	NA	3.91E-30	7.06E-30
RP11-219A15	NA	NA	NA
RP11-219A15	NA	NA	NA
TNFRSF13B	NA	NA	NA
AC104024.1	NA	NA	NA
AC104024.2	NA	NA	NA
RP11-416I2.1	NA	NA	NA
MPRIP	1.81678947	2.29E-48	5.34E-48
MPRIP-AS1	Inf	1.40E-07	1.76E-07
RP11-45M22	NA	NA	NA
PLD6	2.50078449	0.88084139	0.88616561
RP11-45M22	NA	NA	NA
FLCN	0.98556215	1.01E-53	2.53E-53
RP11-45M22	NA	1.39E-91	6.37E-91
COPS3	1.31614786	1.78E-125	1.93E-124

NT5M	1.62289235	5.44E-06	6.59E-06
MED9	2.09727626	6.06E-05	7.15E-05
RASD1	Inf	4.14E-07	5.14E-07
PEMT	2.77639429	3.11E-109	2.05E-108
RP11-524F11	NA	NA	NA
RP11-524F11	NA	1.30E-11	1.76E-11
SMCR2	Inf	2.22E-129	2.90E-128
RAI1	3.1459144	2.81E-121	2.61E-120
RAI1-AS1	NA	NA	NA
SMCR5	2.69649805	1.63E-11	2.20E-11
SREBF1	1.89997153	0.00742064	0.00825871
TOM1L2	1.99740597	1.62E-21	2.59E-21
RP1-253P7.4	NA	4.76E-19	7.32E-19
DRC3	Inf	0.23587366	0.24711093
ATPAF2	1.75340467	1.45E-64	4.28E-64
GID4	1.70233463	1.68E-108	1.08E-107
DRG2	1.17861639	5.31E-124	5.48E-123
MYO15A	NA	7.29E-115	5.55E-114
RP11-258F1.	0.74902724	0.00607305	0.0067718
ALKBH5	1.7800412	0.00228174	0.00257498
LLGL1	2.62159533	5.68E-36	1.11E-35
FLII	2.15629053	4.09E-11	5.50E-11
MIEF2	1.99740597	3.38E-48	7.84E-48
RP1-178F10.	NA	5.77E-98	2.99E-97
TOP3A	3.29571984	1.31E-96	6.63E-96
SMCR8	2.08063121	0.00050248	0.00057828
SHMT1	0.82585054	1.36E-139	3.65E-138
EVPLL	NA	NA	NA
RP1-37N7.1	NA	NA	NA
FLJ35934	1.49805447	2.17E-14	3.10E-14
LGALS9C	NA	2.72E-103	1.57E-102
FAM106A	1.49805447	3.05E-05	3.62E-05
TBC1D28	NA	NA	NA
ZNF286B	0.42801556	7.64E-34	1.45E-33
TRIM16L	5.09338521	1.18E-117	9.68E-117
FBXW10	NA	NA	NA
TVP23B	0.62418936	8.97E-21	1.42E-20
CTD-2145A24	NA	6.96E-25	1.17E-24
PRPSAP2	1.3618677	8.47E-26	1.44E-25
CTD-2145A24	NA	NA	NA
SLC5A10	NA	2.82E-71	9.18E-71
AC090286.4	NA	NA	NA
FAM83G	1.99740597	5.13E-20	8.00E-20
GRAP	1.99740597	0.09673714	0.10332937
RP11-160E2.	NA	NA	NA

RP11-160E2.	NA	NA	NA
RP11-160E2.	0	0.55823384	0.57130244
GRAPL	NA	2.46E-98	1.29E-97
CTC-457L16.2	NA	NA	NA
AC007952.6	NA	NA	NA
RP11-160E2.	NA	NA	NA
AC007952.4	NA	NA	NA
RP11-160E2.	NA	NA	NA
EPN2	0.93628405	5.10E-08	6.45E-08
RP11-744A16	NA	NA	NA
EPN2-AS1	NA	NA	NA
RP11-135L13	Inf	2.30E-28	4.05E-28
B9D1	1.42783317	3.43E-52	8.46E-52
MAPK7	2.62159533	1.31E-113	9.72E-113
MFAP4	NA	NA	NA
RNF112	NA	NA	NA
CTB-187M2.2	NA	NA	NA
AC004448.5	NA	NA	NA
AC004448.2	NA	NA	NA
CTB-187M2.1	NA	NA	NA
SLC47A1	NA	7.34E-77	2.59E-76
RP11-1113L8	NA	NA	NA
ALDH3A2	2.24708171	4.52E-29	8.05E-29
CTD-2104P17	NA	NA	NA
SLC47A2	NA	NA	NA
RP11-311F12	NA	NA	NA
RP11-311F12	NA	NA	NA
ALDH3A1	0	8.28E-51	2.00E-50
ULK2	NA	NA	NA
RP11-78O7.3	NA	NA	NA
RP11-78O7.2	NA	1.69E-68	5.30E-68
AKAP10	2.07084001	2.73E-05	3.25E-05
RP11-209D14	2.24708171	1.80E-07	2.25E-07
SPECC1	1.77893969	5.16E-11	6.93E-11
AC004702.2	NA	1.35E-14	1.94E-14
RP11-434D2.	NA	NA	NA
LGALS9B	NA	8.68E-93	4.07E-92
RP11-434D2.	NA	NA	NA
AC008088.4	NA	1.19E-63	3.44E-63
RP11-434D2.	NA	NA	NA
RP11-434D2.	NA	NA	NA
CDRT15L2	NA	NA	NA
AC087499.10	NA	NA	NA
RP11-283C24	NA	3.33E-76	1.16E-75
CCDC144NL	0	1.07E-06	1.32E-06

RP11-344E13	NA	NA	NA
CCDC144NL-A	NA	NA	NA
RP11-381P6.	NA	NA	NA
USP22	2.70971618	8.54E-130	1.14E-128
RP11-746M1	Inf	9.67E-127	1.11E-125
LINC01563	NA	NA	NA
DHRS7B	1.20575116	1.12E-103	6.49E-103
TMEM11	1.51433768	2.71E-20	4.24E-20
AC087294.2	0.74902724	0.39084614	0.40361627
NATD1	0.74902724	0.00483567	0.00541068
MAP2K3	1.71352806	0.01089622	0.01205141
KCNJ12	Inf	2.28E-34	4.37E-34
RP11-728E14	NA	NA	NA
RP11-728E14	NA	NA	NA
RP11-822E23	NA	NA	NA
C17orf51	1.67090691	1.25E-06	1.54E-06
RP11-822E23	NA	NA	NA
KCNJ18	NA	NA	NA
RP11-1109M	NA	NA	NA
RP11-1109M	NA	NA	NA
FAM27L	NA	NA	NA
FLJ36000	NA	NA	NA
RP11-744K17	NA	NA	NA
MTRNR2L1	Inf	6.90E-44	1.50E-43
RP11-846F4.	NA	NA	NA
RP11-285M2	NA	NA	NA
RP11-285M2	NA	NA	NA
RP11-260A9.	NA	NA	NA
RP11-663N2	NA	NA	NA
WSB1	1.58793774	1.32E-12	1.82E-12
RP11-173M1	NA	0.06664489	0.07160625
KSR1	2.99610895	3.15E-67	9.65E-67
AC015688.3	NA	NA	NA
RP11-19P22.	NA	NA	NA
LGALS9	1.07786846	4.12E-140	1.17E-138
RP11-19P22.	NA	NA	NA
NOS2	NA	NA	NA
RP1-66C13.4	NA	NA	NA
RP11-138P22	Inf	9.04E-32	1.67E-31
LYRM9	1.12354086	8.80E-17	1.31E-16
RP11-218F4.	NA	NA	NA
NLK	1.66450497	4.73E-52	1.16E-51
CTD-2008P7.	NA	NA	NA
CTD-2008P7.	NA	NA	NA
CTD-2008P7.	NA	NA	NA

CTD-2008P7.	NA	NA	NA
CTD-2008P7.	NA	6.59E-30	1.19E-29
TMEM97	1.29933296	9.52E-144	4.76E-142
IFT20	1.99337894	3.17E-11	4.26E-11
TNFAIP1	2.0598249	2.58E-07	3.21E-07
POLDIP2	1.75591631	2.75E-29	4.91E-29
TMEM199	1.34308332	7.19E-120	6.40E-119
CTB-96E2.7	0	6.29E-80	2.34E-79
SEBOX	NA	NA	NA
CTB-96E2.2	NA	NA	NA
SARM1	Inf	2.99E-74	1.02E-73
VTN	NA	NA	NA
SLC46A1	NA	NA	NA
CTD-2350C19	NA	NA	NA
RP11-192H23	2.17898833	1.54E-87	6.52E-87
SLC13A2	NA	NA	NA
FOXN1	NA	NA	NA
UNC119	1.3732166	1.07E-98	5.63E-98
PIGS	1.78177691	5.94E-41	1.25E-40
ALDOC	0.85603113	9.63E-92	4.42E-91
RP11-192H23	NA	NA	NA
SPAG5	1.29377432	4.74E-13	6.60E-13
RP11-192H23	1.95579334	1.76E-10	2.33E-10
SGK494	2.69649805	0.00084395	0.00096538
KIAA0100	2.59278659	7.75E-05	9.11E-05
RP11-192H23	NA	NA	NA
SDF2	1.58013965	2.37E-41	5.01E-41
SUPT6H	2.13693065	1.38E-71	4.52E-71
AC010761.13	NA	NA	NA
PROCA1	1.34824903	0.00140491	0.00159588
RAB34	Inf	1.51E-122	1.48E-121
RPL23A	2.21621028	1.18E-133	1.95E-132
AC010761.8	1.24837873	1.50E-49	3.55E-49
TLCD1	1.61040856	9.59E-17	1.42E-16
NEK8	1.92607004	6.25E-22	1.00E-21
AC010761.14	NA	NA	NA
AC010761.6	NA	NA	NA
TRAF4	1.94312863	0.13149267	0.13951343
AC010761.9	NA	NA	NA
AC010761.10	NA	1.75E-135	3.23E-134
FAM222B	10.4863813	4.16E-73	1.39E-72
ERAL1	1.46181122	9.63E-89	4.19E-88
RP11-20B24.	NA	NA	NA
FLOT2	1.84046693	8.77E-19	1.34E-18
RP11-20B24.	NA	NA	NA

RP11-20B24.	NA	4.05E-06	4.92E-06
DHRS13	1.89040208	1.16E-19	1.80E-19
PHF12	1.53747696	5.37E-51	1.30E-50
RP11-20B24.	NA	0.00013894	0.00016228
RP11-20B24.	NA	NA	NA
PIPOX	NA	1.14E-135	2.14E-134
SEZ6	1.49805447	8.05E-51	1.94E-50
RP11-321A17	NA	NA	NA
RP11-321A17	0	0.62448014	0.6365501
MYO18A	Inf	0.00077846	0.00089097
TIAF1	Inf	5.64E-146	5.81E-144
RP11-321A17	NA	NA	NA
CRYBA1	NA	NA	NA
NUFIP2	1.49805447	0.0001134	0.00013278
RP11-296K13	0	4.22E-46	9.52E-46
RP11-802D6.	NA	2.00E-35	3.89E-35
TAOK1	2.65904669	4.16E-117	3.37E-116
ABHD15	2.45136187	4.29E-74	1.46E-73
ABHD15-AS1	NA	1.03E-130	1.44E-129
TP53I13	1.85136921	0.00014931	0.00017425
GIT1	2.62159533	3.58E-118	2.99E-117
ANKRD13B	2.59278659	2.27E-16	3.35E-16
RP11-68I3.7	0.74902724	1.97E-35	3.83E-35
CORO6	1.12354086	1.49E-20	2.34E-20
SSH2	1.92607004	9.46E-06	1.14E-05
RP11-68I3.5	NA	3.00E-35	5.82E-35
RP11-82O19.	NA	1.75E-05	2.10E-05
RP11-338L22	NA	1.62E-116	1.29E-115
EFCAB5	1.49805447	0.00012351	0.00014451
AC104984.4	NA	NA	NA
RP11-1148O4	NA	NA	NA
NSRP1	1.36805801	1.12E-49	2.64E-49
RP11-354P11	5.24319066	2.87E-25	4.84E-25
RP11-354P11	NA	1.62E-79	5.97E-79
SLC6A4	NA	NA	NA
RP11-354P11	NA	NA	NA
RP11-354P11	NA	NA	NA
BLMH	1.52261274	0.01134163	0.01253793
TMIGD1	NA	NA	NA
CPD	1.85628489	2.46E-06	3.01E-06
GOSR1	2.02368762	7.77E-22	1.24E-21
RP11-218M1	NA	NA	NA
TBC1D29	NA	7.43E-45	1.64E-44
RP11-218M1	NA	NA	NA
RP11-218M1	NA	5.67E-112	4.01E-111

CTD-2349P21	NA	NA	NA
CTD-2349P21	NA	9.67E-127	1.11E-125
CRLF3	2.24708171	0.60302732	0.61572048
CTD-2349P21	1.87256809	4.73E-07	5.86E-07
CTD-2349P21	NA	3.20E-37	6.38E-37
ATAD5	1.18115834	2.54E-88	1.10E-87
CTD-2349P21	NA	NA	NA
RP13-753N3.	NA	4.00E-105	2.40E-104
TEFM	0.93628405	5.80E-138	1.31E-136
RP13-753N3.	3.74513619	6.39E-89	2.79E-88
ADAP2	1.85473411	1.22E-66	3.71E-66
AC091177.1	NA	NA	NA
RP11-848P1.	NA	8.62E-70	2.75E-69
RNF135	1.38882134	1.12E-30	2.03E-30
RP11-848P1.	Inf	5.50E-145	3.61E-143
RP11-848P1.	0.74902724	3.46E-13	4.83E-13
NF1	1.91418072	2.55E-05	3.03E-05
OMG	Inf	2.46E-98	1.29E-97
EVI2B	1.12354086	3.69E-96	1.85E-95
CTD-2370N5.	NA	NA	NA
EVI2A	1.02991245	1.49E-85	6.09E-85
RAB11FIP4	2.24708171	8.01E-06	9.66E-06
RP1-41C23.4	NA	NA	NA
AC003101.1	NA	NA	NA
RP1-41C23.1	NA	NA	NA
RP11-805L22	NA	NA	NA
CTC-542B22.	NA	NA	NA
COPRS	1.75529615	0.01931523	0.02118219
UTP6	0.80943266	3.47E-126	3.92E-125
CTC-542B22.	NA	NA	NA
SUZ12	1.99056553	3.80E-82	1.47E-81
LRRC37B	1.79115209	4.90E-43	1.06E-42
CTD-2095E4.	1.49805447	0.01587351	0.01745951
AC090616.2	3.74513619	1.55E-07	1.94E-07
RHOT1	2.28869434	1.87E-44	4.12E-44
RHBDL3	NA	9.59E-07	1.18E-06
RP11-443G1	NA	NA	NA
C17orf75	0.67412451	5.31E-52	1.30E-51
RP11-227G1	NA	NA	NA
ZNF207	1.66450497	2.76E-27	4.80E-27
RP11-227G1	0	1.29E-43	2.81E-43
RP11-227G1	NA	2.11E-107	1.33E-106
RP11-227G1	0.74902724	4.63E-47	1.06E-46
RP11-227G1	NA	1.50E-75	5.20E-75
RP11-227G1	NA	NA	NA

PSMD11	1.24837873	1.80E-112	1.30E-111
CDK5R1	1.99740597	7.22E-14	1.02E-13
MYO1D	2.82965845	1.16E-44	2.55E-44
RP11-466A1!	NA	NA	NA
RP11-466A1!	NA	NA	NA
RP11-220C2.	NA	NA	NA
AC084809.2	Inf	1.04E-14	1.49E-14
AC084809.3	Inf	6.41E-87	2.69E-86
TMEM98	2.22317659	2.27E-57	6.01E-57
SPACA3	NA	5.28E-112	3.74E-111
ASIC2	Inf	4.08E-37	8.12E-37
RP11-40A13.	NA	NA	NA
RP11-31I22.4	NA	NA	NA
RP11-31I22.3	NA	NA	NA
RP11-31I22.2	NA	NA	NA
RP11-118G2:	NA	NA	NA
RP11-118G2:	NA	NA	NA
RP11-17M24	NA	NA	NA
CTC-304I17.2	NA	NA	NA
CTC-304I17.5	NA	NA	NA
RP11-215E1:	NA	NA	NA
RP11-215E1:	NA	NA	NA
CCL2	Inf	5.81E-138	1.31E-136
CCL7	NA	NA	NA
CCL11	NA	NA	NA
CCL8	NA	NA	NA
CCL13	NA	NA	NA
CCL1	NA	NA	NA
AC011193.1	NA	NA	NA
C17orf102	NA	NA	NA
TMEM132E	NA	2.32E-33	4.37E-33
RP11-212E8.	NA	NA	NA
RP11-642M2	NA	NA	NA
CCT6B	Inf	0.12313831	0.13087143
ZNF830	1.49805447	1.61E-95	7.97E-95
LIG3	1.69516691	0.03400163	0.03693316
RFFL	5.61770428	2.78E-05	3.31E-05
RAD51L3-RF	NA	NA	NA
RP5-837J1.6	NA	0.27339749	0.28535135
RP5-837J1.4	Inf	5.37E-38	1.08E-37
RAD51D	2.04280156	3.36E-99	1.78E-98
FNDC8	0	1.75E-135	3.23E-134
NLE1	1.75553259	2.71E-36	5.34E-36
UNC45B	NA	NA	NA
RP11-799D4.	NA	NA	NA

SLC35G3	NA	NA	NA
RP11-799D4.	NA	NA	NA
SLFN5	Inf	1.15E-08	1.47E-08
RP11-47L3.1	NA	NA	NA
SLFN11	3.22081712	8.84E-84	3.51E-83
RP11-686D2.	NA	3.35E-81	1.27E-80
RP11-686D2.	NA	NA	NA
SLFN12	NA	6.09E-45	1.35E-44
SLFN13	Inf	1.13E-110	7.76E-110
SLFN12L	3.74513619	2.02E-143	9.50E-142
RP11-686D2.	NA	NA	NA
RP11-1094M	NA	NA	NA
SLFN14	NA	9.03E-47	2.05E-46
RP11-1094M	NA	7.20E-126	8.01E-125
RP11-1094M	NA	NA	NA
RP11-1094M	2.99610895	1.37E-131	2.01E-130
PEX12	0.59922179	1.20E-24	2.01E-24
AP2B1	3.89494163	2.88E-144	1.63E-142
TAF15	1.71329219	3.79E-06	4.61E-06
RASL10B	3.1459144	6.59E-139	1.65E-137
GAS2L2	NA	5.79E-45	1.28E-44
MMP28	Inf	2.11E-61	5.93E-61
C17orf50	Inf	5.78E-05	6.82E-05
AC015849.12	NA	NA	NA
AC015849.19	NA	1.11E-12	1.53E-12
HEATR9	0	3.84E-125	4.13E-124
AC015849.2	NA	1.44E-126	1.65E-125
CCL5	7.49027237	0.81035034	0.81801557
AC015849.16	Inf	2.17E-110	1.48E-109
RDM1	1.34824903	3.09E-13	4.32E-13
LYZL6	NA	NA	NA
CCL16	NA	NA	NA
CCL14	NA	NA	NA
CTB-186H2.3	NA	NA	NA
CCL15-CCL14	NA	NA	NA
CCL15	NA	NA	NA
RP11-104J23	NA	NA	NA
RP11-104J23	NA	NA	NA
CCL23	NA	7.97E-25	1.34E-24
CCL18	NA	NA	NA
AC069363.1	NA	2.07E-112	1.48E-111
CCL3	NA	NA	NA
CCL4	NA	NA	NA
CTB-91J4.1	NA	NA	NA
TBC1D3B	NA	NA	NA

AC131056.3	NA	NA	NA
CCL3L3	NA	8.59E-86	3.53E-85
CCL4L2	NA	8.12E-113	5.91E-112
AC131056.5	NA	NA	NA
TBC1D3I	NA	NA	NA
TBC1D3G	NA	NA	NA
TBC1D3H	NA	NA	NA
TBC1D3F	NA	NA	NA
ZNHIT3	1.47868308	4.88E-39	9.99E-39
MYO19	1.95282101	3.87E-56	1.01E-55
PIGW	2.15345331	0.10066015	0.10740319
GGNBP2	1.61508998	3.70E-51	8.96E-51
DHRS11	1.35672858	2.99E-43	6.46E-43
MRM1	1.23369192	1.11E-109	7.39E-109
CTB-75G16.3	NA	1.64E-117	1.35E-116
CTD-3194G1	NA	NA	NA
CTD-3194G1	NA	2.00E-89	8.78E-89
RP11-445F12	1.24837873	1.06E-40	2.22E-40
LHX1	0.74902724	2.05E-43	4.44E-43
RP11-445F12	NA	NA	NA
AATF	1.11660542	1.23E-105	7.43E-105
CTC-268N12.	NA	NA	NA
ACACA	1.94192247	9.22E-26	1.57E-25
C17orf78	NA	1.16E-32	2.17E-32
TADA2A	2.12224384	3.59E-07	4.47E-07
RP11-378E13	NA	NA	NA
DUSP14	1.84046693	2.92E-11	3.94E-11
SYNRG	1.79766537	3.91E-24	6.49E-24
RP11-697E22	NA	NA	NA
DDX52	1.51033361	4.94E-59	1.33E-58
RP11-697E22	NA	NA	NA
RP11-697E22	NA	NA	NA
HNF1B	NA	NA	NA
AC091199.1	NA	NA	NA
TBC1D3K	NA	NA	NA
TBC1D3L	NA	7.22E-27	1.24E-26
TBC1D3C	NA	NA	NA
TBC1D3D	NA	NA	NA
TBC1D3E	NA	NA	NA
TBC1D3	NA	NA	NA
MRPL45	1.15920882	2.36E-106	1.45E-105
GPR179	NA	NA	NA
SOCS7	1.77893969	8.24E-08	1.04E-07
ARHGAP23	2.43433852	0.78051322	0.78903771
SRCIN1	0.74902724	0.00021654	0.0002517

CTB-58E17.2	NA	NA	NA
C17orf96	2.45680934	1.39E-18	2.12E-18
CTB-58E17.1	1.3732166	3.48E-07	4.32E-07
CTB-58E17.3	Inf	2.53E-15	3.67E-15
MLLT6	3.25664016	6.36E-119	5.45E-118
CTB-58E17.9	0	5.84E-97	2.97E-96
AC006449.2	NA	NA	NA
CISD3	1.97153283	1.49E-44	3.29E-44
PCGF2	3.2604715	3.37E-77	1.20E-76
CTB-58E17.5	NA	NA	NA
PSMB3	1.11523107	1.00E-142	4.16E-141
PIP4K2B	2.39688716	3.71E-90	1.65E-89
CWC25	1.46748193	4.35E-46	9.79E-46
C17orf98	NA	1.75E-60	4.84E-60
RPL23	2.10380429	7.44E-142	2.69E-140
LASP1	2.32456729	2.35E-10	3.11E-10
RP1-56K13.2	NA	NA	NA
RP1-56K13.3	NA	NA	NA
LINC00672	NA	4.38E-14	6.22E-14
FBXO47	NA	NA	NA
RP1-56K13.5	NA	NA	NA
CTD-2206N4.	NA	NA	NA
PLXDC1	NA	9.17E-78	3.29E-77
ARL5C	NA	NA	NA
RP5-906A24.	Inf	1.45E-61	4.07E-61
CACNB1	4.49416342	3.68E-55	9.44E-55
RPL19	1.99151559	1.36E-58	3.66E-58
STAC2	NA	2.80E-25	4.73E-25
FBXL20	1.03437095	3.35E-13	4.67E-13
CTB-131K11.	Inf	3.48E-118	2.91E-117
MED1	1.87256809	1.98E-121	1.85E-120
CDK12	1.93713941	1.47E-08	1.88E-08
RP11-390P2	2.24708171	4.11E-55	1.05E-54
NEUROD2	NA	1.63E-24	2.71E-24
AC087491.2	NA	NA	NA
PPP1R1B	NA	NA	NA
STARD3	1.84375935	0.46219516	0.47518671
TCAP	1.49805447	0.00960056	0.01063445
PNMT	NA	NA	NA
PGAP3	0.93628405	6.78E-05	7.99E-05
ERBB2	2.24708171	3.93E-13	5.48E-13
MIEN1	2.05080048	2.79E-33	5.26E-33
GRB7	NA	NA	NA
IKZF3	NA	0.27537372	0.28731482
ZPBP2	NA	NA	NA

GSDMB	1.49805447	0.02697076	0.0294141
ORMDL3	2.09727626	0.05569261	0.06001279
RP11-387H17	0	5.59E-72	1.84E-71
LRRC3C	NA	NA	NA
GSDMA	0	3.63E-156	1.27E-153
PSMD3	1.38000127	2.28E-75	7.90E-75
RP11-387H17	NA	NA	NA
CSF3	NA	NA	NA
MED24	3.05372643	1.61E-49	3.81E-49
THRA	2.47178988	5.25E-78	1.89E-77
NR1D1	2.24708171	3.03E-16	4.46E-16
MSL1	2.0598249	2.69E-56	7.01E-56
CASC3	2.70184825	2.94E-49	6.91E-49
RAPGEFL1	Inf	1.48E-92	6.94E-92
WIPF2	2.24708171	4.36E-15	6.30E-15
CDC6	2.50455982	1.85E-34	3.54E-34
RARA	3.2604715	7.87E-74	2.66E-73
RARA-AS1	NA	NA	NA
CTD-2267D19	Inf	1.06E-154	2.70E-152
GJD3	NA	8.17E-144	4.16E-142
TOP2A	1.66668364	2.26E-53	5.68E-53
IGFBP4	2.26977951	5.53E-10	7.28E-10
TNS4	NA	NA	NA
RP5-1028K7.	Inf	2.84E-87	1.20E-86
CCR7	Inf	5.59E-39	1.14E-38
SMARCE1	2.07230869	1.29E-06	1.58E-06
RP5-1028K7.	NA	NA	NA
RP11-458J1.1	Inf	7.44E-42	1.58E-41
KRT222	NA	NA	NA
KRT24	NA	NA	NA
KRT25	NA	NA	NA
KRT26	NA	NA	NA
KRT27	NA	NA	NA
KRT28	NA	NA	NA
RP11-605F20	NA	NA	NA
KRT10	2.63487596	1.11E-142	4.55E-141
TMEM99	2.14721141	1.12E-65	3.37E-65
RP5-1110E20	NA	NA	NA
KRT12	NA	NA	NA
KRT20	NA	NA	NA
AC004231.2	NA	NA	NA
KRT23	NA	NA	NA
KRT39	NA	NA	NA
KRT40	NA	NA	NA
KRTAP3-3	NA	NA	NA

KRTAP3-2	NA	NA	NA
KRTAP3-1	NA	NA	NA
KRTAP1-5	NA	NA	NA
KRTAP1-4	NA	NA	NA
KRTAP1-3	NA	NA	NA
KRTAP1-1	NA	NA	NA
KRTAP2-1	NA	NA	NA
KRTAP2-2	NA	NA	NA
KRTAP2-3	NA	NA	NA
KRTAP2-4	NA	NA	NA
KRTAP4-7	NA	NA	NA
KRTAP4-8	NA	NA	NA
KRTAP4-16P	NA	NA	NA
KRTAP4-9	NA	NA	NA
KRTAP4-11	NA	NA	NA
KRTAP4-12	NA	NA	NA
KRTAP4-6	NA	NA	NA
KRTAP4-5	NA	NA	NA
KRTAP4-4	NA	NA	NA
KRTAP4-3	NA	NA	NA
KRTAP4-2	NA	NA	NA
KRTAP4-1	NA	NA	NA
KRTAP9-1	NA	NA	NA
KRTAP9-2	NA	NA	NA
KRTAP9-9	NA	NA	NA
KRTAP9-3	NA	NA	NA
KRTAP9-8	NA	NA	NA
KRTAP9-4	NA	NA	NA
KRTAP9-6	NA	NA	NA
KRTAP9-7	NA	NA	NA
KRTAP29-1	NA	NA	NA
KRTAP16-1	NA	NA	NA
KRTAP17-1	NA	NA	NA
KRT33A	NA	NA	NA
KRT33B	NA	NA	NA
KRT34	NA	NA	NA
KRT31	NA	NA	NA
AC003958.2	NA	NA	NA
KRT37	NA	NA	NA
KRT38	NA	NA	NA
KRT32	NA	NA	NA
KRT35	NA	NA	NA
KRT36	NA	NA	NA
KRT13	NA	NA	NA
AC019349.5	NA	NA	NA

KRT15	NA	NA	NA
KRT19	NA	8.68E-41	1.82E-40
LINC00974	NA	NA	NA
KRT9	NA	NA	NA
KRT14	NA	NA	NA
KRT16	NA	NA	NA
KRT17	NA	NA	NA
EIF1	1.52973539	7.04E-101	3.85E-100
GAST	NA	NA	NA
HAP1	0.74902724	8.97E-05	1.05E-04
JUP	2.07084001	7.90E-25	1.32E-24
P3H4	1.7655642	8.53E-07	1.05E-06
FKBP10	2.54017933	6.36E-21	1.01E-20
NT5C3B	2.08745296	2.81E-08	3.58E-08
KLHL10	NA	4.41E-23	7.20E-23
KLHL11	Inf	0.93272588	0.93587262
ACLY	1.96509499	3.31E-33	6.23E-33
RP11-229E13	NA	1.35E-14	1.94E-14
TTC25	NA	9.46E-46	2.12E-45
CNP	1.28404669	5.99E-140	1.68E-138
DNAJC7	1.25032173	3.93E-108	2.52E-107
NKIRAS2	1.97470817	4.30E-09	5.57E-09
ZNF385C	0	9.82E-14	1.38E-13
DHX58	0	3.59E-87	1.51E-86
KAT2A	1.92317804	4.66E-06	5.65E-06
CTD-2132N1	NA	NA	NA
HSPB9	Inf	6.59E-19	1.01E-18
RAB5C	1.35553344	1.41E-87	5.98E-87
RP11-358B2	Inf	1.25E-136	2.53E-135
KCNH4	NA	NA	NA
HCRT	0.74902724	0.1323604	0.14041766
GHDC	2.46108949	0.23918427	0.25052139
STAT5B	1.45863199	0.03266753	0.03553297
RP11-358B2	NA	NA	NA
STAT5A	1.95579334	6.03E-09	7.79E-09
STAT3	2.01661179	8.70E-09	1.12E-08
PTRF	1.49805447	1.68E-25	2.84E-25
ATP6V0A1	2.87127108	1.48E-09	1.94E-09
NAGLU	0.92736706	1.88E-71	6.13E-71
HSD17B1	1.64785992	2.62E-10	3.47E-10
RP11-400F1	4.11964981	1.18E-60	3.28E-60
COASY	1.60746295	4.88E-100	2.63E-99
MLX	1.56262579	4.38E-05	5.19E-05
PSMC3IP	1.08192823	6.83E-45	1.51E-44
FAM134C	2.11089494	0.03426515	0.03721494

TUBG1	1.1180333	4.13E-103	2.37E-102
TUBG2	NA	NA	NA
PLEKHH3	2.40758755	6.71E-22	1.08E-21
CCR10	1.08192823	2.25E-76	7.88E-76
CTD-3193K9.	2.12224384	5.73E-14	8.10E-14
CNTNAP1	0.74902724	1.30E-08	1.67E-08
CTD-3193K9.	NA	NA	NA
EZH1	2.80885214	1.51E-09	1.97E-09
CTD-3193K9.	NA	NA	NA
RAMP2-AS1	NA	1.46E-52	3.61E-52
RAMP2	Inf	3.39E-13	4.73E-13
VPS25	1.4227333	7.85E-91	3.54E-90
WNK4	0	1.46E-36	2.88E-36
COA3	1.9051345	0.00102261	0.00116732
CNTD1	Inf	7.46E-40	1.54E-39
BECN1	2.14721141	2.21E-20	3.46E-20
PSME3	1.57203247	5.84E-17	8.69E-17
AOC2	NA	0.11036839	0.11745781
AOC3	1.49805447	7.24E-56	1.88E-55
LINC00671	NA	NA	NA
G6PC	NA	NA	NA
PTGES3L-AAI	NA	NA	NA
AARSD1	3.27075227	6.21E-87	2.60E-86
PTGES3L	0	1.29E-26	2.23E-26
RUNDC1	1.14557107	2.56E-10	3.40E-10
AC055866.1	NA	NA	NA
RPL27	2.13239562	1.35E-139	3.64E-138
IFI35	1.07003891	7.04E-106	4.28E-105
VAT1	1.82195814	7.49E-09	9.65E-09
RND2	0	2.91E-38	5.88E-38
BRCA1	2.68519198	3.19E-131	4.62E-130
RP11-242D8.	3.91158668	1.51E-122	1.47E-121
NBR1	2.41353221	9.27E-08	1.17E-07
TMEM106A	5.05593385	4.28E-53	1.07E-52
LINC00910	Inf	1.10E-112	7.93E-112
ARL4D	1.3618677	4.28E-47	9.78E-47
MIR2117HG	NA	NA	NA
DHX8	3.99481193	4.34E-118	3.62E-117
ETV4	1.76122621	4.74E-08	6.00E-08
RP11-392O1.	NA	NA	NA
MEOX1	NA	NA	NA
CTC-501O10.	NA	NA	NA
SOST	NA	NA	NA
DUSP3	1.57689945	0.00053404	0.00061409
C17orf105	NA	NA	NA

RP5-905N1.2	NA	NA	NA
MPP3	1.49805447	0.23722157	0.24849435
CD300LG	NA	NA	NA
MPP2	1.49805447	0.00014546	0.0001698
FAM215A	NA	NA	NA
RP11-527L4.(	NA	NA	NA
RP11-527L4.:	0.83225249	3.19E-75	1.10E-74
PPY	NA	NA	NA
PYY	NA	NA	NA
NAGS	NA	1.83E-147	2.08E-145
TMEM101	0.99166986	3.86E-127	4.55E-126
LSM12	2.031844	1.08E-69	3.45E-69
G6PC3	1.09143969	1.93E-103	1.11E-102
HDAC5	2.0130107	0.61082902	0.62323042
RP11-546M2	NA	2.12E-14	3.02E-14
C17orf53	1.87256809	1.18E-73	3.98E-73
ASB16	Inf	9.47E-101	5.18E-100
ASB16-AS1	4.11964981	3.08E-57	8.13E-57
TMUB2	2.16823674	0.00397904	0.00446347
ATXN7L3	2.14007782	8.37E-27	1.44E-26
CTB-175E5.7	NA	NA	NA
UBTF	2.12495771	1.59E-28	2.81E-28
RP5-882C2.2	1.74773022	2.40E-12	3.31E-12
SLC4A1	NA	5.86E-11	7.85E-11
RUNDC3A-AS	0.37451362	1.15E-52	2.85E-52
RUNDC3A	NA	3.60E-30	6.50E-30
SLC25A39	2.09237465	3.06E-28	5.38E-28
RP5-1067M6	NA	NA	NA
GRN	0.7891537	1.81E-145	1.58E-143
FAM171A2	4.04474708	2.00E-66	6.06E-66
ITGA2B	Inf	5.91E-17	8.79E-17
GPATCH8	1.85285685	1.25E-51	3.05E-51
FZD2	1.49805447	1.40E-16	2.08E-16
LINC01180	NA	NA	NA
MEIOC	1.68531128	0.01232635	0.01360827
CCDC43	1.19844358	2.52E-54	6.41E-54
RP11-1072C1	NA	0.44219927	0.45514463
DBF4B	2.56809339	0.58114036	0.59414192
ADAM11	2.66320796	0.00119377	0.00135979
CTC-296K1.3	NA	NA	NA
CTC-296K1.4	NA	NA	NA
GJC1	3.59533074	1.80E-56	4.69E-56
HIGD1B	0	3.38E-72	1.12E-71
EFTUD2	2.19033722	2.21E-12	3.05E-12
CCDC103	NA	NA	NA

FAM187A	NA	NA	NA
AC015936.3	NA	NA	NA
GFAP	Inf	4.72E-63	1.35E-62
KIF18B	2.01661179	3.31E-13	4.62E-13
CTD-2534I21	NA	2.61E-94	1.26E-93
C1QL1	2.24708171	1.86E-05	2.22E-05
CTD-2534I21	NA	NA	NA
DCAKD	1.44455253	5.11E-19	7.85E-19
NMT1	1.73774319	5.46E-26	9.31E-26
PLCD3	1.67090691	4.09E-24	6.79E-24
ACBD4	0.85603113	1.36E-21	2.17E-21
AC142472.6	2.62159533	0.54109253	0.55441662
HEXIM1	1.05418648	1.49E-123	1.52E-122
AC002117.1	Inf	1.19E-25	2.02E-25
HEXIM2	1.34824903	1.09E-56	2.84E-56
RP13-890H1	NA	5.69E-85	2.31E-84
CTD-2020K1	1.99740597	0.00021798	0.00025334
FMNL1	2.11089494	3.75E-32	6.95E-32
CTD-2020K1	NA	2.41E-74	8.22E-74
CTD-2020K1	NA	NA	NA
MAP3K14-AS	NA	1.64E-09	2.14E-09
SPATA32	NA	1.27E-08	1.63E-08
MAP3K14	0.74902724	3.43E-23	5.61E-23
CTB-39G8.2	NA	NA	NA
ARHGAP27	2.2262754	1.47E-11	2.00E-11
CTB-39G8.3	NA	NA	NA
PLEKHM1	1.49805447	0.00846428	0.00939658
AC091132.1	NA	NA	NA
RP11-798G7	NA	2.25E-110	1.52E-109
RP11-798G7	NA	7.65E-06	9.24E-06
RP11-798G7	NA	3.05E-52	7.53E-52
CRHR1	1.49805447	2.31E-28	4.07E-28
RP11-293E1	NA	NA	NA
RP11-293E1	NA	NA	NA
CRHR1	NA	NA	NA
MAPT-AS1	NA	NA	NA
SPPL2C	NA	NA	NA
MAPT	1.87256809	1.14E-05	1.37E-05
RP11-669E1	NA	NA	NA
STH	NA	NA	NA
KANSL1	5.8257674	6.01E-93	2.83E-92
RP11-669E1	NA	NA	NA
KANSL1-AS1	1.12354086	1.02E-17	1.54E-17
ARL17B	0.93628405	0.17047826	0.17986721
LRRC37A	NA	1.75E-05	2.10E-05

LRRC37A2	0	1.35E-135	2.52E-134
ARL17A	1.87256809	0.4998303	0.51309462
FAM215B	1.93713941	0.01813981	0.01990398
NSF	2.43433852	0.67933699	0.69048864
WNT3	Inf	1.87E-17	2.81E-17
WNT9B	NA	NA	NA
RP11-63A1.2	NA	NA	NA
RP11-63A1.1	Inf	6.21E-24	1.03E-23
GOSR2	1.85136921	7.02E-07	8.67E-07
RP11-156P1.	NA	NA	NA
RPRML	0.74902724	1.62E-49	3.84E-49
CDC27	1.49805447	2.21E-09	2.88E-09
RP5-867C24.	NA	NA	NA
MYL4	NA	1.64E-59	4.46E-59
ITGB3	NA	NA	NA
RP11-290H9.	NA	NA	NA
RP11-290H9.	NA	NA	NA
THCAT158	NA	NA	NA
EFCAB13	NA	NA	NA
CTD-2026D2(	NA	3.45E-80	1.29E-79
NPEPPS	2.2105438	1.17E-11	1.59E-11
RP11-580I16	Inf	1.06E-154	2.70E-152
KPNB1	2.34397081	6.31E-130	8.47E-129
RP11-138C9.	NA	1.12E-40	2.35E-40
TBKBP1	1.8385214	0.80833867	0.81616653
TBX21	NA	1.63E-132	2.52E-131
OSBPL7	2.54669261	2.51E-09	3.26E-09
MRPL10	1.16195251	4.89E-81	1.85E-80
LRRC46	NA	3.04E-12	4.19E-12
SCRN2	1.43385214	1.73E-07	2.17E-07
SP6	NA	NA	NA
RP11-6N17.1	NA	NA	NA
RP11-6N17.2	Inf	5.68E-50	1.35E-49
SP2	1.3618677	4.74E-11	6.36E-11
SP2-AS1	4.49416342	2.58E-12	3.55E-12
RP11-6N17.3	NA	NA	NA
PNPO	1.13741173	7.78E-38	1.56E-37
RP11-6N17.6	Inf	4.59E-58	1.22E-57
PRR15L	NA	NA	NA
CDK5RAP3	1.29207198	6.62E-95	3.23E-94
RP11-6N17.1	NA	NA	NA
COPZ2	NA	NA	NA
NFE2L1	2.96044099	6.07E-67	1.86E-66
RP5-890E16.	NA	NA	NA
RP5-890E16.	NA	0.00064728	0.00074271

CBX1	1.64937311	2.50E-49	5.89E-49
SNX11	0.88521401	7.50E-59	2.02E-58
SKAP1	NA	NA	NA
RP11-456D7.	NA	NA	NA
THRA1/BTR	NA	NA	NA
RP11-433M2	NA	1.15E-158	7.43E-156
HOXB1	NA	NA	NA
HOXB2	2.72859922	5.20E-11	6.97E-11
HOXB-AS1	2.66320796	2.39E-08	3.04E-08
HOXB3	1.49805447	5.42E-24	8.97E-24
HOXB-AS3	NA	1.35E-60	3.75E-60
HOXB-AS2	NA	NA	NA
HOXB4	1.54299611	3.74E-74	1.27E-73
HOXB5	1.68531128	2.89E-103	1.66E-102
HOXB6	1.21166171	6.52E-115	4.98E-114
HOXB7	NA	NA	NA
HOXB8	NA	NA	NA
HOXB9	0	8.13E-132	1.21E-130
HOXB-AS4	NA	NA	NA
RP11-357H1	NA	1.24E-56	3.25E-56
CTD-2377D2	NA	NA	NA
PRAC1	NA	NA	NA
PRAC2	NA	NA	NA
HOXB13	NA	NA	NA
CTD-2377D2	NA	NA	NA
TTLL6	NA	NA	NA
CALCOCO2	1.23752326	9.84E-57	2.58E-56
ATP5G1	2.02942851	6.63E-66	1.99E-65
UBE2Z	1.6271971	3.43E-44	7.51E-44
SNF8	1.92004165	1.97E-17	2.95E-17
AC091133.1	NA	9.64E-116	7.50E-115
GIP	NA	NA	NA
RP11-501C1	NA	1.29E-35	2.52E-35
IGF2BP1	0.37451362	1.80E-145	1.58E-143
RP11-501C1	NA	NA	NA
B4GALNT2	NA	NA	NA
GNGT2	Inf	2.67E-08	3.40E-08
ABI3	NA	3.10E-11	4.18E-11
PHOSPHO1	0	4.51E-75	1.55E-74
ZNF652	1.81014916	0.82749885	0.83481533
RP11-1079K	0.74902724	1.16E-18	1.78E-18
RP11-81K2.1	NA	2.03E-87	8.61E-87
RP11-1079K	NA	NA	NA
PHB	1.40451019	2.19E-97	1.13E-96
RP11-1079K	0	1.96E-12	2.71E-12

AC091180.1	NA	NA	NA
RP11-1079K1	NA	NA	NA
NGFR	Inf	1.74E-30	3.16E-30
RP5-1029K1(	NA	NA	NA
RP5-1029K1(	NA	NA	NA
NXPH3	Inf	1.18E-08	1.51E-08
SPOP	1.44043699	1.06E-37	2.13E-37
SLC35B1	1.7223621	4.17E-77	1.48E-76
RP11-613C6.	NA	NA	NA
FAM117A	2.68401427	9.89E-11	1.32E-10
KAT7	2.36534917	7.17E-25	1.20E-24
RP11-304F15	NA	NA	NA
TAC4	NA	5.92E-17	8.81E-17
RP11-304F15	1.49805447	1.14E-84	4.61E-84
RP11-304F15	NA	NA	NA
RP11-304F15	NA	NA	NA
DLX4	1.84375935	0.00077497	0.00088714
DLX3	3.46425097	4.61E-37	9.18E-37
RP11-1094H2	NA	NA	NA
RP11-1094H2	NA	NA	NA
ITGA3	Inf	4.72E-09	6.11E-09
RP5-875H18.	NA	NA	NA
PDK2	1.22568093	7.15E-48	1.65E-47
RP5-875H18.	NA	NA	NA
RP5-875H18.	NA	NA	NA
SAMD14	0.37451362	1.13E-93	5.40E-93
PPP1R9B	2.18194891	1.85E-47	4.24E-47
AC002401.1	NA	NA	NA
RP11-893F2.	NA	NA	NA
RP11-893F2.	NA	NA	NA
SGCA	NA	5.66E-05	6.68E-05
COL1A1	NA	6.92E-139	1.72E-137
RP11-893F2.	NA	NA	NA
RP11-893F2.	NA	NA	NA
RP11-893F2.	NA	NA	NA
TMEM92	1.10382961	1.06E-139	2.90E-138
TMEM92-AS	NA	NA	NA
XYLT2	1.49805447	6.14E-23	1.00E-22
MRPL27	1.62318199	3.07E-76	1.08E-75
EME1	1.08192823	1.53E-41	3.25E-41
LRRC59	1.63353744	6.11E-15	8.81E-15
RP1-117B12.	NA	2.77E-73	9.33E-73
ACSF2	0.85603113	5.33E-39	1.09E-38
CHAD	NA	NA	NA
RP11-94C24.	NA	8.61E-111	5.90E-110

RSAD1	1.67639429	1.29E-12	1.78E-12
RP11-94C24.	NA	NA	NA
MYCBPAP	NA	1.71E-92	8.00E-92
EPN3	NA	2.85E-61	8.01E-61
SPATA20	1.29831388	2.20E-16	3.26E-16
CACNA1G-AS	NA	4.12E-15	5.96E-15
CACNA1G	NA	0.0160227	0.01761507
RP11-94C24.	NA	NA	NA
CTB-22K21.2	NA	NA	NA
ABCC3	NA	NA	NA
ANKRD40	2.11332685	0.52252696	0.53578726
RP11-294J22	2.32198444	1.30E-13	1.83E-13
LUC7L3	1.49805447	5.30E-66	1.60E-65
WFIKKN2	NA	NA	NA
RP11-506D1	NA	NA	NA
TOB1	0.99870298	8.12E-59	2.19E-58
RP11-700H6.	NA	NA	NA
RP11-700H6.	NA	NA	NA
RP11-700H6.	NA	NA	NA
SPAG9	2.22568093	8.20E-33	1.53E-32
RP11-481C4.	NA	NA	NA
NME1	1.54812103	3.00E-22	4.84E-22
NME1-NME2	NA	1.59E-80	5.96E-80
NME2	1.23054475	1.34E-136	2.69E-135
MBTD1	6.24189364	7.34E-72	2.41E-71
UTP18	1.35634662	1.14E-95	5.62E-95
RP11-1018N	NA	5.28E-112	3.74E-111
RP11-1018N	NA	NA	NA
RP11-1018N	NA	NA	NA
RP11-1018N	NA	NA	NA
RP11-1018N	NA	NA	NA
CA10	NA	NA	NA
RP11-429O1	NA	NA	NA
AC102948.2	NA	NA	NA
C17orf112	NA	NA	NA
KIF2B	NA	NA	NA
TOM1L1	1.87256809	0.98043023	0.9816206
COX11	1.52433613	3.58E-12	4.92E-12
RP11-257O5	NA	NA	NA
STXBP4	1.87256809	1.43E-15	2.08E-15
HLF	NA	7.61E-21	1.20E-20
RP11-515O1	NA	1.84E-45	4.11E-45
RP11-515O1	NA	NA	NA
MMD	2.24708171	1.16E-19	1.79E-19
RP11-515E2	NA	NA	NA

RP11-515E23	NA	NA	NA
TMEM100	NA	NA	NA
PCTP	3.99481193	0.14826559	0.15682337
RP11-763E3.	NA	NA	NA
ANKFN1	NA	NA	NA
NOG	1.74773022	1.20E-83	4.75E-83
C17orf67	1.43563554	2.24E-57	5.91E-57
DGKE	2.99610895	1.07E-88	4.67E-88
TRIM25	2.5766537	5.66E-60	1.56E-59
RP11-670E13	NA	NA	NA
RP11-670E13	2.62159533	0.08117079	0.08696118
COIL	1.38222552	2.75E-80	1.03E-79
RP5-1107A17	NA	NA	NA
SCPEP1	0.81144617	1.36E-136	2.74E-135
RP11-166P13	Inf	0.48558982	0.49887142
AKAP1	2.11089494	1.16E-37	2.32E-37
RP11-166P13	1.24837873	0.0039464	0.00442713
MSI2	1.73881323	1.04E-33	1.96E-33
RP11-118E18	NA	NA	NA
RP11-118E18	NA	NA	NA
CCDC182	NA	NA	NA
RP11-60A24.	NA	4.08E-41	8.58E-41
MRPS23	1.62098643	7.35E-75	2.53E-74
CUEDC1	Inf	1.15E-111	8.05E-111
RP11-343K8.	NA	NA	NA
RP11-159D12	NA	NA	NA
VEZF1	1.61040856	3.64E-44	7.95E-44
RP11-159D12	0.37451362	2.91E-09	3.78E-09
RP11-159D12	1.2733463	0.06520238	0.07008956
SRSF1	1.84542943	0.18209618	0.19180149
RP11-159D12	NA	4.78E-26	8.15E-26
DYNLL2	2.32015754	1.49E-58	3.99E-58
OR4D1	NA	NA	NA
OR4D2	NA	NA	NA
EPX	0	9.68E-176	3.57E-172
RP11-506H21	0	2.33E-78	8.44E-78
MKS1	1.44043699	2.31E-70	7.43E-70
LPO	NA	0.05246102	0.05661459
MPO	0.06584855	1.81E-145	1.58E-143
BZRAP1	1.5396671	1.16E-27	2.03E-27
BZRAP1-AS1	2.54669261	7.31E-05	8.60E-05
RP5-1171I10	1.57578372	2.68E-05	3.19E-05
SUPT4H1	1.77042802	9.19E-08	1.16E-07
RNF43	NA	1.18E-05	1.42E-05
HSF5	NA	NA	NA

MTMR4	1.8385214	1.16E-11	1.57E-11
SEPT4-AS1	NA	NA	NA
4-Sep	Inf	3.36E-53	8.40E-53
C17orf47	NA	NA	NA
TEX14	NA	5.60E-48	1.29E-47
RAD51C	1.47535668	5.58E-80	2.07E-79
RP11-579A4.	NA	NA	NA
PPM1E	Inf	9.71E-25	1.63E-24
TRIM37	2.20963035	1.76E-84	7.09E-84
AC099850.1	Inf	7.84E-68	2.43E-67
SKA2	1.91662852	2.41E-55	6.19E-55
PRR11	2.35723278	6.10E-20	9.50E-20
CTD-2510F5.	NA	NA	NA
CTD-2510F5.	NA	NA	NA
SMG8	Inf	7.94E-91	3.58E-90
GDPD1	0.14980545	1.35E-38	2.74E-38
YPEL2	1.99740597	2.63E-21	4.19E-21
RP11-567L7.!	NA	NA	NA
LINC01476	NA	NA	NA
DHX40	2.0598249	3.14E-09	4.07E-09
RP11-758H9.	0.32101167	0.02148111	0.02351761
CLTC	1.65574442	7.12E-08	8.98E-08
PTRH2	1.06239578	1.39E-125	1.53E-124
VMP1	1.26524871	3.82E-67	1.17E-66
TUBD1	1.37978702	7.71E-13	1.07E-12
RPS6KB1	1.83511673	0.13634193	0.14443884
RP11-178C3.	NA	NA	NA
RNFT1	1.13562194	6.52E-129	8.34E-128
TBC1D3P1-DI	NA	NA	NA
RP11-178C3.	Inf	1.09E-20	1.71E-20
HEATR6	1.04863813	1.29E-66	3.92E-66
CTD-2319I12	0.74902724	3.88E-36	7.62E-36
CTD-2319I12	NA	NA	NA
CA4	NA	NA	NA
USP32	1.81405034	7.91E-38	1.59E-37
C17orf64	2.62159533	4.54E-11	6.10E-11
APPBP2	1.68531128	1.42E-11	1.92E-11
RP11-15E18.	NA	3.70E-131	5.31E-130
RP11-15E18.	NA	2.95E-25	4.97E-25
PPM1D	1.15758755	1.55E-21	2.47E-21
AC110602.1	NA	NA	NA
BCAS3	Inf	0.00020015	0.00023291
RP11-264B14	NA	NA	NA
RP11-264B14	NA	NA	NA
RP11-136H1!	NA	NA	NA

RP11-332H18	NA	NA	NA
RP11-332H18	NA	NA	NA
TBX2-AS1	1.59575368	2.75E-16	4.06E-16
TBX2	1.71206226	5.70E-30	1.03E-29
C17orf82	NA	6.67E-90	2.96E-89
TBX4	NA	NA	NA
RP11-15K2.2	NA	NA	NA
NACA2	2.43433852	0.54006206	0.55342329
BRIP1	1.99740597	1.82E-35	3.54E-35
INTS2	2.46108949	1.07E-66	3.25E-66
MED13	1.64072633	7.11E-13	9.88E-13
RP11-51L5.7	NA	NA	NA
EFCAB3	0.74902724	1.08E-38	2.21E-38
METTL2A	0.99135958	4.13E-86	1.71E-85
TLK2	1.94747082	6.73E-09	8.68E-09
CTD-3035K23	NA	3.31E-154	8.11E-152
CTD-3035K23	Inf	3.24E-136	6.31E-135
MRC2	2.20302129	5.99E-21	9.49E-21
RP11-156L14	NA	NA	NA
10-Mar	NA	NA	NA
RP11-453A12	NA	NA	NA
RP11-180P8.	NA	NA	NA
RP11-180P8.	NA	NA	NA
TANC2	1.31079767	2.70E-09	3.51E-09
RP11-556O9.	NA	NA	NA
AC037445.1	NA	NA	NA
RP11-269G24	NA	NA	NA
AC015923.1	NA	NA	NA
RP11-269G24	NA	NA	NA
RP11-269G24	NA	NA	NA
CYB561	2.78638132	3.17E-93	1.50E-92
RP11-269G24	NA	NA	NA
ACE	2.7464332	2.03E-08	2.59E-08
CTD-2501B8.	NA	NA	NA
KCNH6	NA	1.25E-13	1.76E-13
DCAF7	1.45034573	4.62E-55	1.18E-54
TACO1	1.23849058	3.55E-70	1.14E-69
MAP3K3	3.62990123	2.67E-102	1.50E-101
LIMD2	1.72764137	9.45E-08	1.19E-07
STRADA	Inf	8.97E-23	1.46E-22
RP11-51F16.	NA	NA	NA
CCDC47	1.97957198	1.45E-41	3.07E-41
DDX42	2.04585051	1.35E-66	4.11E-66
FTSJ3	0.89400025	1.61E-59	4.39E-59
PSMC5	1.01639118	1.58E-142	6.15E-141

SMARCD2	1.80717683	4.89E-24	8.10E-24
CSH2	NA	NA	NA
GH2	NA	NA	NA
CSH1	NA	NA	NA
CSHL1	NA	NA	NA
GH1	NA	NA	NA
CD79B	1.49805447	1.21E-17	1.82E-17
SCN4A	NA	NA	NA
CTC-264K15.(	NA	NA	NA
PRR29-AS1	NA	3.55E-36	6.99E-36
PRR29	Inf	0.39932794	0.41216404
ICAM2	1.65226596	1.43E-29	2.56E-29
ERN1	2.30950065	1.22E-82	4.73E-82
SNHG25	2.63986429	5.52E-144	2.91E-142
TEX2	2.99610895	2.78E-100	1.50E-99
PECAM1	Inf	5.67E-127	6.63E-126
MILR1	2.24708171	0.0017633	0.00199686
POLG2	1.49805447	2.80E-47	6.41E-47
DDX5	1.22419139	1.18E-135	2.21E-134
CEP95	1.37978702	0.01249001	0.01378643
SMURF2	1.39105058	5.53E-16	8.11E-16
LRRC37A3	0.74902724	3.57E-20	5.58E-20
RP11-927P21	NA	7.02E-82	2.69E-81
RP11-927P21	NA	1.91E-27	3.32E-27
RP11-583F2.	NA	NA	NA
GNA13	1.65856031	1.08E-09	1.42E-09
RP11-583F2.	NA	1.83E-147	2.08E-145
RP11-160O5.	1.49805447	0.00948552	0.01050845
RGS9	0.74902724	1.34E-70	4.33E-70
CRAT40	NA	NA	NA
AXIN2	Inf	0.00198366	0.00224333
CTD-2535L24	NA	NA	NA
CEP112	NA	1.77E-88	7.66E-88
APOH	NA	NA	NA
PRKCA	1.70233463	1.39E-07	1.75E-07
PRKCA-AS1	NA	8.75E-122	8.30E-121
CACNG5	NA	NA	NA
CACNG4	NA	NA	NA
RP11-349A8.	NA	NA	NA
RP11-74H8.1	NA	NA	NA
CACNG1	NA	NA	NA
HELZ	2.00546002	8.56E-72	2.81E-71
RP11-401F2.	NA	NA	NA
RP11-401F2.	2.24708171	2.03E-37	4.06E-37
PSMD12	1.46476438	2.49E-69	7.91E-69

PITPNC1	2.17217899	0.00054548	0.00062709
NOL11	1.45311284	5.85E-10	7.71E-10
BPTF	2.16708851	7.92E-19	1.21E-18
RP11-855A2.	NA	NA	NA
C17orf58	1.76494924	0.06208726	0.06678053
RP11-855A2.	NA	NA	NA
KPNA2	0.9015441	1.30E-133	2.14E-132
AC145343.2	NA	NA	NA
RP11-147L13	1.94747082	7.82E-05	9.20E-05
RP11-147L13	1.49805447	4.95E-20	7.73E-20
RP11-147L13	2.24708171	3.90E-05	4.63E-05
RP11-147L13	NA	NA	NA
RP11-147L13	NA	NA	NA
RP11-147L13	6.74124514	2.72E-64	7.96E-64
RP11-147L13	0.37451362	2.41E-25	4.07E-25
AMZ2	2.32456729	1.86E-17	2.79E-17
ARSG	Inf	2.98E-31	5.46E-31
SLC16A6	NA	NA	NA
WIPI1	0.37451362	1.48E-75	5.13E-75
PRKAR1A	1.46748193	8.28E-65	2.45E-64
FAM20A	NA	NA	NA
RP11-120M1	NA	NA	NA
LINC01482	NA	NA	NA
RP11-118B18	NA	4.68E-128	5.75E-127
ABCA8	NA	NA	NA
ABCA9-AS1	NA	NA	NA
ABCA9	Inf	0.01777059	0.01951065
ABCA6	NA	1.60E-73	5.39E-73
ABCA10	NA	6.37E-150	9.39E-148
ABCA5	1.49805447	4.33E-92	2.01E-91
MAP2K6	Inf	3.25E-106	2.00E-105
RP1-193H18.	NA	NA	NA
RP1-193H18.	Inf	2.96E-151	5.06E-149
LINC01483	0	5.06E-34	9.64E-34
LINC01497	NA	NA	NA
AC002539.1	NA	NA	NA
LINC01028	NA	NA	NA
KCNJ16	NA	NA	NA
KCNJ2-AS1	NA	NA	NA
KCNJ2	NA	NA	NA
CTD-2378E21	NA	NA	NA
RP11-238F2.	NA	NA	NA
CASC17	NA	NA	NA
AC118653.2	NA	NA	NA
AC005152.3	NA	NA	NA

LINC01152	NA	NA	NA
AC007461.2	NA	NA	NA
SOX9	NA	NA	NA
LINC00511	Inf	0.13835758	0.14651432
RP11-1124B1	NA	NA	NA
RP11-57A1.1	NA	NA	NA
CTD-3010D24	NA	NA	NA
SLC39A11	1.49805447	6.19E-80	2.30E-79
RP11-171G2.	NA	NA	NA
SSTR2	2.99610895	4.41E-30	7.96E-30
COG1	3.18336576	2.21E-11	2.98E-11
RP11-143K11	Inf	1.20E-22	1.94E-22
FAM104A	2.16281615	7.37E-60	2.02E-59
C17orf80	2.66320796	2.62E-97	1.35E-96
CPSF4L	NA	NA	NA
CDC42EP4	2.12224384	9.40E-23	1.53E-22
SDK2	NA	NA	NA
RP11-449L23	NA	NA	NA
RP11-449L23	NA	NA	NA
RP11-277J6.2	NA	NA	NA
RP11-277J6.3	NA	NA	NA
CTD-2532D12	NA	NA	NA
LINC00469	NA	NA	NA
AC125421.1	NA	NA	NA
CTD-2532D12	NA	NA	NA
CTD-2582D11	NA	NA	NA
RP11-101O2	NA	NA	NA
RPL38	2.39470022	1.96E-145	1.63E-143
MGC16275	1.49805447	2.53E-49	5.95E-49
TTYH2	1.22568093	0.24704349	0.25854416
CTD-2514K5.	NA	NA	NA
DNAI2	NA	NA	NA
RP11-647F2.	NA	NA	NA
KIF19	NA	NA	NA
BTBD17	NA	NA	NA
GPR142	NA	NA	NA
GPRC5C	0.87386511	1.15E-116	9.20E-116
CD300A	0.51249232	3.55E-105	2.13E-104
CD300LB	NA	4.53E-136	8.68E-135
CD300C	NA	0.91767204	0.9215325
CD300LD	NA	NA	NA
C17orf77	NA	4.07E-40	8.45E-40
CTD-2006K23	NA	NA	NA
CD300E	NA	NA	NA
RAB37	0.42801556	2.32E-48	5.39E-48

CTD-2006K23	NA	NA	NA
CD300LF	NA	0.00073116	0.00083747
RP11-452I5.2	NA	NA	NA
SLC9A3R1	1.79766537	6.99E-24	1.15E-23
NAT9	1.82263294	0.38008379	0.39308404
TMEM104	3.37062257	9.93E-14	1.40E-13
GRIN2C	NA	1.91E-89	8.40E-89
FDXR	1.20238583	4.34E-88	1.86E-87
FADS6	NA	5.92E-18	8.95E-18
USH1G	NA	5.25E-101	2.89E-100
OTOP2	NA	0.38801892	0.40076511
AC087651.1	NA	NA	NA
OTOP3	NA	NA	NA
HID1	Inf	6.03E-58	1.60E-57
HID1-AS1	NA	NA	NA
CDR2L	1.77893969	2.91E-20	4.56E-20
ICT1	1.41519748	1.08E-95	5.36E-95
KCTD2	2.46108949	0.17226622	0.18171136
AC087651.2	NA	NA	NA
ATP5H	1.22555389	1.31E-137	2.85E-136
SLC16A5	1.74773022	1.97E-05	2.35E-05
ARMC7	1.64785992	7.17E-37	1.42E-36
NT5C	1.87603581	4.15E-49	9.74E-49
HN1	1.6284821	5.91E-60	1.62E-59
SUMO2	1.69898578	6.06E-27	1.05E-26
NUP85	1.74773022	7.35E-35	1.42E-34
GGA3	2.99610895	2.08E-22	3.37E-22
MRPS7	1.49575685	6.06E-72	1.99E-71
MIF4GD	1.42996109	3.18E-32	5.91E-32
RP11-649A18	Inf	2.42E-25	4.09E-25
SLC25A19	1.48297339	1.04E-38	2.11E-38
GRB2	1.43747139	1.07E-77	3.83E-77
RP11-16C1.3	NA	NA	NA
RP11-16C1.1	NA	1.65E-46	3.75E-46
TMEM94	1.82575389	2.42E-13	3.39E-13
CASKIN2	1.74773022	2.00E-20	3.14E-20
TSEN54	1.97051781	2.02E-15	2.93E-15
LLGL2	1.22757242	1.43E-45	3.19E-45
MYO15B	Inf	2.09E-40	4.37E-40
RECQL5	2.54669261	0.00148393	0.00168449
SMIM5	0	3.14E-49	7.40E-49
SMIM6	NA	6.04E-52	1.48E-51
SAP30BP	1.27466039	6.30E-101	3.45E-100
RP11-474I11	NA	NA	NA
RP11-474I11	NA	NA	NA

ITGB4	Inf	1.94E-10	2.57E-10
GALK1	0.94335034	1.83E-145	1.58E-143
H3F3B	1.55475375	1.59E-46	3.61E-46
UNK	1.6025699	2.13E-07	2.65E-07
RP11-552F3.	NA	NA	NA
UNC13D	1.06592338	8.46E-87	3.54E-86
WBP2	1.3765906	2.14E-142	8.20E-141
TRIM47	1.22840467	8.36E-91	3.77E-90
RP11-552F3.	NA	2.38E-66	7.22E-66
TRIM65	2.21587224	0.66042591	0.67213323
RP11-552F3.	NA	NA	NA
MRPL38	1.49805447	2.65E-37	5.29E-37
RP11-552F3.	NA	NA	NA
FBF1	2.24708171	0.00044539	0.00051362
FBF1	NA	NA	NA
ACOX1	2.8463035	6.75E-57	1.77E-56
RP11-552F3.	NA	NA	NA
TEN1	NA	NA	NA
TEN1-CDK3	NA	NA	NA
CDK3	NA	NA	NA
EVPL	NA	NA	NA
SRP68	1.45311284	1.62E-33	3.07E-33
ZACN	Inf	1.08E-08	1.39E-08
GALR2	NA	1.15E-15	1.68E-15
EXOC7	1.58722438	0.14055735	0.14883508
FOXJ1	NA	NA	NA
RP11-449J21	NA	NA	NA
RNF157	4.26945525	2.64E-67	8.10E-67
UBALD2	1.57786885	1.73E-09	2.26E-09
QRICH2	0.74902724	0.01131632	0.0125107
PRPSAP1	1.33160398	8.25E-118	6.82E-117
SPHK1	1.15610726	3.95E-36	7.77E-36
UBE2O	2.96489948	8.08E-101	4.42E-100
AANAT	Inf	4.32E-154	1.03E-151
RHBDF2	2.8463035	1.53E-18	2.34E-18
CYGB	NA	NA	NA
PRCD	NA	NA	NA
RP11-666A8.	NA	NA	NA
ST6GALNAC2	4.49416342	9.88E-23	1.61E-22
RP11-666A8.	NA	NA	NA
ST6GALNAC1	NA	4.08E-41	8.58E-41
RP11-318A15	2.99610895	2.14E-18	3.26E-18
MXRA7	1.67782101	2.73E-33	5.15E-33
RP11-318A15	NA	2.00E-92	9.33E-92
JMJD6	0.8827821	4.34E-137	9.04E-136

METTL23	1.36664619	5.84E-84	2.33E-83
RP11-318A1!	NA	NA	NA
SRSF2	1.69730711	7.20E-08	9.08E-08
MFSD11	2.09727626	7.40E-39	1.51E-38
CTD-2246P4.	NA	NA	NA
LINC00868	NA	NA	NA
MGAT5B	Inf	1.66E-31	3.06E-31
RP11-87G24.	NA	NA	NA
SEC14L1	3.43671321	2.14E-37	4.28E-37
RP11-285E9.	NA	NA	NA
RP11-285E9.	NA	NA	NA
9-Sep	2-Jan	0.01204008	0.01329708
RP11-936I5.1	NA	NA	NA
RP11-75C10.	NA	NA	NA
RP11-13K12.	NA	5.11E-124	5.29E-123
RP11-13K12.	NA	NA	NA
RP11-13K12.	NA	NA	NA
RP11-316M2	NA	NA	NA
AC104981.1	NA	NA	NA
FLJ45079	NA	NA	NA
TNRC6C	1.55155642	1.53E-19	2.36E-19
TMC6	1.93353543	9.12E-52	2.23E-51
TMC8	2.70802155	2.32E-68	7.27E-68
C17orf99	NA	NA	NA
SYNGR2	2.13795192	7.55E-86	3.10E-85
TK1	1.06641166	1.76E-144	1.04E-142
AFMID	1.76897922	0.00011114	0.00013014
BIRC5	1.65482762	2.22E-42	4.75E-42
TMEM235	NA	NA	NA
RP11-219G1	Inf	1.41E-89	6.22E-89
AC061992.2	NA	NA	NA
SOCS3	1.12354086	4.09E-30	7.39E-30
RP11-806H1(	NA	2.28E-13	3.19E-13
PGS1	1.9661965	1.55E-119	1.36E-118
DNAH17	NA	NA	NA
CTD-2357A8.	1.94747082	3.99E-14	5.65E-14
CYTH1	14.9805447	7.47E-127	8.71E-126
USP36	1.9926951	1.03E-22	1.67E-22
TIMP2	1.75486381	2.61E-09	3.40E-09
RP11-323N1	NA	1.31E-142	5.18E-141
CEP295NL	Inf	5.50E-20	8.57E-20
CTD-2373H9.	NA	5.48E-110	3.68E-109
LGALS3BP	1.74773022	7.53E-21	1.19E-20
CANT1	1.83690013	0.00122568	0.00139526
C1QTNF1-AS	NA	NA	NA

C1QTNF1	NA	6.44E-57	1.69E-56
ENGASE	1.33754864	4.78E-26	8.16E-26
RBFOX3	NA	5.94E-64	1.73E-63
RP11-398J5.:	NA	NA	NA
HP09025	NA	NA	NA
ENPP7	NA	NA	NA
CBX2	1.88885129	2.06E-07	2.57E-07
CBX8	1.16515348	0.42138339	0.43411342
RP11-353N1	NA	NA	NA
RP11-353N1	NA	NA	NA
CBX4	1.89753567	6.97E-18	1.05E-17
RP11-353N1	NA	NA	NA
RP11-353N1	NA	NA	NA
RP11-353N1	NA	1.75E-09	2.28E-09
TBC1D16	2.17898833	3.33E-32	6.19E-32
RP11-353N1	NA	NA	NA
CTD-2529O2	NA	NA	NA
CCDC40	Inf	2.20E-60	6.10E-60
GAA	1.60505837	2.43E-14	3.47E-14
EIF4A3	0.67769131	1.11E-129	1.47E-128
RP11-334C1	NA	8.05E-14	1.14E-13
CARD14	NA	0.38801892	0.40076511
RP11-334C1	NA	0.10239071	0.10914055
SGSH	Inf	5.83E-98	3.02E-97
SLC26A11	0	3.51E-18	5.34E-18
RNF213	1.63849708	5.74E-47	1.31E-46
CTD-2047H1	NA	NA	NA
CTD-2047H1	NA	4.27E-123	4.26E-122
ENDOV	1.64785992	1.77E-16	2.62E-16
CTD-2526A2.	NA	2.17E-108	1.40E-107
NPTX1	NA	0.00103954	0.00118642
RPTOR	2.88910506	0.00094276	0.00107739
RP11-28G8.1	NA	NA	NA
CTD-2561B2	NA	NA	NA
CTD-2561B2	NA	NA	NA
CTD-2561B2	NA	3.77E-142	1.39E-140
CHMP6	1.79115209	2.12E-08	2.70E-08
CTD-2561B2	NA	NA	NA
CTD-2561B2	Inf	3.70E-84	1.48E-83
CTD-2561B2	NA	NA	NA
BAIAP2-AS1	1.60505837	6.23E-05	7.34E-05
BAIAP2	2.21451531	1.56E-30	2.84E-30
AATK	1.49805447	5.28E-07	6.53E-07
RP11-149I9.:	NA	NA	NA
AATK-AS1	NA	NA	NA

CEP131	1.32520204	1.58E-54	4.03E-54
RP11-455O6.	NA	NA	NA
ENTHD2	1.24837873	8.15E-85	3.30E-84
AC027601.1	NA	1.43E-132	2.23E-131
C17orf89	2.65550501	1.83E-145	1.58E-143
SLC38A10	2.20765923	0.56629008	0.57931852
RP11-455O6.	Inf	4.56E-74	1.55E-73
LINC00482	NA	NA	NA
TMEM105	NA	NA	NA
RP11-455O6.	0	7.50E-77	2.65E-76
RP11-1055B8	3.74513619	4.51E-108	2.89E-107
RP11-1055B8	NA	NA	NA
RP11-1055B8	NA	NA	NA
RP11-1055B8	NA	8.82E-29	1.56E-28
RP11-1055B8	0.74902724	6.00E-47	1.37E-46
BAHCC1	2.8463035	1.87E-122	1.82E-121
RP11-1055B8	NA	NA	NA
RP13-766D2(	NA	6.88E-102	3.85E-101
ACTG1	0.98841568	6.93E-145	4.43E-143
RP13-766D2(	NA	NA	NA
FSCN2	NA	NA	NA
FAAP100	1.523883	1.28E-37	2.57E-37
NPLOC4	2.93001831	1.30E-118	1.10E-117
TSPAN10	3.74513619	3.12E-12	4.29E-12
PDE6G	Inf	6.30E-70	2.02E-69
OXLD1	1.69822555	0.03220504	0.03503411
CCDC137	1.65325832	1.50E-06	1.84E-06
ARL16	1.79875884	0.20577006	0.21628508
HGS	1.4841836	2.54E-63	7.33E-63
RP13-1032I1	Inf	2.46E-25	4.15E-25
RP13-1032I1	2.47178988	2.44E-26	4.18E-26
MRPL12	1.562047	5.43E-24	8.99E-24
RP13-1032I1	NA	NA	NA
SLC25A10	0.97699205	4.70E-143	2.04E-141
GCGR	NA	NA	NA
FAM195B	2.04092743	2.25E-34	4.32E-34
PPP1R27	NA	2.43E-06	2.97E-06
P4HB	1.22785743	1.10E-130	1.54E-129
RP11-498C9.	NA	2.08E-57	5.49E-57
RP11-498C9.	Inf	1.84E-53	4.62E-53
ARHGDIA	1.54381989	1.16E-78	4.23E-78
RP11-498C9.	3.6770428	2.36E-28	4.16E-28
RP11-498C9.	NA	0.22131413	0.23213927
ALYREF	1.59168288	3.48E-38	7.03E-38
ANAPC11	1.986464	2.30E-12	3.17E-12

NPB	0.74902724	4.21E-40	8.74E-40
PCYT2	1.39818418	2.94E-11	3.96E-11
SIRT7	1.46839003	0.95663219	0.95879902
MAFG	2.93849147	1.52E-134	2.66E-133
RP11-498C9.	NA	NA	NA
MAFG-AS1	0	0.0100962	0.01117475
PYCR1	1.21206226	3.53E-133	5.72E-132
RP11-498C9.	Inf	1.66E-72	5.51E-72
MYADML2	NA	NA	NA
AC137723.5	NA	NA	NA
NOTUM	1.12354086	9.72E-114	7.21E-113
RP11-498C9.	NA	NA	NA
ASPSCR1	1.60961172	1.07E-103	6.25E-103
STRA13	2.22548813	6.31E-98	3.27E-97
LRRC45	1.4205689	3.54E-22	5.70E-22
RAC3	2.14007782	0.00016281	0.00018984
DCXR	1.37683201	8.98E-145	5.60E-143
RP13-650J16	Inf	2.53E-103	1.46E-102
RFNG	1.78339818	7.81E-05	9.19E-05
GPS1	1.31671104	5.22E-135	9.35E-134
DUS1L	2.34670602	5.06E-110	3.41E-109
FASN	1.80153965	2.25E-10	2.99E-10
CCDC57	1.6728275	1.55E-35	3.03E-35
RP11-1376P1	NA	NA	NA
RP11-1376P1	NA	5.25E-71	1.70E-70
RP13-516M1	Inf	5.30E-94	2.54E-93
SLC16A3	0.87578569	2.14E-137	4.59E-136
CSNK1D	1.40789379	2.19E-92	1.02E-91
RP13-516M1	NA	2.20E-06	2.69E-06
RP13-516M1	1.24837873	7.54E-13	1.05E-12
RP13-516M1	0.99870298	0.13668374	0.1447925
CD7	NA	1.10E-78	4.01E-78
SECTM1	NA	NA	NA
TEX19	Inf	1.84E-107	1.16E-106
UTS2R	NA	NA	NA
RP13-20L14.	NA	5.48E-101	3.01E-100
OGFOD3	2.18466278	2.83E-26	4.83E-26
HEXDC	1.40817121	3.35E-40	6.97E-40
C17orf62	2.06549935	0.32096702	0.33359976
RP13-20L14.	NA	1.98E-138	4.65E-137
NARF	1.77113732	7.98E-115	6.07E-114
RP13-991F5.	NA	NA	NA
FOXK2	1.93018557	1.62E-11	2.20E-11
RP13-638C3.	NA	NA	NA
RP13-638C3.	0.74902724	1.35E-32	2.52E-32

WDR45B	1.42556797	0.13036746	0.13837622
RAB40B	1.73346304	0.04487324	0.04853576
RP11-388C12	NA	NA	NA
FN3KRP	0.9844358	1.88E-74	6.41E-74
AC024361.1	NA	NA	NA
RP11-388C12	NA	NA	NA
FN3K	1.2733463	5.52E-29	9.80E-29
TBCD	1.34200713	9.18E-66	2.76E-65
RP11-567O1	NA	NA	NA
ZNF750	NA	NA	NA
RP11-497H1	NA	NA	NA
B3GNTL1	2.03049552	1.10E-09	1.44E-09
RP11-1197K1	NA	NA	NA
METRNL	3.25538761	1.08E-62	3.08E-62
AC144831.3	NA	NA	NA
AC144831.1	NA	NA	NA
AC139099.4	NA	NA	NA
AC139099.6	NA	0.00024112	0.00027994
AC139099.5	NA	NA	NA
AP005530.1	NA	NA	NA
AP005530.2	NA	NA	NA
RP11-683L23	NA	NA	NA
USP14	1.47065104	2.94E-16	4.33E-16
THOC1	1.05896954	3.55E-41	7.48E-41
RP11-705O1.	Inf	1.02E-50	2.45E-50
RP11-720L2.	NA	NA	NA
COLEC12	1.49805447	0.18130472	0.19100112
RP11-14P20.	NA	NA	NA
CETN1	NA	NA	NA
CLUL1	NA	NA	NA
RP11-806L2.	NA	1.88E-114	1.42E-113
TYMS	1.14137484	4.64E-131	6.62E-130
ENOSF1	1.57976654	0.00013262	0.00015502
RP11-806L2.	NA	NA	NA
YES1	2.08657588	0.2931962	0.30540079
RP11-769O8.	NA	3.34E-38	6.75E-38
RP11-769O8.	NA	NA	NA
RP11-672L10	Inf	6.40E-43	1.38E-42
RP11-672L10	NA	NA	NA
ADCYAP1	NA	NA	NA
RP11-672L10	NA	NA	NA
RP11-689C9.	NA	1.47E-18	2.24E-18
RP11-78F17.	NA	NA	NA
LINC00470	NA	NA	NA
RP11-476K15	NA	NA	NA

CTD-2015H3.	NA	NA	NA
CTD-2015H3.	NA	NA	NA
RP11-161I6.2	NA	NA	NA
METTL4	2.51945525	1.44E-48	3.36E-48
NDC80	0.91547773	1.65E-132	2.56E-131
SMCHD1	1.27612048	3.04E-103	1.75E-102
RP11-703M2	NA	NA	NA
EMILIN2	0.56177043	3.88E-109	2.55E-108
LPIN2	3.89494163	1.26E-109	8.38E-109
RP11-737O2	NA	NA	NA
RP11-737O2	NA	2.00E-92	9.33E-92
RP11-737O2	NA	NA	NA
MYOM1	NA	0.41834832	0.43115805
RP13-270P17	NA	NA	NA
RP13-270P17	1.12354086	1.48E-88	6.40E-88
MYL12A	1.14744598	1.67E-136	3.31E-135
RP13-270P17	2.24708171	8.72E-08	1.10E-07
MYL12B	1.4405255	2.06E-89	9.07E-89
RP11-838N2.	NA	NA	NA
RP11-838N2.	NA	NA	NA
IGLJCOR18	NA	NA	NA
TGIF1	0.84889754	5.64E-145	3.69E-143
GAPLINC	NA	5.32E-125	5.69E-124
DLGAP1	Inf	0.02763725	0.03012648
DLGAP1-AS1	0.99870298	0.42078394	0.43354511
DLGAP1-AS2	0.74902724	3.09E-40	6.43E-40
RP11-874J12	Inf	3.65E-05	4.33E-05
DLGAP1-AS3	NA	NA	NA
DLGAP1-AS4	NA	NA	NA
DLGAP1-AS5	NA	NA	NA
RP11-172F10	NA	NA	NA
RP11-92G19.	NA	NA	NA
C18orf42	NA	NA	NA
RP11-190I17	NA	NA	NA
RP11-835E18	NA	NA	NA
LINC00526	NA	NA	NA
LINC00667	NA	NA	NA
ZBTB14	2.62159533	4.63E-75	1.59E-74
CTD-3096M3	NA	NA	NA
CTD-3096M3	NA	NA	NA
EPB41L3	3.07495392	4.66E-83	1.83E-82
RP11-286N3.	NA	NA	NA
RP11-286N3.	NA	NA	NA
MIR3976HG	NA	NA	NA
TMEM200C	NA	NA	NA

RP11-945C19	NA	NA	NA
L3MBTL4	NA	5.06E-06	6.13E-06
RP11-793A3.	NA	NA	NA
RP11-793A3.	NA	NA	NA
L3MBTL4-AS	NA	NA	NA
CTD-2124B2(	NA	NA	NA
LINC01387	NA	NA	NA
RP11-91I8.1	NA	NA	NA
RP11-91I8.3	NA	NA	NA
ARHGAP28	NA	NA	NA
RP11-146G7.	NA	NA	NA
LINC00668	NA	NA	NA
LAMA1	NA	NA	NA
RP11-781P6.	NA	NA	NA
RP11-76K13.	NA	NA	NA
LRRC30	NA	NA	NA
PTPRM	NA	9.88E-134	1.64E-132
RP11-678G1!	NA	NA	NA
RP11-789C17	NA	NA	NA
RP11-789C17	NA	NA	NA
RAB12	0.74902724	0.64675409	0.65847762
RP11-661O1:	NA	NA	NA
GACAT2	NA	4.29E-48	9.91E-48
MTCL1	1.49805447	4.87E-16	7.16E-16
NDUFV2	1.69695419	1.39E-23	2.28E-23
RP11-143J12	NA	2.03E-08	2.59E-08
NDUFV2-AS1	Inf	7.87E-20	1.22E-19
ANKRD12	1.69632639	4.50E-60	1.24E-59
RP11-888D1(	NA	NA	NA
TWSG1	2.49675746	9.31E-80	3.45E-79
RP11-61L19.:	Inf	4.66E-85	1.89E-84
RALBP1	2.22018294	3.28E-137	6.92E-136
PPP4R1	1.27008966	3.84E-42	8.20E-42
RP11-881L2.	NA	NA	NA
PPP4R1-AS1	NA	1.53E-50	3.66E-50
RAB31	1.52480545	7.03E-32	1.30E-31
TXNDC2	NA	NA	NA
RP11-474N2	Inf	4.09E-24	6.78E-24
VAPA	1.28986564	3.51E-124	3.66E-123
RP11-419J16	NA	NA	NA
RP11-138E9.	NA	NA	NA
RP11-243E1:	NA	NA	NA
LINC01254	NA	NA	NA
APCDD1	NA	NA	NA
NAPG	1.20379377	3.30E-17	4.93E-17

RP11-883A18	NA	NA	NA
RP11-856M7	NA	NA	NA
RP11-856M7	NA	NA	NA
RP11-856M7	NA	NA	NA
PIEZO2	NA	NA	NA
RP11-856M7	NA	NA	NA
RP11-513M1	NA	NA	NA
RP11-128P17	NA	NA	NA
RP11-128P17	NA	NA	NA
RP11-128P17	NA	NA	NA
RP11-128P17	NA	NA	NA
LINC01255	NA	NA	NA
RP11-677O4.	NA	NA	NA
RP11-677O4.	NA	NA	NA
GNAL	0.74902724	1.47E-54	3.73E-54
RP11-78A19.	NA	NA	NA
CHMP1B	1.82762646	1.03E-12	1.43E-12
RP11-78A19.	NA	1.32E-51	3.23E-51
RP11-78A19.	NA	NA	NA
MPPE1	2.10663911	0.01861523	0.02042032
RP11-820I16	0.74902724	1.14E-24	1.91E-24
RP11-820I16	NA	1.32E-34	2.53E-34
IMPA2	1.39292784	5.48E-106	3.34E-105
RP11-820I16	NA	NA	NA
RP11-703I16	NA	NA	NA
RP11-815J4.1	NA	NA	NA
ANKRD62	NA	2.00E-92	9.33E-92
RP11-64C12.	NA	NA	NA
RP11-64C12.	NA	NA	NA
CIDEA	NA	NA	NA
RP11-64C12.	NA	1.48E-28	2.61E-28
TUBB6	1.36429961	1.98E-106	1.22E-105
AFG3L2	1.85628489	2.28E-21	3.63E-21
PRELID3A	1.49805447	3.95E-13	5.51E-13
RP11-861E21	NA	NA	NA
RP11-861E21	Inf	1.44E-100	7.85E-100
SPIRE1	1.9539841	2.59E-45	5.76E-45
PSMG2	1.19844358	1.97E-139	5.20E-138
CEP76	1.7655642	2.49E-07	3.11E-07
RP11-973H7.	NA	NA	NA
RP11-973H7.	NA	NA	NA
RP11-973H7.	NA	NA	NA
PTPN2	1.60505837	1.72E-19	2.66E-19
SEH1L	1.41815824	2.38E-53	5.95E-53
RP11-773H22	Inf	7.99E-149	1.03E-146

CEP192	1.74773022	4.34E-15	6.27E-15
RP11-794M8	NA	NA	NA
RP11-794M8	NA	NA	NA
LDLRAD4	0.74902724	0.54937144	0.56242293
RP11-756J15	NA	NA	NA
RP11-691H4.	NA	NA	NA
LDLRAD4-AS	NA	NA	NA
RP11-691H4.	NA	NA	NA
RP11-53B2.5	NA	NA	NA
RP11-53B2.3	NA	3.73E-51	9.02E-51
RP11-53B2.4	NA	NA	NA
RP11-701H1	NA	NA	NA
FAM210A	2.01462498	1.26E-05	1.52E-05
RNMT	1.97900881	0.00046266	0.0005333
MC5R	NA	NA	NA
MC2R	NA	NA	NA
ZNF519	1.09473212	0.45636774	0.46930186
RP11-411B1	NA	NA	NA
RP11-411B1	NA	NA	NA
RP11-757O6.	NA	NA	NA
CYP4F35P	Inf	1.56E-146	1.65E-144
CXADRP3	NA	NA	NA
POTEC	NA	NA	NA
ANKRD30B	NA	NA	NA
RP11-1157N	NA	NA	NA
RP11-527H1	NA	NA	NA
RP11-527H1	NA	NA	NA
LINC01443	NA	NA	NA
LINC01444	NA	NA	NA
RP11-527H1	NA	NA	NA
AP005242.1	NA	NA	NA
RP11-805F1	NA	NA	NA
RP11-805F1	NA	NA	NA
ROCK1	2.16385646	0.00934354	0.01035431
RP11-699A5.	NA	NA	NA
GREB1L	0	9.74E-80	3.60E-79
RP11-296E2	NA	NA	NA
ESCO1	1.65856031	1.26E-46	2.87E-46
SNRPD1	1.68046765	0.05245382	0.05661018
ABHD3	1.76021401	1.98E-74	6.75E-74
RP11-13N13.	NA	NA	NA
MIB1	2.72373541	2.82E-13	3.94E-13
MIR133A1H	NA	3.65E-49	8.58E-49
RP11-268I9.	NA	NA	NA
RP11-595B2	NA	NA	NA

RP11-595B24	NA	NA	NA
GATA6-AS1	NA	4.36E-05	5.17E-05
GATA6	1.49805447	0.42725335	0.44008575
RP11-627G1	NA	NA	NA
RP11-627G1	NA	NA	NA
RP11-627G1	NA	NA	NA
RP11-535A5.	NA	NA	NA
CTAGE1	NA	NA	NA
RP11-739L1C	NA	2.03E-70	6.53E-70
RP11-370A5.	0	3.58E-33	6.72E-33
RBBP8	1.97243839	6.46E-34	1.23E-33
CABLES1	1.74773022	3.51E-11	4.72E-11
TMEM241	1.87256809	4.39E-43	9.47E-43
RP11-17J14.:	Inf	7.32E-54	1.85E-53
RIOK3	2.41993415	7.42E-22	1.19E-21
C18orf8	2.16385646	5.10E-14	7.23E-14
NPC1	0.66580199	0.71873061	0.72889545
ANKRD29	NA	2.14E-133	3.47E-132
LAMA3	Inf	3.17E-100	1.71E-99
RP11-403A2	NA	NA	NA
TTC39C	2.48677043	2.70E-132	4.13E-131
TTC39C-AS1	NA	NA	NA
RP11-403A2	NA	NA	NA
RP11-799B1	NA	NA	NA
CABYR	NA	2.85E-96	1.43E-95
RP11-799B1	Inf	3.72E-07	4.62E-07
OSBPL1A	1.74148833	6.30E-61	1.76E-60
RP11-621L6.	NA	NA	NA
RP11-621L6.	NA	1.11E-60	3.08E-60
IMPACT	NA	NA	NA
HRH4	NA	4.00E-42	8.54E-42
RP11-178F1C	NA	NA	NA
RP11-178F1C	NA	NA	NA
RP11-449D8.	Inf	4.89E-75	1.68E-74
RP11-449D8.	NA	NA	NA
RP11-449D8.	NA	NA	NA
RP11-958F21	NA	NA	NA
RP11-958F21	NA	NA	NA
ZNF521	5.49286641	5.75E-07	7.11E-07
CTD-2006O1	NA	1.47E-18	2.24E-18
SS18	2.07230869	3.59E-14	5.10E-14
PSMA8	NA	NA	NA
TAF4B	1.81014916	2.21E-12	3.04E-12
LINC01543	NA	NA	NA
KCTD1	2.24708171	1.01E-16	1.49E-16

AQP4-AS1	NA	NA	NA
PCAT18	NA	NA	NA
RP11-17A19.	NA	NA	NA
RP11-552O4.	NA	NA	NA
AQP4	NA	NA	NA
CHST9	NA	NA	NA
RP11-57J16.1	NA	NA	NA
RP11-739N1(	NA	NA	NA
RP11-26N15.	NA	NA	NA
RP11-612A1.	NA	NA	NA
CDH2	0	3.42E-08	4.35E-08
AC015933.2	NA	NA	NA
RP11-510D2:	NA	NA	NA
CTD-2515C1:	NA	NA	NA
RP11-784B1!	NA	NA	NA
RP11-675P14	NA	NA	NA
RP11-25I11.1	NA	NA	NA
DSC3	NA	NA	NA
DSC2	1.24837873	2.30E-06	2.81E-06
DSCAS	NA	NA	NA
DSC1	NA	NA	NA
RP11-408H2(	NA	NA	NA
DSG1	NA	NA	NA
DSG1-AS1	NA	NA	NA
DSG4	NA	NA	NA
DSG3	NA	NA	NA
RP11-650P1!	NA	NA	NA
DSG2	1.94192247	1.84E-46	4.18E-46
DSG2-AS1	NA	NA	NA
TTR	NA	NA	NA
B4GALT6	3.74513619	6.25E-46	1.41E-45
RP11-549B1{	Inf	7.98E-14	1.13E-13
SLC25A52	NA	NA	NA
TRAPPC8	1.92607004	1.21E-15	1.76E-15
RP11-210K2(	NA	7.74E-138	1.72E-136
RP11-326K1:	1.39105058	9.79E-43	2.11E-42
RNF125	1.56047341	1.10E-19	1.70E-19
RP11-53I6.3	0.74902724	4.98E-15	7.19E-15
RNF138	1.93594732	0.12082247	0.12844025
RP11-53I6.4	NA	NA	NA
GAREM1	1.12354086	0.41452071	0.42733463
MEP1B	NA	NA	NA
RP11-344B2.	NA	NA	NA
AC021224.1	0.74902724	0.24220008	0.25356304
RP11-386P4.	NA	NA	NA

KLHL14	NA	NA	NA
AC012123.1	NA	NA	NA
RP11-746B8.	NA	NA	NA
CCDC178	NA	NA	NA
RP11-680N2(	NA	NA	NA
RP11-258B1(	NA	NA	NA
ASXL3	NA	NA	NA
NOL4	NA	NA	NA
RP11-379L18	NA	NA	NA
RP11-379L18	NA	NA	NA
DTNA	NA	NA	NA
RP11-138H1:	NA	NA	NA
RP11-96A15.	NA	NA	NA
MAPRE2	2.21141375	1.67E-51	4.07E-51
RP11-294N2	NA	1.46E-121	1.36E-120
ZNF397	2.04280156	5.48E-95	2.68E-94
ZSCAN30	5.9922179	1.28E-46	2.90E-46
RP11-158H5.	Inf	1.87E-122	1.82E-121
RP11-158H5.	Inf	1.70E-31	3.13E-31
RP11-54G14.	Inf	2.82E-115	2.17E-114
ZNF24	1.3959144	1.39E-57	3.68E-57
ZNF396	NA	2.71E-106	1.67E-105
RP11-322E11	NA	1.06E-11	1.44E-11
RP11-322E11	NA	7.01E-72	2.30E-71
RP11-322E11	0	5.86E-48	1.35E-47
INO80C	1.56414511	6.32E-65	1.88E-64
GALNT1	2.71522374	1.61E-115	1.24E-114
RP11-873E2(	Inf	3.25E-06	3.96E-06
RP11-905K4.	NA	NA	NA
C18orf21	2.02677958	0.00010537	0.00012346
RPRD1A	1.88127771	2.45E-74	8.36E-74
SLC39A6	2.30256521	0.00265413	0.0029902
ELP2	1.69779507	3.57E-11	4.81E-11
RP11-49I11.1	1.49805447	0.96573412	0.96744055
MOCOS	4.49416342	4.20E-11	5.64E-11
FHOD3	NA	5.27E-109	3.45E-108
TPGS2	1.80014833	0.00014424	0.0001684
KIAA1328	1.28404669	2.50E-09	3.25E-09
RP11-58G13.	NA	NA	NA
RP11-95O2.1	NA	NA	NA
CELF4	NA	NA	NA
RP11-95O2.5	NA	9.15E-47	2.08E-46
RP11-797E24	NA	NA	NA
RP11-687D1!	NA	NA	NA
RP11-244M2	1.49805447	0.04681865	0.05060979

RP11-636O2	NA	NA	NA
LINC01477	NA	NA	NA
KC6	NA	NA	NA
PIK3C3	4.86867704	0.33573731	0.34843238
LINC00907	NA	8.75E-11	1.17E-10
RIT2	NA	NA	NA
SYT4	NA	NA	NA
LINC01478	NA	NA	NA
RP11-456K2:	NA	NA	NA
SETBP1	0	2.29E-119	2.00E-118
RP11-846C1!	NA	NA	NA
SLC14A2	0	1.19E-81	4.55E-81
SLC14A2-AS1	NA	NA	NA
RP11-116O1!	NA	NA	NA
SLC14A1	NA	1.19E-43	2.58E-43
SIGLEC15	NA	5.11E-58	1.36E-57
EPG5	1.71206226	0.00257592	0.00290336
PSTPIP2	2.20546909	2.58E-12	3.56E-12
ATP5A1	0.92601855	3.56E-138	8.17E-137
HAUS1	0.81870419	2.73E-143	1.25E-141
C18orf25	2.04280156	3.21E-07	4.00E-07
RNF165	NA	NA	NA
LOXHD1	NA	NA	NA
ST8SIA5	NA	5.45E-122	5.21E-121
RP11-687F6.	Inf	7.89E-06	9.52E-06
PIAS2	2.41621689	2.57E-78	9.29E-78
KATNAL2	1.87256809	0.00464753	0.00520209
TCEB3CL2	NA	NA	NA
TCEB3CL	NA	NA	NA
RP11-49K24.	NA	NA	NA
TCEB3C	NA	NA	NA
TCEB3B	NA	NA	NA
RP11-49K24.	NA	NA	NA
RP11-49K24.	Inf	2.62E-149	3.57E-147
HDHD2	1.69632639	0.38121199	0.39422835
RP11-49K24.	NA	NA	NA
IER3IP1	1.79219302	1.75E-05	2.09E-05
SKOR2	NA	NA	NA
CTD-2130O1:	NA	NA	NA
RP11-157P2:	NA	NA	NA
SMAD2	1.82762646	6.76E-37	1.34E-36
RP11-380M2	NA	NA	NA
ZBTB7C	Inf	9.57E-141	2.95E-139
CTIF	8.98832685	1.26E-72	4.19E-72
RP11-426J5.:	NA	NA	NA

RP11-484L8.1	NA	NA	NA
SMAD7	Inf	1.14E-99	6.07E-99
RP11-1058N	NA	NA	NA
RP11-15F12.	NA	NA	NA
DYM	2.16682879	0.10694662	0.11388973
RP11-15F12.	NA	NA	NA
RP11-30L3.2	NA	NA	NA
C18orf32	4.49416342	5.88E-06	7.12E-06
RPL17-C18or	NA	1.55E-11	2.10E-11
MIR1539	NA	NA	NA
RPL17	2.56501414	1.76E-80	6.59E-80
RP11-110H1.	NA	NA	NA
LIPG	Inf	0.9404108	0.94342694
RP11-813F2(	NA	NA	NA
ACAA2	0.90255456	1.78E-137	3.85E-136
RP11-886H2.	NA	NA	NA
SNHG22	NA	6.39E-96	3.18E-95
MYO5B	NA	7.93E-26	1.35E-25
CFAP53	Inf	1.68E-29	3.02E-29
MBD1	2.48540856	4.35E-21	6.89E-21
CXXC1	1.23648941	1.63E-127	1.96E-126
RP11-1030E3	NA	NA	NA
SKA1	1.76241703	2.27E-22	3.67E-22
MAPK4	NA	NA	NA
MRO	NA	NA	NA
ME2	1.72852439	1.19E-102	6.73E-102
ELAC1	1.62289235	3.91E-07	4.86E-07
RP11-729L2.	NA	NA	NA
SMAD4	2.80885214	0.75434618	0.7636061
MEX3C	1.81906615	3.56E-08	4.51E-08
RP11-267C16	NA	NA	NA
RP11-25O3.1	NA	NA	NA
DCC	NA	NA	NA
AC011155.1	NA	NA	NA
RP11-671P2.	NA	NA	NA
RP11-202D1.	NA	NA	NA
RP11-202D1.	NA	NA	NA
MBD2	2.11549585	7.95E-133	1.25E-131
RP11-850A1	NA	4.41E-77	1.56E-76
POLI	2.99610895	1.28E-40	2.69E-40
STARD6	NA	3.65E-69	1.15E-68
C18orf54	4.49416342	2.99E-123	3.00E-122
DYNAP	NA	NA	NA
RAB27B	NA	5.56E-54	1.41E-53
RP11-99A1.2	NA	NA	NA

RP11-839G9.	NA	NA	NA
CCDC68	NA	9.42E-102	5.25E-101
CTD-2171N6.	NA	NA	NA
TCF4	1.72276265	0.00574532	0.00641267
TCF4-AS1	NA	NA	NA
TCF4-AS2	Inf	5.81E-73	1.94E-72
RP11-397A1(	NA	NA	NA
LINC01415	NA	NA	NA
RP11-397A1(	NA	NA	NA
LINC01416	NA	NA	NA
CTD-2008L17	NA	NA	NA
CTD-2008L17	NA	NA	NA
RP11-456O1!	NA	NA	NA
RP11-456O1!	NA	NA	NA
LINC01539	NA	NA	NA
RP11-456O1!	NA	NA	NA
TXNL1	1.08090392	4.25E-108	2.73E-107
WDR7	1.74773022	0.67534839	0.68666539
RP11-383D2.	NA	NA	NA
LINC-ROR	NA	NA	NA
CTD-2526M8	NA	NA	NA
BOD1L2	NA	NA	NA
CTD-2526M8	NA	NA	NA
ST8SIA3	NA	NA	NA
RP11-714M2	NA	NA	NA
ONECUT2	2.08063121	1.24E-14	1.78E-14
FECH	1.36429961	1.97E-10	2.61E-10
RP11-706P11	Inf	1.05E-99	5.63E-99
NARS	1.67906939	9.16E-06	1.10E-05
RP11-35G9.3	2.04734112	0.00039987	0.00046171
RP11-35G9.5	1.49805447	4.94E-32	9.15E-32
ATP8B1	NA	6.76E-53	1.68E-52
RP11-693L9.:	NA	NA	NA
NEDD4L	1.74773022	1.66E-58	4.45E-58
RP11-718I15	NA	NA	NA
RP11-845C2:	NA	NA	NA
RP11-1151B:	NA	NA	NA
RP11-1151B:	NA	NA	NA
RP11-1151B:	NA	NA	NA
ALPK2	NA	NA	NA
RP11-1151B:	NA	NA	NA
RP11-126O1.	NA	NA	NA
RP11-126O1.	NA	NA	NA
RP11-126O1.	0.24967575	1.09E-46	2.48E-46
MALT1	2.41353221	0.11230211	0.11948771

RP11-126O1.	NA	NA	NA
RP11-108P2(	NA	NA	NA
RP11-108P2(	NA	NA	NA
RP11-108P2(	NA	NA	NA
ZNF532	NA	NA	NA
RP11-685A2:	NA	NA	NA
SEC11C	2.0364178	1.39E-39	2.87E-39
GRP	NA	NA	NA
RAX	NA	NA	NA
CPLX4	NA	NA	NA
LMAN1	1.23015422	2.21E-64	6.49E-64
RP11-27G24.	NA	NA	NA
CCBE1	Inf	1.35E-45	3.03E-45
RP11-27G24.	NA	NA	NA
RP11-760L24	NA	NA	NA
RP11-2N1.1	NA	NA	NA
RP11-2N1.2	NA	NA	NA
RP11-2N1.3	NA	NA	NA
PMAIP1	1.45756652	1.99E-21	3.17E-21
RP11-866E2(	NA	3.17E-21	5.03E-21
RP11-795H1(	NA	NA	NA
RP11-396N1:	NA	NA	NA
MC4R	NA	NA	NA
RP11-325K1!	NA	NA	NA
CDH20	NA	NA	NA
RP11-879F14	NA	NA	NA
RP11-879F14	NA	1.13E-120	1.03E-119
RP11-879F14	NA	NA	NA
RP11-1096D!	NA	NA	NA
LINC01544	NA	NA	NA
RNF152	0.74902724	1.33E-13	1.87E-13
PIGN	2.88910506	1.02E-57	2.70E-57
KIAA1468	9.73735409	4.78E-88	2.05E-87
RP11-173A1(	NA	NA	NA
TNFRSF11A	2.49675746	4.71E-12	6.46E-12
RP11-640A1.	NA	NA	NA
ZCCHC2	0.91547773	6.30E-06	7.63E-06
RP11-120K1!	NA	3.46E-109	2.28E-108
PHLPP1	2.43433852	1.29E-55	3.33E-55
BCL2	1.93018557	1.20E-05	1.45E-05
RP11-299P2.	NA	1.28E-142	5.08E-141
KDSR	1.6692607	8.21E-64	2.39E-63
RP11-635N1!	2.62159533	8.33E-06	1.00E-05
VPS4B	1.75272374	0.00016146	0.00018829
SERPINB5	NA	NA	NA

SERPINB12	NA	NA	NA
SERPINB13	NA	NA	NA
SERPINB4	NA	NA	NA
SERPINB3	NA	NA	NA
SERPINB11	NA	NA	NA
SERPINB7	NA	NA	NA
SERPINB2	NA	NA	NA
SERPINB10	NA	NA	NA
HMSD	NA	3.74E-17	5.59E-17
SERPINB8	0	1.61E-76	5.64E-76
LINC00305	NA	NA	NA
RP11-146N1	NA	NA	NA
AC100848.1	NA	NA	NA
LINC01538	NA	NA	NA
AC007948.1	NA	NA	NA
AC007631.1	NA	NA	NA
RP11-453M2	NA	NA	NA
RP11-775G2	NA	NA	NA
CDH7	NA	NA	NA
RP11-389J22	NA	NA	NA
CDH19	NA	NA	NA
CTD-2541J13	NA	1.75E-05	2.10E-05
DSEL	2.39688716	9.00E-14	1.27E-13
CTD-2541J13	NA	NA	NA
RP11-638L3.	NA	NA	NA
RP11-28O3.2	NA	NA	NA
RP11-638L3.	NA	NA	NA
RP11-659F2	NA	NA	NA
RP11-526H1	NA	NA	NA
RP11-421N8.	NA	NA	NA
RP11-128D1	NA	NA	NA
TMX3	1.28404669	9.50E-19	1.45E-18
CCDC102B	2.24708171	4.85E-23	7.92E-23
RP11-106E1	NA	0.00048233	0.00055541
RP11-861L17	NA	NA	NA
RP11-674P1	NA	NA	NA
RP11-17E3.1	NA	NA	NA
DOK6	NA	NA	NA
RP11-465I4.2	NA	NA	NA
RP11-543H2	NA	NA	NA
CD226	0	4.75E-36	9.32E-36
RTTN	1.74773022	6.53E-20	1.02E-19
SOCS6	1.81014916	4.02E-14	5.70E-14
RP11-41O4.1	Inf	8.17E-34	1.55E-33
RP11-484N1	NA	NA	NA

RP11-41O4.2	NA	NA	NA
GTSCR1	NA	NA	NA
RP11-384E22	NA	NA	NA
RP11-47G4.2	NA	NA	NA
LINC01541	NA	NA	NA
RP11-723G8.	NA	NA	NA
RP11-723G8.	NA	NA	NA
RP11-736G1.	NA	NA	NA
CBLN2	NA	NA	NA
NETO1	NA	NA	NA
RP11-676J15	NA	NA	NA
RP11-126K15	NA	NA	NA
RP11-25L3.3	NA	NA	NA
FBXO15	NA	4.98E-46	1.12E-45
TIMM21	1.65629967	5.35E-113	3.91E-112
RP11-669I1.1	NA	NA	NA
CYB5A	NA	0.0007698	0.00088128
C18orf63	NA	NA	NA
RP11-231E4.	NA	NA	NA
FAM69C	NA	NA	NA
CNDP2	1.32782101	1.05E-81	4.01E-81
CNDP1	NA	3.26E-141	1.07E-139
RP11-231E4.	NA	NA	NA
LINC00909	1.74773022	3.15E-29	5.61E-29
ZNF407	1.68531128	4.13E-12	5.67E-12
RP11-405M1	NA	3.32E-09	4.31E-09
RP11-405M1	NA	2.64E-126	3.00E-125
ZADH2	1.66450497	0.0666919	0.07165252
TSHZ1	4.28015564	4.48E-62	1.27E-61
RP11-321M2	NA	NA	NA
RP11-321M2	NA	NA	NA
SMIM21	NA	NA	NA
RP11-321M2	NA	NA	NA
RP11-704C2.	NA	NA	NA
RP11-173L6.	NA	NA	NA
RP11-357H3.	NA	NA	NA
RP11-94B19.	NA	NA	NA
RP11-94B19.	NA	NA	NA
RP11-94B19.	NA	NA	NA
RP11-94B19.	NA	NA	NA
RP11-94B19.	NA	NA	NA
RP11-94B19.	NA	NA	NA
RP11-94B19.	NA	NA	NA
ZNF516	0.42801556	8.29E-28	1.45E-27
RP11-504I13	NA	NA	NA

RP11-17M16	Inf	0.00134547	0.00152942
C18orf65	NA	1.74E-21	2.77E-21
LINC00908	NA	NA	NA
LINC00683	Inf	2.60E-122	2.50E-121
RP11-111H3.	NA	NA	NA
AC034110.1	NA	NA	NA
RP11-162A1:	NA	0.03784837	0.04104275
RP11-162A1:	2.24708171	6.30E-58	1.68E-57
ZNF236	2.17217899	2.24E-64	6.58E-64
RP11-862L9.:	NA	1.66E-31	3.06E-31
MBP	1.87256809	1.84E-26	3.16E-26
RP11-4B16.3	NA	NA	NA
RP11-4B16.4	NA	1.48E-28	2.61E-28
RP11-751H1	NA	NA	NA
GALR1	NA	NA	NA
RP11-176N1	NA	NA	NA
RP11-63N3.1	NA	NA	NA
LINC01029	NA	NA	NA
RP11-671C1	NA	NA	NA
RP11-100K1	NA	NA	NA
RP11-451L1	NA	NA	NA
RP11-123I22	NA	NA	NA
RP11-715C4.	NA	NA	NA
CTD-2382H1	NA	NA	NA
CTD-2382H1	NA	NA	NA
RP11-849I19	NA	NA	NA
SALL3	NA	5.76E-32	1.06E-31
ATP9B	2.24708171	0.00022135	0.0002572
RP11-1136J1	NA	NA	NA
RP11-800A1	NA	NA	NA
NFATC1	2.38752432	1.36E-12	1.88E-12
RP11-196B3.	NA	NA	NA
RP11-567M1	NA	NA	NA
RP11-567M1	NA	NA	NA
RP11-567M1	4.49416342	8.04E-47	1.83E-46
CTDP1	1.55353797	1.18E-41	2.51E-41
RP11-567M1	NA	NA	NA
RP11-154H1	NA	NA	NA
RP11-154H1	NA	3.18E-73	1.07E-72
KCNG2	NA	NA	NA
RP11-248M1	Inf	1.09E-70	3.51E-70
PQLC1	2.4663092	1.36E-137	2.96E-136
HSBP1L1	2.24224928	3.74E-117	3.03E-116
TXNL4A	1.99204483	2.48E-79	9.11E-79
RP11-795F1	1.49805447	0.00028195	0.00032697

RBFA	1.31971466	4.42E-97	2.26E-96
RP11-795F19	Inf	3.58E-66	1.08E-65
ADNP2	2.16385646	1.48E-49	3.51E-49
PARD6G-AS1	NA	2.01E-138	4.69E-137
PARD6G	2.58754864	2.22E-55	5.71E-55
AC139100.3	NA	2.10E-127	2.51E-126
DEFB125	NA	NA	NA
DEFB126	NA	NA	NA
DEFB127	NA	NA	NA
DEFB128	NA	NA	NA
DEFB129	NA	NA	NA
DEFB132	NA	NA	NA
RP5-1103G7.	NA	NA	NA
C20orf96	2.56809339	0.08198894	0.08781175
ZCCHC3	1.6719358	6.47E-24	1.07E-23
NRSN2-AS1	2.3540856	3.82E-32	7.08E-32
SOX12	1.98492218	2.22E-41	4.69E-41
NRSN2	0.74902724	2.26E-107	1.42E-106
TRIB3	1.74004789	8.73E-52	2.14E-51
RBCK1	2.44682231	5.55E-143	2.39E-141
TBC1D20	2.52796693	1.61E-64	4.73E-64
CSNK2A1	1.88817283	0.44980319	0.46278736
TCF15	Inf	1.65E-93	7.86E-93
SRXN1	0.74902724	1.20E-105	7.28E-105
RP5-850E9.3	NA	NA	NA
SCRT2	NA	NA	NA
SLC52A3	NA	NA	NA
FAM110A	2.08063121	1.79E-32	3.33E-32
ANGPT4	NA	NA	NA
RSPO4	NA	NA	NA
PSMF1	1.21924989	5.78E-130	7.78E-129
RP4-545L17.	1.49805447	1.39E-05	1.67E-05
TMEM74B	2.99610895	8.10E-18	1.22E-17
C20orf202	NA	NA	NA
RAD21L1	NA	NA	NA
SNPH	NA	NA	NA
SDCBP2	0.74902724	6.82E-41	1.43E-40
RP11-314N1	NA	NA	NA
SDCBP2-AS1	Inf	3.27E-27	5.68E-27
RP11-314N1	NA	8.92E-13	1.24E-12
FKBP1A	1.90581473	1.80E-05	2.15E-05
NSFL1C	1.10625561	1.22E-96	6.18E-96
SIRPB2	NA	1.52E-128	1.91E-127
SIRPD	NA	2.03E-144	1.18E-142
RP4-576H24.	NA	NA	NA

SIRPB1	NA	7.43E-94	3.55E-93
SIRPG	NA	NA	NA
SIRPG-AS1	NA	NA	NA
RP5-968J1.1	NA	NA	NA
RP5-858L17.	Inf	0.00053842	0.0006191
SIRPA	1.63424125	0.01955815	0.02143951
RP4-684O24.	NA	NA	NA
PDYN	NA	NA	NA
STK35	2.90248054	9.73E-66	2.92E-65
RP11-128M1	0	1.18E-41	2.50E-41
TGM3	NA	NA	NA
TGM6	NA	NA	NA
SNRPB	1.4884457	1.71E-138	4.07E-137
RP4-734P14.	NA	NA	NA
ZNF343	2.03307393	1.44E-48	3.36E-48
TMC2	NA	NA	NA
NOP56	2.0716766	7.26E-14	1.03E-13
IDH3B	1.04001624	2.90E-142	1.09E-140
RP4-686C3.7	NA	NA	NA
EBF4	Inf	1.40E-47	3.21E-47
CPXM1	0.74902724	1.01E-19	1.57E-19
RP5-860F19.	NA	NA	NA
C20orf141	NA	NA	NA
TMEM239	NA	NA	NA
PCED1A	0.89883268	1.09E-46	2.47E-46
VPS16	2.02237354	1.84E-71	5.99E-71
PTPRA	1.8385214	8.63E-26	1.47E-25
GNRH2	NA	NA	NA
MRPS26	1.39853337	1.83E-52	4.52E-52
OXT	2.52796693	2.46E-07	3.07E-07
AVP	0	0.00036774	0.00042512
UBOX5-AS1	NA	NA	NA
UBOX5	1.49805447	2.65E-14	3.77E-14
FASTKD5	1.1432521	7.51E-92	3.46E-91
RP5-1187M1	2.99610895	4.52E-98	2.35E-97
DDRGK1	0.98068515	1.10E-119	9.74E-119
ITPA	1.64679747	7.55E-83	2.95E-82
SLC4A11	2.52796693	1.28E-42	2.75E-42
RP4-794I6.4	0.49935149	3.42E-20	5.35E-20
C20orf194	1.49805447	0.02549965	0.02783482
ATRN	0.8642622	5.71E-18	8.65E-18
GFRA4	NA	NA	NA
ADAM33	NA	NA	NA
SIGLEC1	NA	NA	NA
HSPA12B	NA	NA	NA

C20orf27	2.03955901	4.49E-77	1.59E-76
SPEF1	NA	6.69E-24	1.10E-23
CENPB	2.17740476	3.12E-58	8.33E-58
CDC25B	1.25836576	6.01E-61	1.68E-60
RP5-1009E24	NA	1.15E-12	1.60E-12
AP5S1	1.26398346	3.18E-28	5.59E-28
MAVS	2.43869333	5.41E-64	1.58E-63
RP11-119B16	13.4824903	4.68E-87	1.96E-86
PANK2	1.49805447	1.06E-76	3.72E-76
RP4-681N20.	NA	1.48E-51	3.60E-51
RNF24	1.44744453	9.82E-42	2.09E-41
RP11-352D3.	NA	9.81E-47	2.23E-46
SMOX	0.80664472	4.09E-38	8.26E-38
LINC01433	NA	3.00E-35	5.82E-35
ADRA1D	NA	NA	NA
RP11-307O10	NA	NA	NA
RP5-1164C1.	NA	NA	NA
PRNP	1.70468268	0.05990313	0.06448482
PRND	NA	NA	NA
PRNT	NA	NA	NA
RP5-1068H6.	NA	0.00057222	0.00065754
RASSF2	1.78614187	3.55E-13	4.95E-13
SLC23A2	2.69649805	3.98E-91	1.81E-90
RP5-1116H23	NA	NA	NA
RP5-1116H23	Inf	3.53E-127	4.18E-126
RP5-1116H23	Inf	1.30E-92	6.11E-92
RP5-1116H23	NA	NA	NA
TMEM230	1.70209983	2.73E-11	3.68E-11
PCNA	0.81590467	1.27E-140	3.86E-139
CDS2	1.44639742	2.45E-14	3.49E-14
PROKR2	NA	NA	NA
RP5-828H9.4	NA	NA	NA
LINC00658	NA	NA	NA
RP5-828H9.1	NA	NA	NA
LINC00654	NA	1.03E-120	9.42E-120
RP5-1022P6.	NA	NA	NA
RP5-1022P6.	NA	NA	NA
RP5-1022P6.	NA	NA	NA
GPCPD1	2.47178988	0.0957529	0.10229355
C20orf196	2.42684825	7.79E-28	1.36E-27
CHGB	0	0.0004614	0.00053188
TRMT6	1.48903005	9.89E-45	2.18E-44
MCM8	3.02867535	2.08E-117	1.70E-116
MCM8-AS1	NA	NA	NA
RP5-967N21.	Inf	8.11E-46	1.82E-45

CRLS1	2.16763943	3.50E-69	1.11E-68
LRRN4	NA	1.75E-05	2.10E-05
RP5-1056H1.	NA	0.00787581	0.00875401
FERMT1	1.95899431	1.07E-60	2.98E-60
CASC20	NA	NA	NA
RP5-859D4.3	NA	NA	NA
BMP2	NA	8.05E-14	1.14E-13
RP4-764O22.	NA	NA	NA
LINC01428	NA	NA	NA
RP11-19D2.1	NA	NA	NA
RP11-19D2.2	NA	NA	NA
RP5-988G17.	NA	NA	NA
HAO1	NA	NA	NA
TMX4	2.12224384	8.84E-30	1.59E-29
RP5-971N18.	NA	NA	NA
PLCB1	NA	1.25E-48	2.93E-48
PLCB4	NA	NA	NA
LAMP5-AS1	NA	NA	NA
LAMP5	NA	NA	NA
PAK7	NA	NA	NA
RP5-986I17.2	NA	NA	NA
RP5-839B4.8	NA	NA	NA
ANKEF1	0	1.29E-08	1.65E-08
SNAP25-AS1	NA	NA	NA
RP11-416N4.	NA	NA	NA
RP11-416N4.	NA	NA	NA
SNAP25	NA	1.45E-08	1.86E-08
MKKS	2.45015132	5.76E-136	1.10E-134
SLX4IP	1.81014916	3.22E-12	4.43E-12
JAG1	2.99610895	8.28E-99	4.36E-98
RP11-103J8.1	NA	NA	NA
RP1-278O22.	NA	4.81E-139	1.22E-137
RP1-278O22.	NA	NA	NA
RP4-697P8.3	NA	NA	NA
RP11-103J8.2	NA	NA	NA
C20orf187	NA	NA	NA
RP4-734C18.	NA	NA	NA
RP5-1128N1	NA	NA	NA
RP11-268G1	NA	NA	NA
LINC00687	NA	NA	NA
BTBD3	1.31079767	1.25E-08	1.60E-08
RP4-742J24.2	NA	NA	NA
RP4-796I8.1	NA	NA	NA
RP11-102J14	NA	NA	NA
RP5-1069C8.	NA	NA	NA

RP5-1069C8.	NA	NA	NA
RP11-157E14	NA	NA	NA
SPTLC3	NA	NA	NA
ISM1	0	1.63E-118	1.37E-117
ISM1-AS1	NA	NA	NA
RP5-1077I2.3	NA	NA	NA
RP4-585I14.3	NA	0.02658807	0.02900546
TASP1	0.85603113	2.84E-97	1.46E-96
ESF1	1.50356203	2.71E-19	4.19E-19
NDUFAF5	1.49805447	1.02E-26	1.76E-26
SEL1L2	NA	NA	NA
MACROD2	NA	NA	NA
FLRT3	NA	NA	NA
MACROD2-A	NA	NA	NA
RP5-974N19.	NA	NA	NA
RP11-388K2.	NA	NA	NA
KIF16B	0.81144617	4.54E-09	5.88E-09
AL049794.1	2.62159533	3.13E-36	6.17E-36
RP4-777L9.2	NA	5.67E-07	7.01E-07
RP4-777L9.3	NA	4.68E-128	5.75E-127
SNRPB2	1.11692401	2.12E-124	2.22E-123
OTOR	NA	NA	NA
RP5-1195D24	NA	NA	NA
PCSK2	NA	NA	NA
BFSP1	NA	0.09092797	0.09720773
DSTN	1.78893884	1.21E-06	1.49E-06
RRBP1	1.93353543	4.82E-34	9.20E-34
BANF2	NA	NA	NA
RP5-905G11.	NA	NA	NA
SNX5	2.18173034	2.02E-129	2.65E-128
OVOL2	NA	0.00426398	0.00477897
MGME1	1.93713941	3.33E-87	1.40E-86
RP4-726N1.2	NA	NA	NA
PET117	2.42866407	6.30E-102	3.53E-101
CSRP2BP	1.55155642	0.90547019	0.9099845
ZNF133	0.87386511	0.50016023	0.51334617
RP4-568F9.3	NA	NA	NA
RP4-568F9.6	NA	NA	NA
LINC00851	NA	NA	NA
DZANK1	1.49805447	0.09123709	0.09752671
POLR3F	1.14137484	7.89E-21	1.25E-20
RBBP9	0.93628405	1.36E-15	1.99E-15
SEC23B	0.97096123	1.91E-59	5.21E-59
LINC00493	1.67001014	1.87E-08	2.39E-08
DTD1	1.37836456	4.29E-68	1.34E-67

RP11-379J5.5	NA	NA	NA
LINC00652	NA	2.36E-17	3.54E-17
RP5-1068E13	1.49805447	1.78E-28	3.15E-28
SCP2D1	NA	NA	NA
RP11-97N19.	NA	NA	NA
SLC24A3	NA	NA	NA
RP11-97N19.	NA	NA	NA
RP5-1027G4.	NA	NA	NA
RP1-122P22.	NA	NA	NA
RP1-122P22.	NA	NA	NA
RP5-999L4.2	NA	3.36E-115	2.57E-114
RIN2	NA	7.50E-22	1.20E-21
NAA20	1.36404321	9.89E-65	2.92E-64
CRNKL1	1.12354086	2.32E-11	3.13E-11
CFAP61	Inf	4.57E-87	1.92E-86
RP5-1002M8	NA	NA	NA
RP5-1178H5.	NA	NA	NA
RP5-1096J16	NA	NA	NA
INSM1	NA	2.51E-23	4.11E-23
RALGAPA2	1.49805447	7.42E-10	9.76E-10
RP5-1177M2	NA	NA	NA
LINC00237	NA	NA	NA
KIZ	0.92187968	5.20E-84	2.08E-83
RP5-872K7.7	NA	NA	NA
KIZ-AS1	NA	1.64E-85	6.70E-85
RP1-198K11.	2.62159533	3.86E-13	5.38E-13
XRN2	1.44493198	2.77E-102	1.56E-101
NKX2-4	NA	NA	NA
RP11-227D2.	NA	0.38801892	0.40076511
RP5-984P4.6	3.6381323	2.53E-112	1.81E-111
NKX2-2	1.12354086	6.24E-29	1.11E-28
NKX2-2-AS1	NA	NA	NA
RP5-984P4.4	NA	NA	NA
RP1-167O22.	NA	NA	NA
RP5-1065O2.	NA	NA	NA
PAX1	NA	NA	NA
RP5-1185K9.	NA	NA	NA
LINC01432	NA	NA	NA
RP5-1097P24	NA	NA	NA
LINC01427	NA	NA	NA
RP5-1004I9.1	NA	NA	NA
LINC00261	Inf	9.69E-12	1.32E-11
FOXA2	1.49805447	1.02E-28	1.80E-28
LINC01384	NA	NA	NA
RP11-359G2.	NA	5.04E-119	4.33E-118

RP11-359G2:	NA	NA	NA
RP4-753D10.	NA	NA	NA
SSTR4	NA	NA	NA
THBD	Inf	1.04E-24	1.75E-24
CD93	NA	8.45E-29	1.50E-28
LINC00656	NA	NA	NA
RP4-737E23.	NA	NA	NA
RP4-737E23.	NA	NA	NA
RP5-851M4.:	Inf	1.37E-26	2.35E-26
RP3-322G13.	NA	NA	NA
NXT1	1.7984258	0.04529041	0.0489753
LINC01431	1.68531128	5.56E-34	1.06E-33
GZF1	1.49805447	6.57E-07	8.12E-07
NAPB	1.49805447	1.70E-46	3.85E-46
CSTL1	NA	NA	NA
CST11	NA	NA	NA
RP3-333B15.	NA	NA	NA
RP3-333B15.	NA	NA	NA
RP3-333B15.	NA	NA	NA
CST8	NA	NA	NA
CST9L	NA	NA	NA
CST9	NA	NA	NA
CST3	0.65914397	9.77E-144	4.87E-142
RP11-218C1	NA	NA	NA
RP11-218C1	NA	NA	NA
CST4	NA	NA	NA
CST1	NA	NA	NA
XXyac-YX60D	NA	NA	NA
CST2	NA	NA	NA
CST5	NA	NA	NA
GGTLC1	NA	NA	NA
RP5-1100I6.1	NA	NA	NA
FLJ33581	NA	NA	NA
RP4-564O4.1	NA	NA	NA
SYNDIG1	NA	NA	NA
RP11-526K1	NA	NA	NA
RP5-860P4.2	NA	NA	NA
RP4-568C11.	NA	NA	NA
CST7	1.19163424	3.72E-52	9.15E-52
APMAP	0.86803156	7.99E-140	2.21E-138
ACSS1	1.33522247	0.00017544	0.00020436
RP5-1025A1.	NA	NA	NA
VSX1	NA	NA	NA
RP4-738P15.	NA	2.80E-84	1.12E-83
ENTPD6	1.41837073	0.96490647	0.96666481

RP5-965G21.	0	4.57E-08	5.78E-08
RP5-965G21.	NA	5.76E-24	9.52E-24
PYGB	2.0598249	1.49E-33	2.82E-33
RP5-965G21.	NA	NA	NA
RP5-965G21.	Inf	9.60E-122	9.08E-121
ABHD12	1.69345288	0.27671191	0.28864743
GINS1	4.36198215	2.55E-137	5.46E-136
NINL	NA	9.63E-60	2.63E-59
NANP	3.49546044	2.64E-20	4.13E-20
ZNF337-AS1	Inf	6.36E-32	1.17E-31
ZNF337	1.31079767	0.01800053	0.01975474
RP4-694B14.	0.74902724	1.26E-30	2.29E-30
RP13-401N8.	NA	NA	NA
FAM182B	NA	NA	NA
RP4-760C5.5	NA	NA	NA
RP4-760C5.3	NA	NA	NA
RP13-329D4.	NA	NA	NA
RP3-410C9.2	NA	NA	NA
LINC01597	NA	NA	NA
LINC01598	NA	NA	NA
DEFB115	NA	NA	NA
DEFB116	NA	NA	NA
DEFB118	NA	NA	NA
DEFB119	NA	NA	NA
DEFB121	NA	NA	NA
DEFB123	NA	NA	NA
DEFB124	NA	NA	NA
REM1	NA	NA	NA
LINC00028	Inf	2.83E-72	9.37E-72
HM13	1.51925336	3.51E-61	9.83E-61
HM13-AS1	Inf	5.16E-90	2.29E-89
RP3-324O17.	NA	1.03E-24	1.73E-24
ID1	3.02107652	3.52E-128	4.35E-127
COX4I2	NA	NA	NA
BCL2L1	3.4330415	2.60E-101	1.44E-100
RP11-243J16	0	0.03528304	0.03829982
ABALON	Inf	6.52E-19	1.00E-18
TPX2	2.14256628	1.82E-24	3.04E-24
MYLK2	Inf	5.60E-142	2.04E-140
FOXS1	NA	NA	NA
DUSP15	2.7464332	0.20326975	0.21371891
TTLL9	NA	2.29E-41	4.83E-41
PDRG1	1.39757521	2.07E-26	3.55E-26
XKR7	NA	NA	NA
CCM2L	NA	1.25E-18	1.91E-18

RP1-310O13.	NA	NA	NA
HCK	0.82392996	3.37E-80	1.26E-79
TM9SF4	2.19714656	1.05E-11	1.43E-11
PLAGL2	1.9214177	9.98E-07	1.23E-06
POFUT1	2.49675746	9.92E-08	1.25E-07
KIF3B	2.18466278	3.42E-98	1.79E-97
RP11-358N2.	NA	3.18E-113	2.33E-112
ASXL1	1.66088648	2.59E-18	3.94E-18
NOL4L	2.15345331	0.93865296	0.94176761
RP5-1184F4.	NA	NA	NA
RP5-1184F4.	NA	1.78E-10	2.36E-10
RP11-410N8.	NA	NA	NA
RP11-410N8.	NA	NA	NA
RP11-410N8.	NA	NA	NA
C20orf203	NA	4.31E-28	7.56E-28
COMMD7	1.49002918	2.03E-62	5.77E-62
DNMT3B	3.05372643	1.28E-56	3.35E-56
MAPRE1	1.30265606	4.98E-87	2.09E-86
RP5-1085F17	NA	NA	NA
RP5-1085F17	1.12354086	1.05E-48	2.45E-48
EFCAB8	NA	NA	NA
SUN5	NA	NA	NA
BPIFB2	NA	NA	NA
BPIFB6	NA	NA	NA
BPIFB3	NA	NA	NA
BPIFB4	NA	NA	NA
BPIFA2	NA	NA	NA
RP11-49G10.	NA	NA	NA
BPIFA3	NA	NA	NA
BPIFA1	NA	NA	NA
BPIFB1	NA	NA	NA
CDK5RAP1	1.47222595	9.99E-92	4.59E-91
SNTA1	1.53062088	3.98E-107	2.49E-106
CBFA2T2	1.99740597	3.22E-18	4.90E-18
RP1-63M2.7	NA	3.94E-07	4.89E-07
NECAB3	1.80647745	1.61E-22	2.60E-22
C20orf144	NA	1.06E-57	2.82E-57
ACTL10	NA	NA	NA
RP1-63M2.5	NA	NA	NA
E2F1	1.97362732	1.74E-69	5.54E-69
PXMP4	1.63023575	5.21E-16	7.64E-16
RP4-553F4.2	NA	NA	NA
ZNF341	3.74513619	6.34E-35	1.22E-34
ZNF341-AS1	NA	0.01402324	0.01544966
CHMP4B	1.46484637	8.88E-73	2.96E-72

RALY-AS1	1.1770428	0.80673025	0.8146332
RP5-1125A1:	NA	4.97E-27	8.60E-27
RP5-1125A1:	Inf	3.33E-17	4.97E-17
RALY	1.77714408	2.82E-26	4.82E-26
RP5-1125A1:	NA	NA	NA
EIF2S2	1.6435874	1.01E-32	1.88E-32
ASIP	NA	NA	NA
AHCY	1.51048646	2.96E-47	6.78E-47
ITCH	1.49805447	8.96E-17	1.33E-16
ITCH-AS1	NA	2.33E-06	2.85E-06
DYNLRB1	1.79807356	0.00034614	0.00040042
MAP1LC3A	NA	NA	NA
PIGU	1.14434717	3.62E-120	3.25E-119
NCOA6	0.97096123	0.81075283	0.81837633
TP53INP2	1.24837873	0.04288898	0.04643107
GGT7	Inf	4.42E-106	2.70E-105
ACSS2	3.2457847	0.97194261	0.97339127
GSS	1.04863813	1.63E-131	2.39E-130
MYH7B	Inf	1.11E-135	2.09E-134
TRPC4AP	2.51144427	5.08E-48	1.17E-47
EDEM2	0.95330739	6.44E-109	4.21E-108
PROCR	1.29377432	2.98E-24	4.96E-24
RP11-42O4.2	NA	NA	NA
MMP24-AS1	1.62645914	1.45E-33	2.75E-33
MMP24	NA	3.21E-82	1.24E-81
RP4-614O4.1	NA	NA	NA
EIF6	1.50133968	5.87E-66	1.77E-65
FAM83C-AS1	Inf	0.0044458	0.00497936
FAM83C	NA	1.91E-36	3.76E-36
UQCC1	1.32181277	1.34E-24	2.23E-24
GDF5OS	NA	NA	NA
GDF5	NA	NA	NA
CEP250	2.14007782	2.29E-08	2.92E-08
RP3-477O4.1	2.78210117	7.34E-60	2.01E-59
C20orf173	NA	NA	NA
ERGIC3	1.25775222	6.80E-123	6.72E-122
SPAG4	1.3732166	1.15E-25	1.94E-25
CPNE1	1.69280156	3.50E-123	3.51E-122
RP1-309K20.	NA	NA	NA
RBM12	1.81639105	1.46E-07	1.83E-07
NFS1	1.32520204	2.27E-131	3.29E-130
ROMO1	1.55470359	2.38E-37	4.76E-37
RBM39	1.74363718	4.29E-08	5.44E-08
PHF20	1.82021673	3.47E-40	7.21E-40
SCAND1	1.65184026	0.26252567	0.27428822

CNBD2	2.18946423	1.23E-36	2.44E-36
LINC00657	2.23695972	3.48E-128	4.30E-127
RP4-550H1.7	NA	7.24E-120	6.43E-119
RP4-550H1.4	NA	NA	NA
RP4-550H1.5	NA	NA	NA
EPB41L1	5.24319066	2.74E-25	4.62E-25
RP11-234K24	NA	NA	NA
RP11-234K24	NA	NA	NA
AAR2	1.3658732	2.63E-104	1.55E-103
DLGAP4	1.9661965	1.28E-14	1.84E-14
DLGAP4-AS1	0.37451362	8.84E-69	2.78E-68
MYL9	NA	1.11E-31	2.05E-31
TGIF2	2.1021087	8.81E-75	3.02E-74
TGIF2-C20or	NA	NA	NA
C20orf24	2.1306436	4.66E-61	1.30E-60
SLA2	0.74902724	6.87E-52	1.68E-51
NDRG3	1.47724816	1.96E-125	2.13E-124
DSN1	1.34308332	3.33E-89	1.46E-88
SOGA1	4.06614786	3.11E-128	3.85E-127
TLDC2	NA	NA	NA
SAMHD1	1.3732166	0.12774937	0.13566874
RBL1	1.87256809	4.11E-24	6.82E-24
RP4-621N11.	Inf	3.73E-18	5.66E-18
MROH8	3.74513619	1.69E-43	3.67E-43
RPN2	1.1587892	4.88E-119	4.21E-118
GHRH	NA	NA	NA
MANBAL	2.38083658	3.52E-12	4.84E-12
SRC	2.99610895	7.44E-11	9.96E-11
BLCAP	1.90928511	0.82668995	0.83409204
NNAT	NA	NA	NA
RP11-425M5	NA	NA	NA
LINC00489	NA	NA	NA
RP4-640H8.2	NA	1.02E-85	4.20E-85
CTNNBL1	1.14473974	5.39E-133	8.62E-132
VSTM2L	0.49935149	6.43E-29	1.14E-28
TTI1	1.74773022	0.01414334	0.01558009
RPRD1B	3.33657588	8.39E-61	2.34E-60
RP5-1057B2(	NA	NA	NA
TGM2	NA	1.43E-07	1.80E-07
KIAA1755	NA	NA	NA
BPI	0	1.81E-165	1.73E-162
CTD-2308N2.	NA	7.46E-10	9.81E-10
LBP	NA	0.0001285	0.00015024
RP4-564F22.	NA	NA	NA
RP4-564F22.	Inf	2.68E-46	6.05E-46

RP4-564F22.	Inf	5.78E-84	2.30E-83
RALGAPB	2.24708171	4.56E-15	6.59E-15
ADIG	NA	NA	NA
ARHGAP40	NA	NA	NA
SLC32A1	NA	NA	NA
ACTR5	1.09857328	1.82E-57	4.82E-57
PPP1R16B	7.49027237	9.67E-73	3.22E-72
FAM83D	0.7846952	3.64E-124	3.80E-123
RP4-616B8.5	2.24708171	0.00417663	0.00468193
RP4-616B8.4	NA	0.08863044	0.09477387
DHX35	1.49805447	1.26E-88	5.50E-88
RP4-705O1.1	NA	NA	NA
RP5-1031J8.:	NA	NA	NA
LINC01370	NA	NA	NA
RP11-101E14	NA	NA	NA
RP1-191L6.2	NA	NA	NA
MAFB	NA	NA	NA
RP4-644L1.2	NA	NA	NA
TOP1	1.43532806	3.68E-69	1.16E-68
PLCG1-AS1	NA	NA	NA
PLCG1	5.9922179	3.12E-10	4.12E-10
ZHX3	1.94747082	0.33300797	0.34573827
LPIN3	Inf	3.26E-141	1.07E-139
EMILIN3	NA	NA	NA
CHD6	0.74902724	1.18E-117	9.68E-117
RP5-1121H1:	NA	NA	NA
RP5-1121H1:	NA	NA	NA
PTPRT	NA	NA	NA
RP1-232N11.	NA	NA	NA
RP4-753D4.2	NA	NA	NA
RP1-269M15	NA	NA	NA
SRSF6	1.89161116	7.97E-16	1.17E-15
L3MBTL1	NA	NA	NA
RP1-138B7.6	NA	NA	NA
RP1-138B7.5	NA	NA	NA
SGK2	NA	NA	NA
RP1-138B7.7	Inf	3.77E-95	1.85E-94
IFT52	1.82762646	6.43E-07	7.94E-07
MYBL2	2.01738003	9.33E-46	2.09E-45
GTSF1L	NA	1.94E-56	5.06E-56
RP5-1030M6	NA	NA	NA
TOX2	0.74902724	2.05E-33	3.87E-33
JPH2	NA	NA	NA
OSER1	1.21271077	4.56E-78	1.64E-77
OSER1-AS1	0.74902724	0.41096675	0.42376712

GDAP1L1	NA	NA	NA
FITM2	0.83225249	1.46E-21	2.33E-21
R3HDML	NA	NA	NA
RP5-881L22.!	NA	NA	NA
HNF4A	NA	NA	NA
HNF4A-AS1	NA	NA	NA
RP5-881L22.(	NA	NA	NA
LINC01430	NA	NA	NA
LINC01620	NA	NA	NA
TTPAL	1.79766537	5.04E-40	1.05E-39
SERINC3	1.6172179	2.44E-37	4.87E-37
PKIG	0.97373541	0.79029214	0.79865642
ADA	1.26095233	4.28E-134	7.24E-133
LINC01260	NA	0.38801892	0.40076511
KCNK15-AS1	NA	3.49E-78	1.26E-77
WISP2	NA	2.94E-53	7.35E-53
KCNK15	NA	NA	NA
RIMS4	NA	NA	NA
YWHAB	1.20600739	3.80E-125	4.10E-124
PABPC1L	11.2354086	2.79E-117	2.27E-116
TOMM34	1.58232004	3.17E-73	1.06E-72
STK4-AS1	NA	1.04E-30	1.88E-30
STK4	1.84135863	4.68E-58	1.25E-57
KCNS1	NA	NA	NA
WFDC5	NA	NA	NA
WFDC12	NA	NA	NA
PI3	NA	NA	NA
SEMG1	NA	NA	NA
SEMG2	NA	NA	NA
SLPI	0.03404669	3.62E-131	5.22E-130
MATN4	NA	2.82E-115	2.17E-114
RBPJL	NA	NA	NA
SDC4	NA	NA	NA
SYS1	1.7880005	0.04310275	0.04665414
SYS1-DBNDD	NA	1.92E-166	1.94E-163
TP53TG5	NA	NA	NA
DBNDD2	NA	1.24E-42	2.67E-42
PIGT	1.31079767	7.50E-120	6.66E-119
RP3-453C12.	Inf	3.36E-11	4.52E-11
RP3-461P17.	NA	1.85E-46	4.18E-46
WFDC2	NA	7.03E-11	9.42E-11
RP3-461P17.	NA	NA	NA
SPINT3	NA	NA	NA
WFDC6	NA	NA	NA
EPPIN-WFDC	NA	NA	NA

EPPIN	NA	NA	NA
WFDC8	NA	NA	NA
WFDC9	NA	NA	NA
WFDC10A	NA	NA	NA
WFDC11	NA	NA	NA
WFDC10B	NA	NA	NA
WFDC13	NA	NA	NA
SPINT4	NA	NA	NA
WFDC3	0	6.85E-106	4.17E-105
DNTTIP1	0.9192607	4.71E-127	5.54E-126
UBE2C	0	3.12E-33	5.87E-33
TNNC2	NA	NA	NA
SNX21	0.74902724	6.82E-37	1.35E-36
ACOT8	1.34824903	2.29E-120	2.07E-119
ZSWIM3	Inf	0.01226322	0.01354022
ZSWIM1	3.74513619	0.04347901	0.04705018
SPATA25	NA	NA	NA
NEURL2	0.74902724	1.35E-40	2.81E-40
CTSA	0.84176394	1.22E-138	2.94E-137
RP3-337O18.	NA	NA	NA
PLTP	0.56177043	9.59E-134	1.60E-132
PCIF1	1.62289235	8.87E-42	1.89E-41
ZNF335	2.0598249	3.06E-84	1.23E-83
MMP9	1.31079767	1.98E-22	3.20E-22
RP11-465L10	3.74513619	8.67E-40	1.79E-39
SLC12A5	2.20302129	5.01E-24	8.29E-24
NCOA5	2.54669261	2.08E-66	6.31E-66
CD40	1.10725766	1.36E-136	2.73E-135
RP5-998H6.2	NA	NA	NA
CDH22	1.49805447	6.68E-59	1.80E-58
SLC35C2	1.95641443	0.0005497	0.0006319
RP11-394O2.	NA	NA	NA
ELMO2	3.49546044	2.69E-08	3.42E-08
ZNF334	NA	NA	NA
OCSTAMP	NA	5.11E-124	5.29E-123
SLC13A3	NA	6.61E-103	3.77E-102
TP53RK	1.22341115	2.98E-36	5.87E-36
RP1-28H20.3	NA	1.26E-45	2.83E-45
SLC2A10	NA	NA	NA
EYA2	NA	NA	NA
RP11-323C15	NA	NA	NA
ZMYND8	2.54669261	0.02941074	0.03202699
RP4-569M23	0	2.31E-56	6.01E-56
RP4-569M23	NA	2.40E-115	1.85E-114
RP1-148H17.	NA	NA	NA

NCOA3	1.3732166	2.57E-15	3.73E-15
SULF2	NA	NA	NA
CTD-2653D5.	NA	NA	NA
RP11-347D2:	NA	NA	NA
RP11-347D2:	NA	NA	NA
LINC01522	NA	NA	NA
LINC01523	NA	NA	NA
RP11-321P16	NA	NA	NA
LINC00494	NA	NA	NA
RP1-66N13.1	NA	NA	NA
RP1-66N13.4	NA	NA	NA
RP5-991B18.	NA	NA	NA
RP5-991B18.	NA	NA	NA
PREX1	1.49805447	1.03E-15	1.50E-15
RP5-906C1.1	1.19844358	5.22E-47	1.19E-46
ARFGEF2	1.44043699	0.72826319	0.73810898
CSE1L-AS1	NA	NA	NA
CSE1L	1.74230249	1.34E-08	1.72E-08
STAU1	1.93713941	0.70441748	0.71493941
DDX27	1.58036516	6.13E-39	1.25E-38
ZNFX1	2.81986725	2.17E-131	3.15E-130
ZFAS1	2.49472551	4.77E-127	5.60E-126
KCNB1	NA	NA	NA
PTGIS	NA	1.73E-35	3.37E-35
B4GALT5	2.20026751	7.73E-25	1.30E-24
SLC9A8	0.74902724	2.08E-19	3.21E-19
SPATA2	1.91418072	0.01505641	0.01657283
RNF114	1.40918684	1.40E-103	8.11E-103
SNAI1	1.12354086	5.07E-19	7.80E-19
TRERNA1	NA	0.00120379	0.00137077
TMEM189-U	NA	0.13377061	0.14180592
UBE2V1	0.93628405	0.00390098	0.00437726
TMEM189	2.20546909	1.16E-05	1.40E-05
RP11-112L6.:	NA	1.84E-51	4.48E-51
RP11-112L6.:	NA	NA	NA
LINC01273	NA	NA	NA
CEBPB-AS1	NA	1.99E-15	2.89E-15
CEBPB	1.85053788	8.92E-05	1.05E-04
LINC01272	NA	4.04E-37	8.04E-37
LINC01270	NA	1.76E-25	2.97E-25
LINC01271	NA	NA	NA
PTPN1	1.71487815	2.47E-24	4.12E-24
RP4-530I15.9	NA	NA	NA
FAM65C	Inf	4.72E-63	1.35E-62
RP4-530I15.6	NA	NA	NA

PARD6B	1.07003891	3.60E-27	6.23E-27
BCAS4	1.76241703	4.83E-38	9.73E-38
ADNP	3.28250172	2.24E-120	2.03E-119
ADNP-AS1	4.49416342	2.56E-13	3.58E-13
DPM1	0.85826038	8.33E-129	1.06E-127
MOCS3	2.3540856	0.33678084	0.34945539
RP5-955M13	NA	NA	NA
KCNG1	1.12354086	7.97E-23	1.30E-22
NFATC2	1.99740597	1.87E-21	2.98E-21
ATP9A	1.49805447	7.15E-09	9.21E-09
SALL4	NA	NA	NA
LINC01429	NA	3.14E-121	2.90E-120
ZFP64	1.31079767	5.20E-22	8.36E-22
LINC01524	NA	NA	NA
RP4-583K8.1	NA	NA	NA
RP5-1022J11	NA	NA	NA
RP4-715N11.	NA	NA	NA
RP11-80K6.2	NA	NA	NA
TSHZ2	NA	NA	NA
RP11-15M15	NA	NA	NA
RP11-15M15	NA	NA	NA
RP4-678D15.	NA	NA	NA
RP4-669H2.1	NA	NA	NA
RP5-823G15.	NA	NA	NA
RP4-724E16.	NA	7.20E-126	8.01E-125
ZNF217	1.77893969	6.28E-17	9.33E-17
AC005808.3	NA	NA	NA
AC006076.1	NA	NA	NA
BCAS1	Inf	1.59E-80	5.96E-80
AC005220.3	NA	NA	NA
CYP24A1	NA	NA	NA
PFDN4	1.82102952	1.84E-06	2.25E-06
AL133335.1	NA	NA	NA
DOK5	NA	NA	NA
LINC01440	NA	NA	NA
LINC01441	NA	NA	NA
RP5-837G17.	NA	NA	NA
CBLN4	NA	NA	NA
MC3R	NA	NA	NA
RP11-380D1!	NA	NA	NA
FAM210B	3.03015564	7.68E-64	2.24E-63
AURKA	1.52853814	4.90E-43	1.06E-42
CSTF1	1.25318019	3.99E-102	2.24E-101
CASS4	2.24708171	0.05083138	0.05488204
RTFDC1	1.28832685	8.03E-115	6.11E-114

GCNT7	NA	NA	NA
FAM209A	NA	1.96E-34	3.75E-34
FAM209B	1.49805447	2.34E-30	4.25E-30
RP5-843L14.1	NA	NA	NA
TFAP2C	NA	NA	NA
RP5-897D18.	NA	NA	NA
RP11-560A15	NA	NA	NA
RP11-560A15	NA	NA	NA
BMP7	NA	NA	NA
BMP7-AS1	NA	NA	NA
RP4-813D12.	NA	NA	NA
SPO11	NA	NA	NA
RAE1	0.85603113	3.51E-136	6.79E-135
MTRNR2L3	NA	1.04E-06	1.28E-06
RP4-800J21.3	2.24708171	2.16E-36	4.26E-36
RBM38	1.69632639	3.03E-23	4.97E-23
CTCFL	NA	NA	NA
PCK1	NA	NA	NA
RP4-718J7.4	NA	NA	NA
ZBP1	NA	NA	NA
PMEPA1	NA	NA	NA
NKILA	NA	NA	NA
RP13-379L11	NA	NA	NA
RP13-379L11	NA	3.65E-69	1.15E-68
RP13-379L11	NA	NA	NA
C20orf85	NA	NA	NA
ANKRD60	NA	NA	NA
RAB22A	1.37821012	8.47E-07	1.04E-06
VAPB	1.74098223	6.39E-11	8.56E-11
APCDD1L	NA	2.18E-60	6.02E-60
RP5-907D15.	NA	NA	NA
RP5-907D15.	NA	NA	NA
STX16	3.40466926	1.14E-65	3.41E-65
STX16-NPEPl	NA	2.43E-122	2.35E-121
NPEPL1	1.5693904	2.40E-11	3.24E-11
RP4-806M20	NA	NA	NA
RP4-806M20	NA	NA	NA
GNAS-AS1	NA	NA	NA
RP4-806M20	NA	NA	NA
GNAS	1.8526664	0.00144069	0.00163601
RP1-309F20.	NA	NA	NA
RP1-309F20.	NA	NA	NA
NELFCD	2.02237354	1.90E-35	3.71E-35
CTSZ	1.40910749	4.85E-132	7.31E-131
TUBB1	NA	2.40E-83	9.44E-83

ATP5E	2.15796915	4.30E-132	6.52E-131
PRELID3B	1.77822497	1.05E-32	1.95E-32
RP3-492J12.2	NA	NA	NA
ZNF831	NA	NA	NA
EDN3	NA	NA	NA
RP4-614C15.	NA	NA	NA
RP4-614C15.	NA	NA	NA
RP11-164D18	NA	NA	NA
PHACTR3	NA	NA	NA
RP4-719C8.1	NA	NA	NA
SYCP2	NA	2.41E-49	5.69E-49
FAM217B	1.96296793	1.69E-45	3.77E-45
PPP1R3D	2.8463035	1.08E-31	1.98E-31
CDH26	0	4.18E-137	8.72E-136
C20orf197	0.46814202	1.54E-70	4.96E-70
RP5-1017F8.	NA	NA	NA
MIR646HG	0.74902724	1.50E-06	1.84E-06
RP5-1016A21	NA	NA	NA
RP11-151E14	NA	NA	NA
RP5-827L5.2	NA	NA	NA
RP5-827L5.1	NA	NA	NA
CDH4	NA	4.11E-88	1.77E-87
WI2-8325B5.	NA	NA	NA
RP5-827E24.	NA	NA	NA
RP11-429E11	NA	NA	NA
RP11-429E11	NA	NA	NA
TAF4	0.93628405	0.22598261	0.23694022
RP11-11M20	0	6.60E-95	3.22E-94
LSM14B	1.79766537	3.68E-49	8.65E-49
PSMA7	1.30916336	2.91E-127	3.47E-126
SS18L1	2.99610895	9.04E-87	3.78E-86
MTG2	1.2963933	6.90E-34	1.31E-33
HRH3	0.37451362	2.01E-59	5.47E-59
OSBPL2	1.49805447	0.0078936	0.00877271
ADRM1	1.18479959	2.66E-110	1.80E-109
RP11-157P1.	Inf	1.66E-42	3.56E-42
LAMA5	1.92607004	2.65E-17	3.96E-17
LAMA5-AS1	NA	NA	NA
RP5-908M14	1.99740597	1.48E-27	2.57E-27
RPS21	2.55629552	1.85E-145	1.58E-143
CABLES2	2.99610895	1.18E-68	3.72E-68
RBBP8NL	NA	NA	NA
RP5-908M14	NA	NA	NA
GATA5	NA	NA	NA
RP13-379O24	NA	NA	NA

RP13-379O24	NA	NA	NA
C20orf166-AS	NA	NA	NA
MIR1-1HG	NA	NA	NA
WI2-87327B8	NA	NA	NA
WI2-87327B8	NA	1.27E-30	2.31E-30
RP13-30A9.1	NA	NA	NA
RP13-30A9.2	NA	1.18E-54	3.02E-54
RP11-93B14.	NA	1.55E-34	2.97E-34
SLCO4A1	2.12723735	0.02115946	0.02316966
RP11-93B14.	NA	NA	NA
RP11-93B14.	NA	NA	NA
SLCO4A1-AS	NA	1.48E-16	2.19E-16
LINC00686	NA	NA	NA
NTSR1	0	2.63E-05	3.13E-05
RP11-93B14.	NA	NA	NA
LINC00659	Inf	1.07E-155	3.41E-153
MRGBP	2.12544481	7.72E-60	2.12E-59
OGFR-AS1	NA	NA	NA
OGFR	1.14785992	1.45E-91	6.62E-91
COL9A3	2.53516911	1.58E-16	2.33E-16
TCFL5	1.57689945	3.06E-50	7.31E-50
DIDO1	2.92801556	7.13E-81	2.70E-80
RP4-563E14.	Inf	9.38E-142	3.33E-140
GID8	1.39506323	2.51E-18	3.82E-18
SLC17A9	1.9539841	0.15061044	0.15923857
BHLHE23	NA	NA	NA
RP11-305P22	NA	NA	NA
LINC00029	NA	NA	NA
LINC01056	NA	NA	NA
HAR1B	Inf	0.32416273	0.33678618
HAR1A	1.49805447	5.19E-77	1.84E-76
RP5-963E22.	NA	NA	NA
RP5-963E22.	NA	NA	NA
YTHDF1	1.9661965	2.44E-43	5.29E-43
RP5-963E22.	0	0.03300228	0.03588847
BIRC7	NA	NA	NA
NKAIN4	NA	NA	NA
FLJ16779	NA	NA	NA
ARFGAP1	1.73774319	3.22E-12	4.43E-12
COL20A1	NA	NA	NA
CHRNA4	NA	NA	NA
RP11-261N1	NA	NA	NA
KCNQ2	2.24708171	1.63E-44	3.58E-44
RP11-358D14	NA	NA	NA
RP11-358D14	NA	NA	NA

EEF1A2	0.68093385	4.56E-45	1.01E-44
RP4-697K14.	NA	NA	NA
PPDPF	2.60661479	2.69E-130	3.70E-129
PTK6	Inf	0.01709536	0.01878409
SRMS	NA	NA	NA
RP4-697K14.	NA	NA	NA
C20orf195	NA	NA	NA
HELZ2	Inf	9.45E-17	1.40E-16
GMEB2	2.7464332	3.64E-91	1.65E-90
CTD-3184A7.	4.11964981	4.64E-118	3.86E-117
STMN3	3.15894096	0.22370215	0.23458985
RTEL1	NA	3.62E-19	5.57E-19
RTEL1-TNFR	NA	5.23E-26	8.91E-26
TNFRSF6B	NA	NA	NA
ARFRP1	1.82762646	0.23109836	0.24222003
ZGPAT	NA	9.69E-41	2.03E-40
RP4-583P15.	NA	NA	NA
RP4-583P15.	NA	NA	NA
LIME1	NA	NA	NA
RP4-583P15.	NA	NA	NA
SLC2A4RG	2.0681106	0.00016814	0.00019599
ZBTB46	2.24708171	4.99E-29	8.88E-29
RP4-583P15.	Inf	0.00015036	0.00017547
ZBTB46-AS1	NA	NA	NA
RP4-591C20.	4.86867704	1.05E-25	1.78E-25
ABHD16B	NA	NA	NA
TPD52L2	1.74773022	6.47E-10	8.52E-10
DNAJC5	2.57166018	1.54E-57	4.09E-57
UCKL1	2.29701686	9.34E-05	1.10E-04
UCKL1-AS1	1.49805447	1.79E-09	2.33E-09
ZNF512B	1.87256809	0.02051421	0.02247939
SAMD10	0.91547773	0.00062624	0.00071879
PRPF6	1.9935648	4.44E-53	1.11E-52
LINC00176	0.74902724	2.44E-20	3.82E-20
SOX18	2.20302129	4.97E-12	6.80E-12
TCEA2	1.62593717	3.52E-86	1.46E-85
RP13-152O1	NA	NA	NA
RGS19	1.4678923	2.60E-101	1.44E-100
OPRL1	NA	1.42E-07	1.78E-07
LKAAEAR1	0.74902724	2.34E-108	1.51E-107
NPBWR2	NA	NA	NA
MYT1	0.56177043	1.34E-17	2.01E-17
PCMTD2	1.91418072	5.50E-10	7.25E-10
LINC00266-1	0	4.80E-44	1.05E-43
AC008993.3	NA	NA	NA

FAM138F	NA	NA	NA
OR4F17	NA	NA	NA
AC092192.1	NA	NA	NA
CTD-3113P1(	3.2457847	5.81E-17	8.65E-17
PLPP2	NA	1.44E-124	1.51E-123
MIER2	1.69516691	4.45E-13	6.20E-13
CTD-3113P1(	NA	NA	NA
THEG	NA	NA	NA
AC010641.1	NA	NA	NA
C2CD4C	0.74902724	1.23E-16	1.83E-16
SHC2	NA	NA	NA
ODF3L2	NA	2.57E-109	1.70E-108
MADCAM1	0.49935149	7.78E-43	1.68E-42
AC005775.2	NA	NA	NA
TPGS1	1.50619608	1.62E-90	7.27E-90
CDC34	1.97525731	0.73685907	0.74652914
GZMM	Inf	1.44E-124	1.51E-123
AC009005.2	1.67782101	7.13E-10	9.38E-10
BSG	1.06054503	1.60E-141	5.52E-140
HCN2	0.37451362	5.46E-16	8.02E-16
POLRMT	1.65693904	0.00111071	0.00126661
AC004449.6	Inf	3.63E-33	6.82E-33
FGF22	2.24708171	6.17E-08	7.78E-08
RNF126	1.73749761	4.07E-05	4.82E-05
AC004156.3	Inf	1.42E-14	2.04E-14
FSTL3	9.73735409	7.94E-111	5.46E-110
PRSS57	1.12071966	3.16E-143	1.43E-141
PALM	2.00364786	2.15E-32	4.00E-32
MISP	NA	4.62E-92	2.14E-91
AC006273.4	NA	4.29E-48	9.91E-48
AC006273.5	NA	NA	NA
PTBP1	1.87256809	4.88E-07	6.05E-07
PLPPR3	0.79658452	4.62E-120	4.13E-119
AZU1	0.03693428	1.93E-145	1.62E-143
PRTN3	0.0283737	2.16E-140	6.34E-139
ELANE	0.18725681	9.89E-134	1.64E-132
CFD	0.35239667	1.83E-145	1.58E-143
MED16	1.44811933	9.64E-12	1.31E-11
R3HDM4	2.10537386	3.15E-07	3.93E-07
KISS1R	Inf	4.26E-99	2.26E-98
ARID3A	1.52970351	3.55E-105	2.13E-104
AC005391.2	NA	NA	NA
WDR18	1.04551719	1.00E-135	1.89E-134
LLNLR-284B4	NA	5.14E-08	6.50E-08
GRIN3B	0.74902724	0.7541489	0.76344899

TMEM259	1.8799115	0.00036122	0.00041765
LLNLR-284B4	0	6.94E-55	1.78E-54
CNN2	1.63676322	3.02E-30	5.47E-30
ABCA7	2.62159533	4.89E-51	1.18E-50
HMHA1	1.80085272	1.01E-06	1.25E-06
POLR2E	1.46309987	1.47E-54	3.75E-54
GPX4	2.03713931	4.57E-111	3.17E-110
SBNO2	4.36932555	1.26E-21	2.02E-21
STK11	2.31910356	3.21E-79	1.18E-78
CBARP	4.49416342	7.20E-42	1.53E-41
AC004221.2	Inf	1.20E-44	2.63E-44
ATP5D	1.77205042	0.61192118	0.62430964
MIDN	1.67639429	3.36E-05	3.99E-05
CIRBP	1.46646899	1.05E-50	2.52E-50
CIRBP-AS1	Inf	1.07E-157	5.54E-155
C19orf24	1.89967105	5.09E-50	1.21E-49
MUM1	1.94747082	0.26407664	0.27587691
EFNA2	NA	NA	NA
AC005330.2	NA	NA	NA
AC004623.2	NA	1.46E-121	1.36E-120
NDUFS7	1.58926023	1.44E-69	4.57E-69
LLNLR-307A6	NA	2.97E-64	8.70E-64
AC005329.7	Inf	0.10951096	0.11656026
GAMT	1.59408361	2.17E-38	4.40E-38
DAZAP1	1.74623962	6.50E-09	8.38E-09
RPS15	1.85216948	5.11E-09	6.61E-09
CTB-25B13.5	1.58127972	0.2263809	0.2373441
APC2	NA	0.00168489	0.00190938
CTB-25B13.1	1.19844358	1.11E-97	5.71E-97
C19orf25	2.06111633	9.46E-65	2.80E-64
PCSK4	NA	4.25E-36	8.35E-36
REEP6	2.11490043	2.48E-26	4.25E-26
ADAMTSL5	1.12354086	2.69E-21	4.27E-21
CTB-25B13.9	NA	NA	NA
PLK5	NA	0.00015625	0.00018228
MEX3D	3.37062257	8.99E-106	5.46E-105
MBD3	2.68616664	2.59E-82	1.00E-81
AC005943.2	NA	NA	NA
UQCR11	2.39092475	4.46E-145	3.08E-143
TCF3	2.77688147	3.44E-125	3.72E-124
AC005256.1	1.49805447	1.92E-05	2.29E-05
ONECUT3	NA	1.16E-32	2.17E-32
ATP8B3	3.44552529	5.14E-20	8.02E-20
REXO1	2.88375486	1.57E-120	1.43E-119
CTB-31O20.3	NA	3.79E-88	1.63E-87

CTB-31O20.6	NA	4.58E-34	8.74E-34
KLF16	2.76723952	0.23394174	0.24514359
CTB-31O20.8	NA	NA	NA
CTB-31O20.2	2.99610895	2.12E-63	6.12E-63
CTB-31O20.9	NA	NA	NA
ABHD17A	2.30016238	9.60E-101	5.24E-100
SCAMP4	1.70468268	2.02E-30	3.67E-30
ADAT3	Inf	1.87E-61	5.27E-61
CSNK1G2	1.89503891	1.09E-100	5.95E-100
CSNK1G2-AS	NA	NA	NA
BTBD2	2.36534917	2.18E-48	5.08E-48
MKNK2	1.70523222	1.64E-25	2.77E-25
MOB3A	2.46108949	6.94E-25	1.17E-24
IZUMO4	2.39688716	1.91E-05	2.28E-05
AP3D1	1.56970056	1.39E-20	2.19E-20
DOT1L	2.07684825	9.09E-115	6.90E-114
AC004490.1	NA	2.25E-15	3.26E-15
PLEKHJ1	1.93880041	6.02E-65	1.79E-64
SF3A2	1.73508841	4.74E-61	1.33E-60
AMH	2.24708171	5.68E-29	1.01E-28
JSRP1	2.99610895	0.17671426	0.18624086
OAZ1	1.87065062	8.87E-13	1.23E-12
LLfos-48D6.2	NA	NA	NA
C19orf35	0	3.26E-140	9.38E-139
LINGO3	Inf	6.31E-60	1.73E-59
LSM7	2.17223526	1.01E-142	4.18E-141
SPPL2B	3.55787938	4.05E-70	1.30E-69
TMPRSS9	NA	1.35E-88	5.87E-88
TIMM13	1.87800188	2.05E-20	3.22E-20
LMNB2	1.65003102	4.01E-47	9.18E-47
AC005624.2	NA	NA	NA
GADD45B	0.97949716	5.64E-129	7.25E-128
GNG7	1.5729572	0.06813083	0.07317681
CTC-265F19.1	Inf	1.12E-68	3.52E-68
DIRAS1	6.36673152	2.44E-05	2.91E-05
AC006538.4	NA	NA	NA
SLC39A3	1.66934365	9.69E-26	1.65E-25
SGTA	2.10808697	4.51E-77	1.60E-76
THOP1	1.81014916	7.74E-28	1.35E-27
ZNF554	1.49805447	9.76E-06	1.17E-05
ZNF555	0.44941634	5.00E-83	1.96E-82
AC006130.3	NA	NA	NA
ZNF556	NA	8.62E-36	1.69E-35
ZNF57	0.87386511	1.32E-16	1.96E-16
AC006277.2	NA	5.53E-15	7.97E-15

ZNF77	0.99870298	1.22E-07	1.54E-07
TLE6	0	3.94E-46	8.89E-46
TLE2	0.74902724	5.66E-49	1.33E-48
AES	2.26198773	1.18E-112	8.50E-112
AC005944.2	Inf	0.00401652	0.0045044
GNA11	2.87127108	1.11E-104	6.60E-104
AC005262.3	NA	NA	NA
AC005262.2	NA	2.02E-24	3.37E-24
GNA15	2.17351654	0.05405297	0.05827712
AC005264.2	0	3.42E-44	7.48E-44
S1PR4	1.17300492	4.29E-144	2.32E-142
NCLN	2.42412451	7.43E-122	7.08E-121
CELF5	Inf	3.30E-112	2.36E-111
AC010649.1	NA	NA	NA
NFIC	2.11434271	6.42E-41	1.35E-40
SMIM24	1.68531128	2.66E-09	3.46E-09
DOHH	1.54991021	2.15E-39	4.42E-39
FZR1	2.15345331	2.71E-16	3.99E-16
MFSD12	2.00321238	0.00018705	0.00021777
C19orf71	NA	NA	NA
AC005786.7	NA	0.22894546	0.23999124
AC005786.5	NA	NA	NA
HMG20B	1.99740597	4.57E-28	8.01E-28
GIPC3	3.82836144	1.18E-70	3.80E-70
TBXA2R	1.3732166	2.00E-12	2.76E-12
CACTIN-AS1	NA	3.45E-80	1.29E-79
CACTIN	1.9193823	2.89E-18	4.40E-18
PIP5K1C	2.65564202	7.62E-07	9.40E-07
AC004637.1	NA	NA	NA
TJP3	NA	0.78724387	0.79566453
APBA3	1.35761187	3.08E-127	3.67E-126
AC005954.4	NA	2.47E-28	4.35E-28
MRPL54	1.70716505	6.49E-54	1.64E-53
RAX2	NA	NA	NA
MATK	1.67537521	1.13E-05	1.35E-05
ZFR2	NA	NA	NA
ATCAY	Inf	4.83E-137	1.00E-135
NMRK2	NA	NA	NA
DAPK3	1.52146158	1.94E-108	1.25E-107
EEF2	1.91708239	0.70499576	0.71548629
PIAS4	4.28988327	1.27E-102	7.23E-102
ZBTB7A	2.51596328	8.50E-17	1.26E-16
MAP2K2	1.96595075	0.37923899	0.39227743
CREB3L3	NA	NA	NA
SIRT6	1.23277399	8.77E-79	3.20E-78

ANKRD24	NA	7.45E-134	1.25E-132
EBI3	NA	NA	NA
CCDC94	1.14226654	2.32E-123	2.34E-122
SHD	NA	0.00012408	0.00014517
TMIGD2	NA	4.01E-64	1.17E-63
FSD1	5.61770428	5.11E-35	9.88E-35
STAP2	NA	1.23E-18	1.89E-18
AC007292.7	NA	NA	NA
MPND	1.74773022	6.07E-20	9.45E-20
AC007292.4	NA	NA	NA
AC007292.3	0.74902724	9.61E-11	1.28E-10
SH3GL1	1.84963869	2.14E-27	3.71E-27
AC007292.6	Inf	8.47E-15	1.22E-14
CHAF1A	1.6499551	1.91E-08	2.43E-08
CTB-50L17.5	NA	NA	NA
UBXN6	1.66450497	0.01791239	0.01966158
CTB-50L17.7	NA	NA	NA
CTB-50L17.9	Inf	2.62E-149	3.57E-147
CTB-50L17.1(	NA	1.25E-48	2.91E-48
CTB-50L17.1(	1.00869001	1.01E-84	4.07E-84
PLIN4	NA	NA	NA
PLIN5	0	0.61648165	0.62875029
CTB-50L17.1	NA	5.83E-98	3.02E-97
LRG1	0.77898833	9.36E-48	2.15E-47
SEMA6B	1.87256809	3.78E-80	1.41E-79
TNFAIP8L1	2.08063121	1.92E-17	2.88E-17
MYDGF	1.51451661	1.46E-50	3.50E-50
AC005339.2	1.49805447	0.02153705	0.02357743
DPP9	2.42788139	1.16E-19	1.80E-19
DPP9-AS1	NA	5.28E-112	3.74E-111
MIR7-3HG	NA	NA	NA
AC005523.3	1.49805447	0.442652	0.45558476
FEM1A	1.72852439	1.73E-10	2.30E-10
AC005523.2	2.09727626	0.12574863	0.13359871
TICAM1	1.14557107	9.01E-36	1.76E-35
CTC-518P12.(	NA	5.76E-06	6.98E-06
PLIN3	0.92187968	6.35E-78	2.29E-77
ARRDC5	NA	NA	NA
UHRF1	1.33268482	1.32E-15	1.92E-15
KDM4B	1.7655642	3.22E-41	6.79E-41
PTPRS	1.52075227	1.09E-59	2.98E-59
CTC-482H14.	NA	NA	NA
LLNLR-304G!	Inf	1.09E-28	1.94E-28
ZNRF4	NA	NA	NA
TINCR	Inf	3.16E-63	9.12E-63

SAFB2	1.69882466	1.96E-55	5.06E-55
SAFB	1.70091602	5.88E-64	1.72E-63
RPL36	2.3239973	2.26E-140	6.63E-139
C19orf70	1.88570892	1.56E-19	2.41E-19
HSD11B1L	4.49416342	1.61E-07	2.02E-07
LONP1	1.69087337	2.31E-09	3.01E-09
CATSPERD	NA	2.17E-108	1.40E-107
PRR22	2.69649805	3.60E-61	1.01E-60
CTB-54O9.9	NA	NA	NA
DUS3L	1.66718966	6.89E-37	1.37E-36
NRTN	2.49675746	9.62E-68	2.98E-67
FUT6	NA	5.35E-82	2.06E-81
FUT3	NA	NA	NA
AC024592.9	NA	NA	NA
FUT5	NA	NA	NA
AC024592.12	0.74902724	0.1088684	0.11589547
NDUFA11	2.04677151	2.63E-100	1.42E-99
AC104532.3	NA	NA	NA
AC104532.2	NA	NA	NA
VMAC	8.23929961	3.06E-28	5.38E-28
AC104532.4	Inf	1.10E-95	5.46E-95
CAPS	Inf	3.80E-27	6.58E-27
CAPS	NA	NA	NA
RANBP3	1.56318728	1.32E-49	3.13E-49
CTC-232P5.1	Inf	1.91E-96	9.62E-96
RFX2	2.99610895	0.09926847	0.10594321
CTC-232P5.3	NA	NA	NA
CTB-66B24.1	NA	NA	NA
ACSBG2	NA	9.10E-16	1.33E-15
MLLT1	2.4244829	3.34E-76	1.17E-75
CTC-503J8.4	0.99870298	1.07E-09	1.40E-09
ACER1	NA	NA	NA
CTB-180A7.3	NA	NA	NA
CLPP	1.67404368	9.29E-39	1.89E-38
ALKBH7	1.82733698	9.71E-54	2.44E-53
PSPN	4.11964981	6.79E-41	1.43E-40
GTF2F1	1.60086213	2.39E-27	4.16E-27
CTB-180A7.8	NA	NA	NA
KHSRP	2.51025344	3.09E-145	2.30E-143
SLC25A41	2.99610895	0.20484143	0.21534642
SLC25A23	2.6692607	9.04E-132	1.34E-130
CRB3	NA	1.03E-29	1.85E-29
DENND1C	1.49805447	5.49E-65	1.63E-64
CTD-2396E7.	NA	2.10E-59	5.72E-59
TUBB4A	3.13655156	1.33E-64	3.92E-64

CTD-2396E7.	NA	NA	NA
CTD-2396E7.	NA	1.40E-113	1.03E-112
TNFSF9	Inf	0.7934925	0.80175667
CD70	NA	NA	NA
TNFSF14	Inf	1.81E-98	9.51E-98
C3	NA	2.06E-56	5.37E-56
GPR108	1.17239046	7.12E-104	4.16E-103
TRIP10	1.63849708	0.69020827	0.7011063
CTD-3128G1(	Inf	8.67E-109	5.65E-108
SH2D3A	1.9661965	6.80E-34	1.29E-33
VAV1	1.08682383	2.37E-58	6.35E-58
ADGRE1	NA	2.35E-95	1.16E-94
RP11-1137G.	NA	NA	NA
CTB-25J19.1	NA	NA	NA
MBD3L5	NA	NA	NA
MBD3L4	NA	NA	NA
MBD3L2	NA	NA	NA
MBD3L3	NA	NA	NA
ZNF557	1.72276265	1.51E-06	1.86E-06
INSR	1.78614187	1.30E-26	2.23E-26
CTB-133G6.1	NA	7.72E-97	3.92E-96
CTD-2207O2.	NA	NA	NA
CTB-133G6.2	NA	7.02E-39	1.43E-38
ARHGEF18	NA	1.63E-50	3.91E-50
PEX11G	NA	NA	NA
C19orf45	NA	NA	NA
CTD-2207O2.	NA	NA	NA
ZNF358	2.09103437	1.14E-06	1.41E-06
CTD-2207O2.	NA	NA	NA
MCOLN1	1.78614187	3.89E-11	5.24E-11
CTD-2207O2.	NA	NA	NA
PNPLA6	1.87256809	0.00033034	0.00038233
CAMSAP3	2.99610895	1.82E-48	4.23E-48
XAB2	0.88521401	4.41E-130	5.97E-129
PET100	1.65187257	0.29224365	0.30442606
CTD-3214H1!	2.24708171	7.89E-25	1.32E-24
PCP2	NA	NA	NA
CTD-3214H1!	NA	NA	NA
STXBP2	0	1.58E-135	2.95E-134
RETN	0	3.46E-145	2.50E-143
MCEMP1	0.11523496	1.83E-123	1.85E-122
CTD-3214H1!	NA	NA	NA
TRAPPC5	2.24708171	1.19E-08	1.52E-08
FCER2	NA	NA	NA
CLEC4G	NA	NA	NA

CD209	NA	NA	NA
CLEC4M	NA	NA	NA
EVI5L	0.74902724	1.42E-20	2.24E-20
PRR36	0.99870298	4.72E-22	7.59E-22
CTD-3193O1:	NA	NA	NA
LRRC8E	2.99610895	0.06535381	0.07024819
CTD-3193O1:	2.0598249	4.98E-55	1.27E-54
MAP2K7	1.91611619	0.00171411	0.00194189
CTD-3193O1:	NA	0.00021029	0.00024448
TGFBR3L	8.23929961	1.64E-125	1.79E-124
CTD-3193O1:	NA	5.05E-117	4.09E-116
SNAPC2	1.45756652	1.66E-69	5.30E-69
CTXN1	2.58949416	4.00E-105	2.40E-104
TIMM44	1.76144867	6.93E-18	1.05E-17
ELAVL1	1.51719021	6.84E-27	1.18E-26
CTD-3193O1:	NA	NA	NA
CTD-2325M2	1.42315175	1.15E-17	1.73E-17
CCL25	NA	NA	NA
FBN3	NA	NA	NA
CERS4	1.19844358	4.86E-73	1.63E-72
CTD-3020H1:	NA	7.24E-65	2.15E-64
CD320	1.74773022	0.78051322	0.78903771
CTD-2550O8.	NA	NA	NA
NDUFA7	2.97783999	1.31E-117	1.08E-116
RPS28	2.37992168	6.83E-144	3.55E-142
KANK3	Inf	3.36E-53	8.40E-53
ANGPTL4	NA	5.06E-139	1.27E-137
RAB11B-AS1	1.49805447	0.00370928	0.00416421
RAB11B	1.40570865	4.31E-75	1.48E-74
2-Mar	1-Jan	0.0038979	0.00437408
RP11-886P1(	NA	NA	NA
HNRNPM	1.26453422	7.18E-54	1.81E-53
PRAM1	0.65132803	1.85E-145	1.58E-143
ZNF414	2.20302129	1.39E-39	2.87E-39
MYO1F	1.69971565	1.33E-41	2.82E-41
AC092316.1	NA	NA	NA
ADAMTS10	Inf	3.36E-119	2.91E-118
AC130469.2	NA	6.24E-09	8.05E-09
CTD-2586B1(	NA	NA	NA
ACTL9	NA	NA	NA
OR2Z1	NA	NA	NA
ZNF558	1.49805447	4.95E-11	6.64E-11
CTD-2529P6.	NA	NA	NA
MBD3L1	NA	NA	NA
MUC16	NA	NA	NA

OR1M1	NA	NA	NA
OR7G2	NA	NA	NA
OR7G1	NA	NA	NA
OR7G3	NA	NA	NA
ZNF317	2.30950065	2.81E-59	7.64E-59
OR7D2	Inf	1.62E-116	1.29E-115
OR7D4	NA	NA	NA
OR7E24	NA	NA	NA
CTC-325H20.	NA	2.80E-140	8.11E-139
ZNF699	Inf	7.59E-16	1.11E-15
ZNF559	1.12354086	0.02531249	0.02763384
ZNF559-ZNF	Inf	1.29E-40	2.70E-40
ZNF177	NA	1.21E-111	8.50E-111
ZNF266	1.39105058	0.00789968	0.00877893
ZNF560	NA	NA	NA
CTC-543D15.	NA	NA	NA
ZNF426	0	5.10E-15	7.36E-15
CTC-543D15.	NA	NA	NA
ZNF121	1.88170257	2.79E-113	2.05E-112
ZNF561	2.24708171	5.12E-09	6.63E-09
AC008759.1	NA	NA	NA
ZNF562	3.11595331	3.61E-119	3.13E-118
ZNF812P	NA	NA	NA
CTD-3116E22	NA	NA	NA
CTD-3116E22	NA	NA	NA
ZNF846	1.87256809	5.28E-22	8.49E-22
CTD-2623N2.	NA	1.74E-18	2.65E-18
FBXL12	1.0083059	1.93E-84	7.76E-84
UBL5	2.12790373	6.92E-144	3.59E-142
CTD-2623N2.	Inf	3.56E-90	1.58E-89
PIN1	1.76460691	0.64862138	0.6603417
OLFM2	1.49805447	0.32977748	0.34248229
COL5A3	NA	NA	NA
CTD-2553C6.	NA	NA	NA
RDH8	NA	NA	NA
C19orf66	2.12224384	0.00029124	0.00033765
CTD-2240E14	NA	1.39E-25	2.35E-25
ANGPTL6	0.6866083	4.03E-72	1.33E-71
PPAN-P2RY1	NA	NA	NA
PPAN	1.46763712	7.11E-38	1.43E-37
P2RY11	2.33030696	0.00034614	0.00040042
EIF3G	1.19399391	4.22E-129	5.45E-128
DNMT1	1.75553259	2.05E-15	2.98E-15
S1PR2	1.94747082	5.98E-104	3.50E-103
MRPL4	1.56144365	2.85E-43	6.17E-43

CTD-2369P2.	NA	2.83E-10	3.75E-10
CTD-2369P2.	Inf	1.91E-119	1.67E-118
ICAM1	2.62159533	1.16E-41	2.47E-41
CTD-2369P2.	Inf	5.17E-22	8.32E-22
ICAM4	1.06592338	2.89E-145	2.17E-143
ICAM5	3.5311284	3.29E-104	1.94E-103
ZGLP1	2.43433852	0.47992416	0.49316251
CTD-2369P2.	4.19455253	7.03E-21	1.11E-20
FDX1L	2.12723735	5.01E-61	1.40E-60
CTD-2369P2.	NA	NA	NA
RAVER1	3.74513619	3.99E-80	1.49E-79
AC114271.2	2.24708171	6.64E-37	1.32E-36
ICAM3	1.41482923	9.31E-60	2.55E-59
TYK2	2.85992218	1.13E-35	2.20E-35
CDC37	1.2393543	2.25E-110	1.52E-109
PDE4A	2.24708171	1.72E-22	2.78E-22
KEAP1	1.35118639	6.05E-50	1.44E-49
S1PR5	NA	1.07E-121	1.01E-120
ATG4D	2.35039581	2.97E-37	5.94E-37
KRI1	1.37576431	0.0220322	0.02409332
CDKN2D	1.84581712	1.25E-16	1.86E-16
AP1M2	NA	7.50E-78	2.69E-77
SLC44A2	2.49675746	5.88E-07	7.27E-07
ILF3-AS1	2.40552978	2.06E-81	7.86E-81
ILF3	1.752237	8.56E-06	1.03E-05
QTRT1	1.4192095	3.68E-100	1.99E-99
DNM2	2.02514772	0.10365532	0.11044311
TMED1	1.31559912	1.30E-138	3.11E-137
C19orf38	0.74902724	1.01E-123	1.03E-122
CARM1	2.56167315	8.78E-127	1.02E-125
YIPF2	1.47222595	1.97E-134	3.41E-133
C19orf52	1.80743529	3.11E-49	7.32E-49
SMARCA4	1.99317417	0.66607719	0.67750462
CTC-215O4.4	NA	NA	NA
LDLR	1.80447471	1.47E-05	1.76E-05
SPC24	1.61789883	2.62E-76	9.19E-76
KANK2	3.99481193	9.68E-110	6.47E-109
DOCK6	0.56177043	3.47E-92	1.61E-91
CTC-510F12.:	NA	5.68E-50	1.35E-49
CTC-510F12.'	NA	2.58E-134	4.43E-133
ANGPTL8	NA	NA	NA
TSPAN16	NA	NA	NA
RAB3D	2.66320796	2.79E-29	4.98E-29
TMEM205	1.09403372	1.75E-125	1.91E-124
CCDC159	0.89883268	0.01165609	0.01288162

PLPPR2	1.43385214	1.73E-116	1.38E-115
CTD-2342J14	NA	0.03359203	0.03649917
SWSAP1	1.10966998	1.90E-68	5.95E-68
EPOR	1.62289235	1.98E-41	4.19E-41
RGL3	NA	NA	NA
CCDC151	Inf	0.66126274	0.67283391
PRKCSH	1.58127972	1.43E-52	3.54E-52
ELAVL3	NA	9.41E-24	1.55E-23
CTC-398G3.6	NA	NA	NA
ZNF653	2.24708171	4.06E-30	7.34E-30
ECSIT	2.0679665	1.44E-20	2.26E-20
CNN1	NA	NA	NA
ELOF1	1.92714545	4.08E-47	9.31E-47
ZNF627	0.56177043	6.93E-77	2.45E-76
ACP5	NA	NA	NA
CTC-499B15.	1.49805447	9.08E-27	1.56E-26
ZNF823	1.31079767	3.12E-35	6.05E-35
ZNF441	0.49935149	1.95E-10	2.59E-10
CTC-499B15.	NA	NA	NA
ZNF491	NA	3.14E-121	2.90E-120
ZNF440	5.9922179	2.99E-87	1.26E-86
CTC-499B15.	NA	NA	NA
ZNF439	NA	0.43990241	0.45283191
ZNF69	1.49805447	9.60E-27	1.65E-26
ZNF700	2.62159533	3.70E-05	4.39E-05
CTD-2006C1.	NA	NA	NA
ZNF763	Inf	7.83E-41	1.64E-40
ZNF433	NA	NA	NA
CTD-2006C1.	NA	NA	NA
ZNF878	NA	NA	NA
ZNF844	Inf	0.08257186	0.08842564
ZNF788	3.86997406	8.17E-113	5.94E-112
ZNF20	0.74902724	2.99E-12	4.11E-12
ZNF625-ZNF	NA	NA	NA
ZNF625	NA	NA	NA
ZNF136	2.24708171	1.38E-09	1.81E-09
CTD-2666L21	NA	NA	NA
ZNF44	1.49805447	1.25E-75	4.34E-75
ZNF563	1.04863813	1.06E-24	1.77E-24
ZNF442	1.3732166	2.02E-59	5.50E-59
CTD-3105H18	1.49805447	0.45400872	0.46695539
ZNF799	1.49805447	0.30699646	0.31944535
CTD-3105H18	NA	NA	NA
ZNF443	2.49675746	0.00515356	0.0057614
CTD-3105H18	Inf	3.25E-135	5.86E-134

CTD-3105H18	NA	NA	NA
ZNF709	3.74513619	1.25E-34	2.40E-34
CTD-2192J16	NA	NA	NA
ZNF564	0.99870298	0.92688611	0.93032187
CTD-2192J16	NA	NA	NA
CTD-2192J16	NA	NA	NA
ZNF490	Inf	3.74E-29	6.66E-29
ZNF791	2.49675746	8.66E-12	1.18E-11
CTD-2192J16	NA	3.02E-83	1.19E-82
MAN2B1	5.9922179	2.85E-63	8.21E-63
CTD-2192J16	NA	NA	NA
WDR83	1.78444724	4.35E-27	7.52E-27
WDR83OS	1.61382591	4.54E-84	1.81E-83
DHPS	1.00457771	1.45E-143	7.01E-142
CTD-2192J16	Inf	6.30E-14	8.91E-14
FBXW9	1.55155642	4.03E-14	5.71E-14
CTD-2659N19	NA	NA	NA
TNPO2	2.67509728	5.84E-41	1.23E-40
CTD-2659N19	NA	NA	NA
C19orf43	1.74611524	8.36E-91	3.77E-90
ASNA1	1.1267975	1.62E-132	2.52E-131
BEST2	NA	5.63E-61	1.57E-60
HOOK2	1.90293406	1.52E-10	2.03E-10
CTD-2659N19	NA	NA	NA
JUNB	1.36964981	7.12E-116	5.57E-115
PRDX2	1.05483512	6.66E-145	4.28E-143
CTD-2659N19	3.74513619	8.28E-70	2.64E-69
RNASEH2A	0.88166748	2.50E-144	1.42E-142
RTBDN	NA	4.09E-129	5.28E-128
CTD-2265O2	NA	6.20E-55	1.59E-54
MAST1	2.30950065	6.07E-19	9.33E-19
DNASE2	1.23301407	1.52E-142	5.94E-141
CTD-2265O2	NA	NA	NA
KLF1	NA	NA	NA
GCDH	1.15029183	1.63E-126	1.87E-125
SYCE2	0.56177043	5.33E-112	3.77E-111
FARSA	0.83444262	6.36E-137	1.31E-135
FARSA-AS1	NA	NA	NA
CALR	1.35849213	8.45E-93	3.97E-92
CTC-425F1.4	1.49805447	4.00E-06	4.86E-06
RAD23A	1.39986738	1.79E-139	4.74E-138
GADD45GIP1	1.57367742	5.91E-50	1.41E-49
DAND5	1.49805447	5.25E-10	6.92E-10
NFIX	2.0842497	4.27E-68	1.33E-67
CTC-239J10.1	NA	3.16E-06	3.85E-06

AC007787.2	NA	0.02164078	0.0236767
LYL1	1.66780233	3.36E-35	6.51E-35
TRMT1	1.94747082	2.94E-43	6.37E-43
NACC1	1.98426514	6.53E-67	1.99E-66
AC005546.2	NA	NA	NA
CTC-250I14.3	NA	3.75E-93	1.77E-92
STX10	1.81656606	8.61E-07	1.06E-06
IER2	1.42315175	1.24E-75	4.32E-75
CTC-250I14.6	NA	2.07E-168	2.69E-165
CACNA1A	1.12354086	6.12E-06	7.41E-06
CCDC130	1.06440713	4.39E-76	1.53E-75
MRI1	2.24708171	0.00012581	0.00014716
CTB-5E10.3	0	6.05E-28	1.06E-27
C19orf53	2.45234873	1.96E-135	3.61E-134
AC008686.1	NA	NA	NA
ZSWIM4	Inf	5.37E-131	7.64E-130
CTD-3252C9.	NA	NA	NA
CTD-3252C9.	1.97957198	0.04103075	0.04446183
NANOS3	3.74513619	2.74E-56	7.13E-56
C19orf57	Inf	1.86E-09	2.42E-09
CC2D1A	2.82965845	4.03E-80	1.50E-79
PODNL1	2.99610895	1.03E-21	1.65E-21
DCAF15	2.49675746	3.62E-13	5.05E-13
RFX1	3.29571984	8.93E-85	3.61E-84
CTB-55O6.13	2.99610895	9.85E-22	1.58E-21
RLN3	NA	NA	NA
CTB-55O6.4	NA	2.65E-67	8.14E-67
IL27RA	2.11089494	1.27E-11	1.72E-11
PALM3	0.58257674	3.60E-31	6.59E-31
CTB-55O6.8	2.08063121	2.71E-25	4.57E-25
C19orf67	NA	NA	NA
SAMD1	2.36359706	5.45E-109	3.57E-108
PRKACA	2.11089494	3.56E-58	9.52E-58
CTB-55O6.10	NA	9.42E-102	5.25E-101
ASF1B	1.18234878	1.51E-108	9.78E-108
CTB-55O6.12	0.74902724	3.03E-68	9.44E-68
ADGRL1	2.0516833	3.53E-68	1.10E-67
CTD-2189E23	NA	NA	NA
CTD-2189E23	NA	NA	NA
CTC-548K16.[illegible]	NA	7.65E-06	9.24E-06
CTC-548K16.[illegible]	NA	6.27E-37	1.25E-36
ADGRE5	1.93498703	8.49E-27	1.46E-26
CTC-548K16.[illegible]	NA	NA	NA
DDX39A	0.79750796	1.48E-134	2.58E-133
PKN1	2.10413758	2.61E-36	5.15E-36

PTGER1	NA	NA	NA
GIPC1	1.6031811	7.76E-40	1.61E-39
DNAJB1	1.22568093	4.14E-98	2.16E-97
TECR	0.90634644	1.24E-142	5.05E-141
NDUFB7	1.85240528	0.04961469	0.05358753
CLEC17A	NA	NA	NA
ADGRE3	NA	NA	NA
ZNF333	2.12224384	2.44E-07	3.04E-07
ADGRE2	0.99870298	3.95E-124	4.10E-123
OR7A5	NA	NA	NA
OR7C1	NA	NA	NA
OR7A10	NA	NA	NA
OR7A17	NA	NA	NA
OR7C2	NA	NA	NA
SLC1A6	NA	NA	NA
CCDC105	NA	NA	NA
CASP14	NA	NA	NA
OR1I1	NA	NA	NA
SYDE1	3.932393	4.40E-24	7.29E-24
ILVBL	1.54798962	2.90E-29	5.17E-29
AC003956.1	0	5.84E-96	2.91E-95
NOTCH3	1.87256809	4.54E-20	7.09E-20
AC004257.1	NA	NA	NA
EPHX3	NA	9.05E-08	1.14E-07
BRD4	2.50414431	5.65E-122	5.39E-121
LLNLR-246C6	NA	2.23E-42	4.78E-42
AKAP8	2.19357977	8.14E-07	1.00E-06
AKAP8L	1.10814989	1.95E-130	2.70E-129
WIZ	2.01661179	1.16E-46	2.64E-46
RASAL3	1.44811933	8.60E-130	1.14E-128
PGLYRP2	NA	NA	NA
CYP4F22	NA	5.66E-128	6.91E-127
AD000091.2	NA	NA	NA
CYP4F8	NA	3.47E-12	4.76E-12
CYP4F3	NA	NA	NA
CYP4F12	NA	NA	NA
OR10H2	NA	NA	NA
OR10H3	NA	NA	NA
LLNLR-249E1	NA	NA	NA
OR10H5	NA	NA	NA
OR10H1	NA	NA	NA
AC004510.3	NA	3.17E-10	4.20E-10
AC004791.2	1.34824903	8.69E-35	1.68E-34
CYP4F2	NA	NA	NA
CYP4F11	NA	4.74E-71	1.53E-70

OR10H4	NA	NA	NA
CTD-2231E14	NA	NA	NA
TPM4	1.43184212	2.62E-34	5.01E-34
CTD-2231E14	NA	NA	NA
RAB8A	1.3715521	1.16E-86	4.83E-86
CTD-2231E14	NA	0.74621559	0.75568787
HSH2D	1.4730869	2.08E-43	4.52E-43
CIB3	1.61328943	3.43E-39	7.05E-39
FAM32A	1.5080415	2.37E-46	5.35E-46
CTD-2562J15	NA	NA	NA
AP1M1	2.71199517	7.03E-25	1.18E-24
CTD-2562J15	NA	1.17E-136	2.37E-135
KLF2	0.24967575	3.51E-14	4.99E-14
CTD-2013N1	Inf	2.64E-53	6.60E-53
EPS15L1	2.83560311	6.05E-55	1.55E-54
CTD-2013N1	NA	1.82E-65	5.45E-65
CALR3	NA	NA	NA
CTD-3222D19	NA	NA	NA
C19orf44	2.24708171	5.74E-14	8.13E-14
CHERP	2.67509728	1.28E-36	2.54E-36
CTD-3222D19	1.49805447	4.12E-07	5.11E-07
SLC35E1	2.03805086	1.31E-10	1.74E-10
CTD-3222D19	NA	1.54E-23	2.53E-23
CTD-3222D19	NA	NA	NA
CTD-3222D19	0.18725681	6.86E-22	1.10E-21
MED26	1.98271916	1.03E-15	1.51E-15
CTC-429P9.4	NA	NA	NA
CTC-429P9.2	NA	NA	NA
CTC-429P9.1	NA	NA	NA
SMIM7	1.58617533	3.28E-111	2.28E-110
TMEM38A	1.26758456	1.53E-17	2.30E-17
NWD1	NA	2.30E-86	9.54E-86
SIN3B	1.3658732	4.13E-51	9.99E-51
F2RL3	Inf	8.33E-26	1.42E-25
CPAMD8	Inf	1.55E-50	3.72E-50
CTD-2528A14	NA	NA	NA
HAUS8	1.90881135	9.12E-26	1.55E-25
CTD-2528A14	Inf	1.00E-95	4.98E-95
MYO9B	1.88548236	0.42770703	0.44052804
CTD-2528A14	NA	NA	NA
CTD-3032J10	NA	NA	NA
CTD-3032J10	NA	NA	NA
CTD-3032J10	NA	6.24E-09	8.05E-09
USE1	1.72276265	0.02317096	0.02532335
OCEL1	1.72852439	3.70E-49	8.69E-49

NR2F6	1.88170257	6.72E-05	7.92E-05
AC010646.3	NA	NA	NA
USHBP1	Inf	7.38E-148	8.47E-146
BABAM1	1.36669522	9.09E-106	5.52E-105
CTD-2278I10	NA	NA	NA
ANKLE1	1.47724816	1.00E-37	2.02E-37
ABHD8	2.03307393	0.00544653	0.00608517
MRPL34	1.88581456	5.69E-26	9.70E-26
DDA1	2.03599222	1.00E-51	2.45E-51
ANO8	2.24708171	9.69E-12	1.32E-11
GTPBP3	1.33160398	2.68E-29	4.78E-29
PLVAP	Inf	4.86E-05	5.75E-05
CTD-2278I10	NA	NA	NA
CTD-2521M2	0	7.15E-22	1.15E-21
BST2	1.61824722	5.34E-29	9.50E-29
BISPR	1.90661479	0.31580534	0.32838543
MVB12A	1.5219596	1.59E-89	7.00E-89
CTD-2521M2	NA	NA	NA
CTD-2521M2	NA	NA	NA
TMEM221	1.34824903	1.67E-07	2.09E-07
CTD-2521M2	NA	NA	NA
CTD-2521M2	NA	NA	NA
NXNL1	NA	NA	NA
SLC27A1	1.34824903	0.20097444	0.2113546
CTD-3131K8.	2.46108949	1.09E-08	1.40E-08
PGLS	1.57054098	3.93E-103	2.26E-102
CTD-3131K8.	NA	2.65E-67	8.14E-67
FAM129C	Inf	8.61E-36	1.68E-35
COLGALT1	1.9450546	1.14E-82	4.44E-82
UNC13A	0.74902724	0.70133512	0.7119305
CTD-3149D2.	NA	NA	NA
MAP1S	1.18115834	7.23E-69	2.28E-68
CTD-3149D2.	NA	NA	NA
FCHO1	1.49805447	9.04E-07	1.11E-06
B3GNT3	NA	7.49E-147	8.05E-145
INSL3	NA	5.44E-18	8.23E-18
JAK3	1.49805447	4.44E-16	6.52E-16
RPL18A	2.21066936	3.99E-140	1.13E-138
SLC5A5	Inf	1.06E-154	2.70E-152
CCDC124	1.64882227	7.19E-35	1.39E-34
KCNN1	NA	2.41E-26	4.12E-26
ARRDC2	1.36489408	2.57E-25	4.33E-25
IL12RB1	NA	NA	NA
MAST3	3.18336576	1.91E-94	9.21E-94
AC007192.6	NA	NA	NA

PIK3R2	3.49546044	3.49E-32	6.48E-32
AC007192.4	NA	NA	NA
IFI30	8.23929961	3.10E-82	1.20E-81
MPV17L2	1.88326848	1.68E-07	2.10E-07
RAB3A	2.28869434	0.00030928	0.00035832
AC068499.1(	NA	1.35E-11	1.83E-11
PDE4C	NA	1.17E-50	2.82E-50
KIAA1683	NA	1.81E-07	2.26E-07
JUND	2.60772446	7.67E-106	4.66E-105
LSM4	1.98192438	5.45E-62	1.54E-61
PGPEP1	1.74773022	4.23E-14	6.00E-14
GDF15	NA	7.88E-34	1.50E-33
LRRC25	NA	3.41E-18	5.18E-18
SSBP4	2.55370105	4.57E-137	9.51E-136
ISYNA1	2.38752432	6.23E-09	8.04E-09
CTD-3137H5.	NA	0.02164078	0.0236767
ELL	1.76241703	5.35E-05	6.32E-05
AC005387.3	NA	NA	NA
FKBP8	1.40152113	1.90E-145	1.61E-143
AC005387.2	NA	NA	NA
KXD1	2.22710765	3.36E-85	1.37E-84
AC005253.2	2.62159533	1.82E-26	3.12E-26
AC005253.4	NA	6.02E-15	8.68E-15
UBA52	1.6967723	2.33E-20	3.66E-20
CRLF1	1.64785992	4.67E-17	6.97E-17
C19orf60	1.84125425	1.33E-17	2.00E-17
TMEM59L	NA	7.01E-27	1.21E-26
KLHL26	3.37062257	3.26E-32	6.06E-32
CRTC1	2.3151751	6.76E-07	8.35E-07
COMP	3.74513619	0.24115708	0.25248567
UPF1	1.97304736	8.52E-115	6.48E-114
CERS1	1.95768482	5.38E-53	1.34E-52
GDF1	NA	NA	NA
AC005197.2	NA	NA	NA
COPE	0.86336989	3.22E-145	2.35E-143
AC002985.3	NA	NA	NA
DDX49	1.18748221	1.22E-119	1.08E-118
HOMER3	1.11687691	5.16E-123	5.12E-122
AC005932.1	0.74902724	1.10E-48	2.57E-48
SUGP2	2.75861251	3.02E-98	1.58E-97
ARMC6	1.48913748	4.76E-44	1.04E-43
SLC25A42	1.49805447	2.10E-37	4.21E-37
TMEM161A	1.32417315	1.19E-94	5.80E-94
BORCS8-MEI	0	0.01196778	0.01321965
MEF2B	NA	0.22131413	0.23213927

BORCS8	1.74479286	2.19E-10	2.91E-10
RFXANK	1.18380742	1.34E-109	8.92E-109
NR2C2AP	1.93998054	4.41E-05	5.22E-05
NCAN	NA	NA	NA
HAPLN4	Inf	6.01E-96	3.00E-95
TM6SF2	NA	NA	NA
SUGP1	1.1385214	2.24E-36	4.42E-36
MAU2	1.77042802	0.0001088	0.00012742
GATAD2A	2.77764267	6.43E-127	7.51E-126
CTB-184G21.	NA	NA	NA
TSSK6	NA	5.69E-11	7.62E-11
NDUFA13	1.91697682	1.27E-33	2.41E-33
CTC-260F20.:	NA	3.27E-103	1.88E-102
YJEFN3	1.49805447	1.50E-46	3.41E-46
CILP2	NA	3.39E-157	1.43E-154
PBX4	4.49416342	0.0618466	0.06653745
AC002306.1	NA	NA	NA
LPAR2	3.37062257	9.23E-39	1.88E-38
GMIP	2.0598249	2.74E-05	3.26E-05
ATP13A1	1.33363386	2.74E-41	5.78E-41
ZNF101	1.31971466	0.00100578	0.0011484
ZNF14	1.49805447	1.98E-45	4.43E-45
CTC-559E9.1	Inf	1.33E-25	2.26E-25
CTC-559E9.4	NA	NA	NA
ZNF506	1.84760052	0.06082615	0.06545513
ZNF253	NA	NA	NA
CTC-559E9.8	NA	NA	NA
ZNF93	NA	NA	NA
AC007204.2	NA	NA	NA
ZNF682	NA	NA	NA
ZNF90	1.74773022	0.4146693	0.42746352
CTC-260E6.6	NA	NA	NA
ZNF486	NA	NA	NA
CTC-513N18.	NA	NA	NA
ZNF737	NA	NA	NA
CTC-513N18.	NA	NA	NA
CTD-2332E11	NA	NA	NA
ZNF626	NA	NA	NA
CTD-2626G1	NA	NA	NA
ZNF66	NA	NA	NA
ZNF85	NA	NA	NA
CTD-2542C24	NA	NA	NA
ZNF430	NA	NA	NA
ZNF714	NA	8.82E-29	1.56E-28
ZNF431	1.88754864	3.35E-44	7.33E-44

ZNF708	Inf	2.45E-75	8.49E-75
ZNF738	1.05745022	0.38546102	0.39853159
ZNF493	2.24708171	5.87E-108	3.75E-107
CTD-2561J22	NA	NA	NA
LINC00664	NA	5.64E-31	1.03E-30
ZNF429	3.37062257	0.49983015	0.51309462
RP11-678G1	NA	NA	NA
RP11-678G1	NA	NA	NA
RP11-678G1	NA	NA	NA
ZNF100	1.34824903	2.20E-06	2.69E-06
RP11-420K1	NA	NA	NA
ZNF43	NA	NA	NA
ZNF208	NA	NA	NA
AC003973.4	NA	NA	NA
AC003973.5	NA	NA	NA
ZNF257	NA	NA	NA
ZNF676	NA	NA	NA
RP11-157B1	NA	1.11E-60	3.08E-60
RP11-157B1	NA	NA	NA
ZNF729	NA	NA	NA
ZNF98	NA	NA	NA
AC011516.1	NA	NA	NA
AC025811.3	NA	NA	NA
LINC01233	NA	NA	NA
AC011516.2	NA	NA	NA
ZNF492	8.23929961	2.72E-64	7.99E-64
CTC-457E21.	NA	NA	NA
ZNF99	NA	NA	NA
CTD-2579N5	NA	NA	NA
ZNF728	NA	NA	NA
CTD-2291D1	NA	NA	NA
CTD-2291D1	NA	NA	NA
CTD-2291D1	NA	NA	NA
CTD-2291D1	NA	NA	NA
ZNF730	3.74513619	0.08207512	0.08789887
ZNF724P	1.68531128	2.43E-05	2.90E-05
RP11-15H20.	NA	NA	NA
ZNF91	3.55787938	1.48E-113	1.09E-112
CTB-176F20.	NA	NA	NA
LINC01224	NA	8.24E-21	1.30E-20
ZNF675	1.31079767	9.93E-44	2.16E-43
ZNF681	2.24708171	8.00E-25	1.34E-24
RP11-255H2	NA	NA	NA
ZNF726	1.5729572	0.00353781	0.0039754
CTB-92J24.3	NA	NA	NA

ZNF254	2.12224384	0.30149843	0.31381437
CTD-2017D1:	1.99740597	1.91E-91	8.74E-91
CTD-2027I19	NA	2.49E-48	5.78E-48
ERVK-28	NA	NA	NA
LINC00662	1.49805447	1.71E-17	2.57E-17
CTC-459F4.1	0.74902724	5.68E-14	8.04E-14
CTC-459F4.3	2.05502345	3.59E-39	7.36E-39
CTC-459F4.5	NA	NA	NA
CTD-2043I16	NA	NA	NA
CTB-70G10.1	NA	NA	NA
AC005780.1	NA	NA	NA
AC093074.1	NA	NA	NA
AC005307.1	NA	NA	NA
AC005307.3	NA	NA	NA
AC005616.1	NA	NA	NA
AC005616.2	Inf	1.87E-18	2.85E-18
AC005394.1	NA	NA	NA
AC079466.1	Inf	2.28E-79	8.40E-79
AC007795.1	NA	NA	NA
AC011524.1	NA	NA	NA
AC008991.1	NA	NA	NA
AC011524.2	NA	NA	NA
AC011524.3	NA	NA	NA
LINC00906	NA	NA	NA
LINC01532	NA	NA	NA
UQCRFS1	1.24717547	9.26E-139	2.26E-137
CTB-32O4.2	Inf	0.54893093	0.5620671
CTB-32O4.3	NA	1.52E-128	1.91E-127
CTB-32O4.4	NA	NA	NA
CTC-525D6.5	NA	NA	NA
CTC-525D6.2	NA	NA	NA
VSTM2B	Inf	2.04E-122	1.98E-121
POP4	1.20706548	4.88E-120	4.36E-119
PLEKHF1	2.04280156	2.56E-37	5.12E-37
C19orf12	2.76814414	1.13E-118	9.64E-118
CCNE1	1.83952277	4.06E-23	6.63E-23
URI1	1.84290365	0.98809171	0.98896391
CTC-448F2.4	NA	6.27E-37	1.25E-36
AC005597.1	NA	NA	NA
ZNF536	NA	NA	NA
CTC-565M22	NA	NA	NA
CTC-400I9.2	NA	NA	NA
CTC-400I9.1	NA	NA	NA
CTC-400I9.3	NA	NA	NA
CTC-439O9.3	NA	NA	NA

TSHZ3	7.49027237	0.04294234	0.04648606
AC007796.1	NA	NA	NA
AC008992.1	NA	NA	NA
CTD-2050I18	NA	NA	NA
CTD-2050I18	NA	NA	NA
AC011525.2	NA	NA	NA
AC011525.4	NA	NA	NA
CTC-360P9.1	NA	NA	NA
CTC-360P9.5	NA	NA	NA
CTC-360P9.2	NA	NA	NA
CTC-360P9.4	NA	NA	NA
LINC01533	NA	NA	NA
AC011518.1	NA	NA	NA
AC011518.2	NA	NA	NA
ZNF507	4.86867704	1.91E-52	4.72E-52
AC007773.2	2.62159533	3.83E-14	5.44E-14
DPY19L3	4.19455253	3.10E-118	2.60E-117
PDCD5	1.96143573	6.21E-14	8.79E-14
ANKRD27	3.84500649	1.26E-139	3.42E-138
CTC-379B2.4	NA	NA	NA
RGS9BP	1.49805447	3.75E-24	6.23E-24
CTD-2538C1.	1.49805447	8.62E-42	1.83E-41
NUDT19	3.29571984	9.55E-101	5.22E-100
CTD-2538C1.	NA	NA	NA
TDRD12	NA	1.91E-11	2.59E-11
SLC7A9	NA	6.09E-45	1.35E-44
CTD-2085J24	NA	NA	NA
CEP89	1.79766537	1.99E-35	3.88E-35
FAAP24	2.15345331	1.03E-42	2.21E-42
RHPN2	11.2354086	3.06E-72	1.01E-71
GPATCH1	2.8463035	0.00738622	0.00822141
WDR88	NA	NA	NA
LRP3	1.52480545	1.21E-110	8.29E-110
SLC7A10	NA	NA	NA
CTD-2540B1!	NA	NA	NA
CTD-2540B1!	NA	NA	NA
CEBPA	2.34563793	0.12905326	0.13701335
CTD-2540B1!	NA	3.21E-52	7.92E-52
CTD-2540B1!	NA	3.77E-133	6.08E-132
CEBPA-AS1	1.24837873	1.46E-07	1.83E-07
CTD-2540B1!	0	4.99E-22	8.03E-22
CEBPG	1.7131217	0.35835994	0.37118847
PEPD	0.76037613	2.95E-145	2.20E-143
CTD-2329C7.	NA	NA	NA
CHST8	0	7.75E-131	1.09E-129

KCTD15	2.0130107	3.61E-70	1.16E-69
CTC-526N19.	NA	NA	NA
LSM14A	1.81431042	1.04E-05	1.26E-05
KIAA0355	1.49805447	6.33E-08	8.00E-08
AC010504.2	NA	NA	NA
GPI	1.65146969	3.81E-74	1.30E-73
RP11-618P17	NA	NA	NA
PDCD2L	1.64785992	3.54E-31	6.47E-31
CTD-2588C8.	0.74902724	4.15E-10	5.48E-10
UBA2	1.89597519	0.00190699	0.00215756
WTIP	0.37451362	5.03E-61	1.40E-60
SCGB1B2P	NA	NA	NA
SCGB2B2	NA	NA	NA
ZNF302	3.12094682	8.06E-136	1.53E-134
CTD-2553L13	1.12354086	0.37025865	0.38322869
ZNF181	0.87386511	6.72E-26	1.14E-25
ZNF599	1.12354086	0.00021839	0.0002538
CTC-523E23.	NA	NA	NA
CTC-523E23.	NA	NA	NA
CTC-523E23.	NA	NA	NA
CTC-523E23.	NA	NA	NA
LINC00904	NA	NA	NA
CTC-523E23.	NA	NA	NA
CTC-523E23.	NA	NA	NA
CTC-523E23.	NA	NA	NA
ZNF30-AS1	NA	NA	NA
ZNF30	0.62418936	3.04E-79	1.12E-78
ZNF792	NA	2.80E-23	4.58E-23
GRAMD1A	2.17898833	2.25E-49	5.31E-49
CTD-2527I21	NA	1.61E-26	2.76E-26
CTD-2527I21	NA	3.06E-85	1.25E-84
SCN1B	0.59922179	1.43E-47	3.29E-47
HPN	NA	NA	NA
HPN-AS1	NA	NA	NA
CTD-2527I21	NA	NA	NA
FXYD3	NA	NA	NA
LGI4	NA	NA	NA
FXYD1	NA	0.50124829	0.51437562
FXYD7	NA	3.83E-53	9.57E-53
FXYD5	1.10167145	1.84E-145	1.58E-143
FAM187B	NA	NA	NA
LSR	2.08063121	3.04E-121	2.82E-120
AC002128.5	NA	NA	NA
USF2	1.74278615	0.13116095	0.13918589
HAMP	NA	1.55E-34	2.97E-34

MAG	NA	5.14E-86	2.12E-85
CD22	NA	1.95E-13	2.74E-13
FFAR1	NA	NA	NA
FFAR3	NA	NA	NA
GPR42	NA	NA	NA
LINC01531	NA	NA	NA
AC002511.2	NA	NA	NA
AC002511.3	NA	NA	NA
FFAR2	NA	6.59E-30	1.19E-29
KRTDAP	NA	NA	NA
DMKN	NA	NA	NA
SBSN	NA	NA	NA
GAPDHS	NA	NA	NA
TMEM147-A!	1.77042802	5.35E-54	1.35E-53
TMEM147	1.83648539	1.67E-06	2.05E-06
ATP4A	NA	NA	NA
AC002115.5	NA	NA	NA
AC002115.9	NA	NA	NA
HAUS5	2.71522374	0.331972	0.34470215
RBM42	0.94397953	1.03E-109	6.89E-109
ETV2	1.12354086	2.44E-33	4.60E-33
COX6B1	1.99621379	7.14E-45	1.58E-44
UPK1A	0	9.88E-134	1.64E-132
UPK1A-AS1	NA	NA	NA
ZBTB32	NA	NA	NA
KMT2B	1.91418072	0.16166875	0.17067177
IGFLR1	Inf	2.38E-35	4.63E-35
AD000671.6	NA	NA	NA
U2AF1L4	1.79766537	2.54E-32	4.72E-32
PSENEN	1.46768851	8.99E-61	2.50E-60
AC002398.9	NA	NA	NA
AC002398.11	NA	5.63E-61	1.57E-60
LIN37	0.74902724	2.28E-06	2.79E-06
AC002398.12	NA	NA	NA
HSPB6	NA	7.42E-121	6.81E-120
PROSER3	5.05593385	8.98E-59	2.42E-58
AC002398.13	NA	NA	NA
ARHGAP33	2.43433852	3.90E-27	6.76E-27
LINC01529	NA	NA	NA
PRODH2	NA	NA	NA
NPHS1	NA	NA	NA
KIRREL2	5.24319066	9.12E-59	2.46E-58
APLP1	0.99870298	0.01258879	0.01389208
NFKBID	2.36231667	1.04E-13	1.46E-13
HCST	0.75637064	5.34E-137	1.10E-135

TYROBP	0.72931599	2.98E-144	1.67E-142
LRFN3	3.37062257	6.76E-74	2.29E-73
AF038458.3	NA	NA	NA
AF038458.5	NA	NA	NA
SDHAF1	2.46679637	4.64E-107	2.89E-106
SYNE4	Inf	1.59E-96	8.03E-96
ALKBH6	2.03901859	3.03E-14	4.31E-14
AC002116.7	2.80885214	4.50E-14	6.38E-14
CLIP3	NA	4.47E-35	8.65E-35
THAP8	3.37062257	3.90E-36	7.67E-36
WDR62	4.38715953	6.14E-08	7.75E-08
OVOL3	NA	3.77E-67	1.16E-66
POLR2I	2.37154915	8.28E-144	4.21E-142
TBCB	1.36509106	6.21E-81	2.35E-80
CAPNS1	1.59435798	1.32E-72	4.39E-72
AD001527.7	NA	NA	NA
COX7A1	NA	NA	NA
ZNF565	0.99870298	1.06E-21	1.69E-21
ZNF146	2.16536965	5.47E-92	2.52E-91
CTD-3162L1C	NA	NA	NA
LINC00665	NA	2.29E-18	3.49E-18
ZFP14	3.74513619	3.01E-105	1.81E-104
CTD-3162L1C	NA	NA	NA
CTD-3162L1C	NA	NA	NA
ZFP82	NA	3.13E-68	9.76E-68
ZNF566	1.43563554	4.24E-14	6.01E-14
CTD-2630F21	NA	1.41E-105	8.51E-105
ZNF260	4.74383917	6.27E-133	9.95E-132
AC092295.4	NA	1.75E-135	3.23E-134
ZNF529	2.54669261	3.17E-134	5.40E-133
ZNF529-AS1	3.74513619	3.80E-51	9.20E-51
ZNF382	0	6.24E-41	1.31E-40
ZNF461	0.37451362	5.02E-53	1.25E-52
CTD-3234P18	0	0.00038758	0.00044775
LINC01534	3.18336576	2.34E-73	7.88E-73
ZNF567	2.24708171	7.67E-13	1.06E-12
ZNF850	0.56177043	0.17555293	0.18503842
CTD-2162K18	0.74902724	0.00015575	0.00018171
CTD-2162K18	NA	NA	NA
ZNF790-AS1	NA	2.51E-23	4.11E-23
ZNF790	2.99610895	1.83E-112	1.31E-111
ZNF345	1.49805447	6.01E-84	2.40E-83
ZNF829	NA	7.60E-105	4.52E-104
ZNF568	NA	1.11E-44	2.44E-44
ZNF420	0.99870298	1.31E-14	1.88E-14

CTD-2293H3.	NA	NA	NA
CTC-454I21.3	NA	NA	NA
ZNF585A	2.7464332	1.27E-46	2.88E-46
CTC-454I21.4	NA	NA	NA
ZNF585B	Inf	0.01252545	0.01382387
ZNF383	1.40442607	1.16E-07	1.46E-07
LINC01535	NA	NA	NA
CTD-3220F14	NA	NA	NA
HKR1	2.08063121	4.93E-10	6.51E-10
CTD-3220F14	Inf	2.73E-120	2.46E-119
ZNF527	1.04863813	1.31E-22	2.13E-22
ZNF569	3.12094682	7.65E-97	3.89E-96
ZNF570	Inf	0.01797531	0.01972825
ZNF793-AS1	NA	NA	NA
CTD-3064H18	NA	NA	NA
ZNF793	Inf	1.14E-06	1.40E-06
CTD-3064H18	NA	NA	NA
ZNF571-AS1	NA	NA	NA
ZNF540	0.99870298	0.00996911	0.0110361
ZNF571	1.49805447	4.99E-05	5.90E-05
ZFP30	2.62159533	7.06E-22	1.13E-21
ZNF781	Inf	1.84E-145	1.58E-143
CTD-2528L19	Inf	3.13E-68	9.76E-68
ZNF607	0.74902724	8.28E-72	2.72E-71
CTD-2528L19	2.49675746	5.74E-23	9.37E-23
ZNF573	1.49805447	2.49E-54	6.33E-54
CTD-2554C21	NA	NA	NA
AC016582.2	NA	NA	NA
CTD-2554C21	NA	NA	NA
WDR87	NA	NA	NA
SIPA1L3	0.74902724	0.02906461	0.03165386
CTC-450M9.1	NA	NA	NA
DPF1	1.99740597	0.0084191	0.00934758
SPINT2	1.64616721	8.87E-70	2.83E-69
PPP1R14A	2.30828627	5.70E-101	3.13E-100
CTB-102L5.4	NA	NA	NA
C19orf33	NA	1.92E-62	5.46E-62
YIF1B	1.30772788	2.46E-131	3.57E-130
KCNK6	2.92120623	0.26585691	0.27770474
CATSPERG	NA	2.18E-117	1.78E-116
AC005625.1	NA	NA	NA
PSMD8	1.11196881	4.63E-117	3.76E-116
GGN	Inf	1.88E-05	2.24E-05
AC005789.9	NA	NA	NA
SPRED3	Inf	3.81E-14	5.41E-14

FAM98C	1.30544747	1.26E-86	5.27E-86
RASGRP4	Inf	0.00076483	0.00087571
RYR1	NA	3.36E-115	2.57E-114
AC067969.1	NA	NA	NA
AC067969.2	NA	NA	NA
MAP4K1	NA	2.25E-33	4.25E-33
CTB-186G2.1	NA	NA	NA
EIF3K	1.41301208	1.38E-91	6.32E-91
ACTN4	2.03439497	4.75E-32	8.81E-32
CTB-186G2.4	NA	NA	NA
CAPN12	Inf	1.07E-157	5.54E-155
CTD-2540F13	NA	3.36E-24	5.58E-24
LGALS7	NA	NA	NA
LGALS7B	NA	NA	NA
LGALS4	1.49805447	1.43E-69	4.54E-69
ECH1	1.05646379	2.74E-145	2.09E-143
AC104534.3	NA	NA	NA
AC104534.2	NA	NA	NA
HNRNPL	2.22881276	1.42E-05	1.70E-05
RINL	2.43433852	5.46E-05	6.45E-05
CTC-360G5.6	NA	NA	NA
SIRT2	1.35599758	1.08E-45	2.42E-45
NFKBIB	1.71998847	0.00117008	0.00133331
CCER2	2.99610895	0.00920317	0.01020063
SARS2	1.63023575	4.55E-71	1.48E-70
CTC-360G5.8	NA	NA	NA
MRPS12	1.96460958	3.66E-50	8.73E-50
CTC-360G5.9	NA	NA	NA
FBXO17	NA	4.63E-22	7.45E-22
FBXO27	Inf	0.00076726	0.00087843
CTB-189B5.3	NA	NA	NA
ACP7	NA	NA	NA
PAK4	7.19066148	6.91E-65	2.05E-64
CTC-218B8.3	NA	NA	NA
NCCRP1	NA	1.10E-78	4.01E-78
SYCN	NA	NA	NA
IFNL3	NA	NA	NA
IFNL2	NA	NA	NA
IFNL1	NA	2.99E-74	1.02E-73
LRFN1	2.38975357	2.83E-33	5.33E-33
GMFG	1.56495937	1.70E-81	6.48E-81
CTC-246B18.	3.2457847	1.32E-55	3.42E-55
SAMD4B	2.97114137	1.29E-120	1.18E-119
PAF1	1.11062659	1.29E-54	3.29E-54
MED29	1.84135863	1.14E-34	2.20E-34

ZFP36	5.24319066	3.05E-61	8.56E-61
PLEKHG2	3.55787938	3.30E-75	1.14E-74
RPS16	2.14390455	7.15E-142	2.59E-140
SUPT5H	1.76944115	1.13E-07	1.42E-07
TIMM50	1.65271192	1.53E-35	2.99E-35
DLL3	1.10570687	3.22E-122	3.09E-121
SELV	NA	NA	NA
EID2B	1.1523496	0.01145125	0.01265833
CTB-60E11.9	NA	NA	NA
EID2	1.88217101	2.66E-09	3.46E-09
LGALS13	NA	NA	NA
LGALS16	NA	NA	NA
AC093063.3	NA	NA	NA
LGALS14	NA	NA	NA
CLC	NA	NA	NA
LEUTX	NA	NA	NA
LLNLR-268E1	Inf	2.69E-107	1.69E-106
DYRK1B	4.49416342	1.57E-45	3.50E-45
FBL	1.4014058	1.22E-132	1.91E-131
FCGBP	NA	NA	NA
PSMC4	0.68528024	1.52E-131	2.23E-130
ZNF546	Inf	4.77E-15	6.89E-15
ZNF780B	1.87256809	1.71E-11	2.31E-11
ZNF780A	1.49805447	0.0094572	0.01047771
AC005614.3	NA	1.24E-48	2.90E-48
MAP3K10	1.99740597	9.21E-11	1.23E-10
TTC9B	NA	1.09E-129	1.45E-128
CNTD2	NA	NA	NA
AKT2	1.79231518	7.23E-10	9.51E-10
C19orf47	2.71522374	2.64E-100	1.43E-99
PLD3	1.01338979	5.00E-60	1.38E-59
HIPK4	NA	NA	NA
PRX	Inf	7.20E-52	1.76E-51
SERTAD1	1.06112192	8.24E-32	1.52E-31
CTC-492K19.[illegible]	NA	0.00218613	0.00246831
SERTAD3	1.59575368	2.78E-33	5.23E-33
CTC-492K19.[illegible]	NA	NA	NA
BLVRB	1.14480145	2.94E-137	6.26E-136
SPTBN4	Inf	6.37E-20	9.91E-20
SHKBP1	1.30816025	1.67E-134	2.91E-133
LTBP4	2.99610895	2.20E-69	6.99E-69
AC010412.1	NA	NA	NA
NUMBL	1.2067661	0.59545063	0.60818969
ADCK4	1.99740597	4.51E-56	1.17E-55
ITPKC	2.62159533	2.86E-36	5.63E-36

C19orf54	1.82575389	4.55E-06	5.52E-06
SNRPA	1.19789282	4.34E-87	1.82E-86
MIA	Inf	4.62E-92	2.14E-91
MIA-RAB4B	NA	NA	NA
RAB4B	1.52579622	5.97E-40	1.24E-39
RAB4B-EGLN	NA	NA	NA
CTC-490E21.	Inf	5.48E-101	3.01E-100
EGLN2	1.71138502	1.35E-12	1.86E-12
CTC-490E21.	NA	NA	NA
CTC-490E21.	NA	NA	NA
CYP2A6	NA	NA	NA
CYP2A7	NA	NA	NA
CYP2B6	NA	NA	NA
CYP2A13	NA	NA	NA
CYP2F1	NA	NA	NA
CYP2S1	2.02237354	2.83E-11	3.82E-11
AXL	2.24708171	0.01935051	0.0212196
CTD-2195B2	NA	NA	NA
HNRNPUL1	2.05025704	3.67E-86	1.52E-85
TGFB1	2.03416026	0.64414733	0.65600769
CCDC97	2.70802155	0.00722184	0.00803992
CTC-435M10	Inf	7.74E-92	3.56E-91
TMEM91	1.58127972	1.56E-14	2.23E-14
B9D2	1.34200713	3.84E-85	1.56E-84
EXOSC5	1.48055384	3.51E-41	7.40E-41
BCKDHA	Inf	9.47E-39	1.93E-38
CTC-435M10	NA	NA	NA
B3GNT8	1.56614786	6.59E-88	2.82E-87
ATP5SL	1.54061284	1.07E-59	2.93E-59
ERICH4	NA	1.75E-09	2.28E-09
PCAT19	NA	NA	NA
AC006129.1	NA	NA	NA
LINC01480	NA	5.00E-13	6.96E-13
AC006129.2	5.24319066	0.02830046	0.03083831
CEACAM21	2.24708171	2.34E-09	3.05E-09
CEACAM4	NA	NA	NA
CEACAM7	NA	NA	NA
CEACAM5	NA	NA	NA
AC011513.3	NA	NA	NA
CEACAM6	NA	NA	NA
AC011513.4	NA	NA	NA
CEACAM3	NA	NA	NA
AC020956.3	NA	NA	NA
LYPD4	NA	NA	NA
DMRTC2	NA	NA	NA

RPS19	2.3231314	1.01E-142	4.20E-141
CD79A	1.54798962	2.76E-05	3.28E-05
ARHGEF1	1.42603262	1.71E-40	3.57E-40
RABAC1	1.19649805	5.60E-144	2.94E-142
ATP1A3	2.56809339	0.14259623	0.15094999
GRIK5	3.91158668	5.29E-123	5.24E-122
ZNF574	2.59956512	0.00201336	0.00227635
POU2F2	3.74513619	5.04E-57	1.33E-56
CTC-378H22.	Inf	1.34E-111	9.40E-111
DEDD2	1.9193823	6.41E-48	1.48E-47
ZNF526	1.3658732	8.10E-28	1.42E-27
GSK3A	2.74197471	9.29E-89	4.05E-88
AC006486.9	NA	NA	NA
ERF	2.00321238	0.67326275	0.68465991
CIC	1.3959144	0.00114473	0.00130516
PAFAH1B3	1.10928601	2.88E-120	2.59E-119
PRR19	1.49805447	5.69E-57	1.50E-56
TMEM145	Inf	1.98E-11	2.67E-11
MEGF8	1.83095547	2.52E-15	3.66E-15
CNFN	0.74902724	0.00888069	0.00985404
LIPE-AS1	1.94747082	6.01E-15	8.67E-15
LIPE	NA	7.75E-61	2.16E-60
CXCL17	0	4.70E-103	2.69E-102
CEACAM1	Inf	1.28E-134	2.25E-133
CEACAM8	NA	NA	NA
PSG3	NA	NA	NA
PSG8	NA	NA	NA
AC004603.4	NA	NA	NA
PSG1	NA	NA	NA
PSG6	NA	NA	NA
PSG7	NA	NA	NA
CTC-490G23.	NA	NA	NA
PSG11	NA	NA	NA
PSG2	NA	NA	NA
AC005392.13	NA	NA	NA
PSG5	NA	NA	NA
PSG4	NA	NA	NA
PSG9	NA	NA	NA
CTC-490G23.	NA	NA	NA
CD177	NA	NA	NA
CTC-490G23.	NA	NA	NA
TEX101	NA	NA	NA
LYPD3	Inf	2.97E-121	2.76E-120
PHLDB3	2.7464332	0.03648331	0.03957904
ETHE1	1.17194738	2.12E-141	7.16E-140

ZNF575	Inf	2.89E-108	1.86E-107
XRCC1	1.24837873	2.95E-122	2.84E-121
L34079.3	NA	NA	NA
L34079.2	NA	NA	NA
L34079.4	NA	NA	NA
PINLYP	NA	2.74E-07	3.42E-07
IRGQ	1.44811933	1.07E-26	1.83E-26
ZNF576	2.32351306	4.80E-32	8.89E-32
SRRM5	NA	0.02551337	0.02784811
ZNF428	1.85543348	1.11E-13	1.56E-13
CADM4	2.62159533	2.96E-66	8.96E-66
PLAUR	0.77485576	1.22E-125	1.34E-124
IRGC	NA	NA	NA
SMG9	1.59168288	4.98E-89	2.17E-88
KCNN4	1.19163424	3.42E-88	1.47E-87
AC115522.3	NA	NA	NA
LYPD5	NA	NA	NA
ZNF283	3.74513619	2.83E-26	4.83E-26
ZNF404	NA	NA	NA
RP11-15A1.3	NA	2.44E-91	1.11E-90
RP11-15A1.2	NA	NA	NA
ZNF45	1.79766537	1.24E-11	1.69E-11
ZNF221	Inf	2.23E-147	2.47E-145
ZNF155	NA	0.38801892	0.40076511
RP11-15A1.7	Inf	7.52E-48	1.73E-47
ZNF230	3.37062257	8.34E-105	4.96E-104
AC067968.3	NA	NA	NA
ZNF222	0.99870298	2.60E-10	3.44E-10
AC084219.2	NA	NA	NA
ZNF223	NA	NA	NA
ZNF284	NA	1.46E-121	1.36E-120
ZNF224	1.83095547	0.00017167	0.00020004
AC084219.4	Inf	3.38E-28	5.95E-28
ZNF225	0.99870298	0.03934022	0.04264776
ZNF234	Inf	3.37E-155	9.85E-153
ZNF226	1.49805447	8.98E-84	3.57E-83
ZNF227	4.99351492	5.11E-24	8.46E-24
ZNF235	0.99870298	1.62E-28	2.85E-28
ZNF233	Inf	1.03E-98	5.44E-98
ZNF112	NA	3.18E-27	5.51E-27
CTC-512J12.6	NA	NA	NA
ZNF285	NA	NA	NA
ZNF229	NA	NA	NA
ZNF180	2.46108949	1.30E-13	1.83E-13
CEACAM20	NA	NA	NA

AC138472.6	NA	NA	NA
IGSF23	NA	2.07E-112	1.48E-111
CTB-171A8.1	Inf	1.86E-153	4.02E-151
PVR	2.62159533	0.00646339	0.00720219
CEACAM19	3.74513619	6.23E-19	9.57E-19
CEACAM16	NA	NA	NA
BCL3	2.54669261	0.00025726	0.00029863
CBLC	NA	NA	NA
BCAM	1.93170182	3.68E-27	6.37E-27
PVRL2	1.11891723	1.80E-138	4.26E-137
CTB-129P6.4	NA	1.98E-06	2.42E-06
TOMM40	1.49706761	3.31E-39	6.79E-39
APOE	1.12354086	0.17489187	0.18436306
APOC1	2.0598249	2.10E-24	3.50E-24
APOC4-APOC	NA	NA	NA
APOC4	NA	NA	NA
APOC2	NA	3.43E-06	4.17E-06
CTB-129P6.1	NA	3.24E-46	7.31E-46
CLPTM1	1.52649855	3.20E-33	6.02E-33
RELB	1.44043699	6.79E-22	1.09E-21
CLASRP	1.72276265	0.46282763	0.47580999
ZNF296	1.33754864	4.04E-115	3.09E-114
CTB-179K24.	NA	1.15E-12	1.59E-12
GEMIN7	1.72012788	1.16E-06	1.43E-06
MARK4	1.36778887	1.08E-14	1.55E-14
PPP1R37	1.74773022	0.05411778	0.05833658
AC005757.6	NA	NA	NA
NKPD1	NA	NA	NA
AC005757.7	NA	NA	NA
TRAPPC6A	0.8527387	2.49E-143	1.15E-141
BLOC1S3	3.41223519	1.19E-59	3.24E-59
AC005779.2	NA	NA	NA
AC006126.3	NA	NA	NA
EXOC3L2	NA	1.37E-48	3.20E-48
AC006126.4	NA	NA	NA
CKM	0.49935149	1.34E-142	5.26E-141
KLC3	2.24708171	1.23E-36	2.44E-36
ERCC2	1.64785992	0.0026025	0.00293276
PPP1R13L	1.24837873	6.10E-11	8.17E-11
CD3EAP	1.36801502	1.60E-56	4.17E-56
ERCC1	1.36047804	1.28E-105	7.73E-105
FOSB	Inf	1.50E-99	8.02E-99
RTN2	1.2067661	1.06E-128	1.35E-127
PPM1N	2.67509728	6.23E-30	1.12E-29
VASP	1.36466606	3.20E-55	8.23E-55

OPA3	1.4014058	1.50E-36	2.97E-36
GPR4	NA	NA	NA
EML2	2.62159533	0.08713509	0.09319684
GIPR	2.0598249	0.01182055	0.01306099
SNRPD2	1.51334896	8.38E-142	3.00E-140
QPCTL	0.37451362	9.80E-81	3.69E-80
FBXO46	1.64785992	9.27E-14	1.31E-13
BHMG1	0	5.13E-111	3.55E-110
SIX5	2.99610895	0.94856925	0.95119077
AC074212.6	NA	NA	NA
DMPK	1.49805447	3.16E-71	1.03E-70
AC011530.4	NA	NA	NA
DMWD	1.73212549	7.66E-11	1.02E-10
RSPH6A	NA	NA	NA
SYMPK	2.31841764	6.65E-27	1.15E-26
AC092301.3	Inf	4.64E-08	5.87E-08
FOXA3	1.49805447	3.00E-11	4.04E-11
IRF2BP1	3.06744488	1.32E-44	2.89E-44
MYPOP	3.57868569	7.92E-104	4.62E-103
NANOS2	NA	NA	NA
NOVA2	NA	NA	NA
CCDC61	0.66580199	2.79E-129	3.62E-128
PGLYRP1	NA	NA	NA
CTC-344H19.	Inf	3.93E-118	3.27E-117
IGFL4	NA	NA	NA
AC007193.6	NA	NA	NA
IGFL3	NA	NA	NA
IGFL2	Inf	4.83E-114	3.61E-113
AC006262.4	NA	NA	NA
AC006262.5	NA	NA	NA
AC006262.1C	NA	NA	NA
IGFL1	NA	NA	NA
HIF3A	NA	5.41E-10	7.14E-10
AC007193.1C	NA	NA	NA
PPP5C	2.07938905	0.1120675	0.11924507
AC007193.9	NA	NA	NA
AC007193.8	NA	NA	NA
CCDC8	NA	NA	NA
LLNLF-187D8	NA	1.17E-136	2.37E-135
PNMAL1	2.24708171	8.00E-32	1.48E-31
PPP5D1	1.87256809	5.94E-22	9.55E-22
PNMAL2	NA	NA	NA
CTB-158D10.	NA	NA	NA
CALM3	1.87256809	7.34E-05	8.64E-05
CTB-12A17.3	NA	1.80E-118	1.52E-117

PTGIR	0.14980545	2.22E-102	1.25E-101
GNG8	NA	NA	NA
DACT3	5.24319066	0.00012577	0.00014712
DACT3-AS1	NA	NA	NA
PRKD2	1.63849708	1.54E-05	1.84E-05
STRN4	2.33696498	6.47E-13	8.99E-13
CTB-174O21.	NA	NA	NA
FKRP	1.07003891	8.22E-25	1.38E-24
SLC1A5	1.32666689	5.30E-17	7.89E-17
CTB-147N14.	Inf	6.74E-08	8.51E-08
AC008622.1	NA	NA	NA
AP2S1	1.49911467	3.70E-52	9.12E-52
ARHGAP35	4.11964981	1.69E-74	5.78E-74
NPAS1	1.68531128	1.05E-08	1.35E-08
TMEM160	2.0619987	2.01E-110	1.37E-109
ZC3H4	1.97243839	9.75E-133	1.53E-131
SAE1	1.19206888	2.08E-83	8.21E-83
BBC3	2.21451531	6.66E-73	2.22E-72
CCDC9	2.24708171	1.06E-23	1.75E-23
INAFM1	1.46809339	5.25E-17	7.82E-17
C5AR1	0	2.21E-143	1.03E-141
C5AR2	NA	5.98E-05	7.05E-05
DHX34	1.91418072	0.53971879	0.55310277
MEIS3	Inf	1.98E-36	3.92E-36
SLC8A2	NA	5.01E-56	1.30E-55
KPTN	1.1432521	9.17E-47	2.09E-46
NAPA-AS1	0.74902724	9.05E-22	1.45E-21
NAPA	1.34361587	2.67E-98	1.40E-97
ZNF541	0	6.27E-57	1.65E-56
CTD-2571L23	NA	1.13E-131	1.67E-130
CTD-3001H1	1.87256809	1.61E-33	3.04E-33
GLTSCR1	2.24708171	6.27E-06	7.58E-06
GLTSCR1-AS	NA	NA	NA
EHD2	1.45124027	1.60E-28	2.82E-28
GLTSCR2	1.9467039	0.00787539	0.00875401
GLTSCR2-AS	NA	NA	NA
SEPW1	2.25712229	1.81E-60	5.02E-60
TPRX1	NA	NA	NA
CRX	0	2.69E-36	5.30E-36
LINC01595	NA	NA	NA
SULT2A1	NA	NA	NA
BSPH1	NA	NA	NA
ELSPBP1	NA	NA	NA
CABP5	NA	NA	NA
PLA2G4C	NA	0.33499265	0.34767944

PLA2G4C-AS	NA	NA	NA
LIG1	1.60136858	5.72E-104	3.35E-103
CTC-453G23.	NA	NA	NA
CTC-453G23.	NA	NA	NA
C19orf68	1.24837873	3.59E-15	5.19E-15
ZNF114	2.7464332	3.40E-12	4.67E-12
CARD8	1.64460328	3.66E-07	4.54E-07
CTC-453G23.	0	4.35E-104	2.55E-103
CARD8-AS1	2.62159533	8.29E-61	2.31E-60
CTC-241F20.	2.24708171	0.0001002	0.00011748
CCDC114	NA	3.14E-11	4.22E-11
EMP3	1.21017212	2.12E-135	3.89E-134
TMEM143	0.88521401	9.29E-111	6.36E-110
SYNGR4	NA	0.95158603	0.95395225
KDELR1	1.14118809	6.36E-119	5.45E-118
GRIN2D	2.24708171	2.79E-46	6.30E-46
GRWD1	1.32466854	2.29E-57	6.05E-57
KCNJ14	7.49027237	7.44E-121	6.83E-120
CTC-273B12.	NA	6.78E-49	1.59E-48
CTC-273B12.	NA	NA	NA
CYTH2	0.99870298	7.75E-63	2.22E-62
LMTK3	0.74902724	6.41E-76	2.23E-75
CTC-273B12.	2.99610895	1.17E-09	1.53E-09
SULT2B1	1.99740597	5.63E-73	1.88E-72
FAM83E	NA	2.44E-69	7.74E-69
SPACA4	NA	NA	NA
RPL18	2.0382581	4.85E-122	4.64E-121
AC022154.7	NA	5.43E-08	6.86E-08
SPHK2	2.57998271	2.29E-132	3.52E-131
DBP	1.36030234	1.87E-97	9.62E-97
CA11	2.24708171	4.91E-24	8.14E-24
NTN5	NA	8.73E-51	2.10E-50
FUT2	NA	0.08661483	0.09264585
MAMSTR	0.89883268	1.10E-38	2.23E-38
RASIP1	NA	8.33E-64	2.42E-63
IZUMO1	NA	9.59E-07	1.18E-06
FUT1	0.74902724	2.84E-36	5.60E-36
FGF21	NA	NA	NA
BCAT2	1.07003891	2.88E-143	1.31E-141
HSD17B14	2.24708171	8.73E-05	1.03E-04
PLEKHA4	0.99870298	4.59E-84	1.83E-83
PPP1R15A	2.24708171	2.28E-08	2.90E-08
TULP2	NA	NA	NA
NUCB1	1.18179853	1.85E-93	8.79E-93
NUCB1-AS1	NA	NA	NA

DHDH	Inf	0.25793057	0.26958039
BAX	1.8672179	0.00033398	0.00038649
CTD-2639E6.	NA	NA	NA
FTL	2.49029449	1.51E-108	9.78E-108
GYS1	2.04734112	1.21E-10	1.61E-10
RUVBL2	1.06053857	1.06E-125	1.17E-124
LHB	NA	NA	NA
CTB-60B18.1	NA	NA	NA
CGB	NA	NA	NA
CTB-60B18.6	NA	NA	NA
CGB2	NA	NA	NA
CGB1	NA	NA	NA
CGB5	NA	NA	NA
CGB8	NA	NA	NA
CGB7	NA	NA	NA
NTF4	NA	NA	NA
CTB-60B18.1	NA	NA	NA
CTB-60B18.1	NA	NA	NA
KCNA7	NA	1.11E-44	2.44E-44
SNRNP70	1.23182198	1.31E-60	3.63E-60
LIN7B	3.85214008	7.64E-11	1.02E-10
C19orf73	1.49805447	1.64E-18	2.51E-18
PPFIA3	1.68531128	4.60E-12	6.31E-12
HRC	NA	0.00022524	0.00026165
TRPM4	3.74513619	7.12E-22	1.14E-21
SLC6A16	NA	2.40E-11	3.23E-11
CTC-301O7.4	NA	1.61E-77	5.74E-77
CD37	1.40035527	6.59E-33	1.24E-32
TEAD2	NA	NA	NA
AC010524.4	NA	NA	NA
DKKL1	NA	1.24E-87	5.29E-87
AC010524.2	NA	NA	NA
CCDC155	NA	1.19E-43	2.58E-43
PTH2	NA	6.36E-31	1.16E-30
GFY	NA	NA	NA
SLC17A7	Inf	0.07758648	0.0831752
PIH1D1	1.30402935	1.19E-141	4.16E-140
ALDH16A1	0.72931599	6.48E-119	5.55E-118
CTD-3148I10	NA	NA	NA
FLT3LG	1.79766537	5.76E-16	8.44E-16
RPL13A	2.23162217	9.21E-103	5.24E-102
RPS11	2.05609736	6.06E-112	4.28E-111
FCGRT	0.41325641	9.60E-145	5.97E-143
RCN3	0.37451362	1.12E-125	1.23E-124
NOSIP	1.43948856	3.19E-103	1.84E-102

PRRG2	4.49416342	1.10E-21	1.75E-21
PRR12	2.99610895	4.22E-47	9.63E-47
CTB-33G10.1	NA	3.17E-100	1.71E-99
RRAS	1.23054475	3.64E-123	3.64E-122
SCAF1	2.33696498	1.38E-70	4.44E-70
IRF3	1.53747696	4.36E-37	8.68E-37
BCL2L12	1.95463283	8.32E-18	1.26E-17
PRMT1	0.8286784	4.65E-133	7.47E-132
ADM5	2.88910506	3.84E-52	9.44E-52
CTB-33G10.6	Inf	1.91E-119	1.67E-118
CPT1C	NA	8.57E-150	1.22E-147
TSKS	NA	NA	NA
AP2A1	3.08973735	1.05E-105	6.39E-105
FUZ	NA	5.26E-29	9.35E-29
AC006942.4	NA	NA	NA
MED25	0.98148397	7.51E-61	2.09E-60
PTOV1-AS1	0	4.41E-12	6.05E-12
PTOV1	1.11304047	1.58E-143	7.54E-142
AC018766.6	NA	9.79E-32	1.81E-31
PTOV1-AS2	NA	1.55E-11	2.10E-11
AC018766.4	0	4.75E-173	1.08E-169
PNKP	1.59470315	0.09836685	0.1050304
AKT1S1	2.07230869	0.31007943	0.32259784
TBC1D17	1.99740597	1.48E-38	3.00E-38
IL4I1	0	3.34E-42	7.13E-42
NUP62	2.06549935	4.32E-15	6.25E-15
CTC-326K19.(	NA	NA	NA
ATF5	1.64785992	3.39E-06	4.12E-06
SIGLEC11	NA	NA	NA
VRK3	1.37889105	7.04E-68	2.18E-67
ZNF473	1.33160398	0.04167712	0.04515146
CTD-2126E3.	NA	NA	NA
CTD-2126E3.	NA	2.51E-30	4.55E-30
IZUMO2	NA	6.59E-30	1.19E-29
MYH14	NA	NA	NA
CTB-191K22.	NA	NA	NA
KCNC3	1.49805447	4.18E-32	7.74E-32
NR1H2	2.18466278	0.00027225	0.00031579
NAPSA	NA	NA	NA
CTB-191K22.	NA	NA	NA
POLD1	1.29978256	2.45E-45	5.46E-45
CTD-2545M3	NA	NA	NA
SPIB	Inf	8.21E-142	2.95E-140
MYBPC2	NA	5.28E-72	1.74E-71
FAM71E1	NA	NA	NA

EMC10	0.74902724	2.16E-47	4.95E-47
CTD-2545M3	NA	NA	NA
CTD-2545M3	0.37451362	4.08E-33	7.66E-33
JOSD2	2.36231667	0.00148926	0.00169043
ASPDH	NA	NA	NA
LRRC4B	NA	0.00048233	0.00055541
SYT3	NA	1.21E-105	7.31E-105
C19orf81	Inf	7.04E-39	1.44E-38
SHANK1	0	6.67E-162	5.26E-159
CLEC11A	0.85228482	3.96E-144	2.17E-142
GPR32	NA	NA	NA
CTD-2568A17	NA	2.50E-36	4.92E-36
ACPT	NA	NA	NA
CTD-2568A17	0	0.01615594	0.01776047
C19orf48	2.44935067	4.25E-125	4.56E-124
MGC45922	NA	0.00012288	0.00014378
KLK1	2.33030696	6.36E-105	3.80E-104
KLK15	NA	NA	NA
AC011523.2	NA	NA	NA
KLK3	NA	NA	NA
KLK2	NA	5.14E-86	2.12E-85
KLK4	NA	NA	NA
KLK5	NA	NA	NA
CTB-147C22.	NA	NA	NA
KLK6	NA	NA	NA
CTB-147C22.	NA	NA	NA
KLK7	NA	NA	NA
KLK8	NA	NA	NA
CTC-518B2.8	NA	NA	NA
KLK9	NA	NA	NA
KLK10	NA	NA	NA
CTC-518B2.1	NA	NA	NA
KLK11	NA	NA	NA
KLK12	NA	NA	NA
CTC-518B2.9	NA	NA	NA
CTC-518B2.1	NA	NA	NA
KLK13	NA	NA	NA
KLK14	NA	NA	NA
CTU1	1.62118224	6.67E-65	1.98E-64
SIGLEC9	NA	NA	NA
SIGLEC7	NA	NA	NA
CTD-3187F8.	NA	NA	NA
CD33	1.45156313	1.07E-121	1.01E-120
SIGLECL1	NA	9.03E-62	2.55E-61
CTD-3187F8.	NA	NA	NA

CTD-3187F8.	NA	NA	NA
IGLON5	NA	NA	NA
VSIG10L	1.74773022	0.00121018	0.00137796
CTD-2616J11	NA	NA	NA
CTD-2616J11	NA	NA	NA
ETFB	1.14516404	1.82E-143	8.64E-142
CTD-2616J11	NA	NA	NA
CLDND2	3.55787938	5.05E-108	3.23E-107
NKG7	0.8527387	2.76E-141	9.18E-140
LIM2	NA	NA	NA
C19orf84	NA	1.35E-88	5.87E-88
CTD-2616J11	Inf	6.83E-75	2.35E-74
SIGLEC10	1.28404669	0.13819557	0.14635984
CTD-2616J11	NA	NA	NA
CTD-2616J11	0	2.50E-17	3.75E-17
SIGLEC8	0	2.58E-15	3.73E-15
CEACAM18	NA	NA	NA
SIGLEC12	0.57278553	8.17E-64	2.38E-63
SIGLEC6	2.43433852	2.62E-127	3.12E-126
CTD-3073N1	NA	2.09E-82	8.11E-82
ZNF175	3.21011673	6.73E-102	3.77E-101
AC018755.17	NA	NA	NA
SIGLEC5	0.74902724	3.70E-24	6.15E-24
AC018755.18	NA	NA	NA
SIGLEC14	NA	NA	NA
SPACA6P-AS	NA	0.20883562	0.21936748
HAS1	NA	NA	NA
FPR1	NA	NA	NA
FPR2	NA	1.69E-56	4.41E-56
FPR3	NA	NA	NA
AC006272.2	NA	NA	NA
AC006272.1	NA	NA	NA
ZNF577	Inf	1.40E-76	4.91E-76
ZNF613	0.74902724	0.00020647	0.00024019
ZNF649-AS1	NA	NA	NA
ZNF649	2.99610895	1.82E-06	2.23E-06
ZNF350-AS1	NA	NA	NA
ZNF350	NA	2.26E-39	4.64E-39
ZNF615	0.74902724	3.80E-21	6.03E-21
ZNF614	1.41482923	0.0473966	0.05121622
ZNF432	2.62159533	0.01634191	0.01796382
CTC-471J1.11	0.74902724	0.00155446	0.00176355
ZNF841	NA	NA	NA
ZNF616	2.99610895	5.27E-38	1.06E-37
CTC-471J1.9	NA	NA	NA

ZNF836	5.24319066	8.09E-77	2.85E-76
CTD-2525I3.2	NA	5.45E-44	1.19E-43
PPP2R1A	1.03882944	3.01E-132	4.59E-131
CTD-2525I3.3	NA	NA	NA
ZNF766	1.89227934	3.62E-23	5.92E-23
CTD-2525I3.5	NA	1.81E-98	9.50E-98
ZNF480	1.38281952	2.05E-38	4.17E-38
CTD-2525I3.6	NA	NA	NA
ZNF610	NA	0.00596164	0.00664881
ZNF880	NA	NA	NA
ZNF528	NA	NA	NA
ZNF534	NA	1.43E-132	2.23E-131
ZNF578	NA	NA	NA
CTD-3099C6.	NA	NA	NA
ZNF808	1.94747082	2.68E-96	1.35E-95
ZNF701	Inf	7.61E-133	1.20E-131
CTD-3099C6.	NA	NA	NA
ZNF83	1.49805447	0.3128093	0.32536328
ZNF611	2.69649805	5.09E-57	1.34E-56
ZNF600	NA	NA	NA
ZNF28	NA	NA	NA
ZNF468	2.99610895	5.47E-70	1.75E-69
ZNF320	NA	NA	NA
CTD-2331H12	NA	NA	NA
ZNF888	NA	NA	NA
CTD-2331H12	NA	NA	NA
ZNF816-ZNF	NA	1.52E-89	6.70E-89
ZNF816	NA	NA	NA
CTD-2620I22	NA	NA	NA
ERVV-1	NA	NA	NA
ERVV-2	NA	NA	NA
ZNF160	2.03307393	5.96E-09	7.69E-09
ZNF415	NA	NA	NA
ZNF347	NA	2.29E-41	4.83E-41
ZNF665	Inf	3.86E-16	5.68E-16
CTD-2245F17	NA	NA	NA
CTD-2245F17	Inf	8.91E-12	1.21E-11
ZNF677	NA	5.47E-100	2.94E-99
CTD-2245F17	NA	NA	NA
VN1R2	NA	NA	NA
VN1R4	NA	NA	NA
CTD-2245F17	NA	NA	NA
BIRC8	NA	NA	NA
ZNF525	NA	0.0829912	0.08884849
ZNF845	4.11964981	0.01279506	0.01411713

ZNF765	1.87256809	2.47E-17	3.69E-17
ZNF761	3.08973735	1.30E-10	1.73E-10
ZNF813	1.06112192	6.62E-05	7.80E-05
ZNF331	2.54669261	1.29E-108	8.34E-108
DPRX	NA	NA	NA
CTC-339O9.1	NA	NA	NA
CTD-3022G6.	NA	NA	NA
AC008753.4	NA	NA	NA
NLRP12	0.99870298	9.24E-42	1.96E-41
AC008753.6	NA	NA	NA
AC008440.10	NA	NA	NA
MYADM	1.85285685	1.08E-22	1.76E-22
AC008440.5	NA	NA	NA
PRKCG	NA	NA	NA
CACNG7	NA	NA	NA
CACNG8	Inf	5.05E-67	1.54E-66
CACNG6	NA	NA	NA
VSTM1	NA	NA	NA
TARM1	NA	NA	NA
OSCAR	0.24967575	5.71E-85	2.31E-84
NDUFA3	1.86840683	5.07E-17	7.55E-17
TFPT	1.24837873	6.64E-65	1.97E-64
PRPF31	1.05371628	8.62E-115	6.55E-114
AC012314.8	NA	1.69E-55	4.37E-55
CNOT3	1.97304736	1.15E-06	1.42E-06
LENG1	2.11681611	5.15E-70	1.65E-69
TMC4	Inf	1.42E-20	2.24E-20
MBOAT7	1.37724363	2.61E-67	8.03E-67
TSEN34	1.45931169	3.14E-43	6.80E-43
AC012314.20	NA	NA	NA
RPS9	1.79525673	5.00E-11	6.71E-11
LILRB3	NA	1.14E-09	1.50E-09
CTB-83J4.1	NA	NA	NA
LILRA6	NA	NA	NA
LILRB2	2.24708171	0.73568446	0.74538073
LILRB5	NA	NA	NA
LILRA5	NA	0.00668199	0.00744486
LILRA4	NA	6.07E-05	7.16E-05
LAIR1	1.11062659	3.87E-107	2.42E-106
TTYH1	1.58127972	3.81E-60	1.05E-59
AC008746.3	NA	NA	NA
CTD-2587H1	NA	0.05737483	0.06179614
AC008746.12	Inf	1.46E-88	6.34E-88
LENG8-AS1	Inf	1.56E-06	1.92E-06
LENG8	2.38643562	2.35E-111	1.64E-110

LENG9	0.93628405	6.41E-76	2.23E-75
CDC42EP5	NA	6.04E-09	7.80E-09
LAIR2	NA	NA	NA
KIR3DX1	NA	4.66E-51	1.13E-50
LILRA2	1.87256809	6.37E-84	2.54E-83
LILRA1	1.49805447	5.18E-32	9.58E-32
CTB-83J4.2	NA	NA	NA
LILRB1	Inf	4.42E-102	2.49E-101
AC009892.1(	NA	NA	NA
LILRB4	NA	NA	NA
KIR3DL3	NA	NA	NA
KIR2DL3	NA	NA	NA
KIR2DL1	NA	NA	NA
KIR2DL4	NA	NA	NA
KIR3DL1	NA	NA	NA
KIR3DL2	NA	NA	NA
FCAR	NA	5.14E-86	2.12E-85
CTB-61M7.2	NA	7.91E-48	1.82E-47
NCR1	NA	NA	NA
NLRP7	Inf	8.12E-113	5.91E-112
NLRP2	0	3.61E-07	4.49E-07
CTC-550B14.	0.37451362	2.88E-87	1.21E-86
GP6	NA	0.05255704	0.05667775
RDH13	1.51776572	1.89E-42	4.05E-42
EPS8L1	1.33160398	0.93101555	0.93425987
PPP1R12C	1.73458939	1.45E-11	1.97E-11
TNNT1	1.48499005	4.01E-63	1.15E-62
TNNI3	0.18725681	6.12E-53	1.52E-52
CTD-2587H2	NA	NA	NA
DNAAF3	0.93628405	1.45E-51	3.54E-51
CTD-2587H2	NA	NA	NA
SYT5	NA	0.26896263	0.28088419
PTPRH	Inf	3.18E-113	2.33E-112
CTD-2587H2	NA	0.75538377	0.7645711
TMEM86B	2.24708171	0.00400203	0.00448843
PPP6R1	1.98271916	3.15E-39	6.47E-39
HSPBP1	1.18417639	5.01E-139	1.26E-137
BRSK1	NA	7.16E-35	1.38E-34
CTD-2105E1	NA	NA	NA
TMEM150B	NA	0.00069756	0.00079964
CTD-2105E1	NA	8.75E-11	1.17E-10
KMT5C	1.47031273	8.88E-73	2.96E-72
COX6B2	Inf	1.05E-07	1.32E-07
CTD-2105E1	NA	NA	NA
FAM71E2	NA	NA	NA

IL11	NA	1.09E-102	6.19E-102
TMEM190	NA	NA	NA
CTD-2105E13	NA	1.99E-52	4.91E-52
TMEM238	Inf	1.30E-106	8.02E-106
RPL28	2.28002417	1.22E-141	4.26E-140
UBE2S	1.97668787	0.00015979	0.00018635
SHISA7	NA	NA	NA
ISOC2	1.60634757	4.88E-09	6.32E-09
CTD-2537I9.1	NA	NA	NA
ZNF628	1.78614187	2.14E-33	4.04E-33
NAT14	2.12791829	1.23E-11	1.68E-11
SSC5D	1.49805447	1.18E-43	2.58E-43
SBK2	NA	NA	NA
SBK3	NA	NA	NA
ZNF579	2.13184675	7.32E-12	1.00E-11
FIZ1	2.39688716	1.17E-05	1.41E-05
ZNF524	1.14744598	0.00040821	0.00047128
ZNF865	1.07003891	8.72E-11	1.17E-10
CTD-2537I9.1	NA	8.61E-111	5.90E-110
ZNF784	0.83225249	0.1278793	0.13579082
ZNF580	1.41195939	4.71E-133	7.55E-132
ZNF581	1.53249251	3.38E-21	5.36E-21
CCDC106	1.65574442	5.58E-97	2.85E-96
U2AF2	1.77710384	0.00203634	0.00230176
CTD-2537I9.1	NA	NA	NA
EPN1	2.57912471	1.68E-135	3.13E-134
AC010525.4	NA	NA	NA
NLRP9	NA	NA	NA
RFPL4A	NA	NA	NA
RFPL4AL1	NA	NA	NA
NLRP11	Inf	7.97E-61	2.22E-60
NLRP4	NA	NA	NA
NLRP13	NA	NA	NA
NLRP8	NA	6.60E-111	4.55E-110
NLRP5	NA	NA	NA
CTD-2621I17	NA	NA	NA
ZNF787	2.75535019	2.38E-123	2.40E-122
ZNF444	2.11867704	0.95125635	0.95367445
GALP	NA	NA	NA
ZSCAN5B	NA	5.50E-20	8.57E-20
ZSCAN5CP	NA	NA	NA
ZSCAN5A	3.12094682	1.46E-87	6.20E-87
AC006116.17	0	1.11E-16	1.65E-16
AC006116.21	NA	NA	NA
ZNF582	Inf	1.93E-58	5.17E-58

AC006116.12	NA	NA	NA
ZNF582-AS1	NA	6.56E-78	2.36E-77
ZNF583	2.51945525	1.28E-32	2.38E-32
ZNF667	NA	NA	NA
ZNF667-AS1	NA	NA	NA
ZNF471	Inf	2.60E-37	5.19E-37
AC005498.4	NA	NA	NA
AC005498.3	NA	7.71E-42	1.64E-41
ZFP28	5.9922179	7.85E-28	1.37E-27
AC007228.11	1.24837873	0.65123712	0.66293031
ZNF470	0.74902724	8.19E-12	1.12E-11
ZNF71	1.8385214	8.18E-11	1.09E-10
SMIM17	NA	NA	NA
ZNF835	NA	NA	NA
AC007228.5	NA	NA	NA
AC007228.9	1.49805447	9.28E-09	1.19E-08
ZIM2-AS1	NA	NA	NA
ZIM2	NA	NA	NA
PEG3	NA	NA	NA
MIMT1	NA	NA	NA
USP29	NA	NA	NA
ZIM3	NA	NA	NA
DUXA	NA	NA	NA
ZNF264	NA	NA	NA
AURKC	NA	NA	NA
ZNF805	0.74902724	2.26E-59	6.14E-59
CTC-444N24.	1.68531128	1.67E-07	2.09E-07
CTC-444N24.	Inf	5.32E-22	8.55E-22
ZNF460	0	0.24053375	0.25189121
CTC-444N24.	2.65040407	8.33E-129	1.06E-127
ZNF543	2.99610895	4.30E-61	1.20E-60
CTC-444N24.	Inf	1.28E-90	5.75E-90
ZNF304	Inf	1.11E-19	1.72E-19
ZNF547	Inf	3.81E-80	1.42E-79
TRAPPC2P1	1.84760052	3.68E-06	4.48E-06
AC003002.4	NA	NA	NA
ZNF548	2.99610895	8.89E-92	4.09E-91
AC003002.6	NA	NA	NA
AC004076.7	NA	NA	NA
ZNF17	1.94747082	0.11976913	0.12734288
ZNF749	1.49805447	2.37E-51	5.76E-51
AC004076.9	NA	NA	NA
AC004076.5	5.9922179	2.08E-76	7.31E-76
VN1R1	NA	4.31E-45	9.56E-45
ZNF772	Inf	1.02E-130	1.44E-129

AC003005.2	NA	7.54E-79	2.75E-78
ZNF419	0.99870298	2.69E-68	8.39E-68
AC003005.4	NA	NA	NA
ZNF773	3.74513619	1.05E-37	2.10E-37
ZNF549	2.09727626	8.96E-45	1.98E-44
ZNF550	3.49546044	1.27E-77	4.53E-77
ZNF416	4.49416342	1.74E-59	4.75E-59
ZIK1	NA	NA	NA
ZNF530	3.08973735	8.43E-20	1.31E-19
ZNF134	1.29831388	3.13E-81	1.19E-80
AC003682.16	NA	NA	NA
ZNF211	4.49416342	1.58E-22	2.57E-22
ZSCAN4	NA	NA	NA
ZNF551	1.43563554	1.27E-27	2.21E-27
AC003006.7	NA	NA	NA
ZNF154	NA	1.06E-50	2.54E-50
ZNF671	1.12354086	0.33456584	0.34725633
ZNF776	1.49805447	2.69E-06	3.28E-06
ZNF586	NA	1.20E-30	2.19E-30
ZNF552	0.99870298	1.24E-45	2.77E-45
CTD-2583A14	NA	NA	NA
ZNF587B	2.34071012	7.24E-36	1.42E-35
ZNF814	Inf	0.00051629	0.0005939
ZNF587	2.54669261	8.13E-52	1.99E-51
CTD-2583A14	NA	NA	NA
ZNF417	0.59922179	3.65E-23	5.97E-23
ZNF418	NA	NA	NA
ZNF256	NA	NA	NA
C19orf18	NA	NA	NA
ZNF606	NA	1.32E-51	3.23E-51
ZSCAN1	NA	NA	NA
ZNF135	NA	NA	NA
ZSCAN18	0.74902724	1.60E-05	1.91E-05
ZNF329	NA	0.27874926	0.2907197
ZNF274	1.10382961	1.53E-48	3.56E-48
ZNF544	0	6.29E-45	1.39E-44
CTD-3138B18	1.94066148	2.10E-13	2.95E-13
ZNF8	1.87256809	0.00022203	0.00025796
AC010642.1	Inf	4.44E-50	1.06E-49
ERVK3-1	1.5729572	1.02E-06	1.25E-06
LLNLR-245B6	2.18287938	3.21E-22	5.18E-22
ZSCAN22	1.34824903	0.00489068	0.00547122
A1BG	Inf	1.07E-157	5.54E-155
A1BG-AS1	NA	1.00E-152	1.93E-150
ZNF497	2.24708171	2.67E-05	3.18E-05

CTD-2619J13	NA	1.29E-94	6.24E-94
CTD-2619J13	NA	NA	NA
ZNF837	3.74513619	0.02218207	0.02425429
RPS5	1.750269	0.85408673	0.86034656
RNF225	NA	NA	NA
CTD-2619J13	NA	3.31E-14	4.70E-14
ZNF584	2.03901859	7.92E-05	9.31E-05
CTD-2619J13	Inf	3.83E-93	1.81E-92
CTD-2619J13	NA	NA	NA
ZNF132	NA	NA	NA
CTD-2619J13	NA	0.01389341	0.01531128
ZNF324B	3.74513619	8.20E-13	1.14E-12
ZNF324	1.24837873	0.25356777	0.26515802
ZNF446	1.49805447	5.25E-88	2.25E-87
CTD-2619J13	NA	3.98E-26	6.79E-26
SLC27A5	1.11145977	5.24E-21	8.31E-21
CTD-2619J13	NA	NA	NA
ZBTB45	2.46108949	0.29702724	0.309267
TRIM28	1.32684825	6.69E-133	1.06E-131
CHMP2A	1.07487524	7.93E-143	3.35E-141
UBE2M	2.04085603	1.18E-08	1.51E-08
MZF1-AS1	0.37451362	2.62E-34	5.02E-34
MZF1	1.62289235	0.10951216	0.11656026
AC016629.3	NA	NA	NA
SRY	NA	NA	NA
RPS4Y1	NA	NA	NA
RP11-414C2	NA	NA	NA
ZFY	NA	NA	NA
ZFY-AS1	NA	NA	NA
LINC00278	NA	NA	NA
TGIF2LY	NA	NA	NA
AC010084.1	NA	NA	NA
RP11-122L9.	Inf	1.11E-141	3.91E-140
PCDH11Y	NA	4.28E-96	2.14E-95
TTTY23B	NA	NA	NA
TSPY2	NA	NA	NA
LINC00280	NA	NA	NA
TTTY1B	NA	NA	NA
TTTY2B	NA	NA	NA
TTTY21B	NA	NA	NA
TTTY7	NA	NA	NA
TTTY8B	NA	NA	NA
AMELY	NA	NA	NA
TBL1Y	NA	NA	NA
TTTY16	NA	NA	NA

TTTY12	NA	NA	NA
AC064829.1	NA	NA	NA
LINC00279	NA	NA	NA
TTTY18	NA	NA	NA
AC009491.2	NA	NA	NA
TTTY19	NA	NA	NA
AC009491.1	NA	NA	NA
TTTY11	NA	NA	NA
TTTY20	NA	NA	NA
TSPY4	NA	NA	NA
TSPY8	NA	NA	NA
TSPY3	NA	NA	NA
FAM197Y5P	NA	NA	NA
TSPY1	NA	NA	NA
TSPY9P	NA	NA	NA
TSPY10	NA	NA	NA
TTTY8	NA	NA	NA
TTTY7B	NA	NA	NA
TTTY21	NA	NA	NA
TTTY2	NA	NA	NA
TTTY1	NA	NA	NA
TTTY22	NA	NA	NA
AC010891.2	NA	NA	NA
TTTY23	NA	NA	NA
TTTY15	NA	NA	NA
USP9Y	NA	NA	NA
DDX3Y	NA	NA	NA
UTY	NA	NA	NA
TMSB4Y	NA	NA	NA
VCY	NA	NA	NA
VCY1B	NA	NA	NA
AC010723.1	NA	NA	NA
NLGN4Y	NA	NA	NA
NLGN4Y-AS1	NA	NA	NA
FAM41AY1	NA	NA	NA
FAM224B	NA	NA	NA
CDY2B	NA	NA	NA
CDY2A	NA	NA	NA
FAM224A	NA	NA	NA
FAM41AY2	NA	NA	NA
AC022486.1	NA	NA	NA
HSFY1	NA	NA	NA
TTTY9B	NA	NA	NA
HSFY2	NA	NA	NA
TTTY14	NA	NA	NA

AC009977.1	NA	NA	NA
RP11-424G1	NA	NA	NA
KDM5D	NA	NA	NA
TTTY10	NA	NA	NA
EIF1AY	NA	NA	NA
RPS4Y2	NA	NA	NA
RP11-65G9.1	NA	NA	NA
PRORY	NA	NA	NA
RBMY1B	NA	NA	NA
RBMY1A1	NA	NA	NA
TTTY13	NA	NA	NA
RBMY1E	NA	NA	NA
RBMY1D	NA	NA	NA
PRY2	NA	NA	NA
AC007359.6	NA	NA	NA
TTTY6B	NA	NA	NA
RBMY1F	NA	NA	NA
TTTY5	NA	NA	NA
RBMY1J	NA	NA	NA
AC008175.1	NA	NA	NA
TTTY6	NA	NA	NA
PRY	NA	NA	NA
TTTY17A	NA	NA	NA
TTTY4	NA	NA	NA
BPY2	NA	NA	NA
DAZ1	NA	NA	NA
DAZ2	NA	NA	NA
TTTY3B	NA	NA	NA
CDY1B	NA	NA	NA
AC012005.4	NA	NA	NA
AC012005.3	NA	NA	NA
TTTY17B	NA	NA	NA
TTTY4B	NA	NA	NA
BPY2B	NA	NA	NA
DAZ3	NA	NA	NA
DAZ4	NA	NA	NA
BPY2C	NA	NA	NA
TTTY4C	NA	NA	NA
TTTY17C	NA	NA	NA
AC006386.1	NA	NA	NA
LINC00266-4	NA	NA	NA
AC006328.4	NA	NA	NA
CSPG4P1Y	NA	NA	NA
CDY1	NA	NA	NA
TTTY3	NA	NA	NA

CU104787.1	NA	NA	NA
BAGE5	NA	NA	NA
LA16c-60D12	NA	NA	NA
LA16c-60D12	NA	NA	NA
OR11H1	NA	NA	NA
LA16c-23H5.	NA	NA	NA
LA16c-2F2.8	NA	NA	NA
POTEH	NA	NA	NA
POTEH-AS1	NA	NA	NA
LINC01297	NA	0.00050247	0.00057828
LA16c-4G1.3	NA	NA	NA
KB-67B5.17	NA	NA	NA
CCT8L2	NA	NA	NA
TPTEP1	2.79757161	1.06E-140	3.26E-139
KB-7G2.9	NA	NA	NA
AC005301.8	NA	NA	NA
XKR3	Inf	1.30E-67	4.02E-67
AC007064.24	NA	NA	NA
IGKV1OR22-	NA	NA	NA
IGKV2OR22-	NA	NA	NA
IGKV2OR22-	NA	NA	NA
IGKV3OR22-	NA	NA	NA
IGKV1OR22-	NA	NA	NA
GAB4	NA	NA	NA
CECR7	NA	NA	NA
AC006946.16	NA	NA	NA
AC006946.17	NA	NA	NA
IL17RA	1.19844358	3.08E-89	1.35E-88
CECR6	Inf	8.17E-124	8.41E-123
AC006946.15	NA	NA	NA
CECR5	1.45399405	1.80E-117	1.47E-116
CECR5-AS1	NA	NA	NA
CECR1	0	5.32E-19	8.18E-19
CECR3	NA	NA	NA
CECR2	1.12354086	8.26E-48	1.90E-47
SLC25A18	NA	5.19E-10	6.84E-10
AC004019.13	NA	2.37E-47	5.44E-47
ATP6V1E1	1.08633752	9.09E-122	8.61E-121
BCL2L13	1.62289235	1.09E-13	1.54E-13
BID	1.94747082	1.13E-34	2.17E-34
LINC00528	2.09727626	0.88678372	0.89189657
MICAL3	1.67429618	5.94E-13	8.26E-13
FLJ41941	NA	NA	NA
XXbac-B476C	1.49805447	4.87E-10	6.43E-10
PEX26	2.77764267	9.14E-110	6.11E-109

TUBA8	NA	3.39E-84	1.35E-83
AC008079.10	Inf	2.01E-09	2.62E-09
USP18	2.34695201	0.01580583	0.01738824
AC008079.9	NA	NA	NA
AC011718.2	NA	3.03E-46	6.83E-46
AC011718.3	NA	NA	NA
XXbac-B33L1	NA	NA	NA
FAM230A	NA	NA	NA
XXbac-B33L1	NA	NA	NA
GGTLC3	NA	NA	NA
TMEM191B	2.99610895	1.80E-23	2.96E-23
RIMBP3	NA	NA	NA
AC008132.15	NA	NA	NA
AC008103.3	NA	NA	NA
DGCR6	1.1770428	3.38E-27	5.86E-27
PRODH	NA	NA	NA
AC007326.1	NA	NA	NA
AC007326.10	NA	NA	NA
DGCR5	NA	5.60E-91	2.53E-90
DGCR9	NA	NA	NA
DGCR2	1.80647745	1.22E-82	4.73E-82
AC004471.9	0	7.88E-117	6.32E-116
AC004471.10	NA	1.09E-115	8.47E-115
DGCR14	2.10440986	0.01367069	0.01507132
TSSK2	NA	NA	NA
GSC2	NA	NA	NA
LINC01311	1.31079767	2.10E-16	3.10E-16
SLC25A1	1.62883701	1.72E-83	6.80E-83
CLTCL1	2.99610895	2.28E-52	5.64E-52
HIRA	2.60376135	4.16E-103	2.38E-102
C22orf39	2.59789194	2.76E-114	2.07E-113
MRPL40	1.4394986	3.07E-96	1.54E-95
AC000068.5	1.49805447	0.08259076	0.08843543
UFD1L	1.06269929	3.84E-123	3.84E-122
AC000068.9	NA	6.00E-22	9.63E-22
AC000068.10	Inf	3.45E-89	1.51E-88
CDC45	0.89883268	1.58E-120	1.43E-119
CLDN5	NA	NA	NA
LINC00895	NA	NA	NA
AC000067.1	NA	NA	NA
5-Sep	NA	1.23E-30	2.24E-30
GP1BB	NA	NA	NA
TBX1	Inf	7.45E-09	9.59E-09
GNB1L	1.22063698	4.48E-61	1.25E-60
C22orf29	1.84375935	0.04331742	0.04687811

TXNRD2	1.32520204	1.35E-143	6.60E-142
COMT	1.5945786	2.19E-50	5.24E-50
ARVCF	2.54669261	9.84E-28	1.72E-27
TANGO2	2.09727626	0.02872198	0.03129011
AC006547.13	NA	2.03E-87	8.61E-87
AC006547.15	NA	1.46E-121	1.36E-120
DGCR8	2.51144427	1.33E-17	2.00E-17
AC006547.8	0.74902724	4.59E-22	7.39E-22
TRMT2A	1.37145832	2.78E-70	8.93E-70
RANBP1	1.37356241	4.19E-70	1.34E-69
ZDHHC8	4.36932555	5.87E-26	1.00E-25
CCDC188	NA	NA	NA
XXbac-B444F	NA	NA	NA
LINC00896	Inf	2.14E-65	6.40E-65
RTN4R	0.74902724	8.06E-67	2.46E-66
DGCR6L	1.73157473	0.41333412	0.42613556
XXbac-B444F	NA	0.19748049	0.20772836
AC007731.1	NA	NA	NA
XXbac-B33L1	NA	NA	NA
USP41	NA	NA	NA
ZNF74	1.33754864	2.06E-14	2.95E-14
SCARF2	Inf	4.39E-72	1.45E-71
XXbac-B562F	NA	NA	NA
KLHL22	0.74902724	1.77E-62	5.05E-62
MED15	1.55797665	4.33E-55	1.11E-54
AC007050.17	NA	NA	NA
PI4KA	2.15896086	0.00225038	0.00254038
SERPIND1	NA	NA	NA
SNAP29	1.67335872	1.02E-12	1.42E-12
AC007308.7	NA	NA	NA
CRKL	2.45136187	9.83E-102	5.48E-101
XXbac-B135H	2.07422927	0.00350848	0.00394269
AIFM3	4.49416342	4.80E-130	6.48E-129
LZTR1	1.66450497	7.88E-06	9.51E-06
XXbac-B135H	0.74902724	7.48E-06	9.04E-06
THAP7	1.81390933	5.15E-30	9.29E-30
THAP7-AS1	3.99481193	2.49E-18	3.79E-18
P2RX6	NA	NA	NA
SLC7A4	NA	NA	NA
AC002472.11	NA	NA	NA
LRRC74B	NA	1.02E-06	1.26E-06
KB-1592A4.1	NA	NA	NA
POM121L7	NA	NA	NA
KB-1592A4.1	NA	NA	NA
GGT2	NA	NA	NA

KB-1183D5.1	NA	2.87E-69	9.11E-69
KB-1183D5.1	NA	NA	NA
KB-1183D5.1	NA	NA	NA
RIMBP3B	NA	NA	NA
HIC2	2.70482058	2.74E-126	3.11E-125
TMEM191C	2.99610895	1.02E-28	1.81E-28
RIMBP3C	NA	0.73164285	0.74136867
UBE2L3	1.559564	4.06E-28	7.13E-28
YDJC	1.77540654	6.17E-12	8.44E-12
CCDC116	Inf	0.1278107	0.13572593
KB-1440D3.1	NA	7.02E-82	2.69E-81
SDF2L1	1.78819964	3.98E-18	6.03E-18
PPIL2	1.91001946	5.91E-23	9.64E-23
YPEL1	1.49805447	0.60864561	0.62107254
MAPK1	2.24708171	1.79E-11	2.42E-11
PPM1F	1.715514	2.31E-51	5.61E-51
LL22NC03-86	2.24708171	0.00131188	0.00149161
TOP3B	2.24708171	6.17E-05	7.27E-05
IGLVI-70	NA	NA	NA
IGLV4-69	NA	NA	NA
IGLVI-68	NA	NA	NA
LL22NC03-23	NA	NA	NA
IGLV10-67	NA	NA	NA
IGLVIV-66-1	NA	NA	NA
IGLVV-66	NA	NA	NA
IGLVIV-65	NA	NA	NA
IGLVIV-64	NA	NA	NA
IGLVI-63	NA	NA	NA
IGLV1-62	NA	NA	NA
IGLV8-61	NA	NA	NA
IGLV4-60	NA	NA	NA
IGLVIV-59	NA	NA	NA
IGLVV-58	NA	NA	NA
IGLV6-57	NA	NA	NA
IGLVI-56	NA	NA	NA
IGLV11-55	NA	NA	NA
IGLV10-54	NA	NA	NA
IGLVIV-53	NA	NA	NA
VPREB1	NA	NA	NA
CH17-264L24	1.12354086	0.00893486	0.00991172
LL22NC03-2H	3.74513619	4.63E-26	7.90E-26
LL22NC03-2H	NA	4.68E-103	2.68E-102
IGLV5-52	NA	NA	NA
IGLV1-51	NA	0.38801892	0.40076511
IGLV1-50	NA	NA	NA

IGLV9-49	NA	NA	NA
IGLV5-48	NA	NA	NA
IGLV1-47	NA	NA	NA
IGLV7-46	NA	NA	NA
IGLV5-45	NA	NA	NA
IGLV1-44	NA	NA	NA
IGLV7-43	NA	NA	NA
IGLVI-42	NA	NA	NA
IGLVVII-41-1	NA	NA	NA
IGLV1-41	NA	NA	NA
IGLV1-40	NA	NA	NA
IGLVI-38	NA	NA	NA
IGLV5-37	NA	NA	NA
IGLV1-36	NA	NA	NA
IGLV7-35	NA	NA	NA
ZNF280B	0.97373541	0.69633976	0.70709705
ZNF280A	NA	NA	NA
PRAME	4.99351492	5.99E-141	1.89E-139
LL22NC03-63	NA	0.00596164	0.00664881
IGLV2-34	NA	NA	NA
IGLV2-33	NA	NA	NA
IGLV3-32	NA	NA	NA
IGLV3-31	NA	NA	NA
IGLV3-30	NA	NA	NA
GGTLC2	NA	NA	NA
IGLV3-29	NA	NA	NA
IGLV2-28	NA	NA	NA
IGLV3-27	NA	NA	NA
IGLV3-26	NA	NA	NA
IGLVVI-25-1	NA	NA	NA
IGLV3-25	NA	NA	NA
LL22NC03-10	NA	NA	NA
IGLV3-24	NA	NA	NA
IGLV2-23	NA	NA	NA
IGLVVI-22-1	NA	NA	NA
IGLV3-22	NA	NA	NA
IGLV3-21	NA	NA	NA
IGLVI-20	NA	NA	NA
IGLV3-19	NA	NA	NA
IGLV2-18	NA	NA	NA
IGLV3-17	NA	NA	NA
IGLV3-16	NA	NA	NA
IGLV3-15	NA	NA	NA
IGLV2-14	NA	NA	NA
IGLV3-13	NA	NA	NA

IGLV3-12	NA	NA	NA
IGLV2-11	NA	NA	NA
IGLV3-10	NA	NA	NA
IGLV3-9	NA	NA	NA
IGLV2-8	NA	NA	NA
IGLV3-7	NA	NA	NA
IGLV3-6	NA	NA	NA
IGLV2-5	NA	NA	NA
IGLV3-4	NA	NA	NA
IGLV4-3	NA	NA	NA
IGLV3-2	NA	NA	NA
IGLV3-1	NA	NA	NA
IGLL5	NA	NA	NA
IGLJ1	NA	NA	NA
IGLC1	NA	NA	NA
IGLJ2	NA	NA	NA
IGLC2	NA	1.79E-99	9.55E-99
IGLJ3	NA	NA	NA
IGLC3	NA	0.0082408	0.00915129
IGLJ4	NA	NA	NA
IGLC4	NA	NA	NA
IGLJ5	NA	NA	NA
IGLC5	NA	NA	NA
IGLJ6	NA	NA	NA
IGLC6	NA	NA	NA
IGLJ7	NA	NA	NA
IGLC7	NA	NA	NA
RSPH14	NA	NA	NA
GNAZ	NA	1.96E-13	2.75E-13
RAB36	NA	NA	NA
BCR	1.24837873	0.38916682	0.40192784
AP000343.2	NA	NA	NA
AP000344.3	0	2.34E-77	8.34E-77
AP000345.1	NA	NA	NA
AP000345.4	NA	NA	NA
PCAT14	1.49805447	2.84E-06	3.46E-06
AP000345.2	NA	0.02164078	0.0236767
IGLL1	2.65155642	1.64E-107	1.03E-106
KB-208E9.1	Inf	0.07918961	0.0848537
DRICH1	Inf	8.84E-05	1.04E-04
RGL4	NA	1.03E-98	5.44E-98
ZNF70	0.93628405	1.01E-40	2.12E-40
VPREB3	2.99610895	1.21E-43	2.63E-43
C22orf15	NA	NA	NA
CHCHD10	2.06874189	7.85E-21	1.24E-20

MMP11	NA	NA	NA
AP000349.2	NA	NA	NA
SMARCB1	1.42497865	5.72E-118	4.76E-117
DERL3	1.02991245	2.00E-109	1.32E-108
KB-1125A3.1	Inf	8.39E-126	9.31E-125
SLC2A11	2.30058366	3.04E-07	3.78E-07
AP000350.1(	Inf	1.77E-69	5.64E-69
KB-1125A3.1	0	3.30E-86	1.37E-85
MIF	1.3960318	6.57E-145	4.24E-143
MIF-AS1	2.69649805	0.00440458	0.00493381
AP000350.5	NA	NA	NA
GSTT2B	NA	NA	NA
DDTL	1.34824903	3.59E-97	1.84E-96
KB-226F1.2	0.99870298	1.06E-05	1.27E-05
DDT	1.98406451	3.07E-80	1.15E-79
CABIN1	1.70233463	4.98E-30	8.99E-30
SUSD2	NA	5.34E-106	3.25E-105
GGT5	2.90248054	4.16E-69	1.32E-68
SPECC1L	1.45644185	0.15090552	0.15954126
SPECC1L-AD(	NA	NA	NA
ADORA2A	NA	1.86E-104	1.10E-103
ADORA2A-A!	NA	4.48E-66	1.35E-65
UPB1	0.74902724	2.18E-47	5.01E-47
AP000355.2	NA	3.59E-37	7.16E-37
GUCD1	1.47464737	0.00153933	0.00174661
SNRPD3	1.52445191	2.51E-33	4.73E-33
GGT1	0.93628405	1.06E-11	1.45E-11
LRRC75B	1.79766537	4.53E-57	1.19E-56
LL22NC03-N!	NA	2.23E-42	4.78E-42
PIWIL3	NA	NA	NA
SGSM1	0.74902724	0.01100163	0.01216502
TMEM211	NA	NA	NA
KIAA1671	NA	4.53E-56	1.18E-55
CTA-243E7.4	0	1.44E-109	9.59E-109
CTA-243E7.3	NA	NA	NA
CTA-243E7.2	NA	NA	NA
CTA-243E7.1	0	1.80E-31	3.31E-31
CTA-221G9.1	NA	0.00021029	0.00024448
CRYBB3	NA	NA	NA
CTA-221G9.7	NA	NA	NA
CRYBB2	Inf	1.96E-125	2.13E-124
CTA-221G9.1	NA	NA	NA
RP3-462D8.2	NA	NA	NA
LRP5L	0.74902724	5.49E-17	8.17E-17
CTA-246H3.1	Inf	3.02E-83	1.19E-82

CTA-390C10.	NA	NA	NA
IGLVIVOR22-	NA	NA	NA
CTA-407F11.	NA	7.33E-18	1.11E-17
ADRBK2	1.2733463	1.12E-14	1.61E-14
CTA-407F11.	NA	NA	NA
MYO18B	NA	NA	NA
CTA-407F11.	NA	NA	NA
CTA-125H2.2	NA	NA	NA
CTA-125H2.1	NA	NA	NA
CTA-796E4.3	NA	NA	NA
CTA-796E4.4	NA	NA	NA
SEZ6L	NA	3.24E-55	8.32E-55
RP11-259P1.	NA	NA	NA
ASPHD2	2.88910506	3.43E-06	4.17E-06
HPS4	2.18466278	2.58E-06	3.15E-06
SRRD	2.02314573	1.32E-16	1.96E-16
TFIP11	0.8672947	1.47E-103	8.52E-103
CTA-445C9.1	1.99740597	3.32E-34	6.35E-34
TPST2	1.36741019	1.75E-39	3.61E-39
CRYBB1	1.34824903	9.55E-18	1.44E-17
CRYBA4	NA	1.55E-08	1.98E-08
MIAT	0	2.51E-15	3.64E-15
CTA-373H7.7	NA	0.1760171	0.18551689
MIATNB	2.24708171	0.1538057	0.1625411
LINC01422	NA	NA	NA
LINC01422	NA	NA	NA
CTA-992D9.1	NA	5.16E-14	7.31E-14
CTA-992D9.1	NA	NA	NA
CTA-992D9.9	NA	NA	NA
CTA-992D9.6	NA	NA	NA
CTA-992D9.8	NA	NA	NA
CTA-992D9.7	NA	NA	NA
CTA-503F6.2	NA	NA	NA
RP5-1172A2	NA	NA	NA
CTA-929C8.5	NA	NA	NA
CTA-929C8.7	NA	NA	NA
CTA-929C8.6	NA	NA	NA
CTA-929C8.8	NA	NA	NA
RP1-205F14	NA	NA	NA
RP11-46E17.	NA	NA	NA
RP1-231P7P.	NA	NA	NA
RP1-213J1P_	NA	NA	NA
RP1-213J1P_	NA	NA	NA
RP11-375H1	NA	NA	NA
MN1	NA	1.02E-20	1.61E-20

PITPNB	1.52529183	5.00E-14	7.09E-14
TTC28	0.74902724	2.37E-104	1.40E-103
CHEK2	1.23877582	9.98E-46	2.23E-45
HSCB	1.51678016	2.52E-77	8.98E-77
CCDC117	2.43433852	2.00E-34	3.84E-34
XBP1	1.90268632	1.14E-56	2.98E-56
CTA-292E10.	2.99610895	5.26E-11	7.06E-11
ZNRF3	1.71206226	0.00065554	0.00075209
ZNRF3-AS1	3.74513619	8.22E-25	1.38E-24
C22orf31	NA	NA	NA
KREMEN1	Inf	1.40E-73	4.73E-73
CTA-747E2.1	NA	NA	NA
EMID1	1.87256809	5.30E-101	2.92E-100
RHBDD3	2.04109922	4.76E-16	6.99E-16
CTA-984G1.5	NA	NA	NA
EWSR1	1.25836576	3.79E-134	6.43E-133
GAS2L1	2.59662776	2.74E-29	4.89E-29
RASL10A	1.79766537	1.90E-13	2.66E-13
AP1B1	2.05502345	2.11E-42	4.52E-42
RFPL1S	NA	NA	NA
RFPL1	NA	NA	NA
AC000035.3	NA	NA	NA
NEFH	NA	NA	NA
THOC5	1.5729572	3.06E-22	4.94E-22
NIPSNAP1	1.64785992	5.52E-93	2.60E-92
NF2	1.82935498	3.96E-89	1.73E-88
RP1-76B20.1	NA	NA	NA
RP1-76B20.1	NA	NA	NA
CABP7	NA	1.55E-11	2.10E-11
ZMAT5	1.49805447	5.66E-61	1.58E-60
UQCR10	2.18918337	3.29E-113	2.41E-112
ASCC2	1.83690013	2.94E-05	3.50E-05
MTMR3	1.87256809	3.84E-07	4.77E-07
RP3-394A18.	1.49805447	0.48421079	0.49748285
HORMAD2-A	NA	NA	NA
HORMAD2	NA	NA	NA
RP3-438O4.4	NA	NA	NA
RP1-102K2.6	NA	NA	NA
LIF	Inf	1.69E-94	8.20E-94
RP1-102K2.8	NA	2.16E-51	5.26E-51
OSM	4.11964981	4.71E-119	4.07E-118
GATSL3	3.37062257	5.58E-14	7.90E-14
RP1-130H16.	NA	NA	NA
TBC1D10A	2.62159533	0.00014036	0.00016391
SF3A1	2.14007782	1.09E-70	3.53E-70

CCDC157	1.12354086	0.61593248	0.6282255
RNF215	1.49805447	2.44E-05	2.91E-05
SEC14L2	2.24708171	6.12E-45	1.35E-44
RP4-539M6.:	NA	NA	NA
MTFP1	2.12224384	3.99E-134	6.75E-133
RP4-539M6.:	Inf	9.95E-78	3.57E-77
SEC14L3	NA	NA	NA
RP4-539M6.:	NA	NA	NA
SEC14L4	NA	NA	NA
SEC14L6	NA	NA	NA
GAL3ST1	NA	NA	NA
PES1	1.42377078	1.10E-06	1.35E-06
TCN2	0.74902724	6.92E-63	1.98E-62
SLC35E4	1.78614187	0.00136133	0.00154716
DUSP18	1.59168288	3.48E-66	1.05E-65
OSBP2	Inf	4.55E-13	6.33E-13
MORC2-AS1	7.49027237	1.05E-46	2.38E-46
MORC2	2.99610895	3.06E-13	4.27E-13
TUG1	1.69516691	2.22E-93	1.05E-92
RP3-430N8.1	NA	NA	NA
RP3-430N8.1	NA	NA	NA
SMTN	1.5729572	2.50E-25	4.23E-25
RP3-412A9.1	2.99610895	7.00E-61	1.95E-60
SELM	2.22125319	9.47E-29	1.68E-28
INPP5J	0	1.23E-81	4.72E-81
PLA2G3	NA	NA	NA
RNF185	2.52796693	1.45E-14	2.08E-14
RNF185-AS1	NA	NA	NA
LIMK2	2.0598249	5.55E-08	7.02E-08
PIK3IP1	1.49805447	0.0006346	0.00072825
PIK3IP1-AS1	NA	7.20E-126	8.01E-125
PATZ1	3.0793342	1.23E-41	2.60E-41
LINC01521	Inf	4.34E-43	9.37E-43
DRG1	0.91021031	9.44E-125	1.00E-123
EIF4ENIF1	1.97957198	2.47E-12	3.40E-12
RP11-247I13	NA	0.33415619	0.34686342
SFI1	1.33754864	7.02E-05	8.27E-05
PISD	1.52075227	1.25E-17	1.88E-17
PRR14L	1.61951835	0.02380753	0.02600965
DEPDC5	1.60505837	3.02E-22	4.88E-22
YWHAH	1.22165287	2.34E-122	2.27E-121
CTA-342B11.	NA	NA	NA
CTA-342B11.	NA	NA	NA
SLC5A1	NA	NA	NA
RP1-127L4.7	NA	NA	NA

C22orf42	NA	NA	NA
RP1-90G24.8	NA	NA	NA
RFPL2	NA	NA	NA
AL008723.1	NA	NA	NA
IGLCOR22-1	NA	NA	NA
RP1-90G24.1	NA	NA	NA
SLC5A4	NA	NA	NA
RP1-90G24.6	NA	NA	NA
RP1-90G24.1	NA	NA	NA
RP1-149A16.	NA	NA	NA
RFPL3	NA	NA	NA
IGLCOR22-2	NA	NA	NA
RFPL3S	Inf	6.67E-90	2.96E-89
RP1-149A16.	NA	NA	NA
IGLVIVOR22-	NA	NA	NA
RP1-149A16.	NA	NA	NA
RTCB	0.89517889	2.83E-135	5.15E-134
BPIFC	NA	NA	NA
FBXO7	1.70990056	5.38E-36	1.06E-35
SYN3	NA	NA	NA
LL22NC03-10	NA	NA	NA
LL22NC01-11	NA	NA	NA
TIMP3	NA	3.67E-09	4.76E-09
RP1-302D9.1	NA	NA	NA
RP1-302D9.2	NA	NA	NA
LARGE	1.49805447	0.52710536	0.54045131
RP1-302D9.3	NA	NA	NA
SC22CB-1D7.	NA	NA	NA
LL22NC03-32	NA	4.00E-09	5.19E-09
LL22NC03-86	NA	NA	NA
LL22NC03-13	NA	NA	NA
RP1-101G11.	NA	NA	NA
RP1-288L1.5	NA	NA	NA
RP1-288L1.4	NA	NA	NA
ISX-AS1	NA	NA	NA
ISX	NA	NA	NA
LINC01399	NA	NA	NA
CTA-714B7.7	NA	NA	NA
RP3-323A16.	2.99610895	4.03E-10	5.32E-10
HMGXB4	1.53920982	4.05E-77	1.44E-76
RP3-510H16.	NA	8.62E-70	2.75E-69
TOM1	1.33754864	6.44E-100	3.45E-99
CTA-286B10.	NA	5.16E-14	7.31E-14
CTA-286B10.	NA	NA	NA
HMOX1	1.87256809	0.16042777	0.16940112

MCM5	1.51886079	8.55E-57	2.24E-56
RP4-569D19.	NA	NA	NA
RASD2	NA	NA	NA
MB	NA	3.00E-35	5.82E-35
APOL6	2.49675746	5.73E-44	1.25E-43
RP1-41P2.7	NA	NA	NA
APOL5	NA	NA	NA
RBFOX2	1.91418072	0.09841971	0.10508065
RP1-78B3.1	0.74902724	1.36E-16	2.02E-16
CTA-212A2.4	Inf	2.25E-78	8.16E-78
CTA-212A2.3	NA	1.52E-128	1.91E-127
CTA-212A2.2	NA	7.21E-40	1.49E-39
APOL3	Inf	4.74E-73	1.58E-72
APOL4	11.6099222	4.74E-138	1.08E-136
APOL2	1.70233463	1.06E-24	1.78E-24
APOL1	1.99740597	8.37E-10	1.10E-09
MYH9	1.79621095	8.96E-54	2.26E-53
RP4-633O19.	NA	3.76E-60	1.04E-59
RP5-1119A7.	NA	NA	NA
TXN2	1.81431042	3.10E-07	3.86E-07
FOXRED2	3.03177691	4.42E-112	3.15E-111
EIF3D	1.2927922	4.08E-109	2.68E-108
RP5-1119A7.	NA	NA	NA
CACNG2	NA	NA	NA
RP1-293L6.1	NA	NA	NA
IFT27	1.08192823	5.14E-142	1.88E-140
PVALB	NA	NA	NA
CITF22-24E5.	NA	NA	NA
CTA-833B7.2	NA	NA	NA
NCF4	1.47868308	1.13E-80	4.23E-80
CSF2RB	NA	3.36E-43	7.27E-43
LL22NC01-81	NA	NA	NA
TEX33	NA	NA	NA
TST	0.59922179	1.50E-142	5.88E-141
MPST	1.56614786	1.98E-123	2.00E-122
KCTD17	1.81014916	1.51E-14	2.16E-14
TMPRSS6	NA	NA	NA
RP5-1170K4.	NA	NA	NA
IL2RB	0	1.62E-37	3.25E-37
RP1-151B14.	NA	5.09E-119	4.37E-118
C1QTNF6	7.49027237	2.09E-05	2.49E-05
SSTR3	NA	1.28E-39	2.63E-39
RAC2	1.14180981	3.13E-52	7.73E-52
CYTH4	2.7464332	1.22E-11	1.65E-11
ELFN2	NA	3.26E-140	9.38E-139

RP1-63G5.8	NA	NA	NA
MFNG	1.87432637	0.09523685	0.10175423
CARD10	0.89883268	2.86E-51	6.92E-51
RP5-1177I5.3	NA	NA	NA
CDC42EP1	1.49805447	1.80E-08	2.30E-08
LGALS2	NA	NA	NA
GGA1	2.00149901	1.01E-15	1.48E-15
SH3BP1	1.67927074	1.46E-35	2.85E-35
Z83844.1	NA	0.00426888	0.00478416
PDXP	Inf	2.99E-06	3.64E-06
LGALS1	1.46944947	3.65E-47	8.36E-47
NOL12	2.24708171	0.00222326	0.00250992
RP1-37E16.1	0	3.49E-84	1.40E-83
TRIOBP	2.16385646	2.40E-31	4.40E-31
H1F0	NA	NA	NA
GCAT	1.40993362	3.22E-124	3.36E-123
GALR3	Inf	1.34E-57	3.55E-57
ANKRD54	1.43430748	5.82E-25	9.79E-25
EIF3L	1.99594587	0.06832722	0.0733834
MICALL1	1.3658732	2.76E-11	3.73E-11
C22orf23	NA	1.16E-51	2.84E-51
RP5-1039K5.	NA	3.83E-05	4.53E-05
POLR2F	1.84729417	1.43E-16	2.12E-16
SOX10	NA	NA	NA
RP5-1039K5.	0	1.28E-33	2.43E-33
RP5-1039K5.	NA	NA	NA
PICK1	1.24837873	5.31E-50	1.26E-49
RP5-1039K5.	NA	NA	NA
SLC16A8	NA	NA	NA
BAIAP2L2	0	2.73E-07	3.40E-07
CTA-228A9.3	NA	7.31E-62	2.07E-61
PLA2G6	4.49416342	3.49E-14	4.96E-14
CTA-228A9.4	1.49805447	1.31E-24	2.19E-24
MAFF	5.61770428	2.33E-61	6.55E-61
TMEM184B	1.79766537	5.46E-07	6.76E-07
RP1-5O6.5	NA	NA	NA
CSNK1E	2.74197471	1.42E-94	6.88E-94
RP3-434P1.6	NA	NA	NA
KCNJ4	1.49805447	2.46E-16	3.63E-16
KDELR3	1.69779507	6.44E-65	1.91E-64
DDX17	2.05725974	6.55E-30	1.18E-29
DMC1	0.24967575	4.38E-40	9.08E-40
FAM227A	NA	4.15E-10	5.48E-10
CBY1	2.17217899	1.38E-25	2.34E-25
RP3-508I15.1	NA	NA	NA

RP3-508I15.9	1.49805447	8.14E-49	1.91E-48
TOMM22	1.93669077	0.20979804	0.22035292
JOSD1	2.53242542	4.49E-132	6.81E-131
GTPBP1	1.92607004	3.32E-30	6.01E-30
RP3-508I15.1	NA	0.00041438	0.00047823
SUN2	1.94747082	2.69E-54	6.83E-54
RP3-508I15.1	NA	NA	NA
RP3-508I15.1	NA	4.08E-110	2.75E-109
RP3-508I15.2	2.24708171	0.34959927	0.36240386
RP3-508I15.2	0	4.06E-19	6.25E-19
DNAL4	3.99481193	0.19942447	0.20974889
NPTXR	1.49805447	3.20E-38	6.47E-38
CBX6	1.77113732	0.69193072	0.70277724
CTA-150C2.1	NA	NA	NA
APOBEC3A	NA	1.73E-49	4.08E-49
APOBEC3B	0.74902724	9.59E-19	1.47E-18
APOBEC3B-A	NA	9.67E-127	1.11E-125
APOBEC3C	1.28999135	1.62E-136	3.23E-135
APOBEC3D	1.49805447	3.39E-35	6.57E-35
APOBEC3F	4.49416342	2.46E-72	8.14E-72
APOBEC3G	0.59922179	4.67E-21	7.40E-21
APOBEC3H	NA	2.25E-15	3.26E-15
CBX7	4.86867704	2.78E-46	6.28E-46
RP4-742C19.	NA	0.5559708	0.56905062
PDGFB	NA	NA	NA
RP3-333H23.	NA	NA	NA
RPL3	1.90794919	0.005166	0.00577494
SYNGR1	1.52871641	0.00246997	0.00278497
TAB1	1.34824903	2.75E-37	5.50E-37
MGAT3	3.74513619	2.75E-100	1.49E-99
MGAT3-AS1	NA	NA	NA
MIEF1	2.56307758	1.23E-135	2.31E-134
ATF4	1.9177507	0.09926847	0.10594321
RPS19BP1	2.25614253	7.33E-137	1.51E-135
CACNA1I	NA	NA	NA
ENTHD1	NA	NA	NA
GRAP2	NA	NA	NA
FAM83F	NA	NA	NA
RP3-496C20.	NA	5.50E-20	8.57E-20
TNRC6B	1.96296793	5.13E-19	7.89E-19
ADSL	1.11955667	2.47E-126	2.81E-125
RP5-1042K10	NA	NA	NA
SGSM3	1.75633973	1.16E-09	1.51E-09
RP5-1042K10	4.11964981	0.00236598	0.00266955
MKL1	2.03307393	1.32E-14	1.89E-14

RP5-1042K1(	NA	NA	NA
RP4-591N18.	Inf	7.20E-126	8.01E-125
MCHR1	NA	NA	NA
SLC25A17	3.34181383	0.0052961	0.00591892
ST13	1.25318019	5.04E-132	7.59E-131
XPNPEP3	3.10311284	7.58E-114	5.64E-113
DNAJB7	NA	2.16E-05	2.57E-05
RBX1	1.6105765	3.51E-44	7.67E-44
EP300	1.68531128	8.15E-21	1.29E-20
RP1-85F18.6	NA	NA	NA
EP300-AS1	Inf	7.08E-76	2.46E-75
L3MBTL2	1.72276265	1.10E-30	2.01E-30
RP4-756G23.	0	9.50E-38	1.91E-37
CHADL	Inf	2.13E-20	3.35E-20
RANGAP1	1.57554005	4.07E-10	5.37E-10
ZC3H7B	3.21641108	2.16E-124	2.26E-123
TEF	4.49416342	1.11E-56	2.90E-56
CTA-223H9.9	NA	8.61E-150	1.22E-147
TOB2	1.24228908	1.55E-94	7.49E-94
PHF5A	1.81947612	0.00013846	0.00016178
ACO2	2.33097276	2.78E-69	8.82E-69
POLR3H	1.8053477	2.30E-10	3.05E-10
CSDC2	NA	NA	NA
PMM1	1.18028534	6.75E-128	8.24E-127
DESI1	1.76503448	0.0013811	0.00156932
XRCC6	0.90228641	1.04E-138	2.53E-137
SNU13	1.77804405	0.77335067	0.7821456
C22orf46	2.33030696	3.40E-47	7.79E-47
MEI1	0.74902724	1.67E-11	2.26E-11
CCDC134	1.24837873	0.06053454	0.06515679
RP5-821D11.	NA	3.84E-30	6.93E-30
SREBF2	1.73346304	1.95E-23	3.20E-23
SHISA8	0.24967575	1.26E-34	2.42E-34
TNFRSF13C	0.99870298	3.95E-47	9.04E-47
CENPM	1.59565499	8.13E-70	2.60E-69
3-Sep	NA	NA	NA
CTA-250D10.	NA	NA	NA
WBP2NL	NA	NA	NA
NAGA	1.2733463	8.74E-124	8.97E-123
FAM109B	NA	1.29E-139	3.49E-138
SMDT1	2.04124217	1.09E-33	2.06E-33
NDUFA6	1.72572642	8.39E-12	1.14E-11
CYP2D6	NA	2.99E-65	8.90E-65
RP4-669P10.	NA	NA	NA
RP4-669P10.	NA	NA	NA

TCF20	2.66320796	2.66E-32	4.94E-32
OGFRP1	NA	7.02E-39	1.43E-38
Z83851.4	0.74902724	6.71E-35	1.30E-34
CTA-989H11.	NA	1.43E-63	4.13E-63
LINC01315	NA	5.61E-48	1.30E-47
NFAM1	NA	NA	NA
CTA-126B4.7	NA	4.99E-61	1.39E-60
RRP7A	1.35831059	1.57E-74	5.36E-74
SERHL2	0	2.52E-44	5.53E-44
POLDIP3	1.79372312	1.50E-09	1.96E-09
RNU12	NA	3.59E-59	9.74E-59
CYB5R3	1.67750892	3.16E-122	3.04E-121
ATP5L2	2.99610895	2.25E-54	5.73E-54
A4GALT	NA	8.24E-91	3.72E-90
ARFGAP3	0.96303502	1.84E-64	5.42E-64
PACSIN2	2.09103437	2.12E-10	2.82E-10
AL022476.2	NA	NA	NA
TTLL1	5.9922179	1.58E-33	2.99E-33
BIK	1.52237354	5.44E-27	9.40E-27
MCAT	1.02668389	1.15E-112	8.31E-112
TSPO	1.45381763	1.69E-111	1.19E-110
TTLL12	2.15821407	3.22E-30	5.82E-30
SCUBE1	Inf	8.60E-05	1.01E-04
Z82214.3	NA	NA	NA
Z82214.2	NA	NA	NA
Z99756.1	NA	NA	NA
RP4-754E20_	NA	NA	NA
MPPED1	NA	1.21E-47	2.79E-47
EFCAB6-AS1	NA	NA	NA
EFCAB6	NA	NA	NA
RP3-388M5.9	NA	NA	NA
SULT4A1	NA	NA	NA
PNPLA5	NA	NA	NA
PNPLA3	1.74773022	0.00207217	0.00234168
SAMM50	0.74902724	3.94E-140	1.12E-138
PARVB	1.2320448	4.46E-130	6.04E-129
PARVG	0.8527387	3.16E-95	1.56E-94
RP4-671O14.	NA	2.01E-138	4.69E-137
KIAA1644	NA	3.03E-13	4.24E-13
RP1-32I10.1(	NA	NA	NA
CTA-397C4.2	NA	NA	NA
LDOC1L	1.49805447	1.82E-31	3.35E-31
LINC00207	NA	NA	NA
LINC00229	NA	NA	NA
PRR5	1.75591631	0.00886046	0.00983218

PRR5-ARHG/	NA	NA	NA
ARHGAP8	NA	4.27E-60	1.18E-59
PHF21B	NA	NA	NA
NUP50-AS1	2.15345331	1.69E-35	3.30E-35
CTA-217C2.2	0	4.29E-52	1.06E-51
NUP50	2.6406384	2.58E-121	2.40E-120
KIAA0930	1.90928511	7.56E-08	9.52E-08
CTA-268H5.1	NA	NA	NA
UPK3A	3.74513619	4.53E-50	1.08E-49
FAM118A	1.49805447	0.62748525	0.63954143
SMC1B	NA	NA	NA
RIBC2	NA	4.56E-104	2.67E-103
RP1-102D24.	NA	NA	NA
FBLN1	NA	8.15E-05	9.58E-05
LINC01589	Inf	6.12E-05	7.22E-05
ATXN10	1.11891723	8.30E-119	7.07E-118
WI2-85898F:	NA	NA	NA
WI2-85898F:	NA	NA	NA
WNT7B	NA	NA	NA
CITF22-92A6	1.49805447	0.00012621	0.00014761
PRR34	0	8.84E-18	1.33E-17
RP6-109B7.5	7.49027237	4.71E-102	2.64E-101
PRR34-AS1	2.93097615	1.79E-75	6.19E-75
MIRLET7BHC	1.79766537	5.13E-05	6.06E-05
RP6-109B7.2	0	7.74E-138	1.72E-136
RP6-109B7.4	0	1.80E-133	2.95E-132
PPARA	2.3540856	1.77E-28	3.13E-28
CDPF1	2.48540856	1.04E-56	2.72E-56
PKDREJ	NA	NA	NA
TTC38	1.08949416	8.13E-54	2.05E-53
GTSE1-AS1	1.87256809	1.20E-67	3.70E-67
GTSE1	1.79483885	3.55E-37	7.08E-37
TRMU	1.54372687	1.20E-50	2.89E-50
CELSR1	NA	NA	NA
RP3-439F8.1	NA	NA	NA
GRAMD4	2.15345331	1.17E-10	1.56E-10
CERK	2.00875486	6.00E-44	1.31E-43
CTA-29F11.1	1.81678947	3.58E-86	1.48E-85
TBC1D22A	1.66828794	4.07E-39	8.34E-39
U51561.1	NA	NA	NA
FP325331.1	NA	NA	NA
CITF22-49D8	NA	NA	NA
LL22NC03-75	NA	NA	NA
LINC00898	NA	NA	NA
RP11-191L9.4	NA	NA	NA

RP13-455A7.	NA	NA	NA
CTA-280A3.2	NA	NA	NA
CTA-280A3_	NA	NA	NA
LL22NC03-27	NA	NA	NA
LL22NC03-12	NA	NA	NA
LL22NC03-12	NA	NA	NA
RP11-536P6.	NA	NA	NA
FAM19A5	NA	5.25E-101	2.89E-100
LINC01310	NA	NA	NA
RP1-34P24.3	NA	NA	NA
C22orf34	1.49805447	9.86E-41	2.07E-40
CTA-722E9.1	NA	NA	NA
RP4-566L20.	NA	NA	NA
RP1-29C18.1	2.87127108	7.51E-125	8.00E-124
RP1-29C18.9	NA	3.77E-133	6.08E-132
RP5-983L19.	NA	NA	NA
BRD1	1.59168288	3.47E-34	6.64E-34
RP3-522J7.7	Inf	2.07E-71	6.74E-71
RP3-522J7.6	NA	6.88E-28	1.20E-27
RP11-494O1	NA	0.00552414	0.00616998
ZBED4	2.37926299	0.70056672	0.71130969
ALG12	2.29701686	6.93E-76	2.41E-75
CITF22-1A6.3	0.74902724	3.15E-24	5.23E-24
CRELD2	1.68848513	1.87E-49	4.42E-49
CITF22-49E9.	NA	5.26E-29	9.35E-29
PIM3	1.65196418	1.50E-35	2.92E-35
IL17REL	NA	NA	NA
TTLL8	NA	NA	NA
MLC1	3.14975556	8.60E-141	2.68E-139
MOV10L1	NA	3.77E-133	6.08E-132
PANX2	6.74124514	8.01E-103	4.56E-102
TRABD	2.03042094	0.01999659	0.02191748
RP3-402G11.	0	2.91E-45	6.48E-45
RP3-402G11.	2.24708171	1.60E-49	3.79E-49
SELO	2.72373541	6.08E-30	1.10E-29
RP3-402G11.	NA	5.71E-18	8.65E-18
RP3-402G11.	Inf	6.88E-63	1.97E-62
TUBGCP6	3.74513619	1.20E-05	1.44E-05
HDAC10	1.73212549	1.07E-17	1.61E-17
MAPK12	2.65863514	4.21E-144	2.28E-142
MAPK11	2.14007782	4.23E-12	5.80E-12
PLXNB2	2.48115272	7.46E-37	1.48E-36
DENND6B	1.79766537	9.11E-09	1.17E-08
XX-C283C717	NA	NA	NA
XX-C00717C0	NA	NA	NA

PPP6R2	2.0105468	3.13E-14	4.45E-14
SBF1	2.24708171	6.95E-15	1.00E-14
ADM2	NA	NA	NA
MIOX	NA	5.92E-18	8.95E-18
LMF2	2.18194891	1.53E-18	2.33E-18
NCAPH2	1.34824903	9.50E-133	1.50E-131
SCO2	1.30138252	2.45E-61	6.87E-61
CTA-384D8.3	1.24837873	0.20735559	0.21788851
TYMP	1.82575389	0.56015667	0.57320561
ODF3B	NA	NA	NA
CTA-384D8.3	NA	NA	NA
CTA-384D8.3	NA	NA	NA
CTA-384D8.3	NA	NA	NA
KLHDC7B	NA	0.13377061	0.14180592
SYCE3	Inf	6.16E-23	1.00E-22
CPT1B	Inf	8.84E-11	1.18E-10
CHKB-CPT1B	NA	NA	NA
CHKB	Inf	1.66E-123	1.68E-122
CHKB-AS1	2.62159533	2.22E-87	9.38E-87
CTA-384D8.3	NA	NA	NA
MAPK8IP2	1.49805447	0.03486445	0.03785224
ARSA	1.15758755	5.34E-84	2.13E-83
SHANK3	1.49805447	3.09E-24	5.13E-24
AC000036.4	NA	NA	NA
ACR	NA	1.48E-51	3.60E-51
AC002056.5	NA	NA	NA
RABL2B	0.94613967	2.68E-68	8.36E-68
CH507-9B2.2	NA	4.59E-22	7.39E-22
CH507-9B2.1	Inf	3.72E-137	7.80E-136
CH507-9B2.8	NA	NA	NA
CH507-9B2.3	1.12354086	0.01595278	0.01754245
CH507-9B2.4	NA	NA	NA
CH507-9B2.5	2.88910506	5.29E-35	1.02E-34
CH507-9B2.9	0.49935149	3.01E-15	4.36E-15
RP11-717F1.	NA	NA	NA
CH507-24F1.	NA	NA	NA
RP11-717F1.	NA	5.41E-108	3.46E-107
CH507-39O4.	NA	NA	NA
CH507-39O4.	NA	NA	NA
CH507-42P11	NA	NA	NA
CH507-42P11	NA	NA	NA
CH507-42P11	NA	NA	NA
CH507-42P11	NA	3.85E-14	5.47E-14
CH507-24F1.	NA	NA	NA
CH507-145C2	0.74902724	9.74E-12	1.33E-11

CBSL	NA	3.74E-52	9.22E-52
U2AF1L5	2.24708171	4.41E-16	6.48E-16
CH507-152C1	NA	NA	NA
CH507-152C1	NA	NA	NA
CH507-154B1	7.49027237	1.23E-142	5.00E-141
CH507-145C2	NA	NA	NA
CH507-254M	Inf	1.77E-06	2.16E-06
CH507-210P1	NA	NA	NA
CH507-210P1	NA	NA	NA
CH507-210P1	NA	NA	NA
CH507-154B1	0	4.18E-24	6.93E-24
CH507-145C2	NA	NA	NA
CH507-236L2	NA	NA	NA
CH507-254M	NA	NA	NA
CH507-254M	NA	NA	NA
CH507-338C2	NA	0.07775103	0.08333683
SMIM11B	1.49805447	2.80E-53	7.01E-53
CH507-396I9	NA	NA	NA
KCNE1B	NA	NA	NA
CH507-513H4	NA	NA	NA
CH507-513H4	NA	NA	NA
CH507-513H4	NA	NA	NA
CH507-528H1	NA	1.30E-67	4.02E-67
CH507-513H4	NA	NA	NA
CH507-513H4	NA	NA	NA
bP-2189O9.3	NA	1.24E-38	2.51E-38
bP-2171C21.1	NA	NA	NA
bP-21264C1.1	2.24708171	2.33E-14	3.32E-14
MGC39584	NA	NA	NA
RP11-555K2.	NA	NA	NA
CH507-216K1	NA	NA	NA
AP003900.6	NA	NA	NA
TPTE	NA	NA	NA
IGHV1OR21-	NA	NA	NA
AP001464.4	NA	NA	NA
AJ239318.1	NA	NA	NA
AP001465.5	NA	NA	NA
POTED	NA	NA	NA
AL050303.7	NA	NA	NA
AP001347.6	1.87256809	0.00117008	0.00133331
LIPI	NA	NA	NA
RBM11	0	1.06E-63	3.07E-63
HSPA13	1.49805447	1.38E-29	2.48E-29
SAMSN1	2.52481091	2.23E-38	4.51E-38
SAMSN1-AS1	Inf	0.00545934	0.0060991

AF165138.7	NA	6.24E-59	1.68E-58
AF127936.3	Inf	1.07E-157	5.54E-155
AF127936.5	NA	3.35E-107	2.10E-106
AF127936.9	2.24708171	6.47E-15	9.32E-15
AF127936.7	NA	3.57E-39	7.32E-39
AF127577.8	NA	NA	NA
NRIP1	1.53550584	2.33E-73	7.85E-73
AF127577.1(	NA	NA	NA
AF127577.11	NA	NA	NA
AF127577.1Ź	NA	NA	NA
AJ006998.2	NA	3.78E-85	1.54E-84
AJ009632.3	NA	NA	NA
USP25	13.4824903	1.10E-130	1.54E-129
MIR99AHG	Inf	4.84E-139	1.23E-137
AP001172.2	Inf	1.34E-111	9.40E-111
AP001172.3	NA	NA	NA
AP000962.2	NA	NA	NA
AP000473.5	NA	NA	NA
AP000473.8	NA	NA	NA
AP000473.6	NA	NA	NA
AF212831.2	NA	NA	NA
LINC01549	NA	NA	NA
CXADR	1.02991245	2.02E-14	2.88E-14
BTG3	1.41095828	3.46E-128	4.27E-127
AP000432.2	NA	NA	NA
C21orf91-OT	NA	NA	NA
C21orf91	1.44811933	2.37E-05	2.83E-05
AL109761.5	NA	NA	NA
CHODL-AS1	NA	NA	NA
CHODL	NA	NA	NA
AP000998.2	NA	NA	NA
TMPRSS15	NA	NA	NA
MIR548XHG	NA	NA	NA
AF240627.2	NA	NA	NA
AL157359.4	NA	NA	NA
AL157359.3	NA	NA	NA
AP000431.1	NA	NA	NA
AP000431.2	NA	NA	NA
AP000855.4	NA	NA	NA
AP000946.2	NA	NA	NA
AP001171.1	3.17235065	1.59E-12	2.20E-12
LINC00320	NA	NA	NA
NCAM2	Inf	2.43E-148	2.94E-146
AP001136.2	NA	NA	NA
AF241725.4	NA	NA	NA

AF241725.6	NA	NA	NA
LINC00317	NA	NA	NA
LINC01425	NA	NA	NA
AP000472.3	NA	NA	NA
AP000472.2	NA	NA	NA
LINC00308	NA	NA	NA
AP000705.7	NA	NA	NA
AP000959.2	NA	NA	NA
AP001255.2	NA	NA	NA
D21S2088E	NA	NA	NA
AP000459.7	NA	NA	NA
AP000474.1	NA	NA	NA
AP000477.2	NA	NA	NA
AP000477.3	NA	NA	NA
AP000470.2	NA	NA	NA
AP000469.2	NA	NA	NA
AP000476.1	NA	1.97E-49	4.64E-49
AP000235.2	NA	NA	NA
AP000402.3	NA	NA	NA
AP000146.2	NA	NA	NA
AP000235.3	NA	NA	NA
AP000233.2	NA	NA	NA
AP000233.3	NA	NA	NA
AP000233.4	NA	NA	NA
LINC00158	NA	NA	NA
AP000221.1	NA	NA	NA
MIR155HG	2.24708171	1.13E-07	1.42E-07
AP000223.42	Inf	3.10E-51	7.52E-51
MRPL39	0.54341192	4.64E-131	6.62E-130
JAM2	0	1.32E-84	5.30E-84
ATP5J	1.91403522	5.39E-34	1.03E-33
GABPA	3.29571984	3.55E-34	6.79E-34
APP	1.89699289	3.92E-05	4.64E-05
AP001439.2	NA	2.26E-39	4.64E-39
AP000230.1	NA	4.09E-09	5.30E-09
AP001595.1	NA	NA	NA
AP001596.6	NA	3.17E-10	4.20E-10
CYYR1-AS1	NA	NA	NA
KB-1466C5.1	NA	NA	NA
CYYR1	NA	NA	NA
ADAMTS1	0.74902724	6.53E-07	8.06E-07
AP001601.2	NA	NA	NA
ADAMTS5	NA	NA	NA
AP001604.3	NA	NA	NA
AP001605.4	NA	NA	NA

AP001607.1	NA	NA	NA
LINC00113	NA	NA	NA
AJ006995.3	NA	NA	NA
LINC00314	NA	NA	NA
AL035610.2	NA	NA	NA
AF131217.1	NA	NA	NA
LINC00161	NA	NA	NA
N6AMT1	1.81906615	1.08E-11	1.47E-11
LTN1	2.62159533	6.04E-51	1.46E-50
RWDD2B	1.77042802	4.91E-05	5.81E-05
RP1-100J12.1	1.49805447	1.65E-09	2.16E-09
USP16	2.02010376	1.31E-50	3.16E-50
CCT8	0.82197288	5.09E-140	1.43E-138
MAP3K7CL	NA	3.24E-55	8.32E-55
AF124730.4	NA	NA	NA
BACH1	1.49805447	0.68219151	0.69315701
BACH1-AS1	NA	2.80E-150	4.16E-148
BACH1-IT2	Inf	1.21E-40	2.53E-40
GRIK1	NA	2.15E-24	3.58E-24
RP11-313P22	Inf	3.77E-142	1.39E-140
GRIK1-AS1	NA	NA	NA
AF096876.1	NA	NA	NA
CLDN17	NA	NA	NA
LINC00307	NA	NA	NA
CLDN8	NA	NA	NA
KRTAP24-1	NA	NA	NA
KRTAP25-1	NA	NA	NA
KRTAP26-1	NA	NA	NA
KRTAP27-1	NA	NA	NA
KRTAP13-2	NA	NA	NA
KRTAP23-1	NA	NA	NA
KRTAP13-1	NA	NA	NA
KRTAP13-3	NA	NA	NA
KRTAP13-4	NA	NA	NA
KRTAP15-1	NA	NA	NA
KRTAP19-1	NA	NA	NA
KRTAP19-3	NA	NA	NA
KRTAP19-2	NA	NA	NA
KRTAP19-4	NA	NA	NA
KRTAP19-5	NA	NA	NA
KRTAP19-6	NA	NA	NA
KRTAP19-7	NA	NA	NA
KRTAP22-2	NA	NA	NA
KRTAP6-3	NA	NA	NA
KRTAP6-2	NA	NA	NA

KRTAP22-1	NA	NA	NA
KRTAP6-1	NA	NA	NA
KRTAP20-1	NA	NA	NA
KRTAP20-4	NA	NA	NA
KRTAP20-2	NA	NA	NA
KRTAP20-3	NA	NA	NA
KRTAP21-3	NA	NA	NA
KRTAP21-2	NA	NA	NA
KRTAP21-1	NA	NA	NA
KRTAP8-1	NA	NA	NA
KRTAP7-1	NA	NA	NA
KRTAP11-1	NA	NA	NA
KRTAP19-8	NA	NA	NA
TIAM1	NA	9.64E-116	7.50E-115
AP000251.3	NA	NA	NA
AP000253.1	0.99870298	1.59E-20	2.51E-20
SOD1	1.70694082	0.02731344	0.02978066
AP000254.8	1.49805447	0.09894945	0.10563381
SCAF4	2.22787589	2.60E-43	5.64E-43
AP000255.6	NA	7.79E-41	1.63E-40
HUNK	0.64202335	4.26E-81	1.61E-80
HUNK-AS1	NA	NA	NA
LINC00159	NA	NA	NA
AP000265.1	NA	9.36E-17	1.39E-16
MIS18A	1.53747696	6.29E-88	2.69E-87
MIS18A-AS1	1.49805447	0.02206485	0.02412756
MRAP	NA	NA	NA
AP000266.7	NA	NA	NA
URB1	2.31221452	2.46E-106	1.51E-105
URB1-AS1	1.81906615	1.45E-25	2.46E-25
EVA1C	0.93628405	0.00035308	0.00040834
TCP10L	0.74902724	0.01696742	0.01864577
AP000275.65	NA	NA	NA
C21orf59	1.04769	8.15E-119	6.95E-118
SYNJ1	2.99610895	1.69E-34	3.24E-34
PAXBP1-AS1	0.74902724	1.21E-67	3.75E-67
PAXBP1	1.74773022	0.00874656	0.00970639
C21orf62-AS	0.74902724	1.95E-17	2.92E-17
C21orf62	Inf	3.22E-153	6.71E-151
AP000281.2	NA	NA	NA
AP000282.2	NA	3.62E-42	7.73E-42
AP000282.3	NA	NA	NA
OLIG2	2.24708171	8.84E-72	2.90E-71
LINC00945	NA	NA	NA
OLIG1	2.43433852	0.83884235	0.8457888

AP000289.6	NA	NA	NA
AP000290.7	NA	NA	NA
LINC01548	NA	NA	NA
IFNAR2	1.69779507	0.04501193	0.04868287
AP000295.9	NA	NA	NA
IL10RB-AS1	1.31079767	1.05E-05	1.26E-05
IL10RB	1.38910506	6.98E-97	3.55E-96
AP000295.10	NA	1.18E-38	2.40E-38
IFNAR1	1.89459831	5.67E-25	9.53E-25
IFNGR2	2.47463429	5.59E-47	1.27E-46
TMEM50B	1.48973195	3.02E-86	1.25E-85
AP000302.58	NA	NA	NA
DNAJC28	Inf	4.51E-15	6.51E-15
GART	1.13982406	8.76E-96	4.35E-95
SON	1.76759079	1.24E-19	1.93E-19
DONSON	1.11284047	5.97E-127	6.98E-126
AP000304.12	NA	9.17E-24	1.51E-23
CRYZL1	0.99870298	6.24E-84	2.49E-83
ITSN1	2.19357977	8.65E-25	1.45E-24
ATP5O	1.336878	1.47E-114	1.11E-113
LINC00649	0	0.00039295	0.00045386
AP000569.9	Inf	2.83E-123	2.84E-122
MRPS6	1.5431766	1.74E-24	2.90E-24
SLC5A3	2.34695201	0.10294106	0.10970785
LINC00310	Inf	1.65E-39	3.40E-39
AP000318.2	NA	NA	NA
KCNE2	NA	NA	NA
AP000320.6	NA	NA	NA
SMIM11A	1.49805447	1.01E-44	2.24E-44
C21orf140	NA	NA	NA
CMP21-97G8	NA	NA	NA
AP000322.53	NA	NA	NA
CMP21-97G8	NA	NA	NA
KCNE1	NA	1.27E-07	1.59E-07
RCAN1	2.09103437	1.57E-55	4.06E-55
CLIC6	NA	NA	NA
LINC00160	NA	NA	NA
LINC01426	NA	8.42E-10	1.11E-09
RUNX1	2.65726329	1.30E-88	5.66E-88
AF015262.2	NA	NA	NA
AF015720.3	NA	NA	NA
AP000687.1	NA	NA	NA
LINC01436	NA	NA	NA
SETD4	2.39688716	8.47E-05	9.95E-05
AP000688.11	NA	NA	NA

CBR1	1.11401932	1.20E-87	5.11E-87
AP000688.15	NA	NA	NA
AP000688.29	NA	NA	NA
CBR3	1.24837873	4.10E-85	1.67E-84
DOPEY2	1.49805447	4.67E-06	5.66E-06
AP000692.10	0	2.36E-75	8.16E-75
MORC3	1.21716926	0.04982608	0.05381265
CHAF1B	1.60820554	1.60E-45	3.57E-45
AP000695.6	NA	1.09E-148	1.38E-146
AP000695.4	NA	6.49E-73	2.16E-72
CLDN14	NA	NA	NA
AP000696.2	NA	NA	NA
AP000697.6	NA	NA	NA
SIM2	0	0.00938104	0.01039524
HLCS	5.9922179	7.99E-35	1.54E-34
AP000704.5	0	1.88E-07	2.35E-07
RIPPLY3	NA	NA	NA
PIGP	1.52418333	9.86E-71	3.19E-70
TTC3	2.48599557	1.11E-86	4.61E-86
TTC3-AS1	NA	NA	NA
DSCR9	0	4.21E-07	5.22E-07
AP001432.14	NA	3.46E-18	5.25E-18
DSCR3	1.73212549	2.26E-14	3.23E-14
AP001412.1	3.74513619	3.54E-61	9.91E-61
AP001437.1	Inf	2.89E-111	2.01E-110
DYRK1A	2.59662776	3.64E-42	7.78E-42
KCNJ6	NA	NA	NA
DSCR4	NA	NA	NA
DSCR8	NA	NA	NA
KCNJ15	NA	NA	NA
AP001434.2	NA	NA	NA
LINC01423	NA	NA	NA
ERG	2.03307393	6.12E-10	8.06E-10
LINC00114	NA	NA	NA
ETS2	3.2457847	0.0049065	0.00548858
AP001042.1	NA	NA	NA
AF064858.6	NA	1.94E-111	1.35E-110
AP001043.1	NA	NA	NA
AF064858.7	NA	NA	NA
AF064858.8	NA	2.08E-141	7.05E-140
AF064858.11	Inf	1.82E-99	9.66E-99
AF064858.10	NA	NA	NA
PSMG1	1.24051969	2.24E-78	8.12E-78
BRWD1	2.59818823	7.96E-20	1.24E-19
AF129408.17	0.74902724	8.00E-69	2.52E-68

BRWD1-AS2	NA	3.73E-19	5.75E-19
BRWD1-AS1	NA	NA	NA
HMGN1	2.01928477	4.07E-91	1.85E-90
WRB	2.19927146	5.97E-06	7.23E-06
LCA5L	1.12354086	4.98E-19	7.65E-19
SH3BGR	1.49805447	9.54E-19	1.46E-18
B3GALT5	NA	NA	NA
B3GALT5-AS	NA	NA	NA
AF064860.7	NA	NA	NA
IGSF5	NA	NA	NA
PCP4	NA	NA	NA
DSCAM	NA	NA	NA
DSCAM-AS1	NA	NA	NA
LINC00323	NA	NA	NA
BACE2	NA	2.87E-31	5.26E-31
AL773572.7	NA	NA	NA
BACE2-IT1	NA	NA	NA
FAM3B	NA	NA	NA
MX2	NA	3.38E-08	4.29E-08
MX1	Inf	6.22E-28	1.09E-27
AP001610.5	NA	NA	NA
TMPRSS2	NA	NA	NA
AP001610.9	NA	NA	NA
AP006748.1	NA	NA	NA
LINC00111	NA	NA	NA
LINC00479	NA	NA	NA
LINC00112	NA	NA	NA
RIPK4	NA	NA	NA
AP001615.9	NA	NA	NA
PRDM15	2.24708171	6.60E-70	2.11E-69
AP001619.3	NA	NA	NA
C2CD2	3.29571984	3.14E-17	4.69E-17
ZBTB21	1.60505837	2.42E-12	3.34E-12
ZNF295-AS1	NA	NA	NA
UMODL1	NA	NA	NA
ABCG1	NA	5.05E-91	2.29E-90
TFF3	Inf	6.39E-43	1.38E-42
TFF2	NA	NA	NA
TFF1	NA	NA	NA
TMPRSS3	NA	NA	NA
UBASH3A	NA	NA	NA
RSPH1	NA	NA	NA
SLC37A1	1.94747082	7.63E-20	1.19E-19
AP001625.6	NA	NA	NA
AP001626.1	NA	NA	NA

AP001626.2	NA	NA	NA
PDE9A	NA	2.00E-26	3.42E-26
AP001627.1	NA	NA	NA
KB-51A8.1	NA	NA	NA
AP001628.7	NA	NA	NA
AP001628.6	NA	NA	NA
WDR4	1.3087399	1.19E-107	7.54E-107
NDUFV3	1.54185724	2.70E-64	7.91E-64
ERVH48-1	NA	4.16E-06	5.05E-06
AP001630.5	NA	NA	NA
PKNOX1	1.99740597	6.64E-16	9.72E-16
CBS	3.2457847	4.81E-96	2.40E-95
U2AF1	2.96044099	4.08E-79	1.50E-78
FRGCA	NA	NA	NA
AP001631.1C	NA	NA	NA
CRYAA	NA	NA	NA
LINC00322	NA	NA	NA
AP001046.5	NA	NA	NA
AP001046.6	NA	NA	NA
SIK1	Inf	1.05E-123	1.07E-122
LINC00319	NA	NA	NA
LINC00313	NA	NA	NA
AP001048.4	NA	NA	NA
HSF2BP	2.24708171	9.94E-21	1.57E-20
H2BFS	NA	2.23E-120	2.02E-119
RRP1B	1.63131699	7.60E-19	1.16E-18
PDXK	1.34903335	1.16E-98	6.12E-98
CSTB	1.86272481	0.68695914	0.6979231
RRP1	1.15084914	3.50E-66	1.06E-65
AATBC	NA	NA	NA
AGPAT3	2.30058366	2.42E-76	8.47E-76
TRAPPC10	2.3151751	8.94E-06	1.08E-05
PWP2	NA	7.60E-105	4.52E-104
C21orf33	1.49805447	4.52E-05	5.34E-05
AP001055.6	0	3.92E-16	5.76E-16
AP001056.1	NA	NA	NA
AP001057.1	NA	NA	NA
AP001058.3	NA	8.17E-54	2.06E-53
ICOSLG	2.04996928	1.10E-131	1.63E-130
AP001059.6	2.7464332	1.04E-71	3.41E-71
AP001059.7	Inf	1.11E-87	4.73E-87
DNMT3L	0.08322525	4.18E-137	8.72E-136
AP001059.5	0.74902724	6.62E-81	2.50E-80
AIRE	NA	NA	NA
PFKL	1.58365759	1.16E-102	6.58E-102

C21orf2	2.0598249	0.51278316	0.5260043
AP001062.7	1.49805447	0.01946336	0.02133948
AP001062.9	NA	NA	NA
AP001063.1	NA	5.83E-98	3.02E-97
TRPM2	1.19844358	0.57192583	0.58495199
TRPM2-AS	NA	NA	NA
LRRC3-AS1	NA	5.53E-15	7.97E-15
LRRC3	1.24837873	0.02082749	0.02281717
KB-68A7.1	NA	5.11E-124	5.29E-123
AP001065.15	NA	NA	NA
KB-68A7.2	NA	NA	NA
TSPEAR	NA	NA	NA
TSPEAR-AS1	NA	NA	NA
TSPEAR-AS2	NA	NA	NA
KRTAP10-1	NA	NA	NA
KRTAP10-2	NA	NA	NA
KRTAP10-3	NA	NA	NA
KRTAP10-4	NA	NA	NA
KRTAP10-5	NA	NA	NA
KRTAP10-6	NA	NA	NA
KRTAP10-7	NA	NA	NA
KRTAP10-8	NA	NA	NA
KRTAP10-9	NA	NA	NA
KRTAP10-10	NA	NA	NA
KRTAP10-11	NA	NA	NA
KRTAP12-3	NA	NA	NA
KRTAP12-4	NA	NA	NA
KRTAP12-2	NA	NA	NA
KRTAP12-1	NA	NA	NA
KRTAP10-12	NA	NA	NA
UBE2G2	1.72217286	2.03E-07	2.54E-07
LINC01424	1.12354086	0.00122106	0.00139009
SUMO3	2.10625643	1.85E-52	4.58E-52
PTTG1IP	1.5080415	1.36E-10	1.81E-10
ITGB2	1.16296334	1.25E-62	3.56E-62
ITGB2-AS1	Inf	4.60E-12	6.31E-12
LINC01547	1.24837873	6.57E-25	1.10E-24
LL21NC02-1C	NA	NA	NA
FAM207A	1.60136858	2.54E-37	5.08E-37
AP001505.1C	2.69649805	3.96E-107	2.47E-106
LINC00163	NA	NA	NA
LINC00165	NA	NA	NA
LINC00162	NA	NA	NA
ADARB1	2.24708171	3.26E-07	4.06E-07
RP5-1023B2:	NA	NA	NA

LINC00334	NA	NA	NA
POFUT2	2.43433852	3.90E-60	1.08E-59
LINC00205	1.31079767	1.93E-13	2.71E-13
LINC00315	NA	NA	NA
BX322557.13	NA	NA	NA
LINC00316	NA	NA	NA
BX322559.3	NA	NA	NA
LL21NC02-21	NA	NA	NA
COL18A1	Inf	1.65E-14	2.36E-14
COL18A1-AS	NA	NA	NA
COL18A1-AS	NA	NA	NA
SLC19A1	2.03805086	3.25E-20	5.09E-20
AL133493.2	3.37062257	3.55E-29	6.32E-29
PCBP3	Inf	6.83E-138	1.54E-136
RP1-101D8.1	Inf	6.68E-50	1.59E-49
AL592528.1	NA	NA	NA
AJ011932.1	NA	0.00775226	0.00861985
COL6A1	2.43433852	1.38E-12	1.91E-12
AP001476.2	NA	NA	NA
AP001476.4	NA	NA	NA
AP001476.3	NA	NA	NA
AP001471.1	NA	NA	NA
COL6A2	1.23054475	2.80E-55	7.20E-55
FTCD	NA	NA	NA
FTCD-AS1	NA	NA	NA
SPATC1L	NA	NA	NA
AP001468.58	NA	NA	NA
LSS	1.59575368	7.68E-44	1.67E-43
AP001469.5	NA	NA	NA
MCM3AP-AS	6.36673152	1.04E-21	1.67E-21
MCM3AP	2.12723735	2.94E-37	5.88E-37
AP001469.7	NA	3.97E-33	7.46E-33
AP001469.9	2.24708171	5.62E-19	8.63E-19
YBEY	1.74773022	3.48E-11	4.69E-11
C21orf58	2.20302129	3.06E-20	4.78E-20
PCNT	2.49675746	3.64E-16	5.36E-16
DIP2A	3.27699416	1.36E-72	4.52E-72
S100B	NA	2.10E-127	2.51E-126
PRMT2	1.77208883	3.77E-40	7.83E-40
MT-ND1	2.14151308	4.34E-85	1.76E-84
MT-ND2	2.07674132	8.85E-65	2.62E-64
MT-CO1	1.88498019	0.2866894	0.29879468
MT-CO2	2.2297253	1.34E-94	6.49E-94
MT-ATP8	0.83225249	1.90E-09	2.48E-09
MT-ATP6	2.11417799	1.04E-77	3.74E-77

MT-CO3	1.96990361	7.82E-27	1.35E-26
MT-ND3	1.72741012	5.03E-05	5.95E-05
MT-ND4L	1.75633973	8.71E-12	1.19E-11
MT-ND4	2.12697316	6.06E-72	1.99E-71
MT-ND5	2.03910232	1.53E-36	3.02E-36
MT-ND6	1.60878024	2.89E-35	5.62E-35
MT-CYB	2.18360754	1.13E-80	4.23E-80
AC133551.1	NA	NA	NA
AC136612.1	NA	NA	NA
AC136616.1	NA	NA	NA
AC136616.3	NA	NA	NA
AC136616.2	NA	NA	NA
AC141272.1	NA	NA	NA
AC136352.5	NA	NA	NA
AC136352.4	NA	NA	NA
AC171558.2	NA	NA	NA
AC171558.1	NA	NA	NA
BX004987.4	3.74513619	7.29E-91	3.29E-90
AC145212.4	0.74902724	2.34E-79	8.61E-79
AC145212.2	1.49805447	1.31E-09	1.72E-09
AC011841.1	NA	NA	NA
AC011043.1	0.74902724	4.10E-59	1.11E-58
AC011043.2	NA	NA	NA
AL592183.1	Inf	1.10E-26	1.89E-26
AC007325.1	NA	NA	NA
AC007325.4	1.5396671	0.01052913	0.01165108
AC007325.2	NA	NA	NA
BX072566.1	NA	NA	NA
AL354822.1	Inf	2.33E-24	3.89E-24
AC023491.2	NA	NA	NA
AC004556.1	NA	4.31E-45	9.56E-45
AC233755.2	NA	NA	NA
AC233755.1	NA	NA	NA
AC240274.1	1.74773022	0.49014921	0.50341298
AC213203.1	NA	NA	NA
FAM231B	NA	NA	NA

G1S

gene	ratio	p_value	BH_adjusted_p_value
RP11-34P13.	NA	NA	NA
FAM138A	NA	NA	NA
OR4F5	NA	NA	NA
RP11-34P13.	Inf	0	0
RP11-34P13.	NA	NA	NA
RP11-34P13.	NA	NA	NA
RP11-34P13.	NA	NA	NA
FO538757.3	NA	NA	NA
FO538757.2	2.39452364	1.09E-210	2.83E-210
AP006222.2	1.43968479	0	0
RP5-857K21.	NA	NA	NA
RP4-669L17.:	NA	NA	NA
RP4-669L17.	3.86002445	7.05E-48	9.86E-48
OR4F29	NA	NA	NA
RP5-857K21.	NA	1.87E-140	3.72E-140
RP5-857K21.	NA	NA	NA
OR4F16	NA	NA	NA
RP11-206L10	NA	NA	NA
RP11-206L10	1.83351161	2.13E-46	2.96E-46
FAM87B	NA	NA	NA
LINC00115	3.08801956	2.21E-231	6.26E-231
FAM41C	1.33346299	6.32E-112	1.14E-111
RP11-54O7.1	0	0	0
RP11-54O7.1	1.54400978	8.30E-22	1.01E-21
RP11-54O7.2	1.35100856	4.87E-185	1.14E-184
RP11-54O7.3	Inf	1.04E-66	1.57E-66
SAMD11	1.54400978	1.49E-08	1.67E-08
NOC2L	1.76351186	3.82E-64	5.72E-64
KLHL17	1.4153423	2.20E-198	5.43E-198
PLEKHN1	0	3.25E-280	1.15E-279
PERM1	NA	0	0
RP11-54O7.1	0	2.82E-285	1.03E-284
HES4	0.92406646	1.54E-223	4.22E-223
ISG15	1.76535384	3.26E-72	5.05E-72
RP11-54O7.1	NA	NA	NA
AGRN	1.20432763	7.30E-237	2.12E-236
RP11-54O7.1	NA	NA	NA
RNF223	NA	NA	NA
C1orf159	1.57109767	0.32353513	0.32947372
LINC01342	NA	NA	NA
RP11-465B2:	NA	NA	NA
TTLL10-AS1	NA	NA	NA
TTLL10	NA	NA	NA

TNFRSF18	NA	NA	NA
TNFRSF4	0.77200489	1.42E-201	3.56E-201
SDF4	1.809814	1.47E-118	2.71E-118
B3GALT6	1.75113304	1.40E-19	1.69E-19
FAM132A	1.3896088	4.08E-06	4.47E-06
RP5-902P8.1	Inf	0	0
UBE2J2	1.83085563	4.63E-41	6.25E-41
RP5-902P8.1	Inf	9.59E-22	1.17E-21
SCNN1D	5.40403423	8.62E-201	2.15E-200
ACAP3	2.06787024	2.70E-05	2.93E-05
PUSL1	2.10177408	0.18016202	0.18465054
CPSF3L	1.54400978	8.53E-261	2.77E-260
CPTP	1.51487752	1.81E-78	2.87E-78
TAS1R3	NA	NA	NA
DVL1	2.65376681	0	0
MXRA8	0.19300122	0	0
AURKAIP1	1.84265052	1.42E-29	1.81E-29
CCNL2	1.94623082	1.53E-264	5.06E-264
MRPL20	2.13876996	0	0
RP4-758J18.:	2.48757131	3.48E-141	6.96E-141
ANKRD65	NA	5.25E-13	6.08E-13
RP4-758J18.7	NA	NA	NA
TMEM88B	NA	NA	NA
RP4-758J18.:	0.77200489	0.00568822	0.00599139
VWA1	1.99338576	9.29E-38	1.23E-37
ATAD3C	3.08801956	0	0
ATAD3B	1.52642425	3.92E-281	1.40E-280
ATAD3A	1.6847742	1.87E-279	6.61E-279
TMEM240	1.54400978	7.94E-269	2.67E-268
SSU72	2.16652108	5.88E-290	2.20E-289
RP5-832C2.5	2.99151895	3.14E-57	4.58E-57
AL645728.1	NA	NA	NA
C1orf233	1.33432944	1.54E-152	3.21E-152
RP11-345P4.	2.15058505	7.03E-83	1.13E-82
MIB2	2.29111129	6.99E-138	1.38E-137
MMP23B	1.54400978	1.77E-53	2.54E-53
CDK11B	1.72538442	1.65E-14	1.94E-14
RP11-345P4.	4.63202934	1.12E-31	1.45E-31
SLC35E2B	2.52466464	1.54E-224	4.22E-224
RP11-345P4.	NA	NA	NA
CDK11A	1.83351161	1.36E-155	2.87E-155
SLC35E2	1.4153423	5.55E-273	1.90E-272
NADK	2.03279394	9.24E-104	1.62E-103
GNB1	2.06196833	1.14E-290	4.29E-290
RP1-140A9.1	1.54400978	1.14E-11	1.31E-11

CALML6	NA	NA	NA
TMEM52	2.19411916	2.27E-127	4.31E-127
CFAP74	NA	1.62E-271	5.51E-271
RP11-547D2·	NA	NA	NA
GABRD	1.15800733	9.22E-208	2.36E-207
RP11-547D2·	Inf	0	0
PRKCZ	1.52518039	5.91E-296	2.28E-295
RP5-892K4.1	Inf	1.21E-107	2.15E-107
RP11-181G1	3.08801956	5.81E-38	7.72E-38
FAAP20	1.9091311	3.17E-10	3.60E-10
RP11-181G1	NA	NA	NA
SKI	1.78879182	1.30E-132	2.52E-132
MORN1	2.31601467	8.00E-27	1.01E-26
RER1	2.1476678	4.54E-35	5.95E-35
PEX10	1.69069071	2.69E-140	5.34E-140
PLCH2	NA	NA	NA
RP3-395M20	NA	NA	NA
RP3-395M20	NA	NA	NA
PANK4	1.80134474	0.81896479	0.82259214
HES5	NA	NA	NA
RP3-395M20	0.77200489	1.09E-198	2.69E-198
RP3-395M20	NA	9.16E-53	1.31E-52
TNFRSF14	1.54400978	1.16E-68	1.77E-68
RP3-395M20	NA	NA	NA
FAM213B	1.24244537	4.64E-175	1.05E-174
MMEL1	NA	1.24E-61	1.84E-61
RP13-436F16	NA	NA	NA
TTC34	1.54400978	5.36E-90	8.90E-90
RP11-740P5.	NA	NA	NA
RP11-740P5.	NA	NA	NA
ACTRT2	NA	NA	NA
LINC00982	0.77200489	2.25E-57	3.28E-57
PRDM16	1.54400978	1.98E-10	2.25E-10
RP1-163G9.2	NA	NA	NA
RP11-22L13.	NA	NA	NA
ARHGEF16	0	6.03E-149	1.24E-148
RP11-168F9.	NA	NA	NA
MEGF6	1.51184291	6.41E-12	7.37E-12
RP11-46F15.	NA	NA	NA
TPRG1L	1.28667482	0.00130774	0.00139077
WRAP73	1.31312981	1.04E-304	4.24E-304
TP73	0	0	0
RP5-1092A1	NA	NA	NA
RP5-1092A1	NA	NA	NA
TP73-AS1	2.19249389	6.91E-38	9.18E-38

CCDC27	NA	NA	NA
SMIM1	0.38600244	0	0
LRRC47	1.63140656	2.94E-284	1.06E-283
RP1-286D6.5	NA	0	0
CEP104	2.0394756	1.68E-16	2.00E-16
DFFB	1.77561125	1.89E-07	2.09E-07
C1orf174	1.83433641	7.03E-15	8.26E-15
LINC01134	NA	1.44E-41	1.95E-41
LINC01346	NA	NA	NA
RP13-614K11	NA	NA	NA
RP5-1166F10	NA	NA	NA
RP1-37J18.1	NA	NA	NA
RP1-37J18.2	NA	NA	NA
AJAP1	NA	NA	NA
RP11-542C10	NA	NA	NA
RP1-58B11.1	NA	NA	NA
RP11-154H17	NA	NA	NA
NPHP4	3.474022	0.01330375	0.01392871
KCNAB2	2.65352435	4.32E-261	1.41E-260
CHD5	0	0	0
RPL22	2.00106423	1.58E-296	6.10E-296
RP1-120G22.	1.94430861	4.60E-08	5.13E-08
RNF207	3.17884366	6.48E-265	2.15E-264
ICMT	1.81559456	5.70E-65	8.57E-65
LINC00337	1.28667482	5.71E-13	6.62E-13
HES3	NA	NA	NA
GPR153	1.23520782	9.20E-16	1.09E-15
ACOT7	1.11589798	0	0
RP1-202O8.3	1.15800733	5.81E-146	1.18E-145
HES2	NA	8.55E-35	1.12E-34
ESPN	0	1.51E-176	3.44E-176
RP1-202O8.2	NA	NA	NA
TNFRSF25	Inf	4.88E-267	1.63E-266
PLEKHG5	0.48250306	0	0
NOL9	2.7132599	7.00E-306	2.86E-305
TAS1R1	NA	NA	NA
ZBTB48	1.36387531	0	0
KLHL21	2.65912795	2.93E-121	5.45E-121
PHF13	1.9167018	4.81E-33	6.24E-33
THAP3	1.84941831	9.60E-60	1.41E-59
DNAJC11	2.31601467	9.90E-123	1.85E-122
RP11-242F24	Inf	1.44E-242	4.28E-242
RP11-312B8.	2.50901589	0.07590398	0.07849122
CAMTA1	2.08277474	3.62E-114	6.59E-114
RP11-334N17	NA	NA	NA

RP3-453P22.	NA	NA	NA
RP4-549F15.	NA	NA	NA
RP11-338N1(	Inf	0	0
RP11-338N1(	NA	NA	NA
RP11-338N1(	NA	1.09E-22	1.34E-22
VAMP3	1.81053528	8.46E-91	1.41E-90
PER3	3.01620515	0	0
RP3-467L1.4	2.41251528	2.50E-121	4.65E-121
UTS2	NA	0	0
TNFRSF9	Inf	0	0
PARK7	1.81918595	3.60E-116	6.59E-116
ERRFI1	0.38600244	0.00477831	0.00504
RP11-431K2⁄	NA	NA	NA
RP11-431K2⁄	NA	NA	NA
RP11-431K2⁄	NA	NA	NA
SLC45A1	NA	NA	NA
RERE	2.92019241	0	0
RP5-1115A1!	NA	NA	NA
RP4-633I8.4	NA	NA	NA
ENO1	0.8247836	0	0
ENO1-AS1	1.28667482	5.07E-16	6.00E-16
CA6	NA	NA	NA
SLC2A7	NA	NA	NA
SLC2A5	1.62121027	0	0
GPR157	1.77561125	7.73E-32	9.98E-32
MIR34AHG	NA	NA	NA
RP3-510D11.	NA	NA	NA
RP3-510D11.	NA	NA	NA
H6PD	2.8306846	3.87E-159	8.26E-159
SPSB1	1.28667482	1.92E-116	3.52E-116
RP13-392I16	NA	NA	NA
SLC25A33	1.85189268	3.30E-09	3.71E-09
TMEM201	2.55496856	1.45E-274	5.00E-274
PIK3CD	1.81070238	3.17E-120	5.89E-120
PIK3CD-AS1	2.31601467	3.70E-169	8.20E-169
PIK3CD-AS2	2.23023635	0	0
CLSTN1	1.73230366	5.87E-18	7.03E-18
AL357140.1	NA	NA	NA
CTNNBIP1	1.61571774	0	0
RP11-84A14.	NA	NA	NA
LZIC	2.00284287	3.10E-05	3.36E-05
NMNAT1	1.81421149	2.75E-162	5.95E-162
RBP7	0.77200489	2.43E-218	6.50E-218
UBE4B	1.5135359	6.36E-72	9.84E-72
KIF1B	1.87184747	9.00E-94	1.51E-93

PGD	1.36078729	0	0
RP4-736L20.:	Inf	1.46E-192	3.54E-192
APITD1-CORT	2.05867971	3.43E-10	3.89E-10
APITD1	2.2167569	4.89E-264	1.61E-263
CORT	NA	1.98E-17	2.36E-17
DFFA	1.78693399	3.00E-94	5.05E-94
RP5-1113E3.	NA	NA	NA
PEX14	1.23103482	0	0
CASZ1	3.4188788	0	0
RP4-734G22.	NA	5.52E-252	1.71E-251
C1orf127	NA	NA	NA
TARDBP	1.74441223	2.73E-131	5.27E-131
RP4-635E18.	NA	NA	NA
MASP2	Inf	0	0
RP4-635E18.	0	0	0
SRM	1.96059585	2.97E-77	4.68E-77
EXOSC10	1.42703934	1.02E-240	3.02E-240
RP4-635E18.	Inf	2.00E-117	3.68E-117
RP4-635E18.	2.27538283	4.37E-59	6.41E-59
MTOR	2.30292984	1.36E-152	2.84E-152
MTOR-AS1	NA	NA	NA
ANGPTL7	NA	NA	NA
UBIAD1	2.17286145	4.80E-84	7.78E-84
PTCHD2	Inf	0	0
RP1-69M21.:	NA	NA	NA
FBXO2	NA	NA	NA
FBXO44	1.15800733	2.56E-49	3.60E-49
FBXO6	0.65137913	0	0
MAD2L2	1.75014721	3.09E-147	6.31E-147
DRAXIN	0	0	0
AGTRAP	0	0	0
C1orf167	NA	NA	NA
RP11-56N19.	NA	NA	NA
MTHFR	2.63768337	8.78E-222	2.38E-221
CLCN6	2.73710825	9.12E-32	1.18E-31
NPPA-AS1	3.34535452	0	0
NPPA	NA	NA	NA
NPPB	NA	NA	NA
KIAA2013	2.18474258	4.34E-179	9.94E-179
PLOD1	2.29028117	0	0
MFN2	3.26132678	0	0
MIIP	1.67615476	1.25E-18	1.50E-18
TNFRSF8	2.13533267	0.03902555	0.04056153
TNFRSF1B	1.79105134	1.69E-30	2.16E-30
VPS13D	5.30753362	6.10E-299	2.39E-298

RP5-888M10	NA	NA	NA
DHRS3	0	1.05E-147	2.15E-147
RP11-474O2	NA	NA	NA
AADACL4	NA	NA	NA
AADACL3	NA	NA	NA
C1orf158	NA	NA	NA
PRAMEF12	NA	NA	NA
PRAMEF1	NA	NA	NA
RP5-845O24.	NA	NA	NA
PRAMEF11	NA	NA	NA
HNRNPCL1	NA	NA	NA
PRAMEF2	NA	NA	NA
PRAMEF4	NA	NA	NA
PRAMEF10	NA	NA	NA
PRAMEF7	NA	NA	NA
PRAMEF6	NA	NA	NA
PRAMEF27	NA	NA	NA
PRAMEF26	NA	NA	NA
PRAMEF25	NA	NA	NA
HNRNPCL3	NA	1.32E-131	2.54E-131
HNRNPCL2	NA	NA	NA
RP13-221M1	NA	NA	NA
HNRNPCL4	NA	NA	NA
PRAMEF9	NA	NA	NA
PRAMEF18	NA	NA	NA
PRAMEF5	NA	NA	NA
PRAMEF8	NA	NA	NA
PRAMEF15	NA	NA	NA
RP11-219C2	NA	NA	NA
PRAMEF14	NA	NA	NA
PRAMEF19	NA	NA	NA
PRAMEF17	NA	NA	NA
PRAMEF20	NA	NA	NA
LRRC38	NA	NA	NA
RP4-597A16.	NA	NA	NA
PDPN	NA	NA	NA
CTA-520D8.2	NA	NA	NA
PRDM2	2.43478465	1.16E-195	2.83E-195
RP11-344F1	NA	NA	NA
RP4-704D23.	NA	NA	NA
KAZN	1.76996243	5.59E-63	8.34E-63
TMEM51-AS	NA	NA	NA
TMEM51	1.82707824	0.04079873	0.04237537
C1orf195	NA	NA	NA
FHAD1	Inf	3.18E-111	5.72E-111

RP3-467K16.	NA	NA	NA
RP3-467K16.	NA	NA	NA
RP3-467K16.	NA	2.90E-166	6.36E-166
EFHD2	1.79304362	5.56E-12	6.40E-12
CTRC	NA	NA	NA
CELA2A	NA	NA	NA
CELA2B	NA	1.52E-260	4.93E-260
CASP9	1.60577017	0.89251363	0.89448582
DNAJC16	2.10546788	1.08E-36	1.43E-36
RP4-680D5.8	NA	NA	NA
AGMAT	2.31601467	1.99E-40	2.68E-40
RP4-680D5.2	NA	NA	NA
DDI2	2.86593596	5.78E-176	1.31E-175
RSC1A1	NA	NA	NA
RP4-680D5.9	NA	0.37997497	0.38610429
PLEKHM2	2.10057144	1.89E-10	2.15E-10
RP11-288I21	NA	NA	NA
SLC25A34	NA	4.67E-164	1.02E-163
RP11-169K16	NA	NA	NA
TMEM82	NA	NA	NA
FBLIM1	1.6297881	3.66E-05	3.96E-05
UQCRHL	1.7896477	1.10E-11	1.27E-11
FLJ37453	1.91163116	1.39E-37	1.85E-37
SPEN	1.96818829	0.03344854	0.03481089
ZBTB17	1.5191064	9.15E-290	3.42E-289
C1orf64	NA	NA	NA
RP11-5P18.5	NA	NA	NA
HSPB7	NA	NA	NA
CLCNKA	NA	NA	NA
CLCNKB	NA	NA	NA
FAM131C	2.31601467	5.83E-279	2.05E-278
EPHA2	0	0	0
RP11-276H7.	NA	NA	NA
RP11-276H7.	NA	NA	NA
ARHGEF19-A	NA	NA	NA
ARHGEF19	1.74716896	3.84E-26	4.82E-26
RSG1	3.28102078	0.48152063	0.48756752
FBXO42	2.01579055	9.24E-106	1.63E-105
SZRD1	2.19439746	2.22E-304	8.98E-304
SPATA21	0	0	0
NECAP2	1.77159504	4.62E-28	5.85E-28
RP4-798A10.	3.08801956	6.50E-298	2.53E-297
RP4-798A10.	1.54400978	9.76E-71	1.50E-70
RP4-798A10.	NA	NA	NA
RP5-875O13.	NA	NA	NA

FAM231C	NA	NA	NA
NBPF1	1.84093474	9.95E-111	1.79E-110
RP5-1182A14	NA	3.05E-172	6.84E-172
RP1-163M9.8	NA	NA	NA
FAM231A	NA	NA	NA
RP1-163M9.7	NA	NA	NA
FAM231C	NA	NA	NA
RP5-1182A14	NA	NA	NA
CROCC	2.6055165	0.02137613	0.02231719
RP11-108M9	NA	NA	NA
RP11-108M9	NA	NA	NA
RP11-108M9	Inf	4.20E-171	9.38E-171
RP11-108M9	NA	NA	NA
RP11-108M9	Inf	0	0
MFAP2	NA	NA	NA
RP1-37C10.3	NA	NA	NA
ATP13A2	2.04174977	7.88E-15	9.25E-15
SDHB	1.24254249	0	0
PADI2	NA	NA	NA
RP11-380J14	NA	NA	NA
RP11-380J14	NA	NA	NA
PADI1	Inf	0	0
PADI3	NA	5.94E-236	1.71E-235
PADI4	NA	2.50E-85	4.06E-85
AC004824.2	NA	NA	NA
PADI6	NA	NA	NA
RP1-20B21.4	NA	NA	NA
RCC2	2.44134014	0	0
ARHGEF10L	NA	NA	NA
RP11-473A10	NA	NA	NA
ACTL8	1.06892985	0	0
RP11-174G17	NA	NA	NA
IGSF21	NA	NA	NA
RP11-174G17	NA	NA	NA
RP11-422P22	NA	NA	NA
KLHDC7A	Inf	1.16E-289	4.33E-289
PAX7	NA	NA	NA
TAS1R2	NA	NA	NA
RP13-279N23	NA	NA	NA
ALDH4A1	2.61623879	5.10E-116	9.32E-116
IFFO2	1.59226009	5.25E-229	1.47E-228
UBR4	2.39321516	1.38E-192	3.34E-192
RP1-43E13.2	3.08801956	2.80E-273	9.62E-273
EMC1	2.06698083	1.10E-30	1.42E-30
MRTO4	1.79655843	3.17E-93	5.32E-93

AKR7A3	0.77200489	0	0
AKR7A2	1.45003692	0	0
PQLC2	1.40777362	0	0
CAPZB	1.42728052	0	0
RP5-1056L3.[illegible]	NA	NA	NA
MINOS1	2.35200622	0	0
MINOS1-NBI	NA	NA	NA
NBL1	1.8427651	3.96E-15	4.66E-15
HTR6	NA	NA	NA
TMCO4	1.88712306	4.86E-66	7.34E-66
RNF186	NA	NA	NA
RP11-91K11.	NA	NA	NA
OTUD3	2.5917307	0.00092525	0.00098595
PLA2G2E	NA	NA	NA
PLA2G2A	NA	NA	NA
PLA2G5	NA	NA	NA
PLA2G2D	NA	NA	NA
PLA2G2F	NA	NA	NA
RP3-340N1.2	NA	NA	NA
PLA2G2C	NA	NA	NA
UBXN10-AS1	0	2.53E-56	3.66E-56
UBXN10	NA	3.40E-83	5.49E-83
RP3-340N1.6	NA	NA	NA
VWA5B1	NA	NA	NA
RP4-745E8.2	NA	NA	NA
LINC01141	NA	NA	NA
RP4-749H3.2	NA	NA	NA
CAMK2N1	Inf	2.01E-31	2.59E-31
MUL1	1.41367129	0	0
FAM43B	NA	NA	NA
CDA	1.02933985	2.99E-226	8.26E-226
PINK1	1.68024594	3.80E-153	7.94E-153
PINK1-AS	1.54400978	0.07083836	0.07330307
DDOST	1.18472621	0	0
KIF17	0.77200489	0	0
SH2D5	1.96861247	1.57E-99	2.70E-99
RP5-930J4.2	NA	NA	NA
HP1BP3	2.22540377	4.50E-11	5.15E-11
EIF4G3	1.93729529	2.70E-60	3.98E-60
ECE1	1.66463554	2.24E-109	4.02E-109
RP3-329E20.	NA	NA	NA
RP5-1071N3.	0	0	0
RP11-293F5.	NA	NA	NA
NBPF3	Inf	1.20E-89	1.98E-89
ALPL	Inf	0	0

RP11-63N8.3	NA	NA	NA
RAP1GAP	0	1.09E-174	2.45E-174
USP48	1.71669508	0	0
LDLRAD2	1.15800733	0.20843534	0.21325479
HSPG2	1.80134474	1.11E-222	3.01E-222
CELA3B	NA	NA	NA
CELA3A	NA	NA	NA
RP1-224A6.3	Inf	3.38E-262	1.11E-261
LINC00339	1.40777362	2.27E-45	3.13E-45
CDC42	1.68032194	1.86E-284	6.72E-284
RP1-224A6.9	NA	6.38E-188	1.51E-187
WNT4	NA	NA	NA
ZBTB40	2.00721271	5.57E-140	1.11E-139
EPHA8	NA	NA	NA
C1QA	NA	0	0
C1QC	0	0	0
C1QB	0	0	0
EPHB2	1.54400978	0.00028091	0.00030133
RP11-69E9.1	NA	NA	NA
LACTBL1	NA	NA	NA
C1orf234	1.54400978	3.06E-07	3.39E-07
KDM1A	1.9678556	9.00E-40	1.21E-39
RP1-184J9.2	NA	NA	NA
LUZP1	1.70111955	0	0
HTR1D	NA	4.06E-169	8.99E-169
LINC01355	1.81421149	0.00027762	0.00029785
HNRNPR	1.84008638	8.17E-294	3.12E-293
ZNF436	3.21668704	1.45E-89	2.41E-89
ZNF436-AS1	1.69105833	8.78E-43	1.20E-42
RP5-1057J7.1	NA	0	0
TCEA3	2.00721271	1.98E-20	2.40E-20
ASAP3	1.737011	3.35E-51	4.75E-51
E2F2	2.02124917	1.09E-248	3.33E-248
RP1-150O5.3	0	0	0
ID3	3.35542634	0	0
MDS2	NA	NA	NA
RPL11	2.10782236	1.71E-253	5.36E-253
TCEB3	2.26748865	2.71E-55	3.91E-55
TCEB3-AS1	3.74422372	8.00E-135	1.56E-134
PITHD1	2.00890943	0.0014913	0.00158495
LYPLA2	1.5623182	4.14E-248	1.26E-247
GALE	1.59799614	1.07E-220	2.88E-220
HMGCL	1.30338488	0	0
FUCA1	1.33346299	1.06E-99	1.83E-99
CNR2	8.49205379	1.29E-142	2.59E-142

RP11-4M23.3	NA	NA	NA
PNRC2	1.90412868	0.2391751	0.24433328
SRSF10	1.75887198	1.60E-280	5.67E-280
RP11-293P20	NA	NA	NA
MYOM3	NA	0	0
RP11-293P20	NA	NA	NA
IL22RA1	NA	NA	NA
IFNLR1	NA	NA	NA
RP11-10N16.	NA	NA	NA
RP11-10N16.	NA	NA	NA
GRHL3	NA	NA	NA
STPG1	1.93001222	2.65E-98	4.54E-98
NIPAL3	2.26454768	1.77E-87	2.90E-87
RCAN3	2.62481663	3.91E-87	6.40E-87
RP4-594I10.3	1.54400978	2.27E-105	4.00E-105
NCMAP	NA	NA	NA
SRRM1	2.11572011	4.25E-245	1.28E-244
CLIC4	1.737011	3.52E-06	3.85E-06
RUNX3	1.84729742	1.65E-130	3.18E-130
RP11-84D1.1	NA	NA	NA
RP11-84D1.2	NA	NA	NA
RP4-799D16.	NA	NA	NA
RP4-706G24.	NA	0	0
SYF2	1.45864385	0	0
RSRP1	1.6197894	8.05E-17	9.58E-17
RP3-465N24.	3.86002445	1.97E-239	5.76E-239
RHD	NA	NA	NA
TMEM50A	2.03085971	0.03099881	0.03228175
RHCE	Inf	0	0
TMEM57	2.1459119	4.21E-05	4.56E-05
LDLRAP1	2.34514693	6.09E-134	1.18E-133
RP11-70P17.	1.93001222	2.46E-11	2.82E-11
MAN1C1	0.51466993	9.77E-89	1.61E-88
RP1-187B23.	0.38600244	1.75E-38	2.34E-38
SEPN1	2.00506826	3.91E-203	9.84E-203
RP1-317E23.	NA	NA	NA
RP1-317E23.	1.54400978	3.80E-70	5.83E-70
MTFR1L	1.77896779	1.19E-07	1.32E-07
RP1-317E23.	NA	NA	NA
AUNIP	1.59079796	5.84E-199	1.45E-198
RP1-125I3.2	NA	NA	NA
PAQR7	0.57900367	5.90E-211	1.53E-210
STMN1	1.38091674	0	0
PAFAH2	3.86002445	0	0
EXTL1	0	0	0

SLC30A2	0	0	0
TRIM63	NA	NA	NA
PDIK1L	2.08895441	2.27E-29	2.90E-29
FAM110D	1.18071336	1.28E-248	3.92E-248
RP11-96L14.8	NA	NA	NA
RP11-96L14.7	1.54400978	8.95E-108	1.59E-107
ZNF593	2.02533489	1.33E-58	1.95E-58
CNKSR1	Inf	1.62E-06	1.78E-06
CATSPER4	NA	NA	NA
CEP85	1.99929472	1.59E-46	2.21E-46
SH3BGRL3	1.3295182	0	0
UBXN11	1.75185725	3.68E-135	7.19E-135
CD52	0.63294811	0	0
AIM1L	0.38600244	4.53E-196	1.11E-195
ZNF683	NA	NA	NA
LIN28A	NA	NA	NA
DHDDS	2.46206965	2.51E-53	3.59E-53
RP3-476K8.3	Inf	0	0
HMGN2	1.62295382	0	0
RPS6KA1	1.63923505	0	0
RP5-968P14.	NA	0	0
ARID1A	2.27450903	8.96E-231	2.53E-230
PIGV	1.71556642	1.33E-07	1.48E-07
ZDHHC18	1.66053882	1.05E-82	1.70E-82
SFN	1.54400978	3.67E-168	8.11E-168
GPN2	1.25305316	0	0
RP1-50O24.6	NA	NA	NA
GPATCH3	1.85036093	4.34E-05	4.70E-05
NUDC	0.8812795	0	0
NR0B2	NA	NA	NA
KDF1	NA	NA	NA
TRNP1	1.54400978	0	0
FAM46B	4.63202934	6.11E-48	8.54E-48
AL137860.1	NA	NA	NA
SLC9A1	2.22519057	3.28E-22	4.02E-22
RP11-40H20.	NA	NA	NA
WDTC1	1.23786991	1.26E-257	4.01E-257
TMEM222	1.7634217	1.14E-17	1.36E-17
SYTL1	1.04503101	0	0
MAP3K6	4.63202934	6.91E-221	1.86E-220
FCN3	NA	NA	NA
CD164L2	NA	NA	NA
GPR3	1.06892985	1.32E-288	4.91E-288
WASF2	2.19236707	4.89E-155	1.03E-154
RP4-752I6.1	NA	NA	NA

RP1-159A19.	5.40403423	5.29E-84	8.57E-84
AHDC1	2.59674372	1.30E-25	1.62E-25
FGR	NA	NA	NA
RP11-288L9.	Inf	3.81E-75	5.97E-75
IFI6	1.49452229	1.14E-206	2.92E-206
RP11-288L9.	0.77200489	1.03E-37	1.37E-37
FAM76A	1.66968499	0.00093776	0.00099905
STX12	1.47382752	9.63E-288	3.55E-287
PPP1R8	1.55713911	0	0
AL109927.1	Inf	2.29E-254	7.19E-254
THEMIS2	1.4972216	1.13E-59	1.67E-59
RPA2	1.51827628	8.83E-255	2.78E-254
SMPDL3B	1.2008965	1.43E-15	1.68E-15
RP11-460I13	NA	4.37E-187	1.03E-186
XKR8	1.5535407	1.96E-159	4.19E-159
EYA3	2.52537193	0	0
PTAFR	0.25733496	1.47E-224	4.04E-224
DNAJC8	1.53470852	0	0
ATPIF1	1.92603516	0.0474065	0.04920187
RP5-1092A3.	5.01803178	2.26E-158	4.82E-158
RP5-1092A3.	NA	NA	NA
SESN2	2.47041565	3.57E-250	1.10E-249
MED18	1.3268834	4.60E-298	1.79E-297
PHACTR4	2.64525205	4.51E-101	7.80E-101
RCC1	1.5498583	0	0
TRNAU1AP	1.48624071	0	0
SNHG12	2.69236705	5.30E-235	1.52E-234
TAF12	1.94157784	6.66E-26	8.34E-26
RAB42	2.15518032	1.48E-216	3.92E-216
RP11-442N2	Inf	0	0
RNU11	NA	8.95E-273	3.07E-272
GMEB1	2.68016792	9.82E-185	2.30E-184
YTHDF2	1.5661726	0	0
OPRD1	0	0	0
RP1-212P9.3	NA	0	0
EPB41	2.29124446	8.29E-248	2.52E-247
TMEM200B	Inf	0	0
RP11-242O2	NA	NA	NA
SRSF4	1.45247452	0	0
MECR	2.01374821	4.55E-55	6.56E-55
RP11-467D1	NA	NA	NA
PTPRU	3.06228606	0	0
RP3-437I16.1	NA	NA	NA
RP4-656G21.	NA	NA	NA
RP11-111I12	NA	NA	NA

RP5-893G23.	NA	NA	NA
RP4-591L5.1	NA	NA	NA
RP4-591L5.2	NA	NA	NA
MATN1	0	3.31E-128	6.29E-128
MATN1-AS1	1.54400978	0.02227663	0.02325064
LAPTM5	1.17814659	0	0
RP5-1125N1	NA	NA	NA
RP5-1125N1	NA	NA	NA
RP1-65J11.5	NA	NA	NA
SDC3	NA	0.13057547	0.13434943
PUM1	2.50684734	5.68E-182	1.32E-181
NKAIN1	0.48250306	0	0
SNRNP40	1.31353469	0	0
ZCCHC17	1.51473945	2.89E-282	1.03E-281
RP11-266K2	NA	NA	NA
FABP3	0	0	0
SERINC2	0.92640587	0	0
LINC01226	0	0	0
RP11-73M7.	NA	NA	NA
TINAGL1	NA	1.54E-264	5.10E-264
HCRTR1	0	0	0
PEF1	1.71921656	3.18E-45	4.39E-45
RP11-73M7.	NA	NA	NA
RP11-73M7.	NA	NA	NA
COL16A1	NA	NA	NA
ADGRB2	2.31601467	2.21E-148	4.53E-148
RP11-84A19.	NA	NA	NA
SPOCD1	NA	NA	NA
RP11-84A19.	0	7.12E-37	9.42E-37
RP11-84A19.	Inf	1.05E-32	1.36E-32
PTP4A2	2.22449474	0	0
RP4-534N18.	NA	NA	NA
KHDRBS1	1.69186452	0	0
RP11-277A4.	Inf	0	0
TMEM39B	1.53909255	6.41E-221	1.73E-220
KPNA6	2.07324584	0.03416194	0.03554723
TXLNA	2.06659771	0.03046248	0.03173234
CCDC28B	1.58562729	0	0
RP4-622L5.7	NA	2.04E-94	3.45E-94
IQCC	2.23023635	1.47E-30	1.89E-30
DCDC2B	NA	NA	NA
TMEM234	2.31601467	9.52E-198	2.35E-197
EIF3I	1.29476412	0	0
FAM167B	Inf	0	0
LCK	NA	NA	NA

HDAC1	1.12941456	0	0
MARCKSL1	1.08496579	0	0
RP4-811H24.	NA	NA	NA
TSSK3	0.77200489	1.52E-30	1.95E-30
FAM229A	2.31601467	6.22E-217	1.65E-216
BSDC1	1.83126741	1.29E-15	1.52E-15
RP1-27O5.3	NA	NA	NA
ZBTB8B	NA	7.12E-08	7.93E-08
ZBTB8A	1.50112062	3.08E-09	3.48E-09
ZBTB8OS	1.89392257	5.68E-17	6.76E-17
RBBP4	1.66139064	0	0
SYNC	1.83533238	2.71E-102	4.71E-102
RP11-114B7.	NA	NA	NA
KIAA1522	2.03159182	1.44E-67	2.18E-67
YARS	1.41393456	0	0
S100PBP	1.88589766	0.71400353	0.71859825
FNDC5	1.54400978	7.52E-37	9.95E-37
HPCA	NA	NA	NA
TMEM54	0.96136458	8.30E-303	3.33E-302
RNF19B	2.47041565	1.20E-07	1.34E-07
RP1-117O3.2	NA	NA	NA
AK2	1.8583768	5.19E-60	7.65E-60
AZIN2	0.77200489	0	0
TRIM62	1.35100856	0.62749724	0.63286941
ZNF362	1.69247226	2.75E-92	4.61E-92
RP11-415J8.[illegible]	Inf	0	0
A3GALT2	NA	NA	NA
RP11-415J8.[illegible]	NA	NA	NA
PHC2	1.7350213	7.97E-71	1.23E-70
RP11-415J8.[illegible]	NA	NA	NA
ZSCAN20	2.57334963	0.09100575	0.09393106
CSMD2	NA	1.15E-252	3.60E-252
HMGB4	NA	NA	NA
CSMD2-AS1	NA	NA	NA
C1orf94	NA	NA	NA
SMIM12	1.80947615	0.12855112	0.13227693
GJB5	NA	NA	NA
GJB4	NA	NA	NA
RP1-34M23.[illegible]	NA	NA	NA
GJB3	NA	NA	NA
GJA4	NA	NA	NA
RP5-997D16.	NA	NA	NA
DLGAP3	0.87493888	0	0
RP11-244H3.	0.77200489	1.85E-235	5.31E-235
ZMYM6NB	NA	NA	NA

ZMYM6	2.41251528	2.92E-58	4.28E-58
ZMYM1	2.21951406	0	0
SFPQ	1.58467852	0	0
ZMYM4	2.15707249	2.53E-219	6.79E-219
ZMYM4-AS1	NA	NA	NA
KIAA0319L	2.04029864	2.12E-188	5.05E-188
NCDN	2.59908313	6.16E-236	1.78E-235
RP4-728D4.2	2.17847357	2.76E-27	3.48E-27
TFAP2E	2.12301345	4.14E-97	7.05E-97
PSMB2	1.60955736	2.68E-192	6.48E-192
C1orf216	1.98217472	5.90E-44	8.09E-44
CLSPN	1.92238372	3.31E-13	3.85E-13
RP11-435D7.	NA	NA	NA
AGO4	1.59915299	2.73E-276	9.50E-276
AGO1	1.84959505	1.68E-55	2.43E-55
RP4-789D17.	0.77200489	7.09E-12	8.16E-12
AGO3	1.92103542	3.17E-76	4.98E-76
RP4-665N4.8	NA	NA	NA
TEKT2	Inf	1.33E-269	4.50E-269
ADPRHL2	1.63055741	3.57E-223	9.75E-223
COL8A2	NA	NA	NA
TRAPPC3	1.80857794	8.87E-61	1.31E-60
MAP7D1	1.63015625	6.47E-95	1.10E-94
THRAP3	1.35341506	0	0
SH3D21	1.54400978	4.15E-23	5.11E-23
EVA1B	2.1167876	0.00068702	0.0007333
RP11-268J15	1.15800733	3.10E-276	1.08E-275
STK40	2.17421785	5.93E-129	1.13E-128
LSM10	2.16351988	9.16E-236	2.64E-235
OSCP1	Inf	2.64E-08	2.95E-08
MRPS15	1.83168318	3.82E-25	4.77E-25
CSF3R	0	0	0
RP4-614N24.	NA	NA	NA
GRIK3	NA	NA	NA
RP5-1180C18	NA	NA	NA
LINC01137	1.65429619	5.54E-291	2.08E-290
ZC3H12A	1.26829375	1.45E-52	2.07E-52
MEAF6	1.74382281	8.61E-178	1.96E-177
SNIP1	2.48757131	1.18E-41	1.61E-41
DNALI1	NA	NA	NA
GNL2	1.3259626	0	0
RSPO1	NA	NA	NA
C1orf109	1.83351161	7.19E-08	8.01E-08
CDCA8	1.68823047	8.65E-233	2.46E-232
EPHA10	NA	NA	NA

MANEAL	1.53046583	1.17E-142	2.34E-142
RP11-109P14	NA	NA	NA
YRDC	1.73315098	0.03068641	0.0319601
C1orf122	1.70883176	4.75E-43	6.49E-43
MTF1	1.90895755	6.34E-145	1.28E-144
INPP5B	2.49033835	4.33E-213	1.13E-212
RP11-109P14	NA	1.63E-46	2.26E-46
SF3A3	1.21681657	0	0
FHL3	1.55525257	0	0
UTP11L	1.17052113	0	0
POU3F1	1.54400978	4.27E-44	5.86E-44
RP5-884C9.2	Inf	0	0
LINC01343	NA	NA	NA
RP11-329N2	NA	NA	NA
RRAGC	1.93001222	2.08E-17	2.49E-17
RP5-864K19.	1.54400978	2.66E-39	3.57E-39
MYCBP	1.84045966	5.01E-186	1.18E-185
RP5-864K19.	NA	NA	NA
GJA9	NA	NA	NA
RHBDL2	NA	1.25E-224	3.43E-224
AKIRIN1	1.67392646	3.58E-278	1.26E-277
NDUFS5	1.94084762	6.13E-05	6.62E-05
MACF1	1.76882439	6.94E-46	9.60E-46
RP11-420K8.	NA	NA	NA
BMP8A	NA	NA	NA
RP11-69E11.	1.54400978	3.74E-150	7.74E-150
PABPC4	2.00864235	8.75E-86	1.43E-85
RP11-69E11.	NA	NA	NA
HEYL	2.12301345	4.09E-109	7.31E-109
RP1-144F13.	NA	NA	NA
NT5C1A	Inf	0	0
HPCAL4	NA	1.11E-192	2.69E-192
PPIE	1.39040674	0	0
BMP8B	1.39696123	8.80E-182	2.04E-181
OXCT2	2.70201711	2.40E-112	4.34E-112
RP1-118J21.	1.59915299	1.87E-05	2.03E-05
TRIT1	1.90618491	6.81E-47	9.47E-47
MYCL	2.91646292	1.73E-41	2.34E-41
RP1-118J21.	NA	0	0
MFSD2A	2.31601467	8.08E-94	1.36E-93
CAP1	1.35506786	0	0
PPT1	1.23214025	0	0
RLF	2.20164358	3.25E-261	1.06E-260
TMCO2	0	0	0
RP1-39G22.	2.00721271	4.69E-183	1.09E-182

ZMPSTE24	2.10156887	5.43E-55	7.83E-55
COL9A2	5.01803178	1.94E-28	2.46E-28
SMAP2	2.40447356	9.87E-247	2.99E-246
ZFP69B	1.737011	6.67E-19	8.03E-19
ZFP69	2.26776436	6.64E-07	7.33E-07
RP11-656D1(	1.54400978	0.99416216	0.99427177
EXO5	2.52188264	1.87E-170	4.17E-170
RP11-656D1(	NA	NA	NA
RP11-656D1(	NA	NA	NA
ZNF684	2.03159182	0.25926131	0.26461427
RIMS3	3.08801956	1.11E-102	1.94E-102
RP4-739H11.	NA	NA	NA
NFYC-AS1	3.60268949	0	0
NFYC	1.39145792	2.22E-290	8.33E-290
KCNQ4	2.52656146	8.50E-145	1.72E-144
CITED4	1.56974328	1.27E-92	2.13E-92
RP5-1066H1:	NA	NA	NA
CTPS1	1.43894403	0	0
SLFNL1-AS1	2.31601467	3.04E-29	3.88E-29
SLFNL1	0	4.98E-185	1.17E-184
SCMH1	2.15518032	1.68E-87	2.76E-87
RP11-399E6.	NA	NA	NA
FOXO6	0.38600244	1.94E-160	4.16E-160
RP11-399E6.	NA	NA	NA
EDN2	NA	NA	NA
HIVEP3	0.84218715	0	0
RP11-486B1(	NA	NA	NA
GUCA2B	NA	NA	NA
GUCA2A	NA	NA	NA
FOXJ3	2.57334963	0	0
RIMKLA	2.85306155	0	0
ZMYND12	NA	NA	NA
PPCS	1.560915	0	0
CCDC30	4.63202934	2.66E-12	3.07E-12
PPIH	1.86308538	1.32E-101	2.28E-101
RP5-994D16.	1.737011	0.00605814	0.0063788
YBX1	1.92958387	1.19E-06	1.31E-06
CLDN19	NA	NA	NA
P3H1	1.74836402	1.11E-179	2.54E-179
C1orf50	2.01649365	1.32E-144	2.66E-144
RP5-994D16.	NA	NA	NA
RP5-994D16.	NA	NA	NA
SVBP	1.87339853	7.68E-20	9.30E-20
ERMAP	1.45823146	3.51E-107	6.23E-107
RP11-342M1	6.56204156	5.65E-240	1.66E-239

ZNF691	1.48886657	0	0
SLC2A1	1.72469178	6.71E-94	1.13E-93
SLC2A1-AS1	0.77200489	4.66E-05	5.05E-05
FAM183A	NA	NA	NA
EBNA1BP2	1.49166217	4.26E-272	1.46E-271
CFAP57	Inf	0	0
TMEM125	NA	NA	NA
C1orf210	0	0	0
TIE1	2.05867971	1.86E-120	3.46E-120
MPL	NA	NA	NA
RP1-92O14.3	1.23520782	1.09E-54	1.57E-54
CDC20	1.07080412	0	0
ELOVL1	1.54173918	3.03E-298	1.18E-297
MED8	1.54694516	1.47E-235	4.23E-235
RP1-92O14.6	Inf	0	0
SZT2	3.86002445	3.40E-154	7.12E-154
SZT2-AS1	NA	NA	NA
HYI	Inf	6.70E-275	2.32E-274
HYI-AS1	0.77200489	2.68E-250	8.25E-250
PTPRF	2.22165852	1.52E-268	5.09E-268
KDM4A	3.04941932	7.59E-102	1.32E-101
KDM4A-AS1	1.59079796	0.00200517	0.00212636
ST3GAL3	1.85281174	7.10E-93	1.19E-92
RP11-184I16	NA	NA	NA
ARTN	0	1.11E-296	4.29E-296
RP11-7O11.3	0.57900367	0	0
IPO13	2.18734719	7.99E-51	1.13E-50
DPH2	1.82748033	4.73E-119	8.75E-119
ATP6V0B	1.81848757	2.12E-19	2.55E-19
B4GALT2	2.29395739	2.24E-259	7.21E-259
CCDC24	2.41251528	1.32E-61	1.95E-61
SLC6A9	1.737011	1.21E-133	2.35E-133
RP5-1198O2(	3.08801956	6.99E-32	9.02E-32
KLF17	NA	NA	NA
KLF18	NA	NA	NA
DMAP1	1.17045903	0	0
ERI3	1.69261757	6.42E-293	2.44E-292
RNF220	2.3055112	1.94E-266	6.45E-266
TMEM53	0	0	0
C1orf228	1.35851037	0	0
KIF2C	1.48141479	0	0
RP11-269F19	1.54400978	8.91E-37	1.18E-36
RPS8	2.05690871	2.86E-217	7.62E-217
BEST4	NA	9.10E-19	1.10E-18
PLK3	1.43142573	4.66E-78	7.36E-78

TCTEX1D4	NA	5.63E-42	7.64E-42
BTBD19	Inf	4.98E-241	1.47E-240
PTCH2	1.02933985	3.80E-223	1.04E-222
EIF2B3	1.03052029	0	0
HECTD3	2.49766288	1.47E-159	3.14E-159
UROD	1.40258189	9.35E-289	3.47E-288
ZSWIM5	NA	NA	NA
LINC01144	2.31601467	5.13E-31	6.60E-31
HPDL	NA	NA	NA
MUTYH	1.59664648	3.08E-143	6.20E-143
TOE1	1.3757523	0	0
TESK2	2.31601467	2.68E-53	3.83E-53
CCDC163P	2.31601467	3.98E-60	5.87E-60
MMACHC	2.44468215	2.59E-11	2.96E-11
PRDX1	1.10025999	0	0
AKR1A1	1.48797717	0	0
NASP	1.35369327	0	0
CCDC17	1.54400978	0	0
GPBP1L1	2.04932207	7.44E-14	8.68E-14
TMEM69	1.84251834	8.03E-96	1.36E-95
IPP	2.31601467	1.38E-129	2.64E-129
MAST2	2.36142672	1.99E-42	2.70E-42
PIK3R3	2.89501834	6.57E-26	8.23E-26
PIK3R3	NA	NA	NA
RP4-533D7.4	NA	NA	NA
RP4-533D7.5	Inf	6.54E-58	9.56E-58
TSPAN1	NA	NA	NA
RP11-322N2	NA	NA	NA
POMGNT1	1.83476487	9.71E-07	1.07E-06
LURAP1	2.02191757	1.03E-190	2.47E-190
RAD54L	1.817947	7.98E-09	8.96E-09
LRRC41	1.44899379	0	0
UQCRH	2.15898104	0	0
NSUN4	2.17680067	7.50E-72	1.16E-71
FAAH	0.77200489	3.01E-71	4.64E-71
DMBX1	NA	NA	NA
RP11-49P4.7	NA	NA	NA
MKNK1-AS1	NA	NA	NA
KNCN	NA	NA	NA
MKNK1	2.11382291	0	0
MOB3C	2.00721271	0.00250532	0.00265255
ATPAF1	1.98666105	1.57E-30	2.01E-30
TEX38	NA	NA	NA
EFCAB14-AS	NA	NA	NA
EFCAB14	2.63389904	0	0

CYP4B1	NA	NA	NA
CYP4A11	NA	NA	NA
CYP4X1	NA	NA	NA
CYP4Z1	NA	NA	NA
CYP4A22-AS1	NA	1.50E-93	2.53E-93
CYP4A22	NA	NA	NA
LINC00853	2.89501834	3.68E-27	4.63E-27
PDZK1IP1	Inf	4.41E-192	1.06E-191
TAL1	2.1167876	1.10E-27	1.39E-27
RP1-18D14.7	Inf	0	0
STIL	2.07338456	0.00012082	0.00013014
CMPK1	2.06305988	2.52E-19	3.04E-19
LINC01389	Inf	1.98E-48	2.78E-48
FOXE3	NA	8.34E-74	1.30E-73
FOXD2-AS1	2.50129584	1.05E-98	1.80E-98
FOXD2	2.0784747	2.46E-280	8.73E-280
RP11-543D5.	NA	NA	NA
TRABD2B	NA	NA	NA
RP11-543D5.	NA	NA	NA
RP11-330M1	NA	NA	NA
RP4-683M8.2	NA	NA	NA
RP11-193P11	NA	NA	NA
AL356289.1	NA	NA	NA
RP5-1024N4.	NA	NA	NA
SLC5A9	NA	NA	NA
RP5-1024N4.	NA	NA	NA
SPATA6	NA	NA	NA
AGBL4	NA	NA	NA
BEND5	1.28667482	4.28E-197	1.05E-196
RP11-141A19	NA	NA	NA
RP11-141A19	NA	NA	NA
ELAVL4	NA	NA	NA
RP11-567C20	NA	NA	NA
RP11-567C20	NA	NA	NA
DMRTA2	NA	NA	NA
FAF1	1.31165879	0	0
RP5-850O15.	NA	NA	NA
CDKN2C	1.74572719	1.55E-154	3.26E-154
C1orf185	NA	NA	NA
LINC01562	Inf	2.16E-154	4.53E-154
RNF11	1.77561125	9.99E-54	1.43E-53
RP11-296A18	NA	1.03E-46	1.43E-46
TTC39A	1.54400978	4.08E-118	7.53E-118
TTC39A-AS1	0	0	0
EPS15	2.7254112	0	0

RP11-253A2(	NA	0	0
RP11-191G2	2.42630108	1.32E-237	3.84E-237
OSBPL9	1.72395077	1.78E-50	2.52E-50
NRDC	1.85938199	4.06E-104	7.12E-104
RP4-657D16.	NA	0	0
RP4-657D16.	NA	3.23E-202	8.09E-202
RAB3B	1.30021876	1.04E-272	3.56E-272
TXNDC12	1.58786058	0	0
KTI12	1.37183603	0	0
RP11-91A18.	NA	NA	NA
TXNDC12-AS	NA	NA	NA
BTF3L4	1.50829425	0	0
ZFYVE9	2.73710825	1.47E-09	1.66E-09
CC2D1B	3.28102078	1.16E-279	4.10E-279
RP11-155O1	2.31601467	2.73E-143	5.49E-143
ORC1	1.57408789	2.42E-07	2.69E-07
PRPF38A	1.65969927	0	0
ZCCHC11	1.7479356	1.05E-25	1.32E-25
RP11-25O10.	0	1.06E-204	2.70E-204
GPX7	2.01825682	0.00037134	0.00039794
FAM159A	0.38600244	0	0
COA7	1.93302787	9.82E-97	1.67E-96
ZYG11B	2.66692598	0.83751794	0.84066894
ZYG11A	Inf	5.94E-08	6.62E-08
RP4-631H13.	NA	NA	NA
ECHDC2	NA	5.82E-10	6.60E-10
SCP2	1.74764006	0	0
AL445183.1	NA	NA	NA
PODN	NA	NA	NA
RP11-334A1	NA	NA	NA
SLC1A7	NA	NA	NA
RP11-334A1	NA	NA	NA
CPT2	1.36855412	4.81E-107	8.54E-107
RP5-1024G6.	0.77200489	7.66E-05	8.27E-05
C1orf123	1.81932035	0	0
RP5-1024G6.	Inf	0	0
MAGOH	1.80275576	4.31E-58	6.30E-58
RP5-1024G6.	1.93001222	0.19848306	0.20326727
LRP8	1.99284983	3.07E-98	5.26E-98
RP4-784A16.	NA	NA	NA
RP4-784A16.	NA	1.72E-145	3.49E-145
RP4-784A16.	NA	NA	NA
RP4-784A16.	Inf	0	0
RP11-117D2	NA	NA	NA
RP11-117D2	NA	NA	NA

DMRTB1	NA	NA	NA
GLIS1	0	0	0
NDC1	2.26202831	9.64E-197	2.37E-196
YIPF1	1.79409587	8.84E-98	1.51E-97
DIO1	NA	NA	NA
HSPB11	2.02272553	1.68E-16	1.99E-16
LRRC42	1.02265583	0	0
LDLRAD1	NA	NA	NA
TMEM59	1.5910495	1.75E-285	6.38E-285
TCEANC2	2.20879177	4.65E-54	6.68E-54
CDCP2	NA	NA	NA
RP11-446E24	NA	7.47E-57	1.09E-56
CYB5RL	1.79215421	4.66E-07	5.15E-07
MRPL37	1.38235064	0	0
SSBP3	1.88805544	2.06E-105	3.62E-105
SSBP3-AS1	0	0	0
RP5-997D24.	NA	NA	NA
RP4-705F19.	NA	NA	NA
RP5-866L20.	NA	NA	NA
ACOT11	1.24378566	3.50E-59	5.14E-59
FAM151A	NA	NA	NA
MROH7	NA	NA	NA
MROH7-TTC4	NA	NA	NA
TTC4	2.47041565	1.75E-30	2.25E-30
PARS2	2.27925253	1.45E-16	1.73E-16
TTC22	NA	NA	NA
RP11-67L3.2	NA	NA	NA
LEXM	NA	NA	NA
DHCR24	2.29711945	0	0
RP11-67L3.5	Inf	1.26E-187	2.98E-187
RP11-67L3.4	1.737011	5.27E-91	8.78E-91
TMEM61	NA	NA	NA
RP11-12C17.	NA	NA	NA
BSND	NA	NA	NA
PCSK9	NA	NA	NA
USP24	2.08727248	6.94E-27	8.73E-27
RP11-101C11	NA	NA	NA
RP11-90C4.1	NA	NA	NA
RP11-90C4.2	NA	NA	NA
RP11-90C4.3	NA	NA	NA
RP1-158P9.1	NA	NA	NA
RP11-504A18	NA	NA	NA
RP4-710M16	NA	NA	NA
PLPP3	1.54400978	4.54E-254	1.42E-253
PRKAA2	NA	NA	NA

C1orf168	NA	NA	NA
RP11-378I13	NA	NA	NA
C8A	NA	NA	NA
C8B	0	4.96E-08	5.53E-08
RP5-1103B4.	NA	NA	NA
DAB1	NA	NA	NA
RP6-102O10.	NA	NA	NA
DAB1-AS1	NA	NA	NA
RP11-393I23	NA	NA	NA
OMA1	1.30383048	3.11E-235	8.95E-235
TACSTD2	NA	2.59E-47	3.61E-47
MYSM1	1.69841076	5.55E-21	6.76E-21
JUN	0.48250306	0	0
LINC01135	NA	NA	NA
RP11-63G10.	Inf	5.94E-217	1.58E-216
RP11-63G10.	NA	NA	NA
RP4-794H19.	Inf	0.59728523	0.60290149
LINC01358	NA	NA	NA
RP11-145M4	NA	NA	NA
RP11-145M4	NA	NA	NA
RP11-467C18	NA	0	0
FGGY	1.72216475	4.25E-27	5.35E-27
RP4-782L23.:	NA	NA	NA
HOOK1	NA	2.63E-211	6.84E-211
CYP2J2	NA	NA	NA
C1orf87	NA	NA	NA
RP11-575B7.	NA	NA	NA
RP11-776H1:	NA	NA	NA
RP11-436K8.	NA	NA	NA
NFIA	1.72216475	1.74E-109	3.11E-109
NFIA-AS2	NA	NA	NA
TM2D1	1.74210915	2.51E-123	4.70E-123
RP11-430G1	3.73135697	5.01E-297	1.94E-296
INADL	6.94804401	0	0
L1TD1	Inf	2.00E-52	2.85E-52
KANK4	NA	0	0
USP1	1.922994	1.72E-15	2.03E-15
DOCK7	1.4054448	3.67E-11	4.20E-11
ANGPTL3	NA	NA	NA
RP11-230B2:	Inf	4.25E-301	1.69E-300
ATG4C	1.66463554	6.97E-293	2.64E-292
RP4-771M4.:	NA	NA	NA
RP11-5P4.1	NA	NA	NA
RP11-5P4.2	NA	NA	NA
RP11-5P4.3	NA	NA	NA

RP11-335E6.	NA	NA	NA
RP11-335E6.	NA	NA	NA
FOXD3-AS1	NA	NA	NA
FOXD3	NA	1.11E-192	2.69E-192
ALG6	1.84874855	5.81E-13	6.74E-13
ITGB3BP	1.75869653	1.39E-214	3.65E-214
EFCAB7	0.51466993	6.93E-206	1.76E-205
PGM1	1.64051039	1.23E-269	4.14E-269
ROR1	0.77200489	3.17E-129	6.05E-129
ROR1-AS1	NA	NA	NA
UBE2U	NA	NA	NA
CACHD1	3.52916521	3.79E-161	8.16E-161
RAVER2	3.17379788	1.25E-298	4.90E-298
JAK1	1.46129497	0	0
RP4-535B20.	NA	NA	NA
RP4-535B20.	NA	1.10E-21	1.34E-21
AK4	2.35971306	5.62E-138	1.11E-137
DNAJC6	1.54400978	1.65E-13	1.92E-13
RP5-1044H5.	NA	NA	NA
LEPROT	1.55473207	1.68E-154	3.53E-154
LEPR	Inf	1.59E-169	3.53E-169
PDE4B	0.77200489	0	0
RP11-397C1	NA	NA	NA
SGIP1	Inf	0	0
RP4-598P13.	NA	NA	NA
TCTEX1D1	1.42820905	1.22E-203	3.07E-203
INSL5	NA	NA	NA
WDR78	1.02933985	2.51E-07	2.78E-07
RP11-342H2	NA	NA	NA
MIER1	2.02578727	1.11E-90	1.85E-90
SLC35D1	2.58995189	4.16E-126	7.87E-126
C1orf141	NA	NA	NA
IL23R	0.90067237	0.0004354	0.000466
IL12RB2	NA	5.46E-39	7.30E-39
SERBP1	1.55253349	0	0
RP11-393N2	NA	NA	NA
GADD45A	1.49575947	1.14E-141	2.29E-141
GNG12	0.51466993	2.53E-88	4.16E-88
GNG12-AS1	NA	NA	NA
DIRAS3	NA	NA	NA
WLS	NA	NA	NA
RPE65	NA	NA	NA
DEPDC1	1.77561125	6.38E-192	1.54E-191
RP4-694A7.2	Inf	0	0
DEPDC1-AS1	1.25450795	4.07E-149	8.39E-149

RP11-424D14	NA	NA	NA
RP11-74K19.	NA	NA	NA
RP3-380B4.1	NA	NA	NA
LRRC7	0.38600244	8.13E-118	1.50E-117
RP11-181B18	NA	NA	NA
LRRC40	2.15810458	0.00432106	0.00456063
SRSF11	1.54334426	0	0
RP4-677H15.	NA	NA	NA
ANKRD13C	2.75026742	6.46E-08	7.20E-08
HHLA3	3.08801956	1.56E-13	1.82E-13
RP11-180O5.	NA	NA	NA
AL158839.1	NA	NA	NA
CTH	0.68622657	0	0
RP11-42O15.	Inf	0.68239366	0.6873951
RP5-952N6.1	NA	NA	NA
RP11-99H8.1	NA	NA	NA
PTGER3	NA	1.53E-43	2.10E-43
RP3-333A15.	NA	NA	NA
ZRANB2-AS1	NA	5.83E-07	6.43E-07
ZRANB2	2.33531479	4.24E-220	1.14E-219
NEGR1	1.6887607	0	0
RP11-82L20.	NA	NA	NA
RP4-660H19.	NA	NA	NA
RP11-262K1.	NA	NA	NA
LINC01360	NA	NA	NA
RP4-788P17.	NA	NA	NA
LRRIQ3	3.91940944	3.05E-302	1.22E-301
FPGT	2.63768337	1.51E-51	2.15E-51
FPGT-TNNI3	NA	6.77E-165	1.48E-164
TNNI3K	NA	NA	NA
RP11-439H8.	NA	NA	NA
RP4-650F12.	NA	NA	NA
LRRC53	NA	NA	NA
ERICH3	NA	9.19E-115	1.67E-114
ERICH3-AS1	NA	NA	NA
RP11-17E13.	NA	2.44E-23	3.01E-23
CRYZ	1.16557601	0	0
TYW3	2.02312288	4.98E-88	8.19E-88
RP11-93N20.	NA	NA	NA
RP11-510C10	NA	NA	NA
RP11-510C10	NA	NA	NA
LHX8	NA	NA	NA
RP11-510C10	NA	NA	NA
SLC44A5	NA	NA	NA
RP4-682C21.	NA	NA	NA

ACADM	1.41468916	0	0
RABGGTB	2.48757131	0	0
MSH4	NA	NA	NA
ASB17	NA	NA	NA
RP11-550H2.	NA	NA	NA
RP11-550H2.	NA	NA	NA
ST6GALNAC3	NA	NA	NA
RP5-1102E8.	NA	NA	NA
RP11-415A2(	NA	NA	NA
ST6GALNAC5	NA	NA	NA
RP4-564M11	NA	NA	NA
PIGK	1.16433524	0	0
AK5	0.51466993	5.87E-210	1.52E-209
RP11-375A5.	NA	NA	NA
ZZZ3	2.2831634	1.44E-59	2.11E-59
USP33	2.29621967	1.53E-200	3.80E-200
FAM73A	2.75026742	0	0
NEXN-AS1	1.54400978	1.64E-07	1.82E-07
NEXN	2.00532978	2.98E-06	3.27E-06
FUBP1	1.97618477	3.43E-202	8.61E-202
DNAJB4	1.75845558	1.29E-66	1.95E-66
GIPC2	NA	NA	NA
RP11-386I14	1.93001222	4.28E-121	7.97E-121
PTGFR	NA	NA	NA
IFI44L	NA	0.07877509	0.08139988
IFI44	4.63202934	2.08E-102	3.61E-102
ADGRL4	NA	NA	NA
RP4-726F1.1	NA	NA	NA
RP4-726F1.2	NA	NA	NA
RP11-339A1:	NA	NA	NA
RP11-339D2:	NA	NA	NA
RP5-933B4.1	NA	NA	NA
RP5-887A10.	NA	NA	NA
ADGRL2	NA	NA	NA
RP5-837I24.2	NA	NA	NA
RP5-837I24.4	NA	NA	NA
RP11-147G1(	NA	NA	NA
LINC01362	NA	NA	NA
LINC01361	NA	NA	NA
RP11-413G1!	NA	NA	NA
RP11-475O6.	NA	NA	NA
RP5-836J3.1	NA	NA	NA
TTLL7	3.20678954	2.50E-11	2.87E-11
RP11-486G1!	1.40221296	1.26E-05	1.38E-05
PRKACB	2.06563471	4.91E-11	5.61E-11

SAMD13	1.67267726	5.47E-264	1.80E-263
DNASE2B	Inf	3.37E-260	1.09E-259
RPF1	1.31873622	0	0
GNG5	1.89666646	5.04E-40	6.77E-40
CTBS	1.75455657	8.97E-13	1.04E-12
LINC01555	NA	NA	NA
SSX2IP	1.86775377	3.75E-43	5.13E-43
LPAR3	NA	3.54E-35	4.64E-35
MCOLN2	1.29837186	3.77E-09	4.25E-09
WDR63	NA	NA	NA
MCOLN3	2.1378597	1.09E-111	1.97E-111
SYDE2	1.87486902	4.86E-23	5.99E-23
C1orf52	1.62273396	3.86E-86	6.30E-86
BCL10	1.42119082	0	0
RP11-131L23	Inf	2.42E-244	7.25E-244
DDAH1	2.86744673	4.62E-268	1.55E-267
RP11-131L23	NA	NA	NA
RP4-621F18.	Inf	0	0
RP11-290M5	Inf	0.00078262	0.0008348
CYR61	1.3896088	8.21E-17	9.77E-17
RP11-290M5	NA	NA	NA
ZNHIT6	2.16562411	8.15E-68	1.24E-67
COL24A1	4.63202934	2.62E-57	3.82E-57
ODF2L	1.9673673	7.99E-278	2.80E-277
CLCA2	NA	NA	NA
CLCA1	NA	NA	NA
CLCA4	NA	NA	NA
RP4-651E10.	NA	NA	NA
RP4-612B15.	1.54400978	6.29E-05	6.80E-05
SH3GLB1	1.79020032	0.01839275	0.01922238
15-Sep	1.71120046	1.58E-179	3.63E-179
HS2ST1	2.02767549	1.15E-39	1.55E-39
RP4-604K5.3	NA	NA	NA
RP5-1052I5.2	NA	NA	NA
LINC01140	Inf	0	0
LMO4	1.71669508	1.30E-28	1.65E-28
LINC01364	NA	NA	NA
RP5-1027O1	NA	NA	NA
PKN2-AS1	Inf	0.0405526	0.04212455
PKN2	1.62792335	4.99E-88	8.21E-88
GTF2B	1.16352166	0	0
CCBL2	0.77200489	2.20E-232	6.26E-232
RBMXL1	1.76886557	4.83E-57	7.02E-57
GBP3	Inf	3.44E-98	5.89E-98
GBP1	NA	NA	NA

GBP2	NA	NA	NA
GBP7	NA	NA	NA
GBP4	0.96500611	2.61E-134	5.09E-134
GBP5	0	2.50E-58	3.66E-58
RP4-620F22.	NA	NA	NA
GBP6	NA	NA	NA
LRRC8B	1.85281174	1.40E-26	1.76E-26
FLJ27354	1.31240831	1.41E-62	2.10E-62
LRRC8C	1.75697665	3.73E-34	4.87E-34
RP11-302M6	NA	NA	NA
RP5-943J3.2	2.31601467	1.70E-76	2.67E-76
LRRC8D	2.54761614	0	0
RP11-302M6	NA	NA	NA
ZNF326	1.67434827	1.40E-290	5.26E-290
RP5-827O9.1	NA	NA	NA
BARHL2	Inf	0	0
RP4-665J23.:	2.90788509	4.70E-187	1.11E-186
RP4-665J23.:	0	0	0
RP4-665J23.4	NA	NA	NA
ZNF644	1.96569312	4.61E-53	6.59E-53
HFM1	NA	NA	NA
CDC7	1.38148243	7.76E-107	1.38E-106
TGFBR3	0	4.55E-218	1.21E-217
BRDT	Inf	0	0
EPHX4	1.67267726	1.73E-228	4.83E-228
SETSIP	NA	8.32E-97	1.42E-96
BTBD8	3.08801956	1.68E-05	1.82E-05
KIAA1107	1.54400978	7.51E-29	9.54E-29
RP4-621B10.	NA	NA	NA
C1orf146	NA	NA	NA
GLMN	1.15188031	0	0
RPAP2	1.85890651	6.70E-10	7.60E-10
GFI1	2.41894866	3.65E-52	5.20E-52
EVI5	1.16790483	3.34E-218	8.92E-218
RPL5	1.67235587	2.29E-185	5.38E-185
FAM69A	0.25733496	4.41E-77	6.95E-77
MTF2	1.79694048	1.32E-34	1.73E-34
TMED5	1.80992258	7.81E-22	9.55E-22
CCDC18	1.41666877	0	0
DR1	1.81835863	1.10E-07	1.23E-07
FNBP1L	1.01159261	0	0
BCAR3	0	0	0
RP5-1033H2:	NA	NA	NA
RP4-561L24.:	NA	1.11E-17	1.33E-17
DNTTIP2	1.34296684	0	0

GCLM	1.95906617	5.48E-83	8.84E-83
ABCA4	NA	NA	NA
RP11-78O9.1	NA	NA	NA
ARHGAP29	NA	0	0
RP11-148B18	NA	NA	NA
RP11-148B18	NA	NA	NA
ABCD3	1.60924963	0	0
F3	1.45823146	7.64E-08	8.50E-08
RP11-86H7.6	NA	NA	NA
SLC44A3	NA	7.45E-260	2.40E-259
CNN3	0.77200489	2.27E-106	4.01E-106
RP4-639F20.	NA	NA	NA
ALG14	2.32826872	1.90E-47	2.65E-47
RP11-313A24	NA	NA	NA
TMEM56	1.54400978	8.42E-86	1.37E-85
TMEM56-RW	4.37469438	0.32136847	0.32730403
RP11-57H12.	NA	NA	NA
RWDD3	1.15800733	5.48E-87	8.97E-87
RP11-57H12.	NA	NA	NA
RP4-586O15.	NA	NA	NA
RP11-14O19.	NA	NA	NA
RP11-14O19.	NA	NA	NA
FLJ31662	NA	NA	NA
RP11-286B14	NA	NA	NA
RP11-286B14	NA	NA	NA
RP11-147C23	NA	NA	NA
RP5-898J17.1	NA	NA	NA
PTBP2	2.79851773	1.06E-05	1.15E-05
DPYD	0.38600244	4.23E-53	6.05E-53
DPYD-AS1	NA	NA	NA
DPYD-AS2	NA	NA	NA
RP11-272L13	NA	NA	NA
RP11-490G2.	NA	NA	NA
MIR137HG	NA	NA	NA
RP5-1070A16	NA	NA	NA
SNX7	NA	5.01E-114	9.10E-114
PLPPR5	NA	NA	NA
RP5-896L10.1	NA	NA	NA
PLPPR4	NA	NA	NA
RP11-413P11	NA	NA	NA
PALMD	NA	NA	NA
FRRS1	1.54400978	0	0
AGL	2.00238768	3.49E-52	4.96E-52
RP5-884G6.2	0.77200489	1.24E-178	2.84E-178
SLC35A3	2.55821228	0	0

MFSD14A	1.76917787	9.08E-33	1.18E-32
RP4-714D9.2	NA	NA	NA
SASS6	2.1766249	1.69E-112	3.06E-112
TRMT13	1.68536279	7.61E-66	1.15E-65
LRRC39	NA	NA	NA
DBT	2.80209182	0	0
RTCA-AS1	0	4.31E-53	6.16E-53
RTCA	1.56846242	1.22E-184	2.87E-184
RP5-837M10	NA	NA	NA
CDC14A	1.98515543	0.06349732	0.06574786
RP5-837M10	NA	NA	NA
GPR88	NA	NA	NA
LINC01349	NA	NA	NA
VCAM1	NA	NA	NA
EXTL2	1.95271825	7.39E-25	9.20E-25
RP4-549L20.:	2.7792176	0	0
SLC30A7	2.56775539	3.62E-296	1.40E-295
DPH5	1.6733304	4.11E-293	1.56E-292
RP11-421L21	0.77200489	7.05E-182	1.64E-181
RP11-421L21	1.93577346	8.31E-96	1.41E-95
RP4-575N6.4	NA	NA	NA
S1PR1	NA	NA	NA
LINC01307	NA	NA	NA
RP11-411H5.	Inf	0.03067555	0.03195061
OLFM3	NA	NA	NA
RP11-202K23	NA	NA	NA
RP5-936J12.:	NA	NA	NA
COL11A1	NA	NA	NA
RP11-153F1.	NA	NA	NA
RP11-153F1.	NA	NA	NA
RNPC3	1.61932733	1.56E-06	1.72E-06
AMY2B	NA	3.11E-09	3.51E-09
AMY2A	NA	NA	NA
AMY1A	NA	NA	NA
AMY1B	NA	NA	NA
AMY1C	NA	NA	NA
RP11-251P6.	NA	NA	NA
RP11-24P14.	NA	NA	NA
RP5-947P14.	NA	NA	NA
RP11-233E12	NA	NA	NA
PRMT6	1.88894814	4.15E-117	7.62E-117
NTNG1	NA	NA	NA
VAV3	2.11586525	8.46E-15	9.93E-15
VAV3-AS1	1.54400978	0.00093431	0.0009955
RP11-356N1.	0	5.23E-289	1.95E-288

SLC25A24	1.61212786	2.10E-231	5.93E-231
RP11-483I13	2.16161369	2.43E-42	3.31E-42
NBPF4	NA	NA	NA
RP11-483I13	NA	NA	NA
NBPF6	NA	NA	NA
RP11-131J3.1	NA	NA	NA
FAM102B	1.72565799	9.09E-89	1.50E-88
HENMT1	NA	NA	NA
PRPF38B	1.59214865	0	0
FNDC7	NA	NA	NA
RP11-293A1(	NA	NA	NA
STXBP3	1.79491137	2.91E-26	3.64E-26
AKNAD1	NA	NA	NA
RP11-475E11	NA	0	0
SPATA42	NA	NA	NA
GPSM2	1.31240831	6.02E-50	8.50E-50
CLCC1	1.75659084	3.17E-09	3.57E-09
WDR47	2.42630108	1.21E-145	2.46E-145
TAF13	2.75026742	1.85E-263	6.08E-263
RP5-1065J22	2.09544184	4.79E-89	7.91E-89
TMEM167B	2.35277681	6.09E-170	1.35E-169
SCARNA2	NA	NA	NA
C1orf194	NA	NA	NA
KIAA1324	NA	0	0
SARS	1.42010776	0	0
CELSR2	1.7094394	5.99E-69	9.15E-69
PSRC1	1.38052639	1.73E-307	7.14E-307
MYBPHL	NA	0	0
SORT1	1.76748488	0	0
PSMA5	1.76268928	8.05E-72	1.24E-71
SYPL2	0.46320293	6.92E-257	2.20E-256
ATXN7L2	2.26087146	3.16E-51	4.48E-51
CYB561D1	2.01579055	5.64E-31	7.24E-31
AMIGO1	Inf	0	0
GPR61	NA	5.84E-53	8.34E-53
RP5-1160K1.	NA	NA	NA
GNAI3	1.32873919	0	0
GNAT2	NA	1.13E-27	1.42E-27
AMPD2	1.49420301	8.96E-44	1.23E-43
RP5-1160K1.	NA	NA	NA
GSTM4	1.21805216	0	0
GSTM2	NA	NA	NA
GSTM1	NA	NA	NA
AC000032.2	NA	NA	NA
GSTM5	NA	NA	NA

RP4-735C1.4	NA	NA	NA
GSTM3	NA	3.95E-157	8.37E-157
EPS8L3	NA	NA	NA
RP4-735C1.6	NA	NA	NA
RP11-195M1	NA	NA	NA
RP11-195M1	NA	NA	NA
CSF1	2.00721271	2.06E-83	3.33E-83
AHCYL1	2.06599036	1.18E-30	1.51E-30
STRIP1	1.65429619	2.09E-10	2.38E-10
RP4-773N10.	2.12301345	2.03E-243	6.08E-243
ALX3	NA	NA	NA
LINC01397	NA	NA	NA
UBL4B	NA	NA	NA
SLC6A17	NA	NA	NA
RP5-1028L1C	NA	NA	NA
RP5-1028L1C	NA	NA	NA
KCNC4-AS1	NA	0.02351516	0.02453485
KCNC4	2.57334963	7.94E-20	9.62E-20
RP5-1074L1.	5.40403423	2.15E-280	7.63E-280
RBM15	2.20793399	6.03E-113	1.09E-112
SLC16A4	1.14122462	8.26E-97	1.41E-96
RP5-1074L1.	2.12301345	5.67E-263	1.86E-262
LAMTOR5	2.17728992	2.62E-127	4.97E-127
PROK1	NA	NA	NA
RP11-470L1S	NA	NA	NA
RP11-470L1S	NA	NA	NA
RP11-470L1S	NA	NA	NA
KCNA10	NA	NA	NA
KCNA2	NA	NA	NA
RP11-284N8.	NA	NA	NA
KCNA3	NA	NA	NA
CD53	1.13790304	0	0
RP11-96K19.	NA	NA	NA
LRIF1	1.70507555	4.14E-63	6.18E-63
RP11-96K19.	2.57334963	0	0
DRAM2	1.37595429	0	0
CEPT1	1.62348087	8.29E-106	1.46E-105
RP5-1180E21	NA	NA	NA
RP5-1180E21	2.70201711	7.96E-89	1.31E-88
DENND2D	3.86002445	2.36E-125	4.45E-125
CHI3L2	NA	NA	NA
CHIA	NA	NA	NA
RP5-1125M8	NA	NA	NA
PIFO	NA	0	0
OVGP1	Inf	2.47E-160	5.31E-160

WDR77	1.85247826	4.41E-52	6.27E-52
ATP5F1	1.43717113	1.94E-247	5.90E-247
C1orf162	0.77200489	5.75E-18	6.89E-18
TMIGD3	0.51466993	1.19E-193	2.90E-193
ADORA3	0	2.01E-85	3.27E-85
RAP1A	1.79131871	8.81E-83	1.42E-82
LINC01160	Inf	0	0
FAM212B	1.26328073	1.40E-28	1.78E-28
FAM212B-AS	NA	0	0
DDX20	1.44107579	5.38E-216	1.42E-215
KCND3	Inf	1.65E-104	2.90E-104
KCND3-AS1	NA	NA	NA
RP11-88H9.2	NA	NA	NA
RP5-965F6.2	NA	NA	NA
CTTNBP2NL	3.4188788	2.44E-100	4.21E-100
WNT2B	3.86002445	4.42E-218	1.18E-217
RP4-671G15.	NA	2.51E-09	2.83E-09
ST7L	1.98264892	1.03E-40	1.38E-40
CAPZA1	1.73489011	4.72E-162	1.02E-161
MOV10	1.64793352	4.43E-159	9.44E-159
RP11-426L16	3.86002445	6.83E-72	1.06E-71
RHOC	1.7010526	7.41E-166	1.62E-165
RP11-426L16	NA	NA	NA
PPM1J	1.64051039	1.12E-298	4.39E-298
RP11-426L16	NA	NA	NA
FAM19A3	Inf	0	0
LINC01356	0.51466993	2.79E-255	8.81E-255
RP3-522D1.1	1.69841076	0.00014331	0.00015426
SLC16A1	2.30748423	0	0
SLC16A1-AS1	2.32659008	9.06E-258	2.89E-257
RP11-31F15.	0.77200489	2.10E-237	6.10E-237
LRIG2	2.88215159	3.85E-273	1.32E-272
MAGI3	1.98036037	4.42E-80	7.04E-80
PHTF1	2.0093278	3.16E-20	3.83E-20
RSBN1	1.76575587	6.85E-32	8.85E-32
RP5-1073O3.	NA	NA	NA
PTPN22	1.87486902	2.79E-06	3.06E-06
AP4B1-AS1	0.38600244	3.80E-290	1.42E-289
BCL2L15	0.77200489	2.04E-111	3.68E-111
AP4B1	3.97031086	1.18E-20	1.44E-20
DCLRE1B	1.73336947	4.06E-214	1.07E-213
HIPK1-AS1	0.46320293	5.11E-263	1.68E-262
HIPK1	2.31601467	1.59E-60	2.34E-60
OLFML3	NA	NA	NA
SYT6	NA	NA	NA

RP4-543J13.1	NA	NA	NA
TRIM33	2.30866224	0	0
BCAS2	1.70839105	7.90E-170	1.75E-169
DENND2C	1.33346299	1.19E-07	1.32E-07
AMPD1	NA	NA	NA
NRAS	1.80732928	1.09E-11	1.25E-11
CSDE1	2.0866509	7.66E-87	1.25E-86
SIKE1	2.20234524	6.22E-111	1.12E-110
SYCP1	NA	NA	NA
TSHB	NA	NA	NA
TSPAN2	1.93001222	8.47E-21	1.03E-20
RP4-666F24.	NA	NA	NA
RP4-663N10.	NA	NA	NA
NGF	NA	NA	NA
VANGL1	1.54400978	1.43E-09	1.61E-09
CASQ2	NA	NA	NA
NHLH2	NA	NA	NA
RP11-12L8.1	NA	NA	NA
SLC22A15	1.4153423	3.32E-156	7.00E-156
RP11-159M1	NA	NA	NA
MAB21L3	NA	2.63E-156	5.55E-156
RP5-1185H1!	NA	NA	NA
ATP1A1	1.5184313	0	0
RP4-655J12.4	NA	0	0
RP4-655J12.5	NA	NA	NA
RP5-1086K13	0	4.23E-78	6.69E-78
CD58	1.46398488	0	0
IGSF3	3.474022	3.58E-114	6.51E-114
C1orf137	NA	NA	NA
CD2	NA	0	0
RP4-753F5.1	2.05867971	2.51E-91	4.19E-91
PTGFRN	1.88293876	2.86E-80	4.56E-80
CD101	Inf	3.46E-09	3.90E-09
RP11-27K13.	0.77200489	8.90E-177	2.02E-176
TTF2	1.74500514	1.77E-299	6.95E-299
TRIM45	1.80134474	9.04E-59	1.33E-58
VTCN1	NA	NA	NA
LINC01525	NA	NA	NA
RP11-188D8.	Inf	0	0
MAN1A2	2.23366748	2.92E-118	5.39E-118
AL157902.3	Inf	0	0
FAM46C	2.31601467	3.25E-274	1.12E-273
GDAP2	2.31601467	0.00038741	0.00041507
WDR3	1.59625071	4.67E-107	8.28E-107
SPAG17	NA	NA	NA

TBX15	NA	NA	NA
RP4-712E4.1	NA	NA	NA
RP4-712E4.2	NA	NA	NA
WARS2	0.77200489	0	0
WARS2-IT1	NA	NA	NA
RP11-418J17	5.9830379	4.43E-216	1.17E-215
RP11-418J17	NA	NA	NA
RP5-834N19.	NA	NA	NA
HAO2	NA	NA	NA
HSD3B2	NA	NA	NA
HSD3B1	NA	NA	NA
ZNF697	1.31240831	0.28389124	0.28937829
PHGDH	1.00187151	0	0
HMGCS2	NA	NA	NA
REG4	NA	NA	NA
ADAM30	NA	NA	NA
NOTCH2	2.7234617	7.88E-82	1.27E-81
RP11-114O1	1.54400978	8.44E-38	1.12E-37
SEC22B	1.97826253	3.23E-55	4.67E-55
AC253572.1	1.54400978	7.86E-291	2.95E-290
NBPF26	Inf	1.10E-61	1.63E-61
PPIAL4A	NA	NA	NA
AC241377.2	NA	NA	NA
LINC00623	1.26328073	3.02E-302	1.21E-301
CH17-118O6.	NA	NA	NA
FCGR1B	0.48250306	0	0
RP11-439A1	NA	NA	NA
RP11-439A1	NA	1.62E-79	2.58E-79
RP11-439A1	NA	NA	NA
FAM72B	2.00721271	5.03E-32	6.50E-32
SRGAP2C	2.00721271	6.32E-21	7.70E-21
SRGAP2-AS1	NA	NA	NA
CH17-437K3.	NA	NA	NA
RP11-343N1	NA	NA	NA
RP11-344P1	NA	1.41E-201	3.53E-201
RP11-344P1	1.54400978	4.78E-304	1.94E-303
RP11-344P1	NA	NA	NA
RP11-763B2	NA	NA	NA
RP11-403I13	NA	9.75E-80	1.55E-79
RP11-403I13	Inf	6.25E-93	1.05E-92
RP5-998N21.	NA	NA	NA
RP5-998N21.	NA	NA	NA
HIST2H3PS2	NA	NA	NA
FAM72C	1.22084494	0	0
IGKV1OR1-1	NA	NA	NA

RP11-14N7.2	2.20572826	1.90E-242	5.67E-242
PPIAL4E	NA	NA	NA
RP11-763B22	7.7200489	1.75E-110	3.14E-110
NBPF15	4.11735941	0	0
PPIAL4F	NA	NA	NA
CH17-125A10	NA	NA	NA
RP11-289H16	NA	3.49E-41	4.72E-41
SRGAP2B	2.04354236	1.71E-67	2.60E-67
FAM72D	1.83611974	1.72E-87	2.82E-87
AC241585.2	NA	NA	NA
PPIAL4D	NA	NA	NA
CH17-373J23	3.86002445	1.62E-227	4.50E-227
RP11-89F3.2	NA	2.26E-28	2.87E-28
NBPF20	1.54400978	1.33E-41	1.81E-41
GPR89A	1.20947433	0	0
PDZK1	NA	NA	NA
CD160	Inf	4.70E-268	1.58E-267
RNF115	1.79666593	1.54E-74	2.41E-74
POLR3C	0.92640587	0	0
NUDT17	1.68437431	2.41E-25	3.00E-25
PIAS3	1.98515543	1.79E-10	2.04E-10
ANKRD35	2.00174719	5.13E-72	7.94E-72
ITGA10	2.50901589	6.26E-301	2.49E-300
RP11-315I20	NA	NA	NA
PEX11B	2.30543926	4.68E-282	1.67E-281
RBM8A	1.7382856	1.02E-80	1.63E-80
RP11-315I20	4.63202934	7.28E-138	1.44E-137
CH17-270A2.	NA	NA	NA
LIX1L	2.59358946	0	0
ANKRD34A	6.94804401	7.31E-10	8.29E-10
CH17-270A2.	NA	4.18E-29	5.32E-29
POLR3GL	2.20353714	3.70E-10	4.20E-10
TXNIP	1.02933985	0	0
HFE2	NA	NA	NA
RP11-458D21	NA	9.51E-215	2.50E-214
NBPF10	0.51466993	0	0
RP11-458D21	NA	NA	NA
NOTCH2NL	4.63202934	2.33E-13	2.71E-13
CH17-353B19	Inf	0	0
AC243756.1	NA	NA	NA
CH17-408M7	NA	NA	NA
NBPF12	2.50901589	4.46E-155	9.37E-155
RP4-704D21.	NA	NA	NA
PRKAB2	2.02318523	1.22E-26	1.53E-26
RP11-337C18	7.33404645	7.40E-244	2.22E-243

FMO5	0.44114565	6.15E-300	2.43E-299
CHD1L	1.61194621	2.03E-132	3.93E-132
LINC00624	0	0	0
BCL9	1.93001222	8.64E-136	1.69E-135
ACP6	1.48610941	0	0
RP11-533N14	NA	NA	NA
GJA5	NA	NA	NA
RP11-433J22	NA	NA	NA
RP11-433J22	NA	NA	NA
GJA8	NA	NA	NA
GPR89B	1.94839329	4.49E-70	6.90E-70
RP6-74O6.2	NA	NA	NA
XXyac-YX155	1.54400978	8.73E-88	1.43E-87
RP11-94I2.4	1.54400978	1.59E-93	2.67E-93
NBPF11	5.79003667	0.00029153	0.00031268
RP4-565E6.1	NA	NA	NA
XXyac-YX155	NA	2.63E-156	5.55E-156
RP11-495P10	NA	NA	NA
RP11-495P10	NA	NA	NA
RP11-495P10	NA	NA	NA
RP6-74O6.6	NA	NA	NA
LINC01138	1.72216475	0.83468701	0.83792008
RP11-495P10	1.93001222	0.28481704	0.29030568
AC245100.1	0.64333741	0.01274424	0.01334753
PPIAL4G	NA	NA	NA
NBPF14	0.96500611	1.85E-91	3.09E-91
PDE4DIP	3.17379788	0	0
RP4-791M13	NA	NA	NA
RP4-791M13	NA	NA	NA
RP4-791M13	NA	NA	NA
NBPF9	0.77200489	3.89E-14	4.55E-14
CH17-189H20	2.44168988	0	0
RP11-326G2	0	1.85E-128	3.53E-128
NBPF19	2.41894866	1.42E-136	2.80E-136
PPIAL4C	NA	NA	NA
LINC00869	0.9089735	0	0
RP11-353N4	NA	NA	NA
RP11-353N4	NA	NA	NA
FAM231D	5.40403423	0	0
FCGR1A	0.45033619	0	0
HIST2H2BF	NA	0	0
RP11-196G18	NA	NA	NA
HIST2H3D	1.54400978	1.54E-76	2.42E-76
HIST2H4A	NA	NA	NA
HIST2H3C	NA	NA	NA

HIST2H2AA3	NA	NA	NA
HIST2H2BE	5.40403423	0.32527077	0.33120404
RP11-196G1	0.25733496	0	0
HIST2H2AA4	0	0	0
HIST2H3A	NA	NA	NA
HIST2H4B	0	0	0
RP11-196G1	2.63389904	2.46E-260	7.97E-260
HIST2H2AC	1.98939722	0.00747823	0.00786174
HIST2H2AB	1.15800733	3.04E-19	3.67E-19
BOLA1	NA	NA	NA
SV2A	3.08801956	3.95E-130	7.58E-130
SF3B4	1.37689897	0	0
MTMR11	0	1.51E-228	4.20E-228
OTUD7B	2.05867971	5.81E-18	6.96E-18
VPS45	1.30442206	0.1995006	0.20428628
PLEKHO1	1.59577687	8.23E-170	1.83E-169
ANP32E	1.79301001	1.07E-181	2.47E-181
RP4-790G17	NA	0.04932871	0.05118516
CA14	Inf	6.28E-238	1.83E-237
APH1A	2.03074048	2.75E-28	3.48E-28
C1orf54	3.24242054	4.40E-66	6.65E-66
CIART	NA	0	0
MRPS21	2.19628319	0	0
AC242988.1	NA	NA	NA
PRPF3	1.28667482	0	0
RPRD2	2.12013283	0.01957533	0.02045123
TARS2	1.3850676	2.44E-33	3.17E-33
ECM1	4.63202934	0.80705573	0.8108997
FALEC	1.93001222	1.57E-55	2.27E-55
RP11-54A4.2	Inf	0	0
ADAMTSL4	1.46680929	2.43E-286	8.88E-286
MCL1	1.82432108	7.57E-26	9.47E-26
ENSA	1.6117812	0	0
GOLPH3L	1.64597269	0	0
HORMAD1	NA	9.71E-82	1.56E-81
CTSS	0.88342828	0	0
CTSK	0.77200489	1.49E-158	3.16E-158
ARNT	2.31601467	2.86E-13	3.33E-13
SETDB1	2.02927	2.27E-220	6.11E-220
CERS2	2.14445803	1.67E-162	3.61E-162
RP11-316M1	Inf	0	0
RP11-316M1	Inf	0	0
ANXA9	Inf	9.39E-242	2.79E-241
FAM63A	1.83351161	0.00077243	0.00082403
PRUNE	2.52656146	3.57E-191	8.58E-191

BNIPL	1.54400978	1.73E-154	3.63E-154
C1orf56	1.96591943	3.68E-37	4.88E-37
CDC42SE1	1.72389441	0.00010543	0.00011362
MLLT11	2.44257285	1.28E-54	1.84E-54
GABPB2	1.81648209	3.50E-57	5.09E-57
SEMA6C	2.31601467	2.66E-81	4.27E-81
TNFAIP8L2	1.37070256	0	0
SCNM1	1.67086504	3.52E-257	1.12E-256
LYSMD1	3.86002445	4.53E-78	7.16E-78
TMOD4	Inf	8.07E-255	2.54E-254
VPS72	1.33239423	0	0
PIP5K1A	1.00833292	0	0
PSMD4	1.08348211	0	0
RP11-126K1.	2.89501834	3.31E-178	7.57E-178
ZNF687	2.01717407	1.99E-42	2.71E-42
PI4KB	1.69505421	0.32399594	0.32992447
RP11-126K1.	NA	NA	NA
RFX5	1.86717462	3.03E-07	3.35E-07
RP11-126K1.	NA	4.54E-24	5.62E-24
RP11-126K1.	1.85014965	1.27E-100	2.20E-100
SELENBP1	NA	NA	NA
PSMB4	1.71361085	3.50E-106	6.18E-106
POGZ	3.21056002	0	0
CGN	3.60268949	0	0
TUFT1	6.43337408	2.57E-263	8.44E-263
RP11-404E16	NA	NA	NA
SNX27	1.83611974	2.00E-42	2.72E-42
RP11-98D18.	NA	NA	NA
CELF3	NA	NA	NA
RIIAD1	NA	NA	NA
RP11-98D18.	Inf	0	0
MRPL9	1.51635918	0	0
OAZ3	1.42820905	6.38E-189	1.52E-188
RP11-98D18.	NA	NA	NA
RP11-98D18.	NA	NA	NA
TDRKH	2.31601467	1.84E-16	2.18E-16
RP11-98D18.	1.737011	6.78E-18	8.12E-18
RP11-98D18.	Inf	0	0
LINGO4	NA	NA	NA
RORC	NA	NA	NA
C2CD4D	NA	NA	NA
AL450992.2	NA	NA	NA
THEM5	NA	NA	NA
THEM4	2.50316737	0	0
S100A10	1.68751367	7.55E-271	2.56E-270

AL591893.1	NA	NA	NA
S100A11	1.6626809	5.03E-136	9.87E-136
TCHHL1	NA	NA	NA
TCHH	0.25733496	3.71E-133	7.20E-133
AL589986.2	NA	NA	NA
RPTN	NA	NA	NA
FLG-AS1	NA	NA	NA
RP11-107M1	NA	NA	NA
HRNR	NA	NA	NA
FLG	NA	NA	NA
FLG2	Inf	0	0
CRNN	NA	NA	NA
LCE5A	NA	NA	NA
LCE3C	NA	NA	NA
AL135842.1	NA	NA	NA
CRCT1	NA	NA	NA
LCE3E	NA	NA	NA
LCE3D	NA	NA	NA
LCE3B	NA	NA	NA
LCE3A	NA	NA	NA
LINC00302	NA	NA	NA
LCE2D	NA	NA	NA
LCE2C	NA	NA	NA
LCE2B	NA	NA	NA
LCE2A	NA	NA	NA
LCE4A	NA	NA	NA
C1orf68	NA	NA	NA
KPRP	NA	NA	NA
LCE1F	NA	NA	NA
LCE1E	NA	NA	NA
LCE1D	NA	NA	NA
LCE1C	NA	NA	NA
LCE1B	NA	NA	NA
LCE1A	NA	NA	NA
LCE6A	NA	NA	NA
SMCP	NA	NA	NA
IVL	NA	NA	NA
LINC01527	NA	NA	NA
SPRR4	NA	NA	NA
SPRR1A	NA	NA	NA
SPRR3	NA	NA	NA
SPRR1B	NA	NA	NA
SPRR2D	NA	NA	NA
SPRR2A	NA	NA	NA
SPRR2B	NA	NA	NA

SPRR2E	NA	NA	NA
SPRR2F	NA	NA	NA
SPRR2G	NA	NA	NA
RP1-140J1.1	NA	NA	NA
LELP1	NA	NA	NA
PRR9	NA	NA	NA
LOR	NA	NA	NA
PGLYRP3	NA	NA	NA
PGLYRP4	NA	NA	NA
S100A9	0	5.09E-153	1.06E-152
S100A12	NA	NA	NA
S100A8	NA	1.64E-129	3.14E-129
S100A7A	NA	NA	NA
S100A7L2	NA	NA	NA
S100A7	NA	NA	NA
BX470102.3	NA	NA	NA
S100A6	Inf	1.07E-119	1.99E-119
S100A5	0.25733496	8.30E-287	3.04E-286
S100A4	0.78934404	0	0
S100A3	NA	3.12E-241	9.23E-241
S100A2	1.54400978	1.03E-135	2.02E-135
S100A16	NA	NA	NA
S100A14	Inf	1.30E-66	1.97E-66
S100A13	1.44798927	0	0
S100A1	0.77200489	6.54E-218	1.74E-217
RP1-178F15.	3.474022	2.53E-92	4.23E-92
CHTOP	1.60908788	0	0
SNAPIN	2.09676528	2.36E-05	2.56E-05
ILF2	1.1785913	0	0
NPR1	NA	NA	NA
INTS3	2.02124917	2.27E-190	5.44E-190
RP11-216N1	NA	NA	NA
RP11-216N1	NA	NA	NA
AL513523.2	NA	1.96E-16	2.32E-16
SLC27A3	1.63483388	4.06E-225	1.12E-224
GATAD2B	2.21308068	0	0
DENND4B	2.50901589	3.61E-137	7.10E-137
CRTC2	3.474022	7.64E-116	1.40E-115
SLC39A1	1.91293247	0.00170874	0.00181435
RP11-422P2	5.01803178	5.14E-51	7.29E-51
RP11-422P2	Inf	1.30E-181	3.02E-181
CREB3L4	1.10975703	0	0
JTB	2.38397183	0	0
RP11-422P2	5.40403423	7.11E-150	1.47E-149
RAB13	1.53776956	0	0

RPS27	2.37502449	0	0
NUP210L	0	0	0
TPM3	1.74455115	1.48E-129	2.83E-129
C1orf189	NA	NA	NA
C1orf43	1.82979051	2.00E-54	2.87E-54
UBAP2L	1.7896477	1.19E-72	1.84E-72
HAX1	1.83265763	7.06E-65	1.06E-64
AQP10	NA	0	0
ATP8B2	2.54307493	0.79405219	0.79801101
RP11-350G8.	NA	3.22E-163	6.97E-163
IL6R	1.54400978	3.68E-109	6.59E-109
SHE	NA	NA	NA
RP11-350G8.	NA	NA	NA
TDRD10	NA	NA	NA
UBE2Q1	1.749653	2.75E-27	3.47E-27
UBE2Q1-AS1	NA	NA	NA
CHRNB2	NA	NA	NA
RP11-61L14.	NA	NA	NA
ADAR	2.27884406	2.71E-234	7.76E-234
KCNN3	Inf	0	0
PMVK	1.90305332	7.27E-169	1.61E-168
RP11-307C1	NA	8.21E-173	1.84E-172
PBXIP1	1.93001222	2.89E-73	4.49E-73
PYGO2	2.07968664	5.55E-25	6.92E-25
RP11-307C1	3.18452017	0.00313814	0.00331811
SHC1	2.75950684	0	0
CKS1B	1.56034131	0	0
FLAD1	1.95912061	2.12E-57	3.09E-57
LENEP	NA	0.01289391	0.01350117
ZBTB7B	1.67267726	1.98E-22	2.43E-22
DCST2	Inf	0	0
DCST1	NA	NA	NA
RP11-307C1	1.11511817	1.34E-236	3.89E-236
ADAM15	1.65302488	0	0
EFNA4	1.25706991	0	0
RP11-540D1	NA	NA	NA
EFNA3	1.51798714	0	0
EFNA1	1.7094394	1.94E-30	2.48E-30
SLC50A1	1.91354767	2.49E-96	4.24E-96
DPM3	1.86083257	8.81E-179	2.02E-178
KRTCAP2	1.45144797	0	0
RP11-201K1	NA	NA	NA
TRIM46	NA	2.50E-74	3.89E-74
MUC1	2.21951406	5.04E-236	1.45E-235
AC234582.2	NA	NA	NA

AC234582.1	NA	NA	NA
RP11-263K1S	4.63202934	7.78E-55	1.12E-54
THBS3	1.93001222	7.27E-233	2.07E-232
MTX1	1.43807678	3.16E-259	1.02E-258
RP11-263K1S	NA	0	0
GBA	1.58261002	8.34E-81	1.33E-80
FAM189B	1.49818301	2.32E-149	4.78E-149
SCAMP3	1.61156021	1.76E-195	4.31E-195
CLK2	2.00149416	8.92E-76	1.40E-75
HCN3	1.82473883	4.76E-199	1.18E-198
PKLR	NA	NA	NA
FDPS	1.05413088	0	0
RUSC1-AS1	0.61760391	7.40E-247	2.25E-246
RUSC1	1.60680444	0.00017098	0.0001839
ASH1L	2.12301345	3.42E-190	8.18E-190
ASH1L-AS1	3.31318765	0	0
RP11-29H23.	NA	1.32E-06	1.45E-06
MSTO1	1.66148878	0	0
YY1AP1	1.6782715	1.91E-54	2.75E-54
DAP3	1.18106722	0	0
RP11-243J18	NA	NA	NA
GON4L	2.08973737	3.16E-28	4.00E-28
SYT11	NA	0	0
RIT1	2.45225083	1.73E-153	3.62E-153
KIAA0907	1.96510336	2.00E-126	3.79E-126
RXFP4	NA	NA	NA
ARHGEF2	1.90066701	1.99E-156	4.20E-156
RP11-336K24	1.80134474	3.16E-154	6.62E-154
RP11-336K24	0	0	0
SSR2	1.80079693	6.09E-274	2.10E-273
UBQLN4	2.22459304	3.68E-08	4.11E-08
LAMTOR2	2.10254755	7.56E-48	1.06E-47
RAB25	Inf	2.67E-122	4.99E-122
MEX3A	1.2008965	0	0
LMNA	0.56335492	0	0
SEMA4A	NA	NA	NA
SLC25A44	2.14655018	1.10E-56	1.60E-56
PMF1-BGLAI	NA	0.69604901	0.70083923
PMF1	1.60241804	0	0
BGLAP	0.38600244	2.49E-243	7.44E-243
PAQR6	2.89501834	1.80E-210	4.67E-210
SMG5	2.15253128	1.42E-37	1.88E-37
TMEM79	1.7094394	1.13E-92	1.89E-92
GLMP	NA	4.77E-137	9.39E-137
VHLL	NA	1.35E-40	1.82E-40

CCT3	1.16139602	0	0
TSACC	2.79851773	4.46E-69	6.82E-69
RHBG	NA	NA	NA
RP11-139I14	NA	NA	NA
C1orf61	0.72911573	0	0
RP11-98G7.1	2.31601467	1.15E-135	2.25E-135
MEF2D	1.36116652	6.82E-218	1.82E-217
RP11-284F21	Inf	0	0
IQGAP3	2.95935208	5.73E-05	6.19E-05
TTC24	NA	NA	NA
APOA1BP	1.72831302	4.34E-247	1.32E-246
GPATCH4	1.95257477	6.90E-65	1.04E-64
AL365181.2	3.86002445	0.76555416	0.76975467
RP11-284F21	NA	NA	NA
HAPLN2	NA	NA	NA
RP11-284F21	0	0	0
BCAN	5.01803178	0	0
RP11-284F21	Inf	2.66E-304	1.08E-303
RP11-284F21	0	0	0
NES	1.02933985	2.38E-212	6.20E-212
RP11-66D17.	Inf	1.78E-258	5.70E-258
CRABP2	1.54400978	0.0032363	0.00342131
RP11-66D17.	NA	NA	NA
ISG20L2	1.73825617	5.60E-69	8.56E-69
RRNAD1	1.90969631	1.96E-94	3.30E-94
MRPL24	1.83906485	1.87E-80	2.98E-80
HDGF	1.78028174	1.14E-91	1.91E-91
PRCC	1.66033928	1.17E-130	2.25E-130
SH2D2A	0.46320293	7.02E-225	1.93E-224
NTRK1	0.77200489	1.55E-33	2.02E-33
INSRR	NA	NA	NA
PEAR1	Inf	8.28E-228	2.30E-227
LRRC71	NA	NA	NA
ARHGEF11	3.39682152	0.00172577	0.00183222
ETV3L	NA	NA	NA
ETV3	3.08801956	9.54E-33	1.24E-32
RP11-71G12.	NA	NA	NA
RP11-85G21.	NA	NA	NA
RP11-85G21.	NA	NA	NA
RP11-85G21.	NA	NA	NA
FCRL5	NA	NA	NA
FCRL4	NA	NA	NA
FCRL3	NA	NA	NA
RP11-367J7.3	NA	NA	NA
FCRL2	NA	NA	NA

FCRL1	NA	NA	NA
CD5L	NA	NA	NA
KIRREL	1.15800733	1.35E-76	2.13E-76
RP11-404O1:	NA	NA	NA
CD1D	NA	8.93E-54	1.28E-53
RP11-404O1:	NA	NA	NA
CD1A	NA	NA	NA
CD1C	NA	NA	NA
CD1B	NA	NA	NA
CD1E	NA	NA	NA
OR10T2	NA	NA	NA
OR10K2	NA	NA	NA
OR10K1	NA	NA	NA
RP11-144L1.4	NA	NA	NA
OR10R2	NA	NA	NA
OR6Y1	NA	NA	NA
OR6P1	NA	NA	NA
OR10X1	NA	NA	NA
OR10Z1	NA	NA	NA
SPTA1	NA	1.02E-140	2.03E-140
OR6K2	NA	NA	NA
OR6K3	NA	NA	NA
OR6K6	NA	NA	NA
OR6N1	NA	NA	NA
OR6N2	NA	NA	NA
MNDA	0	0	0
PYHIN1	0.77200489	2.98E-39	3.99E-39
IFI16	1.58299993	2.53E-222	6.87E-222
AIM2	NA	NA	NA
CADM3	NA	NA	NA
CADM3-AS1	NA	NA	NA
ACKR1	NA	NA	NA
FCER1A	NA	NA	NA
OR10J3	NA	NA	NA
RP11-550P17	NA	NA	NA
OR10J1	NA	NA	NA
RP11-180D2:	NA	NA	NA
OR10J5	NA	NA	NA
APCS	NA	NA	NA
CRP	NA	NA	NA
DUSP23	1.18006462	1.72E-218	4.59E-218
FCRL6	NA	NA	NA
SLAMF8	NA	NA	NA
C1orf204	NA	NA	NA
VSIG8	NA	NA	NA

RP11-190A1.	NA	NA	NA
RP11-190A1.	NA	NA	NA
CFAP45	1.93001222	8.97E-115	1.63E-114
TAGLN2	1.55274946	0	0
IGSF9	NA	2.35E-138	4.65E-138
SLAMF9	NA	NA	NA
LINC01133	Inf	0	0
PIGM	2.93046754	0	0
KCNJ10	NA	4.17E-189	9.94E-189
RP11-536C5.	NA	NA	NA
KCNJ9	NA	NA	NA
IGSF8	1.71556642	2.81E-160	6.02E-160
ATP1A2	NA	3.67E-103	6.40E-103
ATP1A4	NA	NA	NA
CASQ1	NA	NA	NA
RP11-536C5.	0.77200489	0.21296147	0.21781187
PEA15	2.06740293	0.03888149	0.04041643
DCAF8	2.0844132	0.71013437	0.71474385
RP11-574F21	Inf	2.86E-178	6.53E-178
RP11-574F21	Inf	1.08E-06	1.19E-06
PEX19	1.737011	3.84E-149	7.91E-149
COPA	2.11984949	0.51434794	0.52028438
NCSTN	1.88894814	2.58E-177	5.88E-177
NHLH1	Inf	4.17E-207	1.07E-206
VANGL2	2.39321516	1.60E-153	3.34E-153
SLAMF6	Inf	3.25E-20	3.94E-20
RP11-528G1.	NA	NA	NA
CD84	1.4153423	9.51E-222	2.57E-221
SLAMF1	0.96500611	1.18E-24	1.46E-24
RP11-404F1(	Inf	0	0
CD48	2.6817012	1.91E-47	2.66E-47
SLAMF7	NA	NA	NA
LY9	NA	NA	NA
CD244	1.54400978	4.42E-97	7.53E-97
ITLN1	0	2.62E-295	1.01E-294
RP11-312J18	NA	0	0
RP11-544M2	NA	NA	NA
ITLN2	NA	NA	NA
F11R	1.88369193	2.03E-23	2.50E-23
RP11-544M2	NA	NA	NA
TSTD1	0.77200489	0	0
USF1	1.54400978	2.31E-06	2.53E-06
ARHGAP30	1.49532479	5.43E-263	1.78E-262
AL591806.1	NA	NA	NA
PVRL4	NA	NA	NA

RP11-544M2	NA	NA	NA
KLHDC9	NA	NA	NA
PFDN2	2.03389144	1.96E-17	2.34E-17
NIT1	1.20341939	0	0
DEDD	1.62874202	6.94E-263	2.27E-262
UFC1	1.84472407	1.19E-147	2.43E-147
RP11-297K8.	1.54400978	1.08E-24	1.34E-24
USP21	1.42211427	2.02E-21	2.47E-21
PPOX	1.27380807	3.09E-45	4.27E-45
B4GALT3	1.79756041	9.75E-125	1.83E-124
ADAMTS4	NA	4.75E-231	1.34E-230
NDUFS2	0.72911573	0	0
FCER1G	0.77200489	0	0
APOA2	NA	2.42E-71	3.73E-71
TOMM40L	2.24363921	3.33E-24	4.12E-24
NR1I3	NA	0	0
PCP4L1	0.77200489	1.20E-77	1.90E-77
MPZ	2.70201711	1.51E-220	4.06E-220
SDHC	1.35026107	0	0
CFAP126	NA	NA	NA
FCGR2A	1.10286413	8.02E-283	2.88E-282
HSPA6	0.77200489	1.26E-95	2.13E-95
FCGR3A	NA	5.01E-106	8.85E-106
RP11-5K23.5	NA	NA	NA
FCGR3B	NA	NA	NA
FCGR2B	0.17155664	0	0
RP11-25K21.	NA	NA	NA
FCRLA	NA	NA	NA
FCRLB	3.08801956	7.05E-64	1.06E-63
DUSP12	1.59127539	1.40E-302	5.64E-302
RP11-474I16	Inf	0	0
ATF6	2.21746085	1.60E-14	1.88E-14
OLFML2B	1.54400978	3.83E-35	5.03E-35
RP11-227F8.	NA	NA	NA
NOS1AP	0.38600244	1.47E-177	3.34E-177
RP11-565P22	NA	NA	NA
RP11-565P22	NA	NA	NA
C1orf226	Inf	1.29E-138	2.55E-138
C1orf111	NA	NA	NA
SH2D1B	NA	NA	NA
UHMK1	2.0969322	1.92E-87	3.15E-87
RP11-359K18	NA	NA	NA
UAP1	1.76250173	3.86E-176	8.76E-176
DDR2	Inf	2.13E-153	4.46E-153
HSD17B7	1.07645752	0	0

CCDC190	NA	NA	NA
RGS4	NA	NA	NA
RGS5	0.38600244	0	0
RP11-267N1.	NA	NA	NA
RGS5	2.05867971	2.02E-41	2.74E-41
RP11-77M5.	NA	NA	NA
NUF2	1.01362474	0	0
PBX1	NA	4.64E-294	1.77E-293
RP11-506O2	NA	NA	NA
LMX1A	NA	NA	NA
RP11-38C18.	NA	NA	NA
RP11-38C18.	NA	NA	NA
RXRG	NA	NA	NA
RP11-280O1.	NA	NA	NA
LRRC52	NA	NA	NA
RP11-306I1.2	Inf	0	0
MGST3	1.59627527	0	0
ALDH9A1	1.13656275	0	0
RP11-466F5.	NA	7.57E-268	2.54E-267
TMCO1	1.80298591	8.18E-111	1.47E-110
RP11-466F5.	Inf	4.57E-59	6.71E-59
UCK2	2.11300598	2.02E-58	2.96E-58
RP11-525G1.	NA	NA	NA
FAM78B	NA	NA	NA
RP11-375H1	NA	NA	NA
RP11-479J7.2	NA	NA	NA
RP11-479J7.	NA	NA	NA
RP11-276E17	NA	NA	NA
POGK	1.89141198	1.04E-31	1.34E-31
TADA1	1.71092976	9.57E-65	1.44E-64
ILDR2	3.18452017	3.80E-219	1.02E-218
MAEL	Inf	5.65E-185	1.33E-184
RP11-102C16	NA	NA	NA
GPA33	NA	1.91E-24	2.37E-24
DUSP27	0.25733496	2.01E-107	3.58E-107
LINC01363	NA	NA	NA
RP11-277B1	1.30646981	1.15E-127	2.18E-127
POU2F1	1.99196323	1.15E-24	1.43E-24
CD247	Inf	0	0
RP11-104L21	NA	NA	NA
CREG1	1.60370088	0	0
RP3-455J7.4	4.24602689	2.18E-38	2.91E-38
RCSD1	1.61280229	2.75E-208	7.07E-208
MPZL1	1.94347743	2.02E-08	2.26E-08
ADCY10	0	0	0

RP1-313L4.3	Inf	0	0
MPC2	2.07953738	1.20E-40	1.62E-40
DCAF6	1.82473883	0.72793906	0.7324203
GPR161	1.47219537	3.00E-132	5.80E-132
TIPRL	1.60583048	2.83E-274	9.75E-274
SFT2D2	1.98336216	3.50E-100	6.04E-100
TBX19	Inf	0	0
RP5-968D22.	0	0	0
RP5-968D22.	0	0	0
XCL2	0	0	0
XCL1	Inf	0	0
DPT	NA	NA	NA
RP1-10C16.1	NA	6.70E-218	1.79E-217
LINC00626	NA	NA	NA
LINC00970	NA	NA	NA
RP5-1018K9.	NA	NA	NA
ATP1B1	1.59127539	1.84E-91	3.08E-91
NME7	1.35766377	1.57E-154	3.30E-154
RP4-800F24.	NA	NA	NA
BLZF1	2.70201711	3.22E-63	4.81E-63
CCDC181	NA	1.57E-83	2.55E-83
SLC19A2	4.63202934	1.18E-56	1.71E-56
RP1-206D15.	Inf	0	0
F5	NA	NA	NA
SELP	NA	NA	NA
C1orf112	1.63223891	7.53E-72	1.16E-71
SELL	1.02933985	0	0
SELE	NA	NA	NA
RP1-117P20.	NA	NA	NA
METTL18	1.17832325	0	0
SCYL3	1.0444772	3.95E-54	5.67E-54
KIFAP3	3.17379788	1.62E-148	3.32E-148
RP11-332H1	NA	NA	NA
METTL11B	NA	NA	NA
RP11-297H3.	NA	NA	NA
LINC01142	NA	NA	NA
RP11-576I22	NA	NA	NA
GORAB	4.63202934	1.38E-66	2.09E-66
RP11-545I10	NA	NA	NA
RP1-79C4.1	NA	NA	NA
PRRX1	NA	NA	NA
RP1-79C4.4	NA	NA	NA
MROH9	NA	NA	NA
FMO3	NA	NA	NA
FMO2	NA	NA	NA

RP1-127D3.4	NA	NA	NA
RP1-45C12.1	NA	NA	NA
FMO1	NA	NA	NA
FMO4	0.51466993	5.15E-173	1.16E-172
PRRC2C	1.810022	5.06E-101	8.76E-101
MYOC	NA	NA	NA
VAMP4	1.65429619	5.91E-54	8.49E-54
METTL13	1.58008477	1.75E-143	3.52E-143
DNM3	1.54400978	2.03E-92	3.41E-92
DNM3OS	NA	NA	NA
PIGC	1.63772136	1.65E-72	2.55E-72
C1orf105	NA	NA	NA
SUCO	2.47041565	1.32E-20	1.61E-20
FASLG	NA	NA	NA
RP1-15D23.2	NA	NA	NA
RP3-471M13	NA	NA	NA
TNFSF18	NA	NA	NA
RP3-395P12.	NA	NA	NA
TNFSF4	6.94804401	3.15E-280	1.12E-279
RP11-296O1	1.82473883	2.07E-116	3.80E-116
PRDX6	1.45846011	0	0
SLC9C2	NA	NA	NA
RP3-436N22	NA	NA	NA
ANKRD45	Inf	1.25E-06	1.38E-06
RP11-360D2.	NA	NA	NA
KLHL20	1.6685267	6.57E-15	7.72E-15
CENPL	2.05472071	3.23E-07	3.57E-07
DARS2	2.49033835	4.38E-219	1.17E-218
GAS5-AS1	NA	9.79E-125	1.84E-124
ZBTB37	3.76352384	0	0
SERPINC1	NA	1.31E-110	2.35E-110
RC3H1	3.11661233	1.33E-266	4.42E-266
RP11-160H2	NA	NA	NA
RP11-160H2	Inf	0	0
RABGAP1L	1.96510336	0.00087336	0.00093104
GPR52	NA	NA	NA
RP1-102G20	NA	NA	NA
CACYBP	1.36990779	3.99E-285	1.45E-284
MRPS14	2.05267772	1.69E-13	1.97E-13
TNN	NA	NA	NA
KIAA0040	2.05867971	1.91E-34	2.50E-34
RP5-1114G2	NA	NA	NA
RP3-518E13.	NA	NA	NA
TNR	NA	NA	NA
RP11-222A5.	NA	NA	NA

RP11-318C24	1.54400978	2.99E-10	3.40E-10
RFWD2	1.61146652	8.65E-05	9.34E-05
RP11-492I21	NA	NA	NA
RP11-195C7.	NA	NA	NA
PAPPA2	NA	NA	NA
ASTN1	NA	NA	NA
BRINP2	NA	NA	NA
RP1-35C21.1	NA	NA	NA
RP1-35C21.2	NA	NA	NA
RP11-63B19.	NA	NA	NA
SEC16B	NA	3.51E-213	9.20E-213
RASAL2-AS1	1.69841076	2.14E-19	2.58E-19
RASAL2	1.45043343	8.37E-299	3.28E-298
RP4-593C16.	NA	NA	NA
LINC00083	NA	NA	NA
TEX35	Inf	1.37E-238	4.01E-238
C1orf220	3.08801956	1.11E-106	1.97E-106
RP5-1098D14	1.93001222	3.75E-46	5.20E-46
RP11-428K3.	NA	NA	NA
RALGPS2	1.72929095	1.59E-33	2.07E-33
ANGPTL1	NA	NA	NA
FAM20B	2.08973737	0	0
TOR3A	1.78096178	3.93E-67	5.97E-67
ABL2	2.36142672	0	0
SOAT1	1.9627243	8.66E-07	9.54E-07
AXDND1	0	0	0
RP11-545A16	NA	NA	NA
NPHS2	NA	NA	NA
TDRD5	NA	NA	NA
RP11-12M5.1	NA	NA	NA
FAM163A	NA	0	0
RP11-12M5.3	NA	5.65E-272	1.93E-271
RP11-12M5.4	NA	NA	NA
TOR1AIP2	1.87062723	6.40E-15	7.52E-15
RP11-533E19	4.24602689	0	0
TOR1AIP1	1.59547677	1.82E-84	2.95E-84
RP11-533E19	NA	NA	NA
RP11-533E19	Inf	2.00E-49	2.81E-49
CEP350	2.04711409	0.20517139	0.20997458
RP11-502H18	NA	NA	NA
QSOX1	1.4972216	0	0
LHX4	1.54400978	1.99E-229	5.58E-229
ACBD6	1.6218246	2.77E-115	5.05E-115
OVAAL	0	2.61E-25	3.26E-25
XPR1	2.99719546	1.54E-224	4.21E-224

RP11-46A10.	NA	NA	NA
KIAA1614	NA	6.57E-105	1.15E-104
RP11-46A10.	0.77200489	6.35E-21	7.74E-21
KIAA1614-AS	3.86002445	4.78E-26	5.99E-26
STX6	2.12301345	7.69E-154	1.61E-153
MR1	2.40179299	1.39E-19	1.68E-19
IER5	0.77200489	0	0
RP11-540K16	NA	NA	NA
RP11-540K16	NA	NA	NA
GM140	NA	NA	NA
CACNA1E	NA	NA	NA
RP11-33M22	NA	NA	NA
ZNF648	NA	NA	NA
RP11-538D16	Inf	1.37E-305	5.59E-305
RP11-538D16	NA	NA	NA
GS1-122H1.1	NA	NA	NA
LINC01344	NA	NA	NA
GLUL	1.21255116	0	0
TEDDM1	NA	NA	NA
LINC00272	NA	NA	NA
RGSL1	NA	NA	NA
RNASEL	6.17603912	0	0
RGS16	1.34868324	9.69E-158	2.06E-157
RP11-317P15	NA	NA	NA
RGS8	NA	NA	NA
RP11-317P15	NA	NA	NA
NPL	1.98515543	2.30E-17	2.74E-17
DHX9	2.18382205	0	0
SHCBP1L	NA	NA	NA
LAMC1	1.76218507	3.54E-25	4.41E-25
RP11-181K3.	NA	NA	NA
LAMC2	NA	NA	NA
NMNAT2	NA	NA	NA
SMG7	2.43298511	0	0
NCF2	1.54400978	3.18E-12	3.67E-12
ARPC5	1.85302124	4.30E-66	6.50E-66
RGL1	NA	NA	NA
APOBEC4	NA	NA	NA
RP11-444D13	NA	NA	NA
COLGALT2	NA	3.42E-204	8.65E-204
TSEN15	1.96251769	2.38E-46	3.30E-46
RP11-382D12	NA	NA	NA
RP11-382D12	1.15800733	4.78E-252	1.49E-251
C1orf21	0.6465541	0	0
EDEM3	1.74079534	0.05313984	0.05509561

FAM129A	0.46320293	1.80E-261	5.87E-261
GS1-120K12.	NA	NA	NA
RNF2	1.32998862	3.25E-276	1.13E-275
TRMT1L	1.2008965	0	0
SWT1	Inf	2.08E-283	7.49E-283
IVNS1ABP	1.90339137	0.40584996	0.41198131
GS1-279B7.2	0.77200489	0	0
GS1-279B7.1	NA	NA	NA
LINC01350	NA	NA	NA
GS1-204I12.4	NA	NA	NA
HMCN1	NA	NA	NA
GS1-174L6.4	NA	NA	NA
PRG4	Inf	0	0
TPR	1.60781184	0	0
C1orf27	1.76814023	4.09E-102	7.10E-102
AL596220.1	NA	NA	NA
OCLM	NA	2.37E-104	4.15E-104
RP11-295K2.	NA	1.46E-49	2.06E-49
PDC	NA	NA	NA
GS1-304P7.3	NA	NA	NA
PTGS2	NA	NA	NA
PACERR	NA	1.05E-167	2.31E-167
PLA2G4A	1.737011	2.85E-64	4.27E-64
LINC01036	NA	NA	NA
LINC01037	NA	NA	NA
RP11-445P19	NA	NA	NA
ERVMER61-1	NA	NA	NA
RP11-201A3.	NA	NA	NA
RP11-669M2	NA	NA	NA
RP11-316I3.1	NA	NA	NA
RP11-316I3.2	NA	NA	NA
LINC01035	NA	NA	NA
RP11-398M1	NA	NA	NA
RP11-445J9.1	NA	NA	NA
BRINP3	1.54400978	2.29E-05	2.48E-05
RP11-547I7.1	NA	NA	NA
LINC01351	Inf	0.82728175	0.83071597
RP11-161I10	NA	NA	NA
RP11-463J7.2	NA	NA	NA
RP11-309H21	NA	NA	NA
RP11-309H21	NA	NA	NA
RP11-541F9.	NA	NA	NA
RP11-541F9.	NA	NA	NA
RGS18	1.76967275	2.95E-41	4.00E-41
RGS21	NA	NA	NA

RP5-1011O1.	NA	NA	NA
RP5-1011O1.	NA	NA	NA
RGS1	NA	0	0
RGS13	NA	NA	NA
RGS2	1.93001222	4.13E-62	6.13E-62
LINC01032	NA	3.30E-55	4.76E-55
UCHL5	1.67852578	5.51E-152	1.15E-151
TROVE2	1.91817166	3.20E-33	4.16E-33
RP11-101E13	3.08801956	5.56E-16	6.58E-16
GLRX2	2.14211041	6.56E-74	1.02E-73
CDC73	1.56224612	3.12E-216	8.26E-216
B3GALT2	NA	NA	NA
LINC01031	NA	NA	NA
RP11-21J7.1	NA	NA	NA
RP11-98G13.	NA	NA	NA
RP11-563D1(	NA	NA	NA
RP11-476H2(	NA	NA	NA
RP11-476B1.	NA	NA	NA
KCNT2	NA	NA	NA
CFH	1.54400978	0	0
CFHR3	Inf	0	0
RP4-608O15.	NA	NA	NA
CFHR1	Inf	0	0
CFHR4	NA	NA	NA
CFHR2	NA	NA	NA
CFHR5	NA	NA	NA
F13B	NA	NA	NA
ASPM	1.63976232	9.81E-236	2.83E-235
ZBTB41	1.54400978	2.74E-29	3.49E-29
CRB1	NA	NA	NA
RP11-75C23.	NA	NA	NA
DENND1B	1.73035579	3.69E-71	5.69E-71
RP11-448G4.	NA	NA	NA
C1orf53	2.35192187	1.50E-251	4.64E-251
LHX9	NA	1.11E-157	2.36E-157
NEK7	2.77013519	7.83E-282	2.80E-281
ATP6V1G3	NA	NA	NA
RP11-553K8.	Inf	0	0
PTPRC	2.00721271	2.02E-144	4.07E-144
RP11-553K8.	NA	NA	NA
MIR181A1HG	3.22838409	3.99E-288	1.48E-287
RP11-16L9.2	NA	1.50E-93	2.53E-93
LINC01222	2.31601467	0.35154527	0.3576166
LINC01221	NA	NA	NA
RP11-382E9.	NA	NA	NA

NR5A2	0.77200489	9.88E-124	1.85E-123
RP11-532L16	NA	NA	NA
LINC00862	NA	NA	NA
ZNF281	1.74065253	7.36E-10	8.34E-10
RP11-469A15	NA	0	0
RP11-121P12	NA	NA	NA
KIF14	1.96510336	0.01102947	0.01156294
DDX59	1.78154975	2.39E-277	8.36E-277
RP11-92G12.	0.55143206	4.16E-163	9.01E-163
CAMSAP2	0	3.62E-199	8.97E-199
GPR25	NA	NA	NA
C1orf106	NA	0	0
KIF21B	2.00721271	3.14E-20	3.81E-20
RP11-168O10	Inf	3.04E-57	4.43E-57
RP11-168O10	NA	NA	NA
CACNA1S	NA	NA	NA
ASCL5	NA	NA	NA
TMEM9	1.83085563	0.06696177	0.06931137
IGFN1	NA	NA	NA
RP11-567E21	NA	NA	NA
PKP1	NA	NA	NA
TNNT2	NA	NA	NA
LAD1	NA	NA	NA
RP11-27G14.	NA	NA	NA
TNNI1	NA	NA	NA
PHLDA3	NA	1.21E-06	1.33E-06
CSRP1	2.03232613	1.93E-66	2.92E-66
RP11-134G8.	1.15800733	1.44E-22	1.77E-22
RP11-134G8.	5.40403423	2.42E-303	9.74E-303
NAV1	1.4153423	0.09903961	0.10215343
RP11-25B7.1	NA	NA	NA
IPO9-AS1	3.08801956	1.72E-109	3.07E-109
RP11-90L20.	NA	NA	NA
IPO9	1.88079044	1.16E-41	1.57E-41
SHISA4	NA	1.40E-287	5.16E-287
RP11-307B6.	NA	NA	NA
LMOD1	NA	NA	NA
TIMM17A	1.97352297	1.51E-12	1.74E-12
RNPEP	1.06150672	0	0
RP11-465N4.	0	0	0
RP11-465N4.	NA	NA	NA
ELF3	NA	0	0
GPR37L1	NA	0	0
ARL8A	1.77821351	7.58E-63	1.13E-62
PTPN7	2.3253284	0	0

LGR6	NA	NA	NA
UBE2T	1.57467097	8.87E-262	2.89E-261
PPP1R12B	1.80134474	5.53E-63	8.24E-63
SYT2	NA	NA	NA
RP11-569A1:	0	2.89E-208	7.41E-208
RP11-569A1:	NA	NA	NA
KDM5B	1.8177206	4.85E-61	7.18E-61
RP11-480I12	NA	0	0
PCAT6	10.8080685	2.49E-189	5.94E-189
RABIF	1.81884352	1.86E-75	2.92E-75
KLHL12	1.84285038	0.36205481	0.36814249
ADIPOR1	1.78490565	0.5611374	0.56695003
CYB5R1	1.75697665	4.28E-27	5.39E-27
TMEM183A	2.05386625	7.67E-40	1.03E-39
PPFIA4	Inf	1.48E-208	3.80E-208
MYOG	NA	NA	NA
ADORA1	NA	NA	NA
RP11-335O1:	NA	NA	NA
RP11-335O1:	NA	NA	NA
MYBPH	NA	2.57E-46	3.57E-46
CHI3L1	NA	2.31E-42	3.15E-42
CHIT1	NA	NA	NA
LINC01353	NA	NA	NA
LINC01136	NA	1.63E-127	3.10E-127
BTG2	1.23520782	3.43E-94	5.79E-94
FMOD	NA	NA	NA
PRELP	NA	NA	NA
OPTC	NA	NA	NA
ATP2B4	1.85281174	2.66E-55	3.84E-55
LAX1	NA	0.00593929	0.00625476
ZC3H11A	2.13116843	0.00025756	0.00027646
ZBED6	1.93001222	7.74E-149	1.59E-148
SNRPE	2.16487997	1.28E-288	4.74E-288
LINC00303	NA	NA	NA
SOX13	NA	NA	NA
RP11-74C13.	NA	NA	NA
ETNK2	0.51466993	4.12E-82	6.63E-82
RP11-74C13.	NA	NA	NA
REN	NA	NA	NA
KISS1	NA	0	0
GOLT1A	NA	NA	NA
PLEKHA6	NA	NA	NA
RP11-203F1(	NA	NA	NA
LINC00628	NA	NA	NA
RP11-739N2(	NA	NA	NA

PPP1R15B	2.46384539	2.93E-122	5.46E-122
PIK3C2B	2.06897311	4.21E-16	4.98E-16
MDM4	2.26992483	9.57E-61	1.41E-60
RP11-430C7.	NA	NA	NA
LRRN2	NA	NA	NA
RP11-430C7.	NA	NA	NA
RP11-23I7.1	NA	NA	NA
NFASC	Inf	0	0
CNTN2	NA	0	0
TMEM81	2.31601467	0	0
RBBP5	2.16896612	5.04E-62	7.49E-62
DSTYK	2.8049511	2.86E-86	4.67E-86
TMCC2	1.28667482	0.00030667	0.00032878
RP11-383G1(	NA	NA	NA
NUAK2	NA	NA	NA
KLHDC8A	NA	NA	NA
LEMD1-AS1	NA	NA	NA
LEMD1	NA	NA	NA
BLACAT1	NA	NA	NA
RP11-576D8.	NA	6.81E-144	1.37E-143
CDK18	NA	0	0
MFSD4	Inf	0	0
ELK4	2.26922649	0.00015898	0.00017104
SLC45A3	1.64051039	1.63E-89	2.70E-89
NUCKS1	1.52832103	4.59E-263	1.51E-262
RAB29	2.47205821	0	0
SLC41A1	2.35277681	2.47E-136	4.85E-136
PM20D1	NA	NA	NA
SLC26A9	1.54400978	1.20E-56	1.75E-56
RP4-681L3.2	NA	NA	NA
RAB7B	0.56187338	0	0
CTSE	4.63202934	0.00687168	0.00722785
C1orf186	1.46890245	0	0
AVPR1B	NA	1.11E-157	2.36E-157
RP11-38J22.:	Inf	0	0
RP11-38J22.:	NA	NA	NA
FAM72A	3.36874861	1.74E-47	2.43E-47
SRGAP2	1.49947104	2.06E-38	2.75E-38
IKBKE	1.54400978	4.65E-187	1.10E-186
C1orf147	Inf	0	0
RP11-534L2(	NA	0	0
RASSF5	2.02007946	3.28E-226	9.07E-226
EIF2D	1.7936401	2.00E-42	2.72E-42
RP11-343H5.	Inf	4.26E-28	5.39E-28
DYRK3	1.15800733	0.20863286	0.21344484

MAPKAPK2	1.7687707	4.69E-116	8.57E-116
IL10	NA	NA	NA
IL19	NA	NA	NA
IL20	NA	NA	NA
IL24	NA	NA	NA
FCMR	NA	0	0
PIGR	NA	NA	NA
FCAMR	NA	NA	NA
C1orf116	NA	NA	NA
PFKFB2	NA	NA	NA
YOD1	NA	NA	NA
C4BPB	NA	NA	NA
C4BPA	NA	NA	NA
RP11-164O2	NA	NA	NA
RP11-164O2	NA	NA	NA
RP11-6J21.2	NA	NA	NA
CD55	1.71556642	6.52E-10	7.40E-10
RP11-357P1	NA	NA	NA
RP11-357P1	NA	NA	NA
CR2	NA	NA	NA
CR1	NA	NA	NA
RP11-78B10.	NA	NA	NA
CR1L	NA	NA	NA
CD46	3.11141365	5.01E-70	7.69E-70
C1orf132	2.63768337	1.64E-25	2.05E-25
RP11-328D5.	NA	NA	NA
CD34	0.47478301	0	0
PLXNA2	Inf	1.64E-74	2.56E-74
RP11-565N2	NA	NA	NA
RP11-565N2	NA	NA	NA
RP11-459K2	NA	NA	NA
RP5-1051D1	NA	NA	NA
RP11-385M4	NA	NA	NA
RP11-372M1	NA	NA	NA
RP11-372M1	NA	NA	NA
RP1-272L16.	NA	NA	NA
CAMK1G	NA	NA	NA
LAMB3	NA	NA	NA
RP1-28O10.1	NA	NA	NA
G0S2	NA	NA	NA
HSD11B1	NA	NA	NA
TRAF3IP3	1.13962627	0	0
C1orf74	2.45027639	8.54E-27	1.07E-26
IRF6	NA	NA	NA
DIEXF	2.2874219	8.32E-125	1.57E-124

SYT14	NA	NA	NA
SERTAD4-AS	NA	NA	NA
SERTAD4	NA	NA	NA
HHAT	Inf	7.12E-32	9.19E-32
KCNH1	NA	NA	NA
RP11-543B1(	NA	NA	NA
RCOR3	1.92244355	6.60E-164	1.44E-163
TRAF5	1.71347427	5.34E-88	8.78E-88
LINC00467	1.97826253	3.51E-10	3.98E-10
RD3	NA	NA	NA
RP11-359E8.	NA	NA	NA
SLC30A1	1.85804567	3.48E-203	8.76E-203
RP11-354K1.	NA	NA	NA
RP11-354K1.	NA	NA	NA
NEK2	1.54400978	0	0
RP11-122M1	Inf	2.66E-148	5.46E-148
RP11-122M1	NA	NA	NA
LPGAT1	2.39814285	2.72E-170	6.05E-170
RP11-552D8.	Inf	6.08E-144	1.23E-143
INTS7	2.21308068	1.40E-264	4.62E-264
DTL	2.13332649	2.01E-216	5.34E-216
RP11-15I11.2	NA	NA	NA
RP11-15I11.3	0	4.38E-220	1.18E-219
PPP2R5A	1.65288226	0	0
RP11-384C4.	NA	4.88E-39	6.52E-39
TMEM206	1.27313087	0	0
NENF	2.3605802	0	0
RP11-61J19.2	NA	NA	NA
RP11-61J19.3	NA	NA	NA
RP11-61J19.5	1.54400978	3.58E-147	7.32E-147
ATF3	1.00360636	5.42E-63	8.09E-63
RP11-338C15	NA	NA	NA
FAM71A	NA	NA	NA
RP11-338C15	NA	NA	NA
BATF3	2.80571926	0	0
NSL1	1.59751507	0	0
TATDN3	2.06362846	8.51E-15	9.98E-15
SPATA45	1.15800733	2.60E-46	3.60E-46
FLVCR1-AS1	1.54400978	4.20E-25	5.23E-25
FLVCR1	2.04029864	2.34E-22	2.87E-22
RP11-348H3.	NA	NA	NA
VASH2	1.84093474	2.53E-44	3.47E-44
ANGEL2	2.14445803	3.10E-27	3.90E-27
RPS6KC1	1.54400978	6.49E-168	1.43E-167
RP11-554K11	NA	NA	NA

RP11-323I1.1	NA	NA	NA
LINC00538	NA	NA	NA
PROX1	NA	0	0
RP11-478J18	NA	NA	NA
PROX1-AS1	NA	NA	NA
SMYD2	2.08864337	7.39E-39	9.88E-39
PTPN14	1.69343008	1.72E-227	4.77E-227
CENPF	1.58121483	0	0
KCNK2	NA	NA	NA
RP11-199H2.	NA	NA	NA
KCTD3	2.58689358	2.46E-109	4.41E-109
USH2A	NA	NA	NA
RP5-1111A8.	NA	NA	NA
RP11-22M7.2	NA	NA	NA
RP5-1099E6.	NA	NA	NA
ESRRG	NA	0	0
GPATCH2	1.40431366	5.81E-196	1.42E-195
SPATA17	0	5.00E-207	1.28E-206
SPATA17-AS	NA	NA	NA
LINC00210	NA	NA	NA
RP11-152L7.	NA	NA	NA
RP11-152L7.	NA	NA	NA
RRP15	1.93942692	3.51E-23	4.33E-23
TGFB2-AS1	NA	2.35E-28	2.98E-28
TGFB2	NA	7.57E-268	2.54E-267
C1orf143	NA	NA	NA
RP11-412H9.	NA	NA	NA
LYPLAL1-AS1	NA	4.09E-12	4.72E-12
LYPLAL1	1.74584112	1.54E-160	3.31E-160
RP11-135J2.3	NA	NA	NA
RP11-392O1	2.31601467	5.72E-13	6.63E-13
RP11-392O1	NA	NA	NA
RP11-95P13.	NA	NA	NA
RP11-95P13.	NA	NA	NA
SLC30A10	NA	NA	NA
EPRS	1.73760122	1.66E-300	6.58E-300
BPNT1	1.78879182	1.48E-225	4.06E-225
IARS2	2.24868866	7.87E-91	1.31E-90
RAB3GAP2	1.79144724	5.39E-188	1.28E-187
RP11-302I18	NA	NA	NA
RP11-410C4.	NA	NA	NA
RP11-410C4.	NA	NA	NA
MARK1	NA	NA	NA
C1orf115	0.64333741	1.82E-31	2.34E-31
2-Mar	0	2.84E-55	4.10E-55

1-Mar	NA	NA	NA
RNU6ATAC3!	NA	NA	NA
RP11-295M1	NA	NA	NA
LINC01352	NA	NA	NA
HLX-AS1	0.77200489	0	0
HLX	1.03370146	0	0
C1orf140	NA	NA	NA
RP11-103C3.	NA	NA	NA
DUSP10	1.30646981	0.00377403	0.00398466
RP11-191N8.	NA	NA	NA
RP11-815M8	Inf	0	0
RP11-400N1:	NA	NA	NA
RP11-400N1:	NA	NA	NA
RP11-400N1:	NA	NA	NA
RP11-358H9.	NA	NA	NA
RP11-378J18	Inf	0	0
HHIPL2	NA	NA	NA
TAF1A	1.54400978	1.41E-20	1.71E-20
TAF1A-AS1	1.54400978	0.74585772	0.75024123
MIA3	1.54400978	2.74E-106	4.84E-106
RP11-378J18	2.31601467	6.05E-64	9.05E-64
AIDA	1.84959505	4.47E-34	5.83E-34
BROX	1.85095148	5.13E-39	6.86E-39
FAM177B	NA	NA	NA
RP11-452F1!	1.76703341	0.00133657	0.00142109
RP11-455P21	NA	NA	NA
DISP1	0.38600244	9.03E-79	1.43E-78
TLR5	NA	NA	NA
RP11-239E1(	NA	NA	NA
RP11-239E1(	NA	NA	NA
SUSD4	0	3.74E-220	1.00E-219
CCDC185	NA	NA	NA
CAPN8	NA	NA	NA
CAPN2	1.93240976	3.10E-87	5.07E-87
TP53BP2	1.59664648	2.62E-13	3.04E-13
RP11-504P2⁄	NA	NA	NA
FBXO28	1.93001222	1.07E-46	1.48E-46
DEGS1	1.58345529	5.28E-76	8.29E-76
RP11-365O1(	NA	2.73E-250	8.42E-250
NVL	1.75455657	4.99E-200	1.24E-199
CNIH4	1.96381692	0.00430891	0.00454807
WDR26	2.08295659	0	0
CNIH3	2.93361858	1.56E-201	3.91E-201
RP11-3L21.2	NA	NA	NA
RP11-449J1.:	NA	NA	NA

DNAH14	2.01579055	5.44E-21	6.63E-21
LBR	2.52020437	0	0
RP11-496N1:	NA	NA	NA
ENAH	1.88293876	9.74E-65	1.46E-64
RP11-145A3.	NA	NA	NA
SRP9	1.91060073	3.53E-06	3.87E-06
EPHX1	1.41057684	2.43E-289	9.07E-289
RP11-285F7.	2.31601467	1.77E-46	2.46E-46
TMEM63A	2.29028117	1.77E-11	2.03E-11
LEFTY1	Inf	5.01E-126	9.48E-126
RP4-559A3.7	NA	NA	NA
PYCR2	1.58596657	3.40E-190	8.15E-190
LEFTY2	NA	NA	NA
SDE2	1.80134474	0.12732943	0.13104955
RP11-396C2:	Inf	0	0
H3F3A	1.84289608	0	0
RP11-396C2:	6.94804401	0	0
ACBD3	2.03330865	2.88E-23	3.55E-23
RP11-275I14	NA	NA	NA
MIXL1	Inf	0	0
LIN9	2.38034841	7.37E-177	1.67E-176
PARP1	1.78247917	3.50E-98	5.99E-98
C1orf95	Inf	1.18E-175	2.66E-175
ITPKB	3.44828851	0	0
ITPKB-AS1	2.31601467	4.15E-94	7.00E-94
PSEN2	1.62746977	1.51E-40	2.04E-40
ADCK3	1.30693741	6.24E-288	2.31E-287
CDC42BPA	1.72565799	5.03E-182	1.17E-181
RP5-1087E8.	NA	NA	NA
RP11-1B20.1	NA	NA	NA
CTD-2090I13	1.15800733	5.72E-40	7.69E-40
ZNF678	2.5365875	2.69E-115	4.91E-115
SNAP47	1.77477211	6.24E-43	8.52E-43
JMJD4	1.91756053	0.39120024	0.3972881
SNAP47-AS1	NA	NA	NA
PRSS38	NA	NA	NA
WNT9A	Inf	3.24E-152	6.74E-152
WNT3A	NA	NA	NA
RP5-881P19.	NA	NA	NA
ARF1	1.75892496	1.17E-142	2.34E-142
C1orf35	1.58038697	1.37E-31	1.77E-31
MRPL55	2.18997306	0	0
GUK1	2.22090314	0	0
GJC2	Inf	2.48E-222	6.73E-222
IBA57-AS1	Inf	1.61E-43	2.21E-43

IBA57	1.26608802	3.87E-291	1.46E-290
C1orf145	NA	NA	NA
OBSCN	3.08801956	1.29E-208	3.33E-208
RP5-1139B1.	NA	NA	NA
RP5-1139B1.	NA	NA	NA
RP5-1139B1.	NA	NA	NA
RP11-245P1(	NA	NA	NA
TRIM11	1.63931903	0	0
RP11-245P1(	NA	NA	NA
RP11-245P1(	NA	4.47E-33	5.81E-33
TRIM17	NA	NA	NA
HIST3H3	NA	NA	NA
HIST3H2A	1.35549696	6.34E-100	1.09E-99
HIST3H2BB	0.51466993	1.85E-31	2.39E-31
RNF187	1.63570385	1.80E-245	5.42E-245
RHOU	1.10286413	0	0
RP5-1065P1.	NA	NA	NA
RP5-1065P1.	NA	NA	NA
RP5-1061H2(	NA	NA	NA
TMEM78	Inf	0	0
RP5-1061H2(	0.77200489	1.21E-185	2.85E-185
RAB4A	1.88795748	1.91E-25	2.39E-25
SPHAR	NA	NA	NA
RP4-803J11..	0.77200489	7.77E-74	1.21E-73
CCSAP	1.62746977	2.28E-179	5.24E-179
ACTA1	NA	NA	NA
NUP133	1.33346299	1.05E-195	2.56E-195
RP4-613A2.1	Inf	1.94E-255	6.13E-255
ABCB10	1.71880334	5.50E-06	6.01E-06
TAF5L	2.7792176	2.08E-209	5.35E-209
URB2	2.2719001	9.26E-109	1.65E-108
RP11-286M1	NA	NA	NA
RP11-552D4.	NA	NA	NA
GALNT2	2.23881418	0	0
RP5-956O18.	NA	9.93E-29	1.26E-28
RP5-956O18.	0	0	0
PGBD5	3.08801956	0.3798795	0.38605052
RP4-553F17.	NA	NA	NA
RP11-543E8.	NA	NA	NA
COG2	1.72414425	3.61E-93	6.06E-93
AGT	NA	NA	NA
RP11-99J16_	NA	NA	NA
CAPN9	NA	NA	NA
RP5-858B6.1	NA	NA	NA
C1orf198	1.42338402	3.88E-175	8.77E-175

RP5-858B6.3	NA	NA	NA
TTC13	2.21951406	9.98E-70	1.53E-69
ARV1	1.69519407	3.59E-192	8.66E-192
FAM89A	1.85884976	1.52E-52	2.17E-52
TRIM67	NA	NA	NA
RP5-1097F14	NA	NA	NA
C1orf131	1.87620582	0.00413667	0.00436677
GNPAT	1.96758768	5.70E-27	7.18E-27
EXOC8	3.08801956	1.68E-111	3.02E-111
SPRTN	1.80134474	2.97E-37	3.93E-37
EGLN1	1.36585481	0	0
RP11-295G20	1.88894814	2.27E-35	2.98E-35
TSNAX-DISC1	0	7.35E-143	1.48E-142
TSNAX	1.30066041	2.41E-222	6.53E-222
LINC00582	NA	NA	NA
DISC1	0.97787286	0.12682769	0.13056279
RP5-865N13.	NA	NA	NA
RP5-865N13.	NA	NA	NA
SIPA1L2	0	5.78E-210	1.49E-209
RP11-66A2.1	NA	NA	NA
RP11-66A2.2	NA	NA	NA
MAP10	NA	NA	NA
NTPCR	2.00721271	1.06E-18	1.28E-18
PCNXL2	0.30880196	0	0
RP5-862P8.2	2.57334963	1.93E-277	6.74E-277
KCNK1	NA	3.16E-107	5.61E-107
SLC35F3	Inf	0	0
RP4-799P18.	NA	NA	NA
RP4-799P18.	NA	NA	NA
RP5-827C21.	NA	NA	NA
RP5-827C21.	NA	NA	NA
RP5-827C21.	2.93361858	0	0
COA6	2.03122623	1.85E-63	2.77E-63
TARBP1	1.9375809	4.29E-153	8.96E-153
LINC01354	Inf	5.02E-254	1.58E-253
RP5-855F14.	NA	NA	NA
IRF2BP2	1.86458808	8.07E-34	1.05E-33
RP4-781K5.2	NA	6.19E-156	1.31E-155
LINC00184	NA	NA	NA
RP4-781K5.9	NA	NA	NA
RP4-781K5.4	NA	NA	NA
RP4-781K5.5	NA	NA	NA
RP4-781K5.6	NA	0	0
RP4-781K5.7	NA	NA	NA
LINC01132	1.15800733	2.54E-63	3.80E-63

AL360294.1	NA	NA	NA
RP11-443B7.	NA	3.00E-06	3.29E-06
RP11-443B7.	0.66171848	0	0
RP11-443B7.	1.54400978	9.75E-215	2.57E-214
LINC01348	NA	NA	NA
RP4-597N16.	1.54400978	2.17E-147	4.43E-147
TOMM20	1.90359531	3.35E-48	4.69E-48
RBM34	1.3696861	0	0
ARID4B	1.75336704	3.21E-85	5.22E-85
GGPS1	1.6600628	1.78E-221	4.82E-221
TBCE	0.97123196	0	0
B3GALNT2	1.80902638	0.00010368	0.00011174
GNG4	0.42109358	0	0
LYST	0.85778321	0	0
LYST-AS1	NA	NA	NA
RP5-940F7.2	NA	NA	NA
RP4-580N22.	NA	NA	NA
NID1	1.80134474	1.33E-19	1.61E-19
GPR137B	1.49575947	1.19E-298	4.66E-298
ERO1B	3.474022	1.39E-78	2.20E-78
EDARADD	1.54400978	1.93E-89	3.19E-89
LGALS8	1.06610199	1.66E-278	5.83E-278
LGALS8-AS1	NA	6.98E-110	1.25E-109
RP11-385F5.	0.90067237	2.26E-12	2.61E-12
RP11-385F5.	Inf	0.00223513	0.00236856
HEATR1	1.92162087	9.07E-126	1.71E-125
ACTN2	5.40403423	1.65E-207	4.22E-207
MTR	2.00450392	1.00E-22	1.23E-22
MT1HL1	NA	5.57E-181	1.29E-180
RYR2	0.51466993	3.83E-276	1.33E-275
RP11-193H5.	NA	NA	NA
ZP4	NA	NA	NA
MTRNR2L11	Inf	9.49E-287	3.48E-286
RP11-136B1	NA	NA	NA
LINC01139	NA	NA	NA
RP11-177F1	NA	0.1280168	0.13173459
RP11-544D2	NA	1.67E-130	3.21E-130
CHRM3	3.19830597	1.11E-45	1.53E-45
CHRM3-AS2	0.51466993	1.25E-06	1.38E-06
CHRM3-AS1	NA	NA	NA
RP11-35L17.	NA	NA	NA
FMN2	Inf	9.50E-278	3.33E-277
RP11-567G2	NA	NA	NA
RP11-177F1	NA	NA	NA
GREM2	NA	NA	NA

RP11-467I20	NA	NA	NA
RP11-80B9.4	NA	NA	NA
RP11-80B9.1	NA	NA	NA
RGS7	NA	NA	NA
RP11-527D7.	1.54400978	9.53E-42	1.29E-41
FH	0.7320736	0	0
KMO	3.86002445	1.05E-45	1.45E-45
OPN3	1.15271963	1.43E-286	5.23E-286
CHML	2.39872948	2.09E-24	2.59E-24
WDR64	NA	5.65E-185	1.33E-184
EXO1	1.7878008	1.66E-45	2.29E-45
BECN2	NA	NA	NA
MAP1LC3C	NA	NA	NA
PLD5	NA	NA	NA
RP11-561I11	NA	NA	NA
RP11-561I11	NA	NA	NA
RP11-261C1(	NA	NA	NA
LINC01347	NA	NA	NA
CEP170	1.88460017	2.61E-99	4.50E-99
RP11-261C1(	NA	NA	NA
RP11-261C1(	NA	NA	NA
SDCCAG8	2.62712112	6.61E-45	9.10E-45
AKT3	0	3.77E-106	6.66E-106
RP11-269F2(	NA	NA	NA
RP11-278H7.	NA	NA	NA
ZBTB18	2.86744673	6.33E-107	1.12E-106
RP11-278H7.	NA	NA	NA
RP11-278H7.	NA	NA	NA
C1orf100	NA	NA	NA
RP11-518L1(	NA	0	0
ADSS	1.59249318	0	0
C1orf101	Inf	0.56367218	0.56941593
DESI2	2.64525205	0	0
RP11-173A6.	NA	NA	NA
COX20	2.30385711	2.63E-299	1.03E-298
HNRNPU-AS:	1.85495619	0.00286731	0.00303369
RP11-11N7.5	NA	NA	NA
HNRNPU	2.00238768	7.42E-10	8.41E-10
RP11-11N7.4	NA	1.99E-192	4.81E-192
RP11-156E8.	1.93001222	1.76E-20	2.14E-20
EFCAB2	1.76748488	1.94E-106	3.43E-106
KIF26B	1.02933985	8.08E-35	1.06E-34
RP11-62I21.1	NA	NA	NA
RP11-522M2	NA	NA	NA
RP11-522M2	0	2.21E-05	2.41E-05

SMYD3	1.71199924	0	0
RP11-83A16.	NA	NA	NA
RP11-36N20.	NA	1.83E-09	2.07E-09
RP11-690C23	0	0	0
TFB2M	1.35511497	0	0
CNST	3.1247817	1.47E-256	4.69E-256
RP11-439E19	Inf	0	0
RP11-439E19	NA	2.03E-46	2.82E-46
SCCPDH	1.24737736	0	0
RP11-439E19	NA	0	0
AHCTF1	2.19411916	5.31E-224	1.45E-223
ZNF695	2.95692439	0	0
ZNF670-ZNF	3.86002445	1.74E-129	3.33E-129
ZNF670	1.43372337	1.02E-180	2.35E-180
ZNF669	2.38619693	1.07E-87	1.76E-87
C1orf229	NA	NA	NA
ZNF124	1.737011	7.89E-42	1.07E-41
RP11-488L18	2.57334963	6.14E-288	2.27E-287
RP11-488L18	0	0	0
ZNF496	2.58222325	2.05E-300	8.13E-300
NLRP3	0.77200489	3.36E-28	4.26E-28
OR2B11	NA	NA	NA
GCSAML	10.8080685	0	0
GCSAML-AS1	NA	NA	NA
OR2C3	NA	NA	NA
RP11-978I15	NA	NA	NA
OR2G2	NA	NA	NA
OR2G3	NA	NA	NA
RP11-634B7.	NA	NA	NA
OR13G1	NA	NA	NA
OR6F1	NA	NA	NA
OR14A2	NA	NA	NA
OR14K1	NA	NA	NA
OR1C1	NA	NA	NA
OR14A16	NA	NA	NA
OR11L1	NA	NA	NA
TRIM58	2.40509216	4.55E-147	9.29E-147
OR2W3	NA	1.74E-26	2.18E-26
OR2T8	NA	8.81E-159	1.88E-158
OR2AJ1	NA	NA	NA
OR2L13	NA	NA	NA
OR2L8	NA	NA	NA
OR2AK2	NA	NA	NA
OR2L5	NA	NA	NA
OR2L2	NA	NA	NA

OR2L3	NA	NA	NA
OR2M5	NA	NA	NA
OR2M2	NA	NA	NA
OR2M3	NA	NA	NA
OR2M4	NA	NA	NA
OR2T33	NA	NA	NA
OR2T12	NA	NA	NA
OR2M7	NA	NA	NA
OR14C36	NA	NA	NA
OR2T4	NA	NA	NA
OR2T6	NA	NA	NA
OR2T1	NA	NA	NA
OR2T2	NA	NA	NA
OR2T3	NA	NA	NA
RP11-407H1:	NA	NA	NA
OR2T5	NA	NA	NA
OR2G6	NA	NA	NA
RP11-438F14	NA	NA	NA
OR2T29	NA	NA	NA
OR2T34	NA	NA	NA
OR2T10	NA	NA	NA
OR2T11	NA	NA	NA
OR2T35	NA	NA	NA
OR2T27	NA	NA	NA
OR14I1	NA	NA	NA
XX-CR54.1	NA	NA	NA
LYPD8	NA	NA	NA
SH3BP5L	1.47783793	6.07E-182	1.41E-181
ZNF672	1.62057225	4.34E-303	1.75E-302
ZNF692	1.71445242	1.00E-47	1.40E-47
AL672294.1	NA	NA	NA
PGBD2	0.90067237	0.00029198	0.00031315
FAM110C	NA	NA	NA
AC079779.7	NA	NA	NA
SH3YL1	1.57488998	7.98E-42	1.08E-41
ACP1	2.08742641	3.43E-185	8.06E-185
FAM150B	NA	NA	NA
AC079779.5	NA	NA	NA
RP11-356M6	NA	NA	NA
AC079779.6	NA	NA	NA
AC105393.1	NA	NA	NA
AC105393.2	NA	NA	NA
AC093326.1	NA	NA	NA
AC093326.2	NA	NA	NA
AC093326.3	NA	NA	NA

TMEM18	1.67597643	1.17E-180	2.69E-180
AC092159.2	NA	3.85E-50	5.43E-50
AC092159.1	NA	NA	NA
AC092159.3	NA	NA	NA
AC116609.1	NA	NA	NA
RP13-539J13	NA	NA	NA
AC116609.3	NA	NA	NA
AC116609.2	NA	NA	NA
LINC01115	NA	NA	NA
AC113607.2	NA	NA	NA
AC113607.3	NA	NA	NA
AC116614.1	NA	NA	NA
SNTG2	NA	NA	NA
AC114808.3	NA	NA	NA
AC114808.2	NA	NA	NA
AC108462.1	NA	NA	NA
TPO	NA	NA	NA
AC144450.1	NA	NA	NA
AC141930.2	NA	NA	NA
AC144450.2	Inf	0	0
PXDN	NA	NA	NA
MYT1L	Inf	2.30E-34	3.01E-34
AC093390.1	NA	NA	NA
MYT1L-AS1	NA	NA	NA
AC018685.1	NA	NA	NA
AC018685.2	NA	NA	NA
AC011995.1	NA	NA	NA
AC011995.3	NA	NA	NA
LINC01250	NA	NA	NA
AC019118.3	NA	NA	NA
AC019118.4	NA	NA	NA
TSSC1	1.80512908	3.38E-104	5.92E-104
TRAPPC12	1.15800733	0	0
TRAPPC12-AS	NA	4.72E-265	1.56E-264
RP11-1293J1	Inf	0	0
ADI1	1.69049509	3.25E-190	7.79E-190
AC142528.1	1.96510336	4.71E-39	6.30E-39
AC108488.4	1.54400978	2.85E-17	3.40E-17
RP13-512J5.1	NA	7.20E-96	1.22E-95
RNASEH1	2.23248185	4.06E-138	8.02E-138
RNASEH1-AS	2.11229116	5.37E-44	7.36E-44
RPS7	1.94070585	8.86E-06	9.67E-06
COLEC11	NA	NA	NA
AC010907.2	NA	NA	NA
ALLC	NA	NA	NA

AC010907.5	NA	NA	NA
DCDC2C	NA	NA	NA
AC019172.2	NA	NA	NA
LINC01304	NA	NA	NA
AC012445.1	NA	NA	NA
LINC01249	NA	NA	NA
AC107057.1	NA	NA	NA
LINC01248	NA	NA	NA
SOX11	NA	NA	NA
AC010729.2	NA	NA	NA
AC010729.3	NA	NA	NA
AC017053.1	NA	NA	NA
LINC01247	NA	NA	NA
AC021021.2	NA	NA	NA
MIR7515HG	NA	NA	NA
LINC00487	NA	NA	NA
NRIR	Inf	0	0
CMPK2	2.71143181	0	0
RSAD2	4.96978148	0	0
RP11-439M1	6.17603912	9.96E-257	3.17E-256
RNF144A	3.33564377	0	0
AC019048.1	NA	NA	NA
RP11-557L19	Inf	7.20E-286	2.63E-285
AC092580.3	NA	NA	NA
AC092580.4	NA	NA	NA
AC007463.2	NA	NA	NA
LINC00298	NA	NA	NA
LINC00299	NA	NA	NA
U91324.1	NA	NA	NA
AC011747.3	NA	NA	NA
AC011747.4	0.77200489	3.30E-54	4.73E-54
AC011747.6	NA	NA	NA
ID2-AS1	NA	NA	NA
ID2	2.21951406	1.55E-16	1.84E-16
RP11-434B12	NA	0	0
KIDINS220	2.05099807	0.00654313	0.00688467
MBOAT2	1.28667482	0	0
RP11-734K21	NA	NA	NA
RP11-734K21	NA	NA	NA
RP11-734K21	NA	NA	NA
ASAP2	2.04980609	1.37E-15	1.62E-15
ITGB1BP1	2.30191132	0	0
CPSF3	1.51184291	0	0
IAH1	2.35277681	2.79E-119	5.16E-119
ADAM17	2.08820995	8.76E-17	1.04E-16

RP11-400L8.:	NA	NA	NA
RP11-214N9.	4.63202934	9.38E-81	1.50E-80
YWHAQ	1.44161845	0	0
RP11-521D1:	NA	NA	NA
RP11-521D1:	NA	NA	NA
RP11-521D1:	NA	NA	NA
TAF1B	1.24708482	1.67E-104	2.94E-104
RP11-95D17.	1.93001222	0.00544013	0.0057314
GRHL1	2.70201711	2.31E-140	4.61E-140
AC010969.1	NA	NA	NA
RP11-254F7.	NA	NA	NA
RP11-254F7.	Inf	0.6315441	0.63688011
KLF11	1.76458261	1.44E-19	1.74E-19
RP11-254F7.	NA	NA	NA
CYS1	Inf	1.08E-91	1.80E-91
AC104794.4	NA	NA	NA
RP11-254F7.	1.60834352	3.58E-101	6.19E-101
RRM2	1.29002772	2.21E-280	7.82E-280
C2orf48	1.77107004	1.14E-74	1.78E-74
HPCAL1	2.07819326	2.99E-36	3.94E-36
ODC1	2.12088934	4.47E-260	1.44E-259
RP11-320M2	1.15800733	1.74E-15	2.05E-15
AC007249.3	NA	NA	NA
NOL10	1.46206513	0	0
AC092687.5	NA	NA	NA
RN7SL832P	1.54400978	1.69E-27	2.13E-27
AC092687.3	NA	NA	NA
ATP6V1C2	0.19300122	0	0
RP11-791G1!	Inf	0	0
PDIA6	1.10306538	0	0
AC092687.4	NA	NA	NA
RP11-245G1:	NA	NA	NA
KCNF1	NA	NA	NA
AC062028.1	NA	2.70E-43	3.69E-43
C2orf50	0	0	0
PQLC3	1.73462827	6.53E-31	8.39E-31
ROCK2	2.22284167	1.13E-08	1.27E-08
AC099344.2	NA	NA	NA
AC099344.3	NA	NA	NA
LINC00570	Inf	3.27E-155	6.88E-155
E2F6	1.76567455	1.04E-17	1.25E-17
GREB1	3.86002445	8.09E-56	1.17E-55
NTSR2	NA	NA	NA
RP11-494G9.	NA	NA	NA
LPIN1	1.3979548	1.43E-104	2.50E-104

AC012456.3	NA	NA	NA
AC012456.4	NA	NA	NA
MIR3681HG	0	0	0
AC096559.2	NA	NA	NA
RP11-333O1.	NA	NA	NA
TRIB2	2.88215159	4.86E-09	5.47E-09
AC092635.1	NA	NA	NA
LINC00276	NA	NA	NA
AC016730.1	NA	NA	NA
AC011897.2	NA	NA	NA
FAM84A	NA	7.52E-261	2.44E-260
AC068286.1	NA	NA	NA
NBAS	2.29188952	1.29E-118	2.39E-118
AC008278.2	NA	NA	NA
DDX1	1.81454141	1.78E-196	4.37E-196
AC008278.3	NA	NA	NA
AC008271.1	NA	NA	NA
AC113608.1	NA	NA	NA
MYCNUT	NA	NA	NA
MYCNOS	NA	NA	NA
MYCN	NA	NA	NA
AC010145.4	NA	NA	NA
GACAT3	NA	NA	NA
AC142119.1	NA	NA	NA
AC010745.1	NA	NA	NA
AC010745.2	NA	NA	NA
AC010745.3	NA	NA	NA
AC010745.4	NA	NA	NA
AC010880.1	NA	NA	NA
AC104623.2	NA	NA	NA
FAM49A	1.13530131	1.01E-282	3.62E-282
AC008069.1	NA	NA	NA
AC080094.1	NA	NA	NA
RAD51AP2	NA	NA	NA
VSNL1	NA	NA	NA
SMC6	2.04948917	1.02E-22	1.26E-22
GEN1	1.82193154	2.63E-45	3.63E-45
MSGN1	NA	NA	NA
KCNS3	1.23520782	3.55E-19	4.29E-19
RP11-111J6.:	NA	5.85E-120	1.08E-119
RDH14	1.6260353	4.84E-280	1.71E-279
NT5C1B-RDH	NA	NA	NA
NT5C1B	NA	NA	NA
AC106053.1	NA	NA	NA
LINC01376	Inf	0	0

OSR1	NA	NA	NA
AC010096.1	NA	1.74E-135	3.41E-135
AC010096.2	NA	NA	NA
AC019055.1	NA	NA	NA
LINC00954	1.54400978	1.71E-144	3.45E-144
TTC32	2.16622268	2.11E-33	2.75E-33
RP11-79O8.1	Inf	0	0
WDR35	1.54400978	0.28240589	0.28791277
AC079145.4	NA	NA	NA
MATN3	NA	NA	NA
LAPTM4A	1.45377544	3.20E-248	9.76E-248
RP11-644K8.	Inf	2.96E-275	1.02E-274
AC098828.2	NA	NA	NA
SDC1	1.69841076	0.00045796	0.00048993
PUM2	2.53796608	7.63E-233	2.17E-232
RHOB	NA	NA	NA
AC023137.2	NA	NA	NA
AC012065.4	NA	NA	NA
HS1BP3	1.13615814	0	0
RP11-130L8.:	NA	NA	NA
GDF7	1.83351161	9.30E-145	1.88E-144
RP11-130L8.	1.54400978	0.75020249	0.75452787
LDAH	1.9621791	4.25E-05	4.60E-05
AC012361.1	NA	NA	NA
RP11-116D2.	NA	NA	NA
APOB	NA	NA	NA
TDRD15	NA	0	0
AC067959.1	NA	NA	NA
AC011752.1	NA	NA	NA
AC009411.2	NA	NA	NA
AC009411.1	NA	NA	NA
AC018742.1	NA	NA	NA
AC068490.2	NA	NA	NA
AC068490.1	NA	NA	NA
AC096570.2	NA	NA	NA
AC096570.1	NA	NA	NA
AC016768.1	NA	NA	NA
AC012506.1	NA	NA	NA
AC012506.2	NA	NA	NA
AC012506.3	NA	NA	NA
AC012506.4	NA	NA	NA
KLHL29	NA	NA	NA
AC011239.1	NA	NA	NA
RP11-557N2	NA	3.43E-17	4.09E-17
ATAD2B	2.00721271	1.77E-282	6.33E-282

UBXN2A	2.10908552	1.38E-164	3.02E-164
MFSD2B	1.80134474	1.93E-159	4.13E-159
C2orf44	1.68437431	3.69E-149	7.60E-149
FKBP1B	1.77256386	0.69642001	0.70117386
SF3B6	1.7421304	5.02E-298	1.96E-297
FAM228B	2.04029864	3.14E-20	3.81E-20
TP53I3	1.80649144	1.28E-228	3.59E-228
PFN4	2.70201711	2.10E-52	3.00E-52
RP11-507M3	NA	NA	NA
AC008073.7	NA	NA	NA
FAM228A	2.31601467	3.81E-23	4.70E-23
AC008073.9	NA	1.81E-107	3.23E-107
ITSN2	1.61172951	2.23E-17	2.66E-17
AC009228.1	NA	8.60E-41	1.16E-40
NCOA1	3.16154384	4.52E-135	8.83E-135
PTRHD1	1.79968986	7.51E-17	8.94E-17
CENPO	1.83187601	0.00035094	0.00037615
ADCY3	1.91163116	2.03E-16	2.41E-16
RP11-443B2(	2.57334963	9.05E-198	2.23E-197
DNAJC27	3.98869193	2.65E-102	4.61E-102
DNAJC27-AS	0.77200489	1.14E-88	1.87E-88
EFR3B	NA	1.72E-145	3.49E-145
POMC	NA	NA	NA
LINC01381	NA	NA	NA
DNMT3A	2.60043752	1.05E-55	1.52E-55
AC012074.2	NA	1.53E-28	1.94E-28
DTNB	1.54400978	0	0
AC104699.1	NA	NA	NA
ASXL2	2.06821063	2.16E-77	3.41E-77
KIF3C	1.72565799	0.38111961	0.38722402
RAB10	1.46754923	0	0
GAREM2	2.27804722	4.06E-231	1.15E-230
HADHA	1.48114293	0	0
HADHB	1.07295595	0	0
ADGRF3	Inf	1.80E-307	7.40E-307
EPT1	2.91369588	5.06E-143	1.02E-142
DRC1	NA	NA	NA
OTOF	NA	NA	NA
C2orf70	NA	NA	NA
CIB4	NA	NA	NA
AC015977.6	NA	NA	NA
KCNK3	NA	NA	NA
SLC35F6	1.82251787	5.53E-63	8.25E-63
CENPA	1.58840006	0	0
DPYSL5	NA	5.25E-07	5.80E-07

AC013472.4	NA	NA	NA
MAPRE3	Inf	6.85E-218	1.83E-217
TMEM214	1.61499874	6.79E-91	1.13E-90
AGBL5	1.70173121	4.47E-50	6.32E-50
AGBL5-AS1	1.54400978	0.04000758	0.04156554
RP11-503P1(	Inf	0	0
RP11-195B1	0	0	0
OST4	2.50426482	0	0
EMILIN1	1.958605	7.82E-05	8.44E-05
KHK	1.49989521	2.25E-74	3.52E-74
CGREF1	NA	NA	NA
ABHD1	2.12301345	2.92E-228	8.15E-228
PREB	1.80285848	1.02E-07	1.13E-07
PRR30	NA	NA	NA
TCF23	NA	8.77E-07	9.67E-07
SLC5A6	1.96608722	9.22E-224	2.52E-223
ATRAID	1.6262726	1.68E-157	3.57E-157
CAD	2.23522346	1.65E-136	3.23E-136
SLC30A3	1.3493867	0	0
DNAJC5G	NA	3.54E-35	4.64E-35
TRIM54	NA	NA	NA
UCN	2.87357376	0.14300071	0.1469536
MPV17	1.69953164	0	0
GTF3C2	2.5365875	2.49E-253	7.79E-253
GTF3C2-AS1	0	0	0
AC074117.1(	2.1378597	2.32E-109	4.16E-109
EIF2B4	1.19197555	0	0
SNX17	1.44425634	0	0
ZNF513	2.01908971	6.29E-34	8.21E-34
PPM1G	1.69856081	2.70E-284	9.79E-284
NRBP1	1.13962627	0	0
KRTCAP3	Inf	9.86E-300	3.89E-299
IFT172	0.8822913	8.60E-213	2.25E-212
FNDC4	0.72911573	0	0
GCKR	NA	NA	NA
AC109829.1	NA	NA	NA
AC109829.2	NA	NA	NA
C2orf16	NA	NA	NA
ZNF512	1.88603726	2.32E-54	3.33E-54
CCDC121	0.66171848	3.34E-201	8.34E-201
GPN1	1.62816197	3.79E-120	7.04E-120
SUPT7L	1.27548634	8.79E-251	2.72E-250
SLC4A1AP	1.35685708	0	0
LINC01460	NA	2.68E-166	5.88E-166
MRPL33	1.84462738	0.06163749	0.0638367

RBKS	1.06892985	2.08E-193	5.04E-193
BRE	1.38289572	8.76E-261	2.84E-260
AC093690.1	NA	NA	NA
FLJ31356	NA	NA	NA
FOSL2	1.33814181	1.31E-210	3.38E-210
RP11-373D2:	NA	NA	NA
AC104695.4	NA	NA	NA
PLB1	0	0	0
AC074011.2	NA	NA	NA
AC097724.3	Inf	6.37E-202	1.59E-201
PPP1CB	1.77679895	9.34E-98	1.60E-97
SPDYA	Inf	1.58E-45	2.19E-45
RP11-713D1!	Inf	0.01915006	0.02001039
TRMT61B	1.74222725	2.36E-189	5.64E-189
WDR43	1.89924391	2.59E-104	4.53E-104
FAM179A	NA	NA	NA
C2orf71	NA	NA	NA
AC105398.3	NA	NA	NA
CLIP4	0.25733496	0	0
ALK	NA	NA	NA
AC106870.1	NA	NA	NA
AC106870.2	NA	NA	NA
AC016907.3	NA	0	0
YPEL5	1.89141198	0.00180778	0.00191816
LBH	0.94356153	0	0
AC109642.1	NA	NA	NA
LCLAT1	1.96510336	5.75E-34	7.51E-34
CAPN13	NA	NA	NA
GALNT14	1.68781461	0	0
AC009305.1	NA	NA	NA
CAPN14	NA	NA	NA
EHD3	NA	NA	NA
XDH	NA	NA	NA
SRD5A2	0	0	0
AL133247.2	NA	NA	NA
AL133249.1	NA	NA	NA
RP11-1057B(	0	4.38E-184	1.02E-183
MEMO1	2.18734719	0.38304477	0.38913642
DPY30	2.02767549	1.91E-57	2.78E-57
CTC-336P14.:	Inf	1.93E-81	3.09E-81
SPAST	2.42630108	0.01244091	0.01303286
RP11-563N4.	2.85641809	1.45E-97	2.47E-97
SLC30A6	2.26087146	3.79E-248	1.16E-247
NLRC4	Inf	3.41E-260	1.10E-259
YIPF4	1.56311881	1.09E-15	1.29E-15

AL133245.2	1.737011	2.97E-34	3.88E-34
BIRC6	2.1314048	1.69E-27	2.13E-27
BIRC6-AS1	NA	NA	NA
BIRC6-AS2	NA	NA	NA
TTC27	1.15800733	4.48E-99	7.70E-99
LINC00486	NA	NA	NA
AL121656.5	NA	NA	NA
LTBP1	Inf	2.20E-144	4.45E-144
RASGRP3	1.54400978	7.60E-94	1.28E-93
AC020594.5	Inf	0	0
FAM98A	2.07011682	1.81E-114	3.29E-114
LINC01317	NA	NA	NA
LINC01318	NA	NA	NA
AC073218.1	NA	NA	NA
LINC01320	NA	NA	NA
RP11-529E15	NA	NA	NA
AC073218.3	NA	NA	NA
AC012593.1	NA	5.31E-25	6.61E-25
AC019064.1	NA	NA	NA
RP11-490M8	1.54400978	4.71E-112	8.50E-112
CRIM1	1.18374083	0.00037849	0.00040553
RP11-78I14.1	NA	6.49E-09	7.29E-09
FEZ2	1.41077036	3.84E-250	1.18E-249
VIT	NA	NA	NA
STRN	1.43933115	9.78E-09	1.10E-08
HEATR5B	2.57334963	2.03E-26	2.55E-26
GPATCH11	2.22186773	1.36E-12	1.57E-12
EIF2AK2	2.37343652	0	0
SULT6B1	NA	NA	NA
CEBPZOS	1.72941253	3.09E-72	4.78E-72
CEBPZ	2.4881509	0	0
NDUFAF7	1.50265238	2.48E-201	6.20E-201
PRKD3	2.09708791	0.00698257	0.00734364
AC007391.2	Inf	0	0
QPCT	NA	2.70E-37	3.58E-37
AC006369.2	NA	NA	NA
CDC42EP3	1.19793862	1.34E-187	3.17E-187
AC006369.3	NA	NA	NA
LINC00211	Inf	1.92E-29	2.45E-29
RMDN2	NA	NA	NA
RMDN2-AS1	Inf	1.10E-210	2.86E-210
CYP1B1	3.37149011	0	0
CYP1B1-AS1	0.77200489	9.02E-07	9.94E-07
AC009229.6	NA	NA	NA
RP11-314C9.	NA	NA	NA

AC009229.5	NA	NA	NA
ATL2	1.59226009	1.49E-94	2.52E-94
AC016995.3	NA	NA	NA
AC093822.1	NA	NA	NA
HNRNPLL	1.32818046	0	0
AC011247.3	NA	NA	NA
GALM	0	3.18E-190	7.62E-190
AC074366.3	NA	1.18E-206	3.01E-206
SRSF7	1.84063311	1.85E-42	2.52E-42
GEMIN6	2.04860334	1.36E-22	1.67E-22
DHX57	2.2662079	2.53E-19	3.05E-19
AC018693.6	NA	NA	NA
MORN2	1.99929472	1.04E-55	1.50E-55
ARHGEF33	NA	NA	NA
RP11-173C1.	NA	NA	NA
SOS1	2.36142672	1.61E-113	2.92E-113
CDKL4	1.737011	4.09E-48	5.72E-48
MAP4K3	2.00721271	7.95E-16	9.40E-16
AC007246.3	2.12301345	2.10E-236	6.08E-236
TMEM178A	0	0	0
THUMPD2	2.2166477	1.53E-124	2.87E-124
SLC8A1-AS1	NA	NA	NA
SLC8A1	0	0	0
AC007317.1	NA	NA	NA
AC104654.1	NA	NA	NA
AC104654.2	NA	NA	NA
C2orf91	NA	NA	NA
AC013480.2	NA	NA	NA
PKDCC	1.78154975	2.60E-141	5.19E-141
AC083949.1	NA	NA	NA
EML4	2.18251006	1.18E-207	3.03E-207
COX7A2L	1.84045966	9.39E-92	1.57E-91
KCNG3	0.25733496	9.93E-233	2.83E-232
MTA3	1.04913485	4.50E-275	1.56E-274
OXER1	1.93001222	3.43E-203	8.63E-203
HAAO	1.63483388	0	0
AC016735.1	NA	NA	NA
AC016735.2	NA	NA	NA
AC016735.3	NA	NA	NA
AC093609.1	NA	8.62E-22	1.05E-21
ZFP36L2	1.93729529	1.21E-31	1.57E-31
LINC01126	NA	0.39240116	0.3984408
AC010883.5	6.17603912	7.43E-35	9.74E-35
THADA	2.29621967	1.34E-19	1.62E-19
PLEKHH2	1.02933985	3.77E-230	1.06E-229

C1GALT1C1L	NA	NA	NA
DYNC2LI1	0.93743451	3.36E-224	9.19E-224
ABCG5	NA	NA	NA
ABCG8	NA	NA	NA
LRPPRC	2.04654126	1.83E-156	3.87E-156
RP11-559M2	Inf	4.21E-226	1.16E-225
PPM1B	1.56079249	1.18E-300	4.68E-300
SLC3A1	NA	9.07E-128	1.72E-127
PREPL	1.44183266	2.03E-16	2.41E-16
CAMKMT	1.65257297	1.23E-223	3.37E-223
RP11-89K21.	2.7792176	1.81E-202	4.54E-202
SIX3-AS1	1.69841076	0.07286604	0.07537981
SIX3	2.12934136	5.44E-59	7.99E-59
AC012354.6	1.68437431	4.50E-50	6.35E-50
SIX2	0.38600244	6.07E-97	1.03E-96
AC093702.1	NA	0	0
AC009236.2	1.93001222	4.48E-120	8.31E-120
SRBD1	1.72929095	2.22E-220	5.98E-220
PRKCE	2.07222365	9.16E-82	1.47E-81
U51244.2	NA	NA	NA
AC017006.2	NA	NA	NA
AC017006.3	NA	NA	NA
EPAS1	0	2.96E-219	7.92E-219
AC016912.3	NA	6.44E-29	8.20E-29
TMEM247	0	0	0
ATP6V1E2	2.5572662	8.47E-156	1.79E-155
RP11-417F21	NA	NA	NA
RHOQ	2.11650779	1.17E-74	1.84E-74
RP11-417F21	NA	1.58E-174	3.56E-174
PIGF	1.93001222	4.27E-05	4.63E-05
CRIPT	2.00291052	0.00047622	0.00050935
SOCS5	2.13436646	9.82E-08	1.09E-07
RP11-333I13	NA	0	0
LINC01118	Inf	0	0
LINC01119	Inf	0.01674121	0.0175034
AC016722.3	0	0	0
AC016722.4	NA	8.99E-38	1.19E-37
MCFD2	2.0563403	5.91E-13	6.85E-13
TTC7A	2.08012429	6.27E-23	7.72E-23
RP11-15I20.1	NA	NA	NA
AC093732.1	NA	NA	NA
C2orf61	NA	NA	NA
RP11-761B3.	NA	NA	NA
AC073283.7	NA	0	0
CALM2	1.69179523	0	0

AC073283.4	NA	NA	NA
AC106869.2	NA	NA	NA
BCYRN1	3.52227231	0	0
EPCAM	NA	NA	NA
MSH2	1.54400978	1.38E-161	2.98E-161
KCNK12	1.54400978	7.57E-71	1.17E-70
HCG2040054	Inf	9.87E-05	0.00010639
MSH6	1.76656975	1.19E-57	1.74E-57
FBXO11	1.44439625	1.45E-189	3.47E-189
AC079807.2	Inf	0	0
AC079807.4	NA	NA	NA
FOXN2	2.1471386	0.39790981	0.40401163
RP11-191L17	1.54400978	9.14E-179	2.09E-178
PPP1R21	1.48462479	0	0
STON1	2.18734719	2.67E-34	3.48E-34
STON1-GTF2	NA	NA	NA
GTF2A1L	NA	NA	NA
RP11-310N1	NA	NA	NA
LHCGR	NA	NA	NA
RP11-460M2	NA	NA	NA
FSHR	NA	NA	NA
RP11-152D8.	NA	NA	NA
RP11-443F4.	NA	NA	NA
NRXN1	NA	NA	NA
RP11-358H1	NA	NA	NA
AC007682.1	NA	NA	NA
AC087073.1	NA	NA	NA
AC139712.4	NA	NA	NA
AC010967.2	NA	NA	NA
AC010967.3	NA	NA	NA
ASB3	2.49416964	2.30E-191	5.54E-191
GPR75-ASB3	NA	NA	NA
CHAC2	1.98299295	1.10E-06	1.21E-06
ERLEC1	2.09700619	1.62E-44	2.22E-44
GPR75	NA	2.83E-66	4.27E-66
PSME4	2.12301345	8.04E-146	1.63E-145
ACYP2	1.11093387	1.30E-299	5.11E-299
RP11-477N3.	Inf	0	0
TSPYL6	NA	NA	NA
C2orf73	NA	NA	NA
SPTBN1	1.35277921	1.68E-282	6.02E-282
AC092839.3	NA	NA	NA
AC092839.4	NA	NA	NA
AC093110.3	NA	NA	NA
EML6	NA	0	0

AC104781.1	NA	NA	NA
RTN4	1.41472072	0	0
CLHC1	2.57334963	2.10E-12	2.42E-12
AC012358.7	NA	NA	NA
RPS27A	1.76546471	2.73E-86	4.47E-86
MTIF2	1.830477	3.87E-222	1.05E-221
AC012358.8	1.54400978	2.28E-24	2.83E-24
CCDC88A	1.77234925	0	0
CFAP36	1.48431868	3.27E-216	8.66E-216
PPP4R3B	2.34658912	2.75E-295	1.06E-294
RP11-554J4.1	2.31601467	2.18E-122	4.06E-122
PNPT1	1.71647896	4.78E-31	6.14E-31
EFEMP1	NA	NA	NA
RP11-481J13	NA	NA	NA
MIR217HG	NA	NA	NA
AC011306.1	NA	NA	NA
AC007743.1	NA	NA	NA
CCDC85A	NA	NA	NA
VRK2	1.49154343	8.58E-198	2.12E-197
FANCL	1.6328245	0	0
RP11-334G2	2.57334963	1.89E-12	2.18E-12
AC007250.4	NA	NA	NA
LINC01122	Inf	2.76E-196	6.77E-196
AC007131.1	NA	NA	NA
RP11-444A2	NA	7.69E-227	2.13E-226
AC007131.2	NA	NA	NA
AC007179.1	NA	NA	NA
AC007381.3	NA	NA	NA
MIR4432HG	NA	NA	NA
BCL11A	2.46888693	1.95E-07	2.16E-07
AC009970.1	NA	NA	NA
PAPOLG	1.45318568	4.78E-64	7.16E-64
LINC01185	NA	NA	NA
REL	1.78704836	1.04E-06	1.15E-06
PUS10	3.76352384	1.10E-50	1.56E-50
PEX13	1.59413997	6.05E-31	7.77E-31
KIAA1841	2.50901589	3.16E-155	6.65E-155
C2orf74	0	0	0
RP11-493E12	NA	NA	NA
AHSA2	1.70127004	6.44E-59	9.44E-59
USP34	2.1081672	5.46E-157	1.16E-156
RP11-355B1	0.77200489	1.20E-247	3.65E-247
XPO1	2.0690621	0.03502354	0.03643749
FAM161A	1.3081194	1.43E-210	3.70E-210
CCT4	1.45431208	0	0

AC107081.5	Inf	7.64E-304	3.09E-303
COMMD1	2.03665838	0.00022822	0.00024513
AC107081.1	NA	NA	NA
AC018462.2	NA	NA	NA
B3GNT2	2.01978024	1.40E-06	1.54E-06
AC093159.1	0.57900367	1.36E-99	2.34E-99
TMEM17	2.85047959	0	0
AC092155.4	NA	NA	NA
EHBP1	2.39587724	7.14E-159	1.52E-158
AC007098.1	NA	NA	NA
AC009501.4	0.64333741	3.92E-06	4.29E-06
OTX1	2.44468215	3.62E-245	1.09E-244
WDPCP	1.65429619	7.25E-64	1.08E-63
MDH1	0.84948208	0	0
AC016734.1	NA	NA	NA
UGP2	1.38035897	0	0
VPS54	2.11462209	0.00010128	0.00010916
AC012368.1	NA	NA	NA
PELI1	1.15800733	3.20E-06	3.51E-06
AC074289.1	Inf	8.15E-134	1.58E-133
LINC00309	NA	NA	NA
AC114752.1	NA	NA	NA
AC114752.3	NA	NA	NA
AC008074.3	NA	NA	NA
LGALSL	2.8306846	1.50E-86	2.45E-86
AC008074.4	NA	NA	NA
RP11-568N6.	1.54400978	1.34E-17	1.61E-17
AFTPH	2.77349905	6.91E-91	1.15E-90
AC008074.1	NA	NA	NA
AC007365.1	NA	NA	NA
SERTAD2	1.9981303	2.55E-22	3.12E-22
AC007365.3	0.77200489	2.96E-29	3.77E-29
AC007880.1	Inf	1.75E-125	3.30E-125
AC007386.2	NA	NA	NA
SLC1A4	1.09862234	3.03E-156	6.39E-156
AC007386.4	NA	NA	NA
CEP68	2.46306322	8.65E-227	2.39E-226
RAB1A	1.56068098	1.07E-209	2.76E-209
ACTR2	2.38444238	0	0
SPRED2	1.67581549	9.17E-65	1.38E-64
AC074391.1	NA	0	0
AC012370.3	NA	6.93E-33	8.99E-33
AC007389.3	Inf	0	0
RP11-418H1(	NA	0.00345698	0.00365246
RP11-340F1(	NA	NA	NA

AC118345.1	NA	NA	NA
AC092669.6	NA	NA	NA
AC092669.3	NA	0	0
MEIS1-AS3	NA	NA	NA
MEIS1	1.56891316	0	0
MEIS1-AS2	0.51466993	9.06E-275	3.13E-274
AC007392.3	NA	NA	NA
AC007392.4	NA	NA	NA
AC007403.2	NA	NA	NA
LINC01628	NA	NA	NA
AC007403.3	NA	NA	NA
AC023115.2	NA	NA	NA
AC078941.1	NA	NA	NA
AC023115.4	NA	NA	NA
ETAA1	1.76458261	7.92E-06	8.64E-06
AC007422.1	NA	NA	NA
AC010987.5	NA	NA	NA
AC010987.6	NA	NA	NA
C1D	1.61374972	4.75E-243	1.42E-242
WDR92	1.43544659	7.53E-278	2.64E-277
RP11-474G2	NA	1.63E-15	1.92E-15
PNO1	1.61601024	4.10E-129	7.83E-129
PPP3R1	3.474022	4.28E-97	7.29E-97
RP11-474G2	1.54400978	7.82E-81	1.25E-80
RP11-474G2	2.0844132	3.50E-35	4.60E-35
CNRIP1	1.8725225	6.98E-193	1.69E-192
AC015969.3	NA	NA	NA
PLEK	1.81698388	3.29E-123	6.16E-123
FBXO48	1.74987775	9.15E-103	1.59E-102
APLF	6.17603912	8.00E-151	1.66E-150
PROKR1	NA	0	0
ARHGAP25	2.01579055	9.13E-170	2.03E-169
AC097495.2	Inf	7.15E-156	1.51E-155
AC097495.3	NA	NA	NA
BMP10	NA	NA	NA
GKN2	NA	NA	NA
GKN1	NA	NA	NA
ANTXR1	0.51466993	2.93E-167	6.45E-167
GFPT1	1.28667482	2.57E-170	5.72E-170
NFU1	1.80401974	5.12E-151	1.06E-150
AAK1	2.28632217	0.00289467	0.00306246
ANXA4	1.25031227	0	0
AC092431.3	NA	NA	NA
GMCL1	2.10491958	3.49E-149	7.20E-149
SNRNP27	1.96575319	3.70E-81	5.92E-81

MXD1	1.28667482	1.19E-22	1.46E-22
AC019206.1	NA	NA	NA
ASPRV1	NA	3.33E-41	4.51E-41
PCBP1	1.55084168	0	0
AC016700.2	NA	NA	NA
AC016700.5	1.15800733	7.10E-43	9.68E-43
C2orf42	1.80134474	1.87E-09	2.12E-09
TIA1	1.88211411	0.28120542	0.28672111
PCYOX1	1.51184291	4.66E-130	8.93E-130
SNRPG	2.13196129	8.49E-219	2.27E-218
FAM136A	2.28238122	5.28E-224	1.44E-223
AC022201.5	Inf	3.65E-85	5.94E-85
AC022201.4	NA	NA	NA
TGFA	NA	NA	NA
ADD2	1.95032814	1.83E-133	3.56E-133
AC007395.3	NA	NA	NA
FIGLA	NA	5.79E-176	1.31E-175
CLEC4F	0	2.31E-141	4.62E-141
CD207	NA	1.12E-186	2.65E-186
LINC01143	Inf	0	0
VAX2	1.28152812	1.91E-110	3.44E-110
ATP6V1B1	Inf	2.16E-159	4.61E-159
AC007040.11	4.54625102	0	0
ATP6V1B1-A	2.20572826	5.08E-264	1.67E-263
ANKRD53	NA	NA	NA
TEX261	2.16553914	3.04E-13	3.53E-13
AC007040.6	NA	NA	NA
AC007040.8	NA	NA	NA
NAGK	1.60119533	0	0
RP11-467P9.	NA	1.28E-21	1.56E-21
MCEE	2.46076559	2.27E-24	2.82E-24
MPHOSPH10	1.64398164	7.80E-219	2.09E-218
PAIP2B	1.67267726	1.77E-24	2.20E-24
ZNF638	1.47117913	0	0
DYSF	NA	NA	NA
CYP26B1	NA	4.73E-22	5.79E-22
EXOC6B	8.10605134	3.66E-246	1.11E-245
SPR	1.34508827	0	0
EMX1	NA	NA	NA
RP11-466M2	NA	NA	NA
SFXN5	2.09727995	3.85E-292	1.46E-291
RAB11FIP5	2.06698083	3.67E-253	1.15E-252
NOTO	NA	NA	NA
SMYD5	1.32551783	2.35E-21	2.87E-21
PRADC1	2.24293654	4.32E-281	1.54E-280

CCT7	1.01806796	0	0
FBXO41	2.58139135	1.38E-16	1.65E-16
EGR4	Inf	1.53E-40	2.06E-40
ALMS1	2.36925639	0.12715086	0.13088804
NAT8	NA	NA	NA
TPRKB	1.96730715	4.58E-86	7.48E-86
RP11-434P11	NA	0	0
DUSP11	0.93094707	0	0
C2orf78	NA	NA	NA
STAMBP	1.49821288	2.02E-190	4.83E-190
ACTG2	NA	NA	NA
DGUOK	2.16136919	0	0
DGUOK-AS1	3.564363	0	0
AC073046.25	NA	3.05E-149	6.28E-149
TET3	2.2838478	3.88E-145	7.86E-145
RP11-287D1.	NA	NA	NA
BOLA3	2.76337495	0	0
BOLA3-AS1	2.31601467	3.68E-10	4.18E-10
MOB1A	1.93822504	2.65E-39	3.55E-39
RP11-287D1.	Inf	0	0
MTHFD2	1.72484706	3.02E-199	7.50E-199
RP11-287D1.	NA	NA	NA
SLC4A5	0	0.00125723	0.00133759
DCTN1	1.90558169	2.46E-27	3.10E-27
DCTN1-AS1	NA	NA	NA
C2orf81	0	0	0
WDR54	1.28158915	0	0
RTKN	1.58942183	0.00045367	0.00048543
INO80B	2.93361858	0	0
INO80B-WBI	NA	NA	NA
WBP1	2.31601467	1.18E-241	3.50E-241
MOGS	1.82386155	1.53E-204	3.86E-204
MRPL53	1.93001222	3.23E-274	1.11E-273
CCDC142	1.70373493	1.24E-06	1.37E-06
TTC31	2.72775061	8.09E-147	1.65E-146
LBX2	NA	NA	NA
RP11-523H2(	Inf	0	0
LBX2-AS1	NA	6.13E-212	1.60E-211
PCGF1	1.4826003	4.01E-40	5.39E-40
TLX2	NA	NA	NA
DQX1	1.54400978	1.02E-89	1.69E-89
AUP1	1.68859335	6.27E-204	1.58E-203
HTRA2	1.7595885	1.26E-294	4.84E-294
LOXL3	2.14818752	2.17E-08	2.42E-08
DOK1	1.66575966	0	0

M1AP	1.28667482	8.03E-236	2.32E-235
SEMA4F	3.86002445	5.07E-293	1.92E-292
RP11-259N1!	3.02368582	8.87E-206	2.25E-205
HK2	3.04808827	0	0
LINC01291	Inf	7.54E-100	1.30E-99
AC104135.2	Inf	0	0
LINC01293	NA	NA	NA
POLE4	1.78605943	2.69E-90	4.47E-90
TACR1	NA	NA	NA
RP11-355F1(	0.77200489	5.21E-273	1.79E-272
EVA1A	NA	NA	NA
AC007099.2	NA	NA	NA
AC007099.1	NA	NA	NA
MRPL19	1.52406133	3.69E-270	1.25E-269
GCFC2	2.57111194	0	0
RP11-342K6.	4.24602689	2.54E-22	3.12E-22
RP11-342K6.	NA	NA	NA
RP11-342K6.	NA	5.30E-214	1.39E-213
LRRTM4	NA	NA	NA
AC079117.1	NA	NA	NA
AC084149.2	NA	NA	NA
AC084149.1	0	0	0
AC073628.1	NA	NA	NA
AC012494.1	NA	NA	NA
AC092660.1	NA	NA	NA
REG3G	NA	NA	NA
REG1B	NA	NA	NA
REG1A	NA	NA	NA
REG3A	NA	NA	NA
CTNNA2	NA	0	0
AC010975.1	NA	NA	NA
AC016716.2	NA	NA	NA
LRRTM1	NA	NA	NA
AC008067.2	NA	NA	NA
AC084193.1	NA	NA	NA
AC012075.2	NA	NA	NA
AC079896.1	NA	NA	NA
AC010105.1	NA	NA	NA
AC010744.1	NA	NA	NA
AC106874.1	NA	NA	NA
SUCLG1	1.29051564	0	0
DNAH6	NA	NA	NA
TRABD2A	1.92059753	0.25227928	0.25757503
AC022210.1	NA	NA	NA
TMSB10	1.93299074	1.31E-13	1.52E-13

KCMF1	2.17858972	1.60E-293	6.09E-293
RP11-60H5.1	NA	NA	NA
TCF7L1	1.17479005	5.99E-245	1.80E-244
TGOLN2	2.04817624	0.00014396	0.00015495
RETSAT	2.68363605	0.00116762	0.0012424
ELMOD3	1.85281174	4.62E-223	1.26E-222
RP11-717A5.	NA	NA	NA
CAPG	1.48752162	0	0
SH2D6	NA	NA	NA
MAT2A	2.61990269	0	0
GGCX	2.26367536	2.99E-163	6.48E-163
VAMP8	2.04098793	2.03E-91	3.39E-91
AC016753.2	NA	NA	NA
VAMP5	1.31240831	2.32E-24	2.87E-24
RNF181	1.87617068	1.44E-138	2.86E-138
TMEM150A	1.83260974	0.00056981	0.00060895
USP39	1.53946857	0	0
C2orf68	2.17565014	0.47102067	0.47701557
SFTPB	NA	NA	NA
GNLY	NA	1.13E-07	1.26E-07
ATOH8	0	0	0
AC105053.3	NA	NA	NA
ST3GAL5	1.77561125	0.01115293	0.01169034
ST3GAL5-AS	NA	NA	NA
RP11-548P2.	0	9.69E-64	1.45E-63
POLR1A	2.13854228	2.05E-145	4.16E-145
PTCD3	1.77130056	1.02E-149	2.10E-149
IMMT	1.41848053	0	0
RP11-301O1	0.38600244	2.90E-35	3.82E-35
MRPL35	1.58115091	0	0
REEP1	Inf	8.29E-233	2.36E-232
KDM3A	2.08895441	4.77E-85	7.75E-85
CHMP3	2.1849195	1.67E-188	3.97E-188
RNF103-CHN	NA	3.25E-123	6.09E-123
AC015971.2	NA	0.00098505	0.00104888
RNF103	3.66702323	7.47E-128	1.42E-127
RMND5A	1.86969934	5.01E-69	7.67E-69
CD8A	NA	NA	NA
CD8B	1.18706128	0	0
RGPD1	NA	NA	NA
PLGLB1	1.15800733	1.11E-167	2.44E-167
RP11-223J6.	NA	NA	NA
IGKV3OR2-2	NA	NA	NA
AC133644.2	0	6.07E-228	1.69E-227
LINC00152	2.42187487	0	0

RP11-1399P1	NA	NA	NA
PLGLB2	NA	NA	NA
RGPD2	NA	NA	NA
KRCC1	1.45136919	0	0
SMYD1	NA	NA	NA
FABP1	NA	NA	NA
THNSL2	NA	NA	NA
FOXI3	NA	NA	NA
TEX37	NA	NA	NA
AC104134.2	NA	NA	NA
EIF2AK3	1.19309847	1.07E-50	1.51E-50
AC062029.1	Inf	2.12E-16	2.51E-16
RPIA	1.66991616	1.07E-117	1.97E-117
AC096579.13	NA	NA	NA
IGKC	0.77200489	2.32E-08	2.59E-08
IGKV1-12	NA	NA	NA
IGKV1-8	NA	NA	NA
IGKJ5	NA	NA	NA
IGKJ4	NA	NA	NA
IGKJ3	NA	NA	NA
IGKJ2	NA	NA	NA
IGKJ1	NA	NA	NA
IGKV4-1	NA	NA	NA
IGKV5-2	NA	NA	NA
IGKV7-3	NA	NA	NA
IGKV2-4	NA	NA	NA
IGKV1-5	NA	NA	NA
IGKV1-6	NA	NA	NA
IGKV3-7	NA	NA	NA
IGKV1-9	NA	NA	NA
IGKV2-10	NA	NA	NA
IGKV3-11	NA	NA	NA
IGKV1-13	NA	NA	NA
IGKV2-14	NA	NA	NA
IGKV3-15	NA	NA	NA
IGKV1-16	NA	NA	NA
IGKV1-17	NA	NA	NA
IGKV2-18	NA	NA	NA
IGKV2-19	NA	NA	NA
IGKV3-20	NA	NA	NA
IGKV6-21	NA	NA	NA
IGKV1-22	NA	NA	NA
IGKV2-23	NA	NA	NA
IGKV2-24	NA	NA	NA
IGKV3-25	NA	NA	NA

IGKV2-26	NA	NA	NA
IGKV1-27	NA	NA	NA
IGKV2-28	NA	NA	NA
IGKV2-29	NA	NA	NA
IGKV2-30	NA	NA	NA
IGKV3-31	NA	NA	NA
IGKV1-32	NA	NA	NA
IGKV1-33	NA	NA	NA
IGKV3-34	NA	NA	NA
IGKV1-35	NA	NA	NA
IGKV2-36	NA	NA	NA
IGKV1-37	NA	NA	NA
IGKV2-38	NA	NA	NA
IGKV1-39	NA	NA	NA
IGKV2-40	NA	NA	NA
IGKV2D-40	NA	NA	NA
IGKV1D-39	NA	NA	NA
IGKV2D-38	NA	NA	NA
IGKV1D-37	NA	NA	NA
IGKV2D-36	NA	NA	NA
IGKV1D-35	NA	NA	NA
IGKV3D-34	NA	NA	NA
IGKV1D-33	NA	NA	NA
IGKV1D-32	NA	NA	NA
IGKV3D-31	NA	NA	NA
IGKV2D-30	NA	NA	NA
IGKV2D-29	NA	NA	NA
IGKV2D-28	NA	NA	NA
IGKV1D-27	NA	NA	NA
IGKV2D-26	NA	NA	NA
IGKV3D-25	NA	NA	NA
IGKV2D-24	NA	NA	NA
IGKV2D-23	NA	NA	NA
IGKV1D-22	NA	NA	NA
IGKV6D-21	NA	NA	NA
IGKV3D-20	NA	NA	NA
IGKV2D-19	NA	NA	NA
IGKV2D-18	NA	NA	NA
IGKV6D-41	NA	NA	NA
IGKV1D-17	NA	NA	NA
IGKV1D-16	NA	NA	NA
IGKV3D-15	NA	NA	NA
IGKV3D-11	NA	NA	NA
IGKV2D-14	NA	NA	NA
IGKV1D-13	NA	NA	NA

IGKV1D-12	NA	NA	NA
IGKV2D-10	NA	NA	NA
IGKV1D-42	NA	NA	NA
IGKV1D-43	NA	NA	NA
IGKV1D-8	NA	NA	NA
IGKV3D-7	NA	NA	NA
IGKV1OR2-1	NA	NA	NA
CH17-132F21	NA	NA	NA
RP11-575H3.	NA	1.50E-161	3.24E-161
IGKV1OR2-1	NA	NA	NA
RP11-407P15	NA	NA	NA
TEKT4	NA	NA	NA
AC103563.8	NA	NA	NA
MAL	NA	NA	NA
AC103563.9	NA	NA	NA
MRPS5	1.78023008	5.82E-96	9.89E-96
ZNF514	2.54761614	1.53E-28	1.94E-28
ZNF2	1.80134474	2.29E-74	3.57E-74
AC092835.2	NA	NA	NA
PROM2	NA	NA	NA
KCNIP3	NA	0	0
FAHD2A	1.60541926	1.28E-229	3.59E-229
RP11-440D17	NA	NA	NA
RP11-440D17	NA	NA	NA
TRIM43	NA	NA	NA
AC008268.1	NA	NA	NA
LINC00342	2.23881418	1.16E-06	1.27E-06
ANKRD36C	1.54400978	1.20E-51	1.70E-51
GPAT2	NA	NA	NA
ADRA2B	NA	1.22E-98	2.09E-98
ASTL	NA	NA	NA
DUSP2	1.97290139	8.64E-105	1.52E-104
AC012307.2	NA	NA	NA
STARD7	2.00751665	1.35E-70	2.07E-70
TMEM127	2.02651284	1.12E-12	1.29E-12
CIAO1	1.75845558	3.27E-28	4.14E-28
SNRNP200	2.4224981	4.50E-291	1.69E-290
AC021188.4	4.63202934	0.00765372	0.0080439
ITPRIPL1	1.98515543	3.00E-84	4.86E-84
NCAPH	1.49204791	0	0
NEURL3	NA	NA	NA
RP11-363D14	NA	0	0
ARID5A	1.23011768	0	0
KANSL3	2.57334963	1.22E-229	3.42E-229
FER1L5	NA	NA	NA

LMAN2L	1.33814181	3.06E-156	6.45E-156
CNNM4	4.11735941	8.69E-240	2.55E-239
RP11-353K11	NA	0.00845131	0.00887598
CNNM3	2.05867971	2.67E-14	3.12E-14
ANKRD23	3.86002445	0	0
ANKRD39	1.99599555	2.81E-104	4.92E-104
SEMA4C	1.48224939	8.04E-20	9.74E-20
FAM178B	NA	NA	NA
IGKV2OR2-1	NA	NA	NA
IGKV2OR2-2	NA	NA	NA
IGKV1OR2-3	NA	NA	NA
FAHD2B	1.63931903	0	0
ANKRD36	1.7441592	1.68E-202	4.23E-202
RP11-734K23	NA	NA	NA
AC159540.3	NA	NA	NA
IGKV1OR2-9	NA	NA	NA
IGKV2OR2-10	NA	NA	NA
IGKV2OR2-7	NA	NA	NA
IGKV3OR2-5	NA	NA	NA
IGKV1OR2-6	NA	NA	NA
IGKV2OR2-7	NA	NA	NA
IGKV2OR2-8	NA	NA	NA
IGKV1OR2-1	NA	NA	NA
AC159540.1	0.85778321	7.28E-07	8.03E-07
AC159540.2	NA	NA	NA
ANKRD36B	2.23881418	0.00026068	0.00027977
COX5B	2.02508269	6.72E-118	1.24E-117
ACTR1B	1.48141479	3.72E-132	7.19E-132
LINC01125	0.15440098	7.33E-130	1.41E-129
ZAP70	NA	NA	NA
TMEM131	1.78262947	3.46E-103	6.04E-103
VWA3B	NA	NA	NA
AC092675.3	NA	NA	NA
CNGA3	NA	NA	NA
INPP4A	2.1101467	1.47E-202	3.70E-202
COA5	2.13473417	0	0
UNC50	1.49750346	1.95E-135	3.82E-135
MGAT4A	1.89325009	2.50E-232	7.10E-232
AC109826.1	NA	2.48E-112	4.49E-112
KIAA1211L	0	1.43E-134	2.79E-134
TSGA10	1.6887607	3.28E-216	8.68E-216
C2orf15	0.96500611	3.88E-17	4.62E-17
RP11-111H13	2.31601467	2.82E-43	3.86E-43
LIPT1	1.0444772	0	0
RP11-38C17.	3.86002445	9.51E-106	1.68E-105

MITD1	1.27470575	0	0
MRPL30	1.96114145	0.51114888	0.51710606
LYG2	1.18374083	1.16E-21	1.41E-21
LYG1	2.31601467	3.21E-20	3.89E-20
TXNDC9	1.24268593	0	0
EIF5B	1.78003966	2.08E-263	6.83E-263
REV1	1.9073062	4.33E-103	7.55E-103
RP11-527J8.:	NA	2.48E-05	2.69E-05
AFF3	NA	NA	NA
AC092667.2	NA	NA	NA
LINC01104	NA	NA	NA
LONRF2	NA	NA	NA
CHST10	2.1314048	5.03E-57	7.31E-57
NMS	NA	NA	NA
PDCL3	1.41183774	0	0
AC068538.4	NA	NA	NA
AC092168.3	NA	NA	NA
AC092168.4	NA	NA	NA
NPAS2	NA	NA	NA
AC016738.3	NA	NA	NA
AC016738.4	NA	6.35E-237	1.84E-236
RPL31	2.04240397	3.18E-179	7.30E-179
TBC1D8	1.59915299	9.25E-17	1.10E-16
RP11-299H2:	0.77200489	1.41E-76	2.21E-76
CNOT11	1.27968202	0	0
RNF149	2.07943253	1.09E-139	2.17E-139
CREG2	Inf	1.62E-226	4.46E-226
RFX8	Inf	9.86E-38	1.31E-37
AC092570.2	NA	NA	NA
MAP4K4	2.26732968	2.78E-18	3.34E-18
LINC01127	NA	NA	NA
IL1R2	NA	NA	NA
IL1R1	NA	5.19E-40	6.97E-40
AC007271.3	NA	NA	NA
IL1RL2	NA	NA	NA
IL1RL1	3.21668704	1.28E-141	2.55E-141
IL18R1	Inf	0	0
IL18RAP	1.54400978	4.40E-66	6.64E-66
SLC9A4	NA	NA	NA
SLC9A2	NA	NA	NA
MFSD9	2.25882912	2.76E-08	3.08E-08
TMEM182	1.11511817	1.69E-40	2.28E-40
AC007251.2	NA	NA	NA
AC108051.1	NA	NA	NA
AC108051.2	NA	2.09E-291	7.86E-291

AC108051.3	NA	NA	NA
AC073987.1	NA	NA	NA
AC013727.1	NA	NA	NA
AC013727.2	NA	NA	NA
RP11-76I14.1	NA	NA	NA
AC096554.1	NA	NA	NA
AC068535.2	NA	NA	NA
AC068535.3	NA	NA	NA
LINC01102	NA	NA	NA
LINC01103	NA	NA	NA
AC013402.4	NA	NA	NA
AC013402.3	NA	NA	NA
AC013402.5	NA	NA	NA
AC068057.1	NA	NA	NA
LINC01114	3.08801956	0.00065764	0.00070219
POU3F3	1.93001222	2.99E-90	4.97E-90
AC018730.4	NA	3.64E-92	6.09E-92
AC010884.1	NA	NA	NA
MRPS9	0.99093165	0	0
UTAT33	NA	NA	NA
AC104655.3	NA	NA	NA
GPR45	NA	NA	NA
RP11-332H14	0.77200489	1.17E-65	1.76E-65
TGFBRAP1	2.14680812	3.44E-22	4.21E-22
AC012360.4	Inf	0	0
RP11-332H14	2.09752272	1.26E-38	1.68E-38
C2orf49	1.86838158	0.467092	0.47322182
FHL2	1.10432488	0	0
AC012360.6	NA	NA	NA
NCK2	2.02574083	0	0
AC009505.2	NA	NA	NA
AC009505.4	NA	NA	NA
C2orf40	NA	NA	NA
UXS1	1.53404843	0	0
AC018878.3	NA	NA	NA
RGPD3	1.54400978	9.27E-24	1.15E-23
AC108868.5	NA	1.40E-156	2.95E-156
AC108868.6	1.05273394	0	0
ST6GAL2	NA	NA	NA
AC016994.2	NA	NA	NA
AC006227.1	NA	NA	NA
AC096669.1	NA	NA	NA
AC096669.2	NA	NA	NA
AC096669.3	NA	NA	NA
GACAT1	NA	NA	NA

RGPD4-AS1	NA	0	0
RGPD4	NA	NA	NA
SLC5A7	NA	NA	NA
LINC01593	NA	NA	NA
LINC01594	NA	NA	NA
SULT1C3	NA	NA	NA
SULT1C2	NA	NA	NA
SULT1C4	NA	0	0
GCC2	1.40364525	0	0
GCC2-AS1	0	3.93E-34	5.14E-34
LIMS1	2.15653948	9.84E-233	2.80E-232
LIMS1-AS1	NA	NA	NA
RANBP2	1.56844031	0	0
CCDC138	1.93001222	3.53E-26	4.43E-26
EDAR	NA	NA	NA
SH3RF3-AS1	NA	NA	NA
SH3RF3	NA	NA	NA
AC109344.1	NA	NA	NA
10-Sep	Inf	0	0
SOWAHC	Inf	3.45E-197	8.49E-197
RP11-506F3.	NA	NA	NA
RGPD5	1.54400978	1.62E-47	2.26E-47
LIMS3	NA	NA	NA
LINC01123	1.10286413	6.34E-83	1.02E-82
MALL	NA	NA	NA
NPHP1	3.86002445	9.80E-116	1.79E-115
LINC00116	2.63037677	0	0
RP11-1223D1	NA	NA	NA
LINC01106	1.69841076	1.34E-24	1.67E-24
RP13-1039J1	NA	NA	NA
AC108938.5	NA	NA	NA
LIMS3L	NA	NA	NA
RP13-1039J1	NA	NA	NA
RGPD6	NA	NA	NA
AC123886.2	NA	NA	NA
BUB1	1.52825458	1.27E-272	4.34E-272
AC114776.3	NA	NA	NA
ACOXL	NA	NA	NA
AC096670.3	NA	NA	NA
BCL2L11	2.07943253	8.96E-85	1.45E-84
AC108463.1	NA	NA	NA
MIR4435-2H	2.33495958	0	0
RP11-181E1(	Inf	4.48E-230	1.26E-229
AC068491.2	NA	NA	NA
RP11-803D5.	1.85281174	0.09607136	0.09913131

RP11-68E19.	NA	NA	NA
AC017002.1	NA	2.17E-260	7.02E-260
AC017002.2	NA	9.94E-51	1.41E-50
AC017002.4	NA	NA	NA
ANAPC1	1.67434827	4.82E-15	5.67E-15
MERTK	1.76458261	1.03E-20	1.25E-20
TMEM87B	4.05302567	3.37E-219	9.02E-219
FBLN7	NA	7.71E-288	2.85E-287
ZC3H8	2.01983669	2.24E-10	2.55E-10
ZC3H6	1.61419204	4.95E-184	1.16E-183
RGPD8	1.54400978	8.41E-97	1.43E-96
TTL	2.57849633	4.37E-104	7.65E-104
POLR1B	1.99591508	1.28E-05	1.40E-05
CHCHD5	2.303881	0	0
AC012442.6	1.54400978	4.82E-55	6.95E-55
AC079922.3	1.88712306	2.53E-151	5.25E-151
SLC20A1	1.74522382	1.39E-10	1.58E-10
NT5DC4	NA	3.49E-41	4.72E-41
CKAP2L	1.72092757	8.73E-67	1.32E-66
IL1A	NA	NA	NA
IL1B	0.3216687	0	0
IL37	NA	4.01E-50	5.66E-50
IL36G	NA	6.17E-12	7.10E-12
IL36A	NA	NA	NA
IL36B	NA	NA	NA
IL36RN	NA	NA	NA
IL1F10	NA	NA	NA
IL1RN	NA	3.16E-12	3.65E-12
PSD4	2.09544184	3.28E-185	7.69E-185
AC016683.5	NA	NA	NA
PAX8	1.43372337	2.57E-20	3.12E-20
RP11-65I12.1	1.87988204	2.11E-59	3.10E-59
IGKV1OR2-1(	NA	NA	NA
AC016745.3	NA	NA	NA
RP11-480C16	0.77200489	2.46E-250	7.59E-250
CBWD2	1.68024594	1.46E-174	3.30E-174
FOXD4L1	NA	3.90E-80	6.22E-80
RP11-395L14	NA	7.38E-20	8.94E-20
PGM5P4-AS1	NA	NA	NA
FAM138B	NA	NA	NA
RABL2A	1.87486902	4.69E-17	5.59E-17
AC017074.1	NA	NA	NA
AC017074.2	NA	NA	NA
SLC35F5	1.54400978	1.66E-17	1.98E-17
AC024704.2	NA	1.47E-270	4.99E-270

AC104653.1	0	1.24E-305	5.07E-305
RP11-141B14	1.737011	1.96E-06	2.15E-06
ACTR3	1.37358606	0	0
LINC01191	0	0	0
AC110769.3	NA	NA	NA
AC010982.2	NA	NA	NA
DPP10	0	0	0
DPP10-AS3	NA	NA	NA
DPP10-AS2	NA	NA	NA
DPP10-AS1	2.31601467	2.61E-27	3.29E-27
AC009312.1	NA	NA	NA
DDX18	2.05587649	2.83E-119	5.24E-119
AC009404.2	2.05867971	2.54E-210	6.56E-210
CCDC93	1.97290139	1.15E-141	2.30E-141
AC009303.1	Inf	4.67E-164	1.02E-163
RP11-98C1.2	NA	NA	NA
INSIG2	1.52685412	4.78E-23	5.89E-23
AC093901.1	NA	NA	NA
RP11-19E11.	NA	NA	NA
EN1	NA	NA	NA
MARCO	NA	NA	NA
RP11-77A13.	NA	NA	NA
C1QL2	NA	NA	NA
STEAP3	2.18734719	7.89E-07	8.70E-07
STEAP3-AS1	NA	1.37E-239	4.03E-239
C2orf76	2.20372305	1.86E-194	4.54E-194
DBI	1.85761648	1.88E-59	2.76E-59
TMEM37	1.39925886	0	0
SCTR	NA	NA	NA
AC013275.2	NA	NA	NA
CFAP221	NA	NA	NA
TMEM177	1.69525972	5.75E-30	7.36E-30
PTPN4	2.28513447	1.48E-302	5.95E-302
EPB41L5	1.98515543	3.13E-66	4.74E-66
AC012363.4	NA	NA	NA
TMEM185B	1.3426172	8.53E-302	3.40E-301
RALB	1.62884548	0	0
AC012363.2	NA	NA	NA
AC012363.13	NA	NA	NA
INHBB	NA	NA	NA
AC073257.2	NA	NA	NA
AC073257.1	NA	NA	NA
AC018866.1	NA	NA	NA
GLI2	0.77200489	4.80E-63	7.17E-63
RP11-297J22	NA	NA	NA

AC067960.1	NA	NA	NA
AC079988.3	NA	NA	NA
TFCP2L1	2.5917307	0	0
CLASP1	1.95145681	4.82E-09	5.42E-09
RP11-204L24	Inf	0	0
NIFK-AS1	1.61994469	6.35E-08	7.07E-08
NIFK	1.361766	0	0
TSN	1.60706197	8.03E-283	2.88E-282
AC018737.3	NA	2.57E-131	4.97E-131
AC062020.2	NA	NA	NA
AC062020.1	NA	NA	NA
AC073409.1	NA	NA	NA
AC079154.1	NA	NA	NA
CNTNAP5	NA	NA	NA
AC097499.1	NA	NA	NA
AC114813.1	NA	NA	NA
AC023347.1	NA	NA	NA
GYPC	NA	NA	NA
RP11-521O1(	NA	NA	NA
RP11-521O1(	NA	NA	NA
BIN1	1.24378566	0	0
CYP27C1	NA	2.51E-162	5.42E-162
ERCC3	1.6879429	1.62E-174	3.65E-174
MAP3K2	2.5131223	7.32E-144	1.48E-143
AC068282.3	NA	8.08E-209	2.08E-208
PROC	3.86002445	7.57E-69	1.16E-68
IWS1	1.6297881	2.15E-279	7.57E-279
AC010976.2	NA	NA	NA
MYO7B	NA	NA	NA
RP11-286H1!	NA	NA	NA
LIMS2	NA	0	0
GPR17	NA	NA	NA
WDR33	1.76373425	0	0
SFT2D3	3.6921973	0	0
POLR2D	2.20541846	1.01E-39	1.35E-39
AMMECR1L	3.57052262	7.61E-112	1.37E-111
RP11-395A1:	2.15058505	1.95E-139	3.87E-139
SAP130	2.33940876	6.07E-207	1.55E-206
UGGT1	2.67931109	6.12E-287	2.25E-286
HS6ST1	1.85281174	4.15E-13	4.82E-13
AC012451.1	NA	NA	NA
AC068483.1	NA	NA	NA
AC079586.1	NA	NA	NA
AC079776.1	NA	NA	NA
AC079776.3	NA	NA	NA

RAB6C	NA	NA	NA
POTEF	NA	NA	NA
AC018804.3	NA	NA	NA
CCDC74B	0	1.04E-105	1.84E-105
SMPD4	1.83454925	1.81E-29	2.31E-29
MZT2B	2.12836894	0	0
TUBA3E	NA	NA	NA
CCDC115	1.83415927	1.14E-05	1.24E-05
IMP4	1.74230741	1.21E-168	2.67E-168
PTPN18	2.08261784	0.0106938	0.01121363
POTEI	NA	NA	NA
AC013269.3	NA	NA	NA
CFC1B	NA	NA	NA
AC013269.4	NA	NA	NA
AC013269.5	NA	NA	NA
TISP43	NA	0.00098505	0.00104888
AC140481.4	NA	NA	NA
CFC1	NA	NA	NA
POTEJ	NA	NA	NA
GPR148	NA	NA	NA
AC140481.8	NA	NA	NA
AMER3	NA	NA	NA
AC133785.1	NA	NA	NA
ARHGEF4	NA	0	0
FAM168B	2.26906843	7.51E-232	2.13E-231
PLEKHB2	1.82040659	3.01E-138	5.94E-138
POTEE	NA	NA	NA
LINC01120	NA	NA	NA
RP11-109E12	NA	NA	NA
MZT2A	2.1297384	0	0
TUBA3D	NA	NA	NA
CCDC74A	3.474022	5.02E-123	9.40E-123
LINC01087	NA	NA	NA
C2orf27B	Inf	0.55897942	0.56492701
AC103564.7	NA	NA	NA
AJ239322.3	NA	NA	NA
AJ239322.1	NA	NA	NA
RP1-156L9.1	NA	NA	NA
ANKRD30BL	NA	NA	NA
AC097532.2	NA	NA	NA
RP11-725P16	NA	NA	NA
GPR39	NA	NA	NA
LYPD1	NA	NA	NA
AC010974.3	NA	NA	NA
NCKAP5	NA	0	0

AC016909.1	NA	NA	NA
AC010890.1	NA	NA	NA
AC011243.1	NA	NA	NA
MGAT5	1.94104087	2.77E-13	3.22E-13
TMEM163	1.51312958	2.51E-177	5.71E-177
CCNT2-AS1	3.66702323	1.11E-194	2.70E-194
ACMSD	NA	NA	NA
CCNT2	2.24363921	1.45E-67	2.20E-67
MAP3K19	Inf	0	0
RAB3GAP1	2.04581296	4.64E-12	5.34E-12
ZRANB3	3.37752139	0	0
R3HDM1	2.73510304	1.69E-301	6.75E-301
UBXN4	2.18265725	2.91E-96	4.95E-96
LCT	Inf	0	0
AC011893.3	NA	NA	NA
MCM6	1.80984165	2.10E-156	4.44E-156
DARS	1.40255547	0	0
DARS-AS1	2.00721271	1.11E-69	1.71E-69
AC068492.1	NA	NA	NA
CXCR4	NA	2.74E-112	4.95E-112
THSD7B	NA	NA	NA
HNMT	1.46129497	7.48E-148	1.53E-147
AC069394.1	NA	NA	NA
AC097721.2	2.31601467	3.40E-151	7.06E-151
AC114763.1	NA	NA	NA
SPOPL	1.30021876	0.07667567	0.0792847
RP11-231E19	NA	2.37E-104	4.15E-104
AC092620.3	NA	1.69E-163	3.67E-163
NXPH2	NA	NA	NA
AC062021.1	NA	NA	NA
AC016710.1	NA	NA	NA
AC078851.1	NA	NA	NA
AC073928.2	NA	NA	NA
LRP1B	NA	NA	NA
KYNU	NA	1.48E-90	2.45E-90
ARHGAP15	1.44153125	0	0
RP11-190J23	NA	NA	NA
AC096558.1	NA	NA	NA
RP11-570L15	NA	NA	NA
RP11-570L15	NA	NA	NA
AC092652.1	NA	NA	NA
RP11-434H14	NA	NA	NA
CTD-2252P21	NA	NA	NA
AC079584.2	NA	NA	NA
AC016910.1	NA	NA	NA

GTDC1	1.44750917	2.93E-224	8.03E-224
ZEB2	2.70201711	0	0
RP11-107E5.	NA	NA	NA
ZEB2-AS1	1.21315054	0.03786085	0.03936904
LINC01412	NA	1.90E-266	6.33E-266
TEX41	NA	NA	NA
AC023128.1	NA	NA	NA
RP11-514A9.	NA	NA	NA
AC064865.1	NA	NA	NA
AC092484.1	NA	NA	NA
AC079163.1	NA	NA	NA
AC062031.1	NA	NA	NA
AC103881.1	NA	NA	NA
AC062032.1	NA	NA	NA
ACVR2A	1.23520782	1.99E-176	4.52E-176
AC009480.3	NA	NA	NA
ORC4	1.13962627	0	0
MBD5	1.63308727	3.93E-22	4.81E-22
EPC2	1.81021836	1.53E-50	2.16E-50
AC105402.4	NA	NA	NA
KIF5C	Inf	1.12E-16	1.33E-16
LYPD6B	NA	NA	NA
LYPD6	NA	2.48E-233	7.08E-233
MMADHC	1.71866684	2.48E-79	3.94E-79
AC144449.1	NA	8.84E-86	1.44E-85
AC007364.1	NA	NA	NA
AC016682.1	NA	NA	NA
AC113610.1	NA	NA	NA
RND3	NA	NA	NA
AC104777.2	NA	NA	NA
AC104777.3	NA	NA	NA
AC104777.1	NA	NA	NA
AC104777.4	NA	NA	NA
AC023469.2	NA	NA	NA
AC023469.1	NA	NA	NA
AC018731.3	NA	NA	NA
RBM43	2.31601467	8.17E-24	1.01E-23
NMI	1.28400813	0	0
TNFAIP6	NA	NA	NA
RIF1	2.02795314	5.34E-168	1.18E-167
NEB	Inf	7.23E-109	1.29E-108
ARL5A	2.17191981	1.78E-257	5.68E-257
CACNB4	2.05867971	6.40E-37	8.48E-37
AC079790.2	NA	NA	NA
STAM2	3.17379788	1.71E-119	3.17E-119

FMNL2	2.11462209	3.95E-108	7.05E-108
PRPF40A	1.45575708	3.13E-284	1.13E-283
ARL6IP6	2.04152404	3.69E-161	7.93E-161
AC011901.2	NA	NA	NA
AC079150.3	NA	NA	NA
RPRM	NA	NA	NA
GALNT13	NA	NA	NA
AC009227.2	NA	NA	NA
AC009227.3	NA	NA	NA
AC061961.2	NA	NA	NA
KCNJ3	NA	8.50E-15	9.98E-15
AC092625.1	NA	NA	NA
AC093375.1	NA	NA	NA
RP11-605B1(	NA	NA	NA
NR4A2	0.61760391	5.89E-215	1.55E-214
GPD2	2.09084658	5.10E-71	7.87E-71
GALNT5	NA	NA	NA
ERMN	NA	0	0
CYTIP	0.6829274	0	0
ACVR1C	NA	0	0
AC019186.1	Inf	0	0
ACVR1	1.6297881	0.00097003	0.00103325
UPP2	NA	NA	NA
RP11-546J1.:	Inf	0	0
RP11-1152H:	NA	NA	NA
CCDC148-AS:	NA	NA	NA
CCDC148	NA	NA	NA
PKP4	2.60092124	0	0
AC005042.1	NA	NA	NA
AC005042.4	NA	NA	NA
AC005042.5	NA	NA	NA
DAPL1	2.64197229	0	0
TANC1	3.78984219	3.10E-121	5.78E-121
WDSUB1	1.68437431	3.86E-43	5.28E-43
BAZ2B	2.31601467	4.52E-294	1.73E-293
AC008277.1	NA	NA	NA
AC009506.1	1.33104291	3.79E-45	5.23E-45
AC009961.3	10.8080685	0	0
7-Mar	1.94170927	0.57524488	0.58094481
CD302	2.08335566	1.62E-165	3.54E-165
LY75-CD302	NA	NA	NA
LY75	NA	0	0
PLA2R1	NA	NA	NA
ITGB6	NA	NA	NA
RBMS1	1.27648333	1.86E-297	7.26E-297

AC009313.1	NA	NA	NA
TANK	1.72216475	3.13E-84	5.08E-84
AC009299.2	NA	NA	NA
AC009299.3	NA	3.55E-165	7.76E-165
PSMD14	1.1355173	0	0
TBR1	0.77200489	1.01E-22	1.25E-22
AC009487.4	NA	NA	NA
SLC4A10	NA	NA	NA
DPP4	22.3881418	0	0
AC008063.2	NA	NA	NA
AC008063.3	NA	NA	NA
GCG	NA	NA	NA
AC007750.5	NA	NA	NA
FAP	NA	NA	NA
IFIH1	3.60268949	1.35E-39	1.82E-39
GCA	1.4948822	5.71E-168	1.26E-167
KCNH7	NA	NA	NA
AC007740.1	NA	NA	NA
FIGN	Inf	0	0
AC092684.1	NA	NA	NA
GRB14	NA	NA	NA
COBLL1	4.63202934	5.20E-302	2.08E-301
AC019181.2	NA	NA	NA
SLC38A11	NA	NA	NA
AC013463.2	NA	NA	NA
SCN3A	NA	NA	NA
SCN2A	NA	3.25E-61	4.81E-61
CSRNP3	NA	NA	NA
GALNT3	0.77200489	0.00917793	0.00963409
AC009495.2	NA	NA	NA
AC009495.4	NA	NA	NA
AC009495.3	NA	0	0
TTC21B	1.54400978	9.19E-83	1.48E-82
AC010127.3	NA	NA	NA
SCN1A	NA	NA	NA
SCN9A	5.66136919	0	0
SCN7A	NA	NA	NA
XIRP2	NA	NA	NA
XIRP2-AS1	NA	NA	NA
AC074363.1	NA	NA	NA
AC016723.4	NA	NA	NA
B3GALT1	NA	NA	NA
STK39	2.73170961	0.56594237	0.5716456
CERS6	1.87656573	0.00332601	0.00351512
NOSTRIN	3.08801956	0.05311777	0.05507587

SPC25	1.15962241	0	0
G6PC2	NA	NA	NA
ABCB11	NA	NA	NA
DHRS9	0	0	0
AC007556.3	NA	NA	NA
LRP2	NA	NA	NA
BBS5	Inf	0	0
RP11-724O1(	NA	NA	NA
KLHL41	NA	NA	NA
FASTKD1	2.03159182	0.00066861	0.00071382
PPIG	1.55558407	0	0
CCDC173	0.70182263	2.34E-285	8.54E-285
PHOSPHO2	1.81648209	1.61E-192	3.89E-192
KLHL23	2.77707315	0	0
SSB	1.51773349	4.30E-145	8.70E-145
METTL5	1.9279742	4.18E-13	4.85E-13
UBR3	1.54400978	9.04E-62	1.34E-61
MYO3B	NA	5.55E-141	1.11E-140
AC012594.1	NA	NA	NA
AC007277.3	NA	NA	NA
LINC01124	NA	NA	NA
SP5	NA	1.10E-163	2.39E-163
AC007405.4	0	0	0
AC007405.6	0.44114565	0	0
ERICH2	0	0	0
GAD1	0	0	0
AC007405.8	NA	NA	NA
GORASP2	1.39722012	0	0
TLK1	2.17892969	6.72E-193	1.63E-192
METTL8	1.5804251	1.58E-203	3.98E-203
DCAF17	1.57260255	3.92E-10	4.45E-10
CYBRD1	1.83565607	3.27E-79	5.19E-79
DYNC1I2	1.86410937	2.17E-166	4.76E-166
SLC25A12	1.70484413	5.32E-114	9.66E-114
HAT1	0.97501358	0	0
METAP1D	2.49147033	1.19E-34	1.56E-34
DLX1	1.8969263	0.0011667	0.0012415
DLX2	1.70236976	1.91E-52	2.73E-52
DLX2-AS1	Inf	5.32E-69	8.14E-69
AC104088.1	NA	NA	NA
ITGA6	0.7205379	0	0
AC078883.4	NA	NA	NA
AC078883.3	1.15800733	2.13E-151	4.43E-151
AC093818.1	1.94679494	2.14E-100	3.70E-100
PDK1	1.62012294	7.41E-64	1.11E-63

RAPGEF4-AS	NA	NA	NA
RAPGEF4	3.86002445	1.51E-190	3.62E-190
AC013461.1	1.89492109	6.08E-106	1.07E-105
MLK7-AS1	NA	NA	NA
CDCA7	2.00429448	1.08E-58	1.58E-58
RP11-796E1(	NA	NA	NA
AC106900.6	NA	NA	NA
SP3	2.1730508	7.05E-30	9.01E-30
RP11-394I13	Inf	1.62E-172	3.64E-172
RP11-394I13	NA	NA	NA
RP11-394I13	NA	NA	NA
OLA1	1.45540467	1.16E-280	4.11E-280
LINC01305	NA	NA	NA
SP9	1.66753056	1.11E-10	1.26E-10
CIR1	1.50985027	0	0
SCRN3	1.23520782	0	0
GPR155	1.33965554	1.20E-37	1.59E-37
AC010894.3	2.31601467	0.00023695	0.00025447
AC018890.6	NA	0	0
WIPF1	2.61930231	1.50E-206	3.81E-206
AC010894.5	NA	NA	NA
CHRNA1	NA	NA	NA
AC018890.4	NA	NA	NA
CHN1	3.08801956	1.72E-78	2.72E-78
ATF2	1.61263244	0.06905528	0.07146611
AC096649.2	NA	NA	NA
ATP5G3	2.22765944	0	0
AC096649.3	NA	NA	NA
AC016751.3	NA	NA	NA
AC016751.2	NA	NA	NA
KIAA1715	2.46612673	2.15E-56	3.12E-56
EVX2	NA	1.24E-61	1.84E-61
HOXD13	1.84609865	7.34E-44	1.01E-43
HOXD12	NA	NA	NA
HOXD11	NA	NA	NA
HOXD10	NA	NA	NA
HOXD-AS2	NA	NA	NA
HOXD9	NA	NA	NA
HOXD8	NA	NA	NA
HOXD3	NA	NA	NA
AC009336.19	NA	NA	NA
HOXD4	NA	NA	NA
RP11-387A1.	NA	NA	NA
HAGLR	NA	1.74E-40	2.35E-40
HAGLROS	3.86002445	1.53E-223	4.17E-223

HOXD1	NA	NA	NA
MTX2	1.63634259	7.01E-54	1.01E-53
AC017048.1	NA	NA	NA
AC017048.2	NA	NA	NA
RP11-324L17	NA	NA	NA
LINC01116	5.40403423	6.58E-76	1.03E-75
LINC01117	NA	NA	NA
AC092162.1	NA	NA	NA
AC073636.1	NA	NA	NA
AC079305.11	NA	NA	NA
AC079305.8	NA	NA	NA
HNRNPA3	1.67722152	3.22E-285	1.17E-284
NFE2L2	1.75455657	2.79E-223	7.61E-223
AC079305.10	0.38600244	5.20E-163	1.13E-162
RP11-337N6.	NA	NA	NA
RP11-337N6.	NA	NA	NA
AGPS	1.46106711	4.17E-207	1.07E-206
TTC30B	3.08801956	2.14E-204	5.41E-204
AC073834.3	NA	NA	NA
TTC30A	Inf	0	0
PDE11A	NA	NA	NA
AC012499.1	NA	NA	NA
RBM45	1.70653713	2.75E-82	4.42E-82
OSBPL6	1.52997333	0.16453671	0.16879807
AC009948.5	2.71532754	1.35E-23	1.67E-23
PRKRA	1.6188061	8.14E-301	3.24E-300
DFNB59	Inf	0	0
FKBP7	1.12016396	3.16E-133	6.14E-133
PLEKHA3	2.15282664	7.24E-99	1.24E-98
TTN-AS1	NA	2.45E-206	6.24E-206
TTN	NA	NA	NA
RP11-65L3.4	NA	NA	NA
RP11-65L3.2	NA	NA	NA
RP11-171I2.	NA	NA	NA
RP11-171I2.	NA	NA	NA
RP11-171I2.	NA	NA	NA
RP11-171I2.	NA	NA	NA
RP11-171I2.	NA	NA	NA
CCDC141	NA	NA	NA
SESTD1	2.51820643	6.52E-160	1.40E-159
AC093911.1	NA	NA	NA
ZNF385B	NA	NA	NA
CWC22	1.48886657	8.20E-301	3.26E-300
AC009478.1	NA	NA	NA
SCHLAP1	NA	NA	NA

UBE2E3	1.78947427	5.46E-234	1.56E-233
AC104076.3	NA	NA	NA
AC068196.1	NA	NA	NA
AC104820.2	NA	NA	NA
AC020595.1	NA	NA	NA
ITGA4	1.81460943	9.12E-47	1.27E-46
CERKL	Inf	5.98E-30	7.65E-30
NEUROD1	Inf	0	0
AC013733.3	NA	NA	NA
AC013733.4	NA	NA	NA
RP11-366L5.	1.93001222	1.01E-226	2.79E-226
SSFA2	1.89299829	5.78E-169	1.28E-168
PPP1R1C	Inf	0	0
PDE1A	NA	NA	NA
DNAJC10	2.68870669	0	0
FRZB	NA	NA	NA
NCKAP1	2.81554725	1.46E-163	3.17E-163
DUSP19	2.62481663	1.69E-07	1.87E-07
AC064871.3	NA	NA	NA
NUP35	1.56116544	5.69E-257	1.81E-256
RP11-438L1	3.08801956	1.18E-59	1.74E-59
AC093639.1	NA	NA	NA
ZNF804A	1.43628817	4.09E-130	7.85E-130
AC007966.1	NA	NA	NA
FSIP2	NA	NA	NA
FSIP2-AS1	NA	1.34E-300	5.32E-300
LINC01473	0.57900367	4.05E-26	5.07E-26
RP11-410E4.	NA	4.55E-80	7.26E-80
ZC3H15	1.53090787	1.88E-232	5.34E-232
AC018867.1	NA	NA	NA
ITGAV	1.75845558	0.00092717	0.00098794
AC017101.1	NA	5.99E-19	7.22E-19
FAM171B	1.91163116	3.32E-05	3.60E-05
ZSWIM2	NA	NA	NA
AC007319.1	NA	NA	NA
CALCRL	Inf	1.28E-05	1.39E-05
TFPI	2.31601467	2.65E-91	4.43E-91
LINC01090	NA	NA	NA
GULP1	0.77200489	6.27E-60	9.23E-60
DIRC1	NA	NA	NA
COL3A1	NA	NA	NA
COL5A2	NA	0	0
AC133106.2	NA	NA	NA
WDR75	1.70102772	2.45E-274	8.45E-274
SLC40A1	0	1.51E-11	1.74E-11

ASNSD1	2.18620854	7.47E-43	1.02E-42
ANKAR	2.97773315	2.92E-202	7.31E-202
OSGEPL1	1.4153423	2.33E-230	6.55E-230
OSGEPL1-AS	0.25733496	0	0
RP11-455J20	Inf	0	0
ORMDL1	1.94381629	8.07E-74	1.26E-73
PMS1	1.99691932	0.00496866	0.00523834
C2orf88	2.14297909	3.38E-84	5.47E-84
MSTN	NA	NA	NA
HIBCH	1.15800733	0	0
INPP1	1.31405088	0	0
MFSD6	4.19088369	0	0
RP11-647K16	1.54400978	1.89E-47	2.63E-47
NEMP2	2.50901589	0.3451746	0.35125407
AC093388.3	5.40403423	8.83E-43	1.20E-42
NAB1	1.46414721	2.28E-11	2.61E-11
AC006460.2	NA	NA	NA
AC005540.3	NA	NA	NA
GLS	2.45225083	2.12E-268	7.11E-268
STAT1	2.07037675	2.31E-170	5.15E-170
AC067945.2	NA	NA	NA
STAT4	8.49205379	0	0
AC092614.2	NA	1.97E-108	3.52E-108
MYO1B	2.09332095	1.02E-254	3.22E-254
NABP1	1.28667482	5.09E-175	1.15E-174
AC098872.3	NA	NA	NA
SDPR	NA	NA	NA
AC098617.1	NA	NA	NA
TMEFF2	NA	NA	NA
AC013401.1	NA	NA	NA
AC013401.2	NA	NA	NA
PCGEM1	NA	NA	NA
RP11-764E7.	NA	NA	NA
AC018799.1	NA	NA	NA
AC006196.1	NA	NA	NA
AC010983.1	NA	6.43E-247	1.95E-246
AC064834.1	0.77200489	9.18E-23	1.13E-22
RP11-762E8.	NA	NA	NA
AC064834.2	NA	NA	NA
AC064834.3	0.77200489	3.00E-06	3.29E-06
SLC39A10	1.64051039	0	0
DNAH7	Inf	6.13E-249	1.88E-248
STK17B	1.51541701	0	0
RP11-347P5.	2.00721271	1.73E-225	4.75E-225
HECW2	NA	0	0

AC020571.3	0	4.29E-166	9.41E-166
RP11-369J9.1	NA	1.62E-81	2.60E-81
CCDC150	2.8306846	8.25E-210	2.13E-209
GTF3C3	1.83039869	0.19736107	0.20214106
C2orf66	NA	9.32E-146	1.89E-145
PGAP1	3.66702323	0	0
ANKRD44	1.37245314	1.04E-60	1.53E-60
AC013264.2	NA	NA	NA
AC010746.3	NA	NA	NA
SF3B1	1.45937823	0	0
COQ10B	1.33432944	1.36E-206	3.48E-206
HSPD1	1.16146637	0	0
HSPE1	2.15149953	9.19E-172	2.05E-171
HSPE1-MOB4	NA	NA	NA
MOB4	1.82223232	4.86E-78	7.69E-78
RFTN2	NA	2.48E-44	3.41E-44
AC011997.1	Inf	9.64E-277	3.36E-276
MARS2	2.79284122	7.45E-91	1.24E-90
BOLL	NA	NA	NA
PLCL1	NA	NA	NA
AC109589.1	NA	NA	NA
AC005235.1	NA	NA	NA
AC019330.1	NA	NA	NA
AC018717.1	NA	NA	NA
SATB2	1.94104087	2.45E-29	3.12E-29
RP11-486F17	NA	NA	NA
SATB2-AS1	Inf	0.35954486	0.36563133
AC093590.1	NA	NA	NA
FTCDNL1	1.81972581	3.85E-17	4.58E-17
C2orf69	1.64152619	4.61E-52	6.56E-52
TYW5	2.10769589	0.43059791	0.43683427
C2orf47	1.30167641	1.74E-272	5.96E-272
SPATS2L	2.09611025	1.11E-17	1.33E-17
KCTD18	2.31601467	5.02E-196	1.23E-195
SGOL2	1.4014858	0	0
AOX1	NA	1.06E-189	2.54E-189
AC007163.2	NA	NA	NA
AC007163.3	NA	NA	NA
AC007163.6	NA	NA	NA
BZW1	1.38033895	0	0
CLK1	1.8442339	3.00E-76	4.71E-76
PPIL3	1.84093474	0.00018699	0.00020105
NIF3L1	1.42211427	3.51E-303	1.41E-302
ORC2	2.02265281	1.78E-47	2.48E-47
AC005037.3	1.67267726	1.81E-61	2.69E-61

FAM126B	1.96510336	6.63E-63	9.89E-63
NDUFB3	2.17517199	1.91E-271	6.50E-271
RP11-13J8.1	NA	NA	NA
CFLAR	1.68437431	3.38E-67	5.14E-67
CFLAR-AS1	NA	NA	NA
CASP10	1.87486902	4.72E-165	1.03E-164
CASP8	2.34496485	1.28E-190	3.07E-190
ALS2CR12	2.31601467	0.02515242	0.02622954
TRAK2	3.88273048	0	0
STRADB	1.94609566	0.75730261	0.76158448
ALS2CR11	NA	NA	NA
TMEM237	1.69028439	5.19E-98	8.89E-98
MPP4	Inf	0	0
ALS2	2.41016161	1.49E-06	1.63E-06
CDK15	Inf	5.80E-155	1.22E-154
RP11-107N1!	0.77200489	3.07E-227	8.52E-227
FZD7	2.02651284	4.01E-72	6.21E-72
KIAA2012	Inf	2.96E-133	5.75E-133
AC079354.3	NA	NA	NA
AC079354.5	3.55122249	0	0
SUMO1	1.8605655	3.70E-50	5.22E-50
NOP58	1.41373395	0	0
RP11-686O6.	0.77200489	2.08E-32	2.69E-32
RP11-686O6.	2.05867971	1.54E-07	1.71E-07
BMPR2	3.474022	0	0
FAM117B	3.24242054	0	0
ICA1L	2.8306846	0	0
WDR12	1.36876941	7.03E-216	1.86E-215
CARF	2.31601467	9.62E-272	3.28E-271
NBEAL1	1.93434933	0.01537984	0.01609025
CYP20A1	2.07338456	6.39E-27	8.04E-27
ABI2	1.7045868	5.12E-09	5.76E-09
RP11-363J17	NA	NA	NA
RAPH1	NA	2.30E-13	2.68E-13
CD28	NA	NA	NA
CTLA4	NA	NA	NA
ICOS	NA	NA	NA
AC009965.2	NA	NA	NA
AC009498.1	NA	NA	NA
AC016903.1	NA	NA	NA
AC016903.2	NA	NA	NA
PARD3B	NA	NA	NA
NRP2	1.62527345	4.29E-11	4.91E-11
AC007362.3	NA	NA	NA
AC007679.3	NA	NA	NA

INO80D	1.83683922	0.17768775	0.18214551
AC007383.3	Inf	0	0
NDUFS1	1.97690972	1.21E-170	2.70E-170
AC007383.4	1.54400978	0.00016874	0.00018153
EEF1B2	1.88629416	1.55E-24	1.93E-24
GPR1	NA	NA	NA
ZDBF2	0	0	0
AC017081.1	NA	NA	NA
ADAM23	NA	NA	NA
AC010731.2	NA	NA	NA
AC010731.3	NA	NA	NA
AC010731.4	NA	NA	NA
DYTN	NA	NA	NA
MDH1B	NA	NA	NA
FASTKD2	1.52646421	1.46E-76	2.29E-76
RP11-95H11.	NA	NA	NA
AC008269.2	NA	NA	NA
CPO	NA	NA	NA
KLF7	1.36585481	1.32E-49	1.87E-49
AC007879.1	NA	NA	NA
AC007879.2	NA	NA	NA
AC007879.4	NA	1.69E-17	2.02E-17
AC007879.3	NA	NA	NA
AC007879.7	NA	4.29E-147	8.76E-147
AC007879.5	NA	NA	NA
AC007879.6	NA	NA	NA
CREB1	2.06133264	8.24E-54	1.18E-53
METTL21A	2.05234531	0.0005025	0.00053733
AC079767.4	NA	NA	NA
CCNYL1	2.38034841	1.02E-182	2.37E-182
RP11-801F7.	Inf	1.19E-39	1.59E-39
FZD5	8.49205379	7.95E-254	2.49E-253
AC096772.6	Inf	0	0
PLEKHM3	Inf	2.13E-52	3.04E-52
CRYGD	NA	NA	NA
CRYGC	NA	NA	NA
CRYGB	NA	NA	NA
CRYGA	NA	NA	NA
C2orf80	Inf	0	0
IDH1	1.28280512	0	0
IDH1-AS1	2.70201711	8.54E-264	2.81E-263
PIKFYVE	2.00238768	1.45E-55	2.09E-55
PTH2R	NA	NA	NA
MAP2	NA	8.41E-10	9.53E-10
UNC80	0	1.09E-129	2.10E-129

RPE	1.838933	5.34E-172	1.19E-171
KANSL1L	1.28667482	6.22E-289	2.31E-288
RP11-260M2	1.13769142	2.07E-14	2.42E-14
AC007038.7	1.54400978	0.71864945	0.72319383
AC006994.2	Inf	0	0
ACADL	NA	NA	NA
AC006994.3	NA	NA	NA
MYL1	NA	NA	NA
LANCL1-AS1	NA	NA	NA
LANCL1	2.02651284	1.36E-83	2.20E-83
CPS1	Inf	0	0
ERBB4	NA	NA	NA
AC093381.2	NA	NA	NA
AC108066.1	NA	NA	NA
IKZF2	2.56775539	7.66E-275	2.65E-274
RP11-105N1	1.63483388	8.81E-06	9.62E-06
RP11-105N1	NA	NA	NA
RP11-105N1	NA	NA	NA
AC079610.2	NA	NA	NA
SPAG16	NA	5.88E-145	1.19E-144
AC107218.3	NA	NA	NA
VWC2L	NA	NA	NA
BARD1	2.08556545	6.60E-14	7.71E-14
AC072062.1	Inf	0	0
ABCA12	NA	NA	NA
AC072062.3	NA	NA	NA
AC073284.4	NA	NA	NA
ATIC	1.11807605	0	0
FN1	NA	4.11E-175	9.29E-175
AC012462.1	NA	NA	NA
AC012462.2	NA	NA	NA
AC012462.3	NA	NA	NA
AC012668.1	NA	NA	NA
AC012668.2	NA	NA	NA
AC012668.3	NA	NA	NA
LINC00607	NA	NA	NA
LINC01614	NA	NA	NA
AC122136.2	NA	NA	NA
AC093382.1	NA	1.97E-205	4.99E-205
MREG	1.48224939	2.05E-57	2.98E-57
PECR	1.71411255	3.50E-307	1.44E-306
TMEM169	0	0	0
XRCC5	1.66049069	0	0
PKI55	2.31601467	2.11E-20	2.56E-20
4-Mar	NA	NA	NA

AC012513.6	NA	NA	NA
AC069155.1	NA	NA	NA
AC098820.2	NA	NA	NA
SMARCAL1	1.72782047	0	0
AC098820.4	0	1.60E-51	2.27E-51
RPL37A	2.55119925	0	0
LINC01280	NA	NA	NA
AC073321.4	0	2.99E-130	5.74E-130
IGFBP2	1.55794832	0	0
IGFBP5	NA	NA	NA
AC007563.5	NA	NA	NA
AC007563.3	NA	NA	NA
AC007557.4	NA	NA	NA
TNP1	NA	NA	NA
AC007557.5	NA	NA	NA
AC007557.2	NA	NA	NA
AC007557.3	NA	NA	NA
AC007563.1	NA	NA	NA
AC007557.1	NA	NA	NA
RP11-574O1(	NA	NA	NA
DIRC3-AS1	NA	NA	NA
DIRC3	NA	NA	NA
TNS1	1.15800733	6.56E-138	1.30E-137
AC010136.2	NA	NA	NA
RUFY4	NA	NA	NA
CXCR2	1.54400978	2.12E-50	2.99E-50
CXCR1	NA	NA	NA
ARPC2	1.89929506	0.98041735	0.98090397
GPBAR1	NA	0	0
AAMP	1.06841458	0	0
PNKD	1.92141848	3.99E-15	4.69E-15
TMBIM1	1.99656437	1.13E-19	1.37E-19
CATIP-AS2	NA	NA	NA
CATIP	Inf	0.31989937	0.32582609
SLC11A1	2.57334963	2.41E-51	3.42E-51
CTDSP1	1.82473883	6.75E-32	8.71E-32
RP11-378A1:	0.96500611	2.42E-124	4.55E-124
VIL1	NA	1.54E-51	2.19E-51
USP37	2.54995555	1.39E-146	2.82E-146
RQCD1	2.24708566	0	0
PLCD4	NA	NA	NA
RP11-548H3.	1.47967604	2.87E-49	4.03E-49
ZNF142	2.63183485	0	0
BCS1L	1.82657366	1.44E-130	2.77E-130
RNF25	1.30275825	8.05E-300	3.17E-299

STK36	8.87805623	1.29E-301	5.14E-301
TTLL4	3.4464504	1.32E-231	3.74E-231
CYP27A1	NA	1.44E-142	2.90E-142
RP11-459I19	NA	NA	NA
PRKAG3	NA	NA	NA
WNT6	0.51466993	7.33E-258	2.34E-257
WNT10A	Inf	1.11E-184	2.61E-184
LINC01494	NA	NA	NA
AC097468.7	NA	NA	NA
CDK5R2	0	0	0
LINC00608	NA	NA	NA
FEV	0.61760391	3.58E-179	8.21E-179
CRYBA2	NA	NA	NA
AC097468.4	NA	NA	NA
CFAP65	NA	NA	NA
IHH	NA	NA	NA
RP11-33O4.1	1.54400978	1.11E-13	1.29E-13
NHEJ1	Inf	0	0
RP11-33O4.2	NA	NA	NA
SLC23A3	1.54400978	1.24E-159	2.65E-159
CNPPD1	1.4153423	0	0
FAM134A	2.11261218	2.46E-20	2.98E-20
ZFAND2B	2.09611025	4.52E-189	1.08E-188
ABCB6	NA	NA	NA
ATG9A	2.23179595	0.35616927	0.36225952
ANKZF1	1.67267726	0	0
GLB1L	1.65429619	6.93E-76	1.09E-75
STK16	1.79562619	3.69E-55	5.33E-55
TUBA4A	0.72869108	0	0
TUBA4B	NA	0	0
DNAJB2	1.39925886	5.62E-244	1.68E-243
PTPRN	NA	0	0
AC114803.3	NA	NA	NA
RESP18	NA	NA	NA
DNPEP	1.56997936	8.56E-191	2.06E-190
AC053503.4	0.77200489	2.50E-06	2.74E-06
DES	NA	5.84E-53	8.34E-53
AC053503.6	NA	NA	NA
SPEG	Inf	1.25E-224	3.43E-224
AC053503.11	NA	NA	NA
RP11-316O1	Inf	8.98E-84	1.45E-83
GMPPA	0.9837548	0	0
ASIC4	0	0	0
CHPF	1.24807457	2.21E-255	7.00E-255
TMEM198	0.92640587	1.72E-99	2.96E-99

RP11-256I23	NA	NA	NA
OBSL1	1.48084574	3.26E-82	5.24E-82
RP11-256I23	Inf	0	0
INHA	Inf	0	0
STK11IP	1.50891865	0.47085546	0.47687488
AC009955.8	NA	0	0
SLC4A3	0.38600244	2.25E-51	3.19E-51
RP11-256I23	Inf	0	0
AC009502.4	NA	NA	NA
AC008281.1	NA	NA	NA
AC114765.1	NA	NA	NA
AC114765.2	NA	NA	NA
AC067956.1	NA	NA	NA
AC093843.1	NA	NA	NA
EPHA4	NA	NA	NA
CTD-2308L22	NA	NA	NA
AC068489.1	NA	NA	NA
PAX3	NA	NA	NA
CCDC140	NA	NA	NA
RP11-384O8.	Inf	0	0
SGPP2	0	0	0
RP11-16P6.1	5.40403423	2.69E-117	4.94E-117
FARSB	1.34169126	0	0
MOGAT1	NA	NA	NA
ACSL3	1.57208269	2.61E-286	9.55E-286
AC097461.4	NA	NA	NA
KCNE4	NA	NA	NA
AC013448.1	NA	NA	NA
AC013448.2	NA	NA	NA
SCG2	Inf	0	0
AP1S3	2.1358802	1.00E-07	1.12E-07
WDFY1	3.04260751	4.07E-08	4.54E-08
MRPL44	1.25421102	0	0
AC073641.2	NA	NA	NA
SERPINE2	1.50337794	0	0
FAM124B	3.57052262	6.95E-40	9.33E-40
RP11-92F20.	Inf	0	0
CUL3	1.7579894	8.57E-243	2.55E-242
DOCK10	NA	1.62E-114	2.94E-114
AC067961.1	NA	NA	NA
NYAP2	0	3.48E-89	5.75E-89
RP11-314B1.	NA	NA	NA
AC068138.1	NA	NA	NA
IRS1	NA	NA	NA
RP11-395N3.	NA	NA	NA

RHBDD1	NA	NA	NA
COL4A4	NA	NA	NA
COL4A3	NA	NA	NA
AC097662.2	0	6.95E-109	1.24E-108
MFF	2.39345343	4.11E-282	1.47E-281
TM4SF20	NA	NA	NA
AGFG1	2.54475686	0	0
C2orf83	NA	NA	NA
SLC19A3	NA	NA	NA
CCL20	NA	5.01E-106	8.85E-106
DAW1	NA	NA	NA
SPHKAP	NA	NA	NA
AC009410.1	NA	NA	NA
AC074019.1	NA	NA	NA
PID1	NA	NA	NA
DNER	NA	NA	NA
TRIP12	2.03985032	1.06E-11	1.22E-11
FBXO36	1.67267726	3.06E-12	3.54E-12
SLC16A14	1.08907833	4.06E-262	1.33E-261
AC009950.2	Inf	2.18E-184	5.11E-184
SP110	1.44331349	3.59E-252	1.12E-251
AC009950.1	NA	NA	NA
SP140	NA	1.15E-11	1.32E-11
SP140L	1.93942692	1.25E-05	1.36E-05
SP100	2.0543181	2.32E-29	2.96E-29
AC010149.4	NA	NA	NA
AC098823.3	NA	NA	NA
CAB39	2.66916584	1.55E-223	4.23E-223
ITM2C	1.60031484	0	0
AC012507.3	NA	NA	NA
GPR55	4.63202934	0	0
AC012507.4	NA	NA	NA
SPATA3-AS1	1.15800733	0.78705503	0.79111044
SPATA3	NA	NA	NA
C2orf72	NA	2.01E-61	2.98E-61
PSMD1	1.81608199	0.00227078	0.00240606
HTR2B	NA	NA	NA
ARMC9	1.737011	4.41E-34	5.76E-34
B3GNT7	NA	0	0
AC017104.2	NA	5.85E-122	1.09E-121
NCL	1.36139381	0	0
LINC00471	1.02933985	1.21E-44	1.66E-44
NMUR1	NA	NA	NA
C2orf57	NA	NA	NA
PTMA	2.18745988	5.06E-138	1.00E-137

PDE6D	2.11497173	1.67E-75	2.61E-75
COPS7B	2.00101602	7.74E-29	9.84E-29
NPPC	NA	2.63E-113	4.77E-113
DIS3L2	1.80762121	1.71E-32	2.21E-32
AC105461.1	NA	NA	NA
ALPP	NA	NA	NA
AC068134.8	NA	NA	NA
ALPPL2	NA	NA	NA
AC068134.6	NA	NA	NA
ALPI	NA	NA	NA
ECEL1	NA	NA	NA
PRSS56	0	0	0
CHRND	NA	NA	NA
CHRNG	NA	NA	NA
TIGD1	2.22693718	1.86E-06	2.04E-06
EIF4E2	1.67880428	3.66E-236	1.06E-235
AC073254.1	NA	NA	NA
EFHD1	2.31601467	2.64E-47	3.68E-47
GIGYF2	2.29330864	5.85E-06	6.40E-06
KCNJ13	NA	NA	NA
C2orf82	3.72797098	0	0
NGEF	NA	NA	NA
NEU2	NA	NA	NA
INPP5D	2.1143716	2.28E-250	7.04E-250
ATG16L1	2.47854202	9.19E-120	1.70E-119
SAG	NA	8.72E-23	1.07E-22
RP11-400N9.	NA	5.92E-45	8.16E-45
DGKD	3.29388753	6.89E-214	1.81E-213
USP40	3.49672803	3.33E-300	1.32E-299
UGT1A8	NA	NA	NA
UGT1A10	NA	NA	NA
UGT1A9	NA	NA	NA
UGT1A7	NA	NA	NA
UGT1A6	NA	NA	NA
AC114812.8	NA	NA	NA
UGT1A5	NA	NA	NA
UGT1A4	NA	NA	NA
UGT1A3	NA	NA	NA
UGT1A1	NA	3.09E-242	9.19E-242
MROH2A	NA	NA	NA
HJURP	1.69607135	3.50E-295	1.34E-294
TRPM8	NA	NA	NA
AC005538.5	NA	NA	NA
SPP2	NA	NA	NA
AC006037.2	NA	NA	NA

RP11-309M7	NA	NA	NA
AC097713.3	NA	NA	NA
AC097713.4	NA	NA	NA
ARL4C	NA	1.08E-262	3.55E-262
LINC01173	NA	NA	NA
SH3BP4	1.18374083	4.80E-116	8.78E-116
AC114814.4	NA	NA	NA
AC114814.3	NA	NA	NA
AGAP1	2.72233303	3.24E-274	1.12E-273
AC064874.1	NA	NA	NA
GBX2	1.45823146	1.08E-13	1.26E-13
AC079135.1	1.93001222	6.23E-67	9.46E-67
ASB18	NA	0	0
IQCA1	NA	NA	NA
RP11-785G1	NA	0	0
IQCA1-AS1	NA	NA	NA
ACKR3	NA	NA	NA
AC011286.1	NA	NA	NA
AC105760.3	NA	NA	NA
AC105760.2	0.64333741	8.42E-69	1.29E-68
COPS8	1.60528001	1.75E-117	3.22E-117
AC107079.1	NA	NA	NA
AC112715.2	NA	NA	NA
COL6A3	NA	6.43E-18	7.71E-18
AC112721.1	0	0	0
AC112721.2	NA	NA	NA
MLPH	NA	1.40E-130	2.70E-130
PRLH	NA	0	0
RAB17	0	0	0
AC104667.3	NA	NA	NA
AC096574.5	NA	NA	NA
LRRFIP1	1.91863347	2.20E-20	2.67E-20
RBM44	Inf	7.71E-288	2.85E-287
RAMP1	0.17772774	0	0
UBE2F	1.7598391	2.54E-59	3.73E-59
UBE2F-SCLY	NA	0	0
SCLY	2.34818154	2.18E-28	2.76E-28
ESPNL	NA	1.48E-129	2.84E-129
KLHL30	NA	NA	NA
FAM132B	1.69247226	7.66E-102	1.33E-101
ILKAP	1.30442206	0	0
HES6	1.75430502	0	0
PER2	3.61298289	0.1164138	0.11988982
AC012485.2	NA	NA	NA
TRAF3IP1	2.6038809	0	0

ASB1	1.58942183	6.02E-48	8.43E-48
AC016999.2	NA	NA	NA
LINC01107	NA	NA	NA
AC113618.1	NA	NA	NA
AC144525.1	NA	NA	NA
TWIST2	NA	NA	NA
FLJ43879	NA	NA	NA
HDAC4	2.05867971	2.10E-209	5.40E-209
AC017028.1(	NA	NA	NA
AC062017.1	4.11735941	7.90E-198	1.95E-197
AC079612.1	NA	NA	NA
AC079612.2	NA	NA	NA
RP11-315F2:	NA	NA	NA
AC093802.1	NA	NA	NA
NDUFA10	1.0213245	0	0
OR6B2	NA	NA	NA
PRR21	NA	NA	NA
OR6B3	NA	NA	NA
MYEOV2	2.46034825	0	0
OTOS	NA	2.32E-147	4.73E-147
AC124861.1	NA	NA	NA
GPC1	0.9583509	0	0
ANKMY1	1.70373493	1.99E-81	3.19E-81
DUSP28	1.61293879	2.44E-56	3.54E-56
RNPEPL1	1.46385627	2.29E-174	5.15E-174
CAPN10-AS1	1.21315054	1.24E-15	1.47E-15
CAPN10	1.97186791	8.04E-17	9.57E-17
GPR35	NA	9.55E-107	1.69E-106
AQP12B	NA	NA	NA
AC011298.2	NA	NA	NA
AQP12A	NA	NA	NA
KIF1A	NA	NA	NA
AGXT	NA	NA	NA
C2orf54	NA	NA	NA
CROCC2	NA	NA	NA
AC104809.2	NA	NA	NA
SNED1	NA	NA	NA
AC093585.6	NA	NA	NA
AC005237.4	NA	NA	NA
MTERF4	1.53213278	1.72E-269	5.79E-269
PASK	2.40179299	1.96E-240	5.77E-240
PPP1R7	1.43982919	9.21E-295	3.53E-294
ANO7	1.37858016	8.53E-52	1.21E-51
HDLBP	2.26301662	8.68E-265	2.87E-264
2-Sep	1.752992	1.58E-36	2.09E-36

AC005104.3	NA	NA	NA
FARP2	2.29188952	1.52E-55	2.20E-55
STK25	1.6461869	0	0
BOK-AS1	Inf	0	0
BOK	NA	NA	NA
RP11-367H1.	NA	NA	NA
THAP4	2.22053703	8.14E-306	3.33E-305
ATG4B	1.70504147	2.29E-86	3.75E-86
DTYMK	1.90576076	1.83E-33	2.38E-33
AC133528.2	3.86002445	1.00E-37	1.33E-37
ING5	1.96603912	0	0
AC114730.11	NA	NA	NA
D2HGDH	1.76748488	5.57E-26	6.97E-26
AC114730.8	0.77200489	2.41E-70	3.71E-70
AC114730.7	NA	NA	NA
GAL3ST2	NA	NA	NA
AC114730.5	NA	NA	NA
AC114730.3	NA	NA	NA
NEU4	0	3.13E-306	1.28E-305
AC114730.2	NA	NA	NA
PDCD1	NA	NA	NA
RTP5	NA	NA	NA
AC131097.4	NA	NA	NA
AC093642.3	0.77200489	2.75E-135	5.38E-135
AC093642.4	NA	NA	NA
AC093642.6	NA	NA	NA
RP11-341N2.	Inf	0	0
AY269186.1	NA	NA	NA
AY269186.2	NA	NA	NA
CHL1	NA	NA	NA
CHL1-AS1	NA	NA	NA
LINC01266	NA	NA	NA
AC090044.2	NA	NA	NA
AC087430.1	NA	NA	NA
CNTN6	NA	NA	NA
RP11-204C23	NA	NA	NA
CNTN4	NA	0	0
CNTN4-AS2	NA	NA	NA
CNTN4-AS1	NA	NA	NA
IL5RA	NA	NA	NA
TRNT1	1.49575947	2.47E-161	5.32E-161
AC024060.1	NA	NA	NA
CRBN	1.99714308	1.96E-07	2.17E-07
RP11-97C16.	2.5365875	8.07E-220	2.16E-219
AC026188.1	NA	NA	NA

SUMF1	2.42128806	7.62E-49	1.07E-48
LRRN1	NA	NA	NA
SETMAR	2.54307493	3.99E-203	1.00E-202
ITPR1-AS1	Inf	0	0
ITPR1	2.05867971	5.02E-11	5.74E-11
EGOT	NA	NA	NA
AC018816.3	5.40403423	6.57E-32	8.48E-32
BHLHE40-AS	Inf	0	0
BHLHE40	2.50901589	2.25E-103	3.93E-103
ARL8B	2.09611025	7.30E-30	9.34E-30
AC026202.3	NA	NA	NA
RP11-572B2.	0	0	0
EDEM1	1.86775377	1.97E-05	2.14E-05
AC026202.5	NA	NA	NA
AC027119.1	NA	NA	NA
AC026167.1	NA	NA	NA
AC069277.2	NA	NA	NA
GRM7-AS3	NA	NA	NA
GRM7	NA	1.54E-38	2.06E-38
GRM7-AS2	NA	NA	NA
GRM7-AS1	NA	NA	NA
RP11-157E16	NA	NA	NA
LMCD1-AS1	NA	NA	NA
AC018832.1	NA	NA	NA
AC023481.1	NA	NA	NA
LMCD1	0	5.42E-259	1.74E-258
AC034187.2	NA	NA	NA
SSUH2	NA	1.86E-38	2.49E-38
CAV3	NA	NA	NA
OXTR	3.08801956	0	0
RAD18	2.17035337	4.51E-241	1.33E-240
SRGAP3	1.3896088	3.39E-28	4.30E-28
SRGAP3-AS1	NA	NA	NA
SRGAP3-AS2	NA	NA	NA
SRGAP3-AS3	NA	NA	NA
SRGAP3-AS4	Inf	5.55E-196	1.36E-195
RP11-380O2	Inf	0	0
THUMPD3-AS	2.3419956	0	0
THUMPD3	1.40247555	0	0
SETD5	1.66352518	2.96E-210	7.64E-210
LHFPL4	0.77200489	5.25E-167	1.15E-166
MTMR14	1.54400978	0	0
CPNE9	0.51466993	8.87E-28	1.12E-27
BRPF1	2.17421785	5.49E-15	6.46E-15
OGG1	1.99284983	3.23E-68	4.92E-68

CAMK1	NA	5.88E-145	1.19E-144
TADA3	1.54755109	0	0
ARPC4	2.05557929	2.51E-175	5.68E-175
ARPC4-TTLL3	0.77200489	5.27E-05	5.70E-05
TTLL3	2.41082229	7.19E-103	1.25E-102
RP11-266J6.2	0.38600244	0	0
RPUSD3	1.83390983	1.35E-262	4.41E-262
CIDEC	NA	NA	NA
JAGN1	1.72401591	2.02E-259	6.49E-259
IL17RE	1.77107004	0.58182389	0.5874909
IL17RC	2.26776436	0.00051816	0.00055405
CRELD1	1.85281174	1.25E-266	4.17E-266
PRRT3	1.77561125	1.74E-22	2.14E-22
PRRT3-AS1	1.15800733	1.02E-107	1.82E-107
RP11-1020A1	Inf	6.41E-180	1.47E-179
EMC3	1.39415001	0	0
AC022007.5	0	0	0
FANCD2	2.22742394	5.91E-63	8.82E-63
FANCD2OS	NA	NA	NA
BRK1	1.89248169	3.19E-79	5.07E-79
VHL	2.15789319	0.56026315	0.56616132
IRAK2	3.08801956	1.45E-55	2.10E-55
TATDN2	2.33086092	0	0
RP11-438J1.1	NA	NA	NA
LINC00852	1.80134474	3.27E-109	5.85E-109
GHRL	1.93001222	3.89E-15	4.58E-15
GHRLOS	1.02933985	3.58E-53	5.12E-53
SEC13	1.10117715	0	0
ATP2B2	NA	NA	NA
LINC00606	NA	NA	NA
AC018495.3	NA	NA	NA
SLC6A11	NA	NA	NA
SLC6A1	NA	NA	NA
SLC6A1-AS1	NA	NA	NA
HRH1	NA	1.50E-48	2.10E-48
ATG7	1.85853029	2.47E-06	2.71E-06
VGLL4	2.24888381	0.95159486	0.9523823
RP11-169K17	NA	NA	NA
RP11-169K17	NA	NA	NA
TAMM41	1.16543046	1.59E-290	5.97E-290
SYN2	0	2.00E-296	7.74E-296
TIMP4	NA	NA	NA
PPARG	NA	NA	NA
TSEN2	1.5770957	8.23E-288	3.04E-287
MKRN2OS	NA	1.42E-153	2.97E-153

MKRN2	1.68967108	1.71E-33	2.23E-33
RAF1	2.23238081	8.76E-141	1.75E-140
TMEM40	NA	NA	NA
CAND2	NA	0	0
RP11-767C1.	3.86002445	1.97E-208	5.06E-208
RPL32	2.10789967	1.57E-305	6.41E-305
RP11-767C1.	NA	NA	NA
IQSEC1	1.38242736	1.30E-21	1.59E-21
NUP210	2.87681067	1.73E-208	4.45E-208
HDAC11-AS1	NA	NA	NA
HDAC11	1.98515543	4.95E-94	8.33E-94
FBLN2	NA	NA	NA
LINC00620	NA	NA	NA
WNT7A	NA	NA	NA
TPRXL	NA	NA	NA
CHCHD4	2.14612363	1.71E-26	2.15E-26
TMEM43	1.58261002	1.65E-80	2.64E-80
RP11-434D1.	NA	9.39E-36	1.24E-35
AC093495.4	1.15800733	7.81E-22	9.55E-22
XPC	2.07476314	0.42296909	0.42916696
LSM3	2.06292149	2.62E-190	6.29E-190
RP11-536I6.2	NA	NA	NA
LINC01267	NA	NA	NA
SLC6A6	2.68772073	5.65E-15	6.64E-15
GRIP2	NA	NA	NA
AC090952.5	NA	NA	NA
CCDC174	1.92536159	0.00785686	0.00825501
C3orf20	NA	0	0
AC090957.2	NA	NA	NA
RP11-95M5.1	NA	NA	NA
FGD5	NA	NA	NA
FGD5-AS1	2.00841897	9.66E-305	3.93E-304
NR2C2	1.84285038	4.28E-193	1.04E-192
MRPS25	1.67153678	1.57E-195	3.85E-195
RBSN	3.11771206	1.10E-65	1.65E-65
CAPN7	2.71879983	8.56E-227	2.37E-226
SH3BP5-AS1	0.51466993	3.06E-221	8.27E-221
SH3BP5	0.60312882	0	0
METTL6	1.56487478	3.18E-245	9.56E-245
EAF1	2.18734719	1.51E-05	1.64E-05
EAF1-AS1	4.24602689	6.43E-83	1.04E-82
COLQ	Inf	0.09945015	0.10257104
HACL1	1.11312333	6.85E-261	2.23E-260
BTD	1.31884169	4.49E-279	1.58E-278
ANKRD28	2.66610991	0	0

GALNT15	NA	NA	NA
DPH3	2.17539949	5.42E-141	1.08E-140
OXNAD1	1.55309219	7.89E-54	1.13E-53
RFTN1	2.09253957	2.66E-05	2.88E-05
RP11-415F2:	3.08801956	5.53E-268	1.86E-267
RP11-415F2:	NA	5.27E-233	1.50E-232
RP11-415F2:	Inf	1.14E-22	1.40E-22
RP11-194G1(	NA	NA	NA
LINC00690	NA	NA	NA
DAZL	NA	NA	NA
AC091493.2	NA	NA	NA
PLCL2	1.90427873	0.50150045	0.50745838
PLCL2-AS1	NA	NA	NA
TBC1D5	2.41671096	3.27E-17	3.89E-17
AC132807.1	NA	NA	NA
RP11-320G1(	Inf	3.71E-109	6.64E-109
SATB1	1.57273554	4.90E-95	8.30E-95
KCNH8	NA	NA	NA
EFHB	3.86002445	9.04E-10	1.02E-09
RAB5A	1.43985039	1.17E-114	2.12E-114
PP2D1	NA	NA	NA
KAT2B	2.31601467	4.41E-35	5.79E-35
SGOL1	1.50702751	2.48E-213	6.49E-213
RP11-669C1!	NA	NA	NA
RP11-901H1:	NA	NA	NA
ZNF385D	NA	2.72E-146	5.54E-146
ZNF385D-AS	NA	NA	NA
ZNF385D-AS	NA	NA	NA
UBE2E2-AS1	NA	0	0
UBE2E2	1.56622575	0	0
UBE2E1-AS1	Inf	2.84E-37	3.76E-37
UBE2E1	1.9019636	2.82E-123	5.29E-123
NKIRAS1	1.67911064	1.83E-171	4.08E-171
RPL15	1.91797708	1.93E-12	2.23E-12
NR1D2	1.72565799	1.19E-188	2.82E-188
LINC00691	NA	NA	NA
THRB	NA	NA	NA
RP11-317L1(	NA	NA	NA
AC133680.1	NA	NA	NA
RARB	NA	NA	NA
TOP2B	2.76728323	0	0
NGLY1	2.13718868	1.40E-67	2.13E-67
OXSM	1.31884169	7.52E-304	3.04E-303
AC099754.1	NA	NA	NA
LRRC3B	NA	NA	NA

AC114877.3	NA	NA	NA
NEK10	0.77200489	4.32E-105	7.60E-105
SLC4A7	1.93001222	1.36E-74	2.13E-74
RP11-222K1(	NA	NA	NA
RP11-222K1(	NA	1.22E-109	2.19E-109
EOMES	Inf	1.20E-05	1.31E-05
AC098973.2	NA	NA	NA
AC098973.1	NA	NA	NA
AC092415.1	NA	NA	NA
CMC1	1.97193104	8.78E-45	1.21E-44
AZI2	1.73468569	2.31E-64	3.46E-64
ZCWPW2	0.25733496	0	0
LINC00693	NA	NA	NA
RBMS3-AS3	NA	NA	NA
RBMS3	NA	NA	NA
RBMS3-AS2	NA	NA	NA
RBMS3-AS1	NA	NA	NA
AC116035.1	NA	NA	NA
AC116035.2	NA	NA	NA
TGFBR2	2.66692598	3.27E-94	5.51E-94
GADL1	NA	NA	NA
STT3B	1.774459	6.39E-78	1.01E-77
OSBPL10	1.15800733	5.53E-33	7.18E-33
OSBPL10-AS	NA	NA	NA
ZNF860	NA	NA	NA
GPD1L	2.31601467	0.11281457	0.11622271
CMTM8	1.58185316	3.18E-08	3.55E-08
CMTM7	1.78883998	5.59E-12	6.43E-12
CMTM6	1.91439363	0.82934556	0.83274226
DYNC1LI1	1.28932775	0	0
CNOT10	2.27608338	1.03E-305	4.20E-305
CNOT10-AS1	NA	0	0
TRIM71	1.7896477	2.75E-26	3.45E-26
CCR4	0	0	0
GLB1	1.55416774	0	0
TMPPE	Inf	0	0
CRTAP	2.38884532	0	0
RP11-627J17	NA	NA	NA
SUSD5	NA	NA	NA
FBXL2	5.40403423	0.00021874	0.00023505
UBP1	2.05269517	9.31E-270	3.14E-269
CLASP2	2.14270745	2.12E-18	2.55E-18
RP11-10C24.	0	1.56E-271	5.31E-271
RP11-10C24.	Inf	8.92E-64	1.34E-63
RP11-10C24.	1.54400978	6.41E-18	7.68E-18

PDCD6IP	2.00721271	2.75E-215	7.26E-215
AC018359.1	NA	NA	NA
AC123023.1	NA	NA	NA
AC018359.3	NA	NA	NA
ARPP21	NA	NA	NA
ARPP21-AS1	NA	NA	NA
STAC	NA	NA	NA
DCLK3	NA	NA	NA
RP11-640L9.1	NA	NA	NA
TRANK1	1.69841076	1.36E-11	1.56E-11
RP11-129K12	Inf	4.47E-66	6.76E-66
EPM2AIP1	2.36748166	2.88E-22	3.53E-22
MLH1	1.64694377	7.46E-67	1.13E-66
LRRFIP2	2.39183658	5.19E-212	1.35E-211
RP11-285J16	0.38600244	7.73E-22	9.45E-22
RP11-259K5.	NA	NA	NA
RP11-259K5.	0.77200489	0.23909468	0.24426488
GOLGA4	1.49829896	0	0
C3orf35	0	3.96E-251	1.22E-250
ITGA9	1.15800733	1.28E-262	4.18E-262
CTDSPL	2.78280832	6.06E-163	1.31E-162
VILL	2.70201711	2.99E-299	1.17E-298
PLCD1	0.51466993	1.17E-293	4.47E-293
DLEC1	Inf	0	0
ACAA1	1.12598093	0	0
MYD88	1.18071336	5.02E-212	1.31E-211
OXSR1	2.48027103	3.31E-235	9.52E-235
SLC22A13	NA	0.00064724	0.00069112
SLC22A14	NA	4.43E-14	5.18E-14
XYLB	1.99095998	0.3718228	0.37792645
ACVR2B-AS1	Inf	9.49E-285	3.45E-284
ACVR2B	1.506351	8.76E-136	1.72E-135
EXOG	2.20021394	1.41E-138	2.79E-138
SCN5A	1.64833477	2.30E-163	4.98E-163
SCN10A	NA	NA	NA
RP11-134J21	NA	NA	NA
SCN11A	NA	NA	NA
WDR48	1.89784535	0.93816608	0.93940882
GORASP1	1.72929095	8.38E-11	9.57E-11
TTC21A	2.31601467	7.46E-157	1.58E-156
CSRNP1	0.91236942	2.92E-257	9.32E-257
XIRP1	NA	NA	NA
CX3CR1	NA	NA	NA
CCR8	NA	NA	NA
SLC25A38	1.66655024	1.65E-235	4.76E-235

RPSA	1.99067254	2.14E-101	3.70E-101
MOBP	NA	0	0
MYRIP	Inf	4.76E-202	1.19E-201
EIF1B	1.82637733	1.02E-140	2.03E-140
ENTPD3-AS1	3.25054691	1.99E-119	3.68E-119
ENTPD3	NA	NA	NA
RPL14	2.02732947	6.55E-131	1.26E-130
ZNF619	3.86002445	1.10E-47	1.53E-47
ZNF620	2.26776436	3.45E-78	5.46E-78
ZNF621	1.94679494	3.55E-19	4.28E-19
RP11-528N2	NA	NA	NA
RP11-761N2	NA	NA	NA
RP11-520A2:	NA	NA	NA
RP11-372H2.	NA	NA	NA
CTNNB1	1.60024855	1.92E-66	2.91E-66
ULK4	2.67628362	0	0
TRAK1	2.31601467	4.11E-122	7.68E-122
CCK	NA	NA	NA
RP11-333B1:	NA	NA	NA
LYZL4	NA	NA	NA
VIPR1	1.80134474	8.70E-49	1.22E-48
VIPR1-AS1	NA	6.81E-06	7.44E-06
SEC22C	2.00284287	2.50E-41	3.38E-41
SS18L2	2.10420633	6.38E-177	1.45E-176
NKTR	2.00450392	3.22E-26	4.04E-26
RP4-613B23.	2.31601467	0.00015554	0.00016734
ZBTB47	0.77200489	4.00E-281	1.42E-280
KLHL40	NA	NA	NA
CCDC13	0.77200489	2.84E-272	9.72E-272
HHATL	NA	NA	NA
HHATL-AS1	NA	NA	NA
CCDC13-AS1	NA	NA	NA
RP4-613B23.	NA	NA	NA
RP11-70C1.1	Inf	2.44E-11	2.80E-11
HIGD1A	2.02241102	1.08E-112	1.96E-112
RP11-70C1.3	NA	NA	NA
ACKR2	1.54400978	2.75E-86	4.49E-86
RP11-136C24	NA	NA	NA
KRBOX1	NA	NA	NA
CYP8B1	NA	NA	NA
ZNF662	NA	NA	NA
KRBOX1-AS1	NA	NA	NA
FAM198A	2.80359671	5.90E-16	6.98E-16
POMGNT2	1.85875024	3.64E-43	4.98E-43
RP11-136C24	NA	NA	NA

SNRK	3.04738772	9.35E-169	2.07E-168
RP11-188P2(	NA	1.44E-288	5.35E-288
SNRK-AS1	NA	NA	NA
ANO10	1.65429619	7.10E-181	1.64E-180
ABHD5	2.39452364	1.03E-263	3.38E-263
RP4-672N11.	NA	NA	NA
RP4-555D20.	NA	NA	NA
RP4-555D20.	2.70201711	0.07406035	0.07659785
RP4-555D20.	2.31601467	1.84E-141	3.69E-141
TOPAZ1	NA	NA	NA
RP11-424N2	1.80134474	4.67E-227	1.29E-226
TCAIM	1.89008094	0.06088164	0.06306467
LINC00694	1.82352879	3.08E-05	3.35E-05
RP11-101C2	NA	NA	NA
ZNF445	2.30648375	2.10E-258	6.72E-258
ZNF852	4.37469438	5.49E-12	6.32E-12
ZKSCAN7	NA	2.63E-105	4.63E-105
RP11-944L7.	NA	NA	NA
ZNF660	NA	NA	NA
ZNF197	1.99095998	0.51304977	0.51900016
ZNF197-AS1	NA	NA	NA
ZNF35	Inf	2.19E-21	2.68E-21
RP11-348P1(	NA	2.53E-09	2.85E-09
ZNF502	NA	NA	NA
ZNF501	NA	NA	NA
KIAA1143	1.88286689	4.76E-21	5.81E-21
KIF15	1.7896477	1.76E-234	5.05E-234
TMEM42	4.63202934	4.19E-292	1.58E-291
TGM4	NA	NA	NA
RP11-272D2(	NA	NA	NA
ZDHHC3	2.19858998	3.67E-20	4.46E-20
EXOSC7	1.58235439	0	0
CLEC3B	Inf	8.87E-229	2.48E-228
CDCP1	NA	NA	NA
TMEM158	1.18557894	1.74E-16	2.06E-16
LARS2	3.55980033	0	0
LARS2-AS1	NA	NA	NA
LIMD1	2.24984282	3.47E-79	5.51E-79
LIMD1-AS1	1.54400978	2.32E-20	2.81E-20
SACM1L	2.06311652	1.77E-216	4.70E-216
SLC6A20	NA	NA	NA
LZTFL1	1.19309847	7.44E-295	2.86E-294
CCR9	NA	NA	NA
FYCO1	8.02885086	0	0
CXCR6	NA	2.59E-47	3.61E-47

XCR1	NA	NA	NA
CCR3	NA	NA	NA
CCR1	Inf	0	0
CCR2	NA	NA	NA
RP11-24F11.	0	0	0
CCR5	NA	NA	NA
CCRL2	0.77200489	1.10E-145	2.24E-145
LTF	NA	NA	NA
RTP3	NA	NA	NA
LRRC2	NA	NA	NA
LRRC2-AS1	NA	NA	NA
TDGF1	NA	NA	NA
ALS2CL	7.46271394	0	0
TMIE	2.70201711	2.92E-43	3.99E-43
PRSS50	NA	NA	NA
PRSS46	NA	NA	NA
PRSS45	NA	NA	NA
PRSS42	NA	NA	NA
MYL3	NA	NA	NA
PTH1R	NA	0	0
CCDC12	1.48066028	0	0
NBEAL2	2.73170961	9.96E-146	2.02E-145
SETD2	1.99353161	3.06E-39	4.10E-39
KIF9-AS1	7.7200489	0	0
KIF9	2.18631785	0	0
KLHL18	2.43417868	0	0
RP11-708J19	2.59674372	8.92E-202	2.23E-201
PTPN23	4.01442543	0	0
SCAP	2.2125501	1.96E-165	4.28E-165
ELP6	1.51312958	4.17E-232	1.18E-231
CSPG5	1.06892985	1.99E-300	7.87E-300
SMARCC1	1.74952034	0	0
DHX30	2.15369569	1.58E-189	3.77E-189
MAP4	2.08675261	6.00E-65	9.03E-65
CDC25A	1.86325992	0.82519654	0.82871384
CAMP	0	0	0
ZNF589	1.79491137	0.03208145	0.03339195
NME6	1.97964111	0.00067543	0.00072102
SPINK8	NA	NA	NA
FBXW12	NA	0	0
PLXNB1	2.31601467	2.84E-98	4.86E-98
CCDC51	1.35502942	0	0
TMA7	2.40789746	0	0
RP11-24C3.2	NA	8.13E-75	1.27E-74
ATRIP	NA	NA	NA

ATRIP	0.77200489	9.93E-211	2.57E-210
TREX1	NA	NA	NA
SHISA5	1.99530047	0.01168733	0.01224625
PFKFB4	1.93001222	9.99E-122	1.86E-121
UCN2	NA	3.71E-109	6.64E-109
COL7A1	1.54400978	1.77E-54	2.54E-54
UQCRC1	1.08024641	0	0
TMEM89	NA	7.90E-34	1.03E-33
SLC26A6	1.76814023	0	0
CELSR3	0	0	0
CELSR3-AS1	NA	NA	NA
RP11-572O6.	NA	NA	NA
NCKIPSD	1.99095998	0.00139924	0.00148746
IP6K2	1.6319597	0	0
PRKAR2A	2.0741198	0.45409264	0.46015465
PRKAR2A-AS	Inf	1.40E-22	1.72E-22
SLC25A20	1.49859773	7.33E-276	2.54E-275
ARIH2OS	1.64051039	3.44E-123	6.45E-123
ARIH2	2.04138842	4.61E-284	1.66E-283
RP13-131K1S	1.02933985	1.21E-26	1.53E-26
RP13-131K1S	1.54400978	1.22E-48	1.70E-48
RP13-131K1S	4.63202934	3.50E-114	6.37E-114
P4HTM	1.62121027	2.90E-229	8.13E-229
WDR6	1.99506882	1.38E-33	1.80E-33
DALRD3	1.60684739	0	0
NDUFAF3	1.80233908	1.62E-94	2.73E-94
IMPDH2	1.10009132	0	0
RP13-131K1S	Inf	0	0
QRICH1	1.50112062	2.09E-232	5.95E-232
QARS	1.52572545	3.32E-228	9.24E-228
USP19	2.48144429	7.90E-61	1.17E-60
LAMB2	1.28667482	1.37E-76	2.15E-76
CCDC71	1.55309219	1.49E-253	4.66E-253
KLHDC8B	1.36499415	0	0
C3orf84	NA	NA	NA
CCDC36	NA	NA	NA
RP11-3B7.1	NA	NA	NA
C3orf62	2.73710825	5.49E-286	2.00E-285
USP4	2.16161369	2.11E-151	4.39E-151
GPX1	2.40839738	0	0
RHOA	1.43660378	0	0
TCTA	1.89062422	3.49E-126	6.61E-126
AMT	4.63202934	1.09E-186	2.58E-186
NICN1	1.6297881	2.44E-105	4.30E-105
DAG1	2.25168093	0.00075179	0.00080219

BSN-AS2	Inf	0	0
BSN	0.96500611	2.60E-131	5.02E-131
BSN-AS1	NA	NA	NA
APEH	1.32502762	0	0
MST1	Inf	7.99E-55	1.15E-54
RNF123	2.42630108	1.98E-30	2.54E-30
AMIGO3	Inf	0	0
GMPPB	1.59915299	3.80E-179	8.72E-179
IP6K1	1.95032814	3.35E-14	3.92E-14
CDHR4	Inf	4.63E-210	1.20E-209
FAM212A	1.48010209	0	0
UBA7	1.23520782	0.00014863	0.00015995
TRAIP	1.78526131	1.84E-131	3.56E-131
CAMKV	1.28667482	0	0
MST1R	NA	2.51E-265	8.33E-265
CTD-2330K9.	NA	NA	NA
CTD-2330K9.	NA	NA	NA
MON1A	2.30144854	2.48E-40	3.33E-40
RBM6	1.91571584	1.25E-80	1.99E-80
RBM5	1.86495563	1.60E-10	1.82E-10
RBM5-AS1	Inf	1.96E-251	6.06E-251
SEMA3F-AS1	NA	NA	NA
SEMA3F	Inf	0	0
GNAT1	NA	NA	NA
SLC38A3	NA	0	0
GNAI2	1.4369992	0	0
U73166.2	6.94804401	3.36E-290	1.26E-289
SEMA3B-AS1	NA	NA	NA
SEMA3B	1.08080685	3.44E-19	4.15E-19
LSMEM2	1.15800733	2.40E-89	3.97E-89
IFRD2	1.66934704	2.00E-191	4.82E-191
HYAL3	1.23129894	0	0
NAT6	2.08792232	2.55E-07	2.82E-07
HYAL1	Inf	2.10E-95	3.56E-95
HYAL2	1.75455657	2.90E-119	5.37E-119
TUSC2	1.99904186	0	0
RASSF1	1.65710604	2.34E-228	6.52E-228
RASSF1-AS1	2.70201711	1.83E-230	5.14E-230
ZMYND10-AS	NA	NA	NA
ZMYND10	3.86002445	5.77E-10	6.55E-10
NPRL2	1.28667482	0	0
CYB561D2	1.7457397	1.46E-146	2.97E-146
XXcos-LUCA1	NA	NA	NA
TMEM115	1.66224476	4.44E-211	1.15E-210
CACNA2D2	Inf	0	0

CYB561D2	0.30880196	0	0
C3orf18	Inf	8.46E-37	1.12E-36
HEMK1	1.86630308	1.23E-38	1.64E-38
RP11-804H8.	NA	NA	NA
CISH	2.03202716	6.32E-41	8.53E-41
MAPKAPK3	1.67841175	0	0
RP11-804H8.	1.54400978	0.24703345	0.25224747
DOCK3	0.55143206	1.79E-229	5.01E-229
MANF	1.56305925	2.30E-184	5.39E-184
RBM15B	1.76631485	1.24E-130	2.38E-130
VPRBP	2.22243832	2.24E-43	3.06E-43
RAD54L2	2.78389642	0	0
TEX264	1.51883571	3.31E-270	1.12E-269
GRM2	NA	0	0
IQCF6	NA	NA	NA
IQCF3	NA	NA	NA
IQCF2	NA	NA	NA
IQCF5-AS1	NA	NA	NA
IQCF5	NA	NA	NA
IQCF1	NA	NA	NA
RRP9	1.24901604	0	0
PARP3	Inf	3.86E-226	1.07E-225
GPR62	NA	NA	NA
PCBP4	1.75060264	1.18E-108	2.11E-108
ABHD14B	1.95025467	6.93E-73	1.08E-72
ABHD14A	1.79215421	6.83E-19	8.22E-19
ABHD14A-AC	NA	NA	NA
ACY1	3.60268949	0.04795437	0.04976764
RPL29	2.05200347	3.76E-194	9.14E-194
DUSP7	2.36280285	8.79E-11	1.00E-10
POC1A	1.55258761	1.29E-248	3.94E-248
ALAS1	1.24590888	0	0
TLR9	NA	NA	NA
RP11-330H6.	NA	NA	NA
TWF2	1.01424112	0	0
RP5-1157M2	0.77200489	2.16E-152	4.49E-152
PPM1M	1.78154975	2.44E-102	4.24E-102
WDR82	2.12301345	2.76E-165	6.03E-165
GLYCTK	1.78154975	7.30E-48	1.02E-47
DNAH1	0	2.92E-130	5.62E-130
BAP1	2.09652308	3.20E-263	1.05E-262
PHF7	1.57488998	4.17E-68	6.35E-68
SEMA3G	Inf	3.04E-168	6.72E-168
TNNC1	NA	4.75E-231	1.34E-230
NISCH	1.68697365	1.45E-22	1.78E-22

STAB1	NA	0	0
NT5DC2	2.00905448	4.85E-42	6.59E-42
SMIM4	2.36726811	6.01E-188	1.42E-187
PBRM1	1.96510336	5.59E-141	1.12E-140
GNL3	1.36930074	0	0
GLT8D1	1.58380385	0	0
SPCS1	2.02946694	4.81E-62	7.14E-62
NEK4	1.57617665	2.63E-15	3.09E-15
ITIH1	NA	NA	NA
ITIH3	NA	NA	NA
ITIH4	NA	7.26E-299	2.84E-298
RP5-966M1.(	NA	7.11E-38	9.45E-38
ITIH4-AS1	NA	NA	NA
MUSTN1	NA	NA	NA
TMEM110-M	0.77200489	2.41E-249	7.39E-249
TMEM110	1.737011	2.96E-14	3.46E-14
SFMBT1	2.03723513	0.13675672	0.1406087
RP11-894J14	NA	5.39E-211	1.40E-210
RFT1	1.18800752	0	0
PRKCD	1.08080685	2.97E-252	9.24E-252
TKT	1.26505003	0	0
DCP1A	2.03393596	6.71E-05	7.24E-05
CACNA1D	0	0	0
RP11-884K1(	NA	NA	NA
CHDH	2.62481663	0	0
IL17RB	3.01081907	5.67E-69	8.67E-69
RP11-884K1(	2.20572826	2.10E-24	2.60E-24
ACTR8	1.42338402	9.57E-145	1.94E-144
SELK	1.82614811	0.84938834	0.85215966
CACNA2D3	4.63202934	1.13E-149	2.33E-149
CACNA2D3-A	NA	NA	NA
LRTM1	2.31601467	0	0
RP11-889D3.	NA	NA	NA
RP11-889D3.	NA	NA	NA
RP11-64C1.1	NA	NA	NA
RP11-875H7.	NA	NA	NA
RP11-875H7.	NA	NA	NA
WNT5A	NA	NA	NA
WNT5A-AS1	NA	NA	NA
RP11-875H7.	NA	NA	NA
ERC2	1.93001222	1.33E-23	1.65E-23
RP11-58O15.	NA	NA	NA
CCDC66	2.0966951	1.48E-08	1.66E-08
FAM208A	2.12695225	6.34E-26	7.94E-26
ARHGEF3	3.68846781	3.40E-165	7.42E-165

ARHGEF3-AS	NA	NA	NA
SPATA12	NA	0	0
RP11-157F2(	NA	NA	NA
IL17RD	NA	3.33E-08	3.72E-08
HESX1	2.5365875	9.07E-163	1.96E-162
APPL1	2.63975866	0	0
ASB14	NA	NA	NA
DNAH12	NA	NA	NA
PDE12	2.69098847	3.83E-160	8.21E-160
ARF4	1.60092507	4.86E-162	1.05E-161
ARF4-AS1	3.08801956	0.00042877	0.00045898
DENND6A	1.97826253	0.00452493	0.00477386
DENND6A-A	NA	NA	NA
RP11-755B1(	Inf	0	0
SLMAP	1.8711305	9.08E-81	1.45E-80
FLNB	2.66208583	0	0
FLNB-AS1	NA	0	0
DNASE1L3	NA	NA	NA
ABHD6	2.89501834	8.89E-18	1.06E-17
RPP14	2.44870301	1.01E-28	1.29E-28
RP11-80H18.	Inf	0	0
RP11-80H18.	NA	NA	NA
PXK	1.80611021	1.10E-32	1.43E-32
PDHB	1.12618049	0	0
RP11-802O2	2.70201711	0.27814006	0.28367534
RP11-359I18	NA	NA	NA
KCTD6	2.75348411	1.62E-18	1.95E-18
ACOX2	NA	3.50E-257	1.11E-256
FAM107A	NA	NA	NA
RP11-475O2	NA	NA	NA
FAM3D-AS1	NA	NA	NA
FAM3D	NA	NA	NA
C3orf67	0	0	0
C3orf67-AS1	NA	NA	NA
CTD-2185K1(	NA	NA	NA
RP11-285B2	NA	NA	NA
RP11-719N2	NA	NA	NA
FHIT	NA	NA	NA
PTPRG	1.98515543	1.88E-06	2.07E-06
C3orf14	NA	NA	NA
FEZF2	NA	NA	NA
CADPS	Inf	0	0
LINC00698	NA	NA	NA
SYNPR	NA	NA	NA
SYNPR-AS1	NA	NA	NA

SNTN	NA	NA	NA
C3orf49	0.77200489	0.00623093	0.00655807
THOC7	1.89514162	0.01696094	0.01773212
THOC7-AS1	NA	NA	NA
ATXN7	2.350326	6.93E-133	1.34E-132
SCAANT1	NA	NA	NA
PSMD6-AS2	2.31601467	0.05014572	0.05202102
RP11-245J9.!	3.08801956	1.45E-150	3.01E-150
PSMD6	0.98595544	0	0
RP11-245J9.4	Inf	0	0
RP11-245J9.(	Inf	0	0
PRICKLE2-AS	NA	NA	NA
LINC00994	NA	NA	NA
PRICKLE2	NA	NA	NA
PRICKLE2-AS	NA	NA	NA
PRICKLE2-AS	NA	NA	NA
PRICKLE2-AS	NA	NA	NA
RP11-14D22.	NA	NA	NA
RP11-14D22.	NA	NA	NA
ADAMTS9	NA	3.19E-20	3.87E-20
ADAMTS9-A!	NA	NA	NA
ADAMTS9-A!	NA	NA	NA
RP11-543A1!	NA	NA	NA
MAGI1	1.69105833	0.25102068	0.25630443
MAGI1-AS1	NA	NA	NA
SLC25A26	1.89333326	1.70E-17	2.03E-17
LRIG1	1.14469691	7.54E-215	1.99E-214
KBTBD8	1.97826253	0.60117698	0.60679608
RP11-85I21.1	NA	NA	NA
SUCLG2	1.3081194	0	0
SUCLG2-AS1	0	5.83E-80	9.29E-80
FAM19A1	NA	NA	NA
FAM19A4	NA	NA	NA
EOGT	1.77107004	1.76E-07	1.96E-07
CTD-2013N2	1.54400978	6.35E-81	1.02E-80
TMF1	2.09253957	3.65E-92	6.11E-92
UBA3	1.35308384	2.51E-166	5.51E-166
ARL6IP5	1.9193883	4.99E-13	5.79E-13
LMOD3	6.17603912	7.65E-30	9.78E-30
FRMD4B	2.70201711	4.49E-59	6.58E-59
RP11-62G11.	NA	NA	NA
RP11-444P1(	NA	NA	NA
MITF	6.94804401	0	0
LINC01212	NA	1.32E-131	2.54E-131
RP11-231I13	NA	NA	NA

FOXP1	2.13713549	3.52E-15	4.15E-15
FOXP1-AS1	NA	NA	NA
RP11-154H2:	NA	NA	NA
RP11-154H2:	0	0	0
RP11-154H2:	1.54400978	1.31E-59	1.92E-59
EIF4E3	0	0	0
GPR27	NA	NA	NA
PROK2	NA	NA	NA
LINC00877	NA	NA	NA
RP11-398A8.	NA	NA	NA
RP11-398A8.	NA	NA	NA
LINC00870	NA	NA	NA
RP11-433A1(	NA	NA	NA
RP11-433A1(	NA	NA	NA
RP11-447M4	NA	NA	NA
RYBP	4.16024857	0	0
RP11-654C2:	NA	NA	NA
SHQ1	1.66968499	1.67E-08	1.87E-08
GXYLT2	2.34091805	1.53E-275	5.29E-275
PPP4R2	1.88564413	2.25E-54	3.23E-54
EBLN2	2.44468215	4.68E-139	9.29E-139
PDZRN3	NA	NA	NA
PDZRN3-AS1	NA	NA	NA
RP11-20B7.1	NA	NA	NA
RP11-508O1	NA	NA	NA
CNTN3	NA	NA	NA
RP11-803B1.	1.02933985	1.02E-73	1.59E-73
CTD-2026G6.	7.7200489	3.85E-53	5.51E-53
FRG2C	NA	NA	NA
LINC00960	NA	NA	NA
ZNF717	1.87486902	5.69E-121	1.06E-120
ROBO2	NA	NA	NA
RP11-354H2:	NA	NA	NA
RP11-354H2:	NA	NA	NA
RP11-314M2	NA	NA	NA
ROBO1	2.17977851	2.96E-61	4.38E-61
RP11-47P18.	NA	NA	NA
RP11-47P18.	NA	NA	NA
RP11-6B4.1	NA	NA	NA
RP11-520D1!	NA	NA	NA
RP11-520D1!	NA	NA	NA
GBE1	1.56283917	6.46E-64	9.67E-64
RP11-142L1.	NA	NA	NA
RP11-359D2	NA	NA	NA
RP11-260O1	NA	NA	NA

LINC00971	NA	NA	NA
RP11-735B1:	NA	NA	NA
CADM2	3.86002445	0	0
CADM2-AS2	NA	NA	NA
CADM2-AS1	NA	NA	NA
RP11-789F5.	NA	NA	NA
RP11-331K1!	NA	NA	NA
VGLL3	NA	NA	NA
LINC00506	NA	NA	NA
CHMP2B	1.5925358	4.31E-230	1.21E-229
POU1F1	NA	NA	NA
RP11-451B8.	NA	NA	NA
HTR1F	NA	NA	NA
CGGBP1	2.215099	0	0
RP11-159G9.	2.6055165	2.60E-50	3.67E-50
ZNF654	2.17126375	0	0
C3orf38	1.6887607	7.00E-50	9.88E-50
EPHA3	NA	NA	NA
PROS1	2.05867971	6.49E-08	7.23E-08
ARL13B	1.37390701	1.02E-122	1.91E-122
STX19	NA	NA	NA
DHFRL1	3.60268949	0	0
NSUN3	1.36019909	6.42E-76	1.01E-75
LINC00879	NA	NA	NA
MTRNR2L12	2.47041565	9.09E-11	1.04E-10
EPHA6	NA	NA	NA
RP11-389G6.	NA	NA	NA
ARL6	0.84218715	2.58E-78	4.08E-78
RP11-389G6.	Inf	0	0
CRYBG3	2.31601467	0.5154166	0.5213363
RP11-389G6.	NA	NA	NA
MINA	2.15657888	1.18E-107	2.10E-107
GABRR3	NA	NA	NA
OR5AC2	NA	NA	NA
RP11-343D2.	NA	NA	NA
OR5H1	NA	NA	NA
OR5H14	NA	NA	NA
OR5H15	NA	NA	NA
RP11-325B2:	NA	NA	NA
OR5H6	NA	NA	NA
OR5H2	NA	NA	NA
OR5K4	NA	NA	NA
OR5K3	NA	NA	NA
OR5K1	NA	NA	NA
OR5K2	NA	NA	NA

CLDND1	2.32224052	3.77E-177	8.58E-177
CPOX	1.28319732	4.53E-238	1.32E-237
RP11-227H4.	NA	NA	NA
GPR15	NA	NA	NA
ST3GAL6-AS	2.31601467	2.02E-42	2.75E-42
ST3GAL6	1.68257476	5.53E-294	2.11E-293
DCBLD2	1.54400978	3.20E-102	5.57E-102
CTD-2021J15	NA	NA	NA
LINC00973	NA	NA	NA
RP11-779P15	NA	NA	NA
RP11-779P15	NA	NA	NA
RP11-201E8.	NA	NA	NA
COL8A1	Inf	0.02045642	0.02136314
RP11-383I23	Inf	3.36E-163	7.29E-163
CMSS1	1.51854788	0	0
FILIP1L	Inf	5.07E-06	5.54E-06
TMEM30C	NA	NA	NA
TBC1D23	1.72046804	3.59E-13	4.17E-13
NIT2	1.46321857	0	0
TOMM70A	2.05240324	1.01E-65	1.52E-65
LNP1	0.46320293	0.85944721	0.86196535
TMEM45A	2.70201711	0	0
ADGRG7	NA	NA	NA
TFG	1.27450989	0	0
ABI3BP	NA	NA	NA
IMPG2	NA	1.69E-204	4.27E-204
SENP7	2.4563792	0.42040943	0.42659365
TRMT10C	1.71921656	5.24E-106	9.24E-106
PCNP	1.89790229	2.65E-70	4.08E-70
ZBTB11	2.01774005	4.60E-253	1.44E-252
ZBTB11-AS1	1.63483388	1.43E-52	2.04E-52
RPL24	2.03669264	4.59E-302	1.84E-301
CEP97	2.50901589	3.11E-130	5.97E-130
NXPE3	1.85281174	1.45E-05	1.58E-05
RP11-49I4.3	NA	NA	NA
NFKBIZ	0.84920538	0	0
RP11-221J22	NA	NA	NA
RP11-221J22	NA	NA	NA
ZPLD1	NA	NA	NA
RP11-280H2	NA	NA	NA
ALCAM	2.33317033	8.85E-127	1.68E-126
CBLB	1.98515543	2.45E-87	4.01E-87
LINC00882	Inf	2.01E-158	4.28E-158
DUBR	2.64687391	1.71E-08	1.92E-08
RP11-446H1	NA	NA	NA

RP11-446H18	NA	NA	NA
CCDC54	NA	NA	NA
RP11-115H18	NA	1.00E-256	3.19E-256
BBX	1.65851768	0	0
LINC00635	NA	NA	NA
LINC00636	Inf	0	0
CD47	1.53588341	1.29E-299	5.07E-299
LINC01215	1.76017115	0.00134115	0.00142588
IFT57	1.79066694	3.13E-170	6.97E-170
HHLA2	NA	1.08E-177	2.46E-177
MYH15	0	0	0
KIAA1524	1.13388218	0	0
AC069499.1	NA	NA	NA
DZIP3	2.57334963	0.00179436	0.00190404
RETNLB	NA	NA	NA
TRAT1	NA	NA	NA
GUCA1C	NA	NA	NA
MORC1	NA	NA	NA
MORC1-AS1	NA	NA	NA
FLJ22763	NA	NA	NA
RP11-59E19.	NA	NA	NA
LINC00488	NA	NA	NA
DPPA2	NA	NA	NA
DPPA4	NA	NA	NA
LINC01205	NA	NA	NA
RP11-457K10	NA	NA	NA
RP11-119D18	NA	NA	NA
RP11-553A10	NA	NA	NA
PVRL3-AS1	NA	NA	NA
PVRL3	0.77200489	2.17E-276	7.56E-276
CD96	0.77200489	1.07E-24	1.32E-24
RP11-615J4.3	NA	NA	NA
ZBED2	NA	NA	NA
PLCXD2	0	0	0
PLCXD2-AS1	NA	NA	NA
PHLDB2	NA	NA	NA
ABHD10	2.1081672	5.12E-81	8.19E-81
TAGLN3	NA	NA	NA
TMPRSS7	NA	NA	NA
C3orf52	NA	NA	NA
GCSAM	NA	NA	NA
RP11-757F18	NA	NA	NA
SLC9C1	NA	NA	NA
RP11-90K6.1	NA	NA	NA
CD200	0	0	0

RP11-231E6.	NA	4.43E-14	5.18E-14
BTLA	NA	NA	NA
ATG3	1.6922571	3.73E-174	8.40E-174
SLC35A5	2.12301345	5.06E-75	7.91E-75
CCDC80	NA	0	0
RP11-572C1!	NA	NA	NA
CD200R1L	NA	NA	NA
CD200R1	NA	NA	NA
RP11-484K9.	0.77200489	0.13244016	0.1362246
GTPBP8	1.57182978	2.69E-18	3.23E-18
C3orf17	1.62387235	2.42E-228	6.74E-228
RP11-572M1	NA	4.38E-100	7.56E-100
RP11-572M1	NA	NA	NA
RP11-572M1	NA	NA	NA
BOC	0.51466993	3.22E-34	4.21E-34
CFAP44	3.98869193	3.86E-189	9.22E-189
CFAP44-AS1	NA	0	0
SPICE1	1.72216475	2.85E-120	5.30E-120
SIDT1	0	0	0
SIDT1-AS1	NA	2.89E-242	8.61E-242
USF3	2.04749123	4.71E-46	6.52E-46
NAA50	2.10796589	3.66E-105	6.44E-105
RP11-271C2	Inf	6.22E-75	9.72E-75
ATP6V1A	2.63389904	0	0
GRAMD1C	2.31601467	7.43E-54	1.07E-53
RP11-255E6.	NA	4.61E-66	6.96E-66
ZDHHC23	2.50901589	1.70E-25	2.12E-25
CCDC191	0.38600244	3.12E-259	1.00E-258
QTRTD1	1.69283	5.65E-99	9.71E-99
DRD3	NA	NA	NA
RP11-553L6.	NA	NA	NA
ZNF80	NA	NA	NA
TIGIT	NA	NA	NA
RP11-553L6.	NA	6.77E-133	1.31E-132
ZBTB20	2.31601467	1.78E-77	2.81E-77
ZBTB20-AS1	NA	NA	NA
RP11-197K3.	NA	NA	NA
ZBTB20-AS2	NA	1.35E-40	1.82E-40
ZBTB20-AS3	NA	NA	NA
ZBTB20-AS4	NA	NA	NA
AC069063.1	NA	NA	NA
AC069063.2	NA	NA	NA
RP11-190P1	NA	NA	NA
GAP43	NA	NA	NA
LSAMP	5.69353606	3.03E-296	1.17E-295

LSAMP-AS1	NA	NA	NA
LINC00903	NA	NA	NA
TUSC7	NA	NA	NA
LINC00901	NA	NA	NA
RP11-384F7.	NA	NA	NA
RP11-768G7.	NA	NA	NA
RP11-768G7.	NA	NA	NA
RP11-384F7.	NA	NA	NA
IGSF11	NA	NA	NA
IGSF11-AS1	NA	NA	NA
C3orf30	NA	NA	NA
RP11-484M3	NA	NA	NA
UPK1B	NA	NA	NA
B4GALT4	1.74716896	2.36E-285	8.61E-285
B4GALT4-AS	NA	NA	NA
ARHGAP31	3.60268949	3.84E-300	1.52E-299
ARHGAP31-A	NA	NA	NA
TMEM39A	1.70776839	2.07E-120	3.84E-120
POGLUT1	1.34435334	5.16E-277	1.80E-276
RP11-190C22	2.57334963	0	0
TIMMDC1	1.7687707	1.28E-163	2.78E-163
CD80	Inf	2.99E-170	6.65E-170
RP11-190C22	Inf	0	0
ADPRH	Inf	0	0
PLA1A	NA	NA	NA
POPDC2	0.77200489	3.18E-113	5.77E-113
COX17	2.16865719	5.44E-224	1.49E-223
MAATS1	NA	NA	NA
RP11-169N1	NA	NA	NA
NR1I2	NA	NA	NA
GSK3B	1.5566656	1.10E-152	2.29E-152
RP11-18H7.1	1.02933985	4.53E-52	6.44E-52
GPR156	NA	NA	NA
LRRC58	2.58392504	0	0
RP11-174O3.	NA	NA	NA
FSTL1	1.71288585	2.54E-08	2.84E-08
NDUFB4	2.36983156	0	0
HGD	NA	NA	NA
RABL3	2.11508189	1.13E-20	1.38E-20
GTF2E1	1.88460017	0.16076469	0.165003
RP11-500K19	NA	NA	NA
STXBP5L	0	0.03196789	0.03327566
POLQ	3.68846781	1.88E-233	5.35E-233
ARGFX	NA	NA	NA
FBXO40	NA	NA	NA

HCLS1	1.48802469	0	0
GOLGB1	1.89595319	1.12E-05	1.22E-05
IQCB1	1.74987775	3.87E-62	5.75E-62
EAF2	2.19340213	2.53E-39	3.39E-39
SLC15A2	3.21668704	0	0
ILDR1	NA	NA	NA
CD86	2.58070206	7.64E-236	2.21E-235
CASR	NA	NA	NA
CSTA	0.12866748	0	0
CCDC58	2.35242999	0.0258525	0.02695339
FAM162A	1.91368386	3.23E-31	4.15E-31
WDR5B	1.09367359	1.59E-151	3.31E-151
RP11-299J3.8	0.38600244	2.77E-47	3.86E-47
KPNA1	2.22284167	2.42E-297	9.41E-297
PARP9	2.17565014	0.03672182	0.03818682
DTX3L	2.26922649	6.26E-206	1.59E-205
PARP15	NA	NA	NA
PARP14	2.04627802	8.71E-158	1.85E-157
HSPBAP1	1.96510336	0.58259117	0.58823291
DIRC2	1.49254279	0	0
RP11-67L2.2	1.15800733	9.77E-06	1.07E-05
SEMA5B	NA	NA	NA
PDIA5	1.32098615	1.52E-295	5.85E-295
SEC22A	1.46213047	2.32E-232	6.59E-232
RP11-797D24	NA	NA	NA
ADCY5	NA	NA	NA
RP11-797D24	Inf	0	0
HACD2	2.0557883	4.73E-36	6.23E-36
MYLK-AS1	1.58689894	0.01585806	0.01658864
MYLK	1.4153423	1.70E-60	2.51E-60
MYLK-AS2	NA	NA	NA
RP11-9N20.3	NA	NA	NA
CCDC14	1.77810159	1.00E-181	2.32E-181
ROPN1	NA	NA	NA
KALRN	0.77200489	5.77E-10	6.54E-10
UMPS	1.23183294	0	0
ITGB5	NA	0.039988	0.04154758
ITGB5-AS1	NA	NA	NA
MUC13	NA	NA	NA
HEG1	1.54400978	1.05E-196	2.58E-196
SLC12A8	NA	2.03E-46	2.82E-46
ZNF148	2.05620533	0.03810553	0.03961892
SNX4	1.27932239	0	0
OSBPL11	2.04581296	2.39E-253	7.47E-253
RP11-379B18	NA	1.81E-34	2.36E-34

RP11-666A2(	NA	NA	NA
ALG1L	NA	NA	NA
ROPN1B	NA	NA	NA
SLC41A3	0.96851523	2.00E-178	4.58E-178
RP11-158I23	NA	NA	NA
RP11-124N2.	NA	NA	NA
ALDH1L1	7.7200489	2.37E-115	4.32E-115
ALDH1L1-AS:	NA	NA	NA
ALDH1L1-AS:	NA	NA	NA
RP11-71E19.	NA	NA	NA
RP11-71E19.	NA	NA	NA
KLF15	1.02933985	0.00077079	0.00082233
CCDC37-AS1	NA	NA	NA
CFAP100	NA	NA	NA
ZXDC	1.26328073	7.04E-95	1.19E-94
UROC1	NA	NA	NA
CHST13	1.93001222	3.88E-288	1.44E-287
C3orf22	NA	NA	NA
TXNRD3	0.91236942	1.54E-284	5.59E-284
TXNRD3NB	NA	NA	NA
CHCHD6	1.89926867	4.02E-78	6.35E-78
PLXNA1	2.08727248	1.03E-98	1.77E-98
C3orf56	NA	NA	NA
RP11-88I21.:	NA	NA	NA
LINC01471	NA	NA	NA
RP11-59J16.:	NA	NA	NA
RP11-59J16.:	NA	NA	NA
RP11-153F1(	Inf	2.88E-49	4.05E-49
TPRA1	1.94810609	0	0
MCM2	1.61385784	0.46978241	0.47584124
PODXL2	1.31306815	0	0
ABTB1	1.21315054	4.86E-32	6.28E-32
MGLL	NA	2.23E-60	3.28E-60
KBTBD12	Inf	0	0
SEC61A1	2.38382591	0	0
RUVBL1	0.91700073	0	0
RUVBL1-AS1	NA	NA	NA
EEFSEC	1.01246543	0	0
DNAJB8	NA	NA	NA
DNAJB8-AS1	NA	NA	NA
GATA2	1.85853029	0.03880541	0.04033966
GATA2-AS1	2.8306846	6.41E-101	1.11E-100
LINC01565	Inf	1.78E-258	5.70E-258
RPN1	1.80634154	2.89E-125	5.46E-125
RAB7A	1.69124783	5.03E-150	1.04E-149

RP11-723O4.	Inf	1.24E-307	5.11E-307
RP11-723O4.	NA	7.69E-227	2.13E-226
ACAD9	1.46776238	0	0
KIAA1257	NA	5.08E-16	6.01E-16
RP11-723O4.	NA	NA	NA
EFCC1	NA	NA	NA
GP9	NA	NA	NA
RAB43	0	3.10E-18	3.71E-18
ISY1-RAB43	NA	NA	NA
RP11-434H6.	Inf	0	0
ISY1	1.74065253	4.47E-258	1.43E-257
RP11-434H6.	1.46680929	6.28E-09	7.06E-09
CNBP	1.81654399	9.48E-23	1.17E-22
COPG1	1.9621791	4.02E-69	6.15E-69
HMCES	1.29392369	0	0
H1FX	1.07936047	0	0
H1FX-AS1	2.70201711	2.54E-166	5.57E-166
EFCAB12	Inf	1.88E-70	2.89E-70
MBD4	1.64233116	0	0
IFT122	2.23023635	2.65E-52	3.77E-52
RHO	NA	NA	NA
H1FOO	NA	NA	NA
PLXND1	2.47759709	0	0
TMCC1	2.04029864	8.94E-32	1.15E-31
TMCC1-AS1	2.4046054	5.01E-42	6.81E-42
RP11-93K22.	Inf	0	0
TRH	NA	NA	NA
ALG1L2	Inf	1.75E-235	5.04E-235
RP11-93K22.	NA	0	0
RP11-77P16.	0.25733496	2.11E-241	6.25E-241
COL6A5	NA	NA	NA
COL6A6	NA	NA	NA
PIK3R4	2.72039818	1.33E-239	3.90E-239
ATP2C1	1.73091623	8.77E-152	1.82E-151
AC055733.1	NA	NA	NA
RP11-39E3.3	NA	NA	NA
ASTE1	2.51917385	1.58E-21	1.93E-21
NEK11	1.15800733	3.30E-133	6.40E-133
RP11-265F19	0	0	0
NUDT16	2.02365779	2.25E-105	3.95E-105
MRPL3	1.46047194	1.17E-281	4.16E-281
RP11-517B1:	2.31601467	1.65E-45	2.28E-45
CPNE4	NA	0	0
ACPP	1.67267726	0.12647185	0.13020386
DNAJC13	2.26776436	1.22E-47	1.70E-47

ACAD11	1.54400978	2.44E-06	2.67E-06
NPHP3-ACAD	Inf	0	0
ACKR4	NA	NA	NA
UBA5	1.21315054	1.32E-255	4.18E-255
NPHP3	2.54761614	7.68E-137	1.51E-136
NPHP3-AS1	NA	NA	NA
RP11-469L4.	NA	NA	NA
TMEM108	NA	NA	NA
TMEM108-A	NA	NA	NA
BFSP2	NA	NA	NA
BFSP2-AS1	NA	NA	NA
RP11-91K8.5	NA	NA	NA
CDV3	2.38860471	3.15E-285	1.15E-284
TOPBP1	2.20021394	8.81E-152	1.83E-151
TF	NA	NA	NA
SRPRB	1.57248088	9.75E-216	2.58E-215
RAB6B	0.90067237	0	0
C3orf36	NA	NA	NA
SLCO2A1	NA	0	0
RP11-202A1	NA	NA	NA
RYK	2.45298328	2.40E-175	5.42E-175
RP11-200A1.	NA	NA	NA
RP11-375I20	NA	NA	NA
AMOTL2	6.17603912	0.63883999	0.64409442
ANAPC13	1.77369719	9.80E-185	2.30E-184
CEP63	1.74494256	6.78E-204	1.71E-203
EPHB1	0	0	0
KY	NA	NA	NA
PPP2R3A	1.3896088	2.24E-60	3.31E-60
RP11-305O4	Inf	1.25E-08	1.40E-08
MSL2	1.49076806	0.00252055	0.00266853
PCCB	0.85778321	0	0
STAG1	2.19113153	7.84E-106	1.38E-105
RP11-102M1	NA	2.43E-11	2.78E-11
SLC35G2	1.89141198	1.35E-77	2.13E-77
RP11-731C1	2.05867971	0.53658671	0.54253782
NCK1-AS1	2.54475686	9.21E-72	1.42E-71
NCK1	1.55165339	2.18E-246	6.62E-246
IL20RB	0.33085924	5.48E-294	2.09E-293
IL20RB-AS1	NA	NA	NA
SOX14	NA	NA	NA
LINC01210	NA	NA	NA
RP11-2A4.4	NA	NA	NA
CLDN18	NA	NA	NA
RP11-461M2	NA	4.89E-206	1.24E-205

DZIP1L	1.18769983	5.63E-50	7.95E-50
A4GNT	NA	NA	NA
DBR1	1.40890892	6.51E-195	1.59E-194
ARMC8	1.77990016	2.26E-66	3.42E-66
NME9	NA	NA	NA
MRAS	NA	NA	NA
ESYT3	1.43372337	3.60E-07	3.98E-07
CEP70	1.93407541	2.52E-11	2.89E-11
FAIM	1.9501055	0.0165584	0.01731726
PIK3CB	2.19825121	3.01E-186	7.09E-186
LINC01391	0	0	0
FOXL2	NA	NA	NA
FOXL2NB	NA	NA	NA
PRR23A	NA	NA	NA
MRPS22	1.20515267	0	0
PRR23B	NA	NA	NA
PRR23C	NA	NA	NA
BPESC1	NA	NA	NA
PISRT1	0.77200489	2.96E-07	3.27E-07
RP11-219D1!	Inf	0	0
COPB2	1.32527506	0	0
RP11-319G6.	3.86002445	1.31E-42	1.78E-42
RBP2	NA	NA	NA
RP11-319G6.	NA	NA	NA
RBP1	NA	NA	NA
NMNAT3	2.46612673	0	0
RP11-442N1.	NA	NA	NA
RP11-442N1.	NA	NA	NA
RP11-166D1!	NA	NA	NA
CLSTN2	NA	NA	NA
RP11-68L1.2	NA	NA	NA
RP11-68L1.1	NA	NA	NA
CLSTN2-AS1	NA	NA	NA
TRIM42	NA	NA	NA
RP11-691G1	NA	NA	NA
SLC25A36	1.94088553	2.72E-06	2.98E-06
SPSB4	NA	3.83E-99	6.58E-99
RP11-231L11	NA	NA	NA
PXYLP1	2.33317033	0.36647903	0.37255758
RP11-438D8.	NA	NA	NA
ZBTB38	2.8306846	5.45E-176	1.24E-175
RASA2	3.36874861	4.63E-251	1.43E-250
AC010184.1	NA	NA	NA
RP11-340E6.	NA	NA	NA
RNF7	1.8399044	0.00013221	0.00014236

GRK7	NA	NA	NA
ATP1B3	1.3519245	0	0
ATP1B3-AS1	NA	4.53E-112	8.18E-112
TFDP2	1.80861923	3.30E-34	4.31E-34
GK5	4.10566237	0	0
XRN1	1.737011	2.98E-85	4.85E-85
ATR	1.54400978	2.07E-24	2.57E-24
PLS1	2.02651284	0.7846463	0.788733
PLS1-AS1	NA	NA	NA
TRPC1	5.40403423	0	0
PCOLCE2	1.72029436	7.50E-171	1.67E-170
RP11-372E1.	NA	NA	NA
PAQR9	1.4153423	1.54E-21	1.88E-21
PAQR9-AS1	2.70201711	0	0
U2SURP	1.84475064	8.66E-59	1.27E-58
RP11-91G21.	2.16161369	0.00045497	0.00048679
RP11-80H8.3	NA	NA	NA
CHST2	0.77200489	1.59E-254	5.01E-254
SLC9A9	2.31601467	6.63E-183	1.54E-182
SLC9A9-AS1	NA	NA	NA
SLC9A9-AS2	NA	NA	NA
C3orf58	2.09544184	1.39E-49	1.96E-49
RP11-88H10.	NA	NA	NA
RP11-88H10.	NA	NA	NA
RP11-274H2.	NA	NA	NA
RP11-274H2.	NA	NA	NA
PLOD2	1.54400978	6.25E-258	2.00E-257
PLSCR4	0	7.40E-82	1.19E-81
PLSCR2	NA	NA	NA
PLSCR1	1.59829137	2.50E-181	5.79E-181
PLSCR5	NA	NA	NA
PLSCR5-AS1	NA	NA	NA
RP11-649A1(	NA	NA	NA
RP11-232M2	NA	NA	NA
ZIC4	NA	NA	NA
ZIC4-AS1	NA	NA	NA
ZIC1	NA	NA	NA
RP11-78O22.	NA	NA	NA
RP11-71N10.	Inf	1.80E-307	7.40E-307
RP11-639B1.	NA	NA	NA
RP11-501O2.	NA	NA	NA
RP11-501O2.	NA	NA	NA
RP11-501O2.	NA	NA	NA
RP11-501O2.	NA	NA	NA
AGTR1	NA	NA	NA

CPB1	NA	NA	NA
RP11-680B3.	NA	NA	NA
CPA3	NA	NA	NA
GYG1	1.4608308	0	0
HLTF	1.96374059	0.00328356	0.00347106
HLTF-AS1	NA	NA	NA
HPS3	1.66682874	0	0
CP	NA	NA	NA
RP11-206M1	NA	2.58E-125	4.87E-125
TM4SF18	NA	NA	NA
TM4SF1	0.25733496	1.42E-303	5.71E-303
TM4SF1-AS1	NA	NA	NA
RP11-278L1!	NA	NA	NA
TM4SF4	NA	NA	NA
WWTR1	2.12301345	1.06E-31	1.37E-31
WWTR1-AS1	NA	0.05053723	0.05242418
COMMD2	1.96369029	4.97E-06	5.43E-06
ANKUB1	NA	NA	NA
RNF13	1.73272209	7.18E-148	1.47E-147
PFN2	1.15800733	0	0
RP11-651P2:	NA	NA	NA
RP11-167H9.	NA	NA	NA
RP11-167H9.	NA	NA	NA
RP11-167H9.	NA	NA	NA
LINC01213	NA	NA	NA
LINC01214	NA	NA	NA
TSC22D2	1.5225652	1.16E-35	1.53E-35
SERP1	2.18786098	0	0
EIF2A	1.53547934	0	0
SELT	1.91104405	0.85338798	0.85598301
ERICH6	NA	NA	NA
ERICH6-AS1	3.08801956	3.29E-261	1.07E-260
SIAH2	1.74509012	1.04E-50	1.48E-50
SIAH2-AS1	0	0	0
AC016142.1	NA	NA	NA
CLRN1-AS1	NA	NA	NA
RP11-166N6.	NA	NA	NA
RP11-166N6.	NA	NA	NA
RP11-373I8.1	NA	NA	NA
CLRN1	NA	NA	NA
MED12L	3.474022	1.05E-123	1.97E-123
GPR171	2.08105666	0.76774088	0.77191061
P2RY14	NA	NA	NA
GPR87	NA	NA	NA
P2RY13	NA	0	0

P2RY12	NA	NA	NA
IGSF10	2.39947466	0.07280174	0.07531759
RP11-454C18	NA	NA	NA
AADACL2	NA	NA	NA
AADACL2-AS	NA	NA	NA
RP11-64D22.	NA	NA	NA
AADAC	NA	NA	NA
SUCNR1	1.70331238	3.96E-15	4.66E-15
RP11-246A10	NA	4.53E-11	5.18E-11
MBNL1	3.21421267	0	0
MBNL1-AS1	2.70201711	1.22E-122	2.28E-122
TMEM14EP	NA	NA	NA
RP11-362A9.	NA	NA	NA
RP11-788A4.	NA	NA	NA
P2RY1	1.77561125	4.51E-37	5.97E-37
RP11-529G2	NA	1.69E-204	4.27E-204
RAP2B	2.428991	7.10E-111	1.28E-110
RP11-292E2.	NA	0.00018509	0.00019903
RP11-292E2.	NA	NA	NA
RP11-292E2.	NA	NA	NA
C3orf79	NA	NA	NA
RP11-217E22	NA	NA	NA
ARHGEF26	1.65981051	1.24E-235	3.58E-235
DHX36	1.87989346	0.31801699	0.32394521
GPR149	NA	NA	NA
RP11-656A15	NA	NA	NA
RP11-439C8.	NA	NA	NA
RP11-439C8.	NA	NA	NA
MME	1.54400978	0.00249994	0.00264717
MME-AS1	NA	NA	NA
LINC01487	NA	NA	NA
RP11-451G4.	Inf	0	0
RP11-451G4.	NA	NA	NA
PLCH1	2.234751	1.03E-79	1.65E-79
PLCH1-AS1	NA	NA	NA
PLCH1-AS2	NA	NA	NA
C3orf33	2.06698083	3.24E-08	3.62E-08
SLC33A1	2.66692598	1.77E-182	4.13E-182
GMPS	1.78914877	5.09E-147	1.04E-146
KCNAB1	NA	1.58E-44	2.17E-44
KCNAB1-AS2	NA	NA	NA
KCNAB1-AS1	NA	NA	NA
RP11-305K5.	3.08801956	0.94487845	0.94602565
SSR3	1.98762453	6.74E-88	1.11E-87
TIPARP-AS1	2.00721271	1.04E-69	1.59E-69

TIPARP	2.01579055	6.06E-57	8.80E-57
LINC00886	Inf	0	0
LEKR1	NA	NA	NA
LINC00880	NA	NA	NA
RP11-6F2.5	NA	NA	NA
LINC00881	Inf	0	0
CCNL1	1.54400978	4.80E-54	6.89E-54
RP11-555M1	NA	NA	NA
VEPH1	NA	NA	NA
PTX3	NA	2.31E-143	4.65E-143
PQLC2L	Inf	0	0
RP11-290K4.	NA	NA	NA
RP11-290K4.	NA	NA	NA
SHOX2	1.81070238	0.00330926	0.00349802
RSRC1	1.69804401	0	0
RP11-538P18	2.51673594	9.56E-232	2.71E-231
MLF1	NA	0	0
GFM1	1.97826253	0.60379585	0.60940552
LXN	0.85445201	0	0
RP11-379F4.	NA	NA	NA
RP11-379F4.	0.51466993	2.86E-06	3.13E-06
RARRES1	NA	NA	NA
MFSD1	1.16446223	0	0
RP11-379F4.	NA	6.94E-124	1.30E-123
RP11-379F4.	0.51466993	1.16E-186	2.74E-186
IQCJ-SCHIP1	1.54400978	2.73E-154	5.73E-154
IQCJ	NA	NA	NA
IQCJ-SCHIP1-	NA	NA	NA
SCHIP1	0.77200489	3.18E-32	4.11E-32
IL12A-AS1	6.94804401	3.82E-36	5.05E-36
IL12A	3.474022	9.87E-26	1.23E-25
LINC01100	NA	5.39E-211	1.40E-210
C3orf80	2.90216653	1.00E-283	3.61E-283
RP11-432B6.	NA	NA	NA
IFT80	6.94804401	0	0
SMC4	1.41550333	0	0
TRIM59	Inf	1.19E-220	3.21E-220
KPNA4	2.57145746	0	0
ARL14	NA	NA	NA
RP11-16N11.	3.86002445	0	0
PPM1L	2.00721271	2.82E-60	4.16E-60
B3GALNT1	NA	NA	NA
NMD3	2.16464116	5.02E-63	7.49E-63
SPTSSB	NA	NA	NA
RP11-3P17.4	1.15800733	0	0

RP11-3P17.5	Inf	0	0
OTOL1	NA	NA	NA
RP11-774I5.1	NA	NA	NA
RP11-10O22.	NA	NA	NA
LINC01192	NA	NA	NA
RP11-418B1.	NA	NA	NA
LINC01323	NA	NA	NA
LINC01324	NA	NA	NA
SI	NA	NA	NA
RP11-747D1	NA	NA	NA
SLITRK3	NA	NA	NA
LINC01322	NA	NA	NA
BCHE	Inf	0	0
LINC01326	NA	NA	NA
RP11-12K11.	NA	1.47E-08	1.65E-08
ZBBX	NA	NA	NA
LINC01327	NA	NA	NA
SERPINI2	NA	NA	NA
WDR49	NA	NA	NA
PDCD10	1.66308508	1.07E-104	1.88E-104
SERPINI1	Inf	5.65E-51	8.00E-51
RP11-298O2	NA	NA	NA
RP11-298O2	NA	NA	NA
RP11-298O2	NA	NA	NA
RP11-298O2	NA	NA	NA
LINC01330	NA	NA	NA
GOLIM4	1.23798982	1.60E-274	5.52E-274
RP11-637O1	NA	NA	NA
RP11-368I23	Inf	1.56E-128	2.97E-128
RP11-368I23	NA	NA	NA
RP11-368I23	NA	NA	NA
RP11-152C1	NA	NA	NA
MECOM	3.33365748	1.62E-223	4.43E-223
RP11-641D5.	NA	NA	NA
AC074033.1	NA	NA	NA
TERC	NA	NA	NA
ACTRT3	2.70201711	8.54E-52	1.21E-51
RP11-816J6.3	Inf	0	0
MYNN	1.69247226	6.99E-06	7.63E-06
RP11-362K1	0.77200489	5.29E-49	7.43E-49
LRRC34	2.0392582	2.25E-12	2.60E-12
RP11-362K1	NA	1.41E-201	3.53E-201
RP11-362K1	NA	0.17881584	0.18329154
LRRIQ4	NA	1.06E-238	3.10E-238
LRRC31	NA	NA	NA

SAMD7	NA	NA	NA
RP11-379K17	Inf	0	0
SEC62	2.04354236	0.30684894	0.31263919
SEC62-AS1	NA	NA	NA
GPR160	2.66298316	0	0
PHC3	3.0451304	0	0
RP11-379K17	1.15800733	1.09E-160	2.34E-160
PRKCI	2.12665498	6.74E-26	8.43E-26
RP11-543D10	NA	NA	NA
SKIL	1.31240831	8.20E-26	1.03E-25
RP11-469J4.3	NA	NA	NA
CLDN11	Inf	1.21E-89	2.01E-89
SLC7A14	NA	3.20E-34	4.19E-34
RP11-373E16	NA	NA	NA
RPL22L1	3.11494996	0	0
EIF5A2	2.25375621	6.71E-240	1.97E-239
SLC2A2	NA	NA	NA
TNIK	2.66692598	1.30E-54	1.87E-54
RP11-185E8.	0.77200489	0	0
PLD1	1.25450795	9.18E-273	3.14E-272
TMEM212	NA	NA	NA
TMEM212-AS	NA	NA	NA
FNDC3B	2.16161369	3.53E-35	4.63E-35
GHSR	NA	NA	NA
TNFSF10	1.85982996	1.22E-128	2.33E-128
RP11-408H1.	NA	6.52E-240	1.92E-239
NCEH1	1.25450795	2.18E-58	3.19E-58
ECT2	2.77439257	5.06E-200	1.26E-199
SPATA16	NA	NA	NA
RP11-324C10	NA	NA	NA
NLGN1	NA	NA	NA
NLGN1-AS1	NA	NA	NA
NAALADL2	NA	NA	NA
NAALADL2-A	NA	NA	NA
NAALADL2-A	NA	NA	NA
RP11-809F4.	NA	NA	NA
LINC01208	NA	NA	NA
RP11-644C3.	NA	NA	NA
LINC01209	NA	5.91E-158	1.26E-157
TBL1XR1	2.43141746	1.62E-242	4.82E-242
TBL1XR1-AS1	NA	NA	NA
LINC00501	NA	NA	NA
RP11-706D8.	3.08801956	1.34E-186	3.17E-186
LINC00578	NA	NA	NA
RP11-114M1	NA	NA	NA

RP11-114M1	0.77200489	0	0
KCCAT211	NA	NA	NA
RP11-2L8.1	NA	NA	NA
RP11-2L8.2	NA	NA	NA
RP11-33A14.	NA	NA	NA
KCNMB2	NA	NA	NA
RP11-385J1.3	NA	NA	NA
KCNMB2-AS1	NA	NA	NA
ZMAT3	2.39321516	6.70E-182	1.55E-181
RP11-360P21	NA	NA	NA
PIK3CA	1.50891865	1.56E-38	2.08E-38
KCNMB3	3.66702323	7.26E-199	1.80E-198
ZNF639	1.9550309	0.32573331	0.3316564
RP11-255C15	NA	4.72E-302	1.89E-301
MFN1	1.93001222	0.00157313	0.00167105
GNB4	2.07948551	8.91E-199	2.21E-198
AC007620.3	Inf	0	0
RP11-145M9	NA	3.61E-94	6.09E-94
ACTL6A	1.06439911	0	0
RP11-15L13.4	NA	NA	NA
RP11-145M9	NA	1.21E-244	3.65E-244
MRPL47	1.24400788	0	0
NDUFB5	1.66219945	1.29E-293	4.93E-293
USP13	1.65698611	5.77E-05	6.23E-05
PEX5L	NA	NA	NA
RP11-494H4.	Inf	0	0
PEX5L-AS1	NA	NA	NA
PEX5L-AS2	NA	NA	NA
RP11-420J11	NA	NA	NA
RP11-496B10	NA	NA	NA
TTC14	2.29457009	1.30E-105	2.29E-105
CCDC39	NA	7.98E-164	1.74E-163
CCDC39-AS1	NA	NA	NA
FXR1	1.36320566	0	0
DNAJC19	2.49731885	0	0
RP11-275H4.	NA	NA	NA
RP11-4B14.3	NA	NA	NA
SOX2	NA	NA	NA
LINC01206	NA	NA	NA
RP11-416O18	NA	NA	NA
RP11-338L18	NA	NA	NA
FLJ46066	NA	NA	NA
RP11-139K4.	NA	NA	NA
RP11-646E18	NA	NA	NA
RP11-646E18	NA	NA	NA

RP11-225N1(	NA	NA	NA
ATP11B	1.06150672	1.44E-48	2.02E-48
RP11-531F1(	NA	1.17E-233	3.34E-233
DCUN1D1	2.65376681	1.94E-225	5.33E-225
MCCC1	1.80811672	2.00E-47	2.78E-47
MCCC1-AS1	NA	0	0
LAMP3	NA	NA	NA
MCF2L2	0.77200489	0	0
B3GNT5	1.85281174	8.33E-58	1.22E-57
KLHL6	2.04581296	0.01978702	0.0206712
KLHL6-AS1	NA	NA	NA
KLHL24	1.09367359	8.21E-135	1.60E-134
YEATS2	2.42033966	3.22E-11	3.69E-11
YEATS2-AS1	1.54400978	1.59E-10	1.81E-10
MAP6D1	1.71556642	2.04E-129	3.91E-129
PARL	1.70951874	2.05E-289	7.65E-289
ABCC5	1.94839329	1.51E-155	3.19E-155
ABCC5-AS1	NA	NA	NA
HTR3D	NA	NA	NA
HTR3C	NA	NA	NA
HTR3E-AS1	NA	NA	NA
HTR3E	NA	NA	NA
RP11-778D9.	3.08801956	4.18E-181	9.66E-181
RP11-778D9.	1.93001222	4.04E-199	1.00E-198
EIF2B5	1.57945898	9.54E-20	1.15E-19
DVL3	1.68738212	1.66E-74	2.59E-74
AP2M1	1.4184476	0	0
ABCF3	1.39337468	9.07E-211	2.35E-210
VWA5B2	NA	NA	NA
ALG3	1.48256994	0	0
ECE2	1.90212612	2.99E-05	3.24E-05
CAMK2N2	1.4153423	1.06E-121	1.98E-121
PSMD2	1.08381222	0	0
EIF4G1	1.7678912	1.55E-294	5.93E-294
FAM131A	12.3520782	0	0
CLCN2	1.54400978	5.77E-18	6.91E-18
POLR2H	1.83079495	7.61E-64	1.14E-63
THPO	NA	NA	NA
CHRD	0	4.88E-239	1.43E-238
RP11-433C9.	NA	NA	NA
EIF2B5-AS1	NA	NA	NA
EPHB3	Inf	8.27E-231	2.33E-230
MAGEF1	0.74443329	0	0
RP11-329B9.	NA	0	0
RP11-329B9.	NA	NA	NA

RP11-329B9.	NA	NA	NA
RP11-329B9.	NA	NA	NA
VPS8	2.54552964	2.26E-115	4.12E-115
C3orf70	NA	NA	NA
EHHADH-AS1	NA	NA	NA
EHHADH	NA	NA	NA
MAP3K13	NA	0	0
TMEM41A	2.05867971	7.56E-15	8.88E-15
LIPH	NA	NA	NA
SENP2	1.79808734	6.05E-07	6.68E-07
IGF2BP2	1.91246666	1.26E-64	1.90E-64
IGF2BP2-AS1	NA	NA	NA
TRA2B	1.85056653	0.09985756	0.10298538
ETV5	1.30442206	2.14E-106	3.78E-106
ETV5-AS1	NA	NA	NA
DGKG	5.07317499	0	0
RP11-78H24.	NA	NA	NA
RP11-48F14.	NA	NA	NA
RP11-48F14.	NA	NA	NA
CRYGS	2.00721271	2.71E-169	6.00E-169
TBCCD1	2.40683877	7.17E-136	1.41E-135
DNAJB11	1.17389606	0	0
RP11-573D15	NA	0	0
AHSG	NA	4.91E-97	8.37E-97
FETUB	NA	NA	NA
HRG	NA	NA	NA
KNG1	Inf	2.59E-234	7.42E-234
RP11-573D15	NA	1.27E-52	1.81E-52
EIF4A2	1.7848581	3.79E-260	1.22E-259
RFC4	1.03907685	0	0
RP11-573D15	0	0	0
RP11-573D15	1.48462479	4.76E-53	6.80E-53
ADIPOQ	NA	NA	NA
ADIPOQ-AS1	NA	NA	NA
ST6GAL1	1.1918672	0	0
RPL39L	2.08697771	3.85E-191	9.25E-191
RP11-208N14	NA	NA	NA
RTP1	NA	NA	NA
MASP1	NA	NA	NA
RTP4	NA	0.00093494	0.00099611
RP11-567G11	NA	NA	NA
SST	NA	NA	NA
RTP2	NA	NA	NA
RP11-211G3.	NA	NA	NA
BCL6	Inf	2.41E-215	6.35E-215

RP11-211G3.	NA	NA	NA
RP11-30O15.	NA	NA	NA
RP11-44H4.1	NA	NA	NA
RP11-132N1	NA	NA	NA
RP11-132N1	NA	NA	NA
RP11-132N1	NA	NA	NA
RP11-430L16	NA	NA	NA
LPP-AS2	3.08801956	0	0
LPP	2.8306846	2.75E-18	3.30E-18
LPP-AS1	NA	NA	NA
TPRG1-AS1	NA	NA	NA
TPRG1	NA	NA	NA
TPRG1-AS2	NA	NA	NA
TP63	NA	NA	NA
P3H2	NA	NA	NA
P3H2-AS1	NA	NA	NA
CLDN1	NA	NA	NA
CLDN16	NA	NA	NA
TMEM207	NA	NA	NA
IL1RAP	2.92109958	0	0
RP11-268E2	NA	NA	NA
RP11-95L3.2	NA	NA	NA
GMNC	NA	NA	NA
OSTN	NA	NA	NA
OSTN-AS1	NA	NA	NA
UTS2B	NA	NA	NA
CCDC50	2.06065921	0.01671677	0.01747987
PYDC2	NA	NA	NA
RP11-197K6.	NA	NA	NA
RP11-655G2	NA	NA	NA
FGF12	NA	NA	NA
FGF12-AS1	NA	NA	NA
FGF12-AS2	NA	NA	NA
FGF12-AS3	NA	NA	NA
MB21D2	1.54400978	1.31E-38	1.74E-38
RP11-143P4.	NA	NA	NA
HRASLS	NA	0	0
ATP13A5	NA	NA	NA
ATP13A5-AS	NA	NA	NA
ATP13A4	NA	8.18E-120	1.52E-119
ATP13A4-AS	NA	NA	NA
OPA1	2.05368291	7.65E-81	1.22E-80
OPA1-AS1	NA	NA	NA
RP11-528A4.	NA	NA	NA
RP11-699L21	NA	NA	NA

RP11-699L21	NA	NA	NA
RP11-407B7.	NA	NA	NA
RP11-135A1.	NA	NA	NA
RP11-135A1.	0	0	0
HES1	0.13623616	0	0
RP11-513G1	Inf	1.79E-46	2.48E-46
RP11-513G1	NA	NA	NA
RP11-513G1	NA	NA	NA
LINC00887	0.38600244	2.34E-195	5.71E-195
CPN2	NA	NA	NA
LRRC15	NA	NA	NA
GP5	NA	0	0
ATP13A3	2.51917385	3.60E-278	1.26E-277
LINC00884	1.54400978	2.11E-126	3.99E-126
TMEM44-AS	2.41251528	0.10414263	0.10734975
TMEM44	1.20625764	1.21E-259	3.91E-259
AC046143.3	1.93001222	1.33E-56	1.93E-56
RP11-534C1	3.08801956	1.22E-104	2.15E-104
LSG1	1.57225386	3.15E-215	8.32E-215
FAM43A	1.36387531	0	0
AC106706.1	NA	NA	NA
AC090505.6	NA	6.50E-47	9.04E-47
AC090505.4	NA	8.43E-184	1.97E-183
AC090505.1	NA	NA	NA
XXYLT1	1.4758917	3.14E-108	5.60E-108
XXYLT1-AS1	NA	NA	NA
XXYLT1-AS2	Inf	4.66E-154	9.76E-154
ACAP2	1.99894123	0.00562815	0.00592846
PPP1R2	1.3458481	0	0
AC091633.3	NA	NA	NA
APOD	NA	NA	NA
AC069513.4	3.34535452	1.79E-19	2.16E-19
LINC00969	2.16590261	0	0
MUC20	0.46320293	0	0
MUC4	Inf	4.21E-70	6.47E-70
AC124944.5	NA	NA	NA
TNK2	2.51740725	8.28E-36	1.09E-35
TNK2-AS1	NA	1.05E-264	3.48E-264
AC124944.3	NA	NA	NA
RP11-352G9.	NA	0	0
RP11-480A1	1.28667482	5.13E-165	1.12E-164
TFRC	3.20821194	0	0
LINC00885	NA	0	0
ZDHHC19	Inf	4.51E-13	5.23E-13
SLC51A	0.77200489	7.02E-37	9.29E-37

PCYT1A	1.61753406	3.95E-06	4.33E-06
RP11-447L10	NA	NA	NA
AC069257.8	NA	NA	NA
TCTEX1D2	2.37143086	0	0
TM4SF19-TC	Inf	0	0
TM4SF19-AS	0.77200489	5.16E-95	8.73E-95
TM4SF19	NA	NA	NA
UBXN7	1.72616824	6.52E-10	7.40E-10
UBXN7-AS1	NA	NA	NA
RNF168	2.39727834	0	0
CTD-2002J20	NA	NA	NA
SMCO1	NA	NA	NA
WDR53	1.90969631	4.04E-178	9.23E-178
FBXO45	1.9510669	2.05E-64	3.07E-64
LINC01063	Inf	0	0
NRROS	1.63799298	0	0
PIGX	2.1174076	0.21223484	0.21708093
CEP19	1.30905177	1.27E-58	1.87E-58
PAK2	2.2291136	4.12E-294	1.58E-293
SENP5	2.17275603	1.02E-96	1.74E-96
NCBP2	2.13487386	1.62E-101	2.81E-101
NCBP2-AS1	NA	NA	NA
NCBP2-AS2	2.10786652	6.51E-144	1.31E-143
PIGZ	2.05867971	9.16E-76	1.44E-75
MFI2	1.47629005	1.35E-271	4.59E-271
MFI2-AS1	1.84874855	0.04417208	0.04586332
DLG1	1.94017018	9.51E-239	2.78E-238
DLG1-AS1	0.77200489	3.28E-216	8.70E-216
AC128709.3	NA	NA	NA
AC128709.4	NA	NA	NA
AC128709.2	NA	NA	NA
RP13-616I3.1	NA	1.43E-94	2.41E-94
BDH1	1.90680989	4.59E-74	7.15E-74
RP11-496H1.	2.31601467	3.66E-149	7.54E-149
AC024560.2	NA	6.51E-14	7.59E-14
RUBCN	2.78779544	4.70E-86	7.67E-86
FYTTD1	1.7330722	5.75E-99	9.89E-99
RP11-803P9.	Inf	0	0
LRCH3	1.96677436	8.56E-164	1.86E-163
AC055764.1	NA	NA	NA
IQCG	1.83351161	1.33E-35	1.75E-35
RPL35A	2.31364213	0	0
LMLN	2.8306846	0.00177078	0.00187923
LMLN-AS1	Inf	0	0
ZNF595	2.1378597	0.94564438	0.94674026

ZNF718	2.70201711	3.64E-16	4.31E-16
ZNF732	NA	NA	NA
ZNF141	NA	NA	NA
RP11-2H3.7	NA	NA	NA
RP11-2H3.6	Inf	0	0
ZNF721	2.3595079	2.09E-263	6.88E-263
PIGG	2.48144429	1.74E-92	2.92E-92
RP11-1263C1	NA	0	0
PDE6B	NA	NA	NA
RP11-1191J2	NA	NA	NA
RP11-1191J2	NA	NA	NA
ATP5I	2.44043603	0	0
MYL5	3.19095355	2.54E-65	3.83E-65
MFSD7	NA	NA	NA
PCGF3	2.44468215	1.76E-240	5.18E-240
RP11-1191J2	NA	NA	NA
RP11-440L14	0.38600244	2.07E-128	3.94E-128
RP11-440L14	2.31601467	4.02E-09	4.53E-09
AC139887.4	NA	NA	NA
CPLX1	Inf	3.69E-225	1.01E-224
RP11-440L14	NA	NA	NA
GAK	1.918074	1.63E-104	2.86E-104
TMEM175	1.37618263	0.00191553	0.00203202
DGKQ	2.31601467	2.13E-09	2.40E-09
SLC26A1	Inf	2.49E-146	5.06E-146
IDUA	1.08080685	5.56E-130	1.07E-129
FGFRL1	2.61485527	1.45E-82	2.33E-82
RP11-460I19	NA	NA	NA
RNF212	NA	NA	NA
RP11-20I20.2	NA	NA	NA
AC092535.3	NA	NA	NA
SPON2	2.7792176	2.54E-27	3.21E-27
RP11-20I20.4	NA	NA	NA
CTBP1-AS	3.08801956	1.80E-120	3.34E-120
CTBP1	2.26043032	0	0
CTBP1-AS2	2.20572826	4.93E-78	7.79E-78
MAEA	1.25991198	0	0
UVSSA	1.67267726	0.00371418	0.0039217
CRIPAK	7.7200489	3.44E-11	3.94E-11
NKX1-1	NA	NA	NA
RP13-1016M	NA	NA	NA
RP11-1398P2	1.43372337	5.14E-40	6.90E-40
FAM53A	0.77200489	1.47E-176	3.33E-176
SLBP	1.5478892	2.08E-261	6.77E-261
RP11-572O1	1.84609865	9.59E-171	2.14E-170

TMEM129	1.24273958	0	0
TACC3	1.48724471	0	0
FGFR3	0.92640587	3.89E-213	1.02E-212
LETM1	1.63320556	7.05E-95	1.19E-94
WHSC1	2.23835737	6.72E-256	2.13E-255
NELFA	2.21415291	5.39E-12	6.21E-12
C4orf48	2.2108116	0	0
NAT8L	2.46483489	2.28E-276	7.93E-276
POLN	1.3896088	4.79E-107	8.51E-107
RP11-317B7.	NA	NA	NA
HAUS3	2.08105666	2.51E-07	2.78E-07
MXD4	1.11093387	4.03E-200	1.00E-199
ZFYVE28	1.54400978	3.87E-11	4.43E-11
RP11-478C1.	NA	NA	NA
CFAP99	NA	NA	NA
CFAP99	NA	NA	NA
RNF4	2.07404299	4.45E-188	1.06E-187
FAM193A	1.96309815	1.22E-24	1.51E-24
TNIP2	1.83234896	1.38E-58	2.02E-58
SH3BP2	2.46664977	2.28E-118	4.21E-118
ADD1	2.0139258	0.00233213	0.00247063
MFSD10	1.41723845	0	0
NOP14-AS1	2.57334963	5.78E-51	8.19E-51
NOP14	1.8216273	7.28E-33	9.44E-33
GRK4	1.74987775	2.81E-58	4.11E-58
HTT-AS	NA	NA	NA
HTT	2.2908406	3.19E-228	8.90E-228
MSANTD1	0.77200489	4.94E-61	7.31E-61
RGS12	1.61212786	2.85E-42	3.88E-42
HGFAC	NA	NA	NA
DOK7	NA	NA	NA
LRPAP1	1.01386718	0	0
AL590235.1	NA	NA	NA
RP11-529E1(	NA	0	0
RP3-368B9.2	NA	NA	NA
RP3-513G18.	NA	NA	NA
AC141928.1	NA	5.19E-05	5.62E-05
ADRA2C	2.4563792	3.77E-271	1.28E-270
OTOP1	NA	NA	NA
TMEM128	2.40918767	0.00081111	0.00086499
LYAR	1.68014057	7.57E-69	1.16E-68
ZBTB49	1.23520782	0.0257216	0.02681847
RP11-265O1:	NA	NA	NA
NSG1	NA	NA	NA
STX18	1.15800733	0	0

STX18-AS1	1.02933985	1.34E-36	1.77E-36
LINC01396	NA	NA	NA
MSX1	1.29970444	0	0
CYTL1	0.95519249	0	0
STK32B	1.76017115	0	0
EVC2	NA	NA	NA
EVC	2.18734719	9.61E-46	1.33E-45
CRMP1	NA	0	0
C4orf50	NA	NA	NA
JAKMIP1	NA	NA	NA
RP11-586D19	NA	NA	NA
RP11-586D19	NA	NA	NA
RP11-1406H1	NA	NA	NA
WFS1	0	0	0
PPP2R2C	NA	NA	NA
MAN2B2	5.40403423	2.22E-153	4.65E-153
MRFAP1	1.62270615	1.03E-270	3.49E-270
RP11-539L10	1.65429619	0.03805565	0.03956933
AC093323.3	2.12048505	1.16E-33	1.52E-33
RP11-539L10	Inf	0	0
S100P	4.63202934	0	0
MRFAP1L1	1.98515543	9.05E-11	1.03E-10
BLOC1S4	1.76845849	4.53E-32	5.85E-32
KIAA0232	2.36987548	6.06E-154	1.27E-153
TBC1D14	2.18175295	1.07E-32	1.39E-32
RP11-367J11	Inf	2.59E-234	7.42E-234
CCDC96	3.08801956	4.70E-34	6.13E-34
TADA2B	2.1101467	1.08E-136	2.13E-136
GRPEL1	1.21743671	0	0
RP11-367J11	Inf	0	0
SORCS2	NA	NA	NA
PSAPL1	NA	NA	NA
AFAP1-AS1	NA	NA	NA
AFAP1	1.54400978	4.45E-35	5.84E-35
AC097381.1	NA	NA	NA
ABLIM2	NA	7.27E-155	1.53E-154
RP11-338K13	NA	NA	NA
RP11-1258F1	NA	NA	NA
SH3TC1	0.51466993	0	0
AC104650.2	NA	NA	NA
HTRA3	0.38600244	0	0
RP11-774O3.	NA	NA	NA
RP11-774O3.	NA	NA	NA
RP11-774O3.	2.31601467	2.46E-40	3.31E-40
ACOX3	1.86189415	1.39E-141	2.77E-141

TRMT44	1.98515543	1.66E-44	2.28E-44
RP11-689P11	NA	NA	NA
RP11-689P11	NA	9.28E-153	1.93E-152
GPR78	NA	NA	NA
CPZ	NA	NA	NA
RP11-1338A2	NA	NA	NA
HMX1	NA	NA	NA
FAM90A26	NA	NA	NA
USP17L10	NA	NA	NA
USP17L11	NA	NA	NA
USP17L12	NA	NA	NA
USP17L13	NA	NA	NA
USP17L15	NA	NA	NA
USP17L17	NA	NA	NA
USP17L18	NA	NA	NA
USP17L19	NA	NA	NA
USP17L20	NA	NA	NA
USP17L21	NA	NA	NA
USP17L22	NA	NA	NA
USP17L23	NA	NA	NA
USP17L24	NA	NA	NA
USP17L25	NA	NA	NA
USP17L26	NA	NA	NA
USP17L5	NA	NA	NA
USP17L27	NA	NA	NA
USP17L28	NA	NA	NA
USP17L29	NA	NA	NA
USP17L30	NA	NA	NA
DEFB131	NA	NA	NA
SLC2A9	4.11735941	3.27E-113	5.92E-113
DRD5	NA	NA	NA
RP13-560N1	NA	NA	NA
RP11-448G1	NA	NA	NA
WDR1	1.41214559	0	0
ZNF518B	Inf	0	0
CLNK	NA	2.28E-110	4.10E-110
RP11-61G19.	NA	NA	NA
RP11-61G19.	NA	NA	NA
HS3ST1	NA	NA	NA
RP11-281P2	NA	NA	NA
RP11-281P2	NA	NA	NA
RP11-74M11	NA	NA	NA
RP11-1J7.1	NA	NA	NA
RP11-168E1	NA	NA	NA
RP11-22A3.2	0	5.22E-73	8.11E-73

RP11-22A3.1	Inf	0	0
RAB28	2.17815665	4.14E-63	6.17E-63
LINC01097	Inf	0	0
NKX3-2	1.33814181	1.12E-214	2.96E-214
LINC01096	NA	0.89584826	0.89772863
BOD1L1	1.95970472	7.25E-21	8.83E-21
AC006445.1	NA	NA	NA
LINC01182	NA	NA	NA
AC007126.1	NA	NA	NA
LINC01085	NA	NA	NA
RP11-669M1	NA	NA	NA
RP11-669M1	NA	NA	NA
AC006296.1	NA	NA	NA
AC006296.2	NA	NA	NA
LINC00504	0	0	0
CPEB2-AS1	NA	NA	NA
CPEB2	1.02933985	3.01E-245	9.05E-245
RP11-665G4.	NA	NA	NA
C1QTNF7	NA	NA	NA
RP11-484O2.	NA	NA	NA
CC2D2A	1.05642774	3.11E-227	8.60E-227
RP11-799M1	Inf	0	0
FBXL5	2.31601467	1.71E-110	3.07E-110
FAM200B	2.26684238	8.68E-289	3.23E-288
BST1	1.32664918	0	0
CD38	NA	NA	NA
FGFBP1	NA	NA	NA
FGFBP2	NA	NA	NA
PROM1	NA	NA	NA
TAPT1	1.15800733	9.97E-129	1.90E-128
RP11-452J21	NA	NA	NA
TAPT1-AS1	0.77200489	2.78E-58	4.06E-58
RP11-446J8.:	NA	NA	NA
LDB2	NA	NA	NA
RP11-141E1:	NA	NA	NA
RP11-576E2(	NA	NA	NA
QDPR	1.75158384	5.80E-258	1.86E-257
CLRN2	NA	NA	NA
LAP3	0.9837548	0	0
AC006160.5	NA	1.60E-45	2.21E-45
MED28	1.95831383	0.19932819	0.20412125
FAM184B	1.75455657	4.13E-11	4.73E-11
DCAF16	2.3910707	2.11E-220	5.69E-220
NCAPG	1.2900608	0	0
LCORL	1.69538329	7.07E-27	8.89E-27

RP11-608B3.	NA	NA	NA
RP11-3J1.1	NA	NA	NA
RP11-608O2	NA	NA	NA
RP11-94H6.1	NA	NA	NA
SLIT2	NA	NA	NA
PACRGL	2.80729051	7.52E-176	1.70E-175
KCNIP4	NA	NA	NA
RP11-362J17	NA	NA	NA
RP11-120A1.	NA	NA	NA
RP11-556G2:	NA	NA	NA
RP11-556G2:	NA	NA	NA
KCNIP4-IT1	NA	NA	NA
RP11-17E2.2	NA	NA	NA
ADGRA3	2.16464116	8.28E-241	2.44E-240
RP11-412P11	NA	NA	NA
RP11-552M1	NA	NA	NA
RP11-453O5.	NA	NA	NA
RP11-380P1:	NA	NA	NA
ERVH-1	NA	NA	NA
PPARGC1A	NA	NA	NA
RP13-497K6.	NA	NA	NA
DHX15	1.63902577	5.82E-283	2.09E-282
RP11-496D2	NA	NA	NA
SOD3	NA	NA	NA
CCDC149	1.15800733	1.29E-96	2.20E-96
LGI2	Inf	4.43E-32	5.73E-32
SEPSECS	1.60834352	1.06E-43	1.46E-43
SEPSECS-AS1	2.13955641	8.56E-36	1.13E-35
RP11-717K11	NA	NA	NA
RP11-660M5	NA	0.21412432	0.21893949
PI4K2B	1.22948927	6.20E-207	1.58E-206
ZCCHC4	1.7094394	0.00014202	0.00015288
ANAPC4	1.66682874	4.24E-08	4.73E-08
RP11-302F12	NA	NA	NA
SLC34A2	NA	NA	NA
SEL1L3	4.8893643	0	0
RP11-302F12	NA	NA	NA
SMIM20	2.00871175	8.80E-149	1.81E-148
RP13-494C2:	NA	NA	NA
RBPJ	1.6361523	4.64E-151	9.62E-151
CCKAR	NA	NA	NA
TBC1D19	2.8306846	0.2179055	0.22276804
STIM2	1.94545232	2.20E-36	2.91E-36
RP11-293A2:	NA	1.21E-296	4.70E-296
RP11-415C15	NA	NA	NA

AC024132.1	NA	NA	NA
RP11-415C15	NA	NA	NA
RP11-415C15	NA	NA	NA
AC007106.1	NA	NA	NA
RP11-180C1.	NA	NA	NA
RP11-123O2	NA	NA	NA
RP11-292B1.	NA	NA	NA
RP11-769N2	Inf	4.73E-150	9.79E-150
RP11-472K2	NA	NA	NA
RP11-472K2	NA	NA	NA
PCDH7	0	3.50E-257	1.11E-256
RP11-619J20	NA	NA	NA
RP11-617I14	NA	NA	NA
RP11-315A1	NA	NA	NA
RP11-665I14	NA	NA	NA
RP11-734I18	NA	NA	NA
RP11-240A1	NA	NA	NA
RP11-802H3.	NA	NA	NA
RP11-79E3.3	NA	NA	NA
RP11-79E3.2	NA	NA	NA
RP11-548L2C	NA	NA	NA
RP11-725M2	NA	NA	NA
ARAP2	6.17603912	1.35E-273	4.63E-273
RP11-431M7	0.77200489	1.79E-140	3.57E-140
DTHD1	NA	NA	NA
RP11-431M7	NA	NA	NA
RP11-722M1	NA	NA	NA
RP11-103J17	NA	NA	NA
NWD2	NA	NA	NA
C4orf19	NA	NA	NA
RP11-36B15.	Inf	7.99E-23	9.83E-23
RELL1	1.31240831	1.02E-06	1.12E-06
PGM2	1.6962361	2.41E-235	6.92E-235
AC021106.1	NA	NA	NA
TBC1D1	2.22159681	2.37E-22	2.91E-22
PTTG2	1.15800733	3.78E-162	8.17E-162
RP11-600L4.	NA	NA	NA
RP11-83C7.1	NA	NA	NA
LINC01258	NA	NA	NA
LINC01259	NA	NA	NA
RP11-213G2	NA	NA	NA
RP11-617D2	NA	NA	NA
KLF3	1.67267726	0.01572038	0.01644558
TLR10	0	0	0
TLR1	1.15800733	0	0

TLR6	1.28667482	0.00315501	0.00333556
FAM114A1	NA	NA	NA
TMEM156	1.17832325	4.53E-30	5.79E-30
KLHL5	1.97136963	0.06064164	0.06281966
RP11-360F5.	NA	2.07E-282	7.43E-282
RP11-360F5.	NA	NA	NA
WDR19	1.35100856	1.46E-184	3.41E-184
RFC1	1.79880942	4.66E-208	1.20E-207
KLB	Inf	0	0
RPL9	1.99053419	1.10E-119	2.03E-119
LIAS	1.79789058	5.15E-145	1.04E-144
UGDH	1.7232252	7.81E-05	8.43E-05
UGDH-AS1	0.77200489	3.74E-165	8.16E-165
SMIM14	1.35100856	8.74E-304	3.53E-303
RP11-539G1	Inf	8.75E-67	1.33E-66
UBE2K	2.0726653	3.75E-30	4.80E-30
PDS5A	2.62619521	0	0
N4BP2	1.4153423	5.99E-74	9.32E-74
RHOH	1.50689416	1.23E-68	1.88E-68
RP11-395I6.2	NA	NA	NA
AC195454.1	NA	1.21E-111	2.19E-111
CHRNA9	NA	NA	NA
RBM47	1.02933985	8.20E-228	2.28E-227
RP11-588L15	NA	NA	NA
NSUN7	NA	NA	NA
APBB2	2.17977851	3.87E-232	1.10E-231
RP11-632F7.	NA	NA	NA
UCHL1-AS1	NA	NA	NA
UCHL1	1.35100856	8.11E-30	1.04E-29
LIMCH1	1.54400978	1.98E-98	3.39E-98
RP11-227F19	NA	NA	NA
PHOX2B	NA	NA	NA
RP11-227F19	NA	NA	NA
RP11-227F19	NA	NA	NA
LINC00682	NA	2.52E-293	9.59E-293
RP11-457P14	NA	NA	NA
TMEM33	1.83443067	7.86E-38	1.05E-37
DCAF4L1	4.63202934	2.35E-47	3.28E-47
RP11-814H16	NA	0	0
SLC30A9	1.85763677	1.68E-07	1.87E-07
BEND4	1.76996243	5.75E-83	9.28E-83
RP11-63A11.	NA	NA	NA
SHISA3	2.18175295	0.20523541	0.21002825
ATP8A1	2.14055901	1.43E-30	1.84E-30
RP11-109E24	NA	NA	NA

RP11-109E24	NA	NA	NA
GRXCR1	NA	NA	NA
RP11-395F4.	NA	NA	NA
RP11-324J13	NA	NA	NA
RP11-663P9.	NA	NA	NA
LVCAT1	NA	NA	NA
KCTD8	0	0	0
YIPF7	NA	NA	NA
GUF1	2.12301345	2.75E-163	5.97E-163
GNPDA2	2.29188952	6.97E-10	7.90E-10
RP11-700J17	NA	3.19E-18	3.82E-18
RP11-700J17	2.31601467	4.18E-148	8.57E-148
RP11-362I1.1	NA	NA	NA
GABRG1	NA	NA	NA
GABRA2	NA	NA	NA
RP11-436F23	NA	NA	NA
COX7B2	NA	NA	NA
GABRA4	NA	NA	NA
GABRB1	NA	NA	NA
COMMD8	1.97042403	5.24E-46	7.26E-46
ATP10D	1.08080685	6.67E-242	1.98E-241
AC092597.3	NA	NA	NA
CORIN	NA	7.33E-304	2.96E-303
RP11-121C2.	NA	NA	NA
RP11-121C2.	2.12301345	4.48E-69	6.85E-69
NFXL1	1.80134474	6.86E-42	9.31E-42
NIPAL1	1.54400978	6.32E-11	7.23E-11
CNGA1	NA	NA	NA
TXK	Inf	0	0
TEC	1.97290139	9.35E-77	1.47E-76
SLAIN2	3.0208887	2.40E-299	9.43E-299
SLC10A4	1.15324187	2.42E-305	9.88E-305
ZAR1	2.31601467	2.20E-69	3.37E-69
FRYL	1.74907358	7.62E-33	9.89E-33
OCIAD1	1.58343131	0	0
OCIAD1-AS1	NA	NA	NA
OCIAD2	2.08712479	1.25E-159	2.68E-159
CWH43	NA	NA	NA
DCUN1D4	1.78447032	1.50E-14	1.76E-14
RP11-365H22	2.31601467	3.07E-31	3.96E-31
LRRC66	NA	5.01E-08	5.59E-08
SGCB	1.76748488	4.58E-29	5.83E-29
RP11-535C7.	NA	NA	NA
SPATA18	NA	NA	NA
USP46	1.85648795	1.41E-20	1.71E-20

USP46-AS1	1.43372337	1.93E-181	4.46E-181
RP11-177B4.	NA	NA	NA
ERVMER34-1	NA	0.00098505	0.00104888
LINC01618	NA	NA	NA
RASL11B	0.77200489	4.61E-286	1.68E-285
SCFD2	0.92640587	1.21E-113	2.20E-113
RP11-752D2	NA	NA	NA
FIP1L1	1.44825724	0	0
RP11-231C1	NA	NA	NA
LNX1	1.15800733	1.24E-231	3.52E-231
LNX1-AS1	NA	NA	NA
RP11-571I18	1.54400978	3.61E-50	5.11E-50
CHIC2	1.40827266	0	0
RP11-571I18	NA	NA	NA
GSX2	NA	NA	NA
PDGFRA	0	0	0
RP11-231C1	NA	NA	NA
RP11-231C1	NA	NA	NA
RP11-545H2	NA	NA	NA
AC006552.1	NA	NA	NA
KIT	0.17155664	0	0
RP11-273B1	NA	NA	NA
RP11-273B1	NA	NA	NA
RP11-463H1	NA	NA	NA
RP11-530I17	NA	NA	NA
KDR	NA	NA	NA
SRD5A3	2.20181276	5.93E-10	6.73E-10
RP11-177J6.	2.31601467	1.78E-38	2.38E-38
TMEM165	1.64733739	4.58E-273	1.57E-272
CLOCK	1.64833477	2.79E-05	3.03E-05
RP11-528I4.	2.31601467	1.39E-64	2.09E-64
PDCL2	NA	NA	NA
NMU	5.40403423	0.00127769	0.00135913
RP11-345F1	Inf	0	0
EXOC1	2.62481663	8.23E-09	9.23E-09
CEP135	1.58077192	7.42E-226	2.05E-225
KIAA1211	0.77200489	6.44E-32	8.31E-32
AASDH	1.80839502	5.92E-165	1.29E-164
RP11-646I6.	1.69841076	1.31E-32	1.70E-32
PPAT	1.6876386	0.00893742	0.00938217
RP11-646I6.	Inf	0	0
PAICS	1.63917628	1.38E-293	5.26E-293
SRP72	1.7981937	6.69E-11	7.65E-11
ARL9	NA	3.02E-49	4.24E-49
THEGL	NA	NA	NA

HOPX	0	0	0
SPINK2	0.69783848	0	0
REST	1.72494843	0	0
RP11-738E22	0	0	0
NOA1	1.39878114	3.48E-238	1.01E-237
POLR2B	1.45136919	0	0
IGFBP7	0.74341212	0	0
IGFBP7-AS1	NA	NA	NA
RP11-300M6	NA	NA	NA
RP11-319E12	NA	NA	NA
RP11-319E12	NA	NA	NA
RP11-685F15	NA	NA	NA
RP11-4O3.1	NA	NA	NA
RP11-4O3.2	NA	NA	NA
RP11-577G20	NA	NA	NA
RP11-577G20	NA	NA	NA
RP11-506N2.	NA	NA	NA
RP11-340A13	NA	NA	NA
RP11-340A13	NA	NA	NA
RP11-622J8.1	NA	NA	NA
RP11-725D20	NA	NA	NA
RP11-525J21	NA	NA	NA
RP11-593F5.	NA	NA	NA
RP11-16N2.1	NA	6.17E-12	7.10E-12
ADGRL3	0	1.25E-110	2.25E-110
RP11-798L4.	1.54400978	2.19E-48	3.07E-48
ADGRL3-AS1	NA	NA	NA
RP11-257A22	NA	NA	NA
RP11-12K22.	NA	NA	NA
TECRL	NA	NA	NA
RP11-707A18	NA	NA	NA
EPHA5	NA	NA	NA
EPHA5-AS1	NA	NA	NA
RP11-807H7.	NA	NA	NA
RP11-51P8.1	NA	NA	NA
RP11-25H12.	NA	NA	NA
RP11-19C24.	NA	NA	NA
RP11-584P21	Inf	0	0
CENPC	1.51692189	0	0
STAP1	1.54400978	2.16E-36	2.86E-36
UBA6	1.59009962	1.75E-76	2.75E-76
UBA6-AS1	1.13656275	4.37E-185	1.03E-184
GNRHR	NA	NA	NA
TMPRSS11D	NA	NA	NA
TMPRSS11A	NA	NA	NA

TMPRSS11F	NA	NA	NA
TMPRSS11B	NA	NA	NA
YTHDC1	2.05781905	0.0166293	0.01739041
TMPRSS11E	NA	NA	NA
UGT2B17	NA	NA	NA
UGT2B15	NA	NA	NA
UGT2B10	NA	NA	NA
RP11-468N1	NA	NA	NA
UGT2A3	NA	NA	NA
UGT2B11	0	0	0
UGT2B7	NA	NA	NA
RP11-704M1	0	0	0
UGT2B28	NA	NA	NA
UGT2B4	NA	NA	NA
UGT2A1	NA	NA	NA
UGT2A2	NA	NA	NA
SULT1B1	NA	NA	NA
SULT1E1	NA	NA	NA
CSN1S1	NA	NA	NA
CSN2	NA	NA	NA
STATH	NA	NA	NA
AC063956.1	NA	NA	NA
HTN3	NA	NA	NA
HTN1	NA	NA	NA
PRR27	NA	NA	NA
ODAM	NA	NA	NA
FDCSP	NA	NA	NA
CSN3	NA	NA	NA
CABS1	NA	NA	NA
SMR3A	NA	NA	NA
SMR3B	NA	NA	NA
PROL1	NA	NA	NA
MUC7	NA	NA	NA
AMTN	NA	NA	NA
AMBN	NA	NA	NA
ENAM	NA	NA	NA
JCHAIN	NA	0	0
UTP3	1.39654817	1.22E-209	3.15E-209
RP11-46J23.	1.35100856	8.59E-09	9.64E-09
RUFY3	1.77990016	8.45E-16	9.99E-16
GRSF1	1.47211575	0	0
MOB1B	2.81230353	3.79E-302	1.52E-301
DCK	2.10546788	9.15E-08	1.02E-07
SLC4A4	Inf	2.52E-158	5.36E-158
GC	NA	NA	NA

RP11-545L5.1	NA	NA	NA
NPFFR2	NA	NA	NA
ADAMTS3	2.12301345	1.18E-85	1.92E-85
RP11-373J21	NA	NA	NA
COX18	2.27925253	4.18E-109	7.47E-109
ANKRD17	2.23295085	9.31E-05	0.00010039
RP11-692D12	0	0	0
ALB	NA	NA	NA
AFP	NA	NA	NA
AFM	NA	NA	NA
RP11-622A1.	NA	NA	NA
RASSF6	NA	NA	NA
AC112518.3	NA	NA	NA
CXCL8	0.77200489	2.16E-06	2.37E-06
CXCL6	NA	NA	NA
PF4V1	NA	NA	NA
CXCL1	NA	NA	NA
PF4	NA	NA	NA
PPBP	NA	NA	NA
CXCL5	NA	8.50E-15	9.98E-15
CXCL3	1.54400978	0.18863934	0.19329539
CXCL2	4.24602689	0	0
MTHFD2L	1.97290139	2.26E-70	3.47E-70
RP11-629B11	1.54400978	8.68E-67	1.32E-66
EPGN	NA	NA	NA
EREG	0	0.15097234	0.1550402
RP11-727M1	NA	NA	NA
AREG	Inf	6.30E-295	2.42E-294
AC142293.3	NA	NA	NA
BTC	NA	NA	NA
PARM1	Inf	0	0
RP11-44F21.	NA	NA	NA
RP11-44F21.	NA	NA	NA
RP11-44F21.	NA	NA	NA
RP11-542G1	NA	NA	NA
RP11-542G1	NA	NA	NA
RP11-542G1	NA	NA	NA
RP11-567N4.	NA	NA	NA
RP11-567N4.	NA	NA	NA
RCHY1	1.69841076	2.44E-167	5.36E-167
THAP6	1.48306203	6.25E-143	1.26E-142
C4orf26	NA	0	0
CDKL2	NA	NA	NA
G3BP2	1.74019584	3.38E-07	3.74E-07
USO1	2.26142847	2.72E-62	4.05E-62

RP11-556N4.	Inf	2.34E-276	8.13E-276
PPEF2	NA	NA	NA
NAAA	1.6460564	1.36E-273	4.67E-273
SDAD1	1.65479075	1.72E-20	2.09E-20
AC110615.1	NA	2.34E-255	7.41E-255
RP11-630D6.	NA	NA	NA
AC112719.2	NA	NA	NA
CXCL9	NA	NA	NA
ART3	NA	NA	NA
CXCL10	Inf	0	0
CXCL11	NA	4.56E-130	8.75E-130
NUP54	1.62844781	0	0
SCARB2	2.05283118	0	0
FAM47E	4.63202934	0	0
FAM47E-STB	NA	NA	NA
STBD1	Inf	0	0
CCDC158	NA	NA	NA
SHROOM3	NA	NA	NA
RP11-359D1	NA	NA	NA
RP11-359D1	NA	NA	NA
SOWAHB	NA	NA	NA
11-Sep	1.36626295	0	0
CCNI	1.67717905	0	0
CCNG2	0.41569494	0	0
RP11-625I7.1	NA	NA	NA
CXCL13	NA	NA	NA
CNOT6L	2.5917307	3.00E-19	3.62E-19
MRPL1	1.25349923	0	0
FRAS1	0	0	0
ANXA3	0.57900367	4.64E-301	1.84E-300
LINC01094	0.77200489	2.76E-93	4.63E-93
RP11-109G2	0.51466993	5.30E-43	7.24E-43
BMP2K	1.66380364	3.01E-06	3.30E-06
PAQR3	2.35890383	2.33E-11	2.68E-11
LINC01088	1.68622121	0.62445339	0.62990455
NAA11	NA	NA	NA
GK2	NA	NA	NA
LINC00989	NA	0	0
RP11-234K1	NA	NA	NA
RP11-452C8.	NA	7.11E-38	9.45E-38
PCAT4	NA	NA	NA
ANTXR2	1.737011	9.11E-226	2.51E-225
RP11-377G1	NA	NA	NA
PRDM8	1.78526131	3.24E-21	3.95E-21
FGF5	0.19300122	1.65E-154	3.47E-154

C4orf22	NA	3.02E-246	9.14E-246
BMP3	NA	NA	NA
PRKG2	NA	NA	NA
RP11-100N2(	NA	NA	NA
RASGEF1B	NA	0	0
RP11-689K5.	NA	NA	NA
RP11-127B2(	2.79851773	3.22E-152	6.70E-152
HNRNPD	1.4316452	0	0
RP11-127B2(	1.85281174	3.89E-90	6.46E-90
HNRNPDL	1.44170859	0	0
ENOPH1	1.73006082	1.18E-70	1.81E-70
TMEM150C	1.4153423	1.66E-183	3.88E-183
LINC00575	NA	NA	NA
SCD5	1.54400978	1.23E-195	3.01E-195
SEC31A	2.11315207	1.61E-303	6.48E-303
THAP9-AS1	1.68109476	7.80E-134	1.52E-133
THAP9	1.15800733	2.39E-106	4.24E-106
LIN54	2.72233303	1.05E-146	2.13E-146
COPS4	1.37758956	0	0
PLAC8	1.24244537	1.05E-11	1.21E-11
RP11-576N1	0.25733496	7.26E-298	2.83E-297
COQ2	1.66734784	1.98E-152	4.12E-152
HPSE	1.737011	1.47E-138	2.91E-138
HELQ	1.1763884	7.74E-222	2.09E-221
MRPS18C	2.28485511	2.24E-192	5.43E-192
FAM175A	2.02390471	4.66E-221	1.26E-220
GPAT3	1.67056796	5.77E-22	7.06E-22
RP11-767N1!	NA	NA	NA
RP11-42A4.1	NA	NA	NA
NKX6-1	1.48574526	4.89E-300	1.93E-299
CDS1	1.3896088	3.51E-27	4.42E-27
WDFY3	2.02651284	3.93E-125	7.41E-125
WDFY3-AS1	NA	1.63E-15	1.92E-15
ARHGAP24	1.54400978	5.90E-65	8.87E-65
RP13-514E2:	NA	NA	NA
MAPK10	NA	NA	NA
RP11-778J15	NA	NA	NA
PTPN13	1.81648209	2.94E-13	3.42E-13
SLC10A6	NA	NA	NA
C4orf36	1.64051039	0.00187367	0.00198773
AFF1	1.09705958	0.0268065	0.0279416
KLHL8	1.94980722	4.92E-153	1.03E-152
RP11-529H2.	NA	NA	NA
HSD17B13	Inf	5.72E-281	2.03E-280
HSD17B11	1.22152672	0	0

NUDT9	1.47195599	0	0
RP11-710E1.	NA	NA	NA
SPARCL1	NA	NA	NA
RP11-742B1{	NA	NA	NA
DSPP	Inf	0	0
DMP1	NA	NA	NA
IBSP	NA	NA	NA
MEPE	NA	NA	NA
SPP1	NA	2.29E-29	2.91E-29
PKD2	1.93001222	0.15704526	0.16124024
ABCG2	1.80134474	7.64E-238	2.22E-237
PPM1K	1.89973752	1.12E-134	2.18E-134
RP11-10L7.1	NA	0	0
HERC6	2.21308068	2.65E-112	4.79E-112
HERC5	2.39519466	5.84E-38	7.77E-38
PYURF	1.9102696	0.62591111	0.63130479
HERC3	2.00721271	1.13E-33	1.47E-33
PIGY	NA	NA	NA
NAP1L5	1.49859773	0.19382235	0.19857268
FAM13A-AS1	Inf	0	0
FAM13A	2.41759426	1.59E-89	2.63E-89
TIGD2	1.49420301	0.8782731	0.88035972
RP11-84C13.	Inf	3.10E-77	4.89E-77
GPRIN3	NA	0	0
RP11-115D1!	NA	NA	NA
SNCA	21.9670482	0	0
RP11-115D1!	NA	NA	NA
RP11-115D1!	NA	NA	NA
RP11-115D1!	NA	NA	NA
SNCA-AS1	NA	2.48E-75	3.87E-75
MMRN1	Inf	2.62E-137	5.15E-137
CCSER1	NA	4.67E-164	1.02E-163
AC004054.1	NA	NA	NA
RP11-763F8.	NA	NA	NA
RP11-354O2·	NA	NA	NA
RP11-562F9.	NA	NA	NA
RP11-9B6.1	NA	NA	NA
GRID2	NA	NA	NA
RP11-428L21	NA	NA	NA
RP11-703C1(	NA	NA	NA
ATOH1	Inf	1.43E-47	2.00E-47
RP11-363G1!	NA	NA	NA
SMARCAD1	2.00721271	1.51E-07	1.68E-07
HPGDS	Inf	1.62E-156	3.42E-156
PDLIM5	1.737011	1.39E-86	2.28E-86

RP11-554D1:	NA	NA	NA
BMPR1B-AS:	NA	NA	NA
BMPR1B	NA	NA	NA
UNC5C	NA	NA	NA
RP11-710C1:	NA	NA	NA
PDHA2	NA	NA	NA
RP11-145G2(	Inf	0	0
RP11-431J17	NA	NA	NA
STPG2	NA	NA	NA
STPG2-AS1	NA	NA	NA
RP11-323J4.:	5.01803178	2.88E-86	4.71E-86
RAP1GDS1	1.5811254	4.75E-88	7.81E-88
TSPAN5	3.0315314	0	0
RP11-724M2	NA	NA	NA
RP11-1299A:	NA	NA	NA
EIF4E	1.65309743	6.23E-174	1.40E-173
RP11-571L1!	NA	NA	NA
METAP1	1.74682462	0.10052318	0.10366007
RP11-571L1!	Inf	9.38E-147	1.91E-146
ADH5	1.27371084	0	0
RP11-696N1	NA	1.35E-114	2.45E-114
ADH4	NA	NA	NA
ADH6	NA	2.91E-295	1.12E-294
ADH1A	NA	NA	NA
ADH1B	NA	NA	NA
ADH1C	NA	NA	NA
ADH7	NA	NA	NA
C4orf17	NA	NA	NA
TRMT10A	1.26094132	5.52E-25	6.87E-25
MTTP	NA	0.21412432	0.21893949
RP11-766F1	NA	NA	NA
RP11-766F1	NA	NA	NA
DAPP1	NA	NA	NA
LAMTOR3	1.8131957	3.57E-139	7.10E-139
DNAJB14	2.00721271	6.27E-78	9.91E-78
H2AFZ	1.88878501	2.32E-05	2.52E-05
RP11-15B17.	NA	0	0
DDIT4L	NA	NA	NA
RP11-588P8.	NA	NA	NA
EMCN	Inf	0	0
PPP3CA	2.11749913	1.37E-61	2.04E-61
FLJ20021	1.87966408	0.8172261	0.82092612
BANK1	2.31601467	3.06E-37	4.05E-37
RP11-498M5	NA	NA	NA
SLC39A8	1.97542428	2.56E-45	3.53E-45

RP11-499E18	1.737011	4.11E-41	5.56E-41
AF213884.2	6.94804401	1.64E-290	6.13E-290
NFKB1	2.02469207	1.02E-99	1.75E-99
MANBA	1.89784535	1.03E-258	3.32E-258
UBE2D3	1.81008277	3.24E-117	5.95E-117
RP11-10L12.4	2.73170961	6.68E-180	1.54E-179
CISD2	2.26125618	3.05E-266	1.02E-265
SLC9B1	1.54400978	4.52E-220	1.22E-219
SLC9B2	2.38314553	2.79E-154	5.86E-154
BDH2	1.57488998	4.17E-289	1.55E-288
CENPE	1.9073062	0	0
RP11-328K4.	NA	NA	NA
RP11-297P16	NA	NA	NA
RP11-297P16	NA	NA	NA
TACR3	NA	NA	NA
RP11-703G6.	NA	NA	NA
RP11-729M2	NA	NA	NA
CXXC4	Inf	0	0
AC004053.1	NA	NA	NA
AC004063.1	NA	NA	NA
RP11-556I14	NA	NA	NA
RP11-556I14	NA	NA	NA
AC004069.2	3.08801956	0.00347732	0.00367353
TET2	3.34535452	4.46E-303	1.79E-302
TET2-AS1	NA	NA	NA
PPA2	1.33636709	0	0
AC004066.3	NA	NA	NA
ARHGEF38	NA	1.50E-161	3.24E-161
INTS12	1.22748778	0	0
GSTCD	1.61212786	4.13E-133	8.01E-133
RP11-45L9.1	NA	NA	NA
NPNT	0	3.61E-26	4.52E-26
RP11-710F7.	NA	NA	NA
RP11-710F7.	Inf	2.11E-235	6.09E-235
TBCK	1.36585481	5.81E-163	1.26E-162
AIMP1	1.72701094	1.90E-183	4.43E-183
GIMD1	NA	NA	NA
RP13-612N2	NA	NA	NA
DKK2	NA	6.53E-246	1.97E-245
RP11-713M6	NA	NA	NA
PAPSS1	1.87062723	0.03511265	0.03652811
SGMS2	Inf	0.55272437	0.55869877
RP11-286E11	NA	0	0
CYP2U1	2.12301345	0.01120867	0.01174809
RP11-286E11	0.77200489	1.90E-143	3.82E-143

HADH	1.19029118	0	0
LEF1	0.19300122	0	0
RPL34-AS1	0.77200489	1.76E-10	2.00E-10
RPL34	2.26554786	0	0
OSTC	1.99369986	1.95E-203	4.92E-203
ETNPPL	NA	NA	NA
COL25A1	NA	NA	NA
AC004051.2	NA	NA	NA
SEC24B-AS1	1.54400978	4.65E-63	6.94E-63
SEC24B	2.14445803	8.72E-14	1.02E-13
CCDC109B	2.08509239	8.15E-151	1.69E-150
CASP6	1.77138108	1.84E-129	3.53E-129
AC004067.5	NA	1.82E-123	3.42E-123
PLA2G12A	2.21015447	1.03E-71	1.59E-71
CFI	NA	NA	NA
RP11-602N2	0	1.20E-227	3.34E-227
GAR1	1.54766857	3.26E-253	1.02E-252
RRH	NA	2.08E-68	3.17E-68
LRIT3	Inf	0	0
EGF	Inf	0	0
ELOVL6	1.74870805	0	0
ENPEP	NA	NA	NA
RP11-380D2	NA	NA	NA
RP11-380D2	NA	NA	NA
PITX2	NA	NA	NA
LINC01438	NA	NA	NA
RP11-255I10	NA	NA	NA
RP11-269F2	NA	NA	NA
RP11-269F2	NA	NA	NA
RP11-269F2	NA	NA	NA
C4orf32	3.44766842	0	0
RP11-73K9.2	Inf	0	0
AP1AR	1.93001222	2.43E-163	5.26E-163
RP11-73K9.3	Inf	0	0
TIFA	1.52342298	6.15E-129	1.17E-128
ALPK1	1.54400978	1.41E-158	3.01E-158
NEUROG2	NA	0	0
RP11-402J6.	2.31601467	0.00012099	0.00013031
ZGRF1	1.95574572	5.28E-19	6.36E-19
LARP7	1.31616111	0	0
RP11-148B6.	NA	NA	NA
RP11-148B6.	NA	3.13E-125	5.89E-125
ANK2	1.54400978	4.24E-66	6.41E-66
RP11-119H1	NA	NA	NA
RP11-650J17	NA	NA	NA

RP11-650J17	NA	NA	NA
CAMK2D	NA	NA	NA
ARSJ	NA	NA	NA
RP11-26P13.	NA	NA	NA
UGT8	Inf	2.86E-178	6.53E-178
NDST4	NA	NA	NA
RP11-659O3.	1.69841076	7.95E-31	1.02E-30
MTRNR2L13	NA	NA	NA
RP11-55L3.1	NA	NA	NA
TRAM1L1	0.77200489	6.69E-34	8.73E-34
AC107399.2	NA	NA	NA
AC113617.1	NA	NA	NA
LINC01378	NA	NA	NA
AC108056.1	NA	NA	NA
NDST3	0.22057283	0	0
SNHG8	2.26180609	0	0
PRSS12	0.38600244	0	0
RP11-384K6.	NA	0	0
METTL14	1.71771088	9.51E-150	1.97E-149
SEC24D	1.88460017	8.81E-36	1.16E-35
SYNPO2	NA	2.45E-140	4.87E-140
MYOZ2	Inf	3.13E-158	6.67E-158
USP53	1.20432763	9.02E-256	2.86E-255
C4orf3	2.19783121	9.95E-133	1.93E-132
FABP2	NA	NA	NA
RP11-33B1.4	NA	2.48E-75	3.87E-75
RP11-33B1.3	NA	NA	NA
PDE5A	0.38600244	5.21E-206	1.32E-205
LINC01365	NA	NA	NA
RP11-700N1.	NA	NA	NA
RP11-96A1.5	NA	NA	NA
MAD2L1	1.489834	0	0
RP11-679C8.	Inf	2.73E-250	8.42E-250
RP11-501E14	NA	NA	NA
PRDM5	NA	NA	NA
NDNF	NA	NA	NA
RP11-647P12	NA	NA	NA
TNIP3	NA	NA	NA
QRFPR	NA	NA	NA
ANXA5	0.93365212	0	0
TMEM155	NA	NA	NA
PP12613	NA	NA	NA
EXOSC9	1.20518541	0	0
CCNA2	1.36599039	0	0
BBS7	2.21951406	0.00537229	0.00566158

RP11-63B13.	NA	NA	NA
TRPC3	NA	NA	NA
KIAA1109	2.40179299	1.22E-62	1.82E-62
ADAD1	NA	NA	NA
IL2	NA	NA	NA
IL21	NA	NA	NA
IL21-AS1	NA	NA	NA
BBS12	2.70201711	3.58E-250	1.10E-249
FGF2	NA	6.77E-104	1.19E-103
RP11-170N1(	NA	1.44E-142	2.90E-142
NUDT6	1.44503479	1.42E-234	4.09E-234
SPATA5	1.91571584	2.03E-11	2.33E-11
SPRY1	1.40364525	7.87E-134	1.53E-133
RP11-381N2(	NA	NA	NA
RP11-381N2(	NA	NA	NA
LINC01091	NA	NA	NA
AC096732.2	NA	NA	NA
CTD-2325B1:	NA	NA	NA
RP11-93I21.3	NA	NA	NA
ANKRD50	1.54400978	1.75E-19	2.12E-19
FAT4	NA	NA	NA
RP11-399F2.	NA	NA	NA
RP11-318I4.1	NA	NA	NA
RP11-810P8.	NA	NA	NA
RP11-125O1٤	NA	NA	NA
INTU	2.34570717	0	0
SLC25A31	0	1.12E-06	1.23E-06
HSPA4L	1.54400978	1.09E-84	1.77E-84
RP11-50D9.3	Inf	0	0
PLK4	2.08792232	7.34E-164	1.60E-163
MFSD8	2.44468215	0.00091714	0.00097754
ABHD18	2.63768337	0	0
LARP1B	1.59964977	7.39E-66	1.12E-65
PGRMC2	1.4882022	1.21E-304	4.94E-304
RP11-420A2:	1.15800733	6.97E-16	8.25E-16
RP11-130C6.	NA	NA	NA
RP11-184M1	NA	NA	NA
RP11-184M1	NA	NA	NA
JADRR	NA	NA	NA
JADE1	2.03125877	1.85E-32	2.39E-32
SCLT1	1.55709461	0	0
C4orf33	1.25450795	4.01E-154	8.39E-154
RP11-419L4.:	NA	NA	NA
RP11-519M1	NA	NA	NA
RP11-422J15	NA	NA	NA

RP11-521E5.	NA	NA	NA
RP11-401I19	NA	NA	NA
RP11-314N1	NA	NA	NA
RP11-614F17	NA	NA	NA
RP11-745L13	NA	NA	NA
RP11-789C2.	NA	NA	NA
RP11-1E22.1	NA	NA	NA
RP11-62N21.	NA	NA	NA
LINC01256	NA	NA	NA
RP11-404I7.2	NA	NA	NA
RP11-404I7.1	NA	NA	NA
RP11-9G1.3	NA	NA	NA
PCDH10	NA	NA	NA
RP11-427M2	NA	NA	NA
PABPC4L	NA	6.23E-150	1.29E-149
RP11-400D2.	NA	NA	NA
RP11-400D2.	NA	NA	NA
CTD-2012I17	NA	NA	NA
RP11-192C21	NA	NA	NA
RP11-553P9.	NA	NA	NA
RP11-553P9.	NA	NA	NA
RP11-780O1	NA	NA	NA
LINC00613	NA	NA	NA
RP11-775H9.	NA	NA	NA
RP11-775H9.	NA	NA	NA
RP11-775H9.	NA	NA	NA
RP11-138I17	0	0	0
RP11-785F11	NA	NA	NA
PCDH18	NA	NA	NA
RP11-714L20	NA	1.73E-37	2.30E-37
RP13-884E18	NA	NA	NA
RP13-884E18	NA	NA	NA
RP11-793B23	NA	NA	NA
LINC00616	NA	NA	NA
SLC7A11	1.47382752	7.44E-06	8.12E-06
RP11-733C7.	NA	NA	NA
LINC00498	NA	NA	NA
LINC00499	NA	NA	NA
LINC00500	NA	NA	NA
RP11-173E2.	NA	NA	NA
RP11-98O2.1	NA	NA	NA
RP11-371F15	NA	NA	NA
RP11-577B7.	NA	NA	NA
NOCT	1.98515543	0.01123161	0.01177145
ELF2	1.85880789	1.34E-06	1.47E-06

MGARP	1.93001222	3.77E-34	4.92E-34
NDUFC1	2.38418806	0	0
NAA15	1.7570971	3.18E-246	9.62E-246
RP11-83A24.	1.94839329	6.85E-138	1.35E-137
RAB33B	2.31601467	1.46E-188	3.48E-188
SETD7	2.27676018	3.16E-83	5.11E-83
RP11-342I1.2	Inf	4.71E-06	5.15E-06
RP11-308D13	NA	NA	NA
MGST2	1.88926752	2.30E-53	3.29E-53
MAML3	2.39321516	1.71E-48	2.39E-48
RP11-392B6.	NA	NA	NA
SCOC	1.99095998	6.01E-14	7.02E-14
SCOC-AS1	2.04581296	7.17E-163	1.55E-162
CLGN	1.93001222	1.05E-230	2.95E-230
MGAT4D	NA	NA	NA
ELMOD2	1.69343008	1.12E-20	1.37E-20
UCP1	NA	NA	NA
TBC1D9	19.3001222	0	0
RP11-102N1	NA	NA	NA
RP11-102N1	3.474022	0.08074711	0.0834186
RNF150	7.7200489	6.72E-287	2.47E-286
ZNF330	1.62387235	4.87E-300	1.92E-299
RP11-586L23	NA	NA	NA
RP11-362F19	NA	NA	NA
RP11-362F19	NA	NA	NA
RP11-208N2(	NA	NA	NA
IL15	NA	5.12E-166	1.12E-165
INPP4B	11.5800733	0	0
RP11-223C24	NA	NA	NA
RP11-223C24	NA	NA	NA
RP11-284M1	NA	8.08E-88	1.33E-87
USP38	2.52188264	3.52E-113	6.38E-113
RP11-364L4.	NA	NA	NA
GAB1	1.80134474	0.00170535	0.00181086
SMARCA5	1.82783511	3.05E-114	5.54E-114
SMARCA5-AS	0	0	0
FREM3	NA	NA	NA
RP13-578N3.	NA	NA	NA
GYPE	NA	5.84E-53	8.34E-53
RP11-673E1.	NA	4.60E-09	5.17E-09
RP11-673E1.	NA	NA	NA
GYPB	NA	3.16E-107	5.61E-107
GYPA	NA	NA	NA
RP11-361D14	NA	NA	NA
HHIP-AS1	NA	4.26E-143	8.56E-143

HHIP	NA	NA	NA
RP13-539F13	NA	NA	NA
ANAPC10	1.78611782	1.19E-11	1.37E-11
ABCE1	1.62792335	1.34E-187	3.17E-187
OTUD4	2.0392582	9.79E-62	1.45E-61
RP11-142A22	NA	NA	NA
SMAD1	2.35774466	2.12E-93	3.57E-93
SMAD1-AS2	NA	NA	NA
SMAD1-AS1	NA	9.94E-51	1.41E-50
RP11-557J10	NA	NA	NA
MMAA	1.737011	5.49E-265	1.82E-264
RP11-557J10	NA	NA	NA
C4orf51	NA	NA	NA
ZNF827	3.55122249	0	0
RP11-181K12	NA	NA	NA
RP11-203B7.	NA	NA	NA
RP11-203B7.	NA	NA	NA
LINC01095	NA	NA	NA
LSM6	2.22739325	0	0
RP11-6L6.2	NA	NA	NA
RP11-6L6.7	NA	NA	NA
SLC10A7	2.04029864	9.41E-05	0.0001015
POU4F2	NA	NA	NA
TTC29	0.38600244	4.41E-26	5.53E-26
RP11-292D4.	NA	NA	NA
RP11-292D4.	Inf	5.28E-253	1.65E-252
RP11-292D4.	NA	NA	NA
EDNRA	NA	NA	NA
RP11-752L20	NA	NA	NA
RP11-425A23	NA	3.08E-244	9.25E-244
TMEM184C	1.76175475	4.41E-21	5.38E-21
PRMT9	2.28844307	1.19E-153	2.49E-153
ARHGAP10	1.85281174	0.66509151	0.67011497
NR3C2	Inf	9.64E-111	1.73E-110
RP11-76G10.	NA	NA	NA
RP11-503L19	NA	NA	NA
RP11-563M4	NA	NA	NA
RP11-342A1.	NA	NA	NA
RP11-24I21.1	NA	NA	NA
RP11-526A4.	NA	NA	NA
RP11-707F2.	NA	NA	NA
RP11-423J7.1	NA	NA	NA
DCLK2	0	1.02E-183	2.39E-183
LRBA	1.70102772	7.86E-102	1.36E-101
RP11-1336O1	Inf	2.36E-300	9.36E-300

MAB21L2	NA	NA	NA
RPS3A	1.74403167	5.40E-206	1.37E-205
SH3D19	1.67267726	0	0
RP11-372K14	2.31601467	1.59E-65	2.39E-65
PRSS48	NA	NA	NA
RP11-731D1.	NA	NA	NA
RP11-610P16	NA	NA	NA
FAM160A1	NA	NA	NA
RP11-164P12	NA	NA	NA
GATB	1.3955473	0	0
RP11-164P12	NA	NA	NA
RP11-424M2	NA	NA	NA
RP11-503L23	NA	NA	NA
RP11-73G16.	NA	NA	NA
RP11-73G16.	NA	NA	NA
RP11-18H21.	NA	NA	NA
RP11-18H21.	NA	NA	NA
RP11-18H21.	NA	NA	NA
RP11-461L13	NA	NA	NA
RP11-461L13	NA	NA	NA
RP11-461L13	NA	0	0
FBXW7	2.24583241	0.00245032	0.00259477
DEAR	NA	NA	NA
DKFZP434I07	2.70201711	2.80E-275	9.69E-275
TMEM154	2.70201711	8.63E-45	1.19E-44
RP11-768B22	NA	NA	NA
ARFIP1	1.76458261	2.70E-44	3.71E-44
TIGD4	1.54400978	0	0
FHDC1	NA	NA	NA
RP11-285C1.	NA	NA	NA
TRIM2	3.08801956	2.54E-49	3.57E-49
MND1	1.58427307	1.13E-274	3.90E-274
KIAA0922	1.11259528	1.28E-287	4.71E-287
TLR2	NA	NA	NA
RNF175	0	0	0
RP11-153M7	NA	NA	NA
SFRP2	0	0	0
RP11-290O1:	NA	NA	NA
DCHS2	NA	NA	NA
RP11-9N12.2	NA	NA	NA
RP11-9N12.3	NA	NA	NA
PLRG1	1.4014858	2.87E-221	7.75E-221
FGB	NA	NA	NA
FGA	NA	NA	NA
FGG	NA	NA	NA

LRAT	0.77200489	6.75E-190	1.62E-189
RP11-21G20.	NA	NA	NA
RBM46	NA	NA	NA
AC097467.2	NA	NA	NA
RP11-92A5.2	NA	NA	NA
NPY2R	NA	NA	NA
RP11-27G13.	NA	NA	NA
MAP9	2.05867971	3.03E-122	5.65E-122
RP13-487K5.	NA	NA	NA
GUCY1A3	2.09357258	3.66E-11	4.19E-11
GUCY1B3	2.61810354	1.27E-223	3.47E-223
ASIC5	NA	NA	NA
TDO2	NA	7.47E-57	1.09E-56
CTSO	0.96500611	2.03E-110	3.65E-110
RP11-171N4.	NA	NA	NA
RP11-171N4.	NA	NA	NA
RP11-171N4.	NA	NA	NA
RP11-171N4.	NA	NA	NA
PDGFC	1.1229162	0	0
RP11-154F14	NA	NA	NA
GLRB	4.63202934	0	0
GRIA2	NA	NA	NA
RP11-364P22	NA	NA	NA
RP11-364P22	NA	NA	NA
FAM198B	0.67550428	0	0
RP11-597D13	0.77200489	7.61E-18	9.12E-18
RP11-597D13	NA	NA	NA
TMEM144	1.64928317	2.49E-35	3.27E-35
RXFP1	Inf	0	0
RP11-781M1	NA	NA	NA
C4orf46	2.01166659	0.00014762	0.00015887
ETFDH	1.68536279	0.40211943	0.40824011
PPID	1.0697782	0	0
FNIP2	2.60812463	3.69E-18	4.43E-18
C4orf45	NA	NA	NA
RAPGEF2	4.01442543	3.97E-37	5.26E-37
RP11-138A23	NA	NA	NA
RP11-6C14.1	NA	NA	NA
RP11-502M1	NA	NA	NA
RP11-234O6.	NA	NA	NA
FSTL5	2.96612405	2.32E-241	6.86E-241
RP11-497K21	NA	NA	NA
AC005150.1	NA	NA	NA
RP11-563E2.	NA	NA	NA
NAF1	1.95326538	0.26401521	0.26943601

NPY1R	NA	NA	NA
NPY5R	NA	NA	NA
TKTL2	NA	NA	NA
TMA16	1.79633172	3.16E-100	5.45E-100
1-Mar	NA	5.78E-51	8.19E-51
RP11-218F1(	1.28667482	2.80E-244	8.40E-244
LINC01207	0	0	0
APELA	NA	NA	NA
TRIM61	NA	NA	NA
FAM218A	NA	NA	NA
RP11-366M4	NA	NA	NA
TRIM60	NA	4.38E-100	7.56E-100
TMEM192	2.1806394	3.21E-57	4.68E-57
KLHL2	1.51184291	3.39E-161	7.29E-161
MSMO1	0.99642492	0	0
CPE	0.77200489	6.49E-06	7.09E-06
LINC01179	NA	NA	NA
TLL1	NA	NA	NA
RP11-217C7.	NA	NA	NA
SPOCK3	NA	NA	NA
RP11-436A7.	NA	NA	NA
RP11-310I9.1	NA	NA	NA
ANXA10	NA	NA	NA
DDX60	1.10286413	2.09E-102	3.63E-102
DDX60L	1.72929095	1.90E-28	2.41E-28
PALLD	22.3881418	0	0
RP11-610J23	NA	NA	NA
RP11-635L1.	NA	NA	NA
CBR4	2.11497173	9.60E-69	1.47E-68
RP11-483A2(	NA	NA	NA
SH3RF1	1.60834352	1.95E-243	5.84E-243
RP11-327O1	NA	NA	NA
NEK1	1.65753991	7.88E-104	1.38E-103
CLCN3	2.00149416	5.53E-104	9.69E-104
C4orf27	1.50190042	1.65E-304	6.72E-304
RP11-205M3	NA	NA	NA
RP11-6E9.5	NA	NA	NA
RP11-6E9.4	NA	NA	NA
MFAP3L	0.99257772	3.32E-146	6.75E-146
AADAT	1.90427873	0.00148327	0.00157651
LINC01612	Inf	5.95E-250	1.83E-249
RP11-789C1.	NA	NA	NA
RP11-322J23	NA	NA	NA
RP11-344G1	NA	NA	NA
RP11-717H1	NA	NA	NA

RP11-97E7.1	NA	NA	NA
RP11-97E7.2	NA	NA	NA
GALNTL6	NA	NA	NA
RP11-10K16.	2.31601467	9.14E-210	2.36E-209
GALNT7	2.13317141	2.93E-50	4.14E-50
RP11-798M1	Inf	0.04204803	0.04366545
HMGB2	1.47652018	0	0
RP11-798M1	1.80134474	6.11E-05	6.60E-05
SAP30	1.75372764	6.54E-148	1.34E-147
SCRG1	1.54400978	0.02821193	0.0293981
RP11-489G1	NA	NA	NA
HAND2	NA	NA	NA
HAND2-AS1	NA	0	0
RP11-475B2.	NA	NA	NA
RP11-161D1!	NA	NA	NA
RP11-161D1!	NA	NA	NA
RP11-161D1!	NA	NA	NA
RP11-148L24	NA	NA	NA
RP11-248N2	NA	NA	NA
RP11-248N2	NA	NA	NA
FBXO8	1.59915299	0.00489525	0.00516155
CEP44	1.27932239	5.55E-07	6.12E-07
RP11-51M24	NA	NA	NA
HPGD	NA	3.69E-223	1.01E-222
RP11-440I14	NA	NA	NA
RP11-440I14	NA	NA	NA
GLRA3	NA	NA	NA
ADAM29	NA	NA	NA
RP13-577H1	NA	NA	NA
RP11-287F9.	NA	NA	NA
RP11-598D1	NA	NA	NA
GPM6A	NA	NA	NA
RP11-806K1!	NA	NA	NA
WDR17	1.93001222	0.00178673	0.00189605
SPATA4	NA	NA	NA
ASB5	NA	NA	NA
RP11-87F15.	NA	NA	NA
SPCS3	2.42505491	0	0
RP11-313E1	NA	NA	NA
VEGFC	NA	NA	NA
RP11-624A4.	NA	NA	NA
RP11-765K1	NA	NA	NA
RP11-487E1	NA	NA	NA
NEIL3	2.48757131	2.03E-279	7.17E-279
RP11-376O6.	NA	NA	NA

AGA	1.80134474	1.36E-166	2.98E-166
RP11-130F1(	NA	0	0
RP11-140M1	NA	NA	NA
LINC01098	NA	NA	NA
LINC01099	NA	NA	NA
RP11-404J23	Inf	1.37E-224	3.76E-224
RP11-460H9.	NA	NA	NA
LINC00290	NA	NA	NA
RP11-665C1	NA	NA	NA
RP11-540E1(	NA	NA	NA
AC108142.1	NA	NA	NA
TENM3	NA	NA	NA
RP11-18D7.3	NA	NA	NA
RP11-18D7.2	NA	NA	NA
RP11-188P17	0	1.23E-225	3.40E-225
DCTD	1.77203969	1.98E-63	2.95E-63
RP11-335L23	NA	NA	NA
RP11-335L23	NA	NA	NA
WWC2-AS2	Inf	0	0
WWC2	2.17977851	1.23E-216	3.27E-216
WWC2-AS1	NA	NA	NA
CLDN22	NA	NA	NA
CLDN24	NA	NA	NA
CDKN2AIP	1.47472729	0	0
RP11-367N1	Inf	6.31E-95	1.07E-94
ING2	1.76157479	1.57E-71	2.43E-71
RP11-367N1	2.31601467	2.91E-182	6.76E-182
RWDD4	2.23388649	1.78E-190	4.27E-190
TRAPPC11	2.49247293	2.36E-97	4.03E-97
STOX2	Inf	2.84E-296	1.10E-295
ENPP6	NA	NA	NA
RP11-290F5.	Inf	3.95E-78	6.25E-78
RP11-290F5.	NA	NA	NA
IRF2	1.24573516	0	0
RP11-326I11	2.31601467	1.02E-05	1.11E-05
RP11-326I11	NA	NA	NA
RP11-242J7.:	NA	NA	NA
CASP3	1.74987775	0.00132435	0.00140818
PRIMPOL	2.75026742	5.35E-209	1.38E-208
CENPU	1.48799681	0	0
ACSL1	2.42128806	6.63E-07	7.32E-07
RP11-701P1(	NA	NA	NA
MIR3945HG	1.61419204	0.02622146	0.02733336
LINC01093	NA	NA	NA
RP11-386B1:	NA	NA	NA

HELT	NA	NA	NA
RP11-386B13	NA	NA	NA
SLC25A4	0.3474022	0	0
CFAP97	1.80790941	2.15E-237	6.25E-237
SNX25	1.27153747	3.99E-216	1.06E-215
LRP2BP	6.17603912	8.03E-36	1.06E-35
RP11-714G18	NA	NA	NA
ANKRD37	1.63483388	5.24E-182	1.22E-181
UFSP2	1.26829375	0	0
C4orf47	Inf	0	0
CCDC110	NA	NA	NA
RP11-279O9.	NA	4.55E-72	7.04E-72
PDLIM3	NA	NA	NA
SORBS2	NA	NA	NA
RP11-301L8.1	NA	NA	NA
RP11-626E13	NA	NA	NA
RP11-45I20.1	NA	NA	NA
TLR3	NA	1.02E-306	4.19E-306
FAM149A	3.08801956	1.18E-09	1.33E-09
CYP4V2	1.69841076	2.43E-77	3.84E-77
KLKB1	NA	NA	NA
F11	NA	NA	NA
F11-AS1	NA	NA	NA
RP11-215A19	NA	NA	NA
MTNR1A	NA	NA	NA
FAT1	1.4153423	3.08E-10	3.51E-10
RP11-11N5.1	NA	NA	NA
RP11-11N5.3	NA	NA	NA
RP11-217E13	NA	NA	NA
RP11-696F12	NA	NA	NA
RP11-308K2.	NA	NA	NA
RP11-138B4.	NA	NA	NA
RP11-91J3.2	NA	NA	NA
RP11-91J3.3	NA	NA	NA
RP11-237D3.	NA	NA	NA
RP11-565A3.	NA	NA	NA
RP11-565A3.	NA	NA	NA
ZFP42	Inf	9.26E-257	2.94E-256
TRIML2	NA	NA	NA
TRIML1	NA	NA	NA
RP11-366H4.	NA	NA	NA
RP11-366H4.	NA	NA	NA
LINC01060	NA	NA	NA
RP11-756P10	NA	NA	NA
RP11-756P10	NA	NA	NA

RP11-756P1(	NA	NA	NA
RP11-818C3.	NA	NA	NA
RP11-706F1.	NA	NA	NA
RP11-706F1.	NA	NA	NA
LINC01262	Inf	1.61E-170	3.59E-170
RP11-462G2.	NA	NA	NA
AF146191.4	NA	3.72E-15	4.38E-15
LINC01596	NA	NA	NA
FRG1	1.19826526	0	0
FRG2	NA	NA	NA
DBET	NA	NA	NA
DUX4	NA	NA	NA
CTD-2231H1(	NA	NA	NA
PLEKHG4B	1.54400978	2.71E-20	3.29E-20
LRRC14B	NA	NA	NA
CCDC127	1.57651525	5.23E-129	9.99E-129
SDHA	0.93283924	0	0
CTD-2083E4.	NA	0.00150172	0.00159575
PDCD6	1.77804819	2.39E-123	4.47E-123
AHRR	2.20793399	1.03E-286	3.76E-286
EXOC3-AS1	2.1730508	2.44E-65	3.68E-65
EXOC3	2.22693718	8.00E-15	9.39E-15
PP7080	1.90427873	7.74E-103	1.35E-102
SLC9A3	0	0	0
RP11-310P5.	NA	NA	NA
RP11-310P5.	NA	NA	NA
CEP72	1.58261002	1.41E-68	2.15E-68
TPPP	Inf	0	0
CTD-2589H1!	5.40403423	0	0
ZDHHC11B	3.08801956	6.11E-106	1.08E-105
ZDHHC11	1.93001222	2.44E-77	3.84E-77
BRD9	2.13909688	0.00450024	0.00474809
TRIP13	1.47103419	0	0
RP11-661C8.	NA	NA	NA
RP11-661C8.	NA	1.16E-289	4.33E-289
RP11-43F13.	5.79003667	6.50E-169	1.44E-168
RP11-43F13.	3.86002445	5.78E-124	1.09E-123
NKD2	2.95420538	0	0
SLC12A7	3.95652506	1.18E-136	2.32E-136
CTD-3080P1.	NA	NA	NA
SLC6A19	NA	NA	NA
SLC6A18	NA	NA	NA
TERT	2.14445803	0.61158886	0.61709928
CLPTM1L	2.16717474	5.57E-19	6.71E-19
LINC01511	NA	NA	NA

SLC6A3	NA	NA	NA
LPCAT1	1.88784389	1.85E-129	3.53E-129
CTD-2245E1!	NA	NA	NA
CTD-2012J19	NA	NA	NA
CTD-2587M2	NA	NA	NA
MRPL36	1.84554299	0.27535349	0.28088068
NDUFS6	2.21159197	0	0
CTD-2194D2:	NA	NA	NA
CTD-2194D2:	NA	NA	NA
IRX4	NA	NA	NA
CTD-2194D2:	NA	NA	NA
CTD-2194D2:	NA	NA	NA
RP11-259O2.	NA	NA	NA
RP11-259O2.	NA	NA	NA
RP11-259O2.	NA	NA	NA
LSINCT5	Inf	2.57E-74	4.00E-74
RP11-129I19	NA	NA	NA
IRX2	NA	NA	NA
C5orf38	NA	NA	NA
RP11-468D1:	NA	NA	NA
RP11-35O7.1	NA	NA	NA
LINC01377	NA	NA	NA
LINC01019	NA	NA	NA
RP11-121L11	NA	NA	NA
LINC01017	NA	NA	NA
IRX1	NA	NA	NA
CTD-2012M1	NA	NA	NA
CTD-2287N1	NA	NA	NA
CTD-2383I20	NA	NA	NA
CTD-2008N3.	NA	NA	NA
RP11-445O3.	NA	NA	NA
RP11-445O3.	NA	NA	NA
RP11-445O3.	NA	NA	NA
CTD-2318H2:	NA	NA	NA
CTD-2161F6.	NA	NA	NA
CTD-2161F6.	NA	NA	NA
CTD-2161F6.	NA	NA	NA
CTD-2046I8.1	NA	NA	NA
CTD-2247C11	NA	NA	NA
CTD-2247C11	NA	NA	NA
LINC01020	NA	NA	NA
CTD-2247C11	NA	NA	NA
CTD-2247C11	NA	NA	NA
CTD-2297D1(	NA	NA	NA
ADAMTS16	NA	NA	NA

CTD-2297D1(	NA	NA	NA
ICE1	2.01175392	2.10E-40	2.83E-40
CTC-471C19.:	NA	NA	NA
CTC-471C19.:	NA	NA	NA
FLJ33360	NA	NA	NA
MED10	2.11771915	3.37E-39	4.51E-39
UBE2QL1	0.77200489	3.85E-123	7.21E-123
LINC01018	NA	NA	NA
NSUN2	1.99766214	0.1633441	0.16759353
SRD5A1	2.25341968	0.00067377	0.00071929
CTD-2044J15	Inf	0	0
PAPD7	2.16659437	6.55E-71	1.01E-70
RP11-332J15	NA	NA	NA
RP11-332J15	NA	NA	NA
RP11-332J15	NA	NA	NA
RP11-332J15	NA	NA	NA
RP11-122F2⁄	NA	NA	NA
RP11-404K5.	NA	NA	NA
CTD-2296D1.	NA	NA	NA
CTD-2296D1.	NA	NA	NA
CTD-2296D1.	NA	NA	NA
CTD-2296D1.	NA	NA	NA
CTD-2296D1.	NA	NA	NA
ADCY2	NA	NA	NA
RP11-711G1(	NA	NA	NA
C5orf49	NA	NA	NA
MTRR	2.74264895	7.04E-231	1.98E-230
FASTKD3	1.45597413	1.86E-137	3.66E-137
CTD-2072I24	NA	NA	NA
RP11-480D4.	NA	NA	NA
RP11-480D4.	NA	NA	NA
RP11-480D4.	NA	NA	NA
MIR4458HG	NA	1.94E-14	2.28E-14
RP11-417J1.:	NA	NA	NA
RP11-315A1(	NA	NA	NA
RP11-143A1.	NA	NA	NA
CTD-2215L1C	NA	NA	NA
SEMA5A	NA	NA	NA
CTD-2201E9.	NA	NA	NA
CTD-2201E9.	NA	NA	NA
SNHG18	NA	NA	NA
TAS2R1	NA	1.41E-180	3.26E-180
CTD-2143L24	NA	NA	NA
RP11-447B1{	NA	NA	NA
CTD-2199O4.	NA	2.83E-88	4.67E-88

CTD-2199O4.	NA	NA	NA
CTD-2199O4.	NA	NA	NA
CTD-2199O4.	Inf	0	0
FAM173B	1.75113304	8.21E-63	1.22E-62
CTD-2256P15	2.31601467	1.07E-162	2.32E-162
CCT5	1.06799095	0	0
CTD-2256P15	4.63202934	0.38728138	0.39337432
CTD-2256P15	NA	NA	NA
CMBL	1.39353425	0	0
CTD-2256P15	0.8685055	8.30E-200	2.06E-199
6-Mar	1.90522308	1.48E-09	1.67E-09
ROPN1L-AS1	NA	NA	NA
ROPN1L	1.48886657	2.61E-21	3.19E-21
LINC01513	NA	5.67E-235	1.63E-234
RP11-1C1.6	NA	NA	NA
RP11-1C1.4	NA	4.06E-228	1.13E-227
RP11-1C1.5	NA	NA	NA
ANKRD33B	1.40974806	4.09E-106	7.22E-106
ANKRD33B-A	NA	NA	NA
DAP	1.83575581	1.29E-214	3.40E-214
CTD-2154B17	Inf	0	0
CTD-2154B17	NA	NA	NA
CTNND2	NA	NA	NA
RP11-308B16	NA	NA	NA
LINC01194	NA	NA	NA
RP11-419C19	NA	NA	NA
CTC-305H11.	NA	NA	NA
DNAH5	Inf	0	0
CTB-51A17.1	NA	NA	NA
TRIO	2.24583241	0.01595369	0.01668772
AC016549.1	5.79003667	1.16E-51	1.65E-51
FAM105A	1.76017115	0	0
CTD-2165H16	0	2.48E-93	4.16E-93
OTULIN	2.09406326	5.13E-70	7.88E-70
ANKH	2.76427557	0	0
CTB-40H15.4	Inf	2.54E-257	8.10E-257
RP1-251I12.1	NA	NA	NA
RP1-137K24.	NA	NA	NA
FBXL7	NA	NA	NA
CTD-2350J17	NA	NA	NA
11-Mar	NA	NA	NA
RP11-19O2.1	NA	NA	NA
RP11-19O2.2	NA	NA	NA
RP1-167G20.	NA	NA	NA
RP1-167G20.	NA	NA	NA

ZNF622	1.12876473	0	0
FAM134B	Inf	6.28E-238	1.83E-237
RP11-260E18	NA	NA	NA
CTC-461F20.1	NA	NA	NA
MYO10	1.21315054	0.00242107	0.00256396
BASP1	0.2728839	0	0
CTD-2228A4.	NA	NA	NA
AC091878.1	NA	NA	NA
CTD-2139B15	1.54400978	1.34E-06	1.47E-06
CTD-2139B15	NA	NA	NA
CTD-2139B15	NA	NA	NA
RP11-321E2.	Inf	0	0
RP11-321E2.	NA	NA	NA
RP11-454P21	NA	NA	NA
CTD-2023M8	NA	NA	NA
RP11-124N3.	NA	NA	NA
RP11-124N3.	NA	NA	NA
CDH18	NA	7.04E-139	1.40E-138
RP11-420O1(	NA	NA	NA
RP11-774D14	NA	NA	NA
CTD-2333M2	NA	NA	NA
RP11-374A4.	NA	NA	NA
RP11-360I2.1	Inf	0	0
RP11-804N13	NA	NA	NA
CDH12	NA	4.43E-14	5.18E-14
CTD-2234B2(	NA	NA	NA
PRDM9	NA	NA	NA
C5orf17	NA	NA	NA
CTD-2074D8.	NA	NA	NA
CDH10	NA	NA	NA
RP11-116O17	NA	NA	NA
RP11-730N24	NA	NA	NA
RP11-730N24	NA	NA	NA
RP11-549K2(	0	1.48E-186	3.48E-186
RP11-184E9.	NA	NA	NA
RP11-192H6.	NA	NA	NA
RP11-184E9.	NA	NA	NA
CTD-2306M5	NA	1.02E-45	1.41E-45
RP11-351N6.	NA	NA	NA
CTD-2533K21	NA	NA	NA
CDH9	NA	NA	NA
CTD-3007L5.1	NA	NA	NA
LINC01021	2.5365875	0.00313678	0.00331687
CTD-2061E9.	NA	NA	NA
CTD-2134P3.	NA	NA	NA

CTD-2118P12	NA	0	0
CTD-2010I22	NA	NA	NA
RP11-152K4.	NA	NA	NA
CDH6	NA	NA	NA
DROSHA	1.79105134	7.29E-67	1.11E-66
C5orf22	1.67267726	1.28E-09	1.44E-09
PDZD2	NA	5.81E-207	1.48E-206
RP11-5N11.4	NA	NA	NA
RP11-5N11.5	NA	NA	NA
RP11-5N11.6	NA	NA	NA
CTD-2152M2	NA	NA	NA
GOLPH3	1.75545061	7.83E-13	9.07E-13
CTD-2186M1	1.48462479	6.77E-09	7.60E-09
MTMR12	3.53835575	0	0
ZFR	1.53533557	0	0
SUB1	1.7710771	6.19E-238	1.81E-237
CTD-2004A9.	NA	NA	NA
NPR3	2.0611781	2.60E-52	3.70E-52
CTD-2066L21	NA	NA	NA
CTD-2218G2(	NA	NA	NA
CTD-2066L21	NA	NA	NA
CTD-2066L21	NA	NA	NA
RP11-113I22	NA	NA	NA
CTD-2203K17	0	0	0
TARS	1.981775	1.83E-190	4.38E-190
CTD-2233C11	NA	NA	NA
ADAMTS12	NA	NA	NA
RXFP3	NA	NA	NA
SLC45A2	NA	NA	NA
AMACR	Inf	1.52E-275	5.26E-275
C1QTNF3-AN	NA	NA	NA
C1QTNF3	1.54400978	6.65E-138	1.31E-137
RP11-1250I1	NA	NA	NA
CTD-2024P1(	NA	NA	NA
RAI14	2.14818752	0	0
CTD-2024P1(	NA	NA	NA
CTD-2517O1(	Inf	1.13E-117	2.09E-117
TTC23L	NA	2.59E-63	3.87E-63
RAD1	1.95669442	0.15066514	0.15473348
BRIX1	1.00184873	0	0
DNAJC21	2.07524087	1.48E-106	2.62E-106
AGXT2	NA	NA	NA
PRLR	NA	NA	NA
SPEF2	2.70201711	8.39E-07	9.25E-07
CTD-2113L7.	NA	NA	NA

IL7R	NA	0	0
CAPSL	NA	NA	NA
RP11-79C6.3	NA	NA	NA
UGT3A1	NA	NA	NA
UGT3A2	0	0	0
LMBRD2	2.44468215	6.09E-201	1.52E-200
SKP2	2.18977488	8.97E-31	1.15E-30
NADK2	1.65898923	2.07E-149	4.28E-149
NADK2-AS1	NA	2.11E-40	2.84E-40
RANBP3L	Inf	2.47E-05	2.69E-05
SLC1A3	1.42963869	4.67E-78	7.38E-78
CTD-2282P23	NA	NA	NA
CTD-2353F22	NA	1.11E-192	2.69E-192
CTD-2653M2	NA	NA	NA
NIPBL	1.83509359	8.96E-55	1.29E-54
C5orf42	2.6055165	1.45E-76	2.29E-76
NUP155	1.72782047	2.03E-48	2.84E-48
WDR70	1.41090549	4.01E-228	1.12E-227
GDNF	NA	NA	NA
GDNF-AS1	NA	NA	NA
CTD-2194L12	NA	NA	NA
CTD-2194L12	NA	NA	NA
CTD-2130F23	NA	NA	NA
CTD-2130F23	NA	NA	NA
CTD-2116N24	NA	NA	NA
CTD-2207A17	NA	NA	NA
EGFLAM	NA	NA	NA
EGFLAM-AS4	NA	NA	NA
EGFLAM-AS3	NA	NA	NA
EGFLAM-AS2	NA	NA	NA
EGFLAM-AS1	NA	NA	NA
CTD-2263F21	NA	NA	NA
LIFR	0	0	0
LIFR-AS1	Inf	2.41E-78	3.81E-78
CTD-2196P11	NA	NA	NA
LINC01265	NA	NA	NA
OSMR-AS1	NA	NA	NA
RP11-122C5.	NA	NA	NA
OSMR	0	1.84E-54	2.65E-54
RICTOR	2.22148346	9.87E-17	1.17E-16
FYB	1.3896088	6.26E-05	6.76E-05
C9	NA	NA	NA
DAB2	2.26215386	7.31E-106	1.29E-105
CTD-2078B5.	Inf	0	0
LINC00603	NA	NA	NA

LINC00604	NA	NA	NA
PTGER4	4.63202934	0.00016915	0.00018195
TTC33	1.78526131	1.33E-11	1.52E-11
PRKAA1	2.94326864	5.01E-154	1.05E-153
AC008810.1	NA	NA	NA
RPL37	2.56141901	0	0
CARD6	3.55122249	8.74E-264	2.88E-263
C7	NA	NA	NA
MROH2B	NA	NA	NA
C6	NA	NA	NA
PLCXD3	NA	NA	NA
OXCT1	1.83161944	4.44E-10	5.04E-10
C5orf51	2.39519466	0	0
FBXO4	1.86955401	8.56E-11	9.78E-11
RP11-112L7.	NA	NA	NA
GHR	0.77200489	1.10E-244	3.30E-244
CCDC152	0	0	0
SEPP1	NA	NA	NA
CTD-2325A1	NA	NA	NA
CTD-2201E1	3.86002445	1.33E-271	4.52E-271
CTD-2035E1	1.54400978	1.64E-189	3.91E-189
CTD-2035E1	0	0	0
ANXA2R	1.57757521	0.01209947	0.01267663
AC025171.1	3.474022	3.75E-06	4.11E-06
CTD-2035E1	0.38600244	2.48E-11	2.84E-11
ZNF131	1.80496918	1.82E-29	2.32E-29
NIM1K	NA	5.63E-08	6.27E-08
CTD-2636A2	NA	3.01E-08	3.36E-08
HMGCS1	1.21717559	0	0
CCL28	NA	NA	NA
C5orf28	2.39947466	1.26E-15	1.49E-15
C5orf34	2.31601467	2.10E-08	2.35E-08
RP11-159F2	Inf	0	0
RP11-159F2	NA	0	0
PAIP1	1.55274946	2.12E-231	6.01E-231
NNT-AS1	2.07689811	0.08684736	0.08966451
NNT	2.42033966	1.52E-80	2.43E-80
RP11-8L21.1	NA	NA	NA
FGF10	NA	NA	NA
FGF10-AS1	NA	NA	NA
RP11-473L1	NA	NA	NA
RP11-503D1	NA	NA	NA
RP11-53O19	9.26405868	0	0
RP11-53O19	NA	NA	NA
MRPS30	1.44845217	6.12E-308	2.53E-307

RP11-53O19.	3.28102078	0	0
RP11-357F12	NA	NA	NA
HCN1	NA	NA	NA
CTD-2013M1	NA	NA	NA
EMB	2.03758668	1.61E-162	3.48E-162
RP11-269M2	NA	NA	NA
PARP8	1.6887607	7.98E-56	1.16E-55
RP11-317O2	NA	NA	NA
RP11-317O2	NA	NA	NA
CTD-2089N3.	NA	NA	NA
CTD-2089N3.	NA	NA	NA
CTD-2089N3.	NA	NA	NA
CTD-2314G2	1.15800733	1.20E-09	1.35E-09
ISL1	1.10975703	1.84E-125	3.48E-125
CTD-2335O3.	NA	NA	NA
CTD-2203A3.	NA	NA	NA
CTD-2288O8.	NA	NA	NA
ITGA1	3.08801956	1.89E-15	2.23E-15
PELO	1.3651306	0	0
CTD-2175A2	NA	NA	NA
ITGA2	3.86002445	6.34E-294	2.42E-293
CTD-2366F1	Inf	1.56E-34	2.04E-34
MOCS2	1.75349854	1.05E-161	2.26E-161
CTD-2366F1	2.63768337	2.60E-206	6.63E-206
FST	2.93361858	0.35108365	0.35716703
NDUFS4	1.70060306	1.61E-177	3.68E-177
CTD-2081C1	NA	NA	NA
CTD-2081C1	Inf	2.23E-36	2.95E-36
ARL15	3.18452017	2.22E-300	8.79E-300
LINC01033	Inf	0	0
HSPB3	NA	NA	NA
SNX18	2.39321516	1.94E-38	2.58E-38
RP11-45H22.	NA	NA	NA
ESM1	NA	NA	NA
GZMK	NA	NA	NA
CTD-2313F1	NA	NA	NA
CTD-2313F1	NA	NA	NA
GZMA	NA	NA	NA
CDC20B	NA	NA	NA
GPX8	1.5191064	9.86E-19	1.19E-18
MCIDAS	1.35100856	1.06E-54	1.53E-54
CCNO	1.58411393	1.84E-179	4.22E-179
RP11-506H2	Inf	6.42E-53	9.16E-53
DHX29	1.65959243	5.15E-170	1.15E-169
SKIV2L2	1.82352879	3.01E-36	3.97E-36

PLPP1	0.12866748	0	0
SLC38A9	1.14256724	1.34E-175	3.03E-175
DDX4	NA	NA	NA
IL31RA	NA	NA	NA
AC008914.1	NA	NA	NA
IL6ST	1.69841076	0.08053751	0.08320681
CTD-2031P19	NA	NA	NA
FLJ31104	1.02933985	1.67E-67	2.53E-67
CTD-2227I18	NA	NA	NA
ANKRD55	0.97123196	1.41E-304	5.71E-304
RP11-155L15	NA	NA	NA
CTC-236F12.4	NA	NA	NA
C5orf67	NA	NA	NA
AC008940.1	NA	NA	NA
MAP3K1	1.44750917	7.74E-189	1.84E-188
AC008937.2	NA	NA	NA
AC008937.3	NA	NA	NA
SETD9	1.98939722	4.49E-84	7.28E-84
MIER3	2.35277681	1.45E-06	1.60E-06
CTD-2310F14	Inf	0	0
AC016644.1	NA	NA	NA
GPBP1	1.82707824	1.66E-248	5.07E-248
CTD-2023N9.	NA	NA	NA
ACTBL2	NA	NA	NA
CTD-2023N9.	NA	NA	NA
CTD-2023N9.	NA	NA	NA
RP11-772C9.	NA	NA	NA
RP11-478P10	NA	NA	NA
CTD-2037L6.1	NA	NA	NA
PLK2	1.11511817	1.69E-80	2.69E-80
GAPT	0.92991498	0	0
CTD-2117L12	NA	NA	NA
RAB3C	0.12866748	0	0
RP11-479O16	NA	NA	NA
CTD-2176I21	NA	NA	NA
PDE4D	2.4563792	0	0
RP11-266N13	NA	0	0
CTD-2254N19	NA	NA	NA
AC109486.1	NA	NA	NA
PART1	0.77200489	7.44E-82	1.20E-81
DEPDC1B	1.57086212	0	0
ELOVL7	2.14445803	2.02E-21	2.46E-21
RP11-231G3.	Inf	2.97E-232	8.43E-232
ERCC8	1.70295196	0.03866204	0.04019292
AC104113.3	NA	NA	NA

NDUFAF2	2.07433615	7.45E-58	1.09E-57
SMIM15	NA	NA	NA
CTC-436P18.:	NA	NA	NA
CTC-436P18.:	NA	NA	NA
ZSWIM6	1.62121027	1.72E-46	2.39E-46
RP11-2O17.2	NA	NA	NA
CTD-2170G1.	NA	NA	NA
CTD-2244C2(	NA	NA	NA
KIF2A	2.31601467	8.54E-79	1.35E-78
DIMT1	1.33944066	0	0
IPO11	2.52386214	6.79E-268	2.28E-267
LRRC70	NA	NA	NA
HTR1A	NA	NA	NA
RNF180	NA	NA	NA
RGS7BP	NA	NA	NA
FAM159B	NA	NA	NA
SREK1IP1	2.07246551	0.40186812	0.4080078
CWC27	1.53723781	1.58E-134	3.07E-134
RP11-307L14	3.86002445	1.05E-295	4.06E-295
RP11-307L14	Inf	0	0
ADAMTS6	Inf	0	0
CTD-2116N2(	1.93001222	7.73E-100	1.33E-99
CENPK	1.87922243	4.65E-64	6.96E-64
PPWD1	1.84093474	1.61E-264	5.30E-264
TRIM23	3.08801956	2.32E-41	3.14E-41
TRAPPC13	1.61753406	3.98E-179	9.13E-179
CTC-534A2.2	1.7878008	1.07E-246	3.24E-246
SGTB	1.60049794	1.26E-06	1.38E-06
NLN	2.32673696	6.54E-224	1.79E-223
CTD-2033C11	2.31601467	1.30E-08	1.45E-08
ERBB2IP	2.20372305	1.54E-17	1.84E-17
SREK1	1.62834645	0	0
AC025442.3	NA	NA	NA
CTD-2016O1	NA	NA	NA
RP11-305P14	NA	NA	NA
RP11-5P22.3	NA	NA	NA
MAST4	1.40038096	0	0
MAST4-AS1	2.35664651	0.00126301	0.00134367
CD180	0.77200489	0.00250488	0.00265225
CTD-2187J20	NA	NA	NA
RP11-434D9.	NA	NA	NA
RP11-83M16	Inf	0	0
RP11-83M16	NA	NA	NA
RP11-434D9.	NA	NA	NA
RP11-404L6.:	NA	NA	NA

PIK3R1	2.44468215	1.95E-119	3.61E-119
CTD-2582M2	NA	NA	NA
CTC-537E7.3	NA	NA	NA
CTC-537E7.1	NA	NA	NA
CTC-537E7.2	NA	NA	NA
CTC-340D7.1	NA	NA	NA
RP11-141O1	NA	NA	NA
RP11-141O1	NA	NA	NA
CTC-498J12.1	NA	NA	NA
SLC30A5	2.10579738	2.61E-283	9.38E-283
CTC-498J12.3	NA	1.11E-38	1.48E-38
CCNB1	0.83687925	0	0
CENPH	1.45377544	0	0
MRPS36	1.89559162	2.02E-37	2.67E-37
CDK7	1.60781184	1.28E-304	5.18E-304
CCDC125	1.70653713	2.02E-05	2.19E-05
AK6	2.00580266	0.05149908	0.05341278
TAF9	1.69599602	5.99E-145	1.21E-144
RAD17	2.05867971	3.91E-96	6.64E-96
MARVELD2	Inf	0.45687052	0.46294375
RP11-241G9.	NA	NA	NA
OCLN	2.31601467	7.25E-61	1.07E-60
GTF2H2C	2.07222365	0.02217239	0.02314451
SERF1B	NA	1.53E-28	1.94E-28
SMN2	1.2008965	1.24E-298	4.84E-298
RP11-497H1(	2.70201711	1.72E-11	1.98E-11
SERF1A	1.93001222	1.11E-23	1.38E-23
SMN1	0.85778321	1.34E-293	5.11E-293
NAIP	1.87486902	0.07459062	0.07714189
GTF2H2	1.91592454	9.11E-45	1.25E-44
RP11-136K7.	NA	NA	NA
RP11-136K7.	NA	NA	NA
RP11-136K7.	NA	NA	NA
BDP1	1.68859932	1.26E-100	2.19E-100
MCCC2	1.69412184	8.82E-211	2.29E-210
CARTPT	NA	NA	NA
MAP1B	Inf	2.01E-11	2.31E-11
MRPS27	1.65095418	3.67E-172	8.22E-172
PTCD2	1.94839329	0.00782874	0.00822593
ZNF366	NA	NA	NA
CTC-347C20.1	NA	9.77E-98	1.67E-97
CTC-347C20.1	NA	NA	NA
CTD-2631K1(	0	1.80E-23	2.22E-23
TNPO1	2.14664625	7.64E-197	1.88E-196
CTD-2376I4.1	NA	1.38E-08	1.54E-08

CTD-2376I4.2	Inf	2.74E-14	3.21E-14
FCHO2	1.65429619	0.00345291	0.00364838
TMEM171	0	0	0
RP11-232L2.1	NA	NA	NA
TMEM174	NA	NA	NA
RP11-60A8.1	NA	NA	NA
RP11-60A8.2	NA	NA	NA
FOXD1	1.57428448	6.50E-98	1.11E-97
FOXD1-AS1	NA	NA	NA
LINC01385	NA	NA	NA
LINC01386	NA	NA	NA
RP11-79P5.9	2.24583241	0	0
BTF3	1.77742203	0	0
ANKRA2	1.7878008	4.76E-26	5.97E-26
UTP15	1.51988463	4.54E-174	1.02E-173
ARHGEF28	NA	NA	NA
CTC-575I10.1	NA	NA	NA
RP11-428C6.	NA	NA	NA
CTD-2292M1	NA	NA	NA
LINC01335	NA	NA	NA
CTD-2275D2	NA	NA	NA
LINC01333	NA	NA	NA
LINC01332	NA	NA	NA
LINC01331	NA	NA	NA
ENC1	NA	4.22E-200	1.05E-199
HEXB	1.10286413	0	0
GFM2	1.85281174	1.60E-70	2.46E-70
NSA2	1.78775088	5.35E-199	1.33E-198
FAM169A	4.16882641	1.78E-80	2.84E-80
CTD-2377O1	NA	5.99E-19	7.22E-19
CTD-2503O1	NA	NA	NA
GCNT4	Inf	0	0
LINC01336	NA	NA	NA
ANKRD31	NA	5.94E-217	1.58E-216
CTD-2235C1	NA	NA	NA
HMGCR	1.93990972	1.40E-79	2.22E-79
CTD-2235C1	NA	0	0
COL4A3BP	1.63618947	0	0
POLK	2.99151895	0	0
CTC-366B18.	Inf	0	0
CTC-366B18.	1.54400978	9.66E-11	1.10E-10
ANKDD1B	Inf	0	0
POC5	1.85281174	7.56E-52	1.07E-51
CTC-235G5.3	NA	NA	NA
SV2C	NA	NA	NA

AC113404.1	NA	0	0
RP11-466P24	NA	NA	NA
IQGAP2	1.84251834	1.26E-243	3.77E-243
CTD-2236F14	NA	NA	NA
F2RL2	NA	5.84E-53	8.34E-53
CTD-2384B11	NA	NA	NA
F2R	2.61167612	0	0
F2RL1	2.27925253	2.00E-34	2.61E-34
S100Z	1.90588707	1.12E-250	3.45E-250
CRHBP	NA	8.32E-48	1.16E-47
AGGF1	2.27925253	3.08E-172	6.91E-172
ZBED3	2.57820501	2.79E-59	4.10E-59
CTC-564N23.	NA	NA	NA
ZBED3-AS1	2.79851773	1.14E-09	1.29E-09
PDE8B	NA	0	0
WDR41	1.52061569	0	0
OTP	Inf	0	0
TBCA	2.11166043	3.45E-270	1.17E-269
RP11-107N7.	NA	NA	NA
AP3B1	2.27312551	3.25E-31	4.18E-31
SCAMP1-AS1	2.3837344	5.20E-35	6.82E-35
SCAMP1	1.64326755	2.40E-151	4.98E-151
LHFPL2	1.40974806	8.40E-14	9.79E-14
ARSB	2.87747277	1.53E-124	2.89E-124
DMGDH	0.77200489	0.63415256	0.63943952
BHMT2	NA	NA	NA
BHMT	NA	NA	NA
JMY	1.27276482	4.21E-190	1.01E-189
HOMER1	1.57234023	1.48E-167	3.27E-167
PAPD4	1.29019995	0	0
CMYA5	0	0	0
CTC-431G16.	NA	NA	NA
LINC01455	NA	NA	NA
MTX3	NA	0	0
THBS4	NA	NA	NA
CTD-2201I18	NA	2.71E-12	3.13E-12
SERINC5	1.94104087	1.09E-24	1.36E-24
CTC-458I2.2	NA	NA	NA
SPZ1	NA	NA	NA
ZFYVE16	2.71879983	1.74E-224	4.76E-224
CTD-2015H6.	2.55355464	2.64E-68	4.02E-68
FAM151B	1.11511817	8.09E-146	1.64E-145
ANKRD34B	NA	NA	NA
LINC01337	0.77200489	9.24E-61	1.36E-60
DHFR	1.91760267	4.41E-101	7.64E-101

CTC-325J23.2	0.38600244	1.02E-135	2.01E-135
MSH3	1.51312958	2.03E-215	5.36E-215
RASGRF2-AS	NA	NA	NA
RASGRF2	NA	NA	NA
CTD-2193P3.	NA	NA	NA
CKMT2-AS1	1.90176814	3.81E-31	4.90E-31
CKMT2	NA	NA	NA
ZCCHC9	2.10942181	1.08E-169	2.41E-169
ACOT12	1.54400978	0	0
SSBP2	1.8177206	4.03E-71	6.22E-71
CTD-2249K22	0	1.69E-210	4.38E-210
ATG10	2.01506361	1.27E-85	2.07E-85
ATG10-AS1	NA	NA	NA
RPS23	2.33028108	0	0
ATP6AP1L	0.77200489	4.04E-141	8.07E-141
CTD-2015A6.	NA	NA	NA
CTD-2015A6.	NA	NA	NA
CTD-2218K11	NA	NA	NA
LINC01338	NA	NA	NA
RP11-78C3.1	NA	NA	NA
CTD-2227C6.	NA	NA	NA
CTD-2227C6.	NA	NA	NA
RP11-343L5.1	1.54400978	1.21E-24	1.51E-24
TMEM167A	2.26768381	0	0
XRCC4	0.92814071	0	0
VCAN	2.72614227	0	0
VCAN-AS1	NA	NA	NA
HAPLN1	NA	NA	NA
EDIL3	Inf	1.85E-74	2.89E-74
CTD-2269F5.	NA	1.05E-214	2.76E-214
CTC-384G19.	NA	NA	NA
RP11-1072N1	NA	NA	NA
CTD-2331D11	NA	NA	NA
CTD-2331D11	NA	NA	NA
CTD-2331D11	NA	NA	NA
CTC-480C2.1	NA	NA	NA
CTD-2036A18	NA	NA	NA
CTC-261N6.3	NA	NA	NA
CTC-261N6.1	NA	NA	NA
CTC-261N6.2	NA	NA	NA
COX7C	2.26713334	0	0
CTC-493L21.1	NA	NA	NA
CTC-493L21.2	NA	NA	NA
RP11-72L22.1	NA	NA	NA
CTC-242N15.	NA	NA	NA

RASA1	2.01908971	3.18E-104	5.57E-104
CTC-428H11.	1.69841076	0.01023832	0.01073911
CCNH	1.37640346	2.66E-264	8.77E-264
CTD-2316B1.	NA	NA	NA
CTD-2316B1.	NA	NA	NA
TMEM161B	2.00721271	1.60E-45	2.21E-45
TMEM161B-/	2.30886648	2.20E-182	5.10E-182
CTC-498M16	NA	NA	NA
CTC-498M16	NA	NA	NA
LINC00461	NA	NA	NA
CTC-467M3.1	NA	NA	NA
CTC-467M3.3	NA	NA	NA
CTC-467M3.2	NA	NA	NA
MEF2C	1.8756092	1.80E-10	2.05E-10
MEF2C-AS1	2.840978	1.36E-168	3.01E-168
CTD-2232E5.	NA	NA	NA
RP11-116A1.	NA	NA	NA
AC113167.1	NA	NA	NA
AC113167.2	NA	NA	NA
RP11-61G23.	NA	0	0
CETN3	1.57260255	1.84E-84	2.98E-84
MBLAC2	1.65429619	2.28E-57	3.32E-57
POLR3G	2.87526231	0	0
LYSMD3	2.97773315	2.20E-149	4.54E-149
ADGRV1	NA	NA	NA
RP11-414H23	NA	NA	NA
RP11-414H23	NA	NA	NA
RP11-213H15	NA	NA	NA
LUCAT1	1.54400978	5.01E-110	8.99E-110
RP11-213H15	NA	NA	NA
CTD-2061E19	NA	NA	NA
ARRDC3	2.49416964	5.30E-100	9.15E-100
ARRDC3-AS1	1.80134474	1.58E-46	2.20E-46
RP11-348J24	NA	NA	NA
RP11-348J24	NA	NA	NA
RP11-133F8.	NA	NA	NA
CTC-529L17.1	NA	NA	NA
CTC-529L17.2	NA	NA	NA
CTC-458G6.4	NA	NA	NA
CTC-458G6.2	NA	NA	NA
CTD-2091N23	NA	NA	NA
NR2F1-AS1	1.00594577	1.57E-208	4.05E-208
NR2F1	1.13107693	9.25E-147	1.89E-146
FAM172A	1.77425685	2.47E-38	3.30E-38
POU5F2	3.86002445	1.09E-245	3.30E-245

RP11-185E12	NA	NA	NA
KIAA0825	Inf	0	0
SLF1	1.5061664	0	0
MCTP1	0.77200489	0	0
CTC-484P3.3	NA	NA	NA
CTC-558O19.	NA	NA	NA
FAM81B	NA	NA	NA
TTC37	2.32630807	4.76E-23	5.86E-23
ARSK	4.40042787	1.12E-228	3.13E-228
GPR150	NA	NA	NA
RFESD	1.93001222	5.09E-20	6.17E-20
SPATA9	0.38600244	2.15E-216	5.70E-216
CTD-2154I11	Inf	8.27E-60	1.22E-59
RHOBTB3	1.77337355	1.07E-59	1.57E-59
GLRX	2.13034261	1.18E-08	1.32E-08
AC008592.4	NA	NA	NA
AC008592.8	0	0	0
LINC01554	NA	4.11E-287	1.51E-286
ELL2	2.75716032	0.02472361	0.02578977
CTD-2337A12	NA	NA	NA
AC008592.7	NA	NA	NA
RP11-254I22	NA	NA	NA
RP11-254I22	NA	NA	NA
RP11-254I22	NA	NA	NA
PCSK1	NA	NA	NA
CAST	1.92368432	0.00048907	0.000523
CTC-506B8.1	NA	NA	NA
ERAP1	2.50236068	6.09E-102	1.06E-101
CTD-2260A17	Inf	0	0
CTD-2260A17	NA	NA	NA
CTD-2260A17	1.80134474	1.95E-41	2.64E-41
ERAP2	1.72216475	1.12E-08	1.26E-08
LNPEP	2.39133222	1.01E-306	4.16E-306
CTD-2215E18	NA	NA	NA
LIX1	NA	NA	NA
RIOK2	1.12636779	0	0
CTD-2215E18	NA	NA	NA
CTD-2215E18	NA	NA	NA
LINC01340	NA	NA	NA
RP11-455B3.	NA	NA	NA
AC008834.1	NA	NA	NA
RGMB	0.19300122	1.93E-178	4.41E-178
RGMB-AS1	NA	NA	NA
AC008522.1	NA	2.22E-195	5.43E-195
CHD1	1.62488648	8.79E-112	1.59E-111

CTD-2007H13	0	0	0
CTD-2151A2.	NA	NA	NA
RP11-346J10	NA	NA	NA
CTD-2001C12	4.24602689	2.38E-251	7.37E-251
FAM174A	1.817947	4.00E-39	5.35E-39
CTC-293G12.	NA	NA	NA
ST8SIA4	2.57334963	4.26E-168	9.41E-168
CTD-2333K2.	NA	NA	NA
SLCO4C1	0.57900367	0	0
SLCO6A1	NA	NA	NA
RP11-58B2.1	NA	NA	NA
LINC00492	NA	NA	NA
CTD-2340E1.	NA	NA	NA
LINC00491	NA	NA	NA
PAM	1.15800733	0.98602191	0.98634812
GIN1	1.87486902	2.98E-20	3.62E-20
PPIP5K2	1.42714665	0	0
CTC-503K11.1	NA	NA	NA
C5orf30	1.88828223	1.18E-20	1.44E-20
CTD-2154H6.	NA	NA	NA
CTD-2193G5.	NA	NA	NA
NUDT12	2.07037675	1.20E-17	1.43E-17
RP11-138J23	NA	NA	NA
RP11-6N13.1	NA	NA	NA
RP11-6N13.4	NA	NA	NA
CTD-2374C24	NA	NA	NA
CTC-254B4.1	NA	NA	NA
EFNA5	0	2.04E-112	3.70E-112
RP11-252I13	NA	NA	NA
FBXL17	1.98515543	7.63E-210	1.97E-209
LINC01023	3.43113284	0	0
FER	1.54400978	7.79E-183	1.81E-182
CTD-2197I11	NA	NA	NA
CTD-2587M2	Inf	5.84E-252	1.81E-251
PJA2	2.86285147	1.63E-178	3.73E-178
RP11-231G15	NA	NA	NA
RP11-231G15	NA	NA	NA
RP11-231G15	NA	NA	NA
CTC-332L22.1	3.08801956	1.20E-178	2.75E-178
MAN2A1	1.93001222	7.21E-13	8.36E-13
AC011366.3	NA	NA	NA
TMEM232	NA	0	0
SLC25A46	1.92586166	6.59E-137	1.30E-136
CTC-551A13.	NA	NA	NA
TSLP	2.02651284	2.06E-123	3.85E-123

CTC-551A13.	NA	NA	NA
WDR36	2.11364446	1.05E-63	1.57E-63
CAMK4	NA	4.56E-134	8.89E-134
AC010468.2	NA	NA	NA
CTC-499J9.1	NA	NA	NA
STARD4	2.25662968	7.99E-19	9.61E-19
STARD4-AS1	1.54400978	3.76E-53	5.37E-53
NREP	1.70127004	0	0
NREP-AS1	NA	NA	NA
EPB41L4A	1.54400978	1.97E-06	2.16E-06
EPB41L4A-AS	2.48309036	1.34E-251	4.14E-251
CTC-459M5.1	NA	NA	NA
CTC-459M5.2	NA	NA	NA
RP11-526F3.	NA	NA	NA
EPB41L4A-AS	NA	NA	NA
RP11-159K7.	NA	NA	NA
APC	1.86072973	2.79E-21	3.40E-21
CTC-554D6.1	NA	NA	NA
CTC-487M23	0.77200489	1.69E-88	2.78E-88
SRP19	1.93421704	0.00021496	0.00023101
REEP5	1.52681939	4.12E-182	9.56E-182
ZRSR1	0.77200489	3.36E-88	5.54E-88
CTC-487M23	0.51466993	9.82E-117	1.80E-116
CTC-487M23	NA	NA	NA
DCP2	1.78255062	2.61E-136	5.13E-136
MCC	0	0	0
CTD-2201G3.	NA	NA	NA
TSSK1B	NA	NA	NA
YTHDC2	1.40265677	0	0
RP11-371M2	NA	NA	NA
KCNN2	NA	NA	NA
RP11-492A1(	NA	NA	NA
CTC-313D10.	NA	NA	NA
RP11-438C19	NA	NA	NA
RP11-438C19	NA	NA	NA
TRIM36	2.0784747	1.54E-171	3.45E-171
CTC-428G20.	NA	NA	NA
PGGT1B	1.79438974	1.82E-106	3.22E-106
CTC-428G20.	5.40403423	0	0
CCDC112	2.00907297	6.23E-15	7.32E-15
FEM1C	3.1523533	4.02E-254	1.26E-253
TMED7-TICAI	Inf	0	0
TICAM2	NA	NA	NA
AC010226.4	1.02933985	2.66E-27	3.35E-27
TMED7	1.80414186	0.01723126	0.01801161

CTC-505O3.3	NA	NA	NA
CTC-505O3.2	NA	NA	NA
CDO1	1.88088464	1.22E-69	1.86E-69
ATG12	1.78808005	5.99E-44	8.22E-44
AP3S1	1.93444903	1.19E-284	4.32E-284
AC026449.1	NA	NA	NA
LVRN	NA	9.66E-144	1.95E-143
CTD-2287O1(	NA	NA	NA
ARL14EPL	NA	NA	NA
CTD-2287O1(	NA	NA	NA
CTD-2287O1(	2.99151895	1.18E-144	2.39E-144
COMMD10	1.51583442	5.19E-237	1.51E-236
CTC-339F2.2	NA	NA	NA
SEMA6A	0.38600244	4.36E-215	1.15E-214
SEMA6A-AS1	Inf	0	0
CTB-118N6.2	NA	NA	NA
RP11-249M1	NA	NA	NA
CTC-472C24.	NA	NA	NA
RP11-492L8.	NA	NA	NA
LINC00992	NA	NA	NA
CTD-3179P9.	NA	NA	NA
HNCAT21	NA	NA	NA
CTD-2281M2	NA	NA	NA
RP11-2N5.2	NA	NA	NA
RP11-2N5.1	NA	NA	NA
CTC-448D22.	NA	NA	NA
DTWD2	1.1229162	7.44E-258	2.38E-257
CTB-161M19	NA	NA	NA
DMXL1	1.98515543	1.48E-101	2.57E-101
TNFAIP8	0.74929886	0	0
HSD17B4	0.86391023	0	0
FAM170A	NA	NA	NA
CTC-552D5.1	NA	NA	NA
RP11-574H6.	NA	NA	NA
PRR16	1.54400978	2.51E-115	4.59E-115
CTD-2334D1!	NA	NA	NA
CTC-546K23.	2.1378597	3.48E-200	8.65E-200
FTMT	NA	NA	NA
SRFBP1	1.55278256	0	0
LOX	1.02933985	9.35E-38	1.24E-37
CTC-441N14.	NA	NA	NA
ZNF474	NA	4.50E-79	7.14E-79
CTC-441N14.	NA	NA	NA
CTD-2544H1.	NA	NA	NA
SNCAIP	NA	NA	NA

CTD-2544H1;	NA	NA	NA
CTD-2280E9.	NA	NA	NA
MGC32805	NA	NA	NA
RP11-166A1;	NA	NA	NA
SNX2	0.94854864	0	0
SNX24	3.73135697	1.21E-125	2.28E-125
PPIC	1.56679959	0	0
RP11-359P5.	Inf	5.22E-264	1.72E-263
AC106786.1	0	0	0
PRDM6	1.54400978	0	0
CEP120	1.93001222	4.94E-10	5.61E-10
CSNK1G3	2.55619397	7.67E-139	1.52E-138
LINC01170	NA	NA	NA
CTC-369A16.	NA	NA	NA
CTD-2308B1	NA	NA	NA
CTD-2308B1	NA	NA	NA
CTD-2308B1	NA	NA	NA
CTD-2308B1	NA	NA	NA
ZNF608	NA	NA	NA
RP11-43D2.2	NA	NA	NA
RP11-284A2(	NA	NA	NA
RP11-284A2(	NA	NA	NA
RP11-284A2(	NA	NA	NA
RP11-257I8.2	NA	NA	NA
RP11-508M8	NA	NA	NA
RP11-395P1;	NA	NA	NA
RP11-395P1;	NA	NA	NA
RP11-756H2(	NA	NA	NA
RP11-114J13	NA	NA	NA
CTC-339D2.1	NA	NA	NA
GRAMD3	3.34535452	0	0
RP11-517I3.1	NA	NA	NA
ALDH7A1	NA	NA	NA
PHAX	1.4165915	0	0
TEX43	NA	NA	NA
RP11-434D1;	NA	3.92E-201	9.77E-201
LMNB1	1.70127004	2.29E-199	5.69E-199
3-Mar	1.74907358	9.56E-08	1.06E-07
C5orf63	NA	NA	NA
MEGF10	0.46320293	0	0
CTD-2195M1	NA	NA	NA
PRRC1	2.04817624	2.15E-08	2.40E-08
CTD-2195M1	NA	NA	NA
CTXN3	NA	NA	NA
CTC-548H10.	NA	NA	NA

LINC01183	NA	NA	NA
CTC-228N24.	NA	NA	NA
LINC01184	2.36582144	2.32E-20	2.82E-20
SLC12A2	1.41291461	1.45E-278	5.09E-278
FBN2	2.17815665	7.99E-09	8.97E-09
SLC27A6	NA	5.59E-10	6.34E-10
ISOC1	1.70082327	9.00E-190	2.15E-189
ADAMTS19-/	NA	NA	NA
ADAMTS19	NA	NA	NA
CTC-575N7.1	NA	NA	NA
KIAA1024L	NA	6.79E-280	2.40E-279
CHSY3	Inf	0	0
HINT1	2.24866994	0	0
LYRM7	2.31139189	2.11E-97	3.60E-97
CDC42SE2	2.11822829	1.45E-204	3.68E-204
RAPGEF6	4.17790882	0	0
CTC-432M15	NA	NA	NA
FNIP1	2.05624051	9.30E-281	3.30E-280
MEIKIN	NA	NA	NA
CTB-127M13	NA	NA	NA
AC034228.4	NA	NA	NA
ACSL6	Inf	0	0
AC034228.3	NA	NA	NA
AC034228.2	NA	NA	NA
IL3	NA	NA	NA
CSF2	NA	NA	NA
AC063976.3	NA	NA	NA
P4HA2-AS1	NA	NA	NA
P4HA2	1.06150672	0	0
PDLIM4	NA	NA	NA
SLC22A4	0.77200489	9.98E-60	1.47E-59
SLC22A5	1.33814181	0	0
C5orf56	1.57757521	0.00040509	0.00043386
AC116366.5	0.77200489	0.00029686	0.00031835
AC116366.6	NA	NA	NA
IRF1	1.36447376	3.58E-101	6.20E-101
RP11-89G4.1	NA	NA	NA
IL5	NA	NA	NA
RAD50	1.65981051	0	0
TH2LCRR	NA	NA	NA
IL13	NA	NA	NA
IL4	NA	NA	NA
AC004237.1	NA	NA	NA
KIF3A	1.61871993	2.21E-97	3.78E-97
CCNI2	0	0	0

8-Sep	3.03125449	0	0
SOWAHA	NA	NA	NA
AC004775.5	NA	1.71E-19	2.06E-19
SHROOM1	1.42211427	3.47E-13	4.03E-13
GDF9	Inf	1.80E-301	7.18E-301
UQCRQ	2.32195907	0	0
LEAP2	2.03888471	0.12350027	0.1271518
AFF4	1.74028221	6.92E-250	2.13E-249
CTC-350I8.1	Inf	0	0
ZCCHC10	1.97174222	3.50E-94	5.90E-94
HSPA4	1.61068293	7.89E-234	2.26E-233
RP11-485M7	NA	NA	NA
CTB-49A3.2	NA	NA	NA
FSTL4	NA	NA	NA
CTB-49A3.5	NA	NA	NA
CTB-49A3.4	NA	NA	NA
CTB-3M24.3	NA	NA	NA
WSPAR	NA	NA	NA
C5orf15	1.77081348	1.51E-199	3.76E-199
VDAC1	1.38037513	0	0
CTB-113I20.2	1.90031973	1.41E-105	2.48E-105
TCF7	1.89492109	2.10E-13	2.44E-13
SKP1	1.64240256	1.05E-266	3.52E-266
CTD-2410N1	NA	NA	NA
PPP2CA	1.3054128	0	0
CTD-2410N1	NA	1.02E-306	4.19E-306
CDKL3	3.52916521	0	0
UBE2B	1.73452066	1.50E-225	4.12E-225
RP11-215P8.	1.54400978	1.74E-224	4.77E-224
CDKN2AIPNL	1.96926671	0.05055731	0.05244201
RP11-215P8.	NA	NA	NA
AC005355.1	NA	NA	NA
AC005355.2	NA	NA	NA
JADE2	1.55976498	1.68E-164	3.66E-164
SAR1B	1.887583	5.34E-23	6.58E-23
SEC24A	2.4563792	0.64534615	0.65061794
CAMLG	1.65930921	0	0
DDX46	1.92228685	2.78E-11	3.19E-11
C5orf24	1.76585027	1.06E-08	1.19E-08
TXNDC15	1.96146428	1.36E-46	1.90E-46
PCBD2	2.20912169	6.86E-245	2.06E-244
CATSPER3	2.57334963	5.30E-53	7.59E-53
PITX1	1.29007759	0	0
C5orf66	2.31601467	5.65E-15	6.64E-15
CTC-276P9.4	NA	1.10E-169	2.45E-169

C5orf66-AS1	NA	NA	NA
CTC-276P9.2	NA	NA	NA
CTC-276P9.3	NA	NA	NA
C5orf66-AS2	NA	NA	NA
H2AFY	1.1587903	0	0
CTC-203F4.2	Inf	1.92E-94	3.25E-94
DCANP1	NA	NA	NA
TIFAB	NA	NA	NA
CTB-138E5.1	0	0	0
NEUROG1	NA	NA	NA
CTC-321K16.	NA	9.39E-60	1.38E-59
CXCL14	NA	NA	NA
CTC-321K16.	NA	NA	NA
RP11-119J18	NA	NA	NA
SLC25A48	Inf	0	0
IL9	NA	NA	NA
GS1-39E22.2	NA	NA	NA
GS1-39E22.1	NA	NA	NA
LECT2	NA	NA	NA
TGFBI	NA	NA	NA
SMAD5-AS1	0.25733496	1.06E-210	2.75E-210
SMAD5	2.04284371	4.97E-211	1.29E-210
AC009014.3	NA	NA	NA
TRPC7	NA	NA	NA
TRPC7-AS1	NA	NA	NA
TRPC7-AS2	NA	NA	NA
AC063980.3	NA	NA	NA
RP11-114H2	NA	NA	NA
CTB-1I21.1	NA	NA	NA
SPOCK1	Inf	1.31E-299	5.16E-299
KLHL3	3.474022	0.02040014	0.02130558
HNRNPA0	1.64810759	0	0
RP11-381K2	NA	NA	NA
RP11-381K2	2.25662968	3.38E-216	8.94E-216
MYOT	NA	NA	NA
PKD2L2	Inf	3.88E-71	5.98E-71
FAM13B	2.38953895	3.16E-87	5.18E-87
RP11-325L7.	0.38600244	2.34E-142	4.69E-142
RP11-325L7.	NA	NA	NA
WNT8A	NA	NA	NA
NME5	NA	NA	NA
BRD8	1.38537864	4.66E-187	1.10E-186
KIF20A	1.4153423	0	0
CDC23	1.94430861	2.55E-83	4.12E-83
GFRA3	NA	8.77E-07	9.67E-07

CDC25C	2.00721271	8.94E-67	1.35E-66
FAM53C	2.16562411	3.01E-80	4.80E-80
RP11-256P1.	NA	NA	NA
KDM3B	2.13220398	0.01098015	0.0115119
REEP2	2.18734719	1.42E-150	2.94E-150
EGR1	3.08801956	9.17E-11	1.05E-10
ETF1	1.62796303	1.31E-230	3.70E-230
HSPA9	1.52993677	0	0
CTNNA1	1.63815672	3.82E-36	5.05E-36
AC034243.1	NA	NA	NA
LRRTM2	NA	0	0
SIL1	1.11029917	0	0
CTB-46B19.2	NA	NA	NA
MATR3	3.08801956	3.79E-15	4.47E-15
MATR3	1.85281174	1.75E-41	2.37E-41
PAIP2	1.94872153	0.94649936	0.94754394
CTB-43P18.1	2.31601467	3.08E-197	7.58E-197
SLC23A1	Inf	0	0
MZB1	1.737011	9.12E-17	1.08E-16
PROB1	1.31694952	7.87E-260	2.54E-259
SPATA24	1.45318568	3.41E-122	6.37E-122
DNAJC18	0.83913575	1.36E-120	2.53E-120
ECSCR	NA	NA	NA
ECSCR	0	0	0
TMEM173	0.87546946	0	0
UBE2D2	1.90128972	1.44E-07	1.60E-07
CTD-3224K1!	2.31601467	3.12E-07	3.46E-07
CXXC5	1.95943043	2.89E-47	4.03E-47
CTD-3224K1!	NA	NA	NA
CTB-35F21.1	Inf	0	0
CTB-35F21.2	NA	NA	NA
CTB-35F21.3	NA	NA	NA
CTB-35F21.5	NA	NA	NA
PSD2	NA	NA	NA
NRG2	1.59915299	2.29E-166	5.01E-166
CTB-35F21.4	NA	NA	NA
LINC01024	6.17603912	1.01E-27	1.28E-27
PURA	2.03576632	7.03E-103	1.23E-102
IGIP	Inf	0	0
CYSTM1	1.74366622	1.06E-86	1.73E-86
PFDN1	1.89971836	0.00165934	0.0017622
AC011380.1	NA	NA	NA
HBEGF	1.14371095	2.26E-183	5.27E-183
CTC-329D1.3	NA	NA	NA
SLC4A9	NA	NA	NA

CTC-329D1.2	1.54400978	1.31E-123	2.45E-123
ANKHD1	1.90856764	5.89E-90	9.78E-90
ANKHD1-EIF4	1.54400978	3.22E-11	3.69E-11
SRA1	1.71250291	1.11E-235	3.21E-235
EIF4EBP3	2.31601467	6.59E-128	1.25E-127
APBB3	NA	NA	NA
SLC35A4	NA	NA	NA
CD14	0	0	0
NDUFA2	2.13279472	2.04E-253	6.38E-253
TMCO6	2.73710825	1.20E-281	4.28E-281
IK	1.41054453	0	0
WDR55	1.53621175	5.83E-116	1.07E-115
DND1	1.737011	3.02E-29	3.85E-29
HARS	1.03682052	0	0
HARS2	2.05867971	1.42E-08	1.59E-08
ZMAT2	1.6052326	1.52E-303	6.13E-303
PCDHA1	NA	NA	NA
AC005609.16	NA	NA	NA
PCDHA2	NA	NA	NA
PCDHA3	0.77200489	4.13E-79	6.56E-79
PCDHA4	NA	NA	NA
PCDHA5	NA	0	0
PCDHA6	NA	8.25E-189	1.96E-188
PCDHA7	NA	2.83E-88	4.67E-88
PCDHA8	NA	NA	NA
PCDHA9	NA	2.41E-253	7.54E-253
AC005609.19	NA	NA	NA
PCDHA10	0	0	0
AC005609.20	NA	NA	NA
PCDHA11	NA	NA	NA
PCDHA12	NA	NA	NA
AC005609.18	NA	NA	NA
AC005609.17	NA	NA	NA
PCDHA13	NA	NA	NA
PCDHAC1	Inf	6.60E-241	1.95E-240
CTC-270D5.2	NA	NA	NA
PCDHAC2	NA	NA	NA
CTC-270D5.1	NA	NA	NA
PCDHB1	NA	NA	NA
CH17-140K24	NA	NA	NA
PCDHB2	NA	NA	NA
AC005754.7	NA	NA	NA
PCDHB3	NA	NA	NA
AC005754.8	NA	NA	NA
PCDHB4	NA	NA	NA

PCDHB5	NA	NA	NA
CH17-140K24	NA	NA	NA
PCDHB6	NA	NA	NA
CH17-140K24	NA	NA	NA
PCDHB7	NA	NA	NA
PCDHB8	NA	NA	NA
PCDHB16	NA	3.70E-201	9.24E-201
CH17-140K24	NA	NA	NA
PCDHB10	NA	2.69E-289	1.00E-288
CH17-140K24	NA	NA	NA
PCDHB9	NA	NA	NA
CH17-140K24	NA	NA	NA
PCDHB11	NA	NA	NA
PCDHB12	NA	NA	NA
PCDHB13	Inf	1.44E-288	5.35E-288
PCDHB14	NA	NA	NA
CH17-140K24	NA	NA	NA
PCDHB15	NA	NA	NA
TAF7	1.61150748	0	0
SLC25A2	NA	NA	NA
AC005618.6	NA	3.09E-299	1.21E-298
PCDHGA1	NA	NA	NA
PCDHGA2	NA	NA	NA
PCDHGA3	NA	NA	NA
PCDHGB1	Inf	4.18E-278	1.47E-277
AC005618.8	NA	NA	NA
PCDHGA4	NA	NA	NA
PCDHGB2	NA	NA	NA
PCDHGA5	NA	0	0
PCDHGB3	NA	NA	NA
PCDHGA6	Inf	3.58E-187	8.46E-187
PCDHGA7	NA	NA	NA
PCDHGB4	NA	NA	NA
PCDHGA8	Inf	0.98645326	0.9867252
PCDHGB5	NA	NA	NA
PCDHGA9	NA	NA	NA
PCDHGB6	NA	NA	NA
PCDHGA10	1.23520782	5.03E-107	8.93E-107
PCDHGB7	NA	1.09E-144	2.19E-144
PCDHGA11	NA	0	0
AC005618.9	NA	NA	NA
PCDHGA12	NA	NA	NA
PCDHGC3	NA	NA	NA
PCDHGC4	NA	NA	NA
PCDHGC5	NA	NA	NA

DIAPH1	2.19329713	2.50E-302	1.00E-301
CTD-2024I7.1	NA	NA	NA
AC008781.7	NA	NA	NA
HDAC3	1.8594339	3.45E-52	4.91E-52
RELL2	1.99740948	6.86E-08	7.64E-08
FCHSD1	2.23023635	4.37E-142	8.76E-142
ARAP3	0.92640587	2.61E-228	7.27E-228
PCDH1	Inf	1.14E-09	1.29E-09
KIAA0141	2.26776436	1.91E-71	2.95E-71
PCDH12	Inf	0	0
AC005740.6	NA	0	0
RNF14	2.11586525	2.44E-52	3.47E-52
AC005740.5	NA	NA	NA
GNPDA1	0.77200489	0	0
NDFIP1	1.69680241	2.19E-150	4.53E-150
SPRY4	NA	0.07877509	0.08139988
SPRY4-IT1	Inf	0	0
AC005592.2	NA	NA	NA
FGF1	NA	NA	NA
AC005592.3	NA	NA	NA
AC005592.1	NA	NA	NA
ARHGAP26	1.71556642	3.64E-179	8.34E-179
ARHGAP26-A	NA	NA	NA
NR3C1	1.75309444	0.2097852	0.21459955
CTD-2050E21	NA	NA	NA
CTD-2050E21	NA	NA	NA
HMHB1	NA	NA	NA
YIPF5	1.61721714	1.51E-38	2.01E-38
KCTD16	Inf	4.48E-256	1.42E-255
CTC-367F4.1	NA	NA	NA
CTC-806A22.	Inf	0	0
CTB-73N10.1	NA	NA	NA
PRELID2	2.11326591	4.59E-13	5.32E-13
GRXCR2	NA	NA	NA
SH3RF2	NA	NA	NA
PLAC8L1	3.08801956	0.8306713	0.83402726
RP11-118M9	NA	NA	NA
LARS	1.927781	4.60E-135	9.00E-135
RP11-449H3.	NA	8.60E-41	1.16E-40
RBM27	3.1044452	0	0
CTC-359M8.1	NA	NA	NA
POU4F3	NA	NA	NA
CTB-1H10.1	NA	NA	NA
TCERG1	1.36913574	0	0
GPR151	NA	NA	NA

CTB-99A3.1	NA	NA	NA
PPP2R2B	Inf	0	0
CTC-255N20.	NA	NA	NA
STK32A	NA	NA	NA
DPYSL3	NA	5.59E-10	6.34E-10
CTB-108O6.2	NA	NA	NA
JAKMIP2-AS1	NA	NA	NA
JAKMIP2	NA	NA	NA
SPINK1	NA	NA	NA
SCGB3A2	NA	NA	NA
C5orf46	NA	NA	NA
CTC-327F10.!	NA	NA	NA
CTC-327F10.4	NA	NA	NA
SPINK5	NA	NA	NA
SPINK14	NA	NA	NA
SPINK6	NA	NA	NA
CTC-295J13.3	NA	NA	NA
SPINK13	NA	NA	NA
RP11-373N2	NA	1.66E-106	2.94E-106
SPINK7	NA	NA	NA
SPINK9	NA	NA	NA
FBXO38	2.67232462	2.59E-278	9.10E-278
CTD-2283N1!	0	1.34E-238	3.93E-238
HTR4	NA	NA	NA
ADRB2	0.77200489	0	0
SH3TC2	0	8.49E-183	1.98E-182
RP11-44B19.	NA	NA	NA
CTC-529P8.1	0	0	0
ABLIM3	NA	4.07E-46	5.64E-46
RP11-331K21	NA	NA	NA
AC012613.2	NA	NA	NA
AFAP1L1	NA	6.30E-87	1.03E-86
GRPEL2	2.68363605	3.83E-77	6.03E-77
GRPEL2-AS1	NA	NA	NA
PCYOX1L	1.6887607	2.04E-269	6.87E-269
IL17B	NA	NA	NA
RP11-394O4.	0.77200489	3.14E-12	3.62E-12
CARMN	NA	NA	NA
RP11-394O4.	NA	NA	NA
RP11-394O4.	NA	NA	NA
CSNK1A1	1.73458026	1.37E-94	2.32E-94
ARHGEF37	2.50901589	2.98E-09	3.35E-09
PPARGC1B	2.1152934	1.09E-74	1.70E-74
PDE6A	1.28667482	9.51E-205	2.41E-204
SLC26A2	1.98264892	6.16E-145	1.25E-144

TIGD6	1.98515543	1.79E-71	2.77E-71
HMGXB3	2.24946252	3.22E-44	4.42E-44
CSF1R	1.54400978	1.99E-41	2.70E-41
PDGFRB	NA	0	0
CDX1	NA	NA	NA
SLC6A7	NA	NA	NA
CAMK2A	NA	NA	NA
ARSI	NA	NA	NA
TCOF1	2.09122501	3.87E-07	4.27E-07
CD74	0.87084702	0	0
RPS14	2.21216272	0	0
CTC-367J11.1	Inf	1.99E-24	2.47E-24
NDST1	1.98817698	4.70E-110	8.45E-110
SYNPO	1.02933985	0	0
CTB-140J7.2	NA	NA	NA
MYOZ3	1.80134474	5.11E-29	6.50E-29
CTC-345K18.:	NA	NA	NA
RBM22	1.36728577	0	0
DCTN4	2.98226547	0	0
SMIM3	2.08968392	1.02E-269	3.43E-269
IRGM	4.24602689	0	0
ZNF300	Inf	0	0
AC008641.1	NA	NA	NA
GPX3	NA	NA	NA
TNIP1	1.68125509	6.65E-56	9.63E-56
ANXA6	1.10286413	0	0
CTB-17P3.4	Inf	2.10E-68	3.20E-68
CCDC69	2.03070851	0	0
GM2A	1.98515543	0.00152712	0.00162227
SLC36A3	NA	NA	NA
SLC36A2	NA	NA	NA
SLC36A1	1.22234108	0.94945663	0.95034716
FAT2	NA	NA	NA
CTC-251D13.	Inf	0	0
CTB-113P19.	NA	NA	NA
SPARC	1.15324187	0	0
CTB-113P19.	NA	NA	NA
CTB-113P19.	Inf	7.11E-220	1.91E-219
ATOX1	2.23272457	0	0
CTB-113P19.	NA	NA	NA
RP11-54C4.3	0.77200489	0	0
G3BP1	1.84022269	0	0
GLRA1	NA	NA	NA
CTB-12O2.1	NA	NA	NA
NMUR2	NA	NA	NA

LINC01470	NA	NA	NA
GRIA1	NA	NA	NA
AC091962.3	NA	NA	NA
RP11-461L18	NA	NA	NA
FAM114A2	1.91756053	0.00873348	0.00916914
MFAP3	0.96500611	0	0
GALNT10	1.6887607	1.10E-81	1.77E-81
SAP30L-AS1	0	0	0
SAP30L	2.4792027	0	0
HAND1	NA	NA	NA
CTB-158E9.1	NA	NA	NA
LARP1	2.51396464	0	0
FAXDC2	Inf	0	0
CNOT8	1.91985427	0.01251495	0.01310966
GEMIN5	1.78923486	2.03E-52	2.89E-52
MRPL22	1.76272123	2.61E-116	4.77E-116
KIF4B	NA	4.27E-09	4.81E-09
SGCD	NA	NA	NA
AC016577.1	NA	NA	NA
CTC-340I23.2	NA	NA	NA
TIMD4	NA	NA	NA
HAVCR1	NA	NA	NA
HAVCR2	Inf	7.78E-18	9.32E-18
CTB-120L21.1	NA	NA	NA
MED7	1.79105134	5.19E-242	1.54E-241
ITK	Inf	0	0
FAM71B	NA	NA	NA
CTB-4E7.1	NA	NA	NA
CTC-248O19.	NA	NA	NA
CYFIP2	2.05867971	0.00039767	0.00042601
FNDC9	NA	NA	NA
CTB-47B11.3	NA	NA	NA
ADAM19	Inf	2.18E-184	5.11E-184
NIPAL4	NA	NA	NA
AC106801.1	NA	NA	NA
SOX30	NA	NA	NA
C5orf52	NA	NA	NA
AC008694.3	NA	NA	NA
THG1L	1.19452108	0	0
LSM11	2.41759426	2.63E-261	8.55E-261
CLINT1	2.15965925	9.60E-72	1.48E-71
CTC-436K13.	NA	NA	NA
CTC-436K13.	NA	NA	NA
CTC-436K13.	NA	NA	NA
CTC-436K13.	NA	NA	NA

CTC-436K13.1	NA	NA	NA
RP11-524N5.	NA	NA	NA
RP11-542A14	NA	NA	NA
RP11-542A14	NA	NA	NA
RP11-32D16.	NA	NA	NA
EBF1	Inf	0	0
CTD-2363C16	NA	NA	NA
CTD-2363C16	NA	NA	NA
RP11-175K6.	0	0	0
RNF145	2.16012907	4.01E-153	8.38E-153
CTB-11I22.1	NA	1.71E-19	2.06E-19
CTB-11I22.2	NA	NA	NA
UBLCP1	4.63202934	1.37E-179	3.14E-179
AC008697.1	NA	8.50E-15	9.98E-15
IL12B	NA	NA	NA
AC008703.1	NA	NA	NA
AC008691.1	NA	NA	NA
AC011343.1	NA	NA	NA
ADRA1B	NA	NA	NA
TTC1	1.99861213	7.72E-78	1.22E-77
PWWP2A	1.80530374	2.02E-250	6.22E-250
FABP6	NA	NA	NA
CTB-127C13.	NA	NA	NA
CCNJL	1.11511817	1.75E-202	4.39E-202
C1QTNF2	0.77200489	1.75E-13	2.03E-13
ZBED8	1.15800733	8.01E-88	1.32E-87
SLU7	1.33170844	0	0
PTTG1	1.61501808	0	0
MIR3142HG	1.28667482	2.02E-70	3.10E-70
ATP10B	NA	0	0
CTC-348L5.1	NA	NA	NA
CTC-529G1.1	NA	NA	NA
RP11-109J4.1	NA	NA	NA
GABRB2	2.05867971	2.58E-21	3.15E-21
GABRA6	NA	NA	NA
RP11-348M1	NA	NA	NA
GABRA1	NA	NA	NA
LINC01202	NA	NA	NA
GABRG2	NA	NA	NA
RP11-96G10.	NA	NA	NA
RP11-167P20	NA	NA	NA
RP11-541P9.	Inf	6.42E-239	1.88E-238
CCNG1	1.69441763	4.06E-230	1.14E-229
NUDCD2	2.07627933	7.60E-71	1.17E-70
HMMR	1.35715509	0	0

HMMR-AS1	NA	NA	NA
MAT2B	1.48581846	6.41E-292	2.42E-291
CTC-207P7.1	NA	NA	NA
CTB-32P11.1	NA	NA	NA
CTC-340A15.	Inf	1.41E-48	1.98E-48
CTD-2313P7.	NA	NA	NA
CTB-181F24.	NA	NA	NA
CTC-535M15	NA	NA	NA
RP11-67M9.1	NA	NA	NA
CTB-7E3.1	NA	NA	NA
CTC-264O10.	NA	NA	NA
CTC-264O10.	NA	NA	NA
TENM2	NA	NA	NA
CTB-180C19.	NA	NA	NA
CTC-286N12.	NA	NA	NA
CTB-105L4.2	NA	NA	NA
CTB-78F1.1	NA	NA	NA
CTC-353G13.	NA	NA	NA
CTB-77H17.1	NA	NA	NA
CTB-178M22	NA	NA	NA
CTB-178M22	3.86002445	0	0
WWC1	2.48547916	8.67E-157	1.83E-156
RARS	1.43502085	0	0
FBLL1	2.12301345	1.44E-06	1.58E-06
PANK3	2.18956559	6.37E-87	1.04E-86
CTC-558O2.2	NA	NA	NA
SLIT3	NA	NA	NA
CTC-558O2.1	NA	NA	NA
CTB-174D11.	NA	NA	NA
CTB-174D11.	NA	NA	NA
SPDL1	1.92603282	8.89E-37	1.18E-36
DOCK2	2.01080343	9.31E-18	1.11E-17
CTB-37A13.1	NA	NA	NA
FAM196B	NA	NA	NA
FOXI1	NA	NA	NA
LINC01187	NA	NA	NA
LCP2	2.32826872	6.37E-12	7.33E-12
CTB-114C7.4	NA	NA	NA
LINC01366	0.77200489	4.59E-16	5.44E-16
KCNIP1	NA	NA	NA
KCNMB1	0.77200489	4.52E-161	9.71E-161
CTD-2270F17	NA	NA	NA
CTB-147C13.	NA	NA	NA
CTC-265N9.1	NA	NA	NA
CTC-455F18.1	NA	NA	NA

GABRP	NA	NA	NA
RANBP17	NA	NA	NA
RP11-546B8.	NA	NA	NA
TLX3	NA	NA	NA
NPM1	1.39122759	2.58E-258	8.26E-258
FGF18	NA	NA	NA
CTB-78H18.1	NA	NA	NA
SMIM23	NA	NA	NA
FBXW11	2.16995969	5.69E-129	1.09E-128
STK10	1.7441592	0.00128049	0.00136203
EFCAB9	0.30880196	0	0
UBTD2	2.18057522	1.25E-103	2.18E-103
SH3PXD2B	1.85281174	4.22E-07	4.66E-07
CTB-54I1.1	NA	NA	NA
NEURL1B	0.77200489	1.29E-31	1.66E-31
CTB-79E8.2	NA	NA	NA
RP11-779O1	NA	NA	NA
RP11-779O1	NA	NA	NA
DUSP1	1.93001222	2.35E-141	4.69E-141
RP11-536N1	NA	NA	NA
ERGIC1	1.82127914	2.88E-09	3.25E-09
CTC-308K20.	Inf	0	0
CTC-308K20.	NA	NA	NA
RPL26L1	2.20166423	4.77E-182	1.11E-181
ATP6V0E1	1.9309514	5.71E-13	6.62E-13
CREBRF	Inf	2.68E-186	6.31E-186
CTC-209H22.	NA	NA	NA
BNIP1	1.52562871	8.20E-195	2.00E-194
NKX2-5	NA	NA	NA
STC2	1.76458261	2.01E-248	6.13E-248
CTB-32H22.1	NA	NA	NA
CTB-164N12.	NA	NA	NA
CTB-33O18.1	NA	NA	NA
CTB-33O18.2	NA	NA	NA
CTB-33O18.3	NA	NA	NA
BOD1	1.97476613	1.52E-05	1.65E-05
CTB-43E15.1	NA	NA	NA
CTB-43E15.2	NA	NA	NA
LINC01484	NA	NA	NA
LINC01485	NA	NA	NA
CPEB4	1.62121027	0.67765486	0.68265947
C5orf47	NA	NA	NA
HMP19	NA	NA	NA
RP11-619L12	NA	NA	NA
RP11-619L12	NA	NA	NA

CTC-430J12.2	NA	NA	NA
LINC01411	Inf	1.00E-300	3.98E-300
RP11-267A15	NA	NA	NA
MSX2	0.96500611	7.41E-61	1.10E-60
CTD-2532K18	NA	NA	NA
CTD-2532K18	NA	NA	NA
CTC-281M20	NA	NA	NA
CTC-281M20	NA	NA	NA
DRD1	NA	NA	NA
SFXN1	1.83589855	3.63E-45	5.01E-45
HRH2	1.24807457	1.25E-89	2.07E-89
CPLX2	NA	NA	NA
THOC3	1.59871879	1.23E-114	2.23E-114
RP11-91H12.	NA	NA	NA
RP11-826N14	NA	NA	NA
RP11-826N14	NA	NA	NA
FAM153B	NA	NA	NA
RP11-844P9.	NA	NA	NA
RP11-826N14	NA	NA	NA
RP11-844P9.	NA	0	0
SIMC1	1.25450795	1.67E-08	1.87E-08
KIAA1191	1.27038779	0	0
RP11-843P14	NA	NA	NA
RP11-843P14	NA	2.56E-49	3.60E-49
ARL10	Inf	6.71E-19	8.08E-19
NOP16	1.7942751	8.54E-34	1.11E-33
HIGD2A	1.7957505	5.54E-140	1.10E-139
CLTB	1.84054146	4.21E-43	5.75E-43
FAF2	1.71368118	2.40E-08	2.68E-08
RNF44	2.87747277	2.84E-170	6.32E-170
CDHR2	Inf	1.41E-23	1.75E-23
GPRIN1	1.80134474	4.16E-129	7.95E-129
SNCB	Inf	1.96E-88	3.24E-88
EIF4E1B	NA	NA	NA
TSPAN17	2.31601467	1.11E-160	2.38E-160
RP11-375B1.	NA	NA	NA
RP11-375B1.	NA	NA	NA
LINC01574	NA	NA	NA
UNC5A	0.77200489	1.84E-97	3.15E-97
HK3	NA	NA	NA
UIMC1	2.02191757	3.61E-09	4.07E-09
ZNF346	1.76113616	2.67E-33	3.47E-33
FGFR4	1.48886657	9.65E-228	2.68E-227
NSD1	1.88850659	2.45E-07	2.71E-07
RAB24	2.57334963	6.99E-26	8.74E-26

MXD3	1.4949936	1.44E-258	4.62E-258
PRELID1	2.07478776	0	0
LMAN2	1.48668577	0	0
RGS14	1.66380364	6.27E-85	1.02E-84
SLC34A1	NA	4.30E-58	6.29E-58
PFN3	NA	NA	NA
F12	1.6297881	9.17E-170	2.04E-169
GRK6	1.99830497	9.70E-58	1.42E-57
PRR7-AS1	2.00721271	2.41E-33	3.13E-33
PRR7	1.81389767	6.63E-136	1.30E-135
DBN1	1.32144981	0	0
PDLIM7	1.28259013	0	0
RP11-1334A2	NA	NA	NA
DOK3	2.00721271	4.24E-05	4.60E-05
DDX41	1.52018247	0	0
FAM193B	1.737011	0.37103253	0.37714433
RP11-1277A3	3.86002445	2.87E-254	9.00E-254
TMED9	1.54958516	5.87E-267	1.96E-266
B4GALT7	2.02098095	2.64E-55	3.81E-55
RP11-1277A3	3.08801956	0.64585502	0.65109478
RP11-1101H1	NA	NA	NA
FAM153A	NA	NA	NA
RP11-1026M	NA	NA	NA
RP11-1026M	NA	NA	NA
RP11-423H2.	NA	NA	NA
RP11-1252I4	NA	NA	NA
RP11-423H2.	2.20572826	2.12E-176	4.82E-176
RP11-423H2.	1.15800733	2.70E-52	3.84E-52
PROP1	NA	NA	NA
FAM153C	NA	NA	NA
N4BP3	1.62121027	9.10E-51	1.29E-50
RMND5B	1.65192444	3.01E-06	3.30E-06
NHP2	1.97505372	2.60E-14	3.04E-14
HNRNPAB	1.71662021	1.08E-35	1.42E-35
PHYKPL	1.61684043	3.02E-59	4.43E-59
COL23A1	Inf	1.54E-49	2.17E-49
RP11-1259L2	NA	NA	NA
CLK4	1.68437431	0.30623503	0.31204875
ZNF354A	1.93001222	1.29E-05	1.41E-05
ZNF354B	1.98515543	8.62E-65	1.30E-64
ZFP2	Inf	1.75E-77	2.77E-77
RP11-281O15	NA	NA	NA
ZNF454	Inf	2.77E-08	3.10E-08
RP11-281O15	NA	NA	NA
GRM6	0	0	0

ZNF879	NA	NA	NA
ZNF354C	NA	2.56E-49	3.60E-49
ADAMTS2	2.57334963	7.80E-87	1.28E-86
RP11-443C1(	NA	NA	NA
RP11-443C1(	NA	NA	NA
RP11-798K2:	NA	NA	NA
RP11-798K2:	0	0	0
RUFY1	1.70331238	2.56E-182	5.94E-182
RP11-1379J2	Inf	5.77E-306	2.36E-305
HNRNPH1	1.64371521	1.77E-298	6.90E-298
RP11-1379J2	3.86002445	1.15E-165	2.51E-165
CBY3	NA	NA	NA
CANX	2.31846938	0	0
MAML1	1.97290139	3.24E-187	7.65E-187
LTC4S	NA	5.44E-240	1.60E-239
MGAT4B	1.89932584	2.77E-222	7.52E-222
SQSTM1	1.51279664	0	0
C5orf45	1.4371168	2.18E-92	3.65E-92
CTC-241N9.1	2.70201711	2.59E-52	3.69E-52
TBC1D9B	2.4206933	0	0
RNF130	2.1319212	2.44E-169	5.40E-169
CTC-563A5.2	NA	NA	NA
RASGEF1C	Inf	2.43E-124	4.57E-124
MAPK9	2.04817624	1.19E-42	1.62E-42
CTB-129O4.1	1.37245314	4.27E-35	5.61E-35
GFPT2	0.19300122	8.99E-228	2.50E-227
CTC-573N18.	NA	NA	NA
CNOT6	3.2595762	1.21E-244	3.64E-244
SCGB3A1	NA	NA	NA
FLT4	Inf	0	0
RP11-451H2:	NA	NA	NA
OR2Y1	NA	NA	NA
MGAT1	1.75455657	0.92049034	0.9219641
LINC00847	NA	NA	NA
ZFP62	1.91714548	2.93E-12	3.38E-12
BTNL8	NA	NA	NA
BTNL3	NA	NA	NA
BTNL9	0.46320293	0	0
OR2V1	NA	NA	NA
OR2V2	NA	NA	NA
CTC-338M12	0	0	0
CTC-338M12	2.39321516	2.66E-238	7.76E-238
TRIM7	1.86596698	1.17E-36	1.55E-36
CTC-338M12	1.54400978	1.02E-258	3.28E-258
CTC-338M12	NA	NA	NA

TRIM41	2.09299104	1.96E-10	2.23E-10
CTC-338M12	NA	NA	NA
GNB2L1	1.4621437	0	0
TRIM52	1.28667482	3.58E-86	5.84E-86
CTC-338M12	NA	NA	NA
AC138035.2	NA	NA	NA
AC138035.1	NA	NA	NA
OR4F3	NA	NA	NA
LINC00266-3	NA	NA	NA
RP3-416J7.4	NA	NA	NA
RP3-416J7.5	NA	NA	NA
DUSP22	2.05240324	2.77E-51	3.94E-51
IRF4	0.77200489	2.88E-77	4.54E-77
EXOC2	2.11285549	2.89E-55	4.17E-55
RP1-20B11.2	NA	NA	NA
HUS1B	0.77200489	1.69E-73	2.63E-73
RP11-532F6.	1.54400978	0.28310148	0.28858948
RP11-532F6.	NA	NA	NA
RP11-532F6.	NA	NA	NA
RP11-284J1.:	NA	NA	NA
RP5-1077H2:	NA	NA	NA
RP5-1077H2:	NA	NA	NA
RP5-856G1.1	NA	NA	NA
RP5-856G1.2	NA	NA	NA
FOXQ1	NA	NA	NA
LINC01394	NA	NA	NA
RP4-668J24.:	NA	NA	NA
FOXF2	NA	NA	NA
RP11-157J24	NA	NA	NA
FOXCUT	NA	NA	NA
FOXC1	2.22693718	4.60E-212	1.20E-211
GMDS	0.97495463	0	0
GMDS-AS1	4.32322738	9.96E-66	1.50E-65
RP1-136B1.1	NA	1.88E-133	3.66E-133
LINC01600	NA	NA	NA
RP11-145H9.	NA	NA	NA
MYLK4	NA	6.93E-33	8.99E-33
WRNIP1	1.99949267	6.26E-20	7.58E-20
SERPINB1	1.82266243	1.02E-225	2.80E-225
SERPINB9P1	NA	NA	NA
SERPINB9	Inf	2.63E-211	6.84E-211
RP1-90J20.2	NA	NA	NA
SERPINB6	1.09962773	0	0
LINC01011	2.57334963	3.00E-107	5.33E-107
NQO2	2.05283118	4.09E-13	4.74E-13

RP1-90J20.8	Inf	4.59E-203	1.16E-202
RP1-40E16.2	NA	NA	NA
RIPK1	1.65049321	3.81E-22	4.66E-22
RP1-40E16.1	3.26617453	3.75E-102	6.53E-102
BPHL	1.23520782	0	0
RP1-40E16.1	NA	0	0
TUBB2A	0.98522529	5.65E-270	1.91E-269
RP1-40E16.9	NA	5.31E-25	6.61E-25
TUBB2B	NA	3.43E-102	5.97E-102
PSMG4	2.35580874	0	0
SLC22A23	1.23520782	0.0013149	0.00139829
RP11-506K6.	0	0	0
RP1-223B1.1	NA	NA	NA
PXDC1	NA	2.19E-281	7.81E-281
RP11-420L9.	NA	NA	NA
FAM50B	NA	NA	NA
RP1-140K8.1	NA	NA	NA
RP1-140K8.5	NA	NA	NA
RP3-406P24.	NA	4.38E-100	7.56E-100
RP3-406P24.	NA	NA	NA
PRPF4B	1.93859006	0.39159592	0.39764542
FAM217A	NA	NA	NA
C6orf201	NA	2.10E-128	4.00E-128
ECI2	1.56075939	0	0
RP3-400B16.	NA	NA	NA
RP3-400B16.	NA	NA	NA
RP3-400B16.	NA	NA	NA
RP3-527G5.1	NA	NA	NA
RP11-274H2	0	0	0
RP4-529N6.2	NA	NA	NA
RP4-529N6.1	NA	NA	NA
CDYL	2.11497173	2.45E-98	4.21E-98
RP3-430A16.	NA	NA	NA
RPP40	1.19078113	0	0
RP11-428J1.	1.54400978	2.71E-251	8.38E-251
RP11-428J1.	0	0.82608265	0.82955782
PPP1R3G	Inf	5.89E-191	1.42E-190
LYRM4	2.11412335	8.11E-253	2.53E-252
FARS2	1.10286413	0	0
RP1-232P20.	NA	NA	NA
RP1-256G22.	NA	NA	NA
RP3-380B8.4	Inf	0	0
NRN1	1.64799411	0	0
F13A1	NA	NA	NA
LY86-AS1	Inf	1.84E-125	3.48E-125

LY86	1.14072364	4.33E-145	8.77E-145
RP1-80N2.3	3.08801956	7.15E-59	1.05E-58
RP1-80N2.2	Inf	2.70E-55	3.90E-55
RP3-470L22.1	NA	NA	NA
RP3-429O6.1	NA	NA	NA
RP11-556O15	NA	NA	NA
RREB1	1.98515543	6.37E-08	7.10E-08
SSR1	1.96460678	1.36E-48	1.91E-48
RP11-69L16.4	NA	NA	NA
CAGE1	Inf	1.04E-22	1.27E-22
RIOK1	1.73101718	1.50E-290	5.63E-290
RP3-512B11.	NA	3.86E-21	4.71E-21
DSP	0	7.29E-257	2.32E-256
SNRNP48	1.14631029	0	0
BMP6	2.91646292	9.58E-76	1.50E-75
TXNDC5	2.93361858	0	0
BLOC1S5-TXI	NA	NA	NA
BLOC1S5	2.10371333	3.10E-05	3.36E-05
EEF1E1-BLO(	NA	NA	NA
BLOC1S5	NA	NA	NA
EEF1E1	1.80835139	3.44E-88	5.66E-88
RP11-203H2.	NA	NA	NA
RP11-203H2.	NA	NA	NA
SLC35B3	1.49310836	0	0
HULC	NA	NA	NA
RP11-314C16	NA	NA	NA
RP11-354I10	NA	NA	NA
OFCC1	NA	NA	NA
TFAP2A	4.05302567	8.48E-196	2.08E-195
TFAP2A-AS1	4.8893643	1.59E-176	3.60E-176
RP1-290I10.3	NA	NA	NA
LINC00518	NA	NA	NA
MIR5689HG	0.77200489	9.45E-269	3.18E-268
RP1-290I10.5	NA	NA	NA
GCNT2	3.66702323	3.86E-271	1.31E-270
RP11-360O19	Inf	3.44E-180	7.93E-180
C6orf52	1.74987775	4.00E-87	6.55E-87
PAK1IP1	1.31823476	0	0
TMEM14C	1.72440955	2.20E-293	8.38E-293
RP11-421M1	1.54400978	1.47E-49	2.07E-49
TMEM14B	1.990495	9.96E-271	3.38E-270
RP11-637O19	NA	NA	NA
MAK	Inf	2.02E-161	4.34E-161
GCM2	NA	NA	NA
RP11-637O19	NA	NA	NA

SYCP2L	1.66277976	0.52470696	0.53067419
ELOVL2	1.58689894	8.58E-69	1.31E-68
ELOVL2-AS1	NA	NA	NA
SMIM13	2.28902149	1.28E-176	2.90E-176
ERVFRD-1	NA	0	0
RP3-510L9.1	NA	NA	NA
NEDD9	3.08801956	8.95E-191	2.15E-190
RP11-716O2	NA	NA	NA
RP11-716O2	NA	NA	NA
TMEM170B	1.78154975	1.31E-148	2.68E-148
RP11-679B1	NA	NA	NA
ADTRP	NA	NA	NA
RP3-413H6.2	NA	NA	NA
RP11-456H1	NA	NA	NA
RP11-456H1	NA	NA	NA
HIVEP1	5.91870416	1.69E-163	3.67E-163
EDN1	NA	NA	NA
RP11-125M1	NA	0	0
PHACTR1	1.28181944	0	0
RP1-257A7.4	0.77200489	2.15E-188	5.10E-188
TBC1D7	1.44044815	1.32E-182	3.06E-182
RP1-257A7.5	1.85281174	4.47E-37	5.92E-37
GFOD1	1.56319003	1.05E-06	1.15E-06
GFOD1-AS1	NA	NA	NA
SIRT5	1.67510495	0	0
RP1-223E5.4	2.8306846	1.91E-41	2.59E-41
NOL7	1.42163191	3.08E-230	8.67E-230
RANBP9	2.40760847	1.14E-301	4.54E-301
MCUR1	2.24402708	3.84E-290	1.44E-289
RP3-500L14.	Inf	2.57E-192	6.21E-192
RNF182	NA	0	0
RP3-380E11.	NA	NA	NA
CD83	1.62613796	1.46E-44	2.01E-44
RP11-359N1	NA	NA	NA
LINC01108	NA	NA	NA
RP3-448I9.2	NA	NA	NA
RP3-448I9.1	NA	NA	NA
RP11-330A1	NA	NA	NA
RP11-146I2.	NA	NA	NA
RP11-560J1.	1.28667482	1.39E-212	3.62E-212
JARID2	2.21133604	0.02777489	0.02894435
JARID2-AS1	0.77200489	1.56E-277	5.47E-277
DTNBP1	1.2960666	0	0
RP11-401E1	NA	NA	NA
MYLIP	0.89344386	0	0

GMPR	0.97553345	0	0
RP3-467D16.	NA	NA	NA
ATXN1	0.67550428	0	0
RP1-151F17.	NA	NA	NA
RP1-151F17.	Inf	0	0
STMND1	NA	NA	NA
RBM24	NA	NA	NA
CAP2	NA	NA	NA
FAM8A1	2.77219938	1.04E-192	2.51E-192
NUP153	2.67232462	6.79E-223	1.85E-222
RP11-500C11	2.00721271	0	0
KIF13A	2.59908313	3.34E-67	5.07E-67
NHLRC1	NA	NA	NA
TPMT	1.94125501	8.74E-16	1.03E-15
KDM1B	2.75348411	3.16E-93	5.31E-93
DEK	1.82101153	1.11E-187	2.64E-187
RNF144B	1.54400978	1.38E-11	1.59E-11
RP11-686D16	NA	NA	NA
RP1-167F1.2	NA	NA	NA
RP3-471C18.	NA	NA	NA
RP3-471C18.	NA	NA	NA
RP4-625H18.	NA	NA	NA
ID4	NA	NA	NA
MBOAT1	0.77200489	6.77E-238	1.97E-237
RP11-239H6.	NA	NA	NA
E2F3	2.15120464	6.83E-07	7.53E-07
CDKAL1	2.88215159	1.23E-07	1.37E-07
RP3-348I23.2	NA	NA	NA
RP1-135L22.	NA	NA	NA
LINC00581	NA	NA	NA
RP11-204E9.	NA	NA	NA
SOX4	1.86061853	4.41E-06	4.82E-06
CASC15	0	0	0
NBAT1	NA	NA	NA
RP11-524C21	NA	NA	NA
RP3-404K8.2	NA	NA	NA
PRL	NA	NA	NA
HDGFL1	NA	NA	NA
RP1-309H15.	NA	NA	NA
RP1-209A6.1	NA	NA	NA
RP11-439H9.	NA	NA	NA
NRSN1	NA	NA	NA
DCDC2	NA	NA	NA
KAAG1	NA	NA	NA
MRS2	2.10343361	5.16E-69	7.89E-69

GPLD1	3.08801956	0	0
ALDH5A1	2.47573982	1.10E-188	2.61E-188
KIAA0319	NA	1.49E-225	4.10E-225
TDP2	1.69960766	8.24E-219	2.20E-218
ACOT13	2.19383462	1.08E-96	1.84E-96
RP1-30M3.5	1.28667482	0.09806317	0.10116354
C6orf62	2.21393138	4.37E-256	1.39E-255
RP1-30M3.6	0.77200489	3.12E-44	4.29E-44
RP3-369A17.	NA	NA	NA
GMNN	1.51154228	2.87E-197	7.07E-197
C6orf229	NA	NA	NA
RP3-369A17.	NA	NA	NA
FAM65B	NA	7.92E-29	1.01E-28
RP11-367G6.	8.10605134	7.64E-127	1.45E-126
RP3-425P12.	NA	NA	NA
RP3-425P12.	NA	NA	NA
RP3-425P12.	NA	NA	NA
RP3-522P13.	NA	NA	NA
LRRC16A	0.38600244	0	0
SCGN	NA	NA	NA
HIST1H2AA	NA	NA	NA
HIST1H2BA	NA	NA	NA
SLC17A4	NA	NA	NA
SLC17A1	NA	NA	NA
SLC17A3	NA	NA	NA
SLC17A2	NA	NA	NA
TRIM38	1.31884169	4.55E-203	1.14E-202
U91328.21	NA	NA	NA
U91328.19	2.00721271	3.69E-33	4.79E-33
U91328.22	NA	5.57E-300	2.20E-299
HIST1H1A	Inf	0	0
HIST1H3A	0.77200489	2.61E-154	5.47E-154
HIST1H4A	0.77200489	0	0
HIST1H4B	Inf	8.06E-129	1.54E-128
HIST1H3B	2.31601467	9.74E-41	1.31E-40
HIST1H2AB	0.77200489	1.55E-28	1.97E-28
HIST1H2BB	NA	NA	NA
HIST1H3C	NA	NA	NA
HIST1H1C	1.3896088	5.47E-107	9.70E-107
HFE	NA	NA	NA
HIST1H4C	2.7900181	0	0
HIST1H1T	NA	NA	NA
HIST1H2BC	2.10546788	0.14465698	0.14861356
HIST1H2AC	0.81263673	0	0
HIST1H1E	1.64928317	3.25E-120	6.03E-120

HIST1H2BD	2.12301345	1.43E-156	3.02E-156
RP1-221C16.	NA	NA	NA
HIST1H2BE	2.31601467	4.45E-196	1.09E-195
HIST1H4D	1.54400978	8.81E-08	9.80E-08
RP1-34B20.2	1.27313087	1.52E-283	5.47E-283
HIST1H3D	NA	NA	NA
HIST1H2AD	NA	NA	NA
HIST1H2BF	0.57900367	0.00331308	0.00350186
HIST1H4E	1.54400978	4.39E-27	5.53E-27
HIST1H2BG	0.81489405	0	0
HIST1H2AE	1.737011	3.14E-22	3.84E-22
HIST1H3E	NA	NA	NA
HIST1H1D	3.36594132	0	0
HIST1H4F	NA	NA	NA
HIST1H4G	NA	NA	NA
HIST1H3F	NA	NA	NA
HIST1H2BH	NA	NA	NA
HIST1H3G	3.66702323	0.8902143	0.8922307
HIST1H2BI	2.31601467	1.35E-10	1.53E-10
HIST1H4H	0.61760391	7.83E-13	9.07E-13
BTN3A2	2.05190773	3.63E-154	7.60E-154
BTN2A2	2.52656146	1.97E-148	4.03E-148
BTN3A1	10.0360636	4.44E-233	1.27E-232
BTN3A3	Inf	0	0
BTN2A1	1.81253322	5.79E-14	6.76E-14
BTN1A1	NA	7.40E-30	9.46E-30
HCG11	1.85281174	1.67E-15	1.97E-15
CTA-14H9.5	3.474022	2.81E-56	4.08E-56
HMGN4	1.68437431	1.04E-29	1.33E-29
ABT1	1.80726668	0.38385071	0.38993335
RP11-457M1	NA	NA	NA
ZNF322	2.38463733	0	0
RP11-457M1	1.93001222	6.52E-75	1.02E-74
LINC00240	Inf	1.89E-168	4.18E-168
RP11-239L2(	NA	NA	NA
RP1-86C11.7	NA	NA	NA
HIST1H2BJ	1.39925886	1.72E-101	2.98E-101
HIST1H2AG	2.23881418	1.05E-143	2.12E-143
HIST1H4I	2.7792176	1.45E-110	2.61E-110
HIST1H2BK	5.40403423	0	0
HIST1H2AH	2.05867971	0	0
PRSS16	1.37858016	1.85E-42	2.52E-42
POM121L2	NA	NA	NA
ZNF391	Inf	0	0
RP1-153G14.	NA	NA	NA

ZNF184	NA	NA	NA
LINC01012	NA	NA	NA
HIST1H2BL	NA	NA	NA
HIST1H2AI	NA	NA	NA
HIST1H3H	0.44114565	5.44E-118	1.00E-117
HIST1H2AJ	NA	NA	NA
HIST1H2BM	NA	NA	NA
HIST1H4J	Inf	0	0
HIST1H4K	NA	NA	NA
HIST1H2BN	1.77896779	2.22E-28	2.82E-28
HIST1H2AK	0.25733496	1.58E-208	4.07E-208
HIST1H2AL	NA	NA	NA
HIST1H1B	NA	NA	NA
HIST1H3I	NA	NA	NA
HIST1H4L	NA	NA	NA
HIST1H3J	Inf	4.66E-159	9.95E-159
HIST1H2AM	Inf	3.28E-197	8.08E-197
HIST1H2BO	Inf	2.37E-267	7.95E-267
OR2B2	NA	NA	NA
OR2B6	0	2.13E-290	7.97E-290
RP1-313I6.12	1.95271825	4.07E-69	6.22E-69
ZNF165	1.54400978	2.92E-59	4.29E-59
ZSCAN16-AS	2.26454768	5.37E-258	1.72E-257
ZSCAN16	2.0844132	7.27E-75	1.14E-74
RP1-265C24.	NA	1.78E-287	6.56E-287
ZKSCAN8	2.58848698	1.47E-208	3.79E-208
ZSCAN9	1.29586535	3.35E-189	7.99E-189
ZKSCAN4	1.3426172	0.42756812	0.43378486
NKAPL	0	0	0
ZSCAN26	1.95970472	7.08E-20	8.57E-20
RP5-874C20.	NA	NA	NA
PGBD1	1.63665037	8.57E-11	9.79E-11
ZSCAN31	1.00954486	0	0
ZKSCAN3	2.12301345	1.25E-181	2.89E-181
ZSCAN12	NA	NA	NA
ZSCAN23	NA	1.78E-258	5.70E-258
GPX6	NA	NA	NA
GPX5	NA	NA	NA
ZBED9	NA	NA	NA
RP5-1186N24	1.54400978	2.27E-295	8.73E-295
LINC00533	NA	NA	NA
XXbac-BPG3(	1.93001222	2.68E-07	2.97E-07
LINC01623	NA	6.94E-204	1.75E-203
HCG14	Inf	2.58E-44	3.54E-44
TRIM27	1.59039634	1.44E-279	5.09E-279

LINC01556	1.71556642	7.24E-134	1.41E-133
HCG15	NA	NA	NA
HCG16	NA	NA	NA
ZNF311	NA	NA	NA
XXbac-BPG2!	NA	NA	NA
OR2W1	NA	NA	NA
OR2B3	NA	NA	NA
OR2J3	NA	NA	NA
XXbac-BCX19	NA	NA	NA
OR2J2	NA	NA	NA
XXbac-BPG3(	NA	NA	NA
OR14J1	NA	NA	NA
OR5V1	NA	NA	NA
OR12D3	NA	NA	NA
OR12D2	NA	NA	NA
OR11A1	NA	NA	NA
OR10C1	NA	NA	NA
OR2H1	NA	NA	NA
MAS1L	NA	NA	NA
XXbac-BPG13	NA	2.39E-62	3.56E-62
LINC01015	NA	NA	NA
UBD	NA	NA	NA
GABBR1	2.8306846	1.41E-25	1.76E-25
OR2H2	NA	NA	NA
MOG	Inf	5.21E-271	1.77E-270
ZFP57	NA	NA	NA
HLA-F	NA	NA	NA
XXbac-BPG1	Inf	1.83E-97	3.12E-97
HLA-G	NA	NA	NA
HCP5B	NA	NA	NA
HLA-A	1.06264203	0	0
HCG9	NA	4.92E-136	9.66E-136
ZNRD1-AS1	2.20572826	0.01464179	0.01532076
ZNRD1	2.15522783	1.70E-63	2.54E-63
PPP1R11	1.91874208	6.76E-73	1.05E-72
RNF39	NA	NA	NA
TRIM31	Inf	1.21E-41	1.64E-41
TRIM31-AS1	NA	NA	NA
TRIM40	NA	NA	NA
TRIM10	NA	NA	NA
TRIM15	0	0	0
TRIM26	1.90176814	8.74E-90	1.45E-89
HCG17	Inf	0	0
HCG18	1.86969934	2.65E-22	3.25E-22
TRIM39	2.05867971	0	0

TRIM39-RPP2	NA	NA	NA
RPP21	2.45145412	9.22E-212	2.40E-211
HLA-E	0.98115363	0	0
XXbac-BPG24	NA	0	0
GNL1	1.40303497	0	0
PRR3	1.69343008	1.65E-119	3.05E-119
ABCF1	1.49167047	0	0
PPP1R10	1.65192444	4.95E-300	1.95E-299
MRPS18B	1.40270949	0	0
ATAT1	1.36082218	0	0
C6orf136	1.55607236	1.29E-06	1.42E-06
DHX16	2.06193711	8.20E-32	1.06E-31
PPP1R18	1.97686801	1.33E-105	2.35E-105
NRM	1.76263063	5.21E-138	1.03E-137
MDC1	2.20211231	1.06E-84	1.72E-84
MDC1-AS1	NA	NA	NA
TUBB	1.12050995	0	0
XXbac-BPG25	0.15440098	0	0
FLOT1	1.93878501	4.82E-17	5.74E-17
XXbac-BPG25	0	0	0
IER3	0.77200489	3.14E-137	6.18E-137
HCG20	NA	NA	NA
LINC00243	NA	4.72E-62	7.02E-62
XXbac-BPG27	NA	NA	NA
DDR1-AS1	NA	2.05E-89	3.40E-89
DDR1	2.31601467	1.04E-61	1.55E-61
GTF2H4	NA	6.48E-109	1.16E-108
VARS2	2.16161369	2.03E-297	7.90E-297
SFTA2	NA	NA	NA
DPCR1	NA	NA	NA
HCG21	NA	NA	NA
MUC21	NA	NA	NA
MUC22	NA	NA	NA
HCG22	NA	NA	NA
C6orf15	NA	NA	NA
PSORS1C1	Inf	2.07E-210	5.36E-210
CDSN	NA	NA	NA
PSORS1C2	NA	NA	NA
CCHCR1	1.76113616	6.51E-51	9.23E-51
TCF19	1.89808721	2.17E-06	2.38E-06
POU5F1	NA	1.41E-245	4.27E-245
XXbac-BPG29	2.25882912	1.48E-207	3.78E-207
HCG27	3.08801956	6.90E-134	1.34E-133
XXbac-BPG29	NA	NA	NA
HLA-C	1.16954894	0	0

XXbac-BPG2	NA	NA	NA
HLA-B	1.07460893	0	0
XXbac-BPG1	0.86282899	1.03E-166	2.27E-166
MICA	1.10584484	3.36E-163	7.27E-163
LINC01149	0	0	0
MICB	1.85281174	1.37E-26	1.72E-26
MCCD1	NA	NA	NA
ATP6V1G2-D	NA	NA	NA
DDX39B	1.014635	0	0
DDX39B-AS1	Inf	7.20E-222	1.95E-221
ATP6V1G2	1.93001222	5.02E-11	5.74E-11
NFKBIL1	2.11650779	1.47E-205	3.73E-205
LTA	Inf	0	0
TNF	0	0	0
LTB	0.33100747	0	0
LST1	0.79792622	0	0
NCR3	0.77200489	0	0
AIF1	1.38891173	0	0
PRRC2A	2.20609344	6.92E-237	2.01E-236
BAG6	1.59748198	9.42E-202	2.36E-201
APOM	1.24708482	5.93E-149	1.22E-148
C6orf47	2.07222365	0.01059666	0.0111124
C6orf47-AS1	NA	0	0
GPANK1	1.55871463	2.26E-170	5.04E-170
CSNK2B	2.27090521	7.92E-117	1.45E-116
XXbac-BPG3	NA	NA	NA
LY6G5B	3.08801956	1.72E-90	2.86E-90
LY6G5C	2.23881418	4.04E-16	4.79E-16
ABHD16A	Inf	2.28E-64	3.42E-64
XXbac-BPG3	NA	NA	NA
LY6G6F	NA	NA	NA
XXbac-BPG3	0	0	0
LY6G6E	NA	NA	NA
LY6G6D	NA	NA	NA
C6orf25	NA	NA	NA
LY6G6C	NA	6.48E-109	1.16E-108
DDAH2	1.48860273	0	0
CLIC1	1.49020565	9.22E-303	3.70E-302
MSH5	2.70201711	3.57E-09	4.03E-09
MSH5-SAPCI	NA	NA	NA
SAPCD1	NA	NA	NA
SAPCD1-AS1	NA	0.37997497	0.38610429
VWA7	NA	NA	NA
VARS	1.83285515	2.68E-105	4.72E-105
LSM2	2.23414979	0	0

HSPA1L	NA	0.00078833	0.00084085
HSPA1A	0.83855704	0	0
HSPA1B	1.07409376	0	0
C6orf48	2.59389161	3.68E-302	1.47E-301
NEU1	1.08448306	0	0
SLC44A4	Inf	1.24E-14	1.46E-14
EHMT2	1.8624618	2.61E-127	4.96E-127
EHMT2-AS1	NA	NA	NA
C2	NA	2.97E-85	4.83E-85
ZBTB12	NA	0	0
XXbac-BPG1:	NA	NA	NA
C2-AS1	NA	NA	NA
CFB	NA	NA	NA
NELFE	1.73904259	1.69E-223	4.62E-223
SKIV2L	1.42963869	0	0
DXO	1.72648366	3.25E-99	5.59E-99
STK19	1.63173761	8.31E-104	1.45E-103
C4A	NA	NA	NA
C4A-AS1	NA	NA	NA
AL645922.1	NA	NA	NA
C4B	NA	NA	NA
C4B-AS1	NA	NA	NA
CYP21A2	NA	NA	NA
TNXB	NA	NA	NA
ATF6B	2.32050307	0.31773512	0.32367626
FKBPL	1.83565607	8.01E-161	1.72E-160
PRRT1	3.08801956	1.90E-281	6.77E-281
PPT2	1.72469178	0.90689528	0.90859809
PPT2-EGFL8	2.31601467	5.50E-47	7.65E-47
EGFL8	1.80134474	3.01E-229	8.42E-229
AGPAT1	2.01437038	4.06E-06	4.44E-06
RNF5	1.6606683	0	0
AGER	1.82473883	6.90E-271	2.34E-270
PBX2	1.753627	1.76E-28	2.23E-28
GPSM3	1.72716837	2.34E-61	3.47E-61
NOTCH4	1.54400978	2.33E-68	3.54E-68
XXbac-BPG1!	NA	NA	NA
XXbac-BPG1!	NA	0	0
C6orf10	NA	NA	NA
HCG23	NA	NA	NA
BTNL2	NA	NA	NA
HLA-DRA	0.89924669	0	0
HLA-DRB5	0.77200489	0	0
HLA-DRB1	1.04416933	3.41E-251	1.06E-250
HLA-DQA1	0	2.96E-272	1.01E-271

HLA-DQB1	1.15800733	0	0
HLA-DQB1-A	NA	NA	NA
HLA-DQA2	NA	NA	NA
HLA-DQB2	NA	NA	NA
HLA-DOB	2.16161369	1.18E-32	1.53E-32
XXbac-BPG2	NA	NA	NA
TAP2	1.37245314	5.28E-172	1.18E-171
PSMB8	1.65429619	2.28E-258	7.31E-258
PSMB8-AS1	1.72782047	1.44E-83	2.32E-83
PSMB9	1.72851409	2.06E-210	5.34E-210
TAP1	2.1081672	1.08E-86	1.76E-86
HLA-DMB	Inf	5.29E-111	9.51E-111
XXbac-BPG1	NA	NA	NA
HLA-DMA	1.12831484	0	0
BRD2	1.96006032	1.64E-136	3.23E-136
XXbac-BPG1	NA	NA	NA
HLA-DOA	NA	NA	NA
HLA-DPA1	0.28950183	0	0
HLA-DPB1	0.77200489	0	0
HCG24	NA	NA	NA
COL11A2	Inf	1.80E-307	7.40E-307
RXRB	1.91957973	9.37E-263	3.07E-262
SLC39A7	1.54837139	2.71E-130	5.21E-130
HSD17B8	1.40574025	0	0
RING1	1.4962569	6.16E-267	2.06E-266
XXbac-BPG1	5.40403423	1.75E-70	2.69E-70
HCG25	2.31601467	6.44E-260	2.08E-259
VPS52	2.06698083	2.90E-252	9.03E-252
RPS18	2.1621664	3.67E-256	1.17E-255
B3GALT4	5.01803178	4.05E-98	6.94E-98
WDR46	1.00602728	0	0
PFDN6	2.02300708	2.51E-10	2.86E-10
RGL2	1.49473287	8.47E-72	1.31E-71
TAPBP	2.03357386	1.57E-62	2.34E-62
ZBTB22	2.04029864	7.47E-60	1.10E-59
DAXX	1.79202945	6.81E-162	1.47E-161
KIFC1	1.00360636	0	0
PHF1	1.64051039	2.89E-294	1.11E-293
CUTA	1.89603683	7.41E-53	1.06E-52
SYNGAP1	1.77561125	1.37E-07	1.52E-07
XXbac-BPG2	2.20572826	0.41565054	0.42181194
ZBTB9	0	4.11E-134	8.01E-134
BAK1	1.83013747	9.08E-123	1.70E-122
LINC00336	NA	NA	NA
ITPR3	1.45318568	1.90E-153	3.98E-153

UQCC2	2.05951251	1.80E-53	2.58E-53
IP6K3	NA	NA	NA
LEMD2	1.63854099	2.27E-100	3.92E-100
MLN	NA	NA	NA
LINC01016	NA	NA	NA
RP3-468B3.2	NA	2.56E-49	3.60E-49
GRM4	NA	2.51E-223	6.85E-223
HMGA1	2.00960799	1.01E-210	2.62E-210
C6orf1	1.63758613	2.54E-160	5.44E-160
NUDT3	1.91240176	1.24E-17	1.48E-17
RPS10-NUDT	NA	1.05E-196	2.57E-196
RPS10	2.84867276	0	0
PACSIN1	0.77200489	4.09E-106	7.22E-106
SPDEF	NA	NA	NA
C6orf106	1.85281174	8.46E-14	9.86E-14
RP11-140K17	1.83351161	7.94E-08	8.84E-08
SNRPC	1.74119657	0	0
UHRF1BP1	2.2581143	1.50E-24	1.86E-24
TAF11	1.67556218	0.68263996	0.68760503
ANKS1A	1.71771088	7.60E-33	9.86E-33
TCP11	NA	0	0
SCUBE3	NA	6.20E-147	1.27E-146
RP3-329A5.8	0.96500611	0.11391508	0.11733647
ZNF76	1.817947	5.00E-184	1.17E-183
DEF6	1.56606706	0	0
PPARD	1.54400978	7.88E-78	1.24E-77
FANCE	3.08801956	1.74E-148	3.57E-148
RPL10A	1.78613451	1.24E-112	2.24E-112
TEAD3	1.54400978	0.08658391	0.0893976
TULP1	NA	NA	NA
RP3-340B19.	NA	2.65E-142	5.32E-142
FKBP5	2.23966254	1.62E-129	3.11E-129
RP3-510O8.4	NA	NA	NA
ARMC12	Inf	0	0
CLPSL2	1.54400978	1.28E-116	2.35E-116
CLPSL1	NA	3.22E-72	4.98E-72
CLPS	NA	NA	NA
LHFPL5	NA	NA	NA
SRPK1	1.46610103	1.62E-249	4.97E-249
SLC26A8	NA	0	0
MAPK14	2.47653054	3.83E-154	8.03E-154
MAPK13	Inf	6.33E-179	1.45E-178
RP1-179N16.	1.15800733	2.92E-59	4.28E-59
BRPF3	2.72775061	4.63E-294	1.77E-293
PNPLA1	NA	NA	NA

C6orf222	NA	NA	NA
ETV7	NA	0	0
RP1-50J22.4	NA	NA	NA
PXT1	NA	NA	NA
KCTD20	2.18350637	0	0
STK38	2.46664977	0	0
SRSF3	1.80575559	7.38E-217	1.96E-216
PANDAR	0	0	0
CDKN1A	1.65981051	1.61E-222	4.36E-222
RAB44	1.6297881	1.27E-221	3.43E-221
CPNE5	NA	NA	NA
PPIL1	1.45628195	0	0
C6orf89	2.27789097	1.56E-09	1.77E-09
RP1-90K10.4	NA	NA	NA
PI16	NA	0	0
MTCH1	1.32262602	0	0
FGD2	NA	8.57E-198	2.12E-197
PIM1	2.37656407	1.53E-229	4.29E-229
TMEM217	0.38600244	5.01E-101	8.67E-101
TBC1D22B	1.2778012	2.64E-47	3.68E-47
RNF8	1.87601188	1.16E-138	2.30E-138
CMTR1	2.24888381	8.97E-143	1.80E-142
CCDC167	2.00960036	4.75E-119	8.78E-119
RP1-153P14.	NA	NA	NA
RP1-153P14.	NA	NA	NA
MDGA1	4.24602689	1.69E-70	2.60E-70
RP3-441A12.	NA	1.63E-47	2.28E-47
ZFAND3	2.04513576	4.83E-189	1.15E-188
BTBD9	1.54400978	3.74E-05	4.06E-05
BTBD9-AS1	NA	NA	NA
GLO1	1.4256357	0	0
RP3-503A6.2	NA	NA	NA
DNAH8	NA	NA	NA
RP1-207H1.3	NA	NA	NA
GLP1R	NA	NA	NA
SAYSD1	2.38953895	0.18254427	0.18708158
KCNK5	1.57910091	1.18E-306	4.84E-306
KCNK17	NA	NA	NA
KCNK16	NA	NA	NA
KIF6	NA	1.33E-34	1.74E-34
DAAM2	NA	0	0
RP11-61I13.3	NA	NA	NA
MOCS1	NA	NA	NA
RP11-552E2(	NA	NA	NA
TDRG1	NA	NA	NA

LINC00951	NA	NA	NA
RP11-552E2(	NA	NA	NA
LRFN2	NA	NA	NA
RP11-570K4.	NA	NA	NA
UNC5CL	0.77200489	1.21E-147	2.48E-147
OARD1	1.61361678	0	0
TSPO2	NA	NA	NA
APOBEC2	NA	NA	NA
NFYA	1.56813493	1.74E-199	4.33E-199
TREML1	NA	NA	NA
TREM2	NA	0	0
TREML2	Inf	1.83E-145	3.72E-145
TREML4	NA	NA	NA
TREM1	NA	NA	NA
NCR2	NA	NA	NA
FOXP4-AS1	2.65461331	0	0
LINC01276	NA	NA	NA
FOXP4	2.20278729	6.19E-66	9.35E-66
MDFI	1.79955769	1.51E-62	2.24E-62
TFEB	1.51988463	5.54E-50	7.82E-50
RP11-298J23	NA	NA	NA
PGC	NA	NA	NA
RP11-298J23	NA	NA	NA
FRS3	0.77200489	5.74E-59	8.41E-59
PRICKLE4	2.97773315	3.15E-263	1.04E-262
PRICKLE4	NA	NA	NA
TOMM6	NA	NA	NA
USP49	2.47041565	3.14E-156	6.63E-156
RP11-298J23	NA	NA	NA
RP5-973N23.	NA	2.93E-127	5.56E-127
MED20	1.65607501	3.77E-162	8.14E-162
BYSL	0.95144386	0	0
CCND3	1.369974	7.76E-294	2.96E-293
RP11-533O2(	NA	NA	NA
TAF8	2.25585844	2.20E-40	2.96E-40
RP11-7K24.3	Inf	0	0
C6orf132	1.80134474	6.90E-17	8.21E-17
GUCA1A	NA	NA	NA
RP1-139D8.6	NA	NA	NA
GUCA1B	NA	7.94E-112	1.43E-111
MRPS10	1.75065755	9.71E-61	1.43E-60
TRERF1	2.57334963	2.31E-61	3.42E-61
UBR2	2.37539966	5.10E-09	5.73E-09
PRPH2	NA	NA	NA
TBCC	1.67166413	5.64E-176	1.28E-175

GLTSCR1L	1.69841076	1.52E-09	1.71E-09
RPL7L1	1.46264415	0	0
C6orf226	1.64051039	2.77E-71	4.28E-71
PTCRA	NA	NA	NA
CNPY3	1.87643115	2.89E-111	5.21E-111
RP3-475N16.	1.3896088	0.8937521	0.89567755
GNMT	2.31601467	1.06E-17	1.26E-17
PEX6	1.41768171	4.35E-277	1.52E-276
PPP2R5D	1.82783511	0.15942022	0.1636416
MEA1	1.75933087	2.23E-124	4.19E-124
KLHDC3	1.65239722	0	0
RRP36	1.7890907	1.60E-91	2.67E-91
RP1-20C7.6	Inf	0	0
CUL7	1.21315054	0	0
KLC4	2.03528562	1.26E-08	1.41E-08
MRPL2	1.48329029	0	0
RP11-387M2	3.08801956	1.10E-34	1.44E-34
PTK7	1.44107579	1.22E-246	3.69E-246
SRF	2.19853566	2.76E-17	3.29E-17
CUL9	1.93001222	3.22E-32	4.16E-32
RP3-330M21	NA	1.89E-307	7.80E-307
DNPH1	2.14994385	2.41E-277	8.43E-277
TTBK1	NA	NA	NA
SLC22A7	NA	NA	NA
CRIP3	NA	NA	NA
ZNF318	1.85281174	1.66E-80	2.66E-80
ABCC10	1.93001222	0.00041981	0.00044947
DLK2	1.43372337	0	0
TJAP1	2.25341968	0.08222673	0.08492783
LRRC73	Inf	0.00010697	0.00011527
POLR1C	1.34304343	0	0
YIPF3	1.58573977	0	0
RP3-337H4.9	0.77200489	1.78E-242	5.30E-242
XPO5	2.38806846	2.99E-60	4.41E-60
RP3-337H4.1	NA	NA	NA
POLH	1.4153423	7.85E-251	2.43E-250
RP3-337H4.8	0	0	0
GTPBP2	0.51466993	7.98E-268	2.68E-267
MAD2L1BP	1.99778365	4.13E-208	1.06E-207
RSPH9	Inf	0	0
MRPS18A	1.74596887	1.56E-180	3.60E-180
RP1-261G23.	NA	NA	NA
VEGFA	1.88176192	4.48E-82	7.21E-82
RP1-261G23.	Inf	9.89E-122	1.84E-121
RP11-344J7.2	NA	NA	NA

RP5-1120P11	NA	NA	NA
C6orf223	0.25733496	0	0
RP5-1120P11	NA	1.32E-06	1.45E-06
MRPL14	1.92981498	9.20E-16	1.09E-15
TMEM63B	1.80134474	2.05E-87	3.37E-87
CAPN11	1.54400978	1.29E-194	3.15E-194
RP1-302G2.5	NA	NA	NA
SLC29A1	1.40874397	0	0
HSP90AB1	1.40625489	0	0
SLC35B2	1.65952876	3.30E-133	6.40E-133
NFKBIE	2.13404209	3.98E-77	6.28E-77
TMEM151B	NA	NA	NA
RP11-444E17	NA	NA	NA
TCTE1	NA	NA	NA
AARS2	3.52916521	1.60E-254	5.02E-254
SPATS1	NA	NA	NA
CDC5L	1.22591517	0	0
RP3-449H6.1	NA	NA	NA
SUPT3H	1.3896088	0	0
RUNX2	1.54400978	0.00062705	0.00066988
RP1-244F24.	NA	1.21E-64	1.82E-64
RP1-166H4.2	NA	NA	NA
CLIC5	NA	NA	NA
RP1-8B1.4	5.79003667	1.69E-172	3.78E-172
ENPP4	3.70562347	0.00255014	0.00269969
ENPP5	NA	NA	NA
RCAN2	NA	NA	NA
RP11-795J1.1	NA	NA	NA
CYP39A1	Inf	0	0
SLC25A27	0.77200489	4.09E-186	9.64E-186
RP11-446F17	NA	NA	NA
TDRD6	NA	NA	NA
PLA2G7	NA	NA	NA
ANKRD66	NA	NA	NA
RP11-268F1.	NA	NA	NA
MEP1A	NA	NA	NA
ADGRF5	NA	NA	NA
RP3-365O12.	NA	NA	NA
ADGRF1	NA	1.53E-43	2.10E-43
TNFRSF21	1.65429619	0.10142551	0.10458461
RP11-385F7.	0.61760391	0.31647957	0.32241534
CD2AP	1.97290139	1.10E-99	1.89E-99
ADGRF2	NA	NA	NA
ADGRF4	NA	NA	NA
RP11-550C4.	NA	NA	NA

OPN5	NA	NA	NA
PTCHD4	NA	NA	NA
MUT	1.28667482	1.31E-23	1.61E-23
CENPQ	1.21976773	4.20E-306	1.72E-305
GLYATL3	NA	NA	NA
C6orf141	2.03888471	0.01032988	0.0108339
RHAG	NA	1.10E-253	3.45E-253
CRISP2	NA	5.76E-203	1.45E-202
CRISP3	NA	NA	NA
PGK2	NA	NA	NA
RP3-417L20.4	NA	NA	NA
RP11-719J20	NA	NA	NA
CRISP1	NA	NA	NA
DEFB133	NA	NA	NA
DEFB114	NA	NA	NA
DEFB113	NA	NA	NA
DEFB110	NA	NA	NA
DEFB112	NA	NA	NA
RP11-397G17	NA	NA	NA
RP11-524K22	NA	NA	NA
RP1-28O17.1	NA	NA	NA
TFAP2D	NA	NA	NA
TFAP2B	NA	NA	NA
RP3-335N17.	NA	NA	NA
PKHD1	NA	NA	NA
LINCMD1	NA	NA	NA
IL17A	NA	NA	NA
IL17F	NA	NA	NA
MCM3	1.67091469	6.66E-233	1.90E-232
PAQR8	1.50724764	1.53E-06	1.68E-06
EFHC1	1.23520782	5.65E-26	7.07E-26
TRAM2	1.81421149	0.9266035	0.92803581
TRAM2-AS1	2.91646292	2.88E-152	5.99E-152
TMEM14A	1.92001216	7.32E-72	1.13E-71
GSTA2	NA	NA	NA
GSTA1	NA	NA	NA
GSTA5	NA	NA	NA
GSTA3	NA	NA	NA
GSTA4	0.46320293	6.73E-224	1.84E-223
ICK	2.63183485	2.97E-125	5.60E-125
FBXO9	2.01513071	4.18E-47	5.82E-47
GCM1	NA	NA	NA
ELOVL5	1.75697665	8.57E-33	1.11E-32
RP3-483K16.	Inf	0	0
GCLC	2.28092354	1.29E-29	1.64E-29

RP1-27K12.4	NA	NA	NA
RP1-27K12.2	NA	NA	NA
LINC01564	NA	NA	NA
KLHL31	Inf	0	0
RP11-79N23.	NA	NA	NA
RP13-476E2(	Inf	4.63E-210	1.20E-209
LRRC1	1.54400978	0.00052792	0.00056441
RP3-523E19.	NA	NA	NA
MLIP	NA	NA	NA
RP11-411K7.	NA	NA	NA
MLIP-AS1	NA	NA	NA
TINAG	NA	NA	NA
RP11-124I4.2	NA	NA	NA
RP11-524H1!	NA	NA	NA
FAM83B	NA	NA	NA
HCRTR2	NA	NA	NA
GFRAL	NA	NA	NA
HMGCLL1	NA	NA	NA
BMP5	NA	NA	NA
COL21A1	NA	NA	NA
DST	1.31773248	1.70E-190	4.07E-190
RP11-472M1	NA	NA	NA
BEND6	2.62481663	9.06E-13	1.05E-12
KIAA1586	1.62387235	3.46E-104	6.06E-104
ZNF451	1.78688772	0.10647989	0.10972159
RP11-203B9.	Inf	0	0
BAG2	1.59363867	4.61E-142	9.24E-142
RAB23	2.94765503	7.06E-273	2.42E-272
PRIM2	1.75265975	2.36E-128	4.49E-128
RP1-71H19.2	NA	NA	NA
XXbac-BPGB	NA	1.32E-85	2.14E-85
XXbac-BPGB	NA	5.01E-106	8.85E-106
XXbac-BPGB	2.70201711	1.06E-234	3.04E-234
LINC00680	1.40364525	0.07134265	0.07381228
XXbac-BPG5!	NA	NA	NA
RP11-143A2.	NA	NA	NA
RP11-343D2.	NA	NA	NA
AL356135.1	NA	NA	NA
KHDRBS2	4.63202934	3.17E-65	4.77E-65
FKBP1C	4.63202934	7.61E-21	9.27E-21
LGSN	NA	NA	NA
PTP4A1	1.79614606	8.63E-33	1.12E-32
RP5-1148A2.	NA	0	0
PHF3	1.80489419	0.38111961	0.38722402
EYS	0.77200489	3.06E-18	3.68E-18

RP11-59D5_	Inf	0	0
RP11-349P19	NA	NA	NA
RP11-301G19	1.81331381	3.58E-172	8.02E-172
RP11-542F9.	NA	NA	NA
RP11-406O16	NA	NA	NA
RP3-525N10.	Inf	0	0
ADGRB3	NA	0.01700484	0.01777596
LMBRD1	1.81124224	1.45E-77	2.30E-77
COL19A1	1.54400978	6.39E-05	6.90E-05
COL9A1	NA	NA	NA
RP1-149L1.1	NA	NA	NA
RP11-462G2.	0	0	0
RP11-462G2.	Inf	1.48E-298	5.79E-298
FAM135A	1.69247226	8.76E-05	9.45E-05
SDHAF4	2.29395739	6.89E-05	7.44E-05
RP11-134K13	NA	0	0
SMAP1	2.46955066	0	0
B3GAT2	3.14316277	0	0
OGFRL1	3.04123138	3.57E-272	1.22E-271
RP3-331H24.	NA	NA	NA
RP3-331H24.	NA	NA	NA
LINC00472	NA	NA	NA
LINC01626	NA	NA	NA
RIMS1	NA	NA	NA
KCNQ5	2.26776436	0.00046369	0.000496
KCNQ5-AS1	NA	NA	NA
RP11-257K9.	NA	NA	NA
KHDC1L	2.31601467	5.77E-57	8.39E-57
KHDC1	1.69841076	1.03E-87	1.70E-87
RP11-398K22	2.89501834	0	0
DPPA5	NA	NA	NA
KHDC3L	NA	NA	NA
OOEP	Inf	0	0
OOEP-AS1	NA	NA	NA
DDX43	NA	NA	NA
MB21D1	2.23157664	9.72E-19	1.17E-18
MTO1	1.96407126	5.47E-81	8.75E-81
RP11-505P4.	NA	NA	NA
EEF1A1	1.92597368	2.47E-07	2.74E-07
RP11-505P4.	Inf	2.38E-222	6.47E-222
SLC17A5	1.87062723	1.63E-07	1.81E-07
RP11-553A21	4.63202934	0	0
CD109	6.69070905	0	0
RP11-554D15	NA	NA	NA
RP11-554D15	NA	NA	NA

RP11-554D1!	NA	NA	NA
COL12A1	NA	NA	NA
COX7A2	2.14648507	0	0
TMEM30A	2.06787024	2.42E-17	2.89E-17
RP1-234P15.	6.94804401	9.25E-160	1.98E-159
FILIP1	NA	0	0
RP11-415D1	NA	NA	NA
RP11-415D1	NA	NA	NA
SENP6	2.63053518	4.14E-259	1.33E-258
MYO6	1.08080685	3.57E-16	4.23E-16
IMPG1	NA	NA	NA
RP11-35J1.1	NA	NA	NA
RP11-35J1.2	NA	NA	NA
RP11-354K4.	NA	NA	NA
HTR1B	NA	NA	NA
MEI4	NA	NA	NA
RP3-390M24	NA	NA	NA
RP11-173D1	NA	NA	NA
IRAK1BP1	5.14669927	0	0
PHIP	1.60936469	0	0
HMGN3	1.9926359	5.39E-102	9.36E-102
HMGN3-AS1	2.31601467	3.92E-160	8.39E-160
RP1-232L24.	NA	NA	NA
LCA5	Inf	1.85E-299	7.27E-299
RP11-177G2	Inf	0	0
SH3BGRL2	Inf	0	0
LINC01621	NA	NA	NA
RP1-92C4.2	NA	NA	NA
ELOVL4	5.40403423	2.05E-62	3.05E-62
AL133475.1	NA	NA	NA
TTK	1.21895509	0	0
BCKDHB	1.68697365	6.99E-07	7.71E-07
RP11-250B2.	1.54400978	1.61E-25	2.01E-25
RP11-250B2.	Inf	0	0
RP11-250B2.	Inf	0.07112798	0.07359438
FAM46A	1.38765436	0	0
RP11-244G1	NA	NA	NA
LINC01526	NA	NA	NA
RP11-379B8.	Inf	0	0
RP11-801I18	NA	NA	NA
IBTK	2.15738353	2.84E-205	7.21E-205
TPBG	1.28667482	0	0
UBE3D	1.70264092	1.17E-194	2.85E-194
RP11-445L13	NA	NA	NA
DOPEY1	2.18734719	4.23E-93	7.11E-93

AL139333.1	NA	NA	NA
PGM3	1.65429619	1.90E-147	3.89E-147
RWDD2A	1.44750917	5.90E-48	8.25E-48
ME1	1.57458423	4.05E-291	1.52E-290
PRSS35	0	0	0
SNAP91	NA	NA	NA
RP3-393K13.	NA	NA	NA
RIPPLY2	NA	NA	NA
CYB5R4	1.91457213	0.85107193	0.85375432
RP4-676J13.:	NA	NA	NA
RP11-51G5.1	NA	NA	NA
MRAP2	1.55588678	7.27E-152	1.51E-151
CEP162	1.90427873	6.43E-68	9.79E-68
LINC01611	NA	NA	NA
TBX18	NA	NA	NA
TBX18-AS1	NA	NA	NA
RP11-30P6.6	NA	NA	NA
NT5E	NA	NA	NA
SNX14	1.82076625	5.60E-239	1.64E-238
RP11-321N4.	NA	9.47E-81	1.51E-80
SYNCRIP	1.88581347	6.77E-98	1.16E-97
HTR1E	NA	NA	NA
CGA	NA	NA	NA
RP11-393I2.4	Inf	1.31E-83	2.11E-83
ZNF292	2.1152934	1.49E-06	1.64E-06
GJB7	NA	NA	NA
SMIM8	2.13016164	2.74E-14	3.20E-14
C6orf163	2.31601467	1.53E-104	2.69E-104
CFAP206	NA	NA	NA
RP3-382I10.7	NA	1.53E-28	1.94E-28
SLC35A1	1.75290522	1.29E-82	2.08E-82
RARS2	1.75803094	4.78E-296	1.84E-295
ORC3	1.77210213	0.01051112	0.01102334
AKIRIN2	1.02691216	0	0
SPACA1	NA	NA	NA
CNR1	NA	NA	NA
RP11-459O1.	NA	NA	NA
RNGTT	2.1314048	5.58E-10	6.33E-10
RP11-63L7.5	NA	NA	NA
PNRC1	1.13725452	0	0
SRSF12	1.88938039	2.69E-118	4.96E-118
PM20D2	2.62633036	2.20E-193	5.34E-193
GABRR1	NA	NA	NA
GABRR2	NA	NA	NA
UBE2J1	1.91756053	5.36E-133	1.04E-132

RRAGD	2.06377545	3.99E-124	7.50E-124
ANKRD6	2.40179299	0	0
RP11-16C18.	NA	0	0
LYRM2	1.78638341	4.18E-80	6.65E-80
MDN1	2.30648375	1.20E-06	1.32E-06
RP1-122O8.7	NA	NA	NA
CASP8AP2	1.69431162	2.39E-80	3.82E-80
GJA10	NA	NA	NA
RP11-63K6.7	NA	NA	NA
BACH2	NA	NA	NA
RP3-512E2.2	NA	NA	NA
RP1-45N11.1	NA	NA	NA
MAP3K7	1.59059628	1.07E-81	1.71E-81
RP1-177I10.1	NA	NA	NA
CASC6	NA	NA	NA
RP1-149C7.1	NA	NA	NA
RP11-127B1(	NA	NA	NA
RP1-23E21.2	NA	2.13E-26	2.67E-26
EPHA7	NA	NA	NA
RP11-524K14	NA	NA	NA
RP3-463P15.	NA	NA	NA
MANEA-AS1	1.71556642	1.55E-07	1.72E-07
MANEA	2.16896612	6.81E-141	1.36E-140
FUT9	NA	NA	NA
UFL1-AS1	NA	NA	NA
UFL1	1.52061569	2.97E-173	6.67E-173
FHL5	NA	NA	NA
RP3-417O22.	NA	NA	NA
GPR63	2.24069712	4.57E-131	8.82E-131
NDUFAF4	2.02938955	0.00034705	0.00037201
KLHL32	NA	NA	NA
MMS22L	2.2125501	1.19E-244	3.58E-244
RP11-436D23	NA	NA	NA
RP1-104O17.	NA	NA	NA
RP11-111D3.	NA	NA	NA
RP1-273N12.	NA	NA	NA
POU3F2	0.77200489	6.97E-48	9.74E-48
FBXL4	2.19411916	5.05E-146	1.03E-145
FAXC	2.57334963	7.52E-11	8.59E-11
COQ3	1.7503521	1.08E-148	2.22E-148
PNISR	1.58330344	0	0
RP11-98I9.4	3.08801956	0.00800839	0.00841373
USP45	1.63223891	3.73E-174	8.40E-174
TSTD3	1.95271825	2.04E-05	2.22E-05
CCNC	1.77576473	3.57E-205	9.05E-205

RP1-199J3.7	Inf	7.70E-25	9.59E-25
PRDM13	NA	NA	NA
MCHR2	NA	NA	NA
MCHR2-AS1	NA	NA	NA
SIM1	NA	NA	NA
RP1-121G13.	NA	NA	NA
ASCC3	1.85115151	4.17E-05	4.51E-05
RP3-467N11.	1.54400978	4.48E-10	5.09E-10
GRIK2	NA	NA	NA
HACE1	4.63202934	0	0
LINC00577	NA	2.56E-17	3.06E-17
LIN28B	2.33580967	2.81E-90	4.67E-90
BVES	Inf	5.65E-287	2.08E-286
BVES-AS1	NA	3.80E-111	6.85E-111
POPDC3	2.40469091	0	0
RP3-355L5.5	0	0	0
PREP	1.84285038	2.31E-14	2.71E-14
RP3-355L5.4	NA	NA	NA
RP3-359N14.	NA	NA	NA
PRDM1	0.77200489	1.99E-148	4.09E-148
ATG5	1.93577346	3.48E-120	6.47E-120
RP11-404H1	NA	NA	NA
AIM1	NA	NA	NA
RP3-335E1.1	NA	NA	NA
RTN4IP1	1.52825458	1.35E-203	3.39E-203
QRSL1	1.45823146	4.76E-273	1.63E-272
RP1-60O19.2	NA	0.01284922	0.01345593
RP1-60O19.1	NA	NA	NA
CD24	0.16542962	1.89E-46	2.63E-46
C6orf203	1.31905471	1.17E-259	3.78E-259
BEND3	1.0422066	0.00109162	0.00116188
PDSS2	2.22624666	0	0
SOBP	1.50112062	4.62E-24	5.72E-24
RP1-67A8.3	NA	NA	NA
SCML4	Inf	0	0
SEC63	1.86809374	2.59E-135	5.07E-135
RP1-191J18.	1.28667482	1.98E-16	2.36E-16
RP1-111B22.	Inf	0	0
OSTM1	1.77990016	2.85E-44	3.91E-44
OSTM1-AS1	NA	5.19E-40	6.97E-40
NR2E1	NA	NA	NA
SNX3	1.79466072	5.57E-141	1.11E-140
RP1-128O3.4	NA	NA	NA
LACE1	0.95581558	7.47E-252	2.32E-251
FOXO3	2.46738818	2.58E-38	3.43E-38

LINC00222	NA	NA	NA
ARMC2	1.93001222	4.59E-25	5.73E-25
ARMC2-AS1	NA	NA	NA
SESN1	1.43372337	3.98E-38	5.29E-38
RP11-787I22	NA	NA	NA
CEP57L1	1.60339477	4.33E-07	4.79E-07
CD164	1.58703956	0	0
RP11-425D1(	2.31601467	8.89E-52	1.26E-51
PPIL6	3.86002445	4.65E-16	5.51E-16
SMPD2	1.66968499	3.36E-285	1.22E-284
MICAL1	1.26328073	5.48E-127	1.04E-126
ZBTB24	1.70881981	6.57E-116	1.20E-115
RP5-919F19.	NA	NA	NA
AK9	1.06150672	3.13E-29	3.99E-29
FIG4	1.87486902	1.17E-41	1.58E-41
RP1-249I4.2	NA	3.29E-120	6.11E-120
GPR6	NA	NA	NA
WASF1	2.12301345	2.48E-13	2.89E-13
CDC40	1.67168751	3.47E-80	5.53E-80
METTL24	NA	NA	NA
DDO	NA	NA	NA
SLC22A16	NA	NA	NA
CTA-331P3.1	Inf	4.96E-47	6.90E-47
CDK19	1.72046804	2.81E-18	3.37E-18
AMD1	1.8245979	6.43E-23	7.92E-23
GTF3C6	2.0166283	2.98E-07	3.30E-07
RPF2	1.240532	0	0
SLC16A10	1.79062245	2.08E-157	4.41E-157
RP11-428F8.	NA	NA	NA
KIAA1919	2.57334963	8.54E-122	1.59E-121
RP5-1112D6.	3.474022	3.16E-221	8.52E-221
RP5-1112D6.	Inf	0	0
REV3L	1.73272209	0.00015375	0.00016543
RP5-1112D6.	1.15800733	2.37E-234	6.78E-234
TRAF3IP2-AS	1.88712306	0.00021897	0.00023529
TRAF3IP2	1.83351161	2.09E-260	6.78E-260
C6orf3	3.86002445	1.48E-218	3.95E-218
FYN	0.57900367	1.31E-150	2.72E-150
RP1-97J1.2	NA	NA	NA
WISP3	NA	NA	NA
TUBE1	1.85530207	5.77E-15	6.78E-15
FAM229B	3.18452017	0	0
LAMA4	NA	NA	NA
RP1-142L7.5	NA	NA	NA
RP1-142L7.9	NA	NA	NA

RP1-142L7.8	NA	NA	NA
RP11-506B6.	NA	NA	NA
RP11-506B6.	NA	NA	NA
RFPL4B	NA	NA	NA
RP1-105O18.	NA	NA	NA
RP11-437J19	NA	NA	NA
RP1-124C6.1	NA	NA	NA
RP11-346D1!	NA	NA	NA
RP11-367G1!	NA	NA	NA
RP1-249H1.2	NA	NA	NA
MARCKS	4.53133305	0	0
LINC01268	NA	NA	NA
RP11-544L8_	NA	NA	NA
HDAC2	1.25405489	0	0
RP3-399L15.:	Inf	0	0
HS3ST5	NA	NA	NA
RP3-399L15.:	NA	NA	NA
RP3-399L15.:	NA	NA	NA
RP11-282C5.	NA	NA	NA
FRK	NA	NA	NA
NT5DC1	1.52722706	1.76E-305	7.17E-305
COL10A1	NA	NA	NA
RP3-486I3.5	NA	NA	NA
TSPYL4	2.8306846	0	0
DSE	1.3896088	2.48E-19	2.99E-19
RP3-486I3.7	Inf	1.78E-258	5.70E-258
TSPYL1	2.01643068	3.73E-46	5.16E-46
RP1-93H18.1	NA	NA	NA
RP1-93H18.6	NA	NA	NA
FAM26F	NA	0	0
RP11-259P2(	NA	NA	NA
TRAPPC3L	NA	NA	NA
FAM26E	NA	NA	NA
FAM26D	NA	NA	NA
RWDD1	2.25343748	0	0
RSPH4A	1.54400978	4.01E-46	5.55E-46
ZUFSP	1.08563188	0	0
KPNA5	3.474022	1.40E-254	4.39E-254
FAM162B	NA	NA	NA
GPRC6A	NA	NA	NA
RFX6	NA	NA	NA
VGLL2	NA	NA	NA
ROS1	NA	NA	NA
RP1-179P9.3	NA	NA	NA
RP1-92C8.3	NA	NA	NA

DCBLD1	2.5572662	2.01E-29	2.56E-29
GOPC	2.12243732	4.43E-26	5.55E-26
NUS1	2.04687535	4.81E-120	8.93E-120
SLC35F1	NA	NA	NA
CEP85L	1.83810688	3.40E-255	1.07E-254
PLN	NA	0	0
MCM9	2.18175295	9.83E-112	1.77E-111
ASF1A	1.92863855	1.21E-08	1.35E-08
RP11-351A1	0	1.04E-113	1.89E-113
FAM184A	1.78154975	2.54E-255	8.03E-255
MAN1A1	2.00019449	8.85E-217	2.35E-216
TBC1D32	1.3896088	6.27E-61	9.28E-61
GJA1	0	0	0
HSF2	1.21895509	7.75E-220	2.08E-219
SERINC1	1.99227068	2.19E-262	7.15E-262
PKIB	0.30880196	0	0
FABP7	NA	NA	NA
SMPDL3A	1.43372337	3.13E-05	3.40E-05
CLVS2	NA	NA	NA
TRDN	NA	NA	NA
RP11-532N4	NA	NA	NA
NKAIN2	NA	5.84E-53	8.34E-53
RNF217-AS1	0	0	0
RNF217	2.81230353	2.01E-25	2.51E-25
TPD52L1	5.3325523	0	0
HDDC2	2.05274121	2.00E-218	5.35E-218
RP11-735G4	NA	NA	NA
RP11-138M1	NA	NA	NA
RP11-624M8	NA	NA	NA
RP1-293L8.2	NA	NA	NA
HEY2	NA	2.45E-157	5.20E-157
NCOA7	1.74007451	0.13163949	0.13541639
NCOA7-AS1	NA	NA	NA
HINT3	2.04029864	3.16E-37	4.18E-37
TRMT11	1.47472729	9.28E-223	2.52E-222
RP11-527F1	NA	1.37E-180	3.16E-180
CENPW	2.02958797	5.06E-06	5.53E-06
RSPO3	NA	NA	NA
RNF146	1.67267726	1.48E-64	2.23E-64
ECHDC1	NA	NA	NA
RP3-403A15.	NA	NA	NA
KIAA0408	NA	NA	NA
SOGA3	NA	NA	NA
C6orf58	NA	NA	NA
RP11-325O2	NA	NA	NA

THEMIS	NA	NA	NA
PTPRK	2.00721271	5.32E-26	6.67E-26
RP11-103C16	NA	NA	NA
RP1-86D1.5	NA	NA	NA
LAMA2	NA	1.48E-228	4.14E-228
RP1-69D17.3	NA	NA	NA
RP1-69D17.4	NA	NA	NA
ARHGAP18	3.08801956	8.17E-07	9.01E-07
TMEM244	NA	NA	NA
L3MBTL3	1.58942183	8.54E-67	1.29E-66
RP11-73O6.3	NA	NA	NA
SAMD3	NA	NA	NA
TMEM200A	0	1.30E-298	5.08E-298
RP11-102N1	NA	NA	NA
SMLR1	NA	NA	NA
EPB41L2	1.81627936	1.25E-105	2.21E-105
AKAP7	2.31601467	1.27E-49	1.78E-49
ARG1	NA	NA	NA
MED23	2.63768337	1.15E-206	2.92E-206
ENPP3	Inf	4.17E-189	9.94E-189
OR2A4	NA	NA	NA
CTAGE9	NA	NA	NA
ENPP1	Inf	0	0
RP11-69I8.2	NA	NA	NA
CTGF	Inf	0	0
RP11-69I8.3	NA	NA	NA
RP3-523C21.	NA	NA	NA
LINC01013	NA	NA	NA
MOXD1	NA	NA	NA
STX7	1.75697665	4.07E-62	6.05E-62
TAAR9	NA	NA	NA
TAAR8	NA	NA	NA
TAAR6	NA	NA	NA
TAAR5	NA	NA	NA
TAAR2	NA	NA	NA
TAAR1	NA	NA	NA
VNN1	0.77200489	3.84E-146	7.82E-146
VNN3	0	7.44E-259	2.39E-258
VNN2	0.48250306	0	0
RP1-55C23.7	NA	NA	NA
SLC18B1	2.07222365	0.00636389	0.00669685
RPS12	2.59866932	0	0
LINC00326	NA	NA	NA
EYA4	NA	NA	NA
RP1-283K11.	NA	NA	NA

RP1-283K11.	NA	NA	NA
TARID	NA	NA	NA
RP11-704J17	NA	NA	NA
LINC01312	NA	NA	NA
TCF21	NA	NA	NA
TBPL1	2.11852505	1.42E-73	2.20E-73
SLC2A12	NA	NA	NA
SGK1	Inf	0	0
RP11-557H1!	NA	NA	NA
LINC01010	NA	NA	NA
RP11-557H1!	NA	2.87E-97	4.89E-97
RP11-557H1!	Inf	8.32E-205	2.11E-204
CTA-31J9.2	NA	NA	NA
RP1-287H17.	NA	NA	NA
ALDH8A1	Inf	3.63E-32	4.69E-32
RP11-349J5.:	NA	NA	NA
HBS1L	1.40470063	0	0
CTA-212D2.2	Inf	1.70E-265	5.65E-265
MYB	2.10565911	1.93E-93	3.25E-93
MYB-AS1	NA	NA	NA
RP1-32B1.4	NA	NA	NA
AHI1	2.13105517	1.66E-34	2.17E-34
RP3-388E23.	0.51466993	0	0
LINC00271	0	0	0
RP1-38C16.2	NA	NA	NA
RP11-394G3.	NA	NA	NA
PDE7B	NA	NA	NA
RP13-143G1!	NA	NA	NA
MTFR2	1.13317677	0	0
BCLAF1	1.88627796	6.71E-79	1.06E-78
RP3-406A7.7	Inf	8.44E-215	2.22E-214
MAP7	2.19319571	7.87E-13	9.11E-13
RP3-325F22.	6.69070905	0	0
MAP3K5	2.98508557	2.05E-12	2.37E-12
RP3-325F22.	NA	NA	NA
PEX7	1.54400978	8.67E-212	2.26E-211
SLC35D3	Inf	2.34E-264	7.73E-264
NHEG1	NA	NA	NA
RP11-204P2.	NA	NA	NA
IL20RA	NA	NA	NA
IL22RA2	NA	NA	NA
IFNGR1	1.35100856	2.60E-204	6.57E-204
OLIG3	NA	NA	NA
RP11-95M15	NA	NA	NA
RP11-356I2.1	NA	NA	NA

RP11-356I2.4	0.77200489	7.17E-22	8.77E-22
TNFAIP3	1.54400978	2.85E-116	5.21E-116
RP11-10J5.1	NA	NA	NA
RP11-240M1	NA	NA	NA
PERP	0.77200489	2.39E-100	4.13E-100
ARFGEF3	0.77200489	4.93E-118	9.08E-118
PBOV1	Inf	0	0
RP3-422G23.	NA	NA	NA
HEBP2	2.20678262	0	0
NHSL1	1.93001222	8.50E-27	1.07E-26
RP11-390P2.	2.01717407	9.71E-05	0.00010471
GVQW2	1.02933985	9.56E-99	1.64E-98
CCDC28A	2.3835651	5.03E-134	9.79E-134
ECT2L	NA	NA	NA
REPS1	2.07278025	0.52953059	0.53549297
ABRACL	2.27045776	0	0
HECA	1.82473883	4.96E-59	7.28E-59
RP1-225E12.	NA	NA	NA
TXLNB	1.23520782	2.71E-126	5.12E-126
RP11-445F6.	NA	NA	NA
CITED2	1.23979967	0	0
LINC01625	NA	NA	NA
RP5-899B16.	0	0	0
RP5-899B16.	NA	NA	NA
RP3-332B22.	NA	NA	NA
RP3-460G2.2	2.67610176	0	0
RP11-63E9.1	NA	NA	NA
NMBR	NA	NA	NA
RP11-137J7.2	NA	NA	NA
GJE1	NA	NA	NA
VTA1	1.47967604	5.87E-244	1.76E-243
RP11-753B14	NA	NA	NA
ADGRG6	NA	NA	NA
RP11-440G9.	NA	NA	NA
RP1-67K17.3	NA	NA	NA
HIVEP2	4.63202934	1.09E-05	1.19E-05
RP1-67K17.4	NA	NA	NA
RP11-439L18	NA	NA	NA
RP11-439L18	NA	NA	NA
LINC01277	NA	NA	NA
RP1-45I4.3	NA	NA	NA
AIG1	2.19724469	6.40E-58	9.36E-58
RP1-45I4.2	NA	NA	NA
ADAT2	1.98036037	0.1272178	0.13094952
PEX3	1.04324985	0	0

FUCA2	0.77200489	0	0
PHACTR2	1.95271825	1.35E-97	2.31E-97
PHACTR2-AS	NA	1.80E-78	2.84E-78
RP3-468K18.	NA	NA	NA
LTV1	1.14665432	0	0
RP3-468K18.	NA	NA	NA
ZC2HC1B	NA	NA	NA
PLAGL1	1.32956398	2.30E-43	3.15E-43
SF3B5	2.23930822	1.33E-297	5.19E-297
STX11	0.96500611	1.17E-113	2.13E-113
UTRN	1.44750917	3.11E-142	6.23E-142
RP1-91J24.3	NA	NA	NA
EPM2A	2.57334963	0.21786372	0.22273788
RP3-466P17.	0.51466993	1.08E-114	1.97E-114
RP3-466P17.	NA	NA	NA
AL023806.1	NA	1.53E-141	3.06E-141
FBXO30	2.00019449	3.57E-14	4.18E-14
RP11-545I5.3	1.15800733	1.23E-06	1.36E-06
SHPRH	2.63669362	1.20E-287	4.42E-287
GRM1	NA	NA	NA
RAB32	0	0	0
RP11-715G15	NA	NA	NA
ADGB	NA	NA	NA
STXBP5-AS1	NA	NA	NA
RP11-497D6.	NA	NA	NA
RP11-497D6.	NA	NA	NA
LUADT1	NA	NA	NA
STXBP5	2.99151895	4.96E-238	1.44E-237
SAMD5	NA	NA	NA
RP11-307P5.	NA	NA	NA
RP11-307P5.	NA	NA	NA
RP11-242F11	NA	NA	NA
RP11-631F7.	NA	NA	NA
RP11-631F7.	NA	NA	NA
SASH1	0	0	0
UST	2.31601467	4.04E-258	1.29E-257
UST-AS1	NA	NA	NA
RP11-162J8.3	NA	NA	NA
RP1-111D6.3	NA	NA	NA
TAB2	2.04207745	7.52E-19	9.05E-19
RP1-111D6.2	NA	NA	NA
SUMO4	1.54400978	7.62E-09	8.55E-09
ZC3H12D	3.86002445	0	0
PPIL4	1.197167	0	0
RP1-12G14.5	NA	NA	NA

GINM1	2.26673776	3.55E-84	5.76E-84
RP1-12G14.9	NA	NA	NA
RP1-12G14.7	1.54400978	5.46E-25	6.80E-25
KATNA1	1.68071898	0	0
LATS1	2.44468215	0.00079066	0.00084328
AL358852.1	4.63202934	1.07E-43	1.46E-43
NUP43	1.77644136	6.60E-09	7.41E-09
PCMT1	1.53518687	5.09E-207	1.30E-206
RP11-350J20	0.77200489	3.17E-05	3.44E-05
LRP11	2.62481663	5.16E-178	1.18E-177
RP11-350J20	NA	NA	NA
RAET1E-AS1	NA	NA	NA
RAET1E	Inf	6.77E-31	8.70E-31
RAET1E-AS1	NA	NA	NA
RAET1G	0.25733496	4.56E-307	1.87E-306
ULBP2	1.06150672	2.44E-250	7.52E-250
ULBP1	3.08801956	1.68E-128	3.21E-128
RAET1L	NA	NA	NA
RP11-472G2	NA	NA	NA
ULBP3	Inf	0	0
PPP1R14C	1.61684043	2.78E-10	3.16E-10
IYD	Inf	1.08E-293	4.13E-293
PLEKHG1	NA	NA	NA
RP11-136K1	NA	NA	NA
MTHFD1L	2.19987234	0.44281436	0.44892641
RP1-292B18.	Inf	1.91E-257	6.09E-257
RP1-292B18.	NA	NA	NA
RP1-297M16	NA	NA	NA
AKAP12	1.93001222	2.24E-28	2.84E-28
ZBTB2	2.76377751	6.99E-76	1.10E-75
RMND1	2.49416964	0	0
ARMT1	1.42355512	4.14E-26	5.19E-26
CCDC170	2.62481663	1.04E-16	1.23E-16
ESR1	NA	NA	NA
RP3-443C4.2	NA	NA	NA
SYNE1	Inf	0	0
SYNE1-AS1	NA	NA	NA
RP3-398G3.5	NA	NA	NA
NANOGP11	NA	NA	NA
MYCT1	NA	NA	NA
VIP	NA	NA	NA
RP1-200K18.	NA	NA	NA
FBXO5	1.76205696	1.50E-14	1.75E-14
RP1-101K10.	NA	1.48E-42	2.02E-42
MTRF1L	1.65305002	9.69E-66	1.46E-65

RGS17	1.77561125	3.78E-128	7.19E-128
RP11-306O1:	NA	7.45E-260	2.40E-259
RP11-475C16	NA	NA	NA
OPRM1	NA	5.57E-117	1.02E-116
IPCEF1	1.15800733	4.35E-86	7.10E-86
CNKSR3	2.23023635	1.64E-05	1.79E-05
SCAF8	1.68437431	0.00026968	0.00028936
TIAM2	3.60268949	1.48E-105	2.61E-105
RP11-477D19	4.11735941	6.27E-126	1.19E-125
TFB1M	1.32664918	0	0
CLDN20	NA	0.64613888	0.65134475
RP1-257I9.2	NA	NA	NA
NOX3	NA	NA	NA
RP11-518I13	NA	NA	NA
RP11-230C9.	0	0	0
RP11-230C9.	Inf	0	0
ARID1B	2.54307493	1.71E-174	3.85E-174
RP11-230C9.	NA	NA	NA
RP1-137K2.2	NA	NA	NA
TMEM242	2.08105666	2.96E-05	3.21E-05
RP5-933K21.	NA	7.93E-177	1.80E-176
ZDHHC14	1.97290139	1.96E-68	2.99E-68
SNX9	1.25064792	1.01E-243	3.03E-243
RP11-52J3.3	NA	NA	NA
RP11-52J3.2	NA	NA	NA
RP3-403L10.:	NA	NA	NA
SYNJ2	2.19411916	7.94E-175	1.79E-174
SERAC1	2.21308068	1.10E-18	1.33E-18
GTF2H5	2.14055901	1.05E-211	2.73E-211
TULP4	1.77561125	6.09E-116	1.11E-115
RP11-732M1	NA	NA	NA
TMEM181	2.4582261	0	0
DYNLT1	1.70255739	1.82E-68	2.78E-68
SYTL3	3.08801956	1.80E-10	2.05E-10
EZR	1.88260842	0	0
EZR-AS1	NA	NA	NA
C6orf99	3.08801956	0	0
RSPH3	2.41894866	1.71E-06	1.88E-06
RP1-111C20.	NA	3.66E-160	7.84E-160
TAGAP	1.77107004	2.29E-16	2.71E-16
RP1-111C20.	NA	NA	NA
RP11-13P5.1	NA	NA	NA
RP11-13P5.2	NA	NA	NA
FNDC1	NA	NA	NA
RP11-125D1:	NA	NA	NA

RP11-125D1:	NA	NA	NA
RP3-393E18.	NA	2.16E-290	8.09E-290
SOD2	1.62199007	0	0
WTAP	1.75792232	1.28E-126	2.42E-126
ACAT2	0.89449184	0	0
TCP1	1.60512892	0	0
MRPL18	2.2008036	1.94E-98	3.33E-98
PNLDC1	1.54400978	1.85E-27	2.33E-27
RP1-249F5.3	NA	NA	NA
MAS1	0.77200489	1.65E-147	3.37E-147
IGF2R	2.11166043	1.24E-21	1.52E-21
AIRN	NA	NA	NA
SLC22A1	NA	0	0
SLC22A2	NA	NA	NA
RP1-276N6.2	NA	NA	NA
SLC22A3	NA	NA	NA
LPA	NA	NA	NA
RP1-81D8.3	NA	NA	NA
PLG	NA	NA	NA
RP11-235G2	NA	NA	NA
RP11-235G2	NA	NA	NA
RP3-428L16.	NA	NA	NA
RP3-428L16.	2.54761614	7.73E-47	1.08E-46
MAP3K4	1.737011	8.56E-40	1.15E-39
AGPAT4	2.21951406	8.07E-117	1.48E-116
PARK2	0.77200489	0	0
PACRG	0.77200489	0	0
PACRG-AS2	NA	NA	NA
PACRG-AS3	NA	NA	NA
PACRG-AS1	NA	NA	NA
CAHM	2.20572826	1.99E-52	2.83E-52
QKI	2.12706242	8.14E-240	2.39E-239
RP5-826L7.1	NA	NA	NA
RP1-230L10.	NA	NA	NA
RP1-155D22.	NA	NA	NA
RP1-155D22.	NA	NA	NA
RP11-347L18	NA	NA	NA
RP11-300M2	NA	NA	NA
C6orf118	NA	NA	NA
PDE10A	NA	NA	NA
RP11-252P19	NA	NA	NA
LINC00473	NA	NA	NA
LINC00602	NA	NA	NA
RP11-459F1.	NA	NA	NA
T	NA	NA	NA

RP4-655C5.4	NA	NA	NA
RP4-655C5.9	NA	NA	NA
PRR18	Inf	3.13E-125	5.89E-125
SFT2D1	1.87405208	1.17E-19	1.42E-19
MPC1	2.05824794	0.00182682	0.00193825
RP1-168L15.!	1.93001222	0.00014153	0.00015236
RPS6KA2	1.54400978	2.41E-106	4.27E-106
RP11-514O1:	1.64051039	1.25E-18	1.51E-18
RPS6KA2-AS	NA	NA	NA
RNASET2	1.79381105	4.39E-223	1.20E-222
RP1-167A14.	Inf	1.06E-09	1.20E-09
FGFR1OP	1.62251875	1.19E-262	3.88E-262
CCR6	Inf	0	0
TCP10L2	NA	NA	NA
GPR31	NA	NA	NA
RP11-568A7.	NA	NA	NA
RP11-568A7.	NA	NA	NA
RP11-568A7.	NA	NA	NA
UNC93A	NA	NA	NA
TTLL2	NA	NA	NA
TCP10	NA	NA	NA
RP11-351J23	NA	NA	NA
RP11-351J23	NA	NA	NA
LINC01558	NA	NA	NA
RP3-431P23.	NA	NA	NA
MLLT4-AS1	1.15800733	2.23E-289	8.30E-289
MLLT4	2.31601467	1.91E-39	2.56E-39
RP3-470B24.	NA	NA	NA
KIF25-AS1	NA	NA	NA
KIF25	NA	2.03E-46	2.82E-46
FRMD1	NA	NA	NA
RP11-503C2	NA	NA	NA
RP11-503C2	NA	NA	NA
RP11-503C2	NA	NA	NA
RP11-503C2	NA	NA	NA
DACT2	NA	NA	NA
RP1-39J2.1	NA	NA	NA
SMOC2	NA	NA	NA
RP1-125N5.2	NA	NA	NA
RP3-495K2.3	NA	NA	NA
RP3-495K2.2	NA	NA	NA
RP3-495K2.1	NA	NA	NA
LINC01615	NA	NA	NA
RP11-417E7.	NA	NA	NA
XXyac-YX65C	NA	NA	NA

THBS2	NA	NA	NA
XXyac-YX65C	NA	NA	NA
RP1-137D17.	NA	NA	NA
RP1-137D17.	NA	1.02E-179	2.34E-179
WDR27	2.21951406	1.02E-185	2.41E-185
C6orf120	1.8725225	9.50E-74	1.48E-73
PHF10	2.31601467	1.77E-81	2.84E-81
RP1-266L20.:	0.77200489	3.78E-145	7.65E-145
RP1-266L20.4	NA	NA	NA
TCTE3	1.47967604	3.37E-42	4.59E-42
ERMARD	1.61419204	1.64E-98	2.81E-98
LINC00242	NA	NA	NA
LINC00574	NA	0	0
RP1-182D15.	NA	NA	NA
RP11-302L19	NA	NA	NA
RP11-302L19	NA	NA	NA
RP5-1086L22	NA	NA	NA
RP5-894D12.	NA	NA	NA
LINC01624	NA	NA	NA
DLL1	0.96500611	2.64E-259	8.50E-259
FAM120B	1.72782047	1.91E-120	3.54E-120
RP5-894D12.	NA	9.67E-52	1.37E-51
PSMB1	1.55365553	0	0
TBP	1.93634013	4.16E-08	4.64E-08
PDCD2	1.96119174	2.20E-55	3.17E-55
XX-C2158C12	NA	NA	NA
XX-C2158C12	1.59547677	1.24E-10	1.41E-10
XX-C2158C6.:	NA	NA	NA
XX-C2158C6.:	NA	NA	NA
ABC7-48172:	NA	NA	NA
AC093627.7	NA	NA	NA
AC093627.8	NA	NA	NA
AC093627.9	1.80134474	1.19E-07	1.32E-07
AC093627.1C	NA	2.84E-25	3.54E-25
RP11-90P13.	NA	NA	NA
AC093627.11	NA	NA	NA
AC093627.12	NA	NA	NA
FAM20C	0.30880196	0	0
WI2-2373I1.:	NA	NA	NA
AC226118.1	NA	NA	NA
AC147651.1	NA	NA	NA
PDGFA	1.02933985	3.10E-24	3.84E-24
HRAT92	Inf	2.37E-26	2.97E-26
PRKAR1B	1.78418908	9.74E-104	1.70E-103
AC147651.5	NA	NA	NA

AC147651.4	2.48757131	0.00150284	0.00159685
DNAAF5	2.19640828	3.06E-195	7.47E-195
SUN1	2.26183889	7.17E-63	1.07E-62
GET4	0	0	0
RP11-449P15	1.54400978	4.91E-236	1.42E-235
ADAP1	1.46680929	1.93E-217	5.15E-217
COX19	1.84520866	0.0654556	0.06776395
CYP2W1	NA	NA	NA
C7orf50	2.07785718	4.57E-175	1.03E-174
AC073957.15	NA	NA	NA
GPR146	1.93942692	6.31E-21	7.70E-21
RP11-449P15	0.38600244	0	0
AC091729.7	NA	NA	NA
AC091729.8	NA	NA	NA
GPER1	Inf	0	0
ZFAND2A	1.65429619	0.05398203	0.0559656
AC091729.9	1.72216475	1.07E-200	2.65E-200
UNCX	0.38600244	0	0
AC073094.4	NA	NA	NA
MICALL2	2.00721271	9.87E-29	1.25E-28
AC102953.4	NA	NA	NA
RP11-1246C1	2.21951406	0.72330597	0.72779906
INTS1	2.57713397	3.02E-170	6.72E-170
AC102953.6	NA	NA	NA
MAFK	2.41759426	0.00927066	0.00973087
TMEM184A	NA	0	0
PSMG3	2.04073198	5.80E-124	1.09E-123
PSMG3-AS1	2.23329986	8.45E-168	1.86E-167
AC074389.5	NA	NA	NA
ELFN1	1.32343695	1.84E-103	3.21E-103
AC074389.7	NA	NA	NA
AC074389.6	NA	NA	NA
ELFN1-AS1	2.50129584	2.61E-306	1.07E-305
MAD1L1	3.04909494	0	0
AC110781.3	1.54400978	1.46E-08	1.63E-08
FTSJ2	1.92663611	0.00066446	0.00070943
NUDT1	1.9583094	0.55972085	0.56564481
SNX8	1.49859773	4.12E-135	8.04E-135
EIF3B	1.7749625	6.17E-293	2.34E-292
AC004840.8	NA	NA	NA
CHST12	1.17921626	0	0
GRIFIN	NA	NA	NA
LFNG	1.93001222	1.19E-38	1.59E-38
BRAT1	1.48527028	1.84E-22	2.26E-22
IQCE	2.95935208	0	0

TTYH3	3.51691117	0	0
AMZ1	NA	NA	NA
GNA12	2.19853566	1.86E-41	2.52E-41
CARD11	2.31601467	5.78E-36	7.62E-36
AC004906.3	NA	NA	NA
AC024028.1	NA	NA	NA
AC091801.1	NA	NA	NA
AC073316.2	NA	NA	NA
AC073316.1	NA	NA	NA
SDK1	NA	NA	NA
AC011284.3	NA	NA	NA
FOXK1	1.65236134	4.16E-30	5.32E-30
AP5Z1	1.76458261	2.97E-15	3.50E-15
RADIL	2.16161369	4.38E-79	6.95E-79
PAPOLB	NA	9.78E-168	2.16E-167
MMD2	NA	NA	NA
RBAK-RBAKI	3.02674933	5.34E-250	1.64E-249
RBAK	2.42128806	4.49E-250	1.38E-249
RBAKDN	NA	NA	NA
WIPI2	1.74914251	0	0
SLC29A4	1.61638524	0.0021583	0.00228821
TNRC18	3.52916521	1.13E-34	1.48E-34
AC093620.5	NA	NA	NA
AC092171.2	NA	3.40E-68	5.18E-68
RP11-1275H:	0.64333741	9.79E-25	1.22E-24
RP11-1275H:	2.42630108	1.11E-68	1.69E-68
RP11-1275H:	0.22057283	8.00E-296	3.08E-295
FBXL18	1.75455657	1.00E-31	1.29E-31
AC092171.4	NA	NA	NA
ACTB	1.0208024	0	0
CTB-161C1.1	NA	9.42E-132	1.82E-131
FSCN1	1.47242035	0	0
RNF216	1.93315046	0.86618352	0.86848134
OCM	NA	0	0
CCZ1	1.73296203	7.79E-11	8.90E-11
RSPH10B	0	0	0
PMS2	2.0139258	3.96E-139	7.87E-139
AIMP2	1.46680929	5.54E-164	1.21E-163
EIF2AK1	1.90791781	0	0
ANKRD61	Inf	6.28E-238	1.83E-237
AC004895.4	NA	4.42E-83	7.14E-83
USP42	2.76485472	1.11E-180	2.57E-180
CYTH3	3.60268949	5.33E-06	5.82E-06
FAM220A	2.66692598	1.47E-237	4.27E-237
RAC1	1.80684122	5.94E-18	7.12E-18

DAGLB	2.03357386	0.90062141	0.90246195
KDELR2	1.74367936	7.46E-149	1.53E-148
GRID2IP	NA	NA	NA
ZDHHC4	1.78296367	3.46E-242	1.03E-241
AC079742.4	NA	1.83E-20	2.22E-20
C7orf26	1.44107579	7.57E-249	2.32E-248
ZNF853	NA	0.08629104	0.08910028
ZNF316	1.65123268	9.63E-10	1.09E-09
AC073343.13	NA	NA	NA
ZNF12	1.68697365	0	0
RSPH10B2	NA	NA	NA
CCZ1B	1.50891865	1.41E-89	2.33E-89
C1GALT1	2.35192187	0.00485384	0.00511908
COL28A1	NA	NA	NA
RP5-1159O4.	NA	0	0
RP5-1159O4.	1.02933985	8.68E-14	1.01E-13
MIOS	1.82608849	3.68E-205	9.32E-205
RP5-1007H1(	Inf	0	0
RPA3	2.09193085	2.16E-61	3.20E-61
UMAD1	1.82931593	1.79E-58	2.61E-58
AC007161.5	0	0	0
AC009473.1	1.54400978	7.72E-14	9.01E-14
AC006042.7	NA	NA	NA
GLCCI1	2.62481663	5.95E-40	7.99E-40
ICA1	1.4054448	0.13765595	0.14151721
AC006042.6	NA	NA	NA
AC007009.1	NA	NA	NA
AC007128.1	NA	3.93E-151	8.16E-151
RP4-594A5.1	NA	NA	NA
NXPH1	NA	NA	NA
RP4-668E10.	NA	NA	NA
AC060834.3	NA	NA	NA
AC006373.1	NA	NA	NA
CTD-2060L22	0	0	0
AC004535.2	NA	NA	NA
NDUFA4	2.20418564	2.33E-277	8.16E-277
RP5-855F16.	Inf	5.81E-82	9.33E-82
PHF14	1.51320659	1.12E-253	3.51E-253
THSD7A	0.77200489	1.58E-145	3.20E-145
AC004160.4	NA	NA	NA
TMEM106B	1.82278932	2.90E-47	4.05E-47
VWDE	2.64687391	1.59E-56	2.30E-56
AC005281.1	NA	2.93E-14	3.43E-14
AC005281.2	NA	NA	NA
SCIN	NA	NA	NA

ARL4A	1.7429106	3.19E-299	1.25E-298
AC011288.2	NA	NA	NA
AC006000.5	NA	NA	NA
AC005019.3	NA	NA	NA
ETV1	0.8822913	7.13E-67	1.08E-66
DGKB	NA	1.99E-76	3.12E-76
AC006150.1	NA	NA	NA
AC006458.3	NA	NA	NA
AGMO	NA	NA	NA
MEOX2	NA	NA	NA
AC005550.3	NA	NA	NA
MEOX2-AS1	NA	NA	NA
AC005550.5	NA	NA	NA
AC006041.1	NA	NA	NA
ISPD	1.47967604	1.05E-66	1.60E-66
ISPD-AS1	NA	NA	NA
SOSTDC1	NA	NA	NA
GS1-166A23.	NA	NA	NA
LRRC72	NA	NA	NA
GS1-166A23.	Inf	0	0
ANKMY2	1.19109326	6.98E-144	1.41E-143
BZW2	1.18955561	0	0
AC073333.8	NA	1.11E-57	1.63E-57
TSPAN13	1.84516339	7.89E-66	1.19E-65
AC073333.1	NA	NA	NA
AGR2	0	0	0
AGR3	0	0	0
AC003075.4	2.75716032	1.45E-130	2.79E-130
AHR	4.4604727	1.37E-232	3.89E-232
AC019117.1	NA	NA	NA
AC019117.2	NA	NA	NA
AC017060.1	NA	NA	NA
AC006482.1	NA	NA	NA
SNX13	3.20991507	4.56E-130	8.75E-130
PRPS1L1	NA	NA	NA
HDAC9	NA	NA	NA
AC010082.2	NA	NA	NA
AC004744.3	NA	NA	NA
TWIST1	1.73116248	1.09E-20	1.33E-20
AC003986.6	NA	NA	NA
AC003986.7	NA	NA	NA
AC003986.5	NA	NA	NA
FERD3L	NA	NA	NA
AC007091.1	NA	NA	NA
TWISTNB	1.51184291	2.07E-248	6.32E-248

TMEM196	NA	NA	NA
AC004543.2	NA	NA	NA
RP11-486P11	NA	NA	NA
MACC1	Inf	0.04129019	0.04288337
MACC1-AS1	NA	NA	NA
AC099342.1	NA	NA	NA
CTA-293F17.	NA	4.75E-282	1.70E-281
ITGB8	NA	1.26E-83	2.04E-83
ABCB5	NA	NA	NA
SP8	NA	0.00193735	0.00205504
LINC01162	NA	NA	NA
SP4	1.77107004	1.28E-05	1.39E-05
DNAH11	Inf	0	0
CDCA7L	1.74323685	3.13E-290	1.17E-289
RAPGEF5	NA	NA	NA
STEAP1B	NA	NA	NA
AC002480.3	NA	NA	NA
AC002480.4	NA	NA	NA
AC002480.2	NA	NA	NA
AC073072.5	NA	NA	NA
IL6	NA	NA	NA
AC073072.1	NA	NA	NA
TOMM7	2.62572269	0	0
FAM126A	1.67560152	1.05E-278	3.69E-278
KLHL7-AS1	1.28667482	2.39E-52	3.40E-52
KLHL7	2.01717407	4.68E-91	7.80E-91
NUPL2	1.30021876	0	0
AC005082.12	NA	0	0
GPNMB	Inf	0	0
MALSU1	1.95834268	0.05747475	0.05956623
IGF2BP3	1.51487752	3.36E-266	1.12E-265
TRA2A	1.53056022	0	0
CCDC126	3.02368582	1.01E-96	1.71E-96
AC006026.2	NA	NA	NA
FAM221A	NA	0	0
AC006026.13	NA	NA	NA
STK31	NA	NA	NA
AC009508.1	NA	NA	NA
AC004485.3	NA	NA	NA
NPY	NA	NA	NA
MPP6	2.0414089	1.52E-37	2.01E-37
DFNA5	Inf	1.26E-187	2.98E-187
OSBPL3	2.16161369	1.37E-12	1.59E-12
CYCS	2.05225068	1.25E-46	1.74E-46
C7orf31	1.59226009	3.22E-132	6.22E-132

NPVF	NA	NA	NA
AC003985.1	NA	NA	NA
RP5-978I12.1	NA	NA	NA
AC091705.1	NA	NA	NA
AC003090.1	NA	NA	NA
CTD-2227E11	4.11735941	7.43E-178	1.70E-177
NFE2L3	1.24708482	1.41E-146	2.88E-146
CTB-119C2.1	Inf	4.92E-25	6.14E-25
HNRNPA2B1	1.56222358	0	0
CBX3	2.48689322	0	0
SNX10	2.48144429	0	0
AC004540.4	7.7200489	0	0
AC004540.5	2.70201711	5.54E-153	1.16E-152
KIAA0087	NA	NA	NA
AC004947.2	NA	NA	NA
C7orf71	NA	NA	NA
SKAP2	1.57109767	8.16E-255	2.57E-254
HOXA1	1.98515543	2.09E-64	3.14E-64
HOTAIRM1	2.22742394	4.01E-59	5.89E-59
HOXA2	Inf	2.85E-203	7.16E-203
HOXA3	1.54400978	3.76E-22	4.61E-22
HOXA-AS2	NA	NA	NA
HOXA4	Inf	2.89E-86	4.71E-86
HOXA-AS3	2.31601467	0.19575486	0.20054123
HOXA5	1.40364525	3.79E-11	4.34E-11
HOXA6	5.79003667	0	0
HOXA7	1.72685304	0.57817814	0.58384213
HOXA9	1.82068205	2.36E-89	3.90E-89
HOXA10-HOX	NA	NA	NA
HOXA10-AS	3.08801956	0	0
HOXA10	2.20500743	1.28E-70	1.97E-70
HOXA11	1.87486902	0.0317348	0.03303493
HOXA11-AS	1.52997333	0	0
RP1-170O19.	NA	NA	NA
HOXA13	1.51431728	0.84661892	0.84942819
HOTTIP	1.93001222	7.69E-96	1.30E-95
RP1-170O19.	NA	NA	NA
EVX1-AS	NA	NA	NA
EVX1	NA	NA	NA
AC004009.3	NA	NA	NA
HIBADH	1.54400978	0	0
AC004549.6	Inf	0	0
TAX1BP1	1.69841076	2.23E-177	5.08E-177
JAZF1	2.23881418	2.67E-44	3.67E-44
JAZF1-AS1	Inf	4.76E-202	1.19E-201

CREB5	NA	NA	NA
TRIL	NA	NA	NA
AC005013.5	NA	NA	NA
CTB-113D17.	NA	6.33E-45	8.72E-45
AC005162.5	NA	NA	NA
CPVL	NA	0	0
AC005162.4	NA	NA	NA
CHN2	Inf	1.83E-278	6.45E-278
AC004593.3	NA	NA	NA
AC007255.8	NA	NA	NA
PRR15	0	5.84E-176	1.32E-175
WIPF3	0	0	0
SCRN1	2.00450392	1.03E-21	1.26E-21
AC007285.6	NA	NA	NA
FKBP14	1.95271825	0	0
PLEKHA8	3.37244241	0	0
AC007285.7	NA	NA	NA
MTURN	2.97773315	0	0
AC007036.6	NA	NA	NA
ZNRF2	2.31601467	9.10E-05	9.82E-05
GS1-114I9.3	NA	NA	NA
LINC01176	Inf	0.00078171	0.00083388
NOD1	2.31601467	4.75E-297	1.84E-296
GS1-114I9.1	NA	2.37E-104	4.15E-104
GGCT	1.97264066	1.24E-45	1.72E-45
RP4-777O23.	NA	5.39E-211	1.40E-210
RP4-777O23.	NA	NA	NA
RP4-777O23.	NA	NA	NA
GARS	1.89876441	1.03E-92	1.72E-92
CRHR2	NA	NA	NA
INMT	NA	NA	NA
INMT-FAM18	NA	NA	NA
FAM188B	NA	NA	NA
AC004691.5	NA	NA	NA
RP5-877J2.1	NA	NA	NA
AQP1	NA	NA	NA
GHRHR	NA	NA	NA
ADCYAP1R1	NA	NA	NA
NEUROD6	NA	NA	NA
AC006380.3	NA	NA	NA
CCDC129	NA	NA	NA
PPP1R17	NA	NA	NA
PDE1C	NA	NA	NA
LSM5	2.32285447	0	0
AVL9	1.98299295	2.21E-05	2.40E-05

RP11-225B1	2.31601467	0	0
LINC00997	0.38600244	1.13E-156	2.38E-156
RP11-265E1	NA	NA	NA
KBTBD2	1.59381655	8.62E-34	1.12E-33
FKBP9	1.4972216	3.77E-203	9.48E-203
NT5C3A	1.48205877	0	0
RP9	2.0311081	8.06E-51	1.14E-50
BBS9	4.37469438	0	0
RP11-89N17	NA	NA	NA
RP11-89N17	NA	NA	NA
RP11-89N17	NA	NA	NA
RP11-89N17	NA	NA	NA
BMPER	NA	NA	NA
AC009262.2	NA	NA	NA
NPSR1	NA	NA	NA
DPY19L1	1.90427873	2.27E-130	4.36E-130
TBX20	Inf	7.81E-170	1.73E-169
AC009531.2	NA	NA	NA
AC007652.1	NA	NA	NA
HERPUD2	2.47977328	5.11E-07	5.64E-07
RP11-379H1	1.44107579	1.68E-13	1.96E-13
AC018647.3	NA	NA	NA
SEPT7-AS1	NA	6.80E-250	2.09E-249
7-Sep	1.63620066	1.01E-260	3.29E-260
AC083864.3	NA	NA	NA
AC083864.4	NA	NA	NA
EEPD1	0.96500611	4.87E-150	1.01E-149
AC007327.5	NA	NA	NA
RP11-182J23	NA	1.13E-26	1.42E-26
KIAA0895	9.52139364	0	0
ANLN	1.68622121	1.07E-113	1.95E-113
AC006960.7	NA	NA	NA
AOAH	NA	9.39E-60	1.38E-59
RP11-85E16.	NA	0.01284922	0.01345593
AC007349.4	NA	NA	NA
AC007349.5	NA	NA	NA
AC007349.7	NA	NA	NA
ELMO1	2.55905325	1.75E-38	2.33E-38
ELMO1-AS1	NA	NA	NA
GPR141	NA	6.93E-58	1.01E-57
EPDR1	3.02975504	5.25E-305	2.14E-304
NME8	NA	NA	NA
SFRP4	Inf	0	0
STARD3NL	1.96089242	1.09E-19	1.32E-19
TRGC2	1.13227384	3.93E-202	9.86E-202

TRGJ2	NA	NA	NA
TRGJP2	NA	NA	NA
TRGC1	1.4571282	8.24E-125	1.55E-124
TRGJ1	NA	NA	NA
TRGJP	NA	NA	NA
TRGJP1	NA	NA	NA
TRGV11	NA	NA	NA
TRGVB	NA	NA	NA
TRGV10	NA	NA	NA
TRGV9	NA	8.69E-66	1.31E-65
TRGVA	NA	NA	NA
RP11-121A8.	Inf	1.19E-107	2.12E-107
TRGV8	NA	NA	NA
TRGV7	NA	NA	NA
TRGV6	NA	NA	NA
TRG-AS1	2.03723513	0	0
TRGV5	3.34535452	0.37600693	0.38215786
TRGV5P	NA	NA	NA
TRGV4	NA	NA	NA
TRGV3	NA	5.51E-234	1.57E-233
TRGV2	NA	NA	NA
TRGV1	NA	NA	NA
AMPH	0	1.74E-29	2.22E-29
VPS41	1.86906447	6.45E-33	8.37E-33
POU6F2	NA	NA	NA
POU6F2-AS2	NA	NA	NA
POU6F2-AS1	NA	NA	NA
AC011290.4	NA	NA	NA
YAE1D1	1.89492109	7.73E-84	1.25E-83
AC004837.4	NA	NA	NA
RALA	1.71818161	2.71E-70	4.17E-70
AC004837.5	NA	NA	NA
LINC00265	Inf	9.68E-104	1.69E-103
RP11-467D6.	0.38600244	0.44296049	0.44904947
CDK13	1.54400978	3.57E-183	8.31E-183
MPLKIP	1.7788927	1.65E-23	2.03E-23
SUGCT	1.93001222	2.10E-23	2.59E-23
AC004988.1	NA	NA	NA
LINC01450	NA	NA	NA
LINC01449	NA	NA	NA
INHBA	0	8.44E-115	1.54E-114
INHBA-AS1	NA	NA	NA
GLI3	NA	NA	NA
LINC01448	NA	NA	NA
AC010132.1(	NA	NA	NA

RP11-111K18	NA	3.12E-39	4.17E-39
C7orf25	3.08801956	6.39E-06	6.98E-06
RP11-111K18	NA	NA	NA
PSMA2	2.11494871	7.33E-51	1.04E-50
MRPL32	1.55720644	3.50E-192	8.46E-192
AC005537.2	NA	NA	NA
HECW1	NA	NA	NA
AC004692.4	NA	NA	NA
AC011738.4	NA	NA	NA
STK17A	1.75568854	1.15E-102	2.00E-102
COA1	1.97957674	1.26E-148	2.60E-148
BLVRA	1.51685383	5.32E-139	1.06E-138
MRPS24	1.09367359	0.20392598	0.20874712
URGCP-MRP	NA	NA	NA
URGCP	1.56606706	1.44E-79	2.29E-79
UBE2D4	1.76458261	3.08E-85	5.01E-85
SPDYE1	NA	NA	NA
AC004951.6	2.38256682	5.46E-137	1.07E-136
LINC00957	Inf	4.03E-41	5.45E-41
DBNL	1.40300432	0	0
PGAM2	0	0	0
POLM	1.51541701	4.44E-17	5.28E-17
AEBP1	NA	NA	NA
POLD2	1.64080193	0	0
MYL7	NA	NA	NA
GCK	NA	NA	NA
YKT6	2.34743347	8.09E-278	2.84E-277
CAMK2B	NA	0	0
NUDCD3	1.94679494	1.01E-124	1.90E-124
NPC1L1	NA	NA	NA
DDX56	1.12323234	0	0
TMED4	1.83400146	1.64E-06	1.80E-06
OGDH	1.96642755	4.39E-18	5.26E-18
ZMIZ2	2.18734719	7.89E-57	1.15E-56
AC013436.6	NA	NA	NA
PPIA	1.8264697	1.54E-252	4.80E-252
H2AFV	2.11580049	2.29E-80	3.65E-80
AC004854.4	Inf	1.97E-156	4.16E-156
PURB	2.36962612	4.43E-174	9.98E-174
RP4-673M15	2.31601467	0.3475351	0.35359664
MYO1G	2.41415088	7.95E-283	2.85E-282
SNHG15	2.4153182	0	0
CCM2	2.04647848	3.01E-39	4.02E-39
NACAD	NA	NA	NA
TBRG4	1.33222083	0	0

RAMP3	NA	NA	NA
AC073325.1	NA	NA	NA
ADCY1	NA	2.10E-18	2.52E-18
IGFBP1	NA	NA	NA
IGFBP3	NA	NA	NA
AC073115.6	NA	NA	NA
AC073115.7	0	8.26E-99	1.42E-98
AC023669.1	NA	NA	NA
AC092657.2	NA	NA	NA
AC004869.3	NA	NA	NA
AC004869.2	NA	NA	NA
AC011294.3	NA	NA	NA
AC004901.1	NA	NA	NA
AC004870.3	NA	NA	NA
TNS3	NA	NA	NA
LINC01447	NA	NA	NA
C7orf65	Inf	0	0
HUS1	2.26183889	1.52E-29	1.94E-29
LINC00525	NA	NA	NA
PKD1L1	NA	NA	NA
C7orf69	NA	NA	NA
SUN3	2.31601467	2.70E-102	4.70E-102
C7orf57	Inf	2.21E-192	5.35E-192
UPP1	0.77200489	1.40E-168	3.09E-168
ABCA13	NA	NA	NA
AC091770.3	NA	NA	NA
AC010971.1	NA	NA	NA
VWC2	NA	NA	NA
ZPBP	NA	NA	NA
C7orf72	NA	NA	NA
AC020743.2	NA	NA	NA
AC020743.3	NA	NA	NA
AC020743.4	NA	NA	NA
IKZF1	1.42653077	1.61E-240	4.74E-240
FIGNL1	2.52036891	4.22E-277	1.47E-276
DDC	Inf	8.19E-220	2.20E-219
DDC-AS1	NA	NA	NA
GRB10	2.01445026	0.00314954	0.00332997
AC004920.3	NA	NA	NA
RP4-724E13.	NA	NA	NA
COBL	NA	NA	NA
RP4-718N17.	NA	NA	NA
RP11-153N1	NA	NA	NA
POM121L12	NA	NA	NA
GS1-278J22.	NA	NA	NA

GS1-278J22.:	NA	NA	NA
LINC01446	NA	NA	NA
RP11-806J6.:	NA	NA	NA
HPVC1	NA	NA	NA
LINC01445	NA	NA	NA
VSTM2A	NA	NA	NA
GS1-18A18.2	NA	NA	NA
SEC61G	2.20556942	0	0
RP11-745C15	NA	NA	NA
EGFR	NA	NA	NA
EGFR-AS1	NA	NA	NA
ELDR	NA	NA	NA
LANCL2	1.27276482	1.30E-26	1.64E-26
VOPP1	2.39727834	1.82E-276	6.36E-276
RP11-310H4.	NA	NA	NA
RP11-310H4.	NA	NA	NA
RP11-310H4.	NA	NA	NA
14-Sep	Inf	0	0
RP11-15K19.	NA	NA	NA
ZNF713	2.50901589	8.14E-51	1.15E-50
GBAS	1.73126203	2.02E-275	7.02E-275
MRPS17	2.70481423	0	0
PSPH	1.92599137	6.35E-14	7.41E-14
CCT6A	1.43428605	0	0
SUMF2	1.49729611	0	0
PHKG1	1.46482979	6.50E-114	1.18E-113
CHCHD2	2.14749709	0	0
NUPR2	NA	NA	NA
RP11-760D2.	NA	NA	NA
RP11-760D2.	NA	NA	NA
RP11-760D2.	NA	NA	NA
AC118758.1	NA	NA	NA
RP13-580B18	NA	NA	NA
ZNF479	NA	NA	NA
RP11-1217F2	NA	NA	NA
RP11-114G1	NA	NA	NA
ZNF716	NA	NA	NA
RP11-196D18	NA	NA	NA
RP5-905H7.9	NA	NA	NA
RP5-905H7.8	NA	NA	NA
RP11-73B2.2	NA	NA	NA
RP11-73B2.4	NA	NA	NA
RP11-340I6.3	NA	NA	NA
RP11-340I6.1	NA	NA	NA
AC079355.1	NA	NA	NA

RP11-340I6.7	Inf	1.42E-284	5.14E-284
RP11-340I6.8	NA	9.47E-266	3.15E-265
LINC01005	NA	NA	NA
ZNF727	NA	NA	NA
RP11-321E8.	NA	NA	NA
ZNF735	NA	NA	NA
ZNF679	NA	NA	NA
ZNF736	2.40179299	9.51E-08	1.06E-07
ZNF680	2.15765469	4.10E-70	6.29E-70
ZNF107	2.39321516	3.29E-229	9.22E-229
ZNF138	1.4618816	9.49E-220	2.55E-219
ZNF273	1.98036037	0.6521376	0.65728225
RP11-797H7.	NA	NA	NA
ZNF117	2.35277681	8.57E-62	1.27E-61
ERV3-1	2.18631785	0	0
AC104073.2	NA	NA	NA
RP11-328P23	0	0	0
ZNF92	2.26776436	2.33E-52	3.32E-52
RP11-667F9.	NA	NA	NA
AC104057.1	NA	NA	NA
RP11-479O9.	Inf	9.74E-161	2.09E-160
VKORC1L1	2.28277044	1.39E-160	2.98E-160
GUSB	1.3408506	0	0
ASL	1.47219537	0	0
AC068533.7	NA	NA	NA
CRCP	2.15765469	0.00104579	0.00111342
RP5-1132H15	NA	NA	NA
TPST1	1.11807605	0	0
LINC00174	Inf	1.14E-146	2.32E-146
GS1-124K5.3	6.17603912	2.28E-51	3.23E-51
GS1-124K5.4	2.40179299	3.58E-41	4.85E-41
GS1-124K5.1	1.93001222	1.17E-13	1.36E-13
KCTD7	1.80134474	0.86512757	0.86751848
RABGEF1	0.95015986	0	0
RP11-792A8.	2.20136048	8.90E-246	2.69E-245
RP11-458F8.	Inf	8.19E-92	1.37E-91
TMEM248	1.73317654	1.57E-31	2.03E-31
SBDS	1.13138648	0	0
TYW1	1.60902072	1.15E-19	1.40E-19
RP11-166O4.	3.86002445	3.59E-65	5.40E-65
LINC01372	Inf	0	0
RP4-736H5.3	NA	NA	NA
RP11-358M3	NA	NA	NA
RP5-945F2.2	NA	NA	NA
RP5-945F2.3	NA	NA	NA

RP11-123O1.	NA	NA	NA
RP11-3P22.1	NA	NA	NA
RP11-3P22.2	NA	NA	NA
RP5-942I16.1	NA	0.00351228	0.00371024
AUTS2	1.54400978	2.40E-224	6.58E-224
WBSCR17	NA	NA	NA
CALN1	NA	2.49E-187	5.88E-187
TYW1B	1.23520782	4.51E-238	1.32E-237
POM121	2.58963666	2.17E-284	7.85E-284
RP11-313P13	0.64333741	2.25E-189	5.38E-189
TRIM74	NA	NA	NA
NSUN5	1.44615001	1.59E-281	5.67E-281
TRIM50	NA	NA	NA
FKBP6	NA	NA	NA
FZD9	1.60339477	0.70574062	0.7104004
BAZ1B	1.72553525	1.27E-183	2.97E-183
BCL7B	2.17653479	1.92E-238	5.60E-238
TBL2	1.22481545	0	0
MLXIPL	NA	5.38E-68	8.18E-68
VPS37D	0.38600244	1.37E-133	2.67E-133
DNAJC30	1.42019767	0	0
WBSCR22	1.66000546	7.73E-145	1.56E-144
STX1A	0.38600244	1.36E-49	1.91E-49
ABHD11-AS1	NA	1.08E-215	2.86E-215
ABHD11	1.61483592	5.02E-149	1.03E-148
CLDN3	Inf	4.57E-43	6.25E-43
CLDN4	NA	NA	NA
WBSCR27	Inf	9.51E-146	1.93E-145
WBSCR28	NA	NA	NA
RP11-731K22	NA	NA	NA
ELN	0	0	0
CTB-51J22.1	NA	NA	NA
LIMK1	1.49076806	1.23E-36	1.63E-36
EIF4H	1.87780347	0.14404184	0.14800674
LAT2	1.51643818	0	0
RFC2	1.35930969	0	0
CLIP2	1.94679494	1.07E-52	1.53E-52
GTF2IRD1	1.72782047	6.76E-41	9.13E-41
RP5-1186P1(	Inf	0	0
GTF2I	2.41100409	0	0
NCF1	0.90067237	0	0
GTF2IRD2	2.93361858	5.27E-115	9.61E-115
GATSL2	1.54400978	5.69E-15	6.69E-15
WBSCR16	1.64051039	1.42E-270	4.80E-270
GTF2IRD2B	1.54400978	0.05821615	0.06032427

AC004878.2	1.54400978	2.22E-103	3.87E-103
AC004878.8	NA	NA	NA
TRIM73	1.83351161	0.00396731	0.00418825
POM121C	2.65461331	0	0
SPDYE5	NA	NA	NA
HIP1	1.90244062	5.99E-11	6.85E-11
CCL26	NA	NA	NA
CCL24	NA	NA	NA
RHBDD2	1.63842764	2.26E-269	7.60E-269
POR	1.26643499	0	0
TMEM120A	1.22234108	6.08E-143	1.22E-142
STYXL1	1.62588909	1.00E-185	2.36E-185
MDH2	1.24698942	0	0
SRRM3	0.85413307	0	0
HSPB1	1.68240792	3.38E-287	1.24E-286
YWHAG	2.29704649	0	0
SSC4D	2.70201711	1.23E-52	1.75E-52
ZP3	Inf	0	0
DTX2	1.10286413	0	0
AC007078.4	NA	NA	NA
UPK3B	NA	NA	NA
SPDYE16	0	0	0
POMZP3	1.28667482	3.81E-249	1.17E-248
AC005522.7	NA	NA	NA
CCDC146	0	1.33E-06	1.46E-06
FGL2	NA	NA	NA
AC073635.5	NA	NA	NA
GSAP	0.77200489	1.08E-61	1.60E-61
RP5-899E9.1	NA	NA	NA
PTPN12	2.07364104	6.51E-79	1.03E-78
APTR	2.03159182	8.13E-85	1.32E-84
RSBN1L	2.0510831	1.17E-24	1.46E-24
TMEM60	1.95271825	1.34E-24	1.67E-24
PHTF2	2.50316737	1.14E-149	2.35E-149
RP5-1185I7.1	2.12301345	9.43E-09	1.06E-08
MAGI2	NA	NA	NA
MAGI2-AS1	NA	NA	NA
MAGI2-AS2	NA	NA	NA
RP5-1093G2	NA	NA	NA
GNAI1	1.44220694	0	0
AC003988.1	NA	NA	NA
AC004862.6	NA	NA	NA
CD36	NA	1.40E-64	2.11E-64
GNAT3	NA	NA	NA
SEMA3C	NA	NA	NA

AC005008.2	NA	NA	NA
AC008163.4	NA	NA	NA
AC010091.1	NA	NA	NA
HGF	0.16946449	0	0
CACNA2D1	1.54400978	0.07373731	0.07627243
AC006145.4	NA	NA	NA
PCLO	NA	1.20E-76	1.89E-76
AC079799.2	NA	NA	NA
SEMA3E	0	0	0
SEMA3A	Inf	0	0
AC003984.1	NA	NA	NA
AC074183.4	NA	NA	NA
SEMA3D	3.86002445	0.0059768	0.00629353
LINC00972	NA	1.04E-120	1.94E-120
GRM3	NA	NA	NA
AC005009.1	NA	NA	NA
AC005009.2	NA	NA	NA
KIAA1324L	Inf	0	0
CTA-254O6.1	NA	NA	NA
AC005076.5	2.61011177	1.91E-143	3.85E-143
DMTF1	2.23881418	5.28E-132	1.02E-131
TMEM243	2.0611781	3.29E-220	8.85E-220
TP53TG1	NA	NA	NA
CROT	NA	NA	NA
ABCB4	NA	NA	NA
ABCB1	Inf	0	0
RUNDC3B	13.896088	0	0
SLC25A40	2.39727834	3.68E-204	9.31E-204
DBF4	1.95032814	6.46E-11	7.39E-11
ADAM22	1.10975703	3.28E-134	6.40E-134
SRI	2.20590112	1.36E-105	2.39E-105
CTB-167B5.1	NA	NA	NA
AC003991.3	NA	NA	NA
STEAP4	NA	0	0
AC002127.4	NA	NA	NA
ZNF804B	NA	NA	NA
C7orf62	NA	NA	NA
AC002383.2	NA	NA	NA
RP5-1121E1(	NA	NA	NA
STEAP1	1.63591512	3.48E-06	3.81E-06
STEAP2	Inf	0	0
CFAP69	Inf	0	0
AC002064.4	NA	NA	NA
AC002064.5	NA	NA	NA
GTPBP10	2.12301345	1.03E-14	1.21E-14

CLDN12	1.36585481	5.71E-75	8.92E-75
CDK14	0	1.27E-250	3.91E-250
AC002456.2	NA	NA	NA
FZD1	2.31601467	2.11E-76	3.31E-76
RP11-115N4.	NA	NA	NA
RP11-142A5.	NA	NA	NA
CTB-111F10.	NA	NA	NA
MTERF1	1.30137967	8.58E-253	2.67E-252
AKAP9	1.8802608	0	0
CYP51A1	1.15800733	8.10E-06	8.83E-06
LRRD1	NA	0	0
CYP51A1-AS1	NA	8.20E-251	2.53E-250
KRIT1	2.25341968	4.44E-149	9.15E-149
ANKIB1	2.05867971	1.17E-32	1.52E-32
GATAD1	1.61871993	1.34E-129	2.57E-129
AC007566.10	NA	9.00E-26	1.13E-25
ERVW-1	NA	NA	NA
PEX1	1.737011	2.11E-122	3.94E-122
RBM48	1.51827628	1.35E-264	4.47E-264
FAM133B	1.82118334	3.21E-196	7.88E-196
CDK6	3.58226608	0	0
AC002454.1	19.8147922	0	0
SAMD9	2.31601467	3.44E-12	3.97E-12
SAMD9L	Inf	0	0
HEPACAM2	NA	NA	NA
VPS50	1.76458261	1.01E-104	1.77E-104
CALCR	NA	NA	NA
GNGT1	NA	NA	NA
TFPI2	NA	NA	NA
AC002076.10	NA	NA	NA
GNG11	2.26874906	6.83E-136	1.34E-135
BET1	1.788478	1.02E-157	2.17E-157
AC006378.2	NA	NA	NA
AC003092.1	NA	NA	NA
AC003092.2	NA	NA	NA
COL1A2	NA	0.00150172	0.00159575
CASD1	2.4563792	3.56E-05	3.86E-05
SGCE	0.73983802	0	0
PEG10	1.1743173	7.06E-56	1.02E-55
PPP1R9A	0	2.23E-300	8.82E-300
AC002429.5	NA	NA	NA
PON1	NA	NA	NA
PON3	NA	NA	NA
PON2	1.61871993	2.64E-172	5.91E-172
GS1-293C5.1	NA	NA	NA

AC004012.1	Inf	0	0
ASB4	NA	NA	NA
PDK4	NA	NA	NA
AC002451.3	NA	NA	NA
DYNC1I1	1.93001222	8.03E-46	1.11E-45
SLC25A13	1.73945406	3.08E-32	3.99E-32
SHFM1	2.20843809	0	0
DLX6-AS1	3.08801956	3.56E-223	9.70E-223
DLX6	0.90067237	3.62E-161	7.79E-161
DLX5	NA	NA	NA
SDHAF3	2.09790176	2.94E-129	5.63E-129
TAC1	NA	NA	NA
ASNS	1.08782507	1.67E-145	3.39E-145
OCM2	NA	NA	NA
LMTK2	2.57334963	6.61E-241	1.95E-240
BHLHA15	NA	NA	NA
TECPR1	1.6297881	5.56E-18	6.67E-18
BRI3	1.87635269	1.62E-15	1.91E-15
BAIAP2L1	1.15800733	1.38E-32	1.79E-32
RP11-307C18	0.77200489	0.01179388	0.01235718
NPTX2	NA	NA	NA
TMEM130	NA	5.05E-128	9.60E-128
TRRAP	2.12966866	5.20E-66	7.86E-66
AC004893.11	2.8306846	0.56030836	0.56617546
SMURF1	2.62481663	1.65E-39	2.21E-39
KPNA7	NA	NA	NA
ARPC1A	1.9315666	5.58E-25	6.94E-25
ARPC1B	1.9099045	1.71E-43	2.34E-43
PDAP1	1.79053803	7.57E-109	1.35E-108
BUD31	1.84285038	1.14E-101	1.98E-101
PTCD1	1.62746977	0.00068489	0.00073107
ATP5J2-PTCD	NA	0	0
CPSF4	1.82473883	0.00176513	0.00187334
ATP5J2	2.10565231	8.38E-165	1.83E-164
ZNF789	1.13769142	2.00E-33	2.60E-33
ZNF394	1.45646283	6.25E-226	1.72E-225
ZKSCAN5	3.40590393	0	0
FAM200A	0.99906515	9.13E-200	2.27E-199
ZNF655	1.89075774	1.00E-28	1.27E-28
GS1-259H13.	NA	NA	NA
GS1-259H13.	1.54400978	3.38E-35	4.44E-35
ZSCAN25	2.16161369	5.13E-92	8.58E-92
CYP3A5	2.18734719	0.27321243	0.27875934
CYP3A7-CYP3	NA	NA	NA
CYP3A7	NA	NA	NA

CYP3A4	NA	NA	NA
RP11-757A1:	NA	NA	NA
CYP3A43	NA	NA	NA
OR2AE1	NA	NA	NA
TRIM4	2.14445803	0.00485587	0.00512092
GJC3	0	1.35E-203	3.39E-203
RP4-604G5.1	NA	NA	NA
AZGP1	NA	NA	NA
ZKSCAN1	2.19355872	7.86E-20	9.52E-20
ZSCAN21	1.25958693	0	0
ZNF3	1.60834352	4.98E-120	9.24E-120
COPS6	1.18548548	0	0
MCM7	1.17394086	0	0
AP4M1	1.80902638	1.49E-244	4.48E-244
TAF6	1.50753711	1.76E-100	3.05E-100
RP11-506M1	NA	0	0
CNPY4	1.9073062	1.28E-19	1.54E-19
MBLAC1	2.31601467	1.59E-66	2.41E-66
AC073842.1!	NA	NA	NA
LAMTOR4	1.94857425	0.09368176	0.09667109
C7orf43	1.39925886	9.08E-90	1.51E-89
GAL3ST4	0	9.73E-300	3.83E-299
GPC2	1.54400978	0	0
STAG3	2.16161369	4.72E-66	7.13E-66
GATS	1.26328073	1.20E-99	2.07E-99
PVRIG	NA	NA	NA
SPDYE3	NA	3.80E-43	5.19E-43
PILRB	1.93001222	0.10332528	0.10651933
PILRA	Inf	3.65E-10	4.14E-10
ZCWPW1	1.06150672	0	0
MEPCE	2.0866509	9.69E-25	1.21E-24
RP11-758P17	Inf	0	0
PPP1R35	2.06850165	6.25E-110	1.12E-109
RP11-758P17	Inf	1.90E-17	2.27E-17
C7orf61	1.85281174	5.14E-35	6.74E-35
TSC22D4	1.81064601	5.79E-216	1.53E-215
NYAP1	NA	7.69E-227	2.13E-226
RP11-44M6.:	NA	NA	NA
AGFG2	1.77561125	1.41E-166	3.10E-166
SAP25	2.70201711	0.27574758	0.28126687
LRCH4	1.48886657	1.30E-280	4.62E-280
FBXO24	Inf	0.00017115	0.00018407
PCOLCE-AS1	NA	NA	NA
PCOLCE	1.87486902	2.64E-284	9.57E-284
MOSPD3	1.30947665	2.01E-299	7.91E-299

TFR2	1.93788983	7.66E-05	8.27E-05
ACTL6B	NA	NA	NA
GNB2	1.73193697	6.30E-149	1.29E-148
GIGYF1	1.66919976	6.96E-68	1.06E-67
POP7	2.02974244	1.67E-13	1.95E-13
EPO	NA	3.48E-13	4.04E-13
ZAN	NA	NA	NA
EPHB4	1.98515543	5.21E-140	1.04E-139
SLC12A9	1.86410937	0.00240747	0.00254999
RP11-126L15	1.54400978	0.55293369	0.55887921
TRIP6	1.3584127	0	0
SRRT	1.5157657	0	0
UFSP1	6.56204156	1.05E-41	1.43E-41
ACHE	0.8822913	9.34E-232	2.65E-231
MUC3A	1.54400978	0	0
RP11-395B7.	NA	NA	NA
MUC12	1.87486902	0.01340412	0.01403298
RP11-395B7.	1.93001222	0.00011346	0.00012224
MUC17	NA	NA	NA
TRIM56	2.66341687	6.11E-288	2.26E-287
SERPINE1	Inf	4.45E-12	5.13E-12
AP1S1	1.76634719	6.51E-96	1.10E-95
VGF	1.45318568	5.54E-259	1.78E-258
NAT16	Inf	0	0
MOGAT3	Inf	0	0
PLOD3	1.44883109	2.36E-292	8.93E-292
ZNHIT1	1.88327829	1.19E-62	1.77E-62
CLDN15	1.63260051	1.10E-20	1.33E-20
FIS1	2.08740437	9.17E-169	2.03E-168
RP11-132A1.	NA	NA	NA
RP11-132A1.	NA	NA	NA
IFT22	2.28863861	0	0
COL26A1	Inf	2.10E-164	4.58E-164
LINC01007	NA	NA	NA
MYL10	NA	NA	NA
AC004953.1	NA	NA	NA
CUX1	2.06787024	9.91E-45	1.36E-44
CTA-339C12.	NA	NA	NA
CTA-305I2.1	NA	NA	NA
SH2B2	1.4153423	5.57E-232	1.58E-231
SPDYE6	NA	NA	NA
PRKRIP1	1.86025275	3.04E-45	4.19E-45
RP11-514P8.	NA	NA	NA
ORAI2	1.75113304	0	0
ALKBH4	1.5586681	4.38E-204	1.11E-203

LRWD1	1.01325642	0	0
POLR2J	2.18547566	0	0
RASA4B	NA	0	0
POLR2J3	2.04066626	1.00E-18	1.21E-18
RP11-514P8.	NA	NA	NA
SPDYE2	NA	NA	NA
RASA4	1.54400978	2.89E-07	3.19E-07
RP11-514P8.	NA	3.10E-16	3.67E-16
UPK3BL	NA	1.02E-140	2.03E-140
RP11-577H5.	NA	NA	NA
SPDYE2B	NA	NA	NA
POLR2J2	0.55755909	0	0
AC105052.2	NA	NA	NA
FAM185A	1.31240831	1.66E-174	3.73E-174
FBXL13	1.48462479	1.57E-181	3.64E-181
LRRC17	NA	0	0
NFE4	2.25738139	0	0
RP11-645N1	NA	0	0
ARMC10	2.03451012	1.52E-222	4.12E-222
NAPEPLD	1.60834352	4.52E-306	1.85E-305
PMPCB	1.06497085	0	0
DNAJC2	1.68961924	1.87E-158	3.99E-158
PSMC2	0.88587561	0	0
SLC26A5	NA	NA	NA
CTB-107G13.	NA	NA	NA
RELN	NA	NA	NA
ORC5	1.66277976	3.31E-88	5.45E-88
LHFPL3	NA	NA	NA
RP11-325F22	NA	NA	NA
LINC01004	0.77200489	2.91E-16	3.45E-16
KMT2E-AS1	1.63483388	6.14E-164	1.34E-163
KMT2E	1.717317	2.60E-160	5.58E-160
CTB-152G17.	1.08080685	1.87E-28	2.37E-28
SRPK2	1.59361652	0	0
RP4-778K6.3	NA	NA	NA
PUS7	2.72100084	1.19E-24	1.47E-24
RINT1	1.58359977	2.82E-97	4.82E-97
EFCAB10	NA	NA	NA
RP11-251G2	Inf	0	0
ATXN7L1	1.97826253	1.01E-56	1.47E-56
CDHR3	2.39321516	1.17E-103	2.04E-103
SYPL1	1.41105338	0	0
NAMPT	1.90215638	2.91E-37	3.85E-37
RP11-22N19.	Inf	0	0
CTB-30L5.1	NA	NA	NA

CCDC71L	2.77219938	1.04E-243	3.10E-243
RP5-884M6.:	1.15800733	7.26E-26	9.09E-26
PIK3CG	1.3896088	1.73E-136	3.39E-136
PRKAR2B	2.65376681	1.03E-196	2.53E-196
HBP1	0.90067237	0	0
CTA-363E19.	Inf	0	0
COG5	2.12301345	9.00E-06	9.82E-06
GPR22	NA	0	0
DUS4L	1.87737553	6.32E-53	9.03E-53
RP4-593H12.	1.93001222	0.1334481	0.13723022
BCAP29	2.59747479	3.36E-222	9.12E-222
SLC26A4-AS1	1.15800733	0.91065528	0.91226439
SLC26A4	Inf	0	0
AC002467.7	2.44911896	7.87E-07	8.68E-07
CBLL1	2.44710984	6.67E-26	8.35E-26
SLC26A3	NA	NA	NA
DLD	1.44977444	0	0
LAMB1	2.18734719	4.66E-32	6.03E-32
CTB-13F3.1	1.93001222	3.01E-39	4.03E-39
LAMB4	Inf	8.25E-189	1.96E-188
NRCAM	NA	1.22E-55	1.76E-55
PNPLA8	1.43372337	6.67E-270	2.25E-269
THAP5	2.24032792	9.58E-54	1.37E-53
DNAJB9	1.91898358	4.41E-33	5.73E-33
AC005487.2	NA	NA	NA
C7orf66	NA	NA	NA
AC004014.3	NA	NA	NA
AC002386.1	NA	NA	NA
AC073071.1	NA	NA	NA
AC003088.1	NA	NA	NA
IMMP2L	2.2375057	0.0074563	0.00783914
LRRN3	NA	NA	NA
DOCK4	1.54400978	9.83E-183	2.29E-182
DOCK4-AS1	NA	NA	NA
ZNF277	1.4188198	2.12E-91	3.54E-91
AC004112.4	NA	NA	NA
IFRD1	1.89965248	2.78E-112	5.03E-112
LSMEM1	Inf	1.50E-62	2.24E-62
AC002463.3	NA	NA	NA
TMEM168	NA	6.83E-63	1.02E-62
C7orf60	2.31601467	2.63E-25	3.28E-25
HRAT17	NA	NA	NA
GPR85	1.62844781	3.44E-114	6.25E-114
LINC00998	1.82234528	1.28E-178	2.92E-178
PPP1R3A	NA	NA	NA

FOXP2	1.52314478	0.15762331	0.16181542
AC073626.2	NA	NA	NA
MDFIC	2.03622766	0.99548324	0.99553812
LINC01392	NA	NA	NA
LINC01393	Inf	0.00216935	0.00229966
RP11-458K1(	NA	NA	NA
RP11-458K1(	NA	NA	NA
RP11-458K1(	NA	NA	NA
TFEC	1.59915299	1.67E-15	1.97E-15
TES	3.13090872	2.19E-145	4.45E-145
AC073130.3	NA	NA	NA
AC002066.1	NA	NA	NA
AC073130.2	NA	NA	NA
CAV2	NA	NA	NA
CAV1	1.28667482	0.0066879	0.00703618
AC006159.5	NA	NA	NA
AC006159.4	NA	NA	NA
LINC01510	NA	NA	NA
MET	6.17603912	1.25E-19	1.51E-19
CAPZA2	1.66436689	1.20E-277	4.19E-277
ST7-AS1	0.77200489	6.91E-69	1.06E-68
ST7	1.17832325	0	0
AC106873.4	NA	NA	NA
ST7-AS2	NA	NA	NA
WNT2	NA	NA	NA
AC002465.2	NA	NA	NA
ASZ1	NA	NA	NA
CFTR	NA	NA	NA
AC000111.3	NA	NA	NA
AC000111.6	NA	NA	NA
CTTNBP2	1.03821347	2.61E-293	9.91E-293
AC003084.2	NA	NA	NA
LSM8	2.10335592	4.12E-144	8.31E-144
ANKRD7	NA	NA	NA
RP11-328J2.:	NA	NA	NA
RP4-742N3.1	NA	NA	NA
RP5-1006K1:	NA	NA	NA
KCND2	NA	NA	NA
RP4-797C5.2	NA	NA	NA
TSPAN12	0.25733496	5.55E-216	1.47E-215
ING3	1.70532423	3.70E-240	1.09E-239
CPED1	Inf	0	0
WNT16	NA	NA	NA
FAM3C	2.08390862	3.64E-09	4.09E-09
RP4-745K6.1	NA	NA	NA

PTPRZ1	NA	NA	NA
AASS	5.40403423	0	0
RP11-560I19	NA	NA	NA
FEZF1	NA	NA	NA
FEZF1-AS1	NA	NA	NA
CADPS2	1.80134474	5.69E-09	6.40E-09
RP5-1101C3.	NA	NA	NA
RNF133	NA	NA	NA
RNF148	Inf	8.73E-191	2.10E-190
TAS2R16	NA	NA	NA
RP11-61D3.1	NA	NA	NA
SLC13A1	NA	NA	NA
IQUB	NA	NA	NA
RP11-332K15	NA	NA	NA
NDUFA5	1.92937209	9.30E-42	1.26E-41
ASB15	NA	NA	NA
RP11-390E23	NA	NA	NA
LMOD2	NA	NA	NA
WASL	2.18734719	2.09E-15	2.46E-15
RP11-390E23	1.94679494	3.81E-51	5.40E-51
HYAL4	NA	NA	NA
SPAM1	NA	NA	NA
RP4-630C24.	NA	NA	NA
TMEM229A	NA	NA	NA
RP5-921G16.	NA	NA	NA
RP11-264K23	NA	NA	NA
RP5-921G16.	NA	NA	NA
GPR37	NA	NA	NA
C7orf77	NA	NA	NA
POT1	1.62121027	7.75E-102	1.35E-101
POT1-AS1	Inf	0	0
RP11-3B12.5	NA	NA	NA
RP11-3B12.2	NA	NA	NA
RP11-3B12.3	NA	NA	NA
RP11-807H17	NA	NA	NA
AC005276.1	NA	NA	NA
AC000370.2	NA	NA	NA
GRM8	0.25733496	0	0
AC000099.1	NA	NA	NA
ZNF800	1.99434597	4.28E-149	8.81E-149
AC000123.4	NA	NA	NA
AC000124.1	NA	NA	NA
GCC1	3.31962103	0.20600473	0.21080365
ARF5	1.19777728	0	0
FSCN3	NA	1.88E-236	5.43E-236

PAX4	NA	NA	NA
AC073934.6	NA	NA	NA
SND1	1.7359447	1.95E-126	3.69E-126
SND1-IT1	2.37777506	3.43E-149	7.07E-149
LRRC4	NA	2.25E-231	6.35E-231
LEP	NA	NA	NA
RP11-62J1.4	NA	0	0
RBM28	1.78129267	0	0
PRRT4	3.26966777	0	0
IMPDH1	1.70772288	1.55E-88	2.56E-88
AC010655.1	NA	NA	NA
HILPDA	1.94518263	5.76E-75	9.00E-75
METTL2B	1.85763677	1.86E-100	3.21E-100
RP11-212P7.	2.8306846	0.03150785	0.03280056
RP11-274B2:	0	2.11E-264	6.98E-264
FAM71F2	Inf	0	0
RP11-274B2:	NA	1.22E-109	2.19E-109
FAM71F1	NA	NA	NA
CALU	1.51087652	7.09E-302	2.83E-301
OPN1SW	NA	NA	NA
CCDC136	2.39727834	1.31E-103	2.29E-103
FLNC	1.82783511	1.20E-39	1.61E-39
RP11-309L24	NA	NA	NA
KCP	1.54400978	1.81E-41	2.46E-41
ATP6V1F	2.05514852	1.09E-92	1.83E-92
RP11-309L24	0.77200489	2.04E-198	5.03E-198
IRF5	2.74942092	0	0
RP11-309L24	Inf	0.17751688	0.18198063
TNPO3	2.21757109	4.52E-222	1.23E-221
TSPAN33	2.18734719	1.02E-26	1.28E-26
SMO	2.8108896	8.90E-282	3.18E-281
RP11-286H1	NA	NA	NA
AHCYL2	2.31601467	3.65E-85	5.94E-85
STRIP2	3.08801956	3.13E-125	5.90E-125
SMKR1	2.87747277	0	0
RP11-448A1	3.01081907	0	0
NRF1	2.59674372	1.34E-237	3.90E-237
RP11-738B7.	NA	NA	NA
UBE2H	1.59547677	2.81E-93	4.72E-93
RP11-306G2	2.05867971	1.47E-15	1.74E-15
ZC3HC1	1.22572706	0	0
KLHDC10	1.79342674	0.1053494	0.10857518
RP11-775D2:	NA	NA	NA
TMEM209	3.13782633	1.88E-245	5.67E-245
RP11-775D2:	NA	NA	NA

SSMEM1	NA	NA	NA
CPA2	1.93001222	1.64E-247	5.00E-247
CPA4	0	0	0
CPA5	NA	NA	NA
CPA1	NA	NA	NA
CEP41	2.47519094	5.37E-96	9.11E-96
RP11-2E11.5	NA	NA	NA
MESTIT1	NA	NA	NA
MEST	0.90067237	1.02E-149	2.10E-149
RP11-2E11.6	0.77200489	5.17E-36	6.82E-36
COPG2	0.98255168	8.52E-207	2.17E-206
RP11-2E11.9	NA	9.51E-215	2.50E-214
TSGA13	NA	0	0
KLF14	NA	NA	NA
RP11-36B6.1	NA	NA	NA
AC016831.7	2.50901589	3.06E-21	3.73E-21
AC058791.1	Inf	0	0
RP11-138A9.	4.74231575	8.00E-233	2.28E-232
RP11-138A9.	1.28667482	0.99316243	0.99332669
LINC-PINT	1.77561125	2.53E-158	5.38E-158
MKLN1	2.9379075	2.20E-81	3.52E-81
RP11-180C16	Inf	2.05E-36	2.71E-36
PODXL	2.18057522	1.66E-05	1.81E-05
AC009518.4	NA	NA	NA
PLXNA4	0	0	0
AC018643.4	NA	NA	NA
AC011625.1	NA	NA	NA
FLJ40288	NA	2.62E-20	3.17E-20
AC009365.4	NA	NA	NA
CHCHD3	1.53570865	0	0
AC009518.8	NA	NA	NA
EXOC4	2.5365875	6.66E-171	1.48E-170
LRGUK	NA	1.69E-17	2.02E-17
AC008154.4	NA	NA	NA
SLC35B4	1.91612724	2.62E-91	4.38E-91
AC008154.5	NA	NA	NA
RP11-38M8.:	2.70201711	2.19E-192	5.29E-192
AKR1B1	1.287614	0	0
AKR1B10	NA	NA	NA
AKR1B15	NA	NA	NA
BPGM	1.4101956	8.78E-249	2.69E-248
AC009276.4	NA	NA	NA
CALD1	Inf	0	0
AGBL3	1.737011	9.28E-108	1.65E-107
C7orf49	1.914934	7.70E-08	8.57E-08

TMEM140	0	1.33E-143	2.68E-143
RP11-134L1C	NA	NA	NA
WDR91	1.28667482	4.63E-186	1.09E-185
AC009542.2	NA	NA	NA
STRA8	NA	NA	NA
CNOT4	2.07663331	0.76234579	0.76661368
NUP205	2.01012594	1.18E-38	1.57E-38
C7orf73	2.20572826	0	0
SLC13A4	NA	NA	NA
RP11-644N4.	Inf	1.70E-245	5.12E-245
FAM180A	NA	NA	NA
AC015987.1	NA	3.11E-284	1.12E-283
MTPN	2.29469698	2.74E-292	1.04E-291
LUZP6	NA	NA	NA
AC024084.1	NA	NA	NA
AC009784.3	NA	NA	NA
AC009264.1	NA	NA	NA
CHRM2	0	0	0
PTN	NA	NA	NA
AC078842.4	NA	NA	NA
AC078842.3	NA	NA	NA
DGKI	NA	NA	NA
AC090498.1	1.19660758	0	0
CREB3L2	2.56967342	0	0
AC022173.2	NA	NA	NA
AKR1D1	NA	2.62E-137	5.15E-137
AC083867.4	NA	NA	NA
TRIM24	1.72876309	5.36E-45	7.39E-45
SVOPL	0.6667315	0	0
ATP6V0A4	NA	NA	NA
TMEM213	NA	NA	NA
KIAA1549	2.10156887	0.61030261	0.6158357
ZC3HAV1L	0.96500611	3.85E-213	1.01E-212
ZC3HAV1	2.07404299	1.45E-203	3.66E-203
TTC26	1.14122462	4.59E-95	7.78E-95
UBN2	1.59435792	3.67E-65	5.52E-65
C7orf55	2.00349628	3.90E-44	5.36E-44
LUC7L2	1.69958491	3.67E-207	9.38E-207
C7orf55-LUC	NA	9.71E-82	1.56E-81
RP11-634H2	1.71556642	3.40E-35	4.46E-35
KLRG2	NA	5.05E-128	9.60E-128
CLEC2L	NA	NA	NA
HIPK2	3.20678954	6.69E-127	1.27E-126
TBXAS1	1.97290139	1.94E-89	3.21E-89
PARP12	2.14445803	2.11E-06	2.31E-06

KDM7A	0.77200489	1.92E-218	5.12E-218
JHDM1D-AS1	3.86002445	1.79E-73	2.79E-73
SLC37A3	1.06892985	4.51E-194	1.10E-193
RAB19	NA	NA	NA
MKRN1	1.54400978	0	0
DENND2A	NA	1.99E-184	4.65E-184
ADCK2	1.92603282	7.67E-142	1.53E-141
NDUFB2	2.31742498	0	0
NDUFB2-AS1	1.3896088	2.69E-169	5.97E-169
BRAF	1.83351161	8.86E-79	1.40E-78
MRPS33	1.90680472	0.28220557	0.28772471
TMEM178B	NA	NA	NA
RP5-842K16.	NA	NA	NA
RP5-842K16.	NA	NA	NA
RP11-744I24	NA	NA	NA
RP11-744I24	NA	NA	NA
AGK	1.23786991	0	0
RP5-894A10.	2.21951406	1.11E-68	1.70E-68
KIAA1147	2.24363921	0.10174331	0.10490038
WEE2-AS1	Inf	0	0
WEE2	NA	NA	NA
SSBP1	1.82777274	1.56E-58	2.29E-58
TAS2R3	NA	NA	NA
TAS2R4	2.31601467	2.13E-280	7.56E-280
TAS2R5	NA	1.23E-65	1.85E-65
PRSS37	NA	NA	NA
MGAM	0	1.07E-05	1.17E-05
OR9A4	NA	NA	NA
CLEC5A	0.77200489	3.22E-173	7.24E-173
TAS2R38	NA	NA	NA
MGAM2	NA	NA	NA
PRSS58	NA	NA	NA
U66059.58	NA	NA	NA
TRBV1	NA	NA	NA
TRBV2	NA	NA	NA
TRBV3-1	NA	NA	NA
TRBV4-1	NA	NA	NA
TRBV5-1	NA	NA	NA
TRBV6-1	NA	NA	NA
TRBV7-1	NA	NA	NA
TRBV4-2	NA	NA	NA
TRBV6-2	NA	NA	NA
TRBV7-2	NA	NA	NA
TRBV8-1	NA	NA	NA
TRBV5-2	NA	NA	NA

TRBV6-4	NA	NA	NA
TRBV7-3	NA	NA	NA
TRBV8-2	NA	NA	NA
TRBV5-3	NA	NA	NA
TRBV9	NA	NA	NA
TRBV10-1	NA	NA	NA
TRBV11-1	NA	NA	NA
TRBV12-1	NA	NA	NA
TRBV10-2	NA	NA	NA
TRBV11-2	NA	NA	NA
TRBV12-2	NA	NA	NA
TRBV6-5	NA	NA	NA
TRBV7-4	NA	NA	NA
TRBV5-4	NA	NA	NA
TRBV6-6	NA	NA	NA
TRBV7-5	NA	NA	NA
TRBV5-5	NA	NA	NA
TRBV6-7	NA	NA	NA
TRBV7-6	NA	NA	NA
TRBV5-6	NA	NA	NA
TRBV6-8	NA	NA	NA
TRBV7-7	NA	NA	NA
TRBV5-7	NA	NA	NA
TRBV7-9	NA	NA	NA
TRBV13	NA	NA	NA
TRBV10-3	NA	NA	NA
TRBV11-3	NA	NA	NA
TRBV12-3	NA	NA	NA
TRBV12-4	NA	NA	NA
TRBV12-5	NA	NA	NA
TRBV14	NA	NA	NA
TRBV15	NA	NA	NA
TRBV16	NA	NA	NA
TRBV17	NA	NA	NA
TRBV18	NA	NA	NA
TRBV19	NA	NA	NA
TRBV20-1	NA	NA	NA
TRBV21-1	NA	NA	NA
TRBV22-1	NA	NA	NA
TRBV23-1	NA	NA	NA
TRBV24-1	NA	NA	NA
MTRNR2L6	Inf	8.76E-192	2.11E-191
TRBV25-1	NA	NA	NA
TRBVA	NA	NA	NA
TRBV26	NA	NA	NA

TRBVB	NA	NA	NA
TRBV27	NA	NA	NA
TRBV28	NA	NA	NA
TRBC1	Inf	5.90E-174	1.33E-173
TRBV29-1	NA	NA	NA
PRSS2	Inf	0	0
PRSS1	NA	NA	NA
TRBD1	NA	NA	NA
TRBJ1-1	NA	NA	NA
TRBJ1-2	NA	NA	NA
TRBJ1-3	NA	NA	NA
TRBJ1-4	NA	NA	NA
TRBJ1-5	NA	NA	NA
TRBJ1-6	NA	NA	NA
TRBJ2-1	NA	NA	NA
TRBJ2-2	NA	NA	NA
TRBJ2-2P	NA	NA	NA
TRBJ2-3	NA	NA	NA
TRBJ2-4	NA	NA	NA
TRBJ2-5	NA	NA	NA
TRBJ2-6	NA	NA	NA
TRBJ2-7	NA	NA	NA
TRBC2	NA	NA	NA
TRBV30	NA	NA	NA
EPHB6	1.91163116	6.29E-93	1.06E-92
TRPV6	NA	NA	NA
RP11-114L1(	NA	NA	NA
TRPV5	0	0	0
C7orf34	NA	NA	NA
KEL	NA	NA	NA
OR9A2	NA	NA	NA
OR6V1	NA	NA	NA
PIP	NA	NA	NA
TAS2R39	NA	NA	NA
RP11-556I13	NA	NA	NA
TAS2R40	NA	NA	NA
GSTK1	1.77603895	5.38E-162	1.16E-161
AC073342.12	Inf	1.21E-296	4.70E-296
TMEM139	NA	NA	NA
CASP2	2.34648855	5.49E-39	7.34E-39
CLCN1	NA	NA	NA
FAM131B	NA	6.00E-156	1.26E-155
RP11-563K2:	NA	NA	NA
AC093673.5	1.6297881	8.27E-20	1.00E-19
ZYX	1.26449077	0	0

EPHA1	NA	6.09E-103	1.06E-102
EPHA1-AS1	1.85281174	2.44E-127	4.64E-127
TAS2R60	NA	NA	NA
TAS2R41	NA	NA	NA
CTAGE15	NA	NA	NA
TCAF2	NA	0	0
CTAGE6	NA	NA	NA
TCAF1	2.74690112	0	0
OR2F2	NA	NA	NA
RP4-669B10.	NA	NA	NA
AC004853.1	NA	NA	NA
OR6B1	NA	NA	NA
OR2A5	NA	NA	NA
OR2A25	NA	NA	NA
OR2A12	NA	NA	NA
OR2A2	NA	NA	NA
OR2A14	NA	NA	NA
CTAGE4	NA	NA	NA
ARHGEF35	1.54400978	3.28E-24	4.06E-24
RP4-545C24.	6.17603912	8.37E-06	9.13E-06
OR2A42	NA	NA	NA
OR2A1-AS1	3.34535452	5.09E-78	8.04E-78
OR2A7	NA	NA	NA
CTAGE8	NA	NA	NA
OR2A1	NA	NA	NA
ARHGEF5	Inf	0	0
NOBOX	NA	NA	NA
TPK1	2.16959995	0.23622322	0.24134491
AC006007.1	NA	NA	NA
CNTNAP2	NA	NA	NA
AC006004.1	NA	NA	NA
C7orf33	NA	NA	NA
RP5-1136G1:	1.54400978	2.41E-14	2.82E-14
CUL1	2.00262655	1.21E-42	1.65E-42
EZH2	2.07154645	0.03537724	0.03679492
GHET1	Inf	2.19E-27	2.77E-27
PDIA4	1.45104584	0	0
ZNF786	2.10156887	2.71E-39	3.63E-39
ZNF425	3.08801956	1.12E-18	1.34E-18
ZNF398	1.61419204	6.43E-71	9.91E-71
ZNF282	2.53418996	0	0
ZNF212	1.85281174	3.37E-32	4.36E-32
ZNF783	2.31601467	7.45E-113	1.35E-112
AC004941.3	NA	NA	NA
RP11-143I21	NA	NA	NA

ZNF777	2.58725963	1.37E-150	2.85E-150
ZNF746	2.31601467	3.40E-143	6.83E-143
KRBA1	2.64687391	7.57E-253	2.36E-252
ZNF467	1.65429619	2.87E-224	7.86E-224
SSPO	NA	0	0
ZNF862	0.38600244	2.53E-18	3.04E-18
RP4-751H13.	Inf	4.91E-187	1.16E-186
ATP6V0E2-A!	0.99257772	0.0006428	0.00068663
ATP6V0E2	1.77740661	4.78E-54	6.87E-54
RP11-445N2(	NA	NA	NA
RP11-728K2(	NA	5.99E-108	1.07E-107
RP11-728K2(	NA	NA	NA
RP11-545G3.	NA	NA	NA
ACTR3C	NA	0	0
LRRC61	NA	NA	NA
ZBED6CL	NA	NA	NA
RARRES2	NA	NA	NA
RP4-584D14.	NA	NA	NA
REPIN1	1.84133599	2.53E-08	2.83E-08
ZNF775	1.05078443	5.15E-171	1.15E-170
RP4-584D14.	NA	NA	NA
RP11-511P7.	1.83351161	6.93E-38	9.21E-38
LINC00996	NA	7.87E-86	1.28E-85
GIMAP8	0	3.41E-276	1.19E-275
GIMAP7	NA	NA	NA
GIMAP4	NA	NA	NA
GIMAP6	1.3896088	8.01E-39	1.07E-38
GIMAP2	1.71556642	4.51E-223	1.23E-222
GIMAP1	1.80134474	0.04993225	0.05180253
GIMAP1-GIM	NA	NA	NA
GIMAP5	0.77200489	2.60E-25	3.25E-25
TMEM176B	NA	0	0
TMEM176A	1.54400978	2.89E-05	3.14E-05
AOC1	0.77200489	2.71E-17	3.23E-17
KCNH2	1.45318568	1.22E-43	1.68E-43
NOS3	0.77200489	5.03E-189	1.20E-188
ATG9B	0	0	0
ABCB8	1.33346299	1.81E-133	3.52E-133
RP11-148K1.	NA	NA	NA
ASIC3	1.54400978	2.59E-45	3.57E-45
CDK5	1.40737175	5.33E-207	1.36E-206
SLC4A2	1.86236231	0.0002279	0.0002448
RP11-148K1.	Inf	0	0
FASTK	2.00854952	5.57E-44	7.64E-44
TMUB1	2.09458691	1.94E-10	2.21E-10

AGAP3	2.20079006	4.05E-215	1.07E-214
GBX1	NA	NA	NA
ASB10	NA	NA	NA
IQCA1L	NA	NA	NA
ABCF2	1.72731688	1.77E-41	2.40E-41
CHPF2	2.1282297	7.59E-236	2.19E-235
SMARCD3	0.66171848	0	0
RP4-548D19.	1.6297881	3.97E-92	6.65E-92
RP5-1070G2	NA	NA	NA
NUB1	1.48512805	0	0
WDR86	1.93001222	0.27444853	0.27998905
RP4-555L14.	NA	NA	NA
CRYGN	NA	NA	NA
RHEB	1.92242868	0.15881072	0.16302518
PRKAG2	1.94632219	1.97E-167	4.34E-167
PRKAG2-AS1	1.79851689	3.68E-41	4.98E-41
RP11-796I2.1	NA	NA	NA
GALNTL5	NA	NA	NA
GALNT11	1.83586529	0.00091817	0.00097858
RP5-981O7.2	NA	NA	NA
KMT2C	1.65562494	2.60E-95	4.40E-95
LINC01003	1.9073062	0.57603861	0.58171403
XRCC2	2.61011177	0	0
ACTR3B	1.92179941	2.16E-152	4.50E-152
LINC01287	NA	NA	NA
DPP6	NA	5.48E-168	1.21E-167
RP5-1048B1(	NA	NA	NA
AC006019.3	NA	NA	NA
AC006019.4	NA	NA	NA
RP11-476H2	NA	NA	NA
PAXIP1-AS2	NA	1.18E-81	1.90E-81
PAXIP1	2.63646953	2.93E-301	1.17E-300
RP11-5C23.1	NA	NA	NA
RP11-5C23.2	NA	NA	NA
PAXIP1-AS1	2.15120464	6.36E-116	1.16E-115
HTR5A-AS1	NA	NA	NA
HTR5A	NA	NA	NA
AC099552.4	NA	NA	NA
AC099552.3	NA	NA	NA
AC099552.2	NA	NA	NA
AC144652.1	2.70201711	7.96E-237	2.31E-236
INSIG1	1.08998928	0	0
BLACE	NA	NA	NA
AC008060.7	NA	NA	NA
AC008060.8	NA	NA	NA

EN2	NA	0	0
CNPY1	NA	NA	NA
AC008060.5	NA	NA	NA
AC009403.2	2.56381871	4.43E-283	1.59E-282
RBM33	2.18420896	3.49E-09	3.93E-09
SHH	1.89492109	1.31E-146	2.67E-146
AC021218.2	NA	NA	NA
AC073133.2	NA	NA	NA
AC073133.1	NA	NA	NA
LINC01006	NA	NA	NA
LINC00244	NA	NA	NA
RNF32	NA	NA	NA
AC005534.8	NA	2.05E-89	3.40E-89
LMBR1	2.00506826	9.57E-20	1.16E-19
RP5-1121A1!	NA	NA	NA
NOM1	1.89313301	2.16E-15	2.54E-15
MNX1	1.65753991	7.00E-95	1.18E-94
MNX1-AS2	0.77200489	4.98E-184	1.16E-183
MNX1-AS1	2.03809291	8.04E-53	1.15E-52
RP4-639J15.:	NA	NA	NA
RP4-639J15.:	NA	NA	NA
UBE3C	2.50265321	0	0
RP5-1142J19	NA	NA	NA
DNAJB6	1.61329227	3.51E-242	1.04E-241
AC006372.4	NA	NA	NA
AC006372.5	NA	NA	NA
AC006372.6	NA	NA	NA
PTPRN2	NA	5.22E-115	9.51E-115
AC005481.5	NA	NA	NA
AC011899.9	Inf	2.04E-256	6.47E-256
RP11-452C1:	NA	NA	NA
AC011899.1C	NA	NA	NA
AC078942.1	NA	NA	NA
LINC01022	NA	NA	NA
NCAPG2	2.47392476	3.77E-140	7.49E-140
ESYT2	2.85462273	2.90E-112	5.25E-112
WDR60	1.92512612	0.13830955	0.14218108
VIPR2	NA	NA	NA
PLCXD1	1.25450795	2.81E-264	9.27E-264
GTPBP6	1.51666356	0	0
LINC00685	1.66808199	3.13E-12	3.61E-12
PPP2R3B	2.59908313	9.42E-176	2.13E-175
RP13-465B1	NA	3.76E-21	4.58E-21
RP13-465B1	1.54400978	1.99E-14	2.33E-14
SHOX	NA	NA	NA

RP11-309M2	NA	NA	NA
CRLF2	NA	0	0
CSF2RA	0.87727828	0	0
IL3RA	0.83633863	0	0
SLC25A6	1.68745916	6.55E-150	1.35E-149
LINC00106	1.15800733	2.18E-161	4.69E-161
ASMTL-AS1	Inf	1.08E-183	2.51E-183
ASMTL	0.93743451	0	0
P2RY8	2.21610815	0	0
AKAP17A	1.59879722	8.92E-228	2.48E-227
ASMT	NA	NA	NA
RP13-297E16	NA	NA	NA
RP13-297E16	NA	NA	NA
DHRSX	1.82524013	1.17E-74	1.83E-74
ZBED1	1.80512908	1.01E-15	1.19E-15
LINC00102	NA	NA	NA
CD99	1.8679711	4.71E-95	7.98E-95
XG	NA	NA	NA
GYG2	2.9517834	1.40E-123	2.63E-123
GYG2-AS1	NA	NA	NA
ARSD	2.18734719	4.47E-22	5.47E-22
ARSD-AS1	NA	NA	NA
ARSE	NA	NA	NA
ARSH	NA	NA	NA
ARSF	NA	NA	NA
LINC01546	NA	NA	NA
MXRA5	NA	NA	NA
PRKX	1.77896779	1.44E-124	2.70E-124
PRKX-AS1	NA	NA	NA
RP11-706O15	1.78638341	7.59E-41	1.02E-40
RP11-706O15	Inf	0	0
RP11-706O15	NA	NA	NA
RP11-706O15	NA	NA	NA
RP11-707P20	NA	NA	NA
RP11-733O18	NA	NA	NA
NLGN4X	NA	NA	NA
VCX3A	NA	NA	NA
RP11-1M18.1	NA	NA	NA
PUDP	1.3413585	3.07E-248	9.39E-248
STS	Inf	2.64E-262	8.63E-262
VCX	NA	NA	NA
PNPLA4	NA	NA	NA
RP11-692P14	NA	NA	NA
VCX2	NA	NA	NA
VCX3B	NA	NA	NA

ANOS1	NA	NA	NA
FAM9A	NA	NA	NA
GS1-542M4.4	NA	NA	NA
FAM9B	NA	NA	NA
GS1-519E5.1	NA	NA	NA
TBL1X	1.97325731	1.66E-140	3.32E-140
GPR143	NA	2.99E-196	7.34E-196
SHROOM2	NA	0	0
CLDN34	NA	NA	NA
WWC3	1.83126741	4.92E-67	7.47E-67
WWC3-AS1	NA	3.94E-19	4.75E-19
CLCN4	0.89077487	1.88E-62	2.79E-62
RP6-1O2.1	NA	NA	NA
MID1	0	0	0
RP11-120D5.	5.40403423	1.89E-53	2.71E-53
HCCS	1.34643507	7.83E-246	2.37E-245
ARHGAP6	NA	NA	NA
AMELX	NA	NA	NA
GS1-590J6.3	NA	1.26E-252	3.92E-252
MSL3	1.78228289	3.18E-34	4.15E-34
FRMPD4	NA	NA	NA
FRMPD4-AS1	NA	NA	NA
PRPS2	1.79390732	6.63E-147	1.35E-146
TLR7	NA	6.50E-67	9.86E-67
TLR8-AS1	NA	NA	NA
TLR8	NA	NA	NA
TMSB4X	1.20516002	0	0
FAM9C	NA	NA	NA
RP11-791M2	NA	NA	NA
GS1-600G8.5	NA	NA	NA
GS1-600G8.3	NA	NA	NA
ATXN3L	NA	NA	NA
LINC01203	NA	NA	NA
RP11-1L9.1	NA	NA	NA
EGFL6	NA	NA	NA
TCEANC	2.64687391	1.14E-265	3.79E-265
RAB9A	1.93788983	4.20E-30	5.38E-30
TRAPPC2	2.06181794	4.69E-10	5.32E-10
OFD1	1.78923486	0.24170278	0.24688766
GPM6B	1.9420748	2.25E-22	2.76E-22
RP1-122K4.2	NA	NA	NA
RP1-122K4.3	NA	NA	NA
GEMIN8	1.4351373	0	0
GLRA2	NA	NA	NA
FANCB	2.11716493	5.16E-28	6.53E-28

MOSPD2	2.40179299	3.78E-06	4.14E-06
ASB9	4.67266118	0	0
ASB11	Inf	0	0
PIGA	1.91571584	0.00092052	0.00098103
FIGF	0.77200489	9.10E-10	1.03E-09
PIR	1.64997124	4.90E-108	8.73E-108
BMX	0.60947754	0	0
ACE2	NA	1.63E-127	3.10E-127
GS1-594A7.3	NA	NA	NA
TMEM27	0	1.86E-10	2.11E-10
CA5B	3.60268949	5.09E-21	6.20E-21
INE2	NA	1.46E-35	1.92E-35
ZRSR2	1.62861306	7.46E-141	1.49E-140
AP1S2	2.02309083	8.62E-67	1.31E-66
GRPR	NA	NA	NA
RP11-431J24	NA	NA	NA
MAGEB17	NA	NA	NA
CTPS2	2.12886197	9.75E-52	1.38E-51
S100G	NA	NA	NA
SYAP1	1.60296958	1.70E-250	5.25E-250
TXLNG	1.53028525	0	0
RBBP7	1.23944855	0	0
REPS2	NA	1.08E-79	1.72E-79
NHS	NA	NA	NA
NHS-AS1	NA	NA	NA
SCML1	NA	NA	NA
RAI2	NA	NA	NA
LINC01456	NA	NA	NA
BEND2	NA	NA	NA
SCML2	1.48175132	1.22E-106	2.15E-106
CDKL5	2.70201711	6.79E-06	7.41E-06
RS1	NA	NA	NA
PPEF1	NA	NA	NA
PPEF1-AS1	NA	NA	NA
PHKA2-AS1	NA	NA	NA
PHKA2	2.4563792	0	0
ADGRG2	NA	NA	NA
PDHA1	1.07365963	0	0
MAP3K15	5.24963325	0	0
SH3KBP1	1.41789017	0	0
CXorf23	2.37539966	1.47E-56	2.13E-56
MAP7D2	0	0	0
EIF1AX	2.19427263	1.84E-236	5.32E-236
EIF1AX-AS1	Inf	1.37E-238	4.01E-238
RPS6KA3	2.18734719	8.92E-48	1.25E-47

CNKSR2	1.46274611	1.99E-17	2.37E-17
KLHL34	1.54400978	3.24E-211	8.43E-211
SMPX	NA	NA	NA
MBTPS2	2.00091063	6.90E-80	1.10E-79
YY2	2.72775061	0.62521375	0.63063649
SMS	1.72790937	1.42E-302	5.69E-302
PHEX	Inf	0	0
PHEX-AS1	NA	NA	NA
PTCHD1-AS	NA	NA	NA
ZNF645	NA	NA	NA
RP11-494I9.2	NA	NA	NA
GS1-433O24	NA	NA	NA
DDX53	NA	NA	NA
PTCHD1	NA	0	0
PRDX4	1.83789035	1.24E-68	1.89E-68
ACOT9	1.57408789	0	0
RP13-314C1(	NA	NA	NA
SAT1	1.44180954	0	0
APOO	1.76708201	1.82E-61	2.70E-61
CXorf58	NA	0	0
KLHL15	2.26776436	3.62E-182	8.41E-182
EIF2S3	2.02602299	1.14E-27	1.44E-27
ZFX-AS1	NA	NA	NA
ZFX	1.79073299	3.34E-146	6.78E-146
PDK3	1.30848286	0	0
PCYT1B	NA	NA	NA
PCYT1B-AS1	NA	NA	NA
POLA1	2.15291505	2.12E-165	4.62E-165
ARX	1.37030868	4.05E-220	1.09E-219
RP11-86A5.1	NA	NA	NA
MAGEB18	NA	NA	NA
MAGEB6P1	NA	NA	NA
MAGEB6	NA	NA	NA
MAGEB5	NA	NA	NA
VENTXP1	NA	NA	NA
RP11-268G1	NA	NA	NA
RP11-268G1	NA	8.48E-55	1.22E-54
DCAF8L2	NA	NA	NA
MAGEB10	NA	NA	NA
DCAF8L1	NA	NA	NA
RP11-149B9.	NA	NA	NA
IL1RAPL1	NA	NA	NA
MAGEB2	Inf	0	0
MAGEB3	NA	NA	NA
MAGEB4	NA	NA	NA

MAGEB1	NA	NA	NA
NR0B1	NA	NA	NA
CXorf21	2.09253957	1.59E-92	2.67E-92
GK	1.58518337	0	0
GK-AS1	NA	3.03E-177	6.90E-177
TAB3	1.66463554	4.61E-35	6.05E-35
TAB3-AS1	NA	NA	NA
TAB3-AS2	NA	NA	NA
FTHL17	NA	NA	NA
DMD	2.42128806	1.20E-106	2.13E-106
DMD-AS3	NA	NA	NA
RP11-305F18	NA	NA	NA
FAM47A	NA	NA	NA
RP11-545D19	NA	NA	NA
TMEM47	NA	NA	NA
FAM47B	NA	NA	NA
MAGEB16	NA	NA	NA
CFAP47	NA	NA	NA
RP11-87M18	NA	NA	NA
FAM47C	NA	NA	NA
PRRG1	1.59381655	0	0
RP5-972B16.	NA	NA	NA
LANCL3	0.51466993	0	0
XK	1.3896088	3.80E-117	6.98E-117
CYBB	0.51466993	0	0
DYNLT3	1.11589798	0	0
AL121578.2	NA	NA	NA
HYPM	NA	NA	NA
SYTL5	NA	NA	NA
SRPX	NA	0	0
RP13-43E11.	NA	NA	NA
RPGR	2.05867971	3.60E-08	4.02E-08
OTC	NA	NA	NA
TSPAN7	Inf	4.74E-113	8.59E-113
RP4-646N3.1	NA	NA	NA
MID1IP1	1.66579083	2.87E-112	5.18E-112
MID1IP1-AS1	Inf	1.13E-22	1.39E-22
RP11-265P11	NA	NA	NA
LINC01281	NA	NA	NA
LINC01282	NA	NA	NA
RP11-157D23	NA	NA	NA
RP13-126P21	NA	NA	NA
AC092198.1	NA	NA	NA
BCOR	1.68554401	0.00339625	0.00358872
RP11-320G24	NA	NA	NA

ATP6AP2	1.19694618	0	0
CXorf38	2.03723513	4.19E-189	9.98E-189
MED14	2.28573997	4.06E-107	7.21E-107
MED14OS	2.14445803	5.74E-281	2.04E-280
USP9X	2.52969459	5.28E-210	1.36E-209
RP5-1172N1(	Inf	0	0
DDX3X	1.88342572	0.00041536	0.00044473
NYX	NA	NA	NA
CASK	1.85281174	2.62E-92	4.38E-92
CASK-AS1	NA	NA	NA
GPR34	NA	1.70E-150	3.51E-150
GPR82	3.08801956	9.04E-13	1.05E-12
RP11-524P6.	NA	NA	NA
RP1-154K9.2	NA	NA	NA
AL023875.1	NA	NA	NA
RP3-326I13.1	NA	NA	NA
MAOA	1.59226009	8.25E-25	1.03E-24
MAOB	NA	NA	NA
NDP	NA	NA	NA
NDP-AS1	NA	NA	NA
EFHC2	0.77200489	1.61E-48	2.25E-48
FUNDC1	1.67648399	3.92E-194	9.54E-194
DUSP21	NA	NA	NA
KDM6A	3.21668704	1.16E-194	2.82E-194
CXorf36	NA	2.34E-05	2.54E-05
RP11-342D1	NA	NA	NA
LINC01204	NA	NA	NA
MIR222HG	2.3495801	0	0
RP6-99M1.3	0	3.94E-18	4.73E-18
RP5-1158E12	Inf	0.00030243	0.00032429
RP5-1158E12	NA	NA	NA
LINC01186	NA	NA	NA
KRBOX4	1.62613796	6.15E-94	1.04E-93
ZNF674	3.86002445	1.46E-57	2.14E-57
ZNF674-AS1	2.49247293	1.22E-68	1.86E-68
CHST7	1.83207131	1.07E-133	2.08E-133
SLC9A7	2.82264288	9.00E-291	3.38E-290
RP2	1.76017115	9.90E-17	1.18E-16
LINC01545	Inf	1.27E-07	1.41E-07
JADE3	1.74836402	9.01E-224	2.46E-223
RGN	Inf	1.77E-225	4.87E-225
NDUFB11	2.07035823	1.71E-33	2.22E-33
RBM10	1.78588256	0	0
UBA1	1.62437622	0	0
CDK16	1.70373493	3.62E-148	7.43E-148

USP11	1.5651606	3.02E-183	7.03E-183
RP11-571E6.	NA	NA	NA
ZNF157	Inf	0	0
ZNF41	3.34535452	3.10E-111	5.58E-111
LINC01560	1.1229162	3.49E-260	1.13E-259
ARAF	1.95526472	8.78E-172	1.96E-171
SYN1	0.38600244	6.58E-217	1.75E-216
TIMP1	1.25315355	0	0
CFP	0.99257772	0	0
ELK1	1.99706429	4.87E-08	5.43E-08
UXT	1.96538981	2.37E-116	4.35E-116
UXT-AS1	NA	NA	NA
AL022578.1	0.77200489	5.24E-14	6.13E-14
ZNF81	1.76458261	0.00534789	0.00563619
ZNF182	1.94679494	3.29E-12	3.79E-12
ZNF630	NA	NA	NA
SPACA5	NA	NA	NA
ZNF630-AS1	NA	NA	NA
SPACA5B	NA	NA	NA
SSX5	NA	NA	NA
SSX1	NA	NA	NA
RP11-344N1	NA	NA	NA
SSX3	NA	NA	NA
SSX4	NA	NA	NA
SSX4B	NA	NA	NA
SLC38A5	1.17590149	0	0
FTSJ1	1.62527345	6.62E-195	1.61E-194
AF196972.9	3.474022	5.44E-123	1.02E-122
PORCN	0.9607172	0	0
EBP	1.42106316	0	0
TBC1D25	1.42211427	1.60E-163	3.48E-163
RP11-1148L6	0	1.90E-29	2.42E-29
RBM3	2.16260563	0	0
RP11-1148L6	NA	NA	NA
WDR13	2.19996165	2.74E-80	4.37E-80
WAS	1.47087247	0	0
SUV39H1	1.74855808	3.82E-77	6.02E-77
AF196970.3	NA	0	0
GLOD5	NA	0	0
GATA1	0	1.83E-26	2.29E-26
HDAC6	1.93001222	4.67E-16	5.53E-16
ERAS	Inf	0	0
PCSK1N	NA	NA	NA
TIMM17B	1.83564681	6.43E-196	1.58E-195
PQBP1	1.309709	0	0

SLC35A2	1.69742101	9.74E-44	1.34E-43
PIM2	1.25586711	0	0
OTUD5	1.75971703	9.90E-88	1.63E-87
KCND1	0.77200489	9.68E-146	1.97E-145
GRIPAP1	1.91163116	1.80E-198	4.45E-198
TFE3	2.64687391	2.46E-70	3.78E-70
CCDC120	2.70201711	0	0
PRAF2	2.70201711	2.08E-128	3.96E-128
WDR45	1.55527992	0	0
GPKOW	0.76632838	0	0
AC231657.1	NA	NA	NA
MAGIX	1.25189982	2.60E-137	5.12E-137
PLP2	1.56645009	1.38E-176	3.13E-176
PRICKLE3	1.5643257	0	0
SYP	1.4704855	4.27E-32	5.52E-32
SYP-AS1	NA	NA	NA
CACNA1F	NA	0	0
CCDC22	0.77988249	0	0
FOXP3	NA	0	0
PPP1R3F	1.54400978	3.21E-79	5.09E-79
LL0XNC01-7F	1.93001222	2.14E-05	2.33E-05
GAGE10	NA	NA	NA
GAGE12J	NA	NA	NA
GAGE13	NA	NA	NA
GAGE2E	NA	NA	NA
GAGE12B	NA	NA	NA
GAGE12C	NA	NA	NA
GAGE12D	NA	NA	NA
GAGE12E	NA	NA	NA
GAGE12F	NA	NA	NA
GAGE12G	NA	NA	NA
GAGE12H	NA	NA	NA
GAGE1	NA	NA	NA
GAGE2A	NA	NA	NA
PAGE1	NA	NA	NA
PAGE4	NA	NA	NA
USP27X-AS1	0.57900367	1.26E-106	2.22E-106
USP27X	1.75455657	9.11E-98	1.56E-97
CLCN5	2.19538891	1.34E-41	1.82E-41
AKAP4	NA	NA	NA
CCNB3	NA	2.49E-187	5.88E-187
DGKK	NA	NA	NA
SHROOM4	Inf	0	0
BMP15	NA	NA	NA
LINC01284	NA	NA	NA

NUDT10	NA	3.88E-17	4.63E-17
RP11-348F1.	NA	NA	NA
RP11-348F1.	NA	NA	NA
CXorf67	NA	5.27E-199	1.30E-198
NUDT11	1.52300284	1.10E-233	3.14E-233
LINC01496	NA	NA	NA
CENPVP3	NA	NA	NA
RP11-141O1!	NA	NA	NA
CENPVP1	NA	NA	NA
GSPT2	NA	NA	NA
MAGED1	2.29457009	0.01909364	0.01995258
RP11-114H2(	NA	NA	NA
RP11-33H15.	NA	NA	NA
MAGED4B	NA	NA	NA
MAGED4	NA	NA	NA
RP11-363G1(	NA	NA	NA
XAGE2	NA	NA	NA
XAGE1A	NA	NA	NA
XAGE1B	NA	NA	NA
SSX7	NA	NA	NA
SSX2	NA	NA	NA
SSX2B	NA	NA	NA
SPANXN5	NA	NA	NA
XAGE5	NA	NA	NA
XAGE3	NA	NA	NA
FAM156B	NA	0	0
FAM156A	Inf	0	0
GPR173	NA	NA	NA
TSPYL2	1.37513371	0.98994909	0.99016741
RP11-236P24	NA	NA	NA
KANTR	Inf	0	0
RP11-258C1!	2.05132728	2.89E-14	3.38E-14
KDM5C	1.74870805	3.05E-118	5.62E-118
IQSEC2	1.81648209	7.88E-210	2.03E-209
SMC1A	1.86652978	0	0
RIBC1	0.38600244	0	0
HSD17B10	1.79932264	1.46E-132	2.83E-132
RP3-339A18.	NA	2.11E-11	2.42E-11
HUWE1	1.82150463	7.53E-134	1.46E-133
PHF8	2.27465727	1.48E-222	4.03E-222
FAM120C	2.1896866	1.75E-41	2.36E-41
WNK3	NA	NA	NA
TSR2	2.01979548	5.70E-18	6.83E-18
FGD1	1.80594001	3.90E-27	4.92E-27
GNL3L	2.26313762	3.37E-27	4.25E-27

ITIH6	NA	NA	NA
MAGED2	1.01631023	0	0
TRO	NA	NA	NA
PFKFB1	Inf	1.16E-232	3.31E-232
APEX2	1.36884901	0	0
ALAS2	NA	NA	NA
PAGE2B	Inf	1.99E-294	7.61E-294
PAGE2	NA	NA	NA
FAM104B	2.4450805	4.55E-263	1.49E-262
MTRNR2L10	Inf	8.70E-88	1.43E-87
PAGE5	Inf	0.00241084	0.00255327
PAGE3	NA	NA	NA
MAGEH1	3.474022	6.62E-90	1.10E-89
USP51	NA	NA	NA
FOXR2	NA	NA	NA
RRAGB	2.12730236	5.30E-76	8.31E-76
RP13-188A5.	Inf	0	0
KLF8	NA	NA	NA
UBQLN2	1.92082169	1.68E-32	2.18E-32
RP11-431N1	NA	NA	NA
LINC01420	1.8877424	1.30E-10	1.48E-10
RP11-445O1	NA	NA	NA
SPIN3	0.99717298	3.05E-35	4.01E-35
SPIN2B	1.71076284	4.66E-93	7.83E-93
RP3-323P24.	2.31601467	6.46E-08	7.19E-08
SPIN2A	2.31601467	9.16E-171	2.04E-170
FAAH2	0.77200489	0	0
ZXDB	2.23881418	1.44E-33	1.88E-33
NLRP2P	NA	NA	NA
ZXDA	1.80134474	6.83E-61	1.01E-60
SPIN4	3.42255501	0	0
SPIN4-AS1	NA	9.95E-134	1.93E-133
ARHGEF9	2.84238164	4.26E-189	1.01E-188
AMER1	2.20879177	5.85E-162	1.26E-161
ASB12	Inf	0.00194428	0.00206228
MTMR8	1.54400978	2.22E-69	3.40E-69
ZC4H2	1.56043542	5.69E-216	1.51E-215
ZC3H12B	0.77200489	2.48E-46	3.45E-46
LAS1L	1.49982878	0	0
MSN	2.38660009	1.15E-231	3.26E-231
RP6-159A1.3	Inf	0	0
RP6-159A1.4	2.38847399	6.91E-236	1.99E-235
VSIG4	0.18381069	0	0
HEPH	NA	NA	NA
EDA2R	NA	NA	NA

AR	1.98849744	1.68E-77	2.65E-77
RP6-201G10.	Inf	3.89E-251	1.20E-250
OPHN1	1.93001222	1.82E-09	2.06E-09
YIPF6	1.99486064	6.07E-09	6.82E-09
STARD8	2.70201711	4.42E-201	1.10E-200
EFNB1	1.8467568	1.96E-22	2.41E-22
PJA1	0.96500611	0	0
LINC00269	Inf	3.66E-227	1.01E-226
FAM155B	NA	NA	NA
RP13-57D9.3	NA	NA	NA
EDA	3.86002445	9.91E-05	0.00010686
AWAT2	NA	NA	NA
OTUD6A	NA	NA	NA
IGBP1	1.60889527	9.37E-267	3.13E-266
IGBP1-AS2	NA	NA	NA
IGBP1-AS1	NA	NA	NA
DGAT2L6	NA	NA	NA
AWAT1	NA	NA	NA
P2RY4	NA	NA	NA
ARR3	NA	NA	NA
RAB41	NA	NA	NA
PDZD11	1.79831727	5.52E-135	1.08E-134
KIF4A	1.35604337	0	0
GDPD2	NA	0.00012221	0.0001316
DLG3	1.49859773	5.14E-43	7.02E-43
DLG3-AS1	NA	NA	NA
TEX11	NA	NA	NA
SLC7A3	NA	NA	NA
SNX12	1.72697048	4.27E-45	5.88E-45
FOXO4	1.63223891	8.87E-10	1.00E-09
CXorf65	1.58464162	2.35E-83	3.79E-83
IL2RG	1.45901842	0	0
MED12	2.20829306	1.38E-155	2.90E-155
NLGN3	4.63202934	8.68E-108	1.54E-107
RP5-1091N2.	3.08801956	4.92E-211	1.28E-210
GJB1	NA	NA	NA
ZMYM3	2.52954794	3.25E-150	6.72E-150
NONO	1.99859944	1.40E-16	1.66E-16
ITGB1BP2	1.97290139	3.11E-70	4.77E-70
TAF1	2.11749913	5.77E-279	2.03E-278
OGT	2.20142019	6.53E-201	1.63E-200
ACRC	1.68437431	2.26E-12	2.61E-12
CXCR3	0.25733496	5.84E-258	1.87E-257
LINC00891	0.55143206	0	0
CXorf49	NA	NA	NA

RP11-402P6.	NA	NA	NA
CXorf49B	NA	NA	NA
NHSL2	1.49575947	7.49E-109	1.34E-108
RGAG4	0.19300122	0	0
PIN4	1.87419654	1.82E-57	2.66E-57
ERCC6L	2.26277295	1.73E-25	2.16E-25
RPS4X	1.82536547	4.80E-80	7.65E-80
CITED1	1.7817239	5.66E-180	1.30E-179
HDAC8	1.8269618	4.59E-127	8.71E-127
PHKA1	1.80134474	4.97E-76	7.80E-76
PHKA1-AS1	NA	NA	NA
DMRTC1B	NA	NA	NA
RP1-172N19.	NA	NA	NA
RP11-69A21.	NA	NA	NA
DMRTC1	NA	NA	NA
LINC00684	NA	NA	NA
PABPC1L2B-/	NA	NA	NA
PABPC1L2B	NA	NA	NA
PABPC1L2A	NA	NA	NA
PABPC1L2B-/	NA	NA	NA
NAP1L6	Inf	0	0
NAP1L2	NA	NA	NA
CDX4	NA	NA	NA
CHIC1	0.77200489	1.53E-12	1.77E-12
TSIX	NA	0	0
XIST	NA	NA	NA
JPX	2.31415889	1.53E-214	4.02E-214
RP13-216E22	NA	0	0
FTX	2.17565014	3.11E-113	5.64E-113
ZCCHC13	NA	NA	NA
SLC16A2	0.38600244	5.47E-304	2.22E-303
RLIM	1.98836143	1.85E-18	2.22E-18
KIAA2022	3.14316277	3.69E-106	6.53E-106
ABCB7	1.44628764	1.51E-227	4.19E-227
UPRT	1.74052012	9.58E-211	2.49E-210
ZDHHC15	1.80134474	7.78E-25	9.68E-25
MAGEE2	NA	NA	NA
RP11-212D3.	NA	NA	NA
PBDC1	1.73794338	3.03E-79	4.80E-79
MAGEE1	1.15800733	3.69E-05	4.00E-05
MIR325HG	NA	NA	NA
FGF16	NA	NA	NA
ATRX	1.69610627	0	0
MAGT1	1.86518739	4.69E-74	7.30E-74
COX7B	2.0502067	1.35E-124	2.55E-124

ATP7A	2.8306846	1.40E-19	1.70E-19
PGAM4	NA	NA	NA
PGK1	0.8183345	0	0
TAF9B	2.54452812	3.68E-20	4.46E-20
CYSLTR1	1.22050297	0	0
ZCCHC5	NA	0	0
LPAR4	1.10286413	2.42E-210	6.27E-210
P2RY10	NA	NA	NA
GPR174	NA	NA	NA
ITM2A	0.74612518	0	0
TBX22	NA	NA	NA
FAM46D	NA	NA	NA
BRWD3	1.56545436	5.59E-163	1.21E-162
HMGN5	1.95888395	2.80E-89	4.63E-89
SH3BGRL	1.59460233	0	0
POU3F4	NA	NA	NA
CYLC1	NA	NA	NA
RPS6KA6	Inf	0	0
HDX	NA	NA	NA
APOOL	1.83161944	3.11E-20	3.77E-20
SATL1	NA	NA	NA
RP5-928E24.	NA	NA	NA
ZNF711	1.91957973	2.66E-24	3.29E-24
POF1B	NA	NA	NA
CHM	2.08165604	1.37E-48	1.92E-48
DACH2	NA	NA	NA
KLHL4	1.54400978	0	0
RP1-138A5.3	NA	NA	NA
CPXCR1	NA	NA	NA
RP13-348B1:	NA	NA	NA
TGIF2LX	NA	NA	NA
RP13-212L9.	NA	NA	NA
PABPC5-AS1	NA	NA	NA
PABPC5	NA	NA	NA
PCDH11X	2.50901589	1.71E-06	1.88E-06
NAP1L3	1.02933985	2.23E-104	3.91E-104
FAM133A	NA	4.01E-40	5.39E-40
DIAPH2	1.81972581	7.79E-137	1.53E-136
RPA4	1.87486902	2.00E-27	2.53E-27
DIAPH2-AS1	NA	NA	NA
RP11-485F1:	NA	NA	NA
PCDH19	NA	NA	NA
TNMD	NA	NA	NA
TSPAN6	Inf	0	0
SRPX2	NA	NA	NA

RP11-524D16	NA	NA	NA
SYTL4	1.54400978	7.27E-201	1.81E-200
CSTF2	1.21315054	0	0
NOX1	NA	NA	NA
XKRX	NA	NA	NA
ARL13A	NA	NA	NA
TRMT2B	1.82324559	1.53E-09	1.73E-09
TMEM35	NA	NA	NA
CENPI	1.15800733	3.77E-09	4.25E-09
DRP2	Inf	3.32E-296	1.28E-295
TAF7L	Inf	0	0
TIMM8A	2.25218275	1.57E-113	2.84E-113
BTK	1.91485201	1.52E-41	2.06E-41
RPL36A	2.81408612	0	0
RPL36A-HNR	NA	NA	NA
GLA	0.89201493	0	0
HNRNPH2	1.1988233	0	0
ARMCX4	NA	NA	NA
ARMCX1	NA	NA	NA
ARMCX6	2.32904429	1.94E-31	2.50E-31
ARMCX3	1.89141198	1.30E-239	3.81E-239
ARMCX3-AS1	NA	NA	NA
ARMCX2	NA	NA	NA
NXF5	NA	NA	NA
ZMAT1	Inf	7.46E-72	1.15E-71
TCEAL2	NA	8.71E-23	1.07E-22
TCEAL6	NA	NA	NA
BEX5	2.13116843	7.22E-83	1.16E-82
NXF2	NA	NA	NA
NXF2B	NA	NA	NA
TCP11X2	NA	NA	NA
TMSB15A	1.12694967	0	0
ARMCX5	1.29497594	1.23E-175	2.78E-175
RP4-769N13.	NA	2.89E-196	7.10E-196
GPRASP1	1.54400978	2.96E-15	3.48E-15
GPRASP2	1.83351161	0.20674397	0.21154817
BHLHB9	2.16161369	2.97E-85	4.83E-85
LINC00630	1.23520782	0.00169161	0.00179637
LL0XNC01-23	1.737011	2.38E-257	7.61E-257
RAB40AL	4.63202934	2.23E-26	2.80E-26
LL0XNC01-23	NA	NA	NA
BEX1	NA	4.35E-304	1.76E-303
NXF3	1.91457213	6.01E-47	8.37E-47
BEX4	1.66370046	0	0
TCEAL8	2.08666176	5.78E-42	7.86E-42

TCEAL5	NA	NA	NA
BEX2	NA	NA	NA
TCEAL7	NA	NA	NA
WBP5	NA	NA	NA
NGFRAP1	2.28680894	1.66E-267	5.56E-267
RP13-349O2(	NA	NA	NA
LL0XNC01-25	NA	NA	NA
RAB40A	NA	6.36E-183	1.48E-182
TCEAL4	1.51032229	7.41E-277	2.59E-276
TCEAL3	2.31601467	8.40E-260	2.71E-259
TCEAL3-AS1	NA	NA	NA
TCEAL1	1.86649284	5.20E-12	5.99E-12
MORF4L2	1.32028348	0	0
MORF4L2-AS	3.57052262	1.00E-20	1.22E-20
GLRA4	NA	NA	NA
TMEM31	0.51466993	4.75E-261	1.54E-260
PLP1	NA	NA	NA
RAB9B	Inf	1.26E-138	2.50E-138
LL0XNC01-11	2.70201711	7.52E-42	1.02E-41
TMSB15B	1.54400978	3.99E-27	5.03E-27
H2BFWT	NA	NA	NA
H2BFM	NA	NA	NA
RP11-722G7.	Inf	3.89E-14	4.55E-14
SLC25A53	2.46076559	0	0
ZCCHC18	1.68437431	2.10E-119	3.89E-119
FAM199X	1.92059753	1.90E-07	2.10E-07
ESX1	NA	NA	NA
IL1RAPL2	NA	NA	NA
TEX13A	NA	NA	NA
NRK	NA	NA	NA
SERPINA7	NA	NA	NA
MUM1L1	NA	NA	NA
CXorf57	2.7792176	5.47E-15	6.43E-15
RNF128	NA	NA	NA
TBC1D8B	1.46680929	4.73E-05	5.12E-05
MORC4	1.60971232	0.00221488	0.00234737
RIPPLY1	NA	2.42E-171	5.40E-171
CLDN2	NA	NA	NA
RBM41	1.64833477	1.52E-268	5.10E-268
NUP62CL	1.57260255	7.64E-136	1.50E-135
PIH1D3	NA	8.55E-35	1.12E-34
FRMPD3-AS1	NA	NA	NA
FRMPD3	NA	2.65E-142	5.32E-142
PRPS1	1.40818336	1.18E-256	3.74E-256
TSC22D3	0.99814774	0	0

NCBP2L	NA	NA	NA
MID2	0.77200489	0	0
RP6-191P20.	NA	NA	NA
TEX13B	NA	NA	NA
VSIG1	Inf	7.99E-86	1.30E-85
PSMD10	1.96301615	8.29E-127	1.57E-126
ATG4A	1.5066547	4.48E-155	9.43E-155
COL4A6	NA	NA	NA
COL4A5	NA	NA	NA
RP6-24A23.6	NA	NA	NA
IRS4	NA	NA	NA
RP6-24A23.3	NA	NA	NA
GUCY2F	NA	5.39E-305	2.19E-304
NXT2	2.07404299	4.17E-33	5.42E-33
KCNE5	Inf	1.87E-269	6.31E-269
ACSL4	1.75455657	1.46E-135	2.85E-135
TMEM164	2.03630275	2.23E-12	2.57E-12
AMMECR1	2.1073647	8.02E-100	1.38E-99
RGAG1	NA	NA	NA
CHRDL1	1.72745649	5.43E-14	6.35E-14
PAK3	NA	NA	NA
CAPN6	NA	NA	NA
DCX	NA	NA	NA
LINC00890	NA	NA	NA
ALG13	2.67711373	2.38E-245	7.17E-245
ALG13-AS1	NA	NA	NA
TRPC5	NA	NA	NA
TRPC5OS	Inf	0	0
ZCCHC16	NA	NA	NA
LHFPL1	NA	NA	NA
AMOT	0.38600244	1.58E-97	2.71E-97
XACT	2.24583241	3.33E-152	6.93E-152
HTR2C	NA	NA	NA
IL13RA2	NA	NA	NA
LRCH2	NA	NA	NA
RBMXL3	NA	NA	NA
LUZP4	NA	NA	NA
PLS3-AS1	NA	9.55E-32	1.23E-31
PLS3	2.34460744	6.14E-110	1.10E-109
DANT1	NA	NA	NA
DANT2	2.54761614	5.35E-294	2.04E-293
AGTR2	NA	NA	NA
SLC6A14	NA	NA	NA
CT83	NA	NA	NA
RP11-232D9.	NA	NA	NA

KLHL13	1.72216475	5.78E-206	1.47E-205
WDR44	1.30275825	8.47E-40	1.14E-39
DOCK11	1.75754305	0	0
IL13RA1	1.83351161	2.18E-146	4.43E-146
ZCCHC12	NA	4.91E-132	9.49E-132
LINC01285	3.08801956	1.79E-76	2.81E-76
LONRF3	NA	0	0
RP13-347D8.	NA	NA	NA
KIAA1210	NA	NA	NA
PGRMC1	1.57008337	0	0
RP5-1139I1.1	NA	NA	NA
RP5-1139I1.2	Inf	1.62E-56	2.36E-56
SLC25A43	1.59617227	4.21E-142	8.43E-142
SLC25A5	1.35611705	0	0
CXorf56	1.16633832	0	0
UBE2A	1.86969934	5.49E-57	7.99E-57
NKRF	2.30762331	3.97E-23	4.89E-23
6-Sep	1.71960925	0	0
SOWAHD	1.22455948	0	0
RPL39	2.30819907	0	0
UPF3B	1.84941831	6.90E-186	1.62E-185
RNF113A	1.3043005	0	0
NDUFA1	2.36235904	0	0
AKAP14	NA	1.61E-40	2.17E-40
NKAP	1.69327476	0	0
RHOXF1-AS1	NA	3.76E-78	5.95E-78
RHOXF2B	NA	NA	NA
RHOXF1	NA	NA	NA
LINC01402	NA	NA	NA
RHOXF2	NA	NA	NA
ZBTB33	2.22099868	1.51E-134	2.95E-134
TMEM255A	0.95015986	0	0
ATP1B4	NA	NA	NA
LAMP2	1.61803765	0	0
CUL4B	1.79666593	5.29E-115	9.65E-115
MCTS1	1.59987855	5.45E-297	2.11E-296
C1GALT1C1	1.09283809	0	0
CT47B1	NA	NA	NA
CT47A8	NA	NA	NA
RP1-321E8.5	NA	NA	NA
CT47A12	NA	NA	NA
CT47A11	NA	NA	NA
CT47A10	NA	NA	NA
CT47A9	NA	NA	NA
CT47A6	NA	NA	NA

CT47A7	NA	NA	NA
CT47A5	NA	NA	NA
CT47A4	NA	NA	NA
CT47A3	NA	NA	NA
CT47A2	NA	NA	NA
CT47A1	NA	NA	NA
GLUD2	Inf	0	0
RP13-192B1!	NA	NA	NA
GRIA3	NA	NA	NA
THOC2	1.82559621	2.05E-287	7.56E-287
XIAP	2.41947924	0	0
XIAP-AS1	NA	0	0
RP1-315G1.3	4.24602689	0	0
STAG2	2.24616661	1.59E-109	2.85E-109
SH2D1A	NA	1.18E-206	3.01E-206
TEX13D	NA	NA	NA
TENM1	NA	NA	NA
TEX13C	NA	NA	NA
RP11-13E5.2	NA	NA	NA
DCAF12L2	NA	NA	NA
DCAF12L1	NA	NA	NA
PRR32	NA	NA	NA
CTD-2076M1	NA	NA	NA
ACTRT1	NA	NA	NA
RP1-30E17.2	NA	NA	NA
SMARCA1	Inf	6.71E-126	1.27E-125
OCRL	1.66098022	6.75E-10	7.65E-10
APLN	1.26829375	1.72E-40	2.32E-40
XPNPEP2	0.6948044	0	0
SASH3	1.36090606	4.40E-189	1.05E-188
ZDHHC9	2.62075344	2.70E-67	4.10E-67
RP4-537K23.	NA	NA	NA
UTP14A	1.36509019	0	0
BCORL1	2.71304576	8.41E-95	1.42E-94
ELF4	2.89501834	0	0
AIFM1	1.24273958	0	0
RAB33A	1.27325925	0	0
ZNF280C	2.70201711	6.55E-61	9.69E-61
SLC25A14	1.55687653	5.37E-190	1.28E-189
GPR119	NA	NA	NA
RBMX2	1.99054217	9.65E-107	1.71E-106
RP1-274L7.1	NA	NA	NA
ENOX2	1.19017421	0	0
LINC01201	NA	NA	NA
ARHGAP36	NA	NA	NA

IGSF1	NA	0	0
OR13H1	NA	NA	NA
FIRRE	3.73135697	1.13E-162	2.44E-162
STK26	1.98682013	0.00120072	0.00127755
FRMD7	NA	NA	NA
RAP2C	1.83025878	6.69E-05	7.23E-05
RAP2C-AS1	2.31601467	0.00074898	0.00079925
MBNL3	2.4563792	0.85595114	0.8585065
HS6ST2	NA	1.58E-174	3.56E-174
HS6ST2-AS1	NA	NA	NA
USP26	NA	NA	NA
TFDP3	NA	NA	NA
GPC4	NA	NA	NA
GPC3	NA	NA	NA
CCDC160	Inf	3.97E-273	1.36E-272
PHF6	2.16072633	1.72E-28	2.19E-28
HPRT1	1.65113669	1.66E-178	3.81E-178
MIR503HG	2.55980569	1.93E-73	3.00E-73
LINC00629	1.54400978	6.01E-09	6.76E-09
PLAC1	NA	0	0
RP11-308B5.	NA	NA	NA
FAM122B	2.33317033	1.20E-145	2.45E-145
FAM122C	2.93361858	1.40E-66	2.13E-66
MOSPD1	2.4563792	3.23E-131	6.23E-131
RP3-473B4.3	NA	NA	NA
SMIM10	3.13880936	0	0
FAM127C	NA	NA	NA
FAM127A	NA	7.91E-13	9.16E-13
FAM127B	NA	NA	NA
RP11-85L21.	Inf	0	0
SMIM10L2B	1.35100856	8.13E-66	1.23E-65
LINC00633	NA	NA	NA
RP11-85L21.	NA	NA	NA
CT55	NA	0	0
ZNF75D	3.24242054	0.44015916	0.44628444
RP13-210D1	NA	NA	NA
ZNF449	1.25450795	3.69E-05	4.00E-05
RP11-265D1	NA	NA	NA
SMIM10L2A	NA	5.01E-290	1.87E-289
DDX26B-AS1	0.77200489	0.30322435	0.30899827
DDX26B	1.40777362	2.15E-144	4.34E-144
CT45A1	NA	NA	NA
CT45A3	NA	NA	NA
CT45A5	NA	NA	NA
CT45A6	NA	NA	NA

CT45A2	NA	NA	NA
CT45A7	NA	NA	NA
CT45A8	NA	NA	NA
CT45A9	NA	NA	NA
CT45A10	NA	NA	NA
SAGE1	NA	NA	NA
MMGT1	2.49614914	0	0
SLC9A6	1.77107004	0.0124205	0.01301223
FHL1	0.71686168	0	0
MAP7D3	1.81763177	7.42E-245	2.23E-244
ADGRG4	NA	NA	NA
BRS3	NA	NA	NA
HTATSF1	1.99766214	0.64778899	0.65297188
VGLL1	NA	NA	NA
CD40LG	NA	NA	NA
ARHGEF6	2.27312551	6.13E-130	1.17E-129
RP11-1114A5	2.75716032	9.54E-109	1.70E-108
RBMX	1.60197787	0	0
GPR101	NA	NA	NA
RP1-137H15.	NA	NA	NA
ZIC3	NA	NA	NA
FGF13	1.24072214	9.67E-217	2.57E-216
RP6-27P15.2	NA	NA	NA
FGF13-AS1	NA	NA	NA
F9	NA	NA	NA
MCF2	NA	0	0
ATP11C	2.22916412	9.95E-104	1.74E-103
CXorf66	NA	NA	NA
RP11-364B14	NA	NA	NA
AC004070.1	NA	NA	NA
SOX3	NA	NA	NA
XXyac-YR29II	NA	NA	NA
LINC00632	NA	NA	NA
CDR1	NA	NA	NA
CDR1-AS	NA	NA	NA
RP11-298A8.	NA	NA	NA
SPANXB1	NA	NA	NA
RP3-507I15.2	NA	NA	NA
LDOC1	1.02933985	1.28E-203	3.24E-203
SPANXC	NA	NA	NA
SPANXA2-OT	NA	NA	NA
SPANXA1	NA	NA	NA
SPANXA2	NA	NA	NA
RP1-171K16.	NA	NA	NA
SPANXD	NA	NA	NA

MAGEC3	NA	NA	NA
MAGEC1	NA	NA	NA
MAGEC2	NA	NA	NA
SPANXN4	NA	NA	NA
GS1-256O22	NA	NA	NA
SPANXN3	NA	NA	NA
SLITRK4	NA	NA	NA
SPANXN2	NA	NA	NA
RP3-526F5.2	NA	NA	NA
RP11-143G3.	NA	NA	NA
SPANXN1	NA	NA	NA
SLITRK2	NA	NA	NA
TMEM257	NA	NA	NA
CXorf51B	NA	NA	NA
CXorf51A	NA	NA	NA
L29074.3	NA	NA	NA
FMR1	2.4950303	0	0
FMR1NB	NA	NA	NA
AC002368.4	NA	NA	NA
AFF2	NA	NA	NA
IDS	2.36252099	3.08E-213	8.06E-213
AF011889.5	NA	NA	NA
AF011889.2	NA	NA	NA
LINC00893	2.31601467	1.07E-226	2.95E-226
CXorf40A	1.40007666	0	0
RP5-937E21.	NA	NA	NA
MAGEA9B	NA	NA	NA
HSFX2	NA	NA	NA
TMEM185A	1.84874855	1.53E-52	2.19E-52
MAGEA11	NA	NA	NA
HSFX1	NA	NA	NA
MAGEA9	NA	NA	NA
LINC00850	NA	NA	NA
MAGEA8-AS	NA	NA	NA
MAGEA8	NA	NA	NA
CXorf40B	1.48224939	0	0
XX-FW81066	NA	NA	NA
LINC00894	NA	0	0
MAMLD1	3.66702323	0	0
MTM1	1.40364525	5.37E-41	7.25E-41
MTMR1	2.7792176	1.16E-10	1.33E-10
CD99L2	1.72469178	0.05187017	0.05379458
AF003626.1	NA	NA	NA
HMGB3	1.52480966	0	0
GPR50-AS1	NA	NA	NA

GPR50	NA	NA	NA
AF003625.3	NA	NA	NA
VMA21	2.78253596	0	0
WI2-80269A(	0.77200489	2.14E-22	2.63E-22
XX-FW80269	NA	NA	NA
PASD1	NA	NA	NA
PRRG3	NA	NA	NA
FATE1	NA	NA	NA
CNGA2	NA	NA	NA
RP11-366F6.	NA	NA	NA
MAGEA4	NA	NA	NA
GABRE	NA	NA	NA
MAGEA10	NA	NA	NA
RP11-329E24	NA	NA	NA
GABRA3	NA	NA	NA
GABRQ	NA	NA	NA
MAGEA3	NA	NA	NA
MAGEA2B	NA	NA	NA
CSAG1	Inf	5.48E-94	9.23E-94
MAGEA12	NA	NA	NA
RP1-14D6.7	NA	NA	NA
MAGEA2	NA	NA	NA
MAGEA6	NA	NA	NA
CETN2	1.85223561	9.37E-69	1.43E-68
NSDHL	0.8168889	0	0
ZNF185	2.89501834	0	0
PNMA5	NA	NA	NA
PNMA3	Inf	1.02E-72	1.58E-72
PNMA6A	NA	NA	NA
MAGEA1	NA	NA	NA
RP11-635O1(	NA	NA	NA
LL0XNC01-16	NA	NA	NA
ZNF275	2.41251528	1.06E-127	2.01E-127
U82695.5	NA	NA	NA
ZFP92	0.99257772	3.96E-17	4.72E-17
TREX2	NA	NA	NA
HAUS7	1.53248732	2.17E-211	5.64E-211
U82695.9	NA	NA	NA
BGN	NA	NA	NA
ATP2B3	NA	NA	NA
FAM58A	1.91505089	0.11383385	0.11725947
RP11-66N11.	1.79304362	3.87E-31	4.98E-31
RP11-66N11.	NA	NA	NA
DUSP9	1.5010251	3.32E-231	9.36E-231
PNCK	Inf	9.74E-39	1.30E-38

SLC6A8	2.14909469	2.97E-145	6.01E-145
BCAP31	1.26058672	0	0
ABCD1	1.40221296	4.49E-255	1.41E-254
U52111.14	NA	0.07877509	0.08139988
PLXNB3	NA	NA	NA
SRPK3	NA	NA	NA
IDH3G	1.17217776	0	0
SSR4	1.99251639	4.66E-06	5.10E-06
PDZD4	NA	2.66E-288	9.87E-288
L1CAM	0	4.66E-153	9.73E-153
AVPR2	NA	NA	NA
ARHGAP4	1.70164429	2.80E-255	8.85E-255
NAA10	1.98737109	0.78426796	0.78839637
RENBP	1.31240831	4.71E-260	1.52E-259
HCFC1	2.31601467	4.32E-257	1.38E-256
HCFC1-AS1	0.77200489	1.50E-48	2.11E-48
TMEM187	1.49859773	6.08E-240	1.79E-239
IRAK1	2.04917393	1.77E-87	2.91E-87
MECP2	1.91197473	2.03E-24	2.52E-24
OPN1LW	NA	NA	NA
OPN1MW	NA	NA	NA
OPN1MW2	NA	NA	NA
OPN1MW3	NA	NA	NA
TEX28	NA	NA	NA
TKTL1	NA	NA	NA
XX-FW83128	Inf	2.62E-223	7.15E-223
FLNA	1.78112557	6.06E-37	8.02E-37
EMD	1.96078691	3.34E-106	5.91E-106
RPL10	1.94181768	3.23E-09	3.64E-09
XX-FW83563	1.54400978	1.15E-32	1.50E-32
DNASE1L1	1.30646981	7.24E-158	1.54E-157
TAZ	1.47461608	0	0
CH17-340M2	1.73421388	0.17374771	0.17813682
ATP6AP1	1.22477533	0	0
GDI1	1.64152619	2.79E-57	4.06E-57
FAM50A	1.13664189	0	0
PLXNA3	2.57334963	4.14E-57	6.02E-57
LAGE3	2.25433243	0	0
UBL4A	1.78845498	1.81E-179	4.17E-179
SLC10A3	1.71833346	4.90E-139	9.72E-139
FAM3A	1.85530207	1.37E-07	1.52E-07
G6PD	1.09751441	0	0
IKBKG	1.61280229	1.13E-99	1.94E-99
FAM223A	NA	NA	NA
CTAG1A	NA	NA	NA

CTAG1B	NA	NA	NA
FAM223B	NA	NA	NA
CTAG2	NA	NA	NA
GAB3	1.22026579	1.27E-138	2.52E-138
DKC1	1.45368521	0	0
MPP1	1.23974903	0	0
SMIM9	NA	NA	NA
F8	0.77200489	0	0
H2AFB1	NA	NA	NA
F8A1	1.31061295	4.35E-231	1.23E-230
FUNDC2	1.79181382	7.34E-271	2.49E-270
MTCP1	0.64333741	4.21E-237	1.22E-236
CMC4	0.66171848	5.18E-282	1.85E-281
BRCC3	1.9107121	0.49908219	0.50512408
VBP1	1.56052325	2.22E-293	8.45E-293
RAB39B	2.12301345	8.82E-174	1.98E-173
CLIC2	1.54400978	9.86E-118	1.82E-117
RP13-228J13	NA	NA	NA
H2AFB2	NA	NA	NA
F8A2	NA	NA	NA
F8A3	4.05302567	2.63E-79	4.18E-79
H2AFB3	NA	3.12E-27	3.93E-27
TMLHE-AS1	Inf	6.65E-189	1.58E-188
RP11-218L14	NA	NA	NA
TMLHE	1.62121027	2.34E-59	3.43E-59
SPRY3	Inf	1.65E-79	2.62E-79
VAMP7	1.85963609	2.96E-80	4.71E-80
IL9R	1.54400978	5.58E-149	1.15E-148
WASIR1	NA	NA	NA
AC144568.2	NA	NA	NA
OR4F21	NA	NA	NA
ZNF596	0.77200489	1.93E-205	4.89E-205
RP5-855D21.	NA	NA	NA
RP5-855D21.	Inf	1.16E-76	1.83E-76
RP11-63E5.6	NA	NA	NA
FAM87A	NA	NA	NA
FBXO25	1.20769082	0	0
RP11-91J19.3	NA	NA	NA
TDRP	NA	NA	NA
ERICH1	1.81552528	9.41E-249	2.88E-248
ERICH1-AS1	NA	2.16E-255	6.85E-255
RP11-43A14.	NA	NA	NA
RP11-555E9.	NA	NA	NA
CTD-3212A4.	NA	NA	NA
CTD-2281E23	NA	NA	NA

CTD-2281E23	NA	NA	NA
AF067845.1	NA	NA	NA
AF067845.2	NA	NA	NA
AF067845.3	NA	NA	NA
DLGAP2	NA	NA	NA
DLGAP2-AS1	NA	NA	NA
CLN8	1.63550666	2.90E-169	6.44E-169
CTD-2336O2.	3.86002445	2.41E-12	2.78E-12
CTD-2336O2.	1.7267549	5.14E-66	7.76E-66
ARHGEF10	1.69197738	0.00128349	0.00136514
KBTBD11-OT	1.23520782	1.31E-107	2.33E-107
RP11-439C15	NA	NA	NA
KBTBD11	1.96510336	8.27E-106	1.46E-105
RP11-439C15	2.57334963	5.47E-250	1.68E-249
RP11-1049H7	NA	NA	NA
MYOM2	1.3896088	1.13E-64	1.69E-64
AC133633.2	NA	NA	NA
CH17-31A6.1	NA	NA	NA
CH17-174L20	NA	NA	NA
AC133633.1	NA	NA	NA
RP11-378A12	NA	NA	NA
RP11-134O2	1.9110285	4.06E-94	6.84E-94
GS1-57L11.1	NA	NA	NA
CSMD1	NA	NA	NA
RP5-991O23.	NA	NA	NA
RP11-281H11	NA	NA	NA
RP11-158A14	NA	NA	NA
RP11-124B13	NA	3.63E-148	7.44E-148
RP11-115C21	1.9963564	1.99E-06	2.18E-06
MCPH1	1.66919976	4.40E-51	6.23E-51
ANGPT2	3.474022	0	0
CTD-2541M1	NA	NA	NA
MCPH1-AS1	Inf	0	0
AGPAT5	1.96188399	6.49E-42	8.81E-42
XKR5	NA	NA	NA
GS1-24F4.2	NA	3.31E-40	4.46E-40
DEFB1	NA	NA	NA
DEFA6	NA	NA	NA
GS1-24F4.3	NA	NA	NA
DEFA4	0	2.50E-306	1.03E-305
DEFA1	0	4.54E-24	5.62E-24
DEFA1B	0	9.03E-27	1.14E-26
DEFA3	0.00882291	3.15E-298	1.23E-297
DEFA5	NA	NA	NA
FAM66B	Inf	0	0

USP17L1	NA	NA	NA
USP17L4	NA	NA	NA
ZNF705G	NA	NA	NA
DEFB4B	NA	NA	NA
DEFB103B	NA	NA	NA
SPAG11B	NA	NA	NA
DEFB104B	NA	NA	NA
DEFB106B	NA	NA	NA
DEFB105B	NA	NA	NA
DEFB107B	NA	NA	NA
PRR23D1	NA	NA	NA
PRR23D2	NA	NA	NA
DEFB107A	NA	NA	NA
DEFB105A	NA	NA	NA
DEFB106A	NA	NA	NA
DEFB104A	NA	NA	NA
SPAG11A	NA	NA	NA
DEFB103A	NA	NA	NA
DEFB4A	NA	NA	NA
ZNF705B	NA	NA	NA
FAM66E	NA	NA	NA
USP17L8	NA	NA	NA
USP17L3	NA	NA	NA
FAM85B	0	0	0
RP11-556O5.	NA	NA	NA
SGK223	3.57052262	0	0
CTA-398F10.	NA	NA	NA
CTA-398F10.	NA	3.08E-244	9.25E-244
CTD-3023L14	NA	NA	NA
CTD-3023L14	NA	NA	NA
CTD-3023L14	NA	NA	NA
CLDN23	Inf	2.57E-113	4.65E-113
RP11-211C9.	NA	NA	NA
MFHAS1	2.56714879	5.90E-205	1.49E-204
ERI1	1.85924511	4.07E-41	5.50E-41
PPP1R3B	1.88712306	2.86E-06	3.13E-06
RP11-10A14.	0.38600244	7.93E-243	2.37E-242
RP11-10A14.	NA	NA	NA
RP11-10A14.	NA	NA	NA
RP11-10A14.	NA	NA	NA
RP11-10A14.	NA	NA	NA
RP11-115J16	NA	NA	NA
RP11-10A14.	NA	NA	NA
RP11-115J16	NA	NA	NA
RP11-115J16	NA	NA	NA

RP11-375N1!	1.80134474	2.42E-267	8.12E-267
TNKS	2.04476971	0.31945052	0.3253872
LINC00599	NA	4.20E-85	6.82E-85
RP11-1E4.1	1.28667482	4.62E-12	5.33E-12
MSRA	1.32784841	0	0
LINCR-0001	NA	NA	NA
RP11-981G7.	NA	NA	NA
PRSS51	NA	NA	NA
RP11-981G7.	NA	9.62E-26	1.20E-25
PRSS55	NA	NA	NA
RP1L1	Inf	6.12E-07	6.76E-07
C8orf74	NA	NA	NA
SOX7	1.51431728	0.45067345	0.45676635
CTD-2135J3.4	NA	NA	NA
CTD-2135J3.3	NA	NA	NA
PINX1	1.48091323	3.64E-252	1.13E-251
RP11-177H2.	3.08801956	2.12E-245	6.38E-245
XKR6	1.40364525	2.21E-11	2.54E-11
AF131215.6	NA	6.30E-87	1.03E-86
AF131215.8	0.77200489	2.44E-38	3.25E-38
LINC00529	NA	NA	NA
AF131216.1	5.40403423	3.90E-99	6.72E-99
MTMR9	2.27359682	4.75E-232	1.35E-231
AF131216.6	NA	4.18E-46	5.79E-46
SLC35G5	4.63202934	1.51E-47	2.11E-47
AF131216.5	NA	9.43E-127	1.79E-126
FAM167A	Inf	7.40E-122	1.38E-121
BLK	NA	NA	NA
RP11-148O2	NA	5.25E-216	1.39E-215
RP11-148O2	NA	NA	NA
RP11-148O2	NA	NA	NA
RP11-148O2	NA	NA	NA
LINC00208	NA	NA	NA
GATA4	NA	NA	NA
C8orf49	NA	NA	NA
NEIL2	1.50462178	0	0
FDFT1	1.05255805	0	0
RP11-297N6.	1.02933985	1.74E-220	4.68E-220
CTSB	1.58660315	2.91E-210	7.52E-210
DEFB136	Inf	2.86E-27	3.60E-27
DEFB135	NA	0	0
DEFB134	NA	NA	NA
RP11-1236K1	NA	NA	NA
ZNF705D	NA	NA	NA
FAM66D	NA	NA	NA

USP17L7	NA	NA	NA
USP17L2	NA	NA	NA
FAM86B1	1.89784535	0.00870485	0.00913961
AC145124.2	2.31601467	8.81E-226	2.43E-225
DEFB130	NA	NA	NA
FAM66A	NA	NA	NA
RP11-351I21	NA	8.08E-209	2.08E-208
FAM86B2	Inf	0	0
RP11-419I17	NA	0.00830716	0.00872509
LONRF1	1.50283619	1.22E-45	1.69E-45
RP11-252C15	NA	NA	NA
LINC00681	NA	NA	NA
KIAA1456	NA	7.99E-255	2.52E-254
DLC1	4.51765825	1.47E-283	5.30E-283
RP11-10C8.2	NA	NA	NA
C8orf48	NA	0	0
RP11-480O10	NA	NA	NA
SGCZ	NA	NA	NA
RP11-3G21.1	NA	NA	NA
TUSC3	NA	NA	NA
RP11-77B22.	NA	NA	NA
MSR1	NA	NA	NA
RP11-13N12.	NA	NA	NA
RP11-13N12.	NA	NA	NA
FGF20	NA	NA	NA
MICU3	1.34386036	1.92E-239	5.64E-239
RP11-468H14	Inf	2.22E-132	4.30E-132
ZDHHC2	1.99032511	1.34E-72	2.08E-72
CNOT7	1.8717548	2.63E-36	3.47E-36
VPS37A	1.46895375	8.85E-153	1.84E-152
MTMR7	NA	NA	NA
SLC7A2	2.2662079	3.89E-187	9.20E-187
PDGFRL	0.23160147	0	0
MTUS1	1.76458261	1.20E-216	3.19E-216
RP11-326L2.	NA	NA	NA
RP11-156K13	NA	NA	NA
FGL1	NA	NA	NA
RP11-156K13	NA	NA	NA
RP11-156K13	NA	NA	NA
PCM1	1.58171696	0	0
ASAH1	1.82350274	2.56E-14	2.99E-14
CTD-2547L16	1.54400978	3.88E-13	4.51E-13
NAT1	1.70776839	1.51E-98	2.59E-98
NAT2	NA	NA	NA
PSD3	2.12301345	2.73E-70	4.20E-70

RP11-161I2.1	NA	NA	NA
RP11-420B22	NA	NA	NA
RP11-1080G	NA	NA	NA
RP11-1080G	NA	0	0
RP11-618M2	NA	NA	NA
RP11-618M2	NA	NA	NA
SH2D4A	Inf	0	0
CSGALNACT1	NA	NA	NA
RP11-1105O	NA	NA	NA
INTS10	1.79010741	2.48E-100	4.29E-100
LPL	NA	NA	NA
AC100802.3	NA	NA	NA
SLC18A1	NA	1.95E-159	4.17E-159
ATP6V1B2	1.2380671	0	0
LZTS1	3.97031086	8.17E-214	2.14E-213
LZTS1-AS1	NA	NA	NA
RP11-563N12	NA	NA	NA
RP11-108E14	NA	NA	NA
RP11-369E15	NA	NA	NA
RP11-369E15	NA	NA	NA
RP11-369E15	NA	NA	NA
RP11-369E15	NA	NA	NA
RP11-421P23	NA	NA	NA
RP11-421P23	NA	NA	NA
RP11-24P4.1	NA	NA	NA
GFRA2	NA	3.70E-201	9.24E-201
DOK2	1.17344743	0	0
XPO7	2.27126076	6.12E-160	1.31E-159
NPM2	0.99257772	0	0
FGF17	0.77200489	9.26E-129	1.77E-128
DMTN	0.57900367	3.02E-244	9.07E-244
FAM160B2	2.33086092	1.84E-228	5.14E-228
NUDT18	1.76113616	3.03E-09	3.42E-09
HR	1.15800733	3.47E-175	7.84E-175
REEP4	1.20941202	0	0
LGI3	2.31601467	0.00041305	0.00044231
SFTPC	NA	NA	NA
BMP1	1.8684756	0.00352558	0.00372386
PHYHIP	NA	NA	NA
POLR3D	1.55713911	0	0
CTD-2530N2	NA	NA	NA
PIWIL2	NA	2.87E-97	4.89E-97
SLC39A14	2.63885308	6.24E-240	1.83E-239
PPP3CC	1.76748488	5.16E-55	7.45E-55
RP11-582J16	NA	2.94E-80	4.69E-80

SORBS3	2.09710969	3.79E-196	9.30E-196
RP11-582J16	0.77200489	1.65E-66	2.50E-66
PDLIM2	1.53713223	0	0
AC037459.4	NA	NA	NA
C8orf58	1.42903033	0	0
CCAR2	1.89313301	1.18E-81	1.90E-81
RP11-582J16	1.93001222	2.90E-70	4.46E-70
BIN3	1.75303637	1.59E-109	2.84E-109
EGR3	0.77200489	7.59E-21	9.25E-21
PEBP4	2.31601467	1.93E-29	2.47E-29
RP11-87E22.	NA	NA	NA
RP11-875O1	2.31601467	5.72E-87	9.37E-87
RHOBTB2	2.25425428	2.50E-34	3.26E-34
TNFRSF10B	1.80938646	1.19E-104	2.08E-104
RP11-875O1	NA	3.97E-81	6.35E-81
RP11-875O1	NA	NA	NA
TNFRSF10C	0.90067237	3.48E-77	5.48E-77
TNFRSF10D	0.95015986	1.36E-259	4.39E-259
RP11-1149O	NA	NA	NA
TNFRSF10A	2.09783937	1.71E-06	1.88E-06
RP11-1149O	NA	2.43E-306	9.94E-306
CHMP7	1.55724415	0	0
R3HCC1	1.55646147	0	0
LOXL2	0	0	0
ENTPD4	2.06466424	3.37E-247	1.03E-246
CTC-756D1.2	Inf	9.49E-287	3.48E-286
CTC-756D1.3	NA	NA	NA
SLC25A37	2.10106429	8.94E-139	1.77E-138
NKX3-1	0.46320293	7.31E-64	1.09E-63
NKX2-6	NA	NA	NA
RP11-175E9.	NA	NA	NA
RP11-213G6.	NA	NA	NA
STC1	NA	NA	NA
RP11-203E8.	NA	NA	NA
ADAM28	0.96500611	2.42E-121	4.51E-121
RP11-624C2	NA	NA	NA
ADAMDEC1	NA	NA	NA
ADAM7	NA	NA	NA
RP11-561E1.	NA	NA	NA
GS1-72M22.	NA	NA	NA
NEFM	NA	NA	NA
NEFL	NA	NA	NA
CTD-2168K2	NA	NA	NA
CTD-2168K2	NA	NA	NA
DOCK5	2.46483489	0	0

GNRH1	1.6297881	2.66E-69	4.07E-69
RP11-395I14	NA	NA	NA
KCTD9	1.95413738	7.46E-112	1.35E-111
CDCA2	1.59597165	9.36E-295	3.59E-294
RP11-219J21	NA	NA	NA
RP11-219J21	NA	NA	NA
RP11-299D14	NA	NA	NA
EBF2	NA	5.26E-61	7.79E-61
PPP2R2A	1.52458827	0	0
BNIP3L	1.44578252	2.49E-281	8.87E-281
PNMA2	1.85853029	9.30E-17	1.11E-16
DPYSL2	1.59151777	1.75E-35	2.31E-35
ADRA1A	NA	NA	NA
RP11-521M1	NA	NA	NA
RP11-521M1	NA	NA	NA
STMN4	NA	NA	NA
TRIM35	2.08727248	6.64E-08	7.40E-08
PTK2B	1.22417918	0	0
CHRNA2	NA	NA	NA
EPHX2	NA	2.53E-195	6.17E-195
CLU	1.95032814	0.75009661	0.75446319
SCARA3	2.31601467	1.16E-170	2.58E-170
CCDC25	1.88587227	1.74E-27	2.20E-27
RP11-16P20.	NA	NA	NA
ESCO2	2.14909469	2.29E-89	3.79E-89
PBK	0.92152362	0	0
SCARA5	NA	NA	NA
RP11-597M1	NA	NA	NA
NUGGC	0	0	0
ELP3	1.56397542	8.44E-245	2.54E-244
RP11-380I10	NA	NA	NA
PNOC	Inf	1.37E-143	2.75E-143
ZNF395	2.12301345	1.90E-17	2.28E-17
FBXO16	1.79105134	2.32E-230	6.53E-230
RP11-181B1	NA	NA	NA
FZD3	2.54118276	1.91E-234	5.48E-234
EXTL3	2.92019241	1.67E-256	5.32E-256
EXTL3-AS1	0.38600244	3.43E-173	7.71E-173
INTS9	2.08261784	1.91E-102	3.32E-102
RP11-662B1	NA	NA	NA
HMBOX1	1.23520782	7.22E-269	2.43E-268
CTD-2647L4.	NA	5.13E-91	8.55E-91
KIF13B	2.5572662	3.38E-109	6.05E-109
CTD-2647L4.	NA	NA	NA
DUSP4	1.47687892	1.31E-49	1.84E-49

RP11-486M2	NA	NA	NA
RP11-486M2	NA	NA	NA
LINC00589	NA	NA	NA
AC145110.1	NA	1.48E-31	1.90E-31
RP11-94H18.	NA	NA	NA
RP11-94H18.	NA	NA	NA
RP11-489E7.	NA	NA	NA
FAM183CP	NA	NA	NA
SARAF	1.57336771	0	0
RP11-489E7.	1.54400978	1.66E-108	2.97E-108
LEPROTL1	2.01022752	1.67E-80	2.67E-80
MBOAT4	NA	NA	NA
RP11-51J9.5	1.83075445	1.74E-29	2.22E-29
DCTN6	1.72899498	7.32E-233	2.09E-232
RP11-51J9.4	NA	NA	NA
RP11-51J9.6	Inf	2.29E-08	2.56E-08
RBPMS-AS1	1.43372337	6.35E-13	7.36E-13
RBPMS	1.2281896	0	0
GTF2E2	1.56518291	0	0
CTD-2373N4.	NA	NA	NA
SMIM18	NA	NA	NA
GSR	1.38213779	1.24E-254	3.89E-254
UBXN8	NA	NA	NA
PPP2CB	2.02610566	5.46E-50	7.71E-50
TEX15	2.04029864	6.89E-104	1.20E-103
PURG	NA	2.21E-192	5.35E-192
WRN	1.6782715	4.12E-32	5.33E-32
RP11-363L24	NA	NA	NA
RP11-566H8.	NA	NA	NA
RP11-566H8.	NA	NA	NA
NRG1	NA	NA	NA
RP11-11N9.4	1.97498338	0.28306764	0.2885712
RP11-722E23	2.4563792	2.15E-49	3.02E-49
FUT10	1.68437431	1.17E-24	1.46E-24
TTI2	1.60162209	0	0
MAK16	2.01423094	9.63E-39	1.29E-38
RNF122	0	2.37E-276	8.24E-276
DUSP26	NA	NA	NA
RP11-317N1	NA	NA	NA
RP1-273G13.	NA	NA	NA
RP11-431M3	NA	NA	NA
RP1-84O15.2	NA	NA	NA
RP11-431M3	NA	NA	NA
LINC01288	NA	NA	NA
UNC5D	NA	NA	NA

RP11-473J6.:	NA	NA	NA
RP11-89M20	NA	NA	NA
RP11-962G1!	NA	NA	NA
KCNU1	NA	NA	NA
RP11-419C2:	NA	NA	NA
RP11-527N2:	NA	NA	NA
LINC01605	NA	NA	NA
RP11-150O1:	NA	NA	NA
RP11-150O1:	NA	NA	NA
RP11-150O1:	NA	NA	NA
RP11-150O1:	NA	NA	NA
RP11-346L1.:	NA	NA	NA
ZNF703	1.80134474	3.00E-12	3.46E-12
RP11-863K1(	NA	NA	NA
RP11-863K1(	2.31601467	5.24E-65	7.89E-65
ERLIN2	2.1378597	4.45E-05	4.81E-05
PROSC	1.79525397	2.36E-232	6.70E-232
ADGRA2	2.38256682	2.23E-45	3.09E-45
BRF2	1.47560428	2.15E-147	4.39E-147
RAB11FIP1	2.1183061	5.74E-38	7.64E-38
GOT1L1	NA	NA	NA
ADRB3	NA	NA	NA
EIF4EBP1	2.23428076	0	0
KB-1836B5.4	1.54400978	1.89E-50	2.68E-50
ASH2L	1.01447574	0	0
RP11-90P5.5	NA	0	0
STAR	0.83473029	0	0
RP11-90P5.2	0.77200489	0	0
LSM1	1.97525644	1.21E-06	1.34E-06
BAG4	1.64487474	2.77E-105	4.87E-105
DDHD2	2.05533769	2.62E-138	5.18E-138
PLPP5	2.04690029	5.51E-234	1.57E-233
WHSC1L1	1.98547235	8.88E-69	1.36E-68
RP11-513D5.	NA	NA	NA
RP11-513D5.	NA	NA	NA
RP11-350N1!	2.47041565	0.20510739	0.20992092
LETM2	1.95574572	1.34E-25	1.68E-25
RP11-350N1!	NA	NA	NA
RP11-350N1!	NA	2.35E-91	3.92E-91
FGFR1	1.3496589	5.37E-276	1.87E-275
RP11-350N1!	NA	NA	NA
C8orf86	NA	NA	NA
RP11-675F6.	NA	NA	NA
RP11-675F6.	NA	NA	NA
RP11-495O1(	NA	NA	NA

TACC1	1.75488149	5.64E-27	7.10E-27
RP11-723D2:	NA	0	0
PLEKHA2	2.62254602	2.85E-11	3.26E-11
CTD-2544N1	NA	9.05E-283	3.24E-282
HTRA4	NA	0	0
TM2D2	1.73384705	5.87E-43	8.02E-43
ADAM9	1.76458261	1.63E-48	2.29E-48
ADAM32	NA	1.32E-06	1.45E-06
RP11-122L4.	NA	NA	NA
CTD-2024D2:	NA	NA	NA
ADAM18	0	1.35E-108	2.42E-108
ADAM2	NA	NA	NA
IDO1	NA	0	0
RP11-44K6.3	NA	NA	NA
RP11-44K6.4	NA	NA	NA
IDO2	NA	NA	NA
RP11-359E19	NA	NA	NA
RP11-359E19	NA	NA	NA
C8orf4	NA	NA	NA
RP11-470M1	NA	NA	NA
CTA-392C11.	NA	NA	NA
CTA-392C11.	NA	NA	NA
RP11-301G7.	NA	NA	NA
ZMAT4	NA	9.01E-36	1.19E-35
RP11-465K16	NA	NA	NA
SFRP1	NA	NA	NA
RP11-15G16.	NA	NA	NA
GOLGA7	1.64166195	6.01E-278	2.11E-277
GINS4	1.92172891	8.74E-41	1.18E-40
RP11-360L9.	NA	0	0
RP11-360L9.	Inf	1.77E-71	2.74E-71
GPAT4	1.76903363	0.01838832	0.01921885
RP11-360L9.	Inf	0	0
NKX6-3	NA	NA	NA
ANK1	NA	9.26E-195	2.26E-194
RP11-930P14	NA	NA	NA
RP11-930P14	1.80134474	0.34120992	0.34723904
KAT6A	2.33507652	8.99E-05	9.69E-05
RP11-589C21	Inf	7.70E-270	2.60E-269
RP11-589C21	NA	NA	NA
AP3M2	2.17631855	0.000585	0.00062511
PLAT	1.54400978	1.96E-62	2.92E-62
RP11-231D20	2.31601467	0.00042935	0.00045957
IKBKB	2.46124331	0	0
POLB	1.57225386	1.70E-197	4.18E-197

DKK4	NA	NA	NA
VDAC3	1.27543802	0	0
SLC20A2	1.9238852	1.56E-100	2.69E-100
RP11-503E24	Inf	7.77E-171	1.73E-170
SMIM19	1.95732372	2.88E-78	4.56E-78
CHRNB3	NA	NA	NA
RP11-412B14	NA	NA	NA
CHRNA6	NA	NA	NA
THAP1	2.2181549	3.88E-211	1.01E-210
RNF170	2.34514693	6.14E-43	8.38E-43
HOOK3	1.70653713	2.90E-05	3.15E-05
RP11-598P2(	NA	NA	NA
FNTA	1.36984548	0	0
POMK	0.51466993	0.87183679	0.874053
HGSNAT	1.84093474	0.42338362	0.42956354
LINC00293	NA	NA	NA
RP11-350F1(	NA	NA	NA
RP11-350F1(	NA	NA	NA
RP11-1134I1	Inf	1.76E-32	2.28E-32
IGLV8OR8-1	NA	NA	NA
SPIDR	2.12301345	7.74E-60	1.14E-59
CEBPD	1.63584679	2.29E-203	5.77E-203
PRKDC	2.07464574	1.36E-05	1.48E-05
MCM4	2.35881576	0	0
UBE2V2	1.9564734	0.00304745	0.00322334
RP11-567J20	NA	6.50E-67	9.86E-67
RP11-770E5.	NA	NA	NA
RP11-567J20	NA	NA	NA
AC026904.1	NA	NA	NA
RP11-567J20	NA	NA	NA
RP11-770E5.	NA	NA	NA
RP11-770E5.	NA	NA	NA
EFCAB1	NA	NA	NA
SNAI2	NA	NA	NA
C8orf22	NA	NA	NA
RP11-10H3.1	NA	NA	NA
RP11-738G5.	NA	NA	NA
RP11-738G5.	NA	NA	NA
RP11-109P6.	NA	NA	NA
RP11-401N1:	NA	NA	NA
SNTG1	NA	NA	NA
RP11-401H2.	NA	NA	NA
PXDNL	NA	NA	NA
RP11-11C20.	NA	NA	NA
PCMTD1	3.05585269	1.25E-307	5.17E-307

RP11-110G2	2.48293465	7.86E-264	2.59E-263
RP11-546K22	0	0	0
RP11-546K22	Inf	3.59E-94	6.06E-94
RP11-546K22	NA	NA	NA
ST18	NA	0	0
RP11-26M5.3	NA	NA	NA
RP11-26M5.2	NA	NA	NA
RP11-1023P1	NA	NA	NA
FAM150A	1.15800733	5.75E-191	1.38E-190
RB1CC1	2.16801373	3.70E-93	6.22E-93
NPBWR1	NA	NA	NA
RP11-162D9.	NA	NA	NA
OPRK1	NA	NA	NA
RP11-1081M	NA	NA	NA
RP11-1081M	NA	NA	NA
RP11-400K9.	NA	NA	NA
RP11-400K9.	NA	NA	NA
ATP6V1H	1.27416005	0	0
RGS20	1.52887243	2.95E-37	3.91E-37
RP11-1070A2	0	0	0
TCEA1	1.98638715	2.23E-07	2.47E-07
AC100821.2	NA	NA	NA
RP11-30L15.(	Inf	0	0
LYPLA1	1.86921331	8.58E-32	1.11E-31
MRPL15	1.4307026	0	0
SOX17	NA	NA	NA
RP11-53M11	NA	NA	NA
RP1	NA	NA	NA
XKR4	Inf	3.74E-133	7.25E-133
RP11-386G2	NA	NA	NA
RP11-386G2	NA	NA	NA
RP11-628E19	NA	NA	NA
TMEM68	1.86252928	2.92E-21	3.56E-21
TGS1	2.11344314	0.24550216	0.25069797
LYN	1.99344995	0.86523315	0.8675764
RPS20	2.44727597	0	0
CTA-397H3.3	NA	1.48E-70	2.28E-70
MOS	NA	NA	NA
PLAG1	0.33085924	7.18E-257	2.29E-256
CHCHD7	2.22828684	0	0
RP11-140I16	6.94804401	2.41E-112	4.36E-112
SDR16C5	NA	NA	NA
PENK	NA	NA	NA
RP11-17A4.2	NA	NA	NA
LINC00968	NA	NA	NA

RP11-17A4.3	NA	NA	NA
RP11-17A4.4	NA	NA	NA
IMPAD1	2.00974388	2.37E-37	3.14E-37
LINC01606	NA	NA	NA
RP11-513O1	NA	NA	NA
LINC00588	NA	NA	NA
RP11-756K1	NA	NA	NA
RP11-246K1	2.93361858	1.12E-19	1.36E-19
RP11-388G2	NA	NA	NA
CTD-2339F6.	2.31601467	8.63E-44	1.18E-43
RP11-44D19.	NA	NA	NA
LINC01602	2.48757131	0.02314288	0.0241506
FAM110B	2.00721271	4.09E-14	4.79E-14
RP11-1112C	NA	NA	NA
RP11-648L3.	NA	NA	NA
UBXN2B	2.26725647	2.48E-102	4.32E-102
CYP7A1	NA	NA	NA
SDCBP	1.23439304	0	0
NSMAF	1.66451786	1.58E-20	1.92E-20
TOX	2.31601467	2.12E-23	2.62E-23
RP11-25K19.	2.34720679	6.88E-123	1.29E-122
RP11-379I19	NA	NA	NA
RP11-379I19	NA	NA	NA
RP11-27P7.1	NA	NA	NA
CA8	1.1971292	2.16E-279	7.61E-279
LINC01301	2.31601467	5.21E-176	1.18E-175
RAB2A	1.86490767	9.80E-18	1.17E-17
RP11-91I20.4	NA	0	0
RP11-91I20.3	NA	NA	NA
RP11-91I20.2	NA	NA	NA
RP11-91I20.1	NA	NA	NA
CHD7	1.94340663	5.29E-14	6.18E-14
RP11-33I11.2	NA	NA	NA
AC022182.1	NA	NA	NA
AC022182.3	Inf	0	0
CLVS1	NA	NA	NA
RP11-787D1	NA	NA	NA
RP11-787D1	NA	NA	NA
ASPH	2.65430895	0	0
RP11-280G9.	NA	NA	NA
RP11-705O2	NA	5.84E-179	1.34E-178
RP11-705O2	NA	NA	NA
RP11-705O2	NA	NA	NA
NKAIN3	NA	NA	NA
RP11-577N1	NA	NA	NA

UG0898H09	NA	NA	NA
RP11-659E9.	Inf	6.57E-99	1.13E-98
GGH	1.41435363	0	0
RP11-659E9.	NA	NA	NA
TTPA	5.14669927	3.89E-283	1.40E-282
YTHDF3-AS1	1.89042223	4.94E-47	6.88E-47
YTHDF3	2.32219071	0	0
RP11-16E18.	2.31601467	4.94E-241	1.46E-240
CTD-3046C4.	NA	NA	NA
RP11-45K10.	NA	NA	NA
RP11-573J24	NA	NA	NA
RP11-32K4.1	NA	NA	NA
RP11-579E24	NA	NA	NA
LINC01289	NA	NA	NA
MIR124-2HG	NA	NA	NA
RP11-21C4.1	NA	NA	NA
BHLHE22	NA	9.36E-221	2.52E-220
CYP7B1	NA	NA	NA
RP11-1D12.2	NA	NA	NA
LINC00251	NA	NA	NA
LINC01299	NA	NA	NA
CTD-3025N2(	1.72216475	1.47E-17	1.76E-17
ARMC1	1.83697574	2.65E-116	4.85E-116
MTFR1	1.60735377	0	0
RP11-707M3	1.28667482	1.35E-190	3.23E-190
PDE7A	1.66487923	6.75E-28	8.54E-28
CTD-2532N2(	3.08801956	1.75E-09	1.98E-09
DNAJC5B	0.77200489	2.17E-12	2.51E-12
AC084082.3	Inf	1.62E-114	2.94E-114
TRIM55	Inf	3.87E-87	6.35E-87
CRH	NA	NA	NA
LINC00967	NA	NA	NA
RRS1-AS1	5.40403423	0.14270779	0.1466692
RRS1	1.48258444	0	0
ADHFE1	Inf	0.02927715	0.0305046
C8orf46	NA	NA	NA
MYBL1	1.7094394	4.60E-15	5.42E-15
VCPIP1	1.96861247	4.59E-16	5.43E-16
C8orf44-SGK	NA	1.58E-213	4.15E-213
C8orf44	1.8935969	5.80E-96	9.85E-96
AC090154.1	NA	NA	NA
SGK3	2.67628362	0	0
MCMDC2	Inf	0	0
TCF24	2.40179299	4.85E-84	7.86E-84
PPP1R42	NA	0	0

COPS5	1.42249049	0	0
CSPP1	1.59159912	3.37E-277	1.18E-276
ARFGEF1	1.75153798	3.90E-189	9.31E-189
RP11-7F18.2	1.08080685	9.48E-19	1.14E-18
CPA6	NA	NA	NA
RP11-396J6.1	NA	NA	NA
PREX2	NA	NA	NA
RP11-403D15	NA	NA	NA
C8orf34-AS1	NA	NA	NA
C8orf34	NA	NA	NA
RP11-865I6.2	NA	NA	NA
LINC01592	NA	NA	NA
RP11-21C17.	NA	NA	NA
LINC01603	NA	NA	NA
SULF1	NA	NA	NA
SLCO5A1	1.54400978	7.20E-07	7.94E-07
RP11-102F4.	NA	NA	NA
RP11-159H10	0	1.02E-209	2.64E-209
PRDM14	NA	NA	NA
NCOA2	3.03287635	8.80E-133	1.71E-132
RP11-333A23	NA	NA	NA
RP11-333A23	NA	NA	NA
TRAM1	2.16733225	1.89E-215	5.00E-215
LACTB2-AS1	1.15800733	0.16377524	0.16802638
LACTB2	1.50579172	0	0
XKR9	1.93001222	2.14E-159	4.57E-159
RP11-326E22	NA	NA	NA
EYA1	Inf	0	0
RP11-1102P1	Inf	0	0
RP11-1144P2	NA	NA	NA
RP11-281N10	NA	NA	NA
MSC-AS1	NA	NA	NA
MSC	NA	NA	NA
TRPA1	NA	NA	NA
RP11-142A23	NA	NA	NA
RP11-351E7.	NA	NA	NA
KCNB2	NA	0.1280168	0.13173459
RP11-1145L2	NA	NA	NA
RP11-531A24	NA	NA	NA
TERF1	1.32190785	5.41E-292	2.04E-291
RP11-531A24	NA	NA	NA
RP11-531A24	0.51466993	1.03E-30	1.32E-30
SBSPON	NA	8.87E-229	2.48E-228
C8orf89	NA	NA	NA
RPL7	1.67553861	7.46E-226	2.06E-225

RDH10	2.58661432	0	0
RDH10-AS1	Inf	6.65E-296	2.57E-295
RP11-434I12	NA	NA	NA
RP11-434I12	NA	NA	NA
STAU2-AS1	NA	6.83E-50	9.63E-50
STAU2	1.86138957	6.10E-83	9.85E-83
RP11-463D19	NA	NA	NA
RP11-463D19	NA	NA	NA
UBE2W	1.6123288	0	0
TCEB1	2.17916008	6.08E-130	1.16E-129
TMEM70	1.89661148	5.45E-63	8.13E-63
LY96	1.65981051	1.83E-174	4.11E-174
RP11-6I2.3	NA	NA	NA
RP11-6I2.4	NA	NA	NA
RP1-16A9.1	NA	NA	NA
JPH1	3.84119506	0	0
GDAP1	1.82473883	0.19664646	0.20142052
CTD-2320G14	NA	NA	NA
MIR2052HG	NA	NA	NA
RP11-730G20	NA	NA	NA
RP11-758M4	NA	NA	NA
PI15	NA	NA	NA
RP11-3N13.2	NA	NA	NA
CRISPLD1	1.91571584	8.81E-105	1.55E-104
RP11-300E4.	NA	NA	NA
RP11-697M1	NA	0	0
CASC9	NA	NA	NA
HNF4G	NA	NA	NA
RP11-706J10	NA	NA	NA
LINC01109	NA	NA	NA
LINC01111	NA	NA	NA
ZFHX4-AS1	NA	NA	NA
ZFHX4	NA	NA	NA
RP11-48D4.2	NA	NA	NA
PEX2	1.47285726	0	0
RP11-38H17.	NA	NA	NA
RP11-91P17.	NA	NA	NA
PKIA-AS1	NA	NA	NA
PKIA	2.75716032	3.77E-42	5.13E-42
ZC2HC1A	1.43372337	1.88E-32	2.44E-32
IL7	NA	NA	NA
RP11-79H23.	NA	NA	NA
RP11-1114I9	NA	NA	NA
STMN2	NA	NA	NA
HEY1	NA	0	0

LINC01607	NA	NA	NA
RP11-26J3.1	NA	0	0
MRPS28	1.71608316	0	0
RP11-26J3.4	NA	NA	NA
RP11-92K15.	1.54400978	4.93E-62	7.32E-62
TPD52	2.17050589	2.00E-44	2.75E-44
RP11-92K15.	NA	NA	NA
RP11-941H1!	NA	NA	NA
RP11-48B3.5	3.08801956	0	0
ZBTB10	2.72614227	2.08E-83	3.36E-83
RP11-48B3.3	0.77200489	7.69E-162	1.66E-161
RP11-48B3.4	2.31601467	2.64E-81	4.24E-81
ZNF704	1.28667482	2.69E-36	3.55E-36
PAG1	0.77200489	1.08E-99	1.86E-99
RP11-172E1(	NA	NA	NA
RP11-1149M	NA	NA	NA
RP11-1149M	NA	0.07877509	0.08139988
RP11-363E6.	0.38600244	2.47E-237	7.16E-237
RP11-363E6.	1.02933985	4.20E-82	6.75E-82
FABP5	2.05413616	3.85E-22	4.72E-22
RP11-157I4.4	NA	NA	NA
PMP2	NA	NA	NA
FABP9	NA	NA	NA
FABP4	NA	NA	NA
RP11-257P3.	NA	NA	NA
FABP12	NA	NA	NA
IMPA1	1.58380385	0	0
SLC10A5	5.40403423	3.16E-110	5.67E-110
ZFAND1	1.6904245	0	0
CHMP4C	NA	NA	NA
RP13-923O2:	NA	NA	NA
SNX16	1.54400978	4.31E-88	7.10E-88
RP11-354A1⁄	NA	0	0
RP11-99H20.	NA	NA	NA
RP11-653B1(	NA	NA	NA
RP11-731N1(	NA	NA	NA
CTD-2272D1ᵴ	NA	NA	NA
LINC01419	NA	NA	NA
RP11-120I21	NA	NA	NA
RALYL	NA	NA	NA
LRRCC1	1.86932002	1.85E-76	2.91E-76
RP11-219B4.	Inf	0	0
E2F5	2.01027016	1.00E-40	1.35E-40
RP11-219B4.	NA	NA	NA
C8orf59	1.96682305	2.48E-27	3.13E-27

CA13	1.74987775	3.93E-13	4.56E-13
RP11-219B4.	0.77200489	1.31E-219	3.53E-219
RP11-219B4.	NA	NA	NA
CA1	NA	NA	NA
CA3	NA	7.32E-118	1.35E-117
CA3-AS1	4.63202934	3.35E-142	6.71E-142
CA2	1.00136866	0	0
RP11-317J10	NA	NA	NA
CTA-392E5.1	NA	NA	NA
ATP6V0D2	NA	NA	NA
PSKH2	NA	NA	NA
CTD-3118D1	NA	NA	NA
CTD-3118D1	NA	NA	NA
SLC7A13	NA	NA	NA
WWP1	2.46076559	2.75E-70	4.23E-70
RMDN1	1.58642763	3.19E-306	1.31E-305
CPNE3	1.78598146	3.18E-94	5.37E-94
CNGB3	NA	NA	NA
RP11-386D6.	NA	NA	NA
RP11-386D6.	NA	NA	NA
CNBD1	NA	NA	NA
AF121898.3	NA	NA	NA
DCAF4L2	NA	NA	NA
CTB-118P15.	NA	NA	NA
MMP16	3.08801956	3.71E-48	5.19E-48
RP11-586K2.	NA	NA	NA
RP11-642C5.	NA	NA	NA
RP11-37B2.1	1.66277976	3.29E-106	5.81E-106
RIPK2	1.58464162	1.31E-160	2.82E-160
OSGIN2	2.26922649	7.37E-257	2.35E-256
NBN	1.71399251	0	0
DECR1	1.48154221	0	0
CALB1	NA	3.16E-107	5.61E-107
LINC00534	NA	NA	NA
AB015752.3	NA	NA	NA
LINC01030	NA	1.38E-20	1.67E-20
TMEM64	1.62121027	2.47E-45	3.41E-45
RP11-68L18.	2.31601467	1.74E-242	5.18E-242
NECAB1	1.54400978	6.38E-14	7.45E-14
RP11-662G2	NA	NA	NA
C8orf88	2.03330865	2.13E-251	6.61E-251
TMEM55A	1.3164715	7.68E-206	1.95E-205
GS1-251I9.3	NA	NA	NA
OTUD6B-AS1	1.93368844	4.87E-29	6.20E-29
OTUD6B	2.10647049	9.21E-40	1.24E-39

LRRC69	1.32343695	3.79E-272	1.30E-271
SLC26A7	0	1.25E-110	2.25E-110
RP11-122C21	NA	NA	NA
RUNX1T1	NA	8.09E-171	1.80E-170
GS1-5L10.1	NA	NA	NA
RP11-700E23	NA	NA	NA
RP11-700E23	NA	NA	NA
RP11-587H10	NA	NA	NA
RP11-587H10	NA	NA	NA
RP11-100L22	NA	NA	NA
RP11-100L22	NA	NA	NA
RP11-100L22	NA	NA	NA
CTD-3239E11	NA	NA	NA
TRIQK	1.87486902	9.80E-116	1.79E-115
C8orf87	NA	NA	NA
LINC00535	Inf	0	0
RP11-388K12	NA	NA	NA
RP11-388K12	NA	NA	NA
FAM92A1	2.39894726	0	0
CTD-2006H14	2.8306846	5.83E-53	8.34E-53
RP11-10N23.	0.77200489	1.49E-14	1.75E-14
RBM12B	1.80594001	1.15E-76	1.82E-76
RP11-10N23.	NA	NA	NA
RP11-10N23.	0.77200489	2.85E-33	3.71E-33
TMEM67	2.39587724	1.58E-254	4.96E-254
RP3-388N13.	NA	NA	NA
PDP1	2.53046047	2.38E-261	7.76E-261
RP3-388N13.	NA	1.15E-197	2.84E-197
KB-1184D12.	NA	NA	NA
CDH17	NA	NA	NA
GEM	NA	NA	NA
RAD54B	1.3955473	6.43E-87	1.05E-86
FSBP	NA	NA	NA
KIAA1429	1.95449043	4.04E-91	6.73E-91
RP11-267M2	NA	NA	NA
RP11-267M2	1.80134474	6.61E-214	1.74E-213
RP11-22C11.	2.31601467	8.48E-34	1.11E-33
ESRP1	5.59703545	0	0
DPY19L4	1.93342818	1.28E-12	1.48E-12
KB-1608C10.	NA	NA	NA
INTS8	1.77937712	6.94E-94	1.17E-93
CCNE2	1.54400978	4.30E-297	1.67E-296
RP11-347C18	1.54400978	2.31E-173	5.18E-173
NDUFAF6	2.13930811	4.14E-64	6.20E-64
TP53INP1	NA	4.67E-32	6.03E-32

RP11-320N2	NA	NA	NA
RP11-320N2	NA	NA	NA
PLEKHF2	1.26608802	0	0
C8orf37-AS1	Inf	4.72E-62	7.02E-62
LINC01298	NA	NA	NA
C8orf37	2.02651284	6.27E-108	1.12E-107
RP11-90D11.	NA	0	0
RP11-31K23.	NA	NA	NA
KB-1043D8.8	NA	NA	NA
GDF6	NA	3.92E-201	9.77E-201
UQCRB	1.73314959	0	0
KB-1043D8.6	0	2.67E-63	3.99E-63
MTERF3	1.20017567	0	0
PTDSS1	1.76943521	1.21E-72	1.87E-72
KB-1448A5.1	NA	NA	NA
SDC2	0	0	0
CPQ	0.19300122	0	0
KB-1958F4.2	NA	NA	NA
KB-1000E4.2	NA	NA	NA
TSPYL5	NA	1.52E-274	5.24E-274
MTDH	2.43816323	0	0
LAPTM4B	1.94908001	9.79E-08	1.09E-07
MATN2	2.31601467	3.85E-69	5.89E-69
RPL30	2.19676066	0	0
KB-1208A12.	8.49205379	1.67E-48	2.34E-48
ERICH5	1.54400978	1.46E-54	2.10E-54
HRSP12	2.0150888	0.00306655	0.00324336
POP1	1.90871554	0.03895347	0.04048893
NIPAL2	1.44439625	5.61E-14	6.55E-14
KB-1458E12.	0	0	0
STK3	1.82144904	1.90E-79	3.02E-79
KCNS2	NA	NA	NA
KB-1517D11.	NA	NA	NA
KB-1205A7.2	NA	NA	NA
RP11-44N12.	1.54400978	2.02E-217	5.39E-217
OSR2	1.34444389	0	0
RP11-44N12.	NA	NA	NA
RP11-410L14	2.350326	3.11E-250	9.58E-250
VPS13B	2.75026742	1.27E-16	1.51E-16
CTD-2340D6.	NA	NA	NA
RP11-402L5.	NA	NA	NA
COX6C	2.15735109	2.61E-271	8.88E-271
RGS22	NA	NA	NA
FBXO43	1.76458261	2.91E-111	5.24E-111
POLR2K	1.87808804	9.79E-07	1.08E-06

SPAG1	1.99434597	0.00824495	0.00866026
RNF19A	1.99095998	1.71E-61	2.54E-61
KB-1991G8.1	NA	NA	NA
KB-173C10.2	NA	NA	NA
KB-173C10.1	NA	NA	NA
KB-1615E4.2	NA	NA	NA
KB-1615E4.3	NA	NA	NA
ANKRD46	1.64928317	1.73E-46	2.41E-46
SNX31	NA	NA	NA
KB-1083B1.1	NA	NA	NA
PABPC1	2.1247258	2.81E-242	8.37E-242
YWHAZ	1.94057363	2.03E-09	2.29E-09
FLJ42969	Inf	1.80E-307	7.40E-307
KB-1460A1.1	NA	NA	NA
KB-1460A1.3	NA	NA	NA
KB-1460A1.5	0.77200489	0	0
ZNF706	2.09064797	5.25E-107	9.31E-107
KB-1410C5.5	1.15800733	2.21E-42	3.01E-42
KB-1410C5.2	NA	NA	NA
KB-1410C5.3	NA	NA	NA
KB-1930G5.3	NA	NA	NA
KB-1930G5.4	NA	NA	NA
KB-1562D12.	NA	NA	NA
GRHL2	NA	NA	NA
NCALD	1.50112062	1.13E-152	2.35E-152
KB-1107E3.1	NA	7.65E-30	9.77E-30
RRM2B	1.81021836	1.78E-54	2.56E-54
UBR5-AS1	1.27380807	0.00443999	0.00468506
UBR5	1.4252398	3.78E-223	1.03E-222
KB-431C1.5	Inf	1.23E-235	3.53E-235
KB-1980E6.3	1.02933985	2.93E-72	4.54E-72
ODF1	Inf	2.53E-130	4.86E-130
KLF10	1.57488998	4.30E-74	6.69E-74
KB-1732A1.1	Inf	1.06E-24	1.32E-24
AZIN1	2.08909949	1.00E-25	1.25E-25
KB-1254G8.1	0	0	0
AZIN1-AS1	2.94326864	8.00E-28	1.01E-27
KB-1507C5.3	NA	1.26E-187	2.98E-187
KB-1507C5.4	Inf	1.62E-115	2.96E-115
KB-1639H6.2	NA	NA	NA
ATP6V1C1	1.26197703	0	0
LINC01181	NA	NA	NA
BAALC-AS2	1.15800733	0.10411362	0.10732594
BAALC	1.66277976	3.53E-248	1.08E-247
BAALC-AS1	2.31601467	1.61E-19	1.94E-19

RP11-318M2	NA	NA	NA
FZD6	2.94097101	2.98E-42	4.05E-42
CTHRC1	NA	NA	NA
RP11-1C8.6	2.31601467	0.44108521	0.44719838
SLC25A32	1.75279098	4.04E-211	1.05E-210
DCAF13	0.98950738	0	0
RP11-1C8.5	Inf	0	0
RP11-1C8.7	6.17603912	0.00173604	0.00184291
RP11-1C8.4	0.77200489	1.04E-40	1.40E-40
RIMS2	1.81972581	4.39E-158	9.33E-158
DPYS	NA	3.33E-08	3.72E-08
DCSTAMP	NA	NA	NA
LRP12	2.41894866	1.51E-94	2.55E-94
ZFPM2	NA	1.68E-225	4.63E-225
RP11-574O7.	NA	NA	NA
RP11-642D2:	NA	NA	NA
RP11-152P17	NA	NA	NA
RP11-778D1:	NA	NA	NA
RP11-395G2:	1.9673673	0.58686581	0.59251598
OXR1	1.86236231	1.05E-29	1.34E-29
RP11-649G1!	Inf	9.79E-124	1.84E-123
ABRA	NA	NA	NA
ANGPT1	0.49408313	0	0
RSPO2	NA	NA	NA
EIF3E	1.40332028	0	0
RP11-35G22.	NA	NA	NA
EMC2	1.17757428	0	0
RP11-790J24	NA	NA	NA
TMEM74	1.62121027	1.29E-50	1.83E-50
RP11-1084E!	NA	2.65E-142	5.32E-142
TRHR	NA	NA	NA
NUDCD1	1.48698669	0	0
RP11-122A2:	NA	1.39E-196	3.40E-196
ENY2	1.92732792	1.49E-35	1.97E-35
PKHD1L1	NA	NA	NA
EBAG9	2.18949165	6.13E-88	1.01E-87
SYBU	11.5800733	1.23E-278	4.32E-278
RP11-422N1(	NA	NA	NA
KCNV1	NA	NA	NA
RP11-696P8.	NA	NA	NA
RP11-211F2.	NA	NA	NA
RP11-238I10	NA	NA	NA
KB-1299A7.2	NA	NA	NA
LINC01608	NA	NA	NA
LINC01609	NA	NA	NA

RP11-114O8.	NA	NA	NA
RP11-1101K5	NA	NA	NA
RP11-58O3.2	NA	NA	NA
CSMD3	2.05867971	0.01113152	0.01166857
RP11-67H2.1	NA	NA	NA
RP11-267L5.	NA	NA	NA
TRPS1	NA	3.40E-83	5.49E-83
AF178030.2	NA	NA	NA
LINC00536	NA	NA	NA
RP11-536K17	NA	NA	NA
EIF3H	1.69924553	1.33E-290	4.97E-290
UTP23	1.64816917	1.18E-208	3.03E-208
RAD21	1.74255701	1.60E-248	4.89E-248
AC087350.1	NA	NA	NA
RAD21-AS1	0.38600244	5.51E-111	9.92E-111
AARD	NA	NA	NA
SLC30A8	NA	NA	NA
MED30	1.82426991	2.96E-08	3.31E-08
EXT1	2.17565014	2.05E-44	2.82E-44
SAMD12	NA	5.80E-25	7.23E-25
AC023590.1	0	1.76E-214	4.62E-214
SAMD12-AS1	NA	NA	NA
TNFRSF11B	NA	NA	NA
COLEC10	NA	NA	NA
RP11-278I4.2	NA	NA	NA
MAL2	NA	NA	NA
RP11-4K16.2	NA	NA	NA
NOV	1.15800733	3.73E-48	5.22E-48
RP11-775B15	NA	NA	NA
ENPP2	NA	NA	NA
TAF2	1.94679494	3.51E-18	4.21E-18
DSCC1	1.64422195	1.22E-269	4.12E-269
KB-1471A8.1	3.08801956	4.88E-231	1.38E-230
DEPTOR	1.60339477	0	0
RP11-760H22	4.47762836	9.96E-134	1.94E-133
COL14A1	NA	3.07E-241	9.09E-241
MRPL13	1.65825667	0	0
MTBP	1.45136919	6.36E-31	8.17E-31
SNTB1	3.17884366	5.11E-13	5.92E-13
RP11-713M1	NA	NA	NA
RP11-713M1	2.31601467	1.69E-105	2.97E-105
RP11-369K17	2.31601467	1.16E-192	2.80E-192
HAS2	NA	NA	NA
HAS2-AS1	NA	NA	NA
RP11-3G20.1	NA	NA	NA

RP11-3G20.2	NA	NA	NA
RP11-785H2(	NA	NA	NA
RP11-398G2	NA	NA	NA
RP11-94A24.	NA	NA	NA
RP11-96B2.1	NA	NA	NA
LINC01151	NA	NA	NA
RP11-973F1	NA	NA	NA
RP11-44N11.	0.38600244	9.08E-241	2.68E-240
RP11-44N11.	NA	0	0
ZHX2	1.54400978	1.73E-212	4.51E-212
RP11-557C1	1.54400978	0.00017021	0.00018309
RP11-557C1	NA	NA	NA
DERL1	1.51559242	8.38E-262	2.73E-261
TBC1D31	1.84959505	1.08E-35	1.43E-35
FAM83A	NA	8.65E-258	2.76E-257
RP11-539E1	NA	NA	NA
FAM83A-AS1	Inf	0	0
C8orf76	1.38317543	3.06E-160	6.57E-160
ZHX1-C8orf7	1.60015559	2.03E-06	2.23E-06
ZHX1	2.23360965	1.43E-66	2.16E-66
ATAD2	2.16689501	0.08722722	0.09005157
WDYHV1	1.87420464	1.44E-55	2.08E-55
FBXO32	1.737011	0.94703375	0.94802661
RP11-174I12	NA	NA	NA
CTD-2552K1	NA	NA	NA
KLHL38	NA	NA	NA
ANXA13	NA	NA	NA
FAM91A1	2.79343875	0	0
FER1L6	0.77200489	7.79E-57	1.13E-56
RP11-245A1	NA	NA	NA
FER1L6-AS1	NA	NA	NA
RP11-959I15	NA	NA	NA
FER1L6-AS2	NA	NA	NA
RP11-37N22.	0.64333741	6.02E-197	1.48E-196
RP11-383J24	NA	NA	NA
TMEM65	1.68983293	1.23E-199	3.06E-199
TRMT12	1.82783511	3.85E-81	6.17E-81
RNF139-AS1	2.12301345	1.28E-25	1.60E-25
RNF139	1.68508682	1.87E-215	4.95E-215
TATDN1	1.63360819	0	0
RP11-158K1.	Inf	5.77E-05	6.24E-05
NDUFB9	1.83338761	5.11E-106	9.02E-106
MTSS1	0.77200489	8.29E-247	2.52E-246
RP11-532M2	Inf	0	0
RP11-1082L	NA	NA	NA

RP11-1082L8	NA	1.03E-122	1.93E-122
LINC00964	NA	NA	NA
RP11-1082L8	NA	NA	NA
ZNF572	2.31601467	6.38E-124	1.20E-123
RP11-6D1.3	NA	NA	NA
SQLE	2.25218749	4.72E-227	1.31E-226
KIAA0196	1.91219673	5.03E-41	6.80E-41
KIAA0196-AS	NA	NA	NA
NSMCE2	1.35580958	0	0
RP11-550A5.	NA	NA	NA
TRIB1	1.02933985	3.50E-33	4.54E-33
RP11-136O1	NA	NA	NA
RP11-697B24	NA	NA	NA
LINC00861	NA	NA	NA
RP11-622O1	NA	NA	NA
RP11-65D17.	NA	NA	NA
RP11-103H7.	NA	NA	NA
RP11-103H7.	NA	NA	NA
RP11-103H7.	NA	NA	NA
FAM84B	2.21951406	2.07E-50	2.93E-50
RP11-103H7.	NA	NA	NA
RP11-89K10.	NA	NA	NA
RP11-351C8.	NA	NA	NA
PCAT1	0	0	0
PCAT2	NA	NA	NA
PRNCR1	3.86002445	3.26E-204	8.24E-204
CASC19	NA	NA	NA
CCAT1	NA	NA	NA
CASC8	NA	NA	NA
CASC21	NA	NA	NA
CCAT2	NA	NA	NA
POU5F1B	NA	7.69E-193	1.86E-192
AC108925.1	NA	NA	NA
CASC11	2.31601467	5.13E-17	6.11E-17
MYC	1.45876063	0	0
PVT1	2.08064733	1.12E-58	1.64E-58
AC026248.1	NA	NA	NA
LINC00976	0	0	0
LINC00977	2.31601467	0.13080463	0.13456522
CCDC26	1.737011	0	0
RP11-26E5.1	NA	NA	NA
RP11-419K12	NA	0	0
GSDMC	Inf	0	0
RP11-473O4.	NA	NA	NA
FAM49B	1.86640009	2.67E-86	4.36E-86

RP11-473O4.	NA	6.23E-44	8.54E-44
RP11-473O4.	NA	NA	NA
ASAP1	1.6904245	2.48E-273	8.53E-273
KB-1568E2.1	NA	NA	NA
ADCY8	NA	NA	NA
RP11-737F9.	NA	NA	NA
RP11-381I15	NA	NA	NA
CTD-2501M5	NA	NA	NA
EFR3A	1.17409077	0	0
OC90	NA	NA	NA
RP11-240B1:	NA	NA	NA
HHLA1	NA	NA	NA
KCNQ3	NA	NA	NA
HPYR1	NA	NA	NA
LRRC6	Inf	8.39E-153	1.75E-152
TMEM71	NA	NA	NA
PHF20L1	1.91571584	0	0
AF230666.2	NA	1.80E-12	2.08E-12
TG	NA	NA	NA
SLA	0.80876703	0	0
WISP1	NA	NA	NA
NDRG1	1.99434597	0.55018238	0.55625327
ST3GAL1	1.75455657	1.25E-47	1.75E-47
RP11-629O1.	1.42903033	1.60E-153	3.34E-153
CTD-2588J1.:	NA	NA	NA
CTD-2309H9.	NA	NA	NA
RP11-157E21	NA	NA	NA
RP11-513H8.	NA	NA	NA
ZFAT	2.3511058	5.78E-129	1.10E-128
ZFAT-AS1	NA	NA	NA
CTD-2342N2:	1.15800733	2.31E-255	7.31E-255
AC083843.1	3.29856635	2.03E-215	5.37E-215
AC083843.2	NA	NA	NA
NCRNA0025(	NA	NA	NA
RP11-1057B8	NA	NA	NA
LINC01591	NA	NA	NA
RP11-343P9.	NA	NA	NA
KHDRBS3	1.33465252	0	0
RP11-149P24	NA	NA	NA
RP11-431D1:	NA	NA	NA
RP11-30J20.:	Inf	0	0
RP11-238K6.	NA	NA	NA
RP11-238K6.	NA	NA	NA
RP11-489O1:	NA	NA	NA
FAM135B	NA	NA	NA

COL22A1	NA	NA	NA
RP11-324F11	NA	NA	NA
CTD-2534J5.1	NA	NA	NA
RP11-398H6.	NA	NA	NA
KCNK9	NA	NA	NA
TRAPPC9	1.60049794	3.96E-94	6.67E-94
RP11-284H18	NA	NA	NA
CTA-204B4.2	4.37469438	2.04E-24	2.53E-24
CHRAC1	1.52598632	3.42E-263	1.12E-262
AGO2	2.1264599	1.59E-114	2.88E-114
ERICD	NA	3.46E-187	8.17E-187
PTK2	Inf	4.37E-106	7.71E-106
AC105009.1	NA	NA	NA
RP11-128L5.1	NA	NA	NA
DENND3	2.350326	1.65E-05	1.79E-05
RP11-809O17	Inf	2.59E-43	3.55E-43
SLC45A4	2.42630108	0.84059588	0.84357177
RP11-10J21.4	NA	NA	NA
RP11-10J21.3	NA	NA	NA
RP11-10J21.5	NA	NA	NA
RP11-10J21.6	NA	NA	NA
LINC01300	NA	NA	NA
GPR20	NA	NA	NA
CTD-3064M3	NA	NA	NA
PTP4A3	1.13962627	0	0
CTD-3064M3	NA	NA	NA
AC138647.1	NA	NA	NA
RP11-953B20	NA	NA	NA
LINC00051	NA	NA	NA
TSNARE1	1.93001222	1.66E-39	2.23E-39
RP13-467H17	0	1.83E-12	2.11E-12
ADGRB1	2.27060262	6.80E-162	1.47E-161
ARC	0.57900367	0	0
AP006547.3	NA	NA	NA
AC145123.2	NA	NA	NA
JRK	2.17744969	2.95E-171	6.60E-171
PSCA	2.7792176	0.24419475	0.24939096
LY6K	0	0	0
CTD-2292P10	NA	4.16E-270	1.41E-269
THEM6	1.85281174	1.01E-55	1.46E-55
CTD-2292P10	NA	NA	NA
SLURP1	NA	NA	NA
LYPD2	NA	NA	NA
RP11-706C16	NA	NA	NA
LYNX1	NA	NA	NA

LY6D	NA	NA	NA
RP11-706C16	NA	NA	NA
RP11-706C16	NA	NA	NA
GML	NA	NA	NA
CYP11B1	NA	NA	NA
CYP11B2	NA	NA	NA
RP11-273G15	1.15800733	1.26E-289	4.69E-289
LY6E	1.61961594	0	0
RP11-520P18	NA	NA	NA
LY6H	NA	NA	NA
GPIHBP1	NA	NA	NA
ZFP41	2.31601467	2.70E-23	3.33E-23
RP13-582O9.	NA	NA	NA
GLI4	2.350326	2.78E-69	4.26E-69
MINCR	2.23179595	1.51E-70	2.33E-70
ZNF696	1.88938039	1.16E-49	1.64E-49
RP13-582O9.	3.474022	9.09E-74	1.41E-73
TOP1MT	1.77712703	1.03E-231	2.93E-231
RHPN1-AS1	3.08801956	2.66E-250	8.20E-250
RHPN1	2.07037675	3.16E-59	4.65E-59
RP11-909N17	NA	NA	NA
MAFA-AS1	NA	NA	NA
MAFA	0.77200489	3.43E-123	6.42E-123
ZC3H3	1.41949286	1.03E-244	3.10E-244
RP11-661A12	Inf	0.00310643	0.00328534
RP11-661A12	Inf	2.52E-293	9.59E-293
GSDMD	1.109875	0	0
MROH6	2.18367097	2.62E-14	3.07E-14
RP11-661A12	NA	2.83E-88	4.67E-88
NAPRT	1.24322865	0	0
RP11-661A12	Inf	5.04E-05	5.45E-05
EEF1D	1.74978182	3.09E-216	8.18E-216
TIGD5	2.01908971	6.63E-05	7.16E-05
PYCRL	1.76061547	0	0
TSTA3	1.62236498	1.19E-304	4.83E-304
RP11-661A12	0	0	0
ZNF623	2.52656146	2.34E-13	2.72E-13
ZNF707	1.66590529	5.42E-27	6.83E-27
RP11-429J17	2.12301345	0.00262415	0.00277739
CCDC166	NA	NA	NA
RP11-429J17	NA	NA	NA
MAPK15	NA	NA	NA
RP11-429J17	NA	NA	NA
FAM83H	0	0	0
FAM83H-AS1	NA	1.07E-15	1.27E-15

RP11-429J17	NA	NA	NA
RP11-429J17	NA	NA	NA
SCRIB	1.92407373	1.50E-120	2.79E-120
PUF60	0.89905595	0	0
NRBP2	0.91675581	1.00E-170	2.23E-170
EPPK1	NA	NA	NA
PLEC	1.51312958	0	0
PARP10	1.65429619	9.88E-176	2.24E-175
GRINA	1.39595405	0	0
SPATC1	NA	NA	NA
SMPD5	NA	9.01E-71	1.39E-70
OPLAH	NA	NA	NA
CTD-3065J16	1.44750917	2.48E-193	6.02E-193
EXOSC4	1.66145765	1.61E-187	3.82E-187
GPAA1	1.58837788	0	0
CYC1	1.44860696	0	0
SHARPIN	1.87924764	0	0
MAF1	1.69600418	6.94E-250	2.13E-249
WDR97	4.63202934	4.41E-176	1.00E-175
HGH1	1.95465068	5.07E-43	6.93E-43
MROH1	1.33741692	1.96E-74	3.06E-74
BOP1	1.2013909	0	0
SCX	6.17603912	2.95E-195	7.21E-195
HSF1	1.71918767	0	0
DGAT1	1.56581783	0	0
GS1-393G12	NA	NA	NA
SCRT1	NA	1.09E-281	3.88E-281
TMEM249	Inf	0	0
GS1-393G12	Inf	0	0
SLC52A2	1.65298326	4.34E-189	1.03E-188
FBXL6	1.44883109	0	0
ADCK5	1.32856655	0	0
CPSF1	2.04029864	2.65E-72	4.10E-72
GS1-393G12	4.63202934	1.88E-205	4.76E-205
SLC39A4	1.72360004	8.31E-107	1.47E-106
VPS28	1.39059404	0	0
TONSL	2.15058505	2.89E-126	5.47E-126
TONSL-AS1	NA	NA	NA
CYHR1	1.82321822	7.22E-272	2.46E-271
CTD-2517M2	Inf	0	0
KIFC2	2.01189153	6.16E-39	8.24E-39
FOXH1	Inf	3.29E-57	4.79E-57
PPP1R16A	1.58711665	1.34E-166	2.94E-166
GPT	Inf	3.16E-41	4.27E-41
MFSD3	1.44876242	0	0

RECQL4	2.26529902	0.0058276	0.00613749
CTD-2517M2	0	0	0
LRRC14	1.81857219	4.07E-62	6.06E-62
LRRC24	2.05867971	0.49193524	0.49800173
C8orf82	1.58175224	0	0
ARHGAP39	NA	NA	NA
CTD-2517M2	NA	NA	NA
AF186192.1	NA	NA	NA
AF186192.6	NA	NA	NA
ZNF251	1.90244062	1.44E-34	1.89E-34
ZNF34	0.70487403	0	0
RPL8	1.77367141	0	0
ZNF517	2.18734719	2.93E-07	3.24E-07
ZNF7	1.8513821	4.79E-210	1.24E-209
COMMD5	1.67439283	1.94E-191	4.67E-191
ZNF250	1.46680929	2.31E-219	6.20E-219
ZNF16	2.71879983	6.17E-22	7.55E-22
RP5-1047A1	NA	NA	NA
ZNF252P-AS	0.77200489	1.86E-107	3.31E-107
C8orf33	2.07210588	5.72E-93	9.60E-93
WASH1	2.0784747	3.14E-17	3.75E-17
XXyac-YRM2	3.08801956	4.61E-56	6.67E-56
FAM138C	NA	NA	NA
PGM5P3-AS1	NA	NA	NA
RP11-143M1	2.31601467	0.40262235	0.40872781
LINC01388	0.34311328	1.04E-176	2.36E-176
FOXD4	1.54400978	2.10E-22	2.57E-22
CBWD1	1.98264892	2.87E-31	3.70E-31
C9orf66	2.62481663	9.29E-38	1.23E-37
DOCK8	1.49014897	0	0
RP11-59O6.3	NA	NA	NA
RP11-165F2	NA	NA	NA
KANK1	1.92179941	1.95E-56	2.82E-56
RP11-130C1	NA	NA	NA
DMRT1	NA	NA	NA
DMRT3	NA	NA	NA
LINC01230	NA	NA	NA
DMRT2	NA	NA	NA
SMARCA2	1.69314709	1.51E-294	5.77E-294
RP11-264I13	NA	NA	NA
VLDLR-AS1	4.24602689	5.97E-09	6.71E-09
RP11-91N2.3	NA	NA	NA
VLDLR	2.73710825	3.53E-137	6.95E-137
KCNV2	NA	NA	NA
PUM3	1.79409587	8.71E-48	1.22E-47

LINC01231	NA	NA	NA
RFX3	0.96500611	5.50E-238	1.60E-237
AL365202.1	NA	NA	NA
RFX3-AS1	0	0	0
RP11-509J21	NA	3.53E-279	1.25E-278
RP11-509J21	NA	NA	NA
GLIS3	NA	0	0
RP11-252M1	NA	NA	NA
GLIS3-AS1	NA	NA	NA
RP11-358M1	NA	NA	NA
SLC1A1	NA	NA	NA
SPATA6L	NA	NA	NA
PLPP6	1.30646981	1.14E-189	2.72E-189
CDC37L1-AS1	1.93001222	6.93E-136	1.36E-135
CDC37L1	1.65898923	2.73E-306	1.12E-305
AK3	1.56731559	2.57E-293	9.78E-293
RCL1	1.04248836	0	0
RP11-125K1(	NA	NA	NA
JAK2	2.93361858	2.55E-10	2.90E-10
IGHEP2	NA	8.98E-115	1.63E-114
INSL6	NA	NA	NA
INSL4	NA	NA	NA
RLN2	NA	NA	NA
RLN1	NA	NA	NA
RP11-12D24.	NA	NA	NA
PLGRKT	1.8846921	4.32E-241	1.28E-240
CD274	NA	NA	NA
PDCD1LG2	NA	NA	NA
RP11-574F11	NA	NA	NA
RIC1	1.80134474	2.70E-165	5.90E-165
RP11-207C16	NA	NA	NA
ERMP1	1.58942183	5.47E-207	1.40E-206
KIAA2026	2.18239844	4.36E-36	5.75E-36
MLANA	NA	NA	NA
RANBP6	2.35890383	6.35E-95	1.07E-94
RP11-218I7.2	NA	1.02E-123	1.92E-123
IL33	NA	NA	NA
TPD52L3	NA	NA	NA
UHRF2	2.5390383	0	0
RP11-307L3.4	NA	NA	NA
GLDC	NA	NA	NA
RP11-390F4.	NA	NA	NA
RP11-390F4.	NA	NA	NA
RP11-390F4.	NA	0	0
RP11-146B14	NA	NA	NA

AL513412.1	NA	NA	NA
KDM4C	2.62481663	7.67E-47	1.07E-46
RP11-77E14.	Inf	1.22E-106	2.16E-106
TMEM261	2.17513787	2.53E-268	8.48E-268
RP11-29B9.1	NA	NA	NA
RP11-29B9.2	NA	NA	NA
PTPRD	NA	NA	NA
PTPRD-AS1	Inf	0	0
RP11-75C9.2	NA	NA	NA
RP11-527D1!	NA	NA	NA
PTPRD-AS2	NA	NA	NA
RP11-87N24.	NA	NA	NA
RP11-109M1	NA	NA	NA
RP11-23D5.1	NA	NA	NA
RP11-74C3.1	NA	NA	NA
TYRP1	Inf	3.92E-201	9.77E-201
LURAP1L-AS	NA	NA	NA
LURAP1L	Inf	8.67E-58	1.27E-57
MPDZ	NA	NA	NA
LINC01235	NA	0	0
RP11-536O1!	NA	NA	NA
LINC00583	NA	NA	NA
RP11-284P2(	NA	NA	NA
NFIB	1.60834352	3.12E-55	4.50E-55
RP11-120J1.:	NA	NA	NA
RP11-408A1:	Inf	0	0
RP11-408A1:	Inf	0	0
ZDHHC21	3.73135697	3.81E-257	1.21E-256
CER1	NA	NA	NA
FREM1	NA	NA	NA
TTC39B	1.15800733	0.00174811	0.00185561
SNAPC3	1.37030868	2.85E-170	6.34E-170
PSIP1	1.56727568	0	0
CCDC171	2.8306846	4.80E-148	9.84E-148
C9orf92	NA	NA	NA
BNC2	Inf	0	0
RP11-183I6.2	NA	NA	NA
RP11-62F24.	NA	NA	NA
RP11-62F24.	NA	NA	NA
RP11-132E1:	NA	NA	NA
CNTLN	1.50373126	3.11E-307	1.28E-306
SH3GL2	NA	1.49E-248	4.56E-248
RP11-570H1!	NA	NA	NA
ADAMTSL1	NA	NA	NA
SAXO1	NA	NA	NA

RRAGA	1.30156196	0	0
HAUS6	1.96658088	2.15E-194	5.25E-194
PLIN2	1.61511549	3.72E-246	1.12E-245
DENND4C	2.16161369	6.96E-51	9.86E-51
RP11-146N2:	NA	NA	NA
RP11-513M1	2.70201711	4.02E-102	6.99E-102
RP11-513M1	3.08801956	2.90E-42	3.94E-42
RPS6	1.86300039	1.95E-28	2.47E-28
ACER2	NA	NA	NA
SLC24A2	NA	NA	NA
RP11-378I6.1	NA	NA	NA
MLLT3	1.41291461	0	0
FOCAD	2.1081672	0.03514757	0.03656234
FOCAD-AS1	2.31601467	2.10E-17	2.51E-17
HACD4	1.72461643	2.32E-242	6.92E-242
IFNB1	Inf	0	0
IFNW1	NA	NA	NA
IFNA21	NA	NA	NA
IFNA4	NA	NA	NA
IFNA7	NA	NA	NA
IFNA10	NA	NA	NA
IFNA16	NA	NA	NA
IFNA17	NA	NA	NA
IFNA14	NA	NA	NA
IFNA5	NA	NA	NA
KLHL9	2.28844307	4.15E-68	6.32E-68
IFNA6	NA	NA	NA
IFNA13	NA	NA	NA
IFNA2	NA	NA	NA
RP11-354P17	NA	NA	NA
IFNA8	NA	NA	NA
IFNA1	NA	NA	NA
IFNE	NA	NA	NA
MTAP	1.53617217	1.91E-97	3.25E-97
RP11-145E5.	NA	NA	NA
RP11-70L8.4	NA	5.26E-302	2.10E-301
CDKN2A-AS1	NA	NA	NA
CDKN2A	1.8088155	4.38E-173	9.84E-173
CDKN2B-AS1	3.474022	3.37E-181	7.78E-181
CDKN2B	0.83138988	0	0
RP11-408N1	NA	2.65E-142	5.32E-142
DMRTA1	2.49766288	4.39E-32	5.67E-32
LINC01239	NA	NA	NA
RP11-370B1:	NA	NA	NA
RP11-315I14	NA	NA	NA

ELAVL2	0	0	0
RP11-321L2.1	NA	NA	NA
RP11-321L2.2	NA	NA	NA
IZUMO3	NA	NA	NA
RP11-20A20.	NA	NA	NA
TUSC1	2.02840501	2.19E-22	2.68E-22
LINC01241	NA	0	0
RP11-80I3.1	NA	NA	NA
RP11-18A15.	NA	NA	NA
RP11-337A23	NA	NA	NA
CAAP1	1.80785955	1.76E-291	6.64E-291
PLAA	1.47219537	0	0
IFT74	1.5643257	4.34E-184	1.02E-183
IFT74-AS1	Inf	0.33151418	0.33748557
LRRC19	NA	NA	NA
RP11-56F10.	NA	NA	NA
TEK	NA	NA	NA
LINC00032	NA	NA	NA
EQTN	NA	NA	NA
MOB3B	1.33104291	6.85E-149	1.41E-148
RP11-298E2.	NA	NA	NA
IFNK	NA	NA	NA
C9orf72	2.31601467	4.28E-49	6.01E-49
RP11-575I8.1	NA	NA	NA
LINGO2	4.24602689	4.59E-42	6.24E-42
LINC01242	NA	NA	NA
LINC01243	NA	NA	NA
ACO1	1.98515543	1.86E-34	2.43E-34
DDX58	4.63202934	2.29E-149	4.73E-149
TOPORS	1.81648209	3.97E-90	6.60E-90
TOPORS-AS1	2.02927	9.78E-62	1.45E-61
NDUFB6	1.94771103	0.00929125	0.00975191
GVQW1	2.31601467	1.03E-50	1.46E-50
TAF1L	NA	NA	NA
RP11-555J4.4	NA	NA	NA
RP11-555J4.3	NA	NA	NA
TMEM215	NA	NA	NA
RP11-462B18	NA	NA	NA
APTX	1.99547463	2.51E-83	4.05E-83
DNAJA1	0.90738546	0	0
SMU1	1.72861964	1.27E-116	2.32E-116
B4GALT1	1.83967123	0.41270866	0.41889675
B4GALT1-AS	1.23520782	1.27E-197	3.12E-197
SPINK4	1.29265935	0	0
BAG1	2.02850362	9.16E-119	1.69E-118

CHMP5	1.07262985	0	0
NFX1	2.24318402	0	0
AQP7	NA	0	0
RP11-311H1(	NA	NA	NA
AQP3	1.46274611	6.03E-299	2.36E-298
NOL6	2.3495801	2.89E-84	4.69E-84
RP11-255A1:	Inf	0	0
ANKRD18B	1.42606459	0	0
CYP4F26P	3.86002445	0	0
TRBV20OR9-	NA	NA	NA
TRBV21OR9-	NA	NA	NA
TRBV22OR9-	NA	NA	NA
TRBV23OR9-	NA	NA	NA
TRBV24OR9-	NA	NA	NA
PTENP1-AS	NA	NA	NA
TRBV26OR9-	NA	NA	NA
RP11-384P7.	NA	NA	NA
RP11-384P7.	NA	NA	NA
LINC01251	NA	9.66E-144	1.95E-143
PRSS3	NA	NA	NA
UBE2R2-AS1	NA	NA	NA
TRBV29OR9-	NA	NA	NA
UBE2R2	2.03930207	3.93E-12	4.53E-12
UBAP2	2.08220747	2.44E-56	3.53E-56
RP11-537H1!	NA	NA	NA
DCAF12	2.75182388	1.09E-121	2.04E-121
UBAP1	1.51514978	2.97E-199	7.38E-199
KIF24	2.64687391	1.93E-34	2.52E-34
NUDT2	2.11872453	1.38E-57	2.02E-57
KIAA1161	1.6297881	1.32E-158	2.82E-158
C9orf24	NA	NA	NA
FAM219A	1.3896088	7.57E-292	2.86E-291
DNAI1	NA	NA	NA
ENHO	0.99906515	5.18E-05	5.60E-05
RP11-296L22	NA	NA	NA
CNTFR	NA	NA	NA
CNTFR-AS1	NA	NA	NA
RPP25L	1.79248909	0	0
DCTN3	1.67697921	1.10E-206	2.81E-206
ARID3C	NA	9.77E-09	1.10E-08
SIGMAR1	1.72772486	0	0
GALT	1.63260051	7.14E-101	1.23E-100
RP11-195F1!	NA	NA	NA
IL11RA	1.54400978	1.39E-238	4.05E-238
CCL27	Inf	1.52E-260	4.93E-260

RP11-195F1§	4.63202934	1.87E-68	2.85E-68
RP11-195F1§	1.54400978	4.46E-30	5.70E-30
CCL19	NA	NA	NA
CCL21	NA	NA	NA
FAM205A	NA	NA	NA
FAM205C	NA	NA	NA
PHF24	NA	1.71E-123	3.20E-123
DNAJB5-AS1	NA	NA	NA
DNAJB5	1.00954486	3.48E-192	8.39E-192
C9orf131	NA	NA	NA
VCP	1.71693888	0	0
FANCG	1.54400978	5.18E-36	6.83E-36
PIGO	1.97290139	9.48E-09	1.06E-08
RP11-182N2	Inf	1.42E-284	5.14E-284
STOML2	1.81199546	2.71E-111	4.88E-111
FAM214B	2.50901589	1.18E-114	2.14E-114
UNC13B	2.4318154	6.06E-137	1.19E-136
RUSC2	4.37469438	5.47E-135	1.07E-134
FAM166B	Inf	5.52E-53	7.90E-53
TESK1	2.22519057	1.92E-237	5.59E-237
CD72	Inf	0.04535403	0.04708513
RP11-331F9.	Inf	5.77E-306	2.36E-305
RP11-331F9.	NA	NA	NA
SIT1	0.77200489	4.96E-165	1.08E-164
RMRP	0.77200489	2.00E-118	3.69E-118
CCDC107	1.84355742	1.19E-38	1.59E-38
ARHGEF39	1.21674684	0	0
CA9	NA	NA	NA
TPM2	2.8306846	1.67E-126	3.16E-126
TLN1	2.12388676	5.10E-79	8.09E-79
CREB3	1.7634217	9.72E-38	1.29E-37
GBA2	1.48786397	1.02E-49	1.44E-49
RGP1	2.43580853	0	0
MSMP	1.54400978	1.58E-36	2.09E-36
RP11-112J3.	NA	NA	NA
RP11-112J3.	2.93361858	3.81E-66	5.77E-66
NPR2	3.08801956	0.47712793	0.48314663
SPAG8	10.0360636	1.06E-304	4.33E-304
HINT2	1.77535562	0	0
TMEM8B	1.98515543	0.00044094	0.00047184
FAM221B	NA	NA	NA
LINC00950	NA	NA	NA
OR13J1	NA	NA	NA
HRCT1	NA	NA	NA
LINC00961	NA	NA	NA

RP11-327L3.:	NA	NA	NA
OR2S2	NA	NA	NA
RECK	2.48144429	1.04E-92	1.75E-92
GLIPR2	1.70484413	0	0
CCIN	NA	2.96E-115	5.40E-115
CLTA	1.58407391	1.13E-291	4.25E-291
GNE	1.35100856	2.00E-160	4.29E-160
RNF38	2.60043752	7.35E-98	1.26E-97
MELK	1.68523019	0	0
PAX5	1.81850041	1.12E-94	1.89E-94
RP11-344B2:	NA	NA	NA
RP11-297B1:	NA	NA	NA
LINC01400	0	0	0
RP11-220I1.5	Inf	0	0
EBLN3	2.03723513	0	0
RP11-220I1.2	NA	2.17E-266	7.24E-266
ZCCHC7	1.52901939	5.53E-258	1.77E-257
LINC01627	NA	NA	NA
GRHPR	1.6998144	3.30E-207	8.45E-207
ZBTB5	3.77424613	0	0
POLR1E	1.14835727	0	0
RP11-613M1	NA	1.03E-58	1.51E-58
FBXO10	1.65981051	7.34E-30	9.38E-30
RP11-613M1	NA	3.25E-65	4.90E-65
TOMM5	1.96318431	1.26E-98	2.17E-98
RP11-613M1	NA	NA	NA
FRMPD1	NA	2.01E-61	2.98E-61
TRMT10B	1.54400978	2.55E-138	5.04E-138
EXOSC3	1.63476271	2.22E-184	5.19E-184
DCAF10	1.97430759	0.00105344	0.0011215
SLC25A51	1.56239085	4.34E-294	1.66E-293
SHB	1.99591508	4.52E-36	5.97E-36
RP11-113O2	NA	NA	NA
ALDH1B1	1.73078516	4.32E-80	6.88E-80
IGFBPL1	NA	NA	NA
FAM95C	0.54040342	0	0
ANKRD18A	0.77200489	0	0
FAM201A	0.25733496	0	0
RP11-402N8.	NA	NA	NA
CNTNAP3	NA	NA	NA
SPATA31A1	NA	NA	NA
FAM74A1	NA	NA	NA
RP11-347J14	NA	NA	NA
RP11-347J14	NA	NA	NA
GLIDR	1.93001222	1.46E-29	1.87E-29

RP11-216M2	NA	NA	NA
ANKRD20A2	NA	1.77E-116	3.25E-116
FAM95B1	1.15800733	6.43E-157	1.36E-156
AC129778.2	NA	0	0
BMS1P14	NA	2.30E-69	3.52E-69
CH17-80A12.	NA	NA	NA
IGKV1OR9-2	NA	NA	NA
RP11-318K1	NA	NA	NA
PGM5P2	NA	NA	NA
PGM5-AS1	NA	NA	NA
BX255923.3	0.77200489	0.00011849	0.00012765
FOXD4L6	NA	NA	NA
CBWD7	0.8271481	0	0
RP11-4L24.3	6.17603912	3.36E-26	4.22E-26
RP11-187C1	NA	NA	NA
RP11-157L3.	NA	NA	NA
CNTNAP3B	Inf	2.44E-111	4.39E-111
SPATA31A6	NA	NA	NA
FAM74A7	NA	NA	NA
RP11-286O1	NA	NA	NA
RP11-475I24	NA	NA	NA
RP11-475I24	NA	NA	NA
RP11-175I6.	NA	NA	NA
RP11-459O1	NA	NA	NA
RP11-459O1	NA	NA	NA
FP325317.1	NA	NA	NA
SPATA31A5	NA	NA	NA
RP11-144A1	NA	NA	NA
FAM74A6	NA	9.94E-51	1.41E-50
RP11-144A1	NA	NA	NA
SPATA31A7	NA	NA	NA
RP11-101E5.	NA	NA	NA
RP11-101E5.	NA	NA	NA
RP11-160N1	NA	NA	NA
RP11-160N1	NA	NA	NA
FAM27C	1.77561125	3.69E-233	1.05E-232
RP11-374M1	NA	NA	NA
RP11-374M1	0.77200489	1.44E-54	2.07E-54
RP11-374M1	NA	NA	NA
RP11-449H1	NA	NA	NA
RP11-111F5.	NA	NA	NA
RP11-111F5.	1.28667482	0	0
RP11-111F5.	NA	NA	NA
LINC01189	NA	NA	NA
RP11-211N8.	NA	NA	NA

LINC01410	4.9408313	0	0
RP11-262H14	3.86002445	7.75E-99	1.33E-98
RP11-262H14	1.35100856	9.59E-140	1.90E-139
RP11-381O7.	NA	NA	NA
RP11-149F8.	NA	NA	NA
RP11-764K9.	0	0	0
ANKRD20A4	Inf	3.73E-101	6.46E-101
IGKV1OR-2	NA	NA	NA
IGKV1OR9-1	NA	NA	NA
FOXD4L5	NA	NA	NA
CBWD5	1.65528976	1.07E-37	1.42E-37
FOXD4L4	NA	NA	NA
IGKV1OR-3	NA	NA	NA
RP11-15E1.5	NA	0	0
ANKRD20A3	1.35100856	2.61E-11	2.99E-11
RP11-327I22	NA	NA	NA
ZNF658	2.31601467	1.44E-09	1.62E-09
RP11-395E19	NA	NA	NA
RP11-395E19	NA	NA	NA
SPATA31A3	NA	NA	NA
RP11-118H15	NA	NA	NA
ANKRD20A1	0	0	0
RP11-561O23	NA	2.06E-208	5.30E-208
CBWD3	1.83611974	2.32E-191	5.58E-191
FOXD4L3	NA	NA	NA
RP11-561O23	NA	NA	NA
RP11-561O23	NA	NA	NA
PGM5	NA	NA	NA
PGM5-AS1	NA	NA	NA
RP11-88I18.2	1.35100856	1.05E-207	2.70E-207
TMEM252	NA	NA	NA
RP11-274B18	NA	NA	NA
LINC01506	NA	NA	NA
PIP5K1B	Inf	1.69E-218	4.51E-218
FAM122A	1.4153423	1.72E-94	2.90E-94
RP11-203L2.4	NA	NA	NA
PRKACG	NA	NA	NA
FXN	2.17598514	1.21E-121	2.26E-121
TJP2	2.4037425	0.02224295	0.02321682
BANCR	NA	NA	NA
FAM189A2	NA	NA	NA
APBA1	NA	0	0
RP11-470P21	NA	NA	NA
RP11-109D9.	NA	NA	NA
PTAR1	2.7675647	0	0

C9orf135-AS1	NA	NA	NA
C9orf135	NA	NA	NA
MAMDC2-AS	NA	0	0
MAMDC2	NA	NA	NA
SMC5-AS1	3.86002445	3.95E-85	6.42E-85
SMC5	1.56622575	7.40E-242	2.20E-241
KLF9	1.97290139	0	0
TRPM3	0.77200489	0	0
RP11-141J10	NA	NA	NA
RP11-141J10	NA	NA	NA
TMEM2	1.15800733	1.19E-304	4.85E-304
ABHD17B	1.90031973	0.21873399	0.22360241
AL138751.1	NA	NA	NA
C9orf85	1.79916394	1.19E-121	2.22E-121
C9orf57	NA	NA	NA
GDA	NA	NA	NA
RP11-63P12.	NA	NA	NA
LINC01504	0.77200489	0.97675367	0.97734626
ZFAND5	1.74519893	0.00034392	0.00036869
TMC1	NA	NA	NA
LINC01474	NA	NA	NA
ALDH1A1	NA	NA	NA
ANXA1	0.35630995	0	0
RP11-128I7.1	NA	NA	NA
RP11-171A24	NA	NA	NA
RORB-AS1	NA	NA	NA
RORB	NA	4.02E-60	5.93E-60
TRPM6	0	0	0
C9orf40	1.55478194	1.36E-242	4.05E-242
C9orf41-AS1	Inf	0	0
CARNMT1	2.38143881	4.72E-87	7.73E-87
NMRK1	2.44468215	2.16E-16	2.56E-16
OSTF1	1.21315054	0	0
RP11-470F18	NA	NA	NA
PCSK5	1.54400978	2.51E-229	7.02E-229
RFK	2.60467737	9.52E-289	3.54E-288
GCNT1	1.14256724	5.90E-49	8.29E-49
PRUNE2	NA	0.03125678	0.03254293
PCA3	NA	6.83E-24	8.45E-24
FOXB2	NA	NA	NA
VPS13A-AS1	NA	1.62E-81	2.60E-81
VPS13A	1.75845558	1.44E-131	2.78E-131
GNA14	NA	8.58E-28	1.08E-27
GNA14-AS1	NA	NA	NA
GNAQ	2.57009223	0	0

CEP78	1.5528834	3.94E-115	7.17E-115
PSAT1	1.27163952	0	0
RP11-165H2:	NA	NA	NA
RP11-375O1:	NA	NA	NA
TLE4	1.49859773	5.93E-36	7.82E-36
RP11-403N1(	NA	NA	NA
RP11-403N1(	NA	NA	NA
RP11-327L9.:	NA	NA	NA
LINC01507	NA	NA	NA
RP11-289F5.	NA	NA	NA
TLE1	2.57334963	7.51E-35	9.84E-35
RP11-154D1:	NA	NA	NA
RP11-383M4	NA	NA	NA
SPATA31D4	NA	NA	NA
RP11-383M4	NA	NA	NA
SPATA31D3	NA	NA	NA
SPATA31D1	NA	NA	NA
RP11-15B24.	NA	NA	NA
RP11-15B24.	NA	NA	NA
RP11-15B24.	NA	NA	NA
RASEF	NA	NA	NA
RP11-439K3.	NA	NA	NA
FRMD3	Inf	0	0
RP11-439K3.	NA	NA	NA
IDNK	1.4704855	1.58E-17	1.89E-17
UBQLN1	2.12034523	3.27E-180	7.53E-180
RP11-522I20	2.12301345	0.00765678	0.00804665
GKAP1	1.86410937	1.21E-51	1.72E-51
RP11-575L7.:	NA	NA	NA
RP11-575L7.4	NA	NA	NA
KIF27	0.92640587	9.00E-26	1.13E-25
C9orf64	NA	NA	NA
HNRNPK	1.35939045	0	0
RP11-575L7.8	NA	2.01E-18	2.41E-18
RMI1	1.64694377	0	0
RP11-158D2.	NA	NA	NA
SLC28A3	NA	NA	NA
RP11-380F14	NA	NA	NA
NTRK2	NA	5.65E-272	1.93E-271
AGTPBP1	1.72565799	3.03E-63	4.52E-63
RP11-202I11	NA	NA	NA
RP11-213G2.	NA	NA	NA
NAA35	1.74028221	3.07E-130	5.89E-130
GOLM1	1.85920704	0.07908266	0.08170839
C9orf153	0.77200489	1.35E-58	1.97E-58

ISCA1	2.27797385	1.77E-254	5.56E-254
ZCCHC6	1.77275197	3.92E-66	5.92E-66
RP11-359J6.1	NA	NA	NA
RP11-395D3.	NA	NA	NA
GAS1	1.21315054	0.00553988	0.00583582
GAS1RR	NA	NA	NA
RP11-276H19	NA	NA	NA
C9orf170	NA	NA	NA
DAPK1	2.1314048	5.68E-194	1.38E-193
CTSL	0.91983561	0	0
XXyac-YM21(	NA	NA	NA
XXyac-YM21(	NA	NA	NA
XXyac-YM21(	NA	NA	NA
SPATA31E1	NA	NA	NA
CDK20	NA	NA	NA
SPIN1	2.66262911	7.60E-100	1.31E-99
NXNL2	4.63202934	2.67E-07	2.96E-07
RP13-60M5.2	NA	NA	NA
RP11-791O2.	NA	NA	NA
C9orf47	NA	NA	NA
S1PR3	Inf	0	0
SHC3	NA	NA	NA
CKS2	1.87541708	3.48E-160	7.47E-160
SECISBP2	2.02363835	8.98E-285	3.26E-284
SEMA4D	2.31601467	2.04E-60	3.01E-60
GADD45G	NA	1.32E-06	1.45E-06
UNQ6494	NA	NA	NA
RP5-1050E16	NA	NA	NA
RP5-1050E16	NA	NA	NA
RP11-406A2(	NA	NA	NA
MIR4290HG	NA	NA	NA
LINC01508	NA	NA	NA
LINC01501	NA	NA	NA
DIRAS2	NA	NA	NA
SYK	2.60844076	7.34E-188	1.74E-187
RP11-367F23	NA	NA	NA
RP11-367F23	NA	NA	NA
RP11-305L7.	NA	5.92E-55	8.53E-55
RP11-305L7.(	NA	3.18E-11	3.64E-11
RP11-305L7.:	NA	0	0
LINC00484	NA	0	0
RP11-305L7.	NA	1.77E-240	5.20E-240
AUH	1.25762087	4.12E-108	7.35E-108
NFIL3	1.15800733	2.47E-07	2.74E-07
RP11-440G5.	NA	NA	NA

ROR2	1.54400978	1.20E-65	1.81E-65
SPTLC1	1.737011	2.09E-87	3.43E-87
LINC00475	NA	NA	NA
RP11-100G1!	NA	NA	NA
RP11-62C3.6	NA	NA	NA
IARS	1.68368793	2.10E-116	3.86E-116
AL136097.1	NA	NA	NA
NOL8	1.6264569	0	0
CENPP	1.79750392	3.26E-136	6.39E-136
OGN	NA	NA	NA
OMD	NA	NA	NA
ASPN	0	0	0
ECM2	NA	NA	NA
IPPK	1.84729742	3.58E-29	4.55E-29
BICD2	1.70731851	3.75E-17	4.47E-17
ZNF484	1.54400978	9.98E-96	1.69E-95
FGD3	0.94356153	4.90E-221	1.32E-220
SUSD3	1.50761898	0	0
RP11-274J16	NA	NA	NA
CARD19	1.8423543	1.15E-24	1.43E-24
NINJ1	2.03455455	0.84021102	0.8433255
RP11-370F5.	NA	NA	NA
WNK2	3.02368582	1.87E-294	7.15E-294
C9orf129	NA	NA	NA
RP11-165J3.!	NA	NA	NA
RP11-165J3.(	NA	1.67E-68	2.54E-68
FAM120AOS	2.11554931	2.79E-26	3.50E-26
FAM120A	2.38727666	0	0
PHF2	2.37539966	0.00356627	0.00376662
RP11-53B5.1	NA	NA	NA
BARX1	2.34818154	2.61E-120	4.84E-120
BARX1-AS1	NA	NA	NA
PTPDC1	1.47967604	4.23E-23	5.21E-23
RP11-2B6.3	NA	2.26E-210	5.85E-210
RP11-2B6.2	0.77200489	0.00175573	0.00186359
MIRLET7DHC	NA	0	0
ZNF169	3.17379788	3.10E-283	1.12E-282
NUTM2F	NA	NA	NA
RP11-307E17	Inf	8.18E-32	1.06E-31
MFSD14B	2.44911896	1.11E-217	2.95E-217
PCAT7	NA	4.29E-09	4.83E-09
FBP2	NA	NA	NA
FBP1	NA	NA	NA
C9orf3	2.04354236	7.19E-80	1.14E-79
RP11-54O15.	NA	NA	NA

FANCC	2.31601467	3.03E-16	3.59E-16
RP11-80I15.4	NA	NA	NA
RP11-435O5.	NA	NA	NA
RP11-435O5.	Inf	5.09E-283	1.83E-282
PTCH1	1.15800733	6.34E-250	1.95E-249
RP11-435O5.	NA	NA	NA
RP11-435O5.	NA	1.22E-98	2.09E-98
RP11-435O5.	0.77200489	0.14699259	0.15100451
RP11-332M4	NA	NA	NA
RP11-180I4.2	NA	NA	NA
RP11-180I4.4	NA	NA	NA
ERCC6L2	1.64051039	4.16E-91	6.94E-91
LINC00092	NA	NA	NA
RP11-569G1	NA	NA	NA
RP11-569G1	NA	NA	NA
HSD17B3	NA	NA	NA
RP11-240L7.4	NA	NA	NA
SLC35D2	2.33209811	2.11E-74	3.30E-74
ZNF367	2.65376681	2.38E-213	6.24E-213
HABP4	1.7232252	0.25914795	0.26451345
CDC14B	1.83810688	9.97E-28	1.26E-27
AAED1	2.1981223	1.90E-45	2.63E-45
RP11-535M1	NA	NA	NA
ZNF510	2.05867971	1.83E-43	2.50E-43
ZNF782	3.86002445	5.32E-148	1.09E-147
MFSD14C	1.98515543	1.14E-103	1.99E-103
NUTM2G	NA	NA	NA
CTSV	0.5614581	0	0
RP11-498P14	NA	NA	NA
RP11-498P14	3.86002445	2.07E-129	3.97E-129
RP11-498P14	NA	NA	NA
CCDC180	2.5365875	2.36E-92	3.95E-92
TDRD7	1.737011	6.17E-30	7.89E-30
TMOD1	1.15800733	6.46E-234	1.85E-233
RP11-244N9.	NA	NA	NA
TSTD2	1.71946544	4.24E-180	9.76E-180
NCBP1	1.88512822	0.50056086	0.50656413
RP11-244N9.	NA	NA	NA
XPA	1.99756265	5.94E-123	1.11E-122
RP11-546O6.	NA	NA	NA
PTCSC2	NA	NA	NA
FOXE1	NA	NA	NA
TRMO	1.18964688	0	0
HEMGN	NA	1.76E-29	2.25E-29
ANP32B	1.86719279	1.76E-52	2.50E-52

RP11-535C21	NA	NA	NA
NANS	1.13593467	0	0
TRIM14	2.16437085	0.56370364	0.569416
CORO2A	2.06897311	1.61E-11	1.85E-11
TBC1D2	1.02933985	3.36E-24	4.16E-24
GABBR2	NA	NA	NA
ANKS6	2.32190784	0	0
GALNT12	1.50337794	7.95E-132	1.54E-131
RP11-92C4.3	NA	NA	NA
COL15A1	0	0	0
RP11-92C4.6	NA	NA	NA
TGFBR1	2.11890704	0.00553075	0.00582655
RP11-96L7.2	NA	NA	NA
ALG2	1.90494713	4.76E-42	6.46E-42
SEC61B	2.40627052	0	0
NAMA	NA	NA	NA
RP11-547C13	NA	NA	NA
RP11-554F20	NA	5.48E-41	7.40E-41
NR4A3	NA	8.80E-206	2.24E-205
STX17-AS1	4.63202934	0	0
STX17	2.73170961	1.29E-36	1.71E-36
RP11-60I3.4	NA	NA	NA
ERP44	1.22000773	0	0
INVS	2.42630108	0.02249276	0.02347487
TEX10	1.7025465	1.21E-134	2.35E-134
MSANTD3	2.40683877	7.38E-272	2.52E-271
MSANTD3-TI	NA	NA	NA
TMEFF1	NA	3.53E-31	4.54E-31
MURC	6.94804401	5.27E-239	1.54E-238
PLPPR1	NA	NA	NA
BAAT	NA	7.33E-151	1.52E-150
MRPL50	1.63192493	6.31E-290	2.36E-289
ZNF189	2.88215159	2.83E-24	3.51E-24
ALDOB	NA	NA	NA
TMEM246-AS	NA	NA	NA
TMEM246	NA	NA	NA
RNF20	2.63183485	1.63E-68	2.48E-68
GRIN3A	NA	NA	NA
PPP3R2	NA	NA	NA
LINC00587	NA	NA	NA
CYLC2	NA	NA	NA
LINC01492	NA	NA	NA
RP11-436F21	NA	NA	NA
SMC2-AS1	0.57900367	9.37E-100	1.61E-99
SMC2	1.65860426	6.17E-124	1.16E-123

OR13F1	NA	NA	NA
RP11-317C2(	NA	NA	NA
OR13C4	NA	NA	NA
OR13C3	NA	NA	NA
OR13C8	NA	NA	NA
OR13C5	NA	NA	NA
OR13C2	NA	NA	NA
OR13C9	NA	NA	NA
OR13D1	NA	NA	NA
NIPSNAP3A	1.40015794	0	0
NIPSNAP3B	2.31601467	1.61E-207	4.13E-207
ABCA1	NA	NA	NA
RP11-217B7.	NA	NA	NA
RP11-217B7.	NA	NA	NA
SLC44A1	1.24192091	1.68E-133	3.27E-133
FSD1L	2.25662968	5.54E-49	7.79E-49
RP11-235C2:	NA	2.48E-233	7.08E-233
FKTN	2.87357376	9.61E-219	2.57E-218
TAL2	0	6.39E-238	1.86E-237
TMEM38B	1.4836969	4.45E-283	1.60E-282
RP11-6F6.1	NA	NA	NA
RP11-308N1!	NA	NA	NA
RP11-540H2.	NA	NA	NA
RP11-308N1!	NA	NA	NA
LINC01505	NA	NA	NA
ZNF462	1.87486902	4.48E-23	5.52E-23
RP11-508N1.	NA	NA	NA
RP11-196I18	NA	NA	NA
RAD23B	1.85602842	1.60E-92	2.67E-92
LINC01509	NA	NA	NA
KLF4	0.38600244	3.86E-285	1.40E-284
RP11-505C1:	NA	NA	NA
RP11-339N8.	NA	NA	NA
ACTL7B	NA	NA	NA
ACTL7A	NA	NA	NA
IKBKAP	2.12301345	0.24441237	0.24959917
FAM206A	2.22337408	5.79E-175	1.31E-174
CTNNAL1	2.04684105	3.25E-50	4.60E-50
TMEM245	2.55442794	0	0
FRRS1L	Inf	0	0
EPB41L4B	Inf	1.31E-119	2.43E-119
PTPN3	0	1.04E-128	1.99E-128
PALM2	NA	NA	NA
RP11-406O2.	NA	NA	NA
PALM2-AKAF	NA	NA	NA

AKAP2	NA	NA	NA
C9orf152	NA	NA	NA
TXN	2.0604706	2.00E-147	4.08E-147
TXNDC8	NA	NA	NA
SVEP1	NA	NA	NA
RP11-410K21	NA	NA	NA
MUSK	NA	NA	NA
LPAR1	NA	NA	NA
RP11-202G18	NA	NA	NA
OR2K2	NA	NA	NA
KIAA0368	2.01824136	1.82E-73	2.83E-73
ZNF483	1.54400978	6.65E-71	1.02E-70
PTGR1	Inf	0	0
RP11-16L21.7	NA	NA	NA
DNAJC25	2.26673776	3.92E-187	9.26E-187
DNAJC25-GN	NA	1.45E-136	2.86E-136
GNG10	2.62481663	0	0
C9orf84	NA	NA	NA
UGCG	2.06698083	3.57E-09	4.02E-09
SUSD1	1.17832325	9.12E-178	2.08E-177
PTBP3	2.18350637	2.76E-134	5.38E-134
HSDL2	1.82993752	1.58E-146	3.21E-146
C9orf147	0.38600244	2.71E-155	5.70E-155
KIAA1958	1.02933985	0	0
INIP	2.20844022	5.59E-120	1.04E-119
RP11-276E15	1.54400978	0.44579887	0.45190161
SNX30	3.15820182	2.54E-129	4.86E-129
SLC46A2	NA	NA	NA
RP11-408O19	Inf	0	0
ZNF883	NA	NA	NA
ZFP37	NA	NA	NA
FAM225B	1.02933985	2.45E-26	3.07E-26
FAM225A	3.474022	6.35E-124	1.19E-123
SLC31A2	1.2811996	8.31E-203	2.09E-202
FKBP15	1.95364503	6.62E-41	8.94E-41
SLC31A1	1.94771876	2.93E-62	4.36E-62
CDC26	2.38088903	0	0
PRPF4	1.72152962	5.86E-30	7.49E-30
RNF183	NA	NA	NA
WDR31	1.80134474	2.30E-123	4.31E-123
BSPRY	2.0035365	1.80E-06	1.98E-06
HDHD3	1.91133469	2.91E-148	5.97E-148
ALAD	2.22519057	2.91E-32	3.77E-32
POLE3	2.08369838	2.76E-45	3.81E-45
C9orf43	1.15800733	6.66E-29	8.47E-29

RGS3	0.46320293	0	0
RP11-168K11	NA	NA	NA
RP11-168K11	NA	NA	NA
RP11-18B16.	NA	NA	NA
ZNF618	0.90067237	6.29E-197	1.55E-196
AMBP	NA	NA	NA
KIF12	NA	NA	NA
COL27A1	2.24490896	6.17E-164	1.34E-163
ORM1	NA	NA	NA
ORM2	NA	NA	NA
AKNA	2.17815665	1.76E-27	2.23E-27
RP11-9M16.2	NA	1.26E-92	2.11E-92
DFNB31	0.38600244	8.91E-267	2.97E-266
ATP6V1G1	1.77939912	2.54E-109	4.56E-109
C9orf91	2.24130452	3.33E-90	5.54E-90
RP11-402G3.	NA	NA	NA
RP11-402G3.	NA	NA	NA
TNFSF15	NA	NA	NA
TNFSF8	NA	NA	NA
TNC	NA	NA	NA
RP11-523L1.	NA	NA	NA
1-Dec	NA	NA	NA
RP11-445L6.	NA	NA	NA
RP11-284G1(	NA	NA	NA
LINC00474	NA	NA	NA
RP11-787B4.	NA	NA	NA
PAPPA	NA	4.84E-18	5.80E-18
PAPPA-AS2	NA	NA	NA
RP11-45A16.	NA	NA	NA
PAPPA-AS1	NA	NA	NA
ASTN2	1.7878008	3.90E-24	4.83E-24
ASTN2-AS1	NA	NA	NA
RP11-67K19.	NA	NA	NA
TRIM32	1.90244062	0	0
RP11-388N2.	NA	NA	NA
RP11-500B1.	NA	NA	NA
TLR4	0.51466993	9.69E-242	2.87E-241
RP11-281A2(	0	3.86E-172	8.64E-172
RP11-281A2(	NA	7.41E-186	1.74E-185
RP11-349E4.	NA	NA	NA
RP11-127L21	NA	NA	NA
BRINP1	NA	NA	NA
RP11-295D2.	NA	NA	NA
LINC01613	NA	NA	NA
RP11-360A1.	NA	NA	NA

CDK5RAP2	1.47614122	1.39E-199	3.44E-199
MEGF9	2.97773315	0	0
FBXW2	1.76071291	2.27E-53	3.26E-53
PSMD5	Inf	0	0
PSMD5-AS1	NA	0	0
PHF19	1.92369726	3.06E-31	3.94E-31
TRAF1	1.15800733	0.03671756	0.03818458
C5	0.77200489	5.05E-96	8.58E-96
CNTRL	1.54400978	0	0
RAB14	1.69037502	9.83E-146	2.00E-145
GSN	0.51466993	0	0
RP11-477J21	NA	NA	NA
GSN-AS1	NA	1.44E-96	2.45E-96
STOM	1.73362502	1.09E-82	1.75E-82
RP11-162D1(	NA	NA	NA
DAB2IP	0.77200489	0.02507855	0.02615401
RP11-524G2	NA	NA	NA
TTLL11	2.234751	1.57E-28	2.00E-28
TTLL11-IT1	NA	2.63E-155	5.54E-155
NDUFA8	1.85379832	4.86E-37	6.43E-37
MORN5	NA	NA	NA
LHX6	6.56204156	0.41997066	0.42617226
RBM18	1.84203957	3.93E-28	4.98E-28
MRRF	1.60558072	0	0
PTGS1	1.09574888	3.88E-290	1.45E-289
RP11-542K23	NA	NA	NA
RP11-542K23	0	0	0
RP11-542K23	NA	NA	NA
OR1J1	NA	NA	NA
OR1J2	NA	NA	NA
OR1J4	NA	NA	NA
OR1N1	NA	NA	NA
OR1N2	NA	NA	NA
OR1L8	NA	NA	NA
RP11-64P14.	NA	NA	NA
OR1Q1	NA	NA	NA
OR1B1	NA	NA	NA
OR1L1	NA	NA	NA
OR1L3	NA	NA	NA
OR1L4	NA	NA	NA
OR1L6	NA	NA	NA
OR5C1	NA	NA	NA
PDCL	1.40479578	0	0
OR1K1	NA	NA	NA
RC3H2	1.97826253	9.68E-90	1.61E-89

ZBTB6	2.47041565	1.79E-07	1.99E-07
ZBTB26	2.70201711	1.42E-65	2.14E-65
RABGAP1	2.03528562	2.55E-19	3.07E-19
GPR21	NA	NA	NA
STRBP	1.8736615	9.10E-150	1.88E-149
CRB2	NA	NA	NA
DENND1A	1.93001222	2.36E-103	4.12E-103
RP11-230L22	NA	NA	NA
RP11-417B4.	NA	NA	NA
LHX2	Inf	1.28E-273	4.39E-273
RP11-85O21.	NA	NA	NA
RP11-85O21.	NA	NA	NA
NEK6	1.12492141	0	0
RP11-121A14	NA	NA	NA
PSMB7	1.52031154	1.28E-241	3.80E-241
ADGRD2	Inf	0	0
NR5A1	NA	NA	NA
RP11-10J18.3	NA	NA	NA
NR6A1	1.83161944	2.14E-98	3.68E-98
MIR181A2HC	2.31601467	3.71E-260	1.20E-259
RP11-175D17	Inf	1.74E-116	3.18E-116
OLFML2A	NA	NA	NA
WDR38	NA	NA	NA
RPL35	2.13725916	0	0
ARPC5L	2.13726703	7.39E-83	1.19E-82
GOLGA1	2.41894866	1.29E-183	3.01E-183
SCAI	2.05867971	2.11E-45	2.91E-45
PPP6C	1.67730559	1.70E-289	6.32E-289
RABEPK	1.94987365	0.95997279	0.96071416
HSPA5	1.12751053	0	0
RP11-65N13.	NA	NA	NA
GAPVD1	2.1101467	1.95E-159	4.16E-159
MAPKAP1	2.03649566	2.70E-64	4.05E-64
RP11-12A16.	NA	NA	NA
PBX3	2.27925253	0.45091527	0.45698592
RP11-343J18	NA	NA	NA
RP11-343J18	NA	NA	NA
MVB12B	2.70201711	1.21E-156	2.56E-156
RP11-205K6.	NA	NA	NA
RP11-205K6.	NA	NA	NA
RP11-123K19	0.38600244	0	0
LMX1B	NA	2.20E-197	5.43E-197
ZBTB43	2.03888471	8.65E-62	1.28E-61
ZBTB34	1.78154975	2.47E-215	6.53E-215
RALGPS1	Inf	0	0

ANGPTL2	NA	NA	NA
RP13-225O2	NA	NA	NA
GARNL3	0.77200489	0	0
SLC2A8	1.3268834	9.02E-181	2.08E-180
ZNF79	2.57334963	1.64E-80	2.62E-80
RPL12	2.20337777	9.25E-266	3.08E-265
LRSAM1	2.50901589	2.38E-59	3.50E-59
FAM129B	2.44120465	8.05E-47	1.12E-46
STXBP1	2.02651284	3.03E-17	3.61E-17
RP11-56D16.	2.31601467	2.10E-42	2.86E-42
PTRH1	1.89959991	0.38740425	0.3934771
CFAP157	NA	6.67E-243	1.99E-242
TTC16	NA	0	0
TOR2A	1.59381655	0	0
SH2D3C	0.77200489	0	0
AL162426.1	NA	NA	NA
CDK9	1.44107579	0	0
FPGS	1.65601049	1.62E-250	4.99E-250
ENG	1.44044815	0	0
RP11-228B1!	2.31601467	1.18E-195	2.90E-195
AK1	1.06150672	1.18E-43	1.62E-43
ST6GALNAC(	1.70841823	9.27E-39	1.24E-38
ST6GALNAC4	1.92368432	2.72E-34	3.56E-34
PIP5KL1	1.15800733	2.71E-118	5.00E-118
RP11-203J24	NA	NA	NA
DPM2	2.17177165	1.85E-181	4.27E-181
FAM102A	3.474022	2.37E-07	2.63E-07
RP11-379C1(	NA	NA	NA
NAIF1	1.82473883	3.01E-294	1.15E-293
SLC25A25	1.27548634	2.61E-155	5.49E-155
RP11-379C1(	NA	NA	NA
SLC25A25-AS	2.62481663	1.46E-102	2.54E-102
PTGES2	1.29166193	0	0
PTGES2-AS1	0.77200489	3.19E-35	4.20E-35
LCN2	NA	NA	NA
C9orf16	1.82323344	1.14E-07	1.27E-07
CIZ1	1.90372532	0.13181146	0.13558561
DNM1	1.80134474	0.33368223	0.33961644
GOLGA2	1.87737553	6.12E-08	6.82E-08
SWI5	1.7896477	1.65E-150	3.42E-150
TRUB2	1.369366	0	0
RP11-339B2:	Inf	9.19E-67	1.39E-66
COQ4	1.86022647	0.1727025	0.17708522
SLC27A4	2.10030742	1.68E-07	1.87E-07
URM1	1.92023049	1.23E-80	1.96E-80

RP11-339B2:	NA	NA	NA
MIR219A2	Inf	4.56E-130	8.75E-130
CERCAM	1.2099692	0	0
ODF2	1.67560152	7.35E-36	9.68E-36
ODF2-AS1	NA	NA	NA
GLE1	1.74453053	5.48E-250	1.69E-249
RP11-216B9.	Inf	4.91E-36	6.47E-36
SPTAN1	2.24583241	1.42E-166	3.11E-166
RP11-216B9.	NA	NA	NA
WDR34	1.39494832	0	0
HMGA1P4	2.12301345	3.24E-88	5.34E-88
SET	1.84354768	1.04E-86	1.69E-86
PKN3	1.71226726	1.41E-30	1.81E-30
ZDHHC12	1.80192173	1.43E-279	5.06E-279
RP11-545E17	1.09516973	4.16E-261	1.35E-260
ZER1	1.00954486	6.17E-252	1.91E-251
TBC1D13	1.7957505	3.83E-91	6.40E-91
ENDOG	1.53170364	1.59E-302	6.36E-302
C9orf114	1.34884	0	0
CCBL1	1.31061295	0	0
LRRC8A	1.80811672	1.05E-20	1.28E-20
PHYHD1	NA	NA	NA
RP11-101E3.	NA	NA	NA
DOLK	1.94839329	0.3649137	0.37102866
NUP188	2.17565014	0.38824039	0.39430427
RP11-167N5.	Inf	0	0
SH3GLB2	2.03528562	4.16E-143	8.36E-143
FAM73B	2.06698083	4.66E-88	7.67E-88
DOLPP1	2.41251528	5.92E-98	1.01E-97
CRAT	1.59079796	1.23E-76	1.94E-76
RP11-247A1:	NA	NA	NA
PPP2R4	1.94363584	1.81E-146	3.69E-146
RP11-247A1:	0.38600244	9.16E-259	2.94E-258
IER5L	1.05273394	4.42E-212	1.15E-211
RP11-247A1:	Inf	2.92E-211	7.58E-211
RP11-247A1:	NA	NA	NA
RP11-344B5.	NA	NA	NA
RP11-344B5.	NA	NA	NA
RP11-344B5.	NA	NA	NA
C9orf106	0.38600244	0	0
RP11-65J3.1!	NA	NA	NA
LINC01503	1.93001222	0.01993514	0.02082474
RP11-65J3.3	NA	NA	NA
RP11-65J3.2	NA	NA	NA
RP11-65J3.1‹	NA	0.21412432	0.21893949

RP11-492E3.	Inf	0	0
RP11-492E3.	NA	NA	NA
NTMT1	1.72602719	1.42E-137	2.80E-137
C9orf50	NA	NA	NA
ASB6	1.43678688	1.12E-166	2.46E-166
RP11-483H2(	NA	NA	NA
PRRX2	NA	NA	NA
PRRX2-AS1	NA	NA	NA
PTGES	0	0	0
TOR1B	1.46274611	4.67E-56	6.77E-56
TOR1A	1.47204322	1.74E-222	4.73E-222
C9orf78	1.70500577	4.56E-133	8.85E-133
USP20	1.88176192	3.18E-46	4.41E-46
FNBP1	1.65490551	2.90E-99	4.99E-99
RP11-409K2(	NA	NA	NA
RP11-138E2.	Inf	1.86E-05	2.03E-05
GPR107	2.28092354	2.23E-56	3.24E-56
RP11-88G17.	NA	NA	NA
NCS1	2.59553368	3.98E-85	6.47E-85
HMCN2	NA	NA	NA
ASS1	1.35100856	4.85E-62	7.21E-62
FUBP3	2.28513447	1.27E-178	2.90E-178
PRDM12	3.08801956	2.14E-26	2.68E-26
EXOSC2	1.78635436	5.91E-66	8.91E-66
ABL1	2.06787024	9.32E-117	1.71E-116
QRFP	0	0	0
FIBCD1	2.31601467	2.53E-299	9.95E-299
RP11-83J21.3	NA	NA	NA
LAMC3	NA	1.78E-287	6.56E-287
AIF1L	1.23103482	8.11E-153	1.69E-152
NUP214	1.70073258	1.68E-89	2.78E-89
RP11-544A1.	NA	1.16E-162	2.51E-162
RP11-544A1.	1.10286413	7.97E-47	1.11E-46
FAM78A	1.93315046	0.20354389	0.20836775
PLPP7	NA	0	0
PRRC2B	1.93729529	3.66E-296	1.41E-295
RP11-334J6.7	Inf	1.60E-207	4.08E-207
POMT1	1.55830617	0.60595089	0.61154656
RP11-334J6.6	0	0	0
UCK1	1.82076625	1.96E-39	2.62E-39
RAPGEF1	2.16537957	9.87E-35	1.29E-34
RP11-323H2:	NA	NA	NA
MED27	1.18019138	8.82E-302	3.52E-301
NTNG2	1.54400978	1.40E-77	2.21E-77
SETX	1.55687653	1.32E-119	2.45E-119

TTF1	1.8046229	9.11E-126	1.72E-125
CFAP77	NA	NA	NA
BARHL1	NA	NA	NA
DDX31	1.62844781	1.34E-60	1.98E-60
GTF3C4	2.40990716	8.78E-182	2.04E-181
AK8	1.74987775	4.66E-59	6.84E-59
C9orf9	1.94545232	0.00086206	0.00091905
TSC1	1.93001222	3.63E-82	5.84E-82
GFI1B	0.77200489	1.77E-129	3.39E-129
GTF3C5	1.58077192	0	0
CEL	1.54400978	1.69E-75	2.65E-75
RALGDS	2.18420896	6.32E-115	1.15E-114
GBGT1	1.93001222	0.00107331	0.00114245
OBP2B	NA	NA	NA
ABO	NA	NA	NA
SURF6	1.80736931	0	0
MED22	2.06270057	1.81E-81	2.90E-81
RPL7A	1.62418633	5.89E-146	1.20E-145
SURF1	1.57972531	1.04E-260	3.36E-260
SURF2	1.61827077	0.00456379	0.00481429
SURF4	1.74885584	8.08E-22	9.88E-22
STKLD1	3.86002445	1.48E-27	1.87E-27
REXO4	1.78513977	1.04E-31	1.34E-31
ADAMTS13	1.54400978	3.43E-11	3.93E-11
CACFD1	0.57900367	0	0
SLC2A6	1.27677732	1.64E-230	4.62E-230
TMEM8C	NA	NA	NA
ADAMTSL2	NA	NA	NA
FAM163B	NA	NA	NA
LL09NC01-25	NA	NA	NA
DBH	NA	NA	NA
DBH-AS1	NA	NA	NA
SARDH	NA	NA	NA
VAV2	1.43372337	5.03E-144	1.02E-143
LINC00094	2.11950433	6.68E-21	8.14E-21
BRD3	1.85281174	1.85E-233	5.28E-233
RP11-374P2(	NA	NA	NA
LL09NC01-25	Inf	2.08E-130	4.00E-130
WDR5	1.8139592	0.0062721	0.00660064
LL09NC01-13	Inf	4.68E-255	1.48E-254
RP11-145E17	NA	NA	NA
RXRA	1.93001222	1.52E-09	1.72E-09
RP11-473E2.	NA	NA	NA
RP11-473E2.	NA	NA	NA
RP11-473E2.	NA	NA	NA

COL5A1	NA	NA	NA
COL5A1-AS1	NA	NA	NA
FCN2	NA	NA	NA
FCN1	NA	NA	NA
RP11-447M1	NA	NA	NA
OLFM1	1.15800733	3.00E-76	4.71E-76
RP11-399H1:	NA	NA	NA
RP11-399H1:	NA	NA	NA
C9orf62	NA	NA	NA
RP11-555H7.	NA	NA	NA
PPP1R26-AS:	1.93001222	0.0147419	0.01542462
PPP1R26	1.91163116	1.15E-190	2.77E-190
C9orf116	1.96974777	0.02103474	0.02196203
MRPS2	1.32242399	0	0
RP11-426A6.	NA	4.18E-132	8.09E-132
LCN1	NA	NA	NA
OBP2A	NA	NA	NA
PAEP	NA	NA	NA
LINC01502	NA	0	0
RP11-98L5.5	NA	NA	NA
GLT6D1	NA	NA	NA
LCN9	NA	NA	NA
SOHLH1	NA	NA	NA
KCNT1	NA	NA	NA
CAMSAP1	2.22186773	4.58E-174	1.03E-173
RP11-432J22	2.31601467	2.40E-202	6.03E-202
UBAC1	1.9191005	2.24E-179	5.14E-179
NACC2	3.86002445	8.79E-297	3.41E-296
RP11-83N9.5	NA	0	0
C9orf69	2.06918317	1.56E-13	1.82E-13
RP11-83N9.6	Inf	0	0
LHX3	NA	NA	NA
QSOX2	1.34652016	5.32E-219	1.42E-218
WI2-1959D1!	NA	NA	NA
CR392000.1	NA	NA	NA
CCDC187	NA	NA	NA
DKFZP434A0	NA	0.00098505	0.00104888
GPSM1	1.20625764	7.46E-110	1.34E-109
DNLZ	1.48462479	2.47E-39	3.31E-39
CARD9	1.54400978	0	0
SNAPC4	2.91914349	1.47E-264	4.86E-264
SDCCAG3	1.83509359	8.15E-72	1.26E-71
PMPCA	1.10832385	0	0
INPP5E	1.36855412	0.25841195	0.26379189
SEC16A	2.24662097	1.50E-307	6.20E-307

C9orf163	4.63202934	1.12E-244	3.36E-244
NOTCH1	2.25425428	1.90E-33	2.47E-33
RP11-413M3	NA	NA	NA
NALT1	Inf	0	0
LINC01451	NA	NA	NA
RP11-251M1	1.84093474	2.84E-44	3.89E-44
EGFL7	1.54089581	0	0
AGPAT2	1.74674415	1.35E-287	4.99E-287
FAM69B	1.55026083	0	0
SNHG7	2.05309241	0	0
LCN10	NA	NA	NA
RP11-216L13	NA	NA	NA
LCN6	NA	NA	NA
LCN8	NA	NA	NA
LCN15	Inf	0	0
TMEM141	1.8973196	0.19610402	0.20088758
RP11-216L13	NA	NA	NA
CCDC183	0.38600244	4.69E-247	1.43E-246
CCDC183-AS1	Inf	1.65E-143	3.31E-143
RABL6	1.91716801	1.25E-228	3.48E-228
RABL6	NA	NA	NA
C9orf172	2.57334963	0	0
PHPT1	1.89352443	2.35E-17	2.81E-17
MAMDC4	2.10546788	8.15E-118	1.50E-117
EDF1	1.6750512	0	0
TRAF2	1.3852798	5.73E-276	1.99E-275
FBXW5	1.66401054	5.28E-195	1.29E-194
C8G	1.54400978	1.76E-125	3.32E-125
LCN12	0.77200489	7.92E-222	2.14E-221
PRR31	NA	NA	NA
RP11-229P13	NA	NA	NA
PTGDS	Inf	0	0
LCNL1	NA	NA	NA
C9orf142	2.02975655	2.65E-98	4.55E-98
CLIC3	0.77200489	2.13E-306	8.74E-306
ABCA2	2.00948332	6.24E-50	8.80E-50
C9orf139	1.08080685	0	0
FUT7	2.10546788	5.68E-15	6.68E-15
RP11-229P13	NA	NA	NA
NPDC1	1.90210023	4.16E-07	4.60E-07
ENTPD2	NA	NA	NA
RP11-229P13	NA	NA	NA
RP11-229P13	NA	NA	NA
SAPCD2	1.6368626	8.35E-190	2.00E-189
RP11-229P13	0	0	0

UAP1L1	1.43752635	1.73E-229	4.84E-229
MAN1B1-AS	2.74490628	5.16E-05	5.58E-05
MAN1B1	1.27932239	0	0
DPP7	1.54022915	0	0
GRIN1	NA	0	0
LRRC26	1.12065226	2.31E-229	6.46E-229
RP11-350O1	NA	NA	NA
TMEM210	NA	NA	NA
ANAPC2	1.57910091	0	0
SSNA1	1.91514556	1.44E-65	2.17E-65
TPRN	1.64226495	1.97E-20	2.39E-20
TMEM203	1.85703226	4.62E-98	7.91E-98
NDOR1	2.7792176	0.00064386	0.00068772
RNF208	1.96324422	2.96E-136	5.81E-136
CYSRT1	0.77200489	7.71E-25	9.60E-25
RNF224	NA	NA	NA
SLC34A3	NA	NA	NA
TUBB4B	0.82966733	0	0
FAM166A	0.77200489	3.04E-137	6.00E-137
C9orf173-AS	5.40403423	1.30E-97	2.23E-97
C9orf173	NA	NA	NA
NELFB	1.85123622	1.42E-67	2.16E-67
TOR4A	1.1173755	0	0
NRARP	0.72375458	0	0
EXD3	0.89077487	6.86E-89	1.13E-88
NOXA1	0.15440098	0	0
ENTPD8	NA	0	0
NSMF	1.64454995	2.92E-163	6.34E-163
PNPLA7	NA	0	0
MRPL41	1.81497457	3.57E-10	4.06E-10
DPH7	1.4988633	3.29E-249	1.01E-248
ZMYND19	1.94063615	9.73E-144	1.96E-143
ARRDC1	1.50227979	0	0
ARRDC1-AS1	2.24583241	0	0
EHMT1	1.79898387	6.18E-141	1.23E-140
RP11-188C1	NA	NA	NA
CACNA1B	NA	NA	NA
RP11-885N1	NA	NA	NA
FAM157B	Inf	0	0
RP11-304M2	NA	NA	NA
LINC01001	0.77200489	3.94E-70	6.05E-70
RP11-304M2	NA	NA	NA
BET1L	2.33336309	6.54E-180	1.50E-179
SCGB1C1	NA	NA	NA
ODF3	NA	NA	NA

RP11-304M2	0	0	0
RIC8A	1.63268602	5.37E-149	1.10E-148
SIRT3	1.61114064	1.27E-52	1.81E-52
PSMD13	1.25726511	0	0
NLRP6	NA	NA	NA
RP11-326C3.	NA	NA	NA
ATHL1	1.59547677	1.56E-253	4.89E-253
IFITM5	NA	NA	NA
IFITM2	1.38942505	0	0
RP11-326C3.	NA	NA	NA
IFITM1	0.37134412	0	0
RP11-326C3.	1.15800733	7.99E-19	9.61E-19
IFITM3	0.53075336	0	0
RP11-326C3.	NA	NA	NA
RP11-326C3.	NA	NA	NA
RP11-326C3.	NA	NA	NA
RP11-326C3.	Inf	1.80E-67	2.73E-67
RP11-326C3.	0	3.70E-21	4.52E-21
B4GALNT4	1.09367359	3.05E-180	7.02E-180
PKP3	NA	1.31E-159	2.80E-159
SIGIRR	1.55815647	3.38E-222	9.17E-222
ANO9	NA	NA	NA
PTDSS2	1.09862234	0	0
RP13-317D1:	Inf	4.08E-63	6.09E-63
RNH1	1.05642774	0	0
RP13-46H24.	NA	NA	NA
HRAS	2.13311544	3.28E-180	7.56E-180
LRRC56	0.77200489	0	0
LMNTD2	0.77200489	3.25E-138	6.43E-138
RP11-496I9.1	1.54400978	2.52E-54	3.63E-54
RASSF7	1.89644679	7.32E-06	7.99E-06
MIR210HG	0.8685055	2.40E-63	3.58E-63
PHRF1	1.70731851	2.19E-53	3.14E-53
IRF7	1.07612803	0	0
CDHR5	NA	NA	NA
SCT	NA	3.04E-240	8.96E-240
DRD4	1.54400978	2.70E-09	3.04E-09
DEAF1	1.71538846	0	0
RP11-754B1	NA	NA	NA
EPS8L2	1.80134474	0	0
TMEM80	2.28265643	1.33E-118	2.45E-118
AP006621.9	NA	NA	NA
TALDO1	1.28460232	0	0
PDDC1	2.0865643	1.19E-279	4.20E-279
AP006621.5	2.52954794	1.12E-10	1.27E-10

AP006621.6	NA	NA	NA
CEND1	NA	NA	NA
SLC25A22	1.49805711	1.46E-271	4.96E-271
PANO1	1.93001222	7.42E-208	1.90E-207
PIDD1	2.64985462	1.19E-51	1.70E-51
RPLP2	2.35775183	0	0
PNPLA2	1.07106985	0	0
AP006621.8	Inf	0	0
CRACR2B	0.77200489	8.79E-96	1.49E-95
CD151	1.52619428	0	0
POLR2L	2.16645046	1.20E-303	4.85E-303
TSPAN4	1.64310294	0	0
RP11-1391J7	1.97633252	4.77E-36	6.28E-36
CHID1	1.65992643	6.15E-214	1.61E-213
AP2A2	1.59108325	9.69E-79	1.54E-78
MUC6	NA	NA	NA
RP13-870H1	NA	NA	NA
MUC5AC	NA	NA	NA
MUC5B	NA	NA	NA
RP11-532E4.	NA	NA	NA
TOLLIP	1.37824186	0	0
TOLLIP-AS1	4.11735941	0	0
BRSK2	0.77200489	1.43E-58	2.09E-58
MOB2	1.88480473	1.02E-36	1.34E-36
DUSP8	1.54400978	5.09E-12	5.86E-12
KRTAP5-AS1	NA	NA	NA
AP006285.2	NA	NA	NA
KRTAP5-1	NA	NA	NA
KRTAP5-2	NA	NA	NA
KRTAP5-3	NA	NA	NA
KRTAP5-4	NA	NA	NA
KRTAP5-5	NA	NA	NA
AP006285.7	NA	NA	NA
FAM99A	NA	NA	NA
FAM99B	NA	NA	NA
AP006285.6	NA	NA	NA
KRTAP5-6	NA	NA	NA
IFITM10	0.77200489	2.10E-256	6.65E-256
RP11-295K3.	NA	NA	NA
CTSD	1.40188336	0	0
AC068580.6	NA	NA	NA
AC068580.7	NA	NA	NA
SYT8	NA	NA	NA
TNNI2	0	0	0
LSP1	0.2144458	0	0

PRR33	NA	NA	NA
LINC01150	NA	NA	NA
TNNT3	0	9.07E-64	1.36E-63
MRPL23	1.72109475	0	0
MRPL23-AS1	NA	NA	NA
LINC01219	NA	NA	NA
IGF2	NA	NA	NA
INS-IGF2	NA	NA	NA
IGF2-AS	NA	NA	NA
INS	NA	NA	NA
TH	Inf	5.93E-270	2.00E-269
ASCL2	0.96500611	1.34E-304	5.43E-304
C11orf21	1.80134474	4.24E-16	5.02E-16
TSPAN32	2.97773315	1.60E-72	2.48E-72
CD81-AS1	NA	5.62E-15	6.61E-15
CD81	1.52894627	1.22E-189	2.91E-189
TSSC4	1.55400984	4.67E-153	9.74E-153
AC124057.5	NA	NA	NA
TRPM5	NA	NA	NA
KCNQ1	0.77200489	1.29E-155	2.73E-155
KCNQ1OT1	Inf	0.00043803	0.00046875
KCNQ1-AS1	NA	NA	NA
KCNQ1DN	NA	NA	NA
CDKN1C	0.54040342	0	0
SLC22A18AS	0.77200489	0	0
SLC22A18	0.96500611	7.00E-258	2.24E-257
PHLDA2	0.90426071	1.08E-283	3.91E-283
NAP1L4	1.76428291	2.37E-62	3.53E-62
CTC-343N3.1	NA	NA	NA
CARS	2.09544184	5.72E-183	1.33E-182
CARS-AS1	NA	NA	NA
OSBPL5	0.77200489	0	0
AC108448.3	NA	NA	NA
MRGPRG	NA	NA	NA
MRGPRG-AS	NA	NA	NA
AC109309.4	NA	NA	NA
MRGPRE	NA	NA	NA
RP5-1173A5.	NA	NA	NA
ZNF195	1.68297066	3.30E-81	5.29E-81
RP13-726E6.	NA	NA	NA
RP13-726E6.	0.77200489	5.38E-51	7.63E-51
ART5	1.4153423	0	0
ART1	NA	NA	NA
CHRNA10	Inf	8.68E-213	2.27E-212
NUP98	1.88980364	1.21E-128	2.30E-128

PGAP2	2.02436838	0.14705031	0.15105525
RHOG	2.02275644	1.48E-111	2.67E-111
AC090587.5	1.54400978	1.17E-48	1.63E-48
STIM1	2.42323757	6.18E-282	2.21E-281
RRM1	1.77847052	8.70E-269	2.92E-268
RRM1-AS1	NA	NA	NA
RP11-23F23.	2.57334963	3.87E-20	4.69E-20
AC009758.1	NA	NA	NA
TRIM21	4.82503056	0.78826031	0.79223414
OR52K2	NA	NA	NA
OR52K1	NA	NA	NA
OR52M1	NA	NA	NA
C11orf40	NA	NA	NA
OR52I2	NA	NA	NA
OR52I1	NA	NA	NA
TRIM68	1.737011	2.03E-112	3.67E-112
OR51D1	NA	NA	NA
OR51E1	NA	NA	NA
OR51E2	NA	NA	NA
MMP26	NA	NA	NA
OR51F1	NA	NA	NA
OR52R1	NA	NA	NA
OR51F2	NA	NA	NA
OR51S1	NA	NA	NA
OR51H1	NA	NA	NA
OR51T1	NA	NA	NA
OR51A7	NA	NA	NA
OR51G2	NA	NA	NA
OR51G1	NA	NA	NA
OR51A4	NA	NA	NA
OR51A2	NA	NA	NA
OR51L1	NA	NA	NA
OR52J3	NA	NA	NA
OR52E2	NA	NA	NA
AC113331.9	NA	NA	NA
OR52A5	NA	NA	NA
OR52A1	NA	NA	NA
OR51V1	NA	NA	NA
AC104389.16	NA	NA	NA
HBB	Inf	8.68E-191	2.08E-190
HBD	NA	1.04E-221	2.80E-221
HBG1	NA	NA	NA
HBG2	NA	1.68E-115	3.07E-115
AC104389.2	NA	NA	NA
AC104389.1	NA	NA	NA

HBE1	NA	1.03E-05	1.12E-05
OR51B4	NA	NA	NA
OR51B2	NA	NA	NA
OR51B5	NA	NA	NA
OR51B6	NA	NA	NA
OR51M1	NA	NA	NA
OR51J1	NA	NA	NA
OR51Q1	NA	NA	NA
OR51I1	NA	NA	NA
OR51I2	NA	NA	NA
OR52D1	NA	NA	NA
UBQLN3	NA	NA	NA
UBQLNL	NA	NA	NA
OR52H1	NA	NA	NA
AC015691.13	NA	2.16E-154	4.53E-154
OR52B6	NA	NA	NA
TRIM6	1.81648209	2.42E-14	2.83E-14
TRIM6-TRIM	NA	NA	NA
TRIM34	NA	2.47E-282	8.85E-282
TRIM5	1.65429619	7.88E-06	8.60E-06
TRIM22	2.48757131	1.67E-252	5.19E-252
OR56B1	NA	NA	NA
OR52N4	NA	NA	NA
OR52N5	NA	NA	NA
OR52N1	NA	NA	NA
OR52N2	NA	NA	NA
OR52E6	NA	NA	NA
OR52E8	NA	NA	NA
OR52E4	NA	NA	NA
OR52E5	NA	NA	NA
AC025016.1	NA	NA	NA
OR56A3	NA	NA	NA
OR56A5	NA	NA	NA
OR52L1	NA	NA	NA
OR56A4	NA	NA	NA
OR56A1	NA	NA	NA
OR56B4	NA	NA	NA
OR52B2	NA	NA	NA
OR52W1	NA	NA	NA
RP11-290F24	2.31601467	1.79E-53	2.57E-53
C11orf42	NA	NA	NA
FAM160A2	2.90637135	6.66E-48	9.32E-48
CNGA4	NA	NA	NA
CCKBR	NA	NA	NA
PRKCDBP	0	0	0

RP11-304C12	NA	NA	NA
RP11-304C12	NA	NA	NA
SMPD1	2.02191757	1.85E-224	5.08E-224
APBB1	1.73230366	5.86E-104	1.03E-103
HPX	NA	1.09E-10	1.24E-10
TRIM3	1.43372337	0.11360899	0.11703448
ARFIP2	1.66682874	5.45E-101	9.43E-101
TIMM10B	1.91098394	1.10E-49	1.55E-49
TIMM10B	NA	1.05E-264	3.48E-264
DNHD1	2.8306846	6.39E-187	1.51E-186
RRP8	1.76458261	0	0
RP11-732A19	NA	NA	NA
ILK	1.09023591	0	0
TAF10	2.24077691	0	0
TPP1	1.74761547	0.03045561	0.031727
RP11-732A19	Inf	7.04E-38	9.36E-38
DCHS1	NA	7.72E-175	1.74E-174
RP11-732A19	NA	NA	NA
RP11-732A19	NA	NA	NA
MRPL17	2.0844132	3.99E-27	5.03E-27
OR2AG2	NA	NA	NA
OR2AG1	NA	NA	NA
OR6A2	NA	NA	NA
OR10A5	NA	NA	NA
OR10A2	NA	NA	NA
OR10A4	NA	NA	NA
OR2D2	NA	NA	NA
OR2D3	NA	NA	NA
ZNF215	1.50112062	5.54E-91	9.24E-91
ZNF214	NA	NA	NA
NLRP14	NA	NA	NA
RBMXL2	NA	NA	NA
RP11-324J3.1	NA	NA	NA
SYT9	NA	NA	NA
CTD-2516F10	0	5.57E-248	1.70E-247
OLFML1	NA	NA	NA
PPFIBP2	1.93001222	1.76E-171	3.92E-171
CTD-2516F10	NA	NA	NA
CYB5R2	NA	NA	NA
OVCH2	NA	NA	NA
RP11-35J10.5	NA	NA	NA
RP11-35J10.4	NA	NA	NA
OR5P2	NA	NA	NA
OR5P3	NA	NA	NA
OR10A6	NA	NA	NA

OR10A3	NA	NA	NA
NLRP10	NA	NA	NA
EIF3F	1.74621004	3.41E-105	6.00E-105
CASC23	NA	NA	NA
TUB	2.61293963	9.52E-198	2.35E-197
RP11-236J17	NA	NA	NA
RIC3	NA	NA	NA
RP11-379P15	NA	NA	NA
LMO1	0.77200489	1.48E-97	2.52E-97
STK33	NA	NA	NA
TRIM66	2.99151895	4.65E-47	6.48E-47
RP11-152H18	NA	NA	NA
RPL27A	2.30248211	0	0
ST5	Inf	9.06E-83	1.46E-82
RP11-152H18	NA	1.25E-147	2.55E-147
RP11-318C2.	NA	NA	NA
AKIP1	2.27998778	0.53481832	0.54077996
C11orf16	NA	NA	NA
ASCL3	NA	NA	NA
TMEM9B	1.86884244	1.97E-19	2.38E-19
TMEM9B-AS	Inf	1.58E-64	2.37E-64
NRIP3	3.31962103	9.35E-228	2.60E-227
RP11-467K18	NA	NA	NA
SCUBE2	NA	NA	NA
DENND5A	1.83918812	2.59E-18	3.11E-18
TMEM41B	1.9495073	6.92E-28	8.75E-28
IPO7	2.17774514	0	0
CTD-2371O3.	Inf	2.29E-87	3.75E-87
CTD-2371O3.	1.87486902	1.33E-10	1.51E-10
ZNF143	1.91898358	0.84504225	0.84794007
WEE1	1.85014965	1.43E-42	1.95E-42
SWAP70	1.82324559	2.08E-42	2.83E-42
RP11-540A21	6.17603912	3.25E-231	9.18E-231
SBF2-AS1	1.49859773	1.20E-252	3.72E-252
SBF2	1.64051039	1.18E-09	1.34E-09
RP11-1H15.2	NA	0	0
RP11-748C4.	NA	NA	NA
RP11-351I24	NA	NA	NA
ADM	0.57900367	1.51E-184	3.53E-184
AMPD3	1.27153747	4.24E-43	5.79E-43
MTRNR2L8	1.32343695	0	0
RNF141	2.44666165	1.92E-30	2.46E-30
MRVI1-AS1	Inf	0	0
LYVE1	NA	NA	NA
MRVI1	Inf	0	0

CTR9	2.41119335	9.50E-31	1.22E-30
RP11-685M7	NA	NA	NA
EIF4G2	2.17106421	1.21E-291	4.55E-291
RP11-685M7	NA	4.13E-200	1.03E-199
ZBED5	1.99714308	1.23E-65	1.85E-65
ZBED5-AS1	1.55914713	2.69E-40	3.62E-40
CTD-2003C8.	NA	NA	NA
RP11-179A1(	NA	NA	NA
CTD-3224I3.3	NA	NA	NA
GALNT18	2.16537957	5.98E-06	6.54E-06
CSNK2A3	Inf	8.28E-86	1.35E-85
RP11-567I13	NA	1.53E-44	2.10E-44
RP11-483L5.	NA	1.79E-57	2.62E-57
USP47	1.94332265	9.02E-74	1.40E-73
DKK3	NA	NA	NA
RP13-631K18	NA	NA	NA
RP13-631K18	NA	NA	NA
RP13-631K18	NA	NA	NA
MICAL2	0.99257772	1.71E-85	2.79E-85
RP11-265D17	NA	NA	NA
MICALCL	NA	5.70E-98	9.76E-98
PARVA	NA	NA	NA
RP11-51B23.	NA	NA	NA
TEAD1	Inf	0	0
RP11-47J17.3	NA	NA	NA
RP11-47J17.2	NA	0	0
LINC00958	0.77200489	2.83E-150	5.86E-150
CTC-497E21.3	NA	NA	NA
RASSF10	NA	NA	NA
CTC-497E21.5	NA	NA	NA
ARNTL	1.58464162	3.72E-285	1.35E-284
BTBD10	1.7441592	1.11E-214	2.93E-214
PTH	NA	NA	NA
FAR1	2.32789167	6.22E-25	7.75E-25
RP11-98J9.1	NA	NA	NA
RP11-98J9.2	NA	NA	NA
RP11-98J9.3	NA	NA	NA
RP11-231N3.	Inf	6.49E-29	8.25E-29
SPON1	1.15800733	2.68E-196	6.59E-196
RP11-21L19.	NA	NA	NA
RRAS2	1.71657558	1.78E-20	2.16E-20
COPB1	1.02355705	0	0
RP11-140L24	NA	NA	NA
PSMA1	1.58493618	1.81E-251	5.61E-251
PDE3B	1.54400978	0	0

CYP2R1	0.57900367	0	0
CALCB	NA	NA	NA
CALCA	NA	NA	NA
INSC	NA	NA	NA
RP11-531H8.	NA	NA	NA
RP11-531H8.	NA	NA	NA
RP11-396O2(	NA	NA	NA
RP11-396O2(	NA	NA	NA
RP11-222N1:	NA	NA	NA
SOX6	Inf	4.60E-160	9.87E-160
CTD-3096P4.	NA	NA	NA
C11orf58	1.88769645	1.66E-49	2.33E-49
PLEKHA7	1.54400978	2.64E-19	3.19E-19
RPS13	2.0315516	0	0
PIK3C2A	2.21308068	1.41E-163	3.06E-163
NUCB2	1.79227087	6.41E-133	1.24E-132
NCR3LG1	2.31601467	0	0
RP1-239B22.	2.50430855	0	0
KCNJ11	1.23520782	3.29E-23	4.05E-23
ABCC8	NA	NA	NA
USH1C	NA	NA	NA
OTOG	NA	NA	NA
RP11-358H1{	NA	NA	NA
RP11-358H1{	NA	NA	NA
MYOD1	NA	NA	NA
KCNC1	NA	0.00997327	0.01046412
SERGEF	1.94617047	4.25E-36	5.60E-36
TPH1	1.3896088	9.84E-15	1.16E-14
SAAL1	1.26699626	0	0
MRGPRX3	NA	NA	NA
RP11-113D6.	NA	NA	NA
MRGPRX4	NA	NA	NA
SAA4	NA	NA	NA
SAA2-SAA4	NA	NA	NA
SAA2	NA	NA	NA
SAA1	NA	NA	NA
HPS5	1.50337794	7.03E-69	1.07E-68
GTF2H1	1.93247084	1.66E-09	1.87E-09
LDHA	1.1132745	0	0
LDHC	NA	0	0
LDHAL6A	NA	NA	NA
TSG101	1.10022254	0	0
UEVLD	1.74987775	5.46E-16	6.46E-16
SPTY2D1-AS:	3.39682152	0	0
SPTY2D1	2.5365875	5.13E-234	1.47E-233

TMEM86A	1.54400978	1.05E-29	1.34E-29
IGSF22	NA	NA	NA
RP11-1081L1	NA	NA	NA
PTPN5	NA	NA	NA
MRGPRX1	NA	NA	NA
RP11-583F24	NA	NA	NA
MRGPRX2	NA	NA	NA
ZDHHC13	1.8969263	3.12E-46	4.32E-46
CSRP3	NA	NA	NA
RP11-428C19	NA	NA	NA
E2F8	1.72782047	2.50E-31	3.22E-31
RP11-428C19	NA	NA	NA
NAV2	Inf	1.22E-23	1.50E-23
NAV2-AS5	NA	NA	NA
NAV2-AS4	NA	NA	NA
RP11-359E10	NA	4.21E-229	1.18E-228
NAV2-AS3	Inf	7.95E-147	1.62E-146
NAV2-AS2	NA	NA	NA
NAV2-AS1	NA	NA	NA
DBX1	NA	NA	NA
HTATIP2	NA	NA	NA
PRMT3	2.25168093	1.21E-06	1.33E-06
SLC6A5	NA	NA	NA
NELL1	NA	NA	NA
RP11-701I24	NA	NA	NA
RP11-670N15	NA	NA	NA
CTD-2019O4.	NA	NA	NA
ANO5	2.70201711	6.01E-124	1.13E-123
CTD-3064C13	NA	NA	NA
CTD-2140G10	NA	NA	NA
SLC17A6	NA	NA	NA
CTD-2140G10	NA	NA	NA
LINC01495	NA	NA	NA
RP11-34N19.	NA	NA	NA
FANCF	2.04354236	0.90976834	0.91142621
GAS2	0	9.04E-24	1.12E-23
SVIP	2.58394578	0	0
RP11-17A1.3	Inf	1.67E-91	2.79E-91
CCDC179	NA	NA	NA
RP11-266A24	NA	NA	NA
RP11-945A11	NA	NA	NA
RP11-945A11	NA	NA	NA
RP11-2F20.1	NA	NA	NA
LUZP2	NA	NA	NA
RP11-405K6.	NA	NA	NA

RP11-20J1.1	NA	NA	NA
CTD-2507G9.	NA	NA	NA
RP11-430L3.	NA	NA	NA
ANO3	NA	NA	NA
MUC15	NA	NA	NA
SLC5A12	NA	NA	NA
FIBIN	NA	NA	NA
BBOX1	NA	NA	NA
BBOX1-AS1	NA	NA	NA
CCDC34	2.19306136	1.43E-134	2.79E-134
LGR4	1.8442339	7.45E-17	8.86E-17
RP11-159H2.	Inf	3.92E-06	4.29E-06
LIN7C	1.79215421	2.54E-74	3.96E-74
BDNF-AS	NA	0	0
LINC00678	NA	NA	NA
BDNF	NA	2.32E-147	4.73E-147
RP11-406D1.	NA	0	0
KIF18A	2.31601467	7.39E-12	8.50E-12
METTL15	1.79377607	3.60E-64	5.40E-64
RP11-22P4.2	NA	NA	NA
RP11-960D2	NA	NA	NA
RP11-115J23	NA	NA	NA
RP11-466I1.1	NA	NA	NA
RP11-460B1.	NA	NA	NA
RP11-460B1.	NA	NA	NA
CTD-3012A1	NA	NA	NA
RP5-1027O1	NA	NA	NA
CTD-3138F1	NA	NA	NA
LINC01616	NA	NA	NA
KCNA4	NA	NA	NA
RP11-624D1	NA	NA	NA
FSHB	NA	NA	NA
ARL14EP	2.15490061	6.53E-11	7.46E-11
MPPED2	5.59703545	0	0
RP4-710M3.	NA	NA	NA
RP5-1024C2	NA	4.63E-210	1.20E-209
DCDC1	NA	NA	NA
RP1-296L11.	NA	NA	NA
DNAJC24	3.71396947	0	0
IMMP1L	2.16161369	2.69E-262	8.78E-262
ELP4	0.94892268	0	0
Z83001.1	NA	NA	NA
PAX6	1.49113273	1.17E-260	3.79E-260
RCN1	1.46877202	0	0
PAUPAR	NA	NA	NA

RP1-17K7.2	NA	NA	NA
RP1-17K7.3	NA	NA	NA
RP1-65P5.3	NA	NA	NA
RP1-65P5.5	NA	NA	NA
WT1	1.36116652	1.13E-183	2.64E-183
WT1-AS	1.19936474	1.53E-245	4.63E-245
EIF3M	1.41327179	0	0
CCDC73	1.54400978	0.0005329	0.0005697
PRRG4	1.71226726	6.88E-11	7.87E-11
QSER1	3.27631344	5.38E-179	1.23E-178
DEPDC7	0.73524275	0	0
TCP11L1	1.28667482	3.99E-149	8.21E-149
AC131263.1	NA	NA	NA
LINC00294	2.64687391	1.42E-55	2.06E-55
CSTF3	1.93696722	3.57E-08	3.99E-08
CSTF3-AS1	Inf	1.84E-218	4.93E-218
HIPK3	1.76917787	1.90E-160	4.08E-160
KIAA1549L	Inf	7.41E-292	2.80E-291
RP4-541C22.	NA	NA	NA
C11orf91	NA	0.00064724	0.00069112
CD59	1.60302668	0	0
FBXO3	1.61582419	1.24E-155	2.61E-155
FBXO3-AS1	NA	9.39E-84	1.52E-83
RP11-646J21	NA	NA	NA
RP11-646J21	NA	NA	NA
RP11-646J21	NA	NA	NA
RP11-646J21	NA	NA	NA
LMO2	1.59660379	0	0
RP13-786C16	NA	NA	NA
CAPRIN1	2.1831054	0	0
NAT10	2.20057469	3.34E-174	7.51E-174
ABTB2	3.95963798	5.03E-274	1.73E-273
CAT	2.91892331	5.67E-137	1.12E-136
ELF5	NA	NA	NA
RP4-594L9.2	NA	NA	NA
RP11-350D17	NA	NA	NA
EHF	NA	NA	NA
APIP	1.58761139	0	0
PDHX	1.28667482	5.72E-260	1.85E-259
RP4-607I7.1	Inf	0	0
CD44	0.92608817	0	0
RP1-68D18.4	NA	NA	NA
SLC1A2	NA	NA	NA
RP1-68D18.3	NA	NA	NA
RP4-683L5.1	NA	NA	NA

PAMR1	NA	NA	NA
RP5-945I17.2	NA	NA	NA
FJX1	2.30682414	5.94E-18	7.11E-18
RP1-276E15.	NA	NA	NA
TRIM44	1.84396072	1.08E-10	1.24E-10
RP11-698N1	NA	NA	NA
LDLRAD3	1.02933985	1.31E-296	5.09E-296
COMMD9	1.92663611	5.89E-05	6.37E-05
PRR5L	0.95015986	5.38E-281	1.91E-280
RP11-514F3.	NA	NA	NA
RP11-219O3.	NA	NA	NA
TRAF6	1.31694952	0.43677388	0.44295107
RAG1	9.26405868	0	0
RAG2	Inf	0	0
C11orf74	0.88698434	0	0
RP11-159D8.	NA	NA	NA
RP11-63D14.	NA	NA	NA
RP11-277K23	NA	NA	NA
LINC01493	NA	NA	NA
LRRC4C	NA	NA	NA
RP11-63C8.1	NA	NA	NA
RP11-124G5.	NA	NA	NA
LINC01499	NA	NA	NA
RP11-375D13	NA	NA	NA
RP11-375D13	NA	NA	NA
RP11-148I19	NA	NA	NA
CTD-2572N1	NA	NA	NA
API5	1.91550085	0.00024729	0.0002655
RP11-484D2.	NA	NA	NA
TTC17	2.50316737	2.35E-33	3.06E-33
RP11-484D2.	NA	NA	NA
HSD17B12	1.88176192	2.65E-27	3.34E-27
RP11-613D13	NA	NA	NA
ALKBH3	2.08148154	4.67E-25	5.81E-25
ALKBH3-AS1	NA	0	0
C11orf96	1.61753406	0.00218234	0.00231329
RP11-613D13	NA	NA	NA
ACCSL	NA	NA	NA
ACCS	1.8442339	6.40E-22	7.83E-22
EXT2	3.03287635	7.85E-53	1.12E-52
ALX4	NA	NA	NA
RP11-58K22.	NA	NA	NA
CD82	0.33775214	0	0
RP11-58K22.	NA	NA	NA
RP11-58K22.	NA	NA	NA

RP11-45A12.	NA	NA	NA
RP11-45A12.	NA	NA	NA
TSPAN18	3.0178373	4.76E-07	5.25E-07
TP53I11	1.88176192	1.10E-55	1.59E-55
AC068858.1	NA	NA	NA
PRDM11	3.474022	5.76E-86	9.39E-86
CTD-2560E9.	NA	NA	NA
SYT13	NA	NA	NA
CTD-2560E9.	NA	NA	NA
RP11-430H1(	NA	NA	NA
RP11-430H1(	NA	NA	NA
RP11-430H1(	NA	NA	NA
RP11-430H1(	NA	NA	NA
RP11-958J22	NA	NA	NA
CHST1	Inf	2.49E-281	8.86E-281
RP11-495O1	NA	NA	NA
CTD-2210P24	NA	NA	NA
CTD-2210P24	NA	NA	NA
DKFZp779M(	0	0	0
SLC35C1	2.07078959	3.88E-62	5.77E-62
CTD-2210P24	NA	NA	NA
CRY2	3.53496976	0	0
MAPK8IP1	0.38600244	3.89E-252	1.21E-251
RP11-618K13	NA	6.59E-24	8.16E-24
C11orf94	NA	4.07E-147	8.32E-147
PEX16	1.74079534	1.84E-260	5.98E-260
GYLTL1B	NA	NA	NA
PHF21A	2.57334963	6.23E-40	8.36E-40
RP11-702F3.	NA	NA	NA
RP11-702F3.	Inf	0	0
RP11-702F3.	NA	NA	NA
CTD-2589M5	NA	NA	NA
CTD-2589M5	NA	NA	NA
CREB3L1	0.51466993	6.61E-171	1.47E-170
DGKZ	1.86830923	1.28E-07	1.42E-07
MDK	1.64818867	2.38E-66	3.60E-66
CHRM4	NA	4.59E-225	1.26E-224
AMBRA1	2.08860238	1.14E-16	1.35E-16
HARBI1	1.33814181	0.014872	0.01555985
ATG13	2.0857676	5.97E-10	6.77E-10
ARHGAP1	2.54118276	1.36E-57	1.98E-57
ZNF408	1.59127539	3.34E-285	1.22E-284
F2	NA	NA	NA
CKAP5	1.51249938	0	0
LRP4-AS1	Inf	4.07E-129	7.78E-129

LRP4	2.23023635	1.83E-116	3.35E-116
C11orf49	2.74742917	0.00079669	0.00084966
RP11-390K5.	NA	1.68E-32	2.18E-32
ARFGAP2	1.63017104	7.05E-278	2.47E-277
PACSIN3	1.22890574	0	0
DDB2	1.30583806	0	0
RP11-17G12.	NA	NA	NA
ACP2	1.05273394	0	0
NR1H3	1.48346038	1.09E-67	1.65E-67
MADD	2.41016161	1.35E-134	2.63E-134
RP11-17G12.	NA	NA	NA
MYBPC3	NA	0	0
SPI1	1.74734659	8.11E-240	2.38E-239
RP11-750H9.	NA	NA	NA
SLC39A13	2.28513447	6.43E-96	1.09E-95
PSMC3	0.87155289	0	0
RAPSN	NA	1.07E-15	1.27E-15
CELF1	2.07083726	3.04E-228	8.46E-228
NDUFS3	1.65132166	3.64E-255	1.15E-254
PTPMT1	2.68271699	0	0
KBTBD4	1.54400978	0.27851702	0.28404383
RP11-76I23.7	0.77200489	9.06E-05	9.77E-05
FAM180B	0.12866748	0	0
C1QTNF4	0	0	0
MTCH2	1.92082567	0.72320537	0.72773819
AGBL2	NA	0	0
FNBP4	2.27576797	0.21875075	0.22360694
NUP160	2.01750611	5.06E-06	5.54E-06
PTPRJ	3.16522005	2.33E-108	4.16E-108
PTPRJ-AS1	NA	NA	NA
OR4B1	NA	NA	NA
OR4X2	NA	NA	NA
OR4X1	NA	NA	NA
OR4S1	NA	NA	NA
OR4C3	NA	NA	NA
OR4C5	NA	NA	NA
OR4A47	NA	NA	NA
TRIM49B	NA	NA	NA
TRIM64C	NA	NA	NA
FOLH1	NA	5.03E-99	8.64E-99
OR4C13	NA	NA	NA
OR4C12	NA	NA	NA
RP11-347H15	NA	NA	NA
OR4C46	NA	NA	NA
OR4A5	NA	NA	NA

TRIM48	NA	NA	NA
OR4A16	NA	NA	NA
OR4A15	NA	NA	NA
OR4C15	NA	NA	NA
OR4C16	NA	NA	NA
OR4C11	NA	NA	NA
OR4P4	NA	NA	NA
OR4S2	NA	NA	NA
OR4C6	NA	NA	NA
RP11-674C21	NA	NA	NA
OR5D13	NA	NA	NA
OR5D14	NA	NA	NA
OR5L1	NA	NA	NA
OR5D18	NA	NA	NA
OR5L2	NA	NA	NA
OR5D16	NA	NA	NA
TRIM51	NA	NA	NA
OR5W2	NA	NA	NA
OR5I1	NA	NA	NA
OR10AG1	NA	NA	NA
OR5F1	NA	NA	NA
OR5AS1	NA	NA	NA
OR8I2	NA	NA	NA
OR8H2	NA	NA	NA
OR8H3	NA	NA	NA
OR8J3	NA	NA	NA
OR8K5	NA	NA	NA
OR5J2	NA	NA	NA
OR5T2	NA	NA	NA
OR5T3	NA	NA	NA
OR5T1	NA	NA	NA
OR8H1	NA	NA	NA
OR8K3	NA	NA	NA
OR8K1	NA	NA	NA
OR8J1	NA	NA	NA
OR8U1	NA	NA	NA
OR5R1	NA	NA	NA
OR5M9	NA	NA	NA
OR5M3	NA	NA	NA
OR5M8	NA	NA	NA
OR5M11	NA	NA	NA
OR5M10	NA	NA	NA
OR5M1	NA	NA	NA
OR5AP2	NA	NA	NA
OR5AR1	NA	NA	NA

OR9G1	NA	NA	NA
OR9G4	NA	NA	NA
AP000479.1	NA	NA	NA
OR5AK2	NA	NA	NA
LRRC55	NA	NA	NA
APLNR	NA	NA	NA
TNKS1BP1	1.44750917	0.03485746	0.03626679
RP11-872D1	0	0	0
SSRP1	1.78236324	3.47E-34	4.53E-34
P2RX3	NA	NA	NA
PRG3	NA	NA	NA
PRG2	0	9.04E-24	1.12E-23
RP11-872D1	NA	NA	NA
SLC43A3	1.56793106	0	0
RTN4RL2	1.21016983	3.08E-165	6.74E-165
RP11-624G1	Inf	9.25E-164	2.01E-163
SLC43A1	1.83683922	1.60E-15	1.88E-15
TIMM10	2.58848698	0	0
SMTNL1	NA	NA	NA
UBE2L6	1.85827726	5.15E-184	1.20E-183
SERPING1	0.77200489	2.74E-232	7.77E-232
YPEL4	0	0	0
CLP1	1.30021876	0	0
ZDHHC5	2.21640114	2.31E-262	7.57E-262
MED19	1.60191015	0	0
TMX2	1.53913909	3.18E-213	8.34E-213
TMX2-CTNNI	NA	NA	NA
C11orf31	1.98577916	3.17E-39	4.25E-39
RP11-691N7.	NA	NA	NA
BTBD18	NA	NA	NA
CTNND1	2.00721271	3.73E-24	4.62E-24
OR9Q1	NA	NA	NA
OR6Q1	NA	NA	NA
RP11-659P1	NA	NA	NA
OR9I1	NA	NA	NA
OR9Q2	NA	NA	NA
OR1S2	NA	NA	NA
OR1S1	NA	NA	NA
OR10Q1	NA	NA	NA
OR10W1	NA	NA	NA
OR5B17	NA	NA	NA
OR5B3	NA	NA	NA
OR5B2	NA	NA	NA
OR5B12	NA	NA	NA
RP11-655C2.	NA	NA	NA

OR5B21	NA	NA	NA
LPXN	1.737011	2.69E-179	6.18E-179
ZFP91	1.84093474	0.0339541	0.03533299
ZFP91-CNTF	NA	NA	NA
CMB9-55A18	NA	NA	NA
CNTF	Inf	0	0
GLYAT	NA	NA	NA
GLYATL2	NA	0	0
GLYATL1	NA	NA	NA
RP11-780O24	NA	NA	NA
RP11-142C4.	NA	NA	NA
GLYATL1P3	NA	NA	NA
FAM111B	NA	NA	NA
AP001258.4	2.05867971	0.02700706	0.02814903
FAM111A	1.73972933	5.58E-66	8.43E-66
DTX4	2.31601467	0.50887841	0.51483784
MPEG1	NA	NA	NA
RP11-1036E2	NA	NA	NA
OR5AN1	NA	NA	NA
OR5A2	NA	NA	NA
OR5A1	NA	NA	NA
OR4D6	NA	NA	NA
OR4D10	NA	NA	NA
OR4D11	NA	NA	NA
OR4D9	NA	NA	NA
AP000442.4	NA	2.06E-133	4.00E-133
OSBP	1.76113616	3.35E-201	8.36E-201
AP000442.1	Inf	6.04E-262	1.97E-261
PATL1	2.35461491	5.31E-157	1.12E-156
OR10V1	NA	NA	NA
STX3	1.20947433	0	0
AP000640.10	NA	NA	NA
MRPL16	1.27287041	0	0
GIF	NA	NA	NA
TCN1	0	3.58E-294	1.37E-293
RP11-736I10	NA	0	0
OOSP2	NA	1.04E-217	2.77E-217
MS4A3	0.16375861	0	0
MS4A2	NA	0.00043773	0.00046845
AP001257.1	NA	NA	NA
MS4A6A	0.04288916	0	0
MS4A4E	NA	NA	NA
MS4A4A	0.37269202	0	0
MS4A6E	NA	NA	NA
MS4A7	0.25733496	0	0

MS4A14	NA	0	0
MS4A5	NA	NA	NA
MS4A1	NA	NA	NA
MS4A12	NA	NA	NA
MS4A13	NA	NA	NA
LINC00301	NA	NA	NA
MS4A8	NA	NA	NA
MS4A18	NA	NA	NA
MS4A15	NA	NA	NA
MS4A10	NA	NA	NA
RP11-804A23	NA	NA	NA
RP11-804A23	NA	1.08E-180	2.49E-180
RP11-804A23	Inf	0	0
CCDC86	1.54188304	0	0
PTGDR2	NA	0.21412432	0.21893949
ZP1	NA	NA	NA
PRPF19	1.29466659	0	0
RP11-881M1	NA	NA	NA
RP11-881M1	NA	4.52E-06	4.94E-06
TMEM109	1.97290139	1.84E-43	2.52E-43
RP11-881M1	NA	2.13E-198	5.25E-198
TMEM132A	1.29192655	7.28E-91	1.21E-90
SLC15A3	1.28667482	2.38E-09	2.69E-09
CD6	NA	NA	NA
RP11-881M1	NA	NA	NA
CD5	Inf	0	0
VPS37C	1.46680929	2.71E-221	7.32E-221
PGA3	NA	NA	NA
PGA4	NA	NA	NA
PGA5	NA	NA	NA
VWCE	Inf	0	0
DDB1	2.05132728	3.38E-78	5.35E-78
TKFC	1.33965554	0	0
CYB561A3	2.1568384	2.60E-297	1.01E-296
TMEM138	1.75556881	4.26E-55	6.15E-55
TMEM216	1.76560378	1.78E-21	2.17E-21
CPSF7	1.63591512	5.55E-83	8.96E-83
RP11-286N2	7.7200489	0	0
SDHAF2	1.70075017	0	0
PPP1R32	0.77200489	6.28E-55	9.05E-55
RP11-286N2	NA	NA	NA
LRRC10B	NA	0	0
SYT7	1.15800733	1.08E-181	2.50E-181
RP11-794G2	2.31601467	5.77E-37	7.64E-37
RP11-855O1	NA	NA	NA

RP11-855O1(	NA	NA	NA
DAGLA	2.31601467	1.94E-201	4.86E-201
DKFZP434K0:	NA	NA	NA
MYRF	1.02933985	1.51E-44	2.08E-44
TMEM258	2.28558165	0	0
FEN1	1.18118347	0	0
FADS2	1.48649652	0	0
FADS1	1.13846959	0	0
FADS3	1.24192091	0	0
RAB3IL1	1.14371095	0	0
BEST1	3.23277048	0	0
FTH1	4.09118788	0	0
RP11-810P1:	NA	NA	NA
INCENP	1.09952212	4.51E-139	8.95E-139
SCGB1D1	NA	NA	NA
SCGB2A1	NA	NA	NA
RP11-703H8.	NA	NA	NA
SCGB1D2	NA	NA	NA
SCGB2A2	NA	NA	NA
SCGB1D4	NA	NA	NA
RP11-703H8.	NA	NA	NA
ASRGL1	NA	NA	NA
CTD-2531D1!	NA	NA	NA
SCGB1A1	NA	NA	NA
CTD-2531D1!	NA	NA	NA
AHNAK	1.65192444	3.85E-273	1.32E-272
RP11-864I4.3	NA	NA	NA
RP11-864I4.4	Inf	4.18E-278	1.47E-277
EEF1G	3.39682152	5.80E-256	1.84E-255
RP11-864I4.1	NA	4.03E-41	5.45E-41
TUT1	0.94356153	0	0
MTA2	1.70191987	0.09620554	0.09926411
EML3	1.3780648	4.81E-219	1.29E-218
RP11-831H9.	NA	NA	NA
ROM1	2.05867971	0.19649137	0.20127302
B3GAT3	2.40713656	7.19E-305	2.92E-304
GANAB	2.47094991	0	0
INTS5	1.47967604	8.66E-148	1.77E-147
RP11-831H9.	NA	NA	NA
C11orf98	0.77200489	7.50E-276	2.60E-275
LBHD1	Inf	9.19E-65	1.38E-64
METTL12	2.35354269	2.07E-183	4.83E-183
UQCC3	1.79295818	8.06E-08	8.97E-08
UBXN1	1.8407234	0.00687908	0.00723522
LRRN4CL	0	4.46E-08	4.98E-08

BSCL2	0.77200489	6.17E-220	1.66E-219
HNRNPUL2-E	0	0	0
GNG3	NA	NA	NA
HNRNPUL2	1.66380364	2.24E-127	4.26E-127
TTC9C	1.78154975	2.59E-165	5.67E-165
ZBTB3	1.93001222	0	0
POLR2G	1.59158431	0	0
RP11-727F15	1.85281174	1.02E-115	1.86E-115
TAF6L	1.1494295	0	0
TMEM223	1.77678349	7.98E-157	1.69E-156
RP11-727F15	2.31601467	9.71E-23	1.19E-22
TMEM179B	2.01305922	1.49E-05	1.62E-05
NXF1	1.4292523	5.97E-188	1.42E-187
STX5	1.27654352	0	0
RP11-727F15	0.51466993	0	0
WDR74	1.46693992	0	0
SLC3A2	1.38650304	0	0
CHRM1	NA	NA	NA
AP000438.2	NA	NA	NA
SLC22A6	NA	NA	NA
SLC22A8	NA	NA	NA
SLC22A24	NA	NA	NA
SLC22A10	NA	NA	NA
SLC22A25	NA	NA	NA
SLC22A9	NA	NA	NA
HRASLS5	NA	NA	NA
LGALS12	0.44114565	0	0
RARRES3	0.38600244	2.47E-130	4.75E-130
HRASLS2	NA	NA	NA
PLA2G16	0	1.28E-276	4.45E-276
RP11-697H9.	NA	NA	NA
ATL3	1.47382752	2.62E-103	4.57E-103
RP11-697H9.	NA	NA	NA
RTN3	1.19438917	0	0
C11orf95	1.9167018	0.23172159	0.23678569
C11orf84	1.99056163	5.00E-167	1.10E-166
MARK2	2.21245304	0.00082751	0.00088243
RCOR2	0.83138988	0	0
NAA40	2.41251528	1.37E-17	1.64E-17
COX8A	2.13099272	0	0
AP000721.4	NA	NA	NA
OTUB1	1.34776181	0	0
MACROD1	1.06212138	0	0
RP11-21A7A.	NA	NA	NA
RP11-21A7A.	NA	NA	NA

FLRT1	NA	2.75E-115	5.02E-115
RP11-21A7A.	1.28667482	1.51E-07	1.68E-07
STIP1	1.29512017	0	0
FERMT3	1.75219087	1.25E-17	1.49E-17
TRPT1	1.61472779	7.22E-27	9.09E-27
NUDT22	2.08915358	2.16E-82	3.48E-82
RP11-783K16	0.77200489	1.16E-33	1.51E-33
DNAJC4	1.73167948	0	0
VEGFB	2.35560466	3.46E-286	1.26E-285
FKBP2	1.95253551	0.00054134	0.00057866
PPP1R14B	2.44873172	0	0
RP11-783K16	1.1229162	2.30E-164	5.00E-164
RP11-783K16	Inf	1.24E-196	3.04E-196
PLCB3	1.74987775	3.04E-101	5.26E-101
BAD	2.30851948	1.82E-138	3.59E-138
GPR137	1.57295996	7.47E-16	8.83E-16
KCNK4	Inf	0	0
TEX40	0.80966367	0	0
ESRRA	2.13759576	9.79E-09	1.10E-08
TRMT112	1.94855926	0.08369626	0.08643087
PRDX5	1.66685813	0	0
AP003774.1	0.38600244	5.81E-23	7.15E-23
CCDC88B	1.91322951	1.65E-14	1.93E-14
RPS6KA4	1.51487752	0.13075943	0.13452634
AP003774.6	NA	NA	NA
AP003774.5	NA	NA	NA
AP003774.4	NA	5.80E-123	1.09E-122
AP005273.1	NA	NA	NA
SLC22A11	NA	NA	NA
SLC22A12	NA	NA	NA
NRXN2	1.68437431	1.51E-159	3.23E-159
AP001092.4	NA	NA	NA
RASGRP2	1.7280219	7.75E-293	2.94E-292
PYGM	0	0	0
SF1	2.1692329	9.01E-83	1.45E-82
AP001462.6	1.71556642	3.42E-29	4.35E-29
MAP4K2	1.62527345	0.0611781	0.06336815
MEN1	1.91831518	9.51E-45	1.31E-44
CDC42BPG	7.7200489	0	0
EHD1	1.55687653	1.24E-87	2.04E-87
MIR194-2HG	Inf	4.55E-194	1.11E-193
ATG2A	1.95413738	3.26E-12	3.76E-12
PPP2R5B	1.05273394	1.66E-74	2.59E-74
GPHA2	NA	NA	NA
C11orf85	NA	NA	NA

BATF2	NA	1.23E-216	3.26E-216
ARL2	2.29544616	9.63E-228	2.67E-227
ARL2-SNX15	NA	NA	NA
SNX15	Inf	0	0
SAC3D1	1.73531503	4.12E-132	7.98E-132
NAALADL1	NA	NA	NA
CDCA5	1.8853172	8.30E-19	9.99E-19
ZFPL1	1.68396388	1.13E-167	2.48E-167
TMEM262	0	0	0
VPS51	1.65162258	9.29E-277	3.24E-276
AP003068.9	NA	0	0
TM7SF2	1.36736459	0	0
ZNHIT2	1.62207769	3.55E-227	9.82E-227
AP003068.12	NA	NA	NA
FAU	1.64378256	1.56E-286	5.73E-286
SYVN1	1.89008094	1.63E-10	1.85E-10
MRPL49	1.94147764	6.56E-37	8.68E-37
SPDYC	NA	NA	NA
AP003068.23	1.44750917	6.37E-20	7.71E-20
CAPN1	1.58918028	2.08E-188	4.95E-188
POLA2	1.44750917	0	0
CDC42EP2	1.51827628	8.05E-59	1.18E-58
DPF2	1.98995049	0.02715296	0.02829947
TIGD3	0.88780562	8.48E-208	2.17E-207
RP11-867O8.	NA	NA	NA
SLC25A45	3.00224124	0.01288618	0.01349386
FRMD8	2.4156282	0	0
NEAT1	3.0322412	0	0
CMB9-22P13	2.55355464	7.32E-14	8.54E-14
CMB9-22P13	NA	NA	NA
MALAT1	3.01726215	1.45E-114	2.63E-114
AP000769.7	Inf	8.25E-189	1.96E-188
AP000769.1	4.63202934	1.00E-05	1.09E-05
SCYL1	1.737011	1.32E-241	3.91E-241
LTBP3	2.62481663	8.22E-112	1.48E-111
SSSCA1	2.26474116	1.34E-276	4.66E-276
FAM89B	1.922994	1.34E-20	1.63E-20
EHBP1L1	2.4156282	1.03E-173	2.31E-173
SIPA1	1.87759214	1.67E-21	2.04E-21
KCNK7	0	3.21E-157	6.81E-157
MAP3K11	2.1553436	0	0
PCNXL3	3.05445413	9.01E-253	2.81E-252
RELA	1.74323685	3.78E-73	5.87E-73
KAT5	1.02710216	0	0
RNASEH2C	2.1944298	0	0

RP11-770G2:	NA	NA	NA
AP5B1	1.93966229	1.05E-59	1.55E-59
OVOL1	NA	4.43E-14	5.18E-14
OVOL1-AS1	NA	0	0
RP11-770G2:	NA	NA	NA
CFL1	1.41359074	0	0
SNX32	NA	1.26E-40	1.69E-40
MUS81	1.62900114	0	0
EFEMP2	Inf	2.17E-176	4.92E-176
CTSW	2.05867971	8.58E-23	1.06E-22
FIBP	1.56161691	0	0
CCDC85B	1.98469321	1.28E-17	1.53E-17
FOSL1	1.73179475	2.43E-72	3.76E-72
C11orf68	2.10633433	0.65120245	0.65637618
DRAP1	1.74266672	0	0
TSGA10IP	2.31601467	0.00216712	0.00229743
SART1	1.3377893	0	0
EIF1AD	2.06439826	1.47E-30	1.88E-30
BANF1	1.81460385	4.53E-129	8.64E-129
CST6	NA	NA	NA
CATSPER1	0.77200489	1.54E-08	1.72E-08
GAL3ST3	NA	NA	NA
SF3B2	1.6204061	0	0
RP11-1167A:	NA	2.56E-17	3.06E-17
PACS1	2.31601467	9.19E-177	2.09E-176
RP11-755F1(	Inf	3.73E-240	1.10E-239
KLC2	1.88712306	1.32E-34	1.73E-34
RP11-755F1(	NA	NA	NA
RP11-867G2:	NA	NA	NA
RP11-867G2:	NA	2.59E-47	3.61E-47
RAB1B	2.15058505	8.11E-184	1.90E-183
RP11-867G2:	NA	0	0
RP11-867G2:	1.15800733	1.39E-249	4.28E-249
CNIH2	1.43372337	1.30E-39	1.75E-39
YIF1A	1.62874202	3.51E-160	7.52E-160
TMEM151A	NA	2.50E-300	9.90E-300
RP11-867G2:	NA	NA	NA
CD248	NA	1.13E-62	1.68E-62
RIN1	0.77200489	6.70E-272	2.29E-271
RP11-867G2:	NA	NA	NA
BRMS1	1.58578494	1.98E-169	4.40E-169
B4GAT1	1.60049794	7.78E-73	1.21E-72
RP11-867G2:	1.00360636	1.85E-150	3.84E-150
SLC29A2	1.9997717	0.16055114	0.16479314
NPAS4	NA	NA	NA

MRPL11	2.12332625	1.32E-243	3.93E-243
PELI3	1.42963869	0.34650259	0.35258567
CTD-3074O7.	1.44750917	5.49E-23	6.76E-23
DPP3	1.46345275	0	0
CTD-3074O7.	NA	NA	NA
BBS1	Inf	0	0
ZDHHC24	1.83425968	1.36E-81	2.18E-81
ACTN3	NA	NA	NA
CTD-3074O7.	NA	NA	NA
CTSF	1.21895509	0	0
CCDC87	Inf	0	0
CCS	1.87239992	3.47E-145	7.04E-145
RBM14	1.47876993	0	0
RBM14-RBM	3.08801956	1.35E-196	3.32E-196
RBM4	1.87578048	0.00023891	0.00025656
RBM4B	1.75961475	4.06E-261	1.32E-260
RP11-658F2.	2.50901589	1.08E-22	1.33E-22
SPTBN2	1.38428463	2.57E-30	3.29E-30
C11orf80	1.05793263	0	0
RCE1	1.23520782	0	0
PC	1.15800733	4.78E-41	6.46E-41
LRFN4	2.86744673	0	0
C11orf86	NA	NA	NA
SYT12	NA	9.83E-117	1.80E-116
RHOD	NA	NA	NA
KDM2A	2.65569682	0	0
ADRBK1	2.12736686	3.09E-41	4.18E-41
ANKRD13D	2.07659543	4.67E-08	5.21E-08
SSH3	1.67267726	0.83321996	0.83653992
RAD9A	1.95332781	3.53E-42	4.80E-42
POLD4	1.97826253	1.12E-300	4.46E-300
AP003419.11	NA	NA	NA
CTC-1337H24	Inf	0	0
CLCF1	0	0	0
CTC-1337H24	1.15800733	1.29E-112	2.34E-112
PPP1CA	1.2953465	0	0
TBC1D10C	1.79491137	1.32E-50	1.87E-50
CARNS1	0	3.09E-117	5.69E-117
RPS6KB2	1.68971211	9.95E-115	1.81E-114
AP003419.16	0.77200489	2.27E-173	5.10E-173
CORO1B	0.78119542	0	0
PTPRCAP	Inf	5.03E-128	9.58E-128
GPR152	NA	NA	NA
CABP4	NA	NA	NA
TMEM134	2.15628952	0.65417102	0.65925845

AIP	0.99700097	0	0
PITPNM1	1.68437431	2.37E-67	3.60E-67
CDK2AP2	1.58874516	0	0
CABP2	NA	NA	NA
GSTP1	1.56570248	3.96E-267	1.32E-266
C11orf72	NA	3.64E-92	6.09E-92
RP11-655M1	NA	NA	NA
NDUFV1	1.16310461	0	0
RP11-655M1	Inf	5.02E-145	1.02E-144
NUDT8	1.55904884	1.36E-195	3.32E-195
TBX10	NA	NA	NA
ACY3	NA	3.20E-124	6.01E-124
ALDH3B2	NA	NA	NA
RP11-119D9.	NA	NA	NA
RP5-903G2.2	1.3896088	0.83388355	0.83715983
UNC93B1	1.41970391	9.64E-282	3.44E-281
ALDH3B1	0.28950183	0	0
RP5-901A4.1	NA	2.89E-192	6.97E-192
NDUFS8	1.93905249	0.07905211	0.08168147
TCIRG1	1.42392013	0	0
RP11-802E16	2.31601467	0	0
CHKA	1.42963869	1.54E-288	5.70E-288
CTD-2655K5.	NA	2.00E-144	4.04E-144
KMT5B	1.95881838	4.02E-51	5.70E-51
C11orf24	2.26776436	1.75E-31	2.25E-31
LRP5	3.08801956	4.65E-53	6.65E-53
PPP6R3	2.28052019	1.68E-119	3.11E-119
CTD-2007L18	NA	NA	NA
GAL	NA	NA	NA
MTL5	1.21064403	3.42E-180	7.87E-180
CPT1A	2.47759709	5.94E-188	1.41E-187
RP11-757G1.	0.38600244	1.23E-288	4.57E-288
MRPL21	1.84171101	4.49E-55	6.49E-55
IGHMBP2	2.27060262	3.55E-17	4.23E-17
RP11-757G1.	NA	NA	NA
MRGPRD	NA	NA	NA
RP11-554A1:	NA	NA	NA
MRGPRF	NA	NA	NA
MRGPRF-AS	NA	NA	NA
TPCN2	1.24192091	1.64E-47	2.28E-47
RP11-554A1:	Inf	3.41E-59	5.00E-59
RP11-554A1:	NA	NA	NA
RP11-554A1:	NA	6.31E-291	2.37E-290
MYEOV	NA	1.28E-74	2.00E-74
RP11-211G2:	NA	NA	NA

RP11-211G2:	NA	NA	NA
AP000439.1	NA	NA	NA
AP000439.2	NA	NA	NA
AP000439.5	NA	NA	NA
AP000439.3	NA	NA	NA
CCND1	0.38600244	1.11E-234	3.18E-234
ORAOV1	1.75779575	8.41E-11	9.61E-11
AP001888.1	NA	NA	NA
FGF19	NA	NA	NA
FGF4	NA	NA	NA
FGF3	NA	NA	NA
RP11-643C9.	NA	NA	NA
RP11-643C9.	NA	NA	NA
RP11-626H1:	NA	NA	NA
RP11-626H1:	NA	NA	NA
RP11-626H1:	NA	NA	NA
ANO1-AS2	NA	NA	NA
ANO1	NA	NA	NA
ANO1-AS1	NA	NA	NA
FADD	1.4876274	2.90E-260	9.40E-260
RP11-805J14	0.77200489	0	0
PPFIA1	2.16959995	1.46E-13	1.70E-13
AP000487.6	NA	NA	NA
CTA-797E19.	NA	NA	NA
AP000487.4	NA	NA	NA
AP000487.5	2.31601467	0.10817856	0.11146563
CTTN	2.31601467	1.34E-225	3.68E-225
SHANK2	NA	NA	NA
SHANK2-AS1	NA	NA	NA
SHANK2-AS2	NA	NA	NA
SHANK2-AS3	NA	NA	NA
DHCR7	1.40411628	0	0
RP11-660L16	1.73399536	0.14850329	0.15253053
NADSYN1	2.30983863	2.36E-101	4.10E-101
KRTAP5-7	NA	1.53E-28	1.94E-28
KRTAP5-8	NA	NA	NA
KRTAP5-9	Inf	4.44E-195	1.08E-194
KRTAP5-10	0.77200489	5.90E-86	9.62E-86
KRTAP5-11	NA	NA	NA
RP11-167J8.:	NA	NA	NA
FAM86C1	1.7094394	8.00E-09	8.97E-09
DEFB108B	NA	NA	NA
RP11-849H4.	2.75716032	1.22E-255	3.86E-255
CTD-2313N1:	NA	NA	NA
RNF121	1.06150672	0	0

IL18BP	1.93001222	1.90E-179	4.37E-179
NUMA1	2.32488829	9.00E-49	1.26E-48
RP11-849H4.	Inf	2.16E-16	2.57E-16
LRTOMT	3.24242054	1.69E-88	2.79E-88
LAMTOR1	1.84289441	7.41E-155	1.56E-154
ANAPC15	2.069402	0	0
FOLR3	NA	NA	NA
RP11-807H2.	NA	NA	NA
FOLR1	NA	NA	NA
FOLR2	0.77200489	0	0
INPPL1	2.03528562	1.14E-27	1.44E-27
PHOX2A	NA	NA	NA
CLPB	1.55416774	0	0
AP000593.7	NA	NA	NA
RP11-45F15.	NA	NA	NA
RP11-45F15.	NA	NA	NA
RP11-7N14.1	NA	NA	NA
LINC01537	NA	NA	NA
PDE2A	NA	7.08E-192	1.71E-191
RP11-169D4.	NA	3.14E-07	3.48E-07
RP11-31L22.	NA	NA	NA
ARAP1	3.17884366	0	0
ARAP1-AS1	NA	9.90E-76	1.55E-75
ARAP1-AS2	NA	NA	NA
STARD10	1.55096478	7.64E-86	1.25E-85
ATG16L2	2.36142672	3.34E-66	5.05E-66
FCHSD2	1.77561125	2.82E-45	3.89E-45
RP11-800A3.	NA	NA	NA
RP11-800A3.	Inf	0	0
P2RY2	1.27059138	9.91E-230	2.78E-229
P2RY6	2.16995969	1.66E-13	1.93E-13
RP11-800A3.	NA	2.71E-46	3.76E-46
ARHGEF17	1.25450795	1.58E-63	2.36E-63
RELT	2.05320449	8.35E-26	1.04E-25
RP11-809N8.	NA	NA	NA
FAM168A	2.47041565	2.63E-116	4.82E-116
RP11-809N8.	1.93001222	1.93E-157	4.10E-157
PLEKHB1	Inf	0	0
RAB6A	2.03955346	6.29E-06	6.87E-06
RP11-456I15	NA	NA	NA
RP11-707G1	0	2.55E-91	4.26E-91
MRPL48	1.45744088	0	0
COA4	1.98471311	5.46E-16	6.46E-16
PAAF1	1.40949378	0	0
DNAJB13	Inf	0	0

RP11-167N4.	NA	NA	NA
UCP2	1.75238278	1.47E-193	3.58E-193
UCP3	3.86002445	1.57E-141	3.14E-141
C2CD3	5.24963325	6.56E-49	9.21E-49
PPME1	2.34091805	2.23E-31	2.87E-31
P4HA3	NA	NA	NA
RP11-632K5.	NA	3.19E-18	3.82E-18
PGM2L1	1.41833457	2.58E-05	2.80E-05
RP11-702H2:	NA	NA	NA
KCNE3	2.34460744	1.79E-87	2.93E-87
RP11-702H2:	NA	0	0
LIPT2	2.36142672	0.69711501	0.70183466
AP001372.2	1.69841076	5.82E-257	1.85E-256
POLD3	1.79921801	0.00709697	0.00746352
CHRDL2	NA	NA	NA
RNF169	2.64221392	0	0
XRRA1	1.80811672	7.78E-43	1.06E-42
SPCS2	1.31425351	0	0
NEU3	2.58689358	0	0
OR2AT4	NA	NA	NA
CTD-2562J17	NA	NA	NA
SLCO2B1	NA	NA	NA
CTD-2562J17	NA	NA	NA
TPBGL	NA	1.65E-183	3.86E-183
CTD-2562J17	0	3.50E-247	1.07E-246
ARRB1	1.87084397	3.02E-147	6.17E-147
CTD-2562J17	NA	NA	NA
RPS3	1.96370621	2.81E-13	3.27E-13
KLHL35	1.93001222	0.00358008	0.00378099
GDPD5	1.08497985	1.24E-286	4.56E-286
SERPINH1	1.03963325	0	0
CTD-2530H1:	NA	NA	NA
MAP6	NA	NA	NA
MOGAT2	NA	NA	NA
CTD-2530H1:	NA	NA	NA
DGAT2	0.6389006	0	0
RP11-535A1!	NA	NA	NA
RP11-535A1!	NA	NA	NA
UVRAG	2.73170961	0.00539234	0.00568198
CTD-2011F1	NA	NA	NA
WNT11	NA	3.05E-15	3.59E-15
RP11-619A1	NA	NA	NA
RP11-619A1	0.77200489	6.65E-23	8.19E-23
PRKRIR	2.0844132	6.33E-92	1.06E-91
RP11-111M2	3.34535452	1.66E-230	4.67E-230

RP11-111M2	4.63202934	6.63E-05	7.16E-05
RP11-111M2	Inf	0	0
RP11-111M2	2.18734719	3.16E-37	4.19E-37
EMSY	1.32730665	5.08E-35	6.67E-35
RP11-672A2.	NA	NA	NA
RP11-672A2.	NA	NA	NA
RP11-672A2.	NA	NA	NA
AP001189.4	NA	NA	NA
LRRC32	Inf	3.32E-129	6.34E-129
RP11-672A2.	NA	NA	NA
RP11-672A2.	NA	NA	NA
RP11-21L23.4	NA	NA	NA
TSKU	1.99227068	1.00E-51	1.43E-51
RP11-21L23.3	Inf	4.03E-108	7.17E-108
ACER3	1.97290139	1.12E-40	1.51E-40
CTD-2547H18	NA	NA	NA
B3GNT6	Inf	1.40E-261	4.56E-261
CAPN5	2.57334963	8.88E-14	1.04E-13
OMP	NA	NA	NA
MYO7A	NA	1.43E-209	3.69E-209
GDPD4	NA	NA	NA
PAK1	2.5109654	7.51E-218	2.00E-217
RP11-739B23	NA	NA	NA
CLNS1A	1.85636702	2.36E-118	4.36E-118
AQP11	1.54400978	2.70E-38	3.59E-38
RSF1	1.64226495	0	0
AAMDC	1.62323701	4.90E-265	1.63E-264
RP11-91P24.	Inf	0	0
RP11-91P24.	NA	NA	NA
RP11-91P24.	NA	NA	NA
INTS4	2.04980609	9.38E-09	1.05E-08
KCTD14	0.77200489	0.01414341	0.01480268
RP11-7I15.3	NA	NA	NA
NDUFC2-KCT	NA	NA	NA
RP11-7I15.4	NA	NA	NA
THRSP	NA	3.71E-10	4.22E-10
NDUFC2	2.27593365	2.89E-283	1.04E-282
ALG8	1.5225652	0	0
KCTD21-AS1	5.09523227	3.66E-142	7.34E-142
KCTD21	1.54400978	1.18E-58	1.72E-58
USP35	0.66171848	1.19E-09	1.34E-09
GAB2	2.53314105	2.79E-257	8.91E-257
RP11-452H21	NA	NA	NA
RP11-452H21	NA	NA	NA
RP11-452H21	0	1.04E-89	1.72E-89

NARS2	1.90031973	3.28E-07	3.63E-07
RP11-843A2:	NA	NA	NA
TENM4	NA	NA	NA
RP11-673F1٤	NA	NA	NA
CTD-2337I7.1	NA	NA	NA
RP11-802F5.	NA	NA	NA
RP11-258O1:	NA	NA	NA
CTD-2555I5.1	NA	NA	NA
RP11-686G2:	NA	NA	NA
MIR4300HG	NA	0.07877509	0.08139988
RP11-876F1⁄	NA	NA	NA
RP11-179A1(	NA	NA	NA
FAM181B	1.15800733	2.73E-218	7.30E-218
RP11-718B1:	NA	NA	NA
PRCP	1.49805711	0	0
DDIAS	1.34605981	4.36E-300	1.72E-299
RAB30	0.5614581	1.39E-225	3.82E-225
RAB30-AS1	1.77107004	2.38E-30	3.05E-30
RP11-113K21	Inf	5.01E-290	1.87E-289
PCF11	1.67774291	9.19E-228	2.55E-227
PCF11-AS1	1.54400978	9.31E-07	1.03E-06
ANKRD42	1.30275825	0.00312498	0.00330458
RP11-727A2:	NA	NA	NA
CCDC90B	1.85213454	3.05E-158	6.50E-158
RP11-727A2:	NA	7.69E-227	2.13E-226
DLG2	Inf	2.43E-92	4.07E-92
RP11-160H1:	NA	NA	NA
CTD-2537O9.	NA	NA	NA
AP000857.3	NA	NA	NA
TMEM126B	1.94627637	0.56988559	0.5755965
TMEM126A	2.13195045	1.58E-204	3.99E-204
CREBZF	1.94609566	1.09E-34	1.42E-34
CCDC89	NA	NA	NA
SYTL2	0	2.82E-42	3.83E-42
CCDC83	NA	NA	NA
RP11-90K17.	NA	NA	NA
PICALM	2.00948332	8.95E-40	1.20E-39
RP11-12D16.	NA	0.01997577	0.02086599
EED	1.28667482	0	0
C11orf73	2.29390686	0	0
CCDC81	NA	1.10E-169	2.45E-169
RP11-317J19	NA	NA	NA
ME3	NA	NA	NA
CTD-2005H7.	NA	NA	NA
CTD-2005H7.	NA	NA	NA

PRSS23	NA	5.44E-126	1.03E-125
AP000654.4	NA	NA	NA
RP11-736K2(	1.15800733	2.02E-57	2.94E-57
FZD4	1.08080685	1.90E-222	5.15E-222
RP11-736K2(	NA	NA	NA
TMEM135	1.70653713	1.92E-149	3.96E-149
RP11-720D4.	NA	NA	NA
RP11-164N3.	NA	NA	NA
RP11-164N3.	NA	NA	NA
RP11-164N3.	NA	NA	NA
RAB38	1.87062723	0.95041879	0.95125773
CTSC	2.51880998	0	0
GRM5-AS1	NA	NA	NA
GRM5	NA	NA	NA
TYR	NA	NA	NA
NOX4	NA	NA	NA
RP11-643G5.	NA	NA	NA
TRIM77	NA	NA	NA
RP11-313I2.1	NA	NA	NA
TRIM49	NA	NA	NA
TRIM64B	NA	NA	NA
TRIM49D1	NA	NA	NA
TRIM49D2	NA	NA	NA
TRIM64	NA	NA	NA
TRIM49C	NA	NA	NA
UBTFL1	NA	NA	NA
NAALAD2	NA	NA	NA
CHORDC1	1.78668642	6.32E-43	8.63E-43
RP11-699F1(	NA	NA	NA
RP11-201M2	NA	NA	NA
FAT3	NA	NA	NA
RP11-675M1	NA	NA	NA
RP11-203F8.	NA	NA	NA
RP11-676F2(	NA	NA	NA
RP11-676F2(	NA	NA	NA
RP11-676F2(	NA	NA	NA
MTNR1B	NA	NA	NA
SLC36A4	1.58942183	4.47E-09	5.03E-09
RP11-755E2:	NA	NA	NA
CCDC67	NA	NA	NA
SMCO4	1.99366156	1.18E-82	1.90E-82
CEP295	1.55830617	1.73E-157	3.66E-157
SCARNA9	NA	NA	NA
TAF1D	2.15946497	3.53E-22	4.33E-22
C11orf54	1.47382752	0	0

MED17	2.02007946	7.86E-121	1.46E-120
VSTM5	NA	NA	NA
HEPHL1	NA	NA	NA
PANX1	1.40364525	3.66E-33	4.76E-33
RP11-680H2(	NA	NA	NA
IZUMO1R	NA	NA	NA
GPR83	0	0	0
MRE11A	1.58812435	0	0
RP11-685N1(	Inf	1.05E-87	1.72E-87
ANKRD49	1.55172983	7.36E-265	2.44E-264
C11orf97	NA	NA	NA
PIWIL4	0.38600244	1.58E-240	4.67E-240
FUT4	1.93001222	0.00073871	0.00078838
RP11-867G2.	NA	1.97E-229	5.53E-229
RP11-867G2.	NA	NA	NA
RP11-867G2.	NA	NA	NA
AMOTL1	2.36426498	3.96E-51	5.62E-51
RP11-856F16	NA	NA	NA
CWC15	1.93455343	5.11E-59	7.50E-59
KDM4D	Inf	0	0
KDM4E	NA	NA	NA
SRSF8	1.6764616	6.75E-07	7.45E-07
ENDOD1	2.85306155	0	0
RP11-712B9.	NA	NA	NA
SESN3	0	0	0
RP11-338H14	NA	NA	NA
RP11-644L4.	NA	NA	NA
FAM76B	1.68024594	1.32E-40	1.77E-40
CEP57	1.54400978	0	0
MTMR2	1.6936842	1.04E-42	1.42E-42
MAML2	0.38600244	2.33E-146	4.74E-146
CCDC82	1.54400978	0	0
JRKL	2.93361858	3.56E-296	1.38E-295
JRKL-AS1	NA	NA	NA
RP11-49K4.2	NA	NA	NA
RP11-360K13	NA	NA	NA
RP11-882G5.	NA	NA	NA
RP11-379J13	NA	NA	NA
RP11-115E19	NA	NA	NA
RP11-99C10.	NA	NA	NA
CNTN5	NA	NA	NA
CTD-2383M3	NA	1.02E-45	1.41E-45
ARHGAP42	0.38600244	1.87E-267	6.27E-267
TMEM133	Inf	7.57E-140	1.50E-139
PGR	NA	NA	NA

TRPC6	NA	NA	NA
ANGPTL5	NA	NA	NA
CEP126	Inf	0	0
C11orf70	NA	8.34E-74	1.30E-73
RP11-732A2:	NA	NA	NA
RP11-732A2:	NA	6.26E-212	1.63E-211
YAP1	NA	NA	NA
RP11-864G5.	NA	NA	NA
BIRC3	6.94804401	0	0
BIRC2	2.51524174	1.24E-106	2.20E-106
TMEM123	2.30954897	0	0
RP11-315O6.	2.31601467	6.76E-46	9.36E-46
RP11-315O6.	NA	NA	NA
MMP7	NA	NA	NA
MMP20	NA	NA	NA
RP11-817J15	NA	NA	NA
RP11-817J15	NA	NA	NA
MMP27	NA	NA	NA
MMP8	NA	NA	NA
MMP10	NA	NA	NA
MMP1	NA	NA	NA
MMP3	NA	NA	NA
MMP12	NA	NA	NA
MMP13	NA	NA	NA
DCUN1D5	1.51876349	9.90E-234	2.83E-233
DYNC2H1	2.18734719	2.09E-20	2.53E-20
RP11-563P16	NA	NA	NA
PDGFD	9.26405868	5.77E-279	2.03E-278
RP11-617B3.	NA	NA	NA
DDI1	NA	NA	NA
RP11-886D1!	NA	NA	NA
CASP4	1.67530313	0	0
CASP5	NA	NA	NA
CASP1	Inf	4.66E-222	1.26E-221
CARD16	4.63202934	3.65E-12	4.21E-12
CARD17	NA	NA	NA
CARD18	NA	NA	NA
RP11-94P11.	NA	NA	NA
GRIA4	NA	NA	NA
MSANTD4	1.68257476	7.70E-80	1.23E-79
KBTBD3	1.737011	1.20E-89	2.00E-89
AASDHPPT	1.47083396	4.33E-305	1.76E-304
RP11-677I18	NA	1.42E-127	2.71E-127
RP11-677I18	NA	NA	NA
GUCY1A2	Inf	7.49E-89	1.24E-88

RP11-819C21	3.73135697	0	0
CWF19L2	1.23812105	1.21E-179	2.78E-179
ALKBH8	3.16522005	1.08E-48	1.52E-48
ELMOD1	1.15800733	1.33E-86	2.17E-86
SLN	0.77200489	1.64E-196	4.02E-196
SLC35F2	1.7441592	8.49E-86	1.38E-85
RAB39A	1.02933985	8.20E-214	2.15E-213
CUL5	2.16497023	9.34E-18	1.12E-17
RP11-144G7.	NA	NA	NA
ACAT1	0.98080738	0	0
NPAT	1.59127539	5.21E-153	1.09E-152
ATM	1.55390728	4.29E-88	7.07E-88
C11orf65	Inf	3.04E-10	3.45E-10
KDELC2	2.1030478	0.00017837	0.00019182
EXPH5	0.54040342	4.43E-157	9.39E-157
DDX10	1.63260051	0	0
RP11-25I9.3	NA	NA	NA
RP11-25I9.2	NA	NA	NA
RP11-708B6.	NA	NA	NA
C11orf87	NA	NA	NA
RP11-262A1.	NA	NA	NA
ZC3H12C	2.09544184	5.16E-07	5.70E-07
RDX	1.38685164	0	0
RP11-347E1(	NA	NA	NA
FDX1	2.32264701	0	0
ARHGAP20	NA	NA	NA
RP11-89C3.3	NA	NA	NA
RP11-89C3.4	NA	NA	NA
C11orf53	NA	NA	NA
COLCA1	Inf	0	0
COLCA2	NA	NA	NA
POU2AF1	NA	NA	NA
RP11-794P6.	NA	NA	NA
RP11-794P6.	NA	NA	NA
BTG4	NA	NA	NA
RP11-794P6.	NA	NA	NA
C11orf88	NA	NA	NA
LAYN	0.77200489	3.04E-46	4.22E-46
SIK2	3.44432951	0	0
PPP2R1B	1.61753406	9.28E-11	1.06E-10
RP11-108O1(	NA	NA	NA
ALG9	2.00721271	3.90E-32	5.04E-32
RP11-108O1(	NA	NA	NA
FDXACB1	1.32784841	1.20E-09	1.36E-09
C11orf1	1.92011473	4.51E-174	1.01E-173

CRYAB	Inf	4.11E-287	1.51E-286
HSPB2	NA	NA	NA
HSPB2-C11or	NA	NA	NA
C11orf52	NA	NA	NA
DIXDC1	1.80549531	5.81E-50	8.20E-50
DLAT	1.49463737	0	0
PIH1D2	2.05867971	3.35E-100	5.79E-100
C11orf57	2.13817061	4.71E-236	1.36E-235
TIMM8B	2.0501948	9.37E-128	1.78E-127
SDHD	1.87003013	7.47E-30	9.55E-30
AP002884.2	NA	NA	NA
IL18	4.72852995	0	0
RP11-356J5.4	NA	NA	NA
TEX12	Inf	0	0
BCO2	1.80134474	0.00448763	0.00473505
PTS	2.1030478	1.58E-44	2.17E-44
PLET1	NA	NA	NA
RP11-356J5.1	1.54400978	0	0
RP11-356J5.1	0.77200489	2.40E-90	3.99E-90
RP11-65M17	NA	4.06E-169	8.99E-169
RP11-65M17	NA	NA	NA
RP11-27G22.	NA	NA	NA
RP11-629G1:	NA	NA	NA
NCAM1	NA	NA	NA
NCAM1-AS1	NA	NA	NA
RP11-839D17	NA	NA	NA
TTC12	NA	NA	NA
ANKK1	NA	NA	NA
RP11-159N11	NA	NA	NA
DRD2	NA	NA	NA
TMPRSS5	NA	3.73E-215	9.84E-215
ZW10	1.56239085	9.16E-165	2.00E-164
RP11-667M1	Inf	0	0
CLDN25	NA	NA	NA
USP28	2.42323757	5.16E-155	1.09E-154
RP11-667M1	NA	NA	NA
HTR3B	NA	NA	NA
HTR3A	NA	NA	NA
ZBTB16	0.87727828	0	0
RP11-64D24.	NA	NA	NA
NNMT	NA	2.05E-89	3.40E-89
RP11-64D24.	NA	NA	NA
C11orf71	2.61623879	6.80E-52	9.67E-52
C11orf71	NA	NA	NA
RBM7	1.53465214	4.66E-265	1.54E-264

RP11-212D19	NA	NA	NA
REXO2	1.83883371	9.70E-16	1.15E-15
NXPE1	NA	NA	NA
NXPE4	NA	NA	NA
NXPE2	NA	NA	NA
CADM1	NA	NA	NA
AP000462.3	NA	NA	NA
AP000462.2	NA	NA	NA
RP11-136I14	NA	NA	NA
RP11-136I14	NA	NA	NA
AP000997.1	NA	NA	NA
AP000997.2	NA	NA	NA
AP000797.3	NA	NA	NA
LINC00900	NA	NA	NA
AP000797.4	NA	NA	NA
AP001891.1	NA	NA	NA
AP000770.1	NA	NA	NA
BUD13	1.56283917	6.68E-137	1.31E-136
AP006216.10	Inf	2.60E-128	4.94E-128
ZPR1	1.57208269	4.70E-236	1.36E-235
APOA5	NA	NA	NA
AP006216.5	NA	NA	NA
APOA4	NA	NA	NA
APOC3	NA	NA	NA
APOA1	1.15800733	1.48E-223	4.05E-223
APOA1-AS	NA	NA	NA
SIK3	2.37539966	3.96E-41	5.35E-41
AP000936.4	NA	NA	NA
PAFAH1B2	2.1057812	5.18E-213	1.36E-212
SIDT2	2.07476314	2.72E-16	3.22E-16
TAGLN	3.474022	2.39E-19	2.89E-19
PCSK7	1.82026643	2.75E-202	6.89E-202
RNF214	1.92179941	1.16E-139	2.31E-139
BACE1	2.48547916	8.63E-07	9.51E-07
BACE1-AS	1.11511817	1.51E-270	5.13E-270
CEP164	1.7509966	1.75E-08	1.96E-08
AP000892.4	NA	NA	NA
DSCAML1	NA	NA	NA
RP11-35P15.	NA	NA	NA
FXYD2	NA	NA	NA
RP11-728F11	NA	NA	NA
FXYD6-FXYD2	NA	NA	NA
RP11-728F11	NA	NA	NA
FXYD6	NA	NA	NA
TMPRSS13	Inf	0	0

IL10RA	Inf	0.59476866	0.60039467
TMPRSS4-AS	NA	NA	NA
AP002962.1	NA	NA	NA
TMPRSS4	NA	1.43E-287	5.26E-287
SCN4B	NA	1.35E-173	3.03E-173
SCN2B	NA	NA	NA
JAML	0.92640587	1.37E-59	2.02E-59
MPZL3	0.8822913	8.73E-16	1.03E-15
MPZL2	NA	NA	NA
CD3E	2.31601467	0.00055986	0.00059835
CD3D	0.35983279	0	0
CD3G	NA	NA	NA
UBE4A	2.3783989	1.63E-40	2.19E-40
RP11-770J1.5	1.02933985	1.31E-193	3.18E-193
ATP5L	2.10523313	1.32E-265	4.39E-265
RP11-770J1.4	1.54400978	7.27E-10	8.24E-10
KMT2A	2.00068873	4.56E-144	9.21E-144
RP11-770J1.3	NA	NA	NA
TTC36	NA	4.32E-09	4.86E-09
TMEM25	NA	2.36E-15	2.78E-15
IFT46	1.48073069	4.29E-258	1.37E-257
ARCN1	2.11056176	0.00021008	0.00022581
PHLDB1	2.08895441	6.00E-19	7.23E-19
AP002954.3	NA	2.56E-21	3.12E-21
TREH	NA	9.94E-206	2.52E-205
AP002954.4	NA	NA	NA
DDX6	2.09299104	2.11E-82	3.39E-82
RP11-158I9.8	1.37245314	2.20E-58	3.22E-58
CXCR5	NA	NA	NA
RP11-158I9.5	NA	NA	NA
BCL9L	4.05302567	1.38E-16	1.64E-16
UPK2	0	5.30E-295	2.04E-294
FOXR1	1.54400978	2.59E-146	5.27E-146
RP11-110I1.1	2.44468215	3.38E-42	4.59E-42
CCDC84	2.57334963	3.93E-33	5.10E-33
RP11-110I1.1	NA	4.84E-142	9.70E-142
RPS25	2.08354466	0	0
TRAPPC4	1.73544697	2.77E-89	4.59E-89
SLC37A4	1.64654168	1.03E-247	3.14E-247
RP11-110I1.6	NA	0	0
HYOU1	1.65429619	0.94340035	0.94459789
RP11-110I1.1	3.474022	9.76E-60	1.44E-59
RP11-110I1.1	2.47041565	4.89E-80	7.80E-80
VPS11	1.56740387	0.08918032	0.09205743
HMBS	1.63628128	1.57E-235	4.51E-235

H2AFX	1.96028693	6.80E-20	8.23E-20
DPAGT1	1.69159895	1.49E-103	2.60E-103
C2CD2L	1.88369193	5.92E-253	1.85E-252
HINFP	1.89325009	1.18E-227	3.28E-227
ABCG4	0	0	0
NLRX1	1.29638557	1.59E-205	4.04E-205
PDZD3	NA	1.68E-101	2.91E-101
CCDC153	1.93001222	0.25335609	0.25865989
CBL	1.58942183	1.40E-220	3.76E-220
MCAM	1.84093474	1.15E-08	1.29E-08
RNF26	1.87047951	4.75E-289	1.77E-288
RP11-334E6.	NA	7.44E-276	2.58E-275
C1QTNF5	NA	NA	NA
MFRP	NA	NA	NA
USP2	2.24583241	2.61E-94	4.40E-94
RP11-334E6.	NA	2.45E-140	4.87E-140
USP2-AS1	2.4563792	2.73E-06	2.99E-06
THY1	NA	NA	NA
RP11-196E1.	Inf	0	0
PVRL1	1.51784012	4.88E-92	8.16E-92
CTD-2523D1	NA	NA	NA
CTD-2523D1	NA	NA	NA
RP11-831A1(	NA	NA	NA
RP11-831A1(	NA	NA	NA
TRIM29	NA	NA	NA
AP000679.2	NA	NA	NA
OAF	1.3896088	0	0
POU2F3	NA	3.00E-06	3.29E-06
RP11-778O1	NA	NA	NA
TMEM136	NA	NA	NA
ARHGEF12	1.08080685	4.63E-34	6.05E-34
GRIK4	NA	NA	NA
RP11-640N1	2.31601467	2.94E-79	4.67E-79
TBCEL	1.46680929	1.00E-30	1.29E-30
TECTA	NA	1.40E-207	3.58E-207
SC5D	1.22540459	0	0
RP11-730K1	NA	NA	NA
SORL1	1.30442206	0	0
BLID	NA	NA	NA
MIR100HG	NA	NA	NA
RP11-716H6.	NA	NA	NA
RP11-716H6.	NA	NA	NA
UBASH3B	3.13343161	0	0
CRTAM	NA	NA	NA
C11orf63	2.70201711	2.37E-23	2.92E-23

BSX	NA	NA	NA
HSPA8	1.51626181	4.20E-231	1.19E-230
CLMP	NA	NA	NA
CTD-2216M2	NA	NA	NA
RP11-109E1(	NA	NA	NA
GRAMD1B	2.31601467	9.10E-46	1.26E-45
SCN3B	1.28667482	0.01007692	0.01057164
RP11-634B2.	NA	NA	NA
ZNF202	1.43047965	0.99958938	0.99958938
OR6X1	NA	NA	NA
OR6M1	NA	NA	NA
TMEM225	NA	NA	NA
OR8D4	NA	NA	NA
OR4D5	NA	NA	NA
OR6T1	NA	NA	NA
OR10S1	NA	NA	NA
OR10G6	NA	NA	NA
OR10G4	NA	NA	NA
OR10G9	NA	NA	NA
OR10G8	NA	NA	NA
OR10G7	NA	NA	NA
VWA5A	0.96500611	1.41E-163	3.07E-163
OR10D3	NA	NA	NA
OR8G1	NA	NA	NA
OR8G5	NA	NA	NA
OR8D1	NA	NA	NA
OR8D2	NA	NA	NA
OR8B2	NA	NA	NA
OR8B3	NA	NA	NA
OR8B4	NA	NA	NA
OR8B8	NA	NA	NA
OR8B12	NA	NA	NA
OR8A1	NA	NA	NA
PANX3	NA	NA	NA
TBRG1	1.95758383	5.85E-07	6.46E-07
SIAE	NA	2.61E-261	8.49E-261
SPA17	0.33085924	0	0
NRGN	1.68536279	7.79E-12	8.96E-12
RP11-677M1	NA	NA	NA
VSIG2	NA	NA	NA
ESAM	0.21054679	3.18E-290	1.19E-289
RP11-677M1	Inf	1.56E-70	2.40E-70
MSANTD2	2.20572826	7.08E-249	2.17E-248
AP000866.1	Inf	2.99E-100	5.17E-100
RP11-677M1	NA	8.13E-82	1.31E-81

ROBO3	0.96500611	3.12E-236	9.02E-236
RP11-664I21	NA	NA	NA
ROBO4	NA	NA	NA
RP11-664I21	NA	NA	NA
HEPACAM	NA	NA	NA
HEPN1	NA	NA	NA
CCDC15	1.48462479	3.47E-247	1.06E-246
SLC37A2	1.02933985	7.11E-176	1.61E-175
TMEM218	1.56380478	1.46E-249	4.49E-249
RP11-687M2	NA	NA	NA
PKNOX2-AS1	NA	NA	NA
PKNOX2	NA	NA	NA
RP11-687M2	NA	NA	NA
FEZ1	NA	NA	NA
AP000708.1	NA	NA	NA
EI24	1.90706802	3.94E-05	4.27E-05
STT3A-AS1	NA	9.94E-51	1.41E-50
STT3A	1.73462827	9.09E-264	2.99E-263
CHEK1	1.76137038	2.28E-82	3.66E-82
ACRV1	NA	NA	NA
PATE1	NA	NA	NA
PATE2	NA	1.63E-46	2.26E-46
PATE3	NA	NA	NA
PATE4	NA	NA	NA
RP11-680F2(	NA	NA	NA
HYLS1	1.30646981	1.62E-281	5.78E-281
PUS3	1.83509359	2.68E-296	1.04E-295
RP11-680F2(	NA	NA	NA
DDX25	NA	NA	NA
RP11-680F2(	NA	NA	NA
RP11-680F2(	NA	NA	NA
RP11-680F2(	NA	NA	NA
CDON	Inf	0.00623213	0.00655896
RP11-680F2(	NA	NA	NA
RP11-673E11	NA	NA	NA
RP11-50B3.2	NA	NA	NA
RPUSD4	1.76458261	3.97E-05	4.30E-05
RP11-50B3.4	0	0	0
FAM118B	1.17045903	0	0
SRPRA	1.92665568	0.00110132	0.00117213
FOXRED1	1.37398489	0	0
TIRAP	0.77200489	1.60E-131	3.08E-131
RP11-712L6.7	NA	NA	NA
RP11-712L6.5	5.59703545	1.10E-303	4.44E-303
DCPS	1.69786129	0	0

ST3GAL4-AS	1.94170927	1.20E-72	1.87E-72
ST3GAL4	2.59674372	0	0
KIRREL3	NA	4.81E-211	1.25E-210
KIRREL3-AS1	NA	NA	NA
RP11-115C1(	NA	NA	NA
RP11-688I9.4	NA	NA	NA
KIRREL3-AS2	NA	NA	NA
RP11-688I9.2	NA	NA	NA
KIRREL3-AS3	NA	NA	NA
RP11-168K9.	NA	NA	NA
RP11-168K9.	NA	NA	NA
CTD-2234N1	NA	NA	NA
CTD-2234N1	NA	NA	NA
RP11-480C22	NA	NA	NA
RP11-702B1(	NA	NA	NA
RP11-702B1(	NA	NA	NA
ETS1	NA	9.71E-82	1.56E-81
RP11-1007G!	NA	NA	NA
RP11-264E2(	NA	NA	NA
RP11-264E2(	NA	NA	NA
RP11-744N1:	0.77200489	5.50E-59	8.07E-59
FLI1	2.14671535	2.37E-11	2.72E-11
SENCR	1.60991264	0	0
KCNJ1	NA	NA	NA
KCNJ5	Inf	1.37E-78	2.17E-78
C11orf45	2.12301345	8.57E-12	9.86E-12
TP53AIP1	NA	NA	NA
ARHGAP32	1.50337794	1.13E-06	1.24E-06
BARX2	NA	0	0
RP11-237N1!	NA	NA	NA
LINC01395	NA	NA	NA
TMEM45B	Inf	5.75E-276	2.00E-275
NFRKB	1.60339477	0	0
PRDM10	0.95015986	1.23E-248	3.76E-248
LINC00167	Inf	0.00105363	0.00112164
APLP2	1.6419226	0	0
ST14	NA	0	0
ZBTB44	1.89595319	3.01E-08	3.36E-08
RP11-121M2	NA	5.05E-161	1.08E-160
ADAMTS8	0.51466993	5.78E-253	1.80E-252
ADAMTS15	Inf	4.94E-40	6.64E-40
C11orf44	NA	NA	NA
RP11-890B1!	Inf	0	0
RP11-890B1!	NA	NA	NA
SNX19	NA	NA	NA

AP002856.4	NA	NA	NA
AP002856.6	NA	NA	NA
AP002856.7	NA	NA	NA
AP002856.5	NA	NA	NA
NTM	NA	NA	NA
AP003025.2	NA	NA	NA
RP11-217L21	NA	NA	NA
NTM-AS1	NA	NA	NA
AP004372.1	NA	NA	NA
RP11-697E14	NA	NA	NA
OPCML	NA	NA	NA
RP11-448P19	NA	NA	NA
SPATA19	NA	NA	NA
IGSF9B	0	2.27E-83	3.67E-83
RP11-713P17	NA	NA	NA
RP11-713P17	3.86002445	2.40E-42	3.27E-42
RP11-713P17	NA	NA	NA
JAM3	2.13699904	1.41E-19	1.71E-19
NCAPD3	2.00335269	7.79E-06	8.50E-06
RP11-700F16	NA	9.28E-156	1.96E-155
VPS26B	2.16161369	2.76E-105	4.86E-105
THYN1	1.53318005	1.16E-258	3.71E-258
ACAD8	1.3109517	0	0
GLB1L3	NA	NA	NA
GLB1L2	2.09357258	1.94E-14	2.27E-14
B3GAT1	NA	NA	NA
AP004550.1	NA	NA	NA
RP11-469N6.	NA	NA	NA
RP11-469N6.	NA	NA	NA
RP11-469N6.	NA	NA	NA
RP11-555G19	NA	NA	NA
AP005135.2	NA	NA	NA
RP11-631M2	NA	NA	NA
TUBB8	NA	0	0
ZMYND11	2.37696242	1.20E-22	1.47E-22
DIP2C	NA	0	0
RP11-490E15	NA	NA	NA
RP11-809C18	NA	NA	NA
PRR26	0	0	0
RP11-164C1.	NA	NA	NA
LARP4B	2.12144433	1.12E-232	3.20E-232
RP11-363N2	NA	1.91E-09	2.15E-09
GTPBP4	1.30618837	0	0
IDI2	Inf	5.01E-290	1.87E-289
IDI2-AS1	5.40403423	5.40E-33	7.01E-33

IDI1	1.31197863	0	0
WDR37	2.02191757	2.26E-200	5.62E-200
LINC00200	NA	NA	NA
ADARB2	NA	NA	NA
ADARB2-AS1	NA	NA	NA
LINC00700	NA	NA	NA
RP11-69C17.	NA	NA	NA
RP11-69C17.	NA	NA	NA
RP11-69C17.	NA	NA	NA
LINC00701	NA	NA	NA
RP11-446F3.	NA	NA	NA
RP11-526P5.	NA	NA	NA
RP11-526P5.	NA	NA	NA
RP11-118K6.	NA	NA	NA
RP11-118K6.	Inf	1.85E-12	2.14E-12
PFKP	1.42931191	0	0
RP11-298E9.	NA	NA	NA
PITRM1	1.737011	4.12E-91	6.86E-91
PITRM1-AS1	Inf	0	0
RP11-298E9.	NA	NA	NA
RP11-298E9.	NA	NA	NA
RP11-195B3.	NA	NA	NA
RP11-482E14	NA	NA	NA
RP11-482E14	NA	NA	NA
RP11-184A2.	NA	NA	NA
RP11-184A2.	NA	NA	NA
KLF6	1.50294569	9.35E-37	1.24E-36
RP11-464C19	NA	NA	NA
RP11-464C19	NA	NA	NA
RP11-433J20	NA	NA	NA
RP11-433J20	NA	NA	NA
LINC00702	NA	NA	NA
LINC00703	NA	NA	NA
RP11-34C15.	NA	NA	NA
LINC00704	NA	NA	NA
LINC00705	NA	NA	NA
AKR1E2	NA	4.79E-27	6.03E-27
AKR1C2	NA	NA	NA
AKR1C1	NA	NA	NA
RP11-499O7.	NA	NA	NA
AKR1C3	0.38600244	0	0
AKR1C3	NA	NA	NA
AKR1C4	NA	NA	NA
RP11-445P17	NA	NA	NA
RP11-445P17	NA	NA	NA

UCN3	NA	NA	NA
TUBAL3	NA	NA	NA
NET1	2.17793249	1.48E-14	1.73E-14
CALML5	Inf	4.36E-139	8.66E-139
CALML3-AS1	NA	NA	NA
CALML3	NA	NA	NA
RP11-116G8.	NA	NA	NA
RP13-463N1(	NA	1.47E-112	2.66E-112
RP11-336A1(	NA	NA	NA
RP11-336A1(	NA	NA	NA
ASB13	1.92499921	6.11E-276	2.12E-275
FAM208B	2.55647521	4.75E-176	1.08E-175
RP11-336A1(	NA	NA	NA
GDI2	1.49641249	0	0
RP11-318E3.	Inf	3.49E-06	3.82E-06
ANKRD16	2.04207745	9.28E-139	1.84E-138
FBXO18	1.89141198	0.97981054	0.98035092
RP11-536K7.	6.17603912	3.65E-100	6.30E-100
IL15RA	2.36987548	0	0
IL2RA	4.11735941	5.95E-48	8.33E-48
RP11-536K7.	NA	NA	NA
RBM17	1.97627964	4.91E-76	7.70E-76
PFKFB3	1.43103345	1.15E-61	1.70E-61
RP11-414H1;	NA	NA	NA
RP11-563J2.:	NA	0	0
RP11-563J2.:	NA	NA	NA
PRKCQ	2.16161369	1.24E-08	1.38E-08
RP11-554I8.1	NA	NA	NA
RP11-554I8.2	NA	NA	NA
LINC00706	NA	NA	NA
LINC00707	NA	NA	NA
RP11-799O2	NA	NA	NA
RP11-393K1;	NA	NA	NA
SFMBT2	2.14055901	1.26E-169	2.81E-169
RP5-1031D4.	NA	NA	NA
RP11-385N2:	NA	NA	NA
ITIH5	Inf	1.40E-207	3.58E-207
ITIH2	NA	NA	NA
KIN	1.80423615	3.03E-185	7.12E-185
ATP5C1	1.41223835	0	0
TAF3	2.03031207	2.61E-277	9.12E-277
GATA3-AS1	NA	NA	NA
RP11-379F1;	NA	NA	NA
RP11-379F1;	NA	NA	NA
GATA3	1.66277976	6.25E-09	7.02E-09

LINC00708	NA	NA	NA
RP5-1119O2	NA	NA	NA
RP11-543F8.	NA	NA	NA
RP11-428L9.	NA	NA	NA
RP11-428L9.	NA	NA	NA
LINC00709	NA	NA	NA
RP5-1051H1	NA	NA	NA
RP5-933E2.1	NA	NA	NA
RP11-271F1	NA	NA	NA
RP11-271F1	NA	NA	NA
SFTA1P	NA	5.58E-28	7.05E-28
RP1-251M9.	NA	NA	NA
CELF2	2.35164566	1.35E-205	3.43E-205
RP1-251M9.	NA	NA	NA
RP11-397O4	NA	NA	NA
CELF2-AS2	NA	NA	NA
RP3-323N1.2	Inf	3.56E-208	9.15E-208
CELF2-AS1	NA	NA	NA
USP6NL	1.93729529	0.00046408	0.00049639
RP11-138I18	NA	NA	NA
RP11-138I18	NA	NA	NA
ECHDC3	1.53357728	1.52E-162	3.28E-162
PROSER2	1.72336445	1.05E-90	1.75E-90
PROSER2-AS	NA	1.04E-120	1.94E-120
UPF2	2.25037863	8.73E-289	3.25E-288
DHTKD1	1.95970472	5.95E-45	8.20E-45
SEC61A2	2.03528562	0.0002291	0.00024607
NUDT5	1.56390396	1.39E-243	4.16E-243
CDC123	1.47875511	2.04E-241	6.03E-241
RP11-186N1	0.25733496	0	0
CAMK1D	0.27247231	0	0
CCDC3	4.46950199	0	0
OPTN	2.31601467	2.61E-23	3.22E-23
MCM10	1.59915299	4.15E-153	8.66E-153
UCMA	NA	NA	NA
PHYH	1.08421275	0	0
SEPHS1	1.70719781	1.57E-98	2.69E-98
RP11-445K1	NA	NA	NA
BEND7	1.71833346	8.35E-254	2.62E-253
RP11-214D1	NA	8.49E-267	2.83E-266
PRPF18	1.74987775	5.96E-05	6.44E-05
RP11-295P9.	4.24602689	4.80E-128	9.13E-128
FRMD4A	NA	NA	NA
RP11-295P9.	NA	3.53E-05	3.83E-05
RP11-295P9.	NA	NA	NA

RP11-353M9	NA	NA	NA
RP11-142M1	NA	NA	NA
RP11-397C18	NA	NA	NA
FAM107B	1.83499624	4.22E-61	6.25E-61
RP11-7C6.1	NA	NA	NA
CDNF	1.80134474	0.00090242	0.00096191
HSPA14	1.22183451	0	0
RP11-398C13	1.47382752	1.04E-184	2.44E-184
SUV39H2	1.80456143	1.01E-93	1.70E-93
DCLRE1C	1.71998148	1.31E-284	4.74E-284
MEIG1	0.94356153	8.60E-87	1.41E-86
OLAH	0	0	0
ACBD7	0	1.95E-212	5.09E-212
C10orf111	5.40403423	1.37E-52	1.95E-52
RPP38	1.58920031	0	0
NMT2	1.4252398	3.87E-64	5.79E-64
FAM171A1	1.55570682	4.81E-176	1.09E-175
RP11-25G10.	NA	NA	NA
ITGA8	NA	4.83E-234	1.38E-233
FAM188A	1.9621791	2.69E-89	4.45E-89
RP11-461K13	NA	NA	NA
PTER	2.16659437	3.74E-07	4.14E-07
C1QL3	NA	NA	NA
RSU1	1.35901322	1.25E-275	4.35E-275
CUBN	NA	1.81E-176	4.11E-176
RP11-406H21	1.15800733	3.21E-104	5.63E-104
TRDMT1	2.37539966	5.51E-12	6.35E-12
VIM-AS1	2.48757131	1.47E-97	2.51E-97
VIM	0.92346705	0	0
RP11-124N14	0	1.76E-127	3.33E-127
ST8SIA6	1.54400978	2.13E-229	5.97E-229
ST8SIA6-AS1	NA	NA	NA
HACD1	1.91862199	1.63E-24	2.02E-24
STAM-AS1	2.57334963	2.48E-79	3.94E-79
STAM	1.77990016	1.13E-68	1.72E-68
RP11-390B4.	NA	NA	NA
TMEM236	NA	6.60E-241	1.95E-240
MRC1	NA	NA	NA
RP11-457D2.	NA	NA	NA
SLC39A12	NA	NA	NA
SLC39A12-AS	NA	NA	NA
CACNB2	Inf	0	0
RP11-499P20	2.31601467	8.38E-05	9.04E-05
RP11-383B4.	NA	NA	NA
NSUN6	2.12301345	1.30E-23	1.60E-23

ARL5B	2.04207745	0.00010762	0.00011596
RP11-139J15	NA	NA	NA
MALRD1	NA	NA	NA
RP11-49L2.1	NA	NA	NA
RP11-354E11	Inf	0	0
PLXDC2	2.31601467	2.84E-156	6.01E-156
RP11-575A19	NA	NA	NA
NEBL	NA	NA	NA
C10orf113	NA	NA	NA
NEBL-AS1	NA	NA	NA
RP11-275N1.	NA	NA	NA
CASC10	2.26087146	1.30E-61	1.93E-61
SKIDA1	1.3896088	3.25E-12	3.75E-12
MLLT10	2.21225057	0.00042603	0.00045607
DNAJC1	1.92530488	1.74E-73	2.71E-73
EBLN1	NA	NA	NA
RP11-108B14	NA	NA	NA
RP11-573G6.	NA	NA	NA
COMMD3	1.81581983	4.79E-113	8.67E-113
COMMD3-BM	NA	NA	NA
BMI1	1.91038498	0.09269592	0.09565924
RP11-573G6.	NA	0	0
RP11-573G6.	NA	NA	NA
RP11-573G6.	0.77200489	0.19767142	0.2024475
SPAG6	NA	NA	NA
PIP4K2A	2.3290995	2.21E-25	2.75E-25
ARMC3	NA	NA	NA
MSRB2	2.00846801	1.47E-35	1.94E-35
PTF1A	NA	NA	NA
LINC01552	NA	NA	NA
C10orf67	NA	NA	NA
RP11-371A19	NA	NA	NA
OTUD1	2.7792176	1.40E-92	2.35E-92
KIAA1217	NA	NA	NA
ARHGAP21	2.27312551	1.04E-160	2.23E-160
PRTFDC1	1.40479578	2.10E-304	8.50E-304
RP11-165A20	2.8306846	1.39E-223	3.79E-223
ENKUR	0.77200489	7.49E-165	1.63E-164
THNSL1	1.62121027	1.29E-35	1.69E-35
LINC01516	NA	NA	NA
GPR158-AS1	NA	NA	NA
GPR158	NA	NA	NA
LINC00836	NA	NA	NA
RP11-435M3	NA	NA	NA
MYO3A	NA	NA	NA

GAD2	NA	NA	NA
APBB1IP	2.00127421	2.26E-116	4.15E-116
RP11-128B16	NA	NA	NA
LINC00264	NA	NA	NA
RP13-16H11.	NA	NA	NA
LINC00202-2	NA	NA	NA
RP13-16H11.	NA	NA	NA
PDSS1	1.3436421	0	0
RP13-16H11.	1.15800733	1.59E-19	1.92E-19
ABI1	1.55934763	2.66E-267	8.90E-267
LINC00202-1	NA	4.12E-43	5.63E-43
LINC00614	NA	NA	NA
ANKRD26	2.46384539	4.68E-13	5.42E-13
YME1L1	2.01493276	3.92E-43	5.35E-43
MASTL	1.45493229	6.97E-21	8.49E-21
ACBD5	2.4318154	3.09E-169	6.84E-169
RP11-85G18.	NA	NA	NA
RP11-748L13	NA	NA	NA
PTCHD3	NA	NA	NA
RAB18	1.92508453	3.93E-08	4.38E-08
MKX	NA	NA	NA
MKX-AS1	NA	NA	NA
ARMC4	1.15800733	0.02150264	0.02244798
RP11-218D6.	NA	NA	NA
MPP7	0	0	0
RP11-351M1	NA	NA	NA
RP11-351M1	NA	NA	NA
WAC-AS1	1.737011	2.55E-10	2.90E-10
WAC	1.85281174	4.41E-67	6.70E-67
BAMBI	1.54400978	1.19E-280	4.23E-280
RP11-478H13	NA	NA	NA
LINC00837	NA	NA	NA
C10orf126	NA	NA	NA
LYZL1	NA	NA	NA
SVIL-AS1	2.66692598	0	0
SVIL	2.12639943	6.80E-39	9.09E-39
KIAA1462	0.64333741	8.11E-91	1.35E-90
MTPAP	1.50931293	1.66E-181	3.84E-181
MAP3K8	2.45387269	0	0
RP11-253D19	NA	0	0
RP11-253D19	NA	NA	NA
LYZL2	NA	NA	NA
RP11-14C22.	NA	NA	NA
RP11-14C22.	NA	NA	NA
ZNF438	1.28667482	1.17E-177	2.66E-177

RP11-192P3.	NA	2.20E-162	4.76E-162
ZEB1-AS1	1.4153423	3.39E-17	4.04E-17
ZEB1	1.54400978	1.60E-212	4.17E-212
RP11-472N1:	NA	NA	NA
RP11-472N1:	0.77200489	9.37E-61	1.38E-60
ARHGAP12	1.23520782	1.38E-33	1.79E-33
KIF5B	1.42673031	0	0
RP11-166N1	NA	NA	NA
EPC1	1.87486902	3.91E-13	4.54E-13
RP11-166N1	NA	NA	NA
RP11-135A24	Inf	0	0
RP11-135A24	NA	NA	NA
CCDC7	1.15800733	1.84E-212	4.81E-212
CCDC7	Inf	0	0
RP11-479G2:	0	0	0
ITGB1	2.3807983	9.04E-254	2.83E-253
RP11-462L8.	NA	NA	NA
RP11-342D1:	NA	NA	NA
NRP1	1.06150672	5.05E-166	1.11E-165
RP11-342D1:	NA	NA	NA
RP11-90B22.	NA	NA	NA
RP11-476F14	NA	NA	NA
RP13-259N1:	NA	NA	NA
LINC00838	NA	NA	NA
RP11-490O24	NA	NA	NA
PARD3	2.24984282	0.48573923	0.49175676
PARD3-AS1	Inf	1.62E-105	2.85E-105
RP11-297A16	NA	NA	NA
CUL2	1.68967108	2.38E-54	3.41E-54
RP11-297A16	NA	0	0
CREM	2.22811312	0	0
RP11-324I22	NA	0	0
CCNY	2.44551226	0	0
RP11-324I22	4.24602689	1.00E-54	1.44E-54
GJD4	NA	5.12E-166	1.12E-165
RP11-425A6.	Inf	0	0
FZD8	Inf	1.29E-221	3.48E-221
PCAT5	NA	NA	NA
RP11-309N24	NA	NA	NA
RP11-810B23	NA	NA	NA
RP11-92J19.3	NA	NA	NA
RP11-322I2.1	NA	NA	NA
ANKRD30A	NA	NA	NA
RP11-20F24.	NA	NA	NA
LINC00993	NA	NA	NA

MTRNR2L7	NA	NA	NA
RP11-258F22	Inf	4.43E-143	8.90E-143
ZNF248	2.47854202	1.45E-127	2.75E-127
RP11-258F22	NA	NA	NA
RP11-162G1(	1.93001222	4.57E-69	6.99E-69
ZNF25	3.55122249	0	0
RP11-393J16	NA	NA	NA
ZNF33A	2.49695332	1.08E-32	1.40E-32
ZNF37A	2.15518032	1.66E-06	1.82E-06
RP11-508N2:	0.77200489	2.25E-23	2.78E-23
RP11-508N2:	1.54400978	7.42E-96	1.26E-95
RP11-508N2:	NA	NA	NA
RP11-672F9.	NA	NA	NA
RP11-291L22	NA	NA	NA
IGKV1OR10-:	NA	NA	NA
LINC00839	NA	NA	NA
ZNF33B	2.17744969	8.15E-05	8.80E-05
LINC01518	NA	NA	NA
AL022344.5	NA	NA	NA
AL022344.7	NA	NA	NA
BMS1	1.89587595	2.08E-182	4.84E-182
RP11-124O1:	NA	NA	NA
LINC01264	NA	NA	NA
RET	0	0	0
RP11-351D1(	Inf	4.85E-180	1.12E-179
CSGALNACT2	1.48886657	1.38E-135	2.71E-135
RASGEF1A	NA	NA	NA
RP11-168L22	NA	NA	NA
FXYD4	NA	NA	NA
HNRNPF	1.34151669	0	0
RP11-517P14	1.6297881	4.14E-22	5.07E-22
ZNF487	3.00224124	4.34E-156	9.15E-156
ZNF239	NA	NA	NA
ZNF485	NA	NA	NA
ZNF32-AS3	NA	NA	NA
RP11-402L1.4	NA	NA	NA
ZNF32	2.08944802	5.91E-15	6.94E-15
ZNF32-AS1	NA	NA	NA
ZNF32-AS2	NA	NA	NA
RP11-402L1.:	NA	NA	NA
RP11-272J7.4	NA	NA	NA
LINC00619	NA	NA	NA
LINC00840	NA	NA	NA
RP11-168P8.	NA	NA	NA
RP11-168P8.	NA	NA	NA

LINC00841	NA	NA	NA
RP11-20J15.2	NA	NA	NA
RP11-20J15.3	NA	NA	NA
C10orf142	NA	NA	NA
CXCL12	NA	NA	NA
RP11-733D4.	NA	NA	NA
TMEM72-AS	NA	NA	NA
RP11-285G1.	NA	NA	NA
TMEM72	0	0	0
RP11-285G1.	NA	NA	NA
RASSF4	0.90067237	0	0
C10orf10	1.54400978	0.00129759	0.00138005
C10orf25	3.08801956	2.16E-14	2.53E-14
ZNF22	1.81454141	0	0
RP11-445N1	NA	NA	NA
OR13A1	NA	NA	NA
ALOX5	NA	9.72E-220	2.61E-219
RP11-67C2.2	NA	NA	NA
8-Mar	1.36236157	2.93E-06	3.21E-06
ZFAND4	NA	6.92E-197	1.70E-196
FAM21C	Inf	0	0
AGAP4	3.08801956	4.14E-124	7.78E-124
TIMM23	1.78274222	3.56E-135	6.95E-135
NCOA4	1.71226726	6.67E-33	8.66E-33
MSMB	NA	NA	NA
ANTXRL	NA	NA	NA
AGAP14P	NA	NA	NA
ANXA8L1	NA	NA	NA
NPY4R	NA	NA	NA
GPRIN2	NA	NA	NA
SYT15	NA	NA	NA
RP11-38L15.3	NA	NA	NA
RP11-38L15.2	NA	NA	NA
RP11-38L15.8	NA	NA	NA
PTPN20	NA	NA	NA
GDF10	NA	NA	NA
GDF2	NA	NA	NA
RBP3	NA	NA	NA
ZNF488	5.40403423	1.49E-169	3.30E-169
ANXA8	NA	NA	NA
FAM25G	NA	NA	NA
RP11-144G6.	NA	NA	NA
AGAP9	1.10286413	1.35E-09	1.53E-09
LINC00864	NA	NA	NA
ABC7-42404	NA	NA	NA

CH17-360D5.	NA	NA	NA
CH17-360D5.	NA	NA	NA
CH17-360D5.	NA	NA	NA
FAM25C	NA	NA	NA
FRMPD2	NA	NA	NA
MAPK8	1.64051039	2.12E-41	2.87E-41
ARHGAP22	2.05224633	8.96E-23	1.10E-22
RP11-534L6.:	NA	NA	NA
WDFY4	1.99929472	0.48537526	0.4914157
LRRC18	NA	NA	NA
RP11-523O1:	NA	NA	NA
VSTM4	3.08801956	6.59E-53	9.41E-53
FAM170B-AS	NA	NA	NA
FAM170B	NA	NA	NA
C10orf128	7.52704768	0	0
C10orf71-AS	NA	NA	NA
C10orf71	NA	NA	NA
DRGX	NA	NA	NA
RP11-123B3.	NA	NA	NA
ERCC6	1.87486902	3.84E-21	4.69E-21
PGBD3	NA	NA	NA
ERCC6-PGBD	NA	0	0
CHAT	NA	NA	NA
SLC18A3	NA	NA	NA
C10orf53	NA	NA	NA
OGDHL	1.54400978	6.88E-31	8.84E-31
PARG	2.59393643	5.97E-61	8.84E-61
TIMM23B	1.64362331	0.83611968	0.83931186
LINC00843	NA	2.22E-65	3.34E-65
AGAP6	3.08801956	2.93E-301	1.17E-300
FAM21A	2.39321516	1.13E-229	3.18E-229
ASAH2	2.31601467	1.57E-94	2.65E-94
SGMS1	2.88215159	3.31E-249	1.02E-248
RP11-512N4.	NA	NA	NA
SGMS1-AS1	0.38600244	4.11E-74	6.40E-74
ASAH2B	1.69841076	1.04E-41	1.41E-41
A1CF	NA	NA	NA
RP11-96B5.4	NA	NA	NA
PRKG1	NA	NA	NA
RP11-96B5.3	NA	NA	NA
RP11-40C11.	NA	NA	NA
CSTF2T	1.82993752	2.14E-130	4.11E-130
PRKG1-AS1	NA	NA	NA
DKK1	NA	0	0
LINC01468	NA	NA	NA

RP11-556E13	NA	NA	NA
MBL2	NA	NA	NA
RP11-319F12	0.25733496	0	0
RP11-71J2.1	NA	NA	NA
PCDH15	NA	NA	NA
RP11-257I14	NA	NA	NA
RP11-598C1(	NA	NA	NA
RP11-598C1(	NA	NA	NA
MTRNR2L5	NA	NA	NA
ZWINT	1.35725359	0	0
IPMK	1.04772092	0	0
CISD1	2.16529328	2.61E-29	3.33E-29
RP11-179B15	NA	1.70E-187	4.03E-187
RP11-179B15	NA	NA	NA
UBE2D1	1.64132132	2.22E-166	4.87E-166
TFAM	2.04759098	0.13903724	0.14292105
BICC1	0.57900367	0	0
LINC00844	NA	NA	NA
PHYHIPL	NA	NA	NA
FAM13C	NA	NA	NA
RP11-443O1	NA	NA	NA
RP11-135D1	NA	NA	NA
SLC16A9	2.57334963	5.47E-95	9.25E-95
MRLN	NA	NA	NA
CCDC6	1.91898358	2.40E-14	2.81E-14
LINC01553	NA	NA	NA
ANK3	NA	NA	NA
RP11-388P9.	NA	NA	NA
RP11-388P9.	NA	NA	NA
RP11-131N1	NA	NA	NA
CDK1	1.0042606	0	0
RHOBTB1	0.90067237	7.59E-108	1.35E-107
LINC00845	NA	NA	NA
TMEM26	NA	NA	NA
TMEM26-AS	NA	NA	NA
C10orf107	NA	NA	NA
RP11-63A2.2	NA	1.69E-163	3.67E-163
RP11-491H19	NA	NA	NA
ARID5B	1.35798451	2.73E-267	9.13E-267
RTKN2	1.7878008	1.42E-103	2.48E-103
RP11-120C12	NA	NA	NA
ZNF365	NA	NA	NA
RP11-436D1(	Inf	2.16E-290	8.09E-290
ADO	1.76777931	1.54E-49	2.17E-49
EGR2	0.77200489	0.00596678	0.00628334

RP11-144G1(	NA	NA	NA
NRBF2	1.70308958	1.72E-118	3.18E-118
JMJD1C	1.85281174	3.54E-190	8.47E-190
JMJD1C-AS1	1.77561125	8.18E-12	9.41E-12
REEP3	1.84891927	6.01E-06	6.56E-06
RP11-170M1	NA	NA	NA
RP11-174J11	NA	NA	NA
RP11-179K3.	NA	NA	NA
RP11-252C24	NA	NA	NA
RP11-428G2.	NA	NA	NA
LINC01515	2.14445803	1.23E-60	1.82E-60
CTNNA3	NA	NA	NA
RP11-210G2.	NA	NA	NA
LRRTM3	NA	NA	NA
RP11-344A5.	NA	NA	NA
DNAJC12	Inf	0	0
RP11-57G10.	1.80134474	8.64E-94	1.45E-93
SIRT1	2.16995969	4.81E-37	6.38E-37
HERC4	1.62746977	1.46E-28	1.85E-28
MYPN	NA	NA	NA
ATOH7	NA	NA	NA
RP11-153K11	NA	NA	NA
PBLD	0.64333741	7.20E-55	1.04E-54
HNRNPH3	1.4191362	5.75E-284	2.07E-283
RUFY2	2.91646292	8.33E-95	1.41E-94
DNA2	1.97290139	6.37E-61	9.42E-61
SLC25A16	1.83918812	1.87E-18	2.25E-18
TET1	1.32343695	0.00610123	0.0064238
CCAR1	1.93166889	2.32E-75	3.64E-75
STOX1	1.70373493	0.93464244	0.93598384
DDX50	1.79910705	8.31E-225	2.28E-224
DDX21	1.90424962	1.50E-07	1.66E-07
KIF1BP	1.17832325	0	0
SRGN	0.95144386	0	0
VPS26A	1.37423277	0	0
SUPV3L1	1.57314204	2.92E-235	8.41E-235
RP11-227H1!	NA	NA	NA
HKDC1	NA	NA	NA
RP11-227H1!	NA	NA	NA
HK1	1.10765919	1.54E-115	2.82E-115
TACR2	NA	1.07E-69	1.65E-69
TSPAN15	1.7833313	1.75E-52	2.49E-52
NEUROG3	NA	NA	NA
RP11-343J3.2	NA	NA	NA
C10orf35	0.77200489	3.48E-74	5.43E-74

RP11-242G2(	NA	NA	NA
COL13A1	NA	NA	NA
RP11-262I2.2	NA	NA	NA
H2AFY2	NA	1.83E-265	6.09E-265
AIFM2	1.92536159	4.52E-28	5.73E-28
TYSND1	2.06235592	1.30E-08	1.46E-08
SAR1A	2.08856847	5.03E-12	5.80E-12
PPA1	1.41385977	0	0
NPFFR1	Inf	1.30E-249	3.98E-249
LRRC20	1.63758613	5.00E-29	6.37E-29
EIF4EBP2	1.97686801	5.00E-05	5.41E-05
NODAL	NA	NA	NA
PALD1	2.41894866	2.63E-34	3.44E-34
PRF1	Inf	2.34E-108	4.16E-108
ADAMTS14	1.15800733	5.88E-19	7.08E-19
TBATA	NA	NA	NA
SGPL1	2.40179299	7.38E-22	9.03E-22
PCBD1	2.13849483	3.19E-280	1.13E-279
RP11-432J9.3	NA	NA	NA
RP11-432J9.(	NA	NA	NA
UNC5B	Inf	1.59E-28	2.01E-28
UNC5B-AS1	NA	NA	NA
SLC29A3	1.2281896	0	0
CDH23	0.19300122	1.94E-298	7.58E-298
CDH23-AS1	NA	NA	NA
C10orf105	NA	9.28E-153	1.93E-152
C10orf54	1.56785549	7.94E-243	2.37E-242
PSAP	1.43058726	0	0
RP11-472G2	NA	3.85E-44	5.28E-44
CHST3	2.20021394	7.02E-290	2.62E-289
RP11-150D2(	NA	NA	NA
SPOCK2	1.15800733	1.62E-46	2.26E-46
ASCC1	1.01218419	0	0
ANAPC16	1.92581655	3.01E-29	3.84E-29
DDIT4	1.50090291	4.37E-291	1.64E-290
RP11-442H2:	Inf	2.11E-189	5.03E-189
DNAJB12	1.54400978	4.07E-209	1.05E-208
MICU1	1.63780477	0.84042095	0.84348954
RP11-167P22	NA	NA	NA
MCU	2.07476314	5.90E-220	1.58E-219
OIT3	NA	NA	NA
PLA2G12B	NA	NA	NA
P4HA1	2.15348732	0.00012323	0.00013269
RP11-344N1(	NA	NA	NA
RP11-344N1(	1.93001222	0.00055233	0.00059033

NUDT13	4.63202934	1.45E-137	2.86E-137
RP11-152N1:	2.50901589	1.75E-130	3.36E-130
ECD	1.05559852	0	0
FAM149B1	1.13043573	4.79E-287	1.76E-286
DNAJC9	1.78974971	3.44E-62	5.11E-62
DNAJC9-AS1	0.77200489	1.20E-48	1.68E-48
MRPS16	2.33936453	0	0
RP11-152N1:	1.89141198	3.31E-16	3.92E-16
CFAP70	1.87486902	1.78E-17	2.12E-17
ANXA7	1.41722066	0	0
RP11-537A6.	NA	0	0
MSS51	5.40403423	0.0025972	0.0027492
PPP3CB	1.59033007	0	0
USP54	2.38619693	1.42E-36	1.88E-36
RP11-464F9.	1.67267726	3.85E-34	5.02E-34
RP11-464F9.	4.63202934	2.58E-255	8.17E-255
MYOZ1	NA	0	0
SYNPO2L	0.77200489	2.16E-16	2.57E-16
RP11-464F9.	NA	NA	NA
AGAP5	3.86002445	0	0
RP11-574K11	Inf	0	0
SEC24C	2.6055165	1.05E-284	3.82E-284
FUT11	1.9981303	1.31E-24	1.62E-24
CHCHD1	2.11090884	2.24E-172	5.01E-172
ZSWIM8	2.07476314	4.36E-252	1.36E-251
RP11-574K11	NA	NA	NA
ZSWIM8-AS1	NA	NA	NA
NDST2	4.3011701	0	0
CAMK2G	1.6818678	1.24E-09	1.40E-09
RP11-574K11	NA	NA	NA
RP11-574K11	NA	NA	NA
PLAU	1.49420301	0	0
C10orf55	Inf	1.94E-97	3.31E-97
VCL	1.55960584	0	0
AP3M1	1.89410502	8.62E-73	1.34E-72
ADK	1.2982219	6.79E-278	2.38E-277
RP11-46O21.	NA	NA	NA
RP11-222G7.	NA	5.58E-28	7.05E-28
KAT6B	2.25031213	2.40E-45	3.31E-45
RP11-77G23.	NA	NA	NA
RP11-77G23.	NA	NA	NA
DUPD1	NA	NA	NA
DUSP13	NA	1.40E-64	2.11E-64
SAMD8	2.49416964	1.00E-24	1.24E-24
VDAC2	1.21602026	0	0

COMTD1	2.04622801	5.94E-99	1.02E-98
ZNF503-AS1	NA	NA	NA
RP11-399K21	NA	NA	NA
ZNF503	1.17945192	0	0
ZNF503-AS2	4.82503056	1.88E-151	3.91E-151
RP11-399K21	1.76458261	1.06E-16	1.26E-16
RP11-399K21	Inf	3.87E-179	8.87E-179
RP11-399K21	NA	NA	NA
C10orf11	1.35100856	4.77E-253	1.49E-252
AC013286.1	NA	NA	NA
RP11-369F10	NA	NA	NA
RP11-325D15	NA	NA	NA
KCNMA1	NA	NA	NA
KCNMA1-AS1	NA	NA	NA
KCNMA1-AS2	NA	NA	NA
RP11-328K22	NA	NA	NA
KCNMA1-AS3	NA	NA	NA
RP13-39P12.	NA	6.14E-155	1.29E-154
DLG5	2.14445803	3.66E-284	1.32E-283
RP11-126H7.	NA	NA	NA
POLR3A	3.13213413	0	0
RPS24	1.80512908	2.44E-61	3.62E-61
RP11-157J13	NA	NA	NA
RP11-90J7.3	NA	NA	NA
LINC00856	NA	NA	NA
RP11-90J7.2	Inf	3.07E-132	5.94E-132
RP11-90J7.4	NA	NA	NA
RP11-17G2.1	NA	NA	NA
RP11-31E13.	NA	NA	NA
ZMIZ1-AS1	NA	NA	NA
RP11-481G8.	NA	NA	NA
ZMIZ1	2.94765503	0	0
PPIF	1.65324418	4.61E-283	1.65E-282
ZCCHC24	1.15800733	7.96E-54	1.14E-53
RP11-342M3	NA	NA	NA
RP11-342M3	NA	NA	NA
EIF5AL1	2.30025947	1.98E-17	2.37E-17
SFTPA2	NA	NA	NA
SFTPA1	NA	NA	NA
RP11-589B3.	NA	NA	NA
RP11-506M1	2.31601467	3.83E-123	7.17E-123
NUTM2B-AS	2.01546315	1.82E-296	7.04E-296
NUTM2B	NA	NA	NA
RP11-182L21	2.50901589	9.77E-181	2.26E-180
NUTM2E	NA	2.27E-148	4.65E-148

SFTPD	NA	NA	NA
SFTPD-AS1	NA	0	0
TMEM254-A!	Inf	0	0
TMEM254	2.61293963	2.67E-10	3.03E-10
PLAC9	NA	NA	NA
ANXA11	1.25289062	0	0
LINC00857	NA	NA	NA
MAT1A	0	0	0
RP11-36D19.	NA	NA	NA
DYDC1	NA	NA	NA
DYDC2	NA	7.85E-138	1.55E-137
FAM213A	NA	NA	NA
TSPAN14	1.75754305	2.14E-09	2.42E-09
RP11-137H2.	NA	NA	NA
SH2D4B	NA	NA	NA
RP11-514F8.	NA	NA	NA
NRG3	NA	NA	NA
RP11-202D1ł	NA	NA	NA
RP11-344L13	NA	NA	NA
RP11-219F1(	NA	NA	NA
RP11-338I21	NA	0	0
GHITM	1.39716689	0	0
C10orf99	NA	NA	NA
CDHR1	NA	NA	NA
LRIT2	NA	NA	NA
LRIT1	NA	NA	NA
RGR	NA	NA	NA
LINC00858	NA	NA	NA
CCSER2	2.1386622	0.06751039	0.06987525
LINC01519	NA	NA	NA
RP11-475D1:	NA	NA	NA
RP11-475D1:	NA	NA	NA
LINC01520	NA	NA	NA
GRID1-AS1	NA	NA	NA
GRID1	NA	NA	NA
RP11-93H12.	NA	NA	NA
WAPL	1.75820767	9.34E-109	1.67E-108
RP11-77P6.2	2.64687391	1.74E-76	2.74E-76
OPN4	NA	NA	NA
LDB3	NA	4.61E-66	6.96E-66
RP11-359E3.	3.86002445	4.71E-113	8.53E-113
BMPR1A	1.98515543	0.00750722	0.00789176
MMRN2	NA	NA	NA
SNCG	1.43372337	2.77E-13	3.22E-13
ADIRF	NA	NA	NA

RP11-96C23.	NA	NA	NA
RP11-96C23.	NA	NA	NA
FAM25A	NA	NA	NA
GLUD1	1.8000114	7.60E-45	1.05E-44
FAM35A	1.99317626	1.48E-09	1.67E-09
NUTM2A-AS	1.54400978	1.30E-295	5.02E-295
NUTM2A	NA	NA	NA
LINC00863	0	0	0
NUTM2D	NA	NA	NA
LINC00864	NA	NA	NA
MINPP1	1.03738157	7.60E-219	2.03E-218
RP11-57C13.	NA	NA	NA
RP11-57C13.	NA	NA	NA
RP11-57C13.	NA	NA	NA
PAPSS2	NA	NA	NA
ATAD1	2.02007946	0.01407824	0.01473533
KLLN	0.46320293	2.91E-179	6.68E-179
PTEN	1.71756127	6.56E-95	1.11E-94
RNLS	NA	NA	NA
LIPJ	NA	NA	NA
LIPF	NA	NA	NA
LIPK	NA	NA	NA
LIPN	NA	1.21E-266	4.04E-266
LIPM	NA	NA	NA
ANKRD22	6.94804401	0	0
STAMBPL1	4.86363081	0	0
ACTA2-AS1	NA	5.50E-35	7.21E-35
[truncated: 386,732 more chars]
