# Supplementary material for: Integrated multi-omics analyses reveal homology-directed repair pathway as a unique dependency in near-haploid leukemia
Source: Blood Cancer J. 2023 Jun 8;13(1):92. doi: 10.1038/s41408-023-00863-1 (PMC10247733; doi:10.1038/s41408-023-00863-1)
Supplement: Supplementary file 4 — Supplementary Table 3 [file 41408_2023_863_MOESM4_ESM.pdf]

Raw read counts

Construct Ba	Construct ID	H29 input	H29 output	H29 diploidiz	H14D input	H14D output	H29 FLR 0204 sorted
AAAAAAAAT	BRDN00012	286	227	67	510	438	5
AAAAAAAGG	BRDN00012	316	296	883	510	845	6
AAAAAAATG	BRDN00011	104	51	28	476	376	28
AAAAAAATG	BRDN00012	102	91	0	196	179	15
AAAAAACAC	BRDN00011	191	99	362	327	425	17
AAAAAACAG	BRDN00011	47	7	2	243	100	0
AAAAAACCA	BRDN00011	142	57	30	243	185	0
AAAAAACCC	BRDN00011	49	20	0	158	123	0
AAAAAACCT	BRDN00012	91	31	474	216	21	3
AAAAAACCT	BRDN00011	117	191	171	211	195	36
AAAAAACGA	BRDN00011	405	485	767	607	381	109
AAAAAACTC	BRDN00011	195	185	147	335	354	6
AAAAAACTG	BRDN00011	306	356	867	681	843	64
AAAAAACTT	BRDN00012	113	84	177	128	17	51
AAAAAACTT	BRDN00012	130	123	0	241	329	9
AAAAAACTT	BRDN00011	194	193	1258	356	325	56
AAAAAAGAA	BRDN00012	62	66	2	123	80	0
AAAAAAGAC	BRDN00011	306	279	36	543	682	15
AAAAAAGCA	BRDN00011	277	132	10	367	358	0
AAAAAAGCC	BRDN00012	115	545	2366	292	230	237
AAAAAAGCG	BRDN00012	153	31	6	392	215	7
AAAAAAGGG	BRDN00012	161	119	12	276	268	6
AAAAAAGTC	BRDN00012	171	158	332	264	960	26
AAAAAAGTT	BRDN00012	177	59	101	264	196	6
AAAAAATAT	BRDN00011	191	160	105	290	158	25
AAAAAATGA	BRDN00012	83	78	53	220	125	3
AAAAAATGA	BRDN00012	459	387	168	617	811	86
AAAAAATGT	BRDN00011	303	226	901	501	212	36
AAAAAATTC	BRDN00011	118	13	28	164	323	0
AAAAACACC	BRDN00011	44	26	1	495	493	15
AAAAACACT	BRDN00011	152	133	616	218	170	13
AAAAACACT	BRDN00011	218	148	181	452	605	35
AAAAACACT	BRDN00011	62	117	116	196	183	0
AAAAACACT	BRDN00012	187	144	136	232	453	0
AAAAACCAA	BRDN00011	149	216	14	300	271	5
AAAAACCAC	BRDN00011	316	168	1045	720	588	27
AAAAACCAG	BRDN00012	597	503	2057	888	1040	109
AAAAACCCA	BRDN00012	432	159	368	843	928	17
AAAAACCCT	BRDN00012	341	286	848	510	381	50
AAAAACCTA	BRDN00011	183	229	42	217	213	20
AAAAACCTG	BRDN00011	243	272	204	602	1029	19
AAAAACCTT	BRDN00011	109	69	0	179	171	33
AAAAACGAA	BRDN00012	274	392	412	478	511	14
AAAAACGCA	BRDN00012	184	143	182	374	197	46
AAAAACGCA	BRDN00012	116	35	29	748	691	0
AAAAACGCA	BRDN00012	255	240	289	508	568	6
AAAAACGCG	BRDN00011	67	79	1	201	46	5
AAAAACGGG	BRDN00012	335	297	276	514	661	23
AAAAACGGT	BRDN00012	182	130	15	278	319	37
AAAAACTCA	BRDN00011	199	181	443	276	321	26
AAAAACTCT	BRDN00011	117	71	58	217	278	7
AAAAACTGC	BRDN00012	133	19	0	365	319	0
AAAAAGAAG	BRDN00011	211	134	185	519	485	33
AAAAAGAAG	BRDN00012	76	50	86	123	133	26
AAAAAGACA	BRDN00012	197	194	2149	349	317	126
AAAAAGACA	BRDN00012	337	270	168	579	517	14
AAAAAGACA	BRDN00012	205	149	215	332	855	18
AAAAAGAGA	BRDN00012	125	166	85	179	193	39
AAAAAGAGG	BRDN00011	141	183	38	397	129	24
AAAAAGATA	BRDN00012	104	0	0	214	175	0
AAAAAGATC	BRDN00012	105	28	230	223	300	0
AAAAAGATC	BRDN00012	89	25	0	280	184	0
AAAAAGATG	BRDN00011	146	166	111	414	548	12
AAAAAGCAA	BRDN00011	129	89	0	183	83	0
AAAAAGCAG	BRDN00011	161	12	80	479	396	36
AAAAAGCAG	BRDN00011	133	12	9	236	299	0
AAAAAGCCC	BRDN00012	206	155	182	377	261	13
AAAAAGCCT	BRDN00011	201	171	66	381	294	0
AAAAAGCCT	BRDN00012	102	103	5	189	270	0
AAAAAGCTG	BRDN00012	202	159	539	405	427	18
AAAAAGCTT	BRDN00012	531	479	1785	893	894	42
AAAAAGCTT	BRDN00011	164	12	76	347	359	0
AAAAAGCTT	BRDN00011	226	244	47	435	431	76
AAAAAGGCA	BRDN00012	423	276	323	664	798	24
AAAAAGGTG	BRDN00011	81	103	183	193	263	16
AAAAAGGTG	BRDN00012	153	166	120	363	795	5
AAAAAGTAG	BRDN00011	178	94	183	408	273	9
AAAAAGTAT	BRDN00011	145	151	1404	238	238	51
AAAAAGTCA	BRDN00012	144	2	248	328	593	0
AAAAAGTCC	BRDN00012	199	105	30	381	366	0
AAAAAGTGA	BRDN00012	91	154	520	317	79	10
AAAAAGTGT	BRDN00012	61	71	20	137	101	0
AAAAAGTTA	BRDN00012	130	116	225	323	268	0
AAAAAGTTA	BRDN00011	235	129	25	441	599	29
AAAAAGTTC	BRDN00011	147	160	165	230	306	22
AAAAAGTTC	BRDN00011	146	248	675	324	192	35
AAAAATACA	BRDN00012	160	85	3	306	168	0
AAAAATACT	BRDN00012	281	283	457	534	335	58
AAAAATAGA	BRDN00011	258	313	349	501	528	10
AAAAATCAA	BRDN00011	249	122	32	354	631	0
AAAAATCCC	BRDN00012	280	73	139	496	817	10
AAAAATCCT	BRDN00012	179	260	3838	229	312	73
AAAAATCGA	BRDN00011	216	257	25	437	287	4
AAAAATCGC	BRDN00011	146	192	8	258	454	41
AAAAATCGT	BRDN00012	373	275	929	544	484	40
AAAAATCTC	BRDN00011	333	180	1190	488	506	73
AAAAATGAT	BRDN00011	150	96	471	269	126	23
AAAAATGAT	BRDN00011	106	57	596	233	149	9
AAAAATGCG	BRDN00012	426	346	818	667	745	51
AAAAATGGC	BRDN00012	219	217	439	563	587	42
AAAAATGTA	BRDN00012	366	381	2170	814	614	88
AAAAATGTG	BRDN00012	82	32	0	158	227	0
AAAAATTAA	BRDN00011	208	301	297	404	387	62
AAAAATTCA	BRDN00011	289	245	682	508	600	74
AAAAATTGA	BRDN00011	192	194	298	290	289	26
AAAAATTGG	BRDN00012	320	275	950	623	868	35
AAAAATTGT	BRDN00011	205	34	0	399	182	0
AAAACAAAA	BRDN00011	156	205	0	384	185	8
AAAACAAAG	BRDN00011	205	28	1777	385	333	9
AAAACAACA	BRDN00011	365	366	3741	711	858	129
AAAACAACA	BRDN00012	119	4	0	530	603	0
AAAACAACT	BRDN00011	223	13	0	456	428	6
AAAACAAGA	BRDN00012	367	386	147	735	474	9
AAAACAAGA	BRDN00012	373	292	133	548	398	44
AAAACAAGG	BRDN00011	239	382	550	564	490	50
AAAACAAGT	BRDN00012	164	151	40	591	442	20
AAAACAATA	BRDN00011	137	128	304	237	263	3
AAAACACAT	BRDN00012	28	0	2	238	232	0
AAAACACAT	BRDN00011	213	337	376	353	320	52
AAAACACAT	BRDN00012	326	398	140	496	442	55
AAAACACCA	BRDN00011	309	361	251	471	822	97
AAAACACTA	BRDN00011	74	28	110	160	80	12
AAAACACTG	BRDN00011	199	178	61	407	551	0
AAAACAGCA	BRDN00012	291	182	1047	612	1065	7
AAAACAGCA	BRDN00011	406	267	24	524	620	52
AAAACAGCG	BRDN00012	167	286	6	338	652	64
AAAACAGGA	BRDN00012	391	360	608	701	500	69
AAAACAGTC	BRDN00012	462	290	631	922	845	28
AAAACATAA	BRDN00012	202	153	326	393	223	36
AAAACATAG	BRDN00011	44	23	0	90	62	0
AAAACATCA	BRDN00012	136	145	40	365	424	0
AAAACATCA	BRDN00011	271	192	145	587	626	10
AAAACATCG	BRDN00012	349	325	1012	700	570	78
AAAACATCG	BRDN00012	127	122	46	400	338	2
AAAACATGG	BRDN00011	136	166	34	331	648	0
AAAACATTA	BRDN00011	305	228	44	594	493	13
AAAACATTT	BRDN00012	223	190	297	380	499	14
AAAACCAAA	BRDN00012	180	127	73	303	384	0
AAAACCAAA	BRDN00011	133	191	471	349	388	68
AAAACCAAC	BRDN00012	305	309	854	614	551	28
AAAACCACA	BRDN00012	105	74	15	252	195	14
AAAACCACA	BRDN00012	197	187	96	284	379	42
AAAACCACC	BRDN00012	499	348	1237	851	1223	54
AAAACCACC	BRDN00012	287	345	1202	773	1331	60
AAAACCAGA	BRDN00012	282	175	458	772	694	0
AAAACCAGT	BRDN00011	205	270	260	411	357	33
AAAACCATA	BRDN00011	257	86	101	465	286	2
AAAACCATG	BRDN00012	177	179	1021	352	462	6
AAAACCATT	BRDN00011	40	0	0	420	115	0
AAAACCCAA	BRDN00011	625	659	607	862	1009	52
AAAACCCCA	BRDN00012	374	444	998	669	600	88
AAAACCCCC	BRDN00012	177	15	48	329	266	8
AAAACCCCG	BRDN00011	357	388	656	686	859	28
AAAACCCCT	BRDN00011	203	128	243	386	332	6
AAAACCCTT	BRDN00012	226	120	275	340	417	0
AAAACCGAG	BRDN00012	142	76	1	328	283	0
AAAACCGAT	BRDN00011	238	257	83	463	444	13
AAAACCGCA	BRDN00011	405	440	636	796	808	2
AAAACCGGA	BRDN00011	65	89	169	197	299	0
AAAACCTCA	BRDN00012	125	39	28	177	286	13
AAAACCTGC	BRDN00012	426	434	431	899	731	41
AAAACCTGT	BRDN00012	215	213	315	346	138	25
AAAACCTTA	BRDN00012	237	294	407	579	534	7
AAAACGACA	BRDN00011	139	117	569	203	121	14
AAAACGACG	BRDN00011	146	171	9	211	503	17
AAAACGCAA	BRDN00011	208	204	614	513	466	32
AAAACGCCC	BRDN00012	468	434	2352	836	946	62
AAAACGCCC	BRDN00012	227	205	990	333	216	12
AAAACGCGG	BRDN00011	156	70	281	157	600	0
AAAACGGGC	BRDN00012	301	143	51	388	336	10
AAAACGGTT	BRDN00011	119	104	181	216	134	7
AAAACGTCC	BRDN00011	65	24	612	176	279	4
AAAACGTGA	BRDN00012	266	12	165	398	546	7
AAAACTAAA	BRDN00012	270	261	530	584	580	94
AAAACTACA	BRDN00012	56	75	269	121	51	21
AAAACTACC	BRDN00012	531	397	1933	861	738	42
AAAACTACG	BRDN00012	328	316	335	694	717	142
AAAACTACG	BRDN00011	495	436	640	700	459	44
AAAACTCAA	BRDN00011	181	15	0	273	269	0
AAAACTCTA	BRDN00011	220	242	1158	368	220	22
AAAACTCTG	BRDN00011	156	155	167	313	285	3
AAAACTGAA	BRDN00011	248	291	996	472	763	8
AAAACTGAA	BRDN00011	108	92	13	202	106	0
AAAACTGCA	BRDN00011	217	254	348	596	409	45
AAAACTGCA	BRDN00012	285	111	1	649	726	9
AAAACTGGT	BRDN00011	289	255	342	479	543	20
AAAACTGGT	BRDN00011	215	254	238	378	340	5
AAAACTGTA	BRDN00011	204	193	142	273	236	11
AAAACTGTC	BRDN00012	200	342	276	333	395	17
AAAACTTAG	BRDN00011	100	52	69	151	212	0
AAAACTTAG	BRDN00011	134	0	1	467	1040	0
AAAACTTCG	BRDN00011	222	19	0	265	133	0
AAAACTTGA	BRDN00011	183	159	431	396	338	0
AAAACTTTA	BRDN00012	131	111	289	280	315	7
AAAAGAAAA	BRDN00011	121	60	1	240	162	16
AAAAGAAAC	BRDN00011	314	254	593	536	447	50
AAAAGAACA	BRDN00012	384	236	367	564	473	20
AAAAGAAGA	BRDN00012	191	57	12	400	473	6
AAAAGAAGA	BRDN00011	290	295	366	579	703	0
AAAAGAAGG	BRDN00011	183	182	195	377	346	5
AAAAGAATC	BRDN00011	252	243	301	478	285	30
AAAAGAATG	BRDN00012	193	28	53	245	406	0
AAAAGACAA	BRDN00011	125	121	439	267	252	6
AAAAGACCA	BRDN00012	162	214	884	384	638	47
AAAAGACCT	BRDN00012	328	373	268	523	553	30
AAAAGACGA	BRDN00011	113	118	0	284	213	0
AAAAGAGAA	BRDN00012	48	87	200	124	286	0
AAAAGAGAA	BRDN00011	412	310	3422	839	683	44
AAAAGAGAC	BRDN00012	69	24	57	192	118	0
AAAAGAGAT	BRDN00012	381	225	640	652	618	39
AAAAGAGCA	BRDN00012	110	127	198	184	131	0
AAAAGAGCC	BRDN00011	176	220	33	181	255	24
AAAAGAGCG	BRDN00012	274	298	294	661	813	44
AAAAGATAA	BRDN00012	177	61	27	328	376	0
AAAAGATAA	BRDN00012	198	120	103	245	232	7
AAAAGATAC	BRDN00012	90	0	0	257	239	0
AAAAGATAC	BRDN00012	210	161	500	354	603	0
AAAAGATAT	BRDN00012	91	8	0	172	105	0
AAAAGATCG	BRDN00012	399	272	2239	644	651	103
AAAAGATGA	BRDN00011	205	147	149	354	409	26
AAAAGATGA	BRDN00011	260	204	951	410	479	18
AAAAGATGC	BRDN00011	412	544	799	723	320	32
AAAAGATTC	BRDN00012	229	338	92	546	607	2
AAAAGATTG	BRDN00012	270	117	186	418	467	11
AAAAGATTG	BRDN00011	143	120	261	184	394	10
AAAAGATTG	BRDN00012	119	198	24	552	543	25
AAAAGCAAG	BRDN00011	287	160	83	491	377	6
AAAAGCAAG	BRDN00011	179	128	16	331	191	0
AAAAGCAAG	BRDN00011	312	362	4382	553	512	78
AAAAGCACC	BRDN00012	434	419	500	784	1083	5
AAAAGCACG	BRDN00011	124	79	164	124	114	6
AAAAGCAGA	BRDN00011	62	66	278	153	315	17
AAAAGCAGA	BRDN00011	184	233	438	384	404	122
AAAAGCAGC	BRDN00012	146	155	401	321	255	42
AAAAGCAGT	BRDN00011	352	0	0	721	503	0
AAAAGCATT	BRDN00012	157	112	2	234	327	4
AAAAGCCAA	BRDN00012	117	113	17	359	136	2
AAAAGCCAA	BRDN00011	96	0	0	420	155	0
AAAAGCCAC	BRDN00012	194	107	413	284	139	0
AAAAGCCAT	BRDN00012	170	170	168	268	434	49
AAAAGCCGG	BRDN00011	249	312	402	387	503	27
AAAAGCCGG	BRDN00011	86	52	288	140	78	10
AAAAGCCTC	BRDN00011	171	184	1046	404	128	20
AAAAGCCTG	BRDN00012	415	312	281	820	903	2
AAAAGCCTG	BRDN00012	127	102	26	295	416	0
AAAAGCCTG	BRDN00012	359	326	978	715	409	63
AAAAGCGAA	BRDN00012	371	259	381	499	352	10
AAAAGCGAC	BRDN00012	173	150	97	326	164	0
AAAAGCGCA	BRDN00012	255	26	114	567	337	1
AAAAGCGCA	BRDN00012	184	174	384	417	426	10
AAAAGCGGC	BRDN00011	172	169	81	446	682	7
AAAAGCGTC	BRDN00011	91	170	51	228	137	0
AAAAGCTCT	BRDN00011	91	0	2	451	409	0
AAAAGCTCT	BRDN00012	153	2	64	444	319	0
AAAAGCTGG	BRDN00012	153	108	787	233	219	8
AAAAGCTGT	BRDN00012	58	7	0	260	411	0
AAAAGCTTT	BRDN00012	137	120	1	307	229	0
AAAAGCTTT	BRDN00011	139	162	50	261	183	1
AAAAGGAAC	BRDN00011	251	206	316	755	649	16
AAAAGGAAG	BRDN00012	361	323	550	634	528	52
AAAAGGAAT	BRDN00012	94	87	15	154	85	0
AAAAGGAAT	BRDN00011	94	0	0	305	379	0
AAAAGGACC	BRDN00012	204	124	142	423	536	12
AAAAGGACG	BRDN00011	42	61	14	131	136	0
AAAAGGAGA	BRDN00011	106	53	0	189	113	5
AAAAGGATC	BRDN00012	71	40	8	741	663	19
AAAAGGATC	BRDN00012	150	145	12	387	368	48
AAAAGGATG	BRDN00012	347	39	80	690	498	0
AAAAGGATG	BRDN00012	121	54	691	300	344	40
AAAAGGCAG	BRDN00012	414	251	967	711	1255	69
AAAAGGCAG	BRDN00011	282	194	31	369	365	1
AAAAGGCAT	BRDN00012	434	295	215	959	812	6
AAAAGGCAT	BRDN00012	106	3	0	450	36	0
AAAAGGCCA	BRDN00012	53	56	101	199	88	0
AAAAGGCTC	BRDN00012	33	35	1	215	147	0
AAAAGGCTT	BRDN00011	218	186	626	465	917	18
AAAAGGGAA	BRDN00011	318	281	292	766	532	8
AAAAGGGAA	BRDN00011	165	195	221	210	187	17
AAAAGGGAC	BRDN00011	266	238	1060	396	644	18
AAAAGGGAC	BRDN00012	111	138	1	302	415	20
AAAAGGGAT	BRDN00011	175	126	290	350	479	29
AAAAGGGCA	BRDN00011	166	129	324	233	178	0
AAAAGGGGA	BRDN00012	117	113	83	237	236	0
AAAAGGGTG	BRDN00012	397	355	94	731	632	7
AAAAGGTAA	BRDN00011	221	239	12	503	525	19
AAAAGGTAT	BRDN00012	329	300	351	585	957	38
AAAAGGTCA	BRDN00012	286	65	88	299	249	3
AAAAGGTCC	BRDN00012	265	165	130	404	188	9
AAAAGGTCG	BRDN00011	186	193	401	306	316	80
AAAAGGTGG	BRDN00012	175	174	26	335	406	5
AAAAGGTGG	BRDN00012	300	171	67	528	524	13
AAAAGTAAT	BRDN00012	215	206	392	403	645	15
AAAAGTAAT	BRDN00012	159	157	126	439	203	50
AAAAGTACA	BRDN00011	183	216	292	380	670	42
AAAAGTACA	BRDN00011	85	102	1726	213	94	8
AAAAGTACC	BRDN00011	129	104	59	346	245	14
AAAAGTAGT	BRDN00012	238	307	437	406	249	5
AAAAGTATA	BRDN00012	130	20	56	208	158	12
AAAAGTATT	BRDN00012	212	215	1021	380	477	29
AAAAGTCAG	BRDN00011	88	23	0	312	176	0
AAAAGTCAT	BRDN00012	449	301	358	590	853	35
AAAAGTCCT	BRDN00011	247	116	277	297	258	17
AAAAGTGAA	BRDN00012	183	93	292	318	675	9
AAAAGTGAG	BRDN00011	306	319	128	642	322	31
AAAAGTGAT	BRDN00011	187	61	0	140	129	0
AAAAGTGCT	BRDN00012	208	241	113	407	254	0
AAAAGTGGA	BRDN00011	165	101	66	234	229	0
AAAAGTTAC	BRDN00012	177	215	421	312	436	14
AAAAGTTCT	BRDN00012	274	157	1542	602	637	31
AAAAGTTCT	BRDN00011	343	347	668	648	502	17
AAAAGTTGT	BRDN00011	166	180	112	272	216	15
AAAAGTTTC	BRDN00011	202	116	55	306	252	11
AAAAGTTTG	BRDN00011	280	122	564	616	958	12
AAAAGTTTG	BRDN00011	86	43	0	215	124	13
AAAATAAAT	BRDN00011	82	98	230	171	184	12
AAAATACAA	BRDN00012	87	91	235	166	93	0
AAAATACAG	BRDN00012	167	7	1	323	160	0
AAAATACCA	BRDN00012	119	70	24	169	166	5
AAAATACCT	BRDN00011	95	70	4	127	116	8
AAAATACTA	BRDN00011	107	131	279	140	138	13
AAAATACTG	BRDN00012	357	339	684	594	515	35
AAAATAGCA	BRDN00011	158	103	3	236	261	0
AAAATAGCA	BRDN00012	347	322	213	520	252	38
AAAATAGTT	BRDN00011	85	182	215	156	112	23
AAAATAGTT	BRDN00012	189	145	183	257	89	28
AAAATATCT	BRDN00012	40	43	67	141	18	0
AAAATATGA	BRDN00011	169	34	7	413	131	13
AAAATATGA	BRDN00012	72	99	33	161	193	0
AAAATATTG	BRDN00011	267	176	434	375	235	10
AAAATCAAA	BRDN00011	190	194	510	562	333	23
AAAATCAAA	BRDN00011	176	140	104	345	501	55
AAAATCAAA	BRDN00012	383	524	405	604	572	72
AAAATCAAT	BRDN00012	89	52	86	196	171	5
AAAATCAAC	BRDN00012	148	146	5	217	394	20
AAAATCAAT	BRDN00011	139	94	1	327	277	39
AAAATCAAT	BRDN00011	246	244	545	413	574	67
AAAATCACA	BRDN00012	284	129	212	468	369	0
AAAATCACC	BRDN00012	539	44	1	1005	550	0
AAAATCAGA	BRDN00012	325	366	989	437	443	33
AAAATCAGC	BRDN00011	330	372	8	498	311	37
AAAATCAGG	BRDN00011	219	234	488	480	460	0
AAAATCAGT	BRDN00012	164	141	373	310	311	3
AAAATCAGT	BRDN00011	239	79	224	279	498	6
AAAATCATA	BRDN00011	85	121	108	177	106	9
AAAATCCAC	BRDN00011	282	422	267	583	642	16
AAAATCCAC	BRDN00012	130	157	10	184	256	9
AAAATCCAC	BRDN00012	198	383	1775	587	323	46
AAAATCCAG	BRDN00012	67	38	0	224	141	0
AAAATCCCA	BRDN00011	210	195	221	345	274	17
AAAATCCCC	BRDN00012	226	176	651	378	358	52
AAAATCCCC	BRDN00011	168	183	198	272	191	16
AAAATCCTG	BRDN00012	219	104	39	483	204	0
AAAATCGAA	BRDN00011	138	64	0	224	206	9
AAAATCGAG	BRDN00012	195	218	72	388	429	1
AAAATCGAT	BRDN00012	531	570	1517	906	889	30
AAAATCGCA	BRDN00011	173	115	14	279	185	9
AAAATCGTC	BRDN00012	13	0	0	296	241	0
AAAATCTAC	BRDN00012	106	108	0	131	194	0
AAAATCTAC	BRDN00011	191	59	25	237	188	0
AAAATCTAC	BRDN00011	184	208	242	318	271	7
AAAATCTAT	BRDN00011	118	115	112	252	473	0
AAAATCTCG	BRDN00011	177	140	77	207	226	22
AAAATCTCT	BRDN00012	354	209	41	516	357	12
AAAATCTGC	BRDN00012	327	397	209	802	681	8
AAAATCTTC	BRDN00011	130	134	284	176	140	23
AAAATGAAT	BRDN00012	160	232	12	298	440	26
AAAATGAAT	BRDN00012	120	117	13	206	285	0
AAAATGAGA	BRDN00011	316	202	803	436	232	32
AAAATGAGA	BRDN00011	283	200	1005	481	753	37
AAAATGAGC	BRDN00011	171	155	38	469	232	7
AAAATGAGC	BRDN00012	392	257	291	800	740	7
AAAATGAGT	BRDN00012	92	63	1004	177	69	15
AAAATGAGT	BRDN00012	305	268	33	486	714	12
AAAATGAGT	BRDN00011	140	212	47	342	153	0
AAAATGATG	BRDN00012	424	235	440	504	765	13
AAAATGCAA	BRDN00011	260	260	337	319	394	0
AAAATGCAA	BRDN00012	454	391	349	708	834	46
AAAATGCAG	BRDN00011	470	597	1386	861	830	60
AAAATGCAG	BRDN00012	358	349	851	646	741	13
AAAATGCCA	BRDN00011	162	182	516	335	383	66
AAAATGCCA	BRDN00012	219	85	0	438	398	0
AAAATGGAG	BRDN00011	24	13	0	197	173	0
AAAATGGAG	BRDN00011	194	14	0	420	480	0
AAAATGGCC	BRDN00012	58	2	41	613	416	0
AAAATGGCC	BRDN00011	429	381	316	713	671	35
AAAATGGCG	BRDN00011	133	68	26	195	206	0
AAAATGGCT	BRDN00011	138	127	26	329	301	0
AAAATGGCT	BRDN00011	234	214	456	359	207	91
AAAATGGGC	BRDN00011	237	260	224	513	445	54
AAAATGGTA	BRDN00011	134	173	234	290	248	17
AAAATGGTG	BRDN00011	129	120	582	252	297	12
AAAATGTAA	BRDN00011	226	164	168	444	531	31
AAAATGTAC	BRDN00012	182	338	343	430	677	21
AAAATGTAT	BRDN00012	168	51	295	477	302	15
AAAATGTCA	BRDN00012	171	30	50	391	259	0
AAAATGTCT	BRDN00012	94	64	213	171	90	19
AAAATGTGA	BRDN00012	219	165	406	170	176	5
AAAATGTGA	BRDN00011	250	217	87	529	495	0
AAAATGTTA	BRDN00011	159	111	325	292	578	34
AAAATGTTG	BRDN00012	209	186	330	310	382	59
AAAATGTTG	BRDN00012	211	133	455	535	758	29
AAAATTATC	BRDN00012	391	358	321	636	1147	45
AAAATTCAC	BRDN00012	221	243	1024	480	463	43
AAAATTCAG	BRDN00011	149	103	39	199	282	1
AAAATTCCG	BRDN00012	170	120	224	281	325	24
AAAATTCCT	BRDN00012	328	217	486	516	488	11
AAAATTCTA	BRDN00011	120	78	13	174	112	0
AAAATTCTA	BRDN00012	62	73	546	191	259	7
AAAATTCTT	BRDN00012	106	60	0	316	168	0
AAAATTGAC	BRDN00011	237	312	1010	560	437	40
AAAATTGCG	BRDN00011	73	120	213	127	23	15
AAAATTGGA	BRDN00012	103	164	1	274	288	0
AAAATTGGA	BRDN00012	136	134	9	259	114	0
AAAATTTAT	BRDN00012	165	179	72	339	257	47
AAAATTTCA	BRDN00011	69	0	0	242	450	0
AAAATTTCT	BRDN00011	266	173	1214	394	298	12
AAAATTTGC	BRDN00012	121	67	276	267	249	55
AAAATTTGG	BRDN00011	209	241	193	364	508	10
AAAATTTGG	BRDN00012	267	256	229	541	726	47
AAAATTTGT	BRDN00012	108	23	23	402	532	0
AAAATTTGT	BRDN00011	158	167	422	234	235	17
AAACAAAAC	BRDN00012	265	226	408	481	657	0
AAACAAAAG	BRDN00012	137	0	2	431	376	3
AAACAAAAG	BRDN00011	116	54	44	271	160	15
AAACAAAAG	BRDN00011	296	288	380	483	794	11
AAACAAAAG	BRDN00012	109	44	0	273	249	13
AAACAAAAT	BRDN00011	202	178	734	416	387	17
AAACAAACC	BRDN00012	39	38	0	626	248	0
AAACAAACG	BRDN00012	386	405	145	883	1254	86
AAACAAAGG	BRDN00012	79	9	54	255	110	4
AAACAAAGG	BRDN00011	520	429	1932	737	868	45
AAACAAAGG	BRDN00012	212	116	433	266	245	0
AAACAAATA	BRDN00012	169	163	299	420	343	28
AAACAAATG	BRDN00012	213	281	62	491	458	28
AAACAAATG	BRDN00011	149	244	277	216	150	4
AAACAACAA	BRDN00011	223	243	151	263	309	83
AAACAACAC	BRDN00012	232	223	810	417	514	2
AAACAACAT	BRDN00012	199	81	45	459	517	26
AAACAACCA	BRDN00011	406	374	256	1074	1146	54
AAACAACCC	BRDN00012	498	264	1218	675	709	54
AAACAACCT	BRDN00011	88	76	65	264	160	62
AAACAACGC	BRDN00011	138	139	108	323	240	62
AAACAAGAA	BRDN00012	131	113	1	236	164	0
AAACAAGAA	BRDN00011	214	182	171	482	591	16
AAACAAGAA	BRDN00012	249	272	181	546	534	3
AAACAAGTG	BRDN00012	140	139	15	279	231	0
AAACAAGTG	BRDN00011	48	32	4	152	18	16
AAACAATCA	BRDN00012	384	235	196	591	730	24
AAACAATCA	BRDN00012	243	132	1076	362	602	51
AAACAATCC	BRDN00012	232	238	1012	485	542	224
AAACAATCT	BRDN00012	132	138	0	329	523	28
AAACAATGC	BRDN00012	357	18	6	757	812	1
AAACAATGT	BRDN00011	119	76	0	194	233	0

AAACAATTC	BRDN00011	406	439	3268	888	777	52
AAACAATTCT	BRDN00011	86	169	172	230	193	255
AAACACAAA	BRDN00012	290	75	26	587	570	8
AAACACAACT	BRDN00011	225	24	0	434	452	0
AAACACAATT	BRDN00012	220	120	50	557	291	60
AAACACACAT	BRDN00012	192	146	100	323	483	0
AAACACACAT	BRDN00012	447	353	527	807	850	30
AAACACACTC	BRDN00011	100	108	11	236	379	0
AAACACAGG	BRDN00012	199	141	46	389	266	10
AAACACATA	BRDN00012	390	33	33	982	566	0
AAACACATC	BRDN00012	76	14	1	689	456	0
AAACACATC	BRDN00011	138	172	30	412	441	39
AAACACATCT	BRDN00012	260	220	166	428	499	10
AAACACCAA	BRDN00011	327	204	505	414	404	12
AAACACCAG	BRDN00011	397	464	791	993	867	41
AAACACCATA	BRDN00012	358	334	1664	661	620	42
AAACACCCG	BRDN00011	99	120	65	279	265	0
AAACACCGA	BRDN00012	450	533	138	810	881	93
AAACACGAA	BRDN00011	158	173	335	413	510	61
AAACACGATT	BRDN00011	87	119	26	187	272	0
AAACACGCA	BRDN00011	193	290	297	392	504	85
AAACACGCCT	BRDN00011	406	456	1398	817	880	57
AAACACGGC	BRDN00011	234	170	109	402	493	23
AAACACTCA	BRDN00011	45	1	1	282	133	0
AAACACTCA	BRDN00012	182	132	79	336	322	35
AAACACTCCG	BRDN00011	335	265	736	642	544	23
AAACACTGG	BRDN00011	400	535	347	574	778	54
AAACACTTAC	BRDN00012	146	150	24	231	312	24
AAACACTTGA	BRDN00011	165	141	69	385	532	7
AAACACTTTA	BRDN00012	124	92	118	235	445	27
AAACACTTTC	BRDN00011	350	203	240	630	453	12
AAACAGAGG	BRDN00011	201	198	66	520	396	0
AAACAGATT	BRDN00011	143	162	353	288	190	30
AAACAGCAC	BRDN00012	180	2	0	249	61	0
AAACAGCAG	BRDN00011	144	208	1606	333	359	57
AAACAGCAG	BRDN00011	190	216	230	369	409	17
AAACAGCAT	BRDN00012	191	0	0	459	329	0
AAACAGCAT	BRDN00011	347	479	573	732	728	44
AAACAGCTA	BRDN00011	159	236	155	292	630	10
AAACAGGAA	BRDN00012	294	238	253	491	569	22
AAACAGGAA	BRDN00011	390	276	625	562	567	23
AAACAGGCG	BRDN00011	287	165	43	666	676	16
AAACAGGCT	BRDN00012	126	26	0	299	117	0
AAACAGGGA	BRDN00012	190	224	104	415	488	11
AAACAGGGA	BRDN00011	102	83	351	309	122	24
AAACAGGTG	BRDN00011	262	192	364	710	644	18
AAACAGGTG	BRDN00012	248	236	1714	427	310	43
AAACAGTAG	BRDN00012	138	165	351	258	395	7
AAACAGTCA	BRDN00011	211	59	0	540	324	0
AAACAGTGG	BRDN00012	279	390	351	496	523	63
AAACAGTGT	BRDN00011	232	336	702	574	608	66
AAACAGTTA	BRDN00011	160	164	5	180	203	16
AAACATAAA	BRDN00012	214	211	1	366	498	0
AAACATAAA	BRDN00012	227	168	666	417	391	31
AAACATAAA	BRDN00012	105	63	0	269	147	0
AAACATACG	BRDN00012	271	279	450	409	357	20
AAACATAGG	BRDN00011	85	143	121	263	135	0
AAACATAGT	BRDN00011	156	145	143	318	595	10
AAACATCAC	BRDN00011	244	159	503	540	1361	30
AAACATCCAT	BRDN00011	142	115	152	212	257	0
AAACATCGT	BRDN00011	673	331	1861	1331	1401	47
AAACATCTCA	BRDN00012	234	179	62	270	282	0
AAACATCTCC	BRDN00011	88	14	0	205	153	0
AAACATCTG	BRDN00011	129	112	229	317	142	13
AAACATCTTG	BRDN00012	61	0	0	277	282	0
AAACATGAC	BRDN00012	166	129	277	363	227	24
AAACATGAT	BRDN00012	101	31	1	627	374	0
AAACATGCA	BRDN00012	368	408	985	584	621	23
AAACATGCG	BRDN00012	377	228	128	558	746	22
AAACATGGA	BRDN00012	203	227	674	552	459	10
AAACATGTG	BRDN00011	161	168	2392	277	364	64
AAACATTAC	BRDN00012	413	421	1018	678	867	82
AAACATTCG	BRDN00012	133	64	0	294	91	0
AAACATTTG	BRDN00012	255	89	596	439	467	26
AAACCAAAA	BRDN00011	240	159	750	423	599	8
AAACCAAAG	BRDN00011	200	214	249	455	509	13
AAACCAAAT	BRDN00012	232	243	646	597	821	30
AAACCAAATT	BRDN00011	164	185	424	362	383	38
AAACCAACAT	BRDN00012	285	217	17	475	741	15
AAACCAACCT	BRDN00012	364	260	440	875	884	1
AAACCAACG	BRDN00011	128	92	165	244	250	34
AAACCAACTC	BRDN00012	179	215	146	152	402	15
AAACCAATCC	BRDN00012	167	124	620	295	375	25
AAACCAATG	BRDN00012	85	14	0	265	358	0
AAACCAATG	BRDN00012	368	293	1499	711	586	59
AAACCACAAT	BRDN00011	110	72	0	169	333	0
AAACCACAG	BRDN00012	356	312	1536	790	501	105
AAACCACCAC	BRDN00011	121	135	208	712	485	9
AAACCACCTA	BRDN00011	266	323	78	471	309	53
AAACCACCTC	BRDN00012	133	26	73	509	350	0
AAACCACTAC	BRDN00011	283	256	253	317	352	3
AAACCAGCA	BRDN00012	559	467	1143	1044	1072	80
AAACCAGCA	BRDN00011	168	282	66	304	239	20
AAACCAGGG	BRDN00011	523	330	901	1006	1018	44
AAACCAGGG	BRDN00011	239	136	391	426	582	24
AAACCAGTA	BRDN00012	182	168	16	246	134	9
AAACCATCTG	BRDN00012	5	0	0	0	0	0
AAACCATTCT	BRDN00012	213	209	225	469	537	27
AAACCCAAA	BRDN00011	287	139	276	462	411	32
AAACCCAAAT	BRDN00012	119	101	304	229	138	12
AAACCCAAC	BRDN00012	501	420	949	959	704	73
AAACCCACCT	BRDN00012	111	195	13	264	211	12
AAACCCAGA	BRDN00012	266	6	28	516	436	0
AAACCCATA	BRDN00011	464	433	560	916	1005	34
AAACCCATAT	BRDN00012	227	360	1306	355	507	12
AAACCCATC	BRDN00012	200	310	352	421	321	32
AAACCCATTC	BRDN00012	138	45	518	246	146	22
AAACCCATTT	BRDN00011	175	140	272	285	264	48
AAACCCCAAT	BRDN00011	54	76	13	229	115	22
AAACCCTATG	BRDN00012	601	613	411	942	1020	80
AAACCCTCCG	BRDN00011	122	7	0	621	230	0
AAACCCTGC	BRDN00011	363	369	880	646	905	51
AAACCCTTAC	BRDN00011	349	397	577	646	762	36
AAACCCTTCA	BRDN00012	461	499	798	1003	1319	27
AAACCGAAA	BRDN00011	162	128	415	270	395	14
AAACCGACA	BRDN00012	435	324	631	823	742	23
AAACCGAGA	BRDN00012	105	51	972	216	519	38
AAACCGCTT	BRDN00011	198	146	528	374	316	14
AAACCGGGC	BRDN00012	320	228	359	398	517	32
AAACCGGTA	BRDN00011	127	49	59	169	127	9
AAACCGTAA	BRDN00011	249	122	1427	502	342	28
AAACCGTGA	BRDN00011	312	295	449	607	349	23
AAACCGTTCT	BRDN00012	154	116	8	318	243	9
AAACCTAAA	BRDN00012	375	350	176	601	700	32
AAACCTACCG	BRDN00011	233	187	93	469	350	26
AAACCTACGT	BRDN00011	177	78	13	124	156	26
AAACCTAGC	BRDN00012	585	638	1072	897	679	46
AAACCTAGCT	BRDN00011	86	101	9	318	146	0
AAACCTCAA	BRDN00012	284	255	166	549	532	31
AAACCTCACA	BRDN00012	353	354	76	679	588	32
AAACCTCCAC	BRDN00011	157	93	0	309	230	0
AAACCTCCG	BRDN00011	261	263	199	583	946	19
AAACCTCTAC	BRDN00011	292	41	0	715	1136	1
AAACCTCTTT	BRDN00011	295	102	323	583	544	1
AAACCTGAG	BRDN00011	115	69	1208	186	105	9
AAACCTGGA	BRDN00011	549	504	1433	855	810	121
AAACCTGGT	BRDN00011	131	49	211	368	343	2
AAACCTGTA	BRDN00012	438	397	285	685	700	6
AAACCTGTG	BRDN00012	516	433	223	1010	1123	45
AAACCTGTTC	BRDN00011	197	202	223	314	409	7
AAACCTTCG	BRDN00012	78	57	46	172	288	0
AAACCTTGCC	BRDN00012	338	262	196	563	680	3
AAACCTTGG	BRDN00012	142	0	0	389	113	0
AAACGAAAG	BRDN00011	63	5	0	139	272	0
AAACGAACA	BRDN00012	129	177	734	261	241	50
AAACGAAGC	BRDN00012	99	91	35	241	175	6
AAACGAATG	BRDN00012	164	213	121	365	191	7
AAACGAATT	BRDN00011	136	31	129	367	146	0
AAACGAGAA	BRDN00012	407	539	664	752	522	29
AAACGAGAT	BRDN00012	657	732	1557	990	1485	95
AAACGAGGA	BRDN00011	287	196	108	540	337	40
AAACGAGGC	BRDN00011	150	185	38	336	326	29
AAACGCATC	BRDN00012	218	196	227	338	285	12
AAACGCATC	BRDN00011	138	138	3	236	368	9
AAACGCATG	BRDN00012	112	68	429	247	159	16
AAACGCCCA	BRDN00011	246	281	384	793	591	27
AAACGCCCTT	BRDN00012	158	134	210	276	338	19
AAACGCCGG	BRDN00011	599	612	861	1120	1421	79
AAACGCTGG	BRDN00012	418	484	3504	811	598	55
AAACGCTTC	BRDN00012	212	186	201	353	157	0
AAACGGAAA	BRDN00012	327	379	467	649	1237	49
AAACGGAAC	BRDN00012	162	71	2	81	106	31
AAACGGAAC	BRDN00011	215	183	87	455	334	0
AAACGGAAT	BRDN00011	211	109	226	482	295	4
AAACGGAGG	BRDN00011	230	202	313	372	239	0
AAACGGCAG	BRDN00012	483	416	586	922	901	16
AAACGGGCC	BRDN00012	449	347	1907	771	846	34
AAACGGGCG	BRDN00011	397	325	1161	916	738	28
AAACGGTAC	BRDN00012	598	706	1716	1036	1167	91
AAACGGTCA	BRDN00012	384	37	5	667	736	0
AAACGGTGC	BRDN00012	159	167	38	312	383	33
AAACGGTGT	BRDN00011	121	37	64	104	336	29
AAACGGTGT	BRDN00011	127	141	259	302	444	2
AAACGTAGC	BRDN00012	358	289	417	634	713	9
AAACGTCAT	BRDN00011	145	13	110	334	205	0
AAACGTGAC	BRDN00011	75	70	2	223	271	4
AAACGTGGT	BRDN00011	413	288	3621	753	658	125
AAACGTTTC	BRDN00012	187	237	513	417	216	21
AAACTAACTT	BRDN00012	128	4	0	437	344	0
AAACTAATG	BRDN00012	77	130	32	317	223	0
AAACTAATTC	BRDN00011	192	207	664	332	412	10
AAACTACAA	BRDN00012	105	88	0	304	407	0
AAACTACATT	BRDN00012	173	112	14	267	306	0
AAACTACGG	BRDN00012	419	439	635	592	546	33
AAACTATAG	BRDN00011	168	186	116	329	250	15
AAACTATGC	BRDN00011	137	283	109	271	185	21
AAACTATTGT	BRDN00012	180	191	71	304	147	0
AAACTATTTG	BRDN00012	283	310	407	538	374	106
AAACTCAATA	BRDN00011	334	273	425	685	709	40
AAACTCAATC	BRDN00012	53	0	0	265	285	0
AAACTCACCA	BRDN00012	48	8	0	293	144	0
AAACTCACCC	BRDN00012	454	525	1429	744	608	59
AAACTCACTT	BRDN00012	380	520	738	792	831	30
AAACTCAGG	BRDN00012	204	203	265	436	420	12
AAACTCAGTC	BRDN00011	219	155	57	561	349	6
AAACTCAGTC	BRDN00011	206	124	17	373	233	0
AAACTCATG	BRDN00011	116	54	494	220	238	18
AAACTCCTCA	BRDN00012	464	414	1314	954	862	50
AAACTCTGTC	BRDN00012	215	78	78	649	454	0
AAACTGACA	BRDN00012	318	307	304	559	427	120
AAACTGACC	BRDN00012	301	401	807	491	640	23
AAACTGACCT	BRDN00011	109	159	0	203	196	33
AAACTGAGG	BRDN00012	326	365	810	477	446	61
AAACTGATA	BRDN00011	119	107	744	129	307	15
AAACTGCAA	BRDN00011	167	161	176	324	200	6
AAACTGCAC	BRDN00012	90	74	11	211	378	0
AAACTGCAG	BRDN00012	188	96	310	192	229	9
AAACTGCAG	BRDN00012	492	416	633	965	1121	40
AAACTGCAG	BRDN00011	175	66	318	221	285	25
AAACTGCAT	BRDN00012	193	153	37	374	444	29
AAACTGCCG	BRDN00012	270	182	169	454	533	35
AAACTGCGC	BRDN00012	439	262	981	737	589	24
AAACTGCGG	BRDN00011	179	19	1	432	189	0
AAACTGCTG	BRDN00011	135	126	53	291	124	18
AAACTGGAT	BRDN00011	97	90	54	165	258	20
AAACTGGGA	BRDN00012	258	368	1445	439	357	64
AAACTGTAA	BRDN00011	291	69	122	573	360	13
AAACTGTAG	BRDN00012	555	490	650	839	891	57
AAACTGTCA	BRDN00012	479	408	116	943	1106	31
AAACTGTTG	BRDN00012	148	81	60	262	167	0
AAACTTAAA	BRDN00011	97	39	188	165	234	20
AAACTTACA	BRDN00011	290	173	85	638	628	0
AAACTTACC	BRDN00011	64	37	0	210	308	0
AAACTTACCG	BRDN00012	154	16	1	525	382	1
AAACTTACTA	BRDN00011	22	0	0	239	220	0
AAACTTACTC	BRDN00011	71	19	3	144	61	25
AAACTTCGA	BRDN00011	337	250	1830	576	566	68
AAACTTCTCA	BRDN00012	489	484	1303	676	651	52
AAACTTGCG	BRDN00011	131	107	65	230	65	1
AAACTTGGA	BRDN00012	166	264	24	523	664	27
AAACTTGGC	BRDN00012	163	33	1	905	412	0
AAACTTGTCA	BRDN00011	533	646	370	1007	1717	153
AAACTTGTCC	BRDN00011	251	273	225	495	869	18
AAACTTTACA	BRDN00012	137	79	18	428	360	6
AAACTTTATT	BRDN00012	220	173	212	522	556	25
AAACTTTCTA	BRDN00011	148	147	40	252	295	4
AAAGAAAAA	BRDN00011	152	8	121	239	179	0
AAAGAAAAA	BRDN00012	227	296	2138	443	288	40
AAAGAAAAA	BRDN00011	177	153	689	354	250	22
AAAGAAAAC	BRDN00011	174	270	401	390	414	58
AAAGAAAAC	BRDN00012	115	28	149	224	193	7
AAAGAAAAG	BRDN00011	296	334	121	661	489	25
AAAGAAACA	BRDN00012	104	86	9	134	120	4
AAAGAAACA	BRDN00011	117	124	84	237	254	10
AAAGAAACC	BRDN00011	77	22	0	404	135	0
AAAGAAAGA	BRDN00011	60	55	251	135	251	3
AAAGAAAGA	BRDN00011	108	250	107	251	344	49
AAAGAAAGA	BRDN00012	245	200	776	443	403	15
AAAGAAAGC	BRDN00011	243	216	271	578	643	21
AAAGAAAGC	BRDN00012	253	200	124	535	409	29
AAAGAAAGT	BRDN00011	164	198	38	312	302	14
AAAGAAATG	BRDN00011	124	74	171	264	236	1
AAAGAAATT	BRDN00012	255	163	119	332	835	113
AAAGAAATT	BRDN00012	274	56	125	505	420	12
AAAGAACAA	BRDN00011	194	127	766	193	115	20
AAAGAACAA	BRDN00011	38	18	0	124	192	0
AAAGAACAC	BRDN00011	83	118	91	162	127	63
AAAGAACAG	BRDN00011	95	54	1592	135	176	6
AAAGAACAT	BRDN00012	368	338	1721	510	483	64
AAAGAACAT	BRDN00011	135	123	148	328	307	0
AAAGAACCA	BRDN00012	132	106	243	323	670	16
AAAGAACTT	BRDN00012	69	58	180	195	150	24
AAAGAAGAA	BRDN00011	182	133	413	292	685	15
AAAGAAGAC	BRDN00012	89	5	26	190	440	0
AAAGAAGAG	BRDN00012	276	217	25	515	381	29
AAAGAAGAG	BRDN00012	205	129	129	412	627	2
AAAGAAGCT	BRDN00012	257	234	915	385	406	23
AAAGAAGCT	BRDN00011	297	195	244	499	577	23
AAAGAAGGA	BRDN00012	151	102	161	433	195	26
AAAGAAGTC	BRDN00011	184	121	35	341	396	0
AAAGAATAG	BRDN00011	254	187	524	315	286	26
AAAGAATAG	BRDN00012	85	136	159	218	181	12
AAAGAATCA	BRDN00012	378	150	321	695	1095	21
AAAGAATGA	BRDN00012	210	184	15	469	443	16
AAAGAATTC	BRDN00011	182	321	976	402	429	76
AAAGACAAA	BRDN00012	450	274	302	706	977	11
AAAGACAAA	BRDN00011	347	344	480	488	761	33
AAAGACAAA	BRDN00011	114	97	1234	196	197	32
AAAGACAAG	BRDN00012	444	349	1103	864	722	34
AAAGACAAT	BRDN00011	99	155	279	164	299	8
AAAGACACT	BRDN00012	198	172	301	333	365	11
AAAGACAGA	BRDN00011	145	46	0	339	191	0
AAAGACAGA	BRDN00011	146	13	0	167	98	0
AAAGACAGC	BRDN00012	294	315	77	651	491	18
AAAGACAGG	BRDN00012	146	5	23	460	75	0
AAAGACAGG	BRDN00011	46	10	0	210	157	0
AAAGACAGT	BRDN00011	381	326	521	430	389	6
AAAGACATA	BRDN00012	193	198	202	372	400	31
AAAGACATC	BRDN00011	164	173	310	319	221	126
AAAGACATG	BRDN00012	126	104	101	215	179	18
AAAGACCAA	BRDN00011	132	111	50	249	388	4
AAAGACCAC	BRDN00011	104	145	309	206	197	20
AAAGACCAG	BRDN00012	171	173	1369	324	312	62
AAAGACCAG	BRDN00011	81	174	151	107	12	8
AAAGACCAT	BRDN00012	343	332	1510	688	914	26
AAAGACCCA	BRDN00011	524	578	4489	910	1256	102
AAAGACCGA	BRDN00011	85	126	107	236	163	39
AAAGACCTA	BRDN00012	145	100	230	293	849	0
AAAGACGAC	BRDN00011	248	171	87	487	517	7
AAAGACGAG	BRDN00012	66	5	0	227	46	0
AAAGACGCC	BRDN00011	360	370	89	785	756	0
AAAGACGGA	BRDN00012	277	176	245	472	767	47
AAAGACGGC	BRDN00012	185	197	323	331	261	54
AAAGACGTA	BRDN00012	211	290	256	433	543	17
AAAGACGTG	BRDN00012	253	129	149	420	211	19
AAAGACGTT	BRDN00012	141	136	150	209	426	0
AAAGACTCA	BRDN00011	187	186	280	471	319	0
AAAGACTCT	BRDN00011	288	174	554	689	365	28
AAAGACTGA	BRDN00012	159	123	0	367	588	8
AAAGACTGC	BRDN00011	336	261	228	517	1535	9
AAAGACTGT	BRDN00011	109	123	83	179	105	18
AAAGACTTA	BRDN00011	96	153	442	161	221	11
AAAGACTTC	BRDN00012	321	123	1315	874	785	27
AAAGACTTG	BRDN00012	375	319	436	559	542	73
AAAGAGAAA	BRDN00011	107	69	0	226	194	17
AAAGAGAAA	BRDN00012	58	8	0	89	143	11
AAAGAGAAT	BRDN00011	71	75	935	268	222	0
AAAGAGAAT	BRDN00012	161	130	257	273	301	14
AAAGAGAAT	BRDN00012	314	338	620	608	587	51
AAAGAGACC	BRDN00012	56	7	0	676	365	0
AAAGAGAGT	BRDN00012	141	13	0	384	547	0
AAAGAGCAA	BRDN00012	108	101	165	169	295	0
AAAGAGCAC	BRDN00011	140	144	167	199	211	0
AAAGAGCAC	BRDN00012	375	285	581	649	1571	58
AAAGAGCAG	BRDN00012	336	250	369	633	736	44
AAAGAGCAG	BRDN00012	131	209	65	292	153	3
AAAGAGGAA	BRDN00011	212	155	5040	484	468	69
AAAGAGGAC	BRDN00012	265	217	165	562	494	16
AAAGAGGAT	BRDN00011	73	0	0	204	200	0
AAAGAGGCT	BRDN00012	44	19	58	351	193	0
AAAGAGGGA	BRDN00011	209	3	0	409	240	0
AAAGAGGTA	BRDN00012	213	137	359	389	287	15
AAAGAGGTT	BRDN00011	187	146	324	441	445	18
AAAGAGTAA	BRDN00012	193	212	63	305	194	12
AAAGAGTAT	BRDN00012	223	257	620	374	327	64
AAAGAGTGT	BRDN00011	78	161	0	220	85	0
AAAGAGTTA	BRDN00011	39	18	15	122	60	0
AAAGAGTTA	BRDN00011	120	119	372	139	180	28
AAAGATAAA	BRDN00012	205	131	119	379	628	15
AAAGATAAG	BRDN00011	163	61	39	334	404	0
AAAGATAGG	BRDN00011	276	148	300	560	680	11
AAAGATAGT	BRDN00012	180	70	64	631	241	0
AAAGATATA	BRDN00011	110	0	0	147	183	0
AAAGATATA	BRDN00012	212	198	28	307	375	8
AAAGATATG	BRDN00011	133	196	1201	273	246	3
AAAGATCAA	BRDN00011	199	141	286	393	332	8
AAAGATCAA	BRDN00012	126	232	160	260	345	17
AAAGATCAA	BRDN00011	392	375	277	500	357	2
AAAGATCAC	BRDN00011	350	383	1272	752	870	14
AAAGATCCA	BRDN00011	312	267	282	542	523	0
AAAGATCCT	BRDN00011	230	296	123	422	254	39
AAAGATCTT	BRDN00012	390	418	102	619	430	1
AAAGATGAC	BRDN00011	118	168	0	237	182	5
AAAGATGAC	BRDN00012	466	505	1722	564	503	28
AAAGATGAG	BRDN00011	212	197	4	453	127	18
AAAGATGAT	BRDN00011	56	95	0	115	180	15
AAAGATGAT	BRDN00012	347	91	115	636	607	6
AAAGATGGC	BRDN00012	435	449	618	724	464	61
AAAGATGTA	BRDN00011	153	175	1806	420	337	11
AAAGATGTC	BRDN00012	385	546	856	696	898	64
AAAGATGTG	BRDN00011	153	164	665	201	376	4
AAAGATGTG	BRDN00011	117	90	194	230	314	5
AAAGATTAG	BRDN00011	131	201	8	374	301	15
AAAGATTAT	BRDN00011	134	182	0	190	408	7
AAAGATTCT	BRDN00012	390	173	154	563	547	19
AAAGATTTA	BRDN00012	58	201	60	198	177	10
AAAGATTTG	BRDN00012	150	63	106	322	430	0
AAAGATTTG	BRDN00012	444	406	148	806	1130	57
AAAGCAAAA	BRDN00011	48	55	0	190	103	22
AAAGCAAAC	BRDN00012	139	180	122	355	776	30
AAAGCAAAG	BRDN00011	114	0	0	251	48	0
AAAGCAAAG	BRDN00012	246	205	1063	376	410	43
AAAGCAAAG	BRDN00011	108	131	128	510	520	1
AAAGCAAAT	BRDN00011	458	408	450	691	639	13
AAAGCAACA	BRDN00011	132	264	41	293	423	4
AAAGCAAGA	BRDN00011	84	56	0	218	62	0
AAAGCAAGC	BRDN00012	250	177	294	337	303	17
AAAGCAAGG	BRDN00012	159	186	108	205	216	3
AAAGCAAGT	BRDN00011	284	19	472	471	668	9
AAAGCAATC	BRDN00011	79	94	718	136	80	14
AAAGCACAC	BRDN00011	422	374	515	769	502	21
AAAGCACAG	BRDN00012	237	207	652	425	524	29
AAAGCACAT	BRDN00012	117	149	296	245	177	16
AAAGCACAT	BRDN00011	122	31	35	279	348	0
AAAGCACAT	BRDN00012	184	97	24	352	445	15
AAAGCACCA	BRDN00011	116	130	526	250	640	15
AAAGCACCA	BRDN00011	57	0	0	291	45	0
AAAGCACTA	BRDN00011	105	102	63	216	168	13
AAAGCACTG	BRDN00011	121	4	0	267	186	0
AAAGCAGAA	BRDN00011	94	0	1	349	308	0
AAAGCAGAG	BRDN00012	399	270	1493	773	1043	31
AAAGCAGGA	BRDN00012	297	363	783	637	614	82
AAAGCAGGT	BRDN00011	212	244	457	436	208	18
AAAGCAGTC	BRDN00012	177	167	323	229	350	58
AAAGCAGTG	BRDN00012	424	321	710	652	321	22
AAAGCAGTG	BRDN00012	105	133	138	324	418	0
AAAGCAGTT	BRDN00011	212	28	55	379	472	1
AAAGCATAC	BRDN00011	34	0	0	333	275	0
AAAGCATAT	BRDN00011	78	8	82	182	198	0
AAAGCATAT	BRDN00011	170	170	3533	285	629	70
AAAGCATCA	BRDN00012	167	169	160	326	206	9
AAAGCATCC	BRDN00011	125	153	158	187	260	5
AAAGCATCT	BRDN00012	182	215	695	232	111	42
AAAGCATGT	BRDN00011	108	93	128	326	315	19
AAAGCATTC	BRDN00012	178	130	358	294	165	26
AAAGCATTC	BRDN00011	37	1	8	467	924	0
AAAGCATTG	BRDN00012	209	118	273	292	436	22
AAAGCATTG	BRDN00012	30	38	0	530	169	11
AAAGCATTT	BRDN00012	201	342	165	492	394	3
AAAGCCAAA	BRDN00011	367	337	415	661	781	23
AAAGCCAAA	BRDN00012	130	126	73	251	286	0
AAAGCCAAA	BRDN00011	54	87	38	236	103	0
AAAGCCAAC	BRDN00012	150	0	0	559	471	0
AAAGCCAAT	BRDN00011	169	183	0	280	274	18
AAAGCCACC	BRDN00012	150	154	27	176	220	0
AAAGCCACT	BRDN00011	180	147	94	193	342	34
AAAGCCAGC	BRDN00012	319	326	367	597	993	8
AAAGCCAGC	BRDN00012	448	464	901	849	606	34
AAAGCCAGT	BRDN00011	145	81	356	261	316	18
AAAGCCATA	BRDN00012	317	27	0	451	255	0
AAAGCCCAA	BRDN00011	394	357	228	841	1433	0
AAAGCCCAA	BRDN00011	362	398	301	624	577	21
AAAGCCCAG	BRDN00011	186	97	23	253	232	0
AAAGCCCCC	BRDN00012	183	243	233	248	126	15
AAAGCCCCT	BRDN00011	99	0	0	191	155	0
AAAGCCCGC	BRDN00012	451	231	337	792	497	10
AAAGCCCGG	BRDN00011	318	247	354	468	584	7
AAAGCCCGG	BRDN00011	228	267	1029	472	513	47
AAAGCCCGT	BRDN00011	126	141	39	435	262	18
AAAGCCCTG	BRDN00011	161	99	9	245	592	22
AAAGCCCTT	BRDN00011	84	53	141	122	98	0
AAAGCCGAG	BRDN00012	67	68	96	249	212	22
AAAGCCGCA	BRDN00012	349	363	160	540	521	32
AAAGCCGTC	BRDN00012	271	355	411	605	644	38
AAAGCCTAA	BRDN00011	278	37	0	382	207	0
AAAGCCTAG	BRDN00012	187	307	123	578	560	37
AAAGCCTAT	BRDN00012	144	42	930	576	460	21
AAAGCCTCA	BRDN00011	154	143	80	315	227	0
AAAGCCTCT	BRDN00012	228	273	572	268	292	46
AAAGCCTGG	BRDN00011	239	93	482	407	476	8
AAAGCCTGG	BRDN00011	265	108	0	617	512	0
AAAGCCTTC	BRDN00011	140	97	50	161	204	5
AAAGCCTTC	BRDN00012	534	389	1455	746	833	75
AAAGCCTTG	BRDN00011	88	64	446	213	122	0
AAAGCCTTT	BRDN00011	146	96	8	441	388	0
AAAGCGACG	BRDN00012	376	382	1640	629	333	62
AAAGCGAGT	BRDN00012	139	135	160	367	393	7
AAAGCGATT	BRDN00011	418	393	603	649	1032	49
AAAGCGCAA	BRDN00011	212	252	572	403	436	17
AAAGCGCAA	BRDN00012	150	185	166	284	534	14
AAAGCGCAC	BRDN00012	186	230	1027	384	222	51
AAAGCGCGC	BRDN00011	48	7	1	237	169	0
AAAGCGCTT	BRDN00011	131	95	94	201	321	21
AAAGCGGAG	BRDN00012	352	222	152	957	1180	13
AAAGCGGCG	BRDN00012	199	143	678	253	405	32

AAAGCGGGA	BRDN00012	270	271	773	455	637	44
AAAGCGGGA	BRDN00012	115	150	580	315	308	8
AAAGCGGTC	BRDN00012	344	244	801	600	499	19
AAAGCGTAA	BRDN00012	201	343	248	413	563	36
AAAGCGTCC	BRDN00012	238	190	1507	307	715	79
AAAGCGTGA	BRDN00012	257	151	699	265	309	32
AAAGCGTGA	BRDN00011	390	433	1340	870	798	26
AAAGCGTGA	BRDN00011	148	118	56	308	218	12
AAAGCGTGA	BRDN00012	390	222	1047	672	647	13
AAAGCGTGT	BRDN00011	167	175	1269	248	154	52
AAAGCGTTA	BRDN00011	220	254	320	544	514	0
AAAGCTACC	BRDN00012	200	179	748	343	251	20
AAAGCTACT	BRDN00012	349	346	384	664	983	27
AAAGCTCCA	BRDN00012	326	234	272	442	470	16
AAAGCTCCA	BRDN00012	60	21	0	249	152	0
AAAGCTCCT	BRDN00012	228	153	63	275	568	0
AAAGCTCTA	BRDN00012	199	135	4152	349	394	49
AAAGCTCTTC	BRDN00012	179	173	494	364	285	46
AAAGCTGAA	BRDN00011	281	269	222	782	1033	33
AAAGCTGAC	BRDN00012	285	222	255	378	390	11
AAAGCTGAT	BRDN00012	102	58	2	252	211	24
AAAGCTGGC	BRDN00012	80	19	0	164	177	0
AAAGCTGTC	BRDN00011	249	333	1762	415	460	15
AAAGCTGTG	BRDN00012	254	359	81	413	410	0
AAAGCTGTT	BRDN00012	258	320	378	485	526	65
AAAGCTGTT	BRDN00012	456	506	822	824	901	96
AAAGCTTAA	BRDN00012	243	189	1843	388	626	27
AAAGCTTAC	BRDN00012	180	10	0	569	443	0
AAAGCTTAT	BRDN00012	421	116	309	686	599	9
AAAGCTTCC	BRDN00011	354	390	445	606	673	39
AAAGCTTCG	BRDN00011	160	118	271	314	261	0
AAAGCTTCT	BRDN00011	128	43	16	288	165	4
AAAGCTTGT	BRDN00011	203	161	280	417	786	6
AAAGCTTTA	BRDN00011	187	0	0	628	761	0
AAAGGAAAC	BRDN00012	245	208	192	437	1106	20
AAAGGAAAT	BRDN00011	57	43	118	100	107	7
AAAGGAACT	BRDN00011	333	383	3262	490	483	49
AAAGGAAGC	BRDN00012	373	334	371	786	499	8
AAAGGAATG	BRDN00011	165	206	318	396	651	15
AAAGGAATT	BRDN00012	116	157	203	200	177	11
AAAGGAATT	BRDN00012	264	231	330	506	294	49
AAAGGACAA	BRDN00012	161	166	24	338	542	31
AAAGGACAA	BRDN00012	252	148	4	498	317	0
AAAGGACAG	BRDN00012	219	134	211	674	544	20
AAAGGACCC	BRDN00011	110	202	95	304	201	22
AAAGGACCT	BRDN00012	244	277	177	506	305	9
AAAGGACCT	BRDN00011	168	178	205	363	480	78
AAAGGACGA	BRDN00011	339	296	324	427	651	87
AAAGGACGG	BRDN00011	29	0	0	280	78	0
AAAGGACTC	BRDN00012	361	438	985	675	573	41
AAAGGACTG	BRDN00011	220	200	302	393	386	26
AAAGGACTG	BRDN00012	87	83	71	243	197	10
AAAGGACTT	BRDN00011	98	162	189	214	322	20
AAAGGAGA	BRDN00012	131	53	109	639	421	2
AAAGGAGCA	BRDN00012	173	41	81	498	192	0
AAAGGAGTC	BRDN00011	141	94	38	305	234	0
AAAGGAGTC	BRDN00012	116	175	22	282	796	20
AAAGGATAA	BRDN00011	178	235	284	319	342	20
AAAGGATCC	BRDN00012	320	290	1008	688	834	66
AAAGGATGA	BRDN00012	351	316	2122	610	659	34
AAAGGATGG	BRDN00012	97	0	1	427	195	0
AAAGGATGG	BRDN00011	314	317	707	598	420	24
AAAGGATTC	BRDN00012	356	392	1658	619	257	57
AAAGGATTC	BRDN00012	293	256	117	539	503	10
AAAGGATTG	BRDN00011	350	299	807	613	570	12
AAAGGATTG	BRDN00012	322	264	215	426	452	28
AAAGGATTT	BRDN00011	316	74	57	565	457	0
AAAGGCAAA	BRDN00012	345	360	1372	850	1082	56
AAAGGCAAT	BRDN00011	391	349	920	731	969	54
AAAGGCAAT	BRDN00011	134	141	126	273	245	25
AAAGGCACC	BRDN00011	132	127	207	174	264	18
AAAGGCACT	BRDN00011	77	42	26	176	73	24
AAAGGCAGG	BRDN00011	343	287	419	615	980	23
AAAGGCAGG	BRDN00012	273	132	1922	382	321	39
AAAGGCATA	BRDN00012	97	51	205	259	346	9
AAAGGCCCA	BRDN00012	433	605	443	836	788	9
AAAGGCCGG	BRDN00012	121	108	698	182	121	4
AAAGGCCTC	BRDN00011	208	182	37	384	222	0
AAAGGCGAG	BRDN00011	177	81	464	316	357	0
AAAGGCGAT	BRDN00011	78	10	31	151	114	3
AAAGGCGTC	BRDN00011	187	168	2076	395	408	33
AAAGGCTAA	BRDN00011	119	93	14	192	398	0
AAAGGCTAT	BRDN00011	266	180	9	414	744	51
AAAGGCTCA	BRDN00011	145	161	0	269	205	0
AAAGGCTCA	BRDN00011	488	285	323	849	955	27
AAAGGCTCG	BRDN00011	100	92	170	174	307	8
AAAGGCTCT	BRDN00012	136	136	1	230	238	9
AAAGGCTGA	BRDN00011	185	169	77	390	194	8
AAAGGCTGC	BRDN00011	420	443	399	878	922	40
AAAGGCTGT	BRDN00012	231	269	308	425	316	25
AAAGGCTTA	BRDN00012	115	0	0	360	221	0
AAAGGCTTG	BRDN00011	67	123	93	195	186	5
AAAGGCTTT	BRDN00011	344	561	690	853	1246	17
AAAGGCTTT	BRDN00011	133	151	106	194	189	0
AAAGGGAA	BRDN00012	208	207	371	316	447	16
AAAGGGAA	BRDN00012	12	0	0	314	121	0
AAAGGGATC	BRDN00011	279	241	323	577	692	19
AAAGGGATG	BRDN00011	392	460	298	669	668	25
AAAGGGCAG	BRDN00012	225	187	556	613	475	29
AAAGGGCAT	BRDN00012	300	483	886	542	468	32
AAAGGGCCA	BRDN00011	211	170	420	381	277	51
AAAGGGCCT	BRDN00011	170	193	112	256	614	21
AAAGGGCGA	BRDN00012	98	49	1	268	138	1
AAAGGGCTA	BRDN00012	380	375	450	487	419	17
AAAGGGCTG	BRDN00012	257	355	408	698	1147	4
AAAGGGGA	BRDN00012	180	79	219	223	161	7
AAAGGGGA	BRDN00011	83	78	231	147	130	24
AAAGGGGT	BRDN00011	151	271	105	409	128	95
AAAGGGGTC	BRDN00011	120	101	20	208	178	3
AAAGGGTAT	BRDN00012	111	91	294	250	200	9
AAAGGGTAT	BRDN00012	98	170	0	196	282	19
AAAGGGTCA	BRDN00011	156	122	250	252	220	6
AAAGGGTCA	BRDN00012	190	177	158	434	621	6
AAAGGGTGA	BRDN00012	464	421	797	737	894	49
AAAGGGTGA	BRDN00012	68	33	57	173	203	0
AAAGGGTTC	BRDN00011	242	157	42	348	330	9
AAAGGTAAA	BRDN00012	123	81	494	189	239	0
AAAGGTAAA	BRDN00011	378	387	707	628	920	108
AAAGGTAAG	BRDN00011	437	403	147	840	1465	15
AAAGGTAGG	BRDN00012	169	33	0	316	237	0
AAAGGTATG	BRDN00011	109	109	5	207	249	8
AAAGGTATG	BRDN00011	101	84	0	212	52	0
AAAGGTCAA	BRDN00012	123	109	419	223	229	0
AAAGGTCAG	BRDN00011	123	133	742	270	222	35
AAAGGTCAG	BRDN00011	236	300	164	567	558	28
AAAGGTCCG	BRDN00011	59	30	8	223	269	8
AAAGGTCTT	BRDN00012	17	0	0	648	166	0
AAAGGTGAT	BRDN00011	112	142	136	176	186	11
AAAGGTGGA	BRDN00012	296	229	505	381	548	28
AAAGGTGGC	BRDN00012	75	160	614	164	139	9
AAAGGTGGC	BRDN00012	475	461	2423	801	593	77
AAAGGTTCT	BRDN00012	326	261	172	611	795	0
AAAGGTTGG	BRDN00011	125	86	45	241	279	0
AAAGGTTTA	BRDN00011	323	305	2023	776	1090	56
AAAGGTTTC	BRDN00012	370	349	1503	696	834	30
AAAGTAAAC	BRDN00011	154	140	305	221	477	3
AAAGTAAAC	BRDN00011	146	112	259	318	269	11
AAAGTACAA	BRDN00012	280	276	107	549	524	28
AAAGTACAA	BRDN00012	99	126	110	260	496	18
AAAGTACAC	BRDN00011	252	309	1019	482	516	56
AAAGTACAG	BRDN00012	136	96	33	213	122	0
AAAGTACAG	BRDN00012	256	205	121	665	849	18
AAAGTACAT	BRDN00011	157	206	312	353	478	25
AAAGTACCA	BRDN00011	278	239	404	473	534	67
AAAGTACCT	BRDN00012	322	249	269	586	605	17
AAAGTACTC	BRDN00012	237	29	66	512	505	0
AAAGTACTG	BRDN00012	225	252	695	453	861	70
AAAGTACTG	BRDN00012	8	0	0	426	304	0
AAAGTACTTC	BRDN00011	160	216	1072	244	256	19
AAAGTACTT	BRDN00012	126	78	111	175	236	0
AAAGTAGAG	BRDN00012	217	83	187	337	630	0
AAAGTAGCC	BRDN00012	66	115	1	189	501	7
AAAGTAGTA	BRDN00012	132	72	2	143	287	7
AAAGTAGTA	BRDN00012	104	62	9	251	180	3
AAAGTATAT	BRDN00011	291	286	651	569	554	6
AAAGTATCA	BRDN00012	232	197	188	377	368	12
AAAGTATCG	BRDN00012	300	263	231	527	514	0
AAAGTATCT	BRDN00011	145	171	2438	297	434	51
AAAGTATGG	BRDN00011	159	95	685	290	406	27
AAAGTATTG	BRDN00012	272	322	647	556	490	66
AAAGTCAAA	BRDN00012	79	48	69	505	398	0
AAAGTCAAA	BRDN00012	102	83	527	140	234	26
AAAGTCAAC	BRDN00012	89	108	261	216	80	10
AAAGTCAAG	BRDN00012	213	111	189	468	446	7
AAAGTCACG	BRDN00011	88	155	43	260	428	16
AAAGTCACT	BRDN00012	139	109	0	267	170	1
AAAGTCAGT	BRDN00011	421	418	129	685	685	74
AAAGTCATA	BRDN00011	57	10	9	196	299	0
AAAGTCATA	BRDN00011	141	130	847	261	223	36
AAAGTCATC	BRDN00012	171	199	533	265	327	0
AAAGTCCAT	BRDN00011	114	138	1	268	246	0
AAAGTCCAT	BRDN00011	187	237	672	311	396	20
AAAGTCCGG	BRDN00012	363	218	130	734	491	2
AAAGTCCGT	BRDN00012	306	235	418	737	569	38
AAAGTCCTT	BRDN00012	181	192	162	395	309	22
AAAGTCGAA	BRDN00012	21	0	0	316	75	0
AAAGTCGAA	BRDN00012	105	123	198	222	105	22
AAAGTCGAA	BRDN00012	313	227	380	719	872	33
AAAGTCGAC	BRDN00012	171	243	327	362	298	12
AAAGTCGAG	BRDN00011	302	183	90	430	498	77
AAAGTCGAG	BRDN00011	322	352	153	712	857	4
AAAGTCGAT	BRDN00012	389	330	208	691	338	35
AAAGTCGCT	BRDN00012	290	201	428	374	404	42
AAAGTCGGG	BRDN00012	227	136	19	305	332	20
AAAGTCGGG	BRDN00011	223	114	893	400	352	29
AAAGTCGTA	BRDN00012	433	477	444	508	721	66
AAAGTCTTT	BRDN00012	253	169	579	395	305	0
AAAGTCTTT	BRDN00011	136	111	61	278	227	0
AAAGTGAAA	BRDN00012	173	207	808	404	515	23
AAAGTGAAC	BRDN00012	66	61	1	213	125	0
AAAGTGAAC	BRDN00012	149	77	4	298	369	0
AAAGTGAAG	BRDN00012	361	303	332	656	922	25
AAAGTGAAT	BRDN00012	206	119	581	275	490	34
AAAGTGACA	BRDN00012	44	20	0	416	243	0
AAAGTGACA	BRDN00011	381	327	99	526	490	8
AAAGTGACA	BRDN00011	309	259	173	346	465	50
AAAGTGACA	BRDN00012	428	166	191	751	639	24
AAAGTGACC	BRDN00011	203	205	168	435	557	0
AAAGTGACG	BRDN00011	122	0	1	147	127	0
AAAGTGAGA	BRDN00012	54	128	146	208	107	8
AAAGTGAGA	BRDN00012	241	233	658	569	474	26
AAAGTGAGC	BRDN00012	298	259	296	632	626	12
AAAGTGAGC	BRDN00012	136	163	217	238	168	6
AAAGTGAGG	BRDN00012	7	0	0	450	243	0
AAAGTGATG	BRDN00012	219	46	220	640	450	0
AAAGTGATG	BRDN00011	107	126	674	322	380	12
AAAGTGATG	BRDN00011	148	157	23	279	243	0
AAAGTGATG	BRDN00011	392	445	247	760	1391	71
AAAGTGCCA	BRDN00012	382	67	119	836	902	0
AAAGTGCGA	BRDN00012	130	147	205	262	438	20
AAAGTGCTC	BRDN00012	274	231	926	549	618	69
AAAGTGCTT	BRDN00012	135	176	304	330	192	64
AAAGTGCTT	BRDN00011	106	73	64	223	185	0
AAAGTGGAA	BRDN00012	81	138	1309	189	277	13
AAAGTGGAC	BRDN00011	181	195	295	413	122	4
AAAGTGGAG	BRDN00012	312	297	703	513	696	35
AAAGTGGAT	BRDN00011	116	90	13	239	148	27
AAAGTGGCA	BRDN00012	340	285	1186	528	616	64
AAAGTGGCC	BRDN00012	128	141	145	264	254	23
AAAGTGGCG	BRDN00011	417	233	52	770	480	12
AAAGTGGCT	BRDN00012	272	181	43	387	298	24
AAAGTGGGA	BRDN00011	33	0	0	276	41	0
AAAGTGGGC	BRDN00011	330	252	1053	573	529	29
AAAGTGGTG	BRDN00012	368	113	1	665	352	0
AAAGTGTAG	BRDN00011	136	150	0	176	274	0
AAAGTGTCC	BRDN00012	225	136	527	324	173	9
AAAGTGTCG	BRDN00012	103	82	14	310	119	6
AAAGTTAAC	BRDN00012	210	227	647	413	237	31
AAAGTTAAG	BRDN00012	186	238	99	391	762	1
AAAGTTAGA	BRDN00011	219	281	567	399	1144	14
AAAGTTCAG	BRDN00011	79	58	0	260	104	0
AAAGTTCCA	BRDN00012	314	356	817	710	524	27
AAAGTTCCA	BRDN00011	173	209	230	494	744	5
AAAGTTCCC	BRDN00011	168	254	194	366	96	19
AAAGTTCCC	BRDN00012	111	89	1	253	201	6
AAAGTTCTA	BRDN00011	115	130	80	375	290	10
AAAGTTCTC	BRDN00012	352	278	461	734	906	15
AAAGTTCTG	BRDN00011	103	0	0	535	411	0
AAAGTTGAC	BRDN00012	446	384	1433	754	977	45
AAAGTTGAG	BRDN00011	296	325	253	495	368	29
AAAGTTGCC	BRDN00012	363	87	84	769	1036	0
AAAGTTGGT	BRDN00011	56	0	0	314	148	0
AAAGTTGTA	BRDN00011	78	63	380	127	202	0
AAAGTTTCA	BRDN00012	14	0	0	307	101	0
AAAGTTTCC	BRDN00012	399	341	1047	689	679	95
AAAGTTTGA	BRDN00012	175	131	161	445	201	12
AAAGTTTGA	BRDN00011	149	131	81	315	482	2
AAATAAAAG	BRDN00011	241	135	30	307	251	2
AAATAAAAG	BRDN00011	142	0	0	463	89	0
AAATAAAAG	BRDN00012	171	121	223	406	798	23
AAATAAAAT	BRDN00012	301	206	1307	660	502	76
AAATAAACA	BRDN00012	114	175	4248	211	220	94
AAATAAAGA	BRDN00011	173	32	3	302	294	0
AAATAAAGA	BRDN00012	159	78	50	361	268	5
AAATAAAGC	BRDN00012	125	148	621	218	204	8
AAATAAAGC	BRDN00011	275	367	1252	737	1457	70
AAATAAATG	BRDN00012	242	304	1408	445	204	42
AAATAACAG	BRDN00012	167	155	351	331	358	3
AAATAAGAA	BRDN00012	207	211	212	380	239	11
AAATAAGCA	BRDN00012	31	16	6	341	188	25
AAATAAGGA	BRDN00012	143	55	10	342	327	24
AAATAAGGA	BRDN00012	547	566	1569	799	1254	124
AAATAAGGG	BRDN00012	269	299	627	639	447	30
AAATAATAG	BRDN00012	80	102	199	146	136	18
AAATAATAT	BRDN00012	321	371	510	662	699	54
AAATAATCA	BRDN00012	219	215	463	315	696	37
AAATAATCC	BRDN00011	312	234	1148	581	1186	21
AAATAATCC	BRDN00011	154	76	139	293	271	0
AAATAATGC	BRDN00012	135	128	0	276	101	26
AAATAATGG	BRDN00011	135	156	37	217	377	18
AAATAATGG	BRDN00012	275	328	1227	552	825	31
AAATAATTA	BRDN00012	103	10	1	289	622	0
AAATAATTTC	BRDN00011	63	96	131	184	144	4
AAATACAAA	BRDN00012	164	299	35	417	672	51
AAATACAAA	BRDN00011	257	162	14	430	389	36
AAATACAAG	BRDN00011	401	495	2351	778	827	47
AAATACAAG	BRDN00012	292	220	153	441	421	65
AAATACAAG	BRDN00012	302	251	543	609	740	20
AAATACACA	BRDN00012	327	408	326	552	456	43
AAATACACC	BRDN00012	129	32	31	325	282	0
AAATACACC	BRDN00011	183	90	698	378	468	23
AAATACACC	BRDN00012	119	134	890	329	246	8
AAATACACT	BRDN00012	259	177	94	660	672	27
AAATACAGC	BRDN00012	112	122	291	189	144	6
AAATACAGT	BRDN00011	381	322	203	667	699	34
AAATACAGT	BRDN00012	145	57	0	206	208	0
AAATACAGT	BRDN00011	189	109	1374	408	524	25
AAATACATA	BRDN00011	213	175	93	390	300	23
AAATACATG	BRDN00011	290	458	785	662	588	28
AAATACCAA	BRDN00012	289	297	570	449	473	7
AAATACCAG	BRDN00012	204	274	33	357	368	70
AAATACCAG	BRDN00012	158	113	203	328	144	28
AAATACCAT	BRDN00012	338	223	576	587	767	24
AAATACCTTC	BRDN00011	135	192	73	301	288	4
AAATACGAT	BRDN00012	131	164	70	301	231	129
AAATACGCC	BRDN00011	105	30	751	595	629	5
AAATACGCT	BRDN00012	106	107	8	157	131	11
AAATACGCT	BRDN00011	142	163	14	182	189	0
AAATACTAA	BRDN00011	69	0	0	234	85	0
AAATACTACT	BRDN00011	137	186	352	379	556	35
AAATACTCCT	BRDN00012	301	264	747	537	651	65
AAATACTGC	BRDN00011	70	46	3	258	194	18
AAATACTGG	BRDN00012	269	177	3206	481	627	122
AAATACTTA	BRDN00011	148	153	33	399	388	28
AAATACTTCA	BRDN00012	173	69	47	476	466	1
AAATACTTG	BRDN00012	6	13	0	168	178	0
AAATAGAAT	BRDN00012	417	297	571	428	404	28
AAATAGAGC	BRDN00011	151	90	82	304	229	25
AAATAGAGG	BRDN00012	247	279	160	455	684	17
AAATAGATA	BRDN00012	90	22	0	252	38	11
AAATAGATT	BRDN00012	69	52	222	158	150	0
AAATAGCCA	BRDN00011	210	148	269	376	350	26
AAATAGCTA	BRDN00011	84	0	1	659	557	0
AAATAGCTG	BRDN00012	116	58	1	666	887	0
AAATAGCTTC	BRDN00012	226	240	587	451	342	37
AAATAGGAA	BRDN00011	63	55	0	244	77	8
AAATAGGAC	BRDN00012	90	31	35	192	249	0
AAATAGGAG	BRDN00011	158	231	721	417	445	26
AAATAGTGT	BRDN00012	262	209	691	470	346	40
AAATAGTTA	BRDN00011	272	284	840	509	656	38
AAATAGTTG	BRDN00011	69	26	208	186	160	5
AAATATAAA	BRDN00011	191	282	290	308	549	17
AAATATAAT	BRDN00011	247	205	77	558	531	36
AAATATACA	BRDN00012	199	88	106	283	244	8
AAATATACT	BRDN00012	87	95	308	248	357	17
AAATATAGC	BRDN00011	65	85	4	118	107	0
AAATATATT	BRDN00012	373	262	287	719	930	43
AAATATCCA	BRDN00011	115	157	1106	287	220	15
AAATATCTG	BRDN00012	311	192	140	596	663	18
AAATATCTG	BRDN00012	127	134	7	444	305	11
AAATATCTG	BRDN00012	279	137	85	455	865	0
AAATATCTG	BRDN00011	112	46	39	136	366	21
AAATATGAC	BRDN00011	95	30	53	182	194	6
AAATATGCA	BRDN00012	218	149	970	496	652	66
AAATATGCT	BRDN00012	252	189	131	434	600	12
AAATATGCTC	BRDN00012	174	140	238	289	258	49
AAATATGGA	BRDN00012	51	0	0	378	35	0
AAATATGTA	BRDN00011	46	0	0	307	248	0
AAATATTAC	BRDN00011	151	118	11	199	322	28
AAATATTAT	BRDN00012	228	201	1472	494	484	63
AAATATTCTC	BRDN00012	348	40	79	491	535	0
AAATATTCTT	BRDN00011	200	147	317	317	269	17
AAATATTGA	BRDN00012	299	400	519	620	249	46
AAATATTGA	BRDN00012	303	237	316	527	470	60
AAATATTGCT	BRDN00011	93	138	932	234	288	16
AAATCAAAA	BRDN00012	276	262	66	770	817	0
AAATCAAAA	BRDN00012	204	261	85	618	581	0
AAATCAAAC	BRDN00011	187	132	243	407	481	0
AAATCAAAC	BRDN00011	135	56	7	127	68	0
AAATCAACT	BRDN00011	253	234	55	586	614	85
AAATCAAGC	BRDN00012	157	340	1468	378	520	34
AAATCACAA	BRDN00011	206	185	390	412	275	22
AAATCACAA	BRDN00011	469	491	689	834	929	55
AAATCACAG	BRDN00011	92	90	0	259	131	0
AAATCACATT	BRDN00012	40	204	42	193	413	12
AAATCACCA	BRDN00012	263	327	90	560	674	5
AAATCACCCT	BRDN00011	207	285	184	429	676	23
AAATCACTA	BRDN00011	330	266	1628	737	803	60
AAATCAGAG	BRDN00011	151	112	26	277	341	11
AAATCAGGA	BRDN00011	202	253	246	398	357	50
AAATCAGTG	BRDN00012	322	293	2747	496	723	81
AAATCAGTG	BRDN00012	302	196	364	585	772	21
AAATCATGA	BRDN00012	414	440	764	800	784	8
AAATCATGA	BRDN00012	196	225	1705	294	203	40
AAATCATGG	BRDN00012	146	119	1225	375	630	17
AAATCATTA	BRDN00011	137	6	0	336	604	1
AAATCATTAT	BRDN00012	88	14	21	228	65	0
AAATCATTCT	BRDN00011	130	22	0	370	752	0
AAATCATTTC	BRDN00011	100	58	219	261	121	0
AAATCCAAT	BRDN00012	498	350	1243	906	1016	69
AAATCCACG	BRDN00012	134	57	0	244	168	0
AAATCCAGT	BRDN00011	26	9	1	206	124	0
AAATCCATA	BRDN00011	168	180	659	300	401	28
AAATCCCACT	BRDN00011	277	223	872	792	899	12
AAATCCCAGT	BRDN00012	289	211	249	500	456	3
AAATCCGAC	BRDN00012	430	432	2711	694	768	64
AAATCCGCC	BRDN00011	265	20	0	420	177	0
AAATCCGGC	BRDN00011	523	324	468	935	686	18
AAATCCTACA	BRDN00011	68	62	0	143	131	0
AAATCCTACC	BRDN00011	170	131	307	201	384	0
AAATCCTAG	BRDN00012	410	464	855	651	318	73
AAATCCTCCA	BRDN00011	105	80	400	195	298	5
AAATCCTCTC	BRDN00012	309	437	1492	602	381	34
AAATCCTGA	BRDN00011	17	0	0	299	390	0
AAATCCTGTC	BRDN00012	243	184	1014	287	463	57
AAATCCTTAC	BRDN00012	153	135	67	244	506	77
AAATCCTTAT	BRDN00011	298	242	408	313	692	38
AAATCCTTGT	BRDN00011	143	93	19	359	142	0
AAATCGAAC	BRDN00012	340	418	1922	717	1321	68
AAATCGAAG	BRDN00011	177	155	126	313	196	8
AAATCGACC	BRDN00012	164	111	7	316	397	7
AAATCGAGG	BRDN00012	366	296	1827	679	628	65
AAATCGCTG	BRDN00011	503	436	1659	851	844	31
AAATCGGAT	BRDN00011	222	314	421	454	434	17
AAATCGGGC	BRDN00012	177	21	24	697	354	0
AAATCGGGG	BRDN00012	294	234	299	632	627	9
AAATCGGTC	BRDN00012	163	87	172	346	296	11
AAATCGGTG	BRDN00011	75	122	6	130	28	6
AAATCGTGA	BRDN00011	157	216	95	248	202	0
AAATCGTTC	BRDN00012	233	251	443	287	542	18
AAATCTAAA	BRDN00012	168	176	4966	465	410	56
AAATCTAAA	BRDN00012	329	160	288	640	354	8
AAATCTAAG	BRDN00012	125	127	72	245	259	50
AAATCTAAT	BRDN00012	304	249	497	486	429	29
AAATCTACA	BRDN00011	242	44	45	428	401	0
AAATCTAGA	BRDN00011	210	146	58	336	593	10
AAATCTAGA	BRDN00011	110	65	26	235	208	5
AAATCTCAGA	BRDN00011	184	191	26	269	153	4
AAATCTCAGA	BRDN00012	231	205	1436	417	254	66
AAATCTCCAG	BRDN00012	236	68	18	752	508	0
AAATCTCCGA	BRDN00011	112	121	300	193	172	52
AAATCTCTAA	BRDN00011	129	264	37	295	540	24
AAATCTCTTA	BRDN00011	264	199	348	429	354	31
AAATCTCTTG	BRDN00012	188	1	108	485	505	1
AAATCTGAA	BRDN00011	385	370	806	779	866	139
AAATCTGAG	BRDN00011	374	260	479	669	616	48
AAATCTGCA	BRDN00011	147	83	148	238	319	1
AAATCTTAA	BRDN00011	121	120	128	198	153	0
AAATCTTACT	BRDN00012	312	319	126	681	376	11
AAATCTTAG	BRDN00012	136	93	88	245	329	4
AAATCTTCG	BRDN00011	181	0	1	284	213	0
AAATCTTGC	BRDN00012	394	437	159	651	957	33
AAATCTTGG	BRDN00012	34	1	12	328	207	0
AAATCTTGTC	BRDN00012	154	127	61	253	459	3
AAATGAAAC	BRDN00012	413	100	163	639	429	6
AAATGAAAG	BRDN00012	473	436	4037	811	880	51
AAATGAACA	BRDN00012	147	84	486	318	262	6
AAATGAACC	BRDN00012	198	93	81	282	402	0
AAATGAACG	BRDN00012	145	57	26	316	289	10
AAATGAAGG	BRDN00012	107	166	20	229	212	89
AAATGAAGT	BRDN00011	175	150	42	180	153	11
AAATGAATA	BRDN00012	148	98	997	320	267	22
AAATGACAT	BRDN00012	126	23	5	264	263	0
AAATGACCCT	BRDN00012	135	71	29	231	193	2
AAATGACCG	BRDN00011	104	162	79	225	196	0
AAATGAGAC	BRDN00011	106	47	63	182	303	8
AAATGAGAG	BRDN00012	330	244	1179	638	695	47
AAATGAGCT	BRDN00011	88	192	43	250	237	0
AAATGAGGA	BRDN00012	142	205	336	384	388	45
AAATGAGGT	BRDN00011	266	73	0	454	475	0
AAATGAGTA	BRDN00011	358	270	9	628	311	29
AAATGAGTG	BRDN00011	109	155	108	237	80	24
AAATGAGTT	BRDN00011	318	348	326	777	782	19
AAATGATAC	BRDN00012	212	192	1409	433	616	67
AAATGATAC	BRDN00011	172	73	64	497	443	16
AAATGATAC	BRDN00012	415	349	1302	639	818	69
AAATGATAG	BRDN00012	203	177	17	541	913	28
AAATGATAT	BRDN00011	139	201	153	252	396	11
AAATGATCCT	BRDN00012	238	221	32	574	276	0
AAATGATGA	BRDN00011	127	169	122	289	560	1
AAATGATGA	BRDN00012	125	108	46	253	157	26
AAATGATGG	BRDN00011	180	222	275	447	691	23
AAATGATTC	BRDN00011	273	241	20	704	787	18
AAATGATTCT	BRDN00012	307	424	2030	623	662	72
AAATGATTTC	BRDN00011	194	170	141	272	318	27

AAATGCAAA	BRDN00012	292	368	537	726	893	39
AAATGCAAG	BRDN00012	322	377	237	507	400	35
AAATGCAAT	BRDN00011	108	53	61	309	234	3
AAATGCACA	BRDN00012	400	307	1693	651	619	142
AAATGCAGA	BRDN00012	362	220	362	627	794	43
AAATGCAGA	BRDN00012	464	44	1	756	822	0
AAATGCAGT	BRDN00012	637	593	1427	993	1393	71
AAATGCATC	BRDN00012	575	433	142	897	831	21
AAATGCCAA	BRDN00011	217	290	1320	474	203	49
AAATGCCAA	BRDN00011	174	154	381	424	422	24
AAATGCCAG	BRDN00012	395	349	143	763	612	27
AAATGCCAG	BRDN00012	235	206	196	483	533	0
AAATGCCGG	BRDN00011	83	108	1012	178	75	26
AAATGCCTT	BRDN00012	185	137	142	237	78	16
AAATGCGAA	BRDN00011	490	557	408	1058	998	45
AAATGCGAA	BRDN00012	239	215	154	380	140	5
AAATGCGCC	BRDN00012	44	1	20	534	420	0
AAATGCGCG	BRDN00011	446	312	488	823	347	20
AAATGCTCA	BRDN00012	110	114	329	174	356	46
AAATGCTCA	BRDN00012	169	134	0	330	300	4
AAATGCTCC	BRDN00011	84	91	124	235	649	8
AAATGCTGA	BRDN00012	68	103	3359	323	291	47
AAATGCTGG	BRDN00011	107	3	0	224	33	0
AAATGCTTA	BRDN00011	103	81	324	276	234	0
AAATGCTTC	BRDN00011	85	115	535	205	381	17
AAATGCTTC	BRDN00012	212	209	1066	254	265	5
AAATGGAAA	BRDN00012	97	46	225	245	408	21
AAATGGAAC	BRDN00011	173	165	31	398	186	35
AAATGGAAC	BRDN00012	206	140	454	339	609	31
AAATGGAAT	BRDN00011	98	94	0	253	152	2
AAATGGACG	BRDN00012	120	140	363	253	239	14
AAATGGAGA	BRDN00012	173	112	0	240	369	10
AAATGGAGA	BRDN00011	164	68	138	353	227	1
AAATGGATA	BRDN00011	270	88	34	762	708	11
AAATGGATC	BRDN00012	211	169	505	321	520	6
AAATGGATC	BRDN00012	369	428	315	662	520	38
AAATGGCAC	BRDN00011	97	107	115	198	308	0
AAATGGCAT	BRDN00011	201	100	1679	414	377	40
AAATGGCCT	BRDN00012	466	597	1927	873	961	65
AAATGGCTA	BRDN00011	271	96	30	623	1080	17
AAATGGCTA	BRDN00012	263	147	459	525	245	8
AAATGGCTT	BRDN00011	188	183	456	317	274	47
AAATGGGAA	BRDN00011	245	232	64	446	286	6
AAATGGGAT	BRDN00012	260	1	0	732	311	0
AAATGGGTA	BRDN00011	111	100	134	354	179	3
AAATGGGTA	BRDN00011	227	341	223	502	357	16
AAATGGTAC	BRDN00011	319	256	575	499	632	0
AAATGGTAT	BRDN00011	154	0	0	223	62	0
AAATGGTAT	BRDN00012	448	473	2083	811	1259	69
AAATGGTCT	BRDN00012	219	142	207	355	569	55
AAATGGTCT	BRDN00011	180	51	154	206	189	9
AAATGGTTC	BRDN00012	119	121	17	292	227	0
AAATGTAAT	BRDN00012	247	286	1368	392	294	20
AAATGTACA	BRDN00012	211	325	186	333	257	23
AAATGTACA	BRDN00011	85	59	843	158	95	22
AAATGTACG	BRDN00011	385	234	684	657	873	18
AAATGTAGG	BRDN00012	124	92	524	217	268	0
AAATGTAGG	BRDN00012	278	186	217	488	904	31
AAATGTATG	BRDN00012	89	27	36	192	111	4
AAATGTATG	BRDN00011	177	388	643	352	317	44
AAATGTATG	BRDN00011	133	105	417	281	237	16
AAATGTCAC	BRDN00012	122	65	442	284	298	21
AAATGTCAT	BRDN00011	162	179	481	308	353	16
AAATGTCTA	BRDN00012	206	199	43	299	139	50
AAATGTCTA	BRDN00011	87	134	3	188	198	6
AAATGTCTC	BRDN00012	388	0	1	861	427	1
AAATGTCTT	BRDN00012	296	153	121	453	251	24
AAATGTCTT	BRDN00012	489	389	1034	912	1036	0
AAATGTCTT	BRDN00012	277	242	421	603	376	66
AAATGTGAA	BRDN00011	386	201	255	492	542	17
AAATGTGAA	BRDN00011	101	94	0	199	184	31
AAATGTGAC	BRDN00012	422	453	1470	721	543	76
AAATGTGTG	BRDN00012	66	0	0	301	0	0
AAATGTGTG	BRDN00011	180	261	332	502	305	59
AAATGTTAA	BRDN00012	143	89	364	266	219	32
AAATGTTAA	BRDN00011	251	252	183	420	172	25
AAATGTTAC	BRDN00011	158	98	45	232	217	20
AAATGTTAC	BRDN00012	110	15	0	355	214	0
AAATGTTCA	BRDN00012	364	419	350	661	1080	52
AAATGTTGG	BRDN00012	72	0	0	347	230	0
AAATGTTTA	BRDN00011	235	276	724	443	509	23
AAATTAAAA	BRDN00012	243	393	1284	534	502	49
AAATTACAA	BRDN00011	433	437	1099	730	791	49
AAATTACCA	BRDN00011	220	335	83	554	787	56
AAATTACGA	BRDN00012	123	0	0	133	158	0
AAATTAGAA	BRDN00012	327	248	699	593	504	16
AAATTATAT	BRDN00012	81	5	0	456	283	0
AAATTATCA	BRDN00011	107	119	104	223	259	5
AAATTATCT	BRDN00011	105	110	140	320	220	12
AAATTATGA	BRDN00011	22	24	0	135	264	0
AAATTATGA	BRDN00012	125	123	285	181	287	0
AAATTATGG	BRDN00012	184	63	402	310	238	37
AAATTATTC	BRDN00012	197	111	270	288	522	23
AAATTATTC	BRDN00012	291	217	139	338	356	29
AAATTATTT	BRDN00012	184	269	1232	413	637	52
AAATTCAAA	BRDN00011	247	307	609	646	718	27
AAATTCACC	BRDN00012	390	359	476	627	1075	40
AAATTCACT	BRDN00012	29	9	0	426	172	0
AAATTCAGC	BRDN00012	197	152	77	405	304	18
AAATTCAGG	BRDN00011	252	160	151	447	561	8
AAATTCAGT	BRDN00011	201	39	107	432	397	17
AAATTCATA	BRDN00012	239	199	949	434	475	16
AAATTCATA	BRDN00012	322	294	344	537	596	40
AAATTCATA	BRDN00012	81	10	49	308	239	0
AAATTCATG	BRDN00011	193	101	10	300	310	0
AAATTCCGA	BRDN00011	329	247	607	639	681	11
AAATTCCTC	BRDN00011	116	97	415	290	185	0
AAATTCGAG	BRDN00011	306	224	822	470	377	4
AAATTCGAG	BRDN00011	247	0	0	613	182	0
AAATTCGGC	BRDN00012	105	60	30	142	26	0
AAATTCGGT	BRDN00011	235	150	471	394	531	26
AAATTCTAG	BRDN00012	300	345	1010	599	351	70
AAATTCTGA	BRDN00012	202	227	254	323	195	20
AAATTCTGT	BRDN00012	83	30	0	200	96	5
AAATTGAAA	BRDN00012	299	255	225	420	598	5
AAATTGAAG	BRDN00011	153	76	0	549	478	0
AAATTGACC	BRDN00012	174	206	44	378	490	37
AAATTGACC	BRDN00012	113	150	760	213	351	0
AAATTGACC	BRDN00012	157	53	83	666	517	6
AAATTGATG	BRDN00012	357	286	7	602	498	7
AAATTGCAA	BRDN00012	163	242	174	361	226	12
AAATTGCAG	BRDN00011	157	179	1	395	204	16
AAATTGCCA	BRDN00012	144	125	7	421	505	0
AAATTGCCC	BRDN00011	138	160	32	273	82	1
AAATTGCGT	BRDN00011	372	514	3294	735	892	94
AAATTGCTA	BRDN00012	243	207	162	395	341	25
AAATTGCTC	BRDN00011	116	169	554	282	242	76
AAATTGCTT	BRDN00011	108	95	23	211	224	0
AAATTGGAA	BRDN00012	62	2	0	523	163	0
AAATTGGAG	BRDN00011	259	309	349	599	750	48
AAATTGGAG	BRDN00011	111	75	0	280	238	11
AAATTGGAT	BRDN00011	105	108	281	349	453	0
AAATTGGCG	BRDN00012	160	207	113	369	455	6
AAATTGGCT	BRDN00012	383	454	1436	742	673	252
AAATTGGTG	BRDN00012	158	81	0	324	279	2
AAATTGTAA	BRDN00011	154	107	18	140	62	9
AAATTGTAG	BRDN00012	358	218	448	550	531	9
AAATTGTAT	BRDN00011	281	227	841	384	341	49
AAATTGTAT	BRDN00012	252	5	67	655	471	0
AAATTGTTC	BRDN00012	369	350	1003	829	780	38
AAATTGTTG	BRDN00011	361	344	841	633	387	40
AAATTTAAC	BRDN00012	253	130	1954	822	971	12
AAATTTAGA	BRDN00011	155	220	2571	331	473	53
AAATTTATG	BRDN00012	191	26	15	420	493	0
AAATTTATT	BRDN00011	311	253	91	515	375	0
AAATTTCAC	BRDN00012	147	127	845	262	533	48
AAATTTCTT	BRDN00012	212	227	1007	496	535	26
AAATTTGAC	BRDN00011	275	308	3181	420	414	79
AAATTTGAT	BRDN00011	126	130	584	277	178	10
AAATTTGAT	BRDN00011	160	117	784	498	247	66
AAATTTGCG	BRDN00011	201	58	69	471	317	0
AAATTTGTT	BRDN00011	346	223	11	534	672	14
AACAAAAAC	BRDN00012	217	342	1691	536	464	24
AACAAAAAT	BRDN00011	95	105	0	150	92	0
AACAAAACA	BRDN00011	105	173	86	303	245	19
AACAAAACC	BRDN00012	25	0	0	435	154	0
AACAAAACT	BRDN00012	92	0	0	216	177	0
AACAAAAGA	BRDN00012	165	147	197	256	178	18
AACAAAAGT	BRDN00012	212	325	84	514	587	40
AACAAACAA	BRDN00011	93	139	239	170	196	4
AACAAACCG	BRDN00012	136	205	65	336	226	0
AACAAACTA	BRDN00012	231	313	135	424	593	29
AACAAAGAA	BRDN00011	56	0	1	537	114	0
AACAAAGAA	BRDN00011	51	0	0	673	551	0
AACAAAGAA	BRDN00011	199	210	89	429	463	0
AACAAAGAT	BRDN00012	221	239	18	270	334	8
AACAAAGCC	BRDN00011	158	67	181	376	599	20
AACAAATTC	BRDN00012	291	180	212	462	245	36
AACAACAAT	BRDN00012	111	205	101	333	461	17
AACAACACA	BRDN00012	410	423	729	730	671	51
AACAACAGA	BRDN00011	122	126	41	318	352	0
AACAACAGC	BRDN00012	175	234	416	600	484	4
AACAACCAG	BRDN00011	181	127	59	348	815	0
AACAACCAG	BRDN00012	214	217	332	672	532	12
AACAACCGT	BRDN00011	83	118	50	92	5	23
AACAACCTA	BRDN00011	193	252	693	321	235	16
AACAACCTT	BRDN00012	119	80	140	232	342	4
AACAACGAA	BRDN00011	233	45	56	499	466	0
AACAACGAC	BRDN00012	117	78	0	314	294	0
AACAACTAC	BRDN00012	177	152	945	325	285	14
AACAACTAT	BRDN00011	167	226	73	305	387	22
AACAACTGT	BRDN00012	162	61	48	299	268	0
AACAACTTA	BRDN00011	74	126	54	194	262	12
AACAACTTC	BRDN00011	241	132	0	571	511	17
AACAACTTC	BRDN00011	250	291	627	565	644	33
AACAAGAAA	BRDN00012	74	19	179	214	77	0
AACAAGAAA	BRDN00012	91	85	173	132	137	0
AACAAGAAA	BRDN00012	334	428	1046	476	706	49
AACAAGAAG	BRDN00012	485	329	1293	898	904	41
AACAAGAAT	BRDN00011	237	203	818	428	399	68
AACAAGACC	BRDN00012	381	351	639	661	1089	45
AACAAGAGC	BRDN00012	119	88	235	223	127	16
AACAAGCCA	BRDN00011	366	285	351	719	713	2
AACAAGCCT	BRDN00012	156	139	1542	223	86	0
AACAAGCTA	BRDN00012	97	46	117	300	325	0
AACAAGCTT	BRDN00012	128	74	311	201	459	3
AACAAGGAA	BRDN00012	302	249	948	612	466	27
AACAAGGAT	BRDN00011	84	69	146	198	197	3
AACAAGGCA	BRDN00012	71	60	0	201	275	0
AACAAGTCA	BRDN00011	184	195	987	286	158	47
AACAAGTCA	BRDN00011	43	0	1	435	531	0
AACAAGTCT	BRDN00011	348	263	959	958	819	75
AACAAGTCT	BRDN00012	531	393	1964	862	1133	42
AACAAGTGA	BRDN00012	254	317	1514	556	480	87
AACAATACA	BRDN00012	173	165	363	285	230	0
AACAATATG	BRDN00011	107	102	83	203	56	29
AACAATCCA	BRDN00011	292	267	499	523	496	33
AACAATCTC	BRDN00012	454	439	403	841	1013	61
AACAATGCA	BRDN00012	88	75	0	184	165	0
AACAATGCC	BRDN00012	337	407	896	645	445	66
AACAATGGC	BRDN00012	198	115	6	306	533	0
AACAATGGC	BRDN00011	121	169	2117	241	68	17
AACAATTAT	BRDN00012	136	166	12	475	272	4
AACAATTTC	BRDN00011	153	101	183	289	151	0
AACAATTTG	BRDN00011	289	347	328	393	548	44
AACAATTTG	BRDN00012	219	289	367	629	506	22
AACACAACA	BRDN00012	427	366	310	632	508	58
AACACAAGC	BRDN00011	70	74	184	269	198	6
AACACACAG	BRDN00011	224	301	682	246	348	123
AACACACAT	BRDN00011	161	106	50	211	68	0
AACACACCA	BRDN00011	465	393	1380	831	541	55
AACACACCT	BRDN00012	490	454	1587	1116	4515	91
AACACACGG	BRDN00012	133	89	80	285	213	10
AACACACGT	BRDN00011	212	146	454	336	308	16
AACACAGCA	BRDN00012	564	551	970	1159	1089	100
AACACAGTG	BRDN00012	104	45	96	289	244	36
AACACAGTG	BRDN00011	280	273	1158	362	298	56
AACACATCC	BRDN00011	432	332	491	814	992	24
AACACATCC	BRDN00012	366	327	309	671	721	22
AACACATTG	BRDN00011	281	70	607	670	555	174
AACACCAAA	BRDN00012	304	241	71	685	435	20
AACACCAAA	BRDN00012	82	50	248	234	336	0
AACACCAAG	BRDN00012	319	355	832	619	857	15
AACACCACC	BRDN00011	709	551	1407	1279	951	60
AACACCACC	BRDN00012	177	43	0	435	205	0
AACACCAGC	BRDN00012	168	90	54	374	65	26
AACACCAGG	BRDN00012	460	442	3416	904	689	125
AACACCAGT	BRDN00011	333	448	555	710	596	48
AACACCATG	BRDN00012	168	71	53	281	674	47
AACACCCAA	BRDN00011	147	73	29	397	345	17
AACACCCAA	BRDN00011	132	133	508	270	187	31
AACACCCCG	BRDN00011	232	97	22	514	667	9
AACACCGAA	BRDN00011	343	242	1344	493	402	80
AACACCGAT	BRDN00012	428	510	296	892	723	54
AACACCGTA	BRDN00012	368	355	159	732	691	12
AACACCGTG	BRDN00011	142	129	68	380	232	0
AACACCTAA	BRDN00011	209	228	793	130	165	22
AACACCTGC	BRDN00012	254	269	10	331	452	9
AACACCTGT	BRDN00011	153	165	218	242	234	0
AACACCTTA	BRDN00011	249	147	973	568	705	17
AACACCTTG	BRDN00011	249	181	122	408	386	4
AACACGACC	BRDN00011	95	86	0	163	160	10
AACACGAGC	BRDN00012	175	157	92	264	210	10
AACACGATC	BRDN00012	110	42	171	235	124	18
AACACGATC	BRDN00011	129	1	0	535	145	0
AACACGATG	BRDN00012	326	354	1782	544	554	38
AACACGCAC	BRDN00011	172	223	119	539	555	30
AACACGCCC	BRDN00011	193	209	145	415	350	3
AACACGCTC	BRDN00011	164	138	307	425	143	14
AACACGCTG	BRDN00011	131	107	12	259	311	11
AACACGGAC	BRDN00011	313	290	697	534	580	20
AACACGGGG	BRDN00011	97	71	336	222	324	0
AACACGGTA	BRDN00011	516	489	377	1066	1242	66
AACACGGTC	BRDN00011	49	48	150	254	308	6
AACACGGTG	BRDN00012	376	295	994	555	660	53
AACACTAAT	BRDN00012	113	132	81	181	190	16
AACACTAGG	BRDN00011	201	86	338	443	492	2
AACACTATT	BRDN00012	383	171	320	818	528	0
AACACTCAG	BRDN00011	173	73	5	277	459	0
AACACTCAG	BRDN00012	472	568	382	778	1200	70
AACACTCCA	BRDN00011	157	144	0	266	165	8
AACACTCCA	BRDN00012	179	107	98	287	301	0
AACACTCCA	BRDN00012	84	48	26	114	147	0
AACACTCTG	BRDN00011	129	228	207	408	513	11
AACACTGAC	BRDN00011	115	169	282	222	226	0
AACACTGCA	BRDN00012	275	215	10	656	1542	31
AACACTGCA	BRDN00011	229	211	1230	706	726	41
AACACTGCT	BRDN00011	223	125	220	406	313	27
AACACTGCT	BRDN00011	199	187	48	449	413	44
AACACTGCT	BRDN00012	288	340	334	522	777	77
AACACTGGA	BRDN00011	222	247	616	452	370	12
AACACTGGA	BRDN00011	380	298	648	680	591	49
AACACTGTA	BRDN00012	300	217	138	747	537	60
AACACTGTT	BRDN00011	211	228	373	503	282	35
AACACTTAC	BRDN00011	232	270	80	326	463	81
AACACTTAG	BRDN00011	168	161	109	419	326	22
AACACTTCA	BRDN00012	238	223	1467	412	269	37
AACACTTGG	BRDN00012	152	201	44	290	567	45
AACAGAAAG	BRDN00012	163	106	10	177	407	8
AACAGAACA	BRDN00012	224	99	753	557	597	10
AACAGAACA	BRDN00012	515	532	1097	1013	918	89
AACAGAACC	BRDN00012	218	132	393	408	410	1
AACAGAATA	BRDN00012	257	220	0	717	637	0
AACAGAATC	BRDN00011	192	83	27	380	571	0
AACAGAATC	BRDN00011	261	287	81	470	658	33
AACAGACAG	BRDN00012	400	281	239	597	587	33
AACAGACCA	BRDN00011	162	81	21	257	160	0
AACAGACCT	BRDN00012	301	290	122	547	563	45
AACAGACGT	BRDN00011	110	0	2	1152	420	0
AACAGACTA	BRDN00012	510	270	439	598	507	28
AACAGACTG	BRDN00011	168	19	2	406	152	0
AACAGAGAG	BRDN00012	272	277	276	555	969	60
AACAGAGCA	BRDN00011	105	53	60	321	575	20
AACAGAGGC	BRDN00011	289	128	53	654	725	20
AACAGAGTA	BRDN00011	47	6	80	245	312	0
AACAGAGTT	BRDN00012	144	114	165	374	241	0
AACAGATAT	BRDN00012	250	272	564	550	586	25
AACAGATCC	BRDN00012	169	136	137	363	412	14
AACAGATGT	BRDN00012	202	191	409	386	497	32
AACAGATGT	BRDN00012	347	321	689	603	807	1
AACAGATTA	BRDN00012	59	42	89	119	184	0
AACAGATTT	BRDN00012	152	72	115	251	219	11
AACAGCAAA	BRDN00012	59	72	25	200	134	47
AACAGCAAA	BRDN00012	292	250	32	779	560	17
AACAGCAAG	BRDN00011	175	124	218	260	190	36
AACAGCAAT	BRDN00011	301	227	113	630	353	7
AACAGCAAT	BRDN00012	122	137	246	192	203	5
AACAGCACG	BRDN00011	105	49	46	310	260	18
AACAGCACT	BRDN00012	432	283	919	724	736	35
AACAGCAGA	BRDN00011	268	318	299	435	439	38
AACAGCATG	BRDN00011	205	104	665	427	226	14
AACAGCATT	BRDN00012	106	1	1	492	377	0
AACAGCCAA	BRDN00012	181	179	352	387	459	0
AACAGCCAG	BRDN00012	188	188	146	340	285	40
AACAGCCAT	BRDN00012	434	552	131	786	957	145
AACAGCCCA	BRDN00012	155	79	771	221	39	28
AACAGCCCT	BRDN00011	57	74	57	453	590	17
AACAGCGAA	BRDN00012	99	48	99	187	108	10
AACAGCGCT	BRDN00012	165	181	41	433	540	16
AACAGCTAC	BRDN00012	333	267	956	527	229	25
AACAGCTAC	BRDN00011	388	330	1271	633	712	18
AACAGCTCA	BRDN00011	138	82	119	298	180	34
AACAGCTCA	BRDN00012	539	474	426	899	756	70
AACAGCTCC	BRDN00011	236	271	823	579	396	47
AACAGCTCG	BRDN00012	238	218	4	423	326	23
AACAGCTGG	BRDN00011	180	117	19	223	461	11
AACAGCTGT	BRDN00012	58	0	0	345	299	0
AACAGCTTA	BRDN00012	185	107	0	393	437	33
AACAGCTTA	BRDN00011	199	85	0	356	462	0
AACAGCTTA	BRDN00011	199	39	47	714	651	0
AACAGCTTG	BRDN00011	212	0	0	896	1548	0
AACAGCTTT	BRDN00012	416	405	1630	737	599	76
AACAGGAAA	BRDN00012	401	385	623	532	794	37
AACAGGAAG	BRDN00012	372	353	281	706	636	51
AACAGGAAT	BRDN00012	300	152	194	436	419	49
AACAGGACC	BRDN00012	357	271	265	821	891	22
AACAGGATG	BRDN00012	162	172	10	266	226	42
AACAGGCAT	BRDN00012	91	18	13	237	144	7
AACAGGCTT	BRDN00011	437	158	207	937	327	18
AACAGGGTC	BRDN00012	68	94	41	328	237	0
AACAGGTAA	BRDN00012	723	766	892	1061	921	57
AACAGGTCC	BRDN00011	177	210	9	460	593	25
AACAGGTTA	BRDN00012	134	155	366	303	366	9
AACAGGTTT	BRDN00011	219	298	458	397	391	14
AACAGTAAC	BRDN00011	312	212	1021	610	586	24
AACAGTACT	BRDN00012	326	340	792	591	819	13
AACAGTACT	BRDN00011	248	185	2	576	523	9
AACAGTACT	BRDN00011	145	50	0	252	263	7
AACAGTAGT	BRDN00012	230	34	0	325	178	1
AACAGTATC	BRDN00011	126	107	89	380	150	11
AACAGTATC	BRDN00012	187	107	87	129	117	10
AACAGTCGA	BRDN00011	232	133	1141	418	525	34
AACAGTCGG	BRDN00011	181	167	208	245	272	62
AACAGTCTC	BRDN00012	106	71	44	382	329	0
AACAGTGAA	BRDN00011	291	98	200	741	557	0
AACAGTGAA	BRDN00012	160	154	4515	453	562	44
AACAGTGAT	BRDN00012	288	124	54	471	701	4
AACAGTGCC	BRDN00011	501	387	519	779	1131	3
AACAGTGCC	BRDN00012	187	188	417	376	204	12
AACAGTGCT	BRDN00012	227	261	316	382	526	80
AACAGTGGC	BRDN00012	214	261	834	519	352	52
AACAGTGGG	BRDN00011	213	90	1263	268	274	58
AACAGTTCA	BRDN00012	181	122	76	273	277	4
AACAGTTCT	BRDN00011	201	9	0	531	304	0
AACATAAAA	BRDN00011	87	106	0	74	154	5
AACATAAAC	BRDN00011	286	224	893	350	418	22
AACATAAAT	BRDN00012	121	108	90	298	349	0
AACATAATA	BRDN00011	274	194	728	533	354	27
AACATACAT	BRDN00012	271	247	1844	326	392	51
AACATACCA	BRDN00012	219	248	302	368	405	3
AACATACCA	BRDN00011	269	93	354	590	329	28
AACATACCC	BRDN00012	371	394	268	820	948	19
AACATACTT	BRDN00011	193	110	623	283	243	0
AACATAGAC	BRDN00012	279	293	558	408	272	9
AACATAGCG	BRDN00012	287	143	28	675	337	5
AACATAGGC	BRDN00011	131	163	715	339	549	33
AACATATCA	BRDN00012	125	134	87	305	161	24
AACATATTC	BRDN00012	50	13	0	233	115	0
AACATATTG	BRDN00011	367	242	370	648	361	45
AACATATTT	BRDN00012	168	124	299	318	406	10
AACATCAAC	BRDN00012	103	239	123	414	480	15
AACATCAAC	BRDN00011	235	261	1104	418	693	22
AACATCAGA	BRDN00012	73	51	1	351	112	0
AACATCATA	BRDN00011	86	80	202	229	89	56
AACATCATC	BRDN00012	226	163	50	428	505	0
AACATCATC	BRDN00012	220	139	99	582	631	3
AACATCCAC	BRDN00011	368	623	1483	618	762	188
AACATCCCC	BRDN00012	237	162	224	371	247	21
AACATCCTC	BRDN00011	69	26	0	252	113	0
AACATCCTG	BRDN00012	352	385	536	853	677	18
AACATCCTG	BRDN00011	193	124	60	231	255	2
AACATCGAC	BRDN00012	266	425	463	722	974	103
AACATCGAC	BRDN00012	288	176	236	482	562	36
AACATCGAG	BRDN00012	205	255	418	398	267	4
AACATCTAA	BRDN00012	438	522	588	805	784	44
AACATCTAC	BRDN00011	187	136	947	363	157	22
AACATCTCC	BRDN00011	148	141	2278	309	445	48
AACATCTCG	BRDN00012	462	477	1351	913	662	77
AACATCTGC	BRDN00011	166	179	87	431	603	22
AACATCTGC	BRDN00011	258	261	1491	431	420	76
AACATCTGC	BRDN00012	51	7	0	338	115	0
AACATCTGG	BRDN00012	222	86	2947	404	364	53
AACATCTGT	BRDN00012	195	181	308	356	344	11
AACATCTTC	BRDN00011	327	368	393	558	794	44
AACATGAAA	BRDN00011	327	288	159	403	816	6
AACATGAAG	BRDN00012	267	192	167	498	387	49
AACATGACC	BRDN00011	251	310	1259	503	650	58
AACATGACT	BRDN00011	296	149	19	588	309	1
AACATGAGC	BRDN00012	198	223	47	350	333	0
AACATGAGT	BRDN00011	153	163	154	284	444	6
AACATGATA	BRDN00012	255	236	352	721	891	0
AACATGATG	BRDN00012	120	91	0	232	174	0
AACATGATT	BRDN00012	177	0	0	341	191	0
AACATGCAC	BRDN00012	216	286	139	388	435	232
AACATGCCA	BRDN00011	245	232	7947	334	584	78
AACATGCTA	BRDN00012	46	12	9	647	314	0
AACATGCTC	BRDN00011	102	86	19	202	165	13
AACATGGAA	BRDN00011	125	128	140	181	427	0
AACATGGCC	BRDN00011	157	168	951	324	152	20
AACATGGTA	BRDN00011	255	297	78	639	808	17
AACATGGTC	BRDN00012	249	146	50	450	269	4
AACATGGTG	BRDN00012	511	439	715	1054	896	9
AACATGTAT	BRDN00011	185	78	1450	772	531	40
AACATGTCC	BRDN00011	104	99	60	253	268	24
AACATGTCT	BRDN00011	134	10	8	361	64	38
AACATGTGG	BRDN00011	403	494	351	725	1060	38
AACATGTGT	BRDN00012	141	96	203	312	227	0
AACATTAGT	BRDN00011	339	229	290	500	439	0
AACATTCAG	BRDN00011	215	153	446	558	402	22
AACATTCAG	BRDN00011	12	0	0	509	233	0
AACATTCAG	BRDN00011	132	133	185	298	192	2
AACATTCAG	BRDN00012	119	106	727	220	153	2
AACATTCCA	BRDN00011	145	128	1072	322	630	73
AACATTCCA	BRDN00012	272	272	938	397	642	25
AACATTCCG	BRDN00012	366	343	21	750	637	26
AACATTCCG	BRDN00012	269	184	116	328	937	8
AACATTCCG	BRDN00012	216	226	207	514	520	12
AACATTCCG	BRDN00012	162	218	46	272	189	29
AACATTGTA	BRDN00011	234	222	672	439	424	45
AACATTGTG	BRDN00012	124	96	204	255	401	27
AACATTTGG	BRDN00012	272	243	1138	512	849	22
AACCAAAAG	BRDN00011	207	204	1033	295	310	6
AACCAAAAT	BRDN00011	363	345	274	666	1076	25
AACCAAAGA	BRDN00011	67	22	0	183	137	0
AACCAAAGT	BRDN00011	9	8	0	300	126	0

AACCAACAG	BRDN00012	131	173	42	357	267	39
AACCAAGAT	BRDN00012	290	202	345	593	543	0
AACCAAGAT	BRDN00012	219	178	708	448	331	35
AACCAAGCC	BRDN00012	136	117	94	238	175	3
AACCAAGCG	BRDN00012	173	139	0	311	235	0
AACCAAGCG	BRDN00011	211	177	201	352	337	1
AACCAAGTC	BRDN00011	97	129	827	193	129	17
AACCAATCA	BRDN00011	225	281	276	545	229	5
AACCAATGA	BRDN00011	285	239	349	570	730	3
AACCAATTC	BRDN00011	114	61	33	158	308	0
AACCAATTC	BRDN00012	194	267	441	415	206	18
AACCACACA	BRDN00011	138	35	0	536	466	0
AACCACACA	BRDN00012	104	90	14	147	311	6
AACCACACC	BRDN00011	203	48	60	299	181	16
AACCACACC	BRDN00011	194	380	415	358	222	64
AACCACCAA	BRDN00012	245	138	15	441	452	30
AACCACCATT	BRDN00012	173	159	228	360	648	8
AACCACCTTC	BRDN00011	214	172	94	455	689	5
AACCACGCA	BRDN00012	412	468	2410	643	947	62
AACCACGCC	BRDN00012	107	178	96	184	272	31
AACCACGGC	BRDN00012	681	704	1206	1181	1127	25
AACCACGTG	BRDN00012	233	248	710	484	490	73
AACCACTATG	BRDN00011	329	269	1290	538	415	35
AACCACTCAT	BRDN00011	190	123	92	275	173	16
AACCACTCTC	BRDN00012	310	386	160	488	564	50
AACCACTGT	BRDN00012	263	125	2	502	243	3
AACCAGACA	BRDN00011	298	239	1143	487	637	59
AACCAGACG	BRDN00011	327	283	337	582	497	9
AACCAGAGC	BRDN00012	416	547	443	616	756	22
AACCAGAGT	BRDN00012	132	112	94	352	383	7
AACCAGATG	BRDN00012	191	95	840	283	554	24
AACCAGATG	BRDN00012	390	348	139	822	912	19
AACCAGATT	BRDN00011	158	114	185	420	312	0
AACCAGCAT	BRDN00012	410	376	612	682	863	70
AACCAGCCCT	BRDN00012	187	167	57	288	183	14
AACCAGCCCT	BRDN00012	125	141	0	259	481	4
AACCAGCTA	BRDN00011	379	109	0	660	557	0
AACCAGCTC	BRDN00011	339	286	819	523	574	126
AACCAGGAG	BRDN00011	125	138	30	211	216	15
AACCAGGCA	BRDN00012	142	45	0	276	265	2
AACCAGGCG	BRDN00012	500	416	246	621	498	59
AACCAGGTC	BRDN00012	614	715	1158	1123	992	56
AACCAGTACT	BRDN00012	138	165	1301	253	303	46
AACCAGTCA	BRDN00011	140	138	147	329	421	2
AACCAGTCAT	BRDN00011	208	199	257	316	503	0
AACCAGTGC	BRDN00012	298	132	71	332	211	20
AACCAGTTC	BRDN00011	372	318	304	652	475	23
AACCATAAG	BRDN00012	154	182	267	232	263	30
AACCATACTT	BRDN00011	216	170	454	356	229	11
AACCATAGC	BRDN00011	251	146	1682	650	533	23
AACCATATTG	BRDN00011	170	172	71	202	338	3
AACCATCAA	BRDN00012	503	478	1114	835	1031	84
AACCATCAA	BRDN00012	119	96	49	220	145	24
AACCATCACC	BRDN00012	283	254	114	655	737	24
AACCATCACT	BRDN00011	107	110	0	186	154	10
AACCATCCG	BRDN00012	236	128	453	412	242	32
AACCATCCTA	BRDN00011	246	133	801	328	411	16
AACCATCTTT	BRDN00011	254	208	656	514	677	103
AACCATGAG	BRDN00011	176	149	975	231	316	62
AACCATGAG	BRDN00012	210	135	997	596	719	66
AACCATTAAT	BRDN00012	188	247	125	260	268	50
AACCATTCAG	BRDN00012	147	210	337	294	110	31
AACCATTGG	BRDN00012	408	344	834	731	774	65
AACCCAAAA	BRDN00012	289	207	212	764	789	4
AACCCAAAAT	BRDN00012	303	299	143	473	326	10
AACCCAAGA	BRDN00011	120	112	820	226	498	29
AACCCAAGG	BRDN00012	116	8	0	304	253	0
AACCCAAGT	BRDN00011	427	482	1231	925	780	83
AACCCAATGT	BRDN00012	368	128	119	534	172	0
AACCCAATTT	BRDN00011	355	390	909	750	492	32
AACCCACAA	BRDN00012	243	240	61	324	427	31
AACCCACCCT	BRDN00012	381	321	2409	698	1012	102
AACCCACTTA	BRDN00011	331	392	218	715	845	30
AACCCAGGG	BRDN00011	144	145	565	291	324	16
AACCCATACA	BRDN00011	385	411	1613	718	708	19
AACCCATACG	BRDN00012	429	413	423	726	552	33
AACCCATCAT	BRDN00012	216	291	0	575	583	36
AACCCATGG	BRDN00012	100	83	202	262	423	5
AACCCATTGC	BRDN00012	173	186	293	380	547	10
AACCCATTGT	BRDN00011	252	232	9	567	533	5
AACCCCAAGT	BRDN00012	140	99	0	252	316	8
AACCCCACAC	BRDN00011	829	17	64	1082	949	0
AACCCCAGA	BRDN00011	263	209	214	479	232	19
AACCCCAGC	BRDN00012	545	212	221	1006	561	0
AACCCCAGG	BRDN00011	102	107	21	207	189	0
AACCCCCAG	BRDN00012	375	306	92	724	715	14
AACCCCCAG	BRDN00012	216	272	390	360	401	10
AACCCCGACC	BRDN00012	533	420	854	972	778	86
AACCCCGGA	BRDN00012	762	472	2057	1266	995	50
AACCCCTACC	BRDN00012	429	381	410	825	914	18
AACCCGAGT	BRDN00012	154	134	845	395	243	33
AACCCGCAA	BRDN00012	286	202	34	400	172	2
AACCCGCAT	BRDN00012	273	244	91	444	213	28
AACCCGGAC	BRDN00011	57	48	185	167	116	7
AACCCGGCA	BRDN00011	135	204	785	264	609	30
AACCCGTCTT	BRDN00012	453	335	196	782	572	19
AACCCTACCA	BRDN00012	163	104	0	208	158	3
AACCCTATAT	BRDN00012	147	196	105	395	552	13
AACCCTCACC	BRDN00011	254	213	644	528	903	55
AACCCTCAG	BRDN00012	278	223	165	547	443	55
AACCCTCCAA	BRDN00012	143	139	824	446	345	32
AACCCTCGCT	BRDN00012	339	381	1463	778	662	27
AACCCTGAT	BRDN00012	282	276	657	547	730	21
AACCCTGCA	BRDN00012	223	221	795	463	443	9
AACCCTGTA	BRDN00011	214	214	237	386	200	48
AACCGAAAT	BRDN00012	464	368	1530	934	808	105
AACCGACCA	BRDN00012	394	260	531	901	903	15
AACCGACCG	BRDN00011	163	249	26	315	452	9
AACCGACGA	BRDN00011	343	8	0	590	637	0
AACCGAGCA	BRDN00011	353	282	992	675	538	54
AACCGAGGC	BRDN00012	96	117	179	130	124	12
AACCGAGTG	BRDN00012	247	182	166	589	244	20
AACCGATCC	BRDN00012	148	146	196	193	187	22
AACCGCAAG	BRDN00012	263	12	157	522	580	7
AACCGCAGT	BRDN00012	659	486	1673	1044	713	98
AACCGCCGT	BRDN00012	130	94	88	283	221	5
AACCGCCTA	BRDN00012	152	175	293	306	518	0
AACCGCCTG	BRDN00012	425	422	10598	840	654	155
AACCGCTACC	BRDN00011	187	136	10	500	593	12
AACCGCTCG	BRDN00011	347	224	146	569	515	18
AACCGCTTGT	BRDN00011	194	120	335	445	404	18
AACCGGCAG	BRDN00012	239	286	3971	462	455	97
AACCGGCCA	BRDN00011	289	225	481	478	573	5
AACCGGCCC	BRDN00011	205	241	278	381	365	6
AACCGGCCT	BRDN00011	208	122	3797	513	429	58
AACCGGGAG	BRDN00011	180	214	165	392	370	0
AACCGGGGT	BRDN00012	146	223	203	322	446	40
AACCGGGTT	BRDN00011	169	122	240	374	405	24
AACCGTCAG	BRDN00012	349	320	165	575	815	21
AACCGTGTT	BRDN00011	332	282	131	926	901	4
AACCGTTCAT	BRDN00012	497	556	479	1001	1192	45
AACCTAAAG	BRDN00011	374	98	114	584	689	0
AACCTAACA	BRDN00011	258	248	27	331	294	10
AACCTACAA	BRDN00012	430	530	989	732	853	136
AACCTACCAC	BRDN00012	478	412	733	749	659	18
AACCTACCTG	BRDN00012	182	184	248	367	174	7
AACCTACTCC	BRDN00012	170	181	145	320	339	10
AACCTACTGC	BRDN00012	420	348	306	777	852	39
AACCTAGAA	BRDN00011	374	363	1394	639	612	78
AACCTAGCCT	BRDN00011	180	77	40	420	551	3
AACCTATAA	BRDN00011	170	0	0	570	383	0
AACCTATATC	BRDN00011	58	3	1	489	286	0
AACCTATTCA	BRDN00011	176	163	30	426	636	5
AACCTCAAG	BRDN00012	165	89	32	325	382	19
AACCTCACAT	BRDN00012	509	361	422	822	498	93
AACCTCACG	BRDN00012	249	39	0	734	240	0
AACCTCAGA	BRDN00012	135	121	36	316	173	7
AACCTCCACT	BRDN00012	346	297	1534	744	1034	66
AACCTCCAG	BRDN00011	428	434	844	812	768	160
AACCTCCCCG	BRDN00012	373	318	802	762	606	39
AACCTCCGGT	BRDN00012	187	118	451	365	200	6
AACCTCGAA	BRDN00011	217	164	290	359	305	0
AACCTCGAA	BRDN00012	380	137	449	675	454	6
AACCTCGTG	BRDN00012	210	176	394	252	104	449
AACCTCTACA	BRDN00012	389	326	684	659	542	21
AACCTCTGTA	BRDN00012	192	201	542	256	248	25
AACCTGAAA	BRDN00012	410	393	282	624	631	45
AACCTGAAC	BRDN00012	127	150	745	314	158	14
AACCTGAAC	BRDN00011	188	157	456	216	188	39
AACCTGAAG	BRDN00011	288	229	135	417	614	33
AACCTGAAT	BRDN00011	62	87	19	203	151	15
AACCTGAATT	BRDN00012	470	485	1440	668	422	38
AACCTGAATT	BRDN00012	228	234	1884	417	449	53
AACCTGACA	BRDN00011	392	428	416	689	675	37
AACCTGACAT	BRDN00012	240	303	550	414	465	60
AACCTGACC	BRDN00012	240	96	146	560	245	3
AACCTGACC	BRDN00012	433	435	1245	747	632	21
AACCTGAGG	BRDN00012	373	264	606	606	1134	20
AACCTGCAA	BRDN00012	144	50	1	787	555	4
AACCTGCAG	BRDN00012	163	242	614	364	334	13
AACCTGCTGT	BRDN00011	352	415	562	700	622	14
AACCTGCTTA	BRDN00011	415	516	1818	587	398	40
AACCTGGAG	BRDN00011	453	546	1316	1033	833	20
AACCTGGAG	BRDN00011	356	345	934	827	1187	43
AACCTGGCC	BRDN00011	444	514	723	826	610	52
AACCTGGGA	BRDN00012	371	416	299	928	613	67
AACCTGGGG	BRDN00012	433	371	314	1092	1153	45
AACCTGGGT	BRDN00012	185	215	176	302	277	2
AACCTGTCAT	BRDN00012	145	149	260	160	308	0
AACCTTAGG	BRDN00011	105	4	1	460	152	0
AACCTTGAA	BRDN00011	233	263	203	343	420	37
AACCTTGAG	BRDN00012	231	158	1681	426	592	41
AACCTTGTCA	BRDN00011	426	292	3135	761	861	48
AACCTTTACT	BRDN00011	178	160	481	368	371	21
AACCTTTCAT	BRDN00011	133	236	512	369	292	40
AACGAAAGC	BRDN00011	275	256	1341	363	513	18
AACGAAATA	BRDN00011	149	35	29	446	299	0
AACGAACTG	BRDN00011	195	130	789	349	446	13
AACGAATAC	BRDN00012	216	201	1346	434	258	41
AACGAATGG	BRDN00011	151	61	210	314	744	14
AACGACAAA	BRDN00011	38	113	54	111	23	11
AACGACAAC	BRDN00011	145	58	49	219	79	8
AACGACGAG	BRDN00011	344	286	826	605	589	7
AACGACGCT	BRDN00011	112	162	746	250	314	70
AACGACGTC	BRDN00012	275	26	69	799	861	5
AACGACTTA	BRDN00012	324	275	1323	640	541	25
AACGAGAAG	BRDN00011	103	95	141	180	157	11
AACGAGCAA	BRDN00011	74	215	54	141	209	41
AACGAGCTG	BRDN00012	143	107	1566	209	244	10
AACGAGCTG	BRDN00012	541	664	49591	996	1183	640
AACGAGTCC	BRDN00012	337	285	1510	769	814	60
AACGATGAG	BRDN00012	226	262	77	509	534	40
AACGCAAGC	BRDN00012	375	372	422	764	608	26
AACGCAGTA	BRDN00011	93	36	0	137	61	0
AACGCAGTC	BRDN00011	598	653	1356	762	907	43
AACGCATCA	BRDN00012	59	0	0	333	430	0
AACGCCAGG	BRDN00011	422	355	308	672	785	0
AACGCGAGT	BRDN00012	535	817	1147	1021	817	69
AACGCTACC	BRDN00011	207	174	119	379	275	22
AACGCTGAG	BRDN00012	322	388	854	441	557	85
AACGCTGCC	BRDN00011	229	15	0	539	172	0
AACGCTGGC	BRDN00011	186	112	232	393	285	38
AACGCTGTC	BRDN00012	584	598	837	877	1127	81
AACGGACAC	BRDN00012	127	150	1386	232	456	15
AACGGAGTA	BRDN00012	105	44	350	133	266	3
AACGGAGTT	BRDN00012	210	114	366	352	279	27
AACGGATTG	BRDN00011	129	169	77	315	293	12
AACGGCATC	BRDN00011	371	320	299	820	744	16
AACGGCCAA	BRDN00011	224	280	521	427	764	64
AACGGCCGC	BRDN00011	175	166	200	402	603	0
AACGGCTAC	BRDN00012	330	356	227	441	696	34
AACGGCTAT	BRDN00012	267	233	444	444	202	4
AACGGCTCC	BRDN00012	87	87	580	191	92	8
AACGGCTCC	BRDN00012	380	401	373	763	945	63
AACGGCTTG	BRDN00011	58	0	19	255	235	0
AACGGGAAC	BRDN00011	141	236	44	234	86	14
AACGGGACC	BRDN00012	386	287	273	665	956	18
AACGGGATA	BRDN00011	400	437	285	664	687	30
AACGGGCAC	BRDN00012	207	283	606	348	829	7
AACGGGCAG	BRDN00011	123	0	1	345	7	0
AACGGGCCG	BRDN00011	254	251	1043	512	489	35
AACGGGTCA	BRDN00012	99	139	263	417	251	24
AACGGGTCG	BRDN00011	101	25	18	187	39	3
AACGGGTTT	BRDN00011	84	12	0	340	210	0
AACGGTGTG	BRDN00011	117	85	464	132	107	8
AACGGTGTT	BRDN00012	170	273	135	410	160	6
AACGGTTGC	BRDN00012	138	232	788	262	319	22
AACGGTTGT	BRDN00011	346	277	593	591	866	52
AACGTATTC	BRDN00011	449	424	469	713	792	61
AACGTCAAA	BRDN00012	119	68	48	189	305	78
AACGTCCAT	BRDN00011	436	389	1142	928	803	51
AACGTCCGC	BRDN00011	293	425	177	716	1217	17
AACGTCCTCA	BRDN00012	87	1	0	625	179	0
AACGTCTACT	BRDN00012	236	155	76	328	326	18
AACGTCTTCA	BRDN00012	77	40	251	227	167	0
AACGTCTTG	BRDN00012	293	215	81	626	501	11
AACGTCTTTG	BRDN00012	198	215	277	335	543	21
AACGTCTTTG	BRDN00011	195	149	251	389	267	36
AACGTGACG	BRDN00012	375	357	1133	621	792	56
AACGTGAGT	BRDN00011	100	41	105	194	130	0
AACGTGATT	BRDN00012	202	162	355	383	441	20
AACGTGCCA	BRDN00011	191	157	2	276	323	14
AACGTGCCC	BRDN00012	127	105	769	164	179	39
AACGTGCGC	BRDN00011	24	1	1	369	93	0
AACGTGCGT	BRDN00012	119	122	449	226	719	32
AACGTGCTC	BRDN00012	91	62	80	168	183	15
AACGTGCTC	BRDN00011	145	104	230	331	206	15
AACGTGCTG	BRDN00012	645	682	1041	1211	881	37
AACGTGGAG	BRDN00012	75	157	7	177	135	20
AACGTGGAT	BRDN00011	99	126	125	266	193	5
AACGTGGCA	BRDN00012	365	259	97	674	430	59
AACGTGGGA	BRDN00012	329	385	2212	551	576	50
AACGTGGGC	BRDN00011	247	137	297	315	324	17
AACGTGGGG	BRDN00011	237	233	113	466	834	43
AACGTGTAT	BRDN00012	75	74	0	208	310	0
AACGTGTCC	BRDN00012	487	405	466	831	817	27
AACGTGTGA	BRDN00012	178	274	982	370	192	48
AACGTGTGG	BRDN00011	153	155	116	539	10	4
AACGTGTGT	BRDN00012	488	530	751	839	752	114
AACGTTACC	BRDN00011	278	376	497	530	435	74
AACGTTCAT	BRDN00011	305	271	363	546	334	7
AACGTTCCT	BRDN00011	228	183	222	357	321	0
AACGTTGGT	BRDN00011	122	126	7	199	289	7
AACGTTTAA	BRDN00011	209	118	269	401	1100	6
AACGTTTGA	BRDN00012	258	278	89	486	513	13
AACTAAAAA	BRDN00011	19	27	0	135	12	0
AACTAAAAC	BRDN00012	42	0	1	187	234	0
AACTAAACT	BRDN00011	250	298	520	635	825	8
AACTAACAG	BRDN00012	622	574	765	856	1241	62
AACTAACTG	BRDN00011	371	408	359	535	407	33
AACTAAGCG	BRDN00011	121	61	26	218	330	0
AACTAATGG	BRDN00011	133	115	6	248	132	18
AACTAATTG	BRDN00012	125	190	33	168	301	15
AACTACAAG	BRDN00011	57	32	0	285	125	0
AACTACAAG	BRDN00012	379	400	358	669	813	58
AACTACAGC	BRDN00011	261	334	429	495	377	83
AACTACAGT	BRDN00011	133	218	41	235	176	11
AACTACATG	BRDN00011	125	207	71	210	190	30
AACTACCACG	BRDN00011	528	398	200	809	785	7
AACTACCCA	BRDN00012	158	143	0	252	324	13
AACTACCTAT	BRDN00011	164	77	20	463	359	23
AACTACCTCT	BRDN00011	227	171	967	444	416	36
AACTACCTCT	BRDN00011	170	110	274	188	466	10
AACTACCTTT	BRDN00012	254	334	3502	746	382	70
AACTACCTTT	BRDN00012	339	341	524	441	340	26
AACTACGCC	BRDN00012	407	515	465	727	732	7
AACTACTAAT	BRDN00012	249	202	10	379	338	35
AACTACTGA	BRDN00011	430	437	644	851	671	26
AACTACTGG	BRDN00012	509	476	2152	1090	982	47
AACTACTGT	BRDN00011	288	377	300	529	439	68
AACTACTTAC	BRDN00011	120	162	30	241	237	3
AACTACTTTG	BRDN00011	105	13	0	277	261	0
AACTAGAAC	BRDN00012	378	266	450	649	746	103
AACTAGAAT	BRDN00012	519	538	414	637	517	20
AACTAGAGG	BRDN00012	302	261	508	608	492	64
AACTAGAGG	BRDN00011	179	2	0	333	447	0
AACTAGCACT	BRDN00012	394	409	274	775	923	34
AACTAGCAG	BRDN00012	507	534	1364	915	777	43
AACTAGCCC	BRDN00012	537	398	1316	630	928	33
AACTAGTGT	BRDN00012	429	466	778	641	975	50
AACTATAAA	BRDN00011	292	239	624	494	485	3
AACTATAAG	BRDN00011	97	45	14	213	110	0
AACTATAAT	BRDN00011	80	0	0	141	142	0
AACTATATTG	BRDN00012	152	79	22	175	105	0
AACTATCAA	BRDN00012	269	244	21	526	477	21
AACTATCACC	BRDN00011	102	77	6	226	155	5
AACTATGGG	BRDN00012	178	124	615	291	192	13
AACTATGTC	BRDN00011	107	71	85	190	212	0
AACTCAACA	BRDN00012	200	107	22	223	93	5
AACTCAAGC	BRDN00012	329	388	631	820	755	49
AACTCAAGC	BRDN00012	164	142	164	297	529	80
AACTCACAAT	BRDN00011	222	127	68	330	429	4
AACTCACATG	BRDN00012	162	142	550	460	284	33
AACTCACCAG	BRDN00012	41	0	893	311	46	4
AACTCACTGC	BRDN00011	190	37	117	347	526	0
AACTCAGCC	BRDN00012	486	529	984	773	1016	35
AACTCAGCTC	BRDN00012	190	227	85	321	371	50
AACTCATCCT	BRDN00012	115	20	33	552	303	0
AACTCATCGT	BRDN00011	441	305	631	562	1017	23
AACTCATCTT	BRDN00011	453	471	566	713	779	50
AACTCATTAC	BRDN00012	175	196	758	341	321	18
AACTCCACCG	BRDN00011	82	67	0	255	181	0
AACTCCACG	BRDN00011	101	7	0	202	13	0
AACTCCATAG	BRDN00012	212	223	428	550	391	18
AACTCCATCC	BRDN00012	111	137	8	242	301	16
AACTCCCATA	BRDN00012	458	347	483	943	999	30
AACTCCCCAG	BRDN00011	172	111	595	299	139	26
AACTCCCTAA	BRDN00011	154	278	14	480	499	5
AACTCCCTGA	BRDN00012	221	221	194	395	371	63
AACTCCCTGG	BRDN00011	164	137	623	268	297	6
AACTCCGTAC	BRDN00012	84	5	0	203	152	0
AACTCCGTGC	BRDN00011	383	372	252	557	394	20
AACTCCTCGA	BRDN00012	439	484	251	838	709	49
AACTCCTCGC	BRDN00012	197	199	0	439	499	6
AACTCCTTGA	BRDN00011	14	19	198	375	225	0
AACTCGAAA	BRDN00011	181	101	120	335	282	25
AACTCGATG	BRDN00011	172	247	757	415	270	14
AACTCGCTAT	BRDN00012	106	96	62	228	200	10
AACTCGCTCC	BRDN00011	300	196	865	752	639	37
AACTCGCTCC	BRDN00012	164	132	90	320	152	31
AACTCGGCA	BRDN00011	358	464	694	617	552	47
AACTCGGGT	BRDN00012	178	195	10	426	448	0
AACTCGGTG	BRDN00011	162	133	60	428	509	2
AACTCGTAA	BRDN00012	263	0	0	427	227	1
AACTCGTAG	BRDN00011	443	424	549	973	1294	11
AACTCGTTCA	BRDN00012	83	24	0	403	294	1
AACTCTAAA	BRDN00012	74	141	57	132	435	13
AACTCTACAG	BRDN00011	198	132	36	324	398	3
AACTCTACAG	BRDN00012	197	74	126	343	539	37
AACTCTAGCT	BRDN00012	141	18	14	441	281	0
AACTCTAGG	BRDN00011	140	228	35	286	710	40
AACTCTCAAA	BRDN00012	394	289	461	733	582	122
AACTCTCGAC	BRDN00012	87	81	148	179	81	10
AACTCTCGCT	BRDN00012	113	10	0	350	67	0
AACTCTCGTG	BRDN00011	159	94	84	380	227	0
AACTCTGAA	BRDN00012	158	148	72	274	297	12
AACTCTTCGA	BRDN00012	204	224	20	299	375	57
AACTCTTCTG	BRDN00012	334	344	2752	623	784	106
AACTCTTGGC	BRDN00011	473	305	1826	811	713	22
AACTGAAAC	BRDN00012	141	39	0	243	203	5
AACTGAACA	BRDN00012	60	17	0	438	456	0
AACTGAACA	BRDN00012	140	168	158	232	198	66
AACTGAACA	BRDN00011	391	328	1100	839	886	50
AACTGAATC	BRDN00011	489	371	461	803	833	29
AACTGACACT	BRDN00012	508	633	1503	974	844	105
AACTGACAG	BRDN00012	158	210	9	231	229	13
AACTGACGA	BRDN00012	213	136	493	385	167	36
AACTGACTG	BRDN00012	165	37	17	271	252	32
AACTGAGCG	BRDN00012	466	440	1479	869	901	87
AACTGAGGA	BRDN00011	574	414	452	1152	1631	26
AACTGAGGC	BRDN00011	153	144	111	454	626	0
AACTGAGGC	BRDN00011	213	256	481	367	796	26
AACTGATGA	BRDN00011	98	43	25	251	606	0
AACTGATGA	BRDN00011	218	131	2331	470	508	43
AACTGATGA	BRDN00011	169	111	47	233	414	0
AACTGCAACT	BRDN00012	215	266	583	378	366	24
AACTGCACG	BRDN00012	303	169	583	608	475	11
AACTGCAGG	BRDN00012	145	84	324	280	369	14
AACTGCAGG	BRDN00012	168	199	190	328	588	22
AACTGCATC	BRDN00012	437	265	213	845	881	0
AACTGCCAG	BRDN00012	503	434	2190	822	622	474
AACTGCCAT	BRDN00011	626	752	3082	1001	1241	132
AACTGCGCC	BRDN00012	484	513	1527	837	1031	58
AACTGCGGT	BRDN00011	341	315	6913	762	670	91
AACTGCGTG	BRDN00012	125	80	923	187	286	9
AACTGCTCG	BRDN00012	130	0	1	398	59	0
AACTGCTGG	BRDN00011	121	163	91	198	123	0
AACTGCTGG	BRDN00012	159	183	162	382	298	28
AACTGCTGT	BRDN00011	199	12	0	399	337	0
AACTGGAAA	BRDN00012	574	480	1528	1124	959	42
AACTGGAAG	BRDN00012	441	352	269	715	730	6
AACTGGAAT	BRDN00011	150	212	298	440	461	17
AACTGGACA	BRDN00012	346	321	27	608	1212	11
AACTGGACC	BRDN00012	47	1	0	336	78	0
AACTGGATA	BRDN00011	235	193	15	420	416	6
AACTGGATA	BRDN00011	247	267	149	504	393	0
AACTGGATC	BRDN00011	159	55	11	262	135	0
AACTGGATG	BRDN00012	516	473	251	774	379	17
AACTGGATT	BRDN00011	263	187	254	513	291	4
AACTGGCAC	BRDN00011	19	0	0	367	1	0
AACTGGCCA	BRDN00012	295	258	117	660	773	2
AACTGGCCA	BRDN00012	472	477	326	710	832	21
AACTGGCCG	BRDN00012	169	74	1116	344	343	18
AACTGGGAT	BRDN00012	283	283	588	575	555	66
AACTGGGAT	BRDN00012	290	296	833	775	356	23
AACTGGGCC	BRDN00012	38	31	57	223	114	2
AACTGGTAA	BRDN00011	207	158	100	436	300	0
AACTGGTAG	BRDN00012	176	174	290	241	400	33
AACTGGTCA	BRDN00011	271	230	350	462	225	57
AACTGGTCC	BRDN00012	144	93	107	222	258	33
AACTGGTCT	BRDN00011	135	90	61	255	166	12
AACTGGTGC	BRDN00012	469	470	1831	821	787	126
AACTGGTTC	BRDN00012	28	0	0	143	162	0
AACTGTACT	BRDN00011	109	78	22	302	211	21
AACTGTATC	BRDN00012	283	101	505	468	299	19
AACTGTATG	BRDN00011	153	59	33	390	200	6
AACTGTCAC	BRDN00011	468	339	988	702	645	69
AACTGTCAG	BRDN00011	147	200	429	247	627	21
AACTGTCGG	BRDN00011	191	104	0	265	480	16
AACTGTGAA	BRDN00012	140	55	188	320	270	18
AACTGTGAT	BRDN00012	105	69	109	188	119	0
AACTGTTCA	BRDN00011	218	430	264	424	325	36
AACTGTTGA	BRDN00011	177	128	106	250	234	12
AACTTAATA	BRDN00011	62	176	464	172	167	9
AACTTACAG	BRDN00012	141	165	427	299	240	17
AACTTACATG	BRDN00012	131	194	182	286	200	31
AACTTACCG	BRDN00011	435	443	186	779	784	30
AACTTACCGT	BRDN00012	211	139	251	274	338	33
AACTTACCTA	BRDN00011	113	3	0	179	177	0
AACTTACCTT	BRDN00011	265	244	81	469	716	31
AACTTACTGT	BRDN00012	455	429	416	839	1205	7
AACTTACTTG	BRDN00011	209	175	178	418	335	1
AACTTACTTT	BRDN00012	156	309	973	429	275	19
AACTTAGAA	BRDN00012	293	3	1	658	388	0
AACTTAGATC	BRDN00012	249	267	570	493	447	39
AACTTAGATC	BRDN00011	260	107	147	572	598	34
AACTTAGGT	BRDN00012	190	71	934	349	395	23
AACTTATAG	BRDN00012	242	228	92	411	225	81
AACTTATCG	BRDN00011	145	143	500	273	225	13
AACTTATGA	BRDN00011	171	175	121	208	285	11
AACTTATTCA	BRDN00012	386	430	214	755	1049	13
AACTTCACCA	BRDN00011	327	331	1774	414	408	35
AACTTCACTG	BRDN00011	302	15	8	709	370	0
AACTTCAGCC	BRDN00011	543	534	256	859	792	37
AACTTCATG	BRDN00011	364	268	828	469	344	59

AACTTCCAAT	BRDN00011	394	459	285	551	713	13
AACTTCCAG	BRDN00012	427	420	867	1027	1227	47
AACTTCCCCT	BRDN00012	157	194	2366	344	273	33
AACTTCCTGA	BRDN00011	372	521	261	959	1308	40
AACTTCCTGT	BRDN00011	409	386	522	729	663	6
AACTTCGAC	BRDN00012	202	251	557	499	434	19
AACTTCGGT	BRDN00012	283	200	243	716	564	37
AACTTCGTTG	BRDN00011	257	252	731	417	363	41
AACTTCTCTA	BRDN00011	72	89	0	889	667	0
AACTTCTGCT	BRDN00011	135	200	9	373	157	0
AACTTCTTAA	BRDN00012	195	224	187	411	375	26
AACTTCTTGT	BRDN00012	385	264	412	642	706	66
AACTTGAAC	BRDN00011	394	366	741	649	607	30
AACTTGAAG	BRDN00011	202	260	480	288	157	27
AACTTGAAG	BRDN00012	315	241	119	606	482	11
AACTTGAAG	BRDN00011	123	0	1	257	112	0
AACTTGAGA	BRDN00012	106	96	617	531	442	12
AACTTGATC	BRDN00011	155	186	714	430	332	10
AACTTGCCAT	BRDN00011	400	1043	237206	1079	4802	3540
AACTTGCCTA	BRDN00012	393	410	163	753	665	36
AACTTGCGA	BRDN00012	121	61	14	874	333	15
AACTTGGAC	BRDN00012	141	100	94	274	511	3
AACTTGGAG	BRDN00012	138	147	27263	238	118	338
AACTTGGCT	BRDN00012	193	252	393	385	366	5
AACTTGGGA	BRDN00011	328	289	111	813	442	28
AACTTGGTC	BRDN00012	291	254	133	461	323	15
AACTTGGTT	BRDN00012	374	319	622	530	590	7
AACTTGTAA	BRDN00011	208	116	2	353	279	0
AACTTGTAG	BRDN00011	349	523	1409	575	404	90
AACTTGTAG	BRDN00012	148	110	331	214	373	4
AACTTGTGA	BRDN00012	225	194	90	399	431	13
AACTTGTGCT	BRDN00011	138	141	43	337	313	39
AACTTGTGG	BRDN00012	259	269	76	410	422	15
AACTTGTGT	BRDN00011	101	128	7	264	68	0
AACTTGTTAC	BRDN00011	141	25	0	430	429	0
AACTTGTTG	BRDN00011	85	83	30	251	155	0
AACTTTAACT	BRDN00011	212	181	152	479	244	28
AACTTTAACT	BRDN00011	327	405	525	698	650	13
AACTTTAAG	BRDN00011	215	105	36	322	335	0
AACTTTACAC	BRDN00012	229	171	1140	424	252	29
AACTTTACAC	BRDN00012	112	107	0	791	558	10
AACTTTACTC	BRDN00012	157	93	88	778	480	0
AACTTTCCCA	BRDN00012	421	381	704	867	867	50
AACTTTCGG	BRDN00012	102	72	282	227	427	10
AACTTTCGTG	BRDN00011	66	0	1	234	420	0
AACTTTGCCA	BRDN00012	338	171	153	820	993	0
AACTTTGTTC	BRDN00011	299	45	30	869	825	0
AAGAAAAAG	BRDN00011	376	456	340	669	689	24
AAGAAAAAT	BRDN00011	143	155	50	268	266	15
AAGAAAAGC	BRDN00011	125	79	52	285	151	25
AAGAAAAGG	BRDN00011	110	128	253	322	271	68
AAGAAAAGG	BRDN00011	103	168	174	147	67	66
AAGAAAAGT	BRDN00012	149	146	29	453	595	38
AAGAAAATG	BRDN00012	146	189	157	341	564	0
AAGAAACAA	BRDN00012	236	273	293	467	474	13
AAGAAACAA	BRDN00012	161	87	349	262	118	34
AAGAAACAA	BRDN00011	238	299	705	325	490	146
AAGAAACAC	BRDN00012	390	386	192	724	1200	2
AAGAAACAT	BRDN00012	138	22	17	210	226	5
AAGAAACCA	BRDN00011	359	243	721	512	472	28
AAGAAACGC	BRDN00011	400	300	1239	695	657	43
AAGAAACGC	BRDN00012	270	192	849	506	665	39
AAGAAACTA	BRDN00012	107	83	54	314	291	85
AAGAAACTA	BRDN00012	17	15	1	164	131	0
AAGAAACTG	BRDN00011	162	86	182	305	153	6
AAGAAACTG	BRDN00012	248	236	283	401	447	0
AAGAAAGAA	BRDN00012	84	100	201	270	491	16
AAGAAAGAC	BRDN00011	104	73	29	261	246	9
AAGAAAGAC	BRDN00011	142	41	0	580	241	0
AAGAAAGAC	BRDN00011	164	146	251	267	163	10
AAGAAAGAC	BRDN00011	178	139	31	289	313	9
AAGAAAGAT	BRDN00011	77	60	304	199	269	0
AAGAAAGAT	BRDN00011	234	100	432	405	558	0
AAGAAAGCA	BRDN00012	150	120	68	334	576	0
AAGAAAGCA	BRDN00012	135	121	125	383	270	4
AAGAAAGCC	BRDN00011	69	87	27	309	187	0
AAGAAAGCT	BRDN00012	310	228	1029	566	662	28
AAGAAAGG	BRDN00012	456	407	1152	937	809	50
AAGAAAGTC	BRDN00012	225	194	174	278	188	17
AAGAAAGTG	BRDN00011	145	10	0	304	12	0
AAGAAATAC	BRDN00011	394	338	197	699	686	26
AAGAAATAC	BRDN00011	88	55	654	188	135	16
AAGAAATCA	BRDN00011	397	477	532	676	1127	34
AAGAAATCG	BRDN00011	183	288	477	356	289	10
AAGAAATCT	BRDN00012	233	178	799	363	426	17
AAGAAATGC	BRDN00012	0	0	1	170	10	0
AAGAAATGG	BRDN00011	90	87	1025	187	177	15
AAGAAATGG	BRDN00011	508	362	1907	940	995	35
AAGAAATGT	BRDN00011	160	112	35	196	253	16
AAGAAATTC	BRDN00011	228	62	23	434	118	0
AAGAAATTG	BRDN00012	124	111	35	261	320	17
AAGAAATTT	BRDN00012	91	34	72	311	228	21
AAGAAATTT	BRDN00011	113	9	0	475	441	0
AAGAACAAA	BRDN00012	174	183	879	361	326	40
AAGAACAAG	BRDN00012	121	58	234	306	373	9
AAGAACACG	BRDN00012	151	137	313	278	239	0
AAGAACACT	BRDN00011	83	0	0	312	196	1
AAGAACAGC	BRDN00011	170	180	26	439	488	14
AAGAACAGG	BRDN00012	107	159	110	304	214	34
AAGAACATA	BRDN00011	118	38	23	213	186	41
AAGAACATC	BRDN00011	181	251	12	330	283	4
AAGAACATC	BRDN00012	119	121	227	225	171	13
AAGAACATC	BRDN00011	70	0	0	306	273	0
AAGAACATT	BRDN00012	205	341	136	439	542	25
AAGAACCAA	BRDN00012	139	151	14	291	390	4
AAGAACCAA	BRDN00012	131	64	693	363	273	18
AAGAACCAG	BRDN00011	139	111	26	480	511	1
AAGAACCAG	BRDN00012	344	305	276	818	641	31
AAGAACCCA	BRDN00011	311	420	2011	613	642	101
AAGAACCCA	BRDN00011	283	315	76	349	390	35
AAGAACCCA	BRDN00011	360	481	170	739	1637	23
AAGAACCCA	BRDN00011	99	185	32	176	143	26
AAGAACCGA	BRDN00011	132	127	234	234	206	11
AAGAACCTG	BRDN00011	84	113	289	218	251	5
AAGAACCTG	BRDN00012	365	393	280	535	480	51
AAGAACGGC	BRDN00012	444	420	1361	650	427	25
AAGAACGGG	BRDN00011	97	104	0	220	224	0
AAGAACGTG	BRDN00011	184	378	421	351	462	7
AAGAACTAC	BRDN00011	96	124	189	176	212	26
AAGAACTCA	BRDN00012	151	151	521	266	209	12
AAGAACTCG	BRDN00012	774	802	1155	1344	1893	85
AAGAACTCG	BRDN00012	70	71	431	139	267	0
AAGAACTCG	BRDN00012	163	266	286	445	347	35
AAGAACTGA	BRDN00011	181	175	473	320	364	16
AAGAACTGA	BRDN00012	134	96	125	429	376	3
AAGAACTGC	BRDN00011	294	291	483	431	317	56
AAGAACTGG	BRDN00012	152	87	63	322	226	0
AAGAACTGG	BRDN00011	285	345	786	619	335	32
AAGAACTTA	BRDN00011	148	135	97	322	332	0
AAGAACTTA	BRDN00011	135	98	27	257	419	10
AAGAACTTA	BRDN00011	188	64	101	348	198	3
AAGAACTTG	BRDN00012	128	125	152	202	200	2
AAGAACTTG	BRDN00012	180	167	159	187	262	16
AAGAAGAAA	BRDN00011	95	129	1	114	108	6
AAGAAGAAA	BRDN00011	219	297	1017	588	392	26
AAGAAGAAC	BRDN00011	273	255	1082	567	503	66
AAGAAGAAG	BRDN00011	357	270	1120	653	908	46
AAGAAGAAG	BRDN00011	484	376	384	875	682	54
AAGAAGAAT	BRDN00012	539	512	1604	766	650	56
AAGAAGACC	BRDN00011	161	170	202	246	186	13
AAGAAGACC	BRDN00012	154	209	841	366	536	22
AAGAAGACC	BRDN00012	252	146	823	378	322	21
AAGAAGACG	BRDN00012	172	119	519	329	455	0
AAGAAGACG	BRDN00011	133	109	17	128	78	6
AAGAAGACT	BRDN00012	273	350	575	449	277	21
AAGAAGACT	BRDN00012	274	194	198	433	605	9
AAGAAGAGA	BRDN00011	47	66	0	131	244	0
AAGAAGAGT	BRDN00012	449	413	2779	636	784	66
AAGAAGAGT	BRDN00011	344	278	404	655	523	50
AAGAAGATC	BRDN00012	158	98	2	292	175	0
AAGAAGATG	BRDN00011	206	126	407	249	300	11
AAGAAGATG	BRDN00012	362	382	1018	646	468	304
AAGAAGATG	BRDN00012	424	350	887	904	635	37
AAGAAGATT	BRDN00012	75	7	0	441	72	0
AAGAAGATT	BRDN00012	111	58	293	256	55	11
AAGAAGCAA	BRDN00011	79	96	80	201	191	0
AAGAAGCAG	BRDN00012	69	141	70	237	289	2
AAGAAGCAG	BRDN00012	285	212	112	548	821	24
AAGAAGCAT	BRDN00012	120	97	338	211	142	6
AAGAAGCCA	BRDN00012	86	74	5	263	240	14
AAGAAGCCA	BRDN00012	139	205	233	457	294	27
AAGAAGCCA	BRDN00012	115	116	82	267	230	6
AAGAAGCCC	BRDN00012	375	370	443	811	673	40
AAGAAGCCG	BRDN00011	410	229	890	783	550	29
AAGAAGCGC	BRDN00012	494	298	3282	735	931	76
AAGAAGCGC	BRDN00011	367	321	1073	572	699	10
AAGAAGCGC	BRDN00011	216	135	86	373	417	35
AAGAAGCGT	BRDN00012	194	194	262	460	409	39
AAGAAGCTG	BRDN00012	68	33	4	202	124	0
AAGAAGCTG	BRDN00012	392	406	338	779	976	26
AAGAAGCTT	BRDN00012	207	88	343	304	596	14
AAGAAGGAC	BRDN00012	116	63	294	235	141	4
AAGAAGGAT	BRDN00011	228	246	806	366	477	52
AAGAAGGCA	BRDN00012	184	166	2877	336	220	59
AAGAAGGCC	BRDN00012	173	168	137	467	457	0
AAGAAGGCG	BRDN00012	399	311	811	769	597	57
AAGAAGGG	BRDN00012	462	257	1537	747	748	54
AAGAAGGTC	BRDN00011	327	357	1494	568	826	93
AAGAAGGTC	BRDN00012	179	83	285	320	194	13
AAGAAGGTT	BRDN00011	484	321	121	791	745	43
AAGAAGTAG	BRDN00012	199	150	148	442	605	17
AAGAAGTAT	BRDN00011	121	97	996	263	86	1
AAGAAGTCA	BRDN00012	125	65	0	316	219	0
AAGAAGTCC	BRDN00011	126	43	2304	153	104	47
AAGAAGTCC	BRDN00012	187	189	673	438	311	29
AAGAAGTCT	BRDN00011	92	109	87	179	262	0
AAGAAGTGA	BRDN00011	247	322	51	423	440	12
AAGAAGTTA	BRDN00011	162	195	164	279	236	5
AAGAAGTTC	BRDN00011	81	100	1572	110	64	27
AAGAAGTTC	BRDN00011	197	124	84	357	132	6
AAGAATAAA	BRDN00012	146	287	123	226	280	52
AAGAATAAC	BRDN00011	126	115	536	183	274	18
AAGAATAAG	BRDN00011	76	29	163	177	354	31
AAGAATACC	BRDN00011	130	162	261	335	276	10
AAGAATACG	BRDN00012	125	141	372	224	357	26
AAGAATACG	BRDN00011	124	155	764	214	206	63
AAGAATACG	BRDN00012	116	16	3135	298	68	19
AAGAATAGC	BRDN00012	15	25	0	433	100	0
AAGAATATA	BRDN00012	95	7	0	224	55	0
AAGAATCCG	BRDN00012	327	373	484	782	456	44
AAGAATCCT	BRDN00012	112	4	0	305	72	0
AAGAATCGA	BRDN00011	184	322	485	228	220	16
AAGAATCGA	BRDN00012	224	197	115	320	278	4
AAGAATCTA	BRDN00011	133	79	31	263	478	14
AAGAATGAG	BRDN00012	186	124	227	308	392	3
AAGAATGAT	BRDN00012	294	449	345	522	279	39
AAGAATGCC	BRDN00011	571	360	888	703	677	72
AAGAATGGA	BRDN00012	199	194	102	237	348	7
AAGAATGGT	BRDN00012	90	78	21	198	222	0
AAGAATGTC	BRDN00011	122	139	6	282	274	7
AAGAATTAA	BRDN00011	358	402	600	559	540	32
AAGAATTAC	BRDN00012	45	0	0	413	36	0
AAGAATTAG	BRDN00012	366	269	618	591	313	40
AAGAATTGA	BRDN00011	130	64	208	185	156	32
AAGAATTGC	BRDN00012	127	0	0	292	127	0
AAGAATTTA	BRDN00012	106	69	346	204	122	6
AAGAATTTG	BRDN00012	281	71	0	487	656	0
AAGAATTTG	BRDN00012	111	189	901	262	186	71
AAGACAAAA	BRDN00012	424	340	672	605	522	63
AAGACAAAA	BRDN00011	144	129	307	115	232	4
AAGACAAAC	BRDN00011	224	245	571	448	211	68
AAGACAAAC	BRDN00012	171	156	1	342	316	0
AAGACAAAG	BRDN00012	367	327	316	669	875	14
AAGACAAAT	BRDN00011	93	36	25	297	321	0
AAGACAACA	BRDN00012	115	148	113	235	129	26
AAGACAACA	BRDN00012	201	17	0	382	149	0
AAGACAACA	BRDN00011	215	112	1010	336	600	20
AAGACAACT	BRDN00011	329	491	639	647	801	14
AAGACAAGA	BRDN00011	66	66	158	288	171	0
AAGACAAGC	BRDN00011	419	322	760	1001	1138	41
AAGACAAGT	BRDN00011	84	73	297	282	181	6
AAGACAAGT	BRDN00011	78	37	90	282	146	6
AAGACAATC	BRDN00011	219	229	367	388	265	19
AAGACACAA	BRDN00011	62	37	19	216	213	0
AAGACACAA	BRDN00012	108	132	245	342	300	48
AAGACACCA	BRDN00012	176	144	690	328	203	41
AAGACACCA	BRDN00012	357	232	37	747	540	13
AAGACACCC	BRDN00012	192	174	1034	298	194	20
AAGACACTG	BRDN00012	429	381	351	718	997	16
AAGACACTT	BRDN00011	297	298	236	593	574	54
AAGACAGAA	BRDN00012	200	180	94	221	322	1
AAGACAGAC	BRDN00011	42	17	0	121	73	0
AAGACAGAT	BRDN00011	350	28	1	615	284	0
AAGACAGCG	BRDN00011	293	271	76	549	413	5
AAGACAGCT	BRDN00012	235	308	1398	580	515	22
AAGACAGGA	BRDN00012	128	0	0	438	225	0
AAGACAGTA	BRDN00012	368	412	204	729	784	37
AAGACAGTA	BRDN00011	129	77	726	238	216	10
AAGACAGTG	BRDN00011	261	228	330	337	640	14
AAGACATAG	BRDN00011	159	144	225	247	109	16
AAGACATAG	BRDN00012	136	104	155	214	378	13
AAGACATCA	BRDN00012	381	179	998	578	862	4
AAGACATCT	BRDN00011	158	127	299	420	281	3
AAGACATGA	BRDN00011	222	138	669	356	741	25
AAGACCAAA	BRDN00012	224	337	94	681	640	9
AAGACCAAA	BRDN00012	190	157	70	325	309	19
AAGACCAAC	BRDN00012	115	0	1	717	358	0
AAGACCAAC	BRDN00012	171	114	55	365	244	37
AAGACCAAG	BRDN00012	175	272	644	335	531	12
AAGACCACC	BRDN00011	97	37	23	237	346	5
AAGACCACC	BRDN00012	390	224	1400	675	532	33
AAGACCAGC	BRDN00012	430	433	1480	894	731	40
AAGACCAGC	BRDN00012	252	250	629	681	347	31
AAGACCAGG	BRDN00011	411	289	69	889	782	59
AAGACCAGT	BRDN00012	88	7	987	353	235	28
AAGACCATA	BRDN00012	376	480	470	632	649	86
AAGACCATA	BRDN00012	127	141	127	305	304	20
AAGACCATG	BRDN00011	175	26	0	267	88	0
AAGACCATG	BRDN00012	130	69	108	327	219	22
AAGACCCAC	BRDN00012	161	177	88	350	135	0
AAGACCCCA	BRDN00011	307	335	271	662	679	69
AAGACCCCC	BRDN00011	214	195	222	484	572	0
AAGACCCTC	BRDN00012	257	198	224	467	447	18
AAGACCCTG	BRDN00012	450	466	550	895	703	6
AAGACCCTT	BRDN00011	298	201	151	652	494	35
AAGACCCTT	BRDN00012	285	199	9	368	154	14
AAGACCGAC	BRDN00011	209	432	373	480	249	18
AAGACCGGC	BRDN00012	222	32	0	1069	1110	0
AAGACCTCA	BRDN00011	190	140	11	478	493	19
AAGACCTCA	BRDN00012	497	359	408	1053	1317	9
AAGACCTGA	BRDN00011	100	2	89	295	96	0
AAGACCTGC	BRDN00012	431	428	1173	773	937	201
AAGACCTGC	BRDN00012	450	564	322	749	1026	21
AAGACCTTC	BRDN00012	92	1	1	316	296	0
AAGACCTTG	BRDN00011	313	216	286	437	886	9
AAGACCTTT	BRDN00011	151	88	342	231	209	32
AAGACGACC	BRDN00012	116	78	45	113	213	11
AAGACGACG	BRDN00011	243	172	553	514	410	120
AAGACGACT	BRDN00012	286	290	163	397	304	27
AAGACGAGT	BRDN00012	209	205	53	450	540	42
AAGACGAGT	BRDN00012	365	256	166	712	558	12
AAGACGCCG	BRDN00011	224	147	176	383	274	0
AAGACGCCT	BRDN00011	425	407	674	1008	1349	120
AAGACGCGG	BRDN00012	254	97	864	1088	743	22
AAGACGCGT	BRDN00011	202	156	127	279	436	27
AAGACGCTC	BRDN00011	245	234	647	397	522	38
AAGACGGAA	BRDN00011	463	459	199	772	944	20
AAGACGGGC	BRDN00011	358	264	471	833	1419	77
AAGACGGGC	BRDN00011	227	273	58	362	441	10
AAGACGTAC	BRDN00011	329	289	595	481	382	40
AAGACGTAG	BRDN00012	306	161	66	577	685	0
AAGACGTCA	BRDN00011	134	129	43	249	142	7
AAGACGTCA	BRDN00011	27	40	0	101	4	0
AAGACGTCC	BRDN00011	141	22	13	637	71	25
AAGACGTCC	BRDN00011	117	66	35	274	262	0
AAGACGTGG	BRDN00012	117	78	31	217	587	5
AAGACGTTG	BRDN00011	129	149	160	353	78	40
AAGACTAAG	BRDN00012	316	174	1447	599	805	66
AAGACTAAG	BRDN00011	95	119	530	168	153	2
AAGACTAGT	BRDN00011	269	213	671	440	508	11
AAGACTATG	BRDN00011	144	111	61	222	210	29
AAGACTCAC	BRDN00012	148	194	4	278	302	2
AAGACTCAG	BRDN00012	145	135	223	321	380	14
AAGACTCCC	BRDN00011	73	96	98	202	116	3
AAGACTCCC	BRDN00011	207	189	865	426	170	31
AAGACTCGA	BRDN00011	185	209	159	326	208	14
AAGACTCGT	BRDN00011	140	127	184	447	284	20
AAGACTCTA	BRDN00011	221	227	143	327	658	17
AAGACTCTG	BRDN00011	147	224	86	391	325	59
AAGACTGAG	BRDN00011	114	21	27	300	219	2
AAGACTGCA	BRDN00012	338	334	874	667	454	32
AAGACTGCC	BRDN00012	15	0	0	357	284	0
AAGACTGGA	BRDN00011	166	108	261	312	132	4
AAGACTTGA	BRDN00011	428	535	871	771	973	56
AAGACTTTA	BRDN00012	121	117	616	289	158	6
AAGACTTTG	BRDN00011	130	152	314	296	293	22
AAGAGAAAC	BRDN00011	488	475	857	822	1120	29
AAGAGAAAT	BRDN00012	88	139	170	295	394	22
AAGAGAACG	BRDN00012	352	365	499	505	619	49
AAGAGAACG	BRDN00011	315	189	29	688	535	29
AAGAGAACT	BRDN00011	404	454	354	761	862	47
AAGAGAACT	BRDN00011	242	144	119	394	469	0
AAGAGAAG	BRDN00011	219	262	272	591	559	15
AAGAGAAG	BRDN00011	388	175	13	799	1050	0
AAGAGAATA	BRDN00012	117	145	223	198	332	7
AAGAGAATA	BRDN00012	287	241	298	526	314	21
AAGAGAATA	BRDN00011	139	197	18	221	143	6
AAGAGAATG	BRDN00012	94	0	1	242	346	0
AAGAGACAC	BRDN00012	192	47	19	283	209	0
AAGAGACAG	BRDN00012	133	75	105	301	405	2
AAGAGACAG	BRDN00011	165	214	575	435	548	8
AAGAGACAT	BRDN00011	94	53	0	232	171	0
AAGAGACTA	BRDN00011	15	0	0	369	56	0
AAGAGACTG	BRDN00012	270	216	238	427	430	18
AAGAGAGA	BRDN00012	167	87	2	244	182	23
AAGAGAGCT	BRDN00011	153	112	16	152	97	24
AAGAGAGCT	BRDN00012	96	20	114	379	294	3
AAGAGATAC	BRDN00011	131	103	50	229	358	12
AAGAGATCA	BRDN00011	229	174	955	360	522	59
AAGAGATCA	BRDN00012	261	247	739	672	984	31
AAGAGATCA	BRDN00012	444	424	1196	740	753	44
AAGAGATCA	BRDN00012	354	364	325	671	674	31
AAGAGATCC	BRDN00012	150	127	79	290	317	0
AAGAGATGA	BRDN00011	82	43	29	160	157	0
AAGAGATGC	BRDN00012	401	350	1100	714	630	31
AAGAGATGC	BRDN00011	171	84	11	222	70	0
AAGAGATTA	BRDN00012	208	8	114	390	520	0
AAGAGATTA	BRDN00011	38	62	0	166	94	31
AAGAGATTC	BRDN00011	422	348	1072	764	942	4
AAGAGATTC	BRDN00012	42	8	0	116	24	0
AAGAGATTC	BRDN00012	49	0	87	393	247	11
AAGAGATTC	BRDN00012	233	265	324	385	311	30
AAGAGATTG	BRDN00011	61	39	0	119	61	0
AAGAGATTT	BRDN00011	236	106	30	341	207	0
AAGAGCAAA	BRDN00012	205	225	1434	585	865	21
AAGAGCAAA	BRDN00011	203	153	199	341	344	10
AAGAGCACT	BRDN00011	167	181	151	343	269	1
AAGAGCATC	BRDN00011	259	229	672	470	154	37
AAGAGCATT	BRDN00011	134	151	198	291	236	22
AAGAGCCAA	BRDN00011	241	200	2701	462	363	23
AAGAGCCAC	BRDN00011	94	79	39	156	110	0
AAGAGCCAC	BRDN00011	519	508	2421	980	1175	19
AAGAGCCAC	BRDN00012	436	364	351	726	698	30
AAGAGCCAG	BRDN00012	113	59	212	214	200	0
AAGAGCCCA	BRDN00011	211	138	1078	344	473	10
AAGAGCCGG	BRDN00012	139	32	37	276	312	5
AAGAGCCGG	BRDN00012	457	311	518	687	866	16
AAGAGCCTG	BRDN00012	109	134	40	269	144	4
AAGAGCCTG	BRDN00012	138	70	1	245	222	11
AAGAGCCTT	BRDN00011	185	179	310	374	372	6
AAGAGCGAA	BRDN00012	388	339	495	728	1035	3
AAGAGCGAG	BRDN00011	326	313	1737	454	494	17
AAGAGCGCA	BRDN00011	128	134	225	179	220	13
AAGAGCGGA	BRDN00011	118	60	93	326	220	3
AAGAGCGTG	BRDN00011	132	105	89	264	331	13
AAGAGCTAC	BRDN00012	161	27	0	514	446	0
AAGAGCTCA	BRDN00012	301	322	519	728	714	28
AAGAGCTCG	BRDN00011	298	324	249	608	452	8
AAGAGCTGC	BRDN00012	410	483	409	724	589	40
AAGAGCTGG	BRDN00012	410	430	387	799	601	50
AAGAGCTTC	BRDN00012	74	3	0	179	74	0
AAGAGCTTC	BRDN00012	360	314	664	515	379	53
AAGAGCTTT	BRDN00012	134	172	2	378	255	0
AAGAGCTTT	BRDN00011	175	130	84	225	458	0
AAGAGGAAA	BRDN00012	240	220	39	476	505	11
AAGAGGAAC	BRDN00012	224	104	184	347	322	27
AAGAGGACC	BRDN00011	293	170	258	356	413	4
AAGAGGAG	BRDN00011	499	406	251	792	863	50
AAGAGGAG	BRDN00012	119	178	228	196	210	2
AAGAGGAG	BRDN00012	141	53	2	337	396	0
AAGAGGAG	BRDN00011	113	66	181	226	138	0
AAGAGGAGT	BRDN00012	165	14	0	477	284	0
AAGAGGAGT	BRDN00012	338	303	206	694	500	33
AAGAGGCAT	BRDN00012	177	141	486	249	349	30
AAGAGGCAT	BRDN00012	37	11	0	387	70	0
AAGAGGCCA	BRDN00011	276	145	667	358	202	43
AAGAGGCG	BRDN00012	456	297	1597	719	918	33
AAGAGGCTG	BRDN00012	61	100	488	261	498	8
AAGAGGCTG	BRDN00011	192	172	38	266	289	3
AAGAGGGA	BRDN00011	298	268	1093	243	165	25
AAGAGGGAT	BRDN00012	323	137	356	815	682	29
AAGAGGGG	BRDN00011	120	96	9	235	151	4
AAGAGGGG	BRDN00012	167	141	511	267	313	13
AAGAGGGT	BRDN00011	139	237	79	224	341	51
AAGAGGGT	BRDN00011	101	60	610	103	187	26
AAGAGGTAA	BRDN00011	89	10	0	276	177	0
AAGAGGTAG	BRDN00012	150	67	80	264	348	1
AAGAGGTCA	BRDN00012	189	86	413	301	468	40
AAGAGGTTA	BRDN00011	286	300	720	389	447	52
AAGAGGTTA	BRDN00012	77	2	0	508	314	0
AAGAGGTTC	BRDN00012	157	156	933	374	410	10
AAGAGTAAA	BRDN00012	166	172	32	237	289	11
AAGAGTAAG	BRDN00011	105	53	1	139	212	0
AAGAGTACC	BRDN00012	133	98	587	352	424	22
AAGAGTAGC	BRDN00011	94	84	97	346	374	9
AAGAGTAGT	BRDN00012	373	387	692	688	926	47
AAGAGTATG	BRDN00011	267	325	362	677	690	78
AAGAGTCAC	BRDN00012	260	156	14	505	445	26
AAGAGTCCC	BRDN00012	183	59	357	375	136	0
AAGAGTCGG	BRDN00012	192	21	0	279	156	0
AAGAGTCTT	BRDN00011	83	4	1	373	416	0
AAGAGTTAC	BRDN00011	230	192	926	341	544	15
AAGAGTTCA	BRDN00011	331	331	208	626	310	38
AAGAGTTCA	BRDN00012	237	157	236	357	405	25
AAGAGTTCG	BRDN00011	129	118	697	298	260	12
AAGAGTTTC	BRDN00012	375	215	397	637	780	29
AAGAGTTTG	BRDN00012	268	160	216	422	648	2
AAGAGTTTG	BRDN00011	359	388	738	695	576	32
AAGATAAAA	BRDN00011	137	102	84	228	116	7
AAGATAAAA	BRDN00011	428	381	402	747	984	31
AAGATAAAG	BRDN00011	265	447	3312	562	626	111
AAGATAAAG	BRDN00011	201	229	158	377	166	5
AAGATAAAG	BRDN00011	140	31	37	231	313	0
AAGATACAA	BRDN00011	80	33	33	163	287	0
AAGATACAC	BRDN00012	548	422	822	1007	840	24
AAGATACAG	BRDN00012	98	149	32	145	43	0
AAGATACCT	BRDN00011	124	82	152	311	331	12
AAGATACCT	BRDN00012	333	248	418	736	678	11
AAGATACGA	BRDN00011	13	0	1	329	118	0
AAGATACGA	BRDN00012	100	119	134	214	88	0
AAGATACGG	BRDN00011	106	90	128	285	188	22
AAGATACTT	BRDN00011	323	279	472	678	801	30
AAGATAGCA	BRDN00012	327	226	5	637	670	0
AAGATAGG	BRDN00012	342	112	270	626	508	5
AAGATAGTG	BRDN00012	294	235	418	443	369	18

AAGATATCA	BRDN00011	347	316	289	575	280	22
AAGATATGG	BRDN00011	154	39	0	356	403	0
AAGATATTA	BRDN00012	24	0	0	256	222	0
AAGATCAAA	BRDN00012	268	272	14162	507	734	262
AAGATCAAG	BRDN00012	163	88	23	288	207	0
AAGATCACC	BRDN00011	476	297	737	683	690	54
AAGATCACG	BRDN00011	110	105	40	235	106	7
AAGATCACT	BRDN00012	147	76	11	336	340	26
AAGATCAGT	BRDN00012	255	123	47	439	373	1
AAGATCAGT	BRDN00011	73	86	102	164	283	0
AAGATCCCA	BRDN00012	21	0	1	339	274	0
AAGATCCCA	BRDN00012	202	232	907	287	255	30
AAGATCCCC	BRDN00011	291	228	630	672	659	27
AAGATCCGA	BRDN00012	89	122	147	212	294	0
AAGATCCTG	BRDN00012	169	130	277	352	625	0
AAGATCGAA	BRDN00011	367	210	1786	602	437	25
AAGATCGCC	BRDN00011	74	18	0	236	250	0
AAGATCGCG	BRDN00012	404	350	570	680	806	39
AAGATCGGG	BRDN00012	362	350	422	564	651	20
AAGATCGTA	BRDN00011	404	377	385	915	1128	74
AAGATCGTG	BRDN00012	300	314	1214	503	627	33
AAGATCTCC	BRDN00012	255	255	380	579	685	33
AAGATCTCG	BRDN00011	141	15	7	266	51	0
AAGATCTGA	BRDN00011	347	305	1111	648	750	27
AAGATCTGC	BRDN00011	247	164	159	424	361	5
AAGATCTGC	BRDN00012	212	87	51	365	162	6
AAGATCTGG	BRDN00012	216	228	286	416	308	14
AAGATCTGT	BRDN00011	420	371	985	663	876	70
AAGATCTTC	BRDN00012	32	0	0	317	75	0
AAGATCTTG	BRDN00011	220	209	239	448	581	2
AAGATCTTG	BRDN00011	109	0	15	146	143	0
AAGATCTTT	BRDN00011	376	301	1589	695	767	21
AAGATGAAC	BRDN00012	332	358	136	628	695	27
AAGATGAAC	BRDN00011	500	371	9661	895	1132	135
AAGATGAAC	BRDN00012	120	110	144	307	155	6
AAGATGAAG	BRDN00012	161	170	123	374	534	16
AAGATGAAT	BRDN00012	213	235	41	416	360	20
AAGATGAAT	BRDN00012	304	166	731	461	449	31
AAGATGACA	BRDN00012	140	1	0	541	600	0
AAGATGACA	BRDN00012	362	343	672	733	489	21
AAGATGACC	BRDN00012	155	208	1	372	387	23
AAGATGACC	BRDN00012	64	0	1	190	175	0
AAGATGACG	BRDN00011	349	61	12	1033	1246	0
AAGATGAGA	BRDN00011	83	0	0	199	21	0
AAGATGAGA	BRDN00011	89	68	189	231	235	7
AAGATGAGA	BRDN00012	444	475	1124	757	521	28
AAGATGAGC	BRDN00011	55	1	0	430	258	0
AAGATGATA	BRDN00012	129	67	83	328	456	41
AAGATGATT	BRDN00011	143	65	1	231	275	24
AAGATGCAC	BRDN00012	358	324	59	626	585	4
AAGATGCAG	BRDN00012	115	83	35	248	430	0
AAGATGCAG	BRDN00012	173	78	79	275	361	7
AAGATGCCA	BRDN00012	333	370	524	616	498	24
AAGATGCCC	BRDN00012	197	70	1	376	300	0
AAGATGCCC	BRDN00011	286	146	81	509	498	10
AAGATGCCG	BRDN00011	411	384	386	595	367	25
AAGATGCCT	BRDN00012	316	200	17	691	783	5
AAGATGCGC	BRDN00012	363	64	0	875	581	0
AAGATGCGG	BRDN00011	125	70	12	219	313	7
AAGATGCTA	BRDN00012	143	16	2	554	1188	0
AAGATGCTC	BRDN00011	124	94	147	185	460	19
AAGATGCTC	BRDN00011	107	65	53	150	374	1
AAGATGCTC	BRDN00012	190	273	705	396	238	14
AAGATGCTG	BRDN00012	119	70	376	189	103	0
AAGATGCTT	BRDN00012	429	33	1037	676	610	14
AAGATGCTT	BRDN00011	150	146	305	231	361	4
AAGATGCTT	BRDN00012	114	99	189	207	72	29
AAGATGGAG	BRDN00012	302	262	106	577	574	3
AAGATGGCA	BRDN00011	143	142	85	310	417	6
AAGATGGCA	BRDN00012	155	177	39	367	426	11
AAGATGGCT	BRDN00012	269	292	248	454	352	14
AAGATGGGC	BRDN00012	166	146	2	280	223	6
AAGATGGGT	BRDN00011	114	124	267	216	178	0
AAGATGGTC	BRDN00011	228	146	1076	393	339	21
AAGATGGTG	BRDN00011	214	215	164	469	560	0
AAGATGGTG	BRDN00012	338	403	338	555	350	52
AAGATGTAG	BRDN00012	173	85	8	329	307	6
AAGATGTAG	BRDN00011	343	260	128	843	818	25
AAGATGTAG	BRDN00012	155	217	39	385	469	44
AAGATGTCA	BRDN00012	211	97	344	325	295	11
AAGATGTCC	BRDN00012	232	273	351	448	329	48
AAGATGTCG	BRDN00011	235	208	305	503	471	8
AAGATGTGA	BRDN00011	322	454	817	654	583	40
AAGATGTGG	BRDN00011	145	136	8	188	124	9
AAGATGTTA	BRDN00012	153	107	97	335	179	5
AAGATGTTG	BRDN00012	333	159	268	548	562	11
AAGATGTTG	BRDN00011	405	397	442	519	498	93
AAGATTATG	BRDN00012	132	74	314	164	123	0
AAGATTCAT	BRDN00012	140	161	207	329	400	8
AAGATTCCA	BRDN00011	149	118	9	261	421	0
AAGATTCGT	BRDN00011	108	128	166	196	150	8
AAGATTCTC	BRDN00011	146	11	212	476	347	10
AAGATTGGT	BRDN00011	192	189	1254	499	506	53
AAGATTTAA	BRDN00012	184	238	621	345	506	4
AAGATTTCA	BRDN00011	114	99	21	362	411	0
AAGATTTGG	BRDN00012	269	231	524	592	941	20
AAGCAAAAC	BRDN00012	378	272	541	594	743	36
AAGCAAACG	BRDN00012	128	113	670	240	235	10
AAGCAAACG	BRDN00012	302	233	1203	791	682	28
AAGCAAACT	BRDN00012	350	299	92	545	606	78
AAGCAAACT	BRDN00012	442	331	860	658	851	37
AAGCAAAGG	BRDN00012	406	379	344	788	661	66
AAGCAAAGT	BRDN00012	42	0	0	205	106	0
AAGCAAATC	BRDN00012	60	0	0	306	147	0
AAGCAACTA	BRDN00012	206	172	990	285	338	21
AAGCAACTT	BRDN00011	266	218	231	441	978	35
AAGCAACTT	BRDN00012	239	169	88	470	378	16
AAGCAACTT	BRDN00012	258	390	119	468	609	39
AAGCAAGAA	BRDN00011	188	300	80	334	291	5
AAGCAAGAC	BRDN00012	462	410	310	966	1323	145
AAGCAAGAG	BRDN00011	137	140	337	235	438	14
AAGCAAGCC	BRDN00012	239	149	81	666	561	0
AAGCAAGCC	BRDN00012	529	484	625	971	1041	29
AAGCAAGGC	BRDN00012	152	48	10	255	183	14
AAGCAAGTG	BRDN00011	350	296	155	813	719	24
AAGCAATAA	BRDN00011	65	1	0	259	137	0
AAGCAATAC	BRDN00012	24	0	1	321	203	0
AAGCAATAG	BRDN00012	213	113	87	205	334	16
AAGCAATCG	BRDN00012	182	122	409	329	291	83
AAGCAATGG	BRDN00011	148	162	2	197	203	31
AAGCAATGT	BRDN00011	104	72	100	174	587	43
AAGCACACG	BRDN00011	288	290	107	609	590	19
AAGCACACT	BRDN00011	354	446	438	595	791	67
AAGCACAGA	BRDN00012	226	130	75	698	553	13
AAGCACAGC	BRDN00011	87	10	5	240	123	0
AAGCACATA	BRDN00011	165	66	190	410	160	0
AAGCACATA	BRDN00011	267	245	119	749	546	5
AAGCACATC	BRDN00012	341	399	1290	707	903	32
AAGCACATC	BRDN00012	26	0	0	386	124	0
AAGCACCAA	BRDN00011	168	243	50	416	247	14
AAGCACCAA	BRDN00012	127	149	229	327	147	6
AAGCACCAC	BRDN00011	205	145	62	321	642	13
AAGCACCAG	BRDN00012	178	138	866	280	332	32
AAGCACCTA	BRDN00012	84	0	1	297	266	0
AAGCACGAA	BRDN00012	226	305	13	570	212	17
AAGCACGAC	BRDN00011	155	152	507	232	166	20
AAGCACGCA	BRDN00011	47	31	1	162	314	0
AAGCACGCA	BRDN00012	230	233	155	312	419	9
AAGCACGGA	BRDN00011	176	143	398	378	273	7
AAGCACGGA	BRDN00012	24	23	0	381	149	0
AAGCACGGG	BRDN00012	452	265	473	722	508	10
AAGCACGGG	BRDN00011	267	226	681	468	486	12
AAGCACGTT	BRDN00011	73	185	263	316	217	8
AAGCACTAG	BRDN00012	504	660	741	874	1063	63
AAGCACTCA	BRDN00011	336	328	1076	550	801	32
AAGCACTGA	BRDN00012	428	427	221	675	425	19
AAGCACTGG	BRDN00012	452	396	310	867	609	29
AAGCACTGG	BRDN00011	194	139	324	329	362	17
AAGCACTGG	BRDN00011	280	169	342	434	334	11
AAGCACTGG	BRDN00011	315	220	1887	584	729	53
AAGCACTTT	BRDN00011	237	212	307	306	366	6
AAGCAGAAC	BRDN00011	224	241	1503	481	532	31
AAGCAGAAG	BRDN00012	163	161	248	264	320	21
AAGCAGACA	BRDN00011	269	138	710	427	287	61
AAGCAGAGA	BRDN00011	286	115	513	466	310	0
AAGCAGAGC	BRDN00012	468	354	115	734	583	59
AAGCAGAGG	BRDN00012	275	343	2083	612	841	27
AAGCAGAGG	BRDN00012	92	93	94	262	108	0
AAGCAGAGG	BRDN00012	345	398	924	637	465	65
AAGCAGAGT	BRDN00011	161	7	88	357	103	0
AAGCAGATG	BRDN00011	98	167	449	177	350	10
AAGCAGCAA	BRDN00012	176	173	123	303	197	28
AAGCAGCAG	BRDN00011	48	62	5	159	151	0
AAGCAGCAG	BRDN00011	407	192	706	972	1049	6
AAGCAGCAG	BRDN00011	197	235	703	316	499	19
AAGCAGCAG	BRDN00012	227	0	0	472	62	0
AAGCAGCAT	BRDN00012	394	503	1019	738	558	95
AAGCAGCCA	BRDN00012	222	169	430	420	466	27
AAGCAGCCA	BRDN00012	227	44	30	587	445	3
AAGCAGCCC	BRDN00012	348	281	1312	696	614	35
AAGCAGCCT	BRDN00011	129	149	652	210	234	3
AAGCAGCGT	BRDN00011	333	105	47	709	419	1
AAGCAGCTT	BRDN00011	171	9	1	464	292	0
AAGCAGGAA	BRDN00011	150	125	147	190	125	5
AAGCAGGAC	BRDN00011	93	72	207	79	30	27
AAGCAGGAG	BRDN00012	304	180	85	935	957	0
AAGCAGGCC	BRDN00012	214	181	201	462	360	0
AAGCAGGGC	BRDN00012	404	417	1103	854	673	62
AAGCAGGGT	BRDN00012	164	79	2	252	210	3
AAGCAGGTC	BRDN00011	142	160	167	335	221	35
AAGCAGTAC	BRDN00011	227	176	176	454	719	46
AAGCAGTAT	BRDN00011	169	153	63	274	677	12
AAGCAGTGC	BRDN00012	189	107	438	239	172	10
AAGCAGTGT	BRDN00012	68	80	21	237	202	7
AAGCAGTTA	BRDN00011	170	196	345	334	198	48
AAGCAGTTG	BRDN00012	218	262	199	471	459	67
AAGCAGTTT	BRDN00012	103	69	345	234	547	4
AAGCATAAA	BRDN00011	281	213	162	502	553	11
AAGCATAAT	BRDN00011	139	93	58	292	181	24
AAGCATACT	BRDN00011	331	277	102	489	701	63
AAGCATCAG	BRDN00012	310	259	445	587	757	2
AAGCATCCC	BRDN00012	489	419	616	807	951	118
AAGCATCCC	BRDN00012	128	41	60	236	365	0
AAGCATCTC	BRDN00011	329	422	1937	593	912	140
AAGCATGAT	BRDN00011	123	174	456	281	457	13
AAGCATGGC	BRDN00011	164	88	12	348	271	3
AAGCATGGC	BRDN00011	152	131	0	287	270	12
AAGCATTAT	BRDN00012	172	300	508	451	382	70
AAGCATTGC	BRDN00012	160	67	125	656	470	0
AAGCCAAAA	BRDN00012	284	216	398	606	645	14
AAGCCAAAA	BRDN00012	93	125	108	495	482	0
AAGCCAAAC	BRDN00012	109	124	210	192	158	0
AAGCCAAAC	BRDN00012	283	417	11550	473	695	238
AAGCCAAAC	BRDN00011	388	295	3514	872	1107	55
AAGCCAAAT	BRDN00011	120	104	433	310	428	11
AAGCCAACA	BRDN00012	451	301	178	726	663	77
AAGCCAACA	BRDN00012	421	317	350	541	728	82
AAGCCAACA	BRDN00011	66	104	238	180	183	0
AAGCCAAGG	BRDN00011	113	124	43	180	159	25
AAGCCAAGG	BRDN00011	372	415	877	830	603	27
AAGCCAATC	BRDN00011	192	204	175	190	476	4
AAGCCACAA	BRDN00011	14	0	0	218	200	0
AAGCCACAA	BRDN00011	237	281	19	368	203	9
AAGCCACAC	BRDN00012	398	356	729	847	777	47
AAGCCACAC	BRDN00012	134	115	26	329	515	5
AAGCCACAG	BRDN00012	115	79	556	201	367	15
AAGCCACAG	BRDN00012	357	376	426	712	426	38
AAGCCACCT	BRDN00012	322	339	236	699	810	11
AAGCCACGG	BRDN00012	129	38	92	264	353	0
AAGCCACGG	BRDN00012	207	234	65	473	570	26
AAGCCACTA	BRDN00012	183	254	185	337	778	12
AAGCCAGAC	BRDN00012	292	304	373	588	642	726
AAGCCAGAG	BRDN00012	57	35	22	138	79	0
AAGCCAGGA	BRDN00011	158	25	130	240	124	4
AAGCCAGTG	BRDN00012	175	171	333	302	196	9
AAGCCATAA	BRDN00012	88	138	109	238	349	0
AAGCCATAC	BRDN00011	241	266	408	457	196	50
AAGCCATCC	BRDN00011	346	337	627	612	371	22
AAGCCATCC	BRDN00012	364	325	685	576	950	64
AAGCCATGG	BRDN00011	158	164	37	298	313	49
AAGCCATGT	BRDN00011	268	255	624	401	324	16
AAGCCATTG	BRDN00012	378	290	1761	698	793	55
AAGCCCAAA	BRDN00011	386	298	517	559	455	4
AAGCCCAAC	BRDN00011	10	0	0	344	272	0
AAGCCCACT	BRDN00012	277	215	317	423	436	2
AAGCCCACT	BRDN00012	385	44	2	780	101	0
AAGCCCAGC	BRDN00012	367	354	767	632	362	8
AAGCCCAGG	BRDN00012	394	285	413	727	519	52
AAGCCCAGG	BRDN00012	80	79	71	213	285	5
AAGCCCATC	BRDN00011	113	0	0	230	128	0
AAGCCCCAC	BRDN00011	88	136	45	224	400	11
AAGCCCCAC	BRDN00011	610	521	259	817	636	64
AAGCCCCAG	BRDN00012	514	513	726	979	1360	83
AAGCCCCAT	BRDN00011	388	382	886	619	945	30
AAGCCCCAT	BRDN00012	363	322	140	766	805	17
AAGCCCGAC	BRDN00011	281	206	675	420	647	41
AAGCCCGAG	BRDN00012	161	156	73	301	83	0
AAGCCCGAG	BRDN00011	131	120	126	244	266	10
AAGCCCGCG	BRDN00011	291	241	130	658	445	20
AAGCCCTAT	BRDN00011	255	284	909	717	324	45
AAGCCCTAT	BRDN00012	180	149	160	354	227	2
AAGCCCTCC	BRDN00012	36	0	0	244	190	0
AAGCCCTCT	BRDN00011	151	167	706	304	276	38
AAGCCCTGC	BRDN00011	368	358	92	718	618	65
AAGCCCTGG	BRDN00011	143	54	0	397	249	20
AAGCCGAAT	BRDN00011	410	377	2413	743	934	64
AAGCCGAGA	BRDN00011	98	53	21	299	116	5
AAGCCGATC	BRDN00011	164	227	107	280	260	10
AAGCCGCAA	BRDN00012	422	414	1115	1018	1042	37
AAGCCGCAG	BRDN00012	161	81	1503	228	228	23
AAGCCGCAG	BRDN00011	81	139	126	220	421	23
AAGCCGCCT	BRDN00011	96	90	84	322	412	27
AAGCCGCGG	BRDN00012	295	262	644	657	431	21
AAGCCGGAC	BRDN00011	197	317	332	485	683	20
AAGCCGGGA	BRDN00011	77	194	145	232	79	9
AAGCCGGGC	BRDN00012	132	50	308	215	251	0
AAGCCGGGC	BRDN00011	83	86	464	125	187	0
AAGCCGGTG	BRDN00011	270	275	1063	525	467	55
AAGCCGTAG	BRDN00011	87	79	90	304	483	3
AAGCCGTCA	BRDN00012	336	274	2668	704	637	61
AAGCCGTGA	BRDN00011	278	112	63	391	643	0
AAGCCGTTT	BRDN00012	232	35	8	630	290	0
AAGCCTAAC	BRDN00011	107	60	66	279	373	61
AAGCCTAAG	BRDN00012	203	282	180	349	512	33
AAGCCTAAT	BRDN00011	161	267	709	314	431	54
AAGCCTACT	BRDN00011	424	322	404	765	769	7
AAGCCTAGA	BRDN00012	289	232	288	386	397	21
AAGCCTGAA	BRDN00011	242	210	612	378	362	38
AAGCCTGCA	BRDN00012	129	195	122	283	215	9
AAGCCTGGG	BRDN00012	363	235	481	576	853	49
AAGCCTGGT	BRDN00012	209	120	6	217	232	29
AAGCCTGGT	BRDN00011	295	201	220	542	441	8
AAGCCTGTG	BRDN00012	650	448	1257	964	1052	30
AAGCCTGTT	BRDN00012	243	175	262	312	498	0
AAGCCTTCA	BRDN00012	139	89	179	259	204	12
AAGCCTTCA	BRDN00012	402	526	1136	760	1109	52
AAGCCTTGA	BRDN00012	266	209	2	528	651	8
AAGCGAAAG	BRDN00012	138	210	115	298	251	22
AAGCGAATA	BRDN00011	107	72	0	212	171	0
AAGCGACAG	BRDN00012	340	219	478	599	546	7
AAGCGACCG	BRDN00012	143	245	721	237	230	127
AAGCGACTC	BRDN00011	170	165	35	332	376	16
AAGCGACTG	BRDN00012	126	67	222	307	276	6
AAGCGAGGA	BRDN00012	135	102	1274	287	158	26
AAGCGAGGT	BRDN00011	408	390	1407	555	473	104
AAGCGAGGT	BRDN00011	301	261	424	588	785	8
AAGCGATAA	BRDN00011	2	0	0	196	100	0
AAGCGATAA	BRDN00012	70	87	84	146	190	0
AAGCGATAG	BRDN00012	418	514	950	795	886	44
AAGCGATTG	BRDN00011	121	112	131	210	135	0
AAGCGCAGC	BRDN00011	200	159	1127	359	573	23
AAGCGCAGG	BRDN00011	128	0	0	351	548	0
AAGCGCATG	BRDN00011	208	261	75	411	1109	49
AAGCGCATG	BRDN00011	187	158	2	250	287	0
AAGCGCATT	BRDN00012	306	282	1031	734	440	16
AAGCGCCAG	BRDN00011	327	272	508	648	746	22
AAGCGCCCA	BRDN00012	110	78	246	258	126	19
AAGCGCCCT	BRDN00011	352	320	134	607	632	35
AAGCGCTCA	BRDN00012	341	99	0	595	730	0
AAGCGCTCC	BRDN00012	160	113	32	277	206	9
AAGCGCTGC	BRDN00012	459	531	769	843	680	59
AAGCGCTGG	BRDN00012	444	388	977	852	1328	46
AAGCGCTTC	BRDN00011	89	92	138	163	199	106
AAGCGCTTG	BRDN00011	185	197	34	245	384	13
AAGCGCTTG	BRDN00011	243	149	140	487	200	5
AAGCGCTTT	BRDN00011	123	122	263	262	225	0
AAGCGGAAC	BRDN00012	381	342	1207	552	570	43
AAGCGGAAC	BRDN00011	77	25	0	354	288	0
AAGCGGACA	BRDN00011	93	92	2382	194	341	54
AAGCGGACC	BRDN00012	501	442	781	725	626	29
AAGCGGACC	BRDN00012	48	0	0	267	67	0
AAGCGGACC	BRDN00011	104	161	308	265	653	4
AAGCGGAGA	BRDN00011	693	583	592	1043	1416	51
AAGCGGAGC	BRDN00011	135	129	78	236	391	6
AAGCGGATT	BRDN00011	263	260	182	442	223	12
AAGCGGCCG	BRDN00011	234	203	375	519	302	8
AAGCGGCGT	BRDN00012	111	19	0	149	131	0
AAGCGGCTC	BRDN00011	335	300	308	593	427	115
AAGCGGCTG	BRDN00011	132	101	40	246	177	6
AAGCGGGAC	BRDN00011	129	59	64	240	263	0
AAGCGGGCA	BRDN00012	617	734	2151	1281	1170	104
AAGCGGGCT	BRDN00012	275	258	208	642	557	41
AAGCGGTAA	BRDN00011	195	235	1151	361	460	29
AAGCGGTCA	BRDN00012	376	402	412	602	541	76
AAGCGGTTA	BRDN00012	274	264	594	713	463	23
AAGCGGTTC	BRDN00012	212	200	136	387	231	14
AAGCGTAAG	BRDN00011	289	396	1996	529	352	40
AAGCGTCAA	BRDN00011	477	498	600	838	687	20
AAGCGTGGA	BRDN00012	263	14	0	934	853	0
AAGCGTGGA	BRDN00012	116	146	1114	226	165	16
AAGCGTGGC	BRDN00012	415	351	540	548	641	40
AAGCGTTGA	BRDN00011	66	70	404	142	55	0
AAGCGTTGA	BRDN00011	282	268	1041	468	324	50
AAGCGTTGG	BRDN00012	271	237	117	514	344	49
AAGCTAAAC	BRDN00012	113	98	135	233	175	11
AAGCTAACG	BRDN00012	79	30	0	155	120	10
AAGCTAAGT	BRDN00011	103	103	381	237	128	0
AAGCTACTG	BRDN00011	286	334	429	526	519	11
AAGCTACTT	BRDN00011	240	181	583	262	253	8
AAGCTAGCA	BRDN00011	208	201	1320	441	230	43
AAGCTAGTA	BRDN00011	459	516	1161	683	497	276
AAGCTAGTG	BRDN00012	233	233	125	284	327	0
AAGCTATAA	BRDN00012	331	312	348	595	443	5
AAGCTCAAC	BRDN00012	84	14	342	580	409	13
AAGCTCAAC	BRDN00011	456	333	335	826	782	60
AAGCTCAGC	BRDN00012	204	237	13	415	456	36
AAGCTCATA	BRDN00011	84	33	28	198	151	30
AAGCTCATC	BRDN00011	288	312	2838	600	712	58
AAGCTCCAC	BRDN00012	210	192	6	359	274	0
AAGCTCCAG	BRDN00012	153	93	73	352	320	8
AAGCTCCCC	BRDN00011	316	193	1385	623	485	47
AAGCTCCCC	BRDN00011	334	331	1419	891	1138	42
AAGCTCCGG	BRDN00011	67	42	0	160	237	13
AAGCTCCTC	BRDN00012	296	320	402	588	868	62
AAGCTCGAA	BRDN00011	156	221	911	410	455	21
AAGCTCGCC	BRDN00012	209	188	251	393	385	24
AAGCTCGCG	BRDN00012	169	152	119	212	136	1
AAGCTCGCT	BRDN00011	395	542	512	784	852	59
AAGCTCTAC	BRDN00012	145	164	65	388	349	8
AAGCTCTAG	BRDN00012	189	102	28	266	345	0
AAGCTCTGT	BRDN00012	324	358	57	644	852	109
AAGCTCTTG	BRDN00011	149	95	436	215	129	9
AAGCTGAAC	BRDN00011	104	86	27	130	98	7
AAGCTGAAG	BRDN00011	148	262	575	396	215	23
AAGCTGACA	BRDN00012	378	365	577	584	596	61
AAGCTGACA	BRDN00012	130	58	99	267	295	17
AAGCTGACA	BRDN00012	152	7	0	252	95	0
AAGCTGACT	BRDN00011	92	65	9	226	247	10
AAGCTGAGG	BRDN00011	153	192	59	308	124	34
AAGCTGATA	BRDN00011	125	186	478	360	357	9
AAGCTGATG	BRDN00012	101	50	311	169	223	17
AAGCTGATT	BRDN00012	297	274	1558	650	618	51
AAGCTGCAA	BRDN00012	287	309	959	499	313	43
AAGCTGCAC	BRDN00012	409	405	57	859	1129	35
AAGCTGCAG	BRDN00012	105	128	169	206	217	7
AAGCTGCAG	BRDN00012	161	96	144	280	348	0
AAGCTGCAT	BRDN00011	60	0	0	322	215	0
AAGCTGCTC	BRDN00012	359	284	207	680	701	103
AAGCTGCTG	BRDN00011	113	89	98	227	167	0
AAGCTGCTG	BRDN00011	363	201	96	745	854	5
AAGCTGGAC	BRDN00012	280	251	13	772	574	12
AAGCTGGAG	BRDN00011	180	207	259	367	399	0
AAGCTGGCA	BRDN00012	190	168	186	379	614	0
AAGCTGGCT	BRDN00012	119	134	77	157	383	12
AAGCTGGTC	BRDN00011	155	41	167	480	340	20
AAGCTGTAA	BRDN00012	213	114	171	434	488	9
AAGCTGTAA	BRDN00012	135	89	55	182	313	21
AAGCTGTAT	BRDN00012	175	202	943	286	184	30
AAGCTGTCC	BRDN00012	442	282	645	596	548	36
AAGCTGTCG	BRDN00012	303	305	542	572	574	46
AAGCTGTCG	BRDN00012	82	34	254	132	303	11
AAGCTGTGG	BRDN00011	119	190	387	344	297	71
AAGCTGTTA	BRDN00012	111	85	222	191	206	8
AAGCTGTTT	BRDN00012	154	162	32	223	117	56
AAGCTGTTT	BRDN00012	411	268	896	759	1067	117
AAGCTTAAG	BRDN00012	106	184	198	268	388	14
AAGCTTACA	BRDN00011	461	299	980	666	757	73
AAGCTTAGT	BRDN00011	106	55	126	265	102	12
AAGCTTATC	BRDN00012	322	236	903	670	646	47
AAGCTTATT	BRDN00012	182	191	545	181	499	0
AAGCTTATT	BRDN00011	131	23	0	664	347	0
AAGCTTCCC	BRDN00011	327	385	335	646	482	53
AAGCTTCGC	BRDN00012	93	89	1390	194	156	42
AAGCTTCTG	BRDN00011	277	216	621	647	695	26
AAGCTTGAA	BRDN00011	104	30	112	226	284	9
AAGCTTGAT	BRDN00012	188	217	184	296	173	2
AAGCTTGCT	BRDN00011	65	0	0	166	67	0
AAGCTTGGG	BRDN00011	178	140	104	259	296	0
AAGCTTTCC	BRDN00012	306	220	273	664	579	10
AAGCTTTCT	BRDN00012	363	361	223	834	763	17
AAGCTTTGC	BRDN00011	141	112	81	246	190	2
AAGGAAAAA	BRDN00011	144	112	0	245	261	0
AAGGAAAAG	BRDN00011	302	385	227	686	729	25
AAGGAAACA	BRDN00012	17	0	0	224	93	0
AAGGAAACG	BRDN00011	508	340	610	669	880	31
AAGGAAAGA	BRDN00011	228	66	172	456	189	2
AAGGAAAGA	BRDN00012	183	225	536	324	290	28
AAGGAAAGC	BRDN00012	113	180	1074	305	269	95
AAGGAAAGT	BRDN00012	162	220	1	218	289	16
AAGGAAATC	BRDN00012	345	390	3880	724	732	120
AAGGAAATC	BRDN00012	260	282	927	592	451	29
AAGGAACAC	BRDN00011	100	68	184	181	72	0
AAGGAACCA	BRDN00012	122	122	88	289	474	0
AAGGAACCT	BRDN00012	409	301	284	854	1204	27
AAGGAACGG	BRDN00012	97	18	0	264	342	11
AAGGAACGG	BRDN00012	147	5	0	321	178	0
AAGGAACGT	BRDN00012	305	315	363	683	404	28
AAGGAACTA	BRDN00012	116	104	59	226	240	10
AAGGAACTC	BRDN00011	375	380	5830	840	789	102
AAGGAACTC	BRDN00012	188	106	156	350	652	12
AAGGAAGAT	BRDN00012	251	282	1446	679	704	41
AAGGAAGCA	BRDN00012	101	55	72	405	306	0
AAGGAAGCG	BRDN00012	265	254	139	401	614	30
AAGGAAGCG	BRDN00011	141	9	1	268	270	0
AAGGAAGCT	BRDN00011	107	58	2115	178	453	22
AAGGAAGCT	BRDN00012	320	498	337	643	412	228
AAGGAAGCT	BRDN00012	364	145	261	537	573	0
AAGGAAGGT	BRDN00011	425	329	571	777	532	18
AAGGAATAA	BRDN00012	243	217	573	410	519	59
AAGGAATAA	BRDN00011	202	77	499	357	220	1
AAGGAATAC	BRDN00012	337	404	174	629	864	55
AAGGAATAG	BRDN00012	76	73	6551	119	235	91
AAGGAATAT	BRDN00012	142	3	1	198	251	0
AAGGAATCA	BRDN00012	309	323	308	690	1019	37
AAGGAATGA	BRDN00011	366	221	219	691	741	1

AAGGAATGG	BRDN00012	507	282	155	800	848	2
AAGGACAAA	BRDN00012	303	419	1045	460	948	119
AAGGACAAC	BRDN00012	471	440	1982	922	978	52
AAGGACAAG	BRDN00011	300	421	69	779	1122	41
AAGGACAAG	BRDN00011	158	100	61	316	231	28
AAGGACACG	BRDN00011	176	163	22	383	383	9
AAGGACAGA	BRDN00012	118	155	261	160	83	38
AAGGACAGG	BRDN00012	150	44	0	597	517	3
AAGGACAGG	BRDN00011	117	66	628	301	159	51
AAGGACATG	BRDN00012	244	220	3662	371	268	76
AAGGACCAA	BRDN00011	255	0	123	594	357	0
AAGGACCAA	BRDN00012	363	358	124	614	553	60
AAGGACCAC	BRDN00011	90	74	15	233	350	0
AAGGACCAT	BRDN00011	490	466	1842	771	1213	68
AAGGACCCC	BRDN00011	220	288	481	517	692	29
AAGGACCCG	BRDN00011	118	137	138	204	505	27
AAGGACCGA	BRDN00012	540	441	244	977	1028	14
AAGGACCGG	BRDN00011	225	129	91	512	542	10
AAGGACCTG	BRDN00012	101	6	0	442	243	0
AAGGACGAC	BRDN00011	9	0	0	385	394	0
AAGGACGAG	BRDN00011	86	52	50	252	150	12
AAGGACGAG	BRDN00012	123	103	313	232	132	12
AAGGACGAG	BRDN00012	82	72	91	222	195	0
AAGGACGAT	BRDN00011	237	467	1140	574	417	27
AAGGACTAG	BRDN00012	383	188	52	642	541	6
AAGGACTCA	BRDN00012	377	368	611	462	470	47
AAGGACTCA	BRDN00012	358	271	758	791	974	90
AAGGACTCA	BRDN00011	123	66	13	228	320	0
AAGGACTCA	BRDN00012	239	180	364	565	1063	4
AAGGACTGG	BRDN00012	500	507	1882	835	1139	64
AAGGACTGG	BRDN00011	80	27	0	171	143	0
AAGGACTTA	BRDN00012	113	65	1316	241	429	27
AAGGACTTG	BRDN00011	101	67	757	222	342	8
AAGGACTTT	BRDN00012	247	237	476	518	404	18
AAGGACTTT	BRDN00011	193	178	461	322	226	41
AAGGAGAA	BRDN00011	83	99	30	124	237	4
AAGGAGAAC	BRDN00011	349	253	172	600	667	16
AAGGAGAAC	BRDN00012	500	427	548	929	770	15
AAGGAGAAT	BRDN00012	397	205	73	875	681	0
AAGGAGAAT	BRDN00011	177	253	254	334	383	47
AAGGAGACA	BRDN00011	152	109	424	226	94	4
AAGGAGAGA	BRDN00012	252	217	7	493	536	0
AAGGAGAGC	BRDN00012	188	174	118	185	156	11
AAGGAGAGT	BRDN00011	121	137	41	143	155	33
AAGGAGCAC	BRDN00011	324	6	1	681	417	3
AAGGAGCAG	BRDN00012	54	84	0	191	66	0
AAGGAGCCA	BRDN00011	100	17	1488	290	163	34
AAGGAGCCA	BRDN00011	92	139	10	278	274	12
AAGGAGCCA	BRDN00011	209	172	308	228	491	62
AAGGAGCCG	BRDN00012	214	14	4	645	334	0
AAGGAGCGT	BRDN00011	383	172	1756	938	1387	29
AAGGAGGA	BRDN00012	349	216	948	772	862	72
AAGGAGGA	BRDN00011	433	454	1755	791	538	26
AAGGAGGA	BRDN00011	290	343	69	494	304	87
AAGGAGGAT	BRDN00011	220	22	346	647	569	9
AAGGAGGCT	BRDN00012	15	0	0	300	68	0
AAGGAGGGI	BRDN00012	39	0	0	132	95	0
AAGGAGGGI	BRDN00012	270	241	81	330	690	23
AAGGAGGGI	BRDN00011	166	77	116	186	240	0
AAGGAGGGT	BRDN00012	154	175	458	373	415	30
AAGGAGTAC	BRDN00011	142	228	291	241	64	35
AAGGAGTAT	BRDN00012	226	370	234	578	291	27
AAGGAGTCA	BRDN00012	353	51	339	774	486	23
AAGGAGTCA	BRDN00011	191	133	382	296	310	14
AAGGAGTCA	BRDN00011	171	168	330	250	247	17
AAGGAGTCC	BRDN00012	146	117	12	366	404	15
AAGGAGTGC	BRDN00011	210	230	816	422	325	52
AAGGAGTGT	BRDN00012	228	327	440	451	418	0
AAGGATAAA	BRDN00012	234	177	86	423	413	0
AAGGATACA	BRDN00011	309	5	289	670	287	5
AAGGATAGC	BRDN00012	102	149	0	190	248	0
AAGGATATG	BRDN00012	328	360	2490	593	767	48
AAGGATCAA	BRDN00012	230	82	674	499	821	0
AAGGATCAA	BRDN00012	283	233	36	540	396	12
AAGGATCAC	BRDN00012	131	161	269	298	238	7
AAGGATCCA	BRDN00012	147	199	1015	309	325	40
AAGGATCCC	BRDN00012	178	106	23	373	504	14
AAGGATCCT	BRDN00011	216	213	240	522	443	91
AAGGATGAT	BRDN00011	352	295	155	699	802	11
AAGGATGCA	BRDN00011	443	406	491	925	1039	104
AAGGATGGC	BRDN00012	228	235	180	356	332	17
AAGGATGTA	BRDN00012	199	174	85	391	226	38
AAGGATGTT	BRDN00011	236	206	91	282	261	22
AAGGATTAC	BRDN00011	85	54	40	76	294	0
AAGGATTCA	BRDN00011	107	49	0	175	208	7
AAGGATTCG	BRDN00011	175	206	64	407	442	7
AAGGATTCG	BRDN00012	152	120	175	310	260	32
AAGGATTCT	BRDN00012	120	13	610	452	439	1
AAGGATTGA	BRDN00012	198	161	643	456	418	26
AAGGATTGA	BRDN00011	56	38	23	84	171	0
AAGGATTGG	BRDN00012	288	19	0	706	845	0
AAGGATTGT	BRDN00012	305	309	1310	769	632	46
AAGGATTTC	BRDN00011	157	185	330	219	252	13
AAGGCAAAA	BRDN00011	80	113	0	215	151	9
AAGGCAAAA	BRDN00012	101	43	132	243	272	4
AAGGCAAAG	BRDN00011	219	277	657	543	585	30
AAGGCAACA	BRDN00012	206	268	621	412	430	18
AAGGCAACC	BRDN00012	421	360	1312	815	760	44
AAGGCAACG	BRDN00011	325	322	127	770	905	8
AAGGCAACT	BRDN00011	214	237	26	323	513	5
AAGGCAAGG	BRDN00012	88	87	214	136	84	16
AAGGCAATG	BRDN00012	95	16	3	379	417	0
AAGGCAATT	BRDN00012	364	285	1061	541	835	40
AAGGCAATT	BRDN00012	539	601	1555	819	898	66
AAGGCACAA	BRDN00012	126	143	10	203	208	18
AAGGCACAC	BRDN00012	483	382	1650	708	663	60
AAGGCACAG	BRDN00011	71	69	36	212	272	0
AAGGCACAG	BRDN00012	324	265	961	584	677	25
AAGGCACAT	BRDN00012	179	3	4	891	719	0
AAGGCACCT	BRDN00012	361	341	3171	563	593	160
AAGGCACGC	BRDN00011	202	271	624	525	614	16
AAGGCAGAC	BRDN00012	195	227	364	372	442	13
AAGGCAGAC	BRDN00012	184	199	27	397	443	9
AAGGCAGAC	BRDN00011	132	99	7	194	214	0
AAGGCAGAG	BRDN00011	146	54	0	410	237	5
AAGGCAGAG	BRDN00012	375	372	2642	790	867	72
AAGGCAGAG	BRDN00011	29	27	0	201	111	0
AAGGCAGCA	BRDN00012	98	101	0	198	167	0
AAGGCAGCG	BRDN00011	160	204	17	302	318	24
AAGGCAGGT	BRDN00012	455	434	1355	826	1191	30
AAGGCAGTC	BRDN00012	373	133	432	721	886	7
AAGGCATAT	BRDN00012	222	183	295	359	337	14
AAGGCATGA	BRDN00011	120	89	52	215	95	36
AAGGCATGC	BRDN00012	156	153	252	305	216	21
AAGGCATGG	BRDN00012	73	34	1	261	280	14
AAGGCCAAA	BRDN00011	371	125	204	598	729	4
AAGGCCAAA	BRDN00011	270	10	0	538	460	0
AAGGCCAAA	BRDN00011	133	4	0	204	260	6
AAGGCCAAC	BRDN00011	90	127	11	212	14	55
AAGGCCAAG	BRDN00012	142	110	437	141	137	12
AAGGCCAAG	BRDN00012	247	7	49	726	276	1
AAGGCCAAT	BRDN00012	166	197	137	273	456	57
AAGGCCAAT	BRDN00011	102	11	0	297	90	0
AAGGCCACC	BRDN00012	571	599	2929	900	915	99
AAGGCCACC	BRDN00011	133	94	128	186	142	0
AAGGCCACG	BRDN00012	180	162	155	293	294	4
AAGGCCAGA	BRDN00011	177	87	345	246	466	0
AAGGCCAGA	BRDN00012	90	33	42	228	73	18
AAGGCCAGC	BRDN00012	279	227	101	372	410	2
AAGGCCAGG	BRDN00012	218	105	163	524	476	19
AAGGCCAGG	BRDN00011	97	58	0	135	120	0
AAGGCCAGT	BRDN00012	479	86	283	945	434	0
AAGGCCAGT	BRDN00012	389	510	2531	878	953	78
AAGGCCAGT	BRDN00012	368	291	301	562	811	17
AAGGCCATG	BRDN00012	404	266	145	789	905	62
AAGGCCATG	BRDN00011	109	3	0	297	345	0
AAGGCCATT	BRDN00012	533	426	1193	1119	943	50
AAGGCCCAA	BRDN00011	400	466	884	622	724	51
AAGGCCCAA	BRDN00012	308	269	656	700	1003	52
AAGGCCCAG	BRDN00011	227	249	173	529	576	15
AAGGCCCAG	BRDN00011	448	551	520	752	756	34
AAGGCCCAG	BRDN00011	202	156	2314	413	616	25
AAGGCCCCC	BRDN00011	202	110	224	317	462	3
AAGGCCCCG	BRDN00012	121	115	63	182	174	15
AAGGCCCGA	BRDN00011	170	155	56	365	938	27
AAGGCCCGC	BRDN00011	94	175	53	221	171	40
AAGGCCCGG	BRDN00011	414	406	1285	785	648	19
AAGGCCCGG	BRDN00012	249	231	207	485	241	19
AAGGCCGAA	BRDN00011	97	86	403	269	232	6
AAGGCCGAG	BRDN00011	29	8	57	164	78	0
AAGGCCGCC	BRDN00012	360	390	209	684	644	19
AAGGCCGCC	BRDN00012	175	81	175	307	38	9
AAGGCCGTC	BRDN00012	190	119	1044	329	794	122
AAGGCCTAC	BRDN00011	134	125	31	229	66	20
AAGGCCTAG	BRDN00012	14	17	0	212	63	0
AAGGCCTAT	BRDN00012	396	412	272	746	706	33
AAGGCCTCC	BRDN00011	210	187	70	417	336	13
AAGGCCTCT	BRDN00011	187	161	839	380	263	30
AAGGCCTGG	BRDN00011	60	45	20	155	124	50
AAGGCCTGG	BRDN00012	235	324	157	375	362	30
AAGGCCTGT	BRDN00011	383	340	1371	493	660	26
AAGGCCTTA	BRDN00012	215	260	89	502	835	19
AAGGCCTTA	BRDN00012	139	0	0	198	104	0
AAGGCCTTA	BRDN00012	258	165	646	483	578	27
AAGGCCTTA	BRDN00011	280	341	114	296	413	100
AAGGCCTTA	BRDN00011	314	472	555	651	468	46
AAGGCCTTC	BRDN00012	486	297	135	1012	641	6
AAGGCGAAA	BRDN00012	143	147	23	347	292	12
AAGGCGAAA	BRDN00011	201	239	260	375	234	334
AAGGCGAAG	BRDN00011	102	75	46	202	194	0
AAGGCGAAG	BRDN00011	206	275	1433	426	340	54
AAGGCGAAT	BRDN00012	307	272	42	459	173	50
AAGGCGACC	BRDN00011	295	266	884	548	761	12
AAGGCGAGA	BRDN00012	76	45	0	223	136	0
AAGGCGATT	BRDN00012	65	35	0	264	61	0
AAGGCGCAA	BRDN00011	51	0	98	392	127	0
AAGGCGCGC	BRDN00012	758	829	652	1349	1379	165
AAGGCGCTC	BRDN00011	157	100	26	356	330	27
AAGGCGCTG	BRDN00011	51	62	0	200	180	6
AAGGCGGAC	BRDN00012	265	255	1075	570	765	53
AAGGCGGTC	BRDN00011	92	64	0	181	126	7
AAGGCGTCG	BRDN00012	87	87	183	165	96	33
AAGGCGTTG	BRDN00011	209	347	143	465	346	58
AAGGCTAAA	BRDN00011	259	151	372	462	477	52
AAGGCTACA	BRDN00012	217	321	344	366	287	159
AAGGCTACT	BRDN00011	86	24	224	100	59	46
AAGGCTACT	BRDN00011	150	148	154	291	175	5
AAGGCTACT	BRDN00012	368	332	236	625	504	3
AAGGCTATG	BRDN00012	325	308	2104	527	304	75
AAGGCTATG	BRDN00011	92	25	0	334	199	0
AAGGCTCAA	BRDN00011	88	102	229	216	223	45
AAGGCTCAC	BRDN00011	182	172	237	382	434	50
AAGGCTCAT	BRDN00011	107	87	269	215	291	0
AAGGCTCAT	BRDN00012	359	226	273	936	876	43
AAGGCTCCC	BRDN00012	64	0	0	257	308	0
AAGGCTCCG	BRDN00011	228	196	843	564	672	17
AAGGCTCGT	BRDN00011	93	100	75	170	96	0
AAGGCTCTA	BRDN00011	247	284	1021	476	528	0
AAGGCTCTC	BRDN00012	313	208	1709	433	593	10
AAGGCTCTG	BRDN00012	471	527	272	905	796	35
AAGGCTGAC	BRDN00011	144	107	498	265	189	19
AAGGCTGAC	BRDN00012	158	109	16	267	252	3
AAGGCTGAG	BRDN00012	139	146	0	241	223	0
AAGGCTGCA	BRDN00012	522	456	511	841	641	39
AAGGCTGCC	BRDN00011	199	234	96	286	293	8
AAGGCTGGA	BRDN00011	106	62	21	264	170	12
AAGGCTGGA	BRDN00012	197	88	215	321	181	11
AAGGCTGGC	BRDN00011	182	196	99	454	514	21
AAGGCTGGC	BRDN00011	171	146	72	274	360	6
AAGGCTGTA	BRDN00011	204	14	0	403	246	0
AAGGCTGTG	BRDN00011	548	804	1215	1018	2284	43
AAGGCTGTG	BRDN00011	147	186	151	356	200	22
AAGGCTGTT	BRDN00012	157	10	0	262	146	0
AAGGCTTCC	BRDN00012	215	147	865	281	252	30
AAGGCTTCT	BRDN00012	120	132	72	312	221	36
AAGGCTTTC	BRDN00011	260	178	702	536	617	15
AAGGGAAA	BRDN00011	178	114	202	372	260	17
AAGGGAAAC	BRDN00011	106	60	154	219	204	2
AAGGGAAAC	BRDN00011	137	182	121	374	340	4
AAGGGAAAC	BRDN00012	251	241	152	354	113	0
AAGGGAACC	BRDN00011	99	156	12	271	146	10
AAGGGAACG	BRDN00012	168	121	223	392	378	6
AAGGGAACT	BRDN00012	167	215	635	315	807	0
AAGGGAACT	BRDN00012	85	164	0	213	65	0
AAGGGAAGA	BRDN00011	309	226	418	577	275	9
AAGGGAAGC	BRDN00012	130	176	589	147	273	23
AAGGGACAG	BRDN00012	119	152	254	200	227	6
AAGGGACCA	BRDN00012	345	528	575	593	842	17
AAGGGACCA	BRDN00012	393	307	116	602	680	50
AAGGGACCA	BRDN00012	175	252	96	344	488	14
AAGGGACCC	BRDN00012	226	99	5	662	476	31
AAGGGACGA	BRDN00011	118	35	10	254	176	0
AAGGGACTC	BRDN00012	294	246	417	580	294	82
AAGGGACTC	BRDN00012	292	222	1075	639	567	25
AAGGGAGAA	BRDN00011	258	276	852	560	1014	31
AAGGGAGAC	BRDN00011	157	70	223	301	108	5
AAGGGAGGA	BRDN00012	282	199	160	354	388	42
AAGGGAGGI	BRDN00012	196	266	37	496	421	14
AAGGGAGGI	BRDN00012	243	153	428	840	716	14
AAGGGAGGI	BRDN00011	205	119	965	633	361	18
AAGGGAGTA	BRDN00012	63	94	314	155	39	0
AAGGGAGTC	BRDN00011	457	378	765	1054	1218	45
AAGGGATGA	BRDN00012	304	199	1046	423	957	30
AAGGGATGA	BRDN00011	102	62	570	173	379	11
AAGGGATGA	BRDN00011	89	34	0	216	169	27
AAGGGATGA	BRDN00012	512	364	109	835	1329	32
AAGGGATGA	BRDN00012	220	267	479	436	221	143
AAGGGATGA	BRDN00011	353	346	209	606	456	11
AAGGGATGA	BRDN00012	106	159	593	240	483	28
AAGGGATGT	BRDN00011	133	140	379	184	293	6
AAGGGATGT	BRDN00012	211	167	204	368	309	0
AAGGGATTC	BRDN00012	261	188	542	578	507	22
AAGGGCAAG	BRDN00011	117	92	111	235	53	11
AAGGGCAAT	BRDN00011	268	140	385	545	531	31
AAGGGCATA	BRDN00011	178	119	36	257	323	0
AAGGGCATT	BRDN00012	219	106	233	487	272	14
AAGGGCCTA	BRDN00012	131	67	544	254	260	0
AAGGGCCTA	BRDN00011	426	421	509	777	1102	26
AAGGGCGAC	BRDN00012	114	71	0	93	131	0
AAGGGCGAC	BRDN00012	300	280	74	307	193	37
AAGGGCGGC	BRDN00011	198	211	846	351	297	30
AAGGGCGTG	BRDN00012	578	635	975	906	1052	41
AAGGGCGTG	BRDN00012	182	228	374	277	271	0
AAGGGCTAC	BRDN00011	145	86	245	236	294	22
AAGGGCTAC	BRDN00011	100	59	15	122	50	6
AAGGGCTCC	BRDN00011	172	125	138	445	202	3
AAGGGCTGA	BRDN00012	123	162	287	271	444	0
AAGGGCTGT	BRDN00012	575	434	4268	1006	979	112
AAGGGCTGT	BRDN00011	114	19	43	240	334	0
AAGGGCTTA	BRDN00011	113	106	26	247	309	6
AAGGGCTTG	BRDN00012	149	75	15	182	165	17
AAGGGGAA	BRDN00011	278	224	168	541	237	18
AAGGGGAAC	BRDN00011	200	274	228	340	640	19
AAGGGGACC	BRDN00012	135	105	14	290	284	0
AAGGGGAGI	BRDN00012	116	158	64	183	236	5
AAGGGGATC	BRDN00012	156	120	285	287	347	17
AAGGGGCAT	BRDN00011	153	97	29	330	422	0
AAGGGGCGA	BRDN00011	119	9	0	403	279	0
AAGGGGCGC	BRDN00012	344	333	300	543	487	4
AAGGGGCTC	BRDN00012	354	63	0	672	425	0
AAGGGGCTC	BRDN00011	247	259	288	602	470	26
AAGGGGGCA	BRDN00011	81	94	229	223	148	9
AAGGGTAAA	BRDN00012	154	175	545	328	257	9
AAGGGTAAT	BRDN00011	370	457	650	688	475	42
AAGGGTAGC	BRDN00012	375	422	645	552	645	44
AAGGGTCAC	BRDN00011	292	356	1040	498	699	60
AAGGGTCAT	BRDN00011	393	511	2292	746	1202	86
AAGGGTCTA	BRDN00011	291	168	290	492	275	24
AAGGGTGGC	BRDN00012	257	254	93	345	390	14
AAGGGTGTC	BRDN00011	157	133	43	266	238	11
AAGGGTGTC	BRDN00012	100	121	421	307	686	142
AAGGGTTAG	BRDN00012	435	545	685	859	1420	27
AAGGGTTGA	BRDN00012	158	142	2843	310	239	55
AAGGGTTGA	BRDN00012	117	210	29	245	297	14
AAGGGTTGG	BRDN00012	101	55	80	169	152	5
AAGGGTTTC	BRDN00012	90	123	77	303	404	52
AAGGTAAAA	BRDN00012	112	126	31	255	351	0
AAGGTAAAA	BRDN00011	341	202	465	523	803	71
AAGGTAAAT	BRDN00011	142	171	215	220	232	7
AAGGTAAAT	BRDN00011	183	89	198	369	411	0
AAGGTAACG	BRDN00011	173	155	419	300	104	19
AAGGTAAGA	BRDN00012	538	527	1370	1093	772	67
AAGGTAAGC	BRDN00012	154	116	0	285	317	0
AAGGTACAA	BRDN00012	163	96	529	328	443	64
AAGGTACAC	BRDN00011	218	166	65	347	341	22
AAGGTACAG	BRDN00011	378	267	487	622	720	10
AAGGTACAG	BRDN00011	353	281	563	637	451	21
AAGGTACCC	BRDN00011	350	294	476	493	658	75
AAGGTACGA	BRDN00012	223	187	1270	415	426	13
AAGGTACGT	BRDN00012	345	252	285	634	462	8
AAGGTACTA	BRDN00011	139	115	335	272	169	0
AAGGTAGAG	BRDN00012	334	2	1	698	281	1
AAGGTAGCG	BRDN00012	375	236	472	570	578	38
AAGGTAGCT	BRDN00011	214	228	1962	351	405	58
AAGGTAGTC	BRDN00012	269	183	1669	556	330	41
AAGGTATAA	BRDN00012	118	115	0	216	317	0
AAGGTATCA	BRDN00012	155	220	228	357	592	99
AAGGTATCC	BRDN00011	279	269	92	443	327	43
AAGGTATGT	BRDN00011	165	133	65	274	330	53
AAGGTATTG	BRDN00011	92	147	0	118	88	0
AAGGTCAAA	BRDN00011	104	53	105	202	134	0
AAGGTCAAA	BRDN00012	125	119	1026	322	443	13
AAGGTCAAC	BRDN00011	82	48	0	176	96	0
AAGGTCAAG	BRDN00011	176	11	122	477	319	0
AAGGTCAAT	BRDN00011	319	336	1150	636	455	27
AAGGTCAAT	BRDN00012	452	507	818	646	782	51
AAGGTCACA	BRDN00012	196	187	91	247	144	8
AAGGTCACA	BRDN00012	359	296	956	626	1176	19
AAGGTCACT	BRDN00012	154	129	871	281	181	17
AAGGTCAGC	BRDN00011	297	280	524	578	464	24
AAGGTCAGT	BRDN00011	237	192	611	248	486	31
AAGGTCATC	BRDN00012	75	4	392	255	191	20
AAGGTCATG	BRDN00012	149	151	54	318	236	34
AAGGTCATG	BRDN00011	352	300	234	477	743	30
AAGGTCCAA	BRDN00012	151	171	2014	308	305	61
AAGGTCCAA	BRDN00011	170	120	190	244	195	0
AAGGTCCAG	BRDN00011	160	3	0	396	378	0
AAGGTCCCA	BRDN00011	511	531	434	904	1114	36
AAGGTCCCG	BRDN00011	92	140	299	195	136	20
AAGGTCCCT	BRDN00012	274	194	668	559	485	63
AAGGTCCGC	BRDN00011	195	98	1600	236	342	8
AAGGTCCGG	BRDN00012	291	238	71	502	507	21
AAGGTCCGG	BRDN00012	516	605	330	1217	959	56
AAGGTCCTC	BRDN00011	188	163	854	302	768	67
AAGGTCCTG	BRDN00012	177	123	47	203	249	0
AAGGTCTGA	BRDN00012	252	293	269	606	630	13
AAGGTCTGA	BRDN00011	240	113	78	411	399	0
AAGGTCTGC	BRDN00011	96	1	0	531	62	0
AAGGTCTGG	BRDN00011	418	323	3307	589	852	72
AAGGTCTGG	BRDN00012	138	0	0	372	79	0
AAGGTCTTG	BRDN00012	170	200	409	337	334	16
AAGGTGAAA	BRDN00011	303	253	229	593	436	65
AAGGTGAAC	BRDN00012	102	76	243	165	75	1
AAGGTGAAC	BRDN00012	150	255	871	278	145	39
AAGGTGAAT	BRDN00012	105	155	68	332	273	2
AAGGTGACA	BRDN00012	175	198	284	368	223	15
AAGGTGAGA	BRDN00012	491	451	1131	930	657	52
AAGGTGAGC	BRDN00012	135	123	39	293	385	19
AAGGTGAGC	BRDN00011	387	499	381	675	817	35
AAGGTGAGT	BRDN00011	184	149	643	347	325	12
AAGGTGATC	BRDN00011	203	247	632	447	352	22
AAGGTGATG	BRDN00012	264	260	145	500	366	13
AAGGTGATG	BRDN00011	247	45	0	467	626	0
AAGGTGATG	BRDN00011	72	111	282	164	105	11
AAGGTGCAA	BRDN00012	213	79	486	716	294	0
AAGGTGCAA	BRDN00011	269	208	45	493	670	3
AAGGTGCAA	BRDN00011	67	60	84	168	226	31
AAGGTGCAC	BRDN00011	93	54	63	130	233	15
AAGGTGCAG	BRDN00011	109	89	129	246	519	13
AAGGTGCAG	BRDN00011	154	244	33	192	362	56
AAGGTGCAG	BRDN00011	303	209	23	539	460	51
AAGGTGCAT	BRDN00012	151	190	69	287	362	4
AAGGTGCAT	BRDN00012	389	351	62	760	522	46
AAGGTGCCA	BRDN00011	227	63	14	308	325	13
AAGGTGCCC	BRDN00011	286	282	356	826	444	31
AAGGTGCCC	BRDN00012	108	148	95	213	77	0
AAGGTGCCT	BRDN00012	450	311	726	740	419	78
AAGGTGCGA	BRDN00012	412	368	1227	671	971	119
AAGGTGCTA	BRDN00012	306	227	199	665	656	43
AAGGTGCTG	BRDN00012	349	444	655	713	1132	122
AAGGTGGAC	BRDN00012	74	69	86	149	110	0
AAGGTGGAC	BRDN00012	192	31	18	333	100	0
AAGGTGGAC	BRDN00011	119	185	244	190	72	78
AAGGTGGGA	BRDN00012	180	194	6	270	254	14
AAGGTGGGA	BRDN00011	59	134	73	131	69	7
AAGGTGGTC	BRDN00012	484	485	1475	836	737	186
AAGGTGGTC	BRDN00012	249	209	539	351	283	33
AAGGTGTAA	BRDN00011	102	79	199	207	243	24
AAGGTGTAA	BRDN00011	69	94	35	74	149	50
AAGGTGTAA	BRDN00011	80	27	14	150	268	0
AAGGTGTAT	BRDN00011	17	0	0	249	62	0
AAGGTGTCA	BRDN00011	212	151	1096	334	392	33
AAGGTGTGA	BRDN00011	163	107	19	243	147	0
AAGGTGTGA	BRDN00012	69	81	104	141	103	6
AAGGTGTGA	BRDN00012	60	0	0	215	104	0
AAGGTGTGC	BRDN00012	301	258	50	508	622	8
AAGGTGTGT	BRDN00011	334	285	4360	637	378	59
AAGGTGTGT	BRDN00012	112	56	27	252	390	7
AAGGTGTTA	BRDN00012	308	443	74	415	781	65
AAGGTGTTA	BRDN00012	177	132	433	250	492	18
AAGGTGTTC	BRDN00012	149	57	110	282	218	3
AAGGTGTTG	BRDN00012	106	160	293	241	260	23
AAGGTTAAC	BRDN00012	194	248	539	368	314	48
AAGGTTACC	BRDN00012	159	132	41	222	176	1
AAGGTTACC	BRDN00012	49	36	1	249	324	0
AAGGTTAGG	BRDN00011	256	287	59	552	355	4
AAGGTTATG	BRDN00011	555	496	1502	895	823	63
AAGGTTCAA	BRDN00012	234	154	362	332	489	12
AAGGTTCAG	BRDN00011	436	557	1233	886	1016	73
AAGGTTCCT	BRDN00011	171	242	589	368	413	21
AAGGTTCGG	BRDN00011	143	212	48	454	501	29
AAGGTTCTC	BRDN00011	128	124	729	292	232	23
AAGGTTGAT	BRDN00011	67	60	221	120	141	1
AAGGTTGAT	BRDN00012	173	64	838	270	407	3
AAGGTTGTA	BRDN00012	356	414	1067	636	663	62
AAGGTTTAC	BRDN00011	312	249	671	455	353	11
AAGGTTTAC	BRDN00011	279	263	433	469	505	84
AAGGTTTCG	BRDN00012	130	77	114	402	323	0
AAGGTTTCG	BRDN00012	146	148	269	206	216	10
AAGGTTTCT	BRDN00011	59	20	0	102	13	0
AAGGTTTGA	BRDN00012	184	180	705	292	231	0
AAGGTTTGC	BRDN00012	207	245	160	374	379	42
AAGTAAAAA	BRDN00012	180	115	132	296	209	19
AAGTAAAAT	BRDN00012	114	38	30	239	517	0
AAGTAAAAT	BRDN00012	394	151	1	815	978	8
AAGTAAACA	BRDN00011	265	227	299	532	392	75
AAGTAAAGA	BRDN00011	309	0	3	688	234	0
AAGTAAATG	BRDN00012	268	237	1071	502	212	104
AAGTAAATG	BRDN00011	291	252	244	399	347	10
AAGTAACAC	BRDN00012	142	99	356	286	175	0
AAGTAACCG	BRDN00012	417	220	161	854	511	0
AAGTAAGAG	BRDN00012	476	509	5310	881	755	146
AAGTAAGAT	BRDN00011	121	0	0	145	268	0
AAGTAAGTT	BRDN00011	288	38	1	566	796	0
AAGTAATAT	BRDN00011	66	35	0	82	38	0
AAGTAATGA	BRDN00011	114	148	877	276	237	11
AAGTAATGG	BRDN00011	233	238	1799	388	619	21
AAGTAATGT	BRDN00011	323	176	130	836	556	11
AAGTAATTG	BRDN00012	255	361	786	657	909	30
AAGTAATTG	BRDN00011	223	208	166	366	244	127
AAGTACAAA	BRDN00011	117	58	0	247	228	13
AAGTACAAG	BRDN00012	622	707	2277	843	830	181
AAGTACAAG	BRDN00011	277	278	140	372	474	7
AAGTACACA	BRDN00011	136	204	449	266	287	0
AAGTACACA	BRDN00011	190	21	96	231	409	0

AAGTACAGA	BRDN00011	264	163	499	447	328	35
AAGTACAGA	BRDN00012	104	91	257	302	188	16
AAGTACAGC	BRDN00011	106	117	0	137	76	0
AAGTACATA	BRDN00012	428	324	279	793	879	39
AAGTACCAA	BRDN00012	167	89	373	236	152	25
AAGTACCAG	BRDN00012	158	56	30	490	223	0
AAGTACCAG	BRDN00012	487	420	320	936	1082	12
AAGTACCTA	BRDN00011	159	167	28	461	270	0
AAGTACGAG	BRDN00011	61	0	11	447	596	0
AAGTACGCT	BRDN00012	96	88	223	249	142	0
AAGTACGGC	BRDN00012	515	405	1364	1189	1128	81
AAGTACTAT	BRDN00011	86	83	372	197	102	19
AAGTACTCA	BRDN00011	150	95	23	348	201	15
AAGTACTCG	BRDN00012	141	122	429	332	347	11
AAGTACTCG	BRDN00012	404	142	8	641	339	0
AAGTACTGG	BRDN00012	388	246	390	783	677	14
AAGTACTGG	BRDN00012	122	53	1	257	115	0
AAGTAGAAC	BRDN00011	110	105	61	299	485	0
AAGTAGACA	BRDN00012	147	142	200	350	232	1
AAGTAGACC	BRDN00011	201	118	38	327	224	8
AAGTAGACG	BRDN00011	92	139	113	227	258	30
AAGTAGAGT	BRDN00011	323	289	781	656	437	27
AAGTAGATA	BRDN00012	475	255	99	631	506	4
AAGTAGATG	BRDN00012	431	508	206	1171	751	71
AAGTAGCAA	BRDN00012	197	150	141	343	516	16
AAGTAGCCT	BRDN00012	111	0	258	274	386	0
AAGTAGCCT	BRDN00011	236	46	0	420	326	0
AAGTAGGAA	BRDN00012	400	361	190	721	1250	70
AAGTAGGCA	BRDN00012	118	89	98	171	152	0
AAGTAGGCT	BRDN00011	240	329	978	551	389	18
AAGTAGGTT	BRDN00012	200	190	539	405	291	13
AAGTAGTAC	BRDN00012	245	273	893	467	314	23
AAGTAGTAG	BRDN00012	346	313	606	509	650	25
AAGTAGTTA	BRDN00012	259	227	183	593	985	0
AAGTAGTTT	BRDN00012	104	103	183	139	201	1
AAGTATATA	BRDN00012	174	136	931	198	362	121
AAGTATCAT	BRDN00012	278	230	388	486	328	1
AAGTATCGA	BRDN00012	179	102	390	302	251	9
AAGTATCGG	BRDN00012	471	348	1546	843	1114	68
AAGTATCTG	BRDN00011	169	65	545	267	197	63
AAGTATGAG	BRDN00012	272	299	84	492	427	9
AAGTATGGC	BRDN00011	162	194	92	481	574	4
AAGTATGTC	BRDN00012	124	19	43	182	164	10
AAGTATTAC	BRDN00011	311	335	171	534	424	31
AAGTATTAG	BRDN00011	72	122	2323	133	116	44
AAGTATTCA	BRDN00012	290	313	1123	576	421	62
AAGTATTCC	BRDN00012	408	481	1712	665	746	43
AAGTATTGA	BRDN00012	218	156	238	364	431	14
AAGTCAAAG	BRDN00012	196	126	1480	193	162	26
AAGTCAAAT	BRDN00011	168	120	198	354	437	8
AAGTCAACA	BRDN00012	305	348	203	413	326	44
AAGTCAACC	BRDN00011	511	338	953	773	923	50
AAGTCAACT	BRDN00011	221	217	1	387	557	0
AAGTCAAGC	BRDN00012	465	390	206	707	890	58
AAGTCAATG	BRDN00011	176	131	21	255	380	44
AAGTCACAG	BRDN00012	316	239	513	556	441	31
AAGTCACCA	BRDN00011	275	206	29	507	307	6
AAGTCACCT	BRDN00011	385	186	627	1060	1155	18
AAGTCACGC	BRDN00011	446	516	573	893	1068	19
AAGTCACGC	BRDN00011	212	265	366	511	536	13
AAGTCACTG	BRDN00012	425	559	967	1289	1321	9
AAGTCACTT	BRDN00012	185	156	356	313	220	33
AAGTCAGCA	BRDN00012	321	235	352	438	246	22
AAGTCAGCG	BRDN00011	131	177	350	160	97	0
AAGTCAGCT	BRDN00012	281	221	1	454	418	46
AAGTCAGGC	BRDN00012	206	142	301	213	117	4
AAGTCAGGT	BRDN00012	346	246	37	541	856	26
AAGTCAGTA	BRDN00011	75	0	0	233	66	0
AAGTCATAG	BRDN00012	75	0	0	272	203	0
AAGTCATCA	BRDN00012	142	105	86	343	157	0
AAGTCATCA	BRDN00011	135	86	750	354	118	35
AAGTCATGC	BRDN00011	110	125	0	201	236	7
AAGTCATTA	BRDN00011	268	256	115	451	382	41
AAGTCATTT	BRDN00012	229	104	113	314	757	19
AAGTCCAAA	BRDN00012	288	294	99	576	552	10
AAGTCCAAA	BRDN00011	435	414	150	823	645	57
AAGTCCAAA	BRDN00011	130	172	76	221	300	27
AAGTCCAAG	BRDN00012	410	488	622	884	1072	112
AAGTCCACC	BRDN00012	377	425	1010	884	903	134
AAGTCCACT	BRDN00011	200	117	0	453	266	11
AAGTCCATA	BRDN00012	167	59	59	429	340	8
AAGTCCATG	BRDN00012	404	317	92	570	434	15
AAGTCCATG	BRDN00011	44	58	10	151	107	5
AAGTCCCAA	BRDN00011	191	83	518	414	308	15
AAGTCCCAA	BRDN00011	287	254	368	625	429	9
AAGTCCCCG	BRDN00011	473	415	1401	1008	919	136
AAGTCCCGC	BRDN00012	255	314	540	340	557	57
AAGTCCCGG	BRDN00012	162	108	657	274	101	10
AAGTCCGGC	BRDN00011	142	82	179	283	294	0
AAGTCCTCG	BRDN00011	94	67	107	171	240	24
AAGTCGAAC	BRDN00011	84	5	1	449	270	0
AAGTCGAAG	BRDN00012	77	107	64	177	101	0
AAGTCGAAT	BRDN00011	138	188	182	274	221	14
AAGTCGACA	BRDN00011	59	56	0	194	318	0
AAGTCGAGC	BRDN00012	156	141	1795	285	350	86
AAGTCGATA	BRDN00012	270	292	142	543	668	68
AAGTCGCGA	BRDN00011	530	246	89	1039	1474	3
AAGTCGCGG	BRDN00011	96	62	11	113	199	0
AAGTCGCGG	BRDN00011	165	114	1795	298	393	59
AAGTCGCGT	BRDN00011	111	26	0	416	351	0
AAGTCGCTT	BRDN00012	177	150	16	296	279	38
AAGTCGGAC	BRDN00011	148	117	192	287	140	7
AAGTCGGCG	BRDN00011	334	443	702	763	526	20
AAGTCGGGT	BRDN00011	156	137	585	205	188	32
AAGTCGTTC	BRDN00011	34	10	1	241	280	0
AAGTCTACA	BRDN00012	73	32	0	323	241	0
AAGTCTACG	BRDN00012	412	221	2022	820	885	49
AAGTCTACT	BRDN00012	114	31	35	244	104	0
AAGTCTAGA	BRDN00012	180	153	129	360	289	14
AAGTCTAGT	BRDN00011	329	268	341	512	360	12
AAGTCTCAA	BRDN00011	166	199	653	250	614	19
AAGTCTCAC	BRDN00011	193	161	377	436	565	5
AAGTCTCCC	BRDN00012	315	231	188	562	483	11
AAGTCTGAA	BRDN00012	228	272	1010	725	587	13
AAGTCTGAG	BRDN00011	118	1	1	296	238	0
AAGTCTGGC	BRDN00011	214	169	236	421	256	27
AAGTCTGGT	BRDN00011	155	63	0	242	177	0
AAGTCTTAA	BRDN00011	62	102	81	295	270	6
AAGTCTTCA	BRDN00011	289	289	186	476	365	86
AAGTCTTCG	BRDN00011	136	100	115	396	93	2
AAGTCTTGA	BRDN00011	236	244	21	437	385	0
AAGTCTTGC	BRDN00011	312	306	309	499	414	5
AAGTCTTTG	BRDN00011	139	112	1034	273	172	9
AAGTGAAAG	BRDN00012	190	221	906	468	550	27
AAGTGAAAT	BRDN00012	145	129	591	181	174	67
AAGTGAAAT	BRDN00012	213	57	73	294	193	17
AAGTGAACC	BRDN00011	75	6	0	211	33	0
AAGTGAACC	BRDN00012	180	163	315	330	139	9
AAGTGAAGC	BRDN00012	426	296	2640	701	600	108
AAGTGAATA	BRDN00011	146	184	24	275	277	47
AAGTGACAG	BRDN00012	495	519	1683	965	1376	154
AAGTGACAG	BRDN00011	185	176	397	438	445	60
AAGTGACAT	BRDN00012	381	564	689	794	788	43
AAGTGACCC	BRDN00011	190	107	444	395	545	12
AAGTGACCT	BRDN00011	228	235	65	660	772	35
AAGTGACGC	BRDN00011	270	148	416	559	605	12
AAGTGACGG	BRDN00012	540	513	1020	712	896	74
AAGTGACGT	BRDN00012	137	0	1	656	299	0
AAGTGACTC	BRDN00012	325	381	171	633	940	87
AAGTGACTT	BRDN00012	118	123	195	219	241	39
AAGTGACTT	BRDN00012	137	129	368	312	315	14
AAGTGAGAG	BRDN00012	446	38	366	771	719	0
AAGTGAGCA	BRDN00012	276	290	367	611	502	56
AAGTGAGCA	BRDN00012	322	289	568	597	315	76
AAGTGAGGA	BRDN00011	106	4	102	188	168	0
AAGTGAGGT	BRDN00012	216	1	0	493	529	0
AAGTGAGTA	BRDN00012	127	101	1461	242	334	71
AAGTGAGTT	BRDN00011	232	301	520	353	638	38
AAGTGATAA	BRDN00012	204	209	30	449	419	34
AAGTGATAC	BRDN00012	169	144	739	256	307	13
AAGTGATAT	BRDN00012	373	409	395	718	792	41
AAGTGATAT	BRDN00012	420	444	1142	731	796	37
AAGTGATGA	BRDN00012	364	349	710	411	512	72
AAGTGATGA	BRDN00012	241	161	186	656	721	1
AAGTGCAAA	BRDN00011	82	86	1	277	140	0
AAGTGCAAG	BRDN00012	331	271	90	404	567	63
AAGTGCAAT	BRDN00011	107	71	147	226	145	0
AAGTGCACA	BRDN00011	227	90	221	636	997	0
AAGTGCACG	BRDN00012	209	141	1410	287	360	14
AAGTGCAGG	BRDN00012	234	236	1	524	535	0
AAGTGCAGG	BRDN00011	320	327	13394	595	664	179
AAGTGCATA	BRDN00011	184	0	0	386	105	0
AAGTGCATC	BRDN00011	370	238	279	855	990	24
AAGTGCATC	BRDN00012	302	202	605	567	566	47
AAGTGCCAA	BRDN00011	72	69	0	236	179	0
AAGTGCCAG	BRDN00012	393	377	1533	721	745	59
AAGTGCCCC	BRDN00012	370	394	657	739	868	43
AAGTGCCCT	BRDN00012	464	371	402	870	867	11
AAGTGCCTA	BRDN00011	162	125	182	312	309	0
AAGTGCCTG	BRDN00011	252	314	214	464	689	56
AAGTGCGTG	BRDN00011	89	0	0	163	12	0
AAGTGCTAA	BRDN00011	228	180	422	358	937	13
AAGTGCTAC	BRDN00012	297	284	857	568	269	69
AAGTGCTAC	BRDN00012	235	403	1397	461	662	32
AAGTGCTAG	BRDN00012	191	138	209	450	582	6
AAGTGCTCA	BRDN00011	81	89	35	283	232	3
AAGTGCTGA	BRDN00011	171	175	159	443	322	0
AAGTGCTGC	BRDN00011	328	376	613	524	631	112
AAGTGCTGT	BRDN00012	438	604	1568	1044	1148	85
AAGTGCTTA	BRDN00011	129	6	1	399	496	0
AAGTGCTTG	BRDN00012	147	108	124	249	187	0
AAGTGGAAA	BRDN00011	324	192	494	663	495	45
AAGTGGAAT	BRDN00012	349	392	1555	549	484	33
AAGTGGAGC	BRDN00012	81	100	0	234	122	0
AAGTGGATG	BRDN00012	616	733	726	945	699	63
AAGTGGCCC	BRDN00011	179	152	265	405	538	12
AAGTGGGAC	BRDN00012	169	137	58	322	354	9
AAGTGGGAC	BRDN00011	167	230	109	294	168	8
AAGTGGGAC	BRDN00011	76	104	60	192	18	0
AAGTGGGCA	BRDN00011	113	88	337	189	154	17
AAGTGGGCC	BRDN00012	537	74	200	1327	438	28
AAGTGGGGA	BRDN00011	135	86	46	279	307	4
AAGTGGGTG	BRDN00011	289	336	75	729	732	14
AAGTGGTAG	BRDN00011	180	142	114	318	402	43
AAGTGGTAT	BRDN00012	357	385	539	787	841	52
AAGTGGTCG	BRDN00011	467	236	271	912	513	28
AAGTGGTGA	BRDN00012	111	72	174	198	332	6
AAGTGGTTG	BRDN00011	255	243	1413	416	439	40
AAGTGGTTG	BRDN00012	223	311	672	443	289	28
AAGTGTACC	BRDN00012	205	135	1	500	734	0
AAGTGTACC	BRDN00012	122	177	114	264	184	30
AAGTGTAGT	BRDN00011	99	51	239	168	297	8
AAGTGTATA	BRDN00011	133	81	25	221	102	4
AAGTGTATA	BRDN00011	227	33	0	579	313	0
AAGTGTCAG	BRDN00012	137	219	125	317	347	25
AAGTGTCCA	BRDN00012	236	199	0	400	354	0
AAGTGTCCG	BRDN00012	130	177	644	342	130	0
AAGTGTCCT	BRDN00012	191	118	589	345	289	4
AAGTGTGAG	BRDN00011	245	167	74	410	559	48
AAGTGTGCT	BRDN00011	84	162	414	303	217	28
AAGTGTGTG	BRDN00012	339	463	1026	560	755	72
AAGTGTGTT	BRDN00011	193	139	45	536	660	0
AAGTGTTAA	BRDN00012	271	281	227	485	670	77
AAGTGTTAG	BRDN00012	124	149	16	260	299	0
AAGTGTTCT	BRDN00011	60	68	182	154	253	0
AAGTGTTGG	BRDN00012	170	0	0	380	252	0
AAGTGTTGT	BRDN00012	263	225	191	442	342	32
AAGTGTTGT	BRDN00012	352	221	103	558	647	15
AAGTGTTTC	BRDN00012	107	101	0	316	313	6
AAGTTAACA	BRDN00012	129	124	19	238	224	35
AAGTTAAGA	BRDN00011	171	98	96	267	426	12
AAGTTAAGG	BRDN00012	339	515	365	705	1015	17
AAGTTACAA	BRDN00011	106	117	1284	275	271	11
AAGTTACCA	BRDN00012	310	268	419	684	874	43
AAGTTACCT	BRDN00012	128	140	469	300	212	6
AAGTTACTC	BRDN00011	182	150	405	337	294	15
AAGTTACTG	BRDN00012	265	526	1967	559	507	51
AAGTTACTG	BRDN00011	351	340	53	560	713	46
AAGTTAGTA	BRDN00011	11	3	236	321	218	0
AAGTTAGTT	BRDN00011	132	91	255	260	382	41
AAGTTAGTT	BRDN00012	232	246	173	411	607	44
AAGTTATGT	BRDN00011	163	110	711	310	266	22
AAGTTATTA	BRDN00012	88	73	177	148	77	17
AAGTTATTG	BRDN00012	318	34	148	629	440	0
AAGTTCAAG	BRDN00012	111	15	0	549	545	0
AAGTTCACC	BRDN00011	354	224	170	713	444	0
AAGTTCAGA	BRDN00011	107	174	616	179	72	39
AAGTTCATA	BRDN00011	265	180	85	473	472	22
AAGTTCATG	BRDN00011	126	159	230	365	472	11
AAGTTCCAC	BRDN00012	316	319	1312	552	678	25
AAGTTCCAG	BRDN00011	225	204	171	437	443	53
AAGTTCCTC	BRDN00011	172	36	1	387	155	0
AAGTTCGCA	BRDN00012	146	97	915	230	200	8
AAGTTCTAA	BRDN00012	94	115	405	286	265	5
AAGTTCTAC	BRDN00012	332	366	2924	524	626	48
AAGTTCTGG	BRDN00011	450	375	706	840	761	57
AAGTTCTGT	BRDN00012	98	127	202	317	367	3
AAGTTCTTC	BRDN00011	96	87	242	265	115	40
AAGTTCTTC	BRDN00012	181	82	39	356	170	9
AAGTTGAAA	BRDN00012	276	379	244	522	742	37
AAGTTGAAC	BRDN00011	138	192	124	251	285	31
AAGTTGAAG	BRDN00012	192	186	428	384	268	13
AAGTTGACA	BRDN00012	266	240	276	634	1160	6
AAGTTGAGC	BRDN00011	219	148	282	395	324	8
AAGTTGATG	BRDN00012	398	585	1243	833	1073	58
AAGTTGATG	BRDN00012	462	342	956	888	1281	24
AAGTTGATT	BRDN00011	173	145	117	275	331	17
AAGTTGCAA	BRDN00011	66	53	5	196	148	0
AAGTTGCAA	BRDN00011	191	176	328	318	517	23
AAGTTGCAC	BRDN00011	315	373	106	592	795	40
AAGTTGCGA	BRDN00012	140	53	0	293	111	0
AAGTTGCTT	BRDN00011	383	347	255	679	598	62
AAGTTGGAC	BRDN00011	162	155	412	376	354	44
AAGTTGGCA	BRDN00012	109	14	0	916	471	0
AAGTTGGCC	BRDN00012	294	29	1	705	160	0
AAGTTGGCC	BRDN00012	107	45	25	268	289	0
AAGTTGGCG	BRDN00011	103	62	72	125	121	5
AAGTTGGGG	BRDN00011	81	131	523	183	388	23
AAGTTGGTC	BRDN00011	184	137	3	251	224	13
AAGTTGGTG	BRDN00011	192	186	26	414	147	12
AAGTTGTAG	BRDN00012	113	123	1	213	295	0
AAGTTGTAT	BRDN00012	134	43	74	328	165	0
AAGTTGTAT	BRDN00011	90	103	233	268	272	13
AAGTTGTGA	BRDN00012	296	251	36	508	336	6
AAGTTGTGA	BRDN00011	154	111	716	406	402	2
AAGTTGTGG	BRDN00011	113	159	73	142	80	0
AAGTTGTTA	BRDN00011	190	160	190	294	270	2
AAGTTTAAC	BRDN00012	174	180	8	280	137	8
AAGTTTAAG	BRDN00011	96	91	112	208	245	5
AAGTTTACC	BRDN00011	110	15	0	267	671	0
AAGTTTATC	BRDN00011	266	258	1385	568	395	63
AAGTTTCAT	BRDN00011	97	91	2293	230	215	38
AAGTTTCCG	BRDN00011	161	185	184	327	508	4
AAGTTTCGA	BRDN00012	146	130	272	270	121	8
AAGTTTCTC	BRDN00011	70	38	100	214	89	7
AAGTTTGAT	BRDN00011	247	219	539	477	564	13
AAGTTTGCC	BRDN00011	112	117	266	263	293	10
AAGTTTGGT	BRDN00011	139	179	274	272	165	10
AATAAAAAG	BRDN00012	107	104	75	138	201	4
AATAAAACT	BRDN00012	379	253	2177	623	950	74
AATAAAAGG	BRDN00012	168	190	94	283	517	32
AATAAAAGT	BRDN00012	41	0	1	383	190	0
AATAAAATA	BRDN00011	84	92	10	182	142	0
AATAAAATG	BRDN00012	203	164	64	344	547	16
AATAAACAT	BRDN00011	87	58	71	176	261	7
AATAAACAT	BRDN00011	110	94	20	249	372	0
AATAAACGC	BRDN00011	130	105	115	291	259	0
AATAAACGT	BRDN00011	352	247	400	661	778	78
AATAAACTG	BRDN00012	301	354	1171	606	704	46
AATAAACTT	BRDN00011	205	96	1213	304	461	38
AATAAAGAT	BRDN00012	178	203	206	236	46	40
AATAAAGGT	BRDN00011	149	96	12	185	149	9
AATAAATCA	BRDN00012	167	142	2	415	234	1
AATAACACC	BRDN00011	189	136	836	340	801	37
AATAACCAA	BRDN00011	85	120	111	322	470	0
AATAACCAC	BRDN00011	132	132	328	222	491	20
AATAACCCA	BRDN00012	244	237	120	495	736	66
AATAACGTT	BRDN00012	160	213	186	322	507	12
AATAACTAA	BRDN00012	102	178	537	192	176	22
AATAACTAT	BRDN00012	169	175	0	368	782	1
AATAACTCC	BRDN00011	120	77	137	250	99	11
AATAACTCT	BRDN00011	44	6	0	193	341	0
AATAACTGT	BRDN00012	217	270	609	405	392	30
AATAAGAAG	BRDN00012	122	79	6	264	204	17
AATAAGAGC	BRDN00012	95	94	0	234	173	32
AATAAGAGC	BRDN00011	106	27	211	198	206	0
AATAAGATG	BRDN00012	70	8	34	267	266	0
AATAAGCAA	BRDN00012	310	271	517	477	432	32
AATAAGCAG	BRDN00012	187	196	110	434	340	16
AATAAGCAG	BRDN00012	77	9	0	253	148	0
AATAAGCAG	BRDN00012	317	240	1365	631	547	35
AATAAGCCC	BRDN00011	147	159	609	304	222	23
AATAAGCCC	BRDN00012	325	228	195	438	628	10
AATAAGCCT	BRDN00012	187	134	376	353	276	0
AATAAGCTA	BRDN00012	128	0	0	324	46	0
AATAAGGGA	BRDN00012	91	139	64	256	250	8
AATAAGGGT	BRDN00012	250	280	331	465	982	30
AATAAGTAC	BRDN00012	179	47	149	321	105	12
AATAAGTCA	BRDN00011	100	106	46	245	166	1
AATAAGTCC	BRDN00011	328	299	1068	756	655	49
AATAAGTTG	BRDN00011	337	288	810	681	482	64
AATAATCCC	BRDN00012	349	219	1462	642	771	30
AATAATCCT	BRDN00011	170	107	39	311	232	33
AATAATCCT	BRDN00012	385	264	793	667	878	21
AATAATCTA	BRDN00011	293	256	215	468	413	8
AATAATCTG	BRDN00012	220	175	23	458	236	0
AATAATGAA	BRDN00011	124	121	225	212	143	32
AATAATGAG	BRDN00012	333	362	332	592	665	20
AATAATTCT	BRDN00011	179	228	99	366	215	12
AATAATTCT	BRDN00011	499	435	1591	840	717	112
AATACAAAC	BRDN00011	61	27	48	116	170	18
AATACAAAG	BRDN00012	151	248	18	277	417	24
AATACAACA	BRDN00012	125	0	1	267	323	0
AATACAATG	BRDN00011	40	0	0	284	197	0
AATACACAT	BRDN00012	244	288	705	496	511	8
AATACACCC	BRDN00011	175	181	313	330	404	32
AATACACTT	BRDN00011	44	0	1	682	590	1
AATACAGAT	BRDN00012	229	162	147	656	574	4
AATACAGCC	BRDN00011	264	153	167	538	422	12
AATACAGTA	BRDN00012	158	245	6	334	190	45
AATACATAC	BRDN00012	174	82	35	198	273	12
AATACATAG	BRDN00012	363	241	60	493	328	10
AATACATAT	BRDN00011	178	192	203	375	455	40
AATACATCT	BRDN00011	197	147	4150	329	212	54
AATACATGC	BRDN00011	204	249	1149	429	267	38
AATACATGT	BRDN00012	77	79	57	215	245	46
AATACATTC	BRDN00012	476	541	1164	843	1003	49
AATACATTT	BRDN00011	186	85	214	475	504	6
AATACCAAA	BRDN00012	95	57	32	286	238	0
AATACCAAG	BRDN00011	413	318	1087	672	654	41
AATACCAAT	BRDN00012	131	94	63	212	190	18
AATACCACA	BRDN00011	312	241	74	599	676	68
AATACCACT	BRDN00012	81	72	261	224	186	0
AATACCAGA	BRDN00011	46	59	71	144	219	1
AATACCATC	BRDN00012	460	498	1182	1068	868	81
AATACCCAA	BRDN00012	142	46	0	254	279	0
AATACCCAC	BRDN00012	177	33	1	825	604	0
AATACCCAG	BRDN00012	207	234	3000	453	675	112
AATACCCAG	BRDN00012	320	193	16	658	744	0
AATACCGAT	BRDN00012	178	167	208	364	387	18
AATACCGTC	BRDN00011	141	108	557	256	102	17
AATACCTAC	BRDN00011	294	329	574	584	539	63
AATACCTAC	BRDN00012	162	98	81	302	334	16
AATACCTAG	BRDN00012	213	161	318	345	346	27
AATACCTAG	BRDN00012	113	164	20	263	128	10
AATACCTGA	BRDN00012	172	198	204	369	232	6
AATACCTGA	BRDN00012	127	135	863	213	198	32
AATACCTGG	BRDN00011	194	199	14	643	704	15
AATACCTGG	BRDN00012	89	80	203	266	262	0
AATACCTGG	BRDN00012	306	204	434	586	663	0
AATACCTGT	BRDN00011	272	188	130	528	416	4
AATACCTTG	BRDN00012	151	127	502	224	157	17
AATACGACT	BRDN00011	252	191	98	332	470	11
AATACGAGG	BRDN00012	96	174	1184	209	219	20
AATACGATG	BRDN00011	138	48	172	156	30	0
AATACGCTA	BRDN00012	127	59	60	569	554	13
AATACGGTA	BRDN00012	402	384	1328	868	780	74
AATACGGTG	BRDN00011	82	99	127	207	198	27
AATACGTAC	BRDN00011	84	4	0	344	241	0
AATACTAAA	BRDN00011	146	89	507	184	188	15
AATACTAAC	BRDN00012	127	130	744	235	189	28
AATACTAAC	BRDN00011	117	31	0	191	317	0
AATACTAAT	BRDN00012	103	16	0	261	192	0
AATACTAAT	BRDN00011	280	257	855	477	325	101
AATACTATG	BRDN00012	107	207	1	165	274	28
AATACTCAG	BRDN00011	149	171	826	249	209	22
AATACTCAT	BRDN00012	249	279	612	509	681	40
AATACTCCA	BRDN00012	161	64	1	430	459	0
AATACTCCA	BRDN00011	124	39	128	111	337	4
AATACTCCC	BRDN00012	190	171	507	470	471	0
AATACTCCG	BRDN00012	421	410	1085	782	991	113
AATACTCTT	BRDN00011	161	178	214	221	205	16
AATACTGAA	BRDN00011	159	205	112	293	655	6
AATACTGAT	BRDN00012	252	209	135	488	363	29
AATACTGCA	BRDN00012	281	214	24	641	746	21
AATACTGCC	BRDN00012	110	111	187	274	131	13
AATACTTAC	BRDN00012	194	156	37	361	440	6
AATACTTAC	BRDN00012	88	94	703	240	248	27
AATACTTCA	BRDN00012	363	277	435	671	722	23
AATACTTCA	BRDN00012	288	257	1316	505	643	24
AATACTTCC	BRDN00011	162	143	60	241	197	0
AATACTTGA	BRDN00011	101	114	780	205	115	6
AATACTTTA	BRDN00012	225	177	21	400	328	31
AATAGAAAT	BRDN00012	314	276	2937	339	419	43
AATAGAAAT	BRDN00012	250	234	659	394	294	86
AATAGACAT	BRDN00011	115	139	0	173	290	7
AATAGACCC	BRDN00011	126	124	114	253	258	0
AATAGACCC	BRDN00012	107	85	20	230	323	0
AATAGACTT	BRDN00012	283	316	540	513	745	11
AATAGAGAA	BRDN00011	213	297	356	382	250	43
AATAGAGAC	BRDN00012	136	251	802	270	272	32
AATAGAGGA	BRDN00011	176	80	83	302	141	13
AATAGAGGC	BRDN00012	295	315	426	601	301	57
AATAGAGTA	BRDN00011	126	1	0	447	339	0
AATAGAGTG	BRDN00011	171	141	96	194	158	17
AATAGCAAT	BRDN00011	148	175	2	259	419	57
AATAGCACA	BRDN00012	350	550	389	559	932	47
AATAGCATA	BRDN00012	354	286	905	729	609	29
AATAGCATA	BRDN00011	150	121	53	227	108	13
AATAGCATA	BRDN00012	268	228	388	604	817	54
AATAGCCAC	BRDN00012	474	658	962	1031	942	58
AATAGCCCA	BRDN00011	303	78	263	608	542	0
AATAGCCTA	BRDN00012	318	1	0	653	236	0
AATAGCCTT	BRDN00011	166	135	136	310	480	20
AATAGCGGC	BRDN00012	373	398	292	809	874	118
AATAGCTAC	BRDN00011	234	156	477	425	677	26
AATAGCTAT	BRDN00012	330	240	599	515	664	0
AATAGCTTT	BRDN00011	135	60	631	385	530	22
AATAGGACA	BRDN00011	169	126	313	256	239	28
AATAGGACT	BRDN00012	99	61	0	236	214	6
AATAGGCAC	BRDN00012	425	484	1051	780	733	58
AATAGGCTC	BRDN00011	70	0	1	184	149	0
AATAGGGAT	BRDN00012	32	19	12	739	639	0
AATAGGTAA	BRDN00011	49	92	86	160	104	0
AATAGTAAC	BRDN00012	197	113	206	362	486	15
AATAGTACC	BRDN00012	404	414	494	617	797	113
AATAGTACT	BRDN00012	194	278	192	331	221	45
AATAGTATC	BRDN00012	100	18	40	200	233	3
AATAGTATG	BRDN00011	164	93	1	294	244	0
AATAGTCAG	BRDN00011	121	93	264	183	166	9
AATAGTCGA	BRDN00011	159	143	44	286	405	7
AATAGTCGC	BRDN00011	84	8	1	576	95	0
AATAGTCTT	BRDN00012	396	532	498	631	724	77
AATAGTGGG	BRDN00011	90	63	82	296	153	0
AATAGTTTG	BRDN00011	160	52	55	426	707	0

AATATAAAG	BRDN00011	117	0	0	366	134	0
AATATAATG	BRDN00011	126	233	82	340	624	54
AATATACATC	BRDN00012	314	302	1404	678	453	77
AATATACATT	BRDN00011	165	111	67	182	133	41
AATATACCG	BRDN00012	106	61	1	147	142	0
AATATAGAC	BRDN00012	224	238	499	504	391	18
AATATAGAG	BRDN00011	89	72	1056	165	109	36
AATATATCCA	BRDN00011	31	0	0	129	131	0
AATATATGA	BRDN00011	188	175	41	292	168	30
AATATATGA	BRDN00012	250	227	242	551	788	39
AATATATGC	BRDN00012	102	97	284	276	261	6
AATATATTG	BRDN00011	130	113	279	223	143	12
AATATCAAC	BRDN00011	172	75	58	252	301	0
AATATCAAG	BRDN00011	108	22	2	183	165	7
AATATCAGA	BRDN00011	316	149	801	614	422	36
AATATCAGT	BRDN00011	117	96	219	238	211	4
AATATCCAA	BRDN00012	288	12	0	553	365	0
AATATCCAAT	BRDN00012	39	62	1	402	80	0
AATATCCCAG	BRDN00012	332	380	402	697	846	41
AATATCCCG	BRDN00011	168	160	75	360	542	9
AATATCGTTC	BRDN00011	75	51	208	189	300	13
AATATCTATT	BRDN00012	266	216	1400	537	543	26
AATATCTCTC	BRDN00012	262	459	1489	374	523	57
AATATCTGA	BRDN00011	160	101	706	238	543	23
AATATCTGA	BRDN00011	255	165	589	386	660	11
AATATGAAC	BRDN00012	187	8	0	275	91	0
AATATGAAG	BRDN00012	345	296	130	513	299	34
AATATGAAG	BRDN00011	145	138	49	235	363	9
AATATGAGA	BRDN00011	385	350	348	515	494	33
AATATGAGA	BRDN00012	230	230	1948	445	451	68
AATATGAGG	BRDN00011	87	0	0	528	208	0
AATATGATA	BRDN00012	125	54	871	224	245	26
AATATGATTC	BRDN00012	143	117	102	212	256	0
AATATGCAC	BRDN00012	176	206	20	346	425	1
AATATGCCA	BRDN00011	198	97	169	417	468	6
AATATGCTAC	BRDN00012	191	204	41	347	242	71
AATATGCTA	BRDN00011	40	52	0	164	123	8
AATATGCTA	BRDN00011	220	176	172	526	281	24
AATATGCTTC	BRDN00012	190	96	247	331	427	1
AATATGGAA	BRDN00012	102	40	6	173	261	0
AATATGGAA	BRDN00011	74	2	0	324	182	0
AATATGGCA	BRDN00011	208	235	948	425	410	11
AATATGGGC	BRDN00012	271	180	508	635	246	0
AATATGGTT	BRDN00012	331	367	252	687	852	39
AATATGGTT	BRDN00012	281	238	1649	430	434	85
AATATGTCA	BRDN00011	93	56	63	185	197	1
AATATGTCTT	BRDN00012	213	199	622	388	295	23
AATATGTTCA	BRDN00012	99	160	108	185	363	0
AATATTAAAC	BRDN00012	116	87	2	288	158	6
AATATTAACA	BRDN00011	150	164	75	254	98	43
AATATTACTG	BRDN00011	200	360	630	451	372	23
AATATTAGA	BRDN00011	125	29	0	211	268	0
AATATTAGG	BRDN00011	279	124	285	529	190	0
AATATTATGC	BRDN00011	123	180	70	262	194	17
AATATTCAG	BRDN00012	80	39	136	149	156	0
AATATTCCTA	BRDN00011	113	91	162	179	284	53
AATATTCTAA	BRDN00012	100	127	150	252	263	15
AATATTCTAT	BRDN00012	138	88	185	251	299	25
AATATTGAA	BRDN00011	151	15	0	300	384	0
AATATTGACC	BRDN00012	300	256	92	642	888	20
AATATTGCTC	BRDN00012	61	0	0	282	31	0
AATATTGGC	BRDN00012	240	120	323	481	768	8
AATATTGTTA	BRDN00012	106	148	59	181	368	0
AATATTTATG	BRDN00012	184	79	60	244	233	0
AATATTTCAA	BRDN00011	158	152	614	283	322	7
AATATTTGG	BRDN00012	320	349	970	594	919	14
AATATTTGTA	BRDN00012	144	95	44	490	848	6
AATATTTGTT	BRDN00012	407	474	1251	737	676	34
AATCAAAGT	BRDN00011	168	169	78	269	196	0
AATCAAATT	BRDN00011	180	179	178	388	241	33
AATCAACAC	BRDN00011	274	341	317	769	670	54
AATCAACCCA	BRDN00012	369	125	20	748	387	12
AATCAACCCC	BRDN00012	281	215	35	633	591	57
AATCAACGA	BRDN00011	257	326	451	561	345	12
AATCAACTG	BRDN00011	445	319	1209	781	652	33
AATCAAGAA	BRDN00011	301	267	478	506	316	35
AATCAAGGC	BRDN00012	545	433	328	960	1147	72
AATCAAGTA	BRDN00012	117	94	50	216	434	0
AATCAAGTG	BRDN00011	149	149	977	195	232	18
AATCAAGTTC	BRDN00011	308	270	229	631	327	104
AATCAATAA	BRDN00012	89	2	0	499	500	0
AATCAATAC	BRDN00011	107	4	0	247	165	0
AATCAATACT	BRDN00012	334	308	730	619	361	70
AATCAATGA	BRDN00012	204	65	0	286	465	2
AATCACAAA	BRDN00011	233	79	148	307	167	12
AATCACAAG	BRDN00012	336	295	1451	757	1155	72
AATCACACCA	BRDN00012	173	92	294	403	282	62
AATCACAGA	BRDN00012	383	427	625	766	355	32
AATCACAGA	BRDN00011	134	131	507	433	373	39
AATCACAGC	BRDN00011	210	269	330	423	410	4
AATCACATA	BRDN00011	161	80	218	375	396	0
AATCACATCC	BRDN00011	242	265	125	336	86	107
AATCACATTT	BRDN00011	118	7	8	283	344	16
AATCACCAA	BRDN00012	510	581	551	1040	864	80
AATCACCATC	BRDN00011	202	4	0	654	683	1
AATCACGAA	BRDN00012	116	127	1	200	101	2
AATCACGCA	BRDN00012	387	398	269	668	645	46
AATCACGGA	BRDN00012	355	237	504	691	965	38
AATCACTCAA	BRDN00011	169	76	86	408	261	0
AATCACTTTG	BRDN00011	102	74	58	199	157	2
AATCAGAAT	BRDN00011	52	71	79	199	67	0
AATCAGAGT	BRDN00012	240	191	362	430	208	35
AATCAGATG	BRDN00012	347	216	63	748	1015	12
AATCAGATG	BRDN00011	181	163	409	292	510	8
AATCAGCCTC	BRDN00012	300	261	575	642	517	37
AATCAGCTG	BRDN00011	148	164	94	230	263	36
AATCAGCTG	BRDN00011	141	114	7	253	135	13
AATCAGCTTC	BRDN00011	131	116	244	311	147	0
AATCAGGCG	BRDN00012	222	97	17	714	648	10
AATCAGGCG	BRDN00012	100	33	46	149	165	0
AATCAGGTG	BRDN00012	321	68	0	696	280	11
AATCAGGTG	BRDN00012	151	134	517	400	672	22
AATCAGTAT	BRDN00011	232	300	116	501	686	10
AATCAGTCC	BRDN00011	143	120	440	216	422	0
AATCAGTGA	BRDN00012	341	178	603	544	742	11
AATCAGTGA	BRDN00012	116	51	51	300	316	4
AATCATAAC	BRDN00012	142	142	82	271	215	6
AATCATAAG	BRDN00011	189	143	180	330	833	45
AATCATAAG	BRDN00012	384	249	154	650	972	16
AATCATAATC	BRDN00011	397	387	235	632	887	43
AATCATACCT	BRDN00011	107	189	197	279	324	12
AATCATCAA	BRDN00012	440	377	1176	648	1040	100
AATCATCATT	BRDN00011	247	295	138	477	298	7
AATCATCCAC	BRDN00011	151	184	32	315	197	84
AATCATCCTT	BRDN00011	355	134	176	452	385	8
AATCATCGG	BRDN00011	62	0	1	236	210	0
AATCATCTTG	BRDN00012	144	92	294	418	517	17
AATCATGAC	BRDN00012	318	332	66	583	678	0
AATCATGCA	BRDN00012	156	178	35	247	273	0
AATCATGGT	BRDN00012	112	11	104	277	107	17
AATCATTATT	BRDN00012	326	208	112	598	667	10
AATCATTTAA	BRDN00011	52	36	141	108	164	1
AATCCAAAAC	BRDN00012	227	181	169	390	393	11
AATCCAAGC	BRDN00011	201	193	637	360	194	8
AATCCAAGTC	BRDN00012	236	265	117	418	443	20
AATCCACAA	BRDN00011	184	177	228	422	455	42
AATCCACAAC	BRDN00011	411	433	879	782	804	33
AATCCACAAC	BRDN00011	406	480	298	871	786	51
AATCCACACA	BRDN00012	400	313	911	613	564	15
AATCCACCTC	BRDN00011	152	133	120	250	371	0
AATCCACCTT	BRDN00012	130	125	133	304	205	1
AATCCAGAC	BRDN00011	554	504	758	870	835	5
AATCCAGAG	BRDN00012	112	151	15	189	134	0
AATCCAGAG	BRDN00012	253	46	271	620	976	0
AATCCAGATC	BRDN00012	257	171	32	397	377	1
AATCCAGATT	BRDN00011	192	132	53	623	673	0
AATCCAGCTC	BRDN00011	367	89	12	728	506	0
AATCCAGGA	BRDN00011	151	77	250	258	182	0
AATCCAGGT	BRDN00012	416	392	461	780	441	26
AATCCATATG	BRDN00012	150	21	0	296	243	0
AATCCATGTC	BRDN00012	436	379	229	906	718	35
AATCCATGTT	BRDN00011	115	143	226	345	478	5
AATCCATTCA	BRDN00011	138	83	170	386	195	0
AATCCCAAA	BRDN00011	25	1	0	180	245	0
AATCCCAACC	BRDN00011	222	185	504	341	396	0
AATCCCAATT	BRDN00012	131	155	122	236	158	0
AATCCCACAT	BRDN00012	173	171	213	368	453	2
AATCCCAGTT	BRDN00011	513	490	1562	798	650	49
AATCCCCAG	BRDN00012	78	84	187	143	190	20
AATCCCCATA	BRDN00011	283	235	548	570	665	42
AATCCCCATG	BRDN00011	209	132	147	306	662	1
AATCCCCGCA	BRDN00012	536	529	690	911	853	35
AATCCCCTGA	BRDN00012	201	171	68	326	470	47
AATCCCGGA	BRDN00012	245	211	548	439	379	22
AATCCCTATG	BRDN00012	100	1	1	182	19	0
AATCCGAGA	BRDN00012	151	182	521	267	533	14
AATCCGAGT	BRDN00012	139	70	572	289	208	11
AATCCGCAG	BRDN00011	642	571	1642	1056	1234	67
AATCCGGAA	BRDN00011	60	15	91	340	141	0
AATCCGTATC	BRDN00012	191	151	72	344	292	9
AATCCGTTCA	BRDN00012	98	74	2	271	216	0
AATCCGTTG	BRDN00011	397	346	356	664	528	33
AATCCTAACA	BRDN00012	230	183	1	731	664	15
AATCCTCAAT	BRDN00011	345	260	176	583	390	0
AATCCTCATG	BRDN00012	126	101	0	280	244	4
AATCCTCCAA	BRDN00012	461	405	281	865	531	34
AATCCTCTCG	BRDN00012	298	243	26	473	478	32
AATCCTCTGG	BRDN00012	406	284	1491	537	723	44
AATCCTGAA	BRDN00011	120	16	252	248	195	0
AATCCTGATC	BRDN00011	131	174	226	315	275	13
AATCCTGATC	BRDN00012	455	510	602	991	993	32
AATCCTGATT	BRDN00012	160	176	419	310	325	23
AATCCTGTCC	BRDN00011	215	89	578	675	333	39
AATCCTTCGA	BRDN00011	176	118	42	140	251	15
AATCCTTGG	BRDN00012	123	79	203	233	363	7
AATCCTTTAA	BRDN00011	331	203	220	538	593	16
AATCGAAAT	BRDN00011	157	202	165	184	266	0
AATCGAACT	BRDN00011	164	223	5	332	272	12
AATCGAATA	BRDN00011	395	299	896	483	708	81
AATCGACTC	BRDN00012	569	386	883	756	746	41
AATCGAGAA	BRDN00011	188	127	9	342	227	0
AATCGATCA	BRDN00012	217	211	196	462	336	24
AATCGATCA	BRDN00011	79	36	45	287	164	19
AATCGATGG	BRDN00012	332	363	234	436	355	10
AATCGATGT	BRDN00011	87	31	0	392	230	2
AATCGATTTA	BRDN00012	10	1	0	324	302	0
AATCGCACTC	BRDN00012	113	46	2	216	259	0
AATCGCAGG	BRDN00012	265	247	737	540	667	27
AATCGCCCAC	BRDN00012	331	395	544	637	875	34
AATCGCCTG	BRDN00011	235	278	2017	398	500	28
AATCGGAAC	BRDN00011	512	538	1196	818	848	112
AATCGGACT	BRDN00012	437	364	2030	1008	627	153
AATCGGAGC	BRDN00012	170	113	100	437	443	0
AATCGGATA	BRDN00011	322	231	392	631	837	38
AATCGGCAG	BRDN00011	314	276	815	502	419	22
AATCGGGGT	BRDN00012	105	101	317	261	636	19
AATCGGTAG	BRDN00011	139	87	192	235	339	7
AATCGGTCC	BRDN00012	268	228	48	385	630	8
AATCGGTGC	BRDN00011	273	405	18	638	796	104
AATCGTAGT	BRDN00011	160	135	166	216	315	26
AATCGTCATT	BRDN00011	232	273	199	269	198	0
AATCGTGCA	BRDN00012	135	296	405	324	428	16
AATCGTGGG	BRDN00011	409	279	735	695	573	53
AATCGTTCAT	BRDN00012	160	205	71	407	630	35
AATCGTTCCA	BRDN00011	128	147	175	256	270	10
AATCTAAGTC	BRDN00011	183	254	251	423	478	25
AATCTAATTG	BRDN00012	239	328	1229	634	615	86
AATCTACACC	BRDN00011	107	61	40	125	167	5
AATCTACGA	BRDN00012	182	166	63	302	205	42
AATCTACTCT	BRDN00012	287	194	1590	465	338	62
AATCTAGGT	BRDN00012	411	300	201	704	844	20
AATCTATCG	BRDN00012	131	56	157	193	92	6
AATCTATGA	BRDN00012	154	89	425	273	333	0
AATCTATGAC	BRDN00012	194	32	14	587	378	0
AATCTATGCT	BRDN00011	163	196	215	312	279	16
AATCTCAACA	BRDN00011	234	137	251	314	267	5
AATCTCACCC	BRDN00012	113	0	0	444	456	0
AATCTCACTA	BRDN00012	270	515	564	551	756	35
AATCTCAGC	BRDN00011	172	105	414	326	299	1
AATCTCATCA	BRDN00012	361	403	708	817	540	33
AATCTCATCT	BRDN00012	133	0	0	336	29	0
AATCTCCAAG	BRDN00011	239	189	63	359	373	13
AATCTCCACG	BRDN00012	215	220	457	430	412	23
AATCTCCACT	BRDN00012	306	27	15	621	898	5
AATCTCCCTC	BRDN00011	84	44	206	103	202	5
AATCTCCGTA	BRDN00012	188	162	3734	390	281	73
AATCTCGCA	BRDN00011	28	21	0	274	246	0
AATCTCGTA	BRDN00011	185	193	164	363	367	37
AATCTCGTTA	BRDN00011	485	581	523	857	697	41
AATCTCTAG	BRDN00012	290	309	375	501	712	30
AATCTCTCTA	BRDN00012	422	437	336	821	824	10
AATCTCTGAC	BRDN00011	98	138	2397	157	177	26
AATCTCTGAC	BRDN00011	190	194	469	324	270	68
AATCTCTGCA	BRDN00011	400	421	2240	688	882	35
AATCTGCAG	BRDN00012	232	232	452	246	421	12
AATCTGCGG	BRDN00011	337	366	2275	848	716	60
AATCTGCTCC	BRDN00011	381	338	1711	803	545	20
AATCTGCTGC	BRDN00011	121	151	23	258	310	27
AATCTGGAA	BRDN00011	71	122	22	208	259	44
AATCTGGAT	BRDN00012	177	129	356	362	400	7
AATCTGTACC	BRDN00012	245	97	60	448	593	14
AATCTGTCAC	BRDN00012	350	236	162	601	554	13
AATCTGTGA	BRDN00012	135	87	479	132	309	14
AATCTGTGCC	BRDN00012	440	460	3633	950	805	97
AATCTGTGG	BRDN00012	416	287	560	872	909	79
AATCTGTGG	BRDN00011	236	214	146	417	244	2
AATCTGTTCA	BRDN00012	141	91	930	190	237	62
AATCTTAACA	BRDN00012	218	67	17	444	590	0
AATCTTAACA	BRDN00012	79	16	0	541	335	0
AATCTTACCT	BRDN00012	118	71	29	344	266	0
AATCTTACTC	BRDN00012	515	545	2542	881	1131	74
AATCTTCACG	BRDN00012	185	173	318	442	410	50
AATCTTCCAA	BRDN00011	169	186	134	436	374	0
AATCTTCCAT	BRDN00012	141	106	120	196	339	18
AATCTTCTAG	BRDN00011	402	241	1384	768	758	37
AATCTTCTCT	BRDN00012	200	201	406	320	241	17
AATCTTCTTG	BRDN00011	325	402	200	679	1072	50
AATCTTGAG	BRDN00012	284	378	900	474	287	38
AATCTTGTGT	BRDN00012	439	369	1444	685	556	52
AATCTTGTTG	BRDN00011	121	106	112	248	304	1
AATCTTTGTT	BRDN00011	96	148	176	301	343	5
AATGAAAAC	BRDN00011	30	65	9	155	285	0
AATGAAAAT	BRDN00012	235	217	934	247	165	1
AATGAAAGA	BRDN00012	103	110	112	152	215	8
AATGAAAGC	BRDN00012	487	501	476	876	697	24
AATGAAAGC	BRDN00012	239	137	0	375	331	0
AATGAAAGC	BRDN00012	288	289	419	511	848	15
AATGAAAGG	BRDN00012	102	180	276	255	198	41
AATGAAATC	BRDN00012	182	129	425	445	552	13
AATGAACGA	BRDN00011	392	353	1313	669	558	36
AATGAACGT	BRDN00012	115	213	180	275	455	19
AATGAACTA	BRDN00011	78	13	21	306	621	0
AATGAACTC	BRDN00011	254	374	373	552	641	37
AATGAACTG	BRDN00011	107	1	0	335	269	0
AATGAACTG	BRDN00011	353	154	130	643	661	21
AATGAACTT	BRDN00011	209	195	121	389	290	23
AATGAACTT	BRDN00011	141	161	685	391	224	8
AATGAAGAC	BRDN00011	316	383	321	770	835	37
AATGAAGAG	BRDN00011	90	68	20	169	396	0
AATGAAGAG	BRDN00011	316	339	273	506	632	47
AATGAAGCA	BRDN00012	310	259	221	414	573	2
AATGAAGCT	BRDN00012	114	204	1017	370	284	15
AATGAAGCT	BRDN00012	339	288	68	989	884	7
AATGAAGCT	BRDN00012	372	143	268	639	791	7
AATGAAGCT	BRDN00012	196	123	0	637	363	0
AATGAAGGA	BRDN00012	330	288	1219	630	1030	36
AATGAAGTG	BRDN00012	130	136	10	229	217	5
AATGAATAC	BRDN00012	187	86	438	399	285	15
AATGAATAC	BRDN00011	167	85	307	305	194	8
AATGAATAT	BRDN00012	94	104	77	223	104	26
AATGAATCC	BRDN00012	117	136	179	295	110	11
AATGAATGG	BRDN00011	307	361	768	575	746	22
AATGAATGG	BRDN00011	99	129	414	233	358	0
AATGAATTC	BRDN00011	207	42	0	352	172	0
AATGAATTG	BRDN00011	277	263	1197	485	531	26
AATGAATTT	BRDN00012	74	48	1649	525	320	23
AATGAATTTC	BRDN00012	230	289	91	352	133	39
AATGACAAC	BRDN00011	194	149	1323	290	364	17
AATGACAAG	BRDN00012	200	143	38	272	192	11
AATGACAAT	BRDN00012	149	18	1	642	448	0
AATGACACA	BRDN00012	100	47	29	628	328	3
AATGACACA	BRDN00011	136	80	3943	179	318	57
AATGACACC	BRDN00011	214	0	1	489	477	0
AATGACACC	BRDN00012	348	305	869	838	552	56
AATGACAGC	BRDN00012	110	12	0	195	54	0
AATGACATC	BRDN00012	242	143	827	447	555	24
AATGACCCC	BRDN00012	56	0	1	347	279	0
AATGACCCT	BRDN00011	130	52	120	169	237	22
AATGACCGT	BRDN00012	222	232	143	555	712	22
AATGACCTG	BRDN00012	221	158	532	363	249	6
AATGACCTTT	BRDN00012	334	311	273	604	544	51
AATGACGAC	BRDN00012	98	94	101	207	225	26
AATGACGCT	BRDN00011	175	1	0	246	255	2
AATGACGTC	BRDN00011	133	63	0	337	246	17
AATGACGTT	BRDN00012	115	105	234	169	164	0
AATGACTAA	BRDN00012	311	349	514	531	775	77
AATGACTAC	BRDN00011	120	174	198	304	427	8
AATGACTAT	BRDN00012	82	140	56	165	232	9
AATGACTATC	BRDN00011	111	122	239	229	101	0
AATGACTGA	BRDN00011	395	345	522	613	501	30
AATGACTGA	BRDN00012	238	311	224	384	309	26
AATGACTGT	BRDN00012	375	349	458	548	369	16
AATGACTTCC	BRDN00012	102	77	384	321	202	0
AATGACTTG	BRDN00012	52	17	0	216	224	0
AATGACTTTC	BRDN00011	96	126	126	144	196	19
AATGAGAAA	BRDN00012	154	190	49	319	296	24
AATGAGAAC	BRDN00012	174	26	839	192	156	0
AATGAGAAC	BRDN00011	129	58	67	200	192	0
AATGAGAAG	BRDN00011	312	205	434	475	519	16
AATGAGACA	BRDN00012	163	93	35	223	485	12
AATGAGACT	BRDN00011	3	0	0	224	83	0
AATGAGACT	BRDN00011	318	335	991	622	286	5
AATGAGAGG	BRDN00011	326	322	133	576	761	7
AATGAGATC	BRDN00011	177	210	33	303	350	18
AATGAGCAC	BRDN00011	155	266	702	364	626	9
AATGAGCAG	BRDN00012	303	331	1323	456	432	63
AATGAGCAG	BRDN00012	185	42	10	309	167	12
AATGAGCAT	BRDN00012	426	464	698	796	593	67
AATGAGCCC	BRDN00011	143	99	598	288	219	8
AATGAGCTT	BRDN00012	63	47	28	98	64	23
AATGAGGAG	BRDN00012	294	375	747	447	724	43
AATGAGGAT	BRDN00012	90	14	26	215	113	7
AATGAGGAT	BRDN00012	167	221	166	346	277	4
AATGAGGTG	BRDN00012	228	50	182	652	365	14
AATGAGTAG	BRDN00011	423	572	898	1008	1470	31
AATGAGTTG	BRDN00011	278	198	573	610	492	18
AATGATACA	BRDN00011	81	0	0	153	111	0
AATGATACG	BRDN00011	240	232	1030	386	809	40
AATGATAGA	BRDN00011	15	0	0	114	47	0
AATGATATTC	BRDN00012	159	211	199	240	236	59
AATGATCAG	BRDN00012	115	72	50	351	211	0
AATGATCATC	BRDN00011	202	64	40	383	369	41
AATGATCCAC	BRDN00011	126	104	4	252	281	2
AATGATCCG	BRDN00012	338	296	611	642	828	24
AATGATCTAC	BRDN00011	136	119	285	246	126	3
AATGATCTCA	BRDN00012	138	189	155	273	904	4
AATGATGAA	BRDN00012	131	49	9	477	226	0
AATGATGAC	BRDN00012	318	388	720	558	840	78
AATGATGAG	BRDN00011	353	327	426	615	777	12
AATGATGAT	BRDN00012	125	83	473	356	288	22
AATGATGCG	BRDN00011	208	150	149	488	269	1
AATGATGCT	BRDN00012	166	110	0	325	325	12
AATGATGGG	BRDN00012	285	132	331	295	432	37
AATGATGTC	BRDN00012	96	87	5	174	234	30
AATGATTAA	BRDN00012	177	156	48	189	476	21
AATGATTAC	BRDN00012	167	5	0	237	194	0
AATGATTGC	BRDN00012	402	367	302	710	819	71
AATGATTTAC	BRDN00011	244	205	556	431	359	18
AATGCAAAA	BRDN00011	342	303	2109	588	732	57
AATGCAAAG	BRDN00011	81	82	5	235	848	17
AATGCAAAT	BRDN00012	147	94	16	351	312	20
AATGCAATA	BRDN00011	369	382	943	601	621	56
AATGCAATT	BRDN00012	220	169	34	303	305	23
AATGCAATT	BRDN00011	116	180	228	209	74	16
AATGCACAA	BRDN00012	319	49	127	852	847	0
AATGCACAG	BRDN00011	69	29	197	117	138	0
AATGCACCA	BRDN00011	119	104	63	390	349	0
AATGCACCG	BRDN00012	319	436	221	751	928	12
AATGCACTA	BRDN00012	322	309	120	459	447	1
AATGCACTG	BRDN00011	145	110	41	283	106	7
AATGCACTTC	BRDN00011	210	70	2	380	637	0
AATGCACTTT	BRDN00012	248	209	530	380	457	4
AATGCAGAA	BRDN00011	237	239	818	657	548	121
AATGCAGAG	BRDN00012	202	243	437	334	503	6
AATGCAGAT	BRDN00012	302	325	166	622	604	21
AATGCAGCA	BRDN00011	194	191	365	320	573	6
AATGCAGGC	BRDN00011	277	320	203	473	739	57
AATGCAGTG	BRDN00011	152	52	562	380	576	5
AATGCATATC	BRDN00012	267	530	19	513	606	63
AATGCATCCC	BRDN00012	136	168	554	251	487	4
AATGCATCCT	BRDN00012	165	226	226	422	175	0
AATGCATGA	BRDN00012	233	184	213	442	259	5
AATGCATGA	BRDN00012	311	328	199	530	617	61
AATGCATGC	BRDN00011	262	109	0	532	434	9
AATGCATTTA	BRDN00012	354	137	385	719	463	27
AATGCATTTC	BRDN00011	178	212	1045	326	328	16
AATGCCAAA	BRDN00011	156	164	0	174	194	19
AATGCCAAA	BRDN00012	467	462	1591	799	726	49
AATGCCAAG	BRDN00011	26	10	0	556	326	0
AATGCCAATC	BRDN00011	309	1	0	580	507	1
AATGCCACA	BRDN00011	92	4	0	251	31	4
AATGCCACA	BRDN00011	388	414	391	648	701	22
AATGCCACCC	BRDN00011	138	92	28	193	196	1
AATGCCACTA	BRDN00012	363	328	1058	842	956	23
AATGCCAGA	BRDN00012	137	59	226	300	199	0
AATGCCAGC	BRDN00012	172	201	21	291	400	7
AATGCCAGT	BRDN00011	129	81	5	253	311	17
AATGCCATAC	BRDN00011	186	106	82	200	385	4
AATGCCATCC	BRDN00011	323	254	285	768	625	19
AATGCCATTA	BRDN00012	205	128	5	770	803	22
AATGCCCAAC	BRDN00012	375	306	919	634	590	9
AATGCCCAAC	BRDN00011	136	106	19	191	137	12
AATGCCCAG	BRDN00011	56	3	0	226	105	0
AATGCCCAG	BRDN00012	78	35	47	124	249	9
AATGCCCGG	BRDN00011	265	186	1696	491	637	128
AATGCCCGTT	BRDN00011	307	203	153	417	501	7
AATGCCCTCT	BRDN00012	336	257	286	549	666	23
AATGCCCTGA	BRDN00011	276	301	1591	514	667	29
AATGCCCTTG	BRDN00011	96	51	138	286	124	11
AATGCCGAT	BRDN00012	357	390	697	597	330	83
AATGCCGATT	BRDN00012	154	101	121	191	226	0
AATGCCGTA	BRDN00011	230	198	293	605	702	37
AATGCCTCCG	BRDN00012	154	221	298	212	362	38
AATGCCTGA	BRDN00012	405	440	2905	573	355	60
AATGCCTGA	BRDN00012	151	161	262	182	186	9
AATGCCTGG	BRDN00011	370	403	690	767	668	100
AATGCCTGG	BRDN00012	11	0	0	305	263	0
AATGCCTGG	BRDN00011	68	68	36	162	158	55
AATGCCTTG	BRDN00012	166	29	66	271	379	1
AATGCGACT	BRDN00011	267	270	2305	434	399	44
AATGCGAGT	BRDN00012	450	492	342	625	835	51
AATGCGAGT	BRDN00011	277	296	39	435	499	8
AATGCGATT	BRDN00012	270	270	717	547	514	14
AATGCGCAT	BRDN00011	244	245	1420	477	355	11
AATGCGCTG	BRDN00012	258	256	845	582	574	45
AATGCGGAC	BRDN00012	330	376	890	573	697	61
AATGCGGTA	BRDN00011	206	0	2	556	309	0
AATGCGTTA	BRDN00012	162	122	14	336	251	0
AATGCTATTA	BRDN00011	346	252	678	651	1226	10
AATGCTCAAC	BRDN00011	99	97	176	163	132	0
AATGCTCATC	BRDN00011	356	440	319	591	401	45
AATGCTCCCT	BRDN00012	135	156	276	198	113	36
AATGCTCCTA	BRDN00011	71	27	0	332	443	0
AATGCTCCTG	BRDN00011	113	75	562	273	214	22
AATGCTCGG	BRDN00012	281	234	204	426	287	20

AATGCTGAA	BRDN00011	132	5	0	211	109	0
AATGCTGAA	BRDN00012	112	247	39	199	371	0
AATGCTGAG	BRDN00011	134	109	0	232	110	0
AATGCTGAT	BRDN00012	84	152	100	118	39	8
AATGCTGCC	BRDN00011	98	135	273	165	306	0
AATGCTGCG	BRDN00011	320	286	431	542	491	37
AATGCTGCG	BRDN00012	227	157	271	503	497	9
AATGCTGCT	BRDN00011	279	177	21	586	690	42
AATGCTGGA	BRDN00011	39	0	0	533	301	0
AATGCTGGG	BRDN00011	137	70	186	178	218	32
AATGCTTAC	BRDN00012	132	119	0	316	297	0
AATGCTTAC	BRDN00011	353	358	175	656	484	13
AATGCTTCA	BRDN00011	130	45	253	249	281	12
AATGCTTCG	BRDN00012	71	0	1	396	319	0
AATGCTTCT	BRDN00011	267	316	233	471	515	6
AATGCTTTA	BRDN00012	100	66	1	563	916	0
AATGGAAAA	BRDN00012	106	62	255	236	91	9
AATGGAAAA	BRDN00012	326	299	524	526	561	32
AATGGAAAA	BRDN00011	183	264	191	269	321	15
AATGGAAAA	BRDN00012	165	69	35	233	108	4
AATGGAAAC	BRDN00011	139	128	647	301	365	33
AATGGAACA	BRDN00011	196	169	193	273	490	39
AATGGAAGA	BRDN00011	118	54	0	279	166	0
AATGGAAGA	BRDN00011	181	208	190	302	274	4
AATGGAAGC	BRDN00012	191	124	3	284	268	0
AATGGAAGC	BRDN00012	138	172	73	328	312	25
AATGGAAGT	BRDN00011	104	58	91	285	86	0
AATGGAATA	BRDN00012	236	334	334	470	472	26
AATGGAATT	BRDN00011	161	83	510	351	352	17
AATGGACAC	BRDN00012	215	122	154	308	180	17
AATGGACAG	BRDN00012	299	288	1154	511	410	19
AATGGACAG	BRDN00012	80	168	11	224	221	2
AATGGACAT	BRDN00011	141	139	938	327	507	16
AATGGACTC	BRDN00011	288	289	412	562	603	0
AATGGACTC	BRDN00011	137	170	1482	222	72	29
AATGGACTC	BRDN00011	323	344	503	417	400	84
AATGGACTG	BRDN00012	203	199	147	277	1062	0
AATGGAGAC	BRDN00012	115	82	0	154	197	6
AATGGAGCA	BRDN00011	288	298	1595	539	779	54
AATGGAGTA	BRDN00012	254	259	123	439	459	1
AATGGATAG	BRDN00012	64	39	7	367	386	0
AATGGATCC	BRDN00012	288	232	4	528	512	23
AATGGATCT	BRDN00012	106	151	9	203	209	18
AATGGATGA	BRDN00012	163	167	104	377	485	3
AATGGATGA	BRDN00012	329	333	128	698	582	9
AATGGATGC	BRDN00012	460	436	1149	713	622	21
AATGGATGT	BRDN00012	66	60	15	106	23	20
AATGGATTT	BRDN00012	210	247	446	400	361	116
AATGGCAAA	BRDN00011	154	97	3	399	482	7
AATGGCAAC	BRDN00012	94	76	199	131	63	4
AATGGCAAG	BRDN00011	20	3	0	481	83	0
AATGGCAAT	BRDN00012	227	203	257	541	514	71
AATGGCAGA	BRDN00011	110	84	202	204	117	0
AATGGCAGT	BRDN00012	471	618	739	805	1017	48
AATGGCATA	BRDN00012	138	136	144	376	313	13
AATGGCATA	BRDN00011	226	249	25	364	164	36
AATGGCATG	BRDN00011	174	194	25	322	298	32
AATGGCCAG	BRDN00012	225	194	977	460	330	14
AATGGCCCG	BRDN00011	137	140	222	264	242	1
AATGGCCCT	BRDN00011	81	3	0	275	130	0
AATGGCCTC	BRDN00012	130	125	536	213	214	61
AATGGCGAG	BRDN00012	196	0	0	709	179	0
AATGGCTAA	BRDN00011	358	260	778	597	670	0
AATGGCTAT	BRDN00011	256	261	96	370	312	19
AATGGCTCC	BRDN00011	344	410	619	567	339	14
AATGGCTCT	BRDN00012	155	134	81	225	139	0
AATGGCTGG	BRDN00011	243	94	624	380	290	19
AATGGCTTA	BRDN00011	160	135	38	206	333	0
AATGGCTTA	BRDN00012	162	191	8	327	150	16
AATGGCTTT	BRDN00011	60	70	0	122	75	0
AATGGGAAA	BRDN00011	393	332	280	603	581	39
AATGGGAAC	BRDN00012	129	242	211	299	679	15
AATGGGACA	BRDN00011	68	61	142	225	304	33
AATGGGACA	BRDN00011	204	269	62	392	408	13
AATGGGAGA	BRDN00012	117	133	19	186	144	15
AATGGGAGC	BRDN00012	376	389	553	829	859	30
AATGGGAGT	BRDN00011	103	108	9	162	110	17
AATGGGGAA	BRDN00012	154	111	934	138	339	12
AATGGGGCA	BRDN00012	151	109	62	197	315	33
AATGGGGCC	BRDN00012	182	209	0	272	339	1
AATGGGGCC	BRDN00011	352	277	794	645	351	47
AATGGGGCT	BRDN00011	133	113	42	247	192	31
AATGGGGTG	BRDN00011	62	91	364	212	252	9
AATGGGTCT	BRDN00012	287	271	957	626	417	54
AATGGGTGG	BRDN00011	85	0	0	95	175	0
AATGGGTTG	BRDN00012	462	357	1165	742	837	46
AATGGGTTG	BRDN00012	321	16	4	722	419	0
AATGGTAAT	BRDN00012	397	317	1083	768	838	25
AATGGTCAA	BRDN00012	339	237	390	821	875	7
AATGGTCAG	BRDN00011	187	191	154	409	371	0
AATGGTCAT	BRDN00012	127	223	18	181	207	0
AATGGTCCC	BRDN00011	88	118	94	281	150	12
AATGGTCGA	BRDN00012	389	43	34	635	315	18
AATGGTCGC	BRDN00012	182	121	210	287	338	0
AATGGTCTG	BRDN00012	12	0	0	251	57	0
AATGGTCTT	BRDN00012	306	349	460	422	455	11
AATGGTGAA	BRDN00012	155	82	348	412	1169	4
AATGGTGAA	BRDN00011	117	94	133	290	240	0
AATGGTGAA	BRDN00012	94	19	275	264	191	0
AATGGTGAG	BRDN00012	295	395	1348	551	648	55
AATGGTGGA	BRDN00011	139	228	347	361	267	40
AATGGTGGT	BRDN00012	379	582	544	776	625	25
AATGGTGTA	BRDN00012	414	392	420	695	777	43
AATGGTGTA	BRDN00012	138	100	211	278	415	44
AATGGTGTC	BRDN00011	194	167	27	252	202	0
AATGGTGTC	BRDN00011	145	114	0	248	370	23
AATGGTTCT	BRDN00011	123	198	29	300	238	2
AATGGTTGC	BRDN00011	201	184	1	238	239	0
AATGGTTGT	BRDN00012	172	215	363	509	501	28
AATGGTTTG	BRDN00011	87	131	142	143	111	15
AATGTAAAA	BRDN00012	320	348	1447	528	962	23
AATGTAACC	BRDN00012	361	296	2676	680	808	49
AATGTAACC	BRDN00012	227	404	342	615	565	41
AATGTAACT	BRDN00011	84	0	0	177	129	0
AATGTAAGG	BRDN00011	280	313	266	432	777	53
AATGTAATG	BRDN00012	234	178	160	427	381	38
AATGTACAA	BRDN00012	108	20	0	209	240	0
AATGTACCT	BRDN00012	131	131	579	250	507	54
AATGTACTC	BRDN00012	160	104	237	237	255	22
AATGTAGAA	BRDN00011	99	142	190	226	115	9
AATGTAGAG	BRDN00012	80	1	0	140	94	0
AATGTAGCA	BRDN00012	87	144	33	113	247	6
AATGTAGTA	BRDN00011	176	61	0	418	371	7
AATGTAGTT	BRDN00012	144	105	239	353	325	0
AATGTATAT	BRDN00011	93	0	0	200	356	0
AATGTATAT	BRDN00011	398	506	1279	706	807	53
AATGTATCA	BRDN00012	128	180	466	234	215	6
AATGTATCT	BRDN00011	228	276	395	382	356	18
AATGTATGC	BRDN00012	373	377	69656	635	710	1149
AATGTATGG	BRDN00011	94	58	113	128	115	12
AATGTATTA	BRDN00012	361	319	426	581	483	21
AATGTATTA	BRDN00011	70	64	22	86	131	18
AATGTCACC	BRDN00012	117	44	233	191	193	22
AATGTCACC	BRDN00012	139	158	344	172	116	10
AATGTCAGC	BRDN00011	537	431	607	813	779	39
AATGTCATC	BRDN00012	124	201	144	380	286	37
AATGTCCAC	BRDN00011	269	268	364	519	401	8
AATGTCCCG	BRDN00011	32	50	25	123	230	0
AATGTCCGC	BRDN00011	247	245	259	511	352	31
AATGTCCGC	BRDN00011	75	48	43	264	110	0
AATGTCCGC	BRDN00012	432	458	5359	849	525	227
AATGTCCTG	BRDN00011	59	0	0	321	184	0
AATGTCCTG	BRDN00012	407	389	921	678	728	62
AATGTCGCG	BRDN00011	226	123	115	288	409	0
AATGTCGGC	BRDN00012	301	146	25	590	747	28
AATGTCGGG	BRDN00012	228	260	121	424	460	29
AATGTCGGT	BRDN00011	167	163	165	394	237	0
AATGTCGTT	BRDN00011	162	197	268	272	255	6
AATGTCTAC	BRDN00011	174	165	538	314	440	26
AATGTCTCC	BRDN00011	60	2	110	258	96	5
AATGTCTCG	BRDN00011	187	83	157	426	325	0
AATGTCTGA	BRDN00011	30	21	0	154	85	0
AATGTCTGA	BRDN00012	182	178	430	412	653	0
AATGTCTGA	BRDN00012	149	68	807	405	155	5
AATGTCTGC	BRDN00012	287	242	290	488	357	21
AATGTCTGT	BRDN00012	116	108	0	244	161	11
AATGTCTTA	BRDN00012	164	105	254	281	275	13
AATGTCTTC	BRDN00012	206	181	53	356	255	3
AATGTGAAG	BRDN00011	200	55	1691	377	745	18
AATGTGAAT	BRDN00012	56	47	18	358	276	9
AATGTGACA	BRDN00011	180	205	16	255	611	27
AATGTGACC	BRDN00012	254	95	8	527	163	0
AATGTGACG	BRDN00012	323	260	911	504	814	77
AATGTGACT	BRDN00012	110	37	0	238	105	0
AATGTGACT	BRDN00012	385	350	962	729	785	57
AATGTGAGA	BRDN00012	110	119	273	230	61	51
AATGTGAGG	BRDN00012	343	305	1048	649	747	32
AATGTGAGG	BRDN00011	261	151	314	511	282	38
AATGTGATA	BRDN00011	120	135	0	222	132	8
AATGTGATA	BRDN00011	159	140	214	281	227	63
AATGTGCCA	BRDN00012	211	31	287	264	181	3
AATGTGCTA	BRDN00011	109	144	225	256	387	0
AATGTGCTA	BRDN00011	138	161	24	269	181	17
AATGTGCTG	BRDN00011	334	96	483	591	702	16
AATGTGCTG	BRDN00012	260	289	594	728	890	12
AATGTGGCT	BRDN00012	226	292	234	288	140	67
AATGTGGGA	BRDN00011	282	296	1302	373	474	71
AATGTGGGT	BRDN00012	88	17	27	346	593	2
AATGTGGTG	BRDN00011	220	27	0	435	325	0
AATGTGGTG	BRDN00011	21	1	0	61	29	0
AATGTGGTT	BRDN00012	131	8	0	537	196	0
AATGTGTAA	BRDN00011	85	124	228	267	256	8
AATGTGTAA	BRDN00012	155	161	2036	245	517	34
AATGTGTAG	BRDN00011	464	371	3229	894	807	79
AATGTGTAT	BRDN00012	54	3	0	188	201	0
AATGTGTCA	BRDN00011	219	129	306	493	249	0
AATGTGTCA	BRDN00012	61	5	0	342	243	0
AATGTGTGA	BRDN00011	103	192	0	360	471	6
AATGTGTGC	BRDN00011	389	298	485	736	643	35
AATGTGTTG	BRDN00011	451	467	318	936	827	70
AATGTTAGC	BRDN00011	169	258	1878	283	393	34
AATGTTAGG	BRDN00012	151	174	348	314	231	8
AATGTTATA	BRDN00011	135	34	0	138	249	0
AATGTTATA	BRDN00011	168	150	2	237	624	3
AATGTTATT	BRDN00012	216	149	219	305	408	57
AATGTTCAC	BRDN00012	289	412	940	529	514	11
AATGTTCAG	BRDN00012	178	286	881	560	697	66
AATGTTCAT	BRDN00011	313	349	299	543	711	7
AATGTTCGC	BRDN00011	203	105	553	427	300	7
AATGTTCGG	BRDN00012	84	98	45	206	266	0
AATGTTCTC	BRDN00012	274	219	360	444	286	23
AATGTTGAA	BRDN00012	108	45	206	254	291	4
AATGTTGAC	BRDN00011	262	200	451	551	205	33
AATGTTGAC	BRDN00012	383	387	1011	710	1106	47
AATGTTGAC	BRDN00012	240	189	94	425	344	10
AATGTTGCA	BRDN00011	97	43	1	158	34	0
AATGTTGCA	BRDN00012	323	370	715	573	754	67
AATGTTGGG	BRDN00011	48	7	1	608	697	0
AATGTTGGG	BRDN00012	171	228	88	318	629	16
AATGTTGGG	BRDN00011	192	183	841	339	489	27
AATGTTGTC	BRDN00011	162	164	591	331	502	19
AATGTTGTG	BRDN00011	172	118	47	247	296	5
AATGTTTCC	BRDN00012	219	289	87	528	561	40
AATGTTTCT	BRDN00012	228	180	602	365	166	22
AATGTTTGG	BRDN00011	83	136	288	174	355	4
AATTAAAAA	BRDN00012	125	101	350	253	461	13
AATTAAAAT	BRDN00012	154	140	15	204	106	18
AATTAAACT	BRDN00012	162	16	81	295	534	0
AATTAAGAA	BRDN00011	50	77	7	189	171	15
AATTAAGAG	BRDN00012	370	559	1878	845	768	201
AATTAAGTG	BRDN00011	117	74	91	242	137	1
AATTAATAA	BRDN00012	242	178	941	490	693	15
AATTAATGA	BRDN00012	135	192	1013	277	253	42
AATTAATGT	BRDN00011	199	256	284	419	403	39
AATTAATTC	BRDN00011	159	306	1162	301	796	41
AATTAATTC	BRDN00011	119	99	167	240	294	8
AATTACAAG	BRDN00012	201	198	154	216	203	50
AATTACAAT	BRDN00012	278	315	175	502	389	22
AATTACAAT	BRDN00011	211	128	936	318	537	46
AATTACACA	BRDN00012	373	332	188	587	529	25
AATTACACT	BRDN00011	112	77	504	291	310	0
AATTACACT	BRDN00012	105	131	99	254	165	8
AATTACAGT	BRDN00011	440	409	726	830	1104	60
AATTACATA	BRDN00012	164	145	506	288	205	51
AATTACATA	BRDN00012	319	222	746	513	269	22
AATTACATG	BRDN00012	252	201	533	553	717	18
AATTACATT	BRDN00012	122	102	97	196	180	8
AATTACCAA	BRDN00011	290	134	336	391	518	11
AATTACCAC	BRDN00011	167	81	19	315	115	14
AATTACCAC	BRDN00011	171	138	45	254	456	13
AATTACCGG	BRDN00012	105	86	52	145	144	1
AATTACCTG	BRDN00011	396	298	204	583	688	0
AATTACGAC	BRDN00011	283	231	664	680	607	34
AATTACGCC	BRDN00012	274	205	867	430	454	15
AATTACTAC	BRDN00011	70	95	162	171	217	0
AATTACTCA	BRDN00012	272	169	508	529	457	35
AATTACTGA	BRDN00011	325	45	365	715	728	40
AATTACTGC	BRDN00012	147	180	539	243	344	3
AATTACTGT	BRDN00011	226	131	154	418	538	0
AATTAGCCT	BRDN00011	302	212	500	619	515	22
AATTAGGAT	BRDN00012	193	220	34	345	179	32
AATTAGTAC	BRDN00012	192	238	160	419	675	10
AATTAGTGC	BRDN00011	144	15	139	212	282	0
AATTAGTGT	BRDN00012	135	183	28	316	207	7
AATTAGTTT	BRDN00012	326	383	53	713	612	6
AATTATAAC	BRDN00012	163	160	1089	284	364	21
AATTATACT	BRDN00012	248	132	443	534	707	36
AATTATAGC	BRDN00012	139	106	17	354	402	8
AATTATAGC	BRDN00011	198	44	19	426	197	0
AATTATCAA	BRDN00012	253	249	836	464	891	43
AATTATCAA	BRDN00011	19	8	0	346	279	0
AATTATCTG	BRDN00012	196	189	590	463	616	12
AATTATCTG	BRDN00011	366	487	1045	632	978	35
AATTATCTT	BRDN00011	317	281	419	514	779	7
AATTATCTT	BRDN00012	183	162	1719	323	302	42
AATTATCTT	BRDN00012	170	80	196	336	244	6
AATTATGGA	BRDN00012	111	106	361	150	216	6
AATTATGGC	BRDN00012	102	27	25	474	387	0
AATTATGTC	BRDN00012	322	289	979	507	269	4
AATTATTAC	BRDN00011	196	128	20	265	433	0
AATTATTCC	BRDN00011	412	370	718	767	652	19
AATTATTGT	BRDN00011	227	204	501	366	519	0
AATTCAAAA	BRDN00011	167	114	40	241	375	0
AATTCAAAT	BRDN00012	186	484	11927	341	265	107
AATTCAAAT	BRDN00012	124	87	1088	233	164	15
AATTCAAAG	BRDN00012	149	238	20	177	273	31
AATTCAAAG	BRDN00012	307	224	510	580	595	15
AATTCAACA	BRDN00012	289	321	78	548	485	30
AATTCAACA	BRDN00011	194	190	1320	254	888	28
AATTCAACA	BRDN00012	255	215	34	485	905	9
AATTCAACT	BRDN00011	342	400	411	711	540	18
AATTCAAGA	BRDN00012	262	225	336	553	691	11
AATTCAAGT	BRDN00011	151	113	5	224	123	24
AATTCAATC	BRDN00011	271	369	916	477	585	55
AATTCAATG	BRDN00011	338	22	1	607	491	0
AATTCACAA	BRDN00012	174	179	269	557	939	2
AATTCACAG	BRDN00012	74	10	1	91	156	0
AATTCACAT	BRDN00012	152	53	59	369	226	30
AATTCATAC	BRDN00012	112	113	128	194	432	5
AATTCATAT	BRDN00011	128	111	127	297	325	0
AATTCATCG	BRDN00012	151	0	0	321	95	0
AATTCATCT	BRDN00012	349	158	76	842	801	0
AATTCATCT	BRDN00011	153	50	0	321	365	0
AATTCATGC	BRDN00012	129	122	84	205	191	16
AATTCATGG	BRDN00012	42	12	0	349	199	0
AATTCATGT	BRDN00011	212	198	88	360	348	25
AATTCATGT	BRDN00011	307	289	360	464	274	62
AATTCATTA	BRDN00012	114	88	2866	341	346	29
AATTCATTG	BRDN00012	66	0	7	322	294	0
AATTCCACT	BRDN00012	325	305	1479	825	760	17
AATTCCAGA	BRDN00012	272	209	181	384	578	26
AATTCCAGT	BRDN00012	303	423	595	632	602	61
AATTCCATT	BRDN00012	379	340	377	682	822	50
AATTCCCAA	BRDN00011	72	26	52	207	155	0
AATTCCCAG	BRDN00012	378	415	927	753	1216	46
AATTCCCAG	BRDN00011	142	71	65	307	110	19
AATTCCCAT	BRDN00012	256	237	236	559	699	49
AATTCCCAT	BRDN00012	189	87	461	208	148	5
AATTCCCTA	BRDN00011	170	53	55	361	163	0
AATTCCCTC	BRDN00012	267	253	1510	478	320	17
AATTCCCTT	BRDN00012	265	285	617	859	1395	39
AATTCCGAT	BRDN00011	222	207	148	539	519	42
AATTCCGGT	BRDN00011	99	14	0	303	222	0
AATTCCTAA	BRDN00012	239	168	474	552	270	5
AATTCCTAA	BRDN00011	52	7	0	208	153	0
AATTCCTAC	BRDN00011	350	299	1434	781	850	31
AATTCCTCA	BRDN00011	204	142	418	430	424	15
AATTCCTGA	BRDN00012	271	301	883	500	502	30
AATTCCTTG	BRDN00012	326	321	786	579	485	18
AATTCGACA	BRDN00011	173	30	3	422	538	0
AATTCGCAA	BRDN00012	136	182	157	278	212	4
AATTCGCTT	BRDN00011	303	224	1101	530	692	55
AATTCGGCA	BRDN00011	61	0	1	115	104	0
AATTCGGGT	BRDN00012	408	441	349	718	1043	17
AATTCGTAC	BRDN00011	153	116	4	208	116	7
AATTCGTAG	BRDN00012	171	184	266	503	1387	19
AATTCTAAG	BRDN00011	69	47	0	152	286	0
AATTCTACA	BRDN00011	78	37	6	191	323	0
AATTCTACA	BRDN00012	190	201	233	443	444	0
AATTCTAGA	BRDN00012	94	107	14	263	487	0
AATTCTATA	BRDN00012	99	72	53	209	172	20
AATTCTCAC	BRDN00012	493	458	515	870	975	74
AATTCTCAG	BRDN00012	170	148	226	391	703	86
AATTCTCAT	BRDN00012	175	90	26	306	276	6
AATTCTCTC	BRDN00011	137	85	595	232	296	23
AATTCTCTG	BRDN00012	378	361	98	722	641	36
AATTCTCTT	BRDN00011	141	197	371	278	470	65
AATTCTGAG	BRDN00012	276	288	955	566	570	13
AATTCTGCA	BRDN00011	176	138	10	238	202	11
AATTCTGCA	BRDN00011	308	340	1600	487	591	90
AATTCTGGA	BRDN00012	385	196	447	776	729	52
AATTCTGGA	BRDN00011	69	0	0	402	218	0
AATTCTGGC	BRDN00011	153	110	206	280	164	0
AATTCTGTG	BRDN00012	185	86	300	281	447	6
AATTCTTAC	BRDN00012	349	349	80	471	504	13
AATTCTTAG	BRDN00012	51	3	0	115	113	0
AATTCTTCA	BRDN00011	59	24	0	238	187	0
AATTCTTCT	BRDN00012	206	172	347	315	234	6
AATTCTTGC	BRDN00012	205	292	78	369	418	32
AATTGAAGA	BRDN00011	153	12	0	437	448	0
AATTGAAGG	BRDN00011	256	189	21	495	419	7
AATTGAAGT	BRDN00011	222	196	294	539	685	14
AATTGAATC	BRDN00011	176	191	80	318	201	28
AATTGAATG	BRDN00012	190	115	842	374	299	26
AATTGAATT	BRDN00012	146	100	17	201	548	16
AATTGACTC	BRDN00011	146	151	507	293	381	11
AATTGAGAG	BRDN00011	137	180	193	281	278	11
AATTGAGAT	BRDN00012	148	247	75	402	788	50
AATTGAGCG	BRDN00012	121	160	952	303	315	22
AATTGATGG	BRDN00012	537	466	344	877	1294	24
AATTGATGT	BRDN00011	191	159	61	310	219	41
AATTGATGT	BRDN00012	121	140	288	253	235	5
AATTGCAAA	BRDN00012	467	453	695	653	644	62
AATTGCAAA	BRDN00012	543	459	3926	939	865	61
AATTGCACG	BRDN00011	95	121	835	383	366	13
AATTGCCTT	BRDN00012	168	59	130	334	217	10
AATTGCGAT	BRDN00011	66	6	326	281	435	8
AATTGCGGG	BRDN00012	390	300	415	574	602	13
AATTGCTAG	BRDN00011	43	44	10	128	134	0
AATTGCTCC	BRDN00011	190	240	275	469	577	68
AATTGCTCG	BRDN00012	198	186	1361	465	521	24
AATTGCTGC	BRDN00011	150	93	387	464	520	26
AATTGCTGT	BRDN00011	361	445	316	476	523	55
AATTGGACT	BRDN00012	584	481	631	935	503	107
AATTGGAGG	BRDN00011	167	135	202	341	268	47
AATTGGAGT	BRDN00012	360	300	213	619	920	21
AATTGGATA	BRDN00011	100	39	133	187	265	22
AATTGGCAT	BRDN00012	181	138	800	331	469	12
AATTGGCCA	BRDN00012	107	152	20	352	275	29
AATTGGCGC	BRDN00011	212	150	93	329	363	0
AATTGGCGG	BRDN00011	106	136	14	264	237	17
AATTGGCTC	BRDN00011	329	205	735	759	398	16
AATTGGCTG	BRDN00012	302	166	13	444	561	23
AATTGGCTT	BRDN00011	246	254	984	476	539	45
AATTGGGCA	BRDN00012	294	289	350	670	403	69
AATTGGTAC	BRDN00011	17	1	1	604	574	0
AATTGGTGA	BRDN00011	226	87	162	418	602	9
AATTGGTGG	BRDN00012	108	43	146	147	102	4
AATTGGTGT	BRDN00012	44	0	0	426	275	0
AATTGGTTA	BRDN00012	245	278	361	493	1862	86
AATTGGTTA	BRDN00011	198	155	364	285	581	25
AATTGTAAT	BRDN00011	158	196	532	432	499	0
AATTGTCAA	BRDN00012	213	121	114	406	371	0
AATTGTCCG	BRDN00011	124	283	748	282	229	45
AATTGTCGA	BRDN00011	137	95	117	246	359	3
AATTGTCTT	BRDN00012	446	292	1152	816	788	25
AATTGTGGA	BRDN00012	197	114	15	279	261	7
AATTGTGGT	BRDN00012	86	71	64	210	189	80
AATTGTTCC	BRDN00011	543	495	7485	926	749	112
AATTGTTCG	BRDN00012	201	134	304	511	469	16
AATTTAAAA	BRDN00012	84	119	96	216	362	12
AATTTAACA	BRDN00011	108	142	153	167	87	21
AATTTAACA	BRDN00011	277	155	494	405	494	9
AATTTAAGA	BRDN00011	84	124	1	178	171	3
AATTTAAGC	BRDN00012	92	15	31	164	83	0
AATTTAAGC	BRDN00012	324	386	647	669	917	22
AATTTAAGG	BRDN00012	313	251	443	453	540	24
AATTTAAGG	BRDN00011	174	179	757	304	167	60
AATTTAAGG	BRDN00011	106	130	610	294	744	38
AATTTACAA	BRDN00011	99	87	1	308	319	35
AATTTACAC	BRDN00011	216	177	492	406	554	36
AATTTACAT	BRDN00011	133	75	156	202	313	10
AATTTACGC	BRDN00012	120	21	0	340	196	1
AATTTAGCA	BRDN00011	369	325	333	595	255	25
AATTTAGTC	BRDN00011	166	201	163	307	277	6
AATTTATAT	BRDN00011	122	90	99	169	79	12
AATTTATCG	BRDN00012	305	223	203	444	431	7
AATTTCAAA	BRDN00011	178	126	508	306	261	28
AATTTCAAG	BRDN00011	188	190	848	373	733	16
AATTTCACA	BRDN00012	223	239	1141	717	813	18
AATTTCATC	BRDN00011	24	0	0	214	234	0
AATTTCATC	BRDN00012	248	212	669	367	273	12
AATTTCATG	BRDN00011	158	4	0	545	359	0
AATTTCATG	BRDN00012	398	522	661	636	1034	52
AATTTCCAG	BRDN00012	342	246	855	469	310	39
AATTTCCCA	BRDN00011	214	302	620	382	357	23
AATTTCCGG	BRDN00011	93	179	189	205	143	10
AATTTCGAG	BRDN00011	214	147	512	459	260	18
AATTTCGTG	BRDN00011	127	88	1	148	229	0
AATTTCTAG	BRDN00012	40	180	417	176	75	0
AATTTCTGC	BRDN00012	173	0	1	207	170	0
AATTTGAAG	BRDN00011	107	5	0	220	478	3
AATTTGAGT	BRDN00011	342	403	1012	610	961	45
AATTTGATG	BRDN00011	158	80	270	256	222	15
AATTTGATT	BRDN00011	204	114	120	352	562	1
AATTTGCAG	BRDN00012	307	312	943	734	704	57
AATTTGCCA	BRDN00011	200	61	175	238	221	5
AATTTGGAT	BRDN00011	190	188	854	365	461	18
AATTTGGCC	BRDN00011	166	289	3219	344	200	69
AATTTGGCT	BRDN00011	36	9	7	479	865	0
AATTTGGCT	BRDN00011	79	97	73	169	143	1
AATTTGGTC	BRDN00012	157	140	859	285	255	15
AATTTGGTT	BRDN00012	288	321	619	504	511	48
AATTTGTAG	BRDN00012	111	175	11	221	89	40
AATTTGTTC	BRDN00012	366	355	525	905	707	38
AATTTGTTT	BRDN00011	209	157	261	221	342	7
ACAAAAAAC	BRDN00012	450	345	3501	774	1002	32
ACAAAAAAT	BRDN00012	141	56	26	179	150	0
ACAAAAAGA	BRDN00012	315	383	216	533	405	6
ACAAAAAGC	BRDN00011	456	412	862	770	911	90
ACAAAAATG	BRDN00012	268	15	114	566	325	0
ACAAAACCA	BRDN00012	281	270	429	644	821	19
ACAAAACTA	BRDN00011	130	56	0	769	653	13
ACAAAACTC	BRDN00012	439	56	277	1020	1080	0
ACAAAACTT	BRDN00012	325	245	347	491	802	3

ACAAAAGAA	BRDN00011	219	186	66	352	368	8
ACAAAAGAA	BRDN00011	138	104	397	276	186	9
ACAAAAGAT	BRDN00012	222	252	744	414	517	41
ACAAAAGAT	BRDN00011	125	3	0	310	176	0
ACAAAAGAT	BRDN00011	195	176	9	419	539	0
ACAAAAGCA	BRDN00012	87	10	39	228	235	5
ACAAAAGCA	BRDN00012	117	146	20	282	210	12
ACAAAAGCA	BRDN00012	335	413	300	590	701	96
ACAAAAGCC	BRDN00011	117	132	389	144	98	9
ACAAAAGGT	BRDN00012	451	431	472	633	407	33
ACAAAAGTA	BRDN00012	388	491	58	718	523	69
ACAAAATCT	BRDN00011	415	366	5102	791	961	95
ACAAAATCT	BRDN00012	263	264	251	633	844	0
ACAAAATGA	BRDN00012	175	151	27	478	491	11
ACAAAATGC	BRDN00012	574	652	3654	1035	1302	85
ACAAAATGG	BRDN00012	318	84	15	1215	616	1
ACAAAATTT	BRDN00011	327	280	3079	430	427	75
ACAAACACA	BRDN00012	190	165	189	301	338	21
ACAAACACG	BRDN00012	127	57	233	374	402	13
ACAAACAGG	BRDN00012	138	149	228	207	485	0
ACAAACAGT	BRDN00011	101	5	0	253	108	0
ACAAACATA	BRDN00012	120	53	156	428	334	0
ACAAACCCA	BRDN00012	143	58	117	211	147	6
ACAAACCTA	BRDN00012	322	339	9	568	493	49
ACAAACCTCA	BRDN00011	44	11	6	259	103	0
ACAAACCTG	BRDN00011	178	229	41	476	430	7
ACAAACCTG	BRDN00011	149	39	0	382	277	0
ACAAACCTGT	BRDN00012	364	55	41	812	692	0
ACAAACGAC	BRDN00012	728	844	2089	1108	1753	191
ACAAACGCA	BRDN00012	272	287	790	563	717	17
ACAAACTACT	BRDN00011	208	194	83	253	307	0
ACAAACTAGT	BRDN00011	187	72	420	185	283	7
ACAAACTCCT	BRDN00011	350	313	295	869	924	9
ACAAACTCGT	BRDN00012	152	189	214	389	522	13
ACAAACTCTG	BRDN00011	91	59	23	242	303	0
ACAAACTTAC	BRDN00011	177	185	384	390	522	39
ACAAACTTCA	BRDN00011	200	113	465	355	415	23
ACAAAGAAC	BRDN00012	424	408	550	874	951	25
ACAAAGAGA	BRDN00011	323	290	166	622	333	7
ACAAAGATC	BRDN00011	162	153	76	227	296	14
ACAAAGATC	BRDN00011	422	489	1085	761	819	66
ACAAAGATC	BRDN00012	521	506	2439	1079	772	48
ACAAAGATT	BRDN00011	173	158	57	288	193	63
ACAAAGCAC	BRDN00011	104	82	18	268	222	5
ACAAAGCAC	BRDN00011	145	108	93	310	273	0
ACAAAGCAG	BRDN00011	67	175	1790	244	340	32
ACAAAGCAG	BRDN00012	148	0	1	637	158	0
ACAAAGCCA	BRDN00012	99	91	479	320	222	7
ACAAAGCCC	BRDN00012	187	225	120	378	320	15
ACAAAGCCG	BRDN00012	198	121	74	436	275	7
ACAAAGCCT	BRDN00012	488	653	247	1214	1268	35
ACAAAGCGA	BRDN00012	427	222	141	693	807	18
ACAAAGCGG	BRDN00012	233	217	328	379	462	4
ACAAAGCTA	BRDN00012	248	419	418	554	598	59
ACAAAGGAG	BRDN00012	135	166	2	352	252	46
ACAAAGGCC	BRDN00011	302	132	118	788	1082	39
ACAAAGGCG	BRDN00011	164	80	233	393	510	26
ACAAAGGCT	BRDN00012	100	60	0	222	275	0
ACAAAGGTA	BRDN00011	170	12	0	412	206	47
ACAAAGGTA	BRDN00011	233	239	317	466	235	58
ACAAAGGTC	BRDN00011	456	337	5	814	1040	48
ACAAAGGTG	BRDN00012	224	219	213	427	598	28
ACAAAGTAC	BRDN00011	301	154	121	850	598	0
ACAAAGTCA	BRDN00011	253	111	36	413	379	0
ACAAAGTCT	BRDN00011	315	399	1416	426	434	63
ACAAAGTCT	BRDN00012	346	467	1852	470	686	85
ACAAAGTGC	BRDN00011	298	253	733	547	500	32
ACAAAGTTC	BRDN00012	459	422	733	1145	1049	157
ACAAATACA	BRDN00012	144	31	0	340	283	0
ACAAATACC	BRDN00012	244	199	275	434	634	73
ACAAATACT	BRDN00011	127	79	1	268	327	0
ACAAATATT	BRDN00011	100	137	27	135	86	0
ACAAATCAG	BRDN00012	375	399	300	507	350	3
ACAAATCGA	BRDN00012	145	169	0	347	172	16
ACAAATCTG	BRDN00012	229	253	857	402	806	14
ACAAATCTTC	BRDN00011	416	365	46	572	692	9
ACAAATGAT	BRDN00011	403	521	2477	842	1107	119
ACAAATGAT	BRDN00011	147	62	175	259	235	11
ACAAATGCT	BRDN00012	57	16	2	516	103	0
ACAAATGCT	BRDN00012	146	185	109	300	504	36
ACAAATGGA	BRDN00011	198	177	196	284	256	48
ACAAATGTA	BRDN00012	475	391	223	961	792	41
ACAAATTGA	BRDN00011	203	90	0	511	502	0
ACAAATTGA	BRDN00012	141	185	111	216	321	26
ACAAATTGA	BRDN00012	180	163	34	271	699	10
ACAACAAAA	BRDN00011	250	278	240	612	1068	10
ACAACAAAG	BRDN00012	441	238	66	774	1056	44
ACAACAAAT	BRDN00011	155	225	235	511	631	61
ACAACAACA	BRDN00011	456	579	764	843	704	50
ACAACAACA	BRDN00011	368	395	783	633	754	69
ACAACAACG	BRDN00012	595	623	867	812	1355	46
ACAACAAGT	BRDN00012	369	326	401	494	586	13
ACAACAAGT	BRDN00012	113	219	482	300	547	8
ACAACACCA	BRDN00012	249	242	118	552	649	35
ACAACACTTA	BRDN00012	179	190	46	501	554	34
ACAACAGAC	BRDN00011	332	239	2440	495	624	43
ACAACAGAG	BRDN00012	74	5	1	664	586	0
ACAACAGCC	BRDN00012	241	250	1386	388	365	20
ACAACAGCT	BRDN00012	81	53	1360	261	103	14
ACAACAGGT	BRDN00012	180	38	22	481	360	13
ACAACAGTC	BRDN00011	231	225	1778	521	568	24
ACAACATAC	BRDN00012	41	9	50	188	228	0
ACAACATCA	BRDN00011	96	84	0	407	314	6
ACAACATCA	BRDN00012	413	259	558	653	499	72
ACAACATCA	BRDN00011	259	101	613	400	701	6
ACAACATCAT	BRDN00011	305	275	5587	517	421	78
ACAACATCAT	BRDN00012	794	802	2124	1307	1494	102
ACAACATCCA	BRDN00011	193	139	143	379	301	93
ACAACATCTT	BRDN00011	210	337	212	458	541	15
ACAACATGA	BRDN00011	155	177	0	435	256	0
ACAACATGC	BRDN00011	274	195	243	492	620	0
ACAACCAAAT	BRDN00012	251	192	63	511	367	91
ACAACCAAC	BRDN00011	227	88	44	747	849	0
ACAACCAAG	BRDN00012	225	231	600	384	554	21
ACAACCAAT	BRDN00011	219	117	4	244	267	5
ACAACCACA	BRDN00011	370	597	2893	851	408	104
ACAACCAGA	BRDN00012	351	556	5718	480	984	202
ACAACCAGT	BRDN00011	436	120	331	558	377	23
ACAACCATA	BRDN00012	704	463	144	762	884	30
ACAACCCAA	BRDN00011	117	135	1650	211	247	25
ACAACCCACA	BRDN00011	249	205	430	664	551	7
ACAACCCAG	BRDN00011	289	163	88	514	340	18
ACAACCCATT	BRDN00012	173	184	32	509	577	0
ACAACCCCA	BRDN00012	258	242	60	416	203	9
ACAACCCCAT	BRDN00012	491	270	80	995	1206	0
ACAACCCCTC	BRDN00012	180	55	2628	1065	579	25
ACAACCCGC	BRDN00012	302	275	342	701	976	20
ACAACCCTAC	BRDN00012	145	163	113	386	273	25
ACAACCGAG	BRDN00012	254	321	320	604	878	25
ACAACCGCC	BRDN00011	463	343	302	918	898	67
ACAACCGCCT	BRDN00011	119	118	15	245	113	12
ACAACCGGA	BRDN00011	212	314	729	606	393	140
ACAACCTACT	BRDN00011	200	167	9	405	533	0
ACAACCTCA	BRDN00012	268	382	1744	566	212	41
ACAACCTCAT	BRDN00012	687	586	2541	1146	1423	21
ACAACCTCG	BRDN00012	205	300	175	446	545	0
ACAACCTCTT	BRDN00012	23	0	0	286	58	0
ACAACCTTA	BRDN00012	419	239	1457	704	556	25
ACAACCTTCA	BRDN00011	239	154	41	286	275	13
ACAACGAAC	BRDN00011	166	220	531	360	1125	3
ACAACGAAC	BRDN00012	63	24	99	389	159	81
ACAACGACA	BRDN00012	311	182	149	556	599	43
ACAACGACA	BRDN00011	218	86	355	373	49	0
ACAACGACA	BRDN00011	267	283	1243	612	616	53
ACAACGAGC	BRDN00011	92	65	0	215	79	5
ACAACGATA	BRDN00011	232	484	1654	373	216	16
ACAACGCAG	BRDN00011	281	214	904	530	377	9
ACAACGCAT	BRDN00011	82	111	226	165	144	73
ACAACGCCA	BRDN00011	533	465	355	1288	1726	39
ACAACGCCG	BRDN00011	159	86	141	328	218	67
ACAACGTAA	BRDN00011	484	364	309	1094	828	14
ACAACGTAC	BRDN00012	194	78	6	287	540	0
ACAACGTGC	BRDN00012	479	381	526	1098	571	36
ACAACGTGT	BRDN00011	286	291	1865	666	437	53
ACAACGTTG	BRDN00011	143	184	1	360	682	11
ACAACTAAG	BRDN00011	160	124	119	184	291	16
ACAACTAAT	BRDN00011	22	0	0	631	484	0
ACAACTACTG	BRDN00012	476	38	25	799	382	0
ACAACTACTT	BRDN00011	211	199	664	475	635	16
ACAACTAGC	BRDN00011	184	304	25	375	584	43
ACAACTATA	BRDN00012	285	226	874	461	365	16
ACAACTATA	BRDN00012	207	302	985	219	159	44
ACAACTATCA	BRDN00012	169	61	104	227	340	0
ACAACTATG	BRDN00012	514	353	1054	1068	424	65
ACAACTATGT	BRDN00012	221	263	261	321	305	11
ACAACTCAA	BRDN00012	238	178	778	495	461	37
ACAACTCAA	BRDN00012	324	239	97	656	581	50
ACAACTCACC	BRDN00011	111	19	1	332	307	0
ACAACTCTG	BRDN00011	246	342	387	602	671	39
ACAACTCTG	BRDN00011	150	226	464	278	433	21
ACAACTGGA	BRDN00011	230	289	692	369	407	9
ACAACTGGG	BRDN00012	128	137	210	265	65	34
ACAACTGTA	BRDN00011	206	181	90	339	250	24
ACAACTGTA	BRDN00011	389	283	1055	566	738	36
ACAACTGTT	BRDN00011	175	153	270	393	588	38
ACAACTTACA	BRDN00011	145	143	215	315	553	2
ACAACTTAG	BRDN00012	97	92	488	274	349	36
ACAACTTCAG	BRDN00011	254	291	619	544	449	58
ACAACTTGA	BRDN00012	179	182	624	483	436	2
ACAACTTGAT	BRDN00012	318	104	55	652	447	8
ACAACTTGG	BRDN00012	76	141	518	363	60	81
ACAACTTGTA	BRDN00012	165	115	33	318	393	16
ACAACTTGTT	BRDN00012	349	567	892	872	737	10
ACAAGAAAA	BRDN00011	209	195	22	353	545	10
ACAAGAAAT	BRDN00011	17	0	0	378	2	0
ACAAGAACC	BRDN00011	188	125	18	358	118	16
ACAAGAACC	BRDN00011	76	58	110	201	188	8
ACAAGAACC	BRDN00012	221	264	22	402	837	35
ACAAGAACT	BRDN00012	131	78	158	407	592	6
ACAAGAAGG	BRDN00012	110	201	5029	231	199	106
ACAAGAAGT	BRDN00012	77	0	0	192	13	0
ACAAGAAGT	BRDN00012	475	316	286	537	576	0
ACAAGAATA	BRDN00012	265	156	1268	479	574	25
ACAAGAATG	BRDN00012	296	245	187	425	92	7
ACAAGAATT	BRDN00011	215	263	196	304	473	0
ACAAGACAA	BRDN00012	181	122	928	350	463	39
ACAAGACAC	BRDN00012	226	185	304	476	513	0
ACAAGACAG	BRDN00011	159	176	124	285	219	47
ACAAGACAT	BRDN00012	547	504	2338	1130	763	76
ACAAGACCA	BRDN00011	228	140	14	299	329	0
ACAAGACCG	BRDN00012	547	363	139	815	792	45
ACAAGACGA	BRDN00011	216	194	487	401	219	20
ACAAGACGC	BRDN00012	140	167	216	281	359	11
ACAAGAGAA	BRDN00012	108	118	138	165	44	8
ACAAGAGAA	BRDN00011	107	178	0	163	218	28
ACAAGAGAG	BRDN00011	206	157	97	476	260	0
ACAAGAGAG	BRDN00011	295	295	190	426	543	8
ACAAGAGCC	BRDN00011	177	191	329	356	108	33
ACAAGAGCC	BRDN00011	496	427	3553	897	1219	79
ACAAGAGGG	BRDN00012	9	4	0	183	320	0
ACAAGAGTC	BRDN00011	144	96	274	341	789	0
ACAAGAGTG	BRDN00012	240	315	637	394	475	38
ACAAGATCG	BRDN00011	198	231	123	290	180	11
ACAAGATTA	BRDN00011	162	164	11	213	234	0
ACAAGATTG	BRDN00011	198	93	635	258	293	12
ACAAGCAAG	BRDN00012	333	323	774	632	621	13
ACAAGCACA	BRDN00011	335	328	229	726	584	30
ACAAGCACC	BRDN00012	138	120	56	304	113	18
ACAAGCACC	BRDN00012	151	133	174	471	427	17
ACAAGCAGC	BRDN00011	182	103	205	231	223	5
ACAAGCAGT	BRDN00011	173	141	362	439	263	0
ACAAGCCAT	BRDN00011	287	176	988	510	266	15
ACAAGCCAT	BRDN00012	321	363	684	567	884	60
ACAAGCCCA	BRDN00011	575	426	1633	953	824	127
ACAAGCCGA	BRDN00012	248	31	0	678	560	1
ACAAGCCGT	BRDN00012	177	183	2481	361	309	71
ACAAGCCTC	BRDN00012	226	138	132	282	385	50
ACAAGCCTG	BRDN00011	342	363	250	762	837	21
ACAAGCGAA	BRDN00011	227	191	691	480	443	18
ACAAGCGGG	BRDN00012	141	223	361	369	411	0
ACAAGCTCA	BRDN00012	326	320	591	578	498	12
ACAAGCTCA	BRDN00012	338	414	683	769	528	25
ACAAGCTCA	BRDN00011	101	11	1	223	64	0
ACAAGCTCG	BRDN00011	329	163	330	705	410	13
ACAAGCTGA	BRDN00012	490	205	78	911	1025	0
ACAAGGAAA	BRDN00012	296	200	280	516	639	20
ACAAGGACA	BRDN00012	355	305	553	839	758	5
ACAAGGACA	BRDN00011	328	364	268	578	724	11
ACAAGGACG	BRDN00011	370	320	448	788	1163	3
ACAAGGACT	BRDN00012	463	413	916	889	2331	62
ACAAGGAGC	BRDN00011	208	175	238	434	449	17
ACAAGGAGC	BRDN00012	308	223	750	576	958	88
ACAAGGCAG	BRDN00011	85	22	460	186	256	6
ACAAGGCCC	BRDN00011	127	168	138	208	129	17
ACAAGGCCT	BRDN00011	454	493	954	651	335	12
ACAAGGCGA	BRDN00011	333	169	821	396	631	39
ACAAGGCTA	BRDN00011	101	43	643	242	340	20
ACAAGGGAC	BRDN00012	390	426	141	744	773	48
ACAAGGGCA	BRDN00012	390	406	648	714	634	36
ACAAGGGTG	BRDN00011	151	197	103	226	309	26
ACAAGGTTA	BRDN00012	113	123	494	287	241	4
ACAAGGTTC	BRDN00012	257	90	262	500	425	25
ACAAGTAAA	BRDN00011	68	71	196	110	135	20
ACAAGTACA	BRDN00012	385	491	1774	860	1037	87
ACAAGTACG	BRDN00011	335	131	1	827	612	0
ACAAGTACG	BRDN00011	169	157	10	272	390	0
ACAAGTAGA	BRDN00012	383	276	6877	685	1269	131
ACAAGTATA	BRDN00012	118	20	0	228	106	0
ACAAGTCAA	BRDN00011	156	82	69	353	202	5
ACAAGTCAT	BRDN00011	159	219	27	447	450	0
ACAAGTGAA	BRDN00011	222	144	130	414	307	0
ACAAGTGCA	BRDN00011	173	86	195	508	566	5
ACAAGTGCT	BRDN00012	54	0	0	337	137	0
ACAAGTGGG	BRDN00011	120	60	118	231	73	0
ACAAGTGTG	BRDN00011	276	159	68	392	243	0
ACAAGTGTT	BRDN00011	416	0	0	658	41	0
ACAAGTTAA	BRDN00012	84	20	15	155	265	4
ACAAGTTCA	BRDN00011	109	123	480	131	184	3
ACAAGTTGC	BRDN00012	371	218	459	793	911	5
ACAAGTTGG	BRDN00012	655	601	688	1275	1219	35
ACAATAAAG	BRDN00011	229	213	693	586	231	46
ACAATAACA	BRDN00012	526	452	1181	871	959	59
ACAATAACA	BRDN00011	251	292	778	537	904	250
ACAATAACT	BRDN00011	168	107	279	260	225	7
ACAATAAGA	BRDN00011	268	219	19	376	564	22
ACAATAAGC	BRDN00012	217	255	1274	431	762	36
ACAATAATC	BRDN00012	215	128	100	311	570	13
ACAATACAA	BRDN00012	170	138	339	298	638	14
ACAATACAG	BRDN00012	24	0	0	372	641	0
ACAATACTTG	BRDN00011	188	93	139	201	174	31
ACAATAGGA	BRDN00012	527	352	745	688	1372	14
ACAATAGTA	BRDN00011	100	110	104	162	183	0
ACAATATCA	BRDN00011	500	364	941	989	466	26
ACAATATCA	BRDN00011	355	334	490	818	820	42
ACAATATCA	BRDN00012	451	375	241	673	657	6
ACAATATCA	BRDN00011	218	45	1	499	357	0
ACAATATCCA	BRDN00011	407	481	1220	748	644	54
ACAATATGA	BRDN00012	569	231	130	775	684	23
ACAATATGT	BRDN00012	270	105	342	524	420	0
ACAATATTA	BRDN00012	436	507	1415	652	852	106
ACAATCAAA	BRDN00011	350	21	0	651	714	0
ACAATCATA	BRDN00012	298	284	763	822	583	15
ACAATCATCA	BRDN00012	471	571	305	967	643	41
ACAATCCAG	BRDN00011	183	177	478	330	684	48
ACAATCCAG	BRDN00012	257	211	177	394	522	0
ACAATCCCTA	BRDN00011	244	173	344	539	560	0
ACAATCCGA	BRDN00012	482	656	5603	1230	752	47
ACAATCCGA	BRDN00012	372	219	521	594	934	3
ACAATCCGA	BRDN00012	145	161	516	290	647	30
ACAATCCGG	BRDN00012	335	291	386	807	551	28
ACAATCCGG	BRDN00011	203	292	337	238	314	12
ACAATCCGT	BRDN00012	203	170	149	418	164	15
ACAATCGGT	BRDN00012	462	68	11	981	694	1
ACAATCTCAC	BRDN00012	391	326	288	607	466	38
ACAATCTCAC	BRDN00011	493	422	264	956	785	130
ACAATCTGA	BRDN00011	112	77	1836	228	308	39
ACAATCTGC	BRDN00012	336	402	1635	807	788	102
ACAATCTTA	BRDN00012	74	12	170	166	203	0
ACAATGAAT	BRDN00012	408	12	3	603	478	0
ACAATGACT	BRDN00012	176	80	537	776	296	6
ACAATGACT	BRDN00012	342	221	724	538	581	11
ACAATGAGG	BRDN00012	449	374	635	980	786	59
ACAATGCAA	BRDN00011	235	227	376	471	658	0
ACAATGCAG	BRDN00011	397	487	1018	801	455	30
ACAATGCCA	BRDN00011	294	275	480	462	459	89
ACAATGCCG	BRDN00011	286	97	149	510	238	2
ACAATGCCT	BRDN00011	370	317	1039	795	675	25
ACAATGCGA	BRDN00012	578	522	934	1162	1081	78
ACAATGCTA	BRDN00011	158	169	1706	320	308	46
ACAATGCTG	BRDN00012	215	184	558	348	300	93
ACAATGCTG	BRDN00012	282	254	1117	418	1882	29
ACAATGGAA	BRDN00012	391	36	0	784	681	0
ACAATGGCA	BRDN00012	122	183	377	344	225	9
ACAATGGGA	BRDN00011	164	288	388	353	410	41
ACAATGGGG	BRDN00011	192	219	25	360	351	26
ACAATGTAA	BRDN00012	161	178	1163	282	352	12
ACAATGTCA	BRDN00012	162	182	880	361	263	17
ACAATGTCAT	BRDN00011	50	0	0	264	305	0
ACAATGTCG	BRDN00011	484	422	1585	914	1434	7
ACAATTAGA	BRDN00012	441	341	203	817	636	23
ACAATTATG	BRDN00012	137	194	122	223	105	20
ACAATTCAG	BRDN00011	307	280	355	565	890	37
ACAATTCAG	BRDN00012	169	52	4	553	228	0
ACAATTCCAC	BRDN00011	93	136	13	240	162	21
ACAATTGAT	BRDN00011	286	216	710	532	446	11
ACAATTGGA	BRDN00012	408	515	843	819	698	65
ACAATTTAG	BRDN00012	418	351	349	603	534	10
ACAATTTGGT	BRDN00011	131	196	1556	292	143	26
ACACAAAAG	BRDN00011	331	230	258	472	765	6
ACACAAAAT	BRDN00011	240	210	824	389	313	29
ACACAAACA	BRDN00012	288	195	161	534	316	10
ACACAAACT	BRDN00011	157	151	2959	249	764	32
ACACAAAGA	BRDN00011	258	290	101	430	301	17
ACACAAAGA	BRDN00012	389	510	788	817	537	101
ACACAAAGA	BRDN00012	390	353	890	717	462	16
ACACAAAGA	BRDN00012	385	400	943	786	510	30
ACACAAAGG	BRDN00012	144	73	153	332	410	0
ACACAAATA	BRDN00011	315	284	632	675	949	83
ACACAACAG	BRDN00011	127	78	142	270	555	40
ACACAACCT	BRDN00011	536	314	151	670	1006	34
ACACAACCT	BRDN00012	131	160	13	278	163	0
ACACAACGT	BRDN00011	365	579	3365	765	616	66
ACACAACTTA	BRDN00011	579	535	406	1024	1294	7
ACACAAGAA	BRDN00011	183	135	1066	259	186	12
ACACAAGAC	BRDN00012	437	346	692	718	627	39
ACACAAGAG	BRDN00011	100	125	7	196	25	11
ACACAAGAG	BRDN00011	60	81	218	274	118	5
ACACAAGCG	BRDN00012	230	156	974	938	543	18
ACACAATATA	BRDN00012	77	97	109	81	318	0
ACACAATCCA	BRDN00011	207	194	347	514	409	38
ACACAATCCA	BRDN00012	422	619	1103	990	1001	32
ACACAATCTC	BRDN00011	55	4	0	539	36	0
ACACAATGA	BRDN00012	587	549	472	954	891	43
ACACAATGCT	BRDN00012	441	129	715	761	654	37
ACACAATGG	BRDN00011	96	118	226	274	192	11
ACACAATGT	BRDN00012	340	308	731	741	746	29
ACACAATTCA	BRDN00012	146	0	0	316	241	0
ACACACAAG	BRDN00011	336	297	3204	564	506	38
ACACACAAGT	BRDN00012	207	175	90	464	495	24
ACACACACA	BRDN00011	657	564	2484	990	1148	23
ACACACACA	BRDN00011	157	131	63	188	172	49
ACACACACA	BRDN00011	379	225	768	646	533	13
ACACACACAT	BRDN00012	737	631	2999	1175	921	60
ACACACAGG	BRDN00012	376	235	123	812	894	76
ACACACAGG	BRDN00011	160	177	215	312	189	5
ACACACAGT	BRDN00012	427	520	339	834	903	92
ACACACAGT	BRDN00012	408	467	586	689	1162	91
ACACACATG	BRDN00012	324	312	118	750	542	56
ACACACATG	BRDN00012	295	389	226	440	390	24
ACACACCAA	BRDN00011	111	86	1048	334	195	0
ACACACCATC	BRDN00011	274	131	722	287	559	24
ACACACCATC	BRDN00011	511	385	422	941	1183	138
ACACACCATG	BRDN00012	375	453	5057	892	1295	157
ACACACCCA	BRDN00011	218	220	125	410	780	18
ACACACGCA	BRDN00012	68	0	213	370	139	0
ACACACGTA	BRDN00012	116	67	103	170	225	15
ACACACGTA	BRDN00011	82	110	9	220	174	37
ACACACGTG	BRDN00011	453	325	724	725	968	54
ACACACGTG	BRDN00011	129	197	68	296	315	3
ACACACTCG	BRDN00012	469	437	1361	1200	1391	43
ACACACTCG	BRDN00012	76	0	0	308	6	0
ACACACTTAC	BRDN00012	579	442	505	1107	1604	64
ACACAGAAG	BRDN00012	73	12	2	890	425	0
ACACAGAAG	BRDN00011	103	53	545	223	181	13
ACACAGACA	BRDN00011	263	197	78	366	306	30
ACACAGACC	BRDN00012	277	339	205	696	993	25
ACACAGACG	BRDN00011	145	32	2	699	531	16
ACACAGAGA	BRDN00012	109	0	1	789	744	0
ACACAGAGT	BRDN00012	173	74	132	380	471	3
ACACAGAGT	BRDN00012	26	0	0	174	37	0
ACACAGATG	BRDN00011	193	109	0	401	457	20
ACACAGCAA	BRDN00012	181	291	390	349	409	20
ACACAGCAC	BRDN00012	149	191	225	273	240	10
ACACAGCAC	BRDN00011	129	164	212	198	94	11
ACACAGCAG	BRDN00012	472	262	1669	885	742	45
ACACAGCAT	BRDN00011	21	0	0	549	132	0
ACACAGCAT	BRDN00012	231	149	1511	514	358	10
ACACAGCAT	BRDN00011	280	25	0	558	847	0
ACACAGCCA	BRDN00012	252	156	610	385	1159	63
ACACAGCCA	BRDN00012	205	240	12	429	399	26
ACACAGCCC	BRDN00011	212	159	2	616	1053	0
ACACAGCGT	BRDN00011	601	275	1109	1184	1364	53
ACACAGCTC	BRDN00012	680	494	1240	1028	810	54
ACACAGGAG	BRDN00012	463	328	300	786	802	25
ACACAGGCA	BRDN00011	303	334	907	626	583	53
ACACAGGGA	BRDN00011	123	104	363	278	207	3
ACACAGGGC	BRDN00011	282	190	2130	637	536	18
ACACAGTAA	BRDN00012	276	178	2283	405	357	17
ACACAGTCC	BRDN00011	487	487	435	1002	1089	59
ACACAGTCTT	BRDN00012	217	82	163	368	375	13
ACACAGTGC	BRDN00012	187	17	0	371	283	0
ACACAGTGG	BRDN00012	490	604	2289	1220	1336	140
ACACAGTTG	BRDN00012	235	259	1717	480	363	32
ACACATAAAT	BRDN00012	553	477	1832	949	1057	63
ACACATAAC	BRDN00011	283	374	293	597	476	40
ACACATAAGT	BRDN00012	189	188	81	508	435	9
ACACATACTT	BRDN00011	145	188	65	323	558	0
ACACATACTT	BRDN00012	375	288	84	885	946	39
ACACATAGG	BRDN00012	419	399	248	700	694	39
ACACATAGT	BRDN00011	283	237	392	510	351	27
ACACATCATG	BRDN00011	319	241	562	536	520	13
ACACATCCTC	BRDN00012	121	25	0	395	241	0
ACACATCGC	BRDN00011	553	313	1765	1067	1080	22
ACACATCTG	BRDN00011	581	457	2383	997	1003	31
ACACATGAA	BRDN00011	274	350	385	584	881	39
ACACATGAC	BRDN00012	317	220	541	1063	804	43
ACACATGAG	BRDN00011	454	359	188	752	770	5
ACACATGCTT	BRDN00011	442	52	209	717	778	0
ACACATGGT	BRDN00012	135	64	1282	160	58	30
ACACATTATC	BRDN00011	261	267	780	614	599	3
ACACATTTG	BRDN00012	449	407	1371	909	968	24
ACACATTTG	BRDN00011	199	202	1	350	445	9
ACACCAAAA	BRDN00011	290	302	185	545	634	15
ACACCAAAC	BRDN00011	182	131	688	346	477	22
ACACCAAAG	BRDN00011	278	243	118	450	833	9
ACACCAACA	BRDN00012	114	109	266	297	124	9
ACACCAACCA	BRDN00011	262	347	19	751	753	21
ACACCAACGT	BRDN00012	527	467	969	981	693	73
ACACCAAGA	BRDN00011	90	18	0	196	81	0
ACACCAAGG	BRDN00011	319	255	855	595	345	18
ACACCACAG	BRDN00011	483	470	115	876	676	140
ACACCACCG	BRDN00011	84	156	1	230	139	1
ACACCACCTG	BRDN00011	67	78	196	391	323	21
ACACCACCTG	BRDN00012	541	508	1655	949	868	92
ACACCACCTT	BRDN00012	503	518	379	884	656	19

ACACCACTCC	BRDN00011	466	328	324	926	978	18
ACACCACTGA	BRDN00011	189	182	680	513	506	73
ACACCACTGA	BRDN00012	679	247	790	937	972	4
ACACCACTGT	BRDN00012	156	0	0	359	272	0
ACACCACTTA	BRDN00012	115	96	12	261	166	1
ACACCACTTA	BRDN00012	552	531	777	1215	905	87
ACACCAGAG	BRDN00011	326	312	302	844	693	3
ACACCAGCA	BRDN00012	705	570	802	1167	1200	57
ACACCAGCA	BRDN00012	614	453	804	1084	1331	57
ACACCAGCAT	BRDN00012	586	742	161	1144	885	45
ACACCAGCG	BRDN00012	510	353	464	843	1044	37
ACACCAGCG	BRDN00011	328	386	590	765	1122	46
ACACCAGGA	BRDN00011	387	22	239	963	752	11
ACACCAGGG	BRDN00012	383	188	473	698	432	46
ACACCAGGT	BRDN00011	281	257	109	625	301	16
ACACCAGGT	BRDN00012	120	17	510	263	184	15
ACACCATAA	BRDN00011	217	251	0	332	160	4
ACACCATAAT	BRDN00012	498	480	386	973	1148	33
ACACCATAGT	BRDN00012	172	137	1075	336	379	35
ACACCATCA	BRDN00011	250	103	22	418	207	0
ACACCATCAG	BRDN00011	258	256	4311	493	635	77
ACACCATCCA	BRDN00011	219	242	536	465	163	0
ACACCATCG	BRDN00011	146	130	16	342	336	0
ACACCATGAT	BRDN00012	470	399	434	698	637	62
ACACCATGAT	BRDN00012	193	256	1220	306	379	11
ACACCATGTT	BRDN00011	182	124	116	151	199	39
ACACCCACCT	BRDN00011	318	145	222	437	723	8
ACACCCAGA	BRDN00011	670	524	1604	1044	886	109
ACACCCAGCT	BRDN00012	194	194	191	456	625	2
ACACCCATTC	BRDN00012	499	578	634	1065	883	41
ACACCCCACT	BRDN00011	186	292	158	431	644	23
ACACCCCGAT	BRDN00012	127	38	1	540	645	0
ACACCCCTCC	BRDN00012	241	275	351	350	336	81
ACACCCGAA	BRDN00011	118	79	661	312	357	11
ACACCCGACT	BRDN00011	180	1	0	388	358	0
ACACCCGCCA	BRDN00011	507	473	226	903	623	51
ACACCCGGTT	BRDN00012	259	186	724	349	786	19
ACACCCGTTT	BRDN00011	323	311	476	625	472	15
ACACCCTGG	BRDN00011	289	104	0	589	886	0
ACACCCTTAT	BRDN00012	133	293	1091	289	258	26
ACACCCTTAT	BRDN00011	121	92	594	317	331	18
ACACCGAAG	BRDN00012	708	674	1517	978	825	54
ACACCGACA	BRDN00012	394	399	266	678	1052	82
ACACCGAGA	BRDN00012	454	565	725	961	851	18
ACACCGAGA	BRDN00011	115	230	462	326	220	10
ACACCGAGC	BRDN00011	237	278	805	458	361	61
ACACCGAGG	BRDN00012	496	565	347	836	509	77
ACACCGAGG	BRDN00011	111	78	22	279	144	0
ACACCGAGG	BRDN00012	460	444	1006	861	564	12
ACACCGCAA	BRDN00011	213	296	678	302	470	46
ACACCGGAT	BRDN00011	387	309	123	701	804	56
ACACCGGCA	BRDN00011	574	471	81	1056	752	54
ACACCGGCG	BRDN00012	331	308	240	831	1481	8
ACACCGGCG	BRDN00011	85	117	37	224	210	9
ACACCGGCG	BRDN00012	3	0	0	112	0	0
ACACCGGCTT	BRDN00011	392	358	510	578	546	69
ACACCGGGA	BRDN00011	111	86	79	179	311	8
ACACCGGTG	BRDN00012	14	0	0	111	26	0
ACACCGTACC	BRDN00012	329	276	336	564	378	28
ACACCGTATC	BRDN00012	192	204	1	267	210	19
ACACCGTGA	BRDN00012	331	339	883	692	287	66
ACACCGTGC	BRDN00012	448	265	777	960	1519	36
ACACCGTGG	BRDN00011	562	404	995	1112	867	59
ACACCGTGTT	BRDN00011	185	177	73	398	456	3
ACACCTACAT	BRDN00012	336	343	86	599	748	111
ACACCTACCG	BRDN00011	440	115	3	974	1152	0
ACACCTAGG	BRDN00012	379	423	273	639	413	9
ACACCTATG	BRDN00011	137	228	192	359	255	41
ACACCTATGC	BRDN00011	123	139	616	219	97	21
ACACCTCAG	BRDN00012	211	171	15	353	297	13
ACACCTCGTG	BRDN00011	179	268	149	452	573	43
ACACCTCGTG	BRDN00012	167	105	1	479	597	7
ACACCTCTTT	BRDN00012	266	247	269	374	431	12
ACACCTGAA	BRDN00011	256	145	655	349	494	11
ACACCTGAAC	BRDN00012	325	340	548	782	624	9
ACACCTGAAT	BRDN00011	167	93	13	326	225	7
ACACCTGAC	BRDN00012	547	651	513	1072	1205	105
ACACCTGACC	BRDN00011	230	209	118	353	189	14
ACACCTGATT	BRDN00012	161	134	377	271	241	117
ACACCTGCAC	BRDN00011	349	307	491	698	570	26
ACACCTGGA	BRDN00012	609	607	203	1209	1180	33
ACACCTGGG	BRDN00011	615	631	1289	893	873	72
ACACCTGGT	BRDN00011	9	0	0	406	244	0
ACACCTGGTT	BRDN00012	290	225	853	394	473	155
ACACCTTCGA	BRDN00012	381	464	12	862	870	22
ACACCTTCGC	BRDN00011	379	267	141	753	1127	31
ACACCTTTCT	BRDN00012	433	428	1548	820	367	60
ACACGAAAA	BRDN00011	135	101	28	262	271	5
ACACGAAGA	BRDN00011	302	290	700	619	738	59
ACACGAAGT	BRDN00011	393	298	2673	778	440	224
ACACGACGTT	BRDN00011	55	0	1	421	36	0
ACACGAGAG	BRDN00011	356	319	122	545	841	32
ACACGAGCC	BRDN00012	166	205	1203	360	284	1
ACACGAGTG	BRDN00011	96	59	18	231	255	11
ACACGCACA	BRDN00012	199	211	130	469	175	22
ACACGCACT	BRDN00012	454	548	3214	926	1147	269
ACACGCAGC	BRDN00011	145	109	395	190	433	0
ACACGCAGG	BRDN00012	668	425	314	1163	920	46
ACACGCATT	BRDN00011	267	411	2991	627	833	85
ACACGCTCAC	BRDN00012	483	393	695	834	713	12
ACACGCTCAC	BRDN00011	326	276	745	713	700	83
ACACGCTCCT	BRDN00011	171	97	24	269	134	10
ACACGCTGA	BRDN00012	331	250	282	609	515	0
ACACGCTGA	BRDN00011	220	156	109	350	591	24
ACACGGAAT	BRDN00011	48	0	1	504	111	0
ACACGGACG	BRDN00012	136	80	741	203	174	11
ACACGGACT	BRDN00011	212	147	511	403	509	4
ACACGGACT	BRDN00012	432	440	746	738	392	89
ACACGGAGC	BRDN00011	171	53	5	540	472	0
ACACGGAGT	BRDN00011	20	0	0	186	7	0
ACACGGAGT	BRDN00012	480	408	678	694	994	22
ACACGGATT	BRDN00011	270	300	126	665	351	54
ACACGGCAG	BRDN00011	132	137	431	243	183	36
ACACGGCAG	BRDN00011	292	263	1291	556	403	19
ACACGGCCTC	BRDN00011	518	303	300	837	814	18
ACACGGCGA	BRDN00011	178	131	582	311	326	23
ACACGGCGG	BRDN00012	147	172	86	197	55	2
ACACGGCGT	BRDN00011	76	53	1	146	184	3
ACACGGCTG	BRDN00012	199	91	20	315	215	0
ACACGGCTT	BRDN00011	150	199	431	508	375	14
ACACGGGAG	BRDN00011	148	218	102	221	578	0
ACACGGGCC	BRDN00012	88	9	0	210	320	0
ACACGGGTA	BRDN00012	230	123	927	427	423	21
ACACGGGTC	BRDN00011	62	25	0	248	172	0
ACACGGGTC	BRDN00011	154	171	34	341	242	0
ACACGGTAC	BRDN00011	281	162	113	862	828	0
ACACGGTAG	BRDN00012	130	133	699	289	338	17
ACACGGTAG	BRDN00012	101	131	738	309	346	6
ACACGGTAT	BRDN00012	121	105	889	273	109	1
ACACGGTATT	BRDN00012	428	644	166	785	612	11
ACACGGTCG	BRDN00011	88	157	198	317	274	0
ACACGGTGT	BRDN00012	397	396	904	739	657	28
ACACGTACC	BRDN00012	121	72	31	272	163	0
ACACGTACG	BRDN00012	566	664	802	946	1098	41
ACACGTAGC	BRDN00011	264	360	130	438	309	38
ACACGTAGC	BRDN00012	396	311	265	536	664	8
ACACGTAGT	BRDN00012	353	458	530	724	473	27
ACACGTCCTC	BRDN00011	157	10	34	415	187	0
ACACGTCGA	BRDN00012	538	598	1138	1039	973	46
ACACGTCGT	BRDN00011	417	329	269	759	657	4
ACACGTGAG	BRDN00012	440	311	201	779	846	14
ACACGTGCA	BRDN00012	171	171	365	259	617	43
ACACGTGGA	BRDN00011	224	157	2153	338	542	57
ACACGTTACT	BRDN00011	172	48	977	311	375	3
ACACGTTTG	BRDN00011	258	295	210	588	392	118
ACACGTTTG	BRDN00011	76	53	0	294	62	0
ACACTAACG	BRDN00012	296	147	610	611	278	5
ACACTACAA	BRDN00011	326	409	402	755	1493	22
ACACTACATG	BRDN00011	219	280	195	479	180	5
ACACTACATT	BRDN00012	489	490	2336	1145	1157	118
ACACTACCAG	BRDN00012	617	492	552	959	800	26
ACACTACCTG	BRDN00012	366	418	2333	631	517	98
ACACTAGCA	BRDN00012	509	499	1250	878	790	18
ACACTATATA	BRDN00011	82	37	0	142	224	8
ACACTATCAG	BRDN00011	385	375	433	577	450	18
ACACTCACAG	BRDN00012	357	363	195	703	1100	25
ACACTCACTC	BRDN00012	187	140	122	459	436	17
ACACTCAGG	BRDN00012	409	441	1065	950	759	12
ACACTCCAA	BRDN00012	167	179	22	380	408	9
ACACTCCAAG	BRDN00012	562	294	98	862	505	12
ACACTCCATC	BRDN00012	186	284	255	503	348	32
ACACTCCGG	BRDN00011	262	140	337	528	333	6
ACACTCCTGA	BRDN00011	455	325	1514	825	1308	34
ACACTCGATC	BRDN00011	570	643	696	1084	1036	52
ACACTCGCG	BRDN00012	349	394	492	816	928	75
ACACTCGGA	BRDN00012	123	3	0	679	232	0
ACACTCGTTG	BRDN00012	105	72	94	330	404	0
ACACTCTCGC	BRDN00011	245	91	241	424	316	15
ACACTCTCTC	BRDN00011	112	0	1	536	365	0
ACACTCTTTG	BRDN00011	260	303	648	921	613	23
ACACTGAAT	BRDN00011	387	166	483	720	328	9
ACACTGAATC	BRDN00012	224	174	13	315	281	65
ACACTGACA	BRDN00012	173	141	316	488	392	0
ACACTGACAT	BRDN00011	291	250	985	687	541	58
ACACTGACC	BRDN00012	170	186	6	459	419	17
ACACTGACCC	BRDN00011	110	90	162	266	411	1
ACACTGACCC	BRDN00011	203	191	59	239	245	69
ACACTGAGA	BRDN00012	117	1	0	766	177	0
ACACTGAGG	BRDN00012	386	473	630	799	949	58
ACACTGATG	BRDN00011	141	40	97	142	216	0
ACACTGCAC	BRDN00011	349	418	519	715	843	27
ACACTGCCTC	BRDN00012	206	146	338	596	477	31
ACACTGCCTG	BRDN00011	322	332	281	635	597	31
ACACTGCTG	BRDN00012	178	151	0	381	420	11
ACACTGGAA	BRDN00011	107	134	26	363	407	6
ACACTGGAG	BRDN00012	218	132	127	268	195	5
ACACTGGCTC	BRDN00012	158	171	82	316	337	7
ACACTGGGA	BRDN00012	388	400	0	630	608	42
ACACTGGGC	BRDN00011	98	15	284	141	70	0
ACACTGTCCA	BRDN00012	430	244	138	799	651	13
ACACTGTCCT	BRDN00012	263	145	378	544	448	15
ACACTGTCG	BRDN00011	134	58	0	137	454	10
ACACTGTGTC	BRDN00011	192	122	221	336	324	0
ACACTGTGTC	BRDN00012	425	313	232	641	1051	16
ACACTTAAG	BRDN00011	436	365	393	810	1076	98
ACACTTACAT	BRDN00011	185	60	33	319	403	14
ACACTTACCA	BRDN00011	151	266	1021	214	251	19
ACACTTACCC	BRDN00011	12	80	6	226	221	2
ACACTTACCG	BRDN00012	185	39	130	614	498	11
ACACTTACCT	BRDN00011	299	79	52	656	326	0
ACACTTACG	BRDN00011	294	221	464	367	429	44
ACACTTACTA	BRDN00011	371	350	904	578	585	123
ACACTTCAAC	BRDN00011	181	163	3	368	703	19
ACACTTCATT	BRDN00012	199	101	8	325	160	5
ACACTTCCAG	BRDN00011	222	47	1	438	130	0
ACACTTCGA	BRDN00012	219	211	52	408	401	24
ACACTTCGAT	BRDN00011	321	341	1737	504	517	25
ACACTTGCAC	BRDN00011	196	58	71	349	410	0
ACACTTGCCC	BRDN00011	521	565	950	1039	740	27
ACACTTGCTA	BRDN00011	107	58	21	290	101	0
ACACTTGGCT	BRDN00011	136	92	269	137	255	0
ACACTTGGG	BRDN00011	733	675	81	1341	1465	6
ACACTTTACA	BRDN00011	121	91	104	277	328	6
ACACTTTGTA	BRDN00011	84	29	6	221	63	0
ACAGAAAAG	BRDN00012	179	172	590	287	229	19
ACAGAAAAG	BRDN00011	68	39	29	133	99	0
ACAGAAACA	BRDN00011	339	250	201	593	375	4
ACAGAAACA	BRDN00011	91	232	112	222	377	0
ACAGAAACA	BRDN00012	431	534	234	812	990	15
ACAGAAACA	BRDN00012	397	366	169	812	664	89
ACAGAAACC	BRDN00012	115	62	15	580	469	0
ACAGAAACTT	BRDN00011	413	334	1377	662	896	19
ACAGAAAGA	BRDN00011	96	51	708	217	128	12
ACAGAAAGA	BRDN00011	139	247	1351	273	64	11
ACAGAAAGG	BRDN00011	502	235	581	1056	749	22
ACAGAAAGT	BRDN00012	490	248	160	827	615	19
ACAGAAATC	BRDN00011	128	85	81	254	315	33
ACAGAAATC	BRDN00011	291	248	170	580	683	74
ACAGAAATG	BRDN00012	59	0	0	409	220	0
ACAGAAATT	BRDN00011	113	100	217	190	78	15
ACAGAACAA	BRDN00012	174	91	180	288	188	0
ACAGAACAG	BRDN00011	181	223	960	475	481	45
ACAGAACCA	BRDN00012	125	92	0	182	318	0
ACAGAACCA	BRDN00012	91	235	7	159	149	0
ACAGAACCCT	BRDN00012	425	400	400	853	1055	16
ACAGAACCTC	BRDN00011	216	148	65	300	225	4
ACAGAACGA	BRDN00011	121	164	62	265	177	3
ACAGAACGC	BRDN00011	119	119	1	219	144	0
ACAGAACGG	BRDN00011	88	71	56	225	365	0
ACAGAACTC	BRDN00011	229	25	0	301	291	0
ACAGAACTC	BRDN00012	464	237	1338	551	543	137
ACAGAACTTT	BRDN00012	459	398	627	793	761	36
ACAGAAGAA	BRDN00012	219	197	805	497	384	18
ACAGAAGAA	BRDN00012	215	314	223	439	708	18
ACAGAAGAA	BRDN00011	284	281	738	619	428	42
ACAGAAGAC	BRDN00011	228	209	32	353	566	82
ACAGAAGAC	BRDN00012	58	0	0	737	48	0
ACAGAAGAG	BRDN00011	250	168	155	616	468	4
ACAGAAGAT	BRDN00011	405	440	61	680	834	19
ACAGAAGCG	BRDN00011	224	324	322	436	658	10
ACAGAAGGA	BRDN00011	203	132	275	294	124	63
ACAGAAGGA	BRDN00012	347	306	213	511	511	47
ACAGAAGGA	BRDN00012	285	354	43	577	342	10
ACAGAAGGT	BRDN00011	93	19	0	372	159	0
ACAGAAGGT	BRDN00012	209	210	206	413	580	42
ACAGAAGTA	BRDN00012	172	69	84	307	155	0
ACAGAAGTA	BRDN00012	97	26	36	90	65	0
ACAGAAGTA	BRDN00011	98	41	0	239	152	0
ACAGAAGTT	BRDN00012	320	337	509	573	888	27
ACAGAATAC	BRDN00011	170	134	754	325	420	42
ACAGAATAG	BRDN00011	43	72	695	116	49	25
ACAGAATAT	BRDN00012	381	330	764	788	756	2
ACAGAATCA	BRDN00011	222	173	528	402	373	0
ACAGAATCCT	BRDN00012	241	27	0	540	309	3
ACAGAATGG	BRDN00011	423	322	957	527	747	145
ACAGAATTG	BRDN00011	168	38	100	301	443	0
ACAGAATTG	BRDN00011	359	398	61	780	698	18
ACAGACAAC	BRDN00012	159	124	134	341	142	1
ACAGACAAG	BRDN00012	173	104	136	264	294	15
ACAGACAAG	BRDN00012	214	140	729	326	276	28
ACAGACAAT	BRDN00012	186	99	1563	343	506	11
ACAGACACC	BRDN00012	197	128	51	324	149	0
ACAGACACC	BRDN00012	270	385	945	518	326	33
ACAGACACTC	BRDN00011	159	98	51	367	421	0
ACAGACAGA	BRDN00011	409	314	191	709	910	9
ACAGACAGC	BRDN00012	512	456	331	974	865	6
ACAGACAGC	BRDN00011	440	307	214	668	456	46
ACAGACAGC	BRDN00011	257	199	136	351	287	31
ACAGACAGG	BRDN00011	140	115	107	243	230	0
ACAGACATA	BRDN00012	197	160	1708	403	510	67
ACAGACATG	BRDN00011	217	355	624	303	203	30
ACAGACATTC	BRDN00011	378	266	99	887	565	37
ACAGACCAC	BRDN00012	80	30	67	224	63	5
ACAGACCCA	BRDN00012	180	124	396	288	299	0
ACAGACCCAC	BRDN00012	296	162	320	480	488	28
ACAGACCTC	BRDN00011	171	95	40	269	483	17
ACAGACCTG	BRDN00012	543	428	183	1094	1437	26
ACAGACGAG	BRDN00012	226	137	119	430	304	1
ACAGACGAG	BRDN00012	257	14	2	796	895	0
ACAGACGAT	BRDN00011	170	226	366	466	371	3
ACAGACGAT	BRDN00012	189	154	1	339	144	11
ACAGACGAT	BRDN00011	153	10	0	398	423	0
ACAGACGCT	BRDN00012	467	383	202	873	1044	58
ACAGACGGC	BRDN00012	176	174	717	241	445	12
ACAGACGTA	BRDN00011	334	349	1102	729	499	51
ACAGACTAG	BRDN00011	322	296	438	681	739	15
ACAGACTAT	BRDN00012	253	296	467	470	281	64
ACAGACTCC	BRDN00012	246	234	1276	410	169	40
ACAGACTCTC	BRDN00011	308	227	2	464	818	19
ACAGACTGC	BRDN00011	143	172	375	329	243	0
ACAGACTGT	BRDN00011	258	243	128	475	460	35
ACAGACTTG	BRDN00012	236	115	86	478	429	18
ACAGACTTTC	BRDN00012	121	143	145	96	50	28
ACAGAGAAA	BRDN00011	74	74	38	185	387	11
ACAGAGAAT	BRDN00012	355	424	1332	913	977	59
ACAGAGAGA	BRDN00011	382	369	425	465	556	45
ACAGAGAGC	BRDN00011	129	96	0	181	190	9
ACAGAGAGT	BRDN00012	261	296	42	314	163	17
ACAGAGAGT	BRDN00011	241	207	481	394	146	26
ACAGAGATC	BRDN00011	205	90	33	399	297	8
ACAGAGATG	BRDN00012	382	702	441	619	692	32
ACAGAGCAA	BRDN00011	107	68	138	260	232	4
ACAGAGCAC	BRDN00012	506	459	1106	859	562	60
ACAGAGCAG	BRDN00012	239	133	38	643	523	8
ACAGAGCCA	BRDN00012	545	623	1322	1345	1047	55
ACAGAGCCA	BRDN00012	349	271	117	490	418	37
ACAGAGCGC	BRDN00012	305	320	59	581	788	36
ACAGAGCGT	BRDN00011	130	54	88	258	169	0
ACAGAGCTG	BRDN00011	259	174	1671	403	678	29
ACAGAGCTT	BRDN00011	135	74	710	201	260	5
ACAGAGGAA	BRDN00011	177	140	117	285	164	19
ACAGAGGAC	BRDN00011	154	0	1	489	417	1
ACAGAGGAC	BRDN00012	107	94	57	195	207	0
ACAGAGGAT	BRDN00012	121	105	0	237	271	8
ACAGAGGCC	BRDN00012	77	125	251	150	150	4
ACAGAGGGT	BRDN00011	45	15	0	127	36	0
ACAGAGTAG	BRDN00012	209	167	336	363	320	51
ACAGAGTAT	BRDN00012	73	69	38	199	198	6
ACAGAGTCA	BRDN00012	514	462	375	1101	1622	57
ACAGAGTCC	BRDN00012	249	170	622	357	161	13
ACAGAGTCG	BRDN00012	193	228	122	351	191	2
ACAGAGTCT	BRDN00011	79	82	0	194	59	9
ACAGAGTCT	BRDN00012	53	5	93	410	38	0
ACAGAGTGA	BRDN00012	328	372	32	390	512	85
ACAGAGTTG	BRDN00012	27	34	1	317	84	0
ACAGATAAC	BRDN00012	419	370	1176	1077	992	10
ACAGATAAC	BRDN00012	247	242	253	352	331	33
ACAGATAAC	BRDN00011	141	64	30	252	120	0
ACAGATAAC	BRDN00011	112	115	215	164	167	41
ACAGATAAC	BRDN00011	337	225	1610	570	440	21
ACAGATAAG	BRDN00012	34	0	0	224	107	0
ACAGATAAG	BRDN00011	90	112	1666	165	218	33
ACAGATAAT	BRDN00011	231	232	7283	537	457	114
ACAGATACA	BRDN00011	145	102	462	209	180	18
ACAGATACA	BRDN00012	200	177	367	571	377	12
ACAGATACC	BRDN00012	98	50	134	236	241	0
ACAGATACTC	BRDN00011	76	124	44	128	277	10
ACAGATACT	BRDN00012	140	49	8	617	266	0
ACAGATCAA	BRDN00012	130	21	0	458	160	0
ACAGATCAG	BRDN00012	139	142	0	269	39	0
ACAGATCAT	BRDN00012	62	38	18	138	36	67
ACAGATCCC	BRDN00012	90	174	56	227	200	12
ACAGATCCCT	BRDN00012	134	180	327	279	335	0
ACAGATCCG	BRDN00012	279	165	620	379	507	9
ACAGATCGA	BRDN00011	170	155	1103	245	444	13
ACAGATCGA	BRDN00011	437	273	226	709	813	85
ACAGATCGA	BRDN00011	412	587	1133	634	675	35
ACAGATCTG	BRDN00012	183	85	36	173	117	0
ACAGATCTG	BRDN00012	128	187	1097	281	174	24
ACAGATGAC	BRDN00011	162	55	26	669	359	0
ACAGATGAC	BRDN00012	133	117	137	257	187	0
ACAGATGAG	BRDN00012	158	112	488	238	93	0
ACAGATGAG	BRDN00011	92	111	112	169	163	7
ACAGATGAG	BRDN00011	96	37	47	176	109	17
ACAGATGAT	BRDN00012	156	56	62	474	402	0
ACAGATGCT	BRDN00012	447	542	828	708	775	61
ACAGATGGA	BRDN00011	224	164	1255	510	407	46
ACAGATGGA	BRDN00011	176	123	215	499	266	17
ACAGATGGG	BRDN00011	283	312	449	665	843	17
ACAGATGGG	BRDN00012	151	108	124	303	274	22
ACAGATGGG	BRDN00012	216	74	24	359	364	0
ACAGATGTC	BRDN00011	289	246	68	593	492	0
ACAGATGTG	BRDN00011	155	94	288	288	304	8
ACAGATGTT	BRDN00012	372	357	190	730	1010	13
ACAGATTAA	BRDN00012	194	27	1	452	286	0
ACAGATTAT	BRDN00012	319	347	292	541	467	19
ACAGATTAT	BRDN00012	188	124	35	347	270	5
ACAGATTCTC	BRDN00011	215	5	2	557	1001	0
ACAGATTGG	BRDN00011	197	193	35	527	450	0
ACAGATTGG	BRDN00011	115	59	183	399	368	0
ACAGATTGT	BRDN00011	211	165	41	380	612	13
ACAGATTTG	BRDN00012	184	188	113	459	643	34
ACAGCAAAA	BRDN00011	148	162	494	335	382	7
ACAGCAAAT	BRDN00011	173	138	0	361	395	10
ACAGCAAAT	BRDN00012	164	46	189	316	168	14
ACAGCAACA	BRDN00012	215	265	165	563	362	27
ACAGCAACC	BRDN00011	498	1	1	934	613	0
ACAGCAACG	BRDN00011	221	321	600	387	802	37
ACAGCAAGA	BRDN00012	428	297	29	613	665	21
ACAGCAAGA	BRDN00011	203	301	847	559	267	16
ACAGCAAGA	BRDN00012	274	378	2181	645	647	110
ACAGCAAGC	BRDN00011	517	495	1766	1016	946	30
ACAGCAAGT	BRDN00011	106	136	148	173	68	82
ACAGCAATA	BRDN00011	369	326	515	691	840	14
ACAGCAATA	BRDN00012	161	129	39	283	303	3
ACAGCAATC	BRDN00011	24	0	0	298	293	0
ACAGCAATG	BRDN00012	342	311	37	804	264	18
ACAGCAATG	BRDN00012	298	262	757	702	656	8
ACAGCAATT	BRDN00012	412	319	650	759	857	29
ACAGCACAA	BRDN00011	299	383	622	432	423	39
ACAGCACAA	BRDN00011	326	317	174	639	600	16
ACAGCACACC	BRDN00012	133	26	0	298	190	6
ACAGCACACC	BRDN00012	232	158	397	365	426	18
ACAGCACACC	BRDN00011	30	0	0	237	1	0
ACAGCACACT	BRDN00011	230	137	24	454	348	9
ACAGCACCA	BRDN00011	253	226	273	457	205	9
ACAGCACCAT	BRDN00012	172	146	573	208	97	40
ACAGCACCG	BRDN00011	128	157	233	211	472	47
ACAGCACGA	BRDN00011	289	142	1936	260	153	62
ACAGCACGG	BRDN00011	573	432	457	1107	1036	56
ACAGCACTA	BRDN00011	214	185	15	400	430	20
ACAGCACTG	BRDN00011	285	174	567	403	298	24
ACAGCACTTC	BRDN00011	336	350	862	739	693	67
ACAGCACTTC	BRDN00012	92	87	262	195	167	13
ACAGCAGAA	BRDN00011	274	349	327	537	279	22
ACAGCAGAC	BRDN00012	282	124	258	742	624	45
ACAGCAGAG	BRDN00012	344	292	237	800	945	33
ACAGCAGAT	BRDN00011	149	162	175	355	411	2
ACAGCAGCA	BRDN00011	133	158	77	206	24	12
ACAGCAGCA	BRDN00011	424	266	598	722	739	60
ACAGCAGCC	BRDN00012	428	410	268	875	523	44
ACAGCAGCT	BRDN00011	119	0	0	366	85	0
ACAGCAGCT	BRDN00012	160	222	534	270	389	5
ACAGCAGCT	BRDN00011	270	326	356	606	456	13
ACAGCAGGA	BRDN00012	193	235	440	546	484	35
ACAGCAGGA	BRDN00011	102	59	264	239	153	21
ACAGCAGGA	BRDN00011	193	145	43	347	258	0
ACAGCAGGC	BRDN00012	505	350	671	1047	784	26
ACAGCAGGT	BRDN00011	555	488	1443	796	1148	37
ACAGCAGGT	BRDN00011	404	467	428	681	538	63
ACAGCAGTA	BRDN00011	108	154	760	224	365	5
ACAGCAGTC	BRDN00012	401	276	253	479	452	33
ACAGCAGTG	BRDN00011	229	213	655	394	369	56
ACAGCAGTT	BRDN00012	240	309	135	728	588	61
ACAGCATAG	BRDN00012	161	105	208	249	561	35
ACAGCATATC	BRDN00012	332	366	534	677	1000	34
ACAGCATATC	BRDN00011	131	0	0	162	6	0
ACAGCATCA	BRDN00012	96	95	1336	166	169	34
ACAGCATCAT	BRDN00011	197	43	114	188	519	8
ACAGCATCCT	BRDN00011	455	169	196	937	1287	0
ACAGCATCG	BRDN00012	612	585	617	1207	952	184
ACAGCATCTA	BRDN00012	234	211	30	401	554	8
ACAGCATGA	BRDN00011	179	209	1	447	489	4
ACAGCATGT	BRDN00011	183	150	65	348	549	9
ACAGCATTCA	BRDN00012	121	159	376	124	115	32
ACAGCATTG	BRDN00011	202	299	162	456	518	0
ACAGCCAAC	BRDN00011	173	253	1071	362	279	21
ACAGCCAAG	BRDN00012	179	256	919	269	534	93
ACAGCCAAG	BRDN00011	142	119	66	372	195	0
ACAGCCACA	BRDN00011	307	264	761	791	549	37
ACAGCCACA	BRDN00012	171	95	133	271	320	0
ACAGCCACTC	BRDN00012	336	283	645	642	563	24
ACAGCCACTT	BRDN00011	265	127	191	356	628	46
ACAGCCAGA	BRDN00011	129	87	273	206	60	0
ACAGCCAGC	BRDN00012	150	238	132	278	229	19

ACAGCCAGC	BRDN00012	252	246	326	421	463	0
ACAGCCAGC	BRDN00012	431	445	176	874	507	0
ACAGCCAGG	BRDN00011	190	0	0	398	252	0
ACAGCCAGG	BRDN00012	119	140	49	211	164	11
ACAGCCATA	BRDN00011	188	122	587	210	306	25
ACAGCCATG	BRDN00011	101	121	33	320	255	0
ACAGCCCAA	BRDN00012	287	337	566	555	932	40
ACAGCCCAT	BRDN00011	130	200	254	246	342	16
ACAGCCCAT	BRDN00011	246	304	240	418	346	360
ACAGCCCATT	BRDN00012	216	183	175	385	237	17
ACAGCCCCA	BRDN00011	189	152	938	375	184	4
ACAGCCCCA	BRDN00011	470	595	259	805	956	41
ACAGCCCGC	BRDN00012	148	121	1	499	534	0
ACAGCCCGCT	BRDN00011	246	89	846	593	574	17
ACAGCCCGG	BRDN00011	447	159	2	889	301	1
ACAGCCCTCA	BRDN00012	456	412	421	766	769	77
ACAGCCGAG	BRDN00011	85	63	0	127	55	0
ACAGCCGGA	BRDN00011	284	343	828	548	552	33
ACAGCCGGA	BRDN00012	59	92	0	215	41	11
ACAGCCGGG	BRDN00011	21	0	0	124	14	0
ACAGCCGGT	BRDN00012	550	507	579	770	678	109
ACAGCCGTC	BRDN00012	398	311	534	681	997	15
ACAGCCTCA	BRDN00011	230	129	151	497	283	0
ACAGCCTCCT	BRDN00011	507	464	87	630	464	26
ACAGCCTCG	BRDN00012	244	346	72	457	750	12
ACAGCCTCGT	BRDN00012	303	1	0	619	356	1
ACAGCCTGA	BRDN00012	470	321	1062	920	735	63
ACAGCCTTA	BRDN00012	209	74	66	398	402	8
ACAGCCTTA	BRDN00012	267	242	256	543	398	24
ACAGCCTTCA	BRDN00011	217	141	47	518	425	17
ACAGCCTTCC	BRDN00011	231	271	233	523	1255	18
ACAGCGAAG	BRDN00012	212	0	0	692	312	8
ACAGCGACA	BRDN00011	138	62	11	235	351	18
ACAGCGACG	BRDN00011	58	144	16	274	169	10
ACAGCGACT	BRDN00011	64	49	84	337	187	0
ACAGCGAGC	BRDN00012	208	176	166	327	268	12
ACAGCGAGG	BRDN00012	376	200	466	577	770	36
ACAGCGAGT	BRDN00012	186	173	387	398	476	122
ACAGCGATG	BRDN00011	170	95	257	320	117	13
ACAGCGCAT	BRDN00012	471	170	85	865	706	8
ACAGCGCCA	BRDN00012	127	30	87	189	77	0
ACAGCGCCG	BRDN00012	108	142	8746	219	443	151
ACAGCGCCT	BRDN00012	209	223	532	429	403	43
ACAGCGCCT	BRDN00011	128	60	0	298	340	0
ACAGCGCCT	BRDN00012	225	175	1003	522	402	71
ACAGCGCGG	BRDN00011	247	24	38	522	238	5
ACAGCGCTCT	BRDN00012	557	332	756	771	712	53
ACAGCGCTG	BRDN00012	567	533	800	802	875	83
ACAGCGGAA	BRDN00012	298	305	289	597	643	92
ACAGCGGAA	BRDN00012	148	182	596	275	314	33
ACAGCGGAC	BRDN00012	111	0	5	303	113	34
ACAGCGGCA	BRDN00011	324	365	3818	554	214	107
ACAGCGGCA	BRDN00011	472	458	845	902	876	81
ACAGCGGTA	BRDN00012	509	381	165	823	667	7
ACAGCGGTA	BRDN00012	316	244	125	533	541	3
ACAGCGGTT	BRDN00011	289	317	102	502	215	0
ACAGCGTAT	BRDN00011	122	127	41	217	43	7
ACAGCGTGC	BRDN00011	48	20	86	173	70	0
ACAGCGTGG	BRDN00012	484	229	945	970	910	10
ACAGCTAAT	BRDN00012	244	212	337	289	368	75
ACAGCTAAT	BRDN00011	273	396	24	616	530	21
ACAGCTACA	BRDN00011	328	335	389	565	437	107
ACAGCTAGA	BRDN00011	95	0	0	203	58	1
ACAGCTAGT	BRDN00011	73	24	872	233	93	15
ACAGCTATA	BRDN00011	96	65	24	242	259	0
ACAGCTATG	BRDN00011	439	243	374	740	645	26
ACAGCTATT	BRDN00012	158	63	330	240	314	5
ACAGCTATT	BRDN00011	85	157	749	191	54	54
ACAGCTCAA	BRDN00011	221	266	162	510	460	0
ACAGCTCAA	BRDN00012	328	98	1	813	542	0
ACAGCTCAC	BRDN00011	83	99	477	216	202	3
ACAGCTCAG	BRDN00012	334	327	463	675	784	182
ACAGCTCAT	BRDN00012	217	135	231	302	688	0
ACAGCTCAT	BRDN00011	161	205	74	206	220	20
ACAGCTCCAT	BRDN00011	138	159	746	279	364	56
ACAGCTCCGT	BRDN00012	65	94	8	170	181	0
ACAGCTCGG	BRDN00012	83	90	0	168	68	0
ACAGCTCGG	BRDN00011	139	90	335	267	361	6
ACAGCTCGT	BRDN00011	146	71	327	223	320	0
ACAGCTCGT	BRDN00011	273	261	147	576	337	48
ACAGCTCTA	BRDN00012	180	96	142	189	113	29
ACAGCTCTA	BRDN00012	155	212	456	281	236	14
ACAGCTCTCT	BRDN00011	70	36	430	174	251	0
ACAGCTCTGT	BRDN00011	220	155	304	415	190	19
ACAGCTGAA	BRDN00012	99	129	389	196	260	68
ACAGCTGAC	BRDN00012	168	7	192	389	375	0
ACAGCTGAC	BRDN00012	351	439	858	761	1199	35
ACAGCTGAG	BRDN00011	155	122	1	436	272	1
ACAGCTGAG	BRDN00012	176	231	106	553	448	16
ACAGCTGCA	BRDN00011	254	142	262	337	560	0
ACAGCTGCC	BRDN00011	314	224	148	547	477	19
ACAGCTGCC	BRDN00012	194	124	157	432	478	3
ACAGCTGCT	BRDN00011	215	183	363	355	569	20
ACAGCTGGC	BRDN00011	182	101	55	320	216	0
ACAGCTGGG	BRDN00011	95	72	0	206	165	0
ACAGCTGGT	BRDN00011	255	270	421	514	552	22
ACAGCTGTA	BRDN00012	218	305	11	321	384	17
ACAGCTGTA	BRDN00012	54	14	1	369	463	0
ACAGCTGTC	BRDN00012	309	260	1468	591	609	78
ACAGCTGTG	BRDN00012	364	263	1701	802	484	43
ACAGCTGTG	BRDN00012	409	556	712	871	1069	93
ACAGCTTAA	BRDN00012	225	208	1214	374	568	27
ACAGCTTCG	BRDN00012	214	419	403	685	569	16
ACAGCTTCG	BRDN00012	378	338	560	761	723	15
ACAGCTTCTC	BRDN00011	269	150	128	306	436	35
ACAGCTTGA	BRDN00011	275	275	397	698	857	5
ACAGCTTGG	BRDN00012	134	60	49	218	226	18
ACAGCTTTC	BRDN00012	291	230	5154	555	609	85
ACAGGAAAC	BRDN00011	297	404	1169	576	1160	71
ACAGGAAAT	BRDN00011	130	104	9	296	183	20
ACAGGAACA	BRDN00011	71	43	12	144	95	0
ACAGGAACA	BRDN00011	305	370	146	487	533	76
ACAGGAACG	BRDN00012	60	88	65	156	11	36
ACAGGAACT	BRDN00012	189	73	123	308	223	0
ACAGGAAGA	BRDN00012	319	68	2	560	192	0
ACAGGAAGA	BRDN00012	331	347	587	541	431	81
ACAGGAAGA	BRDN00012	483	385	473	997	542	34
ACAGGAAGC	BRDN00011	146	152	413	368	407	0
ACAGGAAGG	BRDN00011	67	40	0	151	87	0
ACAGGAAGG	BRDN00011	88	90	0	266	411	0
ACAGGAAGT	BRDN00011	163	130	162	346	221	32
ACAGGAAGT	BRDN00011	308	255	1077	654	592	40
ACAGGAAGT	BRDN00012	205	149	53	280	247	9
ACAGGAAGT	BRDN00011	152	66	634	390	223	0
ACAGGAATG	BRDN00011	240	332	398	352	316	35
ACAGGAATT	BRDN00011	177	261	165	409	467	4
ACAGGAATT	BRDN00011	57	80	572	213	363	1
ACAGGACAA	BRDN00011	197	248	474	405	548	8
ACAGGACAG	BRDN00012	120	141	204	288	169	6
ACAGGACAG	BRDN00011	181	261	892	140	83	57
ACAGGACAG	BRDN00012	102	104	0	261	289	20
ACAGGACAT	BRDN00011	246	294	101	581	277	76
ACAGGACTG	BRDN00012	143	58	133	233	105	14
ACAGGACTG	BRDN00011	425	445	670	748	830	3
ACAGGACTT	BRDN00012	174	59	437	373	660	12
ACAGGAGAA	BRDN00011	256	211	19	606	897	17
ACAGGAGAG	BRDN00011	221	221	62	436	433	3
ACAGGAGCC	BRDN00011	184	209	471	580	697	14
ACAGGAGCC	BRDN00011	237	296	17	830	994	29
ACAGGAGCC	BRDN00012	140	92	5	237	503	7
ACAGGAGCT	BRDN00011	140	102	139	263	180	0
ACAGGAGTA	BRDN00012	281	318	406	435	556	5
ACAGGAGTA	BRDN00012	227	283	711	603	769	38
ACAGGATAA	BRDN00012	51	79	5	152	44	0
ACAGGATCA	BRDN00012	455	452	1373	790	640	50
ACAGGATCC	BRDN00012	260	250	745	530	389	66
ACAGGATCT	BRDN00011	425	475	1258	615	851	46
ACAGGATCT	BRDN00012	138	207	557	249	97	42
ACAGGATGC	BRDN00011	136	101	239	354	651	0
ACAGGATGT	BRDN00011	392	270	86	474	475	23
ACAGGATTA	BRDN00012	159	162	241	381	300	0
ACAGGATTA	BRDN00011	240	210	184	377	299	4
ACAGGATTA	BRDN00012	228	132	211	467	591	21
ACAGGATTC	BRDN00012	154	195	1032	314	196	35
ACAGGATTG	BRDN00012	268	249	1055	436	448	31
ACAGGATTG	BRDN00011	254	158	92	579	654	38
ACAGGCAAA	BRDN00011	386	397	839	794	459	34
ACAGGCAAC	BRDN00012	179	0	1	375	203	0
ACAGGCAAC	BRDN00011	398	340	1101	651	524	38
ACAGGCAAT	BRDN00011	413	305	1082	710	661	23
ACAGGCACA	BRDN00012	68	0	1	200	41	0
ACAGGCACA	BRDN00011	206	196	310	366	174	11
ACAGGCACT	BRDN00011	129	0	0	256	117	0
ACAGGCAGC	BRDN00012	569	484	2013	782	1083	69
ACAGGCAGG	BRDN00012	382	245	228	1085	513	0
ACAGGCATA	BRDN00012	87	95	17	213	93	0
ACAGGCATG	BRDN00012	291	344	248	781	507	16
ACAGGCATT	BRDN00011	262	254	334	467	515	31
ACAGGCATT	BRDN00012	276	465	4197	617	985	114
ACAGGCCAA	BRDN00011	533	643	268	810	922	56
ACAGGCCAC	BRDN00011	274	31	14	595	629	0
ACAGGCCAG	BRDN00011	727	794	2232	1284	1317	241
ACAGGCCAG	BRDN00011	37	31	2	171	66	25
ACAGGCCAT	BRDN00011	558	556	1311	994	960	49
ACAGGCCAT	BRDN00011	81	95	633	249	323	0
ACAGGCCAT	BRDN00012	243	176	69	516	530	21
ACAGGCCGG	BRDN00011	80	30	1057	189	320	9
ACAGGCCGT	BRDN00012	175	1	0	691	816	0
ACAGGCCTA	BRDN00012	101	34	1	212	199	0
ACAGGCCTCT	BRDN00012	369	242	366	841	859	17
ACAGGCCTG	BRDN00011	204	10	19	635	382	0
ACAGGCCTG	BRDN00011	332	260	1279	686	551	57
ACAGGCGAA	BRDN00011	363	293	756	803	1185	44
ACAGGCGAC	BRDN00012	357	448	332	670	378	6
ACAGGCGAG	BRDN00011	109	52	0	216	257	0
ACAGGCGAG	BRDN00012	376	358	528	664	801	72
ACAGGCGTA	BRDN00011	222	121	208	331	425	9
ACAGGCTCA	BRDN00011	504	336	622	746	872	58
ACAGGCTCC	BRDN00011	148	163	69	322	264	0
ACAGGCTCT	BRDN00012	428	435	592	707	1069	46
ACAGGCTTG	BRDN00012	190	109	942	792	758	13
ACAGGCTTG	BRDN00011	148	159	204	242	128	13
ACAGGCTTT	BRDN00011	165	110	1	286	464	0
ACAGGGAAC	BRDN00011	120	70	8	179	166	3
ACAGGGAAG	BRDN00011	160	189	1544	345	178	64
ACAGGGAAT	BRDN00011	192	204	279	303	113	0
ACAGGGACA	BRDN00012	522	244	157	1072	558	0
ACAGGGACG	BRDN00012	317	283	1034	611	555	150
ACAGGGAGC	BRDN00011	554	367	1966	1007	1226	33
ACAGGGAGT	BRDN00011	78	80	77	170	32	0
ACAGGGATA	BRDN00011	69	51	0	198	101	0
ACAGGGCAC	BRDN00012	295	316	11	470	300	8
ACAGGGCAG	BRDN00012	93	120	27	256	215	12
ACAGGGCAT	BRDN00012	271	253	704	422	395	32
ACAGGGCCT	BRDN00012	122	247	136	397	1022	62
ACAGGGCGG	BRDN00011	214	247	93	305	220	0
ACAGGGCTG	BRDN00012	495	438	942	899	667	34
ACAGGGGAC	BRDN00011	360	249	833	744	971	65
ACAGGGGCA	BRDN00012	450	272	235	832	561	61
ACAGGGGCT	BRDN00012	207	229	325	403	363	0
ACAGGGGGC	BRDN00011	137	120	23	271	72	0
ACAGGGTAT	BRDN00012	382	359	659	728	630	28
ACAGGGTCA	BRDN00011	132	47	55	304	284	1
ACAGGGTCA	BRDN00012	224	187	109	526	560	0
ACAGGGTCC	BRDN00012	151	197	52	458	373	0
ACAGGGTGC	BRDN00011	490	470	1567	884	950	64
ACAGGGTTG	BRDN00011	108	116	5	175	122	0
ACAGGGTTG	BRDN00012	24	0	0	247	31	0
ACAGGGTTG	BRDN00011	232	209	430	442	180	7
ACAGGTACA	BRDN00012	255	209	16	546	615	5
ACAGGTACA	BRDN00011	141	70	581	247	266	35
ACAGGTACC	BRDN00012	313	270	302	516	430	2
ACAGGTAGG	BRDN00012	286	320	185	627	587	25
ACAGGTAGT	BRDN00012	212	272	198	654	687	9
ACAGGTAGT	BRDN00011	116	0	0	201	158	0
ACAGGTAGT	BRDN00012	68	83	148	200	79	0
ACAGGTCAA	BRDN00011	164	236	71	202	489	38
ACAGGTCAG	BRDN00011	16	0	0	32	49	0
ACAGGTCAG	BRDN00011	233	173	212	439	744	0
ACAGGTCCA	BRDN00012	275	293	229	493	378	17
ACAGGTCCC	BRDN00012	163	159	293	241	131	102
ACAGGTCCG	BRDN00011	346	214	484	1045	562	6
ACAGGTCGT	BRDN00011	156	80	137	173	271	12
ACAGGTGCA	BRDN00011	348	350	207	683	644	6
ACAGGTGCC	BRDN00012	123	189	247	194	371	32
ACAGGTGCT	BRDN00011	142	110	228	202	165	0
ACAGGTGGT	BRDN00011	164	115	30	404	522	22
ACAGGTGGT	BRDN00011	128	68	1	237	70	0
ACAGGTGTA	BRDN00011	157	94	164	280	179	7
ACAGGTGTA	BRDN00012	90	117	623	143	112	10
ACAGGTGTC	BRDN00012	90	241	20	182	460	0
ACAGGTGTG	BRDN00011	80	31	29	212	186	0
ACAGGTTAT	BRDN00012	140	158	2	317	344	4
ACAGGTTCA	BRDN00011	174	97	805	203	231	12
ACAGGTTCA	BRDN00012	328	403	121	513	831	33
ACAGGTTCC	BRDN00011	198	275	705	419	607	100
ACAGGTTCT	BRDN00012	309	154	22	439	654	0
ACAGGTTGC	BRDN00012	843	615	13142	1141	1214	296
ACAGGTTGG	BRDN00011	83	153	367	218	246	6
ACAGGTTGG	BRDN00011	180	169	748	529	614	15
ACAGGTTTA	BRDN00012	422	485	5554	641	787	137
ACAGGTTTA	BRDN00012	209	219	1196	466	157	26
ACAGGTTTA	BRDN00011	332	252	529	568	408	10
ACAGGTTTA	BRDN00011	368	169	143	585	361	0
ACAGTAAAG	BRDN00011	113	101	68	211	238	138
ACAGTAACC	BRDN00011	296	34	55	630	289	0
ACAGTAAGA	BRDN00011	165	197	249	411	573	26
ACAGTAATG	BRDN00011	141	1	0	267	64	0
ACAGTACAA	BRDN00012	462	424	745	878	1333	19
ACAGTACAA	BRDN00011	42	40	36	183	77	11
ACAGTACCA	BRDN00011	83	70	91	293	145	2
ACAGTACCC	BRDN00011	174	119	73	294	344	27
ACAGTACCG	BRDN00012	535	509	736	887	751	137
ACAGTACCT	BRDN00012	157	93	17	232	441	8
ACAGTACCT	BRDN00012	132	81	0	240	137	15
ACAGTACCT	BRDN00012	263	187	251	583	713	10
ACAGTACGT	BRDN00012	184	240	374	310	309	3
ACAGTACTCT	BRDN00012	418	366	1358	683	1056	27
ACAGTACTG	BRDN00011	268	262	593	426	750	0
ACAGTAGAA	BRDN00011	159	156	1242	249	258	19
ACAGTAGAG	BRDN00011	183	17	1	659	150	0
ACAGTAGCC	BRDN00011	186	282	37	430	604	26
ACAGTAGGT	BRDN00011	141	226	68	175	145	30
ACAGTAGTT	BRDN00011	91	55	729	166	114	20
ACAGTAGTT	BRDN00012	374	219	237	589	509	4
ACAGTATAC	BRDN00011	399	555	759	925	1075	43
ACAGTATAT	BRDN00011	84	63	39	156	372	8
ACAGTCAAA	BRDN00011	177	92	0	398	402	0
ACAGTCAAT	BRDN00011	88	42	33	206	260	0
ACAGTCACA	BRDN00011	201	307	549	527	669	58
ACAGTCACC	BRDN00012	203	73	232	390	201	1
ACAGTCACC	BRDN00011	152	43	38	290	187	0
ACAGTCACT	BRDN00011	157	220	937	244	457	0
ACAGTCAGA	BRDN00011	324	409	1154	896	925	96
ACAGTCAGG	BRDN00011	134	93	56	317	473	39
ACAGTCATCT	BRDN00011	425	0	31	759	515	0
ACAGTCCAA	BRDN00011	223	184	307	371	571	80
ACAGTCCAC	BRDN00011	244	400	1075	561	659	27
ACAGTCCAT	BRDN00012	18	0	1	188	153	0
ACAGTCCCA	BRDN00011	185	235	184	349	272	43
ACAGTCCGG	BRDN00012	291	243	446	520	614	21
ACAGTCCTA	BRDN00011	244	236	2	505	274	13
ACAGTCCTCA	BRDN00011	317	266	370	668	791	3
ACAGTCCTCT	BRDN00011	278	289	249	431	306	52
ACAGTCCTG	BRDN00011	430	466	538	928	638	35
ACAGTCGAA	BRDN00012	227	298	267	384	476	85
ACAGTCGAG	BRDN00012	237	83	793	777	610	16
ACAGTCGGT	BRDN00011	238	98	20	361	490	0
ACAGTCTGA	BRDN00012	188	337	488	508	525	75
ACAGTGAAA	BRDN00011	160	99	0	122	65	8
ACAGTGAAA	BRDN00012	226	265	78	412	467	14
ACAGTGAAA	BRDN00011	426	364	741	694	887	24
ACAGTGAAC	BRDN00011	392	368	555	619	833	40
ACAGTGACA	BRDN00012	239	244	184	431	554	8
ACAGTGACA	BRDN00011	124	109	2	365	164	0
ACAGTGACA	BRDN00011	121	142	1003	252	111	13
ACAGTGACC	BRDN00012	533	651	1332	1151	1536	16
ACAGTGACC	BRDN00012	243	192	261	390	307	12
ACAGTGACG	BRDN00012	193	277	3344	404	274	112
ACAGTGAGC	BRDN00011	262	278	1837	405	428	61
ACAGTGAGT	BRDN00012	404	334	1332	725	520	104
ACAGTGATC	BRDN00011	384	401	217	569	739	26
ACAGTGCAA	BRDN00012	316	231	3227	586	391	43
ACAGTGCAC	BRDN00011	341	395	80	539	804	28
ACAGTGCAG	BRDN00011	90	47	143	191	192	0
ACAGTGCAG	BRDN00011	246	188	838	541	937	29
ACAGTGCGA	BRDN00012	302	235	429	442	521	17
ACAGTGCGC	BRDN00012	124	88	0	195	335	10
ACAGTGCTCT	BRDN00012	229	249	6107	536	951	182
ACAGTGCTG	BRDN00011	462	409	524	669	817	38
ACAGTGCTT	BRDN00012	196	119	31	280	185	1
ACAGTGGAG	BRDN00011	91	124	365	226	318	36
ACAGTGGAT	BRDN00012	131	47	418	224	176	12
ACAGTGGCA	BRDN00012	420	327	2186	896	630	51
ACAGTGGCC	BRDN00012	291	366	273	770	755	43
ACAGTGGCG	BRDN00011	263	313	93	475	399	31
ACAGTGGCG	BRDN00012	210	176	87	361	323	0
ACAGTGGCT	BRDN00012	158	187	245	252	1015	13
ACAGTGGGA	BRDN00012	584	494	173	1100	1219	22
ACAGTGGGC	BRDN00012	207	264	2529	405	731	231
ACAGTGGGG	BRDN00012	399	275	962	682	672	11
ACAGTGGGT	BRDN00012	202	256	30	454	329	0
ACAGTGGTA	BRDN00011	162	69	50	261	467	0
ACAGTGGTG	BRDN00011	233	189	629	345	417	9
ACAGTGGTG	BRDN00012	315	278	584	519	323	39
ACAGTGGTG	BRDN00012	236	159	178	410	218	0
ACAGTGTAC	BRDN00011	225	185	14	211	151	32
ACAGTGTAT	BRDN00011	344	292	3699	639	655	64
ACAGTGTAT	BRDN00011	210	158	792	386	228	17
ACAGTGTAT	BRDN00011	139	148	495	361	289	7
ACAGTGTCA	BRDN00012	324	277	11	578	850	2
ACAGTGTCC	BRDN00012	271	269	340	566	672	80
ACAGTGTCC	BRDN00011	151	192	101	278	407	0
ACAGTGTGG	BRDN00011	572	450	754	871	1246	22
ACAGTGTGG	BRDN00011	105	83	25	289	242	13
ACAGTGTGT	BRDN00011	201	0	0	475	176	0
ACAGTGTGT	BRDN00012	128	216	66	202	166	13
ACAGTGTGT	BRDN00011	158	106	0	402	484	3
ACAGTGTTA	BRDN00012	332	190	3	683	1039	4
ACAGTTAAA	BRDN00011	40	6	0	357	279	0
ACAGTTCAC	BRDN00012	136	104	851	253	632	3
ACAGTTCAG	BRDN00011	158	121	229	454	620	11
ACAGTTCAG	BRDN00011	275	279	112	539	696	125
ACAGTTCCTA	BRDN00012	249	1	0	847	198	0
ACAGTTCTCC	BRDN00012	291	131	65	673	642	0
ACAGTTCTCT	BRDN00011	278	263	86	549	542	42
ACAGTTCTG	BRDN00012	398	485	1457	892	1027	73
ACAGTTCTTA	BRDN00011	272	122	34	377	305	0
ACAGTTGAA	BRDN00011	137	82	93	279	118	0
ACAGTTGAC	BRDN00011	286	211	231	372	708	21
ACAGTTGAC	BRDN00012	353	258	254	564	723	69
ACAGTTGAC	BRDN00011	158	102	432	192	222	0
ACAGTTGAT	BRDN00011	166	165	73	406	327	11
ACAGTTGAT	BRDN00011	356	215	658	515	481	113
ACAGTTGAT	BRDN00011	147	51	9	162	254	7
ACAGTTGCC	BRDN00011	149	99	310	335	427	2
ACAGTTGGT	BRDN00011	222	166	1506	328	819	41
ACAGTTTAT	BRDN00011	90	119	186	157	64	69
ACAGTTTCA	BRDN00011	311	260	380	605	691	16
ACAGTTTCCA	BRDN00012	379	265	52	735	968	0
ACAGTTTCCG	BRDN00012	318	172	400	436	266	15
ACAGTTTCG	BRDN00012	177	170	120	476	258	0
ACAGTTTGT	BRDN00012	363	255	1065	641	587	44
ACATAAAAG	BRDN00012	230	207	792	412	263	25
ACATAACAAT	BRDN00012	235	208	48	435	477	48
ACATAACAG	BRDN00012	214	282	675	445	247	42
ACATAACCTA	BRDN00011	160	107	439	177	276	33
ACATAACGC	BRDN00012	133	111	42	184	196	6
ACATAACTG	BRDN00012	150	92	556	907	818	20
ACATAACTG	BRDN00011	146	80	1	333	148	0
ACATAAGAA	BRDN00011	93	182	0	90	264	0
ACATAAGAA	BRDN00011	201	170	562	392	550	14
ACATAAGAT	BRDN00012	219	183	76	699	577	12
ACATAAGAT	BRDN00011	88	28	48	705	417	0
ACATAAGCT	BRDN00012	209	96	207	311	496	0
ACATAAGTC	BRDN00012	118	138	357	245	496	8
ACATAATAG	BRDN00011	44	130	229	179	143	9
ACATAATAT	BRDN00011	175	251	529	513	592	34
ACATAATCCA	BRDN00012	231	186	972	562	575	25
ACATAATCTG	BRDN00012	243	243	595	334	271	16
ACATAATGC	BRDN00012	202	77	650	309	404	14
ACATACACTA	BRDN00012	287	291	1307	497	629	52
ACATACACTG	BRDN00011	150	57	0	475	250	0
ACATACATTA	BRDN00012	161	158	931	314	296	18
ACATACATTT	BRDN00011	190	106	97	409	582	3
ACATACCAA	BRDN00011	295	156	601	338	208	0
ACATACCAA	BRDN00011	219	199	204	440	280	0
ACATACCACT	BRDN00011	194	219	433	494	475	10
ACATACCATC	BRDN00012	453	306	444	848	1146	48
ACATACCATT	BRDN00011	167	130	59	359	583	14
ACATACCCCC	BRDN00012	527	493	832	795	825	53
ACATACCCCT	BRDN00011	166	298	572	227	349	19
ACATACCCCT	BRDN00012	303	249	327	528	595	78
ACATACCCTT	BRDN00011	500	499	404	992	1050	41
ACATACCGCT	BRDN00012	269	257	382	373	712	25
ACATACCGG	BRDN00012	435	333	2249	667	475	14
ACATACCGG	BRDN00011	168	177	284	256	243	0
ACATACCTCA	BRDN00012	242	209	40	369	407	6
ACATACCTG	BRDN00011	199	265	176	385	270	13
ACATACCTG	BRDN00011	188	76	0	419	476	0
ACATACCTG	BRDN00012	484	364	1009	828	353	61
ACATACGTG	BRDN00011	170	90	586	275	251	30
ACATACTACC	BRDN00011	147	33	253	792	1072	17
ACATACTAG	BRDN00011	178	75	120	404	347	19
ACATACTCTA	BRDN00012	188	88	46	844	1147	0
ACATACTCTA	BRDN00012	191	107	79	366	151	11
ACATACTGA	BRDN00012	600	410	1270	880	935	73
ACATACTGC	BRDN00011	218	129	26	286	116	20
ACATACTTAC	BRDN00011	250	243	608	592	1213	23
ACATACTTAC	BRDN00011	500	3	0	1084	1130	6
ACATAGACG	BRDN00012	212	189	103	274	166	0
ACATAGAGC	BRDN00012	198	142	6	475	395	0
ACATAGGAA	BRDN00011	382	261	26	797	793	68
ACATAGGAC	BRDN00012	243	198	282	698	653	20
ACATAGGCA	BRDN00011	180	183	1198	416	326	28
ACATAGGCC	BRDN00012	190	159	498	603	782	20
ACATAGGCG	BRDN00012	222	138	0	400	341	16
ACATAGGGC	BRDN00011	173	93	286	301	378	8
ACATAGGGG	BRDN00012	401	364	3078	703	637	27
ACATAGTAC	BRDN00012	666	518	789	1082	1252	80
ACATAGTAT	BRDN00012	62	150	8	224	43	11
ACATAGTCG	BRDN00012	278	295	1607	567	486	26
ACATAGTGT	BRDN00012	328	233	397	809	878	11
ACATAGTGT	BRDN00011	218	124	1531	385	136	60
ACATAGTGT	BRDN00011	317	29	0	696	730	0
ACATAGTTT	BRDN00011	100	219	556	208	372	10
ACATATAAT	BRDN00011	161	281	756	408	324	51
ACATATACA	BRDN00012	470	254	192	930	705	142
ACATATACTC	BRDN00012	220	116	55	243	154	0
ACATATAGCT	BRDN00012	268	305	683	617	598	37
ACATATATCC	BRDN00011	135	184	47	469	459	21
ACATATATG	BRDN00011	438	394	2546	905	1030	46
ACATATCAA	BRDN00011	200	65	88	517	428	3
ACATATCAG	BRDN00012	228	275	558	503	367	5
ACATATGAT	BRDN00012	414	318	1475	653	573	51
ACATATGCA	BRDN00011	152	128	13	231	356	0
ACATATGCC	BRDN00012	620	482	1059	992	948	72
ACATATGCG	BRDN00012	287	73	2	795	435	21
ACATATGGC	BRDN00011	120	297	171	189	127	28
ACATATGTG	BRDN00011	69	112	0	220	151	28
ACATATTCG	BRDN00012	362	36	0	835	688	0
ACATATTGG	BRDN00012	484	576	2226	1057	590	66
ACATATTTAC	BRDN00012	208	226	69	318	272	0
ACATCAAACT	BRDN00011	147	98	58	299	166	0
ACATCAAAG	BRDN00011	186	192	366	311	410	3

ACATCAAAG	BRDN00011	231	210	787	421	394	27
ACATCAAAG	BRDN00012	411	229	98	734	924	1
ACATCAAAG	BRDN00012	371	379	1467	937	730	55
ACATCAAAGT	BRDN00011	355	403	611	714	1209	18
ACATCAAAGT	BRDN00012	326	423	6	818	839	16
ACATCAAAGT	BRDN00012	176	188	161	473	561	8
ACATCAACCA	BRDN00011	290	108	123	553	377	0
ACATCAACTT	BRDN00011	393	354	409	706	631	16
ACATCAAGA	BRDN00011	124	267	25	270	415	237
ACATCAAGA	BRDN00011	128	165	925	267	96	11
ACATCAAGA	BRDN00012	487	495	811	1227	935	67
ACATCAAGG	BRDN00012	120	18	1	316	234	41
ACATCAAGTT	BRDN00012	97	51	0	266	317	0
ACATCAATG	BRDN00012	343	291	32	663	329	49
ACATCAATG	BRDN00011	156	153	368	368	406	18
ACATCACACA	BRDN00012	183	156	205	255	291	6
ACATCACAG	BRDN00012	649	392	2795	989	812	74
ACATCACAG	BRDN00012	84	35	3	295	177	0
ACATCACCG	BRDN00012	62	9	0	344	332	0
ACATCACTAA	BRDN00012	240	321	729	469	188	21
ACATCAGAC	BRDN00012	452	308	332	819	580	6
ACATCAGAG	BRDN00011	206	123	89	657	596	8
ACATCAGAT	BRDN00011	127	0	618	250	133	0
ACATCAGCA	BRDN00012	164	135	144	405	161	28
ACATCAGGA	BRDN00012	251	179	128	278	422	0
ACATCAGGA	BRDN00012	287	274	265	409	732	37
ACATCAGGG	BRDN00012	548	748	193	922	537	92
ACATCAGTAT	BRDN00011	242	225	270	513	502	10
ACATCAGTCT	BRDN00012	296	370	22	477	330	41
ACATCAGTTT	BRDN00011	525	521	297	972	1030	2
ACATCATAA	BRDN00011	162	168	101	337	209	5
ACATCATACG	BRDN00011	627	480	2428	946	825	94
ACATCATAG	BRDN00012	62	35	0	190	386	0
ACATCATATC	BRDN00011	216	155	680	442	472	15
ACATCATCAA	BRDN00012	127	75	54	317	138	0
ACATCATCCG	BRDN00011	284	317	245	693	656	40
ACATCATCTT	BRDN00012	203	216	165	417	414	5
ACATCATGA	BRDN00011	226	318	949	453	486	49
ACATCATGA	BRDN00011	398	351	97	803	607	69
ACATCATGCT	BRDN00012	67	3	0	412	383	0
ACATCATTG	BRDN00012	141	68	737	214	305	11
ACATCATTG	BRDN00011	214	119	1	409	325	0
ACATCCAACT	BRDN00011	297	31	0	388	422	0
ACATCCAAG	BRDN00011	197	182	984	324	169	23
ACATCCAAGT	BRDN00011	280	328	548	461	853	26
ACATCCACG	BRDN00012	185	216	28	459	433	9
ACATCCACG	BRDN00012	109	118	106	233	400	16
ACATCCACGT	BRDN00012	580	487	4332	987	853	94
ACATCCACTA	BRDN00011	228	158	9	340	411	9
ACATCCAGA	BRDN00012	301	108	261	723	518	1
ACATCCAGCT	BRDN00012	178	18	1	953	914	0
ACATCCAGCT	BRDN00012	160	66	1	349	176	51
ACATCCAGG	BRDN00011	186	146	78	420	303	68
ACATCCATAT	BRDN00012	137	150	102	503	400	72
ACATCCATAT	BRDN00011	281	18	1	662	422	0
ACATCCATCG	BRDN00012	143	11	0	453	313	0
ACATCCCCGT	BRDN00012	523	466	375	1168	887	109
ACATCCCGA	BRDN00011	376	442	539	636	984	68
ACATCCCTGA	BRDN00011	262	353	332	573	465	33
ACATCCCTGT	BRDN00011	629	414	1367	911	1160	29
ACATCCGAA	BRDN00012	520	545	696	1265	1052	13
ACATCCGAG	BRDN00011	179	30	0	469	218	3
ACATCCGCCA	BRDN00011	171	140	46	293	261	0
ACATCCGCCG	BRDN00011	303	321	361	444	486	75
ACATCCGTCC	BRDN00012	217	111	90	672	400	0
ACATCCTACC	BRDN00012	433	373	888	895	1094	37
ACATCCTACT	BRDN00012	181	120	45	165	286	33
ACATCCTCAA	BRDN00011	186	212	7	502	430	6
ACATCCTCAA	BRDN00011	150	38	22	276	320	12
ACATCCTCCA	BRDN00011	385	412	464	829	1017	15
ACATCCTCGA	BRDN00012	246	250	1213	550	612	29
ACATCCTGA	BRDN00011	187	168	27	388	462	18
ACATCCTTAC	BRDN00011	247	235	160	498	481	5
ACATCGAAG	BRDN00011	498	383	983	811	668	82
ACATCGACA	BRDN00011	410	366	1476	643	768	45
ACATCGACA	BRDN00012	202	238	3619	249	198	66
ACATCGACT	BRDN00011	203	151	305	702	686	0
ACATCGAGC	BRDN00011	143	122	777	333	323	3
ACATCGAGG	BRDN00012	603	483	936	1119	641	114
ACATCGAGG	BRDN00011	203	187	56	312	291	36
ACATCGATA	BRDN00012	168	254	617	353	458	0
ACATCGCCTC	BRDN00012	828	678	863	1327	894	92
ACATCGCGA	BRDN00011	313	358	230	519	618	45
ACATCGCTCT	BRDN00012	225	114	5	358	480	14
ACATCGGAC	BRDN00011	118	49	78	264	163	0
ACATCGGCC	BRDN00012	404	397	637	726	762	25
ACATCGGCC	BRDN00011	247	224	207	534	746	0
ACATCGTCG	BRDN00012	384	387	290	606	527	9
ACATCGTGG	BRDN00011	355	460	540	624	545	94
ACATCTACCT	BRDN00011	229	209	0	315	339	2
ACATCTATTA	BRDN00011	83	98	16	196	144	18
ACATCTCCCT	BRDN00012	183	74	40	909	253	0
ACATCTCCGC	BRDN00011	275	142	519	403	317	12
ACATCTCCGT	BRDN00012	386	312	208	548	495	117
ACATCTCTAC	BRDN00012	151	53	14	751	548	0
ACATCTCTCT	BRDN00012	166	7	0	383	374	0
ACATCTGAA	BRDN00011	426	433	1310	889	1266	89
ACATCTGAAT	BRDN00012	341	270	1502	467	667	15
ACATCTGAG	BRDN00012	390	262	322	493	441	58
ACATCTGATC	BRDN00012	258	236	18	364	509	3
ACATCTGGT	BRDN00012	219	93	87	332	314	0
ACATCTGTCA	BRDN00012	391	129	400	691	716	9
ACATCTGTG	BRDN00012	221	78	0	951	952	0
ACATCTTCAG	BRDN00012	247	186	1178	381	584	8
ACATCTTCAT	BRDN00012	347	249	1928	564	533	54
ACATCTTCTC	BRDN00012	523	405	1947	863	1104	84
ACATCTTGCT	BRDN00011	83	10	12	276	26	16
ACATCTTTGT	BRDN00011	300	345	355	414	296	31
ACATGAAAA	BRDN00011	170	116	33	282	367	47
ACATGAAAC	BRDN00012	148	156	80	296	476	0
ACATGAAAG	BRDN00012	149	135	93	319	278	26
ACATGAAAT	BRDN00011	206	113	889	400	405	11
ACATGAACA	BRDN00011	81	67	1	219	141	15
ACATGAACA	BRDN00012	114	110	513	251	198	83
ACATGAACA	BRDN00012	310	290	142	745	1012	41
ACATGAACA	BRDN00011	198	148	737	261	398	0
ACATGAACC	BRDN00012	156	145	206	388	555	34
ACATGAACG	BRDN00012	484	282	203	856	524	52
ACATGAACT	BRDN00011	414	458	1276	655	1029	150
ACATGAACT	BRDN00012	137	17	0	971	520	0
ACATGAAGG	BRDN00011	218	324	68	554	264	5
ACATGAAGT	BRDN00011	124	121	0	384	365	0
ACATGAATA	BRDN00011	145	204	219	257	317	12
ACATGAATG	BRDN00012	295	73	491	810	823	5
ACATGAATT	BRDN00012	199	243	401	394	719	7
ACATGACAC	BRDN00011	472	281	584	612	960	64
ACATGACAC	BRDN00011	469	77	265	1006	723	0
ACATGACAG	BRDN00011	544	384	690	928	1241	49
ACATGACAT	BRDN00011	439	346	1201	786	1191	33
ACATGACCA	BRDN00011	284	227	399	513	968	35
ACATGACCA	BRDN00011	399	379	3253	873	758	66
ACATGACCAT	BRDN00011	164	111	0	233	313	0
ACATGACCC	BRDN00012	235	187	163	428	378	0
ACATGACGA	BRDN00012	502	496	129	655	746	18
ACATGAGCA	BRDN00012	95	6	40	261	356	9
ACATGAGCC	BRDN00011	615	344	936	897	1328	85
ACATGAGGA	BRDN00012	396	481	352	941	896	42
ACATGATGA	BRDN00011	512	378	442	1009	1463	36
ACATGATGC	BRDN00011	439	517	366	846	866	27
ACATGATGG	BRDN00011	264	223	195	477	372	57
ACATGATGT	BRDN00012	194	78	165	282	491	11
ACATGATTA	BRDN00012	508	433	791	918	586	55
ACATGCAAA	BRDN00012	246	183	207	247	452	30
ACATGCAAA	BRDN00011	451	309	3765	720	956	91
ACATGCACA	BRDN00011	325	280	749	560	630	12
ACATGCACA	BRDN00012	471	457	797	1053	1017	74
ACATGCACC	BRDN00011	639	476	1361	1066	1441	59
ACATGCCAA	BRDN00012	492	11	29	996	928	0
ACATGCCATC	BRDN00012	202	115	27	607	258	0
ACATGCCCG	BRDN00011	536	483	385	1005	991	57
ACATGCCCTC	BRDN00011	203	82	249	334	210	0
ACATGCCTCG	BRDN00011	538	472	1580	586	308	30
ACATGCGCTT	BRDN00012	423	325	3028	894	863	32
ACATGCGGA	BRDN00012	470	399	637	1047	1502	107
ACATGCGGC	BRDN00012	268	182	154	477	464	36
ACATGCGGG	BRDN00011	177	81	1266	272	149	57
ACATGCTAA	BRDN00012	226	127	242	511	508	46
ACATGCTAAT	BRDN00012	248	215	691	388	726	0
ACATGCTACT	BRDN00011	236	309	118	408	441	28
ACATGCTATT	BRDN00012	90	50	61	167	134	15
ACATGCTGA	BRDN00011	173	81	248	271	78	0
ACATGCTGA	BRDN00011	249	240	1505	477	733	28
ACATGCTGG	BRDN00011	607	300	277	1040	1000	35
ACATGGAAA	BRDN00011	156	73	276	192	427	0
ACATGGAAC	BRDN00012	386	456	1277	742	788	193
ACATGGAAT	BRDN00011	204	114	15	366	243	0
ACATGGACA	BRDN00011	181	135	89	513	275	6
ACATGGACA	BRDN00012	511	362	979	931	1481	73
ACATGGACA	BRDN00011	153	114	11	326	375	0
ACATGGACT	BRDN00012	321	228	151	565	551	55
ACATGGAGA	BRDN00011	98	78	3282	180	141	58
ACATGGAGG	BRDN00012	539	601	2950	994	1080	82
ACATGGCAA	BRDN00011	165	85	118	288	286	0
ACATGGCAA	BRDN00012	118	169	207	322	131	5
ACATGGCCA	BRDN00012	630	939	4458	1034	752	392
ACATGGCCA	BRDN00012	527	196	659	1168	1233	3
ACATGGCCC	BRDN00011	285	325	92	486	338	0
ACATGGCCG	BRDN00012	65	19	0	199	187	0
ACATGGCGG	BRDN00011	107	80	372	258	348	0
ACATGGCTC	BRDN00012	169	143	174	169	277	0
ACATGGGAG	BRDN00012	457	555	338	1041	1148	40
ACATGGGAG	BRDN00011	40	42	0	197	101	6
ACATGGGCG	BRDN00011	189	94	317	245	79	0
ACATGGGCT	BRDN00011	655	244	2394	1031	846	55
ACATGGGGC	BRDN00012	300	264	886	580	492	84
ACATGGGGC	BRDN00011	161	164	2	267	274	2
ACATGGGGC	BRDN00012	304	125	8	1150	464	12
ACATGGGGC	BRDN00012	269	193	413	502	388	37
ACATGGGTC	BRDN00011	267	110	102	494	417	4
ACATGGTAT	BRDN00011	356	254	27	502	392	26
ACATGGTGG	BRDN00011	274	168	521	477	513	9
ACATGGTGG	BRDN00011	41	48	0	120	39	0
ACATGGTTG	BRDN00012	472	538	374	764	1259	27
ACATGTAAG	BRDN00011	158	134	0	244	89	0
ACATGTAAT	BRDN00012	172	196	97	300	153	60
ACATGTAGA	BRDN00011	235	222	710	531	571	60
ACATGTAGA	BRDN00011	129	176	286	347	269	162
ACATGTAGC	BRDN00012	622	642	1104	941	643	19
ACATGTAGG	BRDN00012	374	441	1298	1010	817	80
ACATGTATA	BRDN00011	296	299	232	517	430	40
ACATGTCAC	BRDN00011	353	323	682	822	769	35
ACATGTCAG	BRDN00012	269	243	392	461	614	25
ACATGTCCG	BRDN00011	564	352	540	1119	1205	63
ACATGTCCG	BRDN00011	236	303	105	521	515	7
ACATGTCCGT	BRDN00012	152	136	364	316	258	4
ACATGTCCTG	BRDN00012	496	355	821	683	524	66
ACATGTCGC	BRDN00012	512	429	190	991	1110	31
ACATGTCTA	BRDN00012	341	407	258	650	885	33
ACATGTCTA	BRDN00012	281	188	307	531	782	40
ACATGTCTCT	BRDN00011	344	211	2251	884	1102	44
ACATGTGAA	BRDN00011	286	316	477	508	558	64
ACATGTGGG	BRDN00012	298	563	2665	467	1034	64
ACATGTGGG	BRDN00012	104	193	113	373	188	12
ACATGTGTA	BRDN00012	246	218	335	515	795	31
ACATGTTCA	BRDN00011	297	320	756	622	706	27
ACATGTTCCA	BRDN00011	416	450	419	623	457	31
ACATGTTCCC	BRDN00011	102	158	329	298	298	15
ACATGTTCTC	BRDN00012	412	699	759	771	889	20
ACATGTTGG	BRDN00012	624	765	515	1085	1070	47
ACATGTTGTC	BRDN00011	254	419	1593	764	895	60
ACATGTTGTT	BRDN00012	196	234	103	420	186	49
ACATGTTTAT	BRDN00011	261	279	493	567	799	31
ACATTAAACC	BRDN00011	112	51	43	333	90	10
ACATTAACAT	BRDN00011	117	133	372	288	182	2
ACATTAATGT	BRDN00011	314	290	64	690	553	43
ACATTACCCA	BRDN00012	284	32	1	750	721	0
ACATTACCTT	BRDN00011	194	228	8	523	407	8
ACATTACGG	BRDN00011	131	134	25	289	190	0
ACATTAGAA	BRDN00012	344	209	352	612	396	26
ACATTAGCA	BRDN00011	292	243	185	531	615	11
ACATTAGCAT	BRDN00012	188	164	1461	334	408	6
ACATTAGTG	BRDN00011	150	43	6	425	183	9
ACATTATTCC	BRDN00011	277	232	895	363	299	23
ACATTCACA	BRDN00011	204	237	106	302	493	8
ACATTCACAC	BRDN00012	496	373	3560	830	1019	95
ACATTCATAA	BRDN00011	252	216	2105	426	527	73
ACATTCCAAT	BRDN00012	509	580	498	974	1086	57
ACATTCCAGT	BRDN00012	450	33	4	1023	315	0
ACATTCCTTA	BRDN00012	310	361	905	577	629	10
ACATTCGCTA	BRDN00012	397	529	376	709	916	90
ACATTCGCTT	BRDN00012	542	310	948	650	711	44
ACATTCGGG	BRDN00012	258	269	589	723	723	22
ACATTCGGG	BRDN00012	45	0	0	648	150	0
ACATTCTAGT	BRDN00012	428	478	867	698	477	115
ACATTCTATT	BRDN00012	292	209	399	572	374	5
ACATTCTGTA	BRDN00012	90	100	280	308	290	9
ACATTGACA	BRDN00011	141	110	16	438	255	0
ACATTGACC	BRDN00012	154	71	1133	330	183	26
ACATTGACC	BRDN00011	82	108	0	228	27	14
ACATTGACG	BRDN00012	542	262	303	855	639	48
ACATTGACTC	BRDN00012	271	133	1	349	276	22
ACATTGAGC	BRDN00011	219	100	118	391	247	0
ACATTGAGT	BRDN00012	182	10	2	796	395	0
ACATTGATTC	BRDN00012	116	100	81	346	359	0
ACATTGCTCA	BRDN00011	187	166	86	315	451	15
ACATTGGAA	BRDN00011	159	174	1	305	563	0
ACATTGGAT	BRDN00012	196	136	338	509	748	17
ACATTGGCG	BRDN00011	147	157	43	349	80	4
ACATTGGGA	BRDN00012	180	129	68	258	261	8
ACATTGGTG	BRDN00011	203	235	763	495	568	41
ACATTGTAG	BRDN00012	154	206	41	334	148	34
ACATTGTCCA	BRDN00012	302	208	1	528	349	15
ACATTGTCG	BRDN00012	137	11	82	644	258	0
ACATTGTCTC	BRDN00012	201	113	28	391	364	0
ACATTGTTG	BRDN00012	206	142	660	472	430	0
ACATTTAAAC	BRDN00011	235	145	491	344	397	25
ACATTTAAAT	BRDN00011	62	32	0	244	262	0
ACATTTAACG	BRDN00011	132	145	29	325	206	0
ACATTTACAA	BRDN00011	111	89	126	269	228	12
ACATTTACTC	BRDN00011	114	118	1	207	560	0
ACATTTAGG	BRDN00012	454	442	168	819	883	57
ACATTTATCT	BRDN00012	336	343	915	613	457	44
ACATTTCTCC	BRDN00012	655	765	1116	1166	1286	49
ACATTTGAG	BRDN00012	318	141	476	472	281	0
ACATTTGCA	BRDN00012	222	321	310	500	644	45
ACATTTGCG	BRDN00011	626	650	3222	1060	1032	64
ACATTTGTCA	BRDN00012	178	184	528	356	170	17
ACCAAAATG	BRDN00012	64	0	1	509	421	0
ACCAAACAA	BRDN00011	211	214	108	444	450	3
ACCAAACAG	BRDN00011	85	128	6	235	224	0
ACCAAACAT	BRDN00012	283	240	1328	545	2532	26
ACCAAACGT	BRDN00012	352	202	102	689	572	17
ACCAAACGTC	BRDN00012	140	7	0	586	259	0
ACCAAAGCC	BRDN00012	73	17	1	100	492	0
ACCAAAGCT	BRDN00011	90	68	0	191	251	42
ACCAAAGGC	BRDN00011	451	421	92	1142	692	63
ACCAAATATC	BRDN00012	495	451	1885	1001	1212	52
ACCAAATGA	BRDN00012	265	207	10	441	195	0
ACCAAATGG	BRDN00012	195	132	521	343	683	11
ACCAAATTG	BRDN00012	220	141	143	457	301	1
ACCAAATTG	BRDN00011	111	135	917	337	418	66
ACCAACACC	BRDN00012	341	79	1	668	334	0
ACCAACACTC	BRDN00012	427	510	581	899	826	24
ACCAACAGC	BRDN00012	96	125	140	197	176	61
ACCAACAGC	BRDN00011	292	292	7	597	510	8
ACCAACAGG	BRDN00011	115	44	4	286	120	0
ACCAACCAAT	BRDN00012	350	287	257	682	590	25
ACCAACCACA	BRDN00012	131	174	529	294	633	22
ACCAACCAG	BRDN00011	232	109	0	300	329	12
ACCAACCTTC	BRDN00011	95	137	1886	245	170	22
ACCAACGAG	BRDN00011	221	219	706	325	844	40
ACCAACGATC	BRDN00012	133	0	0	637	373	0
ACCAACGGA	BRDN00012	407	304	741	580	504	35
ACCAACGTAT	BRDN00012	321	147	568	916	615	124
ACCAACGTC	BRDN00011	400	197	381	901	746	41
ACCAACTCAA	BRDN00012	64	0	0	215	64	0
ACCAACTCCC	BRDN00011	203	166	660	320	443	32
ACCAACTGCA	BRDN00012	411	621	96	906	698	90
ACCAACTGCT	BRDN00012	257	291	1594	301	271	26
ACCAACTGG	BRDN00011	176	0	0	476	232	0
ACCAACTTCT	BRDN00012	230	134	190	565	645	8
ACCAAGAAG	BRDN00012	99	25	160	249	266	0
ACCAAGAAT	BRDN00012	242	193	156	566	641	7
ACCAAGAAT	BRDN00011	146	158	191	169	200	18
ACCAAGAGA	BRDN00012	230	7	2	625	672	0
ACCAAGATC	BRDN00012	128	74	38	139	24	0
ACCAAGCAC	BRDN00011	369	462	1979	846	498	83
ACCAAGCAC	BRDN00012	218	152	180	421	460	27
ACCAAGCAG	BRDN00011	196	267	417	636	367	4
ACCAAGCCA	BRDN00012	251	299	485	477	458	36
ACCAAGCCAT	BRDN00012	261	180	288	374	370	8
ACCAAGCCCT	BRDN00012	171	120	35	274	392	16
ACCAAGCGA	BRDN00011	258	253	308	527	435	18
ACCAAGCGC	BRDN00011	329	367	6093	521	541	89
ACCAAGCTAT	BRDN00011	171	231	639	323	210	6
ACCAAGGAC	BRDN00011	303	256	576	619	342	15
ACCAAGGAT	BRDN00011	385	444	1152	860	553	25
ACCAAGGCA	BRDN00011	524	549	1894	639	536	52
ACCAAGGCC	BRDN00012	579	504	104	1172	1331	31
ACCAAGGCC	BRDN00012	69	28	265	190	110	6
ACCAAGGCG	BRDN00012	144	0	1	387	220	0
ACCAAGGTA	BRDN00012	161	55	604	211	166	32
ACCAAGTAA	BRDN00012	372	396	500	716	560	40
ACCAAGTAG	BRDN00012	110	13	12	244	153	0
ACCAAGTAT	BRDN00012	336	219	44	309	355	17
ACCAAGTAT	BRDN00012	329	226	254	627	473	0
ACCAAGTTG	BRDN00012	69	0	3	297	73	0
ACCAAGTTT	BRDN00012	366	426	553	643	520	68
ACCAATACA	BRDN00011	78	4	0	98	194	0
ACCAATACTC	BRDN00012	5	0	0	219	0	0
ACCAATAGC	BRDN00011	109	7	0	377	18	0
ACCAATCATC	BRDN00011	421	295	551	618	673	6
ACCAATGAC	BRDN00012	209	121	21	267	683	25
ACCAATGGA	BRDN00011	203	91	233	279	454	0
ACCAATGGT	BRDN00011	261	185	208	443	436	20
ACCAATGTA	BRDN00012	438	345	266	705	704	63
ACCAATGTT	BRDN00012	357	193	253	706	1264	5
ACCAATTACA	BRDN00012	78	2	0	200	220	0
ACCAATTGC	BRDN00011	278	303	787	531	735	4
ACCACAAAG	BRDN00012	75	49	3638	80	82	113
ACCACAACCA	BRDN00012	213	157	221	420	399	9
ACCACAACTA	BRDN00012	206	172	0	404	162	32
ACCACAACTT	BRDN00011	96	74	256	266	286	22
ACCACAAGA	BRDN00012	236	309	568	370	550	23
ACCACAAGC	BRDN00011	355	305	311	624	412	86
ACCACAAGG	BRDN00011	300	356	1252	553	276	25
ACCACAATAT	BRDN00012	306	133	47	386	370	11
ACCACAATAT	BRDN00011	173	125	194	405	792	8
ACCACAATTA	BRDN00012	531	351	1033	872	914	108
ACCACACAA	BRDN00012	495	524	876	954	715	93
ACCACACAAT	BRDN00011	95	100	133	187	357	9
ACCACACAGT	BRDN00011	203	1	0	472	621	0
ACCACACCG	BRDN00011	315	285	86	540	433	4
ACCACACGG	BRDN00011	430	405	240	923	985	26
ACCACACTCA	BRDN00012	193	170	179	479	436	1
ACCACACTCA	BRDN00012	303	271	582	450	332	0
ACCACACTG	BRDN00012	509	341	1833	1220	966	48
ACCACACTGC	BRDN00011	325	43	22	539	453	8
ACCACACTTC	BRDN00011	463	504	1212	897	707	82
ACCACACTTC	BRDN00011	288	261	2402	630	353	58
ACCACACTTG	BRDN00012	401	316	2173	655	358	58
ACCACAGAA	BRDN00011	149	90	26	379	277	0
ACCACAGACT	BRDN00011	360	411	6907	776	1076	163
ACCACAGATT	BRDN00012	332	309	506	749	805	27
ACCACAGCAT	BRDN00012	166	103	707	240	488	32
ACCACAGCC	BRDN00012	456	369	1922	844	806	50
ACCACAGCCT	BRDN00011	146	111	7	250	70	53
ACCACAGGA	BRDN00011	125	127	101	217	199	0
ACCACAGGC	BRDN00012	349	284	599	682	862	34
ACCACAGGT	BRDN00012	146	107	46	266	323	0
ACCACAGTC	BRDN00012	195	111	48	243	246	11
ACCACAGTCC	BRDN00012	465	489	495	946	1375	54
ACCACAGTG	BRDN00012	445	368	1092	1045	1296	51
ACCACATACA	BRDN00011	437	383	214	786	1056	54
ACCACATACC	BRDN00012	225	317	495	443	441	65
ACCACATCCT	BRDN00011	154	116	53	231	213	0
ACCACATTCA	BRDN00012	243	182	125	485	452	60
ACCACATTCT	BRDN00012	136	116	182	334	266	12
ACCACCAAA	BRDN00012	486	469	438	888	807	28
ACCACCAAAT	BRDN00011	279	343	196	629	487	20
ACCACCAAC	BRDN00012	217	226	128	419	316	10
ACCACCAAG	BRDN00012	445	173	434	923	872	22
ACCACCACAT	BRDN00012	419	493	638	792	1348	9
ACCACCACG	BRDN00011	162	79	25	452	355	10
ACCACCACG	BRDN00011	529	337	754	1115	1033	7
ACCACCAGA	BRDN00012	273	268	171	510	431	51
ACCACCAGCT	BRDN00011	490	639	1015	986	434	70
ACCACCATAC	BRDN00012	304	162	150	1053	853	0
ACCACCATG	BRDN00011	124	147	504	225	296	34
ACCACCCACG	BRDN00012	220	76	82	325	382	38
ACCACCCAG	BRDN00012	95	107	0	287	119	4
ACCACCCAGC	BRDN00012	266	316	859	755	519	7
ACCACCCAGT	BRDN00012	426	477	732	709	704	54
ACCACCCCAG	BRDN00012	385	236	141	668	614	35
ACCACCCCCA	BRDN00012	213	134	627	398	317	21
ACCACCCCCG	BRDN00012	512	592	1367	974	780	61
ACCACCCGAC	BRDN00012	183	144	94	873	479	24
ACCACCCGG	BRDN00011	307	136	1318	801	585	51
ACCACCGAA	BRDN00012	183	171	136	441	268	5
ACCACCGAAT	BRDN00011	47	55	31	203	230	0
ACCACCGACC	BRDN00012	105	140	298	195	602	21
ACCACCGAG	BRDN00012	155	71	82	237	329	10
ACCACCGCCT	BRDN00012	632	582	1417	1180	1591	146
ACCACCGTGT	BRDN00012	253	186	210	347	226	22
ACCACCTCAA	BRDN00011	137	198	290	293	155	0
ACCACCTCCA	BRDN00011	310	275	190	528	391	33
ACCACGAAC	BRDN00012	471	484	707	902	984	29
ACCACGAGC	BRDN00012	148	71	0	317	234	0
ACCACGAGC	BRDN00011	232	257	2	250	250	0
ACCACGCAG	BRDN00011	172	235	332	221	484	41
ACCACGCCTT	BRDN00011	175	136	1163	582	452	80
ACCACGGAA	BRDN00012	434	349	618	952	975	65
ACCACGGAC	BRDN00012	233	179	64	353	309	5
ACCACGGAG	BRDN00011	351	260	673	475	556	12
ACCACGGCCC	BRDN00012	297	257	1342	543	325	39
ACCACGGGG	BRDN00012	158	42	92	589	294	0
ACCACGGGT	BRDN00012	345	194	476	683	531	0
ACCACGGTTC	BRDN00012	181	136	355	422	198	4
ACCACGTAT	BRDN00012	407	350	18	637	667	19
ACCACGTCCT	BRDN00011	152	104	0	253	171	0
ACCACGTGTC	BRDN00011	105	116	21	189	79	20
ACCACGTTTA	BRDN00011	167	72	1	390	219	0
ACCACTAATG	BRDN00012	432	352	428	830	693	30
ACCACTACAT	BRDN00012	185	109	55	431	432	0
ACCACTACCC	BRDN00011	536	674	1769	876	968	69
ACCACTACTA	BRDN00012	152	169	127	324	208	12
ACCACTAGCC	BRDN00012	293	180	373	467	374	49
ACCACTATCA	BRDN00011	239	187	451	411	242	42
ACCACTCACA	BRDN00012	374	187	133	757	486	8
ACCACTCACT	BRDN00011	347	428	441	662	538	4
ACCACTCCAA	BRDN00011	95	106	0	216	285	3
ACCACTCCAC	BRDN00011	131	59	1	128	117	0
ACCACTCTCT	BRDN00011	166	117	0	420	266	7
ACCACTGAAT	BRDN00011	81	7	0	493	447	0
ACCACTGACA	BRDN00011	359	279	7	827	899	56
ACCACTGACC	BRDN00011	199	178	0	304	245	18
ACCACTGACC	BRDN00012	251	278	445	362	432	31
ACCACTGACT	BRDN00011	161	184	403	369	352	0
ACCACTGAG	BRDN00012	16	0	0	452	12	0
ACCACTGCG	BRDN00012	240	427	1335	509	588	82
ACCACTGGA	BRDN00011	88	86	92	300	40	0
ACCACTGGA	BRDN00012	38	16	0	170	48	0
ACCACTGGCT	BRDN00011	227	269	385	574	250	58
ACCACTGGG	BRDN00012	94	147	20	203	226	6
ACCACTTATA	BRDN00012	311	279	280	519	460	11

ACCACTTCAC	BRDN00012	534	446	753	872	995	62
ACCAGAAATI	BRDN00012	244	244	106	280	393	18
ACCAGAAGA	BRDN00012	520	473	928	1060	1044	19
ACCAGAATC	BRDN00012	240	259	107	386	288	38
ACCAGAATG	BRDN00012	439	362	2016	827	673	65
ACCAGACAC	BRDN00012	435	235	566	773	598	31
ACCAGACAT	BRDN00012	617	448	378	964	989	36
ACCAGACCA	BRDN00011	127	52	38	315	285	0
ACCAGACCCT	BRDN00011	163	88	7	347	481	24
ACCAGACCG	BRDN00012	439	299	3821	926	967	102
ACCAGACCTT	BRDN00012	206	195	70	420	369	1
ACCAGACTTT	BRDN00012	184	138	152	332	530	0
ACCAGAGCA	BRDN00011	108	36	20	209	269	0
ACCAGAGCG	BRDN00012	414	511	4448	910	647	103
ACCAGAGTG	BRDN00012	169	85	137	428	216	0
ACCAGATCCA	BRDN00012	111	12	0	847	100	0
ACCAGATCCC	BRDN00012	313	254	99	412	797	1
ACCAGATGA	BRDN00011	192	151	115	304	148	64
ACCAGATTAT	BRDN00011	142	96	0	209	57	33
ACCAGCAAA	BRDN00011	79	110	32	282	342	9
ACCAGCAAA	BRDN00012	293	154	502	936	917	28
ACCAGCAAC	BRDN00011	186	193	1213	513	206	35
ACCAGCAAG	BRDN00012	364	433	798	538	589	24
ACCAGCAAT	BRDN00012	181	129	158	433	289	9
ACCAGCACA	BRDN00012	171	203	83	324	238	13
ACCAGCACC	BRDN00011	107	1	1	637	1115	0
ACCAGCACCT	BRDN00011	417	108	418	1008	487	6
ACCAGCACTA	BRDN00012	279	330	167	445	383	23
ACCAGCAGA	BRDN00011	113	107	54	205	137	47
ACCAGCAGG	BRDN00012	118	162	77	323	228	19
ACCAGCAGTA	BRDN00012	273	253	307	393	277	0
ACCAGCATTC	BRDN00011	128	157	26	210	321	0
ACCAGCGAG	BRDN00012	186	158	2	369	189	0
ACCAGCGATA	BRDN00012	356	406	1490	472	357	67
ACCAGCGCA	BRDN00012	435	453	255	692	573	33
ACCAGCGCTC	BRDN00012	263	221	528	476	226	64
ACCAGCGGG	BRDN00011	548	434	426	1070	982	132
ACCAGCGGG	BRDN00012	237	152	95	283	268	22
ACCAGCGTG	BRDN00012	291	210	238	351	348	53
ACCAGCTCG	BRDN00011	481	426	585	1058	879	48
ACCAGCTGA	BRDN00012	313	268	1728	761	640	92
ACCAGCTGCA	BRDN00011	264	11	1	335	19	0
ACCAGCTGCT	BRDN00011	308	359	62	509	684	21
ACCAGCTGTC	BRDN00011	591	357	1808	938	901	55
ACCAGCTTGC	BRDN00012	167	190	417	305	262	111
ACCAGGAAC	BRDN00012	121	147	2006	384	490	57
ACCAGGACA	BRDN00012	465	318	1543	1062	906	4
ACCAGGACA	BRDN00012	192	191	597	283	269	20
ACCAGGACC	BRDN00012	158	155	292	291	150	90
ACCAGGACC	BRDN00011	285	335	177	683	800	41
ACCAGGATG	BRDN00011	280	205	258	460	428	6
ACCAGGCAA	BRDN00012	468	390	719	1021	781	54
ACCAGGCAC	BRDN00012	62	0	0	364	11	0
ACCAGGCCTC	BRDN00011	193	204	1050	462	669	6
ACCAGGCGA	BRDN00012	143	84	522	254	231	19
ACCAGGCGG	BRDN00012	191	134	42	484	540	12
ACCAGGCTA	BRDN00012	379	171	247	577	844	49
ACCAGGCTCT	BRDN00011	558	572	1469	938	1345	24
ACCAGGCTG	BRDN00011	379	95	206	887	233	12
ACCAGGGAC	BRDN00011	130	86	121	185	186	0
ACCAGGGAC	BRDN00012	148	130	88	252	258	8
ACCAGGGCT	BRDN00012	209	96	215	364	376	8
ACCAGGGGA	BRDN00012	480	439	408	881	661	24
ACCAGGGGC	BRDN00012	301	299	46	577	594	6
ACCAGGGTC	BRDN00012	163	119	77	772	636	17
ACCAGGTACI	BRDN00012	145	117	10	366	287	30
ACCAGGTATI	BRDN00012	174	24	1	360	490	0
ACCAGGTATI	BRDN00011	125	6	0	403	430	0
ACCAGGTATI	BRDN00011	295	391	161	651	997	34
ACCAGGTATI	BRDN00012	303	289	1677	442	427	11
ACCAGGTCA	BRDN00011	291	193	340	423	444	5
ACCAGGTCG	BRDN00012	85	110	9	193	226	17
ACCAGGTCG	BRDN00011	382	220	1940	638	431	48
ACCAGGTCTA	BRDN00011	320	338	137	732	527	43
ACCAGGTCTC	BRDN00012	513	630	3272	965	723	82
ACCAGGTGA	BRDN00011	155	133	83	230	135	1
ACCAGGTGC	BRDN00011	676	642	1486	1079	1262	108
ACCAGGTTTC	BRDN00011	425	418	318	863	1116	32
ACCAGTAAG	BRDN00012	137	184	1099	197	196	8
ACCAGTACTC	BRDN00012	238	118	86	498	426	0
ACCAGTAGTA	BRDN00012	208	186	115	405	487	52
ACCAGTATCC	BRDN00012	427	403	1568	757	551	92
ACCAGTATCT	BRDN00011	463	273	4927	978	1114	116
ACCAGTCAGA	BRDN00011	418	345	1648	895	867	59
ACCAGTGAA	BRDN00011	153	96	87	343	205	0
ACCAGTGAG	BRDN00012	587	396	1176	1339	1399	56
ACCAGTGATA	BRDN00011	344	231	614	487	496	6
ACCAGTGCA	BRDN00011	211	153	135	515	375	0
ACCAGTGCG	BRDN00012	256	103	412	348	391	0
ACCAGTTAAA	BRDN00011	150	65	23	314	330	17
ACCAGTTAAT	BRDN00012	198	201	255	376	300	4
ACCAGTTATT	BRDN00011	435	318	693	818	546	11
ACCAGTTCAT	BRDN00011	353	416	5446	786	566	88
ACCAGTTCGA	BRDN00011	92	3	9	503	1	0
ACCAGTTGCC	BRDN00012	198	131	468	338	950	41
ACCAGTTGTA	BRDN00012	167	113	30	365	198	5
ACCAGTTTGA	BRDN00012	412	497	125	688	661	50
ACCATAAAGA	BRDN00011	194	295	647	429	625	72
ACCATAATCA	BRDN00011	276	54	1	647	623	0
ACCATAATGC	BRDN00012	148	40	145	383	540	10
ACCATACAAC	BRDN00012	292	184	508	504	662	25
ACCATACAGA	BRDN00012	593	398	160	902	534	17
ACCATACCAA	BRDN00011	467	533	407	865	749	34
ACCATACCAT	BRDN00012	195	361	326	449	249	8
ACCATACCAT	BRDN00011	247	263	345	506	793	20
ACCATACCCC	BRDN00012	513	503	1514	731	911	44
ACCATAGAAT	BRDN00012	466	383	744	857	577	63
ACCATAGCCC	BRDN00012	220	192	442	521	450	34
ACCATAGGA	BRDN00011	91	58	1	145	106	0
ACCATATACA	BRDN00012	98	19	97	682	614	0
ACCATATAGT	BRDN00011	221	178	769	497	430	7
ACCATATCAC	BRDN00012	636	543	1277	1140	1232	36
ACCATATCAT	BRDN00011	236	250	1378	381	313	15
ACCATATGAC	BRDN00011	186	134	461	427	499	13
ACCATATTCG	BRDN00012	434	358	465	709	419	43
ACCATCAACA	BRDN00011	254	160	213	517	484	5
ACCATCACAT	BRDN00011	253	256	102	661	570	0
ACCATCACCC	BRDN00012	498	432	2579	956	701	13
ACCATCAGA	BRDN00011	412	382	128	875	637	36
ACCATCAGTC	BRDN00011	141	10	0	337	141	0
ACCATCATAA	BRDN00012	93	114	237	280	142	0
ACCATCCAGA	BRDN00012	375	361	426	957	1208	30
ACCATCCCGT	BRDN00012	365	360	611	682	789	22
ACCATCCTAC	BRDN00012	543	637	3004	1101	867	88
ACCATCCTTA	BRDN00012	418	487	189	601	540	55
ACCATCGCCA	BRDN00011	144	137	130	327	573	11
ACCATCGCCC	BRDN00012	313	201	468	421	586	36
ACCATCGTCT	BRDN00012	566	480	433	1059	1052	83
ACCATCTAAA	BRDN00011	148	102	81	255	202	0
ACCATCTACC	BRDN00012	472	468	205	919	1152	55
ACCATCTCGA	BRDN00012	492	560	760	952	671	39
ACCATCTGGA	BRDN00012	248	306	447	548	881	0
ACCATCTGGA	BRDN00012	405	348	1037	918	733	75
ACCATCTGTA	BRDN00012	236	240	168	481	583	44
ACCATCTTAC	BRDN00011	242	1	1	621	501	0
ACCATGAATC	BRDN00011	610	467	646	919	1130	8
ACCATGACCA	BRDN00012	291	237	507	625	1076	9
ACCATGACGI	BRDN00012	370	422	1030	814	583	63
ACCATGAGG	BRDN00012	202	163	103	508	341	0
ACCATGATAC	BRDN00012	109	145	620	286	426	20
ACCATGATAT	BRDN00011	52	65	18	133	53	0
ACCATGATGA	BRDN00011	128	10	0	266	176	0
ACCATGATGI	BRDN00012	504	150	125	1094	469	11
ACCATGCATC	BRDN00012	226	183	52	333	336	5
ACCATGCCCC	BRDN00011	367	262	172	682	733	14
ACCATGCTGC	BRDN00012	478	560	1483	952	1101	84
ACCATGGCTA	BRDN00011	268	274	374	490	405	25
ACCATGGCTT	BRDN00011	406	416	631	877	712	32
ACCATGGGC	BRDN00011	162	44	248	390	169	0
ACCATGGGG	BRDN00011	432	397	1955	679	723	45
ACCATGGGG	BRDN00012	340	366	293	663	448	49
ACCATGGTAI	BRDN00011	177	52	212	443	532	48
ACCATGTAAA	BRDN00011	145	105	38	362	92	1
ACCATGTAGT	BRDN00011	172	265	697	249	319	4
ACCATGTCCG	BRDN00011	112	95	27	334	456	29
ACCATGTCTA	BRDN00012	401	292	2021	763	799	54
ACCATGTGTC	BRDN00011	264	347	169	596	704	23
ACCATGTTTG	BRDN00011	346	245	148	643	473	22
ACCATTAGCC	BRDN00012	167	108	241	285	163	3
ACCATTAGCC	BRDN00011	547	414	1696	954	1125	21
ACCATTCTCA	BRDN00012	226	231	704	329	371	35
ACCATTGCAA	BRDN00012	109	59	7	280	181	0
ACCATTGCCC	BRDN00012	263	294	166	409	248	32
ACCATTGCTC	BRDN00012	318	258	876	438	309	18
ACCATTGGG	BRDN00012	105	83	0	324	130	0
ACCCAAACAT	BRDN00012	183	1	0	377	357	0
ACCCAAAGCC	BRDN00011	183	40	101	308	111	0
ACCCAAAGG	BRDN00012	192	85	25	391	420	0
ACCCAACACC	BRDN00012	151	192	269	241	142	12
ACCCAACAGT	BRDN00012	200	258	170	323	252	6
ACCCAACCAG	BRDN00011	226	316	233	527	521	44
ACCCAACCAG	BRDN00012	203	249	84	380	192	23
ACCCAACCGA	BRDN00011	218	149	701	483	511	28
ACCCAAGACA	BRDN00011	96	12	0	218	130	0
ACCCAAGCAC	BRDN00011	478	326	1580	798	729	36
ACCCAAGCCA	BRDN00012	544	695	791	1101	1423	59
ACCCAAGCGT	BRDN00012	514	567	1552	953	982	41
ACCCAAGTAC	BRDN00011	369	277	363	551	615	94
ACCCAATATG	BRDN00011	195	167	187	387	271	0
ACCCAATGCA	BRDN00012	89	130	59	233	201	0
ACCCAATGTC	BRDN00012	577	566	455	905	1160	76
ACCCACAAAC	BRDN00012	131	216	247	385	425	0
ACCCACAAGA	BRDN00011	278	294	98	545	212	22
ACCCACAGCA	BRDN00011	183	177	1033	350	382	6
ACCCACAGCA	BRDN00011	156	131	77	367	279	0
ACCCACAGCC	BRDN00012	193	186	33	259	228	36
ACCCACAGGI	BRDN00012	325	2	0	811	231	0
ACCCACAGGI	BRDN00012	125	38	51	228	175	0
ACCCACATAG	BRDN00011	173	159	35	325	242	16
ACCCACATAG	BRDN00011	181	123	83	410	514	9
ACCCACATGA	BRDN00011	331	298	7605	655	632	185
ACCCACCAGT	BRDN00012	346	55	7	755	749	0
ACCCACCTAA	BRDN00011	544	385	276	856	755	162
ACCCACGAAC	BRDN00011	176	208	518	455	382	19
ACCCACGGCT	BRDN00011	325	194	796	557	485	0
ACCCACGGCT	BRDN00012	211	198	610	444	539	48
ACCCACTACC	BRDN00011	232	185	1068	406	213	17
ACCCACTATA	BRDN00011	376	356	42	660	757	105
ACCCACTTGT	BRDN00012	379	262	582	706	516	35
ACCCAGAAG	BRDN00011	214	156	350	348	468	13
ACCCAGAATA	BRDN00012	163	281	1	283	162	4
ACCCAGACAC	BRDN00012	271	156	158	454	660	7
ACCCAGACAC	BRDN00011	152	55	173	225	92	0
ACCCAGACCC	BRDN00011	399	509	2924	976	689	94
ACCCAGACGI	BRDN00011	545	363	341	1079	1250	40
ACCCAGACTA	BRDN00011	160	0	0	295	133	0
ACCCAGAGTA	BRDN00011	33	133	14	118	90	0
ACCCAGCACT	BRDN00011	134	1	0	1117	1109	0
ACCCAGCAGI	BRDN00011	160	56	82	215	370	0
ACCCAGCATC	BRDN00011	522	498	3185	920	1326	72
ACCCAGCCGA	BRDN00012	289	306	15	506	542	37
ACCCAGCCGA	BRDN00011	613	762	1263	955	591	69
ACCCAGCTGC	BRDN00011	190	163	276	434	415	25
ACCCAGGAC	BRDN00012	551	379	565	1007	801	45
ACCCAGGCAT	BRDN00012	622	409	248	930	922	82
ACCCAGGCCC	BRDN00011	169	153	0	293	190	7
ACCCAGGCCT	BRDN00011	344	377	1768	759	1505	34
ACCCAGGGA	BRDN00011	316	419	329	530	312	29
ACCCAGGGA	BRDN00011	232	143	306	416	445	0
ACCCAGTAAC	BRDN00012	211	298	1213	257	316	25
ACCCAGTCCA	BRDN00011	171	156	41	393	428	0
ACCCAGTCCG	BRDN00011	256	229	0	505	839	15
ACCCAGTCCG	BRDN00012	191	200	398	285	138	6
ACCCAGTCGT	BRDN00012	120	73	41	178	141	28
ACCCAGTTCA	BRDN00011	225	203	372	471	763	50
ACCCAGTTCT	BRDN00012	171	194	89	325	214	66
ACCCATAGGA	BRDN00012	357	344	323	680	272	77
ACCCATATAT	BRDN00012	435	560	979	1115	1277	55
ACCCATCAGC	BRDN00011	474	408	2085	762	748	28
ACCCATCATG	BRDN00011	256	193	153	410	511	11
ACCCATCATT	BRDN00011	265	223	140	555	788	9
ACCCATCGCA	BRDN00011	504	65	58	1362	1242	0
ACCCATCTTA	BRDN00011	81	106	34	253	308	6
ACCCATGAGT	BRDN00012	623	449	967	740	1238	73
ACCCATGCAT	BRDN00012	137	3	0	701	399	0
ACCCATTAAA	BRDN00011	73	0	0	295	207	0
ACCCATTCGC	BRDN00011	329	275	373	443	569	37
ACCCATTGAC	BRDN00012	540	627	843	1094	1122	72
ACCCCAAAGC	BRDN00011	289	259	249	472	387	12
ACCCCAAATC	BRDN00012	689	558	485	1090	1015	60
ACCCCAATAC	BRDN00012	423	476	286	989	1581	152
ACCCCAATGT	BRDN00011	140	162	153	361	200	52
ACCCCACCAC	BRDN00012	390	370	656	609	932	76
ACCCCACCCT	BRDN00012	155	251	110	454	520	37
ACCCCAGAGA	BRDN00012	102	90	0	189	76	23
ACCCCAGCAT	BRDN00012	545	419	1113	891	1091	37
ACCCCAGCGA	BRDN00011	298	371	823	480	376	145
ACCCCAGGAA	BRDN00012	374	228	91	858	632	19
ACCCCAGGGA	BRDN00011	319	348	87	663	517	0
ACCCCATCGT	BRDN00011	336	271	183	603	1161	0
ACCCCATGCC	BRDN00012	187	146	13	512	567	12
ACCCCCACAT	BRDN00011	221	278	582	563	467	36
ACCCCCAGCA	BRDN00012	81	32	41	221	113	0
ACCCCCAGGT	BRDN00011	277	397	189	584	516	108
ACCCCCAGTG	BRDN00012	111	12	0	303	7	0
ACCCCCCAAT	BRDN00011	253	114	244	416	412	19
ACCCCCCAGA	BRDN00012	104	107	707	205	237	40
ACCCCCCTTC	BRDN00011	254	238	885	508	538	35
ACCCCCGAAC	BRDN00011	351	415	3767	704	454	53
ACCCCCTCGA	BRDN00011	455	515	661	790	799	49
ACCCCCTTCT	BRDN00012	162	271	0	422	491	0
ACCCCGATCA	BRDN00012	185	206	35	384	360	41
ACCCCGCCTG	BRDN00012	540	583	999	1071	1337	28
ACCCCGGTCA	BRDN00012	146	82	126	226	273	0
ACCCCGTAGT	BRDN00011	157	148	61	187	131	25
ACCCCTGCAC	BRDN00011	420	212	975	809	831	12
ACCCCTGCAG	BRDN00012	437	499	2981	798	377	130
ACCCCTTACA	BRDN00011	246	205	719	402	606	29
ACCCGAAAAI	BRDN00011	137	188	710	236	220	52
ACCCGAATGI	BRDN00011	311	255	256	589	796	0
ACCCGAATTC	BRDN00011	370	347	1092	794	536	6
ACCCGACGG	BRDN00012	168	156	0	408	240	12
ACCCGAGCAA	BRDN00011	184	127	1392	269	210	28
ACCCGAGCAI	BRDN00012	478	423	3196	770	643	42
ACCCGAGTGA	BRDN00012	374	256	936	677	888	48
ACCCGATAAC	BRDN00012	171	187	28	299	294	0
ACCCGATAAT	BRDN00012	505	402	998	949	1037	26
ACCCGATGGT	BRDN00012	108	42	0	209	147	0
ACCCGCACCT	BRDN00011	370	332	505	635	833	32
ACCCGCAGAA	BRDN00011	195	153	3	310	167	6
ACCCGCAGAI	BRDN00012	142	102	13	243	33	50
ACCCGCCACT	BRDN00011	117	97	158	234	152	0
ACCCGCCAGT	BRDN00012	469	619	1504	1040	990	6
ACCCGCGAAI	BRDN00012	192	30	0	356	219	0
ACCCGCGAAI	BRDN00012	486	398	1387	890	1419	18
ACCCGCGACC	BRDN00011	655	447	1874	920	978	41
ACCCGCGAGT	BRDN00012	177	141	84	297	185	7
ACCCGCGTGA	BRDN00012	129	140	19	303	433	0
ACCCGCTACT	BRDN00012	139	134	4095	405	615	57
ACCCGGAAGT	BRDN00011	377	386	1373	619	725	51
ACCCGGAATI	BRDN00012	627	670	663	1023	679	88
ACCCGGCACT	BRDN00012	78	20	182	748	576	0
ACCCGGCGTI	BRDN00011	166	36	1174	317	318	45
ACCCGGCTGT	BRDN00012	188	122	264	203	278	0
ACCCGGGAA	BRDN00011	493	493	766	1077	1217	72
ACCCGGGCAT	BRDN00011	149	137	319	221	67	31
ACCCGGGCAT	BRDN00012	190	124	85	217	143	14
ACCCGGGCG	BRDN00012	283	190	1166	558	727	21
ACCCGGGCGT	BRDN00012	278	350	1968	783	361	44
ACCCGGGCTA	BRDN00011	89	33	2	192	208	0
ACCCGTAGG	BRDN00012	260	320	352	584	456	49
ACCCGTCTAC	BRDN00011	152	60	164	331	510	7
ACCCGTGAAI	BRDN00011	525	281	275	833	655	36
ACCCGTGATT	BRDN00011	122	98	817	205	309	2
ACCCGTGTAC	BRDN00011	281	281	136	393	405	32
ACCCGTGTCA	BRDN00012	267	189	43	433	652	11
ACCCGTTAGC	BRDN00012	614	373	428	1092	1167	21
ACCCGTTGCA	BRDN00012	220	163	38	487	602	25
ACCCGTTGCT	BRDN00012	140	131	80	226	112	16
ACCCTAATTC	BRDN00012	487	393	23	977	916	26
ACCCTACCTG	BRDN00011	156	27	99	613	343	0
ACCCTACCTT	BRDN00011	202	166	26	319	158	25
ACCCTAGAAA	BRDN00012	298	169	455	442	375	44
ACCCTAGACA	BRDN00012	363	395	243	712	650	41
ACCCTAGAGA	BRDN00012	171	334	228	314	296	27
ACCCTATATT	BRDN00011	50	18	0	153	53	0
ACCCTCACGA	BRDN00011	241	185	318	890	1016	45
ACCCTCACTG	BRDN00011	259	207	793	539	520	13
ACCCTCATCC	BRDN00012	255	153	9	531	704	6
ACCCTCATGA	BRDN00011	367	509	443	729	562	104
ACCCTCCAAT	BRDN00012	406	516	366	674	836	48
ACCCTCCGAA	BRDN00012	513	655	534	730	703	64
ACCCTCCTAT	BRDN00011	192	219	566	334	606	48
ACCCTCGAAC	BRDN00012	303	276	1022	595	434	42
ACCCTCGGAC	BRDN00011	105	150	1485	241	138	18
ACCCTCTCAG	BRDN00012	166	207	86	449	360	21
ACCCTCTGCC	BRDN00011	189	171	58	405	197	15
ACCCTCTTAC	BRDN00011	180	125	221	401	585	32
ACCCTGAAGA	BRDN00011	132	224	348	263	322	25
ACCCTGAAGT	BRDN00012	502	356	787	949	1435	52
ACCCTGAATC	BRDN00012	63	19	6	538	323	0
ACCCTGAGCC	BRDN00012	509	394	581	790	1028	48
ACCCTGAGCC	BRDN00012	272	238	338	559	450	21
ACCCTGCGCT	BRDN00011	180	170	221	338	314	7
ACCCTGCTGC	BRDN00011	79	134	10	248	505	9
ACCCTGCTGC	BRDN00012	84	14	26	256	220	7
ACCCTGGAG	BRDN00011	259	387	0	719	591	22
ACCCTGGCCA	BRDN00012	132	109	256	366	384	20
ACCCTGGCTA	BRDN00012	211	306	229	555	549	32
ACCCTGGGG	BRDN00012	459	392	1710	720	886	78
ACCCTGGTCA	BRDN00012	404	280	632	696	856	35
ACCCTGGTTA	BRDN00011	173	299	928	317	347	0
ACCCTGTATC	BRDN00012	489	494	824	900	565	79
ACCCTGTCGA	BRDN00012	168	96	118	296	421	62
ACCCTGTTGA	BRDN00012	532	626	1425	808	1310	27
ACCCTTACAT	BRDN00011	159	230	2	408	234	39
ACCCTTATAC	BRDN00011	20	6	0	366	128	0
ACCCTTCACC	BRDN00011	321	264	114	690	823	22
ACCCTTGTGT	BRDN00011	162	17	129	1042	391	6
ACCCTTTAAT	BRDN00012	145	1	0	309	453	0
ACCCTTTACC	BRDN00011	277	364	402	481	684	9
ACCCTTTCCG	BRDN00012	495	455	1584	678	1120	49
ACCGAAACAI	BRDN00011	451	349	291	917	944	32
ACCGAAACGT	BRDN00011	124	56	101	145	142	0
ACCGAAAGA	BRDN00012	400	380	834	662	975	81
ACCGAAGCAT	BRDN00011	247	345	893	394	823	99
ACCGAATCAT	BRDN00011	130	124	167	328	269	16
ACCGAATGG	BRDN00012	131	174	97	287	460	10
ACCGACAGC	BRDN00011	414	379	164	802	945	190
ACCGACAGG	BRDN00011	370	349	670	529	425	50
ACCGACCAGI	BRDN00012	192	185	122	436	274	2
ACCGACCCCC	BRDN00011	106	187	104	290	231	0
ACCGACCTAA	BRDN00011	230	40	30	565	617	0
ACCGACGCG	BRDN00012	348	397	864	572	687	18
ACCGACGGT	BRDN00012	137	98	454	271	196	7
ACCGACTCAC	BRDN00012	102	135	939	363	360	17
ACCGACTCGA	BRDN00012	165	168	12	304	843	20
ACCGACTGG	BRDN00012	445	377	264	1073	695	4
ACCGACTGG	BRDN00011	269	209	241	552	510	17
ACCGACTGG	BRDN00012	494	326	2520	944	483	32
ACCGAGAAG	BRDN00012	80	57	93	227	692	20
ACCGAGACA	BRDN00011	182	254	648	430	132	21
ACCGAGACTT	BRDN00011	94	175	8	185	20	30
ACCGAGCAG	BRDN00011	190	101	2933	270	247	66
ACCGAGCGC	BRDN00012	566	372	1851	869	554	80
ACCGAGCTCT	BRDN00012	188	144	344	427	336	12
ACCGAGGGA	BRDN00012	490	495	639	754	918	104
ACCGAGTCAT	BRDN00011	417	184	959	519	370	19
ACCGATATGA	BRDN00012	284	175	113	491	810	36
ACCGATCTAT	BRDN00012	238	89	0	344	336	11
ACCGATGATI	BRDN00012	76	0	0	493	423	0
ACCGATGGC	BRDN00012	334	245	284	697	854	47
ACCGCAAGG	BRDN00011	272	189	658	502	336	11
ACCGCAATAT	BRDN00012	443	332	232	734	501	43
ACCGCACAAT	BRDN00012	79	7	33	532	622	14
ACCGCCAATC	BRDN00012	134	256	95	333	715	12
ACCGCCACCA	BRDN00011	136	123	115	286	171	0
ACCGCCCCAT	BRDN00012	323	287	187	664	650	51
ACCGCCGCCC	BRDN00011	484	502	71	682	568	37
ACCGCCTCCG	BRDN00011	19	3	0	310	54	0
ACCGCGAGC	BRDN00011	435	303	569	608	539	8
ACCGCGAGG	BRDN00011	116	86	135	148	336	5
ACCGCGCCA	BRDN00011	101	128	448	119	256	35
ACCGCTAGGT	BRDN00012	100	0	0	630	223	0
ACCGCTCACC	BRDN00012	354	222	341	822	644	0
ACCGCTCATA	BRDN00012	354	281	749	636	711	32
ACCGCTCTTC	BRDN00012	246	228	1417	511	442	58
ACCGCTGAA	BRDN00011	140	61	53	219	321	0
ACCGGAATCT	BRDN00012	124	19	0	553	229	0
ACCGGACATT	BRDN00011	196	241	298	439	166	15
ACCGGACGT	BRDN00012	224	148	1136	494	448	42
ACCGGAGAT	BRDN00012	232	319	253	451	334	17
ACCGGAGCA	BRDN00012	253	210	114	353	583	47
ACCGGAGCT	BRDN00011	44	0	0	208	0	0
ACCGGATGA	BRDN00012	491	413	1507	943	574	9
ACCGGATTAT	BRDN00012	459	308	37	850	597	30
ACCGGATTGT	BRDN00011	191	0	1	494	678	0
ACCGGCACAI	BRDN00011	210	185	778	370	284	23
ACCGGCACAT	BRDN00011	192	113	1649	260	181	22
ACCGGCAGC	BRDN00012	124	60	121	206	190	33
ACCGGCAGG	BRDN00011	83	35	1040	223	67	23
ACCGGCGCAT	BRDN00012	456	447	851	662	559	12
ACCGGCGCTA	BRDN00012	73	15	0	222	128	0
ACCGGCGGC	BRDN00011	102	83	0	385	261	4
ACCGGCGGC	BRDN00012	118	216	745	217	92	102
ACCGGCGGG	BRDN00011	484	389	1608	824	803	29
ACCGGCGGT	BRDN00012	248	261	97	454	734	11
ACCGGCTTGI	BRDN00012	83	105	10	294	132	71
ACCGGGAAC	BRDN00011	167	109	180	271	219	0
ACCGGGACA	BRDN00011	163	70	12	622	482	0
ACCGGGACA	BRDN00011	192	95	13	235	215	9
ACCGGGAGA	BRDN00012	209	116	1	348	298	4
ACCGGGGAA	BRDN00012	132	205	127	269	142	4
ACCGGTACG	BRDN00012	320	25	32	535	676	0
ACCGGTCCAC	BRDN00012	308	235	810	460	245	21
ACCGGTCCAC	BRDN00012	132	85	122	364	195	0
ACCGGTCCGA	BRDN00012	516	477	522	1029	583	68
ACCGGTGAT	BRDN00011	237	172	205	374	391	14
ACCGGTGTA	BRDN00011	249	168	565	449	646	6
ACCGTAACTA	BRDN00011	136	26	0	239	150	0
ACCGTACAGI	BRDN00011	468	485	198	954	941	19
ACCGTAGCCC	BRDN00011	69	20	6	174	72	0
ACCGTAGCTA	BRDN00011	98	115	1612	287	93	44
ACCGTATCAC	BRDN00011	228	223	102	429	469	10
ACCGTATCTT	BRDN00011	321	198	857	497	407	21
ACCGTATGTC	BRDN00011	193	112	541	518	217	8
ACCGTATGTT	BRDN00011	167	168	136	397	392	38
ACCGTCACAA	BRDN00011	224	209	274	223	284	13
ACCGTCACAA	BRDN00012	83	51	138	178	102	27
ACCGTCACCG	BRDN00012	282	365	758	851	631	27
ACCGTCACTG	BRDN00011	475	362	1320	751	663	39
ACCGTCAGCA	BRDN00011	275	129	0	555	462	4
ACCGTCCACC	BRDN00012	274	245	361	416	512	29
ACCGTCCAGC	BRDN00012	180	170	88	529	617	4
ACCGTCCTTG	BRDN00012	208	199	270	379	374	25
ACCGTCCTTT	BRDN00011	33	28	2	90	88	0
ACCGTCGAAI	BRDN00012	412	320	2169	798	934	61
ACCGTCGATC	BRDN00011	336	250	209	599	695	85
ACCGTCGCTC	BRDN00012	326	367	443	557	523	5
ACCGTCTACG	BRDN00012	160	108	0	337	178	0
ACCGTGACTC	BRDN00011	132	172	21	311	421	1
ACCGTGCAG	BRDN00012	288	279	392	352	187	45
ACCGTGCATT	BRDN00011	95	0	0	342	138	0
ACCGTGCCCT	BRDN00012	290	187	435	372	868	24
ACCGTGCTGI	BRDN00011	185	119	75	386	437	44

ACCGTGGAA	BRDN00012	380	275	124	769	670	0
ACCGTGGCC	BRDN00012	430	454	399	978	1031	26
ACCGTTACTG	BRDN00012	562	427	2943	1125	1170	170
ACCTAAAAA	BRDN00012	253	302	630	659	331	3
ACCTAAAGA	BRDN00012	389	253	940	598	780	13
ACCTAAAGA	BRDN00011	181	106	26	326	219	0
ACCTAAAGG	BRDN00011	118	57	0	152	156	0
ACCTAAGCCC	BRDN00012	175	154	179	318	224	16
ACCTAAGCG	BRDN00012	226	199	325	305	819	0
ACCTAAGGA	BRDN00012	185	108	97	316	260	16
ACCTAATAA	BRDN00011	120	145	280	228	227	16
ACCTAATGA	BRDN00011	63	17	73	676	408	0
ACCTACAATA	BRDN00012	194	1	0	262	16	0
ACCTACAGA	BRDN00012	435	388	475	604	752	48
ACCTACAGAT	BRDN00011	169	173	66	228	187	52
ACCTACAGG	BRDN00011	368	206	1011	534	501	28
ACCTACCAAT	BRDN00011	216	135	10	361	409	13
ACCTACCACG	BRDN00011	185	300	649	384	666	10
ACCTACCATA	BRDN00011	230	136	187	445	422	0
ACCTACCATG	BRDN00011	242	255	370	425	376	37
ACCTACCATG	BRDN00012	60	118	4	175	160	13
ACCTACCCGA	BRDN00011	132	160	232	203	35	16
ACCTACCCTT	BRDN00012	287	285	1406	497	350	45
ACCTACCCTT	BRDN00011	171	101	18	319	480	10
ACCTACCGA	BRDN00012	151	11	15	311	306	0
ACCTACCGTG	BRDN00011	192	209	495	389	649	49
ACCTACCTGG	BRDN00011	388	404	2578	576	340	39
ACCTACGGG	BRDN00012	210	104	370	250	222	108
ACCTACGGTT	BRDN00011	288	209	82	372	543	0
ACCTACGTGT	BRDN00012	343	321	251	833	403	15
ACCTACTATA	BRDN00011	172	168	908	369	451	11
ACCTACTCCC	BRDN00012	439	461	508	744	826	15
ACCTACTGA	BRDN00012	153	167	269	372	455	4
ACCTACTGGT	BRDN00011	156	180	229	352	253	5
ACCTAGAAC	BRDN00012	98	234	2	248	171	0
ACCTAGAACT	BRDN00011	184	122	5	363	520	40
ACCTAGCCAC	BRDN00011	593	554	1727	1196	1297	18
ACCTAGGGG	BRDN00012	202	179	29	424	798	0
ACCTAGGTA	BRDN00011	74	127	228	306	249	0
ACCTAGGTC	BRDN00012	339	381	732	496	644	28
ACCTAGGTCT	BRDN00012	178	166	4149	396	234	148
ACCTATAACT	BRDN00011	332	263	39	728	635	19
ACCTATAATC	BRDN00012	569	587	383	948	811	0
ACCTATACAG	BRDN00011	187	124	12	288	486	0
ACCTATAGCT	BRDN00012	359	478	7251	703	1018	146
ACCTATCACC	BRDN00012	241	128	42	545	519	0
ACCTATGAA	BRDN00012	444	320	876	725	803	119
ACCTATGAC	BRDN00011	98	186	10	188	208	0
ACCTATGACC	BRDN00011	365	163	3222	749	442	31
ACCTATGAG	BRDN00011	409	517	703	739	577	33
ACCTATGAG	BRDN00011	114	65	222	293	204	14
ACCTATGATC	BRDN00012	224	229	226	339	319	12
ACCTATGCAC	BRDN00011	229	163	449	470	68	0
ACCTATGGC	BRDN00011	117	118	1	222	138	0
ACCTATTGTA	BRDN00011	243	189	115	420	406	18
ACCTATTGTC	BRDN00012	603	628	2729	885	946	60
ACCTCAAAA	BRDN00012	175	240	265	361	274	0
ACCTCAAACA	BRDN00011	112	237	4084	273	87	85
ACCTCAACAA	BRDN00011	238	158	404	475	369	16
ACCTCAAGAC	BRDN00011	450	430	420	735	913	34
ACCTCAAGCC	BRDN00012	477	403	315	903	1144	20
ACCTCAATGC	BRDN00011	214	129	289	388	442	25
ACCTCACAGA	BRDN00012	433	411	448	1053	1427	51
ACCTCACGCC	BRDN00011	122	1	0	331	222	0
ACCTCACGGC	BRDN00012	453	433	572	896	1056	25
ACCTCAGAGI	BRDN00012	568	437	829	1059	1270	30
ACCTCAGAGT	BRDN00011	119	103	1	230	541	0
ACCTCAGCAA	BRDN00011	217	163	103	346	234	5
ACCTCAGCGA	BRDN00012	274	418	365	695	476	6
ACCTCAGCGA	BRDN00011	107	122	317	264	394	0
ACCTCAGCTA	BRDN00011	225	276	1855	472	953	12
ACCTCAGGTC	BRDN00011	154	172	1	441	514	7
ACCTCAGTTC	BRDN00012	202	156	116	282	217	9
ACCTCATAAG	BRDN00011	184	83	162	417	277	16
ACCTCATAGA	BRDN00012	231	179	64	482	473	0
ACCTCATAGC	BRDN00011	301	228	3283	640	993	57
ACCTCATCGC	BRDN00011	193	232	376	418	522	9
ACCTCATCTT	BRDN00012	476	574	275	1013	826	32
ACCTCATTAA	BRDN00011	168	154	2515	439	632	54
ACCTCCAAGA	BRDN00011	477	480	1627	946	1075	182
ACCTCCACAA	BRDN00011	148	54	419	613	173	0
ACCTCCACAC	BRDN00012	361	351	477	890	960	38
ACCTCCACGG	BRDN00012	274	202	52	512	369	20
ACCTCCAGAC	BRDN00012	22	3	0	789	59	0
ACCTCCAGAT	BRDN00011	202	256	148	377	215	14
ACCTCCAGGC	BRDN00011	86	146	9	389	486	7
ACCTCCAGGC	BRDN00012	138	80	43	237	298	12
ACCTCCATAG	BRDN00011	443	516	559	920	1125	36
ACCTCCCAAA	BRDN00012	155	183	26	201	180	0
ACCTCCCAAT	BRDN00012	565	444	1977	908	957	27
ACCTCCCAGA	BRDN00012	417	289	874	756	532	49
ACCTCCCCAG	BRDN00012	325	290	430	537	431	34
ACCTCCCCTG	BRDN00012	193	76	11	323	259	0
ACCTCCCGTC	BRDN00011	462	383	1992	750	629	1132
ACCTCCCTAG	BRDN00011	17	50	0	335	159	0
ACCTCCGAAC	BRDN00012	335	329	543	538	409	80
ACCTCCGATA	BRDN00011	102	129	731	167	255	0
ACCTCCGCTC	BRDN00011	216	212	33	541	317	37
ACCTCCGGCC	BRDN00011	118	37	2	291	121	4
ACCTCCGGCC	BRDN00011	268	277	1709	596	419	77
ACCTCCTGGC	BRDN00011	133	113	10	486	292	51
ACCTCGAACT	BRDN00011	264	356	95	632	970	28
ACCTCGAGG	BRDN00012	269	112	1037	330	559	26
ACCTCGATGA	BRDN00012	179	159	326	453	270	44
ACCTCGCCGC	BRDN00011	447	482	835	923	1104	23
ACCTCGCGAC	BRDN00011	76	104	12	129	288	9
ACCTCGCGCC	BRDN00011	122	108	707	264	164	8
ACCTCGCTCA	BRDN00012	280	120	88	491	421	28
ACCTCGCTCC	BRDN00012	188	131	106	290	254	20
ACCTCGGACC	BRDN00011	398	402	864	654	681	92
ACCTCGGCCC	BRDN00012	185	83	146	250	371	24
ACCTCGGTAC	BRDN00011	442	409	1976	637	449	38
ACCTCGTCCA	BRDN00011	192	143	171	266	223	35
ACCTCGTCCC	BRDN00011	596	453	1944	941	1240	87
ACCTCGTCCG	BRDN00012	470	367	2226	914	720	38
ACCTCTACCA	BRDN00012	488	426	527	1154	820	32
ACCTCTACCA	BRDN00012	153	176	308	270	224	6
ACCTCTCAAA	BRDN00011	226	245	137	500	560	23
ACCTCTCAGA	BRDN00011	201	236	4	247	495	0
ACCTCTTATT	BRDN00012	498	369	1179	875	974	19
ACCTCTTCGC	BRDN00011	186	131	270	376	549	26
ACCTCTTGTA	BRDN00012	150	0	0	211	451	1
ACCTGAAAA	BRDN00011	317	152	258	671	490	14
ACCTGAAATA	BRDN00011	150	53	60	304	458	9
ACCTGAACCA	BRDN00011	279	143	92	623	509	5
ACCTGAAGA	BRDN00011	278	320	8521	741	853	401
ACCTGAATCC	BRDN00012	350	300	3662	745	580	97
ACCTGAATGI	BRDN00011	125	87	9398	209	173	134
ACCTGACAGI	BRDN00012	221	14	6	424	350	0
ACCTGACAGT	BRDN00012	151	100	332	361	260	0
ACCTGACCAA	BRDN00012	219	204	205	449	994	39
ACCTGACCTG	BRDN00012	223	166	1057	218	207	3
ACCTGACCTT	BRDN00012	571	561	610	1093	777	52
ACCTGACGTC	BRDN00011	56	36	140	244	209	0
ACCTGACGTC	BRDN00012	538	657	2705	1095	1269	111
ACCTGACTGA	BRDN00012	380	391	694	859	679	107
ACCTGACTTT	BRDN00011	263	0	0	523	440	0
ACCTGAGAA	BRDN00011	249	335	687	453	435	5
ACCTGAGCTA	BRDN00012	89	88	11	200	524	0
ACCTGAGCTC	BRDN00012	191	158	553	457	357	54
ACCTGAGGA	BRDN00011	527	623	1348	929	757	78
ACCTGAGTAT	BRDN00011	190	284	432	347	464	5
ACCTGATCCC	BRDN00012	246	154	53	521	526	22
ACCTGATCGC	BRDN00011	98	92	619	139	704	29
ACCTGATGG	BRDN00011	340	267	333	689	703	41
ACCTGATGG	BRDN00012	109	165	45	275	275	7
ACCTGATGTT	BRDN00011	199	95	113	376	464	17
ACCTGCAAAC	BRDN00011	289	367	497	600	707	57
ACCTGCAACT	BRDN00012	102	102	8	521	335	0
ACCTGCACGA	BRDN00011	338	450	1393	753	995	39
ACCTGCAGAI	BRDN00012	340	297	656	725	848	66
ACCTGCAGAI	BRDN00011	160	3	51	229	360	9
ACCTGCAGCA	BRDN00011	237	281	1474	655	652	24
ACCTGCAGG	BRDN00011	95	34	11	223	152	12
ACCTGCAGGT	BRDN00012	355	284	2121	769	707	106
ACCTGCATCA	BRDN00011	172	19	0	486	107	0
ACCTGCATGC	BRDN00012	816	709	2285	1458	1422	146
ACCTGCCACA	BRDN00011	363	347	268	528	482	65
ACCTGCCAGC	BRDN00012	295	248	660	774	927	33
ACCTGCCAGT	BRDN00012	548	433	583	1333	1375	55
ACCTGCCAGT	BRDN00011	329	312	228	578	451	19
ACCTGCCATA	BRDN00011	179	56	1	605	650	9
ACCTGCCATG	BRDN00011	179	113	500	344	206	15
ACCTGCCCCA	BRDN00012	559	562	1555	1027	992	63
ACCTGCCGGI	BRDN00011	442	393	846	863	1051	60
ACCTGCCTCA	BRDN00011	606	213	103	932	1006	5
ACCTGCGAG	BRDN00011	571	467	993	1052	757	74
ACCTGCGATC	BRDN00011	242	163	57	440	381	14
ACCTGCGCGA	BRDN00011	64	14	23	155	51	0
ACCTGCGTTC	BRDN00012	116	25	277	355	227	7
ACCTGCTACC	BRDN00011	251	166	93	448	475	59
ACCTGCTACT	BRDN00012	123	161	306	396	297	0
ACCTGCTCCC	BRDN00012	339	153	554	465	552	18
ACCTGCTGA	BRDN00012	548	582	313	758	409	37
ACCTGCTGCC	BRDN00012	288	169	0	373	448	39
ACCTGCTGGA	BRDN00011	418	347	326	792	833	20
ACCTGCTGGI	BRDN00012	572	559	625	1034	492	40
ACCTGGAAAT	BRDN00011	184	91	79	325	377	13
ACCTGGAACI	BRDN00012	418	266	769	974	1279	30
ACCTGGACG	BRDN00011	165	144	1265	349	298	74
ACCTGGACG	BRDN00011	208	159	98	257	166	10
ACCTGGACTA	BRDN00012	639	410	868	975	955	47
ACCTGGACTA	BRDN00012	372	410	551	872	1040	7
ACCTGGAGC	BRDN00012	78	0	0	259	64	1
ACCTGGAGT	BRDN00012	213	163	59	301	321	12
ACCTGGATG	BRDN00012	383	299	921	571	720	55
ACCTGGATG	BRDN00012	333	290	475	598	428	26
ACCTGGATTI	BRDN00012	381	255	587	676	514	11
ACCTGGCAG	BRDN00012	136	178	1631	283	151	32
ACCTGGCCTC	BRDN00012	366	259	218	547	613	10
ACCTGGCCTT	BRDN00012	184	131	105	328	136	19
ACCTGGCGG	BRDN00012	210	105	1	372	306	0
ACCTGGCTCA	BRDN00011	245	319	166	433	438	8
ACCTGGGAT	BRDN00011	53	13	2	681	265	0
ACCTGGTAG	BRDN00012	161	132	269	466	513	0
ACCTGGTCAC	BRDN00012	538	47	2	1028	801	0
ACCTGGTCGT	BRDN00012	588	461	560	1228	964	103
ACCTGGTGG	BRDN00012	337	321	1325	601	570	23
ACCTGGTGTI	BRDN00011	326	118	133	530	455	8
ACCTGGTTA	BRDN00011	107	49	134	301	371	19
ACCTGGTTAC	BRDN00012	262	212	87	530	409	0
ACCTGGTTGA	BRDN00011	267	169	99	472	728	11
ACCTGTAAAT	BRDN00012	64	19	0	134	197	0
ACCTGTAATC	BRDN00012	235	230	214	563	647	16
ACCTGTACCA	BRDN00011	112	147	61	321	154	0
ACCTGTAGAI	BRDN00011	200	22	0	559	223	1
ACCTGTATCG	BRDN00012	326	192	81	531	643	9
ACCTGTATGA	BRDN00011	350	251	1093	541	844	13
ACCTGTCAAC	BRDN00011	149	213	591	322	406	27
ACCTGTCAAT	BRDN00012	178	126	277	435	472	6
ACCTGTCACG	BRDN00011	278	188	7	476	511	37
ACCTGTCAGA	BRDN00011	239	216	71	313	490	4
ACCTGTCAGA	BRDN00012	164	134	165	411	157	6
ACCTGTCAGC	BRDN00012	243	202	323	356	563	14
ACCTGTCTTC	BRDN00012	175	131	353	216	230	3
ACCTGTGAAT	BRDN00011	93	59	89	269	106	2
ACCTGTGAG	BRDN00012	171	246	83	424	486	16
ACCTGTGCTC	BRDN00011	293	169	106	535	424	13
ACCTGTGTAC	BRDN00011	294	310	259	352	283	28
ACCTGTTCTC	BRDN00012	453	385	332	786	957	23
ACCTGTTGTA	BRDN00011	232	205	358	497	486	7
ACCTGTTTAC	BRDN00011	131	132	54	251	447	18
ACCTTAACAT	BRDN00011	283	330	155	509	469	30
ACCTTAATAG	BRDN00012	565	412	470	960	1275	93
ACCTTACAAT	BRDN00012	278	165	160	543	651	34
ACCTTACACA	BRDN00012	183	358	131	334	531	21
ACCTTACACA	BRDN00012	209	229	265	537	432	25
ACCTTACCTG	BRDN00011	166	266	179	377	367	0
ACCTTACGGC	BRDN00012	262	226	680	337	532	38
ACCTTAGAGT	BRDN00011	422	431	1757	679	858	18
ACCTTAGTGA	BRDN00011	322	222	89	584	478	0
ACCTTATAAA	BRDN00012	549	468	565	755	1333	100
ACCTTATCAT	BRDN00011	125	23	1	787	506	0
ACCTTATCTA	BRDN00011	153	177	25	209	363	14
ACCTTATTAT	BRDN00011	113	115	320	327	338	7
ACCTTATTGA	BRDN00011	183	35	0	477	78	0
ACCTTCAACT	BRDN00011	328	185	1095	1032	893	50
ACCTTCACGC	BRDN00012	277	193	115	555	842	34
ACCTTCACTA	BRDN00011	196	5	1	545	17	0
ACCTTCACTG	BRDN00012	226	111	1700	290	145	24
ACCTTCATCG	BRDN00011	639	467	1740	933	997	57
ACCTTCCTGT	BRDN00011	116	74	108	264	114	0
ACCTTCGCCA	BRDN00011	462	273	821	848	595	18
ACCTTCGCCT	BRDN00012	141	70	7	213	217	0
ACCTTCGTAG	BRDN00012	513	579	846	898	840	147
ACCTTCGTGA	BRDN00012	74	64	0	218	364	0
ACCTTCTACA	BRDN00012	398	380	695	787	1089	43
ACCTTCTCCA	BRDN00012	312	265	472	494	203	2
ACCTTCTGAT	BRDN00012	467	404	155	873	781	50
ACCTTGACCA	BRDN00012	255	156	359	533	348	41
ACCTTGAGCA	BRDN00012	188	118	372	222	394	57
ACCTTGATGT	BRDN00012	120	166	603	351	315	21
ACCTTGCAAC	BRDN00011	211	209	176	356	471	11
ACCTTGCAGT	BRDN00012	463	380	103	770	556	61
ACCTTGCCAG	BRDN00012	204	110	274	385	308	11
ACCTTGGACA	BRDN00012	322	267	55	917	1098	7
ACCTTGGATA	BRDN00012	218	207	560	399	214	0
ACCTTGGATC	BRDN00011	200	135	200	356	344	13
ACCTTGGGG	BRDN00012	31	21	18	303	308	0
ACCTTGGGTT	BRDN00012	512	529	1054	980	1017	82
ACCTTGGTAC	BRDN00012	694	429	463	1302	1050	41
ACCTTGGTCC	BRDN00012	190	165	448	277	204	0
ACCTTGTAAA	BRDN00012	514	413	590	784	785	48
ACCTTGTACT	BRDN00011	183	52	52	333	236	24
ACCTTGTACT	BRDN00012	152	27	1	848	603	0
ACCTTTACTG	BRDN00012	487	305	1343	717	1110	33
ACCTTTATGA	BRDN00011	178	164	141	291	427	26
ACCTTTATTG	BRDN00012	525	340	1072	755	1179	64
ACCTTTCATG	BRDN00011	161	143	93	400	322	5
ACCTTTCCAG	BRDN00012	88	56	37	230	241	0
ACCTTTGACA	BRDN00011	249	249	1489	395	541	42
ACCTTTGGGA	BRDN00011	348	442	1891	490	497	52
ACCTTTGGGT	BRDN00011	425	351	135	754	971	0
ACGAAAAAA	BRDN00011	175	154	167	469	299	46
ACGAAAACT	BRDN00011	325	325	2755	476	583	92
ACGAAACTTA	BRDN00012	292	225	494	665	773	38
ACGAAAGTG	BRDN00011	175	186	1	274	211	0
ACGAAATAA	BRDN00012	444	320	244	535	694	30
ACGAAATCC	BRDN00011	218	152	81	386	177	9
ACGAAATGC	BRDN00011	160	126	162	326	190	35
ACGAACACTA	BRDN00011	183	100	323	396	602	0
ACGAACAGA	BRDN00012	131	99	89	249	163	0
ACGAACAGG	BRDN00012	583	531	908	932	1280	72
ACGAACAGG	BRDN00012	279	273	1336	559	467	91
ACGAACCTGT	BRDN00011	366	334	597	509	446	6
ACGAACGCA	BRDN00012	215	0	0	383	71	0
ACGAACGGT	BRDN00011	127	0	0	240	8	0
ACGAACTCAC	BRDN00012	276	337	158	385	249	35
ACGAACTGG	BRDN00012	614	474	1053	865	1128	34
ACGAACTGG	BRDN00012	164	100	4145	259	420	25
ACGAAGAAG	BRDN00012	507	460	3530	930	1234	79
ACGAAGACA	BRDN00011	186	99	56	330	436	7
ACGAAGACG	BRDN00012	98	49	17	195	543	18
ACGAAGATA	BRDN00011	246	270	314	353	519	32
ACGAAGATC	BRDN00011	380	408	70	517	929	84
ACGAAGATC	BRDN00011	231	173	473	404	266	21
ACGAAGCAG	BRDN00011	512	81	0	1112	823	0
ACGAAGGTG	BRDN00011	173	311	634	438	376	7
ACGAAGTAG	BRDN00011	113	109	150	237	171	28
ACGAAGTCTT	BRDN00011	93	0	0	211	117	1
ACGAAGTTA	BRDN00012	405	260	546	569	416	65
ACGAATCGCT	BRDN00011	317	384	1021	805	1363	54
ACGAATCTCT	BRDN00012	482	555	2401	1007	1280	139
ACGAATCTGA	BRDN00011	208	148	1450	394	410	27
ACGAATCTGT	BRDN00011	262	296	96	601	341	103
ACGAATGTTT	BRDN00011	283	413	192	439	478	128
ACGAATTGA	BRDN00012	155	199	310	443	282	0
ACGAATTGC	BRDN00011	209	60	118	278	97	27
ACGACAAAA	BRDN00012	277	150	766	496	538	28
ACGACAACTI	BRDN00012	461	316	658	884	870	16
ACGACAAGC	BRDN00012	315	209	709	634	359	56
ACGACAAGG	BRDN00012	220	336	2055	327	375	104
ACGACAAGT	BRDN00012	177	145	825	286	270	16
ACGACAATG	BRDN00012	503	392	171	897	716	96
ACGACACAG	BRDN00011	110	65	207	180	103	8
ACGACACCAC	BRDN00011	275	200	40	524	470	0
ACGACACCTT	BRDN00012	286	402	402	446	257	16
ACGACATCGI	BRDN00012	510	588	1243	993	1159	64
ACGACATGCT	BRDN00012	231	33	45	749	886	21
ACGACATGG	BRDN00012	263	191	25	574	591	13
ACGACCAGA	BRDN00012	418	265	937	771	702	33
ACGACCAGCT	BRDN00011	420	340	6910	880	955	107
ACGACCAGTI	BRDN00012	184	0	0	281	310	0
ACGACCCCGT	BRDN00011	218	242	926	429	260	21
ACGACCGAG	BRDN00011	134	186	433	293	162	6
ACGACCGTAI	BRDN00011	312	182	126	633	1012	35
ACGACCTCAT	BRDN00012	183	260	293	376	520	71
ACGACGACA	BRDN00011	472	345	831	983	810	19
ACGACGAGA	BRDN00012	335	356	289	617	404	21
ACGACGAGG	BRDN00011	332	301	1919	805	479	10
ACGACGAGT	BRDN00011	246	337	1194	645	891	23
ACGACGATA	BRDN00012	60	101	121	233	81	7
ACGACGATG	BRDN00012	107	62	0	288	306	3
ACGACGCAG	BRDN00012	521	293	116	835	698	54
ACGACGCCCA	BRDN00012	193	254	139	315	136	8
ACGACGCCTC	BRDN00012	190	173	150	287	157	8
ACGACGTCAI	BRDN00012	250	325	1058	524	801	32
ACGACTCCTC	BRDN00012	109	131	127	288	133	1
ACGACTCTGC	BRDN00012	103	0	0	921	624	0
ACGACTGAA	BRDN00012	122	161	19	259	361	81
ACGACTGATI	BRDN00012	374	256	1496	887	739	57
ACGACTGCAI	BRDN00012	330	506	1099	681	741	16
ACGACTGCTA	BRDN00011	427	292	232	512	971	33
ACGACTTCAC	BRDN00012	163	147	520	332	301	17
ACGACTTGAI	BRDN00012	351	347	1547	738	383	59
ACGACTTTAC	BRDN00011	125	34	8	601	365	0
ACGAGAAAA	BRDN00012	69	68	1	203	103	24
ACGAGAAAC	BRDN00012	235	205	432	458	719	35
ACGAGAACG	BRDN00011	348	362	97	567	709	15
ACGAGAAGT	BRDN00012	87	112	344	232	232	0
ACGAGAAGT	BRDN00011	37	0	0	179	66	0
ACGAGACAG	BRDN00011	401	303	180	857	757	53
ACGAGAGAA	BRDN00012	85	5	19	187	72	59
ACGAGAGTC	BRDN00012	125	268	328	179	411	40
ACGAGATCC	BRDN00011	379	274	357	589	280	9
ACGAGATCTT	BRDN00011	39	0	0	241	98	0
ACGAGCAAC	BRDN00012	268	216	161	546	264	68
ACGAGCACG	BRDN00012	433	239	254	827	1018	0
ACGAGCAGC	BRDN00012	248	200	417	488	1043	88
ACGAGCAGT	BRDN00011	361	315	775	610	1153	33
ACGAGCAGT	BRDN00011	224	216	380	451	306	6
ACGAGCCAA	BRDN00011	177	217	10	390	891	30
ACGAGCGCA	BRDN00012	55	43	150	236	148	4
ACGAGCGCA	BRDN00011	290	125	95	714	480	3
ACGAGCGCG	BRDN00012	95	48	294	231	458	7
ACGAGCTGA	BRDN00012	3	0	0	252	155	0
ACGAGGAAG	BRDN00012	59	34	1	284	195	0
ACGAGGAGA	BRDN00012	270	217	514	668	309	59
ACGAGGAGC	BRDN00012	124	0	46	194	247	1
ACGAGGATG	BRDN00012	287	378	746	753	739	32
ACGAGGCAG	BRDN00012	108	75	286	281	139	21
ACGAGGCGA	BRDN00011	105	121	231	241	472	0
ACGAGGCGC	BRDN00012	116	0	1	339	16	0
ACGAGGGTG	BRDN00012	418	380	1240	690	812	111
ACGAGGTAC	BRDN00011	290	247	14	566	634	0
ACGAGGTCT	BRDN00011	295	211	128	392	188	15
ACGAGGTGC	BRDN00012	103	106	0	364	304	0
ACGAGTAGA	BRDN00012	252	193	229	297	118	0
ACGAGTAGG	BRDN00012	117	67	356	250	88	16
ACGAGTATG	BRDN00011	260	198	459	331	283	104
ACGAGTCCTT	BRDN00011	329	419	99	1042	358	55
ACGAGTGTA	BRDN00012	86	40	165	354	177	6
ACGAGTGTG	BRDN00011	73	117	199	287	277	0
ACGAGTTGT	BRDN00012	141	16	0	297	165	0
ACGAGTTTA	BRDN00011	391	646	536	790	1033	60
ACGATAAAA	BRDN00011	53	0	0	186	156	0
ACGATACAG	BRDN00011	162	131	0	295	217	5
ACGATACCA	BRDN00012	152	149	248	250	71	16
ACGATACCCT	BRDN00011	266	262	182	678	597	9
ACGATAGCTT	BRDN00012	195	303	41	346	751	9
ACGATAGGC	BRDN00012	140	90	47	255	139	13
ACGATCATTT	BRDN00012	336	380	121	642	652	89
ACGATCCAAC	BRDN00011	347	417	1025	712	1168	40
ACGATCCAGI	BRDN00011	153	187	187	446	508	20
ACGATCCGAI	BRDN00012	434	351	2515	575	906	77
ACGATCGGT	BRDN00012	440	349	734	813	806	33
ACGATCTCCT	BRDN00012	193	170	997	328	334	60
ACGATGACTI	BRDN00012	171	104	866	269	376	33
ACGATGAGA	BRDN00012	217	251	2115	314	629	262
ACGATGGAC	BRDN00011	538	470	1367	808	578	48
ACGATGTAG	BRDN00011	301	140	1576	471	424	30
ACGATGTCAT	BRDN00011	336	302	777	684	428	78
ACGATGTGG	BRDN00012	113	112	73	220	324	28
ACGATTCCA	BRDN00012	251	279	151	599	526	33
ACGATTGAAT	BRDN00011	62	34	129	216	45	12
ACGATTGCA	BRDN00012	53	14	0	449	444	0
ACGCAAAGC	BRDN00011	457	415	1086	936	1475	83
ACGCAAAGC	BRDN00012	36	0	0	433	64	0
ACGCAACGC	BRDN00011	265	252	171	558	859	98
ACGCAACTTT	BRDN00011	218	212	248	417	373	0
ACGCAAGAG	BRDN00011	94	126	12	256	275	0
ACGCACACAC	BRDN00011	410	309	1492	915	1123	61
ACGCACAGA	BRDN00012	229	199	140	648	608	7
ACGCACAGA	BRDN00011	267	297	150	543	456	42
ACGCACAGTA	BRDN00011	71	6	0	158	47	0
ACGCACCAGI	BRDN00011	378	366	373	842	1215	17
ACGCACGTG	BRDN00012	99	215	130	223	140	34
ACGCACGTTC	BRDN00012	433	182	215	776	894	1
ACGCAGAAG	BRDN00011	508	613	534	1020	714	74
ACGCAGACTT	BRDN00011	73	65	314	167	232	5
ACGCAGAGA	BRDN00012	471	464	629	901	869	83
ACGCAGATG	BRDN00011	238	157	40	371	122	17
ACGCAGCCCA	BRDN00011	71	16	55	203	321	3
ACGCAGCCTC	BRDN00011	479	365	755	820	616	60
ACGCAGCGA	BRDN00012	111	79	0	251	297	7
ACGCAGGAA	BRDN00012	449	446	537	704	556	97
ACGCAGGAG	BRDN00011	119	239	2109	386	393	50
ACGCATAAA	BRDN00011	129	152	3	151	289	5
ACGCATAAAI	BRDN00012	102	132	157	260	376	0
ACGCATACCA	BRDN00011	255	250	341	541	414	0
ACGCATAGA	BRDN00012	46	0	0	219	151	0
ACGCATCCGT	BRDN00012	376	302	307	691	745	24
ACGCATCTTC	BRDN00012	473	238	1505	695	933	22
ACGCATGCGT	BRDN00012	263	317	95	664	580	53
ACGCATGCTT	BRDN00012	424	399	864	748	722	53
ACGCCAAAG	BRDN00011	941	748	422	1291	1005	91
ACGCCAACAC	BRDN00011	228	220	786	578	573	32
ACGCCACCCC	BRDN00012	146	122	371	341	464	39
ACGCCACCTG	BRDN00012	124	229	43	248	317	3
ACGCCAGGT	BRDN00011	191	115	25	244	103	12
ACGCCAGTCT	BRDN00011	218	243	192	242	250	7
ACGCCATATT	BRDN00012	529	575	855	951	1275	102
ACGCCATCGT	BRDN00012	366	413	191	557	360	37
ACGCCATCGT	BRDN00012	147	163	228	231	261	41
ACGCCATCTC	BRDN00012	249	176	89	485	580	0
ACGCCATGTA	BRDN00011	173	206	3535	194	191	58
ACGCCATTGT	BRDN00012	418	483	1097	966	1178	54
ACGCCCAATC	BRDN00011	260	131	722	475	495	16
ACGCCCACTC	BRDN00011	119	112	228	217	180	0
ACGCCCACTT	BRDN00011	147	129	87	193	295	21
ACGCCCGCGA	BRDN00011	126	113	53	353	253	13
ACGCCCGGG	BRDN00012	144	155	175	222	149	0
ACGCCCGGG	BRDN00011	483	407	493	881	957	48
ACGCCCTGCA	BRDN00012	105	85	494	169	126	0
ACGCCCTGGA	BRDN00012	118	61	0	668	68	0

ACGCCCTTAC	BRDN00012	391	312	380	912	1115	24
ACGCCGACA	BRDN00012	340	289	1835	588	502	34
ACGCCGAGC	BRDN00012	162	180	141	394	326	7
ACGCCGAGT	BRDN00012	145	107	96	339	335	55
ACGCCGCCC	BRDN00011	141	49	51	476	530	6
ACGCCGCTCT	BRDN00012	188	204	24	389	397	0
ACGCCGGTC	BRDN00011	190	151	125	328	168	17
ACGCCGTCT	BRDN00011	101	13	1	223	64	0
ACGCCGTTCC	BRDN00011	151	7	0	269	39	1
ACGCCTCCTC	BRDN00012	489	416	229	719	796	102
ACGCCTGCA	BRDN00012	346	355	235	627	720	11
ACGCCTGGC	BRDN00011	229	235	412	291	372	18
ACGCCTTACA	BRDN00011	128	31	77	300	465	0
ACGCGACGA	BRDN00011	103	161	48	255	599	12
ACGCGCACA	BRDN00011	547	535	275	826	993	39
ACGCGCATG	BRDN00011	415	512	4564	985	1012	174
ACGCGCCGT	BRDN00012	147	35	153	272	141	0
ACGCGCGCG	BRDN00012	222	241	398	418	791	86
ACGCGCGGA	BRDN00012	148	186	181	219	172	34
ACGCGCTGG	BRDN00012	169	123	15	284	467	0
ACGCGGAAA	BRDN00011	424	492	998	741	505	39
ACGCGGACC	BRDN00011	263	117	40	589	833	8
ACGCGGCAA	BRDN00011	178	83	727	173	223	12
ACGCGGCCT	BRDN00011	245	102	10	447	380	0
ACGCGGGCG	BRDN00011	162	80	1	234	354	13
ACGCGTAGC	BRDN00012	113	180	54	241	108	0
ACGCGTGCT	BRDN00011	153	113	53	315	287	0
ACGCGTTTCT	BRDN00011	102	130	9	184	202	0
ACGCTACCCC	BRDN00012	230	130	171	353	303	11
ACGCTACCCT	BRDN00011	185	340	459	489	506	28
ACGCTACCTT	BRDN00012	87	78	168	229	99	16
ACGCTATGG	BRDN00011	387	302	384	515	401	35
ACGCTATTG	BRDN00011	234	252	371	389	577	17
ACGCTCAAG	BRDN00012	470	398	165	670	649	30
ACGCTCAGC	BRDN00012	380	326	1773	756	554	104
ACGCTCTAAT	BRDN00012	85	0	1	307	116	0
ACGCTCTCCT	BRDN00012	594	498	2092	1021	1141	85
ACGCTGAAT	BRDN00012	152	207	231	328	636	67
ACGCTGACG	BRDN00012	391	297	469	658	630	10
ACGCTGAGG	BRDN00011	307	240	354	690	870	7
ACGCTGATG	BRDN00011	119	112	184	323	184	0
ACGCTGATG	BRDN00011	241	204	0	564	462	0
ACGCTGCAC	BRDN00011	464	296	203	649	379	21
ACGCTGCTG	BRDN00012	456	149	26	955	1062	6
ACGCTGGTG	BRDN00012	365	303	1445	426	310	57
ACGCTGTGC	BRDN00011	596	549	826	720	816	53
ACGCTGTGT	BRDN00012	327	282	257	510	771	26
ACGCTGTTC	BRDN00012	197	217	442	438	238	25
ACGCTGTTTC	BRDN00012	108	59	178	188	199	3
ACGCTTACTT	BRDN00012	398	619	5160	797	825	289
ACGCTTAGTC	BRDN00012	182	114	66	341	174	0
ACGCTTCAAC	BRDN00012	424	204	593	1010	841	5
ACGCTTCATC	BRDN00012	274	150	85	401	446	20
ACGCTTCCAC	BRDN00012	412	300	629	762	1247	15
ACGCTTGAC	BRDN00011	481	467	723	662	1144	17
ACGGAAAAC	BRDN00012	102	49	29	211	325	8
ACGGAAACC	BRDN00011	222	242	48	605	445	6
ACGGAAACT	BRDN00011	133	68	53	299	318	16
ACGGAAAGA	BRDN00012	495	443	698	748	1437	16
ACGGAACAG	BRDN00012	146	142	1	309	302	43
ACGGAACCA	BRDN00011	458	456	529	774	485	51
ACGGAACTA	BRDN00011	318	261	1005	664	494	12
ACGGAACTT	BRDN00012	183	254	2645	429	572	76
ACGGAAGGC	BRDN00011	156	94	0	225	256	6
ACGGACAAG	BRDN00012	367	366	82	846	790	50
ACGGACAAT	BRDN00011	408	402	927	717	821	59
ACGGACAGA	BRDN00011	157	52	263	296	308	4
ACGGACAGC	BRDN00012	361	304	110	527	820	26
ACGGACATC	BRDN00011	200	340	327	433	483	0
ACGGACATC	BRDN00011	365	214	49	545	433	0
ACGGACATG	BRDN00011	291	206	2096	546	464	43
ACGGACCAT	BRDN00012	180	161	226	411	421	49
ACGGACCCG	BRDN00011	160	252	408	286	256	52
ACGGACGCC	BRDN00011	73	68	273	174	228	30
ACGGACGCG	BRDN00011	245	383	1772	706	502	50
ACGGACTCT	BRDN00012	116	209	351	288	434	7
ACGGACTTG	BRDN00011	253	212	64	430	252	1
ACGGAGAAA	BRDN00011	70	85	0	222	149	0
ACGGAGAAT	BRDN00011	434	455	1126	948	1027	34
ACGGAGACA	BRDN00011	220	245	18	380	494	54
ACGGAGACA	BRDN00011	175	257	903	435	594	39
ACGGAGACC	BRDN00012	274	130	528	383	244	9
ACGGAGACT	BRDN00012	173	102	84	535	455	34
ACGGAGACT	BRDN00012	473	435	1182	796	673	12
ACGGAGCCA	BRDN00011	313	306	548	639	726	33
ACGGAGCCT	BRDN00012	101	164	9	185	292	14
ACGGAGCTC	BRDN00012	582	529	2030	1092	1210	136
ACGGAGCTG	BRDN00011	98	52	201	205	216	12
ACGGAGGAC	BRDN00012	308	229	556	673	537	23
ACGGAGGCT	BRDN00012	566	537	659	1139	1520	61
ACGGAGTCA	BRDN00011	201	213	210	321	328	12
ACGGATAAG	BRDN00012	62	22	0	236	112	0
ACGGATACC	BRDN00011	133	88	14	191	329	1
ACGGATATG	BRDN00012	162	88	165	301	342	54
ACGGATCCT	BRDN00011	120	107	54	290	547	0
ACGGATGTG	BRDN00011	400	321	1546	711	948	67
ACGGATGTT	BRDN00012	269	335	96	549	202	64
ACGGATTCA	BRDN00011	339	314	455	579	495	20
ACGGATTCT	BRDN00011	313	475	2839	550	1118	89
ACGGCAAAG	BRDN00012	141	156	644	185	305	66
ACGGCAACA	BRDN00012	145	103	17	349	539	6
ACGGCAATG	BRDN00011	221	223	626	386	612	11
ACGGCACGC	BRDN00012	355	246	192	541	632	32
ACGGCACTT	BRDN00012	253	224	43	492	251	23
ACGGCAGAC	BRDN00011	288	284	359	478	303	41
ACGGCAGCT	BRDN00011	263	324	255	444	665	56
ACGGCATAT	BRDN00012	166	127	408	386	374	4
ACGGCATTC	BRDN00012	550	472	3757	833	963	97
ACGGCCAAG	BRDN00011	297	168	474	609	432	14
ACGGCCACC	BRDN00011	239	115	153	341	134	3
ACGGCCACCT	BRDN00012	92	151	0	271	331	19
ACGGCCACTC	BRDN00012	243	246	73	463	268	0
ACGGCCCATC	BRDN00012	270	266	569	586	737	35
ACGGCCCATC	BRDN00011	282	223	66	523	532	96
ACGGCCCTCA	BRDN00012	223	135	147	360	488	14
ACGGCCCTG	BRDN00011	158	147	82	381	228	15
ACGGCCGAC	BRDN00012	189	252	475	395	257	18
ACGGCCGTA	BRDN00012	235	127	149	351	655	27
ACGGCCGTC	BRDN00012	293	278	771	624	867	9
ACGGCCTAA	BRDN00012	80	0	0	573	192	0
ACGGCCTCG	BRDN00011	62	13	180	130	40	0
ACGGCGAAG	BRDN00012	355	250	616	586	429	39
ACGGCGAAG	BRDN00011	132	253	1193	251	218	21
ACGGCGACC	BRDN00012	132	98	124	289	242	14
ACGGCGACG	BRDN00011	283	185	1090	547	602	24
ACGGCGAGT	BRDN00012	91	79	0	185	195	0
ACGGCGCCT	BRDN00012	165	158	442	386	356	9
ACGGCGCGC	BRDN00011	76	88	8	153	254	0
ACGGCGCTT	BRDN00011	232	159	98	352	413	66
ACGGCGGCG	BRDN00011	98	121	82	112	61	3
ACGGCGGTA	BRDN00012	196	233	577	266	112	17
ACGGCGTAG	BRDN00012	407	337	509	847	565	46
ACGGCGTCG	BRDN00011	162	231	116	232	381	16
ACGGCGTCT	BRDN00011	97	63	112	159	192	25
ACGGCTATG	BRDN00012	297	282	494	473	353	8
ACGGCTCGT	BRDN00011	144	68	72	211	273	0
ACGGCTGAC	BRDN00011	490	541	1041	914	703	59
ACGGCTGCA	BRDN00011	566	61	69	906	523	0
ACGGCTGGT	BRDN00011	262	285	283	586	1167	37
ACGGCTTGG	BRDN00011	128	147	19	328	476	11
ACGGGAACG	BRDN00011	119	131	37	324	295	46
ACGGGAATT	BRDN00011	67	48	0	181	121	0
ACGGGACAA	BRDN00011	206	98	675	398	1223	22
ACGGGACCC	BRDN00011	523	285	507	703	553	22
ACGGGAGA	BRDN00012	179	4	0	302	383	0
ACGGGATCT	BRDN00012	332	377	497	534	452	33
ACGGGATGC	BRDN00011	179	247	672	430	653	0
ACGGGCACA	BRDN00011	189	148	8	399	445	7
ACGGGCAGA	BRDN00012	406	306	623	718	789	4
ACGGGCCAA	BRDN00011	638	669	1431	1044	738	45
ACGGGCCCT	BRDN00011	178	117	408	253	278	4
ACGGGCCGA	BRDN00012	211	209	480	422	253	46
ACGGGCCGG	BRDN00011	75	50	443	112	82	15
ACGGGCGGC	BRDN00012	399	448	272	799	621	27
ACGGGCGGC	BRDN00012	585	494	1261	1061	1190	24
ACGGGCGTC	BRDN00012	57	9	0	226	46	0
ACGGGCTAC	BRDN00012	439	590	1697	816	830	191
ACGGGCTGT	BRDN00011	165	170	1229	454	249	42
ACGGGGAAC	BRDN00012	672	576	2401	1084	1212	74
ACGGGGAAC	BRDN00012	291	106	38	561	178	0
ACGGGGACA	BRDN00011	89	105	89	217	437	5
ACGGGGACA	BRDN00011	273	168	148	540	503	12
ACGGGGACC	BRDN00012	238	174	0	456	402	0
ACGGGGACC	BRDN00012	83	133	14	229	900	0
ACGGGGACT	BRDN00011	193	291	41	605	641	0
ACGGGGAGT	BRDN00012	53	49	62	129	3	0
ACGGGGGCC	BRDN00012	615	722	850	1120	1587	61
ACGGGGGTA	BRDN00011	86	58	0	169	76	0
ACGGGGGTC	BRDN00011	93	73	2	83	268	0
ACGGGGTGA	BRDN00012	505	502	2861	777	998	84
ACGGGTACT	BRDN00012	162	154	201	400	270	20
ACGGGTCCC	BRDN00012	617	500	247	911	1515	28
ACGGGTCGC	BRDN00011	100	48	6	210	69	0
ACGGGTGAA	BRDN00011	175	91	12	525	506	12
ACGGGTGAG	BRDN00011	276	266	422	559	464	33
ACGGGTGGC	BRDN00011	48	69	61	46	49	0
ACGGTAACA	BRDN00012	96	14	4	243	124	0
ACGGTACAT	BRDN00012	586	514	1040	735	660	72
ACGGTACCC	BRDN00012	156	128	117	187	354	22
ACGGTACCG	BRDN00011	194	54	37	537	98	0
ACGGTACCT	BRDN00012	566	256	650	1072	1336	5
ACGGTACGT	BRDN00012	187	187	53	414	213	0
ACGGTAGAA	BRDN00011	100	0	20	271	371	0
ACGGTAGAT	BRDN00011	77	70	102	179	304	0
ACGGTATAC	BRDN00012	321	336	356	568	603	8
ACGGTATAG	BRDN00011	114	83	625	279	365	6
ACGGTATTA	BRDN00012	395	553	1411	928	1251	35
ACGGTCAAA	BRDN00011	303	405	64	469	766	16
ACGGTCACA	BRDN00012	143	39	835	180	190	0
ACGGTCACC	BRDN00012	313	195	396	781	1194	27
ACGGTCACCT	BRDN00011	309	51	117	600	141	0
ACGGTCAGC	BRDN00012	134	37	0	225	206	0
ACGGTCATA	BRDN00011	237	176	1786	376	601	34
ACGGTCATC	BRDN00011	366	267	347	790	955	23
ACGGTCATC	BRDN00012	205	183	321	425	589	13
ACGGTCCAA	BRDN00011	249	266	311	486	1185	20
ACGGTCCCA	BRDN00011	351	195	1411	564	356	53
ACGGTCGAA	BRDN00011	37	0	0	144	6	0
ACGGTCGCA	BRDN00012	329	318	507	705	715	29
ACGGTCTAC	BRDN00012	439	362	266	805	774	12
ACGGTCTGC	BRDN00012	688	713	1744	1048	890	43
ACGGTGAAA	BRDN00011	224	195	427	367	911	15
ACGGTGACC	BRDN00011	539	423	1651	951	1035	16
ACGGTGAGG	BRDN00011	36	30	38	537	173	0
ACGGTGATG	BRDN00012	77	101	11	638	680	0
ACGGTGCCA	BRDN00011	222	198	443	317	572	15
ACGGTGCGC	BRDN00012	391	280	1551	687	648	38
ACGGTGCTA	BRDN00011	302	483	1056	713	873	85
ACGGTGCTC	BRDN00012	153	118	115	353	369	29
ACGGTGCTG	BRDN00011	581	504	545	915	1028	29
ACGGTGGAC	BRDN00011	180	144	469	292	264	10
ACGGTGGAG	BRDN00012	144	124	120	206	36	4
ACGGTGGAT	BRDN00012	375	12	1	727	194	1
ACGGTGGCC	BRDN00012	188	124	161	288	270	8
ACGGTGGCT	BRDN00011	185	166	23	303	349	16
ACGGTGGGC	BRDN00012	595	538	959	875	957	41
ACGGTGGTG	BRDN00011	75	19	0	189	93	1
ACGGTTAAG	BRDN00011	395	350	126	652	669	188
ACGGTTACG	BRDN00012	131	112	804	684	376	34
ACGGTTAGA	BRDN00011	120	116	424	256	159	6
ACGGTTAGG	BRDN00012	344	120	226	871	578	13
ACGGTTATG	BRDN00012	631	641	870	1197	803	40
ACGGTTCCA	BRDN00011	169	71	0	249	350	8
ACGGTTCTA	BRDN00011	201	80	33	373	129	16
ACGGTTTCG	BRDN00012	497	327	1575	847	1071	26
ACGGTTTGG	BRDN00012	190	159	876	316	201	32
ACGTAAACA	BRDN00011	309	407	5756	755	849	34
ACGTAACAT	BRDN00012	193	217	20	567	607	6
ACGTAAGAT	BRDN00012	203	220	0	360	229	11
ACGTAAGGG	BRDN00012	461	530	71	715	1090	74
ACGTAAGTT	BRDN00011	108	154	97	332	203	0
ACGTACACC	BRDN00011	154	24	133	364	270	0
ACGTACACCT	BRDN00011	255	87	154	527	1029	7
ACGTACACTC	BRDN00012	133	107	197	343	249	15
ACGTACAGA	BRDN00012	170	247	42	390	917	8
ACGTACATT	BRDN00012	439	435	571	928	737	29
ACGTACCAA	BRDN00012	322	68	69	653	542	0
ACGTACCAAT	BRDN00011	162	181	1039	409	390	37
ACGTACGCA	BRDN00012	123	145	264	178	322	69
ACGTACTCCA	BRDN00012	166	141	161	249	354	34
ACGTACTGC	BRDN00012	485	479	1410	840	732	8
ACGTAGACA	BRDN00012	213	178	233	340	284	11
ACGTAGAGA	BRDN00011	211	273	184	394	349	0
ACGTAGCCA	BRDN00011	296	180	317	870	498	0
ACGTAGCCA	BRDN00011	174	157	1179	318	531	3
ACGTAGCCG	BRDN00011	250	138	410	536	319	35
ACGTAGCTG	BRDN00012	434	341	1345	983	1047	49
ACGTAGGCA	BRDN00012	376	450	210	683	758	97
ACGTAGGCC	BRDN00011	487	400	828	774	1728	91
ACGTATCGG	BRDN00012	381	378	543	721	507	18
ACGTATTAT	BRDN00011	243	175	244	506	528	31
ACGTCAAAT	BRDN00011	502	427	953	915	676	28
ACGTCAACT	BRDN00012	1171	1010	2271	1818	2123	127
ACGTCACAA	BRDN00011	52	1	0	660	228	0
ACGTCACCTA	BRDN00011	417	225	347	623	456	27
ACGTCACTG	BRDN00012	157	147	223	391	386	13
ACGTCAGAC	BRDN00011	199	250	208	379	422	28
ACGTCAGCT	BRDN00011	110	111	274	353	154	0
ACGTCAGGG	BRDN00012	301	170	89	780	893	7
ACGTCAGTG	BRDN00012	473	301	810	1012	849	74
ACGTCATCA	BRDN00012	586	609	1532	1214	1690	62
ACGTCATTG	BRDN00012	125	97	0	378	315	1
ACGTCCACG	BRDN00012	184	10	0	414	51	0
ACGTCCAGC	BRDN00011	103	62	200	354	751	11
ACGTCCAGG	BRDN00012	847	843	2683	1408	1494	132
ACGTCCAGT	BRDN00011	181	136	655	408	568	862
ACGTCCATA	BRDN00012	741	611	1047	1156	1285	32
ACGTCCCAG	BRDN00012	284	171	183	550	314	5
ACGTCCGACT	BRDN00011	287	274	306	514	738	124
ACGTCCGCG	BRDN00012	195	116	841	339	543	89
ACGTCCGCG	BRDN00011	154	290	88	403	233	66
ACGTCCGGG	BRDN00011	266	4	0	663	250	0
ACGTCCTCAG	BRDN00012	295	14	2	928	937	0
ACGTCCTGTT	BRDN00012	303	282	2205	730	1016	96
ACGTCGAAA	BRDN00012	327	503	2484	614	788	168
ACGTCGAGC	BRDN00011	462	423	3526	968	950	70
ACGTCGATG	BRDN00012	680	608	658	1261	1013	31
ACGTCGCAG	BRDN00012	222	157	457	518	292	3
ACGTCGCCA	BRDN00012	414	333	1196	882	567	42
ACGTCGGAG	BRDN00012	520	523	795	718	1664	86
ACGTCGTAC	BRDN00011	177	32	1192	261	339	7
ACGTCGTTT	BRDN00012	451	371	884	807	579	49
ACGTCTACTT	BRDN00012	445	354	465	888	816	12
ACGTCTATCA	BRDN00011	242	254	1190	562	321	41
ACGTCTTAGT	BRDN00011	227	196	314	433	787	13
ACGTCTTCCT	BRDN00012	517	568	4610	1139	1359	70
ACGTCTTTGC	BRDN00012	128	67	191	252	274	0
ACGTGAAGT	BRDN00011	213	152	11	294	627	0
ACGTGACAA	BRDN00011	129	126	192	237	123	19
ACGTGACGT	BRDN00011	228	178	789	329	548	39
ACGTGACTTT	BRDN00011	339	407	613	688	1118	36
ACGTGAGCC	BRDN00012	434	434	3148	942	1032	46
ACGTGAGGT	BRDN00011	187	143	29	397	370	41
ACGTGAGTG	BRDN00011	209	136	6	253	174	19
ACGTGATCA	BRDN00011	356	305	754	452	389	19
ACGTGCACA	BRDN00011	476	447	1048	792	673	27
ACGTGCACCT	BRDN00011	233	76	1	353	390	0
ACGTGCACTT	BRDN00011	438	338	620	913	1155	53
ACGTGCAGC	BRDN00011	161	102	45	460	275	0
ACGTGCATG	BRDN00012	260	244	103	466	385	0
ACGTGCCAC	BRDN00012	577	374	314	1227	1435	47
ACGTGCCGG	BRDN00011	114	173	120	372	358	11
ACGTGCCTT	BRDN00011	114	133	75	178	220	0
ACGTGCCTT	BRDN00011	264	245	333	597	402	33
ACGTGCGCA	BRDN00011	364	276	2	778	590	12
ACGTGCGCG	BRDN00011	295	239	195	473	267	20
ACGTGCGCT	BRDN00012	360	476	922	852	537	8
ACGTGCGGT	BRDN00012	541	575	940	930	776	18
ACGTGCGTG	BRDN00011	452	319	1365	806	828	102
ACGTGCTAA	BRDN00012	7	0	0	250	38	0
ACGTGCTCG	BRDN00012	138	172	1396	281	326	25
ACGTGCTGA	BRDN00012	376	153	284	891	893	1
ACGTGCTGA	BRDN00011	190	256	271	350	486	18
ACGTGCTGT	BRDN00011	166	116	90	315	250	40
ACGTGCTGT	BRDN00012	445	371	190	969	626	73
ACGTGGAAG	BRDN00011	92	120	0	247	350	1
ACGTGGACA	BRDN00012	371	243	133	682	739	2
ACGTGGACT	BRDN00012	147	95	529	384	416	0
ACGTGGAGT	BRDN00012	125	151	1	278	300	0
ACGTGGCAA	BRDN00012	134	121	278	273	424	6
ACGTGGCAA	BRDN00011	119	97	45	245	306	0
ACGTGGCCC	BRDN00012	335	279	1674	738	906	50
ACGTGGCTG	BRDN00012	79	89	40	251	99	0
ACGTGGCTT	BRDN00012	419	519	1179	791	1245	96
ACGTGGGCA	BRDN00011	237	180	1512	392	608	26
ACGTGGGCT	BRDN00012	65	19	0	456	107	0
ACGTGGGGA	BRDN00012	614	469	302	922	882	16
ACGTGGTCA	BRDN00011	60	24	0	59	88	0
ACGTGGTGA	BRDN00011	469	356	137	796	499	93
ACGTGGTGA	BRDN00011	80	38	0	226	165	0
ACGTGGTGA	BRDN00012	91	0	0	443	223	0
ACGTGTACA	BRDN00012	256	13	0	709	243	0
ACGTGTACA	BRDN00012	587	570	21	1103	882	49
ACGTGTACA	BRDN00011	96	203	1184	226	186	60
ACGTGTACT	BRDN00011	123	167	260	231	217	48
ACGTGTCTG	BRDN00011	94	186	425	314	456	5
ACGTGTTAG	BRDN00012	523	491	2592	848	1017	43
ACGTGTTGT	BRDN00011	159	247	410	311	415	17
ACGTTACCTT	BRDN00012	217	152	143	345	410	42
ACGTTATTAC	BRDN00012	439	379	595	671	627	62
ACGTTCAAG	BRDN00012	459	487	219	773	898	115
ACGTTCACCC	BRDN00012	615	515	492	1351	1452	53
ACGTTCACCT	BRDN00012	580	524	1678	1142	1483	18
ACGTTCATAT	BRDN00011	377	221	552	631	295	4
ACGTTCATCT	BRDN00011	260	339	517	484	726	47
ACGTTCCATT	BRDN00012	444	424	688	1051	616	32
ACGTTCCCTG	BRDN00011	130	143	1	285	214	4
ACGTTCCCTT	BRDN00011	376	281	117	689	715	2
ACGTTCCTCT	BRDN00011	551	104	28	964	290	0
ACGTTCGAG	BRDN00012	568	715	1127	1017	1101	270
ACGTTCTCAG	BRDN00011	288	309	312	642	362	36
ACGTTCTCAT	BRDN00012	147	194	79	249	397	20
ACGTTGATG	BRDN00012	360	304	968	749	628	21
ACGTTGGCC	BRDN00011	290	16	65	753	447	0
ACGTTGGGC	BRDN00012	718	750	977	1383	1392	42
ACGTTGGTC	BRDN00012	91	16	315	737	828	0
ACGTTGTAG	BRDN00012	217	192	87	349	538	12
ACGTTGTATC	BRDN00011	395	480	1665	767	605	99
ACGTTGTGA	BRDN00011	296	364	162	617	947	33
ACGTTGTGC	BRDN00011	412	377	610	790	585	40
ACGTTGTGG	BRDN00012	174	157	9	377	255	0
ACGTTGTTG	BRDN00012	398	420	714	655	388	38
ACGTTTAATC	BRDN00011	140	252	52	243	222	14
ACGTTTACG	BRDN00011	229	256	807	590	506	7
ACGTTTCGG	BRDN00011	209	25	1	355	461	0
ACGTTTCGG	BRDN00012	132	203	1777	326	346	13
ACGTTTGGA	BRDN00012	205	262	71	305	110	18
ACGTTTGTCA	BRDN00012	224	200	404	540	892	18
ACTAAAACG	BRDN00012	75	0	0	352	136	0
ACTAAAATTA	BRDN00011	187	87	51	348	593	13
ACTAAACTGT	BRDN00012	233	181	161	368	344	0
ACTAAAGAA	BRDN00011	29	0	0	508	56	0
ACTAAAGCTC	BRDN00011	80	31	1	770	537	0
ACTAAAGGA	BRDN00011	499	455	687	963	1147	19
ACTAAAGTT	BRDN00012	284	234	367	469	463	3
ACTAAATACA	BRDN00011	209	189	52	304	241	25
ACTAAATCTC	BRDN00011	19	0	0	465	97	0
ACTAAATGA	BRDN00012	221	0	0	464	225	0
ACTAAATGG	BRDN00012	158	131	6	276	464	11
ACTAACAGA	BRDN00011	176	160	109	246	610	31
ACTAACAGG	BRDN00011	13	0	0	405	77	0
ACTAACAGTA	BRDN00012	364	249	297	614	597	39
ACTAACATGT	BRDN00012	284	114	109	557	352	2
ACTAACCAAC	BRDN00012	105	135	86	199	431	0
ACTAACCAGA	BRDN00011	211	204	32	329	388	8
ACTAACCAGA	BRDN00011	441	361	557	920	1007	55
ACTAACCATG	BRDN00012	377	370	807	774	571	41
ACTAACCATT	BRDN00012	176	188	111	359	288	23
ACTAACCGGT	BRDN00012	136	17	98	1068	1225	0
ACTAACCGTC	BRDN00012	218	121	0	533	343	0
ACTAACCTGA	BRDN00012	198	240	49	247	221	32
ACTAACGGG	BRDN00011	295	291	276	559	532	88
ACTAACGGT	BRDN00012	240	246	880	702	478	19
ACTAACGTTA	BRDN00012	540	360	663	957	863	112
ACTAAGACTC	BRDN00011	370	384	2376	669	905	91
ACTAAGATCT	BRDN00012	165	115	44	284	275	18
ACTAAGCAG	BRDN00012	374	117	142	994	715	17
ACTAAGCAG	BRDN00011	502	465	443	967	1138	36
ACTAAGCCGT	BRDN00012	574	534	337	735	939	54
ACTAAGCGT	BRDN00011	187	162	685	209	277	52
ACTAAGCTG	BRDN00011	135	160	7	324	2062	27
ACTAAGGGT	BRDN00011	100	186	1118	307	234	23
ACTAATAACC	BRDN00011	200	77	66	233	150	25
ACTAATACAC	BRDN00012	180	99	553	350	569	27
ACTAATACTC	BRDN00012	224	143	10	397	184	0
ACTAATCCAG	BRDN00012	155	128	137	278	399	11
ACTAATGGA	BRDN00012	269	331	1602	766	436	0
ACTAATGGT	BRDN00011	120	137	177	293	259	38
ACTAATGTAC	BRDN00012	325	285	137	868	598	6
ACTAATTACC	BRDN00012	115	101	150	248	425	0
ACTAATTGA	BRDN00012	364	241	209	433	498	0
ACTAATTGTC	BRDN00012	181	247	72	375	220	8
ACTACAAAAC	BRDN00012	18	0	0	504	240	0
ACTACAAACA	BRDN00012	432	315	627	569	642	54
ACTACAAATC	BRDN00012	236	253	356	590	365	22
ACTACAACA	BRDN00012	92	43	273	344	265	0
ACTACAACTA	BRDN00011	211	184	150	308	308	0
ACTACAACTC	BRDN00011	110	80	58	246	329	0
ACTACAAGCC	BRDN00012	247	223	1605	405	979	8
ACTACAAGTA	BRDN00012	158	96	62	332	549	3
ACTACACACT	BRDN00012	310	205	71	733	1052	16
ACTACACCAA	BRDN00011	163	170	790	365	563	22
ACTACACCAC	BRDN00011	290	19	0	666	505	0
ACTACACCTT	BRDN00011	178	154	421	285	313	40
ACTACACGTC	BRDN00011	239	188	27	495	684	5
ACTACAGAA	BRDN00011	281	218	374	612	701	87
ACTACAGAG	BRDN00011	203	174	906	367	299	10
ACTACAGATC	BRDN00011	183	138	1503	246	496	32
ACTACAGCCA	BRDN00012	546	429	1521	955	949	122
ACTACAGCCA	BRDN00011	230	14	22	432	277	0
ACTACAGCGA	BRDN00011	180	181	247	356	320	0
ACTACAGCTC	BRDN00012	196	29	16	324	203	0
ACTACAGGA	BRDN00012	304	208	201	483	278	14
ACTACAGGC	BRDN00012	417	378	435	693	662	42
ACTACAGTCA	BRDN00011	88	73	17	250	136	0
ACTACAGTGA	BRDN00011	358	306	333	615	499	65
ACTACATAAC	BRDN00012	413	712	4775	772	1743	300
ACTACATCAA	BRDN00012	311	598	259	698	516	55
ACTACATCCA	BRDN00011	273	0	1	636	269	15
ACTACATCTA	BRDN00012	218	126	372	424	458	16
ACTACATGTA	BRDN00012	174	90	364	418	687	24
ACTACCAACC	BRDN00012	349	448	1016	837	470	40
ACTACCAAGC	BRDN00011	479	53	2	1286	1358	1
ACTACCAAGC	BRDN00012	464	210	230	785	772	377
ACTACCACTT	BRDN00012	165	47	30	328	160	0
ACTACCATGT	BRDN00011	123	117	639	243	194	8
ACTACCCAAC	BRDN00012	289	347	310	436	651	9
ACTACCCAAT	BRDN00012	173	124	52	377	510	0
ACTACCCAGA	BRDN00011	84	100	13	178	362	5
ACTACCCGGA	BRDN00011	226	149	1190	423	517	12
ACTACCCGTT	BRDN00011	126	65	171	378	167	0
ACTACCGAAA	BRDN00011	44	10	0	525	237	0
ACTACCGCCT	BRDN00011	101	111	0	244	200	0
ACTACCGGCA	BRDN00011	209	248	517	375	274	2
ACTACCGGG	BRDN00012	215	388	92	434	335	64

ACTACCTGCT	BRDN00011	364	381	418	714	576	24
ACTACGACC	BRDN00012	155	132	303	295	232	167
ACTACGACCT	BRDN00011	198	218	765	409	267	40
ACTACGACT	BRDN00011	163	139	196	198	391	0
ACTACGACT	BRDN00012	311	214	486	537	307	11
ACTACGAGT	BRDN00011	153	192	10	550	801	0
ACTACGATG	BRDN00011	297	225	82	575	667	10
ACTACGGAC	BRDN00011	153	162	35	212	154	44
ACTACGGAC	BRDN00011	442	336	1190	856	551	35
ACTACGGCG	BRDN00011	336	295	448	392	587	64
ACTACTACAG	BRDN00012	333	205	708	674	576	35
ACTACTACAG	BRDN00012	86	90	234	258	258	0
ACTACTACAG	BRDN00012	48	0	0	224	78	0
ACTACTACCA	BRDN00012	390	318	1166	856	592	28
ACTACTCACG	BRDN00011	292	161	347	409	495	14
ACTACTCCAA	BRDN00012	178	145	327	342	294	16
ACTACTCCGG	BRDN00012	464	388	492	720	985	14
ACTACTCGG	BRDN00011	95	93	19	248	331	0
ACTACTCTTA	BRDN00011	246	223	345	416	695	30
ACTACTGGCT	BRDN00012	650	573	742	1063	1609	56
ACTACTGGG	BRDN00012	231	52	29	498	390	8
ACTACTGGT	BRDN00012	184	150	184	261	173	8
ACTACTGTAC	BRDN00012	141	0	0	335	281	0
ACTACTTACA	BRDN00011	161	114	68	298	268	0
ACTACTTACC	BRDN00012	18	0	0	385	0	0
ACTACTTACC	BRDN00011	50	27	0	295	20	1
ACTACTTAGA	BRDN00011	230	232	119	369	220	6
ACTACTTAGT	BRDN00012	260	196	538	680	717	27
ACTACTTCAG	BRDN00012	326	247	712	618	440	16
ACTACTTCGT	BRDN00011	289	178	994	524	727	27
ACTACTTGAC	BRDN00011	243	257	295	421	373	40
ACTACTTGAC	BRDN00011	231	57	357	432	384	16
ACTACTTGTC	BRDN00012	473	651	1954	829	1339	45
ACTAGAACG	BRDN00011	288	119	1	661	413	0
ACTAGAACTC	BRDN00012	43	0	1	594	300	0
ACTAGAAGA	BRDN00012	160	57	127	390	240	6
ACTAGAAGG	BRDN00012	348	107	718	706	726	15
ACTAGAAGT	BRDN00012	326	70	1	663	367	8
ACTAGAATG	BRDN00012	420	304	384	660	704	15
ACTAGAATG	BRDN00012	165	126	41	334	276	0
ACTAGACACT	BRDN00011	239	134	4215	481	397	178
ACTAGACAG	BRDN00011	128	95	42	262	515	8
ACTAGACCAC	BRDN00012	543	447	711	1125	934	61
ACTAGAGCC	BRDN00011	269	358	1684	438	598	88
ACTAGAGTC	BRDN00012	489	235	482	644	605	27
ACTAGATCTT	BRDN00012	27	4	0	219	2	0
ACTAGATGC	BRDN00011	140	117	21	254	252	27
ACTAGCAAA	BRDN00012	117	90	420	321	98	14
ACTAGCAGT	BRDN00011	217	26	0	513	328	0
ACTAGCAGT	BRDN00012	109	45	0	232	218	0
ACTAGCATAT	BRDN00012	139	96	6	259	387	26
ACTAGCCTGT	BRDN00012	408	467	1753	903	1155	50
ACTAGCTCTT	BRDN00012	188	217	81	253	302	18
ACTAGCTGCC	BRDN00011	75	117	54	198	220	9
ACTAGCTTA	BRDN00011	150	174	50	498	663	14
ACTAGGAAC	BRDN00012	90	110	1772	241	93	58
ACTAGGATG	BRDN00012	229	351	216	521	413	23
ACTAGGCGA	BRDN00011	352	263	278	725	732	30
ACTAGGGCA	BRDN00012	619	574	488	1068	1023	84
ACTAGGGCG	BRDN00012	114	120	99	257	370	3
ACTAGGGCT	BRDN00012	352	239	164	499	771	23
ACTAGGTTA	BRDN00012	199	201	22	340	257	0
ACTAGTACAC	BRDN00011	169	210	480	327	261	70
ACTAGTAGG	BRDN00011	217	0	0	465	471	0
ACTAGTATAT	BRDN00012	144	9	135	403	473	0
ACTAGTCCG	BRDN00012	88	64	234	148	90	6
ACTAGTCTCA	BRDN00011	194	163	25	487	506	19
ACTATAAAAT	BRDN00012	289	338	684	514	450	33
ACTATAAAG	BRDN00011	104	126	100	257	208	0
ACTATAAAG	BRDN00012	176	22	2	354	211	0
ACTATAACAC	BRDN00012	90	118	55	143	106	5
ACTATACAG	BRDN00011	184	135	55	521	321	0
ACTATAGGA	BRDN00011	307	414	2145	428	391	29
ACTATAGGC	BRDN00012	332	220	438	678	625	41
ACTATAGGG	BRDN00011	108	244	1430	256	259	11
ACTATAGGT	BRDN00011	219	129	1078	291	182	47
ACTATATACC	BRDN00012	174	149	615	328	191	2
ACTATATTAG	BRDN00011	63	66	0	182	98	26
ACTATATTGA	BRDN00011	258	1	0	864	946	0
ACTATATTGC	BRDN00011	111	41	52	174	107	0
ACTATCATGC	BRDN00012	698	623	652	1160	1646	37
ACTATCCCAA	BRDN00012	497	406	377	934	677	63
ACTATCCCAT	BRDN00011	460	428	1040	846	865	38
ACTATCTAAA	BRDN00011	204	320	366	525	649	43
ACTATCTCTA	BRDN00012	125	95	0	251	593	0
ACTATCTGA	BRDN00011	189	86	631	355	577	27
ACTATCTTCC	BRDN00012	209	110	268	407	285	0
ACTATCTTGA	BRDN00011	143	138	218	196	91	21
ACTATGAAA	BRDN00012	88	95	396	177	131	8
ACTATGACAC	BRDN00012	402	373	447	727	1437	22
ACTATGACCC	BRDN00012	295	120	453	837	610	12
ACTATGAGA	BRDN00011	146	239	679	234	321	35
ACTATGATCC	BRDN00012	203	237	2102	563	456	45
ACTATGATCC	BRDN00012	203	56	0	499	773	0
ACTATGATTC	BRDN00011	307	187	2091	500	409	20
ACTATGCAG	BRDN00012	106	150	104	370	367	0
ACTATGCAT	BRDN00011	245	218	821	296	396	7
ACTATGCATC	BRDN00012	390	217	574	624	561	51
ACTATGCCCT	BRDN00011	285	161	601	533	761	27
ACTATGCCTA	BRDN00012	179	189	1285	278	213	39
ACTATGCTTG	BRDN00011	235	211	533	460	403	22
ACTATGGCA	BRDN00011	153	108	66	121	144	58
ACTATGGCA	BRDN00012	328	436	1297	725	609	16
ACTATGGCA	BRDN00012	217	197	23	481	398	14
ACTATGGCA	BRDN00012	278	365	216	412	419	31
ACTATGGCT	BRDN00011	124	8	0	331	270	0
ACTATGTGCC	BRDN00011	196	274	379	270	311	35
ACTATGTGTC	BRDN00011	321	242	112	622	493	16
ACTATTAAG	BRDN00011	65	9	0	294	60	0
ACTATTAAGC	BRDN00011	94	142	1	195	211	8
ACTATTATAT	BRDN00012	168	203	155	338	293	17
ACTATTCGTC	BRDN00011	231	219	200	381	218	18
ACTATTCTGT	BRDN00012	154	188	1084	380	481	833
ACTATTGACA	BRDN00012	433	268	885	682	569	56
ACTATTGCAC	BRDN00012	330	225	208	823	519	70
ACTATTGCTC	BRDN00011	126	110	103	311	574	6
ACTATTTAAT	BRDN00012	523	554	1501	769	994	69
ACTCAAAAAC	BRDN00011	177	116	2542	218	202	82
ACTCAAAAAC	BRDN00012	162	145	291	294	168	0
ACTCAAAACC	BRDN00011	305	186	55	680	483	5
ACTCAAACCT	BRDN00011	197	123	187	359	626	19
ACTCAAACCT	BRDN00012	557	485	578	811	416	39
ACTCAAACTT	BRDN00012	157	243	26	362	245	8
ACTCAAAGA	BRDN00011	104	118	193	282	46	0
ACTCAAAGA	BRDN00011	160	173	11	190	301	0
ACTCAAAGG	BRDN00012	247	311	543	455	655	5
ACTCAACACA	BRDN00012	366	281	2148	788	828	79
ACTCAACAGC	BRDN00012	264	201	125	446	694	0
ACTCAAGCA	BRDN00011	196	107	39	232	583	25
ACTCAAGCCT	BRDN00011	289	483	1414	470	602	27
ACTCAAGGA	BRDN00012	189	1	0	296	161	0
ACTCAATCGT	BRDN00012	167	97	273	309	615	0
ACTCAATTAC	BRDN00011	289	248	86	517	396	18
ACTCAATTAT	BRDN00011	410	464	467	682	607	28
ACTCAATTCC	BRDN00011	243	182	74	519	503	26
ACTCACAAA	BRDN00012	520	378	4529	846	700	69
ACTCACAAAC	BRDN00012	259	317	109	656	587	8
ACTCACACAA	BRDN00011	393	292	1927	638	1092	57
ACTCACACCA	BRDN00012	489	397	259	955	513	85
ACTCACACCC	BRDN00011	274	256	591	287	392	17
ACTCACACGA	BRDN00012	152	84	0	397	202	4
ACTCACACTG	BRDN00011	697	592	250	1351	1502	34
ACTCACACTT	BRDN00012	388	320	1893	742	445	21
ACTCACACTT	BRDN00011	211	152	103	313	661	11
ACTCACAGCA	BRDN00012	118	190	1090	328	528	36
ACTCACAGCT	BRDN00012	146	42	32	178	237	10
ACTCACAGG	BRDN00012	168	231	1071	530	1025	1
ACTCACATAA	BRDN00012	295	189	403	556	287	39
ACTCACATAT	BRDN00011	213	84	0	341	593	10
ACTCACATCA	BRDN00011	406	292	783	825	764	19
ACTCACATGA	BRDN00012	143	49	493	474	223	45
ACTCACATGA	BRDN00011	364	220	178	759	659	48
ACTCACATGA	BRDN00011	217	187	381	364	461	48
ACTCACATGT	BRDN00012	610	364	548	982	991	14
ACTCACATTC	BRDN00012	154	33	61	286	141	0
ACTCACATTC	BRDN00012	318	274	1591	722	611	38
ACTCACATTT	BRDN00011	175	0	1	558	275	0
ACTCACCAAA	BRDN00012	530	471	1091	1011	1114	36
ACTCACCAAA	BRDN00012	109	184	575	280	506	32
ACTCACCAAA	BRDN00011	107	151	380	237	326	11
ACTCACCAAT	BRDN00012	503	371	606	716	819	13
ACTCACCACA	BRDN00012	246	187	347	347	240	30
ACTCACCACA	BRDN00012	481	396	104	891	823	17
ACTCACCAGC	BRDN00011	501	656	639	1253	1358	94
ACTCACCAGT	BRDN00011	161	153	5	192	381	8
ACTCACCATC	BRDN00011	225	109	59	423	481	7
ACTCACCATC	BRDN00012	408	434	122	651	1028	24
ACTCACCCAC	BRDN00012	607	320	4214	1192	1093	77
ACTCACCCAC	BRDN00011	591	610	562	1019	887	92
ACTCACCCAT	BRDN00011	281	238	472	348	280	35
ACTCACCCGA	BRDN00011	77	39	572	162	161	11
ACTCACCCGG	BRDN00012	29	2	0	1013	256	0
ACTCACCCGG	BRDN00011	241	180	136	668	348	17
ACTCACCCTT	BRDN00012	63	184	111	224	124	0
ACTCACCGA	BRDN00011	482	596	492	966	780	72
ACTCACCGAC	BRDN00011	109	140	533	209	213	0
ACTCACCGAC	BRDN00012	133	139	138	302	350	7
ACTCACCGAC	BRDN00012	148	32	7	344	235	15
ACTCACCGAT	BRDN00012	594	527	811	1026	809	10
ACTCACCGAT	BRDN00011	89	46	8	371	398	12
ACTCACCGCA	BRDN00011	119	141	63	237	259	39
ACTCACCGGT	BRDN00012	343	343	393	617	764	40
ACTCACCGTC	BRDN00012	131	90	710	263	320	19
ACTCACCGTG	BRDN00011	434	550	6642	825	626	198
ACTCACCTAA	BRDN00012	171	229	231	282	356	2
ACTCACCTAA	BRDN00012	186	0	1	433	459	0
ACTCACCTAC	BRDN00012	178	193	69	359	418	18
ACTCACCTCT	BRDN00011	132	129	555	376	275	33
ACTCACCTGC	BRDN00011	379	209	433	411	414	90
ACTCACCTGT	BRDN00011	492	491	728	834	877	8
ACTCACCTTC	BRDN00012	220	254	15	491	213	34
ACTCACCTTC	BRDN00012	102	11	0	218	283	0
ACTCACCTTG	BRDN00012	323	387	218	561	458	122
ACTCACGACC	BRDN00012	423	409	1632	701	770	53
ACTCACGCA	BRDN00011	188	54	148	253	261	34
ACTCACGCCG	BRDN00011	57	100	49	191	94	16
ACTCACGCTC	BRDN00011	216	173	93	434	488	14
ACTCACGCTT	BRDN00011	183	165	60	356	340	14
ACTCACGGG	BRDN00012	246	283	1205	364	359	44
ACTCACGGG	BRDN00011	84	83	460	281	296	25
ACTCACGGG	BRDN00011	222	85	15	493	402	0
ACTCACGGTT	BRDN00012	408	500	1091	809	619	33
ACTCACGTCG	BRDN00012	217	198	731	589	452	77
ACTCACGTGA	BRDN00011	317	263	2662	467	673	32
ACTCACGTGC	BRDN00011	152	147	232	331	480	37
ACTCACTAGT	BRDN00012	135	178	754	389	385	26
ACTCACTATC	BRDN00011	268	326	1467	607	433	52
ACTCACTCAA	BRDN00012	237	129	67	394	199	6
ACTCACTCAT	BRDN00012	173	93	7	344	424	0
ACTCACTCGC	BRDN00012	501	425	611	949	1183	47
ACTCACTTGA	BRDN00011	190	136	236	379	225	7
ACTCACTTTA	BRDN00011	243	517	1041	662	470	69
ACTCACTTTA	BRDN00011	278	173	480	548	475	12
ACTCAGAAG	BRDN00011	354	343	1377	823	567	88
ACTCAGACC	BRDN00012	160	15	0	372	174	0
ACTCAGACG	BRDN00011	563	443	9976	879	1147	93
ACTCAGAGA	BRDN00011	406	305	4007	580	488	58
ACTCAGCACT	BRDN00012	242	398	173	401	458	7
ACTCAGCCAT	BRDN00012	509	540	1116	682	926	58
ACTCAGCTTC	BRDN00011	396	440	865	648	601	62
ACTCAGGAA	BRDN00011	113	43	1	503	226	0
ACTCAGGGA	BRDN00011	339	366	680	692	862	43
ACTCAGGTA	BRDN00011	457	509	328	834	597	36
ACTCAGTATC	BRDN00011	112	84	296	276	100	22
ACTCAGTCCC	BRDN00012	165	98	211	424	196	0
ACTCAGTCG	BRDN00011	323	198	811	484	359	15
ACTCAGTGA	BRDN00012	382	213	295	1015	476	3
ACTCAGTGCT	BRDN00011	280	241	391	623	784	18
ACTCAGTTCA	BRDN00011	58	0	0	448	486	0
ACTCAGTTCA	BRDN00012	394	430	306	909	1957	111
ACTCATAAG	BRDN00012	211	107	228	555	413	21
ACTCATACCT	BRDN00011	403	322	119	719	636	21
ACTCATAGG	BRDN00012	169	153	578	423	263	14
ACTCATAGTC	BRDN00012	172	88	878	403	302	65
ACTCATCAAA	BRDN00011	280	345	140	691	855	75
ACTCATCAGT	BRDN00011	266	318	354	445	879	66
ACTCATCATG	BRDN00012	300	208	747	334	433	54
ACTCATCCAT	BRDN00012	290	189	604	462	853	58
ACTCATGACC	BRDN00012	331	253	169	591	347	12
ACTCATGCTG	BRDN00012	164	0	1	335	311	0
ACTCATTCAA	BRDN00011	194	137	366	293	271	13
ACTCATTCCT	BRDN00011	293	0	0	387	77	0
ACTCATTCGA	BRDN00012	312	276	642	448	489	28
ACTCATTGCC	BRDN00012	254	152	203	619	627	15
ACTCATTGTT	BRDN00011	165	160	129	339	291	31
ACTCCAAACC	BRDN00011	184	2	0	675	622	0
ACTCCAAATT	BRDN00011	98	0	0	374	342	0
ACTCCAACGA	BRDN00012	539	668	3398	905	1151	42
ACTCCAAGA	BRDN00012	182	271	7472	378	298	68
ACTCCAAGA	BRDN00011	207	207	63	422	463	8
ACTCCAAGA	BRDN00011	326	277	1039	523	521	41
ACTCCAAGAC	BRDN00011	121	181	72	361	402	0
ACTCCAAGTA	BRDN00011	298	228	177	405	436	26
ACTCCAATGC	BRDN00011	170	114	7	296	94	1
ACTCCACACG	BRDN00012	465	440	1244	687	1097	50
ACTCCACAGA	BRDN00011	220	192	525	422	224	14
ACTCCACCAC	BRDN00012	186	54	5	729	105	2
ACTCCACCCA	BRDN00012	378	102	346	1212	571	26
ACTCCACCTG	BRDN00012	461	285	429	536	642	35
ACTCCACGTC	BRDN00012	449	373	274	988	777	25
ACTCCACTGA	BRDN00011	144	142	301	249	391	1
ACTCCAGATA	BRDN00012	304	257	342	507	269	85
ACTCCAGATT	BRDN00011	242	210	31	581	795	22
ACTCCAGCA	BRDN00011	149	199	131	300	328	27
ACTCCAGGA	BRDN00011	223	122	294	373	437	11
ACTCCAGGA	BRDN00012	518	355	560	1048	1275	46
ACTCCAGTCC	BRDN00012	134	106	163	241	415	5
ACTCCAGTTA	BRDN00012	152	41	376	380	320	12
ACTCCATAAG	BRDN00011	246	221	2	708	563	0
ACTCCATCCA	BRDN00012	161	177	30	427	619	8
ACTCCATCGC	BRDN00012	523	2	2	1066	1045	0
ACTCCATGGC	BRDN00011	561	585	441	1026	633	34
ACTCCATGTC	BRDN00011	264	176	321	406	643	19
ACTCCATTCG	BRDN00011	329	354	193	730	634	40
ACTCCATTTG	BRDN00012	125	101	84	273	302	11
ACTCCCAAAA	BRDN00012	230	202	239	616	569	30
ACTCCCAAAC	BRDN00011	430	234	246	902	990	19
ACTCCCACAA	BRDN00011	306	183	1390	542	784	59
ACTCCCAGA	BRDN00011	81	156	49	337	154	0
ACTCCCAGA	BRDN00012	57	67	282	320	89	0
ACTCCCATGT	BRDN00012	310	411	354	669	622	30
ACTCCCATTA	BRDN00012	361	285	2534	669	1287	115
ACTCCCATTT	BRDN00011	175	61	0	204	169	0
ACTCCCCGGA	BRDN00012	91	78	14	303	109	13
ACTCCCCTTG	BRDN00011	241	253	43	403	319	87
ACTCCCGACA	BRDN00012	397	245	312	460	215	81
ACTCCCGCTT	BRDN00012	284	170	449	1220	1182	25
ACTCCCGGA	BRDN00012	99	65	0	262	209	6
ACTCCCGGA	BRDN00011	93	100	0	231	249	28
ACTCCCGGAT	BRDN00011	146	113	237	418	521	8
ACTCCCGGTC	BRDN00011	187	162	45	365	221	11
ACTCCCTAAA	BRDN00012	209	223	122	352	99	31
ACTCCCTAAA	BRDN00012	245	239	651	498	475	65
ACTCCCTCAC	BRDN00011	255	216	696	512	576	70
ACTCCGAAAC	BRDN00012	393	278	479	442	434	133
ACTCCGAATT	BRDN00011	277	267	619	536	297	49
ACTCCGACAC	BRDN00011	398	168	36	991	894	0
ACTCCGACAT	BRDN00012	199	233	0	419	203	27
ACTCCGACCT	BRDN00011	181	131	338	206	249	9
ACTCCGATAC	BRDN00011	61	66	0	246	205	0
ACTCCGGGA	BRDN00011	323	257	366	617	367	69
ACTCCGTACC	BRDN00012	414	185	225	1024	780	6
ACTCCGTACG	BRDN00011	48	54	19	254	45	58
ACTCCGTACT	BRDN00011	254	85	19	506	455	0
ACTCCGTCAG	BRDN00012	128	136	341	295	288	0
ACTCCGTCAT	BRDN00012	332	303	111	641	668	10
ACTCCGTGCA	BRDN00012	583	354	1444	952	1079	95
ACTCCTACGG	BRDN00011	150	235	96	281	454	0
ACTCCTACGG	BRDN00011	208	190	121	448	368	2
ACTCCTACTT	BRDN00012	394	388	1041	831	782	44
ACTCCTAGAT	BRDN00011	135	224	44	271	104	13
ACTCCTAGCA	BRDN00012	171	183	58	310	212	21
ACTCCTCACT	BRDN00012	298	289	160	561	512	28
ACTCCTCAGA	BRDN00011	135	212	117	334	341	18
ACTCCTCGAT	BRDN00011	258	287	453	687	521	47
ACTCCTCTTA	BRDN00011	219	244	49	399	311	19
ACTCCTGAAT	BRDN00012	522	483	1147	1042	803	50
ACTCCTGACA	BRDN00011	223	119	0	329	380	23
ACTCCTGCTC	BRDN00011	147	54	381	276	213	6
ACTCCTGGAC	BRDN00012	386	415	1367	1005	1199	59
ACTCCTGGG	BRDN00012	248	216	590	579	682	15
ACTCCTGGG	BRDN00012	189	317	939	337	562	122
ACTCCTGGTT	BRDN00011	253	118	351	281	233	6
ACTCCTGTAC	BRDN00011	174	109	323	224	179	0
ACTCCTTGCT	BRDN00011	177	182	106	393	368	5
ACTCCTTGGC	BRDN00012	337	494	366	490	346	49
ACTCCTTGGC	BRDN00011	221	283	327	418	355	20
ACTCCTTTAT	BRDN00011	192	243	14	390	541	3
ACTCCTTTAT	BRDN00012	341	344	1077	588	271	20
ACTCCTTTAT	BRDN00011	85	84	235	347	272	16
ACTCGAAATC	BRDN00012	592	536	1241	959	539	28
ACTCGAACTC	BRDN00011	647	781	1394	991	859	55
ACTCGAACTC	BRDN00011	230	168	697	264	98	12
ACTCGAACTC	BRDN00012	286	250	1033	710	865	29
ACTCGAAGA	BRDN00011	138	229	472	315	529	69
ACTCGAAGG	BRDN00011	299	263	94	556	396	60
ACTCGAATAT	BRDN00011	227	229	276	436	458	6
ACTCGAATG	BRDN00011	315	216	2123	371	374	129
ACTCGACTAC	BRDN00012	325	155	1157	447	391	50
ACTCGACTTC	BRDN00012	162	100	104	348	321	6
ACTCGAGTA	BRDN00011	374	253	2	727	911	2
ACTCGAGTA	BRDN00011	453	495	804	944	1065	33
ACTCGAGTA	BRDN00012	349	247	944	606	659	24
ACTCGAGTTC	BRDN00012	453	359	1238	884	871	56
ACTCGATCCA	BRDN00012	345	261	83	496	468	9
ACTCGATGT	BRDN00012	297	373	1393	655	315	8
ACTCGATTTA	BRDN00012	191	179	111	398	167	87
ACTCGCAGG	BRDN00012	703	620	612	1265	1114	69
ACTCGCATAT	BRDN00011	183	109	1465	326	728	25
ACTCGCATCC	BRDN00012	376	197	1297	605	430	19
ACTCGCCACT	BRDN00011	431	516	833	622	660	31
ACTCGCCCTC	BRDN00011	319	420	488	757	464	40
ACTCGCCGCT	BRDN00011	140	191	1983	253	200	15
ACTCGCGCCC	BRDN00012	102	110	268	234	109	5
ACTCGCGGG	BRDN00011	148	146	947	218	268	6
ACTCGCTTAG	BRDN00012	374	262	90	626	434	0
ACTCGCTTTA	BRDN00012	58	14	157	544	56	12
ACTCGGAGA	BRDN00012	329	321	853	946	890	15
ACTCGGAGC	BRDN00011	200	174	956	499	555	11
ACTCGGAGG	BRDN00012	454	365	1321	836	518	20
ACTCGGCAG	BRDN00011	163	64	25	210	137	0
ACTCGGCCA	BRDN00012	263	223	21	563	357	9
ACTCGGCCCC	BRDN00011	149	34	1	473	224	0
ACTCGGCCGT	BRDN00012	94	171	10	250	80	13
ACTCGGCCTC	BRDN00012	498	617	1362	1000	838	176
ACTCGGCGC	BRDN00012	90	81	51	166	113	11
ACTCGGGCC	BRDN00012	224	190	811	383	228	7
ACTCGGTGC	BRDN00011	103	43	105	174	143	0
ACTCGGTGT	BRDN00011	122	70	443	324	375	14
ACTCGGTTCA	BRDN00012	473	361	199	822	937	37
ACTCGTAAAC	BRDN00012	565	346	1202	906	807	173
ACTCGTAAGT	BRDN00011	131	107	9	320	327	0
ACTCGTAATC	BRDN00011	339	125	181	452	278	14
ACTCGTACAC	BRDN00011	246	216	119	560	593	53
ACTCGTCGG	BRDN00012	154	21	31	326	540	2
ACTCGTCTAT	BRDN00012	303	256	759	357	625	37
ACTCGTCTGT	BRDN00012	346	276	696	651	834	83
ACTCTACCAT	BRDN00011	257	222	690	431	457	25
ACTCTACCTA	BRDN00012	289	242	51	345	353	0
ACTCTACCTT	BRDN00012	254	283	226	584	852	0
ACTCTACGA	BRDN00011	16	0	0	648	330	0
ACTCTACGG	BRDN00012	179	153	278	280	271	18
ACTCTACTAT	BRDN00011	109	83	570	175	176	26
ACTCTAGCCT	BRDN00012	356	256	311	562	571	69
ACTCTAGTCA	BRDN00011	163	197	1415	257	154	63
ACTCTATACC	BRDN00011	285	239	77	487	413	14
ACTCTATAGC	BRDN00012	198	133	1	483	146	0
ACTCTCAAG	BRDN00011	199	209	243	274	218	10
ACTCTCACGT	BRDN00011	295	131	198	449	494	7
ACTCTCAGG	BRDN00011	259	181	845	627	1041	26
ACTCTCCAGG	BRDN00011	125	108	21	391	259	12
ACTCTCCCAT	BRDN00011	185	56	15	460	348	1
ACTCTCCGTG	BRDN00012	296	435	1487	834	543	11
ACTCTCCTTC	BRDN00012	305	121	172	392	149	0
ACTCTCGAG	BRDN00012	379	317	510	743	660	8
ACTCTCGAGT	BRDN00011	129	74	104	284	319	2
ACTCTCGCCA	BRDN00012	153	126	40	336	473	7
ACTCTCGGA	BRDN00012	163	131	33	247	542	38
ACTCTCGGAC	BRDN00012	286	129	114	850	1017	7
ACTCTCGGCC	BRDN00011	338	170	424	630	749	4
ACTCTCGGG	BRDN00011	78	86	32	137	253	0
ACTCTCGTGT	BRDN00012	309	278	353	597	745	2
ACTCTCGTTC	BRDN00011	199	192	792	315	439	5
ACTCTCTATA	BRDN00011	296	238	28	353	572	42
ACTCTCTCAA	BRDN00011	537	456	592	844	1583	55
ACTCTCTCCA	BRDN00012	408	330	246	827	1183	16
ACTCTCTGAA	BRDN00012	441	271	227	625	724	30
ACTCTCTGAT	BRDN00011	281	336	821	685	573	7
ACTCTCTGAT	BRDN00011	83	60	0	225	127	18
ACTCTCTTAC	BRDN00012	258	270	234	461	479	36
ACTCTCTTGA	BRDN00012	253	215	17	390	432	37
ACTCTGAAG	BRDN00011	128	41	93	385	413	6
ACTCTGAAG	BRDN00012	177	88	254	458	471	22
ACTCTGAATC	BRDN00011	112	4	0	239	239	0
ACTCTGAATT	BRDN00012	418	268	90	786	660	20
ACTCTGAGG	BRDN00011	124	216	1232	421	457	3
ACTCTGATAC	BRDN00012	308	275	297	673	1289	0
ACTCTGCATG	BRDN00011	243	222	231	428	588	1
ACTCTGCCAC	BRDN00012	64	76	60	322	309	0
ACTCTGCGAC	BRDN00011	160	164	297	291	99	10
ACTCTGCGTC	BRDN00011	429	298	729	771	973	6
ACTCTGGAA	BRDN00011	78	0	439	291	182	9
ACTCTGGAA	BRDN00011	113	112	4	219	185	10
ACTCTGGAA	BRDN00011	305	325	646	749	631	24
ACTCTGGAG	BRDN00011	597	469	1049	1102	972	49
ACTCTGGCTT	BRDN00011	107	186	378	343	148	26
ACTCTGGTCT	BRDN00011	420	421	541	717	581	8
ACTCTGTACA	BRDN00012	130	189	67	173	438	9
ACTCTGTAG	BRDN00011	171	13	245	393	405	6
ACTCTGTCTC	BRDN00011	288	251	1823	416	404	60
ACTCTGTCTT	BRDN00011	128	188	1292	267	124	8
ACTCTGTCTT	BRDN00012	312	153	79	248	299	0
ACTCTGTGCA	BRDN00011	182	259	571	530	567	53
ACTCTGTGCT	BRDN00012	1	0	1	205	81	0
ACTCTTAAAT	BRDN00011	100	62	200	229	293	5
ACTCTTAACA	BRDN00011	300	178	306	609	367	15
ACTCTTAAGC	BRDN00012	187	168	186	305	251	2
ACTCTTATAG	BRDN00011	296	346	3651	550	431	56
ACTCTTCAGA	BRDN00012	92	4	1	304	257	0
ACTCTTCAGT	BRDN00011	88	88	0	205	205	0
ACTCTTCCAT	BRDN00012	214	0	0	754	31	0
ACTCTTCCGG	BRDN00011	203	148	233	277	240	0
ACTCTTCGAT	BRDN00011	298	349	815	632	642	28
ACTCTTCGGA	BRDN00012	95	78	453	175	59	0
ACTCTTCGGC	BRDN00012	680	1104	834	1286	1132	184
ACTCTTCGGC	BRDN00011	152	169	45	543	473	5
ACTCTTCTAG	BRDN00012	217	130	4565	335	159	44
ACTCTTCTCC	BRDN00011	132	165	91	272	236	4
ACTCTTCTTC	BRDN00012	177	12	87	386	81	0
ACTCTTGCTT	BRDN00012	289	335	532	554	300	15
ACTCTTGGAC	BRDN00012	339	222	1077	694	602	14
ACTCTTGTCA	BRDN00011	94	71	188	235	168	5
ACTCTTTCAG	BRDN00011	142	94	237	432	391	28
ACTCTTTGGA	BRDN00012	149	68	74	345	513	0
ACTCTTTGTA	BRDN00011	167	128	337	146	333	72

ACTCTTTGTG BRDN00011		445	337	2455	850	1205	54
ACTGAAAAA BRDN00012		290	186	2040	382	521	25
ACTGAAAAT BRDN00011		126	64	0	272	256	0
ACTGAAACC BRDN00011		193	163	186	436	692	7
ACTGAAACT BRDN00011		251	218	1071	626	505	23
ACTGAAACT BRDN00011		223	469	137	360	437	51
ACTGAAACT BRDN00012		494	493	727	1040	1163	41
ACTGAAAGG BRDN00011		276	235	1254	522	704	27
ACTGAAAGG BRDN00012		421	374	1016	726	580	53
ACTGAAAGT BRDN00012		232	109	393	391	227	20
ACTGAAATC BRDN00011		274	210	603	404	472	58
ACTGAAATC BRDN00012		367	29	0	707	551	0
ACTGAACAA BRDN00011		512	417	652	742	762	17
ACTGAACAG BRDN00011		150	118	668	419	281	23
ACTGAACAT BRDN00011		367	556	485	518	405	26
ACTGAACCA BRDN00012		101	121	316	274	268	30
ACTGAACCA BRDN00012		252	290	390	557	552	30
ACTGAACTG BRDN00011		210	120	431	233	313	35
ACTGAAGCA BRDN00012		362	150	1	692	368	0
ACTGAAGCA BRDN00011		197	128	158	332	555	0
ACTGAAGCC BRDN00012		183	131	104	290	587	66
ACTGAAGCG BRDN00011		91	80	60	240	159	0
ACTGAAGCT BRDN00012		164	177	1035	296	286	19
ACTGAAGCT BRDN00012		334	416	591	549	356	43
ACTGAAGTC BRDN00011		232	85	0	412	133	0
ACTGAATAC BRDN00012		498	349	650	923	1317	64
ACTGAATCA BRDN00012		248	139	443	474	195	51
ACTGAATGA BRDN00012		330	347	388	655	417	9
ACTGAATGA BRDN00012		184	136	255	432	448	25
ACTGAATGA BRDN00012		420	259	169	877	756	50
ACTGAATGT BRDN00011		152	107	1	371	70	0
ACTGAATTG BRDN00011		375	284	977	577	592	43
ACTGAATTG BRDN00011		259	254	1137	446	350	17
ACTGACAAT BRDN00011		107	121	671	234	59	30
ACTGACAAT BRDN00012		340	396	458	577	975	54
ACTGACACT BRDN00011		340	393	257	700	1061	122
ACTGACAGA BRDN00012		229	225	207	455	785	30
ACTGACAGC BRDN00011		225	206	475	527	603	4
ACTGACATC BRDN00012		433	506	1962	925	1387	104
ACTGACATG BRDN00012		493	349	559	729	660	16
ACTGACCAA BRDN00012		390	250	784	605	752	57
ACTGACCAA BRDN00011		245	257	261	488	390	33
ACTGACCAA BRDN00011		468	316	414	766	1358	4
ACTGACCAA BRDN00012		222	195	1493	461	495	17
ACTGACCAC BRDN00011		134	163	159	274	481	7
ACTGACCAC BRDN00011		166	199	1	398	290	0
ACTGACCAC BRDN00011		219	343	856	400	528	33
ACTGACCAG BRDN00011		146	183	93	444	604	0
ACTGACCAT BRDN00011		6	0	0	241	42	0
ACTGACCCA BRDN00012		490	353	373	846	869	71
ACTGACCCA BRDN00011		696	638	1696	1341	1526	173
ACTGACCCC BRDN00011		371	291	70	738	566	3
ACTGACCCC BRDN00012		171	112	266	425	1246	38
ACTGACCGA BRDN00011		134	43	134	268	135	12
ACTGACCTG BRDN00011		405	223	233	689	659	19
ACTGACCTG BRDN00012		233	165	52	372	343	5
ACTGACCTG BRDN00011		514	422	515	1014	1123	17
ACTGACCTG BRDN00011		482	328	13	796	986	19
ACTGACCTTC BRDN00012		219	256	761	381	594	42
ACTGACGAG BRDN00012		213	212	882	320	240	17
ACTGACGCC BRDN00011		337	258	542	553	535	128
ACTGACGGC BRDN00011		482	379	4458	716	1005	112
ACTGACGGG BRDN00012		98	77	378	160	213	5
ACTGACGTA BRDN00012		192	169	89	357	722	0
ACTGACGTC BRDN00011		197	97	27	261	293	13
ACTGACTCCT BRDN00011		111	3	1	172	158	0
ACTGACTGC BRDN00012		424	317	348	641	533	13
ACTGACTGT BRDN00012		319	294	308	545	412	94
ACTGAGACC BRDN00012		488	453	1290	690	732	61
ACTGAGAGC BRDN00011		99	76	44	146	73	7
ACTGAGAGT BRDN00011		245	137	152	604	516	17
ACTGAGATT BRDN00011		181	118	214	345	366	12
ACTGAGCCA BRDN00011		213	253	14	453	341	13
ACTGAGCCC BRDN00012		286	290	185	515	329	20
ACTGAGCCT BRDN00011		414	340	262	666	1304	38
ACTGAGCTA BRDN00012		186	11	0	439	145	0
ACTGAGGAC BRDN00011		156	165	214	298	366	0
ACTGAGGAC BRDN00012		179	111	170	280	438	16
ACTGAGGAT BRDN00011		260	202	474	563	1403	0
ACTGAGGCG BRDN00011		122	198	1627	282	221	70
ACTGAGGTC BRDN00011		324	317	19	554	403	75
ACTGAGTAC BRDN00012		119	60	173	182	193	1
ACTGAGTGC BRDN00011		349	429	3981	675	815	94
ACTGAGTGG BRDN00012		591	664	463	1092	1266	36
ACTGATAAA BRDN00011		256	289	227	468	617	5
ACTGATAAG BRDN00011		243	283	140	322	380	26
ACTGATAAG BRDN00011		293	284	1076	457	783	8
ACTGATACT BRDN00011		191	109	857	447	366	19
ACTGATATC BRDN00011		221	245	340	418	312	11
ACTGATCAG BRDN00012		119	146	1125	279	274	35
ACTGATCCTG BRDN00011		163	196	3439	425	644	74
ACTGATGAG BRDN00011		13	0	0	232	45	0
ACTGATGAG BRDN00011		153	98	89	333	313	55
ACTGATGCA BRDN00011		273	283	1312	582	456	47
ACTGATGCC BRDN00011		310	196	194	551	925	9
ACTGATGGG BRDN00012		284	272	209	509	731	32
ACTGATGGG BRDN00011		356	332	136	730	571	9
ACTGATTAC BRDN00012		193	84	0	345	293	0
ACTGATTAT BRDN00012		143	178	0	271	90	0
ACTGATTCTA BRDN00012		132	0	0	365	296	0
ACTGATTGG BRDN00012		458	522	1683	965	766	39
ACTGATTTG BRDN00012		378	428	479	851	780	39
ACTGCAAAC BRDN00012		320	12	0	683	328	0
ACTGCAAAC BRDN00012		258	197	1194	496	906	24
ACTGCAAAT BRDN00012		359	245	366	688	654	5
ACTGCAACA BRDN00011		138	21	3271	157	192	59
ACTGCAACG BRDN00012		155	188	279	389	318	26
ACTGCAACT BRDN00012		554	672	914	1218	1287	59
ACTGCAAGA BRDN00012		464	285	242	816	1166	36
ACTGCAAGG BRDN00012		308	204	306	493	638	18
ACTGCAAGT BRDN00011		78	109	0	134	83	3
ACTGCAAGT BRDN00011		144	167	280	281	235	36
ACTGCAAGT BRDN00012		283	443	10	373	615	17
ACTGCAAGT BRDN00011		303	221	164	657	586	18
ACTGCAATG BRDN00011		151	45	0	679	1261	4
ACTGCAATG BRDN00011		307	210	451	685	398	11
ACTGCAATT BRDN00012		204	0	1	917	601	0
ACTGCACAA BRDN00011		277	275	450	578	690	89
ACTGCACAC BRDN00012		184	217	258	290	311	9
ACTGCACAC BRDN00011		454	366	863	698	772	44
ACTGCACCA BRDN00011		96	98	109	320	404	10
ACTGCACCTT BRDN00012		535	495	829	970	886	31
ACTGCACGC BRDN00011		234	77	32	449	568	33
ACTGCACGT BRDN00011		120	253	34	176	164	16
ACTGCACGT BRDN00012		274	280	244	535	415	130
ACTGCAGAC BRDN00011		219	166	131	425	459	32
ACTGCAGAG BRDN00012		196	165	337	351	126	12
ACTGCAGAG BRDN00011		82	19	4	264	345	10
ACTGCAGCA BRDN00012		80	42	1	521	659	0
ACTGCAGCA BRDN00012		364	236	419	684	849	42
ACTGCAGCA BRDN00012		155	104	14	650	472	0
ACTGCAGCT BRDN00012		304	332	357	733	897	58
ACTGCAGGA BRDN00011		108	41	126	197	109	10
ACTGCAGGC BRDN00012		137	158	6	278	410	36
ACTGCAGGG BRDN00012		133	52	434	265	488	16
ACTGCAGGG BRDN00012		225	189	166	275	293	15
ACTGCAGGG BRDN00011		117	133	45	247	179	32
ACTGCAGTG BRDN00012		449	481	1372	674	639	66
ACTGCAGTG BRDN00011		63	0	0	149	52	0
ACTGCAGTG BRDN00012		79	9	51	275	243	0
ACTGCATAA BRDN00012		395	354	173	590	817	36
ACTGCATAG BRDN00011		145	89	150	300	340	46
ACTGCATAT BRDN00011		195	221	598	242	239	0
ACTGCATAT BRDN00012		515	287	846	764	1203	15
ACTGCATCA BRDN00012		326	154	221	437	347	17
ACTGCATCA BRDN00012		187	200	275	383	501	61
ACTGCATCA BRDN00012		132	32	2	346	367	0
ACTGCATCC BRDN00011		456	372	2334	691	1216	38
ACTGCATCTG BRDN00011		159	204	435	447	1052	38
ACTGCATGC BRDN00012		428	429	800	761	671	68
ACTGCATGC BRDN00012		162	0	23	564	593	0
ACTGCATTA BRDN00011		167	171	354	280	457	56
ACTGCATTG BRDN00012		160	168	138	294	280	5
ACTGCATTG BRDN00011		214	257	3217	482	861	44
ACTGCCAAT BRDN00011		454	166	336	679	719	0
ACTGCCACA BRDN00012		379	231	802	574	775	118
ACTGCCACG BRDN00012		444	381	425	758	667	50
ACTGCCAGG BRDN00012		476	168	214	798	855	27
ACTGCCATG BRDN00012		430	474	480	1006	1190	103
ACTGCCATTC BRDN00011		341	335	191	696	903	58
ACTGCCCAA BRDN00011		631	635	1218	1420	1343	115
ACTGCCCAA BRDN00012		208	116	1020	356	274	7
ACTGCCCATA BRDN00011		245	381	103	376	608	27
ACTGCCCATA BRDN00011		562	582	3741	809	1459	65
ACTGCCCCA BRDN00011		237	106	184	461	432	0
ACTGCCCGC BRDN00011		41	3	0	200	56	0
ACTGCCCGC BRDN00012		576	513	554	862	797	45
ACTGCCCTAC BRDN00011		186	130	287	313	379	9
ACTGCCGAT BRDN00011		216	166	200	277	250	0
ACTGCCTACC BRDN00011		229	174	203	155	195	35
ACTGCCTAG BRDN00012		274	198	60	245	246	29
ACTGCCTATA BRDN00012		245	305	176	457	568	37
ACTGCCTATC BRDN00012		322	288	444	681	736	4
ACTGCCTCAG BRDN00011		219	233	185	525	305	2
ACTGCCTCTG BRDN00011		455	501	240	664	608	22
ACTGCCTCTT BRDN00012		285	266	120	424	446	16
ACTGCCTGA BRDN00012		24	59	0	931	106	0
ACTGCCTGA BRDN00011		170	112	84	219	364	56
ACTGCCTGC BRDN00012		498	471	1048	957	1108	29
ACTGCCTGG BRDN00012		168	36	28	315	331	0
ACTGCCTGG BRDN00011		161	137	60	306	417	21
ACTGCCTTAC BRDN00012		223	0	0	338	231	0
ACTGCGACC BRDN00012		186	180	159	448	572	17
ACTGCGAGG BRDN00011		478	221	989	791	908	8
ACTGCGATA BRDN00012		273	275	741	841	1065	26
ACTGCGCAC BRDN00012		390	306	533	658	440	21
ACTGCGCCC BRDN00012		345	382	55	749	841	17
ACTGCGCGC BRDN00011		365	317	1076	559	663	85
ACTGCGGAA BRDN00012		475	586	620	824	813	20
ACTGCGGAC BRDN00011		350	345	760	562	494	53
ACTGCGGAG BRDN00012		692	625	862	1128	1719	19
ACTGCGGCA BRDN00012		385	367	160	835	1374	41
ACTGCGGCT BRDN00011		295	372	369	559	753	100
ACTGCGGGA BRDN00011		300	654	72506	618	7289	1104
ACTGCGGTC BRDN00012		274	290	420	554	338	16
ACTGCGTCA BRDN00012		110	109	26	222	257	14
ACTGCGTGG BRDN00011		231	244	174	432	452	29
ACTGCGTGT BRDN00012		112	121	48	200	203	0
ACTGCGTTG BRDN00012		600	543	1178	1334	1536	34
ACTGCTAAA BRDN00011		336	403	689	609	723	67
ACTGCTAAA BRDN00011		140	2	19	354	249	0
ACTGCTAAG BRDN00011		100	0	0	250	172	0
ACTGCTAAG BRDN00012		303	117	53	659	551	7
ACTGCTACG BRDN00012		131	30	0	442	216	0
ACTGCTACTC BRDN00012		469	618	1416	853	547	90
ACTGCTAGG BRDN00012		245	311	279	422	531	10
ACTGCTATC BRDN00012		198	74	0	544	480	8
ACTGCTATG BRDN00011		125	31	0	218	348	0
ACTGCTCAA BRDN00011		201	103	230	291	194	26
ACTGCTCAA BRDN00012		106	37	39	606	300	0
ACTGCTCCAT BRDN00012		255	125	774	421	345	43
ACTGCTCCCG BRDN00012		628	622	1093	1137	1349	42
ACTGCTCCCT BRDN00012		199	0	0	432	188	0
ACTGCTCGC BRDN00011		48	5	0	328	65	0
ACTGCTCGG BRDN00012		176	158	80	172	125	9
ACTGCTCGT BRDN00012		73	61	3	221	367	0
ACTGCTCTCC BRDN00011		233	268	112	465	357	13
ACTGCTCTCT BRDN00011		441	356	413	627	929	57
ACTGCTCTG BRDN00011		234	282	825	561	511	17
ACTGCTCTG BRDN00011		120	187	0	174	214	0
ACTGCTGCA BRDN00011		543	383	220	811	1392	75
ACTGCTGCT BRDN00012		826	855	2584	1329	1540	108
ACTGCTGCT BRDN00012		389	286	207	623	807	5
ACTGCTGCT BRDN00012		398	240	1074	700	544	54
ACTGCTGGA BRDN00012		112	108	24	298	261	31
ACTGCTGGC BRDN00012		197	215	22	292	481	4
ACTGCTGGG BRDN00011		261	125	59	737	569	107
ACTGCTGGG BRDN00011		298	393	179	549	453	17
ACTGCTGGT BRDN00012		286	327	111	519	641	4
ACTGCTGGT BRDN00012		96	0	0	230	69	0
ACTGCTGTA BRDN00012		172	170	100	418	207	0
ACTGCTTAA BRDN00012		191	252	16	357	322	32
ACTGCTTAAT BRDN00012		73	0	0	242	237	0
ACTGCTTCG BRDN00012		99	103	157	488	759	0
ACTGCTTCGT BRDN00011		141	141	109	261	279	7
ACTGCTTGC BRDN00012		339	372	656	728	992	29
ACTGCTTGG BRDN00011		157	55	56	316	292	63
ACTGCTTGT BRDN00012		313	259	167	556	361	14
ACTGCTTGT BRDN00012		264	211	125	604	493	18
ACTGGAAAA BRDN00011		160	82	0	232	349	0
ACTGGAAAC BRDN00011		61	3	0	325	31	0
ACTGGAAAC BRDN00011		102	165	84	178	244	0
ACTGGAAAT BRDN00011		115	110	527	158	109	8
ACTGGAACA BRDN00011		110	77	45	250	212	9
ACTGGAACG BRDN00012		454	416	139	807	870	0
ACTGGAACG BRDN00011		293	312	676	557	349	35
ACTGGAAGC BRDN00011		244	288	699	486	483	16
ACTGGAAGT BRDN00012		243	263	2213	570	380	23
ACTGGAATC BRDN00012		164	171	827	303	773	39
ACTGGAATC BRDN00012		368	328	748	598	871	17
ACTGGAATT BRDN00011		123	34	0	197	175	0
ACTGGACAG BRDN00012		176	250	1438	426	611	27
ACTGGACAT BRDN00011		162	131	351	301	546	21
ACTGGACCA BRDN00011		282	190	164	358	528	20
ACTGGACCA BRDN00012		188	262	192	359	542	4
ACTGGACCC BRDN00011		227	191	1095	408	426	3
ACTGGACGA BRDN00012		175	152	665	300	137	19
ACTGGACGC BRDN00011		336	246	225	597	503	0
ACTGGACGT BRDN00012		440	346	1302	901	982	25
ACTGGAGAC BRDN00012		129	0	0	266	103	0
ACTGGAGAC BRDN00012		449	415	1690	564	1267	25
ACTGGAGCA BRDN00012		588	437	1928	1111	1227	66
ACTGGAGCC BRDN00012		175	78	505	430	382	26
ACTGGAGCC BRDN00011		197	168	102	440	529	0
ACTGGAGCG BRDN00011		149	119	0	269	207	0
ACTGGAGCT BRDN00012		92	0	1	159	18	0
ACTGGAGGC BRDN00011		462	411	2905	829	857	97
ACTGGAGGT BRDN00011		389	467	1714	798	698	109
ACTGGAGTC BRDN00012		338	449	974	724	783	164
ACTGGAGTT BRDN00011		224	218	274	436	810	0
ACTGGATCG BRDN00012		51	107	160	213	270	10
ACTGGATCT BRDN00012		119	57	153	318	395	10
ACTGGATGA BRDN00011		372	436	1534	662	711	27
ACTGGCAAA BRDN00011		92	82	61	209	488	0
ACTGGCAAA BRDN00012		420	464	677	906	732	28
ACTGGCAAA BRDN00012		204	12	18	491	525	0
ACTGGCAAG BRDN00012		433	305	573	728	521	24
ACTGGCAAG BRDN00012		130	75	1638	747	727	44
ACTGGCACA BRDN00011		250	232	648	348	154	19
ACTGGCACC BRDN00011		118	106	279	329	394	28
ACTGGCACC BRDN00012		417	566	385	799	803	52
ACTGGCACC BRDN00011		418	406	770	785	1027	75
ACTGGCACG BRDN00012		41	0	0	194	11	0
ACTGGCAGA BRDN00012		118	85	1	190	258	0
ACTGGCAGT BRDN00011		132	45	0	105	118	0
ACTGGCATC BRDN00011		338	357	694	516	483	69
ACTGGCATT BRDN00011		100	110	369	229	187	0
ACTGGCCAA BRDN00011		402	343	219	444	496	9
ACTGGCCAA BRDN00012		104	219	2268	196	175	68
ACTGGCCAC BRDN00012		136	79	156	314	433	11
ACTGGCCAC BRDN00012		96	83	49	365	618	19
ACTGGCCAG BRDN00011		134	116	10	291	98	7
ACTGGCCAG BRDN00012		115	127	0	323	326	0
ACTGGCGGC BRDN00011		68	84	212	121	42	17
ACTGGCGGC BRDN00011		97	70	28	196	250	0
ACTGGCGGT BRDN00012		231	184	83	338	583	20
ACTGGCGTG BRDN00012		146	84	211	273	408	31
ACTGGCGTT BRDN00011		331	344	1739	642	433	46
ACTGGCTAA BRDN00012		74	0	1	416	258	0
ACTGGCTAC BRDN00012		242	178	306	537	527	58
ACTGGCTGA BRDN00011		333	408	1645	739	598	25
ACTGGCTGC BRDN00011		359	270	308	597	593	13
ACTGGGAAA BRDN00011		289	378	1170	678	605	58
ACTGGGAAC BRDN00011		488	472	217	958	954	68
ACTGGGAAC BRDN00011		53	0	1	309	98	0
ACTGGGAAG BRDN00011		243	156	99	365	623	22
ACTGGGAAG BRDN00012		225	219	121	329	311	0
ACTGGGACA BRDN00012		210	118	165	212	450	19
ACTGGGACG BRDN00012		205	158	81	368	566	7
ACTGGGAGA BRDN00012		543	595	1052	833	862	33
ACTGGGAGA BRDN00012		427	325	78	1199	1067	16
ACTGGGCAA BRDN00012		28	7	1	617	364	0
ACTGGGCCA BRDN00011		136	163	426	179	268	7
ACTGGGCCT BRDN00011		128	55	1019	271	193	12
ACTGGGCGA BRDN00012		467	392	213	846	1076	2
ACTGGGCGA BRDN00011		252	113	790	271	225	13
ACTGGGCGG BRDN00011		112	161	34	329	214	64
ACTGGGCTC BRDN00012		288	279	808	543	520	40
ACTGGGGAC BRDN00012		131	139	11	202	639	20
ACTGGGGAC BRDN00012		386	324	1384	733	530	41
ACTGGGGAT BRDN00011		69	43	5	144	102	27
ACTGGGGCA BRDN00011		208	308	4904	387	128	106
ACTGGGGCT BRDN00011		224	11	0	715	636	0
ACTGGGGTC BRDN00011		129	86	103	251	266	0
ACTGGGTAA BRDN00012		435	335	3409	695	518	43
ACTGGGTAT BRDN00012		286	201	376	557	501	32
ACTGGGTGA BRDN00012		173	179	46	448	266	0
ACTGGGTGG BRDN00011		4	0	0	141	70	0
ACTGGGTTA BRDN00011		133	60	704	178	549	4
ACTGGGTTA BRDN00012		285	415	1655	565	888	161
ACTGGGTTG BRDN00011		139	169	2309	334	223	78
ACTGGTAAA BRDN00011		149	40	0	254	298	0
ACTGGTAAA BRDN00012		167	161	47	285	400	0
ACTGGTACA BRDN00011		11	0	0	280	85	0
ACTGGTACC BRDN00012		118	130	142	219	196	23
ACTGGTACG BRDN00011		109	68	0	125	166	12
ACTGGTACT BRDN00012		219	225	929	419	317	35
ACTGGTAGA BRDN00011		69	43	0	172	184	3
ACTGGTATT BRDN00011		215	93	215	202	256	48
ACTGGTATT BRDN00012		379	623	5898	704	646	128
ACTGGTCAA BRDN00012		122	165	79	218	640	8
ACTGGTCAA BRDN00012		75	17	649	191	236	1
ACTGGTCAC BRDN00012		276	187	111	619	597	93
ACTGGTCAG BRDN00011		389	298	79	865	616	40
ACTGGTCAG BRDN00011		247	340	927	383	409	102
ACTGGTCCA BRDN00012		447	533	1608	573	610	54
ACTGGTCCG BRDN00011		187	124	221	301	429	21
ACTGGTCCT BRDN00012		184	129	134	391	432	8
ACTGGTCGA BRDN00012		183	211	269	929	343	14
ACTGGTCGC BRDN00012		173	235	180	275	317	8
ACTGGTCGC BRDN00011		261	158	104	363	234	1
ACTGGTGAA BRDN00011		80	100	23	119	111	0
ACTGGTGAA BRDN00011		169	138	701	225	162	22
ACTGGTGAA BRDN00011		211	201	260	440	378	13
ACTGGTGAC BRDN00012		272	305	10	499	254	18
ACTGGTGGG BRDN00011		107	123	134	227	253	42
ACTGGTGGT BRDN00011		94	58	125	146	167	10
ACTGGTGTA BRDN00011		118	89	146	283	249	4
ACTGGTGTA BRDN00012		217	1	0	618	216	0
ACTGGTGTC BRDN00011		219	166	569	469	352	1
ACTGGTGTC BRDN00012		558	245	1167	974	764	37
ACTGGTTAA BRDN00012		75	146	9	164	246	0
ACTGGTTAC BRDN00012		423	344	105	761	653	64
ACTGGTTAC BRDN00011		157	226	340	372	405	34
ACTGGTTCA BRDN00012		204	203	141	234	511	46
ACTGGTTGT BRDN00011		171	58	150	339	231	18
ACTGTAAAC BRDN00012		196	221	28	432	303	40
ACTGTAAAT BRDN00011		309	251	1161	509	563	16
ACTGTAACT BRDN00012		208	139	86	302	406	17
ACTGTACACT BRDN00011		325	357	25162	591	533	335
ACTGTACAG BRDN00011		233	187	517	629	826	8
ACTGTACCAC BRDN00012		291	152	15	457	354	0
ACTGTACCCA BRDN00012		128	184	12833	275	200	241
ACTGTACCTG BRDN00012		30	24	27	266	117	5
ACTGTACGT BRDN00011		289	213	974	584	665	88
ACTGTACTG BRDN00011		294	672	82375	818	736	1405
ACTGTAGAC BRDN00011		158	90	11	320	431	0
ACTGTAGAC BRDN00012		129	60	40	237	184	15
ACTGTAGAT BRDN00011		203	188	205	372	492	5
ACTGTAGAT BRDN00012		159	203	500	247	160	42
ACTGTAGCA BRDN00012		127	44	76	213	281	0
ACTGTAGGA BRDN00011		137	126	0	245	193	15
ACTGTAGGG BRDN00012		419	455	992	871	728	42
ACTGTAGTA BRDN00011		194	206	491	409	334	41
ACTGTAGTA BRDN00012		21	0	0	350	35	0
ACTGTAGTC BRDN00011		177	216	741	327	339	0
ACTGTATATT BRDN00011		140	113	3	316	111	11
ACTGTATCCT BRDN00012		181	195	548	308	196	24
ACTGTATCG BRDN00011		278	258	3	457	220	0
ACTGTCAAA BRDN00011		385	276	117	746	747	8
ACTGTCAAC BRDN00012		194	174	193	389	202	31
ACTGTCAAC BRDN00011		330	334	384	877	405	33
ACTGTCACC BRDN00011		185	136	51	312	245	25
ACTGTCACTA BRDN00012		265	371	1051	562	449	84
ACTGTCAGC BRDN00012		516	522	520	899	537	103
ACTGTCATCA BRDN00011		178	164	750	406	723	60
ACTGTCATGT BRDN00011		264	214	341	379	788	94
ACTGTCATTT BRDN00011		464	334	1069	815	900	4
ACTGTCCAA BRDN00012		274	374	315	775	474	67
ACTGTCCACA BRDN00012		550	524	404	1059	745	72
ACTGTCCCCT BRDN00012		63	128	803	272	331	33
ACTGTCGCT BRDN00012		478	461	924	861	644	7
ACTGTCGGC BRDN00012		473	437	1744	672	926	57
ACTGTCTACG BRDN00012		151	31	83	212	192	7
ACTGTCTAG BRDN00011		117	117	242	320	204	31
ACTGTCTCAC BRDN00011		161	203	81	372	437	0
ACTGTCTCTG BRDN00011		167	170	1773	270	839	19
ACTGTCTGT BRDN00012		190	253	7	431	183	44
ACTGTCTTAC BRDN00012		406	431	1601	788	525	36
ACTGTCTTTC BRDN00011		175	131	1657	361	306	36
ACTGTCTTTC BRDN00011		400	123	506	616	764	0
ACTGTGAAA BRDN00012		230	223	34	398	208	35
ACTGTGAAC BRDN00012		148	105	15	317	254	18
ACTGTGAAG BRDN00011		124	158	13	296	500	0
ACTGTGAAG BRDN00011		281	286	689	532	475	70
ACTGTGAAG BRDN00012		189	168	288	365	764	8
ACTGTGACC BRDN00011		422	192	62	704	1102	0
ACTGTGAGC BRDN00011		133	106	1653	260	141	0
ACTGTGAGT BRDN00012		465	470	1133	896	958	34
ACTGTGAGT BRDN00012		144	85	350	314	264	0
ACTGTGATA BRDN00012		226	287	1047	465	302	6
ACTGTGCAA BRDN00011		80	0	0	321	233	0
ACTGTGCAG BRDN00012		481	467	64	963	782	44
ACTGTGCCC BRDN00012		576	437	22063	1015	609	326
ACTGTGCCG BRDN00012		539	446	316	1092	1332	64
ACTGTGCCG BRDN00012		143	114	2	266	363	5
ACTGTGCTG BRDN00012		186	212	422	261	658	12
ACTGTGCTG BRDN00012		227	119	122	312	248	7
ACTGTGCTT BRDN00012		263	189	305	519	604	12
ACTGTGCTT BRDN00012		143	156	2119	327	394	69
ACTGTGCTT BRDN00011		350	188	1744	728	1061	97
ACTGTGGAC BRDN00012		252	169	306	327	258	0
ACTGTGGAG BRDN00012		182	277	409	513	365	13
ACTGTGGAG BRDN00012		267	171	1202	588	447	45
ACTGTGGAT BRDN00012		40	21	0	340	22	0
ACTGTGGCA BRDN00011		183	0	1	450	328	0
ACTGTGGCC BRDN00012		247	156	61	418	354	0
ACTGTGGCC BRDN00012		520	290	69	870	1026	21
ACTGTGGCG BRDN00012		87	0	0	150	57	0
ACTGTGGGC BRDN00011		73	94	0	71	112	9
ACTGTGGTG BRDN00011		0	0	0	5	0	0
ACTGTGGTG BRDN00011		92	33	74	190	102	3
ACTGTGTAA BRDN00012		221	74	473	400	412	18
ACTGTGTAA BRDN00012		147	84	42	364	207	0
ACTGTGTAA BRDN00012		203	258	0	315	460	9
ACTGTGTAA BRDN00011		275	232	28	455	460	10
ACTGTGTAA BRDN00012		411	483	168	689	1134	34
ACTGTGTAC BRDN00012		181	202	140	389	680	5
ACTGTGTCA BRDN00011		34	0	0	397	248	0
ACTGTGTCC BRDN00012		237	106	177	225	198	22
ACTGTGTCTT BRDN00011		255	226	83	371	468	10
ACTGTGTCTT BRDN00012		262	161	216	383	532	0
ACTGTGTGA BRDN00011		183	254	286	327	1121	44
ACTGTGTGC BRDN00012		144	65	26	368	178	0
ACTGTGTGG BRDN00012		361	317	1607	703	808	49

ACTGTGTGG	BRDN00011	102	82	409	175	226	11
ACTGTTACA	BRDN00011	144	134	11	285	189	40
ACTGTTAGA	BRDN00012	369	414	1216	778	544	34
ACTGTTATG	BRDN00011	226	115	29	380	1015	0
ACTGTTATTA	BRDN00011	384	346	941	594	509	137
ACTGTTCAC	BRDN00012	513	414	492	634	334	23
ACTGTTCCAT	BRDN00011	223	160	8	311	249	11
ACTGTTCCAT	BRDN00012	313	393	810	616	651	14
ACTGTTCTAC	BRDN00011	212	266	6	383	377	43
ACTGTTCTAG	BRDN00012	160	145	219	154	260	0
ACTGTTCTCC	BRDN00011	338	228	781	404	458	52
ACTGTTCTTC	BRDN00011	111	69	0	357	159	0
ACTGTTGAA	BRDN00011	204	243	365	339	429	2
ACTGTTGCA	BRDN00012	118	121	789	402	161	11
ACTGTTGCC	BRDN00012	607	579	939	960	1059	38
ACTGTTGGC	BRDN00011	259	265	742	534	284	26
ACTGTTGGG	BRDN00011	86	25	0	633	581	0
ACTGTTGTC	BRDN00012	113	140	0	387	357	6
ACTGTTGTG	BRDN00012	223	67	286	350	333	55
ACTGTTTAG	BRDN00011	387	9	2	1068	1065	1
ACTGTTTATT	BRDN00011	88	200	154	193	228	0
ACTGTTTCA	BRDN00012	356	327	355	581	609	57
ACTGTTTCAC	BRDN00011	127	157	232	274	179	37
ACTGTTTCCT	BRDN00012	198	61	29	420	203	24
ACTGTTTCTT	BRDN00012	272	223	78	799	682	47
ACTTAAAAG	BRDN00012	379	355	283	786	1252	35
ACTTAAACTC	BRDN00011	512	311	903	730	730	29
ACTTAAACTC	BRDN00011	423	408	485	761	796	37
ACTTAAAGC	BRDN00012	192	210	30	453	658	13
ACTTAAAGC	BRDN00012	388	301	1497	711	708	27
ACTTAAAGT	BRDN00011	154	151	521	308	277	5
ACTTAAATA	BRDN00012	244	211	24	438	228	5
ACTTAAATTA	BRDN00011	166	38	68	205	242	0
ACTTAACACT	BRDN00012	221	92	42	392	288	3
ACTTAACATT	BRDN00011	278	175	0	560	505	0
ACTTAACCCT	BRDN00012	399	445	795	608	888	75
ACTTAACTAG	BRDN00012	307	224	101	674	426	39
ACTTAAGCA	BRDN00012	407	540	665	874	872	79
ACTTAAGGC	BRDN00011	121	141	277	229	31	32
ACTTAAGTA	BRDN00011	91	43	887	348	181	23
ACTTAATGA	BRDN00011	184	188	745	357	212	167
ACTTAATGA	BRDN00012	73	1	7	299	292	1
ACTTACAAA	BRDN00011	193	151	323	303	150	12
ACTTACAAG	BRDN00011	295	289	205	456	426	24
ACTTACACA	BRDN00011	283	289	748	503	570	81
ACTTACACAT	BRDN00012	455	193	622	805	1043	14
ACTTACAGA	BRDN00011	246	242	2312	336	376	37
ACTTACAGC	BRDN00012	99	98	625	236	174	17
ACTTACAGG	BRDN00011	243	215	207	624	417	37
ACTTACAGG	BRDN00012	237	30	1	475	280	0
ACTTACAGT	BRDN00012	413	383	774	793	342	17
ACTTACAGT	BRDN00011	435	307	280	647	594	53
ACTTACAGTT	BRDN00011	514	765	2610	887	1200	116
ACTTACATAG	BRDN00012	103	48	5	201	163	0
ACTTACATG	BRDN00011	86	106	27	137	164	17
ACTTACATGT	BRDN00011	213	11	375	468	289	0
ACTTACCAA	BRDN00012	354	252	676	693	622	0
ACTTACCAA	BRDN00012	150	185	3	240	312	12
ACTTACCAA	BRDN00012	586	367	511	716	782	46
ACTTACCAAC	BRDN00011	152	156	1155	353	566	24
ACTTACCAAG	BRDN00012	231	248	629	525	384	6
ACTTACCACA	BRDN00011	315	291	1532	567	488	23
ACTTACCACC	BRDN00011	419	338	268	766	1062	32
ACTTACCACC	BRDN00012	217	165	56	311	403	21
ACTTACCAG	BRDN00012	477	404	339	981	908	34
ACTTACCAG	BRDN00011	280	337	98	476	426	9
ACTTACCAG	BRDN00011	430	14	2	782	526	0
ACTTACCAG	BRDN00012	391	226	426	558	345	0
ACTTACCATC	BRDN00012	378	149	219	571	1044	49
ACTTACCATC	BRDN00011	388	228	425	361	449	12
ACTTACCATC	BRDN00011	414	393	2433	675	895	31
ACTTACCATC	BRDN00012	189	159	40	373	547	22
ACTTACCATC	BRDN00011	125	113	35	291	293	21
ACTTACCCAA	BRDN00011	206	231	31	304	339	0
ACTTACCCCA	BRDN00012	154	262	553	315	563	11
ACTTACCCCC	BRDN00012	500	378	609	1020	1128	27
ACTTACCCCT	BRDN00012	187	148	1	251	312	5
ACTTACCCGA	BRDN00011	531	275	450	707	863	3
ACTTACCCGG	BRDN00012	234	212	362	434	516	11
ACTTACCCTC	BRDN00011	287	259	460	561	766	0
ACTTACCGCA	BRDN00011	264	379	169	570	794	50
ACTTACCGG	BRDN00012	220	266	1225	440	451	35
ACTTACCGTA	BRDN00011	215	122	43	448	173	29
ACTTACCGTG	BRDN00011	394	225	2346	668	392	35
ACTTACCTAC	BRDN00011	111	52	0	219	130	0
ACTTACCTAT	BRDN00011	137	40	152	205	132	0
ACTTACCTAT	BRDN00011	163	109	168	263	270	27
ACTTACCTCG	BRDN00011	6	0	0	315	229	0
ACTTACCTCG	BRDN00011	115	127	613	275	169	22
ACTTACCTGC	BRDN00012	420	319	1027	848	527	102
ACTTACCTGT	BRDN00011	397	24	10	847	661	1
ACTTACCTTA	BRDN00012	211	44	21	301	399	0
ACTTACCTTA	BRDN00011	285	151	1295	508	406	17
ACTTACCTTC	BRDN00012	392	243	670	512	718	14
ACTTACCTTT	BRDN00012	316	198	9	466	271	79
ACTTACGAC	BRDN00011	136	84	6	278	524	9
ACTTACGAG	BRDN00011	196	118	99	353	628	6
ACTTACGCC	BRDN00011	498	408	578	900	1289	53
ACTTACGCG	BRDN00011	644	497	125	952	940	22
ACTTACGGC	BRDN00012	595	726	3565	841	1337	202
ACTTACGGC	BRDN00011	161	84	4	356	526	0
ACTTACGTCA	BRDN00011	194	236	12370	437	799	242
ACTTACGTG	BRDN00011	383	372	741	691	369	27
ACTTACGTTC	BRDN00012	288	98	164	579	623	5
ACTTACGTTT	BRDN00011	166	183	21	312	436	23
ACTTACTAAA	BRDN00011	194	111	94	499	425	11
ACTTACTCAA	BRDN00012	159	6	36	511	585	1
ACTTACTCAG	BRDN00012	350	266	232	667	883	102
ACTTACTCTA	BRDN00012	337	275	665	762	694	32
ACTTACTCTG	BRDN00011	249	270	1685	584	624	6
ACTTACTGG	BRDN00011	84	64	78	172	155	39
ACTTACTTAG	BRDN00011	171	196	13	336	520	13
ACTTACTTTA	BRDN00011	60	27	5	244	341	9
ACTTAGATA	BRDN00012	99	45	1169	209	257	2
ACTTAGCAA	BRDN00012	276	219	179	586	607	4
ACTTAGCCA	BRDN00012	122	38	186	489	459	0
ACTTAGGCC	BRDN00012	407	22	1	637	973	0
ACTTAGGGG	BRDN00012	691	640	3737	1134	1532	105
ACTTAGGTA	BRDN00011	62	51	1	159	174	0
ACTTAGTAA	BRDN00011	140	123	103	248	199	0
ACTTATACG	BRDN00011	399	245	308	717	685	8
ACTTATCCAA	BRDN00011	226	245	204	393	241	59
ACTTATCGA	BRDN00011	159	188	187	294	126	14
ACTTATCTCC	BRDN00011	161	194	2394	343	283	28
ACTTATGCTT	BRDN00011	271	281	766	494	508	45
ACTTATGGG	BRDN00011	173	194	2370	418	289	46
ACTTATGGT	BRDN00011	162	179	175	324	157	26
ACTTATTACA	BRDN00012	193	177	100	318	308	47
ACTTATTCAG	BRDN00011	142	145	21	264	494	14
ACTTCAAAC	BRDN00012	547	527	2368	884	709	112
ACTTCAAAG	BRDN00011	355	297	263	526	524	111
ACTTCAAAG	BRDN00011	421	469	8375	899	945	248
ACTTCAAAG	BRDN00011	153	120	119	309	369	34
ACTTCAAATG	BRDN00011	205	214	458	501	666	30
ACTTCAAATT	BRDN00012	182	249	557	218	339	23
ACTTCAACA	BRDN00012	312	221	584	533	427	60
ACTTCAACAG	BRDN00011	238	120	12	296	319	10
ACTTCAACCT	BRDN00012	467	345	878	917	859	81
ACTTCAAGT	BRDN00012	410	102	80	896	470	10
ACTTCAAGT	BRDN00011	613	592	832	1255	973	42
ACTTCAATG	BRDN00012	188	308	228	361	700	21
ACTTCACAG	BRDN00012	292	304	379	459	452	12
ACTTCAGAA	BRDN00011	305	140	1630	567	551	10
ACTTCAGACT	BRDN00012	489	320	877	659	477	41
ACTTCAGAG	BRDN00011	207	225	260	354	221	18
ACTTCAGATC	BRDN00011	354	377	127	601	865	53
ACTTCAGCG	BRDN00012	122	28	741	627	408	20
ACTTCAGCTC	BRDN00012	79	61	0	246	163	9
ACTTCAGGG	BRDN00011	185	190	405	313	290	19
ACTTCAGGG	BRDN00011	123	7	1	581	176	0
ACTTCAGGT	BRDN00011	226	255	523	503	669	31
ACTTCAGGT	BRDN00011	126	54	151	529	417	0
ACTTCAGTTC	BRDN00012	651	453	577	940	814	87
ACTTCATACC	BRDN00012	286	320	143	585	618	17
ACTTCATCTG	BRDN00011	271	212	213	539	456	26
ACTTCATGA	BRDN00012	236	161	501	401	267	0
ACTTCATGG	BRDN00012	95	2	1	319	34	0
ACTTCATTCG	BRDN00011	227	209	334	329	414	43
ACTTCCAAG	BRDN00012	353	190	92	439	450	3
ACTTCCAGA	BRDN00012	216	92	75	356	165	17
ACTTCCAGG	BRDN00011	184	257	127	579	768	0
ACTTCCAGTT	BRDN00011	158	167	18	208	208	0
ACTTCCATCT	BRDN00012	416	390	973	743	821	44
ACTTCCATGA	BRDN00011	77	57	0	526	340	0
ACTTCCATGC	BRDN00011	262	283	488	309	630	18
ACTTCCCACT	BRDN00012	628	597	1602	981	975	125
ACTTCCCCGA	BRDN00012	196	188	192	319	340	16
ACTTCCCCTT	BRDN00012	427	394	601	799	863	16
ACTTCCCTGA	BRDN00011	265	213	1580	355	306	55
ACTTCCTACC	BRDN00012	360	365	1643	575	736	39
ACTTCCTACG	BRDN00012	133	0	0	601	227	0
ACTTCCTGG	BRDN00012	501	478	517	842	967	62
ACTTCCTGG	BRDN00011	473	516	271	860	886	63
ACTTCCTTAG	BRDN00011	369	367	686	495	366	9
ACTTCGAAC	BRDN00011	101	14	0	308	524	0
ACTTCGAAG	BRDN00011	109	125	426	259	350	33
ACTTCGACA	BRDN00012	199	124	1276	354	212	38
ACTTCGACG	BRDN00012	185	140	172	319	426	0
ACTTCGATA	BRDN00011	226	132	556	375	288	11
ACTTCGATG	BRDN00012	371	330	525	782	782	23
ACTTCGCAG	BRDN00011	121	7	0	315	292	0
ACTTCGCAG	BRDN00011	110	96	54	316	237	0
ACTTCGCCA	BRDN00011	0	0	0	7	0	0
ACTTCGCCCC	BRDN00012	434	284	701	1041	1294	34
ACTTCGCTCA	BRDN00011	22	14	0	290	138	0
ACTTCGGAG	BRDN00012	266	104	11	659	641	14
ACTTCGGCA	BRDN00012	144	0	130	513	398	0
ACTTCGGCT	BRDN00012	180	119	920	210	212	28
ACTTCGGCT	BRDN00012	158	259	23	269	291	21
ACTTCGGGG	BRDN00011	174	108	203	254	484	28
ACTTCGGGG	BRDN00012	113	0	0	312	166	9
ACTTCGTCCT	BRDN00011	108	37	37	273	185	0
ACTTCGTGT	BRDN00012	423	18	0	759	662	0
ACTTCGTTCA	BRDN00011	250	191	784	560	384	28
ACTTCTACAA	BRDN00012	487	448	1636	876	1234	103
ACTTCTAGG	BRDN00011	315	284	430	687	562	42
ACTTCTATCT	BRDN00011	105	95	173	196	378	23
ACTTCTATTA	BRDN00011	107	143	281	301	264	6
ACTTCTCAAA	BRDN00011	148	191	85	426	288	20
ACTTCTCAAG	BRDN00012	308	253	289	799	594	37
ACTTCTCCAT	BRDN00011	168	178	283	359	712	10
ACTTCTCCGA	BRDN00012	154	84	1308	163	147	12
ACTTCTCGAA	BRDN00012	331	185	33	609	498	24
ACTTCTCTGA	BRDN00011	337	158	226	562	638	0
ACTTCTGATT	BRDN00011	121	141	1209	257	464	57
ACTTCTGCCC	BRDN00011	105	77	0	285	155	0
ACTTCTGGA	BRDN00012	40	44	0	553	130	12
ACTTCTGTAA	BRDN00011	214	183	594	343	368	53
ACTTCTGTCC	BRDN00012	273	194	85	445	493	14
ACTTCTTATG	BRDN00012	349	309	958	663	450	39
ACTTCTTATT	BRDN00012	256	154	181	471	834	33
ACTTCTTCTC	BRDN00012	361	307	2993	758	824	128
ACTTCTTGG	BRDN00011	175	298	42	348	242	12
ACTTCTTGG	BRDN00011	258	44	429	415	302	0
ACTTCTTGTA	BRDN00012	179	172	448	232	513	6
ACTTCTTTGA	BRDN00012	400	391	55	664	602	25
ACTTGAAAA	BRDN00011	167	202	126	192	139	7
ACTTGAAAC	BRDN00011	126	61	125	288	226	0
ACTTGAAAG	BRDN00012	154	86	53	344	315	40
ACTTGAACT	BRDN00012	371	28	11	744	588	0
ACTTGAAGC	BRDN00012	295	554	1467	528	1407	186
ACTTGAAGT	BRDN00011	255	264	1795	501	544	75
ACTTGACAG	BRDN00011	399	464	1087	627	592	28
ACTTGACAG	BRDN00012	198	247	921	562	388	17
ACTTGACTCA	BRDN00011	266	272	4299	386	323	44
ACTTGACTC	BRDN00011	189	6	0	492	397	0
ACTTGAGAG	BRDN00011	388	368	1439	693	608	39
ACTTGAGCC	BRDN00012	335	336	385	661	790	19
ACTTGAGGA	BRDN00012	436	476	97	837	955	2
ACTTGAGGA	BRDN00011	266	312	673	419	381	16
ACTTGATAA	BRDN00012	316	275	930	568	888	69
ACTTGATAC	BRDN00011	195	176	64	437	549	4
ACTTGATAG	BRDN00012	47	15	900	197	146	0
ACTTGATAT	BRDN00012	170	86	931	344	630	8
ACTTGATAT	BRDN00012	191	239	577	378	332	6
ACTTGATCG	BRDN00012	301	226	288	520	357	26
ACTTGATCTA	BRDN00011	432	483	788	671	948	31
ACTTGATCTA	BRDN00011	169	179	704	390	272	24
ACTTGATGT	BRDN00011	359	230	81	881	790	1
ACTTGCAAA	BRDN00011	117	162	9	301	411	6
ACTTGCAAC	BRDN00011	216	118	401	363	407	4
ACTTGCAAG	BRDN00012	197	17	58	410	355	0
ACTTGCACA	BRDN00012	114	157	0	237	210	13
ACTTGCAGA	BRDN00012	151	84	88	271	281	5
ACTTGCAGG	BRDN00011	153	157	60	284	264	1
ACTTGCCAA	BRDN00012	263	129	0	460	394	0
ACTTGCCAA	BRDN00011	226	178	214	597	485	0
ACTTGCCACA	BRDN00012	170	43	90	302	192	6
ACTTGCCATG	BRDN00011	120	21	39	375	495	6
ACTTGCCATT	BRDN00012	375	501	116	719	868	22
ACTTGCCATT	BRDN00011	344	582	251	691	718	10
ACTTGCCGA	BRDN00012	157	113	54	191	97	0
ACTTGCCGG	BRDN00012	140	101	3864	266	226	53
ACTTGCGAT	BRDN00012	542	471	1332	1069	1235	70
ACTTGCGGT	BRDN00011	205	161	152	334	311	14
ACTTGCGTA	BRDN00011	140	38	8	489	352	9
ACTTGCTCAT	BRDN00012	143	154	89	260	449	28
ACTTGCTGA	BRDN00012	123	0	0	522	393	0
ACTTGCTGT	BRDN00011	103	49	0	182	15	0
ACTTGCTTTC	BRDN00011	113	149	550	208	115	22
ACTTGGAAA	BRDN00012	165	184	217	297	143	57
ACTTGGAAA	BRDN00012	173	304	100	255	314	15
ACTTGGAAG	BRDN00012	520	380	907	1308	1470	29
ACTTGGACG	BRDN00012	184	131	0	351	458	0
ACTTGGACT	BRDN00012	131	227	4211	234	439	106
ACTTGGAGC	BRDN00012	187	326	260	215	240	5
ACTTGGAGG	BRDN00012	124	51	764	378	385	24
ACTTGGAGG	BRDN00011	304	284	646	851	482	34
ACTTGGAGG	BRDN00012	355	416	3657	577	572	59
ACTTGGATA	BRDN00011	343	234	521	670	1035	21
ACTTGGATG	BRDN00012	127	114	37	236	63	0
ACTTGGATG	BRDN00012	33	1	0	227	386	0
ACTTGGCCA	BRDN00011	187	18	417	380	228	21
ACTTGGCGG	BRDN00011	247	59	1255	429	436	12
ACTTGGCTT	BRDN00011	271	489	2551	409	486	80
ACTTGGGAG	BRDN00011	366	325	652	596	527	43
ACTTGGGAG	BRDN00011	258	183	1221	541	757	13
ACTTGGGCA	BRDN00011	183	159	250	339	638	12
ACTTGGGCA	BRDN00012	164	69	91	425	538	18
ACTTGGGTA	BRDN00012	120	134	1	209	302	4
ACTTGGGTA	BRDN00011	182	105	79	400	475	10
ACTTGGTCA	BRDN00011	127	147	0	221	211	0
ACTTGGTCC	BRDN00012	143	172	1645	271	126	41
ACTTGGTGC	BRDN00012	166	192	1232	310	444	46
ACTTGGTGC	BRDN00011	55	0	2	215	230	0
ACTTGGTTCC	BRDN00012	176	233	146	381	427	52
ACTTGTACA	BRDN00011	126	175	40	333	123	27
ACTTGTACTC	BRDN00012	301	332	1190	565	398	39
ACTTGTATTC	BRDN00011	195	170	135	530	650	32
ACTTGTCACA	BRDN00012	318	181	159	465	311	18
ACTTGTCACA	BRDN00011	211	240	995	410	843	57
ACTTGTCACA	BRDN00011	120	105	503	324	328	13
ACTTGTCACC	BRDN00011	132	49	0	412	341	0
ACTTGTCAG	BRDN00011	333	250	2341	462	541	39
ACTTGTCTGT	BRDN00012	152	18	9	213	86	0
ACTTGTGGA	BRDN00012	519	375	2073	1133	1331	46
ACTTGTGGC	BRDN00012	170	204	95	498	410	9
ACTTGTGTCC	BRDN00011	208	225	418	258	197	51
ACTTGTTACA	BRDN00012	343	170	361	602	514	12
ACTTGTTGCC	BRDN00012	322	314	470	435	473	89
ACTTGTTGCT	BRDN00012	240	74	13	691	635	0
ACTTGTTTGC	BRDN00011	115	146	95	197	304	0
ACTTTAATGA	BRDN00012	282	205	12	432	212	8
ACTTTACACT	BRDN00011	315	333	753	417	335	40
ACTTTACTAC	BRDN00011	258	91	114	426	542	0
ACTTTAGAG	BRDN00011	93	85	103	200	208	8
ACTTTATAAC	BRDN00011	54	41	15	139	152	0
ACTTTATAAG	BRDN00012	277	256	36	450	790	41
ACTTTATGAC	BRDN00011	337	312	690	560	908	43
ACTTTCCAGA	BRDN00012	323	39	3	734	416	0
ACTTTCCGTC	BRDN00012	182	176	331	297	215	15
ACTTTCGATA	BRDN00011	136	8	0	510	629	0
ACTTTCGGC	BRDN00012	284	324	336	363	345	25
ACTTTCTACA	BRDN00012	246	234	998	729	1112	21
ACTTTCTCTA	BRDN00011	288	254	238	569	414	23
ACTTTCTGAA	BRDN00012	149	7	0	312	173	0
ACTTTCTGAT	BRDN00012	306	235	434	645	916	5
ACTTTCTGAT	BRDN00012	383	348	912	680	603	47
ACTTTCTTAC	BRDN00012	226	123	1	363	410	21
ACTTTCTTGC	BRDN00012	156	125	188	267	68	15
ACTTTCTTGT	BRDN00011	356	239	1126	779	288	49
ACTTTGAAA	BRDN00011	469	350	802	766	730	47
ACTTTGAAC	BRDN00011	220	235	81	653	617	16
ACTTTGATG	BRDN00011	356	322	485	835	724	96
ACTTTGATG	BRDN00011	172	140	7	366	340	17
ACTTTGCCTG	BRDN00011	144	146	1085	366	133	13
ACTTTGCTAA	BRDN00011	272	182	439	449	671	36
ACTTTGGAG	BRDN00012	241	288	2640	391	283	26
ACTTTGGAG	BRDN00011	344	177	1358	838	893	32
ACTTTGGCA	BRDN00012	230	127	339	380	305	30
ACTTTGGCC	BRDN00011	163	93	35	387	203	9
ACTTTGGCG	BRDN00012	72	38	105	208	206	7
ACTTTGGGA	BRDN00011	74	0	0	260	20	0
ACTTTGGTC	BRDN00012	68	55	1	640	459	0
ACTTTGTAG	BRDN00011	155	69	360	309	138	0
ACTTTGTAG	BRDN00012	321	295	129	579	643	27
ACTTTGTCCG	BRDN00011	166	0	1	364	231	0
ACTTTGTGG	BRDN00012	146	159	1	317	196	20
ACTTTGTGG	BRDN00011	206	94	1	353	494	11
ACTTTGTTTG	BRDN00011	270	243	521	394	186	0
AGAAAAAAG	BRDN00012	359	415	2152	833	879	52
AGAAAAACC	BRDN00012	309	281	229	599	971	62
AGAAAAACC	BRDN00012	439	182	428	734	864	0
AGAAAAACG	BRDN00011	563	456	935	881	1036	190
AGAAAAACG	BRDN00012	16	24	0	525	75	0
AGAAAAACG	BRDN00012	301	25	596	597	300	1
AGAAAAACT	BRDN00011	222	254	1147	433	251	35
AGAAAAAGG	BRDN00012	374	425	386	632	918	11
AGAAAAAGT	BRDN00011	222	297	204	407	631	0
AGAAAAAGT	BRDN00011	105	1	0	138	58	0
AGAAAAATA	BRDN00011	229	236	1356	403	448	11
AGAAAAATA	BRDN00011	147	138	220	179	67	5
AGAAAAATC	BRDN00011	543	482	1858	832	700	59
AGAAAACAA	BRDN00011	242	265	124	408	387	0
AGAAAACAC	BRDN00011	203	210	120	443	414	7
AGAAAACAC	BRDN00011	465	512	1131	895	1248	16
AGAAAACAC	BRDN00012	367	295	64	424	495	23
AGAAAACAG	BRDN00012	269	303	51	502	455	0
AGAAAACAG	BRDN00011	417	436	394	815	1249	107
AGAAAACAT	BRDN00012	425	423	1002	908	762	72
AGAAAACCA	BRDN00012	569	527	1591	990	1109	87
AGAAAACCA	BRDN00011	174	98	16	254	210	3
AGAAAACCA	BRDN00012	357	259	412	733	604	13
AGAAAACCC	BRDN00011	31	0	0	344	256	0
AGAAAACCG	BRDN00011	618	679	811	1141	1492	92
AGAAAACGA	BRDN00011	396	372	605	599	1770	16
AGAAAACTA	BRDN00012	201	14	14	495	308	3
AGAAAACTA	BRDN00012	136	130	1467	121	202	23
AGAAAACTT	BRDN00012	155	168	18	288	214	0
AGAAAAGAA	BRDN00011	161	101	40	363	216	23
AGAAAAGAA	BRDN00012	405	242	242	605	659	17
AGAAAAGAA	BRDN00012	147	203	1021	332	625	3
AGAAAAGAG	BRDN00011	104	105	54	382	522	25
AGAAAAGCA	BRDN00012	361	436	1243	502	765	33
AGAAAAGCA	BRDN00011	218	71	152	297	222	6
AGAAAAGGT	BRDN00012	336	377	893	571	305	49
AGAAAAGTA	BRDN00011	230	155	222	469	265	6
AGAAAAGTC	BRDN00011	397	299	550	705	785	70
AGAAAATAC	BRDN00012	206	117	1	402	226	3
AGAAAATAT	BRDN00012	256	238	80	508	224	3
AGAAAATCA	BRDN00011	327	322	445	613	532	17
AGAAAATCC	BRDN00011	331	253	1066	529	796	25
AGAAAATCT	BRDN00011	484	451	468	775	1279	158
AGAAAATGA	BRDN00012	274	182	1275	568	218	64
AGAAAATGA	BRDN00012	430	359	1156	920	686	19
AGAAAATGA	BRDN00011	295	166	341	499	408	31
AGAAAATGA	BRDN00011	289	270	111	455	400	5
AGAAAATGA	BRDN00011	300	179	63	532	304	0
AGAAAATTA	BRDN00012	61	44	2	139	268	0
AGAAAATTA	BRDN00012	102	22	0	361	372	0
AGAAAATTC	BRDN00012	314	295	211	693	662	9
AGAAACAAT	BRDN00012	329	349	301	845	1119	39
AGAAACACT	BRDN00012	246	381	984	689	816	0
AGAAACACT	BRDN00012	118	124	45	227	433	0
AGAAACAGA	BRDN00012	307	284	2318	555	517	54
AGAAACAGT	BRDN00012	222	219	90	373	538	16
AGAAACAGT	BRDN00011	20	0	0	386	48	0
AGAAACAGT	BRDN00011	245	207	250	475	379	0
AGAAACCAA	BRDN00011	154	81	86	381	369	4
AGAAACCAC	BRDN00011	132	144	46	308	159	0
AGAAACCAC	BRDN00011	203	200	236	400	308	0
AGAAACCAC	BRDN00012	151	179	503	404	455	12
AGAAACCAG	BRDN00011	203	259	294	269	164	11
AGAAACCAG	BRDN00011	43	12	0	247	71	0
AGAAACCAT	BRDN00012	233	149	289	445	672	33
AGAAACCAT	BRDN00011	429	185	317	774	735	41
AGAAACCCA	BRDN00011	196	162	608	545	543	85
AGAAACCCA	BRDN00011	405	308	2294	913	565	40
AGAAACCCC	BRDN00012	299	335	103	550	467	8
AGAAACCCG	BRDN00012	341	243	979	612	857	83
AGAAACCTA	BRDN00011	55	0	0	332	113	0
AGAAACCTG	BRDN00011	614	702	6512	1015	1121	198
AGAAACGAA	BRDN00012	187	214	448	428	639	6
AGAAACGAG	BRDN00011	551	657	1433	874	838	34
AGAAACGAT	BRDN00012	23	0	2	151	68	0
AGAAACGCG	BRDN00011	284	223	1191	480	188	39
AGAAACGCT	BRDN00012	221	110	178	457	277	12
AGAAACGGC	BRDN00012	304	163	147	506	957	23
AGAAACGTA	BRDN00012	122	107	1	299	185	0
AGAAACGTC	BRDN00011	122	104	55	90	142	2
AGAAACGTG	BRDN00011	142	121	964	357	348	47
AGAAACTAA	BRDN00011	133	111	0	446	202	9
AGAAACTAA	BRDN00011	22	75	0	263	204	0
AGAAACTCA	BRDN00011	232	309	499	385	284	25
AGAAACTCA	BRDN00011	169	184	84	308	354	43
AGAAACTCA	BRDN00012	629	552	450	1073	954	54
AGAAACTCT	BRDN00012	193	183	35	378	189	20
AGAAACTGA	BRDN00012	452	324	2285	649	666	83
AGAAACTGC	BRDN00012	301	366	757	570	284	9
AGAAACTGC	BRDN00012	245	338	619	444	442	11
AGAAACTGT	BRDN00011	520	498	3731	993	810	40
AGAAACTTA	BRDN00011	162	220	244	334	327	0
AGAAACTTA	BRDN00011	237	172	975	347	341	26
AGAAACTTC	BRDN00012	381	445	1107	703	395	18
AGAAACTTT	BRDN00012	209	52	1	498	567	0
AGAAAGAAA	BRDN00012	54	0	0	197	85	0
AGAAAGAAG	BRDN00011	210	180	820	443	367	9
AGAAAGAAG	BRDN00011	123	92	214	250	563	10
AGAAAGAAT	BRDN00012	492	503	2643	592	923	106
AGAAAGACA	BRDN00012	118	111	47	350	769	43
AGAAAGACA	BRDN00011	207	215	1475	482	643	41
AGAAAGACA	BRDN00011	224	147	2	469	647	0
AGAAAGACG	BRDN00011	321	306	218	571	596	188
AGAAAGACG	BRDN00012	184	52	714	340	310	0
AGAAAGAGA	BRDN00012	214	195	118	304	286	14
AGAAAGCAA	BRDN00011	208	133	377	343	283	9
AGAAAGCAC	BRDN00012	283	239	336	523	458	23
AGAAAGCAG	BRDN00012	165	253	306	414	276	19
AGAAAGCAT	BRDN00012	86	52	0	160	97	24
AGAAAGCCT	BRDN00012	266	263	415	524	698	0
AGAAAGCGC	BRDN00012	57	24	0	220	158	0
AGAAAGCTG	BRDN00012	369	324	3542	586	440	57
AGAAAGCTT	BRDN00012	202	178	53	323	477	0
AGAAAGGAC	BRDN00011	87	42	22	248	455	0
AGAAAGGAC	BRDN00012	287	78	113	447	417	0

AGAAAGGA	BRDN00011	374	217	2	875	638	0
AGAAAGGA	BRDN00011	209	206	1391	468	611	10
AGAAAGGAT	BRDN00011	176	144	359	285	256	22
AGAAAGGCC	BRDN00012	157	135	215	235	487	15
AGAAAGGCC	BRDN00011	125	142	142	180	84	0
AGAAAGGG	BRDN00012	117	95	30	191	228	0
AGAAAGGG	BRDN00011	335	253	1856	610	722	82
AGAAAGGTC	BRDN00011	348	342	12	469	629	16
AGAAAGGTT	BRDN00011	166	97	13	215	348	4
AGAAAGTAA	BRDN00011	316	49	296	698	623	5
AGAAAGTAT	BRDN00011	213	124	538	372	285	0
AGAAAGTCA	BRDN00011	388	450	216	757	980	67
AGAAAGTCG	BRDN00012	310	121	7	846	1277	21
AGAAAGTGG	BRDN00012	201	196	402	469	344	0
AGAAAGTGT	BRDN00011	82	6	0	259	150	0
AGAAATAAG	BRDN00012	159	47	11	598	645	4
AGAAATACA	BRDN00011	83	38	0	167	104	0
AGAAATACC	BRDN00011	513	287	432	776	613	48
AGAAATACG	BRDN00012	52	0	0	425	333	0
AGAAATAGC	BRDN00012	210	204	1215	496	511	9
AGAAATAGG	BRDN00012	527	559	438	806	1340	12
AGAAATATA	BRDN00011	237	282	101	431	531	7
AGAAATATA	BRDN00012	325	375	402	738	1297	37
AGAAATATC	BRDN00011	218	0	0	452	900	0
AGAAATATC	BRDN00011	330	368	944	684	415	25
AGAAATCAA	BRDN00012	147	145	2249	359	487	71
AGAAATCAA	BRDN00012	172	135	9858	582	325	121
AGAAATCAG	BRDN00011	136	84	53	235	190	19
AGAAATCAG	BRDN00012	495	373	3152	805	528	142
AGAAATCAG	BRDN00012	193	100	801	367	562	12
AGAAATCAG	BRDN00012	582	779	991	981	977	54
AGAAATCAT	BRDN00011	169	222	78	391	321	0
AGAAATCAT	BRDN00012	206	149	790	531	581	0
AGAAATCCA	BRDN00012	139	132	811	232	275	0
AGAAATCCT	BRDN00011	54	51	0	121	62	0
AGAAATCCT	BRDN00012	288	250	570	334	410	23
AGAAATCGC	BRDN00011	461	448	259	830	754	25
AGAAATCTA	BRDN00012	265	285	216	553	493	22
AGAAATCTA	BRDN00012	147	169	732	289	358	1
AGAAATCTA	BRDN00011	183	84	490	381	202	31
AGAAATCTG	BRDN00011	235	158	352	201	100	26
AGAAATCTT	BRDN00012	99	148	54	246	291	16
AGAAATGAA	BRDN00012	379	259	137	591	389	35
AGAAATGAC	BRDN00012	143	25	1	252	186	0
AGAAATGAG	BRDN00011	67	70	1	161	143	0
AGAAATGAG	BRDN00011	151	172	1	313	151	62
AGAAATGCA	BRDN00012	169	94	4	315	283	0
AGAAATGCT	BRDN00011	208	125	82	432	539	1
AGAAATGGC	BRDN00011	226	276	293	315	321	52
AGAAATGGC	BRDN00011	426	403	56	600	633	19
AGAAATGTA	BRDN00011	90	7	1	252	184	0
AGAAATGTA	BRDN00012	430	199	1952	735	582	64
AGAAATGTT	BRDN00011	116	65	16	144	16	0
AGAAATGTT	BRDN00011	175	196	834	261	385	31
AGAAATTAA	BRDN00012	185	99	15	266	372	25
AGAAATTAA	BRDN00012	218	83	200	496	526	0
AGAAATTAA	BRDN00012	132	81	117	201	325	4
AGAAATTAA	BRDN00011	76	152	299	156	155	0
AGAAATTAT	BRDN00011	85	90	19	130	179	28
AGAAATTCA	BRDN00012	176	204	85	354	511	4
AGAAATTCC	BRDN00011	222	240	52	412	384	23
AGAAATTCG	BRDN00011	485	346	2597	747	733	134
AGAAATTGG	BRDN00012	224	178	125	384	323	11
AGAAATTGG	BRDN00012	331	388	494	621	892	3
AGAAATTGG	BRDN00012	209	300	209	276	532	5
AGAAATTGT	BRDN00012	293	240	321	456	426	21
AGAAATTTA	BRDN00011	148	118	55	360	254	11
AGAACAAAC	BRDN00012	614	617	429	1089	1189	19
AGAACAAAG	BRDN00011	343	352	1251	723	682	47
AGAACAACG	BRDN00011	345	459	1229	662	672	85
AGAACAACT	BRDN00011	512	533	1325	1027	1111	127
AGAACAAGA	BRDN00012	92	59	4	191	118	0
AGAACAAGC	BRDN00012	129	112	2	136	134	21
AGAACAAGG	BRDN00011	88	43	347	350	152	21
AGAACAAGT	BRDN00012	320	308	454	627	519	26
AGAACAATA	BRDN00011	236	214	383	305	227	16
AGAACAATG	BRDN00012	24	19	0	206	338	0
AGAACACAA	BRDN00011	201	168	17	403	370	18
AGAACACAA	BRDN00011	161	215	256	398	499	6
AGAACACAC	BRDN00011	132	74	83	284	396	12
AGAACACCA	BRDN00012	148	101	260	172	118	51
AGAACACCG	BRDN00011	52	0	0	173	52	0
AGAACACGA	BRDN00012	227	328	460	392	341	20
AGAACACGG	BRDN00011	155	125	510	178	446	18
AGAACACGT	BRDN00011	482	509	552	1001	869	47
AGAACACTC	BRDN00011	560	474	2995	1085	1041	127
AGAACAGAA	BRDN00012	475	463	493	803	1042	16
AGAACAGAC	BRDN00012	105	106	1920	254	134	31
AGAACAGAT	BRDN00012	448	422	1473	917	1242	37
AGAACAGCA	BRDN00012	369	270	700	920	477	35
AGAACAGCA	BRDN00012	304	377	22	579	513	15
AGAACAGCA	BRDN00011	135	150	265	300	306	0
AGAACAGCC	BRDN00012	516	268	28	884	902	22
AGAACAGCC	BRDN00012	303	197	890	507	694	0
AGAACAGCT	BRDN00011	230	150	212	396	142	0
AGAACAGCT	BRDN00012	74	86	0	357	307	0
AGAACAGGA	BRDN00011	47	63	0	714	367	8
AGAACAGGA	BRDN00012	193	229	719	289	133	24
AGAACAGGG	BRDN00012	221	216	762	354	382	11
AGAACAGTA	BRDN00011	78	179	160	186	154	39
AGAACAGTC	BRDN00012	326	214	318	730	489	16
AGAACATCA	BRDN00011	456	471	1293	814	831	48
AGAACATCC	BRDN00012	146	84	322	331	352	25
AGAACATCT	BRDN00011	66	145	96	153	161	9
AGAACATCT	BRDN00012	447	519	5140	973	738	60
AGAACATCT	BRDN00012	238	81	744	458	500	5
AGAACATGA	BRDN00011	201	213	729	371	364	16
AGAACATGA	BRDN00012	458	288	1103	742	632	54
AGAACATGA	BRDN00012	150	152	442	754	426	6
AGAACATGC	BRDN00011	135	95	242	217	337	17
AGAACATGG	BRDN00012	358	394	595	949	1101	35
AGAACATGG	BRDN00011	523	419	615	1166	586	46
AGAACATGG	BRDN00012	342	375	381	706	620	63
AGAACATGT	BRDN00012	505	408	1168	1152	1254	81
AGAACATTC	BRDN00011	322	329	641	467	410	3
AGAACCAAA	BRDN00011	151	240	327	406	319	0
AGAACCAAT	BRDN00012	547	517	160	977	737	115
AGAACCAAT	BRDN00011	269	177	207	511	512	33
AGAACCACA	BRDN00012	131	11	0	515	410	2
AGAACCACA	BRDN00012	371	458	391	633	587	37
AGAACCACT	BRDN00011	176	142	451	354	392	4
AGAACCACT	BRDN00012	244	240	1097	516	469	8
AGAACCACT	BRDN00012	277	352	485	418	635	23
AGAACCACT	BRDN00012	168	187	20	400	166	27
AGAACCAGA	BRDN00012	158	0	0	262	229	0
AGAACCAGA	BRDN00012	464	608	375	993	977	14
AGAACCAGA	BRDN00011	69	47	10	239	208	0
AGAACCAGA	BRDN00012	124	32	9	206	90	0
AGAACCAGC	BRDN00012	235	177	919	566	415	38
AGAACCAGC	BRDN00012	215	88	192	346	649	0
AGAACCAGC	BRDN00011	221	123	9	430	488	0
AGAACCAGC	BRDN00011	234	435	484	600	800	27
AGAACCAGG	BRDN00011	243	247	279	410	280	96
AGAACCAGG	BRDN00011	376	427	156	699	651	66
AGAACCAGG	BRDN00011	127	88	0	365	388	0
AGAACCAGG	BRDN00011	143	251	98	428	470	12
AGAACCAGT	BRDN00012	426	502	960	721	366	27
AGAACCATG	BRDN00012	583	141	296	1230	1755	5
AGAACCATT	BRDN00011	51	36	0	515	466	0
AGAACCCAC	BRDN00011	307	115	1009	709	712	41
AGAACCCAG	BRDN00011	240	24	0	543	238	23
AGAACCCAG	BRDN00012	563	334	687	801	850	42
AGAACCCAG	BRDN00012	498	409	1847	795	1263	69
AGAACCCCA	BRDN00011	76	43	557	317	525	17
AGAACCCGC	BRDN00012	235	247	16	307	113	9
AGAACCCGT	BRDN00011	261	381	5	814	1187	22
AGAACCCTA	BRDN00012	433	487	634	781	1038	50
AGAACCCTA	BRDN00012	172	214	166	386	547	18
AGAACCCTT	BRDN00012	77	64	0	221	214	0
AGAACCGAA	BRDN00012	216	26	1249	651	606	0
AGAACCGAC	BRDN00012	199	109	257	442	779	21
AGAACCGAC	BRDN00011	199	185	372	261	214	18
AGAACCGAG	BRDN00012	447	126	10084	982	1028	110
AGAACCGAG	BRDN00011	138	124	91	190	224	51
AGAACCGCA	BRDN00012	503	416	916	1006	1032	33
AGAACCGCA	BRDN00011	131	79	247	269	250	0
AGAACCGCG	BRDN00011	408	377	4422	829	642	65
AGAACCGCT	BRDN00011	83	205	482	280	398	23
AGAACCGGG	BRDN00011	199	183	19	426	236	0
AGAACCTGA	BRDN00011	411	25	59	1023	536	0
AGAACCTGC	BRDN00012	161	134	217	340	390	30
AGAACCTGG	BRDN00012	572	634	2115	1021	978	84
AGAACCTTC	BRDN00012	237	248	189	502	416	43
AGAACGACA	BRDN00012	112	31	0	493	202	0
AGAACGACC	BRDN00012	219	260	288	503	520	8
AGAACGACC	BRDN00012	186	156	237	331	304	12
AGAACGAGC	BRDN00011	110	110	35	148	48	0
AGAACGAGC	BRDN00012	497	503	1410	857	555	59
AGAACGATT	BRDN00012	244	162	147	383	503	0
AGAACGCCA	BRDN00012	528	452	337	828	1164	60
AGAACGCCG	BRDN00011	716	789	1782	1101	1566	94
AGAACGCGC	BRDN00011	139	124	50	224	220	12
AGAACGCTG	BRDN00011	493	506	1062	825	1043	64
AGAACGCTT	BRDN00012	71	57	0	187	62	0
AGAACGGGA	BRDN00012	495	326	401	814	900	15
AGAACGTAG	BRDN00011	356	282	13	637	559	0
AGAACGTCC	BRDN00012	44	48	70	411	281	6
AGAACGTGA	BRDN00011	59	86	4221	98	86	61
AGAACGTGA	BRDN00012	588	438	350	877	861	111
AGAACGTGA	BRDN00012	239	263	43	365	348	46
AGAACGTGG	BRDN00011	417	250	274	925	1034	14
AGAACGTTC	BRDN00012	198	139	3390	394	291	50
AGAACGTTG	BRDN00012	220	0	1	403	317	0
AGAACTAAA	BRDN00011	405	372	1473	920	1196	30
AGAACTACA	BRDN00012	294	300	658	798	1359	33
AGAACTACA	BRDN00012	389	338	1921	512	472	48
AGAACTACG	BRDN00011	130	135	40	308	210	0
AGAACTAGA	BRDN00012	244	165	705	391	209	75
AGAACTAGC	BRDN00011	111	188	153	214	265	7
AGAACTAGC	BRDN00011	381	344	155	641	431	38
AGAACTAGG	BRDN00011	391	394	824	583	805	14
AGAACTATG	BRDN00012	362	332	2243	795	954	94
AGAACTATG	BRDN00012	177	240	24	273	282	47
AGAACTATG	BRDN00012	400	184	1759	626	1077	5
AGAACTCAA	BRDN00011	275	303	713	524	558	57
AGAACTCAA	BRDN00011	170	190	220	410	474	9
AGAACTCAA	BRDN00012	181	173	3054	384	268	31
AGAACTCAC	BRDN00011	152	172	45	332	307	19
AGAACTCAG	BRDN00012	522	520	450	1108	1243	73
AGAACTCAT	BRDN00012	374	339	506	742	750	14
AGAACTCCG	BRDN00011	89	64	60	288	146	6
AGAACTCCG	BRDN00011	208	211	9	500	325	5
AGAACTCCG	BRDN00012	135	146	12	281	195	44
AGAACTCGG	BRDN00012	75	0	0	203	97	0
AGAACTCGG	BRDN00012	356	293	803	611	564	43
AGAACTCGT	BRDN00011	234	232	182	449	263	13
AGAACTCTC	BRDN00012	136	0	0	651	495	0
AGAACTCTG	BRDN00011	358	371	182	656	540	38
AGAACTGAA	BRDN00012	492	719	1444	1063	1232	63
AGAACTGAC	BRDN00011	284	172	358	432	306	38
AGAACTGAG	BRDN00011	291	233	41	557	521	54
AGAACTGAT	BRDN00012	196	68	504	418	110	41
AGAACTGAT	BRDN00011	381	367	486	787	755	97
AGAACTGAT	BRDN00011	478	533	510	834	826	52
AGAACTGCC	BRDN00012	450	473	718	727	953	32
AGAACTGCG	BRDN00012	424	424	125	892	1286	20
AGAACTGGA	BRDN00011	205	116	597	524	583	33
AGAACTGGA	BRDN00011	241	193	176	484	656	11
AGAACTGGG	BRDN00012	27	9	0	293	73	0
AGAACTGGT	BRDN00011	166	177	291	327	241	10
AGAACTGTC	BRDN00011	182	126	198	394	474	0
AGAACTGTC	BRDN00012	440	372	106	895	1159	92
AGAACTTAA	BRDN00012	196	42	0	236	84	0
AGAACTTAC	BRDN00011	121	227	4	252	553	26
AGAACTTAC	BRDN00012	125	102	313	286	371	25
AGAACTTAC	BRDN00011	121	58	737	265	126	52
AGAACTTCC	BRDN00011	420	383	1578	803	1290	69
AGAACTTGA	BRDN00012	498	613	2104	840	985	114
AGAACTTGG	BRDN00012	151	157	968	351	864	16
AGAACTTGG	BRDN00011	147	56	113	329	346	0
AGAACTTGG	BRDN00012	240	285	635	486	281	28
AGAACTTGT	BRDN00012	557	1	0	948	594	0
AGAACTTTA	BRDN00011	308	352	215	532	804	25
AGAACTTTC	BRDN00012	237	257	102	431	386	23
AGAACTTTC	BRDN00011	168	122	0	384	442	18
AGAAGAAAA	BRDN00012	283	157	0	564	767	22
AGAAGAAAA	BRDN00012	156	154	275	418	433	28
AGAAGAAAC	BRDN00012	219	197	193	539	566	0
AGAAGAAAG	BRDN00012	328	380	1711	618	859	24
AGAAGAAAT	BRDN00012	118	114	13	174	171	0
AGAAGAAAT	BRDN00012	333	427	929	622	557	47
AGAAGAAGA	BRDN00011	133	151	23	287	397	22
AGAAGAAGC	BRDN00012	259	310	152	481	471	6
AGAAGAAGC	BRDN00011	131	44	198	264	72	0
AGAAGAAGC	BRDN00011	469	29	144	756	824	3
AGAAGAAGC	BRDN00011	192	129	965	414	472	14
AGAAGAAGC	BRDN00012	160	198	348	257	218	0
AGAAGAATC	BRDN00012	205	93	90	266	222	5
AGAAGAATG	BRDN00012	231	56	2	432	407	0
AGAAGAATT	BRDN00011	154	170	271	390	185	13
AGAAGAATT	BRDN00011	597	563	1333	944	965	35
AGAAGACAC	BRDN00012	44	3	0	248	68	0
AGAAGACAC	BRDN00012	386	288	396	583	323	33
AGAAGACAG	BRDN00011	138	83	0	206	301	0
AGAAGACAG	BRDN00012	551	744	1489	1065	1253	51
AGAAGACAT	BRDN00012	264	280	11	489	406	0
AGAAGACCA	BRDN00012	324	362	232	693	747	5
AGAAGACCC	BRDN00012	227	217	548	421	393	15
AGAAGACCG	BRDN00012	598	697	1420	971	1512	91
AGAAGACCG	BRDN00012	694	676	434	1089	1691	33
AGAAGACCG	BRDN00012	238	147	204	406	431	0
AGAAGACGA	BRDN00011	139	22	0	205	71	0
AGAAGACGG	BRDN00011	123	109	33	285	13	19
AGAAGACTA	BRDN00011	283	205	620	360	606	28
AGAAGACTG	BRDN00011	316	197	205	566	697	2
AGAAGACTG	BRDN00011	497	468	1417	916	706	27
AGAAGACTT	BRDN00011	57	0	0	422	188	0
AGAAGACTT	BRDN00011	428	228	1340	759	1759	62
AGAAGAGAA	BRDN00011	138	133	7	267	313	72
AGAAGAGAC	BRDN00011	92	72	1	234	195	0
AGAAGAGAC	BRDN00012	111	67	1	168	117	0
AGAAGAGCA	BRDN00011	147	1	1	325	106	0
AGAAGAGCA	BRDN00012	433	677	592	670	711	122
AGAAGAGCC	BRDN00012	335	330	117	817	1034	36
AGAAGAGCC	BRDN00011	359	515	739	636	772	51
AGAAGAGCC	BRDN00012	218	287	148	365	163	24
AGAAGAGCT	BRDN00011	181	89	606	287	363	7
AGAAGAGGA	BRDN00012	337	321	75	552	540	60
AGAAGAGGT	BRDN00011	280	266	3476	479	288	46
AGAAGAGGT	BRDN00012	618	636	3348	1236	1399	163
AGAAGAGTC	BRDN00012	519	539	172	923	1606	48
AGAAGAGTC	BRDN00011	13	6	0	218	257	0
AGAAGAGTT	BRDN00011	254	100	356	372	348	3
AGAAGATCC	BRDN00011	416	341	188	575	915	10
AGAAGATCC	BRDN00011	532	465	571	1141	650	59
AGAAGATCC	BRDN00012	589	378	3845	1230	1552	87
AGAAGATCG	BRDN00011	382	185	168	772	546	30
AGAAGATGA	BRDN00011	191	108	919	259	177	42
AGAAGATGA	BRDN00012	489	258	221	690	480	40
AGAAGATGC	BRDN00012	479	340	205	778	1321	26
AGAAGATGC	BRDN00012	288	237	230	368	467	8
AGAAGATGC	BRDN00011	177	33	13	402	374	0
AGAAGATGC	BRDN00012	475	490	1057	939	1041	9
AGAAGATGT	BRDN00011	209	140	976	312	536	18
AGAAGATGT	BRDN00012	426	450	1600	914	935	91
AGAAGCACT	BRDN00011	254	225	290	571	596	42
AGAAGCACT	BRDN00011	259	241	230	557	277	12
AGAAGCACT	BRDN00011	533	285	1448	942	627	66
AGAAGCACT	BRDN00011	263	278	596	632	646	9
AGAAGCAGA	BRDN00011	101	88	35	158	206	0
AGAAGCAGC	BRDN00012	252	245	276	621	515	0
AGAAGCAGT	BRDN00012	440	364	327	725	714	48
AGAAGCATA	BRDN00012	292	283	349	541	844	23
AGAAGCATA	BRDN00011	124	73	31	225	127	0
AGAAGCATA	BRDN00012	227	297	68	529	542	38
AGAAGCATC	BRDN00011	72	62	0	316	321	0
AGAAGCCAA	BRDN00011	126	266	397	253	194	19
AGAAGCCAC	BRDN00011	118	99	339	207	255	74
AGAAGCCAG	BRDN00011	215	181	306	376	219	24
AGAAGCCAT	BRDN00011	309	460	1583	632	495	91
AGAAGCCCA	BRDN00011	293	483	1357	598	860	38
AGAAGCCCT	BRDN00012	393	363	802	924	715	23
AGAAGCCGC	BRDN00012	168	64	89	287	273	9
AGAAGCCGG	BRDN00012	108	53	0	144	237	12
AGAAGCCGT	BRDN00012	568	487	1378	868	856	72
AGAAGCGAA	BRDN00012	166	133	84	191	187	0
AGAAGCGAC	BRDN00012	299	183	32	536	488	6
AGAAGCGCA	BRDN00011	221	270	79	356	197	5
AGAAGCGCA	BRDN00011	141	110	1	185	226	0
AGAAGCGCT	BRDN00012	245	245	722	614	859	48
AGAAGCGCT	BRDN00012	570	419	990	1079	1428	45
AGAAGCGGA	BRDN00011	123	14	8	267	218	0
AGAAGCGGC	BRDN00011	95	138	178	266	246	0
AGAAGCGGC	BRDN00011	473	341	308	1165	1095	6
AGAAGCGGT	BRDN00012	471	305	478	1021	824	49
AGAAGCTAA	BRDN00011	384	339	152	815	547	33
AGAAGCTAC	BRDN00012	222	287	530	405	602	19
AGAAGCTAC	BRDN00011	110	76	339	158	156	0
AGAAGCTAG	BRDN00011	278	366	409	471	474	208
AGAAGCTAG	BRDN00011	310	53	119	547	633	6
AGAAGCTAG	BRDN00011	197	285	805	410	197	44
AGAAGCTAT	BRDN00012	87	35	84	862	632	0
AGAAGCTCA	BRDN00011	187	191	188	212	78	0
AGAAGCTCG	BRDN00012	463	531	670	755	796	143
AGAAGCTGA	BRDN00011	140	13	0	529	283	0
AGAAGCTGC	BRDN00011	75	14	0	229	146	0
AGAAGCTGG	BRDN00012	459	312	62	720	726	19
AGAAGCTGG	BRDN00012	105	190	395	241	289	13
AGAAGCTGT	BRDN00011	32	58	20	262	56	0
AGAAGCTGT	BRDN00012	233	245	361	575	529	5
AGAAGCTTA	BRDN00011	236	251	464	308	223	13
AGAAGCTTA	BRDN00011	511	406	412	1127	1255	22
AGAAGCTTA	BRDN00011	205	292	204	346	293	5
AGAAGCTTC	BRDN00011	245	246	1417	421	305	38
AGAAGCTTG	BRDN00011	242	252	570	538	426	70
AGAAGGAAA	BRDN00011	154	182	283	280	250	20
AGAAGGAAC	BRDN00012	97	16	31	195	151	0
AGAAGGAAT	BRDN00011	32	31	0	610	647	0
AGAAGGACA	BRDN00011	179	4	0	474	33	0
AGAAGGACT	BRDN00011	686	634	1491	1343	1248	111
AGAAGGAGC	BRDN00012	320	250	837	601	700	33
AGAAGGATA	BRDN00011	325	263	288	660	506	13
AGAAGGCAA	BRDN00011	274	258	141	360	232	16
AGAAGGCAC	BRDN00012	246	296	176	650	799	20
AGAAGGCAG	BRDN00012	135	175	19	285	266	0
AGAAGGCAG	BRDN00012	431	487	203	885	1155	22
AGAAGGCGA	BRDN00012	184	278	52	554	432	37
AGAAGGCGC	BRDN00011	404	350	97	734	935	37
AGAAGGCGC	BRDN00011	151	68	236	367	240	9
AGAAGGCGC	BRDN00011	554	388	441	1047	981	62
AGAAGGCGC	BRDN00012	406	259	729	702	839	110
AGAAGGCGT	BRDN00012	535	501	388	1051	997	63
AGAAGGCGT	BRDN00011	87	64	270	143	292	10
AGAAGGCGT	BRDN00011	268	219	395	478	532	12
AGAAGGCTT	BRDN00011	273	224	126	431	426	22
AGAAGGGAA	BRDN00012	299	297	7	718	498	63
AGAAGGGAC	BRDN00011	156	0	0	705	470	0
AGAAGGGAC	BRDN00011	139	111	313	265	272	6
AGAAGGGGG	BRDN00011	122	126	1706	278	242	15
AGAAGGGGG	BRDN00011	511	333	339	991	802	7
AGAAGGGTT	BRDN00011	567	785	1092	1096	984	43
AGAAGGGTT	BRDN00011	120	63	191	229	357	0
AGAAGGTAG	BRDN00011	327	454	294	713	491	50
AGAAGGTAG	BRDN00011	163	180	290	525	550	45
AGAAGGTCC	BRDN00012	590	625	1591	1146	892	132
AGAAGGTCT	BRDN00011	143	200	293	384	624	14
AGAAGGTCT	BRDN00011	154	135	0	431	269	0
AGAAGGTGA	BRDN00012	309	164	343	698	813	6
AGAAGGTGA	BRDN00012	107	118	552	167	508	0
AGAAGGTGC	BRDN00012	127	0	62	456	107	0
AGAAGGTTA	BRDN00012	269	152	134	362	207	25
AGAAGGTTC	BRDN00012	170	152	91	359	109	14
AGAAGTAAA	BRDN00011	75	157	12	164	42	2
AGAAGTAAG	BRDN00012	226	188	1065	356	347	50
AGAAGTAAT	BRDN00011	354	510	1059	773	495	73
AGAAGTACT	BRDN00011	139	104	819	147	185	8
AGAAGTAGA	BRDN00011	615	522	220	1006	686	59
AGAAGTAGA	BRDN00011	304	260	399	444	367	11
AGAAGTAGA	BRDN00012	154	115	425	545	242	2
AGAAGTAGC	BRDN00011	242	354	299	625	332	38
AGAAGTAGC	BRDN00012	322	249	193	721	846	28
AGAAGTAGG	BRDN00011	206	116	296	698	818	0
AGAAGTAGG	BRDN00011	521	441	719	896	1053	55
AGAAGTAGG	BRDN00011	122	161	206	260	229	84
AGAAGTATA	BRDN00011	171	129	1169	494	940	35
AGAAGTCAA	BRDN00011	202	182	459	325	510	6
AGAAGTCAG	BRDN00011	118	113	320	319	136	15
AGAAGTCAT	BRDN00011	172	295	142	329	726	0
AGAAGTCCA	BRDN00012	423	404	487	870	783	50
AGAAGTCCC	BRDN00012	183	142	223	379	278	4
AGAAGTCCT	BRDN00012	515	473	464	1059	1586	0
AGAAGTCCT	BRDN00012	331	253	627	695	316	28
AGAAGTCGA	BRDN00011	477	550	988	959	1250	43
AGAAGTCGA	BRDN00011	48	126	427	174	28	15
AGAAGTCGA	BRDN00011	297	128	813	526	252	14
AGAAGTCTG	BRDN00011	134	207	21	312	383	15
AGAAGTGAA	BRDN00011	440	435	112	819	923	65
AGAAGTGAA	BRDN00011	253	158	427	402	482	0
AGAAGTGAA	BRDN00012	135	75	62	257	443	7
AGAAGTGAA	BRDN00011	201	32	55	280	265	1
AGAAGTGAG	BRDN00012	405	316	428	585	647	19
AGAAGTGAT	BRDN00012	372	529	665	912	769	46
AGAAGTGCC	BRDN00011	337	278	372	697	1265	29
AGAAGTGGA	BRDN00011	211	52	409	531	318	4
AGAAGTGGA	BRDN00012	272	312	71	368	429	13
AGAAGTGGA	BRDN00011	106	0	0	294	82	0
AGAAGTGGT	BRDN00011	335	202	155	522	432	31
AGAAGTGGT	BRDN00011	390	340	783	579	961	17
AGAAGTGGT	BRDN00011	361	311	1437	640	1010	23
AGAAGTTAG	BRDN00012	382	285	283	714	482	39
AGAAGTTAG	BRDN00011	264	146	2	694	712	0
AGAAGTTAT	BRDN00011	302	191	199	462	704	18
AGAAGTTAT	BRDN00012	420	384	1389	827	865	124
AGAAGTTCC	BRDN00011	308	116	635	496	311	30
AGAAGTTGA	BRDN00011	311	208	979	429	2358	34
AGAAGTTGC	BRDN00011	361	265	185	381	486	6
AGAAGTTGC	BRDN00012	371	68	161	1117	917	4
AGAAGTTGT	BRDN00011	241	197	34	613	435	11
AGAAGTTTA	BRDN00012	220	259	5602	315	497	79
AGAAGTTTG	BRDN00011	134	127	0	159	306	0
AGAATAAAC	BRDN00011	143	170	71	364	295	20
AGAATAAAT	BRDN00011	248	379	146	518	376	18
AGAATAACA	BRDN00011	174	99	108	301	436	5
AGAATAACC	BRDN00011	124	195	1	330	412	6
AGAATAAGA	BRDN00011	99	77	959	295	228	9
AGAATAAGG	BRDN00011	180	147	73	262	90	42
AGAATAAGG	BRDN00011	265	218	202	441	190	20
AGAATAAGG	BRDN00011	220	184	357	427	497	18
AGAATACAA	BRDN00011	295	275	606	482	381	58
AGAATACAA	BRDN00012	397	468	206	654	716	37
AGAATACAC	BRDN00012	88	114	0	262	73	16
AGAATACAC	BRDN00012	287	249	503	700	342	389
AGAATACAG	BRDN00011	136	175	185	148	94	0
AGAATACAT	BRDN00012	221	210	423	319	192	19
AGAATACCA	BRDN00011	168	81	526	315	163	6
AGAATACCT	BRDN00011	215	66	0	325	429	0
AGAATACGG	BRDN00011	207	158	991	363	596	41
AGAATACGG	BRDN00011	158	89	90	195	271	6
AGAATAGCC	BRDN00011	126	50	94	226	246	0
AGAATAGCG	BRDN00012	101	118	9	210	168	0
AGAATAGTG	BRDN00011	94	16	0	227	72	0
AGAATATAA	BRDN00011	94	40	563	128	102	5
AGAATATAC	BRDN00012	256	248	833	648	939	38
AGAATATAG	BRDN00011	93	85	1501	369	198	58
AGAATATCA	BRDN00011	181	11	0	437	252	0

AGAATATCT BRDN00011	110	108	1117	214	427	70
AGAATATGG BRDN00012	119	24	0	382	338	0
AGAATATGT BRDN00012	69	0	1	241	265	0
AGAATCAGA BRDN00012	176	248	505	275	368	100
AGAATCAGC BRDN00012	199	170	3	276	157	0
AGAATCATA BRDN00011	98	35	0	155	187	0
AGAATCATG BRDN00011	407	425	1325	574	517	83
AGAATCCAC BRDN00011	320	283	130	526	526	24
AGAATCCAT BRDN00011	437	408	4937	682	509	92
AGAATCCGA BRDN00012	163	87	93	227	292	0
AGAATCCTT BRDN00012	299	389	659	709	333	37
AGAATCCTT BRDN00012	87	1	0	190	88	0
AGAATCCTT BRDN00011	182	74	64	316	186	0
AGAATCGTC BRDN00011	325	413	712	536	501	87
AGAATCTGA BRDN00012	251	42	1123	603	370	14
AGAATCTGG BRDN00012	191	155	87	537	638	25
AGAATCTGG BRDN00011	115	172	1	422	448	24
AGAATCTGG BRDN00011	428	170	69	716	955	14
AGAATCTTG BRDN00012	405	308	243	618	715	29
AGAATGAAG BRDN00011	232	143	345	364	472	0
AGAATGACG BRDN00011	105	0	0	346	396	0
AGAATGACT BRDN00011	171	163	108	276	312	16
AGAATGAGA BRDN00012	183	123	405	207	249	11
AGAATGAGC BRDN00011	23	28	1	645	332	0
AGAATGAGT BRDN00012	287	5	1	605	154	0
AGAATGAGT BRDN00011	537	415	240	830	1118	45
AGAATGATC BRDN00012	134	214	735	137	202	51
AGAATGATG BRDN00012	194	233	300	308	239	31
AGAATGATT BRDN00011	152	230	478	204	320	13
AGAATGCAA BRDN00012	366	5	1	1113	896	0
AGAATGCAC BRDN00012	616	422	310	1407	1308	11
AGAATGCAG BRDN00011	109	223	386	399	609	16
AGAATGCAT BRDN00012	141	241	179	297	262	68
AGAATGCAT BRDN00012	291	227	430	413	208	77
AGAATGCCA BRDN00011	75	12	1	349	152	0
AGAATGCCA BRDN00012	863	672	5079	1180	2183	137
AGAATGCCC BRDN00012	381	256	124	779	640	60
AGAATGCGA BRDN00011	338	121	221	689	857	16
AGAATGCGA BRDN00011	245	220	366	574	410	47
AGAATGCTG BRDN00012	631	526	2015	901	1091	86
AGAATGGAC BRDN00011	204	262	227	306	536	19
AGAATGGAG BRDN00011	169	95	1	195	521	0
AGAATGGAG BRDN00012	125	169	186	301	197	6
AGAATGGAT BRDN00012	472	472	1527	827	644	90
AGAATGGAT BRDN00012	151	66	71	254	329	0
AGAATGGCC BRDN00011	114	101	1	216	217	0
AGAATGGCG BRDN00012	257	27	2	910	953	12
AGAATGGGA BRDN00012	596	620	828	951	1325	28
AGAATGGGC BRDN00012	172	133	59	348	406	0
AGAATGGGC BRDN00011	230	90	127	411	254	7
AGAATGGTA BRDN00011	190	221	1246	310	379	44
AGAATGGTA BRDN00011	62	80	11	164	134	13
AGAATGTAA BRDN00011	238	218	114	551	510	0
AGAATGTAC BRDN00012	119	100	59	240	431	0
AGAATGTAG BRDN00012	153	155	2882	315	254	41
AGAATGTCT BRDN00011	116	43	30	286	344	0
AGAATGTGA BRDN00012	124	76	130	210	205	0
AGAATGTGA BRDN00011	211	129	3682	395	408	99
AGAATGTGA BRDN00011	80	24	117	301	181	0
AGAATGTTC BRDN00012	279	248	14	436	456	21
AGAATTAGA BRDN00012	118	160	163	241	107	33
AGAATTATT BRDN00012	38	59	1	228	77	0
AGAATTCAG BRDN00012	441	376	885	707	389	19
AGAATTCAT BRDN00011	168	117	373	307	276	14
AGAATTCCA BRDN00012	144	0	0	558	419	0
AGAATTCCC BRDN00012	233	131	74	648	170	11
AGAATTCCT BRDN00011	230	173	330	332	252	0
AGAATTCGG BRDN00011	230	210	100	261	321	30
AGAATTCTA BRDN00011	214	236	1129	245	258	30
AGAATTCTA BRDN00011	215	77	2	361	316	5
AGAATTCTG BRDN00011	491	656	630	902	1010	42
AGAATTGAA BRDN00012	632	507	3900	778	1235	50
AGAATTGTA BRDN00012	193	245	1016	347	585	50
AGAATTGTT BRDN00011	231	252	840	412	433	17
AGAATTTAA BRDN00012	210	159	3540	296	129	63
AGAATTTAC BRDN00012	332	284	1250	590	1122	30
AGAATTTAT BRDN00011	284	277	570	528	607	22
AGAATTTCC BRDN00012	225	206	13	450	194	19
AGAATTTGC BRDN00012	415	303	373	614	776	56
AGAATTTGC BRDN00012	348	219	779	425	729	26
AGAATTTGG BRDN00012	246	186	550	536	754	25
AGACAAAAT BRDN00012	701	549	2414	955	1047	108
AGACAAACA BRDN00011	319	423	104	678	801	11
AGACAAACA BRDN00012	353	428	1926	770	1010	66
AGACAAACC BRDN00012	248	241	1725	592	676	59
AGACAAACT BRDN00011	197	262	1415	372	483	57
AGACAAAGA BRDN00012	117	66	98	207	296	7
AGACAAAGA BRDN00012	341	202	548	455	422	17
AGACAAAGC BRDN00012	431	347	129	669	378	2
AGACAAAGG BRDN00011	216	73	764	331	651	12
AGACAAAGT BRDN00011	461	298	699	739	1148	20
AGACAAAGT BRDN00011	479	305	131	709	516	17
AGACAAATA BRDN00012	176	163	126	384	362	7
AGACAAATG BRDN00012	199	194	1	483	706	47
AGACAAATT BRDN00011	362	299	20	734	857	5
AGACAACAC BRDN00011	246	203	317	394	449	24
AGACAACAC BRDN00011	220	217	3804	473	491	124
AGACAACAG BRDN00012	509	334	3268	924	700	56
AGACAACAG BRDN00012	221	103	457	394	277	4
AGACAACAG BRDN00012	197	222	36	275	399	21
AGACAACAT BRDN00012	617	385	1369	1166	1059	61
AGACAACGT BRDN00012	351	294	1055	714	581	74
AGACAACTG BRDN00012	365	200	873	723	523	41
AGACAAGAT BRDN00012	146	202	89	226	175	18
AGACAAGAT BRDN00011	100	66	334	182	377	7
AGACAAGCA BRDN00011	180	222	195	239	346	8
AGACAAGCC BRDN00012	10	0	0	279	76	0
AGACAAGCC BRDN00011	66	96	1	164	266	0
AGACAAGCC BRDN00011	302	215	47	412	652	9
AGACAAGCG BRDN00012	233	244	15	244	258	14
AGACAAGGC BRDN00011	339	267	2529	710	575	59
AGACAAGGC BRDN00011	131	125	174	312	286	6
AGACAAGGC BRDN00011	120	22	33	447	228	0
AGACAATTC BRDN00011	157	211	238	361	501	26
AGACAATTC BRDN00012	181	176	23	392	312	5
AGACAATTT BRDN00011	381	336	306	491	783	36
AGACACAAC BRDN00012	172	163	132	313	261	4
AGACACAAG BRDN00011	229	288	1102	625	619	30
AGACACACT BRDN00012	452	463	915	780	1507	39
AGACACAGA BRDN00011	574	564	632	961	886	49
AGACACAGA BRDN00011	409	574	1107	783	716	147
AGACACAGA BRDN00012	253	261	333	372	226	59
AGACACAGG BRDN00011	140	112	121	214	240	18
AGACACAGT BRDN00012	238	12	1	852	639	1
AGACACATA BRDN00012	377	203	54	538	613	0
AGACACATA BRDN00011	242	99	206	496	363	4
AGACACATG BRDN00012	378	297	145	756	592	22
AGACACATG BRDN00012	509	850	1397	1005	750	78
AGACACATG BRDN00011	366	419	656	1051	519	8
AGACACATG BRDN00012	225	197	54	484	313	35
AGACACCAG BRDN00011	92	32	78	436	237	11
AGACACCAG BRDN00011	224	595	28293	647	1322	448
AGACACCCA BRDN00011	238	204	495	291	117	0
AGACACCGC BRDN00012	599	377	8422	891	948	177
AGACACCGG BRDN00012	201	420	293	355	198	49
AGACACCTC BRDN00011	171	217	399	431	277	3
AGACACCTG BRDN00012	177	179	535	512	537	10
AGACACCTG BRDN00011	176	127	63	426	393	74
AGACACCTT BRDN00011	160	164	65	337	252	38
AGACACGAA BRDN00011	152	200	196	486	297	79
AGACACGCA BRDN00012	614	533	1435	908	981	118
AGACACGCT BRDN00012	37	0	0	354	15	0
AGACACGGA BRDN00011	139	76	0	262	312	7
AGACACGGG BRDN00012	341	301	162	635	515	104
AGACACGGT BRDN00012	253	225	172	518	386	20
AGACACGTA BRDN00012	276	276	1010	466	589	75
AGACACGTC BRDN00012	575	549	1624	1100	846	83
AGACACGTG BRDN00012	169	143	10	317	336	23
AGACACTAC BRDN00011	144	108	221	238	241	8
AGACACTCA BRDN00012	122	97	108	266	439	8
AGACACTGC BRDN00011	161	23	0	425	244	0
AGACACTGC BRDN00012	536	401	1758	1016	1347	52
AGACACTGT BRDN00011	158	135	640	333	147	23
AGACACTTA BRDN00012	259	270	252	482	841	0
AGACAGAAT BRDN00012	147	44	61	438	334	0
AGACAGAAT BRDN00012	273	111	1032	403	543	47
AGACAGACC BRDN00012	279	339	289	554	489	0
AGACAGAGA BRDN00012	297	120	46	815	763	20
AGACAGAGC BRDN00011	87	179	1	177	208	2
AGACAGAGG BRDN00012	85	96	0	259	237	6
AGACAGAGT BRDN00012	573	524	971	1079	1334	35
AGACAGATG BRDN00012	388	311	3017	674	625	95
AGACAGATG BRDN00011	96	15	0	233	315	0
AGACAGATT BRDN00011	320	293	1974	540	632	89
AGACAGCAC BRDN00011	427	297	432	646	564	91
AGACAGCAC BRDN00011	85	8	0	284	178	0
AGACAGCAT BRDN00012	476	474	331	701	758	87
AGACAGCCT BRDN00012	474	442	2366	828	602	79
AGACAGCGA BRDN00012	650	663	702	1248	971	123
AGACAGCTA BRDN00012	462	501	543	825	969	37
AGACAGCTC BRDN00012	169	75	0	221	728	0
AGACAGCTC BRDN00011	285	188	487	810	782	8
AGACAGCTG BRDN00012	406	152	125	989	834	0
AGACAGGAT BRDN00012	157	163	14	330	293	10
AGACAGGGC BRDN00012	152	176	79	194	247	48
AGACAGGGC BRDN00012	251	79	431	449	373	8
AGACAGGTG BRDN00011	182	112	127	355	136	71
AGACAGTAC BRDN00011	191	121	28	425	193	14
AGACAGTGA BRDN00011	169	223	736	337	361	161
AGACAGTGC BRDN00012	311	332	318	659	855	10
AGACAGTGC BRDN00012	495	546	1384	1007	919	58
AGACAGTGG BRDN00011	190	59	94	236	330	8
AGACAGTGG BRDN00011	197	222	243	487	558	42
AGACATAAA BRDN00012	361	105	583	771	734	0
AGACATAAG BRDN00012	326	525	599	694	725	0
AGACATAAT BRDN00012	528	431	149	682	803	50
AGACATACC BRDN00012	209	249	183	387	326	16
AGACATATA BRDN00012	98	89	158	303	255	14
AGACATATG BRDN00012	298	179	407	422	419	0
AGACATCAA BRDN00012	160	141	157	318	463	16
AGACATCAA BRDN00011	174	173	0	423	289	15
AGACATCAG BRDN00012	373	401	710	780	988	11
AGACATCCA BRDN00012	139	283	34	501	719	46
AGACATCCT BRDN00011	158	25	976	605	484	15
AGACATCTA BRDN00012	552	605	1038	879	976	40
AGACATCTT BRDN00012	494	382	435	915	953	67
AGACATGAA BRDN00012	413	399	329	705	653	83
AGACATGAA BRDN00011	210	237	0	334	354	0
AGACATGAA BRDN00012	330	447	933	518	565	11
AGACATGAA BRDN00011	29	0	1	457	151	0
AGACATGCA BRDN00011	461	305	720	805	600	25
AGACATGCG BRDN00012	452	412	1257	869	661	27
AGACATGGT BRDN00012	125	13	0	555	337	0
AGACATTAA BRDN00012	383	297	1373	667	756	35
AGACATTAA BRDN00011	192	130	146	345	459	15
AGACATTCA BRDN00011	184	53	650	378	229	11
AGACATTGA BRDN00012	41	0	0	654	223	0
AGACATTGC BRDN00011	147	171	574	366	433	5
AGACATTGT BRDN00012	359	327	772	599	1229	49
AGACCAAAA BRDN00012	251	239	228	351	560	23
AGACCAACA BRDN00012	722	605	2530	1107	1326	95
AGACCAAGC BRDN00012	481	496	235	829	1059	42
AGACCAATC BRDN00011	135	110	27	287	427	6
AGACCAATT BRDN00012	537	455	377	1050	660	38
AGACCAATT BRDN00012	268	346	1354	446	808	156
AGACCACAG BRDN00011	319	155	233	635	524	6
AGACCACAG BRDN00011	424	294	916	1165	1102	28
AGACCACCA BRDN00011	153	0	0	440	184	0
AGACCACGA BRDN00011	215	181	0	287	187	19
AGACCACGA BRDN00011	157	235	90	382	491	4
AGACCACGG BRDN00012	325	196	286	647	307	28
AGACCACGT BRDN00012	59	37	13	516	496	2
AGACCACGT BRDN00011	379	86	160	818	1130	18
AGACCACTC BRDN00012	228	179	241	487	619	13
AGACCAGAC BRDN00012	134	0	1	518	109	0
AGACCAGAG BRDN00011	171	292	88	287	170	27
AGACCAGCC BRDN00011	197	1	2	852	717	0
AGACCAGCC BRDN00012	648	422	39	1143	1257	66
AGACCAGTA BRDN00012	109	69	44	246	217	13
AGACCATAT BRDN00011	167	118	414	253	119	19
AGACCATAT BRDN00011	70	24	0	248	112	0
AGACCATCA BRDN00012	277	324	231	659	586	8
AGACCATGT BRDN00011	459	626	556	939	1134	70
AGACCATTT BRDN00011	353	149	11	724	794	51
AGACCCACA BRDN00011	266	196	224	460	696	21
AGACCCACT BRDN00011	349	363	141	508	362	67
AGACCCACT BRDN00011	116	145	334	190	353	0
AGACCCAGT BRDN00011	68	0	7	587	197	0
AGACCCATC BRDN00012	211	185	95	357	408	38
AGACCCATG BRDN00012	225	147	406	468	352	14
AGACCCATG BRDN00012	579	541	849	1182	1397	31
AGACCCATT BRDN00011	364	130	75	839	478	0
AGACCCCAA BRDN00011	100	50	24	200	236	0
AGACCCCAA BRDN00012	182	212	377	341	296	15
AGACCCCAC BRDN00011	136	189	594	472	268	4
AGACCCCCT BRDN00012	341	438	246	745	1083	50
AGACCCCCT BRDN00012	478	341	261	712	1004	42
AGACCCCGT BRDN00012	825	728	1244	1195	2189	108
AGACCCCGT BRDN00012	214	247	1096	361	267	66
AGACCCCTC BRDN00011	441	434	196	749	872	41
AGACCCCTT BRDN00012	416	353	580	635	740	52
AGACCCCTT BRDN00011	240	254	102	453	800	0
AGACCCGAG BRDN00011	406	341	383	817	439	43
AGACCCGGC BRDN00011	359	281	353	443	318	30
AGACCCGTC BRDN00012	244	273	1700	542	614	31
AGACCCTAA BRDN00011	278	108	674	704	431	45
AGACCCTGG BRDN00011	74	45	299	94	107	0
AGACCGAAT BRDN00012	150	122	26	344	366	1
AGACCGACA BRDN00011	403	350	403	782	867	48
AGACCGAGG BRDN00012	470	303	425	875	496	27
AGACCGAGT BRDN00011	164	190	322	254	279	22
AGACCGATC BRDN00012	167	173	110	397	410	19
AGACCGCAA BRDN00012	162	195	256	439	330	0
AGACCGCAA BRDN00012	307	283	1832	746	597	12
AGACCGGAG BRDN00012	329	238	202	546	442	38
AGACCGGCA BRDN00011	390	485	1135	1317	774	168
AGACCGGCG BRDN00011	174	154	93	401	251	1
AGACCGGCT BRDN00011	142	239	0	236	297	0
AGACCGGGA BRDN00012	68	96	34	291	158	10
AGACCGGGA BRDN00011	111	113	0	217	132	16
AGACCGGGC BRDN00011	206	225	159	266	135	31
AGACCGTAG BRDN00012	142	127	388	318	166	0
AGACCGTGA BRDN00012	187	207	278	421	457	0
AGACCTACA BRDN00011	278	270	3354	400	295	130
AGACCTAGA BRDN00011	193	225	35	315	392	41
AGACCTCAA BRDN00011	164	96	48	268	165	17
AGACCTCAC BRDN00012	478	492	471	1065	917	33
AGACCTCAG BRDN00011	202	262	14	358	259	15
AGACCTCGA BRDN00011	434	421	231	676	423	14
AGACCTCGA BRDN00011	311	208	387	471	528	27
AGACCTCTC BRDN00012	704	679	1534	1377	1384	75
AGACCTCTG BRDN00011	233	191	317	458	419	1
AGACCTCTG BRDN00011	324	338	783	842	490	26
AGACCTGAA BRDN00011	376	89	172	610	483	0
AGACCTGAG BRDN00012	613	609	1638	1243	1193	58
AGACCTGGT BRDN00011	782	187	873	1413	669	29
AGACCTGTC BRDN00011	172	111	36	296	319	0
AGACCTGTC BRDN00011	180	143	35	467	178	0
AGACCTTAA BRDN00011	262	202	23	451	256	10
AGACCTTAT BRDN00011	139	234	9	237	294	6
AGACCTTCA BRDN00012	558	435	1316	1222	1248	41
AGACCTTCA BRDN00012	386	516	2158	879	888	52
AGACCTTCT BRDN00012	410	207	1114	758	696	38
AGACCTTCT BRDN00012	105	0	1	1065	116	0
AGACCTTGC BRDN00012	461	339	376	643	702	127
AGACCTTGT BRDN00011	414	281	1247	769	982	7
AGACGAAAG BRDN00012	17	0	0	2	4	0
AGACGAAGA BRDN00011	24	0	0	61	0	0
AGACGAATT BRDN00011	0	0	0	31	7	0
AGACGAATT BRDN00011	22	0	9	42	17	0
AGACGACCA BRDN00012	12	0	24	7	18	0
AGACGACCT BRDN00012	6	3	0	2	0	0
AGACGACTG BRDN00012	44	14	0	37	12	4
AGACGAGAA BRDN00011	0	0	0	4	0	0
AGACGAGCT BRDN00011	0	3	0	22	23	0
AGACGAGGT BRDN00011	0	0	0	27	0	0
AGACGAGGT BRDN00012	6	20	0	15	30	0
AGACGAGTA BRDN00011	31	37	0	37	87	0
AGACGATCT BRDN00012	0	0	0	28	15	0
AGACGATGA BRDN00011	0	0	0	0	0	0
AGACGATGG BRDN00011	0	0	0	6	0	0
AGACGATGG BRDN00011	0	0	0	21	39	0
AGACGCACC BRDN00011	0	5	0	17	51	0
AGACGCAGA BRDN00011	31	0	0	47	0	0
AGACGCAGT BRDN00011	22	10	0	55	25	0
AGACGCATA BRDN00012	22	0	0	19	0	0
AGACGCATG BRDN00011	25	24	1597	17	0	33
AGACGCCAA BRDN00011	19	4	25	32	14	0
AGACGCCAG BRDN00011	2	9	0	22	0	0
AGACGCCAT BRDN00011	35	21	0	59	5	0
AGACGCCGA BRDN00012	4	0	0	3	0	0
AGACGCGAT BRDN00011	10	11	0	2	63	0
AGACGCGCA BRDN00012	0	14	0	22	0	0
AGACGCGCG BRDN00011	0	0	0	0	0	0
AGACGCGCT BRDN00011	48	32	11	23	0	0
AGACGCGCT BRDN00011	1	0	0	0	0	0
AGACGCGCT BRDN00012	10	13	0	22	173	0
AGACGCGGC BRDN00011	2	0	0	14	0	0
AGACGCGGT BRDN00011	15	7	0	11	0	0
AGACGCGTC BRDN00011	0	0	0	4	0	0
AGACGCTAT BRDN00012	8	5	0	28	0	0
AGACGCTCA BRDN00011	0	5	113	14	0	0
AGACGCTGA BRDN00011	7	23	19	12	0	0
AGACGCTGC BRDN00012	15	0	0	50	19	0
AGACGCTGC BRDN00012	12	23	45	31	69	10
AGACGCTGG BRDN00011	5	18	0	34	53	0
AGACGCTGG BRDN00011	2	0	0	0	0	0
AGACGGAAA BRDN00012	7	0	0	18	0	0
AGACGGAAC BRDN00012	9	0	0	39	51	0
AGACGGAGC BRDN00012	0	0	0	3	0	0
AGACGGAGC BRDN00011	1	0	0	0	0	0
AGACGGAGC BRDN00012	4	7	0	0	0	0
AGACGGAGT BRDN00011	1	0	0	7	0	0
AGACGGAGT BRDN00011	0	0	0	22	40	0
AGACGGATG BRDN00011	61	51	1630	41	131	7
AGACGGCAA BRDN00011	10	11	0	29	9	0
AGACGGCAG BRDN00011	6	0	0	11	0	0
AGACGGCCA BRDN00012	1	4	0	1	0	0
AGACGGCCT BRDN00011	4	22	0	16	0	0
AGACGGCTA BRDN00012	16	26	0	26	0	0
AGACGGGAC BRDN00012	15	9	0	4	0	0
AGACGGGGA BRDN00012	8	0	0	0	37	0
AGACGGGTA BRDN00012	25	21	0	18	0	0
AGACGGGTG BRDN00011	21	4	0	70	72	0
AGACGGTCA BRDN00011	6	11	0	9	0	0
AGACGGTCA BRDN00011	12	0	0	11	0	0
AGACGGTCT BRDN00012	8	0	0	23	12	0
AGACGGTGC BRDN00012	3	0	0	17	6	0
AGACGGTGT BRDN00011	8	0	0	9	1	0
AGACGTAAA BRDN00012	7	34	0	11	81	0
AGACGTAAC BRDN00012	21	4	0	23	0	0
AGACGTAAT BRDN00012	2	1	0	18	0	0
AGACGTACA BRDN00011	15	19	0	14	49	0
AGACGTACC BRDN00012	2	0	0	10	1	0
AGACGTAGA BRDN00011	35	25	12	9	0	0
AGACGTATA BRDN00012	6	0	0	0	0	0
AGACGTCCA BRDN00012	0	0	0	12	17	0
AGACGTCCG BRDN00011	0	0	0	6	0	0
AGACGTCTG BRDN00011	17	0	0	17	0	0
AGACGTGCA BRDN00012	0	0	0	21	0	0
AGACGTGCA BRDN00012	10	0	0	34	42	0
AGACGTGGA BRDN00012	0	0	0	0	0	0
AGACGTGGC BRDN00012	0	0	0	9	0	0
AGACGTGTC BRDN00011	4	0	0	20	0	0
AGACGTGTC BRDN00012	4	7	0	20	12	0
AGACGTTAA BRDN00012	0	0	0	0	0	0
AGACGTTAA BRDN00012	5	4	0	7	0	0
AGACGTTAG BRDN00011	2	12	0	0	0	0
AGACGTTCC BRDN00012	8	1	0	32	0	0
AGACGTTGA BRDN00012	0	0	0	0	0	0
AGACGTTGA BRDN00011	4	36	0	3	21	0
AGACGTTGG BRDN00011	0	0	0	14	0	0
AGACGTTGG BRDN00012	12	0	0	27	13	0
AGACGTTGT BRDN00012	4	0	0	26	39	0
AGACTACAA BRDN00011	255	130	36	478	465	4
AGACTACAG BRDN00011	231	309	130	377	419	12
AGACTACAT BRDN00012	268	143	44	466	712	4
AGACTACTA BRDN00012	164	0	0	312	159	0
AGACTAGGA BRDN00011	129	4	0	167	67	0
AGACTAGTC BRDN00012	423	276	1276	728	939	68
AGACTATAG BRDN00011	150	5	0	272	296	0
AGACTATGA BRDN00012	563	504	337	801	786	11
AGACTATGA BRDN00012	219	170	20	402	284	77
AGACTATGA BRDN00011	290	284	434	604	655	78
AGACTATGT BRDN00011	127	0	0	335	213	0
AGACTCAAG BRDN00011	543	522	519	960	1217	10
AGACTCACA BRDN00012	141	205	25	265	152	0
AGACTCACC BRDN00011	394	340	634	756	885	16
AGACTCACC BRDN00012	169	119	575	225	103	5
AGACTCACG BRDN00011	162	0	0	400	98	0
AGACTCACT BRDN00012	164	146	943	408	257	8
AGACTCCAA BRDN00012	211	120	123	345	323	0
AGACTCCAA BRDN00011	161	138	101	400	98	11
AGACTCCAG BRDN00011	116	80	150	241	204	15
AGACTCCAT BRDN00012	222	170	268	412	373	43
AGACTCCTA BRDN00012	215	286	54	494	822	8
AGACTCCTG BRDN00011	77	63	0	195	198	6
AGACTCGAA BRDN00012	433	72	16	1177	1196	1
AGACTCGGA BRDN00011	142	136	0	230	415	4
AGACTCGGT BRDN00012	245	56	0	571	235	0
AGACTCGGT BRDN00012	632	527	473	999	1496	4
AGACTCGTA BRDN00012	533	578	2547	1053	999	81
AGACTCTAA BRDN00012	427	345	174	932	985	39
AGACTCTGA BRDN00011	552	534	350	971	1062	21
AGACTCTGC BRDN00012	223	120	221	419	489	0
AGACTCTGT BRDN00012	570	690	297	986	1030	92
AGACTCTTA BRDN00011	247	195	125	642	726	3
AGACTGACC BRDN00011	260	176	513	531	586	1
AGACTGAGA BRDN00011	257	294	374	369	505	42
AGACTGAGT BRDN00012	31	4	0	278	135	0
AGACTGCAC BRDN00012	197	182	21	360	333	20
AGACTGCAG BRDN00012	321	305	152	599	833	30
AGACTGCCC BRDN00012	231	224	47	354	598	40
AGACTGCCG BRDN00012	578	435	772	811	579	84
AGACTGCCT BRDN00012	82	72	1733	477	275	8
AGACTGCCT BRDN00011	368	302	514	532	790	45
AGACTGCGG BRDN00012	168	224	87	395	783	9
AGACTGCTG BRDN00012	38	29	0	551	193	0
AGACTGCTG BRDN00012	86	80	0	380	398	11
AGACTGCTG BRDN00011	460	633	675	877	806	49
AGACTGGAA BRDN00011	165	290	11	283	312	12
AGACTGGAA BRDN00012	261	155	73	593	666	31
AGACTGGAC BRDN00012	181	86	875	233	310	11
AGACTGGAT BRDN00011	38	0	0	329	258	0
AGACTGGTC BRDN00011	436	181	261	624	385	0
AGACTGTCG BRDN00011	458	459	2818	794	878	150
AGACTGTGG BRDN00012	310	268	221	616	738	7
AGACTGTGG BRDN00012	190	170	313	351	335	12
AGACTGTTG BRDN00012	227	295	232	329	522	33
AGACTTAAA BRDN00011	451	88	48	1072	1317	0
AGACTTACC BRDN00011	420	345	931	837	757	32
AGACTTCAC BRDN00012	396	584	891	980	878	30
AGACTTCAG BRDN00011	187	243	112	224	200	0
AGACTTCAG BRDN00012	205	152	1161	715	819	20
AGACTTCAT BRDN00011	167	36	1	539	421	8
AGACTTCCA BRDN00012	129	92	104	312	286	19
AGACTTCTC BRDN00011	216	67	166	420	552	34
AGACTTGAA BRDN00012	47	0	0	534	114	0
AGACTTGCA BRDN00011	253	256	631	451	380	6
AGACTTGCT BRDN00011	389	553	450	726	1480	55
AGACTTTAG BRDN00012	273	329	804	387	389	0
AGACTTTCG BRDN00012	419	438	1434	804	975	76
AGACTTTGG BRDN00011	212	252	500	464	337	36
AGACTTTGT BRDN00012	350	305	623	659	548	6
AGAGAAAAG BRDN00011	443	447	383	582	1258	12

AGAGAAAAG	BRDN00012	369	336	135	1029	551	16
AGAGAAAAT	BRDN00012	261	266	94	392	344	0
AGAGAAAAT	BRDN00012	140	137	97	233	417	0
AGAGAAACA	BRDN00012	75	72	0	115	119	5
AGAGAAACA	BRDN00011	135	95	0	250	643	0
AGAGAAACA	BRDN00011	128	135	158	210	397	3
AGAGAAACC	BRDN00012	211	149	683	455	230	23
AGAGAAACC	BRDN00012	121	136	45	399	537	9
AGAGAAAGA	BRDN00011	327	225	417	787	843	50
AGAGAAAGA	BRDN00012	112	102	517	333	207	25
AGAGAAAGA	BRDN00012	64	35	204	236	576	0
AGAGAAAGT	BRDN00012	299	157	129	585	589	6
AGAGAAATT	BRDN00011	316	277	67	591	491	27
AGAGAAATT	BRDN00011	244	190	1	363	354	0
AGAGAACAA	BRDN00012	484	328	556	793	717	31
AGAGAACAT	BRDN00012	636	478	558	775	837	18
AGAGAACAT	BRDN00011	212	184	135	375	181	9
AGAGAACCC	BRDN00011	340	411	308	555	413	1
AGAGAACGG	BRDN00011	198	52	23	586	205	7
AGAGAACTA	BRDN00011	189	62	57	328	964	19
AGAGAACTA	BRDN00011	301	191	717	591	680	45
AGAGAACTT	BRDN00011	418	256	194	787	784	6
AGAGAACTT	BRDN00011	133	84	21	244	223	14
AGAGAAGA	BRDN00011	178	207	85	408	334	3
AGAGAAGA	BRDN00011	130	147	495	315	522	100
AGAGAAGA	BRDN00011	88	66	216	172	52	4
AGAGAAGA	BRDN00012	325	256	408	574	596	12
AGAGAAGCA	BRDN00011	107	0	0	647	181	0
AGAGAAGCA	BRDN00011	77	77	1	505	276	0
AGAGAAGCA	BRDN00012	96	122	0	265	177	0
AGAGAAGCT	BRDN00011	161	212	970	343	393	1160
AGAGAAGCT	BRDN00011	314	232	326	515	711	13
AGAGAAGG	BRDN00012	96	149	856	187	251	0
AGAGAAGG	BRDN00012	355	248	0	707	498	0
AGAGAAGG	BRDN00012	461	407	378	663	766	48
AGAGAAGG	BRDN00011	413	231	431	508	470	31
AGAGAAGG	BRDN00012	331	267	63	612	621	48
AGAGAAGG	BRDN00012	351	175	84	561	423	21
AGAGAAGG	BRDN00012	145	43	14	277	110	0
AGAGAAGG	BRDN00011	128	43	216	269	233	3
AGAGAAGGT	BRDN00012	149	151	153	268	361	14
AGAGAAGTA	BRDN00012	296	209	236	433	443	14
AGAGAAGTC	BRDN00012	110	199	1096	295	213	28
AGAGAAGTC	BRDN00012	16	8	0	254	39	0
AGAGAATAA	BRDN00011	53	32	0	110	163	0
AGAGAATAA	BRDN00011	91	57	49	129	33	0
AGAGAATAC	BRDN00012	333	146	1227	626	653	16
AGAGAATAC	BRDN00011	275	122	404	321	692	14
AGAGAATAT	BRDN00011	271	271	905	518	527	58
AGAGAATCT	BRDN00012	340	310	103	526	430	10
AGAGAATGA	BRDN00012	78	16	0	236	312	0
AGAGACAAA	BRDN00011	405	329	88	741	1107	28
AGAGACAAC	BRDN00011	548	411	798	851	722	42
AGAGACAAT	BRDN00012	267	229	717	613	564	0
AGAGACAAT	BRDN00011	128	45	0	304	217	1
AGAGACACA	BRDN00012	217	215	71	434	388	47
AGAGACACA	BRDN00011	139	111	359	459	533	17
AGAGACACG	BRDN00011	91	13	0	249	93	0
AGAGACAGA	BRDN00011	217	215	316	295	386	12
AGAGACAGC	BRDN00011	208	177	188	227	197	10
AGAGACAGC	BRDN00012	299	268	114	460	815	6
AGAGACAGG	BRDN00012	255	282	700	754	1085	80
AGAGACAGG	BRDN00012	497	542	322	737	1286	83
AGAGACATG	BRDN00011	125	128	46	225	201	13
AGAGACATT	BRDN00011	240	4	74	540	386	0
AGAGACATT	BRDN00012	213	233	394	417	305	70
AGAGACCAA	BRDN00012	162	199	367	401	792	6
AGAGACCAC	BRDN00012	106	149	403	275	129	14
AGAGACCAG	BRDN00011	157	177	93	224	794	2
AGAGACCAG	BRDN00012	577	471	814	1425	865	67
AGAGACCAT	BRDN00012	163	37	0	226	34	1
AGAGACCGG	BRDN00011	209	67	62	376	216	0
AGAGACCGG	BRDN00012	554	557	976	1104	1476	123
AGAGACCTC	BRDN00011	405	378	469	699	1238	10
AGAGACTAT	BRDN00012	176	55	1	334	318	0
AGAGACTCC	BRDN00012	220	124	37	672	415	0
AGAGACTCT	BRDN00012	265	2	2	639	204	0
AGAGACTGA	BRDN00011	139	107	61	405	224	30
AGAGACTGG	BRDN00012	237	221	360	305	331	13
AGAGACTGG	BRDN00011	187	210	194	548	238	11
AGAGACTGT	BRDN00011	304	278	320	573	490	40
AGAGACTGT	BRDN00011	189	195	35	370	492	26
AGAGACTTA	BRDN00011	131	204	157	242	148	15
AGAGACTTG	BRDN00011	151	65	237	233	82	0
AGAGAGAA	BRDN00011	47	54	702	144	47	21
AGAGAGAA	BRDN00011	380	304	252	590	852	0
AGAGAGAA	BRDN00011	600	447	736	822	707	80
AGAGAGACC	BRDN00012	465	602	985	942	1140	89
AGAGAGACT	BRDN00011	90	107	55	161	185	2
AGAGAGAG	BRDN00011	71	30	1	122	228	0
AGAGAGAG	BRDN00011	238	177	1541	421	634	17
AGAGAGAG	BRDN00011	117	143	51	266	1257	0
AGAGAGAGT	BRDN00012	103	116	434	149	75	4
AGAGAGATA	BRDN00011	108	125	90	141	152	39
AGAGAGATG	BRDN00012	108	179	8	327	213	0
AGAGAGCAA	BRDN00012	75	51	408	216	341	20
AGAGAGCAA	BRDN00012	425	268	167	967	1247	33
AGAGAGCAC	BRDN00012	515	504	1229	954	655	54
AGAGAGCAC	BRDN00011	181	182	95	292	251	10
AGAGAGCAC	BRDN00011	278	0	0	439	389	0
AGAGAGCCA	BRDN00011	6	0	0	262	1	0
AGAGAGCCC	BRDN00012	390	384	854	853	981	48
AGAGAGCCT	BRDN00012	378	449	1186	669	634	58
AGAGAGCG	BRDN00011	211	239	462	414	273	26
AGAGAGGA	BRDN00012	84	107	79	217	227	10
AGAGAGGA	BRDN00012	521	589	3850	636	776	190
AGAGAGGC	BRDN00011	50	54	50	232	124	6
AGAGAGGC	BRDN00011	150	117	105	305	475	20
AGAGAGGC	BRDN00012	212	226	1	486	547	0
AGAGAGGC	BRDN00011	184	286	417	298	506	23
AGAGAGGG	BRDN00012	190	0	0	329	233	1
AGAGAGGG	BRDN00012	521	287	324	939	773	18
AGAGAGGT	BRDN00011	138	91	1445	193	191	6
AGAGAGTCG	BRDN00011	147	117	6	285	237	0
AGAGAGTG	BRDN00012	674	522	1518	1111	1279	51
AGAGAGTGT	BRDN00012	78	127	93	221	90	10
AGAGAGTGT	BRDN00012	468	381	295	590	831	8
AGAGAGTTG	BRDN00011	56	68	0	194	223	33
AGAGAGTTG	BRDN00011	296	277	288	548	482	131
AGAGAGTTT	BRDN00012	420	605	3355	642	508	104
AGAGATACA	BRDN00012	596	546	2076	1023	805	91
AGAGATACC	BRDN00012	83	91	223	223	98	0
AGAGATACG	BRDN00012	450	317	615	795	870	6
AGAGATAGT	BRDN00011	156	107	131	269	360	14
AGAGATAGT	BRDN00011	311	199	32355	583	798	377
AGAGATCAA	BRDN00012	284	60	31	663	295	21
AGAGATCAA	BRDN00011	351	242	214	572	436	5
AGAGATCAC	BRDN00011	65	10	0	194	237	0
AGAGATCAG	BRDN00012	293	258	884	530	371	28
AGAGATCAG	BRDN00011	416	9	0	962	341	0
AGAGATCAG	BRDN00011	162	137	424	313	431	15
AGAGATCAT	BRDN00011	259	234	152	415	332	0
AGAGATCCG	BRDN00012	96	0	0	381	10	0
AGAGATCCG	BRDN00012	224	300	834	351	477	6
AGAGATCCG	BRDN00012	160	169	215	287	344	36
AGAGATCCG	BRDN00012	175	152	116	210	253	4
AGAGATCCT	BRDN00011	123	155	372	274	278	3
AGAGATCTG	BRDN00011	233	6	104	363	225	0
AGAGATGAA	BRDN00011	243	196	32	412	339	0
AGAGATGAC	BRDN00012	266	211	169	522	328	31
AGAGATGAC	BRDN00012	159	48	252	437	295	27
AGAGATGAC	BRDN00012	181	69	388	275	168	1
AGAGATGAT	BRDN00012	242	297	217	404	807	14
AGAGATGCA	BRDN00012	88	12	1	269	54	0
AGAGATGCA	BRDN00011	382	40	1	1001	831	1
AGAGATGCT	BRDN00012	253	161	159	516	576	4
AGAGATGG	BRDN00011	116	126	70	274	493	7
AGAGATGG	BRDN00012	571	547	671	1200	1041	49
AGAGATGTA	BRDN00011	285	176	83	383	728	3
AGAGATTAT	BRDN00012	389	242	57	650	743	54
AGAGATTCA	BRDN00011	366	473	431	810	551	62
AGAGATTCC	BRDN00012	315	373	3001	647	1261	153
AGAGATTCC	BRDN00012	210	219	893	323	400	10
AGAGATTCC	BRDN00011	326	0	1	846	163	0
AGAGATTCT	BRDN00011	223	260	486	412	392	13
AGAGATTGT	BRDN00011	75	18	0	196	176	0
AGAGATTGT	BRDN00012	310	305	1110	476	483	10
AGAGATTGT	BRDN00012	332	292	518	694	706	79
AGAGATTTC	BRDN00012	255	184	296	546	523	2
AGAGCAAAA	BRDN00012	192	257	97	668	365	9
AGAGCAAAC	BRDN00011	448	259	1166	874	1317	1
AGAGCAAAG	BRDN00012	366	417	504	659	521	16
AGAGCAAAG	BRDN00012	42	52	96	379	234	8
AGAGCAACC	BRDN00012	164	140	82	303	290	11
AGAGCAACC	BRDN00012	78	85	0	280	239	3
AGAGCAACC	BRDN00011	226	97	42	357	281	0
AGAGCAAGG	BRDN00011	157	154	110	560	421	7
AGAGCAAGT	BRDN00012	116	93	1	235	178	0
AGAGCAATC	BRDN00011	431	357	837	897	1181	55
AGAGCAATC	BRDN00011	137	149	130	187	110	9
AGAGCAATT	BRDN00012	526	331	909	733	1645	59
AGAGCAATT	BRDN00012	81	5	1	202	92	0
AGAGCACAA	BRDN00012	90	154	355	187	84	7
AGAGCACAC	BRDN00012	130	21	8	353	115	0
AGAGCACAG	BRDN00012	504	428	7287	864	1164	63
AGAGCACAG	BRDN00012	208	111	309	480	514	6
AGAGCACAG	BRDN00012	100	112	0	334	44	0
AGAGCACGA	BRDN00012	298	511	187	577	858	5
AGAGCACGC	BRDN00011	147	7	0	561	240	0
AGAGCACTT	BRDN00011	80	75	0	618	204	0
AGAGCACTT	BRDN00011	285	103	109	693	254	11
AGAGCAGAA	BRDN00012	404	235	2112	603	862	65
AGAGCAGAC	BRDN00011	364	353	727	805	849	20
AGAGCAGAC	BRDN00012	262	281	1500	373	298	122
AGAGCAGCA	BRDN00011	219	175	896	442	612	24
AGAGCAGCC	BRDN00011	281	382	643	620	157	17
AGAGCAGCT	BRDN00011	11	0	0	103	17	0
AGAGCAGCT	BRDN00011	280	128	160	533	300	23
AGAGCAGGA	BRDN00012	186	295	431	536	523	36
AGAGCAGG	BRDN00011	109	66	90	261	611	0
AGAGCAGG	BRDN00011	162	31	6	374	700	0
AGAGCAGGT	BRDN00012	533	357	1809	800	687	66
AGAGCATAA	BRDN00011	63	76	85	318	307	30
AGAGCATAC	BRDN00012	153	95	49	344	309	26
AGAGCATAT	BRDN00011	212	90	160	482	276	71
AGAGCATCC	BRDN00012	145	261	52	300	350	16
AGAGCATCC	BRDN00011	268	172	565	306	305	41
AGAGCATCG	BRDN00012	95	136	6	407	176	0
AGAGCATCG	BRDN00012	318	330	374	753	451	13
AGAGCATCT	BRDN00011	171	182	47	350	302	17
AGAGCATGA	BRDN00012	707	523	2025	1032	1234	27
AGAGCATGG	BRDN00012	190	164	811	431	239	49
AGAGCATGT	BRDN00012	383	295	275	752	414	28
AGAGCATTA	BRDN00012	121	93	248	174	457	19
AGAGCATTA	BRDN00012	537	552	1730	1194	1974	65
AGAGCCAAA	BRDN00012	87	18	0	274	66	0
AGAGCCAAA	BRDN00012	218	157	970	517	686	32
AGAGCCAAA	BRDN00012	127	184	146	178	150	5
AGAGCCAAC	BRDN00012	237	303	291	533	439	23
AGAGCCACG	BRDN00012	242	226	11	470	624	0
AGAGCCAGA	BRDN00012	153	137	483	259	255	6
AGAGCCAGG	BRDN00012	190	270	415	339	272	33
AGAGCCATA	BRDN00011	555	457	1029	719	684	18
AGAGCCATA	BRDN00012	316	381	412	656	807	34
AGAGCCATC	BRDN00012	95	16	0	269	159	0
AGAGCCATG	BRDN00012	208	209	5	396	286	1
AGAGCCCAA	BRDN00011	233	149	61	294	276	17
AGAGCCCAC	BRDN00012	74	78	231	228	102	87
AGAGCCCAG	BRDN00011	326	374	2537	638	518	57
AGAGCCCGA	BRDN00011	149	139	39	252	659	19
AGAGCCCGA	BRDN00011	365	298	364	891	901	31
AGAGCCCGA	BRDN00012	54	0	0	274	140	0
AGAGCCCTG	BRDN00012	198	190	610	348	243	5
AGAGCCGAA	BRDN00011	444	302	418	593	470	17
AGAGCCGAA	BRDN00011	163	145	17	359	215	0
AGAGCCGAA	BRDN00011	144	119	147	254	222	10
AGAGCCGCA	BRDN00011	115	100	425	231	187	32
AGAGCCTAC	BRDN00012	357	324	836	641	459	106
AGAGCCTAG	BRDN00011	147	54	0	207	260	8
AGAGCCTAT	BRDN00011	177	143	371	341	573	30
AGAGCCTAT	BRDN00011	115	227	229	295	476	3
AGAGCCTCA	BRDN00011	181	66	116	220	153	2
AGAGCCTCC	BRDN00012	312	435	603	486	547	43
AGAGCCTCG	BRDN00012	538	404	919	1126	1186	57
AGAGCCTCT	BRDN00012	144	115	1047	386	426	31
AGAGCCTCT	BRDN00011	229	193	2416	438	521	44
AGAGCCTGA	BRDN00011	337	243	977	517	770	17
AGAGCCTGA	BRDN00012	110	4	0	282	213	27
AGAGCCTGA	BRDN00012	471	450	515	964	976	30
AGAGCCTGC	BRDN00011	125	146	0	227	249	4
AGAGCCTTA	BRDN00012	538	525	2693	952	773	94
AGAGCCTTA	BRDN00011	144	170	269	398	327	35
AGAGCCTTC	BRDN00012	231	77	2	469	249	0
AGAGCCTTG	BRDN00011	463	674	1226	943	673	31
AGAGCGACT	BRDN00012	372	205	1027	699	740	17
AGAGCGAG	BRDN00012	326	146	75	554	305	95
AGAGCGAGT	BRDN00011	149	120	567	245	127	4
AGAGCGCAC	BRDN00011	475	340	947	604	768	54
AGAGCGCAT	BRDN00012	323	304	303	586	643	66
AGAGCGCGC	BRDN00011	348	345	1013	532	507	65
AGAGCGCGC	BRDN00011	304	6	0	368	11	0
AGAGCGCTC	BRDN00012	199	254	336	298	347	43
AGAGCGGA	BRDN00011	276	311	709	409	342	67
AGAGCGTCC	BRDN00012	450	596	2000	1259	968	104
AGAGCGTCT	BRDN00012	246	221	488	468	565	14
AGAGCGTCT	BRDN00012	556	533	684	924	1233	68
AGAGCGTGA	BRDN00011	171	106	280	293	431	5
AGAGCGTGC	BRDN00011	303	322	377	700	907	6
AGAGCGTGG	BRDN00012	190	89	721	329	77	14
AGAGCGTGT	BRDN00012	172	262	349	356	354	8
AGAGCGTTG	BRDN00011	181	117	103	291	359	6
AGAGCTAAT	BRDN00012	100	67	0	226	46	0
AGAGCTACG	BRDN00011	118	130	741	358	542	30
AGAGCTACG	BRDN00012	79	124	192	233	236	8
AGAGCTACT	BRDN00012	136	62	0	333	345	0
AGAGCTATG	BRDN00012	308	445	191	673	1058	51
AGAGCTATG	BRDN00011	135	87	0	217	442	0
AGAGCTCAA	BRDN00011	114	62	149	258	156	0
AGAGCTCAC	BRDN00011	272	332	40	636	687	19
AGAGCTCAG	BRDN00012	319	446	323	570	502	15
AGAGCTCCA	BRDN00011	354	371	36	786	660	44
AGAGCTCCA	BRDN00012	344	351	1009	625	342	26
AGAGCTCGG	BRDN00011	75	132	20	188	207	17
AGAGCTCGG	BRDN00011	324	289	2542	562	1006	85
AGAGCTCTA	BRDN00011	386	437	392	743	488	55
AGAGCTCTG	BRDN00012	86	32	38	191	165	0
AGAGCTCTT	BRDN00012	200	162	70	403	288	14
AGAGCTGAA	BRDN00012	150	189	449	358	243	3
AGAGCTGAC	BRDN00011	118	36	9	356	469	0
AGAGCTGAC	BRDN00011	260	232	578	558	512	18
AGAGCTGAG	BRDN00011	201	143	8	463	373	4
AGAGCTGAT	BRDN00012	267	286	283	657	435	30
AGAGCTGAT	BRDN00012	270	341	44	494	505	19
AGAGCTGCA	BRDN00011	276	197	355	508	459	9
AGAGCTGCA	BRDN00011	153	56	3	235	144	1
AGAGCTGCG	BRDN00011	213	327	399	637	841	21
AGAGCTGCT	BRDN00011	330	179	489	669	896	16
AGAGCTGCT	BRDN00012	481	395	672	900	1047	56
AGAGCTGGA	BRDN00011	177	147	18	369	248	0
AGAGCTGGA	BRDN00011	380	198	218	668	858	0
AGAGCTGGA	BRDN00011	360	356	2074	828	625	92
AGAGCTGGA	BRDN00012	384	311	516	981	909	56
AGAGCTGGT	BRDN00011	367	415	804	613	560	32
AGAGCTGGT	BRDN00012	196	76	2035	291	123	8
AGAGCTGTA	BRDN00011	92	140	27	211	696	4
AGAGCTGTT	BRDN00012	492	602	154	846	1038	31
AGAGCTGTT	BRDN00011	228	135	26	303	289	58
AGAGCTGTT	BRDN00012	137	112	34	323	114	0
AGAGCTTAA	BRDN00011	182	91	234	489	1050	0
AGAGCTTAA	BRDN00011	191	113	90	639	596	14
AGAGCTTAA	BRDN00011	139	222	37	243	281	30
AGAGCTTAC	BRDN00011	272	265	425	565	813	24
AGAGCTTAC	BRDN00011	147	134	696	185	50	26
AGAGCTTAG	BRDN00012	114	17	199	290	140	2
AGAGCTTAT	BRDN00011	101	43	11	205	392	0
AGAGCTTCA	BRDN00012	140	150	625	298	370	11
AGAGCTTCC	BRDN00011	168	10	0	707	752	0
AGAGCTTCT	BRDN00012	225	161	171	396	596	28
AGAGCTTCT	BRDN00011	415	363	258	778	737	36
AGAGCTTGA	BRDN00012	80	71	0	133	236	0
AGAGCTTGC	BRDN00012	360	187	330	855	1136	4
AGAGCTTGC	BRDN00011	316	321	419	485	636	24
AGAGCTTGG	BRDN00012	241	318	944	607	432	52
AGAGCTTGG	BRDN00012	277	295	172	546	402	13
AGAGGAAA	BRDN00011	445	266	889	659	1236	40
AGAGGAAA	BRDN00012	191	60	1	340	497	28
AGAGGAAA	BRDN00011	224	274	546	408	321	13
AGAGGAAA	BRDN00012	394	365	866	1002	1235	24
AGAGGAAA	BRDN00012	150	124	470	219	414	21
AGAGGAAA	BRDN00012	312	98	53	694	465	0
AGAGGAAA	BRDN00011	167	317	344	462	842	0
AGAGGAAA	BRDN00012	56	0	1	828	421	0
AGAGGAAAT	BRDN00011	125	19	0	234	197	0
AGAGGAACA	BRDN00011	237	363	318	413	255	43
AGAGGAACA	BRDN00012	193	264	407	543	294	45
AGAGGAACC	BRDN00011	249	330	120	637	281	48
AGAGGAACT	BRDN00012	478	398	1307	829	785	32
AGAGGAACT	BRDN00012	173	309	790	486	582	19
AGAGGAAGA	BRDN00012	205	180	17	497	407	4
AGAGGAAGA	BRDN00012	552	385	1349	943	946	25
AGAGGAAGA	BRDN00011	361	330	1689	717	772	0
AGAGGAAGA	BRDN00012	459	332	1794	679	824	37
AGAGGAAGA	BRDN00011	163	52	199	263	304	18
AGAGGAAG	BRDN00012	169	80	182	333	374	7
AGAGGAAG	BRDN00012	174	125	224	386	348	7
AGAGGAAG	BRDN00012	171	207	572	326	186	37
AGAGGAAG	BRDN00011	186	74	533	479	225	6
AGAGGAAG	BRDN00011	255	237	795	616	425	55
AGAGGAAG	BRDN00012	253	259	44	579	566	107
AGAGGAAGT	BRDN00011	173	231	37723	461	360	574
AGAGGAATT	BRDN00012	181	81	779	291	130	63
AGAGGAATT	BRDN00011	200	148	282	423	374	0
AGAGGACAA	BRDN00012	122	107	278	333	31	49
AGAGGACAC	BRDN00011	175	123	84	378	511	55
AGAGGACAC	BRDN00012	260	282	553	446	281	4
AGAGGACAC	BRDN00011	448	246	2941	751	513	80
AGAGGACAT	BRDN00011	212	113	1	415	535	0
AGAGGACCA	BRDN00011	75	83	0	131	307	2
AGAGGACCT	BRDN00012	106	105	66	263	160	0
AGAGGACGA	BRDN00011	79	127	88	220	143	0
AGAGGACG	BRDN00011	307	495	516	575	830	14
AGAGGACG	BRDN00012	263	316	984	637	590	24
AGAGGACTG	BRDN00012	60	51	0	787	253	0
AGAGGACTG	BRDN00011	536	463	631	900	527	85
AGAGGACTT	BRDN00011	235	171	466	549	271	0
AGAGGAGA	BRDN00012	143	86	17	325	157	0
AGAGGAGCA	BRDN00011	32	0	0	188	106	0
AGAGGAGCC	BRDN00011	157	108	873	305	193	24
AGAGGAGCT	BRDN00011	220	41	35	310	179	21
AGAGGAGG	BRDN00011	120	163	535	157	171	55
AGAGGAGG	BRDN00011	528	676	798	1053	1292	62
AGAGGAGG	BRDN00012	91	0	0	359	366	0
AGAGGAGG	BRDN00011	172	105	33	325	72	0
AGAGGAGG	BRDN00011	197	23	0	451	744	0
AGAGGAGG	BRDN00011	224	233	85	417	444	9
AGAGGAGG	BRDN00011	128	22	0	310	248	0
AGAGGAGG	BRDN00011	66	39	20	196	207	0
AGAGGAGG	BRDN00012	147	100	66	407	380	10
AGAGGAGG	BRDN00012	272	312	178	393	488	15
AGAGGAGG	BRDN00012	560	323	809	1116	957	21
AGAGGAGG	BRDN00012	217	125	165	462	578	3
AGAGGAGTA	BRDN00012	141	87	0	294	121	0
AGAGGAGTC	BRDN00012	72	142	197	145	202	8
AGAGGAGTC	BRDN00011	349	284	160	1000	597	51
AGAGGAGTC	BRDN00012	166	274	1017	416	224	8
AGAGGAGTC	BRDN00012	277	196	232	809	594	9
AGAGGATCA	BRDN00012	297	315	500	364	358	45
AGAGGATCC	BRDN00012	123	49	0	527	499	0
AGAGGATCT	BRDN00012	418	467	506	923	1297	51
AGAGGATGA	BRDN00012	179	185	449	384	486	56
AGAGGATGA	BRDN00012	172	98	642	281	179	16
AGAGGATGA	BRDN00012	378	359	1666	754	1148	60
AGAGGATG	BRDN00012	84	39	13	223	450	17
AGAGGATG	BRDN00011	47	23	15	123	57	0
AGAGGATTG	BRDN00012	416	236	514	696	757	15
AGAGGATTT	BRDN00012	130	143	99	262	357	32
AGAGGCAAA	BRDN00011	228	57	717	434	297	30
AGAGGCAAA	BRDN00012	327	246	870	635	778	58
AGAGGCAAC	BRDN00012	205	189	162	297	545	12
AGAGGCACA	BRDN00011	141	79	135	294	253	0
AGAGGCACA	BRDN00011	431	464	2263	901	1074	84
AGAGGCACC	BRDN00011	180	0	0	463	604	0
AGAGGCACC	BRDN00011	214	311	76	424	799	0
AGAGGCACT	BRDN00012	158	256	26	520	700	21
AGAGGCAGA	BRDN00011	343	402	1	782	1020	8
AGAGGCAG	BRDN00012	215	200	32	510	230	7
AGAGGCATC	BRDN00011	109	102	647	199	198	0
AGAGGCATG	BRDN00011	81	0	1	341	142	0
AGAGGCATT	BRDN00011	119	133	132	227	218	0
AGAGGCATT	BRDN00011	198	196	789	238	452	1
AGAGGCATT	BRDN00011	32	21	53	258	352	7
AGAGGCCAA	BRDN00011	264	262	31	427	754	7
AGAGGCCAC	BRDN00012	321	285	2255	656	1106	50
AGAGGCCCA	BRDN00011	215	222	155	416	321	14
AGAGGCCGA	BRDN00012	213	5	0	734	373	0
AGAGGCCGT	BRDN00011	556	661	563	1056	1095	72
AGAGGCCTA	BRDN00012	187	170	1157	381	504	29
AGAGGCCTG	BRDN00011	395	329	644	594	862	17
AGAGGCCTG	BRDN00011	193	245	374	518	305	97
AGAGGCGAA	BRDN00012	176	277	390	469	453	23
AGAGGCGAA	BRDN00011	342	418	616	785	1203	33
AGAGGCGA	BRDN00012	168	19	0	263	149	0
AGAGGCGA	BRDN00011	121	149	174	234	460	16
AGAGGCGA	BRDN00011	250	517	69070	550	905	828
AGAGGCGCC	BRDN00012	407	347	955	839	835	36
AGAGGCGCG	BRDN00012	309	457	129	503	573	15
AGAGGCGCT	BRDN00011	171	100	237	261	355	27
AGAGGCGGA	BRDN00011	220	87	128	418	451	0
AGAGGCGG	BRDN00012	125	89	271	182	194	34
AGAGGCGG	BRDN00011	176	138	77	326	363	5
AGAGGCGG	BRDN00012	259	147	903	440	378	26
AGAGGCGGT	BRDN00011	1	0	0	200	69	0
AGAGGCGTC	BRDN00012	179	263	328	296	326	10
AGAGGCTAC	BRDN00011	96	56	354	308	278	38
AGAGGCTGA	BRDN00011	271	350	366	592	817	62
AGAGGCTGA	BRDN00012	200	75	491	479	320	4
AGAGGCTGC	BRDN00012	145	189	113	332	159	33
AGAGGCTGG	BRDN00011	317	155	51	478	788	13
AGAGGCTGG	BRDN00012	99	63	228	270	188	6
AGAGGGAAA	BRDN00011	72	150	127	267	359	16
AGAGGGAA	BRDN00011	159	62	333	283	298	5
AGAGGGACA	BRDN00012	305	147	186	527	524	4
AGAGGGACC	BRDN00012	112	125	19	343	219	6
AGAGGGACC	BRDN00012	134	218	145	410	366	13
AGAGGGACT	BRDN00012	226	123	282	432	166	6
AGAGGGATA	BRDN00012	61	12	0	426	207	1
AGAGGGATA	BRDN00012	286	41	20	554	205	0
AGAGGGATT	BRDN00012	682	634	5726	1191	1079	125
AGAGGGCAA	BRDN00012	341	184	379	749	879	12
AGAGGGCCA	BRDN00012	587	497	3908	1000	817	98
AGAGGGCG	BRDN00011	132	71	26	350	266	33
AGAGGGCG	BRDN00011	174	273	17	451	358	17
AGAGGGCTG	BRDN00012	274	0	257	765	108	0
AGAGGGGCA	BRDN00011	163	161	32	281	106	0
AGAGGGGG	BRDN00011	192	142	755	232	281	65
AGAGGGGGT	BRDN00012	432	414	248	733	663	76
AGAGGGTAC	BRDN00011	171	79	9	257	264	12
AGAGGGTCA	BRDN00012	194	204	470	558	471	17
AGAGGGTCA	BRDN00011	230	192	275	310	236	5
AGAGGGTCA	BRDN00011	245	202	10	535	676	5
AGAGGGTCA	BRDN00012	257	272	276	564	610	19
AGAGGGTCC	BRDN00012	350	398	472	601	509	41
AGAGGGTGA	BRDN00011	294	175	243	488	521	21
AGAGGGTGC	BRDN00012	439	295	530	659	640	32
AGAGGGTGT	BRDN00012	68	52	20	161	92	6

AGAGGGTGT	BRDN000119	10	0	0	285	27	0
AGAGGGTGT	BRDN000121	191	287	700	468	557	55
AGAGGTAAG	BRDN000116	170	122	671	371	388	19
AGAGGTATG	BRDN000119	402	317	469	805	471	27
AGAGGTATT	BRDN000118	147	259	3515	242	212	51
AGAGGTATT	BRDN000121	254	221	701	477	2247	73
AGAGGTCAA	BRDN000119	300	206	112	382	525	0
AGAGGTCAG	BRDN000116	217	258	74	482	476	15
AGAGGTCAG	BRDN000119	143	174	61	208	427	0
AGAGGTCAT	BRDN000118	65	194	1231	199	308	55
AGAGGTCCC	BRDN000121	517	302	2	819	566	13
AGAGGTCTC	BRDN000121	179	215	221	482	327	19
AGAGGTCTC	BRDN000120	274	130	0	282	299	3
AGAGGTGAA	BRDN000122	346	271	429	570	547	44
AGAGGTGAC	BRDN000119	591	557	705	1029	674	85
AGAGGTGAC	BRDN000119	251	244	271	425	384	4
AGAGGTGAT	BRDN000118	116	157	6	384	341	16
AGAGGTGAT	BRDN000121	337	344	136	638	487	49
AGAGGTGCA	BRDN000121	240	246	398	481	242	42
AGAGGTGCA	BRDN000119	603	59	2011	1082	691	25
AGAGGTGCT	BRDN000116	170	104	30	359	282	0
AGAGGTGGA	BRDN000116	231	218	186	374	242	14
AGAGGTGGC	BRDN000121	276	339	902	430	395	43
AGAGGTGGC	BRDN000119	51	67	0	184	316	0
AGAGGTTAC	BRDN000118	46	12	1	158	69	8
AGAGGTTCA	BRDN000121	103	82	210	127	145	3
AGAGGTTCT	BRDN000120	700	603	243	1147	1215	48
AGAGGTTTG	BRDN000118	137	73	981	221	226	23
AGAGTAACG	BRDN000121	56	58	3694	137	211	134
AGAGTAAGC	BRDN000117	221	102	424	362	498	4
AGAGTAAGC	BRDN000118	132	109	60	191	98	9
AGAGTAAGG	BRDN000118	228	146	164	359	330	18
AGAGTAAGT	BRDN000119	91	82	171	190	167	30
AGAGTAATC	BRDN000119	221	193	955	375	274	45
AGAGTAATT	BRDN000117	63	92	59	137	80	0
AGAGTAATT	BRDN000121	71	78	0	116	131	0
AGAGTACAC	BRDN000122	382	492	977	636	405	36
AGAGTACAG	BRDN000121	459	306	139	778	463	14
AGAGTACAT	BRDN000119	440	327	320	838	1012	12
AGAGTACCA	BRDN000121	33	30	51	390	223	0
AGAGTACCG	BRDN000122	124	115	7	337	63	22
AGAGTACCT	BRDN000118	194	124	917	423	184	49
AGAGTACTT	BRDN000121	198	150	198	623	443	10
AGAGTAGAA	BRDN000122	96	58	129	212	336	0
AGAGTAGCT	BRDN000118	221	135	430	267	303	0
AGAGTAGTG	BRDN000118	97	32	7	178	68	10
AGAGTATAA	BRDN000118	231	147	921	441	347	43
AGAGTATCT	BRDN000117	79	72	2260	122	319	31
AGAGTATGC	BRDN000120	169	199	233	323	443	1
AGAGTATGG	BRDN000116	128	8	0	241	125	0
AGAGTATGG	BRDN000119	123	140	108	338	181	12
AGAGTATTT	BRDN000119	122	157	283	176	252	7
AGAGTCAAG	BRDN000119	141	57	15	209	101	0
AGAGTCACA	BRDN000122	214	199	266	327	367	32
AGAGTCACC	BRDN000121	258	149	106	404	1017	0
AGAGTCATA	BRDN000116	124	56	43	275	275	8
AGAGTCATT	BRDN000117	386	335	900	797	723	33
AGAGTCCAA	BRDN000119	490	578	996	1102	1164	85
AGAGTCCAC	BRDN000121	281	163	0	813	520	0
AGAGTCCAC	BRDN000122	263	116	0	523	550	25
AGAGTCCAT	BRDN000122	147	112	35	313	169	5
AGAGTCCAT	BRDN000121	153	119	992	296	576	8
AGAGTCCGT	BRDN000118	221	314	252	464	328	0
AGAGTCGCC	BRDN000119	431	367	1461	882	1339	69
AGAGTCGGC	BRDN000121	377	343	58	674	904	16
AGAGTCGTG	BRDN000117	335	0	0	763	259	0
AGAGTCGTT	BRDN000120	362	182	204	670	458	13
AGAGTCTAA	BRDN000119	345	363	566	1007	865	52
AGAGTCTCA	BRDN000119	184	240	461	386	228	8
AGAGTCTCA	BRDN000121	296	301	158	640	319	1
AGAGTCTGA	BRDN000119	128	204	16	266	202	4
AGAGTCTGA	BRDN000121	381	418	2168	953	667	51
AGAGTCTGC	BRDN000116	121	165	78	390	121	0
AGAGTCTGC	BRDN000119	571	601	1124	1278	1092	32
AGAGTCTGG	BRDN000121	461	310	199	860	1095	31
AGAGTCTTA	BRDN000119	40	44	25	146	174	21
AGAGTGAAG	BRDN000117	158	127	11	284	256	14
AGAGTGCAC	BRDN000122	521	442	2829	722	936	67
AGAGTGCAG	BRDN000121	227	161	92	301	443	8
AGAGTGCAG	BRDN000122	524	332	315	945	842	14
AGAGTGCAT	BRDN000122	318	288	310	638	620	13
AGAGTGCAT	BRDN000118	430	359	2874	825	759	74
AGAGTGCAT	BRDN000118	80	10	0	484	103	0
AGAGTGCAT	BRDN000121	96	118	152	176	80	4
AGAGTGCCG	BRDN000118	51	22	0	255	202	0
AGAGTGCTG	BRDN000121	374	279	639	683	463	0
AGAGTGCTT	BRDN000121	441	314	340	845	584	46
AGAGTGGAC	BRDN000121	191	199	51	423	397	37
AGAGTGGAC	BRDN000116	112	55	110	311	203	14
AGAGTGGCA	BRDN000118	220	269	1434	492	842	22
AGAGTGGGT	BRDN000120	134	95	65	359	422	24
AGAGTGGGT	BRDN000117	239	111	254	266	285	60
AGAGTGGGT	BRDN000118	357	206	24	560	621	31
AGAGTGGTA	BRDN000119	510	426	424	944	1066	65
AGAGTGTAA	BRDN000121	167	83	390	263	809	11
AGAGTGTAA	BRDN000117	256	289	876	500	784	44
AGAGTGTCA	BRDN000116	117	188	201	231	248	26
AGAGTGTCC	BRDN000117	111	96	128	202	157	19
AGAGTGTCG	BRDN000116	167	228	327	524	303	107
AGAGTGTCG	BRDN000116	78	112	167	181	232	29
AGAGTGTGC	BRDN000120	153	86	298	236	322	0
AGAGTGTGG	BRDN000118	200	97	332	426	495	12
AGAGTGTGG	BRDN000121	194	146	159	279	682	25
AGAGTGTTA	BRDN000117	52	0	0	290	143	0
AGAGTGTTA	BRDN000116	143	99	60	443	508	13
AGAGTGTTC	BRDN000116	124	282	634	275	370	46
AGAGTGTTT	BRDN000120	99	81	30	250	472	0
AGAGTTAAA	BRDN000121	138	71	86	462	349	26
AGAGTTAAG	BRDN000120	468	493	1516	968	1226	57
AGAGTTAAT	BRDN000121	26	0	0	186	32	0
AGAGTTAGT	BRDN000121	383	121	37	753	569	0
AGAGTTATG	BRDN000117	102	79	101	208	59	0
AGAGTTCAC	BRDN000122	776	486	1193	1292	1790	102
AGAGTTCAT	BRDN000118	314	277	2232	635	646	27
AGAGTTCCA	BRDN000122	198	123	142	314	150	25
AGAGTTCCG	BRDN000121	245	242	499	454	556	8
AGAGTTCGG	BRDN000121	451	407	1014	910	833	45
AGAGTTCTA	BRDN000120	269	183	2019	612	646	9
AGAGTTCTC	BRDN000116	223	93	36	234	368	0
AGAGTTCTT	BRDN000120	402	291	353	821	1083	9
AGAGTTGAA	BRDN000121	211	180	57	437	812	26
AGAGTTGAA	BRDN000116	435	369	396	506	598	9
AGAGTTGAT	BRDN000119	78	66	0	259	94	0
AGAGTTGAT	BRDN000121	233	121	32	390	532	25
AGAGTTGCA	BRDN000120	198	197	330	368	124	20
AGAGTTGCG	BRDN000121	442	360	13	701	902	64
AGAGTTGCG	BRDN000119	295	82	32	860	800	0
AGAGTTGCT	BRDN000118	151	144	173	386	349	0
AGAGTTGCT	BRDN000122	215	188	1175	408	231	11
AGAGTTGGA	BRDN000120	148	20	2151	319	458	9
AGAGTTGGC	BRDN000121	384	474	605	748	805	36
AGAGTTGGG	BRDN000121	197	216	1106	304	560	23
AGAGTTGGT	BRDN000116	177	117	536	226	244	18
AGAGTTGTC	BRDN000116	129	138	398	234	110	24
AGAGTTTAA	BRDN000122	294	546	208	651	359	21
AGAGTTTAT	BRDN000120	329	305	825	839	908	11
AGAGTTTAT	BRDN000119	231	163	175	539	634	0
AGAGTTTGA	BRDN000121	443	570	1926	925	809	93
AGAGTTTGG	BRDN000118	331	362	224	617	736	25
AGAGTTTGG	BRDN000121	205	193	132	389	354	0
AGAGTTTGT	BRDN000119	142	138	27	384	212	12
AGAGTTTGT	BRDN000119	76	38	11	168	150	0
AGATAAACA	BRDN000122	252	175	753	398	317	37
AGATAAACC	BRDN000122	381	437	225	789	404	55
AGATAAAGA	BRDN000121	257	50	198	401	644	0
AGATAAATG	BRDN000119	430	473	137	825	930	41
AGATAACAC	BRDN000117	396	335	1598	525	809	73
AGATAACAC	BRDN000119	327	26	230	551	1003	0
AGATAACAG	BRDN000117	70	0	0	171	71	0
AGATAACAG	BRDN000121	270	464	2711	404	368	106
AGATAACAG	BRDN000117	156	147	246	308	437	1
AGATAACAT	BRDN000120	600	475	1170	1092	1169	27
AGATAACCA	BRDN000118	287	245	172	616	580	0
AGATAACTA	BRDN000118	82	44	76	180	119	0
AGATAACTG	BRDN000118	206	242	0	400	594	2
AGATAACTG	BRDN000120	526	449	483	882	934	34
AGATAACTT	BRDN000118	225	199	51	382	344	13
AGATAACTT	BRDN000121	132	67	176	205	154	15
AGATAAGAA	BRDN000116	145	158	876	424	605	28
AGATAAGAG	BRDN000117	597	519	138	845	490	48
AGATAAGAT	BRDN000120	159	84	289	208	188	11
AGATAAGCA	BRDN000121	333	420	0	601	644	4
AGATAAGCA	BRDN000121	310	263	22	434	466	31
AGATAAGTG	BRDN000117	60	133	16	216	278	9
AGATAATAG	BRDN000121	285	214	2028	586	455	65
AGATAATCG	BRDN000119	168	122	920	318	218	14
AGATAATCT	BRDN000118	158	53	0	321	277	0
AGATAATGA	BRDN000121	178	125	169	366	847	0
AGATAATGG	BRDN000121	538	376	50	925	1162	58
AGATAATTA	BRDN000122	119	44	0	238	219	7
AGATAATTA	BRDN000120	245	142	0	462	445	4
AGATACAAA	BRDN000119	415	423	360	817	864	49
AGATACAAC	BRDN000120	507	515	86	869	938	43
AGATACAAG	BRDN000118	262	247	524	408	638	50
AGATACAAG	BRDN000122	168	128	249	307	306	18
AGATACACT	BRDN000120	204	172	207	324	269	0
AGATACAGC	BRDN000118	176	202	887	442	636	21
AGATACATC	BRDN000118	49	56	116	146	132	57
AGATACATT	BRDN000119	313	463	1840	495	352	58
AGATACCCA	BRDN000122	73	62	52	147	187	0
AGATACCGA	BRDN000116	91	16	0	129	581	0
AGATACCGA	BRDN000121	83	26	0	185	208	0
AGATACCGT	BRDN000122	178	47	1	356	173	0
AGATACCTC	BRDN000120	133	96	27	213	279	0
AGATACCTG	BRDN000117	106	55	234	280	261	8
AGATACCTG	BRDN000121	435	398	199	715	550	66
AGATACGCT	BRDN000121	547	518	630	1057	1282	6
AGATACTAG	BRDN000117	168	187	2	173	279	6
AGATACTCA	BRDN000120	556	436	1567	1031	1141	62
AGATACTCC	BRDN000121	452	333	1342	809	704	29
AGATACTCG	BRDN000116	152	121	35	310	312	10
AGATACTTC	BRDN000118	98	86	103	326	218	61
AGATAGAAT	BRDN000116	95	129	237	134	48	13
AGATAGACA	BRDN000117	236	106	207	458	376	88
AGATAGACC	BRDN000119	548	511	1573	820	904	56
AGATAGACC	BRDN000121	537	379	433	1115	907	30
AGATAGACT	BRDN000116	450	357	543	851	1352	63
AGATAGAGC	BRDN000119	167	199	473	228	280	93
AGATAGAGT	BRDN000120	322	227	73	803	581	44
AGATAGAGT	BRDN000116	131	192	6	164	194	61
AGATAGCAG	BRDN000121	342	154	82	813	521	17
AGATAGCTG	BRDN000121	349	451	748	594	586	17
AGATAGGAA	BRDN000121	90	30	1	220	67	5
AGATAGGAG	BRDN000118	155	259	947	437	329	40
AGATAGGTC	BRDN000122	239	130	44	300	423	11
AGATAGTCC	BRDN000116	127	249	628	246	480	36
AGATAGTCG	BRDN000119	193	140	28	424	361	0
AGATAGTGC	BRDN000121	229	174	3145	465	217	57
AGATAGTTA	BRDN000117	195	204	481	352	121	27
AGATATAAC	BRDN000121	381	332	782	886	711	4
AGATATACA	BRDN000118	334	224	1073	465	438	44
AGATATACT	BRDN000120	255	4	0	496	511	0
AGATATATG	BRDN000121	423	529	1214	773	1019	28
AGATATCAA	BRDN000119	216	192	166	610	819	6
AGATATCAC	BRDN000121	254	101	0	344	201	0
AGATATCAT	BRDN000121	329	385	169	692	700	20
AGATATCCA	BRDN000117	174	230	4872	318	205	73
AGATATCCA	BRDN000117	406	390	138	601	638	28
AGATATCGG	BRDN000116	192	187	171	231	349	25
AGATATCGT	BRDN000121	552	516	297	782	680	111
AGATATCTA	BRDN000120	142	1	1	358	268	0
AGATATCTT	BRDN000117	106	15	15	220	58	0
AGATATGAC	BRDN000118	222	146	348	344	551	18
AGATATGAT	BRDN000117	172	259	156	407	302	15
AGATATGCA	BRDN000116	227	268	588	490	476	10
AGATATGCC	BRDN000120	613	340	3753	899	938	52
AGATATGGA	BRDN000121	125	87	11	326	161	15
AGATATGGC	BRDN000119	184	112	155	340	343	4
AGATATGGG	BRDN000118	51	133	0	198	226	16
AGATATGGG	BRDN000122	126	85	1123	185	273	98
AGATATGTA	BRDN000117	141	114	70	344	362	4
AGATATGTT	BRDN000120	500	282	2378	732	1017	39
AGATATTAA	BRDN000122	144	168	9	241	139	0
AGATATTAT	BRDN000121	301	261	599	442	668	40
AGATATTAT	BRDN000117	73	56	521	327	172	9
AGATATTCA	BRDN000122	116	8	0	457	423	0
AGATATTCA	BRDN000118	216	139	82	444	417	20
AGATATTCC	BRDN000121	245	257	156	491	543	57
AGATATTCT	BRDN000120	94	64	166	174	214	0
AGATATTCT	BRDN000121	409	349	860	755	617	151
AGATATTGA	BRDN000117	316	176	269	411	304	22
AGATATTGA	BRDN000119	88	4	18	357	108	1
AGATATTGA	BRDN000118	26	0	0	225	267	0
AGATATTGG	BRDN000120	368	372	173	816	590	42
AGATATTGT	BRDN000118	211	404	4811	576	782	232
AGATATTTG	BRDN000117	207	157	54	454	284	0
AGATATTTG	BRDN000117	130	59	0	126	41	4
AGATCAAAA	BRDN000121	161	144	842	351	228	20
AGATCAAAA	BRDN000122	375	240	585	500	990	11
AGATCAACT	BRDN000120	690	749	3676	1222	1472	128
AGATCAACT	BRDN000116	369	179	1044	677	682	6
AGATCAAGG	BRDN000121	603	601	573	1063	1131	45
AGATCAAGT	BRDN000120	436	553	666	930	883	62
AGATCACAG	BRDN000122	328	209	174	547	507	67
AGATCACAG	BRDN000119	269	404	292	611	610	33
AGATCACAT	BRDN000121	232	173	422	273	275	11
AGATCACCT	BRDN000121	301	257	198	599	460	15
AGATCACTA	BRDN000117	117	73	37	157	63	14
AGATCACTC	BRDN000116	337	290	977	569	593	47
AGATCACTT	BRDN000122	356	403	439	531	647	17
AGATCACTT	BRDN000117	226	217	303	373	448	7
AGATCAGAT	BRDN000118	288	485	177	471	528	1
AGATCAGCC	BRDN000117	314	396	680	431	472	53
AGATCAGCT	BRDN000121	219	230	1019	411	497	0
AGATCAGTA	BRDN000119	305	23	1	789	777	0
AGATCATAT	BRDN000119	226	122	260	328	270	27
AGATCATCT	BRDN000121	150	58	274	322	102	0
AGATCATGG	BRDN000122	438	300	1380	1028	555	175
AGATCATTA	BRDN000120	534	483	1327	1099	1607	72
AGATCCAAA	BRDN000116	165	142	1	477	475	14
AGATCCACC	BRDN000118	81	0	1	434	156	0
AGATCCACC	BRDN000120	193	61	123	426	242	0
AGATCCACG	BRDN000119	220	99	806	414	511	76
AGATCCAGA	BRDN000122	377	438	492	699	1179	24
AGATCCAGG	BRDN000121	347	328	365	669	394	42
AGATCCAGT	BRDN000121	130	74	278	256	187	6
AGATCCATT	BRDN000117	182	195	402	295	406	0
AGATCCCTG	BRDN000117	364	461	971	987	684	87
AGATCCGAA	BRDN000121	308	61	1198	1005	428	6
AGATCCGGG	BRDN000121	367	9	1	641	145	0
AGATCCGGG	BRDN000117	246	7	0	808	97	0
AGATCCTAG	BRDN000121	387	261	1287	611	497	54
AGATCCTGG	BRDN000119	390	86	6	776	251	0
AGATCCTGT	BRDN000121	316	254	2001	535	185	67
AGATCCTTG	BRDN000121	311	336	939	664	555	88
AGATCCTTG	BRDN000117	267	254	273	487	590	29
AGATCCTTT	BRDN000119	585	393	555	910	1005	35
AGATCGAAA	BRDN000120	146	80	40	270	138	0
AGATCGAAA	BRDN000116	202	206	370	556	296	0
AGATCGAAG	BRDN000121	177	26	41	354	50	16
AGATCGACA	BRDN000120	555	484	154	970	856	25
AGATCGACC	BRDN000121	432	493	1126	723	635	57
AGATCGAGT	BRDN000122	320	0	0	1207	790	0
AGATCGATT	BRDN000122	332	269	1479	640	642	48
AGATCGCAC	BRDN000116	176	261	42	290	186	19
AGATCGCTA	BRDN000120	547	108	427	1076	966	0
AGATCGCTG	BRDN000121	284	477	834	566	520	10
AGATCGGAT	BRDN000116	213	261	477	399	188	52
AGATCGTGG	BRDN000116	383	388	465	799	462	68
AGATCGTGT	BRDN000117	240	162	189	483	273	26
AGATCTACC	BRDN000119	191	254	336	414	215	4
AGATCTAGA	BRDN000120	457	393	1423	811	792	48
AGATCTAGA	BRDN000119	436	368	994	985	1610	13
AGATCTAGT	BRDN000121	346	92	51	617	447	1
AGATCTATA	BRDN000116	164	158	11	284	263	0
AGATCTATC	BRDN000119	156	167	211	355	601	24
AGATCTCAT	BRDN000120	548	52	2	1236	1029	0
AGATCTCCA	BRDN000117	227	119	324	520	672	24
AGATCTCCA	BRDN000122	348	402	146	827	852	19
AGATCTCCT	BRDN000122	199	229	31	376	336	1
AGATCTCGA	BRDN000117	204	95	49	460	502	18
AGATCTCTC	BRDN000121	170	30	182	464	421	0
AGATCTCTG	BRDN000122	354	222	24	525	496	21
AGATCTGAT	BRDN000121	284	347	45	722	509	26
AGATCTGCA	BRDN000119	455	460	1925	903	960	31
AGATCTGCT	BRDN000119	134	66	103	334	132	0
AGATCTGCT	BRDN000120	504	352	1043	652	900	74
AGATCTGGG	BRDN000122	80	74	137	274	119	0
AGATCTGTG	BRDN000122	229	160	536	241	414	0
AGATCTTAC	BRDN000120	182	172	853	351	248	7
AGATCTTCT	BRDN000121	207	160	1070	444	635	21
AGATCTTGA	BRDN000121	448	266	0	717	685	13
AGATCTTGC	BRDN000121	123	137	26	290	220	0
AGATCTTTC	BRDN000119	258	198	80	282	298	29
AGATGAAAA	BRDN000120	203	219	639	528	436	4
AGATGAAAA	BRDN000121	409	418	428	799	878	58
AGATGAAAG	BRDN000118	191	122	34	321	236	0
AGATGAACA	BRDN000121	306	193	515	570	751	24
AGATGAACA	BRDN000118	271	114	648	536	474	20
AGATGAACA	BRDN000117	442	490	913	833	1241	110
AGATGAACT	BRDN000122	307	296	1186	608	869	90
AGATGAAGC	BRDN000121	230	328	273	572	1051	0
AGATGAAGC	BRDN000119	204	55	19	548	438	4
AGATGAAGC	BRDN000118	419	413	326	989	1048	18
AGATGAAGG	BRDN000120	164	222	1	237	152	1
AGATGAAGG	BRDN000119	249	211	29	330	279	15
AGATGAAGG	BRDN000122	186	1	2	1110	760	0
AGATGAATC	BRDN000121	229	69	67	442	272	18
AGATGAATG	BRDN000117	192	123	72	245	125	0
AGATGAATG	BRDN000117	203	252	176	367	258	40
AGATGACAG	BRDN000116	122	0	1	471	439	0
AGATGACAG	BRDN000116	158	23	12	325	304	27
AGATGACAT	BRDN000119	317	0	2	766	1077	0
AGATGACCA	BRDN000120	174	197	287	264	288	0
AGATGACCA	BRDN000119	604	530	443	914	1104	36
AGATGACCA	BRDN000121	176	200	282	525	557	15
AGATGACCG	BRDN000117	379	331	103	559	559	5
AGATGACCG	BRDN000121	212	210	836	409	424	44
AGATGACCT	BRDN000120	294	339	117	663	736	1
AGATGACCT	BRDN000117	384	558	119	539	308	62
AGATGACGA	BRDN000120	682	612	1051	1366	1065	83
AGATGACGA	BRDN000121	575	637	1454	1089	770	99
AGATGAGAC	BRDN000119	164	88	0	253	415	0
AGATGAGGA	BRDN000119	107	33	1	367	146	0
AGATGAGGC	BRDN000118	244	173	20	310	434	0
AGATGAGGC	BRDN000122	230	214	1006	446	593	20
AGATGAGGT	BRDN000119	175	93	196	378	487	6
AGATGAGTG	BRDN000120	24	0	0	383	130	0
AGATGAGTT	BRDN000121	62	0	0	559	118	0
AGATGATAA	BRDN000119	509	407	809	764	1109	25
AGATGATAC	BRDN000122	276	222	204	410	413	0
AGATGATAG	BRDN000121	165	0	0	191	144	0
AGATGATCA	BRDN000122	236	196	732	324	347	6
AGATGATGA	BRDN000118	404	367	1379	539	449	52
AGATGATGG	BRDN000122	386	143	79	756	748	0
AGATGATGG	BRDN000122	113	149	147	319	240	12
AGATGATGG	BRDN000121	145	158	240	318	480	31
AGATGATGG	BRDN000119	810	753	2207	1410	1341	69
AGATGATGT	BRDN000120	525	617	890	1071	1201	63
AGATGATTG	BRDN000117	198	0	0	324	122	0
AGATGCAAC	BRDN000117	139	102	236	355	531	0
AGATGCAAC	BRDN000117	273	314	553	434	593	79
AGATGCAAT	BRDN000118	446	462	418	711	906	82
AGATGCACA	BRDN000121	340	399	1614	648	700	58
AGATGCACA	BRDN000119	266	71	258	963	549	10
AGATGCACC	BRDN000122	219	119	189	353	175	20
AGATGCACG	BRDN000119	630	721	1193	1216	809	53
AGATGCAGA	BRDN000120	476	485	1526	843	919	83
AGATGCATA	BRDN000117	156	94	163	212	410	7
AGATGCATA	BRDN000121	200	173	127	376	119	72
AGATGCCAG	BRDN000119	244	205	83	365	462	22
AGATGCCAG	BRDN000122	592	396	576	874	818	13
AGATGCCCC	BRDN000118	163	179	707	293	144	27
AGATGCCCG	BRDN000121	433	370	555	964	1245	33
AGATGCCCT	BRDN000121	195	196	262	344	236	0
AGATGCCGA	BRDN000118	109	169	95	309	238	18
AGATGCCGA	BRDN000122	123	11	184	384	20	10
AGATGCCGG	BRDN000117	330	356	721	413	519	4
AGATGCCTC	BRDN000117	157	178	641	392	145	48
AGATGCGAA	BRDN000121	580	691	1430	1174	1008	125
AGATGCGAC	BRDN000118	286	186	669	608	332	21
AGATGCGGC	BRDN000119	260	401	494	443	666	8
AGATGCGGG	BRDN000121	342	270	1411	574	446	49
AGATGCGGG	BRDN000122	239	262	885	460	403	59
AGATGCTAA	BRDN000120	110	49	45	210	348	12
AGATGCTAC	BRDN000120	653	456	506	1222	938	102
AGATGCTCC	BRDN000121	201	190	273	255	405	10
AGATGCTCC	BRDN000121	126	0	2	382	458	0
AGATGCTCG	BRDN000122	190	161	217	352	472	10
AGATGCTGA	BRDN000120	258	281	586	538	434	18
AGATGCTGC	BRDN000119	29	0	1	304	176	0
AGATGCTGT	BRDN000119	564	401	421	1114	939	3
AGATGCTGT	BRDN000117	477	503	531	940	1406	35
AGATGGAAA	BRDN000116	320	231	526	545	399	12
AGATGGAAA	BRDN000122	129	177	295	201	563	61
AGATGGAAC	BRDN000118	264	0	0	546	506	0
AGATGGAAG	BRDN000121	739	711	1011	1501	1625	44
AGATGGAAG	BRDN000118	307	212	473	527	414	15
AGATGGAAT	BRDN000116	147	23	1	258	415	0
AGATGGAAT	BRDN000119	52	62	11	313	130	1
AGATGGACA	BRDN000117	303	196	401	628	684	34
AGATGGACA	BRDN000120	254	164	521	291	564	39
AGATGGACC	BRDN000121	179	159	33	334	516	21
AGATGGACT	BRDN000120	404	324	668	701	497	34
AGATGGAGA	BRDN000121	193	164	246	392	324	16
AGATGGAGT	BRDN000120	130	35	198	481	600	9
AGATGGATA	BRDN000122	186	102	69	287	497	0
AGATGGCAA	BRDN000121	466	436	1259	633	811	78
AGATGGCAA	BRDN000121	331	51	192	623	387	12
AGATGGCAA	BRDN000121	529	774	1403	1034	1134	120
AGATGGCAC	BRDN000117	277	160	169	498	243	6
AGATGGCAG	BRDN000119	350	356	253	617	659	13
AGATGGCAG	BRDN000118	120	100	20	151	242	12
AGATGGCAT	BRDN000118	91	83	117	262	159	1
AGATGGCAT	BRDN000120	245	240	403	605	398	22
AGATGGCAT	BRDN000116	233	150	160	391	401	5
AGATGGCGA	BRDN000121	444	379	726	689	495	207
AGATGGCGA	BRDN000120	736	533	710	1440	1320	53
AGATGGCGA	BRDN000120	175	182	117	414	489	16
AGATGGCGC	BRDN000121	353	363	241	719	426	11
AGATGGCTA	BRDN000118	57	0	2	605	325	0
AGATGGCTA	BRDN000116	287	171	1895	577	229	79
AGATGGCTA	BRDN000120	123	0	0	857	140	0
AGATGGCTA	BRDN000121	255	213	308	589	614	22
AGATGGCTA	BRDN000117	158	87	0	153	50	7
AGATGGCTG	BRDN000119	27	28	12	122	54	0
AGATGGGAC	BRDN000120	244	241	298	457	490	16
AGATGGGCC	BRDN000122	446	376	693	797	717	37
AGATGGGCT	BRDN000116	299	268	146	598	242	31
AGATGGGGA	BRDN000117	123	84	328	366	331	0
AGATGGGTT	BRDN000118	226	275	399	476	644	3
AGATGGTAC	BRDN000121	164	84	11	219	248	0
AGATGGTAG	BRDN000121	451	271	5421	796	856	75
AGATGGTAG	BRDN000121	129	91	58	248	435	0
AGATGGTAG	BRDN000117	15	0	1	507	520	0
AGATGGTAT	BRDN000117	319	0	59	758	470	0
AGATGGTCA	BRDN000119	479	426	1984	777	997	59
AGATGGTCA	BRDN000121	429	386	148	745	1298	14
AGATGGTCC	BRDN000121	478	504	1252	602	407	65
AGATGGTCT	BRDN000122	104	104	342	367	225	14
AGATGGTGA	BRDN000118	18	0	0	512	196	0
AGATGGTGA	BRDN000117	390	395	1115	558	966	48
AGATGGTGC	BRDN000121	171	59	112	282	387	5
AGATGGTGT	BRDN000122	267	205	1722	568	253	65
AGATGGTGT	BRDN000119	194	156	216	202	247	30

AGATGGTTC	BRDN0001	327	332	589	557	479	32
AGATGGTTT	BRDN0001	184	95	344	199	229	14
AGATGTAAT	BRDN0001	125	143	268	371	321	24
AGATGTACA	BRDN0001	403	446	1506	774	899	74
AGATGTACT	BRDN0001	488	477	1163	985	953	62
AGATGTAGA	BRDN0001	482	357	469	903	1729	3
AGATGTAGA	BRDN0001	99	108	199	212	234	4
AGATGTATG	BRDN0001	222	90	1	408	189	0
AGATGTATG	BRDN0001	198	251	19	445	398	25
AGATGTATG	BRDN0001	189	132	0	332	217	3
AGATGTATT	BRDN0001	240	21	0	363	219	1
AGATGTCAG	BRDN0001	265	279	302	311	139	4
AGATGTCAT	BRDN0001	300	297	473	570	748	5
AGATGTCTA	BRDN0001	169	27	30	219	129	1
AGATGTCTC	BRDN0001	215	243	27	403	716	8
AGATGTGAG	BRDN0001	336	133	10	676	876	0
AGATGTGCA	BRDN0001	204	99	211	533	288	21
AGATGTGCG	BRDN0001	422	454	825	681	680	51
AGATGTGGA	BRDN0001	460	612	549	722	588	40
AGATGTGGA	BRDN0001	254	296	311	497	683	52
AGATGTGGC	BRDN0001	534	46	3	1533	1832	1
AGATGTGGG	BRDN0001	331	172	928	668	492	30
AGATGTGTA	BRDN0001	63	0	19	372	171	0
AGATGTGTG	BRDN0001	512	402	954	848	1162	75
AGATGTGTT	BRDN0001	206	168	264	319	558	30
AGATGTTAC	BRDN0001	252	151	0	663	469	0
AGATGTTCA	BRDN0001	205	195	1648	531	494	58
AGATGTTCC	BRDN0001	293	179	409	493	539	0
AGATGTTCT	BRDN0001	356	223	1366	677	562	76
AGATGTTGC	BRDN0001	439	218	82	1032	208	0
AGATGTTGC	BRDN0001	499	435	538	945	996	16
AGATGTTGT	BRDN0001	195	240	340	353	562	8
AGATGTTTA	BRDN0001	191	190	44	361	210	3
AGATTAAGA	BRDN0001	198	155	216	423	554	20
AGATTAAGG	BRDN0001	131	132	47	160	288	31
AGATTAATT	BRDN0001	143	128	0	207	159	8
AGATTACGC	BRDN0001	491	454	2752	729	972	35
AGATTACGG	BRDN0001	169	9	1	902	56	0
AGATTACTT	BRDN0001	371	409	587	663	1079	22
AGATTAGAA	BRDN0001	310	317	568	598	616	17
AGATTAGAA	BRDN0001	173	279	508	349	587	0
AGATTAGTC	BRDN0001	189	191	103	302	424	19
AGATTATCA	BRDN0001	314	174	1101	443	521	11
AGATTATCA	BRDN0001	125	87	0	472	448	15
AGATTATCT	BRDN0001	164	267	88	405	589	165
AGATTATCT	BRDN0001	410	292	756	606	622	38
AGATTATGC	BRDN0001	448	618	653	1177	1242	55
AGATTATTG	BRDN0001	215	146	423	426	332	48
AGATTATTT	BRDN0001	450	373	2334	865	804	128
AGATTCACA	BRDN0001	319	165	87	596	708	0
AGATTCACA	BRDN0001	199	103	0	299	456	0
AGATTCACT	BRDN0001	266	251	635	347	460	45
AGATTCAGG	BRDN0001	104	121	1598	270	132	45
AGATTCAGG	BRDN0001	194	169	17	350	349	7
AGATTCATT	BRDN0001	563	550	2127	897	914	69
AGATTCCAA	BRDN0001	195	155	251	366	218	20
AGATTCCAG	BRDN0001	440	752	5904	701	1436	115
AGATTCCAG	BRDN0001	260	200	483	596	712	60
AGATTCCCA	BRDN0001	111	269	4758	285	155	82
AGATTCGCA	BRDN0001	205	60	1	588	451	0
AGATTCGCC	BRDN0001	201	221	109	418	249	2
AGATTCGGA	BRDN0001	518	509	259	908	1216	90
AGATTCTAA	BRDN0001	168	209	1049	548	550	3
AGATTCTAA	BRDN0001	228	46	217	313	426	0
AGATTCTAC	BRDN0001	250	207	109	493	442	0
AGATTCTAT	BRDN0001	100	0	0	258	405	0
AGATTCTGA	BRDN0001	462	436	866	763	669	79
AGATTCTTC	BRDN0001	420	380	1130	636	520	82
AGATTCTTC	BRDN0001	337	358	121	542	1045	56
AGATTGAAA	BRDN0001	286	267	17	528	687	0
AGATTGAAA	BRDN0001	269	240	174	462	426	9
AGATTGAAC	BRDN0001	464	355	294	808	934	88
AGATTGACA	BRDN0001	234	176	204	320	350	8
AGATTGACG	BRDN0001	372	278	144	696	668	48
AGATTGAGA	BRDN0001	353	205	434	505	301	28
AGATTGAGT	BRDN0001	133	134	53	351	177	9
AGATTGATC	BRDN0001	109	29	10	591	234	9
AGATTGATG	BRDN0001	161	129	194	227	207	7
AGATTGATG	BRDN0001	435	451	1508	670	910	58
AGATTGCAG	BRDN0001	118	134	137	207	96	1
AGATTGCTA	BRDN0001	381	281	2081	619	1116	12
AGATTGCTC	BRDN0001	198	147	288	302	265	6
AGATTGCTG	BRDN0001	179	97	228	360	377	42
AGATTGGAA	BRDN0001	417	366	1922	848	944	68
AGATTGGAA	BRDN0001	405	377	396	682	749	6
AGATTGGAG	BRDN0001	175	134	128	484	356	22
AGATTGGAG	BRDN0001	419	394	149	643	414	10
AGATTGGCA	BRDN0001	538	555	300	936	1151	23
AGATTGGCT	BRDN0001	434	332	111	927	652	17
AGATTGGGC	BRDN0001	180	10	20	490	239	0
AGATTGGGG	BRDN0001	125	153	98	275	413	30
AGATTGTAA	BRDN0001	267	246	184	538	313	0
AGATTGTAC	BRDN0001	144	117	97	264	256	0
AGATTGTAT	BRDN0001	110	179	311	194	171	16
AGATTGTGC	BRDN0001	123	105	1328	321	225	5
AGATTGTGC	BRDN0001	113	112	353	237	250	19
AGATTGTGG	BRDN0001	603	637	481	1102	1130	13
AGATTGTTG	BRDN0001	408	385	423	610	778	47
AGATTTAAA	BRDN0001	103	66	1	179	137	0
AGATTTAAG	BRDN0001	555	628	1597	966	824	50
AGATTTACA	BRDN0001	132	145	467	305	458	14
AGATTTACC	BRDN0001	126	89	491	293	225	13
AGATTTACT	BRDN0001	156	151	83	302	480	3
AGATTTATT	BRDN0001	187	140	945	488	202	9
AGATTTCAC	BRDN0001	406	184	40	632	787	22
AGATTTCCA	BRDN0001	123	61	1015	380	549	20
AGATTTGAA	BRDN0001	206	132	352	478	259	42
AGATTTGAC	BRDN0001	67	0	1	354	322	1
AGATTTGAG	BRDN0001	117	63	1	261	300	0
AGATTTGAT	BRDN0001	320	186	313	505	444	33
AGATTTGCA	BRDN0001	240	149	339	407	122	16
AGATTTGGA	BRDN0001	174	168	662	382	829	26
AGCAAAAAA	BRDN0001	574	565	1011	877	841	61
AGCAAAAAC	BRDN0001	201	167	454	377	173	8
AGCAAAACA	BRDN0001	246	258	415	336	338	148
AGCAAAACC	BRDN0001	82	113	0	212	152	0
AGCAAAATT	BRDN0001	208	133	219	386	304	26
AGCAAACAT	BRDN0001	230	176	632	501	574	25
AGCAAACCA	BRDN0001	361	369	55	733	607	10
AGCAAACCA	BRDN0001	470	440	225	827	1511	35
AGCAAACTG	BRDN0001	177	0	1	244	31	0
AGCAAAGAA	BRDN0001	118	105	23	226	226	32
AGCAAAGAA	BRDN0001	98	123	333	397	224	22
AGCAAAGAA	BRDN0001	415	295	2128	680	621	60
AGCAAAGCA	BRDN0001	173	105	1	187	211	0
AGCAAAGCT	BRDN0001	136	106	201	321	436	11
AGCAAAGGC	BRDN0001	219	140	254	593	887	0
AGCAAAGTT	BRDN0001	463	459	1253	849	1220	56
AGCAAATGC	BRDN0001	482	457	8551	777	1108	228
AGCAACACA	BRDN0001	145	148	72	211	241	0
AGCAACACA	BRDN0001	560	558	1407	1046	1174	34
AGCAACACA	BRDN0001	183	82	480	1037	915	0
AGCAACACT	BRDN0001	245	258	390	533	362	6
AGCAACAGA	BRDN0001	304	175	2178	373	493	133
AGCAACAGC	BRDN0001	445	338	342	728	653	56
AGCAACAGG	BRDN0001	132	85	0	326	168	0
AGCAACATA	BRDN0001	239	341	652	478	432	11
AGCAACATG	BRDN0001	318	261	2	488	552	12
AGCAACATG	BRDN0001	171	118	83	293	77	0
AGCAACATG	BRDN0001	521	483	992	1199	1102	66
AGCAACCAA	BRDN0001	192	1	0	730	441	1
AGCAACCAC	BRDN0001	231	178	243	354	377	12
AGCAACCAG	BRDN0001	109	9	0	425	342	1
AGCAACCAG	BRDN0001	70	0	11	311	106	0
AGCAACCCC	BRDN0001	84	60	23	243	187	12
AGCAACCTA	BRDN0001	146	255	26	348	245	3
AGCAACCTG	BRDN0001	284	287	786	473	502	31
AGCAACGAG	BRDN0001	281	213	82	481	470	3
AGCAACGCC	BRDN0001	148	71	155	338	278	16
AGCAACGGC	BRDN0001	209	312	124	408	563	21
AGCAACGGC	BRDN0001	76	26	270	165	93	0
AGCAACGGG	BRDN0001	128	130	49	226	192	3
AGCAACTAC	BRDN0001	178	77	1	297	313	4
AGCAACTCA	BRDN0001	257	0	0	476	230	0
AGCAACTCA	BRDN0001	117	247	23	403	106	34
AGCAACTGA	BRDN0001	157	153	455	272	186	4
AGCAACTGG	BRDN0001	364	355	389	649	559	32
AGCAACTGG	BRDN0001	551	471	1313	1062	1239	79
AGCAACTGT	BRDN0001	153	293	919	390	222	103
AGCAAGAAA	BRDN0001	208	209	347	378	482	0
AGCAAGAAA	BRDN0001	140	93	84	331	332	0
AGCAAGAAC	BRDN0001	665	705	574	1292	1118	69
AGCAAGAAT	BRDN0001	224	120	2028	354	197	72
AGCAAGACA	BRDN0001	96	140	154	203	362	14
AGCAAGACA	BRDN0001	350	505	407	736	742	36
AGCAAGACC	BRDN0001	643	270	165	1061	893	3
AGCAAGACG	BRDN0001	165	98	1	475	393	1
AGCAAGACG	BRDN0001	632	582	976	999	692	66
AGCAAGAGC	BRDN0001	508	517	1122	936	804	22
AGCAAGAGG	BRDN0001	16	0	0	114	0	0
AGCAAGAGT	BRDN0001	105	69	0	230	96	17
AGCAAGATC	BRDN0001	10	0	0	596	152	0
AGCAAGCAA	BRDN0001	158	88	426	294	366	42
AGCAAGCCC	BRDN0001	403	307	678	808	818	34
AGCAAGCCT	BRDN0001	94	98	81	222	173	44
AGCAAGCGG	BRDN0001	71	22	0	203	89	0
AGCAAGCTC	BRDN0001	159	105	133	335	398	24
AGCAAGCTG	BRDN0001	364	480	811	688	569	42
AGCAAGGCT	BRDN0001	380	410	320	771	555	36
AGCAAGGGC	BRDN0001	170	209	163	181	219	0
AGCAAGTCG	BRDN0001	241	190	331	374	409	17
AGCAAGTGG	BRDN0001	236	34	1904	485	154	33
AGCAAGTGT	BRDN0001	39	0	0	316	91	0
AGCAATAAT	BRDN0001	132	179	92	227	224	0
AGCAATAAT	BRDN0001	189	156	1442	346	266	31
AGCAATACT	BRDN0001	322	366	159	751	434	21
AGCAATATT	BRDN0001	244	310	115	504	375	0
AGCAATCAA	BRDN0001	207	98	168	299	242	0
AGCAATCAG	BRDN0001	197	224	58	454	634	18
AGCAATGAA	BRDN0001	233	264	1356	485	220	18
AGCAATGAG	BRDN0001	472	301	1583	1039	965	53
AGCAATGAG	BRDN0001	88	181	189	292	266	0
AGCAATGAG	BRDN0001	128	151	6	260	446	41
AGCAATGAT	BRDN0001	27	0	0	657	143	0
AGCAATGCA	BRDN0001	425	364	1081	596	798	79
AGCAATGCA	BRDN0001	202	225	2408	330	331	73
AGCAATGCC	BRDN0001	165	229	116	332	328	38
AGCAATGCT	BRDN0001	200	0	1	364	268	1
AGCAATGCT	BRDN0001	534	566	892	1045	1109	89
AGCAATGGC	BRDN0001	112	102	1	150	50	1
AGCAATGGG	BRDN0001	429	489	2592	571	514	109
AGCAATGGT	BRDN0001	463	527	478	842	860	136
AGCAATGTA	BRDN0001	155	73	18	347	321	9
AGCACAAAA	BRDN0001	174	154	842	281	271	26
AGCACAAAA	BRDN0001	474	433	307	983	823	11
AGCACAAAT	BRDN0001	172	108	0	191	97	0
AGCACAAAT	BRDN0001	306	283	439	408	664	17
AGCACAACC	BRDN0001	354	292	1366	631	1183	58
AGCACAAGA	BRDN0001	99	58	0	218	225	0
AGCACAAGC	BRDN0001	458	668	684	1020	1358	33
AGCACAAGT	BRDN0001	87	52	66	294	97	21
AGCACACAA	BRDN0001	450	250	942	630	951	59
AGCACACAC	BRDN0001	113	79	49	124	120	0
AGCACACAC	BRDN0001	146	92	565	243	526	13
AGCACACAC	BRDN0001	390	343	707	594	604	36
AGCACACAG	BRDN0001	888	787	3122	1353	1296	91
AGCACACAG	BRDN0001	516	487	2269	927	1408	27
AGCACACAT	BRDN0001	139	108	607	306	271	68
AGCACACGG	BRDN0001	109	157	373	265	303	8
AGCACACGT	BRDN0001	181	153	195	434	352	18
AGCACACGT	BRDN0001	563	326	763	1161	565	21
AGCACACTA	BRDN0001	274	110	636	493	509	38
AGCACACTG	BRDN0001	323	221	272	830	661	15
AGCACAGAG	BRDN0001	364	344	187	558	1165	66
AGCACAGAT	BRDN0001	513	380	1680	838	1426	90
AGCACAGCA	BRDN0001	447	441	663	1124	791	36
AGCACAGCA	BRDN0001	198	295	915	514	465	37
AGCACAGCA	BRDN0001	279	241	1069	448	639	77
AGCACAGCA	BRDN0001	257	264	35	464	341	6
AGCACAGCA	BRDN0001	536	507	1570	1144	1091	101
AGCACAGCC	BRDN0001	446	388	52	839	578	34
AGCACAGCG	BRDN0001	191	82	2	286	198	0
AGCACAGGA	BRDN0001	178	131	335	297	186	0
AGCACAGGG	BRDN0001	95	113	830	308	206	9
AGCACAGGG	BRDN0001	216	163	153	483	397	0
AGCACAGTA	BRDN0001	49	21	89	367	183	0
AGCACATAA	BRDN0001	257	221	44	448	247	0
AGCACATGA	BRDN0001	161	18	1	434	179	0
AGCACATGG	BRDN0001	263	241	317	574	498	19
AGCACATGG	BRDN0001	412	488	1173	958	877	88
AGCACATGG	BRDN0001	365	278	1365	680	602	54
AGCACATTC	BRDN0001	166	202	1567	356	269	28
AGCACCAAT	BRDN0001	43	5	0	845	358	0
AGCACCACA	BRDN0001	372	377	807	802	626	60
AGCACCACC	BRDN0001	731	587	441	1578	1665	83
AGCACCACG	BRDN0001	94	84	31	273	271	127
AGCACCAGA	BRDN0001	360	419	1108	718	702	43
AGCACCAGA	BRDN0001	132	52	22	337	266	12
AGCACCAGC	BRDN0001	282	216	735	480	472	25
AGCACCAGT	BRDN0001	309	72	13	568	198	0
AGCACCATA	BRDN0001	192	89	34	265	133	12
AGCACCATA	BRDN0001	143	119	988	205	107	19
AGCACCCAA	BRDN0001	590	558	553	912	406	41
AGCACCCAA	BRDN0001	379	270	158	831	806	15
AGCACCCAA	BRDN0001	316	308	1639	557	705	38
AGCACCCAG	BRDN0001	319	302	86	694	664	36
AGCACCCAC	BRDN0001	342	209	235	510	680	26
AGCACCCCC	BRDN0001	458	441	444	921	1151	28
AGCACCCGA	BRDN0001	167	218	253	282	293	37
AGCACCCGA	BRDN0001	132	0	0	422	34	0
AGCACCCGA	BRDN0001	117	211	324	218	268	0
AGCACCCGG	BRDN0001	369	301	472	771	942	4
AGCACCCGT	BRDN0001	293	279	664	482	380	27
AGCACCGAC	BRDN0001	81	120	9	272	207	0
AGCACCGCC	BRDN0001	512	425	1081	794	646	44
AGCACCGCT	BRDN0001	49	16	1	223	139	0
AGCACCGGA	BRDN0001	476	452	12475	901	1247	175
AGCACCGGC	BRDN0001	260	192	1985	467	251	48
AGCACCGGG	BRDN0001	440	424	1319	884	686	42
AGCACCGGG	BRDN0001	246	203	36	615	544	31
AGCACCGGT	BRDN0001	65	191	14	226	105	29
AGCACCGTC	BRDN0001	229	184	11	413	509	2
AGCACCTAC	BRDN0001	419	526	402	860	751	28
AGCACCTAC	BRDN0001	412	428	616	753	775	70
AGCACCTAG	BRDN0001	388	518	769	780	769	51
AGCACCTAT	BRDN0001	415	154	63	620	577	20
AGCACCTGA	BRDN0001	529	310	711	873	853	19
AGCACCTGA	BRDN0001	263	169	254	377	513	1
AGCACCTGG	BRDN0001	258	196	97	879	762	0
AGCACCTTG	BRDN0001	527	557	2061	1041	906	24
AGCACGACC	BRDN0001	274	301	206	595	555	34
AGCACGACC	BRDN0001	431	415	765	748	504	49
AGCACGCCA	BRDN0001	593	514	1716	1056	974	82
AGCACGCCC	BRDN0001	255	308	1266	386	435	25
AGCACGGAG	BRDN0001	96	77	639	233	239	9
AGCACGGAT	BRDN0001	577	506	1085	1020	622	63
AGCACGGCA	BRDN0001	289	174	100	320	300	15
AGCACGTAA	BRDN0001	482	334	1931	953	979	83
AGCACGTAC	BRDN0001	201	194	1087	318	695	39
AGCACGTCG	BRDN0001	564	537	291	1161	1061	61
AGCACGTGA	BRDN0001	211	223	1566	378	173	20
AGCACGTTC	BRDN0001	129	67	98	205	184	4
AGCACTAAC	BRDN0001	250	22	2	636	472	0
AGCACTAAT	BRDN0001	194	136	39	308	272	16
AGCACTAAT	BRDN0001	256	162	620	480	541	7
AGCACTACA	BRDN0001	279	133	817	298	369	44
AGCACTACC	BRDN0001	349	381	569	879	796	50
AGCACTACC	BRDN0001	427	315	298	728	986	22
AGCACTACG	BRDN0001	166	151	373	317	401	11
AGCACTACG	BRDN0001	140	141	164	248	331	0
AGCACTATA	BRDN0001	405	404	1569	840	638	47
AGCACTCAC	BRDN0001	269	176	391	349	471	16
AGCACTCAT	BRDN0001	418	343	848	853	885	57
AGCACTCCG	BRDN0001	208	135	392	451	582	0
AGCACTCCT	BRDN0001	213	280	7405	415	430	71
AGCACTCGC	BRDN0001	351	422	456	770	1118	21
AGCACTCGC	BRDN0001	205	113	593	346	385	39
AGCACTCGG	BRDN0001	158	2	0	304	107	0
AGCACTGAA	BRDN0001	216	252	1724	496	283	24
AGCACTGAA	BRDN0001	253	354	1310	578	470	51
AGCACTGAA	BRDN0001	258	253	128	412	458	70
AGCACTGAG	BRDN0001	479	309	392	922	967	27
AGCACTGAG	BRDN0001	85	83	0	234	128	26
AGCACTGAG	BRDN0001	112	209	542	202	315	40
AGCACTGAT	BRDN0001	137	280	1427	323	274	62
AGCACTGCA	BRDN0001	202	200	87	486	659	16
AGCACTGCC	BRDN0001	112	182	182	263	355	5
AGCACTGCG	BRDN0001	633	446	1625	1434	1187	34
AGCACTGGC	BRDN0001	200	155	37	232	263	31
AGCACTGGT	BRDN0001	159	189	208	347	335	15
AGCACTGTC	BRDN0001	250	212	561	389	308	59
AGCACTTCT	BRDN0001	189	213	368	361	162	21
AGCACTTGG	BRDN0001	419	402	942	697	1000	75
AGCACTTTA	BRDN0001	319	325	1131	477	428	61
AGCACTTTC	BRDN0001	206	3	2	596	219	0
AGCACTTTG	BRDN0001	69	0	0	177	199	0
AGCACTTTG	BRDN0001	356	282	164	719	1055	30
AGCAGAAAC	BRDN0001	138	59	138	154	193	3
AGCAGAAAG	BRDN0001	344	409	834	742	757	30
AGCAGAACA	BRDN0001	512	508	2869	841	1389	78
AGCAGAACC	BRDN0001	421	468	71	619	840	25
AGCAGAACG	BRDN0001	304	87	100	646	636	9
AGCAGAAGC	BRDN0001	375	352	371	590	559	89
AGCAGAATG	BRDN0001	290	259	290	591	788	59
AGCAGAATG	BRDN0001	162	215	967	317	283	33
AGCAGACAA	BRDN0001	264	146	730	389	564	43
AGCAGACAA	BRDN0001	90	229	9	204	277	0
AGCAGACAA	BRDN0001	56	39	0	331	133	18
AGCAGACAT	BRDN0001	417	19	1	948	702	0
AGCAGACCC	BRDN0001	261	140	205	399	674	21
AGCAGACCT	BRDN0001	223	162	315	400	267	26
AGCAGACGG	BRDN0001	534	464	2930	804	1139	63
AGCAGACGT	BRDN0001	180	169	38	234	173	0
AGCAGACGT	BRDN0001	192	52	157	492	214	21
AGCAGACTC	BRDN0001	195	103	155	659	364	0
AGCAGAGAA	BRDN0001	288	310	32	571	712	34
AGCAGAGCA	BRDN0001	221	244	59	540	252	16
AGCAGAGCA	BRDN0001	249	178	9	357	203	10
AGCAGAGCC	BRDN0001	178	0	0	335	213	0
AGCAGAGCC	BRDN0001	212	195	9	263	237	29
AGCAGAGCC	BRDN0001	168	161	531	335	288	21
AGCAGAGCT	BRDN0001	272	263	147	657	601	18
AGCAGAGGA	BRDN0001	65	37	0	169	283	1
AGCAGAGGC	BRDN0001	432	381	1063	659	447	26
AGCAGAGGT	BRDN0001	129	122	576	253	922	45
AGCAGAGTA	BRDN0001	296	191	182	561	451	4
AGCAGAGTC	BRDN0001	280	213	318	563	638	14
AGCAGAGTG	BRDN0001	235	390	481	534	529	41
AGCAGATAG	BRDN0001	240	209	150	328	262	21
AGCAGATCC	BRDN0001	376	359	204	862	672	55
AGCAGATCG	BRDN0001	245	215	1492	483	523	55
AGCAGATGA	BRDN0001	354	337	477	802	936	53
AGCAGATGG	BRDN0001	132	184	107	259	262	17
AGCAGATGG	BRDN0001	382	320	528	573	367	31
AGCAGATGT	BRDN0001	111	121	1304	230	661	16
AGCAGATGT	BRDN0001	522	463	316	996	987	58
AGCAGATTC	BRDN0001	252	161	112	604	399	14
AGCAGATTC	BRDN0001	143	62	344	363	228	68
AGCAGCAAA	BRDN0001	172	230	68	411	379	9
AGCAGCAAC	BRDN0001	161	35	321	351	316	4
AGCAGCAAC	BRDN0001	386	199	24	1101	388	13
AGCAGCAAG	BRDN0001	402	414	131	920	1075	83
AGCAGCACA	BRDN0001	556	421	2254	1149	576	47
AGCAGCACA	BRDN0001	160	36	0	314	266	0
AGCAGCACC	BRDN0001	428	561	323	887	591	78
AGCAGCACT	BRDN0001	377	279	295	522	505	33
AGCAGCAGA	BRDN0001	113	82	46	377	123	0
AGCAGCAGC	BRDN0001	242	203	209	478	515	8
AGCAGCAGG	BRDN0001	119	287	100	225	245	116
AGCAGCAGG	BRDN0001	109	95	2836	255	365	45
AGCAGCAGT	BRDN0001	83	116	5	267	175	0
AGCAGCATC	BRDN0001	494	339	1143	685	952	19
AGCAGCATT	BRDN0001	192	133	282	375	382	16
AGCAGCCAA	BRDN0001	176	79	138	255	410	184
AGCAGCCAC	BRDN0001	155	208	662	236	191	3
AGCAGCCAC	BRDN0001	132	75	140	326	365	4
AGCAGCCAG	BRDN0001	319	254	229	465	498	3
AGCAGCCAG	BRDN0001	504	545	629	1004	1425	19
AGCAGCCGA	BRDN0001	134	102	107	292	102	47
AGCAGCCGC	BRDN0001	650	695	277	1225	1103	129
AGCAGCCGC	BRDN0001	193	183	41	318	228	28
AGCAGCCGG	BRDN0001	191	117	140	340	225	0
AGCAGCCGG	BRDN0001	236	76	302	197	459	14
AGCAGCCGT	BRDN0001	323	404	269	716	645	33
AGCAGCCTC	BRDN0001	113	69	196	276	208	0
AGCAGCCTG	BRDN0001	31	7	1	638	135	0
AGCAGCCTG	BRDN0001	308	438	773	646	580	73
AGCAGCGAT	BRDN0001	71	54	168	200	71	16
AGCAGCGAT	BRDN0001	432	449	328	872	712	141
AGCAGCGAT	BRDN0001	165	88	1	253	437	0
AGCAGCGCG	BRDN0001	365	271	264	693	419	39
AGCAGCGCG	BRDN0001	125	177	6	265	531	15
AGCAGCGGA	BRDN0001	27	0	1	165	132	0
AGCAGCGGC	BRDN0001	124	174	1	278	101	0
AGCAGCGGC	BRDN0001	271	140	661	258	350	46
AGCAGCGGC	BRDN0001	504	417	808	878	624	32
AGCAGCGGC	BRDN0001	714	455	1145	1034	733	122
AGCAGCGGT	BRDN0001	192	184	346	436	649	12
AGCAGCGGT	BRDN0001	24	1	0	530	51	0
AGCAGCGTA	BRDN0001	105	71	208	229	76	1
AGCAGCGTC	BRDN0001	510	481	1012	869	915	37
AGCAGCGTG	BRDN0001	259	190	387	383	603	15
AGCAGCGTG	BRDN0001	136	209	131	168	175	7
AGCAGCTAC	BRDN0001	253	297	920	443	255	20
AGCAGCTAT	BRDN0001	127	101	14	315	67	0
AGCAGCTCC	BRDN0001	551	467	232	934	925	13
AGCAGCTCG	BRDN0001	98	86	179	260	159	15
AGCAGCTCG	BRDN0001	266	209	113	777	719	9
AGCAGCTGA	BRDN0001	741	809	1839	1377	1327	113
AGCAGCTGG	BRDN0001	109	0	0	286	186	0
AGCAGCTGG	BRDN0001	208	247	65	235	512	60
AGCAGCTTC	BRDN0001	283	399	120	535	737	9
AGCAGCTTG	BRDN0001	169	175	1	319	489	24
AGCAGGAAA	BRDN0001	60	96	21	224	165	0
AGCAGGAAA	BRDN0001	403	470	750	808	859	80
AGCAGGAAA	BRDN0001	156	172	66	390	410	38
AGCAGGAAC	BRDN0001	185	311	410	342	207	51
AGCAGGAAG	BRDN0001	65	36	42	179	109	2
AGCAGGACA	BRDN0001	200	250	15	425	266	0
AGCAGGACA	BRDN0001	594	578	642	952	882	71
AGCAGGACA	BRDN0001	281	50	359	398	543	6
AGCAGGACA	BRDN0001	69	69	0	175	188	0
AGCAGGACT	BRDN0001	159	84	0	494	227	0
AGCAGGAGC	BRDN0001	105	82	10	213	274	9
AGCAGGAGC	BRDN0001	301	366	253	466	696	34
AGCAGGAGT	BRDN0001	139	150	474	318	197	32
AGCAGGATC	BRDN0001	517	439	194	747	831	39
AGCAGGATG	BRDN0001	243	190	518	393	384	13
AGCAGGATT	BRDN0001	69	82	24	183	274	7
AGCAGGCGA	BRDN0001	254	192	154	633	391	60
AGCAGGCGC	BRDN0001	189	138	667	308	378	11
AGCAGGCTG	BRDN0001	162	153	42	325	343	0
AGCAGGGAA	BRDN0001	463	452	987	946	981	44
AGCAGGGAA	BRDN0001	81	70	282	130	17	1
AGCAGGGAC	BRDN0001	276	285	261	481	710	47
AGCAGGGAC	BRDN0001	521	354	429	796	544	52
AGCAGGGCC	BRDN0001	594	350	68	974	1775	14
AGCAGGGCG	BRDN0001	391	436	108	890	977	13
AGCAGGGCT	BRDN0001	496	481	702	876	1117	84
AGCAGGGCT	BRDN0001	171	105	139	384	364	4
AGCAGGTAC	BRDN0001	103	107	154	240	258	0
AGCAGGTAT	BRDN0001	322	68	155	734	710	0
AGCAGGTCT	BRDN0001	307	148	204	427	204	0
AGCAGGTGA	BRDN0001	91	12	0	364	69	0
AGCAGGTGG	BRDN0001	75	77	14	94	121	0

AGCAGGTGG	BRDN00011	300	354	680	649	323	3
AGCAGGTGT	BRDN00011	170	96	570	326	679	43
AGCAGGTTG	BRDN00011	108	152	576	237	330	15
AGCAGGTTG	BRDN00012	187	166	25	351	250	1
AGCAGTAAA	BRDN00012	432	463	1390	833	807	14
AGCAGTAAA	BRDN00012	414	60	36	747	708	0
AGCAGTAAA	BRDN00012	250	147	669	427	381	24
AGCAGTAAC	BRDN00012	193	179	94	296	314	15
AGCAGTAAC	BRDN00012	262	282	202	598	940	40
AGCAGTAAT	BRDN00011	166	127	267	286	203	12
AGCAGTACG	BRDN00012	372	399	249	854	1418	33
AGCAGTAGG	BRDN00011	173	157	565	357	190	20
AGCAGTATG	BRDN00012	426	196	402	971	1021	33
AGCAGTCAA	BRDN00012	260	204	250	340	476	1
AGCAGTCAC	BRDN00012	548	359	580	915	904	48
AGCAGTCAC	BRDN00012	320	206	414	404	409	6
AGCAGTCAG	BRDN00011	339	403	4880	520	937	68
AGCAGTCAG	BRDN00012	154	112	14	399	380	0
AGCAGTCAT	BRDN00011	625	379	851	1252	981	43
AGCAGTCCA	BRDN00012	260	221	460	242	242	29
AGCAGTCCC	BRDN00012	407	560	2123	786	627	47
AGCAGTCCC	BRDN00012	598	516	1532	1003	994	66
AGCAGTCCT	BRDN00011	135	123	42	335	322	6
AGCAGTCGG	BRDN00012	639	497	10	1221	914	0
AGCAGTCGT	BRDN00012	83	100	385	221	181	12
AGCAGTGAG	BRDN00011	374	391	708	940	698	65
AGCAGTGAG	BRDN00011	165	223	252	304	335	37
AGCAGTGCC	BRDN00011	273	223	554	563	387	49
AGCAGTGCG	BRDN00011	95	129	15	253	331	16
AGCAGTGCT	BRDN00012	406	569	569	712	675	63
AGCAGTGGA	BRDN00011	693	632	584	1107	1353	63
AGCAGTGGA	BRDN00011	312	313	5756	658	300	103
AGCAGTGGA	BRDN00011	94	38	0	289	324	0
AGCAGTGGT	BRDN00012	150	163	98	256	111	18
AGCAGTGTG	BRDN00012	680	791	868	1012	1392	26
AGCAGTGTG	BRDN00012	303	279	35	407	371	43
AGCAGTGTT	BRDN00012	486	469	880	756	754	31
AGCAGTTCG	BRDN00011	403	313	747	730	766	41
AGCAGTTGA	BRDN00012	258	219	880	555	726	24
AGCAGTTGA	BRDN00012	246	73	651	513	245	9
AGCAGTTGG	BRDN00011	237	345	7	480	795	4
AGCAGTTGG	BRDN00011	111	18	0	263	274	0
AGCAGTTGT	BRDN00012	342	284	261	667	477	28
AGCATAAAC	BRDN00012	158	125	182	257	241	6
AGCATAATC	BRDN00011	148	143	79	256	201	2
AGCATACAA	BRDN00011	187	268	48	333	398	0
AGCATACAG	BRDN00011	371	401	61	664	520	6
AGCATACGA	BRDN00012	155	215	15	249	105	7
AGCATACGC	BRDN00012	286	293	252	553	870	31
AGCATACTC	BRDN00012	95	144	39	255	146	0
AGCATAGCA	BRDN00011	154	91	44	357	193	0
AGCATAGCC	BRDN00011	156	268	937	309	457	17
AGCATAGCC	BRDN00012	3	0	0	728	69	0
AGCATAGGA	BRDN00012	168	137	11	266	139	0
AGCATAGTA	BRDN00011	331	528	368	736	686	116
AGCATAGTC	BRDN00012	239	268	291	595	253	22
AGCATATAA	BRDN00011	55	0	0	98	143	0
AGCATATGA	BRDN00012	169	12	1017	342	380	4
AGCATATGA	BRDN00012	220	158	949	306	222	8
AGCATATTC	BRDN00011	362	183	288	707	755	18
AGCATATTG	BRDN00011	90	51	2	311	453	0
AGCATCAAA	BRDN00011	210	155	383	428	545	13
AGCATCAAC	BRDN00012	185	127	426	492	530	5
AGCATCAAG	BRDN00012	307	239	1779	642	1268	6
AGCATCAAG	BRDN00011	575	621	829	972	938	36
AGCATCAAG	BRDN00011	280	215	2154	609	681	36
AGCATCACC	BRDN00012	359	302	634	681	933	26
AGCATCACG	BRDN00012	168	226	1351	217	127	12
AGCATCACT	BRDN00011	297	221	75	378	486	20
AGCATCATA	BRDN00012	345	182	492	487	445	32
AGCATCATC	BRDN00012	81	25	97	844	169	30
AGCATCATC	BRDN00011	400	367	3137	554	706	40
AGCATCCAA	BRDN00012	75	144	0	155	94	24
AGCATCCAC	BRDN00011	309	62	290	392	147	5
AGCATCCAT	BRDN00011	383	430	270	732	778	38
AGCATCCCG	BRDN00011	201	371	111	495	327	3
AGCATCGCG	BRDN00012	173	132	42	211	141	13
AGCATCGTC	BRDN00011	332	243	283	883	415	16
AGCATCGTC	BRDN00011	414	230	337	734	902	22
AGCATCGTT	BRDN00012	284	234	696	540	668	46
AGCATCTAA	BRDN00012	269	295	469	568	573	0
AGCATCTAG	BRDN00012	412	379	263	889	826	28
AGCATCTTA	BRDN00012	114	19	2584	427	531	21
AGCATGAAA	BRDN00011	76	66	44	161	158	6
AGCATGAAA	BRDN00012	171	132	305	359	320	33
AGCATGACC	BRDN00011	301	272	1233	665	311	53
AGCATGAGC	BRDN00012	61	44	500	203	149	3
AGCATGAGC	BRDN00011	525	492	535	1018	728	102
AGCATGAGC	BRDN00011	556	382	1054	1069	931	68
AGCATGAGC	BRDN00012	131	95	479	126	282	0
AGCATGCAC	BRDN00011	155	94	304	309	489	3
AGCATGCCG	BRDN00011	137	147	0	250	330	34
AGCATGCGG	BRDN00012	201	112	197	361	207	30
AGCATGCTC	BRDN00012	469	459	335	773	753	115
AGCATGGAC	BRDN00011	549	535	354	947	1096	7
AGCATGGAT	BRDN00012	541	445	1115	915	1132	90
AGCATGGAT	BRDN00012	487	441	924	802	814	63
AGCATGGCA	BRDN00011	191	165	876	484	446	40
AGCATGGCA	BRDN00011	153	279	858	284	261	50
AGCATGGCA	BRDN00011	310	362	132	526	338	37
AGCATGGCC	BRDN00011	603	524	1639	956	829	286
AGCATGGCC	BRDN00011	139	143	102	313	322	0
AGCATGGCT	BRDN00011	439	275	344	675	601	19
AGCATGGGA	BRDN00012	128	44	102	180	292	31
AGCATGGGT	BRDN00011	308	320	270	486	559	20
AGCATGGGT	BRDN00011	155	15	660	438	488	6
AGCATGTAG	BRDN00012	185	156	947	570	907	15
AGCATGTCA	BRDN00012	153	213	400	338	214	16
AGCATGTCT	BRDN00012	406	28	23	706	321	1
AGCATGTCT	BRDN00011	386	493	355	882	694	52
AGCATGTGA	BRDN00012	352	298	479	614	864	37
AGCATGTGC	BRDN00011	166	114	320	395	186	10
AGCATGTGG	BRDN00012	160	105	1	434	186	0
AGCATGTTG	BRDN00011	325	286	50	835	777	15
AGCATTAAA	BRDN00011	50	0	0	201	7	0
AGCATTAAG	BRDN00012	129	131	46	242	202	8
AGCATTAAG	BRDN00012	169	410	34	500	756	19
AGCATTACA	BRDN00012	491	418	275	1008	1143	17
AGCATTAGT	BRDN00012	453	393	332	861	1214	1
AGCATTATC	BRDN00012	153	70	1	199	158	0
AGCATTCAG	BRDN00012	261	167	305	379	375	5
AGCATTCAT	BRDN00011	222	292	732	322	287	67
AGCATTCCA	BRDN00012	178	170	950	398	355	55
AGCATTCGC	BRDN00011	351	317	588	677	475	33
AGCATTCGC	BRDN00012	433	136	69	658	573	7
AGCATTCGG	BRDN00012	480	376	1335	758	722	15
AGCATTCTC	BRDN00012	428	632	1092	933	1200	0
AGCATTGAA	BRDN00011	93	47	0	346	207	0
AGCATTGCC	BRDN00011	360	18	0	1042	277	0
AGCATTGCT	BRDN00011	258	110	1	814	760	5
AGCATTGTA	BRDN00011	81	0	0	524	144	0
AGCATTGTT	BRDN00012	133	135	723	305	246	9
AGCATTTCA	BRDN00011	429	372	564	995	842	4
AGCATTTCC	BRDN00011	132	65	617	166	322	0
AGCATTTGA	BRDN00012	93	83	29	229	93	26
AGCATTTGA	BRDN00012	211	298	71	457	234	43
AGCATTTGC	BRDN00012	658	653	1041	1107	1556	43
AGCCAAAAG	BRDN00012	214	230	937	286	321	20
AGCCAAAAT	BRDN00011	342	226	388	681	842	19
AGCCAAAAT	BRDN00011	184	92	578	280	283	34
AGCCAAAAT	BRDN00011	417	496	725	746	688	35
AGCCAAAAT	BRDN00011	108	0	0	241	95	0
AGCCAAACA	BRDN00012	481	353	6272	1093	1263	145
AGCCAAACG	BRDN00011	242	106	174	449	312	14
AGCCAAACG	BRDN00011	362	370	339	946	554	46
AGCCAAAGA	BRDN00012	232	271	954	362	352	67
AGCCAAAGG	BRDN00011	283	185	141	363	497	31
AGCCAAAGT	BRDN00012	146	113	286	311	268	0
AGCCAAATG	BRDN00012	562	67	0	726	848	0
AGCCAAATT	BRDN00012	445	442	1231	770	561	91
AGCCAACCA	BRDN00011	140	196	649	397	399	6
AGCCAACCA	BRDN00011	119	53	40	190	320	27
AGCCAACCA	BRDN00011	160	177	252	404	408	0
AGCCAACCC	BRDN00012	203	171	170	288	287	7
AGCCAACTG	BRDN00012	151	29	0	373	219	0
AGCCAAGAG	BRDN00011	136	89	84	239	162	6
AGCCAAGAG	BRDN00012	310	254	273	534	475	1
AGCCAAGCA	BRDN00012	159	151	349	310	449	0
AGCCAAGCA	BRDN00011	19	0	0	697	159	1
AGCCAAGGA	BRDN00011	162	66	11	364	316	2
AGCCAAGGG	BRDN00011	195	13	48	831	415	23
AGCCAATAA	BRDN00012	415	321	88	817	375	23
AGCCAATCA	BRDN00012	328	193	348	646	983	31
AGCCAATGA	BRDN00011	263	155	15	343	114	0
AGCCAATGA	BRDN00012	567	437	1151	1243	986	46
AGCCAATGA	BRDN00011	216	235	133	507	161	4
AGCCAATTA	BRDN00011	331	237	0	618	259	0
AGCCACAAA	BRDN00011	394	399	47	912	1169	83
AGCCACAAA	BRDN00012	310	64	29	566	390	0
AGCCACAGA	BRDN00012	93	58	445	278	242	5
AGCCACAGA	BRDN00011	176	159	519	366	510	34
AGCCACAGA	BRDN00011	202	179	417	348	825	38
AGCCACAGA	BRDN00012	166	91	22	292	303	9
AGCCACATA	BRDN00011	276	278	129	575	466	22
AGCCACATA	BRDN00012	366	358	187	598	590	35
AGCCACATC	BRDN00012	127	187	109	295	494	0
AGCCACATC	BRDN00011	151	72	10	264	363	0
AGCCACATG	BRDN00011	84	28	1	773	419	0
AGCCACATT	BRDN00012	351	333	369	578	458	32
AGCCACATT	BRDN00011	112	27	179	497	256	17
AGCCACCAA	BRDN00011	237	201	308	487	639	34
AGCCACCAA	BRDN00011	198	92	10	386	225	0
AGCCACCCA	BRDN00012	529	386	2290	711	617	45
AGCCACCCA	BRDN00011	106	146	547	162	21	37
AGCCACCTA	BRDN00011	292	236	718	771	296	25
AGCCACCTT	BRDN00011	240	227	140	586	459	26
AGCCACGAA	BRDN00012	533	315	109	760	669	57
AGCCACGAA	BRDN00012	612	689	2403	1184	1573	177
AGCCACGAT	BRDN00011	362	231	1747	624	651	38
AGCCACGAT	BRDN00011	642	431	772	882	544	71
AGCCACGGC	BRDN00012	184	273	120	319	49	14
AGCCACGGC	BRDN00012	382	340	406	803	449	30
AGCCACGGC	BRDN00011	230	86	72	436	505	6
AGCCACGTA	BRDN00012	332	306	365	609	673	2
AGCCACTAA	BRDN00012	240	368	676	760	825	11
AGCCACTCG	BRDN00012	169	166	177	294	256	35
AGCCACTGA	BRDN00012	517	500	1389	855	973	75
AGCCACTGA	BRDN00012	98	91	0	256	187	3
AGCCACTGC	BRDN00011	533	487	201	941	1016	46
AGCCACTGC	BRDN00011	233	59	542	443	371	9
AGCCACTGG	BRDN00012	171	165	156	212	154	23
AGCCACTGT	BRDN00012	275	200	1009	481	367	15
AGCCACTGT	BRDN00012	123	65	539	300	362	48
AGCCACTTA	BRDN00012	235	129	2828	523	555	42
AGCCACTTA	BRDN00012	467	337	454	1131	1326	29
AGCCACTTC	BRDN00012	75	124	678	184	185	15
AGCCAGACA	BRDN00012	229	223	10	498	319	3
AGCCAGACC	BRDN00012	163	9	0	576	274	0
AGCCAGACG	BRDN00011	257	284	669	499	350	65
AGCCAGACT	BRDN00012	167	83	0	368	236	0
AGCCAGACT	BRDN00011	226	145	874	531	519	18
AGCCAGACT	BRDN00012	178	229	34	365	342	37
AGCCAGAGC	BRDN00012	104	75	109	384	413	5
AGCCAGAGC	BRDN00012	435	462	196	649	691	9
AGCCAGAGG	BRDN00012	274	183	315	320	671	17
AGCCAGAGG	BRDN00011	238	242	139	440	262	8
AGCCAGATT	BRDN00012	151	0	0	267	204	0
AGCCAGCAG	BRDN00012	510	319	1927	778	574	48
AGCCAGCCA	BRDN00012	327	377	201	640	465	25
AGCCAGCCA	BRDN00011	589	519	426	1167	1059	76
AGCCAGCCA	BRDN00011	358	301	2	649	544	17
AGCCAGCCG	BRDN00012	119	32	0	308	135	0
AGCCAGCGG	BRDN00011	301	131	711	382	236	0
AGCCAGCGG	BRDN00012	107	105	43	173	152	0
AGCCAGCTC	BRDN00012	441	542	1046	701	938	44
AGCCAGGAA	BRDN00011	433	168	871	678	472	58
AGCCAGGAC	BRDN00012	499	513	3881	903	1156	145
AGCCAGGAC	BRDN00012	79	93	197	141	312	182
AGCCAGGCC	BRDN00012	351	278	1106	872	679	26
AGCCAGGCT	BRDN00011	167	238	463	344	506	0
AGCCAGGGA	BRDN00011	286	203	72	378	259	6
AGCCAGGGA	BRDN00012	469	414	407	741	660	62
AGCCAGGGA	BRDN00012	155	195	236	205	220	0
AGCCAGGGC	BRDN00012	175	229	0	271	239	80
AGCCAGGTC	BRDN00012	92	152	4	230	192	0
AGCCAGGTT	BRDN00012	433	386	1107	863	604	66
AGCCAGTAG	BRDN00011	141	138	0	374	362	29
AGCCAGTAT	BRDN00011	339	322	468	591	502	29
AGCCAGTCC	BRDN00012	166	179	182	414	235	12
AGCCAGTGA	BRDN00012	350	380	625	709	840	20
AGCCAGTGG	BRDN00012	437	118	21	840	1639	7
AGCCAGTGT	BRDN00011	532	448	716	891	686	141
AGCCAGTTC	BRDN00012	558	484	4274	1024	1057	102
AGCCATAAA	BRDN00012	365	305	2192	550	652	21
AGCCATAAT	BRDN00011	221	77	144	298	328	37
AGCCATAAT	BRDN00012	118	204	1403	534	356	16
AGCCATAAT	BRDN00012	265	175	133	496	484	0
AGCCATAAT	BRDN00011	226	86	69	407	376	11
AGCCATACC	BRDN00011	502	815	800	942	747	60
AGCCATACT	BRDN00011	357	394	1177	531	517	66
AGCCATAGC	BRDN00011	126	180	172	335	249	0
AGCCATAGG	BRDN00011	555	385	566	911	1088	38
AGCCATCAA	BRDN00012	354	580	192	664	622	87
AGCCATCAC	BRDN00011	447	389	508	679	368	17
AGCCATCAG	BRDN00011	127	68	1184	202	500	18
AGCCATCCA	BRDN00011	161	179	75	293	253	0
AGCCATCCG	BRDN00012	253	248	580	545	1442	22
AGCCATCCT	BRDN00012	209	205	161	358	389	31
AGCCATCGC	BRDN00012	488	464	724	920	1252	19
AGCCATCGG	BRDN00012	593	305	525	1136	1058	19
AGCCATCGT	BRDN00011	156	71	14	298	228	48
AGCCATCTC	BRDN00011	191	136	183	327	559	6
AGCCATCTC	BRDN00012	429	372	3297	795	843	79
AGCCATCTG	BRDN00012	480	334	742	816	743	41
AGCCATGAA	BRDN00012	97	151	1681	287	169	64
AGCCATGAA	BRDN00011	135	6	0	206	140	0
AGCCATGAC	BRDN00012	180	171	32	310	275	10
AGCCATGAG	BRDN00012	204	185	16	307	211	25
AGCCATGAG	BRDN00012	213	161	71	350	249	0
AGCCATGAT	BRDN00011	135	129	60	224	118	28
AGCCATGCC	BRDN00011	434	514	137	827	647	143
AGCCATGGA	BRDN00011	150	130	0	397	252	0
AGCCATGGA	BRDN00012	436	511	198	653	573	25
AGCCATGGG	BRDN00012	137	213	92	269	267	33
AGCCATGGG	BRDN00011	380	394	591	953	720	24
AGCCATGGT	BRDN00012	217	229	153	514	396	0
AGCCATGTT	BRDN00011	205	33	15	290	479	20
AGCCATGTT	BRDN00012	178	224	51	406	408	6
AGCCATTAT	BRDN00012	178	72	14	373	410	0
AGCCATTCA	BRDN00012	559	608	273	1079	558	39
AGCCATTCT	BRDN00012	349	283	986	584	1253	17
AGCCATTGG	BRDN00012	243	146	501	437	421	15
AGCCATTGG	BRDN00011	227	235	2125	391	258	65
AGCCATTGG	BRDN00012	472	392	299	797	1097	19
AGCCATTGG	BRDN00011	388	284	760	459	595	106
AGCCATTGT	BRDN00011	299	310	119	462	406	65
AGCCCAAAC	BRDN00011	436	160	613	572	894	61
AGCCCAAAT	BRDN00011	376	230	596	572	550	13
AGCCCAACA	BRDN00012	391	377	1335	972	1386	18
AGCCCAAGC	BRDN00011	68	1	0	390	45	0
AGCCCAAGT	BRDN00012	46	0	0	263	236	0
AGCCCACAA	BRDN00012	468	501	1048	905	802	50
AGCCCACCC	BRDN00011	521	353	719	1071	665	8
AGCCCACCG	BRDN00012	128	215	758	338	387	31
AGCCCACCT	BRDN00011	329	262	319	608	493	0
AGCCCACTA	BRDN00012	54	9	0	439	424	0
AGCCCACTC	BRDN00012	369	145	92	578	472	0
AGCCCACTC	BRDN00011	136	117	66	331	187	41
AGCCCAGAA	BRDN00011	401	382	707	738	979	27
AGCCCAGCA	BRDN00012	252	232	217	477	518	21
AGCCCAGCA	BRDN00011	248	304	208	434	731	101
AGCCCAGCC	BRDN00012	27	1	0	202	203	3
AGCCCAGGC	BRDN00011	575	447	335	922	650	122
AGCCCAGGG	BRDN00011	154	60	503	468	410	0
AGCCCAGGT	BRDN00012	196	197	164	442	446	28
AGCCCAGTT	BRDN00012	355	344	1251	937	541	35
AGCCCAGTT	BRDN00012	91	0	0	263	187	0
AGCCCATGT	BRDN00012	420	336	399	826	643	40
AGCCCATTG	BRDN00012	389	363	25	926	1117	9
AGCCCCAAA	BRDN00011	111	117	41	216	252	25
AGCCCCAAC	BRDN00011	395	267	468	639	610	14
AGCCCCAAC	BRDN00012	646	491	750	1014	1095	130
AGCCCCACA	BRDN00012	403	385	267	751	596	0
AGCCCCACC	BRDN00011	172	104	9	555	532	0
AGCCCCACC	BRDN00011	365	160	19	978	1234	0
AGCCCCACC	BRDN00011	173	97	219	270	330	0
AGCCCCACC	BRDN00011	150	142	39	221	423	26
AGCCCCACG	BRDN00012	416	558	15485	845	592	465
AGCCCCAGA	BRDN00012	254	241	414	328	504	12
AGCCCCAGG	BRDN00011	39	0	0	231	30	0
AGCCCCAGG	BRDN00012	99	34	0	470	465	8
AGCCCCATA	BRDN00012	419	337	368	823	661	19
AGCCCCATC	BRDN00011	272	89	107	693	323	1
AGCCCCCACA	BRDN00012	57	78	348	184	208	16
AGCCCCCACA	BRDN00012	244	123	132	389	681	6
AGCCCCCATA	BRDN00012	497	353	363	1216	1315	46
AGCCCCCATA	BRDN00012	492	419	400	1043	772	91
AGCCCCCGC	BRDN00012	338	384	1656	709	703	74
AGCCCCGCT	BRDN00011	278	213	69	649	351	12
AGCCCCGAG	BRDN00011	321	292	292	417	361	29
AGCCCCGCA	BRDN00011	305	1	1	614	1	0
AGCCCCGCT	BRDN00011	385	327	3946	494	309	44
AGCCCCGGA	BRDN00011	197	42	0	379	121	7
AGCCCCGGA	BRDN00012	119	42	13	178	220	21
AGCCCCGGA	BRDN00012	343	305	1493	678	586	57
AGCCCCGGG	BRDN00011	98	76	519	225	246	0
AGCCCCGTG	BRDN00011	417	461	530	975	980	76
AGCCCCGTG	BRDN00011	338	111	51	918	682	22
AGCCCCTAG	BRDN00011	228	83	193	280	484	19
AGCCCCTGC	BRDN00012	235	131	32	521	719	0
AGCCCGAAC	BRDN00012	281	437	71	668	1207	25
AGCCCGAAG	BRDN00011	568	380	1195	1009	576	44
AGCCCGACT	BRDN00011	108	110	90	334	216	20
AGCCCGAGA	BRDN00011	175	167	427	301	451	6
AGCCCGAGA	BRDN00012	353	396	427	769	808	61
AGCCCGAGA	BRDN00012	252	101	221	563	911	34
AGCCCGAGA	BRDN00012	396	392	416	786	769	16
AGCCCGAGC	BRDN00011	172	155	436	256	782	12
AGCCCGAGT	BRDN00012	146	82	16	167	75	0
AGCCCGATG	BRDN00011	451	390	431	929	1037	38
AGCCCGCCA	BRDN00012	699	519	704	1057	1295	92
AGCCCGCCA	BRDN00012	179	108	203	429	315	21
AGCCCGCGG	BRDN00012	506	447	481	903	1175	19
AGCCCGCTG	BRDN00011	512	542	368	952	1262	66
AGCCCGCTT	BRDN00012	137	113	409	269	307	2
AGCCCGGAA	BRDN00011	351	252	1094	424	89	39
AGCCCGGAG	BRDN00011	206	125	72	240	290	6
AGCCCGGAG	BRDN00011	170	94	0	288	442	0
AGCCCGGTC	BRDN00011	324	248	116	474	331	21
AGCCCGTGG	BRDN00012	431	495	1027	695	1275	155
AGCCCGTTG	BRDN00011	543	532	11	1126	1237	20
AGCCCGTTT	BRDN00012	448	370	1338	799	624	15
AGCCCTACC	BRDN00012	383	3	109	568	364	0
AGCCCTACC	BRDN00012	345	349	365	791	296	59
AGCCCTACT	BRDN00012	308	320	1481	419	395	19
AGCCCTATC	BRDN00011	404	400	1643	705	832	54
AGCCCTATC	BRDN00011	183	261	1083	410	569	22
AGCCCTCAA	BRDN00012	220	103	74	342	391	0
AGCCCTCTA	BRDN00012	86	96	0	212	194	12
AGCCCTGAT	BRDN00011	100	130	70	194	274	6
AGCCCTGCC	BRDN00011	132	90	166	318	190	78
AGCCCTGGA	BRDN00012	143	105	45	455	348	57
AGCCCTGTT	BRDN00012	178	124	357	329	610	5
AGCCCTTAA	BRDN00012	148	122	37	516	599	87
AGCCCTTAC	BRDN00012	228	0	0	537	311	0
AGCCCTTAG	BRDN00011	171	147	117	290	284	0
AGCCCTTCC	BRDN00012	548	373	355	939	983	28
AGCCCTTCT	BRDN00011	225	231	563	482	624	34
AGCCCTTCT	BRDN00012	170	226	3030	310	221	55
AGCCCTTCT	BRDN00012	370	239	1441	773	694	56
AGCCGAAAT	BRDN00012	321	45	1	587	358	0
AGCCGAACA	BRDN00012	194	130	59	589	531	0
AGCCGAACA	BRDN00012	92	36	1	181	161	21
AGCCGAAGG	BRDN00011	108	80	0	319	345	4
AGCCGAAGG	BRDN00011	659	604	3185	1091	1065	66
AGCCGAATT	BRDN00012	296	239	655	576	714	32
AGCCGACAA	BRDN00012	212	196	162	287	276	23
AGCCGACAA	BRDN00011	113	106	1	212	347	0
AGCCGACAT	BRDN00011	298	233	2061	335	493	42
AGCCGACTT	BRDN00012	82	84	11945	198	101	164
AGCCGAGAC	BRDN00011	183	207	52	386	420	45
AGCCGAGAC	BRDN00011	441	476	585	676	1141	16
AGCCGAGAG	BRDN00011	204	196	340	316	431	23
AGCCGAGCA	BRDN00012	220	196	41	382	549	8
AGCCGAGGC	BRDN00012	199	260	40	408	193	31
AGCCGAGTA	BRDN00012	148	114	247	185	169	87
AGCCGAGTC	BRDN00011	163	182	470	307	178	0
AGCCGATCT	BRDN00012	202	183	368	333	806	59
AGCCGCAAA	BRDN00012	107	86	31	305	316	25
AGCCGCAGC	BRDN00012	697	697	4600	1320	1664	124
AGCCGCAGC	BRDN00012	187	202	86	346	144	0
AGCCGCAGG	BRDN00012	512	434	759	943	884	64
AGCCGCAGT	BRDN00011	182	131	14	289	123	8
AGCCGCCAA	BRDN00011	614	459	1329	1160	1189	15
AGCCGCCAT	BRDN00012	132	106	339	271	169	28
AGCCGCCCC	BRDN00011	402	340	126	884	415	8
AGCCGCCCG	BRDN00012	284	316	617	599	423	42
AGCCGCCTC	BRDN00012	658	346	833	783	1050	76
AGCCGCGAG	BRDN00012	104	105	8	167	275	7
AGCCGCGAT	BRDN00011	232	229	681	244	412	47
AGCCGCGCC	BRDN00011	149	149	44	323	199	4
AGCCGCGGA	BRDN00012	254	414	800	560	501	78
AGCCGCGGT	BRDN00012	239	208	467	396	839	30
AGCCGCGTC	BRDN00012	95	10	16	151	192	26
AGCCGCTAC	BRDN00011	243	252	85	438	559	21
AGCCGCTAC	BRDN00012	181	149	104	225	448	9
AGCCGCTCC	BRDN00012	244	192	1361	639	581	52
AGCCGCTCC	BRDN00012	202	176	125	363	370	7
AGCCGCTTA	BRDN00012	346	350	572	589	383	66
AGCCGCTTA	BRDN00012	125	157	409	247	143	3
AGCCGCTTC	BRDN00011	408	395	754	704	705	39
AGCCGGAAA	BRDN00012	89	12	26	869	908	0
AGCCGGAAG	BRDN00011	81	56	1013	163	108	5
AGCCGGAGA	BRDN00011	388	366	5	780	702	17
AGCCGGAGA	BRDN00011	194	104	282	308	592	12
AGCCGGAGA	BRDN00011	166	55	472	365	215	4
AGCCGGATC	BRDN00012	274	333	2107	665	726	41
AGCCGGATG	BRDN00011	222	147	751	312	315	53
AGCCGGCAA	BRDN00011	220	218	1866	335	483	23
AGCCGGCAT	BRDN00011	175	154	451	382	551	39
AGCCGGCAT	BRDN00012	166	192	168	291	282	0
AGCCGGCCA	BRDN00012	99	93	175	236	154	28
AGCCGGCTG	BRDN00011	362	617	2236	724	893	108
AGCCGGCTG	BRDN00012	216	186	133	749	762	17
AGCCGGCTC	BRDN00012	12	0	0	234	0	0
AGCCGGCTT	BRDN00012	768	710	2269	1181	1521	60
AGCCGGGAA	BRDN00011	67	62	88	150	243	0
AGCCGGGAC	BRDN00011	415	391	502	741	904	115
AGCCGGGCC	BRDN00012	458	286	1894	756	982	83
AGCCGGGCG	BRDN00012	393	356	1154	805	1203	23
AGCCGGGCT	BRDN00011	140	95	83	313	245	2
AGCCGGGTT	BRDN00012	320	525	4627	818	959	81
AGCCGGTCA	BRDN00011	615	112	49	958	608	32
AGCCGGTCC	BRDN00012	393	502	545	928	1360	69
AGCCGGTCC	BRDN00012	93	139	262	306	504	9
AGCCGGTGA	BRDN00012	184	115	100	264	400	36
AGCCGGTGT	BRDN00012	119	7	0	236	199	0
AGCCGTAAT	BRDN00012	194	281	486	340	333	35
AGCCGTACC	BRDN00012	370	328	423	489	827	13
AGCCGTACT	BRDN00012	288	211	1067	604	637	49
AGCCGTAGT	BRDN00011	259	153	480	504	414	21
AGCCGTATG	BRDN00012	209	191	130	319	320	3
AGCCGTCAG	BRDN00011	91	55	27	178	320	0
AGCCGTCCG	BRDN00012	145	137	706	477	181	51
AGCCGTCTG	BRDN00012	234	190	162	412	565	33

AGCCGTCTG	BRDN00011	32	27	1	106	114	22
AGCCGTGAT	BRDN00011	214	137	37	254	192	0
AGCCGTGCA	BRDN00011	140	119	187	246	281	0
AGCCGTGGA	BRDN00011	256	110	155	331	403	7
AGCCGTGGT	BRDN00012	143	85	151	333	327	8
AGCCGTGTA	BRDN00011	241	221	461	371	136	8
AGCCGTGTC	BRDN00012	527	464	335	786	915	57
AGCCGTGTG	BRDN00011	262	209	647	541	298	43
AGCCGTTGC	BRDN00012	141	107	0	193	208	0
AGCCTAACT	BRDN00012	352	23	40	965	937	0
AGCCTAAGA	BRDN00012	180	122	950	337	249	15
AGCCTACAA	BRDN00011	71	66	23	160	49	21
AGCCTACAA	BRDN00012	244	249	321	450	486	8
AGCCTACAG	BRDN00012	324	265	1003	604	615	47
AGCCTACAG	BRDN00011	308	151	655	423	344	12
AGCCTACAG	BRDN00012	561	491	161	786	1145	25
AGCCTACAT	BRDN00011	174	164	410	254	154	8
AGCCTATGG	BRDN00012	173	254	729	266	233	15
AGCCTATGG	BRDN00012	211	187	132	282	310	43
AGCCTATTG	BRDN00012	477	431	927	942	1074	87
AGCCTCAAG	BRDN00012	396	257	691	827	721	31
AGCCTCAGA	BRDN00012	336	194	202	552	393	17
AGCCTCCACA	BRDN00012	204	171	375	494	301	33
AGCCTCCACA	BRDN00011	141	118	150	342	274	58
AGCCTCCACA	BRDN00011	351	289	462	752	780	73
AGCCTCCAG	BRDN00011	194	168	236	315	223	23
AGCCTCCATA	BRDN00011	322	325	227	536	497	33
AGCCTCCATT	BRDN00011	241	180	422	474	318	46
AGCCTCCTG	BRDN00012	307	249	524	528	506	16
AGCCTCCTTA	BRDN00011	108	151	297	324	308	5
AGCCTCGAA	BRDN00012	407	339	326	874	1324	17
AGCCTCGAA	BRDN00011	415	340	100	698	528	6
AGCCTCGAG	BRDN00011	264	325	984	500	1041	50
AGCCTCGCG	BRDN00011	254	248	512	413	377	12
AGCCTCGCT	BRDN00011	476	773	5629	1058	2138	325
AGCCTCGCT	BRDN00012	160	269	292	282	162	38
AGCCTCGGG	BRDN00012	379	315	384	507	432	20
AGCCTCGGG	BRDN00011	307	226	56	528	434	41
AGCCTCTAA	BRDN00011	277	210	123	582	705	28
AGCCTCTAA	BRDN00011	134	143	11	248	142	4
AGCCTCTGG	BRDN00012	72	77	0	240	139	0
AGCCTCTGT	BRDN00011	110	37	16	200	210	0
AGCCTCTTG	BRDN00011	141	102	202	254	261	17
AGCCTCTTG	BRDN00012	265	311	513	296	354	15
AGCCTGAAA	BRDN00011	170	101	25	255	236	0
AGCCTGAAG	BRDN00012	165	93	158	263	271	0
AGCCTGACA	BRDN00012	367	401	46	904	736	18
AGCCTGACA	BRDN00011	181	149	223	346	322	16
AGCCTGACG	BRDN00012	181	140	247	347	243	4
AGCCTGAGC	BRDN00012	82	68	194	300	390	5
AGCCTGATG	BRDN00011	485	654	869	995	684	48
AGCCTGCAA	BRDN00012	344	50	485	808	755	7
AGCCTGCAA	BRDN00011	321	333	490	521	778	4
AGCCTGCAC	BRDN00012	216	211	331	506	511	8
AGCCTGCAG	BRDN00011	128	158	248	276	238	8
AGCCTGCAG	BRDN00011	212	109	141	395	356	51
AGCCTGCAT	BRDN00011	88	159	614	315	473	20
AGCCTGCTA	BRDN00012	180	122	60	257	296	21
AGCCTGCTG	BRDN00011	422	299	1503	811	696	83
AGCCTGGGG	BRDN00011	127	140	360	279	149	20
AGCCTGGTA	BRDN00011	112	149	31	336	196	9
AGCCTGGTG	BRDN00011	269	264	2275	435	629	61
AGCCTGGTT	BRDN00011	197	189	583	377	241	13
AGCCTGTAA	BRDN00012	389	349	507	797	1771	40
AGCCTGTAT	BRDN00012	640	492	1471	914	845	44
AGCCTGTCA	BRDN00012	403	385	405	739	770	62
AGCCTGTCA	BRDN00012	207	198	243	269	404	19
AGCCTGTGA	BRDN00011	112	0	0	1141	489	0
AGCCTGTGG	BRDN00012	174	299	106	278	192	76
AGCCTGTGG	BRDN00012	384	269	1685	673	642	34
AGCCTTAAT	BRDN00011	218	202	166	462	452	87
AGCCTTACA	BRDN00011	181	200	535	278	495	30
AGCCTTATA	BRDN00011	153	43	264	300	476	11
AGCCTTATG	BRDN00012	62	3	0	381	126	0
AGCCTTCAA	BRDN00012	635	591	1069	1267	1443	131
AGCCTTCAA	BRDN00012	125	74	1	309	126	30
AGCCTTCACA	BRDN00011	328	489	744	721	757	18
AGCCTTCAG	BRDN00011	167	98	14	328	310	0
AGCCTTCATG	BRDN00011	186	167	454	395	557	15
AGCCTTCCG	BRDN00012	144	118	490	349	444	10
AGCCTTCCTC	BRDN00012	553	553	2208	1037	850	43
AGCCTTCCTG	BRDN00012	263	204	155	565	605	9
AGCCTTGAA	BRDN00011	87	0	0	197	142	0
AGCCTTGAC	BRDN00011	177	206	174	292	177	8
AGCCTTGAG	BRDN00012	596	629	1018	1026	936	47
AGCCTTGGC	BRDN00011	445	425	422	911	584	68
AGCCTTGGG	BRDN00011	255	229	320	463	276	107
AGCCTTGGG	BRDN00011	150	111	167	167	107	16
AGCCTTGTA	BRDN00011	197	117	446	461	465	23
AGCCTTGTC	BRDN00012	4	7	0	436	18	0
AGCGAAACT	BRDN00011	249	255	123	428	386	0
AGCGAAAGC	BRDN00012	175	287	90	297	411	71
AGCGAACAG	BRDN00012	160	158	236	213	42	21
AGCGAACGC	BRDN00012	458	249	1363	856	479	42
AGCGAATTT	BRDN00012	297	323	482	639	505	3
AGCGACAAC	BRDN00011	493	513	1433	732	489	68
AGCGACACC	BRDN00011	118	123	278	161	168	0
AGCGACACG	BRDN00012	132	51	0	241	380	1
AGCGACCGC	BRDN00011	153	175	495	371	345	12
AGCGACCGG	BRDN00012	334	423	1796	774	880	39
AGCGACGAC	BRDN00012	189	102	1277	381	518	7
AGCGACGAG	BRDN00011	285	265	338	532	571	4
AGCGACGCC	BRDN00011	97	82	504	142	134	40
AGCGACGCT	BRDN00011	200	104	0	458	501	12
AGCGACGCT	BRDN00011	126	129	0	227	146	0
AGCGACTAC	BRDN00011	169	130	1	302	356	0
AGCGAGACA	BRDN00011	184	131	7	147	223	23
AGCGAGAGC	BRDN00011	182	370	420	407	246	81
AGCGAGAGC	BRDN00011	125	86	10	272	213	5
AGCGAGGAC	BRDN00012	283	1	2	803	219	0
AGCGAGGAC	BRDN00012	95	17	18	635	428	0
AGCGATACT	BRDN00012	149	47	89	183	166	19
AGCGATAGA	BRDN00012	233	1	0	587	114	0
AGCGATCTG	BRDN00012	599	565	579	970	1213	65
AGCGATGAT	BRDN00011	251	359	1202	385	540	23
AGCGATGGA	BRDN00011	154	67	523	421	338	26
AGCGATGGT	BRDN00011	130	89	0	327	149	0
AGCGATTCA	BRDN00012	541	517	766	710	749	47
AGCGATTGT	BRDN00011	116	25	0	296	305	0
AGCGATTTA	BRDN00012	259	284	654	444	370	50
AGCGCAAAG	BRDN00011	242	256	183	483	395	2
AGCGCAAGC	BRDN00011	187	127	1330	287	823	14
AGCGCACAC	BRDN00012	94	103	940	251	158	7
AGCGCACCG	BRDN00011	547	490	470	901	961	0
AGCGCACGT	BRDN00011	312	200	364	618	338	20
AGCGCACTG	BRDN00012	150	158	214	259	104	6
AGCGCAGAG	BRDN00012	105	32	234	100	193	6
AGCGCAGAT	BRDN00012	756	778	2314	1011	1290	48
AGCGCAGGA	BRDN00011	259	212	436	370	374	11
AGCGCAGGG	BRDN00011	341	162	720	631	660	25
AGCGCATCA	BRDN00011	495	445	463	976	946	62
AGCGCATGG	BRDN00011	199	96	2686	292	173	111
AGCGCATGG	BRDN00012	183	86	941	369	308	6
AGCGCCAAC	BRDN00012	119	64	41	298	301	5
AGCGCCCCA	BRDN00011	331	212	123	431	871	16
AGCGCCCCT	BRDN00011	130	91	1012	265	188	40
AGCGCCGAA	BRDN00011	104	74	73	169	61	23
AGCGCCGGA	BRDN00012	229	125	27	407	362	0
AGCGCCGGC	BRDN00012	478	607	981	1073	1028	67
AGCGCCGGG	BRDN00011	289	182	81	316	463	27
AGCGCCGGT	BRDN00011	109	69	270	330	201	12
AGCGCCTCC	BRDN00011	195	0	73	412	213	0
AGCGCCTCC	BRDN00012	118	77	1249	208	191	15
AGCGCCTCT	BRDN00012	300	215	97	535	287	0
AGCGCCTGC	BRDN00011	141	129	42	180	147	7
AGCGCGAAG	BRDN00012	324	406	928	574	479	37
AGCGCGAAG	BRDN00011	68	32	12	154	188	0
AGCGCGACG	BRDN00011	295	307	244	634	640	18
AGCGCGACT	BRDN00012	92	13	36	194	116	0
AGCGCGAGC	BRDN00011	420	385	1092	926	501	18
AGCGCGATG	BRDN00011	260	263	292	410	467	30
AGCGCGATG	BRDN00012	396	342	468	691	892	39
AGCGCGCAA	BRDN00011	331	359	300	698	885	66
AGCGCGCGA	BRDN00011	251	265	189	394	475	50
AGCGCGCGT	BRDN00012	47	47	258	168	240	5
AGCGCGCTC	BRDN00012	131	121	1	300	317	0
AGCGCGGAT	BRDN00011	203	163	80	323	245	38
AGCGCGGCG	BRDN00011	114	123	278	284	227	2
AGCGCGGGT	BRDN00012	90	109	305	235	175	7
AGCGCGGTG	BRDN00012	558	422	412	842	756	80
AGCGCGTGA	BRDN00011	237	349	170	532	302	18
AGCGCGTTG	BRDN00012	215	183	201	414	293	13
AGCGCTACA	BRDN00012	162	205	238	215	96	86
AGCGCTAGG	BRDN00011	399	381	1864	788	795	46
AGCGCTCAA	BRDN00011	133	158	1984	188	1292	71
AGCGCTCAA	BRDN00012	149	357	31	222	224	15
AGCGCTCCC	BRDN00012	399	406	470	628	874	22
AGCGCTCGT	BRDN00011	166	197	780	272	212	43
AGCGCTCTC	BRDN00011	121	97	277	237	249	0
AGCGCTCTG	BRDN00012	990	764	826	1440	912	92
AGCGCTGAT	BRDN00011	55	24	1	134	159	0
AGCGCTGGA	BRDN00011	235	213	577	495	373	93
AGCGCTGGC	BRDN00011	250	242	31	476	475	7
AGCGCTGGG	BRDN00011	223	171	70	465	527	5
AGCGCTGGT	BRDN00011	107	65	23	495	469	18
AGCGCTTAT	BRDN00012	118	192	84	161	146	4
AGCGCTTTA	BRDN00011	377	307	311	753	1031	49
AGCGGAAGA	BRDN00012	118	127	481	375	353	14
AGCGGACCC	BRDN00011	250	124	41	415	489	10
AGCGGACGT	BRDN00011	276	324	273	541	896	47
AGCGGAGAC	BRDN00011	53	74	0	301	208	0
AGCGGAGAC	BRDN00011	207	115	8	299	72	0
AGCGGAGAC	BRDN00011	85	108	734	131	111	19
AGCGGCAAC	BRDN00011	38	0	0	162	62	0
AGCGGCACG	BRDN00011	315	286	68	498	597	0
AGCGGCAGA	BRDN00012	467	406	658	761	653	52
AGCGGCAGC	BRDN00011	115	137	52	180	122	181
AGCGGCATT	BRDN00011	178	1	0	555	408	0
AGCGGCCAG	BRDN00012	327	249	548	521	783	39
AGCGGCCCG	BRDN00011	579	538	232	992	906	72
AGCGGCCGA	BRDN00012	238	259	410	418	291	6
AGCGGCGAC	BRDN00011	102	151	0	250	317	0
AGCGGCGAC	BRDN00012	174	89	0	391	325	16
AGCGGCGAT	BRDN00011	228	16	0	426	535	0
AGCGGCGCA	BRDN00011	88	95	10	227	146	16
AGCGGCGCT	BRDN00011	90	62	149	218	186	12
AGCGGCGGA	BRDN00011	393	377	277	767	608	26
AGCGGCGGC	BRDN00012	134	129	571	273	266	7
AGCGGCGGC	BRDN00012	96	98	30	179	65	3
AGCGGCGGC	BRDN00011	603	509	430	1055	944	27
AGCGGCGGC	BRDN00012	208	58	38	364	290	0
AGCGGCTCA	BRDN00011	150	105	689	270	116	5
AGCGGCTCC	BRDN00012	175	129	0	355	204	18
AGCGGCTGG	BRDN00012	178	150	12	279	341	11
AGCGGCTGT	BRDN00012	161	144	38	283	162	11
AGCGGCTTA	BRDN00011	68	63	31	139	132	6
AGCGGCTTA	BRDN00012	250	381	391	614	1068	17
AGCGGCTTG	BRDN00012	245	152	952	292	698	30
AGCGGCTTG	BRDN00011	231	162	315	374	756	0
AGCGGGAAA	BRDN00012	319	346	1648	749	592	58
AGCGGGAAT	BRDN00011	113	147	466	265	222	12
AGCGGGACT	BRDN00011	179	72	140	413	228	4
AGCGGGAGC	BRDN00011	334	297	549	667	915	13
AGCGGGAGT	BRDN00012	162	236	682	551	396	0
AGCGGGGAA	BRDN00011	69	0	0	158	66	0
AGCGGGGAC	BRDN00012	466	221	213	798	594	19
AGCGGGGAC	BRDN00012	336	298	567	559	245	16
AGCGGGGCA	BRDN00012	323	347	76	656	374	60
AGCGGGTCA	BRDN00011	254	227	411	435	496	13
AGCGGGTGA	BRDN00011	159	155	1721	472	458	28
AGCGGGTGA	BRDN00012	336	208	338	471	391	30
AGCGGGTGC	BRDN00012	331	316	335	637	560	20
AGCGGGTTG	BRDN00011	137	92	69	383	408	0
AGCGGTAAC	BRDN00011	33	45	5	138	355	0
AGCGGTACA	BRDN00011	148	89	38	243	245	11
AGCGGTACC	BRDN00011	142	0	0	818	229	0
AGCGGTAGT	BRDN00012	270	264	495	717	760	16
AGCGGTCAA	BRDN00011	152	96	374	282	216	4
AGCGGTCAC	BRDN00011	124	120	240	370	236	0
AGCGGTCGG	BRDN00011	120	128	250	276	134	0
AGCGGTGCC	BRDN00012	442	261	1093	904	549	178
AGCGTAAGC	BRDN00011	274	362	260	641	791	98
AGCGTAAGG	BRDN00011	290	111	39	715	424	42
AGCGTAAGT	BRDN00011	20	7	0	346	185	0
AGCGTACCT	BRDN00012	2	7	0	169	48	0
AGCGTATTC	BRDN00012	69	113	0	668	356	0
AGCGTCACC	BRDN00011	113	102	1	182	179	0
AGCGTCATA	BRDN00012	296	130	136	466	387	10
AGCGTCATC	BRDN00012	172	342	60	466	685	15
AGCGTCCCT	BRDN00012	268	145	109	485	680	5
AGCGTCCGA	BRDN00012	229	107	419	266	585	28
AGCGTCGGG	BRDN00012	239	204	743	594	671	40
AGCGTCGTA	BRDN00011	132	0	2	220	137	0
AGCGTCGTT	BRDN00012	355	326	431	859	806	25
AGCGTCTAC	BRDN00011	151	151	341	342	272	21
AGCGTCTGC	BRDN00011	132	93	690	353	23	0
AGCGTCTTG	BRDN00011	387	549	1476	579	706	38
AGCGTGAAC	BRDN00011	63	18	7	172	281	20
AGCGTGAAG	BRDN00012	72	116	54	201	173	28
AGCGTGAAG	BRDN00012	97	151	204	181	8	19
AGCGTGCCA	BRDN00012	444	567	663	1000	725	61
AGCGTGGAA	BRDN00011	233	137	24	355	496	33
AGCGTGGTA	BRDN00012	203	292	718	448	852	10
AGCGTGGTA	BRDN00011	431	388	1218	606	489	62
AGCGTGTCT	BRDN00012	438	400	756	656	838	38
AGCGTGTGA	BRDN00012	255	182	37	305	94	18
AGCGTTAAA	BRDN00011	310	181	157	308	348	40
AGCGTTCAG	BRDN00012	227	181	319	299	319	13
AGCGTTCGT	BRDN00012	371	496	1378	738	813	34
AGCGTTCTC	BRDN00012	132	171	668	306	275	3
AGCGTTCTC	BRDN00011	148	60	0	253	582	8
AGCGTTGAT	BRDN00011	120	180	29	224	265	43
AGCGTTTCG	BRDN00011	304	262	172	534	453	5
AGCGTTTGC	BRDN00012	663	575	977	923	916	42
AGCTAAAAT	BRDN00011	359	144	24	586	477	0
AGCTAAAGG	BRDN00012	160	181	1	333	170	9
AGCTAAAGT	BRDN00011	300	398	351	456	757	54
AGCTAACAA	BRDN00011	396	426	273	879	678	34
AGCTAACCT	BRDN00011	99	148	62	203	501	0
AGCTAAGCG	BRDN00012	131	109	525	356	265	40
AGCTAAGTG	BRDN00011	155	25	28	494	930	0
AGCTAATAA	BRDN00011	296	20	0	873	739	0
AGCTAATAC	BRDN00011	171	193	92	250	97	0
AGCTAATCG	BRDN00011	155	159	5	358	324	5
AGCTAATGG	BRDN00011	143	245	5336	348	259	152
AGCTAATTA	BRDN00012	329	209	19	566	508	79
AGCTACACT	BRDN00012	174	305	140	522	520	4
AGCTACACTT	BRDN00012	231	200	122	610	432	98
AGCTACAGA	BRDN00011	99	86	0	284	246	0
AGCTACAGG	BRDN00011	210	220	1	406	449	0
AGCTACAGG	BRDN00012	462	547	650	761	877	67
AGCTACAGG	BRDN00011	132	0	0	1034	251	0
AGCTACCAA	BRDN00011	52	23	0	618	447	0
AGCTACCAT	BRDN00012	593	398	626	1001	936	46
AGCTACCCG	BRDN00011	214	126	1708	284	436	32
AGCTACCTG	BRDN00011	176	98	18	315	178	10
AGCTACGGC	BRDN00011	553	454	684	958	1033	71
AGCTACGTC	BRDN00011	276	167	62	543	385	0
AGCTACGTG	BRDN00011	102	140	66	286	472	210
AGCTACGTG	BRDN00012	229	168	24	418	250	6
AGCTACTAC	BRDN00012	490	421	1711	1033	1228	66
AGCTACTAT	BRDN00011	118	181	0	220	77	0
AGCTACTAT	BRDN00012	203	133	9	378	158	6
AGCTACTCA	BRDN00011	117	0	0	210	88	0
AGCTACTGC	BRDN00011	137	109	327	312	224	1
AGCTACTGG	BRDN00011	170	145	140	416	207	24
AGCTACTGG	BRDN00011	151	103	1109	217	232	7
AGCTAGAGA	BRDN00012	139	171	166	351	417	4
AGCTAGCAA	BRDN00011	444	308	112	716	859	5
AGCTAGCAG	BRDN00012	513	451	504	856	735	132
AGCTAGCAG	BRDN00011	123	129	253	193	178	19
AGCTAGCCT	BRDN00012	193	168	384	327	222	48
AGCTAGCGA	BRDN00012	535	576	1083	814	992	91
AGCTAGCTA	BRDN00011	240	13	0	477	366	0
AGCTAGGAT	BRDN00011	97	117	0	200	399	0
AGCTAGGCA	BRDN00011	380	236	625	479	793	25
AGCTAGGTC	BRDN00011	136	15	0	657	184	0
AGCTAGTCT	BRDN00011	233	179	2267	500	407	46
AGCTATAGC	BRDN00012	241	164	5807	285	71	106
AGCTATAGG	BRDN00012	271	249	3469	688	598	75
AGCTATATG	BRDN00012	283	300	558	556	480	24
AGCTATCAA	BRDN00012	72	19	0	496	40	0
AGCTATCCG	BRDN00011	195	30	0	316	15	0
AGCTATCCT	BRDN00012	474	518	353	841	897	31
AGCTATCGA	BRDN00011	227	393	1493	676	790	65
AGCTATCGG	BRDN00012	319	152	25	410	658	14
AGCTATCTA	BRDN00011	147	6	0	289	176	0
AGCTATGAG	BRDN00012	459	390	1703	921	944	85
AGCTATGCC	BRDN00011	235	279	430	268	583	26
AGCTATGCG	BRDN00011	390	14	0	858	949	0
AGCTATGCG	BRDN00012	354	315	949	593	536	12
AGCTATGGA	BRDN00011	148	88	108	296	340	4
AGCTATGGC	BRDN00011	256	0	0	534	422	0
AGCTATGGC	BRDN00011	151	259	92	374	346	47
AGCTATTAA	BRDN00012	166	188	0	281	223	0
AGCTATTCT	BRDN00011	120	222	26	312	304	0
AGCTATTGA	BRDN00011	130	46	13	241	307	0
AGCTATTTC	BRDN00012	134	132	9	267	250	3
AGCTATTTCT	BRDN00011	257	124	293	328	431	0
AGCTCAAAA	BRDN00012	316	185	229	465	661	1
AGCTCAAAG	BRDN00011	175	281	789	461	1553	27
AGCTCAACA	BRDN00011	174	272	3365	390	316	64
AGCTCAACG	BRDN00012	805	585	4748	1193	1264	141
AGCTCAAGA	BRDN00012	470	459	171	738	874	68
AGCTCAAGC	BRDN00012	100	47	469	206	561	0
AGCTCAAGG	BRDN00012	209	251	154	580	365	18
AGCTCAAGG	BRDN00011	205	195	305	471	568	15
AGCTCAATA	BRDN00012	587	556	361	1010	842	48
AGCTCACAG	BRDN00012	458	344	70	1028	1043	41
AGCTCAGAT	BRDN00011	291	463	153	639	489	20
AGCTCAGGA	BRDN00011	161	109	248	350	302	0
AGCTCAGGC	BRDN00011	155	154	119	283	190	15
AGCTCAGGT	BRDN00011	335	469	54	727	644	34
AGCTCATAA	BRDN00012	44	19	1	370	181	0
AGCTCATAG	BRDN00012	134	10	10	259	67	0
AGCTCATCC	BRDN00012	110	124	3594	318	381	171
AGCTCATCG	BRDN00012	321	439	266	620	670	27
AGCTCATCG	BRDN00011	358	288	530	631	668	46
AGCTCATGC	BRDN00012	435	195	64	832	573	50
AGCTCATTG	BRDN00011	155	103	0	317	91	0
AGCTCCAAA	BRDN00012	119	141	1	375	320	0
AGCTCCAAA	BRDN00011	74	0	0	140	205	0
AGCTCCACA	BRDN00012	149	134	34	231	155	5
AGCTCCAGC	BRDN00012	190	187	203	352	420	40
AGCTCCAGC	BRDN00012	89	38	18	438	186	19
AGCTCCAGT	BRDN00012	175	221	50	150	78	42
AGCTCCATC	BRDN00012	746	616	1664	996	1761	55
AGCTCCATG	BRDN00011	166	32	143	278	354	5
AGCTCCCAA	BRDN00011	427	272	285	780	779	29
AGCTCCCCTT	BRDN00011	176	142	5753	384	343	20
AGCTCCCGA	BRDN00012	823	578	816	1392	1045	131
AGCTCCCGG	BRDN00012	514	458	294	780	886	185
AGCTCCCGT	BRDN00012	317	316	637	816	521	48
AGCTCCCTTT	BRDN00011	333	7	1	666	536	0
AGCTCCGCA	BRDN00012	55	0	1	542	445	0
AGCTCCGGG	BRDN00012	215	123	563	1148	545	25
AGCTCCGTC	BRDN00012	391	403	300	617	1019	8
AGCTCCGTG	BRDN00012	291	312	327	565	666	70
AGCTCCTAG	BRDN00011	9	0	0	266	39	0
AGCTCCTGC	BRDN00012	65	157	18	151	138	0
AGCTCCTGG	BRDN00011	138	31	67	286	169	13
AGCTCCTTCA	BRDN00011	72	37	0	509	167	24
AGCTCCTTCT	BRDN00011	160	208	110	364	284	15
AGCTCGAAC	BRDN00011	103	35	0	302	205	0
AGCTCGACG	BRDN00012	274	260	47	478	958	43
AGCTCGACG	BRDN00011	521	228	584	733	598	49
AGCTCGAGT	BRDN00011	137	97	1473	222	337	45
AGCTCGATA	BRDN00012	103	81	136	239	137	4
AGCTCGATC	BRDN00012	507	286	4212	977	1254	29
AGCTCGCAC	BRDN00011	237	196	312	460	297	0
AGCTCGCAG	BRDN00011	179	0	0	437	215	0
AGCTCGCAG	BRDN00011	444	284	561	819	1157	52
AGCTCGCCA	BRDN00012	884	797	870	1451	1234	172
AGCTCGCCA	BRDN00011	139	256	0	384	230	12
AGCTCGCTG	BRDN00012	553	726	2751	1118	1428	113
AGCTCGGAG	BRDN00012	507	499	56	942	1275	39
AGCTCGGAT	BRDN00012	138	21	0	322	155	0
AGCTCGGCC	BRDN00012	204	133	72	358	93	6
AGCTCGGGG	BRDN00011	351	297	381	551	716	21
AGCTCGTAA	BRDN00011	175	177	276	482	333	11
AGCTCTAAA	BRDN00012	128	57	0	257	550	0
AGCTCTACG	BRDN00011	314	208	688	551	439	26
AGCTCTACTC	BRDN00012	357	306	404	858	868	25
AGCTCTACTC	BRDN00011	398	410	620	739	645	84
AGCTCTAGG	BRDN00011	20	5	0	158	48	0
AGCTCTATG	BRDN00012	299	171	89	389	176	4
AGCTCTCACT	BRDN00011	285	261	1452	362	186	22
AGCTCTCAG	BRDN00011	175	92	383	434	174	21
AGCTCTCATA	BRDN00012	393	352	317	742	848	121
AGCTCTCGA	BRDN00011	214	249	174	421	470	11
AGCTCTCGG	BRDN00011	279	249	429	500	433	19
AGCTCTCTG	BRDN00011	403	433	178	801	531	62
AGCTCTCTTA	BRDN00011	593	586	334	885	629	78
AGCTCTGAC	BRDN00011	115	54	1	543	271	0
AGCTCTGCA	BRDN00011	276	105	196	383	330	4
AGCTCTGCG	BRDN00012	247	325	516	438	777	5
AGCTCTGCT	BRDN00011	125	0	0	463	276	0
AGCTCTGCT	BRDN00011	280	195	1265	414	593	15
AGCTCTGCT	BRDN00011	242	300	829	389	482	47
AGCTCTGTG	BRDN00012	58	16	0	143	131	0
AGCTCTTCCA	BRDN00012	260	330	56	482	594	9
AGCTCTTCCA	BRDN00011	435	420	743	776	1268	37
AGCTCTTGG	BRDN00011	345	431	170	871	283	11
AGCTCTTGG	BRDN00011	209	121	36	386	237	4
AGCTCTTTA	BRDN00012	230	121	323	295	250	0
AGCTGAAAA	BRDN00012	263	119	286	548	312	0
AGCTGAAAA	BRDN00012	357	304	80	704	809	1
AGCTGAACA	BRDN00012	137	153	248	310	351	9
AGCTGAACC	BRDN00012	241	271	1247	498	441	27
AGCTGAACG	BRDN00011	324	232	138	795	858	36
AGCTGAAGC	BRDN00012	166	98	214	413	213	4
AGCTGAAGC	BRDN00012	582	565	1429	1309	1116	105
AGCTGAATC	BRDN00011	186	87	635	344	223	0
AGCTGAATG	BRDN00012	436	365	1285	993	1273	56
AGCTGAATT	BRDN00012	388	439	1440	964	592	55
AGCTGAATT	BRDN00012	186	218	143	260	313	0
AGCTGACAA	BRDN00011	176	1	0	632	480	0
AGCTGACAA	BRDN00011	595	553	381	1177	960	125
AGCTGACAG	BRDN00012	393	349	302	817	1047	56
AGCTGACCA	BRDN00011	303	288	19	604	636	14
AGCTGACCC	BRDN00011	80	28	1	192	180	0
AGCTGACCT	BRDN00012	186	308	150	473	300	16
AGCTGACCT	BRDN00011	101	41	1	226	409	0
AGCTGACTT	BRDN00011	287	17	0	650	482	1
AGCTGAGAA	BRDN00012	517	445	3618	1018	644	90
AGCTGAGAA	BRDN00012	66	107	547	297	373	24
AGCTGAGAC	BRDN00011	176	152	266	413	632	4
AGCTGAGAG	BRDN00012	105	70	457	242	80	7
AGCTGAGAG	BRDN00012	122	106	134	243	475	19
AGCTGAGCG	BRDN00011	199	296	193	325	186	57
AGCTGAGGG	BRDN00011	107	83	205	303	387	20
AGCTGAGGG	BRDN00012	174	118	15	361	149	6
AGCTGAGGT	BRDN00011	138	41	182	170	58	8
AGCTGATAT	BRDN00012	199	184	241	444	251	5
AGCTGATCT	BRDN00012	113	60	0	421	605	0
AGCTGATGG	BRDN00011	209	115	16	437	150	0
AGCTGATGG	BRDN00012	229	170	41	333	1050	4
AGCTGATGT	BRDN00011	545	560	225	1002	654	27
AGCTGATTA	BRDN00011	462	384	3387	894	812	60
AGCTGCAAC	BRDN00011	170	81	281	158	128	7
AGCTGCAAC	BRDN00012	617	606	1624	1134	741	45
AGCTGCAAC	BRDN00011	350	80	1	528	728	0
AGCTGCACA	BRDN00012	489	565	1754	824	857	104
AGCTGCACA	BRDN00011	104	243	169	245	253	23
AGCTGCACA	BRDN00012	40	22	1	378	136	0

AGCTGCAGA	BRDN00011	246	152	250	326	246	25
AGCTGCAGT	BRDN00012	57	44	40	171	68	0
AGCTGCCAA	BRDN00012	358	321	271	728	454	19
AGCTGCCAG	BRDN00012	337	454	833	770	756	55
AGCTGCCAG	BRDN00012	642	571	3856	1276	1523	115
AGCTGCCCA	BRDN00011	159	142	125	237	378	0
AGCTGCCCT	BRDN00011	318	356	67	585	736	33
AGCTGCCGA	BRDN00011	132	65	6	318	233	9
AGCTGCCTT	BRDN00011	473	524	359	826	1197	11
AGCTGCGAC	BRDN00011	190	167	611	374	109	60
AGCTGCGAC	BRDN00011	130	25	39	295	127	1
AGCTGCGCA	BRDN00012	510	515	467	1003	949	81
AGCTGCGCA	BRDN00012	244	229	411	276	293	5
AGCTGCGCC	BRDN00012	174	268	1163	444	470	2
AGCTGCGCG	BRDN00012	895	926	1305	1700	2718	43
AGCTGCGCG	BRDN00011	328	221	643	752	608	56
AGCTGCGGG	BRDN00012	484	267	536	939	961	40
AGCTGCGGT	BRDN00012	218	115	127	320	364	29
AGCTGCTAC	BRDN00012	290	134	211	666	390	1
AGCTGCTAG	BRDN00011	439	255	339	600	633	142
AGCTGCTAG	BRDN00011	137	72	132	290	303	1
AGCTGCTCA	BRDN00012	359	274	592	506	747	13
AGCTGCTCC	BRDN00012	414	297	295	1243	1235	25
AGCTGCTGA	BRDN00012	495	395	1351	908	1167	92
AGCTGCTGA	BRDN00011	159	0	1	314	63	26
AGCTGCTGC	BRDN00012	662	483	754	1033	956	50
AGCTGCTGT	BRDN00012	144	99	328	207	195	16
AGCTGCTGT	BRDN00012	385	344	330	794	791	40
AGCTGCTGT	BRDN00012	357	318	294	973	1058	6
AGCTGCTTA	BRDN00011	691	862	3875	1280	1056	71
AGCTGCTTC	BRDN00012	94	93	0	281	201	0
AGCTGGAAA	BRDN00012	160	142	103	348	367	19
AGCTGGAAC	BRDN00012	580	293	3939	1212	1265	86
AGCTGGAAC	BRDN00012	444	410	477	855	815	16
AGCTGGACA	BRDN00011	290	196	806	466	435	11
AGCTGGACA	BRDN00011	206	66	5	802	1153	0
AGCTGGACG	BRDN00011	139	87	983	339	445	5
AGCTGGACG	BRDN00011	104	36	19	272	250	15
AGCTGGACT	BRDN00012	313	321	261	504	742	31
AGCTGGAGA	BRDN00011	272	268	57	614	394	38
AGCTGGAGA	BRDN00012	321	326	855	686	847	89
AGCTGGAGC	BRDN00011	236	7	141	480	414	14
AGCTGGAGC	BRDN00012	490	579	494	852	692	88
AGCTGGAGG	BRDN00012	200	199	420	356	1324	16
AGCTGGAGG	BRDN00012	537	448	293	1032	934	9
AGCTGGAGT	BRDN00011	216	147	15	623	933	12
AGCTGGATA	BRDN00012	123	149	0	200	142	0
AGCTGGATC	BRDN00011	467	176	213	1006	538	12
AGCTGGCAA	BRDN00012	306	455	415	404	440	32
AGCTGGCAC	BRDN00011	70	22	1	340	385	0
AGCTGGCAG	BRDN00011	515	337	7457	789	2092	64
AGCTGGCAG	BRDN00011	205	193	461	448	327	22
AGCTGGCCA	BRDN00011	157	169	2049	233	255	28
AGCTGGCCC	BRDN00011	428	346	776	920	718	33
AGCTGGCCG	BRDN00011	157	161	35	318	288	18
AGCTGGCGT	BRDN00011	171	187	38	210	246	43
AGCTGGCTA	BRDN00012	427	262	1347	712	485	24
AGCTGGGAC	BRDN00012	227	175	15	365	380	7
AGCTGGGAC	BRDN00012	313	287	345	515	239	40
AGCTGGGCA	BRDN00011	533	578	1436	907	3055	103
AGCTGGGCT	BRDN00012	280	292	39	577	585	20
AGCTGGGTA	BRDN00012	131	146	511	379	278	21
AGCTGGGTA	BRDN00012	216	138	1	341	400	4
AGCTGGGTC	BRDN00012	147	68	886	231	230	23
AGCTGGTAG	BRDN00011	441	367	980	769	730	44
AGCTGGTCG	BRDN00012	93	91	876	180	104	35
AGCTGGTCG	BRDN00012	105	167	1769	282	280	22
AGCTGGTCT	BRDN00011	212	41	504	499	394	0
AGCTGGTGA	BRDN00011	101	169	5	239	272	0
AGCTGGTGA	BRDN00011	295	268	22	476	299	32
AGCTGGTGC	BRDN00011	250	306	94	482	421	38
AGCTGGTGG	BRDN00011	26	24	0	99	42	0
AGCTGGTGG	BRDN00012	447	368	108	709	397	0
AGCTGGTGG	BRDN00012	229	106	244	540	263	7
AGCTGGTGT	BRDN00011	113	0	14	269	307	0
AGCTGGTTG	BRDN00011	336	309	57	634	747	22
AGCTGTAAA	BRDN00012	66	64	0	135	134	0
AGCTGTACC	BRDN00011	385	285	743	528	636	0
AGCTGTACT	BRDN00012	133	108	186	249	65	5
AGCTGTAGC	BRDN00012	291	300	203	721	578	14
AGCTGTCAA	BRDN00012	300	197	19	905	394	21
AGCTGTCAG	BRDN00011	146	16	0	350	137	0
AGCTGTCAG	BRDN00011	374	424	651	839	1188	44
AGCTGTCCA	BRDN00011	580	457	415	1281	1314	53
AGCTGTCCC	BRDN00011	225	308	10	321	390	27
AGCTGTCCT	BRDN00012	521	396	116	1192	974	30
AGCTGTCGC	BRDN00012	217	168	960	329	443	14
AGCTGTCTC	BRDN00011	411	426	1232	785	1059	62
AGCTGTCTG	BRDN00011	103	119	34	202	224	11
AGCTGTCTG	BRDN00012	288	226	44	450	303	0
AGCTGTGAA	BRDN00012	297	219	195	425	292	0
AGCTGTGAA	BRDN00012	415	416	1456	653	846	24
AGCTGTGAG	BRDN00011	191	127	0	518	218	0
AGCTGTGAT	BRDN00011	409	400	1467	1004	1200	9
AGCTGTGCA	BRDN00011	195	134	0	489	601	12
AGCTGTGGA	BRDN00011	100	10	600	185	98	11
AGCTGTGGA	BRDN00012	485	411	1042	821	907	48
AGCTGTGGC	BRDN00011	282	282	28	433	313	32
AGCTGTGGC	BRDN00011	114	35	42	450	226	0
AGCTGTGTA	BRDN00012	593	582	1716	938	1301	98
AGCTGTGTC	BRDN00011	153	116	662	244	144	7
AGCTGTTAG	BRDN00012	310	351	262	617	992	57
AGCTGTTCA	BRDN00012	135	136	402	355	548	9
AGCTGTTCA	BRDN00012	684	533	1693	1217	1299	87
AGCTGTTGA	BRDN00011	678	552	978	1232	1115	98
AGCTGTTGT	BRDN00011	197	273	10	320	314	11
AGCTGTTTG	BRDN00011	379	289	275	829	801	37
AGCTGTTTG	BRDN00011	258	162	180	261	263	9
AGCTTAACC	BRDN00012	430	389	1496	851	1293	39
AGCTTAATG	BRDN00012	462	599	218	1070	829	68
AGCTTACCC	BRDN00012	286	352	203	691	698	56
AGCTTACTA	BRDN00012	177	220	91	273	141	40
AGCTTAGAC	BRDN00011	170	121	11	246	370	12
AGCTTAGGG	BRDN00012	130	199	69	450	768	0
AGCTTATGG	BRDN00011	134	113	8	274	250	14
AGCTTCAAA	BRDN00011	232	210	2001	269	239	40
AGCTTCAAG	BRDN00011	406	344	3778	686	460	43
AGCTTCAAT	BRDN00012	525	643	2178	1119	881	77
AGCTTCAGA	BRDN00012	619	421	1260	1144	1184	90
AGCTTCAGA	BRDN00012	119	201	68	146	79	24
AGCTTCAGC	BRDN00012	495	472	1118	990	793	34
AGCTTCAGG	BRDN00011	155	86	121	277	328	6
AGCTTCATA	BRDN00012	265	36	0	590	482	0
AGCTTCATG	BRDN00012	337	92	70	1030	594	0
AGCTTCCAC	BRDN00012	539	98	37	1262	751	4
AGCTTCCAG	BRDN00011	155	211	95	276	259	25
AGCTTCCCC	BRDN00012	173	168	2576	374	505	32
AGCTTCCCT	BRDN00011	542	559	372	707	795	49
AGCTTCCCG	BRDN00011	316	12	1	559	694	0
AGCTTCCTC	BRDN00012	136	109	88	224	357	8
AGCTTCCTG	BRDN00012	85	91	56	239	149	13
AGCTTCGAA	BRDN00012	555	662	199	974	838	73
AGCTTCGAC	BRDN00012	118	139	342	310	240	15
AGCTTCGAT	BRDN00011	280	208	1745	603	604	21
AGCTTCGGC	BRDN00011	473	400	966	997	1047	48
AGCTTCGGG	BRDN00011	222	208	77	591	539	6
AGCTTCGGG	BRDN00011	50	31	0	113	144	0
AGCTTCGGT	BRDN00011	387	418	161	613	1339	10
AGCTTCGTG	BRDN00012	218	183	144	420	527	21
AGCTTCGTG	BRDN00011	276	429	463	502	556	28
AGCTTCTAC	BRDN00011	141	219	64	273	66	12
AGCTTGAAT	BRDN00012	265	241	274	670	519	7
AGCTTGAGA	BRDN00012	103	215	212	286	205	22
AGCTTGAGT	BRDN00012	290	542	146	601	766	14
AGCTTGAGT	BRDN00011	393	272	261	875	673	69
AGCTTGATA	BRDN00011	419	462	2626	656	587	45
AGCTTGATC	BRDN00011	292	281	803	663	493	40
AGCTTGCCA	BRDN00011	208	203	436	289	342	10
AGCTTGCCC	BRDN00012	302	294	289	494	568	35
AGCTTGCCG	BRDN00012	191	248	707	454	931	38
AGCTTGCGA	BRDN00012	456	339	837	900	1490	33
AGCTTGCGT	BRDN00011	179	162	388	269	241	25
AGCTTGGAT	BRDN00011	266	296	153	579	377	55
AGCTTGGCA	BRDN00011	277	225	236	447	594	45
AGCTTGGCC	BRDN00011	115	50	56	265	477	138
AGCTTGGCT	BRDN00012	323	489	1158	886	838	14
AGCTTGGTC	BRDN00012	192	166	245	446	176	18
AGCTTGTAG	BRDN00011	465	152	483	792	816	20
AGCTTGTAG	BRDN00011	529	577	484	1132	921	48
AGCTTGTCA	BRDN00011	598	576	2171	1071	1168	114
AGCTTGTCC	BRDN00011	129	83	169	312	149	56
AGCTTGTCT	BRDN00011	269	170	689	333	358	10
AGCTTGTGG	BRDN00012	665	335	1185	964	1575	41
AGCTTGTGT	BRDN00012	430	556	436	1126	1021	88
AGCTTGTTT	BRDN00012	146	59	106	156	359	0
AGCTTTACT	BRDN00011	423	60	3932	754	621	38
AGCTTTAGC	BRDN00011	115	0	0	322	190	0
AGCTTTATT	BRDN00012	356	310	716	875	1168	49
AGCTTTCCT	BRDN00012	224	186	95	382	130	3
AGCTTTCGA	BRDN00012	400	498	4183	923	999	141
AGCTTTGAT	BRDN00011	254	347	706	347	471	33
AGCTTTGCA	BRDN00011	242	135	50	371	529	21
AGCTTTGCC	BRDN00011	171	93	7	312	259	0
AGCTTTGGA	BRDN00011	286	271	1010	529	691	103
AGCTTTGGC	BRDN00012	289	262	322	975	427	18
AGCTTTGTA	BRDN00012	636	788	1291	1121	1792	128
AGCTTTGTG	BRDN00011	131	49	0	348	108	0
AGGAAAAAA	BRDN00011	166	197	103	345	205	10
AGGAAAAAC	BRDN00012	105	109	186	256	281	19
AGGAAAAAC	BRDN00011	253	62	1	598	426	0
AGGAAAAAG	BRDN00011	277	128	521	479	479	12
AGGAAAAAT	BRDN00011	133	161	174	267	150	21
AGGAAAACC	BRDN00012	338	0	6	791	639	8
AGGAAAACC	BRDN00011	280	218	203	350	326	5
AGGAAAAGT	BRDN00012	481	361	515	793	632	28
AGGAAAATT	BRDN00011	76	98	1	188	141	0
AGGAAACAA	BRDN00011	319	266	52	709	429	29
AGGAAACAG	BRDN00011	151	143	309	441	190	23
AGGAAACAG	BRDN00011	511	401	981	816	902	17
AGGAAACAG	BRDN00011	539	571	767	778	689	133
AGGAAACCT	BRDN00011	333	363	929	599	556	44
AGGAAACGC	BRDN00012	200	138	314	517	423	28
AGGAAACTC	BRDN00012	239	23	0	562	414	1
AGGAAACTG	BRDN00011	423	312	576	734	2064	64
AGGAAACTG	BRDN00011	318	514	4739	669	941	88
AGGAAAGAA	BRDN00012	328	250	979	688	762	64
AGGAAAGAC	BRDN00011	458	467	1067	1026	791	44
AGGAAAGAC	BRDN00011	241	300	482	328	304	24
AGGAAAGAT	BRDN00012	107	101	349	350	299	7
AGGAAAGCA	BRDN00012	526	526	302	1158	835	57
AGGAAAGCC	BRDN00012	472	293	76	975	944	0
AGGAAAGTA	BRDN00011	74	126	7	169	91	51
AGGAAAGTA	BRDN00011	309	215	757	631	342	38
AGGAAAGTG	BRDN00011	173	26	84	372	333	0
AGGAAATAA	BRDN00011	292	285	473	497	596	51
AGGAAATAC	BRDN00011	482	380	2291	648	473	93
AGGAAATCC	BRDN00012	375	179	365	711	455	27
AGGAAATGA	BRDN00012	333	2	1	826	842	0
AGGAAATGC	BRDN00011	493	382	235	931	912	60
AGGAAATGC	BRDN00012	427	457	431	927	835	101
AGGAAATGG	BRDN00011	86	0	0	281	130	0
AGGAAATGG	BRDN00012	223	272	478	376	369	103
AGGAAATTT	BRDN00012	423	406	211	646	447	49
AGGAACAAA	BRDN00012	252	309	32296	470	441	479
AGGAACAAC	BRDN00011	252	266	1294	356	214	66
AGGAACAAG	BRDN00012	207	193	149	331	127	0
AGGAACAAT	BRDN00011	84	158	0	224	342	0
AGGAACACC	BRDN00012	155	87	726	221	76	23
AGGAACACC	BRDN00011	229	91	257	438	541	5
AGGAACAGA	BRDN00012	321	138	149	475	567	38
AGGAACAGG	BRDN00012	67	116	0	121	43	10
AGGAACAGG	BRDN00011	750	548	1342	1556	1267	201
AGGAACATC	BRDN00012	609	439	490	825	393	68
AGGAACCAA	BRDN00012	456	326	848	997	1063	20
AGGAACCAG	BRDN00011	71	105	116	182	99	20
AGGAACCCC	BRDN00012	247	165	386	558	596	57
AGGAACCTG	BRDN00012	96	179	344	277	126	36
AGGAACGCA	BRDN00011	495	646	1349	878	459	46
AGGAACGCC	BRDN00012	251	269	1278	302	363	41
AGGAACGGA	BRDN00011	449	587	570	970	1253	54
AGGAACGGA	BRDN00012	379	243	51	541	769	33
AGGAACGTG	BRDN00012	164	162	34	362	197	14
AGGAACTAC	BRDN00011	140	52	0	331	52	0
AGGAACTAG	BRDN00011	571	366	1580	947	963	43
AGGAACTCA	BRDN00011	185	101	8	257	342	17
AGGAACTCA	BRDN00011	254	328	185	352	433	36
AGGAACTCC	BRDN00012	482	318	459	830	736	35
AGGAACTCT	BRDN00012	166	151	0	211	226	7
AGGAACTGA	BRDN00012	332	268	147	533	342	0
AGGAACTGG	BRDN00012	184	194	90	289	575	18
AGGAACTGG	BRDN00011	220	245	886	464	352	18
AGGAACTTA	BRDN00011	207	180	29	359	95	0
AGGAACTTC	BRDN00012	441	105	0	650	437	0
AGGAACTTG	BRDN00012	499	479	685	1015	1167	15
AGGAACTTG	BRDN00012	130	217	198	356	415	7
AGGAAGAAC	BRDN00011	318	53	142	596	673	7
AGGAAGAAC	BRDN00012	216	233	1903	568	822	6
AGGAAGAAC	BRDN00011	124	226	2	195	197	0
AGGAAGACA	BRDN00012	371	465	2641	889	657	26
AGGAAGACC	BRDN00012	131	0	0	475	254	0
AGGAAGACC	BRDN00011	189	227	54	324	396	3
AGGAAGACT	BRDN00011	204	257	1951	392	101	38
AGGAAGACT	BRDN00011	298	211	285	538	778	37
AGGAAGACT	BRDN00011	114	105	558	222	143	34
AGGAAGAGC	BRDN00012	121	158	223	301	379	8
AGGAAGAGC	BRDN00011	45	1	0	301	128	0
AGGAAGAGC	BRDN00012	301	312	157	419	395	60
AGGAAGAGC	BRDN00012	12	0	0	554	39	0
AGGAAGAGC	BRDN00012	95	39	0	205	135	0
AGGAAGATA	BRDN00012	543	500	919	950	590	37
AGGAAGATA	BRDN00012	40	17	0	194	18	1
AGGAAGATC	BRDN00012	97	44	1622	188	141	18
AGGAAGATG	BRDN00012	202	76	180	275	266	9
AGGAAGATG	BRDN00011	417	253	232	827	847	9
AGGAAGATG	BRDN00012	314	224	114	608	1142	44
AGGAAGCAA	BRDN00012	262	178	466	346	77	11
AGGAAGCAG	BRDN00011	151	35	50	475	180	9
AGGAAGCCA	BRDN00012	99	53	63	158	555	42
AGGAAGCCA	BRDN00011	340	218	19	567	468	0
AGGAAGCCC	BRDN00011	605	599	464	865	719	86
AGGAAGCCC	BRDN00011	261	131	219	513	487	0
AGGAAGCCT	BRDN00012	124	192	114	369	226	4
AGGAAGCGA	BRDN00011	283	193	212	663	914	19
AGGAAGCTC	BRDN00012	590	639	425	977	1088	103
AGGAAGGAA	BRDN00011	164	254	18	341	516	29
AGGAAGGAA	BRDN00012	328	207	129	319	292	0
AGGAAGGAC	BRDN00011	176	210	2131	482	282	32
AGGAAGGAC	BRDN00012	215	150	149	444	903	30
AGGAAGGCA	BRDN00011	334	359	738	436	435	17
AGGAAGGCC	BRDN00011	169	110	622	188	186	18
AGGAAGGTC	BRDN00012	429	500	657	1115	1205	11
AGGAAGGTC	BRDN00011	69	1	0	253	166	0
AGGAAGGTC	BRDN00011	359	307	194	656	1095	10
AGGAAGGTC	BRDN00012	166	126	883	451	202	11
AGGAAGGTC	BRDN00012	323	435	187	716	1128	46
AGGAAGTAA	BRDN00012	233	128	338	449	551	5
AGGAAGTAA	BRDN00012	276	301	186	637	639	38
AGGAAGTAG	BRDN00012	150	137	451	303	652	14
AGGAAGTCA	BRDN00012	264	230	184	556	391	24
AGGAAGTCA	BRDN00011	297	2	0	238	88	0
AGGAAGTCC	BRDN00011	279	261	255	534	448	21
AGGAAGTCC	BRDN00011	191	288	393	452	775	36
AGGAAGTGA	BRDN00011	163	128	22	356	203	49
AGGAAGTGC	BRDN00012	187	0	0	210	74	0
AGGAAGTTC	BRDN00012	208	183	0	350	370	0
AGGAAGTTC	BRDN00011	235	122	40	543	726	42
AGGAATAAA	BRDN00012	124	333	7	255	451	6
AGGAATAAG	BRDN00012	114	84	77	130	176	13
AGGAATAGC	BRDN00012	220	128	68	416	403	56
AGGAATAGG	BRDN00012	462	415	193	906	749	180
AGGAATATG	BRDN00011	57	7	0	116	174	0
AGGAATCAA	BRDN00011	135	113	1	247	244	0
AGGAATCAG	BRDN00011	259	236	26	360	241	0
AGGAATCCA	BRDN00011	171	179	27	245	310	0
AGGAATCCA	BRDN00011	364	219	194	487	1110	20
AGGAATCCA	BRDN00012	283	315	304	627	484	42
AGGAATCCC	BRDN00011	93	167	64	178	33	11
AGGAATCCC	BRDN00012	132	127	261	379	209	5
AGGAATCCC	BRDN00012	385	69	24	823	560	0
AGGAATCCG	BRDN00011	165	208	266	286	424	17
AGGAATCTG	BRDN00012	104	129	241	329	440	10
AGGAATCTG	BRDN00012	132	172	615	315	585	15
AGGAATGAA	BRDN00012	88	0	0	233	223	0
AGGAATGAA	BRDN00012	109	1	1	434	452	0
AGGAATGAC	BRDN00011	458	367	1186	800	1000	77
AGGAATGCA	BRDN00011	210	68	21	688	520	0
AGGAATGCA	BRDN00012	711	522	1962	1351	1157	72
AGGAATGGA	BRDN00011	229	114	625	312	306	14
AGGAATGGT	BRDN00012	364	402	545	650	524	72
AGGAATGTA	BRDN00012	335	244	27	922	461	16
AGGAATGTA	BRDN00011	211	123	219	453	442	21
AGGAATGTG	BRDN00011	372	294	88	989	746	9
AGGAATGTT	BRDN00012	241	38	15	478	488	0
AGGAATGTT	BRDN00012	148	35	1	406	112	0
AGGAATTAC	BRDN00011	65	16	1	163	74	0
AGGAATTCA	BRDN00011	159	146	327	482	523	18
AGGAATTCC	BRDN00012	185	115	193	383	486	32
AGGAATTGA	BRDN00011	62	13	0	274	131	0
AGGAATTGA	BRDN00011	185	234	224	370	269	46
AGGAATTGG	BRDN00011	269	206	132	673	540	5
AGGAATTTG	BRDN00012	245	210	600	572	760	106
AGGACAAAA	BRDN00011	162	202	7	191	346	11
AGGACAAAC	BRDN00012	205	206	138	348	333	20
AGGACAAAC	BRDN00012	222	10	1	644	366	0
AGGACAACA	BRDN00011	284	97	66	809	282	7
AGGACAACC	BRDN00012	672	579	2096	978	897	260
AGGACAACT	BRDN00011	154	72	21	303	200	7
AGGACAACT	BRDN00012	172	117	279	272	239	0
AGGACAAGC	BRDN00011	242	251	554	315	402	13
AGGACAAGC	BRDN00012	644	696	406	1261	1139	56
AGGACAAGG	BRDN00011	106	71	248	247	236	31
AGGACAATG	BRDN00012	104	92	408	204	236	6
AGGACACAA	BRDN00012	574	423	698	947	1063	34
AGGACACAC	BRDN00011	158	79	942	256	245	48
AGGACACAC	BRDN00012	496	660	838	1020	1074	55
AGGACACAG	BRDN00012	123	138	137	247	347	0
AGGACACAG	BRDN00011	112	13	9	139	169	0
AGGACACAG	BRDN00012	641	714	3040	1059	794	96
AGGACACCG	BRDN00012	596	583	924	1098	1063	38
AGGACACCT	BRDN00011	386	306	181	777	478	5
AGGACACGA	BRDN00011	98	129	249	162	270	8
AGGACACGC	BRDN00012	304	265	354	553	590	59
AGGACACGC	BRDN00011	462	401	551	961	759	25
AGGACACGG	BRDN00012	121	207	925	224	410	5
AGGACACGT	BRDN00011	661	595	869	943	893	81
AGGACACTC	BRDN00012	232	281	203	485	410	12
AGGACACTC	BRDN00011	127	107	3142	220	100	21
AGGACACTC	BRDN00012	297	307	288	470	342	35
AGGACACTG	BRDN00011	165	144	13	374	295	20
AGGACACTT	BRDN00011	128	148	67	147	202	11
AGGACAGCT	BRDN00012	96	0	0	331	33	0
AGGACAGGA	BRDN00011	132	199	48	261	541	14
AGGACAGGA	BRDN00012	216	113	142	403	353	37
AGGACAGGC	BRDN00012	91	217	55	296	489	53
AGGACAGGC	BRDN00011	495	381	1256	784	446	29
AGGACAGGT	BRDN00011	585	621	4283	957	807	77
AGGACAGTA	BRDN00012	417	342	556	954	997	68
AGGACAGTG	BRDN00012	178	139	466	201	57	8
AGGACAGTG	BRDN00011	474	292	902	960	861	77
AGGACATAC	BRDN00012	116	172	233	377	362	14
AGGACATAC	BRDN00012	311	350	17	471	605	39
AGGACATCA	BRDN00011	137	164	61	359	213	11
AGGACATCA	BRDN00012	294	214	2980	480	309	58
AGGACATCC	BRDN00012	236	211	710	370	348	13
AGGACATGA	BRDN00011	235	239	1556	309	764	39
AGGACATGA	BRDN00011	323	243	436	586	653	71
AGGACATGA	BRDN00011	411	362	517	730	657	77
AGGACATTC	BRDN00011	453	369	206	908	593	84
AGGACATTG	BRDN00011	118	113	559	255	166	4
AGGACCAAA	BRDN00011	41	6	1	289	421	0
AGGACCAAC	BRDN00011	204	211	2661	451	718	24
AGGACCAAC	BRDN00012	187	93	13	309	264	0
AGGACCAAG	BRDN00012	198	245	2022	359	314	37
AGGACCACA	BRDN00012	562	555	735	796	769	103
AGGACCACA	BRDN00012	225	103	51	378	443	15
AGGACCACG	BRDN00011	375	341	252	561	478	29
AGGACCAGA	BRDN00012	262	239	818	486	305	57
AGGACCAGA	BRDN00011	216	133	213	406	372	14
AGGACCAGT	BRDN00011	166	89	91	207	273	6
AGGACCATC	BRDN00011	528	404	2898	872	719	35
AGGACCATC	BRDN00011	779	539	644	1565	923	83
AGGACCATG	BRDN00011	195	155	34	623	284	2
AGGACCCAA	BRDN00012	515	602	201	1227	2522	3
AGGACCCAA	BRDN00011	449	396	1049	631	507	28
AGGACCCAC	BRDN00011	379	102	943	784	474	8
AGGACCCAG	BRDN00012	96	152	8	300	291	8
AGGACCCAT	BRDN00012	579	626	1200	1332	1280	108
AGGACCCAT	BRDN00012	471	481	405	789	684	99
AGGACCCCA	BRDN00011	214	50	0	257	218	0
AGGACCCCA	BRDN00011	203	116	197	215	175	21
AGGACCCCC	BRDN00011	424	386	31	609	511	42
AGGACCCGC	BRDN00012	445	438	325	774	1042	11
AGGACCCTA	BRDN00012	228	304	50	340	955	11
AGGACCGAC	BRDN00011	253	261	109	529	261	15
AGGACCGAT	BRDN00011	541	426	1102	597	654	19
AGGACCGCA	BRDN00011	232	77	973	486	319	11
AGGACCGCG	BRDN00011	426	0	2	1033	11	0
AGGACCGGG	BRDN00012	329	172	43	707	941	27
AGGACCTCG	BRDN00011	202	204	275	392	267	9
AGGACCTGG	BRDN00012	373	144	794	910	470	16
AGGACCTGG	BRDN00011	297	310	584	690	775	22
AGGACCTTG	BRDN00012	378	393	313	812	655	31
AGGACGAAC	BRDN00011	379	364	1714	555	340	24
AGGACGAAG	BRDN00012	90	116	443	138	198	13
AGGACGACG	BRDN00012	597	512	175	1035	1167	22
AGGACGACG	BRDN00011	352	275	1101	662	616	48
AGGACGACG	BRDN00012	426	457	1887	858	505	106
AGGACGACG	BRDN00011	297	246	664	720	581	22
AGGACGAGC	BRDN00011	147	131	39	354	347	25
AGGACGAGC	BRDN00012	125	107	143	256	129	47
AGGACGAGC	BRDN00012	550	463	1486	1182	1214	77
AGGACGAGC	BRDN00012	627	524	679	1122	992	63
AGGACGAGT	BRDN00011	239	3	0	613	308	0
AGGACGAGT	BRDN00012	450	446	97	939	1101	26
AGGACGATA	BRDN00011	73	58	17	191	106	40
AGGACGATA	BRDN00011	370	271	582	742	826	27
AGGACGATC	BRDN00011	458	404	1366	934	1437	49
AGGACGCAA	BRDN00012	171	107	173	248	203	6
AGGACGCCA	BRDN00012	227	178	433	534	434	26
AGGACGCCC	BRDN00012	329	408	65	673	704	9
AGGACGCCC	BRDN00011	314	232	43	683	599	57
AGGACGCCG	BRDN00011	259	337	2	591	467	24
AGGACGCCT	BRDN00012	184	145	5	250	164	20
AGGACGCCT	BRDN00011	198	187	151	508	496	5
AGGACGCGC	BRDN00012	780	652	1243	1480	1572	176
AGGACGCGT	BRDN00011	292	219	248	413	741	15
AGGACGCGT	BRDN00012	53	50	0	152	123	9
AGGACGGAC	BRDN00012	478	735	5208	635	1087	110
AGGACGGAC	BRDN00011	223	206	664	465	638	52
AGGACGGAC	BRDN00012	102	103	0	192	193	0
AGGACGGCG	BRDN00011	432	251	372	806	656	35
AGGACGGCT	BRDN00012	557	510	401	1256	1256	27
AGGACGGGC	BRDN00012	34	8	0	361	122	0
AGGACGGGT	BRDN00012	429	262	2162	729	1250	23
AGGACGGTG	BRDN00012	154	94	606	256	265	3
AGGACGGTG	BRDN00012	422	542	256	845	838	28
AGGACGGTT	BRDN00012	270	216	1458	607	984	10
AGGACGTAA	BRDN00012	205	132	571	514	399	0
AGGACGTCA	BRDN00012	135	241	772	387	389	58
AGGACGTCA	BRDN00012	452	445	754	757	693	8
AGGACGTCC	BRDN00012	104	190	20	260	192	10
AGGACGTCC	BRDN00011	324	241	199	595	451	82
AGGACGTGC	BRDN00011	185	203	0	283	87	3
AGGACGTGC	BRDN00012	172	135	354	455	404	12
AGGACTAGC	BRDN00011	208	107	18	543	476	5

AGGACTAGT	BRDN00012	453	527	575	770	617	73
AGGACTATC	BRDN00011	355	295	419	745	598	5
AGGACTATC	BRDN00012	193	132	396	392	569	26
AGGACTATG	BRDN00011	192	38	23	471	269	0
AGGACTATT	BRDN00011	355	214	124	681	676	31
AGGACTCAC	BRDN00012	137	108	0	222	214	0
AGGACTCAC	BRDN00012	408	284	384	960	1301	34
AGGACTCAC	BRDN00011	691	502	485	1380	1262	63
AGGACTCAC	BRDN00011	399	250	854	888	1008	43
AGGACTCCA	BRDN00012	612	547	2920	857	941	109
AGGACTCGA	BRDN00012	282	299	1374	545	393	52
AGGACTCGA	BRDN00012	312	193	310	491	540	10
AGGACTCGG	BRDN00012	531	392	316	894	1016	64
AGGACTCTA	BRDN00011	248	179	1306	454	393	26
AGGACTCTA	BRDN00012	294	250	1808	841	739	77
AGGACTCTG	BRDN00012	334	387	5763	811	934	98
AGGACTGCC	BRDN00011	82	82	651	282	106	9
AGGACTGCC	BRDN00011	307	421	88	800	663	37
AGGACTGCC	BRDN00012	184	183	257	353	228	14
AGGACTGCT	BRDN00012	132	164	1	330	351	34
AGGACTGGG	BRDN00012	616	401	298	1148	1095	72
AGGACTGTG	BRDN00011	226	287	634	476	445	63
AGGACTTCA	BRDN00012	140	144	113	316	266	36
AGGACTTCC	BRDN00012	122	206	43	445	211	26
AGGACTTCC	BRDN00012	157	170	185	365	604	12
AGGACTTCG	BRDN00011	147	51	1	315	156	0
AGGACTTGA	BRDN00011	80	139	0	200	230	39
AGGACTTGG	BRDN00011	106	6	0	237	52	0
AGGACTTGG	BRDN00012	119	29	34	403	338	0
AGGACTTGG	BRDN00012	189	277	1221	355	625	25
AGGACTTGG	BRDN00012	109	61	81	146	298	0
AGGACTTTG	BRDN00012	324	207	733	400	559	28
AGGACTTTG	BRDN00012	421	394	1646	1196	1091	105
AGGAGAAAA	BRDN00012	433	457	100	880	725	30
AGGAGAAAC	BRDN00011	283	229	117	600	367	6
AGGAGAAAT	BRDN00011	129	97	171	361	68	0
AGGAGAACC	BRDN00011	127	39	2	197	159	0
AGGAGAACT	BRDN00011	347	321	133	631	789	76
AGGAGAACT	BRDN00011	320	256	244	402	366	6
AGGAGAAGA	BRDN00012	186	206	63	264	310	13
AGGAGAAGC	BRDN00011	436	276	825	629	654	0
AGGAGAAGC	BRDN00011	172	295	62	339	170	19
AGGAGAAGC	BRDN00012	279	301	757	746	450	29
AGGAGAAGC	BRDN00012	409	294	172	941	694	2
AGGAGAAGC	BRDN00012	374	353	268	590	742	57
AGGAGAAGC	BRDN00011	161	98	20	226	270	11
AGGAGAAGT	BRDN00011	490	480	1070	802	676	34
AGGAGACAA	BRDN00012	103	110	1	325	558	0
AGGAGACAC	BRDN00012	285	284	102	396	412	44
AGGAGACAG	BRDN00012	664	459	295	1183	946	47
AGGAGACAT	BRDN00012	332	156	138	330	150	23
AGGAGACCA	BRDN00011	321	156	4	606	553	1
AGGAGACCC	BRDN00012	409	312	424	674	377	53
AGGAGACTA	BRDN00011	282	425	159	715	1190	300
AGGAGACTA	BRDN00011	230	18	72	551	482	0
AGGAGACTC	BRDN00012	206	123	384	669	496	9
AGGAGACTG	BRDN00012	319	273	918	759	1047	18
AGGAGAGAC	BRDN00011	146	165	125	279	440	20
AGGAGAGAC	BRDN00012	510	646	2705	873	1467	71
AGGAGAGCC	BRDN00012	45	0	0	200	107	0
AGGAGAGGA	BRDN00012	494	366	617	870	495	51
AGGAGAGGA	BRDN00012	297	352	981	773	522	44
AGGAGAGGT	BRDN00011	200	264	118	364	279	29
AGGAGAGTT	BRDN00012	155	28	0	267	274	0
AGGAGATCG	BRDN00011	511	704	541	881	316	68
AGGAGATCT	BRDN00012	209	228	242	401	463	32
AGGAGATGA	BRDN00011	83	68	0	233	142	0
AGGAGATGA	BRDN00012	163	14	1	364	34	0
AGGAGATGA	BRDN00012	471	418	2515	711	877	83
AGGAGATGC	BRDN00011	726	316	430	1178	1075	10
AGGAGATGC	BRDN00012	222	279	816	406	424	95
AGGAGATGC	BRDN00012	216	0	1	397	112	0
AGGAGATGT	BRDN00011	447	502	587	684	649	47
AGGAGATTA	BRDN00012	185	42	231	300	208	0
AGGAGATTC	BRDN00011	228	175	465	324	249	54
AGGAGCAAA	BRDN00012	318	328	507	774	932	17
AGGAGCACA	BRDN00012	329	359	1380	916	734	67
AGGAGCACC	BRDN00012	256	191	1175	434	546	3
AGGAGCACC	BRDN00011	197	245	79	443	653	39
AGGAGCAGA	BRDN00012	445	657	1271	1124	1365	265
AGGAGCAGC	BRDN00012	456	414	970	988	721	66
AGGAGCAGC	BRDN00011	211	14	20	536	159	0
AGGAGCAGT	BRDN00011	265	244	517	369	466	25
AGGAGCATA	BRDN00012	236	194	125	410	365	12
AGGAGCATT	BRDN00011	390	199	46	648	577	37
AGGAGCCAA	BRDN00012	177	64	198	366	353	11
AGGAGCCAG	BRDN00011	332	335	89	512	656	20
AGGAGCCAG	BRDN00011	237	172	228	431	327	14
AGGAGCCCA	BRDN00011	87	51	1967	137	215	20
AGGAGCCCC	BRDN00012	541	551	556	966	1122	24
AGGAGCCCG	BRDN00012	201	205	0	371	457	4
AGGAGCCGC	BRDN00011	429	347	1037	719	723	91
AGGAGCCGG	BRDN00011	62	51	0	166	127	0
AGGAGCCGT	BRDN00011	131	138	328	286	428	17
AGGAGCCGT	BRDN00012	374	0	0	791	12	0
AGGAGCCTA	BRDN00012	406	396	178	808	800	190
AGGAGCCTG	BRDN00012	120	107	3	225	252	0
AGGAGCCTG	BRDN00011	136	76	1801	245	208	6
AGGAGCGAA	BRDN00011	79	62	53	215	173	18
AGGAGCGAT	BRDN00011	394	470	1152	763	911	26
AGGAGCGCA	BRDN00012	270	369	296	611	571	104
AGGAGCGGA	BRDN00011	349	345	1057	824	755	151
AGGAGCGGA	BRDN00012	61	35	1	191	95	6
AGGAGCGTT	BRDN00012	378	330	344	517	447	19
AGGAGCTAA	BRDN00011	143	69	10	217	232	10
AGGAGCTAA	BRDN00011	423	356	234	864	1066	77
AGGAGCTAC	BRDN00011	393	351	731	912	941	5
AGGAGCTGA	BRDN00011	271	0	0	696	211	0
AGGAGCTGA	BRDN00012	432	107	123	1007	891	4
AGGAGCTGA	BRDN00012	370	284	177	671	683	10
AGGAGCTGA	BRDN00011	166	14	0	256	506	0
AGGAGCTGA	BRDN00011	155	274	65	444	506	0
AGGAGCTGC	BRDN00011	691	590	1008	1356	1072	94
AGGAGCTGG	BRDN00011	248	172	834	492	701	18
AGGAGCTGG	BRDN00012	118	114	16	200	66	0
AGGAGCTGT	BRDN00012	265	183	366	725	554	2
AGGAGCTTA	BRDN00011	82	1	0	315	175	0
AGGAGGAAA	BRDN00012	313	236	1235	551	515	16
AGGAGGAAA	BRDN00012	205	132	1448	263	209	31
AGGAGGAAA	BRDN00011	230	155	278	309	532	17
AGGAGGAAC	BRDN00011	179	237	306	415	355	12
AGGAGGAAC	BRDN00012	218	203	1313	360	595	30
AGGAGGAAT	BRDN00012	60	4	152	487	267	7
AGGAGGAAT	BRDN00012	120	29	1	387	651	0
AGGAGGACA	BRDN00011	270	186	735	525	393	15
AGGAGGACA	BRDN00012	288	306	272	603	803	8
AGGAGGACT	BRDN00011	52	109	106	179	302	1
AGGAGGAGA	BRDN00011	197	120	31	296	739	41
AGGAGGAGG	BRDN00011	128	235	213	364	325	0
AGGAGGAGG	BRDN00012	139	201	38	299	229	16
AGGAGGAGG	BRDN00011	201	213	967	353	454	9
AGGAGGAGG	BRDN00011	437	566	424	780	746	168
AGGAGGAGG	BRDN00011	216	104	28	387	357	6
AGGAGGAGG	BRDN00012	216	88	107	427	478	20
AGGAGGATC	BRDN00012	185	248	234	287	209	4
AGGAGGCAC	BRDN00011	118	0	0	414	26	0
AGGAGGCAC	BRDN00012	440	146	303	896	1173	22
AGGAGGCAC	BRDN00011	503	486	1100	880	1352	60
AGGAGGCAC	BRDN00011	205	220	35	424	164	69
AGGAGGCCA	BRDN00011	236	208	222	467	571	16
AGGAGGCCA	BRDN00011	501	420	599	847	737	46
AGGAGGCCG	BRDN00011	140	114	836	402	150	32
AGGAGGCCG	BRDN00012	302	206	13	643	516	25
AGGAGGCGT	BRDN00011	335	281	200	600	688	21
AGGAGGCTC	BRDN00012	469	469	529	1036	1153	33
AGGAGGGAA	BRDN00011	446	378	611	650	565	14
AGGAGGGAA	BRDN00012	201	150	278	325	413	58
AGGAGGGAG	BRDN00011	51	27	1	346	321	0
AGGAGGGAG	BRDN00011	238	133	366	455	596	31
AGGAGGGAT	BRDN00011	547	409	963	1032	937	30
AGGAGGGCA	BRDN00011	690	789	833	1031	1271	54
AGGAGGGCC	BRDN00012	174	88	943	354	254	5
AGGAGGGCT	BRDN00011	110	58	465	280	109	2
AGGAGGGGG	BRDN00012	292	314	120	496	631	27
AGGAGGGTC	BRDN00011	216	111	631	492	552	18
AGGAGGTAA	BRDN00012	69	0	0	505	352	0
AGGAGGTCA	BRDN00011	450	214	364	813	1105	48
AGGAGGTGA	BRDN00011	141	179	417	382	387	11
AGGAGGTGA	BRDN00012	165	36	33	338	152	0
AGGAGGTGA	BRDN00011	478	404	473	784	553	16
AGGAGGTGC	BRDN00011	585	423	407	1017	862	115
AGGAGGTGC	BRDN00012	457	363	1082	745	652	60
AGGAGGTGC	BRDN00012	178	112	297	503	489	20
AGGAGTAAG	BRDN00011	183	133	282	326	339	7
AGGAGTAAG	BRDN00012	471	623	1399	1070	923	61
AGGAGTACA	BRDN00012	766	590	1028	1121	881	28
AGGAGTACC	BRDN00012	447	509	1396	928	858	37
AGGAGTACG	BRDN00012	197	149	642	334	361	30
AGGAGTACG	BRDN00012	235	144	267	413	575	12
AGGAGTACT	BRDN00011	364	183	13	624	672	9
AGGAGTAGA	BRDN00012	409	404	130	625	587	0
AGGAGTATA	BRDN00012	116	85	296	212	470	2
AGGAGTCAA	BRDN00012	352	305	741	652	644	21
AGGAGTCAA	BRDN00011	529	549	114	1014	994	120
AGGAGTCAC	BRDN00012	163	134	169	257	713	31
AGGAGTCAT	BRDN00011	428	372	2948	842	919	42
AGGAGTCCA	BRDN00011	233	262	572	487	515	76
AGGAGTCCG	BRDN00012	306	111	10	853	676	4
AGGAGTCCT	BRDN00012	319	198	80	515	374	27
AGGAGTCGG	BRDN00011	90	5	0	353	482	0
AGGAGTCTA	BRDN00012	404	433	18	705	754	45
AGGAGTCTG	BRDN00011	504	46	1	840	456	0
AGGAGTCTG	BRDN00012	119	155	767	417	354	0
AGGAGTCTT	BRDN00012	263	186	165	342	515	87
AGGAGTGAT	BRDN00012	115	125	88	216	343	22
AGGAGTGCT	BRDN00011	175	161	34	302	340	35
AGGAGTGGA	BRDN00012	237	125	50	360	399	11
AGGAGTGGC	BRDN00011	230	200	0	281	265	10
AGGAGTGGT	BRDN00012	229	257	105	324	193	27
AGGAGTGTC	BRDN00011	192	185	587	411	354	17
AGGAGTGTC	BRDN00011	230	260	222	451	347	18
AGGAGTGTT	BRDN00012	454	651	1246	805	1118	100
AGGAGTTAA	BRDN00011	88	98	430	239	219	10
AGGAGTTAA	BRDN00011	249	242	294	684	676	33
AGGAGTTAA	BRDN00012	90	0	1	315	372	0
AGGAGTTAC	BRDN00012	189	197	2461	441	316	57
AGGAGTTCA	BRDN00012	445	414	528	954	602	39
AGGAGTTCG	BRDN00012	389	308	60	763	806	39
AGGAGTTCT	BRDN00012	257	117	277	328	603	31
AGGAGTTGA	BRDN00011	275	180	464	575	374	60
AGGAGTTGG	BRDN00012	377	126	316	894	893	18
AGGAGTTGT	BRDN00012	226	160	182	508	350	19
AGGAGTTTG	BRDN00012	153	0	0	316	644	0
AGGAGTTTG	BRDN00012	389	410	858	639	543	44
AGGATAACA	BRDN00011	215	205	28	418	504	17
AGGATAAGA	BRDN00011	321	290	1645	434	438	96
AGGATAAGT	BRDN00012	331	245	875	569	892	92
AGGATAATC	BRDN00011	208	184	280	375	372	23
AGGATACAC	BRDN00011	112	50	1	279	217	7
AGGATACAG	BRDN00011	121	0	1	228	77	0
AGGATACCC	BRDN00011	113	102	83	153	202	0
AGGATACGA	BRDN00011	495	503	2089	767	466	94
AGGATACGC	BRDN00011	318	270	206	408	581	0
AGGATACGT	BRDN00011	140	94	319	175	165	9
AGGATACTG	BRDN00012	152	231	8	362	294	21
AGGATAGAG	BRDN00012	140	11	0	328	149	0
AGGATAGGC	BRDN00011	96	0	0	235	209	0
AGGATAGGT	BRDN00011	164	222	211	459	136	0
AGGATAGTG	BRDN00012	72	63	221	212	230	0
AGGATATCC	BRDN00011	115	124	29	183	107	30
AGGATATGA	BRDN00012	547	472	432	987	1356	34
AGGATATGC	BRDN00012	245	393	697	412	456	20
AGGATATTC	BRDN00012	161	92	9	230	221	0
AGGATCAAC	BRDN00011	209	155	213	327	334	5
AGGATCAAT	BRDN00011	573	491	1580	907	1058	54
AGGATCACA	BRDN00011	223	127	560	292	533	5
AGGATCAGC	BRDN00012	525	493	334	1039	938	20
AGGATCATA	BRDN00011	1	0	0	264	193	0
AGGATCATA	BRDN00012	102	0	26	265	177	0
AGGATCATA	BRDN00011	65	119	220	143	273	0
AGGATCATG	BRDN00011	164	174	153	498	819	0
AGGATCATG	BRDN00011	196	279	763	316	265	16
AGGATCCAA	BRDN00011	169	135	566	291	283	15
AGGATCCAA	BRDN00012	411	394	417	715	334	28
AGGATCCAA	BRDN00011	105	2	1	248	221	9
AGGATCCAC	BRDN00011	517	419	383	943	1223	60
AGGATCCAC	BRDN00012	536	509	1912	802	988	50
AGGATCCAG	BRDN00011	140	200	13	331	105	26
AGGATCCCA	BRDN00012	831	770	1561	1345	1254	82
AGGATCCTC	BRDN00012	204	248	298	383	222	11
AGGATCCTG	BRDN00012	327	260	700	781	824	54
AGGATCCTG	BRDN00012	548	560	263	1059	1791	53
AGGATCCTG	BRDN00011	301	290	508	629	954	13
AGGATCCTT	BRDN00011	144	0	0	397	239	0
AGGATCGAA	BRDN00011	185	140	139	369	238	6
AGGATCGAC	BRDN00011	348	317	179	673	974	5
AGGATCGAC	BRDN00012	348	130	10	695	752	0
AGGATCGTC	BRDN00011	140	106	2514	288	314	57
AGGATCGTG	BRDN00012	659	487	1612	986	1097	131
AGGATCTCG	BRDN00012	470	456	868	1084	700	47
AGGATCTCG	BRDN00011	175	207	1301	289	514	64
AGGATCTCT	BRDN00011	200	212	327	384	379	28
AGGATCTGC	BRDN00012	74	107	16	122	220	5
AGGATCTGG	BRDN00011	133	110	6	173	215	0
AGGATCTGG	BRDN00012	361	378	189	850	959	14
AGGATCTGT	BRDN00011	282	252	619	431	493	67
AGGATCTGT	BRDN00012	630	90	271	1249	1500	5
AGGATCTGT	BRDN00011	78	80	58	202	480	20
AGGATCTTC	BRDN00011	370	493	458	689	547	15
AGGATCTTC	BRDN00011	22	0	0	333	101	0
AGGATCTTG	BRDN00011	92	6	0	182	324	0
AGGATGAAG	BRDN00011	203	161	1943	266	339	37
AGGATGAAG	BRDN00012	189	180	177	384	164	0
AGGATGAAT	BRDN00011	331	244	96	811	430	12
AGGATGAAT	BRDN00011	316	413	1927	448	1256	87
AGGATGACA	BRDN00012	459	26	1	1341	800	0
AGGATGACA	BRDN00011	198	184	1090	300	266	19
AGGATGACC	BRDN00011	453	443	545	666	840	59
AGGATGACC	BRDN00012	784	800	2866	1314	964	200
AGGATGACC	BRDN00011	496	452	963	1018	738	123
AGGATGACG	BRDN00011	553	650	1743	906	1291	58
AGGATGACT	BRDN00012	475	539	882	973	1008	47
AGGATGACT	BRDN00012	157	121	263	225	176	0
AGGATGAGA	BRDN00011	274	238	226	464	306	8
AGGATGAGC	BRDN00011	85	119	215	191	192	38
AGGATGAGC	BRDN00011	230	157	28	353	371	17
AGGATGAGT	BRDN00012	438	378	846	881	982	126
AGGATGATC	BRDN00012	184	95	272	268	347	2
AGGATGATG	BRDN00012	576	598	851	1064	1549	12
AGGATGATG	BRDN00011	293	234	810	414	441	24
AGGATGCCC	BRDN00012	172	63	175	818	390	20
AGGATGCGA	BRDN00012	204	131	954	485	714	5
AGGATGCGG	BRDN00012	348	425	588	675	842	186
AGGATGCGG	BRDN00011	162	43	0	290	339	0
AGGATGCGG	BRDN00012	131	50	125	695	132	0
AGGATGCGG	BRDN00012	207	193	217	215	368	25
AGGATGCGG	BRDN00011	73	110	172	162	101	0
AGGATGCTG	BRDN00012	558	338	1024	988	1038	30
AGGATGGAA	BRDN00011	345	121	29	577	445	29
AGGATGGAC	BRDN00011	180	203	203	200	207	10
AGGATGGAC	BRDN00011	313	234	811	750	664	34
AGGATGGAT	BRDN00012	564	577	536	1125	1205	55
AGGATGGAT	BRDN00012	139	142	481	260	445	33
AGGATGGCC	BRDN00011	282	221	1096	419	223	67
AGGATGGCG	BRDN00011	552	663	1559	982	935	69
AGGATGGGT	BRDN00011	141	33	1	284	339	0
AGGATGGTG	BRDN00011	167	185	11	254	142	25
AGGATGTAA	BRDN00012	235	56	305	377	81	5
AGGATGTAC	BRDN00012	498	452	739	869	571	46
AGGATGTCA	BRDN00011	413	336	1492	708	914	79
AGGATGTCA	BRDN00011	103	93	347	328	460	2
AGGATGTCC	BRDN00012	428	348	1187	685	653	29
AGGATGTCG	BRDN00012	548	458	442	1011	716	40
AGGATGTCT	BRDN00012	151	202	369	418	326	7
AGGATGTGC	BRDN00011	158	147	199	387	314	9
AGGATGTGT	BRDN00012	362	434	53	684	746	8
AGGATGTTA	BRDN00011	129	128	879	374	429	63
AGGATGTTA	BRDN00011	167	176	625	201	421	21
AGGATGTTC	BRDN00012	319	1	1	899	289	0
AGGATGTTG	BRDN00011	357	286	76	727	408	10
AGGATTAAA	BRDN00011	77	133	416	205	452	4
AGGATTAAC	BRDN00011	249	335	58	532	683	9
AGGATTAGG	BRDN00011	55	34	53	155	193	12
AGGATTCAC	BRDN00011	299	295	330	582	509	11
AGGATTCAT	BRDN00012	370	293	1131	661	361	12
AGGATTCGC	BRDN00012	340	502	1102	768	796	33
AGGATTCGT	BRDN00012	336	313	990	686	695	17
AGGATTCTT	BRDN00011	124	146	799	309	240	6
AGGATTGCC	BRDN00012	522	563	1072	1052	910	40
AGGATTGGT	BRDN00011	288	407	753	911	1012	13
AGGATTGTG	BRDN00011	262	178	220	544	595	18
AGGATTTCT	BRDN00012	436	281	321	799	877	47
AGGATTTGA	BRDN00011	319	314	208	643	462	16
AGGATTTGA	BRDN00012	292	375	470	583	675	32
AGGCAAAAA	BRDN00011	330	110	1143	513	330	38
AGGCAAAAC	BRDN00012	339	217	367	615	648	18
AGGCAAAAC	BRDN00012	236	88	95	463	491	41
AGGCAAAAC	BRDN00011	188	161	369	311	238	6
AGGCAAACG	BRDN00011	493	297	1007	791	1138	55
AGGCAAACG	BRDN00011	395	279	264	588	797	57
AGGCAAACG	BRDN00012	136	194	0	409	250	6
AGGCAAACT	BRDN00012	403	433	6017	816	789	162
AGGCAAACT	BRDN00012	354	284	80	607	638	48
AGGCAAACT	BRDN00012	129	107	0	276	255	15
AGGCAAACT	BRDN00012	280	45	907	766	455	15
AGGCAAAGC	BRDN00012	609	539	4697	940	1092	67
AGGCAAAGT	BRDN00011	184	97	522	258	391	5
AGGCAAATA	BRDN00012	155	212	169	425	610	16
AGGCAAATC	BRDN00012	315	204	1349	384	526	58
AGGCAAATG	BRDN00012	241	235	413	570	743	9
AGGCAACAT	BRDN00011	456	103	29	978	920	0
AGGCAACGT	BRDN00012	460	410	1314	736	521	29
AGGCAACTA	BRDN00011	146	64	368	232	76	41
AGGCAACTA	BRDN00011	403	11	6	892	727	0
AGGCAAGAC	BRDN00012	265	327	1213	741	439	15
AGGCAAGGA	BRDN00012	332	162	208	1005	1153	0
AGGCAAGGT	BRDN00011	291	273	81	640	839	47
AGGCAAGTC	BRDN00011	179	83	1	491	229	17
AGGCAAGTC	BRDN00011	377	281	276	697	752	0
AGGCAAGTT	BRDN00012	95	23	0	204	267	0
AGGCAATAG	BRDN00012	426	461	807	780	926	28
AGGCAATCA	BRDN00012	185	69	1596	424	336	15
AGGCAATTT	BRDN00011	116	92	1122	203	150	6
AGGCACAAA	BRDN00012	307	330	220	665	455	32
AGGCACAAC	BRDN00012	448	408	238	775	846	43
AGGCACAAT	BRDN00012	246	365	1483	646	408	83
AGGCACAGG	BRDN00012	196	203	143	496	255	4
AGGCACAGG	BRDN00012	419	396	392	812	591	51
AGGCACATC	BRDN00011	126	163	65	190	191	48
AGGCACATG	BRDN00012	247	293	281	475	547	12
AGGCACCAA	BRDN00012	215	215	1266	515	130	72
AGGCACCAA	BRDN00012	346	1	28	890	356	1
AGGCACCAC	BRDN00012	153	234	899	431	242	68
AGGCACCAC	BRDN00012	111	22	0	205	273	0
AGGCACCAC	BRDN00012	480	254	555	695	638	30
AGGCACCAG	BRDN00012	300	235	570	677	613	0
AGGCACCCC	BRDN00011	132	114	79	322	411	7
AGGCACCCG	BRDN00012	153	84	682	187	68	0
AGGCACCCG	BRDN00011	234	242	25	327	127	15
AGGCACCGT	BRDN00012	124	100	155	163	209	0
AGGCACGCT	BRDN00011	381	383	2105	647	663	57
AGGCACGTC	BRDN00012	325	296	575	552	876	24
AGGCACGTG	BRDN00012	165	176	276	252	294	20
AGGCACTAG	BRDN00011	297	238	2057	616	692	17
AGGCACTCA	BRDN00012	187	113	149	540	487	13
AGGCACTCG	BRDN00011	291	156	70	597	1087	0
AGGCACTCG	BRDN00011	207	173	742	364	396	17
AGGCACTGA	BRDN00011	243	325	414	471	599	75
AGGCACTGA	BRDN00011	419	317	346	742	624	47
AGGCACTGC	BRDN00012	362	222	234	468	452	12
AGGCACTGG	BRDN00012	178	300	283	450	540	26
AGGCACTGG	BRDN00011	166	209	198	429	1326	14
AGGCACTTA	BRDN00012	212	8	1	591	527	0
AGGCACTTG	BRDN00012	229	118	1884	314	609	141
AGGCAGAAA	BRDN00012	298	350	864	686	347	21
AGGCAGAAG	BRDN00012	175	277	16	273	177	23
AGGCAGACC	BRDN00012	674	615	659	1129	2144	186
AGGCAGAGA	BRDN00011	94	7	133	178	195	26
AGGCAGAGC	BRDN00012	564	432	155	1201	804	34
AGGCAGAGC	BRDN00011	252	339	1181	488	506	9
AGGCAGATG	BRDN00012	108	84	1	313	277	0
AGGCAGATG	BRDN00011	206	266	519	315	337	38
AGGCAGATT	BRDN00011	96	80	0	134	64	13
AGGCAGCAA	BRDN00011	112	7	0	497	215	0
AGGCAGCAC	BRDN00011	283	281	218	494	519	104
AGGCAGCAC	BRDN00011	488	282	1952	959	793	65
AGGCAGCCA	BRDN00011	501	308	2047	836	1116	141
AGGCAGCCA	BRDN00011	524	566	311	1025	617	104
AGGCAGCCC	BRDN00012	603	642	456	1203	1253	73
AGGCAGCCG	BRDN00012	582	454	213	1084	1191	1
AGGCAGCCC	BRDN00012	729	655	1090	1246	1013	67
AGGCAGCGT	BRDN00011	134	239	0	302	306	0
AGGCAGCTG	BRDN00011	298	205	175	580	584	6
AGGCAGGAA	BRDN00012	172	121	339	350	272	0
AGGCAGGAC	BRDN00011	109	129	1	199	178	12
AGGCAGGCA	BRDN00011	110	80	2935	232	255	50
AGGCAGGCC	BRDN00012	694	797	564	1171	1156	152
AGGCAGGGC	BRDN00012	691	555	3321	1328	979	66
AGGCAGGGT	BRDN00011	114	92	62	323	340	26
AGGCAGTAA	BRDN00011	181	132	44	347	481	15
AGGCAGTAT	BRDN00012	272	196	1200	494	207	27
AGGCAGTCA	BRDN00011	277	477	974	472	653	21
AGGCAGTCA	BRDN00012	182	155	100	330	297	23
AGGCAGTGG	BRDN00012	313	503	518	634	1068	76
AGGCAGTGT	BRDN00011	215	171	126	399	331	0
AGGCAGTGT	BRDN00012	320	378	864	425	774	35
AGGCAGTTC	BRDN00011	103	71	647	719	455	24
AGGCAGTTG	BRDN00011	117	128	418	273	408	9
AGGCATAAA	BRDN00012	510	510	975	860	1105	46
AGGCATACA	BRDN00011	176	363	237	283	61	68
AGGCATACT	BRDN00012	127	166	15	395	270	9
AGGCATACT	BRDN00011	193	198	66	319	371	6
AGGCATATA	BRDN00011	345	346	184	567	689	23
AGGCATATC	BRDN00012	200	162	0	289	329	0
AGGCATATG	BRDN00012	167	238	636	422	252	44
AGGCATATG	BRDN00012	49	5	0	394	93	0
AGGCATCAC	BRDN00012	382	313	115	798	843	1
AGGCATCAG	BRDN00012	441	165	85	925	682	0
AGGCATCCG	BRDN00012	223	300	275	640	451	78
AGGCATCGT	BRDN00011	122	123	163	275	82	9
AGGCATCTC	BRDN00012	246	376	15623	431	450	377
AGGCATCTC	BRDN00012	89	8	0	344	277	0
AGGCATCTG	BRDN00012	166	191	378	231	264	6
AGGCATGAC	BRDN00011	402	604	4415	690	1101	403
AGGCATGAC	BRDN00012	149	248	480	473	431	49
AGGCATGAG	BRDN00011	119	0	0	312	260	0
AGGCATGAG	BRDN00011	282	238	130	482	644	26
AGGCATGGA	BRDN00011	164	206	793	257	166	71
AGGCATGGA	BRDN00012	544	543	866	847	1551	56
AGGCATGGT	BRDN00012	55	0	0	562	483	0
AGGCATGGT	BRDN00011	98	10	1	781	233	0
AGGCATTCA	BRDN00012	172	56	1	730	809	0
AGGCATTGA	BRDN00011	118	38	147	179	213	14
AGGCATTGA	BRDN00012	218	222	528	482	287	36
AGGCATTTA	BRDN00011	323	410	1377	775	951	49
AGGCCAAAA	BRDN00012	375	325	707	686	1047	38
AGGCCAAAC	BRDN00011	182	122	21	431	434	0
AGGCCAAAT	BRDN00011	131	78	77	322	256	21
AGGCCAACA	BRDN00011	332	3	0	972	260	0
AGGCCAACC	BRDN00012	591	546	4966	1092	1105	103
AGGCCAACT	BRDN00012	647	573	1070	1252	1680	44
AGGCCAAGG	BRDN00012	534	689	131	974	1145	51
AGGCCAAGG	BRDN00011	166	79	403	207	127	3
AGGCCAAGT	BRDN00012	244	180	330	227	395	24
AGGCCAAGT	BRDN00012	229	307	266	390	704	11

AGGCCACAA	BRDN00012	569	365	1634	874	913	57
AGGCCACAC	BRDN00012	547	709	485	1027	759	90
AGGCCACAC	BRDN00011	330	205	60	606	721	0
AGGCCACAT	BRDN00011	197	150	388	366	450	5
AGGCCACCA	BRDN00012	434	410	669	745	768	9
AGGCCACGA	BRDN00012	238	161	730	474	622	16
AGGCCACGG	BRDN00012	73	23	0	217	249	0
AGGCCACTCT	BRDN00012	437	52	2	1046	963	0
AGGCCACTG	BRDN00012	391	205	421	926	721	39
AGGCCAGAA	BRDN00011	271	205	88	352	290	55
AGGCCAGAC	BRDN00012	159	65	51	501	244	0
AGGCCAGAG	BRDN00011	424	311	149	884	430	32
AGGCCAGCA	BRDN00011	156	103	941	310	387	18
AGGCCAGCG	BRDN00012	329	290	646	692	321	10
AGGCCAGCT	BRDN00012	202	186	467	509	560	71
AGGCCAGGA	BRDN00011	145	107	225	234	232	14
AGGCCAGGA	BRDN00011	511	522	83	936	841	74
AGGCCAGGA	BRDN00011	445	326	530	730	729	32
AGGCCAGGA	BRDN00011	146	181	824	269	253	19
AGGCCAGGC	BRDN00011	350	219	1027	634	781	22
AGGCCAGTC	BRDN00011	430	314	1879	619	676	27
AGGCCAGTC	BRDN00012	135	140	21	293	346	77
AGGCCAGTG	BRDN00011	339	396	265	866	698	14
AGGCCATAC	BRDN00011	130	47	0	306	160	0
AGGCCATAT	BRDN00012	126	213	60	317	304	0
AGGCCATAT	BRDN00012	411	501	1504	599	385	63
AGGCCATCA	BRDN00011	600	424	1636	1195	688	47
AGGCCATCA	BRDN00012	373	392	110	710	785	16
AGGCCATGC	BRDN00011	294	396	413	525	286	55
AGGCCATGG	BRDN00012	44	0	7	320	322	0
AGGCCATGT	BRDN00011	321	259	166	577	746	0
AGGCCATGT	BRDN00012	204	127	296	406	465	27
AGGCCCACG	BRDN00012	370	408	78	922	484	31
AGGCCCAGA	BRDN00011	597	563	710	793	1496	126
AGGCCCAGA	BRDN00011	538	545	121	877	984	44
AGGCCCAGG	BRDN00012	199	215	143	284	279	21
AGGCCCATG	BRDN00012	277	139	488	347	431	12
AGGCCCATG	BRDN00012	238	90	132	459	412	27
AGGCCCCAT	BRDN00012	550	429	1220	1099	1165	25
AGGCCCCCA	BRDN00012	508	485	1695	850	1179	18
AGGCCCCCA	BRDN00012	403	256	498	746	645	31
AGGCCCCCG	BRDN00011	116	96	31	245	46	8
AGGCCCCGG	BRDN00011	163	126	343	259	39	35
AGGCCCCGT	BRDN00011	430	45	0	649	247	0
AGGCCCCGT	BRDN00012	183	288	5978	571	439	127
AGGCCCCTA	BRDN00012	158	178	111	363	328	7
AGGCCCGTA	BRDN00012	157	35	166	314	187	7
AGGCCCTAC	BRDN00011	111	105	2	183	297	4
AGGCCCTAT	BRDN00011	289	172	1284	400	402	23
AGGCCCTCT	BRDN00011	200	146	70	256	108	0
AGGCCCTGA	BRDN00012	335	342	1182	857	654	56
AGGCCCTGA	BRDN00012	405	425	628	556	690	120
AGGCCCTTC	BRDN00012	279	258	102	459	270	3
AGGCCGACT	BRDN00011	460	519	392	956	829	6
AGGCCGAGA	BRDN00011	124	54	332	133	124	7
AGGCCGAGG	BRDN00011	123	149	1837	217	42	19
AGGCCGAGT	BRDN00012	161	156	96	317	231	9
AGGCCGAGT	BRDN00011	139	57	1	215	225	0
AGGCCGAGT	BRDN00012	210	109	2	332	189	2
AGGCCGATC	BRDN00012	79	154	19	174	82	0
AGGCCGATT	BRDN00011	181	205	3619	438	293	84
AGGCCGCGA	BRDN00011	223	205	654	239	111	35
AGGCCGCGC	BRDN00011	483	581	407	864	924	51
AGGCCGCGT	BRDN00011	151	183	1335	282	256	9
AGGCCGGGC	BRDN00012	138	221	55	266	319	27
AGGCCGTAG	BRDN00011	79	39	194	153	14	0
AGGCCGTGC	BRDN00012	96	77	39	274	120	12
AGGCCGTGG	BRDN00012	442	509	1398	1029	973	48
AGGCCGTGG	BRDN00011	166	135	16	283	385	9
AGGCCGTGT	BRDN00012	141	68	804	229	345	7
AGGCCTACG	BRDN00011	143	167	64	345	168	10
AGGCCTAGA	BRDN00011	360	239	269	675	882	56
AGGCCTAGT	BRDN00012	355	681	404	701	695	18
AGGCCTATA	BRDN00011	188	166	4	242	333	22
AGGCCTATG	BRDN00012	141	136	445	285	213	18
AGGCCTCAA	BRDN00011	92	117	316	367	363	0
AGGCCTCAG	BRDN00011	70	17	0	152	81	4
AGGCCTCCA	BRDN00011	174	229	478	380	272	51
AGGCCTCCA	BRDN00011	481	381	1158	860	536	23
AGGCCTCCA	BRDN00012	224	153	231	481	577	5
AGGCCTCCA	BRDN00011	280	176	353	503	607	0
AGGCCTCCA	BRDN00011	388	246	33	478	639	36
AGGCCTCGT	BRDN00012	235	228	143	489	258	28
AGGCCTCTG	BRDN00012	419	333	1392	747	733	24
AGGCCTGAC	BRDN00011	395	318	205	872	550	56
AGGCCTGCA	BRDN00011	390	213	783	769	292	26
AGGCCTGCC	BRDN00011	179	88	302	270	163	0
AGGCCTGCC	BRDN00011	97	102	160	267	190	21
AGGCCTGCG	BRDN00011	239	191	101	372	509	6
AGGCCTGCG	BRDN00012	130	214	192	286	515	0
AGGCCTGCT	BRDN00012	729	822	636	1085	1401	96
AGGCCTTAC	BRDN00011	573	213	3	1043	1182	17
AGGCCTTAG	BRDN00012	24	15	0	501	58	0
AGGCCTTAT	BRDN00012	309	232	1081	805	952	15
AGGCCTTCA	BRDN00011	356	418	369	652	703	17
AGGCCTTCG	BRDN00011	251	206	353	510	396	0
AGGCCTTGA	BRDN00011	573	495	1525	922	1186	77
AGGCCTTGT	BRDN00011	310	256	393	689	623	3
AGGCCTTTA	BRDN00012	555	439	1144	923	844	101
AGGCCTTTA	BRDN00012	492	334	604	961	906	29
AGGCGAAAT	BRDN00011	151	198	13455	246	340	263
AGGCGAACA	BRDN00012	277	177	372	529	630	44
AGGCGAACC	BRDN00012	455	117	49	811	1430	0
AGGCGAAGA	BRDN00011	208	134	434	374	537	9
AGGCGAAGA	BRDN00011	181	116	0	432	344	36
AGGCGAATA	BRDN00011	326	289	618	639	441	42
AGGCGAATT	BRDN00012	439	377	358	683	1121	0
AGGCGACAC	BRDN00011	205	182	277	407	219	21
AGGCGACAG	BRDN00011	175	131	1151	351	221	22
AGGCGACGA	BRDN00011	79	83	87	130	62	0
AGGCGACTG	BRDN00012	810	672	2007	1449	1488	73
AGGCGACTG	BRDN00011	251	119	219	346	194	12
AGGCGACTG	BRDN00011	146	9	946	378	158	9
AGGCGACTT	BRDN00011	131	107	185	290	307	0
AGGCGAGAC	BRDN00012	170	178	3128	388	363	66
AGGCGAGCA	BRDN00012	555	360	3128	829	760	52
AGGCGAGGA	BRDN00011	243	143	436	426	264	19
AGGCGATAG	BRDN00011	566	403	1700	989	794	63
AGGCGATCC	BRDN00011	95	40	327	342	234	3
AGGCGATGA	BRDN00012	151	117	79	366	523	5
AGGCGATGC	BRDN00012	472	529	578	934	1687	63
AGGCGATGT	BRDN00011	175	118	238	321	118	51
AGGCGATTC	BRDN00012	327	292	220	964	1066	8
AGGCGCAAC	BRDN00012	509	507	517	1121	968	59
AGGCGCAAT	BRDN00011	464	380	1773	697	888	86
AGGCGCACC	BRDN00011	169	127	39	256	235	46
AGGCGCAGA	BRDN00012	389	367	108	721	717	26
AGGCGCAGA	BRDN00011	464	497	226	1080	1379	27
AGGCGCAGT	BRDN00011	432	395	1118	628	707	40
AGGCGCATG	BRDN00011	155	199	56	379	309	17
AGGCGCCAA	BRDN00012	699	591	1790	1363	1565	67
AGGCGCCAG	BRDN00011	212	275	200	381	173	7
AGGCGCCCC	BRDN00011	189	255	173	339	294	6
AGGCGCCGG	BRDN00012	418	588	1342	1062	815	91
AGGCGCGGC	BRDN00012	179	35	31	315	20	0
AGGCGCGGT	BRDN00012	191	108	67	264	322	20
AGGCGCGTC	BRDN00012	158	239	0	208	103	5
AGGCGCTAA	BRDN00011	397	351	982	780	641	8
AGGCGCTCA	BRDN00011	187	242	401	264	684	7
AGGCGCTGG	BRDN00011	4	0	0	192	13	0
AGGCGCTGG	BRDN00011	389	274	1013	840	1134	18
AGGCGCTTC	BRDN00011	122	182	662	496	557	34
AGGCGGAAC	BRDN00012	225	72	12	350	333	9
AGGCGGAAC	BRDN00011	178	113	83	308	285	8
AGGCGGACT	BRDN00011	75	53	26	143	155	0
AGGCGGCAA	BRDN00011	360	4	0	667	492	0
AGGCGGCCA	BRDN00012	210	95	1514	409	331	18
AGGCGGCCA	BRDN00011	200	279	18	556	319	0
AGGCGGCCG	BRDN00012	516	520	1083	946	840	81
AGGCGGCGC	BRDN00011	106	5	0	336	66	0
AGGCGGCTC	BRDN00012	193	171	322	437	245	21
AGGCGGCTT	BRDN00012	432	348	615	834	954	59
AGGCGGGAA	BRDN00012	436	292	636	974	542	14
AGGCGGGAT	BRDN00011	106	35	38	131	163	0
AGGCGGGCC	BRDN00012	199	106	177	302	370	19
AGGCGGGGI	BRDN00011	129	163	309	248	403	7
AGGCGGGTC	BRDN00011	123	117	107	224	549	10
AGGCGGTAA	BRDN00011	151	2	26	316	123	0
AGGCGGTAC	BRDN00012	353	229	280	562	879	28
AGGCGGTCT	BRDN00012	57	0	1	317	206	0
AGGCGGTGA	BRDN00012	139	115	291	228	208	41
AGGCGGTGC	BRDN00011	288	465	421	592	560	17
AGGCGTAGA	BRDN00012	449	587	2038	718	670	90
AGGCGTCAA	BRDN00011	148	117	49	207	515	23
AGGCGTCAA	BRDN00012	431	433	254	942	1064	26
AGGCGTGAC	BRDN00011	214	319	1152	604	463	5
AGGCGTGAG	BRDN00012	26	0	1	507	43	0
AGGCGTGCA	BRDN00012	185	201	182	431	396	0
AGGCGTGCA	BRDN00012	386	486	2732	663	718	77
AGGCGTGCG	BRDN00011	261	145	551	419	376	15
AGGCGTGCG	BRDN00011	260	215	1900	510	345	41
AGGCGTGCT	BRDN00011	141	55	1	237	325	5
AGGCGTGGA	BRDN00011	222	284	678	317	281	8
AGGCGTGGC	BRDN00011	179	130	40	260	170	9
AGGCGTGGT	BRDN00011	105	61	48	233	311	0
AGGCGTGTA	BRDN00011	180	83	791	356	204	29
AGGCGTTAT	BRDN00011	521	465	1389	998	680	74
AGGCGTTCT	BRDN00012	326	448	383	690	842	7
AGGCGTTCT	BRDN00012	173	190	0	449	500	0
AGGCGTTGC	BRDN00012	209	66	14	310	338	24
AGGCGTTTG	BRDN00012	362	346	205	540	854	25
AGGCTAAAG	BRDN00011	97	70	30	128	72	0
AGGCTAACT	BRDN00011	489	422	1248	1025	1210	57
AGGCTAAGT	BRDN00012	422	387	299	794	723	19
AGGCTAATC	BRDN00011	149	243	32	319	236	0
AGGCTACCA	BRDN00011	417	211	34	683	802	31
AGGCTACGC	BRDN00012	228	180	6	368	383	6
AGGCTACTC	BRDN00012	295	284	748	657	1350	39
AGGCTACTT	BRDN00011	367	373	509	725	835	83
AGGCTAGCA	BRDN00011	169	59	298	346	194	0
AGGCTATCA	BRDN00012	351	275	1420	652	556	15
AGGCTATCG	BRDN00011	124	11	1	561	548	0
AGGCTATCT	BRDN00012	385	226	164	639	646	0
AGGCTATGC	BRDN00012	137	108	3	224	199	18
AGGCTATGG	BRDN00011	319	442	116	782	960	41
AGGCTATTC	BRDN00011	137	120	19	258	142	20
AGGCTATTC	BRDN00011	320	222	2088	505	561	21
AGGCTCAGC	BRDN00012	229	264	852	357	517	67
AGGCTCAGG	BRDN00011	342	226	371	629	949	43
AGGCTCAGG	BRDN00011	266	192	618	540	431	17
AGGCTCATG	BRDN00011	368	345	1072	852	603	58
AGGCTCCAA	BRDN00012	207	180	401	461	506	0
AGGCTCCAG	BRDN00011	568	361	248	898	784	77
AGGCTCCAG	BRDN00012	540	248	704	836	908	27
AGGCTCCAG	BRDN00011	528	588	593	934	1009	45
AGGCTCCAT	BRDN00012	414	611	2416	906	1090	133
AGGCTCCCC	BRDN00012	570	115	24	872	441	0
AGGCTCGCA	BRDN00012	395	443	420	736	653	53
AGGCTCGCA	BRDN00012	413	343	133	754	636	15
AGGCTCGCT	BRDN00011	141	176	108	421	851	3
AGGCTCGGG	BRDN00011	278	127	243	397	378	58
AGGCTCGTT	BRDN00012	48	5	0	206	120	0
AGGCTCTAG	BRDN00011	95	79	1452	275	155	5
AGGCTCTCA	BRDN00011	289	181	122	981	834	6
AGGCTCTCG	BRDN00011	117	180	1108	312	754	27
AGGCTCTGG	BRDN00011	351	283	809	605	810	31
AGGCTCTGT	BRDN00012	151	157	634	248	138	49
AGGCTCTGT	BRDN00012	242	146	99	289	472	2
AGGCTGAAC	BRDN00012	319	172	55	350	280	28
AGGCTGAAG	BRDN00011	379	407	1003	699	621	59
AGGCTGAAG	BRDN00012	300	33	259	458	8	0
AGGCTGAAT	BRDN00012	499	238	634	711	778	40
AGGCTGACA	BRDN00012	475	363	1542	814	1183	7
AGGCTGACA	BRDN00012	136	126	264	226	175	18
AGGCTGACC	BRDN00011	153	127	130	203	134	9
AGGCTGACT	BRDN00011	344	1	0	874	302	0
AGGCTGAGC	BRDN00012	59	46	810	159	134	8
AGGCTGATA	BRDN00011	377	259	85	634	608	43
AGGCTGATG	BRDN00011	225	187	22	364	420	0
AGGCTGATT	BRDN00012	64	1	0	237	39	0
AGGCTGCAA	BRDN00011	111	118	177	272	150	0
AGGCTGCAA	BRDN00011	232	363	1330	534	830	23
AGGCTGCAC	BRDN00011	69	72	610	156	105	6
AGGCTGCAG	BRDN00012	401	373	3115	982	1213	70
AGGCTGCGG	BRDN00011	267	261	108	387	201	0
AGGCTGCGG	BRDN00011	267	359	81	413	531	25
AGGCTGCTG	BRDN00012	164	177	681	341	460	17
AGGCTGGAA	BRDN00012	569	365	454	1129	1923	51
AGGCTGGAA	BRDN00011	207	216	111	472	650	0
AGGCTGGAC	BRDN00012	181	157	305	319	371	4
AGGCTGGAC	BRDN00012	449	385	1011	845	1074	46
AGGCTGGCA	BRDN00012	533	470	498	765	924	33
AGGCTGGCA	BRDN00011	626	408	2211	948	1300	38
AGGCTGGCC	BRDN00011	275	360	1009	525	845	10
AGGCTGGCC	BRDN00012	309	216	205	540	743	8
AGGCTGGCG	BRDN00012	259	385	337	605	608	44
AGGCTGGCG	BRDN00012	191	130	143	277	147	4
AGGCTGGCG	BRDN00012	652	755	2170	1465	1165	201
AGGCTGGCT	BRDN00012	654	492	2121	1014	1341	25
AGGCTGGCT	BRDN00011	86	2	0	175	0	0
AGGCTGGGC	BRDN00012	273	285	223	759	659	6
AGGCTGGTA	BRDN00011	228	193	323	448	228	7
AGGCTGGTG	BRDN00011	242	247	880	323	185	38
AGGCTGTAG	BRDN00012	289	350	320	467	482	84
AGGCTGTAT	BRDN00012	215	401	235	517	593	15
AGGCTGTCG	BRDN00012	268	351	1117	745	452	33
AGGCTGTGT	BRDN00012	36	0	1	294	246	0
AGGCTGTTA	BRDN00011	167	134	17	201	474	4
AGGCTGTTG	BRDN00012	848	725	2016	1659	1573	107
AGGCTGTTT	BRDN00011	174	160	165	486	405	82
AGGCTGTTT	BRDN00011	112	7	0	646	729	0
AGGCTTAAA	BRDN00012	551	645	248	979	771	52
AGGCTTACA	BRDN00011	288	379	658	505	366	48
AGGCTTACA	BRDN00011	556	649	1084	1077	1032	97
AGGCTTACC	BRDN00011	144	128	1364	333	377	22
AGGCTTACG	BRDN00011	211	330	1270	336	579	49
AGGCTTAGT	BRDN00012	308	194	1174	483	581	7
AGGCTTCAA	BRDN00011	192	71	0	543	111	0
AGGCTTCCC	BRDN00012	482	344	2394	838	773	59
AGGCTTCCT	BRDN00012	155	281	903	432	428	41
AGGCTTCGA	BRDN00011	119	80	1	171	259	0
AGGCTTCTC	BRDN00011	175	130	476	391	310	17
AGGCTTCTT	BRDN00011	254	177	1148	462	300	51
AGGCTTGGA	BRDN00012	21	0	0	273	146	0
AGGCTTGGC	BRDN00012	363	241	578	529	526	5
AGGCTTGGG	BRDN00012	104	65	6	273	256	0
AGGCTTGGT	BRDN00011	288	7	0	622	499	0
AGGCTTGTG	BRDN00011	85	0	1	229	40	0
AGGCTTTCC	BRDN00011	437	385	86	875	884	31
AGGCTTTGG	BRDN00011	509	492	1631	919	1016	18
AGGGAAAAA	BRDN00011	92	108	12	307	223	0
AGGGAAAAC	BRDN00011	137	175	1162	292	320	12
AGGGAAACA	BRDN00011	297	196	625	359	390	7
AGGGAAACC	BRDN00012	1072	1123	405	1702	1699	197
AGGGAAACC	BRDN00011	215	2	0	454	184	0
AGGGAAAGA	BRDN00011	184	136	24	280	252	6
AGGGAAAGA	BRDN00012	362	370	1042	880	914	50
AGGGAAAGA	BRDN00012	133	107	30	213	278	0
AGGGAAAGA	BRDN00011	183	211	2	360	251	4
AGGGAAAGT	BRDN00011	514	353	649	846	958	23
AGGGAAATC	BRDN00011	116	124	0	237	162	0
AGGGAAATG	BRDN00011	251	337	161	501	314	21
AGGGAAATG	BRDN00012	124	138	810	275	550	5
AGGGAAATT	BRDN00012	410	386	1462	735	835	23
AGGGAACAA	BRDN00012	169	39	0	671	239	0
AGGGAACAT	BRDN00011	102	133	123	160	315	6
AGGGAACCA	BRDN00011	177	94	113	287	613	3
AGGGAACCC	BRDN00012	265	159	160	337	272	32
AGGGAACCT	BRDN00011	104	98	96	128	83	26
AGGGAACCT	BRDN00012	454	372	613	784	479	24
AGGGAACGA	BRDN00012	451	30	1	675	779	0
AGGGAACTA	BRDN00012	96	14	1	519	270	0
AGGGAACTG	BRDN00011	189	87	0	472	691	38
AGGGAACTG	BRDN00011	107	97	99	286	120	0
AGGGAAGAA	BRDN00012	256	260	100	526	650	15
AGGGAAGAC	BRDN00012	147	153	113	408	330	5
AGGGAAGCC	BRDN00012	171	196	54	347	119	59
AGGGAAGGA	BRDN00012	361	173	1079	618	371	55
AGGGAAGTC	BRDN00011	95	26	65	209	223	5
AGGGAAGTC	BRDN00012	282	542	1032	522	735	96
AGGGAAGTC	BRDN00012	262	170	97	362	281	51
AGGGAAGTC	BRDN00011	87	104	60	235	207	8
AGGGAAGTC	BRDN00012	433	273	418	838	707	30
AGGGAATGT	BRDN00012	249	356	28	529	538	77
AGGGAATTC	BRDN00011	145	133	0	214	148	0
AGGGAATTC	BRDN00012	249	173	47	411	177	1
AGGGACAAT	BRDN00012	203	272	2512	372	222	36
AGGGACACC	BRDN00011	292	355	68	696	530	36
AGGGACACT	BRDN00011	248	95	565	592	520	19
AGGGACATA	BRDN00012	102	74	8	263	198	10
AGGGACATT	BRDN00012	261	247	1266	441	332	34
AGGGACCAA	BRDN00012	363	283	511	544	369	70
AGGGACCAG	BRDN00012	140	241	1	285	263	23
AGGGACCAT	BRDN00012	173	84	106	413	307	13
AGGGACCGT	BRDN00011	494	394	1468	895	874	69
AGGGACGCT	BRDN00012	105	9	358	493	294	0
AGGGACGGA	BRDN00012	649	494	2164	1127	821	117
AGGGACGTG	BRDN00012	553	589	1378	796	954	90
AGGGACGTT	BRDN00011	116	103	13	314	355	8
AGGGACTAA	BRDN00012	644	523	547	1238	894	32
AGGGACTCA	BRDN00011	251	17	7	343	298	0
AGGGACTCC	BRDN00011	291	258	283	728	718	27
AGGGACTGA	BRDN00011	116	109	9	194	76	1
AGGGACTGC	BRDN00011	514	455	4056	964	948	47
AGGGACTGC	BRDN00012	185	142	266	423	326	12
AGGGACTGC	BRDN00011	99	5	0	252	27	0
AGGGACTGT	BRDN00011	407	263	941	728	535	8
AGGGACTTG	BRDN00012	280	175	503	458	295	14
AGGGAGAAA	BRDN00012	455	619	975	1134	948	163
AGGGAGAAA	BRDN00011	239	220	397	492	514	33
AGGGAGAAC	BRDN00012	482	441	702	913	725	77
AGGGAGAAC	BRDN00011	154	231	198	492	480	18
AGGGAGACA	BRDN00012	157	142	110	218	665	4
AGGGAGACA	BRDN00012	139	158	51	212	602	0
AGGGAGATC	BRDN00011	200	248	291	406	248	0
AGGGAGATC	BRDN00012	206	79	110	304	211	16
AGGGAGCAT	BRDN00012	442	558	2174	966	1449	76
AGGGAGCCA	BRDN00011	120	138	0	208	241	13
AGGGAGCGA	BRDN00012	178	94	75	418	500	0
AGGGAGGAA	BRDN00011	298	173	72	636	513	16
AGGGAGGCC	BRDN00011	231	20	22	447	98	0
AGGGAGGCC	BRDN00012	134	164	0	256	242	8
AGGGAGGCT	BRDN00012	120	138	102	234	188	35
AGGGAGGGA	BRDN00012	529	660	733	1000	855	119
AGGGAGGTT	BRDN00012	324	475	384	621	325	43
AGGGAGTAC	BRDN00011	151	29	8	208	118	19
AGGGAGTCG	BRDN00012	140	81	34	426	427	0
AGGGAGTCT	BRDN00011	377	269	155	652	611	40
AGGGAGTGC	BRDN00011	72	75	0	308	587	0
AGGGAGTGT	BRDN00012	15	9	0	411	141	0
AGGGAGTTC	BRDN00011	219	222	210	274	321	23
AGGGATCCC	BRDN00011	94	1	0	290	40	0
AGGGATCCT	BRDN00011	349	347	83	667	772	24
AGGGATCGT	BRDN00011	375	295	585	578	1087	24
AGGGATCGT	BRDN00012	154	167	476	273	292	9
AGGGATCGT	BRDN00012	789	677	1086	1263	1514	55
AGGGATCTC	BRDN00011	319	176	15	589	497	9
AGGGATGAC	BRDN00012	123	152	34	128	39	11
AGGGATGCC	BRDN00012	492	415	879	870	507	84
AGGGATGGA	BRDN00012	432	432	1418	788	773	55
AGGGATGGC	BRDN00012	188	82	75	265	187	0
AGGGATGGC	BRDN00012	679	374	1660	1138	1013	66
AGGGATTTC	BRDN00011	314	248	420	810	741	6
AGGGCAAAT	BRDN00011	300	229	1013	695	529	22
AGGGCAACA	BRDN00011	282	90	44	416	623	0
AGGGCAACC	BRDN00012	125	102	23	280	253	49
AGGGCAACC	BRDN00012	88	68	283	301	530	9
AGGGCAAGA	BRDN00012	205	169	5481	259	236	71
AGGGCAATA	BRDN00011	200	127	223	317	350	35
AGGGCAATG	BRDN00012	66	26	0	248	122	0
AGGGCACAA	BRDN00012	605	387	564	975	1088	1
AGGGCACCC	BRDN00012	730	733	1193	1328	1764	139
AGGGCACCG	BRDN00011	80	49	69	221	284	4
AGGGCACGC	BRDN00012	166	140	176	239	361	27
AGGGCACGC	BRDN00012	68	42	143	173	113	0
AGGGCACGG	BRDN00012	135	51	0	308	357	0
AGGGCAGAC	BRDN00012	531	351	193	965	759	73
AGGGCAGCC	BRDN00012	175	123	76	389	341	27
AGGGCAGGA	BRDN00011	263	339	1003	629	782	65
AGGGCAGGT	BRDN00011	193	168	935	514	544	13
AGGGCAGTC	BRDN00012	336	384	376	604	880	41
AGGGCATAC	BRDN00011	419	379	451	995	1002	27
AGGGCATAC	BRDN00012	127	30	32	188	55	8
AGGGCATAG	BRDN00012	149	56	66	250	108	9
AGGGCATCG	BRDN00012	364	396	1016	739	1416	83
AGGGCATCG	BRDN00011	478	454	572	887	775	14
AGGGCATGC	BRDN00011	101	0	1	272	90	0
AGGGCATGC	BRDN00011	243	0	1	488	118	0
AGGGCATGT	BRDN00012	107	44	0	268	127	0
AGGGCCACC	BRDN00012	572	499	789	766	878	31
AGGGCCACC	BRDN00011	505	424	1127	985	864	19
AGGGCCACC	BRDN00011	172	72	267	340	174	14
AGGGCCACC	BRDN00011	169	73	15	239	376	16
AGGGCCACT	BRDN00012	71	88	183	132	249	12
AGGGCCAGA	BRDN00011	77	20	0	240	265	0
AGGGCCAGC	BRDN00012	26	0	17	421	345	0
AGGGCCATG	BRDN00011	394	0	1	844	504	0
AGGGCCCCA	BRDN00011	141	93	1387	278	235	21
AGGGCCCGG	BRDN00012	196	62	1	426	576	15
AGGGCCCGG	BRDN00012	51	41	985	31	7	39
AGGGCCGAC	BRDN00012	246	232	396	371	659	20
AGGGCCGAC	BRDN00011	111	90	677	196	207	4
AGGGCCGCA	BRDN00012	585	392	1191	998	1163	10
AGGGCCGGC	BRDN00011	87	122	3	160	184	34
AGGGCCGGC	BRDN00012	76	51	2	129	112	6
AGGGCCGTA	BRDN00012	440	444	5536	905	1151	78
AGGGCCGTG	BRDN00012	386	385	1410	753	632	127
AGGGCCGTG	BRDN00012	128	110	107	291	330	0
AGGGCCTAC	BRDN00011	196	228	123	390	216	52
AGGGCCTCC	BRDN00011	158	23	0	348	46	0
AGGGCCTTT	BRDN00012	732	725	2406	1444	2103	60
AGGGCGAAC	BRDN00012	149	181	326	415	359	25
AGGGCGAAC	BRDN00011	107	55	967	284	222	0
AGGGCGACG	BRDN00011	14	0	0	86	77	0
AGGGCGACT	BRDN00012	110	114	2	159	61	32
AGGGCGAGC	BRDN00012	875	725	3335	1327	1719	42
AGGGCGAGC	BRDN00012	151	41	66	367	139	3
AGGGCGATA	BRDN00011	158	151	48	328	400	37
AGGGCGATG	BRDN00011	167	81	3	331	278	5
AGGGCGCCA	BRDN00011	322	437	294	706	757	32
AGGGCGCGC	BRDN00012	215	231	323	702	1212	0
AGGGCGCGC	BRDN00011	92	103	118	187	22	10
AGGGCGCTA	BRDN00012	558	510	671	1074	802	23
AGGGCGCTA	BRDN00012	313	298	485	601	504	57
AGGGCGTCA	BRDN00012	322	203	174	793	547	21
AGGGCGTCA	BRDN00012	131	67	0	230	253	0
AGGGCGTCC	BRDN00011	235	243	142	476	471	0
AGGGCGTTT	BRDN00012	106	107	254	299	276	11
AGGGCTAAG	BRDN00012	461	380	317	963	1503	11
AGGGCTAAT	BRDN00011	439	362	601	800	585	26
AGGGCTATG	BRDN00012	315	283	145	539	418	56
AGGGCTCAA	BRDN00011	194	162	732	339	410	33
AGGGCTCAC	BRDN00012	258	208	892	446	463	7
AGGGCTCAG	BRDN00012	142	65	325	458	355	97
AGGGCTCCA	BRDN00011	480	217	223	580	520	8
AGGGCTCCA	BRDN00012	284	214	36	450	215	16
AGGGCTCCC	BRDN00011	155	219	138	173	267	49
AGGGCTCCC	BRDN00012	516	482	217	911	972	21
AGGGCTCCC	BRDN00012	184	212	407	401	488	9
AGGGCTGCA	BRDN00012	405	332	870	509	613	3
AGGGCTGGA	BRDN00011	97	0	0	484	36	1
AGGGCTGGA	BRDN00012	297	272	2940	466	600	74
AGGGCTGGC	BRDN00011	118	67	1138	244	155	27
AGGGCTGGC	BRDN00011	128	178	165	189	226	8
AGGGCTGGC	BRDN00012	127	97	6	190	244	0

AGGGCTTCA	BRDN00012	563	378	1898	856	1008	34
AGGGCTTCG	BRDN00011	641	464	3915	1252	1104	72
AGGGCTTCG	BRDN00012	143	88	436	174	126	13
AGGGCTTCT	BRDN00012	145	18	0	359	266	1
AGGGCTTTC	BRDN00011	226	152	466	515	607	8
AGGGGAAA	BRDN00012	113	97	32	183	271	15
AGGGGAAA	BRDN00011	291	566	418	614	872	36
AGGGGAAA	BRDN00011	189	113	211	421	414	21
AGGGGAAA	BRDN00011	308	96	115	529	637	18
AGGGGAAAT	BRDN00011	563	552	7741	874	1089	197
AGGGGAACA	BRDN00011	125	61	102	154	261	59
AGGGGAAC	BRDN00012	181	156	737	437	235	28
AGGGGAACT	BRDN00011	363	9	0	661	744	0
AGGGGAAG	BRDN00012	477	479	738	727	775	21
AGGGGAAG	BRDN00011	385	208	1147	665	299	16
AGGGGAAG	BRDN00011	334	275	225	744	575	12
AGGGGAAT	BRDN00012	108	90	141	170	101	11
AGGGGACA	BRDN00012	204	178	922	298	340	37
AGGGGACA	BRDN00011	128	165	681	277	424	61
AGGGGACA	BRDN00012	114	116	173	151	97	0
AGGGGACA	BRDN00011	304	177	404	1111	326	49
AGGGGACCT	BRDN00011	308	204	1830	743	572	55
AGGGGACCT	BRDN00011	252	203	35	550	478	6
AGGGGACG	BRDN00012	409	334	374	708	727	61
AGGGGACTC	BRDN00012	400	276	474	713	511	22
AGGGGACTT	BRDN00011	283	416	635	491	401	207
AGGGGAGC	BRDN00011	253	326	562	533	844	42
AGGGGAGT	BRDN00011	355	0	0	981	194	0
AGGGGATA	BRDN00011	235	249	122	610	704	0
AGGGGATG	BRDN00012	443	298	1209	862	894	21
AGGGGATG	BRDN00012	182	38	0	352	489	0
AGGGGATG	BRDN00011	171	142	44	398	248	53
AGGGGATG	BRDN00012	142	72	1	206	193	0
AGGGGATT	BRDN00012	49	0	1	186	30	0
AGGGGCAA	BRDN00012	159	134	480	247	319	26
AGGGGCAA	BRDN00012	446	416	139	925	1151	56
AGGGGCAG	BRDN00011	111	135	376	194	208	26
AGGGGCAG	BRDN00011	121	118	38	271	721	0
AGGGGCAG	BRDN00012	75	15	2	353	255	0
AGGGGCAG	BRDN00011	176	252	602	308	153	30
AGGGGCAG	BRDN00012	447	478	2112	773	690	81
AGGGGCAGT	BRDN00012	219	122	826	343	127	37
AGGGGCATG	BRDN00011	104	101	1857	176	369	81
AGGGGCCAT	BRDN00011	252	173	178	386	169	7
AGGGGCCCA	BRDN00012	237	203	356	525	560	28
AGGGGCCCG	BRDN00012	449	504	1444	781	1130	74
AGGGGCCCG	BRDN00012	191	224	203	303	439	7
AGGGGCCCG	BRDN00012	258	167	853	376	513	90
AGGGGCCTC	BRDN00011	455	527	349	997	970	75
AGGGGCGA	BRDN00012	168	1	0	540	13	0
AGGGGCGG	BRDN00012	373	15	76	812	669	0
AGGGGCGG	BRDN00011	414	372	650	954	563	51
AGGGGCTG	BRDN00012	557	560	2553	1208	1019	104
AGGGGCTG	BRDN00011	286	208	187	617	423	6
AGGGGCTG	BRDN00012	429	106	675	1158	813	22
AGGGGCTGT	BRDN00011	343	340	463	599	775	82
AGGGGGAG	BRDN00011	239	199	663	507	374	25
AGGGGGATT	BRDN00012	132	112	413	259	284	9
AGGGGGCC	BRDN00011	541	400	48	916	1137	34
AGGGGGCC	BRDN00011	89	75	1	258	201	0
AGGGGGCG	BRDN00012	99	64	56	176	57	11
AGGGGGCG	BRDN00011	282	153	817	355	854	33
AGGGGGCT	BRDN00012	299	238	111	618	794	0
AGGGGGCT	BRDN00012	219	331	312	503	232	16
AGGGGGGA	BRDN00012	48	88	34	189	145	0
AGGGGGGC	BRDN00011	519	209	114	851	574	31
AGGGGGGC	BRDN00012	123	100	54	129	137	0
AGGGGGGT	BRDN00012	345	250	339	580	602	50
AGGGGGTG	BRDN00012	224	239	21	699	618	37
AGGGGGTG	BRDN00011	267	174	1118	453	581	7
AGGGGGTG	BRDN00012	62	55	408	218	119	19
AGGGGTACA	BRDN00011	161	67	44	329	329	8
AGGGGTACA	BRDN00012	229	518	156	521	696	65
AGGGGTATC	BRDN00012	489	493	305	872	652	44
AGGGGTCAG	BRDN00012	171	161	0	353	247	0
AGGGGTCCA	BRDN00012	244	188	1056	603	507	42
AGGGGTCG	BRDN00011	347	390	382	603	332	83
AGGGGTCTT	BRDN00011	191	60	259	351	320	2
AGGGGTCTT	BRDN00011	100	131	9	280	530	0
AGGGGTGA	BRDN00012	444	347	819	600	752	30
AGGGGTGG	BRDN00011	101	118	136	244	201	5
AGGGGTTAT	BRDN00012	137	132	221	175	186	33
AGGGGTTCT	BRDN00011	213	201	261	183	391	0
AGGGGTTCT	BRDN00011	87	109	0	256	195	0
AGGGGTTG	BRDN00011	104	84	502	131	126	13
AGGGTAACA	BRDN00011	398	325	772	833	1014	60
AGGGTAACT	BRDN00011	254	330	540	454	647	55
AGGGTAAGC	BRDN00011	303	259	758	493	589	38
AGGGTACAA	BRDN00011	543	490	980	983	1147	97
AGGGTACAT	BRDN00012	234	164	77	424	474	10
AGGGTACCA	BRDN00011	421	439	390	889	865	9
AGGGTACGG	BRDN00011	288	213	836	398	437	21
AGGGTACTG	BRDN00012	323	295	351	643	689	14
AGGGTAGCT	BRDN00012	226	27	1	844	835	0
AGGGTAGTG	BRDN00012	464	170	119	699	342	2
AGGGTAGTT	BRDN00011	510	439	280	997	854	64
AGGGTATAA	BRDN00012	250	64	1	759	357	0
AGGGTATCC	BRDN00012	274	152	1121	398	474	35
AGGGTATCG	BRDN00011	459	493	380	794	1013	93
AGGGTCAAT	BRDN00011	30	73	181	104	96	0
AGGGTCACA	BRDN00011	182	158	290	303	266	8
AGGGTCACG	BRDN00012	780	685	2189	1021	900	198
AGGGTCAGT	BRDN00012	437	577	843	742	787	18
AGGGTCAGT	BRDN00012	521	485	136	923	801	107
AGGGTCATA	BRDN00012	298	24	13	390	230	6
AGGGTCCAA	BRDN00012	216	236	26	487	699	39
AGGGTCCAC	BRDN00012	114	84	604	229	123	5
AGGGTCCGA	BRDN00012	471	445	103	809	412	7
AGGGTCCGA	BRDN00011	368	138	1271	709	788	45
AGGGTCGAT	BRDN00011	248	246	1	389	315	56
AGGGTCGGT	BRDN00012	353	390	1843	784	983	47
AGGGTCGGT	BRDN00011	121	217	642	216	302	62
AGGGTCTAA	BRDN00011	145	12	451	550	383	7
AGGGTCTAC	BRDN00011	286	275	90	532	506	9
AGGGTCTAT	BRDN00011	258	48	0	698	488	0
AGGGTCTCG	BRDN00011	210	310	207	353	257	5
AGGGTCTGG	BRDN00011	377	313	3287	782	677	42
AGGGTCTTC	BRDN00011	170	141	12	233	311	1
AGGGTGAA	BRDN00012	414	211	580	812	896	3
AGGGTGAC	BRDN00012	115	89	0	189	57	8
AGGGTGAC	BRDN00011	483	428	782	945	1262	37
AGGGTGACT	BRDN00012	272	15	55	649	206	0
AGGGTGAG	BRDN00011	84	96	312	251	57	5
AGGGTGAG	BRDN00011	203	187	74	238	513	4
AGGGTGAG	BRDN00011	306	247	4827	513	417	329
AGGGTGAGT	BRDN00012	209	298	7	405	324	38
AGGGTGCAA	BRDN00011	138	150	925	390	456	14
AGGGTGCAA	BRDN00011	93	202	59	229	122	18
AGGGTGCAC	BRDN00012	427	434	373	752	626	50
AGGGTGCAT	BRDN00012	145	173	351	384	436	13
AGGGTGGC	BRDN00012	49	40	359	95	179	25
AGGGTGGC	BRDN00012	96	86	0	192	113	0
AGGGTGGC	BRDN00012	108	185	74	228	302	31
AGGGTGGCT	BRDN00011	44	22	0	111	20	0
AGGGTGGG	BRDN00011	80	86	112	298	520	12
AGGGTGGG	BRDN00012	316	343	151	730	540	6
AGGGTGGG	BRDN00012	275	181	795	585	389	22
AGGGTGGT	BRDN00012	178	2	1	305	357	0
AGGGTGTAA	BRDN00011	196	301	185	352	262	20
AGGGTGTCA	BRDN00012	322	214	225	587	622	55
AGGGTGTCC	BRDN00012	250	197	82	387	379	13
AGGGTGTCG	BRDN00012	336	360	669	759	1363	74
AGGGTGTGA	BRDN00012	478	376	122	926	617	9
AGGGTGTGC	BRDN00012	107	88	48	160	156	8
AGGGTGTTC	BRDN00011	42	106	54	80	36	52
AGGGTGTTG	BRDN00012	422	404	296	749	1200	24
AGGGTGTTG	BRDN00011	88	124	40	203	264	1
AGGGTGTTG	BRDN00011	356	449	549	760	411	55
AGGGTTCAG	BRDN00011	83	87	111	182	184	0
AGGGTTCAG	BRDN00011	470	561	492	974	646	43
AGGGTTCAG	BRDN00011	489	509	280	883	560	60
AGGGTTCGT	BRDN00011	39	1	0	417	120	0
AGGGTTGCT	BRDN00011	118	28	0	359	95	0
AGGGTTTGC	BRDN00011	116	190	209	189	102	12
AGGTAAAAA	BRDN00011	149	140	235	255	479	6
AGGTAAAAC	BRDN00011	66	135	57	210	108	0
AGGTAAAAG	BRDN00012	422	448	389	780	591	25
AGGTAAAAT	BRDN00012	292	265	154	573	945	7
AGGTAAACA	BRDN00011	334	288	1159	927	1458	91
AGGTAAAGC	BRDN00012	542	545	580	1140	1382	75
AGGTAAATC	BRDN00011	223	237	2193	490	420	66
AGGTAACAG	BRDN00011	127	119	292	143	105	31
AGGTAACCC	BRDN00011	292	77	195	1270	413	16
AGGTAACCT	BRDN00011	432	482	670	921	971	38
AGGTAACCT	BRDN00012	383	298	468	641	724	54
AGGTAACCT	BRDN00011	171	186	242	367	212	11
AGGTAACGC	BRDN00011	167	249	6634	322	252	118
AGGTAACGG	BRDN00012	123	27	3	294	27	0
AGGTAACTG	BRDN00011	119	61	213	337	218	6
AGGTAAGAG	BRDN00012	113	80	1	337	236	0
AGGTAAGCA	BRDN00012	218	179	296	435	379	78
AGGTAAGCC	BRDN00012	733	770	1534	1263	1453	113
AGGTAAGG	BRDN00012	423	581	442	760	937	41
AGGTAAGTC	BRDN00011	141	194	593	294	539	48
AGGTAAGTG	BRDN00011	271	225	51	357	524	15
AGGTAATAA	BRDN00012	164	104	72	236	308	7
AGGTAATAG	BRDN00012	55	190	165	199	180	5
AGGTAATAT	BRDN00011	94	156	5	218	170	0
AGGTAATCA	BRDN00012	196	250	111	369	583	5
AGGTAATGC	BRDN00012	283	376	426	504	502	26
AGGTAATGT	BRDN00012	408	600	576	800	652	87
AGGTACAAA	BRDN00011	0	0	0	191	53	0
AGGTACACA	BRDN00011	517	349	475	895	770	68
AGGTACACC	BRDN00012	264	154	141	376	349	31
AGGTACACT	BRDN00011	276	146	322	466	478	36
AGGTACAGC	BRDN00012	252	270	1791	476	325	45
AGGTACATG	BRDN00011	304	267	898	567	218	29
AGGTACCAC	BRDN00011	314	317	641	655	786	42
AGGTACCAT	BRDN00012	309	519	1093	791	721	69
AGGTACCAT	BRDN00011	286	436	1	583	649	8
AGGTACCCA	BRDN00012	380	498	344	654	931	144
AGGTACCCC	BRDN00011	196	51	0	427	779	0
AGGTACCCG	BRDN00011	127	127	184	229	229	7
AGGTACCCT	BRDN00012	618	356	94	1201	920	23
AGGTACCTG	BRDN00012	506	122	11	933	232	0
AGGTACCTG	BRDN00012	841	648	2167	1402	1476	134
AGGTACGTG	BRDN00012	135	188	111	448	904	5
AGGTACTCA	BRDN00011	223	241	1064	406	180	54
AGGTACTCC	BRDN00011	241	158	218	453	408	0
AGGTACTCC	BRDN00012	605	663	815	1095	909	20
AGGTACTCG	BRDN00011	137	78	0	345	338	11
AGGTACTGA	BRDN00012	554	631	642	1200	1627	35
AGGTACTGC	BRDN00011	139	202	176	215	148	45
AGGTACTGT	BRDN00012	369	275	1320	736	526	52
AGGTACTTA	BRDN00012	581	237	289	994	1041	10
AGGTACTTG	BRDN00011	232	77	383	389	385	10
AGGTAGAAG	BRDN00012	230	235	698	405	489	41
AGGTAGAAG	BRDN00011	387	541	637	789	681	24
AGGTAGAAT	BRDN00012	131	140	0	185	178	0
AGGTAGACA	BRDN00011	137	165	555	189	280	140
AGGTAGACG	BRDN00012	212	254	119	468	422	5
AGGTAGACG	BRDN00012	176	101	12	298	255	0
AGGTAGAG	BRDN00011	343	322	521	579	801	22
AGGTAGCCT	BRDN00011	389	405	497	682	1168	52
AGGTAGCGC	BRDN00011	117	95	193	175	377	8
AGGTAGCTA	BRDN00012	415	508	1000	598	792	106
AGGTAGGG	BRDN00012	464	514	39	738	576	33
AGGTAGGG	BRDN00011	452	408	297	1006	700	38
AGGTAGGTA	BRDN00011	265	303	686	448	444	12
AGGTAGTAG	BRDN00012	357	87	111	615	619	2
AGGTAGTCA	BRDN00011	452	270	164	908	831	48
AGGTAGTGA	BRDN00012	251	196	366	516	826	0
AGGTAGTGC	BRDN00011	389	352	52	693	742	38
AGGTAGTGC	BRDN00012	155	189	132	367	655	17
AGGTAGTGC	BRDN00012	174	137	1086	303	231	30
AGGTAGTTC	BRDN00012	194	156	704	288	319	23
AGGTAGTTG	BRDN00011	265	348	746	643	640	11
AGGTATAAA	BRDN00012	179	301	242	488	549	33
AGGTATCAT	BRDN00012	132	177	287	323	283	43
AGGTATCCT	BRDN00011	180	161	200	253	137	95
AGGTATCTG	BRDN00011	338	317	1611	640	666	53
AGGTATGAC	BRDN00011	360	345	693	653	793	1
AGGTATGAG	BRDN00012	159	331	65	457	371	10
AGGTATGAG	BRDN00011	178	104	0	413	296	0
AGGTATGAT	BRDN00012	166	108	2843	353	380	154
AGGTATGCA	BRDN00011	125	20	63	387	578	24
AGGTATGCA	BRDN00011	474	417	1132	1084	841	57
AGGTATGGG	BRDN00011	354	378	1727	593	613	30
AGGTATGTA	BRDN00011	133	76	740	320	314	74
AGGTATGTG	BRDN00011	144	156	53	232	209	0
AGGTATTCA	BRDN00011	246	24	0	464	384	0
AGGTATTCG	BRDN00011	298	276	220	425	280	12
AGGTATTCT	BRDN00011	527	397	987	758	953	34
AGGTATTTC	BRDN00012	270	175	120	639	577	0
AGGTCAAAG	BRDN00012	156	137	5	236	177	5
AGGTCAAAG	BRDN00011	263	127	38	385	308	12
AGGTCAACT	BRDN00011	215	165	30	405	335	8
AGGTCAAGA	BRDN00011	175	256	96	298	471	28
AGGTCAAGC	BRDN00012	346	389	514	696	728	71
AGGTCAATA	BRDN00011	474	627	237	721	725	1
AGGTCAATA	BRDN00011	176	114	30	444	227	14
AGGTCAATA	BRDN00012	334	445	1472	707	261	53
AGGTCAATA	BRDN00011	206	102	715	588	681	30
AGGTCACAT	BRDN00011	256	174	536	396	351	25
AGGTCACCT	BRDN00012	688	626	3168	1122	1593	97
AGGTCACGA	BRDN00012	143	63	0	269	171	0
AGGTCACGG	BRDN00012	147	78	1	328	247	0
AGGTCACGT	BRDN00012	255	74	65	550	494	0
AGGTCACTA	BRDN00012	247	244	596	570	707	45
AGGTCACTG	BRDN00012	468	511	221	846	1123	22
AGGTCACTG	BRDN00012	238	213	132	445	318	32
AGGTCAGCA	BRDN00012	332	375	2157	604	607	120
AGGTCAGGC	BRDN00012	517	282	53	959	802	14
AGGTCAGTC	BRDN00012	209	387	782	410	381	19
AGGTCAGTC	BRDN00011	267	163	372	576	1185	10
AGGTCATCA	BRDN00011	148	194	56	261	280	5
AGGTCATCA	BRDN00012	434	391	610	806	1052	45
AGGTCATCA	BRDN00011	184	264	27	452	382	32
AGGTCATCA	BRDN00011	166	1	156	465	488	0
AGGTCATCC	BRDN00012	133	136	719	166	260	18
AGGTCATGA	BRDN00011	443	420	634	1006	1348	11
AGGTCATGC	BRDN00012	154	125	9	245	251	11
AGGTCATTA	BRDN00012	234	136	1192	429	579	99
AGGTCATTC	BRDN00012	120	85	108	164	44	28
AGGTCATTT	BRDN00012	121	55	16	190	214	0
AGGTCCAAA	BRDN00012	38	0	0	417	237	0
AGGTCCAAG	BRDN00011	436	553	2925	843	1212	60
AGGTCCACG	BRDN00012	220	301	456	408	238	12
AGGTCCAGA	BRDN00011	400	370	451	838	804	28
AGGTCCAGG	BRDN00011	467	608	1859	953	748	68
AGGTCCAGG	BRDN00012	545	386	851	1042	1099	27
AGGTCCATG	BRDN00011	503	333	1443	828	687	69
AGGTCCCAA	BRDN00012	190	228	218	321	171	0
AGGTCCCAG	BRDN00012	97	69	0	182	494	0
AGGTCCCAT	BRDN00012	167	159	998	298	166	37
AGGTCCCCG	BRDN00011	246	182	190	463	298	36
AGGTCCCGG	BRDN00012	85	47	789	218	163	248
AGGTCCCTA	BRDN00012	509	450	1707	773	1732	104
AGGTCCCTG	BRDN00011	284	61	0	639	499	0
AGGTCCCTT	BRDN00012	272	86	0	767	352	3
AGGTCCGAC	BRDN00012	200	74	20	319	258	18
AGGTCCGAG	BRDN00011	320	206	12	418	467	14
AGGTCCGGG	BRDN00011	568	635	3076	1029	899	93
AGGTCCTAC	BRDN00012	716	710	473	1315	1262	28
AGGTCCTAC	BRDN00011	170	45	220	331	113	4
AGGTCCTGA	BRDN00011	177	249	229	301	218	32
AGGTCCTGG	BRDN00012	192	85	0	415	231	0
AGGTCCTTC	BRDN00011	84	84	3	511	388	0
AGGTCCTTC	BRDN00012	445	380	278	712	811	46
AGGTCGAGT	BRDN00011	393	451	272	711	737	80
AGGTCGAGT	BRDN00012	211	175	647	353	345	27
AGGTCGATA	BRDN00012	57	0	0	297	65	0
AGGTCGATG	BRDN00011	231	313	862	529	446	57
AGGTCGATG	BRDN00011	178	143	556	254	490	3
AGGTCGCAC	BRDN00011	95	54	89	233	383	0
AGGTCGCTC	BRDN00012	304	303	4242	458	443	72
AGGTCGGAG	BRDN00012	222	250	70	353	96	25
AGGTCGGCC	BRDN00012	427	367	1074	803	557	32
AGGTCGGG	BRDN00012	207	68	331	472	356	7
AGGTCGGG	BRDN00011	222	196	83	283	251	41
AGGTCGTAC	BRDN00012	519	706	728	1267	955	33
AGGTCGTAG	BRDN00012	247	210	321	481	512	10
AGGTCGTCA	BRDN00012	474	555	714	959	616	72
AGGTCGTGA	BRDN00012	430	389	715	749	644	25
AGGTCGTGG	BRDN00012	164	262	569	266	147	25
AGGTCTACG	BRDN00011	118	121	169	226	199	12
AGGTCTAGC	BRDN00012	103	1	1	963	187	0
AGGTCTAGG	BRDN00011	296	277	99	539	434	22
AGGTCTATA	BRDN00011	179	196	112	357	204	11
AGGTCTATT	BRDN00011	311	382	187	493	721	30
AGGTCTCAA	BRDN00012	498	557	213	1082	671	74
AGGTCTCAG	BRDN00012	346	146	170	756	728	0
AGGTCTCAT	BRDN00012	485	610	182	1073	1296	34
AGGTCTCCC	BRDN00012	262	210	579	490	706	9
AGGTCTCGC	BRDN00011	96	157	683	214	339	5
AGGTCTCGG	BRDN00012	170	139	154	344	312	4
AGGTCTGAA	BRDN00012	447	359	117	729	786	47
AGGTCTGAG	BRDN00012	170	196	80	511	506	0
AGGTCTGCG	BRDN00012	402	456	3107	763	758	94
AGGTCTGGT	BRDN00011	193	168	633	255	315	68
AGGTCTTAA	BRDN00011	258	379	1624	714	608	42
AGGTCTTCC	BRDN00011	110	149	188	333	457	0
AGGTCTTGG	BRDN00012	271	280	1026	633	1478	27
AGGTCTTGT	BRDN00012	491	517	297	952	803	28
AGGTCTTGT	BRDN00011	124	62	9	355	323	0
AGGTCTTGT	BRDN00011	267	319	639	507	658	0
AGGTGAACG	BRDN00011	237	261	190	507	407	29
AGGTGAAGA	BRDN00012	480	413	891	758	714	14
AGGTGAAGA	BRDN00011	415	283	3425	789	567	40
AGGTGAAGC	BRDN00012	148	79	0	227	251	12
AGGTGAAGC	BRDN00012	165	239	350	357	191	13
AGGTGAAGT	BRDN00012	356	418	1948	698	437	46
AGGTGAATA	BRDN00012	188	168	6	338	333	20
AGGTGACAA	BRDN00011	263	180	28	292	185	10
AGGTGACAG	BRDN00012	178	121	27	228	239	0
AGGTGACAG	BRDN00012	184	267	1255	362	238	53
AGGTGACAT	BRDN00012	602	702	438	1284	1310	44
AGGTGACCA	BRDN00012	226	229	757	359	811	23
AGGTGACCT	BRDN00012	172	163	92	261	352	45
AGGTGACCT	BRDN00012	197	168	208	542	408	28
AGGTGACGA	BRDN00012	251	99	198	651	1212	18
AGGTGACTG	BRDN00012	281	217	173	465	344	23
AGGTGACTG	BRDN00012	221	212	320	263	312	12
AGGTGACTT	BRDN00011	140	138	403	420	164	48
AGGTGAGA	BRDN00012	221	201	198	452	806	65
AGGTGAGCA	BRDN00011	478	482	377	981	720	1
AGGTGAGG	BRDN00011	181	133	185	279	180	4
AGGTGAGTC	BRDN00012	869	744	2895	1337	1330	57
AGGTGATAA	BRDN00012	522	468	1863	878	586	49
AGGTGATAA	BRDN00012	20	14	11	239	766	0
AGGTGATAA	BRDN00011	271	262	372	307	531	38
AGGTGATAA	BRDN00012	166	132	2	499	572	26
AGGTGATAG	BRDN00012	270	311	565	537	478	33
AGGTGATAT	BRDN00011	154	205	133	281	197	9
AGGTGATCC	BRDN00012	519	465	754	726	986	87
AGGTGATCG	BRDN00011	394	537	9508	857	1034	216
AGGTGATGC	BRDN00011	267	231	162	627	776	23
AGGTGATGC	BRDN00012	233	75	846	510	199	9
AGGTGCAAC	BRDN00012	357	308	536	936	633	49
AGGTGCAAT	BRDN00012	461	483	1232	745	981	54
AGGTGCACC	BRDN00012	336	17	18	698	357	0
AGGTGCAGA	BRDN00011	146	95	222	282	315	49
AGGTGCAGC	BRDN00011	150	130	92	305	342	13
AGGTGCAGC	BRDN00011	147	167	973	154	278	40
AGGTGCAGT	BRDN00011	31	0	0	534	341	0
AGGTGCAGT	BRDN00012	415	424	644	907	1210	66
AGGTGCCAA	BRDN00012	155	1	1	1090	696	0
AGGTGCCAT	BRDN00012	624	559	709	1093	813	34
AGGTGCCAT	BRDN00011	387	424	163	997	841	19
AGGTGCCAT	BRDN00012	611	323	453	993	997	0
AGGTGCCCA	BRDN00012	425	596	242	993	720	107
AGGTGCCTC	BRDN00012	245	163	316	419	357	49
AGGTGCGAA	BRDN00011	221	281	14	320	408	46
AGGTGCGAA	BRDN00012	393	414	486	752	508	44
AGGTGCGAC	BRDN00011	307	214	530	371	201	14
AGGTGCGAC	BRDN00011	246	459	567	684	513	11
AGGTGCGCA	BRDN00011	538	442	390	821	914	17
AGGTGCTAG	BRDN00012	181	206	336	394	142	58
AGGTGCTCA	BRDN00011	353	433	461	698	698	59
AGGTGCTGC	BRDN00012	110	27	0	301	142	1
AGGTGCTGC	BRDN00011	101	57	0	160	184	13
AGGTGCTGC	BRDN00011	460	360	477	701	608	39
AGGTGCTGC	BRDN00011	143	125	427	387	367	0
AGGTGCTGG	BRDN00012	287	150	11	503	490	0
AGGTGGAA	BRDN00011	195	211	67	508	521	39
AGGTGGAA	BRDN00012	412	506	334	1011	1045	42
AGGTGGAAT	BRDN00011	132	25	168	248	227	0
AGGTGGACA	BRDN00011	238	131	1149	404	484	48
AGGTGGACA	BRDN00012	169	171	910	300	157	8
AGGTGGACA	BRDN00012	220	119	1100	443	339	43
AGGTGGACA	BRDN00012	59	38	0	182	9	6
AGGTGGACC	BRDN00012	177	101	41	217	298	1
AGGTGGACC	BRDN00011	108	179	79	262	335	8
AGGTGGACT	BRDN00011	110	103	1	198	114	0
AGGTGGAG	BRDN00012	287	381	14	588	507	38
AGGTGGAG	BRDN00011	174	251	24	341	299	9
AGGTGGAG	BRDN00011	107	168	90	255	56	23
AGGTGGAG	BRDN00012	426	350	668	760	734	51
AGGTGGAGT	BRDN00011	59	55	26	182	172	12
AGGTGGAGT	BRDN00011	151	155	2956	459	457	37
AGGTGGCAA	BRDN00012	539	514	1501	888	1669	68
AGGTGGCAA	BRDN00011	81	36	0	104	102	4
AGGTGGCAA	BRDN00011	185	200	544	243	242	42
AGGTGGCAC	BRDN00012	187	124	853	361	388	9
AGGTGGCAT	BRDN00011	62	116	0	106	38	33
AGGTGGCTC	BRDN00012	365	298	372	818	690	9
AGGTGGCTC	BRDN00012	202	158	854	483	283	16
AGGTGGGA	BRDN00011	110	83	5	217	427	40
AGGTGGGA	BRDN00011	608	535	3284	814	686	127
AGGTGGGA	BRDN00012	222	200	117	518	314	6
AGGTGGGC	BRDN00012	289	282	1139	691	708	130
AGGTGGGC	BRDN00011	145	68	17	362	204	10
AGGTGGGG	BRDN00011	298	264	424	424	471	12
AGGTGGGG	BRDN00012	353	428	614	717	749	8
AGGTGGGT	BRDN00012	425	330	660	1055	1145	10
AGGTGGGT	BRDN00011	243	162	737	483	381	38
AGGTGGGT	BRDN00012	217	290	56	369	595	28
AGGTGGTAA	BRDN00011	79	10	9	98	127	0
AGGTGGTAC	BRDN00011	255	210	1090	712	421	56
AGGTGGTAG	BRDN00012	449	463	1122	920	900	89
AGGTGGTAG	BRDN00011	246	228	971	286	371	22
AGGTGGTCA	BRDN00011	359	376	607	589	1211	32
AGGTGGTCA	BRDN00011	222	323	118	435	570	35
AGGTGGTCG	BRDN00012	510	383	737	1193	1364	24
AGGTGGTCT	BRDN00012	160	110	109	245	87	0
AGGTGGTGA	BRDN00012	200	167	252	596	533	13
AGGTGGTGA	BRDN00011	452	225	61	903	695	33
AGGTGGTGC	BRDN00011	282	147	485	441	195	60
AGGTGGTGC	BRDN00011	87	40	396	614	450	9
AGGTGGTGC	BRDN00011	288	24	17	522	438	0
AGGTGGTGT	BRDN00012	258	265	807	409	269	5
AGGTGGTTA	BRDN00011	107	138	578	318	358	7
AGGTGGTTC	BRDN00012	386	359	1419	722	764	22
AGGTGTAAT	BRDN00011	244	196	282	635	662	19
AGGTGTACA	BRDN00011	200	160	62	483	574	12
AGGTGTCAG	BRDN00012	126	105	625	322	519	8
AGGTGTCGG	BRDN00011	125	75	154	331	291	24
AGGTGTCTG	BRDN00011	339	249	84	694	488	44
AGGTGTCTG	BRDN00011	323	261	1010	539	379	14
AGGTGTCTG	BRDN00012	242	177	59	388	417	25
AGGTGTGAA	BRDN00011	118	81	1330	200	309	4
AGGTGTGAA	BRDN00012	76	15	71	283	378	0
AGGTGTGAA	BRDN00012	309	388	1296	571	477	48
AGGTGTGAA	BRDN00011	296	313	210	593	1054	21
AGGTGTGGT	BRDN00011	339	263	353	588	598	68

AGGTGTGGT	BRDN00012	237	192	2191	556	385	59
AGGTGTTAG	BRDN00011	286	175	387	556	372	4
AGGTGTTAT	BRDN00011	129	118	142	293	422	0
AGGTGTTAT	BRDN00011	224	323	351	459	567	16
AGGTGTTCC	BRDN00011	292	306	334	598	418	12
AGGTGTTCG	BRDN00012	463	397	2370	786	1042	32
AGGTGTTCT	BRDN00011	126	76	257	240	139	7
AGGTGTTTC	BRDN00011	139	99	323	220	225	20
AGGTGTTTC	BRDN00012	310	135	373	568	588	4
AGGTGTTTC	BRDN00012	110	94	123	256	131	25
AGGTGTTTG	BRDN00012	104	119	123	258	482	0
AGGTTAAAG	BRDN00011	249	182	210	488	342	20
AGGTTAACA	BRDN00011	206	219	476	367	260	105
AGGTTAATC	BRDN00011	68	0	1	530	78	1
AGGTTACAA	BRDN00012	113	117	374	283	404	13
AGGTTACCA	BRDN00012	454	483	2273	767	1081	59
AGGTTACCT	BRDN00012	327	335	1074	628	587	115
AGGTTACCT	BRDN00012	431	483	514	718	1039	30
AGGTTACGG	BRDN00011	600	691	1869	1253	883	51
AGGTTACTG	BRDN00011	126	57	32	234	267	0
AGGTTAGAG	BRDN00012	150	87	76	364	224	6
AGGTTATAA	BRDN00011	137	146	514	312	316	77
AGGTTATCC	BRDN00011	196	140	251	345	191	0
AGGTTATCG	BRDN00012	263	126	47	376	187	18
AGGTTATTA	BRDN00012	233	150	79	492	373	13
AGGTTCAGT	BRDN00012	312	216	149	503	645	28
AGGTTCATG	BRDN00012	254	189	470	364	576	12
AGGTTCCAG	BRDN00011	395	538	213	834	469	90
AGGTTCCCG	BRDN00011	169	157	69	364	201	8
AGGTTCCTC	BRDN00012	357	68	0	905	443	1
AGGTTCCTG	BRDN00011	121	141	247	272	305	17
AGGTTCCTG	BRDN00011	265	305	90	435	226	35
AGGTTCGCG	BRDN00011	387	431	350	690	811	51
AGGTTCGGC	BRDN00011	176	225	665	303	225	8
AGGTTCTGG	BRDN00011	2	0	0	285	448	0
AGGTTGAAC	BRDN00012	422	484	1297	983	714	57
AGGTTGAAG	BRDN00011	535	485	2344	1091	1215	61
AGGTTGAAT	BRDN00012	449	606	294	845	847	30
AGGTTGACA	BRDN00012	55	0	156	367	425	6
AGGTTGACA	BRDN00011	242	184	93	326	397	9
AGGTTGACA	BRDN00012	551	382	387	1033	913	42
AGGTTGACC	BRDN00011	602	444	1076	1194	1440	27
AGGTTGACT	BRDN00011	389	347	126	687	813	22
AGGTTGACT	BRDN00011	252	163	531	501	316	45
AGGTTGAGG	BRDN00012	203	312	4	523	294	5
AGGTTGAGG	BRDN00011	105	77	27	310	178	65
AGGTTGAGT	BRDN00012	547	424	1361	801	944	38
AGGTTGATG	BRDN00012	142	104	3	164	312	30
AGGTTGCAA	BRDN00011	175	134	200	422	230	12
AGGTTGCCT	BRDN00011	644	762	1216	1483	1359	46
AGGTTGCCT	BRDN00012	86	124	445	184	310	6
AGGTTGCGC	BRDN00011	421	330	255	639	724	34
AGGTTGCTA	BRDN00012	106	136	281	278	397	7
AGGTTGGAC	BRDN00011	197	132	45	319	336	4
AGGTTGGCG	BRDN00011	237	183	43	385	158	14
AGGTTGGCT	BRDN00011	192	198	273	341	228	17
AGGTTGGGA	BRDN00012	180	97	122	422	367	7
AGGTTGGGT	BRDN00012	314	433	223	694	413	103
AGGTTGGTA	BRDN00012	231	251	397	572	495	29
AGGTTGGTG	BRDN00011	114	0	0	238	168	1
AGGTTGTCA	BRDN00011	107	119	21	251	229	1
AGGTTGTCC	BRDN00012	231	179	3	456	582	7
AGGTTGTCC	BRDN00011	268	225	201	372	349	25
AGGTTGTCT	BRDN00012	132	99	1	335	202	0
AGGTTGTGC	BRDN00011	279	192	1066	459	755	20
AGGTTGTGC	BRDN00011	502	164	1	907	835	0
AGGTTGTGG	BRDN00012	223	228	273	447	471	0
AGGTTGTTG	BRDN00011	235	112	616	455	461	21
AGGTTGTTG	BRDN00011	165	74	313	208	152	18
AGGTTGTTT	BRDN00011	503	527	914	809	796	27
AGGTTTAAA	BRDN00012	361	425	535	701	807	38
AGGTTTAGA	BRDN00012	428	372	161	734	556	16
AGGTTTATA	BRDN00011	301	408	2789	431	402	179
AGGTTTCAG	BRDN00012	559	470	1293	1037	952	191
AGGTTTCAG	BRDN00011	235	325	415	474	334	49
AGGTTTCCC	BRDN00012	669	465	303	977	1194	47
AGGTTTCCT	BRDN00012	202	204	1136	478	690	31
AGGTTTCGG	BRDN00011	287	260	233	651	674	11
AGGTTTCTA	BRDN00011	105	105	80	346	280	52
AGGTTTCTG	BRDN00011	328	303	1085	500	454	44
AGGTTTCTT	BRDN00011	240	190	1261	463	580	41
AGGTTTGTT	BRDN00012	221	113	14	439	698	8
AGTAAAACC	BRDN00011	135	78	164	278	318	7
AGTAAAACG	BRDN00012	87	11	0	256	148	0
AGTAAAATA	BRDN00012	85	16	0	183	92	0
AGTAAACAC	BRDN00011	220	150	56	345	402	29
AGTAAACAT	BRDN00012	134	88	83	194	205	0
AGTAAACCC	BRDN00011	152	0	0	266	272	0
AGTAAACCC	BRDN00012	183	262	474	351	124	21
AGTAAACGT	BRDN00012	350	333	419	714	829	1
AGTAAACTC	BRDN00012	239	169	287	439	183	15
AGTAAACTG	BRDN00012	108	140	567	288	348	47
AGTAAACTT	BRDN00012	224	142	36	360	217	12
AGTAAAGAA	BRDN00011	154	90	47	263	356	12
AGTAAAGCC	BRDN00011	408	423	400	840	698	40
AGTAAAGGC	BRDN00011	225	220	13	326	454	27
AGTAAAGTG	BRDN00012	215	19	27	756	428	0
AGTAAAGTT	BRDN00012	123	183	457	278	319	11
AGTAAATCC	BRDN00012	290	465	1829	665	945	40
AGTAAATCT	BRDN00012	105	65	3	232	30	0
AGTAAATTA	BRDN00012	169	139	38	259	891	15
AGTAAATTG	BRDN00011	149	163	187	409	353	0
AGTAACACA	BRDN00011	157	81	126	340	278	5
AGTAACACA	BRDN00011	101	47	10	112	143	16
AGTAACAGT	BRDN00012	311	224	55	484	370	30
AGTAACAGT	BRDN00012	155	107	238	246	392	8
AGTAACCAA	BRDN00012	484	321	795	695	577	10
AGTAACCCT	BRDN00012	531	630	587	1115	1152	59
AGTAACCGT	BRDN00011	208	231	246	344	363	8
AGTAACCTG	BRDN00012	279	179	365	439	378	38
AGTAACTGA	BRDN00012	228	241	187	561	388	44
AGTAACTGA	BRDN00011	165	234	667	291	568	122
AGTAACTGC	BRDN00011	518	538	372	1053	1143	94
AGTAACTGG	BRDN00012	126	114	32	257	231	5
AGTAACTTA	BRDN00012	232	212	297	363	206	10
AGTAAGAAA	BRDN00011	83	0	0	272	182	0
AGTAAGAGA	BRDN00012	80	49	181	198	97	5
AGTAAGCAT	BRDN00012	170	86	380	353	293	1
AGTAAGGCG	BRDN00011	62	57	90	105	189	14
AGTAAGTAT	BRDN00011	138	129	876	372	277	14
AGTAATAAA	BRDN00012	271	229	961	747	881	19
AGTAATAGT	BRDN00012	136	168	98	319	294	15
AGTAATAGT	BRDN00012	245	121	926	493	477	32
AGTAATATA	BRDN00012	194	81	0	334	298	17
AGTAATCAA	BRDN00012	334	326	808	703	686	61
AGTAATCAC	BRDN00011	71	92	550	183	82	6
AGTAATCAG	BRDN00011	147	107	550	181	387	14
AGTAATCCC	BRDN00011	187	175	300	418	349	7
AGTAATCGT	BRDN00011	452	314	394	686	833	33
AGTAATCTA	BRDN00012	258	174	164	323	203	0
AGTAATGAG	BRDN00012	491	582	282	812	1330	83
AGTAATGAG	BRDN00011	220	171	45	451	1280	62
AGTAATGCT	BRDN00011	207	249	197	373	461	0
AGTAATGGG	BRDN00011	327	460	1636	734	904	22
AGTAATGGG	BRDN00012	214	81	105	468	296	8
AGTAATTAA	BRDN00012	88	95	123	202	154	30
AGTAATTCT	BRDN00012	371	217	2649	648	478	55
AGTAATTGA	BRDN00012	317	253	14	560	827	36
AGTAATTGT	BRDN00011	170	159	80	319	176	40
AGTAATTGT	BRDN00011	256	159	544	502	614	9
AGTAATTTG	BRDN00011	204	14	3	273	196	0
AGTACAACA	BRDN00012	563	583	1525	893	716	99
AGTACAAGT	BRDN00012	521	403	64	878	820	11
AGTACACAA	BRDN00012	176	93	90	367	266	5
AGTACACAG	BRDN00012	99	171	0	232	269	6
AGTACACAG	BRDN00011	116	136	45	313	205	40
AGTACACAT	BRDN00011	308	43	0	855	535	0
AGTACACAT	BRDN00012	432	401	672	683	712	63
AGTACACGG	BRDN00011	133	155	17	245	427	0
AGTACACGT	BRDN00012	341	183	552	542	467	26
AGTACACTC	BRDN00011	55	23	687	326	297	21
AGTACAGCC	BRDN00011	243	271	30	407	321	17
AGTACAGCT	BRDN00012	414	325	1370	672	606	45
AGTACAGCT	BRDN00012	242	336	298	305	446	40
AGTACAGTG	BRDN00011	204	210	28	660	345	11
AGTACAGTG	BRDN00011	594	418	240	763	920	52
AGTACAGTT	BRDN00011	393	276	529	614	645	47
AGTACATCC	BRDN00012	393	374	1377	768	911	8
AGTACATCG	BRDN00012	103	0	45	278	112	0
AGTACATCT	BRDN00011	170	60	866	280	153	24
AGTACATGA	BRDN00012	177	73	372	373	251	10
AGTACATGA	BRDN00011	151	174	661	354	260	4
AGTACATGG	BRDN00011	114	118	0	192	101	28
AGTACATTC	BRDN00012	204	244	119	418	630	63
AGTACCAAA	BRDN00011	151	84	382	219	347	8
AGTACCAGA	BRDN00012	188	61	465	906	437	16
AGTACCAGC	BRDN00012	541	700	454	1206	1850	33
AGTACCAGG	BRDN00011	176	5	0	341	192	0
AGTACCAGT	BRDN00012	158	88	523	276	289	10
AGTACCATT	BRDN00012	519	437	2836	812	694	50
AGTACCCCG	BRDN00011	196	255	993	372	248	21
AGTACCCCG	BRDN00012	129	131	207	383	423	9
AGTACCCCT	BRDN00012	507	552	349	1090	735	66
AGTACCGAG	BRDN00012	257	167	290	400	297	0
AGTACCGAG	BRDN00012	366	336	190	890	1368	20
AGTACCGCA	BRDN00012	139	49	3	950	818	0
AGTACCGCA	BRDN00012	731	565	689	1212	1314	71
AGTACCGGA	BRDN00012	60	0	0	276	98	0
AGTACCGTG	BRDN00012	364	570	536	650	801	53
AGTACCTAT	BRDN00011	81	100	124	173	293	0
AGTACCTCA	BRDN00012	482	415	656	908	1089	61
AGTACCTCG	BRDN00011	300	135	97	340	337	0
AGTACCTGA	BRDN00012	434	554	1449	956	904	63
AGTACCTGA	BRDN00012	494	204	356	797	877	0
AGTACCTGA	BRDN00011	175	208	116	335	394	11
AGTACCTGC	BRDN00011	183	171	206	297	367	21
AGTACCTGC	BRDN00011	96	96	55	233	143	14
AGTACCTGG	BRDN00011	250	137	170	211	150	23
AGTACCTGG	BRDN00011	220	248	79	524	487	40
AGTACCTGG	BRDN00011	365	383	91	615	595	33
AGTACCTGT	BRDN00011	221	247	1829	380	276	47
AGTACCTGT	BRDN00011	134	98	63	208	267	3
AGTACCTGT	BRDN00011	306	384	336	633	758	42
AGTACCTGT	BRDN00011	302	322	554	648	430	26
AGTACCTTC	BRDN00011	223	144	0	316	288	1
AGTACCTTT	BRDN00012	250	337	1284	421	93	37
AGTACGAAG	BRDN00011	626	358	199	926	1150	30
AGTACGACT	BRDN00012	564	129	869	949	694	15
AGTACGAGG	BRDN00012	376	443	141	644	702	20
AGTACGCTT	BRDN00012	152	154	359	604	943	3
AGTACGGGA	BRDN00011	367	239	955	795	727	42
AGTACGGTA	BRDN00011	223	197	216	278	352	11
AGTACTACA	BRDN00011	407	232	647	615	802	17
AGTACTACA	BRDN00011	188	173	181	253	426	24
AGTACTACG	BRDN00011	88	52	125	198	103	3
AGTACTACG	BRDN00011	336	424	191	596	740	21
AGTACTAGT	BRDN00011	208	54	211	388	335	5
AGTACTATA	BRDN00012	82	32	83	563	400	0
AGTACTCAC	BRDN00012	250	125	641	345	452	5
AGTACTCCA	BRDN00011	289	259	776	389	578	16
AGTACTGAC	BRDN00012	60	0	0	285	222	0
AGTACTGCG	BRDN00012	225	192	38	364	219	18
AGTACTGCG	BRDN00012	288	239	1942	628	908	60
AGTACTGCT	BRDN00012	215	247	113	392	217	13
AGTACTGGA	BRDN00011	200	173	265	313	365	17
AGTACTGGT	BRDN00011	188	284	238	590	544	3
AGTACTGTG	BRDN00011	155	93	2	323	326	2
AGTACTTAC	BRDN00012	452	265	459	765	556	46
AGTACTTCA	BRDN00012	355	13	0	776	567	4
AGTACTTCA	BRDN00012	619	542	1621	1136	1127	64
AGTACTTCC	BRDN00012	60	46	0	422	160	11
AGTACTTCG	BRDN00011	184	147	12	251	257	5
AGTACTTCT	BRDN00011	124	271	662	234	170	79
AGTACTTCT	BRDN00012	230	225	652	547	792	20
AGTACTTGA	BRDN00012	427	430	881	943	971	8
AGTACTTGT	BRDN00012	394	329	1017	706	614	15
AGTACTTTG	BRDN00011	504	180	212	1021	649	42
AGTAGAAAA	BRDN00012	328	704	1406	726	648	142
AGTAGAAAG	BRDN00012	131	203	46	338	227	0
AGTAGAACA	BRDN00011	288	386	360	636	526	29
AGTAGAACG	BRDN00012	467	485	2006	784	941	41
AGTAGAACT	BRDN00011	299	247	2103	457	478	69
AGTAGAAGA	BRDN00011	60	4	0	227	158	0
AGTAGAAGC	BRDN00012	420	355	3294	510	486	106
AGTAGAAGC	BRDN00011	127	135	1	206	282	0
AGTAGAAGT	BRDN00012	350	361	847	522	568	59
AGTAGAAGT	BRDN00012	360	78	461	830	594	17
AGTAGACCT	BRDN00012	173	170	1	277	153	24
AGTAGACGG	BRDN00012	697	671	336	1089	1068	13
AGTAGACGG	BRDN00012	150	75	59	141	166	0
AGTAGACGT	BRDN00012	336	176	246	567	556	0
AGTAGACTG	BRDN00012	371	145	1057	592	747	23
AGTAGAGCT	BRDN00012	511	452	417	1101	1214	33
AGTAGAGGC	BRDN00011	555	262	210	712	908	42
AGTAGAGGC	BRDN00011	236	184	1845	382	179	18
AGTAGAGTA	BRDN00011	62	15	66	186	79	0
AGTAGAGTC	BRDN00011	246	33	21	725	102	9
AGTAGATAA	BRDN00012	144	115	1004	141	221	27
AGTAGATGG	BRDN00011	197	221	232	396	378	5
AGTAGATGT	BRDN00012	213	110	77	250	231	19
AGTAGCAAA	BRDN00012	442	445	60	631	864	17
AGTAGCAGA	BRDN00012	283	304	428	686	565	6
AGTAGCAGT	BRDN00011	177	175	237	256	144	20
AGTAGCATT	BRDN00012	94	8	7	252	90	0
AGTAGCCCA	BRDN00012	410	919	10440	643	397	1238
AGTAGCCCC	BRDN00011	114	71	5	147	300	9
AGTAGCCCT	BRDN00012	174	223	35	431	244	9
AGTAGCCCT	BRDN00011	211	117	282	351	301	22
AGTAGCGCG	BRDN00012	246	80	108	759	363	13
AGTAGCGGA	BRDN00011	218	24	129	475	596	50
AGTAGCGGA	BRDN00012	417	413	917	818	994	109
AGTAGCTCC	BRDN00012	473	306	281	899	1243	100
AGTAGGAAT	BRDN00012	355	347	856	572	763	0
AGTAGGACC	BRDN00011	496	456	727	860	705	52
AGTAGGACT	BRDN00012	451	310	2033	874	689	105
AGTAGGATA	BRDN00012	507	450	1038	887	812	11
AGTAGGCAT	BRDN00011	601	533	973	1017	643	115
AGTAGGGCA	BRDN00012	444	225	329	729	780	49
AGTAGGGCC	BRDN00012	370	280	40	757	744	23
AGTAGGGTA	BRDN00012	199	97	393	254	437	0
AGTAGGTCG	BRDN00011	245	35	0	643	515	0
AGTAGGTCT	BRDN00012	369	229	671	886	915	2
AGTAGGTGC	BRDN00011	105	114	4607	145	186	103
AGTAGGTGC	BRDN00011	142	108	398	233	128	39
AGTAGGTTC	BRDN00012	390	368	154	653	642	60
AGTAGTAAT	BRDN00011	139	103	7	252	114	8
AGTAGTACA	BRDN00012	193	60	143	332	340	0
AGTAGTACA	BRDN00012	297	414	1346	689	415	10
AGTAGTACT	BRDN00012	217	59	0	287	129	7
AGTAGTAGT	BRDN00012	207	164	690	242	228	21
AGTAGTATA	BRDN00011	154	146	592	340	664	42
AGTAGTATA	BRDN00011	74	0	122	338	354	0
AGTAGTGAA	BRDN00011	141	10	75	462	349	0
AGTAGTGAC	BRDN00011	264	78	95	480	428	9
AGTAGTTAC	BRDN00012	215	208	405	264	318	77
AGTAGTTCA	BRDN00012	179	137	923	454	406	19
AGTAGTTTC	BRDN00011	253	417	753	534	791	108
AGTAGTTTG	BRDN00011	141	0	1	436	142	0
AGTATAAAA	BRDN00011	146	125	563	264	365	8
AGTATAAGA	BRDN00012	254	203	697	529	358	19
AGTATAAGT	BRDN00011	260	301	980	753	486	30
AGTATAATC	BRDN00011	272	153	401	384	326	38
AGTATACAA	BRDN00012	227	218	753	637	295	37
AGTATACAA	BRDN00011	120	48	1862	160	58	30
AGTATACCA	BRDN00012	316	314	181	722	813	15
AGTATACCA	BRDN00012	493	196	65	758	871	8
AGTATACGC	BRDN00011	164	60	98	326	444	0
AGTATAGCC	BRDN00011	218	190	23	272	289	19
AGTATAGTT	BRDN00011	272	249	1515	420	655	43
AGTATATAC	BRDN00012	225	0	0	504	347	0
AGTATATCC	BRDN00011	79	47	1	252	293	0
AGTATATTC	BRDN00012	151	242	14	360	142	23
AGTATCAAT	BRDN00011	116	205	10	192	275	28
AGTATCACC	BRDN00012	225	244	769	521	276	20
AGTATCAGG	BRDN00012	456	410	858	630	680	25
AGTATCAGG	BRDN00011	255	225	181	504	384	4
AGTATCATA	BRDN00011	311	256	512	509	267	7
AGTATCATC	BRDN00011	123	131	80	245	95	5
AGTATCCAC	BRDN00012	464	395	1393	728	1093	40
AGTATCCCC	BRDN00012	172	104	44	171	332	13
AGTATCCCT	BRDN00012	165	236	210	338	98	9
AGTATCCCT	BRDN00011	235	198	252	390	394	230
AGTATCGGA	BRDN00012	176	216	260	340	375	24
AGTATCGGC	BRDN00012	147	123	19	243	38	0
AGTATCGTT	BRDN00011	302	217	846	692	643	8
AGTATCTAC	BRDN00012	195	172	801	441	487	28
AGTATCTAC	BRDN00012	269	270	346	486	566	9
AGTATCTCT	BRDN00011	249	148	124	270	439	19
AGTATGAAT	BRDN00011	290	304	780	397	180	54
AGTATGAGA	BRDN00012	451	635	1071	986	696	102
AGTATGAGA	BRDN00012	300	376	325	586	705	26
AGTATGAGT	BRDN00012	343	165	1531	629	638	38
AGTATGAGT	BRDN00011	129	154	21	252	392	13
AGTATGATG	BRDN00011	91	44	0	273	190	0
AGTATGATT	BRDN00011	390	463	36	727	813	123
AGTATGCAA	BRDN00012	424	389	281	847	855	59
AGTATGCCA	BRDN00012	533	468	294	1013	1162	6
AGTATGCTG	BRDN00011	247	176	673	454	376	21
AGTATGGCT	BRDN00012	188	229	35	346	574	0
AGTATGGGT	BRDN00011	202	126	11	403	1223	0
AGTATGGTA	BRDN00012	240	157	479	411	518	35
AGTATGTCA	BRDN00012	226	122	753	488	408	6
AGTATGTCT	BRDN00011	407	433	1914	697	477	14
AGTATGTGG	BRDN00011	466	363	826	813	596	6
AGTATGTGT	BRDN00012	112	295	466	511	368	19
AGTATTAGG	BRDN00012	736	734	695	1060	1174	134
AGTATTCAA	BRDN00012	153	65	84	431	382	5
AGTATTCAA	BRDN00012	365	207	606	655	784	42
AGTATTCAG	BRDN00012	33	0	113	305	124	0
AGTATTCTC	BRDN00011	274	281	76	524	654	58
AGTATTGAC	BRDN00012	125	139	31	266	309	0
AGTATTGAC	BRDN00011	218	159	172	631	680	37
AGTATTGAG	BRDN00011	356	286	144	709	800	42
AGTATTGTG	BRDN00011	229	178	170	419	218	29
AGTATTGTG	BRDN00012	508	509	1556	839	725	45
AGTATTTCA	BRDN00011	242	324	453	581	306	16
AGTATTTGA	BRDN00011	113	160	43	331	427	13
AGTATTTGT	BRDN00011	167	53	52	181	327	0
AGTCAAAAA	BRDN00011	92	97	294	292	98	0
AGTCAAAAA	BRDN00011	162	126	78	399	217	15
AGTCAAAGA	BRDN00011	155	108	117	244	47	7
AGTCAAATG	BRDN00011	23	0	1	605	78	0
AGTCAACAC	BRDN00011	242	308	1047	404	274	21
AGTCAACCA	BRDN00011	190	165	276	198	137	0
AGTCAACCA	BRDN00012	529	467	789	1087	1048	16
AGTCAACCA	BRDN00011	256	179	368	411	377	26
AGTCAACCT	BRDN00011	450	311	701	778	917	24
AGTCAACGG	BRDN00011	386	359	1538	799	958	45
AGTCAACGT	BRDN00011	429	278	2400	849	1287	62
AGTCAACTC	BRDN00011	118	136	692	233	364	11
AGTCAACTG	BRDN00011	83	44	85	346	224	6
AGTCAACTG	BRDN00011	158	164	8	225	127	17
AGTCAACTT	BRDN00011	216	219	904	516	411	46
AGTCAAGCA	BRDN00011	184	171	115	266	489	17
AGTCAATAC	BRDN00012	402	354	520	807	1259	28
AGTCAATAT	BRDN00011	252	266	922	474	488	39
AGTCAATGT	BRDN00012	184	101	359	456	609	5
AGTCACAAT	BRDN00012	326	254	271	838	665	18
AGTCACACA	BRDN00011	351	227	385	605	620	42
AGTCACACA	BRDN00012	103	114	3	238	45	0
AGTCACACG	BRDN00011	114	208	1488	301	211	69
AGTCACACG	BRDN00011	174	126	1915	323	451	18
AGTCACAGA	BRDN00011	170	214	213	377	486	3
AGTCACAGC	BRDN00012	177	0	1	593	624	0
AGTCACAGC	BRDN00011	371	421	1956	644	668	33
AGTCACAGG	BRDN00012	455	536	457	1005	1067	62
AGTCACATC	BRDN00012	674	470	696	985	1189	59
AGTCACATG	BRDN00012	319	347	61	673	1567	59
AGTCACATT	BRDN00011	292	227	1144	435	724	59
AGTCACCAT	BRDN00012	155	130	1164	262	137	62
AGTCACCCT	BRDN00012	266	191	13	474	387	65
AGTCACCCT	BRDN00012	249	185	50	400	556	7
AGTCACCGC	BRDN00012	685	13	44	1413	1678	0
AGTCACGCA	BRDN00012	477	467	269	873	787	14
AGTCACGCG	BRDN00011	411	573	442	603	344	31
AGTCACGCT	BRDN00012	544	494	1706	912	1277	136
AGTCACGGA	BRDN00012	163	79	50	182	318	15
AGTCACGTA	BRDN00012	275	162	107	499	223	14
AGTCACTCG	BRDN00012	19	16	0	269	74	0
AGTCACTCT	BRDN00011	166	50	35	284	231	5
AGTCACTGA	BRDN00012	130	178	21	433	689	0
AGTCACTGA	BRDN00011	215	175	254	300	443	40
AGTCACTGC	BRDN00011	133	72	1086	319	374	27
AGTCACTGG	BRDN00012	187	168	758	375	306	15
AGTCACTGG	BRDN00011	251	242	259	476	519	32
AGTCACTTT	BRDN00012	323	353	277	786	787	53
AGTCACTTT	BRDN00011	153	18	25	265	399	0
AGTCAGAAC	BRDN00011	347	405	1627	628	682	57
AGTCAGACA	BRDN00012	364	355	264	620	600	63
AGTCAGACA	BRDN00012	180	189	722	280	192	13
AGTCAGACG	BRDN00012	168	113	151	257	215	1
AGTCAGAGA	BRDN00012	59	102	79	188	306	0
AGTCAGAGG	BRDN00012	114	79	194	218	193	12
AGTCAGATG	BRDN00012	291	182	252	386	546	66
AGTCAGATG	BRDN00011	233	213	1093	461	331	40
AGTCAGATT	BRDN00012	528	364	781	953	943	55
AGTCAGCAA	BRDN00011	247	330	388	618	530	6
AGTCAGCAC	BRDN00012	494	325	1201	884	1135	41
AGTCAGCAT	BRDN00011	115	186	344	236	207	5
AGTCAGCCA	BRDN00011	466	468	3258	853	996	107
AGTCAGGAA	BRDN00011	429	446	236	806	456	41
AGTCAGGGC	BRDN00012	137	207	1104	308	475	7
AGTCAGGGC	BRDN00011	82	106	171	208	301	0
AGTCAGGGT	BRDN00011	396	320	194	755	541	0
AGTCAGGTG	BRDN00011	103	173	0	261	317	17
AGTCAGGTT	BRDN00011	109	169	125	199	205	36
AGTCAGTAC	BRDN00011	321	414	28	876	1158	88
AGTCAGTCC	BRDN00011	150	78	18	461	83	0
AGTCAGTCG	BRDN00012	482	436	1066	1140	839	50
AGTCATAAC	BRDN00011	336	296	1423	633	594	65
AGTCATAAC	BRDN00012	360	371	885	682	649	58
AGTCATACC	BRDN00011	353	242	1334	733	1946	30
AGTCATACC	BRDN00011	436	337	684	751	789	26
AGTCATAGC	BRDN00011	399	461	1458	856	807	54
AGTCATATT	BRDN00012	402	190	98	893	433	5
AGTCATCAA	BRDN00012	310	345	1004	664	1292	3
AGTCATCAG	BRDN00012	153	1	0	324	315	11
AGTCATCAG	BRDN00012	58	1	1	458	693	1
AGTCATCAG	BRDN00012	169	290	1984	291	432	47
AGTCATCAT	BRDN00011	302	247	198	611	523	14
AGTCATCAT	BRDN00011	47	53	58	213	405	0
AGTCATCCC	BRDN00012	200	185	72	254	417	20
AGTCATCCC	BRDN00012	239	255	100	490	335	30
AGTCATCCT	BRDN00011	321	234	684	384	567	7
AGTCATCGC	BRDN00011	44	0	0	290	216	0
AGTCATCGT	BRDN00012	209	31	0	330	424	0
AGTCATCTA	BRDN00011	307	256	426	507	749	40
AGTCATCTT	BRDN00012	65	0	27	324	187	0
AGTCATCTT	BRDN00012	243	133	1	523	507	68
AGTCATGAA	BRDN00012	163	205	9	287	527	0
AGTCATGAG	BRDN00012	420	276	240	706	738	53
AGTCATGAT	BRDN00011	410	393	789	807	760	27
AGTCATGGA	BRDN00012	255	262	339	488	334	65
AGTCATGGT	BRDN00011	207	219	389	427	498	48
AGTCATGTT	BRDN00011	440	531	2488	713	661	219
AGTCATTAA	BRDN00011	104	79	3905	225	122	47
AGTCATTAC	BRDN00012	483	593	1586	930	1434	67
AGTCATTCG	BRDN00012	172	242	22	412	234	14
AGTCATTGT	BRDN00011	133	6	0	1089	375	0
AGTCATTTA	BRDN00012	179	55	166	443	473	0
AGTCCAAAG	BRDN00012	478	262	2116	802	768	19
AGTCCAAAG	BRDN00011	265	282	274	558	233	6
AGTCCAACT	BRDN00011	271	174	487	526	226	6
AGTCCAAGG	BRDN00011	134	250	85	316	298	29
AGTCCAAGG	BRDN00012	224	175	452	451	403	10

AGTCCACACT	BRDN0001	144	161	35	259	186	0
AGTCCACAG	BRDN0001	346	263	220	659	1007	0
AGTCCACAG	BRDN0001	185	200	25	343	205	0
AGTCCACCA	BRDN0001	492	379	415	798	486	62
AGTCCACCAT	BRDN0001	252	168	372	414	435	17
AGTCCACCCA	BRDN0001	462	373	425	987	816	18
AGTCCACCCC	BRDN0001	108	208	372	265	257	28
AGTCCACCG	BRDN0001	152	181	270	341	348	9
AGTCCACCGT	BRDN0001	283	326	2851	739	898	76
AGTCCACCTC	BRDN0001	665	534	3123	1270	656	74
AGTCCACGC	BRDN0001	402	365	165	768	1417	29
AGTCCACTTA	BRDN0001	133	164	228	271	147	14
AGTCCAGAA	BRDN0001	547	541	2129	1021	1589	145
AGTCCAGAA	BRDN0001	113	82	59	295	339	0
AGTCCAGCC	BRDN0001	118	79	91	322	256	23
AGTCCAGCT	BRDN0001	275	254	408	444	388	35
AGTCCAGGA	BRDN0001	290	439	86	611	647	28
AGTCCAGTG	BRDN0001	270	151	207	322	177	11
AGTCCATCCA	BRDN0001	217	190	512	372	488	0
AGTCCATCTT	BRDN0001	436	434	252	1027	1637	13
AGTCCATGG	BRDN0001	216	65	70	330	302	14
AGTCCCAAA	BRDN0001	169	219	1982	444	676	28
AGTCCCAAT	BRDN0001	239	205	91	442	616	20
AGTCCCACA	BRDN0001	190	0	0	907	20	0
AGTCCCACA	BRDN0001	436	575	862	890	663	33
AGTCCCACG	BRDN0001	160	205	0	383	344	0
AGTCCCACTA	BRDN0001	282	219	114	486	579	29
AGTCCCAGC	BRDN0001	399	280	2430	700	851	22
AGTCCCATG	BRDN0001	276	279	1441	443	744	55
AGTCCCCACA	BRDN0001	412	415	292	825	941	54
AGTCCCCAG	BRDN0001	679	442	1881	953	1226	72
AGTCCCCCAT	BRDN0001	416	206	684	795	968	72
AGTCCCCGAT	BRDN0001	399	289	815	848	668	80
AGTCCCCGAT	BRDN0001	190	221	271	360	242	15
AGTCCCCGG	BRDN0001	149	166	46	373	500	64
AGTCCCGCA	BRDN0001	172	127	252	256	140	6
AGTCCCGTTT	BRDN0001	639	475	190	942	1089	21
AGTCCCTCAA	BRDN0001	183	40	51	300	160	0
AGTCCCTCCA	BRDN0001	202	140	729	406	565	17
AGTCCCTCCC	BRDN0001	164	249	1169	262	187	652
AGTCCCTCTG	BRDN0001	255	111	264	305	356	11
AGTCCCTGC	BRDN0001	173	243	222	309	372	0
AGTCCCTGG	BRDN0001	246	166	421	365	588	8
AGTCCGAAA	BRDN0001	408	284	202	773	610	44
AGTCCGAGA	BRDN0001	474	411	305	1094	1334	14
AGTCCGATC	BRDN0001	132	211	329	312	362	11
AGTCCGCAG	BRDN0001	213	176	462	371	232	24
AGTCCGGAC	BRDN0001	280	185	523	517	469	48
AGTCCGGAG	BRDN0001	265	188	564	320	272	97
AGTCCGGGT	BRDN0001	504	501	700	875	816	53
AGTCCGTCC	BRDN0001	250	211	215	552	442	5
AGTCCTAAA	BRDN0001	312	156	854	654	752	35
AGTCCTAAAT	BRDN0001	335	138	66	465	439	7
AGTCCTACC	BRDN0001	67	20	46	328	257	0
AGTCCTACCT	BRDN0001	587	427	1573	941	987	127
AGTCCTCAA	BRDN0001	119	172	0	390	188	0
AGTCCTCACT	BRDN0001	73	73	157	176	158	8
AGTCCTCATA	BRDN0001	224	152	0	373	154	0
AGTCCTCCTG	BRDN0001	218	241	225	389	250	11
AGTCCTGAA	BRDN0001	93	178	39	305	244	32
AGTCCTGAC	BRDN0001	187	214	154	305	372	18
AGTCCTGCG	BRDN0001	515	580	1244	932	1108	87
AGTCCTGCT	BRDN0001	115	135	6	212	353	4
AGTCCTGGA	BRDN0001	174	146	267	371	280	20
AGTCCTGGA	BRDN0001	129	75	0	280	489	33
AGTCCTGGC	BRDN0001	273	23	0	817	422	0
AGTCCTGGG	BRDN0001	35	0	2	725	34	0
AGTCCTGTC	BRDN0001	281	38	396	801	761	0
AGTCCTGTG	BRDN0001	386	238	1250	629	520	6
AGTCCTGTG	BRDN0001	222	294	2413	383	698	40
AGTCCTTCAT	BRDN0001	484	420	691	1111	743	54
AGTCCTTGG	BRDN0001	198	100	19	386	553	0
AGTCCTTGT	BRDN0001	177	141	2	1189	1333	2
AGTCCTTGT	BRDN0001	69	19	0	485	247	6
AGTCGAACT	BRDN0001	135	33	123	293	288	0
AGTCGAACT	BRDN0001	111	36	15	178	137	0
AGTCGAAGT	BRDN0001	306	186	584	614	255	62
AGTCGACAC	BRDN0001	259	301	600	564	514	36
AGTCGAGGG	BRDN0001	360	444	992	858	854	26
AGTCGATGG	BRDN0001	51	89	520	176	192	18
AGTCGATGT	BRDN0001	167	146	412	440	213	39
AGTCGATTA	BRDN0001	397	197	556	764	945	0
AGTCGATTT	BRDN0001	75	49	0	337	128	0
AGTCGCATT	BRDN0001	112	109	537	247	220	20
AGTCGCCAA	BRDN0001	602	449	935	1019	1520	34
AGTCGCCAG	BRDN0001	191	0	14	568	38	0
AGTCGCCAT	BRDN0001	536	322	2952	1090	1083	45
AGTCGCCGT	BRDN0001	307	284	92	557	549	52
AGTCGCCTA	BRDN0001	123	114	202	212	244	0
AGTCGCGGA	BRDN0001	196	197	1104	309	451	14
AGTCGCGGT	BRDN0001	403	431	3643	778	1420	81
AGTCGCTCA	BRDN0001	176	158	396	632	510	0
AGTCGCTGA	BRDN0001	12	0	15	356	10	0
AGTCGCTGG	BRDN0001	14	17	0	335	232	0
AGTCGCTGG	BRDN0001	377	388	107	677	760	23
AGTCGGAAT	BRDN0001	255	236	1443	516	577	22
AGTCGGACA	BRDN0001	219	183	242	380	340	37
AGTCGGAGA	BRDN0001	131	156	590	474	240	45
AGTCGGAGC	BRDN0001	443	326	730	776	1092	34
AGTCGGATA	BRDN0001	308	117	429	652	636	31
AGTCGGCAA	BRDN0001	344	390	510	791	584	86
AGTCGGCAA	BRDN0001	254	254	106	566	463	67
AGTCGGCAG	BRDN0001	141	11	18	290	384	0
AGTCGGCCA	BRDN0001	342	390	184	731	771	123
AGTCGGCCA	BRDN0001	164	131	9	233	103	0
AGTCGGCTA	BRDN0001	607	671	2375	1373	1067	69
AGTCGGCTT	BRDN0001	33	20	0	297	58	0
AGTCGGGGT	BRDN0001	87	0	2	436	311	0
AGTCGGGTG	BRDN0001	187	219	187	373	360	23
AGTCGTAAC	BRDN0001	122	136	150	235	174	6
AGTCGTAAT	BRDN0001	226	134	106	460	498	34
AGTCGTAGC	BRDN0001	631	453	147	1039	743	9
AGTCGTAGC	BRDN0001	172	37	12	360	211	1
AGTCGTCTA	BRDN0001	184	156	171	395	361	40
AGTCGTGGC	BRDN0001	319	245	396	636	430	5
AGTCGTGGG	BRDN0001	90	0	1	286	350	0
AGTCGTGTC	BRDN0001	83	47	1	541	422	0
AGTCGTGTC	BRDN0001	328	387	2033	706	851	77
AGTCGTTCC	BRDN0001	86	65	316	242	240	23
AGTCGTTCT	BRDN0001	352	314	1859	413	533	44
AGTCGTTGA	BRDN0001	159	147	26	214	192	17
AGTCTAACA	BRDN0001	153	23	36	202	146	0
AGTCTAAGC	BRDN0001	252	192	226	489	344	23
AGTCTAAGT	BRDN0001	273	180	154	258	350	3
AGTCTACAT	BRDN0001	380	405	168	901	1593	13
AGTCTACTA	BRDN0001	174	167	152	234	300	21
AGTCTACTG	BRDN0001	526	95	0	933	544	0
AGTCTAGAA	BRDN0001	193	124	46	404	177	0
AGTCTAGGC	BRDN0001	109	90	2	255	49	0
AGTCTAGGG	BRDN0001	428	36	33	740	100	0
AGTCTATGT	BRDN0001	486	610	430	1079	773	76
AGTCTCAAT	BRDN0001	209	170	146	398	698	8
AGTCTCACAT	BRDN0001	163	150	602	311	312	34
AGTCTCACGT	BRDN0001	225	324	634	349	376	57
AGTCTCAGT	BRDN0001	177	268	320	443	560	33
AGTCTCCAA	BRDN0001	191	147	235	281	134	24
AGTCTCCAA	BRDN0001	510	560	556	803	615	50
AGTCTCCAG	BRDN0001	282	236	4006	714	563	81
AGTCTCCAG	BRDN0001	129	7	0	338	141	0
AGTCTCCATA	BRDN0001	329	188	920	419	308	18
AGTCTCCCGA	BRDN0001	261	245	230	517	509	18
AGTCTCCCGC	BRDN0001	487	452	1504	907	708	63
AGTCTCCTAT	BRDN0001	416	349	790	853	658	94
AGTCTCCTCA	BRDN0001	95	62	0	193	218	15
AGTCTCCTCA	BRDN0001	114	155	891	152	66	21
AGTCTCCTCG	BRDN0001	197	216	237	428	325	5
AGTCTCCTGA	BRDN0001	557	409	824	1124	804	13
AGTCTCGGA	BRDN0001	274	175	770	549	240	41
AGTCTCGGG	BRDN0001	167	248	32	332	318	21
AGTCTCGGG	BRDN0001	461	509	180	778	832	71
AGTCTCGTCA	BRDN0001	437	322	425	728	547	12
AGTCTCTCAT	BRDN0001	208	181	45	486	307	1
AGTCTCTGAT	BRDN0001	166	77	815	362	177	7
AGTCTCTGCC	BRDN0001	349	388	823	645	518	9
AGTCTGAAG	BRDN0001	245	272	187	624	322	0
AGTCTGACCT	BRDN0001	249	295	977	547	200	38
AGTCTGAGG	BRDN0001	110	163	261	205	66	9
AGTCTGCAA	BRDN0001	231	263	201	411	512	0
AGTCTGCAA	BRDN0001	450	325	110	721	858	73
AGTCTGCAA	BRDN0001	214	474	967	429	335	36
AGTCTGCAA	BRDN0001	114	67	14	214	93	30
AGTCTGCAT	BRDN0001	282	227	200	496	689	31
AGTCTGCAT	BRDN0001	362	391	470	747	413	21
AGTCTGCCA	BRDN0001	149	145	31	371	260	58
AGTCTGCCG	BRDN0001	423	518	879	647	356	82
AGTCTGCTG	BRDN0001	397	311	271	823	1159	13
AGTCTGCTG	BRDN0001	199	1	36	327	34	0
AGTCTGGAA	BRDN0001	45	0	0	395	69	0
AGTCTGGAG	BRDN0001	238	167	156	478	424	22
AGTCTGGGC	BRDN0001	667	521	1279	1137	856	29
AGTCTGTAA	BRDN0001	270	437	307	612	489	2
AGTCTGTAC	BRDN0001	359	266	159	874	1244	16
AGTCTGTAG	BRDN0001	329	305	500	787	766	68
AGTCTGTCA	BRDN0001	476	204	1248	864	668	26
AGTCTGTCC	BRDN0001	427	91	408	808	495	0
AGTCTGTGG	BRDN0001	302	275	318	729	1214	27
AGTCTGTGG	BRDN0001	114	206	21	268	443	5
AGTCTGTGT	BRDN0001	287	266	923	405	387	76
AGTCTGTTC	BRDN0001	168	44	106	378	528	0
AGTCTTAAA	BRDN0001	370	292	674	848	929	64
AGTCTTAAT	BRDN0001	183	272	0	220	130	0
AGTCTTACCT	BRDN0001	420	392	1477	709	654	52
AGTCTTATGT	BRDN0001	235	215	0	539	545	8
AGTCTTCAA	BRDN0001	245	242	1162	372	322	42
AGTCTTCACA	BRDN0001	298	344	405	661	506	32
AGTCTTCACT	BRDN0001	316	269	576	730	610	47
AGTCTTCATG	BRDN0001	211	160	246	412	312	25
AGTCTTCCAA	BRDN0001	324	378	73	504	454	56
AGTCTTCCAC	BRDN0001	434	418	1018	922	645	22
AGTCTTCCGC	BRDN0001	174	172	763	321	215	5
AGTCTTCCTC	BRDN0001	537	331	1325	818	551	21
AGTCTTGAG	BRDN0001	293	211	1020	506	715	84
AGTCTTGCA	BRDN0001	208	201	214	517	723	21
AGTCTTGGA	BRDN0001	226	269	189	532	890	32
AGTCTTGGA	BRDN0001	324	248	741	642	539	48
AGTCTTGGA	BRDN0001	171	138	27	400	444	14
AGTCTTGGC	BRDN0001	684	702	2880	1472	2008	116
AGTCTTGGG	BRDN0001	299	197	207	691	373	45
AGTCTTGGT	BRDN0001	243	354	811	506	401	31
AGTCTTGTG	BRDN0001	166	175	58	177	242	6
AGTCTTTGG	BRDN0001	210	247	531	444	489	4
AGTCTTTGG	BRDN0001	26	15	0	161	78	0
AGTCTTTGG	BRDN0001	267	125	198	439	214	10
AGTCTTTGTA	BRDN0001	95	48	3	163	156	10
AGTGAAAAA	BRDN0001	36	46	566	466	282	21
AGTGAAACC	BRDN0001	160	87	35	467	592	3
AGTGAAACC	BRDN0001	121	35	0	294	139	0
AGTGAAAGA	BRDN0001	131	180	98	288	281	0
AGTGAAAGT	BRDN0001	303	187	398	628	649	2
AGTGAAATA	BRDN0001	62	57	22	180	267	4
AGTGAACAA	BRDN0001	616	570	594	731	532	65
AGTGAACAA	BRDN0001	434	234	306	817	718	27
AGTGAACAA	BRDN0001	138	164	12	224	398	14
AGTGAACAC	BRDN0001	199	147	140	423	236	12
AGTGAACAT	BRDN0001	27	0	0	51	52	0
AGTGAACAT	BRDN0001	259	42	2	670	233	0
AGTGAACCA	BRDN0001	379	379	255	591	551	32
AGTGAACCT	BRDN0001	264	212	318	358	624	34
AGTGAACTC	BRDN0001	168	178	69	366	367	15
AGTGAACTT	BRDN0001	432	420	953	559	691	58
AGTGAAGAC	BRDN0001	137	164	31	214	264	11
AGTGAAGAG	BRDN0001	211	158	2476	411	374	48
AGTGAAGCG	BRDN0001	178	144	377	391	514	14
AGTGAAGCT	BRDN0001	404	24	30	789	533	0
AGTGAAGGA	BRDN0001	159	194	0	311	196	9
AGTGAAGGC	BRDN0001	213	258	810	517	263	24
AGTGAAGGC	BRDN0001	246	181	266	308	398	11
AGTGAAGTC	BRDN0001	173	182	0	286	151	0
AGTGAAGTG	BRDN0001	106	53	19	161	107	6
AGTGAATAC	BRDN0001	318	323	195	453	645	6
AGTGAATAT	BRDN0001	309	134	886	486	198	18
AGTGAATCT	BRDN0001	364	309	530	639	1043	44
AGTGAATGA	BRDN0001	108	48	35	275	452	1
AGTGAATGA	BRDN0001	313	163	1592	515	629	6
AGTGAATGC	BRDN0001	418	426	2268	868	693	274
AGTGAATGT	BRDN0001	201	140	219	231	311	0
AGTGAATTA	BRDN0001	138	8	1	405	97	7
AGTGAATTC	BRDN0001	294	341	12	489	879	34
AGTGAATTC	BRDN0001	148	264	692	468	705	44
AGTGAATTT	BRDN0001	348	505	2020	657	1103	76
AGTGACAAA	BRDN0001	322	201	240	513	627	0
AGTGACAAT	BRDN0001	90	32	0	198	171	0
AGTGACACA	BRDN0001	279	262	401	695	526	22
AGTGACACA	BRDN0001	262	167	535	590	548	14
AGTGACACC	BRDN0001	159	80	185	265	350	0
AGTGACAGA	BRDN0001	67	50	136	218	149	11
AGTGACATC	BRDN0001	373	379	618	707	754	32
AGTGACATC	BRDN0001	403	416	325	823	812	37
AGTGACATG	BRDN0001	352	361	2042	620	847	8
AGTGACATT	BRDN0001	97	191	468	296	191	4
AGTGACCAA	BRDN0001	155	75	477	168	152	35
AGTGACCCC	BRDN0001	246	273	505	359	416	55
AGTGACCGC	BRDN0001	117	49	44	438	325	11
AGTGACCTA	BRDN0001	285	243	112	408	460	51
AGTGACTAC	BRDN0001	154	212	358	384	477	37
AGTGACTAC	BRDN0001	148	169	1865	323	662	24
AGTGACTAT	BRDN0001	387	465	447	577	716	10
AGTGACTCA	BRDN0001	271	0	0	431	134	0
AGTGACTGA	BRDN0001	103	128	128	300	286	0
AGTGACTGC	BRDN0001	227	211	82	508	577	8
AGTGACTGG	BRDN0001	294	403	1784	535	513	82
AGTGACTGT	BRDN0001	319	423	946	608	712	34
AGTGACTTA	BRDN0001	229	165	370	444	603	8
AGTGACTTC	BRDN0001	235	325	70	526	466	26
AGTGAGAAG	BRDN0001	174	144	193	331	249	34
AGTGAGAAT	BRDN0001	126	60	3	142	179	0
AGTGAGATA	BRDN0001	85	56	0	181	74	0
AGTGAGATG	BRDN0001	203	174	639	497	243	0
AGTGAGATT	BRDN0001	127	93	322	266	290	0
AGTGAGCAA	BRDN0001	373	281	998	597	590	39
AGTGAGCAC	BRDN0001	81	111	164	186	97	15
AGTGAGCAC	BRDN0001	474	422	1604	943	599	74
AGTGAGCAG	BRDN0001	96	81	193	131	74	0
AGTGAGCCA	BRDN0001	372	150	328	575	528	39
AGTGAGCCG	BRDN0001	32	5	326	201	6	0
AGTGAGCTA	BRDN0001	282	301	229	487	334	21
AGTGAGCTC	BRDN0001	16	1	0	280	64	0
AGTGAGGA	BRDN0001	90	119	71	126	72	0
AGTGAGGAC	BRDN0001	306	231	413	480	270	55
AGTGAGGGC	BRDN0001	93	71	179	190	28	20
AGTGAGTAG	BRDN0001	82	17	0	139	404	0
AGTGAGTAT	BRDN0001	113	95	52	293	167	56
AGTGAGTGA	BRDN0001	635	654	1018	1118	1266	86
AGTGAGTTC	BRDN0001	139	129	30	287	266	6
AGTGATAAT	BRDN0001	79	183	367	231	237	4
AGTGATACT	BRDN0001	108	131	36	151	160	28
AGTGATAGC	BRDN0001	289	309	241	514	361	64
AGTGATAGC	BRDN0001	488	499	684	915	773	15
AGTGATATC	BRDN0001	324	309	714	612	585	20
AGTGATATC	BRDN0001	87	28	68	272	194	0
AGTGATATC	BRDN0001	133	8	0	313	71	1
AGTGATATG	BRDN0001	80	43	54	433	395	0
AGTGATCAA	BRDN0001	138	135	2237	283	231	63
AGTGATCGA	BRDN0001	289	0	0	821	224	0
AGTGATCGT	BRDN0001	55	42	0	86	203	19
AGTGATCTA	BRDN0001	359	595	344	948	793	8
AGTGATCTA	BRDN0001	167	138	319	560	726	0
AGTGATCTA	BRDN0001	58	13	0	190	256	0
AGTGATCTC	BRDN0001	64	60	0	149	293	12
AGTGATCTT	BRDN0001	184	313	29	365	222	12
AGTGATGAG	BRDN0001	176	113	15	392	134	13
AGTGATGCT	BRDN0001	228	140	283	274	281	0
AGTGATGGT	BRDN0001	71	113	55	267	231	0
AGTGATGTG	BRDN0001	415	251	437	674	810	24
AGTGATGTT	BRDN0001	175	98	54	374	471	73
AGTGATTAC	BRDN0001	221	170	0	405	264	6
AGTGATTCG	BRDN0001	142	96	30	246	203	0
AGTGATTCG	BRDN0001	231	217	107	487	224	10
AGTGATTGT	BRDN0001	105	0	1	406	171	0
AGTGATTTA	BRDN0001	10	0	0	177	18	0
AGTGATTTA	BRDN0001	113	212	25	243	267	0
AGTGCAAAA	BRDN0001	183	159	588	466	211	16
AGTGCAAAG	BRDN0001	346	283	24	568	638	45
AGTGCAAAG	BRDN0001	112	4	11	296	153	0
AGTGCAACA	BRDN0001	442	519	1079	727	658	57
AGTGCAACA	BRDN0001	292	264	548	753	445	19
AGTGCAACC	BRDN0001	225	70	25	353	271	12
AGTGCAACG	BRDN0001	269	312	1984	573	948	130
AGTGCAAGC	BRDN0001	546	303	176	1057	1178	55
AGTGCAAGG	BRDN0001	148	0	0	547	448	0
AGTGCAATA	BRDN0001	118	10	0	326	239	0
AGTGCAATG	BRDN0001	331	256	251	575	384	13
AGTGCAATG	BRDN0001	231	457	612	519	559	34
AGTGCACAA	BRDN0001	373	245	418	587	481	33
AGTGCACAG	BRDN0001	341	321	1098	469	708	29
AGTGCACAG	BRDN0001	471	386	56	1020	1088	38
AGTGCACCT	BRDN0001	248	336	82	646	795	12
AGTGCACTA	BRDN0001	196	137	143	314	206	0
AGTGCAGAA	BRDN0001	256	183	205	428	414	7
AGTGCAGAA	BRDN0001	333	439	1460	570	568	169
AGTGCAGAG	BRDN0001	509	349	807	892	756	33
AGTGCAGCC	BRDN0001	91	45	667	184	138	6
AGTGCAGCT	BRDN0001	107	116	0	223	310	0
AGTGCAGGA	BRDN0001	224	204	388	286	471	74
AGTGCAGGA	BRDN0001	286	316	750	651	861	48
AGTGCAGGC	BRDN0001	110	87	2	129	173	6
AGTGCAGGC	BRDN0001	385	493	1215	702	839	153
AGTGCAGTA	BRDN0001	188	162	382	468	248	10
AGTGCATAC	BRDN0001	174	36	3	398	147	0
AGTGCATAG	BRDN0001	156	280	65	324	707	52
AGTGCATAG	BRDN0001	162	64	170	281	131	6
AGTGCATCC	BRDN0001	190	199	1846	316	282	69
AGTGCATCC	BRDN0001	217	139	0	443	275	15
AGTGCATGA	BRDN0001	388	366	1479	858	542	60
AGTGCATGC	BRDN0001	287	166	3840	494	472	78
AGTGCATGT	BRDN0001	342	247	757	391	552	31
AGTGCATTG	BRDN0001	106	129	2666	206	144	209
AGTGCCAAC	BRDN0001	426	269	398	486	286	16
AGTGCCAAT	BRDN0001	200	117	88	249	298	6
AGTGCCAAT	BRDN0001	131	33	4	292	160	11
AGTGCCACT	BRDN0001	115	131	504	298	118	25
AGTGCCACT	BRDN0001	155	124	192	452	400	30
AGTGCCACT	BRDN0001	142	63	200	506	968	14
AGTGCCAGC	BRDN0001	146	136	551	293	415	12
AGTGCCAGC	BRDN0001	381	343	311	702	805	18
AGTGCCAGG	BRDN0001	162	136	8	175	356	4
AGTGCCAGG	BRDN0001	660	631	692	1207	1254	54
AGTGCCCAA	BRDN0001	316	228	448	546	368	35
AGTGCCCAC	BRDN0001	56	55	0	217	273	0
AGTGCCCAG	BRDN0001	164	95	76	331	153	0
AGTGCCCAG	BRDN0001	114	103	1348	362	253	17
AGTGCCCAT	BRDN0001	172	204	2958	307	261	100
AGTGCCCGG	BRDN0001	85	76	631	150	264	4
AGTGCCCGG	BRDN0001	625	716	808	1150	899	130
AGTGCCGAG	BRDN0001	191	193	795	278	278	26
AGTGCCGGA	BRDN0001	379	272	99	403	456	27
AGTGCCGGG	BRDN0001	45	78	97	177	359	0
AGTGCCTAA	BRDN0001	229	261	258	430	728	47
AGTGCCTAC	BRDN0001	154	93	225	403	303	8
AGTGCCTGC	BRDN0001	233	160	40	403	447	13
AGTGCCTGG	BRDN0001	55	42	204	305	95	1
AGTGCCTTT	BRDN0001	218	171	166	437	496	31
AGTGCGACG	BRDN0001	176	211	624	332	129	27
AGTGCGACG	BRDN0001	319	359	427	716	274	30
AGTGCGAGA	BRDN0001	377	366	1077	833	574	10
AGTGCGAGT	BRDN0001	173	156	6	406	378	22
AGTGCGCAC	BRDN0001	86	42	30	218	316	27
AGTGCGCAG	BRDN0001	159	107	114	253	178	24
AGTGCGCAG	BRDN0001	403	668	314	636	992	0
AGTGCGCCC	BRDN0001	78	4	47	258	7	0
AGTGCGGAC	BRDN0001	98	87	94	243	144	12
AGTGCGGTG	BRDN0001	131	97	339	175	147	25
AGTGCGTAG	BRDN0001	194	127	1538	291	482	19
AGTGCGTCG	BRDN0001	183	183	363	387	203	12
AGTGCGTGA	BRDN0001	376	380	311	723	546	41
AGTGCGTTC	BRDN0001	177	32	55	289	344	0
AGTGCTACA	BRDN0001	275	329	217	595	492	88
AGTGCTACT	BRDN0001	752	743	1092	1028	1518	136
AGTGCTCAA	BRDN0001	102	190	2228	341	373	54
AGTGCTCAC	BRDN0001	78	14	0	176	30	0
AGTGCTCAC	BRDN0001	391	224	678	777	779	85
AGTGCTCCG	BRDN0001	412	331	185	676	712	78
AGTGCTCGA	BRDN0001	255	259	28	410	562	5
AGTGCTCTA	BRDN0001	163	116	135	287	274	27
AGTGCTCTG	BRDN0001	118	86	16	301	472	0
AGTGCTCTG	BRDN0001	162	158	676	209	155	8
AGTGCTGAA	BRDN0001	223	248	0	368	101	22
AGTGCTGAC	BRDN0001	386	275	112	756	673	1
AGTGCTGAC	BRDN0001	358	293	252	532	801	65
AGTGCTGAG	BRDN0001	164	268	511	289	572	0
AGTGCTGCT	BRDN0001	159	187	47	215	171	6
AGTGCTGCT	BRDN0001	94	159	258	299	261	36
AGTGCTGCT	BRDN0001	504	272	324	989	862	0
AGTGCTGGA	BRDN0001	163	167	277	415	484	5
AGTGCTGGC	BRDN0001	128	79	161	348	316	3
AGTGCTGGG	BRDN0001	68	78	0	153	316	0
AGTGCTGGG	BRDN0001	152	145	16	361	253	4
AGTGCTGGT	BRDN0001	78	79	27	218	135	4
AGTGCTGTA	BRDN0001	348	266	930	622	736	48
AGTGCTGTG	BRDN0001	134	214	917	363	448	110
AGTGCTGTT	BRDN0001	583	372	1786	827	989	47
AGTGCTTAG	BRDN0001	379	590	920	712	894	152
AGTGCTTAT	BRDN0001	350	246	147	707	591	20
AGTGCTTCG	BRDN0001	293	202	784	659	959	12
AGTGCTTCG	BRDN0001	197	122	250	283	255	3
AGTGCTTGA	BRDN0001	300	402	1868	715	507	27
AGTGCTTGC	BRDN0001	300	462	536	956	934	30
AGTGCTTGG	BRDN0001	221	257	1217	554	579	13
AGTGCTTGT	BRDN0001	148	57	12	180	261	4
AGTGCTTTA	BRDN0001	414	502	221	740	990	52
AGTGCTTTG	BRDN0001	129	48	19	296	443	0
AGTGGAAAA	BRDN0001	158	54	91	267	159	4
AGTGGAAAC	BRDN0001	248	181	1602	466	629	26
AGTGGAACA	BRDN0001	208	132	144	254	512	4
AGTGGAACA	BRDN0001	86	111	55	226	141	5
AGTGGAACG	BRDN0001	180	147	279	391	514	25
AGTGGAACT	BRDN0001	380	74	219	922	667	12
AGTGGAATA	BRDN0001	123	149	9	210	571	8
AGTGGAATC	BRDN0001	185	228	65	208	206	11
AGTGGACAC	BRDN0001	313	392	399	822	886	16
AGTGGACAC	BRDN0001	535	438	508	991	1538	36
AGTGGACAT	BRDN0001	80	70	0	202	131	0
AGTGGACAT	BRDN0001	159	194	309	182	151	0
AGTGGACCG	BRDN0001	113	43	112	249	265	0
AGTGGACCG	BRDN0001	385	279	1637	613	603	88
AGTGGACCG	BRDN0001	201	356	558	646	980	9
AGTGGACGG	BRDN0001	452	438	1135	696	674	13
AGTGGACTG	BRDN0001	138	205	0	220	246	26
AGTGGAGAC	BRDN0001	162	135	0	210	202	0
AGTGGAGAC	BRDN0001	314	219	219	380	455	0
AGTGGAGGC	BRDN0001	160	135	10	262	396	22
AGTGGAGGT	BRDN0001	59	56	603	119	163	8
AGTGGAGTA	BRDN0001	215	222	1101	361	240	85
AGTGGATAC	BRDN0001	140	89	408	265	289	12
AGTGGATAG	BRDN0001	117	201	38	388	313	34
AGTGGATAG	BRDN0001	140	119	407	281	178	15
AGTGGATAT	BRDN0001	173	191	721	396	607	47
AGTGGATAT	BRDN0001	217	178	903	459	803	31
AGTGGATCT	BRDN0001	182	128	154	280	60	38
AGTGGATGA	BRDN0001	136	23	44	525	244	0
AGTGGATGA	BRDN0001	159	158	254	229	229	12
AGTGGATGC	BRDN0001	332	192	130	908	826	4
AGTGGATGC	BRDN0001	178	75	254	233	116	17
AGTGGATGT	BRDN0001	104	60	329	228	68	15
AGTGGATTA	BRDN0001	153	116	330	282	196	26

AGTGGATTA	BRDN00011	325	352	93	594	645	15
AGTGGATTG	BRDN00011	324	271	104	535	461	11
AGTGGATTG	BRDN00012	409	315	3537	783	1287	114
AGTGGCAAA	BRDN00012	209	52	2	449	126	0
AGTGGCACA	BRDN00012	117	60	281	158	139	3
AGTGGCACG	BRDN00012	207	328	201	541	567	49
AGTGGCAGA	BRDN00011	107	32	2	328	349	0
AGTGGCAGA	BRDN00011	297	121	1	811	1334	0
AGTGGCAGC	BRDN00012	234	189	709	471	374	49
AGTGGCAGC	BRDN00012	237	115	89	215	407	21
AGTGGCATA	BRDN00012	114	0	0	685	515	0
AGTGGCATA	BRDN00012	84	149	251	181	607	3
AGTGGCCAC	BRDN00011	164	216	670	331	454	0
AGTGGCCAG	BRDN00012	81	52	31	133	101	0
AGTGGCCAG	BRDN00012	372	356	702	561	1109	28
AGTGGCCAG	BRDN00012	225	216	575	517	483	22
AGTGGCCCG	BRDN00011	202	170	496	401	332	20
AGTGGCCCT	BRDN00011	163	200	67	246	359	17
AGTGGCCGT	BRDN00012	477	407	457	606	709	9
AGTGGCCTA	BRDN00012	126	50	0	259	133	16
AGTGGCGCC	BRDN00011	96	202	79	234	199	2
AGTGGCGCC	BRDN00011	185	60	79	575	482	0
AGTGGCGGC	BRDN00012	94	122	29	307	497	7
AGTGGCGTA	BRDN00012	227	195	110	490	598	12
AGTGGCGTG	BRDN00011	362	349	1239	570	260	46
AGTGGCTAA	BRDN00012	141	162	1189	209	406	67
AGTGGCTAC	BRDN00012	232	140	121	390	501	0
AGTGGCTCA	BRDN00011	213	170	273	338	599	5
AGTGGCTCG	BRDN00011	245	221	2007	555	746	29
AGTGGCTGC	BRDN00011	410	416	385	774	808	65
AGTGGCTGT	BRDN00011	116	163	67	349	313	11
AGTGGCTTC	BRDN00012	68	80	0	143	21	0
AGTGGCTTG	BRDN00012	124	93	164	260	174	0
AGTGGCTTG	BRDN00011	77	106	36	247	208	0
AGTGGCTTT	BRDN00011	289	230	50	405	468	31
AGTGGGAAC	BRDN00012	583	105	100	950	971	17
AGTGGGACA	BRDN00012	146	193	369	317	446	21
AGTGGGACC	BRDN00011	121	190	22	310	255	0
AGTGGGAGA	BRDN00011	78	92	1	139	143	0
AGTGGGATT	BRDN00011	113	142	228	187	334	13
AGTGGGCAA	BRDN00012	189	238	63	394	124	7
AGTGGGCCA	BRDN00011	159	185	47	220	110	13
AGTGGGCCC	BRDN00012	111	124	127	278	593	0
AGTGGGCGC	BRDN00012	466	411	771	1013	647	5
AGTGGGCTC	BRDN00011	423	276	418	694	878	4
AGTGGGCTG	BRDN00011	3	0	0	775	272	0
AGTGGGCTG	BRDN00011	360	247	246	447	436	162
AGTGGGCTT	BRDN00012	165	92	0	190	219	25
AGTGGGGAA	BRDN00011	241	351	266	362	278	69
AGTGGGGAC	BRDN00012	326	235	1087	773	574	55
AGTGGGGAC	BRDN00011	368	353	757	680	780	15
AGTGGGGAT	BRDN00011	321	239	1844	615	864	23
AGTGGGGCC	BRDN00012	194	93	79	242	185	7
AGTGGGGCC	BRDN00012	541	503	603	844	1035	38
AGTGGGGGT	BRDN00011	365	388	1252	686	1030	46
AGTGGGGGT	BRDN00011	212	179	62	459	691	32
AGTGGGGTC	BRDN00011	111	144	19	322	355	0
AGTGGGGTT	BRDN00011	89	37	0	236	264	0
AGTGGGTAA	BRDN00012	203	231	293	472	237	6
AGTGGGTAC	BRDN00011	297	307	461	625	462	50
AGTGGGTAC	BRDN00012	479	141	123	655	678	1
AGTGGGTAG	BRDN00011	368	389	1101	504	656	57
AGTGGGTGA	BRDN00012	174	127	350	367	414	5
AGTGGGTGC	BRDN00012	324	348	348	703	798	13
AGTGGGTGC	BRDN00011	222	171	112	416	219	30
AGTGGGTTG	BRDN00012	54	64	152	257	364	3
AGTGGTAAT	BRDN00012	251	253	281	432	702	31
AGTGGTACC	BRDN00011	146	135	373	261	211	40
AGTGGTAGG	BRDN00012	287	151	404	504	692	0
AGTGGTAGG	BRDN00011	325	282	627	720	743	27
AGTGGTATA	BRDN00012	126	35	408	258	137	37
AGTGGTCAA	BRDN00011	369	320	550	780	852	19
AGTGGTCAA	BRDN00011	269	230	425	355	239	6
AGTGGTCAA	BRDN00012	380	316	1772	654	771	57
AGTGGTCAC	BRDN00012	427	640	276	1029	1068	19
AGTGGTCAG	BRDN00011	62	112	689	212	244	6
AGTGGTCAG	BRDN00011	320	296	127	686	425	6
AGTGGTCAG	BRDN00012	312	420	56	738	968	6
AGTGGTCAG	BRDN00011	78	90	0	213	181	9
AGTGGTCAT	BRDN00012	132	185	2139	636	598	6
AGTGGTCAT	BRDN00012	401	295	925	791	1093	64
AGTGGTCTA	BRDN00012	326	419	410	670	692	75
AGTGGTCTA	BRDN00012	172	167	576	247	451	8
AGTGGTCTC	BRDN00011	363	387	2660	643	814	76
AGTGGTGAC	BRDN00011	121	76	47	306	126	39
AGTGGTGAT	BRDN00012	172	157	78	293	273	6
AGTGGTGCC	BRDN00012	144	179	17	320	132	10
AGTGGTGCG	BRDN00012	132	82	170	142	102	0
AGTGGTGCT	BRDN00011	172	125	24	295	158	44
AGTGGTGGA	BRDN00011	58	64	0	176	163	0
AGTGGTGGA	BRDN00012	392	573	1206	773	1641	192
AGTGGTGGA	BRDN00012	372	413	2563	796	929	104
AGTGGTGGC	BRDN00011	20	6	0	41	46	5
AGTGGTGGC	BRDN00012	28	0	0	244	52	0
AGTGGTGGC	BRDN00012	185	53	1	528	636	0
AGTGGTGGC	BRDN00012	357	254	400	754	553	2
AGTGGTGGT	BRDN00011	179	133	135	216	416	0
AGTGGTGTG	BRDN00011	253	235	241	384	407	52
AGTGGTTAC	BRDN00012	109	8	62	627	354	1
AGTGGTTAG	BRDN00012	162	177	48	357	279	12
AGTGGTTCA	BRDN00011	238	153	154	361	236	1
AGTGGTTCG	BRDN00012	126	192	249	185	52	36
AGTGGTTGA	BRDN00011	116	94	548	123	9	14
AGTGGTTGA	BRDN00011	153	153	0	182	139	3
AGTGGTTGC	BRDN00011	187	77	102	313	249	4
AGTGGTTGG	BRDN00011	222	88	464	291	471	2
AGTGGTTGT	BRDN00012	286	212	479	544	515	11
AGTGGTTTC	BRDN00012	352	280	458	507	775	0
AGTGGTTTC	BRDN00012	155	132	1	342	366	11
AGTGTAAAG	BRDN00012	500	406	1906	846	897	92
AGTGTAATC	BRDN00011	116	92	381	192	267	0
AGTGTAATT	BRDN00012	159	220	62	300	583	23
AGTGTACCA	BRDN00012	106	46	2	173	67	12
AGTGTACCT	BRDN00012	185	136	238	341	336	4
AGTGTACGA	BRDN00011	215	179	1981	436	256	39
AGTGTAGAG	BRDN00011	113	33	643	145	142	16
AGTGTAGGC	BRDN00011	148	152	130	309	238	52
AGTGTATAC	BRDN00011	169	186	121	300	242	29
AGTGTATAC	BRDN00012	198	260	248	202	261	11
AGTGTATAG	BRDN00012	243	160	50	451	322	19
AGTGTATCG	BRDN00012	93	84	86	249	204	0
AGTGTATCT	BRDN00012	468	378	2859	579	751	41
AGTGTATGA	BRDN00012	204	201	232	307	378	0
AGTGTATTC	BRDN00011	250	0	36	677	269	0
AGTGTCAAT	BRDN00012	101	119	6	295	134	2
AGTGTCACA	BRDN00012	237	218	220	433	264	15
AGTGTCACT	BRDN00012	144	161	463	239	97	43
AGTGTCACT	BRDN00012	216	125	77	582	429	0
AGTGTCATG	BRDN00012	158	90	134	382	249	35
AGTGTCATT	BRDN00012	160	174	665	256	509	20
AGTGTCCAG	BRDN00012	199	148	175	272	314	3
AGTGTCCAG	BRDN00012	78	79	0	239	440	0
AGTGTCCAT	BRDN00012	286	332	168	587	822	39
AGTGTCCCA	BRDN00011	71	21	0	153	35	0
AGTGTCCCA	BRDN00012	277	255	129	682	1038	0
AGTGTCCCC	BRDN00012	285	194	334	511	458	6
AGTGTCCCC	BRDN00011	323	107	65	802	113	18
AGTGTCCCT	BRDN00011	164	215	395	374	249	14
AGTGTCCGA	BRDN00012	72	81	52	248	182	7
AGTGTCGGA	BRDN00011	297	304	85	631	571	33
AGTGTCGGG	BRDN00011	134	133	2696	243	165	42
AGTGTCGTC	BRDN00012	104	169	39	190	100	30
AGTGTCTAA	BRDN00011	116	188	127	217	134	15
AGTGTCTAC	BRDN00011	103	46	0	162	72	0
AGTGTCTGC	BRDN00012	144	100	416	245	273	15
AGTGTCTTA	BRDN00011	167	107	113	279	296	0
AGTGTCTTC	BRDN00011	377	204	214	774	874	6
AGTGTGAAA	BRDN00011	144	230	593	316	351	35
AGTGTGAAC	BRDN00011	131	73	454	233	78	19
AGTGTGAAT	BRDN00012	119	102	128	286	208	11
AGTGTGACA	BRDN00012	197	188	41	211	154	26
AGTGTGACC	BRDN00011	140	131	231	180	78	1
AGTGTGAGA	BRDN00012	38	57	47	112	178	1
AGTGTGAGA	BRDN00011	44	76	0	180	133	7
AGTGTGATA	BRDN00012	100	97	59	243	271	2
AGTGTGATC	BRDN00011	113	163	41	205	126	0
AGTGTGATG	BRDN00011	233	195	109	420	687	21
AGTGTGATG	BRDN00012	344	386	252	728	850	0
AGTGTGATG	BRDN00011	94	116	165	161	178	28
AGTGTGCAA	BRDN00011	224	123	25	378	301	0
AGTGTGCCT	BRDN00011	114	135	64	207	284	0
AGTGTGCTG	BRDN00011	210	225	192	418	404	38
AGTGTGCTT	BRDN00012	244	285	467	613	613	87
AGTGTGGAA	BRDN00012	452	502	816	731	813	44
AGTGTGGAC	BRDN00012	117	9	1	531	262	0
AGTGTGGAC	BRDN00011	98	119	236	177	334	43
AGTGTGGAC	BRDN00011	124	68	16	247	266	8
AGTGTGGCT	BRDN00011	224	126	162	427	196	41
AGTGTGGGA	BRDN00011	80	106	133	359	767	14
AGTGTGGGC	BRDN00011	51	48	100	51	97	8
AGTGTGGGC	BRDN00011	510	406	1233	1128	1089	44
AGTGTGGTA	BRDN00012	283	329	235	434	586	18
AGTGTGGTA	BRDN00011	440	375	1754	846	1056	38
AGTGTGGTG	BRDN00011	233	247	2242	380	509	36
AGTGTGTAA	BRDN00012	143	199	36	342	405	2
AGTGTGTAC	BRDN00012	185	254	669	564	548	29
AGTGTGTAC	BRDN00012	232	225	190	398	372	45
AGTGTGTAC	BRDN00011	270	304	2067	428	529	47
AGTGTGTGC	BRDN00011	118	135	133	251	221	26
AGTGTGTGG	BRDN00011	335	229	665	603	482	1
AGTGTGTGT	BRDN00012	124	64	148	249	138	34
AGTGTGTTA	BRDN00011	266	253	33	558	685	36
AGTGTGTTC	BRDN00011	474	298	798	730	791	27
AGTGTGTTG	BRDN00012	309	536	1559	518	590	76
AGTGTTAAA	BRDN00011	127	111	92	219	176	13
AGTGTTACC	BRDN00012	129	32	4	316	281	0
AGTGTTACC	BRDN00012	303	270	224	583	490	65
AGTGTTACC	BRDN00011	205	56	36	359	571	0
AGTGTTACG	BRDN00011	53	0	0	362	19	0
AGTGTTACG	BRDN00012	96	92	281	160	111	5
AGTGTTATA	BRDN00012	321	328	128	509	780	47
AGTGTTCAC	BRDN00012	328	430	288	854	1152	51
AGTGTTCAC	BRDN00012	119	48	0	219	79	1
AGTGTTCAC	BRDN00012	283	419	393	747	719	5
AGTGTTCAG	BRDN00011	152	178	1	383	203	0
AGTGTTCCG	BRDN00012	220	168	471	399	309	4
AGTGTTCTT	BRDN00011	113	174	6	248	206	42
AGTGTTGAC	BRDN00011	163	131	103	295	308	43
AGTGTTGCA	BRDN00012	368	339	974	461	830	25
AGTGTTGTA	BRDN00012	275	238	122	355	309	41
AGTGTTTCA	BRDN00012	182	184	188	429	145	11
AGTGTTTCG	BRDN00012	292	206	106	438	401	20
AGTGTTTGA	BRDN00012	399	325	1031	564	427	5
AGTGTTTGA	BRDN00011	31	12	1	350	188	0
AGTGTTTGT	BRDN00012	232	171	192	584	296	4
AGTTAAAAA	BRDN00011	55	44	9	136	132	0
AGTTAAAAC	BRDN00012	527	346	804	938	1424	25
AGTTAAACA	BRDN00011	172	128	595	366	315	31
AGTTAAAGG	BRDN00011	208	183	52	516	473	10
AGTTAAAGT	BRDN00012	272	280	137	398	390	18
AGTTAAATC	BRDN00011	124	8	94	271	240	0
AGTTAAATG	BRDN00012	401	494	1637	825	652	42
AGTTAACAG	BRDN00012	135	125	34	153	282	12
AGTTAACAT	BRDN00011	296	242	97	370	585	52
AGTTAACGC	BRDN00012	405	235	1452	644	576	23
AGTTAACTG	BRDN00012	374	277	1387	538	456	57
AGTTAAGAC	BRDN00012	68	88	0	241	142	21
AGTTAAGCT	BRDN00011	232	304	123	420	415	48
AGTTACAAC	BRDN00012	329	191	110	633	1142	43
AGTTACAAG	BRDN00012	446	306	350	832	492	13
AGTTACAAG	BRDN00011	129	198	33	326	249	24
AGTTACAGT	BRDN00011	202	109	1	369	517	25
AGTTACATG	BRDN00012	40	0	0	444	190	0
AGTTACCAC	BRDN00012	134	51	26	599	863	0
AGTTACCAT	BRDN00012	225	129	4	406	743	41
AGTTACCCG	BRDN00011	249	199	4	404	473	27
AGTTACCGA	BRDN00011	163	64	0	344	317	20
AGTTACCTG	BRDN00011	463	388	294	721	1052	140
AGTTACGCA	BRDN00012	375	267	309	626	544	9
AGTTACGGA	BRDN00011	79	88	86	164	141	8
AGTTACGGA	BRDN00012	105	92	41	297	368	21
AGTTACGGG	BRDN00011	312	317	660	488	572	93
AGTTACGGG	BRDN00012	492	508	1081	1036	687	50
AGTTACTAG	BRDN00012	334	234	62	716	743	29
AGTTACTCA	BRDN00012	26	11	0	450	17	0
AGTTACTCA	BRDN00012	281	214	472	539	316	9
AGTTACTGA	BRDN00012	246	325	630	362	147	26
AGTTACTTC	BRDN00012	202	164	37	461	419	5
AGTTACTTT	BRDN00012	221	143	59	203	157	46
AGTTACTTT	BRDN00012	272	167	1272	651	429	75
AGTTAGAAA	BRDN00012	136	108	108	199	227	1
AGTTAGAGG	BRDN00012	145	136	89	337	285	22
AGTTAGCAA	BRDN00011	88	37	0	268	159	8
AGTTAGCCA	BRDN00011	176	220	278	539	735	0
AGTTAGCTG	BRDN00011	255	158	327	493	489	5
AGTTAGGAA	BRDN00012	148	190	19	365	260	40
AGTTAGTAA	BRDN00011	148	0	2	426	364	0
AGTTAGTGA	BRDN00011	363	278	113	496	463	11
AGTTAGTGG	BRDN00012	259	318	1460	594	604	36
AGTTATAAA	BRDN00012	198	180	99	372	399	7
AGTTATATC	BRDN00012	364	323	1293	680	516	11
AGTTATCAA	BRDN00012	90	7	0	219	75	0
AGTTATCAA	BRDN00012	122	132	1311	170	18	12
AGTTATCAC	BRDN00011	214	113	351	423	204	0
AGTTATCCA	BRDN00011	438	295	698	588	818	68
AGTTATCCA	BRDN00012	95	55	28	324	291	10
AGTTATCTA	BRDN00012	160	135	45	249	294	5
AGTTATCTG	BRDN00011	415	255	2874	917	922	58
AGTTATCTT	BRDN00012	290	244	821	571	767	26
AGTTATGGA	BRDN00012	522	518	160	678	443	43
AGTTATGGA	BRDN00012	338	132	204	705	550	87
AGTTATGGT	BRDN00012	463	411	424	1076	1180	80
AGTTATGTT	BRDN00012	214	277	2186	484	425	52
AGTTATTAA	BRDN00012	294	20	0	619	352	0
AGTTATTCA	BRDN00012	160	142	438	248	221	8
AGTTATTCC	BRDN00012	224	107	40	317	318	35
AGTTATTGA	BRDN00012	160	17	103	416	496	1
AGTTATTGA	BRDN00012	101	122	112	216	89	0
AGTTATTGG	BRDN00011	340	1	1	507	278	0
AGTTATTTA	BRDN00012	169	163	22	399	515	0
AGTTCAAAG	BRDN00012	138	90	43	190	123	5
AGTTCAAAT	BRDN00011	343	327	190	602	594	8
AGTTCAAAT	BRDN00012	171	162	504	292	441	21
AGTTCAACA	BRDN00011	66	27	39	266	232	10
AGTTCAAGG	BRDN00011	227	339	961	467	491	92
AGTTCACAT	BRDN00011	197	155	782	579	492	24
AGTTCACCA	BRDN00012	200	235	564	365	602	60
AGTTCACCA	BRDN00012	187	177	908	299	490	30
AGTTCACGG	BRDN00011	185	80	1	392	274	5
AGTTCAGAA	BRDN00011	302	203	130	512	679	244
AGTTCAGCA	BRDN00011	183	139	235	362	457	0
AGTTCAGCC	BRDN00011	85	36	0	269	97	0
AGTTCAGCC	BRDN00011	167	61	195	209	157	0
AGTTCAGGA	BRDN00012	431	277	583	681	482	29
AGTTCAGGC	BRDN00011	128	127	219	264	240	5
AGTTCAGGT	BRDN00011	254	274	51	413	251	44
AGTTCAGTG	BRDN00012	171	126	9	222	168	7
AGTTCATAT	BRDN00012	234	339	458	404	168	15
AGTTCATCG	BRDN00011	393	17	0	939	263	0
AGTTCATGT	BRDN00012	393	468	125	741	1053	122
AGTTCATTC	BRDN00012	400	368	563	749	641	22
AGTTCATTC	BRDN00011	487	469	1097	960	543	51
AGTTCCAAG	BRDN00012	339	314	1890	913	458	74
AGTTCCAAT	BRDN00011	239	153	493	450	734	73
AGTTCCAAT	BRDN00012	421	272	339	645	1148	15
AGTTCCACG	BRDN00012	178	106	115	314	122	14
AGTTCCAGG	BRDN00012	481	475	2053	747	795	60
AGTTCCATA	BRDN00012	190	145	864	360	566	16
AGTTCCATC	BRDN00012	357	278	71	652	666	25
AGTTCCATT	BRDN00011	196	263	111	517	459	7
AGTTCCATT	BRDN00012	553	473	569	738	1232	27
AGTTCCCAA	BRDN00011	212	145	106	425	579	40
AGTTCCCAC	BRDN00012	228	322	195	482	395	22
AGTTCCCAG	BRDN00012	266	323	334	521	347	43
AGTTCCCCT	BRDN00011	276	260	501	398	356	12
AGTTCCCGA	BRDN00011	357	329	564	542	348	17
AGTTCCCGC	BRDN00012	276	140	1011	568	294	20
AGTTCCCTT	BRDN00011	470	243	4860	863	655	74
AGTTCCGAT	BRDN00012	92	58	1	143	145	0
AGTTCCGTG	BRDN00012	269	5	2	1046	1052	0
AGTTCCTCA	BRDN00011	312	215	33	490	162	3
AGTTCCTCA	BRDN00012	330	178	69	610	534	11
AGTTCCTGC	BRDN00012	680	712	1496	1297	1314	99
AGTTCCTTA	BRDN00011	67	13	33	295	234	0
AGTTCCTTA	BRDN00012	252	167	102	492	479	5
AGTTCGAAC	BRDN00011	386	360	113	758	673	24
AGTTCGAAG	BRDN00011	176	0	0	295	548	0
AGTTCGAAG	BRDN00011	136	88	10	341	132	0
AGTTCGAAG	BRDN00011	269	149	383	428	413	119
AGTTCGAAG	BRDN00012	434	427	417	823	1011	34
AGTTCGATA	BRDN00012	283	192	347	511	752	10
AGTTCGATA	BRDN00011	136	72	448	251	226	10
AGTTCGCCA	BRDN00011	105	136	43	365	368	13
AGTTCGCGA	BRDN00011	312	273	1380	495	371	44
AGTTCGGAG	BRDN00012	519	399	260	824	787	21
AGTTCGGTA	BRDN00011	632	446	448	847	954	92
AGTTCGGTG	BRDN00011	219	210	195	401	450	34
AGTTCGTGA	BRDN00011	142	72	9	231	292	0
AGTTCGTGA	BRDN00012	215	124	108	416	271	17
AGTTCGTGT	BRDN00011	156	47	0	189	483	0
AGTTCTAAT	BRDN00012	141	164	562	229	429	34
AGTTCTACA	BRDN00011	358	276	487	671	531	25
AGTTCTACC	BRDN00012	213	131	237	677	449	13
AGTTCTACG	BRDN00011	358	243	111	361	356	33
AGTTCTACT	BRDN00011	173	213	149	271	298	10
AGTTCTATC	BRDN00011	130	105	16	254	207	0
AGTTCTATG	BRDN00011	282	255	43	642	644	27
AGTTCTCAG	BRDN00011	195	216	235	295	369	0
AGTTCTCCA	BRDN00011	105	69	79	195	181	17
AGTTCTCCA	BRDN00011	234	316	236	534	733	13
AGTTCTCCG	BRDN00012	293	308	143	539	559	22
AGTTCTCGC	BRDN00012	163	129	24	571	403	10
AGTTCTCTA	BRDN00012	448	469	101	817	562	76
AGTTCTGAA	BRDN00011	109	119	4	207	376	17
AGTTCTGCA	BRDN00012	172	100	23	259	615	13
AGTTCTGCA	BRDN00011	130	104	16	287	392	0
AGTTCTGCG	BRDN00011	571	508	1221	918	447	42
AGTTCTGGA	BRDN00012	182	141	194	341	391	0
AGTTCTGGC	BRDN00012	213	189	684	435	598	10
AGTTCTGGG	BRDN00012	122	108	146	225	82	0
AGTTCTGTT	BRDN00012	480	495	278	759	970	84
AGTTCTTCA	BRDN00011	123	40	35	375	631	1
AGTTCTTCA	BRDN00012	106	57	216	289	169	0
AGTTCTTCC	BRDN00011	229	200	12	539	621	17
AGTTCTTGG	BRDN00012	249	255	743	472	492	42
AGTTCTTGG	BRDN00012	385	317	199	809	719	52
AGTTCTTTA	BRDN00011	97	15	0	323	399	0
AGTTGAAAA	BRDN00012	352	238	624	550	943	23
AGTTGAAAT	BRDN00011	351	295	142	580	518	2
AGTTGAACA	BRDN00012	198	174	384	332	276	19
AGTTGAATA	BRDN00012	92	101	67	162	192	0
AGTTGAATG	BRDN00012	204	104	0	279	438	0
AGTTGAATG	BRDN00012	494	335	2343	862	738	57
AGTTGACAA	BRDN00012	359	293	218	689	634	30
AGTTGACAG	BRDN00011	267	15	2	613	341	15
AGTTGACGC	BRDN00011	191	187	1699	379	333	41
AGTTGACTG	BRDN00011	138	34	17	226	409	0
AGTTGACTG	BRDN00012	314	238	2703	464	365	230
AGTTGAGTT	BRDN00012	427	511	2543	963	999	59
AGTTGATAA	BRDN00012	291	196	1036	518	739	18
AGTTGATCC	BRDN00012	151	85	1056	373	521	33
AGTTGATCT	BRDN00011	364	298	555	505	404	55
AGTTGATGA	BRDN00011	420	632	660	781	605	62
AGTTGATGC	BRDN00011	14	0	0	401	379	0
AGTTGCAAT	BRDN00012	207	206	1340	226	357	47
AGTTGCATC	BRDN00012	236	270	763	407	544	78
AGTTGCCAA	BRDN00012	218	168	224	343	309	0
AGTTGCCAG	BRDN00012	336	329	67	644	560	13
AGTTGCCAT	BRDN00011	241	170	280	425	504	16
AGTTGCCAT	BRDN00011	90	76	122	242	197	3
AGTTGCCGG	BRDN00012	280	264	1303	496	245	5
AGTTGCCGT	BRDN00012	182	154	278	390	391	5
AGTTGCCTT	BRDN00011	193	132	121	729	648	0
AGTTGCGAA	BRDN00012	240	191	95	391	320	0
AGTTGCGCA	BRDN00012	89	6	0	309	381	0
AGTTGCGGT	BRDN00012	542	524	251	1006	929	40
AGTTGGAAA	BRDN00012	205	36	218	783	690	10
AGTTGGAAA	BRDN00012	485	680	1232	913	1208	60
AGTTGGAAA	BRDN00012	256	138	67	308	350	5
AGTTGGAAG	BRDN00011	331	411	1273	654	625	95
AGTTGGAAT	BRDN00012	314	295	2531	572	592	7
AGTTGGACA	BRDN00012	423	14	1	710	582	0
AGTTGGACA	BRDN00012	261	110	0	784	260	7
AGTTGGAGA	BRDN00011	93	16	105	184	34	7
AGTTGGAGC	BRDN00011	228	396	1446	349	407	72
AGTTGGCAG	BRDN00012	246	0	1	685	693	0
AGTTGGCAT	BRDN00012	257	96	259	400	758	0
AGTTGGCTA	BRDN00011	378	390	147	825	574	20
AGTTGGCTA	BRDN00012	267	167	926	586	658	9
AGTTGGCTG	BRDN00011	187	178	1034	314	225	84
AGTTGGCTT	BRDN00011	140	125	184	149	127	44
AGTTGGGGA	BRDN00012	34	64	0	123	73	0
AGTTGGGGC	BRDN00012	89	140	420	270	92	27
AGTTGGGGC	BRDN00011	96	81	376	227	131	10
AGTTGGGTA	BRDN00012	247	285	160	561	755	10
AGTTGGGTT	BRDN00012	605	85	625	1034	1252	22
AGTTGGTAA	BRDN00011	110	117	0	246	163	26
AGTTGGTAC	BRDN00012	133	8	0	278	222	0
AGTTGGTGA	BRDN00012	135	129	269	411	187	0
AGTTGGTGA	BRDN00012	75	0	0	285	226	0
AGTTGGTTG	BRDN00011	209	257	386	362	369	13
AGTTGTAAT	BRDN00011	105	9	0	370	231	0
AGTTGTAAT	BRDN00012	437	373	101	593	476	16
AGTTGTACC	BRDN00012	348	413	1261	865	1465	64
AGTTGTACG	BRDN00011	332	403	1721	540	294	73
AGTTGTAGG	BRDN00012	387	244	1254	598	404	16
AGTTGTAGT	BRDN00011	328	262	217	581	430	33
AGTTGTATC	BRDN00012	373	405	258	602	1051	18
AGTTGTCCA	BRDN00011	431	362	254	861	946	38
AGTTGTCCT	BRDN00011	106	8	0	312	526	0
AGTTGTCTA	BRDN00012	163	198	280	413	344	14
AGTTGTCTA	BRDN00012	620	274	97	605	758	9
AGTTGTGAG	BRDN00011	263	259	1422	700	610	60
AGTTGTGAT	BRDN00012	150	16	19	471	279	0
AGTTGTGCA	BRDN00011	132	52	0	168	303	0
AGTTGTGTA	BRDN00012	154	162	577	202	451	57
AGTTGTTAC	BRDN00012	250	363	679	584	516	60
AGTTGTTAG	BRDN00012	60	0	0	207	146	0
AGTTGTTGG	BRDN00011	107	147	104	140	444	36
AGTTGTTGG	BRDN00011	486	458	1233	876	895	35
AGTTGTTTA	BRDN00011	312	229	158	585	690	15
AGTTTAAAA	BRDN00012	107	90	1	92	128	0
AGTTTAAGG	BRDN00012	53	40	14	122	108	0
AGTTTAAGT	BRDN00011	143	187	107	294	315	0
AGTTTACAC	BRDN00011	134	77	87	277	106	0
AGTTTACAT	BRDN00011	432	529	893	804	801	74
AGTTTACCA	BRDN00012	65	9	1	319	255	0
AGTTTACCT	BRDN00012	440	510	1027	718	1045	100
AGTTTACCT	BRDN00011	185	124	305	273	252	0
AGTTTACGC	BRDN00012	418	247	1093	586	707	30
AGTTTACTT	BRDN00012	349	340	372	585	521	11
AGTTTATGA	BRDN00012	211	117	0	347	203	0
AGTTTATGC	BRDN00012	760	597	750	1186	1377	41
AGTTTATGG	BRDN00012	251	118	87	365	400	3
AGTTTATTG	BRDN00012	240	90	0	474	526	0
AGTTTCAAA	BRDN00011	96	102	0	202	402	18
AGTTTCCAT	BRDN00012	149	60	142	345	411	27
AGTTTCCAT	BRDN00012	162	174	51	372	656	10
AGTTTCCCT	BRDN00011	172	155	161	270	827	37
AGTTTCCTG	BRDN00012	328	457	59	677	507	23
AGTTTCGGG	BRDN00011	457	6	12	913	1241	0
AGTTTCTCA	BRDN00011	153	131	638	334	379	38
AGTTTCTCC	BRDN00011	116	137	86	310	350	2
AGTTTCTTT	BRDN00012	224	204	128	290	331	52
AGTTTGAAG	BRDN00011	119	146	927	184	39	1

AGTTTGAAG	BRDN00012	227	217	843	281	276	1
AGTTTGACA	BRDN00011	178	130	44	228	245	0
AGTTTGACC	BRDN00011	572	388	333	974	847	24
AGTTTGACT	BRDN00012	429	347	2351	668	1019	68
AGTTTGAGA	BRDN00012	79	170	85	247	166	0
AGTTTGAGC	BRDN00012	68	32	240	240	262	12
AGTTTGATT	BRDN00011	378	500	205	878	532	167
AGTTTGCAC	BRDN00011	109	175	268	256	138	20
AGTTTGCAG	BRDN00011	85	56	1	175	186	4
AGTTTGCCA	BRDN00012	279	273	462	566	433	39
AGTTTGCTC	BRDN00011	403	391	243	838	1350	15
AGTTTGGGG	BRDN00011	123	112	524	329	568	18
AGTTTGTAA	BRDN00011	339	318	514	469	603	45
AGTTTGTAC	BRDN00012	155	155	889	351	123	30
AGTTTGTACT	BRDN00012	137	117	0	239	333	22
AGTTTGTAG	BRDN00012	108	22	108	739	209	0
AGTTTGTCA	BRDN00011	40	1	0	320	349	0
AGTTTGTCT	BRDN00011	283	313	387	466	385	25
AGTTTGTGA	BRDN00012	533	575	105	778	793	145
AGTTTGTTA	BRDN00011	113	49	333	194	54	13
AGTTTGTTCT	BRDN00011	486	320	537	771	1155	18
ATAAAAAAG	BRDN00012	329	315	1142	816	809	101
ATAAAAACA	BRDN00011	96	91	871	221	163	16
ATAAAAAGG	BRDN00011	191	108	414	235	206	10
ATAAAAATG	BRDN00011	409	289	181	508	468	55
ATAAAACTA	BRDN00011	239	201	456	482	451	36
ATAAAACTT	BRDN00011	178	107	42	439	92	0
ATAAAAGAT	BRDN00012	384	374	745	708	834	18
ATAAAAGCC	BRDN00012	168	146	533	312	204	0
ATAAAAGCT	BRDN00011	164	10	5	411	123	0
ATAAAATAC	BRDN00011	126	129	51	233	131	10
ATAAAATGA	BRDN00011	155	175	91	365	301	19
ATAAAATTCT	BRDN00012	195	92	265	250	348	7
ATAAACAAA	BRDN00012	209	197	250	543	313	30
ATAAACAAC	BRDN00012	241	147	44	411	334	21
ATAAACACC	BRDN00012	177	45	90	356	725	23
ATAAACAGG	BRDN00011	191	80	1588	306	326	38
ATAAACATG	BRDN00011	42	0	0	170	185	0
ATAAACCGC	BRDN00012	125	196	5710	391	336	124
ATAAACCTA	BRDN00011	116	8	0	238	215	0
ATAAACGGT	BRDN00012	438	509	105	744	558	6
ATAAACGTG	BRDN00011	279	199	383	519	440	19
ATAAACGTG	BRDN00011	161	106	20	327	168	0
ATAAACTAA	BRDN00011	81	17	0	595	100	0
ATAAACTAA	BRDN00012	209	40	9	297	512	0
ATAAACTCA	BRDN00012	412	279	417	813	1352	124
ATAAACTGC	BRDN00012	350	191	107	523	1498	37
ATAAACTGG	BRDN00012	131	125	452	271	329	82
ATAAACTGG	BRDN00011	157	2	0	324	186	0
ATAAACTGG	BRDN00011	126	98	34	212	296	0
ATAAACTTG	BRDN00011	191	123	2102	375	130	40
ATAAACTTGT	BRDN00012	326	194	1540	607	994	175
ATAAACTTGT	BRDN00012	444	502	735	851	592	32
ATAAAGACC	BRDN00012	236	36	395	637	638	0
ATAAAGATA	BRDN00012	281	326	678	493	579	41
ATAAAGCAA	BRDN00011	297	279	263	837	1085	21
ATAAAGGAC	BRDN00012	227	166	428	348	570	13
ATAAAGGAG	BRDN00011	213	270	610	665	359	35
ATAAAGGAT	BRDN00012	57	102	0	415	233	9
ATAAAGGAT	BRDN00012	389	216	714	576	670	10
ATAAAGTAA	BRDN00011	87	6	1	360	397	0
ATAAAGTAA	BRDN00012	188	199	228	387	679	10
ATAAAGTCC	BRDN00012	370	345	128	635	831	13
ATAAAGTCG	BRDN00011	192	157	709	361	458	36
ATAAAGTGC	BRDN00011	82	43	39	194	63	5
ATAAAGTGG	BRDN00012	212	220	904	458	524	5
ATAAAGTTA	BRDN00012	188	58	82	673	744	0
ATAAAGTTG	BRDN00011	112	55	2	706	541	0
ATAAATACA	BRDN00012	221	157	206	525	555	6
ATAAATACT	BRDN00011	373	391	582	840	854	51
ATAAATACT	BRDN00011	319	222	3117	456	326	55
ATAAATACT	BRDN00011	584	209	384	629	694	31
ATAAATAGT	BRDN00012	148	156	245	405	634	23
ATAAATAGT	BRDN00011	140	190	508	216	308	6
ATAAATATA	BRDN00011	226	176	82	332	408	12
ATAAATATA	BRDN00011	118	88	201	196	200	19
ATAAATATA	BRDN00011	231	234	181	415	248	1
ATAAATATT	BRDN00011	185	181	666	298	240	28
ATAAATCTA	BRDN00012	227	217	280	451	515	48
ATAAATGAG	BRDN00012	62	0	0	273	248	0
ATAAATGAG	BRDN00012	138	121	57	333	139	16
ATAAATGCA	BRDN00011	177	196	327	388	215	25
ATAAATGGT	BRDN00012	35	0	0	162	74	0
ATAAATGTG	BRDN00012	179	205	6	249	114	40
ATAAATTAC	BRDN00011	183	248	333	454	511	15
ATAAATTAC	BRDN00012	355	326	926	689	740	150
ATAAATTCG	BRDN00011	174	132	381	342	844	26
ATAAATTGC	BRDN00012	295	257	1367	682	601	28
ATAAATTTCA	BRDN00011	172	243	504	180	376	35
ATAACAACA	BRDN00011	259	249	1186	404	332	56
ATAACAACC	BRDN00011	169	173	1049	240	350	13
ATAACAATC	BRDN00012	378	33	136	609	559	5
ATAACACAA	BRDN00011	285	375	309	477	379	19
ATAACACAA	BRDN00011	266	213	379	601	416	0
ATAACACGG	BRDN00011	155	157	1115	228	287	54
ATAACACTG	BRDN00011	469	456	1392	772	1219	93
ATAACAGAA	BRDN00012	132	120	92	401	525	0
ATAACAGCA	BRDN00012	458	229	1210	789	539	24
ATAACAGGT	BRDN00011	214	218	114	432	267	16
ATAACAGTCT	BRDN00012	261	99	276	350	399	16
ATAACATGC	BRDN00011	228	242	66	394	357	12
ATAACCACT	BRDN00012	251	261	502	466	551	25
ATAACCAGA	BRDN00011	110	19	22	358	286	0
ATAACCAGT	BRDN00012	446	376	329	938	475	49
ATAACCCGA	BRDN00012	179	0	0	640	143	0
ATAACCCTCA	BRDN00012	433	290	465	715	676	25
ATAACCGAG	BRDN00012	524	525	2669	851	1096	115
ATAACCGAG	BRDN00012	333	306	1417	623	441	113
ATAACCGTT	BRDN00012	480	626	264	862	1035	61
ATAACCTGC	BRDN00012	137	111	75	313	209	23
ATAACCTGT	BRDN00012	135	140	543	257	312	42
ATAACGAAG	BRDN00012	320	375	121	513	561	3
ATAACGAAG	BRDN00011	34	39	3	131	125	0
ATAACGAAT	BRDN00011	306	196	117	610	431	0
ATAACGACC	BRDN00011	142	178	497	257	150	6
ATAACGATC	BRDN00011	212	226	46	580	569	20
ATAACGCAA	BRDN00012	256	313	379	597	774	110
ATAACGCCG	BRDN00011	193	246	199	285	305	10
ATAACGGCG	BRDN00011	443	424	1113	733	741	34
ATAACGGGT	BRDN00012	139	102	341	384	220	22
ATAACGTGG	BRDN00011	259	216	38	461	406	18
ATAACGTTC	BRDN00011	321	113	879	568	466	2
ATAACTATTG	BRDN00011	113	174	78	326	449	66
ATAACTCACA	BRDN00011	180	208	100	418	475	17
ATAACTCACC	BRDN00011	255	171	523	502	782	0
ATAACTCATA	BRDN00012	205	176	131	248	317	42
ATAACTCATA	BRDN00011	62	37	98	314	113	0
ATAACTCATC	BRDN00011	282	373	1313	489	251	86
ATAACTCATT	BRDN00012	154	175	2	262	250	0
ATAACTGAC	BRDN00011	149	274	77	341	565	39
ATAACTGAG	BRDN00012	478	434	773	769	588	74
ATAACTGTT	BRDN00012	280	228	315	413	532	40
ATAACTTACC	BRDN00011	134	44	1	310	123	0
ATAACTTGT	BRDN00012	298	206	103	549	421	13
ATAACTTGT	BRDN00011	435	5	121	1004	672	0
ATAACTTTG	BRDN00012	161	148	8	381	207	28
ATAACTTTG	BRDN00012	178	124	21	272	547	25
ATAAGAAAA	BRDN00012	140	111	524	195	196	0
ATAAGAAAA	BRDN00012	122	182	780	249	397	8
ATAAGAAAC	BRDN00011	206	45	361	426	659	3
ATAAGAAAT	BRDN00011	60	44	0	158	105	1
ATAAGAACC	BRDN00012	198	197	355	202	250	0
ATAAGAACT	BRDN00012	317	178	136	550	376	49
ATAAGACTA	BRDN00012	142	39	0	329	188	0
ATAAGAGAC	BRDN00012	513	317	368	1166	882	12
ATAAGAGAT	BRDN00011	149	125	403	176	196	33
ATAAGAGGC	BRDN00012	256	185	239	416	372	21
ATAAGAGGG	BRDN00012	128	1	1	235	120	0
ATAAGATAT	BRDN00012	348	281	857	772	1085	56
ATAAGATCG	BRDN00011	165	117	171	253	123	0
ATAAGATGA	BRDN00011	308	361	529	611	469	37
ATAAGCAAA	BRDN00011	74	0	1	601	193	0
ATAAGCAAT	BRDN00012	236	142	377	434	932	79
ATAAGCACC	BRDN00012	267	207	82	757	504	82
ATAAGCAGG	BRDN00012	91	116	8	189	182	7
ATAAGCATA	BRDN00011	196	66	0	360	397	0
ATAAGCATC	BRDN00012	299	375	676	673	541	48
ATAAGCATG	BRDN00012	202	301	63	373	205	0
ATAAGCCAC	BRDN00012	501	589	352	914	464	14
ATAAGCCAG	BRDN00011	310	347	281	638	418	0
ATAAGCCTC	BRDN00011	406	392	465	678	499	13
ATAAGCTAC	BRDN00012	579	588	3911	1184	898	162
ATAAGCTTG	BRDN00011	49	0	0	635	117	0
ATAAGGAAT	BRDN00012	287	192	1448	572	533	26
ATAAGGAGT	BRDN00011	240	287	756	514	693	7
ATAAGGAGT	BRDN00012	299	26	644	561	567	8
ATAAGGCAA	BRDN00012	181	244	25	303	119	12
ATAAGGCCC	BRDN00012	262	199	574	369	294	40
ATAAGGCCT	BRDN00012	239	340	597	509	480	145
ATAAGGGTT	BRDN00011	154	104	227	400	391	0
ATAAGGTAC	BRDN00012	234	309	66	584	874	78
ATAAGGTGT	BRDN00011	296	15	0	892	925	0
ATAAGGTTG	BRDN00011	119	155	199	273	173	25
ATAAGTCAA	BRDN00011	113	199	49	199	277	18
ATAAGTCCC	BRDN00011	432	368	666	665	948	13
ATAAGTGAA	BRDN00012	152	161	119	172	355	10
ATAAGTTCT	BRDN00012	333	330	913	657	452	27
ATAAGTTTG	BRDN00011	152	265	257	267	1709	30
ATAATAAAT	BRDN00011	170	179	223	486	456	15
ATAATAACA	BRDN00012	439	243	236	626	331	27
ATAATAACC	BRDN00012	168	222	221	220	487	4
ATAATAACT	BRDN00012	335	236	1583	669	356	39
ATAATAACT	BRDN00012	236	188	0	351	352	38
ATAATAAGC	BRDN00011	102	154	0	196	301	9
ATAATAAGG	BRDN00011	21	1	1	273	334	0
ATAATAAGT	BRDN00012	210	204	83	349	529	20
ATAATAAGT	BRDN00011	232	171	116	521	227	0
ATAATAATG	BRDN00012	406	435	379	698	816	56
ATAATAATT	BRDN00012	262	153	793	244	344	20
ATAATACAA	BRDN00011	422	468	1984	874	883	68
ATAATACGG	BRDN00011	21	0	0	227	75	0
ATAATACTC	BRDN00011	229	234	552	440	312	25
ATAATACTTA	BRDN00012	145	170	10	299	348	6
ATAATAGTG	BRDN00011	233	186	361	559	632	34
ATAATAGTG	BRDN00012	198	178	2540	355	107	30
ATAATATCG	BRDN00012	327	287	80	765	791	20
ATAATATGT	BRDN00012	123	41	0	217	285	33
ATAATCAAT	BRDN00011	67	144	68	219	72	0
ATAATCAGG	BRDN00011	388	12	29	770	605	0
ATAATCAGT	BRDN00011	161	129	0	273	119	0
ATAATCATA	BRDN00011	276	264	655	563	816	62
ATAATCATC	BRDN00012	318	272	212	406	356	3
ATAATCATTA	BRDN00011	190	48	1	410	604	0
ATAATCCGG	BRDN00012	356	281	3284	595	688	67
ATAATCGAA	BRDN00011	256	309	300	605	572	44
ATAATCGAG	BRDN00012	253	220	221	245	296	5
ATAATCGCC	BRDN00011	620	308	970	947	890	27
ATAATCGTA	BRDN00011	164	107	1466	336	431	38
ATAATCTAA	BRDN00012	126	204	6	149	15	8
ATAATCTCC	BRDN00012	372	167	1215	990	561	28
ATAATCTTCT	BRDN00011	102	59	76	306	281	0
ATAATGAAG	BRDN00012	146	111	105	447	759	0
ATAATGAAT	BRDN00011	48	0	0	769	284	1
ATAATGACA	BRDN00012	85	0	0	194	77	0
ATAATGAGT	BRDN00012	169	128	377	314	984	0
ATAATGATC	BRDN00012	246	0	0	905	323	0
ATAATGATG	BRDN00012	395	400	754	880	1047	23
ATAATGCAC	BRDN00012	186	139	90	365	519	0
ATAATGCAT	BRDN00011	295	193	480	520	745	25
ATAATGGAA	BRDN00012	237	196	668	443	432	27
ATAATGGCT	BRDN00012	451	369	1351	836	669	73
ATAATGGTG	BRDN00012	335	286	146	446	418	11
ATAATGTAT	BRDN00012	404	408	1074	989	1116	73
ATAATGTTC	BRDN00012	317	316	365	563	367	28
ATAATGTTT	BRDN00012	121	62	19	228	170	0
ATAATTACC	BRDN00011	103	206	22	205	207	0
ATAATTATG	BRDN00011	166	112	131	316	316	5
ATAATTCCTA	BRDN00011	640	599	485	1037	1227	62
ATAATTCGG	BRDN00012	80	154	19	171	517	36
ATAATTCTG	BRDN00011	211	127	2990	390	239	22
ATAATTGAC	BRDN00012	322	456	144	732	346	12
ATAATTGGA	BRDN00011	240	214	331	359	97	0
ATAATTGGC	BRDN00011	112	145	307	177	330	17
ATAATTGGG	BRDN00012	162	163	1299	382	329	17
ATAATTGTT	BRDN00012	230	231	638	627	508	33
ATAATTTCCG	BRDN00011	78	79	842	227	55	9
ATAATTTGG	BRDN00012	169	104	452	382	200	25
ATACAAAAC	BRDN00012	179	17	0	405	213	0
ATACAAACA	BRDN00012	128	48	1241	319	83	23
ATACAAACT	BRDN00012	329	131	417	502	562	22
ATACAAAGA	BRDN00012	204	240	61	387	294	37
ATACAACAT	BRDN00011	125	32	1	491	425	0
ATACAACCA	BRDN00012	315	219	1354	448	429	21
ATACAAGCT	BRDN00012	333	440	611	669	390	65
ATACAAGTC	BRDN00012	216	6	0	453	264	0
ATACAATAC	BRDN00011	435	607	258	776	583	6
ATACAATACT	BRDN00012	394	388	1121	620	405	32
ATACAATCA	BRDN00011	146	0	65	553	382	0
ATACAATCC	BRDN00012	171	246	110	440	397	0
ATACAATCTG	BRDN00012	136	59	31	315	198	14
ATACAATGCT	BRDN00011	227	118	153	522	384	11
ATACACAACT	BRDN00011	384	278	477	699	866	56
ATACACACAT	BRDN00011	361	374	393	519	585	24
ATACACAGA	BRDN00012	294	149	531	479	687	1
ATACACAGT	BRDN00011	417	70	0	620	476	0
ATACACATC	BRDN00012	616	25	0	1256	933	2
ATACACCAAT	BRDN00011	191	128	195	392	397	0
ATACACCCAG	BRDN00011	158	208	393	311	260	85
ATACACGTG	BRDN00012	193	236	1239	211	480	11
ATACACTAG	BRDN00012	263	171	1	372	361	30
ATACACTATC	BRDN00012	186	141	34	390	170	11
ATACACTATT	BRDN00012	241	208	260	462	378	71
ATACACTGA	BRDN00012	289	385	425	683	506	39
ATACACTTAA	BRDN00011	544	500	621	918	717	35
ATACACTTAT	BRDN00012	511	378	55	1023	1466	89
ATACAGAAC	BRDN00011	173	277	862	488	455	31
ATACAGAAC	BRDN00012	370	391	561	691	785	23
ATACAGAAG	BRDN00011	186	124	134	603	556	7
ATACAGACT	BRDN00012	226	96	2	1030	1028	0
ATACAGAGT	BRDN00011	85	82	64	254	343	3
ATACAGATG	BRDN00012	417	378	1248	755	695	230
ATACAGATG	BRDN00012	493	499	553	759	855	43
ATACAGCACT	BRDN00012	481	338	4049	785	973	40
ATACAGCGC	BRDN00012	374	341	415	695	817	53
ATACAGCTC	BRDN00012	622	622	566	1254	1392	37
ATACAGCTT	BRDN00011	228	210	497	388	315	9
ATACAGCTT	BRDN00012	225	187	381	406	530	67
ATACAGGAA	BRDN00012	92	91	436	280	211	66
ATACAGGAT	BRDN00011	129	105	31	203	110	34
ATACAGGCA	BRDN00012	621	532	1484	1037	707	136
ATACAGGGT	BRDN00012	281	242	11	356	452	7
ATACAGTAT	BRDN00012	167	0	0	395	25	0
ATACAGTGG	BRDN00011	651	687	1667	876	998	80
ATACAGTGG	BRDN00012	281	388	999	471	574	78
ATACAGTGT	BRDN00011	144	69	1016	268	546	10
ATACATAAG	BRDN00012	217	366	157	470	617	7
ATACATAGA	BRDN00012	127	198	22	502	315	7
ATACATATA	BRDN00011	333	209	217	404	302	45
ATACATATA	BRDN00012	128	148	116	409	276	52
ATACATATAT	BRDN00011	304	235	661	591	246	36
ATACATATG	BRDN00012	149	118	171	415	332	4
ATACATATGT	BRDN00011	80	63	1173	185	373	19
ATACATCAG	BRDN00012	160	242	316	361	384	0
ATACATCAGT	BRDN00011	195	150	31	396	572	0
ATACATCAGT	BRDN00011	197	230	52	464	665	0
ATACATCCG	BRDN00012	470	486	671	1010	468	33
ATACATCGG	BRDN00012	264	329	1632	460	577	16
ATACATCGG	BRDN00011	508	406	1039	907	937	106
ATACATCTA	BRDN00011	215	257	225	463	228	6
ATACATGAA	BRDN00012	131	113	67	276	2032	6
ATACATGAA	BRDN00012	184	221	515	413	308	12
ATACATGAA	BRDN00012	128	215	85	305	238	5
ATACATGCC	BRDN00012	611	612	2763	988	900	37
ATACATGGG	BRDN00012	57	57	0	176	121	0
ATACATGTG	BRDN00012	187	250	80	379	517	7
ATACATTCA	BRDN00011	440	316	316	521	940	62
ATACATTGG	BRDN00011	204	88	134	286	178	4
ATACCAAAA	BRDN00012	106	107	40	304	284	20
ATACCAACTC	BRDN00011	167	124	7	215	245	31
ATACCAAGA	BRDN00011	155	223	332	155	281	19
ATACCAAGT	BRDN00012	6	0	0	406	44	0
ATACCAATCA	BRDN00011	282	117	219	574	468	54
ATACCAGAT	BRDN00012	729	877	2103	1204	1511	116
ATACCAGCA	BRDN00012	452	413	979	784	898	65
ATACCAGCT	BRDN00012	157	180	11	340	274	35
ATACCAGTA	BRDN00011	63	107	641	163	131	31
ATACCATGA	BRDN00011	247	251	16	620	723	5
ATACCATGCT	BRDN00012	239	198	226	388	416	48
ATACCATTTA	BRDN00012	193	200	65	246	294	45
ATACCATTTA	BRDN00011	156	112	40	351	340	0
ATACCCAAG	BRDN00012	161	104	1056	313	327	39
ATACCCAATA	BRDN00011	113	93	227	254	197	0
ATACCCACAA	BRDN00012	368	217	237	788	684	4
ATACCCACCA	BRDN00012	241	283	587	314	313	31
ATACCCACCT	BRDN00012	276	292	623	480	543	12
ATACCCATCC	BRDN00012	229	107	414	502	341	15
ATACCCGAG	BRDN00011	175	91	234	318	356	1
ATACCCTAG	BRDN00012	284	322	184	415	429	14
ATACCCTGC	BRDN00011	125	140	35	261	338	17
ATACCCTTTG	BRDN00012	195	167	141	408	300	33
ATACCGCCC	BRDN00012	375	416	232	668	598	140
ATACCGCCTA	BRDN00012	177	165	132	295	546	0
ATACCGGGC	BRDN00011	385	419	516	672	548	128
ATACCGTAT	BRDN00011	258	36	547	611	330	16
ATACCGTTTG	BRDN00012	178	126	0	319	243	13
ATACCTAGA	BRDN00011	35	0	0	353	363	0
ATACCTATG	BRDN00011	408	405	3637	786	841	64
ATACCTATTG	BRDN00011	112	84	38	234	520	65
ATACCTCAG	BRDN00012	365	256	1902	964	516	6
ATACCTGAA	BRDN00012	109	46	127	216	145	73
ATACCTGAA	BRDN00012	324	283	213	803	886	17
ATACCTGGC	BRDN00011	342	331	112	530	322	15
ATACCTGTA	BRDN00011	140	187	81	336	718	0
ATACCTGTGT	BRDN00012	540	536	383	1270	2004	61
ATACCTTAG	BRDN00012	263	232	211	635	693	1
ATACCTTAG	BRDN00012	408	353	427	749	630	51
ATACCTTAG	BRDN00012	130	127	123	237	357	4
ATACCTTATG	BRDN00011	103	123	19	165	163	32
ATACCTTCTG	BRDN00011	123	179	88	197	419	18
ATACCTTGA	BRDN00012	500	282	482	781	772	19
ATACCTTGTG	BRDN00011	379	98	8	732	540	2
ATACCTTTAA	BRDN00011	249	117	254	579	550	7
ATACGAAAC	BRDN00012	519	443	1542	877	829	71
ATACGAAAT	BRDN00012	132	86	71	296	118	0
ATACGAAGC	BRDN00012	127	112	354	227	186	29
ATACGAGAG	BRDN00012	119	108	189	408	296	14
ATACGAGGC	BRDN00012	611	596	1044	882	849	126
ATACGAGTC	BRDN00011	111	138	13	264	423	13
ATACGATAG	BRDN00012	162	150	733	375	753	30
ATACGATGC	BRDN00011	186	179	293	309	366	0
ATACGCAAG	BRDN00011	415	3	1	803	1247	0
ATACGCATG	BRDN00012	788	705	2845	1244	1696	92
ATACGCCAT	BRDN00012	469	150	9	1161	606	0
ATACGCCCC	BRDN00011	232	221	77	340	154	40
ATACGCTAA	BRDN00012	501	597	1266	817	749	31
ATACGCTCTT	BRDN00011	235	164	601	416	331	42
ATACGGATG	BRDN00011	259	193	73	374	113	1
ATACGGCAT	BRDN00011	123	86	146	269	325	5
ATACGGCCG	BRDN00012	849	721	1608	1622	1451	66
ATACGGGGC	BRDN00011	486	352	498	995	926	115
ATACGGTGG	BRDN00012	68	0	0	244	112	0
ATACGGTTT	BRDN00011	380	251	529	559	494	8
ATACGTAAC	BRDN00012	178	87	10	314	353	0
ATACGTACC	BRDN00011	127	122	192	291	453	0
ATACGTCCTC	BRDN00012	268	203	89	403	707	10
ATACGTGCA	BRDN00011	20	0	0	150	79	0
ATACGTGGA	BRDN00011	273	262	188	435	464	7
ATACGTGGC	BRDN00012	562	647	566	1034	1657	49
ATACGTTCG	BRDN00012	338	330	591	637	670	48
ATACGTTGG	BRDN00011	304	182	234	630	251	9
ATACTAAACT	BRDN00012	522	493	382	1132	982	111
ATACTAACTT	BRDN00012	139	149	287	318	446	16
ATACTAATG	BRDN00012	138	52	29	314	228	0
ATACTACCTA	BRDN00011	36	1	0	347	261	0
ATACTACCTC	BRDN00011	196	237	454	352	638	41
ATACTACTG	BRDN00012	290	72	25	706	546	0
ATACTAGTA	BRDN00012	193	206	104	385	189	7
ATACTATGC	BRDN00012	420	386	204	756	463	36
ATACTCACAA	BRDN00011	186	167	54	308	441	23
ATACTCACAG	BRDN00012	165	50	0	553	329	0
ATACTCACAG	BRDN00012	670	536	1620	1232	1402	245
ATACTCACAT	BRDN00011	479	430	1181	883	829	49
ATACTCACCA	BRDN00011	185	220	281	392	353	26
ATACTCACGT	BRDN00011	343	437	298	921	951	48
ATACTCACTG	BRDN00011	181	110	33	273	166	3
ATACTCACTG	BRDN00011	178	239	89	295	143	9
ATACTCAGA	BRDN00012	373	407	403	744	679	86
ATACTCATCT	BRDN00012	149	130	77	239	96	10
ATACTCGTA	BRDN00011	54	79	1	251	151	0
ATACTCTCAC	BRDN00012	640	906	1594	1188	923	188
ATACTCTCAG	BRDN00012	182	139	207	352	554	13
ATACTGACT	BRDN00011	106	206	1456	238	201	17
ATACTGCAA	BRDN00011	570	456	470	1057	779	4
ATACTGCAC	BRDN00011	197	308	3312	367	243	52
ATACTGCCC	BRDN00011	600	382	2206	1027	1193	45
ATACTGCTC	BRDN00012	219	204	185	325	522	0
ATACTGGAA	BRDN00011	263	289	76	492	187	51
ATACTGGAA	BRDN00012	250	217	1970	270	111	108
ATACTGGAG	BRDN00011	86	33	2	373	367	0
ATACTGTCTA	BRDN00011	419	494	690	1011	812	55
ATACTGTTCA	BRDN00011	563	344	640	823	1187	56
ATACTGTTG	BRDN00011	439	401	403	569	767	28
ATACTTACAA	BRDN00011	308	394	123	715	829	6
ATACTTACAC	BRDN00012	41	0	0	391	265	0
ATACTTACAG	BRDN00012	128	126	43	253	310	30
ATACTTACAT	BRDN00011	173	10	21	288	495	0
ATACTTACCA	BRDN00011	203	141	647	407	307	5
ATACTTACCT	BRDN00012	270	247	111	395	555	8
ATACTTACG	BRDN00011	64	75	157	123	181	0
ATACTTACG	BRDN00011	306	293	219	588	637	36
ATACTTACG	BRDN00012	207	248	178	422	583	0
ATACTTATAT	BRDN00012	203	169	566	462	728	24
ATACTTCACG	BRDN00011	464	388	738	1065	1220	35
ATACTTCAGT	BRDN00012	443	117	54	856	948	0
ATACTTCCG	BRDN00012	135	109	37	423	423	0
ATACTTCTGG	BRDN00011	95	65	41	244	141	0
ATACTTGTA	BRDN00011	241	126	2	447	617	0
ATACTTGTAC	BRDN00011	255	167	419	364	344	34
ATACTTTACT	BRDN00011	151	92	0	298	278	0
ATACTTTGA	BRDN00011	102	78	543	226	440	0
ATACTTTGTC	BRDN00011	262	191	247	433	385	0
ATAGAAAAC	BRDN00011	391	343	941	666	590	59
ATAGAAAAG	BRDN00012	101	74	351	321	161	16
ATAGAAACT	BRDN00011	370	404	219	620	468	131
ATAGAAAGC	BRDN00011	320	244	44	568	623	8
ATAGAAAGC	BRDN00012	387	238	497	783	549	18
ATAGAAAGG	BRDN00011	150	5	1	403	467	0
ATAGAACAC	BRDN00011	79	99	10	220	629	1
ATAGAACAG	BRDN00011	168	151	296	363	326	52
ATAGAACAT	BRDN00012	101	88	70	159	131	15
ATAGAAGAG	BRDN00012	336	499	659	567	454	50
ATAGAAGGC	BRDN00012	345	314	1471	634	290	40
ATAGAAGGT	BRDN00011	199	236	48	349	284	20
ATAGAAGTG	BRDN00012	440	456	2658	720	835	244
ATAGAATCG	BRDN00011	182	145	280	200	329	4
ATAGACAAG	BRDN00011	204	145	272	376	485	3
ATAGACAAT	BRDN00012	302	100	168	637	596	0
ATAGACAGC	BRDN00012	240	238	70	408	364	29
ATAGACATC	BRDN00012	265	293	107	618	417	49
ATAGACCAT	BRDN00011	211	109	27	343	337	0
ATAGACCCA	BRDN00012	160	76	220	434	314	0
ATAGACCCA	BRDN00011	375	407	322	698	849	58
ATAGACCCA	BRDN00011	175	133	624	360	260	9
ATAGACCCC	BRDN00012	138	71	264	198	265	17
ATAGACCGA	BRDN00012	221	155	163	514	545	0

ATAGACCGT	BRDN00012	66	22	0	426	341	0
ATAGACGAG	BRDN00012	287	346	611	581	281	32
ATAGACTTA	BRDN00011	120	70	157	271	131	4
ATAGAGAAA	BRDN00012	277	332	320	501	637	10
ATAGAGAGA	BRDN00012	147	143	405	203	168	0
ATAGAGCAA	BRDN00011	159	160	27	260	514	48
ATAGAGCAC	BRDN00012	215	264	325	372	562	4
ATAGAGCAT	BRDN00012	316	168	1061	873	573	47
ATAGAGCCA	BRDN00012	185	143	43	391	242	0
ATAGAGCCG	BRDN00011	142	0	0	320	89	0
ATAGAGCTG	BRDN00011	175	133	192	413	609	0
ATAGAGGCA	BRDN00012	57	69	851	165	163	7
ATAGAGGGA	BRDN00011	115	1	0	176	62	0
ATAGAGTAC	BRDN00011	65	53	0	124	62	12
ATAGAGTAC	BRDN00011	134	204	0	253	237	0
ATAGAGTCA	BRDN00011	136	23	105	694	437	0
ATAGAGTGG	BRDN00012	311	201	1916	660	493	60
ATAGATAAA	BRDN00012	89	30	0	290	188	6
ATAGATAAG	BRDN00011	168	131	11	347	376	20
ATAGATAGC	BRDN00012	128	147	103	234	408	3
ATAGATATA	BRDN00011	161	147	354	409	369	23
ATAGATCCC	BRDN00011	256	285	420	497	460	29
ATAGATCTG	BRDN00011	165	137	863	273	203	21
ATAGATGAT	BRDN00012	258	219	70	630	787	0
ATAGATGCA	BRDN00012	276	0	0	536	384	0
ATAGATGCG	BRDN00011	150	134	3	300	356	10
ATAGATGCT	BRDN00011	86	22	1	252	226	0
ATAGATGGG	BRDN00012	434	150	320	841	741	5
ATAGATGGG	BRDN00011	191	153	285	341	520	25
ATAGATGGG	BRDN00012	222	283	318	403	353	23
ATAGATGGT	BRDN00011	97	41	459	275	102	11
ATAGATGTC	BRDN00011	415	396	353	773	414	56
ATAGATGTG	BRDN00012	285	234	70	454	515	30
ATAGATTCA	BRDN00011	86	150	1	190	370	0
ATAGCAAAG	BRDN00012	481	494	2066	1047	832	63
ATAGCAATA	BRDN00011	466	353	612	627	640	25
ATAGCAATA	BRDN00011	347	321	109	478	687	31
ATAGCAATC	BRDN00012	102	48	89	203	200	0
ATAGCACAA	BRDN00011	158	98	3211	305	378	33
ATAGCACGC	BRDN00012	204	75	0	311	54	1
ATAGCACTT	BRDN00011	314	336	3976	687	429	90
ATAGCAGAA	BRDN00012	136	261	703	222	444	14
ATAGCAGAC	BRDN00012	31	0	1	349	104	0
ATAGCAGAC	BRDN00012	135	116	90	256	148	0
ATAGCAGAG	BRDN00011	150	20	0	373	108	0
ATAGCAGGA	BRDN00012	488	494	1004	842	525	15
ATAGCAGTG	BRDN00011	212	95	356	486	271	6
ATAGCATAG	BRDN00012	106	14	0	735	434	0
ATAGCATGT	BRDN00011	408	489	425	861	477	12
ATAGCATTG	BRDN00012	417	433	1010	620	539	13
ATAGCCAAA	BRDN00011	62	82	1	131	166	19
ATAGCCAAG	BRDN00011	304	138	382	504	414	18
ATAGCCAAG	BRDN00012	370	634	3944	609	935	229
ATAGCCACA	BRDN00012	182	115	685	457	918	5
ATAGCCACC	BRDN00011	162	67	60	196	137	0
ATAGCCAGA	BRDN00012	126	161	2	226	80	12
ATAGCCAGC	BRDN00011	133	54	68	727	675	0
ATAGCCATA	BRDN00011	361	402	1704	422	438	36
ATAGCCATC	BRDN00011	194	95	374	382	503	15
ATAGCCATG	BRDN00011	77	143	431	300	132	10
ATAGCCATT	BRDN00011	256	310	265	429	322	75
ATAGCCATT	BRDN00012	76	112	253	219	214	5
ATAGCCATT	BRDN00011	341	415	937	762	444	148
ATAGCCCAA	BRDN00012	348	517	456	725	595	53
ATAGCCCAT	BRDN00011	532	551	1542	858	710	24
ATAGCCCAT	BRDN00011	217	218	375	446	440	48
ATAGCCGAT	BRDN00011	237	232	330	316	313	68
ATAGCCGCC	BRDN00012	549	476	717	890	1090	29
ATAGCCTAT	BRDN00012	459	442	3167	776	440	192
ATAGCCTCA	BRDN00012	305	353	1089	477	579	31
ATAGCCTTG	BRDN00012	229	133	147	362	443	0
ATAGCCTTG	BRDN00012	137	75	50	425	241	3
ATAGCGAGC	BRDN00012	124	138	41	367	354	5
ATAGCGATC	BRDN00011	79	12	0	317	162	0
ATAGCGATC	BRDN00012	329	358	348	598	508	101
ATAGCGATC	BRDN00012	113	110	20	409	445	20
ATAGCGATC	BRDN00012	462	487	691	714	1048	55
ATAGCGATC	BRDN00012	183	54	90	344	453	0
ATAGCGCAG	BRDN00012	196	149	1806	265	327	15
ATAGCGCTA	BRDN00012	121	104	1	272	248	0
ATAGCGGAT	BRDN00012	488	390	1105	611	683	43
ATAGCGTAG	BRDN00011	364	323	427	660	754	28
ATAGCGTAG	BRDN00012	330	372	89	629	309	100
ATAGCGTGT	BRDN00011	26	21	0	384	208	0
ATAGCTAAA	BRDN00012	495	360	655	684	533	10
ATAGCTACC	BRDN00012	304	201	107	556	691	14
ATAGCTATC	BRDN00011	143	0	0	606	139	0
ATAGCTATT	BRDN00011	94	113	641	96	26	13
ATAGCTCAG	BRDN00011	207	222	979	371	747	35
ATAGCTCAT	BRDN00012	83	58	474	252	394	0
ATAGCTCCA	BRDN00011	79	76	171	245	273	0
ATAGCTCTG	BRDN00012	304	284	1012	545	372	59
ATAGCTGCA	BRDN00011	154	109	4	257	382	0
ATAGCTGCC	BRDN00012	452	425	1261	706	826	87
ATAGCTGCT	BRDN00012	341	256	169	970	845	0
ATAGCTGTC	BRDN00011	337	357	491	573	431	12
ATAGCTTAC	BRDN00012	91	20	92	344	210	0
ATAGCTTCA	BRDN00012	331	256	770	563	803	28
ATAGCTTGA	BRDN00012	221	177	243	391	379	0
ATAGCTTGT	BRDN00011	328	401	257	677	693	48
ATAGGAAAA	BRDN00012	160	105	84	230	257	18
ATAGGAACT	BRDN00012	354	394	615	547	392	21
ATAGGAATG	BRDN00011	202	174	516	476	507	39
ATAGGACAA	BRDN00012	197	154	1168	323	600	41
ATAGGACAA	BRDN00012	218	190	353	334	99	8
ATAGGACAC	BRDN00012	307	319	281	702	861	18
ATAGGACCA	BRDN00012	173	234	441	311	132	11
ATAGGACGC	BRDN00011	124	79	101	163	124	11
ATAGGAGGC	BRDN00011	155	135	470	458	333	25
ATAGGAGTA	BRDN00012	271	199	83	359	403	36
ATAGGATGC	BRDN00012	389	0	14	703	923	0
ATAGGCACC	BRDN00012	400	349	222	620	680	10
ATAGGCAGT	BRDN00012	496	125	108	852	604	0
ATAGGCCCA	BRDN00011	214	136	55	644	503	28
ATAGGCCCA	BRDN00011	160	69	73	490	157	0
ATAGGCCTC	BRDN00012	328	426	472	569	252	6
ATAGGCGAG	BRDN00012	154	0	1	543	64	0
ATAGGCGGT	BRDN00012	104	65	31	598	134	10
ATAGGCTTA	BRDN00012	116	22	88	247	243	0
ATAGGGCGA	BRDN00011	454	484	5097	896	678	99
ATAGGGCGA	BRDN00012	170	167	140	345	217	73
ATAGGTATA	BRDN00012	170	186	282	342	423	39
ATAGGTATG	BRDN00011	114	118	24	215	151	11
ATAGGTATT	BRDN00012	120	198	1021	146	215	142
ATAGGTCAT	BRDN00012	556	491	399	797	913	21
ATAGGTCCG	BRDN00012	549	489	759	1195	1205	9
ATAGGTGAG	BRDN00012	135	102	46	344	165	30
ATAGGTGCC	BRDN00012	278	317	354	660	680	51
ATAGGTGCT	BRDN00012	239	294	3	374	578	0
ATAGGTGTA	BRDN00011	113	216	336	293	158	13
ATAGGTGTT	BRDN00011	121	82	13	228	109	10
ATAGTAAAA	BRDN00011	136	103	124	268	196	4
ATAGTAAAA	BRDN00012	256	205	156	484	362	23
ATAGTAAAG	BRDN00011	172	263	54	429	588	16
ATAGTAAAG	BRDN00012	171	162	58	409	287	5
ATAGTAACG	BRDN00012	342	257	102	780	777	12
ATAGTAAGT	BRDN00012	117	123	13	251	124	0
ATAGTACCT	BRDN00012	469	496	232	776	638	238
ATAGTAGAA	BRDN00011	122	139	1	257	719	0
ATAGTAGGG	BRDN00011	310	419	20	538	880	22
ATAGTATCA	BRDN00011	224	166	573	364	336	22
ATAGTCAAA	BRDN00011	265	373	207	661	1007	66
ATAGTCAAA	BRDN00011	381	187	18	587	585	0
ATAGTCAAT	BRDN00012	60	2	1	165	19	0
ATAGTCAAT	BRDN00012	266	135	38	1037	869	0
ATAGTCATA	BRDN00012	218	115	577	445	307	9
ATAGTCATG	BRDN00012	292	262	191	715	603	18
ATAGTCCAT	BRDN00011	359	271	394	585	704	59
ATAGTCGAG	BRDN00011	196	219	724	376	351	144
ATAGTCGGA	BRDN00012	154	42	68	183	213	0
ATAGTCGTT	BRDN00011	621	586	658	1017	1544	59
ATAGTCTGT	BRDN00012	319	362	1009	479	579	54
ATAGTCTGT	BRDN00012	115	61	0	301	450	2
ATAGTCTTC	BRDN00011	261	166	43	405	397	48
ATAGTGAAA	BRDN00012	171	108	1010	233	447	27
ATAGTGAAC	BRDN00011	195	205	112	261	165	16
ATAGTGACG	BRDN00012	118	67	693	406	220	52
ATAGTGACG	BRDN00012	172	121	10	278	348	7
ATAGTGAGT	BRDN00012	74	54	0	254	65	0
ATAGTGCAC	BRDN00011	282	203	480	554	655	13
ATAGTGCAG	BRDN00011	169	172	132	353	185	4
ATAGTGCCA	BRDN00011	399	490	628	713	642	69
ATAGTGCCG	BRDN00012	427	361	1037	548	631	71
ATAGTGCCT	BRDN00012	401	486	660	734	1049	84
ATAGTGGAA	BRDN00011	393	427	1151	815	1292	12
ATAGTGGAC	BRDN00012	73	67	100	307	184	0
ATAGTGGAG	BRDN00012	501	453	324	923	781	41
ATAGTGGCA	BRDN00011	138	151	179	324	282	0
ATAGTGGCA	BRDN00011	186	227	6404	392	234	134
ATAGTGGTG	BRDN00011	175	91	11	671	932	1
ATAGTTACA	BRDN00012	284	264	416	560	604	61
ATAGTTACC	BRDN00012	197	190	1931	254	233	6
ATAGTTACT	BRDN00012	247	232	48	434	236	5
ATAGTTATA	BRDN00012	201	201	31	467	240	0
ATAGTTCAC	BRDN00012	371	278	333	667	361	13
ATAGTTCCA	BRDN00011	234	325	691	342	247	30
ATAGTTCCT	BRDN00012	356	516	232	781	435	66
ATAGTTCGG	BRDN00012	136	103	65	199	157	16
ATAGTTGAT	BRDN00012	197	196	43	389	630	54
ATAGTTGCA	BRDN00012	227	305	76	431	234	14
ATAGTTGCG	BRDN00012	188	258	254	423	770	38
ATAGTTTAC	BRDN00012	258	134	666	417	765	49
ATAGTTTCT	BRDN00012	154	317	328	303	315	43
ATAGTTTGA	BRDN00011	294	225	492	456	514	18
ATATAAAAA	BRDN00011	40	31	0	109	16	2
ATATAAACA	BRDN00012	170	102	308	185	30	97
ATATAAACT	BRDN00012	463	356	630	809	580	36
ATATAAAGG	BRDN00012	251	316	74	422	551	15
ATATAAATA	BRDN00012	170	57	89	318	299	12
ATATAAATG	BRDN00011	358	442	2289	735	499	110
ATATAACCA	BRDN00012	163	13	0	454	271	0
ATATAACTT	BRDN00011	379	344	133	622	759	7
ATATAAGGA	BRDN00011	46	8	0	162	93	0
ATATAAGGG	BRDN00012	136	168	7	297	465	6
ATATAATCC	BRDN00011	392	382	348	726	695	35
ATATAATGG	BRDN00011	186	73	1	354	143	0
ATATAATGG	BRDN00011	217	35	21	344	241	0
ATATAATGG	BRDN00012	214	241	488	508	644	63
ATATACAAA	BRDN00011	266	273	1015	408	277	3
ATATACAAG	BRDN00012	291	217	52	398	365	31
ATATACAGA	BRDN00011	275	227	163	530	603	12
ATATACCAT	BRDN00012	41	7	1	333	230	0
ATATACCGT	BRDN00012	367	117	82	693	936	0
ATATACCTG	BRDN00012	109	162	0	244	79	10
ATATACTAA	BRDN00012	239	235	306	286	140	14
ATATACTCC	BRDN00011	106	7	79	254	123	0
ATATACTGA	BRDN00011	440	411	1741	877	450	44
ATATACTTA	BRDN00011	224	210	45	484	639	9
ATATACTTC	BRDN00012	301	146	35	483	597	3
ATATAGAAA	BRDN00011	242	199	428	446	405	5
ATATAGATC	BRDN00012	362	207	720	629	567	42
ATATAGATG	BRDN00011	216	192	0	364	193	21
ATATAGATT	BRDN00011	387	59	221	822	827	5
ATATAGCAA	BRDN00012	368	253	1392	742	1075	19
ATATAGCAG	BRDN00012	167	271	679	268	315	25
ATATAGCAG	BRDN00012	91	120	0	237	97	21
ATATAGCCT	BRDN00012	90	115	8	114	341	0
ATATAGTAT	BRDN00012	364	465	382	837	642	53
ATATAGTCT	BRDN00011	304	249	1138	439	533	5
ATATAGTTC	BRDN00012	297	220	934	589	499	4
ATATATAAC	BRDN00012	152	49	0	394	331	0
ATATATAGG	BRDN00011	285	181	951	375	338	15
ATATATCAA	BRDN00012	376	383	486	518	439	90
ATATATCAC	BRDN00012	201	128	600	413	192	16
ATATATCAC	BRDN00012	259	154	2577	493	343	45
ATATATCAG	BRDN00011	183	233	338	373	419	10
ATATATCCA	BRDN00011	207	148	45	373	452	1
ATATATCCG	BRDN00011	245	172	477	428	477	10
ATATATCCG	BRDN00011	120	91	88	219	184	0
ATATATCTG	BRDN00012	128	99	116	296	162	10
ATATATGCC	BRDN00012	152	32	50	337	54	9
ATATATGCT	BRDN00011	190	218	166	430	560	82
ATATATGGA	BRDN00012	401	484	907	698	689	50
ATATATGTG	BRDN00011	420	280	2447	722	779	29
ATATATTCA	BRDN00011	108	91	732	215	274	59
ATATATTGG	BRDN00011	54	0	1	219	416	0
ATATATTGG	BRDN00011	239	181	740	455	590	29
ATATATTTA	BRDN00012	168	184	1039	368	44	64
ATATATTTC	BRDN00011	236	194	582	472	387	10
ATATCAAAG	BRDN00012	506	73	4	761	648	0
ATATCAAGA	BRDN00012	303	9	2	798	609	0
ATATCAAGT	BRDN00012	276	334	8	334	644	9
ATATCAATA	BRDN00011	167	111	55	312	295	18
ATATCAATG	BRDN00012	312	325	1073	397	251	51
ATATCACAA	BRDN00012	501	193	201	822	1039	36
ATATCACGT	BRDN00012	162	145	37	303	345	0
ATATCACTG	BRDN00011	507	252	33	719	1020	0
ATATCAGAA	BRDN00011	86	98	51	238	105	10
ATATCAGCA	BRDN00012	227	209	110	390	382	50
ATATCAGCG	BRDN00012	465	436	1325	729	597	39
ATATCAGCG	BRDN00012	361	47	72	716	824	0
ATATCAGGT	BRDN00011	182	79	117	314	325	5
ATATCAGTG	BRDN00012	169	96	517	323	313	11
ATATCATAG	BRDN00012	409	336	629	653	1001	19
ATATCATCC	BRDN00012	228	224	204	403	315	14
ATATCATCT	BRDN00012	304	4	2	548	680	24
ATATCCAAA	BRDN00011	406	379	396	821	768	4
ATATCCAAC	BRDN00012	244	256	117	605	341	3
ATATCCACA	BRDN00011	82	0	0	365	163	0
ATATCCACA	BRDN00011	363	335	1437	735	457	15
ATATCCACA	BRDN00012	203	232	844	369	650	27
ATATCCACA	BRDN00012	457	321	955	926	901	21
ATATCCACC	BRDN00011	402	203	707	864	677	22
ATATCCAGA	BRDN00012	65	0	0	445	259	0
ATATCCAGC	BRDN00011	362	421	235	844	799	52
ATATCCAGC	BRDN00011	305	237	1155	551	526	46
ATATCCATC	BRDN00012	568	637	1023	994	682	76
ATATCCATG	BRDN00012	197	321	1294	354	704	62
ATATCCCGA	BRDN00011	130	123	136	381	239	6
ATATCCCTG	BRDN00012	158	96	827	218	258	24
ATATCCGAA	BRDN00011	304	267	275	548	430	67
ATATCCGAC	BRDN00012	56	8	0	275	81	0
ATATCCGTA	BRDN00012	440	529	2100	755	1263	49
ATATCCGTG	BRDN00012	716	581	2646	1556	1744	44
ATATCCTGA	BRDN00012	141	168	389	300	216	4
ATATCCTGG	BRDN00011	368	386	1092	698	1092	96
ATATCCTTT	BRDN00012	155	101	39	301	508	0
ATATCCTTT	BRDN00011	161	134	599	463	289	50
ATATCGAAG	BRDN00012	235	241	1724	427	661	100
ATATCGATC	BRDN00011	332	321	561	499	776	19
ATATCGGCA	BRDN00011	99	125	93	153	201	0
ATATCGGGT	BRDN00011	167	145	0	347	248	1
ATATCGTGT	BRDN00011	318	289	77	639	463	30
ATATCGTTG	BRDN00011	284	248	158	569	418	9
ATATCTATC	BRDN00011	162	0	0	423	35	0
ATATCTATG	BRDN00011	295	112	59	525	850	17
ATATCTATT	BRDN00012	181	0	2	300	261	0
ATATCTCAA	BRDN00011	265	356	793	486	528	39
ATATCTCGT	BRDN00012	181	57	56	487	576	0
ATATCTCTG	BRDN00012	321	204	786	664	751	10
ATATCTGCT	BRDN00012	105	139	26	312	158	6
ATATCTGGC	BRDN00011	276	124	268	686	576	12
ATATCTGGT	BRDN00012	361	0	0	638	387	0
ATATCTTCA	BRDN00012	336	314	327	818	1091	31
ATATCTTCA	BRDN00011	144	88	1	176	208	0
ATATCTTCC	BRDN00011	32	0	0	249	154	0
ATATCTTCT	BRDN00012	131	135	125	248	143	7
ATATGAAAC	BRDN00012	218	53	352	721	561	9
ATATGAAAG	BRDN00011	2	0	0	482	249	0
ATATGAACA	BRDN00011	158	103	2	358	328	13
ATATGAACT	BRDN00012	27	0	0	344	168	0
ATATGAATT	BRDN00011	185	116	545	346	233	82
ATATGACAC	BRDN00012	498	394	1409	1003	948	22
ATATGACAG	BRDN00012	571	582	434	916	655	14
ATATGACCA	BRDN00011	175	194	0	225	349	17
ATATGACCA	BRDN00012	204	307	257	504	324	22
ATATGACGA	BRDN00012	119	35	0	332	371	1
ATATGAGAC	BRDN00012	423	369	275	643	780	6
ATATGAGCA	BRDN00012	432	363	1735	620	532	61
ATATGAGCT	BRDN00012	240	228	226	368	467	0
ATATGAGGG	BRDN00012	159	239	247	341	390	29
ATATGATAC	BRDN00011	195	169	17	346	444	68
ATATGATAT	BRDN00011	228	138	229	373	299	38
ATATGATCA	BRDN00012	344	95	86	801	620	0
ATATGATCA	BRDN00012	153	187	305	435	545	34
ATATGATTA	BRDN00011	181	168	290	263	177	0
ATATGATTG	BRDN00011	205	1	0	337	355	0
ATATGCACA	BRDN00012	359	229	273	620	589	29
ATATGCACT	BRDN00012	157	105	551	313	252	0
ATATGCCCA	BRDN00012	213	378	132	507	436	17
ATATGCCGA	BRDN00011	334	119	55	342	399	0
ATATGCTTG	BRDN00012	420	489	659	858	834	22
ATATGGACA	BRDN00011	174	88	0	411	404	0
ATATGGACG	BRDN00012	298	262	168	498	686	36
ATATGGCTC	BRDN00012	525	562	455	695	655	113
ATATGGCTT	BRDN00011	467	422	739	777	928	122
ATATGGTAC	BRDN00012	169	150	193	288	217	6
ATATGGTTA	BRDN00011	300	275	4028	623	495	60
ATATGTAAA	BRDN00012	111	48	19	195	93	0
ATATGTAAA	BRDN00012	201	147	15	336	319	0
ATATGTACG	BRDN00012	122	65	215	288	694	0
ATATGTCAG	BRDN00012	632	615	1968	1127	907	47
ATATGTCAT	BRDN00012	238	223	662	490	438	9
ATATGTCAT	BRDN00011	128	144	329	251	248	17
ATATGTCCA	BRDN00012	485	505	2507	1018	890	51
ATATGTCCT	BRDN00012	251	172	1152	342	571	15
ATATGTCTG	BRDN00011	146	167	187	275	246	54
ATATGTGAA	BRDN00012	182	311	55	445	690	20
ATATGTGAT	BRDN00011	199	173	143	373	521	0
ATATGTGAT	BRDN00011	224	207	68	438	161	0
ATATGTGCA	BRDN00011	284	249	1486	694	541	22
ATATGTGTC	BRDN00011	181	181	34	373	257	68
ATATGTGTT	BRDN00012	131	22	0	227	510	0
ATATGTGTT	BRDN00011	259	191	247	372	444	22
ATATGTTAA	BRDN00012	123	199	1126	254	226	17
ATATGTTAA	BRDN00012	443	276	1599	686	492	23
ATATGTTGC	BRDN00011	120	49	13	364	140	0
ATATGTTGG	BRDN00012	132	43	0	212	425	7
ATATGTTGG	BRDN00011	153	140	80	422	522	7
ATATGTTGT	BRDN00012	485	415	428	720	710	80
ATATGTTTA	BRDN00012	286	255	119	535	888	13
ATATGTTTC	BRDN00012	491	253	2507	714	714	112
ATATTAAAC	BRDN00012	112	13	20	223	196	0
ATATTAACA	BRDN00012	214	64	519	379	315	9
ATATTAATT	BRDN00011	185	178	522	290	315	5
ATATTAATT	BRDN00011	15	0	0	48	12	0
ATATTACAG	BRDN00012	127	77	0	273	280	0
ATATTACAT	BRDN00011	206	198	204	442	420	5
ATATTACTC	BRDN00012	164	15	2804	412	622	32
ATATTAGAT	BRDN00012	208	261	152	465	266	17
ATATTATCG	BRDN00012	447	101	753	878	500	8
ATATTATTC	BRDN00011	165	189	800	440	354	20
ATATTATTG	BRDN00012	50	7	0	239	512	0
ATATTCAAC	BRDN00012	218	53	2	463	353	0
ATATTCAAG	BRDN00011	289	163	1551	439	396	0
ATATTCAAG	BRDN00012	58	89	136	238	128	14
ATATTCAAT	BRDN00011	117	157	80	351	403	10
ATATTCACG	BRDN00012	437	300	472	759	399	46
ATATTCATG	BRDN00011	361	344	264	543	693	42
ATATTCCGC	BRDN00012	255	116	77	387	1209	10
ATATTCCGT	BRDN00011	448	326	2049	778	506	44
ATATTCCTG	BRDN00012	638	515	390	1123	1264	38
ATATTCCTG	BRDN00011	481	594	3468	1213	1072	86
ATATTCGAA	BRDN00011	386	333	399	784	1488	4
ATATTCGAA	BRDN00012	189	152	23	272	222	5
ATATTCGAC	BRDN00012	166	265	27	405	408	1
ATATTCGAT	BRDN00011	206	779	6785	485	785	248
ATATTCGGC	BRDN00011	331	287	2476	508	320	65
ATATTCGGC	BRDN00011	194	13	95	281	296	12
ATATTCTAG	BRDN00012	196	206	62	327	294	39
ATATTCTGG	BRDN00011	168	113	1490	197	179	0
ATATTCTGT	BRDN00011	137	174	501	348	264	13
ATATTGAAA	BRDN00012	191	264	249	523	522	46
ATATTGATA	BRDN00011	62	14	121	104	222	0
ATATTGATA	BRDN00011	225	207	973	299	493	39
ATATTGATG	BRDN00011	317	147	170	523	491	16
ATATTGATG	BRDN00012	367	296	4027	598	527	48
ATATTGCAT	BRDN00012	163	153	102	294	160	0
ATATTGCTA	BRDN00012	182	172	116	315	142	3
ATATTGCTC	BRDN00012	119	42	30	415	405	2
ATATTGGAC	BRDN00011	189	180	40	417	396	18
ATATTGGAG	BRDN00012	14	2	1	749	493	0
ATATTGGAT	BRDN00012	137	93	828	269	249	41
ATATTGGCA	BRDN00011	344	205	1298	805	2024	15
ATATTGGGG	BRDN00011	260	113	31	435	366	0
ATATTGGTA	BRDN00011	378	491	166	704	699	48
ATATTGTAA	BRDN00011	118	43	312	270	611	23
ATATTGTAG	BRDN00012	374	344	179	666	762	33
ATATTGTTG	BRDN00012	302	253	109	607	461	20
ATATTTAAA	BRDN00012	113	0	1	200	79	0
ATATTTAAT	BRDN00011	258	140	119	489	388	22
ATATTTAAT	BRDN00012	134	98	26	184	87	8
ATATTTAGG	BRDN00012	174	12	0	518	659	0
ATATTTCAC	BRDN00011	74	53	150	157	162	0
ATATTTCAT	BRDN00011	136	75	0	224	360	0
ATATTTCCA	BRDN00012	216	224	178	386	224	16
ATATTTCCC	BRDN00011	409	370	448	722	862	40
ATATTTCGG	BRDN00011	129	121	3	225	195	24
ATATTTCGG	BRDN00012	752	781	1038	1295	3729	174
ATATTTCTC	BRDN00011	121	122	93	224	251	5
ATATTTCTG	BRDN00011	153	116	571	266	391	24
ATATTTCTG	BRDN00011	34	15	0	53	0	6
ATATTTCTT	BRDN00012	201	24	37	397	251	0
ATATTTGAT	BRDN00011	111	51	82	229	71	0
ATATTTGCT	BRDN00011	211	186	478	489	408	26
ATATTTGGG	BRDN00011	409	194	509	719	539	7
ATATTTGTG	BRDN00012	415	277	353	992	1183	21
ATATTTGTT	BRDN00011	223	175	369	394	407	1
ATATTTTAT	BRDN00012	254	302	158	295	625	69
ATCAAAAAT	BRDN00011	141	153	1052	294	477	0
ATCAAAACA	BRDN00012	322	65	1	534	597	0
ATCAAAACG	BRDN00012	259	216	2935	431	785	70
ATCAAAAGT	BRDN00011	145	188	180	375	342	92
ATCAAAATG	BRDN00012	193	141	90	276	219	0
ATCAAACAC	BRDN00011	500	468	510	1139	1063	34
ATCAAACAC	BRDN00012	426	453	950	803	889	52
ATCAAAGAC	BRDN00012	119	95	42	330	564	0
ATCAAAGAG	BRDN00011	52	20	35	228	235	0
ATCAAAGAG	BRDN00011	189	130	2416	368	275	45
ATCAAAGCC	BRDN00011	44	5	652	295	404	19
ATCAAAGCC	BRDN00011	504	464	502	953	586	33
ATCAAAGCC	BRDN00011	440	0	0	914	795	0
ATCAAAGCG	BRDN00011	113	195	5	263	334	9
ATCAAAGGC	BRDN00011	114	77	3	305	326	12
ATCAAAGTG	BRDN00012	249	225	107	702	910	40
ATCAAAGTG	BRDN00012	375	344	229	650	915	78
ATCAAATTA	BRDN00012	128	134	46	291	204	0
ATCAAATTG	BRDN00011	367	20	0	869	748	0
ATCAACAAT	BRDN00011	166	172	65	395	284	12
ATCAACAAT	BRDN00012	6	0	0	362	22	0
ATCAACAGC	BRDN00012	504	526	949	728	862	45
ATCAACAGG	BRDN00011	272	167	106	500	395	19
ATCAACAGG	BRDN00011	325	214	304	789	404	24
ATCAACCAA	BRDN00011	302	321	912	430	404	43
ATCAACCCA	BRDN00012	426	461	101	572	662	70
ATCAACCTT	BRDN00012	82	54	0	335	463	0
ATCAACGAA	BRDN00011	225	193	8	347	400	6
ATCAACGAC	BRDN00012	227	248	26	256	173	19
ATCAACGCC	BRDN00012	499	436	270	941	806	44
ATCAACGGA	BRDN00011	310	383	20	338	359	29
ATCAACGGC	BRDN00011	136	125	13	360	390	0
ATCAACGTA	BRDN00012	377	458	435	811	1034	105
ATCAACGTG	BRDN00012	346	213	987	593	441	26
ATCAACTGA	BRDN00011	182	161	1638	387	628	25
ATCAACTGC	BRDN00012	149	7	1	388	313	0

ATCAACTGT BRDN0001	192	198	1929	500	236	13
ATCAACTTCG BRDN0001	127	36	1137	529	487	17
ATCAAGAAC BRDN0001	213	0	0	449	97	0
ATCAAGAAT BRDN0001	298	33	0	629	509	0
ATCAAGAAT BRDN0001	178	116	161	325	402	40
ATCAAGACA BRDN0001	448	337	500	712	570	22
ATCAAGCGG BRDN0001	172	168	945	442	319	15
ATCAAGCGG BRDN0001	162	132	243	517	179	32
ATCAAGCGT BRDN0001	122	86	178	287	216	0
ATCAAGGAC BRDN0001	245	334	273	472	585	12
ATCAAGGCA BRDN0001	203	181	352	504	808	27
ATCAAGGTA BRDN0001	472	519	1638	853	928	35
ATCAAGTAG BRDN0001	106	113	5	343	194	9
ATCAAGTCA BRDN0001	772	848	2304	1382	1161	157
ATCAAGTCC BRDN0001	424	392	1608	812	704	59
ATCAAGTGG BRDN0001	211	360	169	415	292	9
ATCAATAGA BRDN0001	136	153	749	313	313	10
ATCAATCTAG BRDN0001	252	219	164	469	434	2
ATCAATGAA BRDN0001	148	189	7	220	419	41
ATCAATGAT BRDN0001	351	464	963	734	848	6
ATCAATGGA BRDN0001	151	178	971	244	359	20
ATCAATGGT BRDN0001	117	108	70	398	289	0
ATCAATGTA BRDN0001	216	240	123	353	332	23
ATCAATTGA BRDN0001	411	308	210	689	707	12
ATCAATTGT BRDN0001	20	0	0	302	24	0
ATCAATTTCA BRDN0001	390	344	926	649	713	48
ATCACAAAT BRDN0001	59	54	8	480	155	0
ATCACAAAT BRDN0001	213	325	144	430	369	0
ATCACAACA BRDN0001	312	223	166	619	449	20
ATCACAACCA BRDN0001	171	74	45	420	202	3
ATCACAACTA BRDN0001	153	3	2	444	488	0
ATCACAAGA BRDN0001	283	422	598	625	536	103
ATCACAAGC BRDN0001	195	183	303	572	598	10
ATCACAAGG BRDN0001	236	55	406	602	254	9
ATCACAAGT BRDN0001	154	122	1	137	95	16
ATCACAAGT BRDN0001	131	11	0	594	280	0
ATCACAAGT BRDN0001	202	92	243	383	520	2
ATCACAATTA BRDN0001	201	326	78	324	254	39
ATCACAATTG BRDN0001	106	122	1082	373	454	21
ATCACACACC BRDN0001	421	418	576	782	758	110
ATCACACACG BRDN0001	271	242	4655	573	792	70
ATCACACAG BRDN0001	282	189	1203	725	679	34
ATCACACATA BRDN0001	21	12	0	199	72	0
ATCACACATC BRDN0001	528	526	945	911	1358	47
ATCACAGAC BRDN0001	459	333	54	724	971	7
ATCACAGCA BRDN0001	328	257	329	600	725	45
ATCACAGGC BRDN0001	192	201	47	445	481	10
ATCACAGTA BRDN0001	204	177	140	330	341	4
ATCACAGTC BRDN0001	178	126	187	471	448	0
ATCACAGTG BRDN0001	276	162	244	501	480	103
ATCACATGC BRDN0001	189	125	17	329	312	5
ATCACATGG BRDN0001	125	155	47	270	260	25
ATCACATTCT BRDN0001	287	233	476	630	542	13
ATCACATTG BRDN0001	399	268	844	644	500	41
ATCACCAACC BRDN0001	265	384	320	602	457	22
ATCACCAATA BRDN0001	247	316	683	413	274	34
ATCACCAATG BRDN0001	103	164	0	231	240	0
ATCACCACAG BRDN0001	461	432	868	824	1030	116
ATCACCACCA BRDN0001	286	192	297	404	203	0
ATCACCACCT BRDN0001	215	218	914	528	205	53
ATCACCACTC BRDN0001	195	173	325	407	231	77
ATCACCAGC BRDN0001	436	238	200	785	681	5
ATCACCAGCT BRDN0001	504	643	1881	1149	1211	97
ATCACCAGCT BRDN0001	427	288	21	771	843	34
ATCACCAGG BRDN0001	629	524	1325	1200	1145	92
ATCACCAGG BRDN0001	23	0	0	199	0	0
ATCACCATAA BRDN0001	332	176	1696	560	683	54
ATCACCATCC BRDN0001	244	284	295	429	216	36
ATCACCATCT BRDN0001	409	428	45	888	827	0
ATCACCATG BRDN0001	111	61	7	314	336	0
ATCACCATTA BRDN0001	135	73	67	220	500	0
ATCACCATTC BRDN0001	183	241	671	433	269	14
ATCACCCCAT BRDN0001	476	347	488	803	494	3
ATCACCCCCA BRDN0001	137	110	46	476	447	0
ATCACCCCGC BRDN0001	517	540	362	711	702	60
ATCACCCGTT BRDN0001	142	145	444	333	139	15
ATCACCCTAA BRDN0001	49	19	0	157	12	0
ATCACCGCG BRDN0001	194	205	656	422	373	19
ATCACCTCAG BRDN0001	146	5	0	695	419	0
ATCACCTGA BRDN0001	535	581	855	1003	1439	73
ATCACCTGTC BRDN0001	247	193	412	349	599	81
ATCACCTTCA BRDN0001	337	177	99	532	363	17
ATCACGAAA BRDN0001	257	267	640	471	494	25
ATCACGACA BRDN0001	185	215	229	364	278	12
ATCACGCCA BRDN0001	420	361	1057	609	481	63
ATCACGCCG BRDN0001	144	144	97	298	448	7
ATCACGCTA BRDN0001	283	117	78	283	216	48
ATCACGCTC BRDN0001	259	296	425	565	239	2
ATCACGGAA BRDN0001	139	43	77	589	360	2
ATCACGGCG BRDN0001	128	139	284	234	354	51
ATCACGGTC BRDN0001	322	304	737	539	684	17
ATCACGTAC BRDN0001	125	151	17	299	401	12
ATCACGTGA BRDN0001	604	572	1266	1027	922	82
ATCACGTGG BRDN0001	153	133	165	300	309	13
ATCACTAAG BRDN0001	216	221	66	322	317	58
ATCACTACA BRDN0001	183	241	343	415	345	62
ATCACTAGC BRDN0001	362	262	493	421	269	6
ATCACTAGC BRDN0001	148	38	112	278	146	0
ATCACTGAC BRDN0001	405	344	864	917	729	6
ATCACTGCA BRDN0001	265	280	511	522	379	17
ATCACTGGA BRDN0001	114	174	236	447	496	15
ATCACTGGT BRDN0001	264	365	779	426	607	86
ATCACTGTCT BRDN0001	138	182	599	376	552	6
ATCACTGTTG BRDN0001	128	18	75	266	85	0
ATCACTTCAT BRDN0001	181	22	0	310	341	0
ATCAGAATG BRDN0001	107	119	93	195	99	9
ATCAGAATG BRDN0001	182	193	24	326	285	127
ATCAGACAT BRDN0001	528	484	1059	971	806	66
ATCAGACCA BRDN0001	273	167	799	671	600	45
ATCAGAGCC BRDN0001	143	109	718	278	259	23
ATCAGAGCG BRDN0001	487	336	1072	990	979	28
ATCAGAGTG BRDN0001	463	364	7422	930	1013	174
ATCAGATAA BRDN0001	312	200	83	472	410	0
ATCAGATAG BRDN0001	180	73	787	401	323	26
ATCAGATGG BRDN0001	261	43	93	537	313	0
ATCAGCAAA BRDN0001	477	454	308	864	1044	13
ATCAGCACA BRDN0001	162	238	32	390	172	31
ATCAGCACT BRDN0001	190	244	557	350	246	15
ATCAGCAGC BRDN0001	136	43	1	282	215	0
ATCAGCAGC BRDN0001	264	236	393	392	867	13
ATCAGCAGG BRDN0001	432	563	606	642	746	32
ATCAGCATCT BRDN0001	268	281	1147	625	729	31
ATCAGCATT BRDN0001	295	263	113	635	624	16
ATCAGCCAG BRDN0001	116	80	0	170	429	0
ATCAGCCATT BRDN0001	213	158	146	445	479	0
ATCAGCCCA BRDN0001	175	165	464	203	106	14
ATCAGCCCAT BRDN0001	644	528	974	1197	1409	59
ATCAGCCTG BRDN0001	282	271	586	479	405	390
ATCAGCGAA BRDN0001	154	148	1064	312	238	25
ATCAGCGAC BRDN0001	183	157	257	409	507	8
ATCAGCGAG BRDN0001	631	532	2009	1154	1197	71
ATCAGCGCC BRDN0001	102	61	2743	192	75	27
ATCAGCGTC BRDN0001	127	88	312	249	560	48
ATCAGCGTT BRDN0001	111	109	0	227	599	38
ATCAGCGTT BRDN0001	406	235	2148	804	681	33
ATCAGCTATT BRDN0001	168	156	78	434	400	0
ATCAGCTCTC BRDN0001	173	168	80	421	496	64
ATCAGCTGC BRDN0001	386	436	2811	783	582	40
ATCAGCTTTA BRDN0001	155	150	248	282	141	7
ATCAGCTTTC BRDN0001	349	324	854	689	593	5
ATCAGGAAC BRDN0001	254	268	1032	551	849	31
ATCAGGAAG BRDN0001	475	200	2859	1153	480	50
ATCAGGAAT BRDN0001	428	493	449	853	966	61
ATCAGGAGA BRDN0001	180	143	4	327	167	0
ATCAGGAGG BRDN0001	561	465	1125	716	853	129
ATCAGGATC BRDN0001	73	59	111	151	179	11
ATCAGGATG BRDN0001	137	51	1	256	372	12
ATCAGGCCA BRDN0001	155	325	161	268	322	0
ATCAGGCCG BRDN0001	212	274	206	577	428	63
ATCAGGCCT BRDN0001	575	693	1525	915	947	107
ATCAGGCGA BRDN0001	261	199	1197	643	634	33
ATCAGGGTA BRDN0001	82	154	0	328	207	0
ATCAGGGTC BRDN0001	114	73	35	229	188	0
ATCAGGTAC BRDN0001	97	89	746	322	244	11
ATCAGGTCC BRDN0001	127	149	155	289	123	53
ATCAGGTGG BRDN0001	133	112	20	172	177	2
ATCAGTACT BRDN0001	254	241	706	474	436	38
ATCAGTATG BRDN0001	273	143	16	749	677	0
ATCAGTCAC BRDN0001	220	254	974	497	298	51
ATCAGTCAG BRDN0001	111	166	437	286	501	14
ATCAGTCTCT BRDN0001	84	33	1	279	267	4
ATCAGTGAC BRDN0001	261	262	827	527	241	60
ATCAGTGCA BRDN0001	162	1	117	449	270	9
ATCAGTGGT BRDN0001	152	161	115	388	460	0
ATCAGTGGT BRDN0001	246	260	320	503	453	50
ATCAGTGTG BRDN0001	109	88	14	204	200	0
ATCAGTGTT BRDN0001	172	309	223	444	767	41
ATCAGTGTT BRDN0001	206	269	257	616	661	23
ATCAGTTAT BRDN0001	69	0	1	283	115	0
ATCAGTTATT BRDN0001	448	610	685	709	1027	1541
ATCAGTTCA BRDN0001	148	97	135	555	781	0
ATCAGTTTCT BRDN0001	116	75	18	319	162	0
ATCATAACTC BRDN0001	475	508	851	794	798	93
ATCATACAG BRDN0001	162	27	15	343	181	0
ATCATACGC BRDN0001	600	6	1	1217	179	0
ATCATAGTG BRDN0001	147	138	74	236	178	45
ATCATATAG BRDN0001	237	4	0	608	84	0
ATCATATCCT BRDN0001	398	339	929	850	749	98
ATCATATTCA BRDN0001	523	358	1732	842	625	48
ATCATCAGA BRDN0001	90	51	44	154	390	0
ATCATCAGC BRDN0001	253	172	2202	462	462	64
ATCATCAGTT BRDN0001	216	331	1198	368	527	29
ATCATCATG BRDN0001	204	122	114	266	193	2
ATCATCATTC BRDN0001	222	253	36	473	447	5
ATCATCATTG BRDN0001	259	351	363	431	695	8
ATCATCCAAT BRDN0001	185	131	180	267	617	8
ATCATCCAAT BRDN0001	452	360	717	933	920	122
ATCATCCAG BRDN0001	275	152	502	271	142	23
ATCATCCTAC BRDN0001	27	0	0	225	75	0
ATCATCGAG BRDN0001	430	641	903	707	717	25
ATCATCGCA BRDN0001	445	160	2386	1339	368	21
ATCATCGGA BRDN0001	419	319	1147	703	838	35
ATCATCGGC BRDN0001	215	190	117	586	403	4
ATCATCGGG BRDN0001	173	137	31	281	421	23
ATCATCGGG BRDN0001	474	443	2927	1049	814	8
ATCATCGTCT BRDN0001	285	309	1190	509	325	34
ATCATCTACA BRDN0001	157	121	364	349	423	6
ATCATCTACA BRDN0001	462	511	907	1119	651	76
ATCATCTCAC BRDN0001	369	102	23	754	415	51
ATCATCTCAC BRDN0001	142	0	1	614	95	0
ATCATCTCAG BRDN0001	201	331	487	481	494	67
ATCATCTCCA BRDN0001	378	393	580	646	652	59
ATCATCTCGA BRDN0001	166	249	70	341	250	33
ATCATCTCGG BRDN0001	210	150	124	480	225	23
ATCATCTTAC BRDN0001	214	248	73	718	464	0
ATCATCTTAC BRDN0001	466	443	443	779	940	36
ATCATCTTGA BRDN0001	219	220	367	408	508	37
ATCATCTTGG BRDN0001	183	155	9	411	511	8
ATCATCTTGG BRDN0001	346	418	192	453	648	108
ATCATGAAC BRDN0001	351	423	1047	795	755	40
ATCATGACC BRDN0001	136	44	0	264	314	0
ATCATGACC BRDN0001	147	185	243	294	323	17
ATCATGACC BRDN0001	452	366	1132	841	1010	42
ATCATGAGC BRDN0001	21	11	0	756	224	0
ATCATGATCT BRDN0001	116	192	1	244	367	35
ATCATGATT BRDN0001	100	85	72	151	342	4
ATCATGGAA BRDN0001	156	195	873	380	310	12
ATCATGGAG BRDN0001	127	30	1	150	155	0
ATCATGGAT BRDN0001	137	106	360	257	178	21
ATCATGGTT BRDN0001	165	186	89	332	259	8
ATCATGTAA BRDN0001	355	427	235	782	646	49
ATCATGTCG BRDN0001	211	184	215	291	258	31
ATCATGTGA BRDN0001	267	158	998	763	542	56
ATCATTACCC BRDN0001	221	8	23	761	981	0
ATCATTAGA BRDN0001	339	199	1556	644	499	86
ATCATTAGA BRDN0001	217	206	408	582	539	43
ATCATTAGCT BRDN0001	142	216	164	350	522	0
ATCATTATA BRDN0001	313	3	1	428	675	0
ATCATTCCGT BRDN0001	463	340	1188	714	918	25
ATCATTGATT BRDN0001	247	271	361	512	273	25
ATCATTGCCA BRDN0001	247	264	344	322	665	13
ATCATTGCTC BRDN0001	291	190	40	343	571	6
ATCATTGGCT BRDN0001	111	152	17	230	408	7
ATCATTGGTT BRDN0001	24	41	2	276	275	0
ATCATTGGTT BRDN0001	275	116	0	976	841	18
ATCATTTGG BRDN0001	434	382	342	908	1052	24
ATCCAAAGC BRDN0001	74	0	0	321	248	0
ATCCAAAGT BRDN0001	272	279	297	660	426	0
ATCCAAAGTT BRDN0001	306	436	1048	688	564	36
ATCCAACAAT BRDN0001	543	471	1139	868	1033	47
ATCCAACAG BRDN0001	151	160	374	321	287	31
ATCCAACTCT BRDN0001	219	186	320	364	381	66
ATCCAACTGT BRDN0001	239	177	38	490	641	18
ATCCAAGGT BRDN0001	239	176	235	342	259	17
ATCCAAGTT BRDN0001	145	50	618	194	45	6
ATCCAATAG BRDN0001	135	104	146	190	536	7
ATCCAATCA BRDN0001	154	191	0	270	260	1
ATCCAATGA BRDN0001	242	144	69	530	578	1
ATCCAATGT BRDN0001	184	152	14	259	251	19
ATCCAATTTC BRDN0001	241	315	166	630	538	19
ATCCACAAA BRDN0001	412	422	852	653	654	95
ATCCACAACC BRDN0001	162	155	2	299	306	0
ATCCACAAG BRDN0001	175	135	705	325	254	10
ATCCACACA BRDN0001	106	5	0	952	627	0
ATCCACACG BRDN0001	117	129	301	148	145	36
ATCCACACTG BRDN0001	393	360	272	565	486	15
ATCCACAGA BRDN0001	234	313	2263	512	246	29
ATCCACAGG BRDN0001	125	89	736	471	289	0
ATCCACATTG BRDN0001	212	120	113	411	525	5
ATCCACCAG BRDN0001	132	239	464	276	584	0
ATCCACCTAC BRDN0001	161	103	14	256	188	9
ATCCACGAG BRDN0001	248	133	278	412	528	7
ATCCACGCCC BRDN0001	258	211	1169	415	360	63
ATCCACGCCC BRDN0001	245	123	146	463	496	7
ATCCACGGA BRDN0001	173	125	223	366	291	48
ATCCACTATT BRDN0001	116	39	268	209	259	0
ATCCACTGCC BRDN0001	275	221	186	569	380	25
ATCCACTTAT BRDN0001	352	383	760	689	768	32
ATCCAGAAA BRDN0001	36	2	0	324	283	0
ATCCAGAAA BRDN0001	165	134	144	313	332	84
ATCCAGAAC BRDN0001	129	126	303	239	348	6
ATCCAGAAT BRDN0001	323	37	1	538	436	0
ATCCAGAAT BRDN0001	408	201	30	697	802	33
ATCCAGACA BRDN0001	244	299	316	538	376	23
ATCCAGACC BRDN0001	353	237	3350	657	837	57
ATCCAGACCT BRDN0001	54	35	0	458	182	0
ATCCAGAGC BRDN0001	419	408	363	700	342	19
ATCCAGAGG BRDN0001	572	576	685	1020	508	164
ATCCAGATA BRDN0001	165	81	26	321	315	8
ATCCAGATC BRDN0001	618	555	2418	959	879	166
ATCCAGATC BRDN0001	328	285	9	498	613	7
ATCCAGATG BRDN0001	362	308	258	660	878	137
ATCCAGCAA BRDN0001	467	391	2311	738	788	45
ATCCAGCAG BRDN0001	129	213	172	313	413	3
ATCCAGCCCA BRDN0001	146	151	68	294	197	13
ATCCAGCCCT BRDN0001	282	211	226	415	329	84
ATCCAGCGT BRDN0001	216	10	0	423	69	0
ATCCAGCTA BRDN0001	646	705	197	1053	1948	53
ATCCAGCTCC BRDN0001	296	288	35	545	533	25
ATCCAGCTGT BRDN0001	165	165	698	383	333	4
ATCCAGCTTC BRDN0001	221	142	732	363	231	0
ATCCAGGAT BRDN0001	556	525	958	960	1163	40
ATCCAGGCA BRDN0001	98	83	177	397	310	1
ATCCAGGCG BRDN0001	294	328	1738	796	678	53
ATCCAGGCTT BRDN0001	211	226	171	371	238	25
ATCCAGTATT BRDN0001	306	103	36	808	728	10
ATCCAGTCA BRDN0001	192	232	18	359	684	19
ATCCAGTCCA BRDN0001	211	220	349	381	234	61
ATCCAGTCCC BRDN0001	418	408	301	914	879	27
ATCCAGTGG BRDN0001	272	191	2936	364	836	115
ATCCAGTGG BRDN0001	197	9	6	417	236	0
ATCCAGTTCC BRDN0001	600	443	594	1006	865	56
ATCCAGTTG BRDN0001	510	0	1	1044	807	0
ATCCATAACA BRDN0001	201	210	193	436	475	34
ATCCATAATA BRDN0001	202	93	0	331	460	0
ATCCATACCC BRDN0001	420	358	1460	664	728	105
ATCCATAGA BRDN0001	274	178	68	314	166	14
ATCCATAGCC BRDN0001	177	173	167	387	458	34
ATCCATAGT BRDN0001	236	261	431	496	659	26
ATCCATCAGT BRDN0001	434	512	1208	691	753	45
ATCCATCCCA BRDN0001	99	101	522	190	242	21
ATCCATCGA BRDN0001	107	61	16	196	98	36
ATCCATGAGT BRDN0001	217	174	14	424	285	20
ATCCATGTG BRDN0001	390	486	1609	543	893	15
ATCCATTCAG BRDN0001	450	12	0	893	741	0
ATCCATTCGA BRDN0001	511	459	897	867	661	41
ATCCATTCTC BRDN0001	334	308	624	717	642	83
ATCCATTGCT BRDN0001	321	304	103	430	670	35
ATCCCAAAA BRDN0001	277	194	501	550	525	62
ATCCCAAAG BRDN0001	369	540	762	790	561	63
ATCCCAAAG BRDN0001	239	204	224	376	351	29
ATCCCAAGTT BRDN0001	337	385	461	748	1693	39
ATCCCAATTG BRDN0001	140	18	0	338	482	0
ATCCCACACT BRDN0001	168	87	211	295	195	0
ATCCCACCAA BRDN0001	316	319	453	472	614	0
ATCCCAGAA BRDN0001	502	494	3311	902	634	75
ATCCCAGAA BRDN0001	207	123	389	326	150	4
ATCCCAGAG BRDN0001	163	237	858	320	685	28
ATCCCAGAT BRDN0001	234	168	273	434	254	6
ATCCCAGCAT BRDN0001	368	301	660	643	933	27
ATCCCAGTG BRDN0001	315	193	1025	505	369	50
ATCCCATAAT BRDN0001	400	224	333	549	381	6
ATCCCATTAT BRDN0001	109	19	0	385	184	0
ATCCCATTCA BRDN0001	214	75	45	263	260	4
ATCCCCAACA BRDN0001	348	550	394	879	781	24
ATCCCCAAGA BRDN0001	240	173	155	354	353	0
ATCCCCAAGA BRDN0001	468	584	1202	996	1261	35
ATCCCCACAC BRDN0001	188	184	231	468	676	26
ATCCCCACCA BRDN0001	171	63	49	295	386	1
ATCCCCACTT BRDN0001	336	351	897	783	694	62
ATCCCCAGAT BRDN0001	449	481	1310	832	726	41
ATCCCCAGG BRDN0001	598	492	1570	1235	745	87
ATCCCCATAT BRDN0001	358	310	150	598	524	11
ATCCCCCCAC BRDN0001	259	243	707	646	589	30
ATCCCCCCGA BRDN0001	167	10	0	352	199	0
ATCCCCCTCT BRDN0001	199	69	69	330	269	0
ATCCCCTAGG BRDN0001	247	138	113	367	399	0
ATCCCGAAA BRDN0001	72	113	291	198	65	11
ATCCCGAAC BRDN0001	201	2	1	492	381	0
ATCCCGATG BRDN0001	183	127	288	290	509	0
ATCCCGCGT BRDN0001	141	9	0	269	124	0
ATCCCGTCCA BRDN0001	174	161	227	256	294	24
ATCCCGTCCA BRDN0001	555	457	711	906	922	39
ATCCCTAAAG BRDN0001	179	138	166	498	492	6
ATCCCTAAGC BRDN0001	172	156	836	296	186	56
ATCCCTAGA BRDN0001	202	157	139	303	426	18
ATCCCTCAGC BRDN0001	255	173	77	546	292	1
ATCCCTCCGT BRDN0001	595	579	858	922	1129	99
ATCCCTCTAG BRDN0001	102	74	799	229	118	22
ATCCCTGCTA BRDN0001	717	758	1766	1005	1302	225
ATCCCTGGC BRDN0001	362	330	637	1161	543	18
ATCCCTGGC BRDN0001	189	59	8	365	438	57
ATCCCTTCCA BRDN0001	224	169	595	260	218	7
ATCCCTTTGA BRDN0001	289	236	555	422	559	25
ATCCGAAAA BRDN0001	399	464	267	699	946	42
ATCCGAAAA BRDN0001	196	115	155	441	373	13
ATCCGAAAGT BRDN0001	247	115	730	225	190	18
ATCCGAACA BRDN0001	326	211	788	946	1016	46
ATCCGAACC BRDN0001	601	394	445	1305	1058	37
ATCCGAAGA BRDN0001	170	225	1018	326	205	64
ATCCGAAGA BRDN0001	356	240	851	843	480	78
ATCCGAAGC BRDN0001	203	24	0	835	1177	0
ATCCGAAGC BRDN0001	126	67	72	355	404	6
ATCCGACCA BRDN0001	347	276	181	524	399	54
ATCCGACGC BRDN0001	468	501	1716	695	902	71
ATCCGAGAA BRDN0001	136	152	258	229	244	22
ATCCGAGAG BRDN0001	135	120	231	253	248	13
ATCCGAGCC BRDN0001	355	348	541	625	487	13
ATCCGCAAC BRDN0001	391	666	7853	661	1165	374
ATCCGCAAG BRDN0001	84	59	698	249	199	27
ATCCGCAGG BRDN0001	203	200	120	419	570	23
ATCCGCATCC BRDN0001	161	96	82	212	292	0
ATCCGCATTC BRDN0001	123	0	10	708	607	0
ATCCGCCATA BRDN0001	86	30	0	405	501	3
ATCCGCTCCA BRDN0001	201	220	558	312	369	28
ATCCGCTGG BRDN0001	433	174	192	749	487	27
ATCCGCTGT BRDN0001	513	448	301	857	752	17
ATCCGGACC BRDN0001	148	130	962	280	103	10
ATCCGGGAC BRDN0001	158	157	182	279	236	14
ATCCGGGAG BRDN0001	232	46	67	408	672	13
ATCCGGGCG BRDN0001	242	280	758	372	310	63
ATCCGGGGG BRDN0001	341	288	1591	515	488	50
ATCCGGGTA BRDN0001	74	0	1	513	282	0
ATCCGGGTA BRDN0001	359	273	1147	817	808	90
ATCCGGGTC BRDN0001	200	148	114	315	388	36
ATCCGGTCA BRDN0001	293	242	409	513	333	45
ATCCGGTTT BRDN0001	132	104	498	181	236	12
ATCCGTCTGC BRDN0001	503	507	672	916	860	49
ATCCGTGAC BRDN0001	215	311	1531	494	556	110
ATCCGTGATT BRDN0001	577	529	670	968	1369	45
ATCCGTGCG BRDN0001	70	55	68	150	106	7
ATCCGTGTA BRDN0001	389	308	1829	800	827	81
ATCCGTGTCC BRDN0001	88	1	0	900	375	1
ATCCGTTGC BRDN0001	457	483	1488	782	623	63
ATCCTAAGA BRDN0001	115	66	1268	250	117	70
ATCCTACCGA BRDN0001	168	102	690	415	420	41
ATCCTAGGG BRDN0001	211	183	32	438	203	7
ATCCTAGGT BRDN0001	354	337	1427	679	820	30
ATCCTATCCA BRDN0001	314	317	272	688	767	0
ATCCTATGA BRDN0001	415	375	498	756	790	27
ATCCTCAAAC BRDN0001	382	266	340	557	567	13
ATCCTCAAAG BRDN0001	256	197	1006	513	563	38
ATCCTCACAG BRDN0001	216	149	217	375	416	38
ATCCTCACAG BRDN0001	403	422	3260	816	531	102
ATCCTCACCT BRDN0001	227	140	10	366	202	0
ATCCTCAGG BRDN0001	148	169	393	202	133	40
ATCCTCATAC BRDN0001	404	325	78	977	771	30
ATCCTCATCC BRDN0001	212	19	0	946	231	0
ATCCTCATGC BRDN0001	231	305	729	518	289	44
ATCCTCCAAG BRDN0001	540	675	805	936	811	87
ATCCTCCACA BRDN0001	454	449	6873	805	639	244
ATCCTCCACC BRDN0001	453	18	65	757	710	0
ATCCTCCAGA BRDN0001	438	501	1104	959	1087	149
ATCCTCCCAG BRDN0001	314	250	31	503	304	62
ATCCTCCCTG BRDN0001	436	489	184	852	995	41
ATCCTCCTCG BRDN0001	314	168	119	606	867	45
ATCCTCGAAC BRDN0001	513	554	856	848	697	41
ATCCTCGCAA BRDN0001	457	128	677	799	1239	0
ATCCTCGCAC BRDN0001	553	641	3744	1068	1422	65
ATCCTCGGAT BRDN0001	538	695	501	1118	811	64
ATCCTCGTCC BRDN0001	130	99	0	288	410	0
ATCCTCTAGC BRDN0001	178	154	376	346	252	12
ATCCTCTGAT BRDN0001	516	509	560	818	773	31
ATCCTCTGCC BRDN0001	297	139	51	755	979	44
ATCCTCTTGA BRDN0001	270	330	884	410	597	39
ATCCTCTTGA BRDN0001	178	150	260	441	168	0
ATCCTGAAC BRDN0001	338	322	239	738	406	0
ATCCTGACA BRDN0001	242	168	743	549	780	87
ATCCTGACA BRDN0001	164	191	117	359	546	9
ATCCTGACA BRDN0001	551	401	526	1006	812	13
ATCCTGACAT BRDN0001	185	110	568	413	335	9
ATCCTGAGA BRDN0001	571	542	199	979	567	31
ATCCTGAGC BRDN0001	345	282	241	490	488	38
ATCCTGAGC BRDN0001	304	253	363	736	522	11
ATCCTGATA BRDN0001	215	174	53	266	196	6
ATCCTGCCAA BRDN0001	160	153	589	283	385	7
ATCCTGCCAA BRDN0001	488	396	276	713	762	38
ATCCTGCGA BRDN0001	642	583	470	1109	997	120
ATCCTGCTCG BRDN0001	197	121	103	281	469	12
ATCCTGCTCT BRDN0001	461	374	577	697	1141	143
ATCCTGGAC BRDN0001	168	134	1848	326	532	36
ATCCTGGAG BRDN0001	325	196	378	725	524	0
ATCCTGGAT BRDN0001	297	329	766	408	1013	99
ATCCTGGCA BRDN0001	263	214	104	383	414	32
ATCCTGGGA BRDN0001	656	699	5178	1139	1650	135

ATCCTGGGT	BRDN00012	268	21	27	1344	740	0
ATCCTGGTG	BRDN00012	185	122	493	304	317	10
ATCCTGGTT	BRDN00011	296	340	215	589	625	0
ATCCTGTCA	BRDN00012	413	0	1	680	1163	0
ATCCTGTCA	BRDN00011	627	391	411	844	855	41
ATCCTGTGG	BRDN00012	113	133	14	425	598	0
ATCCTGTTCT	BRDN00011	267	149	163	440	405	0
ATCCTTCAGA	BRDN00011	376	262	2917	759	661	46
ATCCTTCCCT	BRDN00012	413	201	1064	823	599	19
ATCCTTCCGA	BRDN00012	302	217	369	532	328	11
ATCCTTCTAG	BRDN00011	53	11	42	195	182	0
ATCCTTGCTA	BRDN00011	292	22	20	454	492	0
ATCCTTGCTC	BRDN00012	568	431	1043	933	1117	59
ATCGAAATT	BRDN00011	249	115	575	558	718	16
ATCGACAAA	BRDN00011	192	177	947	545	287	57
ATCGACAGA	BRDN00012	128	190	360	271	116	14
ATCGACAGG	BRDN00011	99	142	75	288	62	86
ATCGACATG	BRDN00012	126	166	113	294	251	192
ATCGACCAT	BRDN00012	502	433	888	884	956	112
ATCGACCTCA	BRDN00012	264	183	371	530	792	24
ATCGACGTCT	BRDN00011	373	594	1682	828	890	116
ATCGAGAAC	BRDN00011	343	412	278	599	631	21
ATCGAGAAG	BRDN00011	342	340	382	666	819	19
ATCGAGACT	BRDN00012	116	73	197	510	229	5
ATCGAGCCA	BRDN00011	451	306	1045	506	553	15
ATCGAGCTA	BRDN00012	394	417	243	754	668	62
ATCGAGGTC	BRDN00011	549	706	2554	991	1408	173
ATCGAGTTA	BRDN00012	33	0	0	129	78	0
ATCGATACA	BRDN00012	141	107	0	232	307	22
ATCGATATA	BRDN00012	252	266	408	590	612	84
ATCGATGGA	BRDN00012	426	447	195	705	1047	11
ATCGCAAAC	BRDN00012	369	298	19	797	445	24
ATCGCAAGG	BRDN00012	449	481	683	889	953	20
ATCGCAAGT	BRDN00012	331	0	0	572	265	0
ATCGCAGAG	BRDN00011	58	105	0	112	112	0
ATCGCAGAG	BRDN00011	237	240	271	346	635	38
ATCGCAGCC	BRDN00011	223	103	226	276	168	0
ATCGCCACCA	BRDN00011	463	574	1295	1045	1288	43
ATCGCCAGC	BRDN00011	334	11	52	858	836	0
ATCGCCAGG	BRDN00011	160	181	1430	266	337	45
ATCGCCTCTA	BRDN00012	154	120	53	260	272	32
ATCGCCTCTT	BRDN00012	377	318	3237	704	286	66
ATCGCGTCA	BRDN00011	298	204	29	438	264	8
ATCGCGTCC	BRDN00012	188	234	61	328	276	4
ATCGCTCACA	BRDN00011	314	398	54	449	178	11
ATCGCTTGT	BRDN00012	123	64	181	277	189	7
ATCGGACCC	BRDN00012	238	115	181	344	164	21
ATCGGATCC	BRDN00011	524	215	85	986	760	4
ATCGGCAGA	BRDN00012	80	5	0	522	39	13
ATCGGCATT	BRDN00011	353	278	867	485	799	13
ATCGGCGCA	BRDN00012	83	77	16	217	447	26
ATCGGCTGT	BRDN00012	158	61	10	237	358	0
ATCGGGAGC	BRDN00012	187	105	299	255	197	29
ATCGGGGTA	BRDN00012	286	277	30	472	435	30
ATCGGTACC	BRDN00012	460	346	1374	747	720	21
ATCGGTCTT	BRDN00011	126	130	2369	320	304	59
ATCGGTTTG	BRDN00012	207	138	171	266	164	0
ATCGTAAAC	BRDN00011	249	183	426	431	297	23
ATCGTAAGG	BRDN00011	201	165	925	347	250	17
ATCGTAGCC	BRDN00011	444	494	716	859	660	8
ATCGTATCAT	BRDN00012	390	472	1541	770	749	29
ATCGTATTG	BRDN00012	232	20	79	601	447	0
ATCGTCACTT	BRDN00011	330	194	292	568	433	24
ATCGTCATA	BRDN00012	205	225	131	513	412	0
ATCGTCCATA	BRDN00011	321	211	188	564	534	18
ATCGTCCGA	BRDN00012	210	138	105	264	234	0
ATCGTCGAG	BRDN00011	143	117	38	199	622	0
ATCGTCGCA	BRDN00011	111	12	0	214	58	0
ATCGTCGTA	BRDN00012	345	327	288	589	297	24
ATCGTCGTC	BRDN00012	604	599	387	965	879	20
ATCGTCTACT	BRDN00012	188	156	31	291	320	5
ATCGTCTGA	BRDN00012	154	109	139	395	255	27
ATCGTGACT	BRDN00011	125	76	77	182	208	4
ATCGTGCAG	BRDN00012	252	279	640	501	455	26
ATCGTGCGG	BRDN00012	387	301	248	540	540	31
ATCGTGGAG	BRDN00012	287	228	63	488	516	100
ATCGTGGCG	BRDN00012	134	188	276	207	245	28
ATCGTGTCT	BRDN00012	464	503	1028	718	568	96
ATCGTTGAG	BRDN00011	342	351	808	615	893	43
ATCGTTGCT	BRDN00012	550	429	689	768	711	39
ATCGTTGCT	BRDN00012	502	260	138	989	702	10
ATCGTTGGG	BRDN00011	99	82	1	173	91	12
ATCGTTTCCG	BRDN00012	538	597	1461	879	1024	31
ATCTAAACA	BRDN00012	268	226	585	548	470	2
ATCTAAACA	BRDN00011	177	112	1520	303	110	45
ATCTAACACT	BRDN00011	546	444	1064	872	717	46
ATCTAAGAG	BRDN00012	182	219	74	297	256	78
ATCTAAGAG	BRDN00012	88	82	10	157	147	12
ATCTAAGCA	BRDN00012	166	7	2	1001	535	0
ATCTAATTCA	BRDN00012	179	62	1047	260	326	2
ATCTACAAAT	BRDN00011	167	90	0	413	502	38
ATCTACAATC	BRDN00012	653	451	1254	1151	1122	9
ATCTACACAG	BRDN00011	201	192	133	411	207	11
ATCTACACCC	BRDN00012	187	135	735	391	565	4
ATCTACAGC	BRDN00012	658	505	2420	1167	1301	26
ATCTACATCC	BRDN00011	151	146	477	300	451	7
ATCTACCGAT	BRDN00011	149	139	0	354	272	40
ATCTACGAG	BRDN00012	380	277	597	817	639	1
ATCTACGCA	BRDN00011	254	177	169	543	531	9
ATCTACGCCT	BRDN00011	392	412	200	823	679	16
ATCTACTGAT	BRDN00012	180	211	38	454	838	26
ATCTACTGTC	BRDN00011	476	417	770	1000	933	42
ATCTAGACTT	BRDN00011	516	396	1041	1061	1177	39
ATCTAGGTA	BRDN00011	76	76	10	181	307	11
ATCTAGTGT	BRDN00012	75	8	0	205	146	0
ATCTATCTGA	BRDN00012	145	168	39	346	355	24
ATCTATGATT	BRDN00012	109	107	1441	251	162	23
ATCTATGGT	BRDN00011	267	232	324	417	365	18
ATCTATTTGA	BRDN00012	111	119	201	260	379	15
ATCTCAAATG	BRDN00011	225	170	56	379	369	55
ATCTCAACAG	BRDN00012	188	128	38	358	655	23
ATCTCAACCA	BRDN00012	407	499	782	1017	786	97
ATCTCAACTA	BRDN00011	373	281	320	582	496	54
ATCTCACCAA	BRDN00011	461	405	733	710	795	67
ATCTCACCAC	BRDN00012	699	546	986	1132	1507	61
ATCTCACCCA	BRDN00012	100	93	435	242	206	7
ATCTCACTGT	BRDN00012	473	426	701	1090	731	23
ATCTCAGAA	BRDN00012	177	143	13	266	407	0
ATCTCAGCAT	BRDN00011	416	616	661	747	970	30
ATCTCAGGA	BRDN00012	260	15	95	325	420	0
ATCTCATAAC	BRDN00012	464	372	1593	803	1184	51
ATCTCATAG	BRDN00011	207	155	95	476	506	18
ATCTCCAACA	BRDN00012	606	655	590	1388	1713	99
ATCTCCAAG	BRDN00011	55	14	0	180	29	0
ATCTCCATCG	BRDN00012	157	216	42	386	341	0
ATCTCCATGC	BRDN00011	128	138	151	409	283	31
ATCTCCATGT	BRDN00012	178	137	69	376	830	59
ATCTCCCACA	BRDN00011	195	277	268	350	181	51
ATCTCCCATT	BRDN00011	759	594	3901	1008	580	294
ATCTCCCGG	BRDN00012	202	82	154	360	138	12
ATCTCCCGTG	BRDN00011	132	14	19	416	339	0
ATCTCCGGG	BRDN00011	130	134	0	169	81	0
ATCTCCGTG	BRDN00011	95	95	540	206	285	27
ATCTCCTACA	BRDN00011	141	125	369	291	267	10
ATCTCCTTCA	BRDN00011	186	82	160	244	312	1
ATCTCGAAT	BRDN00011	130	72	0	320	205	1
ATCTCGAGC	BRDN00012	375	255	517	654	1047	21
ATCTCGAGG	BRDN00011	680	1038	1982	1495	1952	270
ATCTCGATG	BRDN00012	438	287	1470	772	639	38
ATCTCGCCAT	BRDN00011	231	261	1768	394	458	33
ATCTCGCCTA	BRDN00012	406	391	216	753	880	32
ATCTCGCGC	BRDN00011	171	132	57	225	176	0
ATCTCGGAG	BRDN00011	148	106	495	284	505	8
ATCTCGGCG	BRDN00011	351	329	543	527	655	59
ATCTCGGGG	BRDN00012	606	533	805	1127	865	41
ATCTCGGGT	BRDN00012	766	879	3586	1258	1771	148
ATCTCGGTA	BRDN00012	230	47	1	1067	893	0
ATCTCGTAAT	BRDN00012	280	52	1028	891	576	4
ATCTCGTCCC	BRDN00012	87	5	257	233	87	0
ATCTCTAAAA	BRDN00012	188	56	428	315	495	25
ATCTCTAGAT	BRDN00011	206	280	99	570	268	11
ATCTCTATAC	BRDN00012	496	476	549	940	805	35
ATCTCTATCT	BRDN00011	82	23	14	162	90	0
ATCTCTATGA	BRDN00011	172	30	1	260	349	0
ATCTCTCAGA	BRDN00012	67	32	0	1148	1385	32
ATCTCTCGG	BRDN00012	244	188	294	602	412	0
ATCTCTCTGA	BRDN00012	560	318	440	1127	1559	34
ATCTCTGACA	BRDN00012	352	293	17	608	621	27
ATCTCTGACT	BRDN00012	348	340	181	1049	1110	40
ATCTCTGAG	BRDN00012	221	156	707	257	234	31
ATCTCTGCAG	BRDN00011	127	137	2867	472	286	35
ATCTCTGCCC	BRDN00012	96	136	190	319	286	21
ATCTCTGGC	BRDN00011	54	0	0	224	30	0
ATCTCTGTAC	BRDN00012	697	522	618	1093	1019	54
ATCTCTTCTC	BRDN00012	410	335	2099	700	710	36
ATCTCTTTGG	BRDN00012	341	296	388	718	887	23
ATCTGAAACT	BRDN00011	143	159	244	303	319	42
ATCTGAACT	BRDN00012	166	218	10	222	825	5
ATCTGAAGA	BRDN00012	336	277	621	663	529	4
ATCTGAAGA	BRDN00012	187	281	1114	406	437	85
ATCTGAATA	BRDN00012	513	444	563	794	446	103
ATCTGACAA	BRDN00011	409	389	1489	646	447	66
ATCTGACAG	BRDN00012	140	72	871	333	542	9
ATCTGACCTA	BRDN00011	279	353	43	567	472	50
ATCTGACGA	BRDN00012	180	126	197	264	349	15
ATCTGACGA	BRDN00012	255	93	114	687	596	14
ATCTGACGT	BRDN00012	216	143	129	387	314	24
ATCTGAGAG	BRDN00011	353	317	433	615	628	4
ATCTGAGAG	BRDN00011	108	54	0	220	229	3
ATCTGAGCA	BRDN00011	245	272	784	555	719	23
ATCTGAGCG	BRDN00012	648	464	744	1191	944	192
ATCTGAGGA	BRDN00011	87	36	280	624	661	0
ATCTGATAG	BRDN00011	96	45	0	345	186	0
ATCTGATCTG	BRDN00011	322	356	38	400	873	36
ATCTGATGG	BRDN00011	367	67	1275	683	915	32
ATCTGCAAT	BRDN00012	108	162	110	344	138	0
ATCTGCACA	BRDN00012	224	136	6	352	313	0
ATCTGCACAT	BRDN00012	508	405	782	916	803	60
ATCTGCACG	BRDN00011	213	378	303	384	97	9
ATCTGCAGT	BRDN00011	123	144	500	238	276	12
ATCTGCATA	BRDN00011	256	207	423	676	639	35
ATCTGCCAG	BRDN00012	416	447	4318	864	696	97
ATCTGCCATG	BRDN00012	859	932	2296	1559	1751	98
ATCTGCCATT	BRDN00012	273	182	730	578	390	53
ATCTGCGCA	BRDN00011	82	0	0	463	607	0
ATCTGCGCC	BRDN00012	323	184	339	709	749	31
ATCTGCGTC	BRDN00012	507	463	450	828	915	23
ATCTGCGTT	BRDN00012	286	225	24	619	682	69
ATCTGCTACT	BRDN00012	167	201	366	348	361	35
ATCTGCTATC	BRDN00011	515	444	1274	812	1255	50
ATCTGCTCAG	BRDN00012	343	441	1058	846	968	20
ATCTGCTCAT	BRDN00012	301	422	783	649	1296	54
ATCTGCTGG	BRDN00011	162	19	12	117	85	26
ATCTGGAAC	BRDN00012	281	366	3542	700	974	100
ATCTGGAAC	BRDN00012	532	374	2228	671	538	95
ATCTGGAAT	BRDN00011	65	35	163	283	248	0
ATCTGGACC	BRDN00012	321	306	2208	656	808	44
ATCTGGACC	BRDN00011	132	81	75	231	99	0
ATCTGGAGC	BRDN00012	79	1	0	399	278	0
ATCTGGATC	BRDN00012	365	87	77	669	609	0
ATCTGGGAC	BRDN00011	270	163	109	542	560	7
ATCTGGGAG	BRDN00012	507	330	1856	865	955	27
ATCTGGGGA	BRDN00011	190	127	643	272	357	5
ATCTGGGTA	BRDN00012	229	203	132	549	899	20
ATCTGGTAC	BRDN00012	259	225	181	612	493	22
ATCTGGTGG	BRDN00011	290	566	6403	312	249	276
ATCTGGTTT	BRDN00011	302	343	19	397	314	0
ATCTGTAAC	BRDN00012	237	91	357	333	382	7
ATCTGTATCA	BRDN00011	89	8	0	295	189	1
ATCTGTCAG	BRDN00012	330	181	148	458	405	17
ATCTGTCCAA	BRDN00012	458	523	1831	753	865	63
ATCTGTCCTA	BRDN00012	593	452	1061	691	888	45
ATCTGTCCTT	BRDN00011	287	220	164	506	556	9
ATCTGTGCA	BRDN00011	424	443	361	788	722	40
ATCTGTGCC	BRDN00012	191	5	0	722	747	0
ATCTGTGGC	BRDN00012	27	0	0	204	297	0
ATCTGTGTG	BRDN00012	843	706	2649	1327	1007	58
ATCTGTTACC	BRDN00012	329	272	547	835	738	10
ATCTGTTCCC	BRDN00011	306	59	101	721	1024	1
ATCTGTTGT	BRDN00012	351	251	678	683	879	63
ATCTTAACTA	BRDN00012	246	23	0	640	404	0
ATCTTAATCT	BRDN00011	120	19	10	246	182	0
ATCTTACTTA	BRDN00012	57	71	0	272	273	0
ATCTTAGATC	BRDN00011	155	238	416	379	431	59
ATCTTAGCTC	BRDN00011	182	209	1493	389	443	12
ATCTTATAG	BRDN00012	82	129	0	274	263	0
ATCTTATCAC	BRDN00012	152	88	681	328	297	7
ATCTTCACAG	BRDN00012	175	322	148	361	374	44
ATCTTCAGAC	BRDN00011	164	2	0	399	271	0
ATCTTCAGCT	BRDN00011	116	146	383	309	353	7
ATCTTCAGG	BRDN00012	352	426	783	687	489	44
ATCTTCCCCA	BRDN00011	193	185	495	350	284	23
ATCTTCCGAT	BRDN00012	476	460	436	955	552	16
ATCTTCCTCA	BRDN00012	161	122	62	486	693	5
ATCTTCCTTC	BRDN00012	29	0	0	293	83	0
ATCTTCGAG	BRDN00011	93	86	222	194	610	0
ATCTTCGCAC	BRDN00011	155	0	0	374	41	0
ATCTTCTCCG	BRDN00012	329	10	1	863	1671	0
ATCTTCTCGA	BRDN00012	358	429	390	837	733	41
ATCTTCTGGA	BRDN00011	227	247	219	451	183	26
ATCTTCTTAT	BRDN00012	271	210	293	558	376	36
ATCTTCTTCA	BRDN00012	269	145	1618	390	225	65
ATCTTCTTTC	BRDN00012	296	263	777	902	1110	43
ATCTTGAACT	BRDN00012	202	17	0	492	142	10
ATCTTGAATC	BRDN00011	78	75	865	199	113	32
ATCTTGACCA	BRDN00012	563	343	734	1043	1137	64
ATCTTGCCAA	BRDN00011	190	163	885	385	219	38
ATCTTGCCAG	BRDN00011	560	491	560	824	1148	29
ATCTTGCGC	BRDN00011	422	302	181	446	727	18
ATCTTGCTAT	BRDN00011	152	205	394	255	455	15
ATCTTGGAA	BRDN00012	120	150	108	281	234	26
ATCTTGGACT	BRDN00012	258	164	414	445	306	20
ATCTTGGCA	BRDN00012	453	320	464	877	1267	49
ATCTTGGCA	BRDN00011	0	0	0	197	56	0
ATCTTGTATA	BRDN00012	167	202	15	296	203	20
ATCTTGTTAT	BRDN00012	90	181	544	276	189	22
ATCTTGTTGC	BRDN00012	359	437	68	840	623	85
ATCTTTACAG	BRDN00011	134	60	96	386	156	0
ATCTTTACCC	BRDN00012	155	225	38	485	438	4
ATCTTTATAC	BRDN00012	186	55	4	372	553	0
ATCTTTATTA	BRDN00011	75	45	0	123	145	9
ATCTTTGAGT	BRDN00012	246	148	1523	362	170	43
ATCTTTGGA	BRDN00011	271	259	303	416	470	10
ATCTTTGGTA	BRDN00011	143	121	128	347	553	0
ATGAAAAAC	BRDN00011	137	219	409	329	431	81
ATGAAAAAG	BRDN00011	123	13	801	315	644	22
ATGAAAAAT	BRDN00012	110	111	115	234	216	7
ATGAAAACT	BRDN00012	191	243	652	479	550	31
ATGAAAAGC	BRDN00011	153	81	52	227	271	0
ATGAAAAGC	BRDN00012	251	166	194	457	12934	9
ATGAAAAGG	BRDN00012	433	366	477	746	743	21
ATGAAACAA	BRDN00011	315	160	585	612	859	19
ATGAAACAG	BRDN00012	121	4	0	359	304	0
ATGAAACAT	BRDN00012	243	112	319	475	192	3
ATGAAACCCT	BRDN00012	464	309	48	865	493	26
ATGAAAGCG	BRDN00011	208	247	199	395	575	77
ATGAAAGCT	BRDN00011	212	156	431	448	569	20
ATGAAAGCT	BRDN00011	441	333	284	649	815	12
ATGAAAGGC	BRDN00012	106	73	155	227	251	0
ATGAAAGGG	BRDN00011	125	90	6228	232	393	111
ATGAAAGTG	BRDN00012	64	85	55	305	188	12
ATGAAATAG	BRDN00011	75	76	1	153	47	0
ATGAAATTG	BRDN00011	143	173	705	275	309	48
ATGAACAAA	BRDN00012	160	138	0	292	198	11
ATGAACACA	BRDN00012	40	5	1	420	443	0
ATGAACACC	BRDN00012	280	405	135	519	484	31
ATGAACACCT	BRDN00012	203	189	114	391	362	11
ATGAACACG	BRDN00012	142	146	527	202	355	12
ATGAACAGG	BRDN00011	106	0	13	383	456	0
ATGAACATG	BRDN00011	209	215	28	327	362	7
ATGAACATG	BRDN00012	199	93	2	537	547	12
ATGAACCAG	BRDN00011	367	219	33	610	690	83
ATGAACCAT	BRDN00011	270	315	715	411	369	87
ATGAACCAT	BRDN00011	261	262	193	406	93	0
ATGAACCGG	BRDN00011	106	28	46	287	161	3
ATGAACCTC	BRDN00012	121	203	246	185	202	23
ATGAACGAA	BRDN00012	440	408	364	974	703	32
ATGAACGAA	BRDN00012	415	314	583	484	710	8
ATGAACGAG	BRDN00012	160	115	6	192	685	0
ATGAACGAG	BRDN00012	286	19	54	325	347	0
ATGAACGTA	BRDN00012	355	350	506	547	440	74
ATGAACGTA	BRDN00012	267	260	198	542	524	36
ATGAACTCA	BRDN00012	474	495	683	981	1080	87
ATGAACTCA	BRDN00011	67	119	34	212	335	22
ATGAACTCA	BRDN00012	217	236	323	455	783	27
ATGAACTGA	BRDN00012	155	186	197	389	176	5
ATGAACTGG	BRDN00011	179	236	232	274	270	0
ATGAACTGG	BRDN00012	139	17	38	938	494	0
ATGAACTTC	BRDN00012	333	190	552	562	397	28
ATGAAGAAT	BRDN00012	53	20	0	451	273	0
ATGAAGACT	BRDN00012	233	235	988	293	175	3
ATGAAGAGA	BRDN00012	330	294	218	794	612	40
ATGAAGAGG	BRDN00012	468	441	727	903	1196	33
ATGAAGATA	BRDN00012	186	210	4399	251	295	61
ATGAAGATG	BRDN00012	409	386	606	1169	952	40
ATGAAGCCA	BRDN00012	458	511	713	1066	1029	109
ATGAAGCCC	BRDN00012	355	223	1468	736	1045	88
ATGAAGCCC	BRDN00012	235	262	786	445	356	47
ATGAAGCCG	BRDN00012	444	428	4160	873	929	78
ATGAAGCCG	BRDN00012	290	281	597	428	329	26
ATGAAGCCT	BRDN00012	408	281	1357	648	988	70
ATGAAGCGG	BRDN00012	266	72	76	705	482	0
ATGAAGCTG	BRDN00011	265	189	90	734	563	17
ATGAAGCTG	BRDN00012	357	466	750	646	1305	53
ATGAAGGAG	BRDN00012	56	15	53	269	225	0
ATGAAGGCG	BRDN00011	322	222	581	577	343	34
ATGAAGGGA	BRDN00011	209	140	247	398	403	0
ATGAAGGTA	BRDN00011	426	359	263	830	735	53
ATGAAGTCG	BRDN00011	125	221	47	246	428	65
ATGAAGTGA	BRDN00012	147	94	1	209	238	0
ATGAAGTGC	BRDN00011	244	201	2	460	502	4
ATGAAGTGG	BRDN00012	130	212	786	291	250	13
ATGAAGTGG	BRDN00012	152	4	10	603	558	1
ATGAAGTGT	BRDN00011	359	278	735	490	697	13
ATGAAGTTA	BRDN00011	311	349	270	547	596	22
ATGAAGTTG	BRDN00011	316	302	697	556	483	31
ATGAAGTTG	BRDN00011	132	0	1	289	46	0
ATGAAGTTT	BRDN00012	97	2	0	300	214	0
ATGAAGTTT	BRDN00011	332	293	106	612	1001	62
ATGAATACT	BRDN00011	58	0	0	306	201	0
ATGAATAGA	BRDN00012	301	315	437	612	330	49
ATGAATATG	BRDN00011	217	274	108	348	310	14
ATGAATCAA	BRDN00011	297	359	73	655	659	25
ATGAATCAC	BRDN00012	165	232	834	330	415	24
ATGAATCAT	BRDN00012	245	165	449	435	591	5
ATGAATCAT	BRDN00011	283	222	209	456	366	38
ATGAATCCA	BRDN00012	138	138	45	432	295	44
ATGAATCGA	BRDN00012	163	77	46	249	214	0
ATGAATCTA	BRDN00011	201	110	789	297	505	11
ATGAATGAA	BRDN00012	444	389	532	754	544	44
ATGAATGAC	BRDN00011	162	195	363	262	200	35
ATGAATGAG	BRDN00011	142	202	234	387	219	0
ATGAATGCA	BRDN00012	439	369	3141	788	764	72
ATGAATGGG	BRDN00011	100	1	7	316	74	0
ATGAATGTA	BRDN00011	347	160	596	556	397	3
ATGAATGTC	BRDN00011	516	349	1433	836	1002	32
ATGACAAAA	BRDN00012	493	242	217	874	589	31
ATGACAAAC	BRDN00011	329	355	1048	710	463	63
ATGACAAAG	BRDN00011	76	26	1	297	256	14
ATGACAAAG	BRDN00012	426	410	1152	679	940	55
ATGACAAAT	BRDN00012	415	597	810	888	806	120
ATGACAACC	BRDN00012	299	335	6247	484	1075	103
ATGACAACT	BRDN00012	171	90	35	291	174	0
ATGACAACT	BRDN00012	671	607	367	1216	1306	84
ATGACAAGG	BRDN00012	553	404	1371	943	908	173
ATGACAAGG	BRDN00012	528	293	575	1077	1222	63
ATGACAATA	BRDN00011	179	95	151	395	153	87
ATGACAATG	BRDN00012	175	223	13	320	320	4
ATGACAATG	BRDN00012	198	207	282	404	182	9
ATGACACAA	BRDN00011	114	1	1	374	238	0
ATGACACAT	BRDN00011	204	100	9	332	334	59
ATGACACGG	BRDN00011	259	233	2	604	443	0
ATGACAGAA	BRDN00011	420	453	509	887	464	45
ATGACAGAA	BRDN00011	332	168	394	375	410	13
ATGACAGAT	BRDN00012	350	358	224	739	663	114
ATGACAGCA	BRDN00012	133	139	24	311	321	0
ATGACAGCC	BRDN00012	638	494	627	1088	981	34
ATGACAGCT	BRDN00012	260	230	121	516	449	54
ATGACAGGC	BRDN00012	223	126	1163	338	256	30
ATGACAGGG	BRDN00012	336	299	362	678	798	16
ATGACAGTA	BRDN00012	281	296	115	462	520	32
ATGACAGTA	BRDN00012	663	528	891	1043	1095	78
ATGACAGTG	BRDN00011	191	134	965	345	210	7
ATGACAGTT	BRDN00012	151	66	237	416	161	29
ATGACATCC	BRDN00012	224	173	87	431	260	29
ATGACATTC	BRDN00012	353	354	801	819	754	49
ATGACATTG	BRDN00012	471	545	856	762	894	108
ATGACATTG	BRDN00011	406	396	175	849	732	9
ATGACATTT	BRDN00011	324	371	383	621	485	34
ATGACCAAA	BRDN00011	435	437	437	600	761	5
ATGACCAAA	BRDN00012	354	262	176	571	402	10
ATGACCACA	BRDN00011	133	167	47	384	184	8
ATGACCACA	BRDN00012	376	342	379	493	487	37
ATGACCACC	BRDN00012	230	267	86	413	340	12
ATGACCACG	BRDN00011	438	394	891	883	1083	69
ATGACCAGA	BRDN00012	73	3	2	163	142	6
ATGACCATA	BRDN00012	241	196	392	438	484	3
ATGACCATC	BRDN00011	170	198	2101	427	588	21
ATGACCATG	BRDN00011	210	86	190	404	392	1
ATGACCATG	BRDN00012	514	433	945	977	605	69
ATGACCCAT	BRDN00012	250	146	636	473	342	26
ATGACCCTA	BRDN00012	371	74	51	667	676	4
ATGACCGGC	BRDN00011	314	0	0	910	1092	0
ATGACCGTA	BRDN00012	431	396	2016	800	963	109
ATGACCTACT	BRDN00012	61	71	255	477	849	4
ATGACCTCA	BRDN00012	195	209	741	400	552	33
ATGACCTCCA	BRDN00012	69	0	9	786	543	0
ATGACCTCTT	BRDN00011	340	292	176	579	741	47
ATGACCTGA	BRDN00011	210	211	708	376	260	17
ATGACCTGG	BRDN00012	196	40	0	703	220	13
ATGACCTTG	BRDN00012	204	115	18	495	532	2
ATGACGACC	BRDN00011	302	281	637	418	460	23
ATGACGACG	BRDN00012	256	0	0	416	31	0
ATGACGAGC	BRDN00012	438	547	1222	942	767	116
ATGACGCCG	BRDN00011	134	102	817	201	269	31
ATGACGCGA	BRDN00011	78	0	0	401	315	0
ATGACGGAA	BRDN00012	348	306	617	645	714	79
ATGACGGCA	BRDN00011	372	174	122	717	765	17
ATGACGGCC	BRDN00012	268	90	468	351	352	28
ATGACGGCT	BRDN00011	338	296	81	710	804	43
ATGACGGTA	BRDN00011	180	134	103	436	337	15
ATGACGGTG	BRDN00011	166	240	162	315	195	48
ATGACGGTG	BRDN00012	146	119	1	432	277	2
ATGACGTCA	BRDN00012	661	427	713	980	828	91
ATGACGTGC	BRDN00012	248	318	1126	503	343	22
ATGACGTTA	BRDN00011	352	300	430	523	727	4
ATGACGTTG	BRDN00012	523	666	7535	887	896	198
ATGACGTTG	BRDN00011	343	371	972	753	1106	62
ATGACGTTG	BRDN00011	172	179	109	342	608	30
ATGACTACA	BRDN00011	204	125	454	280	335	12
ATGACTACA	BRDN00011	27	0	1	416	1	0
ATGACTACCT	BRDN00011	92	0	1	342	101	0
ATGACTACT	BRDN00011	174	102	145	318	353	35
ATGACTCAT	BRDN00011	194	229	296	350	495	0
ATGACTCCCC	BRDN00012	286	234	60	509	892	5
ATGACTCCG	BRDN00012	165	21	28	289	110	0
ATGACTCGG	BRDN00011	183	167	628	543	489	9
ATGACTCGTT	BRDN00012	433	186	1015	828	1378	95
ATGACTCTA	BRDN00012	267	208	99	585	601	4
ATGACTGAA	BRDN00011	137	127	277	273	234	8
ATGACTGAA	BRDN00011	251	299	1549	622	813	122
ATGACTGAG	BRDN00011	197	144	0	423	387	0
ATGACTGCA	BRDN00012	493	437	759	1056	1099	41
ATGACTGCG	BRDN00011	461	450	595	1042	830	28
ATGACTGGA	BRDN00012	159	85	85	309	286	0

ATGACTGGC	BRDN00011	136	113	330	159	159	6
ATGACTGTA	BRDN00012	88	127	280	275	263	10
ATGACTGTG	BRDN00011	219	108	88	288	416	29
ATGACTGTG	BRDN00012	179	224	249	442	252	36
ATGACTTCA	BRDN00012	134	179	832	540	622	12
ATGACTTCTA	BRDN00011	169	92	362	281	384	7
ATGACTTGG	BRDN00011	506	422	657	985	974	19
ATGAGAAAA	BRDN00011	124	77	4	244	294	27
ATGAGAAAT	BRDN00011	127	16	0	261	211	0
ATGAGAAAT	BRDN00012	218	145	2160	370	409	47
ATGAGAACC	BRDN00011	146	177	93	340	247	8
ATGAGAACG	BRDN00012	121	31	241	358	565	0
ATGAGAAGC	BRDN00011	280	253	584	576	848	44
ATGAGACAA	BRDN00012	185	228	178	368	517	8
ATGAGACCT	BRDN00012	184	94	1336	284	174	33
ATGAGACTG	BRDN00011	322	234	74	548	714	14
ATGAGACTG	BRDN00012	237	31	1	563	632	0
ATGAGAGAA	BRDN00011	70	59	159	283	177	9
ATGAGAGAT	BRDN00012	272	121	41	557	391	21
ATGAGAGCA	BRDN00011	415	202	1059	679	799	5
ATGAGAGCG	BRDN00012	125	53	535	249	150	12
ATGAGAGGC	BRDN00011	112	73	20	204	301	0
ATGAGAGGC	BRDN00012	203	297	1292	368	494	17
ATGAGATAA	BRDN00011	228	136	32	507	464	6
ATGAGATCA	BRDN00011	218	197	2641	298	421	51
ATGAGATCT	BRDN00012	474	358	1238	765	1014	42
ATGAGATGA	BRDN00012	333	445	433	651	639	0
ATGAGATGG	BRDN00011	177	146	1383	348	207	28
ATGAGATTA	BRDN00012	220	189	130	430	581	11
ATGAGATTC	BRDN00012	711	529	511	1150	1145	51
ATGAGCAAT	BRDN00011	288	402	92	618	690	15
ATGAGCACA	BRDN00011	165	224	140	354	251	76
ATGAGCACG	BRDN00012	245	192	585	463	365	38
ATGAGCACG	BRDN00011	357	341	315	760	602	90
ATGAGCAGG	BRDN00011	83	10	34	321	85	7
ATGAGCAGG	BRDN00011	115	45	133	327	139	0
ATGAGCATA	BRDN00012	344	167	116	688	524	15
ATGAGCATC	BRDN00011	130	240	911	441	92	35
ATGAGCATG	BRDN00011	87	89	665	247	570	6
ATGAGCATG	BRDN00011	112	58	496	318	256	4
ATGAGCCAG	BRDN00012	168	121	189	297	446	0
ATGAGCCCA	BRDN00012	342	455	429	779	788	130
ATGAGCCGT	BRDN00012	194	77	11	314	290	17
ATGAGCCTC	BRDN00012	72	0	1	379	257	0
ATGAGCGAG	BRDN00012	137	112	196	185	82	15
ATGAGCGAG	BRDN00012	135	144	309	273	511	7
ATGAGCGCA	BRDN00011	137	151	1441	171	215	67
ATGAGCTCG	BRDN00012	215	0	0	361	529	0
ATGAGCTCG	BRDN00012	223	21	31	288	133	0
ATGAGCTTA	BRDN00012	165	135	306	337	313	31
ATGAGCTTC	BRDN00011	381	390	1768	624	619	62
ATGAGGAAG	BRDN00011	255	330	1559	352	500	56
ATGAGGACA	BRDN00011	107	0	0	307	263	0
ATGAGGACA	BRDN00012	179	152	192	295	299	23
ATGAGGACT	BRDN00011	307	205	508	612	704	22
ATGAGGAGA	BRDN00011	145	185	585	320	283	42
ATGAGGAGA	BRDN00012	233	169	1120	500	606	19
ATGAGGAGA	BRDN00012	36	6	0	271	38	0
ATGAGGATG	BRDN00012	252	64	19	918	837	0
ATGAGGATG	BRDN00011	89	37	37	274	90	0
ATGAGGCAA	BRDN00012	201	184	91	333	248	65
ATGAGGCCA	BRDN00011	414	335	728	1159	1112	26
ATGAGGCCC	BRDN00012	137	97	143	162	58	8
ATGAGGCGA	BRDN00012	142	189	383	173	129	15
ATGAGGCTG	BRDN00011	167	194	2935	356	625	50
ATGAGGCTG	BRDN00012	387	552	941	941	1050	66
ATGAGGCTG	BRDN00012	326	360	2133	655	597	57
ATGAGGGTA	BRDN00011	244	103	5	171	138	39
ATGAGGGTC	BRDN00012	518	600	459	848	548	43
ATGAGGGTG	BRDN00012	131	212	1105	322	348	15
ATGAGGTAA	BRDN00012	108	159	78	203	119	18
ATGAGGTCG	BRDN00012	439	566	1078	800	867	87
ATGAGGTGA	BRDN00011	117	202	1	303	323	0
ATGAGTACG	BRDN00011	367	318	186	684	1228	35
ATGAGTAGA	BRDN00011	132	79	59	289	320	0
ATGAGTAGA	BRDN00011	117	144	86	239	316	8
ATGAGTAGA	BRDN00012	167	103	145	255	211	12
ATGAGTCTG	BRDN00011	306	291	2330	483	736	54
ATGAGTCTT	BRDN00011	108	120	50	337	340	75
ATGAGTGAA	BRDN00011	146	118	0	307	285	0
ATGAGTGAG	BRDN00012	164	112	371	317	431	8
ATGAGTGCA	BRDN00011	119	117	42	191	78	3
ATGAGTGCT	BRDN00011	68	23	1	888	474	0
ATGAGTGGA	BRDN00011	202	154	1122	478	734	0
ATGAGTGGC	BRDN00011	173	55	78	613	142	4
ATGAGTTAA	BRDN00011	184	126	262	287	501	7
ATGAGTTCT	BRDN00012	344	224	123	669	713	73
ATGAGTTTC	BRDN00011	284	211	60	390	256	0
ATGATAAAG	BRDN00012	256	354	102	413	544	29
ATGATAAGG	BRDN00012	194	186	248	405	209	39
ATGATAATA	BRDN00011	335	341	75	869	706	15
ATGATAATT	BRDN00011	129	117	66	310	372	7
ATGATACGA	BRDN00011	293	274	1429	638	978	48
ATGATACTC	BRDN00011	217	372	1627	531	395	41
ATGATACTG	BRDN00011	408	391	652	695	774	22
ATGATAGAC	BRDN00012	381	424	935	891	469	66
ATGATAGTA	BRDN00011	75	61	263	324	300	5
ATGATAGTG	BRDN00011	283	237	234	627	795	90
ATGATAGTG	BRDN00012	166	197	254	295	164	26
ATGATATCT	BRDN00012	510	675	3606	850	949	176
ATGATATCT	BRDN00012	495	386	891	794	903	40
ATGATATGG	BRDN00012	246	347	130	492	212	61
ATGATATTA	BRDN00011	59	70	514	156	203	14
ATGATCAAA	BRDN00011	204	501	750	378	210	101
ATGATCACG	BRDN00011	108	97	88	286	243	0
ATGATCAGA	BRDN00012	47	0	0	117	20	0
ATGATCAGA	BRDN00012	56	0	0	691	524	9
ATGATCAGT	BRDN00011	103	115	0	206	165	0
ATGATCATT	BRDN00011	145	166	249	344	228	0
ATGATCCAA	BRDN00011	211	221	1257	491	353	18
ATGATCCAG	BRDN00011	251	324	509	514	390	28
ATGATCCAG	BRDN00012	532	340	321	1016	906	5
ATGATCCAT	BRDN00012	65	80	1	235	190	14
ATGATCCCA	BRDN00011	103	103	769	314	240	35
ATGATCCGC	BRDN00012	266	303	266	650	747	11
ATGATCCTG	BRDN00012	183	1	0	426	451	0
ATGATCGAC	BRDN00011	274	96	241	1124	413	16
ATGATCGCA	BRDN00011	416	251	261	562	577	24
ATGATCGGT	BRDN00011	333	497	3631	733	475	110
ATGATCGTG	BRDN00012	10	0	18	418	89	0
ATGATCTAC	BRDN00011	105	114	55	202	221	0
ATGATCTCA	BRDN00012	148	166	171	300	240	3
ATGATCTGC	BRDN00011	164	0	0	259	95	0
ATGATCTGT	BRDN00012	135	143	8	380	555	40
ATGATCTTCT	BRDN00012	273	184	42	408	698	27
ATGATGAAA	BRDN00011	169	201	262	225	394	39
ATGATGAAC	BRDN00011	90	102	505	134	49	0
ATGATGAAC	BRDN00011	187	179	71	210	313	10
ATGATGAAC	BRDN00011	113	179	734	481	332	36
ATGATGAAT	BRDN00011	191	167	74	469	481	1
ATGATGACA	BRDN00011	295	280	353	545	393	71
ATGATGACA	BRDN00012	114	70	494	264	866	10
ATGATGACA	BRDN00011	199	98	0	242	389	6
ATGATGACA	BRDN00011	405	428	1147	857	905	22
ATGATGACG	BRDN00011	293	368	669	444	556	54
ATGATGACT	BRDN00011	267	366	95	366	594	16
ATGATGACT	BRDN00012	422	203	247	859	859	34
ATGATGAGA	BRDN00012	486	771	653	1122	771	56
ATGATGAGC	BRDN00012	145	104	85	270	256	0
ATGATGAGG	BRDN00012	341	292	171	695	438	44
ATGATGAGG	BRDN00012	506	321	366	933	840	36
ATGATGATA	BRDN00011	170	211	175	263	576	23
ATGATGATG	BRDN00011	313	139	2120	508	262	12
ATGATGCAG	BRDN00012	533	497	173	1023	1194	4
ATGATGCCA	BRDN00011	577	470	487	1207	1244	24
ATGATGCCA	BRDN00011	347	249	177	939	678	52
ATGATGCTG	BRDN00012	294	302	446	426	331	22
ATGATGCTG	BRDN00011	196	101	135	341	307	9
ATGATGCTT	BRDN00012	163	100	554	365	274	77
ATGATGGAC	BRDN00012	222	192	305	364	350	5
ATGATGGAT	BRDN00011	335	335	1439	793	420	51
ATGATGGAT	BRDN00011	306	271	1054	505	862	29
ATGATGGAT	BRDN00011	109	116	1633	186	230	54
ATGATGGAT	BRDN00011	92	62	59	201	214	0
ATGATGGAT	BRDN00011	110	148	2032	389	280	34
ATGATGGCA	BRDN00011	527	606	1866	1206	1765	40
ATGATGGCA	BRDN00012	231	337	240	635	486	44
ATGATGGCC	BRDN00012	151	179	1	370	163	9
ATGATGGCC	BRDN00011	123	54	9	245	256	25
ATGATGGCC	BRDN00012	293	378	5075	455	351	119
ATGATGGGA	BRDN00012	355	106	1981	794	1028	25
ATGATGGGC	BRDN00012	309	147	138	654	605	15
ATGATGGGG	BRDN00012	151	236	72	287	131	33
ATGATGGGG	BRDN00012	460	490	1003	933	1298	37
ATGATGGTA	BRDN00012	140	148	67	270	363	12
ATGATGGTG	BRDN00012	167	243	235	300	261	17
ATGATGTAA	BRDN00012	451	624	1181	973	972	25
ATGATGTAA	BRDN00011	367	288	788	828	461	20
ATGATGTAC	BRDN00011	67	0	0	498	855	0
ATGATGTAC	BRDN00012	555	426	2577	906	1441	151
ATGATGTCG	BRDN00012	241	20	4	597	621	0
ATGATGTCT	BRDN00011	355	317	103	558	657	30
ATGATGTGG	BRDN00011	116	53	1	289	220	0
ATGATGTTG	BRDN00011	429	430	1110	891	565	40
ATGATGTTG	BRDN00011	203	123	476	404	339	21
ATGATGTTT	BRDN00011	93	67	219	216	278	32
ATGATGTTT	BRDN00012	55	20	0	440	143	10
ATGATTAAT	BRDN00012	432	441	3429	859	694	64
ATGATTACT	BRDN00011	204	185	455	467	383	1252
ATGATTATG	BRDN00012	204	147	368	305	421	9
ATGATTCAA	BRDN00012	14	0	1	353	64	0
ATGATTCAG	BRDN00012	132	95	116	288	525	0
ATGATTGCA	BRDN00012	289	264	811	546	839	26
ATGATTGCC	BRDN00011	227	395	1921	408	423	128
ATGATTGGA	BRDN00012	482	393	352	957	1046	56
ATGATTGGA	BRDN00011	178	11	100	563	670	1
ATGATTGGC	BRDN00011	297	326	31	609	793	20
ATGATTGTG	BRDN00012	757	600	182	1093	1193	97
ATGATTGTG	BRDN00012	402	381	364	532	634	30
ATGATTTAA	BRDN00011	27	7	0	141	72	0
ATGCAAACC	BRDN00012	360	346	238	730	621	35
ATGCAAAGG	BRDN00011	117	118	383	295	184	2
ATGCAAAGT	BRDN00012	281	219	227	357	273	0
ATGCAAATA	BRDN00011	164	0	0	293	20	1
ATGCAAATA	BRDN00012	149	147	280	275	167	26
ATGCAACAA	BRDN00011	215	152	2577	295	327	89
ATGCAACAG	BRDN00011	128	82	76	121	243	21
ATGCAACTG	BRDN00012	181	179	33	293	564	58
ATGCAAGAC	BRDN00012	510	631	1175	946	754	26
ATGCAAGAG	BRDN00012	280	93	45	1106	1258	0
ATGCAAGAT	BRDN00012	238	206	827	401	203	9
ATGCAAGCT	BRDN00011	294	112	26	413	162	20
ATGCAAGTG	BRDN00011	192	91	124	266	471	0
ATGCAATAT	BRDN00011	232	201	520	619	721	4
ATGCAATCT	BRDN00012	327	332	103	590	980	28
ATGCAATGA	BRDN00012	133	2	0	552	468	0
ATGCAATGA	BRDN00011	93	125	23	278	185	0
ATGCAATGT	BRDN00011	237	119	308	439	145	0
ATGCAATTT	BRDN00012	464	305	381	670	1036	48
ATGCACAAA	BRDN00011	308	162	603	417	244	26
ATGCACAAG	BRDN00011	73	53	446	233	122	3
ATGCACAAT	BRDN00011	218	172	32	317	534	0
ATGCACAAT	BRDN00011	158	118	681	330	264	1
ATGCACAGA	BRDN00011	158	201	63	404	571	0
ATGCACAGC	BRDN00011	472	449	2744	991	650	84
ATGCACCTTA	BRDN00012	364	261	1132	498	563	43
ATGCACGCA	BRDN00012	184	141	9	398	296	0
ATGCACTAG	BRDN00011	315	286	931	746	896	19
ATGCACTCA	BRDN00012	683	646	2218	1202	1632	68
ATGCACTGG	BRDN00011	454	0	286	925	439	0
ATGCACTTG	BRDN00011	49	9	1	282	115	0
ATGCACTTG	BRDN00012	108	48	72	136	74	9
ATGCAGAAC	BRDN00012	174	169	144	211	194	24
ATGCAGAAC	BRDN00012	195	182	132	403	512	110
ATGCAGAAG	BRDN00012	220	35	0	484	318	8
ATGCAGAGT	BRDN00011	178	145	689	298	474	24
ATGCAGAGT	BRDN00012	200	210	152	454	368	8
ATGCAGATA	BRDN00011	114	81	0	222	317	0
ATGCAGATC	BRDN00011	197	161	233	407	415	7
ATGCAGCAG	BRDN00011	364	311	644	690	477	12
ATGCAGCAG	BRDN00012	96	167	5	190	268	5
ATGCAGCCA	BRDN00012	234	1	1	707	442	0
ATGCAGCTA	BRDN00012	156	86	165	268	389	0
ATGCAGCTC	BRDN00012	254	147	98	389	472	5
ATGCAGCTG	BRDN00012	178	202	478	491	433	3
ATGCAGCTG	BRDN00012	423	372	415	1445	1436	0
ATGCAGCTG	BRDN00012	94	137	158	168	164	5
ATGCAGGAC	BRDN00011	185	39	252	384	232	12
ATGCAGGAC	BRDN00012	522	9	4	1280	326	0
ATGCAGGCC	BRDN00011	114	162	0	168	279	20
ATGCAGGTC	BRDN00011	89	0	0	341	66	0
ATGCAGTAG	BRDN00012	149	82	308	652	655	10
ATGCAGTAT	BRDN00012	242	191	586	365	360	25
ATGCAGTCA	BRDN00012	401	310	593	881	670	52
ATGCAGTCC	BRDN00012	452	322	302	780	757	0
ATGCAGTCT	BRDN00012	228	31	2	807	936	0
ATGCAGTGA	BRDN00012	447	339	620	921	982	32
ATGCAGTGC	BRDN00012	398	542	509	779	1005	54
ATGCATACC	BRDN00012	372	315	539	786	884	25
ATGCATACC	BRDN00011	254	227	342	305	220	20
ATGCATACT	BRDN00012	601	557	1016	931	1268	49
ATGCATCAC	BRDN00012	450	269	99	850	907	22
ATGCATCAG	BRDN00011	511	377	1075	908	721	75
ATGCATCCA	BRDN00011	165	167	389	298	215	31
ATGCATCTA	BRDN00011	264	1	62	736	681	0
ATGCATGAA	BRDN00011	273	235	657	394	840	41
ATGCATGAT	BRDN00011	257	188	240	540	629	10
ATGCATGGA	BRDN00012	240	259	696	423	684	39
ATGCATTGC	BRDN00012	255	257	1468	557	170	67
ATGCATTTG	BRDN00012	148	51	720	607	514	27
ATGCCAAAT	BRDN00011	270	248	459	298	465	2
ATGCCAAAT	BRDN00011	227	103	14	334	192	31
ATGCCAACA	BRDN00011	160	131	56	224	185	3
ATGCCAACT	BRDN00012	484	406	4636	1070	992	137
ATGCCAACT	BRDN00011	184	106	355	295	163	6
ATGCCAAGC	BRDN00011	534	527	2338	1205	1402	81
ATGCCACAA	BRDN00012	132	263	3098	454	868	148
ATGCCACTG	BRDN00011	211	240	14	379	625	9
ATGCCAGAA	BRDN00012	139	218	132	397	93	38
ATGCCAGCC	BRDN00011	77	63	34	199	232	36
ATGCCAGCT	BRDN00011	375	420	1294	956	1122	62
ATGCCAGTA	BRDN00011	176	99	109	397	283	62
ATGCCAGTA	BRDN00011	171	259	16	367	444	5
ATGCCAGTG	BRDN00012	284	212	188	497	454	5
ATGCCAGTT	BRDN00011	559	470	303	747	628	39
ATGCCATAC	BRDN00012	642	682	1701	1352	1508	69
ATGCCATAG	BRDN00012	114	49	1036	472	513	6
ATGCCATAT	BRDN00011	178	242	686	325	261	12
ATGCCATCA	BRDN00011	399	320	2140	685	662	93
ATGCCATCC	BRDN00011	403	446	104	637	759	26
ATGCCATGA	BRDN00011	173	211	2115	432	381	78
ATGCCATTG	BRDN00012	189	177	0	359	297	1
ATGCCCAAA	BRDN00011	194	139	942	359	94	30
ATGCCCAAA	BRDN00012	242	288	261	425	471	25
ATGCCCAAG	BRDN00011	132	79	19	226	132	5
ATGCCCAAT	BRDN00012	652	178	157	1109	1149	8
ATGCCCATC	BRDN00011	388	277	259	689	585	17
ATGCCCATG	BRDN00011	485	369	2760	794	1057	46
ATGCCCCATC	BRDN00011	178	137	1099	263	340	18
ATGCCCCGT	BRDN00011	159	252	104	501	974	35
ATGCCCCTTA	BRDN00011	528	594	6570	1019	685	166
ATGCCCGAG	BRDN00012	29	20	0	384	195	0
ATGCCCGAT	BRDN00012	166	104	57	329	255	18
ATGCCCGAT	BRDN00012	329	312	181	678	567	43
ATGCCCGCC	BRDN00012	216	151	96	461	348	49
ATGCCCGGG	BRDN00011	83	81	1111	240	251	15
ATGCCCGTA	BRDN00011	348	375	1127	709	1012	44
ATGCCCTCTA	BRDN00011	129	24	0	368	370	0
ATGCCGAGT	BRDN00012	430	341	1086	601	811	48
ATGCCGCAA	BRDN00012	245	177	70	405	309	0
ATGCCGCAG	BRDN00011	126	174	7	353	412	12
ATGCCGTAA	BRDN00011	142	4	0	186	256	0
ATGCCGTGC	BRDN00011	381	429	235	635	578	30
ATGCCGTGC	BRDN00011	432	481	527	764	438	8
ATGCCGTGT	BRDN00011	100	0	0	397	385	0
ATGCCTAAC	BRDN00011	139	0	0	516	345	0
ATGCCTAAG	BRDN00011	375	356	827	465	629	64
ATGCCTACC	BRDN00012	210	6	1	573	338	0
ATGCCTAGC	BRDN00012	471	381	1316	855	596	23
ATGCCTATG	BRDN00011	267	266	159	776	982	40
ATGCCTCACT	BRDN00012	381	365	1071	660	896	41
ATGCCTCCTC	BRDN00011	281	80	1595	734	664	56
ATGCCTCTA	BRDN00011	369	229	454	507	944	25
ATGCCTCTG	BRDN00011	136	0	1	223	290	1
ATGCCTTAA	BRDN00012	395	567	820	918	1144	206
ATGCCTTAG	BRDN00012	554	472	264	1092	947	25
ATGCCTTCA	BRDN00012	455	338	115	754	751	0
ATGCCTTCAT	BRDN00011	287	234	410	522	431	4
ATGCCTTCC	BRDN00012	44	58	0	221	164	23
ATGCCTTCG	BRDN00012	214	183	287	385	455	15
ATGCCTTGA	BRDN00012	157	47	0	528	304	0
ATGCCTTGG	BRDN00012	214	168	307	400	659	19
ATGCCTTTCA	BRDN00012	143	170	351	408	263	19
ATGCCTTTG	BRDN00012	181	185	185	347	582	21
ATGCGAAAC	BRDN00012	338	367	490	586	468	49
ATGCGAGAC	BRDN00011	280	208	816	491	547	22
ATGCGATCC	BRDN00012	192	119	297	224	264	3
ATGCGCAGC	BRDN00011	535	191	156	1073	697	1
ATGCGCAGC	BRDN00012	475	437	372	839	1072	74
ATGCGCCCT	BRDN00012	538	107	522	891	1206	28
ATGCGCGCG	BRDN00012	523	438	1098	930	602	21
ATGCGCTCC	BRDN00012	175	48	388	424	476	3
ATGCGCTGG	BRDN00012	92	84	51	993	770	6
ATGCGCTGG	BRDN00012	536	307	885	1172	845	9
ATGCGCTTT	BRDN00012	519	524	780	792	812	0
ATGCGGATG	BRDN00012	205	226	316	364	426	0
ATGCGGATG	BRDN00011	99	90	11	263	172	0
ATGCGGATG	BRDN00012	96	7	0	320	139	0
ATGCGGCGC	BRDN00011	162	81	161	296	713	18
ATGCGGGAC	BRDN00012	212	131	44	273	368	7
ATGCGGGAC	BRDN00011	146	134	330	329	345	35
ATGCGGGGT	BRDN00011	269	16	6	605	204	0
ATGCGGGTC	BRDN00011	144	53	1	261	118	5
ATGCGGTCT	BRDN00012	240	167	460	386	454	46
ATGCGGTGG	BRDN00011	10	0	0	209	91	0
ATGCGTAAT	BRDN00011	136	209	10	355	702	33
ATGCGTACC	BRDN00012	135	205	386	375	550	28
ATGCGTATC	BRDN00012	253	0	6	647	978	12
ATGCGTCAC	BRDN00012	223	271	222	467	220	18
ATGCGTCCA	BRDN00012	538	482	853	974	967	51
ATGCGTGAT	BRDN00011	369	324	348	667	673	22
ATGCGTGGC	BRDN00012	117	150	325	209	242	141
ATGCGTTGT	BRDN00012	259	299	785	317	324	33
ATGCTAAAA	BRDN00011	125	111	177	253	479	0
ATGCTAAAT	BRDN00011	131	24	0	163	151	0
ATGCTAAAG	BRDN00011	284	27	0	575	1405	0
ATGCTAAGA	BRDN00011	172	188	238	284	266	13
ATGCTAAGT	BRDN00011	219	91	54	264	123	0
ATGCTAATT	BRDN00011	49	0	0	143	87	4
ATGCTACAC	BRDN00011	272	201	1015	483	610	129
ATGCTACGT	BRDN00012	128	150	211	250	136	7
ATGCTAGAA	BRDN00012	160	128	24	205	227	4
ATGCTAGAC	BRDN00011	181	190	25	220	269	10
ATGCTAGGA	BRDN00011	155	113	1273	329	368	34
ATGCTAGGA	BRDN00011	278	277	1360	561	499	55
ATGCTAGGT	BRDN00012	217	63	0	359	275	0
ATGCTAGGT	BRDN00012	126	41	93	222	258	9
ATGCTATGA	BRDN00012	106	79	683	194	180	4
ATGCTATGT	BRDN00012	151	18	0	419	267	0
ATGCTATTA	BRDN00011	219	44	3878	236	186	58
ATGCTATTTA	BRDN00012	129	128	55	260	596	0
ATGCTCAAC	BRDN00011	448	439	372	888	660	0
ATGCTCAGA	BRDN00011	181	43	122	819	743	0
ATGCTCAGA	BRDN00011	184	188	120	312	301	30
ATGCTCAGG	BRDN00012	147	143	185	401	643	8
ATGCTCATTC	BRDN00011	148	165	134	260	310	11
ATGCTCCAG	BRDN00012	175	134	0	266	260	5
ATGCTCCTTA	BRDN00012	197	136	181	267	247	0
ATGCTCCTTA	BRDN00011	69	17	25	288	258	0
ATGCTCGAA	BRDN00011	147	126	37	304	189	15
ATGCTCGAT	BRDN00012	247	209	368	690	531	17
ATGCTCTCC	BRDN00011	308	192	146	488	376	12
ATGCTGAAA	BRDN00011	512	266	986	1031	806	9
ATGCTGAAC	BRDN00011	386	377	860	797	788	18
ATGCTGAAC	BRDN00011	191	125	1332	397	681	62
ATGCTGAAG	BRDN00011	148	69	94	354	231	4
ATGCTGAAT	BRDN00011	159	49	49	285	348	0
ATGCTGAAT	BRDN00011	132	208	43	299	103	8
ATGCTGACA	BRDN00012	348	312	565	585	639	37
ATGCTGAGG	BRDN00011	334	350	818	683	781	173
ATGCTGATG	BRDN00012	128	80	7	260	415	4
ATGCTGCAG	BRDN00012	322	197	10	349	583	5
ATGCTGCAG	BRDN00012	711	602	1225	1309	1039	77
ATGCTGCCC	BRDN00012	460	480	763	965	1178	13
ATGCTGCCC	BRDN00012	183	86	166	259	226	25
ATGCTGCCT	BRDN00011	130	19	0	228	211	7
ATGCTGCGA	BRDN00012	383	404	269	576	419	96
ATGCTGCTG	BRDN00011	656	343	3333	1368	1370	74
ATGCTGGAC	BRDN00012	607	58	224	1388	1188	4
ATGCTGGAC	BRDN00011	249	177	784	325	332	12
ATGCTGGCC	BRDN00012	227	138	87	351	281	8
ATGCTGGGC	BRDN00012	222	0	31	842	224	0
ATGCTGGGG	BRDN00011	193	228	119	433	864	10
ATGCTGGGT	BRDN00012	484	502	320	738	1071	84
ATGCTGTAA	BRDN00011	139	87	0	217	249	0
ATGCTGTAC	BRDN00011	222	63	78	402	293	40
ATGCTGTAT	BRDN00011	164	316	137	270	344	3
ATGCTGTCA	BRDN00011	259	145	153	394	189	51
ATGCTGTCG	BRDN00011	198	110	233	432	334	33
ATGCTGTGG	BRDN00011	209	121	69	230	349	16
ATGCTGTGG	BRDN00012	159	0	0	792	149	0
ATGCTGTGT	BRDN00011	382	346	1091	545	1169	111
ATGCTTCCCT	BRDN00011	466	464	1045	843	636	40
ATGCTTCTTC	BRDN00011	193	268	355	325	408	25
ATGCTTGAA	BRDN00012	392	397	482	558	663	65
ATGCTTGCA	BRDN00012	386	483	460	864	870	53
ATGCTTGGC	BRDN00012	105	70	10	300	236	0
ATGCTTGTG	BRDN00011	288	32	27	675	407	0
ATGCTTTAAT	BRDN00011	268	246	130	393	329	49
ATGCTTTCTC	BRDN00012	181	134	184	356	240	13
ATGGAAAAC	BRDN00011	332	362	174	675	556	6
ATGGAAAAT	BRDN00011	293	267	832	564	605	41
ATGGAAACA	BRDN00012	357	190	628	472	823	64
ATGGAAACG	BRDN00012	425	327	390	919	816	27
ATGGAAAGA	BRDN00012	476	292	844	867	563	58
ATGGAAATA	BRDN00011	182	146	543	318	377	42
ATGGAAATC	BRDN00011	518	509	697	750	764	58
ATGGAAATC	BRDN00011	31	2	0	335	38	0
ATGGAACAA	BRDN00011	536	422	364	967	1252	16
ATGGAACAG	BRDN00012	187	151	111	326	221	14
ATGGAACAT	BRDN00012	87	25	18	198	37	1
ATGGAACTA	BRDN00011	111	124	102	263	181	14
ATGGAACTG	BRDN00012	422	418	1191	768	715	30
ATGGAACTG	BRDN00012	440	586	1719	946	1035	44
ATGGAAGAG	BRDN00012	517	585	700	878	1199	167
ATGGAAGGA	BRDN00012	145	22	1	205	105	0
ATGGAAGGC	BRDN00012	375	117	129	667	347	10
ATGGAAGTG	BRDN00012	114	151	63	199	167	13
ATGGAAGTG	BRDN00011	129	117	3633	271	228	20
ATGGAAGTT	BRDN00012	430	320	109	682	1065	28
ATGGAATCA	BRDN00011	203	130	483	405	509	0
ATGGAATCT	BRDN00012	197	179	144	399	450	30
ATGGAATGG	BRDN00012	237	162	439	230	299	2
ATGGAATGT	BRDN00012	98	0	0	326	224	0
ATGGACAAA	BRDN00011	193	157	631	471	437	0
ATGGACAAC	BRDN00012	656	645	1098	1106	897	89
ATGGACAAC	BRDN00012	262	273	263	434	338	40
ATGGACAAG	BRDN00011	91	164	183	208	119	0
ATGGACAAG	BRDN00012	526	599	3344	1284	858	123
ATGGACAAG	BRDN00011	116	230	373	372	232	19
ATGGACAAT	BRDN00012	294	340	363	487	432	29
ATGGACACA	BRDN00011	592	550	358	985	755	57
ATGGACACA	BRDN00012	184	181	80	447	416	0
ATGGACATA	BRDN00012	259	227	723	508	402	18
ATGGACATC	BRDN00012	197	124	13	437	370	0

ATGGACATG	BRDN0001	826	619	1933	1580	1461	60
ATGGACCAT	BRDN0001	244	189	1497	451	535	28
ATGGACCAT	BRDN0001	247	0	44	637	459	1
ATGGACCCC	BRDN0001	254	168	194	556	291	4
ATGGACCCC	BRDN0001	541	244	463	896	1190	8
ATGGACCTA	BRDN0001	129	223	502	289	162	18
ATGGACCTG	BRDN0001	179	198	1616	342	517	32
ATGGACGAA	BRDN0001	70	25	46	860	410	12
ATGGACGAG	BRDN0001	471	437	1210	798	1014	29
ATGGACGCA	BRDN0001	577	356	1266	1157	1089	11
ATGGACTCA	BRDN0001	530	380	835	821	677	93
ATGGACTGG	BRDN0001	249	134	1362	521	479	16
ATGGACTGG	BRDN0001	212	265	510	382	426	1
ATGGACTGT	BRDN0001	198	136	762	246	119	57
ATGGAGAAA	BRDN0001	186	131	97	358	945	0
ATGGAGAAC	BRDN0001	210	244	186	308	340	65
ATGGAGACA	BRDN0001	166	48	698	344	109	0
ATGGAGACA	BRDN0001	196	161	135	407	386	25
ATGGAGACT	BRDN0001	96	0	1	381	73	0
ATGGAGAGA	BRDN0001	73	0	0	331	78	0
ATGGAGAGA	BRDN0001	162	78	0	405	322	0
ATGGAGATA	BRDN0001	137	88	31	220	296	9
ATGGAGATA	BRDN0001	63	60	1	153	40	0
ATGGAGATC	BRDN0001	365	101	70	683	524	11
ATGGAGATG	BRDN0001	314	300	312	415	493	4
ATGGAGATG	BRDN0001	211	291	1101	372	547	6
ATGGAGCCA	BRDN0001	203	177	814	449	361	3
ATGGAGCCA	BRDN0001	326	357	803	573	450	82
ATGGAGCCC	BRDN0001	130	194	1289	238	84	51
ATGGAGCCC	BRDN0001	169	129	847	245	335	24
ATGGAGCCG	BRDN0001	388	249	1675	596	428	61
ATGGAGCCG	BRDN0001	202	216	117	535	351	6
ATGGAGCCT	BRDN0001	654	553	1971	1125	909	137
ATGGAGCGG	BRDN0001	170	143	114	324	330	0
ATGGAGGAC	BRDN0001	117	112	28	211	305	18
ATGGAGGAC	BRDN0001	233	123	111	452	468	0
ATGGAGGCA	BRDN0001	541	443	2414	953	1079	96
ATGGAGGCC	BRDN0001	179	127	788	358	247	23
ATGGAGGCT	BRDN0001	543	551	2892	808	935	118
ATGGAGGGA	BRDN0001	134	122	3	336	416	0
ATGGAGGTA	BRDN0001	257	23	1	639	809	0
ATGGAGGTA	BRDN0001	182	9	0	441	246	0
ATGGAGGTG	BRDN0001	312	262	259	456	260	101
ATGGAGTAA	BRDN0001	328	319	1398	520	748	36
ATGGAGTCA	BRDN0001	189	165	324	231	401	3
ATGGAGTCC	BRDN0001	160	178	337	268	277	41
ATGGAGTTC	BRDN0001	293	248	626	490	657	47
ATGGATAAA	BRDN0001	347	310	77	579	863	65
ATGGATACC	BRDN0001	393	447	1260	851	883	28
ATGGATACT	BRDN0001	441	437	294	761	863	28
ATGGATAGA	BRDN0001	382	351	919	765	636	56
ATGGATAGT	BRDN0001	197	319	157	427	585	20
ATGGATATA	BRDN0001	250	310	20	431	392	26
ATGGATATA	BRDN0001	175	179	350	292	412	3
ATGGATCAA	BRDN0001	467	457	315	863	1157	20
ATGGATCAC	BRDN0001	203	218	1	425	391	4
ATGGATCGA	BRDN0001	245	9	122	613	298	21
ATGGATCGC	BRDN0001	442	438	1317	776	1196	46
ATGGATCGT	BRDN0001	144	178	438	255	305	4
ATGGATCTG	BRDN0001	181	55	0	494	450	0
ATGGATGAC	BRDN0001	261	115	301	760	430	59
ATGGATGCA	BRDN0001	164	18	84	835	300	0
ATGGATGCC	BRDN0001	150	92	1	188	109	4
ATGGATGCG	BRDN0001	192	162	16	252	347	16
ATGGATGCG	BRDN0001	374	489	16060	756	820	172
ATGGATGCT	BRDN0001	338	10	2	977	699	0
ATGGATGGT	BRDN0001	377	469	2334	845	762	36
ATGGATGGT	BRDN0001	218	223	2674	309	300	39
ATGGATGTC	BRDN0001	388	389	1233	767	528	132
ATGGATGTG	BRDN0001	136	142	120	298	348	10
ATGGATGTG	BRDN0001	106	102	49	992	412	0
ATGGATTAA	BRDN0001	167	129	19	308	203	5
ATGGATTAC	BRDN0001	134	122	100	221	91	0
ATGGATTAC	BRDN0001	36	11	0	445	255	0
ATGGATTAT	BRDN0001	320	303	243	581	627	17
ATGGATTGA	BRDN0001	74	0	0	210	136	0
ATGGATTGC	BRDN0001	313	134	905	943	738	12
ATGGATTGC	BRDN0001	123	0	0	286	69	0
ATGGATTGG	BRDN0001	171	4	0	245	298	0
ATGGATTTA	BRDN0001	305	312	1608	705	680	76
ATGGATTTC	BRDN0001	341	271	230	615	612	37
ATGGCAAAA	BRDN0001	210	192	64	508	675	4
ATGGCAAAA	BRDN0001	436	333	227	805	760	146
ATGGCAAAG	BRDN0001	227	148	509	404	225	0
ATGGCAAAT	BRDN0001	174	121	31	262	90	1
ATGGCAAAT	BRDN0001	252	52	87	295	352	0
ATGGCAACA	BRDN0001	263	161	251	457	564	0
ATGGCAACT	BRDN0001	228	198	261	440	413	0
ATGGCAAGA	BRDN0001	218	0	0	428	287	1
ATGGCAAGG	BRDN0001	163	209	594	349	402	19
ATGGCAAGT	BRDN0001	322	164	227	646	694	2
ATGGCAAGT	BRDN0001	199	132	21	283	362	43
ATGGCAATC	BRDN0001	155	156	142	342	230	6
ATGGCAATG	BRDN0001	152	218	7	365	674	11
ATGGCAATG	BRDN0001	367	230	1060	659	639	38
ATGGCAATG	BRDN0001	419	414	4859	763	950	147
ATGGCACAC	BRDN0001	290	263	541	503	929	45
ATGGCACAG	BRDN0001	119	73	55	159	241	0
ATGGCACAT	BRDN0001	133	121	447	233	308	67
ATGGCACGA	BRDN0001	294	226	107	363	414	1
ATGGCACGG	BRDN0001	97	87	9	227	248	23
ATGGCAGAG	BRDN0001	154	224	224	414	465	19
ATGGCAGCA	BRDN0001	203	319	359	437	493	8
ATGGCAGCC	BRDN0001	623	685	1167	1024	1041	64
ATGGCAGCT	BRDN0001	107	49	164	214	575	4
ATGGCATAC	BRDN0001	184	157	345	347	385	13
ATGGCATCA	BRDN0001	383	429	223	658	843	17
ATGGCATCC	BRDN0001	141	78	11	182	141	0
ATGGCATCG	BRDN0001	241	92	82	547	440	10
ATGGCATGT	BRDN0001	121	136	496	277	180	19
ATGGCATTA	BRDN0001	105	104	135	368	412	6
ATGGCATTC	BRDN0001	362	253	659	612	669	72
ATGGCCAAA	BRDN0001	150	98	115	315	225	0
ATGGCCAAA	BRDN0001	98	68	0	178	166	2
ATGGCCAAA	BRDN0001	173	200	115	378	183	15
ATGGCCAAC	BRDN0001	152	133	1252	324	126	40
ATGGCCAAT	BRDN0001	402	409	1132	513	674	79
ATGGCCACG	BRDN0001	309	331	939	429	476	46
ATGGCCAGG	BRDN0001	325	1	1	993	1257	1
ATGGCCAGT	BRDN0001	178	177	415	274	272	50
ATGGCCAGT	BRDN0001	360	272	244	604	892	75
ATGGCCATA	BRDN0001	434	52	16	773	701	0
ATGGCCATA	BRDN0001	415	102	282	1007	687	1
ATGGCCATA	BRDN0001	533	661	860	1034	841	22
ATGGCCATG	BRDN0001	131	169	1394	308	231	93
ATGGCCATG	BRDN0001	193	367	5201	514	491	170
ATGGCCATT	BRDN0001	157	173	177	373	348	28
ATGGCCCAT	BRDN0001	128	23	85	265	97	0
ATGGCCCTG	BRDN0001	181	203	390	417	344	18
ATGGCCCTT	BRDN0001	189	160	138	462	418	11
ATGGCCGAA	BRDN0001	184	210	33	475	317	0
ATGGCCGCT	BRDN0001	535	601	284	1057	1174	81
ATGGCCGGG	BRDN0001	244	15	1	447	446	0
ATGGCCGTT	BRDN0001	142	114	178	182	150	5
ATGGCCGTT	BRDN0001	311	387	361	806	633	62
ATGGCCTAG	BRDN0001	225	208	573	516	692	12
ATGGCCTGG	BRDN0001	232	123	161	314	232	17
ATGGCCTTA	BRDN0001	411	329	2281	668	554	44
ATGGCGAAG	BRDN0001	233	41	8	361	238	0
ATGGCGACA	BRDN0001	217	92	399	447	327	0
ATGGCGACA	BRDN0001	299	207	259	450	638	0
ATGGCGACT	BRDN0001	61	0	0	201	35	0
ATGGCGATC	BRDN0001	315	341	122	806	1469	22
ATGGCGATG	BRDN0001	241	57	1	658	158	0
ATGGCGCAA	BRDN0001	88	98	60504	528	299	809
ATGGCGCAC	BRDN0001	392	337	374	992	950	10
ATGGCGCCT	BRDN0001	109	160	596	388	225	63
ATGGCGCTG	BRDN0001	313	247	703	521	471	98
ATGGCGGCC	BRDN0001	153	254	219	288	381	0
ATGGCGGGA	BRDN0001	277	182	169	450	938	17
ATGGCGGTT	BRDN0001	127	90	56	252	283	20
ATGGCGTAC	BRDN0001	68	6	140	357	230	0
ATGGCGTAG	BRDN0001	298	314	340	542	519	27
ATGGCGTCA	BRDN0001	124	134	76	252	210	21
ATGGCGTCC	BRDN0001	621	793	583	1305	964	56
ATGGCGTCG	BRDN0001	186	141	70	303	256	2
ATGGCGTGA	BRDN0001	193	201	204	353	184	6
ATGGCGTGC	BRDN0001	288	374	1604	516	602	62
ATGGCGTGG	BRDN0001	320	292	520	614	558	0
ATGGCGTGT	BRDN0001	49	83	332	256	251	6
ATGGCGTTG	BRDN0001	417	504	713	607	871	122
ATGGCTAAA	BRDN0001	108	16	0	309	68	1
ATGGCTAAG	BRDN0001	165	81	1	153	317	0
ATGGCTATT	BRDN0001	25	60	0	132	57	0
ATGGCTCAA	BRDN0001	212	51	140	307	185	1
ATGGCTCAG	BRDN0001	371	755	1320	855	557	48
ATGGCTCGC	BRDN0001	304	189	488	410	491	14
ATGGCTCGG	BRDN0001	362	231	645	576	691	24
ATGGCTCGG	BRDN0001	209	150	64	510	440	25
ATGGCTCGT	BRDN0001	198	55	87	426	525	6
ATGGCTCTC	BRDN0001	408	360	387	749	486	16
ATGGCTCTG	BRDN0001	413	388	362	693	669	59
ATGGCTGAG	BRDN0001	81	129	0	216	182	9
ATGGCTGAG	BRDN0001	93	0	0	618	388	0
ATGGCTGGG	BRDN0001	97	91	54	152	109	16
ATGGCTGGT	BRDN0001	273	184	332	303	314	11
ATGGCTGTG	BRDN0001	436	442	492	898	1130	52
ATGGCTTAG	BRDN0001	222	50	0	478	348	0
ATGGCTTCA	BRDN0001	304	298	531	470	540	28
ATGGCTTCA	BRDN0001	447	351	994	726	688	53
ATGGCTTTA	BRDN0001	181	98	67	378	577	0
ATGGCTTTC	BRDN0001	297	257	73	568	315	54
ATGGCTTTG	BRDN0001	240	26	0	686	697	0
ATGGGAAAG	BRDN0001	128	97	29	274	695	0
ATGGGAAAG	BRDN0001	481	213	53	797	1125	0
ATGGGAAAG	BRDN0001	113	44	41	281	123	0
ATGGGAAAT	BRDN0001	90	71	91	285	395	17
ATGGGAAGC	BRDN0001	186	296	2172	384	434	5
ATGGGAATG	BRDN0001	588	513	184	858	860	55
ATGGGACCC	BRDN0001	347	230	676	648	420	31
ATGGGACCC	BRDN0001	364	27	71	751	417	0
ATGGGACGC	BRDN0001	229	136	9	495	280	88
ATGGGAGAC	BRDN0001	238	164	242	903	538	0
ATGGGAGAC	BRDN0001	222	161	138	520	477	4
ATGGGAGAT	BRDN0001	431	320	994	712	813	25
ATGGGAGCA	BRDN0001	135	110	103	269	268	0
ATGGGATGA	BRDN0001	94	68	1	348	338	14
ATGGGATGC	BRDN0001	108	128	1697	214	125	30
ATGGGATGC	BRDN0001	219	91	19	363	201	15
ATGGGCAAG	BRDN0001	211	178	345	328	204	0
ATGGGCAAG	BRDN0001	94	113	4	269	514	0
ATGGGCACA	BRDN0001	135	86	1462	367	269	22
ATGGGCACA	BRDN0001	156	29	0	287	197	0
ATGGGCAGC	BRDN0001	217	155	463	703	533	25
ATGGGCAGC	BRDN0001	64	29	73	199	64	0
ATGGGCCAA	BRDN0001	502	409	41	903	918	29
ATGGGCCAC	BRDN0001	177	132	172	554	506	111
ATGGGCCAG	BRDN0001	140	94	494	261	167	16
ATGGGCCCA	BRDN0001	289	360	738	605	567	39
ATGGGCCCC	BRDN0001	332	245	866	615	741	126
ATGGGCCGG	BRDN0001	207	153	161	394	363	4
ATGGGCCTC	BRDN0001	43	88	102	422	80	0
ATGGGCGAC	BRDN0001	389	396	337	475	621	1
ATGGGCGAG	BRDN0001	181	30	5	440	364	0
ATGGGCGAT	BRDN0001	205	206	148	390	267	37
ATGGGCTAT	BRDN0001	345	435	545	768	516	19
ATGGGCTGC	BRDN0001	635	760	164	1132	987	23
ATGGGCTGG	BRDN0001	109	79	0	260	136	7
ATGGGCTTG	BRDN0001	162	185	1096	279	184	65
ATGGGGAAC	BRDN0001	138	95	5	223	158	0
ATGGGGAGC	BRDN0001	357	396	833	737	366	56
ATGGGGAGC	BRDN0001	443	468	218	993	894	5
ATGGGGATG	BRDN0001	53	0	0	355	64	0
ATGGGGATG	BRDN0001	354	439	915	547	354	86
ATGGGGCCT	BRDN0001	235	374	241	419	590	2
ATGGGGCTT	BRDN0001	267	238	445	611	417	14
ATGGGGGAC	BRDN0001	415	355	342	915	1052	36
ATGGGGGGG	BRDN0001	114	102	1038	278	234	51
ATGGGGTCA	BRDN0001	109	63	1	229	131	0
ATGGGGTCG	BRDN0001	227	144	1662	457	406	25
ATGGGGTGC	BRDN0001	103	103	448	143	186	52
ATGGGGTGC	BRDN0001	551	618	1107	1163	1103	374
ATGGGGTTC	BRDN0001	230	180	552	480	543	33
ATGGGTACG	BRDN0001	360	410	1043	611	296	64
ATGGGTATT	BRDN0001	278	321	545	601	571	46
ATGGGTCCA	BRDN0001	67	67	63	179	183	4
ATGGGTCCC	BRDN0001	147	85	51	312	215	5
ATGGGTCCT	BRDN0001	202	201	44	409	494	12
ATGGGTCGT	BRDN0001	203	273	392	305	433	89
ATGGGTCTT	BRDN0001	233	72	5	433	242	0
ATGGGTGAC	BRDN0001	233	223	502	383	409	19
ATGGGTGGA	BRDN0001	81	50	0	183	184	22
ATGGGTTAC	BRDN0001	291	322	610	434	494	25
ATGGGTTAC	BRDN0001	373	267	751	624	663	29
ATGGGTTAC	BRDN0001	242	157	901	534	516	25
ATGGGTTCC	BRDN0001	373	374	1058	1014	1112	29
ATGGTAAAA	BRDN0001	250	290	289	431	216	5
ATGGTACAA	BRDN0001	449	333	799	794	783	50
ATGGTACAC	BRDN0001	399	386	716	803	597	26
ATGGTACAT	BRDN0001	251	309	113	329	300	172
ATGGTAGAT	BRDN0001	341	3	0	902	751	0
ATGGTAGCG	BRDN0001	211	176	1103	388	384	24
ATGGTAGTG	BRDN0001	163	111	472	296	225	13
ATGGTATCA	BRDN0001	80	92	0	234	129	0
ATGGTCAAG	BRDN0001	510	178	63	1133	948	0
ATGGTCAAG	BRDN0001	237	95	635	415	214	30
ATGGTCAAG	BRDN0001	445	257	214	632	598	0
ATGGTCATG	BRDN0001	131	16	19	524	35	0
ATGGTCATT	BRDN0001	310	144	11	349	244	0
ATGGTCCAA	BRDN0001	456	464	721	762	577	117
ATGGTCCAA	BRDN0001	93	88	216	216	460	36
ATGGTCCAG	BRDN0001	76	75	93	148	156	8
ATGGTCCGC	BRDN0001	138	45	219	421	269	0
ATGGTCCTG	BRDN0001	435	322	398	818	757	6
ATGGTCGAC	BRDN0001	158	195	317	289	242	26
ATGGTCGTC	BRDN0001	288	424	350	749	750	11
ATGGTCGTG	BRDN0001	602	519	1154	926	1279	41
ATGGTCGTG	BRDN0001	207	148	83	289	187	6
ATGGTCTAC	BRDN0001	125	60	348	217	187	9
ATGGTCTCA	BRDN0001	237	258	291	438	621	2
ATGGTCTGC	BRDN0001	424	311	580	650	769	8
ATGGTCTGG	BRDN0001	162	144	308	203	454	16
ATGGTGAAA	BRDN0001	141	54	0	138	68	0
ATGGTGAAA	BRDN0001	314	369	657	694	434	32
ATGGTGAAA	BRDN0001	380	270	313	615	480	21
ATGGTGAAC	BRDN0001	313	156	1947	622	744	52
ATGGTGAAC	BRDN0001	257	294	545	570	607	37
ATGGTGAAG	BRDN0001	296	372	1661	541	562	35
ATGGTGAAT	BRDN0001	122	169	425	338	122	38
ATGGTGAAT	BRDN0001	134	218	18	268	243	22
ATGGTGAAT	BRDN0001	318	187	168	389	380	52
ATGGTGACA	BRDN0001	230	263	82	469	192	28
ATGGTGACC	BRDN0001	91	83	112	275	179	6
ATGGTGACT	BRDN0001	310	285	612	723	860	11
ATGGTGATC	BRDN0001	99	58	1	235	250	0
ATGGTGCCG	BRDN0001	207	157	210	641	362	13
ATGGTGCTA	BRDN0001	227	201	634	418	492	49
ATGGTGCTA	BRDN0001	680	462	251	1182	1229	42
ATGGTGCTG	BRDN0001	593	379	736	937	684	21
ATGGTGGAA	BRDN0001	201	270	9	419	395	5
ATGGTGGAC	BRDN0001	199	145	231	229	169	9
ATGGTGGCA	BRDN0001	115	45	148	250	193	0
ATGGTGGCC	BRDN0001	272	263	885	422	494	25
ATGGTGGCC	BRDN0001	58	49	0	76	28	0
ATGGTGGCT	BRDN0001	526	442	1145	1093	1154	15
ATGGTGGGA	BRDN0001	478	241	1465	757	740	50
ATGGTGGGT	BRDN0001	441	330	278	660	754	73
ATGGTGGGT	BRDN0001	343	323	365	639	664	28
ATGGTGGTA	BRDN0001	301	255	630	487	512	14
ATGGTGGTG	BRDN0001	220	211	148	405	873	43
ATGGTGTAA	BRDN0001	329	320	759	560	741	90
ATGGTGTAT	BRDN0001	261	158	507	426	295	8
ATGGTGTCA	BRDN0001	115	176	31	200	222	17
ATGGTGTCC	BRDN0001	406	289	654	508	923	23
ATGGTGTGG	BRDN0001	241	147	1536	453	470	11
ATGGTGTGT	BRDN0001	286	222	245	534	474	56
ATGGTGTGT	BRDN0001	95	20	13	341	330	0
ATGGTGTTA	BRDN0001	128	54	585	247	360	11
ATGGTGTTG	BRDN0001	310	122	233	534	823	3
ATGGTGTTT	BRDN0001	155	155	151	267	189	45
ATGGTTAAA	BRDN0001	240	170	259	282	206	7
ATGGTTATA	BRDN0001	287	271	464	469	666	77
ATGGTTATG	BRDN0001	298	174	716	366	553	21
ATGGTTCAG	BRDN0001	284	139	598	419	471	14
ATGGTTCCT	BRDN0001	172	158	48	290	292	25
ATGGTTCCT	BRDN0001	109	187	670	286	460	0
ATGGTTCTT	BRDN0001	170	174	373	398	244	25
ATGGTTCTT	BRDN0001	30	0	0	314	193	0
ATGGTTGGA	BRDN0001	129	112	52	309	362	19
ATGGTTGTA	BRDN0001	132	118	50	199	233	9
ATGGTTGTA	BRDN0001	108	162	108	273	162	3
ATGGTTTAG	BRDN0001	204	167	509	201	251	8
ATGGTTTGA	BRDN0001	73	34	111	287	116	0
ATGGTTTGC	BRDN0001	186	129	387	337	99	26
ATGGTTTGC	BRDN0001	335	266	182	674	683	34
ATGTAAAAT	BRDN0001	156	109	556	275	276	9
ATGTAAAAT	BRDN0001	81	172	91	144	233	1
ATGTAAAGC	BRDN0001	380	247	646	726	487	53
ATGTAAAGT	BRDN0001	282	292	66	367	273	16
ATGTAACAA	BRDN0001	119	72	48	168	243	66
ATGTAACAT	BRDN0001	90	9	0	445	374	0
ATGTAACCA	BRDN0001	99	167	416	320	102	2
ATGTAACCC	BRDN0001	313	352	87	684	550	1
ATGTAACGA	BRDN0001	730	469	1008	962	993	50
ATGTAAGCA	BRDN0001	120	65	113	202	178	0
ATGTAAGGG	BRDN0001	103	98	73	211	127	15
ATGTAATAG	BRDN0001	67	30	7	117	104	7
ATGTAATGA	BRDN0001	419	378	1353	656	857	32
ATGTAATGA	BRDN0001	110	147	4	250	165	63
ATGTAATGA	BRDN0001	333	357	1087	658	595	57
ATGTAATTC	BRDN0001	222	197	433	261	253	11
ATGTACAAC	BRDN0001	161	151	493	267	330	21
ATGTACACG	BRDN0001	449	84	492	860	1107	1
ATGTACAGC	BRDN0001	339	339	879	680	401	34
ATGTACAGC	BRDN0001	148	168	379	445	214	21
ATGTACAGG	BRDN0001	339	408	1134	692	693	89
ATGTACAGT	BRDN0001	610	646	596	1182	1777	55
ATGTACCAA	BRDN0001	429	615	1710	708	718	130
ATGTACCAG	BRDN0001	384	402	266	521	539	15
ATGTACCGA	BRDN0001	325	305	773	863	1048	72
ATGTACCTC	BRDN0001	323	274	68	514	545	38
ATGTACCTT	BRDN0001	315	237	953	711	1012	4
ATGTACGAG	BRDN0001	195	187	298	373	507	48
ATGTACGCA	BRDN0001	239	149	32	576	372	0
ATGTACGCT	BRDN0001	235	191	315	599	627	48
ATGTACGGC	BRDN0001	309	380	326	486	494	180
ATGTACGGG	BRDN0001	216	38	2	550	370	0
ATGTACTAC	BRDN0001	163	169	9	212	304	0
ATGTACTAG	BRDN0001	385	188	486	811	674	18
ATGTACTAT	BRDN0001	211	229	353	333	364	66
ATGTACTGA	BRDN0001	107	5	10	210	71	13
ATGTACTGC	BRDN0001	137	129	320	344	262	23
ATGTACTTG	BRDN0001	171	177	256	463	336	14
ATGTAGAGA	BRDN0001	123	233	1367	285	476	36
ATGTAGATC	BRDN0001	154	95	51	291	161	8
ATGTAGCAA	BRDN0001	454	691	567	943	1043	56
ATGTAGCCC	BRDN0001	177	81	590	353	237	5
ATGTAGCTG	BRDN0001	154	117	7	230	122	13
ATGTAGTCC	BRDN0001	180	114	273	379	351	12
ATGTAGTGG	BRDN0001	139	144	68	206	113	37
ATGTAGTGG	BRDN0001	93	93	4	252	93	9
ATGTAGTGT	BRDN0001	437	302	306	685	592	13
ATGTAGTTC	BRDN0001	154	139	43	307	181	0
ATGTATAAA	BRDN0001	74	31	167	188	136	0
ATGTATAAT	BRDN0001	109	135	932	328	296	27
ATGTATATA	BRDN0001	215	147	24	499	786	1
ATGTATCCA	BRDN0001	38	0	0	418	502	0
ATGTATCTG	BRDN0001	339	235	10	603	272	22
ATGTATGAT	BRDN0001	334	28	2	589	564	0
ATGTATGCA	BRDN0001	117	66	99	214	87	6
ATGTATGCA	BRDN0001	200	154	1023	377	237	22
ATGTATGGC	BRDN0001	169	55	0	529	348	0
ATGTATGTA	BRDN0001	252	99	237	427	300	6
ATGTATGTA	BRDN0001	639	518	1984	1120	1085	71
ATGTATGTC	BRDN0001	249	16	1	530	466	1
ATGTATTGC	BRDN0001	190	251	836	335	330	53
ATGTATTTCT	BRDN0001	150	119	0	300	193	1
ATGTATTTG	BRDN0001	267	225	433	597	834	23
ATGTCAAAA	BRDN0001	452	529	1890	826	1141	85
ATGTCAAAG	BRDN0001	197	151	34	351	386	48
ATGTCAACA	BRDN0001	647	448	1309	1188	1106	56
ATGTCAACC	BRDN0001	229	60	16	657	480	21
ATGTCAAGA	BRDN0001	296	210	67	405	491	6
ATGTCAATA	BRDN0001	350	466	301	702	543	33
ATGTCAATG	BRDN0001	165	166	1521	334	289	33
ATGTCAATG	BRDN0001	284	325	1331	543	403	58
ATGTCACAC	BRDN0001	571	666	727	907	1289	38
ATGTCACCT	BRDN0001	142	200	377	228	148	10
ATGTCAGAA	BRDN0001	40	0	0	297	70	0
ATGTCAGAC	BRDN0001	323	122	145	530	801	0
ATGTCAGGA	BRDN0001	241	173	7526	439	355	127
ATGTCAGTG	BRDN0001	269	184	1145	633	629	8
ATGTCATAG	BRDN0001	78	62	100	192	107	5
ATGTCATCTA	BRDN0001	158	246	47	497	468	38
ATGTCATTA	BRDN0001	240	324	106	573	520	19
ATGTCCAAA	BRDN0001	298	258	398	498	511	22
ATGTCCAAG	BRDN0001	238	188	234	450	742	23
ATGTCCACA	BRDN0001	599	415	2946	700	694	89
ATGTCCACA	BRDN0001	114	120	112	309	277	7
ATGTCCACC	BRDN0001	211	294	611	311	354	29
ATGTCCACC	BRDN0001	270	195	63	423	467	4
ATGTCCAGA	BRDN0001	418	514	479	869	646	15
ATGTCCAGA	BRDN0001	147	130	302	241	261	0
ATGTCCAGT	BRDN0001	267	14	0	988	296	0
ATGTCCATG	BRDN0001	331	61	31	727	655	0
ATGTCCCGG	BRDN0001	95	124	0	279	60	7
ATGTCCCGT	BRDN0001	283	493	1209	891	885	49
ATGTCCCTA	BRDN0001	125	57	229	244	417	0
ATGTCCGCT	BRDN0001	494	608	602	1172	1159	51
ATGTCCGTG	BRDN0001	101	50	290	272	470	25
ATGTCCTCG	BRDN0001	442	542	868	908	619	44
ATGTCCTGC	BRDN0001	250	199	445	368	298	42
ATGTCCTTG	BRDN0001	753	756	1166	1284	1189	41
ATGTCGAAA	BRDN0001	67	0	0	447	61	0
ATGTCGAGT	BRDN0001	220	185	291	328	288	40
ATGTCGCAG	BRDN0001	108	171	13	182	161	12
ATGTCGGAG	BRDN0001	262	175	161	421	550	4
ATGTCGGCG	BRDN0001	152	86	0	170	202	8
ATGTCGGGA	BRDN0001	101	62	0	227	281	0
ATGTCGTAG	BRDN0001	184	97	323	268	411	0
ATGTCGTCC	BRDN0001	373	403	814	614	538	23
ATGTCGTCG	BRDN0001	151	83	19	312	105	7
ATGTCGTCT	BRDN0001	86	11	19	180	31	0
ATGTCGTGG	BRDN0001	57	77	763	664	87	30
ATGTCTAAA	BRDN0001	224	174	80	367	484	11
ATGTCTAGA	BRDN0001	160	149	325	254	656	30
ATGTCTAGA	BRDN0001	677	469	1077	994	1158	82
ATGTCTATA	BRDN0001	256	120	556	360	273	22
ATGTCTATTC	BRDN0001	244	28	1446	329	532	53
ATGTCTCATG	BRDN0001	462	327	374	908	677	96
ATGTCTCCA	BRDN0001	256	148	234	361	551	7
ATGTCTCTG	BRDN0001	287	226	37	645	931	13
ATGTCTGAA	BRDN0001	876	589	7920	1150	891	125
ATGTCTGAG	BRDN0001	494	279	784	1065	875	35
ATGTCTGAG	BRDN0001	366	303	475	767	823	37
ATGTCTGCA	BRDN0001	209	146	174	393	365	51
ATGTCTGGC	BRDN0001	274	183	136	593	558	4
ATGTCTGGC	BRDN0001	101	20	6	308	165	0
ATGTCTGGG	BRDN0001	549	428	2131	1097	1125	75
ATGTCTGTC	BRDN0001	159	149	38	297	636	7
ATGTCTGTG	BRDN0001	236	272	464	477	596	5
ATGTCTTCA	BRDN0001	94	53	0	158	192	2
ATGTCTTCG	BRDN0001	291	235	89	428	685	13
ATGTCTTGA	BRDN0001	400	276	871	572	836	37
ATGTCTTGC	BRDN0001	499	790	518	1067	826	369

ATGTCTTTAG	BRDN00012	185	185	25	316	385	0
ATGTCTTTCT	BRDN00012	436	315	301	733	857	47
ATGTGAAAA	BRDN00011	284	167	7	366	551	9
ATGTGAAAG	BRDN00012	250	199	370	350	503	4
ATGTGAACA	BRDN00011	196	62	106	316	142	9
ATGTGAACA	BRDN00012	502	395	371	793	688	39
ATGTGAACG	BRDN00011	265	249	301	522	539	0
ATGTGAACG	BRDN00011	238	121	142	413	348	0
ATGTGAACT	BRDN00012	264	268	1208	471	562	128
ATGTGAAGC	BRDN00011	255	284	383	426	517	25
ATGTGACAA	BRDN00011	125	35	0	280	668	0
ATGTGACAA	BRDN00012	274	313	860	531	633	18
ATGTGACAG	BRDN00012	511	413	1314	832	915	61
ATGTGACCA	BRDN00011	316	364	512	625	564	137
ATGTGACCC	BRDN00012	94	18	17	433	421	0
ATGTGAGAA	BRDN00011	198	123	30	505	709	7
ATGTGAGAA	BRDN00011	101	113	1146	205	338	14
ATGTGAGCA	BRDN00012	180	363	344	401	314	14
ATGTGAGCC	BRDN00011	205	202	293	446	339	28
ATGTGAGTA	BRDN00011	198	88	114	351	216	0
ATGTGAGTG	BRDN00011	250	270	128	335	263	10
ATGTGATCA	BRDN00012	509	630	397	1048	1306	52
ATGTGATGA	BRDN00012	492	485	1825	1114	1012	136
ATGTGATTA	BRDN00012	224	252	113	526	708	17
ATGTGATTG	BRDN00011	178	168	161	227	137	7
ATGTGATTG	BRDN00011	171	202	339	249	368	7
ATGTGATTT	BRDN00011	158	237	139	341	288	37
ATGTGATTT	BRDN00011	364	495	896	680	598	34
ATGTGCAAG	BRDN00012	147	149	102	278	218	22
ATGTGCACA	BRDN00011	109	80	0	267	378	15
ATGTGCACA	BRDN00012	287	227	1776	576	523	28
ATGTGCACA	BRDN00012	275	220	884	484	777	55
ATGTGCACG	BRDN00011	366	341	550	639	842	25
ATGTGCACT	BRDN00011	440	1	2	894	889	0
ATGTGCACT	BRDN00011	233	283	22	425	283	18
ATGTGCAGC	BRDN00011	89	116	530	300	411	26
ATGTGCAGG	BRDN00012	176	169	247	396	346	4
ATGTGCAGG	BRDN00012	165	150	335	402	255	59
ATGTGCCAA	BRDN00012	415	514	1251	875	581	35
ATGTGCCAG	BRDN00012	401	396	210	667	609	22
ATGTGCCTG	BRDN00012	569	639	799	1019	1007	35
ATGTGCGAT	BRDN00012	151	167	227	269	363	8
ATGTGCGCT	BRDN00012	35	0	0	233	34	0
ATGTGCGGC	BRDN00012	447	450	1528	943	1531	43
ATGTGCGGG	BRDN00011	123	107	272	186	171	17
ATGTGCTAC	BRDN00012	269	412	505	697	794	41
ATGTGCTCA	BRDN00012	465	520	1070	761	960	88
ATGTGCTCA	BRDN00012	626	752	230	1182	1769	160
ATGTGCTGA	BRDN00011	455	537	713	1087	717	78
ATGTGCTGC	BRDN00012	533	604	248	983	944	35
ATGTGGAAA	BRDN00012	211	107	36	295	377	4
ATGTGGAAA	BRDN00012	186	153	385	405	168	11
ATGTGGAAT	BRDN00011	257	114	31	763	528	13
ATGTGGACA	BRDN00011	167	194	63	291	15	19
ATGTGGACC	BRDN00012	152	112	33	262	514	0
ATGTGGACC	BRDN00012	243	183	51	352	268	20
ATGTGGACT	BRDN00011	260	223	227	453	406	48
ATGTGGAGT	BRDN00012	342	335	2025	547	710	65
ATGTGGAGT	BRDN00012	219	282	120	299	397	40
ATGTGGATA	BRDN00011	465	401	898	763	386	1
ATGTGGATA	BRDN00012	467	497	1222	881	931	63
ATGTGGCAA	BRDN00012	168	189	179	374	319	59
ATGTGGCAC	BRDN00011	171	365	1	351	370	9
ATGTGGCAT	BRDN00012	204	156	2482	392	287	26
ATGTGGCTC	BRDN00012	576	510	92	1113	1250	61
ATGTGGCTG	BRDN00011	21	9	0	1218	149	0
ATGTGGGAA	BRDN00011	409	349	1628	709	623	91
ATGTGGGAA	BRDN00011	125	19	302	340	93	9
ATGTGGGAG	BRDN00012	435	438	390	724	1080	47
ATGTGGGAG	BRDN00012	348	256	323	540	525	13
ATGTGGGG	BRDN00012	270	383	322	505	355	48
ATGTGGTAA	BRDN00011	254	391	816	503	902	43
ATGTGGTAG	BRDN00012	94	104	12	244	63	5
ATGTGGTTC	BRDN00011	150	107	0	309	357	0
ATGTGTAAG	BRDN00011	271	127	472	435	567	9
ATGTGTACC	BRDN00011	157	143	299	205	130	60
ATGTGTACC	BRDN00012	380	363	86	640	313	41
ATGTGTCAA	BRDN00012	316	320	1086	502	461	86
ATGTGTCAG	BRDN00012	614	469	1360	1070	1327	68
ATGTGTCAT	BRDN00012	545	413	462	974	1369	59
ATGTGTCCG	BRDN00011	294	197	148	535	474	11
ATGTGTCGC	BRDN00012	246	186	117	317	688	0
ATGTGTCTA	BRDN00012	275	94	9	343	332	5
ATGTGTCTT	BRDN00012	475	418	1292	796	449	130
ATGTGTGAA	BRDN00011	128	68	103	451	374	0
ATGTGTGAT	BRDN00012	264	193	0	559	386	0
ATGTGTGAT	BRDN00011	134	234	382	347	324	26
ATGTGTGAT	BRDN00011	170	121	49	330	348	3
ATGTGTGTA	BRDN00012	351	302	831	618	434	58
ATGTGTGTA	BRDN00011	117	2	0	198	224	0
ATGTGTGTA	BRDN00012	407	355	186	745	560	0
ATGTGTGTA	BRDN00012	452	384	1090	731	810	65
ATGTGTGTC	BRDN00012	301	210	69	435	462	18
ATGTGTGTG	BRDN00012	215	303	393	372	434	8
ATGTGTGTT	BRDN00012	230	268	80	371	493	6
ATGTGTTTG	BRDN00012	386	301	1004	520	616	18
ATGTGTTTG	BRDN00011	282	421	388	617	599	37
ATGTTAACG	BRDN00012	104	41	6	577	556	0
ATGTTAACT	BRDN00012	680	462	1125	999	623	9
ATGTTAAGG	BRDN00012	442	129	61	821	861	0
ATGTTACCC	BRDN00012	85	1	0	471	480	0
ATGTTACCG	BRDN00011	224	6	1	531	954	0
ATGTTACGC	BRDN00011	114	112	0	238	375	7
ATGTTACTG	BRDN00011	204	208	146	492	277	24
ATGTTAGAT	BRDN00011	342	323	205	713	535	21
ATGTTAGAT	BRDN00011	165	150	63	417	458	12
ATGTTATAA	BRDN00012	118	86	144	280	180	12
ATGTTATTA	BRDN00012	581	451	1320	881	969	20
ATGTTCAAT	BRDN00012	155	174	121	302	211	4
ATGTTCACG	BRDN00012	284	393	187	641	776	46
ATGTTCAGA	BRDN00012	175	142	54	373	158	37
ATGTTCATG	BRDN00011	83	0	0	251	732	0
ATGTTCCCG	BRDN00012	201	195	218	529	443	1
ATGTTCCGG	BRDN00011	90	55	11	251	56	14
ATGTTCCTG	BRDN00011	186	168	388	201	187	7
ATGTTCGCA	BRDN00012	418	325	1950	619	657	37
ATGTTCTCA	BRDN00011	500	343	2096	759	577	31
ATGTTCTCAT	BRDN00011	538	457	689	1122	1204	26
ATGTTCTGT	BRDN00012	218	147	229	362	239	6
ATGTTCTTC	BRDN00012	533	501	736	752	1087	27
ATGTTCTTG	BRDN00011	137	0	0	290	194	0
ATGTTGAAT	BRDN00012	184	157	519	343	490	0
ATGTTGACA	BRDN00012	38	0	0	618	236	0
ATGTTGACT	BRDN00012	440	442	883	987	878	113
ATGTTGAGA	BRDN00011	256	377	1510	428	271	15
ATGTTGATG	BRDN00011	180	118	351	370	517	9
ATGTTGCCA	BRDN00012	581	602	1051	1211	1558	58
ATGTTGCCC	BRDN00012	449	429	41	791	685	37
ATGTTGCGG	BRDN00012	291	380	322	563	712	24
ATGTTGCTA	BRDN00012	144	105	31	309	266	0
ATGTTGCTA	BRDN00011	524	342	1855	775	856	92
ATGTTGCTG	BRDN00012	144	66	0	255	52	7
ATGTTGGAA	BRDN00012	58	0	0	213	154	0
ATGTTGGAA	BRDN00011	158	195	1325	265	314	39
ATGTTGGCA	BRDN00012	436	589	1006	907	927	102
ATGTTGGCT	BRDN00011	280	216	854	686	481	1
ATGTTGGGG	BRDN00012	215	26	104	493	290	0
ATGTTGGTG	BRDN00012	196	156	1172	259	185	9
ATGTTGTAA	BRDN00011	191	117	145	308	163	22
ATGTTGTCC	BRDN00011	205	220	987	369	355	26
ATGTTGTGT	BRDN00012	237	232	21	611	474	24
ATGTTGTGT	BRDN00011	303	245	3162	606	600	25
ATGTTGTTG	BRDN00012	401	517	780	774	849	43
ATGTTTACA	BRDN00011	471	421	1254	895	1290	61
ATGTTTACA	BRDN00011	281	392	622	432	304	37
ATGTTTACG	BRDN00011	216	147	283	401	287	5
ATGTTTATA	BRDN00011	100	121	430	220	135	0
ATGTTTCACC	BRDN00012	286	186	535	524	692	14
ATGTTTCAG	BRDN00012	403	437	740	606	727	20
ATGTTTCATT	BRDN00012	393	514	1318	970	1271	15
ATGTTTCCTC	BRDN00012	412	334	1318	773	554	55
ATGTTTCTCT	BRDN00011	235	229	44	381	456	19
ATGTTTGCA	BRDN00012	129	105	3	180	214	0
ATGTTTGCA	BRDN00011	238	200	205	384	318	20
ATGTTTGTC	BRDN00011	343	322	537	536	564	32
ATGTTTGTG	BRDN00011	381	298	172	565	587	0
ATTAAAAAA	BRDN00011	148	118	88	177	191	5
ATTAAAACA	BRDN00011	179	197	75	305	387	24
ATTAAAAGT	BRDN00011	82	53	49	115	137	11
ATTAAACGA	BRDN00012	551	553	852	704	836	72
ATTAAACGG	BRDN00012	223	246	233	378	672	32
ATTAAACTTA	BRDN00012	142	125	149	263	273	16
ATTAAAGAT	BRDN00012	168	181	11344	562	437	148
ATTAAATGT	BRDN00012	37	8	99	169	153	6
ATTAAATTCA	BRDN00012	128	6	512	273	462	14
ATTAACAAA	BRDN00012	207	136	350	306	266	17
ATTAACACTC	BRDN00012	309	299	2241	690	589	31
ATTAACAGG	BRDN00011	231	180	148	469	774	0
ATTAACAGT	BRDN00011	186	208	2652	309	185	28
ATTAACATA	BRDN00011	259	253	382	329	494	26
ATTAACTACT	BRDN00012	175	152	88	202	372	0
ATTAACTCG	BRDN00011	384	227	225	369	315	26
ATTAACTGA	BRDN00012	125	117	553	156	207	6
ATTAACTGG	BRDN00012	154	91	352	680	530	4
ATTAACTTG	BRDN00011	227	137	243	477	308	35
ATTAAGATT	BRDN00012	438	368	285	550	739	65
ATTAAGCGT	BRDN00012	185	87	296	298	194	8
ATTAAGGAG	BRDN00012	327	103	391	558	546	8
ATTAAGGCC	BRDN00012	544	28	0	1016	595	0
ATTAAGTAG	BRDN00012	148	50	42	411	488	0
ATTAAGTCG	BRDN00012	84	153	13	200	331	3
ATTAAGTGT	BRDN00012	321	306	356	582	994	18
ATTAAGTTA	BRDN00011	104	10	0	543	596	0
ATTAATAAC	BRDN00011	131	1	0	613	491	0
ATTAATAAG	BRDN00012	259	276	142	543	538	8
ATTAATACC	BRDN00012	341	278	192	596	738	57
ATTAATCCAT	BRDN00011	93	58	92	372	508	4
ATTAATCGA	BRDN00011	169	301	2197	291	282	73
ATTAATCTTA	BRDN00011	37	73	1236	163	45	23
ATTAATGAA	BRDN00012	353	283	1040	471	608	48
ATTAATTCCT	BRDN00011	291	227	611	499	517	54
ATTAATTGA	BRDN00011	199	1	0	431	295	0
ATTAATTTAC	BRDN00012	505	434	784	573	858	60
ATTAATTTCT	BRDN00011	174	187	30	261	200	5
ATTAATTTCT	BRDN00012	463	582	81	754	893	88
ATTACAAAA	BRDN00011	193	282	202	412	474	2
ATTACAAAC	BRDN00011	120	103	104	211	179	0
ATTACAAAG	BRDN00011	314	316	276	634	643	3
ATTACAAAG	BRDN00012	224	81	217	332	100	156
ATTACAAAG	BRDN00012	169	64	606	291	284	24
ATTACAAAT	BRDN00011	125	81	56	245	223	11
ATTACAAAT	BRDN00011	208	370	83	336	192	0
ATTACAAGA	BRDN00012	178	112	0	424	150	3
ATTACAAGA	BRDN00011	391	385	216	706	571	50
ATTACACATA	BRDN00011	164	348	6721	330	199	137
ATTACACCA	BRDN00011	366	268	251	505	648	11
ATTACACTG	BRDN00011	201	117	9	505	516	9
ATTACAGAC	BRDN00011	230	215	304	357	172	66
ATTACAGAC	BRDN00012	345	426	365	750	718	75
ATTACAGAG	BRDN00012	370	287	2309	527	245	18
ATTACAGAT	BRDN00012	159	126	215	305	250	28
ATTACAGCA	BRDN00011	105	57	517	194	185	31
ATTACAGGA	BRDN00011	253	95	279	627	636	10
ATTACAGGA	BRDN00011	236	295	403	553	798	18
ATTACAGGG	BRDN00011	36	0	0	332	273	0
ATTACAGGG	BRDN00012	194	214	1264	383	470	12
ATTACAGTA	BRDN00011	304	178	112	492	478	11
ATTACATACA	BRDN00012	71	9	0	289	165	0
ATTACATCAT	BRDN00012	365	445	523	629	583	31
ATTACCAAA	BRDN00012	230	8	1	429	545	0
ATTACCAAAT	BRDN00012	225	232	0	298	174	0
ATTACCAGA	BRDN00011	217	245	336	544	866	21
ATTACCAGC	BRDN00012	325	63	121	1166	394	1
ATTACCAGG	BRDN00012	83	5	1	491	429	0
ATTACCATTA	BRDN00011	394	58	0	1020	637	0
ATTACCCACA	BRDN00012	158	95	21	290	205	8
ATTACCTAAG	BRDN00011	384	338	218	605	678	57
ATTACCTATA	BRDN00011	52	6	0	239	309	0
ATTACCTCCA	BRDN00012	434	341	517	782	866	0
ATTACCTCTC	BRDN00012	360	253	325	671	976	8
ATTACCTGA	BRDN00011	145	238	1129	329	528	78
ATTACCTTAA	BRDN00012	204	246	1331	256	482	18
ATTACCTTAA	BRDN00011	161	186	0	292	162	6
ATTACCTTGC	BRDN00012	403	450	526	661	878	44
ATTACGAAG	BRDN00012	31	19	71	288	71	7
ATTACGAAG	BRDN00012	320	283	613	781	833	5
ATTACGACC	BRDN00012	503	387	3525	849	752	86
ATTACGATG	BRDN00011	137	99	148	272	209	0
ATTACGCAT	BRDN00011	148	80	0	303	228	0
ATTACGCTTA	BRDN00012	175	171	107	266	155	0
ATTACGGCT	BRDN00012	151	232	561	305	313	5
ATTACTACA	BRDN00011	243	220	305	555	478	12
ATTACTACAT	BRDN00012	172	161	90	225	164	1
ATTACTATG	BRDN00012	267	140	51	602	487	0
ATTACTGAG	BRDN00011	413	335	407	634	646	38
ATTACTGCG	BRDN00011	307	286	386	640	469	17
ATTACTGCG	BRDN00011	167	312	1281	460	280	105
ATTACTGGG	BRDN00012	207	255	126	349	400	15
ATTACTGGG	BRDN00011	138	59	11	298	224	0
ATTACTGGG	BRDN00012	214	226	1125	240	132	18
ATTACTTACA	BRDN00012	226	81	267	377	341	20
ATTACTTACT	BRDN00012	155	194	228	314	271	1
ATTAGAACC	BRDN00011	163	213	1	335	210	0
ATTAGAAGA	BRDN00011	201	147	99	427	332	0
ATTAGAAGC	BRDN00011	130	149	0	315	222	14
ATTAGAATA	BRDN00011	487	337	3205	427	600	51
ATTAGAATC	BRDN00012	109	136	20	292	220	18
ATTAGACAA	BRDN00011	130	101	16	184	220	45
ATTAGACAA	BRDN00011	438	383	735	507	508	7
ATTAGACCA	BRDN00012	91	0	24	313	382	0
ATTAGAGAA	BRDN00011	343	115	61	776	576	50
ATTAGAGAG	BRDN00012	316	229	3092	674	582	51
ATTAGAGAT	BRDN00011	206	63	15	259	347	6
ATTAGATCA	BRDN00012	165	245	23	245	263	26
ATTAGATGC	BRDN00012	367	353	221	649	487	2
ATTAGCACG	BRDN00012	605	616	1085	832	898	29
ATTAGCAGA	BRDN00011	187	104	381	346	658	4
ATTAGCAGC	BRDN00012	289	348	191	405	635	21
ATTAGCCAG	BRDN00012	78	100	33	813	917	17
ATTAGCCGA	BRDN00012	219	174	413	417	569	7
ATTAGCCGA	BRDN00012	160	163	260	300	598	7
ATTAGCCGT	BRDN00012	514	416	164	819	449	24
ATTAGCCTG	BRDN00011	122	95	176	315	144	16
ATTAGCTAC	BRDN00012	211	70	41	665	506	0
ATTAGCTTTA	BRDN00012	266	312	1575	563	1013	30
ATTAGGAAA	BRDN00011	164	163	13	290	284	27
ATTAGGACA	BRDN00012	385	525	1052	675	627	40
ATTAGGACC	BRDN00011	96	88	183	185	237	0
ATTAGGACC	BRDN00012	478	525	856	801	852	124
ATTAGGCCT	BRDN00012	217	271	58	391	399	23
ATTAGGGTA	BRDN00012	477	378	169	898	1289	64
ATTAGGTGG	BRDN00012	460	434	325	862	477	49
ATTAGTAAG	BRDN00011	342	237	299	508	457	49
ATTAGTAGT	BRDN00011	288	215	233	564	308	18
ATTAGTATC	BRDN00011	94	55	114	195	110	0
ATTAGTCCA	BRDN00011	130	80	2013	237	222	13
ATTAGTGCG	BRDN00012	339	29	53	626	190	0
ATTAGTGTG	BRDN00012	188	107	10	498	823	5
ATTAGTTCA	BRDN00012	200	189	14	441	695	24
ATTATAAAT	BRDN00012	131	157	301	288	382	11
ATTATAAAT	BRDN00012	107	122	776	160	153	30
ATTATAGAA	BRDN00012	372	240	299	655	608	19
ATTATAGGT	BRDN00012	408	318	4666	628	527	88
ATTATATAA	BRDN00011	129	49	0	168	160	0
ATTATATCTT	BRDN00012	85	110	15	229	200	0
ATTATATGA	BRDN00011	75	51	22	188	123	0
ATTATATGA	BRDN00011	93	159	122	262	327	25
ATTATATGG	BRDN00011	127	109	176	272	617	30
ATTATATTCA	BRDN00012	81	76	739	302	265	10
ATTATATTCT	BRDN00011	162	203	46	249	275	7
ATTATATTG	BRDN00011	236	345	388	519	338	32
ATTATCCAA	BRDN00011	197	75	84	366	399	1
ATTATCCAAC	BRDN00012	253	299	191	711	1060	29
ATTATCCGTG	BRDN00011	224	217	152	386	627	9
ATTATCGAG	BRDN00012	447	470	1959	1024	1689	63
ATTATCGCA	BRDN00011	251	221	755	706	679	41
ATTATCGGA	BRDN00012	194	6	1	883	731	1
ATTATCGTTC	BRDN00012	328	208	584	724	716	57
ATTATCTCCA	BRDN00012	480	309	436	559	825	48
ATTATCTCCT	BRDN00012	113	54	130	240	269	27
ATTATCTCTG	BRDN00012	189	267	197	348	560	22
ATTATCTGCA	BRDN00011	309	222	980	509	359	63
ATTATGAAC	BRDN00011	147	171	43	370	147	1
ATTATGAAC	BRDN00012	349	319	664	731	848	55
ATTATGACC	BRDN00011	184	105	2	377	861	0
ATTATGAGC	BRDN00012	76	94	89	202	101	9
ATTATGCAT	BRDN00011	195	220	507	349	312	10
ATTATGCCA	BRDN00012	198	132	62	309	264	9
ATTATGCCA	BRDN00012	227	204	1246	427	956	32
ATTATGCCG	BRDN00011	329	478	461	612	410	26
ATTATGCTTG	BRDN00012	250	62	18	764	753	9
ATTATGGAC	BRDN00011	108	88	52	170	125	0
ATTATGTCA	BRDN00012	250	237	472	338	366	16
ATTATGTGA	BRDN00012	111	111	31	311	216	41
ATTATGTGG	BRDN00012	249	291	65	581	442	30
ATTATGTTG	BRDN00012	293	199	173	531	374	14
ATTATTAATT	BRDN00012	89	30	1	198	105	0
ATTATTACCA	BRDN00012	188	139	304	301	366	0
ATTATTAGC	BRDN00011	409	255	945	895	1210	13
ATTATTATAG	BRDN00012	132	288	313	314	279	36
ATTATTATG	BRDN00011	171	206	9	320	328	8
ATTATTCAGT	BRDN00011	91	127	90	263	217	152
ATTATTCGTG	BRDN00012	157	154	223	312	332	8
ATTATTTACA	BRDN00012	283	258	661	473	340	49
ATTATTTCCG	BRDN00012	86	81	39	153	196	14
ATTCAAAAGT	BRDN00012	154	207	22	345	385	0
ATTCAAACG	BRDN00012	173	160	46	357	650	0
ATTCAAAGG	BRDN00012	137	104	545	269	227	21
ATTCAAATAT	BRDN00011	160	148	98	232	164	0
ATTCAACCAG	BRDN00012	402	721	398	931	1061	69
ATTCAACTTC	BRDN00012	80	51	21	309	58	13
ATTCAAGAA	BRDN00012	214	155	499	297	152	2
ATTCAAGAT	BRDN00011	451	432	478	703	595	15
ATTCAATAA	BRDN00011	99	33	23	342	282	0
ATTCAATTCT	BRDN00011	202	266	1327	375	281	24
ATTCAATTTA	BRDN00012	320	270	241	674	499	0
ATTCACAAGT	BRDN00012	220	280	257	560	639	20
ATTCACACTT	BRDN00011	492	145	236	857	783	8
ATTCACACTT	BRDN00012	403	307	87	811	852	20
ATTCACCAAC	BRDN00012	464	403	950	638	890	22
ATTCACCACC	BRDN00011	169	247	292	565	138	3
ATTCACCAG	BRDN00012	277	243	128	384	647	37
ATTCACCAGT	BRDN00011	77	25	5	297	241	16
ATTCACCCAG	BRDN00012	172	151	7	424	485	34
ATTCACGGA	BRDN00011	139	155	87	177	702	2
ATTCACTCGG	BRDN00012	192	218	355	567	547	6
ATTCACTGA	BRDN00011	148	201	1082	229	156	18
ATTCACTGG	BRDN00011	237	270	347	368	409	24
ATTCACTTCA	BRDN00011	385	364	1006	766	960	41
ATTCACTTTG	BRDN00011	210	268	845	432	464	0
ATTCAGAAG	BRDN00011	366	371	266	665	675	41
ATTCAGACA	BRDN00011	252	215	235	552	280	9
ATTCAGACAT	BRDN00012	493	642	707	722	1307	312
ATTCAGCAA	BRDN00011	332	452	1662	625	674	68
ATTCAGCAT	BRDN00011	245	333	254	320	375	23
ATTCAGCGC	BRDN00012	571	773	1278	1177	1564	62
ATTCAGCTG	BRDN00012	690	554	4411	1338	1508	43
ATTCAGGAT	BRDN00011	463	313	483	848	808	27
ATTCAGGGG	BRDN00011	93	117	370	272	71	30
ATTCAGGTA	BRDN00011	408	487	2913	720	1305	90
ATTCAGGTG	BRDN00011	191	2	221	493	486	1
ATTCAGGTT	BRDN00011	395	448	522	604	588	68
ATTCAGTAC	BRDN00011	83	0	0	512	189	0
ATTCAGTCTT	BRDN00011	77	60	13	197	245	8
ATTCAGTGT	BRDN00012	200	119	535	391	426	8
ATTCATACCA	BRDN00012	207	218	787	418	332	90
ATTCATACCG	BRDN00011	99	38	261	185	130	0
ATTCATACTA	BRDN00011	170	115	230	364	317	5
ATTCATAGA	BRDN00011	46	0	0	253	222	0
ATTCATAGA	BRDN00012	404	396	1637	767	1088	128
ATTCATAGT	BRDN00011	103	88	409	192	176	6
ATTCATCAAC	BRDN00012	285	46	102	707	667	0
ATTCATCCTG	BRDN00011	131	85	66	308	251	6
ATTCATCGA	BRDN00012	209	84	395	360	207	4
ATTCATCGTG	BRDN00011	200	94	521	506	419	0
ATTCATGCG	BRDN00012	586	607	1204	1119	1442	73
ATTCATGCTA	BRDN00012	102	106	50	300	370	0
ATTCATGGG	BRDN00012	206	192	1324	408	551	14
ATTCATGTG	BRDN00012	144	89	37	347	254	0
ATTCATTGG	BRDN00012	255	199	519	468	567	0
ATTCATTGG	BRDN00012	226	234	3506	364	432	53
ATTCATTTAC	BRDN00012	261	325	685	458	561	9
ATTCATTTAG	BRDN00012	11	0	25	149	63	0
ATTCCAAAA	BRDN00012	160	90	51	310	259	0
ATTCCAAAG	BRDN00012	313	374	516	801	920	25
ATTCCAAGA	BRDN00011	142	81	770	276	586	27
ATTCCAAGG	BRDN00012	335	440	3871	783	859	59
ATTCCAATAC	BRDN00011	161	145	408	252	168	6
ATTCCAATAG	BRDN00012	443	311	785	709	1401	151
ATTCCAATG	BRDN00011	188	278	424	408	312	37
ATTCCACACA	BRDN00011	126	89	275	251	329	1
ATTCCAGAA	BRDN00011	227	127	828	375	426	14
ATTCCAGAAT	BRDN00011	128	121	110	186	321	0
ATTCCAGAG	BRDN00011	278	210	140	322	326	11
ATTCCAGAT	BRDN00012	179	190	753	327	349	15
ATTCCAGAT	BRDN00011	106	38	10	287	128	13
ATTCCAGCTA	BRDN00011	358	447	1706	751	934	45
ATTCCAGCTC	BRDN00011	192	96	65	390	337	10
ATTCCAGCTG	BRDN00011	221	313	6458	402	541	138
ATTCCATCAA	BRDN00012	86	114	249	179	162	6
ATTCCCACAA	BRDN00012	238	136	307	548	441	42
ATTCCCACTA	BRDN00012	249	175	154	468	521	59
ATTCCCAGA	BRDN00012	114	127	0	244	261	34
ATTCCCAGAT	BRDN00011	626	440	613	954	774	27
ATTCCCAGCC	BRDN00012	191	126	628	290	198	9
ATTCCCAGG	BRDN00011	309	272	4575	528	552	93
ATTCCCATAA	BRDN00011	493	543	615	791	700	45
ATTCCCATTA	BRDN00012	161	210	1460	258	428	32
ATTCCCCACT	BRDN00012	295	491	128	535	734	16
ATTCCCCCAA	BRDN00012	351	256	48	663	668	51
ATTCCCGACT	BRDN00012	267	454	543	540	736	33
ATTCCCGCAA	BRDN00012	144	122	138	245	305	31
ATTCCCGCG	BRDN00011	406	40	122	893	240	4
ATTCCCTACC	BRDN00011	231	210	320	383	675	41
ATTCCGAAG	BRDN00011	122	67	63	162	128	27
ATTCCGACTT	BRDN00012	125	108	144	289	356	6
ATTCCGAGA	BRDN00011	14	0	1	231	229	0
ATTCCGATG	BRDN00012	326	351	1427	858	825	23
ATTCCGCCTT	BRDN00012	228	114	82	526	67	10
ATTCCGCGT	BRDN00012	149	175	1229	281	147	8
ATTCCGTACT	BRDN00011	400	364	1367	707	705	52
ATTCCGTGT	BRDN00011	443	430	1507	973	718	77
ATTCCGTTAT	BRDN00011	147	239	237	371	460	54
ATTCCTACAA	BRDN00011	168	152	16	237	291	13
ATTCCTACTA	BRDN00012	387	502	290	648	379	58
ATTCCTACTT	BRDN00012	196	274	835	460	321	31
ATTCCTAGG	BRDN00012	152	136	121	324	441	0
ATTCCTCACA	BRDN00012	260	386	424	767	1290	95
ATTCCTCAGT	BRDN00012	405	403	2193	731	835	109
ATTCCTCATA	BRDN00011	171	126	1295	202	139	35
ATTCCTCATC	BRDN00011	271	1	1	548	371	0
ATTCCTCCCC	BRDN00012	171	159	274	318	197	6
ATTCCTCCTT	BRDN00012	150	0	0	509	616	0
ATTCCTCCTT	BRDN00012	160	165	15	497	383	31
ATTCCTCGAC	BRDN00011	237	207	259	469	470	24
ATTCCTCTCC	BRDN00012	208	168	1332	255	235	41
ATTCCTGATC	BRDN00012	126	11	0	207	150	0
ATTCCTGCTG	BRDN00011	344	386	1267	631	567	31
ATTCCTGCTG	BRDN00011	146	194	133	331	247	0
ATTCCTGGG	BRDN00012	319	352	246	671	657	9
ATTCCTGGTC	BRDN00011	167	93	1396	253	333	70
ATTCCTGTGT	BRDN00012	268	232	1210	520	411	44
ATTCCTTCGG	BRDN00012	471	495	965	843	927	88
ATTCCTTGG	BRDN00011	140	196	258	345	600	12
ATTCCTTGG	BRDN00011	101	71	87	201	155	0

ATTCCTTTAG	BRDN00011	255	92	109	502	296	1
ATTCCTTTAG	BRDN00011	146	45	1	234	202	0
ATTCCTTTCT	BRDN00012	221	86	137	351	507	0
ATTCCTTTGC	BRDN00012	231	142	70	375	409	0
ATTCGAACTT	BRDN00012	247	191	72	452	477	72
ATTCGAATG	BRDN00012	208	244	229	359	321	26
ATTCGAATTC	BRDN00011	357	340	701	660	823	25
ATTCGACATC	BRDN00012	232	285	230	426	742	7
ATTCGACCA	BRDN00012	292	228	64	771	490	1
ATTCGACCG	BRDN00011	325	388	474	669	642	120
ATTCGACGA	BRDN00011	277	272	467	463	346	37
ATTCGATAAT	BRDN00012	41	11	0	300	581	0
ATTCGATCA	BRDN00011	257	348	1166	528	419	14
ATTCGATGC	BRDN00012	296	331	466	469	517	0
ATTCGATGTC	BRDN00011	117	71	211	276	295	30
ATTCGATTG	BRDN00012	424	373	1133	865	608	94
ATTCGCAAA	BRDN00012	301	331	1545	466	474	87
ATTCGCACG	BRDN00011	307	299	350	463	561	26
ATTCGCAGA	BRDN00012	595	556	3083	1026	764	119
ATTCGCATAT	BRDN00012	53	17	0	661	295	0
ATTCGCATCT	BRDN00011	152	164	234	290	312	7
ATTCGCCAAC	BRDN00011	123	80	37	306	245	6
ATTCGCCATT	BRDN00012	568	462	2256	963	563	35
ATTCGCCGTT	BRDN00011	119	113	133	261	261	8
ATTCGCGGA	BRDN00012	276	178	26	703	270	0
ATTCGCGTCC	BRDN00012	207	154	745	368	212	11
ATTCGGAGC	BRDN00012	344	359	268	538	274	11
ATTCGGAGC	BRDN00011	319	165	1381	509	1225	45
ATTCGGATG	BRDN00012	143	50	15	254	206	0
ATTCGGCAG	BRDN00012	438	392	467	749	737	5
ATTCGGCCA	BRDN00011	332	496	535	501	647	21
ATTCGGCGC	BRDN00011	371	254	418	613	867	23
ATTCGGCGT	BRDN00011	402	301	607	536	527	57
ATTCGGGCA	BRDN00011	137	101	1993	338	279	31
ATTCGGGGG	BRDN00012	229	296	190	424	581	0
ATTCGGTGC	BRDN00012	307	332	264	449	310	13
ATTCGTAGTC	BRDN00011	174	158	200	309	165	43
ATTCGTCCAT	BRDN00011	309	253	397	504	320	4
ATTCGTGCTC	BRDN00012	294	199	211	472	451	28
ATTCTAAACT	BRDN00012	307	336	35	631	390	0
ATTCTAGACC	BRDN00012	20	27	0	422	531	0
ATTCTAGACC	BRDN00011	126	151	359	239	262	40
ATTCTAGAGT	BRDN00012	23	38	0	308	255	0
ATTCTAGCA	BRDN00012	153	276	459	380	350	54
ATTCTAGGA	BRDN00012	108	56	1013	290	229	11
ATTCTATGCC	BRDN00012	160	20	0	401	414	0
ATTCTATTAG	BRDN00012	161	203	233	449	418	10
ATTCTCAAAA	BRDN00012	228	118	364	395	69	26
ATTCTCAACA	BRDN00012	241	220	1084	542	483	118
ATTCTCAGTA	BRDN00011	225	154	212	411	695	6
ATTCTCAGTG	BRDN00011	324	332	931	662	708	39
ATTCTCATGC	BRDN00012	503	485	456	990	1168	97
ATTCTCATTG	BRDN00011	165	103	88	265	331	0
ATTCTCCACC	BRDN00012	115	165	297	317	352	0
ATTCTCCCTA	BRDN00012	287	440	1100	687	509	54
ATTCTCTATG	BRDN00011	431	533	3123	849	627	16
ATTCTCTCTC	BRDN00012	413	331	1640	632	640	37
ATTCTCTGAT	BRDN00012	154	91	135	160	209	8
ATTCTCTTAG	BRDN00012	320	10	0	768	469	0
ATTCTGAAAC	BRDN00011	68	9	2	74	71	22
ATTCTGACAC	BRDN00012	212	253	538	336	462	6
ATTCTGACTA	BRDN00011	409	449	106	675	703	76
ATTCTGATAC	BRDN00012	353	269	266	687	584	0
ATTCTGCAAC	BRDN00012	604	512	1294	1069	1160	51
ATTCTGCACA	BRDN00011	449	359	534	711	1069	141
ATTCTGCACA	BRDN00012	166	198	607	408	297	13
ATTCTGCATT	BRDN00012	631	596	1175	1014	691	32
ATTCTGCCTC	BRDN00011	172	116	680	313	152	39
ATTCTGGAA	BRDN00011	234	455	516	455	301	117
ATTCTGGCAT	BRDN00011	89	99	162	220	207	22
ATTCTGGGA	BRDN00012	32	20	608	256	72	14
ATTCTGTAGC	BRDN00011	211	73	191	299	483	0
ATTCTGTGAC	BRDN00012	213	0	20	526	173	0
ATTCTGTGG	BRDN00011	328	276	375	535	460	107
ATTCTGTGG	BRDN00012	307	254	199	347	302	9
ATTCTGTTAA	BRDN00011	398	435	292	844	495	20
ATTCTGTTAC	BRDN00012	394	85	95	784	934	0
ATTCTGTTAG	BRDN00011	182	124	723	234	331	27
ATTCTTAAAA	BRDN00011	291	220	140	461	553	73
ATTCTTAATT	BRDN00011	134	70	73	408	344	5
ATTCTTACAT	BRDN00011	317	387	2275	752	583	57
ATTCTTACGA	BRDN00012	378	349	133	601	429	11
ATTCTTACGT	BRDN00012	312	302	191	534	493	5
ATTCTTAGCC	BRDN00012	412	437	355	782	740	36
ATTCTTATAA	BRDN00011	52	17	196	292	140	0
ATTCTTCAGA	BRDN00012	234	182	80	388	421	7
ATTCTTCATA	BRDN00011	327	291	582	521	613	60
ATTCTTCCAA	BRDN00012	140	109	0	195	80	12
ATTCTTCCAC	BRDN00012	389	435	464	855	1067	82
ATTCTTCCGA	BRDN00011	283	156	269	346	319	10
ATTCTTCGAC	BRDN00011	120	91	24	247	156	0
ATTCTTGAAC	BRDN00012	218	215	529	416	376	52
ATTCTTGACA	BRDN00011	85	40	90	196	185	1
ATTCTTGGGT	BRDN00012	145	24	8	216	299	0
ATTCTTGGTT	BRDN00012	154	298	297	463	448	36
ATTCTTGTGA	BRDN00011	181	0	0	637	381	0
ATTGAAAAG	BRDN00012	410	464	2348	520	430	49
ATTGAAACGT	BRDN00011	96	38	1	242	327	0
ATTGAAACTA	BRDN00011	127	130	265	201	379	4
ATTGAAACTT	BRDN00011	187	224	571	493	426	39
ATTGAACTGA	BRDN00011	130	17	0	611	556	0
ATTGAACTTT	BRDN00012	296	235	384	555	882	6
ATTGAAGAC	BRDN00011	101	17	0	303	317	0
ATTGAAGAG	BRDN00011	208	272	2299	444	439	68
ATTGAAGCG	BRDN00011	126	260	289	436	274	12
ATTGAAGGA	BRDN00011	346	368	1347	566	650	27
ATTGAAGTG	BRDN00012	208	199	147	341	158	4
ATTGAATACT	BRDN00011	182	150	291	368	301	16
ATTGAATAG	BRDN00011	100	124	119	170	264	24
ATTGAATATC	BRDN00012	6	0	0	164	29	0
ATTGAATATC	BRDN00012	173	126	325	338	182	38
ATTGACAACC	BRDN00012	456	367	903	684	519	20
ATTGACAATC	BRDN00011	274	281	430	668	832	45
ATTGACACAC	BRDN00012	455	479	1415	775	1066	25
ATTGACACTT	BRDN00011	323	325	566	737	579	13
ATTGACAGA	BRDN00012	39	0	0	200	77	0
ATTGACAGC	BRDN00011	157	80	23	267	255	13
ATTGACAGC	BRDN00011	247	206	2384	486	560	34
ATTGACAGCT	BRDN00011	355	277	2645	645	854	84
ATTGACATCA	BRDN00012	213	191	444	341	331	22
ATTGACATGT	BRDN00011	110	24	84	624	442	0
ATTGACCACT	BRDN00011	424	468	268	472	481	138
ATTGACCAG	BRDN00012	372	354	600	720	712	43
ATTGACCGA	BRDN00012	98	111	184	213	148	1
ATTGACCTCT	BRDN00012	231	254	135	433	735	46
ATTGACGTCA	BRDN00011	169	231	186	276	107	21
ATTGACGTTC	BRDN00012	136	133	144	284	460	10
ATTGACTCCT	BRDN00011	216	270	249	372	294	25
ATTGACTTTA	BRDN00012	194	239	737	423	628	47
ATTGAGAATT	BRDN00012	393	463	1215	605	579	46
ATTGAGCAA	BRDN00011	88	191	147	357	161	4
ATTGAGCAG	BRDN00011	178	167	93	416	508	29
ATTGAGCGG	BRDN00011	85	98	44	207	62	27
ATTGAGGAA	BRDN00011	262	175	351	465	406	13
ATTGAGGAC	BRDN00011	332	279	1408	460	470	60
ATTGAGTAAT	BRDN00012	182	156	913	284	374	20
ATTGAGTACT	BRDN00011	367	322	990	448	157	36
ATTGAGTAG	BRDN00012	150	92	121	257	104	6
ATTGAGTCC	BRDN00012	260	220	331	500	213	0
ATTGATAAAT	BRDN00012	92	47	335	225	568	17
ATTGATAACC	BRDN00012	270	207	48	449	390	62
ATTGATACGA	BRDN00011	222	196	359	641	761	0
ATTGATACTA	BRDN00011	227	152	9	318	331	29
ATTGATACTT	BRDN00011	250	200	709	452	996	80
ATTGATAGC	BRDN00011	133	167	232	287	214	53
ATTGATCAAT	BRDN00012	121	36	118	460	567	8
ATTGATCACC	BRDN00012	101	99	67	286	104	4
ATTGATCAG	BRDN00011	109	174	848	228	243	6
ATTGATCCA	BRDN00012	89	1	1	265	325	0
ATTGATGAA	BRDN00012	201	28	1	442	417	0
ATTGATGAA	BRDN00011	404	289	403	698	869	82
ATTGATGAC	BRDN00011	222	178	416	402	320	6
ATTGATGACT	BRDN00012	202	164	927	351	581	9
ATTGATGAG	BRDN00012	75	139	38	243	199	0
ATTGATGCA	BRDN00011	100	106	21	229	168	27
ATTGATGTA	BRDN00011	119	91	295	278	398	7
ATTGATGTTC	BRDN00012	411	414	334	701	528	71
ATTGCAACAT	BRDN00011	352	319	189	825	1072	50
ATTGCAACTA	BRDN00011	102	60	0	183	169	0
ATTGCACTCA	BRDN00011	279	318	2501	570	558	74
ATTGCAGAA	BRDN00012	116	173	520	314	309	3
ATTGCAGCA	BRDN00012	98	0	47	303	191	0
ATTGCAGCA	BRDN00012	251	242	269	573	285	32
ATTGCAGCC	BRDN00011	141	57	274	231	216	0
ATTGCAGCG	BRDN00012	30	0	0	187	99	0
ATTGCAGGA	BRDN00012	135	116	80	287	327	6
ATTGCAGGT	BRDN00012	172	80	35	435	473	38
ATTGCATAA	BRDN00011	67	48	28	110	180	0
ATTGCATAT	BRDN00012	231	234	421	466	480	22
ATTGCATCAC	BRDN00012	209	328	168	375	910	23
ATTGCATCAT	BRDN00012	462	303	62	845	870	18
ATTGCATCAT	BRDN00012	166	148	111	293	330	31
ATTGCATCTA	BRDN00011	124	139	0	276	133	0
ATTGCATTCG	BRDN00012	449	403	2215	874	790	77
ATTGCCAAAT	BRDN00012	256	241	180	426	462	35
ATTGCCAACA	BRDN00012	215	252	1238	529	799	40
ATTGCCAATT	BRDN00012	198	186	229	294	252	14
ATTGCCACA	BRDN00012	114	14	31	602	724	0
ATTGCCACAC	BRDN00011	187	230	761	369	182	17
ATTGCCACCT	BRDN00011	184	179	73	267	364	9
ATTGCCAGC	BRDN00012	428	450	1727	759	503	73
ATTGCCATGC	BRDN00012	224	274	287	375	520	30
ATTGCCATTC	BRDN00011	97	6	28	222	352	0
ATTGCCCAA	BRDN00012	272	249	557	356	733	12
ATTGCCCATT	BRDN00011	316	0	0	627	308	0
ATTGCCCCCC	BRDN00011	21	0	0	379	265	0
ATTGCCCTGC	BRDN00012	118	156	231	282	107	13
ATTGCCGCG	BRDN00012	356	391	1671	456	1118	51
ATTGCCGTTA	BRDN00011	121	11	19	289	212	0
ATTGCCTAGA	BRDN00011	78	151	299	239	549	0
ATTGCCTGG	BRDN00011	40	33	38	230	70	0
ATTGCGAGG	BRDN00012	91	78	166	315	430	0
ATTGCGATTC	BRDN00012	124	112	126	284	206	0
ATTGCGCAAT	BRDN00012	452	356	1471	823	1863	70
ATTGCGCACC	BRDN00011	96	0	0	286	120	0
ATTGCGGTCT	BRDN00012	150	135	4	342	95	0
ATTGCGTACT	BRDN00012	107	129	1	264	432	8
ATTGCGTTA	BRDN00012	117	11	102	286	230	9
ATTGCGTTG	BRDN00012	187	96	129	330	345	0
ATTGCTACAC	BRDN00011	255	275	1134	531	418	4
ATTGCTACCA	BRDN00011	377	379	785	683	674	22
ATTGCTACCT	BRDN00012	671	774	4196	817	1096	159
ATTGCTCAA	BRDN00012	188	46	10	309	1244	0
ATTGCTCCAG	BRDN00011	142	34	371	367	504	8
ATTGCTCCGA	BRDN00012	253	158	55	382	566	4
ATTGCTCTCA	BRDN00011	155	124	903	354	310	20
ATTGCTCTGT	BRDN00012	330	442	1522	830	499	103
ATTGCTGCTC	BRDN00012	237	316	50	569	406	117
ATTGCTGGG	BRDN00011	133	151	439	184	182	12
ATTGCTGGT	BRDN00012	312	379	319	606	334	53
ATTGCTGTTA	BRDN00012	442	496	1473	677	863	14
ATTGCTTATA	BRDN00012	153	105	392	332	227	0
ATTGCTTCCA	BRDN00011	158	174	61	250	156	12
ATTGCTTCTG	BRDN00012	125	79	0	203	234	0
ATTGCTTGTC	BRDN00011	166	154	7	391	187	21
ATTGCTTTGT	BRDN00012	121	185	60	252	309	31
ATTGGAAAA	BRDN00012	236	256	196	424	514	8
ATTGGAAAC	BRDN00012	218	202	607	400	329	17
ATTGGAAAG	BRDN00012	251	208	298	638	375	2
ATTGGAACA	BRDN00011	167	124	177	334	327	42
ATTGGAATC	BRDN00011	108	6	0	426	0	0
ATTGGAATG	BRDN00012	261	157	41	385	316	0
ATTGGACAA	BRDN00012	46	18	0	256	190	0
ATTGGACTCA	BRDN00012	143	150	380	302	439	5
ATTGGACTCC	BRDN00011	168	88	1161	255	390	34
ATTGGACTCT	BRDN00011	129	173	712	354	226	211
ATTGGACTGT	BRDN00012	176	403	67	473	630	38
ATTGGAGCC	BRDN00011	148	87	0	255	80	0
ATTGGAGGA	BRDN00012	325	469	76	710	624	23
ATTGGAGGC	BRDN00012	57	0	1	321	26	0
ATTGGAGTA	BRDN00012	416	431	1826	819	916	15
ATTGGAGTA	BRDN00012	88	0	76	364	146	0
ATTGGATAAT	BRDN00011	36	0	0	172	14	0
ATTGGATAG	BRDN00011	149	308	98	355	349	12
ATTGGATAT	BRDN00011	209	91	10	409	700	0
ATTGGATCAT	BRDN00011	83	86	87	346	462	42
ATTGGATGC	BRDN00012	533	277	652	1188	1070	62
ATTGGATTCT	BRDN00012	130	9	0	338	316	0
ATTGGATTTC	BRDN00012	324	440	1588	525	444	82
ATTGGCAAG	BRDN00011	198	167	124	381	282	6
ATTGGCAAG	BRDN00011	153	180	44	213	288	23
ATTGGCACA	BRDN00011	127	45	0	553	433	22
ATTGGCACG	BRDN00011	52	99	1	145	228	0
ATTGGCAGA	BRDN00011	193	188	405	213	70	16
ATTGGCATA	BRDN00011	120	138	16	185	198	12
ATTGGCCAGT	BRDN00011	95	66	20	189	147	12
ATTGGCGAC	BRDN00012	234	179	134	346	399	14
ATTGGCGCA	BRDN00012	248	224	32	498	371	81
ATTGGCTACC	BRDN00011	282	201	153	576	400	0
ATTGGCTCG	BRDN00011	252	219	34	514	254	3
ATTGGCTGC	BRDN00011	481	380	504	909	983	10
ATTGGCTGG	BRDN00012	43	29	1	539	517	0
ATTGGCTGG	BRDN00012	144	196	118	333	369	1
ATTGGGAAA	BRDN00011	268	2	2	570	434	0
ATTGGGACC	BRDN00011	105	279	99	254	376	74
ATTGGGAGC	BRDN00011	157	190	0	371	357	5
ATTGGGATA	BRDN00012	102	58	0	204	208	0
ATTGGGATT	BRDN00012	255	283	541	581	623	23
ATTGGGCAG	BRDN00012	99	32	61	320	145	3
ATTGGGCAG	BRDN00012	169	109	160	324	383	27
ATTGGGCAT	BRDN00011	470	497	221	543	753	31
ATTGGGGAG	BRDN00011	120	95	8	228	236	35
ATTGGGGCA	BRDN00012	73	1	0	298	148	0
ATTGGGGTA	BRDN00011	176	118	151	201	101	30
ATTGGGGTC	BRDN00012	178	198	817	324	391	15
ATTGGGTTC	BRDN00012	153	151	113	265	378	20
ATTGGGTTG	BRDN00012	238	366	586	721	822	21
ATTGGTAAAT	BRDN00011	199	230	10	399	545	30
ATTGGTACCA	BRDN00011	101	64	194	218	359	5
ATTGGTCAA	BRDN00011	112	216	120	177	100	49
ATTGGTCCCC	BRDN00011	453	406	385	780	644	105
ATTGGTGAA	BRDN00012	66	135	17	173	161	10
ATTGGTGAA	BRDN00011	106	91	6	145	124	16
ATTGGTGCA	BRDN00012	200	118	348	362	306	0
ATTGGTGCC	BRDN00012	381	387	163	730	808	15
ATTGGTGGA	BRDN00011	367	318	1044	796	1356	34
ATTGGTGGC	BRDN00012	33	20	9	68	126	46
ATTGGTGGT	BRDN00012	185	74	212	235	338	16
ATTGGTGTT	BRDN00011	395	384	434	679	935	44
ATTGGTTACT	BRDN00011	154	163	118	228	191	7
ATTGGTTATA	BRDN00011	282	264	632	460	506	51
ATTGGTTCGA	BRDN00011	183	103	5	350	420	14
ATTGGTTGG	BRDN00011	170	258	1323	274	307	20
ATTGGTTGG	BRDN00011	420	216	670	485	469	71
ATTGTAAAA	BRDN00012	233	171	78	564	414	41
ATTGTAACTC	BRDN00012	176	222	862	227	308	27
ATTGTAAGA	BRDN00012	130	62	36	346	381	12
ATTGTAAGTA	BRDN00011	184	58	245	378	601	4
ATTGTAATTT	BRDN00011	181	120	0	310	238	38
ATTGTACCA	BRDN00012	134	130	720	223	192	48
ATTGTACCAC	BRDN00012	183	145	34	300	135	18
ATTGTACCAC	BRDN00012	166	100	0	231	161	5
ATTGTACCGT	BRDN00011	175	119	975	267	263	23
ATTGTAGAC	BRDN00012	110	108	587	209	273	0
ATTGTAGAC	BRDN00011	375	464	2606	707	708	36
ATTGTAGGT	BRDN00012	114	149	27	247	361	41
ATTGTATCAT	BRDN00012	387	430	813	658	624	49
ATTGTATGCC	BRDN00011	164	93	10	198	96	1
ATTGTATGG	BRDN00011	370	264	461	736	857	21
ATTGTATTTG	BRDN00012	174	320	78	331	435	44
ATTGTCAGAT	BRDN00011	92	237	32	228	162	9
ATTGTCCAAC	BRDN00011	184	109	128	309	313	7
ATTGTCCAGA	BRDN00011	149	218	236	231	278	149
ATTGTCCCCA	BRDN00011	380	356	304	644	772	39
ATTGTCCTGT	BRDN00012	193	238	1455	412	405	75
ATTGTCCTGT	BRDN00011	190	35	332	562	737	0
ATTGTCCTTT	BRDN00012	113	142	275	300	534	41
ATTGTCGAA	BRDN00011	188	90	170	252	504	0
ATTGTCGATC	BRDN00012	320	233	311	567	545	10
ATTGTCGCAC	BRDN00012	269	277	719	510	198	7
ATTGTCGTAC	BRDN00011	339	326	277	528	861	75
ATTGTCTACT	BRDN00012	295	431	939	613	781	15
ATTGTCTACT	BRDN00012	253	238	501	543	692	34
ATTGTCTCAG	BRDN00012	128	114	1	284	264	8
ATTGTCTTCA	BRDN00011	129	121	42	311	245	0
ATTGTGACA	BRDN00011	73	33	112	225	206	0
ATTGTGACTA	BRDN00012	355	323	289	703	624	84
ATTGTGATG	BRDN00011	128	129	0	334	118	0
ATTGTGATG	BRDN00011	328	41	12	588	568	0
ATTGTGCAA	BRDN00011	228	273	858	576	594	24
ATTGTGCAG	BRDN00011	154	11	0	439	111	0
ATTGTGCAG	BRDN00011	215	173	124	497	583	5
ATTGTGCATC	BRDN00012	201	146	257	275	244	19
ATTGTGCGCT	BRDN00012	279	204	1034	489	1058	66
ATTGTGCGG	BRDN00011	146	174	383	338	480	32
ATTGTGCTAT	BRDN00012	133	59	59	391	412	0
ATTGTGGAA	BRDN00011	88	193	96	246	465	0
ATTGTGGAA	BRDN00012	186	93	0	368	307	33
ATTGTGGAG	BRDN00011	313	230	771	391	418	19
ATTGTGGAT	BRDN00011	81	108	385	186	206	18
ATTGTGGGC	BRDN00012	407	343	551	644	1268	27
ATTGTGGGC	BRDN00011	180	54	1	264	245	0
ATTGTGGGT	BRDN00012	137	87	84	181	104	8
ATTGTGGGT	BRDN00012	672	588	2533	1160	1096	53
ATTGTGGGT	BRDN00011	183	146	1424	280	377	36
ATTGTGGTG	BRDN00011	199	166	4949	490	493	86
ATTGTGGTG	BRDN00011	55	75	0	127	99	0
ATTGTGGTT	BRDN00012	165	0	0	247	325	0
ATTGTGTAA	BRDN00011	183	160	4	463	478	0
ATTGTGTAAT	BRDN00011	279	303	479	454	460	15
ATTGTGTATC	BRDN00012	183	62	833	493	641	7
ATTGTGTGTT	BRDN00012	458	311	1098	713	1164	12
ATTGTGTTG	BRDN00012	518	344	115	918	1190	9
ATTGTGTTG	BRDN00012	285	282	894	608	466	61
ATTGTTAACA	BRDN00012	37	29	435	308	331	0
ATTGTTAAGT	BRDN00012	116	3	1	220	203	0
ATTGTTACTA	BRDN00011	229	8	0	434	299	0
ATTGTTACTT	BRDN00011	308	260	1093	623	664	28
ATTGTTAGAT	BRDN00011	36	12	12	302	347	9
ATTGTTATAC	BRDN00012	93	88	101	217	273	32
ATTGTTCAGC	BRDN00011	132	176	57	327	359	41
ATTGTTCCAA	BRDN00012	533	401	7241	867	807	67
ATTGTTCCCA	BRDN00012	198	211	262	369	203	0
ATTGTTCTTA	BRDN00011	342	254	478	681	900	62
ATTGTTGATC	BRDN00011	189	215	14	318	227	0
ATTGTTGGC	BRDN00012	283	230	1	392	403	28
ATTGTTGTAC	BRDN00012	183	121	342	358	286	20
ATTGTTGTG	BRDN00012	120	177	120	292	266	0
ATTGTTTCTC	BRDN00011	89	102	60	244	173	0
ATTGTTTGAC	BRDN00012	146	40	20	456	439	0
ATTGTTTGCC	BRDN00011	356	306	165	599	512	41
ATTTAAAATC	BRDN00011	99	71	35	124	137	8
ATTTAAACCG	BRDN00012	327	315	914	588	517	78
ATTTAAAGA	BRDN00011	219	191	102	477	489	2
ATTTAAATAC	BRDN00012	145	88	118	237	577	11
ATTTAACTCC	BRDN00012	307	309	675	641	719	10
ATTTAAGACC	BRDN00012	480	298	393	776	727	49
ATTTAAGATA	BRDN00011	158	50	21	330	357	6
ATTTAAGCG	BRDN00011	269	139	245	428	301	20
ATTTAAGTAA	BRDN00011	409	228	530	782	783	73
ATTTAATAGC	BRDN00011	207	139	182	345	364	5
ATTTAATGGA	BRDN00011	368	105	690	490	889	10
ATTTAATGG	BRDN00011	206	227	103	340	477	42
ATTTAATGGT	BRDN00012	155	0	0	731	258	0
ATTTAATTCC	BRDN00012	349	180	36	462	461	23
ATTTACAACT	BRDN00012	453	158	987	888	1178	12
ATTTACACAC	BRDN00011	119	31	10	271	327	1
ATTTACAGA	BRDN00012	76	117	144	266	216	9
ATTTACAGA	BRDN00011	325	327	757	422	562	8
ATTTACATAC	BRDN00012	145	143	201	333	254	15
ATTTACATTA	BRDN00011	174	248	1338	356	300	19
ATTTACCCTT	BRDN00011	321	186	198	481	618	10
ATTTACCTAA	BRDN00011	91	106	15	164	203	7
ATTTACGGA	BRDN00011	178	156	44	210	297	7
ATTTACTCAG	BRDN00012	473	449	994	928	915	60
ATTTACTGGC	BRDN00012	70	7	30	159	152	0
ATTTACTTAC	BRDN00011	95	151	26	297	136	0
ATTTACTTCA	BRDN00012	338	394	1146	799	703	113
ATTTACTTGC	BRDN00012	153	169	135	295	478	55
ATTTACTTTC	BRDN00011	252	199	587	427	575	0
ATTTAGAAA	BRDN00011	190	220	137	443	556	18
ATTTAGAAG	BRDN00012	97	73	1	184	102	0
ATTTAGACTC	BRDN00012	433	376	240	817	920	54
ATTTAGAGA	BRDN00011	163	101	783	228	124	36
ATTTAGATCA	BRDN00012	370	185	217	694	1022	10
ATTTAGATGA	BRDN00011	206	244	241	430	539	12
ATTTAGCATT	BRDN00011	272	289	649	468	839	21
ATTTAGCTTC	BRDN00011	262	248	0	619	489	11
ATTTAGGAAT	BRDN00011	244	175	799	387	754	20
ATTTAGGAT	BRDN00011	183	153	135	260	175	7
ATTTAGGGCT	BRDN00011	215	113	608	400	402	2
ATTTAGGGT	BRDN00011	108	59	150	213	79	5
ATTTAGTAAC	BRDN00012	475	488	1959	856	701	71
ATTTAGTAAT	BRDN00012	534	456	483	687	1189	72
ATTTAGTAGA	BRDN00012	519	521	442	852	823	64
ATTTAGTGA	BRDN00012	390	163	581	469	666	6
ATTTATAAAC	BRDN00012	336	458	292	555	304	126
ATTTATAAAC	BRDN00011	62	59	119	132	135	0
ATTTATATAG	BRDN00011	357	290	482	654	778	28
ATTTATATGC	BRDN00012	125	43	87	324	308	8
ATTTATCAAT	BRDN00012	342	342	120	613	414	46
ATTTATCAGA	BRDN00011	200	95	314	328	210	18
ATTTATCAGT	BRDN00011	243	8	0	520	495	0
ATTTATCATC	BRDN00011	116	166	451	281	414	25
ATTTATCATG	BRDN00012	505	639	433	922	1319	48
ATTTATCCCA	BRDN00012	325	394	204	606	373	16
ATTTATCCTA	BRDN00011	178	278	799	405	453	18
ATTTATCGTT	BRDN00012	221	161	676	417	332	25
ATTTATGGA	BRDN00011	177	181	263	221	195	33
ATTTATGTGC	BRDN00012	182	227	828	333	393	74
ATTTATTCCA	BRDN00011	345	355	1023	761	423	61
ATTTCAAAAC	BRDN00012	394	369	381	676	755	27
ATTTCAAGA	BRDN00012	232	271	143	692	505	21
ATTTCAAGA	BRDN00012	135	93	99	189	486	46
ATTTCACAGA	BRDN00012	181	112	249	469	631	12
ATTTCACCAG	BRDN00011	147	144	46	432	334	7
ATTTCACCCC	BRDN00012	100	82	376	240	538	12
ATTTCACCCT	BRDN00011	80	108	10	266	91	15
ATTTCACGCA	BRDN00012	768	490	1934	1157	1244	122
ATTTCAGATC	BRDN00011	316	159	378	530	685	14
ATTTCAGCCT	BRDN00011	277	245	329	642	537	9
ATTTCAGGA	BRDN00012	207	229	51	314	176	4
ATTTCAGGT	BRDN00012	131	92	187	205	224	13
ATTTCATCAA	BRDN00012	338	255	70	679	944	1
ATTTCATCAC	BRDN00012	323	203	1214	488	431	61
ATTTCATCTG	BRDN00012	203	250	827	372	284	9
ATTTCATGAC	BRDN00012	178	92	247	313	565	3
ATTTCATGCT	BRDN00012	148	186	166	268	337	0
ATTTCATGTA	BRDN00012	201	212	241	395	447	33
ATTTCATTGA	BRDN00011	338	276	1375	657	933	8
ATTTCCAAGA	BRDN00011	398	315	121	613	761	11
ATTTCCAAGT	BRDN00011	449	391	753	687	785	23
ATTTCCACAA	BRDN00011	165	220	4841	327	363	220
ATTTCCAGA	BRDN00011	105	61	141	239	315	11
ATTTCCAGAC	BRDN00011	170	133	495	359	329	31
ATTTCCAGG	BRDN00012	205	158	152	333	276	9
ATTTCCAGG	BRDN00011	311	265	884	553	601	2
ATTTCCAGTA	BRDN00011	229	328	513	412	375	34
ATTTCCATTC	BRDN00012	325	159	85	586	627	0
ATTTCCCACA	BRDN00011	206	138	479	422	394	21
ATTTCCCAGT	BRDN00012	126	91	196	102	261	35
ATTTCCCTAC	BRDN00012	540	540	1463	812	649	14
ATTTCCCTTT	BRDN00011	224	260	854	565	442	33
ATTTCCGTTC	BRDN00011	407	48	0	1058	594	0
ATTTCCTAGA	BRDN00012	232	152	915	431	412	16

ATTTCCTATA BRDN00012	289	357	1003	726	741	22
ATTTCCTGCT BRDN00011	474	369	268	765	1033	23
ATTTCCTTCA BRDN00011	49	0	0	435	151	0
ATTTCCTTCC BRDN00012	456	403	412	827	675	72
ATTTCCTTGG BRDN00011	37	0	0	321	22	0
ATTTCGGCCT BRDN00011	303	19	0	434	275	0
ATTTCGGTAC BRDN00012	303	304	63	758	575	26
ATTTCGTAAT BRDN00012	203	393	21	418	188	58
ATTTCTAAAG BRDN00012	202	200	101	439	558	19
ATTTCTACAG BRDN00012	491	316	418	741	815	37
ATTTCTACCG BRDN00012	231	309	148	288	346	57
ATTTCTAGA BRDN00012	216	46	23	378	382	9
ATTTCTATCC BRDN00012	353	283	918	604	409	35
ATTTCTCAGC BRDN00011	372	400	984	654	652	26
ATTTCTCCTA BRDN00011	150	116	54	239	101	18
ATTTCTCCTT BRDN00011	368	400	770	516	534	22
ATTTCTCGGA BRDN00012	167	38	282	554	496	20
ATTTCTCGGA BRDN00012	289	251	386	535	595	124
ATTTCTCGTA BRDN00011	184	81	58	348	392	13
ATTTCTCTTG BRDN00012	147	223	282	525	489	4
ATTTCTGAA BRDN00012	134	206	470	476	418	23
ATTTCTGACT BRDN00012	129	64	198	201	307	4
ATTTCTGCCA BRDN00012	207	121	132	621	521	35
ATTTCTGCTG BRDN00011	157	179	20	356	216	9
ATTTCTGGA BRDN00011	107	143	768	274	591	0
ATTTCTGGC BRDN00011	178	669	17	266	150	0
ATTTCTGTGA BRDN00012	184	148	229	321	548	9
ATTTCTGTGT BRDN00012	193	77	172	298	506	1
ATTTCTGTTG BRDN00012	290	315	775	666	515	82
ATTTCTTACC BRDN00012	230	277	147	453	302	35
ATTTCTTCAA BRDN00012	682	548	136	1085	1089	34
ATTTCTTGCG BRDN00012	464	497	1843	921	791	61
ATTTGAACG BRDN00012	461	425	412	733	962	77
ATTTGACAG BRDN00012	243	159	72	469	508	5
ATTTGACCG BRDN00011	287	61	251	662	319	0
ATTTGACCG BRDN00011	410	424	405	654	1192	6
ATTTGACGT BRDN00011	222	242	174	649	320	2
ATTTGACTA BRDN00012	188	89	79	293	387	5
ATTTGAGACT BRDN00011	188	3	15	311	177	0
ATTTGAGCA BRDN00011	117	128	27	279	277	4
ATTTGAGCC BRDN00011	315	176	181	414	290	6
ATTTGAGCC BRDN00011	503	331	981	1009	1151	41
ATTTGAGCC BRDN00011	391	379	234	800	1087	7
ATTTGAGCGT BRDN00011	255	106	467	494	464	23
ATTTGAGTGT BRDN00011	125	133	227	329	287	4
ATTTGATACC BRDN00012	177	167	0	389	485	0
ATTTGATCA BRDN00012	281	344	566	507	821	29
ATTTGATGA BRDN00011	563	477	1095	998	1069	9
ATTTGATGG BRDN00011	545	323	1644	927	831	49
ATTTGCACC BRDN00012	307	166	248	536	568	18
ATTTGCACC BRDN00012	378	440	642	622	626	36
ATTTGCACG BRDN00011	177	33	0	412	530	0
ATTTGCATCC BRDN00011	248	294	1977	646	396	55
ATTTGCATCC BRDN00011	114	165	438	233	491	25
ATTTGCATCG BRDN00012	531	740	1943	1043	965	50
ATTTGCCAG BRDN00012	298	59	105	348	507	0
ATTTGCCATA BRDN00012	576	596	459	993	1043	30
ATTTGCCCCA BRDN00012	159	107	252	341	345	12
ATTTGCCGA BRDN00011	101	19	37	231	169	0
ATTTGCCGTT BRDN00011	265	223	376	377	515	25
ATTTGCTATA BRDN00011	96	141	171	262	253	23
ATTTGCTATG BRDN00012	267	217	1023	529	281	57
ATTTGCTCCT BRDN00012	264	7	64	433	479	0
ATTTGCTCGT BRDN00011	455	368	457	994	925	18
ATTTGCTCTT BRDN00012	49	5	1	361	324	0
ATTTGCTGAT BRDN00011	188	98	8	320	237	8
ATTTGCTGG BRDN00012	563	506	1917	992	1232	98
ATTTGCTGG BRDN00012	211	170	91	367	476	0
ATTTGCTGT BRDN00011	129	105	156	268	455	14
ATTTGGAAG BRDN00012	252	268	95	596	395	38
ATTTGGAAT BRDN00011	287	234	185	454	465	17
ATTTGGAGT BRDN00011	196	132	115	518	540	0
ATTTGGCAT BRDN00012	322	423	702	642	533	33
ATTTGGCGA BRDN00011	375	419	288	861	932	40
ATTTGGCTA BRDN00011	123	113	71	287	269	10
ATTTGGGGA BRDN00011	405	425	155	813	1069	32
ATTTGGGGA BRDN00012	185	204	425	290	229	14
ATTTGGTGC BRDN00012	198	220	1001	510	510	94
ATTTGGTGT BRDN00012	409	433	884	861	1002	68
ATTTGGTTG BRDN00011	185	0	1	341	303	0
ATTTGTAAC BRDN00012	199	106	197	414	326	70
ATTTGTAAT BRDN00011	106	78	302	222	823	0
ATTTGTAGT BRDN00012	189	15	18	348	349	0
ATTTGTAGT BRDN00011	152	187	16	266	537	25
ATTTGTATG BRDN00012	172	69	78	460	593	6
ATTTGTATG BRDN00012	285	235	362	431	325	278
ATTTGTCAG BRDN00011	118	174	212	172	93	0
ATTTGTCGT BRDN00012	180	199	36	230	216	40
ATTTGTGAC BRDN00011	261	284	271	339	289	0
ATTTGTGAC BRDN00012	241	344	811	594	603	45
ATTTGTGAT BRDN00011	146	70	451	286	179	15
ATTTGTGCG BRDN00012	214	197	45	453	623	0
ATTTGTTACC BRDN00011	304	49	53	935	628	0
ATTTGTTGA BRDN00011	193	167	116	262	252	9
ATTTGTTGA BRDN00011	215	107	1	337	261	0
ATTTGTTTCA BRDN00011	252	143	7	397	328	44
ATTTGTTTGT BRDN00011	183	193	1	240	327	0
ATTTTCGAA BRDN00012	589	799	1945	996	2887	74
CAAAAAAGC BRDN00011	110	112	19	239	140	0
CAAAAAATA BRDN00012	254	279	858	425	445	61
CAAAAACAA BRDN00011	359	251	156	686	430	11
CAAAAACAA BRDN00011	324	290	608	583	627	64
CAAAAACAA BRDN00011	330	320	10	593	706	26
CAAAAACAC BRDN00012	361	473	852	571	760	14
CAAAAACAG BRDN00012	129	204	13	298	213	9
CAAAAACAG BRDN00012	144	101	6922	379	263	57
CAAAAACAG BRDN00011	174	273	472	377	408	26
CAAAAACCCT BRDN00011	131	127	725	309	240	21
CAAAAACCG BRDN00012	198	189	537	250	143	14
CAAAAACCTT BRDN00011	169	234	261	347	411	27
CAAAAACGT BRDN00011	201	77	5	265	580	12
CAAAAACTCA BRDN00011	167	243	455	262	476	3
CAAAAACTG BRDN00011	25	9	0	273	66	0
CAAAAAGAA BRDN00011	192	186	1437	359	593	43
CAAAAAGAA BRDN00011	119	94	2	134	166	0
CAAAAAGAA BRDN00011	187	132	858	337	344	56
CAAAAAGAA BRDN00011	196	464	818	538	411	62
CAAAAAGAA BRDN00011	157	43	46	375	363	0
CAAAAAGAA BRDN00011	270	223	307	505	372	22
CAAAAAGAA BRDN00012	225	109	72	457	316	17
CAAAAAGCC BRDN00011	152	228	47	375	371	41
CAAAAAGGC BRDN00012	597	413	1294	915	1290	39
CAAAAAGTA BRDN00012	271	190	592	486	355	97
CAAAAAGTG BRDN00011	138	129	544	212	366	3
CAAAAAGTT BRDN00011	323	210	97	632	412	11
CAAAAAGTT BRDN00012	121	130	16	128	119	5
CAAAAAGTT BRDN00012	139	226	29	413	204	53
CAAAAATGC BRDN00012	178	112	395	256	213	15
CAAAAATTG BRDN00012	378	72	89	900	813	17
CAAAACAAA BRDN00012	357	274	330	592	522	51
CAAAACACG BRDN00012	294	241	749	389	455	26
CAAAACAGA BRDN00012	288	312	776	579	266	56
CAAAACAGA BRDN00012	247	471	478	505	505	15
CAAAACAGC BRDN00012	183	100	302	303	347	32
CAAAACAGG BRDN00012	125	89	156	235	402	20
CAAAACATA BRDN00012	366	171	376	777	876	18
CAAAACATT BRDN00011	142	10	0	399	151	0
CAAAACCAA BRDN00012	460	296	409	753	567	27
CAAAACCAG BRDN00011	317	272	868	611	490	48
CAAAACCCTA BRDN00012	554	186	204	664	657	15
CAAAACCTA BRDN00012	563	376	977	944	781	22
CAAAACCTCA BRDN00012	441	438	562	738	513	68
CAAAACGAA BRDN00011	226	185	691	483	331	62
CAAAACGAT BRDN00011	328	332	497	657	813	64
CAAAACGCA BRDN00011	245	117	339	404	300	57
CAAAACGGG BRDN00012	388	519	784	692	921	73
CAAAACGGG BRDN00011	98	142	2282	197	555	54
CAAAACGGT BRDN00011	27	28	1	134	48	0
CAAAACTAAT BRDN00012	73	106	136	239	578	0
CAAAACTCA BRDN00012	527	293	42	708	557	24
CAAAACTCCT BRDN00011	162	121	830	319	164	16
CAAAACTGA BRDN00012	183	235	67	362	367	20
CAAAACTGA BRDN00012	28	0	0	178	125	0
CAAAACTTA BRDN00012	438	326	270	800	1228	24
CAAAACTTTA BRDN00011	159	127	122	300	156	0
CAAAAGACA BRDN00012	114	123	72	94	131	0
CAAAAGATA BRDN00012	260	126	144	447	357	17
CAAAAGCAA BRDN00012	268	417	1694	456	494	106
CAAAAGCAA BRDN00012	234	223	107	343	295	6
CAAAAGCAC BRDN00011	210	140	138	265	154	420
CAAAAGCAG BRDN00011	126	23	0	149	154	1
CAAAAGCAT BRDN00012	146	45	0	121	29	0
CAAAAGCAT BRDN00011	57	18	0	218	292	0
CAAAAGCCA BRDN00012	150	51	8	248	197	0
CAAAAGCCA BRDN00011	396	398	332	902	725	9
CAAAAGCCG BRDN00012	147	96	67	245	181	0
CAAAAGCCG BRDN00011	117	149	141	172	191	15
CAAAAGCGT BRDN00012	322	216	217	356	235	19
CAAAAGCTC BRDN00011	277	221	400	646	625	63
CAAAAGGAA BRDN00012	162	191	0	489	416	42
CAAAAGGAA BRDN00011	213	141	348	372	400	7
CAAAAGGAT BRDN00012	333	375	726	620	1032	16
CAAAAGGCC BRDN00011	77	99	349	109	158	4
CAAAAGGGA BRDN00012	313	231	67	685	837	0
CAAAAGGTA BRDN00011	359	419	2701	846	976	45
CAAAAGTAA BRDN00011	197	169	251	247	515	16
CAAAAGTCA BRDN00011	258	318	205	597	550	0
CAAAAGTCT BRDN00012	637	425	443	1025	1698	47
CAAAAGTGA BRDN00012	131	368	49148	306	285	1230
CAAAAGTGT BRDN00012	168	31	0	195	297	10
CAAAAGTGT BRDN00011	38	67	7	123	156	26
CAAAAGTTA BRDN00011	218	215	658	374	362	2
CAAAATAAG BRDN00012	130	0	0	286	248	0
CAAAATAAT BRDN00011	224	210	13	270	526	0
CAAAATAAT BRDN00012	310	344	537	566	413	33
CAAAATACA BRDN00011	120	53	125	238	408	5
CAAAATAGA BRDN00012	219	154	391	331	379	6
CAAAATAGG BRDN00011	117	82	0	195	114	0
CAAAATAGT BRDN00011	149	48	84	202	249	0
CAAAATATA BRDN00011	112	55	331	99	75	19
CAAAATATT BRDN00011	160	123	191	432	149	0
CAAAATCAA BRDN00011	176	257	17	338	729	64
CAAAATCACT BRDN00011	319	246	283	444	368	28
CAAAATCAG BRDN00011	119	112	160	253	561	32
CAAAATCAG BRDN00011	171	171	769	401	302	0
CAAAATCGA BRDN00011	60	11	2	182	306	0
CAAAATCTA BRDN00012	280	478	2241	592	428	68
CAAAATCTAT BRDN00012	264	167	287	444	391	6
CAAAATCTCA BRDN00012	112	29	0	251	670	21
CAAAATCTTG BRDN00012	381	563	369	730	850	43
CAAAATGAT BRDN00012	488	279	329	857	746	21
CAAAATGCA BRDN00012	340	295	147	595	516	20
CAAAATGGG BRDN00012	149	192	164	266	189	12
CAAAATGGT BRDN00011	21	0	0	730	867	0
CAAAATGTT BRDN00012	506	684	2742	667	1134	1108
CAAAATTAG BRDN00012	250	218	433	382	668	30
CAAAATTCA BRDN00011	210	167	64	382	255	40
CAAAATTCCA BRDN00012	199	25	13	403	471	0
CAAAATTGC BRDN00012	137	93	104	192	171	0
CAAACAAAA BRDN00011	293	195	173	422	586	34
CAAACAAAC BRDN00012	314	283	1355	567	481	36
CAAACAAAC BRDN00012	501	542	541	857	855	54
CAAACAAAT BRDN00012	195	289	118	471	475	34
CAAACAACAT BRDN00012	68	110	48	221	165	6
CAAACAACAT BRDN00012	153	109	2178	401	364	29
CAAACAACTT BRDN00011	221	21	73	388	372	0
CAAACAAGA BRDN00011	101	82	140	203	147	5
CAAACAAGA BRDN00011	149	91	743	325	315	30
CAAACAAGG BRDN00012	175	134	121	380	384	9
CAAACACAA BRDN00012	131	148	152	401	275	0
CAAACACAC BRDN00012	409	535	98	626	687	82
CAAACACACC BRDN00011	192	0	0	716	930	0
CAAACACAG BRDN00012	141	237	664	271	233	26
CAAACACAG BRDN00011	301	196	293	527	284	13
CAAACACCA BRDN00012	213	200	1144	536	374	4
CAAACACCA BRDN00011	166	161	429	343	122	1
CAAACACCA BRDN00012	219	201	238	319	119	0
CAAACACCAT BRDN00012	132	28	0	621	360	0
CAAACACGA BRDN00012	87	84	188	288	126	67
CAAACACGG BRDN00012	142	163	169	282	312	0
CAAACACGT BRDN00012	195	86	85	296	67	0
CAAACACGT BRDN00012	124	153	11	211	204	17
CAAACACTA BRDN00012	127	175	0	221	717	0
CAAACACTAT BRDN00011	157	84	228	364	104	35
CAAACACTCA BRDN00011	219	283	521	512	443	15
CAAACACTG BRDN00012	380	417	198	616	532	98
CAAACACTTA BRDN00012	307	288	284	593	474	13
CAAACAGAC BRDN00011	262	153	462	487	202	62
CAAACAGAT BRDN00012	158	120	176	343	218	6
CAAACAGCA BRDN00011	258	206	115	757	771	18
CAAACAGCG BRDN00012	78	85	78	106	52	1
CAAACAGCT BRDN00011	185	83	327	294	223	1
CAAACAGGC BRDN00012	204	187	1060	165	131	37
CAAACAGGG BRDN00012	75	106	21	202	71	5
CAAACAGGG BRDN00012	255	87	15	602	706	0
CAAACAGGT BRDN00012	106	65	6	171	314	1
CAAACAGGT BRDN00012	216	75	25	248	359	9
CAAACAGTA BRDN00012	165	148	19	223	258	62
CAAACAGTA BRDN00011	255	472	203	735	785	34
CAAACAGTA BRDN00012	317	116	130	674	605	13
CAAACAGTCT BRDN00012	301	198	408	581	572	10
CAAACAGTT BRDN00011	274	189	159	519	669	26
CAAACAGTTT BRDN00011	56	12	0	389	223	0
CAAACATAA BRDN00012	76	31	832	103	240	20
CAAACATAAT BRDN00011	145	97	206	284	202	0
CAAACATAC BRDN00011	129	7	0	300	149	0
CAAACATAT BRDN00011	329	240	909	705	631	57
CAAACATCG BRDN00012	405	327	1065	725	1056	55
CAAACATCT BRDN00012	373	451	745	713	660	24
CAAACATGG BRDN00011	182	144	80	340	133	0
CAAACCAAA BRDN00012	327	236	605	660	825	21
CAAACCAACT BRDN00011	244	213	43	384	317	0
CAAACCAACT BRDN00012	285	106	315	417	473	4
CAAACCAAT BRDN00012	256	270	691	524	713	37
CAAACCACCA BRDN00012	224	161	773	495	389	62
CAAACCACG BRDN00012	209	209	358	281	394	7
CAAACCAGA BRDN00012	149	80	98	187	286	9
CAAACCAGA BRDN00012	118	71	159	138	276	0
CAAACCAGA BRDN00011	152	195	71	451	380	11
CAAACCAGG BRDN00012	376	316	733	612	631	8
CAAACCAGG BRDN00012	362	139	36	597	275	7
CAAACCATG BRDN00012	62	10	0	205	42	0
CAAACCCAA BRDN00012	139	157	689	225	340	0
CAAACCCAG BRDN00012	191	240	964	560	213	71
CAAACCCAG BRDN00012	215	170	0	245	164	0
CAAACCCAG BRDN00011	95	66	92	198	200	0
CAAACCCCAA BRDN00012	375	314	966	773	689	44
CAAACCCCAC BRDN00012	243	239	610	667	295	22
CAAACCCCAG BRDN00011	88	0	0	138	70	0
CAAACCCGCT BRDN00011	243	141	163	517	619	33
CAAACCGGA BRDN00012	444	374	303	657	1091	25
CAAACCGGC BRDN00012	90	4	9	509	375	0
CAAACCGTA BRDN00011	141	135	1878	254	106	69
CAAACCGTT BRDN00012	210	119	0	280	301	2
CAAACCTAA BRDN00011	209	227	477	378	552	66
CAAACCTAA BRDN00012	366	278	436	654	979	26
CAAACCTCAC BRDN00011	95	28	0	192	118	0
CAAACCTCGT BRDN00012	135	176	634	223	183	15
CAAACCTCTC BRDN00011	127	44	105	250	382	0
CAAACCTCTC BRDN00011	38	0	0	262	121	0
CAAACCTGA BRDN00011	136	201	0	190	121	14
CAAACCTGG BRDN00011	131	47	115	195	273	2
CAAACGAAA BRDN00012	46	0	1	169	126	0
CAAACGAAC BRDN00012	62	39	12	201	208	0
CAAACGACA BRDN00012	278	180	812	532	514	33
CAAACGACA BRDN00012	252	209	19	511	521	0
CAAACGACT BRDN00011	319	157	253	547	366	103
CAAACGATC BRDN00012	77	75	64	202	72	7
CAAACGATG BRDN00011	251	384	618	433	349	34
CAAACGCAT BRDN00011	196	126	152	339	548	4
CAAACGCAT BRDN00011	99	82	43	164	180	0
CAAACGCGA BRDN00012	205	248	84	387	259	14
CAAACGCTCA BRDN00011	221	27	152	471	451	23
CAAACGGAC BRDN00012	227	400	457	668	328	90
CAAACGGGG BRDN00012	239	134	125	449	274	20
CAAACGTAA BRDN00011	129	95	0	188	113	17
CAAACGTACT BRDN00012	189	181	93	235	500	17
CAAACGTAG BRDN00011	280	261	466	518	504	6
CAAACGTCA BRDN00011	229	173	624	479	450	10
CAAACGTCAT BRDN00011	287	184	1037	492	844	12
CAAACGTGA BRDN00012	29	25	0	206	115	0
CAAACGTGC BRDN00012	365	171	245	469	825	51
CAAACGTGT BRDN00011	37	91	2	156	68	16
CAAACTAAT BRDN00011	103	120	173	196	330	8
CAAACTATA BRDN00012	121	174	74	281	260	30
CAAACTATAT BRDN00012	218	122	83	294	199	13
CAAACTATCA BRDN00011	173	202	783	388	503	5
CAAACTCAA BRDN00012	161	24	613	340	231	0
CAAACTCACT BRDN00012	509	393	633	923	1227	51
CAAACTCACT BRDN00012	381	387	1206	631	498	41
CAAACTCATG BRDN00012	70	70	31	275	262	0
CAAACTCCAT BRDN00012	219	142	746	363	401	44
CAAACTCCTC BRDN00011	436	315	2928	873	820	111
CAAACTCCTG BRDN00012	161	238	0	237	490	12
CAAACTCCTG BRDN00011	126	119	884	213	135	33
CAAACTCGC BRDN00011	48	69	322	147	438	17
CAAACTCTCT BRDN00012	296	222	106	715	630	5
CAAACTCTGA BRDN00011	203	246	73	443	654	20
CAAACTCTTC BRDN00011	219	117	52	349	286	0
CAAACTGAA BRDN00012	128	4	31	230	151	0
CAAACTGAA BRDN00012	404	304	587	799	649	60
CAAACTGAG BRDN00012	86	87	184	267	301	0
CAAACTGCAT BRDN00011	41	72	191	166	115	0
CAAACTGCCT BRDN00011	369	501	635	629	559	136
CAAACTGCT BRDN00012	121	10	0	502	1000	5
CAAACTGGA BRDN00012	266	248	323	407	887	12
CAAACTGGA BRDN00012	422	266	463	515	705	19
CAAACTGGC BRDN00012	168	12	0	351	224	0
CAAACTGGG BRDN00011	135	133	323	342	427	46
CAAACTGGT BRDN00012	112	56	39	258	408	15
CAAACTGGT BRDN00012	238	136	538	684	898	20
CAAACTGTA BRDN00011	109	120	0	399	503	0
CAAACTGTA BRDN00012	67	62	0	182	138	0
CAAACTGTA BRDN00011	350	194	185	638	907	24
CAAACTGTTT BRDN00011	104	96	122	169	220	0
CAAACTTACC BRDN00011	206	96	1	455	488	6
CAAACTTACC BRDN00011	140	105	62	219	328	40
CAAACTTACT BRDN00012	231	299	411	507	803	47
CAAACTTCTC BRDN00012	93	164	425	238	414	10
CAAACTTGA BRDN00012	244	233	680	468	579	3
CAAACTTGC BRDN00011	23	13	1	143	442	0
CAAACTTGCT BRDN00012	209	138	190	427	496	0
CAAACTTGCT BRDN00011	320	446	314	414	454	39
CAAACTTGTA BRDN00012	311	185	64	638	741	10
CAAACTTGTA BRDN00011	185	186	1744	415	276	3
CAAACTTTCT BRDN00011	163	205	70	262	348	58
CAAAGAAAG BRDN00011	174	139	8758	364	306	132
CAAAGAACC BRDN00012	327	317	1191	507	416	77
CAAAGAACT BRDN00012	340	459	964	555	620	209
CAAAGAAGA BRDN00011	81	161	268	242	232	5
CAAAGAAGC BRDN00011	232	114	202	391	341	81
CAAAGAAGC BRDN00011	136	280	43	287	212	33
CAAAGAAGG BRDN00012	108	125	453	316	451	11
CAAAGAAGG BRDN00011	123	128	702	234	287	3
CAAAGAAGT BRDN00011	58	55	431	150	198	0
CAAAGAATG BRDN00012	97	82	60	169	212	0
CAAAGACAA BRDN00012	296	331	252	657	744	62
CAAAGACAA BRDN00012	78	29	1	148	194	7
CAAAGACAA BRDN00012	244	213	1112	427	477	56
CAAAGACAG BRDN00011	75	37	0	206	129	11
CAAAGACAT BRDN00011	340	523	255	698	783	58
CAAAGACCA BRDN00012	128	99	58	158	103	0
CAAAGACCA BRDN00012	169	221	147	363	374	13
CAAAGACCA BRDN00012	45	0	0	310	372	0
CAAAGACCT BRDN00012	440	573	135	532	671	4
CAAAGACGA BRDN00011	126	43	154	137	174	5
CAAAGACGA BRDN00012	103	87	19	359	241	0
CAAAGACGA BRDN00011	233	225	414	412	527	5
CAAAGACGG BRDN00012	314	524	495	673	513	129
CAAAGACGG BRDN00012	281	331	987	477	331	46
CAAAGACTC BRDN00012	100	14	24	200	137	21
CAAAGACTT BRDN00011	89	30	22	205	320	18
CAAAGAGAA BRDN00011	357	201	628	650	641	35
CAAAGAGAA BRDN00012	294	216	112	568	464	12
CAAAGAGAT BRDN00011	113	39	1034	270	183	8
CAAAGAGCG BRDN00011	316	152	43	261	465	25
CAAAGAGCT BRDN00012	181	0	0	378	318	0
CAAAGAGGC BRDN00012	430	303	347	622	853	23
CAAAGAGGG BRDN00012	176	204	2530	257	303	43
CAAAGAGGT BRDN00012	466	345	133	916	660	25
CAAAGATCG BRDN00012	294	331	893	579	533	60
CAAAGATGA BRDN00012	181	152	0	340	272	0
CAAAGATGG BRDN00011	107	90	31	245	549	0
CAAAGATGG BRDN00011	113	173	90	236	199	17
CAAAGATGG BRDN00011	171	215	150	295	466	1
CAAAGATTA BRDN00012	31	0	0	176	164	0
CAAAGCAAA BRDN00011	245	99	155	586	839	0
CAAAGCAAT BRDN00011	331	274	675	781	787	44
CAAAGCACT BRDN00012	104	119	16	266	97	31
CAAAGCAGA BRDN00011	111	97	16	161	352	29
CAAAGCAGA BRDN00011	97	131	156	223	277	0
CAAAGCAGT BRDN00011	33	0	0	322	214	0
CAAAGCATA BRDN00012	282	227	371	529	1053	8
CAAAGCATA BRDN00011	90	0	0	318	148	0
CAAAGCATG BRDN00012	265	296	1343	419	336	53
CAAAGCCAA BRDN00011	460	420	1471	674	458	50
CAAAGCCAC BRDN00011	99	75	128	115	159	0
CAAAGCCACT BRDN00011	46	0	0	430	197	0
CAAAGCCAT BRDN00012	239	265	126	281	531	28
CAAAGCCCAT BRDN00011	80	162	472	183	193	44
CAAAGCCTG BRDN00011	240	130	29	406	473	13
CAAAGCCTT BRDN00012	230	210	156	384	214	4
CAAAGCCTT BRDN00012	166	84	246	381	420	9
CAAAGCGGC BRDN00012	137	222	0	215	196	79
CAAAGCGGG BRDN00011	55	100	0	121	128	21
CAAAGCGTT BRDN00011	429	408	1880	838	589	144
CAAAGCTAA BRDN00012	149	115	61	222	262	0
CAAAGCTAC BRDN00011	323	401	828	562	1170	36
CAAAGCTAT BRDN00012	117	39	30	329	384	0
CAAAGCTCA BRDN00012	184	285	237	390	530	14
CAAAGCTGT BRDN00011	397	327	204	592	782	0
CAAAGGAAA BRDN00012	179	184	908	307	617	29
CAAAGGAAA BRDN00012	259	359	268	441	548	9
CAAAGGAGA BRDN00012	337	594	2478	585	762	46
CAAAGGCAA BRDN00011	245	275	1075	370	473	24
CAAAGGCCC BRDN00011	34	76	0	73	197	0
CAAAGGGAA BRDN00012	286	243	191	527	536	6
CAAAGGGAA BRDN00011	167	134	1000	477	477	6
CAAAGGGAC BRDN00011	157	211	1128	319	190	41
CAAAGGGAC BRDN00012	359	250	620	693	1015	27
CAAAGGGAG BRDN00012	191	179	101	365	448	32
CAAAGGGAT BRDN00011	168	41	33	291	213	0
CAAAGGGGC BRDN00011	360	234	375	788	649	60
CAAAGGGGC BRDN00012	607	648	1708	860	761	135
CAAAGGTAA BRDN00012	114	83	29	154	61	24
CAAAGGTAT BRDN00011	64	71	30	180	225	0
CAAAGGTCC BRDN00011	477	570	773	867	897	50
CAAAGGTCG BRDN00012	325	324	320	560	315	21
CAAAGGTGA BRDN00011	258	286	1091	573	867	38
CAAAGGTGT BRDN00011	192	260	633	309	415	56
CAAAGTAAA BRDN00012	268	182	349	475	539	6
CAAAGTACA BRDN00012	224	29	1	476	286	0
CAAAGTACG BRDN00012	195	81	26	737	525	0
CAAAGTAGA BRDN00012	238	269	129	432	449	7
CAAAGTAGA BRDN00011	134	87	64	249	212	53
CAAAGTAGG BRDN00012	126	132	29	338	318	14
CAAAGTCAA BRDN00011	360	419	485	827	997	69
CAAAGTCACT BRDN00012	391	342	589	759	841	6
CAAAGTCAT BRDN00012	130	82	15	239	265	6
CAAAGTCCA BRDN00011	316	382	130	508	510	69
CAAAGTCCA BRDN00012	366	233	120	652	868	12
CAAAGTCCCA BRDN00011	151	115	392	324	358	18
CAAAGTCCCT BRDN00011	131	0	0	334	154	0
CAAAGTCCG BRDN00012	244	220	1235	478	876	54
CAAAGTCCG BRDN00011	97	191	41	191	416	25

CAAAGTCCT	BRDN00011	97	76	393	171	291	22
CAAAGTCCTT	BRDN00011	150	110	633	351	372	9
CAAAGTCGA	BRDN00012	234	247	1211	305	394	91
CAAAGTCGG	BRDN00012	353	440	179	745	656	45
CAAAGTCTG	BRDN00011	239	169	926	581	436	37
CAAAGTCTG	BRDN00012	350	338	1395	551	610	82
CAAAGTGAG	BRDN00012	204	155	97	354	292	25
CAAAGTGAG	BRDN00012	178	112	0	347	352	0
CAAAGTGAG	BRDN00011	70	46	64	173	180	0
CAAAGTGAG	BRDN00011	46	16	0	251	100	0
CAAAGTGAT	BRDN00011	112	92	291	260	78	6
CAAAGTGCC	BRDN00012	142	141	107	261	382	0
CAAAGTGCT	BRDN00012	334	315	261	688	972	65
CAAAGTGGG	BRDN00011	146	27	1	180	161	0
CAAAGTGGT	BRDN00011	246	272	802	425	486	57
CAAAGTGTC	BRDN00011	156	150	0	285	42	20
CAAAGTGTC	BRDN00011	51	14	0	132	61	0
CAAAGTGTG	BRDN00011	71	88	252	194	77	8
CAAAGTGTG	BRDN00011	66	43	0	255	158	0
CAAAGTTACT	BRDN00012	300	382	307	480	459	25
CAAAGTTCA	BRDN00012	184	218	219	520	564	28
CAAAGTTGC	BRDN00012	273	232	286	569	549	21
CAAAGTTGG	BRDN00012	62	128	467	244	368	89
CAAAGTTGT	BRDN00012	179	212	661	354	409	34
CAAATAAAG	BRDN00011	404	344	2613	510	479	149
CAAATAAAT	BRDN00012	126	132	230	270	145	6
CAAATAATC	BRDN00012	132	10	0	192	131	0
CAAATACAA	BRDN00012	369	345	489	739	1320	55
CAAATACAAT	BRDN00012	285	245	1448	321	301	59
CAAATACAG	BRDN00012	345	371	246	511	453	90
CAAATACCA	BRDN00011	113	95	78	151	122	5
CAAATACCA	BRDN00012	213	245	332	483	250	51
CAAATACTA	BRDN00011	82	84	852	211	111	20
CAAATACTAT	BRDN00012	228	17	2	460	397	0
CAAATACTCT	BRDN00011	92	100	441	472	345	18
CAAATACTG	BRDN00012	92	89	390	120	179	0
CAAATAGTG	BRDN00012	347	270	295	510	911	87
CAAATATAA	BRDN00011	29	8	1	103	83	0
CAAATATAT	BRDN00011	258	224	19	413	590	47
CAAATATCA	BRDN00011	144	65	135	265	132	32
CAAATATCG	BRDN00011	209	203	197	406	406	1
CAAATATGA	BRDN00011	175	99	57	293	136	8
CAAATATGG	BRDN00011	165	106	305	422	271	8
CAAATATTAT	BRDN00011	115	160	396	330	280	15
CAAATCAAA	BRDN00012	178	165	7	230	147	6
CAAATCAAC	BRDN00012	344	289	120	627	642	5
CAAATCAGG	BRDN00011	203	116	132	480	371	28
CAAATCCAG	BRDN00011	122	283	784	189	111	127
CAAATCCAG	BRDN00011	145	138	449	337	322	0
CAAATCCCA	BRDN00012	70	150	25	131	80	29
CAAATCCCAG	BRDN00012	207	125	205	363	411	14
CAAATCCCCA	BRDN00012	246	152	31	515	651	0
CAAATCCCCA	BRDN00011	184	164	8	285	529	30
CAAATCTAG	BRDN00012	54	45	0	136	329	0
CAAATCTATG	BRDN00012	195	98	44	307	291	0
CAAATCTCA	BRDN00011	98	182	630	121	212	22
CAAATCTGA	BRDN00011	148	107	122	184	307	19
CAAATGAAG	BRDN00011	213	138	120	351	264	31
CAAATGAGC	BRDN00011	47	142	27	97	84	0
CAAATGCAA	BRDN00011	80	9	1	146	63	0
CAAATGCAA	BRDN00012	125	155	57	221	365	29
CAAATGCAA	BRDN00011	201	138	247	324	305	0
CAAATGCAA	BRDN00011	349	439	810	796	715	92
CAAATGCAT	BRDN00011	192	294	178	377	248	0
CAAATGCAT	BRDN00011	218	0	0	398	285	0
CAAATGCAT	BRDN00011	331	364	397	641	974	33
CAAATGCAT	BRDN00012	218	0	0	605	30	0
CAAATGCCAT	BRDN00012	128	222	9	317	196	11
CAAATGCCAT	BRDN00012	341	315	43	761	836	60
CAAATGCGA	BRDN00011	244	307	413	581	967	33
CAAATGCGA	BRDN00012	289	338	50	526	517	22
CAAATGCTG	BRDN00012	160	131	1119	372	435	47
CAAATGCTT	BRDN00012	63	99	468	145	95	14
CAAATGGAA	BRDN00011	73	116	589	206	191	0
CAAATGGAA	BRDN00012	386	314	245	558	833	35
CAAATGGAA	BRDN00012	205	175	219	484	549	8
CAAATGGAG	BRDN00011	75	78	0	136	111	6
CAAATGGAT	BRDN00011	290	255	184	620	673	8
CAAATGGAT	BRDN00011	276	204	1041	248	444	0
CAAATGTAA	BRDN00011	275	125	489	456	436	16
CAAATGTAA	BRDN00012	112	107	7	460	309	0
CAAATGTAA	BRDN00012	102	105	704	252	163	1
CAAATGTAC	BRDN00011	312	293	161	593	381	40
CAAATGTAG	BRDN00012	124	82	26	203	267	0
CAAATGTAT	BRDN00011	191	230	108	288	476	26
CAAATGTCTT	BRDN00012	379	256	471	674	854	7
CAAATGTGC	BRDN00011	116	0	0	187	354	0
CAAATGTTA	BRDN00012	192	187	268	455	503	7
CAAATGTTA	BRDN00012	412	511	1046	825	659	59
CAAATTATA	BRDN00011	344	340	61	535	820	0
CAAATTCCA	BRDN00011	110	144	58	211	226	0
CAAATTCGG	BRDN00011	106	37	157	175	158	0
CAAATTCTAG	BRDN00012	293	215	194	670	658	33
CAAATTCTG	BRDN00011	137	110	34	70	210	0
CAAATTGAC	BRDN00011	198	123	1	583	651	0
CAAATTTACA	BRDN00011	281	146	7527	334	405	112
CAAATTTCAT	BRDN00012	93	107	105	203	333	19
CAAATTTGT	BRDN00011	187	99	137	297	291	11
CAACAAAAA	BRDN00012	224	219	445	376	556	0
CAACAAAAT	BRDN00011	178	230	321	477	374	32
CAACAAACC	BRDN00012	353	419	791	598	664	36
CAACAAAGA	BRDN00012	329	458	1175	477	1346	98
CAACAAAGC	BRDN00011	256	321	153	319	264	29
CAACAAAGG	BRDN00012	432	345	2502	923	974	49
CAACAAAGG	BRDN00012	323	411	2378	724	777	45
CAACAAATA	BRDN00012	146	75	234	274	126	7
CAACAAATTT	BRDN00011	214	140	705	372	246	20
CAACAACAA	BRDN00012	440	391	676	703	674	27
CAACAACAA	BRDN00012	273	176	26	659	285	28
CAACAACAC	BRDN00011	304	227	241	555	742	39
CAACAACAG	BRDN00011	342	29	1	383	156	0
CAACAACCTT	BRDN00011	320	175	407	467	606	18
CAACAACGC	BRDN00011	417	353	12951	704	805	107
CAACAACGG	BRDN00011	457	505	3148	984	1235	80
CAACAACTC	BRDN00012	100	92	16	580	320	10
CAACAACTG	BRDN00012	131	22	0	544	32	0
CAACAAGAA	BRDN00011	93	0	21	220	92	0
CAACAAGAA	BRDN00011	186	176	2452	390	311	32
CAACAAGAG	BRDN00012	300	147	370	609	623	21
CAACAAGAG	BRDN00012	157	3	0	263	82	0
CAACAAGAT	BRDN00011	73	43	0	412	71	0
CAACAAGGA	BRDN00012	127	3	0	291	403	0
CAACAAGGG	BRDN00012	142	136	245	166	98	21
CAACAAGGT	BRDN00011	202	86	186	513	476	5
CAACAAGGT	BRDN00012	277	189	106	760	1138	25
CAACAATAA	BRDN00011	62	12	76	219	502	0
CAACAATCA	BRDN00011	105	155	1874	259	178	18
CAACAATCCA	BRDN00011	245	236	330	552	887	0
CAACAATGA	BRDN00012	213	191	572	436	338	19
CAACAATGA	BRDN00012	131	137	209	279	273	7
CAACAATGA	BRDN00012	480	542	885	711	1380	77
CAACACAAT	BRDN00012	77	37	1	553	159	0
CAACACAAT	BRDN00012	243	257	1005	547	1261	54
CAACACAATT	BRDN00011	214	91	278	307	137	8
CAACACACG	BRDN00011	202	193	247	500	566	10
CAACACACTA	BRDN00011	267	27	0	417	313	0
CAACACAGA	BRDN00012	395	468	2177	630	548	52
CAACACAGA	BRDN00012	209	161	410	375	190	45
CAACACAGT	BRDN00011	611	581	1631	1252	639	57
CAACACCAAT	BRDN00011	191	204	754	523	407	83
CAACACCACC	BRDN00011	223	208	171	435	693	8
CAACACCAGT	BRDN00012	184	152	438	462	310	10
CAACACCATA	BRDN00012	358	225	228	657	665	195
CAACACCATG	BRDN00012	589	390	655	823	538	131
CAACACCCCG	BRDN00012	476	472	1600	916	806	60
CAACACCCCT	BRDN00011	175	63	249	173	255	0
CAACACCCGC	BRDN00012	126	83	31	189	180	12
CAACACCCTC	BRDN00011	168	104	1	273	166	7
CAACACCTCT	BRDN00011	67	0	0	235	207	0
CAACACCTTC	BRDN00011	233	238	295	416	722	9
CAACACGAA	BRDN00012	102	127	1817	231	297	63
CAACACGAC	BRDN00012	182	178	40	414	454	0
CAACACGACC	BRDN00012	599	579	1060	997	870	37
CAACACGCG	BRDN00011	234	118	378	265	417	13
CAACACGCG	BRDN00011	151	176	2619	408	565	31
CAACACGCTT	BRDN00011	248	231	159	590	615	1
CAACACGGG	BRDN00012	244	119	35	558	649	4
CAACACTATC	BRDN00012	408	431	2312	798	560	55
CAACACTCAG	BRDN00011	193	301	83	413	660	0
CAACACTGG	BRDN00012	336	262	270	613	1039	27
CAACACTGT	BRDN00012	474	432	1692	893	1054	15
CAACACTTTC	BRDN00012	717	491	2435	1048	1441	83
CAACAGAGA	BRDN00012	100	185	44	137	111	96
CAACAGATG	BRDN00012	143	142	11	229	354	2
CAACAGATT	BRDN00011	272	106	335	450	769	15
CAACAGCAA	BRDN00012	131	1	0	354	317	0
CAACAGCAA	BRDN00012	149	24	0	547	455	0
CAACAGCAG	BRDN00012	342	192	176	661	671	7
CAACAGCAT	BRDN00012	372	329	852	909	867	43
CAACAGGAA	BRDN00011	70	61	16	315	329	4
CAACAGGAG	BRDN00012	448	326	204	524	570	55
CAACAGGAT	BRDN00012	241	279	1505	565	492	16
CAACAGGGA	BRDN00012	105	124	0	235	4898	1
CAACAGGTC	BRDN00012	541	479	343	840	1161	30
CAACAGTAA	BRDN00012	232	228	407	310	135	32
CAACAGTAC	BRDN00012	165	170	3	346	181	15
CAACAGTGA	BRDN00011	126	202	88	318	175	12
CAACAGTGA	BRDN00011	145	186	200	306	501	26
CAACAGTGA	BRDN00011	379	369	28	936	975	13
CAACAGTGC	BRDN00012	372	391	459	655	639	35
CAACAGTGT	BRDN00012	196	197	141	374	274	1
CAACATAAA	BRDN00012	306	112	46	471	403	0
CAACATAAT	BRDN00011	243	311	167	342	648	21
CAACATAAT	BRDN00011	159	86	83	275	182	6
CAACATACG	BRDN00012	194	251	435	498	488	14
CAACATAGA	BRDN00011	291	127	126	569	673	0
CAACATAGC	BRDN00011	93	0	1	859	423	0
CAACATAGT	BRDN00012	97	28	7	238	78	0
CAACATATAT	BRDN00011	151	91	75	132	283	0
CAACATCAA	BRDN00011	101	132	378	359	464	9
CAACATCAC	BRDN00011	81	38	0	86	101	0
CAACATCCAC	BRDN00012	254	279	237	495	502	27
CAACATCCTC	BRDN00011	396	392	122	542	460	11
CAACATCGT	BRDN00011	172	98	2958	337	148	40
CAACATCGTT	BRDN00012	444	633	390	710	1679	156
CAACATCTCA	BRDN00011	317	204	56	399	951	23
CAACATCTGT	BRDN00012	312	320	505	521	338	35
CAACATCTTC	BRDN00011	254	247	47	323	543	84
CAACATCTTG	BRDN00012	56	0	29	238	302	0
CAACATGAG	BRDN00011	61	31	0	221	258	0
CAACATGAT	BRDN00011	63	47	0	240	207	0
CAACATGCT	BRDN00012	196	146	726	332	251	21
CAACATGGA	BRDN00012	110	183	44	231	443	6
CAACATGGC	BRDN00012	275	345	232	635	538	72
CAACATGTC	BRDN00012	208	282	200	361	843	43
CAACATGTG	BRDN00012	184	166	448	231	338	0
CAACATTAA	BRDN00012	58	9	0	237	47	0
CAACATTAA	BRDN00011	125	0	0	207	44	0
CAACATTAG	BRDN00011	414	424	745	552	678	112
CAACATTGA	BRDN00012	342	40	2	529	367	0
CAACATTGA	BRDN00012	374	350	167	638	693	42
CAACATTTAC	BRDN00012	92	32	225	477	414	0
CAACCAAAA	BRDN00011	356	413	250	518	532	89
CAACCAAGG	BRDN00011	94	70	208	183	74	0
CAACCAATG	BRDN00012	352	415	1692	873	511	147
CAACCAATG	BRDN00012	50	11	0	631	125	7
CAACCAATGT	BRDN00011	435	396	432	589	613	47
CAACCACAA	BRDN00012	290	258	513	637	580	36
CAACCACAAT	BRDN00012	312	247	20	925	811	11
CAACCACCCA	BRDN00011	180	164	503	268	224	44
CAACCACCTG	BRDN00012	175	163	495	371	271	20
CAACCACCTT	BRDN00011	454	398	1817	669	639	61
CAACCACTTC	BRDN00011	477	418	2162	891	564	35
CAACCAGAG	BRDN00011	215	210	31	307	208	10
CAACCAGAT	BRDN00011	184	161	144	665	548	50
CAACCAGGC	BRDN00012	114	88	0	201	123	5
CAACCAGTA	BRDN00012	379	352	1529	738	771	60
CAACCAGTT	BRDN00012	47	8	0	277	105	0
CAACCAGTT	BRDN00012	276	419	121	671	898	9
CAACCATAG	BRDN00011	225	132	293	411	434	6
CAACCATCA	BRDN00011	201	23	0	711	276	0
CAACCATGG	BRDN00012	156	137	46	303	327	18
CAACCCAGA	BRDN00012	70	168	319	187	106	12
CAACCCAGA	BRDN00011	209	237	1	290	246	13
CAACCCAGT	BRDN00012	477	250	1017	764	672	38
CAACCCAGT	BRDN00012	168	251	85	472	326	56
CAACCCATC	BRDN00011	514	440	531	810	787	39
CAACCCCGG	BRDN00012	403	379	360	675	526	24
CAACCCGCTA	BRDN00012	10	0	0	313	36	0
CAACCCTAAG	BRDN00012	128	137	180	330	300	1
CAACCCTAGT	BRDN00012	242	202	361	358	585	94
CAACCCTCGC	BRDN00012	80	96	1	215	175	0
CAACCCTGCC	BRDN00012	158	256	2123	230	280	19
CAACCCTTAA	BRDN00012	266	203	36	296	450	3
CAACCGAAA	BRDN00012	116	123	1	337	378	24
CAACCGCCA	BRDN00012	158	24	81	464	270	0
CAACCGCGA	BRDN00011	78	104	89	191	162	8
CAACCGCGT	BRDN00012	527	358	1712	709	707	25
CAACCGGCA	BRDN00011	155	170	133	236	264	17
CAACCGGCG	BRDN00012	429	441	376	613	698	22
CAACCGGCT	BRDN00011	466	414	1166	993	816	29
CAACCGGGA	BRDN00011	277	149	307	391	810	47
CAACCGGTA	BRDN00011	271	104	9	649	184	8
CAACCTAATG	BRDN00011	166	31	132	380	203	0
CAACCTACCA	BRDN00012	148	273	901	229	730	73
CAACCTATGT	BRDN00011	162	204	173	303	497	61
CAACCTCAAG	BRDN00012	220	229	3	451	643	0
CAACCTCATC	BRDN00012	377	296	1960	765	610	45
CAACCTCATT	BRDN00011	201	161	6	246	528	0
CAACCTCCAG	BRDN00011	86	16	0	595	90	9
CAACCTCCCG	BRDN00012	110	63	93	227	77	16
CAACCTCTCG	BRDN00011	92	67	1	214	285	0
CAACCTCTGT	BRDN00012	134	92	1	224	107	7
CAACCTGAA	BRDN00012	283	119	582	927	1050	84
CAACCTGGA	BRDN00012	183	230	5434	263	263	43
CAACCTGGT	BRDN00012	213	288	50	349	246	18
CAACCTGTG	BRDN00012	256	338	188	473	323	5
CAACGAAAA	BRDN00011	238	185	1157	439	367	12
CAACGAACC	BRDN00011	110	68	102	250	411	15
CAACGAACC	BRDN00012	364	298	842	856	1217	40
CAACGACAA	BRDN00011	71	88	487	207	189	13
CAACGACAT	BRDN00012	66	11	2	725	226	1
CAACGACCG	BRDN00011	241	231	521	652	399	11
CAACGACCT	BRDN00011	56	10	16	192	83	5
CAACGACCT	BRDN00012	245	197	225	488	283	0
CAACGACGG	BRDN00012	447	475	2099	1055	1142	26
CAACGACGG	BRDN00012	344	290	149	616	993	1
CAACGAGAA	BRDN00011	237	88	36	645	541	4
CAACGAGAG	BRDN00011	244	118	518	690	579	0
CAACGAGGC	BRDN00011	660	433	782	1046	924	23
CAACGAGGT	BRDN00011	236	234	240	362	289	0
CAACGATCA	BRDN00011	121	104	15	255	211	2
CAACGATGC	BRDN00012	422	14	1	881	311	0
CAACGATTC	BRDN00012	178	99	222	249	276	0
CAACGCACT	BRDN00012	294	155	36	499	518	47
CAACGCAGG	BRDN00012	45	75	9	253	208	0
CAACGCATA	BRDN00011	450	271	51	702	580	0
CAACGCCATC	BRDN00012	475	515	446	923	1047	94
CAACGCCCTC	BRDN00011	135	131	18	369	339	5
CAACGCGAG	BRDN00012	289	103	431	549	364	15
CAACGCGGC	BRDN00012	115	148	60	192	162	0
CAACGCGGG	BRDN00011	127	141	13	228	558	0
CAACGCGTC	BRDN00011	170	77	147	260	505	48
CAACGCTAA	BRDN00012	329	245	159	526	359	0
CAACGCTGG	BRDN00012	129	50	0	238	253	0
CAACGCTGT	BRDN00012	264	255	1035	320	194	63
CAACGGAAG	BRDN00011	429	396	1206	477	821	58
CAACGGATA	BRDN00011	148	115	219	239	151	0
CAACGGATA	BRDN00011	253	178	184	292	381	22
CAACGGCAC	BRDN00012	198	237	920	364	225	25
CAACGGCTA	BRDN00011	290	281	1627	730	387	44
CAACGGCTT	BRDN00012	121	61	35	240	108	10
CAACGGGAC	BRDN00011	194	254	210	401	452	7
CAACGGGAC	BRDN00012	570	512	972	794	1244	33
CAACGGGCA	BRDN00011	192	4	210	143	242	35
CAACGGGGA	BRDN00011	247	139	421	451	386	0
CAACGGGTT	BRDN00012	546	509	3459	879	728	34
CAACGGTAC	BRDN00011	100	170	6	235	255	9
CAACGGTAT	BRDN00012	274	228	96	418	589	14
CAACGGTCT	BRDN00011	201	230	45	403	414	0
CAACGTACG	BRDN00011	133	183	23	378	365	17
CAACGTACT	BRDN00012	117	126	56	228	168	0
CAACGTCAA	BRDN00011	90	90	55	145	134	0
CAACGTCTAT	BRDN00012	102	200	0	282	463	9
CAACGTCTAT	BRDN00011	585	474	1948	1085	1026	67
CAACGTGCA	BRDN00012	481	373	1245	522	620	28
CAACGTGCC	BRDN00011	75	68	710	203	474	35
CAACGTGGA	BRDN00012	143	53	18	353	179	17
CAACGTGGA	BRDN00011	294	285	313	458	468	35
CAACGTGGG	BRDN00011	449	390	491	776	966	24
CAACGTGGG	BRDN00012	164	148	387	243	606	26
CAACGTGTA	BRDN00012	438	320	607	724	778	10
CAACGTTTG	BRDN00011	70	86	5	149	0	19
CAACTACATC	BRDN00011	239	200	8	293	410	6
CAACTACATC	BRDN00011	348	329	82	551	715	2
CAACTACCCT	BRDN00011	282	345	191	599	389	27
CAACTACGC	BRDN00011	120	24	0	212	52	0
CAACTACTCC	BRDN00011	90	55	77	124	59	4
CAACTAGCAC	BRDN00012	118	116	0	203	193	0
CAACTAGTT	BRDN00012	257	251	32	328	535	23
CAACTATAA	BRDN00012	304	380	529	403	554	6
CAACTATAAT	BRDN00012	103	42	96	254	307	9
CAACTATCTG	BRDN00012	159	79	1	162	144	6
CAACTATGT	BRDN00012	230	170	538	427	545	26
CAACTCACGT	BRDN00011	109	149	597	340	460	28
CAACTCAGC	BRDN00012	133	164	1	382	386	7
CAACTCAGTT	BRDN00011	334	327	506	594	601	44
CAACTCATTG	BRDN00011	101	138	6582	225	158	85
CAACTCATTG	BRDN00011	95	122	37	197	331	25
CAACTCCAAG	BRDN00012	394	365	334	699	793	48
CAACTCCACC	BRDN00011	6	0	0	177	67	0
CAACTCCAGT	BRDN00011	276	152	405	493	453	20
CAACTCCTGA	BRDN00011	188	204	377	232	232	15
CAACTCCTTA	BRDN00011	92	41	68	195	157	1
CAACTCGAAT	BRDN00011	202	174	7	518	608	5
CAACTCGAG	BRDN00012	131	19	0	480	181	0
CAACTCGAT	BRDN00011	124	66	0	230	294	0
CAACTCGCTC	BRDN00012	175	87	235	210	676	0
CAACTCGTCC	BRDN00011	170	217	465	335	425	49
CAACTCTGA	BRDN00011	300	349	707	421	429	190
CAACTCTGTA	BRDN00011	217	227	63	313	267	21
CAACTGAAG	BRDN00012	229	177	613	451	755	1
CAACTGACCT	BRDN00011	111	150	37	259	285	6
CAACTGACTA	BRDN00011	228	185	191	382	472	14
CAACTGAGA	BRDN00011	211	89	1141	150	19	65
CAACTGATCC	BRDN00012	139	137	324	270	353	18
CAACTGCCAT	BRDN00011	206	245	624	174	121	24
CAACTGCCGC	BRDN00011	495	508	1170	824	1170	25
CAACTGCGG	BRDN00011	101	128	25	119	124	7
CAACTGCTCG	BRDN00012	390	504	103	919	773	55
CAACTGGAA	BRDN00011	124	274	1212	298	695	24
CAACTGGAC	BRDN00011	217	202	183	537	350	23
CAACTGGCA	BRDN00012	217	107	172	277	312	7
CAACTGGGG	BRDN00012	248	175	426	266	288	56
CAACTGGTA	BRDN00011	448	372	1217	918	871	10
CAACTGTAA	BRDN00012	256	198	184	380	279	30
CAACTGTAA	BRDN00011	137	113	1344	267	183	6
CAACTGTCA	BRDN00011	224	163	660	245	176	44
CAACTGTCA	BRDN00012	192	169	36	307	371	64
CAACTGTGC	BRDN00011	400	0	1	767	340	0
CAACTTACAA	BRDN00011	144	0	0	302	315	0
CAACTTACCG	BRDN00012	124	74	2	445	397	7
CAACTTACTC	BRDN00011	232	288	74	536	328	19
CAACTTATGA	BRDN00012	166	100	71	282	344	0
CAACTTCAAG	BRDN00011	168	358	566	333	265	76
CAACTTCCGA	BRDN00012	170	185	388	354	344	33
CAACTTCTCA	BRDN00012	256	160	634	358	268	17
CAACTTCTCG	BRDN00012	238	215	2	478	564	37
CAACTTCTCG	BRDN00012	328	379	408	640	471	54
CAACTTCTGC	BRDN00012	318	310	653	725	666	35
CAACTTCTGT	BRDN00012	279	281	60	596	550	17
CAACTTGGA	BRDN00011	265	237	1140	338	425	16
CAACTTTAAA	BRDN00012	174	185	748	407	483	60
CAACTTTAGC	BRDN00012	101	175	242	249	288	0
CAAGAAAAG	BRDN00012	337	333	357	644	571	45
CAAGAAAAT	BRDN00012	148	116	658	289	105	2
CAAGAAAAT	BRDN00011	93	39	47	145	139	0
CAAGAAAAT	BRDN00011	128	102	51	156	69	7
CAAGAAACA	BRDN00011	464	283	222	672	701	20
CAAGAAAGA	BRDN00011	38	32	0	196	37	0
CAAGAAAGA	BRDN00011	123	72	407	239	159	18
CAAGAAAGA	BRDN00012	163	85	96	261	432	0
CAAGAAAGA	BRDN00011	206	179	539	382	149	24
CAAGAAAGG	BRDN00012	109	40	177	187	247	7
CAAGAAAGG	BRDN00012	166	131	22	356	594	17
CAAGAAAGT	BRDN00012	282	263	3714	435	143	64
CAAGAAATA	BRDN00011	93	92	14	200	255	0
CAAGAACAA	BRDN00011	178	126	19	256	173	0
CAAGAACAA	BRDN00011	110	175	879	241	85	53
CAAGAACAA	BRDN00012	214	219	181	392	252	2
CAAGAACAA	BRDN00011	82	103	417	149	162	17
CAAGAACAA	BRDN00011	111	56	0	127	53	0
CAAGAACAG	BRDN00012	304	172	159	408	474	8
CAAGAACAT	BRDN00011	238	274	2128	429	241	73
CAAGAACAT	BRDN00011	118	148	1101	231	117	0
CAAGAACCA	BRDN00012	109	18	0	125	188	0
CAAGAACCC	BRDN00011	23	28	0	138	472	0
CAAGAACCC	BRDN00011	90	130	793	127	115	9
CAAGAACCCT	BRDN00012	84	185	963	227	120	26
CAAGAACGA	BRDN00012	59	0	1	127	5	0
CAAGAACGA	BRDN00012	296	214	54	524	700	0
CAAGAACGA	BRDN00012	433	264	407	644	550	217
CAAGAACTA	BRDN00012	77	84	112	214	72	12
CAAGAACTA	BRDN00011	98	46	67	232	137	0
CAAGAACTG	BRDN00012	182	166	1571	239	847	36
CAAGAACTT	BRDN00011	284	279	63	453	450	62
CAAGAACTT	BRDN00011	187	128	154	358	106	25
CAAGAAGAA	BRDN00011	373	411	1156	674	858	41
CAAGAAGAC	BRDN00011	214	158	1921	441	809	33
CAAGAAGAG	BRDN00011	300	194	706	490	901	32
CAAGAAGAG	BRDN00011	2	0	0	135	49	0
CAAGAAGAT	BRDN00012	257	390	400	711	698	84
CAAGAAGAT	BRDN00011	111	180	102	184	189	55
CAAGAAGCA	BRDN00011	291	407	678	490	937	58
CAAGAAGCA	BRDN00011	375	276	793	769	528	33
CAAGAAGCA	BRDN00011	404	456	1348	618	553	99
CAAGAAGCG	BRDN00012	75	317	0	148	246	11
CAAGAAGCC	BRDN00011	33	62	0	107	272	7
CAAGAAGCT	BRDN00012	73	95	29	87	93	8
CAAGAAGCT	BRDN00012	93	77	41	206	104	0
CAAGAAGGA	BRDN00012	107	94	293	151	192	15
CAAGAAGGA	BRDN00012	131	138	692	258	196	20
CAAGAAGGG	BRDN00012	300	258	12	500	470	19
CAAGAAGGG	BRDN00011	146	189	432	248	153	69
CAAGAAGTA	BRDN00011	100	39	0	156	155	0
CAAGAAGTT	BRDN00012	161	64	106	259	577	13
CAAGAAGTT	BRDN00011	61	89	14	118	100	6
CAAGAATAA	BRDN00012	209	119	53	448	223	0
CAAGAATAC	BRDN00011	41	2	17	171	63	0
CAAGAATCG	BRDN00012	250	281	412	498	340	6
CAAGAATCT	BRDN00011	252	152	126	201	365	1
CAAGAATGC	BRDN00012	176	209	210	196	213	26
CAAGAATGG	BRDN00011	153	46	12	247	296	0
CAAGACAAA	BRDN00012	92	60	271	123	73	20
CAAGACAAC	BRDN00011	113	124	0	212	208	6
CAAGACACT	BRDN00011	246	220	473	594	506	135
CAAGACAGC	BRDN00011	388	325	2131	1028	1313	34
CAAGACAGG	BRDN00012	332	313	148	568	730	81
CAAGACATA	BRDN00011	128	121	0	138	148	0
CAAGACATC	BRDN00012	301	350	754	629	779	16
CAAGACATG	BRDN00012	219	215	64	360	316	13
CAAGACCAA	BRDN00012	144	0	1	284	123	0
CAAGACCAA	BRDN00012	291	258	64	501	432	36
CAAGACCAA	BRDN00011	186	346	2483	388	330	138
CAAGACCAC	BRDN00011	162	271	20	427	154	22

CAAGACCAC	BRDN00011	217	201	398	458	659	9
CAAGACCAT	BRDN00012	89	44	21	795	852	3
CAAGACCAT	BRDN00011	61	76	32	163	169	0
CAAGACCTC	BRDN00011	205	43	0	603	206	13
CAAGACCTG	BRDN00011	218	219	76	472	388	1
CAAGACCTT	BRDN00012	313	279	218	565	761	9
CAAGACCTT	BRDN00011	183	169	53	784	958	23
CAAGACGAA	BRDN00011	220	215	440	285	382	22
CAAGACGAT	BRDN00012	409	266	902	614	743	41
CAAGACGCA	BRDN00012	135	26	54	220	84	10
CAAGACGCA	BRDN00011	148	85	9	301	340	6
CAAGACGGG	BRDN00012	91	107	0	174	247	10
CAAGACGGG	BRDN00012	167	157	140	215	302	0
CAAGACGTG	BRDN00011	247	216	2099	650	688	30
CAAGACTCG	BRDN00012	135	137	66	160	316	6
CAAGACTCT	BRDN00011	138	42	1	463	338	3
CAAGACTGG	BRDN00012	316	182	39	521	366	18
CAAGACTTA	BRDN00011	93	80	288	234	75	19
CAAGACTTT	BRDN00011	149	89	0	335	104	0
CAAGAGAAA	BRDN00012	161	132	245	238	199	9
CAAGAGAAC	BRDN00011	136	75	96	191	187	18
CAAGAGAAT	BRDN00012	199	297	408	414	522	23
CAAGAGACA	BRDN00012	444	451	523	732	822	27
CAAGAGACG	BRDN00011	126	243	0	256	148	18
CAAGAGAGA	BRDN00011	343	31	0	552	503	1
CAAGAGCAT	BRDN00011	63	110	0	155	225	9
CAAGAGCCA	BRDN00012	84	65	1	127	58	0
CAAGAGCCG	BRDN00012	156	34	728	307	240	0
CAAGAGCCG	BRDN00012	98	98	1	253	132	0
CAAGAGCCT	BRDN00012	69	130	12	167	302	7
CAAGAGCGA	BRDN00011	84	48	208	115	122	16
CAAGAGCGA	BRDN00011	263	176	230	389	249	25
CAAGAGCGG	BRDN00011	64	5	0	821	71	0
CAAGAGCTT	BRDN00012	132	37	1	166	130	0
CAAGAGGAA	BRDN00012	91	149	202	172	151	16
CAAGAGGAA	BRDN00012	221	226	143	395	355	60
CAAGAGGAA	BRDN00012	71	54	0	178	246	0
CAAGAGGCA	BRDN00011	186	195	75	398	173	32
CAAGAGGCA	BRDN00011	117	130	0	241	128	6
CAAGAGGCA	BRDN00012	123	112	285	196	222	27
CAAGAGGGC	BRDN00011	117	94	25	174	68	0
CAAGAGGTA	BRDN00012	224	245	279	480	378	43
CAAGAGGTA	BRDN00012	270	339	311	402	380	88
CAAGAGGTG	BRDN00011	274	42	0	541	359	0
CAAGAGGTG	BRDN00011	249	231	132	493	421	40
CAAGAGTAG	BRDN00011	285	242	46	331	544	29
CAAGAGTCG	BRDN00011	67	94	151	233	110	28
CAAGAGTCT	BRDN00011	358	80	1063	604	623	21
CAAGAGTGA	BRDN00011	282	408	1588	232	287	52
CAAGAGTGA	BRDN00012	163	154	306	313	206	16
CAAGAGTGG	BRDN00011	81	60	149	186	153	1
CAAGAGTTA	BRDN00012	84	21	950	249	158	34
CAAGAGTTT	BRDN00012	278	275	727	418	559	28
CAAGATAAC	BRDN00011	111	23	61	226	54	0
CAAGATAAG	BRDN00012	409	352	251	703	1128	16
CAAGATAAT	BRDN00012	59	69	337	197	205	13
CAAGATAAT	BRDN00011	79	117	1	131	152	12
CAAGATAAT	BRDN00012	126	310	25	222	217	36
CAAGATACC	BRDN00011	212	134	19	410	399	17
CAAGATACC	BRDN00011	153	130	2297	253	214	45
CAAGATACG	BRDN00011	148	116	584	307	88	6
CAAGATACT	BRDN00012	424	395	220	619	637	7
CAAGATACT	BRDN00012	170	143	0	272	260	0
CAAGATAGA	BRDN00011	330	350	438	584	540	53
CAAGATCAA	BRDN00012	229	283	10	476	307	8
CAAGATCAA	BRDN00012	187	0	0	293	171	0
CAAGATCAC	BRDN00011	113	58	0	686	498	6
CAAGATCAT	BRDN00012	225	105	3576	556	468	41
CAAGATCCA	BRDN00012	218	169	357	430	493	18
CAAGATCCA	BRDN00011	85	161	247	209	266	49
CAAGATCGA	BRDN00011	247	326	258	419	393	4
CAAGATCGC	BRDN00011	175	167	15	374	423	12
CAAGATCGT	BRDN00012	23	0	0	139	0	0
CAAGATCTA	BRDN00012	301	314	632	514	338	15
CAAGATCTA	BRDN00012	73	20	207	291	73	6
CAAGATCTC	BRDN00011	213	270	141	423	537	10
CAAGATCTT	BRDN00012	161	131	117	299	271	7
CAAGATGAC	BRDN00011	129	140	145	391	721	6
CAAGATGAG	BRDN00011	106	133	157	253	363	10
CAAGATGAG	BRDN00011	287	170	261	410	314	2
CAAGATGAT	BRDN00012	106	79	12	286	198	1
CAAGATGCT	BRDN00012	118	0	0	351	339	0
CAAGATGGA	BRDN00012	173	177	16	514	750	6
CAAGATGGC	BRDN00011	223	275	908	357	889	23
CAAGATGGC	BRDN00012	390	319	136	897	717	34
CAAGATGGC	BRDN00011	245	39	18	327	489	0
CAAGATGGG	BRDN00012	133	77	34	119	265	0
CAAGATGGG	BRDN00011	139	91	180	211	182	0
CAAGATGTG	BRDN00011	120	16	21	271	156	0
CAAGATGTG	BRDN00012	134	96	1273	266	302	6
CAAGATTAG	BRDN00011	67	85	720	282	232	4
CAAGATTCC	BRDN00011	270	171	93	486	386	32
CAAGATTCG	BRDN00011	66	49	1	133	112	0
CAAGATTGA	BRDN00011	495	521	980	753	911	53
CAAGCAAAA	BRDN00011	394	376	2100	696	327	55
CAAGCAAAC	BRDN00011	277	339	235	448	394	45
CAAGCAAAC	BRDN00012	335	479	238	596	909	13
CAAGCAAAG	BRDN00012	53	154	212	290	288	13
CAAGCAACA	BRDN00012	53	1	0	130	111	0
CAAGCAACT	BRDN00011	233	176	255	554	294	10
CAAGCAAGA	BRDN00011	81	87	0	186	221	0
CAAGCAATA	BRDN00011	290	304	904	474	163	0
CAAGCAATA	BRDN00012	174	189	900	484	706	20
CAAGCAATC	BRDN00011	155	226	27	342	177	17
CAAGCACAA	BRDN00012	128	134	360	323	153	18
CAAGCACAT	BRDN00011	246	186	86	631	516	8
CAAGCACCG	BRDN00012	234	100	106	563	430	0
CAAGCACTG	BRDN00012	101	113	38	380	228	13
CAAGCAGAG	BRDN00012	101	91	10	245	432	0
CAAGCAGAT	BRDN00012	171	71	184	305	38	0
CAAGCAGCC	BRDN00012	162	199	355	386	344	23
CAAGCAGCC	BRDN00012	52	19	112	163	27	0
CAAGCAGCG	BRDN00012	55	79	0	281	15	0
CAAGCAGCT	BRDN00011	114	43	264	243	345	14
CAAGCAGCT	BRDN00012	101	128	0	358	339	4
CAAGCAGGC	BRDN00011	271	240	1499	398	234	5
CAAGCAGGG	BRDN00011	181	237	52	318	167	0
CAAGCAGTA	BRDN00011	60	47	0	177	118	0
CAAGCAGTC	BRDN00012	89	63	522	187	163	11
CAAGCAGTG	BRDN00012	237	203	302	457	412	134
CAAGCATAA	BRDN00012	257	209	49	327	263	7
CAAGCATCA	BRDN00012	398	402	113	766	796	19
CAAGCATCT	BRDN00012	419	237	220	648	781	5
CAAGCATGT	BRDN00012	118	67	245	244	239	0
CAAGCATTG	BRDN00012	73	25	0	154	134	0
CAAGCATTT	BRDN00012	270	57	279	499	396	5
CAAGCATTT	BRDN00012	351	321	938	678	504	7
CAAGCCAAA	BRDN00011	116	129	1600	241	250	17
CAAGCCAAC	BRDN00012	249	210	113	276	456	54
CAAGCCAGA	BRDN00012	140	92	1470	194	380	38
CAAGCCAGA	BRDN00011	204	228	49	321	301	1
CAAGCCAGA	BRDN00012	112	93	266	202	208	6
CAAGCCAGC	BRDN00011	188	218	97	301	511	27
CAAGCCAGT	BRDN00012	48	0	0	380	143	0
CAAGCCAGT	BRDN00011	242	211	1215	508	455	29
CAAGCCAGT	BRDN00011	184	145	0	143	134	0
CAAGCCATG	BRDN00012	62	25	0	247	371	0
CAAGCCATT	BRDN00012	108	133	440	238	346	5
CAAGCCCAA	BRDN00012	144	80	1452	344	823	125
CAAGCCCAA	BRDN00012	298	228	40	577	399	4
CAAGCCCAT	BRDN00011	98	159	48	209	50	28
CAAGCCCCA	BRDN00011	255	194	54	436	379	7
CAAGCCCCC	BRDN00011	340	340	661	525	748	44
CAAGCCCGA	BRDN00011	84	70	52	248	343	0
CAAGCCCGA	BRDN00012	91	39	504	248	122	14
CAAGCCCGA	BRDN00011	96	137	40	187	255	8
CAAGCCCTA	BRDN00012	351	386	1508	594	1001	85
CAAGCCCTA	BRDN00011	323	394	883	769	1308	36
CAAGCCCTT	BRDN00011	257	345	63	340	401	17
CAAGCCGAA	BRDN00012	252	210	145	556	810	14
CAAGCCGAG	BRDN00012	121	124	40	219	318	8
CAAGCCGCC	BRDN00012	268	312	506	709	497	22
CAAGCCGGA	BRDN00012	169	122	266	230	122	26
CAAGCCGGC	BRDN00012	448	568	2969	655	729	53
CAAGCCGGT	BRDN00011	290	310	752	493	764	21
CAAGCCTTT	BRDN00011	100	101	543	200	234	14
CAAGCGAAG	BRDN00011	184	153	20	321	172	0
CAAGCGAAG	BRDN00011	171	125	32	557	235	31
CAAGCGACG	BRDN00011	100	0	0	488	358	0
CAAGCGACT	BRDN00012	183	203	589	419	424	40
CAAGCGACT	BRDN00012	349	229	424	742	1278	89
CAAGCGAGC	BRDN00011	353	175	409	607	390	11
CAAGCGCAG	BRDN00011	330	470	916	666	484	39
CAAGCGCCA	BRDN00012	155	118	409	271	273	37
CAAGCGCGT	BRDN00012	231	236	970	488	603	51
CAAGCGCTA	BRDN00011	201	185	535	390	260	12
CAAGCGGGG	BRDN00011	154	107	1418	350	362	29
CAAGCGTGA	BRDN00011	173	257	549	437	283	9
CAAGCTAAA	BRDN00012	71	52	0	81	130	0
CAAGCTAAC	BRDN00011	76	35	0	188	202	0
CAAGCTACT	BRDN00012	132	32	1	330	198	0
CAAGCTACT	BRDN00012	252	229	183	850	805	51
CAAGCTATG	BRDN00012	380	217	670	585	748	12
CAAGCTCAA	BRDN00012	140	48	456	277	602	0
CAAGCTCAA	BRDN00012	106	108	11	199	227	2
CAAGCTCAA	BRDN00011	157	154	54	235	81	30
CAAGCTCAC	BRDN00011	264	114	262	590	357	0
CAAGCTCAC	BRDN00012	249	215	822	345	233	13
CAAGCTCAG	BRDN00012	140	186	576	368	223	1
CAAGCTCAG	BRDN00011	163	107	213	188	318	3
CAAGCTCCC	BRDN00011	108	119	6	302	193	19
CAAGCTCGA	BRDN00012	45	56	241	144	22	0
CAAGCTCGG	BRDN00011	89	5	0	146	157	0
CAAGCTCTA	BRDN00011	162	189	808	295	278	33
CAAGCTCTC	BRDN00011	198	179	33	262	380	12
CAAGCTGAG	BRDN00012	102	101	59	264	212	8
CAAGCTGAG	BRDN00012	368	167	326	605	622	57
CAAGCTGCA	BRDN00012	527	407	574	1058	681	39
CAAGCTGCC	BRDN00012	469	336	75	842	701	19
CAAGCTGCC	BRDN00012	397	396	724	793	939	21
CAAGCTGCT	BRDN00012	90	21	61	268	76	0
CAAGCTGGA	BRDN00011	262	228	744	730	744	8
CAAGCTGGA	BRDN00012	328	276	1029	750	671	8
CAAGCTGGG	BRDN00012	257	225	444	494	390	43
CAAGCTGGG	BRDN00011	203	138	1740	363	565	38
CAAGCTGTC	BRDN00012	148	143	87	305	260	4
CAAGCTGTT	BRDN00012	313	323	665	644	777	22
CAAGCTTAA	BRDN00011	204	182	896	227	144	72
CAAGCTTAG	BRDN00011	241	253	129	392	670	22
CAAGCTTAT	BRDN00012	71	81	243	214	240	0
CAAGCTTGA	BRDN00012	316	247	76	555	338	5
CAAGCTTTC	BRDN00012	79	102	41	203	252	9
CAAGCTTTG	BRDN00012	364	389	737	645	654	40
CAAGGAACG	BRDN00011	115	53	7	180	148	0
CAAGGAACT	BRDN00011	114	33	0	350	594	0
CAAGGAAGA	BRDN00012	230	185	511	219	211	22
CAAGGAAGA	BRDN00011	184	236	85	366	190	0
CAAGGAAGA	BRDN00011	106	49	0	197	45	0
CAAGGAAGC	BRDN00012	100	43	3589	208	190	40
CAAGGAAGT	BRDN00012	108	52	0	123	76	0
CAAGGAAGT	BRDN00012	217	224	496	422	413	0
CAAGGAATA	BRDN00011	45	53	0	155	116	0
CAAGGAATA	BRDN00011	107	140	7	213	114	0
CAAGGAATA	BRDN00012	66	8	1	264	81	0
CAAGGAATG	BRDN00012	101	75	187	183	199	0
CAAGGAATT	BRDN00011	202	219	146	289	308	0
CAAGGACAC	BRDN00012	361	310	386	782	520	91
CAAGGACAC	BRDN00011	116	195	169	195	359	29
CAAGGACAG	BRDN00012	179	98	23	206	150	13
CAAGGACAG	BRDN00011	176	227	868	493	419	63
CAAGGACAT	BRDN00012	249	147	473	422	182	51
CAAGGACCA	BRDN00011	84	58	0	166	172	17
CAAGGACCG	BRDN00011	288	75	1	555	258	0
CAAGGACCG	BRDN00011	49	78	199	161	76	7
CAAGGACGT	BRDN00012	283	235	623	595	803	9
CAAGGAGAA	BRDN00011	54	21	1	119	106	0
CAAGGAGAC	BRDN00012	62	63	7	55	128	0
CAAGGAGAT	BRDN00012	125	48	79	239	214	9
CAAGGAGCC	BRDN00012	143	106	425	233	267	9
CAAGGAGGA	BRDN00011	29	37	0	168	96	0
CAAGGAGGA	BRDN00011	347	219	227	288	534	0
CAAGGAGGC	BRDN00012	62	62	1	189	129	0
CAAGGAGGC	BRDN00012	322	278	572	833	614	9
CAAGGAGGC	BRDN00012	359	463	587	594	818	53
CAAGGAGGC	BRDN00011	127	105	398	193	154	0
CAAGGAGGT	BRDN00012	288	177	55	887	611	28
CAAGGAGGT	BRDN00012	163	147	29	511	698	45
CAAGGAGGT	BRDN00012	427	42	5	625	984	0
CAAGGAGTC	BRDN00011	179	141	13	189	398	15
CAAGGAGTC	BRDN00011	69	124	5	186	142	0
CAAGGATAA	BRDN00011	293	185	192	390	315	8
CAAGGATAT	BRDN00012	542	389	1693	804	939	42
CAAGGATCG	BRDN00012	29	7	14	233	373	0
CAAGGATCT	BRDN00012	124	15	17	390	234	0
CAAGGATGA	BRDN00012	86	62	668	201	247	12
CAAGGATGA	BRDN00012	352	393	3244	823	1046	59
CAAGGATGG	BRDN00011	91	63	1037	213	109	38
CAAGGATGG	BRDN00012	97	70	57	169	147	5
CAAGGATTC	BRDN00011	147	120	519	323	418	24
CAAGGATTG	BRDN00012	128	31	106	277	110	14
CAAGGCACA	BRDN00011	217	262	1401	412	561	68
CAAGGCACA	BRDN00011	247	297	470	458	576	5
CAAGGCACT	BRDN00012	308	417	187	533	209	116
CAAGGCAGG	BRDN00011	5	1	0	41	0	0
CAAGGCATG	BRDN00012	111	134	86	128	167	0
CAAGGCATT	BRDN00012	442	337	579	704	459	91
CAAGGCCAA	BRDN00011	94	0	0	395	193	0
CAAGGCCAA	BRDN00011	105	130	181	161	267	13
CAAGGCCAA	BRDN00011	257	212	166	377	312	20
CAAGGCCAA	BRDN00011	371	337	913	793	732	49
CAAGGCCAC	BRDN00011	128	140	23	217	86	3
CAAGGCCAG	BRDN00011	312	274	913	424	452	19
CAAGGCCAG	BRDN00011	57	173	31	267	127	18
CAAGGCCAT	BRDN00011	105	66	728	190	158	8
CAAGGCCCT	BRDN00012	155	175	260	313	307	40
CAAGGCCCT	BRDN00011	276	8	6	485	145	0
CAAGGCCGG	BRDN00011	110	209	2961	226	311	132
CAAGGCCTG	BRDN00012	172	81	593	263	177	18
CAAGGCGAC	BRDN00012	397	268	270	817	1359	11
CAAGGCGAG	BRDN00012	274	141	140	498	362	8
CAAGGCGCA	BRDN00011	237	304	348	596	431	40
CAAGGCGCC	BRDN00012	181	171	14	232	129	21
CAAGGCGGA	BRDN00011	118	113	215	182	57	0
CAAGGCGGC	BRDN00011	528	501	562	964	840	91
CAAGGCTAA	BRDN00012	115	149	0	313	194	4
CAAGGCTCC	BRDN00012	141	166	193	171	63	13
CAAGGCTGT	BRDN00012	96	81	318	196	248	0
CAAGGCTTA	BRDN00011	59	51	80	122	111	0
CAAGGCTTC	BRDN00012	424	342	886	577	576	78
CAAGGGAAC	BRDN00012	392	475	218	521	401	25
CAAGGGAGA	BRDN00012	88	56	46	499	459	0
CAAGGGATA	BRDN00011	395	391	555	773	770	62
CAAGGGCAA	BRDN00012	376	370	570	620	480	53
CAAGGGCAA	BRDN00011	77	73	0	118	95	8
CAAGGGCAG	BRDN00011	100	90	0	300	210	0
CAAGGGCAT	BRDN00012	209	225	670	393	458	54
CAAGGGCCT	BRDN00012	299	298	365	588	734	36
CAAGGGCTA	BRDN00011	116	115	448	158	90	14
CAAGGGGAC	BRDN00012	91	176	729	237	197	18
CAAGGGGCA	BRDN00012	176	148	40	189	159	16
CAAGGGGCC	BRDN00011	301	266	0	541	535	0
CAAGGGGCC	BRDN00012	21	0	0	155	11	0
CAAGGGGTA	BRDN00011	87	31	0	186	188	0
CAAGGGGTT	BRDN00011	112	17	93	286	356	0
CAAGGGTCT	BRDN00012	219	302	164	407	302	9
CAAGGGTGA	BRDN00011	179	108	120	369	197	4
CAAGGTACA	BRDN00012	57	34	0	355	140	6
CAAGGTAGA	BRDN00011	112	70	1028	215	209	40
CAAGGTAGC	BRDN00012	115	108	0	279	243	0
CAAGGTAGG	BRDN00011	274	213	1410	513	601	25
CAAGGTAGT	BRDN00011	191	97	154	219	254	16
CAAGGTCAA	BRDN00011	323	316	372	478	602	43
CAAGGTCAA	BRDN00011	141	101	0	378	344	3
CAAGGTCAC	BRDN00012	443	458	2393	786	558	59
CAAGGTCAG	BRDN00012	320	225	45	744	505	12
CAAGGTCGC	BRDN00011	170	145	1227	485	531	42
CAAGGTCTT	BRDN00011	91	117	6207	228	290	46
CAAGGTGAA	BRDN00012	99	65	68	246	92	35
CAAGGTGCT	BRDN00011	318	386	163	770	888	59
CAAGGTGGA	BRDN00011	316	313	1802	599	740	40
CAAGGTGTA	BRDN00012	168	237	158	325	416	0
CAAGGTGTA	BRDN00012	141	241	36	512	414	22
CAAGGTGTC	BRDN00011	30	0	0	110	74	0
CAAGGTTAA	BRDN00012	280	357	681	401	361	33
CAAGGTTCC	BRDN00011	189	216	441	484	430	34
CAAGGTTCC	BRDN00012	87	115	48	271	215	0
CAAGGTTCT	BRDN00011	144	124	0	359	496	3
CAAGTAAAT	BRDN00012	197	68	1096	539	603	30
CAAGTAACC	BRDN00012	334	367	662	542	756	31
CAAGTAAGT	BRDN00011	259	283	253	467	296	27
CAAGTAATT	BRDN00012	138	36	11	201	145	0
CAAGTACAA	BRDN00011	99	129	0	106	175	0
CAAGTACAC	BRDN00012	107	10	0	246	167	0
CAAGTACAG	BRDN00011	63	52	77	167	55	0
CAAGTACAG	BRDN00011	253	268	800	565	819	80
CAAGTACAT	BRDN00012	495	400	2063	745	663	49
CAAGTACCA	BRDN00012	108	122	9	267	417	14
CAAGTACCG	BRDN00012	222	231	577	460	503	31
CAAGTACCT	BRDN00012	85	123	178	215	424	14
CAAGTACGA	BRDN00012	189	132	469	346	310	2
CAAGTACGT	BRDN00011	119	118	0	212	111	12
CAAGTACTC	BRDN00011	357	141	20	655	603	0
CAAGTACTT	BRDN00012	355	343	2164	564	491	72
CAAGTACTT	BRDN00011	44	59	0	480	390	0
CAAGTACTT	BRDN00012	253	251	70	522	464	15
CAAGTAGGC	BRDN00012	279	258	1428	575	702	34
CAAGTATAA	BRDN00012	182	5	105	647	33	0
CAAGTATCG	BRDN00012	204	152	395	388	380	18
CAAGTATCT	BRDN00011	67	42	157	186	258	0
CAAGTATGG	BRDN00012	219	215	291	563	733	10
CAAGTATGT	BRDN00012	235	216	429	484	348	59
CAAGTCACA	BRDN00011	145	235	519	432	541	44
CAAGTCACC	BRDN00012	209	326	151	734	354	21
CAAGTCACT	BRDN00012	188	229	81	450	622	14
CAAGTCAGT	BRDN00011	115	51	17	222	48	0
CAAGTCAGT	BRDN00012	170	69	92	254	283	0
CAAGTCCAA	BRDN00011	178	91	23	213	125	10
CAAGTCCAA	BRDN00011	438	311	461	793	945	8
CAAGTCCAA	BRDN00011	86	9	114	237	182	10
CAAGTCCAC	BRDN00011	197	156	165	347	273	0
CAAGTCCAT	BRDN00012	306	396	1373	550	412	30
CAAGTCCCA	BRDN00012	96	85	306	250	124	12
CAAGTCCCA	BRDN00011	108	23	7	159	467	0
CAAGTCCCG	BRDN00012	9	0	0	268	46	0
CAAGTCCTG	BRDN00011	179	113	1259	317	321	25
CAAGTCCTG	BRDN00012	455	573	435	925	1012	28
CAAGTCCTT	BRDN00012	375	417	161	740	929	31
CAAGTCGCA	BRDN00011	265	184	18	410	355	27
CAAGTCGGG	BRDN00012	232	127	76	374	303	0
CAAGTCGTA	BRDN00011	174	152	24	331	312	14
CAAGTCTAT	BRDN00011	123	0	13	401	169	0
CAAGTCTCC	BRDN00011	414	421	925	917	663	45
CAAGTCTCG	BRDN00012	418	307	529	805	918	69
CAAGTCTGA	BRDN00012	115	153	89	332	599	35
CAAGTCTGG	BRDN00011	12	0	0	191	214	0
CAAGTCTTG	BRDN00011	79	78	16	162	176	13
CAAGTGAAA	BRDN00011	261	121	36	391	760	17
CAAGTGACA	BRDN00012	361	181	500	492	345	6
CAAGTGACT	BRDN00012	246	107	121	398	244	18
CAAGTGAGA	BRDN00011	301	258	1	480	523	0
CAAGTGAGC	BRDN00012	199	100	0	386	149	0
CAAGTGAGC	BRDN00011	409	431	19	1003	1253	2
CAAGTGAGC	BRDN00012	392	309	186	612	717	5
CAAGTGAGC	BRDN00012	63	15	0	167	53	0
CAAGTGATA	BRDN00011	189	228	5	393	323	30
CAAGTGCAA	BRDN00012	152	189	52	261	162	29
CAAGTGCAA	BRDN00011	40	102	0	65	225	0
CAAGTGCAA	BRDN00012	135	101	16	311	158	0
CAAGTGCAA	BRDN00012	300	339	552	684	608	42
CAAGTGCAC	BRDN00011	160	131	24	182	293	3
CAAGTGCCA	BRDN00012	72	132	38	50	65	45
CAAGTGCCC	BRDN00012	13	15	148	18	0	0
CAAGTGCTC	BRDN00011	148	185	56	337	215	93
CAAGTGGAG	BRDN00012	48	49	0	162	185	12
CAAGTGGAG	BRDN00011	111	137	196	178	370	18
CAAGTGGCG	BRDN00011	96	51	0	137	118	0
CAAGTGGCT	BRDN00012	83	137	457	148	64	0
CAAGTGGCT	BRDN00011	201	56	35	251	496	8
CAAGTGGGT	BRDN00012	452	501	868	627	874	64
CAAGTGGTC	BRDN00011	179	119	233	429	404	59
CAAGTGGTG	BRDN00012	296	253	901	608	597	27
CAAGTGTAA	BRDN00011	93	127	2604	215	449	53
CAAGTGTAA	BRDN00012	129	141	260	354	239	0
CAAGTGTAC	BRDN00012	127	88	0	333	111	0
CAAGTGTAC	BRDN00011	395	267	846	717	851	18
CAAGTGTCA	BRDN00012	260	180	720	644	557	35
CAAGTGTCC	BRDN00011	389	344	125	570	512	0
CAAGTGTCC	BRDN00011	256	151	1061	447	315	16
CAAGTGTGA	BRDN00011	30	41	178	170	181	14
CAAGTGTGA	BRDN00012	121	84	0	205	258	3
CAAGTGTGA	BRDN00012	341	325	252	590	238	32
CAAGTGTGC	BRDN00011	217	158	342	378	325	22
CAAGTGTGG	BRDN00012	690	560	1254	972	832	404
CAAGTGTTC	BRDN00011	102	122	442	181	171	10
CAAGTGTTC	BRDN00011	15	0	0	191	65	0
CAAGTGTTG	BRDN00011	227	178	1674	536	403	45
CAAGTTAAT	BRDN00011	268	131	12	391	502	6
CAAGTTACA	BRDN00011	450	356	48	828	1034	42
CAAGTTACT	BRDN00012	135	0	0	281	139	0
CAAGTTAGT	BRDN00011	111	144	14	348	417	16
CAAGTTATC	BRDN00012	145	0	0	484	99	0
CAAGTTATT	BRDN00012	246	214	81	391	595	33
CAAGTTCAA	BRDN00011	252	163	118	507	413	29
CAAGTTCAA	BRDN00011	176	150	301	303	219	19
CAAGTTCAA	BRDN00011	127	61	27	250	204	0
CAAGTTCAC	BRDN00011	138	93	156	453	234	31
CAAGTTCAG	BRDN00012	342	336	833	621	542	31
CAAGTTCCA	BRDN00011	139	360	935	352	188	19
CAAGTTCCA	BRDN00012	499	380	249	753	722	33
CAAGTTCCA	BRDN00011	218	271	1839	384	149	65
CAAGTTCCG	BRDN00011	135	109	221	140	156	39
CAAGTTCGC	BRDN00012	157	67	66	164	278	11
CAAGTTCTC	BRDN00012	374	352	589	845	632	129
CAAGTTCTG	BRDN00011	110	34	21	209	231	17
CAAGTTGAA	BRDN00012	242	242	143	305	601	54
CAAGTTTCA	BRDN00011	147	45	178	214	159	16
CAAGTTTCC	BRDN00012	241	292	570	398	190	32
CAAGTTTGA	BRDN00011	107	46	1	236	97	0
CAAGTTTGG	BRDN00012	143	0	1	372	276	0
CAAGTTTGG	BRDN00011	326	183	430	567	513	5
CAAGTTTGT	BRDN00012	249	206	188	349	131	41
CAAGTTTTC	BRDN00012	430	411	1252	780	736	33
CAATAAAAA	BRDN00012	41	0	0	168	170	0
CAATAAAAC	BRDN00012	364	384	72	622	352	27
CAATAAAAT	BRDN00012	104	44	196	227	349	44
CAATAAAGA	BRDN00011	80	53	0	209	430	0
CAATAAATC	BRDN00012	213	154	560	329	346	6
CAATAAATC	BRDN00011	330	410	514	577	1097	13
CAATAAATC	BRDN00011	139	153	240	212	280	59
CAATAACAC	BRDN00012	183	110	21	259	261	7
CAATAACAT	BRDN00012	192	233	375	430	369	22
CAATAACAT	BRDN00012	79	132	169	217	50	0
CAATAACTC	BRDN00012	162	334	734	513	523	12
CAATAAGAG	BRDN00011	156	144	32	418	486	69
CAATAAGCC	BRDN00011	277	231	42	487	344	11
CAATAAGTT	BRDN00012	237	190	2361	282	257	68
CAATAATAG	BRDN00011	240	221	82	596	397	44
CAATAATAG	BRDN00011	114	150	63	256	224	268
CAATAATGA	BRDN00011	199	156	64	296	704	0
CAATAATGC	BRDN00011	108	99	1	295	396	0
CAATAATTA	BRDN00012	181	280	85	276	211	36
CAATACAAT	BRDN00012	330	84	242	520	320	0
CAATACACT	BRDN00011	83	98	806	381	396	22
CAATACAGT	BRDN00012	211	124	634	312	238	14
CAATACCTC	BRDN00012	360	191	13	539	489	19

CAATACTTAC BRDN00012	135	153	19	166	93	16
CAATAGAGG BRDN00012	485	443	170	732	284	7
CAATAGAGT BRDN00012	180	76	80	213	319	8
CAATAGCCTT BRDN00011	150	172	864	241	450	3
CAATAGGGA BRDN00012	184	273	1042	499	432	17
CAATAGTAC BRDN00012	273	288	854	452	1127	12
CAATAGTGA BRDN00011	208	191	138	270	160	33
CAATATACTG BRDN00012	302	235	434	466	222	17
CAATATAGT BRDN00011	125	195	11	217	214	7
CAATATCATA BRDN00011	83	48	2	158	350	7
CAATATCATC BRDN00011	373	161	1268	526	327	24
CAATATCCAT BRDN00011	0	0	0	0	0	0
CAATATCGG BRDN00012	759	606	369	1361	968	33
CAATATCTAA BRDN00011	222	22	0	424	383	0
CAATATCTAA BRDN00012	196	125	87	250	332	15
CAATATCTAT BRDN00011	256	276	1014	564	383	42
CAATATCTAT BRDN00012	159	188	49	263	195	12
CAATATCTGA BRDN00011	110	96	161	198	167	20
CAATATCTTG BRDN00011	268	218	376	335	117	34
CAATATGAC BRDN00012	94	83	8	152	181	0
CAATATGTC BRDN00011	139	111	30	266	250	0
CAATATGTG BRDN00011	302	402	428	784	397	42
CAATATTCTG BRDN00012	431	286	1503	813	1208	37
CAATATTGA BRDN00011	66	46	893	178	348	17
CAATATTGT BRDN00012	299	332	912	591	682	9
CAATATTTAC BRDN00012	178	206	39	242	179	0
CAATCAAGA BRDN00012	291	202	423	759	470	37
CAATCAAGT BRDN00011	150	157	26	233	252	19
CAATCAATA BRDN00012	170	67	348	290	500	0
CAATCAATCA BRDN00011	290	214	122	578	549	6
CAATCACACC BRDN00011	125	179	12	321	412	8
CAATCACAGT BRDN00012	370	455	243	733	833	76
CAATCACTTC BRDN00012	263	301	1264	524	483	33
CAATCAGCAT BRDN00012	187	112	715	314	463	0
CAATCAGTG BRDN00011	138	216	89	358	557	29
CAATCATCAG BRDN00012	473	327	839	683	636	23
CAATCATCAT BRDN00012	181	159	3042	352	246	73
CAATCATGCA BRDN00011	166	36	0	319	391	0
CAATCCACCC BRDN00012	255	277	987	520	297	17
CAATCCAGAT BRDN00012	234	180	131	385	708	8
CAATCCAGCC BRDN00012	149	75	1	345	696	0
CAATCCATTC BRDN00011	115	54	121	336	182	13
CAATCCCGA BRDN00011	186	4	0	546	261	0
CAATCCTCGG BRDN00012	357	320	411	667	403	25
CAATCCTCTG BRDN00011	196	206	170	372	248	15
CAATCGCATC BRDN00012	94	0	1	241	256	1
CAATCGCTAC BRDN00012	160	22	234	691	731	6
CAATCGCTAC BRDN00011	153	183	211	368	291	49
CAATCGGCG BRDN00012	353	519	1784	686	1195	30
CAATCTAGG BRDN00011	245	144	564	381	427	36
CAATCTAGG BRDN00012	322	194	686	505	515	32
CAATCTCTAA BRDN00011	128	207	328	354	237	1
CAATCTCTC BRDN00011	284	233	2081	470	658	29
CAATCTGGC BRDN00012	340	237	493	519	370	6
CAATCTGTC BRDN00011	88	158	176	275	442	0
CAATCTGTTC BRDN00011	183	166	162	335	221	0
CAATCTTCT BRDN00011	294	259	347	562	471	23
CAATGAAAC BRDN00011	296	209	479	387	395	76
CAATGAACA BRDN00012	268	76	513	595	458	0
CAATGAACG BRDN00012	326	126	61	633	736	3
CAATGAAGA BRDN00012	104	102	52	265	274	14
CAATGAAGC BRDN00011	207	363	280	509	265	38
CAATGAAGG BRDN00011	110	82	16	228	189	9
CAATGAAGG BRDN00012	408	251	995	803	634	21
CAATGACAA BRDN00011	194	273	91	338	355	8
CAATGACAA BRDN00011	342	225	259	510	394	19
CAATGACAA BRDN00012	120	122	143	270	394	0
CAATGACAT BRDN00012	170	143	10	294	318	0
CAATGACCT BRDN00011	332	305	624	518	370	44
CAATGACCTT BRDN00012	113	150	0	254	255	11
CAATGACGA BRDN00012	467	546	377	664	480	113
CAATGACTT BRDN00012	327	233	1027	582	847	7
CAATGAGAC BRDN00011	212	232	73	452	106	1
CAATGAGAT BRDN00011	365	260	928	744	904	57
CAATGAGCG BRDN00011	95	114	236	189	126	12
CAATGATAC BRDN00012	167	159	477	245	219	35
CAATGATAG BRDN00011	141	49	178	186	108	9
CAATGATCA BRDN00012	176	229	117	318	118	26
CAATGATGT BRDN00011	244	325	1340	631	541	20
CAATGATGT BRDN00012	332	53	10	743	590	0
CAATGATTA BRDN00011	244	201	46	528	666	0
CAATGCAAC BRDN00011	139	154	136	189	202	0
CAATGCAGG BRDN00012	369	362	719	703	1298	28
CAATGCAGG BRDN00012	70	62	106	211	267	0
CAATGCATG BRDN00011	289	292	787	556	506	46
CAATGCCAC BRDN00012	148	150	454	518	455	17
CAATGCCAG BRDN00012	549	522	420	1307	1237	97
CAATGCCAT BRDN00011	383	286	1124	848	2139	51
CAATGCCCCC BRDN00012	207	64	0	261	203	14
CAATGCCCC BRDN00012	194	233	487	487	229	18
CAATGCCGA BRDN00012	94	137	0	190	169	6
CAATGCCGG BRDN00011	283	476	2923	678	1141	74
CAATGCCTA BRDN00012	392	468	876	769	1304	46
CAATGCGGC BRDN00011	148	189	309	255	375	58
CAATGCTAC BRDN00012	547	452	2046	1053	1292	114
CAATGCTCA BRDN00012	439	340	291	675	1208	62
CAATGCTCTG BRDN00012	455	326	1061	830	1077	53
CAATGCTGT BRDN00011	519	467	516	824	1039	24
CAATGCTTA BRDN00011	198	151	177	502	496	8
CAATGGAAG BRDN00011	273	324	41	414	176	18
CAATGGACC BRDN00012	127	93	282	341	136	0
CAATGGACG BRDN00012	236	116	629	333	279	16
CAATGGAGC BRDN00012	403	334	341	734	414	22
CAATGGATT BRDN00012	28	14	14	156	112	0
CAATGGCAA BRDN00011	188	129	178	338	426	5
CAATGGCAA BRDN00011	153	94	343	265	461	41
CAATGGCAA BRDN00011	236	121	154	463	369	31
CAATGGCCC BRDN00012	90	143	50	287	550	12
CAATGGCGA BRDN00012	12	0	0	375	7	0
CAATGGCGG BRDN00012	338	223	151	562	457	18
CAATGGCGT BRDN00012	587	610	2003	824	856	107
CAATGGGAA BRDN00011	166	136	875	393	186	3
CAATGGGAA BRDN00011	242	335	556	547	430	40
CAATGGGAC BRDN00012	66	22	150	369	434	14
CAATGGGCC BRDN00011	168	200	906	318	312	17
CAATGGGTC BRDN00012	399	513	346	992	972	47
CAATGGGTG BRDN00012	205	168	286	332	158	25
CAATGGTAC BRDN00012	38	22	1	225	226	0
CAATGGTCA BRDN00012	179	163	114	283	269	12
CAATGTAGA BRDN00011	143	89	86	227	295	5
CAATGTAGA BRDN00012	419	334	1624	718	697	36
CAATGTATA BRDN00012	20	0	0	89	71	0
CAATGTATT BRDN00012	259	417	408	549	660	35
CAATGTCAA BRDN00012	233	148	158	624	473	20
CAATGTCAT BRDN00012	394	344	237	721	823	41
CAATGTCTA BRDN00012	136	138	1	250	273	14
CAATGTCTG BRDN00011	188	58	1	208	246	1
CAATGTGAA BRDN00012	224	301	334	343	407	156
CAATGTGAT BRDN00012	324	294	777	533	520	27
CAATGTGAT BRDN00011	78	75	8	283	258	0
CAATGTGGC BRDN00012	418	426	428	656	767	50
CAATGTGGG BRDN00012	227	168	384	377	669	0
CAATGTGTC BRDN00012	188	253	0	400	210	0
CAATGTTAC BRDN00012	384	584	844	846	950	19
CAATGTTAG BRDN00011	196	183	1291	443	389	34
CAATGTTATT BRDN00012	265	215	274	591	627	31
CAATGTTGG BRDN00011	156	79	16	286	309	0
CAATGTTGG BRDN00012	439	370	702	736	848	20
CAATGTTTC BRDN00012	262	60	7	477	538	0
CAATGTTTG BRDN00011	112	59	73	193	197	0
CAATTAAAT BRDN00012	261	261	158	526	515	10
CAATTAAGA BRDN00011	129	140	270	207	672	8
CAATTACATT BRDN00012	323	282	641	761	771	20
CAATTACTG BRDN00011	262	353	365	423	401	0
CAATTATAGT BRDN00012	121	87	904	473	912	6
CAATTATCAC BRDN00012	250	126	2165	411	577	13
CAATTATGA BRDN00011	196	153	456	350	617	9
CAATTCCAAT BRDN00012	294	249	27	506	608	6
CAATTCTAAT BRDN00012	290	96	20	329	332	6
CAATTCTAG BRDN00012	192	0	0	547	354	0
CAATTCTCAT BRDN00012	81	77	0	177	450	0
CAATTCTGA BRDN00012	175	125	85	290	261	31
CAATTGAAT BRDN00012	165	121	13	303	224	0
CAATTGAGA BRDN00011	175	117	101	332	449	11
CAATTGAGA BRDN00011	145	109	10	163	194	12
CAATTGATA BRDN00011	161	7	1	830	508	0
CAATTGATA BRDN00012	292	156	170	677	515	15
CAATTGCGA BRDN00012	81	76	237	127	28	16
CAATTGGAG BRDN00011	211	35	0	522	479	0
CAATTGGAT BRDN00011	144	97	0	301	274	21
CAATTGGGT BRDN00012	98	85	169	196	326	16
CAATTGTAA BRDN00012	124	163	93	300	309	0
CAATTGTAG BRDN00012	140	74	217	173	78	0
CAATTGTCA BRDN00011	95	109	101	175	171	11
CAATTGTCA BRDN00011	189	54	458	311	62	0
CAATTTATAC BRDN00011	225	157	1097	300	219	63
CAATTTCACC BRDN00011	350	396	3132	686	693	28
CAATTTCAG BRDN00012	266	98	179	354	773	5
CAATTTCAG BRDN00011	527	372	2105	731	476	86
CAATTTCCCT BRDN00012	96	122	39	302	156	0
CAATTTCGA BRDN00011	106	39	0	262	172	0
CAATTTGAC BRDN00012	105	0	0	244	236	0
CAATTTGAG BRDN00011	214	304	1205	394	294	43
CAATTTGAG BRDN00011	46	73	29	42	164	0
CAATTTGCCT BRDN00011	385	465	939	978	1950	128
CAATTTGGT BRDN00011	166	183	378	342	704	0
CAATTTGTAT BRDN00011	140	84	60	292	436	21
CACAAAAAA BRDN00012	101	56	512	283	334	8
CACAAAAAC BRDN00012	486	267	270	1090	1250	0
CACAAAAACT BRDN00012	209	248	17	257	246	14
CACAAAAGA BRDN00012	170	122	1635	268	409	29
CACAAAATA BRDN00012	162	138	39	254	337	10
CACAAAATT BRDN00011	291	317	139	569	401	22
CACAAACAG BRDN00011	313	262	363	636	845	18
CACAAACAT BRDN00012	96	100	154	173	77	28
CACAAACCA BRDN00011	294	297	249	486	429	5
CACAAACCTG BRDN00011	156	10	6	351	3	1
CACAAACCTG BRDN00011	349	487	584	657	677	19
CACAAACCTG BRDN00011	414	379	1528	566	661	27
CACAAACGT BRDN00012	196	253	112	359	535	25
CACAAAGAT BRDN00012	228	152	1455	400	649	55
CACAAAGCG BRDN00011	98	150	124	192	172	0
CACAAAGCTT BRDN00012	104	71	103	214	571	30
CACAAAGTC BRDN00012	106	144	0	210	178	0
CACAAAGTC BRDN00012	167	132	23	291	399	61
CACAAATAA BRDN00012	112	174	11	205	321	23
CACAAATTG BRDN00011	241	179	1917	435	327	10
CACAACAAG BRDN00012	123	170	773	328	339	79
CACAACAGA BRDN00012	336	257	327	788	775	52
CACAACAGC BRDN00011	131	181	236	274	810	81
CACAACAGG BRDN00012	170	106	45	291	207	0
CACAACAGG BRDN00012	142	38	139	274	156	23
CACAACATA BRDN00012	270	272	116	286	298	33
CACAACATCT BRDN00012	420	279	804	829	731	46
CACAACATG BRDN00011	397	128	132	582	208	0
CACAACCAA BRDN00011	169	59	6983	247	188	89
CACAACCAC BRDN00011	112	104	393	217	98	0
CACAACCCAC BRDN00011	396	289	202	708	552	31
CACAACCCCA BRDN00012	342	315	23	556	890	23
CACAACCGC BRDN00012	118	37	16	129	135	0
CACAACGCT BRDN00012	107	94	13	171	226	14
CACAACGGA BRDN00011	134	74	197	256	667	52
CACAACGGA BRDN00012	191	207	201	520	555	27
CACAACGGA BRDN00012	326	35	0	668	872	1
CACAACGGT BRDN00012	356	406	3290	578	736	54
CACAACTCAC BRDN00012	228	267	306	500	447	39
CACAACTGT BRDN00011	175	16	0	408	528	0
CACAACTGT BRDN00011	112	0	0	307	3	0
CACAACTTCA BRDN00011	151	95	6	218	223	4
CACAACTTGT BRDN00011	160	22	1021	199	148	44
CACAAGAAA BRDN00011	75	119	1216	179	170	15
CACAAGAAG BRDN00012	80	5	0	415	307	0
CACAAGACA BRDN00011	100	65	168	261	263	0
CACAAGAGG BRDN00012	52	158	108	195	126	0
CACAAGATG BRDN00011	279	279	355	522	470	13
CACAAGCAA BRDN00011	156	107	415	290	394	0
CACAAGCAC BRDN00012	226	280	210	358	293	67
CACAAGCACT BRDN00011	102	112	33	170	143	11
CACAAGCCT BRDN00012	402	335	176	750	588	42
CACAAGCGG BRDN00012	261	379	1963	638	747	91
CACAAGGCC BRDN00012	175	123	378	316	733	59
CACAAGGCG BRDN00011	94	77	41	233	508	0
CACAAGTAT BRDN00011	405	519	556	697	549	12
CACAAGTCCT BRDN00011	600	458	941	871	808	63
CACAAGTGA BRDN00011	261	269	101	646	519	4
CACAAGTTC BRDN00011	167	113	869	278	171	12
CACAATACG BRDN00011	135	107	14	168	74	19
CACAATCAA BRDN00011	132	170	193	226	425	15
CACAATCACA BRDN00012	146	207	1177	212	187	16
CACAATCAG BRDN00012	243	275	0	593	505	26
CACAATCTTA BRDN00012	261	199	125	429	413	2
CACAATGAA BRDN00011	250	232	148	527	411	55
CACAATGAC BRDN00012	250	295	320	371	215	11
CACAATGGC BRDN00011	172	160	672	315	341	89
CACAATGGT BRDN00012	138	87	671	209	401	9
CACAATGTCT BRDN00012	169	239	110	372	161	22
CACAATTCTG BRDN00012	310	282	29	672	909	17
CACACAAGC BRDN00012	172	135	162	585	898	0
CACACAAGT BRDN00011	94	220	172	230	201	32
CACACAAGT BRDN00012	529	428	1487	619	839	135
CACACACAA BRDN00012	146	104	115	223	344	0
CACACACACA BRDN00012	267	316	491	579	672	44
CACACACAG BRDN00011	24	1	0	267	323	0
CACACACAG BRDN00011	163	281	208	406	677	44
CACACACATA BRDN00012	415	313	52	569	645	41
CACACACCAG BRDN00012	134	151	73	313	417	21
CACACACCCA BRDN00012	131	78	104	209	166	21
CACACACCCC BRDN00011	330	308	175	635	841	27
CACACACCCG BRDN00012	357	372	2255	636	465	117
CACACACGA BRDN00012	409	430	10262	693	746	110
CACACACGAT BRDN00011	127	30	5	287	176	0
CACACACGG BRDN00011	409	363	87	767	754	47
CACACACTAC BRDN00011	73	74	3	279	606	0
CACACACTCG BRDN00012	344	318	262	676	949	21
CACACACTGA BRDN00011	134	113	1310	255	321	21
CACACACTGC BRDN00011	181	77	62	380	238	0
CACACACTGC BRDN00011	35	19	20	30	18	27
CACACAGGA BRDN00012	193	261	376	354	342	25
CACACAGGA BRDN00011	166	145	944	321	267	30
CACACAGGG BRDN00012	173	221	96	333	248	0
CACACAGGT BRDN00011	123	0	0	305	63	0
CACACAGGT BRDN00012	366	278	323	435	402	32
CACACAGGT BRDN00012	100	86	40	290	222	40
CACACAGTA BRDN00011	223	50	0	384	259	0
CACACAGTAT BRDN00012	386	211	461	433	734	61
CACACAGTCA BRDN00012	152	71	54	396	270	9
CACACAGTCC BRDN00011	412	1	0	666	591	0
CACACAGTCC BRDN00011	250	180	134	398	398	44
CACACAGTGT BRDN00011	11	10	0	390	199	0
CACACAGTTT BRDN00012	224	68	1	391	471	52
CACACATAAT BRDN00011	118	0	0	274	304	0
CACACATATG BRDN00011	396	356	307	499	609	21
CACACATCCA BRDN00012	211	196	1	494	473	0
CACACATCTT BRDN00012	461	395	527	912	685	74
CACACATGAC BRDN00011	214	81	129	453	710	10
CACACCAAAC BRDN00011	420	1203	8317	802	1048	364
CACACCAACT BRDN00011	487	436	408	691	1151	56
CACACCAAGC BRDN00012	246	370	324	475	473	70
CACACCAAGC BRDN00012	34	8	0	302	271	0
CACACCAATG BRDN00011	481	638	650	952	792	86
CACACCACAG BRDN00011	361	11	1	775	174	1
CACACCACCT BRDN00012	259	300	150	624	382	8
CACACCACTA BRDN00011	303	278	306	455	622	14
CACACCACTG BRDN00012	302	254	293	667	442	49
CACACCAGAA BRDN00012	221	321	921	535	533	24
CACACCAGAC BRDN00012	498	391	1648	907	1068	38
CACACCAGCA BRDN00011	187	261	190	360	552	76
CACACCAGTC BRDN00012	394	645	2719	652	475	200
CACACCAGTT BRDN00012	427	301	0	758	396	11
CACACCATAC BRDN00012	156	102	45	281	216	26
CACACCATAG BRDN00012	273	271	801	550	344	64
CACACCCAAT BRDN00011	87	204	106	133	247	32
CACACCCACA BRDN00012	128	137	523	262	343	31
CACACCCACC BRDN00012	420	364	254	671	505	24
CACACCCAGC BRDN00011	83	26	67	113	98	0
CACACCCAGC BRDN00011	67	28	0	78	139	0
CACACCCATA BRDN00011	123	181	640	217	782	43
CACACCCCAG BRDN00012	221	220	1014	329	438	34
CACACCCCGG BRDN00011	172	150	128	251	107	33
CACACCGCAC BRDN00011	201	223	155	391	381	57
CACACCGCTG BRDN00012	122	159	1494	290	573	8
CACACCGGCC BRDN00011	225	163	525	344	488	51
CACACCTATC BRDN00011	182	42	21	361	501	0
CACACCTCTG BRDN00012	567	501	869	915	742	605
CACACCTGAC BRDN00012	365	388	203	809	766	47
CACACCTGCC BRDN00011	123	24	29	201	280	27
CACACCTGGT BRDN00011	277	221	1990	330	307	150
CACACCTTAC BRDN00012	98	84	104	244	53	0
CACACGAATC BRDN00011	327	316	661	728	854	8
CACACGAATC BRDN00011	305	321	120	590	496	14
CACACGACTC BRDN00012	79	36	74	183	151	2
CACACGAGTT BRDN00011	160	169	373	300	277	19
CACACGATCC BRDN00012	130	187	70	341	83	14
CACACGATGT BRDN00011	496	562	560	951	748	64
CACACGCACC BRDN00011	159	4	0	257	156	0
CACACGCACC BRDN00012	406	518	750	668	555	91
CACACGCATC BRDN00012	440	465	3169	748	791	103
CACACGCCTA BRDN00011	366	304	417	789	596	8
CACACGCGG BRDN00011	357	380	510	669	715	76
CACACGCGG BRDN00011	149	186	378	245	153	28
CACACGCTAC BRDN00011	357	139	54	571	384	1
CACACGCTAT BRDN00012	270	395	619	661	618	25
CACACGCTGC BRDN00012	75	54	15	261	610	2
CACACGGATT BRDN00011	285	278	659	557	549	8
CACACGGCTC BRDN00012	80	104	0	89	26	50
CACACGGGC BRDN00012	209	65	2	557	468	0
CACACGGTCC BRDN00011	102	99	7	193	135	9
CACACGGTG BRDN00012	319	409	1235	604	399	69
CACACGGTTC BRDN00011	454	373	946	953	798	13
CACACGTAG BRDN00012	18	17	0	225	139	0
CACACGTCTG BRDN00012	285	220	27	392	664	6
CACACGTGA BRDN00012	191	263	79	399	493	31
CACACGTGC BRDN00012	89	38	0	231	229	0
CACACGTGG BRDN00012	177	242	470	337	212	7
CACACTAACT BRDN00011	191	221	161	251	181	32
CACACTAGA BRDN00012	460	465	252	680	740	56
CACACTCACC BRDN00011	214	206	898	487	528	46
CACACTCACT BRDN00012	128	28	291	331	158	0
CACACTCACT BRDN00012	320	8	568	742	422	7
CACACTCAGA BRDN00011	143	218	1428	352	392	20
CACACTCCAG BRDN00011	511	353	517	917	966	42
CACACTCGCT BRDN00012	188	53	1	297	379	0
CACACTCTAT BRDN00012	273	198	732	422	535	9
CACACTCTCA BRDN00011	144	137	837	245	242	33
CACACTCTGA BRDN00012	45	24	0	173	26	0
CACACTCTGA BRDN00011	309	155	51	681	526	12
CACACTCTGC BRDN00011	192	257	604	243	188	32
CACACTCTTC BRDN00012	184	240	576	372	401	26
CACACTGAA BRDN00012	205	204	1047	264	142	17
CACACTGAG BRDN00012	232	212	320	520	478	11
CACACTGCA BRDN00011	247	236	225	343	262	26
CACACTGCA BRDN00012	219	148	302	355	339	7
CACACTGGTA BRDN00012	243	223	374	625	225	30
CACACTGGTC BRDN00012	251	211	0	220	342	18
CACACTTACA BRDN00012	9	13	0	229	103	0
CACACTTACA BRDN00011	166	143	515	450	474	10
CACACTTACC BRDN00011	149	149	43	212	133	22
CACACTTACT BRDN00011	289	115	228	461	367	18
CACACTTCCC BRDN00012	152	196	2087	264	379	25
CACACTTCTG BRDN00011	273	261	1	354	608	11
CACACTTGCT BRDN00011	146	115	37	249	354	0
CACACTTGGC BRDN00011	261	229	77	421	356	0
CACACTTGGC BRDN00011	50	25	29	141	174	0
CACACTTGGC BRDN00011	203	126	9	334	395	0
CACACTTGTG BRDN00012	333	197	40	577	173	11
CACAGAAAC BRDN00011	185	67	9	276	129	5
CACAGAAAG BRDN00012	269	329	2235	629	712	59
CACAGAAAG BRDN00012	157	77	194	212	181	0
CACAGAAAG BRDN00011	138	52	94	232	282	14
CACAGAAGA BRDN00011	125	44	11	166	79	12
CACAGAAGA BRDN00012	170	217	44	619	341	9
CACAGAAGA BRDN00011	382	356	937	608	274	42
CACAGAAGG BRDN00011	335	318	1428	570	773	41
CACAGAAGG BRDN00012	305	372	969	768	510	23
CACAGAAGG BRDN00011	136	11	0	268	174	0
CACAGAAGG BRDN00011	375	379	526	588	822	18
CACAGAAGT BRDN00012	225	133	85	345	373	34
CACAGAATG BRDN00012	112	71	1382	265	128	46
CACAGAATT BRDN00011	202	92	132	386	483	22
CACAGACAA BRDN00011	151	59	127	218	165	0
CACAGACAC BRDN00012	201	249	20	422	300	35
CACAGACAC BRDN00011	262	258	188	357	628	79
CACAGACAC BRDN00012	66	43	12	133	252	44
CACAGACCA BRDN00012	101	140	69	202	174	22
CACAGACCCC BRDN00012	116	74	12	212	187	22
CACAGACGT BRDN00012	103	154	49	177	140	71
CACAGACTG BRDN00011	107	109	4	212	189	8
CACAGACTG BRDN00011	174	183	279	307	261	15
CACAGACTTT BRDN00011	80	51	51	172	116	20
CACAGAGAC BRDN00011	222	86	6	454	376	0
CACAGAGAG BRDN00012	76	69	24	150	166	29
CACAGAGAG BRDN00012	275	257	28	511	488	103
CACAGAGAT BRDN00012	137	126	39	217	241	0
CACAGAGAT BRDN00011	131	26	11	260	231	0
CACAGAGCC BRDN00012	607	633	1025	888	717	67
CACAGAGCG BRDN00011	144	80	193	311	586	10
CACAGAGCT BRDN00011	152	71	53	331	190	0
CACAGAGGG BRDN00012	333	287	145	762	796	7
CACAGAGTT BRDN00012	332	343	410	557	320	17
CACAGATAT BRDN00012	115	40	98	301	245	0
CACAGATGC BRDN00011	372	261	113	827	784	26
CACAGATGC BRDN00011	87	44	34	300	141	0
CACAGATGC BRDN00011	124	9	2	262	225	0
CACAGATTC BRDN00012	17	0	0	190	10	0
CACAGCACTA BRDN00012	218	23	158	575	254	7
CACAGCACTC BRDN00012	446	417	891	882	1133	77
CACAGCAGG BRDN00012	279	448	1273	613	748	35
CACAGCAGG BRDN00011	137	68	0	299	616	0
CACAGCATA BRDN00011	150	46	257	254	167	7
CACAGCATC BRDN00011	532	492	549	684	1153	58
CACAGCCAAT BRDN00012	98	32	0	237	233	0
CACAGCCAAT BRDN00011	185	177	1012	331	371	10
CACAGCCACC BRDN00011	153	240	203	289	166	13
CACAGCCAG BRDN00012	282	119	544	386	167	51
CACAGCCATA BRDN00012	176	183	416	326	331	36
CACAGCCATA BRDN00012	148	54	359	326	255	11
CACAGCCATA BRDN00012	455	451	925	646	658	37
CACAGCCCAC BRDN00012	98	177	88	158	69	8
CACAGCCCAC BRDN00011	331	370	334	690	567	90
CACAGCCCCG BRDN00011	497	550	198	853	1128	3
CACAGCCCGC BRDN00012	136	100	1	377	321	0
CACAGCCCGC BRDN00011	361	384	304	665	764	25
CACAGCCGA BRDN00011	180	247	570	329	201	21
CACAGCCGCC BRDN00011	267	334	1101	623	547	83
CACAGCCTGC BRDN00011	408	271	661	622	786	15
CACAGCCTTC BRDN00012	184	187	1368	476	283	33
CACAGCCTTT BRDN00011	171	271	395	277	212	57
CACAGCGAG BRDN00011	47	0	0	161	112	0
CACAGCGAT BRDN00012	394	303	657	608	576	15
CACAGCGAT BRDN00012	296	394	120	530	512	15
CACAGCGCA BRDN00011	245	19	2	676	133	0
CACAGCGCC BRDN00012	107	121	136	224	219	5
CACAGCGGG BRDN00012	144	196	658	373	220	20
CACAGCGTG BRDN00011	205	4	0	599	238	0
CACAGCTACA BRDN00011	131	107	32	323	234	1
CACAGCTACC BRDN00011	152	192	62	330	369	4
CACAGCTATA BRDN00012	229	246	168	584	855	0
CACAGCTCCT BRDN00011	144	125	887	167	213	1
CACAGCTCGA BRDN00011	206	152	612	284	338	13
CACAGCTGA BRDN00012	131	104	122	301	143	0
CACAGCTGCT BRDN00012	266	180	190	449	608	64
CACAGCTGTA BRDN00012	140	98	0	235	189	16
CACAGCTGTC BRDN00011	94	131	1627	257	261	23
CACAGCTGTC BRDN00011	214	262	821	444	260	2
CACAGCTGT BRDN00012	239	253	1021	548	743	89

CACAGCTTA	BRDN00011	323	369	2383	586	310	13
CACAGCTTCT	BRDN00012	128	121	116	277	384	4
CACAGGAAT	BRDN00012	381	292	455	662	346	61
CACAGGACA	BRDN00011	114	71	25	142	300	0
CACAGGAGT	BRDN00011	260	6	82	478	294	0
CACAGGCAC	BRDN00011	22	0	0	389	145	0
CACAGGCAC	BRDN00012	148	170	0	331	461	47
CACAGGCAC	BRDN00011	331	259	1591	695	447	50
CACAGGCAG	BRDN00011	119	69	0	148	55	9
CACAGGCAG	BRDN00012	222	172	740	419	428	15
CACAGGCCC	BRDN00012	90	50	64	291	167	21
CACAGGCCG	BRDN00012	90	93	784	143	206	37
CACAGGCTA	BRDN00012	172	191	146	239	216	0
CACAGGCTA	BRDN00011	109	45	1	143	184	0
CACAGGCTA	BRDN00012	316	85	113	595	580	0
CACAGGCTC	BRDN00011	107	92	0	184	167	0
CACAGGGAG	BRDN00011	92	97	0	215	236	0
CACAGGGAT	BRDN00012	284	239	126	512	431	21
CACAGGGCA	BRDN00011	141	169	560	532	286	7
CACAGGGCC	BRDN00011	219	216	30	367	544	15
CACAGGGGA	BRDN00011	145	164	233	306	38	18
CACAGGGGA	BRDN00012	65	54	42	146	83	0
CACAGGGTC	BRDN00012	129	51	73	365	276	0
CACAGGGTC	BRDN00012	258	283	197	423	399	69
CACAGGGTG	BRDN00012	68	61	247	166	225	16
CACAGGGTG	BRDN00012	379	201	469	877	1165	44
CACAGGGTG	BRDN00012	291	316	552	566	396	11
CACAGGTAA	BRDN00012	49	3	1	537	372	0
CACAGGTAC	BRDN00011	347	194	530	594	393	13
CACAGGTAT	BRDN00011	165	71	140	190	187	0
CACAGGTCA	BRDN00012	317	292	308	699	607	45
CACAGGTGA	BRDN00012	72	76	1	129	77	0
CACAGGTGA	BRDN00011	318	355	651	632	472	26
CACAGGTGG	BRDN00011	373	379	652	635	702	51
CACAGGTGT	BRDN00012	98	70	121	198	233	4
CACAGGTTT	BRDN00011	188	153	56	218	275	29
CACAGTAAA	BRDN00012	409	407	211	812	740	44
CACAGTAAT	BRDN00012	101	9	1	205	4	0
CACAGTACC	BRDN00012	308	339	225	710	725	30
CACAGTACG	BRDN00011	431	367	533	793	1139	39
CACAGTAGA	BRDN00012	508	557	977	706	841	15
CACAGTATA	BRDN00012	182	122	445	234	634	23
CACAGTCAA	BRDN00012	447	444	1072	673	1048	33
CACAGTCAG	BRDN00011	83	54	13	147	63	0
CACAGTCAT	BRDN00012	182	138	1232	575	542	20
CACAGTCATT	BRDN00011	117	176	304	276	400	42
CACAGTCTG	BRDN00012	154	206	84	217	193	15
CACAGTCTG	BRDN00012	260	274	57	607	587	7
CACAGTCTTA	BRDN00011	111	74	73	332	127	0
CACAGTCTTG	BRDN00011	196	118	23	231	141	0
CACAGTGAC	BRDN00011	225	300	769	460	411	17
CACAGTGAC	BRDN00012	263	399	486	646	854	33
CACAGTGAG	BRDN00012	71	99	27	159	185	38
CACAGTGAT	BRDN00011	6	0	1	280	84	0
CACAGTGCA	BRDN00012	121	165	42	373	498	5
CACAGTGGG	BRDN00012	396	408	775	617	741	26
CACAGTGTG	BRDN00011	165	136	179	338	362	8
CACAGTGTG	BRDN00012	64	59	0	209	217	0
CACAGTGTG	BRDN00011	63	83	0	175	66	0
CACAGTTAT	BRDN00011	165	189	171	379	150	1
CACAGTTCA	BRDN00012	124	130	687	184	272	24
CACAGTTGC	BRDN00012	324	326	277	549	759	49
CACAGTTGT	BRDN00012	187	37	206	221	405	0
CACAGTTTCA	BRDN00011	224	165	143	282	123	8
CACATAACAT	BRDN00012	392	353	3572	726	629	57
CACATAATG	BRDN00011	254	194	19	426	654	13
CACATACAG	BRDN00012	15	0	0	215	139	0
CACATACAG	BRDN00011	381	350	304	695	513	52
CACATACCCG	BRDN00012	320	299	1332	588	282	28
CACATACCTT	BRDN00011	280	199	957	636	613	26
CACATACGA	BRDN00011	153	259	33	256	352	6
CACATACGCT	BRDN00012	403	559	720	710	907	99
CACATACTAG	BRDN00012	227	242	306	387	253	181
CACATACTTC	BRDN00011	179	224	118	294	352	4
CACATACTTG	BRDN00012	180	151	17	325	666	16
CACATAGAG	BRDN00011	78	159	7	199	65	0
CACATAGAT	BRDN00011	323	358	2913	475	574	6127
CACATAGCG	BRDN00011	263	267	446	389	407	62
CACATAGCG	BRDN00011	171	3	0	598	327	0
CACATAGGA	BRDN00012	331	337	1624	582	578	31
CACATAGGC	BRDN00011	249	267	473	344	231	37
CACATAGTA	BRDN00012	135	138	292	215	447	9
CACATATAG	BRDN00011	104	127	142	173	160	7
CACATATGA	BRDN00012	264	184	10	465	498	27
CACATATTAC	BRDN00011	96	141	532	266	265	2
CACATATTCT	BRDN00012	271	274	150	474	459	0
CACATCAACA	BRDN00011	175	78	30	286	323	0
CACATCAGC	BRDN00011	411	363	464	649	598	11
CACATCAGCT	BRDN00011	245	179	7482	516	388	91
CACATCATG	BRDN00011	285	216	179	462	575	42
CACATCATTG	BRDN00011	265	96	310	445	629	59
CACATCATTT	BRDN00012	329	301	258	579	498	33
CACATCCAAC	BRDN00011	189	92	108	370	296	27
CACATCCACC	BRDN00012	123	249	9	224	407	24
CACATCCAGT	BRDN00011	261	170	88	465	230	0
CACATCCATA	BRDN00012	329	285	330	743	939	34
CACATCCATC	BRDN00012	224	173	278	498	581	39
CACATCCATC	BRDN00012	382	292	294	895	1028	18
CACATCCCTG	BRDN00011	179	32	0	369	66	0
CACATCCGA	BRDN00012	164	143	1040	354	327	38
CACATCCTCT	BRDN00011	119	86	3	214	110	10
CACATCCTGA	BRDN00012	205	238	43	346	176	66
CACATCGATC	BRDN00011	191	173	52	445	572	0
CACATCGATC	BRDN00012	240	335	1664	465	535	42
CACATCGCA	BRDN00011	205	141	663	475	483	22
CACATCGGC	BRDN00012	190	147	852	321	237	30
CACATCGTC	BRDN00011	235	246	3604	468	406	46
CACATCTACC	BRDN00011	108	132	245	237	1041	0
CACATGAGA	BRDN00012	335	316	111	602	644	20
CACATGAGC	BRDN00011	97	115	0	204	229	23
CACATGAGG	BRDN00012	107	27	0	220	339	0
CACATGCCA	BRDN00012	283	270	411	509	548	25
CACATGCCA	BRDN00011	556	449	1500	1083	956	25
CACATGCCCA	BRDN00011	430	281	560	576	750	53
CACATGCTG	BRDN00011	251	271	159	443	615	31
CACATGGAT	BRDN00012	385	351	173	564	741	2
CACATGGAT	BRDN00012	362	420	759	652	659	15
CACATGGCG	BRDN00012	140	183	16	364	182	33
CACATGGCG	BRDN00011	27	0	0	123	1	0
CACATGGCT	BRDN00012	274	129	113	637	463	1
CACATGGGG	BRDN00012	394	291	842	645	750	0
CACATGTACA	BRDN00012	208	111	166	290	431	18
CACATGTAC	BRDN00011	257	71	33	253	76	0
CACATGTAC	BRDN00011	342	360	3772	753	314	18
CACATGTCC	BRDN00012	161	158	8	248	244	0
CACATGTCC	BRDN00012	496	366	275	790	878	83
CACATGTTA	BRDN00012	331	416	952	717	959	86
CACATTAAA	BRDN00012	108	187	243	247	95	18
CACATTCAAC	BRDN00011	461	511	755	926	1102	130
CACATTCACA	BRDN00011	410	206	98	526	337	103
CACATTCCAA	BRDN00012	139	106	95	242	437	6
CACATTCCGG	BRDN00012	91	103	920	245	485	25
CACATTCCTG	BRDN00012	120	266	1105	271	283	27
CACATTCTAA	BRDN00012	370	201	1585	631	327	28
CACATTCTAT	BRDN00012	237	303	109	346	280	19
CACATTCTTG	BRDN00012	309	186	800	457	456	48
CACATTGTG	BRDN00011	410	394	833	661	586	38
CACATTGTTG	BRDN00012	281	384	115	717	1033	35
CACATTTGA	BRDN00011	115	99	203	196	309	24
CACATTTGAT	BRDN00012	127	138	252	215	76	5
CACATTTGC	BRDN00011	287	151	111	385	379	0
CACATTTGG	BRDN00012	304	297	104	527	565	16
CACATTTGGT	BRDN00011	104	105	0	230	140	0
CACCAAAAG	BRDN00012	133	5	1	590	193	0
CACCAAACA	BRDN00012	231	184	34	420	248	22
CACCAAACTC	BRDN00012	145	77	0	431	433	0
CACCAAAGG	BRDN00012	270	273	0	651	670	12
CACCAACAAC	BRDN00011	118	0	2	836	404	0
CACCAACACC	BRDN00012	66	0	0	703	188	0
CACCAACACT	BRDN00012	198	203	61	560	406	3
CACCAACAG	BRDN00011	375	240	1609	689	780	39
CACCAACAG	BRDN00011	97	38	212	352	296	0
CACCAACAG	BRDN00012	400	423	1048	700	758	70
CACCAACATC	BRDN00012	225	121	268	365	591	9
CACCAACATC	BRDN00012	120	134	226	250	296	12
CACCAACATT	BRDN00011	180	200	560	292	171	26
CACCAACCG	BRDN00011	140	101	175	184	63	0
CACCAACCG	BRDN00011	107	40	20	255	146	14
CACCAACGA	BRDN00011	301	282	955	639	731	11
CACCAACTG	BRDN00011	167	170	4619	453	270	36
CACCAAGAA	BRDN00012	156	122	228	171	221	40
CACCAAGCT	BRDN00011	145	63	24	308	127	95
CACCAAGGA	BRDN00011	519	532	1841	969	1249	119
CACCAAGGT	BRDN00011	224	231	185	552	833	41
CACCAAGTA	BRDN00011	145	114	1	567	214	10
CACCAAGTG	BRDN00012	457	307	499	818	1041	10
CACCAATAA	BRDN00011	144	15	0	277	289	0
CACCAATACT	BRDN00011	268	243	74	521	572	18
CACCAATCTT	BRDN00011	297	202	89	459	306	20
CACCAATGA	BRDN00011	234	128	420	312	350	1
CACCAATGC	BRDN00012	165	119	380	373	486	8
CACCAATGT	BRDN00011	68	50	4	233	193	0
CACCAATGTT	BRDN00011	198	283	3293	681	375	43
CACCACAAA	BRDN00011	188	95	1830	412	225	32
CACCACAAA	BRDN00011	241	213	419	410	277	5
CACCACAATC	BRDN00012	242	167	65	472	523	0
CACCACAATC	BRDN00011	134	56	468	232	123	5
CACCACACAG	BRDN00012	165	176	48	325	213	101
CACCACACCT	BRDN00012	243	203	330	401	174	27
CACCACAGA	BRDN00012	183	180	432	231	231	21
CACCACAGC	BRDN00012	249	252	230	503	996	5
CACCACAGC	BRDN00012	43	8	0	413	92	0
CACCACAGT	BRDN00011	313	271	270	499	709	75
CACCACATAC	BRDN00012	482	409	227	723	571	23
CACCACATAT	BRDN00011	131	234	293	333	366	9
CACCACCAAT	BRDN00012	103	6	298	362	51	7
CACCACCAAT	BRDN00012	276	253	1842	458	652	75
CACCACCACA	BRDN00011	155	189	91	470	313	0
CACCACCAG	BRDN00012	162	193	476	224	365	9
CACCACCAG	BRDN00011	203	229	219	439	437	7
CACCACCATG	BRDN00012	276	362	1577	666	434	76
CACCACCCAC	BRDN00012	472	499	703	885	814	32
CACCACCCAT	BRDN00011	436	471	846	574	403	32
CACCACCCCG	BRDN00011	224	142	1240	287	435	328
CACCACCTTT	BRDN00012	201	169	385	332	95	16
CACCACGAA	BRDN00011	354	310	299	567	821	77
CACCACGAC	BRDN00011	82	4	0	157	273	0
CACCACGCCC	BRDN00011	81	88	367	197	79	11
CACCACGCCT	BRDN00011	233	295	618	461	562	8
CACCACGCTT	BRDN00012	518	571	716	952	2430	26
CACCACGGT	BRDN00011	169	108	7	472	450	0
CACCACGTTG	BRDN00011	256	232	861	461	342	48
CACCACTAAT	BRDN00012	157	111	0	291	413	25
CACCACTCGG	BRDN00011	167	128	12	323	339	14
CACCACTCGG	BRDN00012	292	271	258	421	275	33
CACCACTGAC	BRDN00011	282	306	1362	413	318	94
CACCACTGG	BRDN00012	440	449	1504	740	1223	89
CACCAGAAGT	BRDN00012	222	120	46	272	239	8
CACCAGAAGT	BRDN00011	164	135	1774	485	707	34
CACCAGAGA	BRDN00011	89	119	13	192	263	0
CACCAGATGT	BRDN00012	181	99	340	471	230	11
CACCAGCAA	BRDN00011	132	48	0	275	200	0
CACCAGCAAT	BRDN00011	454	503	945	687	760	25
CACCAGCAC	BRDN00012	145	68	835	263	109	22
CACCAGCAC	BRDN00012	151	21	0	352	29	0
CACCAGCAG	BRDN00011	229	172	264	625	804	72
CACCAGCAG	BRDN00011	103	58	45	162	230	0
CACCAGCATT	BRDN00011	318	219	1777	611	966	55
CACCAGCGC	BRDN00012	543	417	370	793	683	45
CACCAGCGGT	BRDN00011	109	123	17	246	174	6
CACCAGCTG	BRDN00012	205	209	43	335	168	7
CACCAGCTG	BRDN00011	121	190	610	223	69	44
CACCAGGGA	BRDN00012	112	107	38	227	306	0
CACCAGGGA	BRDN00012	67	48	323	263	412	4
CACCAGGGG	BRDN00012	410	259	1325	676	604	42
CACCAGGTC	BRDN00011	481	374	2768	680	1210	149
CACCAGGTC	BRDN00011	138	178	143	298	444	6
CACCAGGTT	BRDN00012	322	275	138	419	363	28
CACCAGTACC	BRDN00011	467	516	4522	948	558	93
CACCAGTAG	BRDN00011	300	285	113	365	759	0
CACCAGTATC	BRDN00012	304	284	299	421	489	0
CACCAGTCA	BRDN00012	223	113	89	595	663	34
CACCAGTCA	BRDN00011	23	14	0	63	19	0
CACCAGTCCC	BRDN00012	269	219	547	465	490	19
CACCAGTGA	BRDN00011	236	71	0	316	137	0
CACCAGTGA	BRDN00011	385	300	1104	610	612	44
CACCAGTGG	BRDN00011	221	330	2	553	726	5
CACCAGTGG	BRDN00011	85	77	28	215	225	0
CACCAGTGTC	BRDN00012	244	143	2832	439	282	74
CACCATAAG	BRDN00011	168	0	1	438	239	0
CACCATAATT	BRDN00012	145	120	1	182	200	5
CACCATAGA	BRDN00012	267	221	831	464	568	6
CACCATAGTT	BRDN00011	159	75	126	377	363	47
CACCATATA	BRDN00011	282	266	190	586	791	48
CACCATCACT	BRDN00011	234	195	75	445	348	39
CACCATCAGT	BRDN00012	322	192	107	610	570	124
CACCATCATG	BRDN00012	417	355	750	787	1034	27
CACCATCCAC	BRDN00011	350	58	2	722	942	0
CACCATCCAT	BRDN00011	72	9	0	188	94	0
CACCATCGAC	BRDN00011	185	129	1387	404	555	47
CACCATCTCC	BRDN00011	301	176	0	486	377	5
CACCATCTCG	BRDN00012	221	205	611	327	381	39
CACCATCTTG	BRDN00012	171	181	2122	280	346	18
CACCATGAA	BRDN00011	130	23	237	226	279	8
CACCATGAC	BRDN00012	296	575	2587	574	496	61
CACCATGGA	BRDN00011	69	27	0	177	24	0
CACCATGGC	BRDN00012	543	632	1051	1108	1061	83
CACCATGGG	BRDN00012	228	159	1646	915	1013	76
CACCATGGG	BRDN00011	471	501	861	913	414	29
CACCATGTAC	BRDN00012	424	232	971	718	384	44
CACCATGTAC	BRDN00012	257	40	52	636	757	0
CACCATGTCT	BRDN00011	170	146	221	268	638	14
CACCATTGCT	BRDN00011	181	97	222	322	281	0
CACCATTTCA	BRDN00012	269	354	1386	432	552	65
CACCATTTCT	BRDN00011	328	362	645	587	487	17
CACCCAAGCT	BRDN00011	116	100	161	194	147	0
CACCCAAGG	BRDN00012	316	363	2308	632	1259	45
CACCCAAGGT	BRDN00011	275	173	496	638	312	36
CACCCAATCA	BRDN00011	39	0	0	241	139	0
CACCCACAG	BRDN00011	210	183	131	195	357	10
CACCCACCAA	BRDN00012	106	238	123	277	284	32
CACCCACCAC	BRDN00012	129	12	763	394	659	0
CACCCACGCC	BRDN00012	291	503	5432	845	348	85
CACCCAGAAT	BRDN00011	171	109	185	343	186	0
CACCCAGAC	BRDN00012	113	107	47	331	164	40
CACCCAGAG	BRDN00011	121	197	538	217	215	11
CACCCAGAG	BRDN00012	120	134	38	251	527	26
CACCCAGCCT	BRDN00012	229	300	18	402	308	33
CACCCAGCTT	BRDN00011	68	39	17	181	159	2
CACCCAGTG	BRDN00011	444	318	1549	642	1586	43
CACCCATAG	BRDN00011	151	103	34	278	386	0
CACCCATCTT	BRDN00011	214	72	682	337	273	73
CACCCATGAC	BRDN00011	130	125	23	314	330	9
CACCCCAAAG	BRDN00011	268	0	1	600	681	0
CACCCCAAG	BRDN00012	288	267	608	831	386	29
CACCCCACAG	BRDN00012	138	139	57	254	206	14
CACCCCACCA	BRDN00012	529	285	296	746	766	23
CACCCCAGG	BRDN00011	317	308	590	530	490	27
CACCCCATAA	BRDN00011	171	192	1	243	259	3
CACCCCATTC	BRDN00011	301	307	531	523	248	30
CACCCCCAAT	BRDN00011	282	217	17	588	773	20
CACCCCCATC	BRDN00012	284	272	383	688	453	43
CACCCCCCAA	BRDN00012	385	341	336	604	545	52
CACCCCCCCA	BRDN00011	223	89	1808	261	308	10
CACCCCCGAT	BRDN00012	95	87	182	220	242	11
CACCCCCGCG	BRDN00011	135	104	581	277	239	20
CACCCCCGGT	BRDN00012	112	159	352	131	151	54
CACCCCGACC	BRDN00012	290	106	1403	565	451	11
CACCCCGACT	BRDN00012	523	511	2471	947	750	31
CACCCCGCAC	BRDN00011	39	18	0	696	119	0
CACCCCGCCC	BRDN00012	201	152	121	371	365	0
CACCCCGCTT	BRDN00012	305	213	1666	530	358	81
CACCCCGTAA	BRDN00011	86	137	0	195	247	8
CACCCCTACA	BRDN00012	76	158	2	179	534	0
CACCCCTCGA	BRDN00011	373	318	145	491	683	12
CACCCCTGTC	BRDN00011	134	100	7	184	303	13
CACCCCTTAC	BRDN00011	214	104	348	451	370	13
CACCCGAACC	BRDN00012	182	154	1197	403	303	63
CACCCGATTC	BRDN00011	212	135	2904	365	427	36
CACCCGCATA	BRDN00012	246	158	1040	426	343	20
CACCCGCGA	BRDN00011	143	134	91	276	362	0
CACCCGCGG	BRDN00011	233	122	94	429	301	0
CACCCGGAA	BRDN00012	331	438	24	722	665	54
CACCCGGCA	BRDN00011	280	335	786	576	619	48
CACCCGGCTT	BRDN00012	159	107	251	317	485	25
CACCCGGTG	BRDN00011	124	177	135	257	287	7
CACCCGTAG	BRDN00012	62	72	528	233	171	0
CACCCGTGA	BRDN00012	106	103	0	183	224	1
CACCCGTGA	BRDN00011	275	272	23	466	784	35
CACCCTATAT	BRDN00012	133	173	32	173	72	23
CACCCTATTA	BRDN00011	124	158	96	264	328	8
CACCCTCGCA	BRDN00012	523	527	301	856	928	16
CACCCTCGCC	BRDN00011	142	183	379	219	285	26
CACCCTCTGA	BRDN00012	371	140	1037	436	313	7
CACCCTGAA	BRDN00012	210	237	198	297	331	8
CACCCTGAG	BRDN00011	311	178	98	761	908	3
CACCCTGCAC	BRDN00011	322	343	1595	584	578	91
CACCCTGGC	BRDN00012	310	227	312	549	412	0
CACCCTGTG	BRDN00012	216	138	0	326	250	11
CACCCTTATA	BRDN00012	274	208	488	475	557	16
CACCCTTCAA	BRDN00012	143	158	2809	352	238	101
CACCCTTCAC	BRDN00012	361	419	814	607	1074	61
CACCCTTGGT	BRDN00012	125	193	54	266	168	28
CACCCTTGTC	BRDN00012	455	427	323	865	816	25
CACCCTTTAC	BRDN00011	149	19	0	224	99	0
CACCGAACC	BRDN00011	226	357	297	390	336	23
CACCGAATA	BRDN00012	303	263	584	496	562	15
CACCGAATT	BRDN00011	144	121	660	208	276	24
CACCGACAA	BRDN00012	285	143	198	525	393	7
CACCGACAC	BRDN00011	115	83	6	227	179	0
CACCGACAT	BRDN00012	314	372	3063	789	574	66
CACCGACCA	BRDN00012	216	262	5	310	329	29
CACCGACGG	BRDN00012	591	561	2604	933	501	161
CACCGACTCC	BRDN00011	146	102	215	199	147	0
CACCGAGAC	BRDN00011	497	439	555	814	826	98
CACCGAGCC	BRDN00012	57	110	0	134	135	7
CACCGAGCG	BRDN00012	179	147	64	265	223	19
CACCGAGGA	BRDN00012	238	217	209	496	572	4
CACCGAGGA	BRDN00011	75	99	149	218	61	13
CACCGAGGC	BRDN00011	93	62	213	159	160	0
CACCGATAT	BRDN00011	211	125	21	372	566	22
CACCGATAT	BRDN00012	128	55	23	223	409	0
CACCGATCTT	BRDN00012	420	333	69	667	791	32
CACCGATCTT	BRDN00011	380	473	2023	673	588	98
CACCGCACTG	BRDN00011	326	274	540	410	472	0
CACCGCCCAG	BRDN00012	147	73	14	249	276	0
CACCGCCCTA	BRDN00012	217	262	60	581	254	29
CACCGCCGA	BRDN00012	91	123	19	186	232	19
CACCGCCGC	BRDN00012	174	136	14	266	191	38
CACCGCCGT	BRDN00012	213	104	353	574	259	8
CACCGCCTCA	BRDN00011	301	305	896	465	949	64
CACCGCCTTA	BRDN00012	247	187	5	392	513	39
CACCGCCTTG	BRDN00012	233	220	263	344	323	3
CACCGCGACT	BRDN00012	164	152	0	232	341	0
CACCGCGAT	BRDN00012	145	117	47	276	178	6
CACCGCGCA	BRDN00012	547	377	788	1139	969	33
CACCGCGGC	BRDN00011	351	322	574	670	833	26
CACCGCGGC	BRDN00011	378	334	657	591	991	46
CACCGCTTCA	BRDN00011	19	0	1	242	16	0
CACCGCTTTG	BRDN00012	424	460	331	811	582	37
CACCGGAAG	BRDN00011	93	49	154	485	448	17
CACCGGAGT	BRDN00012	146	103	535	239	190	10
CACCGGAGT	BRDN00012	155	140	5961	335	183	56
CACCGGAGT	BRDN00011	153	88	0	179	78	0
CACCGGATC	BRDN00012	417	432	925	863	1119	22
CACCGGCAG	BRDN00012	314	170	397	610	381	14
CACCGGCGC	BRDN00012	269	120	225	470	365	11
CACCGGCTCA	BRDN00011	304	182	232	611	578	20
CACCGGGAC	BRDN00011	292	291	1036	506	945	85
CACCGGGCC	BRDN00012	358	398	2316	705	671	58
CACCGGGTC	BRDN00011	296	205	581	444	451	32
CACCGGGTG	BRDN00011	126	107	1	282	262	8
CACCGGGTT	BRDN00012	186	216	96	289	352	37
CACCGTACA	BRDN00011	221	223	1	360	637	21
CACCGTCAA	BRDN00011	91	39	119	180	80	6
CACCGTCAA	BRDN00012	122	46	0	198	251	4
CACCGTCACC	BRDN00012	32	19	0	82	0	0
CACCGTCAG	BRDN00011	192	144	158	481	321	20
CACCGTCGC	BRDN00012	247	225	1229	422	529	19
CACCGTCGC	BRDN00011	104	148	37	189	327	31
CACCGTCGG	BRDN00012	292	252	92	518	428	78
CACCGTCTGA	BRDN00011	148	79	2276	269	241	26
CACCGTCTTG	BRDN00011	99	144	735	181	277	37
CACCGTGAG	BRDN00012	395	375	1433	802	453	49
CACCGTGCA	BRDN00012	456	0	1	834	318	0
CACCGTGCT	BRDN00011	123	270	328	322	122	52
CACCGTGTG	BRDN00012	354	282	259	334	481	53
CACCGTTAAT	BRDN00012	126	58	362	169	115	0
CACCGTTGA	BRDN00012	247	358	2279	416	532	89
CACCGTTGA	BRDN00011	189	214	568	459	399	22
CACCGTTTG	BRDN00011	159	134	2514	274	297	28
CACCTACACA	BRDN00011	367	363	2716	537	1158	62
CACCTACAG	BRDN00011	172	29	0	456	130	0
CACCTACCAA	BRDN00012	352	294	391	675	370	23
CACCTACCTA	BRDN00012	330	492	878	499	395	24
CACCTACGCA	BRDN00011	210	186	581	499	512	33
CACCTACTCG	BRDN00012	518	175	2955	847	699	49
CACCTACTGT	BRDN00012	198	176	1	304	287	9
CACCTACTTC	BRDN00012	356	263	605	677	661	75
CACCTACTTG	BRDN00012	259	287	556	576	347	38
CACCTAGTA	BRDN00011	86	71	17	168	223	0
CACCTATAAA	BRDN00011	94	230	961	250	291	0
CACCTATAGA	BRDN00011	333	360	798	596	518	45
CACCTATGA	BRDN00011	243	187	141	382	502	24
CACCTCAACA	BRDN00012	180	281	439	299	410	43
CACCTCAATA	BRDN00011	81	69	515	170	744	24
CACCTCACAC	BRDN00012	324	445	568	842	627	47
CACCTCACTT	BRDN00011	238	326	827	526	336	27
CACCTCAGCT	BRDN00011	430	212	70	938	975	50
CACCTCATAT	BRDN00012	469	294	1408	816	796	110
CACCTCATCA	BRDN00011	190	119	282	444	459	56
CACCTCCAAC	BRDN00011	144	196	724	257	241	24
CACCTCCAAT	BRDN00011	146	120	373	233	248	12
CACCTCCAGG	BRDN00012	160	185	174	264	341	0
CACCTCCTCA	BRDN00011	82	176	1239	149	82	31
CACCTCGATG	BRDN00012	320	173	238	927	597	9
CACCTCGGA	BRDN00011	126	23	34	183	239	0
CACCTCGGT	BRDN00012	166	194	60	409	446	0
CACCTCGTAC	BRDN00011	129	106	123	282	313	98
CACCTCGTCC	BRDN00011	73	77	41	160	113	0
CACCTCTACA	BRDN00011	261	220	65	270	416	23
CACCTCTCCG	BRDN00011	87	165	0	296	94	4
CACCTCTTAT	BRDN00011	136	205	328	290	643	6
CACCTGAAA	BRDN00012	399	201	888	770	539	57
CACCTGAAC	BRDN00012	145	172	400	414	529	0
CACCTGAAC	BRDN00012	12	0	0	258	7	0
CACCTGAAG	BRDN00012	37	0	0	443	220	0
CACCTGAAG	BRDN00012	126	182	79	320	424	13
CACCTGACA	BRDN00011	131	94	157	236	426	13
CACCTGACCG	BRDN00012	135	92	115	263	208	0
CACCTGACGT	BRDN00012	108	85	447	144	113	9
CACCTGCACA	BRDN00011	255	244	316	570	278	18
CACCTGCACC	BRDN00012	60	31	135	238	254	13
CACCTGCACC	BRDN00012	405	540	699	775	603	34
CACCTGCAGA	BRDN00012	202	101	161	312	218	153
CACCTGCAGA	BRDN00012	352	435	1112	484	368	52
CACCTGCAG	BRDN00011	254	204	295	338	485	65
CACCTGCATA	BRDN00012	318	359	1390	786	667	39
CACCTGCATC	BRDN00012	145	141	313	300	355	0
CACCTGCATC	BRDN00011	496	395	1382	1065	1256	54
CACCTGCATT	BRDN00012	394	146	50	789	886	8
CACCTGCCAG	BRDN00012	46	82	114	158	61	0
CACCTGCCCG	BRDN00011	503	579	2310	881	593	181
CACCTGCGA	BRDN00012	175	7	0	417	77	0
CACCTGCGC	BRDN00012	186	255	13	235	490	37

CACCTGCGC	BRDN00012	414	482	2503	767	969	92
CACCTGCGG	BRDN00012	81	53	593	245	65	4
CACCTGCGG	BRDN00012	19	0	0	330	33	0
CACCTGCGG	BRDN00012	365	314	342	704	518	13
CACCTGCTCG	BRDN00012	56	0	0	248	54	0
CACCTGCTCT	BRDN00011	230	164	745	546	355	53
CACCTGCTGT	BRDN00012	142	175	116	366	222	33
CACCTGCTTA	BRDN00011	198	238	31	350	356	63
CACCTGGACT	BRDN00012	157	207	57	265	556	30
CACCTGGAG	BRDN00012	202	169	54	299	313	0
CACCTGGAT	BRDN00011	189	264	627	422	539	48
CACCTGGAT	BRDN00011	102	156	119	230	503	8
CACCTGGCA	BRDN00012	265	191	285	457	583	27
CACCTGGCA	BRDN00011	153	109	0	210	168	1
CACCTGGCA	BRDN00011	115	27	101	417	42	1
CACCTGGCC	BRDN00012	107	24	1	410	238	0
CACCTGGCG	BRDN00011	199	209	30	488	515	16
CACCTGGCT	BRDN00011	72	66	0	147	239	0
CACCTGGCT	BRDN00012	478	386	1093	919	1009	60
CACCTGGGC	BRDN00012	174	136	169	230	319	13
CACCTGGGG	BRDN00011	540	500	2365	1054	867	72
CACCTGGGT	BRDN00012	78	81	18	268	110	0
CACCTGGTA	BRDN00011	75	35	31	167	140	0
CACCTGGTA	BRDN00011	213	199	1033	275	285	32
CACCTGGTG	BRDN00012	120	153	258	162	142	21
CACCTGGTG	BRDN00011	170	219	765	263	204	1
CACCTGGTG	BRDN00011	309	289	174	451	476	83
CACCTGTATT	BRDN00011	177	172	224	453	463	23
CACCTGTGA	BRDN00011	76	132	164	129	77	6
CACCTGTGA	BRDN00011	301	363	143	708	774	31
CACCTGTGC	BRDN00012	385	338	654	507	289	29
CACCTGTGT	BRDN00011	42	109	285	106	61	5
CACCTGTTCC	BRDN00011	84	156	425	203	142	7
CACCTGTTG	BRDN00012	189	172	362	305	269	17
CACCTTAAAG	BRDN00011	146	103	12	395	228	26
CACCTTAAGT	BRDN00012	223	113	164	443	254	0
CACCTTAGG	BRDN00012	469	397	1861	619	1164	44
CACCTTATAC	BRDN00012	311	314	1024	720	787	114
CACCTTATCG	BRDN00011	155	60	0	311	330	19
CACCTTCAAC	BRDN00012	399	300	503	539	657	9
CACCTTCACG	BRDN00011	95	0	158	253	93	0
CACCTTCAGA	BRDN00012	127	79	281	105	14	0
CACCTTCAGC	BRDN00011	348	247	468	621	427	70
CACCTTCATA	BRDN00012	206	0	0	457	321	0
CACCTTCATG	BRDN00012	95	60	1	182	249	19
CACCTTCCAG	BRDN00012	168	321	695	359	360	121
CACCTTCCTG	BRDN00011	229	285	213	522	577	50
CACCTTCCTG	BRDN00012	218	119	787	226	184	2
CACCTTCGCC	BRDN00012	133	145	400	291	303	20
CACCTTCGTC	BRDN00012	88	93	104	195	362	25
CACCTTCTCC	BRDN00011	189	142	101	358	232	0
CACCTTCTCC	BRDN00012	158	137	336	353	466	7
CACCTTCTTA	BRDN00012	109	29	610	267	197	5
CACCTTGAA	BRDN00012	188	176	65	323	397	0
CACCTTGCTA	BRDN00012	378	259	855	566	806	36
CACCTTGTGT	BRDN00012	251	244	374	600	285	11
CACGAAACA	BRDN00011	109	100	66	258	74	0
CACGAAATT	BRDN00012	205	144	145	271	540	32
CACGAACAC	BRDN00011	104	82	2193	236	883	37
CACGAACCA	BRDN00011	167	57	3152	243	169	13
CACGAACTC	BRDN00012	415	349	1425	599	932	36
CACGAACTCT	BRDN00012	117	50	0	180	191	0
CACGAACTG	BRDN00011	253	347	26	510	906	27
CACGAAGGG	BRDN00012	446	358	680	560	554	25
CACGAAGTA	BRDN00012	217	113	641	564	469	31
CACGACACG	BRDN00012	73	9	0	215	163	0
CACGACAGT	BRDN00012	122	91	295	307	313	0
CACGACATG	BRDN00011	189	86	417	354	478	19
CACGACATT	BRDN00011	197	122	68	285	173	20
CACGACGAC	BRDN00012	339	459	576	595	575	72
CACGACGGC	BRDN00012	241	248	85	426	214	30
CACGACGTG	BRDN00011	180	0	0	384	140	0
CACGACTAA	BRDN00012	119	145	117	182	293	6
CACGACTAA	BRDN00012	97	76	0	167	63	0
CACGACTTCT	BRDN00012	284	105	274	462	396	14
CACGAGAAA	BRDN00011	75	80	603	184	57	0
CACGAGAAG	BRDN00011	189	102	768	355	318	8
CACGAGACC	BRDN00012	242	210	101	544	656	18
CACGAGACC	BRDN00011	200	227	115	337	212	0
CACGAGATA	BRDN00012	55	55	0	99	17	0
CACGAGATA	BRDN00011	133	203	1781	234	520	36
CACGAGCAG	BRDN00012	171	207	33	222	164	0
CACGAGCAT	BRDN00011	99	38	46	152	254	0
CACGAGCAT	BRDN00011	184	167	292	368	270	36
CACGAGGTT	BRDN00012	228	154	210	373	209	24
CACGATCAC	BRDN00012	240	278	118	364	362	18
CACGATCAT	BRDN00011	109	111	444	210	81	0
CACGATGAG	BRDN00012	262	165	13	534	479	18
CACGATGAG	BRDN00011	36	11	0	200	105	0
CACGATTTCG	BRDN00011	148	153	156	176	284	0
CACGCACAA	BRDN00011	310	336	230	555	815	36
CACGCACAA	BRDN00012	134	89	21	286	468	8
CACGCACAA	BRDN00012	382	457	550	688	866	11
CACGCACAA	BRDN00011	168	130	1888	247	603	36
CACGCACAC	BRDN00012	205	94	224	277	219	3
CACGCACAG	BRDN00012	122	159	2991	160	70	187
CACGCACCG	BRDN00012	178	195	36	133	60	20
CACGCACCTG	BRDN00011	349	191	65	855	543	0
CACGCACCTT	BRDN00011	340	281	117	495	426	24
CACGCACGA	BRDN00011	466	432	532	810	910	59
CACGCACGT	BRDN00011	78	34	0	215	82	12
CACGCACGT	BRDN00012	143	91	12	209	121	0
CACGCACTCA	BRDN00011	236	200	180	619	898	11
CACGCAGAT	BRDN00011	238	212	465	416	626	14
CACGCAGCA	BRDN00012	318	363	2764	544	398	245
CACGCAGCA	BRDN00012	279	285	195	562	394	30
CACGCAGCA	BRDN00012	278	229	175	350	360	23
CACGCAGCA	BRDN00012	212	225	616	559	281	40
CACGCCAAA	BRDN00011	189	158	433	338	341	1
CACGCCAAC	BRDN00012	123	52	32	76	24	0
CACGCCAAC	BRDN00012	165	144	170	437	267	7
CACGCCAACT	BRDN00012	421	355	661	755	622	17
CACGCCAAT	BRDN00011	184	214	825	463	387	24
CACGCCACG	BRDN00012	123	56	1	224	188	18
CACGCCAGC	BRDN00012	72	90	659	208	577	1
CACGCCATTC	BRDN00012	198	262	124	333	280	180
CACGCCCAGT	BRDN00011	120	46	124	271	337	0
CACGCCCTCG	BRDN00012	184	213	56	248	152	64
CACGCCGAT	BRDN00012	112	7	0	245	192	0
CACGCCGCT	BRDN00011	146	93	25	358	297	0
CACGCCGGC	BRDN00011	112	148	206	214	378	10
CACGCCGTA	BRDN00012	374	10	3	977	872	0
CACGCCGTC	BRDN00012	296	207	980	509	631	0
CACGCCGTTT	BRDN00012	94	59	0	165	122	0
CACGCCTGC	BRDN00012	295	269	404	412	470	10
CACGCCTTCC	BRDN00012	357	265	1900	664	998	44
CACGCGAGA	BRDN00011	53	25	0	116	101	0
CACGCGCCC	BRDN00012	109	10	0	173	65	0
CACGCGCTC	BRDN00012	102	58	0	199	348	0
CACGCGGTA	BRDN00011	143	128	988	260	169	10
CACGCGGTA	BRDN00011	63	36	51	98	43	0
CACGCGTACT	BRDN00011	213	241	990	420	414	43
CACGCTCACA	BRDN00011	253	54	49	525	230	0
CACGCTCTGT	BRDN00011	241	126	169	462	385	23
CACGCTCTTC	BRDN00011	105	117	0	355	412	21
CACGCTGAC	BRDN00011	139	59	100	223	135	20
CACGCTGAT	BRDN00012	130	73	39	231	384	0
CACGCTGCC	BRDN00012	371	297	213	619	770	132
CACGCTGCT	BRDN00012	373	454	223	626	736	98
CACGCTGTG	BRDN00012	438	520	382	926	939	26
CACGCTGTG	BRDN00012	116	50	20	245	317	0
CACGCTTCTG	BRDN00012	336	279	1040	590	604	23
CACGCTTTA	BRDN00011	133	84	125	152	85	18
CACGGAAAT	BRDN00011	86	67	19	90	27	1
CACGGAACA	BRDN00011	373	455	534	637	992	11
CACGGAACC	BRDN00011	382	371	615	742	960	40
CACGGAAGT	BRDN00011	91	61	273	199	173	0
CACGGAATT	BRDN00011	150	178	3450	414	442	64
CACGGACCCT	BRDN00012	100	2	0	306	201	0
CACGGACGT	BRDN00011	375	42	18	526	247	25
CACGGACGT	BRDN00012	215	172	171	191	164	32
CACGGACTC	BRDN00011	93	104	38	196	308	0
CACGGAGCG	BRDN00011	75	26	256	131	177	2
CACGGAGGA	BRDN00012	307	252	87	653	437	8
CACGGAGGT	BRDN00011	54	61	87	179	124	0
CACGGATAG	BRDN00011	104	219	98	342	280	12
CACGGATCA	BRDN00012	170	139	24	220	295	0
CACGGATGG	BRDN00012	314	294	178	491	981	27
CACGGATGT	BRDN00011	300	234	721	506	584	3
CACGGATTCT	BRDN00011	54	37	0	78	31	0
CACGGCACC	BRDN00011	25	5	24	269	62	0
CACGGCCCT	BRDN00012	136	125	35	189	119	15
CACGGCCCT	BRDN00012	282	253	691	563	597	36
CACGGCCTA	BRDN00012	402	344	642	672	900	268
CACGGCCTG	BRDN00012	138	94	138	271	117	21
CACGGCGTA	BRDN00012	289	256	351	457	540	38
CACGGCGTC	BRDN00012	340	327	788	438	344	16
CACGGCTAA	BRDN00011	319	249	933	591	434	26
CACGGCTAG	BRDN00012	208	181	327	467	448	21
CACGGCTGT	BRDN00011	45	129	10	112	168	0
CACGGGACT	BRDN00012	119	61	745	140	160	10
CACGGGCAC	BRDN00011	197	161	425	362	389	6
CACGGGCAG	BRDN00012	69	72	0	190	112	0
CACGGGCAG	BRDN00011	178	232	309	406	257	29
CACGGGGCT	BRDN00012	343	304	937	640	369	39
CACGGGGTC	BRDN00012	119	123	30	193	121	8
CACGGGTCC	BRDN00012	62	75	0	194	119	0
CACGGGTGG	BRDN00011	7	0	0	26	0	0
CACGGTAAC	BRDN00012	169	174	223	383	182	25
CACGGTACA	BRDN00012	116	0	0	170	164	0
CACGGTACT	BRDN00012	39	56	0	120	54	0
CACGGTCGG	BRDN00012	98	123	87	186	152	5
CACGGTGCG	BRDN00012	422	218	825	704	998	20
CACGGTGCT	BRDN00012	323	430	339	747	592	10
CACGGTTCG	BRDN00011	150	73	46	149	212	3
CACGTACCAC	BRDN00012	122	188	561	374	252	43
CACGTACCCC	BRDN00012	124	107	87	253	231	31
CACGTAGAA	BRDN00011	133	96	94	190	63	8
CACGTAGTC	BRDN00012	354	334	529	623	642	38
CACGTATCA	BRDN00012	226	16	0	537	421	0
CACGTATTG	BRDN00012	195	193	160	352	461	4
CACGTCACA	BRDN00011	40	20	0	70	26	0
CACGTCACTA	BRDN00012	70	61	70	156	82	14
CACGTCACTC	BRDN00011	112	148	72	214	219	0
CACGTCCGA	BRDN00012	90	127	179	225	351	11
CACGTCCGA	BRDN00012	82	101	3	148	120	28
CACGTCCTCA	BRDN00011	97	107	144	208	386	0
CACGTCGCT	BRDN00012	240	228	73	660	423	0
CACGTCTGC	BRDN00012	276	220	229	663	717	23
CACGTGAAC	BRDN00012	93	171	246	182	138	8
CACGTGACT	BRDN00012	137	92	35	203	210	0
CACGTGAGA	BRDN00012	100	69	78	238	261	0
CACGTGATA	BRDN00012	244	194	355	353	389	9
CACGTGCAG	BRDN00011	223	278	144	387	374	72
CACGTGCCG	BRDN00012	334	406	1372	563	663	70
CACGTGCCG	BRDN00011	377	408	55	689	788	29
CACGTGCTT	BRDN00011	277	302	836	646	469	37
CACGTGCTT	BRDN00011	102	89	44	236	335	0
CACGTGGAC	BRDN00011	127	96	28	117	105	8
CACGTGGAC	BRDN00011	198	99	0	203	140	11
CACGTGGAT	BRDN00012	81	86	233	164	137	32
CACGTGGGC	BRDN00012	291	305	566	433	576	9
CACGTGGGC	BRDN00012	106	105	534	139	251	14
CACGTGGTC	BRDN00012	231	241	620	494	374	38
CACGTGGTC	BRDN00012	323	314	377	577	480	75
CACGTGTAT	BRDN00012	188	24	12	557	386	6
CACGTGTGG	BRDN00011	261	24	234	577	419	13
CACGTTACA	BRDN00011	168	55	876	251	267	20
CACGTTGAA	BRDN00011	110	98	0	253	388	5
CACGTTTGC	BRDN00012	156	48	501	294	468	9
CACTAAAGC	BRDN00011	143	244	809	429	182	6
CACTAAAGG	BRDN00011	182	201	84	300	249	12
CACTAAATCA	BRDN00011	225	236	436	515	474	18
CACTAACAG	BRDN00011	174	103	313	390	262	42
CACTAACCCC	BRDN00012	305	37	0	554	585	0
CACTAACCG	BRDN00012	132	14	0	184	187	0
CACTAACGA	BRDN00011	217	129	160	585	427	5
CACTAACTAT	BRDN00011	151	126	27	242	328	21
CACTAAGGA	BRDN00011	70	8	35	352	557	0
CACTAAGGC	BRDN00012	439	566	1624	1004	1033	146
CACTAATAA	BRDN00011	83	106	7	235	48	2
CACTAATATA	BRDN00012	80	98	118	259	148	0
CACTAATCAG	BRDN00012	71	61	0	159	176	2
CACTAATCGT	BRDN00011	142	163	580	364	546	66
CACTAATGC	BRDN00012	227	142	129	329	499	5
CACTAATGT	BRDN00011	214	97	2522	348	205	25
CACTAATTAG	BRDN00011	71	30	5	202	436	3
CACTACAAG	BRDN00012	73	54	0	120	126	9
CACTACAAG	BRDN00012	474	374	347	671	734	63
CACTACAAG	BRDN00011	210	195	1322	315	342	6
CACTACACCA	BRDN00012	475	612	786	874	671	73
CACTACACTA	BRDN00011	159	76	0	518	229	6
CACTACAGC	BRDN00012	161	141	352	230	174	16
CACTACAGT	BRDN00011	259	263	407	425	337	16
CACTACATAG	BRDN00011	137	263	238	258	186	18
CACTACATCC	BRDN00011	278	183	288	524	410	35
CACTACATTG	BRDN00011	119	39	0	296	302	0
CACTACCATG	BRDN00012	227	254	852	380	505	45
CACTACCTAC	BRDN00012	208	41	13	303	309	0
CACTACCTGA	BRDN00011	114	221	369	262	328	1
CACTACGAC	BRDN00012	351	254	530	656	607	30
CACTACGAC	BRDN00011	174	6	2	334	28	0
CACTACGAC	BRDN00011	63	53	83	174	121	0
CACTACGGC	BRDN00011	325	274	226	502	255	6
CACTACGGT	BRDN00012	146	65	134	182	172	30
CACTACGTG	BRDN00011	125	91	17	302	194	17
CACTACTCCT	BRDN00011	399	421	312	453	507	52
CACTAGAAA	BRDN00012	244	205	13	310	397	0
CACTAGAAG	BRDN00011	101	111	148	163	190	0
CACTAGACG	BRDN00012	222	170	628	571	795	8
CACTAGACT	BRDN00012	151	186	4262	262	317	63
CACTAGCAA	BRDN00012	416	253	509	639	422	21
CACTAGCAG	BRDN00012	352	246	156	524	392	73
CACTAGCGG	BRDN00012	278	167	220	567	541	17
CACTAGTGT	BRDN00012	182	154	95	422	171	7
CACTATCAA	BRDN00011	204	170	39	262	355	32
CACTATCGG	BRDN00012	496	310	265	1033	629	8
CACTATCTTA	BRDN00011	66	89	13	172	244	8
CACTATGAT	BRDN00011	372	293	747	785	677	29
CACTATGCTC	BRDN00012	269	320	328	480	635	13
CACTATGTA	BRDN00011	161	219	23	403	483	14
CACTATTGA	BRDN00012	305	229	1873	549	689	56
CACTATTGA	BRDN00011	236	369	1066	414	806	20
CACTCAACCA	BRDN00011	417	386	312	802	747	57
CACTCAACCC	BRDN00011	351	334	572	794	748	70
CACTCAAGT	BRDN00012	362	385	953	734	493	23
CACTCAATA	BRDN00012	300	310	322	491	728	14
CACTCAATAG	BRDN00012	181	154	49	380	313	27
CACTCACATT	BRDN00011	103	250	817	358	521	46
CACTCACCAG	BRDN00012	137	214	103	201	335	0
CACTCACCAG	BRDN00012	225	277	407	460	270	7
CACTCACCAT	BRDN00012	101	93	265	240	209	0
CACTCACCCA	BRDN00012	240	90	89	430	208	15
CACTCACCCA	BRDN00012	319	399	634	573	309	21
CACTCACCGG	BRDN00011	160	90	2946	263	540	53
CACTCACCTA	BRDN00012	283	93	315	361	315	0
CACTCACCTG	BRDN00011	341	286	472	477	208	6
CACTCACGAT	BRDN00012	401	150	143	944	630	3
CACTCACGCC	BRDN00012	476	549	991	797	1020	108
CACTCACGG	BRDN00011	319	422	529	463	817	54
CACTCACGTG	BRDN00011	209	205	298	654	687	48
CACTCACGTT	BRDN00012	184	177	583	291	185	57
CACTCACTCG	BRDN00012	244	129	42	438	477	24
CACTCACTGT	BRDN00011	203	171	882	429	325	44
CACTCAGCCA	BRDN00011	369	270	1024	630	943	16
CACTCAGCCT	BRDN00011	70	168	75	274	296	89
CACTCAGCG	BRDN00012	164	108	2308	387	706	198
CACTCAGCTT	BRDN00012	436	308	1060	700	652	39
CACTCAGTTT	BRDN00012	204	144	392	342	198	43
CACTCCAGA	BRDN00011	13	0	0	127	0	0
CACTCCAGA	BRDN00011	12	0	0	235	76	0
CACTCCAGA	BRDN00011	215	128	375	318	272	7
CACTCCATTC	BRDN00012	427	265	600	745	913	15
CACTCCCAAA	BRDN00012	198	75	611	272	146	71
CACTCCCCTC	BRDN00011	100	110	188	305	143	20
CACTCCGGCT	BRDN00011	119	163	252	266	50	7
CACTCCTACC	BRDN00012	293	289	763	787	492	30
CACTCCTGTA	BRDN00012	83	137	167	189	35	6
CACTCCTTCT	BRDN00012	525	397	1223	857	1060	86
CACTCGAGC	BRDN00012	311	286	594	766	870	31
CACTCGATG	BRDN00012	159	187	200	448	320	27
CACTCGGGA	BRDN00011	115	235	117	224	295	0
CACTCGTAG	BRDN00011	143	143	662	326	322	18
CACTCGTCG	BRDN00011	413	381	385	703	669	93
CACTCGTTAC	BRDN00012	312	317	751	695	605	87
CACTCTACTT	BRDN00012	88	23	505	541	236	7
CACTCTCAGA	BRDN00011	100	58	274	203	103	0
CACTCTCAGA	BRDN00011	148	143	143	267	225	6
CACTCTCATT	BRDN00012	135	144	367	228	124	10
CACTCTCCAG	BRDN00012	131	180	0	251	112	12
CACTCTGAA	BRDN00012	318	164	2280	496	504	45
CACTCTGACT	BRDN00012	160	169	127	285	151	0
CACTCTTGTC	BRDN00012	376	369	45	805	427	6
CACTCTTTGG	BRDN00012	103	10	0	784	300	0
CACTGAACA	BRDN00012	169	45	1	542	515	0
CACTGAACA	BRDN00011	189	53	75	310	360	3
CACTGAAGA	BRDN00011	105	164	221	244	487	28
CACTGAATG	BRDN00011	307	419	443	732	618	57
CACTGACAG	BRDN00011	246	324	480	723	491	12
CACTGACCCG	BRDN00012	150	85	314	366	286	7
CACTGACTCC	BRDN00012	338	255	17	831	821	24
CACTGACTCG	BRDN00011	219	182	47	451	490	4
CACTGACTCG	BRDN00012	60	66	0	252	235	0
CACTGAGAC	BRDN00011	250	242	470	472	872	20
CACTGAGACT	BRDN00012	94	73	84	284	107	4
CACTGAGAG	BRDN00012	242	254	9	316	543	0
CACTGAGAG	BRDN00011	214	71	93	352	181	7
CACTGAGCA	BRDN00012	123	152	13	328	264	25
CACTGAGCA	BRDN00012	172	182	12	351	167	0
CACTGAGCT	BRDN00012	364	426	175	737	784	23
CACTGAGGG	BRDN00012	107	21	0	287	257	0
CACTGAGTC	BRDN00011	173	184	336	338	312	8
CACTGATAA	BRDN00011	189	52	13	286	702	0
CACTGATGA	BRDN00012	514	413	1786	828	731	82
CACTGATGG	BRDN00012	123	92	585	259	300	89
CACTGCAAG	BRDN00012	397	550	1511	666	893	42
CACTGCAAG	BRDN00012	210	102	30	326	274	7
CACTGCACA	BRDN00012	153	149	149	346	305	13
CACTGCACCA	BRDN00011	377	525	293	810	794	75
CACTGCACCC	BRDN00011	155	98	15	350	301	17
CACTGCACTC	BRDN00011	245	169	564	463	376	13
CACTGCAGA	BRDN00012	600	511	2294	953	1071	55
CACTGCAGC	BRDN00012	599	479	356	906	1308	49
CACTGCAGT	BRDN00012	462	398	662	965	928	15
CACTGCCCCG	BRDN00012	563	488	1600	767	954	56
CACTGCCCGT	BRDN00012	252	261	211	513	553	7
CACTGCCCTC	BRDN00011	127	181	313	224	151	14
CACTGCCCTT	BRDN00011	247	294	663	561	709	67
CACTGCCGA	BRDN00011	190	263	20	367	545	8
CACTGCGAA	BRDN00011	89	181	1	142	126	11
CACTGCGAA	BRDN00012	290	256	692	718	849	9
CACTGCGGA	BRDN00012	136	103	42	171	206	6
CACTGCGGC	BRDN00011	91	105	3	129	189	0
CACTGCGGG	BRDN00012	101	60	286	154	202	0
CACTGCGTG	BRDN00012	447	298	335	709	542	36
CACTGCTCA	BRDN00012	176	117	29	606	130	14
CACTGCTGA	BRDN00012	108	185	68	295	594	5
CACTGCTGC	BRDN00012	485	361	104	1007	1414	61
CACTGCTTTA	BRDN00012	158	119	169	285	267	4
CACTGGAAC	BRDN00011	82	83	0	270	97	7
CACTGGAAG	BRDN00012	49	43	100	88	317	0
CACTGGAAT	BRDN00012	448	353	189	582	856	27
CACTGGAGG	BRDN00012	118	93	17	276	333	3
CACTGGATG	BRDN00012	238	148	2740	561	550	22
CACTGGCAT	BRDN00012	147	184	7	325	171	8
CACTGGCTA	BRDN00012	238	170	1642	405	533	12
CACTGGGAA	BRDN00012	215	166	456	449	310	16
CACTGGGAC	BRDN00012	103	0	0	421	109	0
CACTGGGCA	BRDN00012	154	170	5	243	553	26
CACTGGGCA	BRDN00011	169	272	743	433	788	52
CACTGGGGC	BRDN00012	291	337	642	619	719	12
CACTGGTAG	BRDN00012	36	57	936	173	104	21
CACTGTAACT	BRDN00011	289	223	1031	445	662	43
CACTGTACTG	BRDN00012	113	65	10	340	713	11
CACTGTACTG	BRDN00012	254	213	81	495	481	19
CACTGTCTG	BRDN00012	206	189	2134	414	293	29
CACTGTCTG	BRDN00011	450	383	768	846	468	3
CACTGTGAA	BRDN00012	9	0	0	31	0	2
CACTGTGAT	BRDN00011	318	224	291	429	615	29
CACTGTGGC	BRDN00012	143	111	84	357	224	8
CACTGTGGC	BRDN00011	158	225	399	379	461	0
CACTGTTAA	BRDN00011	312	192	6302	595	737	130
CACTGTTGC	BRDN00012	223	179	95	370	372	4
CACTGTTGT	BRDN00012	278	285	550	456	398	59
CACTTAACAG	BRDN00011	83	103	1	215	101	0
CACTTAACTG	BRDN00011	97	89	43	174	385	23
CACTTACAAT	BRDN00012	105	139	144	270	237	293
CACTTACACA	BRDN00012	463	558	468	814	1711	30
CACTTACAG	BRDN00011	54	0	0	198	56	0
CACTTACCCT	BRDN00012	121	33	0	198	947	0
CACTTACCTA	BRDN00012	139	194	43	304	261	12
CACTTACCTT	BRDN00012	210	335	52	313	241	73
CACTTACGGT	BRDN00012	199	25	0	359	255	0
CACTTACTAT	BRDN00011	253	381	22	561	451	22
CACTTAGAT	BRDN00012	62	50	16	140	273	0
CACTTAGCGT	BRDN00011	81	0	0	112	42	0
CACTTCACCT	BRDN00011	355	234	334	633	562	14
CACTTCACTC	BRDN00012	340	192	1617	829	890	183
CACTTCCAAC	BRDN00012	133	227	842	247	113	10
CACTTCCAGA	BRDN00012	545	393	1126	977	1205	50
CACTTCCCGG	BRDN00012	183	135	585	590	569	0
CACTTCGACC	BRDN00012	109	0	1	385	84	0
CACTTCGCAC	BRDN00011	147	156	22	216	283	0
CACTTCGGT	BRDN00012	231	208	249	493	917	76
CACTTCTCAG	BRDN00012	346	345	902	756	590	4
CACTTCTCCT	BRDN00011	223	175	58	370	193	22
CACTTGAAG	BRDN00012	382	603	507	740	839	37
CACTTGACA	BRDN00012	148	41	1	328	146	0
CACTTGCCTT	BRDN00012	350	286	660	574	789	25
CACTTGCGC	BRDN00012	113	76	0	276	346	0
CACTTGGCCT	BRDN00012	234	210	279	416	205	24
CACTTGGGA	BRDN00012	78	40	15	280	337	0
CACTTGGGG	BRDN00012	407	344	33	896	807	33
CACTTGGGT	BRDN00011	259	366	504	559	461	16
CACTTTCAGC	BRDN00011	131	167	192	249	153	5
CACTTTCCGA	BRDN00011	234	163	231	315	139	10
CACTTTCTCT	BRDN00012	140	87	910	240	194	12
CACTTTGACG	BRDN00012	471	207	100	537	363	0
CACTTTGGCC	BRDN00012	86	31	550	126	134	20
CAGAAAAAG	BRDN00011	404	439	449	872	612	47
CAGAAAAAG	BRDN00012	73	179	55	262	132	83
CAGAAAACA	BRDN00011	296	377	1251	489	508	39
CAGAAAACT	BRDN00011	142	75	41	431	622	0
CAGAAAACT	BRDN00012	162	51	19	350	422	8
CAGAAAAGA	BRDN00012	218	219	362	379	510	10
CAGAAAAGC	BRDN00011	59	44	119	97	39	5
CAGAAAAGC	BRDN00012	275	161	102	596	441	71
CAGAAAAGG	BRDN00012	358	470	408	636	925	21
CAGAAAAGG	BRDN00012	263	211	237	417	228	21
CAGAAAAGT	BRDN00011	309	244	431	527	549	65
CAGAAACAA	BRDN00012	102	68	7	247	304	46
CAGAAACAC	BRDN00012	140	80	103	176	361	0
CAGAAACAG	BRDN00011	172	204	114	295	268	27
CAGAAACAG	BRDN00012	83	94	498	176	84	0
CAGAAACCA	BRDN00012	132	173	192	299	242	5
CAGAAACCA	BRDN00012	54	54	179	175	238	15
CAGAAACCCT	BRDN00012	316	258	590	528	609	38
CAGAAACCT	BRDN00011	102	77	295	194	134	0
CAGAAACGA	BRDN00012	84	126	523	231	176	7
CAGAAACGC	BRDN00012	246	268	401	568	247	8
CAGAAACTA	BRDN00011	95	137	318	209	92	23
CAGAAACTA	BRDN00012	223	144	175	313	272	43
CAGAAACTG	BRDN00011	289	247	175	848	744	81
CAGAAACTG	BRDN00011	77	83	14	201	244	39
CAGAAACTG	BRDN00012	75	85	38	245	133	0

CAGAAACTT	BRDN00012	178	261	242	480	463	21
CAGAAACTT	BRDN00011	174	100	276	316	346	0
CAGAAAGAG	BRDN00011	134	83	228	159	169	12
CAGAAAGCA	BRDN00011	207	7	340	374	124	17
CAGAAAGCA	BRDN00012	128	128	137	152	56	17
CAGAAAGCG	BRDN00012	167	110	100	234	172	4
CAGAAAGGG	BRDN00012	224	259	193	359	538	0
CAGAAAGTA	BRDN00011	98	73	78	234	303	0
CAGAAAGTG	BRDN00011	194	454	15411	235	381	207
CAGAAATAG	BRDN00012	118	177	31	196	306	0
CAGAAATCT	BRDN00012	18	0	0	334	146	0
CAGAAATGA	BRDN00011	207	181	50	391	302	25
CAGAAATGG	BRDN00011	112	88	58	162	221	0
CAGAAATGG	BRDN00012	203	270	2052	497	966	45
CAGAAATGG	BRDN00011	326	474	336	770	1244	0
CAGAAATGT	BRDN00011	190	122	23	260	228	8
CAGAACAAG	BRDN00012	196	179	622	358	381	12
CAGAACACA	BRDN00011	512	299	1470	740	795	28
CAGAACACA	BRDN00012	425	374	907	776	1109	71
CAGAACACC	BRDN00012	525	311	204	536	426	47
CAGAACACT	BRDN00012	132	67	21	336	334	0
CAGAACAGG	BRDN00012	211	128	617	379	305	2
CAGAACAGT	BRDN00012	286	337	34	557	718	87
CAGAACATG	BRDN00011	115	47	0	170	301	1
CAGAACCAA	BRDN00012	238	159	66	234	349	2
CAGAACCAC	BRDN00012	374	381	399	677	1205	13
CAGAACCAC	BRDN00011	262	203	43	529	252	22
CAGAACCAG	BRDN00011	128	104	402	359	403	7
CAGAACCAG	BRDN00012	572	582	559	926	860	13
CAGAACCAG	BRDN00012	110	179	558	289	341	33
CAGAACCCA	BRDN00011	187	88	1251	577	518	20
CAGAACCCC	BRDN00011	113	60	12	346	554	6
CAGAACCCCT	BRDN00011	138	101	48	262	136	0
CAGAACCTA	BRDN00011	88	67	349	206	153	8
CAGAACCTA	BRDN00012	80	70	218	245	162	16
CAGAACCTC	BRDN00012	160	134	358	395	596	3
CAGAACGAA	BRDN00012	365	330	118	880	1113	106
CAGAACGGG	BRDN00011	125	46	665	251	219	14
CAGAACGTA	BRDN00012	175	154	3806	420	491	62
CAGAACTCC	BRDN00011	122	30	1	277	125	0
CAGAACTCC	BRDN00012	453	489	858	712	227	63
CAGAACTCG	BRDN00011	90	52	114	195	280	11
CAGAACTCT	BRDN00011	170	198	770	307	921	18
CAGAACTGA	BRDN00011	441	414	2432	861	669	84
CAGAACTGT	BRDN00012	307	62	2	653	545	5
CAGAACTGT	BRDN00012	553	498	863	718	507	127
CAGAAGACG	BRDN00012	88	42	0	209	224	0
CAGAAGACG	BRDN00012	99	82	19	161	181	0
CAGAAGACG	BRDN00011	54	40	0	386	87	0
CAGAAGAGA	BRDN00012	178	155	190	279	250	15
CAGAAGATG	BRDN00012	215	246	49	283	326	47
CAGAAGATG	BRDN00012	351	519	794	497	605	32
CAGAAGATG	BRDN00012	55	34	46	173	166	0
CAGAAGATT	BRDN00012	291	243	904	528	678	33
CAGAAGCAA	BRDN00011	199	1	0	706	344	9
CAGAAGCAG	BRDN00012	136	82	63	424	271	26
CAGAAGCAG	BRDN00012	319	261	642	779	818	69
CAGAAGCCA	BRDN00011	148	158	88	242	655	0
CAGAAGCCA	BRDN00011	82	0	0	660	362	0
CAGAAGCCC	BRDN00012	387	254	2846	803	576	104
CAGAAGCCC	BRDN00012	449	9	2	784	724	1
CAGAAGCTA	BRDN00012	161	123	120	248	223	5
CAGAAGCTG	BRDN00011	114	147	285	244	196	5
CAGAAGCTG	BRDN00012	316	364	514	520	393	35
CAGAAGCTG	BRDN00012	347	344	714	858	508	82
CAGAAGCTT	BRDN00011	53	0	0	251	127	0
CAGAAGGAA	BRDN00012	133	276	0	244	384	4
CAGAAGGAC	BRDN00011	203	56	807	340	348	28
CAGAAGGAC	BRDN00011	153	75	96	262	72	6
CAGAAGGAG	BRDN00011	296	464	324	682	480	44
CAGAAGGAG	BRDN00012	295	218	91	652	452	0
CAGAAGGCG	BRDN00012	76	31	24	251	131	0
CAGAAGGCT	BRDN00011	338	243	2025	696	1734	99
CAGAAGGCT	BRDN00011	62	73	42	232	375	4
CAGAAGGGA	BRDN00012	203	147	588	366	237	0
CAGAAGGGG	BRDN00011	184	187	50	329	696	0
CAGAAGGGG	BRDN00011	375	402	2546	756	963	65
CAGAAGGTA	BRDN00012	356	343	294	532	672	9
CAGAAGGTA	BRDN00012	337	191	1109	699	635	17
CAGAAGGTA	BRDN00011	210	264	2584	414	423	33
CAGAAGGTG	BRDN00011	182	152	234	248	132	54
CAGAAGTCA	BRDN00011	298	293	453	506	508	30
CAGAAGTGA	BRDN00011	158	105	434	308	166	20
CAGAAGTGG	BRDN00012	278	191	5700	364	295	81
CAGAAGTGT	BRDN00011	290	0	83	706	725	0
CAGAAGTTA	BRDN00011	372	236	120	557	736	8
CAGAAGTTT	BRDN00011	63	3	0	143	51	21
CAGAATAAG	BRDN00012	93	32	535	442	249	3
CAGAATACA	BRDN00011	220	188	40	329	288	15
CAGAATACA	BRDN00011	149	164	29	170	172	11
CAGAATACC	BRDN00011	196	258	62	452	355	12
CAGAATAGA	BRDN00011	133	16	0	519	180	0
CAGAATATT	BRDN00012	205	232	266	534	182	34
CAGAATCAA	BRDN00011	104	207	112	180	214	34
CAGAATCAT	BRDN00012	515	369	337	777	538	39
CAGAATCCC	BRDN00012	288	231	30	391	584	10
CAGAATCCT	BRDN00011	252	328	239	560	956	14
CAGAATCGC	BRDN00011	263	199	3335	491	387	48
CAGAATCGC	BRDN00012	155	99	154	145	67	5
CAGAATCGT	BRDN00012	180	70	649	319	204	53
CAGAATCTA	BRDN00011	138	122	8	143	217	18
CAGAATCTC	BRDN00012	158	151	57	292	220	0
CAGAATCTG	BRDN00011	40	0	21	226	30	0
CAGAATCTT	BRDN00012	180	207	995	406	437	10
CAGAATGAG	BRDN00011	105	47	35	248	199	0
CAGAATGAC	BRDN00012	97	50	31	191	102	0
CAGAATGAG	BRDN00011	78	32	0	157	168	0
CAGAATGCT	BRDN00011	338	266	310	714	1010	15
CAGAATGGA	BRDN00011	254	157	342	477	487	23
CAGAATGGC	BRDN00011	349	179	858	605	802	22
CAGAATGGG	BRDN00012	56	43	0	491	114	0
CAGAATTCA	BRDN00012	58	0	0	211	128	0
CAGAATTCA	BRDN00012	165	120	502	251	353	0
CAGAATTCT	BRDN00011	266	241	236	490	458	4
CAGAATTGA	BRDN00011	275	170	162	347	433	17
CAGAATTGG	BRDN00012	438	421	265	645	1372	62
CAGACAAAG	BRDN00012	162	112	268	268	354	0
CAGACAACA	BRDN00011	41	162	6	168	188	9
CAGACAACC	BRDN00012	340	314	519	565	501	31
CAGACAACT	BRDN00011	121	39	292	108	180	0
CAGACAAGA	BRDN00011	246	203	881	452	584	20
CAGACAAGG	BRDN00011	118	115	0	126	8	0
CAGACAATA	BRDN00011	239	157	156	595	277	17
CAGACACAG	BRDN00012	402	382	8983	595	691	111
CAGACACAG	BRDN00012	281	39	61	671	728	5
CAGACACAG	BRDN00012	467	71	111	922	739	28
CAGACACAG	BRDN00012	344	253	1422	545	991	49
CAGACACAG	BRDN00012	139	186	147	245	137	167
CAGACACAT	BRDN00011	113	107	10	195	237	5
CAGACACAT	BRDN00011	74	66	150	109	98	21
CAGACACCC	BRDN00012	395	419	820	774	580	76
CAGACACCG	BRDN00011	12	8	0	188	12	0
CAGACACCT	BRDN00011	219	185	477	322	299	6
CAGACACCT	BRDN00012	140	97	11	255	333	9
CAGACACGA	BRDN00012	352	388	136	828	703	1
CAGACACGC	BRDN00011	134	100	45	263	181	0
CAGACACGG	BRDN00012	276	196	3	504	446	0
CAGACACGT	BRDN00011	168	35	338	228	318	44
CAGACACGT	BRDN00011	82	47	163	155	147	4
CAGACACGT	BRDN00011	106	119	292	187	291	12
CAGACACTC	BRDN00012	183	100	313	371	204	18
CAGACACTC	BRDN00011	125	88	658	221	247	34
CAGACACTC	BRDN00012	230	151	2356	370	491	110
CAGACACTC	BRDN00011	171	154	310	335	385	25
CAGACACTC	BRDN00011	251	142	901	279	543	3
CAGACACTG	BRDN00012	156	184	0	247	274	13
CAGACACTG	BRDN00011	334	142	151	602	956	4
CAGACAGAG	BRDN00011	21	28	10	311	115	0
CAGACAGCA	BRDN00012	150	114	62	153	414	33
CAGACAGCC	BRDN00011	106	166	14	275	284	21
CAGACAGCT	BRDN00012	179	194	911	465	551	35
CAGACAGCT	BRDN00011	299	380	853	576	688	37
CAGACAGGC	BRDN00012	331	313	459	688	580	185
CAGACAGGC	BRDN00011	164	148	354	390	372	0
CAGACAGGG	BRDN00011	158	147	109	185	181	0
CAGACAGTG	BRDN00011	378	462	1313	770	670	87
CAGACATAA	BRDN00012	139	89	154	302	299	0
CAGACATCC	BRDN00012	200	189	0	502	698	6
CAGACATCT	BRDN00011	103	150	1035	293	395	311
CAGACATGA	BRDN00011	177	116	32	261	225	30
CAGACATGG	BRDN00012	105	22	0	319	207	0
CAGACATGG	BRDN00012	325	285	837	506	424	21
CAGACATGT	BRDN00011	212	74	173	663	499	2
CAGACATTC	BRDN00012	108	168	2	159	270	0
CAGACATTC	BRDN00012	327	402	460	804	769	32
CAGACATTCT	BRDN00011	177	159	392	319	645	31
CAGACCAAA	BRDN00012	148	166	292	205	309	7
CAGACCAAA	BRDN00011	265	110	130	517	476	0
CAGACCAAA	BRDN00012	297	189	214	492	437	9
CAGACCAAC	BRDN00012	126	191	0	266	37	20
CAGACCAAC	BRDN00011	131	155	644	223	155	35
CAGACCAAG	BRDN00012	388	227	185	679	567	20
CAGACCAAG	BRDN00011	141	171	402	340	743	46
CAGACCAAG	BRDN00012	119	86	328	200	264	0
CAGACCAAG	BRDN00012	194	203	803	236	184	13
CAGACCACG	BRDN00012	138	85	562	199	207	12
CAGACCAGA	BRDN00011	165	114	8	238	270	0
CAGACCAGA	BRDN00011	90	163	160	233	149	106
CAGACCAGC	BRDN00011	91	70	10	262	102	17
CAGACCAGG	BRDN00011	481	406	1153	831	977	97
CAGACCAGT	BRDN00012	369	280	823	719	638	32
CAGACCATA	BRDN00011	89	59	111	178	121	35
CAGACCATG	BRDN00011	372	531	296	773	649	22
CAGACCATT	BRDN00011	520	431	145	806	780	1
CAGACCCAA	BRDN00012	163	76	79	300	306	24
CAGACCCAA	BRDN00012	96	92	1261	126	135	55
CAGACCCAC	BRDN00012	491	327	652	903	921	75
CAGACCCAG	BRDN00012	260	167	10	279	185	8
CAGACCCAG	BRDN00012	404	263	42	725	606	14
CAGACCCAG	BRDN00011	68	13	0	269	219	0
CAGACCCAG	BRDN00012	706	432	1833	889	826	73
CAGACCCCA	BRDN00011	347	216	1449	758	430	45
CAGACCCCA	BRDN00012	367	277	68	608	626	65
CAGACCCCC	BRDN00012	118	97	1455	402	213	11
CAGACCCGT	BRDN00011	307	247	100	476	439	13
CAGACCCTC	BRDN00012	107	196	118	269	257	15
CAGACCCTG	BRDN00012	218	242	20	557	663	13
CAGACCGGA	BRDN00012	212	222	227	531	732	37
CAGACCGGC	BRDN00012	105	80	311	216	145	2
CAGACCGTA	BRDN00012	326	284	810	661	605	27
CAGACCTAG	BRDN00012	631	682	937	1100	1439	160
CAGACCTCG	BRDN00012	245	291	3212	593	487	71
CAGACCTGA	BRDN00011	463	351	2736	642	431	78
CAGACCTGA	BRDN00012	88	68	69	170	386	0
CAGACCTGG	BRDN00012	130	91	91	154	206	0
CAGACCTTA	BRDN00012	310	271	37	695	343	8
CAGACCTTC	BRDN00012	463	560	891	841	976	20
CAGACGAAG	BRDN00012	419	180	215	484	497	0
CAGACGAAG	BRDN00012	26	0	0	508	23	0
CAGACGACT	BRDN00011	68	70	4	201	237	0
CAGACGACT	BRDN00011	263	118	86	478	397	4
CAGACGAGA	BRDN00012	135	122	103	316	433	25
CAGACGAGC	BRDN00011	55	0	0	320	615	0
CAGACGAGG	BRDN00011	74	92	52	289	419	0
CAGACGAGT	BRDN00012	98	19	1	129	123	8
CAGACGATG	BRDN00011	114	159	61	167	254	31
CAGACGCAC	BRDN00012	65	0	0	145	148	8
CAGACGCCC	BRDN00012	345	348	331	596	311	12
CAGACGCCT	BRDN00012	137	30	0	157	138	14
CAGACGCGA	BRDN00011	348	216	962	475	451	14
CAGACGCGA	BRDN00011	192	143	195	265	275	26
CAGACGCTC	BRDN00011	120	77	11	273	522	0
CAGACGCTT	BRDN00012	232	236	353	527	667	27
CAGACGGAC	BRDN00012	101	56	8	191	318	0
CAGACGGAC	BRDN00012	119	71	125	231	168	0
CAGACGGAG	BRDN00011	98	75	113	235	221	10
CAGACGGGC	BRDN00012	216	197	146	504	610	0
CAGACGGGC	BRDN00011	208	191	400	418	375	8
CAGACGGGT	BRDN00011	31	12	1	645	258	0
CAGACGGGT	BRDN00011	256	289	382	359	589	20
CAGACGGTT	BRDN00011	184	150	133	286	274	15
CAGACGGTT	BRDN00012	540	482	473	928	1252	70
CAGACGTGA	BRDN00011	105	92	357	201	363	29
CAGACGTTC	BRDN00012	73	122	237	286	276	8
CAGACTAAA	BRDN00012	52	0	0	582	390	0
CAGACTAAA	BRDN00012	142	123	12	199	161	0
CAGACTAAG	BRDN00011	131	71	1	199	399	0
CAGACTAAG	BRDN00011	106	60	18	327	117	71
CAGACTAAG	BRDN00011	92	117	87	216	225	12
CAGACTACC	BRDN00012	516	566	1430	891	833	92
CAGACTACG	BRDN00011	291	171	137	498	881	0
CAGACTAGT	BRDN00012	16	0	0	315	50	0
CAGACTATC	BRDN00012	70	137	63	186	166	17
CAGACTCAA	BRDN00012	130	17	2	630	216	0
CAGACTCAC	BRDN00011	176	235	211	413	191	36
CAGACTCAC	BRDN00011	289	235	2131	635	531	23
CAGACTCAC	BRDN00011	114	113	33	200	129	8
CAGACTCACT	BRDN00011	316	301	1349	788	592	69
CAGACTCACT	BRDN00011	342	192	296	541	1161	11
CAGACTCAT	BRDN00012	166	148	17	329	165	0
CAGACTCAT	BRDN00012	89	103	69	118	289	6
CAGACTCCT	BRDN00012	223	250	129	277	252	68
CAGACTCCT	BRDN00012	162	317	483	379	334	41
CAGACTCGA	BRDN00012	46	97	14	159	217	36
CAGACTCGA	BRDN00011	102	194	883	168	103	104
CAGACTCGG	BRDN00011	168	133	23	137	340	0
CAGACTCTG	BRDN00012	445	474	15358	945	441	466
CAGACTCTG	BRDN00012	342	147	88	444	260	0
CAGACTGAA	BRDN00012	157	144	490	341	182	0
CAGACTGAC	BRDN00011	87	134	96	220	174	24
CAGACTGAG	BRDN00012	134	97	90	223	328	0
CAGACTGCA	BRDN00011	304	322	318	764	688	27
CAGACTGCA	BRDN00011	199	297	935	367	305	11
CAGACTGGC	BRDN00012	154	219	588	290	216	12
CAGACTGTA	BRDN00012	92	177	8	130	22	28
CAGACTGTC	BRDN00011	357	332	303	619	813	0
CAGACTGTG	BRDN00012	158	105	494	339	82	9
CAGACTTAC	BRDN00011	262	125	148	326	347	0
CAGACTTCG	BRDN00011	88	88	43	137	105	0
CAGACTTGA	BRDN00011	414	313	114	705	802	11
CAGACTTGC	BRDN00011	313	321	1356	631	413	36
CAGACTTGG	BRDN00011	320	256	222	674	218	9
CAGACTTGT	BRDN00012	319	365	357	674	456	46
CAGACTTTA	BRDN00011	142	69	27	205	228	18
CAGACTTTG	BRDN00012	91	0	12	209	221	0
CAGACTTTG	BRDN00012	336	268	111	570	533	21
CAGAGAAAA	BRDN00012	124	36	349	119	248	39
CAGAGAAAA	BRDN00012	85	144	18	269	176	16
CAGAGAAAA	BRDN00012	251	253	1904	505	530	27
CAGAGAAAC	BRDN00011	68	9	0	179	191	0
CAGAGAAAG	BRDN00011	121	121	85	273	276	0
CAGAGAACA	BRDN00011	354	286	317	647	595	26
CAGAGAACA	BRDN00011	400	343	1186	883	858	95
CAGAGAACA	BRDN00012	228	401	269	437	751	31
CAGAGAACG	BRDN00012	90	9	0	443	186	0
CAGAGAACT	BRDN00012	319	438	642	682	720	99
CAGAGAAGA	BRDN00012	250	269	133	658	417	52
CAGAGAAGA	BRDN00011	131	116	39	449	175	0
CAGAGAAGC	BRDN00011	247	344	38	595	440	15
CAGAGAAGC	BRDN00012	137	264	510	272	156	50
CAGAGAAGT	BRDN00011	136	258	61	427	371	44
CAGAGAATG	BRDN00012	440	399	684	736	928	37
CAGAGACAA	BRDN00011	25	9	0	167	115	0
CAGAGACAA	BRDN00012	142	15	0	138	184	0
CAGAGACAA	BRDN00012	113	111	1	284	352	43
CAGAGACAA	BRDN00012	709	636	681	1084	1005	81
CAGAGACAC	BRDN00011	155	133	0	334	304	0
CAGAGACAG	BRDN00011	178	158	27	389	210	19
CAGAGACAG	BRDN00012	252	211	257	647	725	4
CAGAGACAT	BRDN00012	124	102	120	125	159	0
CAGAGACCA	BRDN00011	88	86	37	181	91	0
CAGAGACCA	BRDN00011	315	285	661	467	558	25
CAGAGACCG	BRDN00011	233	95	931	113	178	5
CAGAGACCT	BRDN00012	122	109	136	237	257	19
CAGAGACTA	BRDN00011	293	189	338	454	580	0
CAGAGACTA	BRDN00012	215	211	177	431	421	6
CAGAGACTC	BRDN00011	309	213	745	551	694	38
CAGAGACTC	BRDN00011	191	189	363	418	341	10
CAGAGACTG	BRDN00012	171	93	43	431	429	6
CAGAGACTG	BRDN00012	109	144	927	221	247	30
CAGAGAGAG	BRDN00011	74	17	6	226	176	0
CAGAGAGCC	BRDN00011	208	276	121	434	369	0
CAGAGAGGC	BRDN00012	169	56	275	254	354	11
CAGAGAGTA	BRDN00012	258	200	226	551	562	12
CAGAGATAT	BRDN00011	2	2	0	180	48	0
CAGAGATCC	BRDN00012	168	193	471	421	180	19
CAGAGATGA	BRDN00012	249	131	2	483	300	0
CAGAGATGC	BRDN00012	167	81	17	378	402	3
CAGAGATGG	BRDN00012	110	89	80	206	272	10
CAGAGATGG	BRDN00012	150	118	126	351	106	0
CAGAGATTC	BRDN00012	304	350	420	563	442	18
CAGAGCAAA	BRDN00012	213	114	851	253	119	2
CAGAGCAAA	BRDN00011	103	99	263	233	184	9
CAGAGCAAA	BRDN00012	288	306	199	598	385	0
CAGAGCAAT	BRDN00011	306	281	48	565	595	30
CAGAGCACG	BRDN00012	348	413	607	768	485	32
CAGAGCACG	BRDN00011	147	67	701	478	457	4
CAGAGCACT	BRDN00011	385	128	597	851	1024	2
CAGAGCAGA	BRDN00012	135	320	194	395	191	153
CAGAGCAGC	BRDN00011	298	406	591	848	810	17
CAGAGCAGG	BRDN00012	322	114	77	589	668	67
CAGAGCATA	BRDN00011	18	0	0	183	96	0
CAGAGCATC	BRDN00012	344	267	1106	529	457	17
CAGAGCATC	BRDN00012	59	0	0	238	130	0
CAGAGCATG	BRDN00011	149	197	50	409	283	44
CAGAGCATT	BRDN00012	345	312	162	633	636	15
CAGAGCATT	BRDN00011	257	213	308	403	258	6
CAGAGCCAC	BRDN00012	97	39	59	147	171	5
CAGAGCCAC	BRDN00011	170	87	45	266	309	0
CAGAGCCAG	BRDN00011	229	279	141	583	360	9
CAGAGCCAT	BRDN00011	190	307	272	337	340	99
CAGAGCCAT	BRDN00011	203	186	116	293	181	14
CAGAGCCGG	BRDN00011	68	52	12	165	85	0
CAGAGCCGT	BRDN00012	436	222	420	742	809	3
CAGAGCCTC	BRDN00011	93	78	175	184	162	0
CAGAGCCTT	BRDN00012	93	119	237	184	177	30
CAGAGCCTT	BRDN00012	467	373	1397	701	339	45
CAGAGCGAA	BRDN00012	101	113	67	186	473	6
CAGAGCGAT	BRDN00012	339	275	271	516	851	84
CAGAGCGAT	BRDN00012	226	205	260	256	358	17
CAGAGCGCC	BRDN00012	254	237	42	680	534	29
CAGAGCGCG	BRDN00011	131	139	12	156	218	7
CAGAGCGCT	BRDN00011	149	88	1	184	331	16
CAGAGCGGC	BRDN00011	70	10	0	222	56	0
CAGAGCGTG	BRDN00012	162	164	121	142	224	9
CAGAGCGTG	BRDN00011	55	91	183	187	148	10
CAGAGCTAA	BRDN00011	68	56	0	67	137	0
CAGAGCTAA	BRDN00012	276	38	0	368	320	0
CAGAGCTAC	BRDN00012	248	149	825	616	330	14
CAGAGCTCA	BRDN00012	124	106	372	244	294	7
CAGAGCTCT	BRDN00012	57	60	0	133	251	0
CAGAGCTCT	BRDN00012	109	181	45	280	279	0
CAGAGCTGA	BRDN00011	147	78	0	232	92	0
CAGAGCTGC	BRDN00012	173	247	445	256	703	238
CAGAGCTGG	BRDN00012	222	263	1234	493	647	15
CAGAGCTGG	BRDN00012	102	23	0	325	259	0
CAGAGCTGT	BRDN00012	73	0	0	263	35	0
CAGAGCTTA	BRDN00011	121	32	363	200	239	30
CAGAGCTTC	BRDN00012	136	156	0	302	98	26
CAGAGCTTG	BRDN00012	299	320	720	504	694	117
CAGAGGAAA	BRDN00011	35	81	73	207	124	9
CAGAGGAAC	BRDN00011	57	101	387	179	126	11
CAGAGGAAG	BRDN00011	177	168	123	450	394	15
CAGAGGAAT	BRDN00011	70	19	0	276	97	1
CAGAGGAAT	BRDN00011	107	58	0	127	204	0
CAGAGGACT	BRDN00012	124	174	97	287	164	262
CAGAGGAGC	BRDN00012	108	87	408	203	233	10
CAGAGGAGT	BRDN00012	74	0	8	309	137	0
CAGAGGAGT	BRDN00012	62	0	0	132	17	0
CAGAGGATA	BRDN00012	428	285	287	618	744	25
CAGAGGCAC	BRDN00012	579	496	784	837	545	51
CAGAGGCCG	BRDN00011	243	204	124	655	648	75
CAGAGGCGA	BRDN00012	408	388	2435	676	983	32
CAGAGGCGC	BRDN00011	51	19	1	188	51	0
CAGAGGCGC	BRDN00012	114	79	154	236	268	0
CAGAGGCGC	BRDN00011	68	54	0	200	164	0
CAGAGGCTG	BRDN00011	66	56	0	177	41	0
CAGAGGGAA	BRDN00012	246	231	129	487	475	6
CAGAGGGCA	BRDN00011	124	76	159	320	524	7
CAGAGGGCC	BRDN00011	167	157	66	212	99	0
CAGAGGGCG	BRDN00011	204	249	396	450	308	25
CAGAGGGCT	BRDN00012	510	542	2837	753	780	101
CAGAGGGTC	BRDN00012	243	311	500	405	408	23
CAGAGGTAA	BRDN00011	62	42	190	80	14	0
CAGAGGTCA	BRDN00012	112	94	6	151	132	23
CAGAGGTCA	BRDN00011	465	300	1188	910	1383	47
CAGAGGTCG	BRDN00012	401	326	8915	779	633	125
CAGAGGTCT	BRDN00011	301	304	848	665	592	45
CAGAGGTGG	BRDN00011	102	196	23	180	105	15
CAGAGGTTC	BRDN00011	215	318	744	471	413	14
CAGAGTAAA	BRDN00011	114	204	4142	218	189	160
CAGAGTAGA	BRDN00011	82	60	241	240	213	6
CAGAGTAGG	BRDN00012	401	416	128	696	656	27
CAGAGTATC	BRDN00011	67	129	6	131	193	6
CAGAGTATG	BRDN00012	159	0	1	268	111	0
CAGAGTCAA	BRDN00012	265	159	33	529	253	9
CAGAGTCAG	BRDN00012	220	295	1414	392	281	59
CAGAGTCAT	BRDN00012	146	96	257	238	167	23
CAGAGTCAT	BRDN00012	13	2	0	125	126	0
CAGAGTCGC	BRDN00012	227	150	104	501	321	0
CAGAGTCTG	BRDN00012	136	98	299	274	378	9
CAGAGTCTG	BRDN00012	113	107	0	248	230	10
CAGAGTGAG	BRDN00011	299	313	6013	535	725	311
CAGAGTGCC	BRDN00012	39	0	4	80	69	0
CAGAGTGCG	BRDN00012	452	527	164	735	745	30
CAGAGTGCT	BRDN00012	128	60	0	152	80	0
CAGAGTGGA	BRDN00012	107	94	15	197	80	5
CAGAGTGGA	BRDN00011	159	192	456	153	251	5
CAGAGTGGG	BRDN00012	158	146	638	312	148	9
CAGAGTGGT	BRDN00012	93	16	149	204	148	0
CAGAGTGTG	BRDN00011	358	171	46	796	499	44
CAGAGTTAA	BRDN00012	330	306	954	550	729	29
CAGAGTTCA	BRDN00012	131	126	847	198	237	2
CAGAGTTCG	BRDN00011	242	106	460	467	677	9
CAGAGTTGG	BRDN00011	362	408	309	521	435	52
CAGAGTTGG	BRDN00011	113	124	141	197	161	13
CAGATAAAA	BRDN00012	365	199	152	570	403	0
CAGATAAAG	BRDN00012	230	280	147	274	470	6
CAGATAAAG	BRDN00012	190	11	193	271	186	0
CAGATAACC	BRDN00012	293	183	324	551	647	43
CAGATAACT	BRDN00011	257	199	396	487	644	20
CAGATACAA	BRDN00012	217	138	386	538	610	23
CAGATACAG	BRDN00012	406	388	1218	715	393	56
CAGATACAT	BRDN00011	6	0	1	469	432	0
CAGATACAT	BRDN00012	351	424	1242	545	790	21
CAGATACAT	BRDN00012	54	70	30	104	51	39
CAGATACGA	BRDN00011	137	94	62	407	328	27
CAGATACGG	BRDN00012	161	157	139	241	336	8
CAGATACTG	BRDN00011	438	488	410	806	1394	62
CAGATACTG	BRDN00011	125	68	0	198	92	0
CAGATACTG	BRDN00012	177	154	70	369	511	0
CAGATACTT	BRDN00011	82	109	8	249	166	0
CAGATAGAG	BRDN00011	111	168	165	296	218	4
CAGATAGAT	BRDN00012	260	241	1070	715	644	50
CAGATAGGA	BRDN00011	235	176	119	469	528	0
CAGATAGTG	BRDN00011	225	132	1080	413	214	34
CAGATAGTT	BRDN00011	381	382	154	707	685	23
CAGATATAT	BRDN00012	345	209	4	697	415	21
CAGATATGT	BRDN00011	47	17	0	95	63	0
CAGATATTA	BRDN00011	224	246	210	334	232	14
CAGATATTG	BRDN00011	136	142	160	290	216	31
CAGATCAAT	BRDN00012	100	252	48	246	210	0
CAGATCAAT	BRDN00011	232	300	2079	564	323	29
CAGATCACA	BRDN00012	483	329	407	575	472	60
CAGATCACC	BRDN00011	34	17	0	175	247	5

CAGATCAGA	BRDN0001	215	139	85	336	256	0
CAGATCAGA	BRDN0001	137	176	84	278	195	3
CAGATCAGT	BRDN0001	466	399	145	835	636	7
CAGATCCAA	BRDN0001	375	324	284	672	499	73
CAGATCCAT	BRDN0001	181	101	78	342	347	19
CAGATCCAT	BRDN0001	548	445	495	842	905	71
CAGATCCAT	BRDN0001	420	318	598	776	640	30
CAGATCCCT	BRDN0001	232	292	249	225	181	30
CAGATCCGA	BRDN0001	198	203	148	517	690	44
CAGATCCGC	BRDN0001	130	162	447	279	106	15
CAGATCCGG	BRDN0001	95	44	13	134	186	2
CAGATCCTG	BRDN0001	164	138	909	371	586	35
CAGATCCTG	BRDN0001	94	4	0	195	110	0
CAGATCGAA	BRDN0001	85	150	344	298	165	23
CAGATCGAC	BRDN0001	350	360	184	769	1023	26
CAGATCGAG	BRDN0001	128	88	0	264	257	5
CAGATCGAT	BRDN0001	95	44	28	148	509	4
CAGATCGGA	BRDN0001	381	258	236	611	765	10
CAGATCGGG	BRDN0001	331	283	283	623	617	40
CAGATCGTG	BRDN0001	181	125	3909	229	331	93
CAGATCTAA	BRDN0001	116	130	45	210	114	0
CAGATCTAA	BRDN0001	219	198	1953	292	316	58
CAGATCTGA	BRDN0001	76	48	138	215	319	0
CAGATCTGT	BRDN0001	481	388	338	839	671	28
CAGATCTTA	BRDN0001	361	367	1068	497	509	26
CAGATCTTC	BRDN0001	146	309	498	246	451	1
CAGATCTTG	BRDN0001	372	320	845	533	370	77
CAGATCTTT	BRDN0001	190	78	106	331	348	32
CAGATGAAA	BRDN0001	159	217	287	533	654	17
CAGATGAAA	BRDN0001	289	383	34	625	726	17
CAGATGAAA	BRDN0001	107	130	559	204	233	0
CAGATGAAA	BRDN0001	29	27	0	209	206	0
CAGATGAAC	BRDN0001	161	37	0	247	166	0
CAGATGAAG	BRDN0001	187	135	74	499	338	23
CAGATGAAG	BRDN0001	268	224	529	464	549	0
CAGATGACA	BRDN0001	218	252	2894	411	253	28
CAGATGACC	BRDN0001	471	304	457	754	648	44
CAGATGAGA	BRDN0001	190	173	369	534	310	31
CAGATGAGG	BRDN0001	66	87	95	171	155	13
CAGATGAGG	BRDN0001	113	148	100	232	142	19
CAGATGAGT	BRDN0001	186	91	1	246	568	0
CAGATGATA	BRDN0001	62	110	147	120	107	29
CAGATGATA	BRDN0001	208	146	22	408	321	0
CAGATGATA	BRDN0001	498	416	961	924	846	112
CAGATGATG	BRDN0001	456	288	400	876	1021	31
CAGATGATT	BRDN0001	132	54	441	211	86	29
CAGATGCAC	BRDN0001	97	84	88	118	298	35
CAGATGCAT	BRDN0001	127	111	281	179	257	8
CAGATGCCA	BRDN0001	291	297	196	481	405	64
CAGATGCCA	BRDN0001	124	102	69	366	302	5
CAGATGCCA	BRDN0001	85	114	28	135	141	18
CAGATGCCA	BRDN0001	422	347	151	662	509	18
CAGATGCCG	BRDN0001	158	202	76	377	222	9
CAGATGCCT	BRDN0001	126	101	2146	133	124	14
CAGATGCGA	BRDN0001	144	133	587	304	252	38
CAGATGCGA	BRDN0001	170	104	90	248	261	1
CAGATGCGC	BRDN0001	142	82	22	188	192	32
CAGATGCTG	BRDN0001	139	0	0	347	18	0
CAGATGCTG	BRDN0001	128	64	25	258	563	8
CAGATGGAA	BRDN0001	292	281	375	580	588	9
CAGATGGAA	BRDN0001	135	0	0	216	7	0
CAGATGGAA	BRDN0001	78	105	896	116	172	0
CAGATGGAT	BRDN0001	301	163	361	585	470	16
CAGATGGCA	BRDN0001	162	125	361	316	491	85
CAGATGGCA	BRDN0001	57	44	187	82	115	0
CAGATGGCC	BRDN0001	169	16	0	275	84	0
CAGATGGCT	BRDN0001	198	121	299	328	337	19
CAGATGGCT	BRDN0001	21	0	0	214	102	0
CAGATGGCT	BRDN0001	125	234	209	334	536	19
CAGATGGGC	BRDN0001	418	516	1146	814	581	55
CAGATGGGC	BRDN0001	213	185	1867	318	311	24
CAGATGGGC	BRDN0001	144	146	213	522	462	53
CAGATGGGC	BRDN0001	307	300	1016	565	269	8
CAGATGGTA	BRDN0001	216	206	421	283	245	5
CAGATGGTC	BRDN0001	193	134	166	271	304	10
CAGATGGTC	BRDN0001	131	132	520	123	182	16
CAGATGGTG	BRDN0001	302	310	286	618	543	37
CAGATGGTT	BRDN0001	115	147	57	453	181	0
CAGATGTAA	BRDN0001	152	112	198	177	192	18
CAGATGTCC	BRDN0001	155	223	78	325	373	0
CAGATGTCC	BRDN0001	105	72	14	151	328	0
CAGATGTCC	BRDN0001	310	233	1559	493	482	81
CAGATGTCG	BRDN0001	345	313	38	758	519	68
CAGATGTGA	BRDN0001	80	64	0	146	159	4
CAGATGTGC	BRDN0001	34	0	0	464	125	0
CAGATGTGT	BRDN0001	273	282	173	402	429	0
CAGATGTTG	BRDN0001	256	217	1219	365	248	24
CAGATGTTG	BRDN0001	142	160	287	288	278	14
CAGATGTTG	BRDN0001	236	257	126	403	207	30
CAGATTAAG	BRDN0001	79	0	1	334	114	0
CAGATTATG	BRDN0001	140	243	224	378	271	40
CAGATTCAT	BRDN0001	164	233	328	228	157	8
CAGATTCTG	BRDN0001	177	206	190	325	283	12
CAGATTGAA	BRDN0001	334	385	1087	563	590	25
CAGATTGAA	BRDN0001	453	175	24	811	245	25
CAGATTGAC	BRDN0001	116	182	47	250	201	12
CAGATTGAG	BRDN0001	238	191	31	481	686	27
CAGATTGCC	BRDN0001	115	132	837	203	264	21
CAGATTGTG	BRDN0001	256	113	60	579	414	0
CAGATTGTT	BRDN0001	268	344	245	612	437	21
CAGATTTAC	BRDN0001	173	57	107	241	294	0
CAGATTTCA	BRDN0001	136	104	14	213	212	12
CAGATTTCA	BRDN0001	301	289	683	558	611	53
CAGCAAAAG	BRDN0001	176	83	10	265	632	0
CAGCAAAAT	BRDN0001	109	68	2	263	238	17
CAGCAAACA	BRDN0001	172	76	484	225	136	0
CAGCAAACA	BRDN0001	169	140	98	259	385	22
CAGCAAACC	BRDN0001	124	23	0	371	47	0
CAGCAAAGG	BRDN0001	57	37	0	151	9	14
CAGCAAAGG	BRDN0001	139	119	16	270	266	6
CAGCAAAGG	BRDN0001	495	639	1573	928	692	37
CAGCAAAGG	BRDN0001	202	110	1	353	1582	8
CAGCAAAGT	BRDN0001	298	340	848	535	640	36
CAGCAAATC	BRDN0001	445	294	9	752	612	5
CAGCAAATG	BRDN0001	92	45	32	249	485	0
CAGCAAATG	BRDN0001	100	15	994	176	160	3
CAGCAAATG	BRDN0001	193	190	77	388	402	8
CAGCAAATT	BRDN0001	76	138	19	205	366	10
CAGCAAATT	BRDN0001	178	79	749	284	134	10
CAGCAACAA	BRDN0001	102	94	189	227	432	10
CAGCAACAG	BRDN0001	133	182	2043	410	516	36
CAGCAACAG	BRDN0001	195	217	674	239	495	25
CAGCAACAT	BRDN0001	334	251	412	488	1135	11
CAGCAACCA	BRDN0001	158	233	18	394	398	6
CAGCAACCC	BRDN0001	398	235	1373	401	642	20
CAGCAACGC	BRDN0001	92	120	257	191	132	68
CAGCAACGG	BRDN0001	254	320	146	633	616	66
CAGCAACGT	BRDN0001	95	0	0	230	117	0
CAGCAACTC	BRDN0001	107	56	378	332	581	16
CAGCAACTG	BRDN0001	217	152	725	357	355	13
CAGCAACTT	BRDN0001	201	97	11	449	338	0
CAGCAACTT	BRDN0001	146	103	335	246	172	3
CAGCAAGCC	BRDN0001	128	79	115	211	195	11
CAGCAAGCG	BRDN0001	315	342	357	656	674	32
CAGCAAGCG	BRDN0001	169	180	0	438	362	4
CAGCAAGCT	BRDN0001	88	0	0	147	70	0
CAGCAAGCT	BRDN0001	102	167	99	166	198	12
CAGCAAGCT	BRDN0001	360	216	93	680	493	4
CAGCAAGCT	BRDN0001	88	35	23	274	136	0
CAGCAAGGA	BRDN0001	180	218	288	484	415	32
CAGCAAGGC	BRDN0001	365	42	0	523	474	8
CAGCAAGGC	BRDN0001	42	0	0	167	144	0
CAGCAAGGG	BRDN0001	349	349	775	529	272	27
CAGCAAGTA	BRDN0001	165	148	183	264	199	17
CAGCAAGTC	BRDN0001	88	205	2	277	253	8
CAGCAAGTC	BRDN0001	235	88	21	612	331	0
CAGCAAGTT	BRDN0001	136	95	139	219	278	9
CAGCAAGTT	BRDN0001	65	116	15	253	165	0
CAGCAAGTT	BRDN0001	115	108	5	164	570	0
CAGCAATAC	BRDN0001	345	566	2499	534	894	101
CAGCAATAT	BRDN0001	288	402	1593	447	321	117
CAGCAATCA	BRDN0001	137	32	293	372	199	0
CAGCAATGA	BRDN0001	305	246	643	544	762	7
CAGCAATGC	BRDN0001	526	297	238	976	1089	67
CAGCAATTA	BRDN0001	350	274	513	755	1200	80
CAGCAATTA	BRDN0001	136	114	270	170	162	58
CAGCACAAC	BRDN0001	114	4	0	254	66	0
CAGCACAAG	BRDN0001	160	217	82	444	131	20
CAGCACACA	BRDN0001	106	84	42	235	239	0
CAGCACACA	BRDN0001	336	523	33	790	933	50
CAGCACACA	BRDN0001	182	60	41	388	250	6
CAGCACACC	BRDN0001	83	222	29	285	178	0
CAGCACACT	BRDN0001	325	286	691	591	470	18
CAGCACACG	BRDN0001	13	0	0	234	8	0
CAGCACAGA	BRDN0001	118	103	469	229	172	0
CAGCACAGA	BRDN0001	374	319	1821	779	1665	42
CAGCACAGC	BRDN0001	455	565	1831	747	516	69
CAGCACAGG	BRDN0001	214	197	38	341	512	18
CAGCACAGT	BRDN0001	343	51	47	587	293	0
CAGCACAGT	BRDN0001	158	141	235	357	299	4
CAGCACAGT	BRDN0001	65	68	0	242	242	0
CAGCACATA	BRDN0001	65	126	151	207	113	18
CAGCACATA	BRDN0001	410	249	357	518	472	98
CAGCACATA	BRDN0001	76	177	81	194	201	5
CAGCACATC	BRDN0001	358	313	740	820	934	138
CAGCACATC	BRDN0001	128	196	5	231	203	1
CAGCACCAA	BRDN0001	221	247	557	259	224	18
CAGCACCAA	BRDN0001	97	93	70	198	206	17
CAGCACCAC	BRDN0001	191	108	67	271	281	0
CAGCACCAC	BRDN0001	120	92	57	133	154	0
CAGCACCAC	BRDN0001	196	171	222	510	570	0
CAGCACCAG	BRDN0001	167	121	381	264	343	9
CAGCACCAG	BRDN0001	110	76	109	189	85	0
CAGCACCAG	BRDN0001	196	113	85	271	238	67
CAGCACCAG	BRDN0001	137	59	129	250	103	0
CAGCACCAT	BRDN0001	33	13	22	288	166	11
CAGCACCAT	BRDN0001	139	162	85	218	624	23
CAGCACCCA	BRDN0001	84	100	78	160	123	5
CAGCACCCG	BRDN0001	80	87	180	105	226	0
CAGCACCCG	BRDN0001	369	217	955	552	1033	47
CAGCACCCG	BRDN0001	150	65	50	478	360	33
CAGCACCGG	BRDN0001	212	276	109	304	248	30
CAGCACCGT	BRDN0001	369	428	483	689	823	60
CAGCACCGT	BRDN0001	69	117	322	154	145	0
CAGCACCGT	BRDN0001	316	202	79	880	483	0
CAGCACCTC	BRDN0001	289	287	536	385	643	6
CAGCACCTG	BRDN0001	146	169	303	278	135	14
CAGCACGAA	BRDN0001	103	28	190	156	199	0
CAGCACGAG	BRDN0001	148	204	317	279	268	1
CAGCACGAG	BRDN0001	106	136	24	220	1258	23
CAGCACGCA	BRDN0001	128	134	270	220	134	31
CAGCACGCA	BRDN0001	614	452	847	1050	1293	78
CAGCACGCG	BRDN0001	184	158	507	381	263	13
CAGCACGGA	BRDN0001	403	390	79	655	316	12
CAGCACGGC	BRDN0001	326	176	1807	551	547	28
CAGCACGGT	BRDN0001	86	96	169784	254	28	2185
CAGCACGTA	BRDN0001	299	252	366	479	254	20
CAGCACGTT	BRDN0001	296	181	430	541	532	13
CAGCACTAC	BRDN0001	227	270	1096	471	736	84
CAGCACTAG	BRDN0001	225	153	118	362	405	13
CAGCACTCA	BRDN0001	113	52	24	248	252	0
CAGCACTCA	BRDN0001	235	288	326	463	177	11
CAGCACTCC	BRDN0001	373	278	566	826	629	0
CAGCACTCC	BRDN0001	458	376	173	821	836	34
CAGCACTGA	BRDN0001	343	102	1288	423	735	21
CAGCACTGC	BRDN0001	286	360	864	507	354	26
CAGCACTGG	BRDN0001	78	43	8	183	59	0
CAGCACTGT	BRDN0001	407	390	2309	703	475	243
CAGCACTGT	BRDN0001	124	101	240	211	523	1
CAGCACTTA	BRDN0001	218	148	710	474	211	5
CAGCACTTA	BRDN0001	219	316	245	575	934	18
CAGCAGAAA	BRDN0001	509	377	2629	928	959	50
CAGCAGAAA	BRDN0001	111	40	154	386	391	3
CAGCAGAAC	BRDN0001	227	174	147	362	226	10
CAGCAGAAC	BRDN0001	178	103	243	225	260	16
CAGCAGAAT	BRDN0001	295	152	350	506	275	54
CAGCAGACC	BRDN0001	89	0	0	200	25	0
CAGCAGAGC	BRDN0001	304	383	545	616	510	10
CAGCAGAGG	BRDN0001	359	230	183	482	345	39
CAGCAGAGG	BRDN0001	163	124	0	225	365	0
CAGCAGAGT	BRDN0001	95	121	40	206	234	0
CAGCAGATA	BRDN0001	54	1	0	342	11	0
CAGCAGATG	BRDN0001	141	78	7	258	298	0
CAGCAGCAA	BRDN0001	90	47	9	137	132	0
CAGCAGCAA	BRDN0001	311	405	900	599	781	54
CAGCAGCAC	BRDN0001	222	224	315	453	435	10
CAGCAGCAC	BRDN0001	137	145	418	236	159	11
CAGCAGCAC	BRDN0001	87	97	361	191	350	42
CAGCAGCAC	BRDN0001	131	243	544	447	349	45
CAGCAGCAG	BRDN0001	157	180	74	380	372	31
CAGCAGCAG	BRDN0001	151	105	0	373	471	36
CAGCAGCAG	BRDN0001	87	34	217	304	412	5
CAGCAGCAG	BRDN0001	55	57	15	173	162	28
CAGCAGCAG	BRDN0001	147	149	42	313	183	5
CAGCAGCAG	BRDN0001	105	48	37	193	199	53
CAGCAGCAG	BRDN0001	333	482	165	850	549	22
CAGCAGCCA	BRDN0001	486	314	993	651	699	30
CAGCAGCCA	BRDN0001	146	109	100	250	392	26
CAGCAGCCA	BRDN0001	162	99	337	315	220	9
CAGCAGCCC	BRDN0001	272	292	165	798	664	0
CAGCAGCCC	BRDN0001	268	290	13881	575	1143	240
CAGCAGCCT	BRDN0001	150	191	198	341	191	33
CAGCAGCGC	BRDN0001	345	352	423	726	885	24
CAGCAGCGG	BRDN0001	97	71	173	200	224	3
CAGCAGCGG	BRDN0001	106	126	149	130	76	1
CAGCAGCGT	BRDN0001	138	292	512	336	446	17
CAGCAGCGT	BRDN0001	140	145	32	249	122	6
CAGCAGCGT	BRDN0001	173	175	332	384	349	20
CAGCAGCTA	BRDN0001	101	71	512	247	262	35
CAGCAGCTG	BRDN0001	287	367	7795	680	713	171
CAGCAGCTG	BRDN0001	90	33	46	196	194	12
CAGCAGCTG	BRDN0001	578	604	561	1085	963	136
CAGCAGCTG	BRDN0001	121	111	13	300	660	23
CAGCAGCTT	BRDN0001	299	15	0	412	532	0
CAGCAGGAA	BRDN0001	201	294	17	326	344	38
CAGCAGGAA	BRDN0001	191	125	215	405	232	3
CAGCAGGAC	BRDN0001	62	58	95	155	101	0
CAGCAGGAG	BRDN0001	75	78	389	101	118	10
CAGCAGGCC	BRDN0001	119	87	48	234	315	28
CAGCAGGCC	BRDN0001	395	309	526	650	444	22
CAGCAGGCG	BRDN0001	434	446	116	1095	1331	24
CAGCAGGCT	BRDN0001	368	434	1816	832	807	54
CAGCAGGTA	BRDN0001	377	345	311	794	544	72
CAGCAGGTC	BRDN0001	71	22	0	137	126	0
CAGCAGGTG	BRDN0001	131	181	0	127	152	46
CAGCAGTAC	BRDN0001	169	228	139	238	118	20
CAGCAGTAC	BRDN0001	137	158	9	207	150	0
CAGCAGTAG	BRDN0001	130	84	166	263	191	6
CAGCAGTCA	BRDN0001	370	332	665	630	612	47
CAGCAGTCA	BRDN0001	115	86	84	203	86	6
CAGCAGTCC	BRDN0001	184	178	575	373	566	91
CAGCAGTCC	BRDN0001	61	39	495	277	178	7
CAGCAGTCG	BRDN0001	178	173	58	425	314	43
CAGCAGTCT	BRDN0001	101	8	0	330	245	0
CAGCAGTCT	BRDN0001	79	137	412	237	209	4
CAGCAGTGG	BRDN0001	215	199	846	320	249	21
CAGCAGTGG	BRDN0001	200	97	2	538	522	0
CAGCAGTTA	BRDN0001	103	100	11	184	171	4
CAGCAGTTA	BRDN0001	414	562	71	783	1031	0
CAGCATAAA	BRDN0001	144	0	5	500	125	0
CAGCATACA	BRDN0001	216	14	12	409	124	4
CAGCATAGC	BRDN0001	82	34	63	173	143	0
CAGCATATG	BRDN0001	131	91	4	176	6	19
CAGCATATG	BRDN0001	103	63	19	182	83	21
CAGCATCAA	BRDN0001	143	170	2692	274	333	36
CAGCATCAC	BRDN0001	217	197	133	546	1019	0
CAGCATCAC	BRDN0001	125	126	1	276	255	16
CAGCATCAC	BRDN0001	126	87	205	229	216	4
CAGCATCAC	BRDN0001	360	279	406	368	368	28
CAGCATCAG	BRDN0001	4	0	0	458	21	0
CAGCATCAG	BRDN0001	87	117	184	193	122	1
CAGCATCAG	BRDN0001	447	272	570	1019	1345	72
CAGCATCAG	BRDN0001	268	99	598	353	722	30
CAGCATCAT	BRDN0001	337	235	146	408	735	19
CAGCATCCA	BRDN0001	123	62	500	297	396	0
CAGCATCCC	BRDN0001	227	221	516	359	542	45
CAGCATCCC	BRDN0001	137	124	242	233	525	4
CAGCATCGA	BRDN0001	125	117	368	321	220	1
CAGCATCGA	BRDN0001	147	136	22	349	428	4
CAGCATCGA	BRDN0001	86	74	0	177	514	0
CAGCATCGG	BRDN0001	130	94	60	491	355	0
CAGCATCGT	BRDN0001	174	56	708	332	857	14
CAGCATCGT	BRDN0001	103	141	200	236	77	0
CAGCATCTA	BRDN0001	396	325	1639	566	578	39
CAGCATCTC	BRDN0001	476	215	5499	777	1150	88
CAGCATCTG	BRDN0001	602	438	845	812	531	31
CAGCATCTG	BRDN0001	505	339	381	682	814	35
CAGCATCTG	BRDN0001	197	202	53	228	122	20
CAGCATCTG	BRDN0001	214	334	153	498	344	20
CAGCATCTT	BRDN0001	161	146	49	325	78	35
CAGCATGAC	BRDN0001	225	438	780	430	310	80
CAGCATGAC	BRDN0001	480	382	740	817	915	44
CAGCATGAG	BRDN0001	510	589	589	956	1091	52
CAGCATGAT	BRDN0001	68	51	50	142	252	0
CAGCATGCG	BRDN0001	387	462	1225	714	763	154
CAGCATGCT	BRDN0001	477	485	1131	925	1136	13
CAGCATGGA	BRDN0001	553	497	303	845	773	47
CAGCATGGT	BRDN0001	122	125	34	244	311	7
CAGCATGGT	BRDN0001	301	429	1815	595	672	25
CAGCATGTG	BRDN0001	146	148	16	212	209	30
CAGCATGTG	BRDN0001	243	202	187	455	469	17
CAGCATGTG	BRDN0001	462	533	690	938	1016	42
CAGCATGTT	BRDN0001	43	23	0	52	38	6
CAGCATTAA	BRDN0001	265	186	2	410	398	0
CAGCATTAA	BRDN0001	176	149	443	267	89	8
CAGCATTAA	BRDN0001	179	35	18	247	419	3
CAGCATTAC	BRDN0001	178	131	238	368	384	13
CAGCATTCC	BRDN0001	171	50	1211	748	453	8
CAGCATTCG	BRDN0001	295	20	0	488	964	0
CAGCATTCG	BRDN0001	173	306	426	574	425	57
CAGCATTCG	BRDN0001	207	46	0	371	563	0
CAGCATTGC	BRDN0001	251	286	2544	341	351	50
CAGCATTGC	BRDN0001	102	42	0	251	439	11
CAGCATTGT	BRDN0001	54	0	1	204	96	0
CAGCATTTC	BRDN0001	399	370	121	594	402	0
CAGCCAAAA	BRDN0001	264	197	22	377	283	0
CAGCCAAAC	BRDN0001	402	275	262	653	560	33
CAGCCAAAG	BRDN0001	81	169	268	201	109	6
CAGCCAAAG	BRDN0001	404	218	1846	587	694	28
CAGCCAACA	BRDN0001	387	426	229	665	744	32
CAGCCAACG	BRDN0001	61	30	0	147	125	0
CAGCCAACT	BRDN0001	340	278	6124	567	754	62
CAGCCAAGA	BRDN0001	140	65	0	665	449	0
CAGCCAAGC	BRDN0001	173	139	346	379	425	12
CAGCCAAGT	BRDN0001	104	77	491	136	345	11
CAGCCAATC	BRDN0001	284	454	1367	679	522	66
CAGCCAATG	BRDN0001	42	35	1	158	91	13
CAGCCAATT	BRDN0001	158	99	2	288	488	1
CAGCCACAC	BRDN0001	476	426	1171	687	773	13
CAGCCACAT	BRDN0001	194	128	497	398	193	36
CAGCCACCA	BRDN0001	102	121	241	268	309	15
CAGCCACCA	BRDN0001	409	515	528	568	811	34
CAGCCACCA	BRDN0001	195	128	36	632	856	77
CAGCCACCT	BRDN0001	279	283	573	574	347	10
CAGCCACTG	BRDN0001	299	308	1587	457	407	66
CAGCCAGAA	BRDN0001	183	132	2	287	176	0
CAGCCAGAG	BRDN0001	465	349	416	802	865	6
CAGCCAGAG	BRDN0001	211	130	377	569	635	35
CAGCCAGCA	BRDN0001	122	40	171	281	180	39
CAGCCAGGA	BRDN0001	52	52	362	150	140	7
CAGCCAGGA	BRDN0001	98	0	1	553	112	0
CAGCCAGGA	BRDN0001	401	337	193	709	403	34
CAGCCAGGA	BRDN0001	92	117	184	304	212	34
CAGCCAGGC	BRDN0001	286	378	629	545	365	0
CAGCCAGGG	BRDN0001	143	139	150	353	572	34
CAGCCAGGT	BRDN0001	114	110	92	340	125	5
CAGCCAGTA	BRDN0001	249	202	396	318	486	15
CAGCCAGTA	BRDN0001	114	123	0	291	342	0
CAGCCAGTG	BRDN0001	99	84	119	195	272	9
CAGCCATAG	BRDN0001	136	101	0	192	462	24
CAGCCATAT	BRDN0001	190	222	28	489	295	67
CAGCCATCA	BRDN0001	251	206	520	501	412	23
CAGCCATCA	BRDN0001	350	150	452	700	483	70
CAGCCATCC	BRDN0001	306	326	43	515	305	0
CAGCCATGA	BRDN0001	101	125	692	257	145	0
CAGCCATGA	BRDN0001	488	54	307	806	790	6
CAGCCATGC	BRDN0001	208	168	1036	312	379	74
CAGCCATGG	BRDN0001	252	158	74	463	479	0
CAGCCATTT	BRDN0001	413	309	817	561	623	61
CAGCCCAAA	BRDN0001	54	9	0	407	170	0
CAGCCCAAC	BRDN0001	289	251	2916	592	697	30
CAGCCCAAG	BRDN0001	102	28	462	180	71	9
CAGCCCAAT	BRDN0001	186	47	89	219	210	0
CAGCCCACC	BRDN0001	399	331	1565	802	971	37
CAGCCCACG	BRDN0001	137	142	1268	173	59	79
CAGCCCACG	BRDN0001	280	243	1040	454	784	13
CAGCCCAGA	BRDN0001	297	272	386	534	552	19
CAGCCCATA	BRDN0001	496	501	212	841	783	10
CAGCCCATA	BRDN0001	142	129	167	344	502	18
CAGCCCATT	BRDN0001	251	380	146	531	483	0
CAGCCCCAC	BRDN0001	286	296	1403	373	410	14
CAGCCCCAG	BRDN0001	511	261	798	855	774	73
CAGCCCCCA	BRDN0001	56	9	0	232	226	0
CAGCCCCCC	BRDN0001	157	130	5	382	366	6
CAGCCCCGA	BRDN0001	386	310	423	789	477	19
CAGCCCCGT	BRDN0001	331	331	48	549	600	0
CAGCCCCTC	BRDN0001	388	467	1054	725	653	92
CAGCCCCTC	BRDN0001	178	45	2	610	676	0
CAGCCCGAC	BRDN0001	90	156	162	219	286	0
CAGCCCGAG	BRDN0001	228	229	1100	378	330	37
CAGCCCGCC	BRDN0001	265	201	362	482	314	2
CAGCCCGCG	BRDN0001	95	43	537	174	458	33
CAGCCCGCT	BRDN0001	255	214	295	371	352	52
CAGCCCGGA	BRDN0001	190	77	0	522	712	0
CAGCCCGTC	BRDN0001	126	168	20	158	123	31
CAGCCCGTG	BRDN0001	381	272	521	741	556	38
CAGCCCTAA	BRDN0001	305	137	790	646	855	45
CAGCCCTAG	BRDN0001	115	185	112	160	23	0
CAGCCCTAG	BRDN0001	265	283	395	576	494	26
CAGCCCTCA	BRDN0001	292	228	251	488	435	53
CAGCCCTTA	BRDN0001	310	237	1598	722	833	60
CAGCCGAAA	BRDN0001	74	62	0	188	204	1
CAGCCGAAT	BRDN0001	259	161	2533	420	316	69
CAGCCGACT	BRDN0001	216	340	579	368	399	90
CAGCCGAGT	BRDN0001	222	304	682	606	556	31
CAGCCGATT	BRDN0001	119	165	131	249	187	21
CAGCCGCAC	BRDN0001	280	334	388	510	390	42
CAGCCGCAG	BRDN0001	384	330	2042	474	610	38
CAGCCGCAT	BRDN0001	77	63	356	131	193	8
CAGCCGCCA	BRDN0001	235	146	839	452	265	30
CAGCCGCCA	BRDN0001	276	231	386	740	471	28
CAGCCGCGG	BRDN0001	96	34	0	226	116	0
CAGCCGGAC	BRDN0001	147	274	322	409	262	9
CAGCCGGAG	BRDN0001	333	240	75	676	666	43
CAGCCGGAT	BRDN0001	91	86	0	114	113	0
CAGCCGGCC	BRDN0001	43	46	16	130	138	6
CAGCCGGGA	BRDN0001	215	249	8	568	934	14
CAGCCGGGG	BRDN0001	122	248	2040	378	370	80
CAGCCGGGG	BRDN0001	321	270	745	588	584	71
CAGCCGGGT	BRDN0001	192	159	151	279	248	14
CAGCCGGTT	BRDN0001	123	137	57	168	138	28
CAGCCGTAT	BRDN0001	503	300	1271	791	631	26
CAGCCGTGC	BRDN0001	473	460	1572	688	926	27
CAGCCGTTG	BRDN0001	211	282	364	300	481	98
CAGCCTAAC	BRDN0001	109	135	263	170	67	27
CAGCCTACC	BRDN0001	15	0	0	184	42	0
CAGCCTACT	BRDN0001	107	102	7	255	196	30
CAGCCTATT	BRDN0001	353	192	3480	490	553	104
CAGCCTCAG	BRDN0001	96	66	733	303	151	0
CAGCCTCAG	BRDN0001	263	272	33	372	401	15
CAGCCTCCG	BRDN0001	200	169	399	324	169	13
CAGCCTCCG	BRDN0001	53	42	2	661	138	1
CAGCCTCCT	BRDN0001	399	227	13	533	511	2
CAGCCTCTA	BRDN0001	166	224	31	296	348	7
CAGCCTCTA	BRDN0001	72	19	2	139	427	0

CAGCCTCTGA	BRDN00011	364	413	144	736	419	136
CAGCCTCTGA		145	83	0	271	115	5
CAGCCTCTG	BRDN00012	207	143	5654	325	478	66
CAGCCTGAA	BRDN00011	568	533	428	970	888	89
CAGCCTGAA	BRDN00012	174	89	136	252	564	5
CAGCCTGAC	BRDN00012	176	114	103	272	198	13
CAGCCTGAG	BRDN00011	259	217	718	546	371	7
CAGCCTGAT	BRDN00012	123	157	606	263	372	21
CAGCCTGCA	BRDN00011	452	518	286	702	631	0
CAGCCTGGT	BRDN00012	390	121	650	672	445	13
CAGCCTGTC	BRDN00011	60	25	0	280	87	0
CAGCCTTAA	BRDN00012	230	184	69	338	363	15
CAGCCTTAA	BRDN00012	164	110	110	230	241	16
CAGCCTTAG	BRDN00012	91	72	0	135	222	0
CAGCCTTCA	BRDN00011	141	56	97	282	87	0
CAGCCTTGA	BRDN00012	125	61	9	244	122	0
CAGCCTTGC	BRDN00012	120	115	43	202	122	17
CAGCGAACG	BRDN00011	140	7	0	227	92	0
CAGCGAAGT	BRDN00012	388	271	1010	539	861	23
CAGCGAAGT	BRDN00012	181	88	774	250	227	24
CAGCGAAGT	BRDN00012	151	86	89	314	355	13
CAGCGAATG	BRDN00012	197	147	2590	438	362	41
CAGCGACAA	BRDN00011	48	41	57	125	127	13
CAGCGACAA	BRDN00012	152	131	712	214	825	8
CAGCGACAG	BRDN00012	340	339	336	758	716	35
CAGCGACAT	BRDN00012	155	194	18	292	383	12
CAGCGACCC	BRDN00011	220	144	1013	426	258	20
CAGCGACGC	BRDN00011	244	133	182	394	263	41
CAGCGACGC	BRDN00011	114	17	0	235	55	0
CAGCGACGG	BRDN00011	256	169	492	475	417	17
CAGCGACGT	BRDN00012	332	204	1856	603	699	54
CAGCGACGT	BRDN00011	79	0	0	251	0	0
CAGCGACTT	BRDN00012	150	161	182	301	148	30
CAGCGAGAA	BRDN00012	288	154	611	603	436	45
CAGCGAGAA	BRDN00011	75	32	0	249	128	22
CAGCGAGAC	BRDN00011	218	227	507	436	131	9
CAGCGAGAG	BRDN00012	82	80	962	172	154	33
CAGCGAGGC	BRDN00012	365	411	1733	701	714	70
CAGCGAGGT	BRDN00012	156	159	123	169	202	13
CAGCGAGTA	BRDN00011	228	231	549	547	403	31
CAGCGAGTT	BRDN00011	143	158	824	105	211	9
CAGCGATCA	BRDN00011	233	235	29	437	408	56
CAGCGATGA	BRDN00011	125	109	0	170	53	0
CAGCGATGT	BRDN00012	134	144	31	169	241	20
CAGCGATTC	BRDN00012	261	205	348	517	279	26
CAGCGCAAA	BRDN00011	195	165	15	322	184	4
CAGCGCAAG	BRDN00011	143	118	156	302	901	0
CAGCGCAGC	BRDN00011	273	282	1	615	475	0
CAGCGCAGT	BRDN00011	240	306	690	576	1239	25
CAGCGCCAC	BRDN00012	416	277	265	708	470	37
CAGCGCCAC	BRDN00012	54	76	32	189	161	0
CAGCGCCAG	BRDN00012	108	26	32	200	156	6
CAGCGCCGA	BRDN00012	428	402	1052	730	1102	61
CAGCGCCTG	BRDN00011	299	175	564	394	479	30
CAGCGCCTT	BRDN00012	209	141	639	405	314	6
CAGCGCGCA	BRDN00012	122	33	60	185	163	17
CAGCGCGCA	BRDN00012	102	132	98	181	128	8
CAGCGCGCG	BRDN00011	125	110	931	224	134	30
CAGCGCGGT	BRDN00012	112	139	11	145	86	44
CAGCGCGTA	BRDN00012	95	66	59	232	192	10
CAGCGCGTC	BRDN00012	226	265	46	447	457	9
CAGCGCGTC	BRDN00012	150	127	609	289	301	8
CAGCGCGTG	BRDN00011	175	54	107	263	423	1
CAGCGCTAT	BRDN00012	125	124	30	267	181	53
CAGCGCTCA	BRDN00011	219	139	215	344	331	5
CAGCGCTCC	BRDN00012	195	196	228	425	120	8
CAGCGCTGA	BRDN00011	79	39	0	288	51	0
CAGCGCTGA	BRDN00011	140	30	221	213	118	35
CAGCGCTGG	BRDN00012	294	295	64	497	727	46
CAGCGCTGT	BRDN00011	143	52	4	242	112	14
CAGCGCTTG	BRDN00011	300	196	147	385	211	17
CAGCGGAAG	BRDN00012	401	359	411	997	778	32
CAGCGGAGA	BRDN00012	416	334	46	764	861	72
CAGCGGAGA	BRDN00012	129	57	3	184	13	0
CAGCGGAGC	BRDN00012	111	104	12	216	130	4
CAGCGGAGC	BRDN00012	63	0	0	584	337	0
CAGCGGAGC	BRDN00012	195	258	23	347	240	78
CAGCGGATG	BRDN00012	184	89	290	216	69	0
CAGCGGCAA	BRDN00012	305	335	110	725	542	66
CAGCGGCAC	BRDN00012	327	344	973	802	554	5
CAGCGGCAC	BRDN00012	501	374	1051	919	761	26
CAGCGGCAG	BRDN00011	101	181	900	323	237	17
CAGCGGCAT	BRDN00012	83	74	261	145	132	60
CAGCGGCCA	BRDN00012	193	189	964	512	693	112
CAGCGGCCA	BRDN00011	115	6	0	228	34	0
CAGCGGCCG	BRDN00012	516	476	3499	669	2715	229
CAGCGGCCT	BRDN00012	308	307	405	542	442	55
CAGCGGCGC	BRDN00011	64	74	325	271	115	12
CAGCGGCGC	BRDN00012	108	102	0	183	205	0
CAGCGGCGG	BRDN00012	461	341	1949	618	474	64
CAGCGGCTA	BRDN00012	335	407	990	650	811	42
CAGCGGCTA	BRDN00011	272	147	81	593	306	13
CAGCGGCTG	BRDN00011	366	261	46	701	802	8
CAGCGGCTG	BRDN00011	126	60	246	227	59	3
CAGCGGGAC	BRDN00012	539	539	960	1039	1080	100
CAGCGGGGA	BRDN00011	229	111	145	629	582	2
CAGCGGGTA	BRDN00011	90	94	87	171	329	6
CAGCGGGTT	BRDN00012	67	35	0	160	46	0
CAGCGGGTT	BRDN00011	232	187	395	421	313	0
CAGCGGTAG	BRDN00012	452	262	413	822	880	16
CAGCGGTCA	BRDN00012	101	144	159	256	322	0
CAGCGGTCA	BRDN00012	235	156	92	604	795	2
CAGCGGTCC	BRDN00011	139	171	51	295	347	1
CAGCGGTCG	BRDN00012	212	166	1922	369	414	16
CAGCGGTGA	BRDN00012	108	24	0	208	50	12
CAGCGGTGC	BRDN00012	335	353	2096	728	721	66
CAGCGGTTG	BRDN00011	59	74	313	132	130	25
CAGCGTAAG	BRDN00012	213	187	502	400	397	14
CAGCGTACA	BRDN00011	165	58	162	209	226	14
CAGCGTATC	BRDN00011	147	152	434	251	313	22
CAGCGTCAA	BRDN00011	358	422	1272	1061	1435	26
CAGCGTCCA	BRDN00011	201	136	8	273	357	18
CAGCGTCCT	BRDN00011	41	0	0	111	280	0
CAGCGTCGG	BRDN00011	240	165	198	309	485	8
CAGCGTCTT	BRDN00011	96	90	0	202	143	5
CAGCGTGAA	BRDN00011	100	95	58	191	145	0
CAGCGTGCG	BRDN00012	130	80	576	151	196	8
CAGCGTGGA	BRDN00011	163	154	1647	278	436	30
CAGCGTGTC	BRDN00011	172	100	359	390	506	14
CAGCGTGTG	BRDN00012	326	412	2304	784	737	29
CAGCGTGTG	BRDN00011	313	100	11	719	570	0
CAGCGTTCT	BRDN00012	268	364	1	609	386	5
CAGCGTTTA	BRDN00012	189	85	37	386	197	0
CAGCGTTTG	BRDN00012	182	63	133	255	412	8
CAGCGTTTG	BRDN00011	285	287	2436	536	1172	74
CAGCTAAAA	BRDN00011	73	46	0	200	139	1
CAGCTAATG	BRDN00012	324	302	104	536	845	12
CAGCTACAC	BRDN00012	252	267	1313	440	369	8
CAGCTACAC	BRDN00012	214	293	2330	361	275	21
CAGCTACAC	BRDN00011	295	268	393	398	442	78
CAGCTACCA	BRDN00012	220	281	0	362	337	0
CAGCTACCC	BRDN00012	102	82	43	171	116	9
CAGCTACCT	BRDN00011	205	348	1320	356	514	52
CAGCTACGG	BRDN00012	157	144	1809	208	173	48
CAGCTACGT	BRDN00011	165	245	488	330	245	14
CAGCTACTA	BRDN00012	160	216	82	254	97	13
CAGCTACTA	BRDN00012	177	172	24	247	227	7
CAGCTACTG	BRDN00012	58	38	0	321	175	0
CAGCTACTT	BRDN00011	272	184	822	527	513	39
CAGCTAGAC	BRDN00012	158	49	14	285	384	0
CAGCTAGAG	BRDN00011	217	187	171	393	306	16
CAGCTATGA	BRDN00012	304	156	657	635	550	22
CAGCTATGA	BRDN00012	291	248	695	532	582	16
CAGCTATGG	BRDN00011	29	65	169	119	160	37
CAGCTCAAA	BRDN00012	216	299	521	436	594	49
CAGCTCAAC	BRDN00011	186	286	949	544	381	32
CAGCTCAAC	BRDN00011	196	96	743	396	514	30
CAGCTCACA	BRDN00011	245	239	368	514	601	192
CAGCTCACA	BRDN00012	101	65	231	204	73	5
CAGCTCACC	BRDN00011	164	125	1353	308	498	36
CAGCTCAGC	BRDN00012	432	300	758	778	792	0
CAGCTCAGC	BRDN00012	168	0	0	335	2	0
CAGCTCATC	BRDN00012	121	130	144	300	361	319
CAGCTCATG	BRDN00011	118	197	29	263	185	11
CAGCTCATT	BRDN00012	180	0	2	765	581	0
CAGCTCCAA	BRDN00012	329	253	1024	571	356	36
CAGCTCCAC	BRDN00012	263	236	30	322	311	0
CAGCTCCAC	BRDN00011	98	32	12	198	175	0
CAGCTCCAC	BRDN00011	133	123	426	364	1085	26
CAGCTCCAG	BRDN00011	78	116	212	203	301	4
CAGCTCCAG	BRDN00011	265	143	1	593	397	7
CAGCTCCAT	BRDN00012	238	382	77	657	677	15
CAGCTCCAT	BRDN00012	138	122	5	327	293	25
CAGCTCCCG	BRDN00012	340	340	582	818	959	68
CAGCTCCCT	BRDN00012	92	104	46	161	153	70
CAGCTCCCT	BRDN00011	242	134	1	332	162	9
CAGCTCCGA	BRDN00011	181	203	351	565	1941	231
CAGCTCCGA	BRDN00011	341	141	113	651	670	31
CAGCTCCGC	BRDN00012	146	132	279	300	240	133
CAGCTCCGT	BRDN00012	373	435	295	690	751	52
CAGCTCCTC	BRDN00011	250	112	104	412	386	0
CAGCTCCTC	BRDN00011	154	218	151	240	223	33
CAGCTCCTG	BRDN00012	147	89	223	278	126	9
CAGCTCCTT	BRDN00011	112	76	68	166	200	44
CAGCTCGAA	BRDN00011	87	61	86	323	209	6
CAGCTCGAC	BRDN00012	122	78	99	151	252	16
CAGCTCGAT	BRDN00012	328	407	2176	678	814	26
CAGCTCGCG	BRDN00012	292	226	2033	841	695	76
CAGCTCGCT	BRDN00012	134	144	91	317	325	6
CAGCTCGGA	BRDN00012	148	73	267	292	357	6
CAGCTCGTC	BRDN00012	192	286	760	244	138	32
CAGCTCGTC	BRDN00012	137	104	467	211	267	0
CAGCTCGTG	BRDN00012	195	205	307	259	398	11
CAGCTCTAC	BRDN00012	194	272	89	362	384	29
CAGCTCTAG	BRDN00011	224	272	415	391	478	16
CAGCTCTCT	BRDN00012	360	252	900	635	469	20
CAGCTCTGA	BRDN00012	221	181	70	212	416	30
CAGCTCTGG	BRDN00012	5	0	1	355	92	0
CAGCTCTTA	BRDN00011	82	95	203	170	142	19
CAGCTCTTC	BRDN00012	392	428	96	812	756	16
CAGCTGAAA	BRDN00011	405	144	1199	629	402	0
CAGCTGAAG	BRDN00012	140	102	0	279	332	16
CAGCTGACC	BRDN00012	151	76	50	309	280	0
CAGCTGACC	BRDN00011	439	316	101	621	654	12
CAGCTGACT	BRDN00011	348	346	1060	609	443	35
CAGCTGACT	BRDN00011	218	269	29	668	583	21
CAGCTGACT	BRDN00012	274	259	380	647	639	169
CAGCTGACT	BRDN00011	96	41	311	154	192	102
CAGCTGAGC	BRDN00011	186	88	4	387	739	8
CAGCTGAGG	BRDN00012	130	93	929	238	99	46
CAGCTGAGG	BRDN00011	165	216	505	409	364	2
CAGCTGAGG	BRDN00012	551	677	932	1035	946	55
CAGCTGAGG	BRDN00012	180	184	963	481	229	0
CAGCTGAGT	BRDN00011	192	102	819	316	173	0
CAGCTGATG	BRDN00011	399	491	632	657	581	68
CAGCTGATG	BRDN00012	91	0	0	222	119	0
CAGCTGCAA	BRDN00011	157	165	8	277	369	40
CAGCTGCAA	BRDN00011	272	230	39	494	255	1
CAGCTGCAC	BRDN00011	178	177	149	262	265	27
CAGCTGCAG	BRDN00011	118	117	228	221	72	37
CAGCTGCAG	BRDN00011	212	148	26	554	424	29
CAGCTGCAG	BRDN00011	350	77	14	667	503	0
CAGCTGCAG	BRDN00012	277	209	20	488	398	0
CAGCTGCAT	BRDN00011	251	156	4	331	392	6
CAGCTGCAT	BRDN00012	101	8	0	133	87	1
CAGCTGCCA	BRDN00012	472	338	1220	832	982	31
CAGCTGCCA	BRDN00011	435	361	829	746	815	44
CAGCTGCCA	BRDN00011	264	213	181	706	460	9
CAGCTGCCC	BRDN00012	451	364	543	925	1010	21
CAGCTGCCT	BRDN00011	206	91	422	405	845	0
CAGCTGCGC	BRDN00012	168	127	234	330	323	48
CAGCTGCGG	BRDN00012	134	63	11	366	167	0
CAGCTGCTG	BRDN00011	113	156	418	255	421	24
CAGCTGCTG	BRDN00012	246	294	128	432	758	37
CAGCTGCTT	BRDN00011	211	170	81	293	572	30
CAGCTGCTT	BRDN00011	303	249	364	817	570	95
CAGCTGGAA	BRDN00011	143	11	131	371	209	10
CAGCTGGAA	BRDN00011	204	189	2176	492	454	56
CAGCTGGAC	BRDN00011	296	253	598	723	259	37
CAGCTGGAC	BRDN00011	106	166	2156	252	178	15
CAGCTGGAC	BRDN00012	615	425	870	809	864	65
CAGCTGGAG	BRDN00011	73	96	144	148	11	0
CAGCTGGCA	BRDN00011	237	319	44	434	345	16
CAGCTGGCG	BRDN00011	138	64	151	270	248	7
CAGCTGGGA	BRDN00012	114	163	198	411	239	21
CAGCTGGGA	BRDN00012	16	13	0	243	62	0
CAGCTGGGC	BRDN00012	337	384	180	674	2616	78
CAGCTGGGG	BRDN00012	191	91	137	629	1267	3
CAGCTGGGG	BRDN00012	104	79	0	142	163	0
CAGCTGGGG	BRDN00012	277	349	470	523	428	44
CAGCTGGGT	BRDN00011	194	90	17	279	283	0
CAGCTGGTC	BRDN00011	244	163	624	375	279	48
CAGCTGGTG	BRDN00011	156	86	121	329	543	8
CAGCTGGTG	BRDN00011	242	318	45	424	366	15
CAGCTGGTG	BRDN00011	115	120	143	262	152	6
CAGCTGGTG	BRDN00011	151	94	1	288	206	0
CAGCTGGTT	BRDN00011	287	346	103	785	362	18
CAGCTGTAA	BRDN00011	258	573	1381	661	743	148
CAGCTGTAA	BRDN00012	173	134	1012	197	136	18
CAGCTGTAG	BRDN00011	110	171	0	195	283	24
CAGCTGTCA	BRDN00011	301	154	425	456	470	15
CAGCTGTCC	BRDN00012	246	122	786	541	313	7
CAGCTGTCG	BRDN00011	68	86	37	99	108	0
CAGCTGTCT	BRDN00012	173	167	128	383	273	14
CAGCTGTGA	BRDN00012	253	289	1351	440	467	29
CAGCTGTGC	BRDN00011	238	149	241	387	252	7
CAGCTGTGT	BRDN00012	77	51	0	202	131	5
CAGCTGTGT	BRDN00012	97	71	1764	232	280	26
CAGCTGTTA	BRDN00011	80	57	58	137	215	0
CAGCTGTTG	BRDN00011	400	355	119	554	690	141
CAGCTGTTT	BRDN00011	66	121	47	246	133	0
CAGCTGTTT	BRDN00012	345	396	1456	685	596	17
CAGCTGTTT	BRDN00011	347	340	827	712	668	19
CAGCTTACC	BRDN00011	201	16	0	461	13	1
CAGCTTAGA	BRDN00012	222	192	353	433	227	21
CAGCTTAGG	BRDN00011	146	165	262	215	180	3
CAGCTTAGG	BRDN00012	244	205	112	488	304	2
CAGCTTCAC	BRDN00011	176	142	139	421	474	13
CAGCTTCAG	BRDN00011	150	75	355	204	244	29
CAGCTTCCG	BRDN00012	191	72	380	306	198	17
CAGCTTCCG	BRDN00011	134	84	38	189	291	0
CAGCTTCCG	BRDN00011	217	263	269	439	329	82
CAGCTTCGA	BRDN00012	289	253	8420	512	268	104
CAGCTTCTA	BRDN00012	326	403	989	659	460	44
CAGCTTCTG	BRDN00011	204	121	51	351	437	7
CAGCTTGAC	BRDN00011	221	265	84	277	267	0
CAGCTTGAG	BRDN00011	218	232	610	446	557	69
CAGCTTGGC	BRDN00011	379	195	350	637	708	10
CAGCTTTAG	BRDN00011	119	0	0	316	156	0
CAGCTTTCT	BRDN00011	290	337	303	678	596	91
CAGCTTTCT	BRDN00011	215	193	243	399	629	0
CAGCTTTGA	BRDN00012	282	251	269	629	302	12
CAGCTTTGC	BRDN00011	247	204	176	547	495	5
CAGCTTTGC	BRDN00012	134	255	728	270	73	1311
CAGCTTTGG	BRDN00011	44	19	0	110	81	0
CAGGAAAAA	BRDN00011	217	0	0	343	68	0
CAGGAAAAC	BRDN00011	156	12	0	242	59	0
CAGGAAAAG	BRDN00012	349	287	641	504	727	17
CAGGAAAAG	BRDN00012	157	94	96	155	246	8
CAGGAAACC	BRDN00011	467	350	405	648	601	48
CAGGAAAGA	BRDN00012	99	55	665	136	104	8
CAGGAAATG	BRDN00011	109	153	199	255	226	53
CAGGAAATC	BRDN00011	18	0	1	57	0	0
CAGGAAATG	BRDN00012	93	119	556	163	183	4
CAGGAACAG	BRDN00012	138	33	244	331	284	11
CAGGAACAG	BRDN00011	396	531	546	730	565	19
CAGGAACAC	BRDN00011	301	293	216	454	428	88
CAGGAACAG	BRDN00011	89	16	0	146	7	0
CAGGAACAG	BRDN00012	256	216	167	606	510	30
CAGGAACAT	BRDN00011	96	74	73	204	137	0
CAGGAACCA	BRDN00012	87	31	105	154	57	0
CAGGAACCA	BRDN00011	43	0	0	133	40	0
CAGGAACCG	BRDN00012	31	11	1	303	0	0
CAGGAACCG	BRDN00011	91	62	168	179	133	0
CAGGAACGA	BRDN00011	314	191	50	537	704	16
CAGGAACTC	BRDN00012	119	134	69	226	170	13
CAGGAACTC	BRDN00012	277	356	762	363	484	75
CAGGAACTC	BRDN00011	125	56	73	258	333	0
CAGGAACTC	BRDN00011	169	93	0	340	222	0
CAGGAACTG	BRDN00011	238	131	235	426	520	87
CAGGAACTG	BRDN00011	358	292	435	669	866	17
CAGGAAGAA	BRDN00012	282	203	903	570	432	62
CAGGAAGAA	BRDN00011	111	67	176	291	233	14
CAGGAAGAC	BRDN00011	411	494	837	602	739	28
CAGGAAGAC	BRDN00011	114	62	0	279	92	0
CAGGAAGAC	BRDN00012	289	219	324	552	271	13
CAGGAAGAT	BRDN00011	284	295	915	869	867	68
CAGGAAGAT	BRDN00011	95	135	453	287	196	29
CAGGAAGAT	BRDN00012	197	138	0	308	310	16
CAGGAAGCA	BRDN00011	188	210	20	341	376	0
CAGGAAGCA	BRDN00011	315	289	1422	727	894	61
CAGGAAGCA	BRDN00012	192	209	201	316	283	56
CAGGAAGCC	BRDN00011	304	296	276	607	549	20
CAGGAAGGA	BRDN00012	399	381	305	862	475	69
CAGGAAGGC	BRDN00012	94	79	75	139	80	0
CAGGAAGGC	BRDN00012	76	124	145	134	11	32
CAGGAAGGT	BRDN00012	300	416	3925	486	348	106
CAGGAAGTA	BRDN00012	147	7	1	223	80	0
CAGGAAGTG	BRDN00012	407	343	670	738	592	33
CAGGAATAA	BRDN00011	273	324	172	529	538	17
CAGGAATAC	BRDN00012	107	115	640	163	213	57
CAGGAATAT	BRDN00012	97	0	0	140	0	0
CAGGAATCA	BRDN00012	134	153	125	233	300	27
CAGGAATCC	BRDN00012	160	161	619	314	392	21
CAGGAATCC	BRDN00012	213	220	464	549	539	23
CAGGAATCT	BRDN00012	107	92	151	116	55	23
CAGGAATCT	BRDN00012	127	105	65	259	240	13
CAGGAATGG	BRDN00012	389	424	211	499	642	16
CAGGAATTC	BRDN00012	128	133	47	265	221	86
CAGGACAAA	BRDN00012	258	41	0	337	127	0
CAGGACAAG	BRDN00011	85	35	584	197	273	4
CAGGACAAC	BRDN00012	154	151	12	283	435	0
CAGGACAAG	BRDN00012	119	55	71	299	369	1
CAGGACAAG	BRDN00012	158	111	76	246	180	0
CAGGACAAG	BRDN00012	246	282	75	552	669	0
CAGGACAAG	BRDN00012	92	86	76	171	232	0
CAGGACACA	BRDN00011	298	194	178	478	440	12
CAGGACACA	BRDN00011	144	16	486	422	429	4
CAGGACACC	BRDN00012	228	266	293	524	734	3
CAGGACACC	BRDN00011	176	360	440	493	463	33
CAGGACACG	BRDN00012	315	234	514	565	652	33
CAGGACAGG	BRDN00012	142	113	740	186	192	27
CAGGACAGT	BRDN00012	113	91	356	282	257	8
CAGGACATC	BRDN00012	115	72	0	228	211	0
CAGGACATC	BRDN00011	75	0	0	279	36	0
CAGGACATG	BRDN00011	219	122	45	292	101	62
CAGGACATG	BRDN00011	152	127	231	235	389	0
CAGGACCAA	BRDN00012	260	194	543	382	238	23
CAGGACCAC	BRDN00012	152	180	0	314	141	14
CAGGACCAC	BRDN00011	73	180	0	175	395	3
CAGGACCAG	BRDN00012	340	206	112	529	603	0
CAGGACCAG	BRDN00011	387	357	2494	671	1150	47
CAGGACCAT	BRDN00011	193	221	385	493	751	9
CAGGACCCA	BRDN00011	113	45	485	170	301	0
CAGGACCCC	BRDN00011	43	72	0	121	53	0
CAGGACCCT	BRDN00011	196	208	1493	311	199	19
CAGGACCCT	BRDN00012	118	105	2180	182	250	49
CAGGACCGA	BRDN00012	275	283	1290	501	681	38
CAGGACCGG	BRDN00012	255	226	294	483	296	17
CAGGACCTA	BRDN00011	357	36	63	585	325	1
CAGGACCTC	BRDN00012	400	431	1641	566	409	70
CAGGACCTG	BRDN00011	79	111	588	204	165	0
CAGGACGAG	BRDN00012	92	98	99	127	49	26
CAGGACGCA	BRDN00012	131	60	1	284	271	0
CAGGACGCT	BRDN00012	250	121	47	269	261	0
CAGGACGCT	BRDN00012	62	48	705	239	167	40
CAGGACGGA	BRDN00011	269	158	30	493	388	0
CAGGACGGA	BRDN00012	315	185	213	573	641	11
CAGGACGGT	BRDN00011	99	11	37	393	370	0
CAGGACGGT	BRDN00012	183	131	367	262	200	0
CAGGACGGT	BRDN00012	311	309	1390	437	630	27
CAGGACGTA	BRDN00012	92	49	0	176	228	6
CAGGACGTC	BRDN00012	162	248	0	507	472	9
CAGGACGTG	BRDN00012	195	99	45	220	190	0
CAGGACTCA	BRDN00012	312	260	394	757	663	35
CAGGACTCA	BRDN00012	68	10	100	242	35	0
CAGGACTCC	BRDN00011	172	148	543	218	111	27
CAGGACTGA	BRDN00012	140	116	1	358	371	7
CAGGACTGG	BRDN00011	63	16	0	163	266	0
CAGGACTGT	BRDN00011	332	283	74	496	750	8
CAGGACTTA	BRDN00012	117	67	0	235	128	0
CAGGACTTA	BRDN00012	171	14	1620	567	289	0
CAGGAGAAA	BRDN00011	137	209	38	486	253	0
CAGGAGAAA	BRDN00011	139	88	1	283	296	10
CAGGAGAAC	BRDN00011	92	78	240	250	176	21
CAGGAGAAG	BRDN00012	81	61	973	164	56	6
CAGGAGACA	BRDN00012	452	526	646	863	1237	89
CAGGAGACA	BRDN00011	103	135	217	168	136	0
CAGGAGACT	BRDN00012	3	0	0	21	0	0
CAGGAGATA	BRDN00012	74	30	656	168	178	27
CAGGAGATG	BRDN00012	346	263	1128	638	596	40
CAGGAGATT	BRDN00011	52	72	149	109	213	4
CAGGAGCAA	BRDN00011	150	197	85	435	306	76
CAGGAGCAC	BRDN00011	111	112	40	309	347	58
CAGGAGCAC	BRDN00012	223	168	22	394	312	28
CAGGAGCAC	BRDN00011	131	156	41	280	380	15
CAGGAGCAG	BRDN00011	225	283	219	572	1032	44
CAGGAGCAG	BRDN00012	82	73	1	260	215	4
CAGGAGCAT	BRDN00012	158	135	141	247	362	6
CAGGAGCCA	BRDN00011	290	234	640	640	717	38
CAGGAGCCG	BRDN00012	147	173	11	182	279	0
CAGGAGCCG	BRDN00012	298	273	2542	586	537	129
CAGGAGCGC	BRDN00012	307	224	197	518	441	11
CAGGAGCGT	BRDN00011	27	5	12	64	23	0
CAGGAGCGT	BRDN00012	343	334	191	816	926	15
CAGGAGCGT	BRDN00011	224	166	3952	406	329	70
CAGGAGCTC	BRDN00012	98	52	82	219	459	0
CAGGAGCTG	BRDN00011	169	96	82	481	468	8
CAGGAGCTG	BRDN00012	108	209	13	303	261	29
CAGGAGCTG	BRDN00011	209	217	186	546	413	0
CAGGAGCTT	BRDN00011	171	185	267	392	349	43
CAGGAGCTT	BRDN00011	65	31	44	211	257	41
CAGGAGGAC	BRDN00011	121	217	337	187	252	41
CAGGAGGAC	BRDN00011	92	81	0	149	284	5
CAGGAGGAT	BRDN00011	137	165	111	232	270	0
CAGGAGGCT	BRDN00012	141	119	524	274	144	17
CAGGAGGGA	BRDN00012	79	0	0	433	193	0
CAGGAGGGC	BRDN00011	157	238	565	291	142	47
CAGGAGGGT	BRDN00011	110	93	322	130	190	29
CAGGAGGGT	BRDN00011	238	35	0	726	850	20
CAGGAGGTA	BRDN00011	128	99	100	261	376	11
CAGGAGGTC	BRDN00011	491	380	246	677	957	37
CAGGAGGTC	BRDN00011	241	170	194	474	524	19
CAGGAGGTC	BRDN00011	209	200	158	513	583	7
CAGGAGGTC	BRDN00012	101	103	74	190	224	0
CAGGAGTAA	BRDN00011	228	27	17	441	696	0
CAGGAGTAC	BRDN00011	275	206	1008	544	471	50
CAGGAGTAG	BRDN00011	247	121	1608	352	430	10
CAGGAGTCA	BRDN00012	242	198	195	440	559	7
CAGGAGTCA	BRDN00012	91	99	2	324	213	22
CAGGAGTCA	BRDN00011	109	83	59	141	99	0
CAGGAGTCG	BRDN00012	354	372	461	558	496	77
CAGGAGTGC	BRDN00012	294	400	1385	467	511	36
CAGGAGTGT	BRDN00012	204	139	290	357	205	5
CAGGATAAA	BRDN00012	193	292	319	640	411	29
CAGGATAAT	BRDN00011	132	207	263	188	398	28
CAGGATACA	BRDN00011	176	263	889	621	761	13
CAGGATACA	BRDN00011	95	73	83	153	34	7
CAGGATAGA	BRDN00011	69	88	0	99	513	0
CAGGATAGC	BRDN00011	138	149	263	216	189	0
CAGGATATC	BRDN00012	109	82	546	213	89	18
CAGGATATG	BRDN00012	119	150	32	336	240	4
CAGGATCAG	BRDN00012	103	100	81	217	162	11
CAGGATCAG	BRDN00012	103	94	302	243	350	12
CAGGATCAG	BRDN00012	408	501	922	661	715	39

CAGGATCAG	BRDN00012	104	155	0	193	320	0
CAGGATCAT	BRDN00011	228	327	1100	426	277	18
CAGGATCAT	BRDN00012	109	15	166	196	143	15
CAGGATCCA	BRDN00011	156	124	2510	351	303	51
CAGGATCCA	BRDN00011	102	10	0	314	159	0
CAGGATCCA	BRDN00012	295	423	831	470	343	18
CAGGATCCT	BRDN00011	159	164	338	231	125	44
CAGGATCGA	BRDN00012	165	137	96	205	171	3
CAGGATCGG	BRDN00012	213	79	630	249	240	31
CAGGATCTG	BRDN00012	155	174	35	200	278	0
CAGGATCTG	BRDN00011	69	46	7	178	79	0
CAGGATCTG	BRDN00012	445	454	453	820	1071	22
CAGGATCTG	BRDN00011	265	116	47	560	416	15
CAGGATCTT	BRDN00012	89	117	304	202	176	21
CAGGATGAA	BRDN00011	125	99	36	256	167	0
CAGGATGAG	BRDN00012	339	446	3219	611	527	96
CAGGATGAT	BRDN00012	383	185	373	573	526	8
CAGGATGAT	BRDN00011	104	95	0	171	122	10
CAGGATGAT	BRDN00012	323	160	191	665	818	21
CAGGATGCC	BRDN00012	131	0	0	560	276	0
CAGGATGCC	BRDN00012	353	309	1109	721	738	58
CAGGATGGA	BRDN00012	168	325	67	430	476	54
CAGGATGGA	BRDN00012	420	426	43	677	733	0
CAGGATGTA	BRDN00011	52	60	0	125	82	0
CAGGATGTC	BRDN00012	366	328	690	808	748	83
CAGGATGTC	BRDN00011	156	124	9	282	275	27
CAGGATGTG	BRDN00012	170	335	364	355	197	94
CAGGATGTT	BRDN00012	60	0	0	140	60	0
CAGGATTAA	BRDN00012	162	135	88	284	171	5
CAGGATTAC	BRDN00012	247	310	1045	486	401	53
CAGGATTCA	BRDN00012	99	140	118	351	279	0
CAGGATTCA	BRDN00012	301	203	315	526	442	16
CAGGATTCC	BRDN00011	140	199	492	382	303	22
CAGGATTCT	BRDN00012	69	26	62	205	112	0
CAGGATTGG	BRDN00012	185	182	557	294	229	48
CAGGATTGG	BRDN00011	87	195	13	246	260	27
CAGGATTGT	BRDN00011	313	150	437	648	996	12
CAGGCAAAG	BRDN00011	130	212	0	201	145	0
CAGGCAACA	BRDN00011	135	219	370	309	344	13
CAGGCAACG	BRDN00012	45	14	50	544	173	0
CAGGCAAGA	BRDN00011	211	64	892	361	441	21
CAGGCAAGC	BRDN00011	6	0	0	140	55	0
CAGGCAAGT	BRDN00011	121	136	48	394	466	21
CAGGCAAGT	BRDN00012	99	98	53	370	155	24
CAGGCAAGT	BRDN00012	278	442	375	590	650	45
CAGGCAAGT	BRDN00012	379	328	110	758	800	32
CAGGCAATC	BRDN00011	121	130	288	301	203	15
CAGGCAATC	BRDN00012	34	37	0	109	27	4
CAGGCAATC	BRDN00012	142	192	5	153	179	9
CAGGCAATT	BRDN00011	311	304	377	500	658	59
CAGGCACAA	BRDN00011	88	94	70	165	101	0
CAGGCACAA	BRDN00012	176	267	382	281	494	72
CAGGCACAC	BRDN00011	138	0	0	473	115	0
CAGGCACAC	BRDN00012	126	194	109	296	309	0
CAGGCACAG	BRDN00012	157	74	44	211	150	0
CAGGCACAG	BRDN00011	361	273	940	472	481	7
CAGGCACAG	BRDN00012	75	68	83	231	155	0
CAGGCACAT	BRDN00011	136	28	98	284	206	24
CAGGCACAT	BRDN00012	106	94	202	159	283	18
CAGGCACCA	BRDN00012	131	77	210	327	349	0
CAGGCACCA	BRDN00012	443	344	286	895	855	62
CAGGCACCT	BRDN00012	169	15	125	271	140	0
CAGGCACGA	BRDN00011	64	12	0	99	192	1
CAGGCACGC	BRDN00012	224	274	1039	376	292	34
CAGGCACGC	BRDN00012	113	55	206	180	35	4
CAGGCACGT	BRDN00011	339	320	749	563	608	67
CAGGCACGT	BRDN00011	166	168	8	214	250	32
CAGGCACGT	BRDN00011	400	586	5696	538	478	164
CAGGCACTC	BRDN00012	274	346	133	601	350	19
CAGGCACTC	BRDN00011	42	66	8	145	209	53
CAGGCACTC	BRDN00012	255	176	950	364	2905	21
CAGGCACTG	BRDN00012	167	134	501	213	199	34
CAGGCACTT	BRDN00012	213	6	64	554	273	0
CAGGCACTT	BRDN00011	269	307	900	473	201	56
CAGGCAGAA	BRDN00011	168	216	23	305	433	7
CAGGCAGAA	BRDN00011	123	117	20	174	389	0
CAGGCAGAA	BRDN00011	67	47	41	83	154	0
CAGGCAGAC	BRDN00011	380	307	552	696	437	47
CAGGCAGAC	BRDN00011	178	61	21	265	220	3
CAGGCAGAT	BRDN00012	85	92	29	270	241	0
CAGGCAGCC	BRDN00012	95	0	0	235	170	0
CAGGCAGTA	BRDN00012	178	161	1023	284	261	52
CAGGCAGTC	BRDN00011	125	276	301	266	223	0
CAGGCAGTG	BRDN00011	137	95	158	313	418	0
CAGGCATAC	BRDN00012	242	150	362	375	284	0
CAGGCATAC	BRDN00012	163	87	130	313	200	12
CAGGCATAG	BRDN00012	175	144	2	223	71	17
CAGGCATAT	BRDN00012	290	327	217	711	809	39
CAGGCATCA	BRDN00012	175	149	139	350	357	58
CAGGCATCC	BRDN00012	124	82	35	694	227	32
CAGGCATGA	BRDN00012	135	161	1289	282	228	191
CAGGCATTC	BRDN00012	134	79	536	276	271	7
CAGGCCAAC	BRDN00012	380	393	1217	559	419	41
CAGGCCAAG	BRDN00012	139	89	139	312	296	10
CAGGCCAAT	BRDN00011	152	114	169	307	254	27
CAGGCCAAT	BRDN00012	279	384	676	508	553	85
CAGGCCACA	BRDN00012	429	368	361	647	1156	19
CAGGCCACC	BRDN00011	259	95	17	270	569	25
CAGGCCACT	BRDN00012	402	108	163	797	850	15
CAGGCCAGA	BRDN00012	248	132	26	336	522	46
CAGGCCAGA	BRDN00011	52	16	194	201	137	4
CAGGCCAGC	BRDN00011	133	118	700	301	353	11
CAGGCCAGT	BRDN00012	60	60	26	98	58	0
CAGGCCATA	BRDN00011	373	394	124	675	431	91
CAGGCCATG	BRDN00012	139	147	97	341	266	23
CAGGCCATT	BRDN00011	451	483	1319	699	629	65
CAGGCCCAA	BRDN00012	323	221	290	497	310	3
CAGGCCCAA	BRDN00012	115	130	20	272	262	5
CAGGCCCAC	BRDN00011	121	131	214	173	168	0
CAGGCCCAG	BRDN00011	154	131	41	243	462	0
CAGGCCCCG	BRDN00011	177	149	857	209	235	20
CAGGCCCGG	BRDN00012	286	246	232	435	718	68
CAGGCCCGT	BRDN00012	82	96	46	286	252	0
CAGGCCCTC	BRDN00011	89	137	0	231	217	7
CAGGCCCTC	BRDN00012	324	231	66	604	510	57
CAGGCCCTG	BRDN00011	132	52	67	211	264	0
CAGGCCGAC	BRDN00012	466	500	1032	729	522	13
CAGGCCGAG	BRDN00012	158	175	1023	285	373	23
CAGGCCGAG	BRDN00011	260	326	68	443	400	45
CAGGCCGCG	BRDN00012	146	45	2	341	260	0
CAGGCCGGA	BRDN00012	234	54	0	845	450	48
CAGGCCGGT	BRDN00012	82	83	38	132	204	0
CAGGCCGTG	BRDN00012	107	117	2188	256	156	7
CAGGCCTAG	BRDN00011	67	118	41	112	205	0
CAGGCCTCA	BRDN00011	226	127	705	422	415	32
CAGGCCTCG	BRDN00012	132	80	4	447	437	0
CAGGCCTCT	BRDN00012	116	8	0	229	168	0
CAGGCCTGA	BRDN00011	321	353	375	712	712	0
CAGGCCTGC	BRDN00011	1093	1101	3276	2079	2779	150
CAGGCCTGC	BRDN00012	307	513	966	559	652	30
CAGGCCTGC	BRDN00011	155	108	228	148	287	34
CAGGCCTGC	BRDN00011	135	45	8	264	181	0
CAGGCCTGG	BRDN00012	104	122	639	200	102	10
CAGGCCTGG	BRDN00011	107	80	171	297	141	18
CAGGCGACA	BRDN00012	229	117	88	386	255	1
CAGGCGACG	BRDN00012	115	42	0	459	306	0
CAGGCGAGA	BRDN00011	309	366	177	545	1301	19
CAGGCGAGC	BRDN00011	58	44	44	131	18	0
CAGGCGCAA	BRDN00011	242	353	1869	535	461	88
CAGGCGCAG	BRDN00011	274	187	680	436	411	51
CAGGCGCGA	BRDN00011	201	138	17	239	150	0
CAGGCGCGG	BRDN00011	92	86	16	248	385	29
CAGGCGCGT	BRDN00011	224	251	775	413	615	10
CAGGCGCTC	BRDN00012	418	357	846	469	323	7
CAGGCGCTG	BRDN00012	160	186	202	350	601	0
CAGGCGGAG	BRDN00011	280	337	233	613	501	16
CAGGCGGCC	BRDN00012	202	115	44	550	312	10
CAGGCGGCC	BRDN00012	39	25	0	68	85	0
CAGGCGGCG	BRDN00011	242	109	180	460	336	25
CAGGCGGGA	BRDN00012	270	239	440	490	352	14
CAGGCGGTA	BRDN00011	131	70	1323	163	99	33
CAGGCGGTA	BRDN00012	116	101	448	179	152	0
CAGGCGGTA	BRDN00011	121	114	2364	167	142	35
CAGGCGGTC	BRDN00011	74	44	229	171	25	0
CAGGCGGTG	BRDN00011	347	326	599	563	945	83
CAGGCGTAC	BRDN00011	313	139	320	431	428	11
CAGGCGTCA	BRDN00011	151	74	20	142	108	0
CAGGCGTCA	BRDN00012	121	100	81	310	189	0
CAGGCGTCA	BRDN00011	325	336	15768	619	522	378
CAGGCGTCA	BRDN00012	313	326	1470	616	861	51
CAGGCGTCC	BRDN00011	211	186	438	213	198	44
CAGGCGTCT	BRDN00012	82	0	0	181	159	0
CAGGCGTTC	BRDN00012	158	162	162	251	591	12
CAGGCTACA	BRDN00012	131	67	1281	228	193	4
CAGGCTACA	BRDN00012	175	143	160	321	359	10
CAGGCTACC	BRDN00012	389	427	931	510	321	81
CAGGCTATC	BRDN00012	488	327	2752	950	879	91
CAGGCTCAC	BRDN00011	186	129	135	574	942	35
CAGGCTCAC	BRDN00011	271	193	73	642	479	27
CAGGCTCAG	BRDN00012	247	182	648	571	694	15
CAGGCTCAG	BRDN00011	186	183	2506	400	91	38
CAGGCTCCA	BRDN00011	161	56	149	322	428	27
CAGGCTCCC	BRDN00011	184	161	452	460	528	27
CAGGCTCGA	BRDN00011	109	33	146	142	411	10
CAGGCTCTA	BRDN00012	104	135	149	356	183	16
CAGGCTCTC	BRDN00012	146	90	323	276	236	0
CAGGCTCTG	BRDN00012	183	204	35	356	334	12
CAGGCTCTG	BRDN00012	253	229	551	460	438	27
CAGGCTGAA	BRDN00012	272	138	332	439	380	28
CAGGCTGCC	BRDN00012	243	285	1679	509	246	44
CAGGCTGCG	BRDN00012	328	291	108	750	353	19
CAGGCTGCG	BRDN00012	399	605	744	799	616	56
CAGGCTGCG	BRDN00011	250	132	123	373	220	13
CAGGCTGCT	BRDN00012	401	310	354	726	572	12
CAGGCTGCT	BRDN00012	463	465	544	838	1018	43
CAGGCTGCT	BRDN00012	172	188	239	223	193	10
CAGGCTTAC	BRDN00012	237	164	443	460	394	10
CAGGCTTAG	BRDN00011	160	98	670	195	209	26
CAGGCTTAG	BRDN00012	334	312	1576	624	472	61
CAGGCTTCA	BRDN00011	129	65	300	325	366	9
CAGGCTTGC	BRDN00012	168	216	683	319	287	15
CAGGCTTGG	BRDN00012	247	308	2389	437	500	150
CAGGCTTTA	BRDN00011	132	211	291	209	279	45
CAGGGAAAC	BRDN00012	12	0	0	179	8	0
CAGGGAAAG	BRDN00011	271	285	1514	546	405	20
CAGGGAACA	BRDN00012	311	391	1065	722	871	86
CAGGGAACT	BRDN00012	163	136	452	188	267	17
CAGGGAAGA	BRDN00012	92	30	0	212	96	0
CAGGGAAGA	BRDN00011	137	136	75	175	65	20
CAGGGAAGA	BRDN00012	158	70	295	283	216	3
CAGGGAAGC	BRDN00012	175	171	65	311	185	44
CAGGGAAGC	BRDN00011	152	247	73	335	432	30
CAGGGAATC	BRDN00012	156	232	1152	475	596	15
CAGGGACAA	BRDN00012	404	547	722	872	669	61
CAGGGACAA	BRDN00011	116	226	64	111	56	11
CAGGGACAC	BRDN00011	65	86	159	186	88	0
CAGGGACCA	BRDN00011	135	154	135	193	176	6
CAGGGACCC	BRDN00011	341	432	789	537	740	13
CAGGGACCT	BRDN00012	198	61	558	352	234	22
CAGGGACGA	BRDN00012	244	231	194	459	592	50
CAGGGACGC	BRDN00012	29	35	0	161	357	0
CAGGGACGC	BRDN00012	106	111	224	184	113	9
CAGGGACTA	BRDN00012	282	208	384	506	466	19
CAGGGACTG	BRDN00012	196	61	887	305	397	11
CAGGGAGAA	BRDN00012	243	223	198	363	298	31
CAGGGAGAC	BRDN00012	62	0	0	186	77	0
CAGGGAGAC	BRDN00012	172	239	484	330	152	20
CAGGGAGCA	BRDN00012	206	218	38	331	572	0
CAGGGAGCC	BRDN00011	114	90	141	160	158	0
CAGGGAGCC	BRDN00011	302	372	254	559	779	111
CAGGGAGCC	BRDN00011	266	130	0	414	295	22
CAGGGAGGC	BRDN00012	230	132	87	530	473	34
CAGGGAGTC	BRDN00011	67	15	55	203	1	1
CAGGGAGTC	BRDN00012	108	100	335	390	552	7
CAGGGAGTC	BRDN00012	446	334	14	830	588	32
CAGGGAGTT	BRDN00011	409	324	747	616	670	3
CAGGGATAA	BRDN00011	46	3	0	115	76	0
CAGGGATAA	BRDN00011	137	91	29	192	96	7
CAGGGATAC	BRDN00011	85	66	2025	127	77	16
CAGGGATAG	BRDN00012	54	153	75	274	121	7
CAGGGATCT	BRDN00012	172	171	37	483	321	0
CAGGGATGA	BRDN00011	94	121	94	239	286	0
CAGGGCAAA	BRDN00012	366	359	431	791	696	54
CAGGGCAAC	BRDN00012	102	224	214	237	222	19
CAGGGCAAG	BRDN00011	186	123	1798	333	194	73
CAGGGCAAG	BRDN00011	367	265	1700	566	703	93
CAGGGCAAT	BRDN00012	131	211	33	271	230	43
CAGGGCAAT	BRDN00011	71	36	0	286	331	0
CAGGGCAGA	BRDN00011	75	145	516	290	615	13
CAGGGCAGC	BRDN00012	60	30	1	154	149	0
CAGGGCATG	BRDN00011	110	168	34	258	174	22
CAGGGCCAG	BRDN00011	353	290	1935	846	600	109
CAGGGCCAG	BRDN00011	145	135	650	268	232	0
CAGGGCCAT	BRDN00012	269	437	2605	446	369	84
CAGGGCCCA	BRDN00011	84	115	400	223	21	23
CAGGGCCGG	BRDN00011	286	291	177	670	528	19
CAGGGCCTC	BRDN00012	147	116	1	255	173	0
CAGGGCCTG	BRDN00011	152	171	1	355	124	19
CAGGGCCTG	BRDN00012	556	627	341	675	705	46
CAGGGCCTG	BRDN00011	157	77	2	359	186	0
CAGGGCCTG	BRDN00011	514	492	1207	751	817	50
CAGGGCGAG	BRDN00011	29	0	0	134	8	0
CAGGGCGCC	BRDN00011	306	208	366	636	439	14
CAGGGCGGA	BRDN00011	40	81	0	134	536	39
CAGGGCGGT	BRDN00012	321	681	2930	675	1098	101
CAGGGCGTA	BRDN00011	199	262	17	319	678	34
CAGGGCTAC	BRDN00011	151	47	0	216	163	0
CAGGGCTAG	BRDN00011	277	271	144	606	754	8
CAGGGCTCA	BRDN00011	78	13	125	247	662	0
CAGGGCTCA	BRDN00012	107	111	82	238	151	9
CAGGGCTCC	BRDN00012	208	135	49	274	382	15
CAGGGCTCC	BRDN00012	380	342	708	772	750	28
CAGGGCTCG	BRDN00011	116	58	1	251	416	0
CAGGGCTGA	BRDN00012	357	249	2216	722	820	62
CAGGGCTGG	BRDN00012	386	256	485	740	729	42
CAGGGCTGT	BRDN00011	74	137	442	106	44	2
CAGGGCTGT	BRDN00012	163	179	236	409	309	7
CAGGGCTGT	BRDN00011	134	70	764	216	136	0
CAGGGCTTA	BRDN00011	501	418	781	967	930	32
CAGGGCTTA	BRDN00011	136	92	33	269	279	0
CAGGGGAAC	BRDN00012	332	268	981	625	281	51
CAGGGGACA	BRDN00011	192	162	523	389	745	11
CAGGGGAGC	BRDN00012	143	92	0	326	481	0
CAGGGGAGC	BRDN00011	119	86	430	209	108	13
CAGGGGATA	BRDN00012	410	181	298	618	1397	7
CAGGGGATT	BRDN00012	251	257	138	425	530	8
CAGGGGCAC	BRDN00011	172	204	788	372	290	72
CAGGGGCAG	BRDN00012	156	88	335	250	274	34
CAGGGGCAG	BRDN00011	186	163	34	182	235	15
CAGGGGCCA	BRDN00011	311	354	92	638	869	46
CAGGGGCCT	BRDN00011	165	62	25	359	235	16
CAGGGGCCT	BRDN00012	144	108	213	247	130	0
CAGGGGCGC	BRDN00012	18	0	0	235	36	0
CAGGGGCGC	BRDN00011	117	32	9	358	197	0
CAGGGGCGC	BRDN00012	99	77	232	279	267	34
CAGGGGCTC	BRDN00012	200	156	78	407	328	16
CAGGGGCTG	BRDN00011	147	226	179	581	414	20
CAGGGGCTT	BRDN00011	74	42	343	220	914	6
CAGGGGGGC	BRDN00011	94	147	248	151	290	0
CAGGGGGCC	BRDN00012	293	285	629	606	467	27
CAGGGGGCC	BRDN00011	163	196	553	544	525	40
CAGGGGGTA	BRDN00011	175	213	1137	371	323	100
CAGGGGGTC	BRDN00011	69	133	299	230	180	5
CAGGGGTCT	BRDN00012	89	59	2	298	94	0
CAGGGGTGA	BRDN00011	496	305	1702	689	673	100
CAGGGGTGA	BRDN00011	125	25	34	172	149	5
CAGGGGTTG	BRDN00012	426	442	322	663	435	16
CAGGGTAAA	BRDN00011	24	85	9	170	162	7
CAGGGTAAA	BRDN00011	228	251	396	302	321	21
CAGGGTACA	BRDN00011	364	353	239	586	740	81
CAGGGTAGC	BRDN00012	302	300	267	680	695	14
CAGGGTATA	BRDN00012	105	37	757	212	28	19
CAGGGTCAG	BRDN00012	183	238	1974	333	364	25
CAGGGTCAG	BRDN00011	243	148	683	363	515	39
CAGGGTCAT	BRDN00012	36	20	0	211	246	0
CAGGGTCTC	BRDN00012	297	257	442	578	634	70
CAGGGTCTC	BRDN00012	278	207	677	757	571	29
CAGGGTGAA	BRDN00012	237	266	1037	320	271	44
CAGGGTGAA	BRDN00012	97	97	257	191	213	15
CAGGGTGAA	BRDN00011	128	136	22	224	99	0
CAGGGTGAC	BRDN00012	316	381	1709	454	747	108
CAGGGTGAC	BRDN00011	201	123	46	390	505	0
CAGGGTGAT	BRDN00011	173	144	19	363	303	36
CAGGGTGCA	BRDN00011	257	248	1189	398	526	30
CAGGGTGGA	BRDN00011	40	47	0	41	76	5
CAGGGTGGT	BRDN00012	172	91	907	240	144	22
CAGGGTGTA	BRDN00012	54	127	879	128	79	33
CAGGGTGTG	BRDN00012	586	336	589	633	504	56
CAGGGTTCA	BRDN00011	321	359	542	621	308	7
CAGGGTTGC	BRDN00012	502	349	619	848	954	57
CAGGGTTTG	BRDN00011	76	41	0	194	276	11
CAGGTAACG	BRDN00012	187	168	152	357	449	21
CAGGTAACT	BRDN00011	68	18	73	268	122	9
CAGGTAAGA	BRDN00012	117	109	0	230	254	0
CAGGTAAGA	BRDN00011	300	250	99	534	500	57
CAGGTAAGG	BRDN00011	192	139	0	392	300	0
CAGGTAAGG	BRDN00011	185	188	154	322	274	0
CAGGTAAGT	BRDN00011	231	112	65	414	251	21
CAGGTAAGT	BRDN00012	342	240	1178	591	215	2
CAGGTACAC	BRDN00012	72	0	0	111	14	0
CAGGTACAT	BRDN00012	168	11	0	375	58	0
CAGGTACCA	BRDN00011	185	150	117	244	351	8
CAGGTACCA	BRDN00011	279	337	535	742	590	10
CAGGTACGA	BRDN00011	146	129	261	315	249	7
CAGGTACGC	BRDN00011	63	106	35	152	142	44
CAGGTACGC	BRDN00012	317	208	4	659	458	16
CAGGTACTC	BRDN00012	115	10	6	161	271	0
CAGGTACTG	BRDN00012	112	39	261	260	29	3
CAGGTAGAA	BRDN00012	91	178	352	232	304	198
CAGGTAGAC	BRDN00011	244	223	269	433	462	118
CAGGTAGAG	BRDN00012	257	119	1369	365	204	16
CAGGTAGAG	BRDN00011	219	155	884	494	715	4
CAGGTAGAG	BRDN00012	101	193	208	276	332	19
CAGGTAGCT	BRDN00011	391	333	1119	785	1473	93
CAGGTAGGA	BRDN00011	122	160	445	342	224	10
CAGGTAGGC	BRDN00012	112	49	0	180	380	0
CAGGTAGGC	BRDN00012	166	6	26	843	398	0
CAGGTAGTC	BRDN00011	267	165	636	246	277	53
CAGGTATAA	BRDN00011	175	135	618	308	262	18
CAGGTATCA	BRDN00012	229	31	12	254	175	0
CAGGTATCC	BRDN00011	163	132	1402	369	437	45
CAGGTATGA	BRDN00011	142	189	93	250	82	15
CAGGTATGC	BRDN00011	141	225	104	248	450	7
CAGGTATGT	BRDN00011	176	198	283	335	256	5
CAGGTCAAT	BRDN00012	383	318	470	758	566	53
CAGGTCACA	BRDN00012	305	236	773	517	841	25
CAGGTCACC	BRDN00012	137	117	241	266	123	14
CAGGTCACC	BRDN00012	247	245	237	318	475	35
CAGGTCACG	BRDN00011	88	108	238	191	46	3
CAGGTCACG	BRDN00011	298	214	37	662	387	3
CAGGTCAGA	BRDN00011	131	112	639	172	160	15
CAGGTCATA	BRDN00012	226	126	33	409	252	8
CAGGTCCAG	BRDN00012	144	111	125	189	96	0
CAGGTCCAG	BRDN00012	142	91	239	223	169	24
CAGGTCCAG	BRDN00011	405	199	998	598	789	5
CAGGTCCAT	BRDN00012	27	0	1	512	163	0
CAGGTCCAT	BRDN00012	285	297	155	501	388	29
CAGGTCCAT	BRDN00011	148	113	0	160	112	25
CAGGTCCCA	BRDN00011	93	87	158	227	361	12
CAGGTCCCA	BRDN00011	236	319	29	336	392	38
CAGGTCCCA	BRDN00011	197	267	272	389	595	26
CAGGTCCCC	BRDN00012	400	251	426	569	223	43
CAGGTCCCG	BRDN00012	135	63	3	194	82	0
CAGGTCCCG	BRDN00012	512	603	1703	872	1344	63
CAGGTCCCG	BRDN00011	381	66	0	779	358	0
CAGGTCCCG	BRDN00011	91	146	132	193	128	24
CAGGTCCGA	BRDN00012	94	122	4	149	427	0
CAGGTCCTC	BRDN00011	94	126	751	220	353	30
CAGGTCCTC	BRDN00012	122	0	14	158	24	0
CAGGTCTCA	BRDN00012	317	221	366	665	691	5
CAGGTCTCC	BRDN00011	249	183	530	426	331	21
CAGGTCTCG	BRDN00012	103	0	1	434	226	0
CAGGTCTCG	BRDN00012	272	121	602	516	273	23
CAGGTCTGA	BRDN00011	352	372	271	531	734	8
CAGGTCTGC	BRDN00011	269	116	36	402	315	0
CAGGTCTGG	BRDN00011	40	34	737	256	129	6
CAGGTCTTG	BRDN00011	79	212	99	264	369	4
CAGGTGACA	BRDN00011	301	272	104	649	609	15
CAGGTGACC	BRDN00012	100	131	38	262	162	13
CAGGTGACC	BRDN00011	126	88	47	313	126	0
CAGGTGACG	BRDN00011	239	105	193	434	404	6
CAGGTGACT	BRDN00011	138	160	1201	277	274	38
CAGGTGAGA	BRDN00011	280	193	1400	468	403	20
CAGGTGAGC	BRDN00012	206	172	305	454	598	6
CAGGTGAGC	BRDN00012	266	223	174	476	246	13
CAGGTGAGC	BRDN00011	333	296	458	719	837	15
CAGGTGAGT	BRDN00011	134	90	1	211	157	0
CAGGTGATA	BRDN00011	137	143	264	269	132	3
CAGGTGATA	BRDN00012	185	150	576	435	725	0
CAGGTGCAG	BRDN00011	69	95	35	147	176	0
CAGGTGCAG	BRDN00012	154	115	212	222	299	0
CAGGTGCAT	BRDN00012	126	157	269	252	234	0
CAGGTGCCA	BRDN00011	345	82	294	786	805	13
CAGGTGCCC	BRDN00011	102	126	492	167	148	9
CAGGTGCCC	BRDN00011	195	200	499	286	316	8
CAGGTGCGA	BRDN00012	80	22	146	123	36	0
CAGGTGCGC	BRDN00012	138	175	82	156	200	0
CAGGTGCGG	BRDN00012	95	37	23	313	171	0
CAGGTGCTC	BRDN00012	90	114	269	195	135	11
CAGGTGCTG	BRDN00011	206	199	401	379	392	47
CAGGTGGAC	BRDN00012	282	325	1050	620	566	81
CAGGTGGAC	BRDN00012	186	113	219	262	282	90
CAGGTGGAC	BRDN00012	234	170	11	239	628	22
CAGGTGGAT	BRDN00011	121	34	6	101	71	0
CAGGTGGCA	BRDN00012	202	165	141	331	530	7
CAGGTGGCA	BRDN00011	8	19	0	40	9	0
CAGGTGGCA	BRDN00012	220	251	98	429	304	45
CAGGTGGCC	BRDN00012	106	125	7	251	229	0
CAGGTGGCG	BRDN00011	113	94	184	240	518	0
CAGGTGGGC	BRDN00012	117	58	4	195	98	0
CAGGTGGGC	BRDN00011	166	116	1	180	141	31
CAGGTGGGC	BRDN00011	116	102	95	194	476	20
CAGGTGGGC	BRDN00011	143	92	104	339	277	19
CAGGTGGGT	BRDN00011	275	449	321	587	594	65
CAGGTGGTA	BRDN00012	353	268	365	659	1118	15
CAGGTGGTA	BRDN00012	421	436	3572	916	660	83
CAGGTGGTA	BRDN00012	104	74	25	191	93	0
CAGGTGGTC	BRDN00011	494	647	258	734	622	51
CAGGTGGTC	BRDN00012	224	238	28	629	856	0
CAGGTGGTT	BRDN00011	190	172	598	282	241	14
CAGGTGTAG	BRDN00012	52	0	0	124	13	0
CAGGTGTAG	BRDN00012	159	103	179	277	234	0
CAGGTGTAT	BRDN00011	45	63	3	145	160	0
CAGGTGTCG	BRDN00012	157	210	177	310	247	8
CAGGTGTCT	BRDN00011	207	184	5940	432	424	88
CAGGTGTGA	BRDN00012	195	149	491	386	484	49
CAGGTGTGG	BRDN00011	142	23	0	346	195	0
CAGGTGTGT	BRDN00011	55	37	40	138	92	0
CAGGTGTTG	BRDN00012	200	121	33	358	206	4
CAGGTTAAA	BRDN00011	175	216	69	345	264	8
CAGGTTATA	BRDN00011	236	162	649	438	280	17
CAGGTTCAC	BRDN00011	251	334	134	656	971	0
CAGGTTCAC	BRDN00012	128	10	165	193	193	29
CAGGTTCAG	BRDN00011	127	55	55	167	129	13
CAGGTTCAG	BRDN00011	398	144	260	704	660	17
CAGGTTCAG	BRDN00012	114	63	99	167	247	0
CAGGTTCCC	BRDN00011	140	144	83	293	213	2
CAGGTTCGT	BRDN00012	97	79	3	170	529	0
CAGGTTCTA	BRDN00011	199	70	1	312	223	22
CAGGTTCTG	BRDN00011	78	146	481	169	344	58
CAGGTTGAC	BRDN00012	238	233	2678	292	203	31
CAGGTTGAG	BRDN00012	313	198	48	740	905	0
CAGGTTGCG	BRDN00012	59	43	57	124	43	0
CAGGTTGCG	BRDN00011	127	94	0	188	210	19
CAGGTTGCG	BRDN00011	218	194	94	495	382	1

CAGGTTGGA	BRDN0001[illegible]	46	2	428	149	85	2
CAGGTTGGC	BRDN0001[illegible]	115	91	117	253	364	7
CAGGTTGGG	BRDN0001[illegible]	419	295	136	540	774	47
CAGGTTGGT	BRDN0001[illegible]	179	160	572	227	206	11
CAGGTTGGT	BRDN0001[illegible]	434	424	882	783	482	15
CAGGTTGTC	BRDN0001[illegible]	123	57	424	244	135	15
CAGGTTGTC	BRDN0001[illegible]	475	734	5903	685	888	291
CAGGTTGTG	BRDN0001[illegible]	151	113	74	204	215	27
CAGGTTTAG	BRDN0001[illegible]	138	65	92	189	111	0
CAGGTTTAT	BRDN0001[illegible]	108	64	1	242	585	2
CAGGTTTGA	BRDN0001[illegible]	135	75	0	325	417	0
CAGGTTTGA	BRDN0001[illegible]	126	235	595	260	320	32
CAGGTTTGC	BRDN0001[illegible]	462	343	397	780	883	100
CAGTAAAAG	BRDN0001[illegible]	105	30	16	289	175	0
CAGTAAACT	BRDN0001[illegible]	235	161	267	432	288	7
CAGTAAACT	BRDN0001[illegible]	179	113	68	242	253	0
CAGTAAAGA	BRDN0001[illegible]	218	346	1175	435	1237	33
CAGTAAATT	BRDN0001[illegible]	156	122	473	249	361	1
CAGTAACCA	BRDN0001[illegible]	118	64	15	170	132	6
CAGTAACCC	BRDN0001[illegible]	381	371	465	627	387	15
CAGTAACCC	BRDN0001[illegible]	392	387	762	742	717	16
CAGTAACCCT	BRDN0001[illegible]	138	208	27	209	154	2
CAGTAACTG	BRDN0001[illegible]	241	208	1760	370	520	81
CAGTAACTT	BRDN0001[illegible]	181	140	532	352	658	26
CAGTAAGAA	BRDN0001[illegible]	263	394	406	643	398	56
CAGTAAGAC	BRDN0001[illegible]	200	76	440	321	231	11
CAGTAAGAC	BRDN0001[illegible]	161	168	541	190	163	0
CAGTAAGAT	BRDN0001[illegible]	209	242	646	246	571	46
CAGTAAGCT	BRDN0001[illegible]	116	161	221	334	185	9
CAGTAAGGT	BRDN0001[illegible]	308	123	1473	518	604	7
CAGTAAGTC	BRDN0001[illegible]	125	117	43	231	132	33
CAGTAATAA	BRDN0001[illegible]	235	177	301	375	264	43
CAGTAATAG	BRDN0001[illegible]	236	128	22	357	430	8
CAGTAATGG	BRDN0001[illegible]	103	0	0	163	78	0
CAGTAATGG	BRDN0001[illegible]	325	423	457	626	499	77
CAGTACAAC	BRDN0001[illegible]	158	93	28	235	203	0
CAGTACAGA	BRDN0001[illegible]	364	431	940	498	734	43
CAGTACCAA	BRDN0001[illegible]	412	472	2192	809	480	71
CAGTACCAT	BRDN0001[illegible]	327	434	7447	623	1005	114
CAGTACCAT	BRDN0001[illegible]	184	156	20	206	279	84
CAGTACCCA	BRDN0001[illegible]	375	327	574	660	677	45
CAGTACCTCC	BRDN0001[illegible]	176	217	395	324	231	8
CAGTACCTTA	BRDN0001[illegible]	99	99	726	194	98	29
CAGTACTAG	BRDN0001[illegible]	211	231	189	393	429	10
CAGTACTCA	BRDN0001[illegible]	555	479	655	748	992	71
CAGTACTCTT	BRDN0001[illegible]	252	229	203	372	599	72
CAGTACTGC	BRDN0001[illegible]	150	84	40	345	351	4
CAGTACTGCT	BRDN0001[illegible]	309	298	93	776	779	28
CAGTACTTA	BRDN0001[illegible]	177	256	485	370	278	106
CAGTAGAAG	BRDN0001[illegible]	92	167	16	182	108	41
CAGTAGACA	BRDN0001[illegible]	254	228	560	449	1099	19
CAGTAGACC	BRDN0001[illegible]	314	169	60	544	232	69
CAGTAGATA	BRDN0001[illegible]	180	182	308	341	158	0
CAGTAGATG	BRDN0001[illegible]	157	91	29	274	267	3
CAGTAGATT	BRDN0001[illegible]	140	99	18	161	187	0
CAGTAGCAA	BRDN0001[illegible]	114	76	7	219	400	6
CAGTAGCAC	BRDN0001[illegible]	124	91	1	202	287	0
CAGTAGCAC	BRDN0001[illegible]	120	116	496	190	291	80
CAGTAGCAT	BRDN0001[illegible]	238	218	52	171	108	24
CAGTAGCGA	BRDN0001[illegible]	392	494	322	508	288	41
CAGTAGCTA	BRDN0001[illegible]	122	33	1	342	360	6
CAGTAGGCA	BRDN0001[illegible]	311	272	558	764	1120	11
CAGTAGGGT	BRDN0001[illegible]	97	156	217	153	138	29
CAGTAGTAG	BRDN0001[illegible]	230	331	429	490	631	72
CAGTATAAG	BRDN0001[illegible]	211	291	65	455	627	7
CAGTATACA	BRDN0001[illegible]	271	189	696	554	643	19
CAGTATAGC	BRDN0001[illegible]	142	188	353	236	394	29
CAGTATAGG	BRDN0001[illegible]	28	69	13	121	30	10
CAGTATCAG	BRDN0001[illegible]	459	310	325	668	686	0
CAGTATCAT	BRDN0001[illegible]	118	118	84	354	332	9
CAGTATCATT	BRDN0001[illegible]	85	101	0	181	218	0
CAGTATCCA	BRDN0001[illegible]	260	296	1656	478	1021	119
CAGTATCTCA	BRDN0001[illegible]	118	124	95	305	188	9
CAGTATGAG	BRDN0001[illegible]	160	122	212	286	86	0
CAGTATGAG	BRDN0001[illegible]	442	474	326	706	942	24
CAGTATGAG	BRDN0001[illegible]	16	1	0	241	17	0
CAGTATGCA	BRDN0001[illegible]	90	217	72	165	268	12
CAGTATGCA	BRDN0001[illegible]	137	138	188	358	388	0
CAGTATGTC	BRDN0001[illegible]	267	196	386	540	451	44
CAGTATGTCT	BRDN0001[illegible]	214	365	197	478	549	38
CAGTATGTT	BRDN0001[illegible]	250	338	1393	363	234	46
CAGTATTAG	BRDN0001[illegible]	135	113	64	211	136	0
CAGTATTCA	BRDN0001[illegible]	305	186	66	403	511	42
CAGTCAAAC	BRDN0001[illegible]	71	7	0	95	82	0
CAGTCAAAG	BRDN0001[illegible]	155	111	856	226	286	29
CAGTCAACA	BRDN0001[illegible]	316	234	0	378	207	0
CAGTCAACT	BRDN0001[illegible]	84	19	0	668	387	1
CAGTCAAGG	BRDN0001[illegible]	272	338	192	619	599	8
CAGTCACAA	BRDN0001[illegible]	271	270	222	563	521	0
CAGTCACAC	BRDN0001[illegible]	194	154	346	447	588	9
CAGTCACAC	BRDN0001[illegible]	532	382	1463	809	876	39
CAGTCACAG	BRDN0001[illegible]	223	192	475	638	398	45
CAGTCACAG	BRDN0001[illegible]	338	318	224	566	827	17
CAGTCACAG	BRDN0001[illegible]	164	103	155	195	160	0
CAGTCACCA	BRDN0001[illegible]	60	15	374	683	411	8
CAGTCACCCA	BRDN0001[illegible]	225	167	81	536	616	12
CAGTCACTCA	BRDN0001[illegible]	99	6	13	313	171	0
CAGTCAGAC	BRDN0001[illegible]	196	196	127	258	162	0
CAGTCAGAG	BRDN0001[illegible]	162	209	908	280	1474	12
CAGTCAGGA	BRDN0001[illegible]	380	329	124	595	662	20
CAGTCAGGG	BRDN0001[illegible]	96	157	0	385	383	6
CAGTCAGGT	BRDN0001[illegible]	105	109	11	249	290	0
CAGTCATAG	BRDN0001[illegible]	398	371	80	642	1162	40
CAGTCATCG	BRDN0001[illegible]	115	76	1249	193	100	36
CAGTCATGA	BRDN0001[illegible]	139	220	352	388	503	5
CAGTCATGG	BRDN0001[illegible]	392	313	736	730	778	9
CAGTCATTCC	BRDN0001[illegible]	368	191	680	539	520	0
CAGTCATTG	BRDN0001[illegible]	173	143	528	416	459	2
CAGTCCAAA	BRDN0001[illegible]	108	215	847	281	275	33
CAGTCCAAG	BRDN0001[illegible]	101	95	1042	197	349	22
CAGTCCAAG	BRDN0001[illegible]	179	47	11	707	486	2
CAGTCCACG	BRDN0001[illegible]	157	197	39	311	277	0
CAGTCCAGG	BRDN0001[illegible]	139	134	122	372	253	12
CAGTCCATG	BRDN0001[illegible]	171	153	38	302	390	22
CAGTCCCAA	BRDN0001[illegible]	380	301	2733	763	598	37
CAGTCCCACT	BRDN0001[illegible]	403	166	915	892	625	18
CAGTCCCAG	BRDN0001[illegible]	172	106	700	382	307	18
CAGTCCCAG	BRDN0001[illegible]	625	455	1493	1010	937	65
CAGTCCCCA	BRDN0001[illegible]	403	193	1256	826	627	36
CAGTCCCCTG	BRDN0001[illegible]	81	64	250	167	108	0
CAGTCCCTA	BRDN0001[illegible]	296	275	557	596	781	55
CAGTCCCTCA	BRDN0001[illegible]	170	208	396	566	438	2
CAGTCCCTG	BRDN0001[illegible]	161	31	96	206	223	1
CAGTCCGAA	BRDN0001[illegible]	87	183	104	269	220	7
CAGTCCGAG	BRDN0001[illegible]	229	245	342	430	244	0
CAGTCCGAT	BRDN0001[illegible]	207	110	14	270	196	18
CAGTCCGCA	BRDN0001[illegible]	218	224	347	438	272	17
CAGTCCGGC	BRDN0001[illegible]	258	301	2513	484	343	40
CAGTCCGGT	BRDN0001[illegible]	105	142	69	179	94	15
CAGTCCTAG	BRDN0001[illegible]	440	529	1307	883	1382	83
CAGTCCTAG	BRDN0001[illegible]	437	273	1475	659	634	313
CAGTCCTATA	BRDN0001[illegible]	125	73	0	143	199	0
CAGTCCTCTC	BRDN0001[illegible]	277	246	106	454	481	1
CAGTCCTGT	BRDN0001[illegible]	66	89	0	232	233	0
CAGTCCTTCT	BRDN0001[illegible]	354	302	1433	672	608	22
CAGTCGGGC	BRDN0001[illegible]	173	102	240	261	392	5
CAGTCGGGG	BRDN0001[illegible]	60	57	39	182	49	8
CAGTCGGTC	BRDN0001[illegible]	190	184	685	404	162	37
CAGTCGTAA	BRDN0001[illegible]	118	141	598	362	218	41
CAGTCGTGA	BRDN0001[illegible]	205	184	650	293	400	36
CAGTCGTGG	BRDN0001[illegible]	129	168	0	297	287	0
CAGTCGTTT	BRDN0001[illegible]	502	477	1323	1113	873	148
CAGTCTAAA	BRDN0001[illegible]	283	291	582	516	334	11
CAGTCTAAC	BRDN0001[illegible]	86	101	348	331	404	22
CAGTCTAACT	BRDN0001[illegible]	373	284	1492	365	291	70
CAGTCTAAG	BRDN0001[illegible]	286	292	122	410	578	64
CAGTCTAGC	BRDN0001[illegible]	34	40	72	81	21	8
CAGTCTATA	BRDN0001[illegible]	240	310	1102	569	467	6
CAGTCTATA	BRDN0001[illegible]	203	131	80	296	387	10
CAGTCTCAA	BRDN0001[illegible]	426	386	372	849	1047	158
CAGTCTCATC	BRDN0001[illegible]	339	446	778	771	1362	51
CAGTCTCCAT	BRDN0001[illegible]	123	111	208	232	277	19
CAGTCTCCAT	BRDN0001[illegible]	226	240	147	725	1128	0
CAGTCTCTG	BRDN0001[illegible]	65	48	590	214	86	14
CAGTCTGAA	BRDN0001[illegible]	201	155	65	290	331	15
CAGTCTGACT	BRDN0001[illegible]	213	250	966	373	633	100
CAGTCTGAT	BRDN0001[illegible]	146	94	36	233	272	0
CAGTCTGCT	BRDN0001[illegible]	187	174	29	504	722	0
CAGTCTGGC	BRDN0001[illegible]	315	387	2426	582	556	42
CAGTCTGGG	BRDN0001[illegible]	152	98	46	273	121	4
CAGTCTGTG	BRDN0001[illegible]	172	100	240	287	50	24
CAGTCTGTG	BRDN0001[illegible]	138	111	106	309	172	8
CAGTCTGTG	BRDN0001[illegible]	403	211	516	810	1009	81
CAGTCTGTT	BRDN0001[illegible]	298	102	159	574	467	18
CAGTCTTCAG	BRDN0001[illegible]	340	270	175	503	684	17
CAGTCTTCAG	BRDN0001[illegible]	146	102	520	266	196	23
CAGTGAAAA	BRDN0001[illegible]	23	0	0	229	40	0
CAGTGAAAG	BRDN0001[illegible]	242	212	160	371	262	21
CAGTGAAAT	BRDN0001[illegible]	120	40	9	219	108	0
CAGTGAAGG	BRDN0001[illegible]	142	137	0	275	239	17
CAGTGACAG	BRDN0001[illegible]	136	68	58	304	226	18
CAGTGACCC	BRDN0001[illegible]	171	185	12	370	431	0
CAGTGACCG	BRDN0001[illegible]	138	174	315	213	142	0
CAGTGACCT	BRDN0001[illegible]	118	126	89	226	169	13
CAGTGACGA	BRDN0001[illegible]	66	2	142	212	200	8
CAGTGACGG	BRDN0001[illegible]	290	201	2035	538	893	32
CAGTGACGT	BRDN0001[illegible]	269	311	28	652	600	12
CAGTGACGT	BRDN0001[illegible]	270	171	656	771	816	10
CAGTGACTA	BRDN0001[illegible]	364	280	2636	780	515	50
CAGTGACTC	BRDN0001[illegible]	87	75	0	159	229	0
CAGTGACTG	BRDN0001[illegible]	203	205	220	460	649	9
CAGTGACTG	BRDN0001[illegible]	172	149	646	344	517	36
CAGTGAGAC	BRDN0001[illegible]	97	39	558	86	139	8
CAGTGAGAC	BRDN0001[illegible]	130	131	283	330	155	36
CAGTGAGAG	BRDN0001[illegible]	116	133	0	207	244	12
CAGTGAGAT	BRDN0001[illegible]	183	90	407	184	43	1
CAGTGAGAT	BRDN0001[illegible]	136	184	390	324	396	32
CAGTGAGCG	BRDN0001[illegible]	242	70	1512	269	243	36
CAGTGAGCT	BRDN0001[illegible]	390	264	1638	791	823	51
CAGTGAGGA	BRDN0001[illegible]	154	189	133	176	148	10
CAGTGAGGT	BRDN0001[illegible]	207	56	716	451	532	15
CAGTGAGTA	BRDN0001[illegible]	117	50	0	709	174	0
CAGTGAGTG	BRDN0001[illegible]	178	302	1829	271	227	26
CAGTGAGTG	BRDN0001[illegible]	118	198	672	223	270	4
CAGTGATAA	BRDN0001[illegible]	208	156	581	387	575	12
CAGTGATCC	BRDN0001[illegible]	157	171	72	377	363	0
CAGTGATGA	BRDN0001[illegible]	363	310	273	882	862	96
CAGTGATGA	BRDN0001[illegible]	190	54	67	256	150	0
CAGTGATGA	BRDN0001[illegible]	171	157	70	421	319	6
CAGTGATGG	BRDN0001[illegible]	186	207	48	259	259	0
CAGTGATGT	BRDN0001[illegible]	199	95	75	301	323	0
CAGTGATGT	BRDN0001[illegible]	123	74	19	412	150	0
CAGTGCAAG	BRDN0001[illegible]	176	270	96	554	329	16
CAGTGCAAT	BRDN0001[illegible]	186	0	1	321	342	0
CAGTGCACA	BRDN0001[illegible]	538	456	610	1089	1329	120
CAGTGCAGA	BRDN0001[illegible]	237	57	0	307	321	0
CAGTGCAGG	BRDN0001[illegible]	101	181	678	211	203	223
CAGTGCAGG	BRDN0001[illegible]	435	423	1858	954	648	125
CAGTGCATA	BRDN0001[illegible]	162	40	29	275	84	0
CAGTGCATC	BRDN0001[illegible]	133	184	4	266	51	57
CAGTGCATG	BRDN0001[illegible]	508	506	669	919	1235	43
CAGTGCCAA	BRDN0001[illegible]	449	410	706	790	559	32
CAGTGCCAA	BRDN0001[illegible]	158	74	0	298	237	17
CAGTGCCAA	BRDN0001[illegible]	377	541	981	651	967	9
CAGTGCCAC	BRDN0001[illegible]	389	386	734	751	871	39
CAGTGCCAC	BRDN0001[illegible]	213	309	293	400	654	78
CAGTGCCAG	BRDN0001[illegible]	119	26	171	125	48	16
CAGTGCCAG	BRDN0001[illegible]	241	164	15	343	226	26
CAGTGCCCA	BRDN0001[illegible]	155	165	2055	316	199	36
CAGTGCCCA	BRDN0001[illegible]	110	121	939	175	210	13
CAGTGCCCA	BRDN0001[illegible]	176	46	0	304	205	34
CAGTGCCCA	BRDN0001[illegible]	78	77	566	189	75	11
CAGTGCCCC	BRDN0001[illegible]	176	262	1576	269	273	51
CAGTGCCCG	BRDN0001[illegible]	173	79	887	663	271	24
CAGTGCCCG	BRDN0001[illegible]	333	234	104	579	349	4
CAGTGCCGC	BRDN0001[illegible]	279	293	34	461	944	4
CAGTGCCTC	BRDN0001[illegible]	75	95	383	113	228	9
CAGTGCGAC	BRDN0001[illegible]	320	336	435	579	795	110
CAGTGCGAC	BRDN0001[illegible]	82	147	83	240	124	0
CAGTGCGAT	BRDN0001[illegible]	110	50	0	302	188	0
CAGTGCGCT	BRDN0001[illegible]	266	307	2751	494	471	44
CAGTGCGGA	BRDN0001[illegible]	134	106	1119	367	233	15
CAGTGCGTG	BRDN0001[illegible]	240	218	55	312	294	37
CAGTGCTAA	BRDN0001[illegible]	105	73	2264	274	322	30
CAGTGCTAC	BRDN0001[illegible]	188	135	2055	312	314	26
CAGTGCTAG	BRDN0001[illegible]	41	35	28	281	262	0
CAGTGCTAG	BRDN0001[illegible]	126	181	54	396	515	8
CAGTGCTCA	BRDN0001[illegible]	209	149	152	472	724	29
CAGTGCTGA	BRDN0001[illegible]	133	15	1	240	57	0
CAGTGCTGA	BRDN0001[illegible]	320	227	910	712	586	13
CAGTGCTGC	BRDN0001[illegible]	353	245	423	790	711	41
CAGTGCTTC	BRDN0001[illegible]	143	143	69	246	108	21
CAGTGCTTG	BRDN0001[illegible]	444	564	1492	1009	1345	43
CAGTGCTTG	BRDN0001[illegible]	91	58	1365	143	192	27
CAGTGCTTT	BRDN0001[illegible]	195	189	739	298	281	50
CAGTGCTTT	BRDN0001[illegible]	179	116	690	216	241	0
CAGTGGAAG	BRDN0001[illegible]	107	42	786	253	381	4
CAGTGGACA	BRDN0001[illegible]	203	250	267	566	862	5
CAGTGGACG	BRDN0001[illegible]	519	490	1539	848	1094	42
CAGTGGACT	BRDN0001[illegible]	68	110	49	109	103	0
CAGTGGAGC	BRDN0001[illegible]	280	352	226	672	554	43
CAGTGGATT	BRDN0001[illegible]	219	167	1611	481	674	16
CAGTGGATT	BRDN0001[illegible]	65	28	1	248	139	0
CAGTGGCAC	BRDN0001[illegible]	180	183	149	225	328	7
CAGTGGCAG	BRDN0001[illegible]	122	50	41	203	189	0
CAGTGGCAG	BRDN0001[illegible]	255	306	1783	469	379	78
CAGTGGCCA	BRDN0001[illegible]	98	78	0	191	151	8
CAGTGGCCC	BRDN0001[illegible]	421	304	2491	736	861	39
CAGTGGCCG	BRDN0001[illegible]	145	75	161	346	278	12
CAGTGGCGC	BRDN0001[illegible]	101	101	126	296	445	13
CAGTGGCGC	BRDN0001[illegible]	88	99	33	236	203	7
CAGTGGCGG	BRDN0001[illegible]	35	34	632	146	71	9
CAGTGGCTC	BRDN0001[illegible]	38	0	0	136	58	0
CAGTGGCTG	BRDN0001[illegible]	159	73	149	319	402	11
CAGTGGGAA	BRDN0001[illegible]	174	109	391	255	137	19
CAGTGGGAC	BRDN0001[illegible]	279	172	901	407	487	27
CAGTGGGGA	BRDN0001[illegible]	158	114	215	322	143	0
CAGTGGGGC	BRDN0001[illegible]	78	136	13	193	260	9
CAGTGGGGC	BRDN0001[illegible]	185	187	123	358	162	0
CAGTGGGTA	BRDN0001[illegible]	157	174	274	332	252	72
CAGTGGGTT	BRDN0001[illegible]	219	261	229	443	386	35
CAGTGGTAA	BRDN0001[illegible]	325	275	760	484	415	28
CAGTGGTGC	BRDN0001[illegible]	57	22	42	90	218	1
CAGTGGTGC	BRDN0001[illegible]	444	341	1870	976	787	93
CAGTGGTGG	BRDN0001[illegible]	370	259	360	575	616	33
CAGTGGTTG	BRDN0001[illegible]	131	74	36	284	218	2
CAGTGGTTG	BRDN0001[illegible]	92	98	248	167	202	25
CAGTGTAAG	BRDN0001[illegible]	151	220	3176	229	148	94
CAGTGTACT	BRDN0001[illegible]	266	227	75	544	575	34
CAGTGTATA	BRDN0001[illegible]	393	238	137	477	411	0
CAGTGTCAA	BRDN0001[illegible]	161	72	268	269	274	0
CAGTGTCACT	BRDN0001[illegible]	62	23	178	199	172	0
CAGTGTCAG	BRDN0001[illegible]	62	50	108	155	148	12
CAGTGTCAG	BRDN0001[illegible]	145	173	105	352	221	15
CAGTGTCCC	BRDN0001[illegible]	244	250	42	346	440	11
CAGTGTCCC	BRDN0001[illegible]	87	135	0	230	152	20
CAGTGTCCG	BRDN0001[illegible]	433	437	476	668	865	23
CAGTGTCGC	BRDN0001[illegible]	119	309	271	342	347	31
CAGTGTCGT	BRDN0001[illegible]	302	237	10554	575	332	95
CAGTGTCTC	BRDN0001[illegible]	170	117	210	347	471	3
CAGTGTCTT	BRDN0001[illegible]	215	252	730	291	336	25
CAGTGTGAA	BRDN0001[illegible]	85	68	0	124	34	0
CAGTGTGAG	BRDN0001[illegible]	194	210	136	382	665	0
CAGTGTGCT	BRDN0001[illegible]	85	15	135	399	324	0
CAGTGTGGG	BRDN0001[illegible]	400	353	151	497	557	11
CAGTGTGTA	BRDN0001[illegible]	259	339	703	354	301	0
CAGTGTTAA	BRDN0001[illegible]	59	84	455	184	376	6
CAGTGTTAA	BRDN0001[illegible]	217	60	45	530	605	15
CAGTGTTCA	BRDN0001[illegible]	236	263	470	370	409	9
CAGTGTTCA	BRDN0001[illegible]	37	24	0	208	117	0
CAGTGTTCA	BRDN0001[illegible]	131	66	0	243	299	1
CAGTGTTCT	BRDN0001[illegible]	208	232	89	383	744	30
CAGTGTTGA	BRDN0001[illegible]	305	237	430	570	612	76
CAGTTAAAA	BRDN0001[illegible]	38	20	92	252	280	0
CAGTTAAAT	BRDN0001[illegible]	439	361	727	591	957	35
CAGTTAACA	BRDN0001[illegible]	238	223	605	543	408	30
CAGTTAACA	BRDN0001[illegible]	160	255	175	325	528	27
CAGTTAACA	BRDN0001[illegible]	155	32	34	429	369	0
CAGTTACAC	BRDN0001[illegible]	153	214	427	274	218	7
CAGTTACAG	BRDN0001[illegible]	228	242	80	363	294	32
CAGTTACAG	BRDN0001[illegible]	186	168	168	371	473	11
CAGTTACCA	BRDN0001[illegible]	246	88	196	405	1573	0
CAGTTACCTT	BRDN0001[illegible]	266	107	66	466	370	0
CAGTTAGCG	BRDN0001[illegible]	330	206	376	581	1094	14
CAGTTAGTG	BRDN0001[illegible]	363	263	441	488	322	25
CAGTTATAA	BRDN0001[illegible]	177	215	82	381	199	5
CAGTTATAT	BRDN0001[illegible]	132	103	58	205	264	0
CAGTTATCA	BRDN0001[illegible]	83	0	0	267	239	0
CAGTTATCTT	BRDN0001[illegible]	268	203	618	420	237	18
CAGTTATTCA	BRDN0001[illegible]	72	58	259	116	77	11
CAGTTCAAA	BRDN0001[illegible]	232	112	55	336	281	39
CAGTTCAAC	BRDN0001[illegible]	339	104	14	689	822	5
CAGTTCAACT	BRDN0001[illegible]	161	61	0	438	198	34
CAGTTCACA	BRDN0001[illegible]	149	77	58	271	175	14
CAGTTCACTA	BRDN0001[illegible]	246	253	156	521	625	25
CAGTTCAGG	BRDN0001[illegible]	74	8	0	187	146	12
CAGTTCATCC	BRDN0001[illegible]	149	0	0	491	182	0
CAGTTCCAAT	BRDN0001[illegible]	134	57	35	224	372	0
CAGTTCCAG	BRDN0001[illegible]	323	35	120	806	495	8
CAGTTCCCAG	BRDN0001[illegible]	102	154	324	220	385	26
CAGTTCCCAG	BRDN0001[illegible]	467	426	344	858	552	100
CAGTTCCCG	BRDN0001[illegible]	133	167	1094	232	255	81
CAGTTCCCTG	BRDN0001[illegible]	179	201	471	281	301	13
CAGTTCCGA	BRDN0001[illegible]	431	179	359	966	507	22
CAGTTCCTGT	BRDN0001[illegible]	223	173	115	226	305	1
CAGTTCCTTA	BRDN0001[illegible]	96	42	177	215	240	0
CAGTTCGTT	BRDN0001[illegible]	254	254	246	351	324	12
CAGTTCTCCA	BRDN0001[illegible]	569	151	999	900	1314	57
CAGTTCTCTT	BRDN0001[illegible]	241	212	286	451	556	18
CAGTTCTGA	BRDN0001[illegible]	108	0	0	188	86	0
CAGTTCTGA	BRDN0001[illegible]	302	295	46	431	591	21
CAGTTCTGC	BRDN0001[illegible]	427	255	291	623	487	49
CAGTTCTGCT	BRDN0001[illegible]	199	204	1102	204	441	19
CAGTTCTGG	BRDN0001[illegible]	254	209	299	486	629	22
CAGTTGAAG	BRDN0001[illegible]	82	10	0	277	115	0
CAGTTGACT	BRDN0001[illegible]	40	9	0	223	240	0
CAGTTGATG	BRDN0001[illegible]	252	145	241	313	311	25
CAGTTGCAG	BRDN0001[illegible]	244	151	1217	342	223	16
CAGTTGCCA	BRDN0001[illegible]	53	11	183	90	308	0
CAGTTGCCA	BRDN0001[illegible]	244	180	195	520	553	3
CAGTTGCGA	BRDN0001[illegible]	127	73	58	195	321	14
CAGTTGCTA	BRDN0001[illegible]	382	365	657	664	500	49
CAGTTGCTA	BRDN0001[illegible]	242	14	172	454	1303	0
CAGTTGCTG	BRDN0001[illegible]	407	345	372	780	596	26
CAGTTGGAA	BRDN0001[illegible]	85	36	0	420	823	0
CAGTTGGAA	BRDN0001[illegible]	333	335	104	719	712	6
CAGTTGGCC	BRDN0001[illegible]	247	222	831	442	847	41
CAGTTGGCT	BRDN0001[illegible]	638	384	1471	1027	973	30
CAGTTGGGC	BRDN0001[illegible]	80	98	79	157	56	38
CAGTTGGGT	BRDN0001[illegible]	241	249	710	426	289	10
CAGTTGGGT	BRDN0001[illegible]	186	116	65	570	460	25
CAGTTGGTA	BRDN0001[illegible]	233	70	1	225	293	5
CAGTTGTAG	BRDN0001[illegible]	119	72	17	323	806	0
CAGTTGTAG	BRDN0001[illegible]	135	75	24	286	105	0
CAGTTGTAT	BRDN0001[illegible]	50	65	100	150	130	0
CAGTTTAGC	BRDN0001[illegible]	117	233	464	317	525	49
CAGTTTAGG	BRDN0001[illegible]	301	213	494	511	410	42
CAGTTTATA	BRDN0001[illegible]	87	71	5	164	150	29
CAGTTTATC	BRDN0001[illegible]	200	161	214	396	480	27
CAGTTTCAG	BRDN0001[illegible]	296	149	161	492	502	15
CAGTTTCAG	BRDN0001[illegible]	97	29	233	237	170	10
CAGTTTCCAT	BRDN0001[illegible]	295	340	1075	649	877	29
CAGTTTCGCT	BRDN0001[illegible]	149	86	32	307	185	22
CAGTTTGAA	BRDN0001[illegible]	203	174	152	304	239	0
CAGTTTGAT	BRDN0001[illegible]	447	369	292	762	465	0
CAGTTTGAT	BRDN0001[illegible]	158	59	484	133	220	7
CAGTTTGCA	BRDN0001[illegible]	152	65	22	247	352	0
CAGTTTGCG	BRDN0001[illegible]	166	79	103	326	546	0
CAGTTTGCT	BRDN0001[illegible]	141	180	1343	225	290	26
CAGTTTGGG	BRDN0001[illegible]	103	99	413	198	184	8
CAGTTTGGT	BRDN0001[illegible]	134	142	623	314	190	5
CAGTTTGGT	BRDN0001[illegible]	163	0	0	430	394	0
CAGTTTGTC	BRDN0001[illegible]	337	242	381	798	646	41
CATAAAAAA	BRDN0001[illegible]	49	24	2	187	85	0
CATAAAAAT	BRDN0001[illegible]	51	24	0	112	86	0
CATAAAACC	BRDN0001[illegible]	405	513	222	814	607	36
CATAAAACT	BRDN0001[illegible]	170	162	115	328	284	7
CATAAAAGT	BRDN0001[illegible]	207	197	260	400	484	29
CATAAACACT	BRDN0001[illegible]	238	314	1080	495	463	23
CATAAACGCT	BRDN0001[illegible]	125	61	1398	264	155	3
CATAAACTG	BRDN0001[illegible]	129	132	289	263	275	11
CATAAATCG	BRDN0001[illegible]	244	174	43	512	469	2
CATAAATCTC	BRDN0001[illegible]	22	0	0	228	144	0
CATAACAAG	BRDN0001[illegible]	102	137	466	291	128	36
CATAACAAG	BRDN0001[illegible]	196	149	432	359	291	14
CATAACAGT	BRDN0001[illegible]	195	157	194	287	372	3
CATAACGAC	BRDN0001[illegible]	86	52	93	207	45	6
CATAACGCT	BRDN0001[illegible]	26	26	74	217	92	0
CATAACGGT	BRDN0001[illegible]	319	411	662	513	415	86
CATAACTATC	BRDN0001[illegible]	81	71	0	252	388	0
CATAACTGA	BRDN0001[illegible]	105	95	605	192	283	43
CATAACTGG	BRDN0001[illegible]	113	120	16	227	310	17
CATAAGCTG	BRDN0001[illegible]	156	133	1	219	273	30
CATAAGCTG	BRDN0001[illegible]	220	156	2581	342	307	66
CATAAGGAG	BRDN0001[illegible]	169	88	294	414	353	0
CATAAGGTT	BRDN0001[illegible]	414	469	3769	745	983	149
CATAAGTGG	BRDN0001[illegible]	180	113	1332	367	315	16
CATAATCAT	BRDN0001[illegible]	274	271	10	506	381	25
CATAATCCAT	BRDN0001[illegible]	530	469	1814	891	603	37
CATAATCCCT	BRDN0001[illegible]	90	83	239	277	181	6
CATAATCGT	BRDN0001[illegible]	380	247	1879	773	453	30
CATAATCTCA	BRDN0001[illegible]	213	193	627	348	432	33
CATAATGCT	BRDN0001[illegible]	162	137	14	271	223	17
CATAATGTA	BRDN0001[illegible]	172	168	63	233	159	2
CATAATTGC	BRDN0001[illegible]	226	102	0	274	402	0
CATACAAAG	BRDN0001[illegible]	267	267	44	405	515	35
CATACAACA	BRDN0001[illegible]	151	155	782	358	408	9
CATACAACA	BRDN0001[illegible]	202	119	9	408	618	13
CATACAACA	BRDN0001[illegible]	117	161	1	252	193	7
CATACAATG	BRDN0001[illegible]	111	102	0	212	156	0
CATACACAA	BRDN0001[illegible]	149	53	122	397	928	14
CATACACAG	BRDN0001[illegible]	218	150	1770	468	292	58
CATACACAT	BRDN0001[illegible]	65	0	0	183	151	0
CATACACCAT	BRDN0001[illegible]	90	116	254	226	131	25
CATACACCCA	BRDN0001[illegible]	279	237	60	608	416	22
CATACACCTG	BRDN0001[illegible]	63	0	1	697	202	0
CATACACGA	BRDN0001[illegible]	128	143	210	208	664	22
CATACACGT	BRDN0001[illegible]	294	381	357	538	545	29
CATACACTTC	BRDN0001[illegible]	208	268	186	420	358	61
CATACAGAA	BRDN0001[illegible]	321	348	1356	797	457	52
CATACAGCA	BRDN0001[illegible]	331	243	738	539	493	76
CATACAGCG	BRDN0001[illegible]	156	43	77	225	225	12
CATACAGCG	BRDN0001[illegible]	69	63	2159	317	85	32
CATACAGTG	BRDN0001[illegible]	318	219	862	525	305	35
CATACATGA	BRDN0001[illegible]	204	118	268	474	684	16
CATACATGG	BRDN0001[illegible]	166	319	49	397	191	18
CATACCAATA	BRDN0001[illegible]	194	150	124	351	312	6
CATACCAATT	BRDN0001[illegible]	96	101	484	267	52	25
CATACCACAT	BRDN0001[illegible]	141	182	51	201	207	19
CATACCATGA	BRDN0001[illegible]	121	196	93	331	242	22
CATACCATGA	BRDN0001[illegible]	249	244	447	560	529	0
CATACCATG	BRDN0001[illegible]	208	98	319	479	512	11
CATACCATTG	BRDN0001[illegible]	238	381	66	629	341	0
CATACCCAG	BRDN0001[illegible]	200	163	58	399	406	9
CATACCCGTC	BRDN0001[illegible]	172	133	209	476	311	17
CATACCCTGT	BRDN0001[illegible]	166	69	846	293	208	21
CATACCGAG	BRDN0001[illegible]	162	66	373	194	121	11
CATACCGTCT	BRDN0001[illegible]	97	65	0	158	114	0
CATACCTATC	BRDN0001[illegible]	130	87	105	273	347	21
CATACCTCGA	BRDN0001[illegible]	81	20	0	254	149	0
CATACCTGG	BRDN0001[illegible]	348	440	277	754	594	17
CATACCTGTA	BRDN0001[illegible]	84	125	396	301	268	0
CATACCTGTT	BRDN0001[illegible]	90	179	168	210	191	76
CATACCTTAT	BRDN0001[illegible]	298	229	16	618	623	13
CATACCTTTA	BRDN0001[illegible]	110	115	146	256	256	55

CATACGACTT BRDN00011	164	170	432	442	428	28
CATACGATTC BRDN00011	83	39	0	142	225	0
CATACGCGG BRDN00011	44	74	28	47	188	19
CATACGCGTA BRDN00011	123	0	2	411	167	0
CATACGCGTA BRDN00011	112	12	0	406	295	0
CATACGGAC BRDN00011	17	45	1	144	0	0
CATACGGCA BRDN00012	143	111	0	350	331	0
CATACGGCG BRDN00012	176	41	1459	422	258	29
CATACGGGC BRDN00012	238	38	10	375	59	0
CATACTACAG BRDN00012	163	82	0	270	333	0
CATACTACGA BRDN00011	392	386	958	720	836	45
CATACTACTA BRDN00011	145	99	250	337	265	0
CATACTACTA BRDN00011	201	213	104	321	755	17
CATACTAGA BRDN00012	79	62	69	221	18	3
CATACTAGG BRDN00011	68	87	339	288	142	12
CATACTCAAA BRDN00011	204	218	787	467	506	6
CATACTCAAC BRDN00011	112	98	573	132	182	18
CATACTCAAG BRDN00011	237	168	21	419	580	1
CATACTCACA BRDN00012	180	268	126	320	417	17
CATACTCATG BRDN00011	169	164	70	462	491	8
CATACTCCCC BRDN00012	179	93	101	258	90	71
CATACTCCTT BRDN00011	171	165	51	345	249	0
CATACTCGGA BRDN00012	130	131	545	180	102	25
CATACTCTAT BRDN00011	248	455	1071	373	344	13
CATACTGACC BRDN00012	259	211	388	658	610	14
CATACTGAG BRDN00012	237	221	43860	353	311	313
CATACTGGA BRDN00011	211	116	715	363	365	52
CATACTGTGA BRDN00012	141	210	540	266	244	8
CATACTTACG BRDN00012	331	196	407	559	736	57
CATACTTAGC BRDN00012	128	213	17	228	281	8
CATACTTAGC BRDN00011	160	104	1	249	64	7
CATACTTCAA BRDN00011	375	300	1780	550	544	74
CATACTTCGA BRDN00011	253	272	93	428	246	25
CATACTTCTC BRDN00011	303	92	0	690	469	1
CATACTTCTG BRDN00011	227	109	90	340	316	0
CATACTTGTA BRDN00012	248	207	38	465	240	5
CATACTTGTC BRDN00011	204	137	17	444	472	23
CATAGAAAA BRDN00012	137	56	18	181	75	0
CATAGAATTC BRDN00012	204	256	423	398	669	22
CATAGACAA BRDN00012	89	33	1	262	128	0
CATAGACCAC BRDN00012	80	85	33	227	240	66
CATAGACGA BRDN00011	270	236	1696	538	533	23
CATAGACGG BRDN00011	60	133	437	233	270	6
CATAGACTTC BRDN00011	277	259	270	604	823	1
CATAGAGAC BRDN00012	215	0	20	624	317	10
CATAGAGCA BRDN00011	75	96	2586	185	139	45
CATAGAGGA BRDN00011	276	134	237	488	735	8
CATAGAGGT BRDN00011	260	190	197	459	420	11
CATAGAGGT BRDN00012	245	232	747	493	628	10
CATAGAGTT BRDN00012	255	117	661	648	659	0
CATAGATAG BRDN00012	234	193	336	364	204	13
CATAGATCTT BRDN00012	164	73	71	446	394	0
CATAGATCTT BRDN00012	303	307	88	528	858	0
CATAGATGA BRDN00012	100	130	34	233	587	8
CATAGATGT BRDN00011	77	50	0	148	106	0
CATAGATGT BRDN00012	54	101	242	153	163	7
CATAGATTCC BRDN00011	209	174	0	293	146	17
CATAGATTCC BRDN00012	190	178	973	241	177	4
CATAGCAAC BRDN00012	198	167	1158	357	177	19
CATAGCATA BRDN00012	129	131	2677	249	356	46
CATAGCATTT BRDN00012	206	271	114	574	346	54
CATAGCCAC BRDN00011	309	348	199	693	951	15
CATAGCCATT BRDN00011	155	133	20	586	436	0
CATAGCCGCC BRDN00011	94	78	0	240	163	0
CATAGCCGG BRDN00011	542	647	795	876	825	160
CATAGCCGG BRDN00012	101	62	0	223	380	0
CATAGCGCC BRDN00011	49	102	6	219	101	0
CATAGCGCTC BRDN00012	143	95	131	241	237	15
CATAGCGCTC BRDN00012	130	89	1772	285	201	22
CATAGCGGT BRDN00011	73	37	0	85	57	0
CATAGCTGCA BRDN00011	333	328	648	625	370	53
CATAGCTGTT BRDN00012	238	187	511	663	555	18
CATAGGAAC BRDN00012	150	56	15	238	544	0
CATAGGAAG BRDN00011	148	0	1	356	235	0
CATAGGATC BRDN00011	421	506	628	703	746	62
CATAGGATG BRDN00011	293	274	627	638	714	22
CATAGGCCA BRDN00011	107	49	101	404	414	0
CATAGGCCA BRDN00012	396	296	76	568	708	12
CATAGGCCA BRDN00011	111	90	56	217	67	0
CATAGGCCA BRDN00012	389	284	134	603	801	8
CATAGGCCC BRDN00011	192	117	9	485	574	10
CATAGGCCC BRDN00012	333	416	405	856	610	27
CATAGGCCCC BRDN00011	294	163	279	460	401	0
CATAGGCCG BRDN00011	231	145	421	415	622	38
CATAGGGAT BRDN00011	275	184	195	663	290	1
CATAGGGGG BRDN00012	129	60	177	252	163	4
CATAGGGGT BRDN00011	114	196	125	331	500	0
CATAGGGTT BRDN00011	138	204	19	295	290	0
CATAGGTAG BRDN00012	141	108	101	235	475	41
CATAGGTCA BRDN00011	258	274	10	525	686	20
CATAGGTCCC BRDN00012	429	317	410	798	1060	38
CATAGGTTCC BRDN00012	81	26	464	191	157	22
CATAGTAAA BRDN00012	356	239	2845	529	454	109
CATAGTAGC BRDN00011	383	321	2245	688	151	28
CATAGTAGG BRDN00012	115	47	40	194	390	0
CATAGTAGT BRDN00011	99	0	0	366	200	0
CATAGTATG BRDN00011	99	38	62	187	228	20
CATAGTCACA BRDN00012	200	169	161	398	314	3
CATAGTCATC BRDN00011	342	208	671	485	271	41
CATAGTCTCC BRDN00011	293	244	413	505	525	8
CATAGTGAG BRDN00012	313	424	311	777	669	51
CATATACACC BRDN00011	55	139	0	144	595	0
CATATAGCGA BRDN00012	176	246	348	388	428	4
CATATAGGTA BRDN00012	112	1	0	247	310	0
CATATATAAC BRDN00011	110	145	1	265	182	12
CATATATCCC BRDN00011	93	104	5	272	143	17
CATATCAATT BRDN00011	250	185	20	453	376	0
CATATCACAG BRDN00011	57	44	0	240	96	1
CATATCACCC BRDN00012	409	273	280	823	1178	26
CATATCCCAT BRDN00012	396	408	1358	749	689	38
CATATCCGTT BRDN00012	321	409	263	616	729	50
CATATCCTAG BRDN00012	418	355	539	593	434	9
CATATCCTTC BRDN00011	179	45	38	317	184	0
CATATCCTTC BRDN00012	271	167	8	447	242	14
CATATCGCGA BRDN00011	152	132	334	223	239	0
CATATCGGTC BRDN00011	49	0	1	335	47	0
CATATCTACT BRDN00012	333	246	300	459	709	20
CATATCTCCA BRDN00011	333	270	1134	878	875	29
CATATCTCCG BRDN00012	57	51	5	94	18	0
CATATGAAA BRDN00011	114	18	23	251	107	0
CATATGAAC BRDN00012	120	104	298	239	307	11
CATATGAAG BRDN00011	189	252	0	236	406	16
CATATGAAG BRDN00011	165	198	386	365	240	26
CATATGACAT BRDN00011	372	397	897	743	554	29
CATATGCATC BRDN00011	252	266	451	300	373	45
CATATGCATT BRDN00012	118	0	0	252	159	1
CATATGCCAT BRDN00011	166	158	340	312	553	41
CATATGCGG BRDN00012	139	160	393	321	258	16
CATATGCTGT BRDN00012	186	339	67	387	253	43
CATATGGAC BRDN00012	311	240	526	659	884	30
CATATGGCA BRDN00011	174	78	49	230	241	0
CATATGGCTC BRDN00011	168	21	0	265	154	1
CATATGGGC BRDN00012	358	338	577	660	552	19
CATATGGGG BRDN00012	333	255	309	591	541	56
CATATGTATC BRDN00012	290	278	429	526	498	21
CATATGTTGC BRDN00011	193	321	45	340	485	26
CATATTAGG BRDN00011	317	445	97	485	488	16
CATATTCACT BRDN00012	370	396	536	566	346	91
CATATTCAGT BRDN00012	371	321	2360	526	910	103
CATATTCATA BRDN00012	176	257	601	415	409	19
CATATTCGAT BRDN00011	125	0	0	360	85	0
CATATTCGCA BRDN00012	174	132	73	292	61	9
CATATTCTCA BRDN00012	289	243	819	408	822	32
CATATTGAG BRDN00012	31	12	23	312	135	0
CATATTGGA BRDN00011	258	147	1160	309	371	41
CATATTTCAT BRDN00012	366	279	74	675	598	116
CATATTTGA BRDN00012	186	178	75	288	231	0
CATATTTGTC BRDN00011	186	181	60	302	628	51
CATCAAATC BRDN00012	95	83	84	325	322	0
CATCAAACTT BRDN00012	505	407	1410	665	539	103
CATCAAAGG BRDN00012	87	32	27	128	83	4
CATCAAAGG BRDN00011	366	425	996	730	961	82
CATCAAAGTT BRDN00012	118	107	1623	274	197	7
CATCAAATAC BRDN00011	134	194	16	365	184	16
CATCAAATGC BRDN00012	131	0	0	654	284	0
CATCAACAA BRDN00011	103	0	0	483	293	0
CATCAACAAC BRDN00011	86	78	698	192	103	17
CATCAACAAC BRDN00012	162	110	0	347	241	10
CATCAACACC BRDN00012	211	120	15	352	375	17
CATCAACCAA BRDN00011	157	131	17	270	436	10
CATCAACCCA BRDN00012	14	1	0	426	162	0
CATCAACCCC BRDN00012	354	107	572	409	388	15
CATCAACCTG BRDN00012	254	111	119	330	320	0
CATCAACCTT BRDN00012	250	224	0	602	400	4
CATCAACGAC BRDN00012	160	208	284	303	512	0
CATCAACTAT BRDN00011	125	74	66	232	357	0
CATCAACTCC BRDN00012	133	1	1	764	542	0
CATCAAGATA BRDN00011	167	173	350	436	315	0
CATCAAGCCC BRDN00012	74	27	0	185	91	0
CATCAAGCGA BRDN00012	143	191	38	229	348	14
CATCAAGGA BRDN00011	262	198	1334	538	740	13
CATCAAGGA BRDN00011	133	53	0	227	120	0
CATCAAGGA BRDN00011	140	102	77	255	310	0
CATCAAGGA BRDN00011	134	129	0	286	132	0
CATCAAGGC BRDN00011	265	250	367	376	538	0
CATCAAGGC BRDN00012	537	490	650	864	854	69
CATCAAGGC BRDN00011	96	41	75	254	172	0
CATCAAGGC BRDN00012	211	134	590	273	368	38
CATCAAGGC BRDN00011	294	169	57	538	629	23
CATCAAGGG BRDN00011	96	134	53	160	104	0
CATCAAGGTA BRDN00012	201	225	756	504	690	3
CATCAAGGT BRDN00012	49	0	10	202	26	0
CATCAAGTAC BRDN00012	95	119	198	260	298	8
CATCAAGTG BRDN00011	339	359	613	586	602	46
CATCAAGTTC BRDN00011	202	201	325	243	62	21
CATCAATAAC BRDN00011	218	191	226	448	875	22
CATCAATACA BRDN00011	357	264	2598	726	542	71
CATCAATCCA BRDN00012	84	112	1	143	120	0
CATCAATCCA BRDN00011	291	305	755	981	706	69
CATCAATGAT BRDN00011	48	0	0	307	43	0
CATCAATGCC BRDN00011	315	306	141	622	897	0
CATCAATGTC BRDN00012	122	133	101	229	440	96
CATCAATGTC BRDN00011	179	250	84	482	530	14
CATCACAAA BRDN00011	92	0	0	187	111	0
CATCACAAAC BRDN00011	155	338	536	491	547	0
CATCACAAAT BRDN00012	137	128	151	267	417	31
CATCACACAC BRDN00012	348	189	193	576	947	6
CATCACACGC BRDN00011	82	0	1	624	448	0
CATCACAGAC BRDN00011	160	126	0	249	332	0
CATCACAGAC BRDN00012	90	27	8859	158	238	139
CATCACAGCA BRDN00012	526	801	1861	759	1037	69
CATCACAGCT BRDN00011	203	166	646	441	243	1
CATCACAGTA BRDN00011	193	132	60	309	289	6
CATCACCAAC BRDN00012	272	260	48	434	440	4
CATCACCACA BRDN00011	61	57	1	135	117	0
CATCACCATC BRDN00012	145	184	186	185	113	11
CATCACCATG BRDN00012	161	81	18	254	310	0
CATCACCCAC BRDN00012	373	223	354	644	587	27
CATCACCCAC BRDN00012	457	375	1177	655	505	30
CATCACCCTG BRDN00012	222	383	361	454	936	27
CATCACGCAC BRDN00012	487	592	382	938	839	71
CATCACGCGA BRDN00011	102	71	60	176	111	0
CATCACGCTC BRDN00011	191	79	210	271	98	10
CATCACGCTT BRDN00011	250	240	315	534	383	26
CATCACGGA BRDN00011	378	69	42	629	405	0
CATCACGGCC BRDN00012	250	106	497	288	172	9
CATCACGGG BRDN00011	163	105	277	422	110	5
CATCACGGTT BRDN00011	252	149	88	357	181	3
CATCACGTCC BRDN00012	279	300	158	707	660	104
CATCACTAAC BRDN00012	462	226	37	1030	1567	4
CATCACTAGT BRDN00012	249	285	1402	391	454	23
CATCACTCAG BRDN00011	113	5	0	345	204	0
CATCACTCCT BRDN00012	470	305	116	431	468	103
CATCACTCTT BRDN00011	328	338	251	736	651	57
CATCACTGCA BRDN00012	156	78	127	309	192	0
CATCACTGGA BRDN00012	438	190	56	856	809	5
CATCACTTAT BRDN00011	242	225	24	443	715	0
CATCACTTCA BRDN00012	76	149	27	258	298	31
CATCAGAAA BRDN00012	333	458	957	698	573	5
CATCAGAAA BRDN00012	111	129	0	209	220	8
CATCAGAAG BRDN00012	367	304	224	626	507	34
CATCAGAAG BRDN00012	128	207	1194	215	141	19
CATCAGAGA BRDN00011	152	54	1417	211	222	32
CATCAGAGA BRDN00011	194	208	39	350	201	10
CATCAGATCC BRDN00012	231	170	163	260	157	21
CATCAGCAAC BRDN00011	140	162	635	445	468	0
CATCAGCACC BRDN00011	110	69	91	204	203	0
CATCAGCACC BRDN00011	301	359	671	609	956	28
CATCAGCAG BRDN00011	192	232	262	272	137	19
CATCAGCCTT BRDN00011	73	6	0	283	383	0
CATCAGCTA BRDN00011	281	305	569	588	324	62
CATCAGCTCC BRDN00012	378	197	23	604	559	1
CATCAGCTTC BRDN00012	294	311	6018	499	594	92
CATCAGGAA BRDN00012	212	187	570	297	509	72
CATCAGGACT BRDN00012	183	84	0	216	346	0
CATCAGGGC BRDN00011	222	107	146	544	1031	8
CATCAGTAG BRDN00012	127	66	22	322	302	0
CATCAGTCCC BRDN00012	179	215	70	397	326	12
CATCAGTGA BRDN00012	285	183	793	643	433	29
CATCAGTGA BRDN00011	60	166	2	127	138	0
CATCAGTGA BRDN00012	92	115	6	69	375	21
CATCAGTGCT BRDN00011	286	222	0	437	434	20
CATCATAAAT BRDN00012	451	442	708	748	1420	18
CATCATAATA BRDN00012	116	101	61	286	158	16
CATCATACAC BRDN00011	126	98	81	254	333	0
CATCATACTG BRDN00011	160	200	277	333	379	3
CATCATAGG BRDN00011	252	310	121	439	608	34
CATCATAGG BRDN00012	120	111	14	168	147	0
CATCATAGTT BRDN00011	235	114	2070	443	289	45
CATCATATAG BRDN00012	75	51	639	128	24	18
CATCATCAGC BRDN00011	394	342	354	1017	1149	34
CATCATCAGT BRDN00012	118	21	56	265	228	30
CATCATCATA BRDN00011	168	0	1	233	209	0
CATCATCATG BRDN00011	515	638	1701	1054	990	154
CATCATCCAT BRDN00012	413	283	846	724	570	31
CATCATCCCT BRDN00012	365	298	1565	849	745	38
CATCATCCGG BRDN00011	134	9	115	256	376	7
CATCATCCTC BRDN00011	147	111	112	245	378	18
CATCATCGAC BRDN00011	177	209	85	354	437	41
CATCATCTTG BRDN00011	41	21	0	726	419	0
CATCATGAAC BRDN00011	93	56	336	259	188	11
CATCATGAAC BRDN00011	335	227	1249	659	328	20
CATCATGAG BRDN00011	385	349	115	781	919	56
CATCATGATC BRDN00011	365	499	58	588	1066	54
CATCATGATC BRDN00011	430	437	832	921	338	56
CATCATGCA BRDN00011	355	423	385	527	464	17
CATCATGCAT BRDN00011	196	8	0	537	442	0
CATCATGGA BRDN00011	96	136	75	258	352	9
CATCATGGTA BRDN00012	137	83	48	315	88	8
CATCATGTAC BRDN00011	250	235	259	400	335	10
CATCATGTAC BRDN00012	476	458	1996	799	727	29
CATCATGTCC BRDN00012	105	109	135	217	185	0
CATCATGTCC BRDN00012	451	407	1343	972	1243	41
CATCATGTCT BRDN00012	278	342	2118	700	177	39
CATCATGTTA BRDN00011	92	89	36	229	106	0
CATCATTATT BRDN00012	341	327	578	564	540	57
CATCATTCGA BRDN00011	141	34	207	322	131	10
CATCATTGCC BRDN00011	160	163	50	257	311	0
CATCATTTAA BRDN00012	343	288	516	485	295	130
CATCCAAACC BRDN00012	391	248	383	594	536	42
CATCCAAGA BRDN00011	228	269	942	243	333	13
CATCCAAGAC BRDN00012	519	338	1157	823	748	40
CATCCAAGG BRDN00011	473	361	292	769	528	9
CATCCAAGTC BRDN00011	129	1	184	413	518	0
CATCCAATTA BRDN00011	123	154	62	221	44	44
CATCCACAAT BRDN00011	115	132	1	137	131	0
CATCCACATG BRDN00011	371	330	417	706	1031	223
CATCCACCAC BRDN00011	189	114	282	349	428	0
CATCCACCCA BRDN00012	367	338	432	825	635	27
CATCCACGTG BRDN00012	295	202	129	395	256	13
CATCCACTGC BRDN00012	405	341	2366	689	704	83
CATCCACTTC BRDN00011	171	75	2	188	178	14
CATCCACTTG BRDN00012	234	204	403	470	353	39
CATCCAGATC BRDN00011	204	157	484	313	182	4
CATCCAGCAC BRDN00011	107	0	0	185	211	0
CATCCAGCCA BRDN00011	174	137	448	391	1023	0
CATCCAGCCT BRDN00011	440	217	1924	642	802	52
CATCCAGCGC BRDN00011	166	166	33	249	152	0
CATCCAGGG BRDN00012	232	67	697	314	298	0
CATCCAGTCC BRDN00012	412	435	222	738	637	60
CATCCAGTGA BRDN00011	79	19	0	229	362	0
CATCCAGTGA BRDN00012	91	97	330	233	254	21
CATCCAGTTC BRDN00012	110	88	308	302	333	6
CATCCATAAA BRDN00012	180	212	782	375	375	14
CATCCATACT BRDN00012	167	166	251	370	316	31
CATCCATGAC BRDN00011	239	131	331	395	245	0
CATCCATGAC BRDN00012	208	135	201	452	403	52
CATCCATGTT BRDN00012	412	208	1785	661	610	41
CATCCCAAAG BRDN00011	100	127	49	242	225	0
CATCCCAACG BRDN00011	226	382	806	555	1960	34
CATCCCACGG BRDN00012	116	132	0	202	309	49
CATCCCAGAT BRDN00011	197	54	40	382	534	1
CATCCCAGTC BRDN00012	313	284	329	690	945	3
CATCCCATAT BRDN00012	158	86	14	211	192	15
CATCCCATTT BRDN00011	236	188	310	423	437	3
CATCCCCAAG BRDN00012	244	169	674	464	433	62
CATCCCCACT BRDN00011	527	515	1316	1012	1231	23
CATCCCCAGC BRDN00012	109	147	74	196	100	5
CATCCCCGAC BRDN00011	91	148	0	231	211	0
CATCCCCGTT BRDN00011	79	12	0	201	161	0
CATCCCCTAG BRDN00011	94	76	385	281	148	0
CATCCCCTCC BRDN00011	287	248	3560	433	383	39
CATCCCGGG BRDN00012	78	30	0	205	69	0
CATCCCGGG BRDN00012	58	19	211	210	75	0
CATCCCGGG BRDN00011	257	172	590	451	717	59
CATCCCTAGA BRDN00011	175	233	109	327	397	52
CATCCCTGAC BRDN00012	400	263	223	511	727	4
CATCCCTGCC BRDN00012	433	23	13	902	636	0
CATCCCTGTT BRDN00012	270	226	327	344	437	0
CATCCCTTAC BRDN00011	533	478	894	786	771	35
CATCCGAACT BRDN00012	501	373	1801	701	566	65
CATCCGAAGT BRDN00011	325	268	843	589	581	141
CATCCGAATC BRDN00011	87	85	10	152	197	0
CATCCGACAC BRDN00012	112	90	57	251	195	10
CATCCGCAGA BRDN00012	336	352	1347	524	597	57
CATCCGCCGA BRDN00012	109	0	0	261	81	0
CATCCGCCTC BRDN00012	309	85	37	539	658	0
CATCCGCGTC BRDN00012	357	343	483	777	748	60
CATCCGGAGA BRDN00012	457	345	1152	785	878	19
CATCCGGCAC BRDN00012	226	118	509	397	235	6
CATCCGGCCC BRDN00012	75	93	134	192	214	10
CATCCGGCCC BRDN00012	91	89	229	136	195	8
CATCCGGCG BRDN00011	118	117	70	419	503	8
CATCCGGCTT BRDN00011	244	121	147	377	280	31
CATCCGGGG BRDN00012	205	250	158	742	564	13
CATCCGGGT BRDN00011	176	155	271	263	456	47
CATCCGTAAC BRDN00012	235	282	28	579	742	54
CATCCGTCAG BRDN00012	189	233	76	364	678	21
CATCCGTCCA BRDN00012	309	289	1536	784	651	38
CATCCGTCCA BRDN00012	370	302	2197	881	647	68
CATCCGTGCC BRDN00012	189	292	208	330	244	35
CATCCGTGTC BRDN00012	155	263	359	313	361	14
CATCCTAAAC BRDN00012	396	497	1394	592	718	183
CATCCTACCG BRDN00012	133	110	180	234	121	0
CATCCTAGA BRDN00012	35	0	0	208	206	0
CATCCTAGAC BRDN00012	226	170	99	368	276	12
CATCCTAGGC BRDN00012	146	174	510	399	509	8
CATCCTAGTC BRDN00011	229	209	1161	674	672	26
CATCCTAGTC BRDN00011	195	175	368	247	115	16
CATCCTATGA BRDN00012	114	157	172	184	129	81
CATCCTATTT BRDN00012	79	49	0	198	69	5
CATCCTCAAA BRDN00012	148	176	1392	219	241	37
CATCCTCAAA BRDN00011	372	587	623	697	786	36
CATCCTCACG BRDN00012	181	93	163	302	334	5
CATCCTCACG BRDN00011	212	276	295	302	522	32
CATCCTCACT BRDN00011	200	160	437	417	263	29
CATCCTCCCT BRDN00011	58	0	0	543	652	0
CATCCTCCTG BRDN00011	196	277	279	406	642	61
CATCCTCGCT BRDN00011	352	184	847	622	593	26
CATCCTCTAG BRDN00011	139	72	6	265	187	0
CATCCTCTAG BRDN00012	151	168	212	290	250	54
CATCCTGAAC BRDN00011	69	0	0	228	71	0
CATCCTGAAC BRDN00012	298	275	114	573	310	9
CATCCTGACA BRDN00011	319	252	2070	550	356	56
CATCCTGAGA BRDN00012	115	66	0	229	408	6
CATCCTGAGA BRDN00011	292	111	662	435	432	54
CATCCTGAGC BRDN00012	209	32	0	201	115	7
CATCCTGCTC BRDN00011	190	250	781	295	203	29
CATCCTGGCC BRDN00012	374	355	2068	660	536	43
CATCCTGGGA BRDN00012	243	189	434	622	599	38
CATCCTGGG BRDN00011	338	249	137	577	949	0
CATCCTGGTC BRDN00011	245	115	537	571	273	9
CATCCTTAAA BRDN00012	80	98	4	315	179	34
CATCCTTAGT BRDN00012	207	142	2727	410	644	39
CATCCTTATA BRDN00011	64	5	0	160	0	0
CATCGAACAC BRDN00012	209	115	1283	483	516	13
CATCGAACGT BRDN00012	155	40	233	200	565	12
CATCGAACTC BRDN00011	150	95	3008	189	229	50
CATCGAAGA BRDN00012	135	155	431	155	233	26
CATCGAATAA BRDN00012	160	216	4	173	638	9
CATCGAATCC BRDN00012	175	269	71	382	253	11
CATCGAATCC BRDN00012	105	62	163	219	90	11
CATCGACACC BRDN00011	101	0	0	230	174	0
CATCGACACT BRDN00011	53	31	33	167	173	6
CATCGACATC BRDN00011	151	6	0	302	255	0
CATCGACATT BRDN00012	297	433	1189	617	419	31
CATCGACCGA BRDN00012	190	160	2384	275	130	51
CATCGACCTC BRDN00011	390	377	1149	562	646	30
CATCGACGCC BRDN00012	376	376	269	808	838	62
CATCGACTGC BRDN00012	246	210	277	586	462	6
CATCGACTGT BRDN00012	230	146	89	363	124	11
CATCGACTGT BRDN00012	472	467	1158	780	696	37
CATCGACTTA BRDN00012	189	124	282	303	314	33
CATCGACTTC BRDN00012	351	351	165	615	693	9
CATCGAGAA BRDN00012	5	0	0	151	32	0
CATCGAGGA BRDN00011	128	87	79	621	843	13
CATCGAGGA BRDN00012	206	227	147	444	572	11
CATCGAGGA BRDN00011	221	96	288	441	478	12
CATCGAGGA BRDN00011	98	41	80	113	45	0
CATCGAGGT BRDN00011	274	396	42	409	478	10
CATCGAGTTT BRDN00012	140	59	0	285	228	0
CATCGATGA BRDN00011	137	60	1091	152	69	0
CATCGATGCC BRDN00011	268	187	464	406	635	12
CATCGCAACC BRDN00012	206	156	342	412	215	60
CATCGCATCA BRDN00011	328	286	1977	515	418	32
CATCGCCACC BRDN00012	200	110	98	390	282	7
CATCGCCAGA BRDN00012	157	63	88	754	369	0
CATCGCCGCC BRDN00012	122	7	41	401	375	0
CATCGCCTAC BRDN00011	323	257	330	606	278	7
CATCGCGAG BRDN00012	398	276	2169	650	862	27
CATCGCTCAG BRDN00012	158	80	13	315	416	0
CATCGCTCAT BRDN00012	438	331	377	707	1034	16
CATCGCTGAT BRDN00011	61	0	0	158	89	0
CATCGCTGGT BRDN00012	287	437	1082	625	684	23
CATCGCTTCT BRDN00011	220	192	392	479	671	65
CATCGGAAG BRDN00012	242	121	182	310	214	43
CATCGGAGT BRDN00011	94	170	38	152	191	0
CATCGGATCT BRDN00012	235	213	903	602	792	14
CATCGGCACA BRDN00012	221	286	15	336	490	63
CATCGGCATC BRDN00012	208	197	112	334	240	16
CATCGGCGA BRDN00011	218	156	147	352	458	0
CATCGGGAA BRDN00011	112	112	0	222	213	0
CATCGGGAG BRDN00012	93	80	46	170	231	11
CATCGGGGG BRDN00011	83	113	0	198	96	5
CATCGGGTT BRDN00012	349	211	141	592	624	1
CATCGGTTC BRDN00011	11	7	0	138	8	0
CATCGTAAAC BRDN00011	107	106	409	144	141	6
CATCGTACTT BRDN00012	364	342	175	515	723	29
CATCGTAGG BRDN00011	324	391	98	421	267	15
CATCGTAGG BRDN00011	501	349	270	891	1078	13
CATCGTATAT BRDN00011	168	119	990	261	248	24
CATCGTCACC BRDN00011	170	133	50	298	249	1
CATCGTCAGA BRDN00011	178	154	812	340	212	27
CATCGTCAGA BRDN00012	399	386	10008	660	554	245
CATCGTCCGA BRDN00012	448	438	1551	746	573	106

CATCGTCCTG	BRDN0001	80	0	0	278	0	0
CATCGTCGG	BRDN0001	93	103	28	177	105	0
CATCGTCTAT	BRDN0001	139	9	0	471	337	0
CATCGTCTG	BRDN0001	133	120	160	220	218	2
CATCGTCTG	BRDN0001	222	145	438	414	523	10
CATCGTCTTA	BRDN0001	20	67	0	190	136	0
CATCGTGAT	BRDN0001	139	46	0	494	504	0
CATCGTGCCT	BRDN0001	218	458	255	490	380	34
CATCGTGGG	BRDN0001	197	151	1220	263	361	15
CATCGTGGT	BRDN0001	378	341	297	535	520	76
CATCGTGTC	BRDN0001	234	263	204	497	476	20
CATCGTGTG	BRDN0001	172	92	14	409	381	39
CATCGTGTG	BRDN0001	293	272	619	562	640	31
CATCGTTAA	BRDN0001	49	62	38	125	79	4
CATCGTTCTT	BRDN0001	138	90	29	273	214	17
CATCGTTTAA	BRDN0001	341	317	718	575	433	32
CATCTAACAG	BRDN0001	266	203	347	634	522	57
CATCTAATAT	BRDN0001	101	9	0	177	152	0
CATCTACAAA	BRDN0001	165	124	525	210	556	27
CATCTACAAG	BRDN0001	177	177	269	366	756	54
CATCTACAAG	BRDN0001	103	99	58	226	302	19
CATCTACCAA	BRDN0001	95	55	68	160	170	0
CATCTACCTA	BRDN0001	144	196	29	318	211	12
CATCTACGA	BRDN0001	83	103	360	327	206	8
CATCTACTAC	BRDN0001	47	0	0	274	353	0
CATCTACTGC	BRDN0001	332	143	120	438	507	4
CATCTACTGT	BRDN0001	479	405	623	679	813	51
CATCTACTTT	BRDN0001	193	188	487	471	270	12
CATCTAGAT	BRDN0001	405	476	78	840	801	50
CATCTAGCA	BRDN0001	313	371	336	354	665	31
CATCTAGGA	BRDN0001	386	256	7157	771	534	82
CATCTAGGG	BRDN0001	161	89	248	380	103	16
CATCTAGTA	BRDN0001	251	289	42	444	593	0
CATCTAGTG	BRDN0001	216	215	1345	317	648	11
CATCTATAAC	BRDN0001	103	165	36	167	151	0
CATCTATACG	BRDN0001	211	171	124	440	391	16
CATCTATCCA	BRDN0001	284	392	16	575	353	22
CATCTATCGA	BRDN0001	71	78	593	163	205	7
CATCTATCTG	BRDN0001	252	302	7	474	266	19
CATCTATGCC	BRDN0001	327	193	4612	548	566	59
CATCTATTGA	BRDN0001	141	75	37	232	147	121
CATCTCAACG	BRDN0001	88	93	73	172	142	0
CATCTCAAG	BRDN0001	300	293	2003	510	854	17
CATCTCAGAT	BRDN0001	225	241	1517	441	339	44
CATCTCAGG	BRDN0001	167	61	886	291	278	12
CATCTCAGTC	BRDN0001	63	92	0	161	103	6
CATCTCATAC	BRDN0001	176	219	1120	316	282	24
CATCTCATAG	BRDN0001	158	228	240	289	301	119
CATCTCCAAT	BRDN0001	117	10	17	406	31	0
CATCTCCAAT	BRDN0001	174	209	27	345	431	15
CATCTCCAGC	BRDN0001	151	55	62	325	282	0
CATCTCCAT	BRDN0001	461	343	440	861	732	7
CATCTCCATT	BRDN0001	256	188	49	442	381	0
CATCTCCCCA	BRDN0001	268	266	338	559	602	99
CATCTCCCTT	BRDN0001	182	153	372	332	542	7
CATCTCCGG	BRDN0001	122	114	1	255	263	14
CATCTCCTAC	BRDN0001	44	58	7	262	180	0
CATCTCCTCT	BRDN0001	180	196	6	344	280	24
CATCTCGAA	BRDN0001	317	412	533	485	469	101
CATCTCGACA	BRDN0001	131	169	920	190	577	9
CATCTCGCAC	BRDN0001	302	197	267	769	1050	12
CATCTCGCG	BRDN0001	104	143	631	179	79	18
CATCTCGGA	BRDN0001	131	170	0	181	180	14
CATCTCGGC	BRDN0001	60	147	7	277	492	1
CATCTCTATG	BRDN0001	287	132	8	439	463	0
CATCTCTCAG	BRDN0001	105	91	183	162	203	3
CATCTCTGAT	BRDN0001	145	4	0	309	231	0
CATCTCTTCG	BRDN0001	583	475	5059	1107	952	74
CATCTCTTCT	BRDN0001	157	92	1	329	551	0
CATCTCTTGG	BRDN0001	68	0	0	219	366	0
CATCTGAATT	BRDN0001	133	152	341	319	399	12
CATCTGACCC	BRDN0001	218	163	316	591	861	3
CATCTGAGA	BRDN0001	76	12	0	141	174	0
CATCTGAGG	BRDN0001	209	195	105	300	416	3
CATCTGATCT	BRDN0001	6	0	0	148	30	0
CATCTGCAA	BRDN0001	121	131	26	198	337	0
CATCTGCACA	BRDN0001	168	194	394	329	258	37
CATCTGCAG	BRDN0001	324	197	77	542	436	9
CATCTGCAG	BRDN0001	379	320	315	703	1276	88
CATCTGCATC	BRDN0001	183	86	32	400	362	8
CATCTGCCTC	BRDN0001	33	0	0	435	44	0
CATCTGCGG	BRDN0001	93	56	153	162	190	0
CATCTGCTG	BRDN0001	114	108	1182	175	201	30
CATCTGGAA	BRDN0001	166	80	49	335	250	62
CATCTGGATT	BRDN0001	402	355	253	878	660	4
CATCTGGCA	BRDN0001	279	287	232	559	708	8
CATCTGGCC	BRDN0001	139	102	851	281	106	5
CATCTGGCG	BRDN0001	368	260	1092	742	570	26
CATCTGGGT	BRDN0001	80	25	17	250	97	0
CATCTGGTCA	BRDN0001	438	593	1918	796	860	54
CATCTGGTC	BRDN0001	41	5	0	180	146	0
CATCTGTACA	BRDN0001	274	308	438	421	385	10
CATCTGTAG	BRDN0001	465	520	229	880	876	54
CATCTGTGT	BRDN0001	254	90	0	660	576	2
CATCTGTGT	BRDN0001	83	0	0	232	148	0
CATCTGTTAC	BRDN0001	248	157	54	615	442	24
CATCTTAAAA	BRDN0001	266	148	420	503	434	2
CATCTTACCT	BRDN0001	324	205	1409	634	678	10
CATCTTACCT	BRDN0001	237	181	200	476	408	6
CATCTTAGG	BRDN0001	127	72	70	256	399	0
CATCTTAGG	BRDN0001	252	133	216	535	289	14
CATCTTATAA	BRDN0001	154	0	0	565	331	0
CATCTTCAC	BRDN0001	193	307	773	464	402	35
CATCTTCACT	BRDN0001	124	128	267	278	310	43
CATCTTCAGG	BRDN0001	197	114	56	366	664	38
CATCTTCCCG	BRDN0001	188	254	326	344	163	8
CATCTTCCCT	BRDN0001	48	0	1	268	142	0
CATCTTCCGG	BRDN0001	155	128	22	384	300	21
CATCTTCGTG	BRDN0001	234	248	25	530	572	33
CATCTTCTAC	BRDN0001	330	215	726	687	386	24
CATCTTCTTC	BRDN0001	139	147	72	250	487	0
CATCTTCTTT	BRDN0001	497	478	1607	674	940	43
CATCTTGCAT	BRDN0001	204	276	1284	356	422	44
CATCTTGCAT	BRDN0001	189	354	3505	367	245	94
CATCTTGGA	BRDN0001	330	264	128	601	390	13
CATCTTGGA	BRDN0001	171	126	9	263	87	0
CATCTTGGG	BRDN0001	207	132	87	520	557	0
CATCTTGGG	BRDN0001	131	110	53	216	123	2
CATCTTGGG	BRDN0001	186	159	420	479	625	22
CATCTTGGT	BRDN0001	289	246	239	563	515	4
CATCTTGTAT	BRDN0001	122	131	607	120	97	36
CATCTTTAGC	BRDN0001	190	208	368	515	681	24
CATCTTTCAT	BRDN0001	197	0	0	304	167	0
CATCTTTGCC	BRDN0001	377	496	766	508	1103	73
CATCTTTGG	BRDN0001	180	112	2173	267	292	55
CATCTTTGTA	BRDN0001	71	23	53	218	368	11
CATGAAAAA	BRDN0001	173	211	51	243	193	1
CATGAAAAG	BRDN0001	122	97	13	190	100	8
CATGAAAAT	BRDN0001	319	175	411	591	501	5
CATGAAAAT	BRDN0001	96	59	1	212	179	0
CATGAAACG	BRDN0001	149	39	0	335	432	0
CATGAAAGA	BRDN0001	209	108	86	334	332	15
CATGAAATG	BRDN0001	224	196	36	446	522	5
CATGAACAA	BRDN0001	256	244	636	461	700	54
CATGAACAC	BRDN0001	189	205	24	307	398	46
CATGAACACT	BRDN0001	251	123	1	291	503	56
CATGAACAT	BRDN0001	401	417	276	668	516	39
CATGAACAT	BRDN0001	323	414	919	738	332	61
CATGAACCC	BRDN0001	87	1	0	261	102	0
CATGAACCG	BRDN0001	316	233	220	518	400	10
CATGAACCG	BRDN0001	442	420	926	619	593	46
CATGAACCG	BRDN0001	321	332	1342	717	1049	11
CATGAACCT	BRDN0001	196	180	1718	267	265	27
CATGAACCT	BRDN0001	68	103	4	201	126	56
CATGAACGC	BRDN0001	159	147	390	292	356	44
CATGAAGAA	BRDN0001	197	219	0	255	151	0
CATGAAGAG	BRDN0001	87	40	0	225	129	0
CATGAAGAG	BRDN0001	152	102	2	216	188	0
CATGAAGCC	BRDN0001	213	474	666	649	420	120
CATGAAGCG	BRDN0001	190	0	1	486	67	0
CATGAAGCG	BRDN0001	351	448	269	790	900	22
CATGAAGCT	BRDN0001	273	328	190	540	379	9
CATGAAGCT	BRDN0001	212	210	259	451	127	44
CATGAAGTT	BRDN0001	216	207	662	402	317	13
CATGAAGTT	BRDN0001	217	147	51	383	361	0
CATGAATAA	BRDN0001	80	33	53	156	220	8
CATGAATAA	BRDN0001	160	122	267	365	313	18
CATGAATGA	BRDN0001	213	166	1601	365	206	69
CATGAATGG	BRDN0001	213	161	202	256	316	0
CATGAATGT	BRDN0001	183	391	942	329	546	24
CATGACAAT	BRDN0001	309	244	599	527	447	33
CATGACAAT	BRDN0001	163	97	456	393	419	7
CATGACAAT	BRDN0001	109	48	1	243	401	0
CATGACACA	BRDN0001	38	6	0	632	109	0
CATGACACA	BRDN0001	250	247	127	534	427	14
CATGACACT	BRDN0001	388	440	273	616	519	40
CATGACAGA	BRDN0001	255	76	21	458	309	0
CATGACAGA	BRDN0001	97	96	40	192	140	19
CATGACAGA	BRDN0001	312	322	71	403	470	16
CATGACAGC	BRDN0001	188	161	487	278	159	14
CATGACAGC	BRDN0001	89	46	299	154	157	0
CATGACAGG	BRDN0001	137	209	27	291	287	4
CATGACAGT	BRDN0001	135	25	0	204	180	0
CATGACATA	BRDN0001	126	93	443	268	353	34
CATGACATG	BRDN0001	63	4	0	163	150	0
CATGACCAT	BRDN0001	307	216	191	491	446	33
CATGACCAT	BRDN0001	239	313	34	577	522	6
CATGACCCG	BRDN0001	193	211	231	404	321	1
CATGACCCTA	BRDN0001	20	15	16	174	185	0
CATGACCCT	BRDN0001	141	238	55	325	748	19
CATGACCGA	BRDN0001	212	320	560	537	575	4
CATGACCGC	BRDN0001	407	496	203	699	374	20
CATGACCTAT	BRDN0001	210	239	19	371	520	0
CATGACGAC	BRDN0001	139	76	0	328	239	8
CATGACGGA	BRDN0001	324	390	358	679	528	40
CATGACGGT	BRDN0001	387	229	299	657	607	24
CATGACGGT	BRDN0001	91	51	545	140	201	2
CATGACGTC	BRDN0001	258	222	1694	561	719	40
CATGACTAC	BRDN0001	326	200	542	560	470	14
CATGACTAC	BRDN0001	264	228	1168	370	208	44
CATGACTCAT	BRDN0001	288	398	343	545	513	47
CATGACTCAT	BRDN0001	156	131	305	257	415	19
CATGACTCCC	BRDN0001	288	131	163	658	577	129
CATGACTCG	BRDN0001	71	57	82	172	119	0
CATGACTCG	BRDN0001	130	54	265	202	197	0
CATGACTGT	BRDN0001	125	111	23	322	478	5
CATGACTTCC	BRDN0001	145	69	77	190	158	0
CATGACTTG	BRDN0001	69	97	20	213	290	0
CATGAGAAG	BRDN0001	359	307	311	552	482	6
CATGAGAAT	BRDN0001	291	401	777	538	219	36
CATGAGACC	BRDN0001	76	8	0	101	86	12
CATGAGACT	BRDN0001	154	100	157	206	271	27
CATGAGAGC	BRDN0001	70	88	66	119	53	0
CATGAGATA	BRDN0001	95	99	2895	202	140	72
CATGAGATG	BRDN0001	223	123	73	282	147	4
CATGAGATT	BRDN0001	328	201	54	412	700	5
CATGAGCAC	BRDN0001	102	165	2479	183	260	383
CATGAGCAG	BRDN0001	303	213	28	443	207	0
CATGAGCAG	BRDN0001	508	334	823	826	651	63
CATGAGCAT	BRDN0001	204	83	81	278	211	21
CATGAGCAT	BRDN0001	113	51	0	365	219	0
CATGAGCCA	BRDN0001	168	127	17	314	456	4
CATGAGCGA	BRDN0001	137	219	40	299	281	31
CATGAGCGA	BRDN0001	61	0	0	149	19	0
CATGAGCGC	BRDN0001	343	294	1036	511	1091	66
CATGAGCTA	BRDN0001	279	238	266	675	704	15
CATGAGCTCT	BRDN0001	20	0	0	292	73	0
CATGAGCTT	BRDN0001	66	4	0	177	86	0
CATGAGGCT	BRDN0001	87	0	0	321	75	0
CATGAGGCT	BRDN0001	141	81	660	404	440	7
CATGAGGGA	BRDN0001	333	326	826	774	907	46
CATGAGGTA	BRDN0001	49	70	73	168	214	0
CATGAGGTG	BRDN0001	439	332	902	912	755	47
CATGAGTAA	BRDN0001	158	134	0	362	369	12
CATGAGTAT	BRDN0001	130	203	17	208	626	0
CATGAGTCC	BRDN0001	110	36	0	116	80	0
CATGAGTGT	BRDN0001	89	120	793	264	365	8
CATGATAGA	BRDN0001	160	120	233	277	297	0
CATGATAGA	BRDN0001	255	286	139	515	466	3
CATGATAGG	BRDN0001	261	199	211	319	534	30
CATGATCAA	BRDN0001	215	138	82	460	320	37
CATGATCAG	BRDN0001	204	233	1022	272	388	53
CATGATCAG	BRDN0001	292	324	252	712	606	39
CATGATCAT	BRDN0001	171	49	51	261	243	0
CATGATCGA	BRDN0001	309	339	312	673	610	15
CATGATCTCA	BRDN0001	59	120	0	127	64	16
CATGATCTTC	BRDN0001	307	315	75	611	609	109
CATGATGAC	BRDN0001	103	76	423	312	137	12
CATGATGAG	BRDN0001	258	313	67	818	790	48
CATGATGAG	BRDN0001	190	111	147	411	451	7
CATGATGCA	BRDN0001	277	355	1076	564	463	16
CATGATGCC	BRDN0001	372	288	296	687	619	10
CATGATGCC	BRDN0001	222	197	64	300	814	25
CATGATGCT	BRDN0001	130	227	53	199	148	13
CATGATGGA	BRDN0001	181	100	154	257	249	0
CATGATGGC	BRDN0001	532	314	2554	820	783	33
CATGATGGG	BRDN0001	465	503	485	718	1136	35
CATGATGGT	BRDN0001	71	55	0	158	364	0
CATGATGGT	BRDN0001	196	307	70	615	837	21
CATGATGTA	BRDN0001	182	152	210	357	274	52
CATGATTAA	BRDN0001	97	108	628	185	200	39
CATGATTGC	BRDN0001	260	255	424	449	760	63
CATGCAAAG	BRDN0001	94	101	14	181	210	13
CATGCAACA	BRDN0001	133	208	25	171	129	17
CATGCAACT	BRDN0001	167	153	57	303	561	52
CATGCAAGA	BRDN0001	108	98	53	222	113	0
CATGCAAGA	BRDN0001	53	35	121	102	40	0
CATGCAATG	BRDN0001	360	368	446	721	1265	50
CATGCACAC	BRDN0001	168	205	1283	279	284	16
CATGCACAT	BRDN0001	118	162	33	292	328	0
CATGCACAT	BRDN0001	83	68	145	194	192	27
CATGCACATT	BRDN0001	345	200	34	557	367	9
CATGCACGT	BRDN0001	151	0	0	239	58	0
CATGCACTA	BRDN0001	147	81	331	286	345	7
CATGCAGAC	BRDN0001	194	257	1216	307	250	27
CATGCAGAG	BRDN0001	201	349	757	389	439	23
CATGCAGAT	BRDN0001	390	404	4250	691	406	85
CATGCAGCA	BRDN0001	129	174	451	302	182	62
CATGCAGCA	BRDN0001	44	78	26	189	116	29
CATGCAGCC	BRDN0001	263	88	0	623	1177	0
CATGCATGG	BRDN0001	284	160	1256	344	468	16
CATGCCAAA	BRDN0001	90	59	7	129	100	0
CATGCCAAG	BRDN0001	81	0	0	258	225	0
CATGCCAAG	BRDN0001	251	268	132	403	437	21
CATGCCACA	BRDN0001	183	211	372	284	154	19
CATGCCACAT	BRDN0001	149	134	1413	247	101	47
CATGCCACG	BRDN0001	109	321	499	161	146	51
CATGCCAGA	BRDN0001	304	259	341	489	712	3
CATGCCAGA	BRDN0001	126	132	377	271	131	0
CATGCCAGT	BRDN0001	126	137	11	237	124	0
CATGCCATA	BRDN0001	376	348	494	771	928	32
CATGCCATA	BRDN0001	472	442	382	621	686	100
CATGCCATA	BRDN0001	188	239	130	270	247	35
CATGCCATTA	BRDN0001	207	177	108	272	211	6
CATGCCCATG	BRDN0001	52	42	0	225	34	44
CATGCCCCAC	BRDN0001	153	86	23	425	532	0
CATGCCCGC	BRDN0001	279	192	5179	598	976	81
CATGCCCTCT	BRDN0001	154	199	355	269	212	13
CATGCCCTCT	BRDN0001	255	186	361	538	300	44
CATGCCCTG	BRDN0001	89	229	2	230	120	14
CATGCCCTTC	BRDN0001	226	242	396	474	383	45
CATGCCGAA	BRDN0001	249	201	226	524	510	0
CATGCCGCT	BRDN0001	172	0	1	528	315	0
CATGCCGCT	BRDN0001	226	117	111	221	241	0
CATGCCTAA	BRDN0001	195	249	114	343	355	16
CATGCCTCAG	BRDN0001	183	175	545	420	258	32
CATGCCTGTT	BRDN0001	267	203	949	481	650	56
CATGCCTTCA	BRDN0001	136	55	1	209	233	6
CATGCCTTGT	BRDN0001	191	224	636	301	242	56
CATGCCTTTA	BRDN0001	209	172	10	273	268	26
CATGCCTTTC	BRDN0001	80	33	2015	134	114	25
CATGCGCAA	BRDN0001	148	165	42	277	154	2
CATGCGCAT	BRDN0001	78	90	16	228	106	0
CATGCGCCA	BRDN0001	138	178	9	299	212	6
CATGCGCGT	BRDN0001	376	11	0	485	118	0
CATGCGCTC	BRDN0001	110	92	412	209	215	20
CATGCGCTC	BRDN0001	101	118	387	181	261	19
CATGCGCTT	BRDN0001	64	168	7779	101	43	47
CATGCGCTTT	BRDN0001	73	0	0	246	97	0
CATGCGGAA	BRDN0001	57	26	0	114	9	0
CATGCGGCG	BRDN0001	2	0	0	192	42	0
CATGCGGGA	BRDN0001	282	263	352	509	617	25
CATGCGGGT	BRDN0001	264	206	1372	346	716	33
CATGCGGGT	BRDN0001	293	310	341	608	414	8
CATGCTAAT	BRDN0001	415	355	4158	657	298	134
CATGCTAGA	BRDN0001	282	279	14	499	433	0
CATGCTAGC	BRDN0001	234	121	102	508	276	20
CATGCTAGT	BRDN0001	250	260	154	412	519	7
CATGCTATG	BRDN0001	293	283	178	558	694	8
CATGCTCAA	BRDN0001	353	282	1009	755	654	30
CATGCTCACT	BRDN0001	347	287	618	654	496	0
CATGCTCAG	BRDN0001	146	131	123	194	351	0
CATGCTCAG	BRDN0001	221	238	5	315	396	20
CATGCTCATC	BRDN0001	386	270	485	818	1010	17
CATGCTCGG	BRDN0001	119	96	104	267	107	32
CATGCTCTCA	BRDN0001	286	249	123	539	469	9
CATGCTCTCC	BRDN0001	274	259	158	423	600	14
CATGCTCTTC	BRDN0001	185	225	154	285	262	11
CATGCTGAA	BRDN0001	184	125	969	385	396	37
CATGCTGAC	BRDN0001	188	309	827	464	294	45
CATGCTGGC	BRDN0001	170	174	960	283	191	4
CATGCTGGT	BRDN0001	61	22	0	87	36	0
CATGCTGTA	BRDN0001	171	206	238	561	279	6
CATGCTGTT	BRDN0001	312	309	16	563	650	0
CATGCTTCTC	BRDN0001	342	314	117	495	649	72
CATGCTTGT	BRDN0001	92	62	0	198	102	0
CATGCTTTAT	BRDN0001	333	446	31	518	234	51
CATGGAAAA	BRDN0001	239	429	873	546	298	30
CATGGAAAG	BRDN0001	183	246	241	530	813	22
CATGGAAA	BRDN0001	197	290	86	320	500	23
CATGGAAAC	BRDN0001	154	94	200	208	165	7
CATGGAAGA	BRDN0001	116	60	126	283	555	1
CATGGAAGA	BRDN0001	267	358	1100	473	406	37
CATGGAATA	BRDN0001	59	75	0	135	336	0
CATGGAATG	BRDN0001	265	201	746	468	639	26
CATGGACAA	BRDN0001	299	259	72	405	139	0
CATGGACAC	BRDN0001	113	26	66	176	114	0
CATGGACAG	BRDN0001	306	227	11	528	505	23
CATGGACAG	BRDN0001	129	183	203	276	585	0
CATGGACAG	BRDN0001	347	341	432	538	497	23
CATGGACCA	BRDN0001	157	75	13	269	102	19
CATGGACCA	BRDN0001	123	189	13	328	311	0
CATGGACCA	BRDN0001	165	158	951	394	337	24
CATGGACCG	BRDN0001	19	0	0	109	11	0
CATGGACTA	BRDN0001	132	102	2	177	423	1
CATGGACTC	BRDN0001	216	81	366	284	136	8
CATGGACTG	BRDN0001	272	334	216	608	499	16
CATGGACTG	BRDN0001	48	41	298	128	88	0
CATGGAGAA	BRDN0001	252	246	1047	483	454	71
CATGGAGAG	BRDN0001	133	61	394	252	356	20
CATGGAGAG	BRDN0001	127	53	0	239	237	0
CATGGAGCG	BRDN0001	229	250	534	396	432	53
CATGGAGCG	BRDN0001	162	99	23	470	174	13
CATGGAGCG	BRDN0001	72	123	92	149	196	45
CATGGAGGA	BRDN0001	195	148	9	294	228	0
CATGGAGGA	BRDN0001	79	98	97	256	424	17
CATGGAGGT	BRDN0001	118	91	66	189	71	6
CATGGAGTT	BRDN0001	135	116	0	336	204	7
CATGGATAA	BRDN0001	141	4	48	380	218	0
CATGGATAA	BRDN0001	242	38	79	447	434	7
CATGGATAT	BRDN0001	255	276	609	425	564	81
CATGGATCA	BRDN0001	78	28	183	179	112	0
CATGGATCC	BRDN0001	355	347	263	674	487	0
CATGGATGA	BRDN0001	181	199	63	315	490	20
CATGGATGA	BRDN0001	299	181	188	550	405	30
CATGGATGA	BRDN0001	109	43	44	135	117	0
CATGGATTA	BRDN0001	244	316	245	444	656	4
CATGGCAAA	BRDN0001	357	304	646	555	651	22
CATGGCAAT	BRDN0001	140	290	305	288	476	22
CATGGCACC	BRDN0001	495	437	1160	583	866	51
CATGGCACC	BRDN0001	193	220	95	282	287	0
CATGGCACT	BRDN0001	309	297	127	492	593	39
CATGGCAGT	BRDN0001	132	62	1	146	153	8
CATGGCAGT	BRDN0001	348	341	194	742	737	97
CATGGCAGT	BRDN0001	237	177	558	529	527	0
CATGGCAGT	BRDN0001	122	20	81	447	95	0
CATGGCAGT	BRDN0001	255	44	43	571	426	0
CATGGCATA	BRDN0001	276	265	738	543	716	28
CATGGCATA	BRDN0001	141	114	12	236	124	0
CATGGCATC	BRDN0001	488	462	794	1212	1389	57
CATGGCATG	BRDN0001	472	348	832	872	787	51
CATGGCATG	BRDN0001	62	4	0	240	55	0
CATGGCATG	BRDN0001	79	68	0	131	95	0
CATGGCCAC	BRDN0001	84	73	1	164	123	0
CATGGCCAC	BRDN0001	223	259	585	280	369	30
CATGGCCAG	BRDN0001	163	173	918	322	417	12
CATGGCCAT	BRDN0001	269	254	402	576	509	15
CATGGCCCC	BRDN0001	136	25	0	331	222	0
CATGGCCCT	BRDN0001	94	24	108	244	17	0
CATGGCCGA	BRDN0001	60	21	1	326	89	0
CATGGCCGA	BRDN0001	142	96	20	301	166	15
CATGGCCGG	BRDN0001	142	148	737	352	230	8
CATGGCCTA	BRDN0001	114	15	0	125	128	0
CATGGCCTA	BRDN0001	453	486	637	608	696	26
CATGGCCTA	BRDN0001	441	352	82	641	465	19
CATGGCCTT	BRDN0001	50	39	17	140	270	0
CATGGCCTT	BRDN0001	18	0	0	126	58	0
CATGGCCTTT	BRDN0001	91	56	149	314	224	10
CATGGCGAC	BRDN0001	57	71	66	204	225	0
CATGGCGAG	BRDN0001	217	156	84	354	327	0
CATGGCGAT	BRDN0001	90	234	757	291	359	71
CATGGCGGA	BRDN0001	21	0	0	53	40	0
CATGGCGGC	BRDN0001	39	37	19	92	111	0
CATGGCGGG	BRDN0001	93	49	1	227	105	9
CATGGCGGG	BRDN0001	161	257	33	298	114	40
CATGGCGGT	BRDN0001	96	76	27	231	126	97
CATGGCGTT	BRDN0001	108	104	0	276	174	0
CATGGCTATT	BRDN0001	76	153	37	205	310	0
CATGGCTCT	BRDN0001	112	114	0	195	241	0
CATGGCTGA	BRDN0001	210	288	395	462	495	10
CATGGCTGA	BRDN0001	167	82	78	149	584	10
CATGGCTGC	BRDN0001	85	0	0	387	32	0
CATGGCTGG	BRDN0001	323	167	314	748	550	14
CATGGCTGT	BRDN0001	98	59	34	214	302	5
CATGGCTTA	BRDN0001	152	196	425	207	147	12
CATGGGAAA	BRDN0001	24	0	0	394	185	0
CATGGGAAT	BRDN0001	98	146	648	247	258	0
CATGGGAAT	BRDN0001	468	395	11438	748	635	169
CATGGGAGA	BRDN0001	321	244	557	591	407	119
CATGGGATA	BRDN0001	43	134	11	172	145	19
CATGGGCAA	BRDN0001	327	274	1556	416	542	39
CATGGGCAC	BRDN0001	152	179	21	208	100	22
CATGGGCCA	BRDN0001	85	68	1	161	119	0
CATGGGCGA	BRDN0001	241	290	168	504	243	18
CATGGGCGC	BRDN0001	68	90	106	104	135	22
CATGGGCTA	BRDN0001	258	280	374	610	715	23
CATGGGCTG	BRDN0001	295	281	663	680	668	26
CATGGGCTT	BRDN0001	243	116	410	412	353	3
CATGGGGAA	BRDN0001	202	273	748	420	452	9
CATGGGGAA	BRDN0001	175	0	0	435	216	0
CATGGGGAC	BRDN0001	332	325	566	585	881	59
CATGGGGCA	BRDN0001	351	419	519	838	766	42
CATGGGGCT	BRDN0001	125	63	0	265	162	4
CATGGGGGT	BRDN0001	85	69	0	195	233	0
CATGGGGTC	BRDN0001	126	60	269	144	117	13
CATGGGGTT	BRDN0001	340	254	364	651	961	32
CATGGGTAC	BRDN0001	207	246	177	415	277	14
CATGGGTCA	BRDN0001	98	49	58	195	102	0
CATGGGTGA	BRDN0001	149	161	799	295	464	43
CATGGGTGT	BRDN0001	86	90	172	240	112	11
CATGGGTGT	BRDN0001	133	65	135	297	86	34
CATGGGTTC	BRDN0001	80	127	41	245	307	5
CATGGGTTG	BRDN0001	325	278	794	590	480	9
CATGGTAAA	BRDN0001	98	83	12	235	304	35
CATGGTACA	BRDN0001	313	250	449	557	507	8

CATGGTACA	BRDN00011	136	154	0	211	109	0
CATGGTACG	BRDN00011	188	130	1	329	159	0
CATGGTACT	BRDN00011	239	363	317	476	627	13
CATGGTATC	BRDN00011	172	141	546	345	292	20
CATGGTCAA	BRDN00011	211	170	42	460	776	1
CATGGTCGT	BRDN00012	74	67	0	282	344	0
CATGGTCGT	BRDN00012	114	71	95	506	235	11
CATGGTCTT	BRDN00011	52	48	77	220	15	0
CATGGTGAA	BRDN00012	131	29	0	536	133	0
CATGGTGAA	BRDN00011	428	376	132	711	734	0
CATGGTGAA	BRDN00012	386	205	1576	583	377	42
CATGGTGAC	BRDN00012	284	183	560	575	379	23
CATGGTGCC	BRDN00011	51	113	134	203	124	50
CATGGTGGA	BRDN00011	108	0	0	200	280	0
CATGGTGGT	BRDN00011	201	211	200	394	1005	8
CATGGTGTG	BRDN00012	170	198	274	269	225	9
CATGGTGTT	BRDN00011	140	165	6	221	250	16
CATGGTGTT	BRDN00012	147	133	421	446	523	34
CATGGTTAA	BRDN00012	141	127	335	241	309	0
CATGGTTAA	BRDN00012	108	121	673	319	89	55
CATGGTTAC	BRDN00011	85	132	77	214	230	0
CATGGTTAT	BRDN00012	182	174	446	414	605	34
CATGGTTCT	BRDN00011	152	188	41	266	499	32
CATGGTTGT	BRDN00012	150	124	20	548	610	18
CATGGTTTG	BRDN00011	107	111	36	155	282	12
CATGGTTTG	BRDN00012	59	19	0	186	87	0
CATGTAACT	BRDN00011	247	248	266	316	291	18
CATGTAATA	BRDN00012	55	1	0	383	248	0
CATGTAATG	BRDN00012	94	0	0	331	259	0
CATGTAATT	BRDN00012	257	181	54	616	486	12
CATGTACAA	BRDN00012	89	92	206	216	264	12
CATGTACAA	BRDN00011	119	57	231	305	271	9
CATGTACAA	BRDN00012	226	245	1029	505	255	18
CATGTACAA	BRDN00011	280	335	634	409	329	4
CATGTACAG	BRDN00012	90	73	115	319	300	13
CATGTACCAT	BRDN00011	126	0	0	242	176	0
CATGTACCTC	BRDN00012	151	164	15	244	114	4
CATGTACGA	BRDN00011	97	139	4	248	172	9
CATGTACGT	BRDN00011	226	393	52	361	38	68
CATGTACTA	BRDN00012	22	0	0	191	249	0
CATGTACTA	BRDN00012	185	118	688	425	561	29
CATGTACTCA	BRDN00012	132	82	20	223	173	0
CATGTAGAT	BRDN00012	291	288	243	570	677	57
CATGTAGGC	BRDN00012	91	85	4	151	353	11
CATGTATAA	BRDN00011	159	146	753	165	362	8
CATGTATAG	BRDN00012	246	254	176	479	499	16
CATGTATCTT	BRDN00011	112	3	0	265	321	0
CATGTATGA	BRDN00011	106	80	75	227	47	0
CATGTATGT	BRDN00011	126	110	237	268	255	10
CATGTATTCG	BRDN00011	161	205	115	368	279	10
CATGTCAAT	BRDN00011	122	98	0	200	143	6
CATGTCACCA	BRDN00012	307	241	54	561	657	0
CATGTCAGA	BRDN00012	106	53	84	120	60	17
CATGTCAGC	BRDN00011	74	93	25	165	361	44
CATGTCAGC	BRDN00011	83	0	0	198	145	0
CATGTCATCT	BRDN00012	75	15	150	234	67	9
CATGTCATG	BRDN00011	221	151	90	555	572	84
CATGTCCAA	BRDN00011	5	0	0	177	97	0
CATGTCCACC	BRDN00011	211	159	232	254	288	19
CATGTCCAGT	BRDN00012	270	95	86	399	571	0
CATGTCCATC	BRDN00011	286	272	84	671	812	40
CATGTCCATC	BRDN00011	117	158	538	252	97	13
CATGTCCTCA	BRDN00011	103	81	0	308	225	0
CATGTCGCT	BRDN00012	171	272	198	436	511	3
CATGTCGCT	BRDN00012	158	181	693	284	577	47
CATGTCGGA	BRDN00012	101	174	0	130	199	13
CATGTCTATC	BRDN00011	132	141	497	265	122	16
CATGTCTATG	BRDN00011	161	77	258	279	247	35
CATGTCTGA	BRDN00012	149	63	0	525	469	0
CATGTCTGA	BRDN00011	122	62	35	182	99	0
CATGTCTGA	BRDN00011	143	89	60	269	198	2
CATGTCTGT	BRDN00012	222	269	503	550	755	14
CATGTCTGT	BRDN00011	67	67	18	93	237	16
CATGTCTGT	BRDN00011	218	305	326	584	948	22
CATGTGAAG	BRDN00011	130	124	562	293	200	24
CATGTGACA	BRDN00011	150	135	15	250	252	7
CATGTGACT	BRDN00012	139	81	158	260	362	12
CATGTGACT	BRDN00012	168	204	196	217	205	0
CATGTGAGG	BRDN00011	158	165	186	255	201	25
CATGTGATC	BRDN00011	208	162	294	377	274	0
CATGTGATG	BRDN00012	340	386	1854	664	627	34
CATGTGATG	BRDN00011	101	0	0	152	161	0
CATGTGCAA	BRDN00012	117	0	0	268	283	0
CATGTGCAA	BRDN00012	54	10	100	398	310	7
CATGTGCAC	BRDN00011	297	210	189	457	323	8
CATGTGCAG	BRDN00012	309	327	83	533	1023	24
CATGTGCCG	BRDN00012	111	65	0	221	289	23
CATGTGCGA	BRDN00011	405	354	382	660	654	4
CATGTGGAA	BRDN00012	130	75	20	215	115	7
CATGTGGAA	BRDN00012	100	61	273	184	172	0
CATGTGGAA	BRDN00011	98	79	351	366	295	11
CATGTGGAC	BRDN00011	203	119	334	411	328	53
CATGTGGAG	BRDN00011	282	211	73	527	599	0
CATGTGGAT	BRDN00011	183	170	154	333	247	37
CATGTGGAT	BRDN00012	235	193	703	443	582	0
CATGTGGAT	BRDN00011	91	61	0	207	275	15
CATGTGGCA	BRDN00011	218	256	660	379	456	12
CATGTGGCC	BRDN00011	221	180	301	347	267	33
CATGTGGCG	BRDN00012	409	276	553	739	399	9
CATGTGGCT	BRDN00012	213	134	18	318	384	31
CATGTGGGA	BRDN00012	200	127	16	339	198	0
CATGTGGGC	BRDN00012	28	0	5	5	1	7
CATGTGGTC	BRDN00012	409	425	2867	762	1268	67
CATGTGGTG	BRDN00012	125	101	316	140	377	42
CATGTGGTT	BRDN00011	224	350	82	582	390	43
CATGTGTAA	BRDN00011	45	94	0	168	615	15
CATGTGTAA	BRDN00011	369	391	211	572	598	13
CATGTGTAC	BRDN00011	213	160	4167	378	370	109
CATGTGTAC	BRDN00012	413	391	231	557	676	41
CATGTGTCT	BRDN00011	158	133	97	288	455	38
CATGTGTCT	BRDN00012	416	302	274	611	1042	2
CATGTGTGC	BRDN00012	164	0	1	372	271	0
CATGTGTTG	BRDN00012	98	8	0	231	85	0
CATGTGTTT	BRDN00012	153	118	837	246	221	11
CATGTGTTT	BRDN00011	324	216	141	437	396	27
CATGTTACA	BRDN00012	300	216	199	579	606	11
CATGTTACG	BRDN00011	99	98	27	282	125	49
CATGTTATA	BRDN00012	234	334	198	406	396	9
CATGTTCAA	BRDN00011	138	184	93	287	481	5
CATGTTCAG	BRDN00011	318	286	236	495	524	7
CATGTTCAG	BRDN00012	358	201	523	748	390	10
CATGTTCATT	BRDN00011	146	181	602	325	203	0
CATGTTCCG	BRDN00012	242	200	193	452	349	47
CATGTTCGA	BRDN00011	171	226	625	262	456	32
CATGTTCGC	BRDN00011	112	57	0	146	123	8
CATGTTCTTC	BRDN00011	247	209	1148	388	897	55
CATGTTGAT	BRDN00011	392	310	716	650	455	32
CATGTTGCT	BRDN00011	160	255	11	353	337	38
CATGTTGGA	BRDN00012	230	208	65	371	251	31
CATGTTGGA	BRDN00011	346	412	231	780	574	166
CATGTTGGG	BRDN00011	222	136	146	274	213	26
CATGTTGTA	BRDN00012	85	61	245	148	75	25
CATGTTGTG	BRDN00012	276	247	633	555	386	36
CATGTTTAG	BRDN00011	238	114	15	397	793	0
CATGTTTCCC	BRDN00011	132	20	0	273	379	0
CATGTTTCTG	BRDN00012	251	208	149	404	492	9
CATGTTTGA	BRDN00012	238	291	593	309	187	86
CATTAAAAT	BRDN00012	168	153	106	309	191	0
CATTAAACCA	BRDN00012	150	76	751	397	349	70
CATTAAACCT	BRDN00011	213	167	331	339	336	10
CATTAAACCT	BRDN00012	336	267	694	665	1095	19
CATTAAACTG	BRDN00011	58	3	0	376	123	0
CATTAAAGA	BRDN00012	111	60	69	209	138	0
CATTAAATA	BRDN00012	87	164	0	115	274	0
CATTAACATA	BRDN00012	185	197	32	283	87	10
CATTAACCAC	BRDN00012	222	144	59	580	435	10
CATTAAGAC	BRDN00011	170	238	19	330	350	27
CATTAAGAT	BRDN00011	112	86	259	125	108	8
CATTAAGCA	BRDN00012	282	220	190	384	418	0
CATTAAGCA	BRDN00011	168	248	513	490	479	62
CATTAAGGT	BRDN00012	554	542	803	941	1480	50
CATTAAGTG	BRDN00011	292	204	86	321	100	8
CATTAATGC	BRDN00012	229	180	292	441	517	21
CATTAATGC	BRDN00011	201	146	2251	339	197	36
CATTAATGG	BRDN00012	369	362	742	584	226	15
CATTAATGT	BRDN00011	206	270	1034	327	353	30
CATTAATTAC	BRDN00011	278	198	58	469	800	15
CATTACAACA	BRDN00011	200	203	222	344	385	24
CATTACAATT	BRDN00011	184	147	842	244	219	18
CATTACACAT	BRDN00011	190	290	227	322	151	38
CATTACACTA	BRDN00011	288	119	203	774	931	0
CATTACAGA	BRDN00011	129	73	108	224	110	31
CATTACAGC	BRDN00012	382	179	248	751	850	22
CATTACAGT	BRDN00012	117	11	0	306	189	0
CATTACCAG	BRDN00012	258	234	40	607	391	0
CATTACCCGC	BRDN00011	140	158	55	424	560	12
CATTACCGA	BRDN00011	193	200	129	243	249	133
CATTACCTGG	BRDN00012	263	90	55	504	587	0
CATTACCTGG	BRDN00012	86	64	197	185	88	12
CATTACCTTG	BRDN00011	375	63	0	800	372	0
CATTACGAA	BRDN00012	195	192	1300	519	603	31
CATTACGCA	BRDN00012	455	365	1960	728	834	46
CATTACGCTG	BRDN00011	186	208	464	478	306	16
CATTACGGA	BRDN00011	92	21	595	240	62	12
CATTACGGT	BRDN00012	269	24	1	634	233	0
CATTACTCAC	BRDN00011	142	107	11	256	194	9
CATTACTGA	BRDN00012	159	213	479	112	422	14
CATTACTTGA	BRDN00012	167	126	90	296	415	0
CATTAGACT	BRDN00011	149	124	3494	231	189	74
CATTAGAGA	BRDN00012	309	325	447	526	381	26
CATTAGCAA	BRDN00012	152	169	297	399	349	4
CATTAGCAG	BRDN00012	545	527	2954	941	594	64
CATTAGCCTT	BRDN00011	324	212	12	500	318	50
CATTAGCCTT	BRDN00011	269	306	288	590	871	16
CATTAGCTG	BRDN00011	164	87	514	305	182	34
CATTAGCTG	BRDN00011	177	257	10	301	251	55
CATTAGGAT	BRDN00012	235	270	819	470	709	24
CATTAGGGA	BRDN00011	391	235	794	753	602	22
CATTAGGGG	BRDN00011	217	379	286	494	584	23
CATTAGTAT	BRDN00012	318	447	793	620	1363	96
CATTAGTCTG	BRDN00012	323	245	980	425	425	16
CATTATAAA	BRDN00012	310	168	252	357	313	22
CATTATATGT	BRDN00012	305	291	131	713	822	227
CATTATCACC	BRDN00011	371	437	1372	674	1288	9
CATTATCACG	BRDN00011	468	671	645	889	888	7
CATTATCATT	BRDN00012	166	109	2	459	299	7
CATTATCCCT	BRDN00011	163	122	5	304	167	8
CATTATCTTT	BRDN00011	334	268	604	595	772	98
CATTATGATT	BRDN00012	80	8	47	262	175	0
CATTATGGA	BRDN00011	185	71	234	257	168	8
CATTATGGC	BRDN00012	261	124	11	237	221	1
CATTATGTGT	BRDN00011	165	115	39	236	118	68
CATTATTACT	BRDN00012	17	1	0	286	163	0
CATTATTAG	BRDN00012	324	187	74	453	448	51
CATTATTAG	BRDN00012	166	128	8191	270	297	185
CATTATTGA	BRDN00012	268	212	490	628	738	19
CATTCAAAG	BRDN00011	86	116	0	259	201	3
CATTCAAGC	BRDN00012	257	213	993	431	387	30
CATTCAAGG	BRDN00011	87	16	18	164	117	13
CATTCAATA	BRDN00012	192	124	75	283	238	38
CATTCAATAC	BRDN00012	226	229	71	529	691	0
CATTCAATAT	BRDN00012	211	338	76	426	604	36
CATTCACACA	BRDN00012	264	365	1089	574	778	30
CATTCACAG	BRDN00011	106	155	260	318	379	0
CATTCACAG	BRDN00011	194	185	429	437	455	0
CATTCACCAG	BRDN00011	287	307	369	675	812	16
CATTCACCAG	BRDN00012	393	403	3983	645	581	83
CATTCACGA	BRDN00012	215	218	132	401	188	15
CATTCACTAC	BRDN00012	199	161	110	395	454	3
CATTCAGAA	BRDN00011	73	79	0	187	130	0
CATTCAGAA	BRDN00011	510	497	494	687	551	32
CATTCAGAT	BRDN00011	109	89	252	170	164	15
CATTCAGCCT	BRDN00012	40	8	1	726	552	1
CATTCAGGA	BRDN00011	103	109	425	145	281	33
CATTCAGGC	BRDN00012	200	92	303	326	208	20
CATTCAGGG	BRDN00012	49	0	0	195	207	0
CATTCAGGG	BRDN00011	35	0	0	288	172	0
CATTCAGGG	BRDN00012	184	253	539	348	628	52
CATTCAGTA	BRDN00012	125	6	34	290	328	0
CATTCATAG	BRDN00012	189	103	538	288	167	48
CATTCATATC	BRDN00011	116	100	620	232	131	17
CATTCATATT	BRDN00012	215	153	717	275	463	54
CATTCATCCA	BRDN00012	298	157	320	475	778	20
CATTCATCTT	BRDN00012	83	84	131	126	82	17
CATTCATGA	BRDN00011	93	0	69	310	36	0
CATTCATGCA	BRDN00011	80	135	0	197	212	28
CATTCATGCC	BRDN00012	514	407	1094	800	1283	33
CATTCATTCT	BRDN00012	163	122	40	431	361	11
CATTCATTCT	BRDN00011	100	215	0	224	222	4
CATTCCAAAG	BRDN00012	325	43	227	697	613	18
CATTCCAAAG	BRDN00012	291	254	58	310	518	4
CATTCCAAAT	BRDN00012	147	26	0	275	156	0
CATTCCAACA	BRDN00011	160	120	888	409	244	8
CATTCCAACA	BRDN00011	161	142	122	359	347	13
CATTCCAACA	BRDN00011	253	133	353	395	270	0
CATTCCAATC	BRDN00011	300	227	321	455	466	10
CATTCCACGG	BRDN00012	495	391	1548	858	507	43
CATTCCAGA	BRDN00011	200	139	148	360	308	96
CATTCCAGAT	BRDN00011	93	45	198	170	187	0
CATTCCAGG	BRDN00012	193	0	0	482	317	0
CATTCCCAAT	BRDN00011	199	204	22	308	351	25
CATTCCCAGC	BRDN00012	307	251	105	574	427	0
CATTCCCAGT	BRDN00012	454	492	3088	820	1072	131
CATTCCCCAG	BRDN00012	491	317	784	797	1170	26
CATTCCCCCA	BRDN00011	193	145	26570	302	492	347
CATTCCCTAG	BRDN00012	47	0	23	374	167	0
CATTCCGAA	BRDN00012	433	252	919	911	1082	21
CATTCCGAA	BRDN00011	382	304	1392	1110	1476	77
CATTCCGCTC	BRDN00011	126	170	547	295	435	15
CATTCCGGA	BRDN00012	77	91	1238	207	209	4
CATTCCGGA	BRDN00011	44	0	0	135	29	0
CATTCCGGCT	BRDN00012	246	270	457	568	367	4
CATTCCGGT	BRDN00012	241	214	50	518	432	11
CATTCCTAAC	BRDN00012	202	3	1	418	563	0
CATTCCTAAG	BRDN00011	80	134	709	278	526	10
CATTCCTAGA	BRDN00012	65	56	564	121	242	8
CATTCCTCTA	BRDN00011	100	125	156	233	228	22
CATTCCTGG	BRDN00012	93	48	105	159	96	0
CATTCCTTAA	BRDN00011	298	254	1097	563	662	28
CATTCCTTCT	BRDN00011	117	106	0	184	174	10
CATTCCTTCT	BRDN00012	42	0	0	203	120	0
CATTCGACA	BRDN00012	97	104	631	143	45	57
CATTCGACTT	BRDN00012	172	83	66	182	200	6
CATTCGATG	BRDN00011	174	184	426	305	254	9
CATTCGCAAT	BRDN00011	413	375	712	720	646	4
CATTCGGAG	BRDN00011	106	279	2121	260	414	29
CATTCGGGG	BRDN00012	177	70	25	324	224	9
CATTCGGGT	BRDN00011	91	176	410	170	287	38
CATTCTACAG	BRDN00011	61	116	116	107	100	10
CATTCTAGA	BRDN00012	270	267	371	564	447	46
CATTCTAGA	BRDN00011	45	37	0	165	84	0
CATTCTAGAT	BRDN00012	395	339	460	688	657	24
CATTCTAGCT	BRDN00012	238	91	32	344	344	0
CATTCTCAGA	BRDN00012	457	473	815	792	706	15
CATTCTCCAA	BRDN00011	192	125	63	374	487	47
CATTCTCCCC	BRDN00011	114	159	17	249	187	58
CATTCTCTCA	BRDN00012	98	111	73	175	268	35
CATTCTGAG	BRDN00011	282	202	98	411	320	58
CATTCTGCAC	BRDN00012	135	131	2142	280	259	50
CATTCTGGC	BRDN00012	104	144	39	243	320	19
CATTCTGGC	BRDN00012	351	372	405	656	886	23
CATTCTGGC	BRDN00012	382	465	156	791	887	23
CATTCTGGG	BRDN00012	132	105	1077	195	366	44
CATTCTGGT	BRDN00012	234	202	672	539	226	6
CATTCTGTAA	BRDN00011	68	132	119	202	215	18
CATTCTGTCA	BRDN00011	177	312	140	462	914	40
CATTCTGTG	BRDN00012	170	86	2	403	125	0
CATTCTTCAG	BRDN00012	430	382	190	851	797	45
CATTCTTGG	BRDN00012	294	150	594	589	720	20
CATTGAACT	BRDN00011	243	328	503	550	558	33
CATTGAAGG	BRDN00011	97	70	128	142	273	0
CATTGAAGT	BRDN00012	175	99	287	419	361	2
CATTGAATA	BRDN00011	67	31	146	200	230	44
CATTGACAA	BRDN00012	255	207	2636	242	126	65
CATTGACCA	BRDN00011	115	0	0	357	213	0
CATTGACCCA	BRDN00012	136	126	915	309	300	27
CATTGACCTC	BRDN00011	302	409	4662	644	903	57
CATTGACGT	BRDN00012	393	349	389	721	897	6
CATTGAGAA	BRDN00012	264	341	769	419	701	97
CATTGAGAG	BRDN00012	145	131	29	454	242	16
CATTGAGCT	BRDN00011	208	92	343	544	336	5
CATTGAGCT	BRDN00011	87	57	192	180	68	4
CATTGAGGT	BRDN00011	194	148	286	372	240	16
CATTGAGTG	BRDN00011	173	167	126	301	210	1
CATTGAGTG	BRDN00011	187	214	493	348	479	51
CATTGATAC	BRDN00011	234	220	121	377	228	32
CATTGATAG	BRDN00011	211	211	725	413	300	11
CATTGATCG	BRDN00011	134	179	232	494	347	13
CATTGATGA	BRDN00012	66	24	0	190	97	0
CATTGATGA	BRDN00011	363	442	364	611	530	31
CATTGATGCT	BRDN00012	186	94	7	342	441	0
CATTGATTTA	BRDN00012	291	420	353	580	561	98
CATTGATTTA	BRDN00011	203	85	201	487	326	6
CATTGATTTC	BRDN00012	82	191	385	222	135	3
CATTGCACA	BRDN00012	259	137	20	620	331	4
CATTGCACA	BRDN00011	266	248	21	516	302	15
CATTGCACG	BRDN00012	665	581	1077	960	956	48
CATTGCACTC	BRDN00012	481	453	391	988	1705	83
CATTGCAGG	BRDN00012	130	108	24	344	360	18
CATTGCAGT	BRDN00012	252	161	979	480	435	18
CATTGCAGT	BRDN00012	223	251	856	525	445	9
CATTGCATA	BRDN00012	166	146	222	256	205	63
CATTGCATTT	BRDN00011	336	445	169	452	808	8
CATTGCCAGT	BRDN00011	260	300	1123	436	621	55
CATTGCCATG	BRDN00012	366	233	444	755	755	0
CATTGCCCAC	BRDN00012	131	134	242	194	413	4
CATTGCCGT	BRDN00011	98	50	0	262	110	0
CATTGCCTCA	BRDN00011	107	168	40	419	256	0
CATTGCCTG	BRDN00011	134	103	8	280	146	0
CATTGCGAA	BRDN00011	237	246	216	297	486	27
CATTGCGAG	BRDN00012	135	55	164	273	110	0
CATTGCGAT	BRDN00011	227	171	242	364	315	22
CATTGCGGA	BRDN00011	236	183	486	565	1362	5
CATTGCGGA	BRDN00012	294	230	632	477	384	13
CATTGCGGC	BRDN00011	197	0	0	273	357	0
CATTGCGGC	BRDN00012	131	6	0	299	77	0
CATTGCTAA	BRDN00011	108	56	26	220	359	0
CATTGCTACT	BRDN00011	148	189	57	311	350	0
CATTGCTCAT	BRDN00011	332	369	719	619	610	28
CATTGCTCG	BRDN00012	367	368	204	626	632	7
CATTGCTGC	BRDN00012	125	218	675	292	251	8
CATTGCTTTC	BRDN00012	199	129	535	377	333	18
CATTGGAAA	BRDN00011	73	55	114	190	29	0
CATTGGACA	BRDN00011	402	335	721	633	482	17
CATTGGACT	BRDN00011	187	125	161	232	144	0
CATTGGACT	BRDN00012	311	161	235	672	603	13
CATTGGAGA	BRDN00012	153	155	136	294	297	9
CATTGGAGA	BRDN00011	172	107	187	390	182	21
CATTGGAGC	BRDN00012	95	130	20	226	61	60
CATTGGAGT	BRDN00012	182	161	5553	344	217	51
CATTGGATG	BRDN00011	252	300	229	579	622	2
CATTGGATG	BRDN00012	130	90	154	314	116	6
CATTGGCAA	BRDN00012	133	84	23	276	157	8
CATTGGCAA	BRDN00011	120	105	474	145	352	17
CATTGGCAA	BRDN00012	354	137	345	965	901	0
CATTGGCAA	BRDN00011	62	74	562	181	317	21
CATTGGCCA	BRDN00012	428	504	761	754	731	54
CATTGGCCG	BRDN00012	268	310	923	638	755	47
CATTGGCCT	BRDN00011	300	175	1586	496	670	42
CATTGGCGC	BRDN00012	424	536	4854	804	2538	130
CATTGGCGG	BRDN00011	78	137	0	278	333	0
CATTGGCTC	BRDN00012	206	131	0	297	609	6
CATTGGGAA	BRDN00011	123	211	666	262	42	23
CATTGGGAG	BRDN00011	350	275	639	800	553	56
CATTGGGAT	BRDN00011	526	476	381	814	616	37
CATTGGGAT	BRDN00011	155	177	74	306	197	0
CATTGGGCC	BRDN00011	85	54	53	123	129	4
CATTGGGGT	BRDN00012	329	219	148	643	732	7
CATTGGGGT	BRDN00012	252	171	824	464	540	23
CATTGGTAA	BRDN00011	9	5	0	97	32	0
CATTGGTAC	BRDN00011	147	154	94	318	326	5
CATTGGTGG	BRDN00011	144	92	12	261	42	14
CATTGGTGG	BRDN00012	94	70	273	248	378	9
CATTGGTGT	BRDN00011	92	41	292	197	181	0
CATTGTAAT	BRDN00012	203	305	8	376	389	11
CATTGTACA	BRDN00012	373	403	255	605	478	71
CATTGTAGT	BRDN00012	176	187	872	327	148	10
CATTGTATG	BRDN00012	248	193	209	574	1177	10
CATTGTCCAC	BRDN00011	35	47	0	210	354	0
CATTGTCCTC	BRDN00011	521	436	650	889	1150	46
CATTGTCGA	BRDN00011	67	43	129	259	93	0
CATTGTCGT	BRDN00012	112	70	439	262	245	7
CATTGTGCA	BRDN00012	273	315	384	529	398	32
CATTGTGCA	BRDN00011	321	267	1050	664	411	49
CATTGTGCT	BRDN00012	393	262	286	830	812	0
CATTGTGTG	BRDN00012	168	16	10	208	131	0
CATTGTTCAA	BRDN00011	171	122	89	282	354	0
CATTGTTGA	BRDN00012	315	246	305	598	527	4
CATTGTTGA	BRDN00012	342	515	394	715	763	4
CATTGTTTCA	BRDN00012	151	226	206	237	398	11
CATTTAAAG	BRDN00012	275	287	610	542	442	51
CATTTAAAG	BRDN00012	121	98	121	177	244	0
CATTTAAATA	BRDN00011	166	151	35	241	128	0
CATTTACAAG	BRDN00011	216	192	138	406	439	44
CATTTAGTA	BRDN00011	99	147	123	225	332	49
CATTTATGA	BRDN00012	312	357	1190	569	790	41
CATTTATGA	BRDN00011	183	124	1253	197	170	7
CATTTATTAC	BRDN00012	165	13	0	371	130	0
CATTTATTGA	BRDN00011	334	268	502	552	437	12
CATTTCAAAT	BRDN00011	169	284	0	472	432	0
CATTTCAAG	BRDN00012	113	98	1535	252	453	55
CATTTCAGG	BRDN00012	160	116	1325	264	236	30
CATTTCATAC	BRDN00011	91	53	46	192	170	0
CATTTCATAG	BRDN00011	170	159	56	455	218	19
CATTTCCAAA	BRDN00011	144	71	29	210	345	4
CATTTCCAAC	BRDN00011	367	270	95	396	485	29
CATTTCCAGC	BRDN00012	153	60	6	170	190	15
CATTTCCGCA	BRDN00011	79	0	0	286	226	0
CATTTCCTTC	BRDN00012	90	52	300	142	30	0
CATTTCGAGT	BRDN00011	0	0	0	0	14	0
CATTTCGGA	BRDN00011	297	199	114	403	479	29
CATTTCTAAT	BRDN00012	110	16	0	483	593	0
CATTTCTAAT	BRDN00011	230	338	2436	237	337	41
CATTTGAATT	BRDN00011	99	3	1	169	5	0
CATTTGCAA	BRDN00011	332	350	1249	507	638	35
CATTTGCAAT	BRDN00012	245	394	433	529	454	131
CATTTGCCAC	BRDN00012	139	100	341	267	263	0
CATTTGCCAG	BRDN00012	316	358	1323	670	780	103
CATTTGCCCC	BRDN00012	153	170	37	193	102	22
CATTTGCCG	BRDN00012	369	268	165	850	658	38
CATTTGCTG	BRDN00012	124	60	20	231	96	8
CATTTGGAA	BRDN00011	267	337	1	493	347	35
CATTTGGAG	BRDN00012	186	206	2522	394	338	63
CATTTGGAG	BRDN00012	355	447	342	816	1385	14
CATTTGGGG	BRDN00011	141	177	500	321	468	32
CATTTGGTG	BRDN00011	150	114	430	242	287	17
CATTTGGTT	BRDN00012	323	366	225	538	534	58
CATTTGTGA	BRDN00011	156	162	737	291	506	28
CATTTGTGA	BRDN00012	571	540	542	893	554	87
CATTTGTGG	BRDN00011	236	291	244	517	361	28
CATTTGTGT	BRDN00012	53	8	0	208	62	0
CCAAAAAAC	BRDN00011	76	17	0	111	160	0
CCAAAAAGA	BRDN00012	438	269	775	636	1138	71
CCAAAAATA	BRDN00012	421	366	339	653	986	26
CCAAAACAG	BRDN00012	45	52	517	132	75	5
CCAAAACAT	BRDN00011	159	214	534	430	488	12
CCAAAACCCC	BRDN00011	148	66	30	413	315	22
CCAAAACGA	BRDN00012	365	289	415	451	401	25
CCAAAACTTC	BRDN00011	172	12	15	892	489	0
CCAAAAGAA	BRDN00012	114	109	460	309	292	9
CCAAAAGCA	BRDN00011	333	25	10	912	862	0
CCAAAAGCT	BRDN00011	142	82	624	233	168	17
CCAAAAGGA	BRDN00011	518	348	324	752	592	20
CCAAAAGGA	BRDN00012	198	167	273	340	322	1
CCAAAAGTC	BRDN00011	99	31	44	647	371	0

CCAAAATAC	BRDN00012	230	49	76	469	522	37
CCAAAATATT	BRDN00011	82	25	98	184	328	20
CCAAAATCG	BRDN00012	247	307	4651	518	738	124
CCAAAATGA	BRDN00012	356	298	131	785	1230	34
CCAAAATGG	BRDN00011	179	188	898	448	604	34
CCAAACAACT	BRDN00012	98	160	366	383	455	55
CCAAACACA	BRDN00011	260	129	184	355	497	6
CCAAACACTC	BRDN00012	372	318	285	982	408	22
CCAAACCACA	BRDN00012	178	181	827	566	376	19
CCAAACCAG	BRDN00011	154	107	0	247	284	14
CCAAACCAG	BRDN00011	277	164	82	387	381	25
CCAAACCATT	BRDN00012	156	190	139	309	583	101
CCAAACCCAA	BRDN00012	449	445	1074	794	587	70
CCAAACGAT	BRDN00011	95	41	210	206	130	0
CCAAACGTTT	BRDN00012	146	164	1226	190	320	22
CCAAACTATG	BRDN00011	187	177	305	249	371	7
CCAAACTATT	BRDN00011	69	86	18	192	101	17
CCAAACTCCT	BRDN00011	328	466	323	537	294	11
CCAAACTGA	BRDN00012	399	352	376	658	1072	5
CCAAACTGCT	BRDN00012	409	266	67	562	204	117
CCAAACTGG	BRDN00011	120	139	25	172	195	0
CCAAACTTAC	BRDN00012	299	254	535	526	423	60
CCAAAGACT	BRDN00011	151	156	269	282	315	39
CCAAAGACT	BRDN00012	399	436	876	714	680	47
CCAAAGAGA	BRDN00012	49	69	0	166	128	0
CCAAAGAGG	BRDN00011	94	18	0	401	91	0
CCAAAGAGT	BRDN00011	273	105	26	504	566	33
CCAAAGATA	BRDN00012	73	81	7	317	347	19
CCAAAGATA	BRDN00011	232	224	576	297	301	24
CCAAAGATC	BRDN00012	172	97	13	274	144	4
CCAAAGATC	BRDN00011	332	226	415	571	539	0
CCAAAGCAC	BRDN00012	120	154	172	450	531	0
CCAAAGCCA	BRDN00012	407	244	530	747	840	30
CCAAAGCCT	BRDN00012	76	10	0	488	84	0
CCAAAGCTA	BRDN00011	166	196	102	252	237	3
CCAAAGGAA	BRDN00011	137	113	18	167	356	0
CCAAAGGAC	BRDN00012	137	143	121	216	151	0
CCAAAGGAT	BRDN00011	143	155	1188	293	459	4
CCAAAGGCG	BRDN00011	190	131	212	409	656	19
CCAAAGGCT	BRDN00012	426	302	409	646	409	55
CCAAAGGTA	BRDN00011	81	41	2	568	314	0
CCAAAGTCC	BRDN00011	141	201	424	325	148	119
CCAAAGTGT	BRDN00012	263	367	168	537	380	75
CCAAAGTGT	BRDN00012	131	152	565	334	286	52
CCAAAGTTA	BRDN00012	110	216	296	299	356	7
CCAAAGTTA	BRDN00012	74	165	98	282	144	24
CCAAATAAG	BRDN00011	264	284	202	470	484	47
CCAAATACA	BRDN00011	173	256	629	455	752	66
CCAAATAGT	BRDN00011	131	46	855	193	82	88
CCAAATATTA	BRDN00012	119	93	0	159	786	18
CCAAATCAG	BRDN00012	99	188	206	345	109	8
CCAAATCAG	BRDN00011	110	43	112	201	47	0
CCAAATCCAT	BRDN00012	140	0	0	307	136	0
CCAAATCCAT	BRDN00012	182	219	21	308	376	0
CCAAATCTCC	BRDN00012	172	77	929	251	77	32
CCAAATCTG	BRDN00011	235	255	1543	862	775	34
CCAAATCTGT	BRDN00011	242	195	19	364	373	0
CCAAATGAA	BRDN00011	188	241	316	200	380	28
CCAAATGAC	BRDN00012	187	236	695	371	301	15
CCAAATGGA	BRDN00011	602	492	498	896	1327	36
CCAAATGGG	BRDN00011	74	74	50	114	101	0
CCAAATGGT	BRDN00011	203	128	120	490	1193	14
CCAAATGTA	BRDN00011	130	226	784	272	185	83
CCAAATTCAG	BRDN00012	233	129	365	408	365	7
CCAAATTTCA	BRDN00011	142	0	1	436	191	0
CCAACAACG	BRDN00012	123	126	147	164	154	3
CCAACAACG	BRDN00012	241	145	24	230	201	26
CCAACAAGA	BRDN00012	33	0	0	178	289	0
CCAACAAGA	BRDN00012	536	496	750	981	1871	81
CCAACAAGG	BRDN00012	92	42	155	170	100	8
CCAACAATG	BRDN00011	142	191	746	329	510	25
CCAACACAG	BRDN00012	384	317	2595	731	579	49
CCAACACATT	BRDN00012	331	221	159	628	627	20
CCAACACCAC	BRDN00011	325	165	127	510	381	3
CCAACACCG	BRDN00011	180	0	1	481	244	0
CCAACACCGT	BRDN00011	106	147	197	287	377	39
CCAACACCTA	BRDN00011	116	114	78	467	246	25
CCAACACCTC	BRDN00011	224	159	9	662	243	41
CCAACACGA	BRDN00012	244	293	737	618	476	0
CCAACACGA	BRDN00012	208	116	232	344	229	0
CCAACACGC	BRDN00011	118	186	864	342	173	0
CCAACACTAC	BRDN00012	364	370	2186	678	682	74
CCAACACTCA	BRDN00012	359	285	444	834	564	19
CCAACACTCC	BRDN00011	218	136	179	370	489	28
CCAACACTGC	BRDN00011	199	115	1067	425	704	34
CCAACAGCG	BRDN00012	75	134	61	145	156	0
CCAACAGCG	BRDN00012	183	188	218	331	179	13
CCAACAGCG	BRDN00012	113	60	31	130	112	12
CCAACAGGA	BRDN00012	390	252	52	706	864	14
CCAACAGGA	BRDN00011	278	231	184	699	653	28
CCAACAGGA	BRDN00012	165	156	199	240	658	2
CCAACAGTCC	BRDN00011	83	82	0	179	320	0
CCAACATAA	BRDN00011	162	128	1	122	99	18
CCAACATAG	BRDN00012	283	287	1031	671	498	40
CCAACATATA	BRDN00011	134	183	92	388	395	16
CCAACATCAT	BRDN00012	302	238	763	459	808	6
CCAACATCCA	BRDN00012	308	419	137	611	625	0
CCAACATCG	BRDN00012	487	592	775	890	964	51
CCAACATGA	BRDN00012	116	29	15	309	165	25
CCAACATGA	BRDN00012	98	85	36	274	258	0
CCAACATGG	BRDN00012	110	151	19	292	160	35
CCAACCAACA	BRDN00012	370	277	120	939	642	3
CCAACCAATC	BRDN00012	407	483	765	701	544	28
CCAACCACAC	BRDN00011	117	133	0	294	274	23
CCAACCATAG	BRDN00011	157	0	0	327	53	0
CCAACCATCC	BRDN00011	90	19	0	186	78	1
CCAACCATGC	BRDN00011	357	332	1871	684	687	108
CCAACCCAAG	BRDN00011	221	70	36	778	883	1
CCAACCCATC	BRDN00012	207	183	95	429	243	35
CCAACCCATC	BRDN00011	316	429	235	605	793	88
CCAACCCATC	BRDN00012	411	483	662	756	596	24
CCAACCCCAA	BRDN00011	171	98	394	455	285	13
CCAACCCCAA	BRDN00011	517	451	1672	724	844	97
CCAACCCCAT	BRDN00012	256	242	63	541	526	40
CCAACCCCCA	BRDN00011	83	79	33	265	97	0
CCAACCCCGC	BRDN00012	99	71	33	246	202	19
CCAACCCCGT	BRDN00011	187	246	23	255	430	20
CCAACCCCTA	BRDN00012	270	476	240	450	486	49
CCAACCCTCC	BRDN00011	324	356	473	544	586	0
CCAACCCTGG	BRDN00012	160	143	9	290	302	14
CCAACCGAG	BRDN00012	144	285	369	295	422	31
CCAACCGCAT	BRDN00011	312	373	350	556	283	35
CCAACCGCCT	BRDN00012	220	15	0	455	212	1
CCAACCGTGT	BRDN00011	275	1	0	424	242	0
CCAACCTATA	BRDN00012	341	246	286	528	653	53
CCAACCTGA	BRDN00011	195	163	299	532	442	15
CCAACCTTCC	BRDN00012	389	164	120	1131	664	7
CCAACGAAC	BRDN00011	120	114	114	129	165	9
CCAACGAAG	BRDN00012	384	264	1340	775	612	65
CCAACGAGA	BRDN00011	317	301	111	508	242	37
CCAACGAGT	BRDN00012	227	192	30	557	264	11
CCAACGATC	BRDN00011	134	0	0	307	331	0
CCAACGATC	BRDN00012	116	0	0	352	117	0
CCAACGGAC	BRDN00012	173	209	1147	373	287	8
CCAACGGCA	BRDN00012	467	548	961	780	1070	39
CCAACGGCT	BRDN00011	83	15	9	172	332	0
CCAACGGCT	BRDN00011	482	405	326	640	552	20
CCAACGGCTT	BRDN00011	207	259	709	366	464	8
CCAACGGGA	BRDN00012	178	67	21	166	129	7
CCAACGGTG	BRDN00012	151	87	234	254	228	9
CCAACGTAG	BRDN00012	112	203	96	272	312	22
CCAACGTTTG	BRDN00011	478	404	282	837	677	218
CCAACTACCA	BRDN00011	135	116	27	272	267	19
CCAACTACCT	BRDN00011	162	69	242	358	226	0
CCAACTAGCC	BRDN00011	186	260	111	401	234	2
CCAACTATCT	BRDN00011	303	177	2801	444	279	44
CCAACTCACA	BRDN00012	243	196	172	493	430	14
CCAACTCACC	BRDN00011	157	153	251	292	226	4
CCAACTCAG	BRDN00012	108	41	50	289	167	1
CCAACTCATT	BRDN00011	193	157	84	374	123	11
CCAACTCCAA	BRDN00011	97	230	1516	317	342	36
CCAACTCCAA	BRDN00012	248	267	1445	335	393	112
CCAACTCGA	BRDN00011	483	536	1208	969	777	34
CCAACTCTGC	BRDN00012	187	390	445	538	599	11
CCAACTGCCC	BRDN00012	231	162	852	320	409	32
CCAACTTACA	BRDN00011	95	102	582	370	322	4
CCAACTTACG	BRDN00011	274	245	1796	591	504	67
CCAACTTCAT	BRDN00012	358	236	811	651	575	52
CCAACTTCTA	BRDN00012	81	0	82	318	169	0
CCAACTTCTG	BRDN00012	370	229	42	761	451	0
CCAACTTGCC	BRDN00011	214	13	24	407	510	0
CCAACTTTGA	BRDN00011	227	234	353	402	558	24
CCAAGAAAA	BRDN00012	135	2	0	1081	631	0
CCAAGAAAC	BRDN00012	267	219	530	641	532	7
CCAAGAAAT	BRDN00012	119	160	243	251	444	6
CCAAGAACA	BRDN00011	232	66	530	330	358	13
CCAAGAACA	BRDN00011	349	356	95	486	480	19
CCAAGAACC	BRDN00012	166	75	569	313	442	21
CCAAGAACC	BRDN00011	220	242	183	346	286	63
CCAAGAACTT	BRDN00012	92	94	0	241	461	19
CCAAGAAGG	BRDN00011	154	0	0	334	28	0
CCAAGAAGT	BRDN00011	87	57	6	138	195	0
CCAAGAATA	BRDN00011	179	92	14	244	153	8
CCAAGAATG	BRDN00011	284	484	552	609	576	48
CCAAGAATT	BRDN00011	130	129	429	217	126	3
CCAAGACAA	BRDN00012	199	268	762	316	331	20
CCAAGACAC	BRDN00012	338	241	395	525	913	57
CCAAGACAT	BRDN00012	120	71	82	168	174	11
CCAAGACATC	BRDN00011	102	117	19	235	99	9
CCAAGACCA	BRDN00012	59	40	283	293	165	16
CCAAGACGC	BRDN00012	416	395	1543	878	748	36
CCAAGACGT	BRDN00011	111	46	119	282	195	4
CCAAGACGT	BRDN00011	248	261	284	439	404	10
CCAAGACTC	BRDN00011	53	34	10	379	175	7
CCAAGACTC	BRDN00012	383	356	232	774	601	137
CCAAGAGAA	BRDN00012	84	50	77	203	111	0
CCAAGAGAA	BRDN00012	70	85	14	131	125	10
CCAAGAGCA	BRDN00011	276	50	519	551	202	7
CCAAGAGGA	BRDN00012	155	206	375	255	349	27
CCAAGAGGA	BRDN00011	87	129	5	190	135	0
CCAAGATCA	BRDN00011	50	26	0	194	160	0
CCAAGATCA	BRDN00011	202	168	1	353	306	0
CCAAGATCCT	BRDN00011	93	92	636	166	172	0
CCAAGATCTT	BRDN00012	190	196	1162	531	379	49
CCAAGATGA	BRDN00011	192	251	166	446	1035	56
CCAAGATGG	BRDN00011	161	113	3201	271	141	40
CCAAGATGG	BRDN00011	247	97	311	317	150	7
CCAAGATGT	BRDN00011	16	35	0	216	78	0
CCAAGATGT	BRDN00011	104	72	274	221	115	17
CCAAGATGT	BRDN00012	280	45	5	611	422	0
CCAAGATTTC	BRDN00012	99	90	180	180	218	71
CCAAGCAAT	BRDN00012	254	223	1040	337	392	25
CCAAGCAATT	BRDN00012	86	35	0	321	363	0
CCAAGCAGC	BRDN00012	240	283	604	407	541	44
CCAAGCAGG	BRDN00012	89	2	11	144	119	0
CCAAGCATC	BRDN00011	180	204	550	265	420	33
CCAAGCCAA	BRDN00012	90	103	41	137	184	0
CCAAGCCCA	BRDN00012	86	109	327	163	273	23
CCAAGCCCG	BRDN00011	415	412	836	934	828	32
CCAAGCCCGT	BRDN00012	200	177	26	216	204	1
CCAAGCCGCT	BRDN00012	415	480	781	774	523	6
CCAAGCCGCT	BRDN00011	171	143	247	450	441	20
CCAAGCGCC	BRDN00012	464	685	1244	866	1385	44
CCAAGCGCCT	BRDN00012	244	315	60	267	434	0
CCAAGCGGG	BRDN00012	81	167	251	128	653	27
CCAAGCGTC	BRDN00012	244	233	240	347	473	20
CCAAGCTAG	BRDN00012	208	30	10	529	268	0
CCAAGCTTCC	BRDN00012	112	18	0	296	162	0
CCAAGCTTG	BRDN00011	77	0	0	406	169	0
CCAAGCTTTC	BRDN00012	257	279	990	593	754	20
CCAAGGAAA	BRDN00011	14	0	0	55	26	0
CCAAGGAAA	BRDN00012	165	116	24	342	831	42
CCAAGGAAT	BRDN00012	221	208	1030	456	660	68
CCAAGGACA	BRDN00012	252	144	536	465	328	0
CCAAGGACC	BRDN00012	139	141	186	356	279	26
CCAAGGAGA	BRDN00012	70	0	0	383	112	0
CCAAGGAGC	BRDN00012	174	76	1032	289	148	10
CCAAGGAGC	BRDN00012	198	155	1	332	311	0
CCAAGGAGC	BRDN00012	149	111	0	311	79	11
CCAAGGCCA	BRDN00012	302	328	2122	528	844	80
CCAAGGCCT	BRDN00012	152	250	173	290	260	25
CCAAGGCTA	BRDN00011	54	138	2657	68	92	53
CCAAGGCTA	BRDN00011	297	239	341	570	516	24
CCAAGGCTA	BRDN00012	167	78	0	422	900	6
CCAAGGGAT	BRDN00011	159	192	65	221	90	0
CCAAGGGCT	BRDN00012	102	115	1	291	202	25
CCAAGGGGC	BRDN00012	273	230	382	412	431	0
CCAAGGTAA	BRDN00012	685	367	351	1104	719	50
CCAAGGTAC	BRDN00011	134	220	8	374	196	4
CCAAGGTCA	BRDN00012	121	115	744	327	133	19
CCAAGGTCT	BRDN00011	140	133	236	346	184	4
CCAAGGTCT	BRDN00011	126	75	4	217	197	0
CCAAGGTGA	BRDN00012	141	84	0	277	184	62
CCAAGGTGC	BRDN00012	151	119	0	112	36	5
CCAAGGTGG	BRDN00011	434	251	290	744	720	39
CCAAGGTGT	BRDN00011	169	189	249	316	289	0
CCAAGGTTC	BRDN00011	66	55	3	158	77	5
CCAAGTACC	BRDN00011	121	161	158	324	603	7
CCAAGTACT	BRDN00011	238	152	856	366	389	31
CCAAGTAGC	BRDN00011	267	148	217	523	406	14
CCAAGTATG	BRDN00011	114	22	467	125	101	0
CCAAGTATG	BRDN00012	210	160	161	546	436	46
CCAAGTCAA	BRDN00012	40	65	245	257	141	0
CCAAGTCAC	BRDN00011	238	341	514	554	601	14
CCAAGTCAG	BRDN00012	124	150	44	223	141	70
CCAAGTCAT	BRDN00012	93	158	1214	182	28	26
CCAAGTCATC	BRDN00012	212	244	813	345	451	84
CCAAGTCCG	BRDN00011	415	363	1212	738	653	73
CCAAGTCTAC	BRDN00012	220	177	44	331	210	0
CCAAGTCTGC	BRDN00011	356	313	1031	763	1154	5
CCAAGTCTTC	BRDN00012	291	218	23	539	681	12
CCAAGTGCC	BRDN00011	103	75	74	220	268	0
CCAAGTGCG	BRDN00012	124	58	2	208	37	0
CCAAGTGCTC	BRDN00012	247	160	1281	473	520	43
CCAAGTGTA	BRDN00012	204	165	148	326	354	15
CCAAGTGTTC	BRDN00012	176	0	65	337	139	0
CCAAGTTACT	BRDN00012	192	97	86	303	364	0
CCAAGTTCCA	BRDN00011	136	112	233	220	129	7
CCAAGTTCTA	BRDN00012	217	25	87	448	606	11
CCAAGTTTA	BRDN00012	182	139	77	370	326	8
CCAATAAAA	BRDN00012	238	306	213	411	420	6
CCAATAACG	BRDN00011	119	43	371	172	162	115
CCAATAATA	BRDN00012	294	393	204	549	379	50
CCAATACAG	BRDN00011	202	209	447	173	94	0
CCAATACAG	BRDN00011	129	0	1	464	526	0
CCAATACCGC	BRDN00012	185	61	10	360	295	0
CCAATACCTA	BRDN00012	213	208	175	303	136	45
CCAATACTGT	BRDN00011	169	69	0	328	165	0
CCAATAGCA	BRDN00012	134	4	88	286	242	0
CCAATAGCA	BRDN00011	61	109	0	143	83	3
CCAATAGTA	BRDN00011	151	85	0	359	241	0
CCAATAGTG	BRDN00012	149	144	830	296	225	58
CCAATATAG	BRDN00012	236	193	651	403	651	25
CCAATATCGT	BRDN00011	200	356	16	459	347	10
CCAATATGA	BRDN00011	236	252	395	374	409	3
CCAATATGA	BRDN00011	127	0	2	283	110	0
CCAATATGA	BRDN00011	234	232	159	409	507	0
CCAATATTCA	BRDN00012	142	195	217	397	600	19
CCAATCACAC	BRDN00012	180	94	146	282	1049	3
CCAATCAGA	BRDN00012	125	0	0	652	315	0
CCAATCCAAC	BRDN00011	196	166	72	188	405	37
CCAATCCAAC	BRDN00012	103	153	148	292	240	52
CCAATCCAAT	BRDN00011	143	165	29	216	436	13
CCAATCCAAT	BRDN00012	119	178	167	341	368	33
CCAATCCAG	BRDN00011	407	394	343	688	534	39
CCAATCCATA	BRDN00012	211	214	206	462	344	50
CCAATCCATC	BRDN00012	173	152	381	380	152	44
CCAATCGGA	BRDN00011	107	72	39	257	241	1
CCAATCGGC	BRDN00011	421	628	3560	964	923	98
CCAATCTCAG	BRDN00012	436	424	1384	750	902	28
CCAATCTCAG	BRDN00012	327	281	333	805	1028	24
CCAATCTGA	BRDN00011	403	362	312	620	690	11
CCAATCTGAT	BRDN00011	135	92	37	311	479	13
CCAATCTGTT	BRDN00011	233	251	458	452	506	21
CCAATCTTGA	BRDN00012	451	371	468	651	981	50
CCAATGAAC	BRDN00012	237	143	168	476	433	41
CCAATGAAT	BRDN00012	256	238	583	490	970	7
CCAATGACA	BRDN00012	296	19	1	575	326	0
CCAATGACTT	BRDN00012	154	154	9	224	416	0
CCAATGATA	BRDN00012	631	600	1078	789	684	104
CCAATGATA	BRDN00011	315	369	2227	391	413	49
CCAATGATG	BRDN00011	144	16	0	447	456	0
CCAATGATG	BRDN00012	216	99	358	414	361	0
CCAATGCAA	BRDN00012	66	40	44	430	582	0
CCAATGCAG	BRDN00012	147	88	40	207	267	1
CCAATGCTG	BRDN00012	344	258	3644	608	497	34
CCAATGGAG	BRDN00011	42	12	0	283	290	0
CCAATGGGA	BRDN00012	108	85	944	409	262	14
CCAATGGGA	BRDN00012	128	105	88	249	402	30
CCAATGGGC	BRDN00012	127	86	14	163	285	8
CCAATGGTTC	BRDN00012	514	485	465	645	978	96
CCAATGTCCA	BRDN00012	275	204	25	470	267	34
CCAATGTGG	BRDN00012	252	108	366	389	190	35
CCAATGTGG	BRDN00011	86	52	0	308	119	0
CCAATGTTG	BRDN00012	137	77	171	251	295	0
CCAATTACCT	BRDN00011	85	135	24	203	367	7
CCAATTACCT	BRDN00012	345	128	215	357	176	13
CCAATTATAT	BRDN00012	227	298	6027	553	254	76
CCAATTCAAA	BRDN00011	124	170	259	227	155	0
CCAATTCAG	BRDN00011	509	884	21713	941	990	541
CCAATTCCAG	BRDN00012	114	135	423	282	399	2
CCAATTCCGC	BRDN00012	413	379	1797	995	659	51
CCAATTGAA	BRDN00011	258	181	94	409	316	48
CCAATTGCGT	BRDN00012	226	203	283	504	586	21
CCAATTGCTT	BRDN00011	113	153	646	269	291	15
CCAATTGGT	BRDN00011	349	210	863	524	458	17
CCAATTTAG	BRDN00012	141	54	544	320	333	26
CCAATTTCTA	BRDN00011	102	73	0	227	74	0
CCACAAAAG	BRDN00012	346	173	346	467	646	34
CCACAAAAG	BRDN00011	110	143	10	257	203	0
CCACAAACA	BRDN00012	296	240	866	520	468	28
CCACAAACCC	BRDN00012	272	403	1568	371	328	48
CCACAAACTC	BRDN00012	365	275	3013	827	742	55
CCACAAACTG	BRDN00011	172	247	0	315	223	0
CCACAAAGA	BRDN00012	230	147	25	569	667	48
CCACAAAGA	BRDN00012	145	45	4	275	165	0
CCACAAAGG	BRDN00012	112	188	37	262	153	0
CCACAAAGT	BRDN00011	94	32	304	208	194	11
CCACAAATAT	BRDN00011	117	134	406	338	300	32
CCACAAATAT	BRDN00012	230	203	85	402	264	20
CCACAAATTG	BRDN00011	113	189	129	179	147	14
CCACAACACT	BRDN00011	98	166	2818	146	273	23
CCACAACCG	BRDN00011	79	55	16	325	275	46
CCACAACTAT	BRDN00011	162	75	0	347	250	1
CCACAACTCA	BRDN00012	404	372	1781	855	922	20
CCACAAGCA	BRDN00011	126	203	35	375	248	0
CCACAAGCA	BRDN00011	149	64	36	173	306	61
CCACAAGGA	BRDN00011	196	183	716	335	290	60
CCACAAGGA	BRDN00011	37	9	0	165	192	0
CCACAAGGG	BRDN00012	184	125	22	294	458	15
CCACAAGTA	BRDN00011	235	272	3809	392	391	107
CCACAATCAC	BRDN00011	401	438	315	781	762	134
CCACAATCTC	BRDN00011	165	133	5331	210	201	59
CCACAATCTT	BRDN00012	224	9	32	619	247	0
CCACAATGCC	BRDN00011	106	115	11	161	57	12
CCACAATGG	BRDN00012	354	491	83	698	619	20
CCACAATGG	BRDN00011	283	140	311	517	471	14
CCACAATGTC	BRDN00012	142	62	62	267	46	0
CCACAATGTC	BRDN00011	63	5	1	885	237	0
CCACACAAA	BRDN00011	105	104	1073	299	191	0
CCACACAACG	BRDN00012	431	380	1720	649	1190	134
CCACACAAG	BRDN00011	137	216	43	351	498	42
CCACACACAG	BRDN00012	207	190	97	445	315	0
CCACACACAT	BRDN00011	125	0	0	247	207	0
CCACACACCC	BRDN00012	104	111	24	283	117	9
CCACACACTC	BRDN00011	446	532	227	659	718	30
CCACACAGA	BRDN00011	330	343	427	603	556	16
CCACACAGG	BRDN00011	173	7	1	496	713	0
CCACACATTC	BRDN00011	63	24	1	454	625	0
CCACACCAAA	BRDN00011	173	70	609	352	163	13
CCACACCACG	BRDN00012	262	236	364	374	295	0
CCACACCAG	BRDN00011	219	127	179	540	511	0
CCACACCATG	BRDN00011	367	399	2877	741	566	126
CCACACCATT	BRDN00012	163	119	217	272	158	0
CCACACCCAC	BRDN00012	131	85	22	224	156	0
CCACACCCGC	BRDN00011	163	48	0	297	221	0
CCACACCCTG	BRDN00012	432	524	1263	605	676	48
CCACACCCTG	BRDN00012	328	313	128	392	317	25
CCACACCGCA	BRDN00012	118	124	57	241	159	0
CCACACCTCG	BRDN00012	64	0	295	163	1	0
CCACACCTCC	BRDN00012	112	110	150	326	167	30
CCACACCTGT	BRDN00012	145	117	281	267	377	5
CCACACCTGT	BRDN00012	444	477	629	939	849	62
CCACACGAA	BRDN00011	362	449	1877	704	616	68
CCACACGAC	BRDN00012	351	197	168	630	377	0
CCACACGAG	BRDN00011	333	480	937	749	804	77
CCACACGATT	BRDN00011	178	198	33	278	483	14
CCACACGCA	BRDN00011	502	465	284	1081	698	35
CCACACGGT	BRDN00011	365	328	570	579	529	21
CCACACGTTT	BRDN00011	273	259	231	492	338	37
CCACACTCAC	BRDN00011	217	122	1260	267	623	37
CCACACTCAG	BRDN00011	98	48	0	151	238	0
CCACACTGGT	BRDN00012	183	169	418	299	331	15
CCACACTTAC	BRDN00012	431	240	302	829	1229	27
CCACAGACA	BRDN00011	344	277	347	468	461	15
CCACAGACT	BRDN00011	130	115	567	231	359	19
CCACAGAGA	BRDN00011	83	34	14	151	146	0
CCACAGAGC	BRDN00011	292	208	635	609	913	30
CCACAGAGG	BRDN00012	284	314	838	621	614	10
CCACAGAGG	BRDN00012	320	109	99	601	472	27
CCACAGAGG	BRDN00011	103	104	117	232	340	6
CCACAGAGT	BRDN00012	137	142	8330	268	228	70
CCACAGATA	BRDN00011	72	11	128	214	247	0
CCACAGATG	BRDN00012	78	15	1	167	111	0
CCACAGATG	BRDN00011	201	226	0	368	400	7
CCACAGATG	BRDN00011	84	71	143	159	196	4
CCACAGATG	BRDN00011	165	116	50	315	194	13
CCACAGATT	BRDN00012	303	268	414	584	587	41
CCACAGCAG	BRDN00011	180	54	36	167	44	2
CCACAGCAG	BRDN00011	148	97	8	419	394	0
CCACAGCAG	BRDN00011	156	1	1	528	369	0
CCACAGCCA	BRDN00011	319	361	137	600	512	23
CCACAGCCC	BRDN00011	214	84	191	467	265	36
CCACAGCCG	BRDN00011	101	83	37	276	302	9
CCACAGCCGT	BRDN00011	211	137	73	490	811	20
CCACAGCCTG	BRDN00012	245	133	365	622	772	23
CCACAGCCTG	BRDN00012	144	198	173	409	379	8
CCACAGCCTG	BRDN00011	286	288	164	575	395	32
CCACAGCGA	BRDN00012	112	83	16	313	250	8
CCACAGCTCC	BRDN00011	104	120	85	203	434	8
CCACAGCTG	BRDN00012	263	157	349	840	677	0
CCACAGGAA	BRDN00012	462	429	6441	660	553	124
CCACAGGAA	BRDN00012	112	75	91	261	186	0
CCACAGGAC	BRDN00011	149	101	655	352	296	4
CCACAGGAC	BRDN00012	469	630	849	859	689	50
CCACAGGACT	BRDN00011	222	178	62	406	314	0
CCACAGGAG	BRDN00012	144	160	122	278	139	0
CCACAGGAT	BRDN00011	151	124	844	363	473	12
CCACAGGCC	BRDN00012	29	35	0	459	134	1
CCACAGGTA	BRDN00012	411	495	793	857	1019	502
CCACAGGTCT	BRDN00012	480	475	467	983	817	55
CCACAGTCG	BRDN00011	306	246	82	587	623	26
CCACAGTCTC	BRDN00012	325	374	315	773	946	16
CCACAGTGG	BRDN00011	234	133	252	436	620	13
CCACAGTGG	BRDN00011	165	145	145	153	121	7
CCACAGTGT	BRDN00011	71	75	0	322	224	1
CCACAGTTTC	BRDN00011	295	333	444	592	428	89
CCACATAAA	BRDN00012	123	108	173	179	157	16
CCACATAAA	BRDN00011	305	93	31	322	421	0
CCACATAATC	BRDN00011	35	135	30	163	161	6
CCACATACAC	BRDN00012	99	129	147	267	481	22
CCACATACAC	BRDN00012	409	326	356	663	838	17
CCACATACAG	BRDN00012	148	164	1245	254	433	13
CCACATAGA	BRDN00011	109	118	88	221	209	0
CCACATAGA	BRDN00012	166	79	25	249	214	6

CCACATAGC	BRDN00012	50	109	40	147	183	14
CCACATAGT	BRDN00012	476	434	855	740	828	100
CCACATAGT	BRDN00012	404	432	1687	734	742	83
CCACATATG	BRDN00011	218	368	458	467	616	107
CCACATATTC	BRDN00012	82	98	128	220	162	11
CCACATCAAG	BRDN00011	114	71	65	223	142	0
CCACATCAG	BRDN00012	153	108	271	290	183	0
CCACATCCAA	BRDN00012	71	72	0	251	116	0
CCACATCCAT	BRDN00012	421	353	264	733	1121	31
CCACATCGA	BRDN00011	129	88	359	154	98	1
CCACATCGAT	BRDN00011	378	251	151	584	508	31
CCACATCTAT	BRDN00011	63	1	1	170	68	0
CCACATCTCA	BRDN00012	177	96	10	304	348	19
CCACATCTCA	BRDN00012	213	197	70	407	497	10
CCACATCTCA	BRDN00012	217	356	614	557	514	23
CCACATGAG	BRDN00011	207	254	147	363	214	39
CCACATGCA	BRDN00012	138	139	0	292	362	4
CCACATGCTT	BRDN00012	269	324	208	711	587	50
CCACATTACC	BRDN00011	358	347	39	752	616	6
CCACATTAG	BRDN00011	101	122	21	182	274	0
CCACATTCAT	BRDN00012	450	403	470	765	889	28
CCACATTCGT	BRDN00012	376	330	166	707	494	47
CCACATTCGT	BRDN00012	125	96	27	250	192	8
CCACATTCTT	BRDN00011	521	485	5195	779	740	98
CCACATTCTT	BRDN00012	294	395	318	489	245	99
CCACATTGG	BRDN00011	285	166	431	439	682	24
CCACATTTAT	BRDN00011	195	135	123	303	294	15
CCACCAAAAT	BRDN00011	147	129	360	310	161	2
CCACCAAACC	BRDN00011	123	120	45	255	133	7
CCACCAAACG	BRDN00011	65	187	405	154	47	19
CCACCAAAG	BRDN00012	137	121	30	359	335	91
CCACCAAAG	BRDN00011	270	296	80	559	452	27
CCACCAAGA	BRDN00011	132	62	1497	278	255	11
CCACCACAAA	BRDN00012	172	120	3216	264	603	63
CCACCACAAC	BRDN00012	118	141	161	243	303	0
CCACCACAAT	BRDN00012	200	247	641	293	186	21
CCACCACAG	BRDN00012	210	157	363	274	250	25
CCACCACCAA	BRDN00012	93	86	116	171	90	0
CCACCACCAC	BRDN00011	285	263	302	547	955	24
CCACCACCAT	BRDN00011	70	75	23	274	238	7
CCACCACCCT	BRDN00011	347	257	447	624	899	14
CCACCACCTA	BRDN00011	249	174	15	425	377	41
CCACCACCT	BRDN00012	352	371	180	554	410	10
CCACCACCTC	BRDN00011	362	293	1341	517	556	47
CCACCACCTG	BRDN00011	494	190	265	838	690	15
CCACCACGCA	BRDN00012	161	122	172	226	119	2
CCACCACTAC	BRDN00011	98	145	112	136	115	0
CCACCACTCA	BRDN00012	208	266	101	523	371	0
CCACCACTGA	BRDN00012	82	0	0	219	105	0
CCACCAGAC	BRDN00011	372	281	391	908	862	11
CCACCAGCA	BRDN00011	172	130	4	354	180	38
CCACCAGCA	BRDN00012	204	138	48	369	464	37
CCACCAGCA	BRDN00012	132	105	26	570	424	10
CCACCAGCA	BRDN00011	196	124	445	269	526	5
CCACCAGCA	BRDN00011	369	540	194	601	475	29
CCACCAGCA	BRDN00011	178	136	868	325	559	21
CCACCAGCTT	BRDN00011	344	173	169	678	403	5
CCACCAGGA	BRDN00011	180	253	940	350	511	21
CCACCAGGA	BRDN00011	75	148	0	217	240	0
CCACCAGGT	BRDN00011	253	278	897	550	448	0
CCACCAGTCT	BRDN00012	123	154	0	193	260	0
CCACCAGTG	BRDN00012	221	182	372	548	335	32
CCACCAGTG	BRDN00011	444	400	196	859	1155	8
CCACCATACT	BRDN00011	130	0	0	276	87	0
CCACCATCAT	BRDN00012	67	41	1179	233	266	15
CCACCATGA	BRDN00012	49	88	21	154	4	0
CCACCATTGA	BRDN00011	175	17	0	321	203	0
CCACCCAAAA	BRDN00012	303	101	745	521	584	0
CCACCCAAAC	BRDN00011	578	440	785	798	810	86
CCACCCAACT	BRDN00012	330	312	577	662	923	44
CCACCCAAG	BRDN00011	164	6	187	250	97	0
CCACCCAGA	BRDN00011	456	443	1380	685	919	41
CCACCCAGAT	BRDN00011	422	517	329	916	800	78
CCACCCAGCT	BRDN00012	199	157	68	364	243	46
CCACCCAGTT	BRDN00011	151	151	249	350	451	13
CCACCCCATA	BRDN00012	191	94	21	487	344	0
CCACCCCATG	BRDN00012	336	298	5073	665	538	77
CCACCCCATG	BRDN00012	168	181	298	402	343	22
CCACCCCCCC	BRDN00012	139	15	0	342	236	0
CCACCCCGTT	BRDN00011	287	227	248	563	423	42
CCACCCGAAT	BRDN00011	207	347	107	549	374	21
CCACCCGAG	BRDN00011	294	98	534	485	293	0
CCACCCGAGT	BRDN00011	109	162	100	248	283	26
CCACCCGCCA	BRDN00012	152	223	97	254	113	26
CCACCCGGC	BRDN00011	34	0	0	209	30	0
CCACCCGGTT	BRDN00011	102	56	133	236	282	10
CCACCCTAGT	BRDN00012	340	218	533	544	401	0
CCACCCTCTG	BRDN00012	184	193	1502	296	169	199
CCACCCTGAC	BRDN00011	0	0	0	158	35	0
CCACCCTGTT	BRDN00012	334	360	215	581	507	64
CCACCCTTAG	BRDN00011	150	109	562	309	355	25
CCACCGAAA	BRDN00012	174	152	174	208	228	7
CCACCGAAA	BRDN00012	339	338	606	447	482	50
CCACCGAAAT	BRDN00012	225	203	1131	472	235	48
CCACCGAAC	BRDN00012	235	232	7	316	179	12
CCACCGAAG	BRDN00012	81	65	88	254	180	0
CCACCGACA	BRDN00011	122	27	9	191	349	0
CCACCGACGT	BRDN00012	173	150	123	321	494	14
CCACCGAGA	BRDN00012	519	401	212	820	1293	18
CCACCGAGCT	BRDN00011	611	565	3568	1305	1276	68
CCACCGAGG	BRDN00011	169	142	43	376	554	8
CCACCGAGG	BRDN00012	29	0	0	190	0	0
CCACCGCATA	BRDN00011	330	138	477	725	400	8
CCACCGCATC	BRDN00012	108	87	329	220	270	0
CCACCGCCAA	BRDN00011	91	116	1480	177	138	22
CCACCGCCTC	BRDN00012	227	207	268	491	573	24
CCACCGCTAC	BRDN00012	392	403	792	746	575	112
CCACCGGAC	BRDN00011	249	294	169	381	272	14
CCACCGGAG	BRDN00012	173	59	0	391	236	0
CCACCGGAG	BRDN00012	307	501	4303	789	699	88
CCACCGGCTT	BRDN00011	204	185	2	250	114	0
CCACCGTACC	BRDN00011	105	47	0	92	84	0
CCACCGTCAT	BRDN00012	352	335	31	715	586	14
CCACCGTCTG	BRDN00012	75	26	62	312	118	0
CCACCGTGCT	BRDN00012	245	87	62	729	922	0
CCACCGTGG	BRDN00012	409	495	1531	887	964	64
CCACCGTTCG	BRDN00012	455	447	1492	734	906	63
CCACCTAACT	BRDN00012	224	0	1	867	628	0
CCACCTACCA	BRDN00011	156	177	270	299	272	3
CCACCTACCT	BRDN00011	185	232	3093	408	440	95
CCACCTACTT	BRDN00012	153	274	119	291	500	16
CCACCTAGA	BRDN00012	148	71	9219	243	168	129
CCACCTAGCA	BRDN00012	289	174	58	513	372	8
CCACCTATAT	BRDN00012	68	62	218	206	367	0
CCACCTATGA	BRDN00011	86	9	556	183	315	0
CCACCTATGA	BRDN00011	243	112	162	414	366	0
CCACCTCACT	BRDN00012	391	399	295	729	595	45
CCACCTCAGC	BRDN00012	183	164	673	418	218	27
CCACCTCCCT	BRDN00011	149	79	24	271	238	6
CCACCTCCGG	BRDN00012	82	31	0	667	234	0
CCACCTCCTT	BRDN00011	434	199	83	791	591	39
CCACCTCTTC	BRDN00011	358	223	930	688	1135	26
CCACCTCTTG	BRDN00011	282	214	771	659	438	17
CCACCTGAAT	BRDN00012	146	24	0	348	181	0
CCACCTGCCA	BRDN00012	142	165	146	382	167	19
CCACCTGCGC	BRDN00011	242	286	582	288	375	106
CCACCTGCTC	BRDN00011	288	279	108	534	734	60
CCACCTGGA	BRDN00012	173	129	220	303	270	25
CCACCTGGC	BRDN00012	125	110	1189	259	161	43
CCACCTGGC	BRDN00012	452	39	1	815	1602	0
CCACCTGGG	BRDN00011	102	68	1088	298	190	0
CCACCTGGG	BRDN00011	42	35	72	102	196	12
CCACCTGTG	BRDN00011	454	373	2587	920	550	92
CCACCTGTG	BRDN00012	324	297	2717	579	215	34
CCACCTTACC	BRDN00011	177	172	31	163	184	0
CCACCTTATA	BRDN00011	269	300	1968	378	420	40
CCACCTTGAA	BRDN00011	125	119	126	232	422	0
CCACGAAAC	BRDN00011	330	237	365	534	720	27
CCACGAACG	BRDN00012	80	81	0	195	150	0
CCACGAAGA	BRDN00012	326	159	205	650	610	27
CCACGAAGC	BRDN00011	443	324	822	978	700	56
CCACGAAGT	BRDN00011	145	119	358	279	157	4
CCACGAAGT	BRDN00012	204	162	1074	340	169	14
CCACGAATT	BRDN00012	396	422	122	812	457	55
CCACGAATT	BRDN00011	141	121	258	259	239	11
CCACGACAA	BRDN00011	44	72	0	132	39	6
CCACGACAT	BRDN00011	582	656	697	952	993	127
CCACGACAT	BRDN00012	326	231	850	569	476	6
CCACGACCA	BRDN00011	353	448	841	626	645	22
CCACGACGT	BRDN00011	539	594	1420	923	587	43
CCACGACTAT	BRDN00012	35	75	0	150	114	0
CCACGACTG	BRDN00012	200	221	1382	375	242	59
CCACGAGAT	BRDN00012	327	11	5	626	747	0
CCACGAGGA	BRDN00012	344	299	550	607	480	43
CCACGAGG	BRDN00012	113	14	16	267	250	0
CCACGAGTA	BRDN00012	328	264	228	634	325	13
CCACGATAC	BRDN00012	113	112	125	267	199	15
CCACGATCAT	BRDN00012	129	90	82	328	250	0
CCACGATGA	BRDN00012	335	33	4	741	392	0
CCACGATGC	BRDN00012	649	615	790	1021	952	71
CCACGATGG	BRDN00011	202	190	9	473	433	0
CCACGATGTT	BRDN00012	347	291	351	662	694	63
CCACGCACG	BRDN00012	152	178	72	343	228	0
CCACGCACTC	BRDN00012	55	45	237	223	243	0
CCACGCACTG	BRDN00011	101	51	20	120	112	0
CCACGCAGCT	BRDN00012	257	220	664	508	430	20
CCACGCAGCT	BRDN00011	182	74	28	275	233	12
CCACGCAGG	BRDN00012	402	503	626	672	621	146
CCACGCCCAC	BRDN00011	248	104	78	610	545	6
CCACGCCCAC	BRDN00012	193	156	196	538	638	13
CCACGCCCGT	BRDN00012	206	139	294	644	393	8
CCACGCCGA	BRDN00012	170	0	0	570	9	0
CCACGCGGA	BRDN00011	185	120	254	270	448	11
CCACGCGTG	BRDN00011	109	210	5	193	188	0
CCACGCTGA	BRDN00011	83	33	382	283	188	0
CCACGCTTCA	BRDN00012	146	97	1	305	122	0
CCACGGAAA	BRDN00011	192	67	1	520	1117	1
CCACGGACA	BRDN00012	496	345	805	776	757	23
CCACGGAGA	BRDN00012	205	181	12	422	205	16
CCACGGAGA	BRDN00011	227	303	16	452	453	19
CCACGGCAA	BRDN00012	89	86	122	162	301	0
CCACGGCAA	BRDN00012	314	302	2450	586	455	96
CCACGGCAT	BRDN00012	157	114	2413	226	224	40
CCACGGCGA	BRDN00012	112	0	0	230	82	3
CCACGGCGA	BRDN00011	225	173	458	403	591	38
CCACGGCGA	BRDN00011	332	403	2337	665	797	81
CCACGGCGG	BRDN00011	76	0	0	140	1	0
CCACGGCGT	BRDN00012	117	101	125	242	111	1
CCACGGCGT	BRDN00012	309	200	187	618	753	21
CCACGGCTA	BRDN00011	110	129	135	204	149	19
CCACGGCTG	BRDN00011	217	313	805	419	757	12
CCACGGGAA	BRDN00011	418	344	1373	645	622	57
CCACGGGCG	BRDN00011	109	93	13	236	135	6
CCACGGGGT	BRDN00011	49	53	0	86	21	0
CCACGGTCA	BRDN00012	583	478	917	1022	748	63
CCACGGTGA	BRDN00011	145	143	190	212	171	46
CCACGGTGA	BRDN00011	213	122	732	340	382	34
CCACGGTGC	BRDN00012	254	249	552	528	675	45
CCACGGTTG	BRDN00012	135	113	22	240	267	0
CCACGGTTG	BRDN00011	189	139	658	420	593	14
CCACGTACA	BRDN00011	192	229	507	437	350	22
CCACGTACA	BRDN00011	308	299	374	869	523	24
CCACGTACA	BRDN00012	93	17	0	196	99	0
CCACGTACGT	BRDN00011	90	186	91	346	754	22
CCACGTCAA	BRDN00012	104	190	15	354	235	27
CCACGTCACG	BRDN00011	306	170	17115	630	586	149
CCACGTCATC	BRDN00011	50	22	13	231	174	0
CCACGTCCAC	BRDN00012	244	210	407	432	590	26
CCACGTCCG	BRDN00012	249	215	202	301	179	1
CCACGTCCTG	BRDN00011	267	238	178	494	419	51
CCACGTCGTC	BRDN00011	92	55	0	204	99	14
CCACGTCTTG	BRDN00012	116	0	0	292	89	0
CCACGTGAA	BRDN00012	376	265	128	752	647	15
CCACGTGAA	BRDN00012	119	119	118	229	277	0
CCACGTGCG	BRDN00012	254	259	1646	362	391	63
CCACGTGCG	BRDN00011	159	20	0	248	68	0
CCACGTGCT	BRDN00012	92	124	241	237	52	13
CCACGTGGA	BRDN00011	442	254	231	699	981	24
CCACGTGGG	BRDN00011	169	113	139	260	519	0
CCACGTGTCT	BRDN00011	552	533	203	774	704	103
CCACGTTCCG	BRDN00011	513	446	831	963	768	50
CCACGTTGCA	BRDN00012	448	276	825	906	851	56
CCACGTTGG	BRDN00012	447	415	2822	902	1424	29
CCACGTTTCA	BRDN00011	116	80	0	250	331	0
CCACTAAGA	BRDN00011	245	275	10	454	280	0
CCACTAAGT	BRDN00012	94	85	0	126	76	0
CCACTACAAT	BRDN00012	114	183	199	403	192	14
CCACTACCCT	BRDN00011	174	106	152	213	475	0
CCACTACGTC	BRDN00012	77	7	0	278	233	0
CCACTCAACG	BRDN00012	224	73	491	750	529	17
CCACTCACC	BRDN00011	178	268	119	299	58	8
CCACTCAGTA	BRDN00011	245	197	356	312	206	49
CCACTCAGTA	BRDN00012	232	2	2	441	378	0
CCACTCCAAG	BRDN00012	438	518	1385	839	850	71
CCACTCCAGA	BRDN00011	107	123	503	212	99	7
CCACTCCATG	BRDN00012	162	80	0	371	285	4
CCACTCCCTG	BRDN00012	187	1	41	270	14	0
CCACTCCGAT	BRDN00012	279	195	812	362	659	36
CCACTCGAA	BRDN00011	85	47	78	96	282	0
CCACTCGCAG	BRDN00011	359	317	75	753	665	27
CCACTCGCTC	BRDN00012	288	167	920	610	547	34
CCACTCTTA	BRDN00012	40	8	1	570	392	0
CCACTCTTGG	BRDN00012	82	54	27	247	342	0
CCACTCTTTG	BRDN00012	229	262	128	478	1118	17
CCACTGAAG	BRDN00011	78	40	1	199	70	0
CCACTGAAG	BRDN00012	153	165	37	310	341	18
CCACTGAGT	BRDN00011	61	85	81	128	344	0
CCACTGCACC	BRDN00011	105	49	91	178	386	0
CCACTGCACG	BRDN00011	164	151	51	386	253	10
CCACTGCACT	BRDN00012	476	596	929	803	851	81
CCACTGCCTT	BRDN00012	317	318	187	608	407	9
CCACTGCGC	BRDN00012	171	112	0	235	195	13
CCACTGGAT	BRDN00011	248	208	1036	449	402	16
CCACTGGCT	BRDN00011	197	157	595	265	289	6
CCACTGGGA	BRDN00012	291	213	627	446	436	106
CCACTGGGG	BRDN00011	21	5	839	235	67	8
CCACTGGTA	BRDN00012	494	398	792	803	690	74
CCACTGGTA	BRDN00011	222	215	465	403	458	0
CCACTGTAAT	BRDN00011	58	92	34	241	243	53
CCACTGTACA	BRDN00011	89	164	0	274	133	7
CCACTGTCG	BRDN00011	155	110	0	228	320	4
CCACTGTGCT	BRDN00011	607	517	167	1102	930	63
CCACTGTGG	BRDN00012	69	76	20	296	310	30
CCACTGTGT	BRDN00011	396	317	433	627	972	21
CCACTTACCG	BRDN00012	73	102	0	158	144	0
CCACTTACTG	BRDN00011	142	106	0	149	211	15
CCACTTACTT	BRDN00011	308	273	735	517	474	19
CCACTTATTA	BRDN00012	293	196	371	430	602	6
CCACTTCACA	BRDN00012	269	3	2	750	389	0
CCACTTCATG	BRDN00012	333	363	754	764	548	185
CCACTTCCGA	BRDN00012	238	211	210	460	351	8
CCACTTCCTC	BRDN00011	151	213	0	379	148	0
CCACTTCGAC	BRDN00011	247	315	36	471	370	6
CCACTTCGG	BRDN00011	442	559	575	828	758	60
CCACTTCTA	BRDN00012	443	353	2186	856	953	46
CCACTTGAG	BRDN00012	125	158	0	241	212	0
CCACTTGATG	BRDN00012	150	121	1	272	320	0
CCACTTGCCC	BRDN00011	104	141	12	210	191	13
CCACTTGGC	BRDN00012	88	71	20	227	83	1
CCACTTGTTA	BRDN00012	249	259	647	665	420	12
CCACTTTGAA	BRDN00012	194	81	37	583	769	0
CCACTTTGAC	BRDN00011	243	260	81	463	319	60
CCACTTTGAG	BRDN00012	187	228	6	458	180	45
CCAGAAAAA	BRDN00012	141	120	143	504	562	7
CCAGAAAAA	BRDN00012	99	0	0	147	85	0
CCAGAAAAG	BRDN00012	230	247	172	341	514	29
CCAGAAAAT	BRDN00011	203	125	60	495	512	3
CCAGAAACA	BRDN00011	518	318	691	685	810	28
CCAGAAACA	BRDN00012	311	236	2521	508	668	65
CCAGAAACG	BRDN00011	260	402	3225	485	605	167
CCAGAAACT	BRDN00011	276	267	21	463	742	24
CCAGAAAGA	BRDN00012	404	556	5761	750	798	152
CCAGAAAG	BRDN00012	274	154	118	447	479	0
CCAGAAAGC	BRDN00011	90	43	0	250	127	0
CCAGAAAGG	BRDN00011	283	177	205	423	450	21
CCAGAAAGT	BRDN00011	62	13	0	267	61	0
CCAGAAAGT	BRDN00012	230	275	5000	388	501	73
CCAGAAAGT	BRDN00012	66	3	0	271	49	0
CCAGAAAGT	BRDN00012	207	276	516	408	573	29
CCAGAAATA	BRDN00011	32	9	1	167	143	0
CCAGAAATTT	BRDN00012	334	200	82	520	331	18
CCAGAACAA	BRDN00012	268	340	136	440	655	0
CCAGAACAA	BRDN00011	157	134	255	288	181	0
CCAGAACACT	BRDN00011	167	130	4	294	178	14
CCAGAACAT	BRDN00011	107	45	126	195	170	0
CCAGAACCA	BRDN00012	451	426	184	755	655	67
CCAGAACCA	BRDN00011	148	123	3	204	240	19
CCAGAACCCT	BRDN00011	65	15	0	254	108	0
CCAGAACCT	BRDN00012	146	157	95	329	354	4
CCAGAACGA	BRDN00012	118	177	129	288	170	0
CCAGAACTC	BRDN00011	240	234	804	311	247	43
CCAGAACTCT	BRDN00012	376	325	803	566	416	34
CCAGAACTG	BRDN00012	122	88	268	194	119	57
CCAGAAGAG	BRDN00011	125	99	0	126	46	0
CCAGAAGA	BRDN00011	199	138	809	148	60	17
CCAGAAGAC	BRDN00011	251	279	108	509	515	50
CCAGAAGAG	BRDN00011	210	215	83	289	131	32
CCAGAAGCT	BRDN00011	342	328	210	675	468	18
CCAGAAGTA	BRDN00011	310	285	192	447	699	21
CCAGAAGTG	BRDN00011	120	153	295	242	271	20
CCAGAAGTT	BRDN00011	301	12	0	416	285	0
CCAGAATAG	BRDN00011	84	98	10	197	478	35
CCAGAATAT	BRDN00012	226	271	18	497	687	17
CCAGAATCG	BRDN00011	241	40	0	357	214	0
CCAGAATCT	BRDN00011	229	178	972	483	341	75
CCAGAATCT	BRDN00012	133	124	1085	277	579	11
CCAGAATCT	BRDN00012	152	119	200	246	238	0
CCAGAATCT	BRDN00011	150	156	60	212	265	5
CCAGAATCTT	BRDN00011	127	114	163	134	88	3
CCAGAATGG	BRDN00011	97	86	1431	138	112	7
CCAGAATGT	BRDN00012	186	116	12	478	201	0
CCAGAATTA	BRDN00011	177	123	147	276	333	0
CCAGAATTG	BRDN00011	235	218	298	310	201	35
CCAGACAAC	BRDN00012	102	80	691	272	225	2
CCAGACACA	BRDN00012	109	116	355	233	279	36
CCAGACACC	BRDN00012	373	283	720	543	556	26
CCAGACACC	BRDN00012	97	187	55	161	218	0
CCAGACACC	BRDN00012	245	198	830	426	477	16
CCAGACAGG	BRDN00012	236	188	30	532	451	45
CCAGACAGG	BRDN00012	345	461	201	587	857	8
CCAGACAGT	BRDN00012	100	5	0	236	185	0
CCAGACATC	BRDN00011	130	19	0	339	179	0
CCAGACCAA	BRDN00011	142	131	282	319	188	16
CCAGACCAAT	BRDN00011	115	62	88	218	83	8
CCAGACCAC	BRDN00011	281	221	678	528	389	25
CCAGACCACT	BRDN00012	139	84	354	236	242	0
CCAGACCAG	BRDN00012	57	0	0	391	540	0
CCAGACCAT	BRDN00011	214	291	291	278	325	44
CCAGACCCA	BRDN00011	309	335	30	437	539	43
CCAGACCCA	BRDN00011	241	254	1130	407	499	33
CCAGACCCAT	BRDN00012	163	170	141	271	513	0
CCAGACCCCT	BRDN00012	80	32	0	318	144	0
CCAGACCGG	BRDN00012	113	231	254	121	86	16
CCAGACCTA	BRDN00011	84	128	14	138	570	8
CCAGACCTCT	BRDN00011	116	123	143	215	135	27
CCAGACCTG	BRDN00012	134	138	507	329	245	11
CCAGACCTG	BRDN00011	500	397	706	837	683	51
CCAGACCTTG	BRDN00011	295	371	1065	595	263	23
CCAGACCTTT	BRDN00012	168	86	500	217	306	1
CCAGACGGA	BRDN00011	185	206	19	277	180	27
CCAGACGGC	BRDN00012	82	25	0	173	86	0
CCAGACGG	BRDN00012	335	402	34	488	541	6
CCAGACGGT	BRDN00012	161	175	129	411	536	36
CCAGACTCCA	BRDN00012	263	225	165	327	328	9
CCAGACTCG	BRDN00012	458	362	2060	627	393	11
CCAGACTCTC	BRDN00012	183	214	424	218	369	0
CCAGACTGA	BRDN00011	103	83	0	150	121	1
CCAGACTGG	BRDN00011	229	82	235	297	356	0
CCAGACTGG	BRDN00012	131	220	56	270	164	19
CCAGACTTG	BRDN00012	375	434	1021	849	1057	78
CCAGAGAAA	BRDN00012	45	18	0	139	110	0
CCAGAGAAA	BRDN00011	127	90	294	298	255	42
CCAGAGAAA	BRDN00012	104	77	15	169	316	9
CCAGAGAA	BRDN00011	124	78	35	217	29	17
CCAGAGAA	BRDN00011	255	0	0	486	139	0
CCAGAGAAG	BRDN00011	85	106	142	117	188	7
CCAGAGAAT	BRDN00011	73	65	8	100	128	4
CCAGAGACA	BRDN00011	358	324	141	574	653	7
CCAGAGACA	BRDN00011	321	369	1708	566	405	55
CCAGAGACC	BRDN00011	228	238	122	411	568	46
CCAGAGACT	BRDN00012	364	201	590	524	267	0
CCAGAGAGG	BRDN00011	68	9	0	154	187	0
CCAGAGATG	BRDN00011	195	34	10	506	232	0
CCAGAGATG	BRDN00012	118	82	412	220	280	13
CCAGAGATG	BRDN00012	272	172	19	435	590	85
CCAGAGCAA	BRDN00012	261	184	207	548	301	10
CCAGAGCAA	BRDN00011	92	45	335	269	212	5
CCAGAGCAC	BRDN00011	197	233	714	185	284	32
CCAGAGCAG	BRDN00011	99	81	67	237	92	5
CCAGAGCAT	BRDN00011	448	399	1266	649	886	168
CCAGAGCCC	BRDN00011	66	70	43	185	304	2
CCAGAGCCG	BRDN00011	121	0	3	333	135	0
CCAGAGCCG	BRDN00012	156	212	255	351	248	3
CCAGAGCCT	BRDN00012	328	270	75	533	655	42
CCAGAGCCT	BRDN00012	332	388	348	500	505	36
CCAGAGCCT	BRDN00011	461	391	468	1065	610	14
CCAGAGCCT	BRDN00012	79	2	5	363	354	10
CCAGAGCCT	BRDN00012	308	227	360	611	538	39
CCAGAGCGG	BRDN00012	136	229	185	138	107	33
CCAGAGCTC	BRDN00012	260	233	352	441	377	6
CCAGAGCTT	BRDN00011	245	172	3252	489	422	55
CCAGAGGAC	BRDN00011	364	413	217	766	484	40
CCAGAGGCA	BRDN00011	93	127	351	112	280	56
CCAGAGGGA	BRDN00012	29	13	0	462	180	0
CCAGAGGGC	BRDN00011	24	14	1	185	147	0
CCAGAGGGT	BRDN00011	162	252	636	356	273	6
CCAGAGGTA	BRDN00011	146	185	1176	230	319	6
CCAGAGTAA	BRDN00011	317	200	784	748	1060	25
CCAGAGTAT	BRDN00011	196	198	125	346	81	6
CCAGAGTCA	BRDN00011	369	226	576	979	755	46
CCAGAGTCC	BRDN00011	148	146	18	514	740	31
CCAGAGTGA	BRDN00012	263	144	51	636	1066	10
CCAGAGTGC	BRDN00011	60	52	0	126	119	0
CCAGAGTGC	BRDN00012	264	255	142	574	288	167
CCAGAGTGT	BRDN00011	25	0	0	123	152	0
CCAGAGTTC	BRDN00011	99	125	13	130	189	12
CCAGATAAC	BRDN00012	62	49	0	198	276	0
CCAGATACA	BRDN00011	62	61	13	132	189	0
CCAGATACC	BRDN00011	377	273	1406	612	625	18
CCAGATACG	BRDN00012	192	188	1210	356	318	104
CCAGATACT	BRDN00012	146	23	13	221	257	0
CCAGATAGA	BRDN00011	39	0	0	227	69	0
CCAGATAGG	BRDN00012	192	104	124	249	213	4
CCAGATATA	BRDN00011	156	109	120	235	743	96
CCAGATATC	BRDN00011	128	151	37	234	106	5
CCAGATCAA	BRDN00012	179	300	5691	309	138	52
CCAGATCAG	BRDN00012	121	106	20	290	142	15
CCAGATCCA	BRDN00012	166	94	0	493	331	11
CCAGATCCA	BRDN00011	34	12	12	161	127	0
CCAGATCCCT	BRDN00012	413	323	1510	666	731	35
CCAGATCCG	BRDN00012	240	29	4	819	1009	0
CCAGATCCTT	BRDN00012	133	140	286	290	133	0
CCAGATCGA	BRDN00011	98	0	0	213	147	0
CCAGATCGT	BRDN00011	120	94	264	307	205	0
CCAGATCTA	BRDN00012	507	398	1225	653	1126	49
CCAGATCTCA	BRDN00011	327	352	2157	544	223	50
CCAGATCTCC	BRDN00012	338	307	1506	561	600	17
CCAGATCTCG	BRDN00012	440	405	2174	690	480	47
CCAGATCTCT	BRDN00011	71	70	780	235	306	18
CCAGATCTG	BRDN00011	401	192	53	629	993	1
CCAGATCTG	BRDN00012	73	77	0	189	244	0
CCAGATCTTC	BRDN00012	351	354	112	570	731	14
CCAGATCTTT	BRDN00011	175	207	137	525	317	20
CCAGATGAA	BRDN00012	642	578	770	975	724	54
CCAGATGAC	BRDN00012	85	129	208	227	200	0

CCAGATGAG	BRDN00011	163	287	75	396	403	8
CCAGATGAT	BRDN00011	170	156	29	343	157	49
CCAGATGAT	BRDN00011	230	14	440	571	420	8
CCAGATGCC	BRDN00012	195	296	160	732	480	9
CCAGATGCG	BRDN00011	126	86	161	166	160	30
CCAGATGCG	BRDN00011	84	55	257	153	108	10
CCAGATGGC	BRDN00012	295	153	58	401	385	32
CCAGATGGC	BRDN00012	102	45	10	115	61	6
CCAGATGGC	BRDN00011	267	216	118	269	312	0
CCAGATGGG	BRDN00012	265	324	113	529	534	18
CCAGATTAC	BRDN00012	419	528	2553	627	452	46
CCAGATTAT	BRDN00012	256	165	4055	300	341	67
CCAGATTCC	BRDN00011	287	292	1676	483	536	1
CCAGATTCC	BRDN00011	75	52	0	168	91	15
CCAGATTCG	BRDN00012	136	154	0	286	285	19
CCAGATTGA	BRDN00012	73	106	30	232	219	5
CCAGATTGG	BRDN00012	633	495	221	1039	974	48
CCAGATTTC	BRDN00012	216	170	217	402	413	49
CCAGATTTG	BRDN00011	153	34	342	264	462	16
CCAGCAAAA	BRDN00011	436	477	114	578	878	22
CCAGCAAAT	BRDN00011	100	126	54	201	185	0
CCAGCAACA	BRDN00011	356	280	253	878	862	86
CCAGCAACA	BRDN00012	346	269	1039	659	672	58
CCAGCAACT	BRDN00011	75	4	0	256	12	0
CCAGCAAGA	BRDN00012	107	55	34	178	128	8
CCAGCAAGC	BRDN00011	289	290	808	593	374	25
CCAGCAATA	BRDN00012	423	448	746	681	1091	53
CCAGCAATC	BRDN00012	439	483	769	714	559	77
CCAGCAATC	BRDN00011	210	50	1	433	288	0
CCAGCAATG	BRDN00011	376	361	232	683	1152	55
CCAGCAATT	BRDN00012	208	87	0	322	1105	0
CCAGCACAC	BRDN00012	189	96	101	221	363	6
CCAGCACAC	BRDN00011	219	340	109	475	535	82
CCAGCACAT	BRDN00012	146	59	144	255	336	0
CCAGCACCA	BRDN00011	196	134	194	342	193	6
CCAGCACCC	BRDN00011	376	329	1208	480	518	30
CCAGCACCC	BRDN00012	223	157	735	400	435	92
CCAGCACCC	BRDN00012	106	93	89	247	389	8
CCAGCACCG	BRDN00011	83	42	62	165	202	0
CCAGCACCG	BRDN00011	311	241	21	659	705	46
CCAGCACGA	BRDN00011	50	23	0	120	55	7
CCAGCACGG	BRDN00012	224	180	1037	256	662	40
CCAGCACGT	BRDN00011	160	171	950	226	419	59
CCAGCACGT	BRDN00012	397	337	2225	742	607	36
CCAGCACGT	BRDN00011	168	77	148	206	118	0
CCAGCACTA	BRDN00012	238	344	354	427	920	66
CCAGCACTG	BRDN00011	264	239	2224	437	565	72
CCAGCACTG	BRDN00012	194	139	22	313	210	25
CCAGCACTT	BRDN00012	210	92	201	463	559	0
CCAGCACTT	BRDN00011	232	128	98	410	388	4
CCAGCAGAA	BRDN00012	258	267	98	496	362	0
CCAGCAGAA	BRDN00011	66	80	0	176	180	0
CCAGCAGAA	BRDN00011	131	88	50	242	205	0
CCAGCAGAA	BRDN00012	400	551	424	846	881	56
CCAGCAGAC	BRDN00012	122	78	73	243	199	10
CCAGCAGAG	BRDN00011	126	83	111	223	186	23
CCAGCAGAT	BRDN00012	231	292	459	417	318	9
CCAGCAGAT	BRDN00012	296	198	100	685	628	37
CCAGCAGAT	BRDN00012	196	104	102	320	324	7
CCAGCAGCA	BRDN00012	157	321	672	456	296	15
CCAGCAGCA	BRDN00012	178	67	149	287	282	9
CCAGCAGCC	BRDN00012	26	0	0	274	131	5
CCAGCAGCT	BRDN00011	392	501	602	665	1067	16
CCAGCAGCT	BRDN00012	169	128	24	203	49	50
CCAGCAGCT	BRDN00012	524	449	840	817	589	23
CCAGCAGGA	BRDN00012	195	200	97	346	286	55
CCAGCAGGA	BRDN00011	148	100	562	522	182	47
CCAGCAGGC	BRDN00012	332	638	9581	899	788	145
CCAGCAGGG	BRDN00011	145	165	0	287	178	6
CCAGCAGGG	BRDN00012	293	265	118	667	721	35
CCAGCAGGG	BRDN00011	188	227	453	199	134	0
CCAGCAGGT	BRDN00012	110	96	0	143	162	0
CCAGCAGTA	BRDN00012	152	182	334	225	84	15
CCAGCAGTC	BRDN00011	265	128	576	405	242	0
CCAGCAGTC	BRDN00012	97	105	6	147	117	0
CCAGCAGTG	BRDN00012	39	31	0	264	90	0
CCAGCAGTG	BRDN00011	312	162	570	336	460	4
CCAGCAGTT	BRDN00011	239	122	249	376	594	34
CCAGCATAA	BRDN00011	87	110	1871	129	141	26
CCAGCATAC	BRDN00012	409	459	829	967	432	0
CCAGCATCT	BRDN00011	344	298	866	508	1234	15
CCAGCATCT	BRDN00011	274	201	52	419	445	15
CCAGCATGC	BRDN00011	227	201	5791	508	305	110
CCAGCATGG	BRDN00012	293	165	2179	543	413	45
CCAGCATGG	BRDN00012	275	151	55	345	190	8
CCAGCCAAC	BRDN00012	228	215	58	503	373	8
CCAGCCAAG	BRDN00012	292	242	609	540	460	62
CCAGCCAAG	BRDN00011	50	4	0	123	57	0
CCAGCCACA	BRDN00011	165	8	20	468	953	1
CCAGCCACA	BRDN00012	121	125	1334	184	210	10
CCAGCCACA	BRDN00011	237	6	0	376	152	0
CCAGCCACT	BRDN00012	184	153	45	258	275	0
CCAGCCAGA	BRDN00012	274	359	15	654	708	0
CCAGCCAGC	BRDN00012	151	183	147	154	179	31
CCAGCCAGC	BRDN00011	55	30	0	123	159	0
CCAGCCAGG	BRDN00012	326	353	255	853	421	39
CCAGCCATA	BRDN00011	100	0	1	278	45	1
CCAGCCATC	BRDN00012	86	105	1	215	116	25
CCAGCCATG	BRDN00012	331	379	251	596	284	13
CCAGCCATT	BRDN00012	341	221	495	611	558	34
CCAGCCCAC	BRDN00011	175	184	53	270	258	7
CCAGCCCAT	BRDN00011	92	0	0	268	313	0
CCAGCCCAT	BRDN00011	298	269	59	595	415	17
CCAGCCCAT	BRDN00011	411	279	569	771	853	60
CCAGCCCCA	BRDN00011	112	81	15	278	315	0
CCAGCCCCG	BRDN00012	135	99	178	216	43	15
CCAGCCCCT	BRDN00011	168	250	345	348	402	37
CCAGCCCGA	BRDN00012	104	111	878	147	135	13
CCAGCCCTA	BRDN00011	264	219	303	615	822	84
CCAGCCCTA	BRDN00011	272	236	229	451	531	38
CCAGCCCTC	BRDN00011	168	154	56	365	450	13
CCAGCCCTG	BRDN00011	254	384	139	702	627	16
CCAGCCGAG	BRDN00011	196	104	1	410	355	0
CCAGCCGAT	BRDN00011	303	363	425	638	858	37
CCAGCCGAT	BRDN00012	192	59	1	635	430	0
CCAGCCGCA	BRDN00011	188	185	58	385	374	25
CCAGCCGCC	BRDN00012	110	48	1	572	118	0
CCAGCCGCC	BRDN00011	193	193	1212	266	442	8
CCAGCCGCT	BRDN00011	120	174	140	189	184	12
CCAGCCGCT	BRDN00011	298	434	1506	351	248	25
CCAGCCGGG	BRDN00011	218	245	74	411	315	22
CCAGCCGTA	BRDN00012	87	59	0	172	163	0
CCAGCCTAA	BRDN00012	97	60	30	273	384	11
CCAGCCTAC	BRDN00011	146	180	711	274	428	26
CCAGCCTCA	BRDN00012	197	247	1014	412	559	46
CCAGCCTCA	BRDN00012	114	105	298	251	328	9
CCAGCCTCA	BRDN00012	85	44	13	114	114	7
CCAGCCTCA	BRDN00012	211	121	147	380	258	11
CCAGCCTCA	BRDN00012	178	123	141	278	223	19
CCAGCCTCA	BRDN00012	453	266	452	592	879	4
CCAGCCTCC	BRDN00012	80	130	7	265	187	1
CCAGCCTGC	BRDN00011	202	221	1733	350	448	58
CCAGCCTGC	BRDN00011	353	336	252	754	716	33
CCAGCCTGG	BRDN00012	231	336	1609	526	385	138
CCAGCCTTA	BRDN00011	158	101	291	280	110	57
CCAGCCTTA	BRDN00011	209	259	94	405	371	3
CCAGCCTTA	BRDN00011	271	17	46	483	737	0
CCAGCGAAT	BRDN00011	151	134	165	302	305	36
CCAGCGACC	BRDN00012	512	410	857	834	903	33
CCAGCGACG	BRDN00011	171	241	292	342	638	18
CCAGCGAGA	BRDN00012	187	0	0	496	164	0
CCAGCGAGA	BRDN00012	367	191	546	563	776	18
CCAGCGAGT	BRDN00012	246	365	126	366	266	37
CCAGCGCAC	BRDN00011	127	83	22	219	251	8
CCAGCGCAG	BRDN00011	204	196	534	442	316	30
CCAGCGCAT	BRDN00012	294	266	781	627	424	33
CCAGCGCAT	BRDN00012	134	20	0	866	94	29
CCAGCGCAT	BRDN00011	142	122	17	238	460	5
CCAGCGCCA	BRDN00012	97	133	318	147	82	0
CCAGCGCCC	BRDN00012	160	190	198	275	139	31
CCAGCGCCC	BRDN00012	227	157	131	408	447	4
CCAGCGCGA	BRDN00012	181	100	36	303	269	0
CCAGCGCGC	BRDN00011	13	0	0	90	0	0
CCAGCGCGG	BRDN00012	121	87	15	198	98	4
CCAGCGCGT	BRDN00012	34	4	53	168	78	0
CCAGCGCTC	BRDN00012	193	220	183	409	341	18
CCAGCGCTG	BRDN00012	167	169	1028	242	398	8
CCAGCGCTT	BRDN00012	299	363	3240	501	343	120
CCAGCGGAA	BRDN00012	64	78	14	149	83	0
CCAGCGGAA	BRDN00012	334	347	313	732	582	6
CCAGCGGAA	BRDN00012	341	178	405	554	398	27
CCAGCGGAA	BRDN00012	268	556	2496	564	1240	123
CCAGCGGGA	BRDN00012	154	63	185	246	540	112
CCAGCGGGA	BRDN00011	17	0	0	92	31	0
CCAGCGGGC	BRDN00012	263	386	396	566	881	67
CCAGCGGGT	BRDN00012	467	594	847	935	632	30
CCAGCGGGT	BRDN00012	194	2	0	159	79	0
CCAGCGGTA	BRDN00011	65	17	705	107	121	8
CCAGCGTAT	BRDN00011	78	102	43	174	409	0
CCAGCGTCC	BRDN00012	69	100	155	161	49	10
CCAGCGTCG	BRDN00011	355	217	698	687	668	36
CCAGCGTGA	BRDN00012	274	178	616	533	572	27
CCAGCGTGG	BRDN00012	240	230	345	332	57	20
CCAGCGTGG	BRDN00011	248	272	140	548	376	6
CCAGCGTGG	BRDN00011	248	389	257	407	367	33
CCAGCTAAA	BRDN00011	125	47	972	236	43	9
CCAGCTACC	BRDN00011	121	240	509	211	107	0
CCAGCTAGA	BRDN00011	127	228	1	290	264	15
CCAGCTATA	BRDN00011	288	192	288	383	161	26
CCAGCTATA	BRDN00011	367	388	775	584	1190	16
CCAGCTATA	BRDN00012	344	286	345	590	469	21
CCAGCTATT	BRDN00012	102	32	1	341	58	0
CCAGCTCAG	BRDN00012	136	23	14	294	210	5
CCAGCTCAG	BRDN00012	113	0	0	264	95	0
CCAGCTCAT	BRDN00012	75	192	643	170	105	0
CCAGCTCAT	BRDN00011	219	234	388	475	794	12
CCAGCTCCG	BRDN00012	320	184	410	536	281	31
CCAGCTCCG	BRDN00011	406	343	1972	869	1237	82
CCAGCTCGA	BRDN00011	144	79	48	247	188	0
CCAGCTCGC	BRDN00011	138	186	511	244	307	17
CCAGCTCGG	BRDN00012	80	106	202	275	263	7
CCAGCTCGG	BRDN00011	66	32	38	174	125	0
CCAGCTCGT	BRDN00011	373	0	0	667	227	0
CCAGCTCTA	BRDN00011	77	17	14	195	175	0
CCAGCTCTA	BRDN00011	85	98	2599	174	427	24
CCAGCTCTC	BRDN00012	110	62	19	372	469	0
CCAGCTCTC	BRDN00012	193	1	0	545	140	0
CCAGCTCTG	BRDN00012	239	151	587	349	637	48
CCAGCTGAG	BRDN00012	153	209	382	181	287	0
CCAGCTGAT	BRDN00012	220	35	18	587	945	0
CCAGCTGAT	BRDN00012	102	171	4	280	153	16
CCAGCTGCA	BRDN00012	194	97	25	225	114	3
CCAGCTGCA	BRDN00011	67	19	0	170	26	0
CCAGCTGGA	BRDN00012	371	272	713	576	985	11
CCAGCTGGA	BRDN00011	49	15	127	177	105	0
CCAGCTGGA	BRDN00011	103	171	262	198	196	0
CCAGCTGGC	BRDN00011	207	140	1247	260	276	20
CCAGCTGGG	BRDN00011	259	281	1156	493	407	26
CCAGCTGGG	BRDN00012	327	274	725	600	1104	23
CCAGCTGTC	BRDN00012	86	71	0	181	156	0
CCAGCTGTG	BRDN00011	139	63	83	293	265	0
CCAGCTTAG	BRDN00012	73	74	9	158	164	16
CCAGCTTCA	BRDN00011	128	0	2	415	82	0
CCAGCTTCC	BRDN00012	188	212	2705	490	338	26
CCAGCTTCC	BRDN00012	430	483	1068	690	758	35
CCAGCTTGA	BRDN00011	344	409	568	539	774	18
CCAGCTTGA	BRDN00012	137	108	35	233	107	14
CCAGCTTGA	BRDN00012	235	3	0	679	283	1
CCAGCTTGC	BRDN00012	322	289	740	469	529	5
CCAGCTTGT	BRDN00011	134	164	382	343	488	29
CCAGCTTTA	BRDN00012	267	229	224	429	281	59
CCAGGAAAG	BRDN00011	262	288	94	456	646	7
CCAGGAAAT	BRDN00011	309	339	1184	523	763	24
CCAGGAACC	BRDN00011	147	20	0	301	251	0
CCAGGAACT	BRDN00011	357	283	441	509	574	28
CCAGGAAGA	BRDN00012	261	127	2430	533	475	42
CCAGGAAGA	BRDN00011	548	324	738	1001	692	57
CCAGGAAGA	BRDN00011	310	598	411	476	412	133
CCAGGAAGC	BRDN00011	116	24	127	175	360	8
CCAGGAAGG	BRDN00011	80	75	969	240	393	8
CCAGGAATG	BRDN00011	64	41	0	139	61	1
CCAGGACAA	BRDN00012	115	62	7	226	322	6
CCAGGACAA	BRDN00012	271	266	389	470	372	0
CCAGGACAC	BRDN00011	199	117	9	227	467	9
CCAGGACAC	BRDN00011	231	354	237	534	539	35
CCAGGACAG	BRDN00011	105	148	40	257	288	7
CCAGGACAG	BRDN00012	106	27	303	183	172	5
CCAGGACAT	BRDN00012	95	119	631	174	296	0
CCAGGACCA	BRDN00012	387	284	114	665	656	42
CCAGGACCA	BRDN00012	163	44	1	266	327	0
CCAGGACCA	BRDN00012	53	34	145	213	209	0
CCAGGACCA	BRDN00011	187	170	474	367	810	34
CCAGGACCC	BRDN00012	176	174	113	278	239	18
CCAGGACCT	BRDN00012	136	87	393	282	332	0
CCAGGACCG	BRDN00011	35	6	0	235	17	5
CCAGGACCT	BRDN00011	191	230	1300	290	262	32
CCAGGACCT	BRDN00012	175	240	1377	313	131	32
CCAGGACGA	BRDN00011	189	371	176	344	316	15
CCAGGACGA	BRDN00012	126	183	43	455	310	29
CCAGGACGT	BRDN00011	189	149	5	474	178	31
CCAGGACTA	BRDN00011	167	135	133	367	415	0
CCAGGACTA	BRDN00011	106	128	132	217	56	10
CCAGGACTG	BRDN00012	431	277	282	890	985	55
CCAGGACTG	BRDN00011	243	206	206	454	359	45
CCAGGACTG	BRDN00011	204	170	790	454	492	33
CCAGGAGAA	BRDN00012	176	214	212	522	283	0
CCAGGAGAA	BRDN00011	21	7	0	138	124	0
CCAGGAGAG	BRDN00012	91	152	83	101	41	25
CCAGGAGAG	BRDN00011	107	125	14	207	226	4
CCAGGAGAT	BRDN00011	114	18	107	183	75	2
CCAGGAGAT	BRDN00012	9	37	0	310	218	0
CCAGGAGCA	BRDN00011	224	200	0	531	617	0
CCAGGAGCC	BRDN00012	295	370	722	618	538	70
CCAGGAGCG	BRDN00012	113	81	12	263	410	14
CCAGGAGCG	BRDN00012	273	322	468	608	585	55
CCAGGAGCG	BRDN00012	260	225	776	664	649	115
CCAGGAGCT	BRDN00011	106	19	58	821	258	0
CCAGGAGGC	BRDN00012	260	350	1965	508	461	74
CCAGGAGGC	BRDN00012	154	68	383	400	407	47
CCAGGAGGC	BRDN00011	92	74	42	208	146	0
CCAGGAGGC	BRDN00012	105	107	54	123	203	19
CCAGGAGGT	BRDN00011	129	133	491	195	308	13
CCAGGAGGT	BRDN00012	302	261	1514	698	767	24
CCAGGAGTA	BRDN00011	89	3	0	283	97	0
CCAGGAGTA	BRDN00012	524	187	61	859	1471	0
CCAGGAGTG	BRDN00011	122	116	14	282	33	0
CCAGGAGTG	BRDN00012	251	155	1085	461	255	12
CCAGGATAA	BRDN00012	342	297	449	622	773	3
CCAGGATAC	BRDN00012	67	80	10	79	112	11
CCAGGATAT	BRDN00012	96	23	11	185	18	8
CCAGGATCA	BRDN00011	264	101	124	424	237	0
CCAGGATCC	BRDN00011	196	159	395	192	142	4
CCAGGATCT	BRDN00012	354	270	506	379	429	25
CCAGGATCT	BRDN00011	101	92	6	106	84	9
CCAGGATGA	BRDN00011	165	198	334	395	431	0
CCAGGATGA	BRDN00011	146	142	149	310	586	19
CCAGGATGA	BRDN00012	189	295	407	423	640	17
CCAGGATGC	BRDN00011	105	72	287	276	401	0
CCAGGATGG	BRDN00011	347	308	163	608	394	39
CCAGGATGG	BRDN00012	281	403	1047	517	357	38
CCAGGATGT	BRDN00011	151	48	130	199	109	5
CCAGGATGT	BRDN00012	53	11	7	317	213	0
CCAGGATTA	BRDN00012	144	21	40	183	134	0
CCAGGATTT	BRDN00011	100	148	1756	422	192	81
CCAGGCAAA	BRDN00012	158	101	19	237	214	0
CCAGGCAAT	BRDN00012	111	151	187	234	417	0
CCAGGCACT	BRDN00012	462	470	445	817	472	35
CCAGGCAGG	BRDN00012	69	120	269	257	168	32
CCAGGCAGG	BRDN00012	163	203	17	305	270	82
CCAGGCAGG	BRDN00011	103	0	0	391	54	0
CCAGGCATC	BRDN00011	148	72	360	197	214	3
CCAGGCATC	BRDN00011	179	188	1	360	262	0
CCAGGCATG	BRDN00012	72	20	0	255	212	0
CCAGGCCAC	BRDN00012	74	124	0	114	98	0
CCAGGCCAC	BRDN00012	177	146	61	259	197	0
CCAGGCCAC	BRDN00011	213	106	106	320	169	0
CCAGGCCAT	BRDN00012	278	369	267	654	414	23
CCAGGCCCA	BRDN00011	234	181	219	395	183	33
CCAGGCCCC	BRDN00011	56	10	0	240	433	0
CCAGGCCGT	BRDN00012	148	277	400	323	421	49
CCAGGCCTA	BRDN00011	413	537	1291	681	613	51
CCAGGCCTA	BRDN00011	105	0	0	320	93	0
CCAGGCCTG	BRDN00011	251	0	0	543	78	2
CCAGGCGAA	BRDN00012	143	153	293	399	286	6
CCAGGCGAA	BRDN00012	346	290	434	613	715	17
CCAGGCGAC	BRDN00012	16	6	0	555	126	0
CCAGGCGAC	BRDN00011	140	129	377	248	230	0
CCAGGCGCC	BRDN00012	259	231	405	323	737	11
CCAGGCGCG	BRDN00012	319	159	2199	435	307	28
CCAGGCGCT	BRDN00011	134	0	63	224	697	0
CCAGGCGCT	BRDN00012	76	111	4	132	77	2
CCAGGCGGA	BRDN00011	74	85	18	166	203	0
CCAGGCGGC	BRDN00011	217	258	150	421	126	84
CCAGGCGGG	BRDN00011	132	39	54	241	200	0
CCAGGCGTC	BRDN00012	99	54	0	211	541	0
CCAGGCTAC	BRDN00012	78	11	0	298	174	0
CCAGGCTAC	BRDN00011	149	101	0	160	207	0
CCAGGCTCA	BRDN00012	238	329	43	455	637	9
CCAGGCTCC	BRDN00011	104	73	62	171	143	48
CCAGGCTCC	BRDN00012	242	209	31	389	339	36
CCAGGCTGA	BRDN00012	505	573	925	696	1381	80
CCAGGCTGA	BRDN00012	126	161	1290	193	257	55
CCAGGCTGA	BRDN00011	194	140	146	375	240	0
CCAGGCTGG	BRDN00011	151	40	159	230	106	0
CCAGGCTGT	BRDN00012	162	74	547	367	518	8
CCAGGCTTA	BRDN00011	325	333	1262	589	566	59
CCAGGCTTC	BRDN00012	89	4	0	375	84	0
CCAGGGAAA	BRDN00011	10	19	1	158	135	0
CCAGGGAAA	BRDN00012	88	163	0	237	242	28
CCAGGGAAG	BRDN00011	108	217	355	298	419	0
CCAGGGACA	BRDN00012	146	143	517	277	285	0
CCAGGGACA	BRDN00012	103	109	360	215	700	24
CCAGGGACT	BRDN00012	155	139	767	253	174	25
CCAGGGAGC	BRDN00011	226	106	1	455	240	13
CCAGGGATG	BRDN00012	115	109	1154	131	131	22
CCAGGGATG	BRDN00012	194	163	109	411	421	23
CCAGGGCAA	BRDN00011	272	181	89	555	298	4
CCAGGGCAA	BRDN00011	79	0	0	280	32	1
CCAGGGCAC	BRDN00011	289	346	1949	552	796	17
CCAGGGCAC	BRDN00012	47	12	1	646	945	0
CCAGGGCAG	BRDN00011	158	196	0	406	252	1
CCAGGGCAG	BRDN00012	279	215	31	625	655	5
CCAGGGCAT	BRDN00011	44	68	0	197	322	0
CCAGGGCAT	BRDN00011	263	114	448	620	563	47
CCAGGGCCA	BRDN00011	214	233	410	457	142	3
CCAGGGCCA	BRDN00012	35	29	15	141	254	14
CCAGGGCCA	BRDN00012	155	155	1	257	207	0
CCAGGGCCG	BRDN00012	42	0	0	693	315	0
CCAGGGCCG	BRDN00012	116	63	12	176	171	0
CCAGGGCCG	BRDN00011	148	104	169	213	149	5
CCAGGGCCG	BRDN00011	137	4	0	275	117	0
CCAGGGCCT	BRDN00011	347	481	102	755	589	58
CCAGGGCCT	BRDN00011	109	71	751	177	394	12
CCAGGGCGA	BRDN00011	181	120	271	259	279	30
CCAGGGCGA	BRDN00011	99	81	0	61	76	3
CCAGGGCGC	BRDN00012	70	106	0	113	106	0
CCAGGGCGT	BRDN00011	232	256	354	312	337	10
CCAGGGCGT	BRDN00012	203	227	249	377	616	35
CCAGGGCTA	BRDN00011	302	95	70	399	563	0
CCAGGGCTA	BRDN00012	89	128	140	171	102	4
CCAGGGCTC	BRDN00012	347	312	748	561	638	38
CCAGGGGAT	BRDN00012	106	73	311	344	62	2
CCAGGGGCA	BRDN00012	117	86	35	216	254	7
CCAGGGTAA	BRDN00012	136	117	43	311	168	5
CCAGGGTAC	BRDN00011	319	173	39	453	239	12
CCAGGGTAT	BRDN00011	92	0	0	309	102	0
CCAGGGTAT	BRDN00011	161	229	307	382	544	22
CCAGGGTCA	BRDN00011	100	122	655	501	422	11
CCAGGGTCT	BRDN00011	237	303	1	357	294	10
CCAGGGTGA	BRDN00012	315	237	116	694	971	86
CCAGGGTGA	BRDN00011	74	33	215	299	121	0
CCAGGGTGA	BRDN00011	138	97	108	189	297	0
CCAGGGTGA	BRDN00012	157	278	226	212	110	38
CCAGGGTGC	BRDN00012	149	122	588	290	455	13
CCAGGGTGC	BRDN00012	461	328	605	682	720	89
CCAGGGTGG	BRDN00011	165	226	50	458	313	4
CCAGGGTGG	BRDN00012	114	65	0	157	126	17
CCAGGGTGT	BRDN00011	42	55	57	98	125	0
CCAGGGTTG	BRDN00011	114	16	36	236	281	0
CCAGGGTTT	BRDN00011	7	1	0	389	154	0
CCAGGTAAA	BRDN00011	249	0	0	495	601	0
CCAGGTAAC	BRDN00011	217	181	129	363	693	0
CCAGGTACC	BRDN00012	138	0	0	252	153	0
CCAGGTACT	BRDN00012	188	163	693	218	202	48
CCAGGTACT	BRDN00011	246	326	239	404	395	56
CCAGGTACT	BRDN00012	425	327	810	452	675	29
CCAGGTACT	BRDN00011	62	0	0	136	72	0
CCAGGTACT	BRDN00011	158	77	0	315	202	0
CCAGGTAGA	BRDN00012	170	148	442	313	344	67
CCAGGTAGA	BRDN00011	70	65	8	153	44	0
CCAGGTAGG	BRDN00011	143	96	25	236	417	0
CCAGGTAGG	BRDN00011	558	469	338	943	979	54
CCAGGTAGG	BRDN00012	260	231	685	479	466	29
CCAGGTCAC	BRDN00012	108	34	779	272	271	24
CCAGGTCAC	BRDN00011	56	71	652	205	63	15
CCAGGTCAG	BRDN00011	136	13	1	417	230	0
CCAGGTCAT	BRDN00012	305	252	705	585	451	24
CCAGGTCCA	BRDN00012	385	435	1911	592	956	39
CCAGGTCCG	BRDN00011	73	26	0	194	58	8
CCAGGTCGA	BRDN00011	231	412	133	468	178	45
CCAGGTGAC	BRDN00011	292	276	647	601	601	14
CCAGGTGAG	BRDN00012	102	136	120	207	368	1
CCAGGTGAT	BRDN00012	324	355	191	614	608	56
CCAGGTGCC	BRDN00011	502	594	220	940	387	26
CCAGGTGCG	BRDN00012	101	118	168	197	240	4
CCAGGTGGA	BRDN00011	38	5	1	84	153	0
CCAGGTGGA	BRDN00012	405	322	443	754	1142	30
CCAGGTGGA	BRDN00011	89	51	55	114	151	15
CCAGGTGGA	BRDN00012	304	321	1527	669	840	60
CCAGGTGGC	BRDN00011	76	9	86	86	79	19
CCAGGTGGC	BRDN00011	0	0	0	9	4	0
CCAGGTGGG	BRDN00011	222	333	449	540	494	16
CCAGGTGGG	BRDN00012	461	362	3180	751	673	64
CCAGGTGGT	BRDN00011	53	45	30	137	195	0
CCAGGTGTA	BRDN00012	118	78	16	211	292	16
CCAGGTGTC	BRDN00012	118	121	1	181	256	0
CCAGGTTAA	BRDN00011	80	59	283	176	91	48
CCAGGTTAA	BRDN00012	82	126	96	257	110	5
CCAGGTTAA	BRDN00012	156	133	403	228	245	39
CCAGGTTAT	BRDN00011	97	65	1416	128	106	4
CCAGGTTCG	BRDN00011	100	31	266	153	327	0
CCAGGTTCT	BRDN00012	274	319	392	599	669	59
CCAGGTTGA	BRDN00011	295	204	519	647	596	14
CCAGGTTGG	BRDN00012	84	86	25	214	210	14
CCAGGTTGT	BRDN00012	496	417	388	599	340	55
CCAGGTTTA	BRDN00012	78	53	61	253	495	10
CCAGTAAAC	BRDN00011	296	263	588	510	408	10
CCAGTAAAT	BRDN00011	75	30	23	127	84	8
CCAGTAACC	BRDN00011	243	262	26	332	346	67
CCAGTAATA	BRDN00012	238	181	350	405	456	13
CCAGTAATT	BRDN00011	274	484	681	546	495	97
CCAGTACAA	BRDN00012	321	256	101	477	348	46
CCAGTACAC	BRDN00011	111	70	373	135	285	40
CCAGTACAC	BRDN00011	498	484	632	624	712	76
CCAGTACAG	BRDN00012	94	58	15	143	197	16
CCAGTACAG	BRDN00012	467	458	215	537	666	32
CCAGTACCA	BRDN00011	13	0	0	100	38	0
CCAGTACCA	BRDN00011	155	0	1	369	148	0
CCAGTACCA	BRDN00012	129	185	176	304	171	6
CCAGTACCC	BRDN00012	428	369	472	840	1258	90
CCAGTACCG	BRDN00012	159	128	0	491	265	6
CCAGTACGC	BRDN00011	181	128	389	227	183	12
CCAGTACGC	BRDN00011	146	64	912	118	64	54
CCAGTACGT	BRDN00011	224	0	4	633	366	0
CCAGTACTA	BRDN00012	246	164	5	434	402	13

CCAGTACTG	BRDN00012	204	211	147	277	215	31
CCAGTAGCA	BRDN00012	65	108	342	215	275	23
CCAGTAGCA	BRDN00011	251	374	682	462	344	5
CCAGTAGGG	BRDN00011	240	222	36748	416	357	413
CCAGTATGC	BRDN00012	225	178	75	461	163	11
CCAGTATGT	BRDN00011	102	144	567	268	144	10
CCAGTCAACT	BRDN00011	141	0	1	290	147	0
CCAGTCAAT	BRDN00011	244	105	1136	293	172	26
CCAGTCAAT	BRDN00011	190	125	346	272	365	8
CCAGTCACA	BRDN00012	153	192	558	369	475	27
CCAGTCACA	BRDN00012	185	178	297	324	158	19
CCAGTCACCC	BRDN00011	158	209	318	250	318	13
CCAGTCACG	BRDN00012	439	407	935	891	916	139
CCAGTCAGT	BRDN00011	157	59	215	181	183	12
CCAGTCATCA	BRDN00011	109	5	4	646	435	0
CCAGTCATCA	BRDN00012	104	91	838	247	455	47
CCAGTCCAA	BRDN00012	116	106	146	325	545	8
CCAGTCCAG	BRDN00011	99	15	1	504	358	0
CCAGTCCAG	BRDN00012	3	0	0	320	142	0
CCAGTCCCAC	BRDN00012	68	0	1	350	376	0
CCAGTCCCAG	BRDN00012	341	396	271	602	1019	19
CCAGTCCCG	BRDN00012	185	190	889	366	570	37
CCAGTCCGA	BRDN00011	435	125	121	807	557	0
CCAGTCCTG	BRDN00012	213	268	420	353	440	6
CCAGTCCTG	BRDN00011	351	435	2768	591	693	29
CCAGTCGAC	BRDN00011	404	295	308	743	749	46
CCAGTCGAG	BRDN00012	138	64	0	199	141	0
CCAGTCGCG	BRDN00012	257	16	0	466	49	0
CCAGTCGGC	BRDN00012	115	26	47	208	289	5
CCAGTCGGG	BRDN00012	45	32	82	220	18	0
CCAGTCTAA	BRDN00011	196	172	110	524	384	1
CCAGTCTCAG	BRDN00012	123	75	15	215	359	0
CCAGTCTCG	BRDN00011	318	294	255	533	572	0
CCAGTCTGCC	BRDN00012	384	0	0	643	571	0
CCAGTCTGG	BRDN00012	294	1	5	689	194	0
CCAGTCTTCT	BRDN00012	284	248	50	621	657	40
CCAGTGAAA	BRDN00011	268	224	0	488	229	13
CCAGTGAAA	BRDN00011	296	158	587	400	653	10
CCAGTGAAG	BRDN00011	194	282	241	338	361	55
CCAGTGACA	BRDN00012	505	367	69	997	1137	49
CCAGTGACC	BRDN00012	198	152	531	460	819	50
CCAGTGACC	BRDN00011	228	149	314	416	423	35
CCAGTGACCT	BRDN00012	46	46	8	594	371	0
CCAGTGAGA	BRDN00011	126	76	42	324	110	0
CCAGTGAGC	BRDN00011	369	352	67	608	522	17
CCAGTGATA	BRDN00012	221	186	125	611	306	7
CCAGTGATT	BRDN00012	418	600	788	664	595	101
CCAGTGCAA	BRDN00011	309	287	626	486	978	22
CCAGTGCAG	BRDN00011	324	315	829	662	532	31
CCAGTGCAG	BRDN00012	209	278	38	528	186	17
CCAGTGCAT	BRDN00012	97	54	9	211	250	0
CCAGTGCCA	BRDN00012	130	194	113	273	371	6
CCAGTGCCA	BRDN00012	443	545	414	718	592	46
CCAGTGCCCT	BRDN00012	506	506	413	807	983	80
CCAGTGCGC	BRDN00012	64	8	0	162	21	0
CCAGTGCTA	BRDN00011	135	24	5	289	54	3
CCAGTGCTG	BRDN00012	344	307	1397	598	837	18
CCAGTGCTT	BRDN00012	132	50	31	209	85	0
CCAGTGGAA	BRDN00011	292	254	408	556	878	17
CCAGTGGAA	BRDN00012	110	144	112	261	449	0
CCAGTGGAC	BRDN00011	270	215	414	529	397	12
CCAGTGGAC	BRDN00012	469	369	421	893	828	21
CCAGTGGAT	BRDN00011	28	21	62	444	178	0
CCAGTGGAT	BRDN00012	190	237	121	340	331	0
CCAGTGGCA	BRDN00011	103	8	0	301	88	0
CCAGTGGCA	BRDN00011	60	181	64	239	99	29
CCAGTGGCA	BRDN00011	198	175	252	362	344	7
CCAGTGGCG	BRDN00012	259	234	735	365	423	0
CCAGTGGCT	BRDN00012	128	255	173	351	178	0
CCAGTGGCT	BRDN00011	182	181	549	165	208	0
CCAGTGGGA	BRDN00012	308	249	754	766	918	56
CCAGTGGGC	BRDN00011	259	177	120	389	262	7
CCAGTGGGC	BRDN00011	96	17	59	103	367	3
CCAGTGGTG	BRDN00011	17	0	1	50	86	0
CCAGTGGTT	BRDN00012	112	18	178	187	156	9
CCAGTGTAA	BRDN00011	42	38	0	145	59	0
CCAGTGTAA	BRDN00012	119	163	0	219	182	0
CCAGTGTAT	BRDN00011	207	130	633	327	599	14
CCAGTGTAT	BRDN00011	167	197	2080	199	377	44
CCAGTGTCA	BRDN00012	278	243	305	470	605	7
CCAGTGTCC	BRDN00012	397	309	586	774	1010	19
CCAGTGTGC	BRDN00011	88	114	2097	261	125	5
CCAGTGTGG	BRDN00011	142	192	142	211	292	8
CCAGTGTTCT	BRDN00011	145	40	197	259	208	0
CCAGTGTTG	BRDN00011	127	55	238	225	166	22
CCAGTTAAT	BRDN00012	248	274	95	611	592	49
CCAGTTAAT	BRDN00012	104	48	1091	195	147	16
CCAGTTACA	BRDN00012	124	4	186	400	123	4
CCAGTTACAT	BRDN00011	37	1	0	169	197	0
CCAGTTACCA	BRDN00011	206	22	3573	512	202	42
CCAGTTACC	BRDN00011	315	248	104	599	585	33
CCAGTTACCT	BRDN00011	239	181	10049	604	694	199
CCAGTTATA	BRDN00012	346	255	890	601	394	62
CCAGTTATCA	BRDN00012	255	148	29	362	456	39
CCAGTTATG	BRDN00011	103	162	21	279	128	0
CCAGTTATTA	BRDN00011	98	51	0	360	190	0
CCAGTTATTG	BRDN00011	17	9	0	121	45	0
CCAGTTCAA	BRDN00011	337	358	641	681	1180	38
CCAGTTCAA	BRDN00011	219	128	152	452	407	13
CCAGTTCATC	BRDN00012	384	160	401	662	407	5
CCAGTTCCAC	BRDN00012	182	151	109	314	176	5
CCAGTTCCAG	BRDN00011	446	451	2153	808	527	94
CCAGTTCCAT	BRDN00011	118	101	340	280	354	0
CCAGTTCTTC	BRDN00011	228	150	97	339	243	17
CCAGTTGAT	BRDN00012	475	525	1240	863	967	86
CCAGTTGAT	BRDN00011	204	162	819	246	415	40
CCAGTTGCT	BRDN00012	587	549	354	932	1464	343
CCAGTTGCT	BRDN00012	311	215	0	623	654	75
CCAGTTGTG	BRDN00012	244	120	59	519	595	16
CCAGTTGTG	BRDN00011	327	275	610	467	656	77
CCAGTTTAA	BRDN00012	76	72	454	175	90	19
CCAGTTTCTT	BRDN00011	299	148	188	434	515	13
CCAGTTTGCT	BRDN00011	92	103	256	201	188	19
CCAGTTTGG	BRDN00011	123	99	119	200	485	9
CCATAAAGG	BRDN00012	449	492	723	608	655	32
CCATAAATAT	BRDN00012	78	100	228	216	298	0
CCATAAATG	BRDN00012	109	158	1536	163	173	56
CCATAACAA	BRDN00011	230	191	130	291	405	17
CCATAACACA	BRDN00011	170	190	3443	264	227	120
CCATAACCCA	BRDN00011	414	398	1449	905	918	54
CCATAACGC	BRDN00011	246	125	108	654	516	0
CCATAAGAT	BRDN00011	281	234	2	523	587	0
CCATAAGATT	BRDN00011	98	126	15	216	194	3
CCATAAGCA	BRDN00012	171	273	256	316	232	13
CCATAAGCC	BRDN00012	112	22	0	164	75	0
CCATAAGGG	BRDN00012	301	257	688	454	338	5
CCATAAGGG	BRDN00011	63	41	19	82	196	0
CCATAAGTG	BRDN00012	547	443	1379	764	547	35
CCATAATAA	BRDN00012	165	179	7	351	384	0
CCATAATAG	BRDN00012	148	153	16	279	145	0
CCATAATAGT	BRDN00012	159	212	21	329	359	1
CCATAATATC	BRDN00012	130	130	657	291	384	10
CCATAATCCA	BRDN00012	418	424	822	561	331	23
CCATACAAAT	BRDN00011	339	250	190	682	480	8
CCATACAATA	BRDN00012	421	303	358	539	640	80
CCATACACAA	BRDN00011	132	230	481	254	560	23
CCATACACTG	BRDN00011	399	148	207	609	323	6
CCATACACTT	BRDN00012	103	51	122	352	276	10
CCATACAGA	BRDN00011	177	131	26	416	728	0
CCATACCCTC	BRDN00012	90	73	0	252	192	0
CCATACCGG	BRDN00011	113	198	270	180	277	24
CCATACCTCC	BRDN00011	227	285	154	410	207	29
CCATACCTTA	BRDN00012	218	143	255	457	543	34
CCATACCTTC	BRDN00011	26	4	0	135	1	1
CCATACCTTT	BRDN00011	70	68	46	445	178	7
CCATACGAA	BRDN00012	104	86	282	156	230	0
CCATACGAG	BRDN00011	366	337	1025	733	658	49
CCATACTCAA	BRDN00012	283	318	120	488	466	37
CCATACTCAC	BRDN00012	133	228	63	311	184	11
CCATACTCCG	BRDN00012	279	206	234	870	594	14
CCATACTCGT	BRDN00012	55	8	43	249	97	6
CCATACTGTG	BRDN00012	464	415	2808	830	953	66
CCATACTTAC	BRDN00011	56	0	0	322	274	0
CCATACTTGA	BRDN00012	208	217	264	422	300	7
CCATAGAATT	BRDN00011	210	114	421	265	331	24
CCATAGACG	BRDN00012	84	62	70	202	125	21
CCATAGAGA	BRDN00012	188	124	154	291	125	24
CCATAGATA	BRDN00012	185	134	141	296	560	15
CCATAGATAT	BRDN00011	257	287	443	521	372	12
CCATAGCCAT	BRDN00012	102	150	136	240	184	0
CCATAGCCGT	BRDN00011	185	114	213	544	338	0
CCATAGCGA	BRDN00012	173	163	97	308	168	0
CCATAGCTCT	BRDN00012	342	357	563	537	576	39
CCATAGCTTT	BRDN00012	131	15	1	227	130	0
CCATAGGAC	BRDN00011	295	320	226	619	937	8
CCATAGGCG	BRDN00012	385	319	577	681	1014	74
CCATAGGGA	BRDN00012	108	162	371	257	313	36
CCATAGTAA	BRDN00012	158	149	11	318	212	1
CCATAGTCTT	BRDN00011	142	270	367	347	482	390
CCATAGTTGT	BRDN00011	211	185	285	291	253	52
CCATATAATG	BRDN00011	75	92	39	163	213	56
CCATATACAC	BRDN00011	158	85	1	278	340	0
CCATATACAG	BRDN00011	255	209	738	435	739	44
CCATATACG	BRDN00011	131	91	38	391	251	0
CCATATAGA	BRDN00012	24	12	0	303	40	0
CCATATCATA	BRDN00011	222	180	849	306	486	23
CCATATCCCC	BRDN00011	167	133	17	197	257	88
CCATATCGA	BRDN00011	186	164	381	419	473	178
CCATATCGG	BRDN00012	650	413	3005	931	976	37
CCATATCTAC	BRDN00011	119	174	0	282	376	30
CCATATCTGT	BRDN00012	143	123	63	317	269	56
CCATATGAA	BRDN00012	245	197	30	605	606	11
CCATATGAA	BRDN00011	148	69	679	187	88	24
CCATATGAAT	BRDN00012	351	326	520	610	676	52
CCATATGAT	BRDN00012	331	340	466	488	440	5
CCATATGAT	BRDN00012	417	237	486	777	408	29
CCATATGCA	BRDN00011	101	85	126	189	115	15
CCATATTACA	BRDN00011	67	0	0	191	116	0
CCATATTCGC	BRDN00011	378	426	901	812	616	27
CCATATTCTG	BRDN00011	142	104	178	270	199	0
CCATATTGGT	BRDN00011	10	0	1	369	108	0
CCATATTTGT	BRDN00011	351	161	1367	452	860	38
CCATCAACAA	BRDN00012	140	103	45	227	244	0
CCATCAACAC	BRDN00012	241	229	246	451	248	19
CCATCAACCT	BRDN00011	365	277	191	870	729	154
CCATCAACTT	BRDN00011	177	252	1519	164	157	76
CCATCAAGA	BRDN00011	76	24	228	222	128	6
CCATCAAGAT	BRDN00012	129	37	111	296	114	5
CCATCAAGG	BRDN00011	439	366	114	781	1212	91
CCATCACAAT	BRDN00011	388	136	142	1042	914	21
CCATCACACA	BRDN00012	419	527	490	840	960	29
CCATCACATC	BRDN00012	229	207	122	434	494	14
CCATCACCAT	BRDN00012	232	172	325	367	108	31
CCATCACCGA	BRDN00012	487	545	231	961	1179	34
CCATCACCGA	BRDN00012	751	579	626	1197	1279	108
CCATCACCGT	BRDN00011	119	137	200	339	936	4
CCATCACGTC	BRDN00011	485	436	226	967	722	15
CCATCACTCA	BRDN00011	184	99	685	754	374	0
CCATCACTCA	BRDN00011	116	48	23	163	82	17
CCATCACTGA	BRDN00011	132	18	77	341	414	0
CCATCAGATC	BRDN00011	129	126	34	302	209	36
CCATCAGCG	BRDN00011	273	0	0	644	344	0
CCATCAGCTC	BRDN00012	396	532	533	777	737	25
CCATCAGGA	BRDN00012	178	48	54	252	341	90
CCATCAGTG	BRDN00011	114	101	315	485	249	5
CCATCATAAG	BRDN00012	323	268	285	654	494	18
CCATCATAGT	BRDN00012	424	444	202	668	613	31
CCATCATCAG	BRDN00011	141	100	0	310	300	7
CCATCATCCA	BRDN00011	150	329	69	293	371	65
CCATCATGAC	BRDN00011	321	149	139	555	1074	52
CCATCATGTC	BRDN00011	219	167	988	365	458	12
CCATCATGTG	BRDN00012	390	245	304	462	412	76
CCATCATTCA	BRDN00012	244	233	0	409	278	14
CCATCCAAGT	BRDN00011	265	111	3	429	286	0
CCATCCACAT	BRDN00011	198	214	637	313	611	12
CCATCCACCT	BRDN00011	175	321	1840	328	425	30
CCATCCACCT	BRDN00012	523	572	461	853	960	6
CCATCCAGAC	BRDN00011	127	120	94	390	249	0
CCATCCAGG	BRDN00011	158	146	6	214	182	21
CCATCCAGTA	BRDN00012	189	213	0	469	750	16
CCATCCAGTG	BRDN00011	175	142	172	231	244	17
CCATCCCAGA	BRDN00011	357	303	84	863	691	11
CCATCCCAGA	BRDN00012	319	179	279	629	325	27
CCATCCCAGC	BRDN00012	237	84	1901	504	400	22
CCATCCCCGA	BRDN00011	83	47	150	239	117	0
CCATCCCTAG	BRDN00011	162	151	12	491	430	0
CCATCCCTGG	BRDN00011	476	251	574	744	346	12
CCATCCGCCC	BRDN00011	87	148	383	108	52	9
CCATCCGGTC	BRDN00011	187	213	1188	322	269	20
CCATCGAAG	BRDN00011	185	217	426	388	403	0
CCATCGAGA	BRDN00012	297	404	416	735	424	44
CCATCGAGA	BRDN00012	144	137	581	394	281	4
CCATCGAGG	BRDN00011	143	183	176	278	187	11
CCATCGATTT	BRDN00012	220	240	913	329	234	74
CCATCGCAG	BRDN00011	209	105	39	251	246	25
CCATCGGCA	BRDN00011	293	305	445	442	588	6
CCATCGGCAT	BRDN00011	163	86	82	281	225	5
CCATCGGCG	BRDN00012	67	70	0	148	10	0
CCATCGGTTC	BRDN00011	165	164	449	259	495	9
CCATCGTAG	BRDN00011	134	94	23	264	296	8
CCATCGTCAG	BRDN00011	252	117	201	540	731	22
CCATCTACAC	BRDN00012	219	215	506	327	921	19
CCATCTACGA	BRDN00011	210	287	761	456	245	41
CCATCTAGAC	BRDN00012	263	176	335	440	565	0
CCATCTCAAA	BRDN00011	249	156	489	533	533	46
CCATCTCACA	BRDN00011	250	124	387	881	696	0
CCATCTCATC	BRDN00011	164	154	206	321	238	9
CCATCTCATC	BRDN00012	466	338	340	848	454	23
CCATCTCCCA	BRDN00012	324	1	2	662	213	1
CCATCTCGAA	BRDN00012	151	166	272	257	551	76
CCATCTCGTA	BRDN00012	296	123	0	418	347	0
CCATCTCTGA	BRDN00011	237	305	188	498	428	22
CCATCTGCAA	BRDN00012	215	161	396	258	540	17
CCATCTGGAC	BRDN00012	148	82	0	330	387	0
CCATCTGGCT	BRDN00012	174	175	29	348	385	9
CCATCTGTAG	BRDN00012	345	252	301	842	1687	56
CCATCTTAAT	BRDN00011	420	343	1427	563	340	66
CCATCTTACG	BRDN00012	227	24	1	403	481	0
CCATCTTCGA	BRDN00012	428	385	260	707	686	149
CCATCTTGCA	BRDN00011	336	247	797	437	413	92
CCATCTTGGA	BRDN00011	347	287	165	770	830	12
CCATCTTGGC	BRDN00011	178	57	1715	275	603	28
CCATCTTTCA	BRDN00011	170	156	165	333	260	46
CCATGAAAA	BRDN00011	108	97	8	262	67	0
CCATGAAAG	BRDN00011	230	145	457	382	452	18
CCATGAACA	BRDN00012	282	200	778	565	827	25
CCATGAACG	BRDN00011	104	93	97	178	76	4
CCATGAACG	BRDN00011	188	206	1075	326	220	32
CCATGAAGA	BRDN00012	74	62	91	413	134	0
CCATGAAGC	BRDN00012	150	84	168	250	91	34
CCATGAAGC	BRDN00012	497	426	154	852	1125	54
CCATGAATA	BRDN00011	215	171	57	593	641	0
CCATGAATG	BRDN00012	108	150	1521	234	285	35
CCATGACAC	BRDN00012	252	207	7	233	542	44
CCATGACATT	BRDN00012	231	192	442	582	585	36
CCATGACCG	BRDN00011	245	260	160	534	522	5
CCATGACCTG	BRDN00012	279	93	175	383	413	27
CCATGACGA	BRDN00012	113	78	38	193	159	0
CCATGACGCT	BRDN00012	170	274	2028	370	307	65
CCATGACGG	BRDN00012	313	295	435	571	705	30
CCATGACTAT	BRDN00011	69	81	131	232	221	0
CCATGACTGC	BRDN00012	355	318	1288	483	300	21
CCATGACTTC	BRDN00011	211	205	2074	381	334	36
CCATGACTTC	BRDN00011	291	365	181	654	668	8
CCATGACTTT	BRDN00011	56	0	0	262	110	0
CCATGAGAG	BRDN00011	101	66	418	229	126	4
CCATGAGCA	BRDN00012	259	120	17	338	207	15
CCATGAGCCT	BRDN00011	203	175	279	345	431	0
CCATGAGGA	BRDN00011	262	202	531	422	808	32
CCATGAGGC	BRDN00011	380	424	734	937	1122	49
CCATGAGTA	BRDN00011	93	159	41	178	84	20
CCATGAGTC	BRDN00012	78	89	8	174	187	0
CCATGAGTTC	BRDN00012	335	367	121	462	636	20
CCATGATCAT	BRDN00011	316	330	881	560	710	41
CCATGATCCG	BRDN00011	120	107	112	256	193	8
CCATGATGAT	BRDN00012	128	78	40	301	216	18
CCATGATGCA	BRDN00012	353	108	533	465	545	9
CCATGATTAC	BRDN00011	148	48	0	299	192	0
CCATGATTCC	BRDN00011	290	142	0	466	335	12
CCATGATTGT	BRDN00011	156	294	1020	237	159	30
CCATGCACTC	BRDN00011	278	243	1328	607	471	42
CCATGCAGA	BRDN00012	376	226	254	674	842	10
CCATGCAGCT	BRDN00011	172	140	1614	324	320	64
CCATGCATCC	BRDN00012	391	362	218	760	626	16
CCATGCCAGT	BRDN00011	541	320	442	799	774	34
CCATGCCCAA	BRDN00012	170	171	106	257	79	1
CCATGCCCAG	BRDN00012	137	179	35	369	251	0
CCATGCCCGC	BRDN00011	220	192	2236	414	490	48
CCATGCCGCC	BRDN00011	161	174	576	286	154	11
CCATGCCGTT	BRDN00011	288	223	795	537	620	40
CCATGCCTCA	BRDN00012	234	202	219	582	375	0
CCATGCGAG	BRDN00011	265	240	578	444	512	30
CCATGCGGC	BRDN00011	409	357	391	782	444	10
CCATGCGTAC	BRDN00012	206	318	421	399	330	38
CCATGCTAA	BRDN00011	325	339	1435	742	553	91
CCATGCTCTC	BRDN00011	536	377	505	841	843	25
CCATGCTGA	BRDN00012	392	448	1127	668	1024	18
CCATGCTTG	BRDN00012	126	89	3	219	141	31
CCATGGAGA	BRDN00012	250	303	202	449	553	99
CCATGGAGC	BRDN00012	54	60	32	242	318	2
CCATGGAGT	BRDN00012	162	165	67	382	205	1
CCATGGCAA	BRDN00011	135	146	482	280	165	24
CCATGGCAT	BRDN00011	134	83	76	159	67	18
CCATGGCCC	BRDN00012	267	317	864	654	470	92
CCATGGCCC	BRDN00011	221	301	352	388	295	77
CCATGGCCT	BRDN00011	120	137	50	272	254	0
CCATGGCCTT	BRDN00012	375	451	1647	758	810	80
CCATGGCTG	BRDN00012	69	29	396	210	48	6
CCATGGGAA	BRDN00011	392	261	338	695	543	61
CCATGGGCA	BRDN00011	243	253	1092	529	557	0
CCATGGGGG	BRDN00012	66	49	40	145	95	2
CCATGGTAC	BRDN00012	90	84	4	203	191	5
CCATGGTAG	BRDN00011	407	439	924	876	698	174
CCATGGTCA	BRDN00012	106	21	0	178	126	0
CCATGGTCA	BRDN00012	137	175	305	329	341	33
CCATGGTCC	BRDN00011	441	467	932	869	751	60
CCATGGTGC	BRDN00011	437	167	265	651	426	10
CCATGGTGG	BRDN00011	138	131	881	237	122	55
CCATGTAAA	BRDN00011	290	267	144	589	564	46
CCATGTAAAT	BRDN00012	207	108	743	439	459	29
CCATGTAAC	BRDN00012	235	6	184	618	603	3
CCATGTACCA	BRDN00011	125	164	32	332	456	13
CCATGTAGTT	BRDN00011	237	288	871	517	819	16
CCATGTATTC	BRDN00012	327	198	182	675	646	7
CCATGTCAA	BRDN00012	157	143	17	234	389	0
CCATGTCACC	BRDN00012	370	335	401	844	380	25
CCATGTCAG	BRDN00011	315	197	157	348	297	0
CCATGTCAG	BRDN00011	140	74	1766	275	202	36
CCATGTCAG	BRDN00011	102	112	21	279	159	8
CCATGTCCG	BRDN00011	331	349	209	697	572	69
CCATGTGAA	BRDN00012	299	248	396	646	646	54
CCATGTGAA	BRDN00011	270	199	32	536	415	0
CCATGTGAC	BRDN00012	132	103	59	343	263	1
CCATGTGCC	BRDN00011	162	264	227	232	1926	53
CCATGTGGA	BRDN00011	143	211	72	319	169	21
CCATGTGTA	BRDN00011	498	649	734	774	730	145
CCATGTTCAC	BRDN00011	340	382	622	576	595	11
CCATGTTCAC	BRDN00012	313	197	1503	687	1114	29
CCATGTTCCT	BRDN00012	429	299	905	875	1331	69
CCATGTTCG	BRDN00012	327	340	950	622	870	40
CCATGTTGG	BRDN00012	331	354	118	775	567	7
CCATGTTGG	BRDN00012	398	592	403	719	1166	21
CCATGTTGTC	BRDN00011	222	285	353	475	306	5
CCATTAAACT	BRDN00011	312	346	3865	590	835	65
CCATTAACAA	BRDN00012	322	313	576	611	306	38
CCATTAAGTC	BRDN00012	186	193	147	400	562	0
CCATTACCAG	BRDN00011	76	97	106	151	123	0
CCATTACTAC	BRDN00011	253	315	366	716	611	0
CCATTAGCG	BRDN00012	147	161	340	271	342	23
CCATTAGGTT	BRDN00011	233	176	70	464	723	26
CCATTATCCT	BRDN00012	148	265	2217	304	103	59
CCATTATCCT	BRDN00011	486	441	149	858	784	53
CCATTATTAA	BRDN00012	309	58	34	597	550	11
CCATTCAATA	BRDN00012	276	345	265	646	549	34
CCATTCACAA	BRDN00012	594	642	1295	1205	1481	72
CCATTCACGA	BRDN00012	60	144	774	211	80	0
CCATTCCAAG	BRDN00011	117	51	7	204	186	0
CCATTCCACA	BRDN00011	55	40	396	152	97	0
CCATTCCCGG	BRDN00012	103	128	41	201	143	6
CCATTCCGTA	BRDN00012	698	736	824	1173	1214	74
CCATTCCTAT	BRDN00012	178	114	0	245	274	0
CCATTCGTGT	BRDN00012	500	234	66	959	1161	0
CCATTCTCAA	BRDN00012	487	486	2248	898	839	56
CCATTCTCCA	BRDN00012	429	289	38	694	683	1
CCATTCTGGC	BRDN00011	183	105	50	310	175	0
CCATTGAAGT	BRDN00012	101	0	0	276	96	0
CCATTGACA	BRDN00012	317	350	100	592	311	20
CCATTGACCA	BRDN00012	257	130	185	363	370	7
CCATTGACCT	BRDN00012	407	445	420	797	1299	8
CCATTGACTA	BRDN00012	186	229	77	393	468	5
CCATTGAGTC	BRDN00012	94	102	0	159	67	0
CCATTGATAC	BRDN00012	210	165	55	237	325	6
CCATTGCATA	BRDN00012	200	113	222	302	193	11
CCATTGCCAC	BRDN00012	250	232	3099	527	333	41
CCATTGCCAG	BRDN00012	133	116	208	191	111	0
CCATTGCCTC	BRDN00012	443	441	178	1032	993	5
CCATTGGGC	BRDN00012	376	261	183	652	481	16
CCATTGGTT	BRDN00012	407	520	429	479	589	28
CCATTGTACA	BRDN00012	461	397	1528	939	1157	64
CCATTGTATG	BRDN00012	241	244	616	535	427	13
CCATTGTTCA	BRDN00011	179	261	400	371	290	26
CCATTTATAC	BRDN00011	57	0	0	241	197	0
CCATTTCATC	BRDN00011	418	49	104	927	587	0
CCATTTCCAG	BRDN00011	353	321	1043	733	698	7
CCATTTCCCC	BRDN00011	167	167	570	469	556	14
CCATTTCTCA	BRDN00012	222	244	289	409	455	14
CCATTTGAG	BRDN00011	500	411	279	904	851	57
CCATTTGGT	BRDN00012	357	372	1955	799	819	24
CCCAAAAAAC	BRDN00011	82	170	0	106	102	0
CCCAAAACCA	BRDN00011	239	294	924	603	499	46
CCCAAAACCT	BRDN00012	145	90	85	316	240	30
CCCAAAACGC	BRDN00012	95	94	0	160	153	5
CCCAAAAGCA	BRDN00012	256	254	234	596	413	33
CCCAAAATAC	BRDN00011	166	73	57	282	106	1
CCCAAACACC	BRDN00012	499	357	469	734	647	9
CCCAAACATC	BRDN00012	140	64	115	238	444	19
CCCAAACCCC	BRDN00012	106	116	823	189	247	37
CCCAAACTCG	BRDN00011	172	291	57	422	262	18
CCCAAAGCG	BRDN00012	259	200	504	578	520	0
CCCAAATATT	BRDN00012	259	239	191	366	368	37
CCCAAATCCA	BRDN00011	129	166	110	312	124	7
CCCAAATCGA	BRDN00011	236	118	277	620	342	3
CCCAAATCGT	BRDN00011	251	223	273	410	574	22
CCCAACAACA	BRDN00011	73	74	1	280	224	0
CCCAACACAA	BRDN00011	139	134	425	243	240	28
CCCAACACCA	BRDN00012	339	362	1489	677	906	64
CCCAACACCT	BRDN00012	193	232	167	327	189	31
CCCAACACTC	BRDN00011	316	204	910	737	553	57
CCCAACAGAC	BRDN00012	331	300	248	666	674	14
CCCAACATAA	BRDN00012	245	203	431	589	672	43
CCCAACATAG	BRDN00012	178	235	333	242	253	12
CCCAACCAGC	BRDN00011	68	23	0	159	209	0
CCCAACCCCA	BRDN00011	419	58	187	795	513	0
CCCAACCCCT	BRDN00012	250	23	16	691	506	0
CCCAACCTCA	BRDN00012	273	541	639	674	916	30
CCCAACCTCC	BRDN00012	309	332	15	435	319	65
CCCAACCTCC	BRDN00012	187	177	211	431	334	11
CCCAACCTCC	BRDN00012	204	97	827	351	670	13
CCCAACCTCT	BRDN00011	502	495	730	736	994	34
CCCAACGGA	BRDN00011	379	440	46	688	258	3
CCCAACGTG	BRDN00011	171	102	0	298	266	17
CCCAACTACA	BRDN00012	144	34	0	214	89	0
CCCAACTCAA	BRDN00012	89	152	0	247	208	5
CCCAACTCCG	BRDN00012	571	653	642	1179	966	81
CCCAACTGAC	BRDN00012	154	119	194	389	126	0

CCCAACTGG	BRDN00011	113	137	4089	260	158	33
CCCAACTGGT	BRDN00011	104	165	673	149	861	5
CCCAAGAAA	BRDN00011	26	0	0	93	374	0
CCCAAGACCT	BRDN00012	168	87	13	235	370	9
CCCAAGACG	BRDN00011	81	98	410	151	15	3
CCCAAGAGA	BRDN00011	345	288	840	713	829	42
CCCAAGAGG	BRDN00011	328	206	53	492	528	53
CCCAAGAGG	BRDN00011	210	207	360	352	285	10
CCCAAGATA	BRDN00011	77	86	91	256	382	6
CCCAAGCATT	BRDN00011	362	490	402	609	794	12
CCCAAGCCG	BRDN00011	59	90	0	94	164	0
CCCAAGGAA	BRDN00011	117	159	43	396	283	0
CCCAAGGAC	BRDN00012	176	4	0	580	578	0
CCCAAGGGC	BRDN00012	269	270	372	527	396	11
CCCAAGTAT	BRDN00011	108	143	102	256	217	0
CCCAAGTCCA	BRDN00011	289	148	364	418	393	25
CCCAATACCT	BRDN00012	167	273	1281	307	311	32
CCCAATACTA	BRDN00011	142	125	320	198	271	11
CCCAATACTT	BRDN00011	304	35	27	586	385	0
CCCAATAGG	BRDN00012	111	80	3	211	78	17
CCCAATCCGA	BRDN00011	215	124	36	532	759	14
CCCAATCTGA	BRDN00011	72	66	235	186	115	0
CCCAATCTTG	BRDN00012	321	21	48	410	478	9
CCCAATGAA	BRDN00011	148	124	214	282	328	0
CCCAATGGCT	BRDN00012	459	467	2232	681	1012	56
CCCACAAACT	BRDN00011	180	152	393	394	175	11
CCCACAACTC	BRDN00011	207	207	409	535	1011	9
CCCACAAGA	BRDN00012	161	138	44	187	260	0
CCCACAAGG	BRDN00011	66	0	1	165	99	0
CCCACAAGG	BRDN00012	109	58	245	163	316	4
CCCACAAGG	BRDN00012	295	315	564	539	364	42
CCCACAATG	BRDN00012	340	219	742	693	597	0
CCCACACAA	BRDN00011	404	359	1950	698	885	84
CCCACACAG	BRDN00012	362	350	505	795	759	47
CCCACACATG	BRDN00012	403	339	35	626	607	19
CCCACACCCA	BRDN00011	293	247	161	590	686	6
CCCACACCGC	BRDN00011	316	0	25	481	710	0
CCCACACCGG	BRDN00012	364	274	1139	655	835	1
CCCACACGCT	BRDN00011	93	79	554	160	399	25
CCCACACTAG	BRDN00012	115	83	142	152	402	17
CCCACAGAA	BRDN00012	349	291	598	844	914	18
CCCACAGAA	BRDN00012	105	126	64	352	476	2
CCCACAGAA	BRDN00012	346	302	941	622	223	72
CCCACAGAG	BRDN00012	361	349	271	534	181	6
CCCACAGAT	BRDN00012	149	94	932	212	319	23
CCCACAGCCA	BRDN00012	361	211	217	845	904	5
CCCACAGCCT	BRDN00012	335	371	709	486	268	106
CCCACAGCCT	BRDN00011	86	105	0	197	145	0
CCCACAGCCT	BRDN00012	69	101	81	195	35	35
CCCACAGCGT	BRDN00012	324	365	412	732	1032	24
CCCACAGCTA	BRDN00012	192	105	364	198	124	29
CCCACAGCTC	BRDN00012	76	0	71	340	161	0
CCCACAGCTT	BRDN00011	138	0	0	375	143	0
CCCACAGGA	BRDN00012	121	95	15	190	101	0
CCCACAGGA	BRDN00012	20	0	0	306	2	0
CCCACAGGC	BRDN00012	173	174	2070	247	440	17
CCCACAGGC	BRDN00011	120	77	375	253	174	10
CCCACAGTGT	BRDN00011	128	52	117	233	207	11
CCCACATATA	BRDN00012	168	222	667	376	518	0
CCCACATATC	BRDN00011	188	124	259	381	910	61
CCCACATCAT	BRDN00012	203	200	398	314	248	16
CCCACATCGT	BRDN00012	56	55	181	143	193	0
CCCACATGA	BRDN00012	166	43	706	476	410	7
CCCACATGCT	BRDN00012	80	148	177	282	274	16
CCCACATTTA	BRDN00011	182	27	24	271	143	0
CCCACCAAAT	BRDN00012	289	404	332	489	538	48
CCCACCAACC	BRDN00012	171	245	527	512	278	24
CCCACCAATT	BRDN00011	230	239	28	431	352	7
CCCACCACAC	BRDN00012	308	277	288	478	382	6
CCCACCACAG	BRDN00012	112	111	51	137	159	3
CCCACCACGA	BRDN00012	116	61	203	124	158	13
CCCACCAGAT	BRDN00012	262	194	2	464	665	7
CCCACCAGCA	BRDN00011	223	532	245	415	425	12
CCCACCAGCT	BRDN00012	88	0	7	338	324	0
CCCACCAGCT	BRDN00011	171	171	79	392	350	54
CCCACCAGTA	BRDN00011	245	148	312	376	362	12
CCCACCATAA	BRDN00011	211	144	145	317	294	0
CCCACCATAG	BRDN00011	130	74	8	227	274	19
CCCACCCACA	BRDN00012	285	147	1524	455	995	7
CCCACCCACT	BRDN00011	141	205	794	343	269	7
CCCACCCAGG	BRDN00011	142	65	319	207	335	10
CCCACCCAGG	BRDN00011	171	81	1846	258	160	35
CCCACCCAGC	BRDN00011	306	173	263	555	465	13
CCCACCCATG	BRDN00011	360	264	61	545	642	0
CCCACCCCAA	BRDN00011	430	266	2475	628	653	28
CCCACCCCTA	BRDN00012	571	471	1092	1052	1287	36
CCCACCCGAC	BRDN00011	298	413	1006	465	536	46
CCCACCCGCA	BRDN00012	75	60	0	106	92	0
CCCACCCGCA	BRDN00012	84	82	284	161	250	24
CCCACCCTGC	BRDN00011	183	166	436	313	488	37
CCCACCCTGT	BRDN00011	128	45	0	164	89	0
CCCACCGAG	BRDN00011	350	362	78	671	475	14
CCCACCTAAA	BRDN00012	111	127	1056	194	108	8
CCCACCTCAA	BRDN00012	279	254	1165	884	502	38
CCCACCTCAC	BRDN00012	182	101	839	374	453	126
CCCACCTCAG	BRDN00012	319	300	1395	709	569	28
CCCACCTCTC	BRDN00012	336	319	844	529	423	11
CCCACCTGAT	BRDN00011	72	27	0	123	100	3
CCCACCTGCA	BRDN00012	222	216	392	519	516	23
CCCACCTGCA	BRDN00012	293	267	1414	770	627	21
CCCACCTGGT	BRDN00011	210	217	154	433	260	0
CCCACCTGGT	BRDN00011	157	140	1	270	293	13
CCCACCTTCA	BRDN00011	153	209	266	346	534	0
CCCACCTTGT	BRDN00011	131	118	126	244	213	98
CCCACGAACT	BRDN00011	638	342	1141	1076	679	72
CCCACGAGG	BRDN00011	56	44	6	124	63	0
CCCACGATG	BRDN00012	248	215	332	512	267	14
CCCACGATG	BRDN00012	88	69	0	157	287	0
CCCACGCAA	BRDN00011	204	170	211	253	379	0
CCCACGCAG	BRDN00011	378	290	1104	560	647	20
CCCACGCAG	BRDN00012	144	92	20	397	354	0
CCCACGCAG	BRDN00011	388	454	973	695	477	27
CCCACGCATG	BRDN00011	224	102	332	436	776	20
CCCACGCTAC	BRDN00012	473	323	1003	876	550	52
CCCACGCTCT	BRDN00012	294	205	1947	462	613	173
CCCACGCTTA	BRDN00011	129	50	194	259	303	6
CCCACGGAC	BRDN00012	381	386	1874	613	731	78
CCCACGGGT	BRDN00011	313	197	811	578	117	69
CCCACGTACC	BRDN00012	168	141	10	202	42	9
CCCACGTGCT	BRDN00011	96	44	13	126	83	17
CCCACGTGTC	BRDN00011	99	183	326	239	281	19
CCCACTAAG	BRDN00012	217	217	722	437	325	27
CCCACTAAG	BRDN00011	224	362	203	410	575	5
CCCACTAATA	BRDN00012	242	280	262	569	330	30
CCCACTAATG	BRDN00012	66	0	0	123	0	0
CCCACTACCG	BRDN00011	98	114	479	197	263	10
CCCACTAGA	BRDN00012	135	154	18	219	168	40
CCCACTCAGA	BRDN00011	161	125	434	310	351	64
CCCACTCATG	BRDN00011	133	32	75	458	167	0
CCCACTCCAT	BRDN00012	172	166	305	344	429	36
CCCACTCCTT	BRDN00012	447	45	26	904	835	7
CCCACTGCAA	BRDN00012	66	17	0	165	198	0
CCCACTGCAC	BRDN00012	353	252	2131	503	491	79
CCCACTGCTA	BRDN00012	182	12	65	288	571	0
CCCACTGGTT	BRDN00011	120	60	21	181	111	0
CCCACTGTAC	BRDN00011	136	116	12	268	205	5
CCCACTTACC	BRDN00011	156	130	27	335	226	50
CCCACTTCAG	BRDN00011	291	258	382	573	730	30
CCCACTTCCG	BRDN00012	172	127	7	264	205	6
CCCACTTGCG	BRDN00011	293	356	650	720	548	60
CCCACTTTAG	BRDN00012	132	21	123	686	422	1
CCCAGAAACT	BRDN00011	317	238	200	483	470	21
CCCAGAAAG	BRDN00012	58	41	132	127	158	0
CCCAGAAAGT	BRDN00011	115	70	22	217	303	0
CCCAGAACA	BRDN00011	208	147	1096	471	497	17
CCCAGAACA	BRDN00012	153	153	91	360	123	14
CCCAGAACT	BRDN00012	302	255	70	513	551	71
CCCAGAACT	BRDN00012	188	212	5	537	675	3
CCCAGAAGA	BRDN00012	112	83	581	210	148	44
CCCAGAAGG	BRDN00012	302	318	335	632	416	72
CCCAGAAGT	BRDN00012	100	154	545	226	267	15
CCCAGAAGT	BRDN00011	114	54	624	210	312	16
CCCAGAATA	BRDN00012	149	163	1502	305	407	31
CCCAGAATG	BRDN00012	242	193	180	329	489	14
CCCAGAATG	BRDN00011	450	357	1095	778	826	40
CCCAGACAA	BRDN00012	259	234	454	523	607	32
CCCAGACAA	BRDN00012	74	45	119	186	176	0
CCCAGACAC	BRDN00011	250	272	488	641	638	60
CCCAGACAC	BRDN00012	139	158	87	352	490	12
CCCAGACAC	BRDN00012	468	245	578	776	795	9
CCCAGACAG	BRDN00011	253	160	91	446	470	4
CCCAGACCCC	BRDN00012	480	499	49	893	987	43
CCCAGACGA	BRDN00011	340	401	1703	708	485	28
CCCAGACGT	BRDN00011	237	201	410	411	385	7
CCCAGAGAT	BRDN00012	143	216	111	329	171	14
CCCAGAGCC	BRDN00012	413	460	2104	796	925	96
CCCAGAGCCT	BRDN00011	228	152	427	316	166	6
CCCAGAGCG	BRDN00011	185	115	106	294	355	9
CCCAGAGCG	BRDN00012	317	262	312	445	379	58
CCCAGAGCT	BRDN00012	242	268	386	455	453	5
CCCAGAGCT	BRDN00011	67	0	0	121	72	0
CCCAGAGCTT	BRDN00012	377	290	1903	722	843	149
CCCAGAGGG	BRDN00011	142	176	76	175	188	4
CCCAGAGGG	BRDN00012	108	41	0	174	216	0
CCCAGAGTCT	BRDN00011	435	237	63	722	966	15
CCCAGAGTT	BRDN00011	297	165	0	460	453	38
CCCAGATGA	BRDN00012	291	210	247	684	862	63
CCCAGATGA	BRDN00011	95	111	324	149	250	63
CCCAGATTAT	BRDN00012	201	267	303	555	591	76
CCCAGATTG	BRDN00011	208	112	355	264	182	12
CCCAGATTG	BRDN00011	522	248	178	867	722	0
CCCAGCAAA	BRDN00011	366	320	455	564	864	14
CCCAGCACAT	BRDN00012	148	278	315	162	100	5
CCCAGCACCG	BRDN00012	105	87	38	232	97	19
CCCAGCACG	BRDN00012	45	0	0	588	46	0
CCCAGCACG	BRDN00012	271	257	250	486	473	16
CCCAGCAGA	BRDN00012	142	104	859	370	326	12
CCCAGCAGG	BRDN00011	148	157	1662	311	304	58
CCCAGCAGG	BRDN00011	275	309	1553	453	433	18
CCCAGCAGG	BRDN00011	245	194	149	456	230	0
CCCAGCATA	BRDN00012	191	248	35	275	221	15
CCCAGCATCA	BRDN00012	316	162	1300	547	405	33
CCCAGCCCAG	BRDN00012	142	148	0	165	187	4
CCCAGCCCTG	BRDN00012	247	272	1654	575	603	80
CCCAGCCTTG	BRDN00011	44	82	193	132	50	0
CCCAGCGAG	BRDN00012	342	367	1280	710	871	72
CCCAGCGAT	BRDN00011	108	50	5	244	203	17
CCCAGCGCAT	BRDN00011	183	124	2	332	474	0
CCCAGCGCAT	BRDN00012	269	235	1387	519	560	4
CCCAGCGGT	BRDN00011	63	18	10	495	100	0
CCCAGCGTA	BRDN00011	141	78	8	167	136	8
CCCAGCGTCT	BRDN00012	85	71	11	196	145	18
CCCAGCTACA	BRDN00012	186	158	482	290	313	5
CCCAGCTAG	BRDN00012	118	92	174	227	215	38
CCCAGCTCTG	BRDN00011	221	235	134	493	373	13
CCCAGCTCTG	BRDN00011	186	234	4	366	217	21
CCCAGCTGA	BRDN00012	545	287	1671	1187	920	20
CCCAGCTGCC	BRDN00012	444	618	567	885	1183	36
CCCAGCTTTC	BRDN00011	363	56	0	534	291	0
CCCAGGAACT	BRDN00011	70	0	0	157	27	0
CCCAGGAAT	BRDN00011	184	174	20	340	530	32
CCCAGGAAT	BRDN00011	48	31	0	134	90	0
CCCAGGACA	BRDN00012	186	79	391	369	409	0
CCCAGGACC	BRDN00011	138	168	16	278	282	11
CCCAGGACC	BRDN00011	276	263	101	423	395	62
CCCAGGAGT	BRDN00012	563	486	317	1022	930	43
CCCAGGATA	BRDN00011	82	51	0	224	143	0
CCCAGGATC	BRDN00011	230	166	34	299	201	12
CCCAGGCAC	BRDN00012	330	358	495	718	2054	52
CCCAGGCAC	BRDN00012	52	13	0	289	111	0
CCCAGGCAC	BRDN00012	149	95	7	295	417	11
CCCAGGCAC	BRDN00011	397	312	1394	742	700	51
CCCAGGCAG	BRDN00011	90	41	6	404	232	0
CCCAGGCAG	BRDN00012	171	58	1	239	597	0
CCCAGGCCA	BRDN00012	230	280	331	732	682	2
CCCAGGCCAT	BRDN00012	212	176	434	580	490	3
CCCAGGCCC	BRDN00012	256	172	586	504	369	7
CCCAGGCTG	BRDN00012	448	342	492	970	1130	44
CCCAGGGAA	BRDN00011	91	0	1	188	182	0
CCCAGGGAG	BRDN00011	141	179	1	458	500	2
CCCAGGGCA	BRDN00011	370	284	1413	678	681	21
CCCAGGGCA	BRDN00012	145	98	29	259	262	1
CCCAGGGGA	BRDN00012	236	411	186	376	485	11
CCCAGGGTA	BRDN00012	215	169	295	390	862	0
CCCAGGGTG	BRDN00011	202	115	273	331	437	13
CCCAGGGTG	BRDN00012	47	93	0	140	319	0
CCCAGGTAA	BRDN00012	196	134	20	318	293	12
CCCAGGTCG	BRDN00011	0	0	0	165	35	0
CCCAGGTGC	BRDN00011	61	14	1	197	492	0
CCCAGGTGG	BRDN00011	80	83	183	112	178	0
CCCAGGTGT	BRDN00012	332	359	281	519	624	32
CCCAGGTTC	BRDN00012	217	117	307	432	388	5
CCCAGGTTG	BRDN00011	72	1	0	337	340	0
CCCAGTATCA	BRDN00012	415	287	58	679	744	43
CCCAGTCTG	BRDN00011	100	71	1	176	96	0
CCCAGTGAA	BRDN00011	397	432	1139	855	563	35
CCCAGTGAA	BRDN00011	333	276	130	486	230	6
CCCAGTGAG	BRDN00011	53	0	0	127	0	0
CCCAGTGAT	BRDN00011	352	278	478	767	1392	55
CCCAGTGGA	BRDN00012	209	164	436	616	308	24
CCCAGTGGG	BRDN00011	226	263	133	394	400	19
CCCAGTTCTA	BRDN00012	201	311	165	598	991	28
CCCATAAATA	BRDN00011	100	115	27	219	59	1
CCCATAACTT	BRDN00011	150	44	52	240	409	0
CCCATACACT	BRDN00012	123	60	170	300	74	0
CCCATACATC	BRDN00012	270	272	931	635	324	50
CCCATACATG	BRDN00012	253	232	458	444	406	50
CCCATACCCT	BRDN00012	520	507	1459	866	979	92
CCCATACCCT	BRDN00012	376	297	183	658	801	28
CCCATACTAC	BRDN00012	382	291	3669	708	669	202
CCCATACTTA	BRDN00011	202	156	782	447	156	20
CCCATAGAA	BRDN00011	139	179	625	264	221	21
CCCATAGGA	BRDN00011	115	55	208	204	93	19
CCCATAGGG	BRDN00011	117	112	815	238	291	8
CCCATAGGG	BRDN00012	346	473	742	675	642	23
CCCATCACAA	BRDN00011	289	134	206	358	372	7
CCCATCACGG	BRDN00012	226	2	0	521	57	0
CCCATCAGA	BRDN00012	107	76	267	310	506	1
CCCATCAGCA	BRDN00012	201	109	8	365	257	13
CCCATCCACT	BRDN00012	335	234	375	555	580	13
CCCATCCATA	BRDN00011	120	95	702	215	107	0
CCCATCCGAT	BRDN00011	192	246	731	468	601	44
CCCATCCTCA	BRDN00012	75	89	230	199	264	1
CCCATCCTTA	BRDN00011	189	193	10	410	493	1
CCCATCCTTC	BRDN00012	115	154	35	260	294	14
CCCATCGAA	BRDN00012	159	172	53	388	591	0
CCCATCGACA	BRDN00012	135	162	129	366	143	0
CCCATCGATC	BRDN00012	151	118	1	229	386	0
CCCATCGGA	BRDN00012	112	85	0	199	353	0
CCCATCGGG	BRDN00011	157	116	504	222	332	54
CCCATCGGG	BRDN00012	208	322	153	427	643	37
CCCATCGTTA	BRDN00011	148	80	25	225	268	27
CCCATCTACA	BRDN00012	95	0	0	273	145	0
CCCATCTACG	BRDN00012	437	476	251	1018	884	28
CCCATCTACT	BRDN00012	206	218	14	364	308	0
CCCATCTCTG	BRDN00012	341	340	133	567	1250	7
CCCATCTGCT	BRDN00012	212	157	319	549	435	0
CCCATCTGCT	BRDN00012	246	0	1	588	102	0
CCCATGACGT	BRDN00011	189	95	1022	178	61	36
CCCATGCACA	BRDN00011	232	234	91	328	202	8
CCCATGCACC	BRDN00012	92	91	1268	161	157	2
CCCATGCACG	BRDN00011	407	143	53	576	583	9
CCCATGCCGC	BRDN00011	119	61	736	137	87	22
CCCATGCCGC	BRDN00012	295	271	609	579	828	30
CCCATGCCGC	BRDN00011	70	134	494	214	89	17
CCCATGCGA	BRDN00012	473	773	703	749	1339	191
CCCATGCGCC	BRDN00011	329	314	1390	643	410	22
CCCATGGAC	BRDN00011	337	412	833	692	567	27
CCCATGGAG	BRDN00012	332	288	284	483	371	27
CCCATGGCC	BRDN00012	108	123	333	208	145	7
CCCATGGGC	BRDN00011	171	26	0	514	468	0
CCCATGGTG	BRDN00011	99	71	42	167	223	0
CCCATGTACT	BRDN00011	84	172	3289	187	380	68
CCCATGTGCC	BRDN00011	180	145	231	227	200	66
CCCATTAACA	BRDN00012	390	504	145	833	897	73
CCCATTCAAA	BRDN00011	352	403	225	642	445	6
CCCATTCCCC	BRDN00012	221	0	0	668	97	0
CCCATTCCTC	BRDN00011	129	158	251	178	230	0
CCCATTCCTG	BRDN00012	98	84	96	194	163	23
CCCATTCCTT	BRDN00012	60	72	100	146	109	14
CCCATTCGAA	BRDN00012	188	188	245	384	382	0
CCCATTGAA	BRDN00012	517	445	1867	763	504	24
CCCATTGACC	BRDN00012	428	112	99	704	589	5
CCCATTGGG	BRDN00012	91	138	576	244	103	31
CCCATTGGGT	BRDN00011	90	120	85	245	182	0
CCCATTGGTC	BRDN00012	128	154	2	209	390	18
CCCATTGTAC	BRDN00011	379	305	323	674	718	43
CCCATTTACT	BRDN00012	273	400	1920	613	291	16
CCCCAAAAAT	BRDN00011	145	143	109	214	217	14
CCCCAAAACT	BRDN00012	87	25	0	140	19	0
CCCCAAACCC	BRDN00011	380	412	344	515	521	4
CCCCAAACTG	BRDN00011	244	305	424	403	482	10
CCCCAAAGA	BRDN00012	99	99	317	192	334	95
CCCCAAAGA	BRDN00012	155	177	14	365	341	32
CCCCAAAGG	BRDN00011	229	255	277	623	628	16
CCCCAAATAA	BRDN00012	295	427	732	505	353	19
CCCCAAATTC	BRDN00011	136	64	300	307	187	4
CCCCAACACT	BRDN00011	188	193	34	464	226	4
CCCCAACAG	BRDN00011	188	260	431	381	296	27
CCCCAACCAG	BRDN00011	559	242	424	874	771	12
CCCCAACCAG	BRDN00012	478	536	216	696	837	115
CCCCAACCTG	BRDN00012	94	0	0	208	22	0
CCCCAACGA	BRDN00012	199	167	15	384	213	5
CCCCAACGC	BRDN00011	187	193	495	356	211	4
CCCCAACTCA	BRDN00012	466	437	1239	821	879	48
CCCCAACTTT	BRDN00012	514	386	422	638	679	12
CCCCAAGAA	BRDN00012	281	332	584	766	633	22
CCCCAAGAA	BRDN00012	76	59	0	111	20	12
CCCCAAGCA	BRDN00011	171	125	335	406	574	51
CCCCAAGCA	BRDN00011	128	88	260	346	227	20
CCCCAAGCTC	BRDN00012	133	34	332	200	180	5
CCCCAAGGG	BRDN00011	274	350	2560	442	242	60
CCCCAAGGT	BRDN00012	167	221	134	439	683	10
CCCCAAGGT	BRDN00011	143	84	63	259	312	6
CCCCAAGTCA	BRDN00012	333	203	584	678	473	60
CCCCAAGTTC	BRDN00011	154	296	0	450	757	0
CCCCAATGAT	BRDN00011	73	50	75	312	292	0
CCCCACAAAC	BRDN00011	336	230	937	726	486	223
CCCCACAACA	BRDN00011	143	61	1608	386	713	30
CCCCACAAG	BRDN00012	105	71	56	248	103	4
CCCCACAAG	BRDN00011	66	160	149	206	207	0
CCCCACAATG	BRDN00012	263	74	98	474	183	14
CCCCACACAA	BRDN00012	262	240	767	484	418	54
CCCCACAGA	BRDN00012	134	159	204	224	439	6
CCCCACAGAT	BRDN00011	149	229	8	311	177	46
CCCCACAGCC	BRDN00011	309	482	941	532	516	36
CCCCACAGG	BRDN00011	170	141	115	203	278	41
CCCCACAGG	BRDN00012	462	408	910	788	818	17
CCCCACAGTC	BRDN00012	106	0	0	362	158	0
CCCCACCAAA	BRDN00011	129	67	0	224	281	0
CCCCACCACG	BRDN00011	261	7	0	583	318	0
CCCCACCAGG	BRDN00012	396	509	541	824	555	52
CCCCACCCCA	BRDN00011	279	212	1449	523	1064	23
CCCCACCCCC	BRDN00011	152	161	1	303	472	22
CCCCACCGCA	BRDN00011	148	159	458	423	193	8
CCCCACCGCG	BRDN00011	278	218	343	424	353	30
CCCCACCGG	BRDN00011	2	0	0	171	29	0
CCCCACCGG	BRDN00012	148	123	1489	364	333	11
CCCCACCGGT	BRDN00011	202	96	235	513	267	8
CCCCACCGTT	BRDN00011	285	46	15	466	463	10
CCCCACGACG	BRDN00011	132	93	490	345	396	15
CCCCACGACG	BRDN00012	487	337	74	765	777	25
CCCCACGGCC	BRDN00012	309	383	1462	456	511	40
CCCCACGTTG	BRDN00011	100	20	0	217	123	0
CCCCACTCAG	BRDN00011	169	230	342	347	623	2
CCCCACTGGT	BRDN00012	156	95	191	261	260	0
CCCCACTGTG	BRDN00012	130	109	281	198	306	26
CCCCACTTCC	BRDN00011	292	298	450	391	424	1053
CCCCACTTCT	BRDN00012	156	104	151	438	133	7
CCCCAGAAC	BRDN00012	299	574	453	829	470	45
CCCCAGAACC	BRDN00011	232	138	12	330	245	0
CCCCAGAAGT	BRDN00012	113	154	36	192	339	23
CCCCAGACG	BRDN00012	311	325	554	497	523	9
CCCCAGACG	BRDN00011	233	139	300	429	503	15
CCCCAGAGG	BRDN00012	264	314	740	620	609	16
CCCCAGAGG	BRDN00011	168	140	106	362	500	0
CCCCAGAGTC	BRDN00011	99	0	1	179	135	0
CCCCAGAGTC	BRDN00012	196	52	492	360	49	0
CCCCAGATCC	BRDN00012	261	170	208	557	543	19
CCCCAGCAA	BRDN00012	99	81	31	353	261	0
CCCCAGCACG	BRDN00011	193	161	578	375	295	3
CCCCAGCACG	BRDN00011	114	81	39	234	307	0
CCCCAGCAG	BRDN00012	265	320	161	689	755	39
CCCCAGCAGT	BRDN00012	206	182	3176	241	387	73
CCCCAGCATC	BRDN00011	80	91	29	174	161	0
CCCCAGCCAC	BRDN00012	44	39	27	146	205	0
CCCCAGCCTA	BRDN00011	132	49	11	282	152	0
CCCCAGGAA	BRDN00012	166	51	49	318	270	0
CCCCAGGACT	BRDN00011	227	251	592	517	859	58
CCCCAGGATC	BRDN00011	149	43	25	316	99	0
CCCCAGGCCC	BRDN00011	199	102	312	466	335	0
CCCCAGGCCC	BRDN00012	165	82	226	258	127	9
CCCCAGGGC	BRDN00012	168	129	273	318	492	1
CCCCAGGTCC	BRDN00012	320	304	293	471	214	36
CCCCAGTAA	BRDN00011	297	215	393	481	277	65
CCCCAGTCAA	BRDN00011	86	54	559	150	200	17
CCCCAGTCTC	BRDN00011	31	0	0	395	1	0
CCCCAGTGA	BRDN00011	92	7	98	157	121	0
CCCCAGTGCC	BRDN00012	364	401	1387	698	814	73
CCCCAGTGG	BRDN00012	280	240	531	579	742	47
CCCCAGTTAG	BRDN00011	137	83	100	256	268	0
CCCCAGTTTG	BRDN00012	228	135	352	358	259	14
CCCCATAACA	BRDN00011	277	293	361	470	306	64
CCCCATAACT	BRDN00012	252	244	1344	227	572	19
CCCCATCCAG	BRDN00011	99	130	1	211	151	3
CCCCATCGTA	BRDN00012	99	36	466	228	176	8
CCCCATCTCG	BRDN00012	230	267	81	561	600	30
CCCCATGCAG	BRDN00011	145	93	100	301	211	17
CCCCATGGA	BRDN00012	149	127	1822	148	44	32
CCCCATGGCT	BRDN00011	232	96	196	449	432	5
CCCCATTGG	BRDN00012	31	37	244	67	53	23
CCCCCAAAAA	BRDN00012	294	200	448	423	451	12
CCCCCAAAAT	BRDN00011	240	203	346	334	211	12
CCCCCAAAG	BRDN00012	106	134	110	365	507	9
CCCCCAAAGC	BRDN00011	273	320	994	452	560	29
CCCCCAACAC	BRDN00011	171	121	112	328	215	0
CCCCCAATAA	BRDN00012	255	155	30	510	758	24
CCCCCACAAG	BRDN00011	186	240	1147	374	280	47
CCCCCACAGA	BRDN00012	203	213	313	515	609	31
CCCCCACAGG	BRDN00011	390	395	1042	822	630	0
CCCCCACTCA	BRDN00011	139	54	1	213	221	0
CCCCCACTCG	BRDN00012	176	193	15	296	278	0
CCCCCAGTGC	BRDN00012	118	129	13	327	447	7
CCCCCATATC	BRDN00012	422	345	1204	1017	986	39
CCCCCATATC	BRDN00011	271	47	5	705	642	1
CCCCCATCAG	BRDN00012	220	196	1389	382	320	30
CCCCCATGGC	BRDN00012	411	402	200	581	558	19
CCCCCCAAAA	BRDN00011	243	126	20	368	566	9
CCCCCCAAAA	BRDN00011	104	74	1088	141	129	14
CCCCCCAAGC	BRDN00011	482	390	544	865	789	40
CCCCCCACCC	BRDN00012	215	78	2390	245	392	22
CCCCCCAGAT	BRDN00011	75	157	355	333	125	14
CCCCCCCCAA	BRDN00011	244	190	569	404	328	12
CCCCCCGATA	BRDN00012	123	165	0	233	325	0
CCCCCCGGCG	BRDN00011	120	118	129	179	98	5
CCCCCCGGGC	BRDN00011	273	102	350	373	494	5
CCCCCCGGTC	BRDN00011	208	173	130	389	436	28
CCCCCGAAAT	BRDN00012	138	170	634	353	325	0
CCCCCGACTC	BRDN00011	405	287	862	783	1060	57
CCCCCGCAAG	BRDN00012	364	174	54	832	529	0
CCCCCGCACG	BRDN00011	408	381	488	1007	942	33
CCCCCGCGCT	BRDN00011	285	234	172	460	702	50
CCCCCGCTGA	BRDN00012	55	9	0	213	93	0
CCCCCGGAA	BRDN00011	313	307	2126	464	395	45
CCCCCGGAA	BRDN00012	352	242	857	649	307	55

CCCCCGGAA	BRDN00012	169	64	53	347	393	15
CCCCCTAAGA	BRDN00012	134	119	393	283	313	45
CCCCCTACCA	BRDN00011	373	116	83	768	902	13
CCCCCTACTT	BRDN00012	260	265	1286	438	1029	20
CCCCCTATAT	BRDN00011	27	0	0	232	164	0
CCCCCTATCT	BRDN00011	110	129	7	241	131	0
CCCCCTCCGT	BRDN00012	431	69	1	678	586	0
CCCCCTCGCC	BRDN00012	146	108	0	256	144	10
CCCCCTGTAC	BRDN00012	131	116	169	289	306	0
CCCCCTTCAG	BRDN00012	166	189	532	363	158	31
CCCCGAAAA	BRDN00012	270	333	486	625	461	15
CCCCGAAAC	BRDN00012	215	251	484	383	354	0
CCCCGAAGA	BRDN00011	563	429	1413	941	689	48
CCCCGAAGC	BRDN00012	340	401	964	642	466	40
CCCCGACACT	BRDN00012	157	144	581	251	356	17
CCCCGACAG	BRDN00011	239	0	18	514	50	0
CCCCGACATC	BRDN00011	197	103	375	300	115	4
CCCCGACATT	BRDN00011	266	211	489	526	503	52
CCCCGACCAA	BRDN00011	225	125	255	311	230	0
CCCCGACCAT	BRDN00011	412	346	648	655	506	42
CCCCGACGA	BRDN00012	147	236	26	194	283	29
CCCCGACGC	BRDN00012	158	126	217	249	299	28
CCCCGACGC	BRDN00012	244	178	117	552	319	47
CCCCGACGTT	BRDN00011	182	231	45	292	453	0
CCCCGACTCG	BRDN00011	126	145	159	307	434	11
CCCCGACTTA	BRDN00012	93	151	162	159	315	0
CCCCGAGAA	BRDN00012	252	280	399	739	514	20
CCCCGAGCT	BRDN00012	495	374	798	958	1320	49
CCCCGAGGT	BRDN00011	176	47	127	303	282	8
CCCCGAGTA	BRDN00012	298	317	56	545	588	30
CCCCGAGTG	BRDN00011	326	158	871	411	306	66
CCCCGAGTT	BRDN00012	398	414	902	831	557	74
CCCCGCACCT	BRDN00011	96	42	37	255	330	3
CCCCGCACG	BRDN00012	443	277	328	732	696	64
CCCCGCACGT	BRDN00012	111	139	627	234	420	9
CCCCGCACTG	BRDN00011	86	138	68	254	425	3
CCCCGCACTT	BRDN00012	131	170	0	226	243	12
CCCCGCAGC	BRDN00011	209	0	0	413	128	0
CCCCGCAGT	BRDN00012	116	209	107	373	127	69
CCCCGCAGT	BRDN00012	163	71	0	397	338	0
CCCCGCATCT	BRDN00012	405	344	656	675	367	52
CCCCGCATTT	BRDN00011	475	22	149	730	306	0
CCCCGCCAAA	BRDN00011	289	156	818	591	623	37
CCCCGCCAAG	BRDN00011	145	79	22	267	362	12
CCCCGCCACA	BRDN00012	120	145	413	166	304	17
CCCCGCCACG	BRDN00011	131	55	217	278	145	10
CCCCGCCCAC	BRDN00012	250	115	299	518	390	6
CCCCGCCCTA	BRDN00011	186	68	5	623	679	0
CCCCGCCGCC	BRDN00012	486	316	761	746	544	38
CCCCGCCGTA	BRDN00012	100	146	333	253	206	35
CCCCGCCTCT	BRDN00012	244	272	897	533	509	6
CCCCGCCTCT	BRDN00012	407	374	436	968	555	144
CCCCGCGATC	BRDN00011	258	182	232	349	642	7
CCCCGCGCCT	BRDN00012	121	69	113	165	65	34
CCCCGCGCGA	BRDN00011	120	86	335	179	442	0
CCCCGCGGCA	BRDN00012	371	127	135	799	338	0
CCCCGCGTTG	BRDN00011	245	172	1725	670	1082	26
CCCCGCTCAC	BRDN00011	210	195	151	421	1029	28
CCCCGCTCAC	BRDN00011	257	228	271	585	627	4
CCCCGCTCAC	BRDN00012	82	169	78	257	230	0
CCCCGCTGCT	BRDN00011	433	402	377	971	1058	19
CCCCGCTTCG	BRDN00011	316	233	222	669	361	27
CCCCGGAATC	BRDN00011	232	169	76	783	612	22
CCCCGGAGC	BRDN00011	198	108	107	335	272	0
CCCCGGATAC	BRDN00011	406	329	518	755	799	9
CCCCGGATG	BRDN00011	67	104	346	165	202	4
CCCCGGCAA	BRDN00011	140	202	122	304	247	20
CCCCGGCAG	BRDN00012	102	73	117	142	246	8
CCCCGGCATA	BRDN00012	380	215	389	566	226	12
CCCCGGCCCC	BRDN00011	363	304	573	595	954	34
CCCCGGCCGA	BRDN00011	344	514	1696	633	789	143
CCCCGGGCCC	BRDN00012	228	232	425	404	509	22
CCCCGGGCTA	BRDN00011	95	247	683	172	179	31
CCCCGGGCTC	BRDN00011	136	69	43	298	146	0
CCCCGGGTCA	BRDN00011	67	185	358	210	77	29
CCCCGGGTTT	BRDN00011	285	372	2367	549	1067	89
CCCCGGTGG	BRDN00011	531	578	491	672	947	94
CCCCGTACAC	BRDN00011	287	259	388	438	767	54
CCCCGTACTT	BRDN00011	171	84	17	249	293	0
CCCCGTAGCT	BRDN00012	363	336	321	626	549	28
CCCCGTAGTA	BRDN00011	132	153	137	414	835	19
CCCCGTCATG	BRDN00012	167	111	31	268	348	0
CCCCGTCCCA	BRDN00011	194	147	79	299	288	4
CCCCGTGCAC	BRDN00011	422	323	166	719	848	19
CCCCGTGCCC	BRDN00012	205	112	3	442	512	25
CCCCGTGCGC	BRDN00011	138	144	109	236	328	26
CCCCGTGGCC	BRDN00011	344	354	435	684	693	55
CCCCGTGGTA	BRDN00011	62	37	299	94	466	3
CCCCGTGGTC	BRDN00011	128	94	212	224	193	11
CCCCGTGGTC	BRDN00011	447	331	482	819	335	44
CCCCGTGTGC	BRDN00011	90	64	53	111	46	0
CCCCTAAGGC	BRDN00011	60	49	0	120	132	0
CCCCTAATTA	BRDN00011	245	287	304	307	232	9
CCCCTACACG	BRDN00011	355	275	309	652	594	5
CCCCTACCCG	BRDN00011	179	259	108	414	575	7
CCCCTACGCC	BRDN00012	87	61	29	229	323	0
CCCCTACGTC	BRDN00012	349	367	228	629	511	22
CCCCTACTCA	BRDN00011	138	120	83	395	466	33
CCCCTACTCG	BRDN00012	328	265	198	730	697	0
CCCCTAGGTC	BRDN00012	90	33	124	242	122	0
CCCCTATCCC	BRDN00011	149	211	35	238	172	0
CCCCTATCCT	BRDN00011	268	113	14	472	256	9
CCCCTATGCA	BRDN00012	448	346	343	752	638	9
CCCCTATTAG	BRDN00012	51	0	2	286	118	0
CCCCTCAAAG	BRDN00012	69	87	411	170	110	22
CCCCTCATAT	BRDN00011	212	156	203	451	443	37
CCCCTCATTT	BRDN00012	205	92	114	324	280	0
CCCCTCCCGA	BRDN00011	441	333	1175	730	1254	50
CCCCTCCGAC	BRDN00012	135	127	40	170	388	10
CCCCTCGACA	BRDN00012	342	410	225	718	525	2
CCCCTCGCTG	BRDN00011	272	175	51	347	180	20
CCCCTCTCGC	BRDN00011	122	180	149	204	208	3
CCCCTCTTGG	BRDN00011	178	188	690	387	248	43
CCCCTGAATT	BRDN00011	307	265	480	440	261	9
CCCCTGCACA	BRDN00011	222	198	1467	262	319	65
CCCCTGCATA	BRDN00011	336	289	3004	606	440	40
CCCCTGCTTC	BRDN00012	381	18	0	811	66	0
CCCCTGGAAC	BRDN00012	195	238	35	247	259	127
CCCCTGGCAC	BRDN00012	51	66	55	77	58	28
CCCCTGGCCA	BRDN00012	169	196	270	298	464	0
CCCCTGGCGT	BRDN00012	148	253	106	192	128	2
CCCCTGGCTA	BRDN00011	418	367	1331	555	439	1
CCCCTGGGCC	BRDN00012	156	92	8	164	102	0
CCCCTGTCAC	BRDN00012	209	189	103	336	379	3
CCCCTGTCCA	BRDN00011	255	365	447	580	1325	27
CCCCTTACAC	BRDN00012	128	32	32	256	132	0
CCCCTTATGA	BRDN00012	44	16	0	112	99	0
CCCCTTCAAC	BRDN00011	298	345	176	607	469	28
CCCCTTCCCT	BRDN00012	367	216	357	678	608	42
CCCCTTCCGA	BRDN00011	260	342	335	319	325	20
CCCCTTCCGG	BRDN00012	364	297	354	480	374	55
CCCCTTGGTA	BRDN00012	77	86	665	169	126	10
CCCCTTTACA	BRDN00012	131	147	723	252	228	30
CCCCTTTCAG	BRDN00012	338	363	717	529	356	11
CCCGAAACAT	BRDN00011	343	452	64	801	880	2
CCCGAAATAC	BRDN00012	51	82	569	219	248	37
CCCGAAGAA	BRDN00011	68	59	36	203	63	62
CCCGAAGAG	BRDN00012	337	212	1012	550	325	26
CCCGAAGAT	BRDN00012	122	152	730	139	161	70
CCCGAAGCAT	BRDN00012	93	26	0	262	117	0
CCCGAAGCCA	BRDN00012	78	47	219	178	105	0
CCCGAAGCCT	BRDN00011	347	232	719	526	521	30
CCCGAAGGC	BRDN00011	216	136	131	371	359	0
CCCGAAGGT	BRDN00011	153	28	0	287	122	0
CCCGAAGTCA	BRDN00012	271	232	394	398	1109	19
CCCGAAGTG	BRDN00011	210	109	296	410	297	5
CCCGAATCAT	BRDN00012	227	288	192	534	577	16
CCCGAATGTC	BRDN00012	12	0	0	171	31	0
CCCGACACAC	BRDN00011	277	135	694	495	542	62
CCCGACATGA	BRDN00012	225	86	110	382	631	0
CCCGACCTCC	BRDN00012	245	222	116	494	451	0
CCCGACCTGC	BRDN00011	564	437	339	794	963	67
CCCGACGAG	BRDN00011	86	52	185	132	121	0
CCCGACGCAC	BRDN00012	84	57	14	158	111	0
CCCGACGCGT	BRDN00011	210	263	165	359	227	59
CCCGACTAAC	BRDN00011	129	75	102	188	152	8
CCCGACTCCT	BRDN00012	119	64	0	207	94	4
CCCGACTGTC	BRDN00012	397	307	186	689	704	39
CCCGAGACA	BRDN00012	420	293	1863	772	879	48
CCCGAGATG	BRDN00012	113	87	1267	138	122	36
CCCGAGCACT	BRDN00012	245	256	437	534	682	20
CCCGAGCAGT	BRDN00012	115	94	1040	186	121	30
CCCGAGCTCA	BRDN00012	81	41	9	99	37	7
CCCGAGCTG	BRDN00012	264	174	587	378	210	22
CCCGAGGAC	BRDN00011	90	110	66	308	325	0
CCCGAGGAG	BRDN00012	117	0	0	183	38	0
CCCGAGGAG	BRDN00012	435	247	285	521	540	32
CCCGAGGCC	BRDN00012	557	541	2371	783	557	89
CCCGAGTACA	BRDN00011	118	117	0	219	110	0
CCCGAGTGC	BRDN00012	308	237	513	675	692	14
CCCGATAGTT	BRDN00012	308	176	1646	388	374	45
CCCGATATCT	BRDN00011	99	92	32	246	273	0
CCCGATCCTG	BRDN00011	98	103	73	290	94	19
CCCGATGACT	BRDN00011	320	377	543	554	899	20
CCCGATGATC	BRDN00011	149	99	309	361	360	86
CCCGATGGA	BRDN00012	276	283	348	339	823	32
CCCGCACCGC	BRDN00012	178	134	176	378	343	0
CCCGCACCTA	BRDN00012	364	255	147	845	1422	51
CCCGCACGGT	BRDN00011	122	54	617	217	266	4
CCCGCAGCGA	BRDN00012	93	95	153	162	216	8
CCCGCAGCG	BRDN00011	252	294	587	633	560	73
CCCGCAGGA	BRDN00011	394	233	344	644	421	19
CCCGCAGTAA	BRDN00012	103	93	536	171	111	9
CCCGCAGTAC	BRDN00012	397	324	391	731	751	66
CCCGCATCTA	BRDN00012	204	173	236	469	452	15
CCCGCCAGCA	BRDN00012	56	89	38	213	169	7
CCCGCCCCGC	BRDN00011	43	30	0	42	0	0
CCCGCCCGCT	BRDN00012	258	215	41	422	887	17
CCCGCCCTTG	BRDN00012	80	70	0	163	189	0
CCCGCCCTTG	BRDN00011	222	115	142	374	341	13
CCCGCCGAAC	BRDN00012	428	405	299	815	874	60
CCCGCCGCCC	BRDN00012	355	232	786	550	420	37
CCCGCCGCGC	BRDN00012	59	110	38	185	97	6
CCCGCCGCGC	BRDN00012	397	230	674	779	824	15
CCCGCCGGCT	BRDN00011	130	105	97	208	368	0
CCCGCCGTAT	BRDN00011	81	110	0	212	203	0
CCCGCCTGGA	BRDN00011	19	20	0	347	188	0
CCCGCCTGGC	BRDN00012	252	317	155	531	535	46
CCCGCGAGTA	BRDN00012	68	160	406	217	330	1
CCCGCGCACT	BRDN00012	118	94	138	191	262	0
CCCGCGCAGT	BRDN00011	128	73	29	224	362	20
CCCGCGCCCC	BRDN00011	229	159	813	266	242	94
CCCGCGGCA	BRDN00011	162	160	52	414	381	0
CCCGCGGCCC	BRDN00011	54	35	60	195	314	0
CCCGCGGTGT	BRDN00011	70	0	0	110	19	0
CCCGCGTAGA	BRDN00011	202	330	224	388	538	15
CCCGCGTCTA	BRDN00011	232	214	191	285	468	0
CCCGCTAACA	BRDN00012	461	288	513	771	825	44
CCCGCTCACC	BRDN00012	212	123	122	259	158	14
CCCGCTCTCT	BRDN00012	128	0	0	229	233	0
CCCGGAAAA	BRDN00011	95	37	298	157	327	12
CCCGGAAAG	BRDN00011	190	184	242	542	417	0
CCCGGAAGA	BRDN00011	272	247	233	639	724	79
CCCGGAAGC	BRDN00011	53	29	0	90	129	0
CCCGGAAGG	BRDN00012	117	74	540	194	48	7
CCCGGACAA	BRDN00011	193	32	26	470	217	10
CCCGGACAAT	BRDN00011	65	7	13	130	103	0
CCCGGACACA	BRDN00011	79	63	0	119	278	0
CCCGGACACC	BRDN00012	209	248	2051	379	389	32
CCCGGACCCC	BRDN00012	97	28	33	117	162	25
CCCGGACGA	BRDN00011	233	124	56	386	256	0
CCCGGACGT	BRDN00012	480	470	350	1001	951	102
CCCGGACTTC	BRDN00012	367	257	350	635	703	36
CCCGGAGCCT	BRDN00011	95	107	0	160	163	0
CCCGGAGGT	BRDN00011	110	24	10	252	52	1
CCCGGATCTA	BRDN00011	116	172	30	316	218	0
CCCGGATGCT	BRDN00011	81	40	15	110	60	9
CCCGGCACCA	BRDN00012	359	206	14	558	626	0
CCCGGCACCT	BRDN00011	30	23	0	70	73	0
CCCGGCAGA	BRDN00012	20	68	19	168	115	0
CCCGGCCACA	BRDN00012	335	387	259	573	649	101
CCCGGCCAGA	BRDN00011	89	113	704	145	235	5
CCCGGCCATT	BRDN00012	327	375	284	583	482	30
CCCGGCCCCC	BRDN00011	285	341	605	560	357	65
CCCGGCCGA	BRDN00012	137	153	17	365	217	0
CCCGGCCTCA	BRDN00012	50	10	114	96	65	29
CCCGGCGCA	BRDN00011	238	248	855	432	400	137
CCCGGCGCGA	BRDN00011	153	29	1	704	269	0
CCCGGCGGA	BRDN00011	186	329	385	421	376	28
CCCGGGACA	BRDN00011	60	38	8	123	53	17
CCCGGGACT	BRDN00011	121	50	20	252	179	1
CCCGGGAGA	BRDN00012	0	0	0	79	12	0
CCCGGGAGC	BRDN00011	89	38	183	98	116	5
CCCGGGCAAT	BRDN00011	58	102	289	178	383	8
CCCGGGCCA	BRDN00012	57	91	58	127	108	0
CCCGGGCCG	BRDN00011	49	62	0	56	60	12
CCCGGGCGG	BRDN00011	59	96	0	156	146	0
CCCGGGCTTC	BRDN00011	172	172	699	414	360	69
CCCGGGGGT	BRDN00011	44	9	292	108	75	0
CCCGGGTCG	BRDN00011	125	232	0	285	638	22
CCCGGTACGA	BRDN00012	151	54	159	262	149	1
CCCGGTATGA	BRDN00012	240	319	1420	704	359	52
CCCGGTCATT	BRDN00012	163	110	2	380	271	10
CCCGGTCTAC	BRDN00012	115	137	204	302	211	0
CCCGGTGAC	BRDN00012	84	64	125	266	214	2
CCCGGTGTTC	BRDN00012	442	309	3483	711	658	45
CCCGGTTTAC	BRDN00012	84	0	0	146	572	0
CCCGTAAAAC	BRDN00011	323	200	761	481	416	49
CCCGTAAACA	BRDN00012	411	260	208	643	671	8
CCCGTACGA	BRDN00011	99	82	32	182	482	11
CCCGTAGCCC	BRDN00012	141	94	258	217	252	22
CCCGTAGTCT	BRDN00011	244	72	865	422	290	36
CCCGTAGTG	BRDN00011	229	315	528	371	811	23
CCCGTCAGTC	BRDN00012	165	59	140	234	200	0
CCCGTCATGT	BRDN00012	114	120	147	185	310	48
CCCGTCCAGC	BRDN00011	181	168	353	262	68	34
CCCGTCCCTA	BRDN00011	198	197	765	431	540	95
CCCGTCGGCC	BRDN00012	227	167	58	564	411	10
CCCGTGACAT	BRDN00012	351	336	798	769	616	7
CCCGTGCCAT	BRDN00012	77	88	0	118	178	16
CCCGTGGCGT	BRDN00012	491	573	1017	658	723	87
CCCGTGGGT	BRDN00012	89	81	53	173	117	9
CCCGTGGGT	BRDN00011	85	115	101	224	240	12
CCCGTGTCCT	BRDN00011	153	53	104	266	83	0
CCCGTGTGA	BRDN00011	127	106	93	263	134	0
CCCGTTCTTC	BRDN00011	145	101	60	179	139	4
CCCGTTGAGT	BRDN00011	235	235	211	370	398	35
CCCGTTGCGC	BRDN00012	171	167	98	299	106	18
CCCTAAAAAA	BRDN00012	86	86	14	174	278	0
CCCTAAAAAC	BRDN00012	134	227	307	186	110	25
CCCTAACAGC	BRDN00012	212	223	283	551	329	0
CCCTAACCGA	BRDN00011	197	175	242	405	592	0
CCCTAAGCCA	BRDN00012	127	152	1417	266	238	50
CCCTACAAAT	BRDN00011	72	27	63	376	411	0
CCCTACAATA	BRDN00012	329	278	112	739	832	30
CCCTACACAA	BRDN00011	179	154	314	423	336	7
CCCTACACAG	BRDN00012	173	99	71	317	302	4
CCCTACACAT	BRDN00012	443	342	221	823	464	56
CCCTACACGA	BRDN00012	223	163	564	335	422	25
CCCTACAGCG	BRDN00012	228	228	893	607	560	15
CCCTACAGCG	BRDN00012	182	161	3	311	528	16
CCCTACCAAG	BRDN00012	358	229	637	622	954	27
CCCTACCAAT	BRDN00011	467	301	337	733	607	14
CCCTACCCAA	BRDN00011	237	248	196	515	406	16
CCCTACCCGT	BRDN00012	137	143	247	287	376	58
CCCTACCGAT	BRDN00011	172	142	34	397	194	4
CCCTACCTAT	BRDN00012	538	524	599	770	549	46
CCCTACGACG	BRDN00012	355	398	263	655	718	10
CCCTACGGAC	BRDN00012	312	301	136	593	751	66
CCCTACTATG	BRDN00012	396	458	2685	758	969	29
CCCTAGACAC	BRDN00011	326	112	665	610	777	13
CCCTAGAGGT	BRDN00011	107	151	8	274	429	27
CCCTAGCCCG	BRDN00012	387	370	530	713	1084	45
CCCTAGCGCC	BRDN00012	99	97	22	159	184	10
CCCTAGGAA	BRDN00011	249	315	2455	480	663	49
CCCTATCAAG	BRDN00011	101	91	142	162	456	0
CCCTATCAGC	BRDN00012	92	69	0	119	140	0
CCCTATCTAG	BRDN00012	262	343	12	508	765	10
CCCTATGGTC	BRDN00012	86	61	355	309	419	6
CCCTATTAGC	BRDN00011	206	150	15	447	622	18
CCCTCAAACG	BRDN00012	349	109	40	572	375	28
CCCTCAATGT	BRDN00011	259	244	789	538	697	17
CCCTCACAGC	BRDN00012	147	43	437	267	376	1
CCCTCACCAG	BRDN00012	106	127	104	237	426	16
CCCTCACCGA	BRDN00012	88	108	428	246	329	22
CCCTCACCTA	BRDN00011	137	132	125	269	350	3
CCCTCACCTG	BRDN00012	489	689	813	1052	1023	88
CCCTCAGAAC	BRDN00012	483	307	307	562	629	15
CCCTCAGACA	BRDN00011	201	172	457	317	269	0
CCCTCAGCCC	BRDN00012	208	184	173	327	423	4
CCCTCAGCGC	BRDN00011	100	145	69	156	187	0
CCCTCAGGAC	BRDN00011	38	40	0	180	352	4
CCCTCAGGAC	BRDN00012	441	552	319	827	1327	57
CCCTCAGGGT	BRDN00012	165	101	407	422	347	16
CCCTCAGTCA	BRDN00012	359	136	0	784	722	0
CCCTCAGTGA	BRDN00012	372	319	318	649	506	20
CCCTCATACA	BRDN00012	295	188	105	574	925	6
CCCTCATCAA	BRDN00012	332	273	736	707	913	31
CCCTCATCAC	BRDN00011	391	416	2445	863	816	58
CCCTCCAAAG	BRDN00012	328	277	122	595	733	47
CCCTCCAACC	BRDN00011	124	25	35	199	128	0
CCCTCCAGCA	BRDN00011	65	0	0	202	74	0
CCCTCCAGGC	BRDN00011	201	273	771	432	778	63
CCCTCCCCAA	BRDN00012	301	331	537	551	847	22
CCCTCCCGGA	BRDN00012	164	69	20	269	331	4
CCCTCCCTGG	BRDN00011	145	189	1	268	460	16
CCCTCCGGAC	BRDN00012	187	172	378	403	523	13
CCCTCCGGGT	BRDN00011	185	82	288	408	282	18
CCCTCCTCCG	BRDN00011	145	131	470	226	155	59
CCCTCCTCTA	BRDN00012	106	46	59	319	167	0
CCCTCCTGCT	BRDN00012	120	25	12	294	248	0
CCCTCCTGGA	BRDN00012	80	83	13	297	161	28
CCCTCCTTAG	BRDN00012	140	174	1252	228	99	15
CCCTCGACCC	BRDN00011	184	206	0	484	370	0
CCCTCGACTC	BRDN00011	199	111	61	406	744	1
CCCTCGATTC	BRDN00012	238	353	560	553	520	2
CCCTCGCCGA	BRDN00012	169	109	480	297	348	47
CCCTCGCCGC	BRDN00012	101	82	193	227	106	21
CCCTCGCGCT	BRDN00011	103	58	25	119	183	22
CCCTCGGAGA	BRDN00012	319	242	242	660	646	10
CCCTCGGATA	BRDN00012	514	502	256	783	826	2
CCCTCGGGTA	BRDN00012	463	523	378	924	721	70
CCCTCTACAA	BRDN00012	104	75	129	258	232	15
CCCTCTCCCG	BRDN00012	296	223	348	442	289	15
CCCTCTCGCA	BRDN00012	489	450	1414	931	934	16
CCCTCTTTGA	BRDN00012	106	50	2168	397	702	19
CCCTGAAACT	BRDN00011	56	70	328	195	311	15
CCCTGAACAC	BRDN00012	54	17	34	156	517	0
CCCTGAACCA	BRDN00012	162	108	188	289	409	19
CCCTGAAGA	BRDN00011	132	85	31	308	155	5
CCCTGAAGA	BRDN00011	450	382	387	613	445	26
CCCTGACAAA	BRDN00011	85	112	53	190	450	0
CCCTGACCAA	BRDN00012	75	103	792	292	277	20
CCCTGACCAA	BRDN00012	326	229	197	589	339	33
CCCTGACCAC	BRDN00011	73	149	1586	301	297	48
CCCTGACCCA	BRDN00011	309	299	215	434	1118	86
CCCTGACGCC	BRDN00011	136	197	210	240	418	9
CCCTGACTCC	BRDN00012	164	55	19	192	216	0
CCCTGAGAA	BRDN00011	177	233	16	401	179	11
CCCTGAGCCA	BRDN00012	566	598	634	1144	1773	125
CCCTGAGTAC	BRDN00011	196	76	720	257	226	2
CCCTGATCAA	BRDN00012	342	474	1153	807	841	32
CCCTGATCAG	BRDN00012	282	231	1308	615	570	21
CCCTGATGA	BRDN00012	91	68	2	210	248	6
CCCTGATGCA	BRDN00012	114	109	210	324	326	6
CCCTGCAATA	BRDN00012	194	1	0	462	354	0
CCCTGCACAG	BRDN00012	190	144	207	248	307	64
CCCTGCACCA	BRDN00011	194	187	105	359	570	15
CCCTGCACTG	BRDN00012	142	138	279	348	287	4
CCCTGCAGCA	BRDN00011	167	98	326	300	217	13
CCCTGCAGCA	BRDN00011	84	99	156	173	84	11
CCCTGCAGTA	BRDN00012	309	322	919	571	623	74
CCCTGCATAA	BRDN00012	165	170	52	177	197	7
CCCTGCATTG	BRDN00012	212	169	17	361	312	23
CCCTGCCAGC	BRDN00011	353	477	847	1086	936	63
CCCTGCCATC	BRDN00012	345	410	1118	730	973	23
CCCTGCCCAC	BRDN00011	133	85	159	180	740	13
CCCTGCCCAG	BRDN00012	482	557	300	894	1265	50
CCCTGCCCTA	BRDN00012	13	0	0	337	123	0
CCCTGCCCTA	BRDN00012	100	87	25	232	53	0
CCCTGCCCTC	BRDN00012	145	144	221	280	282	11
CCCTGCCGCA	BRDN00012	81	94	138	734	542	58
CCCTGCCGCC	BRDN00012	389	395	435	551	499	56
CCCTGCCTTA	BRDN00012	242	181	634	347	306	18
CCCTGCGACT	BRDN00011	308	264	1077	653	670	40
CCCTGCGGA	BRDN00011	48	19	0	137	69	0
CCCTGCGGCT	BRDN00011	243	257	835	375	690	35
CCCTGCGTTT	BRDN00011	108	95	1290	266	135	31
CCCTGCTCAA	BRDN00011	337	245	62	668	639	6
CCCTGCTCAA	BRDN00011	100	69	29	181	69	0
CCCTGCTGCT	BRDN00012	143	104	260	313	302	7
CCCTGCTTGT	BRDN00012	129	235	11	198	162	50
CCCTGGAACT	BRDN00011	421	237	814	659	567	76
CCCTGGAATA	BRDN00011	52	40	148	131	53	0
CCCTGGACCT	BRDN00012	80	45	7	140	34	0
CCCTGGACTT	BRDN00011	291	215	2757	408	1067	41
CCCTGGAGA	BRDN00011	395	425	245	862	734	23
CCCTGGATAC	BRDN00012	131	151	6	276	466	5
CCCTGGATTA	BRDN00012	357	340	262	524	448	4
CCCTGGCACA	BRDN00011	79	140	557	138	172	14
CCCTGGCACA	BRDN00012	112	2	0	236	384	0
CCCTGGCGTT	BRDN00011	332	366	113	680	505	24
CCCTGGGAA	BRDN00012	215	179	721	498	585	67
CCCTGGGAC	BRDN00011	237	273	351	303	201	22
CCCTGGGAG	BRDN00011	114	31	895	126	116	25
CCCTGGGCCC	BRDN00011	299	247	225	561	438	32
CCCTGGGCG	BRDN00012	62	65	128	121	385	37
CCCTGGGGA	BRDN00012	79	124	480	176	112	13
CCCTGGGGC	BRDN00011	30	41	46	143	66	0
CCCTGGTCAC	BRDN00012	148	127	121	291	358	9
CCCTGGTCGC	BRDN00011	397	359	261	865	407	14
CCCTGGTCTA	BRDN00011	135	136	46	226	218	4
CCCTGGTGA	BRDN00012	70	47	1530	135	323	13
CCCTGTAACC	BRDN00012	107	111	511	173	125	0
CCCTGTAGCA	BRDN00012	247	270	550	520	810	9
CCCTGTCGGC	BRDN00011	181	121	667	437	222	29
CCCTGTCTGC	BRDN00012	307	377	48	749	790	32
CCCTGTGAAC	BRDN00012	218	242	526	403	259	19
CCCTGTGAAC	BRDN00012	352	285	366	672	733	11
CCCTGTGCAC	BRDN00012	359	209	32	618	359	0
CCCTGTGCTT	BRDN00011	342	220	1060	604	618	88
CCCTGTGGG	BRDN00012	94	126	0	225	79	0
CCCTGTGTCA	BRDN00012	530	455	383	780	546	12
CCCTGTGTCA	BRDN00011	572	597	2021	977	1112	77
CCCTGTGTGA	BRDN00011	282	71	20	494	618	8
CCCTGTTTAG	BRDN00011	214	220	871	405	418	51
CCCTTAACTA	BRDN00012	205	140	605	384	340	3
CCCTTACCGG	BRDN00011	141	98	167	271	99	3
CCCTTACCTG	BRDN00012	26	18	0	150	25	0
CCCTTATAAT	BRDN00012	101	110	10	242	279	11
CCCTTATCAG	BRDN00012	413	342	666	728	532	9
CCCTTCAACA	BRDN00012	538	483	449	876	905	65
CCCTTCAATC	BRDN00011	133	17	14	228	67	0
CCCTTCACAT	BRDN00011	100	148	5	166	238	35
CCCTTCAGAT	BRDN00011	342	224	1085	568	229	5
CCCTTCATTT	BRDN00011	379	454	49	762	1047	4
CCCTTCCCTA	BRDN00012	104	46	158	124	146	0
CCCTTCGAGA	BRDN00012	274	338	101	467	554	45
CCCTTCGGCT	BRDN00011	407	352	393	672	502	24
CCCTTCTCGA	BRDN00011	108	119	248	282	162	26
CCCTTCTGGC	BRDN00012	380	396	468	569	813	31
CCCTTGAAAA	BRDN00011	131	29	0	129	180	0
CCCTTGACGT	BRDN00012	44	41	411	185	142	0
CCCTTGACTA	BRDN00012	258	206	58	328	304	0
CCCTTGATAA	BRDN00012	129	135	1	155	98	0

CCCTTGCTCG	BRDN00012	270	149	519	250	216	0
CCCTTGGAA	BRDN00011	380	340	2629	631	1082	42
CCCTTGGAC	BRDN00011	467	463	1508	848	693	36
CCCTTGGGC	BRDN00011	143	120	523	238	434	0
CCCTTTAGAC	BRDN00011	73	71	560	157	83	8
CCCTTTATTG	BRDN00011	110	118	0	205	592	0
CCCTTTCAAT	BRDN00012	164	125	81	252	311	15
CCCTTTCCTG	BRDN00011	161	163	137	335	271	16
CCCTTTCGAG	BRDN00012	322	283	303	660	763	100
CCCTTTGAG	BRDN00012	208	156	831	349	551	37
CCCTTTGCCC	BRDN00011	408	408	3368	785	348	70
CCCTTTGCTC	BRDN00011	220	180	469	472	245	6
CCCTTTGCTG	BRDN00012	321	283	262	580	775	17
CCCTTTGGTG	BRDN00011	270	252	1466	705	584	56
CCGAAAAAT	BRDN00012	227	142	112	440	479	21
CCGAAAAGA	BRDN00011	232	192	1420	361	410	53
CCGAAACAC	BRDN00012	181	76	399	224	409	61
CCGAAACCC	BRDN00011	61	50	0	169	23	0
CCGAAAGGG	BRDN00011	191	153	387	225	168	25
CCGAAATGT	BRDN00011	230	126	9	587	890	0
CCGAACAGA	BRDN00012	256	178	225	354	450	0
CCGAACAGC	BRDN00012	271	14	59	503	329	8
CCGAACCCCT	BRDN00012	217	132	357	390	344	20
CCGAACCCGA	BRDN00012	116	95	0	222	168	24
CCGAACCGG	BRDN00011	122	118	1417	350	435	30
CCGAACCTTC	BRDN00011	118	104	525	414	190	25
CCGAAGAGT	BRDN00012	102	96	136	92	93	24
CCGAAGCAC	BRDN00011	82	156	972	160	147	5
CCGAAGCCC	BRDN00012	63	1	132	169	82	6
CCGAAGCGG	BRDN00012	12	12	15	66	56	6
CCGAAGGCC	BRDN00011	103	89	6784	283	131	123
CCGAAGTCA	BRDN00011	320	268	458	573	558	13
CCGAAGTTG	BRDN00011	324	258	309	733	742	8
CCGAATACA	BRDN00012	374	305	1190	718	403	33
CCGAATAGA	BRDN00012	178	105	95	286	419	0
CCGAATATG	BRDN00011	295	89	299	925	496	9
CCGAATGAA	BRDN00012	82	87	128	161	33	5
CCGAATGCT	BRDN00012	104	61	0	336	460	15
CCGAATGTA	BRDN00012	405	360	136	477	632	59
CCGAATTCTG	BRDN00012	227	133	929	490	416	44
CCGACAAAG	BRDN00011	202	91	205	606	661	1
CCGACAAGA	BRDN00011	510	471	428	950	1072	39
CCGACAAGG	BRDN00011	128	94	81	314	368	0
CCGACAATA	BRDN00011	261	178	116	574	514	1
CCGACAATT	BRDN00011	220	114	695	356	687	26
CCGACACCCA	BRDN00012	451	304	697	819	881	18
CCGACACGA	BRDN00011	28	4	0	298	118	0
CCGACACGC	BRDN00012	113	87	537	148	387	6
CCGACACGC	BRDN00012	464	542	1142	797	735	61
CCGACACGCT	BRDN00011	103	17	0	143	17	0
CCGACACTCA	BRDN00012	499	592	238	972	511	56
CCGACAGAA	BRDN00012	163	240	1836	291	369	56
CCGACAGAT	BRDN00011	40	0	0	125	119	0
CCGACAGCG	BRDN00012	95	193	104	268	164	0
CCGACAGCT	BRDN00011	275	343	3914	894	658	47
CCGACAGCT	BRDN00011	449	427	371	863	1193	53
CCGACAGGC	BRDN00011	520	309	736	796	1053	62
CCGACAGGG	BRDN00012	311	277	423	500	699	20
CCGACAGGT	BRDN00011	106	27	138	58	61	0
CCGACAGGT	BRDN00011	221	86	104	603	395	20
CCGACAGTG	BRDN00011	159	258	39	545	550	30
CCGACATGG	BRDN00011	238	283	1335	467	415	61
CCGACCACAT	BRDN00011	149	89	152	282	482	0
CCGACCAGC	BRDN00012	105	109	430	242	117	70
CCGACCAGT	BRDN00011	63	62	7	97	61	5
CCGACCATGT	BRDN00011	136	160	1268	240	421	10
CCGACCCCGA	BRDN00011	87	61	119	114	76	0
CCGACCCTAT	BRDN00011	110	124	94	183	40	0
CCGACCGTA	BRDN00011	272	291	94	413	487	30
CCGACCTCGA	BRDN00011	200	335	370	507	371	47
CCGACGACA	BRDN00011	59	60	0	173	103	0
CCGACGACG	BRDN00011	230	185	68	495	369	0
CCGACGCTG	BRDN00012	505	369	582	789	1026	31
CCGACGGAA	BRDN00011	89	49	0	61	197	0
CCGACGGAG	BRDN00012	110	86	1	223	342	7
CCGACGGCA	BRDN00012	358	294	3831	518	987	86
CCGACGGCA	BRDN00012	540	503	2340	1000	1097	53
CCGACGGGC	BRDN00012	35	0	0	135	82	0
CCGACGTACT	BRDN00011	167	81	8	322	343	25
CCGACTACA	BRDN00011	141	154	460	257	157	0
CCGACTCGG	BRDN00012	298	284	775	471	349	47
CCGACTCGT	BRDN00011	227	270	711	360	356	31
CCGACTGAA	BRDN00011	298	320	130	686	257	28
CCGACTGCA	BRDN00011	131	63	1400	257	212	21
CCGACTGGC	BRDN00011	184	180	13	258	132	22
CCGACTTCCA	BRDN00012	96	96	200	219	204	0
CCGACTTCCC	BRDN00011	267	396	413	620	856	18
CCGACTTCCT	BRDN00011	348	218	268	699	612	2
CCGACTTCGA	BRDN00011	249	246	273	663	258	5
CCGAGAAAG	BRDN00012	158	198	104	342	340	0
CCGAGAACA	BRDN00011	156	48	186	371	227	28
CCGAGAAGC	BRDN00012	352	254	382	671	307	22
CCGAGACAC	BRDN00012	157	89	76	277	333	22
CCGAGACCA	BRDN00012	415	534	2996	778	693	62
CCGAGACCG	BRDN00011	291	240	788	648	844	50
CCGAGAGCA	BRDN00012	312	366	471	610	250	0
CCGAGAGGA	BRDN00011	246	227	268	489	412	13
CCGAGATTG	BRDN00011	136	17	2	360	268	12
CCGAGCAAA	BRDN00012	80	104	1	164	190	20
CCGAGCAACT	BRDN00012	316	291	109	632	695	41
CCGAGCCCA	BRDN00012	363	416	12327	985	451	237
CCGAGCCCCT	BRDN00012	129	88	754	257	304	8
CCGAGCCTG	BRDN00011	167	153	119	484	440	32
CCGAGCGTG	BRDN00012	590	621	889	1078	1103	86
CCGAGCTAA	BRDN00011	46	0	0	213	213	0
CCGAGCTGT	BRDN00011	151	160	6	348	773	0
CCGAGGAAC	BRDN00012	130	128	5	185	229	21
CCGAGGACA	BRDN00011	185	265	1858	319	344	42
CCGAGGACG	BRDN00012	81	0	0	237	1	0
CCGAGGACT	BRDN00012	418	369	144	772	551	44
CCGAGGACT	BRDN00011	103	158	11	283	106	0
CCGAGGAGC	BRDN00011	279	236	870	576	795	35
CCGAGGAGG	BRDN00011	136	154	0	275	165	0
CCGAGGATT	BRDN00011	159	266	147	387	364	128
CCGAGGCAA	BRDN00011	178	200	90	217	168	0
CCGAGGCAC	BRDN00011	105	141	416	450	223	20
CCGAGGCCC	BRDN00012	150	177	112	225	170	7
CCGAGGCCG	BRDN00011	220	172	118	414	350	23
CCGAGGCCG	BRDN00011	320	11	25	661	280	0
CCGAGGCGC	BRDN00012	246	67	24	680	380	16
CCGAGGCGG	BRDN00011	57	79	130	133	79	0
CCGAGGCTA	BRDN00011	296	175	17	524	267	47
CCGAGGTCA	BRDN00011	217	164	2465	540	744	28
CCGAGGTCA	BRDN00012	166	77	29	290	218	0
CCGAGGTCC	BRDN00012	75	60	1326	115	258	15
CCGAGGTGA	BRDN00011	123	195	0	214	444	15
CCGAGGTGG	BRDN00011	149	123	57	118	123	0
CCGAGGTTG	BRDN00011	188	121	0	272	273	0
CCGAGTAGT	BRDN00012	217	196	1032	270	228	54
CCGAGTCAG	BRDN00012	207	220	0	494	504	0
CCGAGTCAG	BRDN00011	116	137	15	322	121	44
CCGAGTCAG	BRDN00012	245	188	152	472	1791	35
CCGAGTCAT	BRDN00012	183	153	499	351	277	28
CCGAGTCCG	BRDN00011	119	104	11	251	206	42
CCGAGTCGA	BRDN00012	404	439	734	795	866	17
CCGAGTGAA	BRDN00012	145	142	147	292	337	9
CCGAGTGCA	BRDN00011	319	184	393	635	325	54
CCGAGTGGT	BRDN00012	270	332	615	569	398	48
CCGAGTGTA	BRDN00011	159	183	279	334	187	5
CCGAGTTCC	BRDN00011	378	382	1543	520	220	45
CCGAGTTTG	BRDN00011	78	18	1	158	245	0
CCGATACAA	BRDN00012	184	134	351	401	397	9
CCGATCAGC	BRDN00012	245	209	824	390	586	5
CCGATCGCA	BRDN00011	212	202	1488	464	465	3
CCGATCGGC	BRDN00012	287	228	112	513	379	12
CCGATCGTA	BRDN00012	413	325	684	653	610	35
CCGATGAAG	BRDN00011	197	214	542	425	153	61
CCGATGACA	BRDN00012	97	30	200	693	385	3
CCGATGACC	BRDN00012	138	161	271	275	286	12
CCGATGATA	BRDN00011	149	55	572	283	369	0
CCGATGCCA	BRDN00011	140	110	155	281	555	0
CCGATGCCG	BRDN00011	115	156	33	261	148	36
CCGATGCGT	BRDN00012	240	139	151	400	440	11
CCGATGCTG	BRDN00012	462	16	52	1181	754	0
CCGATGGAA	BRDN00012	383	293	2214	551	620	46
CCGATGGAC	BRDN00012	319	403	114	718	892	6
CCGATGGGT	BRDN00011	351	308	406	585	406	47
CCGATGGTA	BRDN00012	360	593	3868	779	1112	130
CCGATGTTCT	BRDN00011	46	14	25	430	74	0
CCGATTACA	BRDN00011	115	78	14	254	278	0
CCGATTATGT	BRDN00012	279	356	76	653	595	19
CCGATTCCGT	BRDN00011	249	285	88	400	516	37
CCGATTCTTC	BRDN00011	167	132	205	338	290	6
CCGCAAAAG	BRDN00012	94	129	247	346	330	0
CCGCAAAAG	BRDN00012	109	33	0	209	64	1
CCGCAAAAT	BRDN00011	283	329	681	605	1020	45
CCGCAAAGA	BRDN00011	261	264	170	535	779	9
CCGCAAATG	BRDN00011	30	0	38	116	97	0
CCGCAACAC	BRDN00011	86	73	336	227	225	29
CCGCAACCA	BRDN00012	261	183	9	310	317	16
CCGCAACCTG	BRDN00012	415	387	501	688	1064	38
CCGCAACGG	BRDN00012	478	438	472	1029	832	14
CCGCAACGG	BRDN00011	147	143	11	298	208	13
CCGCAACTCA	BRDN00012	176	163	287	314	809	0
CCGCAACTTC	BRDN00011	242	278	1761	571	840	13
CCGCAAGAT	BRDN00011	151	136	2061	273	239	35
CCGCAAGTT	BRDN00011	50	56	0	231	100	0
CCGCAAGTT	BRDN00011	128	115	32	219	169	0
CCGCAATTCT	BRDN00012	95	70	108	161	312	0
CCGCACACA	BRDN00012	331	238	510	700	879	13
CCGCACATGT	BRDN00012	277	324	646	519	393	42
CCGCACCAA	BRDN00012	132	138	348	179	325	40
CCGCACCGA	BRDN00011	53	21	0	179	156	0
CCGCACCTGT	BRDN00012	537	233	155	842	623	19
CCGCACGAG	BRDN00011	208	174	473	447	500	26
CCGCACGCCT	BRDN00012	347	311	1431	608	461	67
CCGCACGGA	BRDN00011	55	89	35	94	47	0
CCGCACGGC	BRDN00012	403	308	1014	624	553	106
CCGCACGTA	BRDN00011	462	533	24	1023	1055	10
CCGCACTCAC	BRDN00011	273	461	109	742	608	24
CCGCACTCAG	BRDN00011	171	138	133	205	308	28
CCGCACTGT	BRDN00012	212	322	2889	369	271	65
CCGCACTTCG	BRDN00012	304	148	1171	408	639	27
CCGCAGACA	BRDN00011	151	124	153	387	280	0
CCGCAGACT	BRDN00012	280	205	3093	539	423	71
CCGCAGAGC	BRDN00011	207	371	12	476	667	14
CCGCAGAGC	BRDN00011	98	104	255	238	28	0
CCGCAGAGT	BRDN00012	337	306	1592	522	537	45
CCGCAGATC	BRDN00011	138	99	16	189	83	19
CCGCAGCAA	BRDN00012	329	321	389	677	824	26
CCGCAGCAC	BRDN00011	422	258	7	653	380	15
CCGCAGCAG	BRDN00011	110	30	116	248	898	0
CCGCAGCAG	BRDN00012	132	220	954	189	287	17
CCGCAGCAT	BRDN00012	369	274	538	776	608	13
CCGCAGCCC	BRDN00011	80	68	287	192	61	57
CCGCAGCCCT	BRDN00011	243	267	221	473	163	21
CCGCAGCCG	BRDN00012	280	225	290	671	450	61
CCGCAGCCT	BRDN00012	460	562	385	662	1112	24
CCGCAGCGA	BRDN00011	49	0	5	513	165	0
CCGCAGCGA	BRDN00011	163	227	1228	320	179	6
CCGCAGCGA	BRDN00011	91	105	30	151	150	0
CCGCAGCTC	BRDN00012	169	203	768	202	147	35
CCGCAGGAC	BRDN00011	319	208	639	513	901	20
CCGCAGGAC	BRDN00011	310	278	899	481	804	23
CCGCAGGAC	BRDN00011	176	7	57	518	206	3
CCGCAGGAT	BRDN00012	204	86	268	387	168	0
CCGCAGGCG	BRDN00011	204	237	86	458	193	6
CCGCAGGGA	BRDN00011	178	204	132	298	399	0
CCGCAGGGT	BRDN00012	235	174	137	391	200	3
CCGCAGGGT	BRDN00011	268	290	432	587	624	30
CCGCAGGGT	BRDN00011	95	5	0	129	35	0
CCGCAGGTC	BRDN00012	104	113	89	136	154	14
CCGCAGTAC	BRDN00011	328	283	1695	502	802	13
CCGCAGTAT	BRDN00011	124	6	150	266	145	0
CCGCAGTCCT	BRDN00012	79	22	0	201	105	9
CCGCATACCT	BRDN00011	287	521	12526	566	1078	312
CCGCATAGT	BRDN00011	298	202	484	397	540	92
CCGCATCAAT	BRDN00011	281	184	827	371	524	31
CCGCATCCAG	BRDN00012	137	42	565	126	115	29
CCGCATCGT	BRDN00012	88	112	0	267	538	25
CCGCATCTTG	BRDN00011	2	0	0	225	37	0
CCGCATGCA	BRDN00012	118	142	79	182	150	0
CCGCATGCAT	BRDN00012	187	129	21	246	162	39
CCGCCAAAA	BRDN00012	52	49	105	195	109	0
CCGCCAACC	BRDN00011	187	221	76	385	308	23
CCGCCAACG	BRDN00011	297	252	631	565	287	20
CCGCCAAGG	BRDN00012	185	218	6277	331	250	84
CCGCCAATTA	BRDN00011	292	242	230	603	409	25
CCGCCACCCT	BRDN00011	118	79	460	182	139	4
CCGCCACTAT	BRDN00011	336	181	179	596	479	0
CCGCCAGCAT	BRDN00011	149	115	78	223	83	0
CCGCCAGCG	BRDN00012	477	395	430	1117	1018	37
CCGCCAGGG	BRDN00012	151	53	52	205	132	0
CCGCCATAAC	BRDN00012	60	12	0	172	51	6
CCGCCATACA	BRDN00011	121	69	1	176	62	0
CCGCCATCCC	BRDN00012	134	0	1	672	416	0
CCGCCATGG	BRDN00011	192	117	32	244	324	0
CCGCCATTTC	BRDN00012	420	99	104	722	936	6
CCGCCCAAG	BRDN00012	102	69	174	187	110	4
CCGCCCCAG	BRDN00011	238	285	810	394	498	42
CCGCCCCATC	BRDN00011	141	106	801	192	64	5
CCGCCCCCGA	BRDN00012	232	223	463	341	579	56
CCGCCCCCGC	BRDN00011	285	72	396	487	691	109
CCGCCCCGA	BRDN00012	112	72	35	210	253	0
CCGCCCGATC	BRDN00012	379	334	794	526	438	61
CCGCCCGCCG	BRDN00012	196	125	13	304	291	9
CCGCCCGGC	BRDN00012	375	182	257	509	1748	25
CCGCCCGGCT	BRDN00011	123	114	7	215	170	44
CCGCCCGTG	BRDN00012	134	171	491	306	206	46
CCGCCCTACA	BRDN00011	84	4	0	401	192	0
CCGCCCTCGA	BRDN00012	62	93	281	244	219	9
CCGCCCTTAC	BRDN00011	63	12	0	200	126	0
CCGCCGAAG	BRDN00012	86	87	1068	186	73	24
CCGCCGAGG	BRDN00012	336	281	0	643	560	11
CCGCCGCAT	BRDN00012	148	107	26	260	513	28
CCGCCGCCG	BRDN00012	156	115	336	321	264	3
CCGCCGCTG	BRDN00011	313	329	388	584	568	42
CCGCCGCTTC	BRDN00012	113	76	93	190	175	30
CCGCCGGAA	BRDN00011	258	235	182	359	272	41
CCGCCGGCC	BRDN00012	291	319	1124	377	853	33
CCGCCGGCG	BRDN00011	177	214	274	352	548	4
CCGCCGGGA	BRDN00011	345	415	492	674	768	65
CCGCCGGGC	BRDN00011	362	346	1407	573	298	59
CCGCCGGGT	BRDN00011	364	400	1257	701	716	5
CCGCCGTCA	BRDN00011	72	0	0	266	78	0
CCGCCGTTC	BRDN00011	298	306	2062	543	541	32
CCGCCTACCA	BRDN00011	402	563	114	872	611	52
CCGCCTCACG	BRDN00011	268	278	11	517	836	29
CCGCCTGAA	BRDN00012	380	511	464	605	623	23
CCGCCTGCA	BRDN00011	374	401	1168	594	706	47
CCGCCTGGT	BRDN00011	237	250	33	416	240	20
CCGCCTTCAG	BRDN00012	448	369	1057	820	979	73
CCGCCTTCCT	BRDN00011	232	152	106	416	418	60
CCGCCTTCGT	BRDN00012	420	466	2407	868	998	58
CCGCGACCA	BRDN00011	104	104	124	149	334	20
CCGCGACGC	BRDN00012	319	116	287	582	462	22
CCGCGACTG	BRDN00012	78	66	275	121	192	8
CCGCGAGTA	BRDN00011	117	81	1	218	281	0
CCGCGCACC	BRDN00012	177	140	2018	417	769	44
CCGCGCAGC	BRDN00012	265	14	17	392	493	0
CCGCGCAGC	BRDN00012	510	454	317	770	887	23
CCGCGCAGG	BRDN00012	326	319	66	527	720	35
CCGCGCATG	BRDN00011	140	126	241	446	444	14
CCGCGCATT	BRDN00012	567	552	2551	858	783	110
CCGCGCCCCT	BRDN00012	75	27	212	156	148	4
CCGCGCCCTT	BRDN00011	132	227	12	402	523	20
CCGCGCGAA	BRDN00012	137	84	0	193	228	0
CCGCGCGCT	BRDN00012	147	34	38	228	223	0
CCGCGCGTA	BRDN00011	79	50	27	156	62	0
CCGCGCTCA	BRDN00011	145	197	18	371	128	57
CCGCGCTCA	BRDN00012	95	129	215	271	323	23
CCGCGCTGG	BRDN00012	194	125	29	407	679	6
CCGCGCTTC	BRDN00012	240	232	867	342	271	14
CCGCGGAGC	BRDN00012	418	305	1418	712	711	87
CCGCGGCAA	BRDN00012	95	108	15	237	192	0
CCGCGGCCG	BRDN00012	67	178	3777	135	24	120
CCGCGGCGC	BRDN00012	110	84	164	203	94	0
CCGCGGCGG	BRDN00011	127	113	297	232	78	0
CCGCGGCTG	BRDN00012	91	132	36	220	164	8
CCGCGGCTG	BRDN00011	102	184	542	248	146	13
CCGCGGGAA	BRDN00012	311	345	4107	958	610	110
CCGCGGGAC	BRDN00011	214	193	128	457	630	27
CCGCGGGAC	BRDN00012	173	41	0	698	138	8
CCGCGGGCA	BRDN00012	244	7	17	385	128	0
CCGCGGGCC	BRDN00011	27	13	0	118	38	0
CCGCGGGCT	BRDN00012	37	97	11	99	334	0
CCGCGGGTG	BRDN00012	138	168	1212	336	349	40
CCGCGGTAA	BRDN00012	173	138	113	354	195	13
CCGCGGTAC	BRDN00011	155	205	179	431	505	27
CCGCGTAGG	BRDN00011	79	50	183	214	107	9
CCGCGTCAC	BRDN00011	119	113	275	237	352	6
CCGCGTCCTG	BRDN00012	393	377	51	702	563	34
CCGCGTCGA	BRDN00012	241	89	17	478	212	5
CCGCGTGCA	BRDN00011	383	194	52	648	516	35
CCGCGTGCT	BRDN00012	432	512	1565	665	894	62
CCGCGTGGA	BRDN00011	103	60	21	236	186	10
CCGCGTTCA	BRDN00011	415	233	33	709	546	0
CCGCTAAAC	BRDN00011	301	170	462	611	745	55
CCGCTACAGT	BRDN00011	451	424	1042	771	732	8
CCGCTACCG	BRDN00011	57	40	0	82	82	0
CCGCTACCG	BRDN00012	8	0	0	0	0	0
CCGCTACCG	BRDN00012	85	50	59	536	270	4
CCGCTACCTG	BRDN00011	195	89	366	278	449	55
CCGCTATGG	BRDN00011	174	157	1664	234	60	62
CCGCTATTGA	BRDN00012	534	505	1182	782	728	225
CCGCTCAATT	BRDN00012	82	55	74	217	107	0
CCGCTCACCC	BRDN00011	256	154	0	489	505	6
CCGCTCACTT	BRDN00012	320	127	340	712	613	9
CCGCTCAGA	BRDN00012	419	111	2	780	489	10
CCGCTCCAGA	BRDN00011	103	95	116	212	187	6
CCGCTCCGAT	BRDN00011	145	144	120	366	381	0
CCGCTCCGCA	BRDN00011	141	105	42	263	638	0
CCGCTCGCGT	BRDN00011	113	242	147	218	311	20
CCGCTCTACT	BRDN00012	349	289	645	582	685	10
CCGCTCTCAC	BRDN00012	137	176	518	278	360	16
CCGCTCTGCG	BRDN00011	38	0	0	323	90	0
CCGCTGAAG	BRDN00011	297	311	2578	443	415	17
CCGCTGAAG	BRDN00011	188	212	81	461	619	0
CCGCTGACT	BRDN00012	541	486	86	745	548	75
CCGCTGCAG	BRDN00011	160	8	184	243	67	0
CCGCTGCAT	BRDN00011	373	346	618	880	1399	33
CCGCTGCCA	BRDN00011	307	413	1573	544	752	12
CCGCTGCCCC	BRDN00012	271	292	555	453	321	29
CCGCTGCGA	BRDN00011	99	88	220	165	219	16
CCGCTGCTCG	BRDN00012	403	236	1633	688	664	32
CCGCTGGAA	BRDN00012	256	251	204	700	817	0
CCGCTGGAA	BRDN00012	496	591	501	854	562	23
CCGCTGGAA	BRDN00012	127	146	118	316	256	35
CCGCTGGAC	BRDN00012	237	195	515	381	408	29
CCGCTGGAG	BRDN00011	316	306	833	736	270	35
CCGCTGGCCT	BRDN00011	531	666	6497	1064	1178	80
CCGCTGGCG	BRDN00011	339	225	437	527	342	20
CCGCTGGCTT	BRDN00011	79	43	185	180	223	5
CCGCTGGGG	BRDN00012	174	93	215	376	199	12
CCGCTGGTA	BRDN00012	183	193	49	400	815	0
CCGCTGGTG	BRDN00012	86	48	0	326	116	0
CCGCTGTCTC	BRDN00012	634	555	328	1144	1000	71
CCGCTTCAAA	BRDN00012	191	265	328	289	422	34
CCGCTTCCAC	BRDN00011	147	167	492	311	211	21
CCGCTTCTAC	BRDN00012	303	311	1675	519	336	59
CCGCTTCTCC	BRDN00012	157	275	455	267	167	17
CCGCTTGCTG	BRDN00012	226	206	52	356	172	12
CCGCTTGTGT	BRDN00011	156	95	178	472	471	50
CCGCTTTAGA	BRDN00012	346	244	450	714	1051	8
CCGCTTTCCA	BRDN00012	408	641	5805	503	365	164
CCGGAAAAG	BRDN00011	112	115	53	238	275	5
CCGGAAAAG	BRDN00011	90	97	38	143	28	0
CCGGAAACC	BRDN00011	249	213	135	502	530	25
CCGGAAAGA	BRDN00012	62	58	5	142	408	12
CCGGAAAGT	BRDN00011	316	268	271	523	421	1
CCGGAAATG	BRDN00011	164	124	49	312	603	0
CCGGAACGT	BRDN00011	110	52	166	240	145	8
CCGGAACTA	BRDN00012	408	89	3	595	534	5
CCGGAACTC	BRDN00012	494	477	999	801	630	67
CCGGAAGAA	BRDN00011	34	67	23	157	187	21
CCGGAAGAG	BRDN00011	317	414	731	500	726	9
CCGGAAGAG	BRDN00011	53	7	0	170	177	0
CCGGAAGGT	BRDN00011	233	121	44	385	627	50
CCGGAAGTA	BRDN00011	269	405	301	443	325	26
CCGGAATAC	BRDN00012	255	181	821	421	318	81
CCGGACAAG	BRDN00011	95	79	125	208	94	4
CCGGACACA	BRDN00011	373	381	575	690	806	132
CCGGACACT	BRDN00011	235	232	231	474	278	34
CCGGACACT	BRDN00011	41	80	5	186	132	8
CCGGACAGG	BRDN00011	145	102	703	262	205	72
CCGGACATA	BRDN00012	385	324	437	528	726	0
CCGGACCCT	BRDN00011	137	117	119	399	300	8
CCGGACCGA	BRDN00012	110	95	0	155	81	0
CCGGACCGC	BRDN00012	49	59	0	158	141	0
CCGGACCGT	BRDN00012	270	295	362	533	441	14
CCGGACCTA	BRDN00012	134	173	1466	257	135	46
CCGGACGAA	BRDN00011	70	1	545	278	297	5
CCGGACGAC	BRDN00012	63	24	1	222	244	0
CCGGACGCG	BRDN00012	159	164	12	194	239	0
CCGGACGGC	BRDN00011	129	106	1243	329	185	17
CCGGACGGT	BRDN00011	7	8	0	116	29	0
CCGGACTCAT	BRDN00012	230	23	10	552	139	10
CCGGACTGA	BRDN00012	288	201	425	468	440	26
CCGGACTGA	BRDN00011	285	473	1268	732	831	22
CCGGACTGG	BRDN00012	202	189	205	256	168	56
CCGGACTTG	BRDN00012	648	749	753	1105	982	40
CCGGAGAAC	BRDN00012	160	212	907	510	327	10
CCGGAGAAT	BRDN00011	157	133	1670	404	446	11
CCGGAGACA	BRDN00011	154	180	399	292	536	4
CCGGAGAGT	BRDN00011	312	426	1111	715	835	65
CCGGAGATC	BRDN00011	283	307	74	412	501	19
CCGGAGCAC	BRDN00011	132	151	63	189	370	9
CCGGAGCCA	BRDN00011	107	71	42	232	424	0
CCGGAGCGG	BRDN00011	107	101	57	225	215	0
CCGGAGCTG	BRDN00011	133	57	676	204	489	17
CCGGAGGG	BRDN00011	87	232	0	201	289	24
CCGGAGTCA	BRDN00012	350	319	842	707	1051	19
CCGGAGTCT	BRDN00011	103	80	290	156	140	0
CCGGAGTGT	BRDN00011	47	14	24	252	298	0
CCGGAGTTA	BRDN00012	61	64	57	55	3	0
CCGGAGTTC	BRDN00011	279	293	829	493	448	32
CCGGAGTTT	BRDN00011	102	51	8	137	69	3
CCGGAGTTT	BRDN00011	18	0	0	223	79	0
CCGGATAGA	BRDN00011	222	205	46	337	419	34
CCGGATCCC	BRDN00011	158	51	65	162	164	20
CCGGATCCC	BRDN00011	155	100	1096	298	532	12
CCGGATCCT	BRDN00012	167	129	6	448	517	3
CCGGATCTC	BRDN00012	77	1	0	147	116	0
CCGGATGCA	BRDN00011	77	91	67	135	103	12
CCGGATGTA	BRDN00012	77	105	60	164	15	1
CCGGCAACT	BRDN00011	487	518	1371	1022	1413	122
CCGGCAAGA	BRDN00012	442	655	738	903	588	23
CCGGCAAGG	BRDN00012	346	425	172	622	886	65
CCGGCAATA	BRDN00011	168	17	0	290	116	0
CCGGCACAA	BRDN00012	309	221	322	597	266	69
CCGGCACCA	BRDN00012	199	278	398	345	239	87
CCGGCACCG	BRDN00011	110	106	130	163	172	13
CCGGCACGT	BRDN00012	189	240	104	378	463	12
CCGGCACTCA	BRDN00011	83	61	269	141	128	0
CCGGCACTG	BRDN00012	155	92	59	216	262	0
CCGGCAGCA	BRDN00012	136	93	95	249	146	0
CCGGCAGCA	BRDN00011	100	185	7	291	177	0
CCGGCAGCA	BRDN00012	392	355	649	755	732	65
CCGGCAGCA	BRDN00011	39	57	172	95	250	12
CCGGCAGGT	BRDN00011	144	120	393	234	215	4
CCGGCCAAG	BRDN00012	0	0	0	1	0	0
CCGGCCAAG	BRDN00012	262	259	128	410	469	24
CCGGCCACC	BRDN00011	126	121	65	317	182	0
CCGGCCACG	BRDN00012	107	51	450	208	228	16
CCGGCCACT	BRDN00011	71	88	24	132	271	0

CCGGCCACT	BRDN00012	77	0	0	165	125	0
CCGGCCAGG	BRDN00011	67	102	1	332	489	26
CCGGCCAGT	BRDN00011	268	316	606	605	648	31
CCGGCCCAG	BRDN00012	412	439	334	641	631	47
CCGGCCCCC	BRDN00012	264	168	606	341	455	53
CCGGCCCGG	BRDN00012	37	33	6	106	69	0
CCGGCCCGT	BRDN00012	67	0	0	161	41	0
CCGGCCGAT	BRDN00012	343	290	553	659	844	62
CCGGCCGCG	BRDN00012	298	167	662	464	393	0
CCGGCCGGC	BRDN00011	205	262	47	367	475	110
CCGGCCGGT	BRDN00012	31	34	0	140	312	0
CCGGCCGGT	BRDN00011	98	35	0	378	413	0
CCGGCCGGT	BRDN00012	182	156	591	271	129	9
CCGGCCGTG	BRDN00011	206	60	104	573	239	0
CCGGCCTCA	BRDN00012	418	388	2206	740	820	56
CCGGCCTCC	BRDN00011	218	134	227	479	532	6
CCGGCCTGA	BRDN00012	66	11	86	276	128	0
CCGGCCTGA	BRDN00011	203	0	2	615	226	0
CCGGCCTGG	BRDN00011	303	386	1505	809	298	74
CCGGCGAAG	BRDN00011	277	209	135	669	626	45
CCGGCGCAA	BRDN00012	474	412	851	755	789	10
CCGGCGCCA	BRDN00011	113	69	1	207	293	0
CCGGCGCCA	BRDN00011	239	123	518	422	434	19
CCGGCGCCA	BRDN00012	113	73	113	240	209	0
CCGGCGCCC	BRDN00011	87	101	1	155	169	0
CCGGCGCTA	BRDN00011	181	169	0	373	163	0
CCGGCGCTT	BRDN00012	266	251	445	369	176	12
CCGGCGGAA	BRDN00012	170	100	127	324	122	18
CCGGCGGGA	BRDN00012	351	198	438	809	469	11
CCGGCGGGG	BRDN00011	57	42	0	163	94	0
CCGGCTATC	BRDN00012	81	155	1714	183	179	42
CCGGCTCAT	BRDN00011	142	115	45	256	198	5
CCGGCTCCC	BRDN00011	165	152	2369	262	44	67
CCGGCTCCC	BRDN00012	275	52	0	520	739	12
CCGGCTCCG	BRDN00012	511	409	1207	957	834	54
CCGGCTCGG	BRDN00012	31	4	0	152	91	0
CCGGCTCTA	BRDN00011	160	104	24	208	209	1
CCGGCTCTC	BRDN00012	158	95	354	259	311	10
CCGGCTGAA	BRDN00011	162	158	0	175	220	24
CCGGCTGCT	BRDN00012	485	363	622	780	1003	7
CCGGCTTCA	BRDN00012	74	0	0	257	18	0
CCGGCTTCT	BRDN00012	374	284	249	624	759	53
CCGGGAACA	BRDN00012	369	280	563	777	547	51
CCGGGAACA	BRDN00012	140	137	560	154	162	30
CCGGGAACA	BRDN00012	232	117	609	581	683	15
CCGGGAACT	BRDN00012	295	267	731	468	307	47
CCGGGAAGA	BRDN00011	447	72	157	879	565	0
CCGGGAAGA	BRDN00011	13	0	0	115	19	0
CCGGGAAGA	BRDN00012	83	13	0	86	140	0
CCGGGAAGG	BRDN00012	127	41	0	260	237	0
CCGGGAAGT	BRDN00011	91	43	166	166	289	9
CCGGGACAC	BRDN00012	130	32	11	195	354	0
CCGGGACAC	BRDN00011	51	70	0	161	55	0
CCGGGACCA	BRDN00011	135	139	4	279	409	10
CCGGGACCC	BRDN00011	285	327	501	659	675	40
CCGGGACCG	BRDN00011	198	172	358	424	278	0
CCGGGACGA	BRDN00012	397	554	951	776	912	48
CCGGGACGC	BRDN00012	221	229	894	407	228	20
CCGGGAGAC	BRDN00012	136	195	25	186	205	0
CCGGGAGAC	BRDN00011	282	94	842	705	873	26
CCGGGAGAC	BRDN00011	156	149	0	331	148	0
CCGGGAGAT	BRDN00012	142	102	113	256	478	0
CCGGGAGCC	BRDN00012	158	104	231	233	188	6
CCGGGAGCC	BRDN00011	192	166	996	273	315	17
CCGGGAGCT	BRDN00011	178	212	332	367	294	19
CCGGGATAG	BRDN00011	39	17	0	72	81	0
CCGGGATGA	BRDN00011	30	0	0	169	145	0
CCGGGATTG	BRDN00012	441	222	134	858	629	0
CCGGGCACT	BRDN00011	98	78	201	184	60	13
CCGGGCAGC	BRDN00012	249	199	52	571	403	21
CCGGGCATA	BRDN00012	117	150	139	259	285	0
CCGGGCATC	BRDN00012	114	111	0	296	162	0
CCGGGCCCG	BRDN00011	174	25	0	446	321	0
CCGGGCCGA	BRDN00011	143	107	22	200	158	19
CCGGGCCGC	BRDN00011	73	54	62	134	63	9
CCGGGCGCC	BRDN00011	112	161	5	162	330	25
CCGGGCGCC	BRDN00012	355	210	412	520	329	2
CCGGGCGTT	BRDN00011	153	80	419	346	267	7
CCGGGCTAT	BRDN00011	361	207	36	392	214	11
CCGGGCTCC	BRDN00012	293	197	353	469	337	11
CCGGGCTCC	BRDN00011	89	63	180	386	137	0
CCGGGCTGA	BRDN00011	406	316	636	677	381	39
CCGGGCTGA	BRDN00012	413	225	1040	602	494	20
CCGGGCTGG	BRDN00011	106	53	56	265	154	0
CCGGGCTGG	BRDN00012	120	158	99	319	401	0
CCGGGCTGT	BRDN00011	44	11	0	71	0	0
CCGGGCTTC	BRDN00011	96	146	64	127	36	19
CCGGGGAAG	BRDN00012	33	0	0	95	15	0
CCGGGGACA	BRDN00012	53	48	11	117	15	2
CCGGGGACA	BRDN00012	127	126	1	230	327	0
CCGGGGACC	BRDN00011	155	141	306	370	498	17
CCGGGGACT	BRDN00012	123	67	3	208	207	21
CCGGGGCAA	BRDN00012	233	0	1	525	256	0
CCGGGGCAG	BRDN00012	197	193	436	325	131	43
CCGGGGCCA	BRDN00012	316	182	583	563	601	6
CCGGGGCTG	BRDN00012	361	164	285	516	228	27
CCGGGGGCC	BRDN00011	99	69	2045	177	160	38
CCGGGGGTG	BRDN00012	86	101	321	94	352	51
CCGGGGGTT	BRDN00012	209	269	22	385	342	29
CCGGGGGTT	BRDN00011	85	87	274	224	304	19
CCGGGGTCT	BRDN00011	364	104	502	519	772	20
CCGGGGTGC	BRDN00012	174	314	66	377	254	37
CCGGGGTGG	BRDN00011	170	261	1338	415	319	24
CCGGGTATC	BRDN00012	116	175	375	256	174	6
CCGGGTATT	BRDN00011	47	49	50	137	138	0
CCGGGTCCA	BRDN00011	117	164	411	240	90	0
CCGGGTCCT	BRDN00011	161	103	1	322	328	0
CCGGGTCGA	BRDN00011	147	196	83	250	229	44
CCGGGTCTC	BRDN00011	96	106	9	240	172	14
CCGGGTGAC	BRDN00012	360	238	1833	686	745	36
CCGGGTGTA	BRDN00011	87	41	0	74	29	0
CCGGGTTCT	BRDN00011	84	0	0	235	49	0
CCGGTAACC	BRDN00012	263	149	509	219	211	3
CCGGTACAA	BRDN00011	121	0	0	175	264	0
CCGGTACAT	BRDN00011	69	37	0	160	20	0
CCGGTACCA	BRDN00012	120	97	0	267	208	35
CCGGTACCA	BRDN00011	185	147	120	410	511	17
CCGGTAGAA	BRDN00012	92	77	267	338	297	15
CCGGTAGAT	BRDN00012	330	184	607	599	1103	28
CCGGTAGGG	BRDN00011	41	45	804	115	169	33
CCGGTATGC	BRDN00012	317	360	85	790	821	31
CCGGTCAAA	BRDN00012	165	269	166	370	342	12
CCGGTCATA	BRDN00012	144	110	491	253	252	7
CCGGTCATA	BRDN00011	284	285	250	379	328	8
CCGGTCATA	BRDN00012	152	222	106	294	201	0
CCGGTCCAG	BRDN00011	138	177	343	305	318	8
CCGGTCCCC	BRDN00011	125	56	0	186	211	11
CCGGTCCCT	BRDN00011	125	204	238	338	43	13
CCGGTCCGC	BRDN00012	399	424	505	816	824	39
CCGGTCCTA	BRDN00012	230	17	0	489	488	0
CCGGTCGAA	BRDN00011	205	153	880	392	317	40
CCGGTCGCT	BRDN00012	125	83	105	156	53	0
CCGGTCGGG	BRDN00011	91	83	24	188	188	18
CCGGTCGTC	BRDN00012	119	79	137	205	272	9
CCGGTCTAT	BRDN00011	119	43	43	205	144	0
CCGGTCTGG	BRDN00012	15	24	0	178	47	4
CCGGTGACC	BRDN00011	129	146	337	263	291	16
CCGGTGAGG	BRDN00012	139	191	104	401	396	13
CCGGTGAGT	BRDN00011	99	129	298	162	93	0
CCGGTGATG	BRDN00011	51	147	70	129	119	39
CCGGTGCAG	BRDN00012	299	279	5463	580	1213	114
CCGGTGCCC	BRDN00011	81	43	137	172	74	20
CCGGTGCGA	BRDN00011	67	38	25	185	245	125
CCGGTGCGT	BRDN00011	81	73	14	152	202	0
CCGGTGCTG	BRDN00011	58	4	14	144	29	0
CCGGTGCTG	BRDN00011	205	169	17	411	353	23
CCGGTGGCC	BRDN00012	162	86	349	325	218	5
CCGGTGGCC	BRDN00011	63	8	26	150	162	0
CCGGTGTAC	BRDN00012	89	96	823	181	172	18
CCGGTGTGA	BRDN00012	201	132	257	360	1027	27
CCGGTGTGA	BRDN00012	63	23	145	127	62	21
CCGGTGTGG	BRDN00012	104	102	0	172	119	0
CCGGTTACA	BRDN00012	189	164	606	293	299	51
CCGGTTCAG	BRDN00011	214	219	196	338	202	9
CCGGTTCCA	BRDN00012	150	110	59	454	650	10
CCGGTTCGC	BRDN00011	143	111	744	237	139	27
CCGGTTCTG	BRDN00011	166	37	0	338	252	0
CCGGTTGCG	BRDN00011	87	87	385	238	219	0
CCGTAAACG	BRDN00011	130	140	108	229	157	0
CCGTAACAG	BRDN00011	108	138	0	159	260	0
CCGTAACTC	BRDN00012	137	8	146	251	93	14
CCGTAAGAA	BRDN00012	123	50	608	278	224	36
CCGTAAGGA	BRDN00012	58	2	0	543	38	0
CCGTACACT	BRDN00012	290	41	0	654	282	0
CCGTACAGG	BRDN00011	164	188	413	293	531	11
CCGTACCCA	BRDN00012	135	83	624	160	334	50
CCGTACCCC	BRDN00011	120	32	79	236	573	0
CCGTACCGT	BRDN00012	132	28	43	165	174	0
CCGTACCTC	BRDN00011	24	10	0	282	741	0
CCGTACGAT	BRDN00011	97	3	13	154	1	0
CCGTACGCG	BRDN00011	321	342	1871	374	447	40
CCGTACTCA	BRDN00011	232	158	51	517	299	69
CCGTACTGG	BRDN00012	197	195	491	490	382	17
CCGTACTTA	BRDN00011	75	139	0	146	80	0
CCGTAGAAC	BRDN00011	203	69	1	348	383	5
CCGTAGACT	BRDN00012	64	47	11	189	109	0
CCGTAGCAA	BRDN00011	70	125	163	206	136	54
CCGTAGCCG	BRDN00011	97	127	36	139	54	3
CCGTAGCCG	BRDN00011	120	262	115	271	147	16
CCGTAGGAC	BRDN00011	123	148	109	257	192	11
CCGTAGGAG	BRDN00011	348	36	66	722	259	6
CCGTAGGTT	BRDN00011	94	100	68	176	130	15
CCGTAGTCG	BRDN00012	151	260	669	329	322	71
CCGTATCAC	BRDN00012	182	52	252	347	300	44
CCGTATGGG	BRDN00011	186	13	0	356	91	0
CCGTATGTG	BRDN00011	310	335	153	444	394	4
CCGTATTGC	BRDN00011	182	107	106	372	231	5
CCGTCAAAA	BRDN00011	184	156	57	368	260	0
CCGTCACAG	BRDN00011	56	101	3	175	310	0
CCGTCACCC	BRDN00012	419	233	1874	680	582	101
CCGTCACTT	BRDN00012	263	249	73	396	221	22
CCGTCACTT	BRDN00012	272	133	984	497	556	31
CCGTCAGAG	BRDN00012	327	305	590	737	943	27
CCGTCAGCC	BRDN00011	313	355	184	512	337	0
CCGTCAGGG	BRDN00012	334	197	449	1028	481	38
CCGTCAGTG	BRDN00011	41	0	0	190	243	0
CCGTCATCA	BRDN00012	132	136	192	207	273	18
CCGTCATTC	BRDN00011	428	578	504	869	820	91
CCGTCCATA	BRDN00011	143	110	0	367	132	33
CCGTCCCAA	BRDN00012	193	191	427	238	330	0
CCGTCCCTG	BRDN00012	192	175	7	335	577	4
CCGTCCGTG	BRDN00011	110	72	8	164	319	3
CCGTCCGTG	BRDN00012	463	404	238	846	937	117
CCGTCCTCG	BRDN00012	163	147	16	254	181	1
CCGTCCTTA	BRDN00011	193	64	64	387	533	0
CCGTCCTTA	BRDN00012	392	313	723	637	994	83
CCGTCGAAC	BRDN00012	137	170	211	301	487	23
CCGTCGAGC	BRDN00011	83	94	13	166	101	18
CCGTCGCCC	BRDN00011	48	62	45	193	95	0
CCGTCGCCT	BRDN00012	362	308	199	521	423	19
CCGTCGCGT	BRDN00011	55	22	1	281	10	0
CCGTCGGAC	BRDN00011	135	187	89	294	363	15
CCGTCGGAG	BRDN00011	126	122	912	327	186	79
CCGTCGTCC	BRDN00011	144	142	308	320	194	0
CCGTCTCCG	BRDN00012	0	0	0	0	0	0
CCGTCTGAA	BRDN00012	336	377	498	799	1066	14
CCGTCTGAA	BRDN00012	359	431	76	750	712	28
CCGTCTGCA	BRDN00012	316	242	597	494	481	17
CCGTCTGCC	BRDN00011	275	225	69	442	345	76
CCGTCTGCG	BRDN00012	428	347	661	834	935	53
CCGTCTGGC	BRDN00011	289	341	3242	569	818	68
CCGTCTTAC	BRDN00011	188	164	170	489	497	7
CCGTCTTGG	BRDN00012	217	251	1520	535	741	39
CCGTGAAAG	BRDN00011	141	69	113	400	273	19
CCGTGACAA	BRDN00012	203	171	111	476	749	9
CCGTGAGAA	BRDN00011	163	138	345	287	408	9
CCGTGAGAG	BRDN00012	289	198	845	504	408	24
CCGTGCAGA	BRDN00011	94	44	262	223	110	8
CCGTGCCCG	BRDN00011	152	86	185	288	341	5
CCGTGCCGC	BRDN00012	239	205	174	392	421	0
CCGTGCCGT	BRDN00011	126	79	544	289	402	0
CCGTGCCGT	BRDN00012	394	407	2174	678	686	51
CCGTGCGAG	BRDN00011	102	57	11	194	162	23
CCGTGCTGA	BRDN00012	344	255	3482	509	685	48
CCGTGGAAC	BRDN00012	114	56	296	226	262	4
CCGTGGACT	BRDN00012	108	50	123	311	112	0
CCGTGGCCA	BRDN00012	138	98	192	270	235	30
CCGTGGCCA	BRDN00011	185	312	9	454	230	28
CCGTGGCCG	BRDN00011	233	378	302	471	167	11
CCGTGGCCG	BRDN00012	194	247	115	618	639	282
CCGTGGGCG	BRDN00011	388	380	1059	559	265	90
CCGTGGGGC	BRDN00012	409	268	3984	839	662	63
CCGTGTAAG	BRDN00012	362	230	1022	519	436	43
CCGTGTAGC	BRDN00011	218	299	525	492	584	49
CCGTGTAGT	BRDN00012	238	157	2511	491	433	5
CCGTGTCAT	BRDN00012	35	5	0	157	0	0
CCGTGTCCG	BRDN00011	222	139	492	272	157	0
CCGTGTCTC	BRDN00012	413	539	1310	716	510	73
CCGTGTGAC	BRDN00011	104	98	101	231	238	51
CCGTGTTCC	BRDN00012	566	583	5574	777	708	96
CCGTTAATG	BRDN00012	332	231	253	430	415	4
CCGTTACCG	BRDN00012	125	97	134	274	184	5
CCGTTACCG	BRDN00012	11	73	0	34	90	0
CCGTTCAGA	BRDN00011	318	368	1228	495	395	26
CCGTTCATG	BRDN00012	343	316	195	645	745	14
CCGTTCGCA	BRDN00012	150	115	65	262	190	6
CCGTTCGCG	BRDN00011	37	4	14	207	305	0
CCGTTCGTT	BRDN00012	163	171	141	231	128	24
CCGTTCTGC	BRDN00012	96	2	87	862	298	3
CCGTTGAGG	BRDN00012	225	173	291	388	265	8
CCGTTGATC	BRDN00012	428	418	1217	804	953	22
CCGTTGGAC	BRDN00012	344	391	586	609	691	21
CCGTTGTAC	BRDN00011	114	232	2204	185	512	46
CCGTTTCCA	BRDN00011	434	71	123	898	585	0
CCGTTTGAG	BRDN00012	188	89	1604	434	509	42
CCTAAAATC	BRDN00011	64	41	27	187	353	14
CCTAAACAG	BRDN00012	185	180	198	373	505	40
CCTAAACTC	BRDN00012	318	312	1910	714	382	13
CCTAAAGCT	BRDN00011	253	355	1502	327	231	123
CCTAAAGGG	BRDN00012	180	137	36	248	166	0
CCTAAAGTC	BRDN00011	77	76	41	140	286	9
CCTAAATCC	BRDN00012	280	240	11	399	215	14
CCTAAATTA	BRDN00012	187	335	33	314	446	85
CCTAACAAG	BRDN00011	240	308	48	387	307	16
CCTAACAGT	BRDN00011	215	205	748	524	471	34
CCTAACCAC	BRDN00011	71	82	37	217	123	10
CCTAACCAT	BRDN00012	126	79	209	361	353	1
CCTAACCCC	BRDN00012	657	602	513	1010	1100	38
CCTAACTAG	BRDN00011	216	1	1	541	109	0
CCTAAGAAC	BRDN00011	39	130	11	185	256	0
CCTAAGACA	BRDN00011	60	152	21	99	32	6
CCTAAGCCT	BRDN00011	78	144	43	170	310	17
CCTAAGGGG	BRDN00012	496	604	516	863	794	55
CCTAAGGTC	BRDN00011	378	368	209	620	613	50
CCTAAGTAG	BRDN00011	207	289	249	431	518	50
CCTAAGTCC	BRDN00011	108	142	179	244	302	4
CCTAAGTCT	BRDN00012	306	464	1008	501	1455	247
CCTAAGTGA	BRDN00011	141	109	0	299	195	24
CCTAATACT	BRDN00012	126	108	425	224	345	0
CCTAATAGC	BRDN00012	212	217	118	469	439	59
CCTAATGAC	BRDN00011	256	215	184	476	614	33
CCTAATGTC	BRDN00012	182	151	665	376	316	24
CCTAATGTG	BRDN00012	120	98	317	233	60	0
CCTACAAAA	BRDN00012	124	106	361	237	106	4
CCTACAAAT	BRDN00011	74	132	3588	278	197	105
CCTACAAAT	BRDN00012	160	289	98	394	387	62
CCTACAACT	BRDN00012	133	301	530	352	587	29
CCTACAAGC	BRDN00012	172	103	322	255	157	0
CCTACAATG	BRDN00011	179	243	831	346	473	11
CCTACAATG	BRDN00012	79	106	1	181	151	24
CCTACACAC	BRDN00012	406	412	149	560	646	204
CCTACACAG	BRDN00011	169	8	157	402	304	7
CCTACACAT	BRDN00012	131	101	378	226	237	5
CCTACACGC	BRDN00012	255	262	526	675	543	32
CCTACAGAT	BRDN00011	139	80	88	224	90	0
CCTACAGCA	BRDN00012	87	72	90	149	136	1
CCTACAGCT	BRDN00012	266	259	465	472	793	42
CCTACAGGC	BRDN00012	275	235	166	668	629	3
CCTACAGGC	BRDN00011	104	40	55	78	116	0
CCTACAGGG	BRDN00012	55	120	250	248	76	0
CCTACAGTA	BRDN00012	186	71	93	402	183	0
CCTACATCA	BRDN00011	420	344	361	806	693	45
CCTACATCG	BRDN00012	181	100	301	203	184	36
CCTACATGC	BRDN00012	268	40	1088	780	358	54
CCTACATGG	BRDN00012	399	459	57	789	912	122
CCTACATTA	BRDN00011	319	297	212	574	612	57
CCTACCACA	BRDN00011	400	504	2318	591	633	103
CCTACCACA	BRDN00011	121	157	291	146	347	15
CCTACCAGA	BRDN00011	166	60	615	333	178	10
CCTACCAGA	BRDN00011	285	189	96	378	304	63
CCTACCAGC	BRDN00012	21	0	5	194	559	0
CCTACCAGG	BRDN00012	322	227	359	675	213	15
CCTACCAGT	BRDN00011	311	183	87	464	618	31
CCTACCCAG	BRDN00012	132	264	459	277	191	8
CCTACCCAT	BRDN00012	195	107	1258	274	260	50
CCTACCCTG	BRDN00012	168	153	102	220	125	32
CCTACCCTT	BRDN00011	153	0	1	370	69	0
CCTACCGTG	BRDN00011	104	179	56	131	200	388
CCTACCGTG	BRDN00011	210	157	133	340	313	13
CCTACCTAA	BRDN00012	76	53	39	135	25	5
CCTACCTAA	BRDN00012	413	334	3753	869	835	174
CCTACCTAG	BRDN00012	5	0	0	191	125	0
CCTACCTCA	BRDN00011	299	219	155	755	794	13
CCTACCTCG	BRDN00012	102	21	1362	488	311	17
CCTACCTGA	BRDN00011	155	180	106	210	262	8
CCTACCTGA	BRDN00011	92	0	0	205	131	0
CCTACCTGG	BRDN00012	319	319	82	718	622	138
CCTACCTGT	BRDN00012	330	199	761	418	643	63
CCTACCTTC	BRDN00012	274	305	1511	555	559	75
CCTACCTTG	BRDN00012	150	54	20	169	91	8
CCTACGAGT	BRDN00011	110	92	152	153	262	12
CCTACGCCA	BRDN00012	288	419	844	825	978	89
CCTACGCCT	BRDN00012	311	232	325	414	524	30
CCTACGCGG	BRDN00012	284	206	1961	628	829	21
CCTACGGCG	BRDN00012	62	33	148	128	90	0
CCTACGGGA	BRDN00011	316	346	487	531	411	27
CCTACGGGG	BRDN00011	63	127	24	248	82	0
CCTACGGTT	BRDN00012	174	87	38	275	303	5
CCTACGTGG	BRDN00011	262	341	293	513	294	12
CCTACGTGG	BRDN00011	78	108	99	207	452	8
CCTACGTGG	BRDN00011	255	87	338	511	263	8
CCTACTACT	BRDN00011	269	126	282	541	886	0
CCTACTATT	BRDN00012	254	289	113	411	1137	27
CCTACTCAA	BRDN00011	201	567	78	322	294	64
CCTACTCAG	BRDN00011	227	152	370	331	339	4
CCTACTCCC	BRDN00012	387	571	797	844	482	33
CCTACTCGC	BRDN00011	170	145	1890	293	373	21
CCTACTCTA	BRDN00011	346	571	1484	719	438	97
CCTACTGCC	BRDN00011	107	52	45	139	145	0
CCTACTGTG	BRDN00012	315	231	247	438	732	17
CCTACTGTG	BRDN00011	135	84	121	109	26	34
CCTACTTAC	BRDN00011	134	70	156	201	80	8
CCTACTTAC	BRDN00012	270	218	22	481	759	22
CCTACTTAG	BRDN00012	482	494	580	859	764	136
CCTACTTAT	BRDN00012	372	282	235	469	359	13
CCTACTTCT	BRDN00012	109	6	10	168	64	0
CCTACTTTC	BRDN00011	215	112	320	363	467	0
CCTAGAAAT	BRDN00011	108	232	242	256	252	8
CCTAGAAGA	BRDN00012	137	88	67	320	271	28
CCTAGAAGC	BRDN00011	97	104	519	271	91	15
CCTAGAATT	BRDN00012	96	103	7	250	74	18
CCTAGACTG	BRDN00011	214	229	87	331	534	23
CCTAGACTT	BRDN00012	191	82	1291	343	256	41
CCTAGACTT	BRDN00011	171	122	321	224	258	13
CCTAGAGCA	BRDN00012	97	37	20	207	150	0
CCTAGAGGT	BRDN00012	539	438	367	822	386	0
CCTAGAGTT	BRDN00012	139	242	556	242	244	14
CCTAGATAG	BRDN00012	7	13	202	145	19	0
CCTAGCAGT	BRDN00012	399	378	756	587	693	72
CCTAGCATG	BRDN00011	224	12	0	583	234	0
CCTAGCCTG	BRDN00012	79	39	1	163	241	0
CCTAGCTTG	BRDN00012	312	29	0	664	64	0
CCTAGGAAC	BRDN00012	194	149	514	416	340	12
CCTAGGACA	BRDN00012	160	165	448	215	82	4
CCTAGGCAA	BRDN00012	120	36	0	266	173	0
CCTAGGCCA	BRDN00011	358	511	130	590	565	115
CCTAGGCCG	BRDN00012	154	49	265	256	199	1
CCTAGGCTT	BRDN00012	106	126	145	367	336	11
CCTAGGGCA	BRDN00012	104	0	0	825	370	0
CCTAGGGCT	BRDN00011	162	146	2	231	191	1
CCTAGGTGA	BRDN00012	156	122	0	331	201	10
CCTAGTAAC	BRDN00012	190	232	1	337	610	0
CCTAGTAAG	BRDN00011	113	82	177	157	202	36
CCTAGTCCG	BRDN00012	526	383	80	775	484	5
CCTAGTGCC	BRDN00012	283	250	1049	549	487	61
CCTAGTTCA	BRDN00012	493	559	1263	715	741	14
CCTATAAAC	BRDN00012	354	154	31	453	559	63
CCTATACAA	BRDN00011	161	111	0	223	452	11
CCTATACCT	BRDN00011	104	145	32	249	49	4
CCTATAGTG	BRDN00011	377	398	61	718	465	9
CCTATATAA	BRDN00011	369	247	4	746	362	0
CCTATATGT	BRDN00012	87	9	0	391	261	0
CCTATCACA	BRDN00011	173	85	618	327	160	7
CCTATCACC	BRDN00012	187	148	12	255	115	0
CCTATCAGC	BRDN00012	270	312	1	222	141	1
CCTATCCAA	BRDN00012	215	276	77	302	240	27
CCTATCCTC	BRDN00011	158	62	0	275	140	0
CCTATCGGA	BRDN00011	219	213	27	336	721	63
CCTATCTAT	BRDN00012	152	134	12	372	209	5
CCTATCTGA	BRDN00012	282	260	241	518	343	21
CCTATGAAC	BRDN00012	156	166	84	258	347	56
CCTATGAAC	BRDN00011	130	108	90	222	224	17
CCTATGAAT	BRDN00011	204	166	494	452	255	17
CCTATGACC	BRDN00011	204	179	148	451	515	29
CCTATGAGA	BRDN00012	364	441	417	538	696	42
CCTATGAGT	BRDN00011	16	13	0	78	65	0
CCTATGCAG	BRDN00011	208	186	299	286	466	28
CCTATGCGT	BRDN00011	60	82	409	228	122	0
CCTATGCTA	BRDN00011	229	197	348	280	207	0
CCTATGGCT	BRDN00012	111	43	47	169	71	0
CCTATGGGA	BRDN00011	124	249	214	215	87	6
CCTATGGGA	BRDN00012	417	434	132	675	818	14
CCTATGGTT	BRDN00012	181	136	1500	310	937	60
CCTATGTAC	BRDN00012	360	421	687	565	755	36
CCTATGTGT	BRDN00011	174	143	30	309	395	20
CCTATGTTG	BRDN00012	224	352	441	544	772	15
CCTATTAAA	BRDN00012	91	14	0	233	339	1
CCTATTAAT	BRDN00011	111	40	39	259	229	39
CCTATTATT	BRDN00011	139	186	501	293	127	11
CCTATTCAC	BRDN00011	115	121	289	176	199	43
CCTATTGTG	BRDN00011	107	20	1	136	75	0
CCTATTTGG	BRDN00011	211	247	39	264	224	22
CCTCAAAAT	BRDN00012	351	440	437	785	724	39
CCTCAAAAT	BRDN00012	336	317	828	649	633	20
CCTCAAACA	BRDN00011	175	57	803	406	435	21
CCTCAAACT	BRDN00011	50	31	0	145	241	5
CCTCAAACT	BRDN00011	255	229	1515	374	221	37
CCTCAAAGA	BRDN00012	121	53	0	298	321	0
CCTCAAAGA	BRDN00011	104	53	49	275	278	0
CCTCAAAGA	BRDN00011	89	84	0	98	94	0
CCTCAAAGG	BRDN00011	361	243	180	607	775	48
CCTCAAAGG	BRDN00011	93	20	79	212	59	0
CCTCAAAGT	BRDN00011	191	218	60	417	536	0
CCTCAACAA	BRDN00011	336	375	1298	663	633	62
CCTCAACAC	BRDN00012	481	437	133	825	621	17
CCTCAACAC	BRDN00012	487	400	1744	823	363	54
CCTCAACAT	BRDN00012	111	147	596	220	86	1
CCTCAACCA	BRDN00012	458	214	24	849	315	3
CCTCAACCG	BRDN00011	259	331	157	407	533	41
CCTCAACCT	BRDN00012	343	304	720	646	316	45
CCTCAACTC	BRDN00012	415	348	1190	857	802	21
CCTCAACTT	BRDN00011	314	255	517	448	495	4
CCTCAAGAA	BRDN00012	58	37	0	121	20	0
CCTCAAGAA	BRDN00011	132	154	764	312	259	0
CCTCAAGGC	BRDN00012	115	96	57	199	96	0

CCTCAAGGG	BRDN00012	662	563	193	1119	1159	119
CCTCAAGTA	BRDN00012	321	291	305	367	279	77
CCTCAAGTCA	BRDN00012	120	163	196	363	319	0
CCTCAAGTG	BRDN00012	178	220	91	493	562	21
CCTCAAGTG	BRDN00011	638	424	1501	1069	1412	109
CCTCAAGTTA	BRDN00012	257	206	1103	664	693	13
CCTCAATGAT	BRDN00012	142	142	625	245	221	0
CCTCAATGG	BRDN00011	509	441	264	797	742	56
CCTCACAAAC	BRDN00011	264	102	17	354	263	0
CCTCACAAAC	BRDN00011	249	41	0	422	114	0
CCTCACAAAG	BRDN00012	234	186	895	499	613	20
CCTCACAAG	BRDN00012	171	174	119	240	135	2
CCTCACACAG	BRDN00011	305	406	567	476	414	47
CCTCACACCA	BRDN00011	205	105	337	343	338	49
CCTCACACCT	BRDN00012	65	212	377	209	167	8
CCTCACACGG	BRDN00011	149	211	435	543	311	10
CCTCACAGAT	BRDN00012	256	207	609	427	425	28
CCTCACAGCC	BRDN00012	184	180	291	359	371	21
CCTCACAGCT	BRDN00012	16	0	0	466	26	0
CCTCACAGG	BRDN00012	18	0	0	216	61	0
CCTCACAGG	BRDN00011	205	220	351	350	406	1
CCTCACATTT	BRDN00011	124	113	484	338	140	32
CCTCACCAAC	BRDN00011	457	503	599449	1324	707	6952
CCTCACCAAT	BRDN00011	126	143	491	312	327	17
CCTCACCACG	BRDN00011	186	169	210	370	428	13
CCTCACCCA	BRDN00011	158	116	521	369	270	28
CCTCACCCA	BRDN00011	246	309	124	656	525	74
CCTCACCCCT	BRDN00012	467	548	1429	873	965	72
CCTCACCCGG	BRDN00011	116	181	102	228	158	22
CCTCACCCTG	BRDN00012	236	195	13	439	155	0
CCTCACCCTT	BRDN00012	107	0	1	306	142	0
CCTCACCGAT	BRDN00012	155	105	0	364	74	0
CCTCACCGG	BRDN00012	212	315	18	372	269	0
CCTCACCTA	BRDN00011	71	62	63	278	310	0
CCTCACCTCG	BRDN00011	335	199	379	646	781	9
CCTCACCTGA	BRDN00012	280	203	509	320	162	48
CCTCACCTGC	BRDN00011	161	92	14	445	404	0
CCTCACCTGG	BRDN00011	223	233	117	411	241	49
CCTCACCTTC	BRDN00012	346	276	314	658	725	9
CCTCACCTTG	BRDN00012	286	215	431	527	300	14
CCTCACCTTG	BRDN00011	127	168	141	314	424	82
CCTCACGCAG	BRDN00012	311	298	335	546	701	26
CCTCACGCTA	BRDN00011	208	179	133	326	371	19
CCTCACGGC	BRDN00012	170	166	421	266	304	15
CCTCACTAT	BRDN00012	135	138	17811	447	730	232
CCTCACTCCC	BRDN00012	107	102	0	237	116	6
CCTCACTCGT	BRDN00011	157	164	120	257	273	27
CCTCACTCTA	BRDN00012	202	202	14	239	198	4
CCTCACTCTT	BRDN00011	181	143	391	286	83	3
CCTCACTCTT	BRDN00012	101	164	53	371	611	8
CCTCACTGAG	BRDN00011	143	105	179	137	64	5
CCTCACTGTC	BRDN00011	274	134	74	734	446	12
CCTCACTTAG	BRDN00011	182	187	254	453	378	8
CCTCACTTA	BRDN00012	72	11	0	182	196	0
CCTCACTTCA	BRDN00012	47	0	0	656	145	0
CCTCAGAAC	BRDN00011	98	0	2	440	285	0
CCTCAGAAG	BRDN00011	89	141	4	225	71	4
CCTCAGAATT	BRDN00012	362	242	1196	812	521	59
CCTCAGACA	BRDN00012	108	146	131	291	696	10
CCTCAGACA	BRDN00012	186	187	26	878	378	7
CCTCAGACAT	BRDN00011	181	174	164	347	151	45
CCTCAGATCT	BRDN00011	396	273	73	532	235	73
CCTCAGCAAT	BRDN00011	117	149	43	262	296	0
CCTCAGCAG	BRDN00011	388	336	763	671	499	8
CCTCAGCCAC	BRDN00011	233	182	684	486	208	10
CCTCAGCCAG	BRDN00011	15	0	0	667	10	0
CCTCAGCTAC	BRDN00012	359	344	419	523	293	20
CCTCAGGAA	BRDN00012	165	182	164	181	264	0
CCTCAGGAC	BRDN00012	132	145	108	332	253	46
CCTCAGGAG	BRDN00011	115	107	766	172	128	23
CCTCAGGGA	BRDN00011	165	31	13	505	407	0
CCTCAGGGA	BRDN00011	148	153	80	215	32	0
CCTCAGGGG	BRDN00011	405	229	74	644	486	12
CCTCAGGTG	BRDN00011	165	173	0	169	157	4
CCTCAGGTG	BRDN00011	80	44	60	89	71	0
CCTCAGTAAT	BRDN00012	110	148	3	192	303	0
CCTCAGTCAC	BRDN00011	111	53	699	214	255	8
CCTCAGTCCC	BRDN00011	151	181	101	228	279	0
CCTCAGTCG	BRDN00012	169	198	278	341	512	6
CCTCAGTGA	BRDN00011	121	24	53	197	267	0
CCTCAGTGT	BRDN00011	66	80	34	299	157	0
CCTCAGTTG	BRDN00012	347	256	1354	717	795	0
CCTCATAAAG	BRDN00012	175	145	485	310	263	23
CCTCATAAAT	BRDN00012	101	71	48	230	381	3
CCTCATAATA	BRDN00012	275	353	70	658	704	14
CCTCATACC	BRDN00011	163	211	32	282	309	0
CCTCATAGTC	BRDN00012	92	96	468	211	215	0
CCTCATATTT	BRDN00011	130	127	10	340	104	0
CCTCATCAAC	BRDN00012	492	643	854	879	844	13
CCTCATCATG	BRDN00011	81	50	49	214	101	24
CCTCATCAT	BRDN00011	392	342	1548	616	537	67
CCTCATCAT	BRDN00012	229	231	275	546	392	10
CCTCATCATC	BRDN00012	472	342	2419	654	1066	78
CCTCATCATG	BRDN00012	328	210	482	722	482	11
CCTCATCATT	BRDN00012	322	330	751	500	883	78
CCTCATCCCG	BRDN00011	167	157	113	324	155	0
CCTCATCCGA	BRDN00012	207	143	326	421	348	4
CCTCATCCGG	BRDN00011	218	231	86	523	528	74
CCTCATCGAG	BRDN00012	91	53	166	225	378	0
CCTCATCTTC	BRDN00012	125	268	182	331	274	17
CCTCATGATC	BRDN00012	174	128	0	431	297	11
CCTCATGCTG	BRDN00012	610	615	1399	879	998	85
CCTCATGCTT	BRDN00012	173	164	837	365	391	34
CCTCATGGTT	BRDN00011	190	213	1029	332	395	14
CCTCATTAT	BRDN00011	241	234	147	647	485	0
CCTCATTATG	BRDN00011	153	199	396	300	236	18
CCTCATTCGA	BRDN00012	116	68	40	699	173	0
CCTCATTGAA	BRDN00011	80	72	3	185	107	4
CCTCATTGCA	BRDN00011	312	222	492	324	358	15
CCTCCAAAGT	BRDN00011	239	0	0	281	90	0
CCTCCAACAC	BRDN00012	336	166	316	657	629	47
CCTCCAACAT	BRDN00011	391	281	2482	657	438	104
CCTCCAACAT	BRDN00011	168	99	93	225	328	16
CCTCCAAGCA	BRDN00012	354	466	590	675	764	3
CCTCCAAGCA	BRDN00011	238	250	411	453	769	68
CCTCCAAGCT	BRDN00012	252	267	25	250	428	3
CCTCCAAGGA	BRDN00011	256	237	57	606	329	7
CCTCCAAGGA	BRDN00011	501	225	9	676	1102	10
CCTCCAAGGA	BRDN00011	205	181	30	275	267	110
CCTCCAAGGT	BRDN00012	358	402	509	536	573	16
CCTCCAAGTG	BRDN00011	138	137	1	238	199	27
CCTCCAATCT	BRDN00012	98	108	19	114	166	12
CCTCCACAAG	BRDN00011	14	9	0	145	83	0
CCTCCACACG	BRDN00011	540	487	321	875	745	52
CCTCCACAGT	BRDN00012	401	436	2585	604	649	42
CCTCCACCAA	BRDN00012	229	370	66	467	483	115
CCTCCACCAA	BRDN00012	102	77	3	145	83	0
CCTCCACCTG	BRDN00011	109	125	55	260	167	112
CCTCCACGGA	BRDN00011	158	101	14	195	253	14
CCTCCACGG	BRDN00011	171	86	0	441	286	17
CCTCCACTAT	BRDN00012	349	303	672	577	465	43
CCTCCACTCA	BRDN00012	207	121	116	506	254	9
CCTCCACTGG	BRDN00012	59	14	2	151	162	0
CCTCCAGAA	BRDN00011	305	208	420	576	602	48
CCTCCAGAAC	BRDN00011	158	88	189	324	390	7
CCTCCAGACA	BRDN00012	101	22	0	310	192	0
CCTCCAGAGA	BRDN00011	129	40	11	234	196	28
CCTCCAGAGT	BRDN00012	170	20	72	464	189	5
CCTCCAGCAC	BRDN00012	143	141	43	204	312	19
CCTCCAGCAG	BRDN00012	114	80	2	234	292	0
CCTCCAGCCA	BRDN00011	285	320	170	548	580	45
CCTCCAGCG	BRDN00012	131	186	409	222	126	53
CCTCCAGCTC	BRDN00012	377	282	1811	469	736	14
CCTCCAGCTC	BRDN00012	86	119	0	257	95	58
CCTCCAGGAC	BRDN00011	264	323	1032	406	374	20
CCTCCAGGG	BRDN00012	282	158	316	503	469	36
CCTCCAGGTC	BRDN00012	395	418	54	774	699	25
CCTCCAGTCG	BRDN00011	156	160	68	211	77	8
CCTCCAGTGC	BRDN00011	130	143	0	212	322	0
CCTCCAGTTC	BRDN00012	167	170	150	348	351	0
CCTCCATAGA	BRDN00011	198	84	221	362	613	0
CCTCCATAT	BRDN00012	333	331	146	584	498	21
CCTCCATGAA	BRDN00012	138	44	0	322	385	0
CCTCCATGTA	BRDN00012	125	198	126	296	312	3
CCTCCATTAC	BRDN00011	139	69	195	296	422	6
CCTCCATTGC	BRDN00011	363	206	322	797	639	30
CCTCCATTGG	BRDN00012	391	199	1297	843	485	55
CCTCCCAAAG	BRDN00011	199	263	94	466	619	19
CCTCCCAACG	BRDN00011	297	193	1196	652	541	72
CCTCCCAAGA	BRDN00012	246	72	1222	373	229	20
CCTCCCAAGA	BRDN00011	457	487	1384	831	670	68
CCTCCCACCC	BRDN00011	131	85	133	308	646	9
CCTCCCACTC	BRDN00012	303	313	1880	752	671	87
CCTCCCAGG	BRDN00011	281	181	1621	506	793	41
CCTCCCAGGT	BRDN00012	252	386	992	555	596	43
CCTCCCAGTT	BRDN00012	208	129	325	472	482	12
CCTCCCATAA	BRDN00012	132	52	158	264	140	8
CCTCCCATAG	BRDN00011	118	148	0	305	111	0
CCTCCCATGA	BRDN00011	128	48	0	275	803	0
CCTCCCCACT	BRDN00011	311	127	34	563	363	0
CCTCCCCAGA	BRDN00011	234	242	483	380	296	36
CCTCCCCCGC	BRDN00011	335	336	505	680	700	63
CCTCCCCGTC	BRDN00012	273	139	251	607	599	19
CCTCCCGAAT	BRDN00011	172	140	636	324	154	51
CCTCCCGAGA	BRDN00011	244	364	1766	630	326	128
CCTCCCGCCA	BRDN00012	248	133	331	492	390	1
CCTCCCGCGG	BRDN00011	183	137	14	264	355	11
CCTCCCGCTG	BRDN00012	289	340	321	518	413	37
CCTCCCGTGT	BRDN00012	81	129	208	219	405	15
CCTCCCTCTA	BRDN00012	61	0	0	267	281	0
CCTCCCTGCA	BRDN00011	617	558	1012	954	711	58
CCTCCCTTCC	BRDN00011	403	353	309	664	382	137
CCTCCGAAAA	BRDN00012	334	290	1820	580	416	13
CCTCCGAAGA	BRDN00012	57	31	0	123	84	0
CCTCCGACAC	BRDN00012	238	177	100	360	211	32
CCTCCGACTA	BRDN00012	357	380	118	561	627	29
CCTCCGATAA	BRDN00012	159	0	81	405	128	1
CCTCCGATAC	BRDN00011	119	73	0	161	145	0
CCTCCGCAAA	BRDN00011	148	183	34	383	146	1
CCTCCGCAAA	BRDN00012	180	65	382	220	354	14
CCTCCGCAAT	BRDN00012	123	8	0	225	375	0
CCTCCGCCCT	BRDN00012	131	223	516	331	170	20
CCTCCGCCTC	BRDN00011	262	161	229	382	314	8
CCTCCGCGCC	BRDN00012	371	256	926	598	387	0
CCTCCGCGCC	BRDN00011	432	332	1351	851	540	58
CCTCCGCGGA	BRDN00011	405	491	1166	859	652	67
CCTCCGGATC	BRDN00012	114	72	18	231	133	0
CCTCCGGGG	BRDN00012	435	440	269	783	898	63
CCTCCGGGG	BRDN00012	326	292	1002	666	971	25
CCTCCGTAGC	BRDN00011	280	171	130	876	565	0
CCTCCGTGCT	BRDN00012	558	553	843	703	712	49
CCTCCTAGGA	BRDN00012	103	24	0	261	172	0
CCTCCTCAGA	BRDN00012	353	111	64	578	517	10
CCTCCTCAGG	BRDN00011	26	0	0	124	36	0
CCTCCTCATA	BRDN00011	438	196	495	916	514	12
CCTCCTCCAA	BRDN00011	168	256	187	222	329	11
CCTCCTCCAC	BRDN00011	385	390	557	488	830	75
CCTCCTCCAT	BRDN00012	108	76	57	252	291	6
CCTCCTCCCA	BRDN00011	190	107	827	294	280	15
CCTCCTCCGA	BRDN00011	168	78	1	140	77	13
CCTCCTCGGT	BRDN00012	147	38	830	351	265	36
CCTCCTCTAC	BRDN00012	217	91	168	253	562	1
CCTCCTCTAT	BRDN00012	35	0	0	361	81	0
CCTCCTCTTC	BRDN00012	137	138	309	309	253	6
CCTCCTGAAG	BRDN00011	226	184	609	271	439	11
CCTCCTGAGT	BRDN00011	234	169	19	226	271	11
CCTCCTGATA	BRDN00012	123	116	9	231	211	0
CCTCCTGCAC	BRDN00011	193	132	194	391	375	13
CCTCCTGGA	BRDN00012	349	243	215	754	719	30
CCTCCTGGCA	BRDN00011	467	319	349	860	696	9
CCTCCTGGG	BRDN00012	402	593	321	739	1077	106
CCTCCTGTAA	BRDN00011	82	20	0	203	177	0
CCTCCTTAGT	BRDN00012	175	56	85	374	310	0
CCTCCTTCCA	BRDN00012	96	55	0	182	151	0
CCTCCTTCCT	BRDN00012	116	148	287	226	294	0
CCTCCTTCTG	BRDN00012	257	246	188	354	385	0
CCTCCTTTAG	BRDN00012	198	167	670	338	258	22
CCTCCTTTGA	BRDN00011	107	44	41	151	81	17
CCTCCTTTGA	BRDN00011	128	111	32	173	91	13
CCTCCTTTGC	BRDN00011	56	0	0	149	320	0
CCTCCTTTGC	BRDN00012	174	171	83	287	198	10
CCTCGAAACC	BRDN00011	201	239	64	468	346	8
CCTCGAACTC	BRDN00012	125	95	123	412	255	8
CCTCGAACTG	BRDN00011	60	20	0	197	212	0
CCTCGAAGA	BRDN00011	353	407	670	503	325	53
CCTCGAAGC	BRDN00012	161	43	647	260	286	0
CCTCGAAGG	BRDN00012	72	139	0	201	606	22
CCTCGAATAT	BRDN00011	175	131	193	413	454	39
CCTCGACATA	BRDN00011	317	212	102	554	881	31
CCTCGACTCG	BRDN00012	50	12	84	108	38	0
CCTCGAGATA	BRDN00012	71	52	0	200	120	0
CCTCGAGCA	BRDN00012	31	6	0	137	102	0
CCTCGAGCAT	BRDN00012	216	171	289	587	675	18
CCTCGAGGC	BRDN00012	64	57	34	188	86	0
CCTCGAGGT	BRDN00012	314	268	1460	617	782	10
CCTCGATACC	BRDN00012	486	375	1042	708	364	47
CCTCGATCTA	BRDN00011	419	276	1075	770	827	36
CCTCGATCTC	BRDN00012	491	484	2041	996	1030	86
CCTCGATGA	BRDN00011	295	183	89	548	807	70
CCTCGATGG	BRDN00012	714	540	1193	1338	1094	93
CCTCGATTTG	BRDN00012	285	239	319	491	429	63
CCTCGCACCC	BRDN00012	426	281	523	991	741	36
CCTCGCATAA	BRDN00011	104	96	2177	221	92	41
CCTCGCCAAC	BRDN00011	110	30	0	269	156	0
CCTCGCCAAG	BRDN00011	163	21	0	490	156	0
CCTCGCCAAT	BRDN00012	107	131	28	288	179	7
CCTCGCCCTT	BRDN00012	80	87	1	203	280	8
CCTCGCCCTT	BRDN00012	391	404	843	666	318	12
CCTCGCCGAC	BRDN00011	100	117	37	294	516	24
CCTCGCCGCT	BRDN00012	61	162	314	213	204	9
CCTCGCGCAC	BRDN00011	280	190	922	415	668	48
CCTCGCGCAT	BRDN00011	143	58	2297	266	112	48
CCTCGCGCGT	BRDN00012	248	282	1126	450	386	38
CCTCGCGGA	BRDN00012	156	167	92	286	326	11
CCTCGCGGC	BRDN00012	82	84	95	208	84	17
CCTCGCGTAC	BRDN00012	155	84	191	376	346	2
CCTCGCTATA	BRDN00012	483	403	524	783	1247	42
CCTCGCTGCA	BRDN00011	168	112	240	303	148	0
CCTCGCTGTA	BRDN00012	107	3	0	253	141	0
CCTCGCTGTC	BRDN00012	355	418	255	633	747	35
CCTCGGAAA	BRDN00011	290	156	1166	453	335	8
CCTCGGAACT	BRDN00011	356	40	118	721	342	0
CCTCGGAATC	BRDN00012	267	295	392	383	178	9
CCTCGGACTT	BRDN00011	134	121	20	143	94	27
CCTCGGAGG	BRDN00012	356	296	771	819	505	31
CCTCGGAGT	BRDN00011	342	195	715	594	544	35
CCTCGGAGTT	BRDN00011	406	388	1580	741	858	21
CCTCGGAGTT	BRDN00011	99	13	10	80	48	5
CCTCGGCAG	BRDN00011	121	102	1	186	221	0
CCTCGGCCAC	BRDN00011	174	169	677	407	334	0
CCTCGGCGCT	BRDN00011	189	217	599	477	532	3
CCTCGGCGCT	BRDN00012	79	148	12	165	154	49
CCTCGGCGTC	BRDN00011	132	96	211	437	397	19
CCTCGGCTCC	BRDN00012	279	241	454	576	565	13
CCTCGGCTGA	BRDN00012	167	131	52	275	320	4
CCTCGGGAA	BRDN00011	113	157	66	407	253	28
CCTCGGGAG	BRDN00012	64	92	3	161	247	1
CCTCGGGCG	BRDN00012	127	23	95	231	367	0
CCTCGGGCG	BRDN00012	457	401	677	688	725	34
CCTCGGGCTT	BRDN00012	461	533	513	704	829	27
CCTCGGGGA	BRDN00011	549	476	465	848	961	87
CCTCGGGGT	BRDN00012	135	91	132	217	206	4
CCTCGGTATT	BRDN00011	209	98	542	548	544	17
CCTCGGTCCT	BRDN00011	279	217	689	523	593	11
CCTCGGTGG	BRDN00012	64	57	28	364	406	5
CCTCGTACCC	BRDN00011	324	67	501	571	144	4
CCTCGTAGTC	BRDN00011	98	75	8	291	389	17
CCTCGTCCAG	BRDN00012	374	289	208	550	556	5
CCTCGTCCGA	BRDN00011	77	122	49	262	93	31
CCTCGTCCTC	BRDN00012	58	27	0	152	66	0
CCTCGTCGTG	BRDN00011	59	106	5	198	93	15
CCTCGTGAAC	BRDN00012	232	145	554	332	383	12
CCTCGTGACA	BRDN00012	283	203	296	556	256	0
CCTCGTTACC	BRDN00011	158	83	1	437	553	0
CCTCGTTGCC	BRDN00012	318	136	351	652	937	15
CCTCGTTTAT	BRDN00012	189	294	559	349	235	22
CCTCTAAAGT	BRDN00012	494	522	708	672	808	19
CCTCTAAATA	BRDN00011	130	130	96	242	289	0
CCTCTACAAC	BRDN00012	211	126	188	405	825	23
CCTCTACAAT	BRDN00011	186	84	0	256	122	0
CCTCTACAGG	BRDN00012	143	109	41334	247	119	594
CCTCTACAGT	BRDN00012	141	37	0	234	321	0
CCTCTACAT	BRDN00011	138	66	818	252	161	2
CCTCTACCAG	BRDN00011	128	116	158	238	559	29
CCTCTACCGC	BRDN00012	56	150	0	194	320	35
CCTCTACGAA	BRDN00012	99	139	996	156	112	16
CCTCTACGGA	BRDN00012	431	280	446	926	544	1
CCTCTAGAGT	BRDN00011	293	246	2349	590	959	26
CCTCTAGCAG	BRDN00012	257	140	21	434	582	4
CCTCTAGGAT	BRDN00012	151	106	680	488	318	0
CCTCTAGTTC	BRDN00012	136	98	0	219	237	0
CCTCTATCCA	BRDN00012	319	0	0	654	334	0
CCTCTATGAG	BRDN00011	156	233	202	445	461	24
CCTCTATGCA	BRDN00011	308	276	560	666	730	8
CCTCTATGCC	BRDN00011	182	119	2	450	761	1
CCTCTATTCC	BRDN00011	79	68	0	136	104	0
CCTCTCAATG	BRDN00012	372	339	343	507	482	0
CCTCTCACCG	BRDN00012	401	393	262	619	948	95
CCTCTCATAC	BRDN00012	185	199	365	380	378	6
CCTCTCCAAT	BRDN00012	249	274	1036	394	361	32
CCTCTCCACA	BRDN00012	86	74	3	162	230	0
CCTCTCCACC	BRDN00012	486	411	796	797	686	36
CCTCTCCAGG	BRDN00011	256	171	125	568	643	17
CCTCTCCATA	BRDN00011	256	121	15	351	300	10
CCTCTCCCAA	BRDN00012	411	531	1715	637	943	102
CCTCTCCCAT	BRDN00011	305	372	777	602	602	26
CCTCTCCCTA	BRDN00012	76	15	0	334	284	0
CCTCTCCCTA	BRDN00012	114	173	99	263	287	19
CCTCTCCGAT	BRDN00011	260	502	1230	385	679	63
CCTCTCCTCC	BRDN00012	137	122	24	311	241	7
CCTCTCGCCC	BRDN00012	70	17	35	281	53	0
CCTCTCGCCG	BRDN00012	203	241	273	390	161	47
CCTCTCGGAT	BRDN00011	548	307	284	1084	707	12
CCTCTCTGGC	BRDN00012	93	74	12	188	241	0
CCTCTCTTAC	BRDN00012	288	189	102	530	761	4
CCTCTGAACA	BRDN00011	259	214	111	485	523	26
CCTCTGACAA	BRDN00012	69	39	0	296	55	0
CCTCTGACAC	BRDN00012	126	182	109	283	373	0
CCTCTGAGG	BRDN00011	113	81	128	180	341	0
CCTCTGCAAG	BRDN00011	308	38	1	755	231	11
CCTCTGCACA	BRDN00012	153	141	68	381	227	89
CCTCTGCCAG	BRDN00011	351	315	2083	558	870	27
CCTCTGCCGT	BRDN00011	224	264	3769	337	803	82
CCTCTGCGAC	BRDN00011	425	309	859	741	791	27
CCTCTGCGCC	BRDN00011	167	96	11	300	425	0
CCTCTGCGCC	BRDN00012	197	119	8	448	304	0
CCTCTGCGTA	BRDN00012	161	123	0	300	289	0
CCTCTGCGTC	BRDN00011	319	275	212	648	661	17
CCTCTGCGTC	BRDN00011	191	128	40	462	526	21
CCTCTGCTAA	BRDN00012	454	391	2108	816	810	51
CCTCTGCTGC	BRDN00011	195	175	82	429	341	31
CCTCTGCTGC	BRDN00011	279	312	772	565	434	60
CCTCTGGAA	BRDN00012	561	566	576	917	1154	42
CCTCTGGACT	BRDN00012	382	211	368	718	1299	81
CCTCTGGGA	BRDN00012	152	169	94	371	378	0
CCTCTGGGG	BRDN00011	314	351	230	595	417	13
CCTCTGGTCT	BRDN00012	388	343	804	672	384	6
CCTCTGGTCT	BRDN00012	204	219	384	402	572	24
CCTCTGGTG	BRDN00011	69	126	163	224	243	14
CCTCTGTACA	BRDN00012	173	43	1	208	637	0
CCTCTGTCCA	BRDN00012	73	162	80	318	472	22
CCTCTGTGTA	BRDN00012	191	241	161	343	420	45
CCTCTGTGTT	BRDN00011	123	111	1	286	417	0
CCTCTGTTCT	BRDN00012	211	243	126	305	514	0
CCTCTTAAAC	BRDN00011	220	254	161	433	870	0
CCTCTTACCA	BRDN00012	235	125	174	504	401	11
CCTCTTACCG	BRDN00011	184	64	302	425	348	5
CCTCTTAGAC	BRDN00011	117	64	0	196	305	0
CCTCTTATAG	BRDN00011	209	98	336	410	289	20
CCTCTTATGG	BRDN00011	169	142	307	265	354	0
CCTCTTCATC	BRDN00011	135	179	58	265	332	0
CCTCTTCCAG	BRDN00012	564	466	253	821	685	35
CCTCTTCTCC	BRDN00011	161	189	104	393	524	13
CCTCTTCTCT	BRDN00012	336	222	140	467	499	58
CCTCTTCTTC	BRDN00011	181	280	2	334	584	13
CCTCTTCTTC	BRDN00012	273	236	587	534	483	93
CCTCTTGATC	BRDN00012	489	334	326	887	696	8
CCTCTTGCCA	BRDN00012	339	237	30	799	666	7
CCTCTTGGA	BRDN00012	21	14	0	490	87	0
CCTCTTGGCA	BRDN00011	308	248	493	497	556	57
CCTCTTGTCA	BRDN00011	211	263	760	414	298	136
CCTGAAAAA	BRDN00011	137	121	81	360	217	25
CCTGAAAACT	BRDN00012	0	0	0	66	16	0
CCTGAAAAG	BRDN00012	225	114	221	324	398	0
CCTGAAACG	BRDN00011	305	112	120	549	874	8
CCTGAAAGA	BRDN00012	216	268	349	433	426	33
CCTGAAAGG	BRDN00012	153	132	520	185	135	0
CCTGAAAGT	BRDN00011	69	0	0	282	30	0
CCTGAAATA	BRDN00012	231	121	162	417	573	33
CCTGAAATG	BRDN00011	161	130	158	333	299	0
CCTGAAATTC	BRDN00012	128	120	9914	224	165	171
CCTGAAATTC	BRDN00011	148	121	106	229	457	0
CCTGAACACC	BRDN00012	327	227	1148	733	554	56
CCTGAACAG	BRDN00011	180	99	225	262	352	0
CCTGAACCTG	BRDN00012	51	12	0	118	0	0
CCTGAACCTG	BRDN00012	114	34	14	209	31	0
CCTGAACCTG	BRDN00011	272	256	222	457	358	0
CCTGAACGCC	BRDN00011	105	103	1	191	252	0
CCTGAACGCT	BRDN00012	121	148	2530	223	245	29
CCTGAACGTA	BRDN00011	190	173	319	256	212	8
CCTGAACTAC	BRDN00012	393	344	329	584	573	3
CCTGAACTAC	BRDN00011	288	284	346	545	358	69
CCTGAACTCT	BRDN00011	277	364	1233	640	797	61
CCTGAAGAA	BRDN00011	154	88	164	310	296	10
CCTGAAGAG	BRDN00011	183	182	52	364	325	1
CCTGAAGAG	BRDN00012	110	97	47	128	227	11
CCTGAAGAT	BRDN00012	295	154	1268	455	311	52
CCTGAAGCA	BRDN00012	129	165	464	241	277	0
CCTGAAGCCC	BRDN00011	152	233	0	173	179	8
CCTGAAGCTA	BRDN00011	90	85	6	131	262	0
CCTGAAGGA	BRDN00012	197	231	3130	394	567	28
CCTGAAGGA	BRDN00012	89	56	1	155	246	0
CCTGAAGGC	BRDN00012	202	168	127	428	450	46
CCTGAAGGG	BRDN00012	130	111	33	300	294	24
CCTGAAGGG	BRDN00012	393	217	194	625	513	31
CCTGAAGTA	BRDN00011	70	15	0	280	139	0
CCTGAAGTA	BRDN00011	84	115	0	75	44	9
CCTGAAGTCA	BRDN00011	184	91	84	319	461	0
CCTGAAGTTC	BRDN00012	146	163	197	238	125	0
CCTGAATAAT	BRDN00011	116	50	523	361	213	9
CCTGAATATC	BRDN00011	2	0	0	177	23	0
CCTGAATCAC	BRDN00011	144	196	93	224	143	0
CCTGAATCTT	BRDN00012	122	54	11	250	111	5
CCTGAATGCC	BRDN00012	128	126	519	270	353	28
CCTGAATGTC	BRDN00011	109	86	0	176	134	0
CCTGAATTAC	BRDN00012	174	148	133	243	328	17
CCTGACAACT	BRDN00011	222	11	2	351	280	0
CCTGACAATC	BRDN00012	276	176	600	688	575	12
CCTGACAATC	BRDN00011	533	459	471	789	826	7
CCTGACACAC	BRDN00011	26	37	0	79	20	7
CCTGACACAT	BRDN00012	211	119	256	318	514	31
CCTGACACCA	BRDN00012	149	136	403	249	401	31
CCTGACAGAT	BRDN00012	106	8	0	227	280	25
CCTGACAGGT	BRDN00011	334	239	177	673	522	182
CCTGACAGTC	BRDN00012	403	410	605	703	546	146
CCTGACATCC	BRDN00012	311	485	112	722	671	32
CCTGACATGC	BRDN00012	402	293	64	636	615	146
CCTGACATGT	BRDN00012	255	239	16	559	767	18
CCTGACCAAA	BRDN00012	425	454	429	710	726	22
CCTGACCACC	BRDN00012	71	100	0	161	214	69
CCTGACCGGA	BRDN00012	366	370	518	618	709	47
CCTGACCGG	BRDN00012	64	53	30	155	151	3
CCTGACCGTA	BRDN00011	227	218	398	430	384	7
CCTGACCGTC	BRDN00012	98	0	0	298	0	0
CCTGACCTCG	BRDN00012	393	250	366	886	545	39

CCTGACCTG	BRDN00011	179	151	54	361	540	0
CCTGACCTG	BRDN00012	101	87	1415	193	118	18
CCTGACGCG	BRDN00011	170	143	13	204	257	0
CCTGACGGA	BRDN00011	286	379	124	602	981	4
CCTGACGGC	BRDN00011	211	137	611	329	414	66
CCTGACGTA	BRDN00011	53	12	0	192	250	0
CCTGACTAA	BRDN00012	79	1	1	765	365	0
CCTGACTATG	BRDN00011	240	336	345	498	797	0
CCTGACTCA	BRDN00012	135	131	56	247	146	7
CCTGACTCA	BRDN00012	132	133	0	213	143	0
CCTGACTCCA	BRDN00011	190	225	530	396	312	38
CCTGACTGG	BRDN00011	401	365	1168	534	288	10
CCTGACTTGT	BRDN00011	77	0	0	147	29	0
CCTGAGAAC	BRDN00011	79	2	0	749	260	0
CCTGAGAAC	BRDN00012	302	99	486	501	1068	30
CCTGAGAAG	BRDN00011	374	282	873	737	789	33
CCTGAGAAG	BRDN00011	212	231	125	436	385	25
CCTGAGAAG	BRDN00012	411	436	6101	689	1416	138
CCTGAGACA	BRDN00011	35	1	0	329	160	0
CCTGAGACA	BRDN00011	26	14	0	251	145	0
CCTGAGACA	BRDN00012	139	226	46	313	149	17
CCTGAGAGC	BRDN00012	352	224	267	481	336	21
CCTGAGAGT	BRDN00011	618	502	1210	731	372	75
CCTGAGAGT	BRDN00011	57	27	206	105	51	0
CCTGAGATA	BRDN00011	273	286	540	400	372	12
CCTGAGATA	BRDN00012	311	246	435	681	703	19
CCTGAGATG	BRDN00011	124	193	0	164	212	19
CCTGAGCAA	BRDN00011	188	84	9	158	339	11
CCTGAGCAC	BRDN00012	280	340	382	591	752	51
CCTGAGCAG	BRDN00011	38	30	7	115	56	0
CCTGAGCAT	BRDN00012	107	201	198	271	225	0
CCTGAGCATT	BRDN00012	385	375	61	708	798	0
CCTGAGCCA	BRDN00011	66	79	15	247	381	0
CCTGAGCCG	BRDN00011	44	5	499	586	390	0
CCTGAGCGA	BRDN00011	297	166	228	540	819	0
CCTGAGCGA	BRDN00011	353	152	435	529	442	28
CCTGAGCGC	BRDN00012	295	238	80	436	410	0
CCTGAGCTC	BRDN00012	460	464	383	677	740	178
CCTGAGCTT	BRDN00011	123	91	460	338	341	0
CCTGAGGAA	BRDN00011	233	358	71	448	420	15
CCTGAGGAG	BRDN00012	151	39	0	434	152	0
CCTGAGGAT	BRDN00012	138	152	95	174	77	9
CCTGAGGCT	BRDN00011	224	183	1187	405	227	15
CCTGAGGGC	BRDN00011	141	51	0	206	163	0
CCTGAGGGG	BRDN00011	180	79	810	288	377	47
CCTGAGGTG	BRDN00011	111	69	375	245	206	0
CCTGAGTCA	BRDN00011	131	162	20	260	267	19
CCTGAGTGA	BRDN00011	117	66	50	326	243	0
CCTGAGTTA	BRDN00012	214	238	700	510	191	36
CCTGATAAA	BRDN00011	94	145	113	305	219	0
CCTGATAAG	BRDN00012	111	82	117	269	91	0
CCTGATACTT	BRDN00011	152	0	0	501	159	0
CCTGATATA	BRDN00012	149	172	259	304	391	7
CCTGATATTT	BRDN00012	114	98	166	276	399	12
CCTGATCAAT	BRDN00012	47	15	0	143	210	0
CCTGATCATT	BRDN00011	112	180	0	247	344	11
CCTGATCCA	BRDN00012	265	336	428	691	798	11
CCTGATCCCC	BRDN00011	222	102	581	456	452	36
CCTGATCCG	BRDN00011	138	106	342	326	250	11
CCTGATCGA	BRDN00012	53	0	0	226	163	0
CCTGATCGT	BRDN00011	529	471	2543	783	536	131
CCTGATCGT	BRDN00012	103	101	264	136	106	18
CCTGATGAC	BRDN00011	135	99	0	187	216	0
CCTGATGAG	BRDN00011	359	0	2	825	398	0
CCTGATGCA	BRDN00012	312	439	909	800	842	7
CCTGATGCC	BRDN00012	22	0	0	594	85	0
CCTGATGCC	BRDN00011	330	303	454	489	727	9
CCTGATGGA	BRDN00012	100	30	1	479	254	0
CCTGATGTG	BRDN00011	114	107	303	198	136	9
CCTGATTAAT	BRDN00012	352	396	745	657	801	40
CCTGATTCAC	BRDN00012	69	71	63	148	55	44
CCTGATTGAT	BRDN00012	89	230	48	170	130	37
CCTGATTGT	BRDN00012	175	121	1	240	536	12
CCTGCAAAA	BRDN00011	121	111	0	233	318	2
CCTGCAAAA	BRDN00011	237	174	15	242	252	0
CCTGCAAAG	BRDN00012	445	270	286	754	525	0
CCTGCAAATT	BRDN00012	125	125	105	249	184	18
CCTGCAAGT	BRDN00012	314	292	257	567	689	98
CCTGCAATAT	BRDN00012	72	42	0	169	119	0
CCTGCAATTA	BRDN00012	259	414	686	404	642	79
CCTGCACAA	BRDN00012	263	309	1030	414	366	6
CCTGCACAAT	BRDN00012	279	35	0	462	145	0
CCTGCACACA	BRDN00011	128	77	549	258	72	63
CCTGCACAG	BRDN00012	76	98	65	133	93	8
CCTGCACATA	BRDN00011	86	31	65	164	139	0
CCTGCACCAA	BRDN00011	81	179	181	207	321	0
CCTGCACCCG	BRDN00012	117	74	1581	304	305	46
CCTGCACCTA	BRDN00011	94	103	67	107	86	0
CCTGCACGA	BRDN00012	211	108	97	316	175	12
CCTGCACGA	BRDN00012	129	77	185	367	315	6
CCTGCACGC	BRDN00012	300	277	967	486	284	21
CCTGCACGT	BRDN00011	160	297	173	438	419	29
CCTGCACTCG	BRDN00012	438	266	317	722	888	29
CCTGCACTG	BRDN00011	133	125	36	202	239	13
CCTGCAGAA	BRDN00012	215	141	135	402	600	38
CCTGCAGAA	BRDN00012	253	247	662	381	435	57
CCTGCAGAG	BRDN00012	74	107	39	136	210	3
CCTGCAGAG	BRDN00011	224	291	1108	420	397	106
CCTGCAGAG	BRDN00012	250	240	230	598	259	18
CCTGCAGCG	BRDN00012	79	75	27	163	99	50
CCTGCAGCG	BRDN00011	189	115	200	155	118	10
CCTGCAGCG	BRDN00011	66	29	0	195	112	6
CCTGCAGCG	BRDN00012	253	258	225	392	581	4
CCTGCAGCT	BRDN00011	163	113	23	215	425	12
CCTGCAGCT	BRDN00012	29	18	1	128	59	0
CCTGCAGCT	BRDN00012	225	99	118	341	441	7
CCTGCAGGA	BRDN00011	187	151	102	338	191	0
CCTGCAGGA	BRDN00011	97	76	85	306	466	35
CCTGCAGGC	BRDN00011	154	194	50	227	455	22
CCTGCAGGG	BRDN00012	302	419	2143	466	519	87
CCTGCAGGG	BRDN00011	205	220	15	309	235	94
CCTGCAGGG	BRDN00011	87	84	204	343	384	9
CCTGCAGGG	BRDN00011	97	111	0	249	412	17
CCTGCAGGT	BRDN00012	238	348	225	493	343	32
CCTGCAGGT	BRDN00011	221	266	139	428	320	66
CCTGCAGGT	BRDN00011	191	114	12	282	248	16
CCTGCAGTA	BRDN00012	200	146	127	405	574	0
CCTGCAGTA	BRDN00011	86	80	101	166	400	12
CCTGCAGTCT	BRDN00011	327	397	1298	656	791	26
CCTGCAGTG	BRDN00011	326	310	455	636	903	27
CCTGCAGTG	BRDN00012	260	196	96	353	236	18
CCTGCAGTTT	BRDN00012	455	494	1579	911	1190	81
CCTGCATATA	BRDN00011	450	319	206	723	598	12
CCTGCATCAC	BRDN00011	336	233	129	774	568	44
CCTGCATCAC	BRDN00011	183	147	0	318	406	13
CCTGCATCAG	BRDN00012	278	173	438	474	458	9
CCTGCATCAT	BRDN00012	312	397	281	639	852	66
CCTGCATCGC	BRDN00012	72	18	68	320	74	0
CCTGCATGCC	BRDN00012	246	187	591	469	400	67
CCTGCATTCT	BRDN00012	406	263	440	823	636	7
CCTGCATTGA	BRDN00012	167	138	313	398	516	0
CCTGCCAAAC	BRDN00011	532	522	3867	779	403	133
CCTGCCAAGA	BRDN00012	111	31	91	319	81	0
CCTGCCACCC	BRDN00011	338	193	43	706	1035	34
CCTGCCACCG	BRDN00012	211	125	1043	696	265	25
CCTGCCAGA	BRDN00011	55	34	0	239	143	0
CCTGCCAGAC	BRDN00011	461	380	1799	698	688	33
CCTGCCAGAC	BRDN00011	79	0	0	322	116	0
CCTGCCAGCC	BRDN00011	284	316	138	504	347	65
CCTGCCAGCT	BRDN00011	192	406	1192	431	273	41
CCTGCCAGG	BRDN00012	144	232	1900	230	234	61
CCTGCCAGG	BRDN00011	66	0	1	149	27	0
CCTGCCAGTC	BRDN00011	226	290	39	415	309	5
CCTGCCATAT	BRDN00011	82	63	3037	100	151	42
CCTGCCATCA	BRDN00011	160	210	0	343	540	0
CCTGCCATCA	BRDN00012	163	138	290	323	302	14
CCTGCCCAAA	BRDN00011	164	201	561	222	630	24
CCTGCCCAAA	BRDN00012	419	430	629	797	839	57
CCTGCCCAGA	BRDN00011	398	385	4201	722	704	119
CCTGCCCAGC	BRDN00012	400	19	2	769	298	0
CCTGCCCATG	BRDN00012	119	70	28	255	252	11
CCTGCCCCCA	BRDN00011	129	175	102	245	417	27
CCTGCCCCCC	BRDN00011	442	247	689	750	594	30
CCTGCCCCTA	BRDN00012	161	147	281	254	268	3
CCTGCCCTAC	BRDN00012	102	4	53	235	204	0
CCTGCCCTCG	BRDN00011	116	128	27	172	29	34
CCTGCCCTTA	BRDN00011	55	20	0	194	69	0
CCTGCCCTTC	BRDN00012	374	278	129	591	748	50
CCTGCCCTTT	BRDN00012	248	271	62	584	669	39
CCTGCCGCAC	BRDN00012	582	303	176	1005	886	19
CCTGCCGCCT	BRDN00012	309	288	380	687	857	12
CCTGCCGCGA	BRDN00012	119	116	0	323	181	0
CCTGCCGCTT	BRDN00012	177	136	18	394	226	41
CCTGCCGGA	BRDN00012	348	360	786	759	699	21
CCTGCCTAAA	BRDN00012	256	302	542	456	706	19
CCTGCCTAGC	BRDN00011	223	193	596	400	318	51
CCTGCCTCAC	BRDN00011	128	0	0	452	77	0
CCTGCCTCTT	BRDN00012	138	1	0	299	27	0
CCTGCCTGAC	BRDN00011	10	0	0	450	120	0
CCTGCGAATC	BRDN00012	169	212	94	397	368	69
CCTGCGACCA	BRDN00012	152	156	124	262	304	0
CCTGCGACCA	BRDN00012	226	203	41	364	457	6
CCTGCGATAC	BRDN00012	301	255	80	426	343	0
CCTGCGCACT	BRDN00012	296	159	300	560	241	30
CCTGCGCAGA	BRDN00012	44	40	1	62	50	0
CCTGCGCAG	BRDN00012	110	7	0	174	174	0
CCTGCGCAG	BRDN00012	395	545	333	731	561	16
CCTGCGCATT	BRDN00011	136	139	344	300	349	36
CCTGCGCCCA	BRDN00011	125	84	0	260	387	4
CCTGCGCCCC	BRDN00012	120	144	59	204	322	24
CCTGCGCCGC	BRDN00011	183	89	88	270	230	6
CCTGCGCCTC	BRDN00011	233	75	574	425	378	12
CCTGCGCGA	BRDN00012	82	37	0	138	125	0
CCTGCGCGC	BRDN00012	133	90	162	243	587	1
CCTGCGCGTA	BRDN00012	387	287	81	469	552	50
CCTGCGCGTC	BRDN00012	173	164	489	361	426	2
CCTGCGCTCC	BRDN00012	63	51	15	226	242	13
CCTGCGCTTC	BRDN00011	87	50	0	237	150	12
CCTGCGGAA	BRDN00012	306	67	15	574	183	2
CCTGCGGAC	BRDN00012	46	75	286	189	117	0
CCTGCGGAC	BRDN00012	70	31	610	153	6	7
CCTGCGGAG	BRDN00011	225	213	639	414	190	10
CCTGCGGCCA	BRDN00012	131	170	459	164	145	18
CCTGCGGCCA	BRDN00012	88	46	0	246	168	39
CCTGCGGCG	BRDN00011	330	263	895	524	608	23
CCTGCGGGA	BRDN00011	430	194	2095	734	285	5
CCTGCGGGG	BRDN00011	216	168	107	438	370	30
CCTGCGGTG	BRDN00012	175	362	115	438	281	76
CCTGCGTCAT	BRDN00012	147	83	168	227	114	39
CCTGCGTCG	BRDN00011	252	274	383	484	277	8
CCTGCGTGA	BRDN00011	461	321	345	736	906	5
CCTGCGTGC	BRDN00011	113	119	131	233	148	18
CCTGCGTGTA	BRDN00012	315	445	2814	634	462	15
CCTGCGTTCT	BRDN00011	139	239	201	218	227	69
CCTGCTACAA	BRDN00012	166	128	83	315	683	0
CCTGCTACGC	BRDN00012	89	79	37	185	207	1
CCTGCTACTC	BRDN00012	180	257	19	388	420	1
CCTGCTAGGA	BRDN00012	92	101	287	170	58	16
CCTGCTAGTC	BRDN00012	190	146	4128	317	443	22
CCTGCTATAT	BRDN00011	140	89	416	244	187	40
CCTGCTCAAA	BRDN00011	64	13	144	247	364	0
CCTGCTCAAC	BRDN00011	236	227	1090	396	431	0
CCTGCTCACA	BRDN00011	308	161	254	432	658	27
CCTGCTCACA	BRDN00012	51	25	25	266	149	0
CCTGCTCAGA	BRDN00011	58	127	113	203	253	0
CCTGCTCAGC	BRDN00012	156	155	228	235	219	31
CCTGCTCAGC	BRDN00011	183	163	32	296	221	11
CCTGCTCATA	BRDN00012	79	0	0	203	90	0
CCTGCTCATC	BRDN00012	297	81	55	816	687	0
CCTGCTCATG	BRDN00012	200	124	1088	327	336	29
CCTGCTCCAA	BRDN00011	60	53	20	139	174	31
CCTGCTCCAC	BRDN00012	120	117	60	319	336	3
CCTGCTCCAC	BRDN00011	90	51	1	208	389	0
CCTGCTCCAG	BRDN00012	106	89	104	280	167	40
CCTGCTCCAG	BRDN00011	194	141	195	269	227	24
CCTGCTCCAG	BRDN00012	533	400	497	961	973	59
CCTGCTCCGC	BRDN00011	236	203	220	362	298	0
CCTGCTCCTC	BRDN00012	482	346	1435	714	668	31
CCTGCTCGAC	BRDN00011	328	263	4971	511	508	67
CCTGCTCGTT	BRDN00011	222	123	153	497	294	9
CCTGCTGAAT	BRDN00012	87	51	55	138	185	0
CCTGCTGACA	BRDN00011	185	196	354	362	130	8
CCTGCTGACA	BRDN00011	260	224	141	610	817	0
CCTGCTGACC	BRDN00011	120	101	91	200	114	5
CCTGCTGAG	BRDN00012	172	70	0	330	161	0
CCTGCTGAGT	BRDN00011	89	81	105	109	80	39
CCTGCTGATC	BRDN00012	309	401	227	663	344	34
CCTGCTGATC	BRDN00011	298	320	55	420	787	84
CCTGCTGCAC	BRDN00011	251	195	270	497	243	7
CCTGCTGCCG	BRDN00012	325	307	316	601	400	9
CCTGCTGCTC	BRDN00012	108	140	98	231	292	13
CCTGCTGCTG	BRDN00011	306	226	1010	616	726	79
CCTGCTGCTG	BRDN00012	173	245	0	397	294	30
CCTGCTGCTG	BRDN00011	267	339	99	550	522	0
CCTGCTGGA	BRDN00011	431	386	334	931	1101	20
CCTGCTGGAT	BRDN00012	435	414	378	799	1688	42
CCTGCTGGG	BRDN00011	166	161	201	295	184	11
CCTGCTGGGT	BRDN00011	157	145	1	396	226	27
CCTGCTTCCA	BRDN00011	291	282	3520	482	449	9
CCTGCTTGCC	BRDN00012	316	287	2569	674	635	39
CCTGCTTGGT	BRDN00012	80	156	376	193	77	8
CCTGCTTTCT	BRDN00011	176	166	117	387	507	68
CCTGGAAAA	BRDN00012	59	60	0	136	192	0
CCTGGAAAG	BRDN00012	158	65	649	373	766	0
CCTGGAAAG	BRDN00012	256	193	58	449	704	80
CCTGGAACA	BRDN00012	144	112	294	262	273	3
CCTGGAACA	BRDN00011	161	63	51	328	151	0
CCTGGAACG	BRDN00012	291	390	1038	669	709	9
CCTGGAACG	BRDN00012	10	37	0	162	47	0
CCTGGAACTC	BRDN00011	166	9	12	685	880	0
CCTGGAAGC	BRDN00012	218	183	117	399	495	11
CCTGGAAGC	BRDN00011	118	57	1599	272	265	5
CCTGGAAGT	BRDN00011	48	94	29	110	202	0
CCTGGAATA	BRDN00011	67	98	0	161	140	0
CCTGGACAA	BRDN00012	106	119	238	221	431	11
CCTGGACAA	BRDN00012	76	12	0	276	221	0
CCTGGACAA	BRDN00011	34	129	341	153	20	33
CCTGGACACC	BRDN00011	150	171	1	374	519	14
CCTGGACAGT	BRDN00011	146	83	3	312	477	0
CCTGGACAGT	BRDN00011	111	141	166	270	327	26
CCTGGACATA	BRDN00011	183	337	278	262	446	135
CCTGGACATC	BRDN00012	325	384	358	615	609	26
CCTGGACCA	BRDN00012	89	59	6	228	290	0
CCTGGACCG	BRDN00012	370	334	712	598	507	75
CCTGGACCGT	BRDN00012	110	151	463	233	91	23
CCTGGACCTA	BRDN00012	258	234	97	312	829	21
CCTGGACCTA	BRDN00011	187	192	400	384	364	13
CCTGGACCTA	BRDN00012	113	71	165	114	268	14
CCTGGACGC	BRDN00012	162	126	2410	240	138	30
CCTGGACGC	BRDN00012	180	100	874	291	312	28
CCTGGACGC	BRDN00012	136	135	10	235	158	5
CCTGGACGG	BRDN00012	306	240	269	402	354	0
CCTGGACGG	BRDN00012	104	0	0	120	60	0
CCTGGACTG	BRDN00011	78	152	1499	266	276	42
CCTGGAGAA	BRDN00011	134	188	266	372	306	24
CCTGGAGAA	BRDN00011	298	288	1088	560	921	44
CCTGGAGAG	BRDN00011	225	204	214	395	243	14
CCTGGAGCA	BRDN00011	516	492	186	987	897	37
CCTGGAGCG	BRDN00011	189	303	558	283	439	56
CCTGGAGCT	BRDN00011	59	42	0	157	96	2
CCTGGAGCT	BRDN00012	33	0	0	189	88	0
CCTGGAGCT	BRDN00011	430	295	362	855	585	31
CCTGGAGGA	BRDN00012	162	143	56	270	329	50
CCTGGAGGA	BRDN00011	379	269	1229	670	433	48
CCTGGAGGA	BRDN00012	434	156	531	672	456	37
CCTGGAGGC	BRDN00011	322	401	1078	577	656	21
CCTGGAGGC	BRDN00011	165	165	164	321	548	0
CCTGGAGTA	BRDN00012	272	212	76	272	504	0
CCTGGAGTA	BRDN00011	112	120	10	237	285	0
CCTGGAGTA	BRDN00012	317	237	2188	480	632	23
CCTGGAGTA	BRDN00012	211	128	1	436	436	23
CCTGGATAA	BRDN00011	5	0	0	305	14	0
CCTGGATAA	BRDN00012	383	298	1202	777	890	23
CCTGGATACT	BRDN00011	172	120	94	259	168	8
CCTGGATACT	BRDN00012	236	270	69	574	472	58
CCTGGATATC	BRDN00012	125	150	153	216	227	28
CCTGGATATC	BRDN00012	185	319	176	316	400	58
CCTGGATCCC	BRDN00012	130	106	719	352	512	0
CCTGGATCCC	BRDN00012	138	96	503	222	779	0
CCTGGATCG	BRDN00012	252	351	550	618	394	48
CCTGGATCTC	BRDN00012	308	205	164	610	361	0
CCTGGATGA	BRDN00011	195	107	0	415	417	1
CCTGGATGA	BRDN00011	212	143	809	336	600	34
CCTGGATGC	BRDN00012	189	175	206	413	474	4
CCTGGATTAT	BRDN00011	137	171	133	227	315	0
CCTGGATTCC	BRDN00012	478	424	1279	724	2404	51
CCTGGCAAA	BRDN00011	133	160	455	175	190	0
CCTGGCAAA	BRDN00012	313	269	663	737	643	0
CCTGGCAAGT	BRDN00011	256	237	311	339	272	21
CCTGGCACCC	BRDN00011	357	219	252	660	378	0
CCTGGCACG	BRDN00012	104	162	9	142	391	43
CCTGGCACG	BRDN00012	43	62	0	158	221	0
CCTGGCAGA	BRDN00011	210	63	70	512	264	0
CCTGGCAGA	BRDN00011	267	335	65	433	281	106
CCTGGCAGG	BRDN00012	439	308	908	964	689	45
CCTGGCAGG	BRDN00011	60	50	0	246	279	0
CCTGGCATG	BRDN00012	315	0	2	702	148	0
CCTGGCATG	BRDN00012	101	89	8	219	428	19
CCTGGCCAA	BRDN00012	150	67	2	260	149	37
CCTGGCCAAC	BRDN00012	80	144	0	262	164	14
CCTGGCCAAC	BRDN00011	120	66	38	306	205	4
CCTGGCCACA	BRDN00012	353	298	378	640	768	15
CCTGGCCAG	BRDN00012	125	243	9	258	230	65
CCTGGCCAG	BRDN00012	168	171	53	377	375	36
CCTGGCCAG	BRDN00011	259	192	577	504	515	12
CCTGGCCATT	BRDN00011	189	256	34	471	512	19
CCTGGCCCA	BRDN00012	377	269	155	621	564	14
CCTGGCCCA	BRDN00012	133	150	95	240	191	12
CCTGGCCCCC	BRDN00012	110	43	9	158	114	0
CCTGGCCGA	BRDN00011	101	29	6	203	199	0
CCTGGCCGG	BRDN00011	323	327	564	620	710	24
CCTGGCCTAC	BRDN00012	107	83	321	215	288	0
CCTGGCCTAC	BRDN00012	409	217	3433	548	330	33
CCTGGCCTCC	BRDN00011	62	10	0	95	122	0
CCTGGCCTGT	BRDN00011	93	126	112	233	126	11
CCTGGCCTTA	BRDN00012	143	59	241	319	511	5
CCTGGCGAG	BRDN00012	319	350	378	572	426	1
CCTGGCGAG	BRDN00011	175	74	10	351	347	1
CCTGGCGAG	BRDN00012	287	239	5	441	459	3
CCTGGCGCG	BRDN00011	224	270	50	570	407	0
CCTGGCGTC	BRDN00012	87	71	110	121	258	4
CCTGGCTAA	BRDN00012	508	467	562	687	696	32
CCTGGCTAA	BRDN00012	361	303	2316	439	402	31
CCTGGCTAA	BRDN00012	304	300	410	711	1093	5
CCTGGCTCTC	BRDN00011	60	69	0	237	212	54
CCTGGCTGC	BRDN00011	88	54	7	342	171	6
CCTGGCTGCT	BRDN00012	231	0	19	476	332	0
CCTGGCTGG	BRDN00011	305	207	1796	693	554	50
CCTGGCTTG	BRDN00012	259	267	9	552	444	26
CCTGGCTTG	BRDN00012	400	311	422	688	830	33
CCTGGCTTGT	BRDN00011	274	142	405	395	411	37
CCTGGGAAC	BRDN00011	136	145	0	263	379	0
CCTGGGAAG	BRDN00012	343	274	236	574	899	26
CCTGGGAAG	BRDN00012	69	11	554	321	66	7
CCTGGGAAT	BRDN00012	119	109	225	209	86	15
CCTGGGAAT	BRDN00012	55	141	36	135	538	1
CCTGGGACA	BRDN00012	201	53	10	568	611	0
CCTGGGAGA	BRDN00012	178	185	0	250	228	20
CCTGGGAGC	BRDN00011	131	142	37	388	815	38
CCTGGGAGC	BRDN00012	200	180	77	419	403	16
CCTGGGAGG	BRDN00012	78	57	9	224	58	0
CCTGGGATA	BRDN00011	181	222	206	393	682	0
CCTGGGATC	BRDN00012	184	135	78	326	490	58
CCTGGGCAA	BRDN00012	135	159	253	161	144	81
CCTGGGCAA	BRDN00011	353	337	693	562	832	0
CCTGGGCAC	BRDN00011	166	140	350	364	225	5
CCTGGGCAC	BRDN00012	148	76	46	524	267	0
CCTGGGCAG	BRDN00012	212	33	0	506	161	0
CCTGGGCAG	BRDN00012	200	126	229	498	696	10
CCTGGGCCA	BRDN00011	85	76	34	122	190	12
CCTGGGCCA	BRDN00012	119	127	528	288	403	0
CCTGGGCCC	BRDN00012	281	321	933	422	589	61
CCTGGGCTG	BRDN00011	93	69	0	165	120	24
CCTGGGGAT	BRDN00011	117	211	194	202	45	17
CCTGGGGCA	BRDN00011	366	292	737	578	778	85
CCTGGGGCA	BRDN00011	128	98	625	227	486	68
CCTGGGGCA	BRDN00011	342	257	347	565	396	16
CCTGGGGGA	BRDN00012	146	171	137	238	390	7
CCTGGGGTA	BRDN00012	185	211	105	349	449	54
CCTGGGGTC	BRDN00012	166	172	52	404	517	10
CCTGGGTAA	BRDN00012	463	453	833	754	829	104
CCTGGGTAC	BRDN00012	120	129	0	279	185	0
CCTGGGTCA	BRDN00012	100	105	110	176	44	0
CCTGGGTCC	BRDN00011	57	126	1010	241	104	25
CCTGGGTCG	BRDN00011	166	135	258	344	445	0
CCTGGGTCT	BRDN00012	78	28	0	124	169	2
CCTGGGTGA	BRDN00012	46	56	574	169	75	0
CCTGGGTGT	BRDN00011	167	251	196	318	305	7
CCTGGGTTC	BRDN00012	149	177	1	401	288	20
CCTGGGTTG	BRDN00012	152	108	52	174	142	53
CCTGGTAAA	BRDN00012	387	256	1449	486	666	60
CCTGGTAAG	BRDN00012	203	78	296	256	263	18
CCTGGTAAG	BRDN00011	90	113	17	178	153	13
CCTGGTAAT	BRDN00012	218	104	124	393	500	0
CCTGGTACA	BRDN00011	136	265	513	279	282	4
CCTGGTACCT	BRDN00011	191	112	393	327	361	54
CCTGGTAGA	BRDN00011	266	184	285	482	872	36
CCTGGTAGC	BRDN00012	215	194	739	355	320	8
CCTGGTAGC	BRDN00012	112	129	201	291	387	0
CCTGGTAGG	BRDN00012	80	111	38	208	39	15
CCTGGTATA	BRDN00012	255	188	532	565	394	26
CCTGGTATA	BRDN00011	298	353	114	520	334	40
CCTGGTATC	BRDN00012	194	185	92	441	241	30
CCTGGTCAA	BRDN00011	200	115	22	380	488	36
CCTGGTCAC	BRDN00012	113	70	60	189	206	0
CCTGGTCAG	BRDN00011	77	17	0	117	27	0
CCTGGTCAT	BRDN00012	149	72	301	199	218	5
CCTGGTCAT	BRDN00012	137	186	62	412	260	26
CCTGGTCCA	BRDN00012	210	252	0	443	360	6
CCTGGTCCG	BRDN00012	138	0	0	251	58	0
CCTGGTCCG	BRDN00011	256	217	846	564	365	16
CCTGGTCGA	BRDN00012	188	213	212	266	275	17
CCTGGTCTA	BRDN00011	198	193	784	401	378	51
CCTGGTCTTG	BRDN00011	206	246	260	499	260	44
CCTGGTGAA	BRDN00012	130	53	1159	219	163	20
CCTGGTGAG	BRDN00011	207	151	116	610	380	18
CCTGGTGCA	BRDN00011	67	40	45	124	131	0
CCTGGTGCCT	BRDN00011	111	0	0	214	55	0
CCTGGTGCG	BRDN00012	137	19	0	199	7	0
CCTGGTGCG	BRDN00011	98	79	0	118	422	0
CCTGGTGCG	BRDN00011	35	43	85	172	267	0
CCTGGTGCG	BRDN00011	221	230	681	539	473	31
CCTGGTGCT	BRDN00011	369	362	515	454	446	16
CCTGGTGGT	BRDN00011	46	61	0	129	9	0
CCTGGTGGT	BRDN00011	195	194	242	427	171	22
CCTGGTGGT	BRDN00012	33	26	0	332	0	0
CCTGGTGGT	BRDN00011	112	126	0	136	192	11
CCTGGTGTC	BRDN00011	82	71	78	188	299	7
CCTGGTGTCT	BRDN00011	285	294	283	722	315	15
CCTGGTTAT	BRDN00011	235	120	254	436	300	11
CCTGGTTCCA	BRDN00011	336	244	791	456	822	46
CCTGGTTGG	BRDN00012	362	355	409	761	726	63
CCTGGTTTCA	BRDN00011	109	50	9	167	123	0
CCTGGTTTCC	BRDN00012	180	225	474	235	129	13
CCTGGTTTGT	BRDN00011	137	93	467	274	351	10
CCTGTAAAAT	BRDN00012	53	3	150	138	75	6
CCTGTAAAG	BRDN00012	186	37	0	211	227	0
CCTGTAATTG	BRDN00011	278	230	27	489	663	19
CCTGTACAA	BRDN00012	143	137	0	317	249	0
CCTGTACACA	BRDN00012	136	193	33	274	183	9
CCTGTACAG	BRDN00012	118	0	0	490	1	0
CCTGTACATC	BRDN00012	137	0	0	308	189	0
CCTGTACCAC	BRDN00011	357	285	255	518	640	0
CCTGTACCAT	BRDN00011	187	272	540	335	477	9
CCTGTACCCA	BRDN00012	34	34	0	113	133	0

CCTGTACCCG	BRDN00012	375	268	1058	777	477	214
CCTGTACCCG	BRDN00012	159	99	3	162	169	1
CCTGTACGCT	BRDN00012	40	0	0	932	200	0
CCTGTACGG	BRDN00011	194	152	99	340	213	20
CCTGTACGG	BRDN00012	240	163	80	595	761	5
CCTGTACGG	BRDN00012	366	199	418	526	605	0
CCTGTACTTC	BRDN00012	471	537	609	689	918	29
CCTGTAGAG	BRDN00011	148	17	1	330	79	0
CCTGTAGAT	BRDN00012	107	84	7	217	40	65
CCTGTAGCA	BRDN00011	109	121	0	243	201	0
CCTGTAGCA	BRDN00012	228	115	778	365	116	6
CCTGTAGCC	BRDN00011	138	130	284	157	170	9
CCTGTAGTC	BRDN00012	133	78	151	214	75	11
CCTGTATATC	BRDN00011	152	196	529	317	384	13
CCTGTATCAC	BRDN00012	60	73	0	164	99	6
CCTGTATGA	BRDN00012	188	92	7	220	263	0
CCTGTATTG	BRDN00012	102	40	598	151	107	6
CCTGTCACCA	BRDN00011	194	234	1168	385	239	71
CCTGTCACTG	BRDN00012	384	215	237	760	723	10
CCTGTCAGA	BRDN00012	24	35	1	338	151	0
CCTGTCAGA	BRDN00012	195	187	259	268	281	0
CCTGTCATG	BRDN00011	96	46	0	92	229	12
CCTGTCCAG	BRDN00012	451	250	121	767	717	15
CCTGTCCATT	BRDN00012	113	106	136	194	231	4
CCTGTCCCAC	BRDN00012	124	31	0	355	377	0
CCTGTCCCGC	BRDN00012	101	95	0	224	184	20
CCTGTCCGA	BRDN00012	206	15	0	735	631	0
CCTGTCCTTT	BRDN00012	226	217	351	560	457	28
CCTGTCGAA	BRDN00011	141	118	216	293	345	0
CCTGTCGCCC	BRDN00012	103	109	367	156	82	7
CCTGTCGCTA	BRDN00012	373	299	729	805	568	52
CCTGTCGCTC	BRDN00011	385	367	457	671	941	64
CCTGTCGGA	BRDN00012	319	214	2158	629	695	33
CCTGTCGGA	BRDN00011	164	160	624	403	236	53
CCTGTCGGC	BRDN00011	408	250	126	513	509	5
CCTGTCTAG	BRDN00011	102	76	285	233	275	0
CCTGTCTCGA	BRDN00012	376	273	859	511	807	34
CCTGTCTCGC	BRDN00012	269	218	771	425	667	11
CCTGTCTTCA	BRDN00012	90	125	411	214	233	0
CCTGTGAAG	BRDN00012	130	5	0	473	11	0
CCTGTGACA	BRDN00011	91	121	0	123	189	0
CCTGTGACCT	BRDN00012	151	145	498	399	520	3
CCTGTGACT	BRDN00011	113	64	173	232	260	0
CCTGTGAGA	BRDN00011	90	102	86	179	54	0
CCTGTGAGC	BRDN00012	471	464	316	957	821	30
CCTGTGAGG	BRDN00011	30	4	0	77	86	0
CCTGTGAGG	BRDN00012	189	182	357	388	323	3
CCTGTGATA	BRDN00012	265	62	3	643	550	0
CCTGTGATG	BRDN00012	101	125	110	190	267	34
CCTGTGATG	BRDN00011	281	220	87	776	750	8
CCTGTGCAA	BRDN00012	230	113	379	315	180	0
CCTGTGCAC	BRDN00011	359	232	686	618	548	35
CCTGTGCAC	BRDN00011	397	258	736	566	439	82
CCTGTGCACT	BRDN00011	196	154	141	398	332	3
CCTGTGCAG	BRDN00012	170	89	45	505	355	23
CCTGTGCCA	BRDN00011	65	82	156	265	127	16
CCTGTGCCA	BRDN00011	79	17	1	244	58	0
CCTGTGCCCA	BRDN00011	140	174	51	281	93	0
CCTGTGCCG	BRDN00011	229	273	692	443	273	100
CCTGTGCCTG	BRDN00012	316	118	374	651	593	16
CCTGTGCCTG	BRDN00012	165	110	40	151	167	14
CCTGTGCGA	BRDN00012	424	527	1008	852	479	24
CCTGTGCTG	BRDN00012	133	245	42	245	668	0
CCTGTGCTG	BRDN00011	119	66	151	125	341	22
CCTGTGCTTG	BRDN00012	126	69	49	258	379	0
CCTGTGGAA	BRDN00012	66	4	1	282	158	0
CCTGTGGAA	BRDN00012	3	0	0	167	0	0
CCTGTGGAT	BRDN00011	153	68	0	262	257	16
CCTGTGGCA	BRDN00012	276	82	65	241	333	60
CCTGTGGCA	BRDN00011	143	54	79	213	245	0
CCTGTGGCC	BRDN00012	66	33	37	130	62	215
CCTGTGGCG	BRDN00011	128	51	190	355	160	24
CCTGTGGCG	BRDN00011	275	265	1393	393	652	50
CCTGTGGCT	BRDN00012	295	178	413	557	631	5
CCTGTGGGG	BRDN00012	155	95	50	278	207	0
CCTGTGGGG	BRDN00011	172	320	190	362	328	56
CCTGTGGGG	BRDN00012	259	243	2587	304	395	172
CCTGTGGGG	BRDN00011	197	124	310	325	512	12
CCTGTGGTA	BRDN00012	348	313	143	587	743	34
CCTGTGTAT	BRDN00011	132	161	196	313	216	37
CCTGTGTCCC	BRDN00011	315	10	1	553	441	0
CCTGTGTCTG	BRDN00012	241	216	241	440	524	0
CCTGTGTGG	BRDN00012	372	181	897	651	426	18
CCTGTGTGG	BRDN00011	139	113	49	330	500	39
CCTGTGTTG	BRDN00012	325	269	146	402	275	38
CCTGTGTTTA	BRDN00011	74	0	0	182	44	0
CCTGTTAAG	BRDN00011	287	173	232	541	252	27
CCTGTTACAA	BRDN00012	220	269	140	474	410	24
CCTGTTACCT	BRDN00011	79	75	0	286	162	0
CCTGTTAGA	BRDN00012	88	74	46	207	124	0
CCTGTTAGG	BRDN00012	105	112	256	267	118	24
CCTGTTATCC	BRDN00011	159	126	10	246	285	8
CCTGTTCAAG	BRDN00012	176	88	707	211	71	16
CCTGTTCATC	BRDN00011	42	26	18	192	298	10
CCTGTTCCCA	BRDN00012	154	180	5	290	326	21
CCTGTTCCCA	BRDN00011	235	348	550	422	731	32
CCTGTTCCTA	BRDN00011	112	97	0	211	154	16
CCTGTTGAA	BRDN00012	302	454	120	466	517	55
CCTGTTGAA	BRDN00012	145	0	0	335	287	0
CCTGTTGACC	BRDN00011	299	197	25	529	132	5
CCTGTTGACT	BRDN00012	327	370	1181	506	641	150
CCTGTTGAG	BRDN00011	305	290	1557	547	573	58
CCTGTTGATT	BRDN00011	65	71	90	191	61	12
CCTGTTGCTA	BRDN00012	261	260	8	388	332	9
CCTGTTGGA	BRDN00012	119	176	120	355	1509	8
CCTGTTGTCA	BRDN00011	347	286	155	708	401	19
CCTGTTTCAC	BRDN00012	105	213	1203	324	90	87
CCTGTTTCTA	BRDN00011	169	86	159	407	334	19
CCTGTTTGA	BRDN00012	116	202	75	366	331	35
CCTGTTTGTA	BRDN00012	98	192	115	396	265	22
CCTTAAAATC	BRDN00011	340	268	389	656	785	13
CCTTAAAGA	BRDN00011	114	135	105	288	286	11
CCTTAAAGA	BRDN00011	117	221	910	281	352	68
CCTTAAAGA	BRDN00011	134	112	1047	309	580	4
CCTTAAAGA	BRDN00011	268	216	12	309	264	44
CCTTAAAGA	BRDN00011	115	152	590	175	220	28
CCTTAAATAA	BRDN00012	201	286	541	428	276	0
CCTTAAATAC	BRDN00012	200	217	23	364	551	30
CCTTAAATGA	BRDN00011	269	70	187	648	559	5
CCTTAAATGC	BRDN00011	110	58	1328	318	396	17
CCTTAACAGA	BRDN00012	132	0	111	230	151	0
CCTTAACAGC	BRDN00012	167	0	0	474	330	0
CCTTAAGAA	BRDN00012	256	210	551	554	372	11
CCTTAAGCTG	BRDN00012	307	301	1126	392	389	27
CCTTAAGGT	BRDN00012	173	139	211	296	351	20
CCTTAAGTG	BRDN00012	261	318	1358	594	755	84
CCTTAATAGA	BRDN00011	128	15	0	196	145	0
CCTTAATCTG	BRDN00011	451	246	301	587	683	42
CCTTACAAGA	BRDN00011	147	180	235	276	223	68
CCTTACACAA	BRDN00012	260	136	148	455	590	0
CCTTACAGGT	BRDN00011	249	286	257	494	540	19
CCTTACATAC	BRDN00011	376	317	377	656	841	45
CCTTACCACT	BRDN00012	180	40	194	449	414	0
CCTTACCAGG	BRDN00011	230	193	394	572	662	8
CCTTACCCTA	BRDN00012	144	171	0	324	200	0
CCTTACCCTA	BRDN00012	311	261	65	636	681	25
CCTTACCGCT	BRDN00011	172	296	1705	393	224	66
CCTTACCTAC	BRDN00011	328	514	8282	518	673	288
CCTTACCTGA	BRDN00011	258	116	33	537	421	12
CCTTACCTGT	BRDN00011	264	323	420	516	456	16
CCTTACCTTC	BRDN00011	162	92	209	342	492	0
CCTTACCTTG	BRDN00012	138	1	0	244	302	0
CCTTACGAG	BRDN00012	101	118	74	127	39	6
CCTTACGGG	BRDN00012	296	167	329	398	817	22
CCTTACTCTC	BRDN00012	187	233	67	414	311	4
CCTTACTCTT	BRDN00012	133	103	2	282	425	12
CCTTACTGAT	BRDN00012	346	320	42	627	289	93
CCTTACTGTC	BRDN00011	266	192	430	463	328	38
CCTTAGAAC	BRDN00012	130	118	281	282	308	5
CCTTAGAAG	BRDN00012	96	67	80	204	262	0
CCTTAGACCC	BRDN00011	150	45	72	215	253	0
CCTTAGACTG	BRDN00012	201	250	504	446	434	19
CCTTAGAGA	BRDN00011	146	85	0	300	239	0
CCTTAGATCG	BRDN00011	21	0	0	174	27	0
CCTTAGATG	BRDN00011	136	41	0	165	156	0
CCTTAGCAA	BRDN00011	138	38	27	219	237	0
CCTTAGCGA	BRDN00012	289	286	737	457	528	22
CCTTAGCTAG	BRDN00012	324	419	629	540	413	27
CCTTAGCTGT	BRDN00011	186	176	203	296	184	0
CCTTAGGGT	BRDN00012	584	441	235	929	1077	10
CCTTAGGTT	BRDN00012	153	307	93	428	389	9
CCTTAGTAA	BRDN00011	307	265	312	629	856	15
CCTTAGTCAG	BRDN00012	90	208	53	196	644	9
CCTTATAATG	BRDN00012	99	150	394	206	450	25
CCTTATCAGT	BRDN00012	194	290	209	398	424	14
CCTTATCCAA	BRDN00011	93	70	0	193	621	0
CCTTATCCTA	BRDN00012	228	297	468	448	380	0
CCTTATCTAT	BRDN00011	112	88	328	158	158	21
CCTTATCTCT	BRDN00012	470	315	52	987	600	11
CCTTATCTTG	BRDN00011	287	120	281	642	345	0
CCTTATGAAT	BRDN00012	377	208	201	660	641	13
CCTTATGCAA	BRDN00012	173	106	188	288	117	26
CCTTATGGA	BRDN00012	336	366	843	556	745	52
CCTTATGGCT	BRDN00012	118	47	101	191	173	9
CCTTATTACA	BRDN00012	158	86	1130	189	370	47
CCTTATTGCA	BRDN00012	350	299	76	590	503	25
CCTTCAAACA	BRDN00012	272	223	141	335	499	12
CCTTCAAACA	BRDN00012	429	596	1088	723	685	106
CCTTCAAATT	BRDN00012	122	232	126	327	211	8
CCTTCAACAA	BRDN00011	103	97	99	401	190	29
CCTTCAACGA	BRDN00012	228	159	71	472	465	3
CCTTCAAGAT	BRDN00011	266	275	135	477	554	13
CCTTCAAGG	BRDN00011	154	65	0	208	137	7
CCTTCAAGG	BRDN00012	377	291	518	555	598	37
CCTTCAATAA	BRDN00011	148	105	423	423	357	24
CCTTCAATAT	BRDN00011	103	43	0	187	146	0
CCTTCAATCT	BRDN00011	267	216	327	492	531	1
CCTTCAATGC	BRDN00011	210	7	0	570	211	0
CCTTCAATGT	BRDN00011	168	178	40	211	290	19
CCTTCACAAT	BRDN00011	161	122	2072	310	124	40
CCTTCACAGA	BRDN00012	158	134	292	310	398	15
CCTTCACCGT	BRDN00011	96	45	255	133	37	5
CCTTCACTAG	BRDN00011	345	120	647	447	546	41
CCTTCACTGA	BRDN00011	95	115	0	319	563	23
CCTTCACTTC	BRDN00011	319	355	256	521	590	6
CCTTCAGAA	BRDN00012	321	157	16	561	833	21
CCTTCAGAA	BRDN00011	171	131	243	445	339	29
CCTTCAGAA	BRDN00012	209	78	55	501	193	15
CCTTCAGACT	BRDN00011	166	65	0	272	100	0
CCTTCAGAGT	BRDN00011	261	229	83	625	582	9
CCTTCAGCAG	BRDN00012	206	298	1026	432	597	3
CCTTCAGCAG	BRDN00012	308	23	219	789	934	4
CCTTCAGCAT	BRDN00012	296	226	59	599	557	0
CCTTCAGCGT	BRDN00012	368	348	1083	647	414	24
CCTTCAGCTA	BRDN00012	124	112	101	277	263	0
CCTTCAGGCT	BRDN00012	483	324	413	745	516	32
CCTTCAGGG	BRDN00011	321	271	657	653	661	36
CCTTCAGGG	BRDN00011	138	82	10	182	178	0
CCTTCAGTCG	BRDN00011	109	92	222	368	261	0
CCTTCATACA	BRDN00012	466	608	1373	899	837	58
CCTTCATAGA	BRDN00012	247	238	773	570	416	388
CCTTCATATG	BRDN00012	261	226	279	683	535	40
CCTTCATCAG	BRDN00011	169	217	91	349	270	0
CCTTCATCCG	BRDN00012	217	286	523	336	169	14
CCTTCATCTA	BRDN00011	209	102	1000	211	99	12
CCTTCATGG	BRDN00011	257	263	840	538	528	12
CCTTCATGG	BRDN00012	43	0	0	215	58	0
CCTTCATGG	BRDN00011	412	313	972	691	674	31
CCTTCATTGT	BRDN00012	240	154	829	278	271	2
CCTTCCAAAC	BRDN00012	308	135	151	358	426	14
CCTTCCAAGC	BRDN00012	122	85	12	288	245	0
CCTTCCACAC	BRDN00011	154	75	39	237	267	33
CCTTCCAGAA	BRDN00011	30	0	0	207	31	0
CCTTCCAGG	BRDN00011	151	74	0	288	459	0
CCTTCCCACA	BRDN00012	115	127	125	185	213	42
CCTTCCCAGC	BRDN00012	343	291	214	735	964	34
CCTTCCCAGT	BRDN00012	100	75	477	161	68	18
CCTTCCCCCA	BRDN00012	77	44	0	129	55	0
CCTTCCCCCA	BRDN00011	103	37	0	223	150	0
CCTTCCCGGG	BRDN00012	307	297	352	535	450	13
CCTTCCCGGG	BRDN00011	211	196	52	570	419	27
CCTTCCGAAG	BRDN00011	126	122	314	337	387	0
CCTTCCGACC	BRDN00011	414	391	2458	759	817	34
CCTTCCGATC	BRDN00012	108	0	2	587	768	0
CCTTCCGATT	BRDN00011	206	242	245	386	548	17
CCTTCCGGTT	BRDN00012	200	139	36	228	65	0
CCTTCCTAAA	BRDN00012	230	247	95	451	385	14
CCTTCCTAAC	BRDN00011	285	219	226	648	739	9
CCTTCCTCAA	BRDN00011	304	205	623	568	653	0
CCTTCCTCAC	BRDN00012	420	229	1888	692	583	35
CCTTCCTCAC	BRDN00011	149	33	106	252	175	0
CCTTCCTCTA	BRDN00011	329	293	190	668	832	11
CCTTCCTGAA	BRDN00012	313	213	717	567	571	6
CCTTCCTGAG	BRDN00011	132	189	1217	303	184	19
CCTTCCTGCC	BRDN00012	331	221	76	747	520	0
CCTTCCTGCG	BRDN00011	257	266	10	282	311	9
CCTTCCTGGC	BRDN00011	363	360	233	555	619	54
CCTTCGAAG	BRDN00011	385	188	61	765	480	22
CCTTCGAAG	BRDN00011	334	254	475	862	652	78
CCTTCGAATG	BRDN00012	527	771	1895	657	668	180
CCTTCGACAA	BRDN00012	230	262	60	532	419	0
CCTTCGACTT	BRDN00011	121	150	903	161	190	5
CCTTCGAGT	BRDN00012	193	252	5	273	381	23
CCTTCGATCC	BRDN00011	165	163	408	228	290	29
CCTTCGATGA	BRDN00012	185	197	1521	357	413	83
CCTTCGATGC	BRDN00012	296	304	286	730	1266	5
CCTTCGCGCG	BRDN00012	234	530	1323	383	362	109
CCTTCGGACC	BRDN00011	231	57	32	616	152	0
CCTTCGGATT	BRDN00011	184	240	3	416	233	11
CCTTCGGCCA	BRDN00012	82	41	14	687	527	15
CCTTCGGGA	BRDN00011	179	127	130	479	524	5
CCTTCGTCCT	BRDN00012	294	318	90	614	1062	10
CCTTCTACTA	BRDN00011	198	175	1	528	372	11
CCTTCTAGCT	BRDN00012	352	332	12	445	552	21
CCTTCTATAA	BRDN00011	353	323	365	620	635	31
CCTTCTCAAA	BRDN00011	107	102	2231	315	224	28
CCTTCTCAAA	BRDN00011	78	100	19	191	179	0
CCTTCTCCGA	BRDN00011	219	149	23	542	375	9
CCTTCTCCTA	BRDN00012	252	233	1337	700	444	21
CCTTCTCTAG	BRDN00011	344	346	324	630	540	10
CCTTCTCTAG	BRDN00011	561	539	6290	1077	738	88
CCTTCTCTGG	BRDN00012	14	0	0	266	158	0
CCTTCTCTTC	BRDN00012	178	171	121	455	444	23
CCTTCTGACT	BRDN00012	357	222	804	557	383	36
CCTTCTGATA	BRDN00011	100	176	17	336	152	2
CCTTCTGCAG	BRDN00011	37	0	0	59	122	0
CCTTCTGCGT	BRDN00012	203	150	494	302	201	4
CCTTCTGCTT	BRDN00012	119	166	122	400	420	18
CCTTCTGGAT	BRDN00012	65	91	9	184	126	0
CCTTCTGGCT	BRDN00011	486	402	1395	699	921	24
CCTTCTGGG	BRDN00012	248	82	925	535	512	55
CCTTCTGGG	BRDN00012	316	331	40	643	601	9
CCTTCTGGG	BRDN00012	57	57	0	520	372	0
CCTTCTTAGT	BRDN00012	159	12	2	692	442	0
CCTTCTTATT	BRDN00012	280	41	1	467	423	0
CCTTCTTCAG	BRDN00011	494	482	261	869	689	40
CCTTCTTCCG	BRDN00012	220	303	623	289	299	31
CCTTCTTCTT	BRDN00011	531	506	855	641	644	124
CCTTCTTCTT	BRDN00011	203	131	159	418	257	0
CCTTCTTGGA	BRDN00012	425	330	272	577	1156	30
CCTTCTTGTG	BRDN00011	305	583	1639	628	359	148
CCTTGAAAC	BRDN00012	121	49	0	173	1	0
CCTTGAAAT	BRDN00012	260	246	2473	574	358	33
CCTTGAACA	BRDN00011	98	104	429	376	200	0
CCTTGAACA	BRDN00012	265	360	437	666	626	56
CCTTGAACTT	BRDN00012	166	175	116	294	184	0
CCTTGAAGA	BRDN00012	196	189	667	227	388	17
CCTTGAAGC	BRDN00011	114	106	386	289	414	22
CCTTGAAGT	BRDN00012	192	96	571	210	325	16
CCTTGAAGT	BRDN00012	195	210	296	318	254	8
CCTTGAATTC	BRDN00012	321	80	216	590	670	16
CCTTGACAA	BRDN00012	122	176	61	309	308	12
CCTTGACAG	BRDN00011	161	123	0	320	201	0
CCTTGACCAG	BRDN00011	493	459	270	736	528	0
CCTTGACCG	BRDN00011	186	130	311	327	161	17
CCTTGACCTA	BRDN00011	224	271	570	353	290	20
CCTTGACGCT	BRDN00012	206	127	312	314	260	0
CCTTGACGT	BRDN00011	246	63	80	589	276	4
CCTTGAGCAT	BRDN00012	183	183	1042	438	589	29
CCTTGAGCCT	BRDN00012	539	567	4152	914	1271	160
CCTTGAGGA	BRDN00011	129	142	19	194	78	8
CCTTGAGGA	BRDN00011	105	157	109	309	818	14
CCTTGAGGT	BRDN00011	179	44	0	404	272	1
CCTTGATCAC	BRDN00012	187	162	97	377	180	10
CCTTGATGA	BRDN00012	112	121	64	361	388	12
CCTTGATGG	BRDN00012	226	245	652	356	262	4
CCTTGATGT	BRDN00012	329	229	137	779	545	35
CCTTGCAAA	BRDN00012	195	161	1298	324	163	30
CCTTGCAAG	BRDN00011	100	159	309	387	303	2
CCTTGCAATT	BRDN00012	201	90	130	446	222	0
CCTTGCACAG	BRDN00011	118	51	1263	324	378	12
CCTTGCACCT	BRDN00012	177	122	284	418	170	44
CCTTGCACTT	BRDN00012	125	129	18	154	368	0
CCTTGCCAAA	BRDN00011	100	114	11	283	180	19
CCTTGCCAAA	BRDN00012	374	271	1899	411	308	36
CCTTGCCACA	BRDN00012	110	115	13	233	177	0
CCTTGCCCCT	BRDN00012	446	414	2182	753	722	32
CCTTGCCCTG	BRDN00011	114	73	66	155	284	0
CCTTGCCGA	BRDN00011	305	284	463	550	796	69
CCTTGCCGCC	BRDN00012	119	22	1	283	108	0
CCTTGCCTAG	BRDN00011	244	295	685	521	571	16
CCTTGCCTCC	BRDN00012	275	323	314	405	295	49
CCTTGCCTGA	BRDN00011	237	178	223	387	562	15
CCTTGCCTGC	BRDN00011	341	263	1721	541	442	56
CCTTGCGAC	BRDN00012	415	336	205	831	685	25
CCTTGCGGA	BRDN00011	99	68	9	189	146	8
CCTTGCTAAA	BRDN00012	382	233	187	878	444	2
CCTTGCTCGG	BRDN00012	306	256	1685	710	493	39
CCTTGCTGCT	BRDN00011	197	67	1	403	288	35
CCTTGCTGG	BRDN00012	203	351	1065	426	171	18
CCTTGCTTTA	BRDN00011	109	155	1559	243	261	24
CCTTGGAAG	BRDN00012	363	353	1459	852	1291	15
CCTTGGACA	BRDN00012	174	133	148	246	265	0
CCTTGGACC	BRDN00011	92	192	136	140	160	14
CCTTGGACG	BRDN00011	119	62	0	224	93	0
CCTTGGACG	BRDN00012	316	369	2270	646	396	27
CCTTGGAGA	BRDN00012	88	68	9	212	364	13
CCTTGGATA	BRDN00011	256	296	421	461	573	7
CCTTGGCAC	BRDN00011	175	174	59	358	271	26
CCTTGGCAG	BRDN00011	79	17	1499	363	182	16
CCTTGGCAT	BRDN00012	201	242	740	486	395	27
CCTTGGCCA	BRDN00012	140	225	211	239	518	0
CCTTGGCCA	BRDN00011	155	161	231	301	291	4
CCTTGGCCA	BRDN00011	395	510	207	597	694	153
CCTTGGCGA	BRDN00012	2	0	0	0	0	0
CCTTGGCGT	BRDN00012	230	191	1423	475	601	16
CCTTGGCTG	BRDN00011	351	362	3140	637	675	31
CCTTGGGAG	BRDN00012	241	265	847	421	779	58
CCTTGGGCTT	BRDN00012	110	99	688	248	329	0
CCTTGGGTC	BRDN00011	76	78	0	212	277	11
CCTTGGGTT	BRDN00011	98	146	31	270	196	2
CCTTGGTAA	BRDN00012	142	180	392	229	25	0
CCTTGGTCCT	BRDN00011	112	69	847	264	322	16
CCTTGGTGA	BRDN00011	318	210	302	497	728	12
CCTTGGTGT	BRDN00011	139	186	93	340	245	8
CCTTGGTGTT	BRDN00012	591	636	2803	1050	912	115
CCTTGGTTA	BRDN00011	433	492	205	1014	888	0
CCTTGTACAG	BRDN00011	130	92	81	296	425	29
CCTTGTAGG	BRDN00011	134	131	523	257	486	6
CCTTGTAGG	BRDN00012	291	277	312	627	712	46
CCTTGTAGG	BRDN00011	190	150	29	232	337	0
CCTTGTATTT	BRDN00012	214	277	478	347	404	47
CCTTGTCAAT	BRDN00012	191	68	88	297	235	1
CCTTGTCATA	BRDN00012	141	229	19	336	431	7
CCTTGTCCAG	BRDN00012	325	281	160	507	397	17
CCTTGTCCAT	BRDN00012	307	180	645	551	476	9
CCTTGTCCTC	BRDN00012	134	143	75	282	172	4
CCTTGTCCTG	BRDN00011	106	8	0	569	141	0
CCTTGTCTAC	BRDN00012	186	238	703	478	408	17
CCTTGTCTAT	BRDN00012	208	109	0	402	309	0
CCTTGTGAA	BRDN00012	175	112	117	247	126	0
CCTTGTGAT	BRDN00011	278	288	548	469	397	37
CCTTGTGGA	BRDN00012	148	164	32	277	379	1
CCTTGTGTA	BRDN00011	167	161	572	331	245	33
CCTTGTGTG	BRDN00011	305	224	818	360	831	119
CCTTGTTAAG	BRDN00012	155	253	290	442	422	37
CCTTGTTCCA	BRDN00012	120	73	8	275	41	9
CCTTGTTCCT	BRDN00012	357	257	104	722	461	65
CCTTGTTCTC	BRDN00011	18	12	0	311	193	0
CCTTGTTGA	BRDN00011	47	74	246	215	142	16
CCTTGTTGCA	BRDN00011	124	128	82	335	240	0
CCTTTAAAAC	BRDN00011	128	110	54	202	113	4
CCTTTAAGA	BRDN00011	59	96	0	143	327	0
CCTTTAATAA	BRDN00011	152	82	582	359	487	15
CCTTTACAAT	BRDN00011	173	120	665	178	167	19
CCTTTACATG	BRDN00011	278	193	314	537	616	14
CCTTTACCAA	BRDN00012	308	117	545	448	495	29
CCTTTACTGT	BRDN00011	175	225	228	387	755	1
CCTTTAGCCT	BRDN00012	83	81	36	225	199	7
CCTTTAGCG	BRDN00011	203	97	116	337	762	9
CCTTTAGGG	BRDN00011	33	1	0	155	125	0
CCTTTAGTAA	BRDN00011	66	54	0	158	71	0
CCTTTAGTAA	BRDN00012	126	33	0	260	289	0
CCTTTAGTG	BRDN00011	363	90	8083	633	754	82
CCTTTATAAA	BRDN00011	108	112	123	192	170	11
CCTTTATAGA	BRDN00011	103	97	6	160	77	2
CCTTTATATT	BRDN00011	74	63	1	138	258	0
CCTTTATCAG	BRDN00011	313	345	538	543	662	6
CCTTTATGAA	BRDN00012	171	209	843	481	425	59
CCTTTCAAAT	BRDN00012	97	114	106	162	193	46
CCTTTCAAAT	BRDN00011	201	129	221	369	255	12
CCTTTCAACT	BRDN00011	118	133	10	182	206	6
CCTTTCAAGT	BRDN00011	115	169	327	273	165	22
CCTTTCATCA	BRDN00012	294	176	64	402	172	20
CCTTTCCAAG	BRDN00012	269	202	579	469	364	42
CCTTTCCCAA	BRDN00011	165	78	81	302	257	25
CCTTTCCCAT	BRDN00011	288	302	1731	390	360	51
CCTTTCCGGT	BRDN00012	83	18	0	264	48	0
CCTTTCCTAA	BRDN00011	24	2	0	267	382	0
CCTTTCCTCG	BRDN00011	271	286	146	436	271	11
CCTTTCCTGA	BRDN00012	91	65	2	185	101	7
CCTTTCGAG	BRDN00011	217	377	1288	594	644	26
CCTTTCGCAC	BRDN00012	157	115	30	289	300	7
CCTTTCGTCG	BRDN00012	47	21	170	269	192	0
CCTTTCGTGG	BRDN00012	325	283	341	719	702	20
CCTTTCTTGA	BRDN00012	211	133	384	346	280	15
CCTTTGAAA	BRDN00012	413	355	256	681	490	31
CCTTTGAACA	BRDN00011	280	408	402	463	296	20
CCTTTGACCA	BRDN00012	404	420	1029	745	511	58
CCTTTGAGC	BRDN00011	87	33	0	172	207	0
CCTTTGATAG	BRDN00011	37	0	0	173	117	0
CCTTTGATCT	BRDN00012	356	312	2277	688	797	63
CCTTTGATTC	BRDN00011	295	565	5512	473	930	611
CCTTTGCAAA	BRDN00012	194	274	58	460	6944	5
CCTTTGCAG	BRDN00012	151	141	61	312	448	13
CCTTTGCATC	BRDN00011	136	122	408	289	252	101
CCTTTGCCAA	BRDN00011	197	140	311	260	234	5
CCTTTGCCAC	BRDN00012	98	45	204	239	195	4
CCTTTGCCAG	BRDN00011	115	134	46	294	429	8
CCTTTGCCAG	BRDN00011	119	126	20	257	113	0
CCTTTGCCCT	BRDN00012	149	151	21	404	504	7
CCTTTGCCTA	BRDN00012	331	405	569	802	804	20
CCTTTGCTCG	BRDN00011	176	145	1302	354	369	63
CCTTTGCTCT	BRDN00011	138	93	124	306	304	1
CCTTTGGAG	BRDN00011	137	122	209	216	258	0
CCTTTGGAT	BRDN00011	127	99	221	194	107	0
CCTTTGGCCA	BRDN00012	176	114	70	229	153	36
CCTTTGGGA	BRDN00012	100	101	516	208	414	5
CCTTTGGTG	BRDN00011	262	235	730	546	715	25
CCTTTGGTG	BRDN00012	119	149	58	201	198	0
CCTTTGTAG	BRDN00011	89	92	10	280	198	3
CCTTTGTAG	BRDN00012	240	276	233	605	924	19
CCTTTGTCAA	BRDN00012	221	251	274	440	592	17
CCTTTGTCAG	BRDN00012	179	249	425	212	207	31
CCTTTGTGA	BRDN00012	239	179	363	334	636	32

CCTTTGTGA	BRDN00011	237	4	1	652	341	0
CGAAAAAGC	BRDN00012	191	120	70	361	275	9
CGAAAACCT	BRDN00011	250	142	6	519	887	23
CGAAAAGAC	BRDN00011	157	0	0	512	264	0
CGAAAAGAG	BRDN00011	67	143	257	158	284	12
CGAAAAGGT	BRDN00011	344	8	1	756	410	0
CGAAAATCC	BRDN00012	173	266	224	287	246	5
CGAAAATCT	BRDN00011	90	206	357	263	174	0
CGAAAATCT	BRDN00012	320	167	519	688	283	0
CGAAAATGC	BRDN00012	244	266	826	443	402	67
CGAAACCAG	BRDN00012	511	347	705	657	1086	22
CGAAACCCT	BRDN00012	292	250	377	383	295	21
CGAAACCGG	BRDN00012	170	219	359	339	444	20
CGAAACCTC	BRDN00012	358	440	373	605	760	25
CGAAACCTT	BRDN00011	290	165	575	634	761	0
CGAAACGAA	BRDN00011	79	79	8	177	77	7
CGAAACGCC	BRDN00012	81	153	115	302	337	25
CGAAACGTT	BRDN00012	260	254	967	525	560	38
CGAAACTCA	BRDN00012	384	357	810	564	741	24
CGAAACTGC	BRDN00012	296	130	381	361	455	3
CGAAAGAAC	BRDN00012	164	142	23	354	872	50
CGAAAGAAG	BRDN00012	442	613	1209	774	579	37
CGAAAGCAA	BRDN00012	186	154	50	457	604	0
CGAAAGTAG	BRDN00012	116	86	129	175	93	0
CGAAAGTCC	BRDN00012	94	158	149	214	333	0
CGAAAGTGG	BRDN00012	177	40	40	515	426	0
CGAAATACC	BRDN00011	95	82	650	183	136	11
CGAAATACT	BRDN00012	126	32	359	159	94	42
CGAAATCTA	BRDN00011	133	95	114	192	325	0
CGAAATGCC	BRDN00012	338	175	312	515	338	42
CGAAATTGA	BRDN00011	157	74	21	229	394	0
CGAACAAAA	BRDN00011	143	133	6	210	278	3
CGAACAAGA	BRDN00011	462	426	482	732	1090	85
CGAACAAGT	BRDN00012	109	168	84	216	142	15
CGAACACAG	BRDN00012	107	73	64	298	353	11
CGAACACGT	BRDN00011	270	160	464	409	891	16
CGAACAGCC	BRDN00012	150	29	0	313	127	0
CGAACAGGA	BRDN00012	298	290	1167	581	1503	84
CGAACATGA	BRDN00012	333	209	840	436	625	18
CGAACATGT	BRDN00011	399	421	790	571	727	24
CGAACATGT	BRDN00012	285	253	1138	536	361	18
CGAACATTA	BRDN00011	214	269	474	289	362	54
CGAACATTT	BRDN00012	531	555	298	728	750	129
CGAACCACA	BRDN00011	95	93	341	225	294	22
CGAACCCAC	BRDN00012	171	7	0	544	206	13
CGAACCGAA	BRDN00011	61	1	0	293	188	0
CGAACCGAG	BRDN00012	97	150	3	248	171	0
CGAACCTAG	BRDN00012	230	259	639	561	331	17
CGAACGCAA	BRDN00012	150	60	1	337	183	0
CGAACGCAG	BRDN00012	350	274	101	422	619	32
CGAACGCGC	BRDN00011	122	118	679	218	408	9
CGAACGTAG	BRDN00011	158	155	160	257	257	5
CGAACGTCA	BRDN00012	518	367	246	937	729	45
CGAACGTGC	BRDN00012	149	148	146	387	350	74
CGAACGTGG	BRDN00012	182	155	689	438	420	29
CGAACTAAA	BRDN00011	329	146	1109	671	389	24
CGAACTAGT	BRDN00011	182	159	99	306	268	0
CGAACTCAT	BRDN00011	200	262	1711	348	502	31
CGAACTCGA	BRDN00011	596	413	1265	1120	1656	57
CGAACTCGC	BRDN00011	92	113	739	250	26	0
CGAACTCTA	BRDN00011	140	133	36	275	110	7
CGAACTCTC	BRDN00012	179	171	224	252	147	22
CGAACTGAA	BRDN00011	66	0	0	219	60	0
CGAACTTAA	BRDN00012	322	383	565	675	488	14
CGAACTTAT	BRDN00011	104	63	223	143	118	19
CGAACTTCA	BRDN00012	118	142	138	161	146	49
CGAACTTCA	BRDN00011	103	0	0	246	79	0
CGAACTTCT	BRDN00012	427	303	248	605	824	7
CGAACTTCT	BRDN00012	497	554	646	1116	1660	60
CGAACTTGA	BRDN00011	184	201	2452	292	179	72
CGAAGAAAT	BRDN00011	81	111	311	217	312	1
CGAAGAACA	BRDN00011	70	0	0	529	233	0
CGAAGAACT	BRDN00012	149	87	74	256	260	5
CGAAGAACT	BRDN00011	72	16	0	124	100	0
CGAAGACAG	BRDN00012	116	143	135	278	144	26
CGAAGACCA	BRDN00011	556	582	190	758	1333	29
CGAAGACGT	BRDN00011	130	52	66	184	331	0
CGAAGACTT	BRDN00011	68	28	178	153	214	0
CGAAGACTT	BRDN00011	69	54	0	163	127	0
CGAAGACTT	BRDN00011	145	47	1	189	141	0
CGAAGAGAA	BRDN00012	62	32	524	141	212	6
CGAAGATGT	BRDN00011	63	0	0	219	130	0
CGAAGCACT	BRDN00011	107	58	39	182	359	0
CGAAGCATA	BRDN00012	431	526	1624	816	1102	71
CGAAGCCAT	BRDN00011	346	448	3575	711	977	72
CGAAGCCCA	BRDN00012	437	309	1551	747	916	66
CGAAGCCCG	BRDN00011	330	247	85	441	493	7
CGAAGCCGC	BRDN00011	88	131	469	208	112	18
CGAAGCGAC	BRDN00011	332	393	572	529	561	22
CGAAGCGTA	BRDN00011	93	82	112	226	224	34
CGAAGCGTG	BRDN00012	27	60	138	86	200	12
CGAAGCTAA	BRDN00012	138	129	4	353	469	0
CGAAGCTGT	BRDN00012	23	0	0	315	83	0
CGAAGCTGT	BRDN00012	317	244	2158	507	800	48
CGAAGGACT	BRDN00011	39	24	42	91	8	0
CGAAGGAGA	BRDN00011	188	246	115	293	193	8
CGAAGGCAG	BRDN00012	122	128	360	262	239	0
CGAAGGCCA	BRDN00012	195	182	13	295	232	43
CGAAGGCGA	BRDN00011	149	185	664	297	248	7
CGAAGGCGT	BRDN00011	164	98	2640	167	288	63
CGAAGGCTG	BRDN00011	419	365	659	769	815	34
CGAAGGGAC	BRDN00011	265	259	1433	544	568	18
CGAAGGGCA	BRDN00011	96	75	637	272	227	25
CGAAGGTCT	BRDN00011	163	214	331	165	234	1
CGAAGGTGC	BRDN00012	336	376	479	771	454	63
CGAAGTACA	BRDN00011	232	162	107	489	227	29
CGAAGTAGC	BRDN00011	69	64	33	140	190	4
CGAAGTATA	BRDN00012	123	79	0	372	759	11
CGAAGTCCG	BRDN00011	178	164	0	287	334	3
CGAAGTCTT	BRDN00012	406	367	838	734	556	81
CGAAGTGAC	BRDN00012	222	276	237	408	380	40
CGAAGTGAT	BRDN00011	66	0	0	114	260	0
CGAAGTGGA	BRDN00011	159	139	2	256	457	0
CGAAGTTGA	BRDN00012	401	383	667	936	1425	47
CGAAGTTGG	BRDN00011	170	93	260	138	1013	0
CGAATAAGG	BRDN00012	371	307	167	603	628	23
CGAATACCT	BRDN00012	247	202	112	351	208	36
CGAATATTA	BRDN00012	286	353	565	447	191	54
CGAATCATC	BRDN00011	299	170	1331	556	674	55
CGAATCCAA	BRDN00012	313	262	64	509	478	24
CGAATCCGG	BRDN00011	233	210	1253	762	522	19
CGAATCGGA	BRDN00012	600	402	789	888	1126	1
CGAATCGGC	BRDN00012	183	79	1	402	390	23
CGAATCGTA	BRDN00012	107	163	30	190	101	0
CGAATCTGG	BRDN00012	152	36	1	342	283	0
CGAATCTTC	BRDN00011	179	202	1332	318	372	23
CGAATGCAA	BRDN00011	293	173	247	647	581	35
CGAATGCCA	BRDN00012	214	42	238	386	745	12
CGAATGCCC	BRDN00011	147	96	248	329	157	0
CGAATGCGG	BRDN00012	85	100	15	137	153	0
CGAATGGAA	BRDN00011	214	269	3	403	383	25
CGAATGGAG	BRDN00012	263	243	253	471	163	13
CGAATGTCA	BRDN00012	77	0	1	322	330	0
CGAATGTCT	BRDN00012	283	278	144	547	689	38
CGAATTAGA	BRDN00012	170	207	160	294	333	25
CGAATTATC	BRDN00012	100	135	76	356	148	19
CGAATTCAA	BRDN00011	295	216	1981	523	588	45
CGAATTCCA	BRDN00012	163	182	626	310	298	39
CGAATTCCA	BRDN00011	108	67	12	241	354	40
CGAATTGAG	BRDN00012	341	161	3286	428	321	88
CGAATTGGA	BRDN00012	257	280	135	331	356	29
CGAATTGTG	BRDN00011	153	91	737	290	312	201
CGACAAAAT	BRDN00012	177	102	321	267	490	4
CGACAAACT	BRDN00012	338	300	489	541	647	67
CGACAACGC	BRDN00012	321	344	833	512	315	32
CGACAACGT	BRDN00012	293	345	239	614	578	89
CGACAACTA	BRDN00011	159	102	169	201	254	17
CGACAAGAA	BRDN00011	139	135	412	177	185	6
CGACAAGGC	BRDN00011	433	212	331	824	822	24
CGACACAGA	BRDN00012	316	276	257	565	363	55
CGACACCGA	BRDN00011	197	61	55	269	303	0
CGACACCGG	BRDN00012	305	347	31	515	255	29
CGACACCGG	BRDN00011	42	11	0	179	84	0
CGACACGAG	BRDN00011	244	161	532	605	221	59
CGACACGCT	BRDN00011	192	197	130	395	498	24
CGACACGTA	BRDN00012	140	88	1568	275	185	35
CGACACTAA	BRDN00011	87	0	9	238	75	0
CGACAGAAT	BRDN00012	94	1	0	210	74	0
CGACAGCAG	BRDN00011	391	440	581	679	954	48
CGACAGCGA	BRDN00012	179	189	838	202	150	10
CGACAGCTA	BRDN00011	295	201	193	812	977	24
CGACAGCTT	BRDN00012	235	179	244	346	195	18
CGACAGGGT	BRDN00012	100	167	234	237	252	11
CGACAGGGT	BRDN00012	35	0	0	310	226	0
CGACAGGTC	BRDN00012	168	157	40	224	175	18
CGACAGTAC	BRDN00011	111	121	314	312	344	89
CGACAGTGT	BRDN00012	510	18	120	644	796	0
CGACATAGG	BRDN00011	15	0	0	545	136	0
CGACATATT	BRDN00012	79	48	412	184	119	14
CGACATCTA	BRDN00012	160	328	224	202	87	13
CGACATCTC	BRDN00011	271	379	698	551	336	32
CGACATCTT	BRDN00012	533	273	652	819	962	85
CGACATCTT	BRDN00011	120	48	141	350	285	17
CGACATGAA	BRDN00011	177	46	0	341	192	0
CGACATGCG	BRDN00011	326	183	2873	452	587	63
CGACATTGT	BRDN00011	137	189	107	184	150	3
CGACCAACA	BRDN00012	235	265	1474	402	623	6
CGACCACAA	BRDN00012	117	113	247	211	194	17
CGACCACAA	BRDN00011	208	261	847	518	737	22
CGACCACAT	BRDN00011	165	183	386	293	195	11
CGACCACCC	BRDN00012	276	253	25	528	353	12
CGACCACCG	BRDN00011	226	157	59	727	279	5
CGACCACGG	BRDN00011	462	437	517	835	1124	13
CGACCAGCT	BRDN00012	326	9	1	1018	537	0
CGACCAGCT	BRDN00011	319	428	340	683	1190	69
CGACCAGCT	BRDN00011	172	140	365	291	776	0
CGACCAGGA	BRDN00012	362	241	534	680	463	216
CGACCATAC	BRDN00012	341	238	745	584	741	33
CGACCATCT	BRDN00012	109	101	18	182	113	0
CGACCATCT	BRDN00011	81	56	40	204	127	0
CGACCATGA	BRDN00012	349	26	70	883	304	0
CGACCCAGA	BRDN00011	159	132	1010	229	111	13
CGACCCATG	BRDN00012	391	308	313	815	724	10
CGACCCCAC	BRDN00011	103	60	106	186	272	32
CGACCCGAC	BRDN00012	220	117	480	270	241	0
CGACCCGGA	BRDN00012	368	278	159	825	832	21
CGACCCGGC	BRDN00012	618	613	410	952	1208	22
CGACCCTGC	BRDN00012	421	261	486	659	519	45
CGACCGATT	BRDN00012	306	196	653	536	390	57
CGACCGCCC	BRDN00012	81	82	135	239	60	0
CGACCGCCT	BRDN00012	289	276	242	648	658	37
CGACCGCGC	BRDN00011	255	199	236	737	609	18
CGACCGCTA	BRDN00012	137	120	19	276	208	0
CGACCGGAA	BRDN00012	412	277	417	702	817	30
CGACCGTGC	BRDN00011	94	114	21	144	69	11
CGACCTACA	BRDN00011	536	339	1815	787	707	159
CGACCTATC	BRDN00011	132	30	1	579	139	0
CGACCTGCT	BRDN00011	205	137	368	440	343	1
CGACCTGGA	BRDN00012	206	141	169	240	551	0
CGACCTTGG	BRDN00011	303	437	1354	749	609	51
CGACGAACG	BRDN00012	77	137	203	352	303	0
CGACGAAGA	BRDN00012	173	115	921	257	153	26
CGACGAATA	BRDN00011	269	213	167	302	229	10
CGACGACAC	BRDN00012	256	248	138	549	545	54
CGACGACCA	BRDN00011	186	138	70	295	261	13
CGACGACCC	BRDN00012	296	367	392	624	868	88
CGACGACGT	BRDN00012	548	397	687	1164	1038	69
CGACGAGCA	BRDN00012	186	85	215	227	134	15
CGACGAGCT	BRDN00011	415	461	104	844	755	56
CGACGAGGC	BRDN00011	133	109	465	248	558	105
CGACGAGGC	BRDN00012	649	539	726	1036	1169	75
CGACGATGA	BRDN00012	41	1	11	341	104	0
CGACGATGT	BRDN00012	449	116	893	819	982	17
CGACGATTC	BRDN00011	106	92	113	159	210	0
CGACGCACT	BRDN00012	67	92	1053	215	91	32
CGACGCCAT	BRDN00011	135	115	1909	317	153	21
CGACGCCGA	BRDN00012	269	13	1	937	529	0
CGACGCCGA	BRDN00011	231	2	0	495	449	0
CGACGCCTA	BRDN00012	261	256	487	423	587	5
CGACGCGAC	BRDN00011	101	112	69	199	321	28
CGACGCGAC	BRDN00012	516	550	295	781	1025	15
CGACGCGCT	BRDN00012	271	264	1061	453	478	39
CGACGCGGA	BRDN00011	99	33	463	167	184	5
CGACGCGTT	BRDN00011	58	56	19	221	313	0
CGACGCTAG	BRDN00012	302	347	552	523	621	18
CGACGCTGA	BRDN00012	450	484	1315	692	933	62
CGACGCTGG	BRDN00011	175	66	497	322	416	7
CGACGGAAT	BRDN00011	246	16	1	403	364	0
CGACGGACT	BRDN00012	234	262	89	397	213	10
CGACGGAGT	BRDN00012	230	198	21	426	637	62
CGACGGCAC	BRDN00011	273	82	2690	403	584	46
CGACGGCGG	BRDN00012	239	303	956	521	643	42
CGACGGGGA	BRDN00012	490	435	692	1029	1188	46
CGACGGTAA	BRDN00012	481	648	3070	630	879	159
CGACGGTGA	BRDN00011	140	69	46	285	89	0
CGACGGTGG	BRDN00011	120	219	13	221	283	15
CGACGTCAT	BRDN00011	105	123	271	193	228	0
CGACGTCCA	BRDN00012	342	336	67	644	590	14
CGACGTCTT	BRDN00012	364	557	2092	588	820	26
CGACGTGCG	BRDN00012	97	86	200	142	169	48
CGACGTGGC	BRDN00012	123	204	117	264	388	0
CGACGTGGG	BRDN00012	514	604	616	755	1044	124
CGACGTGGG	BRDN00011	31	11	0	27	0	0
CGACGTGTC	BRDN00011	419	299	538	593	355	13
CGACGTTCT	BRDN00012	129	133	0	319	464	0
CGACTAACC	BRDN00012	142	187	342	245	235	24
CGACTAACC	BRDN00012	296	297	1008	540	837	49
CGACTACAC	BRDN00011	50	70	0	95	91	0
CGACTACGA	BRDN00011	348	447	241	573	592	21
CGACTACGC	BRDN00011	220	131	213	496	826	9
CGACTACGT	BRDN00012	181	83	554	209	252	10
CGACTACGT	BRDN00011	47	32	0	138	582	0
CGACTACTA	BRDN00012	54	23	135	92	31	11
CGACTACTA	BRDN00011	76	131	0	138	116	0
CGACTATAC	BRDN00012	67	81	6	138	91	27
CGACTCACA	BRDN00012	265	264	824	833	668	36
CGACTCACC	BRDN00011	244	159	1176	367	605	48
CGACTCATC	BRDN00011	401	314	43	594	1051	79
CGACTCCTA	BRDN00011	325	312	99	633	761	44
CGACTCTAC	BRDN00011	132	117	24	199	697	10
CGACTCTGG	BRDN00011	68	6	0	170	291	0
CGACTGACC	BRDN00012	463	365	529	896	1282	37
CGACTGATG	BRDN00012	162	194	365	242	788	11
CGACTGCAA	BRDN00012	253	309	62	495	624	102
CGACTGGGA	BRDN00012	236	21	69	641	533	0
CGACTGTGA	BRDN00011	106	169	147	257	191	0
CGACTTACT	BRDN00012	167	69	544	203	418	3
CGACTTAGA	BRDN00012	82	44	62	213	189	0
CGACTTCAA	BRDN00012	424	431	259	607	622	31
CGACTTCTA	BRDN00011	330	451	485	513	282	11
CGACTTCTA	BRDN00012	316	203	348	650	454	24
CGACTTGAT	BRDN00012	335	429	1638	691	790	33
CGAGAAACA	BRDN00012	126	143	328	313	309	1
CGAGAACAA	BRDN00012	233	238	121	587	558	0
CGAGAACAC	BRDN00011	144	127	10	249	341	37
CGAGAACGT	BRDN00012	122	173	603	341	71	63
CGAGAACTT	BRDN00012	199	227	576	395	443	10
CGAGAAGCC	BRDN00011	351	346	3237	762	885	107
CGAGAAGGA	BRDN00012	223	223	423	393	699	14
CGAGAAGGC	BRDN00012	144	116	2001	294	248	32
CGAGAAGGC	BRDN00011	223	315	745	390	379	15
CGAGAAGTC	BRDN00011	92	155	45	179	35	8
CGAGAAGTT	BRDN00012	190	187	49	315	140	8
CGAGAATGA	BRDN00012	145	55	447	254	64	25
CGAGAATGG	BRDN00012	161	102	148	311	272	86
CGAGACAAG	BRDN00012	335	187	223	555	370	12
CGAGACACG	BRDN00012	167	309	241	389	455	70
CGAGACAGC	BRDN00012	289	304	506	583	701	16
CGAGACATA	BRDN00012	308	200	2532	528	491	32
CGAGACCAA	BRDN00011	155	244	0	315	408	17
CGAGACCCA	BRDN00011	143	124	306	283	386	50
CGAGACCCC	BRDN00012	100	68	9	342	370	43
CGAGACCCC	BRDN00011	152	186	2063	294	652	41
CGAGACCGT	BRDN00011	252	157	709	589	210	7
CGAGACTAA	BRDN00012	425	286	1102	522	709	70
CGAGACTAC	BRDN00012	78	84	67	194	166	31
CGAGAGACA	BRDN00011	184	212	490	517	495	20
CGAGAGACA	BRDN00011	108	169	182	246	231	5
CGAGAGCCA	BRDN00012	85	89	16	134	67	32
CGAGAGCGA	BRDN00011	325	421	4521	523	528	77
CGAGAGCTC	BRDN00011	70	71	57	113	28	0
CGAGAGTAC	BRDN00011	102	132	35	265	59	38
CGAGAGTAT	BRDN00012	291	160	490	532	366	5
CGAGAGTCG	BRDN00012	156	75	16	150	174	0
CGAGATAAC	BRDN00012	40	78	0	170	89	0
CGAGATAAC	BRDN00012	244	245	274	485	675	0
CGAGATAGA	BRDN00012	130	111	60	141	104	0
CGAGATCAT	BRDN00011	324	60	72	681	774	0
CGAGATCCG	BRDN00011	246	162	46	585	722	22
CGAGATCGA	BRDN00011	130	76	1	340	418	8
CGAGATCGA	BRDN00011	248	275	799	482	405	16
CGAGATGAA	BRDN00012	168	16	26	278	151	0
CGAGATGGA	BRDN00011	250	262	494	462	545	31
CGAGATGGA	BRDN00012	244	188	102	540	408	52
CGAGATGGA	BRDN00012	273	112	68	251	225	23
CGAGATTGA	BRDN00012	445	421	452	827	1048	18
CGAGATTGT	BRDN00011	134	110	80	294	232	31
CGAGCAAAG	BRDN00012	278	259	541	570	700	28
CGAGCAACA	BRDN00011	191	1	1	439	16	0
CGAGCAAGG	BRDN00011	98	73	13	116	84	62
CGAGCACAA	BRDN00012	136	134	84	291	272	0
CGAGCACAA	BRDN00012	113	95	1	157	213	1
CGAGCACGG	BRDN00012	391	348	197	787	1053	109
CGAGCACGT	BRDN00012	295	260	482	422	226	17
CGAGCAGCC	BRDN00011	142	174	21	199	292	0
CGAGCAGCC	BRDN00011	442	414	6345	726	1589	67
CGAGCAGTG	BRDN00012	201	230	175	399	662	22
CGAGCATGT	BRDN00011	138	11	39	227	191	0
CGAGCCATA	BRDN00011	73	70	0	176	250	0
CGAGCCATT	BRDN00011	107	54	154	169	227	0
CGAGCCCAC	BRDN00012	200	31	34	403	98	1
CGAGCCCAG	BRDN00012	80	61	0	142	64	0
CGAGCCCTT	BRDN00012	155	148	77	385	199	0
CGAGCCGCC	BRDN00012	56	113	4	194	66	51
CGAGCCGGA	BRDN00011	126	11	220	388	291	10
CGAGCCGTA	BRDN00011	236	269	428	565	576	4
CGAGCCTAC	BRDN00012	356	228	33	646	695	39
CGAGCCTCC	BRDN00012	86	26	0	180	108	0
CGAGCCTGG	BRDN00012	350	373	4747	727	669	137
CGAGCGACA	BRDN00011	400	92	0	610	241	0
CGAGCGCAA	BRDN00012	238	176	3047	385	385	766
CGAGCGCCA	BRDN00011	88	213	0	230	116	0
CGAGCGCCA	BRDN00012	119	120	17	204	212	0
CGAGCGCCC	BRDN00011	255	167	274	435	403	18
CGAGCGCTA	BRDN00011	57	57	91	150	188	0
CGAGCGCTG	BRDN00012	185	229	33	344	198	4
CGAGCGGCA	BRDN00011	328	376	2070	575	616	43
CGAGCGGCA	BRDN00011	72	143	146	203	186	50
CGAGCGGCT	BRDN00011	199	103	30	431	283	26
CGAGCGGGT	BRDN00012	314	196	145	748	745	30
CGAGCTACA	BRDN00012	47	173	22	178	53	18
CGAGCTACC	BRDN00011	108	139	123	190	234	0
CGAGCTCAC	BRDN00012	84	260	49	158	152	35
CGAGCTCCC	BRDN00012	377	249	550	748	848	23
CGAGCTCGA	BRDN00011	212	124	1	371	325	25
CGAGCTCTG	BRDN00011	236	127	295	568	455	0
CGAGCTCTT	BRDN00012	109	61	32	129	156	39
CGAGCTGCA	BRDN00012	427	112	466	603	472	2
CGAGCTGCG	BRDN00011	182	114	151	263	148	7
CGAGCTGCG	BRDN00011	183	131	1195	350	205	23
CGAGCTGGA	BRDN00011	142	63	102	371	75	5
CGAGCTGGC	BRDN00012	194	157	118	386	293	43
CGAGCTGGG	BRDN00012	153	75	341	264	519	25
CGAGCTGTA	BRDN00011	90	66	150	160	72	27
CGAGCTGTC	BRDN00012	318	333	1260	650	395	58
CGAGCTTCC	BRDN00011	113	8	1	303	244	0
CGAGGAAAA	BRDN00011	173	144	188	322	180	11
CGAGGAACA	BRDN00012	45	0	0	140	21	0
CGAGGAAGA	BRDN00011	118	153	195	385	125	65
CGAGGAAGC	BRDN00011	83	59	155	204	96	18
CGAGGAAGT	BRDN00011	478	276	1845	786	849	47
CGAGGAATA	BRDN00011	358	270	2484	579	607	107
CGAGGAATG	BRDN00011	367	292	1201	617	502	68
CGAGGAATG	BRDN00012	202	275	604	337	303	172
CGAGGAATT	BRDN00011	149	167	295	360	232	38
CGAGGACAC	BRDN00012	341	302	791	625	949	40
CGAGGACAC	BRDN00012	214	309	550	408	207	15
CGAGGACAG	BRDN00012	258	220	1313	324	379	58
CGAGGACAT	BRDN00011	137	62	0	325	745	0
CGAGGACCT	BRDN00011	133	234	321	261	214	10
CGAGGACTC	BRDN00011	95	61	604	242	360	0
CGAGGACTT	BRDN00012	603	456	245	1113	889	98
CGAGGAGAA	BRDN00011	219	230	291	484	1307	124
CGAGGAGAC	BRDN00011	218	280	15034	343	151	308
CGAGGAGCA	BRDN00012	232	253	163	558	812	13
CGAGGAGCA	BRDN00011	12	7	0	230	140	0
CGAGGAGCC	BRDN00011	84	34	317	149	165	16
CGAGGAGGC	BRDN00012	110	63	24	239	120	28
CGAGGAGTC	BRDN00012	126	57	22	190	167	0
CGAGGAGTG	BRDN00012	104	80	261	196	106	17
CGAGGATCA	BRDN00011	234	345	707	552	434	33
CGAGGCACA	BRDN00011	129	100	1	153	139	9
CGAGGCAGA	BRDN00012	279	224	235	717	753	53
CGAGGCAGA	BRDN00011	71	0	0	263	317	0
CGAGGCAGA	BRDN00011	417	214	320	792	782	16
CGAGGCAGG	BRDN00012	117	54	566	205	184	0
CGAGGCCAA	BRDN00012	158	183	90	340	244	6
CGAGGCCCA	BRDN00012	173	3	0	489	483	0
CGAGGCCCA	BRDN00011	58	58	26	148	157	6
CGAGGCCCT	BRDN00012	271	372	419	695	427	30
CGAGGCCGG	BRDN00011	289	28	1	677	429	0
CGAGGCGAG	BRDN00011	88	43	1	99	120	0
CGAGGCGCA	BRDN00012	188	138	86	326	260	122
CGAGGCGCC	BRDN00012	413	464	604	569	318	147
CGAGGCGCG	BRDN00011	88	48	0	195	232	0
CGAGGCGCT	BRDN00012	338	313	138	644	431	52
CGAGGCTAC	BRDN00011	97	0	0	250	37	0
CGAGGCTCT	BRDN00012	290	282	1187	530	420	19
CGAGGCTGG	BRDN00011	110	47	277	298	333	9
CGAGGGAAC	BRDN00012	313	257	385	556	548	26
CGAGGGACC	BRDN00011	176	152	331	357	511	36
CGAGGGACT	BRDN00012	455	438	1554	778	529	85
CGAGGGATT	BRDN00012	247	210	405	423	432	50
CGAGGGCAA	BRDN00011	88	165	217	167	158	4
CGAGGGCCG	BRDN00012	242	0	3	609	83	0
CGAGGGCGA	BRDN00012	417	347	1061	675	281	11
CGAGGGCGA	BRDN00012	211	353	5	517	768	0
CGAGGGCGA	BRDN00012	80	15	0	166	85	0
CGAGGGCGC	BRDN00011	90	99	911	143	179	31
CGAGGGCTG	BRDN00012	230	201	225	483	485	49
CGAGGGGCC	BRDN00012	372	286	1423	642	760	47
CGAGGGGCC	BRDN00012	231	70	0	450	267	0
CGAGGTAAC	BRDN00011	113	91	118	228	146	3
CGAGGTCAA	BRDN00011	92	14	34	198	239	0
CGAGGTCCA	BRDN00012	253	273	442	619	404	10
CGAGGTCTT	BRDN00011	310	268	878	469	372	11
CGAGGTCTT	BRDN00012	306	282	154	588	729	27
CGAGGTGAA	BRDN00011	191	58	4421	291	441	70
CGAGGTGCC	BRDN00011	100	128	0	138	70	0
CGAGGTGCT	BRDN00012	135	261	29	310	407	0
CGAGGTGGA	BRDN00011	128	19	1	139	316	23
CGAGGTGTA	BRDN00011	96	108	403	305	618	11
CGAGGTTAA	BRDN00012	96	187	24	169	144	22
CGAGGTTAC	BRDN00011	124	136	63	268	225	0
CGAGTACAA	BRDN00011	52	43	0	84	75	5
CGAGTACAT	BRDN00012	112	56	234	224	135	6
CGAGTATGT	BRDN00012	102	111	121	367	317	2
CGAGTATTA	BRDN00011	120	73	64	155	400	0
CGAGTCACG	BRDN00012	122	174	413	223	161	16
CGAGTCCAC	BRDN00012	93	256	364	207	152	12
CGAGTCCTC	BRDN00011	198	101	551	322	458	57
CGAGTCCTT	BRDN00012	108	92	78	127	331	0
CGAGTCCTT	BRDN00012	248	232	713	504	670	39
CGAGTCGCC	BRDN00011	295	119	357	510	329	0
CGAGTCGCC	BRDN00011	199	0	0	343	7	0

CGAGTCGGC	BRDN00012	165	216	153	354	397	0
CGAGTCGGC	BRDN00012	75	34	17	180	163	0
CGAGTCGTC	BRDN00012	124	88	191	226	110	0
CGAGTGAGA	BRDN00012	130	195	564	113	167	8
CGAGTGCAA	BRDN00012	123	169	238	172	90	14
CGAGTGCAA	BRDN00011	221	136	16	420	508	9
CGAGTGCAA	BRDN00011	90	8	0	240	252	0
CGAGTGCAC	BRDN00011	423	263	949	585	874	57
CGAGTGCCA	BRDN00011	159	188	859	244	216	36
CGAGTGCCC	BRDN00012	241	218	789	521	598	57
CGAGTGCGA	BRDN00011	53	15	193	155	219	0
CGAGTGCGC	BRDN00011	183	167	336	337	409	4
CGAGTGCGT	BRDN00012	256	179	229	315	302	0
CGAGTGCGT	BRDN00011	509	449	298	618	544	46
CGAGTGCTA	BRDN00012	95	161	77	188	177	0
CGAGTGCTT	BRDN00012	69	59	6	87	109	0
CGAGTGGAA	BRDN00012	136	62	665	224	132	84
CGAGTGGAG	BRDN00011	82	125	5445	192	361	52
CGAGTGGAG	BRDN00011	327	256	2402	578	851	11
CGAGTGGCG	BRDN00012	178	74	32	193	310	16
CGAGTGGGA	BRDN00012	520	390	1205	799	734	47
CGAGTGGGA	BRDN00012	221	138	285	436	634	11
CGAGTGTCT	BRDN00011	184	175	116	397	317	38
CGAGTGTGC	BRDN00012	78	139	51	191	158	6
CGAGTGTTA	BRDN00012	136	82	108	228	189	0
CGAGTGTTA	BRDN00012	237	182	236	455	381	4
CGAGTGTTG	BRDN00011	184	264	239	515	815	9
CGAGTTACA	BRDN00012	153	28	132	256	156	16
CGAGTTACC	BRDN00011	345	271	336	600	655	1
CGAGTTCAA	BRDN00011	154	96	89	233	43	3
CGAGTTCAA	BRDN00011	167	131	27	181	229	16
CGAGTTCAG	BRDN00011	105	12	109	342	311	7
CGAGTTCCG	BRDN00011	134	39	0	619	419	0
CGAGTTCGA	BRDN00011	158	130	0	247	218	5
CGAGTTGAA	BRDN00011	152	77	140	344	341	4
CGAGTTGAT	BRDN00011	185	147	446	403	291	29
CGAGTTGGA	BRDN00011	29	5	0	244	237	0
CGAGTTGGC	BRDN00012	246	96	316	225	262	7
CGAGTTGGT	BRDN00012	61	59	0	108	173	0
CGAGTTTGA	BRDN00011	139	152	96	236	110	20
CGATAAAAA	BRDN00012	51	0	1	343	358	1
CGATAAAAG	BRDN00011	415	474	1235	719	958	8
CGATAAACC	BRDN00011	133	137	47	282	272	10
CGATAAAGG	BRDN00011	69	136	191	232	114	126
CGATAACCA	BRDN00012	319	161	125	750	388	7
CGATAACGA	BRDN00012	314	416	1277	561	338	50
CGATAACTA	BRDN00012	163	97	20	402	416	17
CGATAAGCA	BRDN00012	77	72	0	122	41	0
CGATAAGTC	BRDN00011	136	147	270	163	209	10
CGATAATGG	BRDN00012	509	354	354	815	901	76
CGATACAGG	BRDN00011	194	133	2	285	357	0
CGATACCGA	BRDN00012	102	61	80	306	236	25
CGATACCGT	BRDN00012	119	111	194	208	297	11
CGATACGTG	BRDN00011	147	174	1332	238	130	12
CGATACTTA	BRDN00011	381	279	392	635	741	21
CGATAGACG	BRDN00012	207	0	1	490	2	0
CGATAGATG	BRDN00011	346	314	78	506	638	7
CGATAGCGA	BRDN00012	83	98	5	198	150	0
CGATAGTAC	BRDN00012	287	455	751	482	468	41
CGATATAAG	BRDN00011	110	0	119	221	157	0
CGATATGCT	BRDN00012	192	75	736	393	202	25
CGATATGTG	BRDN00012	335	351	312	730	578	87
CGATATTTA	BRDN00012	133	41	0	185	256	0
CGATCAAGG	BRDN00011	148	245	85	482	424	12
CGATCACAG	BRDN00012	350	489	2135	780	389	713
CGATCACGT	BRDN00011	125	167	160	275	239	12
CGATCAGCA	BRDN00012	168	174	14	461	487	4
CGATCAGCG	BRDN00011	248	214	842	348	326	11
CGATCAGGA	BRDN00012	63	0	0	338	101	0
CGATCATGT	BRDN00011	321	307	1911	682	371	80
CGATCCAAA	BRDN00011	121	10	0	284	111	0
CGATCCAAT	BRDN00012	341	476	1547	678	1796	73
CGATCCCAT	BRDN00012	255	222	95	406	286	45
CGATCCCGG	BRDN00011	62	94	105	258	229	0
CGATCCGGG	BRDN00011	129	111	66	151	351	0
CGATCGAGC	BRDN00012	185	194	1	273	283	0
CGATCGCAA	BRDN00011	519	447	1818	803	822	178
CGATCGGGA	BRDN00012	254	288	133	488	378	39
CGATCTCAC	BRDN00011	141	114	27	237	187	7
CGATCTCCT	BRDN00011	319	362	148	431	254	11
CGATCTCCT	BRDN00011	103	29	217	225	213	8
CGATCTTCT	BRDN00011	17	12	0	146	44	0
CGATCTTGC	BRDN00011	371	338	710	787	649	58
CGATCTTGG	BRDN00012	276	316	827	491	142	86
CGATCTTTG	BRDN00011	189	128	466	295	364	10
CGATGAAAA	BRDN00012	108	76	0	153	146	10
CGATGAAGA	BRDN00012	87	0	1	381	60	0
CGATGACAA	BRDN00011	123	118	110	305	161	7
CGATGACAA	BRDN00011	163	91	166	252	509	16
CGATGACAC	BRDN00012	363	305	207	551	363	34
CGATGACAT	BRDN00011	381	263	603	652	635	55
CGATGACCA	BRDN00011	203	195	63	241	210	67
CGATGACGA	BRDN00012	381	332	3923	710	745	25
CGATGAGCC	BRDN00012	191	154	919	352	327	0
CGATGAGGC	BRDN00011	81	61	0	157	154	14
CGATGATGA	BRDN00012	78	78	112	240	113	10
CGATGATGT	BRDN00011	217	210	6	316	339	9
CGATGATTC	BRDN00011	306	173	391	477	819	31
CGATGCCAA	BRDN00012	145	237	199	337	264	25
CGATGCCAA	BRDN00012	469	539	93	883	808	118
CGATGCCAA	BRDN00011	136	219	10	388	127	25
CGATGCCAA	BRDN00011	153	135	655	185	208	11
CGATGCCCA	BRDN00011	255	316	102	466	853	22
CGATGCCCG	BRDN00012	529	536	1245	797	1102	64
CGATGCCTA	BRDN00011	408	256	356	695	569	49
CGATGCGCC	BRDN00012	169	198	1296	370	238	16
CGATGCGGT	BRDN00012	466	356	209	688	703	34
CGATGGAAA	BRDN00011	97	70	1	161	529	5
CGATGGACA	BRDN00012	280	191	95	606	709	12
CGATGGACG	BRDN00012	307	257	296	458	468	15
CGATGGATC	BRDN00012	595	580	1310	1126	1034	66
CGATGGCAG	BRDN00012	205	174	100	412	276	0
CGATGGCCT	BRDN00011	245	333	47	406	247	14
CGATGGCGA	BRDN00012	342	321	114	640	680	105
CGATGGTCA	BRDN00012	197	205	288	652	1282	8
CGATGGTCT	BRDN00011	150	108	340	315	252	0
CGATGGTTG	BRDN00011	182	96	116	224	277	0
CGATGTAAG	BRDN00011	156	42	2605	246	294	15
CGATGTAGT	BRDN00011	235	287	545	551	925	26
CGATGTAGT	BRDN00012	447	552	1946	555	592	24
CGATGTCAT	BRDN00012	177	38	17	400	425	0
CGATGTCCG	BRDN00012	329	313	849	602	797	10
CGATGTGAC	BRDN00011	409	375	1056	613	672	14
CGATGTGCG	BRDN00012	114	0	0	260	97	0
CGATGTGGA	BRDN00011	130	47	29	221	486	29
CGATGTGGC	BRDN00011	238	159	183	408	175	13
CGATGTGGG	BRDN00012	41	55	282	147	146	6
CGATGTGGG	BRDN00012	77	168	213	225	223	12
CGATGTTCA	BRDN00011	110	129	29	140	84	5
CGATGTTGA	BRDN00011	202	157	594	322	281	17
CGATGTTGA	BRDN00012	397	298	408	625	1152	26
CGATTAAAA	BRDN00012	116	103	481	269	154	3
CGATTAGTA	BRDN00012	73	51	0	86	46	0
CGATTCCCT	BRDN00011	147	196	94	326	301	8
CGATTCCTA	BRDN00011	103	62	56	218	326	0
CGATTCCTC	BRDN00011	84	45	29	167	79	25
CGATTCCTG	BRDN00012	152	183	28	289	452	6
CGATTCGCT	BRDN00011	126	62	188	308	559	6
CGATTCGTA	BRDN00012	241	196	1	785	489	20
CGATTCTAT	BRDN00011	307	276	142	440	256	0
CGATTCTCT	BRDN00012	75	20	0	251	194	0
CGATTCTGA	BRDN00011	12	20	0	303	153	1
CGATTGAGG	BRDN00012	125	71	314	213	239	36
CGATTGCTA	BRDN00011	111	68	3604	264	107	29
CGATTGGCT	BRDN00011	269	206	354	407	657	57
CGATTGGTC	BRDN00012	87	63	0	201	122	0
CGATTTCAT	BRDN00011	169	152	75	385	734	6
CGATTTCCC	BRDN00012	119	150	714	341	204	8
CGATTTGGT	BRDN00011	258	180	130	390	332	0
CGCAAACGG	BRDN00011	215	149	2075	458	328	77
CGCAACAGT	BRDN00011	200	187	970	490	531	39
CGCAACCTG	BRDN00011	275	274	1480	680	707	101
CGCAACGAA	BRDN00012	183	221	40	423	229	43
CGCAACTAC	BRDN00011	499	350	1568	932	1021	79
CGCAACTCT	BRDN00011	259	319	179	551	891	0
CGCAAGAGG	BRDN00012	72	45	334	157	82	0
CGCAAGCAT	BRDN00011	165	108	687	275	136	40
CGCAAGCGC	BRDN00012	88	102	43	101	51	37
CGCAAGGTC	BRDN00011	65	64	0	241	351	0
CGCAAGGTG	BRDN00012	272	245	12	422	288	70
CGCAAGGTG	BRDN00012	364	525	2763	885	678	126
CGCAAGTTC	BRDN00011	80	75	0	116	289	0
CGCAATATG	BRDN00011	54	6	9	213	120	0
CGCAATCAC	BRDN00011	83	62	1493	205	71	17
CGCAATCAT	BRDN00011	262	473	1868	553	788	170
CGCAATCCT	BRDN00012	458	442	1419	795	986	82
CGCAATCGC	BRDN00012	84	120	0	83	52	0
CGCAATTGT	BRDN00012	95	97	478	231	155	2
CGCACAACT	BRDN00011	459	452	667	697	721	21
CGCACACAG	BRDN00011	195	236	1050	314	350	45
CGCACACAG	BRDN00012	265	317	204	502	568	6
CGCACACGT	BRDN00012	166	0	1	248	474	0
CGCACAGCG	BRDN00012	153	99	929	270	190	59
CGCACAGGT	BRDN00012	81	106	2	184	298	0
CGCACATCC	BRDN00011	156	167	49	339	487	7
CGCACATCT	BRDN00012	344	369	408	479	482	30
CGCACCAAG	BRDN00011	201	129	178	357	228	1
CGCACCACC	BRDN00011	86	116	31	265	233	0
CGCACCAGC	BRDN00012	409	356	2175	776	719	29
CGCACCATA	BRDN00011	79	75	0	189	86	11
CGCACCCAC	BRDN00011	88	32	3	129	119	0
CGCACCCAG	BRDN00011	172	76	9	468	261	8
CGCACCCAG	BRDN00012	93	43	885	172	186	4
CGCACCCGA	BRDN00011	4	3	0	164	113	0
CGCACCCGA	BRDN00011	116	152	110	264	110	42
CGCACCCTC	BRDN00012	37	57	592	187	92	8
CGCACCGAG	BRDN00012	61	60	39	208	103	4
CGCACCGGG	BRDN00012	8	0	1	162	71	0
CGCACCGTC	BRDN00012	193	86	0	714	485	0
CGCACCGTG	BRDN00011	273	159	515	543	489	15
CGCACCTGG	BRDN00012	170	135	9	409	507	13
CGCACGACC	BRDN00012	765	578	3808	1204	1552	72
CGCACGACT	BRDN00011	92	131	27	108	260	10
CGCACGCAG	BRDN00012	254	341	1213	493	618	25
CGCACGCCC	BRDN00012	103	94	734	140	268	26
CGCACGCCC	BRDN00012	290	232	657	759	770	50
CGCACGCGC	BRDN00011	307	433	2325	417	462	94
CGCACGCTC	BRDN00012	171	167	118	325	478	9
CGCACGGCG	BRDN00011	23	45	9	45	149	7
CGCACGGCG	BRDN00012	80	90	45	143	44	11
CGCACGGGA	BRDN00012	125	182	18	284	178	5
CGCACGTTG	BRDN00011	290	415	192	757	690	157
CGCACTCAC	BRDN00011	54	52	30	197	409	11
CGCACTGGA	BRDN00012	78	93	0	230	83	0
CGCACTGGC	BRDN00012	75	84	1	135	98	0
CGCACTGGT	BRDN00011	251	146	434	303	250	2
CGCACTGTT	BRDN00012	449	377	679	777	464	14
CGCACTTAC	BRDN00011	93	25	679	189	234	8
CGCACTTAC	BRDN00011	152	188	650	295	261	9
CGCACTTCG	BRDN00011	308	195	225	560	308	38
CGCAGAATG	BRDN00012	107	0	0	196	0	0
CGCAGACAT	BRDN00011	323	268	52	452	270	10
CGCAGAGAC	BRDN00011	101	72	526	263	105	18
CGCAGAGAC	BRDN00011	198	164	657	234	519	12
CGCAGAGCG	BRDN00011	91	15	0	131	34	0
CGCAGATAC	BRDN00012	243	224	919	393	113	21
CGCAGATCC	BRDN00012	60	0	0	143	0	0
CGCAGCAAG	BRDN00011	76	80	103	192	145	8
CGCAGCACC	BRDN00012	272	244	312	482	397	90
CGCAGCACC	BRDN00012	444	384	251	902	1175	20
CGCAGCACC	BRDN00012	306	252	529	454	349	29
CGCAGCAGG	BRDN00012	101	58	0	147	352	0
CGCAGCATA	BRDN00011	176	66	0	342	276	0
CGCAGCCAC	BRDN00011	138	68	60	497	383	12
CGCAGCCAC	BRDN00011	174	79	181	421	262	32
CGCAGCCCG	BRDN00011	167	217	110	215	38	0
CGCAGCCGA	BRDN00012	79	96	12	215	96	0
CGCAGCGCA	BRDN00012	409	333	979	770	1034	45
CGCAGCGCC	BRDN00012	206	17	0	558	226	0
CGCAGCGGC	BRDN00011	118	122	1	194	75	0
CGCAGCGGC	BRDN00011	121	30	193	62	15	12
CGCAGCGTA	BRDN00012	28	0	45	203	48	9
CGCAGCTGG	BRDN00012	226	277	793	333	714	24
CGCAGCTGT	BRDN00011	254	0	0	486	334	0
CGCAGCTGT	BRDN00011	135	29	12	344	346	1
CGCAGGAAA	BRDN00011	114	10	212	149	169	0
CGCAGGAAG	BRDN00012	146	117	57	219	370	32
CGCAGGAAG	BRDN00011	104	92	61	230	380	0
CGCAGGACA	BRDN00011	95	139	222	230	257	12
CGCAGGATC	BRDN00011	320	325	2563	851	300	73
CGCAGGCCC	BRDN00012	148	129	27	280	420	13
CGCAGGCTA	BRDN00012	393	366	2534	668	767	12
CGCAGGGCT	BRDN00012	91	0	1	177	94	0
CGCAGGTAC	BRDN00012	331	293	371	645	898	82
CGCAGGTGC	BRDN00011	46	44	49	60	9	0
CGCAGGTTG	BRDN00012	382	374	439	656	439	7
CGCAGTACG	BRDN00012	275	243	367	345	336	34
CGCAGTCAC	BRDN00011	359	238	1182	659	681	37
CGCAGTCAT	BRDN00012	392	487	522	597	547	48
CGCAGTCCG	BRDN00012	170	55	38061	314	125	334
CGCATAAGT	BRDN00012	249	285	400	527	650	21
CGCATACAG	BRDN00011	146	99	102	230	145	42
CGCATCCGC	BRDN00011	323	212	359	553	687	29
CGCATCGAC	BRDN00012	101	71	31	287	221	6
CGCATCGAC	BRDN00012	48	24	0	549	574	0
CGCATCGTG	BRDN00012	134	119	40	339	345	24
CGCATCTCA	BRDN00012	261	258	12	432	405	23
CGCATGACC	BRDN00011	261	178	127	464	724	0
CGCATGACC	BRDN00012	534	395	887	1024	766	24
CGCATGCAG	BRDN00012	169	28	0	394	117	0
CGCATGCAT	BRDN00011	393	366	938	742	891	12
CGCATGCTT	BRDN00011	232	271	1549	474	512	24
CGCATGGAC	BRDN00011	320	338	480	602	583	51
CGCATGTCA	BRDN00011	89	109	1038	247	390	44
CGCATTCAG	BRDN00012	68	34	115	238	201	1
CGCATTCTT	BRDN00011	335	255	394	669	523	0
CGCATTGAC	BRDN00012	89	148	63	283	445	6
CGCATTTCC	BRDN00011	132	18	1	356	182	0
CGCCAAACG	BRDN00011	116	129	94	154	134	63
CGCCAAATA	BRDN00011	87	1	1	226	48	0
CGCCAACAA	BRDN00012	339	263	807	621	731	25
CGCCAAGCA	BRDN00012	273	254	471	658	910	0
CGCCAAGCA	BRDN00012	247	285	311	397	1306	209
CGCCAATCT	BRDN00011	82	90	111	180	76	35
CGCCACAAT	BRDN00012	161	140	6	268	221	15
CGCCACCCG	BRDN00012	230	188	830	353	327	55
CGCCACCGA	BRDN00011	316	196	1621	340	493	18
CGCCACCGC	BRDN00012	129	106	1954	203	105	44
CGCCACCTG	BRDN00011	173	291	1229	279	171	54
CGCCACGAA	BRDN00011	102	110	0	281	286	0
CGCCACGAC	BRDN00011	147	175	1414	260	150	42
CGCCACGAG	BRDN00011	217	146	316	339	151	11
CGCCACGCA	BRDN00011	78	144	157	234	200	0
CGCCACGGG	BRDN00012	318	279	744	732	843	52
CGCCAGAAA	BRDN00011	117	145	1452	268	102	46
CGCCAGACG	BRDN00011	147	163	199	201	298	17
CGCCAGATA	BRDN00012	140	153	991	414	216	22
CGCCAGCAC	BRDN00011	138	102	28	296	236	0
CGCCAGCAG	BRDN00011	217	169	88	395	294	19
CGCCAGCGG	BRDN00012	203	308	177	365	153	56
CGCCAGGCA	BRDN00012	221	149	49	354	318	13
CGCCAGGCC	BRDN00012	333	191	343	618	749	57
CGCCAGGGT	BRDN00011	298	258	79	602	701	33
CGCCAGGGT	BRDN00012	196	208	458	462	254	65
CGCCAGGTA	BRDN00011	250	221	169	449	418	3
CGCCAGGTT	BRDN00011	136	74	32	178	195	5
CGCCATAAA	BRDN00012	55	5	0	78	35	0
CGCCATCAC	BRDN00012	256	80	22	489	549	8
CGCCATCCG	BRDN00012	177	102	9	341	185	54
CGCCATCCT	BRDN00011	149	113	352	140	51	2
CGCCATCGG	BRDN00011	39	43	0	46	137	2
CGCCATCTT	BRDN00012	306	171	156	514	594	0
CGCCATGAT	BRDN00011	211	231	70	375	432	25
CGCCATGGA	BRDN00011	277	295	849	519	741	17
CGCCATTGC	BRDN00012	100	104	128	313	405	21
CGCCCAACA	BRDN00012	197	142	250	352	182	4
CGCCCAAGC	BRDN00012	114	36	52	380	286	0
CGCCCACAT	BRDN00012	157	172	375	414	359	36
CGCCCACCT	BRDN00011	335	407	1718	645	608	72
CGCCCAGGA	BRDN00012	75	13	0	637	457	0
CGCCCAGGC	BRDN00011	6	5	0	69	78	0
CGCCCAGTC	BRDN00011	177	219	48	334	517	59
CGCCCCATG	BRDN00012	309	233	412	453	1048	0
CGCCCCCAA	BRDN00012	134	140	22	238	485	0
CGCCCCCAG	BRDN00012	81	122	436	146	388	21
CGCCCCCGA	BRDN00012	86	49	0	143	103	14
CGCCCCCGG	BRDN00011	31	0	0	184	77	0
CGCCCCGGA	BRDN00011	190	211	4158	450	291	41
CGCCCCTCG	BRDN00011	154	136	229	321	296	9
CGCCCGCAT	BRDN00011	133	95	246	152	543	0
CGCCCGTGA	BRDN00012	85	123	600	180	145	16
CGCCCGTGC	BRDN00012	125	85	81	163	56	0
CGCCCGTTT	BRDN00012	381	228	4411	602	1018	116
CGCCCTACC	BRDN00012	266	236	4454	728	756	35
CGCCCTCGA	BRDN00012	6	0	0	449	49	0
CGCCCTCGG	BRDN00011	375	408	481	711	602	130
CGCCCTGAT	BRDN00012	83	104	0	238	122	0
CGCCCTGGA	BRDN00012	88	55	0	255	335	0
CGCCCTGGA	BRDN00011	94	158	23	245	287	8
CGCCCTGGT	BRDN00012	359	322	635	623	787	46
CGCCCTTGT	BRDN00011	192	0	0	388	45	0
CGCCGAAAG	BRDN00011	181	201	88	219	87	20
CGCCGAAGC	BRDN00011	129	81	1	211	197	0
CGCCGAGCC	BRDN00011	201	231	151	484	430	21
CGCCGAGCT	BRDN00011	177	151	356	260	257	4
CGCCGAGGG	BRDN00011	154	155	220	262	159	7
CGCCGAGTA	BRDN00011	47	57	54	141	165	0
CGCCGATGT	BRDN00012	206	92	360	241	114	39
CGCCGCAAG	BRDN00012	314	255	201	685	492	12
CGCCGCACA	BRDN00011	81	109	31	98	276	22
CGCCGCATC	BRDN00012	106	0	0	185	5	0
CGCCGCCCG	BRDN00012	263	272	445	549	500	41
CGCCGCCTA	BRDN00012	59	20	102	150	124	50
CGCCGCCTT	BRDN00011	81	41	287	174	161	5
CGCCGCCTT	BRDN00012	336	193	449	588	140	6
CGCCGCGAA	BRDN00012	354	332	767	617	437	24
CGCCGCGGG	BRDN00011	113	149	432	212	369	20
CGCCGCGTT	BRDN00011	158	110	111	209	101	6
CGCCGCTCT	BRDN00011	467	568	116	589	481	123
CGCCGGACA	BRDN00012	241	157	333	400	381	1
CGCCGGAGA	BRDN00012	84	110	338	135	134	9
CGCCGGCAC	BRDN00011	80	23	1	284	98	0
CGCCGGCAC	BRDN00011	46	38	34	181	259	37
CGCCGGCGA	BRDN00011	315	300	1628	607	366	34
CGCCGGGAC	BRDN00012	203	22	0	628	286	1
CGCCGGGAC	BRDN00012	410	359	2316	702	697	83
CGCCGGGCC	BRDN00011	99	162	1070	211	461	14
CGCCGGGCT	BRDN00012	529	479	651	735	1228	22
CGCCGTCAA	BRDN00011	204	196	155	348	939	0
CGCCGTCCT	BRDN00011	162	239	328	296	217	19
CGCCGTCGC	BRDN00012	116	147	110	242	324	27
CGCCGTGTA	BRDN00011	104	192	1	245	327	9
CGCCGTTCC	BRDN00012	250	324	705	670	461	51
CGCCGTTCT	BRDN00012	164	57	344	360	140	7
CGCCTAAGA	BRDN00011	125	47	88	274	141	0
CGCCTAATT	BRDN00012	274	266	243	419	420	26
CGCCTACAA	BRDN00011	163	39	0	109	114	0
CGCCTACAC	BRDN00012	133	71	86	230	312	6
CGCCTACAG	BRDN00011	121	181	140	250	114	8
CGCCTACAG	BRDN00011	192	208	1115	343	230	36
CGCCTACAG	BRDN00011	126	122	82	232	78	22
CGCCTACCA	BRDN00012	151	110	678	243	127	36
CGCCTACGA	BRDN00012	93	188	2739	154	196	95
CGCCTACGT	BRDN00011	129	168	52	421	234	0
CGCCTCAAC	BRDN00011	313	286	290	545	608	13
CGCCTCATA	BRDN00011	84	76	1	195	216	0
CGCCTCCGG	BRDN00012	153	151	409	301	744	14
CGCCTCCTC	BRDN00011	123	139	66	247	54	0
CGCCTCCTT	BRDN00012	242	194	21	403	530	5
CGCCTCGAG	BRDN00012	323	271	2562	600	251	57
CGCCTCGGA	BRDN00011	176	137	1	424	279	6
CGCCTCGTC	BRDN00012	112	47	6	270	80	10
CGCCTCTCA	BRDN00012	467	426	771	906	1244	114
CGCCTCTCG	BRDN00012	117	81	112	260	551	0
CGCCTCTGC	BRDN00012	121	90	3975	229	179	12
CGCCTGAAG	BRDN00012	270	183	1102	672	579	8
CGCCTGAGA	BRDN00011	245	167	1397	394	470	6
CGCCTGCAC	BRDN00012	99	52	2	129	311	0
CGCCTGCCC	BRDN00012	118	0	0	193	60	0
CGCCTGCGA	BRDN00012	274	258	246	655	620	36
CGCCTGGCA	BRDN00011	457	428	1282	700	636	74
CGCCTGGGG	BRDN00012	279	0	1	1072	1346	1
CGCCTGGTA	BRDN00011	245	228	21	582	550	6
CGCCTTCCC	BRDN00012	38	30	240	156	63	4
CGCCTTCTG	BRDN00012	173	171	497	275	292	28
CGCCTTGAG	BRDN00012	351	398	244	619	525	67
CGCCTTGAG	BRDN00011	545	365	132	625	663	10
CGCCTTGGG	BRDN00012	140	112	85	255	230	0
CGCGAAAGG	BRDN00011	103	75	51	83	168	0
CGCGAACGC	BRDN00011	180	244	88	195	426	33
CGCGAAGTA	BRDN00011	84	54	46	166	111	0
CGCGACCAC	BRDN00012	303	298	644	430	663	23
CGCGACGAC	BRDN00012	339	354	323	539	718	0
CGCGACGAG	BRDN00011	273	243	1065	440	160	5
CGCGACGAG	BRDN00012	56	87	94	104	58	34
CGCGACGGC	BRDN00012	95	0	1	454	460	0
CGCGACGTG	BRDN00012	152	93	781	218	158	10
CGCGACTCG	BRDN00012	224	239	66	411	360	30
CGCGATATG	BRDN00011	131	119	220	202	92	5
CGCGATGAT	BRDN00011	192	270	136	323	583	4
CGCGCACAG	BRDN00011	128	38	268	159	224	29
CGCGCACCA	BRDN00012	234	324	54	321	343	18
CGCGCACCG	BRDN00011	77	48	0	64	18	0
CGCGCACGG	BRDN00011	150	108	0	289	358	0
CGCGCAGAA	BRDN00012	667	664	946	821	1329	126
CGCGCAGCC	BRDN00012	149	160	185	373	252	12
CGCGCAGCG	BRDN00012	92	95	44	172	150	3
CGCGCCAGG	BRDN00012	146	83	535	316	108	4
CGCGCCCAG	BRDN00012	233	122	244	361	223	0
CGCGCCCGC	BRDN00011	84	124	0	229	157	4
CGCGCCGGT	BRDN00012	49	18	54	126	125	6
CGCGCCTAC	BRDN00012	288	277	47	458	340	38
CGCGCGCAC	BRDN00012	58	31	0	63	123	0
CGCGCGCAG	BRDN00011	107	87	97	173	189	11
CGCGCGCAG	BRDN00011	155	105	11	220	85	0
CGCGCGCAG	BRDN00012	154	22	78	213	300	0
CGCGCGCCC	BRDN00011	34	18	0	127	46	0
CGCGCGCCC	BRDN00011	70	42	1416	151	212	17
CGCGCGGCG	BRDN00011	147	218	18	313	192	0
CGCGCTCAT	BRDN00012	330	251	14	500	614	16
CGCGCTGGC	BRDN00011	155	130	1	306	260	6
CGCGCTGTA	BRDN00011	90	117	325	180	205	17
CGCGCTGTG	BRDN00011	88	10	7	171	232	0
CGCGGAAAT	BRDN00012	413	317	4481	534	696	121
CGCGGAAGA	BRDN00011	234	104	116	610	684	0
CGCGGACCC	BRDN00012	56	39	15	137	71	0
CGCGGACCT	BRDN00012	22	1	1	652	277	0
CGCGGACGA	BRDN00011	261	184	48	445	521	34
CGCGGAGAT	BRDN00012	71	84	1	129	31	0
CGCGGATAC	BRDN00011	106	136	46	138	88	4
CGCGGCCCA	BRDN00012	478	438	1262	795	915	67
CGCGGCCGC	BRDN00011	103	109	0	159	120	11
CGCGGGAAC	BRDN00011	92	72	838	140	154	3
CGCGGGCTA	BRDN00012	76	123	294	204	302	10
CGCGGGGCA	BRDN00011	265	382	79	556	457	6
CGCGGGTAG	BRDN00011	114	67	17	205	197	0
CGCGGGTAG	BRDN00012	69	113	627	125	83	12
CGCGGTAGG	BRDN00011	105	95	0	105	167	27
CGCGGTCGA	BRDN00011	69	48	0	157	50	4
CGCGGTGAG	BRDN00011	85	38	0	182	256	0
CGCGTAACA	BRDN00011	93	119	0	206	97	7
CGCGTACAG	BRDN00011	83	155	27	214	170	38
CGCGTACCG	BRDN00012	124	10	0	518	198	0
CGCGTCAAG	BRDN00011	112	66	182	143	63	0
CGCGTCCCA	BRDN00011	162	191	0	366	233	38

CGCGTCCCC	BRDN00011	62	145	781	168	245	21
CGCGTCCGA	BRDN00011	123	62	0	273	118	0
CGCGTCGCT	BRDN00012	227	213	662	451	323	10
CGCGTGCAT	BRDN00012	395	392	108	641	621	47
CGCGTGTAG	BRDN00012	400	330	1709	562	660	74
CGCGTTCTA	BRDN00011	169	195	23	173	217	0
CGCTAAATT	BRDN00012	387	433	2743	593	826	54
CGCTAACAC	BRDN00011	149	215	73	355	262	1
CGCTAAGGA	BRDN00011	109	72	723	172	255	15
CGCTACAAA	BRDN00012	339	626	4402	841	385	72
CGCTACAAC	BRDN00012	70	75	13	154	285	10
CGCTACACA	BRDN00012	43	20	0	335	24	0
CGCTACAGC	BRDN00011	110	99	70	236	277	20
CGCTACAGG	BRDN00012	167	2	12	330	173	0
CGCTACAGG	BRDN00012	104	109	23	179	47	16
CGCTACAGT	BRDN00012	415	242	139	668	696	68
CGCTACATCT	BRDN00012	254	104	25	279	405	15
CGCTACCCCT	BRDN00011	189	110	13	166	121	21
CGCTACCTCA	BRDN00012	430	363	716	646	538	21
CGCTAGGTC	BRDN00012	427	503	176	730	743	73
CGCTAGGTT	BRDN00012	187	298	15	504	423	0
CGCTAGTAC	BRDN00012	505	289	1642	740	629	12
CGCTATGAG	BRDN00011	102	177	316	252	77	10
CGCTATTAG	BRDN00012	304	444	360	504	421	14
CGCTCAAAG	BRDN00012	193	138	364	430	602	8
CGCTCAACC	BRDN00011	89	42	24	117	80	17
CGCTCAAGG	BRDN00011	360	269	201	635	873	0
CGCTCAAGG	BRDN00012	218	93	100	366	417	0
CGCTCACAG	BRDN00012	361	261	102	638	670	34
CGCTCACCTG	BRDN00011	120	112	183	245	187	53
CGCTCAGGG	BRDN00011	360	200	415	560	1215	8
CGCTCAGTG	BRDN00012	289	180	432	662	736	53
CGCTCATCG	BRDN00012	453	480	394	683	1015	110
CGCTCCGCCT	BRDN00012	134	189	213	326	343	6
CGCTCCTCGA	BRDN00012	186	64	5	332	208	37
CGCTCGCTCA	BRDN00011	287	272	57	495	356	56
CGCTCGGAT	BRDN00012	409	377	752	579	707	6
CGCTCGGGC	BRDN00012	390	253	200	768	546	12
CGCTCGTCTG	BRDN00012	431	384	1307	775	661	37
CGCTCTACTT	BRDN00012	138	149	198	190	245	0
CGCTCTATG	BRDN00011	258	296	162	428	295	26
CGCTCTCGTA	BRDN00012	392	241	4713	580	297	59
CGCTGAAGT	BRDN00012	134	68	5	137	246	0
CGCTGAGCA	BRDN00012	107	65	64	185	344	23
CGCTGATCA	BRDN00011	357	291	716	728	1087	11
CGCTGATGC	BRDN00011	461	508	911	754	1381	29
CGCTGCAAG	BRDN00011	251	26	58	422	251	0
CGCTGCACA	BRDN00011	159	219	1361	338	285	73
CGCTGCCAA	BRDN00011	193	287	141	387	339	10
CGCTGCCAT	BRDN00011	193	155	627	354	310	13
CGCTGCGAA	BRDN00011	202	132	145	255	222	22
CGCTGCGCC	BRDN00012	117	55	8	227	227	0
CGCTGCTCTA	BRDN00012	147	179	1195	402	141	17
CGCTGCTCTG	BRDN00011	104	137	74	173	662	0
CGCTGCTGG	BRDN00012	464	431	226	803	722	44
CGCTGGCCC	BRDN00012	145	92	58	318	379	39
CGCTGGCCC	BRDN00012	95	86	133	154	167	0
CGCTGGCCCT	BRDN00012	236	258	141	378	362	45
CGCTGGCGA	BRDN00011	134	107	340	142	124	7
CGCTGGTTC	BRDN00011	66	58	4	95	442	0
CGCTGTAGT	BRDN00011	104	67	817	158	318	9
CGCTGTCCAT	BRDN00011	154	212	317	227	245	8
CGCTGTCCCA	BRDN00012	119	134	179	209	117	0
CGCTGTCGC	BRDN00012	138	291	1511	239	343	49
CGCTGTGCC	BRDN00012	210	127	403	434	219	54
CGCTGTGCT	BRDN00011	66	123	444	180	149	8
CGCTGTGGT	BRDN00012	68	68	257	113	152	46
CGCTGTGGT	BRDN00012	354	553	2000	605	587	77
CGCTGTGTA	BRDN00011	105	65	162	238	386	14
CGCTGTGTG	BRDN00011	317	351	322	531	326	42
CGCTGTTCA	BRDN00012	116	112	25	234	59	0
CGCTTAGCA	BRDN00011	133	151	316	347	636	4
CGCTTATCAT	BRDN00011	162	93	1269	420	258	5
CGCTTCAGT	BRDN00012	84	55	1	162	50	0
CGCTTCCCAC	BRDN00012	301	176	891	627	336	7
CGCTTCCGC	BRDN00012	464	513	1311	771	894	53
CGCTTCCTGA	BRDN00011	55	70	0	141	76	1
CGCTTCGGC	BRDN00012	226	443	103	655	802	14
CGCTTCGGG	BRDN00011	159	196	252	401	354	0
CGCTTCGTG	BRDN00012	194	211	122	412	470	70
CGCTTCTACT	BRDN00011	147	117	108	401	376	4
CGCTTCTGG	BRDN00011	9	0	0	56	39	0
CGCTTGAAA	BRDN00011	89	80	56	108	42	11
CGCTTGAGG	BRDN00012	185	247	902	231	312	60
CGCTTGCCC	BRDN00011	190	1	0	359	20	0
CGCTTGCTCA	BRDN00011	210	188	273	361	513	15
CGCTTGCTG	BRDN00011	171	217	173	345	216	22
CGCTTGGCA	BRDN00012	76	97	0	168	416	0
CGCTTGTAG	BRDN00011	82	95	98	193	187	69
CGCTTGTTA	BRDN00012	211	241	2217	431	846	71
CGCTTGTTTA	BRDN00011	145	102	536	333	261	46
CGCTTTCAA	BRDN00011	57	6	0	171	20	0
CGCTTTGTG	BRDN00012	289	344	347	519	586	61
CGGAAAACA	BRDN00012	88	45	419	193	333	9
CGGAAAAGT	BRDN00012	171	198	373	286	118	25
CGGAAAATT	BRDN00012	160	129	508	221	204	23
CGGAAACAG	BRDN00012	471	431	612	912	950	63
CGGAAAGCG	BRDN00012	72	57	256	153	124	0
CGGAAAGGA	BRDN00012	183	24	0	289	147	0
CGGAAATGG	BRDN00012	300	250	326	591	421	6
CGGAACAAG	BRDN00012	308	65	184	528	400	0
CGGAACACC	BRDN00011	257	143	254	312	177	49
CGGAACACG	BRDN00012	96	95	67	170	224	3
CGGAACAGA	BRDN00011	211	216	1181	295	227	37
CGGAACATC	BRDN00012	235	202	106	361	427	7
CGGAACATG	BRDN00012	225	236	680	558	372	34
CGGAACCAA	BRDN00011	313	189	213	450	743	7
CGGAACCAC	BRDN00011	48	6	184	104	0	0
CGGAACCAG	BRDN00011	85	26	0	233	150	0
CGGAACCGA	BRDN00012	102	158	14	231	267	0
CGGAACCGC	BRDN00011	98	93	1	155	213	2
CGGAACGAA	BRDN00012	35	52	0	203	215	21
CGGAACGTC	BRDN00011	115	186	46	182	238	15
CGGAACGTG	BRDN00012	656	510	1694	1198	1458	48
CGGAACTGT	BRDN00012	363	319	50	656	695	10
CGGAACTTA	BRDN00012	130	167	1330	287	236	277
CGGAACTTC	BRDN00012	250	132	207	506	572	0
CGGAACTTG	BRDN00011	224	120	1173	245	192	2
CGGAAGAAT	BRDN00011	267	273	316	495	1102	38
CGGAAGACA	BRDN00012	182	155	145	319	351	6
CGGAAGACG	BRDN00012	114	44	65	344	197	0
CGGAAGATT	BRDN00011	222	214	473	260	130	7
CGGAAGCAC	BRDN00011	309	295	450	491	538	23
CGGAAGCAC	BRDN00012	115	165	83	245	77	9
CGGAAGCCA	BRDN00011	300	243	11	556	384	14
CGGAAGCCA	BRDN00011	121	86	7	262	171	0
CGGAAGCGG	BRDN00012	20	10	282	122	186	1
CGGAAGGAC	BRDN00012	105	8	0	187	120	0
CGGAAGGAC	BRDN00011	80	62	55	159	222	10
CGGAAGGCC	BRDN00011	262	153	161	441	378	36
CGGAAGGCT	BRDN00012	339	256	280	630	915	18
CGGAAGTAC	BRDN00011	175	188	7	270	275	9
CGGAAGTCA	BRDN00012	173	260	1572	283	476	29
CGGAAGTCC	BRDN00011	100	83	157	224	282	0
CGGAAGTGG	BRDN00011	189	143	35	323	357	11
CGGAAGTGG	BRDN00012	144	79	138	203	180	18
CGGAATACA	BRDN00012	285	239	312	474	470	74
CGGAATCCT	BRDN00011	316	294	66	534	653	28
CGGAATCGA	BRDN00011	265	59	28	690	531	1
CGGAATCGC	BRDN00012	72	98	28	160	125	0
CGGACAAGA	BRDN00011	97	61	252	313	194	4
CGGACACCC	BRDN00011	152	188	2126	317	333	28
CGGACACCT	BRDN00012	333	314	2586	705	664	59
CGGACACCT	BRDN00011	57	35	26	150	155	0
CGGACACGC	BRDN00012	124	103	1407	195	223	49
CGGACACGT	BRDN00012	374	286	325	815	676	12
CGGACACTC	BRDN00011	332	317	140	630	586	0
CGGACACTT	BRDN00011	267	12	0	607	492	0
CGGACAGAC	BRDN00012	315	198	793	479	228	62
CGGACAGAC	BRDN00011	446	427	24	716	339	36
CGGACAGAT	BRDN00012	249	269	305	600	540	13
CGGACAGCA	BRDN00011	184	149	8	599	304	0
CGGACAGCA	BRDN00011	116	177	20	156	174	50
CGGACAGGT	BRDN00011	341	51	368	605	145	6
CGGACAGTG	BRDN00011	222	402	443	341	210	88
CGGACATCA	BRDN00012	90	69	336	301	316	43
CGGACATGT	BRDN00012	121	84	108	211	347	41
CGGACCACC	BRDN00012	478	465	655	1058	1203	63
CGGACCAGA	BRDN00011	190	110	491	332	393	12
CGGACCAGC	BRDN00012	203	271	1115	540	395	39
CGGACCATT	BRDN00011	208	118	133	448	140	0
CGGACCCCC	BRDN00012	178	91	245	341	160	0
CGGACCCTA	BRDN00012	74	130	522	236	210	0
CGGACCCTC	BRDN00011	138	140	221	268	370	41
CGGACCGCT	BRDN00011	122	132	195	204	140	19
CGGACCTAC	BRDN00012	174	145	402	277	251	10
CGGACCTGA	BRDN00012	182	113	56	249	149	51
CGGACCTGC	BRDN00012	192	189	152	289	319	25
CGGACGACT	BRDN00012	545	643	4960	939	1190	130
CGGACGACT	BRDN00012	326	230	78	576	518	8
CGGACGCCA	BRDN00011	100	106	0	209	247	0
CGGACGCGC	BRDN00011	320	206	603	514	458	16
CGGACGCGG	BRDN00011	59	19	0	55	40	0
CGGACGGCA	BRDN00011	100	162	6	257	49	8
CGGACGGCG	BRDN00012	103	141	628	191	200	11
CGGACGTGG	BRDN00012	305	168	111	446	522	9
CGGACTACA	BRDN00012	77	113	68	132	137	0
CGGACTCCCT	BRDN00011	544	763	1366	874	824	48
CGGACTCCT	BRDN00012	233	182	333	427	389	22
CGGACTCCT	BRDN00011	185	96	318	372	238	42
CGGACTCTG	BRDN00011	58	0	1	209	36	0
CGGACTGCC	BRDN00012	432	237	368	848	1079	7
CGGACTTGG	BRDN00012	153	72	0	326	249	0
CGGACTTGG	BRDN00011	109	158	171	153	407	18
CGGAGAACA	BRDN00012	230	60	27	523	260	15
CGGAGAACG	BRDN00012	109	54	1	263	252	0
CGGAGAAGA	BRDN00011	88	74	21	128	199	5
CGGAGAAGA	BRDN00011	111	114	277	214	104	14
CGGAGAAGA	BRDN00011	150	138	4	330	235	12
CGGAGACAT	BRDN00011	150	73	0	218	197	7
CGGAGACCA	BRDN00012	294	304	2078	690	383	107
CGGAGACCC	BRDN00012	283	320	2582	781	806	66
CGGAGAGAA	BRDN00012	307	239	212	595	652	41
CGGAGAGCC	BRDN00011	149	141	1183	267	234	48
CGGAGAGCT	BRDN00012	48	19	248	82	135	15
CGGAGAGGA	BRDN00012	97	55	0	149	146	0
CGGAGAGTC	BRDN00011	452	332	406	826	760	47
CGGAGATAC	BRDN00012	274	232	1354	346	362	48
CGGAGATAC	BRDN00012	229	204	76	299	816	5
CGGAGATGA	BRDN00012	432	308	591	759	418	43
CGGAGATTA	BRDN00012	82	0	0	300	205	0
CGGAGCACA	BRDN00012	283	277	1435	524	208	114
CGGAGCAGC	BRDN00011	196	253	1417	388	762	29
CGGAGCATT	BRDN00011	499	339	119	940	1077	13
CGGAGCCCA	BRDN00012	99	58	15	256	83	0
CGGAGCCGA	BRDN00011	287	200	1670	579	431	45
CGGAGCCTG	BRDN00011	50	8	89	149	135	13
CGGAGCCTT	BRDN00012	328	360	321	729	439	0
CGGAGCCTT	BRDN00011	84	78	15	243	960	0
CGGAGCGGC	BRDN00011	102	67	61	186	177	17
CGGAGCGTC	BRDN00012	268	187	443	588	317	0
CGGAGCTAA	BRDN00011	30	53	18	70	43	0
CGGAGCTAC	BRDN00012	151	82	156	256	145	0
CGGAGCTTA	BRDN00012	481	575	1605	690	824	92
CGGAGGAAC	BRDN00012	121	148	363	191	483	54
CGGAGGACA	BRDN00011	157	142	45	431	425	28
CGGAGGAGA	BRDN00012	81	166	18	336	61	4
CGGAGGAGC	BRDN00012	376	589	1536	665	316	50
CGGAGGAGT	BRDN00012	428	323	220	775	624	9
CGGAGGCCG	BRDN00012	156	36	18	315	262	0
CGGAGGCGT	BRDN00011	31	5	16	136	143	0
CGGAGGCTG	BRDN00011	343	321	269	701	577	55
CGGAGGGCT	BRDN00011	173	161	45	268	109	15
CGGAGGTCT	BRDN00011	127	257	108	221	349	0
CGGAGGTCT	BRDN00012	232	296	15	658	788	30
CGGAGGTGC	BRDN00011	190	102	175	346	333	4
CGGAGTAAC	BRDN00012	326	306	398	694	817	41
CGGAGTAGT	BRDN00012	137	143	16	215	235	13
CGGAGTCCT	BRDN00011	353	325	1434	795	1151	117
CGGAGTGAT	BRDN00012	208	47	168	285	206	32
CGGAGTGGC	BRDN00012	210	118	190	534	285	8
CGGAGTTAA	BRDN00012	509	556	266	621	829	8
CGGAGTTGG	BRDN00012	177	102	152	326	296	0
CGGATACGC	BRDN00012	166	181	40	395	182	6
CGGATACGC	BRDN00012	338	418	455	645	455	69
CGGATCATG	BRDN00011	169	145	217	370	238	7
CGGATCCGG	BRDN00011	73	5	1	196	70	0
CGGATCCTC	BRDN00012	195	87	0	200	281	3
CGGATCCTG	BRDN00012	208	171	504	353	543	21
CGGATCGAC	BRDN00012	109	58	91	284	284	0
CGGATCGAG	BRDN00012	156	70	1	333	308	0
CGGATCGCC	BRDN00011	442	363	1183	651	842	48
CGGATCGGA	BRDN00012	118	117	31	255	255	30
CGGATCGGC	BRDN00012	212	274	107	257	227	4
CGGATCTGC	BRDN00011	183	136	112	303	286	5
CGGATCTGC	BRDN00011	76	82	27	260	102	0
CGGATCTTC	BRDN00011	67	38	1	157	173	5
CGGATGAAG	BRDN00012	186	157	0	275	141	0
CGGATGACA	BRDN00011	355	172	589	470	466	15
CGGATGACG	BRDN00012	120	28	5	326	78	0
CGGATGATA	BRDN00012	115	73	244	199	230	0
CGGATGCCG	BRDN00012	177	228	217	407	510	15
CGGATGGCA	BRDN00011	136	123	44	173	143	18
CGGATGGCC	BRDN00012	246	164	454	444	671	0
CGGATGGCG	BRDN00011	551	523	1367	1207	1019	72
CGGATTATG	BRDN00012	198	28	62	322	183	0
CGGATTCAA	BRDN00012	332	215	829	503	389	22
CGGATTGAA	BRDN00012	216	191	137	577	664	14
CGGATTTCC	BRDN00011	380	425	431	689	752	0
CGGCAAAGT	BRDN00011	84	0	1	219	172	0
CGGCAACAT	BRDN00012	561	488	1172	774	917	77
CGGCAACTA	BRDN00011	273	238	537	544	274	24
CGGCAAGAC	BRDN00012	94	88	23	170	100	10
CGGCAAGGT	BRDN00012	359	294	742	771	399	34
CGGCAATGC	BRDN00011	81	0	89	219	213	0
CGGCACAAT	BRDN00012	138	64	18	249	126	32
CGGCACACC	BRDN00012	586	586	1310	1027	1570	30
CGGCACAGA	BRDN00012	131	87	1	235	202	4
CGGCACAGC	BRDN00012	195	260	752	178	190	54
CGGCACAGC	BRDN00012	388	265	1467	804	575	27
CGGCACAGC	BRDN00012	453	459	1688	689	1143	73
CGGCACATG	BRDN00011	109	78	0	231	220	0
CGGCACCAC	BRDN00012	164	79	122	208	255	5
CGGCACCAT	BRDN00012	399	435	1438	835	630	22
CGGCACCCC	BRDN00011	168	226	522	323	230	26
CGGCACCCG	BRDN00011	519	474	1662	1010	1507	45
CGGCACCTG	BRDN00012	553	392	536	780	913	71
CGGCACGCC	BRDN00011	199	0	62	466	106	0
CGGCACGCG	BRDN00011	171	99	151	207	165	44
CGGCACGTG	BRDN00011	296	378	474	624	654	26
CGGCACTAG	BRDN00012	380	372	1592	658	846	39
CGGCACTGC	BRDN00012	382	156	1345	575	413	36
CGGCAGAAG	BRDN00011	203	140	379	465	369	0
CGGCAGATG	BRDN00011	117	135	9	216	347	38
CGGCAGCAA	BRDN00012	312	164	329	590	796	12
CGGCAGCAA	BRDN00012	175	47	115	431	528	1
CGGCAGCAC	BRDN00011	169	160	447	236	455	36
CGGCAGCAG	BRDN00011	51	63	0	95	265	19
CGGCAGCAT	BRDN00011	126	51	48	213	248	7
CGGCAGCCA	BRDN00011	301	370	1024	470	624	43
CGGCAGCGA	BRDN00011	334	221	517	599	605	24
CGGCAGCGT	BRDN00012	45	65	265	238	34	24
CGGCAGCTC	BRDN00011	247	168	39	418	391	25
CGGCAGCTG	BRDN00012	7	0	0	214	93	0
CGGCAGCTT	BRDN00012	277	184	94	560	576	0
CGGCAGGAC	BRDN00011	141	120	381	262	93	10
CGGCAGGGC	BRDN00011	351	468	91	477	327	48
CGGCAGGGT	BRDN00012	170	182	1818	343	187	59
CGGCAGGTT	BRDN00012	80	76	13	180	195	0
CGGCATCAC	BRDN00012	255	328	1089	548	559	18
CGGCATCAT	BRDN00011	281	342	1342	478	546	29
CGGCATCCA	BRDN00011	114	290	147	177	136	16
CGGCATCCG	BRDN00012	409	236	129	622	657	58
CGGCATCGC	BRDN00012	422	416	358	564	500	24
CGGCATCTA	BRDN00012	491	407	352	808	943	38
CGGCATCTC	BRDN00011	248	129	58	523	565	0
CGGCATCTG	BRDN00012	238	303	258	514	657	23
CGGCATGAA	BRDN00011	265	257	639	525	296	41
CGGCATGAT	BRDN00011	382	426	1172	578	583	27
CGGCATGGA	BRDN00011	213	145	1716	566	411	21
CGGCCAAAG	BRDN00012	95	68	0	212	165	0
CGGCCAAAG	BRDN00012	292	335	132	660	418	112
CGGCCAAGC	BRDN00011	384	388	983	645	942	95
CGGCCACAG	BRDN00011	202	129	29	381	350	35
CGGCCACAG	BRDN00012	303	270	460	649	458	34
CGGCCACGC	BRDN00012	302	111	108	525	720	0
CGGCCACTG	BRDN00012	160	160	678	410	308	2
CGGCCACTG	BRDN00012	91	45	25	304	232	0
CGGCCACTT	BRDN00012	110	141	259	144	195	26
CGGCCAGGG	BRDN00011	373	300	720	519	308	18
CGGCCAGTA	BRDN00012	342	309	1061	719	449	39
CGGCCATCA	BRDN00012	215	109	9	445	227	24
CGGCCATCT	BRDN00012	69	49	0	180	202	0
CGGCCCAAG	BRDN00012	134	162	26	111	115	22
CGGCCCAGT	BRDN00012	133	105	199	229	468	2
CGGCCCCAA	BRDN00012	471	244	423	731	852	60
CGGCCCCAA	BRDN00012	54	0	1	674	56	0
CGGCCCCCA	BRDN00011	380	343	626	685	596	45
CGGCCCCGA	BRDN00011	128	62	102	173	131	0
CGGCCCCTA	BRDN00012	273	232	1178	352	285	63
CGGCCCGAT	BRDN00011	196	253	2409	212	266	35
CGGCCCGCG	BRDN00011	97	60	1	94	116	0
CGGCCCGCT	BRDN00011	113	37	0	177	290	0
CGGCCCGGA	BRDN00012	210	197	640	577	250	38
CGGCCCGGC	BRDN00011	12	0	1	197	23	0
CGGCCCGGC	BRDN00012	119	139	48	236	97	11
CGGCCCTCA	BRDN00012	254	238	543	464	417	17
CGGCCGAAT	BRDN00011	216	171	645	439	742	36
CGGCCGACT	BRDN00012	357	107	569	662	250	41
CGGCCGAGA	BRDN00011	140	118	489	343	441	8
CGGCCGCAA	BRDN00012	130	109	574	215	226	0
CGGCCGCAG	BRDN00011	155	93	887	220	135	14
CGGCCGCAT	BRDN00012	441	493	188	584	823	63
CGGCCGCAT	BRDN00011	43	62	0	125	60	0
CGGCCGCCC	BRDN00012	235	182	27	316	218	0
CGGCCGCGA	BRDN00011	108	162	0	249	275	23
CGGCCGCGA	BRDN00012	196	270	61	482	115	12
CGGCCGCGC	BRDN00012	70	182	27	170	23	7
CGGCCGCTC	BRDN00011	196	161	1006	430	392	56
CGGCCGCTG	BRDN00011	177	129	268	360	384	15
CGGCCGGAA	BRDN00012	224	194	125	476	273	8
CGGCCGGCC	BRDN00012	104	163	9	259	242	53
CGGCCGGCT	BRDN00011	153	125	46	340	409	25
CGGCCGTCA	BRDN00012	70	153	48	120	268	13
CGGCCGTGG	BRDN00012	99	73	14	184	212	35
CGGCCTAGA	BRDN00012	115	31	0	231	440	0
CGGCCTAGC	BRDN00011	316	473	5785	700	826	74
CGGCCTCAA	BRDN00012	66	102	13	209	282	4
CGGCCTCAC	BRDN00012	443	469	72	954	719	12
CGGCCTCCA	BRDN00012	359	217	1850	731	467	36
CGGCCTCCT	BRDN00011	58	133	160	174	221	0
CGGCCTCGG	BRDN00011	381	366	889	806	762	101
CGGCCTGCT	BRDN00011	287	130	390	436	283	9
CGGCCTGTT	BRDN00012	200	249	221	512	501	30
CGGCCTTCG	BRDN00011	84	5	14	384	252	0
CGGCCTTGC	BRDN00011	281	140	126	497	503	18
CGGCGAATC	BRDN00012	4	30	0	164	60	0
CGGCGACCA	BRDN00012	137	187	108	231	210	43
CGGCGACCC	BRDN00011	118	76	233	208	146	10
CGGCGACGA	BRDN00011	237	298	1425	334	455	97
CGGCGACGA	BRDN00012	225	202	29	435	419	3
CGGCGACGG	BRDN00012	85	34	27	149	335	0
CGGCGACGT	BRDN00011	181	143	228	317	315	15
CGGCGAGCC	BRDN00012	100	69	0	174	129	0
CGGCGATAC	BRDN00011	240	220	52	549	667	1
CGGCGATGT	BRDN00011	217	26	1	385	389	0
CGGCGCACA	BRDN00011	110	81	0	290	382	10
CGGCGCACC	BRDN00011	95	97	167	152	313	20
CGGCGCAGG	BRDN00012	167	203	0	474	270	0
CGGCGCATC	BRDN00011	132	70	1	351	105	0
CGGCGCCAG	BRDN00012	52	68	275	96	41	9
CGGCGCCCG	BRDN00011	147	166	162	309	451	5
CGGCGCCGT	BRDN00011	114	160	523	342	307	10
CGGCGCCGT	BRDN00011	163	88	729	285	282	23
CGGCGCCTC	BRDN00012	106	94	245	175	390	0
CGGCGCGCC	BRDN00012	209	2	0	497	26	0
CGGCGCGCC	BRDN00011	163	131	155	182	109	10
CGGCGCGCC	BRDN00012	150	95	157	368	302	10
CGGCGCGCT	BRDN00011	466	647	686	675	1022	19
CGGCGCTCA	BRDN00012	597	481	845	873	911	60
CGGCGCTGG	BRDN00012	264	243	1268	513	432	55
CGGCGCTTG	BRDN00011	320	218	166	428	666	0
CGGCGGCAC	BRDN00012	136	82	137	205	485	3
CGGCGGCCG	BRDN00011	347	430	167	597	977	137
CGGCGGCGA	BRDN00012	92	84	0	152	295	8
CGGCGGCGA	BRDN00011	84	116	222	168	122	17
CGGCGGCGG	BRDN00012	431	200	1482	721	680	26
CGGCGGCGG	BRDN00011	47	100	0	118	149	14
CGGCGGCTG	BRDN00011	376	543	410	625	1019	90
CGGCGGCTG	BRDN00011	375	403	1262	659	271	56
CGGCGGGAC	BRDN00011	438	323	414	682	224	15
CGGCGGGTA	BRDN00011	88	90	612	126	34	31
CGGCGGTAA	BRDN00012	265	369	403	756	879	7
CGGCGGTGC	BRDN00011	350	389	1365	524	562	76
CGGCGGTGG	BRDN00011	70	20	0	74	121	0
CGGCGGTGG	BRDN00012	264	308	117	511	177	83
CGGCGGTTG	BRDN00012	103	150	483	200	444	31
CGGCGGTTG	BRDN00011	224	156	60	394	603	9
CGGCGTAAA	BRDN00012	278	294	32	413	508	15
CGGCGTCAC	BRDN00011	363	204	376	502	430	47
CGGCGTCAG	BRDN00012	197	254	680	486	462	45
CGGCGTCCA	BRDN00012	195	161	223	272	192	4
CGGCGTCCG	BRDN00012	488	361	984	862	652	41
CGGCGTCGA	BRDN00012	191	136	461	324	334	5
CGGCGTCGG	BRDN00011	454	335	626	765	737	62
CGGCGTCTA	BRDN00012	73	49	0	161	104	0
CGGCGTGAG	BRDN00011	226	190	245	414	288	8
CGGCGTGAT	BRDN00011	112	12	1	165	47	0
CGGCGTGCA	BRDN00011	105	102	394	256	251	8
CGGCGTGCG	BRDN00012	186	40	309	243	112	7
CGGCGTGCG	BRDN00012	102	112	762	201	123	21
CGGCGTGGC	BRDN00011	544	554	627	912	906	28
CGGCGTTAT	BRDN00011	141	162	1354	155	193	51
CGGCGTTCA	BRDN00011	338	523	1587	699	688	21
CGGCGTTCG	BRDN00011	137	103	0	173	284	0
CGGCGTTGT	BRDN00011	335	429	122	641	663	70
CGGCTACAT	BRDN00011	284	95	411	399	409	34
CGGCTACCC	BRDN00012	292	238	322	497	920	69
CGGCTATGG	BRDN00011	92	0	0	276	131	0
CGGCTCAAG	BRDN00012	97	1	0	388	7	0
CGGCTCACA	BRDN00012	158	129	96	400	257	32
CGGCTCAGC	BRDN00011	69	112	1671	153	155	5
CGGCTCAGC	BRDN00011	306	282	720	581	728	24
CGGCTCAGG	BRDN00012	311	220	206	510	485	82
CGGCTCCAT	BRDN00011	150	84	104	202	216	9
CGGCTCCCC	BRDN00012	249	169	619	589	413	42
CGGCTCCGA	BRDN00012	107	250	352	213	291	108
CGGCTCGCT	BRDN00011	120	85	19	220	510	0
CGGCTCGCT	BRDN00012	229	242	868	443	461	40
CGGCTCGTT	BRDN00012	897	820	2050	1036	1495	145
CGGCTCTAG	BRDN00011	148	124	9	381	240	43
CGGCTCTCG	BRDN00012	71	72	198	144	92	1
CGGCTGACC	BRDN00011	147	113	39	190	179	9
CGGCTGAGA	BRDN00012	457	325	623	827	1014	78
CGGCTGAGG	BRDN00012	108	105	100	158	510	0
CGGCTGAGG	BRDN00011	320	67	132	559	360	0
CGGCTGAGG	BRDN00012	681	666	1851	1377	1504	29
CGGCTGCAC	BRDN00012	245	209	554	440	699	0
CGGCTGCCA	BRDN00012	149	107	0	288	352	0
CGGCTGCCA	BRDN00012	124	9	1	786	698	0
CGGCTGCCC	BRDN00011	116	129	22	233	539	10
CGGCTGCCT	BRDN00011	153	253	263	423	301	19
CGGCTGCTC	BRDN00012	232	199	1117	515	412	63
CGGCTGCTG	BRDN00012	2	0	0	307	58	0
CGGCTGGAC	BRDN00012	207	1	0	579	192	0

CGGCTGGAT	BRDN0001	520	350	551	654	916	101
CGGCTGGCT	BRDN0001	135	132	329	226	258	19
CGGCTGGTG	BRDN0001	81	0	0	119	82	0
CGGCTGTAG	BRDN0001	163	155	550	185	121	0
CGGCTGTCA	BRDN0001	170	100	94	377	325	85
CGGCTGTGT	BRDN0001	181	139	39	309	291	16
CGGCTGTTA	BRDN0001	140	53	0	257	270	0
CGGCTTCAC	BRDN0001	253	231	1667	375	842	32
CGGCTTCAG	BRDN0001	325	278	770	427	480	36
CGGCTTGGA	BRDN0001	79	131	74	142	163	0
CGGCTTGTT	BRDN0001	345	295	486	649	357	46
CGGCTTTGT	BRDN0001	327	279	270	655	367	6
CGGGAACAC	BRDN0001	120	108	1	215	357	0
CGGGAACAG	BRDN0001	130	0	1	360	256	0
CGGGAACCG	BRDN0001	470	420	1248	821	555	25
CGGGAACGT	BRDN0001	509	415	217	765	1072	26
CGGGAACGT	BRDN0001	223	41	330	413	446	19
CGGGAACTG	BRDN0001	106	57	21	110	62	0
CGGGAACTT	BRDN0001	102	0	0	399	545	0
CGGGAAGAC	BRDN0001	187	224	245	378	434	7
CGGGAAGGA	BRDN0001	40	0	0	97	5	0
CGGGAAGTA	BRDN0001	237	475	1075	568	358	39
CGGGAATAA	BRDN0001	225	101	14	450	471	27
CGGGAATAG	BRDN0001	121	42	86	185	261	0
CGGGAATCA	BRDN0001	359	321	959	514	803	27
CGGGAATCT	BRDN0001	256	319	57	512	617	38
CGGGAATTA	BRDN0001	82	173	798	136	79	4
CGGGACAAG	BRDN0001	128	123	1274	239	168	12
CGGGACACG	BRDN0001	165	120	38	296	201	0
CGGGACACG	BRDN0001	176	37	15	247	286	0
CGGGACACG	BRDN0001	419	250	65	560	563	13
CGGGACATT	BRDN0001	207	184	524	257	225	24
CGGGACCAA	BRDN0001	240	217	301	424	276	18
CGGGACCGG	BRDN0001	122	112	634	206	61	59
CGGGACCTC	BRDN0001	110	110	819	227	196	36
CGGGACGAC	BRDN0001	96	100	178	200	131	11
CGGGACGCC	BRDN0001	681	654	541	1290	913	28
CGGGACGTC	BRDN0001	297	307	175	543	746	56
CGGGACGTT	BRDN0001	267	183	44	548	473	14
CGGGACTCG	BRDN0001	228	267	757	272	383	19
CGGGACTGG	BRDN0001	62	96	8	229	478	16
CGGGACTTG	BRDN0001	329	568	1075	603	704	118
CGGGAGAAC	BRDN0001	432	328	251	609	555	35
CGGGAGAGC	BRDN0001	144	172	24	229	92	6
CGGGAGATA	BRDN0001	153	112	42	440	418	0
CGGGAGATC	BRDN0001	101	120	342	208	326	23
CGGGAGATG	BRDN0001	220	157	929	329	423	15
CGGGAGATT	BRDN0001	100	40	0	166	160	0
CGGGAGCAG	BRDN0001	230	198	763	296	171	41
CGGGAGCCG	BRDN0001	98	8	0	162	59	7
CGGGAGCCT	BRDN0001	65	137	0	147	38	0
CGGGAGCTG	BRDN0001	295	224	136	564	513	8
CGGGAGGCA	BRDN0001	274	313	343	565	585	35
CGGGAGGCA	BRDN0001	121	120	7	243	246	0
CGGGAGGCC	BRDN0001	128	92	139	220	328	4
CGGGAGGCT	BRDN0001	565	506	2750	888	1017	66
CGGGAGTAA	BRDN0001	65	108	1	137	192	10
CGGGAGTCA	BRDN0001	291	179	479	587	679	36
CGGGAGTCC	BRDN0001	178	259	86	288	275	17
CGGGAGTCG	BRDN0001	173	134	0	319	292	13
CGGGATAGA	BRDN0001	187	79	1	229	398	0
CGGGATCAG	BRDN0001	187	91	86	325	43	27
CGGGATCAT	BRDN0001	95	91	105	102	18	0
CGGGATCTT	BRDN0001	202	341	306	644	651	4
CGGGATGAA	BRDN0001	124	137	31	151	167	0
CGGGATGCA	BRDN0001	631	487	300	990	979	5
CGGGATGGT	BRDN0001	592	563	651	949	1016	61
CGGGATGTA	BRDN0001	295	298	474	499	794	37
CGGGATGTT	BRDN0001	99	58	0	174	232	11
CGGGATTCA	BRDN0001	115	110	1	201	231	0
CGGGATTGT	BRDN0001	60	53	0	199	300	8
CGGGCAACA	BRDN0001	150	98	700	253	256	29
CGGGCAACG	BRDN0001	399	503	636	713	438	11
CGGGCACAA	BRDN0001	338	336	280	794	786	2
CGGGCACAC	BRDN0001	436	432	332	688	1215	37
CGGGCACCA	BRDN0001	51	30	179	152	132	4
CGGGCACCG	BRDN0001	65	44	376	133	70	19
CGGGCACGC	BRDN0001	44	94	14	184	288	18
CGGGCACTC	BRDN0001	265	340	339	633	1593	7
CGGGCACTG	BRDN0001	182	169	68	501	388	0
CGGGCACTG	BRDN0001	98	46	135	159	140	3
CGGGCACTG	BRDN0001	127	64	0	217	168	11
CGGGCAGCG	BRDN0001	97	103	163	174	184	20
CGGGCAGCG	BRDN0001	387	347	3193	808	819	68
CGGGCAGGC	BRDN0001	171	121	419	304	107	52
CGGGCAGGT	BRDN0001	78	122	1111	189	327	117
CGGGCAGTG	BRDN0001	141	115	230	234	154	19
CGGGCATAT	BRDN0001	201	124	29	379	319	35
CGGGCATCA	BRDN0001	548	451	259	804	798	2
CGGGCATCG	BRDN0001	358	230	378	606	681	13
CGGGCATGA	BRDN0001	330	289	1153	488	666	20
CGGGCCAAG	BRDN0001	113	94	0	304	274	67
CGGGCCAAT	BRDN0001	270	289	881	492	554	40
CGGGCCAGA	BRDN0001	349	407	209	938	586	47
CGGGCCAGT	BRDN0001	142	12	50	251	35	0
CGGGCCATG	BRDN0001	91	80	0	197	176	20
CGGGCCCAA	BRDN0001	392	505	1180	623	557	237
CGGGCCCCA	BRDN0001	381	341	504	817	965	36
CGGGCCCCA	BRDN0001	77	82	71	226	272	8
CGGGCCCCA	BRDN0001	88	106	2	223	214	18
CGGGCCCGG	BRDN0001	8	0	0	10	0	0
CGGGCCCTT	BRDN0001	463	389	782	772	805	43
CGGGCCGGG	BRDN0001	457	280	3630	880	638	54
CGGGCCGTC	BRDN0001	454	387	1596	748	821	116
CGGGCCTAT	BRDN0001	403	437	224	700	761	40
CGGGCCTCA	BRDN0001	23	13	0	12	4	0
CGGGCGAGC	BRDN0001	46	12	62	152	102	0
CGGGCGAGT	BRDN0001	104	93	227	178	79	0
CGGGCGCAA	BRDN0001	107	50	41	199	314	1
CGGGCGCGC	BRDN0001	138	56	273	163	126	3
CGGGCGCGT	BRDN0001	393	549	1284	802	1291	77
CGGGCGCTA	BRDN0001	85	17	168	161	140	3
CGGGCGGTA	BRDN0001	168	128	307	341	428	73
CGGGCGTAC	BRDN0001	487	174	1286	797	617	41
CGGGCTAAT	BRDN0001	73	83	197	134	81	0
CGGGCTACT	BRDN0001	227	231	64	557	802	0
CGGGCTACT	BRDN0001	329	521	525	519	394	59
CGGGCTAGC	BRDN0001	57	51	1	91	122	0
CGGGCTCAG	BRDN0001	129	68	1150	147	136	19
CGGGCTCCC	BRDN0001	168	30	101	446	551	0
CGGGCTCCG	BRDN0001	110	66	1	226	358	0
CGGGCTCGT	BRDN0001	257	298	367	533	282	86
CGGGCTGTA	BRDN0001	146	183	64	192	167	21
CGGGCTGTT	BRDN0001	150	224	36	374	464	98
CGGGCTTCA	BRDN0001	97	0	0	247	1	0
CGGGCTTCG	BRDN0001	367	319	106	483	408	19
CGGGGAAAC	BRDN0001	255	170	194	482	516	4
CGGGGAACC	BRDN0001	149	0	0	276	304	0
CGGGGAACC	BRDN0001	109	92	34	238	219	3
CGGGGAATT	BRDN0001	427	349	589	762	894	43
CGGGGAGAA	BRDN0001	353	382	492	791	714	17
CGGGGAGCT	BRDN0001	256	227	9	514	568	6
CGGGGAGTA	BRDN0001	76	80	192	71	88	0
CGGGGAGTT	BRDN0001	167	85	393	334	632	18
CGGGGAGTT	BRDN0001	113	81	247	175	71	0
CGGGGATCA	BRDN0001	323	429	3460	709	1015	84
CGGGGATCC	BRDN0001	113	101	2765	199	395	62
CGGGGATGC	BRDN0001	50	132	510	173	81	10
CGGGGCAGC	BRDN0001	126	94	7	221	176	46
CGGGGCATT	BRDN0001	257	157	745	489	811	4
CGGGGCCCA	BRDN0001	139	108	905	271	135	18
CGGGGCCCG	BRDN0001	180	181	2215	293	303	61
CGGGGCCCG	BRDN0001	37	25	0	47	99	0
CGGGGCCCG	BRDN0001	93	58	1449	178	194	13
CGGGGCCCT	BRDN0001	429	390	426	824	665	20
CGGGGCCTG	BRDN0001	262	227	351	462	371	30
CGGGGCGCC	BRDN0001	76	54	93	246	121	22
CGGGGCGCC	BRDN0001	45	102	194	88	113	27
CGGGGCTGC	BRDN0001	69	94	67	184	124	11
CGGGGCTTT	BRDN0001	89	128	197	236	385	18
CGGGGGAGT	BRDN0001	186	247	147	416	590	104
CGGGGGCAC	BRDN0001	252	117	83	338	354	4
CGGGGGCAC	BRDN0001	23	25	105	124	11	0
CGGGGGCCC	BRDN0001	121	56	73	538	378	0
CGGGGGCGA	BRDN0001	40	20	0	81	21	0
CGGGGGCGC	BRDN0001	38	19	659	59	121	5
CGGGGGCGT	BRDN0001	281	328	13	630	481	28
CGGGGGGCC	BRDN0001	63	58	57	132	124	16
CGGGGTACT	BRDN0001	119	43	1	163	109	39
CGGGGTCAC	BRDN0001	196	138	236	269	130	3
CGGGGTGAA	BRDN0001	82	120	12	147	166	12
CGGGGTGAT	BRDN0001	235	0	1	515	33	0
CGGGGTGCA	BRDN0001	131	48	66	290	207	7
CGGGGTGGC	BRDN0001	145	98	8	256	207	21
CGGGGTGGT	BRDN0001	214	146	41	404	397	6
CGGGGTTAA	BRDN0001	102	20	15	314	461	0
CGGGTACCC	BRDN0001	246	253	166	473	484	17
CGGGTACGG	BRDN0001	218	0	0	492	342	0
CGGGTACGG	BRDN0001	265	68	28	451	511	24
CGGGTACTC	BRDN0001	182	237	360	292	412	12
CGGGTATAA	BRDN0001	143	138	107	206	185	6
CGGGTATCG	BRDN0001	165	158	2	328	231	23
CGGGTATGT	BRDN0001	311	267	56	545	461	15
CGGGTCAGG	BRDN0001	185	131	31	280	234	17
CGGGTCAGT	BRDN0001	212	186	91	403	222	21
CGGGTCCAG	BRDN0001	193	170	308	220	126	7
CGGGTCCCG	BRDN0001	163	127	658	288	181	14
CGGGTCCTC	BRDN0001	17	0	1	563	128	0
CGGGTCCTC	BRDN0001	49	44	0	213	92	0
CGGGTCCTT	BRDN0001	48	30	0	209	271	0
CGGGTCGGG	BRDN0001	129	110	405	289	291	77
CGGGTCGGG	BRDN0001	514	309	4991	709	759	142
CGGGTCTCC	BRDN0001	457	339	118	642	658	20
CGGGTCTGC	BRDN0001	164	169	642	271	343	14
CGGGTCTGT	BRDN0001	358	138	231	560	737	0
CGGGTGAAG	BRDN0001	206	238	172	540	409	44
CGGGTGATG	BRDN0001	112	54	1379	166	131	13
CGGGTGCAC	BRDN0001	363	62	219	653	489	20
CGGGTGCAG	BRDN0001	353	297	137	658	561	16
CGGGTGCGC	BRDN0001	103	98	14	188	158	0
CGGGTGCTT	BRDN0001	146	162	278	184	132	37
CGGGTGGCC	BRDN0001	56	65	356	105	67	6
CGGGTGGCT	BRDN0001	293	354	694	496	337	16
CGGGTGGTA	BRDN0001	228	233	1426	373	299	35
CGGGTGGTC	BRDN0001	184	143	45	792	559	17
CGGGTGGTT	BRDN0001	73	58	70	94	66	26
CGGGTTACA	BRDN0001	199	110	26	396	194	10
CGGGTTATG	BRDN0001	518	493	854	782	747	34
CGGGTTCAA	BRDN0001	164	61	0	212	514	6
CGGGTTCCT	BRDN0001	378	181	673	573	488	0
CGGGTTCGG	BRDN0001	40	60	17	143	316	58
CGGGTTCGG	BRDN0001	286	415	350	794	668	8
CGGGTTCTA	BRDN0001	104	129	2	289	178	1
CGGGTTGAG	BRDN0001	90	128	0	141	108	0
CGGTAAATA	BRDN0001	353	205	22	636	814	11
CGGTAACCC	BRDN0001	166	52	2	355	228	0
CGGTAACGA	BRDN0001	128	179	1009	215	39	11
CGGTAATGA	BRDN0001	232	87	567	382	364	14
CGGTACACG	BRDN0001	95	77	1015	166	91	30
CGGTACAGG	BRDN0001	543	464	358	783	877	12
CGGTACCCA	BRDN0001	189	188	242	277	583	34
CGGTACCCG	BRDN0001	133	92	87	138	163	22
CGGTACCGC	BRDN0001	266	272	673	729	367	27
CGGTACCTA	BRDN0001	308	97	683	684	700	0
CGGTACCTC	BRDN0001	145	121	0	190	239	0
CGGTACTCA	BRDN0001	68	132	77	198	187	0
CGGTAGCAG	BRDN0001	59	54	58	141	162	27
CGGTAGCAT	BRDN0001	498	476	2007	814	1675	76
CGGTAGCGC	BRDN0001	154	127	669	299	314	32
CGGTAGCTG	BRDN0001	149	45	28	171	134	0
CGGTAGGAG	BRDN0001	66	51	26	94	122	0
CGGTAGGAG	BRDN0001	92	74	5	130	170	18
CGGTAGTAG	BRDN0001	197	135	1	342	271	0
CGGTAGTAT	BRDN0001	216	225	96	489	482	24
CGGTAGTCC	BRDN0001	395	333	702	853	1668	20
CGGTAGTGC	BRDN0001	185	179	40	307	367	1
CGGTAGTTG	BRDN0001	180	177	28	301	202	1
CGGTATCAG	BRDN0001	170	160	677	396	204	39
CGGTATGTC	BRDN0001	65	64	58	194	273	0
CGGTATTGA	BRDN0001	174	51	57	333	214	0
CGGTCAAAA	BRDN0001	43	6	0	321	229	44
CGGTCAAAG	BRDN0001	369	288	19	616	548	14
CGGTCAATG	BRDN0001	88	105	43	181	213	0
CGGTCACAG	BRDN0001	189	278	57	323	140	24
CGGTCACCG	BRDN0001	173	55	13	331	56	0
CGGTCACCT	BRDN0001	154	0	0	282	248	0
CGGTCACTC	BRDN0001	236	94	33	523	407	0
CGGTCAGGA	BRDN0001	132	198	438	409	260	3
CGGTCAGTA	BRDN0001	63	52	0	180	121	3
CGGTCATCA	BRDN0001	221	358	294	282	1289	19
CGGTCATTG	BRDN0001	152	14	0	325	370	0
CGGTCCAAA	BRDN0001	93	93	0	180	128	13
CGGTCCAAC	BRDN0001	379	556	919	859	725	87
CGGTCCAGA	BRDN0001	195	178	572	269	272	9
CGGTCCAGG	BRDN0001	101	64	888	203	502	0
CGGTCCCAC	BRDN0001	411	290	1555	769	637	52
CGGTCCCCT	BRDN0001	175	128	409	372	361	66
CGGTCGCCA	BRDN0001	150	143	268	253	359	31
CGGTCGCCC	BRDN0001	115	0	0	727	217	0
CGGTCGCTA	BRDN0001	73	55	0	85	157	0
CGGTCGGCC	BRDN0001	341	290	106	565	503	13
CGGTCGTTC	BRDN0001	282	234	291	511	469	44
CGGTCGTTT	BRDN0001	46	107	66	212	175	0
CGGTCTCAC	BRDN0001	119	64	1	263	89	0
CGGTCTCAG	BRDN0001	192	207	416	417	227	15
CGGTCTCCA	BRDN0001	235	230	112	374	238	49
CGGTCTCGT	BRDN0001	92	23	1	129	52	0
CGGTCTCTA	BRDN0001	212	237	1023	514	384	44
CGGTGAAGC	BRDN0001	63	33	0	132	53	8
CGGTGAATC	BRDN0001	225	232	1418	448	443	39
CGGTGACAA	BRDN0001	39	0	0	176	36	0
CGGTGACCA	BRDN0001	187	11	0	314	348	0
CGGTGACGT	BRDN0001	100	96	20	199	244	0
CGGTGAGCC	BRDN0001	563	399	1587	1008	826	41
CGGTGAGCC	BRDN0001	218	53	1	403	253	0
CGGTGATAG	BRDN0001	4	0	0	119	16	0
CGGTGATGG	BRDN0001	192	188	1181	348	231	96
CGGTGCAAT	BRDN0001	285	215	165	559	443	20
CGGTGCAGC	BRDN0001	163	131	133	255	65	0
CGGTGCAGT	BRDN0001	222	228	99	440	418	76
CGGTGCATG	BRDN0001	355	375	563	602	555	24
CGGTGCCCA	BRDN0001	266	303	649	593	703	24
CGGTGCCGA	BRDN0001	407	323	726	645	984	96
CGGTGCCGA	BRDN0001	529	556	482	788	464	79
CGGTGCCGC	BRDN0001	291	23	125	722	242	1
CGGTGCCGG	BRDN0001	369	335	690	628	638	20
CGGTGCGGT	BRDN0001	72	56	193	137	128	12
CGGTGCGTA	BRDN0001	284	508	802	694	611	72
CGGTGCTAT	BRDN0001	500	517	2075	674	756	182
CGGTGCTCA	BRDN0001	208	243	27	454	270	37
CGGTGCTCG	BRDN0001	149	107	6	241	138	0
CGGTGCTCG	BRDN0001	289	284	371	627	623	97
CGGTGCTGG	BRDN0001	72	26	0	115	86	0
CGGTGCTGT	BRDN0001	482	361	337	836	787	29
CGGTGGCAC	BRDN0001	118	111	11	283	312	28
CGGTGGCCA	BRDN0001	136	133	114	223	520	14
CGGTGGCGC	BRDN0001	192	227	1021	452	284	13
CGGTGGCGC	BRDN0001	256	362	299	561	415	55
CGGTGGGAA	BRDN0001	117	116	210	311	208	17
CGGTGGGAC	BRDN0001	265	133	106	367	338	14
CGGTGGGCA	BRDN0001	93	26	1	204	275	0
CGGTGGGGA	BRDN0001	132	195	603	378	350	24
CGGTGGTGC	BRDN0001	18	0	0	460	104	0
CGGTGGTTG	BRDN0001	0	1	0	99	50	0
CGGTGTCCA	BRDN0001	172	99	375	288	408	20
CGGTGTCCA	BRDN0001	431	242	103	531	506	0
CGGTGTCTA	BRDN0001	54	40	0	297	107	0
CGGTGTGCC	BRDN0001	445	522	596	765	919	45
CGGTGTTCC	BRDN0001	143	146	38	266	143	46
CGGTTACCT	BRDN0001	441	399	533	776	702	25
CGGTTCAAA	BRDN0001	73	40	0	189	323	18
CGGTTCACC	BRDN0001	211	244	61	410	610	0
CGGTTCTCA	BRDN0001	131	57	96	199	53	0
CGGTTCTCA	BRDN0001	337	374	358	766	658	20
CGGTTCTCT	BRDN0001	532	554	1465	940	636	25
CGGTTGAAA	BRDN0001	251	253	253	467	332	18
CGGTTGAGC	BRDN0001	378	527	122	726	1072	20
CGGTTGATT	BRDN0001	145	180	939	300	311	38
CGGTTGCAG	BRDN0001	182	108	1127	453	423	7
CGGTTGCTG	BRDN0001	167	118	49	486	532	1
CGGTTGGGC	BRDN0001	87	108	155	120	227	5
CGGTTGGGT	BRDN0001	169	155	103	173	63	4
CGGTTTACA	BRDN0001	546	539	1669	1092	1282	65
CGTAAATCT	BRDN0001	115	113	42	254	699	0
CGTAACCAT	BRDN0001	279	258	401	386	374	26
CGTAACCTG	BRDN0001	102	101	121	200	140	26
CGTAACGGG	BRDN0001	151	135	21	270	179	0
CGTAAGCAT	BRDN0001	222	228	543	612	612	4
CGTAAGGGA	BRDN0001	153	134	5	422	375	14
CGTAAGGGT	BRDN0001	235	121	153	428	419	35
CGTAAGGTA	BRDN0001	165	291	80	133	149	123
CGTAAGGTG	BRDN0001	392	335	4028	746	643	112
CGTAATCGC	BRDN0001	221	110	1662	406	606	12
CGTAATTCA	BRDN0001	178	194	31	428	239	59
CGTAATTGA	BRDN0001	128	46	124	359	368	7
CGTAATTTG	BRDN0001	237	174	1720	479	912	90
CGTACAAAA	BRDN0001	169	84	103	435	251	0
CGTACACAA	BRDN0001	85	119	9	137	95	42
CGTACACCA	BRDN0001	48	76	14	128	80	12
CGTACACCG	BRDN0001	134	115	12	260	191	0
CGTACAGCG	BRDN0001	111	173	125	397	237	34
CGTACAGCG	BRDN0001	177	5	0	367	250	0
CGTACAGCG	BRDN0001	363	313	1498	577	609	80
CGTACAGGG	BRDN0001	86	1	0	200	101	0
CGTACATGT	BRDN0001	89	0	0	285	53	0
CGTACCAGT	BRDN0001	271	260	377	701	281	45
CGTACCCAC	BRDN0001	54	32	0	139	101	0
CGTACCCCT	BRDN0001	217	175	388	391	345	10
CGTACCGAA	BRDN0001	185	130	2116	411	892	18
CGTACCGCG	BRDN0001	68	19	58	95	56	0
CGTACCGGT	BRDN0001	248	161	198	501	940	0
CGTACCTAG	BRDN0001	295	167	556	709	749	50
CGTACCTCG	BRDN0001	103	114	146	255	353	6
CGTACCTGA	BRDN0001	168	47	10	440	487	0
CGTACCTGG	BRDN0001	175	144	243	186	683	16
CGTACCTTG	BRDN0001	353	277	35	628	1203	0
CGTACGAAG	BRDN0001	277	320	216	707	451	37
CGTACGCAG	BRDN0001	211	200	188	368	227	19
CGTACGCCA	BRDN0001	193	269	8	361	483	30
CGTACGCGC	BRDN0001	232	204	90	611	395	28
CGTACGGCG	BRDN0001	139	108	138	183	137	0
CGTACGGCT	BRDN0001	364	340	645	634	377	21
CGTACGGGA	BRDN0001	75	0	0	97	0	0
CGTACGTGT	BRDN0001	140	258	140	264	163	185
CGTACTCAT	BRDN0001	117	67	10	204	316	0
CGTACTCCA	BRDN0001	161	142	41	252	329	15
CGTACTCCA	BRDN0001	71	68	655	220	166	32
CGTACTCGT	BRDN0001	80	149	135	240	377	20
CGTACTCGT	BRDN0001	141	98	385	155	219	24
CGTACTGGC	BRDN0001	90	88	152	350	176	0
CGTACTTAC	BRDN0001	200	37	19	673	423	11
CGTACTTCG	BRDN0001	68	122	102	143	64	4
CGTACTTCT	BRDN0001	146	101	644	244	109	22
CGTACTTTC	BRDN0001	291	300	90	424	221	24
CGTAGAAAC	BRDN0001	275	286	3485	436	264	81
CGTAGAACT	BRDN0001	480	263	135	754	833	89
CGTAGACAG	BRDN0001	170	164	72	482	508	0
CGTAGACCA	BRDN0001	231	253	557	437	490	26
CGTAGACTG	BRDN0001	187	221	270	376	194	60
CGTAGAGCG	BRDN0001	99	203	109	215	271	0
CGTAGCATT	BRDN0001	60	78	0	231	139	0
CGTAGCCGT	BRDN0001	111	98	114	247	229	0
CGTAGCGAG	BRDN0001	269	329	1110	334	105	19
CGTAGCGCC	BRDN0001	17	0	0	99	0	0
CGTAGCGCC	BRDN0001	674	495	2044	1150	1748	51
CGTAGCGGC	BRDN0001	250	177	176	449	481	15
CGTAGCTGC	BRDN0001	54	38	16	96	137	0
CGTAGCTGC	BRDN0001	270	255	547	640	562	91
CGTAGGAAC	BRDN0001	125	137	28	192	142	8
CGTAGGACG	BRDN0001	140	160	19	367	234	13
CGTAGGCCA	BRDN0001	213	166	117	431	274	0
CGTAGGGAA	BRDN0001	153	135	93	429	633	10
CGTAGGTGA	BRDN0001	28	79	0	123	118	0
CGTAGTAAA	BRDN0001	275	238	583	355	374	53
CGTAGTAGC	BRDN0001	124	87	11	241	50	27
CGTAGTAGT	BRDN0001	75	16	127	157	111	9
CGTAGTCAG	BRDN0001	112	20	5	174	113	0
CGTAGTGGG	BRDN0001	69	0	0	241	84	0
CGTAGTTGT	BRDN0001	250	271	153	572	686	6
CGTATCAAG	BRDN0001	72	25	0	155	98	0
CGTATCCTTC	BRDN0001	176	165	4531	462	733	31
CGTATCGTA	BRDN0001	95	37	57	86	235	0
CGTATCTCCA	BRDN0001	279	159	0	399	82	0
CGTATGCTA	BRDN0001	125	90	3694	234	148	57
CGTATGCTCT	BRDN0001	37	6	53	218	38	0
CGTATGGAT	BRDN0001	332	284	787	603	470	12
CGTATGGGA	BRDN0001	69	58	0	208	194	0
CGTATGTCCT	BRDN0001	130	97	132	264	96	0
CGTATGTGC	BRDN0001	264	318	176	488	436	25
CGTATTCGA	BRDN0001	322	242	106	688	861	17
CGTATTTGA	BRDN0001	79	104	67	247	91	19
CGTCAAACA	BRDN0001	113	118	925	329	191	24
CGTCAAAGC	BRDN0001	336	397	351	609	585	18
CGTCAACAG	BRDN0001	297	210	17	569	381	150
CGTCAACCTG	BRDN0001	134	132	20	308	444	5
CGTCAAGAA	BRDN0001	280	303	116	406	490	84
CGTCAAGAA	BRDN0001	0	0	0	105	0	0
CGTCAAGTA	BRDN0001	58	0	1	451	233	0
CGTCAAGTA	BRDN0001	293	295	111	617	492	6
CGTCAATAG	BRDN0001	200	209	881	503	552	24
CGTCAATTAT	BRDN0001	161	107	41	206	205	0
CGTCACAAC	BRDN0001	16	16	0	253	22	0
CGTCACAAT	BRDN0001	76	16	0	549	309	0
CGTCACACA	BRDN0001	134	10	0	418	609	0
CGTCACATG	BRDN0001	180	83	5	184	231	0
CGTCACATTC	BRDN0001	193	182	886	290	369	17
CGTCACCAG	BRDN0001	155	62	94	321	413	0
CGTCACCGA	BRDN0001	321	126	198	588	387	0
CGTCACCTG	BRDN0001	67	33	0	151	84	0
CGTCACGCC	BRDN0001	110	107	464	243	192	16
CGTCACGGC	BRDN0001	283	247	998	552	785	6
CGTCACGTG	BRDN0001	188	159	66	371	693	166
CGTCACTGG	BRDN0001	68	92	65	126	62	8
CGTCACTGG	BRDN0001	213	114	627	314	339	10
CGTCACTGG	BRDN0001	116	208	0	290	176	0
CGTCAGACA	BRDN0001	101	40	0	109	6	0
CGTCAGACG	BRDN0001	283	316	835	656	609	13
CGTCAGCCTT	BRDN0001	355	253	284	605	247	9
CGTCAGCGC	BRDN0001	265	295	335	469	355	0
CGTCAGCGT	BRDN0001	174	278	224	409	677	10
CGTCAGGCA	BRDN0001	476	398	1121	820	655	30
CGTCAGTGA	BRDN0001	134	261	507	356	237	13
CGTCAGTTTC	BRDN0001	93	50	553	175	267	16
CGTCATAATA	BRDN0001	55	19	0	125	210	0
CGTCATAGC	BRDN0001	235	328	117	380	298	19
CGTCATATAC	BRDN0001	250	295	353	402	280	6
CGTCATCACA	BRDN0001	97	115	0	132	117	0
CGTCATCACC	BRDN0001	171	215	62	486	913	4
CGTCATCCAC	BRDN0001	178	198	92	267	206	3
CGTCATCCTG	BRDN0001	97	38	216	131	70	21
CGTCATGCCT	BRDN0001	248	59	653	514	270	14
CGTCATGGT	BRDN0001	219	303	21	410	1104	27
CGTCATGTG	BRDN0001	236	180	132	464	712	15
CGTCATTCGA	BRDN0001	6	0	0	419	68	0
CGTCATTGA	BRDN0001	108	268	1445	333	450	24
CGTCATTGA	BRDN0001	47	1	0	162	193	0
CGTCCAACTC	BRDN0001	89	129	8	236	103	25
CGTCCAATTT	BRDN0001	105	188	82	244	171	28
CGTCCACAGC	BRDN0001	138	132	44	228	401	11
CGTCCACCAC	BRDN0001	54	9	0	288	242	0
CGTCCACGTC	BRDN0001	67	16	0	197	241	0
CGTCCAGAA	BRDN0001	409	228	66	830	788	16
CGTCCAGAG	BRDN0001	71	103	0	170	73	7
CGTCCAGAT	BRDN0001	145	228	183	451	281	15
CGTCCAGCA	BRDN0001	106	97	511	162	152	2

CGTCCAGGA	BRDN00012	113	100	239	160	88	9
CGTCCAGGG	BRDN00011	132	120	181	211	237	39
CGTCCATGC	BRDN00012	82	21	1982	192	164	6
CGTCCATGC	BRDN00011	56	71	22	225	229	6
CGTCCATGG	BRDN00011	225	65	101	581	422	1
CGTCCCACTC	BRDN00012	183	157	530	349	456	17
CGTCCCCAAA	BRDN00012	110	89	201	186	268	0
CGTCCCCGCC	BRDN00012	186	150	41	235	425	11
CGTCCCCGG	BRDN00012	284	259	562	522	158	6
CGTCCCCTGT	BRDN00012	362	185	248	805	694	4
CGTCCCCTGT	BRDN00011	121	133	65	343	563	18
CGTCCCGCA	BRDN00011	133	72	521	325	183	26
CGTCCCTCAG	BRDN00012	265	233	1	557	650	7
CGTCCCTCCG	BRDN00012	104	0	0	289	631	0
CGTCCCTGA	BRDN00012	237	216	489	452	418	34
CGTCCCTTCG	BRDN00012	3	4	0	0	0	0
CGTCCGCAA	BRDN00011	64	45	0	45	314	0
CGTCCGCAC	BRDN00011	167	115	1414	259	392	32
CGTCCGCACC	BRDN00012	216	164	43	404	156	46
CGTCCGCCCG	BRDN00012	133	52	0	176	129	4
CGTCCGCTG	BRDN00012	343	333	11	440	445	0
CGTCCGCTGT	BRDN00011	93	183	1068	162	133	59
CGTCCGTGA	BRDN00012	109	146	11	242	313	22
CGTCCTAGG	BRDN00011	224	129	182	334	334	37
CGTCCTCAAA	BRDN00012	217	267	219	498	409	71
CGTCCTCAAA	BRDN00011	118	32	0	344	155	0
CGTCCTCTTA	BRDN00011	156	176	649	225	288	12
CGTCCTGAA	BRDN00011	217	177	210	542	283	8
CGTCGAGCT	BRDN00012	49	102	6	112	68	0
CGTCGAGTG	BRDN00012	235	192	641	542	446	45
CGTCGATGA	BRDN00011	148	78	163	99	195	0
CGTCGATGC	BRDN00011	140	164	48	117	123	0
CGTCGCACC	BRDN00011	257	230	341	510	474	24
CGTCGCAGG	BRDN00011	262	321	642	401	772	48
CGTCGCAGG	BRDN00011	259	151	207	529	283	90
CGTCGCCAT	BRDN00012	206	230	137	530	607	23
CGTCGCCCA	BRDN00012	68	75	510	92	63	18
CGTCGCCGG	BRDN00012	252	244	315	440	221	53
CGTCGCCGG	BRDN00011	124	233	760	192	74	0
CGTCGCGAT	BRDN00011	145	111	27	293	57	16
CGTCGCGGT	BRDN00011	118	58	92	214	277	0
CGTCGCTGA	BRDN00012	69	47	270	180	379	5
CGTCGCTGG	BRDN00011	49	64	208	198	136	19
CGTCGCTGT	BRDN00011	36	13	0	71	0	1
CGTCGGACG	BRDN00011	104	105	11	137	0	0
CGTCGGAGG	BRDN00012	125	84	11	196	198	32
CGTCGGATC	BRDN00012	266	448	14687	595	626	114
CGTCGGCCA	BRDN00011	27	5	0	165	116	0
CGTCGGCCA	BRDN00012	260	250	275	525	373	82
CGTCGGCGA	BRDN00011	94	66	0	319	287	0
CGTCGGCGC	BRDN00012	128	46	7	274	414	0
CGTCGGCGT	BRDN00012	109	92	14	169	74	30
CGTCGGGCT	BRDN00012	273	110	162	579	618	12
CGTCGGGCT	BRDN00011	61	105	4	245	178	29
CGTCGGGGA	BRDN00012	110	64	117	154	168	0
CGTCGGGTA	BRDN00012	256	238	120	473	480	1
CGTCGTAGG	BRDN00012	82	152	0	209	52	18
CGTCGTCCCC	BRDN00012	47	13	0	81	53	0
CGTCGTCTTC	BRDN00012	155	42	70	285	374	29
CGTCGTTGA	BRDN00011	57	29	0	146	136	0
CGTCGTTGC	BRDN00012	189	256	523	552	574	0
CGTCGTTGT	BRDN00011	172	222	192	318	100	450
CGTCTAAAG	BRDN00012	224	258	633	356	328	12
CGTCTAAGC	BRDN00011	152	79	85	249	226	0
CGTCTACAG	BRDN00011	168	216	3869	547	512	90
CGTCTACCTG	BRDN00012	57	10	1	143	83	0
CGTCTACTAC	BRDN00012	229	234	179	438	480	27
CGTCTGAGC	BRDN00012	162	69	58	230	99	0
CGTCTGCAA	BRDN00012	182	215	1186	288	119	41
CGTCTGCAT	BRDN00012	136	41	1	306	108	0
CGTCTGGAA	BRDN00012	18	0	0	405	105	0
CGTCTGTCCG	BRDN00012	120	193	1	254	269	0
CGTCTTAAGT	BRDN00012	113	63	548	119	32	5
CGTCTTAGG	BRDN00012	101	116	347	326	287	23
CGTCTTCAG	BRDN00011	239	267	112	388	835	33
CGTCTTCATC	BRDN00012	71	147	1	320	164	0
CGTCTTCCCA	BRDN00012	160	145	387	229	206	7
CGTCTTGATT	BRDN00012	214	278	20	325	193	100
CGTCTTTACA	BRDN00012	95	114	2219	182	41	22
CGTCTTTCCA	BRDN00012	120	90	99	154	219	0
CGTCTTTGG	BRDN00011	184	247	0	351	341	0
CGTGAAAAG	BRDN00012	209	61	168	608	534	15
CGTGAACCA	BRDN00012	286	281	408	540	677	6
CGTGAACTA	BRDN00012	250	321	54	418	217	21
CGTGAACTA	BRDN00011	306	211	269	469	846	19
CGTGAACTC	BRDN00011	266	361	482	576	472	13
CGTGAACTT	BRDN00011	118	64	0	185	112	0
CGTGAAGGC	BRDN00011	172	224	46	477	285	29
CGTGAATAA	BRDN00012	256	299	735	494	860	16
CGTGAATGA	BRDN00011	105	68	0	206	171	0
CGTGACAGG	BRDN00011	301	241	297	484	516	16
CGTGACATT	BRDN00012	125	114	81	226	105	1
CGTGACCCT	BRDN00011	121	29	289	502	326	1
CGTGACCGG	BRDN00012	65	12	0	429	165	0
CGTGACGCC	BRDN00011	298	195	363	582	714	35
CGTGACGCG	BRDN00012	323	263	752	582	761	98
CGTGACGGA	BRDN00012	215	222	302	464	421	33
CGTGACTAT	BRDN00011	57	84	86	216	304	11
CGTGACTCA	BRDN00012	110	76	299	224	430	23
CGTGACTCG	BRDN00012	49	54	1	233	343	0
CGTGACTGA	BRDN00011	296	242	56	493	666	12
CGTGACTGA	BRDN00011	131	226	67	161	59	0
CGTGACTGA	BRDN00011	287	255	2159	451	487	34
CGTGAGCAA	BRDN00012	89	101	0	170	59	12
CGTGAGCAC	BRDN00012	385	306	1082	545	670	38
CGTGAGCCT	BRDN00011	107	60	0	206	110	0
CGTGAGCGG	BRDN00012	97	57	94	87	135	0
CGTGAGGCC	BRDN00012	326	276	220	668	917	11
CGTGAGGGA	BRDN00011	68	72	0	212	108	0
CGTGAGGTC	BRDN00012	31	16	0	91	177	0
CGTGAGTCT	BRDN00012	145	190	103	239	315	6
CGTGAGTGA	BRDN00011	244	314	334	540	446	47
CGTGAGTGT	BRDN00012	266	331	996	555	561	32
CGTGATCAC	BRDN00011	125	47	274	196	239	3
CGTGATCAC	BRDN00011	271	268	502	339	364	0
CGTGATCAC	BRDN00012	72	38	37	150	56	0
CGTGATCGT	BRDN00012	382	269	1760	637	882	17
CGTGATGAC	BRDN00011	119	75	113	205	191	0
CGTGATGAT	BRDN00012	219	141	2326	245	305	119
CGTGATGGA	BRDN00012	71	61	89	325	482	6
CGTGATTAA	BRDN00012	73	98	1263	179	34	44
CGTGATTCC	BRDN00012	101	94	15	256	274	0
CGTGATTGT	BRDN00011	195	202	300	280	386	21
CGTGCAACA	BRDN00011	98	5	0	190	117	0
CGTGCAACG	BRDN00011	152	126	114	256	369	0
CGTGCAAGC	BRDN00012	286	321	104	340	446	8
CGTGCACAA	BRDN00011	110	142	30	298	202	6
CGTGCACAT	BRDN00012	21	17	147	218	155	0
CGTGCACGA	BRDN00011	167	132	217	370	530	0
CGTGCACGC	BRDN00012	368	287	78	521	435	0
CGTGCACGT	BRDN00012	214	313	971	559	290	10
CGTGCACTA	BRDN00012	54	29	0	224	29	0
CGTGCACTCT	BRDN00012	129	84	976	270	615	14
CGTGCACTG	BRDN00011	405	344	170	672	1005	78
CGTGCACTT	BRDN00011	119	96	0	300	600	19
CGTGCAGAA	BRDN00012	336	302	1169	606	767	16
CGTGCAGAA	BRDN00011	431	468	648	861	936	40
CGTGCAGAG	BRDN00011	24	0	0	138	190	0
CGTGCAGAT	BRDN00011	182	211	17	343	346	7
CGTGCAGCA	BRDN00011	69	44	40	120	28	11
CGTGCAGCA	BRDN00012	424	351	1151	697	747	11
CGTGCAGCG	BRDN00012	85	159	779	225	334	33
CGTGCAGGA	BRDN00011	96	88	0	155	163	1
CGTGCAGGA	BRDN00012	105	154	159	146	300	37
CGTGCAGGG	BRDN00012	116	129	80	252	89	0
CGTGCAGGT	BRDN00012	211	91	1131	465	513	74
CGTGCATGC	BRDN00011	182	182	399	427	352	25
CGTGCCACA	BRDN00012	411	432	553	728	427	22
CGTGCCACG	BRDN00012	126	62	154	283	449	31
CGTGCCAGT	BRDN00012	117	43	543	242	220	35
CGTGCCATA	BRDN00011	227	146	56	478	437	0
CGTGCCATG	BRDN00011	69	164	0	204	304	10
CGTGCCCGA	BRDN00011	231	169	304	351	342	10
CGTGCCCGCT	BRDN00011	240	245	77	389	1353	21
CGTGCCCGG	BRDN00011	278	496	1437	521	816	101
CGTGCCGCA	BRDN00012	361	306	432	516	195	69
CGTGCCGCC	BRDN00012	219	165	0	370	466	4
CGTGCCGTC	BRDN00011	134	94	23	478	254	2
CGTGCCTAA	BRDN00012	130	66	198	188	186	16
CGTGCCTCC	BRDN00012	172	282	36	238	260	50
CGTGCCTCTT	BRDN00012	207	50	4142	535	504	103
CGTGCCTGA	BRDN00012	358	601	530	553	568	97
CGTGCCTGT	BRDN00012	194	116	222	256	486	8
CGTGCCTTTA	BRDN00012	394	328	195	557	692	20
CGTGCGCAC	BRDN00012	121	116	255	290	224	0
CGTGCGCCA	BRDN00012	78	69	12	173	238	0
CGTGCGCGT	BRDN00011	291	317	914	546	553	52
CGTGCGCTG	BRDN00011	107	71	0	195	183	3
CGTGCGGCT	BRDN00011	84	110	0	136	173	29
CGTGCGGGA	BRDN00012	30	0	0	197	0	0
CGTGCGGGA	BRDN00011	192	240	42	393	408	0
CGTGCGGGT	BRDN00011	161	147	58	323	60	7
CGTGCGGGT	BRDN00011	116	166	2	214	266	6
CGTGCGGGT	BRDN00011	161	101	142	297	203	8
CGTGCGGTA	BRDN00012	272	219	668	381	180	37
CGTGCGGTA	BRDN00012	259	311	994	481	296	39
CGTGCGTGA	BRDN00012	353	280	3303	500	230	103
CGTGCTAAC	BRDN00011	50	34	0	138	24	0
CGTGCTCAA	BRDN00012	127	114	54	337	416	0
CGTGCTCAC	BRDN00011	102	177	730	121	31	11
CGTGCTCAG	BRDN00011	281	158	810	563	499	25
CGTGCTCCCT	BRDN00011	66	40	0	187	135	0
CGTGCTCTTC	BRDN00011	165	67	435	282	436	14
CGTGCTGAT	BRDN00012	116	147	41	299	244	0
CGTGCTGCA	BRDN00012	109	94	52	143	38	0
CGTGCTGCC	BRDN00012	56	67	66	172	230	20
CGTGCTGGT	BRDN00011	58	167	0	170	182	0
CGTGCTGGT	BRDN00012	162	173	40	382	353	11
CGTGCTGGT	BRDN00012	182	236	258	426	329	2
CGTGCTGTT	BRDN00011	286	345	309	497	761	70
CGTGCTTGT	BRDN00012	142	141	591	205	270	11
CGTGCTTGT	BRDN00011	85	69	8	136	181	0
CGTGGAAAA	BRDN00011	262	277	486	508	501	15
CGTGGAACA	BRDN00011	124	114	118	274	177	1
CGTGGAAGA	BRDN00011	20	1	0	54	7	0
CGTGGAAGA	BRDN00012	242	141	80	361	308	17
CGTGGACAA	BRDN00011	185	189	174	250	267	10
CGTGGACAT	BRDN00011	283	185	984	595	1167	47
CGTGGACCA	BRDN00012	28	10	0	135	161	0
CGTGGACCC	BRDN00012	249	132	28	539	242	3
CGTGGACGA	BRDN00012	64	48	139	135	81	5
CGTGGACGA	BRDN00011	33	39	2	88	45	26
CGTGGACGG	BRDN00011	148	97	265	232	245	0
CGTGGACGG	BRDN00012	144	78	469	441	308	24
CGTGGACGT	BRDN00012	230	21	819	394	500	3
CGTGGAGAA	BRDN00011	62	76	80	120	180	0
CGTGGAGCA	BRDN00011	197	153	11	392	141	15
CGTGGAGGA	BRDN00011	237	82	335	594	320	2
CGTGGAGGA	BRDN00011	1	0	0	142	75	0
CGTGGAGGC	BRDN00011	79	35	674	321	279	6
CGTGGATCT	BRDN00012	238	48	10	311	60	0
CGTGGATGA	BRDN00012	103	36	39	189	89	8
CGTGGATGA	BRDN00011	150	0	1	388	173	0
CGTGGCACT	BRDN00011	94	54	97	167	130	6
CGTGGCAGC	BRDN00011	28	47	0	195	236	0
CGTGGCAGG	BRDN00011	157	191	33155	262	257	369
CGTGGCAGT	BRDN00012	39	84	73	198	81	12
CGTGGCATA	BRDN00012	144	85	68	224	155	7
CGTGGCATT	BRDN00011	134	49	83	237	299	0
CGTGGCCAT	BRDN00011	581	541	1352	858	739	82
CGTGGCCAT	BRDN00011	213	0	1	386	69	0
CGTGGCCGA	BRDN00011	222	49	2	442	352	7
CGTGGCCGA	BRDN00011	179	160	791	366	242	12
CGTGGCCGG	BRDN00012	537	519	716	753	1287	21
CGTGGCCTA	BRDN00011	104	167	67	318	116	0
CGTGGCGAA	BRDN00012	305	130	126	664	615	19
CGTGGCGGC	BRDN00011	175	217	189	563	603	6
CGTGGCGTG	BRDN00011	67	89	66	134	85	8
CGTGGCTGT	BRDN00012	328	184	1632	492	472	28
CGTGGCTGT	BRDN00012	229	169	24	283	296	39
CGTGGGACG	BRDN00012	184	241	21	472	191	15
CGTGGGCAA	BRDN00011	215	73	363	211	422	17
CGTGGGCAA	BRDN00011	145	108	64	255	162	3
CGTGGGCAC	BRDN00011	334	248	192	578	519	24
CGTGGGCCA	BRDN00012	399	227	563	626	704	75
CGTGGGCCG	BRDN00012	181	98	33	353	210	0
CGTGGGCGG	BRDN00012	163	89	413	254	240	15
CGTGGGCTG	BRDN00011	77	70	69	99	38	22
CGTGGGCTT	BRDN00011	188	306	232	445	379	29
CGTGGGCTT	BRDN00011	103	84	264	144	169	1
CGTGGGGAC	BRDN00011	98	5	0	168	83	0
CGTGGGGGA	BRDN00012	259	239	176	446	349	5
CGTGGGGGT	BRDN00012	169	124	109	439	329	0
CGTGGGGGT	BRDN00012	121	65	1277	183	196	13
CGTGGGTAA	BRDN00012	61	172	93	205	102	14
CGTGGGTAA	BRDN00012	272	142	637	371	845	0
CGTGGGTCG	BRDN00012	114	69	78	189	166	52
CGTGGTAAA	BRDN00011	89	57	244	75	11	0
CGTGGTAAA	BRDN00012	78	83	108	174	143	0
CGTGGTAAA	BRDN00012	239	298	205	376	703	0
CGTGGTACC	BRDN00012	66	59	43	257	251	8
CGTGGTACC	BRDN00012	341	267	255	514	250	15
CGTGGTAGG	BRDN00012	264	261	267	558	420	15
CGTGGTAGG	BRDN00012	346	413	978	616	713	28
CGTGGTCAT	BRDN00011	363	178	423	529	629	50
CGTGGTCCC	BRDN00012	155	96	203	451	651	15
CGTGGTCTT	BRDN00011	93	97	52	112	50	19
CGTGGTGCC	BRDN00012	154	106	0	195	169	8
CGTGGTGCT	BRDN00012	105	43	58	255	254	24
CGTGGTGCT	BRDN00012	131	189	51	390	408	29
CGTGGTGGC	BRDN00011	55	116	292	109	46	45
CGTGGTGTA	BRDN00011	126	89	82	199	292	0
CGTGGTGTA	BRDN00011	83	43	0	211	109	0
CGTGGTGTG	BRDN00011	138	85	1279	416	568	2
CGTGGTTAA	BRDN00012	261	125	56	439	401	28
CGTGGTTCA	BRDN00011	191	265	137	396	339	0
CGTGGTTGA	BRDN00011	79	84	8	191	504	0
CGTGGTTGT	BRDN00012	183	143	21	436	591	24
CGTGTAAAA	BRDN00012	285	179	137	450	795	18
CGTGTAATG	BRDN00012	111	64	453	94	145	10
CGTGTACAA	BRDN00011	138	227	106	350	380	52
CGTGTACAA	BRDN00012	86	10	0	224	237	0
CGTGTACAT	BRDN00011	108	197	232	231	331	0
CGTGTACCA	BRDN00011	154	120	741	324	279	21
CGTGTATGC	BRDN00011	88	122	0	152	130	0
CGTGTATGT	BRDN00012	81	64	6	265	197	0
CGTGTCACA	BRDN00012	366	286	296	793	1183	43
CGTGTCAGG	BRDN00011	110	114	38	264	859	0
CGTGTCATT	BRDN00011	140	124	1066	215	313	19
CGTGTCATT	BRDN00011	290	359	747	681	737	85
CGTGTCCAT	BRDN00011	96	159	45	218	72	62
CGTGTCCCT	BRDN00011	106	30	0	282	170	0
CGTGTCCGA	BRDN00012	260	243	324	358	355	0
CGTGTCGGC	BRDN00012	234	128	0	245	412	0
CGTGTCTAG	BRDN00011	279	107	1012	431	614	30
CGTGTCTGT	BRDN00011	112	55	211	171	194	34
CGTGTCTGT	BRDN00012	229	287	1627	362	191	29
CGTGTCTGT	BRDN00012	35	0	0	403	7	0
CGTGTGATC	BRDN00011	88	17	0	116	203	0
CGTGTGCAC	BRDN00011	271	123	62	637	496	0
CGTGTGCCC	BRDN00011	115	159	364	353	399	13
CGTGTGCGT	BRDN00011	96	110	170	180	132	21
CGTGTGGAC	BRDN00011	282	212	523	655	451	0
CGTGTGGCC	BRDN00011	144	48	44	299	273	0
CGTGTGGCG	BRDN00012	17	0	0	153	52	0
CGTGTGGTA	BRDN00012	63	20	13	157	61	44
CGTGTGTGG	BRDN00012	438	429	503	662	1023	52
CGTGTTAGT	BRDN00011	115	118	254	296	232	76
CGTGTTCAA	BRDN00012	54	0	21	155	92	0
CGTGTTCAA	BRDN00011	87	128	415	275	352	0
CGTGTTCAT	BRDN00011	162	255	631	221	280	19
CGTGTTGAA	BRDN00011	69	70	0	159	33	0
CGTGTTGAC	BRDN00011	83	36	429	190	166	7
CGTGTTGGC	BRDN00012	203	231	754	432	328	12
CGTGTTTCT	BRDN00011	123	110	0	241	335	13
CGTGTTTGG	BRDN00012	343	337	630	645	678	5
CGTTAAACC	BRDN00012	388	505	187	644	391	19
CGTTAACCG	BRDN00011	135	151	1	248	170	0
CGTTAAGAC	BRDN00012	393	273	1025	729	1034	37
CGTTAATGA	BRDN00011	318	153	22	640	799	20
CGTTACCAA	BRDN00011	260	349	2213	544	385	54
CGTTACCCAG	BRDN00012	324	92	1	519	401	1
CGTTACCCAG	BRDN00011	261	219	892	430	702	63
CGTTACCGT	BRDN00011	197	171	42	281	107	6
CGTTACCTAC	BRDN00011	80	77	433	174	155	15
CGTTACCTG	BRDN00011	92	108	0	267	155	0
CGTTAGCAA	BRDN00012	253	190	190	488	508	14
CGTTAGGTA	BRDN00011	65	23	142	68	35	0
CGTTATCATC	BRDN00012	138	146	380	242	116	17
CGTTATCCA	BRDN00012	189	188	111	361	563	0
CGTTCAACA	BRDN00011	327	231	572	624	458	10
CGTTCAAGT	BRDN00011	73	89	0	211	130	0
CGTTCACCCA	BRDN00012	324	13	0	593	104	0
CGTTCACGA	BRDN00012	341	396	152	656	761	17
CGTTCAGGG	BRDN00011	133	209	206	153	293	41
CGTTCATCG	BRDN00011	313	244	815	566	492	8
CGTTCCAACT	BRDN00012	439	449	350	738	529	22
CGTTCCCAAG	BRDN00012	418	343	764	502	385	22
CGTTCCCAAT	BRDN00011	288	227	214	430	324	11
CGTTCCCGCT	BRDN00012	97	118	1098	280	102	26
CGTTCCCTAC	BRDN00011	88	31	321	153	141	0
CGTTCCGCG	BRDN00012	55	45	0	144	81	0
CGTTCCTCTT	BRDN00011	86	69	0	214	232	9
CGTTCGAGA	BRDN00011	110	20	1	247	103	0
CGTTCGATG	BRDN00012	94	46	101	115	267	0
CGTTCGGGC	BRDN00012	261	249	145	534	351	3
CGTTCGTAG	BRDN00011	138	164	300	171	263	5
CGTTCTCAG	BRDN00011	77	98	456	246	223	6
CGTTCTCCC	BRDN00012	183	78	0	382	346	1
CGTTCTCGG	BRDN00012	70	39	0	175	3	0
CGTTCTCGTA	BRDN00011	172	47	0	346	244	0
CGTTCTCTGG	BRDN00012	259	137	131	482	533	6
CGTTCTGTG	BRDN00011	202	226	356	322	737	9
CGTTGAAGA	BRDN00011	283	280	327	685	704	13
CGTTGAAGA	BRDN00011	221	130	97	328	198	2
CGTTGACAA	BRDN00012	144	259	7664	414	291	91
CGTTGAGGC	BRDN00011	51	42	0	182	488	2
CGTTGCCAC	BRDN00011	256	170	36	477	196	69
CGTTGCCCA	BRDN00011	118	92	8	623	488	0
CGTTGCCGT	BRDN00011	112	66	0	234	392	6
CGTTGCGAA	BRDN00012	94	128	926	266	300	13
CGTTGCGCA	BRDN00012	184	161	110	427	611	49
CGTTGCGCT	BRDN00011	189	164	1643	326	195	34
CGTTGCTCA	BRDN00011	252	258	50	501	338	21
CGTTGCTCCA	BRDN00012	54	1	0	506	439	0
CGTTGCTGG	BRDN00012	86	31	42	166	112	0
CGTTGGCAA	BRDN00012	92	68	444	229	505	0
CGTTGGCGG	BRDN00011	217	197	80	387	738	44
CGTTGGCTA	BRDN00011	316	282	356	629	631	23
CGTTGGCTG	BRDN00011	173	188	1795	392	414	13
CGTTGGGCA	BRDN00011	204	324	2941	422	1283	106
CGTTGGGCA	BRDN00012	403	313	332	583	538	0
CGTTGGGGC	BRDN00011	108	95	258	242	312	28
CGTTGTAGG	BRDN00011	138	16	0	230	92	0
CGTTGTAGG	BRDN00012	479	442	326	627	442	39
CGTTGTCCTC	BRDN00011	49	0	145	317	283	12
CGTTGTGCA	BRDN00012	297	220	244	470	207	14
CGTTTAAGC	BRDN00012	241	35	0	645	577	0
CGTTTACGA	BRDN00011	81	0	0	94	17	0
CGTTTACTG	BRDN00012	116	89	11563	289	49	147
CGTTTCACCT	BRDN00011	180	143	516	323	487	29
CGTTTCATAG	BRDN00012	127	87	92	270	295	0
CGTTTCTTCT	BRDN00011	182	134	23	352	89	2
CGTTTGACG	BRDN00012	203	225	39	235	302	8
CGTTTGGGG	BRDN00012	235	330	152	418	579	9
CGTTTGTCAT	BRDN00012	101	15	1	634	439	0
CTAAAAAAG	BRDN00011	132	207	63	229	160	52
CTAAAAATG	BRDN00011	328	240	184	532	518	32
CTAAAACAG	BRDN00012	354	376	361	875	552	40
CTAAAACCTC	BRDN00011	175	298	280	489	263	20
CTAAAACTTC	BRDN00011	340	376	1160	528	509	7
CTAAAAGTG	BRDN00011	126	98	1	187	52	0
CTAAAATTTT	BRDN00012	317	278	648	622	749	76
CTAAACAACT	BRDN00011	193	254	72	505	1108	15
CTAAACATCA	BRDN00012	189	141	167	358	383	0
CTAAACCAG	BRDN00012	192	161	241	308	143	0
CTAAACCGTC	BRDN00011	87	10	108	226	215	0
CTAAACTACA	BRDN00011	198	255	679	450	210	98
CTAAACTCAC	BRDN00011	434	347	368	840	956	61
CTAAAGATG	BRDN00012	294	145	214	420	542	13
CTAAAGCTA	BRDN00011	30	25	279	108	107	18
CTAAAGCTG	BRDN00011	186	193	166	272	122	9
CTAAAGGAA	BRDN00012	180	169	4	221	108	0
CTAAAGGCT	BRDN00012	155	177	405	214	206	57
CTAAAGTAA	BRDN00012	116	121	184	153	135	0
CTAAAGTAG	BRDN00012	335	331	321	551	456	26
CTAAAGTCT	BRDN00012	451	481	1450	1079	751	133
CTAAAGTTCT	BRDN00011	148	167	1361	251	118	6
CTAAATAACT	BRDN00012	190	0	0	440	283	1
CTAAATACA	BRDN00012	203	389	280	308	382	38
CTAAATGAA	BRDN00011	104	2	23	155	124	0
CTAAATGCT	BRDN00012	155	83	1342	235	257	29
CTAAATGGG	BRDN00012	82	25	0	152	60	0
CTAAATGTG	BRDN00011	168	223	726	443	336	48
CTAACAATAC	BRDN00011	181	13	239	518	676	0
CTAACACACA	BRDN00011	298	399	182	641	734	9
CTAACACTCA	BRDN00011	222	212	1132	359	489	124
CTAACACTGA	BRDN00011	136	132	22	285	361	19
CTAACAGAA	BRDN00012	109	169	1	218	81	0
CTAACAGGA	BRDN00012	123	0	1	375	8	0
CTAACAGGA	BRDN00011	114	0	0	271	388	0
CTAACAGGT	BRDN00011	145	86	28	152	93	0
CTAACAGTAC	BRDN00011	319	305	509	557	621	6
CTAACAGTG	BRDN00011	137	75	0	164	85	0
CTAACATATC	BRDN00011	334	365	262	626	756	17
CTAACATCAA	BRDN00011	267	285	552	335	270	8
CTAACATCAA	BRDN00011	152	171	401	447	462	22
CTAACATGAC	BRDN00012	420	398	216	556	549	31
CTAACATTGA	BRDN00012	157	202	12	280	351	5
CTAACCAGA	BRDN00012	218	75	147	353	209	0
CTAACCCCTG	BRDN00011	315	351	1192	666	680	80
CTAACCGTTA	BRDN00012	328	258	82	574	553	23
CTAACCGTTC	BRDN00012	301	350	2135	430	447	42
CTAACCTGCC	BRDN00012	240	179	507	308	319	17
CTAACCTGTC	BRDN00012	440	525	264	889	640	109
CTAACCTGTT	BRDN00012	245	194	34	278	315	17
CTAACGCAG	BRDN00011	222	197	128	485	603	14
CTAACGGAC	BRDN00012	501	267	106	747	497	108
CTAACTCCTT	BRDN00011	78	70	16	205	285	16
CTAACTCTGC	BRDN00012	32	0	0	212	70	0
CTAACTTGG	BRDN00012	109	64	10	284	137	31
CTAACTTGGT	BRDN00012	197	123	103	490	411	34
CTAAGAAAG	BRDN00012	120	53	202	212	135	0
CTAAGAAAG	BRDN00012	352	398	771	536	515	39
CTAAGAATT	BRDN00011	229	198	1176	425	843	56
CTAAGACCA	BRDN00011	185	42	0	488	121	0
CTAAGAGAG	BRDN00011	129	107	10	211	222	11
CTAAGAGCC	BRDN00012	89	217	1096	237	347	15
CTAAGAGGA	BRDN00011	60	81	38	126	29	0
CTAAGAGGG	BRDN00011	169	159	1009	341	381	22
CTAAGATGA	BRDN00011	271	227	1776	426	671	23
CTAAGCAAA	BRDN00011	144	82	26	234	200	0
CTAAGCAGA	BRDN00012	120	108	680	280	272	9
CTAAGCAGC	BRDN00012	408	116	203	787	768	0
CTAAGGAAG	BRDN00012	278	219	38	521	432	0
CTAAGGCGG	BRDN00011	71	56	24	188	199	3
CTAAGGCTA	BRDN00011	108	89	233	217	170	68
CTAAGGCTC	BRDN00012	54	57	0	269	279	0
CTAAGGGCA	BRDN00011	261	293	1181	451	847	68
CTAAGGTAG	BRDN00011	24	0	1	243	40	0
CTAAGGTAT	BRDN00012	248	215	1375	563	434	7
CTAAGTACT	BRDN00011	171	111	53	156	172	22
CTAAGTATC	BRDN00012	399	242	349	676	419	12
CTAAGTCCAT	BRDN00012	455	385	911	792	868	100
CTAAGTGAC	BRDN00012	243	143	51	435	300	3

CTAAGTGTG	BRDN0001	103	68	2	294	421	23
CTAAGTTTGT	BRDN0001	358	293	100	731	541	12
CTAATACCTC	BRDN0001	93	53	0	219	101	0
CTAATATCTG	BRDN0001	167	211	125	433	603	27
CTAATATTGA	BRDN0001	196	189	146	330	283	27
CTAATCAAG	BRDN0001	404	424	237	861	411	10
CTAATCACGA	BRDN0001	507	573	699	881	909	51
CTAATCCATG	BRDN0001	164	184	104	319	159	0
CTAATCCATG	BRDN0001	157	166	865	272	265	7
CTAATCCTGA	BRDN0001	338	231	845	626	321	0
CTAATCGGT	BRDN0001	124	173	177	280	163	7
CTAATCGTGA	BRDN0001	186	198	176	430	199	41
CTAATGACA	BRDN0001	14	9	0	202	91	0
CTAATGACTT	BRDN0001	164	96	650	326	209	12
CTAATGAGA	BRDN0001	85	105	364	119	106	41
CTAATGAGG	BRDN0001	204	181	1203	335	262	21
CTAATGTCCG	BRDN0001	93	62	41	210	111	16
CTAATGTTAT	BRDN0001	181	113	231	274	250	0
CTAATGTTAT	BRDN0001	68	50	77	201	190	0
CTAATTACTT	BRDN0001	159	110	1	186	212	27
CTAATTAGTA	BRDN0001	213	77	912	466	422	38
CTAATTCCCA	BRDN0001	270	218	1026	413	335	60
CTAATTCCGT	BRDN0001	263	233	1042	508	643	18
CTACAAAAGA	BRDN0001	247	168	64	376	149	35
CTACAAAAGI	BRDN0001	219	293	1958	407	582	111
CTACAAACGA	BRDN0001	152	132	74	231	370	4
CTACAAACTA	BRDN0001	124	36	1248	358	500	36
CTACAAAGA	BRDN0001	214	126	311	360	298	41
CTACAAAGG	BRDN0001	322	292	2858	583	557	61
CTACAAATAC	BRDN0001	375	458	199	686	869	8
CTACAACAAC	BRDN0001	137	171	294	327	459	22
CTACAACATG	BRDN0001	351	297	777	660	430	42
CTACAACCCC	BRDN0001	297	0	1	745	200	0
CTACAACGGI	BRDN0001	407	420	664	833	1242	59
CTACAACTTA	BRDN0001	328	139	81	421	423	0
CTACAAGAAI	BRDN0001	70	91	1	306	149	0
CTACAAGACC	BRDN0001	365	316	1569	822	441	39
CTACAAGAGI	BRDN0001	157	125	132	174	384	0
CTACAAGCTA	BRDN0001	317	399	461	584	441	0
CTACAAGTAC	BRDN0001	385	448	123	522	500	43
CTACAAGTTT	BRDN0001	210	125	504	316	271	14
CTACAATAGA	BRDN0001	172	92	31	222	261	0
CTACAATCAA	BRDN0001	288	342	882	548	1000	39
CTACAATGAC	BRDN0001	161	0	352	770	544	0
CTACAATGAC	BRDN0001	41	33	0	632	532	0
CTACAATGTT	BRDN0001	352	527	1699	601	478	33
CTACACAAAA	BRDN0001	66	68	91	233	244	12
CTACACAAGA	BRDN0001	133	121	571	291	234	30
CTACACAATT	BRDN0001	328	298	40	697	493	59
CTACACACCC	BRDN0001	149	65	7	185	305	28
CTACACATTA	BRDN0001	197	103	210	309	261	0
CTACACCACT	BRDN0001	381	472	165	752	1156	86
CTACACCATC	BRDN0001	453	335	1254	1022	1079	47
CTACACCGAT	BRDN0001	310	140	908	434	451	21
CTACACCGCC	BRDN0001	139	54	1	337	157	7
CTACACCTGG	BRDN0001	143	222	0	249	693	0
CTACACGCGT	BRDN0001	368	301	619	589	545	12
CTACACGCTA	BRDN0001	92	193	479	301	193	21
CTACACGTGT	BRDN0001	89	36	30	66	203	14
CTACACGTTC	BRDN0001	91	95	14	149	129	0
CTACACTAGA	BRDN0001	98	90	501	251	152	3
CTACACTCCA	BRDN0001	409	467	927	814	662	46
CTACACTTGG	BRDN0001	120	58	540	288	629	18
CTACAGAACC	BRDN0001	140	193	55	271	487	12
CTACAGAAGA	BRDN0001	131	57	133	229	173	8
CTACAGAAGI	BRDN0001	118	74	12	235	144	37
CTACAGAATT	BRDN0001	202	156	13	387	492	0
CTACAGACAC	BRDN0001	311	227	5983	788	511	73
CTACAGACCT	BRDN0001	199	169	554	405	236	37
CTACAGAGCT	BRDN0001	459	355	595	869	894	34
CTACAGCCAA	BRDN0001	111	94	265	227	375	8
CTACAGCCAT	BRDN0001	452	385	334	1076	1282	25
CTACAGCCTA	BRDN0001	210	199	1611	410	440	26
CTACAGCGAI	BRDN0001	95	143	6	256	174	12
CTACAGCTAT	BRDN0001	119	128	1462	161	236	60
CTACAGGCCA	BRDN0001	326	580	541	709	488	46
CTACAGGGCI	BRDN0001	150	78	375	220	417	1
CTACAGGGG	BRDN0001	414	492	6765	697	892	222
CTACAGGTAI	BRDN0001	233	204	302	623	489	32
CTACAGTACC	BRDN0001	231	178	60	319	397	24
CTACAGTACC	BRDN0001	180	144	926	425	465	32
CTACAGTCAT	BRDN0001	292	253	410	567	429	0
CTACAGTGAT	BRDN0001	472	305	574	851	1064	29
CTACATACAA	BRDN0001	123	103	1262	297	389	19
CTACATCAAC	BRDN0001	124	5	1	584	794	0
CTACATCAAG	BRDN0001	326	448	116	534	748	49
CTACATCAGC	BRDN0001	158	111	117	338	535	41
CTACATCAGC	BRDN0001	317	215	391	388	665	0
CTACATCCAC	BRDN0001	132	53	81	327	216	0
CTACATCCAG	BRDN0001	251	214	589	414	434	25
CTACATCGAC	BRDN0001	230	25	1	421	96	0
CTACATCGTG	BRDN0001	94	132	3	312	485	0
CTACATCGTG	BRDN0001	237	40	187	301	299	3
CTACATGCAC	BRDN0001	250	74	0	549	330	0
CTACATGGA	BRDN0001	212	139	282	393	255	19
CTACATGGAI	BRDN0001	136	64	0	259	194	0
CTACATGGG	BRDN0001	359	297	34	692	579	18
CTACATGGTT	BRDN0001	122	204	50	207	109	9
CTACATGTCA	BRDN0001	352	52	146	592	660	0
CTACATTCAA	BRDN0001	305	342	8	749	473	3
CTACATTTAT	BRDN0001	177	186	441	425	639	54
CTACCAAAAT	BRDN0001	165	205	590	234	370	42
CTACCAAAGC	BRDN0001	30	37	61	447	326	0
CTACCAACAA	BRDN0001	416	347	728	586	387	27
CTACCAACCT	BRDN0001	17	6	0	212	185	0
CTACCAACGA	BRDN0001	220	80	190	412	366	0
CTACCAAGAC	BRDN0001	329	328	400	415	593	47
CTACCAAGG	BRDN0001	107	18	129	166	177	0
CTACCAAGTA	BRDN0001	203	175	32	302	521	0
CTACCAATGC	BRDN0001	330	354	323	571	1002	47
CTACCACCCG	BRDN0001	56	113	113	220	453	0
CTACCACCGG	BRDN0001	104	51	154	210	89	0
CTACCACTAC	BRDN0001	149	111	37	224	232	19
CTACCAGAAC	BRDN0001	305	282	646	634	422	35
CTACCAGATA	BRDN0001	114	54	0	302	250	0
CTACCAGCAC	BRDN0001	473	421	1954	693	353	58
CTACCAGCAC	BRDN0001	100	57	110	194	253	10
CTACCAGTAA	BRDN0001	408	360	1240	710	1368	50
CTACCAGTTC	BRDN0001	94	24	465	242	326	5
[truncated: 14,554,422 more chars]
